# Increased reporting of interstitial pneumonitis with combined EGFR-TKI and PD-1/PD-L1 inhibitor therapy in NSCLC: a pharmacovigilance analysis of 67,818 reports

**DOI:** 10.3389/fimmu.2026.1901390

**Published:** 2026-08-28

**Authors:** Jinyi Tao, Zhiwen Fu

**Affiliations:** Department of Pharmacy, Union Hospital, Tongji Medical College, Huazhong University of Science and Technology, Wuhan, Hubei, China

**Keywords:** EGFR-TKI, interstitial pneumonitis, NSCLC, PD-1/PD-L1 inhibitor, pharmacovigilance analysis

## Abstract

**Importance:**

Both epidermal growth factor receptor tyrosine kinase inhibitors (EGFR-TKIs) and PD-1/PD-L1 inhibitors are widely used in non–small cell lung cancer (NSCLC). The safety of their combination, particularly the risk of interstitial pneumonitis (IP), remains unclear but has critical implications for treatment strategies.

**Objective:**

To evaluate reporting patterns of IP associated with EGFR-TKI monotherapy, PD-1/PD-L1 inhibitor monotherapy, and their combination among NSCLC cases.

**Design:**

Retrospective, observational pharmacovigilance study using reports from the US Food and Drug Administration Adverse Event Reporting System (FAERS) submitted from January 1, 2015, to December 31, 2024. Multivariable logistic regression was applied to estimate adjusted odds ratios (aORs) with 95% CIs.

**Setting:**

Spontaneous adverse event reporting system capturing voluntary reports submitted globally by healthcare professionals, patients, and manufacturers; it is not a population-based registry, and the total number of patients exposed to each drug is unknown.

**Participants:**

A total of 67,818 NSCLC-related FAERS reports were identified, including 13,678 reporting EGFR-TKI monotherapy, 30,722 reporting PD-1/PD-L1 inhibitor monotherapy, and 307 reporting co-reported combination therapy. Reports were included if they indicated NSCLC and exposure to at least one EGFR-TKI or PD-1/PD-L1 inhibitor. IP was defined using standardized MedDRA preferred terms.

**Results:**

Among 67,818 NSCLC reports, 3,970 (5.85%) included IP-related preferred terms. The reporting proportions were 4.88% (668/13,678) for EGFR-TKIs, 10.59% (3,254/30,722) for PD-1/PD-L1 inhibitors, and 15.64% (48/307) for combination therapy. Compared with EGFR-TKIs, PD-1/PD-L1 inhibitors were associated with higher adjusted reporting odds (aOR, 1.77; 95% CI, 1.60–1.96), and combination therapy showed the highest adjusted reporting odds (aOR, 3.46; 95% CI, 2.46–4.88). Agent-specific variation was observed: durvalumab plus EGFR-TKI (IP reporting proportion, 47.22%; aOR, 16.58; 95% CI, 7.89–35.51) and nivolumab plus EGFR-TKI (IP reporting proportion, 21.05%; aOR, 4.26; 95% CI, 2.57–6.79) showed the highest reporting odds, whereas pembrolizumab plus EGFR-TKI (3.85%) and atezolizumab plus EGFR-TKI (5.06%) did not show significantly increased reporting odds; these small combination subgroups are exploratory.

**Conclusions and relevance:**

In this large FAERS-based pharmacovigilance analysis, combination therapy with EGFR-TKIs and PD-1/PD-L1 inhibitors was associated with markedly increased reporting of IP, with apparent heterogeneity across agents. As a spontaneous-reporting study without a defined denominator, these findings reflect a reporting association and safety signal rather than a confirmed clinical risk estimate. They support continued pharmacovigilance and prospective studies to clarify the safety of combining these therapies in NSCLC.

## Highlights

- Question: Is combining EGFR-TKIs with PD-1/PD-L1 inhibitors associated with disproportionately increased reporting of interstitial pneumonitis (IP) among NSCLC cases in the FAERS database?
- Findings: In this pharmacovigilance analysis of 67,818 FAERS reports, combination therapy was associated with a markedly higher proportion of IP reports (15.64%) than EGFR-TKI (4.88%) or PD-1/PD-L1 inhibitor monotherapy (10.59%), with correspondingly higher adjusted reporting odds. Durvalumab- and nivolumab-based regimens showed the greatest excess reporting, whereas pembrolizumab- and atezolizumab-based combinations showed no significant increase; these agent-specific combination findings are exploratory given small subgroup numbers.
- Meaning: Combining EGFR-TKIs with PD-1/PD-L1 inhibitors is associated with disproportionately increased reporting of IP, with apparent agent-specific variation. These findings represent a pharmacovigilance signal rather than a confirmed causal risk estimate and warrant vigilant clinical monitoring and further prospective study.

## Introduction

Non-small cell lung cancer (NSCLC) accounts for approximately 85% of all lung cancer cases and remains a leading cause of cancer-related mortality worldwide (1, 2). In recent years, the treatment landscape for NSCLC has undergone a paradigm shift with the advent of molecular targeted therapies and immune checkpoint inhibitors (ICIs) (3, 4). Among targeted agents, epidermal growth factor receptor tyrosine kinase inhibitors (EGFR-TKIs), including gefitinib, erlotinib, afatinib, and osimertinib, have significantly improved outcomes in patients harboring EGFR mutations (5). Concurrently, ICIs (such as nivolumab, pembrolizumab, and atezolizumab) targeting the programmed cell death 1 (PD-1) or its ligand (PD-L1) have demonstrated durable clinical benefits in a broad range of NSCLC patients, regardless of the presence of driver mutations (6).

Given their distinct mechanisms of action and non-overlapping indications, there has been growing interest in combining or sequencing EGFR-TKIs with ICIs to overcome resistance and broaden therapeutic efficacy (7). However, accumulating evidence suggests that such combinations may carry unexpected toxicities. One particularly severe and potentially fatal complication is interstitial pneumonitis (IP), an inflammatory lung condition characterized by alveolar and interstitial infiltration, often leading to respiratory failure (8). IP is a known adverse event associated with both EGFR-TKIs and ICIs at varying frequencies. The incidence of EGFR-TKI-induced IP ranges from 0.3% to 4.3%, with higher rates observed in East Asian populations (9, 10). Similarly, ICI-associated pneumonitis has been reported in approximately 3%-5% of patients in clinical trials, although the rates may be higher in real-world settings (11, 12).

While these risks are manageable when the drugs are used independently, the safety of combination or sequential therapy involving EGFR-TKIs and PD-1/PD-L1 inhibitors remains insufficiently understood; concurrent combination and sequential administration may carry distinct mechanisms and risks and, where possible, should be considered separately. Several case series and early-phase trials have reported a heightened risk of severe pneumonitis in patients receiving both EGFR-TKIs and ICIs, particularly when EGFR-TKIs are administered shortly after immunotherapy. For example, the TATTON trial, which evaluated osimertinib in combination with durvalumab, was prematurely halted due to unacceptably high rates of pneumonitis (13). Consistent with this, a retrospective cohort study of patients with EGFR-mutant NSCLC found that severe immune-related adverse events, including pneumonitis, were common when osimertinib was started shortly after prior PD-(L)1 blockade, whereas no such events were observed when osimertinib preceded PD-(L)1 inhibitor therapy, highlighting that treatment sequence may be an important determinant of risk (14). Moreover, post-marketing surveillance in Japan has identified fatal cases of interstitial lung disease in patients who received EGFR-TKIs following prior treatment with ICIs such as nivolumab (15).

Despite these alarming signals, comprehensive real-world data on the occurrence and risk of IP associated with EGFR-TKI, when combined with PD-1/PD-L1 inhibitors, remain scarce. Most existing studies are limited by small sample sizes, single-center designs, or a lack of control groups. The U.S. Food and Drug Administration Adverse Event Reporting System (FAERS) provides a comprehensive, publicly accessible pharmacovigilance database that captures real-world toxicity signals across diverse populations and treatment regimens (16, 17). In this study, we used the FAERS database to conduct a retrospective pharmacovigilance analysis of 67,818 NSCLC-related reports. We aimed to describe the reporting proportion and reporting odds of IP among cases treated with EGFR-TKIs, PD-1/PD-L1 inhibitors, or both, and to evaluate whether combination therapy was associated with disproportionately increased reporting of IP.

## Methods

### Study design and data source

This retrospective observational study was conducted using data from the FAERS database, a publicly available post-marketing safety surveillance database. FAERS collects spontaneous reports of adverse drug events from healthcare professionals, patients, and pharmaceutical manufacturers worldwide through the FDA MedWatch program (18). This study included data collected between January 1, 2015, and December 31, 2024. The study protocol for our observational, retrospective, cross-sectional pharmacovigilance study of the FAERS database was registered on ClinicalTrials.gov (No. NCT07036016) and followed the Strengthening the Reporting of Observational Studies in Epidemiology (STROBE) reporting guideline. The institutional review board approval and informed consent were exempt since data in FAERS are anonymized and publicly available.

### Study population and case identification

We identified NSCLC reports using the following MedDRA preferred terms (PTs): “Non-small cell lung cancer”, “Lung neoplasm malignant”, “Adenocarcinoma of lung”, “Squamous cell carcinoma of lung”, “Lung cancer metastatic”, and related NSCLC indication terms. Study drugs were searched using generic names, brand names, and common spelling variants: gefitinib (Iressa), erlotinib (Tarceva), afatinib (Gilotrif), osimertinib (Tagrisso), nivolumab (Opdivo), pembrolizumab (Keytruda), atezolizumab (Tecentriq), and durvalumab (Imfinzi). Drug involvement was determined from the FAERS drug file using the role codes Primary Suspect (PS), Secondary Suspect (SS), Concomitant (C), and Interacting (I); PS and SS roles were used to define exposure. Reports were categorized into mutually exclusive groups based on treatment exposure: EGFR-TKI monotherapy, PD-1/PD-L1 inhibitor monotherapy, or combination therapy, the last defined as co-reporting of both an EGFR-TKI and a PD-1/PD-L1 inhibitor on the same FAERS case. Reports were considered not classifiable if they contained incomplete drug information, multiple drugs within the same therapeutic class precluding unique assignment, or non-suspect drug roles only. These reports were excluded from treatment-group comparisons. Because FAERS does not capture treatment timing or dosing schedules, this combination category cannot reliably distinguish concurrent (simultaneous) administration from closely sequential use (e.g., an EGFR-TKI initiated shortly before or after an immune checkpoint inhibitor); we therefore use “combination” to denote co-reported exposure rather than confirmed concurrent dosing, and we discuss this limitation further below. Duplicate case reports were removed by retaining the latest record for each CASEID according to FDA_DT and, when necessary, the highest PRIMARYID.

### Outcome measure

The primary outcome was the reporting proportion of IP in the NSCLC cases reported to the FAERS database, defined by the presence of at least one preferred term related to pneumonitis in the MedDRA preferred term, including: “Interstitial lung disease”, “Pneumonitis”, “Pulmonary fibrosis”, “Organizing pneumonia”, “Diffuse alveolar damage”. Reports containing more than one of these preferred terms were counted once as a single IP case rather than multiple times. All candidate IP reports were independently reviewed by two investigators (J.T. and Z.F.) for consistency in event coding and for plausibility of the NSCLC indication; discordant cases were resolved by consensus discussion.

### Statistical analysis

Descriptive statistics were used to summarize patient demographics, including age, sex, and country of origin. Because FAERS is a spontaneous reporting system without a defined population denominator, all percentages in this study represent reporting proportions among included cases rather than true clinical incidence or absolute risk. The reporting proportion of IP was calculated for each treatment group. Categorical variables were reported as frequencies and percentages, whereas continuous variables were expressed as mean (standard deviation). Multivariable logistic regression was performed to estimate the adjusted odds ratios (aORs) and 95% confidence intervals (CIs) for the association between the treatment group and the reporting of IP. Covariates included in the model were age, sex, and treatment category (EGFR-TKI monotherapy, PD-1/PD-L1 inhibitor monotherapy, or combination therapy); reporting country, reporting year, and reporter type were not included in this primary model. We also did not additionally perform formal disproportionality (signal-detection) analyses, such as the reporting odds ratio or proportional reporting ratio, in this version of the manuscript; our primary aim was to compare reporting patterns between prespecified, clinically defined exposure groups using multivariable-adjusted logistic regression rather than to screen broadly for signals across the FAERS database. All analyses were performed using R software (version 4.3.3, R Foundation for Statistical Computing). A two-sided P-value < 0.05 was considered statistically significant, and odds ratios from this analysis should be interpreted as measures of reporting association rather than clinical risk.

## Results

**Figure 1:**
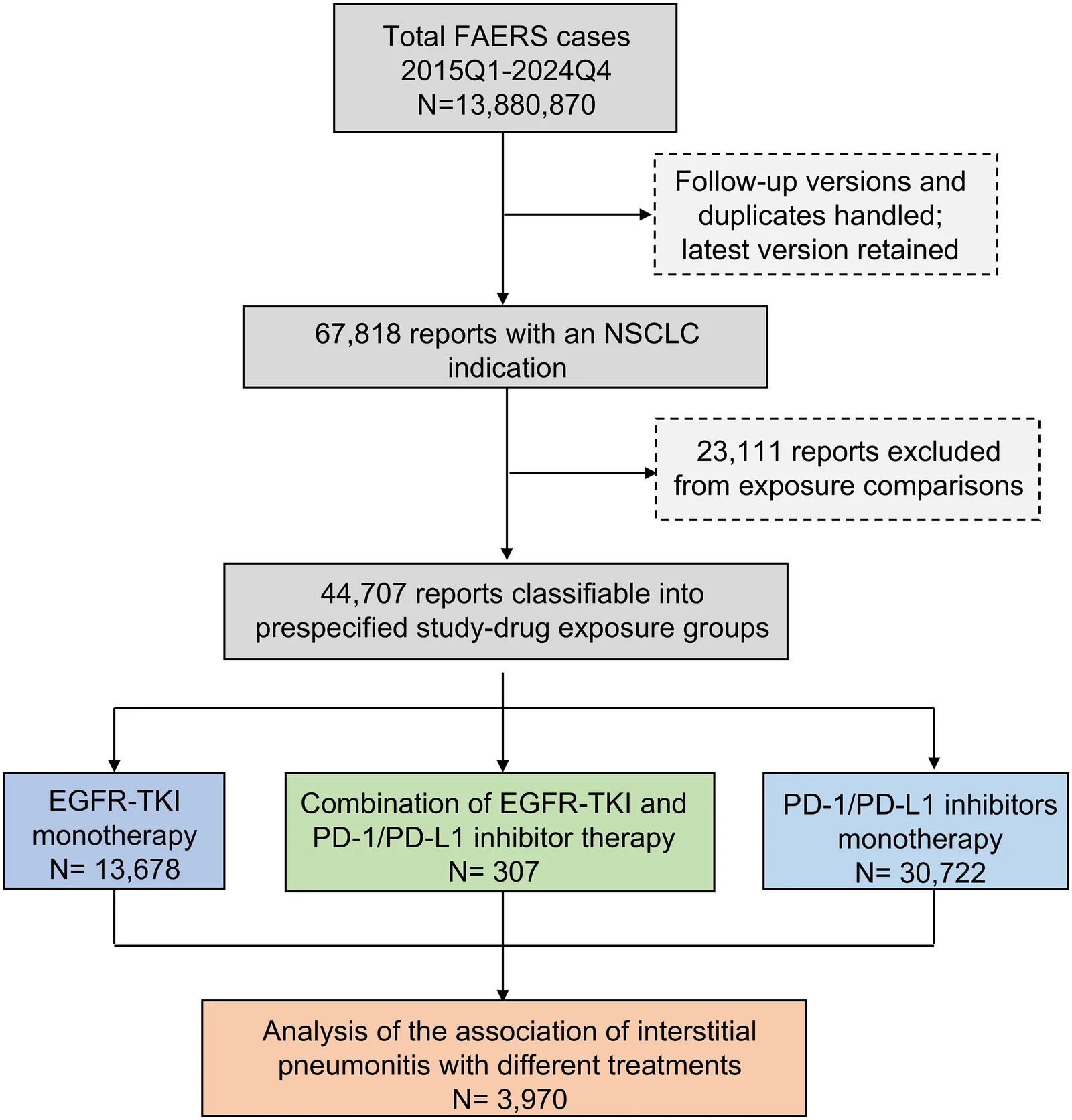
Detailed report-selection flow diagram. In this study, 13,880,870 reports were retrieved from the FAERS database from the first quarter of 2015 to the fourth quarter of 2024. By using NSCLC-related MedDRA preferred terms, 67,818 NSCLC-related reports remained after excluding duplicate reports and reports not meeting drug-exposure criteria. Of 67,818 NSCLC reports, 44,707 were classifiable as EGFR-TKI-only (n = 13,678), PD-1/PD-L1 inhibitor-only (n = 30,722), or dual-class exposure (n = 307), whereas 23,111 reports were not classifiable under the prespecified study-drug criteria and were excluded from exposure comparisons. Among these, 3,970 cases were reported to have interstitial pneumonitis and were ultimately enrolled.

A total of 67,818 NSCLC-related reports were identified in the FAERS database from the first quarter of 2015 to the fourth quarter of 2024. Among these, 13,678 (20.17%) reports involved EGFR-TKI monotherapy, 30,722 (45.30%) involved PD-1/PD-L1 inhibitor monotherapy, and 307 (0.45%) reports involved combination therapy with an EGFR-TKI and a PD-1/PD-L1 inhibitor, whereas 23,111 reports were not classifiable under the prespecified study-drug criteria and were excluded from exposure comparisons because of incomplete drug information, multiple drugs within the same therapeutic class precluding unique assignment, or non-suspect drug roles only (**Figure 1**). Patient demographics and baseline characteristics are summarized in **Table 1**. The mean (SD) age across the study population was 65.6 (11.40) years, and predominantly male (47.91%). Geographically, the top five countries with the largest number were Japan (18,443 cases, 27.19%), the United States (14,201 cases, 20.94%), China (4,316 cases, 6.36%), Germany (4,282 cases, 6.31%), and France (3,295 cases, 4.86%).

**Table 1:** Characteristics of 67,818 NSCLC-related FAERS reports and 3,970 reports with interstitial pneumonitis.

Characteristic	All NSCLC cases (N = 67,818)	IP-coded reports (N = 3,970)
Age, mean (SD), y	65.6 (11.40) (age not reported, n=19,445)	68.8 (9.78) (age not reported, n=755)
Sex, no. (%)		
Female	25,181 (37.10)	1,065 (26.80)
Male	32,475 (47.91)	2,449 (61.70)
Not reported	10,162 (15.01)	456 (11.50)
Reporters, no. (%)		
Healthcare professional	50,131 (73.95)	3,121 (78.62)
Non-healthcare professional	15,639 (23.03)	645 (16.24)
Not reported	2,048 (3.02)	204 (5.14)
Reporting countries (top 5), no. (%)		
Japan	18,443 (27.19)	2,318 (58.39)
United States	14,201 (20.94)	601 (15.14)
China	4,316 (6.36)	202 (5.09)
Germany	4,282 (6.31)	127 (3.20)
France	3,295 (4.86)	106 (2.67)
Reporting year, no. (%)		
2015	3,674 (5.42)	84 (2.12)
2016	5,267 (7.77)	293 (7.38)
2017	6,279 (9.26)	330 (8.31)
2018	6,869 (10.13)	495 (12.51)
2019	7,970 (11.75)	881 (22.23)
2020	7,453 (10.99)	562 (14.22)
2021	7,397 (10.91)	427 (10.81)
2022	8,373 (12.35)	374 (9.45)
2023	6,729 (9.92)	243 (6.12)
2024	7,807 (11.51)	280 (7.05)
Treatments, no. (%)		
EGFR-TKIs	13,678 (20.17)	668 (16.83)
PD-1/PD-L1 inhibitor	30,722 (45.30)	3,254 (81.96)
Combination therapy	307 (0.45)	48 (1.21)

NSCLC, non–small cell lung cancer; EGFR-TKI, epidermal growth factor receptor–tyrosine kinase inhibitor; PD-1/PD-L1, programmed death-1/programmed death-ligand 1; SD, standard deviation.

Among these NSCLC-related reports, 3,970 cases included IP, corresponding to an overall crude reporting proportion of 5.85%. The number of reports that included IP-related preferred terms increased during the 2015–2024 period, with 881 (22.23%) cases reported in 2019. Among reports involving EGFR-TKI monotherapy, 668 of 13,678 (4.88%) included IP. In the PD-1/PD-L1 inhibitor monotherapy group, 3,254 of 30,722 (10.59%) included IP. In contrast, 48 of 307 reports involving both drug classes included IP-related preferred terms, resulting in a significantly higher reporting proportion of 15.64% (95% CI, 11.76%-20.19%; P <.001) compared with either monotherapy group.

**Figure 2:**
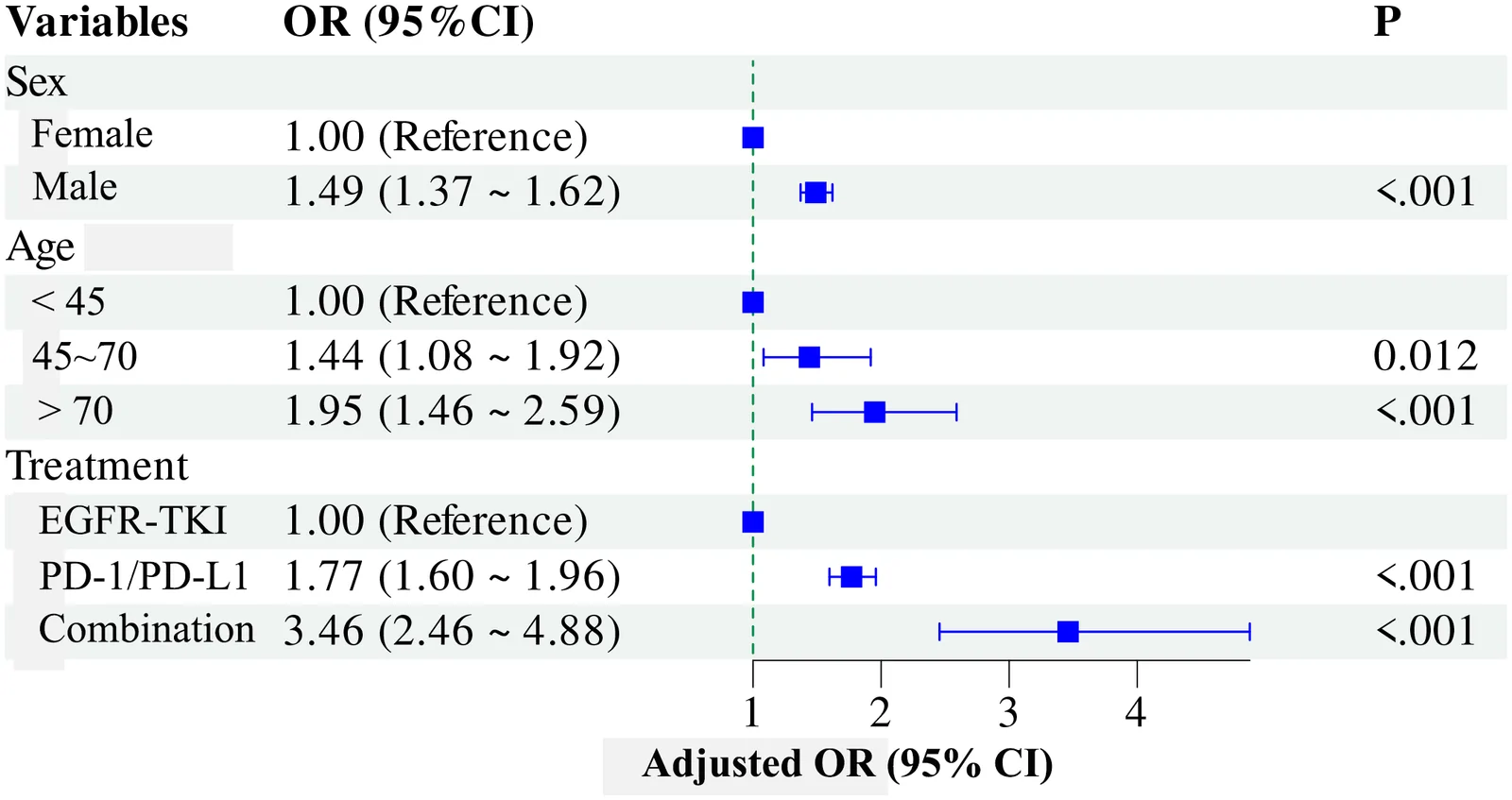
Forest plot showing adjusted odds ratios (aORs) with 95% confidence intervals (CIs) from the multivariable logistic regression model for the reporting of interstitial pneumonitis, by sex, age group, and treatment category (EGFR-TKI monotherapy as reference). P-values indicate statistical significance.

To confirm this observation, we conducted a multivariable logistic regression analysis of the interaction between PD-1/PD-L1 inhibitor and EGFR-TKI. The multivariable logistic regression adjusting for age, sex, and treatment category demonstrated increased adjusted reporting odds of IP associated with PD-1/PD-L1 inhibitor and combination therapy reports (EGFR-TKI monotherapy as the reference) (**Table 2**). As shown in **Figure 2**, the forest plot illustrates the adjusted odds ratios (aOR) for IP across demographic and treatment subgroups. Male patients had significantly higher adjusted reporting odds than female patients (aOR, 1.49; 95% CI, 1.37–1.62; P <0.001). Age was also associated with higher adjusted reporting odds: patients aged 45–70 years had an aOR of 1.44 (95% CI, 1.08–1.92; P = 0.012), whereas those older than 70 years had an aOR of 1.95 (95% CI, 1.46–2.59; P <0.001) relative to those younger than 45 years. Treatment category showed the strongest association: PD-1/PD-L1 inhibitor monotherapy was associated with 1.77-fold higher adjusted reporting odds (95% CI, 1.60–1.96; P <0.001), and combination therapy showed the highest adjusted reporting odds (aOR, 3.46; 95% CI, 2.46–4.88; P <0.001) compared with EGFR-TKI monotherapy. Collectively, these data indicate that both patient demographics and treatment category were independently associated with the reporting of pneumonitis in FAERS. However, it should be noted that these associations should not be interpreted as direct evidence of causal clinical risk because reporting country, reporting year, and reporter type were not adjusted, and clinical confounders such as smoking history, underlying lung disease, and disease stage were unavailable.

**Table 2:** Association between EGFR-TKIs and PD-1/PD-L1 inhibitors with reporting of interstitial pneumonitis among NSCLC cases in FAERS.

Variable	Cases (N = 67,818)	IP cases (N = 3,970)	Proportion of IP (%, 95% CI)	Crude OR (95% CI)	Adjusted OR (95% CI)	*P* value
Sex						
Female	25,181	1,065	4.23 (3.98–4.49)	1 [reference]	1 [reference]	
Male	32,475	2,449	7.54 (7.26–7.83)	1.83 (1.70–1.97)	1.49 (1.37–1.62)	<0.001
Age						
< 45	2,225	53	2.38 (1.79–3.10)	1 [reference]	1 [reference]	
45~70	28,739	1,711	5.95 (5.68–6.23)	2.49 (1.88–3.28)	1.44 (1.08–1.92)	0.012
>70	17,409	1,451	8.33 (7.93–8.76)	3.67 (2.78–4.84)	1.95 (1.46–2.59)	<0.001
Treatment						
EGFR-TKIs	13,678	668	4.88 (4.53–5.26)	1 [reference]	1 [reference]	
PD-1/PD-L1 inhibitors	30,722	3,254	10.59 (10.25–10.94)	2.31 (2.12–2.51)	1.77 (1.60–1.96)	<0.001
Combination	307	48	15.64 (11.76–20.19)	3.61 (2.63–4.96)	3.46 (2.46–4.88)	<0.001

IP, interstitial pneumonitis; CI, confidence interval; EGFR-TKI, epidermal growth factor receptor–tyrosine kinase inhibitor; PD-1/PD-L1, programmed death-1/programmed death-ligand 1.

Among reports involving PD-1/PD-L1 inhibitors and combination therapy, the reporting association with IP varied substantially across different PD-1/PD-L1 inhibitors, and these agent-specific estimates should be considered exploratory given the small size of some subgroups (**Table 3**). Among PD-1/PD-L1 inhibitor monotherapies, durvalumab-associated reports had the highest adjusted odds ratio (aOR, 7.60; 95% CI, 6.93–8.33; P <0.001), followed by pembrolizumab (aOR, 1.41; 95% CI, 1.28–1.55; P <0.001) and nivolumab (aOR, 1.37; 95% CI, 1.26–1.49; P <0.001), whereas atezolizumab was associated with a modest but statistically significant reduction in adjusted reporting odds (aOR, 0.83; 95% CI, 0.72–0.95; P = 0.01). Among combination reports, the highest reporting association was observed for durvalumab plus EGFR-TKI, with an IP reporting proportion of 47.22% and an adjusted odds ratio of 16.58 (95% CI, 7.89–35.51; P <0.001), based on 36 reports; the durvalumab-combination estimate in particular should be interpreted with caution, as durvalumab is frequently used after chemoradiotherapy in stage III NSCLC, where prior thoracic radiotherapy is an important potential confounder for pneumonitis that could not be assessed with these data. Similarly, nivolumab plus EGFR-TKI was associated with an IP reporting proportion of 21.05% and an adjusted odds ratio of 4.26 (95% CI, 2.57–6.79; P <0.001), based on 114 reports. In contrast, pembrolizumab plus EGFR-TKI (IP reporting proportion 3.85%, n=78) and atezolizumab plus EGFR-TKI (IP reporting proportion 5.06%, n=79) did not show significantly increased reporting odds relative to EGFR-TKI monotherapy. Given the small numbers of reports in several combination subgroups, these agent-specific findings should be regarded as hypothesis-generating rather than definitive evidence of differential drug-specific pneumonitis risk.

**Table 3:** Association between EGFR-TKIs combined with different PD-1/PD-L1 inhibitors and reporting of interstitial pneumonitis among NSCLC cases in FAERS.

Variable	Cases (N = 67,818)	IP cases (N = 3,970)	Proportion of IP (95% CI)	Crude OR (95% CI)	Adjusted OR (95% CI)	*P* value
EGFR-TKIs	13,678	668	4.88 (4.53–5.26)	1 [reference]	1 [reference]	
PD-1/PD-L1 inhibitors						
Pembrolizumab (PD-1)	8,907	811	9.11 (8.52–9.72)	1.95 (1.75–2.17)	1.41 (1.28–1.55)	<0.001
Nivolumab (PD-1)	12,533	999	7.96 (7.49–8.45)	1.68 (1.52–1.86)	1.37 (1.26–1.49)	<0.001
Durvalumab (PD-L1)	4,152	1,122	27.02 (25.68–28.40)	7.21 (6.50–8.00)	7.60 (6.93–8.33)	<0.001
Atezolizumab (PD-L1)	5,110	322	6.30 (5.65–7.00)	1.31 (1.14–1.50)	0.83 (0.72–0.95)	0.01
Combination						
Pembrolizumab plus EGFR-TKI	78	3	3.85 (0.80–10.93)	0.78 (0.25–2.48)	0.65 (0.11–2.10)	0.55
Nivolumab plus EGFR-TKI	114	24	21.05 (13.98–29.69)	5.19 (3.29–8.20)	4.26 (2.57–6.79)	<0.001
Durvalumab plus EGFR-TKI	36	17	47.22 (30.41–64.51)	17.43 (9.02–33.68)	16.58 (7.89–35.51)	<0.001
Atezolizumab plus EGFR-TKI	79	4	5.06 (1.40–12.46)	1.04 (0.38–2.85)	0.84 (0.2–2.28)	0.76

IP, interstitial pneumonitis; CI, confidence interval; EGFR-TKI, epidermal growth factor receptor–tyrosine kinase inhibitor; PD-1/PD-L1, programmed death-1/programmed death-ligand 1.

## Discussion

Based on the 67,818 NSCLC reports in the FAERS database from the first quarter of 2015 to the fourth quarter of 2024, this study systematically analyzed the reporting association between EGFR-TKI monotherapy, PD-1/PD-L1 inhibitor monotherapy, their combination therapy, and the reporting association of IP. It suggests the independent effects of patient demographic characteristics, treatment strategies, and drug types on IP reporting, providing pharmacovigilance signals that may help inform clinical treatment decisions and safety management in NSCLC.

The most pivotal finding of this study is the higher reporting association of IP with the combination of EGFR-TKIs and PD-1/PD-L1 inhibitors. Data showed that the reporting proportion of IP in the combination therapy group (15.64%) was significantly higher than that in the EGFR-TKI monotherapy group (4.88%) and the PD-1/PD-L1 inhibitor monotherapy group (10.59%). In the multivariable logistic regression analysis, the adjusted odds ratio (aOR) for combination therapy compared with EGFR-TKI monotherapy was as high as 3.46 (95% CI, 2.46–4.88; P <0.001). Our results are consistent with prior reports that raised concerns about excessive pulmonary toxicity when EGFR-TKIs were combined with ICIs. The TATTON trial, which included osimertinib combined with durvalumab arms, was discontinued due to unexpectedly high rates of interstitial lung disease (13). Similarly, a Japanese post-marketing series reported severe and sometimes fatal IP when EGFR-TKIs were administered following prior PD-1 inhibitor therapy (15). By analyzing over 67,000 FAERS reports, our study extends these observations and describes the reporting patterns across multiple drug classes, thereby strengthening the pharmacovigilance signal warranting clinical caution. It should be emphasized, however, that FAERS cannot distinguish concurrent combination therapy from sequential ICI-then-EGFR-TKI exposure, and the present signal therefore reflects combined or overlapping drug exposure rather than a confirmed effect of concurrent administration.

Mechanistically, the increased IP reporting observed with combined exposure may plausibly be explained by a compounding, rather than formally demonstrated synergistic, effect of the two drug classes on the pulmonary microenvironment; this study did not perform a formal statistical interaction analysis, and the following discussion is hypothesis-generating. EGFR-TKIs exert antitumor effects by inhibiting the EGFR signaling pathway, but EGFR also plays a crucial role in maintaining epithelial cell homeostasis and promoting tissue repair in the lungs (19, 20). Inhibition of EGFR can increase apoptosis of lung epithelial cells and impair the epithelial barrier, laying the foundation for inflammatory responses (21). Similarly, PD-1/PD-L1 inhibitors activate antitumor immune responses by releasing T cells from checkpoint inhibition but may also trigger “off-target” immune-related adverse events (22, 23). IP associated with PD-1/PD-L1 inhibitors has been linked to excessive T-cell infiltration into lung tissue and the release of proinflammatory cytokines (e.g., IFN-γ and TNF-α), which may induce interstitial inflammation and fibrosis (22, 24). It is plausible that when the two drug classes are combined, EGFR-TKI-induced lung epithelial damage may enhance the “immunogenicity” of lung tissue, further amplifying the immune-mediated inflammatory response triggered by PD-1/PD-L1 inhibitors (25, 26). This may plausibly contribute to a cycle of “epithelial damage–immune activation–inflammation exacerbation” that could help explain the significantly higher IP reporting in the combination group; these mechanistic hypotheses require experimental confirmation.

Additionally, the analysis of demographic characteristics provides context for considering reporting patterns. Male patients had significantly higher adjusted reporting odds for IP than female patients (aOR, 1.49; 95% CI, 1.37–1.62; P <0.001), and age was positively associated with reporting odds: patients aged 45–70 years had an aOR of 1.44 (95% CI, 1.08–1.92; P = 0.012), whereas those older than 70 years had an aOR of 1.95 (95% CI, 1.46–2.59; P <0.001) (reference: patients younger than 45 years). This finding is not isolated, and several similar sex- and age-related differences in cancer treatment–associated toxicities have been observed in previous studies (27–30). Plausible explanations include higher smoking exposure and more severe baseline lung damage in male patients, as well as dysregulated immune function and impaired lung repair capacity in elderly patients (31, 32). In clinical practice, for male and elderly NSCLC patients, particularly those receiving combined or overlapping EGFR-TKI and PD-1/PD-L1 inhibitor therapy, enhanced monitoring for early signs of IP may be warranted to enable early detection and intervention.

Another aspect of this study is the observation of heterogeneity in IP reporting among different PD-1/PD-L1 inhibitors, which may inform—though not determine—individualized drug selection (33, 34). In monotherapy, durvalumab had the highest IP reporting proportion (aOR, 7.60; 95% CI, 6.93–8.33; P <0.001), followed by pembrolizumab (aOR, 1.41; 95% CI, 1.28–1.55; P <0.001) and nivolumab (aOR, 1.37; 95% CI, 1.26–1.49; P <0.001). In contrast, atezolizumab was associated with a modest but statistically significant reduction in adjusted reporting odds (aOR, 0.83; 95% CI, 0.72–0.95; P = .01). This difference appeared more pronounced in combination therapy: the reporting proportion of IP with durvalumab plus EGFR-TKI was 47.22% (aOR, 16.58; 95% CI, 7.89–35.51; P <0.001, n=36 reports), and nivolumab plus EGFR-TKI was associated with an IP reporting proportion of 21.05% (aOR, 4.26; 95% CI, 2.57–6.79; P <0.001, n=114 reports). In contrast, pembrolizumab plus EGFR-TKI (3.85%) and atezolizumab plus EGFR-TKI (5.06%) showed no significant difference in reporting odds compared with EGFR-TKI monotherapy. Given the small number of reports underlying several of these combination-specific estimates, they should be regarded as exploratory.

From a pharmacologic perspective, these differences in IP reporting may plausibly be related to several factors, which remain speculative in the absence of direct mechanistic testing: first, variations in target affinity and selectivity. As a PD-L1 inhibitor, durvalumab has been reported to have higher affinity for PD-L1 than other agents in its class and may exhibit cross-reactivity with other immune-related molecules (e.g., B7-H1), which could plausibly contribute to excessive immune activation (35). In contrast, beyond PD-L1 inhibition, atezolizumab may modulate the expression of other immunosuppressive molecules (e.g., IDO and TIM-3) in the tumor microenvironment, balancing immune activation and inflammation control to reduce IP reporting (36). Second is differences in pharmacokinetics and tissue distribution. PD-1/PD-L1 inhibitors vary in half-life, clearance rate, and accumulation in lung tissue (37). For example, durvalumab’s long half-life may result in sustained drug action in the lungs, increasing the cumulative risk of inflammation, whereas atezolizumab has lower lung tissue penetration, potentially reducing immune-mediated damage to normal lung tissue (38). These mechanisms are proposed as possible explanations and require dedicated pharmacologic studies for confirmation.

These reporting patterns may have potential clinical relevance, but they should not be used to guide specific regimen selection without prospective confirmation. Given the disproportionately high IP reporting observed with durvalumab- and nivolumab-based regimens, clinicians caring for patients receiving these combinations may wish to maintain heightened awareness for early pulmonary symptoms while recognizing that our data cannot establish comparative safety between individual PD-1/PD-L1 inhibitors when combined with EGFR-TKIs. Whether strategies such as dose adjustment or prophylactic anti-inflammatory measures could mitigate this signal is an open question that would need to be explored (39).

Several limitations also warrant discussion. First, as with any spontaneous reporting system, FAERS is subject to underreporting, reporting bias, and the absence of a verified denominator of all treated patients; because unaffected patients are not systematically captured, none of the percentages reported here should be interpreted as a true clinical incidence or absolute risk. Selective reporting may distort results in either direction: severe or fatal IP events may be reported disproportionately more often than mild events, which would tend to inflate the apparent reporting proportion, whereas general underreporting to FAERS and differences in reporting practices across countries/regions (e.g., higher reporting volumes from Japan and the United States than from China and Germany) would tend to reduce it. Because the net direction and magnitude of this bias cannot be determined from the available data, we emphasize that the reporting proportions of IP should be interpreted as relative reporting patterns rather than as estimates of true clinical incidence. Second, the FAERS database lacks granular clinical information such as smoking history, baseline interstitial lung disease or chronic obstructive pulmonary disease, prior thoracic radiotherapy, concurrent infection, disease stage, prior chemotherapy, EGFR mutation subtype (e.g., 19del and L858R), and treatment dose, duration, and sequence. Any of these factors could independently affect pneumonitis reporting, and their absence means we could not fully adjust for confounding; the reported associations should therefore be interpreted cautiously (40). Third, causality cannot be definitively established from FAERS data through a retrospective analysis. As an observational study, this research only identifies associations between treatment regimens and IP reporting, rather than establishing causality. For example, IP may be associated with tumor progression (e.g., lung metastases) or concurrent infections rather than drug effects alone (40, 41). Prospective cohort studies or mechanistic experiments are needed to verify causal relationships. Fourth, as noted in the Methods, our combination-therapy group reflects co-reported exposure to an EGFR-TKI and a PD-1/PD-L1 inhibitor and could not fully distinguish concurrent from closely sequential administration; the case-identification process and the manual review of IP cases also relied on criteria that, while described in the Methods, carry inherent subjectivity. Finally, this analysis adjusted only for age, sex, and treatment category and did not additionally adjust for reporting country, reporting year, or reporter type, nor did it include formal disproportionality (signal-detection) analyses such as the reporting odds ratio or proportional reporting ratio; incorporating these approaches represents an important direction for future analysis of this dataset.

## Conclusions

In conclusion, using large-scale FAERS pharmacovigilance data, this study identifies disproportionately high reporting of IP with EGFR-TKI and PD-1/PD-L1 inhibitor combinations, apparent agent-specific differences in PD-1/PD-L1 inhibitor–related IP reporting, and associations with demographic factors. As a spontaneous-reporting analysis without a defined denominator or full adjustment for clinical confounders, these findings represent a hypothesis-generating pharmacovigilance signal rather than a confirmed measure of clinical risk and should not by themselves be used to guide treatment selection. Future prospective cohort studies, additional pharmacovigilance analyses (including formal disproportionality methods), and mechanistic studies are needed to confirm these observations and clarify the safety of combining EGFR-TKIs with PD-1/PD-L1 inhibitors in NSCLC.

## Data Availability

The original contributions presented in the study are included in the article/**Supplementary Material**. Further inquiries can be directed to the corresponding author.
