# Supplementary material for: Increased reporting of interstitial pneumonitis with combined EGFR-TKI and PD-1/PD-L1 inhibitor therapy in NSCLC: a pharmacovigilance analysis of 67,818 reports
Source: Front Immunol. 2026 Aug 28;17:1901390. doi: 10.3389/fimmu.2026.1901390 (PMC13562000; doi:10.3389/fimmu.2026.1901390)
Supplement: Supplementary file 1 [file DataSheet1.pdf]

caseid	sex	occp_cod	ter_co	year	tki	-1/pd-	pem	dur	nivo	ate	:ki_pd:	em_tk	vo_tı	r_tk	te_tk	ip	age
10057390	f	md	US	2015	0	1	0	0	0	1	0	0	0	0	0	0	71
10070361	m	ph	JP	2015	0	0	0	0	0	0	0	0	0	0	0	0	70
10144968	m	md	CR	2015	0	0	0	0	0	0	0	0	0	0	0	0	66
10176376	f	Not repo	PL	2015	0	0	0	0	0	0	0	0	0	0	0	0	59
10221445	f	md	JP	2015	0	0	0	0	0	0	0	0	0	0	0	0	54
10252754	f	md	Not re	2015	1	0	0	0	0	0	0	0	0	0	0	0	54
10252758	f	md	JP	2015	1	0	0	0	0	0	0	0	0	0	0	0	59
10253071	f	md	JP	2015	0	0	0	0	0	0	0	0	0	0	0	0	52
10262050	f	md	TH	2015	0	0	0	0	0	0	0	0	0	0	0	0	69
10270012	f	md	Not re	2015	1	0	0	0	0	0	0	0	0	0	0	0	51
10276894	m	ot	TW	2015	0	0	0	0	0	0	0	0	0	0	0	0	56
10284054	f	ot	US	2015	0	0	0	0	0	0	0	0	0	0	0	0	61
10289515	m	ot	GB	2015	0	0	0	0	0	0	0	0	0	0	0	0	29
10296256	m	md	JP	2015	0	0	0	0	0	0	0	0	0	0	0	0	52
10296486	m	cn	GB	2015	1	0	0	0	0	0	0	0	0	0	0	0	
10298307	f	md	JP	2015	0	0	0	0	0	0	0	0	0	0	0	0	53
10304827	f	md	FR	2015	0	0	0	0	0	0	0	0	0	0	0	0	64
10306418	f	md	Not re	2015	1	0	0	0	0	0	0	0	0	0	0	0	74
10308729	f	md	US	2015	1	0	0	0	0	0	0	0	0	0	0	0	72
10330447	m	ot	DE	2015	0	0	0	0	0	0	0	0	0	0	0	0	71
10333512	f	md	DK	2015	0	1	1	0	0	0	0	0	0	0	0	1	53
10334937	m	md	KR	2015	0	1	1	0	0	0	0	0	0	0	0	0	77
10335142	m	md	ES	2015	0	1	1	0	0	0	0	0	0	0	0	0	59
10343748	f	md	US	2015	1	0	0	0	0	0	0	0	0	0	0	0	75
10344225	f	md	JP	2015	0	0	0	0	0	0	0	0	0	0	0	0	40
10359057	m	cn	TH	2015	0	0	0	0	0	0	0	0	0	0	0	0	56
10359613	m	cn	GB	2015	1	0	0	0	0	0	0	0	0	0	0	0	76
10360668	m	cn	IE	2015	0	0	0	0	0	0	0	0	0	0	0	0	46
10387600	f	md	JP	2015	0	0	0	0	0	0	0	0	0	0	0	0	46
10391315	m	cn	GB	2015	1	0	0	0	0	0	0	0	0	0	0	0	
10392670	m	cn	US	2015	0	0	0	0	0	0	0	0	0	0	0	0	59
10396122	Not	cn	GB	2015	1	0	0	0	0	0	0	0	0	0	0	0	
10402554	f	md	DE	2015	0	0	0	0	0	0	0	0	0	0	0	0	60

10404740	m	cn	TH	2015	0	0	0	0	0	0	0	0	0	0	0	0	70
10408766	m	ot	ES	2015	0	0	0	0	0	0	0	0	0	0	0	0	77
10410037	f	md	FR	2015	0	0	0	0	0	0	0	0	0	0	0	0	60
10414311	f	ot	SE	2015	0	0	0	0	0	0	0	0	0	0	0	0	67
10416445	m	cn	GB	2015	1	0	0	0	0	0	0	0	0	0	0	0	
10416640	m	md	US	2015	0	0	0	0	0	0	0	0	0	0	0	0	42
10419417	f	md	AU	2015	0	0	0	0	0	0	0	0	0	0	0	0	55
10441676	f	md	JP	2015	0	0	0	0	0	0	0	0	0	0	0	0	69
10451052	m	ot	GB	2015	0	0	0	0	0	0	0	0	0	0	0	0	67
10452551	m	ot	US	2015	0	0	0	0	0	0	0	0	0	0	0	0	56
10452700	m	md	DE	2015	0	1	1	0	0	0	0	0	0	0	0	1	62
10462277	m	cn	GB	2015	0	0	0	0	0	0	0	0	0	0	0	0	67
10464719	m	cn	GB	2015	1	0	0	0	0	0	0	0	0	0	0	0	83
10466431	m	ot	GB	2015	0	0	0	0	0	0	0	0	0	0	0	0	67
10466847	f	cn	US	2015	0	0	0	0	0	0	0	0	0	0	0	0	79
10466955	f	md	BE	2015	0	0	0	0	0	0	0	0	0	0	0	0	65
10468719	m	cn	US	2015	0	1	0	0	1	0	0	0	0	0	0	0	65
10473373	f	ot	US	2015	0	1	1	0	0	0	0	0	0	0	0	0	64
10490424	f	cn	CN	2015	1	0	0	0	0	0	0	0	0	0	0	0	76
10510886	m	md	US	2015	0	0	0	0	0	0	0	0	0	0	0	0	49
10516247	m	md	JP	2015	0	0	0	0	0	0	0	0	0	0	0	0	71
10517757	m	cn	US	2015	0	1	0	0	1	0	0	0	0	0	0	1	62
10518233	m	md	DE	2015	0	0	0	0	0	0	0	0	0	0	0	0	68
10518244	f	md	JP	2015	0	0	0	0	0	0	0	0	0	0	0	0	55
10529115	m	md	JP	2015	1	0	0	0	0	0	0	0	0	0	0	0	79
10537230	m	ot	US	2015	0	0	0	0	0	0	0	0	0	0	0	0	52
10539466	m	md	US	2015	0	0	0	0	0	0	0	0	0	0	0	0	75
10540132	f	ot	JP	2015	0	0	0	0	0	0	0	0	0	0	0	0	67
10543441	f	md	Not re	2015	1	0	0	0	0	0	0	0	0	0	0	0	76
10543582	m	ph	JP	2015	0	0	0	0	0	0	0	0	0	0	0	0	61
10554976	f	ot	US	2015	0	0	0	0	0	0	0	0	0	0	0	0	59
10558499	m	cn	US	2015	0	1	0	0	1	0	0	0	0	0	0	0	64
10559149	m	md	JP	2015	0	0	0	0	0	0	0	0	0	0	0	0	68
10564392	f	md	Not re	2015	1	0	0	0	0	0	0	0	0	0	0	0	68

10564732	m	md	JP	2015	0	0	0	0	0	0	0	0	0	0	0	0	65
10570676	f	cn	Not re	2015	0	0	0	0	0	0	0	0	0	0	0	0	
10571238	m	cn	IT	2015	0	0	0	0	0	0	0	0	0	0	0	0	76
10574120	m	md	US	2015	0	0	0	0	0	0	0	0	0	0	0	0	67
10576850	f	md	HK	2015	0	0	0	0	0	0	0	0	0	0	0	0	58
10577096	f	md	US	2015	0	1	1	0	0	0	0	0	0	0	0	0	72
10578579	f	cn	CA	2015	0	0	0	0	0	0	0	0	0	0	0	0	83
10592513	f	md	FR	2015	0	0	0	0	0	0	0	0	0	0	0	0	59
10593390	m	md	US	2015	0	1	1	0	0	0	0	0	0	0	0	0	67
10593522	f	cn	JP	2015	0	0	0	0	0	0	0	0	0	0	0	0	77
10596211	m	md	JP	2015	0	0	0	0	0	0	0	0	0	0	0	0	59
10599250	f	md	US	2015	0	0	0	0	0	0	0	0	0	0	0	0	71
10599738	m	ot	US	2015	0	1	1	0	0	0	0	0	0	0	0	0	68
10603301	f	md	Not re	2015	1	0	0	0	0	0	0	0	0	0	0	0	75
10606356	m	md	JP	2015	0	0	0	0	0	0	0	0	0	0	0	0	50
10609196	f	md	JP	2015	0	0	0	0	0	0	0	0	0	0	0	0	61
10612871	f	md	Not re	2015	0	0	0	0	0	0	0	0	0	0	0	0	61
10614005	m	cn	US	2015	0	0	0	0	0	0	0	0	0	0	0	0	64
10616385	f	ot	NO	2015	0	1	1	0	0	0	0	0	0	0	0	0	47
10620657	m	md	JP	2015	1	0	0	0	0	0	0	0	0	0	0	0	69
10622069	m	md	DE	2015	0	0	0	0	0	0	0	0	0	0	0	0	62
10636578	f	ot	CN	2015	0	0	0	0	0	0	0	0	0	0	0	0	
10640269	f	md	CA	2015	0	0	0	0	0	0	0	0	0	0	0	0	64
10640998	m	md	NL	2015	0	0	0	0	0	0	0	0	0	0	0	0	38
10646428	m	md	JP	2015	0	0	0	0	0	0	0	0	0	0	0	0	58
10651202	f	md	FR	2015	0	1	1	0	0	0	0	0	0	0	0	0	60
10651569	m	md	US	2015	0	1	1	0	0	0	0	0	0	0	0	1	46
10651572	m	md	AU	2015	0	1	1	0	0	0	0	0	0	0	0	0	44
10652473	f	md	JP	2015	0	0	0	0	0	0	0	0	0	0	0	0	79
10652474	f	md	JP	2015	0	0	0	0	0	0	0	0	0	0	0	0	64
10653302	f	md	Not re	2015	1	0	0	0	0	0	0	0	0	0	0	0	59
10654472	f	cn	GB	2015	1	0	0	0	0	0	0	0	0	0	0	0	82
10657613	m	md	US	2015	0	0	0	0	0	0	0	0	0	0	0	0	65
10659491	f	md	Not re	2015	1	0	0	0	0	0	0	0	0	0	0	0	70

10659625	f	md	AU	2015	0	0	0	0	0	0	0	0	0	0	0	0	62
10660614	m	md	JP	2015	1	0	0	0	0	0	0	0	0	0	0	0	55
10661098	f	md	JP	2015	0	0	0	0	0	0	0	0	0	0	0	0	70
10663271	m	md	Not re	2015	1	0	0	0	0	0	0	0	0	0	0	0	60
10663308	f	cn	AU	2015	1	0	0	0	0	0	0	0	0	0	0	0	62
10663620	f	md	DE	2015	0	0	0	0	0	0	0	0	0	0	0	0	42
10664150	m	md	JP	2015	1	0	0	0	0	0	0	0	0	0	0	0	62
10665480	m	md	FR	2015	0	0	0	0	0	0	0	0	0	0	0	0	72
10666718	m	md	JP	2015	0	0	0	0	0	0	0	0	0	0	0	0	86
10667720	f	md	JP	2015	0	0	0	0	0	0	0	0	0	0	0	0	74
10668127	m	cn	Not re	2015	0	0	0	0	0	0	0	0	0	0	0	0	
10669758	m	md	JP	2015	1	0	0	0	0	0	0	0	0	0	0	0	76
10670906	m	ot	DE	2015	0	0	0	0	0	0	0	0	0	0	0	0	43
10671120	m	ot	KR	2015	0	0	0	0	0	0	0	0	0	0	0	0	69
10675832	f	cn	DE	2015	0	0	0	0	0	0	0	0	0	0	0	0	57
10676629	m	cn	JP	2015	0	0	0	0	0	0	0	0	0	0	0	0	70
10676705	Not	ot	GR	2015	1	0	0	0	0	0	0	0	0	0	0	0	
10677205	m	cn	US	2015	0	0	0	0	0	0	0	0	0	0	0	0	78
10678514	f	ot	BE	2015	0	0	0	0	0	0	0	0	0	0	0	0	50
10678548	f	ot	BE	2015	0	0	0	0	0	0	0	0	0	0	0	0	33
10682883	f	md	FR	2015	0	0	0	0	0	0	0	0	0	0	0	0	53
10683972	m	md	SE	2015	0	0	0	0	0	0	0	0	0	0	0	0	73
10684628	m	md	PL	2015	0	0	0	0	0	0	0	0	0	0	0	0	70
10685912	m	cn	JP	2015	0	0	0	0	0	0	0	0	0	0	0	0	63
10686794	m	md	JP	2015	1	0	0	0	0	0	0	0	0	0	0	1	73
10686831	m	ot	CN	2015	0	0	0	0	0	0	0	0	0	0	0	0	
10686988	f	md	CN	2015	0	0	0	0	0	0	0	0	0	0	0	0	45
10688242	f	md	DE	2015	0	0	0	0	0	0	0	0	0	0	0	0	51
10688686	m	md	Not re	2015	1	0	0	0	0	0	0	0	0	0	0	0	71
10691142	m	md	JP	2015	0	0	0	0	0	0	0	0	0	0	0	0	66
10691867	m	md	Not re	2015	1	0	0	0	0	0	0	0	0	0	0	0	
10692230	m	cn	US	2015	0	1	0	0	1	0	0	0	0	0	0	0	57
10693048	f	md	AU	2015	0	0	0	0	0	0	0	0	0	0	0	0	42
10693426	f	md	Not re	2015	1	0	0	0	0	0	0	0	0	0	0	0	76

10693517	f	ph	ES	2015	0	0	0	0	0	0	0	0	0	0	0	0	57
10693944	f	md	JP	2015	0	0	0	0	0	0	0	0	0	0	0	0	66
10694335	f	cn	US	2015	0	1	0	0	1	0	0	0	0	0	0	0	72
10697228	m	ot	IE	2015	0	0	0	0	0	0	0	0	0	0	0	0	69
10698387	m	md	FR	2015	0	0	0	0	0	0	0	0	0	0	0	0	62
10698940	m	cn	US	2015	0	1	0	0	1	0	0	0	0	0	0	0	61
10700128	f	cn	GB	2015	1	0	0	0	0	0	0	0	0	0	0	0	84
10700353	f	md	JP	2015	1	0	0	0	0	0	0	0	0	0	0	0	63
10700437	m	ot	KR	2015	0	0	0	0	0	0	0	0	0	0	0	0	64
10700584	f	md	JP	2015	0	0	0	0	0	0	0	0	0	0	0	0	66
10702577	m	md	Not re	2015	0	0	0	0	0	0	0	0	0	0	0	0	56
10702756	m	cn	US	2015	0	1	0	0	1	0	0	0	0	0	0	0	70
10702966	m	cn	US	2015	0	1	0	0	1	0	0	0	0	0	0	1	70
10704062	f	md	US	2015	0	1	1	0	0	0	0	0	0	0	0	0	45
10705156	f	cn	GB	2015	1	0	0	0	0	0	0	0	0	0	0	0	73
10706713	f	ot	US	2015	0	0	0	0	0	0	0	0	0	0	0	0	58
10706802	m	ot	SI	2015	0	0	0	0	0	0	0	0	0	0	0	0	
10706803	m	ot	SI	2015	0	0	0	0	0	0	0	0	0	0	0	0	
10706905	f	cn	AR	2015	0	0	0	0	0	0	0	0	0	0	0	0	67
10706959	f	md	US	2015	0	0	0	0	0	0	0	0	0	0	0	0	73
10707611	f	md	FR	2015	0	0	0	0	0	0	0	0	0	0	0	0	34
10707965	m	md	US	2015	0	1	1	0	0	0	0	0	0	0	0	0	70
10708060	f	cn	US	2015	0	0	0	0	0	0	0	0	0	0	0	0	67
10708874	f	md	US	2015	0	0	0	0	0	0	0	0	0	0	0	0	62
10709797	m	cn	US	2015	0	1	0	0	1	0	0	0	0	0	0	0	70
10710125	f	cn	GB	2015	1	0	0	0	0	0	0	0	0	0	0	0	
10710169	m	md	US	2015	0	0	0	0	0	0	0	0	0	0	0	0	
10711077	m	md	Not re	2015	1	0	0	0	0	0	0	0	0	0	0	0	61
10711554	Not	md	JP	2015	0	0	0	0	0	0	0	0	0	0	0	0	
10711559	Not	ot	SI	2015	0	0	0	0	0	0	0	0	0	0	0	0	
10712186	f	ot	US	2015	0	0	0	0	0	0	0	0	0	0	0	0	50
10713053	f	cn	GB	2015	1	0	0	0	0	0	0	0	0	0	0	0	51
10713221	m	md	GR	2015	0	0	0	0	0	0	0	0	0	0	0	0	57
10713896	f	md	Not re	2015	0	0	0	0	0	0	0	0	0	0	0	0	49

10714049	f	cn	GB	2015	1	0	0	0	0	0	0	0	0	0	0	0	
10714657	m	cn	US	2015	0	1	0	0	1	0	0	0	0	0	0	0	66
10714724	f	md	JP	2015	1	0	0	0	0	0	0	0	0	0	0	0	68
10716277	m	md	US	2015	1	0	0	0	0	0	0	0	0	0	0	0	59
10716782	m	md	DE	2015	0	0	0	0	0	0	0	0	0	0	0	0	7
10717281	f	md	Not re	2015	1	0	0	0	0	0	0	0	0	0	0	0	73
10717488	f	cn	US	2015	1	0	0	0	0	0	0	0	0	0	0	0	40
10717494	f	ot	US	2015	1	0	0	0	0	0	0	0	0	0	0	0	34
10717498	f	cn	US	2015	1	0	0	0	0	0	0	0	0	0	0	0	45
10717500	f	md	US	2015	1	0	0	0	0	0	0	0	0	0	0	0	
10717512	m	cn	US	2015	1	0	0	0	0	0	0	0	0	0	0	0	66
10717513	f	md	US	2015	1	0	0	0	0	0	0	0	0	0	0	0	
10717521	f	cn	US	2015	1	0	0	0	0	0	0	0	0	0	0	0	
10720479	Not	ot	CN	2015	0	0	0	0	0	0	0	0	0	0	0	0	
10721848	Not	ot	CN	2015	0	0	0	0	0	0	0	0	0	0	0	0	
10721890	m	cn	GB	2015	1	0	0	0	0	0	0	0	0	0	0	0	74
10721916	Not	ot	CN	2015	0	0	0	0	0	0	0	0	0	0	0	0	
10722202	m	cn	IE	2015	0	0	0	0	0	0	0	0	0	0	0	0	68
10723428	Not	ot	CN	2015	0	0	0	0	0	0	0	0	0	0	0	0	
10723523	Not	ot	JP	2015	0	0	0	0	0	0	0	0	0	0	0	0	
10724440	m	md	US	2015	0	0	0	0	0	0	0	0	0	0	0	0	67
10724490	m	cn	US	2015	0	0	0	0	0	0	0	0	0	0	0	0	
10724730	f	ot	US	2015	0	0	0	0	0	0	0	0	0	0	0	0	61
10724758	m	md	MX	2015	0	0	0	0	0	0	0	0	0	0	0	0	
10726245	m	cn	GB	2015	1	0	0	0	0	0	0	0	0	0	0	0	80
10726246	f	md	FR	2015	1	0	0	0	0	0	0	0	0	0	0	0	76
10727458	m	cn	GB	2015	0	0	0	0	0	0	0	0	0	0	0	0	70
10727497	Not	ot	CN	2015	0	0	0	0	0	0	0	0	0	0	0	0	
10727596	f	md	Not re	2015	1	0	0	0	0	0	0	0	0	0	0	0	62
10727645	Not	md	Not re	2015	1	0	0	0	0	0	0	0	0	0	0	0	
10727850	m	md	JP	2015	0	0	0	0	0	0	0	0	0	0	0	0	84
10727992	f	md	JP	2015	1	0	0	0	0	0	0	0	0	0	0	0	43
10729760	f	md	JP	2015	0	0	0	0	0	0	0	0	0	0	0	0	60
10730069	f	cn	AU	2015	0	0	0	0	0	0	0	0	0	0	0	0	45

10730149	f	cn	GB	2015	1	0	0	0	0	0	0	0	0	0	0	0	
10730201	m	cn	FR	2015	0	1	0	0	1	0	0	0	0	0	0	0	52
10731124	f	cn	KP	2015	0	1	0	0	1	0	0	0	0	0	0	0	68
10732186	m	cn	US	2015	1	0	0	0	0	0	0	0	0	0	0	0	67
10732569	m	md	IN	2015	0	0	0	0	0	0	0	0	0	0	0	0	59
10732833	f	md	DK	2015	0	0	0	0	0	0	0	0	0	0	0	0	66
10735085	m	cn	AU	2015	0	1	0	0	1	0	0	0	0	0	0	0	101
10735646	m	md	JP	2015	0	0	0	0	0	0	0	0	0	0	0	0	56
10735892	m	cn	IE	2015	0	0	0	0	0	0	0	0	0	0	0	0	56
10736914	m	md	CN	2015	0	0	0	0	0	0	0	0	0	0	0	0	59
10736915	m	md	US	2015	0	0	0	0	0	0	0	0	0	0	0	0	66
10738320	m	cn	US	2015	0	0	0	0	0	0	0	0	0	0	0	0	57
10738810	m	cn	US	2015	0	0	0	0	0	0	0	0	0	0	0	0	64
10739599	m	cn	US	2015	0	1	0	0	1	0	0	0	0	0	0	0	75
10740473	Not	ot	JP	2015	0	0	0	0	0	0	0	0	0	0	0	0	
10740603	Not	ot	JP	2015	0	0	0	0	0	0	0	0	0	0	0	0	
10741065	m	md	ES	2015	0	1	1	0	0	0	0	0	0	0	0	1	63
10742298	f	md	US	2015	1	0	0	0	0	0	0	0	0	0	0	0	
10742366	m	cn	AT	2015	0	1	0	0	1	0	0	0	0	0	0	0	74
10742386	m	md	JP	2015	1	0	0	0	0	0	0	0	0	0	0	0	49
10742515	m	cn	GB	2015	1	0	0	0	0	0	0	0	0	0	0	0	86
10742978	m	ot	NL	2015	0	1	0	0	1	0	0	0	0	0	0	0	50
10743030	m	md	Not re	2015	0	0	0	0	0	0	0	0	0	0	0	0	66
10743880	Not	ot	JP	2015	0	0	0	0	0	0	0	0	0	0	0	0	
10743981	Not	ot	JP	2015	0	0	0	0	0	0	0	0	0	0	0	0	
10744153	Not	ot	JP	2015	0	0	0	0	0	0	0	0	0	0	0	0	
10744164	Not	Not repo	AU	2015	0	0	0	0	0	0	0	0	0	0	0	0	
10744194	Not	ot	JP	2015	0	0	0	0	0	0	0	0	0	0	0	0	
10744242	Not	ot	AU	2015	0	0	0	0	0	0	0	0	0	0	0	0	
10744561	Not	Not repo	AU	2015	0	0	0	0	0	0	0	0	0	0	0	0	
10744588	Not	Not repo	AU	2015	0	0	0	0	0	0	0	0	0	0	0	0	
10744619	Not	Not repo	AU	2015	0	0	0	0	0	0	0	0	0	0	0	0	
10744641	m	cn	US	2015	0	0	0	0	0	0	0	0	0	0	0	0	64
10744663	Not	ot	AU	2015	0	0	0	0	0	0	0	0	0	0	0	0	

10744682	Not	Not repo	AU	2015	0	0	0	0	0	0	0	0	0	0	0	0	
10744695	Not	Not repo	AU	2015	0	0	0	0	0	0	0	0	0	0	0	0	
10744752	Not	ot	AU	2015	0	0	0	0	0	0	0	0	0	0	0	0	
10744769	Not	Not repo	AU	2015	0	0	0	0	0	0	0	0	0	0	0	0	
10744786	m	cn	GB	2015	1	0	0	0	0	0	0	0	0	0	0	0	
10744787	Not	Not repo	AU	2015	0	0	0	0	0	0	0	0	0	0	0	0	
10744799	Not	Not repo	AU	2015	0	0	0	0	0	0	0	0	0	0	0	0	
10745864	m	Not repo	US	2015	1	0	0	0	0	0	0	0	0	0	0	0	66
10746633	f	md	Not re	2015	1	0	0	0	0	0	0	0	0	0	0	0	65
10748546	Not	ot	US	2015	0	0	0	0	0	0	0	0	0	0	0	0	
10748708	m	ph	US	2015	0	0	0	0	0	0	0	0	0	0	0	0	65
10748853	m	ot	AU	2015	0	1	1	0	0	0	0	0	0	0	0	0	
10749400	m	cn	GB	2015	1	0	0	0	0	0	0	0	0	0	0	0	72
10749419	m	cn	JP	2015	0	0	0	0	0	0	0	0	0	0	0	0	71
10750186	m	cn	DE	2015	0	0	0	0	0	0	0	0	0	0	0	0	63
10750187	m	cn	DE	2015	0	0	0	0	0	0	0	0	0	0	0	0	63
10750468	Not	ot	US	2015	0	0	0	0	0	0	0	0	0	0	0	0	
10750470	Not	ot	US	2015	0	0	0	0	0	0	0	0	0	0	0	0	
10750484	Not	ot	US	2015	0	0	0	0	0	0	0	0	0	0	0	0	
10750497	f	md	FR	2015	0	0	0	0	0	0	0	0	0	0	0	0	
10750522	Not	ot	US	2015	0	0	0	0	0	0	0	0	0	0	0	0	
10750553	f	cn	US	2015	0	1	0	0	1	0	0	0	0	0	0	0	55
10751138	f	ot	US	2015	0	0	0	0	0	0	0	0	0	0	0	0	40
10751546	f	cn	IE	2015	0	0	0	0	0	0	0	0	0	0	0	0	69
10751690	m	cn	DK	2015	0	0	0	0	0	0	0	0	0	0	0	0	56
10751935	f	cn	US	2015	0	1	0	0	1	0	0	0	0	0	0	0	66
10753021	f	ot	US	2015	0	1	0	0	1	0	0	0	0	0	0	0	80
10753234	m	md	JP	2015	1	0	0	0	0	0	0	0	0	0	0	0	69
10753698	m	ot	DK	2015	0	0	0	0	0	0	0	0	0	0	0	0	56
10754097	m	md	FR	2015	0	0	0	0	0	0	0	0	0	0	0	0	
10755808	Not	md	US	2015	0	0	0	0	0	0	0	0	0	0	0	0	
10755825	Not	md	US	2015	0	0	0	0	0	0	0	0	0	0	0	0	
10755877	Not	md	US	2015	0	0	0	0	0	0	0	0	0	0	0	0	
10755883	Not	md	US	2015	0	0	0	0	0	0	0	0	0	0	0	0	

10755886	Not	md	US	2015	0	0	0	0	0	0	0	0	0	0	0	0	
10755891	Not	md	US	2015	0	0	0	0	0	0	0	0	0	0	0	0	
10755954	Not	md	US	2015	0	0	0	0	0	0	0	0	0	0	0	0	
10755959	Not	md	US	2015	0	0	0	0	0	0	0	0	0	0	0	0	
10755980	Not	md	US	2015	0	0	0	0	0	0	0	0	0	0	0	0	
10756007	Not	ot	JP	2015	1	0	0	0	0	0	0	0	0	0	0	0	
10756023	m	cn	JP	2015	0	1	0	0	1	0	0	0	0	0	0	0	40
10756723	Not	Not repo	Not re	2015	1	0	0	0	0	0	0	0	0	0	0	0	
10758490	f	md	US	2015	0	0	0	0	0	0	0	0	0	0	0	0	50
10759765	f	md	DE	2015	1	0	0	0	0	0	0	0	0	0	0	0	
10759793	f	md	FR	2015	0	0	0	0	0	0	0	0	0	0	0	0	52
10761212	f	ot	US	2015	0	0	0	0	0	0	0	0	0	0	0	0	48
10761603	f	md	BE	2015	0	0	0	0	0	0	0	0	0	0	0	0	
10761903	m	md	FR	2015	0	0	0	0	0	0	0	0	0	0	0	0	78
10762354	m	ot	US	2015	0	1	0	0	1	0	0	0	0	0	0	1	56
10762612	m	cn	JP	2015	0	0	0	0	0	0	0	0	0	0	0	0	76
10762631	f	ot	ES	2015	0	0	0	0	0	0	0	0	0	0	0	0	65
10763011	m	cn	GB	2015	1	0	0	0	0	0	0	0	0	0	0	0	55
10763511	Not	ot	CA	2015	0	0	0	0	0	0	0	0	0	0	0	0	
10763530	Not	ot	CA	2015	0	0	0	0	0	0	0	0	0	0	0	0	
10763531	f	md	Not re	2015	1	0	0	0	0	0	0	0	0	0	0	0	79
10763549	Not	ot	CA	2015	0	0	0	0	0	0	0	0	0	0	0	0	
10763804	f	cn	AU	2015	1	0	0	0	0	0	0	0	0	0	0	0	84
10763903	m	md	JP	2015	1	0	0	0	0	0	0	0	0	0	0	1	83
10764187	f	md	US	2015	0	0	0	0	0	0	0	0	0	0	0	0	63
10764926	m	cn	TH	2015	0	0	0	0	0	0	0	0	0	0	0	0	72
10765981	m	cn	GB	2015	1	0	0	0	0	0	0	0	0	0	0	0	
10766353	f	md	JP	2015	0	0	0	0	0	0	0	0	0	0	0	0	67
10767199	f	md	JP	2015	0	0	0	0	0	0	0	0	0	0	0	0	85
10767204	f	ot	GB	2015	0	0	0	0	0	0	0	0	0	0	0	0	27
10767393	Not	ot	DE	2015	0	0	0	0	0	0	0	0	0	0	0	0	
10768543	f	cn	US	2015	0	1	0	0	1	0	0	0	0	0	0	0	50
10769067	f	cn	US	2015	0	0	0	0	0	0	0	0	0	0	0	0	48
10769468	m	md	JP	2015	0	0	0	0	0	0	0	0	0	0	0	0	75

10769742	f	cn	GB	2015	1	0	0	0	0	0	0	0	0	0	0	0	
10771795	f	ot	DE	2015	0	0	0	0	0	0	0	0	0	0	0	0	54
10775190	Not	ot	FR	2015	0	0	0	0	0	0	0	0	0	0	0	0	
10776150	m	cn	GB	2015	1	0	0	0	0	0	0	0	0	0	0	0	
10777181	m	cn	US	2015	0	1	0	0	1	0	0	0	0	0	0	0	68
10778070	m	md	US	2015	0	0	0	0	0	0	0	0	0	0	0	0	52
10778077	m	cn	US	2015	0	1	0	0	1	0	0	0	0	0	0	0	74
10778456	Not	ot	FR	2015	0	0	0	0	0	0	0	0	0	0	0	0	
10778468	m	cn	US	2015	0	1	0	0	1	0	0	0	0	0	0	0	66
10778602	Not	ot	FR	2015	0	0	0	0	0	0	0	0	0	0	0	0	
10779988	f	md	Not re	2015	1	0	0	0	0	0	0	0	0	0	0	0	59
10780682	f	ph	US	2015	0	0	0	0	0	0	0	0	0	0	0	0	58
10781274	m	md	US	2015	0	0	0	0	0	0	0	0	0	0	0	0	68
10781613	f	ot	FR	2015	0	0	0	0	0	0	0	0	0	0	0	0	70
10783200	f	md	TR	2015	0	0	0	0	0	0	0	0	0	0	0	0	58
10784300	Not	ot	KP	2015	0	0	0	0	0	0	0	0	0	0	0	0	
10784302	Not	ot	US	2015	0	0	0	0	0	0	0	0	0	0	0	0	
10784394	Not	ot	KP	2015	0	0	0	0	0	0	0	0	0	0	0	0	
10784426	Not	ot	KP	2015	0	0	0	0	0	0	0	0	0	0	0	0	
10784439	Not	ot	KP	2015	0	0	0	0	0	0	0	0	0	0	0	0	
10784443	Not	ot	KP	2015	0	0	0	0	0	0	0	0	0	0	0	0	
10784452	Not	ot	KP	2015	0	0	0	0	0	0	0	0	0	0	0	0	
10784456	Not	ot	KP	2015	0	0	0	0	0	0	0	0	0	0	0	0	
10784589	Not	ot	KP	2015	0	0	0	0	0	0	0	0	0	0	0	0	
10784651	Not	ot	US	2015	0	0	0	0	0	0	0	0	0	0	0	0	
10784704	Not	ot	KP	2015	0	0	0	0	0	0	0	0	0	0	0	0	
10786135	m	md	US	2015	0	0	0	0	0	0	0	0	0	0	0	0	49
10786300	m	ot	JP	2015	0	0	0	0	0	0	0	0	0	0	0	0	46
10786404	f	cn	GB	2015	1	0	0	0	0	0	0	0	0	0	0	0	78
10788829	f	cn	GB	2015	1	0	0	0	0	0	0	0	0	0	0	0	50
10790509	f	ot	JP	2015	0	0	0	0	0	0	0	0	0	0	0	0	68
10790591	m	md	MX	2015	1	0	0	0	0	0	0	0	0	0	0	0	
10790691	m	ot	KR	2015	0	0	0	0	0	0	0	0	0	0	0	0	68
10790693	m	ot	JP	2015	0	0	0	0	0	0	0	0	0	0	0	0	52

10791406	f	cn	US	2015	0	1	1	0	0	0	0	0	0	0	0	0	
10792264	f	cn	US	2015	0	0	0	0	0	0	0	0	0	0	0	0	70
10792376	m	cn	GB	2015	1	0	0	0	0	0	0	0	0	0	0	0	82
10792417	m	md	GB	2015	0	0	0	0	0	0	0	0	0	0	0	0	
10792775	f	md	ES	2015	0	0	0	0	0	0	0	0	0	0	0	0	53
10793653	f	cn	GB	2015	1	0	0	0	0	0	0	0	0	0	0	0	88
10793752	f	ot	DE	2015	0	0	0	0	0	0	0	0	0	0	0	0	54
10793911	f	md	FR	2015	0	1	1	0	0	0	0	0	0	0	0	0	68
10794204	f	md	US	2015	1	0	0	0	0	0	0	0	0	0	0	0	
10794609	f	md	RU	2015	1	0	0	0	0	0	0	0	0	0	0	0	59
10795932	m	ot	IT	2015	0	0	0	0	0	0	0	0	0	0	0	0	58
10801545	Not	ot	DE	2015	0	0	0	0	0	0	0	0	0	0	0	0	
10801759	m	md	Not re	2015	1	0	0	0	0	0	0	0	0	0	0	0	67
10802038	Not	cn	GB	2015	1	0	0	0	0	0	0	0	0	0	0	0	
10802481	m	cn	US	2015	0	0	0	0	0	0	0	0	0	0	0	0	
10803152	m	ot	US	2015	0	1	1	0	0	0	0	0	0	0	0	0	72
10803795	Not	ot	US	2015	0	0	0	0	0	0	0	0	0	0	0	0	
10803912	m	cn	IT	2015	0	0	0	0	0	0	0	0	0	0	0	0	64
10803957	m	cn	Not re	2015	0	0	0	0	0	0	0	0	0	0	0	0	
10803972	f	md	US	2015	0	0	0	0	0	0	0	0	0	0	0	0	
10818058	m	md	US	2015	0	1	1	0	0	0	0	0	0	0	0	1	85
10823698	m	ot	JP	2015	0	0	0	0	0	0	0	0	0	0	0	0	84
10825405	m	cn	US	2015	0	0	0	0	0	0	0	0	0	0	0	0	66
10825816	m	ot	US	2015	0	1	0	0	1	0	0	0	0	0	0	0	64
10830310	f	md	Not re	2015	1	0	0	0	0	0	0	0	0	0	0	0	44
10830569	f	cn	US	2015	0	0	0	0	0	0	0	0	0	0	0	0	62
10833766	m	ot	US	2015	0	0	0	0	0	0	0	0	0	0	0	0	61
10834088	f	md	JP	2015	1	0	0	0	0	0	0	0	0	0	0	0	85
10835209	m	md	JP	2015	1	0	0	0	0	0	0	0	0	0	0	0	46
10839633	f	ph	FR	2015	0	0	0	0	0	0	0	0	0	0	0	0	70
10839795	Not	Not repo	JP	2015	0	0	0	0	0	0	0	0	0	0	0	0	
10840760	f	ot	DE	2015	0	0	0	0	0	0	0	0	0	0	0	0	54
10841071	m	Not repo	GB	2015	0	0	0	0	0	0	0	0	0	0	0	0	
10841262	m	md	DE	2015	0	0	0	0	0	0	0	0	0	0	0	0	81

10844434	m	cn	CA	2015	0	0	0	0	0	0	0	0	0	0	0	0	66
10846390	m	ot	US	2015	1	0	0	0	0	0	0	0	0	0	0	0	81
10846517	m	ph	DE	2015	0	0	0	0	0	0	0	0	0	0	0	0	66
10847129	f	md	FR	2015	0	0	0	0	0	0	0	0	0	0	0	0	41
10847238	f	ot	US	2015	0	1	0	0	1	0	0	0	0	0	0	0	0
10847360	m	cn	US	2015	0	1	0	0	1	0	0	0	0	0	0	0	64
10853063	f	ot	US	2015	1	0	0	0	0	0	0	0	0	0	0	0	47
10853071	f	ot	US	2015	1	0	0	0	0	0	0	0	0	0	0	0	47
10853189	m	md	JP	2015	1	0	0	0	0	0	0	0	0	0	0	0	
10855856	f	cn	AR	2015	0	0	0	0	0	0	0	0	0	0	0	0	40
10856451	m	md	Not re	2015	1	0	0	0	0	0	0	0	0	0	0	0	76
10857865	m	cn	US	2015	0	0	0	0	0	0	0	0	0	0	0	0	53
10861355	Not	ot	JP	2015	0	0	0	0	0	0	0	0	0	0	0	0	
10861360	Not	md	IT	2015	0	0	0	0	0	0	0	0	0	0	0	0	
10861428	Not	md	SG	2015	0	0	0	0	0	0	0	0	0	0	0	0	
10862430	Not	Not repo	JP	2015	0	0	0	0	0	0	0	0	0	0	0	0	
10862858	f	md	GR	2015	0	1	1	0	0	0	0	0	0	0	0	0	51
10866306	Not	ot	TR	2015	0	0	0	0	0	0	0	0	0	0	0	0	
10866375	Not	ot	FR	2015	0	0	0	0	0	0	0	0	0	0	0	0	
10866954	m	cn	US	2015	0	0	0	0	0	0	0	0	0	0	0	0	
10867270	m	md	JP	2015	0	0	0	0	0	0	0	0	0	0	0	0	76
10867531	Not	ot	NL	2015	1	0	0	0	0	0	0	0	0	0	0	0	
10867554	Not	ot	NL	2015	1	0	0	0	0	0	0	0	0	0	0	0	
10867584	Not	ot	NL	2015	1	0	0	0	0	0	0	0	0	0	0	0	
10867587	Not	ot	NL	2015	1	0	0	0	0	0	0	0	0	0	0	0	
10867595	Not	ot	NL	2015	1	0	0	0	0	0	0	0	0	0	0	0	
10867601	Not	ot	NL	2015	1	0	0	0	0	0	0	0	0	0	0	0	
10867624	Not	ot	NL	2015	1	0	0	0	0	0	0	0	0	0	0	0	
10867919	m	ot	GB	2015	0	0	0	0	0	0	0	0	0	0	0	0	55
10868054	f	ot	AU	2015	0	0	0	0	0	0	0	0	0	0	0	0	70
10868212	m	md	FR	2015	1	0	0	0	0	0	0	0	0	0	0	0	70
10868366	m	md	JP	2015	0	0	0	0	0	0	0	0	0	0	0	0	76
10870734	f	cn	GB	2015	1	0	0	0	0	0	0	0	0	0	0	0	
10870884	f	md	NO	2015	0	0	0	0	0	0	0	0	0	0	0	0	70

10871558	f	md	IT	2015	0	0	0	0	0	0	0	0	0	0	0	0	67
10871608	m	ot	KR	2015	0	0	0	0	0	0	0	0	0	0	0	0	68
10871919	f	md	NL	2015	0	0	0	0	0	0	0	0	0	0	0	0	62
10872127	m	cn	DE	2015	1	0	0	0	0	0	0	0	0	0	0	0	74
10872506	Not	ot	JP	2015	0	0	0	0	0	0	0	0	0	0	0	0	
10872636	f	cn	US	2015	0	1	0	0	1	0	0	0	0	0	0	0	66
10873100	Not	ot	JP	2015	0	0	0	0	0	0	0	0	0	0	0	0	
10873632	m	cn	GB	2015	1	0	0	0	0	0	0	0	0	0	0	0	42
10873788	m	md	SE	2015	0	0	0	0	0	0	0	0	0	0	0	0	
10873883	f	cn	US	2015	0	0	0	0	0	0	0	0	0	0	0	0	
10874716	m	md	JP	2015	1	0	0	0	0	0	0	0	0	0	0	0	70
10874724	f	md	ES	2015	0	0	0	0	0	0	0	0	0	0	0	0	61
10876432	f	ot	TH	2015	0	0	0	0	0	0	0	0	0	0	0	0	75
10876735	f	md	US	2015	0	1	0	0	1	0	0	0	0	0	0	0	
10877837	f	md	AT	2015	0	0	0	0	0	0	0	0	0	0	0	0	68
10879030	f	md	US	2015	0	0	0	0	0	0	0	0	0	0	0	0	68
10879950	m	md	US	2015	0	0	0	0	0	0	0	0	0	0	0	0	62
10880029	m	md	US	2015	0	0	0	0	0	0	0	0	0	0	0	0	66
10880037	f	md	FR	2015	0	0	0	0	0	0	0	0	0	0	0	0	72
10881422	Not	md	GR	2015	0	0	0	0	0	0	0	0	0	0	0	0	
10882334	m	md	DE	2015	0	0	0	0	0	0	0	0	0	0	0	0	
10882598	m	Not repo	US	2015	0	0	0	0	0	0	0	0	0	0	0	0	70
10882800	m	cn	GB	2015	1	0	0	0	0	0	0	0	0	0	0	0	
10882818	m	cn	GB	2015	1	0	0	0	0	0	0	0	0	0	0	0	70
10884717	f	md	JP	2015	0	0	0	0	0	0	0	0	0	0	0	0	57
10886259	m	ph	US	2015	0	0	0	0	0	0	0	0	0	0	0	0	82
10886431	m	cn	GB	2015	1	0	0	0	0	0	0	0	0	0	0	0	67
10889015	m	md	US	2015	0	0	0	0	0	0	0	0	0	0	0	0	65
10889402	f	md	Not re	2015	1	0	0	0	0	0	0	0	0	0	0	0	72
10889423	m	cn	Not re	2015	1	0	0	0	0	0	0	0	0	0	0	0	60
10889663	m	cn	US	2015	0	1	0	0	1	0	0	0	0	0	0	1	61
10889818	f	md	US	2015	1	0	0	0	0	0	0	0	0	0	0	0	66
10890034	Not	ot	JP	2015	0	0	0	0	0	0	0	0	0	0	0	0	
10890303	Not	ot	JP	2015	0	0	0	0	0	0	0	0	0	0	0	0	

10891408	Not	ot	GR	2015	0	0	0	0	0	0	0	0	0	0	0	0	
10891412	f	cn	US	2015	0	0	0	0	0	0	0	0	0	0	0	0	73
10891932	f	md	US	2015	0	0	0	0	0	0	0	0	0	0	0	0	
10893139	f	md	Not re	2015	1	0	0	0	0	0	0	0	0	0	0	0	
10896043	Not	ot	CN	2015	0	0	0	0	0	0	0	0	0	0	0	0	
10896518	Not	md	GR	2015	0	0	0	0	0	0	0	0	0	0	0	0	
10897312	Not	ot	JP	2015	0	0	0	0	0	0	0	0	0	0	0	0	
10897371	Not	cn	GB	2015	1	0	0	0	0	0	0	0	0	0	0	0	
10897372	Not	cn	GB	2015	1	0	0	0	0	0	0	0	0	0	0	0	
10897373	Not	cn	GB	2015	1	0	0	0	0	0	0	0	0	0	0	0	
10897422	Not	cn	GB	2015	1	0	0	0	0	0	0	0	0	0	0	0	
10897424	Not	cn	GB	2015	1	0	0	0	0	0	0	0	0	0	0	0	
10897425	Not	cn	GB	2015	1	0	0	0	0	0	0	0	0	0	0	0	
10897426	Not	cn	GB	2015	1	0	0	0	0	0	0	0	0	0	0	0	
10897427	Not	cn	GB	2015	1	0	0	0	0	0	0	0	0	0	0	0	
10897458	Not	cn	GB	2015	1	0	0	0	0	0	0	0	0	0	0	0	
10897462	Not	cn	GB	2015	1	0	0	0	0	0	0	0	0	0	0	0	
10897560	Not	ot	JP	2015	0	0	0	0	0	0	0	0	0	0	0	0	
10897665	Not	cn	GB	2015	1	0	0	0	0	0	0	0	0	0	0	0	
10897672	Not	cn	GB	2015	1	0	0	0	0	0	0	0	0	0	0	0	
10897678	Not	cn	GB	2015	1	0	0	0	0	0	0	0	0	0	0	0	
10897686	Not	cn	GB	2015	1	0	0	0	0	0	0	0	0	0	0	0	
10897714	m	md	US	2015	0	0	0	0	0	0	0	0	0	0	0	0	
10897733	Not	cn	GB	2015	1	0	0	0	0	0	0	0	0	0	0	0	
10897738	Not	cn	GB	2015	1	0	0	0	0	0	0	0	0	0	0	0	
10898916	m	ph	DE	2015	0	0	0	0	0	0	0	0	0	0	0	0	56
10898927	Not	ot	GR	2015	0	0	0	0	0	0	0	0	0	0	0	0	
10899123	m	cn	CN	2015	1	0	0	0	0	0	0	0	0	0	0	0	81
10899898	Not	ot	JP	2015	0	0	0	0	0	0	0	0	0	0	0	0	
10900060	f	md	US	2015	0	0	0	0	0	0	0	0	0	0	0	0	
10900081	m	cn	US	2015	0	0	0	0	0	0	0	0	0	0	0	0	76
10900534	m	md	US	2015	0	0	0	0	0	0	0	0	0	0	0	0	
10900637	m	Not repo	KR	2015	0	0	0	0	0	0	0	0	0	0	0	0	68
10900648	f	md	US	2015	0	0	0	0	0	0	0	0	0	0	0	0	83

10901169	f	md	Not re	2015	1	0	0	0	0	0	0	0	0	0	0	0	65
10901653	Not	Not repo	US	2015	0	0	0	0	0	0	0	0	0	0	0	0	
10902025	m	cn	GB	2015	0	0	0	0	0	0	0	0	0	0	0	0	
10902053	f	cn	GB	2015	1	0	0	0	0	0	0	0	0	0	0	0	61
10902214	m	cn	US	2015	0	0	0	0	0	0	0	0	0	0	0	0	75
10902936	f	cn	US	2015	0	0	0	0	0	0	0	0	0	0	0	0	52
10902937	m	md	US	2015	0	0	0	0	0	0	0	0	0	0	0	0	
10902940	f	md	US	2015	0	0	0	0	0	0	0	0	0	0	0	0	
10903757	Not	md	VN	2015	0	0	0	0	0	0	0	0	0	0	0	0	
10904734	Not	cn	GB	2015	1	0	0	0	0	0	0	0	0	0	0	0	
10904764	m	cn	US	2015	0	1	0	0	1	0	0	0	0	0	0	0	42
10905069	f	md	Not re	2015	1	0	0	0	0	0	0	0	0	0	0	0	45
10905101	f	cn	US	2015	0	0	0	0	0	0	0	0	0	0	0	0	65
10905437	f	cn	US	2015	0	1	0	0	1	0	0	0	0	0	0	0	42
10906177	Not	ot	US	2015	0	0	0	0	0	0	0	0	0	0	0	0	
10907022	f	ph	US	2015	0	0	0	0	0	0	0	0	0	0	0	0	61
10907215	Not	ot	CN	2015	0	0	0	0	0	0	0	0	0	0	0	0	
10910880	f	ot	NO	2015	0	0	0	0	0	0	0	0	0	0	0	0	70
10912053	m	ot	SE	2015	0	0	0	0	0	0	0	0	0	0	0	0	68
10912705	m	cn	GB	2015	1	0	0	0	0	0	0	0	0	0	0	0	75
10912789	f	md	AU	2015	0	0	0	0	0	0	0	0	0	0	0	0	51
10912872	f	cn	CA	2015	0	0	0	0	0	0	0	0	0	0	0	0	71
10913683	Not	cn	GB	2015	1	0	0	0	0	0	0	0	0	0	0	0	
10914001	m	cn	US	2015	0	0	0	0	0	0	0	0	0	0	0	0	49
10914043	m	md	Not re	2015	0	0	0	0	0	0	0	0	0	0	0	0	62
10917322	m	md	IL	2015	1	0	0	0	0	0	0	0	0	0	0	0	70
10917920	f	md	US	2015	0	0	0	0	0	0	0	0	0	0	0	0	57
10920588	m	cn	AU	2015	0	0	0	0	0	0	0	0	0	0	0	0	73
10920618	f	ot	ES	2015	0	0	0	0	0	0	0	0	0	0	0	0	61
10920812	m	cn	GB	2015	0	0	0	0	0	0	0	0	0	0	0	0	42
10920820	m	cn	GB	2015	1	0	0	0	0	0	0	0	0	0	0	0	79
10921950	f	md	Not re	2015	1	0	0	0	0	0	0	0	0	0	0	0	
10922832	f	md	US	2015	0	0	0	0	0	0	0	0	0	0	0	0	51
10922833	f	cn	BR	2015	0	0	0	0	0	0	0	0	0	0	0	0	

10925594	f	cn	AR	2015	0	0	0	0	0	0	0	0	0	0	0	0		74
10925815	f	Not repo	TW	2015	1	0	0	0	0	0	0	0	0	0	0	0		42
10926611	Not	md	US	2015	0	0	0	0	0	0	0	0	0	0	0	0		
10926728	m	ot	US	2015	0	0	0	0	0	0	0	0	0	0	0	0		64
10926985	Not	Not repo	US	2015	0	0	0	0	0	0	0	0	0	0	0	0		
10927003	Not	md	US	2015	0	0	0	0	0	0	0	0	0	0	0	0		
10927031	Not	md	US	2015	0	0	0	0	0	0	0	0	0	0	0	0		
10927238	Not	md	US	2015	0	0	0	0	0	0	0	0	0	0	0	0		
10927687	m	cn	US	2015	0	1	0	0	1	0	0	0	0	0	0	0		69
10927974	m	ph	US	2015	0	0	0	0	0	0	0	0	0	0	0	0		52
10929169	f	Not repo	US	2015	0	0	0	0	0	0	0	0	0	0	0	0		82
10929798	m	cn	US	2015	0	0	0	0	0	0	0	0	0	0	0	0		
10930073	f	cn	GB	2015	1	0	0	0	0	0	0	0	0	0	0	0		
10933799	f	md	MY	2015	1	0	0	0	0	0	0	0	0	0	0	0		50
10934364	Not	cn	GB	2015	1	0	0	0	0	0	0	0	0	0	0	0		
10934514	m	md	JP	2015	1	0	0	0	0	0	0	0	0	0	0	0		78
10935301	f	cn	US	2015	0	1	0	0	1	0	0	0	0	0	0	0		57
10944112	m	cn	US	2015	0	1	0	0	1	0	0	0	0	0	0	0		64
10944479	Not	ot	JP	2015	0	0	0	0	0	0	0	0	0	0	0	0		
10944640	Not	md	US	2015	0	0	0	0	0	0	0	0	0	0	0	0		
10944774	Not	md	US	2015	0	0	0	0	0	0	0	0	0	0	0	0		
10944821	Not	md	US	2015	0	0	0	0	0	0	0	0	0	0	0	0		
10945030	Not	ot	US	2015	0	0	0	0	0	0	0	0	0	0	0	0		
10945071	Not	md	US	2015	0	0	0	0	0	0	0	0	0	0	0	0		
10945291	Not	md	US	2015	0	0	0	0	0	0	0	0	0	0	0	0		
10945308	Not	md	US	2015	0	0	0	0	0	0	0	0	0	0	0	0		
10945452	m	cn	NO	2015	0	0	0	0	0	0	0	0	0	0	0	0		65
10946573	f	cn	KR	2015	0	0	0	0	0	0	0	0	0	0	0	0		57
10946772	f	md	DE	2015	0	1	1	0	0	0	0	0	0	0	0	0		71
10947416	f	md	DE	2015	1	0	0	0	0	0	0	0	0	0	0	0		54
10949363	f	md	Not re	2015	1	0	0	0	0	0	0	0	0	0	0	0		73
10950170	f	md	JP	2015	0	0	0	0	0	0	0	0	0	0	0	0		79
10953076	Not	ot	JP	2015	0	0	0	0	0	0	0	0	0	0	0	0		
10953080	Not	ot	JP	2015	0	0	0	0	0	0	0	0	0	0	0	0		

10953081	Not	ot	JP	2015	0	0	0	0	0	0	0	0	0	0	0	0	
10953103	m	md	CA	2015	0	0	0	0	0	0	0	0	0	0	0	0	44
10953172	Not	ot	KR	2015	0	0	0	0	0	0	0	0	0	0	0	0	
10953188	Not	ot	KR	2015	0	0	0	0	0	0	0	0	0	0	0	0	
10954373	m	cn	US	2015	0	0	0	0	0	0	0	0	0	0	0	0	76
10954378	m	ot	JP	2015	0	0	0	0	0	0	0	0	0	0	0	0	75
10954809	m	md	US	2015	0	0	0	0	0	0	0	0	0	0	0	0	48
10955382	Not	ot	JP	2015	0	0	0	0	0	0	0	0	0	0	0	0	
10955516	m	md	CL	2015	0	1	1	0	0	0	0	0	0	0	0	0	58
10955524	f	md	DE	2015	0	0	0	0	0	0	0	0	0	0	0	0	
10955924	f	ot	US	2015	1	0	0	0	0	0	0	0	0	0	0	0	76
10956255	f	cn	GB	2015	1	0	0	0	0	0	0	0	0	0	0	0	57
10956296	m	md	JP	2015	0	0	0	0	0	0	0	0	0	0	0	0	61
10957862	m	cn	GB	2015	1	0	0	0	0	0	0	0	0	0	0	0	73
10957930	f	cn	US	2015	0	1	0	0	1	0	0	0	0	0	0	0	67
10958277	f	md	Not re	2015	1	0	0	0	0	0	0	0	0	0	0	0	
10961843	m	cn	GB	2015	1	0	0	0	0	0	0	0	0	0	0	0	
10963203	m	md	GB	2015	1	0	0	0	0	0	0	0	0	0	0	0	60
10963468	Not	Not repo	JP	2015	0	0	0	0	0	0	0	0	0	0	0	0	
10963518	Not	Not repo	US	2015	0	0	0	0	0	0	0	0	0	0	0	0	
10963520	Not	Not repo	JP	2015	0	0	0	0	0	0	0	0	0	0	0	0	
10966750	f	md	US	2015	0	0	0	0	0	0	0	0	0	0	0	0	81
10968068	Not	md	TW	2015	1	0	0	0	0	0	0	0	0	0	0	0	
10969334	Not	ot	JP	2015	0	0	0	0	0	0	0	0	0	0	0	0	
10970409	f	cn	GB	2015	1	0	0	0	0	0	0	0	0	0	0	0	72
10971180	Not	Not repo	JP	2015	0	0	0	0	0	0	0	0	0	0	0	0	
10971691	m	ot	US	2015	0	0	0	0	0	0	0	0	0	0	0	0	76
10977160	m	ot	KR	2015	0	0	0	0	0	0	0	0	0	0	0	0	
6267062	m	md	US	2015	0	0	0	0	0	0	0	0	0	0	0	0	43
6537999	m	md	US	2015	0	0	0	0	0	0	0	0	0	0	0	0	59
6965777	m	ot	GR	2015	1	0	0	0	0	0	0	0	0	0	0	0	73
6965856	m	md	GR	2015	1	0	0	0	0	0	0	0	0	0	0	0	66
6968634	f	md	IT	2015	1	0	0	0	0	0	0	0	0	0	0	0	53
7017742	m	md	FR	2015	0	0	0	0	0	0	0	0	0	0	0	0	70

7031826	m	md	US	2015	1	0	0	0	0	0	0	0	0	0	0	1	81
7106607	f	Not repo	DE	2015	1	0	0	0	0	0	0	0	0	0	0	0	50
7131598	m	ot	GR	2015	1	0	0	0	0	0	0	0	0	0	0	0	61
7294271	m	md	DE	2015	1	0	0	0	0	0	0	0	0	0	0	0	57
7294523	f	ot	GB	2015	1	0	0	0	0	0	0	0	0	0	0	0	65
7356850	f	md	BE	2015	1	0	0	0	0	0	0	0	0	0	0	0	65
7398216	m	ph	US	2015	1	0	0	0	0	0	0	0	0	0	0	0	
8027143	f	ot	FR	2015	0	0	0	0	0	0	0	0	0	0	0	0	36
8070952	Not	md	JP	2015	0	0	0	0	0	0	0	0	0	0	0	0	
8134000	m	ot	US	2015	1	0	0	0	0	0	0	0	0	0	0	0	79
8336155	f	md	US	2015	0	0	0	0	0	0	0	0	0	0	0	0	
8340571	m	cn	US	2015	0	0	0	0	0	0	0	0	0	0	0	0	
8385371	f	ot	US	2015	0	0	0	0	0	0	0	0	0	0	0	0	
8389512	m	md	US	2015	0	0	0	0	0	0	0	0	0	0	0	0	
8447717	Not	ot	US	2015	0	0	0	0	0	0	0	0	0	0	0	0	
8533312	m	ot	GB	2015	1	0	0	0	0	0	0	0	0	0	0	0	83
8566333	f	cn	GB	2015	0	0	0	0	0	0	0	0	0	0	0	0	51
8583080	f	cn	GB	2015	1	0	0	0	0	0	0	0	0	0	0	0	46
8601121	f	md	US	2015	0	0	0	0	0	0	0	0	0	0	0	0	50
8682326	f	md	JP	2015	0	0	0	0	0	0	0	0	0	0	0	0	72
8742402	m	md	JP	2015	0	0	0	0	0	0	0	0	0	0	0	0	42
8773013	Not	ot	JP	2015	0	0	0	0	0	0	0	0	0	0	0	0	
8786149	f	md	US	2015	1	0	0	0	0	0	0	0	0	0	0	0	
8786674	f	md	US	2015	0	0	0	0	0	0	0	0	0	0	0	0	58
8788017	m	md	US	2015	0	0	0	0	0	0	0	0	0	0	0	0	54
8800448	m	md	US	2015	0	0	0	0	0	0	0	0	0	0	0	0	57
8801304	m	md	US	2015	0	0	0	0	0	0	0	0	0	0	0	0	
8802531	f	md	US	2015	0	0	0	0	0	0	0	0	0	0	0	0	49
8815720	m	md	US	2015	0	0	0	0	0	0	0	0	0	0	0	0	
8839871	f	cn	US	2015	0	1	0	0	1	0	0	0	0	0	0	0	52
8890494	m	cn	US	2015	0	0	0	0	0	0	0	0	0	0	0	0	67
9013408	f	md	JP	2015	0	0	0	0	0	0	0	0	0	0	0	0	92
9100640	m	md	JP	2015	0	0	0	0	0	0	0	0	0	0	0	0	63
9140093	m	md	JP	2015	0	0	0	0	0	0	0	0	0	0	0	0	79

9171048	f	md	JP	2015	0	0	0	0	0	0	0	0	0	0	0	0	75
9191298	m	cn	US	2015	0	1	0	0	1	0	0	0	0	0	0	1	56
9222377	f	md	JP	2015	0	0	0	0	0	0	0	0	0	0	0	0	76
9229014	m	cn	US	2015	0	1	0	0	1	0	0	0	0	0	0	1	84
9310355	f	md	JP	2015	0	0	0	0	0	0	0	0	0	0	0	0	64
9311210	m	md	JP	2015	0	0	0	0	0	0	0	0	0	0	0	0	77
9315243	m	cn	SG	2015	0	0	0	0	0	0	0	0	0	0	0	0	59
9320926	Not	ot	SE	2015	0	0	0	0	0	0	0	0	0	0	0	0	
9332839	f	md	JP	2015	0	0	0	0	0	0	0	0	0	0	0	0	37
9412297	f	md	JP	2015	0	0	0	0	0	0	0	0	0	0	0	0	83
9415038	f	md	US	2015	0	0	0	0	0	0	0	0	0	0	0	0	74
9437562	f	md	JP	2015	0	0	0	0	0	0	0	0	0	0	0	0	80
9459235	f	md	JP	2015	0	0	0	0	0	0	0	0	0	0	0	0	51
9459308	f	md	US	2015	0	0	0	0	0	0	0	0	0	0	0	0	54
9477803	f	ot	US	2015	0	0	0	0	0	0	0	0	0	0	0	0	51
9491252	f	md	JP	2015	0	0	0	0	0	0	0	0	0	0	0	0	43
9493497	f	md	JP	2015	0	0	0	0	0	0	0	0	0	0	0	0	75
9663889	m	cn	GB	2015	0	0	0	0	0	0	0	0	0	0	0	0	92
9677166	m	cn	US	2015	0	0	0	0	0	0	0	0	0	0	0	0	71
9723243	f	ot	AU	2015	0	0	0	0	0	0	0	0	0	0	0	0	70
9728495	f	cn	CN	2015	1	0	0	0	0	0	0	0	0	0	0	0	83
9741591	m	md	DE	2015	0	0	0	0	0	0	0	0	0	0	0	0	75
9782658	f	md	JP	2015	0	0	0	0	0	0	0	0	0	0	0	0	74
9791746	f	md	CA	2015	0	0	0	0	0	0	0	0	0	0	0	0	68
9793017	m	md	JP	2015	0	0	0	0	0	0	0	0	0	0	0	0	66
9801652	Not	cn	US	2015	0	1	0	0	1	0	0	0	0	0	0	0	
9802196	m	cn	AR	2015	0	0	0	0	0	0	0	0	0	0	0	0	74
9846198	f	md	US	2015	0	0	0	0	0	0	0	0	0	0	0	0	67
9846211	f	md	JP	2015	0	0	0	0	0	0	0	0	0	0	0	0	70
9870232	m	md	JP	2015	0	0	0	0	0	0	0	0	0	0	0	0	59
9882491	f	md	Not re	2015	1	0	0	0	0	0	0	0	0	0	0	0	78
9883350	f	md	JP	2015	0	0	0	0	0	0	0	0	0	0	0	0	50
9920904	f	ot	DE	2015	0	0	0	0	0	0	0	0	0	0	0	0	48
9921203	m	md	US	2015	0	0	0	0	0	0	0	0	0	0	0	0	63

9937390	f	cn	US	2015	0	0	0	0	0	0	0	0	0	0	0	0		
9956368	m	cn	US	2015	0	0	0	0	0	0	0	0	0	0	0	0		
9980673	m	md	DE	2015	0	0	0	0	0	0	0	0	0	0	0	0	56	
9996460	m	cn	PT	2015	0	0	0	0	0	0	0	0	0	0	0	0	52	
10003329	f	md	Not re	2015	1	0	0	0	0	0	0	0	0	0	0	0	79	
10017832	m	cn	GB	2015	1	0	0	0	0	0	0	0	0	0	0	0	55	
10041187	f	md	DK	2015	0	0	0	0	0	0	0	0	0	0	0	0	56	
10086729	m	cn	US	2015	0	0	0	0	0	0	0	0	0	0	0	0	67	
10128566	Not	ot	US	2015	0	0	0	0	0	0	0	0	0	0	0	0		
10146180	m	ph	AU	2015	0	0	0	0	0	0	0	0	0	0	0	0	76	
10161653	f	md	CA	2015	0	0	0	0	0	0	0	0	0	0	0	0	50	
10169676	f	ph	AU	2015	0	0	0	0	0	0	0	0	0	0	0	0	58	
10216775	f	md	JP	2015	0	0	0	0	0	0	0	0	0	0	0	0	49	
10219207	Not	Not repo	Not re	2015	1	0	0	0	0	0	0	0	0	0	0	0		
10219215	f	cn	GB	2015	1	0	0	0	0	0	0	0	0	0	0	0	72	
10224480	f	md	FR	2015	0	0	0	0	0	0	0	0	0	0	0	0	66	
10247671	f	cn	GB	2015	1	0	0	0	0	0	0	0	0	0	0	0	53	
10296807	m	ot	US	2015	0	1	0	0	1	0	0	0	0	0	0	0	54	
10298384	f	md	JP	2015	0	0	0	0	0	0	0	0	0	0	0	0	64	
10305122	m	cn	GB	2015	1	0	0	0	0	0	0	0	0	0	0	0		
10329169	f	md	JP	2015	0	0	0	0	0	0	0	0	0	0	0	0	57	
10338263	m	ot	US	2015	0	0	0	0	0	0	0	0	0	0	0	0	50	
10349871	f	cn	GB	2015	1	0	0	0	0	0	0	0	0	0	0	0		
10366472	m	ot	JP	2015	0	0	0	0	0	0	0	0	0	0	0	0	68	
10366712	m	md	Not re	2015	1	0	0	0	0	0	0	0	0	0	0	1	71	
10371147	m	cn	GB	2015	1	0	0	0	0	0	0	0	0	0	0	0		
10375360	f	md	JP	2015	0	0	0	0	0	0	0	0	0	0	0	0	63	
10375411	m	cn	GB	2015	1	0	0	0	0	0	0	0	0	0	0	0	74	
10375421	f	cn	GB	2015	1	0	0	0	0	0	0	0	0	0	0	0		
10381330	f	cn	GB	2015	1	0	0	0	0	0	0	0	0	0	0	0	86	
10383221	m	cn	US	2015	0	1	0	0	1	0	0	0	0	0	0	0	72	
10391840	f	md	Not re	2015	1	0	0	0	0	0	0	0	0	0	0	0	87	
10401924	m	md	FR	2015	0	0	0	0	0	0	0	0	0	0	0	0	61	
10401968	m	cn	GB	2015	1	0	0	0	0	0	0	0	0	0	0	0		

10403214	f	md	US	2015	0	0	0	0	0	0	0	0	0	0	0	0	76
10409631	f	cn	GB	2015	1	0	0	0	0	0	0	0	0	0	0	0	
10409642	f	cn	GB	2015	1	0	0	0	0	0	0	0	0	0	0	0	
10419016	f	md	JP	2015	0	0	0	0	0	0	0	0	0	0	0	0	41
10423090	m	cn	GB	2015	1	0	0	0	0	0	0	0	0	0	0	0	
10423091	f	cn	GB	2015	1	0	0	0	0	0	0	0	0	0	0	0	55
10433057	m	md	Not re	2015	1	0	0	0	0	0	0	0	0	0	0	0	49
10441373	f	cn	GB	2015	1	0	0	0	0	0	0	0	0	0	0	0	
10445103	f	cn	US	2015	0	0	0	0	0	0	0	0	0	0	0	0	70
10446945	m	cn	GB	2015	1	0	0	0	0	0	0	0	0	0	0	0	
10447618	m	ot	GB	2015	0	1	1	0	0	0	0	0	0	0	0	0	72
10457024	f	md	FR	2015	0	1	1	0	0	0	0	0	0	0	0	0	70
10457644	m	md	BE	2015	0	1	1	0	0	0	0	0	0	0	0	1	47
10457691	m	ot	US	2015	0	1	1	0	0	0	0	0	0	0	0	0	84
10462657	m	ot	AU	2015	0	1	1	0	0	0	0	0	0	0	0	0	70
10464712	f	cn	GB	2015	1	0	0	0	0	0	0	0	0	0	0	0	
10469067	f	md	JP	2015	0	0	0	0	0	0	0	0	0	0	0	0	42
10475075	m	md	JP	2015	0	0	0	0	0	0	0	0	0	0	0	0	55
10476097	m	ot	AU	2015	0	1	1	0	0	0	0	0	0	0	0	0	76
10485059	m	md	RU	2015	0	1	1	0	0	0	0	0	0	0	0	0	61
10488038	f	md	JP	2015	1	0	0	0	0	0	0	0	0	0	0	0	30
10490421	m	cn	GB	2015	1	0	0	0	0	0	0	0	0	0	0	0	68
10495473	f	ot	Not re	2015	1	0	0	0	0	0	0	0	0	0	0	0	60
10511477	m	ot	Not re	2015	1	0	0	0	0	0	0	0	0	0	0	0	75
10512723	f	cn	US	2015	0	1	0	0	1	0	0	0	0	0	0	1	60
10520737	f	md	JP	2015	1	0	0	0	0	0	0	0	0	0	0	0	49
10529217	m	md	AU	2015	0	1	1	0	0	0	0	0	0	0	0	0	51
10530439	f	md	JP	2015	0	0	0	0	0	0	0	0	0	0	0	0	71
10531879	f	md	US	2015	0	1	1	0	0	0	0	0	0	0	0	0	74
10543393	m	ot	US	2015	0	0	0	0	0	0	0	0	0	0	0	0	62
10548157	f	md	US	2015	0	0	0	0	0	0	0	0	0	0	0	0	53
10548361	f	md	JP	2015	0	0	0	0	0	0	0	0	0	0	0	0	60
10549971	f	md	Not re	2015	1	0	0	0	0	0	0	0	0	0	0	0	
10577997	m	md	JP	2015	0	0	0	0	0	0	0	0	0	0	0	0	84

10581611	m	ph	JP	2015	0	0	0	0	0	0	0	0	0	0	0	0	62
10586414	m	md	FR	2015	0	0	0	0	0	0	0	0	0	0	0	0	56
10587231	f	cn	GB	2015	1	0	0	0	0	0	0	0	0	0	0	0	59
10590023	m	md	Not re	2015	1	0	0	0	0	0	0	0	0	0	0	0	73
10602226	f	md	Not re	2015	1	0	0	0	0	0	0	0	0	0	0	0	72
10608607	m	md	Not re	2015	1	0	0	0	0	0	0	0	0	0	0	0	71
10608976	f	cn	GB	2015	1	0	0	0	0	0	0	0	0	0	0	0	58
10612094	m	md	JP	2015	0	0	0	0	0	0	0	0	0	0	0	0	50
10615577	m	ot	US	2015	0	0	0	0	0	0	0	0	0	0	0	0	61
10618172	m	cn	CN	2015	0	0	0	0	0	0	0	0	0	0	0	0	72
10634005	m	md	FR	2015	0	1	1	0	0	0	0	0	0	0	0	0	76
10634840	f	md	JP	2015	0	0	0	0	0	0	0	0	0	0	0	0	59
10636021	m	md	DE	2015	0	0	0	0	0	0	0	0	0	0	0	0	45
10637748	f	cn	GB	2015	1	0	0	0	0	0	0	0	0	0	0	0	80
10637756	f	cn	GB	2015	1	0	0	0	0	0	0	0	0	0	0	0	47
10649038	m	md	AU	2015	0	0	0	0	0	0	0	0	0	0	0	0	66
10652125	m	md	JP	2015	0	0	0	0	0	0	0	0	0	0	0	0	48
10659617	m	ph	AU	2015	0	0	0	0	0	0	0	0	0	0	0	0	40
10659780	m	md	FR	2015	0	0	0	0	0	0	0	0	0	0	0	0	42
10660274	f	ot	US	2015	0	0	0	0	0	0	0	0	0	0	0	0	56
10670837	m	cn	US	2015	0	0	0	0	0	0	0	0	0	0	0	0	78
10682546	m	cn	GB	2015	1	0	0	0	0	0	0	0	0	0	0	0	
10685252	m	cn	GB	2015	1	0	0	0	0	0	0	0	0	0	0	0	70
10686980	f	md	JP	2015	0	0	0	0	0	0	0	0	0	0	0	0	73
10689531	f	md	US	2015	0	0	0	0	0	0	0	0	0	0	0	0	34
10695988	f	md	Not re	2015	1	0	0	0	0	0	0	0	0	0	0	0	
10700172	m	cn	US	2015	0	0	0	0	0	0	0	0	0	0	0	0	49
10701689	m	md	US	2015	0	1	1	0	0	0	0	0	0	0	0	0	37
10702527	f	md	US	2015	0	0	0	0	0	0	0	0	0	0	0	0	73
10704719	f	md	JP	2015	0	0	0	0	0	0	0	0	0	0	0	0	73
10705710	f	md	FR	2015	0	0	0	0	0	0	0	0	0	0	0	0	57
10706795	m	cn	PH	2015	0	0	0	0	0	0	0	0	0	0	0	0	
10708546	f	md	Not re	2015	1	0	0	0	0	0	0	0	0	0	0	0	66
10722407	m	md	JP	2015	1	0	0	0	0	0	0	0	0	0	0	1	83

10723595	f	md	TH	2015	0	0	0	0	0	0	0	0	0	0	0	0	55
10725480	m	cn	US	2015	0	1	0	0	1	0	0	0	0	0	0	0	50
10726370	m	md	US	2015	0	0	0	0	0	0	0	0	0	0	0	0	34
10734096	f	md	US	2015	0	0	0	0	0	0	0	0	0	0	0	0	62
10734132	m	ot	US	2015	0	1	1	0	0	0	0	0	0	0	0	0	73
10745660	m	cn	DE	2015	0	0	0	0	0	0	0	0	0	0	0	0	72
10747264	f	md	ES	2015	1	0	0	0	0	0	0	0	0	0	0	0	63
10754648	f	md	JP	2015	0	0	0	0	0	0	0	0	0	0	0	0	42
10758228	m	md	JP	2015	0	0	0	0	0	0	0	0	0	0	0	0	53
10758351	f	cn	GB	2015	1	0	0	0	0	0	0	0	0	0	0	0	63
10766352	m	md	JP	2015	0	0	0	0	0	0	0	0	0	0	0	0	83
10768570	f	cn	US	2015	0	1	0	0	1	0	0	0	0	0	0	0	70
10769466	f	md	FR	2015	0	0	0	0	0	0	0	0	0	0	0	0	64
10771807	Not	ph	AU	2015	0	0	0	0	0	0	0	0	0	0	0	0	60
10776157	f	cn	GB	2015	0	0	0	0	0	0	0	0	0	0	0	0	49
10777565	f	cn	EG	2015	0	0	0	0	0	0	0	0	0	0	0	0	
10777962	f	cn	US	2015	0	0	0	0	0	0	0	0	0	0	0	0	56
10786711	m	md	TW	2015	0	1	1	0	0	0	0	0	0	0	0	0	45
10787397	m	md	FR	2015	0	0	0	0	0	0	0	0	0	0	0	0	35
10788193	f	md	SK	2015	0	0	0	0	0	0	0	0	0	0	0	0	23
10790395	f	md	JP	2015	1	0	0	0	0	0	0	0	0	0	0	0	73
10791121	f	md	JP	2015	1	0	0	0	0	0	0	0	0	0	0	0	51
10793028	f	cn	GB	2015	1	0	0	0	0	0	0	0	0	0	0	0	
10802358	m	md	US	2015	0	1	1	0	0	0	0	0	0	0	0	0	64
10818626	f	md	FR	2015	0	0	0	0	0	0	0	0	0	0	0	0	60
10824723	m	cn	DE	2015	0	0	0	0	0	0	0	0	0	0	0	0	78
10833286	m	md	JP	2015	0	0	0	0	0	0	0	0	0	0	0	0	60
10839010	m	md	JP	2015	0	0	0	0	0	0	0	0	0	0	0	0	63
10844060	f	md	US	2015	0	0	0	0	0	0	0	0	0	0	0	0	74
10855281	m	md	JP	2015	0	0	0	0	0	0	0	0	0	0	0	0	68
10861929	f	md	CH	2015	0	0	0	0	0	0	0	0	0	0	0	0	82
10862385	f	cn	GB	2015	1	0	0	0	0	0	0	0	0	0	0	0	74
10872509	Not	ot	GR	2015	0	0	0	0	0	0	0	0	0	0	0	0	
10873879	f	cn	US	2015	0	0	0	0	0	0	0	0	0	0	0	0	53

10874091	f	md	JP	2015	0	0	0	0	0	0	0	0	0	0	0	0	75
10874356	m	cn	US	2015	0	1	0	0	1	0	0	0	0	0	0	0	65
10875250	f	md	DE	2015	1	0	0	0	0	0	0	0	0	0	0	0	75
10877379	m	md	JP	2015	0	0	0	0	0	0	0	0	0	0	0	0	49
10883267	f	md	KR	2015	0	1	1	0	0	0	0	0	0	0	0	0	72
10883881	f	cn	US	2015	0	1	0	0	1	0	0	0	0	0	0	0	54
10889010	Not	ph	Not re	2015	1	0	0	0	0	0	0	0	0	0	0	0	70
10891315	f	md	JP	2015	0	0	0	0	0	0	0	0	0	0	0	0	73
10892050	f	md	US	2015	0	0	0	0	0	0	0	0	0	0	0	0	65
10901380	f	md	GB	2015	0	1	1	0	0	0	0	0	0	0	0	0	52
10901495	f	md	JP	2015	0	0	0	0	0	0	0	0	0	0	0	0	77
10902764	f	md	Not re	2015	1	0	0	0	0	0	0	0	0	0	0	0	94
10903714	f	md	Not re	2015	1	0	0	0	0	0	0	0	0	0	0	0	
10904762	m	cn	GB	2015	1	0	0	0	0	0	0	0	0	0	0	0	79
10905815	f	cn	GB	2015	1	0	0	0	0	0	0	0	0	0	0	0	37
10910907	Not	md	JP	2015	0	0	0	0	0	0	0	0	0	0	0	0	
10914002	m	cn	US	2015	0	1	0	0	1	0	0	0	0	0	0	0	72
10914040	Not	md	PH	2015	1	0	0	0	0	0	0	0	0	0	0	0	
10914041	Not	md	PH	2015	1	0	0	0	0	0	0	0	0	0	0	0	
10914763	m	cn	GB	2015	1	0	0	0	0	0	0	0	0	0	0	0	
10916310	m	md	JP	2015	0	0	0	0	0	0	0	0	0	0	0	0	79
10920534	m	md	FR	2015	0	0	0	0	0	0	0	0	0	0	0	0	46
10921633	f	md	SK	2015	1	0	0	0	0	0	0	0	0	0	0	0	
10922304	f	ot	BE	2015	0	0	0	0	0	0	0	0	0	0	0	0	52
10922396	f	cn	US	2015	0	0	0	0	0	0	0	0	0	0	0	0	59
10922536	m	md	JP	2015	0	1	1	0	0	0	0	0	0	0	0	0	50
10922810	Not	ot	JP	2015	0	0	0	0	0	0	0	0	0	0	0	0	
10922811	Not	ot	JP	2015	0	0	0	0	0	0	0	0	0	0	0	0	
10922814	f	cn	US	2015	0	0	0	0	0	0	0	0	0	0	0	0	59
10923995	Not	md	US	2015	0	0	0	0	0	0	0	0	0	0	0	0	
10923996	Not	md	US	2015	0	0	0	0	0	0	0	0	0	0	0	0	
10923997	Not	md	US	2015	0	0	0	0	0	0	0	0	0	0	0	0	
10926128	f	md	Not re	2015	1	0	0	0	0	0	0	0	0	0	0	0	84
10934618	f	ot	US	2015	0	0	0	0	0	0	0	0	0	0	0	0	69

10934665	f	cn	SK	2015	0	0	0	0	0	0	0	0	0	0	0	0	67
10935300	m	cn	US	2015	0	0	0	0	0	0	0	0	0	0	0	0	56
10943994	m	md	US	2015	0	1	1	0	0	0	0	0	0	0	0	0	62
10949591	f	md	US	2015	0	1	1	0	0	0	0	0	0	0	0	0	77
10949663	m	ot	US	2015	0	0	0	0	0	0	0	0	0	0	0	0	62
10949940	f	md	Not re	2015	1	0	0	0	0	0	0	0	0	0	0	0	
10951159	f	md	DE	2015	1	0	0	0	0	0	0	0	0	0	0	0	
10951402	m	cn	CN	2015	0	0	0	0	0	0	0	0	0	0	0	0	71
10953231	m	md	JP	2015	0	0	0	0	0	0	0	0	0	0	0	0	55
10955431	f	md	JP	2015	0	0	0	0	0	0	0	0	0	0	0	0	73
10957563	m	cn	GB	2015	1	0	0	0	0	0	0	0	0	0	0	0	47
10958206	f	md	GB	2015	1	0	0	0	0	0	0	0	0	0	0	0	69
10960506	f	cn	US	2015	0	1	1	0	0	0	0	0	0	0	0	0	70
10962192	m	ot	US	2015	0	0	0	0	0	0	0	0	0	0	0	0	64
10962302	f	md	Not re	2015	1	0	0	0	0	0	0	0	0	0	0	0	
10963924	f	md	US	2015	1	0	0	0	0	0	0	0	0	0	0	0	65
10963926	f	md	US	2015	1	0	0	0	0	0	0	0	0	0	0	0	65
10963928	f	ot	US	2015	0	0	0	0	0	0	0	0	0	0	0	0	47
10965200	f	ph	US	2015	0	0	0	0	0	0	0	0	0	0	0	0	68
10966564	m	md	JP	2015	0	1	1	0	0	0	0	0	0	0	0	0	74
10968948	f	md	Not re	2015	1	0	0	0	0	0	0	0	0	0	0	0	47
10974216	m	cn	US	2015	0	0	0	0	0	0	0	0	0	0	0	0	
10974355	m	md	JP	2015	0	0	0	0	0	0	0	0	0	0	0	0	66
10974488	m	cn	US	2015	0	0	0	0	0	0	0	0	0	0	0	0	69
10974747	f	md	HK	2015	0	0	0	0	0	0	0	0	0	0	0	0	63
10974748	f	cn	US	2015	0	0	0	0	0	0	0	0	0	0	0	0	
10974855	m	cn	US	2015	0	0	0	0	0	0	0	0	0	0	0	0	69
10974856	f	cn	US	2015	0	0	0	0	0	0	0	0	0	0	0	0	64
10974859	m	ph	US	2015	0	0	0	0	0	0	0	0	0	0	0	0	
10975095	m	cn	GB	2015	1	0	0	0	0	0	0	0	0	0	0	0	
10976288	f	md	Not re	2015	1	0	0	0	0	0	0	0	0	0	0	0	72
10977932	m	cn	CN	2015	1	0	0	0	0	0	0	0	0	0	0	0	
10977996	f	md	Not re	2015	1	0	0	0	0	0	0	0	0	0	0	0	62
10978852	Not	md	US	2015	0	0	0	0	0	0	0	0	0	0	0	0	

10978862	m	md	Not re	2015	1	0	0	0	0	0	0	0	0	0	0	0	59
10978863	f	md	ES	2015	1	0	0	0	0	0	0	0	0	0	0	0	
10978870	f	md	JP	2015	1	0	0	0	0	0	0	0	0	0	0	0	71
10978965	f	md	US	2015	0	0	0	0	0	0	0	0	0	0	0	0	73
10981116	f	md	US	2015	0	0	0	0	0	0	0	0	0	0	0	0	
10981985	m	md	BR	2015	0	1	1	0	0	0	0	0	0	0	0	0	59
10982110	f	md	JP	2015	0	0	0	0	0	0	0	0	0	0	0	0	70
10982302	f	ot	US	2015	1	0	0	0	0	0	0	0	0	0	0	0	66
10983233	m	cn	US	2015	0	0	0	0	0	0	0	0	0	0	0	0	59
10983625	m	md	US	2015	0	0	0	0	0	0	0	0	0	0	0	0	85
10983798	f	cn	GB	2015	1	0	0	0	0	0	0	0	0	0	0	0	
10984300	m	cn	GB	2015	1	0	0	0	0	0	0	0	0	0	0	0	54
10984301	m	cn	GB	2015	1	0	0	0	0	0	0	0	0	0	0	0	72
10985964	f	md	US	2015	0	0	0	0	0	0	0	0	0	0	0	0	
10989867	Not	ot	US	2015	0	1	0	0	1	0	0	0	0	0	0	1	
10990715	m	cn	AR	2015	0	0	0	0	0	0	0	0	0	0	0	0	64
10991143	m	md	GR	2015	0	0	0	0	0	0	0	0	0	0	0	0	68
10992148	m	md	US	2015	0	0	0	0	0	0	0	0	0	0	0	0	53
10993259	Not	ot	GR	2015	0	0	0	0	0	0	0	0	0	0	0	0	
10995038	f	md	Not re	2015	1	0	0	0	0	0	0	0	0	0	0	0	
10995326	m	cn	GB	2015	1	0	0	0	0	0	0	0	0	0	0	0	78
10995843	f	md	DE	2015	1	0	0	0	0	0	0	0	0	0	0	0	74
10996311	Not	ot	US	2015	0	0	0	0	0	0	0	0	0	0	0	0	
10996884	m	md	ES	2015	0	1	1	0	0	0	0	0	0	0	0	0	67
10997094	f	md	JP	2015	0	0	0	0	0	0	0	0	0	0	0	0	75
10997891	f	cn	GB	2015	1	0	0	0	0	0	0	0	0	0	0	0	46
11002634	m	md	US	2015	0	1	1	0	0	0	0	0	0	0	0	0	62
11002706	f	md	JP	2015	0	1	1	0	0	0	0	0	0	0	0	0	45
11003045	m	md	FR	2015	0	0	0	0	0	0	0	0	0	0	0	0	
11004136	f	cn	GB	2015	1	0	0	0	0	0	0	0	0	0	0	1	72
11004137	f	cn	GB	2015	1	0	0	0	0	0	0	0	0	0	0	0	
11004579	m	md	US	2015	1	0	0	0	0	0	0	0	0	0	0	0	
11004650	m	md	DE	2015	0	0	0	0	0	0	0	0	0	0	0	0	54
11004697	m	ph	JP	2015	0	0	0	0	0	0	0	0	0	0	0	0	52

11004743	m	md	JP	2015	0	0	0	0	0	0	0	0	0	0	0	0	65
11004772	f	md	JP	2015	0	0	0	0	0	0	0	0	0	0	0	0	58
11005200	f	cn	ES	2015	0	0	0	0	0	0	0	0	0	0	0	0	63
11005745	f	md	JP	2015	0	0	0	0	0	0	0	0	0	0	0	0	65
11009285	Not	ph	US	2015	1	0	0	0	0	0	0	0	0	0	0	0	
11009979	f	md	MX	2015	1	0	0	0	0	0	0	0	0	0	0	0	87
11010002	f	cn	GB	2015	0	0	0	0	0	0	0	0	0	0	0	0	50
11010055	f	md	US	2015	0	0	0	0	0	0	0	0	0	0	0	0	44
11018368	Not	md	US	2015	0	0	0	0	0	0	0	0	0	0	0	0	
11020348	m	md	JP	2015	0	0	0	0	0	0	0	0	0	0	0	0	64
11020621	f	cn	US	2015	0	1	0	0	1	0	0	0	0	0	0	1	72
11020815	m	cn	US	2015	0	1	0	0	1	0	0	0	0	0	0	0	
11021246	f	md	JP	2015	0	0	0	0	0	0	0	0	0	0	0	0	74
11021760	m	cn	GB	2015	1	0	0	0	0	0	0	0	0	0	0	0	77
11021773	m	cn	GB	2015	1	0	0	0	0	0	0	0	0	0	0	0	52
11021775	m	cn	GB	2015	1	0	0	0	0	0	0	0	0	0	0	0	75
11022296	m	cn	SG	2015	0	0	0	0	0	0	0	0	0	0	0	0	46
11022326	f	cn	GB	2015	0	0	0	0	0	0	0	0	0	0	0	0	65
11023366	f	cn	US	2015	0	0	0	0	0	0	0	0	0	0	0	0	
11025008	Not	Not repo	Not re	2015	1	0	0	0	0	0	0	0	0	0	0	0	
11025861	m	ot	US	2015	0	0	0	0	0	0	0	0	0	0	0	0	58
11025989	m	cn	US	2015	0	0	0	0	0	0	0	0	0	0	0	0	
11026263	f	Not repo	US	2015	1	0	0	0	0	0	0	0	0	0	0	0	73
11026531	m	ot	US	2015	0	1	0	0	1	0	0	0	0	0	0	0	
11026578	f	md	FR	2015	0	0	0	0	0	0	0	0	0	0	0	0	58
11026691	Not	cn	GB	2015	1	0	0	0	0	0	0	0	0	0	0	0	
11028043	f	md	US	2015	0	0	0	0	0	0	0	0	0	0	0	0	75
11029211	m	cn	AU	2015	0	0	0	0	0	0	0	0	0	0	0	0	73
11030277	m	cn	GB	2015	1	0	0	0	0	0	0	0	0	0	0	0	79
11030285	f	cn	GB	2015	1	0	0	0	0	0	0	0	0	0	0	0	88
11030376	m	md	FR	2015	0	0	0	0	0	0	0	0	0	0	0	0	71
11030398	f	md	AT	2015	0	0	0	0	0	0	0	0	0	0	0	0	49
11030857	f	ot	US	2015	0	0	0	0	0	0	0	0	0	0	0	0	65
11038488	f	cn	US	2015	0	0	0	0	0	0	0	0	0	0	0	0	82

11038644	m	md	FR	2015	0	1	0	0	1	0	0	0	0	0	0	0	58
11039274	f	cn	US	2015	0	0	0	0	0	0	0	0	0	0	0	0	
11039763	f	md	GB	2015	0	0	0	0	0	0	0	0	0	0	0	0	54
11043560	m	cn	US	2015	0	1	0	0	1	0	0	0	0	0	0	1	82
11043962	m	md	JP	2015	0	0	0	0	0	0	0	0	0	0	0	0	79
11045220	f	md	KR	2015	0	0	0	0	0	0	0	0	0	0	0	0	61
11045745	m	md	TR	2015	0	0	0	0	0	0	0	0	0	0	0	0	72
11046243	f	cn	AU	2015	1	0	0	0	0	0	0	0	0	0	0	0	61
11046251	f	md	BE	2015	1	0	0	0	0	0	0	0	0	0	0	0	69
11046449	f	cn	US	2015	0	1	0	0	1	0	0	0	0	0	0	0	
11047559	m	md	DE	2015	0	0	0	0	0	0	0	0	0	0	0	0	56
11047739	f	cn	DE	2015	1	0	0	0	0	0	0	0	0	0	0	0	54
11047977	Not	md	CA	2015	0	1	1	0	0	0	0	0	0	0	0	0	
11048768	Not	ot	AU	2015	0	0	0	0	0	0	0	0	0	0	0	0	
11048983	m	cn	US	2015	0	0	0	0	0	0	0	0	0	0	0	0	66
11049078	m	cn	US	2015	0	0	0	0	0	0	0	0	0	0	0	0	45
11049437	m	md	SE	2015	0	0	0	0	0	0	0	0	0	0	0	0	41
11049454	m	md	US	2015	0	0	0	0	0	0	0	0	0	0	0	0	63
11052038	Not	ot	TR	2015	0	0	0	0	0	0	0	0	0	0	0	0	
11052047	f	md	US	2015	0	1	1	0	0	0	0	0	0	0	0	0	72
11052367	m	cn	GB	2015	1	0	0	0	0	0	0	0	0	0	0	0	69
11052371	f	ot	AT	2015	0	0	0	0	0	0	0	0	0	0	0	0	40
11052854	m	md	FR	2015	0	0	0	0	0	0	0	0	0	0	0	0	66
11053776	m	md	DE	2015	0	0	0	0	0	0	0	0	0	0	0	0	4
11055164	m	md	US	2015	0	0	0	0	0	0	0	0	0	0	0	0	58
11055849	f	cn	GB	2015	1	0	0	0	0	0	0	0	0	0	0	0	57
11056084	f	cn	US	2015	0	0	0	0	0	0	0	0	0	0	0	0	50
11056170	m	md	Not re	2015	0	0	0	0	0	0	0	0	0	0	0	0	81
11057093	m	cn	GB	2015	1	0	0	0	0	0	0	0	0	0	0	0	69
11057474	f	md	US	2015	0	1	1	0	0	0	0	0	0	0	0	0	63
11057578	m	md	US	2015	0	0	0	0	0	0	0	0	0	0	0	0	
11058778	Not	ot	SI	2015	0	0	0	0	0	0	0	0	0	0	0	0	
11058779	Not	ot	SI	2015	0	0	0	0	0	0	0	0	0	0	0	0	
11058902	f	cn	GB	2015	1	0	0	0	0	0	0	0	0	0	0	0	

11059011	Not	ot	SI	2015	0	0	0	0	0	0	0	0	0	0	0	0	
11059029	Not	ot	SI	2015	0	0	0	0	0	0	0	0	0	0	0	0	
11059565	Not	md	US	2015	0	1	0	0	1	0	0	0	0	0	0	0	
11059881	Not	ot	SI	2015	0	0	0	0	0	0	0	0	0	0	0	0	
11059882	Not	ot	SI	2015	0	0	0	0	0	0	0	0	0	0	0	0	
11059883	Not	ot	SI	2015	0	0	0	0	0	0	0	0	0	0	0	0	
11060619	f	md	Not re	2015	1	0	0	0	0	0	0	0	0	0	0	0	69
11061928	f	md	DE	2015	0	0	0	0	0	0	0	0	0	0	0	0	62
11062729	f	md	IT	2015	0	0	0	0	0	0	0	0	0	0	0	0	73
11063853	f	cn	US	2015	1	0	0	0	0	0	0	0	0	0	0	0	56
11064229	f	md	Not re	2015	1	0	0	0	0	0	0	0	0	0	0	0	89
11064281	f	cn	GB	2015	1	0	0	0	0	0	0	0	0	0	0	0	60
11065743	Not	ot	JP	2015	0	0	0	0	0	0	0	0	0	0	0	0	
11066622	f	md	US	2015	0	0	0	0	0	0	0	0	0	0	0	0	51
11067670	m	md	FR	2015	0	1	0	0	1	0	0	0	0	0	0	0	72
11068328	m	cn	GB	2015	1	0	0	0	0	0	0	0	0	0	0	0	
11069172	f	md	JP	2015	1	0	0	0	0	0	0	0	0	0	0	0	61
11069186	m	md	DE	2015	0	0	0	0	0	0	0	0	0	0	0	0	73
11069353	Not	cn	US	2015	0	1	1	0	0	0	0	0	0	0	0	0	
11069354	Not	cn	US	2015	0	1	1	0	0	0	0	0	0	0	0	1	
11070147	m	ot	RU	2015	0	1	1	0	0	0	0	0	0	0	0	0	53
11070422	m	ot	Not re	2015	1	0	0	0	0	0	0	0	0	0	0	0	78
11070535	f	md	Not re	2015	1	0	0	0	0	0	0	0	0	0	0	0	83
11070669	m	md	US	2015	0	0	0	0	0	0	0	0	0	0	0	0	
11070746	m	md	JP	2015	0	0	0	0	0	0	0	0	0	0	0	0	51
11070816	m	md	US	2015	0	1	0	0	1	0	0	0	0	0	0	0	
11071670	m	ot	US	2015	1	0	0	0	0	0	0	0	0	0	0	0	59
11073213	m	ot	CH	2015	0	1	1	0	0	0	0	0	0	0	0	0	68
11073514	m	cn	GB	2015	1	0	0	0	0	0	0	0	0	0	0	0	70
11073516	m	cn	GB	2015	1	0	0	0	0	0	0	0	0	0	0	0	70
11073749	m	ph	FR	2015	0	0	0	0	0	0	0	0	0	0	0	0	75
11073848	m	ot	US	2015	0	1	0	0	1	0	0	0	0	0	0	0	72
11074075	f	md	DE	2015	0	0	0	0	0	0	0	0	0	0	0	0	63
11074396	f	cn	ES	2015	0	0	0	0	0	0	0	0	0	0	0	0	

11075223	f	md	JP	2015	0	0	0	0	0	0	0	0	0	0	0	0	58
11075563	m	md	GB	2015	0	0	0	0	0	0	0	0	0	0	0	0	
11077681	m	md	FR	2015	0	0	0	0	0	0	0	0	0	0	0	0	
11077918	m	md	FR	2015	0	0	0	0	0	0	0	0	0	0	0	0	68
11078519	m	cn	GB	2015	1	0	0	0	0	0	0	0	0	0	0	0	38
11078941	f	Not repo	US	2015	0	0	0	0	0	0	0	0	0	0	0	0	61
11078987	m	md	JP	2015	0	0	0	0	0	0	0	0	0	0	0	0	67
11079081	m	md	KR	2015	0	0	0	0	0	0	0	0	0	0	0	0	64
11079183	f	ot	US	2015	0	0	0	0	0	0	0	0	0	0	0	0	69
11079603	m	ot	US	2015	0	1	0	0	1	0	0	0	0	0	0	1	70
11079897	Not	ot	CN	2015	0	0	0	0	0	0	0	0	0	0	0	0	
11079898	Not	ot	CN	2015	0	0	0	0	0	0	0	0	0	0	0	0	
11080327	m	md	KR	2015	0	0	0	0	0	0	0	0	0	0	0	0	64
11080648	m	md	FR	2015	0	0	0	0	0	0	0	0	0	0	0	0	62
11080821	m	ot	KR	2015	0	0	0	0	0	0	0	0	0	0	0	0	58
11081698	m	cn	GB	2015	1	0	0	0	0	0	0	0	0	0	0	0	
11081989	m	md	Not re	2015	1	0	0	0	0	0	0	0	0	0	0	0	
11083698	m	md	JP	2015	1	0	0	0	0	0	0	0	0	0	0	0	66
11084001	f	md	Not re	2015	1	0	0	0	0	0	0	0	0	0	0	0	56
11086324	m	md	JP	2015	0	0	0	0	0	0	0	0	0	0	0	0	
11086757	Not	ot	CN	2015	0	0	0	0	0	0	0	0	0	0	0	0	
11086758	Not	ot	CN	2015	0	0	0	0	0	0	0	0	0	0	0	0	
11086759	Not	ot	CN	2015	0	0	0	0	0	0	0	0	0	0	0	0	
11087348	Not	cn	GB	2015	1	0	0	0	0	0	0	0	0	0	0	0	
11087388	f	md	Not re	2015	1	0	0	0	0	0	0	0	0	0	0	0	
11087563	f	ot	US	2015	1	0	0	0	0	0	0	0	0	0	0	0	72
11088063	m	cn	US	2015	0	0	0	0	0	0	0	0	0	0	0	0	32
11088151	f	md	TR	2015	0	0	0	0	0	0	0	0	0	0	0	0	34
11088339	m	md	KR	2015	0	1	1	0	0	0	0	0	0	0	0	0	72
11088477	m	ot	US	2015	0	0	0	0	0	0	0	0	0	0	0	0	62
11089994	Not	Not repo	Not re	2015	1	0	0	0	0	0	0	0	0	0	0	0	
11091381	m	cn	GB	2015	1	0	0	0	0	0	0	0	0	0	0	0	50
11092221	Not	md	US	2015	0	1	0	0	1	0	0	0	0	0	0	0	
11094690	m	md	US	2015	0	0	0	0	0	0	0	0	0	0	0	0	58

11094817	m	cn	US	2015	0	1	0	0	1	0	0	0	0	0	0	0	77
11095373	f	cn	US	2015	0	0	0	0	0	0	0	0	0	0	0	0	
11095615	f	ot	AU	2015	0	0	0	0	0	0	0	0	0	0	0	0	29
11095636	m	cn	DE	2015	0	0	0	0	0	0	0	0	0	0	0	0	62
11095787	m	md	US	2015	0	0	0	0	0	0	0	0	0	0	0	0	67
11095857	f	md	AU	2015	0	0	0	0	0	0	0	0	0	0	0	0	50
11097220	f	md	AU	2015	0	1	1	0	0	0	0	0	0	0	0	0	62
11098345	f	md	CH	2015	0	0	0	0	0	0	0	0	0	0	0	0	34
11098826	f	md	VN	2015	0	0	0	0	0	0	0	0	0	0	0	0	40
11098899	Not	ot	NO	2015	0	0	0	0	0	0	0	0	0	0	0	0	
11098901	Not	ot	NO	2015	0	0	0	0	0	0	0	0	0	0	0	0	
11098902	Not	ot	JP	2015	0	0	0	0	0	0	0	0	0	0	0	0	
11099561	Not	ot	US	2015	0	0	0	0	0	0	0	0	0	0	0	0	
11099616	Not	ot	JP	2015	0	0	0	0	0	0	0	0	0	0	0	0	
11099799	f	ot	BE	2015	0	0	0	0	0	0	0	0	0	0	0	0	74
11100141	m	cn	GB	2015	1	0	0	0	0	0	0	0	0	0	0	0	
11100162	m	ot	US	2015	0	1	0	0	1	0	0	0	0	0	0	0	
11100447	Not	ot	NO	2015	0	0	0	0	0	0	0	0	0	0	0	0	
11100736	m	cn	FR	2015	0	1	0	0	1	0	0	0	0	0	0	0	63
11101110	f	md	Not re	2015	1	0	0	0	0	0	0	0	0	0	0	0	
11102071	Not	ot	NO	2015	0	0	0	0	0	0	0	0	0	0	0	0	
11102072	Not	ot	NO	2015	0	0	0	0	0	0	0	0	0	0	0	0	
11102073	Not	ot	NO	2015	0	0	0	0	0	0	0	0	0	0	0	0	
11102074	Not	ot	NO	2015	0	0	0	0	0	0	0	0	0	0	0	0	
11102080	Not	ot	US	2015	0	0	0	0	0	0	0	0	0	0	0	0	
11102199	m	ot	US	2015	0	1	0	0	1	0	0	0	0	0	0	0	
11102271	m	ot	JP	2015	0	0	0	0	0	0	0	0	0	0	0	0	81
11103003	f	ot	CN	2015	0	0	0	0	0	0	0	0	0	0	0	0	46
11103332	m	md	US	2015	0	1	1	0	0	0	0	0	0	0	0	0	67
11104344	m	md	JP	2015	0	0	0	0	0	0	0	0	0	0	0	0	81
11105303	Not	ot	FR	2015	1	0	0	0	0	0	0	0	0	0	0	0	
11105628	Not	ot	JP	2015	1	0	0	0	0	0	0	0	0	0	0	1	
11105629	Not	ot	JP	2015	1	0	0	0	0	0	0	0	0	0	0	1	
11105642	Not	ot	JP	2015	1	0	0	0	0	0	0	0	0	0	0	1	

11105643	Not	ot	JP	2015	1	0	0	0	0	0	0	0	0	0	0	1	
11105644	Not	ot	JP	2015	1	0	0	0	0	0	0	0	0	0	0	1	
11105667	m	cn	US	2015	0	1	0	0	1	0	0	0	0	0	0	0	69
11105729	Not	ot	JP	2015	1	0	0	0	0	0	0	0	0	0	0	1	
11107497	m	cn	JP	2015	0	0	0	0	0	0	0	0	0	0	0	0	
11108462	Not	ot	JP	2015	1	0	0	0	0	0	0	0	0	0	0	1	
11108465	Not	ot	JP	2015	1	0	0	0	0	0	0	0	0	0	0	1	
11108471	Not	ot	JP	2015	1	0	0	0	0	0	0	0	0	0	0	0	
11108472	Not	ot	JP	2015	1	0	0	0	0	0	0	0	0	0	0	0	
11110053	Not	md	FR	2015	0	0	0	0	0	0	0	0	0	0	0	0	30
11110435	m	ot	US	2015	0	0	0	0	0	0	0	0	0	0	0	0	68
11110777	f	cn	US	2015	0	1	0	0	1	0	0	0	0	0	0	0	68
11111161	m	cn	US	2015	0	0	0	0	0	0	0	0	0	0	0	0	
11111549	Not	ot	UA	2015	0	0	0	0	0	0	0	0	0	0	0	0	
11111626	f	md	CZ	2015	0	0	0	0	0	0	0	0	0	0	0	0	41
11112071	Not	md	GR	2015	0	0	0	0	0	0	0	0	0	0	0	0	
11112073	Not	md	GR	2015	0	0	0	0	0	0	0	0	0	0	0	0	
11112964	m	ot	US	2015	0	0	0	0	0	0	0	0	0	0	0	0	
11112977	m	Not repo	US	2015	0	0	0	0	0	0	0	0	0	0	0	0	
11113007	m	ot	US	2015	0	0	0	0	0	0	0	0	0	0	0	0	
11113063	f	Not repo	US	2015	0	0	0	0	0	0	0	0	0	0	0	0	54
11113158	m	ot	US	2015	0	0	0	0	0	0	0	0	0	0	0	0	
11113193	f	md	DE	2015	0	0	0	0	0	0	0	0	0	0	0	0	59
11113287	m	Not repo	US	2015	0	0	0	0	0	0	0	0	0	0	0	0	
11113358	m	ot	US	2015	0	0	0	0	0	0	0	0	0	0	0	0	68
11113641	m	md	JP	2015	1	0	0	0	0	0	0	0	0	0	0	0	62
11114119	f	md	Not re	2015	1	0	0	0	0	0	0	0	0	0	0	0	
11115468	f	Not repo	US	2015	0	0	0	0	0	0	0	0	0	0	0	0	70
11115590	f	cn	US	2015	0	1	0	0	1	0	0	0	0	0	0	0	45
11115788	m	Not repo	US	2015	0	0	0	0	0	0	0	0	0	0	0	0	
11115861	m	ot	US	2015	0	0	0	0	0	0	0	0	0	0	0	0	68
11115870	f	Not repo	US	2015	0	0	0	0	0	0	0	0	0	0	0	0	
11116094	m	ot	US	2015	0	0	0	0	0	0	0	0	0	0	0	0	63
11116132	m	md	FI	2015	0	0	0	0	0	0	0	0	0	0	0	0	60

11116411	m	cn	US	2015	0	0	0	0	0	0	0	0	0	0	0	0	
11116644	m	ot	JP	2015	0	1	0	0	1	0	0	0	0	0	0	0	64
11117508	f	md	Not re	2015	1	0	0	0	0	0	0	0	0	0	0	0	64
11118631	m	Not repo	MX	2015	1	0	0	0	0	0	0	0	0	0	0	0	86
11118718	f	md	JP	2015	0	0	0	0	0	0	0	0	0	0	0	0	71
11118720	f	md	JP	2015	0	0	0	0	0	0	0	0	0	0	0	0	41
11118723	f	md	JP	2015	0	0	0	0	0	0	0	0	0	0	0	0	69
11118724	m	md	JP	2015	0	0	0	0	0	0	0	0	0	0	0	0	40
11119331	f	md	JP	2015	0	0	0	0	0	0	0	0	0	0	0	0	48
11119506	f	cn	GB	2015	1	0	0	0	0	0	0	0	0	0	0	0	
11119764	f	cn	US	2015	0	0	0	0	0	0	0	0	0	0	0	0	49
11119942	m	cn	JP	2015	0	1	0	0	1	0	0	0	0	0	0	0	73
11120140	m	cn	US	2015	1	0	0	0	0	0	0	0	0	0	0	0	73
11121691	f	md	JP	2015	0	0	0	0	0	0	0	0	0	0	0	0	63
11122261	m	cn	GB	2015	1	0	0	0	0	0	0	0	0	0	0	0	
11122965	m	cn	FR	2015	0	1	0	0	1	0	0	0	0	0	0	0	73
11123650	m	md	JP	2015	1	0	0	0	0	0	0	0	0	0	0	0	65
11124773	Not	ot	GR	2015	0	0	0	0	0	0	0	0	0	0	0	0	
11124850	m	cn	FR	2015	0	0	0	0	0	0	0	0	0	0	0	0	68
11126406	Not	cn	GB	2015	1	0	0	0	0	0	0	0	0	0	0	0	73
11126414	Not	cn	GB	2015	1	0	0	0	0	0	0	0	0	0	0	0	65
11126415	Not	cn	GB	2015	1	0	0	0	0	0	0	0	0	0	0	0	81
11126744	f	md	Not re	2015	1	0	0	0	0	0	0	0	0	0	0	0	74
11126798	m	ot	KR	2015	0	0	0	0	0	0	0	0	0	0	0	0	65
11126805	m	ot	US	2015	0	0	0	0	0	0	0	0	0	0	0	0	75
11127950	m	md	Not re	2015	1	0	0	0	0	0	0	0	0	0	0	0	64
11128042	Not	cn	GB	2015	1	0	0	0	0	0	0	0	0	0	0	0	68
11128052	m	cn	GB	2015	1	0	0	0	0	0	0	0	0	0	0	0	
11128678	Not	md	GR	2015	0	0	0	0	0	0	0	0	0	0	0	0	
11128822	m	md	FR	2015	0	0	0	0	0	0	0	0	0	0	0	0	78
11128937	m	cn	US	2015	0	0	0	0	0	0	0	0	0	0	0	0	65
11130052	f	md	FR	2015	0	0	0	0	0	0	0	0	0	0	0	0	70
11130182	m	ph	IN	2015	1	0	0	0	0	0	0	0	0	0	0	0	70
11130346	Not	ot	JP	2015	0	0	0	0	0	0	0	0	0	0	0	0	

11130477	f	md	US	2015	0	0	0	0	0	0	0	0	0	0	0	0	52
11131360	f	cn	US	2015	0	1	0	0	1	0	0	0	0	0	0	1	81
11131459	m	md	KR	2015	0	0	0	0	0	0	0	0	0	0	0	0	
11132319	f	ot	US	2015	0	0	0	0	0	0	0	0	0	0	0	0	67
11132761	m	ot	CN	2015	0	0	0	0	0	0	0	0	0	0	0	0	73
11133167	Not	ot	GR	2015	0	0	0	0	0	0	0	0	0	0	0	0	
11133235	f	md	FR	2015	1	0	0	0	0	0	0	0	0	0	0	0	
11133243	m	md	JP	2015	0	0	0	0	0	0	0	0	0	0	0	0	
11134314	f	md	DE	2015	0	0	0	0	0	0	0	0	0	0	0	0	73
11134865	m	md	Not re	2015	0	0	0	0	0	0	0	0	0	0	0	0	51
11135323	f	md	CN	2015	0	0	0	0	0	0	0	0	0	0	0	0	49
11135359	m	md	US	2015	0	0	0	0	0	0	0	0	0	0	0	0	
11135425	Not	Not repo	CN	2015	0	0	0	0	0	0	0	0	0	0	0	0	
11135525	Not	ot	CN	2015	0	0	0	0	0	0	0	0	0	0	0	0	
11135831	f	md	IT	2015	0	0	0	0	0	0	0	0	0	0	0	0	50
11135865	Not	md	US	2015	0	1	0	0	1	0	0	0	0	0	0	1	
11137228	m	cn	GB	2015	1	0	0	0	0	0	0	0	0	0	0	0	
11137489	f	cn	US	2015	0	1	0	0	1	0	0	0	0	0	0	0	55
11137531	f	ot	US	2015	0	0	0	0	0	0	0	0	0	0	0	0	56
11138565	m	cn	US	2015	0	1	0	0	1	0	0	0	0	0	0	0	79
11139514	Not	ot	CN	2015	0	0	0	0	0	0	0	0	0	0	0	0	
11140481	Not	cn	GB	2015	1	0	0	0	0	0	0	0	0	0	0	0	56
11141605	Not	ot	US	2015	0	0	0	0	0	0	0	0	0	0	0	0	
11142487	f	ot	AU	2015	0	1	0	0	1	0	0	0	0	0	0	0	71
11142769	m	cn	US	2015	0	1	0	0	1	0	0	0	0	0	0	0	
11142973	m	cn	US	2015	0	1	0	0	1	0	0	0	0	0	0	0	73
11143335	m	md	US	2015	0	1	0	0	1	0	0	0	0	0	0	0	53
11143564	f	md	JP	2015	0	0	0	0	0	0	0	0	0	0	0	0	83
11143565	f	md	JP	2015	0	0	0	0	0	0	0	0	0	0	0	0	83
11143617	f	ph	US	2015	1	0	0	0	0	0	0	0	0	0	0	0	76
11145118	f	cn	GB	2015	1	0	0	0	0	0	0	0	0	0	0	0	
11145174	f	md	CL	2015	0	1	1	0	0	0	0	0	0	0	0	0	58
11145246	f	ot	US	2015	0	0	0	0	0	0	0	0	0	0	0	0	67
11145276	f	cn	Not re	2015	0	0	0	0	0	0	0	0	0	0	0	0	36

11146532	Not	cn	GB	2015	1	0	0	0	0	0	0	0	0	0	0	0	60
11146674	m	md	FR	2015	0	1	1	0	0	0	0	0	0	0	0	0	62
11146819	m	cn	GB	2015	1	0	0	0	0	0	0	0	0	0	0	0	64
11146820	m	cn	GB	2015	1	0	0	0	0	0	0	0	0	0	0	0	
11147808	f	ot	ES	2015	0	1	0	0	1	0	0	0	0	0	0	0	50
11148420	m	ot	NL	2015	0	0	0	0	0	0	0	0	0	0	0	0	67
11148530	Not	ph	BN	2015	0	0	0	0	0	0	0	0	0	0	0	0	
11149019	m	cn	GB	2015	1	0	0	0	0	0	0	0	0	0	0	0	73
11149095	m	md	Not re	2015	1	0	0	0	0	0	0	0	0	0	0	0	76
11149449	f	cn	GB	2015	1	0	0	0	0	0	0	0	0	0	0	0	59
11149503	m	md	Not re	2015	1	0	0	0	0	0	0	0	0	0	0	0	72
11149929	Not	ot	CN	2015	0	0	0	0	0	0	0	0	0	0	0	0	
11150108	f	md	FR	2015	1	0	0	0	0	0	0	0	0	0	0	0	
11151022	f	cn	CN	2015	1	0	0	0	0	0	0	0	0	0	0	0	64
11151131	f	cn	US	2015	0	0	0	0	0	0	0	0	0	0	0	0	
11151349	f	md	JP	2015	1	0	0	0	0	0	0	0	0	0	0	0	74
11151949	f	md	JP	2015	0	0	0	0	0	0	0	0	0	0	0	0	57
11152287	f	cn	US	2015	0	0	0	0	0	0	0	0	0	0	0	0	66
11152304	m	md	JP	2015	0	1	1	0	0	0	0	0	0	0	0	0	74
11152812	Not	cn	BR	2015	1	0	0	0	0	0	0	0	0	0	0	0	75
11152818	f	cn	GB	2015	1	0	0	0	0	0	0	0	0	0	0	0	44
11152823	m	ot	US	2015	0	0	0	0	0	0	0	0	0	0	0	0	55
11152846	m	cn	US	2015	0	0	0	0	0	0	0	0	0	0	0	0	
11153001	Not	cn	GB	2015	1	0	0	0	0	0	0	0	0	0	0	0	69
11153163	f	md	US	2015	0	1	1	0	0	0	0	0	0	0	0	0	61
11154926	Not	ot	GR	2015	0	0	0	0	0	0	0	0	0	0	0	0	
11156080	m	md	JP	2015	0	1	1	0	0	0	0	0	0	0	0	0	82
11156523	f	cn	US	2015	0	1	0	0	1	0	0	0	0	0	0	0	61
11156621	m	cn	IT	2015	0	0	0	0	0	0	0	0	0	0	0	0	71
11156622	f	cn	GB	2015	0	0	0	0	0	0	0	0	0	0	0	0	63
11156646	f	md	JP	2015	1	0	0	0	0	0	0	0	0	0	0	0	84
11156959	Not	ot	US	2015	0	0	0	0	0	0	0	0	0	0	0	0	
11158043	m	cn	US	2015	1	0	0	0	0	0	0	0	0	0	0	0	89
11158103	f	md	US	2015	1	0	0	0	0	0	0	0	0	0	0	0	48

11158374	Not	cn	GB	2015	1	0	0	0	0	0	0	0	0	0	0	0	47
11158381	Not	cn	GB	2015	1	0	0	0	0	0	0	0	0	0	0	0	57
11160060	m	md	TW	2015	1	0	0	0	0	0	0	0	0	0	0	0	50
11163545	f	md	JP	2015	0	0	0	0	0	0	0	0	0	0	0	0	76
11164171	m	cn	GB	2015	1	0	0	0	0	0	0	0	0	0	0	0	77
11165400	f	cn	GB	2015	1	0	0	0	0	0	0	0	0	0	0	0	78
11165950	m	md	DE	2015	0	0	0	0	0	0	0	0	0	0	0	0	70
11166232	Not	md	JP	2015	1	0	0	0	0	0	0	0	0	0	0	0	
11166773	Not	Not repo	GR	2015	0	0	0	0	0	0	0	0	0	0	0	0	
11166930	Not	ot	GR	2015	0	0	0	0	0	0	0	0	0	0	0	0	
11166937	f	md	GB	2015	0	0	0	0	0	0	0	0	0	0	0	0	74
11166999	Not	Not repo	GR	2015	0	0	0	0	0	0	0	0	0	0	0	0	
11167958	f	md	PH	2015	1	0	0	0	0	0	0	0	0	0	0	0	
11169023	m	cn	US	2015	0	0	0	0	0	0	0	0	0	0	0	0	79
11169467	f	md	Not re	2015	1	0	0	0	0	0	0	0	0	0	0	0	73
11169495	m	cn	US	2015	0	0	0	0	0	0	0	0	0	0	0	0	61
11171113	Not	ot	GB	2015	0	0	0	0	0	0	0	0	0	0	0	0	
11171543	Not	cn	GB	2015	1	0	0	0	0	0	0	0	0	0	0	0	
11171836	f	md	BE	2015	0	0	0	0	0	0	0	0	0	0	0	0	69
11171842	Not	md	US	2015	0	0	0	0	0	0	0	0	0	0	0	0	
11173550	Not	md	US	2015	0	0	0	0	0	0	0	0	0	0	0	0	
11173821	f	md	US	2015	1	0	0	0	0	0	0	0	0	0	0	0	
11174536	m	md	US	2015	0	0	0	0	0	0	0	0	0	0	0	0	
11174553	f	cn	CA	2015	0	0	0	0	0	0	0	0	0	0	0	0	53
11174597	f	ot	GB	2015	0	0	0	0	0	0	0	0	0	0	0	0	74
11174623	f	Not repo	GB	2015	0	0	0	0	0	0	0	0	0	0	0	0	62
11175073	f	cn	GB	2015	0	0	0	0	0	0	0	0	0	0	0	0	62
11176229	m	cn	FR	2015	0	1	0	0	1	0	0	0	0	0	0	0	
11177068	m	ot	FR	2015	0	0	0	0	0	0	0	0	0	0	0	0	62
11177154	f	cn	GB	2015	1	0	0	0	0	0	0	0	0	0	0	0	85
11177525	f	md	JP	2015	0	0	0	0	0	0	0	0	0	0	0	0	78
11178147	f	md	Not re	2015	1	0	0	0	0	0	0	0	0	0	0	0	75
11178446	f	cn	GB	2015	1	0	0	0	0	0	0	0	0	0	0	0	
11179088	m	md	DE	2015	0	0	0	0	0	0	0	0	0	0	0	0	57

11179283	Not	ot	US	2015	0	1	0	0	1	0	0	0	0	0	0	0	
11179381	m	md	ZA	2015	0	0	0	0	0	0	0	0	0	0	0	0	64
11179424	f	md	US	2015	0	0	0	0	0	0	0	0	0	0	0	0	58
11179676	Not	md	US	2015	0	0	0	0	0	0	0	0	0	0	0	0	
11180637	f	cn	US	2015	0	0	0	0	0	0	0	0	0	0	0	0	70
11181275	f	md	GB	2015	0	0	0	0	0	0	0	0	0	0	0	0	74
11181400	f	cn	US	2015	0	0	0	0	0	0	0	0	0	0	0	0	
11181970	Not	cn	GB	2015	1	0	0	0	0	0	0	0	0	0	0	0	85
11182485	Not	cn	GB	2015	1	0	0	0	0	0	0	0	0	0	0	0	56
11183190	m	ot	US	2015	0	1	0	0	1	0	0	0	0	0	0	0	61
11183519	Not	ot	CN	2015	0	0	0	0	0	0	0	0	0	0	0	0	
11183521	Not	ot	CN	2015	0	0	0	0	0	0	0	0	0	0	0	0	
11183531	Not	ot	CN	2015	0	0	0	0	0	0	0	0	0	0	0	0	
11183534	Not	ot	CN	2015	0	0	0	0	0	0	0	0	0	0	0	0	
11183797	f	md	FR	2015	0	0	0	0	0	0	0	0	0	0	0	0	70
11183962	Not	ot	CN	2015	0	0	0	0	0	0	0	0	0	0	0	0	
11183991	Not	ot	CN	2015	0	0	0	0	0	0	0	0	0	0	0	0	
11183992	Not	ot	CN	2015	0	0	0	0	0	0	0	0	0	0	0	0	
11184090	f	cn	GB	2015	1	0	0	0	0	0	0	0	0	0	0	0	74
11184357	m	md	US	2015	0	1	0	0	1	0	0	0	0	0	0	0	
11184838	Not	cn	GB	2015	1	0	0	0	0	0	0	0	0	0	0	0	79
11184915	Not	cn	GB	2015	1	0	0	0	0	0	0	0	0	0	0	0	67
11184925	Not	cn	GB	2015	1	0	0	0	0	0	0	0	0	0	0	0	84
11185914	f	cn	GB	2015	1	0	0	0	0	0	0	0	0	0	0	0	
11185916	Not	cn	GB	2015	1	0	0	0	0	0	0	0	0	0	0	0	76
11185967	Not	cn	GB	2015	1	0	0	0	0	0	0	0	0	0	0	0	74
11185968	Not	cn	GB	2015	1	0	0	0	0	0	0	0	0	0	0	0	84
11185970	Not	cn	GB	2015	1	0	0	0	0	0	0	0	0	0	0	0	79
11186732	m	cn	US	2015	0	1	0	0	1	0	0	0	0	0	0	0	82
11189092	f	md	Not re	2015	0	0	0	0	0	0	0	0	0	0	0	0	71
11189369	f	md	DE	2015	0	0	0	0	0	0	0	0	0	0	0	0	
11189920	Not	cn	GB	2015	1	0	0	0	0	0	0	0	0	0	0	0	51
11189921	Not	cn	GB	2015	1	0	0	0	0	0	0	0	0	0	0	0	71
11189923	Not	cn	GB	2015	1	0	0	0	0	0	0	0	0	0	0	0	67

11190196	f	md	HR	2015	0	0	0	0	0	0	0	0	0	0	0	0	56
11190421	Not	md	US	2015	0	1	0	0	1	0	0	0	0	0	0	0	
11190649	f	cn	CA	2015	0	0	0	0	0	0	0	0	0	0	0	0	
11191120	f	md	Not re	2015	1	0	0	0	0	0	0	0	0	0	0	0	74
11192943	m	cn	US	2015	0	0	0	0	0	0	0	0	0	0	0	0	84
11192944	m	cn	US	2015	0	0	0	0	0	0	0	0	0	0	0	0	
11193564	f	cn	FR	2015	0	0	0	0	0	0	0	0	0	0	0	0	64
11195184	f	cn	AT	2015	0	0	0	0	0	0	0	0	0	0	0	0	
11195432	m	cn	GB	2015	1	0	0	0	0	0	0	0	0	0	0	0	
11195982	m	ot	US	2015	0	0	0	0	0	0	0	0	0	0	0	0	
11196256	Not	md	US	2015	0	1	0	0	1	0	0	0	0	0	0	0	
11197411	f	cn	US	2015	0	1	0	0	1	0	0	0	0	0	0	0	
11199794	m	md	FR	2015	0	0	0	0	0	0	0	0	0	0	0	0	59
11200017	f	cn	MX	2015	0	1	0	0	1	0	0	0	0	0	0	0	54
11200621	f	md	Not re	2015	1	0	0	0	0	0	0	0	0	0	0	0	55
11201159	f	cn	US	2015	0	0	0	0	0	0	0	0	0	0	0	0	62
11201191	f	md	FR	2015	0	0	0	0	0	0	0	0	0	0	0	0	66
11201204	m	md	ES	2015	0	0	0	0	0	0	0	0	0	0	0	0	81
11201301	m	md	US	2015	0	1	0	0	1	0	0	0	0	0	0	0	77
11201448	Not	Not repo	JP	2015	0	0	0	0	0	0	0	0	0	0	0	0	
11201488	Not	Not repo	JP	2015	0	0	0	0	0	0	0	0	0	0	0	0	
11202078	m	cn	DE	2015	0	0	0	0	0	0	0	0	0	0	0	0	57
11202308	m	cn	GB	2015	1	0	0	0	0	0	0	0	0	0	0	0	88
11202321	m	cn	GB	2015	1	0	0	0	0	0	0	0	0	0	0	0	
11202851	f	md	US	2015	0	0	0	0	0	0	0	0	0	0	0	0	66
11203546	f	md	CN	2015	0	0	0	0	0	0	0	0	0	0	0	0	32
11208217	Not	ot	US	2015	0	0	0	0	0	0	0	0	0	0	0	0	
11208230	Not	ot	US	2015	0	0	0	0	0	0	0	0	0	0	0	0	
11208231	Not	ot	NL	2015	0	0	0	0	0	0	0	0	0	0	0	0	
11208232	Not	ot	US	2015	0	0	0	0	0	0	0	0	0	0	0	0	
11208233	Not	ot	US	2015	0	0	0	0	0	0	0	0	0	0	0	0	
11208234	Not	ot	US	2015	0	0	0	0	0	0	0	0	0	0	0	0	
11208235	Not	ot	NL	2015	0	0	0	0	0	0	0	0	0	0	0	0	
11209235	f	cn	US	2015	0	0	0	0	0	0	0	0	0	0	0	0	53

11211149	Not	ph	FR	2015	0	1	0	0	1	0	0	0	0	0	0	0	
11211883	Not	ot	FR	2015	0	0	0	0	0	0	0	0	0	0	0	0	
11212179	f	cn	CN	2015	1	0	0	0	0	0	0	0	0	0	0	0	
11212247	Not	ot	NL	2015	0	0	0	0	0	0	0	0	0	0	0	0	
11212272	Not	ot	NL	2015	0	0	0	0	0	0	0	0	0	0	0	0	
11212407	Not	ot	NL	2015	0	0	0	0	0	0	0	0	0	0	0	0	
11213021	m	md	MX	2015	1	0	0	0	0	0	0	0	0	0	0	0	80
11213356	m	ot	BE	2015	0	0	0	0	0	0	0	0	0	0	0	0	68
11213414	Not	ph	FR	2015	0	1	0	0	1	0	0	0	0	0	0	0	
11213599	Not	cn	GB	2015	1	0	0	0	0	0	0	0	0	0	0	0	78
11214163	Not	ot	US	2015	0	0	0	0	0	0	0	0	0	0	0	0	
11214324	f	ot	US	2015	0	1	0	0	1	0	0	0	0	0	0	0	69
11214633	f	md	IL	2015	0	0	0	0	0	0	0	0	0	0	0	0	
11214742	m	ot	CH	2015	0	1	0	0	1	0	0	0	0	0	0	0	59
11215049	Not	Not repo	ES	2015	0	0	0	0	0	0	0	0	0	0	0	0	
11216127	Not	cn	GB	2015	1	0	0	0	0	0	0	0	0	0	0	0	
11216772	m	ot	US	2015	0	1	0	0	1	0	0	0	0	0	0	0	60
11216850	f	cn	GB	2015	1	0	0	0	0	0	0	0	0	0	0	0	
11217104	f	ot	US	2015	0	1	0	0	1	0	0	0	0	0	0	0	
11217412	f	cn	DE	2015	0	0	0	0	0	0	0	0	0	0	0	0	62
11217577	m	ot	AU	2015	0	1	0	0	1	0	0	0	0	0	0	0	56
11218483	Not	md	US	2015	0	0	0	0	0	0	0	0	0	0	0	0	
11218544	f	md	US	2015	0	0	0	0	0	0	0	0	0	0	0	0	
11218812	m	md	FR	2015	0	0	0	0	0	0	0	0	0	0	0	0	59
11219169	Not	cn	US	2015	0	0	0	0	0	0	0	0	0	0	0	0	
11219215	Not	cn	GB	2015	1	0	0	0	0	0	0	0	0	0	0	0	78
11219219	Not	cn	GB	2015	1	0	0	0	0	0	0	0	0	0	0	0	72
11219221	Not	cn	GB	2015	1	0	0	0	0	0	0	0	0	0	0	0	67
11219223	Not	cn	GB	2015	1	0	0	0	0	0	0	0	0	0	0	0	81
11219227	Not	cn	GB	2015	1	0	0	0	0	0	0	0	0	0	0	0	75
11219230	Not	cn	GB	2015	1	0	0	0	0	0	0	0	0	0	0	0	83
11219231	Not	cn	GB	2015	1	0	0	0	0	0	0	0	0	0	0	0	79
11219232	Not	cn	GB	2015	1	0	0	0	0	0	0	0	0	0	0	0	84
11221545	Not	cn	GB	2015	1	0	0	0	0	0	0	0	0	0	0	0	72

11221549	Not	cn	GB	2015	1	0	0	0	0	0	0	0	0	0	0	0	78
11221795	m	md	JP	2015	0	0	0	0	0	0	0	0	0	0	0	0	68
11222353	f	cn	GB	2015	1	0	0	0	0	0	0	0	0	0	0	0	
11222361	Not	cn	GB	2015	1	0	0	0	0	0	0	0	0	0	0	0	62
11222669	f	cn	GB	2015	1	0	0	0	0	0	0	0	0	0	0	0	77
11222673	Not	cn	GB	2015	1	0	0	0	0	0	0	0	0	0	0	0	67
11222760	m	md	IT	2015	0	0	0	0	0	0	0	0	0	0	0	0	58
11223014	m	md	Not re	2015	1	0	0	0	0	0	0	0	0	0	0	0	73
11223133	m	ph	JP	2015	1	0	0	0	0	0	0	0	0	0	0	0	72
11223315	f	ot	NO	2015	0	1	0	0	1	0	0	0	0	0	0	0	
11223527	m	ot	US	2015	0	0	0	0	0	0	0	0	0	0	0	0	60
11224023	Not	cn	CA	2015	0	0	0	0	0	0	0	0	0	0	0	0	
11224128	m	ot	US	2015	0	0	0	0	0	0	0	0	0	0	0	0	
11224787	f	cn	GB	2015	1	0	0	0	0	0	0	0	0	0	0	0	36
11224793	m	cn	GB	2015	1	0	0	0	0	0	0	0	0	0	0	0	67
11224799	m	cn	GB	2015	1	0	0	0	0	0	0	0	0	0	0	0	81
11224805	f	cn	GB	2015	1	0	0	0	0	0	0	0	0	0	0	0	66
11224808	f	cn	GB	2015	1	0	0	0	0	0	0	0	0	0	0	0	68
11224809	f	cn	GB	2015	1	0	0	0	0	0	0	0	0	0	0	0	60
11224817	m	cn	GB	2015	1	0	0	0	0	0	0	0	0	0	0	0	68
11224818	f	cn	GB	2015	1	0	0	0	0	0	0	0	0	0	0	0	70
11225288	Not	cn	GB	2015	1	0	0	0	0	0	0	0	0	0	0	0	86
11225297	m	cn	GB	2015	1	0	0	0	0	0	0	0	0	0	0	0	
11225313	f	ot	US	2015	0	0	0	0	0	0	0	0	0	0	0	0	66
11225322	f	ot	US	2015	0	0	0	0	0	0	0	0	0	0	0	0	66
11225451	Not	ph	FR	2015	0	1	0	0	1	0	0	0	0	0	0	0	
11226342	m	md	IT	2015	1	0	0	0	0	0	0	0	0	0	0	0	
11227393	Not	cn	GB	2015	1	0	0	0	0	0	0	0	0	0	0	0	71
11227400	Not	cn	GB	2015	1	0	0	0	0	0	0	0	0	0	0	0	79
11227402	Not	cn	GB	2015	1	0	0	0	0	0	0	0	0	0	0	0	62
11227404	Not	cn	GB	2015	1	0	0	0	0	0	0	0	0	0	0	0	85
11227405	Not	cn	GB	2015	1	0	0	0	0	0	0	0	0	0	0	0	70
11227419	f	md	DE	2015	0	0	0	0	0	0	0	0	0	0	0	0	46
11227654	f	cn	US	2015	0	0	0	0	0	0	0	0	0	0	0	0	

11228065	Not	ot	JP	2015	0	0	0	0	0	0	0	0	0	0	0	0	
11228076	Not	cn	GB	2015	1	0	0	0	0	0	0	0	0	0	0	0	68
11228080	Not	cn	GB	2015	1	0	0	0	0	0	0	0	0	0	0	0	74
11228081	Not	cn	GB	2015	1	0	0	0	0	0	0	0	0	0	0	0	76
11228085	Not	cn	GB	2015	1	0	0	0	0	0	0	0	0	0	0	0	71
11228086	Not	cn	GB	2015	1	0	0	0	0	0	0	0	0	0	0	0	65
11228087	Not	cn	GB	2015	1	0	0	0	0	0	0	0	0	0	0	0	69
11228088	Not	cn	GB	2015	1	0	0	0	0	0	0	0	0	0	0	0	86
11228089	Not	cn	GB	2015	1	0	0	0	0	0	0	0	0	0	0	0	82
11228090	Not	cn	GB	2015	1	0	0	0	0	0	0	0	0	0	0	0	79
11228094	Not	cn	GB	2015	1	0	0	0	0	0	0	0	0	0	0	0	61
11228095	f	ot	US	2015	1	0	0	0	0	0	0	0	0	0	0	0	58
11228626	Not	cn	GB	2015	1	0	0	0	0	0	0	0	0	0	0	0	75
11228629	Not	cn	GB	2015	1	0	0	0	0	0	0	0	0	0	0	0	73
11229018	Not	md	JP	2015	0	0	0	0	0	0	0	0	0	0	0	0	
11229758	Not	cn	GB	2015	1	0	0	0	0	0	0	0	0	0	0	0	83
11229759	Not	cn	GB	2015	1	0	0	0	0	0	0	0	0	0	0	0	
11229766	Not	cn	GB	2015	1	0	0	0	0	0	0	0	0	0	0	0	68
11229767	Not	cn	GB	2015	1	0	0	0	0	0	0	0	0	0	0	0	85
11229768	Not	cn	GB	2015	1	0	0	0	0	0	0	0	0	0	0	0	59
11229769	Not	cn	GB	2015	1	0	0	0	0	0	0	0	0	0	0	0	81
11229799	Not	cn	GB	2015	1	0	0	0	0	0	0	0	0	0	0	0	89
11229801	Not	cn	GB	2015	1	0	0	0	0	0	0	0	0	0	0	0	69
11229802	Not	cn	GB	2015	1	0	0	0	0	0	0	0	0	0	0	0	87
11229803	Not	cn	GB	2015	1	0	0	0	0	0	0	0	0	0	0	0	92
11229804	Not	cn	GB	2015	1	0	0	0	0	0	0	0	0	0	0	0	76
11229806	Not	cn	GB	2015	1	0	0	0	0	0	0	0	0	0	0	0	87
11229865	f	cn	US	2015	1	0	0	0	0	0	0	0	0	0	0	0	83
11266066	m	ot	US	2015	0	0	0	0	0	0	0	0	0	0	0	0	56
11266124	m	cn	US	2015	0	0	0	0	0	0	0	0	0	0	0	0	
11275631	f	md	US	2015	0	0	0	0	0	0	0	0	0	0	0	0	
11275678	m	md	US	2015	0	0	0	0	0	0	0	0	0	0	0	0	59
11275686	f	md	US	2015	0	0	0	0	0	0	0	0	0	0	0	0	
11275695	m	md	US	2015	0	0	0	0	0	0	0	0	0	0	0	0	54

11275743	m	Not repo	US	2015	0	0	0	0	0	0	0	0	0	0	0	0	
8000337	Not	md	US	2015	0	0	0	0	0	0	0	0	0	0	0	0	62
8426256	m	cn	DE	2015	0	0	0	0	0	0	0	0	0	0	0	0	57
8466235	m	cn	GB	2015	1	0	0	0	0	0	0	0	0	0	0	0	69
8513789	m	cn	GB	2015	1	0	0	0	0	0	0	0	0	0	0	0	84
8515541	m	cn	GB	2015	1	0	0	0	0	0	0	0	0	0	0	0	
8748245	m	md	US	2015	1	0	0	0	0	0	0	0	0	0	0	0	
8762175	f	md	JP	2015	0	0	0	0	0	0	0	0	0	0	0	0	64
8762800	f	md	JP	2015	0	0	0	0	0	0	0	0	0	0	0	0	82
8792156	f	md	US	2015	1	0	0	0	0	0	0	0	0	0	0	0	
8800069	Not	md	US	2015	0	0	0	0	0	0	0	0	0	0	0	0	
8800681	f	md	US	2015	0	0	0	0	0	0	0	0	0	0	0	0	
8809629	m	md	US	2015	0	0	0	0	0	0	0	0	0	0	0	0	
8812067	m	cn	US	2015	0	0	0	0	0	0	0	0	0	0	0	0	74
8821820	m	cn	US	2015	0	0	0	0	0	0	0	0	0	0	0	0	67
8937091	f	cn	GB	2015	1	0	0	0	0	0	0	0	0	0	0	0	
8956227	m	cn	GB	2015	1	0	0	0	0	0	0	0	0	0	0	0	73
8998695	f	ph	JP	2015	0	0	0	0	0	0	0	0	0	0	0	0	61
9002229	m	cn	US	2015	1	0	0	0	0	0	0	0	0	0	0	0	
9061266	f	cn	GB	2015	1	0	0	0	0	0	0	0	0	0	0	0	74
9088710	f	ot	US	2015	0	1	0	0	1	0	0	0	0	0	0	0	70
9132677	m	cn	US	2015	0	0	0	0	0	0	0	0	0	0	0	0	64
9182483	m	md	JP	2015	0	0	0	0	0	0	0	0	0	0	0	0	92
9197892	m	cn	US	2015	0	0	0	0	0	0	0	0	0	0	0	0	59
9201411	f	md	US	2015	1	0	0	0	0	0	0	0	0	0	0	0	
9205705	f	md	JP	2015	0	0	0	0	0	0	0	0	0	0	0	0	62
9318903	f	ph	JP	2015	0	0	0	0	0	0	0	0	0	0	0	0	89
9373055	m	md	JP	2015	0	0	0	0	0	0	0	0	0	0	0	0	46
9396801	m	cn	CL	2015	0	0	0	0	0	0	0	0	0	0	0	0	67
9413574	m	md	US	2015	0	0	0	0	0	0	0	0	0	0	0	0	46
9454023	f	md	JP	2015	0	0	0	0	0	0	0	0	0	0	0	0	70
9604510	f	cn	GB	2015	1	0	0	0	0	0	0	0	0	0	0	0	62
9615916	m	md	JP	2015	0	0	0	0	0	0	0	0	0	0	0	0	62
9643683	f	cn	US	2015	0	1	0	0	1	0	0	0	0	0	0	0	62

9648888	m	ph	JP	2015	0	0	0	0	0	0	0	0	0	0	0	0	76
9680307	f	cn	US	2015	0	0	0	0	0	0	0	0	0	0	0	0	55
9687493	m	cn	US	2015	0	0	0	0	0	0	0	0	0	0	0	0	55
9715660	m	ot	DE	2015	0	0	0	0	0	0	0	0	0	0	0	0	71
9788835	m	cn	US	2015	1	0	0	0	0	0	0	0	0	0	0	0	54
9818908	m	md	JP	2015	0	0	0	0	0	0	0	0	0	0	0	0	76
9867954	m	md	JP	2015	0	0	0	0	0	0	0	0	0	0	0	0	68
9886148	m	md	JP	2015	0	0	0	0	0	0	0	0	0	0	0	0	63
9901194	f	cn	GB	2015	1	0	0	0	0	0	0	0	0	0	0	0	63
9913417	m	cn	GB	2015	1	0	0	0	0	0	0	0	0	0	0	0	60
9935142	f	md	JP	2015	0	0	0	0	0	0	0	0	0	0	0	0	47
9937521	f	md	JP	2015	0	0	0	0	0	0	0	0	0	0	0	0	57
9973360	f	md	JP	2015	0	0	0	0	0	0	0	0	0	0	0	0	81
10006200	f	cn	GB	2015	1	0	0	0	0	0	0	0	0	0	0	0	
10008097	f	md	JP	2015	0	0	0	0	0	0	0	0	0	0	0	0	69
10017830	m	cn	GB	2015	1	0	0	0	0	0	0	0	0	0	0	0	
10021884	m	md	JP	2015	0	0	0	0	0	0	0	0	0	0	0	0	58
10047448	m	cn	GB	2015	1	0	0	0	0	0	0	0	0	0	0	1	76
10050200	f	md	JP	2015	0	0	0	0	0	0	0	0	0	0	0	0	68
10058548	m	cn	GB	2015	1	0	0	0	0	0	0	0	0	0	0	0	49
10082143	f	md	Not re	2015	1	0	0	0	0	0	0	0	0	0	0	0	63
10128408	Not	md	IT	2015	0	0	0	0	0	0	0	0	0	0	0	0	
10141822	Not	Not repo	Not re	2015	1	0	0	0	0	0	0	0	0	0	0	0	
10142163	Not	cn	GB	2015	1	0	0	0	0	0	0	0	0	0	0	0	
10142633	f	md	JP	2015	0	0	0	0	0	0	0	0	0	0	0	0	70
10149854	f	cn	GB	2015	0	0	0	0	0	0	0	0	0	0	0	0	67
10187248	f	md	HK	2015	0	0	0	0	0	0	0	0	0	0	0	0	70
10216558	f	md	Not re	2015	1	0	0	0	0	0	0	0	0	0	0	0	74
10224042	f	md	JP	2015	0	0	0	0	0	0	0	0	0	0	0	0	60
10224120	f	md	NL	2015	0	0	0	0	0	0	0	0	0	0	0	0	37
10252594	f	md	Not re	2015	1	0	0	0	0	0	0	0	0	0	0	0	79
10268332	m	md	Not re	2015	1	0	0	0	0	0	0	0	0	0	0	0	72
10272325	m	ot	IT	2015	0	0	0	0	0	0	0	0	0	0	0	0	63
10272724	f	md	Not re	2015	1	0	0	0	0	0	0	0	0	0	0	0	45

10304832	f	md	NL	2015	0	0	0	0	0	0	0	0	0	0	0	0	54
10332197	f	ot	US	2015	0	1	0	0	1	0	0	0	0	0	0	0	81
10333515	m	md	CA	2015	0	1	1	0	0	0	0	0	0	0	0	0	67
10336928	m	md	JP	2015	0	1	1	0	0	0	0	0	0	0	0	1	61
10343910	f	md	DE	2015	0	0	0	0	0	0	0	0	0	0	0	0	51
10358583	f	md	CN	2015	0	0	0	0	0	0	0	0	0	0	0	0	51
10365810	m	md	JP	2015	0	0	0	0	0	0	0	0	0	0	0	0	64
10371891	f	cn	GB	2015	1	0	0	0	0	0	0	0	0	0	0	0	48
10375412	m	cn	US	2015	1	0	0	0	0	0	0	0	0	0	0	0	
10391320	f	cn	GB	2015	1	0	0	0	0	0	0	0	0	0	0	0	
10396118	f	cn	US	2015	1	0	0	0	0	0	0	0	0	0	0	0	79
10396119	f	cn	GB	2015	0	0	0	0	0	0	0	0	0	0	0	0	62
10400116	m	md	JP	2015	0	0	0	0	0	0	0	0	0	0	0	0	54
10401382	m	ot	US	2015	0	1	0	0	1	0	0	0	0	0	0	1	67
10403630	m	cn	US	2015	0	1	0	0	1	0	0	0	0	0	0	1	76
10405952	m	cn	GB	2015	1	0	0	0	0	0	0	0	0	0	0	0	
10409723	f	cn	GB	2015	1	0	0	0	0	0	0	0	0	0	0	0	
10427791	f	md	JP	2015	0	0	0	0	0	0	0	0	0	0	0	0	83
10429786	f	md	US	2015	1	0	0	0	0	0	0	0	0	0	0	0	84
10439799	f	cn	GB	2015	1	0	0	0	0	0	0	0	0	0	0	0	
10444157	m	cn	GB	2015	1	0	0	0	0	0	0	0	0	0	0	0	67
10450003	f	cn	GB	2015	1	0	0	0	0	0	0	0	0	0	0	0	
10450301	m	md	CA	2015	0	1	1	0	0	0	0	0	0	0	0	0	73
10457212	f	ot	US	2015	0	0	0	0	0	0	0	0	0	0	0	0	73
10457593	m	md	BE	2015	0	1	1	0	0	0	0	0	0	0	0	0	71
10457693	f	ot	TW	2015	0	1	1	0	0	0	0	0	0	0	0	0	40
10463882	m	md	JP	2015	0	1	1	0	0	0	0	0	0	0	0	0	70
10464424	f	ot	US	2015	0	1	1	0	0	0	0	0	0	0	0	0	74
10464680	m	cn	GB	2015	0	0	0	0	0	0	0	0	0	0	0	0	
10464696	f	ot	US	2015	1	0	0	0	0	0	0	0	0	0	0	0	
10482233	m	md	JP	2015	0	1	1	0	0	0	0	0	0	0	0	1	75
10483892	m	cn	GB	2015	1	0	0	0	0	0	0	0	0	0	0	0	82
10495382	m	md	JP	2015	0	1	1	0	0	0	0	0	0	0	0	0	63
10496961	m	md	JP	2015	0	0	0	0	0	0	0	0	0	0	0	0	71

10502823	f	cn	GB	2015	1	0	0	0	0	0	0	0	0	0	0	0	
10530797	m	cn	GB	2015	1	0	0	0	0	0	0	0	0	0	0	0	61
10533535	f	cn	GB	2015	1	0	0	0	0	0	0	0	0	0	0	0	
10534255	m	md	JP	2015	0	0	0	0	0	0	0	0	0	0	0	0	61
10538907	m	md	US	2015	0	1	1	0	0	0	0	0	0	0	0	0	54
10541090	m	md	JP	2015	0	1	1	0	0	0	0	0	0	0	0	0	66
10550241	m	md	Not re	2015	1	0	0	0	0	0	0	0	0	0	0	0	72
10566531	m	cn	US	2015	0	0	0	0	0	0	0	0	0	0	0	0	70
10577670	m	md	Not re	2015	1	0	0	0	0	0	0	0	0	0	0	0	59
10579944	m	md	US	2015	0	1	1	0	0	0	0	0	0	0	0	0	65
10583810	m	md	JP	2015	0	0	0	0	0	0	0	0	0	0	0	0	64
10588076	m	md	US	2015	0	1	1	0	0	0	0	0	0	0	0	1	69
10588859	m	ot	DE	2015	0	0	0	0	0	0	0	0	0	0	0	0	54
10595937	m	md	RU	2015	0	0	0	0	0	0	0	0	0	0	0	0	53
10609890	f	md	KR	2015	0	0	0	0	0	0	0	0	0	0	0	0	58
10618534	m	md	ES	2015	0	1	1	0	0	0	0	0	0	0	0	0	49
10637551	f	md	US	2015	0	0	0	0	0	0	0	0	0	0	0	0	64
10645822	f	md	Not re	2015	1	0	0	0	0	0	0	0	0	0	0	0	75
10664152	m	md	FR	2015	0	0	0	0	0	0	0	0	0	0	0	0	62
10667707	f	md	FR	2015	0	0	0	0	0	0	0	0	0	0	0	0	62
10684951	m	md	JP	2015	0	0	0	0	0	0	0	0	0	0	0	0	62
10698907	f	md	Not re	2015	1	0	0	0	0	0	0	0	0	0	0	0	
10701541	m	md	US	2015	0	1	1	0	0	0	0	0	0	0	0	0	70
10718305	f	ot	NL	2015	0	1	0	0	1	0	0	0	0	0	0	0	
10726778	f	md	US	2015	0	0	0	0	0	0	0	0	0	0	0	0	69
10727523	m	md	US	2015	0	1	1	0	0	0	0	0	0	0	0	0	74
10727880	m	md	ES	2015	0	1	1	0	0	0	0	0	0	0	0	1	60
10732634	m	md	Not re	2015	1	0	0	0	0	0	0	0	0	0	0	0	
10755280	m	cn	AR	2015	0	0	0	0	0	0	0	0	0	0	0	0	63
10771519	f	md	NL	2015	0	1	1	0	0	0	0	0	0	0	0	0	66
10789644	m	md	US	2015	0	1	1	0	0	0	0	0	0	0	0	0	21
10832019	f	md	IL	2015	0	1	1	0	0	0	0	0	0	0	0	0	61
10857179	m	md	JP	2015	0	0	0	0	0	0	0	0	0	0	0	0	79
10869250	m	cn	AU	2015	0	0	0	0	0	0	0	0	0	0	0	0	59

10873715	f	md	Not re	2015	1	0	0	0	0	0	0	0	0	0	0	0	
10878723	m	md	JP	2015	0	1	1	0	0	0	0	0	0	0	0	0	57
10892533	f	ot	IE	2015	0	0	0	0	0	0	0	0	0	0	0	0	44
10902409	m	ot	US	2015	0	0	0	0	0	0	0	0	0	0	0	0	59
10902412	m	ot	US	2015	0	0	0	0	0	0	0	0	0	0	0	0	53
10913983	f	md	Not re	2015	1	0	0	0	0	0	0	0	0	0	0	0	79
10920435	f	md	DE	2015	0	0	0	0	0	0	0	0	0	0	0	0	58
10923161	m	ot	DE	2015	0	0	0	0	0	0	0	0	0	0	0	0	68
10930392	m	md	JP	2015	0	1	1	0	0	0	0	0	0	0	0	0	42
10955430	f	md	JP	2015	0	0	0	0	0	0	0	0	0	0	0	0	86
10957982	m	md	JP	2015	0	1	1	0	0	0	0	0	0	0	0	0	61
10970799	m	md	JP	2015	0	1	1	0	0	0	0	0	0	0	0	0	69
10971432	m	ot	US	2015	0	0	0	0	0	0	0	0	0	0	0	0	
10974786	f	ot	CZ	2015	0	0	0	0	0	0	0	0	0	0	0	0	
10983186	m	md	US	2015	0	0	0	0	0	0	0	0	0	0	0	0	73
10992357	m	ot	FR	2015	0	0	0	0	0	0	0	0	0	0	0	0	30
10993997	m	cn	DE	2015	0	0	0	0	0	0	0	0	0	0	0	0	67
10995869	m	md	US	2015	0	1	1	0	0	0	0	0	0	0	0	0	76
10995963	f	md	Not re	2015	1	0	0	0	0	0	0	0	0	0	0	0	62
11001772	m	md	JP	2015	0	0	0	0	0	0	0	0	0	0	0	0	55
11020547	f	cn	US	2015	0	1	0	0	1	0	0	0	0	0	0	1	71
11022225	m	md	JP	2015	0	1	1	0	0	0	0	0	0	0	0	0	70
11022233	f	md	Not re	2015	1	0	0	0	0	0	0	0	0	0	0	0	45
11050152	f	md	JP	2015	0	1	1	0	0	0	0	0	0	0	0	0	54
11050174	m	cn	FR	2015	0	1	0	0	1	0	0	0	0	0	0	0	64
11059411	m	ot	CN	2015	0	0	0	0	0	0	0	0	0	0	0	0	60
11060701	m	md	JP	2015	1	0	0	0	0	0	0	0	0	0	0	0	65
11063340	f	md	NL	2015	0	0	0	0	0	0	0	0	0	0	0	0	56
11067404	m	ot	US	2015	0	1	0	0	1	0	0	0	0	0	0	1	54
11074138	m	md	JP	2015	0	0	0	0	0	0	0	0	0	0	0	0	68
11075325	f	md	JP	2015	0	0	0	0	0	0	0	0	0	0	0	0	52
11077218	m	ot	CN	2015	0	0	0	0	0	0	0	0	0	0	0	0	57
11079194	m	md	FR	2015	0	1	1	0	0	0	0	0	0	0	0	0	67
11079847	m	md	NL	2015	0	1	1	0	0	0	0	0	0	0	0	0	61

11081850	f	md	DE	2015	0	1	1	0	0	0	0	0	0	0	0	0	71
11088982	f	ph	US	2015	0	1	0	0	1	0	0	0	0	0	0	0	65
11090117	m	ot	US	2015	0	1	0	0	1	0	0	0	0	0	0	0	53
11093537	m	md	JP	2015	0	1	1	0	0	0	0	0	0	0	0	0	78
11095568	f	cn	US	2015	0	1	0	0	1	0	0	0	0	0	0	1	57
11097328	f	md	Not re	2015	1	0	0	0	0	0	0	0	0	0	0	0	67
11099874	m	md	DE	2015	0	0	0	0	0	0	0	0	0	0	0	0	76
11099968	f	md	GB	2015	0	0	0	0	0	0	0	0	0	0	0	0	69
11101728	m	ot	CN	2015	0	0	0	0	0	0	0	0	0	0	0	0	52
11102206	m	ph	US	2015	0	1	0	0	1	0	0	0	0	0	0	0	63
11105019	f	md	US	2015	0	0	0	0	0	0	1	1	0	0	0	0	67
11108256	f	md	US	2015	0	1	1	0	0	0	0	0	0	0	0	0	61
11110325	m	md	Not re	2015	1	0	0	0	0	0	0	0	0	0	0	0	67
11111762	m	ot	US	2015	0	1	0	0	1	0	0	0	0	0	0	1	65
11128307	m	md	JP	2015	0	0	0	0	0	0	0	0	0	0	0	0	79
11128435	f	md	GB	2015	0	0	0	0	0	0	0	0	0	0	0	0	49
11128442	f	md	US	2015	0	1	1	0	0	0	0	0	0	0	0	0	77
11128628	f	md	JP	2015	0	0	0	0	0	0	0	0	0	0	0	0	67
11129578	m	cn	GB	2015	0	0	0	0	0	0	0	0	0	0	0	0	66
11130083	m	ot	NL	2015	0	0	0	0	0	0	0	0	0	0	0	0	44
11130098	f	md	IN	2015	0	0	0	0	0	0	0	0	0	0	0	0	
11130166	f	md	Not re	2015	1	0	0	0	0	0	0	0	0	0	0	0	76
11132233	f	ot	IT	2015	0	0	0	0	0	0	0	0	0	0	0	0	76
11134356	m	ot	IT	2015	0	0	0	0	0	0	0	0	0	0	0	0	55
11137198	m	md	US	2015	0	0	0	0	0	0	0	0	0	0	0	0	63
11140175	m	md	Not re	2015	1	0	0	0	0	0	0	0	0	0	0	0	51
11146731	f	ot	US	2015	0	1	0	0	1	0	0	0	0	0	0	0	60
11146763	f	md	FR	2015	0	0	0	0	0	0	0	0	0	0	0	0	53
11147601	m	ot	HU	2015	0	0	0	0	0	0	0	0	0	0	0	0	72
11148771	m	md	US	2015	0	0	0	0	0	0	0	0	0	0	0	0	53
11149680	m	md	JP	2015	0	1	1	0	0	0	0	0	0	0	0	0	65
11151158	m	md	Not re	2015	0	0	0	0	0	0	0	0	0	0	0	0	52
11152711	f	md	Not re	2015	1	0	0	0	0	0	0	0	0	0	0	0	56
11153191	f	md	GB	2015	0	0	0	0	0	0	0	0	0	0	0	0	37

11156545	m	md	US	2015	0	0	0	0	0	0	0	0	0	0	0	0	52
11157465	m	md	US	2015	0	0	0	0	0	0	0	0	0	0	0	0	63
11159791	m	md	Not re	2015	1	0	0	0	0	0	0	0	0	0	0	1	62
11160063	f	md	SK	2015	1	0	0	0	0	0	0	0	0	0	0	0	73
11165906	f	md	FR	2015	0	0	0	0	0	0	0	0	0	0	0	0	53
11167453	f	md	Not re	2015	1	0	0	0	0	0	0	0	0	0	0	0	52
11172780	m	md	ZA	2015	0	1	1	0	0	0	0	0	0	0	0	0	79
11173056	m	md	FR	2015	0	1	1	0	0	0	0	0	0	0	0	0	70
11174351	Not	ot	FR	2015	0	0	0	0	0	0	0	0	0	0	0	0	
11174356	m	md	JP	2015	1	0	0	0	0	0	0	0	0	0	0	0	70
11177905	f	md	Not re	2015	1	0	0	0	0	0	0	0	0	0	0	0	61
11178307	f	cn	US	2015	0	0	0	0	0	0	0	0	0	0	0	0	67
11178449	m	cn	GB	2015	1	0	0	0	0	0	0	0	0	0	0	0	55
11180510	f	md	JP	2015	0	0	0	0	0	0	0	0	0	0	0	0	78
11181489	m	ot	CN	2015	0	0	0	0	0	0	0	0	0	0	0	0	57
11181490	m	cn	DE	2015	0	0	0	0	0	0	0	0	0	0	0	0	58
11183495	m	ot	FR	2015	0	0	0	0	0	0	0	0	0	0	0	0	76
11183856	m	cn	US	2015	0	0	0	0	0	0	0	0	0	0	0	0	65
11186245	m	md	BE	2015	0	0	0	0	0	0	0	0	0	0	0	0	62
11190606	f	cn	US	2015	0	1	0	0	1	0	0	0	0	0	0	0	69
11200253	f	ot	US	2015	0	1	0	0	1	0	0	0	0	0	0	0	83
11200359	f	md	DE	2015	0	1	1	0	0	0	0	0	0	0	0	0	52
11202352	f	md	IT	2015	0	0	0	0	0	0	0	0	0	0	0	0	
11203541	m	md	AR	2015	0	1	1	0	0	0	0	0	0	0	0	1	66
11206323	f	cn	GB	2015	1	0	0	0	0	0	0	0	0	0	0	0	75
11212205	f	cn	GB	2015	1	0	0	0	0	0	0	0	0	0	0	0	
11212234	f	md	US	2015	0	0	0	0	0	0	0	0	0	0	0	0	66
11215352	f	ot	ES	2015	0	0	0	0	0	0	0	0	0	0	0	0	60
11215365	m	ot	ES	2015	0	0	0	0	0	0	0	0	0	0	0	0	56
11215800	Not	ot	ES	2015	0	0	0	0	0	0	0	0	0	0	0	0	61
11216071	m	md	ES	2015	0	1	1	0	0	0	0	0	0	0	0	0	46
11217388	f	cn	DE	2015	0	0	0	0	0	0	0	0	0	0	0	0	55
11217417	f	cn	DE	2015	0	0	0	0	0	0	0	0	0	0	0	0	59
11217443	m	cn	DE	2015	0	0	0	0	0	0	0	0	0	0	0	0	57

11219145	f	md	DE	2015	0	0	0	0	0	0	0	0	0	0	0	0	58
11221339	m	ph	JP	2015	0	0	0	0	0	0	0	0	0	0	0	0	76
11223278	m	ot	GB	2015	0	0	0	0	0	0	0	0	0	0	0	0	64
11223392	f	md	Not re	2015	0	0	0	0	0	0	0	0	0	0	0	0	69
11228252	f	md	IL	2015	0	1	1	0	0	0	0	0	0	0	0	0	61
11230090	Not	ot	GB	2015	0	0	0	0	0	0	0	0	0	0	0	0	
11230091	Not	ot	GB	2015	0	0	0	0	0	0	0	0	0	0	0	0	
11230145	Not	ot	GB	2015	0	0	0	0	0	0	0	0	0	0	0	0	
11230146	Not	ot	GB	2015	0	0	0	0	0	0	0	0	0	0	0	0	
11230147	Not	ot	GB	2015	0	0	0	0	0	0	0	0	0	0	0	0	
11231023	m	md	IL	2015	0	1	0	0	1	0	0	0	0	0	0	0	53
11231231	f	md	JP	2015	0	0	0	0	0	0	0	0	0	0	0	0	68
11231383	f	md	JP	2015	0	0	0	0	0	0	0	0	0	0	0	0	63
11231633	m	md	US	2015	0	0	0	0	0	0	0	0	0	0	0	0	60
11231650	Not	cn	GB	2015	1	0	0	0	0	0	0	0	0	0	0	0	84
11231664	Not	cn	GB	2015	1	0	0	0	0	0	0	0	0	0	0	0	88
11232020	Not	ot	Not re	2015	1	0	0	0	0	0	0	0	0	0	0	0	
11233895	f	cn	US	2015	0	1	1	0	0	0	0	0	0	0	0	0	57
11233914	Not	ot	JP	2015	0	0	0	0	0	0	0	0	0	0	0	0	
11234532	f	cn	US	2015	1	0	0	0	0	0	0	0	0	0	0	0	
11234538	Not	cn	GB	2015	1	0	0	0	0	0	0	0	0	0	0	0	73
11234540	Not	cn	GB	2015	1	0	0	0	0	0	0	0	0	0	0	0	87
11234707	m	md	KR	2015	0	0	0	0	0	0	0	0	0	0	0	0	37
11234828	m	ot	GR	2015	0	0	0	0	0	0	0	0	0	0	0	0	74
11234847	m	cn	JP	2015	0	0	0	0	0	0	0	0	0	0	0	0	68
11234935	m	md	JP	2015	0	0	0	0	0	0	0	0	0	0	0	0	40
11235091	m	md	JP	2015	0	1	1	0	0	0	0	0	0	0	0	0	50
11235437	f	cn	JP	2015	0	0	0	0	0	0	0	0	0	0	0	0	64
11236493	f	ot	CN	2015	0	0	0	0	0	0	0	0	0	0	0	0	49
11236836	m	ot	US	2015	0	1	0	0	1	0	0	0	0	0	0	0	60
11237046	f	md	US	2015	0	1	0	0	1	0	0	0	0	0	0	0	
11237105	Not	ot	FR	2015	0	0	0	0	0	0	0	0	0	0	0	0	
11237548	m	ot	AT	2015	0	1	0	0	1	0	0	0	0	0	0	0	
11237657	f	cn	GB	2015	1	0	0	0	0	0	0	0	0	0	0	0	70

11238403	Not	ot	JP	2015	0	0	0	0	0	0	0	0	0	0	0	0	
11239793	m	md	IL	2015	0	0	0	0	0	0	0	0	0	0	0	0	70
11239963	f	cn	AR	2015	0	0	0	0	0	0	0	0	0	0	0	0	65
11240432	m	cn	AR	2015	0	0	0	0	0	0	0	0	0	0	0	0	67
11241240	m	md	FR	2015	0	1	0	0	1	0	0	0	0	0	0	0	
11241628	m	md	IT	2015	0	0	0	0	0	0	0	0	0	0	0	0	66
11242698	m	md	CN	2015	0	0	0	0	0	0	0	0	0	0	0	0	29
11243223	m	md	IE	2015	0	0	0	0	0	0	0	0	0	0	0	0	58
11243375	Not	ot	US	2015	0	0	0	0	0	0	0	0	0	0	0	0	
11244116	m	md	US	2015	0	0	0	0	0	0	0	0	0	0	0	0	
11244274	f	md	Not re	2015	0	0	0	0	0	0	0	0	0	0	0	0	55
11245378	f	md	Not re	2015	1	0	0	0	0	0	0	0	0	0	0	0	65
11246126	f	cn	US	2015	0	1	0	0	1	0	0	0	0	0	0	0	
11248633	f	md	Not re	2015	1	0	0	0	0	0	0	0	0	0	0	0	68
11248867	f	cn	US	2015	0	0	0	0	0	0	0	0	0	0	0	0	70
11248928	f	cn	US	2015	0	0	0	0	0	0	0	0	0	0	0	0	55
11248938	m	cn	US	2015	0	0	0	0	0	0	0	0	0	0	0	0	80
11248939	m	cn	US	2015	0	0	0	0	0	0	0	0	0	0	0	0	49
11248951	m	cn	US	2015	0	0	0	0	0	0	0	0	0	0	0	0	65
11248952	f	cn	US	2015	0	0	0	0	0	0	0	0	0	0	0	0	
11248953	f	cn	US	2015	0	0	0	0	0	0	0	0	0	0	0	0	
11248954	m	cn	US	2015	0	0	0	0	0	0	0	0	0	0	0	0	
11248960	m	cn	Not re	2015	0	0	0	0	0	0	0	0	0	0	0	0	
11248970	f	cn	US	2015	0	0	0	0	0	0	0	0	0	0	0	0	59
11248971	m	cn	US	2015	0	0	0	0	0	0	0	0	0	0	0	0	70
11248972	m	cn	US	2015	0	0	0	0	0	0	0	0	0	0	0	0	69
11248973	f	cn	US	2015	0	0	0	0	0	0	0	0	0	0	0	0	59
11248982	f	cn	US	2015	0	0	0	0	0	0	0	0	0	0	0	0	89
11248986	f	cn	US	2015	0	0	0	0	0	0	0	0	0	0	0	0	
11248988	f	cn	US	2015	0	0	0	0	0	0	0	0	0	0	0	0	50
11248989	m	cn	US	2015	0	0	0	0	0	0	0	0	0	0	0	0	50
11248990	f	cn	US	2015	0	0	0	0	0	0	0	0	0	0	0	0	60
11248991	m	cn	US	2015	0	0	0	0	0	0	0	0	0	0	0	0	
11248995	f	cn	US	2015	0	0	0	0	0	0	0	0	0	0	0	0	83

11248996	f	cn	US	2015	0	0	0	0	0	0	0	0	0	0	0	0	
11248997	m	cn	US	2015	0	0	0	0	0	0	0	0	0	0	0	0	80
11249000	m	cn	US	2015	0	0	0	0	0	0	0	0	0	0	0	0	70
11249001	m	cn	US	2015	0	0	0	0	0	0	0	0	0	0	0	0	75
11249002	f	cn	US	2015	0	0	0	0	0	0	0	0	0	0	0	0	70
11249004	m	cn	US	2015	0	0	0	0	0	0	0	0	0	0	0	0	
11249005	m	cn	US	2015	0	0	0	0	0	0	0	0	0	0	0	0	51
11249006	f	cn	US	2015	0	0	0	0	0	0	0	0	0	0	0	0	
11249007	m	cn	US	2015	0	0	0	0	0	0	0	0	0	0	0	0	
11249008	m	cn	US	2015	0	0	0	0	0	0	0	0	0	0	0	0	
11249010	m	cn	US	2015	0	0	0	0	0	0	0	0	0	0	0	0	
11249011	m	cn	US	2015	0	0	0	0	0	0	0	0	0	0	0	0	
11249012	f	cn	US	2015	0	0	0	0	0	0	0	0	0	0	0	0	
11249015	m	cn	US	2015	0	0	0	0	0	0	0	0	0	0	0	0	
11249016	m	cn	US	2015	0	0	0	0	0	0	0	0	0	0	0	0	48
11249018	m	cn	US	2015	0	0	0	0	0	0	0	0	0	0	0	0	77
11249019	f	cn	US	2015	0	0	0	0	0	0	0	0	0	0	0	0	
11249020	m	cn	US	2015	0	0	0	0	0	0	0	0	0	0	0	0	
11249021	m	cn	US	2015	0	0	0	0	0	0	0	0	0	0	0	0	65
11249022	m	cn	US	2015	0	0	0	0	0	0	0	0	0	0	0	0	65
11249023	m	cn	US	2015	0	0	0	0	0	0	0	0	0	0	0	0	65
11249027	f	cn	DE	2015	0	0	0	0	0	0	0	0	0	0	0	0	67
11249034	f	cn	US	2015	0	0	0	0	0	0	0	0	0	0	0	0	70
11249040	f	cn	US	2015	0	0	0	0	0	0	0	0	0	0	0	0	
11249042	m	cn	US	2015	0	0	0	0	0	0	0	0	0	0	0	0	
11249043	m	cn	US	2015	0	0	0	0	0	0	0	0	0	0	0	0	
11249044	f	cn	US	2015	0	0	0	0	0	0	0	0	0	0	0	0	
11249046	m	cn	US	2015	0	0	0	0	0	0	0	0	0	0	0	0	
11249047	f	cn	US	2015	0	0	0	0	0	0	0	0	0	0	0	0	74
11249048	m	cn	US	2015	0	0	0	0	0	0	0	0	0	0	0	0	74
11249051	m	cn	US	2015	0	0	0	0	0	0	0	0	0	0	0	0	57
11249055	f	cn	US	2015	0	0	0	0	0	0	0	0	0	0	0	0	66
11249056	m	cn	US	2015	0	0	0	0	0	0	0	0	0	0	0	0	
11249057	f	cn	US	2015	0	0	0	0	0	0	0	0	0	0	0	0	65

11249058	f	cn	US	2015	0	0	0	0	0	0	0	0	0	0	0	0	59
11249059	f	cn	US	2015	0	0	0	0	0	0	0	0	0	0	0	0	65
11249060	m	cn	US	2015	0	0	0	0	0	0	0	0	0	0	0	0	
11249061	m	cn	US	2015	0	0	0	0	0	0	0	0	0	0	0	0	
11249062	f	cn	US	2015	0	0	0	0	0	0	0	0	0	0	0	0	61
11249063	f	cn	US	2015	0	0	0	0	0	0	0	0	0	0	0	0	68
11249065	m	cn	US	2015	0	0	0	0	0	0	0	0	0	0	0	0	
11249070	m	cn	US	2015	0	0	0	0	0	0	0	0	0	0	0	0	
11249071	f	cn	US	2015	0	0	0	0	0	0	0	0	0	0	0	0	70
11249074	f	cn	US	2015	0	0	0	0	0	0	0	0	0	0	0	0	82
11249076	f	cn	US	2015	0	0	0	0	0	0	0	0	0	0	0	0	63
11249077	m	cn	US	2015	0	0	0	0	0	0	0	0	0	0	0	0	87
11249082	f	cn	US	2015	0	0	0	0	0	0	0	0	0	0	0	0	53
11249083	f	cn	US	2015	0	0	0	0	0	0	0	0	0	0	0	0	
11249084	m	cn	US	2015	0	0	0	0	0	0	0	0	0	0	0	0	75
11249085	m	cn	US	2015	0	0	0	0	0	0	0	0	0	0	0	0	
11249087	m	cn	US	2015	0	0	0	0	0	0	0	0	0	0	0	0	48
11249088	m	cn	US	2015	0	0	0	0	0	0	0	0	0	0	0	0	66
11249091	m	cn	US	2015	0	0	0	0	0	0	0	0	0	0	0	0	
11249092	f	cn	US	2015	0	0	0	0	0	0	0	0	0	0	0	0	
11249093	f	cn	US	2015	0	0	0	0	0	0	0	0	0	0	0	0	
11249094	f	cn	US	2015	0	0	0	0	0	0	0	0	0	0	0	0	75
11249113	f	cn	US	2015	0	0	0	0	0	0	0	0	0	0	0	0	80
11249125	f	cn	US	2015	0	0	0	0	0	0	0	0	0	0	0	0	
11249126	m	cn	US	2015	0	0	0	0	0	0	0	0	0	0	0	0	70
11249128	m	cn	US	2015	0	0	0	0	0	0	0	0	0	0	0	0	68
11249129	f	cn	US	2015	0	0	0	0	0	0	0	0	0	0	0	0	83
11249131	m	cn	US	2015	0	0	0	0	0	0	0	0	0	0	0	0	70
11249132	m	cn	US	2015	0	0	0	0	0	0	0	0	0	0	0	0	
11249133	f	cn	US	2015	0	0	0	0	0	0	0	0	0	0	0	0	
11249134	m	cn	US	2015	0	0	0	0	0	0	0	0	0	0	0	0	79
11249135	m	cn	US	2015	0	0	0	0	0	0	0	0	0	0	0	0	
11249136	f	cn	US	2015	0	0	0	0	0	0	0	0	0	0	0	0	65
11249141	m	cn	US	2015	0	0	0	0	0	0	0	0	0	0	0	0	65

11249142	f	cn	US	2015	0	0	0	0	0	0	0	0	0	0	0	0	
11249146	f	cn	US	2015	0	0	0	0	0	0	0	0	0	0	0	0	54
11249147	m	cn	US	2015	0	0	0	0	0	0	0	0	0	0	0	0	62
11249148	m	cn	US	2015	0	0	0	0	0	0	0	0	0	0	0	0	75
11249149	m	cn	US	2015	0	0	0	0	0	0	0	0	0	0	0	0	22
11249150	f	cn	US	2015	0	0	0	0	0	0	0	0	0	0	0	0	76
11249154	m	cn	US	2015	0	0	0	0	0	0	0	0	0	0	0	0	65
11249155	f	cn	US	2015	0	0	0	0	0	0	0	0	0	0	0	0	73
11249157	m	cn	US	2015	0	0	0	0	0	0	0	0	0	0	0	0	75
11249160	m	cn	US	2015	0	0	0	0	0	0	0	0	0	0	0	0	63
11249161	f	cn	US	2015	0	0	0	0	0	0	0	0	0	0	0	0	50
11249191	m	md	US	2015	0	0	0	0	0	0	0	0	0	0	0	0	77
11249203	m	cn	US	2015	0	0	0	0	0	0	0	0	0	0	0	0	
11249204	f	cn	US	2015	0	0	0	0	0	0	0	0	0	0	0	0	79
11249205	f	cn	US	2015	0	0	0	0	0	0	0	0	0	0	0	0	63
11249206	f	cn	US	2015	0	0	0	0	0	0	0	0	0	0	0	0	
11249207	f	cn	US	2015	0	0	0	0	0	0	0	0	0	0	0	0	69
11249212	m	cn	US	2015	0	0	0	0	0	0	0	0	0	0	0	0	58
11249213	f	cn	US	2015	0	0	0	0	0	0	0	0	0	0	0	0	
11249216	f	cn	US	2015	0	0	0	0	0	0	0	0	0	0	0	0	53
11249221	m	cn	US	2015	0	0	0	0	0	0	0	0	0	0	0	0	
11249225	f	cn	US	2015	0	0	0	0	0	0	0	0	0	0	0	0	59
11249227	f	cn	US	2015	0	0	0	0	0	0	0	0	0	0	0	0	63
11249230	m	cn	US	2015	1	0	0	0	0	0	0	0	0	0	0	0	51
11249231	f	cn	US	2015	0	0	0	0	0	0	0	0	0	0	0	0	69
11249232	m	cn	US	2015	0	0	0	0	0	0	0	0	0	0	0	0	64
11249233	m	cn	US	2015	0	0	0	0	0	0	0	0	0	0	0	0	62
11249235	m	cn	US	2015	0	0	0	0	0	0	0	0	0	0	0	0	68
11249236	m	cn	US	2015	1	0	0	0	0	0	0	0	0	0	0	0	73
11249237	f	cn	US	2015	0	0	0	0	0	0	0	0	0	0	0	0	62
11249239	f	cn	US	2015	0	0	0	0	0	0	0	0	0	0	0	0	
11249240	f	cn	US	2015	0	0	0	0	0	0	0	0	0	0	0	0	
11249241	f	cn	US	2015	0	0	0	0	0	0	0	0	0	0	0	0	68
11249242	f	cn	US	2015	0	0	0	0	0	0	0	0	0	0	0	0	66

11249244	f	cn	US	2015	0	0	0	0	0	0	0	0	0	0	0	0	
11249245	f	cn	US	2015	0	0	0	0	0	0	0	0	0	0	0	0	65
11249246	m	cn	US	2015	0	0	0	0	0	0	0	0	0	0	0	0	68
11249247	f	cn	US	2015	0	0	0	0	0	0	0	0	0	0	0	0	
11249251	m	cn	US	2015	0	0	0	0	0	0	0	0	0	0	0	0	
11249252	f	cn	US	2015	0	0	0	0	0	0	0	0	0	0	0	0	
11249255	f	cn	US	2015	0	0	0	0	0	0	0	0	0	0	0	0	80
11249256	f	cn	US	2015	0	0	0	0	0	0	0	0	0	0	0	0	
11249259	f	cn	US	2015	0	0	0	0	0	0	0	0	0	0	0	0	
11249262	m	cn	US	2015	0	0	0	0	0	0	0	0	0	0	0	0	81
11249263	f	cn	US	2015	0	0	0	0	0	0	0	0	0	0	0	0	
11249264	m	cn	US	2015	0	0	0	0	0	0	0	0	0	0	0	0	58
11249265	f	cn	US	2015	0	0	0	0	0	0	0	0	0	0	0	0	65
11249266	f	cn	US	2015	0	0	0	0	0	0	0	0	0	0	0	0	55
11249267	f	cn	US	2015	0	0	0	0	0	0	0	0	0	0	0	0	
11249271	m	cn	US	2015	0	0	0	0	0	0	0	0	0	0	0	0	
11249272	f	cn	US	2015	0	0	0	0	0	0	0	0	0	0	0	0	71
11249273	f	cn	US	2015	0	0	0	0	0	0	0	0	0	0	0	0	
11249275	m	cn	US	2015	0	0	0	0	0	0	0	0	0	0	0	0	55
11249278	m	cn	US	2015	0	0	0	0	0	0	0	0	0	0	0	0	
11249279	f	cn	US	2015	0	0	0	0	0	0	0	0	0	0	0	0	
11249280	m	cn	US	2015	0	0	0	0	0	0	0	0	0	0	0	0	67
11249281	f	cn	US	2015	0	0	0	0	0	0	0	0	0	0	0	0	70
11249282	m	cn	US	2015	0	0	0	0	0	0	0	0	0	0	0	0	
11249284	m	cn	US	2015	0	0	0	0	0	0	0	0	0	0	0	0	81
11249285	m	cn	US	2015	0	0	0	0	0	0	0	0	0	0	0	0	56
11249286	f	cn	US	2015	0	0	0	0	0	0	0	0	0	0	0	0	70
11249290	f	cn	US	2015	0	0	0	0	0	0	0	0	0	0	0	0	60
11249291	m	cn	US	2015	0	0	0	0	0	0	0	0	0	0	0	0	33
11249296	m	cn	US	2015	0	0	0	0	0	0	0	0	0	0	0	0	81
11249298	f	cn	US	2015	0	0	0	0	0	0	0	0	0	0	0	0	
11249299	f	cn	US	2015	0	0	0	0	0	0	0	0	0	0	0	0	
11249300	m	cn	US	2015	0	0	0	0	0	0	0	0	0	0	0	0	
11249307	f	cn	US	2015	0	0	0	0	0	0	0	0	0	0	0	0	35

11249310	m	cn	US	2015	0	0	0	0	0	0	0	0	0	0	0	0	54
11249312	f	cn	US	2015	0	0	0	0	0	0	0	0	0	0	0	0	58
11249318	f	cn	US	2015	0	0	0	0	0	0	0	0	0	0	0	0	65
11249319	f	cn	US	2015	0	0	0	0	0	0	0	0	0	0	0	0	65
11249320	f	cn	US	2015	0	0	0	0	0	0	0	0	0	0	0	0	65
11249322	m	cn	US	2015	0	0	0	0	0	0	0	0	0	0	0	0	54
11249327	m	cn	US	2015	0	0	0	0	0	0	0	0	0	0	0	0	59
11249331	m	cn	US	2015	0	0	0	0	0	0	0	0	0	0	0	0	77
11249335	f	cn	US	2015	0	0	0	0	0	0	0	0	0	0	0	0	58
11249341	m	cn	US	2015	0	0	0	0	0	0	0	0	0	0	0	0	
11249343	f	cn	US	2015	0	0	0	0	0	0	0	0	0	0	0	0	81
11249346	f	cn	US	2015	0	0	0	0	0	0	0	0	0	0	0	0	70
11249350	f	cn	US	2015	0	0	0	0	0	0	0	0	0	0	0	0	66
11249351	m	cn	US	2015	0	0	0	0	0	0	0	0	0	0	0	0	73
11249352	f	cn	US	2015	0	0	0	0	0	0	0	0	0	0	0	0	45
11249354	f	cn	US	2015	0	0	0	0	0	0	0	0	0	0	0	0	
11249355	f	cn	US	2015	0	0	0	0	0	0	0	0	0	0	0	0	68
11249357	f	cn	US	2015	0	0	0	0	0	0	0	0	0	0	0	0	59
11249364	f	cn	US	2015	0	0	0	0	0	0	0	0	0	0	0	0	
11249371	f	cn	US	2015	0	0	0	0	0	0	0	0	0	0	0	0	68
11249383	m	cn	US	2015	0	0	0	0	0	0	0	0	0	0	0	0	
11249388	f	cn	US	2015	0	0	0	0	0	0	0	0	0	0	0	0	65
11249389	f	cn	US	2015	0	0	0	0	0	0	0	0	0	0	0	0	43
11249391	m	cn	US	2015	0	0	0	0	0	0	0	0	0	0	0	0	66
11249393	m	cn	US	2015	0	0	0	0	0	0	0	0	0	0	0	0	66
11249395	m	cn	US	2015	0	0	0	0	0	0	0	0	0	0	0	0	66
11249398	m	cn	US	2015	0	0	0	0	0	0	0	0	0	0	0	0	
11249399	m	cn	US	2015	0	0	0	0	0	0	0	0	0	0	0	0	79
11249401	f	cn	US	2015	0	0	0	0	0	0	0	0	0	0	0	0	
11249402	f	cn	US	2015	0	0	0	0	0	0	0	0	0	0	0	0	75
11249406	f	cn	US	2015	0	0	0	0	0	0	0	0	0	0	0	0	
11249412	m	cn	US	2015	0	0	0	0	0	0	0	0	0	0	0	0	72
11249413	f	cn	US	2015	0	0	0	0	0	0	0	0	0	0	0	0	86
11249456	f	cn	US	2015	0	0	0	0	0	0	0	0	0	0	0	0	64

11249464	m	cn	US	2015	0	0	0	0	0	0	0	0	0	0	0	0	57
11249466	f	cn	US	2015	0	0	0	0	0	0	0	0	0	0	0	0	
11249467	f	cn	US	2015	0	0	0	0	0	0	0	0	0	0	0	0	56
11249468	f	cn	US	2015	1	0	0	0	0	0	0	0	0	0	0	0	84
11249469	m	cn	US	2015	0	0	0	0	0	0	0	0	0	0	0	0	
11249471	m	cn	US	2015	0	0	0	0	0	0	0	0	0	0	0	0	72
11249474	f	cn	US	2015	0	0	0	0	0	0	0	0	0	0	0	0	
11249477	m	cn	US	2015	0	0	0	0	0	0	0	0	0	0	0	0	
11249478	m	cn	US	2015	0	0	0	0	0	0	0	0	0	0	0	0	61
11249526	f	cn	US	2015	0	0	0	0	0	0	0	0	0	0	0	0	54
11249527	f	cn	US	2015	0	0	0	0	0	0	0	0	0	0	0	0	45
11249530	f	cn	US	2015	0	0	0	0	0	0	0	0	0	0	0	0	
11249533	m	cn	US	2015	0	0	0	0	0	0	0	0	0	0	0	0	
11249534	m	cn	US	2015	0	0	0	0	0	0	0	0	0	0	0	0	
11249535	m	cn	US	2015	0	0	0	0	0	0	0	0	0	0	0	0	
11249541	m	cn	US	2015	1	0	0	0	0	0	0	0	0	0	0	0	
11249548	m	cn	US	2015	0	0	0	0	0	0	0	0	0	0	0	0	
11249549	f	cn	US	2015	0	0	0	0	0	0	0	0	0	0	0	0	
11249554	m	cn	US	2015	0	0	0	0	0	0	0	0	0	0	0	0	
11249556	m	cn	US	2015	0	0	0	0	0	0	0	0	0	0	0	0	59
11249558	Not	cn	US	2015	0	0	0	0	0	0	0	0	0	0	0	0	
11249559	m	cn	US	2015	0	0	0	0	0	0	0	0	0	0	0	0	50
11249560	f	cn	US	2015	0	0	0	0	0	0	0	0	0	0	0	0	52
11249561	m	cn	US	2015	0	0	0	0	0	0	0	0	0	0	0	0	
11249562	f	cn	US	2015	0	0	0	0	0	0	0	0	0	0	0	0	
11249567	f	cn	US	2015	0	0	0	0	0	0	0	0	0	0	0	0	
11249568	f	cn	Not re	2015	0	0	0	0	0	0	0	0	0	0	0	0	73
11249569	m	cn	US	2015	0	0	0	0	0	0	0	0	0	0	0	0	54
11249570	f	cn	US	2015	0	0	0	0	0	0	0	0	0	0	0	0	58
11249572	f	cn	US	2015	0	0	0	0	0	0	0	0	0	0	0	0	69
11249573	m	cn	US	2015	0	0	0	0	0	0	0	0	0	0	0	0	65
11249581	m	cn	US	2015	0	0	0	0	0	0	0	0	0	0	0	0	
11249591	m	cn	US	2015	0	0	0	0	0	0	0	0	0	0	0	0	
11249593	f	cn	US	2015	0	0	0	0	0	0	0	0	0	0	0	0	

11249594	m	cn	US	2015	0	0	0	0	0	0	0	0	0	0	0	0	
11249598	f	cn	US	2015	0	0	0	0	0	0	0	0	0	0	0	0	
11249599	m	cn	US	2015	0	0	0	0	0	0	0	0	0	0	0	0	
11249602	f	cn	US	2015	0	0	0	0	0	0	0	0	0	0	0	0	
11249608	f	cn	US	2015	0	0	0	0	0	0	0	0	0	0	0	0	
11249610	f	cn	US	2015	0	0	0	0	0	0	0	0	0	0	0	0	
11249617	f	cn	US	2015	0	0	0	0	0	0	0	0	0	0	0	0	
11249623	m	cn	US	2015	0	0	0	0	0	0	0	0	0	0	0	0	
11249653	f	cn	US	2015	0	0	0	0	0	0	0	0	0	0	0	0	
11249658	m	cn	US	2015	0	0	0	0	0	0	0	0	0	0	0	0	66
11249660	m	cn	US	2015	0	0	0	0	0	0	0	0	0	0	0	0	
11249684	f	cn	US	2015	0	0	0	0	0	0	0	0	0	0	0	0	65
11249712	m	cn	US	2015	0	0	0	0	0	0	0	0	0	0	0	0	
11249716	m	cn	US	2015	0	0	0	0	0	0	0	0	0	0	0	0	60
11249723	m	cn	US	2015	0	0	0	0	0	0	0	0	0	0	0	0	
11249739	f	cn	US	2015	0	0	0	0	0	0	0	0	0	0	0	0	81
11249749	m	cn	US	2015	0	0	0	0	0	0	0	0	0	0	0	0	68
11249827	f	cn	US	2015	0	0	0	0	0	0	0	0	0	0	0	0	65
11249879	m	cn	US	2015	0	0	0	0	0	0	0	0	0	0	0	0	
11249901	m	cn	US	2015	0	0	0	0	0	0	0	0	0	0	0	0	45
11249903	f	cn	US	2015	0	0	0	0	0	0	0	0	0	0	0	0	
11249909	f	cn	US	2015	0	0	0	0	0	0	0	0	0	0	0	0	
11249910	f	cn	US	2015	0	0	0	0	0	0	0	0	0	0	0	0	
11249928	f	cn	US	2015	0	0	0	0	0	0	0	0	0	0	0	0	
11249931	m	cn	US	2015	0	0	0	0	0	0	0	0	0	0	0	0	70
11249932	m	cn	US	2015	0	0	0	0	0	0	0	0	0	0	0	0	60
11249933	m	cn	US	2015	0	0	0	0	0	0	0	0	0	0	0	0	
11250428	f	cn	US	2015	0	0	0	0	0	0	0	0	0	0	0	0	
11250449	f	cn	US	2015	0	0	0	0	0	0	0	0	0	0	0	0	
11250455	m	cn	US	2015	0	0	0	0	0	0	0	0	0	0	0	0	
11250462	m	cn	US	2015	0	0	0	0	0	0	0	0	0	0	0	0	
11250465	m	cn	US	2015	0	0	0	0	0	0	0	0	0	0	0	0	
11250474	f	cn	US	2015	0	0	0	0	0	0	0	0	0	0	0	0	42
11250475	m	cn	US	2015	0	0	0	0	0	0	0	0	0	0	0	0	50

11250477	m	cn	US	2015	0	0	0	0	0	0	0	0	0	0	0	0	65
11250481	m	cn	US	2015	0	0	0	0	0	0	0	0	0	0	0	0	
11250487	m	cn	US	2015	0	0	0	0	0	0	0	0	0	0	0	0	
11250496	m	cn	US	2015	0	0	0	0	0	0	0	0	0	0	0	0	
11250502	f	cn	US	2015	0	0	0	0	0	0	0	0	0	0	0	0	
11250508	f	cn	US	2015	0	0	0	0	0	0	0	0	0	0	0	0	
11250511	f	cn	US	2015	0	0	0	0	0	0	0	0	0	0	0	0	66
11250517	f	cn	US	2015	0	0	0	0	0	0	0	0	0	0	0	0	
11250595	m	cn	US	2015	0	0	0	0	0	0	0	0	0	0	0	0	78
11250596	m	cn	US	2015	0	0	0	0	0	0	0	0	0	0	0	0	
11250612	f	cn	US	2015	0	0	0	0	0	0	0	0	0	0	0	0	55
11250613	m	cn	US	2015	0	0	0	0	0	0	0	0	0	0	0	0	65
11250619	m	cn	US	2015	0	0	0	0	0	0	0	0	0	0	0	0	62
11250622	f	cn	US	2015	0	0	0	0	0	0	0	0	0	0	0	0	73
11250624	Not	cn	US	2015	1	0	0	0	0	0	0	0	0	0	0	0	65
11250639	f	cn	US	2015	0	0	0	0	0	0	0	0	0	0	0	0	
11250656	m	cn	US	2015	0	0	0	0	0	0	0	0	0	0	0	0	52
11250671	f	cn	US	2015	0	0	0	0	0	0	0	0	0	0	0	0	
11250688	f	cn	US	2015	0	0	0	0	0	0	0	0	0	0	0	0	
11250720	m	cn	US	2015	0	0	0	0	0	0	0	0	0	0	0	0	
11250723	f	cn	US	2015	0	0	0	0	0	0	0	0	0	0	0	0	40
11250727	f	cn	US	2015	0	0	0	0	0	0	0	0	0	0	0	0	55
11250773	f	cn	US	2015	0	0	0	0	0	0	0	0	0	0	0	0	
11250780	m	cn	US	2015	0	0	0	0	0	0	0	0	0	0	0	0	
11250782	m	cn	US	2015	0	0	0	0	0	0	0	0	0	0	0	0	
11250794	f	cn	US	2015	0	0	0	0	0	0	0	0	0	0	0	0	
11250798	m	cn	US	2015	0	0	0	0	0	0	0	0	0	0	0	0	46
11250870	f	cn	US	2015	0	0	0	0	0	0	0	0	0	0	0	0	
11250920	m	cn	US	2015	0	0	0	0	0	0	0	0	0	0	0	0	77
11250941	m	cn	US	2015	0	0	0	0	0	0	0	0	0	0	0	0	
11250958	f	cn	US	2015	0	0	0	0	0	0	0	0	0	0	0	0	
11250963	f	cn	US	2015	0	0	0	0	0	0	0	0	0	0	0	0	
11250970	f	cn	US	2015	0	0	0	0	0	0	0	0	0	0	0	0	60
11250976	m	cn	US	2015	0	0	0	0	0	0	0	0	0	0	0	0	

11250978	m	cn	US	2015	0	0	0	0	0	0	0	0	0	0	0	0	70
11251012	f	cn	US	2015	0	0	0	0	0	0	0	0	0	0	0	0	
11251023	f	cn	US	2015	0	0	0	0	0	0	0	0	0	0	0	0	62
11251026	f	cn	US	2015	0	0	0	0	0	0	0	0	0	0	0	0	76
11251032	m	cn	US	2015	0	0	0	0	0	0	0	0	0	0	0	0	47
11251044	f	cn	US	2015	0	0	0	0	0	0	0	0	0	0	0	0	75
11251049	f	cn	US	2015	0	0	0	0	0	0	0	0	0	0	0	0	
11251067	f	cn	US	2015	0	0	0	0	0	0	0	0	0	0	0	0	
11251091	m	cn	US	2015	0	0	0	0	0	0	0	0	0	0	0	0	
11251099	f	cn	US	2015	0	0	0	0	0	0	0	0	0	0	0	0	72
11251101	m	cn	US	2015	0	0	0	0	0	0	0	0	0	0	0	0	70
11251116	m	cn	US	2015	0	0	0	0	0	0	0	0	0	0	0	0	
11251158	f	cn	US	2015	0	0	0	0	0	0	0	0	0	0	0	0	
11251169	f	cn	US	2015	0	0	0	0	0	0	0	0	0	0	0	0	
11251174	m	cn	US	2015	0	0	0	0	0	0	0	0	0	0	0	0	
11251183	m	cn	US	2015	0	0	0	0	0	0	0	0	0	0	0	0	
11251188	f	cn	US	2015	0	0	0	0	0	0	0	0	0	0	0	0	
11251216	m	cn	US	2015	0	0	0	0	0	0	0	0	0	0	0	0	
11251223	m	cn	US	2015	0	0	0	0	0	0	0	0	0	0	0	0	
11251275	m	ot	US	2015	0	0	0	0	0	0	0	0	0	0	0	0	
11251277	m	cn	US	2015	0	0	0	0	0	0	0	0	0	0	0	0	
11251278	m	cn	US	2015	0	0	0	0	0	0	0	0	0	0	0	0	
11251287	f	cn	US	2015	0	0	0	0	0	0	0	0	0	0	0	0	83
11251288	f	cn	US	2015	0	0	0	0	0	0	0	0	0	0	0	0	
11251295	f	cn	US	2015	0	0	0	0	0	0	0	0	0	0	0	0	60
11251299	f	cn	US	2015	0	0	0	0	0	0	0	0	0	0	0	0	78
11251303	f	cn	US	2015	0	0	0	0	0	0	0	0	0	0	0	0	80
11251305	f	cn	US	2015	0	0	0	0	0	0	0	0	0	0	0	0	71
11251331	f	cn	US	2015	0	0	0	0	0	0	0	0	0	0	0	0	
11251350	f	cn	US	2015	0	0	0	0	0	0	0	0	0	0	0	0	
11251362	f	cn	US	2015	0	0	0	0	0	0	0	0	0	0	0	0	
11251380	m	cn	US	2015	0	0	0	0	0	0	0	0	0	0	0	0	
11251397	f	cn	US	2015	0	0	0	0	0	0	0	0	0	0	0	0	
11251433	m	cn	US	2015	0	0	0	0	0	0	0	0	0	0	0	0	

11251441	m	cn	US	2015	0	0	0	0	0	0	0	0	0	0	0	0	69
11251449	m	cn	US	2015	0	0	0	0	0	0	0	0	0	0	0	0	
11251451	Not	cn	US	2015	0	0	0	0	0	0	0	0	0	0	0	0	
11251458	m	cn	US	2015	0	0	0	0	0	0	0	0	0	0	0	0	86
11251460	f	cn	US	2015	0	0	0	0	0	0	0	0	0	0	0	0	
11251464	m	cn	US	2015	0	0	0	0	0	0	0	0	0	0	0	0	
11251473	f	cn	US	2015	0	0	0	0	0	0	0	0	0	0	0	0	
11251476	f	cn	US	2015	0	0	0	0	0	0	0	0	0	0	0	0	
11251481	f	cn	US	2015	0	0	0	0	0	0	0	0	0	0	0	0	66
11251486	f	cn	US	2015	0	0	0	0	0	0	0	0	0	0	0	0	70
11251494	m	cn	US	2015	0	0	0	0	0	0	0	0	0	0	0	0	
11251506	f	cn	US	2015	0	0	0	0	0	0	0	0	0	0	0	0	
11251513	f	cn	US	2015	0	0	0	0	0	0	0	0	0	0	0	0	61
11251519	Not	cn	US	2015	0	0	0	0	0	0	0	0	0	0	0	0	56
11251521	Not	cn	US	2015	0	0	0	0	0	0	0	0	0	0	0	0	59
11251535	f	cn	US	2015	0	0	0	0	0	0	0	0	0	0	0	0	67
11251538	f	cn	US	2015	0	0	0	0	0	0	0	0	0	0	0	0	
11251548	m	cn	US	2015	0	0	0	0	0	0	0	0	0	0	0	0	
11251550	Not	cn	US	2015	0	0	0	0	0	0	0	0	0	0	0	0	
11251551	Not	cn	US	2015	0	0	0	0	0	0	0	0	0	0	0	0	
11251554	f	cn	US	2015	0	0	0	0	0	0	0	0	0	0	0	0	
11251555	f	cn	US	2015	0	0	0	0	0	0	0	0	0	0	0	0	
11251562	f	cn	US	2015	0	0	0	0	0	0	0	0	0	0	0	0	
11251584	m	cn	US	2015	0	0	0	0	0	0	0	0	0	0	0	0	
11251589	m	cn	US	2015	0	0	0	0	0	0	0	0	0	0	0	0	62
11251596	m	cn	US	2015	0	0	0	0	0	0	0	0	0	0	0	0	
11251599	m	cn	US	2015	0	0	0	0	0	0	0	0	0	0	0	0	65
11251607	f	cn	US	2015	0	0	0	0	0	0	0	0	0	0	0	0	52
11251635	m	cn	US	2015	0	0	0	0	0	0	0	0	0	0	0	0	70
11251636	m	cn	US	2015	0	0	0	0	0	0	0	0	0	0	0	0	
11251644	m	cn	US	2015	0	0	0	0	0	0	0	0	0	0	0	0	50
11251647	m	cn	US	2015	0	0	0	0	0	0	0	0	0	0	0	0	
11251662	f	cn	US	2015	0	0	0	0	0	0	0	0	0	0	0	0	68
11251663	m	cn	US	2015	0	0	0	0	0	0	0	0	0	0	0	0	77

11251665	m	cn	US	2015	0	0	0	0	0	0	0	0	0	0	0	0	60
11251670	f	cn	US	2015	0	0	0	0	0	0	0	0	0	0	0	0	73
11251688	Not	cn	US	2015	0	0	0	0	0	0	0	0	0	0	0	0	45
11251707	f	cn	US	2015	0	0	0	0	0	0	0	0	0	0	0	0	68
11251733	f	cn	US	2015	0	0	0	0	0	0	0	0	0	0	0	0	
11251740	m	cn	US	2015	0	0	0	0	0	0	0	0	0	0	0	0	
11251743	f	cn	US	2015	0	0	0	0	0	0	0	0	0	0	0	0	40
11251763	m	cn	US	2015	0	0	0	0	0	0	0	0	0	0	0	0	
11251796	m	cn	US	2015	0	0	0	0	0	0	0	0	0	0	0	0	64
11251807	f	cn	US	2015	0	0	0	0	0	0	0	0	0	0	0	0	
11251818	m	cn	US	2015	0	0	0	0	0	0	0	0	0	0	0	0	
11251819	f	cn	US	2015	0	0	0	0	0	0	0	0	0	0	0	0	
11251842	f	cn	US	2015	0	0	0	0	0	0	0	0	0	0	0	0	
11251864	m	cn	US	2015	0	0	0	0	0	0	0	0	0	0	0	0	
11251865	f	cn	US	2015	0	0	0	0	0	0	0	0	0	0	0	0	75
11251870	f	cn	US	2015	0	0	0	0	0	0	0	0	0	0	0	0	78
11251879	m	cn	US	2015	0	0	0	0	0	0	0	0	0	0	0	0	82
11251882	f	cn	US	2015	0	0	0	0	0	0	0	0	0	0	0	0	
11251919	m	cn	US	2015	0	0	0	0	0	0	0	0	0	0	0	0	
11251957	m	cn	US	2015	0	0	0	0	0	0	0	0	0	0	0	0	
11251962	m	cn	US	2015	0	0	0	0	0	0	0	0	0	0	0	0	
11251964	m	cn	US	2015	0	0	0	0	0	0	0	0	0	0	0	0	
11251984	m	cn	US	2015	0	0	0	0	0	0	0	0	0	0	0	0	76
11251986	f	cn	US	2015	0	0	0	0	0	0	0	0	0	0	0	0	
11251994	m	cn	US	2015	0	0	0	0	0	0	0	0	0	0	0	0	
11252003	m	cn	US	2015	0	0	0	0	0	0	0	0	0	0	0	0	
11252013	f	cn	US	2015	0	0	0	0	0	0	0	0	0	0	0	0	
11252014	m	ph	US	2015	0	0	0	0	0	0	0	0	0	0	0	0	
11252019	m	cn	US	2015	0	0	0	0	0	0	0	0	0	0	0	0	70
11252034	m	cn	US	2015	0	0	0	0	0	0	0	0	0	0	0	0	45
11252036	f	ot	US	2015	0	0	0	0	0	0	0	0	0	0	0	0	
11252040	Not	md	US	2015	0	0	0	0	0	0	0	0	0	0	0	0	
11252042	f	ot	US	2015	0	0	0	0	0	0	0	0	0	0	0	0	
11252043	m	md	US	2015	0	0	0	0	0	0	0	0	0	0	0	0	

11252048	m	cn	US	2015	0	0	0	0	0	0	0	0	0	0	0	0	57
11252062	f	md	US	2015	0	0	0	0	0	0	0	0	0	0	0	0	58
11252065	m	cn	US	2015	0	0	0	0	0	0	0	0	0	0	0	0	78
11252071	f	cn	US	2015	0	0	0	0	0	0	0	0	0	0	0	0	
11252073	f	md	US	2015	0	0	0	0	0	0	0	0	0	0	0	0	
11252075	f	cn	US	2015	0	0	0	0	0	0	0	0	0	0	0	0	
11252080	m	cn	US	2015	0	0	0	0	0	0	0	0	0	0	0	0	
11252090	m	cn	US	2015	0	0	0	0	0	0	0	0	0	0	0	0	
11252092	m	ot	US	2015	0	0	0	0	0	0	0	0	0	0	0	0	
11252096	m	cn	US	2015	0	0	0	0	0	0	0	0	0	0	0	0	
11252106	f	cn	US	2015	0	0	0	0	0	0	0	0	0	0	0	0	
11252112	f	cn	US	2015	0	0	0	0	0	0	0	0	0	0	0	0	85
11252115	m	cn	US	2015	0	0	0	0	0	0	0	0	0	0	0	0	76
11252120	m	cn	US	2015	0	0	0	0	0	0	0	0	0	0	0	0	
11252121	f	cn	US	2015	0	0	0	0	0	0	0	0	0	0	0	0	
11252130	m	cn	US	2015	0	0	0	0	0	0	0	0	0	0	0	0	66
11252131	f	cn	US	2015	0	0	0	0	0	0	0	0	0	0	0	0	67
11252138	m	cn	US	2015	0	0	0	0	0	0	0	0	0	0	0	0	
11252163	m	cn	US	2015	0	0	0	0	0	0	0	0	0	0	0	0	
11252167	m	ot	US	2015	0	0	0	0	0	0	0	0	0	0	0	0	76
11252168	m	cn	US	2015	0	0	0	0	0	0	0	0	0	0	0	0	60
11252169	f	cn	US	2015	0	0	0	0	0	0	0	0	0	0	0	0	80
11252171	f	ot	US	2015	0	0	0	0	0	0	0	0	0	0	0	0	77
11252177	f	cn	US	2015	0	0	0	0	0	0	0	0	0	0	0	0	
11252179	f	cn	US	2015	0	0	0	0	0	0	0	0	0	0	0	0	
11252187	f	cn	US	2015	0	0	0	0	0	0	0	0	0	0	0	0	
11252188	f	cn	US	2015	0	0	0	0	0	0	0	0	0	0	0	0	60
11252197	f	cn	US	2015	0	0	0	0	0	0	0	0	0	0	0	0	63
11252211	m	ot	US	2015	0	0	0	0	0	0	0	0	0	0	0	0	
11252222	Not	cn	US	2015	0	0	0	0	0	0	0	0	0	0	0	0	
11252231	f	cn	US	2015	0	0	0	0	0	0	0	0	0	0	0	0	71
11252235	f	ph	US	2015	0	0	0	0	0	0	0	0	0	0	0	0	
11252242	m	cn	US	2015	0	0	0	0	0	0	0	0	0	0	0	0	76
11252258	f	cn	US	2015	0	0	0	0	0	0	0	0	0	0	0	0	

11252261	f	ot	US	2015	0	0	0	0	0	0	0	0	0	0	0	0	70
11252268	m	cn	US	2015	0	0	0	0	0	0	0	0	0	0	0	0	
11252275	m	cn	US	2015	0	0	0	0	0	0	0	0	0	0	0	0	
11252305	m	cn	US	2015	0	0	0	0	0	0	0	0	0	0	0	0	
11252320	f	cn	US	2015	0	0	0	0	0	0	0	0	0	0	0	0	65
11252330	m	cn	US	2015	0	0	0	0	0	0	0	0	0	0	0	0	
11252333	f	cn	US	2015	0	0	0	0	0	0	0	0	0	0	0	0	52
11252341	m	cn	US	2015	0	0	0	0	0	0	0	0	0	0	0	0	52
11252348	f	md	US	2015	0	0	0	0	0	0	0	0	0	0	0	0	53
11252349	m	cn	US	2015	0	0	0	0	0	0	0	0	0	0	0	0	90
11252356	f	cn	US	2015	0	0	0	0	0	0	0	0	0	0	0	0	
11252360	f	cn	US	2015	0	0	0	0	0	0	0	0	0	0	0	0	71
11252367	f	cn	US	2015	0	0	0	0	0	0	0	0	0	0	0	0	
11252372	m	cn	US	2015	0	0	0	0	0	0	0	0	0	0	0	0	82
11252382	m	cn	US	2015	0	0	0	0	0	0	0	0	0	0	0	0	58
11252383	m	ot	US	2015	0	0	0	0	0	0	0	0	0	0	0	0	
11252439	m	cn	US	2015	0	0	0	0	0	0	0	0	0	0	0	0	61
11252440	m	cn	US	2015	0	0	0	0	0	0	0	0	0	0	0	0	74
11252441	m	cn	US	2015	0	0	0	0	0	0	0	0	0	0	0	0	
11252458	f	cn	US	2015	0	0	0	0	0	0	0	0	0	0	0	0	
11252466	f	cn	US	2015	0	0	0	0	0	0	0	0	0	0	0	0	53
11252473	m	ot	US	2015	0	0	0	0	0	0	0	0	0	0	0	0	
11252474	f	cn	US	2015	0	0	0	0	0	0	0	0	0	0	0	0	
11252500	f	cn	US	2015	0	0	0	0	0	0	0	0	0	0	0	0	
11252512	m	cn	US	2015	0	0	0	0	0	0	0	0	0	0	0	0	
11252678	f	cn	US	2015	0	0	0	0	0	0	0	0	0	0	0	0	
11252713	f	cn	US	2015	0	0	0	0	0	0	0	0	0	0	0	0	51
11252721	m	cn	US	2015	0	0	0	0	0	0	0	0	0	0	0	0	
11252908	f	ot	ES	2015	0	0	0	0	0	0	0	0	0	0	0	0	68
11254907	m	md	US	2015	0	0	0	0	0	0	0	0	0	0	0	0	
11254944	Not	ot	US	2015	0	0	0	0	0	0	0	0	0	0	0	0	
11255676	Not	cn	GB	2015	1	0	0	0	0	0	0	0	0	0	0	0	65
11256022	f	cn	US	2015	0	0	0	0	0	0	0	0	0	0	0	0	61
11256063	Not	ot	US	2015	0	0	0	0	0	0	0	0	0	0	0	0	

11257622	Not	md	US	2015	0	0	0	0	0	0	0	0	0	0	0	0	
11258174	Not	md	JP	2015	0	0	0	0	0	0	0	0	0	0	0	0	
11259014	f	ph	JP	2015	0	0	0	0	0	0	0	0	0	0	0	0	81
11259484	m	md	US	2015	0	1	1	0	0	0	0	0	0	0	0	0	79
11261322	f	cn	GB	2015	1	0	0	0	0	0	0	0	0	0	0	0	49
11261732	m	md	US	2015	0	0	0	0	0	0	0	0	0	0	0	0	77
11262080	f	cn	US	2015	0	0	0	0	0	0	0	0	0	0	0	0	
11262786	f	md	AR	2015	1	0	0	0	0	0	0	0	0	0	0	0	72
11263180	f	md	US	2015	1	0	0	0	0	0	0	0	0	0	0	0	
11264118	f	md	IT	2015	0	0	0	0	0	0	0	0	0	0	0	0	67
11265476	Not	cn	GB	2015	1	0	0	0	0	0	0	0	0	0	0	0	87
11265485	Not	cn	GB	2015	1	0	0	0	0	0	0	0	0	0	0	0	89
11267341	Not	md	DE	2015	0	0	0	0	0	0	0	0	0	0	0	0	
11269533	Not	Not repo	Not re	2015	1	0	0	0	0	0	0	0	0	0	0	1	
11269911	f	cn	GB	2015	1	0	0	0	0	0	0	0	0	0	0	0	60
11270518	m	md	JP	2015	0	0	0	0	0	0	0	0	0	0	0	0	83
11270555	f	ot	US	2015	0	0	0	0	0	0	0	0	0	0	0	0	67
11271045	Not	ot	Not re	2015	1	0	0	0	0	0	0	0	0	0	0	0	
11271205	f	md	Not re	2015	1	0	0	0	0	0	0	0	0	0	0	0	82
11271562	f	ot	US	2015	0	1	0	0	1	0	0	0	0	0	0	0	
11272181	Not	md	US	2015	0	0	0	0	0	0	0	0	0	0	0	0	
11272615	m	ot	US	2015	1	0	0	0	0	0	0	0	0	0	0	0	79
11272617	m	cn	CN	2015	1	0	0	0	0	0	0	0	0	0	0	0	62
11272632	f	cn	GB	2015	1	0	0	0	0	0	0	0	0	0	0	0	86
11272768	m	md	Not re	2015	0	0	0	0	0	0	0	0	0	0	0	0	77
11273133	f	cn	US	2015	0	0	0	0	0	0	1	0	1	0	0	0	70
11273262	Not	cn	GB	2015	1	0	0	0	0	0	0	0	0	0	0	0	68
11273854	f	md	JP	2015	0	0	0	0	0	0	0	0	0	0	0	0	50
11275371	Not	md	JP	2015	0	0	0	0	0	0	0	0	0	0	0	0	
11275375	m	md	JP	2015	0	0	0	0	0	0	0	0	0	0	0	0	
11275489	Not	md	JP	2015	0	0	0	0	0	0	0	0	0	0	0	0	
11276463	m	cn	GB	2015	1	0	0	0	0	0	0	0	0	0	0	0	
11276944	Not	cn	GB	2015	1	0	0	0	0	0	0	0	0	0	0	0	87
11277974	Not	cn	Not re	2015	0	0	0	0	0	0	0	0	0	0	0	0	

11278386	Not	md	KR	2015	0	0	0	0	0	0	0	0	0	0	0	0	
11278641	m	ph	JP	2015	0	0	0	0	0	0	0	0	0	0	0	0	61
11278782	m	cn	US	2015	0	1	0	0	1	0	0	0	0	0	0	0	
11279078	m	cn	FR	2015	0	0	0	0	0	0	0	0	0	0	0	0	
11279252	m	md	AU	2015	0	0	0	0	0	0	0	0	0	0	0	0	
11279775	Not	cn	GB	2015	1	0	0	0	0	0	0	0	0	0	0	0	88
11280068	m	cn	US	2015	0	1	0	0	1	0	0	0	0	0	0	0	61
11281584	f	ot	Not re	2015	1	0	0	0	0	0	0	0	0	0	0	0	43
11281798	Not	ot	US	2015	0	0	0	0	0	0	0	0	0	0	0	0	
11281855	m	ot	HK	2015	1	0	0	0	0	0	0	0	0	0	0	0	70
11282118	m	md	JP	2015	1	0	0	0	0	0	0	0	0	0	0	0	63
11283130	m	cn	US	2015	0	0	0	0	0	0	0	0	0	0	0	0	67
11283991	Not	cn	GB	2015	1	0	0	0	0	0	0	0	0	0	0	0	67
11283992	Not	cn	GB	2015	1	0	0	0	0	0	0	0	0	0	0	0	89
11283994	Not	cn	GB	2015	1	0	0	0	0	0	0	0	0	0	0	0	88
11284012	Not	cn	GB	2015	1	0	0	0	0	0	0	0	0	0	0	0	89
11284094	Not	cn	GB	2015	1	0	0	0	0	0	0	0	0	0	0	0	
11284841	f	md	TW	2015	1	0	0	0	0	0	0	0	0	0	0	1	93
11285162	Not	ot	DE	2015	1	0	0	0	0	0	0	0	0	0	0	0	
11286812	Not	cn	Not re	2015	0	0	0	0	0	0	0	0	0	0	0	0	
11287762	f	cn	GB	2015	1	0	0	0	0	0	0	0	0	0	0	0	63
11287939	m	md	TR	2015	0	0	0	0	0	0	0	0	0	0	0	0	36
11288079	f	md	ID	2015	1	0	0	0	0	0	0	0	0	0	0	0	
11288215	f	md	KR	2015	1	0	0	0	0	0	0	0	0	0	0	0	52
11289661	m	md	IN	2015	0	0	0	0	0	0	0	0	0	0	0	0	56
11290360	m	ph	US	2015	0	1	0	0	1	0	0	0	0	0	0	0	63
11291191	Not	cn	Not re	2015	0	0	0	0	0	0	0	0	0	0	0	0	
11291544	Not	cn	GB	2015	1	0	0	0	0	0	0	0	0	0	0	0	88
11291545	Not	cn	GB	2015	1	0	0	0	0	0	0	0	0	0	0	0	89
11291546	Not	cn	GB	2015	1	0	0	0	0	0	0	0	0	0	0	0	74
11291559	m	cn	GB	2015	1	0	0	0	0	0	0	0	0	0	0	0	
11291763	m	ph	JP	2015	0	0	0	0	0	0	0	0	0	0	0	0	71
11292185	Not	cn	GB	2015	1	0	0	0	0	0	0	0	0	0	0	0	87
11292314	m	cn	US	2015	0	1	0	0	1	0	0	0	0	0	0	0	

11292831	Not	cn	GB	2015	1	0	0	0	0	0	0	0	0	0	0	0	87
11292833	Not	cn	GB	2015	1	0	0	0	0	0	0	0	0	0	0	0	88
11292836	Not	cn	GB	2015	1	0	0	0	0	0	0	0	0	0	0	0	87
11294711	f	Not repo	US	2015	0	0	0	0	0	0	0	0	0	0	0	0	73.4
11294950	f	ot	CA	2015	0	0	0	0	0	0	0	0	0	0	0	0	50
11295018	f	md	US	2015	0	1	0	0	1	0	0	0	0	0	0	0	70
11302772	m	cn	GB	2015	1	0	0	0	0	0	0	0	0	0	0	0	53
11303230	f	md	US	2015	0	0	0	0	0	0	0	0	0	0	0	0	66
11305792	m	ot	US	2015	0	1	0	0	1	0	0	0	0	0	0	0	
11306448	Not	cn	GB	2015	1	0	0	0	0	0	0	0	0	0	0	0	88
11307170	f	md	Not re	2015	1	0	0	0	0	0	0	0	0	0	0	0	69
11308248	f	ot	ES	2015	0	1	0	0	1	0	0	0	0	0	0	0	
11308279	f	cn	Not re	2015	0	0	0	0	0	0	0	0	0	0	0	0	49
11308334	f	md	US	2015	0	0	0	0	0	0	0	0	0	0	0	0	
11309386	Not	md	FR	2015	0	1	0	0	1	0	0	0	0	0	0	0	48
11309387	Not	md	FR	2015	0	1	0	0	1	0	0	0	0	0	0	0	55
11309826	Not	md	FR	2015	0	1	0	0	1	0	0	0	0	0	0	0	60
11310386	f	ot	US	2015	0	0	0	0	0	0	0	0	0	0	0	0	77
11310580	f	ot	US	2015	0	0	0	0	0	0	0	0	0	0	0	0	
11310854	m	md	Not re	2015	0	0	0	0	0	0	0	0	0	0	0	0	72
11313479	m	cn	IN	2015	1	0	0	0	0	0	0	0	0	0	0	0	70
11314622	Not	ot	US	2015	0	0	0	0	0	0	0	0	0	0	0	0	
11315710	f	md	JP	2015	0	0	0	0	0	0	0	0	0	0	0	0	72
11315898	m	md	JP	2015	0	0	0	0	0	0	0	0	0	0	0	0	72
11316498	m	ph	JP	2015	1	0	0	0	0	0	0	0	0	0	0	0	78
11316841	f	ot	AU	2015	0	0	0	0	0	0	0	0	0	0	0	0	
11317283	Not	cn	GB	2015	1	0	0	0	0	0	0	0	0	0	0	0	78
11318089	m	cn	ES	2015	0	1	0	0	1	0	0	0	0	0	0	0	61
11318176	Not	cn	GB	2015	1	0	0	0	0	0	0	0	0	0	0	0	
11319248	f	md	JP	2015	0	0	0	0	0	0	0	0	0	0	0	0	62
11319450	f	cn	US	2015	0	1	0	0	1	0	0	0	0	0	0	0	57
11320354	m	cn	FR	2015	0	0	0	0	0	0	0	0	0	0	0	0	61
11321510	f	md	US	2015	0	0	0	0	0	0	0	0	0	0	0	0	51
11321815	m	cn	FR	2015	0	0	0	0	0	0	0	0	0	0	0	0	

11321869	m	ph	JP	2015	1	0	0	0	0	0	0	0	0	0	0	0	69
11322132	f	md	Not re	2015	1	0	0	0	0	0	0	0	0	0	0	0	
11322674	f	md	FR	2015	0	1	0	0	1	0	0	0	0	0	0	0	47
11323625	f	md	NL	2015	0	0	0	0	0	0	0	0	0	0	0	0	62
11324504	m	md	FR	2015	0	1	0	0	1	0	0	0	0	0	0	0	
11325049	f	md	FR	2015	0	0	0	0	0	0	0	0	0	0	0	0	76
11325070	f	ot	US	2015	1	0	0	0	0	0	0	0	0	0	0	0	
11325313	f	ot	Not re	2015	1	0	0	0	0	0	0	0	0	0	0	0	70
11325323	f	ot	Not re	2015	1	0	0	0	0	0	0	0	0	0	0	0	59
11326042	Not	md	US	2015	0	0	0	0	0	0	0	0	0	0	0	0	66
11326155	f	Not repo	US	2015	0	0	0	0	0	0	0	0	0	0	0	0	67
11327128	m	md	JP	2015	1	0	0	0	0	0	0	0	0	0	0	0	76
11327682	Not	cn	GB	2015	1	0	0	0	0	0	0	0	0	0	0	0	78
11327684	Not	cn	GB	2015	1	0	0	0	0	0	0	0	0	0	0	0	51
11327685	Not	cn	GB	2015	1	0	0	0	0	0	0	0	0	0	0	0	69
11327688	Not	cn	GB	2015	1	0	0	0	0	0	0	0	0	0	0	0	59
11327803	m	md	FR	2015	0	1	0	0	1	0	0	0	0	0	0	0	74
11327933	m	cn	FR	2015	0	1	0	0	1	0	0	0	0	0	0	0	64
11328132	m	md	FR	2015	0	1	0	0	1	0	0	0	0	0	0	0	68
11328210	m	ot	JP	2015	0	0	0	0	0	0	0	0	0	0	0	0	66
11328456	m	ot	Not re	2015	1	0	0	0	0	0	0	0	0	0	0	0	67
11328458	m	ot	Not re	2015	1	0	0	0	0	0	0	0	0	0	0	0	62
11328511	m	md	LB	2015	0	0	0	0	0	0	0	0	0	0	0	0	73
11328779	f	md	JP	2015	1	0	0	0	0	0	0	0	0	0	0	1	70
11329042	Not	cn	Not re	2015	0	0	0	0	0	0	0	0	0	0	0	0	
11331170	Not	cn	GB	2015	1	0	0	0	0	0	0	0	0	0	0	0	86
11331171	f	ot	US	2015	0	0	0	0	0	0	0	0	0	0	0	0	69
11331556	f	md	US	2015	0	1	0	0	1	0	0	0	0	0	0	0	
11331659	f	ot	US	2015	0	1	0	0	1	0	0	0	0	0	0	0	
11333898	m	cn	US	2015	0	0	0	0	0	0	0	0	0	0	0	0	
11334276	m	md	Not re	2015	0	0	0	0	0	0	0	0	0	0	0	0	62
11334403	f	md	US	2015	0	0	0	0	0	0	0	0	0	0	0	0	
11335146	f	cn	GB	2015	0	0	0	0	0	0	0	0	0	0	0	0	66
11335438	f	md	Not re	2015	0	0	0	0	0	0	0	0	0	0	0	0	63

11335677	m	md	JP	2015	0	0	0	0	0	0	0	0	0	0	0	0	55
11335726	f	md	LB	2015	0	0	0	0	0	0	0	0	0	0	0	0	75
11336668	m	cn	FR	2015	0	0	0	0	0	0	0	0	0	0	0	0	75
11336673	m	cn	JP	2015	0	0	0	0	0	0	0	0	0	0	0	0	65
11339518	Not	md	US	2015	1	0	0	0	0	0	0	0	0	0	0	0	
11340276	f	ot	GB	2015	0	0	0	0	0	0	0	0	0	0	0	0	66
11341162	m	md	AU	2015	0	1	0	0	1	0	0	0	0	0	0	0	66
11341596	m	md	FR	2015	0	0	0	0	0	0	0	0	0	0	0	0	
11341791	m	ph	US	2015	0	0	0	0	0	0	0	0	0	0	0	0	59
11341814	Not	cn	GB	2015	1	0	0	0	0	0	0	0	0	0	0	0	78
11341815	Not	cn	GB	2015	1	0	0	0	0	0	0	0	0	0	0	0	94
11341821	f	cn	GB	2015	1	0	0	0	0	0	0	0	0	0	0	0	69
11341826	Not	cn	GB	2015	1	0	0	0	0	0	0	0	0	0	0	0	74
11341837	Not	cn	GB	2015	1	0	0	0	0	0	0	0	0	0	0	0	62
11341838	Not	cn	GB	2015	1	0	0	0	0	0	0	0	0	0	0	0	77
11341843	Not	cn	GB	2015	1	0	0	0	0	0	0	0	0	0	0	0	67
11342794	m	md	JP	2015	1	0	0	0	0	0	0	0	0	0	0	0	44
11342822	m	md	BE	2015	0	0	0	0	0	0	0	0	0	0	0	0	63
11343094	m	md	CH	2015	0	1	0	0	1	0	0	0	0	0	0	0	59
11343705	f	ot	US	2015	0	0	0	0	0	0	0	0	0	0	0	0	44
11344072	f	md	Not re	2015	1	0	0	0	0	0	0	0	0	0	0	0	73
11344163	m	ot	CN	2015	1	0	0	0	0	0	0	0	0	0	0	0	57
11344241	Not	cn	GB	2015	1	0	0	0	0	0	0	0	0	0	0	0	87
11344262	Not	cn	GB	2015	1	0	0	0	0	0	0	0	0	0	0	0	60
11344733	m	md	LB	2015	0	0	0	0	0	0	0	0	0	0	0	0	52
11344910	m	md	FR	2015	0	1	0	0	1	0	0	0	0	0	0	0	57
11346326	m	ot	US	2015	0	0	0	0	0	0	0	0	0	0	0	0	64
11348875	f	md	FR	2015	0	0	0	0	0	0	0	0	0	0	0	0	59
11349063	f	ot	GB	2015	0	0	0	0	0	0	0	0	0	0	0	0	66
11349230	m	md	DE	2015	0	0	0	0	0	0	0	0	0	0	0	0	65
11349253	f	ot	US	2015	0	0	0	0	0	0	0	0	0	0	0	0	73
11349820	Not	cn	GB	2015	1	0	0	0	0	0	0	0	0	0	0	0	74
11350124	Not	cn	GB	2015	1	0	0	0	0	0	0	0	0	0	0	0	68
11350125	Not	cn	GB	2015	1	0	0	0	0	0	0	0	0	0	0	0	88

11350513	Not	cn	GB	2015	1	0	0	0	0	0	0	0	0	0	0	0	69
11355105	f	md	JP	2015	0	0	0	0	0	0	0	0	0	0	0	0	48
11355256	Not	md	JP	2015	1	0	0	0	0	0	0	0	0	0	0	0	
11356334	m	ot	KR	2015	0	0	0	0	0	0	0	0	0	0	0	0	67
11359059	m	cn	US	2015	0	1	0	0	1	0	0	0	0	0	0	0	56
11359063	f	ph	US	2015	0	1	0	0	1	0	0	0	0	0	0	0	66
11359916	f	cn	Not re	2015	0	0	0	0	0	0	0	0	0	0	0	0	
11360089	f	ot	GB	2015	0	0	0	0	0	0	0	0	0	0	0	0	66
11360739	m	cn	GB	2015	1	0	0	0	0	0	0	0	0	0	0	0	
11361125	Not	cn	GB	2015	1	0	0	0	0	0	0	0	0	0	0	0	71
11361220	m	md	LB	2015	0	0	0	0	0	0	0	0	0	0	0	0	52
11361857	m	md	CA	2015	0	0	0	0	0	0	0	0	0	0	0	0	62
11362264	m	cn	GB	2015	1	0	0	0	0	0	0	0	0	0	0	0	96
11362520	m	cn	GB	2015	1	0	0	0	0	0	0	0	0	0	0	0	
11362522	Not	cn	GB	2015	1	0	0	0	0	0	0	0	0	0	0	0	83
11362687	m	ot	US	2015	0	0	0	0	0	0	0	0	0	0	0	0	74
11362953	f	md	Not re	2015	1	0	0	0	0	0	0	0	0	0	0	0	52
11363933	f	cn	GB	2015	1	0	0	0	0	0	0	0	0	0	0	0	
11364161	m	ph	CA	2015	0	0	0	0	0	0	0	0	0	0	0	0	82
11364207	m	ph	FR	2015	0	0	0	0	0	0	0	0	0	0	0	0	78
11364479	Not	ot	GB	2015	0	0	0	0	0	0	0	0	0	0	0	0	
11364802	f	cn	GB	2015	0	0	0	0	0	0	0	0	0	0	0	0	
11364805	Not	cn	GB	2015	1	0	0	0	0	0	0	0	0	0	0	0	80
11364825	m	md	NL	2015	0	0	0	0	0	0	0	0	0	0	0	0	76
11364947	Not	cn	GB	2015	1	0	0	0	0	0	0	0	0	0	0	0	75
11365498	f	md	FR	2015	0	0	0	0	0	0	0	0	0	0	0	0	48
11365894	f	cn	US	2015	0	0	0	0	0	0	0	0	0	0	0	0	68
11369089	f	md	FR	2015	0	1	0	0	1	0	0	0	0	0	0	0	35
11370166	f	md	JP	2015	1	0	0	0	0	0	0	0	0	0	0	0	77
11370304	f	md	Not re	2015	1	0	0	0	0	0	0	0	0	0	0	0	54
11370665	f	cn	US	2015	0	0	0	0	0	0	0	0	0	0	0	0	65
11371450	m	cn	US	2015	0	1	0	0	1	0	0	0	0	0	0	0	
11371563	m	cn	GB	2015	1	0	0	0	0	0	0	0	0	0	0	0	
11371566	m	cn	GB	2015	1	0	0	0	0	0	0	0	0	0	0	0	

11371819	m	ot	ES	2015	0	1	0	0	1	0	0	0	0	0	0	0	77
11374388	m	md	JP	2015	0	0	0	0	0	0	0	0	0	0	0	0	
11374399	f	md	DE	2015	0	0	0	0	0	0	0	0	0	0	0	0	63
11374789	m	md	US	2015	0	1	0	0	1	0	0	0	0	0	0	0	67
11374962	Not	cn	GB	2015	1	0	0	0	0	0	0	0	0	0	0	0	53
11375291	f	md	KR	2015	1	0	0	0	0	0	0	0	0	0	0	0	56
11375353	Not	cn	GB	2015	1	0	0	0	0	0	0	0	0	0	0	0	79
11375402	f	cn	DE	2015	0	1	0	0	1	0	0	0	0	0	0	0	
11375793	f	md	KR	2015	1	0	0	0	0	0	0	0	0	0	0	0	87
11375826	f	cn	GB	2015	1	0	0	0	0	0	0	0	0	0	0	0	66
11376023	m	cn	GB	2015	1	0	0	0	0	0	0	0	0	0	0	0	95
11376156	f	ot	US	2015	0	1	0	0	1	0	0	0	0	0	0	0	83
11376398	m	cn	US	2015	0	1	0	0	1	0	0	0	0	0	0	0	
11376556	m	md	Not re	2015	1	0	0	0	0	0	0	0	0	0	0	1	70
11376711	Not	cn	GB	2015	1	0	0	0	0	0	0	0	0	0	0	0	78
11376713	Not	cn	GB	2015	1	0	0	0	0	0	0	0	0	0	0	0	68
11376714	Not	cn	GB	2015	1	0	0	0	0	0	0	0	0	0	0	0	81
11376717	Not	cn	GB	2015	1	0	0	0	0	0	0	0	0	0	0	0	69
11376722	Not	cn	GB	2015	1	0	0	0	0	0	0	0	0	0	0	0	81
11376723	Not	cn	GB	2015	1	0	0	0	0	0	0	0	0	0	0	0	80
11376724	Not	cn	GB	2015	1	0	0	0	0	0	0	0	0	0	0	0	79
11376725	Not	cn	GB	2015	1	0	0	0	0	0	0	0	0	0	0	0	71
11376730	Not	cn	GB	2015	1	0	0	0	0	0	0	0	0	0	0	0	78
11376768	m	ph	AU	2015	0	1	0	0	1	0	0	0	0	0	0	0	71
11380074	f	ph	FR	2015	0	0	0	0	0	0	0	0	0	0	0	0	
11380246	f	cn	US	2015	1	0	0	0	0	0	0	0	0	0	0	0	68
11381856	f	cn	GB	2015	1	0	0	0	0	0	0	0	0	0	0	0	
11382892	m	ot	RU	2015	0	0	0	0	0	0	0	0	0	0	0	0	63
11383097	f	cn	GB	2015	1	0	0	0	0	0	0	0	0	0	0	0	69
11383448	f	ot	US	2015	0	0	0	0	0	0	0	0	0	0	0	0	39
11383471	m	md	IT	2015	0	0	0	0	0	0	0	0	0	0	0	0	49
11383660	m	cn	IN	2015	0	0	0	0	0	0	0	0	0	0	0	0	67
11383918	m	cn	US	2015	0	1	0	0	1	0	0	0	0	0	0	0	
11383919	m	ot	US	2015	0	0	0	0	0	0	0	0	0	0	0	0	34

11385640	m	cn	US	2015	0	1	0	0	1	0	0	0	0	0	0	0	71
11387513	f	md	IL	2015	1	0	0	0	0	0	0	0	0	0	0	0	
11387692	m	cn	IN	2015	0	0	0	0	0	0	0	0	0	0	0	0	32
11387699	m	cn	IN	2015	0	0	0	0	0	0	0	0	0	0	0	0	33
11388758	Not	cn	GB	2015	1	0	0	0	0	0	0	0	0	0	0	0	81
11388759	Not	cn	GB	2015	1	0	0	0	0	0	0	0	0	0	0	0	73
11389049	f	md	Not re	2015	1	0	0	0	0	0	0	0	0	0	0	1	
11389141	m	ot	CH	2015	0	0	0	0	0	0	0	0	0	0	0	0	51
11389193	m	md	US	2015	0	0	0	0	0	0	0	0	0	0	0	0	57
11389988	f	cn	Not re	2015	0	0	0	0	0	0	0	0	0	0	0	0	
11390897	f	ot	GB	2015	0	0	0	0	0	0	0	0	0	0	0	0	66
11394921	f	cn	GB	2015	1	0	0	0	0	0	0	0	0	0	0	0	
11394946	Not	ot	AU	2015	0	0	0	0	0	0	0	0	0	0	0	0	
11395036	m	cn	GB	2015	1	0	0	0	0	0	0	0	0	0	0	0	
11396975	f	md	Not re	2015	1	0	0	0	0	0	0	0	0	0	0	0	63
11397135	m	ot	IT	2015	0	0	0	0	0	0	0	0	0	0	0	0	60
11397309	m	ot	IL	2015	0	1	0	0	1	0	0	0	0	0	0	1	
11398154	f	md	CH	2015	0	0	0	0	0	0	0	0	0	0	0	0	80
11399033	m	cn	GB	2015	1	0	0	0	0	0	0	0	0	0	0	0	63
11399040	f	cn	GB	2015	1	0	0	0	0	0	0	0	0	0	0	0	56
11399308	Not	md	BE	2015	0	0	0	0	0	0	0	0	0	0	0	0	
11399381	m	cn	GB	2015	1	0	0	0	0	0	0	0	0	0	0	0	
11399385	m	cn	GB	2015	1	0	0	0	0	0	0	0	0	0	0	0	91
11399493	m	ot	US	2015	0	0	0	0	0	0	0	0	0	0	0	0	73
11399624	Not	md	JP	2015	0	0	0	0	0	0	0	0	0	0	0	0	
11399627	Not	md	JP	2015	0	0	0	0	0	0	0	0	0	0	0	0	
11399629	Not	md	JP	2015	0	0	0	0	0	0	0	0	0	0	0	0	
11399708	m	ot	JP	2015	0	0	0	0	0	0	0	0	0	0	0	0	68
11399851	m	cn	GB	2015	1	0	0	0	0	0	0	0	0	0	0	0	84
11399895	m	ot	US	2015	0	0	0	0	0	0	0	0	0	0	0	0	74
11400770	m	cn	JP	2015	0	0	0	0	0	0	0	0	0	0	0	0	73
11401228	f	md	Not re	2015	1	0	0	0	0	0	0	0	0	0	0	0	85
11401322	f	md	US	2015	1	0	0	0	0	0	0	0	0	0	0	0	65
11401347	f	md	Not re	2015	1	0	0	0	0	0	0	0	0	0	0	0	62

11403986	m	md	JP	2015	0	0	0	0	0	0	0	0	0	0	0	0	80
11404002	f	md	AU	2015	0	0	0	0	0	0	0	0	0	0	0	0	76
11405015	f	md	US	2015	0	1	0	0	1	0	0	0	0	0	0	0	69
11406227	f	md	ES	2015	0	1	0	0	1	0	0	0	0	0	0	0	61
11406465	f	md	CA	2015	0	1	0	0	1	0	0	0	0	0	0	0	57
11406874	f	md	HU	2015	0	0	0	0	0	0	0	0	0	0	0	0	67
11407222	m	md	BE	2015	0	0	0	0	0	0	0	0	0	0	0	0	79
11408783	m	ot	Not re	2015	1	0	0	0	0	0	0	0	0	0	0	0	68
11409819	Not	ot	US	2015	0	1	0	0	1	0	0	0	0	0	0	0	
11409879	Not	cn	GB	2015	0	0	0	0	0	0	0	0	0	0	0	0	
11414261	m	ot	GB	2015	0	0	0	0	0	0	0	0	0	0	0	0	65
11414357	f	md	Not re	2015	1	0	0	0	0	0	0	0	0	0	0	0	79
11414362	f	md	GB	2015	1	0	0	0	0	0	0	0	0	0	0	1	
11415202	Not	cn	GB	2015	1	0	0	0	0	0	0	0	0	0	0	0	
11415504	f	cn	JP	2015	0	0	0	0	0	0	0	0	0	0	0	0	48
11415942	m	md	JP	2015	0	0	0	0	0	0	0	0	0	0	0	0	75
11417059	f	ph	TH	2015	1	0	0	0	0	0	0	0	0	0	0	0	
11417345	m	md	Not re	2015	1	0	0	0	0	0	0	0	0	0	0	1	72
11417459	Not	md	US	2015	0	1	0	0	1	0	0	0	0	0	0	0	
11417755	m	cn	JP	2015	0	1	0	0	1	0	0	0	0	0	0	0	45
11418666	m	md	GB	2015	0	0	0	0	0	0	0	0	0	0	0	0	65
11418917	Not	md	US	2015	1	0	0	0	0	0	0	0	0	0	0	0	
11419512	f	cn	AR	2015	0	0	0	0	0	0	0	0	0	0	0	0	60
11419884	f	md	JP	2015	1	0	0	0	0	0	0	0	0	0	0	0	60.1
11420043	m	md	JP	2015	1	0	0	0	0	0	0	0	0	0	0	0	58
11420307	m	md	JP	2015	1	0	0	0	0	0	0	0	0	0	0	1	69
11420988	Not	md	GB	2015	0	0	0	0	0	0	0	0	0	0	0	0	
11421929	f	md	US	2015	0	1	0	0	1	0	0	0	0	0	0	0	
11422118	Not	md	JP	2015	0	0	0	0	0	0	0	0	0	0	0	0	
11422125	Not	md	JP	2015	0	0	0	0	0	0	0	0	0	0	0	0	
11422250	m	Not repo	GB	2015	0	0	0	0	0	0	0	0	0	0	0	0	65
11422264	f	cn	US	2015	0	1	0	0	1	0	0	0	0	0	0	0	
11422266	Not	md	US	2015	0	0	0	0	0	0	0	0	0	0	0	0	
11422288	Not	md	JP	2015	0	0	0	0	0	0	0	0	0	0	0	0	

11422635	f	cn	GB	2015	1	0	0	0	0	0	0	0	0	0	0	0	
11422637	f	cn	GB	2015	0	0	0	0	0	0	0	0	0	0	0	0	
11422924	m	md	US	2015	0	1	0	0	1	0	0	0	0	0	0	0	
11422952	m	cn	GB	2015	1	0	0	0	0	0	0	0	0	0	0	0	66
11423009	Not	md	MY	2015	0	0	0	0	0	0	0	0	0	0	0	0	
11424109	f	md	JP	2015	1	0	0	0	0	0	0	0	0	0	0	1	75.3
11424247	f	md	FR	2015	1	0	0	0	0	0	0	0	0	0	0	0	53.4
11427697	Not	md	US	2015	0	0	0	0	0	0	0	0	0	0	0	0	
11427711	Not	md	US	2015	0	0	0	0	0	0	0	0	0	0	0	0	
11427821	m	md	FR	2015	0	1	0	0	1	0	0	0	0	0	0	0	71
11427875	Not	Not repo	JP	2015	0	0	0	0	0	0	0	0	0	0	0	0	
11427881	m	md	US	2015	0	0	0	0	0	0	0	0	0	0	0	0	79
11431561	f	cn	US	2015	0	0	0	0	0	0	0	0	0	0	0	0	66
11432221	m	md	FR	2015	0	0	0	0	0	0	0	0	0	0	0	0	73
11432378	Not	ot	AT	2015	1	0	0	0	0	0	0	0	0	0	0	0	
11433267	Not	md	DE	2015	1	0	0	0	0	0	0	0	0	0	0	0	
11434442	m	ph	US	2015	0	0	0	0	0	0	0	0	0	0	0	0	59
11435073	f	md	Not re	2015	1	0	0	0	0	0	0	0	0	0	0	0	
11435080	f	cn	GB	2015	1	0	0	0	0	0	0	0	0	0	0	0	80
11436155	f	ot	US	2015	0	1	0	0	1	0	0	0	0	0	0	0	
11439208	m	ot	AU	2015	0	0	0	0	0	0	0	0	0	0	0	0	77
11440828	f	cn	IT	2015	0	0	0	0	0	0	0	0	0	0	0	0	49
11442047	Not	md	US	2015	1	0	0	0	0	0	0	0	0	0	0	0	
11442687	f	ot	JP	2015	0	0	0	0	0	0	0	0	0	0	0	0	73
11442903	m	ot	US	2015	0	1	0	0	1	0	0	0	0	0	0	1	76
11443247	m	md	NL	2015	0	0	0	0	0	0	0	0	0	0	0	0	76
11444893	m	md	FR	2015	0	0	0	0	0	0	0	0	0	0	0	0	65
11445615	f	cn	GB	2015	0	0	0	0	0	0	0	0	0	0	0	0	
11445651	Not	md	US	2015	1	0	0	0	0	0	0	0	0	0	0	0	
11446367	f	cn	GB	2015	1	0	0	0	0	0	0	0	0	0	0	0	52
11449154	m	md	Not re	2015	1	0	0	0	0	0	0	0	0	0	0	0	62
11449322	m	cn	US	2015	0	1	0	0	1	0	0	0	0	0	0	0	81
11449412	f	md	FR	2015	0	1	0	0	1	0	0	0	0	0	0	0	51
11452062	m	cn	GB	2015	1	0	0	0	0	0	0	0	0	0	0	0	

11455266	f	cn	US	2015	1	0	0	0	0	0	0	0	0	0	0	0	
11455881	m	ot	BR	2015	0	0	0	0	0	0	0	0	0	0	0	0	32
11455906	f	md	IN	2015	1	0	0	0	0	0	0	0	0	0	0	0	
11457074	Not	ot	US	2015	0	1	0	0	1	0	0	0	0	0	0	0	
11457728	m	ph	US	2015	0	1	0	0	1	0	0	0	0	0	0	0	76
11458029	m	md	Not re	2015	0	0	0	0	0	0	0	0	0	0	0	0	65
11458145	f	md	JP	2015	0	0	0	0	0	0	0	0	0	0	0	0	75
11458159	f	ot	US	2015	0	0	0	0	0	0	0	0	0	0	0	0	60
11458666	Not	md	IT	2015	0	0	0	0	0	0	0	0	0	0	0	0	
11459050	f	ot	US	2015	0	1	0	0	1	0	0	0	0	0	0	0	
11459912	f	ot	IT	2015	0	0	0	0	0	0	0	0	0	0	0	0	55
11459949	f	cn	GB	2015	1	0	0	0	0	0	0	0	0	0	0	0	84
11460946	Not	md	US	2015	0	0	0	0	0	0	0	0	0	0	0	0	
11461047	Not	ot	IT	2015	0	1	0	0	1	0	0	0	0	0	0	0	
11463034	m	md	CZ	2015	0	1	0	0	1	0	0	0	0	0	0	0	83
11464667	f	md	US	2015	0	1	0	0	1	0	0	0	0	0	0	0	
11464847	f	md	FR	2015	0	1	0	0	1	0	0	0	0	0	0	0	
11465125	m	cn	US	2015	0	1	0	0	1	0	0	0	0	0	0	0	
11465968	m	cn	CY	2015	0	1	1	0	0	0	0	0	0	0	0	0	
11466385	f	cn	US	2015	0	1	0	0	1	0	0	0	0	0	0	0	71
11466741	m	ph	AU	2015	0	1	1	0	0	0	0	0	0	0	0	0	
11466935	m	md	JP	2015	0	0	0	0	0	0	0	0	0	0	0	0	64
11467166	f	ot	US	2015	0	0	0	0	0	0	0	0	0	0	0	0	
11467829	m	md	JP	2015	0	0	0	0	0	0	0	0	0	0	0	0	75
11468069	f	cn	US	2015	0	0	0	0	0	0	0	0	0	0	0	0	
11471819	f	md	Not re	2015	0	0	0	0	0	0	0	0	0	0	0	0	
11471820	f	md	ID	2015	1	0	0	0	0	0	0	0	0	0	0	0	80
11472504	f	cn	GB	2015	1	0	0	0	0	0	0	0	0	0	0	0	82
11472860	Not	ot	Not re	2015	1	0	0	0	0	0	0	0	0	0	0	0	
11473699	f	md	FR	2015	1	0	0	0	0	0	0	0	0	0	0	0	58
11476553	f	md	FI	2015	0	1	0	0	1	0	0	0	0	0	0	0	55
11480035	m	ot	NL	2015	0	1	0	0	1	0	0	0	0	0	0	0	75
11480200	m	md	FR	2015	0	1	0	0	1	0	0	0	0	0	0	0	67
11480720	m	cn	US	2015	0	1	0	0	1	0	0	0	0	0	0	0	51

11480935	f	md	JP	2015	0	0	0	0	0	0	0	0	0	0	0	0	65
11482317	m	ot	JP	2015	1	0	0	0	0	0	0	0	0	0	0	0	59
11485264	f	md	IT	2015	0	1	0	0	1	0	0	0	0	0	0	0	73
11485289	f	md	US	2015	0	1	0	0	1	0	0	0	0	0	0	0	
11486959	f	ph	JP	2015	1	0	0	0	0	0	0	0	0	0	0	0	58
11489460	m	md	US	2015	1	0	0	0	0	0	0	0	0	0	0	0	
11489479	f	cn	GB	2015	1	0	0	0	0	0	0	0	0	0	0	0	73
11489929	f	md	TW	2015	1	0	0	0	0	0	0	0	0	0	0	0	57.8
11491673	m	md	CN	2015	1	0	0	0	0	0	0	0	0	0	0	0	69.3
11491801	m	md	JP	2015	0	0	0	0	0	0	0	0	0	0	0	0	70
11492155	f	ot	JP	2015	0	0	0	0	0	0	0	0	0	0	0	0	
11492380	f	md	Not re	2015	1	0	0	0	0	0	0	0	0	0	0	0	
11492923	m	md	JP	2015	0	0	0	0	0	0	0	0	0	0	0	0	57
11492938	Not	md	JP	2015	0	0	0	0	0	0	0	0	0	0	0	0	
11492943	Not	md	JP	2015	0	0	0	0	0	0	0	0	0	0	0	0	
11492944	Not	md	JP	2015	0	0	0	0	0	0	0	0	0	0	0	0	
11493450	f	ph	US	2015	0	1	0	0	1	0	0	0	0	0	0	0	
11494753	Not	cn	GB	2015	1	0	0	0	0	0	0	0	0	0	0	0	
11496404	f	md	US	2015	0	0	0	0	0	0	0	0	0	0	0	0	62
11497235	m	ot	JP	2015	0	0	0	0	0	0	0	0	0	0	0	0	72
11497440	m	ph	US	2015	0	1	0	0	1	0	0	0	0	0	0	0	58
11497687	f	md	FR	2015	0	1	0	0	1	0	0	0	0	0	0	0	57
11497945	m	ot	US	2015	0	1	0	0	1	0	0	0	0	0	0	0	63
11498054	m	ot	US	2015	0	1	0	0	1	0	0	0	0	0	0	0	70
11498378	m	md	BE	2015	0	0	0	0	0	0	0	0	0	0	0	0	57
11500037	f	md	Not re	2015	1	0	0	0	0	0	0	0	0	0	0	0	74
11501692	f	md	RU	2015	0	0	0	0	0	0	0	0	0	0	0	0	43
11501790	f	md	JP	2015	1	0	0	0	0	0	0	0	0	0	0	0	74.2
11501969	m	md	IT	2015	0	0	0	0	0	0	0	0	0	0	0	0	60
11501977	m	md	IE	2015	0	0	0	0	0	0	0	0	0	0	0	0	65
11505626	m	cn	US	2015	1	0	0	0	0	0	0	0	0	0	0	0	63
11506728	m	ot	IT	2015	0	0	0	0	0	0	0	0	0	0	0	0	60
11511218	m	md	DE	2015	0	0	0	0	0	0	0	0	0	0	0	0	78
11511931	f	ot	CA	2015	0	0	0	0	0	0	0	0	0	0	0	0	42

11511932	m	cn	IN	2015	0	0	0	0	0	0	0	0	0	0	0	0	60
11512856	m	md	Not re	2015	1	0	0	0	0	0	0	0	0	0	0	0	86
11513991	m	cn	FR	2015	0	0	0	0	0	0	0	0	0	0	0	0	79
11514010	m	cn	CA	2015	0	0	0	0	0	0	0	0	0	0	0	0	76
11516167	f	ot	US	2015	0	1	0	0	1	0	0	0	0	0	0	0	
11516188	f	ph	GB	2015	0	0	0	0	0	0	0	0	0	0	0	0	68
11516566	f	cn	GB	2015	0	0	0	0	0	0	0	0	0	0	0	0	68
11516662	m	ot	JP	2015	0	0	0	0	0	0	0	0	0	0	0	0	67
11517207	f	md	AT	2015	0	1	0	0	1	0	0	0	0	0	0	0	71
11517241	m	cn	GB	2015	1	0	0	0	0	0	0	0	0	0	0	0	58
11518052	m	md	Not re	2015	1	0	0	0	0	0	0	0	0	0	0	0	69
11518353	m	cn	GB	2015	1	0	0	0	0	0	0	0	0	0	0	0	63
11519911	m	md	FR	2015	0	1	0	0	1	0	0	0	0	0	0	0	58
11520821	m	ph	US	2015	0	0	0	0	0	0	0	0	0	0	0	0	
11525491	f	md	AU	2015	0	1	0	0	1	0	0	0	0	0	0	0	
11525501	m	md	AU	2015	0	1	0	0	1	0	0	0	0	0	0	0	
11525636	m	ot	KR	2015	0	1	0	0	1	0	0	0	0	0	0	0	70
11525739	m	ot	US	2015	0	1	0	0	1	0	0	0	0	0	0	0	75
11526030	f	md	CH	2015	0	1	0	0	1	0	0	0	0	0	0	0	63
11526247	m	md	US	2015	0	1	0	0	1	0	0	0	0	0	0	0	
11527311	Not	ot	CN	2015	0	0	0	0	0	0	0	0	0	0	0	0	
11527312	Not	ot	CN	2015	0	0	0	0	0	0	0	0	0	0	0	0	
11527313	Not	ot	CN	2015	0	0	0	0	0	0	0	0	0	0	0	0	
11527830	m	md	IT	2015	0	0	0	0	0	0	0	0	0	0	0	0	60
11528412	Not	cn	US	2015	0	1	0	0	1	0	0	0	0	0	0	0	
11530985	m	ot	US	2015	0	1	0	0	1	0	0	0	0	0	0	0	75
11532586	f	cn	GB	2015	1	0	0	0	0	0	0	0	0	0	0	0	
11532688	m	ot	US	2015	0	1	0	0	1	0	0	0	0	0	0	1	75
11533717	m	ot	US	2015	0	1	0	0	1	0	0	0	0	0	0	0	
11533975	f	ph	JP	2015	0	0	0	0	0	0	0	0	0	0	0	0	72
11534098	m	md	IT	2015	0	0	0	0	0	0	0	0	0	0	0	0	60
11534516	m	md	JP	2015	0	0	0	0	0	0	0	0	0	0	0	0	57
11537044	f	ot	US	2015	0	0	0	0	0	0	0	0	0	0	0	0	78
11537656	m	cn	US	2015	0	0	0	0	0	0	0	0	0	0	0	0	

11537748	m	ot	IT	2015	0	0	0	0	0	0	0	0	0	0	0	0	60
11537949	f	cn	US	2015	0	0	0	0	0	0	0	0	0	0	0	0	
11539253	f	ph	US	2015	0	0	0	0	0	0	0	0	0	0	0	0	77
11541045	m	cn	US	2015	0	0	0	0	0	0	0	0	0	0	0	0	
11541058	Not	md	JP	2015	1	0	0	0	0	0	0	0	0	0	0	0	
11541576	Not	md	JP	2015	1	0	0	0	0	0	0	0	0	0	0	0	
11542684	m	cn	GB	2015	1	0	0	0	0	0	0	0	0	0	0	0	59
11543023	f	cn	US	2015	0	0	0	0	0	0	0	0	0	0	0	0	
11543453	f	ot	US	2015	1	0	0	0	0	0	0	0	0	0	0	0	
11544134	m	md	DE	2015	1	0	0	0	0	0	0	0	0	0	0	0	74
11544505	m	md	IN	2015	0	0	0	0	0	0	0	0	0	0	0	0	59
11544687	f	cn	US	2015	0	1	0	0	1	0	0	0	0	0	0	0	
11545336	Not	cn	US	2015	0	0	0	0	0	0	0	0	0	0	0	0	
11548116	m	ot	US	2015	1	0	0	0	0	0	0	0	0	0	0	0	
11548617	f	md	US	2015	1	0	0	0	0	0	0	0	0	0	0	0	
11550088	f	md	Not re	2015	0	0	0	0	0	0	0	0	0	0	0	0	55
11551727	m	md	DE	2015	0	0	0	0	0	0	0	0	0	0	0	0	36
11552453	f	ph	US	2015	0	1	0	0	1	0	0	0	0	0	0	0	
11553742	m	md	DE	2015	0	0	0	0	0	0	0	0	0	0	0	0	56
11554359	m	cn	JP	2015	0	1	0	0	1	0	0	0	0	0	0	0	66
11554748	m	md	JP	2015	0	0	0	0	0	0	0	0	0	0	0	0	73
11554767	f	md	BE	2015	0	0	0	0	0	0	0	0	0	0	0	0	58
11555450	m	md	IT	2015	0	1	0	0	1	0	0	0	0	0	0	0	72
11556831	f	md	JP	2015	1	0	0	0	0	0	0	0	0	0	0	0	60
11557463	Not	ot	US	2015	0	1	0	0	1	0	0	0	0	0	0	0	85
11557919	m	ot	US	2015	0	1	0	0	1	0	0	0	0	0	0	0	
11558604	f	ot	US	2015	0	0	0	0	0	0	0	0	0	0	0	0	72
11561447	f	cn	AU	2015	1	0	0	0	0	0	0	0	0	0	0	0	75
11561452	Not	md	Not re	2015	1	0	0	0	0	0	0	0	0	0	0	0	
11561615	m	cn	GB	2015	1	0	0	0	0	0	0	0	0	0	0	0	74
11562769	m	ph	JP	2015	0	0	0	0	0	0	0	0	0	0	0	0	69
11563225	m	ot	JP	2015	1	0	0	0	0	0	0	0	0	0	0	0	70
11569019	Not	ot	CN	2015	0	0	0	0	0	0	0	0	0	0	0	0	
11569023	Not	ot	CN	2015	0	0	0	0	0	0	0	0	0	0	0	0	

11569025	Not	ot	CN	2015	0	0	0	0	0	0	0	0	0	0	0	0	
11570290	Not	ot	US	2015	0	0	0	0	0	0	0	0	0	0	0	0	
11573098	m	ot	US	2015	0	1	0	0	1	0	0	0	0	0	0	0	75
11573771	f	md	HU	2015	0	0	0	0	0	0	0	0	0	0	0	0	57
11577950	m	md	ES	2015	0	0	0	0	0	0	0	0	0	0	0	0	74
11578116	m	md	FR	2015	0	1	0	0	1	0	0	0	0	0	0	0	86
7160020	m	ot	SG	2015	1	0	0	0	0	0	0	0	0	0	0	0	47
8175559	f	ot	US	2015	0	0	0	0	0	0	0	0	0	0	0	0	70
8735019	m	cn	IE	2015	0	0	0	0	0	0	0	0	0	0	0	0	67
8770249	m	ot	KR	2015	0	0	0	0	0	0	0	0	0	0	0	0	52
8827524	m	md	US	2015	0	0	0	0	0	0	0	0	0	0	0	0	76
8922609	m	md	JP	2015	0	0	0	0	0	0	0	0	0	0	0	0	72
8931716	m	cn	GB	2015	1	0	0	0	0	0	0	0	0	0	0	0	
8952925	f	md	US	2015	0	0	0	0	0	0	0	0	0	0	0	0	54
8986482	m	md	US	2015	0	0	0	0	0	0	0	0	0	0	0	0	61
9055137	f	cn	US	2015	1	0	0	0	0	0	0	0	0	0	0	0	
9156849	m	ot	JP	2015	0	0	0	0	0	0	0	0	0	0	0	0	71
9188706	Not	cn	GB	2015	1	0	0	0	0	0	0	0	0	0	0	0	
9308178	f	md	JP	2015	0	0	0	0	0	0	0	0	0	0	0	0	64
9308447	f	cn	IE	2015	0	0	0	0	0	0	0	0	0	0	0	0	55
9311948	m	cn	GB	2015	1	0	0	0	0	0	0	0	0	0	0	0	66
9346794	Not	md	GR	2015	0	0	0	0	0	0	0	0	0	0	0	0	
9450514	f	cn	GB	2015	1	0	0	0	0	0	0	0	0	0	0	0	65
9511968	m	md	JP	2015	0	0	0	0	0	0	0	0	0	0	0	0	64
9580892	f	md	JP	2015	0	0	0	0	0	0	0	0	0	0	0	0	66
9681282	m	md	JP	2015	0	0	0	0	0	0	0	0	0	0	0	0	70
9687923	m	md	JP	2015	0	0	0	0	0	0	0	0	0	0	0	0	56
9723174	f	md	JP	2015	0	0	0	0	0	0	0	0	0	0	0	0	63
9764235	m	ot	JP	2015	0	0	0	0	0	0	0	0	0	0	0	0	61
9804891	m	cn	US	2015	0	0	0	0	0	0	0	0	0	0	0	0	59
9838577	m	ot	RO	2015	0	0	0	0	0	0	0	0	0	0	0	0	71
9863992	m	ph	JP	2015	0	0	0	0	0	0	0	0	0	0	0	0	43
9882837	f	md	Not re	2015	1	0	0	0	0	0	0	0	0	0	0	0	80
9901635	m	md	US	2015	0	0	0	0	0	0	0	0	0	0	0	0	73

9904667	m	md	US	2015	0	0	0	0	0	0	0	0	0	0	0	0	57
9909120	f	md	JP	2015	0	0	0	0	0	0	0	0	0	0	0	0	84
9915254	f	md	DE	2015	0	0	0	0	0	0	0	0	0	0	0	0	57
9924022	m	ot	DE	2015	0	0	0	0	0	0	0	0	0	0	0	0	70
9937603	f	md	JP	2015	0	0	0	0	0	0	0	0	0	0	0	0	47
9980822	m	md	DE	2015	0	0	0	0	0	0	0	0	0	0	0	0	56
9981099	m	cn	GB	2015	1	0	0	0	0	0	0	0	0	0	0	0	76
10004833	f	md	JP	2015	0	0	0	0	0	0	0	0	0	0	0	0	50
10015493	f	md	JP	2015	0	0	0	0	0	0	0	0	0	0	0	0	65
10027319	f	cn	GB	2015	1	0	0	0	0	0	0	0	0	0	0	0	72
10150324	f	md	Not re	2015	1	0	0	0	0	0	0	0	0	0	0	0	72
10151781	f	md	JP	2015	0	0	0	0	0	0	0	0	0	0	0	0	75
10185075	f	md	Not re	2015	1	0	0	0	0	0	0	0	0	0	0	0	57
10191218	f	md	JP	2015	0	0	0	0	0	0	0	0	0	0	0	0	46
10235422	m	md	JP	2015	0	0	0	0	0	0	0	0	0	0	0	0	71
10300382	m	md	NL	2015	0	0	0	0	0	0	0	0	0	0	0	0	
10301170	m	md	JP	2015	0	0	0	0	0	0	0	0	0	0	0	0	75
10347722	Not	cn	GB	2015	1	0	0	0	0	0	0	0	0	0	0	0	
10395885	m	md	JP	2015	0	0	0	0	0	0	0	0	0	0	0	0	72
10406293	f	cn	GB	2015	1	0	0	0	0	0	0	0	0	0	0	0	69
10495367	f	md	Not re	2015	1	0	0	0	0	0	0	0	0	0	0	0	73
10534259	f	md	JP	2015	0	0	0	0	0	0	0	0	0	0	0	0	58
10534682	f	ot	US	2015	0	1	1	0	0	0	0	0	0	0	0	0	51
10571220	m	md	CH	2015	0	0	0	0	0	0	0	0	0	0	0	0	62
10652468	f	cn	GB	2015	1	0	0	0	0	0	0	0	0	0	0	0	76
10653611	Not	md	RU	2015	0	0	0	0	0	0	0	0	0	0	0	0	
10665465	f	ph	US	2015	0	0	0	0	0	0	0	0	0	0	0	0	
10668926	f	md	US	2015	1	0	0	0	0	0	0	0	0	0	0	0	76
10731349	m	md	Not re	2015	0	0	0	0	0	0	0	0	0	0	0	0	61
10815322	f	md	Not re	2015	1	0	0	0	0	0	0	0	0	0	0	0	67
10883539	m	ot	GB	2015	0	0	0	0	0	0	0	0	0	0	0	0	47
10903234	f	ot	US	2015	0	1	0	0	1	0	0	0	0	0	0	0	
10973790	m	md	CN	2015	0	0	0	0	0	0	0	0	0	0	0	0	37
11030550	f	cn	US	2015	0	0	0	0	0	0	0	0	0	0	0	0	69

11071060	m	cn	US	2015	0	1	0	0	1	0	0	0	0	0	0	0	67
11074890	m	ot	IT	2015	1	0	0	0	0	0	0	0	0	0	0	0	63
11087487	f	md	Not re	2015	1	0	0	0	0	0	0	0	0	0	0	0	68
11088146	f	cn	IE	2015	0	0	0	0	0	0	0	0	0	0	0	0	61
11095000	m	md	Not re	2015	1	0	0	0	0	0	0	0	0	0	0	0	82
11095962	m	md	US	2015	0	0	0	0	0	0	0	0	0	0	0	0	73
11105180	f	ph	FR	2015	0	0	0	0	0	0	0	0	0	0	0	0	59
11111837	f	md	JP	2015	0	0	0	0	0	0	0	0	0	0	0	0	72
11167597	f	md	JP	2015	0	0	0	0	0	0	0	0	0	0	0	0	52
11193373	f	ot	CN	2015	0	0	0	0	0	0	0	0	0	0	0	0	63
11203304	f	ot	US	2015	0	1	0	0	1	0	0	0	0	0	0	0	65
11223085	f	md	Not re	2015	1	0	0	0	0	0	0	0	0	0	0	0	70
11228024	f	md	TH	2015	0	0	0	0	0	0	0	0	0	0	0	0	60
11237723	m	md	JP	2015	0	0	0	0	0	0	0	0	0	0	0	0	67
11242924	f	ot	US	2015	0	1	0	0	1	0	0	0	0	0	0	0	71
11243611	f	md	Not re	2015	1	0	0	0	0	0	0	0	0	0	0	0	
11243792	f	md	Not re	2015	1	0	0	0	0	0	0	0	0	0	0	0	60
11263660	Not	ot	JP	2015	0	0	0	0	0	0	0	0	0	0	0	0	
11267121	Not	ot	US	2015	0	0	0	0	0	0	0	0	0	0	0	0	
11267144	f	md	Not re	2015	1	0	0	0	0	0	0	0	0	0	0	0	
11284047	m	md	JP	2015	0	0	0	0	0	0	0	0	0	0	0	0	68
11293081	m	md	CN	2015	0	0	0	0	0	0	0	0	0	0	0	0	69
11309120	f	md	FR	2015	0	1	0	0	1	0	0	0	0	0	0	0	
11309246	m	md	FR	2015	0	1	0	0	1	0	0	0	0	0	0	0	58
11310405	f	md	JP	2015	1	0	0	0	0	0	0	0	0	0	0	0	80
11321867	f	md	Not re	2015	1	0	0	0	0	0	0	0	0	0	0	0	
11329043	m	cn	Not re	2015	0	0	0	0	0	0	0	0	0	0	0	0	
11339435	f	md	FR	2015	0	1	0	0	1	0	0	0	0	0	0	0	62
11339615	m	md	JP	2015	0	0	0	0	0	0	0	0	0	0	0	0	82
11341246	m	md	FR	2015	0	1	0	0	1	0	0	0	0	0	0	0	57
11343082	m	md	FR	2015	0	1	0	0	1	0	0	0	0	0	0	0	63
11345395	m	ot	BE	2015	0	1	0	0	1	0	0	0	0	0	0	0	64
11345996	m	md	FR	2015	0	0	0	0	0	0	0	0	0	0	0	0	64
11350254	m	md	JP	2015	0	0	0	0	0	0	0	0	0	0	0	0	73

11356513	f	md	FR	2015	0	1	0	0	1	0	0	0	0	0	0	0	59
11359322	m	md	FR	2015	0	0	0	0	0	0	0	0	0	0	0	0	79
11360128	m	ot	US	2015	0	1	0	0	1	0	0	0	0	0	0	0	60
11360842	f	cn	AR	2015	0	0	0	0	0	0	0	0	0	0	0	0	48
11371311	m	cn	US	2015	0	0	0	0	0	0	0	0	0	0	0	0	
11383824	Not	md	JP	2015	0	0	0	0	0	0	0	0	0	0	0	0	
11383861	f	ot	JP	2015	1	0	0	0	0	0	0	0	0	0	0	0	77
11388959	f	md	JP	2015	0	0	0	0	0	0	0	0	0	0	0	0	71
11394715	m	md	LB	2015	0	0	0	0	0	0	0	0	0	0	0	0	83
11397232	m	md	FR	2015	0	1	0	0	1	0	0	0	0	0	0	0	68
11400999	m	md	JP	2015	0	0	0	0	0	0	0	0	0	0	0	0	55
11405522	m	md	CA	2015	0	1	0	0	1	0	0	0	0	0	0	0	
11405565	m	md	US	2015	0	0	0	0	0	0	0	0	0	0	0	0	61
11406396	f	md	FR	2015	0	1	0	0	1	0	0	0	0	0	0	0	61
11421646	m	cn	IT	2015	0	1	0	0	1	0	0	0	0	0	0	0	63
11421963	f	md	JP	2015	0	0	0	0	0	0	0	0	0	0	0	0	61
11424531	f	md	Not re	2015	0	0	0	0	0	0	0	0	0	0	0	0	60
11424616	f	md	Not re	2015	1	0	0	0	0	0	0	0	0	0	0	0	84
11429100	m	ot	CH	2015	0	1	0	0	1	0	0	0	0	0	0	0	77
11431565	m	md	JP	2015	0	0	0	0	0	0	0	0	0	0	0	0	69
11431945	f	ot	US	2015	0	0	0	0	0	0	0	0	0	0	0	0	68
11434041	m	md	JP	2015	0	0	0	0	0	0	0	0	0	0	0	0	74
11435294	m	md	FR	2015	0	1	0	0	1	0	0	0	0	0	0	0	64
11436771	f	md	Not re	2015	1	0	0	0	0	0	0	0	0	0	0	0	79
11440564	m	md	FR	2015	0	1	0	0	1	0	0	0	0	0	0	0	62
11441952	Not	md	JP	2015	0	0	0	0	0	0	0	0	0	0	0	0	
11441968	f	ot	SG	2015	0	0	0	0	0	0	0	0	0	0	0	0	70
11442900	f	md	JP	2015	1	0	0	0	0	0	0	0	0	0	0	1	
11454556	m	md	JP	2015	0	0	0	0	0	0	0	0	0	0	0	0	77
11455264	m	md	US	2015	1	0	0	0	0	0	0	0	0	0	0	0	57
11458825	f	md	AU	2015	0	1	0	0	1	0	0	0	0	0	0	0	
11459710	m	ot	DE	2015	0	0	0	0	0	0	0	0	0	0	0	0	64
11459934	m	cn	US	2015	0	1	0	0	1	0	0	0	0	0	0	0	58
11460785	f	md	CH	2015	0	1	0	0	1	0	0	0	0	0	0	0	66

11464875	m	cn	IT	2015	0	1	0	0	1	0	0	0	0	0	0	0	
11466776	m	md	BE	2015	0	1	0	0	1	0	0	0	0	0	0	0	58
11467096	f	md	US	2015	0	0	0	0	0	0	0	0	0	0	0	0	74
11468085	m	md	US	2015	1	0	0	0	0	0	0	0	0	0	0	0	84
11469239	m	ot	US	2015	0	1	0	0	1	0	0	0	0	0	0	0	59
11480685	m	cn	GB	2015	1	0	0	0	0	0	0	0	0	0	0	0	66
11481382	f	ph	US	2015	1	0	0	0	0	0	0	0	0	0	0	0	69
11481606	m	md	DE	2015	0	1	0	0	1	0	0	0	0	0	0	0	75
11481790	m	ot	ES	2015	0	1	0	0	1	0	0	0	0	0	0	0	66
11485326	f	md	NL	2015	0	0	0	0	0	0	0	0	0	0	0	0	59
11486030	m	cn	US	2015	0	1	0	0	1	0	0	0	0	0	0	0	69
11497198	m	md	JP	2015	0	0	0	0	0	0	0	0	0	0	0	0	85
11497891	f	md	FR	2015	0	1	0	0	1	0	0	0	0	0	0	0	68
11500216	f	md	JP	2015	0	0	0	0	0	0	0	0	0	0	0	0	54
11503949	f	md	KR	2015	0	0	0	0	0	0	0	0	0	0	0	0	67
11504040	m	md	JP	2015	0	0	0	0	0	0	0	0	0	0	0	0	51
11504520	m	md	JP	2015	0	0	0	0	0	0	0	0	0	0	0	0	77
11506805	f	cn	GB	2015	1	0	0	0	0	0	0	0	0	0	0	0	53
11507511	m	ot	US	2015	0	0	0	0	0	0	0	0	0	0	0	0	63
11510799	m	md	JP	2015	0	0	0	0	0	0	0	0	0	0	0	0	78
11512106	m	cn	IT	2015	0	1	0	0	1	0	0	0	0	0	0	0	68
11512645	f	md	JP	2015	0	0	0	0	0	0	0	0	0	0	0	0	64
11515437	f	ot	US	2015	0	1	0	0	1	0	0	0	0	0	0	1	68
11517186	m	ot	NL	2015	0	1	0	0	1	0	0	0	0	0	0	0	62
11518068	m	cn	US	2015	1	0	0	0	0	0	0	0	0	0	0	0	89
11518566	m	md	JP	2015	0	1	0	0	1	0	0	0	0	0	0	0	71
11518905	m	md	Not re	2015	1	0	0	0	0	0	0	0	0	0	0	1	67
11526038	m	md	FR	2015	0	1	0	0	1	0	0	0	0	0	0	0	60
11533726	m	ot	BE	2015	0	1	0	0	1	0	0	0	0	0	0	0	60
11533979	f	cn	AR	2015	0	0	0	0	0	0	0	0	0	0	0	0	71
11534514	m	cn	DE	2015	0	0	0	0	0	0	0	0	0	0	0	0	54
11537230	m	cn	GB	2015	1	0	0	0	0	0	0	0	0	0	0	0	67
11537796	m	ot	IT	2015	0	1	0	0	1	0	0	0	0	0	0	0	50
11537993	m	md	CH	2015	0	0	0	0	0	0	0	0	0	0	0	0	57

11538089	f	ot	AU	2015	0	1	0	0	1	0	0	0	0	0	0	0	75
11538727	m	md	JP	2015	0	0	0	0	0	0	0	0	0	0	0	0	70
11539005	f	cn	IT	2015	0	1	0	0	1	0	0	0	0	0	0	0	
11539833	f	cn	US	2015	0	0	0	0	0	0	0	0	0	0	0	0	31
11544415	f	cn	US	2015	0	0	0	0	0	0	0	0	0	0	0	0	58
11546230	m	ot	ES	2015	0	1	0	0	1	0	0	0	0	0	0	0	68
11553032	m	ot	IT	2015	0	1	0	0	1	0	0	0	0	0	0	0	
11553092	m	cn	GB	2015	1	0	0	0	0	0	0	0	0	0	0	0	77
11553421	f	cn	DE	2015	0	0	0	0	0	0	0	0	0	0	0	0	58
11555599	m	md	FR	2015	0	1	0	0	1	0	0	0	0	0	0	0	67
11564574	m	md	CH	2015	1	0	0	0	0	0	0	0	0	0	0	0	53
11567032	m	md	FR	2015	0	1	0	0	1	0	0	0	0	0	0	0	56
11567969	m	md	FR	2015	0	1	0	0	1	0	0	0	0	0	0	0	61
11568034	m	md	DE	2015	0	0	0	0	0	0	0	0	0	0	0	0	64
11572557	f	cn	FR	2015	0	1	0	0	1	0	0	0	0	0	0	0	
11573560	f	md	Not re	2015	1	0	0	0	0	0	0	0	0	0	0	0	58
11575381	Not	ot	JP	2015	1	0	0	0	0	0	0	0	0	0	0	0	
11576095	m	md	JP	2015	0	0	0	0	0	0	0	0	0	0	0	0	45
11576160	Not	ot	JP	2015	1	0	0	0	0	0	0	0	0	0	0	1	
11576467	m	md	Not re	2015	0	0	0	0	0	0	0	0	0	0	0	0	74
11580812	f	ot	IT	2015	0	1	0	0	1	0	0	0	0	0	0	1	51
11581253	m	ot	US	2015	0	0	0	0	0	0	0	0	0	0	0	0	46
11582825	m	ot	GB	2015	0	0	0	0	0	0	0	0	0	0	0	0	53
11583652	m	cn	GB	2015	0	0	0	0	0	0	0	0	0	0	0	0	53
11583673	f	cn	GB	2015	1	0	0	0	0	0	0	0	0	0	0	0	58
11584543	m	md	CA	2015	0	1	0	0	1	0	0	0	0	0	0	0	
11584860	Not	cn	GB	2015	1	0	0	0	0	0	0	0	0	0	0	0	
11584889	m	md	US	2015	0	1	1	0	0	0	0	0	0	0	0	0	74
11585443	Not	ot	US	2015	0	0	0	0	0	0	0	0	0	0	0	0	
11585616	m	ot	GB	2015	0	0	0	0	0	0	0	0	0	0	0	0	53
11586637	f	ot	US	2015	0	0	0	0	0	0	0	0	0	0	0	0	56
11586816	f	ph	US	2015	0	0	0	0	0	0	0	0	0	0	0	0	
11587894	f	md	DE	2015	0	1	0	0	1	0	0	0	0	0	0	0	55
11588113	m	md	ES	2015	0	0	0	0	0	0	0	0	0	0	0	0	64

11588170	f	md	DE	2015	0	0	0	0	0	0	0	0	0	0	0	0	51
11588392	m	cn	CH	2015	0	1	0	0	1	0	0	0	0	0	0	0	59
11588612	f	md	CA	2015	0	1	0	0	1	0	0	0	0	0	0	1	59
11588642	m	md	DE	2015	0	1	0	0	1	0	0	0	0	0	0	0	66
11588946	m	cn	US	2015	0	0	0	0	0	0	0	0	0	0	0	0	61
11589185	m	md	FR	2015	0	1	0	0	1	0	0	0	0	0	0	0	68
11590886	m	md	JP	2015	1	0	0	0	0	0	0	0	0	0	0	0	72.6
11590983	Not	ot	IT	2015	0	0	0	0	0	0	0	0	0	0	0	0	
11591374	m	md	GB	2015	0	0	0	0	0	0	0	0	0	0	0	0	53
11591930	m	ot	GB	2015	0	0	0	0	0	0	0	0	0	0	0	0	53
11592886	m	md	Not re	2015	0	0	0	0	0	0	0	0	0	0	0	0	76
11593119	Not	cn	GB	2015	1	0	0	0	0	0	0	0	0	0	0	0	
11593183	f	md	Not re	2015	1	0	0	0	0	0	0	0	0	0	0	0	76
11594073	Not	cn	GB	2015	1	0	0	0	0	0	0	0	0	0	0	1	
11594080	Not	cn	GB	2015	1	0	0	0	0	0	0	0	0	0	0	0	
11594205	Not	ot	GB	2015	0	0	0	0	0	0	0	0	0	0	0	0	
11594360	Not	ot	JP	2015	0	0	0	0	0	0	0	0	0	0	0	0	
11594363	Not	cn	GB	2015	1	0	0	0	0	0	0	0	0	0	0	0	
11594377	Not	ot	JP	2015	0	0	0	0	0	0	0	0	0	0	0	0	
11595080	f	ot	US	2015	0	0	0	0	0	0	0	0	0	0	0	0	58
11595652	m	ph	US	2015	0	0	0	0	0	0	0	0	0	0	0	0	73
11595743	m	ot	GB	2015	0	0	0	0	0	0	0	0	0	0	0	0	53
11595949	m	cn	US	2015	0	0	0	0	0	0	0	0	0	0	0	0	28
11596222	f	md	GB	2015	1	0	0	0	0	0	0	0	0	0	0	0	
11596228	f	md	CH	2015	1	0	0	0	0	0	0	0	0	0	0	0	73
11596233	m	md	TW	2015	1	0	0	0	0	0	0	0	0	0	0	0	63
11596653	m	md	JP	2015	1	0	0	0	0	0	0	0	0	0	0	0	77
11598241	m	cn	JP	2015	0	0	0	0	0	0	0	0	0	0	0	0	72
11599036	Not	ot	US	2015	0	1	0	0	1	0	0	0	0	0	0	0	
11603178	f	md	AU	2015	0	1	0	0	1	0	0	0	0	0	0	0	78
11603270	f	md	FR	2015	0	1	0	0	1	0	0	0	0	0	0	0	58
11603709	f	md	FR	2015	1	0	0	0	0	0	0	0	0	0	0	0	53
11603751	m	ot	US	2015	0	0	0	0	0	0	0	0	0	0	0	0	76
11603992	Not	ot	NL	2015	0	0	0	0	0	0	0	0	0	0	0	0	

11603995	Not	ot	NL	2015	0	0	0	0	0	0	0	0	0	0	0	0	
11605068	f	cn	US	2015	0	0	0	0	0	0	0	0	0	0	0	0	49
11605833	f	md	US	2015	0	0	0	0	0	0	0	0	0	0	0	0	74
11606219	f	md	Not re	2015	1	0	0	0	0	0	0	0	0	0	0	0	71
11607238	m	md	JP	2015	0	0	0	0	0	0	0	0	0	0	0	0	48
11607461	m	md	FR	2015	1	0	0	0	0	0	0	0	0	0	0	0	57
11607959	Not	md	US	2015	0	1	0	0	1	0	0	0	0	0	0	0	
11608222	Not	md	US	2015	0	1	0	0	1	0	0	0	0	0	0	0	
11608521	m	cn	US	2015	0	1	0	0	1	0	0	0	0	0	0	0	73
11608642	f	ot	US	2015	0	1	0	0	1	0	0	0	0	0	0	0	65
11609242	m	md	JP	2015	0	0	0	0	0	0	0	0	0	0	0	0	56
11610910	m	md	GB	2015	1	0	0	0	0	0	0	0	0	0	0	0	58
11610989	f	cn	US	2015	0	0	0	0	0	0	0	0	0	0	0	0	38
11611159	f	md	JP	2015	0	0	0	0	0	0	0	0	0	0	0	0	48
11611501	m	cn	US	2015	0	0	0	0	0	0	0	0	0	0	0	0	
11611518	Not	ot	JP	2015	0	0	0	0	0	0	0	0	0	0	0	0	
11611525	Not	ot	JP	2015	0	0	0	0	0	0	0	0	0	0	0	0	
11611529	Not	ot	JP	2015	0	0	0	0	0	0	0	0	0	0	0	0	
11611532	Not	ot	JP	2015	0	0	0	0	0	0	0	0	0	0	0	0	
11611533	Not	ot	JP	2015	0	0	0	0	0	0	0	0	0	0	0	0	
11611534	Not	ot	JP	2015	0	0	0	0	0	0	0	0	0	0	0	0	
11611540	Not	ot	JP	2015	0	0	0	0	0	0	0	0	0	0	0	0	
11611551	Not	ot	JP	2015	0	0	0	0	0	0	0	0	0	0	0	0	
11611559	Not	ot	JP	2015	0	0	0	0	0	0	0	0	0	0	0	0	
11611915	m	ot	US	2015	0	0	0	0	0	0	0	0	0	0	0	0	64
11611937	Not	ot	US	2015	0	1	0	0	1	0	0	0	0	0	0	0	
11611994	m	cn	FR	2015	0	1	0	0	1	0	0	0	0	0	0	0	81
11612238	f	md	IT	2015	0	0	0	0	0	0	0	0	0	0	0	0	58
11613438	m	cn	US	2015	0	1	0	0	1	0	0	0	0	0	0	0	82
11613478	m	ot	FR	2015	0	1	0	0	1	0	0	0	0	0	0	0	54
11613686	m	md	FR	2015	1	0	0	0	0	0	0	0	0	0	0	0	
11613806	m	md	FR	2015	0	1	0	0	1	0	0	0	0	0	0	0	81
11614169	m	md	CA	2015	0	1	0	0	1	0	0	0	0	0	0	0	68
11614395	f	ph	FR	2015	0	1	0	0	1	0	0	0	0	0	0	0	75

11614625	f	md	JP	2015	0	0	0	0	0	0	0	0	0	0	0	0	51
11615385	m	cn	GB	2015	1	0	0	0	0	0	0	0	0	0	0	0	65
11615863	m	md	US	2015	0	0	0	0	0	0	0	0	0	0	0	0	67
11616267	f	ot	US	2015	0	0	0	0	0	0	0	0	0	0	0	0	59
11616557	m	md	NL	2015	0	1	0	0	1	0	0	0	0	0	0	0	67
11616983	f	ot	AU	2015	0	0	0	0	0	0	0	0	0	0	0	0	76
11617429	Not	cn	GB	2015	1	0	0	0	0	0	0	0	0	0	0	0	
11618291	f	md	US	2015	0	0	0	0	0	0	0	0	0	0	0	0	
11619481	m	md	IT	2015	0	1	0	0	1	0	0	0	0	0	0	0	69
11619593	m	md	FR	2015	1	0	0	0	0	0	0	0	0	0	0	0	67.2
11620404	f	ot	Not re	2015	1	0	0	0	0	0	0	0	0	0	0	0	68
11620428	f	md	JP	2015	0	0	0	0	0	0	0	0	0	0	0	0	85
11620521	m	cn	GB	2015	1	0	0	0	0	0	0	0	0	0	0	0	73
11620879	m	md	FR	2015	0	1	0	0	1	0	0	0	0	0	0	0	61
11620904	f	ph	FR	2015	0	1	0	0	1	0	0	0	0	0	0	0	
11620915	m	md	GB	2015	1	0	0	0	0	0	0	0	0	0	0	0	48
11620960	m	md	US	2015	0	0	0	0	0	0	0	0	0	0	0	0	
11621098	m	ot	CH	2015	0	1	0	0	1	0	0	0	0	0	0	0	68
11621335	m	ot	US	2015	0	1	0	0	1	0	0	0	0	0	0	0	67
11621341	f	md	FR	2015	0	1	0	0	1	0	0	0	0	0	0	0	
11621664	Not	ot	JP	2015	1	0	0	0	0	0	0	0	0	0	0	0	
11624286	Not	ot	US	2015	0	0	0	0	0	0	0	0	0	0	0	0	
11625088	m	cn	GB	2015	1	0	0	0	0	0	0	0	0	0	0	0	69
11625403	f	md	FR	2015	0	0	0	0	0	0	0	0	0	0	0	0	77
11625572	Not	ot	NL	2015	0	0	0	0	0	0	0	0	0	0	0	0	
11625573	Not	ot	NL	2015	0	0	0	0	0	0	0	0	0	0	0	0	
11626179	f	md	BE	2015	0	0	0	0	0	0	0	0	0	0	0	0	48
11626541	m	md	Not re	2015	1	0	0	0	0	0	0	0	0	0	0	0	45
11626563	m	ot	US	2015	0	0	0	0	0	0	0	0	0	0	0	0	
11626795	m	ot	IT	2015	0	1	0	0	1	0	0	0	0	0	0	0	
11627158	f	md	US	2015	1	0	0	0	0	0	0	0	0	0	0	0	87
11627935	m	md	US	2015	0	0	0	0	0	0	0	0	0	0	0	0	71
11628017	Not	ph	US	2015	0	1	0	0	1	0	0	0	0	0	0	0	
11628041	Not	ot	US	2015	1	0	0	0	0	0	0	0	0	0	0	0	

11628454	m	md	JP	2015	1	0	0	0	0	0	0	0	0	0	0	1	83
11628834	m	md	Not re	2015	1	0	0	0	0	0	0	0	0	0	0	0	62
11629422	f	md	US	2015	0	1	0	0	1	0	0	0	0	0	0	0	73
11629450	f	md	Not re	2015	1	0	0	0	0	0	0	0	0	0	0	0	60
11629901	f	md	JP	2015	1	0	0	0	0	0	0	0	0	0	0	0	64
11630298	f	md	CH	2015	1	0	0	0	0	0	0	0	0	0	0	0	
11630448	f	ot	US	2015	0	1	0	0	1	0	0	0	0	0	0	0	73
11630684	m	ot	ES	2015	0	0	0	0	0	0	0	0	0	0	0	0	70
11631064	f	cn	US	2015	1	0	0	0	0	0	0	0	0	0	0	0	67
11631347	m	md	FR	2015	0	1	0	0	1	0	0	0	0	0	0	0	63
11631357	m	ot	KR	2015	0	0	0	0	0	0	0	0	0	0	0	0	45
11631492	m	cn	CH	2015	0	1	1	0	0	0	0	0	0	0	0	0	60
11631799	Not	md	CN	2015	0	0	0	0	0	0	0	0	0	0	0	0	
11632168	m	cn	US	2015	0	0	0	0	0	0	0	0	0	0	0	0	70
11632170	f	md	JP	2015	0	0	0	0	0	0	0	0	0	0	0	0	49
11632367	f	ot	US	2015	1	0	0	0	0	0	0	0	0	0	0	0	60
11632368	f	ot	US	2015	1	0	0	0	0	0	0	0	0	0	0	0	68
11632549	Not	ot	CN	2015	0	0	0	0	0	0	0	0	0	0	0	0	
11633505	m	cn	US	2015	0	0	0	0	0	0	0	0	0	0	0	0	49
11634436	f	md	US	2015	0	1	0	0	1	0	0	0	0	0	0	0	80
11634472	Not	cn	GB	2015	1	0	0	0	0	0	0	0	0	0	0	0	
11634536	f	md	FR	2015	0	1	0	0	1	0	0	0	0	0	0	0	70
11634544	Not	md	US	2015	0	1	0	0	1	0	0	0	0	0	0	1	
11634962	m	md	IT	2015	0	1	0	0	1	0	0	0	0	0	0	0	72
11636604	f	md	FR	2015	1	0	0	0	0	0	0	0	0	0	0	0	59
11636699	f	md	FR	2015	0	1	0	0	1	0	0	0	0	0	0	0	60
11638717	m	md	JP	2015	1	0	0	0	0	0	0	0	0	0	0	0	61.4
11638795	f	md	FR	2015	0	1	0	0	1	0	0	0	0	0	0	0	
11638824	m	md	FR	2015	0	0	0	0	0	0	0	0	0	0	0	0	63
11640099	f	cn	US	2015	0	0	0	0	0	0	0	0	0	0	0	0	70
11641308	m	cn	US	2015	1	0	0	0	0	0	0	0	0	0	0	0	
11641482	f	ot	KR	2015	0	0	0	0	0	0	0	0	0	0	0	0	57
11642062	m	md	GB	2015	0	1	0	0	1	0	0	0	0	0	0	0	86
11642129	m	md	CZ	2015	0	1	0	0	1	0	0	0	0	0	0	0	

11642484	m	ot	NL	2015	0	1	0	0	1	0	0	0	0	0	0	0	59
11643283	f	md	FR	2015	0	0	0	0	0	0	0	0	0	0	0	0	60
11643691	Not	md	CA	2015	0	0	0	0	0	0	0	0	0	0	0	0	
11644257	m	md	NZ	2015	0	0	0	0	0	0	0	0	0	0	0	0	52
11644559	m	md	TR	2015	0	1	0	0	1	0	0	0	0	0	0	0	56
11644825	m	ot	CA	2015	0	0	0	0	0	0	0	0	0	0	0	0	
11645524	m	ot	US	2015	0	1	0	0	1	0	0	0	0	0	0	0	60
11645663	f	ot	US	2015	0	0	0	0	0	0	0	0	0	0	0	0	72
11647846	m	ot	NL	2015	0	1	0	0	1	0	0	0	0	0	0	0	46
11647975	m	cn	GB	2015	1	0	0	0	0	0	0	0	0	0	0	1	84
11648232	f	md	US	2015	0	0	0	0	0	0	0	0	0	0	0	0	
11648605	f	md	US	2015	0	0	0	0	0	0	0	0	0	0	0	0	51
11649128	f	md	US	2015	0	1	0	0	1	0	0	0	0	0	0	0	
11649496	f	md	GB	2015	0	0	0	0	0	0	0	0	0	0	0	0	64
11649664	Not	ot	GB	2015	0	0	0	0	0	0	0	0	0	0	0	0	
11650357	m	md	AT	2015	0	1	0	0	1	0	0	0	0	0	0	0	70
11650481	m	md	TR	2015	0	1	0	0	1	0	0	0	0	0	0	0	50
11652368	f	md	FR	2015	0	1	0	0	1	0	0	0	0	0	0	0	69
11652742	f	ot	NL	2015	0	1	0	0	1	0	0	0	0	0	0	0	69
11652868	f	md	Not re	2015	1	0	0	0	0	0	0	0	0	0	0	0	
11653176	m	md	FR	2015	0	1	0	0	1	0	0	0	0	0	0	0	39
11653178	m	cn	FR	2015	0	1	0	0	1	0	0	0	0	0	0	0	75
11653718	m	md	Not re	2015	1	0	0	0	0	0	0	0	0	0	0	1	
11654135	f	cn	US	2015	0	0	0	0	0	0	0	0	0	0	0	0	
11654450	f	md	AT	2015	0	1	0	0	1	0	0	0	0	0	0	0	54
11654480	m	md	DE	2015	0	0	0	0	0	0	0	0	0	0	0	0	
11654550	m	md	US	2015	0	0	0	0	0	0	0	0	0	0	0	0	75
11654582	Not	ot	NL	2015	0	0	0	0	0	0	0	0	0	0	0	0	
11654596	Not	ot	NL	2015	0	0	0	0	0	0	0	0	0	0	0	0	
11655126	f	md	NO	2015	0	0	0	0	0	0	0	0	0	0	0	0	79
11655299	m	md	PT	2015	0	1	0	0	1	0	0	0	0	0	0	0	
11655377	f	ot	US	2015	0	0	0	0	0	0	0	0	0	0	0	0	
11655674	Not	md	US	2015	1	0	0	0	0	0	0	0	0	0	0	0	
11656969	m	cn	Not re	2015	0	1	0	0	1	0	0	0	0	0	0	0	

11657172	m	ot	CN	2015	0	0	0	0	0	0	0	0	0	0	0	0	45
11657851	m	md	Not re	2015	1	0	0	0	0	0	0	0	0	0	0	0	74
11658180	m	md	FR	2015	0	1	0	0	1	0	0	0	0	0	0	0	61
11658181	m	md	FR	2015	0	1	0	0	1	0	0	0	0	0	0	0	48
11658979	f	ph	US	2015	1	0	0	0	0	0	0	0	0	0	0	0	77
11659310	f	cn	US	2015	0	0	0	0	0	0	0	0	0	0	0	0	76
11660891	f	cn	CA	2015	0	0	0	0	0	0	0	0	0	0	0	0	
11661053	m	md	US	2015	0	1	0	0	1	0	0	0	0	0	0	0	74
11661234	f	ot	SK	2015	0	1	0	0	1	0	0	0	0	0	0	0	50
11661559	m	md	FR	2015	0	1	0	0	1	0	0	0	0	0	0	0	72
11662388	f	ot	US	2015	0	1	0	0	1	0	0	0	0	0	0	0	
11662843	m	cn	AR	2015	0	0	0	0	0	0	0	0	0	0	0	0	67
11663181	Not	md	US	2015	0	0	0	0	0	0	0	0	0	0	0	0	
11663184	f	ot	US	2015	0	0	0	0	0	0	0	0	0	0	0	0	70
11663187	Not	md	US	2015	0	0	0	0	0	0	0	0	0	0	0	0	
11663194	m	md	US	2015	0	0	0	0	0	0	0	0	0	0	0	0	
11663195	m	ot	US	2015	0	0	0	0	0	0	0	0	0	0	0	0	
11663201	f	ot	US	2015	0	0	0	0	0	0	0	0	0	0	0	0	
11663202	m	cn	US	2015	0	0	0	0	0	0	0	0	0	0	0	0	
11663203	Not	ph	US	2015	0	0	0	0	0	0	0	0	0	0	0	0	
11663211	f	md	US	2015	0	0	0	0	0	0	0	0	0	0	0	0	
11663213	f	ot	US	2015	0	0	0	0	0	0	0	0	0	0	0	0	60
11663236	m	md	US	2015	0	0	0	0	0	0	0	0	0	0	0	0	60
11665325	f	ot	DE	2015	0	0	0	0	0	0	0	0	0	0	0	0	65
11665463	f	md	DE	2015	0	0	0	0	0	0	0	0	0	0	0	0	70
11665804	f	md	US	2015	0	0	0	0	0	0	0	0	0	0	0	0	80
11668033	m	md	FR	2015	1	0	0	0	0	0	0	0	0	0	0	0	
11668136	m	md	US	2015	0	1	0	0	1	0	0	0	0	0	0	1	
11668375	m	ot	GR	2015	0	1	0	0	1	0	0	0	0	0	0	0	72
11669352	f	md	GB	2015	1	0	0	0	0	0	0	0	0	0	0	0	69
11669400	Not	md	US	2015	1	0	0	0	0	0	0	0	0	0	0	0	
11669807	f	md	ES	2015	1	0	0	0	0	0	0	0	0	0	0	0	66.8
11672562	m	cn	US	2015	0	0	0	0	0	0	0	0	0	0	0	0	50
11672983	f	cn	CN	2015	1	0	0	0	0	0	0	0	0	0	0	0	

11674102	f	ot	IT	2015	0	1	0	0	1	0	0	0	0	0	0	0	67
11674573	f	md	CH	2015	0	0	0	0	0	0	0	0	0	0	0	0	48
11675048	f	md	US	2015	0	0	0	0	0	0	0	0	0	0	0	0	
11675718	m	md	TH	2015	0	0	0	0	0	0	0	0	0	0	0	0	64
11677017	m	ot	GB	2015	0	0	0	0	0	0	0	0	0	0	0	0	50
11677513	m	ot	US	2015	0	0	0	0	0	0	0	0	0	0	0	0	49
11677880	m	md	US	2015	0	0	0	0	0	0	0	0	0	0	0	0	75
11678114	m	ot	NL	2015	0	1	0	0	1	0	0	0	0	0	0	0	69
11678435	m	md	Not re	2015	1	0	0	0	0	0	0	0	0	0	0	0	76
11681019	m	md	GB	2015	0	0	0	0	0	0	0	0	0	0	0	0	50
11682100	Not	ot	IT	2015	0	1	0	0	1	0	0	0	0	0	0	0	
11682370	m	cn	US	2015	0	0	0	0	0	0	0	0	0	0	0	0	88
11682415	m	md	US	2015	1	0	0	0	0	0	0	0	0	0	0	0	85
11682757	f	ot	MT	2015	0	0	0	0	0	0	0	0	0	0	0	0	
11682873	m	cn	US	2015	1	0	0	0	0	0	0	0	0	0	0	0	
11683131	f	md	FR	2015	0	0	0	0	0	0	0	0	0	0	0	0	64
11683828	f	md	BG	2015	1	0	0	0	0	0	0	0	0	0	0	0	
11684281	f	cn	CH	2015	0	0	0	0	0	0	0	0	0	0	0	0	46
11684318	f	cn	US	2015	1	0	0	0	0	0	0	0	0	0	0	0	
11684319	Not	md	US	2015	1	0	0	0	0	0	0	0	0	0	0	0	
11684331	f	md	NL	2015	0	0	0	0	0	0	0	0	0	0	0	0	62
11684387	m	md	BE	2015	0	0	0	0	0	0	0	0	0	0	0	0	39
11685376	f	md	CA	2015	0	1	0	0	1	0	0	0	0	0	0	0	
11685464	m	md	FR	2015	0	0	0	0	0	0	0	0	0	0	0	0	64
11685465	m	md	FR	2015	0	0	0	0	0	0	0	0	0	0	0	0	79
11685667	f	ot	US	2015	0	0	0	0	0	0	0	0	0	0	0	0	80
11686025	m	ot	IT	2015	0	1	0	0	1	0	0	0	0	0	0	0	67
11686191	m	cn	GB	2015	1	0	0	0	0	0	0	0	0	0	0	0	64
11686381	m	md	US	2015	1	0	0	0	0	0	0	0	0	0	0	0	49
11686529	m	cn	JP	2015	0	0	0	0	0	0	0	0	0	0	0	0	72
11686678	f	md	FR	2015	0	1	0	0	1	0	0	0	0	0	0	0	68
11686698	m	md	JP	2015	0	0	0	0	0	0	0	0	0	0	0	0	67
11686950	Not	md	IT	2015	0	0	0	0	0	0	0	0	0	0	0	0	70
11687515	m	md	US	2015	1	0	0	0	0	0	0	0	0	0	0	0	

11687606	m	md	Not re	2015	0	0	0	0	0	0	0	0	0	0	0	0	55
11687921	Not	ot	US	2015	0	0	0	0	0	0	0	0	0	0	0	0	55
11688088	m	md	DE	2015	0	1	0	0	1	0	0	0	0	0	0	1	72
11688134	f	md	FR	2015	1	0	0	0	0	0	0	0	0	0	0	0	
11688412	Not	ot	JP	2015	0	0	0	0	0	0	0	0	0	0	0	0	
11688909	m	ot	GB	2015	0	0	0	0	0	0	0	0	0	0	0	0	50
11689078	m	md	FR	2015	0	0	0	0	0	0	0	0	0	0	0	0	59
11689226	f	ot	US	2015	1	0	0	0	0	0	0	0	0	0	0	0	58
11690211	Not	cn	GB	2015	1	0	0	0	0	0	0	0	0	0	0	1	
11690212	Not	cn	GB	2015	1	0	0	0	0	0	0	0	0	0	0	0	
11690700	Not	ot	NL	2015	0	0	0	0	0	0	0	0	0	0	0	0	
11690879	f	cn	US	2015	0	0	0	0	0	0	0	0	0	0	0	0	
11690891	Not	md	DE	2015	0	0	0	0	0	0	0	0	0	0	0	0	
11690894	m	md	AU	2015	0	0	0	0	0	0	0	0	0	0	0	0	52
11690914	m	ot	IT	2015	0	1	0	0	1	0	0	0	0	0	0	0	52
11691161	m	md	US	2015	0	0	0	0	0	0	0	0	0	0	0	0	57
11691178	Not	ot	US	2015	1	0	0	0	0	0	0	0	0	0	0	0	
11692325	m	md	FR	2015	0	0	0	0	0	0	0	0	0	0	0	0	59
11692702	Not	ot	GB	2015	0	0	0	0	0	0	0	0	0	0	0	0	
11692740	f	Not repo	US	2015	1	0	0	0	0	0	0	0	0	0	0	0	76
11693154	f	md	US	2015	1	0	0	0	0	0	0	0	0	0	0	0	
11693263	m	cn	AR	2015	0	0	0	0	0	0	0	0	0	0	0	0	73
11694022	Not	ot	NL	2015	0	0	0	0	0	0	0	0	0	0	0	0	
11694023	Not	ot	NL	2015	0	0	0	0	0	0	0	0	0	0	0	0	
11694264	m	ot	US	2015	1	0	0	0	0	0	0	0	0	0	0	0	51
11694344	f	md	US	2015	0	1	0	0	1	0	0	0	0	0	0	0	
11694510	m	cn	US	2015	0	1	0	0	1	0	0	0	0	0	0	0	70
11695277	f	md	US	2015	0	1	0	0	1	0	0	0	0	0	0	0	90
11695973	m	ot	US	2015	1	0	0	0	0	0	0	0	0	0	0	0	
11696253	m	md	JP	2015	0	0	0	0	0	0	0	0	0	0	0	0	
11696259	f	md	JP	2015	0	0	0	0	0	0	0	0	0	0	0	0	73
11696278	f	cn	GB	2015	1	0	0	0	0	0	0	0	0	0	0	0	
11696304	f	md	GB	2015	0	0	0	0	0	0	0	0	0	0	0	0	35
11696368	f	md	FR	2015	1	0	0	0	0	0	0	0	0	0	0	0	76.6

11696374	m	cn	US	2015	0	0	0	0	0	0	0	0	0	0	0	0	36
11696485	f	ot	US	2015	1	0	0	0	0	0	0	0	0	0	0	0	
11696929	Not	ot	NL	2015	0	0	0	0	0	0	0	0	0	0	0	0	
11698412	m	md	FR	2015	0	1	0	0	1	0	0	0	0	0	0	0	82
11698442	Not	ot	NL	2015	0	0	0	0	0	0	0	0	0	0	0	0	
11698499	f	md	TR	2015	0	1	0	0	1	0	0	0	0	0	0	0	57
11698882	f	md	JP	2015	1	0	0	0	0	0	0	0	0	0	0	0	69
11698906	Not	ot	NL	2015	0	0	0	0	0	0	0	0	0	0	0	0	
11698910	Not	ot	NL	2015	0	0	0	0	0	0	0	0	0	0	0	0	
11699007	Not	ot	NL	2015	0	0	0	0	0	0	0	0	0	0	0	0	
11699112	f	md	CH	2015	0	1	0	0	1	0	0	0	0	0	0	0	66
11699136	f	md	AT	2015	1	0	0	0	0	0	0	0	0	0	0	0	51
11700124	f	ot	GB	2015	0	0	0	0	0	0	0	0	0	0	0	0	67
11700730	f	cn	US	2015	0	1	1	0	0	0	0	0	0	0	0	0	
11700879	m	cn	DE	2015	1	0	0	0	0	0	0	0	0	0	0	0	52
11700887	m	ot	IT	2015	0	1	0	0	1	0	0	0	0	0	0	0	74
11700929	m	md	LB	2015	0	1	0	0	1	0	0	0	0	0	0	0	
11701393	m	md	US	2015	0	1	0	0	1	0	0	0	0	0	0	0	70
11701860	f	ot	GB	2015	0	0	0	0	0	0	0	0	0	0	0	0	67
11702549	m	md	US	2015	0	0	0	0	0	0	0	0	0	0	0	0	70
11703056	m	ot	IL	2015	0	0	0	0	0	0	0	0	0	0	0	0	
11703816	f	md	GB	2015	0	0	0	0	0	0	0	0	0	0	0	0	67
11704137	Not	ot	KR	2015	1	0	0	0	0	0	0	0	0	0	0	0	
11704666	f	ot	AU	2015	0	0	0	0	0	0	0	0	0	0	0	0	58
11705063	m	md	DE	2015	0	0	0	0	0	0	0	0	0	0	0	0	52
11705148	m	cn	GB	2015	1	0	0	0	0	0	0	0	0	0	0	0	
11705495	m	cn	US	2015	0	1	0	0	1	0	0	0	0	0	0	0	72
11705761	f	md	FR	2015	1	0	0	0	0	0	0	0	0	0	0	0	
11707143	f	md	IT	2015	0	1	0	0	1	0	0	0	0	0	0	0	84
11707184	m	ot	US	2015	1	0	0	0	0	0	0	0	0	0	0	0	75
11707316	f	ot	IT	2015	0	0	0	0	0	0	0	0	0	0	0	0	61
11707375	f	md	CA	2015	0	1	0	0	1	0	0	0	0	0	0	0	64
11707917	Not	ot	KR	2015	0	0	0	0	0	0	0	0	0	0	0	0	
11707927	Not	ot	KR	2015	1	0	0	0	0	0	0	0	0	0	0	0	

11713277	m	ot	DE	2015	0	0	0	0	0	0	0	0	0	0	0	0	43
11713742	m	ot	US	2015	1	0	0	0	0	0	0	0	0	0	0	0	75
11713743	Not	ot	GB	2015	0	0	0	0	0	0	0	0	0	0	0	0	
11714766	m	md	TH	2015	0	0	0	0	0	0	0	0	0	0	0	0	68
11715117	m	md	FR	2015	0	1	0	0	1	0	0	0	0	0	0	0	
11715198	f	cn	AU	2015	0	0	0	0	0	0	0	0	0	0	0	0	61
11715203	Not	md	US	2015	0	1	0	0	1	0	0	0	0	0	0	1	
11715204	f	md	US	2015	0	1	0	0	1	0	0	0	0	0	0	0	
11715447	f	md	Not re	2015	1	0	0	0	0	0	0	0	0	0	0	0	59
11716171	f	md	Not re	2015	1	0	0	0	0	0	0	0	0	0	0	0	80
11716862	m	cn	US	2015	0	1	0	0	1	0	0	0	0	0	0	0	
11717136	Not	cn	US	2015	0	1	0	0	1	0	0	0	0	0	0	0	
11717355	f	ot	BE	2015	0	0	0	0	0	0	0	0	0	0	0	0	72
11717723	Not	md	US	2015	0	0	0	0	0	0	0	0	0	0	0	0	
11717736	Not	md	US	2015	0	0	0	0	0	0	0	0	0	0	0	0	
11718481	f	ph	US	2015	0	0	0	0	0	0	0	0	0	0	0	0	67
11718551	f	cn	GB	2015	0	0	0	0	0	0	0	0	0	0	0	0	75
11718829	f	ot	GB	2015	0	0	0	0	0	0	0	0	0	0	0	0	74
11718839	m	md	GB	2015	0	0	0	0	0	0	0	0	0	0	0	0	73
11718840	Not	md	CN	2015	0	0	0	0	0	0	0	0	0	0	0	0	
11719911	m	cn	GB	2015	1	0	0	0	0	0	0	0	0	0	0	0	56
11719913	f	cn	GB	2015	1	0	0	0	0	0	0	0	0	0	0	0	82
11719914	m	cn	GB	2015	1	0	0	0	0	0	0	0	0	0	0	0	81
11719916	m	cn	GB	2015	1	0	0	0	0	0	0	0	0	0	0	0	81
11719918	Not	cn	GB	2015	1	0	0	0	0	0	0	0	0	0	0	0	
11719930	m	cn	GB	2015	0	0	0	0	0	0	0	0	0	0	0	0	58
11720065	m	ph	GB	2015	0	0	0	0	0	0	0	0	0	0	0	0	45
11720230	f	cn	GB	2015	1	0	0	0	0	0	0	0	0	0	0	0	75
11720249	m	cn	GB	2015	1	0	0	0	0	0	0	0	0	0	0	0	76
11720251	m	cn	GB	2015	1	0	0	0	0	0	0	0	0	0	0	0	58
11720334	f	ot	US	2015	1	0	0	0	0	0	0	0	0	0	0	0	
11720684	f	md	Not re	2015	1	0	0	0	0	0	0	0	0	0	0	0	
11720754	f	md	US	2015	0	0	0	0	0	0	0	0	0	0	0	0	
11721264	f	md	US	2015	0	0	0	0	0	0	0	0	0	0	0	0	68

11721332	m	ot	US	2015	0	0	0	0	0	0	0	0	0	0	0	0	75
11722322	Not	md	US	2015	0	0	0	0	0	0	0	0	0	0	0	0	
11722323	Not	md	US	2015	0	0	0	0	0	0	0	0	0	0	0	0	
11722327	Not	md	US	2015	0	0	0	0	0	0	0	0	0	0	0	0	
11722347	Not	md	US	2015	0	0	0	0	0	0	0	0	0	0	0	0	
11722349	Not	md	US	2015	0	0	0	0	0	0	0	0	0	0	0	0	
11722360	f	md	US	2015	0	0	0	0	0	0	0	0	0	0	0	0	81
11722803	m	cn	GB	2015	1	0	0	0	0	0	0	0	0	0	0	0	76
11722808	m	cn	GB	2015	1	0	0	0	0	0	0	0	0	0	0	0	79
11722810	m	cn	GB	2015	1	0	0	0	0	0	0	0	0	0	0	0	77
11722815	f	cn	GB	2015	1	0	0	0	0	0	0	0	0	0	0	0	71
11722821	m	cn	GB	2015	1	0	0	0	0	0	0	0	0	0	0	0	80
11722822	f	cn	GB	2015	1	0	0	0	0	0	0	0	0	0	0	0	74
11722823	m	cn	GB	2015	1	0	0	0	0	0	0	0	0	0	0	0	67
11722825	m	cn	GB	2015	1	0	0	0	0	0	0	0	0	0	0	0	81
11722827	f	cn	GB	2015	1	0	0	0	0	0	0	0	0	0	0	0	74
11722831	m	cn	GB	2015	1	0	0	0	0	0	0	0	0	0	0	0	15
11722833	m	cn	GB	2015	1	0	0	0	0	0	0	0	0	0	0	0	81
11722836	f	cn	GB	2015	1	0	0	0	0	0	0	0	0	0	0	0	69
11723044	f	ph	JP	2015	1	0	0	0	0	0	0	0	0	0	0	0	92
11723124	f	ph	DE	2015	0	0	0	0	0	0	0	0	0	0	0	0	65
11723132	f	md	JP	2015	0	0	0	0	0	0	0	0	0	0	0	0	58
11723827	Not	ot	GB	2015	1	0	0	0	0	0	0	0	0	0	0	1	
11724149	m	cn	FR	2015	0	0	0	0	0	0	0	0	0	0	0	0	
11724404	m	md	Not re	2015	0	0	0	0	0	0	0	0	0	0	0	0	43
11726602	m	md	JP	2015	1	0	0	0	0	0	0	0	0	0	0	0	73
11726641	m	md	DE	2015	0	0	0	0	0	0	0	0	0	0	0	0	81
11726973	m	cn	GB	2015	1	0	0	0	0	0	0	0	0	0	0	0	64
11726976	m	cn	GB	2015	1	0	0	0	0	0	0	0	0	0	0	0	75
11726979	m	cn	GB	2015	1	0	0	0	0	0	0	0	0	0	0	0	80
11726981	f	cn	GB	2015	1	0	0	0	0	0	0	0	0	0	0	0	80
11726982	m	cn	GB	2015	1	0	0	0	0	0	0	0	0	0	0	0	
11726992	m	cn	GB	2015	1	0	0	0	0	0	0	0	0	0	0	0	56
11727504	f	md	IT	2015	0	0	0	0	0	0	0	0	0	0	0	0	63

11727530	m	md	JP	2015	1	0	0	0	0	0	0	0	0	0	0	0	69.8
11727534	m	ot	US	2015	0	1	0	0	1	0	0	0	0	0	0	1	76
11727967	f	ot	VN	2015	1	0	0	0	0	0	0	0	0	0	0	0	70
11728574	m	ot	IT	2015	0	1	0	0	1	0	0	0	0	0	0	0	73
11729227	m	md	US	2015	0	0	0	0	0	0	0	0	0	0	0	0	69
11731043	m	md	FR	2015	0	0	0	0	0	0	0	0	0	0	0	0	78
11733787	f	md	FR	2015	1	0	0	0	0	0	0	0	0	0	0	0	
11734051	m	md	JP	2015	1	0	0	0	0	0	0	0	0	0	0	0	62.9
11734095	f	ph	US	2015	0	0	0	0	0	0	0	0	0	0	0	0	
11734159	m	cn	JP	2015	0	0	0	0	0	0	0	0	0	0	0	0	62
11734631	m	ot	US	2015	1	0	0	0	0	0	0	0	0	0	0	0	75
11735461	Not	ot	NL	2015	1	0	0	0	0	0	0	0	0	0	0	0	
11735566	m	md	PL	2015	0	0	0	0	0	0	0	0	0	0	0	0	72
11736900	m	ot	US	2015	0	1	0	0	1	0	0	0	0	0	0	0	
11736965	f	cn	US	2015	0	1	0	0	1	0	0	0	0	0	0	0	
11736978	f	ot	AU	2015	0	1	0	0	1	0	0	0	0	0	0	0	68
11737931	m	ot	IT	2015	0	1	0	0	1	0	0	0	0	0	0	0	
11738518	f	md	FR	2015	1	0	0	0	0	0	0	0	0	0	0	0	72.2
11739901	m	ph	JP	2015	1	0	0	0	0	0	0	0	0	0	0	0	58
11739906	m	md	IL	2015	1	0	0	0	0	0	0	0	0	0	0	0	37
11740150	m	md	FR	2015	0	1	0	0	1	0	0	0	0	0	0	0	74
11740828	m	md	FR	2015	0	1	0	0	1	0	0	0	0	0	0	0	61
11740853	m	ot	NL	2015	0	1	0	0	1	0	0	0	0	0	0	0	65
11740862	m	ot	GR	2015	0	1	0	0	1	0	0	0	0	0	0	0	72
11740976	f	cn	PE	2015	0	0	0	0	0	0	0	0	0	0	0	0	62
11741035	f	md	ES	2015	0	0	0	0	0	0	0	0	0	0	0	0	36
11742278	f	ot	US	2015	1	0	0	0	0	0	0	0	0	0	0	0	58
11743285	m	ot	US	2015	0	1	0	0	1	0	0	0	0	0	0	1	
11743545	f	ot	IT	2015	0	1	0	0	1	0	0	0	0	0	0	0	78
11743748	m	ot	IT	2015	0	1	0	0	1	0	0	0	0	0	0	0	63
11744654	f	md	Not re	2015	1	0	0	0	0	0	0	0	0	0	0	0	66
11747200	m	md	SG	2015	0	1	0	0	1	0	0	0	0	0	0	0	57
11748051	m	cn	ID	2015	0	0	0	0	0	0	0	0	0	0	0	0	41
11748289	f	md	JP	2015	1	0	0	0	0	0	0	0	0	0	0	0	74.4

11748444	m	md	DE	2015	0	0	0	0	0	0	0	0	0	0	0	0	62
11748959	m	cn	FR	2015	0	0	0	0	0	0	0	0	0	0	0	0	65
11749912	m	md	GB	2015	0	0	0	0	0	0	0	0	0	0	0	0	
11750329	f	ot	US	2015	1	0	0	0	0	0	0	0	0	0	0	0	58
11750830	f	md	JP	2015	1	0	0	0	0	0	0	0	0	0	0	1	76
11751758	f	md	NL	2015	0	1	0	0	1	0	0	0	0	0	0	0	77
11751970	f	md	GB	2015	1	0	0	0	0	0	0	0	0	0	0	0	
11752290	m	ot	GB	2015	0	0	0	0	0	0	0	0	0	0	0	0	58
11752401	m	cn	US	2015	1	0	0	0	0	0	0	0	0	0	0	0	72
11752402	m	ot	US	2015	1	0	0	0	0	0	0	0	0	0	0	0	76
11752404	f	ot	US	2015	1	0	0	0	0	0	0	0	0	0	0	0	85
11752409	f	cn	US	2015	1	0	0	0	0	0	0	0	0	0	0	0	67
11752448	f	cn	US	2015	1	0	0	0	0	0	0	0	0	0	0	0	70
11752450	f	md	PL	2015	1	0	0	0	0	0	0	0	0	0	0	0	68
11753116	Not	md	US	2015	0	1	0	0	1	0	0	0	0	0	0	0	
11753607	m	ph	US	2015	0	0	0	0	0	0	1	0	1	0	0	0	32
11753792	Not	cn	GB	2015	0	0	0	0	0	0	0	0	0	0	0	0	
11753807	Not	cn	GB	2015	1	0	0	0	0	0	0	0	0	0	0	0	63
11753811	f	ph	US	2015	0	1	0	0	1	0	0	0	0	0	0	0	58
11753923	f	md	FR	2015	1	0	0	0	0	0	0	0	0	0	0	0	61
11754211	m	cn	GB	2015	1	0	0	0	0	0	0	0	0	0	0	0	65
11754212	m	cn	GB	2015	1	0	0	0	0	0	0	0	0	0	0	0	68
11754214	m	cn	GB	2015	1	0	0	0	0	0	0	0	0	0	0	0	75
11754216	m	cn	GB	2015	1	0	0	0	0	0	0	0	0	0	0	0	73
11754217	m	cn	GB	2015	1	0	0	0	0	0	0	0	0	0	0	0	80
11754229	f	cn	GB	2015	1	0	0	0	0	0	0	0	0	0	0	0	73
11754257	m	md	FR	2015	0	1	0	0	1	0	0	0	0	0	0	0	57
11754366	m	ph	US	2015	0	0	0	0	0	0	0	0	0	0	0	0	64
11754513	m	cn	GB	2015	1	0	0	0	0	0	0	0	0	0	0	0	69
11754516	f	cn	GB	2015	1	0	0	0	0	0	0	0	0	0	0	0	74
11754850	f	md	AU	2015	0	1	0	0	1	0	0	0	0	0	0	0	62
11755071	f	ph	FR	2015	1	0	0	0	0	0	0	0	0	0	0	0	76.5
11755208	f	cn	US	2015	0	0	0	0	0	0	0	0	0	0	0	0	58
11755241	f	ot	NL	2015	1	0	0	0	0	0	0	0	0	0	0	0	30

11755321	f	md	FR	2015	0	0	0	0	0	0	0	0	0	0	0	0	56
11755384	m	cn	US	2015	1	0	0	0	0	0	0	0	0	0	0	0	
11755438	Not	ot	KR	2015	0	0	0	0	0	0	0	0	0	0	0	0	
11755616	f	md	FR	2015	1	0	0	0	0	0	0	0	0	0	0	0	86
11757756	f	cn	US	2015	0	1	1	0	0	0	0	0	0	0	0	0	56
11758011	f	cn	US	2015	0	0	0	0	0	0	0	0	0	0	0	0	63
11758198	Not	ph	HK	2015	1	0	0	0	0	0	0	0	0	0	0	0	
11758236	m	cn	US	2015	0	0	0	0	0	0	0	0	0	0	0	0	55
11758373	Not	md	US	2015	0	0	0	0	0	0	0	0	0	0	0	0	
11758447	m	ot	PL	2015	0	0	0	0	0	0	0	0	0	0	0	0	66
11758595	m	ot	GB	2015	0	0	0	0	0	0	0	0	0	0	0	0	58
11760067	f	Not repo	IT	2015	0	0	0	0	0	0	0	0	0	0	0	0	63
11760130	m	md	JP	2015	1	0	0	0	0	0	0	0	0	0	0	0	67
11760196	m	md	IT	2015	0	1	0	0	1	0	0	0	0	0	0	0	82
11760679	m	ot	US	2015	1	0	0	0	0	0	0	0	0	0	0	0	75
11760726	m	ot	US	2015	0	1	0	0	1	0	0	0	0	0	0	1	52
11760782	f	cn	US	2015	1	0	0	0	0	0	0	0	0	0	0	0	69
11760897	f	cn	IT	2015	0	1	0	0	1	0	0	0	0	0	0	0	
11761434	m	cn	US	2015	0	0	0	0	0	0	0	0	0	0	0	0	
11761700	m	md	IT	2015	0	0	0	0	0	0	0	0	0	0	0	0	79
11764949	f	md	GB	2015	0	1	0	0	1	0	0	0	0	0	0	0	
11766027	Not	cn	Not re	2015	0	0	0	0	0	0	0	0	0	0	0	0	
11766136	m	md	KR	2015	0	0	0	0	0	0	0	0	0	0	0	0	
11766646	m	cn	GB	2015	0	0	0	0	0	0	0	0	0	0	0	0	58
11767009	f	ot	US	2015	0	1	0	0	1	0	0	0	0	0	0	0	
11767768	m	md	FR	2015	0	0	0	0	0	0	0	0	0	0	0	0	
11767817	m	md	FR	2015	0	0	0	0	0	0	0	0	0	0	0	0	
11768708	m	md	FR	2015	0	1	0	0	1	0	0	0	0	0	0	0	73
11769300	f	md	IL	2015	1	0	0	0	0	0	0	0	0	0	0	0	
11769325	m	cn	GB	2015	1	0	0	0	0	0	0	0	0	0	0	0	63
11769337	m	ot	US	2015	1	0	0	0	0	0	0	0	0	0	0	0	78
11769365	f	ot	US	2015	1	0	0	0	0	0	0	0	0	0	0	0	
11769493	f	md	FR	2015	0	1	0	0	1	0	0	0	0	0	0	0	
11769626	m	md	Not re	2015	1	0	0	0	0	0	0	0	0	0	0	0	87

11770650	f	md	Not re	2015	1	0	0	0	0	0	0	0	0	0	0	0	58
11770819	f	cn	US	2015	0	1	0	0	1	0	0	0	0	0	0	0	
11771328	Not	md	NL	2015	0	0	0	0	0	0	0	0	0	0	0	0	
11771329	Not	md	NL	2015	0	0	0	0	0	0	0	0	0	0	0	0	
11771348	Not	md	NL	2015	0	0	0	0	0	0	0	0	0	0	0	0	
11771349	Not	md	NL	2015	0	0	0	0	0	0	0	0	0	0	0	0	
11771351	Not	md	NL	2015	0	0	0	0	0	0	0	0	0	0	0	0	
11771357	Not	md	NL	2015	0	0	0	0	0	0	0	0	0	0	0	0	
11771358	Not	md	NL	2015	0	0	0	0	0	0	0	0	0	0	0	0	
11771361	Not	md	NL	2015	0	0	0	0	0	0	0	0	0	0	0	0	
11771369	Not	md	NL	2015	0	0	0	0	0	0	0	0	0	0	0	0	
11771372	Not	md	NL	2015	0	0	0	0	0	0	0	0	0	0	0	0	
11771373	Not	md	NL	2015	0	0	0	0	0	0	0	0	0	0	0	0	
11771641	f	md	US	2015	0	0	0	0	0	0	0	0	0	0	0	0	76
11771670	f	cn	GB	2015	1	0	0	0	0	0	0	0	0	0	0	0	58
11771723	f	Not repo	US	2015	0	1	0	0	1	0	0	0	0	0	0	0	57
11772574	m	md	LB	2015	0	0	0	0	0	0	0	0	0	0	0	0	63
11772733	Not	ph	US	2015	0	1	0	0	1	0	0	0	0	0	0	1	
11772947	f	ot	DE	2015	0	0	0	0	0	0	0	0	0	0	0	0	50
11773036	f	md	US	2015	1	0	0	0	0	0	0	0	0	0	0	0	
11773455	m	md	Not re	2015	1	0	0	0	0	0	0	0	0	0	0	0	46
11773492	m	ot	NL	2015	0	0	0	0	0	0	0	0	0	0	0	0	66
11773500	f	ph	NL	2015	0	0	0	0	0	0	0	0	0	0	0	0	61
11773815	f	ot	IT	2015	0	1	0	0	1	0	0	0	0	0	0	0	64
11773816	m	ot	NL	2015	0	1	0	0	1	0	0	0	0	0	0	0	50
11774094	m	md	JP	2015	1	0	0	0	0	0	0	0	0	0	0	0	77
11774295	f	md	DE	2015	1	0	0	0	0	0	0	0	0	0	0	0	60
11774493	m	ot	KR	2015	0	0	0	0	0	0	0	0	0	0	0	0	72
11775031	m	md	FR	2015	0	1	0	0	1	0	0	0	0	0	0	0	54
11775059	f	md	FR	2015	0	1	0	0	1	0	0	0	0	0	0	0	78
11775093	Not	ph	US	2015	0	1	0	0	1	0	0	0	0	0	0	1	
11775235	m	ot	ZA	2015	0	0	0	0	0	0	0	0	0	0	0	0	53
11775237	f	md	IT	2015	0	1	0	0	1	0	0	0	0	0	0	0	67
11775272	f	md	FR	2015	0	1	0	0	1	0	0	0	0	0	0	0	71

11776301	f	cn	US	2015	0	0	0	0	0	0	0	0	0	0	0	0	82
11776839	f	cn	GB	2015	1	0	0	0	0	0	0	0	0	0	0	0	50
11777413	m	cn	JP	2015	0	0	0	0	0	0	0	0	0	0	0	0	65
11777497	m	md	TH	2015	0	0	0	0	0	0	0	0	0	0	0	0	73
11777717	f	ot	Not re	2015	1	0	0	0	0	0	0	0	0	0	0	0	60
11777723	Not	ot	Not re	2015	1	0	0	0	0	0	0	0	0	0	0	0	
11777968	m	md	LB	2015	0	0	0	0	0	0	0	0	0	0	0	0	51
11778121	f	md	JP	2015	1	0	0	0	0	0	0	0	0	0	0	0	51
11778185	Not	md	US	2015	0	0	0	0	0	0	0	0	0	0	0	0	
11778575	f	ot	GB	2015	0	0	0	0	0	0	0	0	0	0	0	0	
11778781	Not	cn	GB	2015	1	0	0	0	0	0	0	0	0	0	0	0	
11778815	m	ot	GB	2015	0	0	0	0	0	0	0	0	0	0	0	0	58
11779056	m	md	DE	2015	0	0	0	0	0	0	0	0	0	0	0	0	65
11779226	f	ot	IT	2015	0	1	0	0	1	0	0	0	0	0	0	0	
11779627	f	ot	GB	2015	0	0	0	0	0	0	0	0	0	0	0	0	
11779833	m	ot	KR	2015	0	0	0	0	0	0	0	0	0	0	0	0	72
11779852	f	ot	BE	2015	0	0	0	0	0	0	0	0	0	0	0	0	65
11780906	m	md	JP	2015	1	0	0	0	0	0	0	0	0	0	0	0	65
11780940	Not	md	US	2015	0	0	0	0	0	0	0	0	0	0	0	0	
11781042	m	md	FR	2015	0	1	0	0	1	0	0	0	0	0	0	0	75
11781546	f	md	JP	2015	1	0	0	0	0	0	0	0	0	0	0	0	64.4
11781759	m	md	FR	2015	0	1	0	0	1	0	0	0	0	0	0	0	71
11781807	Not	cn	US	2015	0	1	0	0	1	0	0	0	0	0	0	0	
11781829	f	md	FR	2015	0	1	0	0	1	0	0	0	0	0	0	0	65
11782129	Not	md	US	2015	0	0	0	0	0	0	0	0	0	0	0	0	45
11782151	Not	ot	US	2015	0	0	0	0	0	0	0	0	0	0	0	0	55
11782304	m	md	ES	2015	0	0	0	0	0	0	0	0	0	0	0	0	64
11782356	m	md	JP	2015	0	0	0	0	0	0	0	0	0	0	0	0	66
11782500	f	md	US	2015	0	0	0	0	0	0	0	0	0	0	0	0	63
11782848	f	md	Not re	2015	0	0	0	0	0	0	0	0	0	0	0	0	59
11782965	m	md	Not re	2015	0	0	0	0	0	0	0	0	0	0	0	0	
11783217	m	md	IL	2015	0	0	0	0	0	0	0	0	0	0	0	0	59
11783376	f	ot	NL	2015	0	0	0	0	0	0	0	0	0	0	0	0	70
11783615	m	ot	US	2015	0	1	0	0	1	0	0	0	0	0	0	0	

11783656	f	ot	US	2015	0	1	0	0	1	0	0	0	0	0	0	0	84
11784327	m	md	Not re	2015	1	0	0	0	0	0	0	0	0	0	0	0	77
11784482	m	md	ES	2015	1	0	0	0	0	0	0	0	0	0	0	0	67
11784520	f	cn	US	2015	0	0	0	0	0	0	0	0	0	0	0	0	82
11784712	m	md	US	2015	0	0	0	0	0	0	0	0	0	0	0	0	67
11784754	Not	md	US	2015	0	0	0	0	0	0	0	0	0	0	0	0	58
11784769	Not	ot	JP	2015	0	0	0	0	0	0	0	0	0	0	0	0	
11784790	f	md	US	2015	0	0	0	0	0	0	0	0	0	0	0	0	80
11785544	f	ot	US	2015	1	0	0	0	0	0	0	0	0	0	0	0	58
11785680	m	md	FR	2015	0	1	0	0	1	0	0	0	0	0	0	0	74
11785747	f	md	GB	2015	0	0	0	0	0	0	0	0	0	0	0	0	70
11785811	f	md	FR	2015	0	1	0	0	1	0	0	0	0	0	0	0	77
11786189	Not	cn	GB	2015	1	0	0	0	0	0	0	0	0	0	0	0	
11787601	f	cn	BR	2015	0	1	1	0	0	0	0	0	0	0	0	1	
11787911	m	md	FR	2015	0	1	0	0	1	0	0	0	0	0	0	0	75
11787916	f	ph	US	2015	0	1	0	0	1	0	0	0	0	0	0	0	61
11787928	m	ot	FR	2015	0	1	0	0	1	0	0	0	0	0	0	0	
11787938	Not	md	US	2015	0	1	0	0	1	0	0	0	0	0	0	0	
11788195	m	md	US	2015	0	0	0	0	0	0	0	0	0	0	0	0	66
11788405	m	md	US	2015	0	0	0	0	0	0	0	0	0	0	0	0	71
11788589	Not	md	US	2015	0	0	0	0	0	0	0	0	0	0	0	0	63
11788610	m	md	US	2015	0	0	0	0	0	0	0	0	0	0	0	0	84
11788622	f	md	US	2015	0	0	0	0	0	0	0	0	0	0	0	0	62
11788657	m	md	US	2015	0	0	0	0	0	0	0	0	0	0	0	0	63
11789175	m	cn	US	2015	0	1	0	0	1	0	0	0	0	0	0	0	
11789852	f	md	DE	2015	0	0	0	0	0	0	0	0	0	0	0	0	48
11789897	m	ot	BE	2015	0	0	0	0	0	0	0	0	0	0	0	0	49
11789988	m	md	AU	2015	0	1	0	0	1	0	0	0	0	0	0	0	
11790032	m	md	IN	2015	1	0	0	0	0	0	0	0	0	0	0	0	59
11790141	f	md	DE	2015	1	0	0	0	0	0	0	0	0	0	0	0	62
11790768	m	md	LB	2015	0	0	0	0	0	0	0	0	0	0	0	0	77
11791107	m	md	TR	2015	0	1	0	0	1	0	0	0	0	0	0	0	72
11791537	Not	md	IN	2015	1	0	0	0	0	0	0	0	0	0	0	0	
11791886	f	md	CH	2015	1	0	0	0	0	0	0	0	0	0	0	0	71

11791891	f	md	JP	2015	1	0	0	0	0	0	0	0	0	0	0	0	75
11791948	m	cn	US	2015	0	1	0	0	1	0	0	0	0	0	0	0	69
11792100	m	md	AR	2015	1	0	0	0	0	0	0	0	0	0	0	0	
11792101	f	md	JP	2015	1	0	0	0	0	0	0	0	0	0	0	0	70
11792243	m	ot	US	2015	0	0	0	0	0	0	0	0	0	0	0	0	
11792756	Not	md	US	2015	0	0	0	0	0	0	0	0	0	0	0	0	71
11792779	f	md	GB	2015	0	0	0	0	0	0	0	0	0	0	0	0	
11792833	Not	ot	JP	2015	0	0	0	0	0	0	0	0	0	0	0	0	
11792967	Not	Not repo	Not re	2015	1	0	0	0	0	0	0	0	0	0	0	0	
11792972	Not	Not repo	Not re	2015	1	0	0	0	0	0	0	0	0	0	0	0	
11792978	Not	Not repo	Not re	2015	1	0	0	0	0	0	0	0	0	0	0	0	
11794557	m	cn	JP	2015	0	0	0	0	0	0	0	0	0	0	0	0	
11795183	m	md	Not re	2015	1	0	0	0	0	0	0	0	0	0	0	0	59
11795232	m	cn	US	2015	0	1	0	0	1	0	0	0	0	0	0	0	
11795235	f	md	IT	2015	0	1	0	0	1	0	0	0	0	0	0	0	75
11795310	f	cn	PL	2015	1	0	0	0	0	0	0	0	0	0	0	0	55
11795810	f	md	US	2015	1	0	0	0	0	0	0	0	0	0	0	0	
11795895	m	md	US	2015	0	0	0	0	0	0	0	0	0	0	0	0	65
11796423	f	md	GB	2015	0	0	0	0	0	0	0	0	0	0	0	0	
11796443	Not	cn	GB	2015	0	0	0	0	0	0	0	0	0	0	0	0	
11796951	m	md	NZ	2015	0	0	0	0	0	0	0	0	0	0	0	0	53
11797054	m	md	US	2015	0	0	0	0	0	0	0	0	0	0	0	0	
11797180	f	md	US	2015	1	0	0	0	0	0	0	0	0	0	0	0	76.7
11797204	f	cn	US	2015	0	1	1	0	0	0	0	0	0	0	0	0	
11797634	m	md	Not re	2015	0	0	0	0	0	0	0	0	0	0	0	0	73
11797858	f	ot	Not re	2015	1	0	0	0	0	0	0	0	0	0	0	0	60
11800233	m	ot	IE	2015	0	0	0	0	0	0	0	0	0	0	0	0	75
11800341	m	md	US	2015	1	0	0	0	0	0	0	0	0	0	0	0	76.9
11800499	Not	cn	IT	2015	0	1	0	0	1	0	0	0	0	0	0	0	
11800535	m	md	FR	2015	0	1	0	0	1	0	0	0	0	0	0	0	77
11800547	Not	md	US	2015	0	0	0	0	0	0	0	0	0	0	0	0	72
11800703	f	Not repo	US	2015	1	0	0	0	0	0	0	0	0	0	0	0	83
11801031	f	ot	US	2015	0	0	0	0	0	0	0	0	0	0	0	0	63
11801165	m	ot	US	2015	0	0	0	0	0	0	0	0	0	0	0	0	

11801818	f	ph	US	2015	0	1	0	0	1	0	0	0	0	0	0	0	61
11801928	f	ot	US	2015	1	0	0	0	0	0	0	0	0	0	0	0	60
11801989	Not	ot	DK	2015	0	0	0	0	0	0	0	0	0	0	0	0	
11802899	f	md	MX	2015	0	0	0	0	0	0	0	0	0	0	0	0	55
11803257	m	md	Not re	2015	0	0	0	0	0	0	0	0	0	0	0	0	56
11803274	f	md	Not re	2015	0	0	0	0	0	0	0	0	0	0	0	0	66
11803368	m	ot	US	2015	0	0	0	0	0	0	0	0	0	0	0	0	67
11803708	f	ot	JP	2015	0	0	0	0	0	0	0	0	0	0	0	0	63
11804555	m	md	FR	2015	0	1	0	0	1	0	0	0	0	0	0	0	70
11804844	f	md	US	2015	1	0	0	0	0	0	0	0	0	0	0	0	80.7
11804907	f	cn	ZA	2015	0	1	0	0	1	0	0	0	0	0	0	0	
11804967	f	md	US	2015	0	1	0	0	1	0	0	0	0	0	0	0	77
11805054	m	md	TR	2015	0	1	0	0	1	0	0	0	0	0	0	0	68
11805671	f	cn	US	2015	0	1	0	0	1	0	0	0	0	0	0	0	
11806258	f	cn	GB	2015	1	0	0	0	0	0	0	0	0	0	0	0	
11806431	m	ot	JP	2015	1	0	0	0	0	0	0	0	0	0	0	0	73
11806434	Not	ot	US	2015	0	0	0	0	0	0	0	0	0	0	0	0	
11806438	Not	ot	US	2015	0	0	0	0	0	0	0	0	0	0	0	0	
11806442	Not	ot	US	2015	0	0	0	0	0	0	0	0	0	0	0	0	
11807065	m	ot	US	2015	0	1	0	0	1	0	0	0	0	0	0	0	56
11807156	m	cn	GB	2015	1	0	0	0	0	0	0	0	0	0	0	0	79
11807442	m	md	FR	2015	0	1	0	0	1	0	0	0	0	0	0	0	66
11807443	m	md	FR	2015	0	1	0	0	1	0	0	0	0	0	0	0	
11807650	m	cn	GB	2015	1	0	0	0	0	0	0	0	0	0	0	0	64
11807651	f	cn	GB	2015	1	0	0	0	0	0	0	0	0	0	0	0	52
11808399	Not	md	US	2015	0	0	0	0	0	0	0	0	0	0	0	0	67
11809986	m	cn	Not re	2015	0	0	0	0	0	0	0	0	0	0	0	0	74
11810054	m	cn	GB	2015	1	0	0	0	0	0	0	0	0	0	0	1	75
11811265	Not	md	US	2015	0	0	0	0	0	0	0	0	0	0	0	0	
11811474	m	cn	GB	2015	1	0	0	0	0	0	0	0	0	0	0	0	73
11811544	f	ot	NL	2015	0	1	0	0	1	0	0	0	0	0	0	0	71
11811547	Not	cn	GB	2015	1	0	0	0	0	0	0	0	0	0	0	0	
11811549	m	md	US	2015	1	0	0	0	0	0	0	0	0	0	0	0	79
11811653	m	ot	NL	2015	0	1	0	0	1	0	0	0	0	0	0	0	60

11811958	m	ot	IT	2015	0	1	0	0	1	0	0	0	0	0	0	0	72
11812308	m	cn	GR	2015	0	1	1	0	0	0	0	0	0	0	0	0	
11812416	f	md	Not re	2015	1	0	0	0	0	0	0	0	0	0	0	0	78
11812656	f	ot	NL	2015	0	1	0	0	1	0	0	0	0	0	0	0	48
11812742	Not	md	US	2015	0	0	0	0	0	0	0	0	0	0	0	0	
11812743	Not	md	US	2015	0	0	0	0	0	0	0	0	0	0	0	0	
11813128	m	md	FR	2015	0	0	0	0	0	0	0	0	0	0	0	0	65
11814218	m	md	CO	2015	0	0	0	0	0	0	0	0	0	0	0	0	74
11814241	Not	ot	US	2015	0	1	0	0	1	0	0	0	0	0	0	0	
11814589	m	cn	GB	2015	1	0	0	0	0	0	0	0	0	0	0	0	
11814673	m	md	US	2015	0	0	0	0	0	0	0	0	0	0	0	0	
11815289	m	cn	US	2015	0	1	0	0	1	0	0	0	0	0	0	0	73
11815820	m	cn	US	2015	0	1	0	0	1	0	0	0	0	0	0	0	76
11815972	Not	ot	IT	2015	0	1	0	0	1	0	0	0	0	0	0	0	
11816462	m	md	US	2015	0	0	0	0	0	0	0	0	0	0	0	0	61
11820014	f	cn	US	2015	0	1	0	0	1	0	0	0	0	0	0	0	
11820845	Not	cn	GB	2015	1	0	0	0	0	0	0	0	0	0	0	0	
11821455	Not	md	US	2015	0	0	0	0	0	0	0	0	0	0	0	0	
11822405	m	md	DE	2015	0	0	0	0	0	0	0	0	0	0	0	0	60
11822938	f	md	NL	2015	0	0	0	0	0	0	0	0	0	0	0	0	56
11823310	m	md	LB	2015	0	0	0	0	0	0	0	0	0	0	0	0	66
11823369	f	ot	NL	2015	0	0	0	0	0	0	0	0	0	0	0	0	
11823382	f	cn	GB	2015	1	0	0	0	0	0	0	0	0	0	0	0	66
11823385	m	cn	GB	2015	1	0	0	0	0	0	0	0	0	0	0	0	60
11824088	m	md	TR	2015	0	1	0	0	1	0	0	0	0	0	0	0	
11824255	f	ot	US	2015	0	0	0	0	0	0	0	0	0	0	0	0	63
11824519	m	ot	NL	2015	0	0	0	0	0	0	0	0	0	0	0	0	
11824798	m	cn	US	2015	0	1	0	0	1	0	0	0	0	0	0	0	
11824850	m	cn	US	2015	0	1	0	0	1	0	0	0	0	0	0	0	
11824981	m	md	AT	2015	0	1	0	0	1	0	0	0	0	0	0	0	75
11825181	f	md	AT	2015	1	0	0	0	0	0	0	0	0	0	0	0	60.8
11825457	f	md	DE	2015	0	0	0	0	0	0	0	0	0	0	0	0	62
11825745	m	ot	DK	2015	0	0	0	0	0	0	0	0	0	0	0	0	33
11826018	f	cn	US	2015	1	0	0	0	0	0	0	0	0	0	0	0	66

11826113	m	md	FR	2015	0	1	0	0	1	0	0	0	0	0	0	0	65
11826289	f	cn	US	2015	0	0	0	0	0	0	0	0	0	0	0	0	
11826401	f	cn	US	2015	0	0	0	0	0	0	0	0	0	0	0	0	
11826625	f	md	FR	2015	0	1	0	0	1	0	0	0	0	0	0	0	69
11827458	f	ph	DE	2015	0	1	0	0	1	0	0	0	0	0	0	0	
11827471	m	ot	US	2015	0	1	0	0	1	0	0	0	0	0	0	0	
11827770	m	ot	US	2015	0	1	0	0	1	0	0	0	0	0	0	0	29
11827967	m	md	Not re	2015	0	0	0	0	0	0	0	0	0	0	0	0	64
11828033	f	md	CA	2015	0	0	0	0	0	0	0	0	0	0	0	0	52
11828718	m	md	DE	2015	0	1	0	0	1	0	0	0	0	0	0	0	42
11828870	m	cn	US	2015	0	1	0	0	1	0	0	0	0	0	0	0	
11828885	m	md	Not re	2015	0	0	0	0	0	0	0	0	0	0	0	0	69
11828925	f	cn	US	2015	0	1	0	0	1	0	0	0	0	0	0	0	
11829874	f	md	JP	2015	1	0	0	0	0	0	0	0	0	0	0	0	73
11829883	Not	md	JP	2015	1	0	0	0	0	0	0	0	0	0	0	0	
11829920	f	md	FR	2015	1	0	0	0	0	0	0	0	0	0	0	0	
11831389	m	cn	JP	2015	0	0	0	0	0	0	0	0	0	0	0	0	66
11831642	f	ot	NL	2015	0	1	0	0	1	0	0	0	0	0	0	0	53
11831820	f	md	GB	2015	0	0	0	0	0	0	0	0	0	0	0	0	
11831853	m	ot	AU	2015	0	1	0	0	1	0	0	0	0	0	0	0	
11831900	f	md	FR	2015	1	0	0	0	0	0	0	0	0	0	0	0	95.7
11832135	f	md	US	2015	1	0	0	0	0	0	0	0	0	0	0	0	
11832522	f	cn	US	2015	0	0	0	0	0	0	0	0	0	0	0	0	53
11832525	m	cn	US	2015	0	0	0	0	0	0	0	0	0	0	0	0	
11832531	m	cn	US	2015	0	0	0	0	0	0	0	0	0	0	0	0	54
11832532	f	cn	US	2015	0	0	0	0	0	0	0	0	0	0	0	0	
11832612	m	cn	BR	2015	0	1	0	0	1	0	0	0	0	0	0	0	
11832876	m	cn	AR	2015	0	0	0	0	0	0	0	0	0	0	0	0	67
11833773	f	ot	IL	2015	0	0	0	0	0	0	0	0	0	0	0	0	19
11836491	m	ot	KR	2015	0	0	0	0	0	0	0	0	0	0	0	0	72
11836655	m	md	US	2015	0	0	0	0	0	0	0	0	0	0	0	0	
11836789	m	ot	BR	2015	0	1	0	0	1	0	0	0	0	0	0	0	68
11836798	f	md	AU	2015	0	1	0	0	1	0	0	0	0	0	0	0	
11836839	Not	cn	US	2015	1	0	0	0	0	0	0	0	0	0	0	1	

11836849	f	ph	US	2015	0	0	0	0	0	0	0	0	0	0	0	0	
11837529	m	ot	AU	2015	0	0	0	0	0	0	0	0	0	0	0	0	61
11837786	f	cn	AR	2015	0	0	0	0	0	0	0	0	0	0	0	0	59
11837996	m	md	PL	2015	0	0	0	0	0	0	0	0	0	0	0	0	65
11838675	m	md	US	2015	0	0	0	0	0	0	0	0	0	0	0	0	54
11838677	Not	md	US	2015	0	0	0	0	0	0	0	0	0	0	0	0	
11838681	Not	ot	US	2015	0	0	0	0	0	0	0	0	0	0	0	0	
11839163	f	ot	US	2015	0	1	0	0	1	0	0	0	0	0	0	0	51
11841819	f	cn	US	2015	0	1	0	0	1	0	0	0	0	0	0	0	
11841820	f	cn	US	2015	0	1	0	0	1	0	0	0	0	0	0	0	
11842743	Not	md	US	2015	1	0	0	0	0	0	0	0	0	0	0	0	
11842801	m	cn	DE	2015	1	0	0	0	0	0	0	0	0	0	0	0	69
11843214	m	ot	ES	2015	0	1	0	0	1	0	0	0	0	0	0	0	74
11843216	f	md	IT	2015	0	1	0	0	1	0	0	0	0	0	0	0	56
11844789	f	md	US	2015	1	0	0	0	0	0	0	0	0	0	0	0	83
11847385	f	ot	US	2015	1	0	0	0	0	0	0	0	0	0	0	0	
11848739	m	cn	US	2015	0	0	0	0	0	0	0	0	0	0	0	0	60
11848822	f	md	US	2015	0	0	0	0	0	0	0	0	0	0	0	0	
11849256	f	ph	US	2015	0	0	0	0	0	0	0	0	0	0	0	0	53
11849741	f	ph	US	2015	0	1	0	0	1	0	0	0	0	0	0	0	73
11849743	f	md	BE	2015	0	1	0	0	1	0	0	0	0	0	0	0	70
11851962	f	ot	US	2015	1	0	0	0	0	0	0	0	0	0	0	0	68
11852954	m	md	DE	2015	0	1	0	0	1	0	0	0	0	0	0	0	70
11852991	f	md	HK	2015	0	0	0	0	0	0	0	0	0	0	0	0	45
11853020	m	md	US	2015	1	0	0	0	0	0	0	0	0	0	0	0	63.4
11853475	f	cn	US	2015	0	1	0	0	1	0	0	0	0	0	0	0	
11853733	f	ot	US	2015	0	1	0	0	1	0	0	0	0	0	0	0	
11853827	m	cn	US	2015	0	0	0	0	0	0	0	0	0	0	0	0	65
11853841	m	cn	IT	2015	0	1	0	0	1	0	0	0	0	0	0	0	67
11853908	f	cn	US	2015	0	1	0	0	1	0	0	0	0	0	0	0	
11853952	Not	md	US	2015	0	1	0	0	1	0	0	0	0	0	0	0	
11853967	m	md	TR	2015	0	1	0	0	1	0	0	0	0	0	0	0	
11854884	m	md	IT	2015	0	1	0	0	1	0	0	0	0	0	0	0	
11854889	f	md	JP	2015	0	0	0	0	0	0	0	0	0	0	0	0	52

11854982	m	md	JP	2015	0	0	0	0	0	0	0	0	0	0	0	0	64
11855520	f	md	Not re	2015	1	0	0	0	0	0	0	0	0	0	0	0	80
11856009	Not	ot	IT	2015	0	1	0	0	1	0	0	0	0	0	0	0	
11856375	f	cn	GB	2015	1	0	0	0	0	0	0	0	0	0	0	0	85
11856904	m	md	US	2015	0	0	0	0	0	0	0	0	0	0	0	0	69
11857320	m	md	FR	2015	0	1	0	0	1	0	0	0	0	0	0	0	
11857418	m	md	ES	2015	0	0	0	0	0	0	0	0	0	0	0	0	58
11858051	m	md	FR	2015	0	0	0	0	0	0	0	0	0	0	0	0	77
11858938	m	md	US	2015	0	1	0	0	1	0	0	0	0	0	0	0	
11859176	Not	md	FR	2015	0	1	0	0	1	0	0	0	0	0	0	0	
11860116	f	md	CN	2015	0	0	0	0	0	0	0	0	0	0	0	0	64
11860447	m	cn	US	2015	0	0	0	0	0	0	0	0	0	0	0	0	
11860793	m	md	IT	2015	0	0	0	0	0	0	0	0	0	0	0	0	45
11860877	Not	md	LB	2015	0	1	0	0	1	0	0	0	0	0	0	0	
11860976	f	ph	US	2015	0	1	0	0	1	0	0	0	0	0	0	0	
11861029	f	cn	DE	2015	1	0	0	0	0	0	0	0	0	0	0	0	
11861210	m	cn	CN	2015	1	0	0	0	0	0	0	0	0	0	0	0	63
11862411	f	md	US	2015	0	1	0	0	1	0	0	0	0	0	0	0	
11862622	Not	md	US	2015	0	1	0	0	1	0	0	0	0	0	0	0	
11863520	m	cn	US	2015	0	1	0	0	1	0	0	0	0	0	0	0	
11863656	Not	cn	JP	2015	0	0	0	0	0	0	0	0	0	0	0	0	55
11863767	m	md	HU	2015	0	1	0	0	1	0	0	0	0	0	0	0	
11865228	Not	ot	HR	2015	1	0	0	0	0	0	0	0	0	0	0	0	
11865335	Not	md	NL	2015	0	0	0	0	0	0	0	0	0	0	0	0	
11865359	m	md	AU	2015	0	1	0	0	1	0	0	0	0	0	0	0	74
11865360	f	ot	US	2015	0	1	0	0	1	0	0	0	0	0	0	0	71
11865397	Not	md	NL	2015	0	0	0	0	0	0	0	0	0	0	0	0	
11865398	Not	md	NL	2015	0	0	0	0	0	0	0	0	0	0	0	0	
11865416	Not	md	NL	2015	0	0	0	0	0	0	0	0	0	0	0	0	
11865417	Not	md	NL	2015	0	0	0	0	0	0	0	0	0	0	0	0	
11865442	Not	md	NL	2015	0	0	0	0	0	0	0	0	0	0	0	0	
11865444	Not	md	NL	2015	0	0	0	0	0	0	0	0	0	0	0	0	
11865695	Not	md	NL	2015	0	0	0	0	0	0	0	0	0	0	0	0	
11866490	f	md	FR	2015	0	1	0	0	1	0	0	0	0	0	0	0	70

11867178	f	md	IT	2015	0	1	0	0	1	0	0	0	0	0	0	0	71
11867218	m	md	RU	2015	0	1	0	0	1	0	0	0	0	0	0	0	
11867292	m	md	FR	2015	0	1	0	0	1	0	0	0	0	0	0	0	50
11867716	m	Not repo	DE	2015	1	0	0	0	0	0	0	0	0	0	0	0	80
11867733	f	md	FR	2015	1	0	0	0	0	0	0	0	0	0	0	0	81.3
11868043	m	md	DE	2015	0	0	0	0	0	0	0	0	0	0	0	0	61
11868066	m	md	JP	2015	1	0	0	0	0	0	0	0	0	0	0	0	80
11868281	m	md	Not re	2015	1	0	0	0	0	0	0	0	0	0	0	0	73
11869218	f	ot	CH	2015	0	1	0	0	1	0	0	0	0	0	0	0	55
11869286	f	ph	FR	2015	0	0	0	0	0	0	0	0	0	0	0	0	53
11869309	m	md	FR	2015	0	1	0	0	1	0	0	0	0	0	0	0	59
11869882	f	cn	US	2015	0	0	0	0	0	0	0	0	0	0	0	0	
11870099	m	md	AU	2015	0	1	0	0	1	0	0	0	0	0	0	0	
11870191	f	cn	US	2015	0	1	0	0	1	0	0	0	0	0	0	0	
11870299	f	md	FR	2015	0	0	0	0	0	0	0	0	0	0	0	0	60
11870897	f	md	FR	2015	0	0	0	0	0	0	0	0	0	0	0	0	57
11871294	Not	md	US	2015	1	0	0	0	0	0	0	0	0	0	0	0	
11871359	f	Not repo	Not re	2015	1	0	0	0	0	0	0	0	0	0	0	0	
11872698	m	cn	US	2015	0	1	0	0	1	0	0	0	0	0	0	0	
11873871	m	md	JP	2015	0	0	0	0	0	0	0	0	0	0	0	0	85
11874319	f	ot	US	2015	0	1	0	0	1	0	0	0	0	0	0	0	83
11874603	m	md	JP	2015	0	0	0	0	0	0	0	0	0	0	0	0	48
11874991	m	md	US	2015	0	1	0	0	1	0	0	0	0	0	0	0	69
11875102	m	md	AU	2015	0	1	0	0	1	0	0	0	0	0	0	0	
11876080	m	md	FR	2015	0	1	0	0	1	0	0	0	0	0	0	0	72
11876886	Not	Not repo	Not re	2015	1	0	0	0	0	0	0	0	0	0	0	0	
11877057	m	ot	US	2015	1	0	0	0	0	0	0	0	0	0	0	0	75
11877144	f	ot	US	2015	1	0	0	0	0	0	0	0	0	0	0	0	
11877463	m	cn	US	2015	0	1	0	0	1	0	0	0	0	0	0	0	60
11877655	f	md	US	2015	1	0	0	0	0	0	0	0	0	0	0	0	
11877661	f	md	US	2015	1	0	0	0	0	0	0	0	0	0	0	0	
11878632	Not	ot	CN	2015	0	0	0	0	0	0	0	0	0	0	0	0	
11878792	Not	cn	GB	2015	1	0	0	0	0	0	0	0	0	0	0	0	
11878996	f	md	IT	2015	0	1	0	0	1	0	0	0	0	0	0	0	67

11880349	f	md	FR	2015	1	0	0	0	0	0	0	0	0	0	0	0	64.9
11881700	f	cn	US	2015	0	0	0	0	0	0	0	0	0	0	0	0	
11881810	f	md	PH	2015	0	0	0	0	0	0	0	0	0	0	0	0	62
11882245	m	md	RU	2015	1	0	0	0	0	0	0	0	0	0	0	0	
11882498	m	cn	US	2015	0	1	0	0	1	0	0	0	0	0	0	0	
11882999	f	ot	US	2015	0	1	0	0	1	0	0	0	0	0	0	0	62
11883377	Not	ot	JP	2015	0	0	0	0	0	0	0	0	0	0	0	0	
11883402	f	cn	US	2015	0	0	0	0	0	0	0	0	0	0	0	0	85
8335639	m	cn	GB	2015	1	0	0	0	0	0	0	0	0	0	0	0	67
8527201	f	cn	GB	2015	1	0	0	0	0	0	0	0	0	0	0	0	
8572870	m	cn	GB	2015	1	0	0	0	0	0	0	0	0	0	0	0	81
8648092	f	ot	US	2015	0	0	0	0	0	0	0	0	0	0	0	0	71
8828673	m	cn	GB	2015	1	0	0	0	0	0	0	0	0	0	0	0	69
8881121	f	md	JP	2015	0	0	0	0	0	0	0	0	0	0	0	0	71
8926960	f	ot	US	2015	0	0	0	0	0	0	0	0	0	0	0	0	65
8976616	m	md	JP	2015	0	0	0	0	0	0	0	0	0	0	0	0	62
8993146	f	md	US	2015	0	0	0	0	0	0	0	0	0	0	0	0	52
9120208	m	md	JP	2015	0	0	0	0	0	0	0	0	0	0	0	0	75
9220178	m	md	JP	2015	0	0	0	0	0	0	0	0	0	0	0	0	75
9236285	f	ph	JP	2015	0	0	0	0	0	0	0	0	0	0	0	0	68
9260689	m	ph	JP	2015	0	0	0	0	0	0	0	0	0	0	0	0	72
9260693	m	md	JP	2015	0	0	0	0	0	0	0	0	0	0	0	0	55
9271523	f	ot	US	2015	0	0	0	0	0	0	0	0	0	0	0	0	69
9288547	f	md	DE	2015	0	0	0	0	0	0	0	0	0	0	0	0	73
9326064	m	md	DE	2015	0	0	0	0	0	0	0	0	0	0	0	0	71
9354823	f	md	JP	2015	0	0	0	0	0	0	0	0	0	0	0	0	61
9387676	f	md	US	2015	1	0	0	0	0	0	0	0	0	0	0	0	63
9411448	m	md	JP	2015	0	0	0	0	0	0	0	0	0	0	0	0	68
9419119	f	md	US	2015	1	0	0	0	0	0	0	0	0	0	0	0	
9518724	m	cn	US	2015	1	0	0	0	0	0	0	0	0	0	0	0	81
9563926	Not	md	IT	2015	0	0	0	0	0	0	0	0	0	0	0	0	
9639964	m	md	JP	2015	0	0	0	0	0	0	0	0	0	0	0	0	37
9688776	f	md	Not re	2015	1	0	0	0	0	0	0	0	0	0	0	0	47
9735165	f	cn	GB	2015	1	0	0	0	0	0	0	0	0	0	0	0	

9779491	f	cn	GB	2015	1	0	0	0	0	0	0	0	0	0	0	0	52
9824218	Not	ot	JP	2015	0	0	0	0	0	0	0	0	0	0	0	0	
9827893	f	ph	JP	2015	0	0	0	0	0	0	0	0	0	0	0	0	61
10034666	f	md	US	2016	1	0	0	0	0	0	0	0	0	0	0	0	57
10094730	Not	cn	GB	2016	1	0	0	0	0	0	0	0	0	0	0	0	
10121293	m	cn	GB	2016	0	0	0	0	0	0	0	0	0	0	0	0	62
10149975	f	md	Not re	2016	1	0	0	0	0	0	0	0	0	0	0	0	77
10243351	f	ot	KP	2016	0	0	0	0	0	0	0	0	0	0	0	0	70
10248831	m	md	SG	2016	0	0	0	0	0	0	0	0	0	0	0	0	48
10373476	m	md	FR	2016	0	0	0	0	0	0	0	0	0	0	0	0	68
10400817	f	md	Not re	2016	1	0	0	0	0	0	0	0	0	0	0	0	62
10401979	m	md	US	2016	1	0	0	0	0	0	0	0	0	0	0	0	73
10434107	m	md	GB	2016	0	0	0	0	0	0	0	0	0	0	0	0	59.2
10450970	m	md	JP	2016	0	0	0	0	0	0	0	0	0	0	0	0	48
10510629	f	md	JP	2016	0	0	0	0	0	0	0	0	0	0	0	0	39
10592563	f	cn	US	2016	0	0	0	0	0	0	0	0	0	0	0	0	67
10664900	f	md	JP	2016	0	0	0	0	0	0	0	0	0	0	0	0	74
10665417	m	md	JP	2016	0	0	0	0	0	0	0	0	0	0	0	0	45
10914517	m	md	Not re	2016	1	0	0	0	0	0	0	0	0	0	0	0	65
11133234	f	ot	Not re	2016	1	0	0	0	0	0	0	0	0	0	0	0	71
11149353	f	ot	CN	2016	0	0	0	0	0	0	0	0	0	0	0	0	63
11254714	f	md	JP	2016	0	0	0	0	0	0	0	0	0	0	0	0	61
11305918	f	md	US	2016	0	1	0	0	0	1	0	0	0	0	0	0	73
11350214	f	ot	FR	2016	0	1	0	0	1	0	0	0	0	0	0	0	64
11383250	m	md	DE	2016	0	1	0	0	1	0	0	0	0	0	0	0	79
11389873	f	cn	Not re	2016	0	0	0	0	0	0	0	0	0	0	0	0	31
11443582	f	cn	IT	2016	0	1	0	0	1	0	0	0	0	0	0	0	70
11447614	m	cn	FR	2016	0	1	0	0	1	0	0	0	0	0	0	0	65
11458781	m	cn	US	2016	1	0	0	0	0	0	0	0	0	0	0	0	81
11485292	m	md	FR	2016	0	1	0	0	1	0	0	0	0	0	0	0	66
11499791	m	md	US	2016	0	1	0	0	0	1	0	0	0	0	0	0	61
11501987	m	md	FR	2016	0	1	0	0	1	0	0	0	0	0	0	0	71
11525761	f	ot	US	2016	0	1	0	0	1	0	0	0	0	0	0	0	61
11536553	m	ot	GB	2016	0	1	0	0	1	0	0	0	0	0	0	0	53

11556835	f	md	Not re	2016	1	0	0	0	0	0	0	0	0	0	0	0	80
11585474	Not	ot	JP	2016	0	0	0	0	0	0	0	0	0	0	0	0	
11588440	m	ot	NL	2016	0	1	0	0	1	0	0	0	0	0	0	0	66
11603701	m	md	JP	2016	0	0	0	0	0	0	0	0	0	0	0	0	61
11620842	f	md	Not re	2016	1	0	0	0	0	0	0	0	0	0	0	0	
11625957	m	cn	GB	2016	1	0	0	0	0	0	0	0	0	0	0	0	58
11626816	f	cn	US	2016	0	0	0	0	0	0	0	0	0	0	0	0	
11628441	f	md	JP	2016	0	0	0	0	0	0	0	0	0	0	0	0	71
11628443	Not	md	JP	2016	1	0	0	0	0	0	0	0	0	0	0	0	
11634417	f	md	Not re	2016	1	0	0	0	0	0	0	0	0	0	0	0	68
11637279	f	ot	US	2016	0	1	0	0	1	0	0	0	0	0	0	0	76
11638516	f	md	US	2016	0	0	0	0	0	0	0	0	0	0	0	0	57
11641349	m	md	US	2016	0	0	0	0	0	0	0	0	0	0	0	0	67
11641508	f	md	JP	2016	0	0	0	0	0	0	0	0	0	0	0	0	65
11647044	m	cn	IT	2016	0	1	0	0	1	0	0	0	0	0	0	0	62
11652334	f	md	FR	2016	1	0	0	0	0	0	0	0	0	0	0	0	46.4
11667623	f	cn	US	2016	1	0	0	0	0	0	0	0	0	0	0	0	52
11682727	f	md	FR	2016	0	1	0	0	1	0	0	0	0	0	0	0	56
11686297	m	cn	IT	2016	0	1	0	0	1	0	0	0	0	0	0	0	65
11694967	f	md	Not re	2016	1	0	0	0	0	0	0	0	0	0	0	0	62
11698488	m	md	Not re	2016	1	0	0	0	0	0	0	0	0	0	0	0	60
11698511	f	md	TR	2016	0	1	0	0	1	0	0	0	0	0	0	0	56
11718823	f	cn	AR	2016	0	0	0	0	0	0	0	0	0	0	0	0	67
11718833	f	md	DE	2016	0	0	0	0	0	0	0	0	0	0	0	0	61
11718875	m	md	JP	2016	0	0	0	0	0	0	0	0	0	0	0	0	75
11735846	f	cn	GB	2016	1	0	0	0	0	0	0	0	0	0	0	0	66
11740840	f	md	IT	2016	0	1	0	0	1	0	0	0	0	0	0	0	58
11752311	f	md	FR	2016	1	0	0	0	0	0	0	0	0	0	0	0	
11754961	m	ot	DE	2016	0	1	0	0	1	0	0	0	0	0	0	0	80
11756551	m	md	FR	2016	1	0	0	0	0	0	0	0	0	0	0	0	
11758446	m	md	FR	2016	0	1	0	0	1	0	0	0	0	0	0	0	71
11767863	f	md	SK	2016	0	0	0	0	0	0	0	0	0	0	0	0	
11769679	f	ot	BE	2016	0	0	0	0	0	0	0	0	0	0	0	0	64
11773456	m	md	Not re	2016	1	0	0	0	0	0	0	0	0	0	0	0	65

11774314	f	cn	US	2016	0	0	0	0	0	0	0	0	0	0	0	0	
11775090	m	md	US	2016	0	1	0	0	1	0	0	0	0	0	0	0	61
11781274	f	md	JP	2016	1	0	0	0	0	0	0	0	0	0	0	1	75.3
11782002	m	ot	IT	2016	0	1	0	0	1	0	0	0	0	0	0	0	75
11783678	f	md	PH	2016	1	0	0	0	0	0	0	0	0	0	0	0	62
11784197	m	md	US	2016	0	1	0	0	0	1	0	0	0	0	0	0	61
11785547	f	cn	GB	2016	1	0	0	0	0	0	0	0	0	0	0	0	
11785891	m	md	IT	2016	0	0	0	0	0	0	0	0	0	0	0	0	56
11785898	m	md	AU	2016	0	0	0	0	0	0	0	0	0	0	0	0	55
11789996	m	md	CH	2016	0	0	0	0	0	0	1	0	1	0	0	0	45
11790629	f	md	US	2016	0	0	0	0	0	0	0	0	0	0	0	0	56
11791060	m	md	PT	2016	0	1	0	0	1	0	0	0	0	0	0	0	80
11791364	f	md	FR	2016	0	0	0	0	0	0	0	0	0	0	0	0	61
11792592	f	md	CA	2016	0	0	0	0	0	0	0	0	0	0	0	0	56
11795566	m	cn	IT	2016	0	1	0	0	1	0	0	0	0	0	0	0	49
11796325	f	md	US	2016	0	0	0	0	0	0	0	0	0	0	0	0	74
11804943	m	md	US	2016	0	0	0	0	0	0	0	0	0	0	0	0	78
11809468	f	md	JP	2016	0	0	0	0	0	0	0	0	0	0	0	0	67
11809960	m	md	FR	2016	0	0	0	0	0	0	0	0	0	0	0	0	63
11810206	m	ot	NL	2016	0	1	0	0	1	0	0	0	0	0	0	0	74
11812005	m	ot	JP	2016	0	1	0	0	0	1	0	0	0	0	0	0	60
11812627	m	ot	KR	2016	0	0	0	0	0	0	0	0	0	0	0	0	63
11812902	f	md	JP	2016	0	0	0	0	0	0	0	0	0	0	0	0	70
11815822	f	ot	US	2016	0	1	0	0	1	0	0	0	0	0	0	0	79
11820311	m	md	TR	2016	0	1	0	0	1	0	0	0	0	0	0	0	58
11822955	Not	ot	IT	2016	0	1	0	0	1	0	0	0	0	0	0	0	
11823626	m	cn	US	2016	0	0	0	0	0	0	0	0	0	0	0	0	
11824285	m	cn	US	2016	0	1	0	0	1	0	0	0	0	0	0	0	
11824759	f	ot	IT	2016	0	1	0	0	1	0	0	0	0	0	0	0	81
11825455	f	md	DE	2016	0	0	0	0	0	0	0	0	0	0	0	0	48
11827132	f	cn	US	2016	0	1	0	0	1	0	0	0	0	0	0	0	77
11827464	m	ot	US	2016	0	1	0	0	1	0	0	0	0	0	0	0	
11828569	f	md	JP	2016	0	0	0	0	0	0	0	0	0	0	0	0	74
11828853	m	md	DE	2016	0	1	0	0	1	0	0	0	0	0	0	0	62

11828982	f	ot	US	2016	0	1	0	0	1	0	0	0	0	0	0	0	74
11829170	f	cn	AR	2016	0	0	0	0	0	0	0	0	0	0	0	0	55
11829742	f	ot	US	2016	0	1	1	0	0	0	0	0	0	0	0	0	
11832325	m	ot	US	2016	1	0	0	0	0	0	0	0	0	0	0	0	79
11832513	m	cn	US	2016	0	0	0	0	0	0	0	0	0	0	0	0	
11832958	m	md	JP	2016	0	0	0	0	0	0	0	0	0	0	0	0	62
11836407	m	md	Not re	2016	1	0	0	0	0	0	0	0	0	0	0	0	79
11837361	f	md	CN	2016	1	0	0	0	0	0	0	0	0	0	0	0	61.2
11837704	m	md	NZ	2016	0	0	0	0	0	0	0	0	0	0	0	0	50
11838222	m	md	FR	2016	1	0	0	0	0	0	0	0	0	0	0	1	62.6
11840385	f	ot	US	2016	0	1	1	0	0	0	0	0	0	0	0	0	
11841287	m	ot	BE	2016	0	1	0	0	1	0	0	0	0	0	0	0	56
11843173	Not	ot	Not re	2016	1	0	0	0	0	0	0	0	0	0	0	0	
11849267	f	md	GB	2016	0	0	0	0	0	0	0	0	0	0	0	0	
11852693	f	md	JP	2016	1	0	0	0	0	0	0	0	0	0	0	0	
11853130	m	ot	JP	2016	1	0	0	0	0	0	0	0	0	0	0	0	80
11853655	f	ph	US	2016	0	0	0	0	0	0	0	0	0	0	0	0	67
11853951	m	cn	US	2016	0	1	0	0	1	0	0	0	0	0	0	1	72
11855136	m	md	SG	2016	0	0	0	0	0	0	0	0	0	0	0	0	66
11855349	f	md	JP	2016	1	0	0	0	0	0	0	0	0	0	0	0	61
11856372	f	md	US	2016	1	0	0	0	0	0	0	0	0	0	0	0	68
11856690	m	cn	FR	2016	0	1	0	0	1	0	0	0	0	0	0	0	60
11856807	m	ot	Not re	2016	1	0	0	0	0	0	0	0	0	0	0	1	79
11858382	f	md	GB	2016	0	0	0	0	0	0	0	0	0	0	0	0	
11860791	m	ph	CA	2016	0	1	0	0	1	0	0	0	0	0	0	0	72
11860903	f	ot	GB	2016	0	0	0	0	0	0	0	0	0	0	0	0	
11860938	f	md	JP	2016	1	0	0	0	0	0	0	0	0	0	0	0	72
11861033	m	md	JP	2016	1	0	0	0	0	0	0	0	0	0	0	0	51
11864506	m	cn	GB	2016	1	0	0	0	0	0	0	0	0	0	0	0	64
11865095	f	ot	US	2016	0	0	0	0	0	0	0	0	0	0	0	0	63
11865515	Not	md	CZ	2016	0	1	0	0	1	0	0	0	0	0	0	0	70
11868280	m	md	ES	2016	1	0	0	0	0	0	0	0	0	0	0	0	60.2
11868812	f	cn	GB	2016	0	0	0	0	0	0	0	0	0	0	0	0	
11869213	m	cn	IT	2016	0	1	0	0	1	0	0	0	0	0	0	0	68

11869321	m	md	DE	2016	0	1	0	0	1	0	0	0	0	0	0	0	75
11869328	m	md	FR	2016	0	1	0	0	1	0	0	0	0	0	0	0	74
11869349	m	md	RU	2016	0	1	0	0	1	0	0	0	0	0	0	0	
11870052	Not	md	GB	2016	0	0	0	0	0	0	0	0	0	0	0	0	
11870817	f	md	GB	2016	0	0	0	0	0	0	0	0	0	0	0	0	
11870934	f	ot	GB	2016	0	0	0	0	0	0	0	0	0	0	0	0	
11871190	f	md	GB	2016	0	0	0	0	0	0	0	0	0	0	0	0	
11871466	f	md	AU	2016	0	0	0	0	0	0	0	0	0	0	0	0	46
11872694	m	cn	IT	2016	0	1	0	0	1	0	0	0	0	0	0	0	72
11872717	f	ot	IT	2016	0	1	0	0	1	0	0	0	0	0	0	0	75
11873864	f	cn	GB	2016	0	0	0	0	0	0	0	0	0	0	0	0	
11874611	f	ot	IE	2016	0	0	0	0	0	0	0	0	0	0	0	0	62
11874670	Not	md	GB	2016	0	0	0	0	0	0	0	0	0	0	0	0	
11875030	m	md	UA	2016	0	0	0	0	0	0	0	0	0	0	0	0	65
11875867	f	md	DE	2016	1	0	0	0	0	0	0	0	0	0	0	0	69
11875868	m	ph	JP	2016	1	0	0	0	0	0	0	0	0	0	0	0	80
11876765	m	md	JP	2016	1	0	0	0	0	0	0	0	0	0	0	0	77.4
11876885	f	md	CH	2016	0	1	0	0	1	0	0	0	0	0	0	0	61
11877657	Not	cn	GB	2016	0	0	0	0	0	0	0	0	0	0	0	0	
11880812	f	md	JP	2016	0	0	0	0	0	0	0	0	0	0	0	0	70
11880876	f	md	JP	2016	0	0	0	0	0	0	0	0	0	0	0	0	50
11882230	m	md	CZ	2016	0	1	0	0	1	0	0	0	0	0	0	0	55
11883164	m	md	Not re	2016	1	0	0	0	0	0	0	0	0	0	0	0	53
11883819	m	md	IT	2016	0	1	0	0	1	0	0	0	0	0	0	0	47
11883831	m	ot	IT	2016	0	1	0	0	1	0	0	0	0	0	0	0	
11883884	m	md	JP	2016	1	0	0	0	0	0	0	0	0	0	0	0	37.1
11883982	f	ph	US	2016	0	1	0	0	1	0	0	0	0	0	0	0	73
11884396	m	md	CH	2016	0	0	0	0	0	0	0	0	0	0	0	0	
11884630	f	md	IT	2016	0	0	0	0	0	0	0	0	0	0	0	0	82
11884661	m	md	FR	2016	0	0	0	0	0	0	0	0	0	0	0	0	69
11884676	m	ot	CA	2016	0	0	0	0	0	0	0	0	0	0	0	0	67
11884775	f	ph	JP	2016	0	0	0	0	0	0	0	0	0	0	0	0	47
11885471	f	cn	GB	2016	1	0	0	0	0	0	0	0	0	0	0	0	
11885518	f	md	Not re	2016	1	0	0	0	0	0	0	0	0	0	0	0	57

11886401	m	cn	AR	2016	0	0	0	0	0	0	0	0	0	0	0	0	65
11886889	f	ot	SG	2016	0	1	0	0	1	0	0	0	0	0	0	1	50
11887703	f	cn	US	2016	0	0	0	0	0	0	1	0	1	0	0	0	
11889371	m	ph	GB	2016	1	0	0	0	0	0	0	0	0	0	0	0	54
11889779	m	cn	AU	2016	0	1	0	0	1	0	0	0	0	0	0	0	75
11889813	f	md	JP	2016	1	0	0	0	0	0	0	0	0	0	0	0	
11890015	m	md	US	2016	0	0	0	0	0	0	0	0	0	0	0	0	49
11891229	m	md	FR	2016	0	0	0	0	0	0	0	0	0	0	0	0	59
11891287	f	md	US	2016	0	0	0	0	0	0	0	0	0	0	0	0	76
11891296	f	ot	US	2016	0	0	0	0	0	0	0	0	0	0	0	0	52
11891493	m	cn	CA	2016	0	0	0	0	0	0	0	0	0	0	0	0	78
11891497	m	cn	CA	2016	0	0	0	0	0	0	0	0	0	0	0	0	58
11891501	m	cn	CA	2016	0	0	0	0	0	0	0	0	0	0	0	0	72
11891758	m	ot	ES	2016	0	0	0	0	0	0	0	0	0	0	0	0	82
11891893	f	ph	US	2016	0	1	0	0	1	0	0	0	0	0	0	0	61
11892457	f	md	US	2016	0	0	0	0	0	0	0	0	0	0	0	0	74
11893160	m	cn	US	2016	0	1	0	0	1	0	0	0	0	0	0	0	
11893281	Not	ot	IT	2016	0	0	0	0	0	0	0	0	0	0	0	0	
11893288	Not	ot	IT	2016	0	0	0	0	0	0	0	0	0	0	0	0	
11893289	Not	ot	IT	2016	0	0	0	0	0	0	0	0	0	0	0	0	
11893292	Not	ot	IT	2016	0	0	0	0	0	0	0	0	0	0	0	0	
11894059	m	md	FR	2016	0	1	0	0	1	0	0	0	0	0	0	0	
11894527	m	cn	US	2016	0	1	0	0	1	0	0	0	0	0	0	0	
11896889	m	md	DE	2016	1	0	0	0	0	0	0	0	0	0	0	0	64
11896892	f	md	BE	2016	0	0	0	0	0	0	0	0	0	0	0	0	55
11897288	m	cn	US	2016	0	1	0	0	1	0	0	0	0	0	0	0	57
11897289	f	ot	US	2016	0	1	0	0	1	0	0	0	0	0	0	0	
11897594	f	ot	US	2016	0	0	0	0	0	0	0	0	0	0	0	0	52
11897714	f	cn	US	2016	0	1	0	0	1	0	0	0	0	0	0	0	80
11897999	m	md	JP	2016	0	0	0	0	0	0	0	0	0	0	0	0	81
11898524	m	ot	DE	2016	0	0	0	0	0	0	0	0	0	0	0	0	63
11898696	m	md	US	2016	0	0	0	0	0	0	0	0	0	0	0	0	58
11899241	f	cn	US	2016	0	1	0	0	1	0	0	0	0	0	0	0	
11900568	m	md	TR	2016	0	1	0	0	1	0	0	0	0	0	0	0	

11901670	f	cn	US	2016	0	1	0	0	1	0	0	0	0	0	0	0	
11902013	Not	cn	GB	2016	1	0	0	0	0	0	0	0	0	0	0	0	
11902237	m	cn	US	2016	0	1	0	0	1	0	0	0	0	0	0	1	73
11902437	f	md	GB	2016	1	0	0	0	0	0	0	0	0	0	0	0	68
11902476	m	ot	NL	2016	0	1	0	0	1	0	0	0	0	0	0	0	67
11904422	m	ot	FR	2016	0	0	0	0	0	0	0	0	0	0	0	0	70
11905570	f	md	AU	2016	0	1	0	0	1	0	0	0	0	0	0	0	
11905571	m	cn	CN	2016	0	1	0	0	1	0	0	0	0	0	0	0	73
11905607	m	md	FR	2016	0	1	0	0	1	0	0	0	0	0	0	0	70
11905948	f	ph	US	2016	0	0	0	0	0	0	0	0	0	0	0	0	74
11906221	m	cn	US	2016	0	0	0	0	0	0	0	0	0	0	0	0	
11906831	Not	ot	IT	2016	0	0	0	0	0	0	0	0	0	0	0	0	
11907065	m	cn	GB	2016	1	0	0	0	0	0	0	0	0	0	0	0	66
11907875	m	md	TR	2016	0	1	0	0	1	0	0	0	0	0	0	0	53
11908389	m	md	US	2016	0	1	0	0	1	0	0	0	0	0	0	0	64
11908620	f	ot	AU	2016	0	0	0	0	0	0	0	0	0	0	0	0	62
11908635	m	ot	AU	2016	0	0	0	0	0	0	0	0	0	0	0	0	72
11908871	f	md	Not re	2016	0	0	0	0	0	0	0	0	0	0	0	0	63
11909142	m	md	JP	2016	0	0	0	0	0	0	0	0	0	0	0	0	58
11909917	m	ot	US	2016	0	1	0	0	1	0	0	0	0	0	0	0	85
11910064	f	ot	US	2016	0	0	0	0	0	0	0	0	0	0	0	0	52
11910158	m	ot	FR	2016	1	0	0	0	0	0	0	0	0	0	0	0	
11910399	m	md	TR	2016	0	1	0	0	1	0	0	0	0	0	0	0	39
11910400	m	md	TR	2016	0	1	0	0	1	0	0	0	0	0	0	0	47
11910408	m	ot	BE	2016	0	1	0	0	1	0	0	0	0	0	0	0	65
11910477	f	cn	Not re	2016	0	0	0	0	0	0	0	0	0	0	0	0	
11910796	f	md	US	2016	0	0	0	0	0	0	0	0	0	0	0	0	74
11910866	m	cn	US	2016	0	1	0	0	1	0	0	0	0	0	0	0	
11910874	m	md	TR	2016	0	1	0	0	1	0	0	0	0	0	0	0	68
11911444	f	cn	US	2016	0	0	0	0	0	0	0	0	0	0	0	0	65
11912058	Not	ot	AU	2016	0	0	0	0	0	0	0	0	0	0	0	0	
11912060	Not	ot	AU	2016	0	0	0	0	0	0	0	0	0	0	0	0	
11912311	Not	md	TR	2016	0	1	0	0	1	0	0	0	0	0	0	0	
11912602	m	ph	US	2016	0	0	0	0	0	0	0	0	0	0	0	0	

11912611	f	md	TH	2016	0	0	0	0	0	0	0	0	0	0	0	0	
11912744	m	cn	Not re	2016	0	0	0	0	0	0	0	0	0	0	0	0	
11913064	m	md	US	2016	0	0	0	0	0	0	0	0	0	0	0	0	67
11913073	m	ot	US	2016	0	1	0	0	1	0	0	0	0	0	0	0	60
11913169	m	cn	US	2016	0	1	0	0	1	0	0	0	0	0	0	0	
11913209	f	md	Not re	2016	1	0	0	0	0	0	0	0	0	0	0	0	74
11913227	m	cn	GB	2016	1	0	0	0	0	0	0	0	0	0	0	0	63
11913260	f	cn	AR	2016	0	0	0	0	0	0	0	0	0	0	0	0	76
11913263	m	ph	US	2016	0	0	0	0	0	0	0	0	0	0	0	0	53
11913425	m	cn	AR	2016	0	0	0	0	0	0	0	0	0	0	0	0	43
11914050	f	cn	GB	2016	1	0	0	0	0	0	0	0	0	0	0	0	78
11914051	m	cn	GB	2016	1	0	0	0	0	0	0	0	0	0	0	0	
11914342	f	cn	US	2016	0	0	0	0	0	0	0	0	0	0	0	0	70
11914848	Not	ot	DK	2016	0	0	0	0	0	0	0	0	0	0	0	0	
11914850	Not	ot	DK	2016	0	0	0	0	0	0	0	0	0	0	0	0	
11914851	m	ot	US	2016	0	1	0	0	1	0	0	0	0	0	0	0	
11914852	Not	ot	DK	2016	0	0	0	0	0	0	0	0	0	0	0	0	
11914853	Not	ot	DK	2016	0	0	0	0	0	0	0	0	0	0	0	0	
11914854	Not	ot	DK	2016	0	0	0	0	0	0	0	0	0	0	0	0	
11914856	Not	ot	DK	2016	0	0	0	0	0	0	0	0	0	0	0	0	
11914859	Not	ot	DK	2016	0	0	0	0	0	0	0	0	0	0	0	0	
11914892	Not	ot	DK	2016	0	0	0	0	0	0	0	0	0	0	0	0	
11914896	f	ot	US	2016	0	0	0	0	0	0	0	0	0	0	0	0	63
11915022	f	cn	US	2016	0	1	0	0	1	0	0	0	0	0	0	0	63
11915319	f	md	US	2016	0	1	0	0	1	0	0	0	0	0	0	0	
11915344	Not	md	US	2016	0	1	0	0	1	0	0	0	0	0	0	1	
11915483	f	md	US	2016	0	1	0	0	1	0	0	0	0	0	0	0	
11915499	f	md	TR	2016	0	1	0	0	1	0	0	0	0	0	0	0	56
11915704	f	cn	US	2016	0	0	0	0	0	0	0	0	0	0	0	0	66
11916132	Not	ot	FR	2016	0	0	0	0	0	0	0	0	0	0	0	0	
11917351	f	Not repo	US	2016	1	0	0	0	0	0	0	0	0	0	0	0	53.2
11917567	Not	cn	GB	2016	1	0	0	0	0	0	0	0	0	0	0	0	
11917646	f	Not repo	US	2016	1	0	0	0	0	0	0	0	0	0	0	0	75.9
11918305	f	ot	US	2016	1	0	0	0	0	0	0	0	0	0	0	0	

11918401	m	md	FR	2016	1	0	0	0	0	0	0	0	0	0	0	0	77.5
11918443	m	cn	CA	2016	0	0	0	0	0	0	0	0	0	0	0	0	
11918757	f	md	US	2016	1	0	0	0	0	0	0	0	0	0	0	0	
11920120	m	md	PT	2016	0	1	0	0	1	0	0	0	0	0	0	0	
11922177	m	cn	GB	2016	0	1	0	0	1	0	0	0	0	0	0	0	54
11922262	f	md	US	2016	1	0	0	0	0	0	0	0	0	0	0	0	84
11922514	f	md	FR	2016	1	0	0	0	0	0	0	0	0	0	0	0	76
11922851	f	md	SI	2016	0	1	0	0	1	0	0	0	0	0	0	0	71
11922870	f	ph	US	2016	0	1	0	0	1	0	0	0	0	0	0	0	85
11922957	f	md	CH	2016	0	1	0	0	1	0	0	0	0	0	0	1	76
11923382	m	ot	CN	2016	0	0	0	0	0	0	0	0	0	0	0	0	34
11923504	Not	ph	US	2016	1	0	0	0	0	0	0	0	0	0	0	0	
11923794	Not	ot	CN	2016	1	0	0	0	0	0	0	0	0	0	0	0	
11924155	f	cn	US	2016	0	0	0	0	0	0	0	0	0	0	0	0	39
11924771	Not	cn	GB	2016	1	0	0	0	0	0	0	0	0	0	0	0	44
11925049	f	cn	IT	2016	0	1	0	0	1	0	0	0	0	0	0	0	67
11925171	m	cn	GB	2016	1	0	0	0	0	0	0	0	0	0	0	0	
11925280	m	md	US	2016	0	0	0	0	0	0	0	0	0	0	0	0	
11925700	m	cn	AU	2016	0	0	0	0	0	0	0	0	0	0	0	0	
11925937	f	cn	US	2016	0	1	1	0	0	0	0	0	0	0	0	0	
11925982	Not	cn	IT	2016	0	1	0	0	1	0	0	0	0	0	0	0	
11926050	m	md	JP	2016	0	0	0	0	0	0	0	0	0	0	0	0	58
11926760	Not	ph	US	2016	0	1	0	0	1	0	0	0	0	0	0	0	
11926788	Not	md	HK	2016	0	0	0	0	0	0	0	0	0	0	0	0	
11926829	m	md	US	2016	0	1	0	0	1	0	0	0	0	0	0	0	
11927527	f	md	FR	2016	0	1	0	0	1	0	0	0	0	0	0	0	69
11928083	m	md	US	2016	0	0	0	0	0	0	0	0	0	0	0	0	61
11928424	Not	md	US	2016	1	0	0	0	0	0	0	0	0	0	0	0	
11928920	m	md	TR	2016	0	1	0	0	1	0	0	0	0	0	0	0	71
11928928	m	cn	US	2016	0	0	0	0	0	0	0	0	0	0	0	0	69
11929082	m	ph	JP	2016	0	0	0	0	0	0	0	0	0	0	0	0	73
11929087	m	ot	CA	2016	0	1	0	0	0	1	0	0	0	0	0	0	79
11929178	f	ot	US	2016	0	0	0	0	0	0	0	0	0	0	0	0	52
11929292	m	cn	JP	2016	0	1	0	0	1	0	0	0	0	0	0	0	49

11929501	f	cn	US	2016	0	1	1	0	0	0	0	0	0	0	0	0		55
11929626	m	md	NL	2016	0	1	0	0	1	0	0	0	0	0	0	0		57
11930025	m	cn	GB	2016	1	0	0	0	0	0	0	0	0	0	0	0		
11930028	f	cn	GB	2016	1	0	0	0	0	0	0	0	0	0	0	0		52
11930097	m	ot	US	2016	0	1	0	0	1	0	0	0	0	0	0	0		
11930274	f	ot	Not re	2016	0	0	0	0	0	0	0	0	0	0	0	0		58
11930314	f	ot	IT	2016	0	0	0	0	0	0	0	0	0	0	0	0		62
11930454	f	md	NL	2016	0	0	0	0	0	0	0	0	0	0	0	0		59
11930556	f	ot	US	2016	0	0	0	0	0	0	0	0	0	0	0	0		52
11930748	m	ot	IT	2016	0	0	0	0	0	0	0	0	0	0	0	0		69
11930763	f	ot	GB	2016	0	0	0	0	0	0	0	0	0	0	0	0		44
11930764	m	ot	US	2016	0	0	0	0	0	0	0	0	0	0	0	0		
11930833	m	ot	IT	2016	0	0	0	0	0	0	0	0	0	0	0	0		50
11931683	f	cn	CN	2016	1	0	0	0	0	0	0	0	0	0	0	0		
11932162	f	ot	US	2016	0	1	0	0	1	0	0	0	0	0	0	0		73
11932848	f	ot	US	2016	0	0	0	0	0	0	0	0	0	0	0	0		67
11932939	m	ot	CH	2016	0	1	0	0	1	0	0	0	0	0	0	0		61
11933082	f	md	US	2016	0	1	0	0	1	0	0	0	0	0	0	0		
11933206	Not	cn	US	2016	0	1	1	0	0	0	0	0	0	0	0	0		
11933407	f	md	CH	2016	0	1	0	0	1	0	0	0	0	0	0	0		58
11933878	m	md	US	2016	1	0	0	0	0	0	0	0	0	0	0	0		66.6
11934421	f	md	US	2016	1	0	0	0	0	0	0	0	0	0	0	0		65
11935085	m	md	FR	2016	0	0	0	0	0	0	0	0	0	0	0	0		70
11935095	m	cn	US	2016	0	1	1	0	0	0	0	0	0	0	0	1		62
11935307	f	md	FR	2016	1	0	0	0	0	0	0	0	0	0	0	0		
11935638	f	ot	US	2016	0	0	0	0	0	0	0	0	0	0	0	0		55
11935669	m	cn	GB	2016	1	0	0	0	0	0	0	0	0	0	0	0		73
11936247	f	md	US	2016	0	0	0	0	0	0	0	0	0	0	0	0		
11936647	Not	ot	JP	2016	0	0	0	0	0	0	0	0	0	0	0	0		66
11936919	m	ot	US	2016	1	0	0	0	0	0	0	0	0	0	0	0		
11937588	m	ot	IT	2016	0	0	0	0	0	0	0	0	0	0	0	0		69
11937733	m	ot	IT	2016	0	0	0	0	0	0	0	0	0	0	0	0		50
11937735	f	ot	IT	2016	0	0	0	0	0	0	0	0	0	0	0	0		62
11937852	m	cn	JP	2016	0	1	0	0	1	0	0	0	0	0	0	0		70

11937877	f	ot	IT	2016	0	1	0	0	1	0	0	0	0	0	0	0	70
11938410	Not	cn	GB	2016	1	0	0	0	0	0	0	0	0	0	0	0	
11938411	f	md	US	2016	1	0	0	0	0	0	0	0	0	0	0	0	
11938485	f	cn	US	2016	0	0	0	0	0	0	0	0	0	0	0	0	
11938633	f	ot	Not re	2016	1	0	0	0	0	0	0	0	0	0	0	0	52
11938917	f	md	US	2016	1	0	0	0	0	0	0	0	0	0	0	0	75.9
11939069	m	md	JP	2016	0	0	0	0	0	0	0	0	0	0	0	0	41
11939584	m	md	CY	2016	0	1	1	0	0	0	0	0	0	0	0	0	37
11939597	f	cn	GB	2016	1	0	0	0	0	0	0	0	0	0	0	0	
11940301	f	md	IT	2016	0	0	0	0	0	0	0	0	0	0	0	0	67
11941029	m	ot	US	2016	0	0	0	0	0	0	0	0	0	0	0	0	52
11941544	f	ot	US	2016	0	1	0	0	1	0	0	0	0	0	0	0	74
11941564	m	ot	BE	2016	0	1	0	0	1	0	0	0	0	0	0	0	
11941656	m	ot	IT	2016	0	1	0	0	1	0	0	0	0	0	0	1	50
11941730	f	ot	GB	2016	0	1	0	0	1	0	0	0	0	0	0	0	72
11941833	m	ot	ES	2016	0	1	0	0	1	0	0	0	0	0	0	1	66
11942020	f	cn	US	2016	0	1	0	0	1	0	0	0	0	0	0	0	
11942112	m	cn	US	2016	0	1	0	0	1	0	0	0	0	0	0	0	
11944108	f	ot	US	2016	0	1	1	0	0	0	0	0	0	0	0	0	84
11944970	m	md	ES	2016	0	0	0	0	0	0	0	0	0	0	0	0	
11945096	Not	cn	GB	2016	1	0	0	0	0	0	0	0	0	0	0	0	
11945414	f	md	US	2016	0	0	0	0	0	0	0	0	0	0	0	0	80
11945888	f	cn	US	2016	0	0	0	0	0	0	0	0	0	0	0	0	61
11946686	f	cn	US	2016	0	1	0	0	1	0	0	0	0	0	0	0	
11947806	f	ot	IT	2016	0	1	0	0	1	0	0	0	0	0	0	0	70
11947818	m	md	PT	2016	0	1	0	0	1	0	0	0	0	0	0	0	50
11948091	m	md	TR	2016	0	1	0	0	1	0	0	0	0	0	0	0	65
11949276	f	cn	GB	2016	0	1	0	0	1	0	0	0	0	0	0	0	72
11950514	f	ot	US	2016	0	0	0	0	0	0	0	0	0	0	0	0	72
11951699	m	cn	AR	2016	0	0	0	0	0	0	0	0	0	0	0	0	65
11953842	m	ot	US	2016	0	0	0	0	0	0	0	0	0	0	0	0	47
11953960	m	md	IT	2016	0	0	0	0	0	0	0	0	0	0	0	0	71
11953978	m	cn	US	2016	0	0	0	0	0	0	0	0	0	0	0	0	65
11954004	m	ot	DE	2016	0	1	0	0	1	0	0	0	0	0	0	0	

11954072	m	ph	FR	2016	1	0	0	0	0	0	0	0	0	0	0	0	
11954126	f	ph	FR	2016	1	0	0	0	0	0	0	0	0	0	0	0	73
11956476	f	ot	NL	2016	0	1	0	0	1	0	0	0	0	0	0	0	67
11956768	f	cn	US	2016	0	1	0	0	1	0	0	0	0	0	0	0	60
11959370	m	cn	GB	2016	1	0	0	0	0	0	0	0	0	0	0	0	
11960897	m	cn	US	2016	0	0	0	0	0	0	0	0	0	0	0	0	73
11962047	m	cn	IT	2016	0	1	0	0	1	0	0	0	0	0	0	0	
11962113	m	cn	DK	2016	0	0	0	0	0	0	0	0	0	0	0	0	78
11966999	m	md	Not re	2016	1	0	0	0	0	0	0	0	0	0	0	0	65
11967360	m	cn	IT	2016	0	1	0	0	1	0	0	0	0	0	0	0	60
11968378	m	ot	NL	2016	0	1	0	0	1	0	0	0	0	0	0	0	52
11968697	f	md	FR	2016	1	0	0	0	0	0	0	0	0	0	0	0	
11968758	Not	Not repo	GB	2016	0	0	0	0	0	0	0	0	0	0	0	0	
11968933	f	cn	US	2016	1	0	0	0	0	0	0	0	0	0	0	0	73
11969455	Not	ot	IT	2016	0	1	0	0	1	0	0	0	0	0	0	0	48
11969463	f	md	US	2016	0	1	0	0	1	0	0	0	0	0	0	0	
11969483	m	cn	CA	2016	0	0	0	0	0	0	0	0	0	0	0	0	72
11970094	f	md	LB	2016	1	0	0	0	0	0	0	0	0	0	0	0	
11970118	f	ot	AU	2016	0	0	0	0	0	0	0	0	0	0	0	0	71
11971318	m	md	US	2016	0	0	0	0	0	0	0	0	0	0	0	0	64
11972991	m	md	US	2016	0	1	1	0	0	0	0	0	0	0	0	0	
11975715	m	md	TR	2016	0	1	0	0	1	0	0	0	0	0	0	0	64
11976482	f	cn	US	2016	0	0	0	0	0	0	0	0	0	0	0	0	
11976644	m	ot	GB	2016	0	1	0	0	1	0	0	0	0	0	0	0	81
11979810	f	md	FR	2016	1	0	0	0	0	0	0	0	0	0	0	0	
11980614	m	ot	IT	2016	0	0	0	0	0	0	0	0	0	0	0	0	67
11980659	m	cn	US	2016	0	1	0	0	1	0	0	0	0	0	0	0	52
11980695	m	cn	CA	2016	0	0	0	0	0	0	0	0	0	0	0	0	73
11980735	f	md	FR	2016	1	0	0	0	0	0	0	0	0	0	0	0	
11980987	m	md	FR	2016	1	0	0	0	0	0	0	0	0	0	0	0	
11981490	m	ot	BG	2016	0	1	0	0	1	0	0	0	0	0	0	0	81
11981518	Not	ot	US	2016	0	1	0	0	1	0	0	0	0	0	0	0	
11982128	m	cn	AR	2016	0	0	0	0	0	0	0	0	0	0	0	0	67
11982349	Not	md	KR	2016	0	0	0	0	0	0	0	0	0	0	0	0	44

11982600	f	md	FR	2016	0	0	0	0	0	0	0	0	0	0	0	0	68
11982635	f	md	MY	2016	0	0	0	0	0	0	0	0	0	0	0	0	
11983471	m	md	JP	2016	0	0	0	0	0	0	0	0	0	0	0	0	77
11984200	f	md	DE	2016	1	0	0	0	0	0	0	0	0	0	0	0	82
11984266	f	md	JP	2016	0	0	0	0	0	0	0	0	0	0	0	0	57
11984349	m	md	US	2016	0	0	0	0	0	0	1	0	1	0	0	0	
11984391	Not	cn	IT	2016	0	1	0	0	1	0	0	0	0	0	0	0	
11984668	f	md	Not re	2016	1	0	0	0	0	0	0	0	0	0	0	0	53
11984741	m	ot	CH	2016	0	1	0	0	1	0	0	0	0	0	0	0	68
11984776	Not	ot	US	2016	0	0	0	0	0	0	0	0	0	0	0	0	
11984953	f	md	US	2016	0	1	0	0	1	0	0	0	0	0	0	0	
11985028	m	ot	CN	2016	0	0	0	0	0	0	0	0	0	0	0	0	63
11985755	m	md	FR	2016	0	1	0	0	0	1	0	0	0	0	0	0	60
11985767	m	ot	US	2016	0	1	0	0	1	0	0	0	0	0	0	0	
11986261	m	ph	US	2016	0	1	0	0	1	0	0	0	0	0	0	0	68
11987558	m	cn	JP	2016	0	1	0	0	1	0	0	0	0	0	0	0	39
11987580	f	md	FR	2016	1	0	0	0	0	0	0	0	0	0	0	0	
11987584	Not	md	KR	2016	0	0	0	0	0	0	0	0	0	0	0	0	
11987638	f	md	FR	2016	1	0	0	0	0	0	0	0	0	0	0	0	
11987671	Not	md	KR	2016	0	0	0	0	0	0	0	0	0	0	0	0	
11987682	m	md	DE	2016	0	0	0	0	0	0	0	0	0	0	0	0	55
11988052	f	cn	US	2016	0	0	0	0	0	0	0	0	0	0	0	0	71
11988193	m	ot	US	2016	0	0	0	0	0	0	0	0	0	0	0	0	50
11988251	Not	ot	US	2016	0	0	0	0	0	0	0	0	0	0	0	0	
11988451	m	ot	US	2016	0	1	0	0	1	0	0	0	0	0	0	0	
11989269	f	md	FR	2016	1	0	0	0	0	0	0	0	0	0	0	0	
11989331	f	md	FR	2016	1	0	0	0	0	0	0	0	0	0	0	0	
11989332	f	md	BE	2016	1	0	0	0	0	0	0	0	0	0	0	0	84.3
11990115	m	md	DE	2016	0	1	0	0	1	0	0	0	0	0	0	0	
11990314	f	md	JP	2016	0	0	0	0	0	0	0	0	0	0	0	0	71
11990619	m	md	IT	2016	0	0	0	0	0	0	0	0	0	0	0	0	69
11990690	f	md	IT	2016	0	0	0	0	0	0	0	0	0	0	0	0	62
11990699	f	md	IT	2016	0	0	0	0	0	0	0	0	0	0	0	0	58
11990773	Not	md	FR	2016	1	0	0	0	0	0	0	0	0	0	0	0	

11990844	m	md	IT	2016	0	0	0	0	0	0	0	0	0	0	0	0	50
11991811	f	ot	ES	2016	0	1	0	0	1	0	0	0	0	0	0	0	64
11992404	f	md	IT	2016	0	1	0	0	1	0	0	0	0	0	0	0	61
11993073	Not	ot	IT	2016	0	1	0	0	1	0	0	0	0	0	0	0	72
11993440	f	ot	FR	2016	0	1	0	0	0	1	0	0	0	0	0	0	74
11993676	m	cn	US	2016	0	0	0	0	0	0	0	0	0	0	0	0	
11993791	f	md	FR	2016	1	0	0	0	0	0	0	0	0	0	0	0	
11993794	f	md	FR	2016	1	0	0	0	0	0	0	0	0	0	0	0	
11994089	f	cn	AR	2016	0	0	0	0	0	0	0	0	0	0	0	0	66
11994831	f	md	US	2016	1	0	0	0	0	0	0	0	0	0	0	0	72
11995496	f	md	RU	2016	1	0	0	0	0	0	0	0	0	0	0	0	
11995887	f	md	FR	2016	1	0	0	0	0	0	0	0	0	0	0	0	
11997541	f	md	Not re	2016	1	0	0	0	0	0	0	0	0	0	0	0	71
11998959	m	md	DE	2016	0	1	0	0	1	0	0	0	0	0	0	0	
11999114	f	md	IT	2016	0	1	0	0	1	0	0	0	0	0	0	0	
11999171	f	md	JP	2016	0	1	0	0	1	0	0	0	0	0	0	0	64
11999192	m	md	US	2016	0	1	0	0	1	0	0	0	0	0	0	0	
11999309	m	md	IT	2016	0	1	0	0	1	0	0	0	0	0	0	0	66
11999388	m	ot	IT	2016	0	1	0	0	1	0	0	0	0	0	0	0	77
11999529	m	md	DE	2016	0	1	0	0	1	0	0	0	0	0	0	0	61
11999559	m	md	FI	2016	0	0	0	0	0	0	0	0	0	0	0	0	61
11999628	m	md	JP	2016	0	1	0	0	1	0	0	0	0	0	0	0	74
12000625	Not	ot	US	2016	1	0	0	0	0	0	0	0	0	0	0	0	
12000848	m	cn	GB	2016	1	0	0	0	0	0	0	0	0	0	0	0	77
12000863	f	cn	GB	2016	1	0	0	0	0	0	0	0	0	0	0	0	
12001328	f	md	US	2016	0	0	0	0	0	0	0	0	0	0	0	0	
12001484	f	md	FR	2016	1	0	0	0	0	0	0	0	0	0	0	0	
12001556	m	ot	AU	2016	0	0	0	0	0	0	0	0	0	0	0	0	72
12001558	m	ot	AU	2016	0	0	0	0	0	0	0	0	0	0	0	0	77
12001907	f	md	FR	2016	1	0	0	0	0	0	0	0	0	0	0	0	
12003253	f	ot	AU	2016	0	0	0	0	0	0	0	0	0	0	0	0	62
12004438	m	md	GB	2016	0	0	0	0	0	0	0	0	0	0	0	0	73
12005222	m	cn	US	2016	0	0	0	0	0	0	0	0	0	0	0	0	66
12005588	Not	ot	SE	2016	0	0	0	0	0	0	0	0	0	0	0	0	

12005667	f	ot	IT	2016	0	0	0	0	0	0	0	0	0	0	0	0	67
12005670	m	md	IT	2016	0	0	0	0	0	0	0	0	0	0	0	0	71
12006694	m	ot	NL	2016	0	1	0	0	1	0	0	0	0	0	0	0	60
12007874	m	md	FR	2016	1	0	0	0	0	0	0	0	0	0	0	0	
12008019	m	ph	US	2016	0	1	0	0	1	0	0	0	0	0	0	0	69
12009454	Not	md	US	2016	0	0	0	0	0	0	0	0	0	0	0	0	
12009455	Not	md	US	2016	0	0	0	0	0	0	0	0	0	0	0	0	
12009641	f	md	US	2016	0	0	0	0	0	0	0	0	0	0	0	0	
12009694	Not	md	US	2016	0	0	0	0	0	0	0	0	0	0	0	0	
12009695	Not	md	US	2016	0	0	0	0	0	0	0	0	0	0	0	0	
12009702	m	cn	GB	2016	1	0	0	0	0	0	0	0	0	0	0	0	67
12009721	Not	md	US	2016	0	0	0	0	0	0	0	0	0	0	0	0	
12009723	Not	md	US	2016	0	0	0	0	0	0	0	0	0	0	0	0	
12009732	Not	md	US	2016	0	0	0	0	0	0	0	0	0	0	0	0	
12009768	f	md	FR	2016	1	0	0	0	0	0	0	0	0	0	0	0	
12009769	Not	md	US	2016	0	0	0	0	0	0	0	0	0	0	0	0	
12009861	Not	md	US	2016	0	0	0	0	0	0	0	0	0	0	0	0	
12009873	Not	md	GR	2016	0	0	0	0	0	0	0	0	0	0	0	0	
12009883	Not	md	US	2016	0	0	0	0	0	0	0	0	0	0	0	0	
12009902	m	md	CN	2016	0	0	0	0	0	0	0	0	0	0	0	0	63
12009927	f	md	DE	2016	1	0	0	0	0	0	0	0	0	0	0	0	40
12009967	Not	md	US	2016	0	0	0	0	0	0	0	0	0	0	0	0	
12010374	f	Not repo	US	2016	0	0	0	0	0	0	0	0	0	0	0	0	54
12010378	m	ot	US	2016	0	0	0	0	0	0	0	0	0	0	0	0	
12010532	m	md	US	2016	0	0	0	0	0	0	0	0	0	0	0	0	40
12010725	Not	ph	US	2016	0	1	0	0	1	0	0	0	0	0	0	0	
12011122	f	cn	US	2016	0	0	0	0	0	0	0	0	0	0	0	0	53
12011393	f	ot	GB	2016	0	1	0	0	1	0	0	0	0	0	0	0	69
12011403	f	md	US	2016	0	0	0	0	0	0	0	0	0	0	0	0	40
12012728	f	md	BR	2016	0	0	0	0	0	0	0	0	0	0	0	0	31
12012752	f	md	FR	2016	0	0	0	0	0	0	0	0	0	0	0	0	80
12013562	m	md	FR	2016	1	0	0	0	0	0	0	0	0	0	0	0	
12013612	f	cn	FR	2016	0	0	0	0	0	0	0	0	0	0	0	0	57
12013847	m	cn	US	2016	0	1	0	0	1	0	0	0	0	0	0	0	

12014105	f	cn	US	2016	0	1	0	0	1	0	0	0	0	0	0	0	
12016655	m	cn	US	2016	0	0	0	0	0	0	0	0	0	0	0	0	69
12020236	f	md	JP	2016	0	1	0	0	1	0	0	0	0	0	0	1	59
12035706	f	ot	IT	2016	0	1	0	0	1	0	0	0	0	0	0	0	55
12036835	m	md	FR	2016	0	0	0	0	0	0	0	0	0	0	0	0	66
12037067	m	md	IT	2016	0	1	0	0	1	0	0	0	0	0	0	1	79
12037188	m	md	JP	2016	0	1	0	0	1	0	0	0	0	0	0	0	62
12037218	m	md	FR	2016	0	0	0	0	0	0	0	0	0	0	0	0	73
12037358	m	cn	FR	2016	0	1	0	0	1	0	0	0	0	0	0	0	80
12040317	f	cn	TH	2016	0	0	0	0	0	0	0	0	0	0	0	0	30
12045985	f	md	US	2016	0	0	0	0	0	0	0	0	0	0	0	0	50
12046911	m	ot	US	2016	0	1	0	0	1	0	0	0	0	0	0	0	78
12047099	f	md	Not re	2016	1	0	0	0	0	0	0	0	0	0	0	0	
12047473	m	md	FR	2016	1	0	0	0	0	0	0	0	0	0	0	0	51
12047907	m	md	US	2016	1	0	0	0	0	0	0	0	0	0	0	0	62
12047920	f	md	US	2016	0	1	0	0	1	0	0	0	0	0	0	0	51
12051994	f	md	US	2016	0	1	0	0	1	0	0	0	0	0	0	0	34
12053045	f	md	FR	2016	1	0	0	0	0	0	0	0	0	0	0	0	
12054196	m	ph	FR	2016	1	0	0	0	0	0	0	0	0	0	0	0	
12055357	f	md	BE	2016	0	0	0	0	0	0	0	0	0	0	0	0	54
12055367	Not	ot	US	2016	0	0	0	0	0	0	0	0	0	0	0	0	
12056007	f	ot	HU	2016	0	1	0	0	1	0	0	0	0	0	0	0	46
12056737	Not	md	FR	2016	1	0	0	0	0	0	0	0	0	0	0	0	
12058986	Not	Not repo	NL	2016	1	0	0	0	0	0	0	0	0	0	0	0	
12059541	f	md	FR	2016	1	0	0	0	0	0	0	0	0	0	0	0	
12059753	f	ph	US	2016	0	0	0	0	0	0	0	0	0	0	0	0	53
12060079	m	cn	GB	2016	1	0	0	0	0	0	0	0	0	0	0	0	79
12060134	f	ph	JP	2016	1	0	0	0	0	0	0	0	0	0	0	0	65
12060709	f	md	JP	2016	0	0	0	0	0	0	0	0	0	0	0	0	78
12060740	f	md	JP	2016	1	0	0	0	0	0	0	0	0	0	0	0	51
12060978	m	ot	US	2016	0	0	0	0	0	0	0	0	0	0	0	0	59
12061030	m	cn	US	2016	0	1	0	0	1	0	0	0	0	0	0	0	
12061034	f	md	TR	2016	0	1	0	0	1	0	0	0	0	0	0	0	54
12061113	Not	md	US	2016	0	1	0	0	1	0	0	0	0	0	0	0	

12061267	Not	ot	KR	2016	0	0	0	0	0	0	0	0	0	0	0	0	44
12063265	f	ot	US	2016	0	0	0	0	0	0	0	0	0	0	0	0	
12063926	f	ot	US	2016	1	0	0	0	0	0	0	0	0	0	0	0	
12065608	f	md	FR	2016	1	0	0	0	0	0	0	0	0	0	0	0	
12066869	m	md	DE	2016	1	0	0	0	0	0	0	0	0	0	0	0	59
12067703	m	ot	BG	2016	0	1	0	0	1	0	0	0	0	0	0	0	
12068531	f	Not repo	US	2016	1	0	0	0	0	0	0	0	0	0	0	0	
12068861	Not	ot	KR	2016	0	0	0	0	0	0	0	0	0	0	0	0	
12069243	m	md	JP	2016	0	1	0	0	1	0	0	0	0	0	0	1	63
12069547	f	md	FR	2016	1	0	0	0	0	0	0	0	0	0	0	0	
12069762	f	ot	US	2016	0	0	0	0	0	0	0	0	0	0	0	0	52
12069822	f	ph	FR	2016	1	0	0	0	0	0	0	0	0	0	0	0	57.4
12070516	m	md	FR	2016	1	0	0	0	0	0	0	0	0	0	0	0	
12070586	f	md	FR	2016	1	0	0	0	0	0	0	0	0	0	0	0	
12070682	f	md	JP	2016	0	1	0	0	1	0	0	0	0	0	0	0	56
12070880	m	md	US	2016	0	0	0	0	0	0	0	0	0	0	0	0	74
12070970	Not	md	GB	2016	0	0	0	0	0	0	0	0	0	0	0	0	
12070971	Not	md	GB	2016	0	0	0	0	0	0	0	0	0	0	0	0	
12070974	f	md	FR	2016	1	0	0	0	0	0	0	0	0	0	0	0	
12071627	m	md	FR	2016	0	0	0	0	0	0	0	0	0	0	0	0	54
12071646	m	md	IT	2016	0	1	0	0	1	0	0	0	0	0	0	0	56
12072040	f	ot	BG	2016	0	1	0	0	1	0	0	0	0	0	0	0	62
12072409	m	cn	GB	2016	1	0	0	0	0	0	0	0	0	0	0	1	76
12072520	m	cn	GB	2016	0	0	0	0	0	0	0	0	0	0	0	0	79
12072769	f	ot	NL	2016	0	0	0	0	0	0	0	0	0	0	0	0	82
12072933	m	md	FR	2016	1	0	0	0	0	0	0	0	0	0	0	0	
12073145	f	md	JP	2016	0	0	0	0	0	0	0	0	0	0	0	0	
12073473	m	md	JP	2016	0	1	0	0	1	0	0	0	0	0	0	0	70
12073604	f	ph	JP	2016	0	0	0	0	0	0	0	0	0	0	0	0	75
12073943	m	cn	GB	2016	0	1	0	0	1	0	0	0	0	0	0	0	70
12074772	m	ot	GB	2016	0	1	0	0	1	0	0	0	0	0	0	0	
12074775	f	cn	SG	2016	1	0	0	0	0	0	0	0	0	0	0	0	59.3
12075185	f	ot	JP	2016	0	0	0	0	0	0	0	0	0	0	0	0	52
12075192	f	md	IT	2016	1	0	0	0	0	0	0	0	0	0	0	0	64

12075241	m	md	MX	2016	1	0	0	0	0	0	0	0	0	0	0	0	83
12075275	f	md	US	2016	1	0	0	0	0	0	0	0	0	0	0	0	69.7
12075320	m	md	IT	2016	0	1	0	0	1	0	0	0	0	0	0	1	58
12075397	Not	ot	IT	2016	0	0	0	0	0	0	0	0	0	0	0	0	
12075399	f	md	AT	2016	0	1	0	0	1	0	0	0	0	0	0	0	
12075632	f	md	DE	2016	0	1	0	0	1	0	0	0	0	0	0	0	54
12075639	Not	md	ES	2016	0	1	0	0	1	0	0	0	0	0	0	0	
12075642	Not	md	US	2016	0	1	0	0	1	0	0	0	0	0	0	0	
12075648	m	ot	NL	2016	0	1	0	0	1	0	0	0	0	0	0	0	54
12075725	f	ph	FR	2016	0	1	0	0	1	0	0	0	0	0	0	0	55
12075730	Not	ot	IT	2016	0	1	0	0	1	0	0	0	0	0	0	1	
12076016	m	md	NL	2016	0	1	0	0	1	0	0	0	0	0	0	0	65
12076060	f	ot	NL	2016	0	0	0	0	0	0	0	0	0	0	0	0	82
12076273	m	ot	GB	2016	0	1	0	0	1	0	0	0	0	0	0	0	73
12076477	Not	cn	US	2016	0	1	1	0	0	0	0	0	0	0	0	0	
12076705	m	cn	US	2016	0	1	1	0	0	0	0	0	0	0	0	0	68
12077030	f	cn	GB	2016	1	0	0	0	0	0	0	0	0	0	0	0	74
12077078	f	md	CH	2016	0	0	0	0	0	0	0	0	0	0	0	0	
12077533	Not	ot	IT	2016	0	1	0	0	1	0	0	0	0	0	0	0	
12078200	f	ot	US	2016	0	0	0	0	0	0	0	0	0	0	0	0	65
12079774	f	md	US	2016	1	0	0	0	0	0	0	0	0	0	0	0	
12079778	m	md	IT	2016	0	1	0	0	1	0	0	0	0	0	0	0	
12080052	m	md	TR	2016	0	1	0	0	1	0	0	0	0	0	0	0	36
12080327	f	md	SK	2016	1	0	0	0	0	0	0	0	0	0	0	0	72
12080997	f	md	SK	2016	1	0	0	0	0	0	0	0	0	0	0	0	82
12081008	m	md	JP	2016	1	0	0	0	0	0	0	0	0	0	0	1	
12081178	m	md	US	2016	1	0	0	0	0	0	0	0	0	0	0	0	
12081454	f	ph	US	2016	1	0	0	0	0	0	0	0	0	0	0	0	72
12081464	f	cn	US	2016	1	0	0	0	0	0	0	0	0	0	0	0	54
12082590	m	ph	US	2016	0	1	0	0	1	0	0	0	0	0	0	0	77
12082636	m	ot	IT	2016	0	1	0	0	1	0	0	0	0	0	0	0	78
12083011	m	ot	US	2016	0	0	0	0	0	0	0	0	0	0	0	0	54
12085202	f	md	US	2016	1	0	0	0	0	0	0	0	0	0	0	0	
12085466	f	cn	US	2016	1	0	0	0	0	0	0	0	0	0	0	0	

12085476	f	cn	US	2016	1	0	0	0	0	0	0	0	0	0	0	0	
12085783	f	cn	US	2016	1	0	0	0	0	0	0	0	0	0	0	0	70
12085803	m	cn	US	2016	1	0	0	0	0	0	0	0	0	0	0	0	61
12085922	f	md	US	2016	1	0	0	0	0	0	0	0	0	0	0	0	
12088186	f	md	ES	2016	1	0	0	0	0	0	0	0	0	0	0	0	71
12088363	Not	md	NL	2016	0	0	0	0	0	0	0	0	0	0	0	0	
12088368	Not	md	NL	2016	0	0	0	0	0	0	0	0	0	0	0	0	
12089040	m	ot	BE	2016	0	1	0	0	1	0	0	0	0	0	0	1	61
12089964	f	md	US	2016	1	0	0	0	0	0	0	0	0	0	0	0	
12090031	f	cn	US	2016	1	0	0	0	0	0	0	0	0	0	0	0	
12090221	m	md	US	2016	0	1	0	0	1	0	0	0	0	0	0	0	
12090259	m	cn	IT	2016	0	1	0	0	1	0	0	0	0	0	0	0	74
12090754	m	md	FR	2016	1	0	0	0	0	0	0	0	0	0	0	0	
12090975	Not	ot	US	2016	0	0	0	0	0	0	0	0	0	0	0	0	
12091189	Not	ot	CN	2016	0	0	0	0	0	0	0	0	0	0	0	0	
12091201	Not	ot	CN	2016	0	0	0	0	0	0	0	0	0	0	0	0	
12091202	Not	md	KR	2016	0	0	0	0	0	0	0	0	0	0	0	0	
12091322	m	md	FR	2016	1	0	0	0	0	0	0	0	0	0	0	0	
12091455	m	md	IT	2016	0	0	0	0	0	0	0	0	0	0	0	0	43
12091617	f	cn	JP	2016	0	1	0	0	1	0	0	0	0	0	0	0	67
12092802	m	md	DE	2016	1	0	0	0	0	0	0	0	0	0	0	0	67
12093716	m	cn	AR	2016	0	0	0	0	0	0	0	0	0	0	0	0	46
12093763	f	md	Not re	2016	0	0	0	0	0	0	0	0	0	0	0	0	65
12094037	m	Not repo	Not re	2016	0	0	0	0	0	0	0	0	0	0	0	0	71
12094124	m	md	TR	2016	0	1	0	0	1	0	0	0	0	0	0	0	51
12095475	m	md	JP	2016	1	0	0	0	0	0	0	0	0	0	0	1	77
12095560	f	md	AU	2016	0	0	0	0	0	0	0	0	0	0	0	0	32
12095571	m	cn	BR	2016	0	1	0	0	1	0	0	0	0	0	0	0	
12095816	m	md	US	2016	0	1	0	0	1	0	0	0	0	0	0	0	
12096368	m	md	DE	2016	1	0	0	0	0	0	0	0	0	0	0	0	49
12096914	f	md	JP	2016	0	1	0	0	1	0	0	0	0	0	0	0	64
12096915	f	cn	JP	2016	0	1	0	0	1	0	0	0	0	0	0	0	70
12096929	m	cn	US	2016	0	1	0	0	1	0	0	0	0	0	0	1	70
12096930	m	md	HR	2016	0	1	0	0	1	0	0	0	0	0	0	0	61

12099441	Not	md	US	2016	0	0	0	0	0	0	0	0	0	0	0	0	
12099833	m	cn	GB	2016	1	0	0	0	0	0	0	0	0	0	0	0	72
12099969	m	cn	AR	2016	0	0	0	0	0	0	0	0	0	0	0	0	55
12100021	f	cn	AR	2016	0	0	0	0	0	0	0	0	0	0	0	0	67
12100612	Not	ot	CN	2016	1	0	0	0	0	0	0	0	0	0	0	0	
12100763	Not	ot	US	2016	0	1	0	0	1	0	0	0	0	0	0	0	
12100845	m	cn	GB	2016	1	0	0	0	0	0	0	0	0	0	0	0	60
12100997	m	md	TR	2016	0	1	0	0	1	0	0	0	0	0	0	0	55
12101257	m	cn	FR	2016	0	0	0	0	0	0	0	0	0	0	0	0	
12101581	f	cn	JP	2016	0	1	0	0	1	0	0	0	0	0	0	0	68
12104488	m	md	EC	2016	0	0	0	0	0	0	0	0	0	0	0	0	58
12104820	f	cn	US	2016	0	0	0	0	0	0	0	0	0	0	0	0	
12105311	m	md	US	2016	0	0	0	0	0	0	0	0	0	0	0	0	44
12105347	f	cn	US	2016	0	1	1	0	0	0	0	0	0	0	0	0	
12105480	Not	md	FR	2016	0	0	0	0	0	0	0	0	0	0	0	0	
12105486	f	cn	US	2016	0	0	0	0	0	0	0	0	0	0	0	0	
12105805	m	cn	US	2016	0	0	0	0	0	0	0	0	0	0	0	0	
12105820	f	cn	GB	2016	0	0	0	0	0	0	0	0	0	0	0	0	57
12106070	f	md	Not re	2016	1	0	0	0	0	0	0	0	0	0	0	0	80
12106274	m	ot	US	2016	0	0	0	0	0	0	0	0	0	0	0	0	68
12108737	f	md	FR	2016	1	0	0	0	0	0	0	0	0	0	0	0	56.2
12108886	f	md	US	2016	0	0	0	0	0	0	0	0	0	0	0	0	44
12109149	m	cn	JP	2016	0	1	0	0	1	0	0	0	0	0	0	0	41
12109438	Not	md	US	2016	0	0	0	0	0	0	0	0	0	0	0	0	
12109448	f	cn	GB	2016	1	0	0	0	0	0	0	0	0	0	0	0	71
12109997	m	md	FR	2016	0	1	0	0	1	0	0	0	0	0	0	0	
12110202	f	cn	Not re	2016	0	0	0	0	0	0	0	0	0	0	0	0	
12110233	m	md	CH	2016	0	0	0	0	0	0	0	0	0	0	0	0	74
12110295	m	md	Not re	2016	1	0	0	0	0	0	0	0	0	0	0	0	77
12110464	m	ot	IT	2016	0	1	0	0	1	0	0	0	0	0	0	0	70
12110625	Not	cn	US	2016	0	0	0	0	0	0	0	0	0	0	0	0	
12110779	f	md	AU	2016	0	0	0	0	0	0	0	0	0	0	0	0	
12111007	Not	md	US	2016	0	0	0	0	0	0	0	0	0	0	0	0	
12111145	m	cn	IT	2016	0	1	0	0	1	0	0	0	0	0	0	0	66

12111386	f	md	FR	2016	1	0	0	0	0	0	0	0	0	0	0	0	
12112608	m	md	JP	2016	0	0	0	0	0	0	0	0	0	0	0	0	64
12112774	m	ot	US	2016	0	0	0	0	0	0	0	0	0	0	0	0	47
12113714	f	md	FR	2016	1	0	0	0	0	0	0	0	0	0	0	0	
12114651	f	md	CH	2016	0	0	0	0	0	0	0	0	0	0	0	0	74
12114990	f	cn	US	2016	0	1	0	0	1	0	0	0	0	0	0	0	
12115201	f	md	JP	2016	1	0	0	0	0	0	0	0	0	0	0	0	70
12115385	f	cn	JP	2016	0	1	0	0	1	0	0	0	0	0	0	0	64
12116180	f	ot	US	2016	0	1	0	0	1	0	0	0	0	0	0	0	66
12117935	m	md	PH	2016	0	1	1	0	0	0	0	0	0	0	0	0	
12118141	f	md	FR	2016	1	0	0	0	0	0	0	0	0	0	0	0	
12118300	m	cn	JP	2016	0	1	0	0	1	0	0	0	0	0	0	0	68
12118775	m	md	Not re	2016	1	0	0	0	0	0	0	0	0	0	0	0	70
12119454	m	md	AU	2016	0	0	0	0	0	0	0	0	0	0	0	0	78
12119780	f	cn	US	2016	0	1	1	0	0	0	0	0	0	0	0	1	70
12119867	f	ot	KR	2016	0	0	0	0	0	0	0	0	0	0	0	0	59
12120682	f	cn	DE	2016	0	0	0	0	0	0	0	0	0	0	0	0	78
12120957	f	md	JP	2016	1	0	0	0	0	0	0	0	0	0	0	0	72.3
12121151	f	ot	NL	2016	0	1	0	0	1	0	0	0	0	0	0	0	
12121323	f	md	Not re	2016	1	0	0	0	0	0	0	0	0	0	0	0	80
12122300	m	ot	KR	2016	0	0	0	0	0	0	0	0	0	0	0	0	45
12122347	m	ot	CN	2016	0	0	0	0	0	0	0	0	0	0	0	0	58
12122448	f	md	CN	2016	0	0	0	0	0	0	0	0	0	0	0	0	51.9
12122931	m	cn	US	2016	0	1	0	0	1	0	0	0	0	0	0	0	
12123152	f	ot	US	2016	0	1	0	0	1	0	0	0	0	0	0	0	73
12123155	m	cn	IT	2016	0	1	0	0	1	0	0	0	0	0	0	0	41
12123187	m	ot	GB	2016	0	1	0	0	1	0	0	0	0	0	0	0	62
12123904	m	md	US	2016	0	1	0	0	1	0	0	0	0	0	0	0	
12123908	f	cn	GB	2016	1	0	0	0	0	0	0	0	0	0	0	0	
12123972	m	cn	Not re	2016	0	0	0	0	0	0	0	0	0	0	0	0	68
12124312	m	md	FR	2016	1	0	0	0	0	0	0	0	0	0	0	0	
12124320	Not	md	JP	2016	0	0	0	0	0	0	0	0	0	0	0	0	
12124392	f	ot	US	2016	0	0	0	0	0	0	0	0	0	0	0	0	54
12124631	m	ot	NL	2016	0	1	0	0	1	0	0	0	0	0	0	0	

12124682	f	md	US	2016	0	0	0	0	0	0	0	0	0	0	0	0	
12126345	m	ot	IT	2016	0	1	0	0	1	0	0	0	0	0	0	0	68
12127005	m	cn	JP	2016	0	1	0	0	1	0	0	0	0	0	0	0	72
12127262	f	md	PL	2016	1	0	0	0	0	0	0	0	0	0	0	0	64
12128064	m	cn	US	2016	0	0	0	0	0	0	0	0	0	0	0	0	
12128214	f	ot	US	2016	1	0	0	0	0	0	0	0	0	0	0	0	
12130310	f	md	FR	2016	1	0	0	0	0	0	0	0	0	0	0	0	50.8
12130611	f	cn	GB	2016	1	0	0	0	0	0	0	0	0	0	0	0	71
12130624	m	cn	GB	2016	1	0	0	0	0	0	0	0	0	0	0	0	
12131869	m	md	AU	2016	0	0	0	0	0	0	0	0	0	0	0	0	52
12133379	f	md	BE	2016	0	0	0	0	0	0	0	0	0	0	0	0	66
12133435	m	md	JP	2016	0	0	0	0	0	0	0	0	0	0	0	0	73
12133674	m	cn	JP	2016	0	1	0	0	1	0	0	0	0	0	0	0	42
12133932	Not	ot	AU	2016	1	0	0	0	0	0	0	0	0	0	0	0	
12134032	m	ot	ES	2016	0	0	0	0	0	0	0	0	0	0	0	0	54
12134383	m	ot	US	2016	0	1	0	0	1	0	0	0	0	0	0	0	65
12135280	f	md	ES	2016	1	0	0	0	0	0	0	0	0	0	0	0	57.4
12135798	f	md	AU	2016	0	1	0	0	1	0	0	0	0	0	0	0	43
12135846	m	md	FR	2016	1	0	0	0	0	0	0	0	0	0	0	0	
12135972	Not	ot	US	2016	0	0	0	0	0	0	0	0	0	0	0	0	
12136014	f	md	FR	2016	1	0	0	0	0	0	0	0	0	0	0	0	
12136438	Not	cn	US	2016	0	0	0	0	0	0	0	0	0	0	0	0	
12136866	m	ot	US	2016	0	0	0	0	0	0	0	0	0	0	0	0	32
12137339	m	md	AU	2016	0	1	0	0	1	0	0	0	0	0	0	0	68
12137382	m	cn	GB	2016	1	0	0	0	0	0	0	0	0	0	0	0	
12137699	f	cn	GB	2016	1	0	0	0	0	0	0	0	0	0	0	0	
12138337	m	md	IT	2016	0	0	0	0	0	0	0	0	0	0	0	0	70
12138456	m	cn	JP	2016	0	1	0	0	1	0	0	0	0	0	0	0	74
12139209	m	md	AU	2016	0	1	0	0	1	0	0	0	0	0	0	0	
12139294	Not	ot	US	2016	0	0	0	0	0	0	0	0	0	0	0	0	
12139457	m	md	AU	2016	0	1	0	0	1	0	0	0	0	0	0	0	76
12140197	f	md	FR	2016	1	0	0	0	0	0	0	0	0	0	0	0	
12140479	f	cn	GB	2016	0	0	0	0	0	0	0	0	0	0	0	0	60
12140836	m	md	AU	2016	0	1	0	0	1	0	0	0	0	0	0	0	67

12141034	f	cn	GB	2016	1	0	0	0	0	0	0	0	0	0	0	0	
12141040	m	cn	GB	2016	1	0	0	0	0	0	0	0	0	0	0	0	72
12141259	m	cn	TH	2016	0	0	0	0	0	0	0	0	0	0	0	0	64
12141940	f	cn	US	2016	0	1	0	0	1	0	0	0	0	0	0	1	61
12142054	m	cn	JP	2016	0	1	0	0	1	0	0	0	0	0	0	0	67
12142055	Not	ot	US	2016	0	1	0	0	1	0	0	0	0	0	0	0	
12142388	m	ot	ES	2016	0	0	0	0	0	0	0	0	0	0	0	0	74
12142677	f	md	ES	2016	1	0	0	0	0	0	0	0	0	0	0	0	78
12145137	f	md	DE	2016	0	0	0	0	0	0	0	0	0	0	0	0	62
12145138	f	md	FR	2016	0	0	0	0	0	0	0	0	0	0	0	0	72
12145325	f	md	NL	2016	0	0	0	0	0	0	0	0	0	0	0	0	62
12145513	m	cn	US	2016	0	1	0	0	1	0	0	0	0	0	0	0	
12146563	m	ot	US	2016	0	0	0	0	0	0	0	0	0	0	0	0	57
12147040	f	ot	US	2016	0	1	0	0	1	0	0	0	0	0	0	0	
12147115	f	md	FR	2016	1	0	0	0	0	0	0	0	0	0	0	0	68
12147337	f	md	FR	2016	1	0	0	0	0	0	0	0	0	0	0	0	
12147739	m	ph	US	2016	0	0	0	0	0	0	0	0	0	0	0	0	52
12147786	m	ot	KR	2016	0	0	0	0	0	0	0	0	0	0	0	0	49
12149058	f	cn	JP	2016	0	1	0	0	1	0	0	0	0	0	0	0	59
12152826	m	md	FR	2016	1	0	0	0	0	0	0	0	0	0	0	0	51.9
12152867	m	ot	JP	2016	0	1	0	0	1	0	0	0	0	0	0	0	69
12153024	m	ot	US	2016	0	1	0	0	1	0	0	0	0	0	0	0	60
12153582	f	md	AU	2016	0	1	0	0	1	0	0	0	0	0	0	0	60
12153603	Not	md	IT	2016	0	1	0	0	1	0	0	0	0	0	0	0	
12153683	m	md	IT	2016	0	1	0	0	1	0	0	0	0	0	0	0	63
12154004	f	ph	US	2016	0	0	0	0	0	0	0	0	0	0	0	0	
12154167	m	ot	US	2016	0	0	0	0	0	0	0	0	0	0	0	0	
12154305	Not	md	IT	2016	0	1	0	0	1	0	0	0	0	0	0	0	
12154795	f	md	DE	2016	0	0	0	0	0	0	0	0	0	0	0	0	60
12155333	f	ot	US	2016	0	0	0	0	0	0	0	0	0	0	0	0	57
12155933	m	cn	AU	2016	0	1	0	0	1	0	0	0	0	0	0	0	70
12156135	f	md	FR	2016	0	0	0	0	0	0	0	0	0	0	0	0	72
12156688	f	md	FR	2016	0	0	0	0	0	0	0	0	0	0	0	0	72
12156715	f	md	LB	2016	1	0	0	0	0	0	0	0	0	0	0	0	70

12157404	m	cn	JP	2016	0	1	0	0	1	0	0	0	0	0	0	0	69
12157823	f	ot	FR	2016	1	0	0	0	0	0	0	0	0	0	0	0	
12159344	m	cn	CN	2016	0	0	0	0	0	0	0	0	0	0	0	0	
12159455	Not	md	LB	2016	0	1	0	0	1	0	0	0	0	0	0	0	
12159456	f	md	IT	2016	0	1	0	0	1	0	0	0	0	0	0	0	
12160010	m	md	FR	2016	1	0	0	0	0	0	0	0	0	0	0	0	
12160116	Not	ot	DE	2016	0	0	0	0	0	0	0	0	0	0	0	0	
12160119	Not	ot	DE	2016	0	0	0	0	0	0	0	0	0	0	0	0	
12160299	m	cn	GB	2016	0	1	0	0	1	0	0	0	0	0	0	0	61
12160422	f	md	AU	2016	0	1	0	0	1	0	0	0	0	0	0	0	
12160443	m	cn	GB	2016	1	0	0	0	0	0	0	0	0	0	0	0	78
12161422	m	md	VN	2016	0	0	0	0	0	0	0	0	0	0	0	0	74
12161969	Not	md	SG	2016	0	0	0	0	0	0	0	0	0	0	0	0	
12162840	f	ph	US	2016	0	1	0	0	1	0	0	0	0	0	0	0	84
12163581	m	cn	CA	2016	0	0	0	0	0	0	0	0	0	0	0	0	76
12163826	f	md	Not re	2016	1	0	0	0	0	0	0	0	0	0	0	0	46
12164752	f	cn	AU	2016	1	0	0	0	0	0	0	0	0	0	0	0	
12165508	m	ot	US	2016	0	0	0	0	0	0	0	0	0	0	0	0	66
12165536	f	md	FR	2016	1	0	0	0	0	0	0	0	0	0	0	0	77
12166661	m	md	JP	2016	0	0	0	0	0	0	0	0	0	0	0	0	70
12166750	f	md	FR	2016	1	0	0	0	0	0	0	0	0	0	0	0	
12166919	m	cn	US	2016	0	1	0	0	1	0	0	0	0	0	0	0	
12167031	Not	cn	IT	2016	0	1	0	0	1	0	0	0	0	0	0	0	76
12167070	m	md	US	2016	0	0	0	0	0	0	0	0	0	0	0	0	69
12167147	Not	ot	KR	2016	0	0	0	0	0	0	0	0	0	0	0	0	
12167754	f	md	FR	2016	1	0	0	0	0	0	0	0	0	0	0	0	
12168321	m	md	Not re	2016	1	0	0	0	0	0	0	0	0	0	0	0	70
12168798	f	md	JP	2016	0	1	0	0	1	0	0	0	0	0	0	0	50
12168856	m	ot	US	2016	0	1	0	0	1	0	0	0	0	0	0	0	73
12168860	m	md	IT	2016	1	0	0	0	0	0	0	0	0	0	0	0	78
12168862	f	cn	GB	2016	1	0	0	0	0	0	0	0	0	0	0	0	
12168921	f	cn	GB	2016	1	0	0	0	0	0	0	0	0	0	0	0	
12169226	f	cn	JP	2016	0	0	0	0	0	0	1	0	1	0	0	0	70
12169261	m	cn	GR	2016	0	1	1	0	0	0	0	0	0	0	0	1	59

12169570	f	md	Not re	2016	0	0	0	0	0	0	0	0	0	0	0	0	61
12169727	f	md	US	2016	1	0	0	0	0	0	0	0	0	0	0	0	80
12170139	m	ot	IT	2016	0	1	0	0	1	0	0	0	0	0	0	0	78
12170691	f	ot	KR	2016	1	0	0	0	0	0	0	0	0	0	0	0	35
12170780	m	ot	US	2016	0	0	0	0	0	0	0	0	0	0	0	0	74
12170786	m	md	KR	2016	1	0	0	0	0	0	0	0	0	0	0	1	46.9
12171216	f	cn	JP	2016	0	0	0	0	0	0	1	0	1	0	0	0	76
12171552	f	md	FR	2016	1	0	0	0	0	0	0	0	0	0	0	0	
12171633	Not	ot	JP	2016	0	0	0	0	0	0	0	0	0	0	0	0	
12172224	m	md	BE	2016	0	1	0	0	1	0	0	0	0	0	0	0	
12172379	f	ot	Not re	2016	0	0	0	0	0	0	0	0	0	0	0	0	65
12172516	m	cn	CN	2016	1	0	0	0	0	0	0	0	0	0	0	0	
12172681	m	md	IT	2016	0	1	0	0	1	0	0	0	0	0	0	0	73
12172876	m	md	JP	2016	0	1	0	0	1	0	0	0	0	0	0	1	86
12172886	m	md	IT	2016	0	1	0	0	1	0	0	0	0	0	0	0	67
12173020	m	cn	GR	2016	0	1	1	0	0	0	0	0	0	0	0	0	70
12173146	m	md	CZ	2016	1	0	0	0	0	0	0	0	0	0	0	0	80
12173534	f	md	FR	2016	1	0	0	0	0	0	0	0	0	0	0	0	74
12173856	m	md	JP	2016	0	0	0	0	0	0	0	0	0	0	0	0	38
12174373	Not	md	JP	2016	0	1	0	0	1	0	0	0	0	0	0	0	
12174378	m	cn	JP	2016	0	1	0	0	1	0	0	0	0	0	0	0	72
12174419	Not	ot	KR	2016	0	0	0	0	0	0	0	0	0	0	0	0	
12174466	m	md	CZ	2016	0	0	0	0	0	0	0	0	0	0	0	0	62
12174512	m	cn	IT	2016	0	1	0	0	1	0	0	0	0	0	0	0	46
12174517	f	cn	US	2016	0	1	0	0	1	0	0	0	0	0	0	0	
12175045	f	ot	FR	2016	0	0	0	0	0	0	0	0	0	0	0	0	80
12175439	m	cn	AR	2016	0	0	0	0	0	0	0	0	0	0	0	0	67
12176096	f	ph	AU	2016	0	1	0	0	1	0	0	0	0	0	0	0	82
12176397	f	ph	AU	2016	0	1	0	0	1	0	0	0	0	0	0	0	72
12176400	m	ph	AU	2016	0	1	0	0	1	0	0	0	0	0	0	0	78
12176403	f	ph	AU	2016	0	1	0	0	1	0	0	0	0	0	0	0	69
12176404	m	ph	AU	2016	0	1	0	0	1	0	0	0	0	0	0	0	67
12176405	f	ph	AU	2016	0	1	0	0	1	0	0	0	0	0	0	0	77
12176408	m	md	AU	2016	0	1	0	0	1	0	0	0	0	0	0	0	57

12176411	m	ph	AU	2016	0	1	0	0	1	0	0	0	0	0	0	0	65
12176413	f	ph	AU	2016	0	1	0	0	1	0	0	0	0	0	0	0	63
12176555	m	md	AU	2016	0	1	0	0	1	0	0	0	0	0	0	0	
12176557	m	md	AU	2016	0	1	0	0	1	0	0	0	0	0	0	0	66
12176708	m	md	IL	2016	0	1	0	0	1	0	0	0	0	0	0	0	84
12178212	m	cn	JP	2016	0	1	0	0	1	0	0	0	0	0	0	0	75
12178220	f	ph	AU	2016	0	1	0	0	1	0	0	0	0	0	0	0	48
12178510	m	ph	AU	2016	0	1	0	0	1	0	0	0	0	0	0	0	67
12178513	m	ph	AU	2016	0	1	0	0	1	0	0	0	0	0	0	0	58
12178631	m	md	FR	2016	1	0	0	0	0	0	0	0	0	0	0	0	
12178792	m	md	FR	2016	0	0	0	0	0	0	0	0	0	0	0	0	
12179577	m	ph	US	2016	0	1	0	0	1	0	0	0	0	0	0	0	68
12179675	m	md	US	2016	0	0	0	0	0	0	0	0	0	0	0	0	32
12180659	f	md	BE	2016	1	0	0	0	0	0	0	0	0	0	0	0	84
12181515	m	ot	ES	2016	0	1	0	0	1	0	0	0	0	0	0	0	61
12181517	f	ot	IT	2016	0	1	0	0	1	0	0	0	0	0	0	0	
12181518	m	ph	AU	2016	0	1	0	0	1	0	0	0	0	0	0	0	68
12181528	m	ph	AU	2016	0	1	0	0	1	0	0	0	0	0	0	0	84
12182178	f	md	AU	2016	0	1	0	0	1	0	0	0	0	0	0	0	65
12182971	f	ot	ZA	2016	0	1	0	0	1	0	0	0	0	0	0	0	58
12183024	m	ot	US	2016	0	0	0	0	0	0	0	0	0	0	0	0	67
12183142	m	ot	JP	2016	0	1	0	0	1	0	0	0	0	0	0	0	61
12183399	m	md	US	2016	0	0	0	0	0	0	0	0	0	0	0	0	32
12183934	f	cn	GB	2016	1	0	0	0	0	0	0	0	0	0	0	0	78
12184890	f	md	FR	2016	1	0	0	0	0	0	0	0	0	0	0	0	
12185004	f	md	US	2016	0	1	0	0	1	0	0	0	0	0	0	0	
12185113	f	md	AT	2016	0	0	0	0	0	0	0	0	0	0	0	0	63
12185209	f	ot	US	2016	0	1	0	0	1	0	0	0	0	0	0	0	
12185422	f	md	Not re	2016	1	0	0	0	0	0	0	0	0	0	0	0	70
12185517	f	ot	DE	2016	0	0	0	0	0	0	0	0	0	0	0	0	59
12185531	f	md	JP	2016	1	0	0	0	0	0	0	0	0	0	0	0	73
12185829	m	md	Not re	2016	1	0	0	0	0	0	0	0	0	0	0	0	51
12186097	Not	ph	JP	2016	0	0	0	0	0	0	0	0	0	0	0	0	
12186355	f	md	US	2016	0	0	0	0	0	0	0	0	0	0	0	0	

12186476	m	ot	US	2016	0	0	0	0	0	0	0	0	0	0	0	0	83
12186809	f	ot	US	2016	0	0	0	0	0	0	0	0	0	0	0	0	65
12187418	m	ph	AU	2016	0	1	0	0	1	0	0	0	0	0	0	0	78
12187469	m	ot	GB	2016	0	0	0	0	0	0	0	0	0	0	0	0	53
12187839	f	cn	GB	2016	1	0	0	0	0	0	0	0	0	0	0	0	58
12187989	m	ot	IT	2016	0	1	0	0	1	0	0	0	0	0	0	0	64
12189344	f	md	FR	2016	1	0	0	0	0	0	0	0	0	0	0	0	
12189442	m	ot	JP	2016	0	1	0	0	1	0	0	0	0	0	0	1	67
12189481	m	md	JP	2016	0	1	0	0	1	0	0	0	0	0	0	0	
12189793	f	ph	CA	2016	1	0	0	0	0	0	0	0	0	0	0	0	71.2
12190764	m	cn	GB	2016	1	0	0	0	0	0	0	0	0	0	0	0	83
12191599	f	Not repo	US	2016	0	0	0	0	0	0	0	0	0	0	0	0	65.5
12192745	f	cn	JP	2016	0	1	0	0	1	0	0	0	0	0	0	0	52
12192916	f	ph	US	2016	0	1	0	0	1	0	0	0	0	0	0	0	
12193124	f	cn	US	2016	1	0	0	0	0	0	0	0	0	0	0	0	61.8
12193136	m	md	IE	2016	0	1	0	0	1	0	0	0	0	0	0	0	64
12193147	m	ot	IE	2016	0	1	0	0	1	0	0	0	0	0	0	0	67
12193182	f	ot	US	2016	0	0	0	0	0	0	0	0	0	0	0	0	63
12193202	f	md	US	2016	0	0	0	0	0	0	0	0	0	0	0	0	83
12193219	m	md	IT	2016	0	1	0	0	1	0	0	0	0	0	0	0	67
12193637	f	md	DE	2016	0	0	0	0	0	0	0	0	0	0	0	0	53
12193775	Not	ot	US	2016	0	0	0	0	0	0	0	0	0	0	0	0	
12194611	f	ot	US	2016	0	0	0	0	0	0	0	0	0	0	0	0	51
12194721	Not	cn	GB	2016	1	0	0	0	0	0	0	0	0	0	0	0	
12195225	m	md	JP	2016	0	0	0	0	0	0	0	0	0	0	0	0	67
12195846	f	cn	US	2016	0	0	0	0	0	0	0	0	0	0	0	0	74
12195959	f	ot	US	2016	0	0	0	0	0	0	0	0	0	0	0	0	65
12196305	m	md	IT	2016	0	1	0	0	1	0	0	0	0	0	0	0	58
12196621	Not	ot	US	2016	0	0	0	0	0	0	0	0	0	0	0	0	60
12197354	m	ot	CZ	2016	0	1	0	0	1	0	0	0	0	0	0	0	69
12197624	f	md	BG	2016	1	0	0	0	0	0	0	0	0	0	0	0	85
12197695	f	md	US	2016	0	0	0	0	0	0	0	0	0	0	0	0	
12197703	m	md	IT	2016	0	1	0	0	1	0	0	0	0	0	0	0	81
12199005	f	ot	US	2016	0	0	0	0	0	0	0	0	0	0	0	0	62

12199533	f	ot	JP	2016	0	0	0	0	0	0	0	0	0	0	0	0	59
12202170	m	ot	BE	2016	0	1	0	0	1	0	0	0	0	0	0	0	45
12202173	m	ot	BE	2016	0	1	0	0	1	0	0	0	0	0	0	0	75
12202754	m	ph	FR	2016	1	0	0	0	0	0	0	0	0	0	0	0	75.4
12202784	m	ot	NL	2016	0	1	0	0	1	0	0	0	0	0	0	0	51
12202965	m	ot	CH	2016	0	1	0	0	1	0	0	0	0	0	0	0	
12203027	f	md	FR	2016	1	0	0	0	0	0	0	0	0	0	0	0	
12203049	f	md	FR	2016	1	0	0	0	0	0	0	0	0	0	0	0	
12203200	Not	ot	US	2016	0	0	0	0	0	0	0	0	0	0	0	0	
12203341	f	md	US	2016	0	1	0	0	1	0	0	0	0	0	0	0	
12203356	f	cn	Not re	2016	0	0	0	0	0	0	0	0	0	0	0	0	57
12203358	m	cn	CN	2016	0	0	0	0	0	0	0	0	0	0	0	0	
12203449	f	cn	CN	2016	0	0	0	0	0	0	0	0	0	0	0	0	62
12204219	f	md	FR	2016	1	0	0	0	0	0	0	0	0	0	0	0	
12204436	f	cn	GB	2016	1	0	0	0	0	0	0	0	0	0	0	0	67
12205553	m	md	AU	2016	0	1	0	0	1	0	0	0	0	0	0	1	76
12206251	f	md	NL	2016	0	0	0	0	0	0	0	0	0	0	0	0	43
12206643	Not	md	US	2016	0	1	0	0	1	0	0	0	0	0	0	0	
12206729	m	cn	GB	2016	0	0	0	0	0	0	0	0	0	0	0	0	32
12206840	m	cn	US	2016	0	0	0	0	0	0	0	0	0	0	0	0	
12207382	Not	md	TH	2016	0	0	0	0	0	0	0	0	0	0	0	0	
12207664	f	md	FR	2016	1	0	0	0	0	0	0	0	0	0	0	0	
12208002	m	ot	IT	2016	0	1	0	0	1	0	0	0	0	0	0	0	60
12208130	f	md	FR	2016	1	0	0	0	0	0	0	0	0	0	0	0	
12208200	m	md	AU	2016	0	0	0	0	0	0	0	0	0	0	0	0	65
12209747	f	cn	CN	2016	0	0	0	0	0	0	0	0	0	0	0	0	69
12209760	f	cn	JP	2016	0	1	0	0	1	0	0	0	0	0	0	0	72
12209843	f	md	FR	2016	1	0	0	0	0	0	0	0	0	0	0	0	
12210054	m	ph	US	2016	0	1	0	0	1	0	0	0	0	0	0	0	
12210439	f	cn	DE	2016	0	0	0	0	0	0	0	0	0	0	0	0	53
12211379	Not	md	FR	2016	1	0	0	0	0	0	0	0	0	0	0	0	
12211386	f	md	FR	2016	1	0	0	0	0	0	0	0	0	0	0	0	68
12211778	m	ot	CN	2016	0	0	0	0	0	0	0	0	0	0	0	0	
12211917	m	md	JP	2016	1	0	0	0	0	0	0	0	0	0	0	1	60

12212016	f	md	CL	2016	0	0	0	0	0	0	0	0	0	0	0	0	48
12212123	f	cn	JP	2016	0	1	0	0	1	0	0	0	0	0	0	0	48
12212390	Not	cn	US	2016	0	1	0	0	1	0	0	0	0	0	0	0	
12212449	f	md	AU	2016	0	1	0	0	1	0	0	0	0	0	0	0	73
12212559	m	ot	IT	2016	0	1	0	0	1	0	0	0	0	0	0	0	71
12212707	m	cn	CN	2016	0	0	0	0	0	0	0	0	0	0	0	0	66
12212826	Not	md	US	2016	0	0	0	0	0	0	0	0	0	0	0	0	
12213017	m	md	US	2016	0	0	0	0	0	0	0	0	0	0	0	0	55
12213080	f	cn	CN	2016	0	0	0	0	0	0	0	0	0	0	0	0	56
12213135	m	ot	NL	2016	0	1	0	0	1	0	0	0	0	0	0	0	45
12213136	Not	md	AU	2016	0	1	0	0	1	0	0	0	0	0	0	0	72
12213737	m	cn	GB	2016	1	0	0	0	0	0	0	0	0	0	0	0	82
12213741	m	cn	GB	2016	1	0	0	0	0	0	0	0	0	0	0	1	
12214186	m	md	Not re	2016	1	0	0	0	0	0	0	0	0	0	0	0	41
12214319	Not	md	GB	2016	0	0	0	0	0	0	0	0	0	0	0	0	
12214534	Not	cn	GB	2016	1	0	0	0	0	0	0	0	0	0	0	0	
12214914	f	ot	NZ	2016	0	0	0	0	0	0	0	0	0	0	0	0	52
12215007	Not	ot	Not re	2016	1	0	0	0	0	0	0	0	0	0	0	0	
12215540	m	ot	JP	2016	0	0	0	0	0	0	0	0	0	0	0	0	68
12215960	f	cn	US	2016	0	1	0	0	1	0	0	0	0	0	0	0	
12215989	m	cn	CN	2016	0	0	0	0	0	0	0	0	0	0	0	0	46
12216185	Not	ot	JP	2016	0	0	0	0	0	0	0	0	0	0	0	0	
12216210	m	md	JP	2016	0	0	0	0	0	0	0	0	0	0	0	0	60
12216247	m	md	TR	2016	0	1	0	0	1	0	0	0	0	0	0	0	58
12217898	m	ot	US	2016	0	0	0	0	0	0	0	0	0	0	0	0	
12218239	Not	md	IT	2016	0	0	0	0	0	0	0	0	0	0	0	0	79
12218253	f	md	DE	2016	1	0	0	0	0	0	0	0	0	0	0	1	46
12218930	f	md	US	2016	1	0	0	0	0	0	0	0	0	0	0	1	
12219607	m	md	DE	2016	0	1	0	0	1	0	0	0	0	0	0	0	53
12219610	m	cn	JP	2016	0	1	0	0	1	0	0	0	0	0	0	0	72
12220041	Not	cn	CN	2016	0	0	0	0	0	0	0	0	0	0	0	0	
12220052	m	cn	GB	2016	0	0	0	0	0	0	0	0	0	0	0	0	32
12220198	f	cn	JP	2016	0	1	0	0	1	0	0	0	0	0	0	0	62
12220289	m	cn	CN	2016	0	0	0	0	0	0	0	0	0	0	0	0	38

12220301	f	ot	IT	2016	0	1	0	0	1	0	0	0	0	0	0	0	73
12220741	f	md	IT	2016	0	1	0	0	1	0	0	0	0	0	0	0	73
12220744	m	cn	JP	2016	0	1	0	0	1	0	0	0	0	0	0	1	68
12221605	m	md	FR	2016	1	0	0	0	0	0	0	0	0	0	0	0	
12223133	f	cn	GB	2016	1	0	0	0	0	0	0	0	0	0	0	0	83
12223146	f	cn	GB	2016	1	0	0	0	0	0	0	0	0	0	0	0	63
12223153	m	cn	GB	2016	1	0	0	0	0	0	0	0	0	0	0	0	85
12223255	f	md	JP	2016	0	1	0	0	1	0	0	0	0	0	0	0	63
12223270	m	cn	RO	2016	0	0	0	0	0	0	0	0	0	0	0	0	
12223312	f	md	DE	2016	1	0	0	0	0	0	0	0	0	0	0	0	65
12223534	f	md	US	2016	0	1	0	0	0	1	0	0	0	0	0	0	69
12223600	f	md	IE	2016	1	0	0	0	0	0	0	0	0	0	0	0	73.8
12223713	m	cn	US	2016	0	1	1	0	0	0	0	0	0	0	0	0	
12223840	Not	md	US	2016	1	0	0	0	0	0	0	0	0	0	0	0	
12224258	f	cn	CN	2016	0	0	0	0	0	0	0	0	0	0	0	0	
12224328	m	cn	CN	2016	0	0	0	0	0	0	0	0	0	0	0	0	61
12224358	m	cn	US	2016	0	0	0	0	0	0	0	0	0	0	0	0	59
12224362	m	ot	GB	2016	0	0	0	0	0	0	0	0	0	0	0	0	60
12224502	m	cn	JP	2016	0	1	0	0	1	0	0	0	0	0	0	1	72
12224864	Not	md	US	2016	0	0	0	0	0	0	0	0	0	0	0	0	70
12225177	m	ot	TN	2016	0	0	0	0	0	0	0	0	0	0	0	0	67
12225533	Not	ot	TN	2016	0	0	0	0	0	0	0	0	0	0	0	0	
12226347	f	md	BE	2016	0	0	0	0	0	0	0	0	0	0	0	0	54
12226368	m	cn	GB	2016	0	0	0	0	0	0	0	0	0	0	0	0	67
12226597	f	md	Not re	2016	1	0	0	0	0	0	0	0	0	0	0	0	56
12226903	m	md	JP	2016	0	1	0	0	1	0	0	0	0	0	0	0	
12227108	f	cn	US	2016	1	0	0	0	0	0	0	0	0	0	0	0	
12227110	f	cn	US	2016	1	0	0	0	0	0	0	0	0	0	0	0	
12227469	Not	cn	GB	2016	0	0	0	0	0	0	0	0	0	0	0	0	
12227534	m	cn	JP	2016	0	1	0	0	1	0	0	0	0	0	0	0	77
12230261	f	ot	US	2016	1	0	0	0	0	0	0	0	0	0	0	0	62
12230427	m	ph	US	2016	0	0	0	0	0	0	0	0	0	0	0	0	71
6997551	f	cn	US	2016	0	0	0	0	0	0	0	0	0	0	0	0	47
8410775	m	cn	CN	2016	1	0	0	0	0	0	0	0	0	0	0	0	49

8663469	m	ot	JP	2016	0	0	0	0	0	0	0	0	0	0	0	0	62
8911609	m	md	JP	2016	0	0	0	0	0	0	0	0	0	0	0	0	66
8911620	m	md	JP	2016	0	0	0	0	0	0	0	0	0	0	0	0	77
8922648	f	md	US	2016	0	0	0	0	0	0	0	0	0	0	0	0	47
8936422	f	md	JP	2016	0	0	0	0	0	0	0	0	0	0	0	0	62
8950228	f	ot	US	2016	1	0	0	0	0	0	0	0	0	0	0	0	78
8956755	Not	md	TW	2016	1	0	0	0	0	0	0	0	0	0	0	0	
9028910	m	cn	GB	2016	0	0	0	0	0	0	0	0	0	0	0	0	77
9171037	m	md	JP	2016	0	0	0	0	0	0	0	0	0	0	0	0	46
9202861	f	md	JP	2016	0	0	0	0	0	0	0	0	0	0	0	0	41
9204198	f	md	JP	2016	0	0	0	0	0	0	0	0	0	0	0	0	41
9300819	m	md	JP	2016	0	0	0	0	0	0	0	0	0	0	0	0	33
9371549	m	cn	GB	2016	1	0	0	0	0	0	0	0	0	0	0	0	70
9374700	f	md	JP	2016	0	0	0	0	0	0	0	0	0	0	0	0	69
9391371	m	cn	GB	2016	1	0	0	0	0	0	0	0	0	0	0	0	87
9411538	f	md	US	2016	1	0	0	0	0	0	0	0	0	0	0	0	95
9452952	f	cn	GB	2016	1	0	0	0	0	0	0	0	0	0	0	0	
9465886	f	cn	GB	2016	1	0	0	0	0	0	0	0	0	0	0	0	54
9582014	f	md	JP	2016	0	0	0	0	0	0	0	0	0	0	0	0	54
9586560	f	Not repo	Not re	2016	1	0	0	0	0	0	0	0	0	0	0	0	76
9697585	f	cn	GB	2016	1	0	0	0	0	0	0	0	0	0	0	0	
9741564	f	md	US	2016	0	0	0	0	0	0	0	0	0	0	0	0	76
9746871	m	cn	GB	2016	0	0	0	0	0	0	0	0	0	0	0	0	78
9776925	m	md	GB	2016	0	0	0	0	0	0	0	0	0	0	0	0	67.6
9816092	f	md	JP	2016	0	0	0	0	0	0	0	0	0	0	0	0	70
9858710	m	md	JP	2016	0	0	0	0	0	0	0	0	0	0	0	0	61
9973363	m	md	JP	2016	0	0	0	0	0	0	0	0	0	0	0	0	44
10114483	Not	ot	JP	2016	0	0	0	0	0	0	0	0	0	0	0	0	
10149376	m	cn	GB	2016	1	0	0	0	0	0	0	0	0	0	0	0	61
10261683	f	md	JP	2016	0	0	0	0	0	0	0	0	0	0	0	0	54
10272273	Not	ot	JP	2016	0	0	0	0	0	0	0	0	0	0	0	0	
10362294	m	ot	US	2016	0	0	0	0	0	0	0	0	0	0	0	0	55
10384541	f	md	US	2016	0	0	0	0	0	0	0	0	0	0	0	0	67
10450824	f	md	US	2016	0	0	0	0	0	0	0	0	0	0	0	0	85

10515023	m	md	AT	2016	0	0	0	0	0	0	0	0	0	0	0	0	56
10532711	f	ot	Not re	2016	1	0	0	0	0	0	0	0	0	0	0	0	75
11427162	f	cn	US	2016	0	1	0	0	1	0	0	0	0	0	0	0	70
11701035	f	md	US	2016	1	0	0	0	0	0	0	0	0	0	0	0	58
11739164	f	md	Not re	2016	1	0	0	0	0	0	0	0	0	0	0	0	66
11750606	f	md	NL	2016	0	0	0	0	0	0	0	0	0	0	0	0	57
11767194	f	ot	US	2016	0	1	0	0	1	0	0	0	0	0	0	0	67
11784783	m	md	CA	2016	0	0	0	0	0	0	0	0	0	0	0	0	73
11826319	m	ot	US	2016	0	0	0	0	0	0	0	0	0	0	0	0	82
11829349	m	md	US	2016	0	0	0	0	0	0	0	0	0	0	0	0	91
11832940	f	md	JP	2016	0	0	0	0	0	0	0	0	0	0	0	0	61
11882828	m	ot	BE	2016	0	1	0	0	1	0	0	0	0	0	0	0	59
11901678	f	ot	JP	2016	0	1	0	0	1	0	0	0	0	0	0	1	41
11906301	m	ot	IT	2016	0	0	0	0	0	0	0	0	0	0	0	0	50
11906302	f	ot	IT	2016	0	0	0	0	0	0	0	0	0	0	0	0	58
11906303	m	ot	IT	2016	0	0	0	0	0	0	0	0	0	0	0	0	69
11907061	f	md	IT	2016	0	0	0	0	0	0	0	0	0	0	0	0	62
11912800	m	ph	FR	2016	1	0	0	0	0	0	0	0	0	0	0	0	71.1
11915502	m	cn	JP	2016	0	1	0	0	1	0	0	0	0	0	0	0	50
11916004	m	cn	IT	2016	0	1	0	0	1	0	0	0	0	0	0	0	71
11917547	f	cn	GB	2016	1	0	0	0	0	0	0	0	0	0	0	0	64
11923396	m	md	US	2016	0	0	0	0	0	0	0	0	0	0	0	0	67
11928216	f	md	DE	2016	1	0	0	0	0	0	0	0	0	0	0	0	60
11928298	f	cn	FR	2016	0	0	0	0	0	0	0	0	0	0	0	0	
11930569	f	ot	US	2016	0	1	0	0	1	0	0	0	0	0	0	0	
11933990	m	ot	NL	2016	0	0	0	0	0	0	0	0	0	0	0	0	67
11937840	m	md	UA	2016	0	1	0	0	0	1	0	0	0	0	0	0	42
11939370	f	md	ES	2016	0	0	0	0	0	0	0	0	0	0	0	0	70
11945097	m	cn	GB	2016	1	0	0	0	0	0	0	0	0	0	0	0	72
11951638	m	md	AT	2016	0	0	0	0	0	0	0	0	0	0	0	0	60
11952460	m	md	Not re	2016	0	0	0	0	0	0	0	0	0	0	0	0	67
11965274	f	md	Not re	2016	1	0	0	0	0	0	0	0	0	0	0	0	74
11966209	f	cn	GR	2016	0	1	1	0	0	0	0	0	0	0	0	0	71
11975922	f	md	US	2016	0	0	0	0	0	0	0	0	0	0	0	0	66

11979665	f	md	FR	2016	1	0	0	0	0	0	0	0	0	0	0	0	
11980931	f	md	CN	2016	1	0	0	0	0	0	0	0	0	0	0	0	63.7
11981525	m	ot	BR	2016	0	1	0	0	1	0	0	0	0	0	0	0	61
11984099	m	md	CN	2016	1	0	0	0	0	0	0	0	0	0	0	0	72.3
11984268	m	cn	GB	2016	1	0	0	0	0	0	0	0	0	0	0	0	
11985522	m	md	Not re	2016	1	0	0	0	0	0	0	0	0	0	0	0	61
11990028	f	cn	US	2016	0	0	0	0	0	0	0	0	0	0	0	0	
12001827	m	md	US	2016	0	0	0	0	0	0	0	0	0	0	0	0	80
12004146	m	md	US	2016	0	1	0	0	1	0	0	0	0	0	0	0	
12007451	f	md	ZA	2016	0	1	0	0	1	0	0	0	0	0	0	0	72
12007987	m	md	JP	2016	0	1	0	0	0	1	0	0	0	0	0	0	68
12039096	f	cn	Not re	2016	0	0	0	0	0	0	0	0	0	0	0	0	59
12051260	f	ot	US	2016	0	0	0	0	0	0	0	0	0	0	0	0	54
12057589	f	md	JP	2016	1	0	0	0	0	0	0	0	0	0	0	1	
12057590	f	md	Not re	2016	1	0	0	0	0	0	0	0	0	0	0	1	65
12060465	f	md	JP	2016	0	0	0	0	0	0	0	0	0	0	0	0	70
12067106	Not	md	JP	2016	0	1	0	0	1	0	0	0	0	0	0	0	45
12069103	f	md	US	2016	0	0	0	0	0	0	1	0	1	0	0	0	
12070141	Not	ot	CN	2016	0	0	0	0	0	0	0	0	0	0	0	0	
12071364	f	md	US	2016	0	0	0	0	0	0	0	0	0	0	0	0	53
12074099	f	md	PT	2016	0	1	0	0	1	0	0	0	0	0	0	0	54
12075188	f	cn	GR	2016	0	1	1	0	0	0	0	0	0	0	0	1	67
12081155	m	ot	NL	2016	0	0	0	0	0	0	0	0	0	0	0	0	68
12082341	m	md	US	2016	0	0	0	0	0	0	0	0	0	0	0	0	50
12085375	m	md	AT	2016	1	0	0	0	0	0	0	0	0	0	0	0	57
12086178	m	cn	JP	2016	0	1	0	0	1	0	0	0	0	0	0	1	77
12090129	m	cn	GR	2016	0	1	1	0	0	0	0	0	0	0	0	0	70
12091391	f	md	IT	2016	1	0	0	0	0	0	0	0	0	0	0	1	63.1
12093069	m	cn	US	2016	0	0	0	0	0	0	0	0	0	0	0	0	68
12093709	f	ot	US	2016	0	0	0	0	0	0	0	0	0	0	0	0	68
12095253	f	md	FR	2016	0	0	0	0	0	0	0	0	0	0	0	0	85
12101435	f	md	US	2016	0	0	0	0	0	0	0	0	0	0	0	0	69
12101449	m	cn	JP	2016	0	0	0	0	0	0	0	0	0	0	0	0	75
12101639	m	ot	ES	2016	0	1	0	0	1	0	0	0	0	0	0	0	68

12103422	m	md	US	2016	0	0	0	0	0	0	0	0	0	0	0	0	67
12113018	f	ot	US	2016	0	1	0	0	0	1	0	0	0	0	0	0	71
12113629	m	ph	FR	2016	0	0	0	0	0	0	0	0	0	0	0	0	51
12117846	m	ot	US	2016	0	1	0	0	1	0	0	0	0	0	0	0	63
12118919	m	md	JP	2016	0	1	0	0	0	1	0	0	0	0	0	0	62
12122493	m	md	CN	2016	1	0	0	0	0	0	0	0	0	0	0	0	62.5
12123118	f	md	JP	2016	0	1	0	0	1	0	0	0	0	0	0	0	
12127016	f	md	Not re	2016	1	0	0	0	0	0	0	0	0	0	0	0	56
12127257	f	md	Not re	2016	1	0	0	0	0	0	0	0	0	0	0	0	69
12138357	m	ot	NL	2016	0	1	0	0	1	0	0	0	0	0	0	0	69
12138388	f	md	GB	2016	0	0	0	0	0	0	0	0	0	0	0	0	36
12138527	f	md	JP	2016	1	0	0	0	0	0	0	0	0	0	0	0	
12138605	m	cn	US	2016	0	0	0	0	0	0	0	0	0	0	0	0	68
12140383	f	cn	GB	2016	0	1	0	0	1	0	0	0	0	0	0	0	66
12143101	m	cn	JP	2016	0	1	0	0	1	0	0	0	0	0	0	0	66
12147941	m	cn	IT	2016	0	1	0	0	1	0	0	0	0	0	0	0	66
12147944	m	md	US	2016	0	1	0	0	0	1	0	0	0	0	0	0	65
12151373	m	md	US	2016	1	0	0	0	0	0	0	0	0	0	0	1	76
12154302	m	ot	JP	2016	0	1	0	0	1	0	0	0	0	0	0	1	71
12159754	m	md	Not re	2016	1	0	0	0	0	0	0	0	0	0	0	0	71
12160161	m	ot	ES	2016	0	1	0	0	1	0	0	0	0	0	0	0	69
12161863	f	md	US	2016	0	1	1	0	0	0	0	0	0	0	0	0	82
12162202	f	md	US	2016	0	0	0	0	0	0	0	0	0	0	0	0	62
12162388	m	cn	IT	2016	0	1	0	0	1	0	0	0	0	0	0	0	
12162482	m	cn	GB	2016	1	0	0	0	0	0	0	0	0	0	0	1	81
12162895	m	md	IT	2016	0	1	0	0	1	0	0	0	0	0	0	0	
12165492	m	cn	JP	2016	0	1	0	0	1	0	0	0	0	0	0	0	63
12166822	m	md	AT	2016	0	1	0	0	1	0	0	0	0	0	0	0	72
12170296	m	md	US	2016	0	0	0	0	0	0	0	0	0	0	0	0	50
12171074	f	md	CH	2016	0	0	0	0	0	0	0	0	0	0	0	0	43
12171712	m	md	CZ	2016	0	0	0	0	0	0	0	0	0	0	0	0	44
12172486	f	md	US	2016	0	0	0	0	0	0	0	0	0	0	0	0	76
12172511	f	md	FR	2016	1	0	0	0	0	0	0	0	0	0	0	0	60.8
12172851	f	ph	US	2016	0	0	0	0	0	0	0	0	0	0	0	0	

12172991	m	cn	ES	2016	0	1	0	0	1	0	0	0	0	0	0	0	68
12173148	m	md	Not re	2016	1	0	0	0	0	0	0	0	0	0	0	0	77
12174152	m	md	US	2016	0	1	0	0	1	0	0	0	0	0	0	0	77
12174526	m	md	DE	2016	0	1	0	0	1	0	0	0	0	0	0	1	80
12174637	m	cn	JP	2016	0	1	0	0	1	0	0	0	0	0	0	0	62
12175708	m	md	AU	2016	0	1	0	0	1	0	0	0	0	0	0	0	43
12177178	m	md	US	2016	0	0	0	0	0	0	0	0	0	0	0	0	65
12178076	m	cn	JP	2016	0	1	0	0	1	0	0	0	0	0	0	1	83
12179476	f	md	BE	2016	0	1	0	0	1	0	0	0	0	0	0	0	47
12180109	f	ot	US	2016	0	0	0	0	0	0	0	0	0	0	0	0	59
12180884	f	md	BE	2016	1	0	0	0	0	0	0	0	0	0	0	0	77
12181672	m	md	JP	2016	0	1	0	0	1	0	0	0	0	0	0	0	80
12182848	Not	ot	BE	2016	0	1	0	0	1	0	0	0	0	0	0	0	56
12185764	m	md	Not re	2016	1	0	0	0	0	0	0	0	0	0	0	0	73
12185883	f	cn	JP	2016	0	1	0	0	1	0	0	0	0	0	0	1	63
12188619	m	md	ES	2016	0	0	0	0	0	0	0	0	0	0	0	0	45
12189533	f	ot	BE	2016	0	1	0	0	1	0	0	0	0	0	0	0	
12189690	m	cn	JP	2016	0	1	0	0	1	0	0	0	0	0	0	0	72
12192445	f	md	BE	2016	0	1	0	0	1	0	0	0	0	0	0	0	48
12192991	m	ot	CN	2016	1	0	0	0	0	0	0	0	0	0	0	0	75
12193204	m	md	AU	2016	0	1	0	0	1	0	0	0	0	0	0	0	66
12193273	m	cn	GB	2016	0	1	0	0	1	0	0	0	0	0	0	0	32
12196103	f	ot	BE	2016	0	1	0	0	0	1	0	0	0	0	0	0	58
12196367	m	md	CA	2016	0	1	0	0	1	0	0	0	0	0	0	0	
12196670	m	md	FR	2016	0	1	0	0	1	0	0	0	0	0	0	0	67
12197312	m	cn	JP	2016	0	1	0	0	1	0	0	0	0	0	0	0	
12203773	f	ot	PL	2016	0	0	0	0	0	0	0	0	0	0	0	0	69
12203939	m	md	BR	2016	0	0	0	0	0	0	0	0	0	0	0	0	
12204677	f	cn	IT	2016	0	1	0	0	1	0	0	0	0	0	0	0	69
12205134	f	cn	JP	2016	0	1	0	0	1	0	0	0	0	0	0	0	54
12205646	m	cn	JP	2016	0	1	0	0	1	0	0	0	0	0	0	0	60
12206025	m	cn	IT	2016	0	1	0	0	1	0	0	0	0	0	0	0	61
12206181	f	md	ES	2016	0	0	0	0	0	0	0	0	0	0	0	0	67
12207379	f	md	BE	2016	0	0	0	0	0	0	0	0	0	0	0	0	60

12209067	m	ph	ZA	2016	0	0	0	0	0	0	0	0	0	0	0	0	
12209513	m	cn	JP	2016	0	1	0	0	1	0	0	0	0	0	0	0	66
12209692	f	ot	BE	2016	0	1	0	0	1	0	0	0	0	0	0	0	69
12209807	m	cn	CN	2016	0	0	0	0	0	0	0	0	0	0	0	0	45
12209848	f	ot	US	2016	0	1	1	0	0	0	0	0	0	0	0	0	87
12211454	f	ph	JP	2016	0	1	0	0	1	0	0	0	0	0	0	0	50
12212448	m	md	DE	2016	0	1	0	0	1	0	0	0	0	0	0	0	
12214871	m	ot	CA	2016	0	1	0	0	1	0	0	0	0	0	0	1	64
12218345	m	md	Not re	2016	1	0	0	0	0	0	0	0	0	0	0	0	67
12218445	m	md	IT	2016	0	1	0	0	1	0	0	0	0	0	0	0	68
12218471	f	md	IT	2016	1	0	0	0	0	0	0	0	0	0	0	0	78.2
12218695	m	md	AE	2016	1	0	0	0	0	0	0	0	0	0	0	0	70
12218936	f	cn	US	2016	0	0	0	0	0	0	0	0	0	0	0	0	61
12219306	f	cn	US	2016	1	0	0	0	0	0	0	0	0	0	0	0	66
12219997	m	ot	PT	2016	0	1	0	0	1	0	0	0	0	0	0	0	
12220919	m	cn	JP	2016	0	1	0	0	1	0	0	0	0	0	0	1	53
12221151	m	md	JP	2016	0	0	0	0	0	0	0	0	0	0	0	0	77
12222458	m	ot	Not re	2016	1	0	0	0	0	0	0	0	0	0	0	0	61
12222628	m	md	JP	2016	0	1	0	0	1	0	0	0	0	0	0	0	
12222876	m	ot	CH	2016	0	1	0	0	1	0	0	0	0	0	0	0	73
12223174	f	md	Not re	2016	0	0	0	0	0	0	0	0	0	0	0	0	66
12224186	f	cn	JP	2016	0	1	0	0	1	0	0	0	0	0	0	0	49
12227616	m	md	US	2016	0	0	0	0	0	0	0	0	0	0	0	0	
12227852	m	md	US	2016	0	0	0	0	0	0	0	0	0	0	0	0	48
12227856	m	ot	TN	2016	0	0	0	0	0	0	0	0	0	0	0	0	67
12228390	f	md	FR	2016	1	0	0	0	0	0	0	0	0	0	0	0	
12228418	m	ot	JP	2016	0	1	0	0	1	0	0	0	0	0	0	0	68
12228606	m	ot	JP	2016	0	0	0	0	0	0	0	0	0	0	0	0	76
12230252	m	md	JP	2016	0	1	0	0	1	0	0	0	0	0	0	0	38
12230449	m	cn	JP	2016	0	1	0	0	1	0	0	0	0	0	0	0	51
12230654	Not	md	MY	2016	0	0	0	0	0	0	0	0	0	0	0	0	
12230886	f	cn	Not re	2016	0	0	0	0	0	0	0	0	0	0	0	0	53
12230926	f	md	FR	2016	1	0	0	0	0	0	0	0	0	0	0	0	56
12231161	m	cn	GB	2016	1	0	0	0	0	0	0	0	0	0	0	0	

12231773	f	ot	IT	2016	0	1	0	0	1	0	0	0	0	0	0	0	71
12231891	m	ot	GB	2016	0	1	0	0	1	0	0	0	0	0	0	0	52
12231902	m	ot	US	2016	0	1	0	0	1	0	0	0	0	0	0	0	
12231940	m	md	FR	2016	1	0	0	0	0	0	0	0	0	0	0	0	
12232052	m	md	JP	2016	0	1	0	0	1	0	0	0	0	0	0	0	69
12232090	Not	md	TH	2016	1	0	0	0	0	0	0	0	0	0	0	0	
12232093	Not	md	TH	2016	1	0	0	0	0	0	0	0	0	0	0	0	
12232097	f	ot	Not re	2016	1	0	0	0	0	0	0	0	0	0	0	0	59
12232426	m	ot	JP	2016	0	1	0	0	1	0	0	0	0	0	0	0	62
12232692	f	md	CN	2016	1	0	0	0	0	0	0	0	0	0	0	0	78
12232771	f	cn	US	2016	0	1	0	0	1	0	0	0	0	0	0	0	
12232786	f	cn	US	2016	0	1	0	0	1	0	0	0	0	0	0	0	
12232816	m	cn	JP	2016	0	1	0	0	1	0	0	0	0	0	0	0	73
12232822	m	cn	JP	2016	0	1	0	0	1	0	0	0	0	0	0	0	75
12232833	f	cn	JP	2016	0	1	0	0	1	0	0	0	0	0	0	0	67
12232904	f	cn	JP	2016	0	1	0	0	1	0	0	0	0	0	0	1	63
12232967	m	ot	IT	2016	0	1	0	0	1	0	0	0	0	0	0	0	
12233245	m	cn	JP	2016	0	1	0	0	1	0	0	0	0	0	0	0	79
12234069	Not	ot	JP	2016	1	0	0	0	0	0	0	0	0	0	0	0	
12234706	m	cn	CA	2016	0	0	0	0	0	0	0	0	0	0	0	0	66
12234799	m	cn	GR	2016	0	1	1	0	0	0	0	0	0	0	0	1	61
12235125	f	ph	AU	2016	0	1	0	0	1	0	0	0	0	0	0	0	60
12235304	m	ot	JP	2016	0	1	0	0	1	0	0	0	0	0	0	0	59
12236191	f	ot	US	2016	0	1	1	0	0	0	0	0	0	0	0	0	76
12236492	f	ot	US	2016	0	1	0	0	1	0	0	0	0	0	0	0	64
12237630	m	ot	GB	2016	0	0	0	0	0	0	0	0	0	0	0	0	67
12238356	m	ot	IT	2016	1	0	0	0	0	0	0	0	0	0	0	0	84
12238369	f	cn	GB	2016	1	0	0	0	0	0	0	0	0	0	0	0	73
12238549	f	md	US	2016	0	0	0	0	0	0	0	0	0	0	0	0	83
12238813	f	ot	US	2016	0	0	0	0	0	0	0	0	0	0	0	0	67
12239009	m	ot	US	2016	0	0	0	0	0	0	0	0	0	0	0	0	39
12239084	f	md	EC	2016	1	0	0	0	0	0	0	0	0	0	0	0	
12239222	m	md	JP	2016	1	0	0	0	0	0	0	0	0	0	0	0	69
12239510	f	md	US	2016	0	1	0	0	1	0	0	0	0	0	0	0	

12239526	m	ot	US	2016	1	0	0	0	0	0	0	0	0	0	0	0	
12239771	f	md	TH	2016	1	0	0	0	0	0	0	0	0	0	0	0	70
12239816	f	ot	US	2016	0	0	0	0	0	0	0	0	0	0	0	0	46
12239869	m	ot	US	2016	0	0	0	0	0	0	0	0	0	0	0	0	49
12239879	f	md	US	2016	0	0	0	0	0	0	0	0	0	0	0	0	
12239937	f	ot	US	2016	0	0	0	0	0	0	0	0	0	0	0	0	
12240030	f	cn	US	2016	0	0	0	0	0	0	0	0	0	0	0	0	
12240471	f	md	Not re	2016	1	0	0	0	0	0	0	0	0	0	0	0	79
12240553	m	md	US	2016	0	1	0	0	1	0	0	0	0	0	0	1	80
12240740	m	md	DE	2016	0	1	0	0	0	1	0	0	0	0	0	0	70
12241108	f	md	CA	2016	0	1	0	0	0	1	0	0	0	0	0	0	71
12241207	f	md	FR	2016	1	0	0	0	0	0	0	0	0	0	0	0	
12241212	m	ph	US	2016	0	0	0	0	0	0	0	0	0	0	0	0	50
12242507	f	md	TH	2016	1	0	0	0	0	0	0	0	0	0	0	0	
12242521	Not	md	US	2016	1	0	0	0	0	0	0	0	0	0	0	0	
12242578	f	md	RU	2016	1	0	0	0	0	0	0	0	0	0	0	0	69
12242719	f	cn	CH	2016	1	0	0	0	0	0	0	0	0	0	0	0	
12242749	f	md	FR	2016	1	0	0	0	0	0	0	0	0	0	0	0	63
12242846	f	cn	ES	2016	0	1	0	0	1	0	0	0	0	0	0	0	
12242923	f	md	IL	2016	0	0	0	0	0	0	0	0	0	0	0	0	
12243003	m	ot	HR	2016	0	1	0	0	1	0	0	0	0	0	0	0	71
12243827	m	md	Not re	2016	1	0	0	0	0	0	0	0	0	0	0	1	50
12244009	f	md	Not re	2016	1	0	0	0	0	0	0	0	0	0	0	0	70
12244011	m	ot	GB	2016	1	0	0	0	0	0	0	0	0	0	0	0	
12244280	f	cn	JP	2016	0	1	0	0	1	0	0	0	0	0	0	1	62
12244316	m	cn	JP	2016	0	1	0	0	1	0	0	0	0	0	0	0	68
12244607	m	ot	TN	2016	0	0	0	0	0	0	0	0	0	0	0	0	67
12244666	f	md	IL	2016	1	0	0	0	0	0	0	0	0	0	0	0	57
12245897	Not	ot	GR	2016	0	0	0	0	0	0	0	0	0	0	0	0	
12245899	Not	ot	GR	2016	0	0	0	0	0	0	0	0	0	0	0	0	
12246314	m	md	Not re	2016	1	0	0	0	0	0	0	0	0	0	0	0	81
12246432	f	md	Not re	2016	1	0	0	0	0	0	0	0	0	0	0	0	79
12246738	m	Not repo	US	2016	1	0	0	0	0	0	0	0	0	0	0	0	56.8
12246874	m	ot	ES	2016	0	1	0	0	1	0	0	0	0	0	0	0	60

12246899	m	ot	CH	2016	0	1	0	0	1	0	0	0	0	0	0	0	71
12246944	f	md	US	2016	0	1	0	0	1	0	0	0	0	0	0	0	70
12247094	f	ot	IT	2016	0	1	0	0	1	0	0	0	0	0	0	0	
12247142	f	ot	US	2016	0	1	0	0	1	0	0	0	0	0	0	0	56
12247814	m	md	JP	2016	0	1	0	0	1	0	0	0	0	0	0	0	67
12248100	m	ot	US	2016	0	0	0	0	0	0	0	0	0	0	0	0	49
12248105	m	md	JP	2016	0	0	0	0	0	0	0	0	0	0	0	0	77
12248333	m	ph	US	2016	0	0	0	0	0	0	0	0	0	0	0	0	50
12248363	Not	cn	IT	2016	0	1	0	0	1	0	0	0	0	0	0	0	
12248369	f	md	US	2016	0	1	0	0	1	0	0	0	0	0	0	0	57
12248546	f	md	US	2016	0	1	0	0	1	0	0	0	0	0	0	0	70
12248643	f	md	US	2016	0	0	0	0	0	0	0	0	0	0	0	0	83
12248674	m	ot	US	2016	0	0	0	0	0	0	0	0	0	0	0	0	
12248793	m	md	JP	2016	0	1	0	0	1	0	0	0	0	0	0	0	70
12249366	m	ot	JP	2016	0	1	0	0	1	0	0	0	0	0	0	0	62
12249373	m	md	FR	2016	1	0	0	0	0	0	0	0	0	0	0	0	74
12249627	Not	md	JP	2016	1	0	0	0	0	0	0	0	0	0	0	0	
12249761	f	ot	GB	2016	0	0	0	0	0	0	0	0	0	0	0	0	80
12249858	m	cn	JP	2016	0	1	0	0	1	0	0	0	0	0	0	0	78
12250179	m	ot	CA	2016	0	1	0	0	1	0	0	0	0	0	0	0	74
12250421	f	ot	US	2016	0	0	0	0	0	0	0	0	0	0	0	0	47
12250663	f	md	JP	2016	0	1	0	0	1	0	0	0	0	0	0	0	75
12251013	f	ph	JP	2016	0	0	0	0	0	0	0	0	0	0	0	0	68
12251721	m	md	JP	2016	0	0	0	0	0	0	0	0	0	0	0	0	68
12251816	Not	ot	GR	2016	0	0	0	0	0	0	0	0	0	0	0	0	
12251836	Not	ot	GR	2016	0	0	0	0	0	0	0	0	0	0	0	0	
12251843	Not	ot	GR	2016	0	0	0	0	0	0	0	0	0	0	0	0	
12251845	Not	ot	GR	2016	0	0	0	0	0	0	0	0	0	0	0	0	
12251847	Not	ot	GR	2016	0	0	0	0	0	0	0	0	0	0	0	0	
12251857	Not	ot	GR	2016	0	0	0	0	0	0	0	0	0	0	0	0	
12251873	Not	ot	GR	2016	0	0	0	0	0	0	0	0	0	0	0	0	
12251875	f	md	HU	2016	0	0	0	0	0	0	0	0	0	0	0	0	61
12251892	m	md	IT	2016	0	1	0	0	1	0	0	0	0	0	0	0	44
12252517	m	ph	US	2016	0	0	0	0	0	0	0	0	0	0	0	0	50

12252709	f	ot	NL	2016	0	1	0	0	1	0	0	0	0	0	0	0	63
12252803	m	ot	US	2016	0	0	0	0	0	0	0	0	0	0	0	0	42
12253070	m	md	JP	2016	0	1	0	0	1	0	0	0	0	0	0	1	70
12253077	m	cn	JP	2016	0	1	0	0	1	0	0	0	0	0	0	0	70
12253456	m	ot	US	2016	0	1	0	0	1	0	0	0	0	0	0	0	
12253813	f	md	US	2016	0	1	0	0	0	1	0	0	0	0	0	0	69
12253960	m	cn	JP	2016	0	0	0	0	0	0	0	0	0	0	0	0	61
12253967	m	cn	FI	2016	0	0	0	0	0	0	0	0	0	0	0	0	60
12254731	f	md	JP	2016	0	0	0	0	0	0	0	0	0	0	0	0	77
12254747	Not	ot	US	2016	0	0	0	0	0	0	0	0	0	0	0	0	
12255290	f	md	FR	2016	0	0	0	0	0	0	0	0	0	0	0	0	76
12255447	m	cn	US	2016	0	0	0	0	0	0	0	0	0	0	0	0	
12255469	m	ot	DE	2016	0	1	0	0	1	0	0	0	0	0	0	0	67
12256038	Not	ot	GR	2016	0	0	0	0	0	0	0	0	0	0	0	0	
12257105	f	cn	US	2016	0	0	0	0	0	0	0	0	0	0	0	0	
12257305	f	ph	US	2016	1	0	0	0	0	0	0	0	0	0	0	0	46
12261098	m	md	FR	2016	1	0	0	0	0	0	0	0	0	0	0	0	
12261441	f	md	US	2016	1	0	0	0	0	0	0	0	0	0	0	0	
12261509	f	Not repo	US	2016	1	0	0	0	0	0	0	0	0	0	0	0	59.7
12261529	f	ot	NO	2016	0	0	0	0	0	0	0	0	0	0	0	0	66
12261589	f	md	FR	2016	1	0	0	0	0	0	0	0	0	0	0	0	87.2
12262389	Not	ot	IN	2016	0	0	0	0	0	0	0	0	0	0	0	0	
12262633	f	md	Not re	2016	1	0	0	0	0	0	0	0	0	0	0	0	79
12262996	m	ot	US	2016	0	0	0	0	0	0	0	0	0	0	0	0	59
12263189	m	md	FR	2016	1	0	0	0	0	0	0	0	0	0	0	0	
12263391	m	cn	JP	2016	0	1	0	0	1	0	0	0	0	0	0	0	80
12263627	m	md	NL	2016	0	0	0	0	0	0	0	0	0	0	0	0	70
12263769	f	ot	US	2016	0	1	0	0	1	0	0	0	0	0	0	0	
12263864	m	ot	ES	2016	0	0	0	0	0	0	0	0	0	0	0	0	65
12264339	f	ot	US	2016	0	1	0	0	1	0	0	0	0	0	0	0	59
12264367	m	ot	TN	2016	0	0	0	0	0	0	0	0	0	0	0	0	67
12265604	m	ot	RO	2016	0	0	0	0	0	0	0	0	0	0	0	0	48
12265619	m	ot	ES	2016	0	0	0	0	0	0	0	0	0	0	0	0	74
12265694	m	md	JP	2016	0	0	0	0	0	0	0	0	0	0	0	0	69

12265750	f	cn	GB	2016	1	0	0	0	0	0	0	0	0	0	0	0	79
12265834	Not	md	JP	2016	0	1	0	0	1	0	0	0	0	0	0	0	
12265863	m	cn	US	2016	0	1	0	0	1	0	0	0	0	0	0	0	
12266592	m	md	FR	2016	1	0	0	0	0	0	0	0	0	0	0	0	
12267388	m	cn	RO	2016	0	0	0	0	0	0	0	0	0	0	0	0	48
12267563	f	md	TH	2016	1	0	0	0	0	0	0	0	0	0	0	0	84
12267731	f	cn	Not re	2016	0	0	0	0	0	0	0	0	0	0	0	0	
12267767	m	Not repo	US	2016	1	0	0	0	0	0	0	0	0	0	0	0	
12268037	f	md	IL	2016	0	0	0	0	0	0	0	0	0	0	0	0	
12268753	m	ot	NL	2016	0	1	0	0	1	0	0	0	0	0	0	0	74
12269084	m	md	JP	2016	0	0	0	0	0	0	0	0	0	0	0	0	78
12269494	m	ph	US	2016	0	1	0	0	1	0	0	0	0	0	0	0	83
12269785	m	cn	US	2016	0	1	1	0	0	0	0	0	0	0	0	0	66
12269879	m	cn	JP	2016	0	1	0	0	1	0	0	0	0	0	0	0	73
12270048	m	cn	NL	2016	0	1	1	0	0	0	0	0	0	0	0	0	
12270195	f	cn	IE	2016	0	0	0	0	0	0	0	0	0	0	0	0	69
12270208	Not	md	VN	2016	0	0	0	0	0	0	0	0	0	0	0	0	
12270225	m	md	IT	2016	0	1	0	0	1	0	0	0	0	0	0	0	
12270359	f	ph	JP	2016	0	0	0	0	0	0	0	0	0	0	0	0	44
12270529	m	cn	US	2016	0	0	0	0	0	0	0	0	0	0	0	0	
12270764	m	cn	GR	2016	0	1	1	0	0	0	0	0	0	0	0	1	62
12270926	m	ot	US	2016	0	1	0	0	0	1	0	0	0	0	0	0	74
12271718	m	ot	FR	2016	0	0	0	0	0	0	0	0	0	0	0	0	65
12272172	m	md	Not re	2016	1	0	0	0	0	0	0	0	0	0	0	0	
12272181	f	md	Not re	2016	1	0	0	0	0	0	0	0	0	0	0	0	76
12272246	m	md	FR	2016	1	0	0	0	0	0	0	0	0	0	0	0	40
12272293	f	md	Not re	2016	1	0	0	0	0	0	0	0	0	0	0	0	85
12272351	m	cn	JP	2016	0	1	0	0	1	0	0	0	0	0	0	0	72
12272596	f	cn	JP	2016	0	1	0	0	1	0	0	0	0	0	0	1	79
12272653	m	md	FR	2016	1	0	0	0	0	0	0	0	0	0	0	0	
12272693	m	ot	JP	2016	0	0	0	0	0	0	0	0	0	0	0	0	
12273074	m	cn	GB	2016	1	0	0	0	0	0	0	0	0	0	0	0	
12273229	m	md	FR	2016	1	0	0	0	0	0	0	0	0	0	0	0	
12273345	m	cn	JP	2016	0	1	0	0	1	0	0	0	0	0	0	1	80

12273346	m	cn	AU	2016	0	1	0	0	1	0	0	0	0	0	0	0	67
12273388	f	ph	IE	2016	0	0	0	0	0	0	0	0	0	0	0	0	72
12273424	f	md	Not re	2016	1	0	0	0	0	0	0	0	0	0	0	0	
12273593	f	md	FR	2016	1	0	0	0	0	0	0	0	0	0	0	0	
12273609	m	cn	CA	2016	0	1	0	0	1	0	0	0	0	0	0	0	68
12273611	m	md	US	2016	0	1	0	0	1	0	0	0	0	0	0	0	
12273709	m	ot	US	2016	0	1	0	0	1	0	0	0	0	0	0	1	60
12273753	m	md	TH	2016	0	0	0	0	0	0	0	0	0	0	0	0	
12273817	m	md	GB	2016	1	0	0	0	0	0	0	0	0	0	0	0	83
12273856	m	ot	Not re	2016	0	0	0	0	0	0	0	0	0	0	0	0	56
12274150	m	cn	CH	2016	0	1	0	0	1	0	0	0	0	0	0	0	72
12274401	f	ph	US	2016	0	1	0	0	1	0	0	0	0	0	0	0	
12274954	f	md	FR	2016	1	0	0	0	0	0	0	0	0	0	0	0	
12275257	m	ot	PT	2016	0	1	0	0	1	0	0	0	0	0	0	0	73
12275622	Not	md	JP	2016	0	1	0	0	1	0	0	0	0	0	0	1	
12275631	m	cn	JP	2016	0	1	0	0	1	0	0	0	0	0	0	0	67
12275635	m	md	IT	2016	0	1	0	0	1	0	0	0	0	0	0	0	61
12275639	m	cn	JP	2016	0	1	0	0	1	0	0	0	0	0	0	0	67
12275746	f	ot	FR	2016	0	0	0	0	0	0	0	0	0	0	0	0	60
12275804	m	ot	BE	2016	0	0	0	0	0	0	0	0	0	0	0	0	65
12276156	Not	md	JP	2016	0	0	0	0	0	0	0	0	0	0	0	0	
12276258	Not	ot	ES	2016	0	0	0	0	0	0	0	0	0	0	0	0	
12276482	Not	ot	CN	2016	0	0	0	0	0	0	0	0	0	0	0	0	
12276553	Not	ot	CO	2016	0	0	0	0	0	0	0	0	0	0	0	0	
12276812	f	md	FR	2016	0	1	0	0	1	0	0	0	0	0	0	0	55
12276943	m	cn	US	2016	1	0	0	0	0	0	0	0	0	0	0	0	40
12276959	Not	md	FR	2016	0	0	0	0	0	0	0	0	0	0	0	0	
12276962	Not	md	FR	2016	0	0	0	0	0	0	0	0	0	0	0	0	
12276963	Not	md	FR	2016	0	0	0	0	0	0	0	0	0	0	0	0	
12276965	Not	md	FR	2016	0	0	0	0	0	0	0	0	0	0	0	0	
12276970	Not	md	FR	2016	0	0	0	0	0	0	0	0	0	0	0	0	
12277101	f	md	US	2016	1	0	0	0	0	0	0	0	0	0	0	0	76.5
12277381	m	cn	US	2016	1	0	0	0	0	0	0	0	0	0	0	0	72
12277980	f	md	US	2016	0	1	0	0	1	0	0	0	0	0	0	0	60

12278009	m	md	MX	2016	1	0	0	0	0	0	0	0	0	0	0	0	
12278120	Not	ot	US	2016	1	0	0	0	0	0	0	0	0	0	0	0	
12278132	m	ot	BE	2016	0	1	0	0	1	0	0	0	0	0	0	0	70
12278200	m	md	DE	2016	0	0	0	0	0	0	0	0	0	0	0	0	62
12278403	m	md	JP	2016	0	0	0	0	0	0	0	0	0	0	0	0	
12279078	Not	ot	US	2016	0	0	0	0	0	0	0	0	0	0	0	0	64
12279916	f	cn	JP	2016	0	1	0	0	1	0	0	0	0	0	0	1	62
12280838	m	cn	US	2016	0	0	0	0	0	0	0	0	0	0	0	0	
12281027	Not	ot	CN	2016	1	0	0	0	0	0	0	0	0	0	0	0	
12281351	f	md	Not re	2016	1	0	0	0	0	0	0	0	0	0	0	0	
12281947	f	cn	PL	2016	1	0	0	0	0	0	0	0	0	0	0	0	
12282327	f	md	Not re	2016	1	0	0	0	0	0	0	0	0	0	0	0	76
12282386	m	md	FR	2016	1	0	0	0	0	0	0	0	0	0	0	0	
12282400	f	md	US	2016	1	0	0	0	0	0	0	0	0	0	0	0	
12282505	Not	ot	US	2016	0	0	0	0	0	0	0	0	0	0	0	0	55
12282599	f	ot	US	2016	0	1	0	0	1	0	0	0	0	0	0	0	67
12282913	Not	ot	BR	2016	1	0	0	0	0	0	0	0	0	0	0	1	
12283010	m	md	Not re	2016	1	0	0	0	0	0	0	0	0	0	0	0	70
12283030	f	md	FR	2016	1	0	0	0	0	0	0	0	0	0	0	0	
12283044	m	md	FR	2016	1	0	0	0	0	0	0	0	0	0	0	0	
12283508	f	ot	ES	2016	0	0	0	0	0	0	0	0	0	0	0	0	69
12287476	f	md	JP	2016	1	0	0	0	0	0	0	0	0	0	0	0	64
12287483	m	ot	KR	2016	0	1	0	0	1	0	0	0	0	0	0	0	63
12287841	m	cn	JP	2016	0	1	0	0	1	0	0	0	0	0	0	1	52
12287847	m	cn	JP	2016	0	1	0	0	1	0	0	0	0	0	0	0	71
12287848	m	md	JP	2016	0	1	0	0	1	0	0	0	0	0	0	0	50
12287976	m	md	JP	2016	1	0	0	0	0	0	0	0	0	0	0	1	72.2
12288081	f	md	FR	2016	1	0	0	0	0	0	0	0	0	0	0	0	59
12288122	f	md	FR	2016	1	0	0	0	0	0	0	0	0	0	0	0	
12288137	f	md	AT	2016	0	0	0	0	0	0	0	0	0	0	0	0	
12288319	f	md	FR	2016	1	0	0	0	0	0	0	0	0	0	0	1	70
12288565	Not	md	JP	2016	0	0	0	0	0	0	0	0	0	0	0	0	
12288576	Not	ot	JP	2016	0	0	0	0	0	0	0	0	0	0	0	0	
12289039	Not	md	US	2016	1	0	0	0	0	0	0	0	0	0	0	0	

12289355	Not	md	JP	2016	1	0	0	0	0	0	0	0	0	0	0	0	77
12289431	f	md	US	2016	1	0	0	0	0	0	0	0	0	0	0	0	67
12289499	f	md	CA	2016	0	0	0	0	0	0	0	0	0	0	0	0	66
12289500	m	ot	US	2016	0	1	0	0	1	0	0	0	0	0	0	0	65
12289565	f	cn	GB	2016	1	0	0	0	0	0	0	0	0	0	0	0	
12290379	m	cn	GB	2016	1	0	0	0	0	0	0	0	0	0	0	0	
12290877	f	md	Not re	2016	1	0	0	0	0	0	0	0	0	0	0	0	63
12291038	f	ph	FR	2016	1	0	0	0	0	0	0	0	0	0	0	0	61.9
12291052	f	md	GB	2016	0	0	0	0	0	0	0	0	0	0	0	0	
12291131	f	ot	KR	2016	0	0	0	0	0	0	0	0	0	0	0	0	63
12293937	m	md	AT	2016	0	1	0	0	1	0	0	0	0	0	0	0	73
12294033	m	md	JP	2016	0	1	0	0	1	0	0	0	0	0	0	0	63
12295705	m	md	Not re	2016	1	0	0	0	0	0	0	0	0	0	0	0	66
12295712	f	ot	US	2016	1	0	0	0	0	0	0	0	0	0	0	0	
12296310	Not	md	AU	2016	0	1	0	0	1	0	0	0	0	0	0	0	
12296463	f	md	Not re	2016	1	0	0	0	0	0	0	0	0	0	0	0	59
12296491	m	ot	PT	2016	0	1	0	0	0	1	0	0	0	0	0	0	57
12296795	m	md	Not re	2016	1	0	0	0	0	0	0	0	0	0	0	0	90
12297128	f	md	Not re	2016	1	0	0	0	0	0	0	0	0	0	0	0	56
12297303	m	md	JP	2016	0	1	0	0	1	0	0	0	0	0	0	0	38
12298051	m	md	FR	2016	1	0	0	0	0	0	0	0	0	0	0	0	
12298110	m	cn	JP	2016	0	1	0	0	1	0	0	0	0	0	0	0	64
12298347	f	cn	MX	2016	0	0	0	0	0	0	0	0	0	0	0	0	54
12298960	f	md	DE	2016	0	0	0	0	0	0	0	0	0	0	0	0	77
12298978	m	ot	BE	2016	0	1	0	0	1	0	0	0	0	0	0	0	66
12299543	m	md	IT	2016	0	1	0	0	1	0	0	0	0	0	0	0	37
12299684	f	ph	US	2016	0	0	0	0	0	0	0	0	0	0	0	0	67
12300660	m	cn	GB	2016	1	0	0	0	0	0	0	0	0	0	0	0	
12301017	m	md	DE	2016	0	0	0	0	0	0	0	0	0	0	0	0	68
12301451	f	md	JP	2016	1	0	0	0	0	0	0	0	0	0	0	0	80.1
12301693	m	ot	GB	2016	0	1	0	0	1	0	0	0	0	0	0	0	59
12302113	f	cn	US	2016	0	0	0	0	0	0	0	0	0	0	0	0	66
12302471	f	md	Not re	2016	1	0	0	0	0	0	0	0	0	0	0	0	66
12302503	m	ot	US	2016	0	0	0	0	0	0	0	0	0	0	0	0	65

12302632	m	md	US	2016	0	0	0	0	0	0	0	0	0	0	0	0	51
12302792	Not	ph	US	2016	0	1	0	0	1	0	0	0	0	0	0	0	
12303437	f	ot	NL	2016	0	1	0	0	1	0	0	0	0	0	0	0	71
12304588	f	cn	Not re	2016	0	0	0	0	0	0	0	0	0	0	0	0	
12304853	f	cn	JP	2016	0	1	0	0	1	0	0	0	0	0	0	0	74
12305348	f	md	FR	2016	1	0	0	0	0	0	0	0	0	0	0	0	
12305407	m	cn	IT	2016	0	0	0	0	0	0	0	0	0	0	0	0	71
12305448	m	cn	JP	2016	0	1	0	0	1	0	0	0	0	0	0	0	51
12305615	f	md	Not re	2016	1	0	0	0	0	0	0	0	0	0	0	0	
12305737	f	md	TH	2016	1	0	0	0	0	0	0	0	0	0	0	0	40
12305860	f	cn	US	2016	1	0	0	0	0	0	0	0	0	0	0	0	67.4
12306369	f	cn	NL	2016	0	0	0	0	0	0	0	0	0	0	0	0	55
12306475	m	md	Not re	2016	1	0	0	0	0	0	0	0	0	0	0	0	65
12306512	m	md	JP	2016	0	1	0	0	1	0	0	0	0	0	0	1	75
12306525	m	md	JP	2016	1	0	0	0	0	0	0	0	0	0	0	0	89
12306599	m	cn	US	2016	0	0	0	0	0	0	0	0	0	0	0	0	70
12306632	f	md	Not re	2016	1	0	0	0	0	0	0	0	0	0	0	0	69
12306868	m	ot	GB	2016	0	0	0	0	0	0	0	0	0	0	0	0	
12308130	f	cn	NL	2016	0	1	0	0	1	0	0	0	0	0	0	0	65
12308135	f	ot	NL	2016	0	1	0	0	1	0	0	0	0	0	0	1	60
12308155	f	ot	BR	2016	0	1	0	0	1	0	0	0	0	0	0	0	68
12308264	m	ot	NZ	2016	0	1	0	0	1	0	0	0	0	0	0	0	66
12308283	f	md	Not re	2016	1	0	0	0	0	0	0	0	0	0	0	0	62
12308400	m	md	Not re	2016	1	0	0	0	0	0	0	0	0	0	0	0	73
12308615	Not	md	DE	2016	0	1	0	0	1	0	0	0	0	0	0	0	
12308808	m	ot	US	2016	1	0	0	0	0	0	0	0	0	0	0	0	75
12309024	f	md	JP	2016	1	0	0	0	0	0	0	0	0	0	0	0	
12309611	Not	md	US	2016	0	0	0	0	0	0	0	0	0	0	0	0	
12310083	m	md	US	2016	0	0	0	0	0	0	0	0	0	0	0	0	64
12310161	f	md	FR	2016	1	0	0	0	0	0	0	0	0	0	0	0	59.4
12311030	f	md	Not re	2016	1	0	0	0	0	0	0	0	0	0	0	0	81
12311033	f	md	Not re	2016	1	0	0	0	0	0	0	0	0	0	0	0	70
12311272	f	ot	AT	2016	0	1	0	0	1	0	0	0	0	0	0	0	57
12311512	m	md	US	2016	0	0	0	0	0	0	0	0	0	0	0	0	77

12311852	m	ot	AT	2016	0	1	0	0	1	0	0	0	0	0	0	0	66
12311881	f	ot	US	2016	0	1	0	0	1	0	0	0	0	0	0	0	77
12311882	f	ot	BE	2016	0	1	0	0	1	0	0	0	0	0	0	0	62
12311937	f	md	Not re	2016	1	0	0	0	0	0	0	0	0	0	0	0	60
12311951	m	ot	US	2016	0	1	0	0	1	0	0	0	0	0	0	0	
12311994	m	ph	ES	2016	1	0	0	0	0	0	0	0	0	0	0	0	85
12312388	m	cn	JP	2016	0	1	0	0	1	0	0	0	0	0	0	1	70
12312840	Not	md	US	2016	0	0	0	0	0	0	0	0	0	0	0	0	
12313218	f	md	FR	2016	1	0	0	0	0	0	0	0	0	0	0	0	70.1
12313378	f	cn	GB	2016	1	0	0	0	0	0	0	0	0	0	0	0	77
12313578	m	md	US	2016	0	0	0	0	0	0	0	0	0	0	0	0	64
12313704	f	cn	GT	2016	0	0	0	0	0	0	0	0	0	0	0	0	54
12314156	m	cn	GB	2016	1	0	0	0	0	0	0	0	0	0	0	0	65
12314159	m	cn	GB	2016	0	0	0	0	0	0	0	0	0	0	0	0	68
12314187	f	md	FR	2016	1	0	0	0	0	0	0	0	0	0	0	0	
12314351	m	cn	JP	2016	0	1	0	0	1	0	0	0	0	0	0	0	64
12314573	f	md	Not re	2016	0	0	0	0	0	0	0	0	0	0	0	0	67
12314906	f	md	SG	2016	0	1	0	0	1	0	0	0	0	0	0	0	36
12315367	f	md	GR	2016	0	0	0	0	0	0	0	0	0	0	0	0	56
12315933	Not	md	JP	2016	0	1	0	0	1	0	0	0	0	0	0	0	
12316035	f	cn	JP	2016	0	1	0	0	1	0	0	0	0	0	0	0	85
12316078	m	cn	JP	2016	0	1	0	0	1	0	0	0	0	0	0	0	
12316158	f	cn	JP	2016	0	1	0	0	1	0	0	0	0	0	0	0	72
12316196	m	cn	US	2016	0	1	0	0	1	0	0	0	0	0	0	0	
12316257	f	md	JP	2016	0	1	0	0	1	0	0	0	0	0	0	0	67
12316269	m	ph	US	2016	0	1	0	0	1	0	0	0	0	0	0	0	
12316980	m	ot	GB	2016	0	0	0	0	0	0	0	0	0	0	0	0	64
12317276	f	cn	US	2016	1	0	0	0	0	0	0	0	0	0	0	0	
12317457	Not	ot	IN	2016	0	0	0	0	0	0	0	0	0	0	0	0	
12317600	f	md	JP	2016	0	0	0	0	0	0	0	0	0	0	0	0	84
12317795	f	md	US	2016	0	0	0	0	0	0	0	0	0	0	0	0	
12317807	f	cn	AR	2016	0	0	0	0	0	0	0	0	0	0	0	0	74
12317884	m	md	US	2016	0	0	0	0	0	0	0	0	0	0	0	0	71
12318460	f	md	US	2016	1	0	0	0	0	0	0	0	0	0	0	0	

12318536	m	cn	JP	2016	0	1	0	0	1	0	0	0	0	0	0	0	72
12319177	m	md	US	2016	0	0	0	0	0	0	0	0	0	0	0	0	69
12319205	f	cn	US	2016	0	1	0	0	1	0	0	0	0	0	0	0	61
12319210	Not	md	US	2016	0	0	0	0	0	0	0	0	0	0	0	0	
12319330	f	md	US	2016	0	0	0	0	0	0	0	0	0	0	0	0	57
12319347	Not	cn	JP	2016	0	1	0	0	1	0	0	0	0	0	0	0	68
12319377	f	ot	US	2016	0	0	0	0	0	0	0	0	0	0	0	0	
12319380	m	ot	US	2016	0	0	0	0	0	0	0	0	0	0	0	0	
12319394	m	md	US	2016	0	0	0	0	0	0	0	0	0	0	0	0	
12319438	m	cn	US	2016	0	0	0	0	0	0	0	0	0	0	0	0	
12319450	f	md	US	2016	0	0	0	0	0	0	0	0	0	0	0	0	61
12319524	f	ot	US	2016	0	0	0	0	0	0	0	0	0	0	0	0	67
12319533	f	md	JP	2016	0	0	0	0	0	0	0	0	0	0	0	0	70
12320053	f	md	US	2016	0	0	0	0	0	0	0	0	0	0	0	0	
12320386	f	cn	US	2016	0	1	1	0	0	0	0	0	0	0	0	0	
12320423	f	cn	US	2016	0	1	0	0	1	0	0	0	0	0	0	0	
12320766	m	md	US	2016	0	0	0	0	0	0	0	0	0	0	0	0	58
12320786	f	md	US	2016	0	0	0	0	0	0	0	0	0	0	0	0	
12320806	f	cn	US	2016	0	0	0	0	0	0	0	0	0	0	0	0	
12320810	m	ph	US	2016	0	0	0	0	0	0	0	0	0	0	0	0	
12320831	f	cn	US	2016	0	0	0	0	0	0	0	0	0	0	0	0	
12320840	f	md	US	2016	0	0	0	0	0	0	0	0	0	0	0	0	
12320871	f	md	US	2016	0	0	0	0	0	0	0	0	0	0	0	0	
12320883	m	md	US	2016	0	0	0	0	0	0	0	0	0	0	0	0	
12320887	m	md	US	2016	0	0	0	0	0	0	0	0	0	0	0	0	
12320893	f	ot	US	2016	0	0	0	0	0	0	0	0	0	0	0	0	
12320939	m	md	US	2016	0	0	0	0	0	0	0	0	0	0	0	0	
12321023	m	md	JP	2016	0	0	0	0	0	0	0	0	0	0	0	0	66
12321425	Not	md	JP	2016	0	1	0	0	1	0	0	0	0	0	0	0	
12321572	m	ot	PL	2016	0	1	0	0	1	0	0	0	0	0	0	0	73
12322311	m	cn	GB	2016	1	0	0	0	0	0	0	0	0	0	0	0	75
12322320	Not	cn	GB	2016	1	0	0	0	0	0	0	0	0	0	0	0	
12322337	f	md	US	2016	0	1	0	0	1	0	0	0	0	0	0	0	
12322545	m	cn	GB	2016	1	0	0	0	0	0	0	0	0	0	0	0	

12325009	m	ot	TN	2016	0	0	0	0	0	0	0	0	0	0	0	0	67
12325271	f	md	TH	2016	1	0	0	0	0	0	0	0	0	0	0	0	71
12325313	f	ot	NL	2016	0	0	0	0	0	0	0	0	0	0	0	0	77
12325772	Not	ot	ES	2016	0	1	0	0	1	0	0	0	0	0	0	0	
12326030	m	cn	JP	2016	0	1	0	0	1	0	0	0	0	0	0	1	77
12326189	f	ot	GB	2016	0	1	0	0	1	0	0	0	0	0	0	0	75
12326727	f	md	US	2016	1	0	0	0	0	0	0	0	0	0	0	0	66
12326728	Not	cn	GB	2016	1	0	0	0	0	0	0	0	0	0	0	0	
12326732	Not	cn	GB	2016	1	0	0	0	0	0	0	0	0	0	0	0	
12326778	f	cn	US	2016	0	0	0	0	0	0	0	0	0	0	0	0	
12327256	Not	ot	US	2016	0	0	0	0	0	0	0	0	0	0	0	0	
12327703	f	md	AU	2016	0	0	0	0	0	0	0	0	0	0	0	0	37
12327710	f	md	DE	2016	0	0	0	0	0	0	0	0	0	0	0	0	57
12327714	f	md	JP	2016	0	0	0	0	0	0	0	0	0	0	0	0	58
12327833	f	cn	IE	2016	1	0	0	0	0	0	0	0	0	0	0	0	76
12327996	m	cn	NL	2016	0	0	0	0	0	0	0	0	0	0	0	0	
12328241	f	ot	US	2016	0	1	0	0	0	1	0	0	0	0	0	0	43
12328260	f	ot	US	2016	0	0	0	0	0	0	0	0	0	0	0	0	55
12328313	f	cn	JP	2016	0	1	0	0	1	0	0	0	0	0	0	0	77
12329208	f	cn	US	2016	0	0	0	0	0	0	0	0	0	0	0	0	
12329331	f	md	US	2016	0	0	0	0	0	0	0	0	0	0	0	0	46
12329407	m	ot	GB	2016	0	1	0	0	1	0	0	0	0	0	0	0	59
12329408	m	cn	JP	2016	0	1	0	0	1	0	0	0	0	0	0	0	59
12329437	m	md	US	2016	0	0	0	0	0	0	0	0	0	0	0	0	74
12329472	f	md	TR	2016	0	1	0	0	1	0	0	0	0	0	0	0	51
12329897	m	cn	JP	2016	0	1	0	0	1	0	0	0	0	0	0	0	74
12330005	Not	ot	US	2016	0	1	0	0	1	0	0	0	0	0	0	0	
12330993	Not	cn	GB	2016	1	0	0	0	0	0	0	0	0	0	0	0	
12330996	f	cn	GB	2016	1	0	0	0	0	0	0	0	0	0	0	0	66
12331042	m	cn	Not re	2016	0	0	0	0	0	0	0	0	0	0	0	0	
12331410	f	md	US	2016	1	0	0	0	0	0	0	0	0	0	0	0	84
12333161	Not	md	AU	2016	0	1	0	0	1	0	0	0	0	0	0	0	78
12334390	m	ot	SE	2016	0	0	0	0	0	0	0	0	0	0	0	0	
12334426	f	cn	DE	2016	0	1	0	0	1	0	0	0	0	0	0	0	80

12335559	f	md	FR	2016	1	0	0	0	0	0	0	0	0	0	0	0	
12335648	f	ot	US	2016	1	0	0	0	0	0	0	0	0	0	0	0	68
12335691	m	cn	US	2016	0	1	0	0	1	0	0	0	0	0	0	0	
12336113	f	cn	US	2016	0	0	0	0	0	0	0	0	0	0	0	0	
12336133	f	cn	US	2016	0	0	0	0	0	0	0	0	0	0	0	0	
12336277	m	cn	JP	2016	0	1	0	0	1	0	0	0	0	0	0	0	59
12336468	m	ph	US	2016	0	0	0	0	0	0	0	0	0	0	0	0	70
12336816	f	cn	GB	2016	1	0	0	0	0	0	0	0	0	0	0	0	73
12336823	f	cn	GB	2016	1	0	0	0	0	0	0	0	0	0	0	0	81
12336830	f	ot	US	2016	1	0	0	0	0	0	0	0	0	0	0	0	60
12337392	f	md	US	2016	0	1	0	0	1	0	0	0	0	0	0	0	
12337710	f	cn	IT	2016	0	1	0	0	1	0	0	0	0	0	0	0	69
12337947	m	md	BE	2016	0	1	0	0	1	0	0	0	0	0	0	0	
12338160	f	ot	KR	2016	0	0	0	0	0	0	0	0	0	0	0	0	33
12338509	f	cn	US	2016	1	0	0	0	0	0	0	0	0	0	0	0	
12338560	f	ot	Not re	2016	1	0	0	0	0	0	0	0	0	0	0	0	59
12339034	f	md	EC	2016	1	0	0	0	0	0	0	0	0	0	0	0	60
12339980	f	md	FR	2016	0	1	0	0	1	0	0	0	0	0	0	0	60
12340043	f	md	SE	2016	0	0	0	0	0	0	0	0	0	0	0	0	57
12340184	m	md	FR	2016	1	0	0	0	0	0	0	0	0	0	0	0	
12341494	f	cn	GB	2016	1	0	0	0	0	0	0	0	0	0	0	0	69
12341496	f	ot	IT	2016	1	0	0	0	0	0	0	0	0	0	0	0	70
12341716	f	cn	IT	2016	0	1	0	0	1	0	0	0	0	0	0	0	55
12341783	m	ot	ES	2016	0	0	0	0	0	0	0	0	0	0	0	0	66
12341959	m	ph	US	2016	0	0	0	0	0	0	0	0	0	0	0	0	71
12342047	m	cn	BE	2016	0	0	0	0	0	0	0	0	0	0	0	0	57
12342161	f	md	AU	2016	0	1	0	0	1	0	0	0	0	0	0	0	
12342162	m	md	NZ	2016	0	1	0	0	1	0	0	0	0	0	0	0	57
12342166	m	ot	IT	2016	0	1	0	0	1	0	0	0	0	0	0	0	
12342361	f	md	IT	2016	0	0	0	0	0	0	0	0	0	0	0	0	49
12342455	m	ot	US	2016	0	1	0	0	1	0	0	0	0	0	0	0	57
12342491	m	md	IL	2016	1	0	0	0	0	0	0	0	0	0	0	0	
12342568	m	ot	CN	2016	0	1	0	0	1	0	0	0	0	0	0	1	57
12343011	m	ot	CA	2016	0	1	0	0	0	1	0	0	0	0	0	0	79

12343197	m	md	KR	2016	0	0	0	0	0	0	0	0	0	0	0	0	74
12343232	m	ot	US	2016	0	0	0	0	0	0	0	0	0	0	0	0	74
12343300	f	md	Not re	2016	0	0	0	0	0	0	0	0	0	0	0	0	59
12345679	Not	ot	US	2016	0	0	0	0	0	0	0	0	0	0	0	0	
12345800	f	ot	BE	2016	0	0	0	0	0	0	0	0	0	0	0	0	36
12346347	m	ot	GB	2016	0	0	0	0	0	0	0	0	0	0	0	0	
12346475	m	cn	GB	2016	0	0	0	0	0	0	0	0	0	0	0	0	
12346488	m	md	US	2016	0	0	0	0	0	0	0	0	0	0	0	0	77
12347249	f	md	AU	2016	0	0	0	0	0	0	0	0	0	0	0	0	56
12347645	Not	md	US	2016	0	0	0	0	0	0	0	0	0	0	0	0	
12347646	Not	md	US	2016	0	0	0	0	0	0	0	0	0	0	0	0	
12347650	Not	md	US	2016	0	0	0	0	0	0	0	0	0	0	0	0	
12347656	Not	md	US	2016	0	0	0	0	0	0	0	0	0	0	0	0	
12347657	Not	md	US	2016	0	0	0	0	0	0	0	0	0	0	0	0	
12348285	Not	md	US	2016	1	0	0	0	0	0	0	0	0	0	0	0	
12348643	m	cn	JP	2016	0	1	0	0	1	0	0	0	0	0	0	0	66
12348712	m	cn	IT	2016	0	1	0	0	1	0	0	0	0	0	0	0	42
12348714	m	ot	US	2016	0	1	0	0	1	0	0	0	0	0	0	0	56
12348881	Not	md	FR	2016	0	1	0	0	1	0	0	0	0	0	0	0	
12348882	f	cn	US	2016	0	1	0	0	1	0	0	0	0	0	0	0	
12349420	m	cn	IT	2016	0	1	0	0	1	0	0	0	0	0	0	1	79
12349827	f	ot	BE	2016	0	0	0	0	0	0	0	0	0	0	0	0	60
12350215	f	md	US	2016	1	0	0	0	0	0	0	0	0	0	0	0	62
12350268	f	cn	Not re	2016	0	0	0	0	0	0	0	0	0	0	0	0	38
12350432	m	ot	US	2016	0	0	0	0	0	0	0	0	0	0	0	0	50
12350988	f	cn	GB	2016	1	0	0	0	0	0	0	0	0	0	0	0	38
12351447	f	md	JP	2016	0	1	0	0	1	0	0	0	0	0	0	0	68
12351676	m	ot	US	2016	1	0	0	0	0	0	0	0	0	0	0	0	66
12351898	m	cn	JP	2016	0	1	0	0	1	0	0	0	0	0	0	0	58
12352229	m	md	NL	2016	0	0	0	0	0	0	0	0	0	0	0	0	76
12352496	m	cn	US	2016	0	0	0	0	0	0	0	0	0	0	0	0	
12352731	f	md	AU	2016	0	1	0	0	1	0	0	0	0	0	0	0	56
12352801	Not	ot	US	2016	0	1	0	0	1	0	0	0	0	0	0	0	
12352934	f	md	MX	2016	0	0	0	0	0	0	0	0	0	0	0	0	81

12353018	f	ot	US	2016	1	0	0	0	0	0	0	0	0	0	0	0	73.7
12353151	m	md	Not re	2016	0	0	0	0	0	0	0	0	0	0	0	0	78
12353499	m	cn	CN	2016	0	0	0	0	0	0	0	0	0	0	0	0	
12353561	m	ot	GB	2016	0	0	0	0	0	0	0	0	0	0	0	0	
12354132	f	cn	IE	2016	0	0	0	0	0	0	0	0	0	0	0	0	87
12354698	Not	cn	GB	2016	1	0	0	0	0	0	0	0	0	0	0	0	
12354993	m	ot	NL	2016	0	1	0	0	0	1	0	0	0	0	0	0	68
12355090	f	ot	FR	2016	0	0	0	0	0	0	0	0	0	0	0	0	72
12355229	f	md	FR	2016	0	0	0	0	0	0	0	0	0	0	0	0	66
12355756	f	md	JP	2016	0	1	0	0	1	0	0	0	0	0	0	0	
12356013	Not	md	Not re	2016	1	0	0	0	0	0	0	0	0	0	0	0	
12356929	f	md	US	2016	0	0	0	0	0	0	0	0	0	0	0	0	68
12357735	f	ot	US	2016	0	0	0	0	0	0	0	0	0	0	0	0	53
12358035	m	cn	US	2016	0	1	0	0	1	0	0	0	0	0	0	0	
12358264	m	cn	Not re	2016	0	0	0	0	0	0	0	0	0	0	0	0	
12358589	m	ot	GB	2016	0	0	0	0	0	0	0	0	0	0	0	0	
12358767	m	md	DE	2016	0	1	0	0	1	0	0	0	0	0	0	0	
12360168	m	ot	US	2016	0	0	0	0	0	0	0	0	0	0	0	0	72
12364191	m	cn	US	2016	0	1	0	0	1	0	0	0	0	0	0	0	
12364407	m	ot	BR	2016	0	0	0	0	0	0	0	0	0	0	0	0	80
12364677	f	cn	GB	2016	1	0	0	0	0	0	0	0	0	0	0	0	37
12365359	f	md	TW	2016	0	0	0	0	0	0	0	0	0	0	0	0	60
12365798	m	md	US	2016	1	0	0	0	0	0	0	0	0	0	0	0	
12365821	m	ot	US	2016	0	1	0	0	1	0	0	0	0	0	0	0	89
12366250	m	md	JP	2016	0	1	0	0	1	0	0	0	0	0	0	1	76
12366437	f	cn	US	2016	0	1	0	0	1	0	0	0	0	0	0	0	85
12366653	f	cn	GB	2016	1	0	0	0	0	0	0	0	0	0	0	0	
12367744	m	cn	US	2016	0	0	0	0	0	0	0	0	0	0	0	0	45
12367784	m	ot	AU	2016	0	1	0	0	1	0	0	0	0	0	0	0	63
12368354	m	md	IL	2016	0	1	0	0	1	0	0	0	0	0	0	0	78
12368377	f	cn	US	2016	0	0	0	0	0	0	0	0	0	0	0	0	59
12368489	f	cn	US	2016	0	0	0	0	0	0	0	0	0	0	0	0	
12369110	f	cn	US	2016	0	0	0	0	0	0	0	0	0	0	0	0	
12369246	m	cn	Not re	2016	0	0	0	0	0	0	0	0	0	0	0	0	

12370035	f	md	US	2016	0	1	0	0	1	0	0	0	0	0	0	0	80
12370241	m	cn	JP	2016	0	1	0	0	1	0	0	0	0	0	0	0	65
12370251	Not	ot	KR	2016	1	0	0	0	0	0	0	0	0	0	0	0	
12370362	Not	cn	US	2016	0	1	0	0	1	0	0	0	0	0	0	0	
12370410	m	ot	JP	2016	0	1	0	0	1	0	0	0	0	0	0	0	64
12370772	f	md	IL	2016	1	0	0	0	0	0	0	0	0	0	0	0	67
12370852	Not	ot	JP	2016	0	0	0	0	0	0	0	0	0	0	0	0	
12370854	Not	ot	JP	2016	0	0	0	0	0	0	0	0	0	0	0	0	
12370858	Not	ot	JP	2016	0	0	0	0	0	0	0	0	0	0	0	0	
12370859	Not	ot	JP	2016	0	0	0	0	0	0	0	0	0	0	0	0	
12372990	m	ot	FR	2016	0	1	0	0	1	0	0	0	0	0	0	0	69
12373109	m	md	PL	2016	1	0	0	0	0	0	0	0	0	0	0	0	68
12373864	Not	md	JP	2016	0	0	0	0	0	0	0	0	0	0	0	0	
12373871	m	ot	IT	2016	0	1	0	0	1	0	0	0	0	0	0	0	75
12373955	Not	ot	DE	2016	0	0	0	0	0	0	0	0	0	0	0	0	
12374107	Not	ot	JP	2016	1	0	0	0	0	0	0	0	0	0	0	0	
12374115	Not	ot	JP	2016	1	0	0	0	0	0	0	0	0	0	0	0	
12374122	Not	ot	JP	2016	1	0	0	0	0	0	0	0	0	0	0	0	
12374124	Not	ot	JP	2016	1	0	0	0	0	0	0	0	0	0	0	0	
12374138	Not	ot	JP	2016	1	0	0	0	0	0	0	0	0	0	0	0	
12374142	Not	ot	JP	2016	1	0	0	0	0	0	0	0	0	0	0	0	
12374169	Not	ot	JP	2016	1	0	0	0	0	0	0	0	0	0	0	0	
12374200	Not	ot	JP	2016	1	0	0	0	0	0	0	0	0	0	0	0	
12374206	Not	ot	JP	2016	1	0	0	0	0	0	0	0	0	0	0	0	
12374211	Not	ot	JP	2016	1	0	0	0	0	0	0	0	0	0	0	0	
12374216	Not	ot	JP	2016	1	0	0	0	0	0	0	0	0	0	0	0	
12374801	m	cn	US	2016	0	0	0	0	0	0	0	0	0	0	0	0	
12375536	m	md	DE	2016	0	0	0	0	0	0	0	0	0	0	0	0	80
12376004	m	cn	GB	2016	1	0	0	0	0	0	0	0	0	0	0	0	
12376397	m	cn	JP	2016	0	1	0	0	1	0	0	0	0	0	0	0	59
12376488	f	cn	US	2016	0	0	0	0	0	0	0	0	0	0	0	0	
12376507	f	cn	GB	2016	1	0	0	0	0	0	0	0	0	0	0	0	74
12378707	m	cn	GT	2016	0	1	1	0	0	0	0	0	0	0	0	0	64
12379005	m	md	Not re	2016	0	0	0	0	0	0	0	0	0	0	0	0	57

12379066	f	md	JP	2016	1	0	0	0	0	0	0	0	0	0	0	0	59
12379500	f	md	JP	2016	0	1	0	0	1	0	0	0	0	0	0	0	73
12379653	m	ot	IT	2016	0	1	0	0	1	0	0	0	0	0	0	0	59
12379762	m	cn	AU	2016	0	1	0	0	1	0	0	0	0	0	0	0	
12381086	m	md	US	2016	0	0	0	0	0	0	0	0	0	0	0	0	
12381092	m	cn	US	2016	0	0	0	0	0	0	0	0	0	0	0	0	77
12381093	m	cn	US	2016	0	0	0	0	0	0	0	0	0	0	0	0	
12381186	m	cn	GB	2016	1	0	0	0	0	0	0	0	0	0	0	0	80
12381363	f	md	CZ	2016	0	1	0	0	1	0	0	0	0	0	0	1	50
12381365	m	ot	IT	2016	0	1	0	0	1	0	0	0	0	0	0	0	80
12383013	m	md	US	2016	1	0	0	0	0	0	0	0	0	0	0	0	91
12383588	f	ot	UY	2016	0	1	0	0	1	0	0	0	0	0	0	0	
12383594	f	md	FR	2016	1	0	0	0	0	0	0	0	0	0	0	0	71
12383724	m	ot	NL	2016	0	1	0	0	1	0	0	0	0	0	0	0	65
12383762	m	cn	US	2016	0	1	0	0	1	0	0	0	0	0	0	0	62
12383925	m	cn	JP	2016	0	1	0	0	1	0	0	0	0	0	0	0	76
12384536	f	cn	JP	2016	0	1	0	0	1	0	0	0	0	0	0	1	63
12384746	f	cn	GB	2016	1	0	0	0	0	0	0	0	0	0	0	0	67
12385509	f	md	GB	2016	0	0	0	0	0	0	0	0	0	0	0	0	53
12386115	f	md	JP	2016	0	1	0	0	1	0	0	0	0	0	0	0	72
12386158	f	ot	US	2016	1	0	0	0	0	0	0	0	0	0	0	0	
12386184	f	ot	US	2016	1	0	0	0	0	0	0	0	0	0	0	0	58.7
12386219	f	ph	US	2016	1	0	0	0	0	0	0	0	0	0	0	0	
12386237	m	ph	US	2016	1	0	0	0	0	0	0	0	0	0	0	0	
12386238	f	ot	US	2016	1	0	0	0	0	0	0	0	0	0	0	0	87
12386239	f	ph	US	2016	1	0	0	0	0	0	0	0	0	0	0	0	
12386694	f	ot	US	2016	0	1	0	0	0	1	0	0	0	0	0	0	71
12387034	f	md	Not re	2016	1	0	0	0	0	0	0	0	0	0	0	0	54
12387242	f	md	TH	2016	1	0	0	0	0	0	0	0	0	0	0	0	86.1
12388122	m	md	KR	2016	0	1	0	0	1	0	0	0	0	0	0	0	
12388723	m	md	JP	2016	0	1	0	0	1	0	0	0	0	0	0	0	77
12389767	f	md	US	2016	1	0	0	0	0	0	0	0	0	0	0	0	
12390575	Not	cn	JP	2016	0	0	0	0	0	0	0	0	0	0	0	0	65
12391165	m	md	KR	2016	1	0	0	0	0	0	0	0	0	0	0	1	82.9

12391313	m	md	JP	2016	0	1	0	0	1	0	0	0	0	0	0	1	68
12391340	m	md	FR	2016	0	1	0	0	1	0	0	0	0	0	0	0	
12391384	m	ot	TW	2016	1	0	0	0	0	0	0	0	0	0	0	0	83
12391769	f	cn	JP	2016	0	1	0	0	1	0	0	0	0	0	0	0	53
12391970	Not	md	FR	2016	0	1	0	0	1	0	0	0	0	0	0	0	
12393378	m	cn	GB	2016	1	0	0	0	0	0	0	0	0	0	0	1	70
12393380	m	cn	GB	2016	1	0	0	0	0	0	0	0	0	0	0	1	70
12393393	m	cn	GB	2016	1	0	0	0	0	0	0	0	0	0	0	1	88
12393720	f	md	AR	2016	1	0	0	0	0	0	0	0	0	0	0	0	39
12394085	m	md	IT	2016	0	1	0	0	1	0	0	0	0	0	0	0	62
12394095	m	md	AT	2016	0	0	0	0	0	0	0	0	0	0	0	0	52
12394112	m	md	TR	2016	0	0	0	0	0	0	0	0	0	0	0	0	80
12394478	m	md	GB	2016	0	0	0	0	0	0	0	0	0	0	0	0	72
12394495	f	md	JP	2016	0	0	0	0	0	0	0	0	0	0	0	0	60
12395152	m	cn	US	2016	0	1	0	0	1	0	0	0	0	0	0	1	
12396472	f	md	JP	2016	0	1	0	0	1	0	0	0	0	0	0	0	73
12397675	m	md	US	2016	0	0	0	0	0	0	0	0	0	0	0	0	
12398508	Not	ph	US	2016	0	1	0	0	1	0	0	0	0	0	0	1	
12398516	f	cn	GB	2016	1	0	0	0	0	0	0	0	0	0	0	0	
12398953	f	cn	JP	2016	0	1	0	0	1	0	0	0	0	0	0	0	69
12399205	Not	ph	JP	2016	0	0	0	0	0	0	0	0	0	0	0	0	
12399268	f	ot	IT	2016	0	1	0	0	1	0	0	0	0	0	0	0	68
12399269	f	cn	JP	2016	0	1	0	0	1	0	0	0	0	0	0	1	60
12400385	m	md	FR	2016	0	1	0	0	0	1	0	0	0	0	0	0	64
12400415	m	md	SG	2016	0	1	0	0	0	1	0	0	0	0	0	0	75
12400432	f	cn	JP	2016	0	1	0	0	1	0	0	0	0	0	0	0	84
12401092	f	md	US	2016	0	0	0	0	0	0	0	0	0	0	0	0	
12403279	m	ot	US	2016	0	1	0	0	1	0	0	0	0	0	0	0	
12403553	f	md	US	2016	0	0	0	0	0	0	0	0	0	0	0	0	86
12403594	m	md	FR	2016	0	0	0	0	0	0	0	0	0	0	0	0	60
12404619	f	cn	GB	2016	1	0	0	0	0	0	0	0	0	0	0	0	56
12404738	m	ot	US	2016	1	0	0	0	0	0	0	0	0	0	0	0	
12405575	f	cn	JP	2016	0	1	0	0	1	0	0	0	0	0	0	0	63
12405581	f	md	US	2016	0	1	0	0	0	1	0	0	0	0	0	0	77

12405601	m	cn	JP	2016	0	1	0	0	1	0	0	0	0	0	0	0	64
12405870	m	md	DE	2016	0	1	0	0	0	1	0	0	0	0	0	0	77
12405983	f	md	JP	2016	0	1	0	0	0	1	0	0	0	0	0	0	73
12406538	Not	cn	IT	2016	0	1	0	0	1	0	0	0	0	0	0	0	73
12406916	f	cn	GB	2016	1	0	0	0	0	0	0	0	0	0	0	0	53
12406918	m	cn	GB	2016	1	0	0	0	0	0	0	0	0	0	0	0	66
12408520	Not	cn	GB	2016	1	0	0	0	0	0	0	0	0	0	0	0	
12409084	m	ot	US	2016	0	0	0	0	0	0	0	0	0	0	0	0	
12409260	f	md	JP	2016	0	1	0	0	1	0	0	0	0	0	0	0	
12409315	f	md	CN	2016	1	0	0	0	0	0	0	0	0	0	0	0	54.6
12409465	m	md	JP	2016	1	0	0	0	0	0	0	0	0	0	0	0	72
12410036	m	md	JP	2016	0	0	0	0	0	0	0	0	0	0	0	0	73
12410502	m	md	ES	2016	0	1	0	0	0	1	0	0	0	0	0	0	56
12410637	m	md	CA	2016	0	1	0	0	1	0	0	0	0	0	0	0	
12410654	f	md	IT	2016	0	0	0	0	0	0	0	0	0	0	0	0	
12410658	f	md	JP	2016	0	1	0	0	0	1	0	0	0	0	0	0	63
12410699	Not	cn	US	2016	0	1	0	0	1	0	0	0	0	0	0	0	
12410707	m	cn	US	2016	0	1	0	0	1	0	0	0	0	0	0	0	
12410737	f	ot	US	2016	0	0	0	0	0	0	0	0	0	0	0	0	
12410843	Not	md	US	2016	0	1	1	0	0	0	0	0	0	0	0	0	
12411013	m	md	Not re	2016	1	0	0	0	0	0	0	0	0	0	0	0	
12411257	f	md	ES	2016	0	1	0	0	0	1	0	0	0	0	0	0	61
12411668	f	md	Not re	2016	1	0	0	0	0	0	0	0	0	0	0	0	
12411898	m	ph	US	2016	0	0	0	0	0	0	0	0	0	0	0	0	
12411967	m	md	NL	2016	0	1	0	0	0	1	0	0	0	0	0	0	68
12412119	f	md	US	2016	0	0	0	0	0	0	0	0	0	0	0	0	76
12412826	m	cn	US	2016	0	0	0	0	0	0	0	0	0	0	0	0	
12413507	Not	md	JP	2016	0	1	0	0	1	0	0	0	0	0	0	0	
12414104	f	ot	NL	2016	1	0	0	0	0	0	0	0	0	0	0	0	77
12414639	f	cn	NL	2016	0	1	0	0	1	0	0	0	0	0	0	1	70
12414650	m	cn	US	2016	0	1	0	0	1	0	0	0	0	0	0	0	
12414664	m	md	JP	2016	0	1	0	0	1	0	0	0	0	0	0	0	74
12414884	m	ot	NL	2016	0	1	0	0	1	0	0	0	0	0	0	0	53
12415725	f	cn	US	2016	0	1	0	0	1	0	0	0	0	0	0	0	

12415727	m	cn	US	2016	0	1	0	0	1	0	0	0	0	0	0	0	
12415732	m	md	GB	2016	0	1	0	0	1	0	0	0	0	0	0	0	
12415753	m	cn	US	2016	0	1	0	0	1	0	0	0	0	0	0	0	73
12416224	m	cn	US	2016	0	1	0	0	1	0	0	0	0	0	0	0	
12416368	m	cn	DE	2016	0	1	0	0	1	0	0	0	0	0	0	0	64
12416401	m	cn	JP	2016	0	1	0	0	1	0	0	0	0	0	0	0	70
12416456	m	md	US	2016	1	0	0	0	0	0	0	0	0	0	0	0	
12416787	f	ot	AU	2016	0	0	0	0	0	0	0	0	0	0	0	0	
12417038	f	ot	US	2016	0	0	0	0	0	0	0	0	0	0	0	0	33
12418421	m	ot	ES	2016	0	1	0	0	1	0	0	0	0	0	0	0	54
12418751	f	cn	US	2016	0	1	0	0	1	0	0	0	0	0	0	0	
12418958	m	ph	JP	2016	1	0	0	0	0	0	0	0	0	0	0	1	70
12419231	m	cn	US	2016	0	0	0	0	0	0	0	0	0	0	0	0	
12419639	m	md	Not re	2016	1	0	0	0	0	0	0	0	0	0	0	0	63
12420062	Not	cn	GB	2016	1	0	0	0	0	0	0	0	0	0	0	0	
12420095	Not	md	US	2016	0	0	0	0	0	0	0	0	0	0	0	0	
12421243	m	ot	JP	2016	0	1	0	0	1	0	0	0	0	0	0	0	64
12421623	f	cn	BE	2016	0	1	0	0	1	0	0	0	0	0	0	0	
12421690	f	cn	US	2016	0	1	0	0	1	0	0	0	0	0	0	0	65
12421839	m	md	TR	2016	0	0	0	0	0	0	0	0	0	0	0	0	60
12421943	f	md	GB	2016	0	0	0	0	0	0	0	0	0	0	0	0	72
12421949	f	ot	FR	2016	0	0	0	0	0	0	0	0	0	0	0	0	63
12422251	m	md	US	2016	0	1	0	0	1	0	0	0	0	0	0	0	
12422689	m	ot	FR	2016	1	0	0	0	0	0	0	0	0	0	0	0	61
12422814	m	cn	GB	2016	0	1	0	0	1	0	0	0	0	0	0	0	61
12422842	f	md	ES	2016	0	1	0	0	0	1	0	0	0	0	0	0	72
12422892	m	md	IT	2016	0	1	0	0	0	1	0	0	0	0	0	0	75
12422897	m	md	US	2016	1	0	0	0	0	0	0	0	0	0	0	0	51
12423230	f	cn	GB	2016	1	0	0	0	0	0	0	0	0	0	0	0	50
12423956	m	ph	US	2016	0	1	0	0	1	0	0	0	0	0	0	0	81
12424084	f	ph	US	2016	0	0	0	0	0	0	0	0	0	0	0	0	64
12424165	f	ot	US	2016	1	0	0	0	0	0	0	0	0	0	0	0	78
12424636	f	md	JP	2016	0	1	0	0	1	0	0	0	0	0	0	0	79
12424854	m	md	FR	2016	0	0	0	0	0	0	0	0	0	0	0	0	

12425344	Not	cn	GB	2016	1	0	0	0	0	0	0	0	0	0	0	0	
12426037	Not	ot	TR	2016	0	0	0	0	0	0	0	0	0	0	0	0	
12426650	f	md	US	2016	0	1	0	0	1	0	0	0	0	0	0	0	58
12426677	Not	md	JP	2016	1	0	0	0	0	0	0	0	0	0	0	0	
12426722	m	ot	FR	2016	0	0	0	0	0	0	0	0	0	0	0	0	65
12426847	f	md	Not re	2016	1	0	0	0	0	0	0	0	0	0	0	0	
12427206	Not	md	JP	2016	0	1	0	0	1	0	0	0	0	0	0	0	
12427373	m	md	JP	2016	0	1	0	0	1	0	0	0	0	0	0	0	56
12427471	m	cn	DE	2016	0	1	0	0	1	0	0	0	0	0	0	0	
12427673	m	md	IT	2016	0	1	0	0	1	0	0	0	0	0	0	0	67
12427782	m	md	FR	2016	0	1	0	0	0	1	0	0	0	0	0	0	74
12427783	f	ph	AU	2016	0	1	0	0	1	0	0	0	0	0	0	0	59
12428184	f	md	ES	2016	0	1	0	0	0	1	0	0	0	0	0	0	75
12428417	f	md	US	2016	0	1	0	0	0	1	0	0	0	0	0	0	53
12428519	m	md	IT	2016	0	1	0	0	1	0	0	0	0	0	0	0	
12429186	f	md	JP	2016	1	0	0	0	0	0	0	0	0	0	0	0	71
12429268	m	md	JP	2016	0	1	0	0	1	0	0	0	0	0	0	0	76
12430058	m	md	JP	2016	0	1	0	0	1	0	0	0	0	0	0	0	49
12430941	Not	ph	US	2016	0	1	0	0	1	0	0	0	0	0	0	0	
12431474	m	md	JP	2016	0	1	0	0	1	0	0	0	0	0	0	0	65
12431523	m	cn	IT	2016	0	1	0	0	1	0	0	0	0	0	0	0	75
12431850	f	ph	AU	2016	0	1	0	0	1	0	0	0	0	0	0	0	70
12432912	f	ph	US	2016	0	0	0	0	0	0	0	0	0	0	0	0	
12433206	f	ot	IE	2016	1	0	0	0	0	0	0	0	0	0	0	0	76
12433303	m	md	US	2016	0	1	0	0	1	0	0	0	0	0	0	0	
12434048	f	cn	US	2016	0	1	0	0	1	0	0	0	0	0	0	0	
12434062	f	md	US	2016	0	1	1	0	0	0	0	0	0	0	0	0	70
12434128	m	md	US	2016	0	1	0	0	1	0	0	0	0	0	0	0	
12434133	Not	cn	JP	2016	0	1	0	0	1	0	0	0	0	0	0	1	64
12434306	m	ot	JP	2016	0	1	0	0	1	0	0	0	0	0	0	1	90
12434308	f	cn	JP	2016	0	1	0	0	1	0	0	0	0	0	0	0	68
12437363	f	ot	AR	2016	0	1	0	0	1	0	0	0	0	0	0	0	65
12438284	m	md	MX	2016	0	0	0	0	0	0	0	0	0	0	0	0	52
12438706	m	ot	US	2016	1	0	0	0	0	0	0	0	0	0	0	0	81

12438822	m	md	Not re	2016	1	0	0	0	0	0	0	0	0	0	0	0	78
12439621	Not	md	GR	2016	0	1	0	0	1	0	0	0	0	0	0	0	
12440876	m	ot	JP	2016	0	1	0	0	1	0	0	0	0	0	0	0	75
12441809	m	md	HR	2016	0	1	0	0	1	0	0	0	0	0	0	0	
12443140	f	cn	CN	2016	1	0	0	0	0	0	0	0	0	0	0	0	65
12443447	m	ot	IT	2016	0	0	0	0	0	0	0	0	0	0	0	0	78
12443795	m	cn	GB	2016	1	0	0	0	0	0	0	0	0	0	0	0	
12444778	Not	ot	JP	2016	0	0	0	0	0	0	0	0	0	0	0	0	
12444842	f	md	RU	2016	0	0	0	0	0	0	0	0	0	0	0	0	74
12444871	m	ot	US	2016	0	1	0	0	1	0	0	0	0	0	0	0	78
12444905	f	ot	US	2016	0	0	0	0	0	0	0	0	0	0	0	0	57
12445347	m	md	SG	2016	0	1	0	0	0	1	0	0	0	0	0	0	69
12446159	m	cn	US	2016	0	1	0	0	1	0	0	0	0	0	0	0	53
12446201	Not	ot	CO	2016	0	0	0	0	0	0	0	0	0	0	0	0	
12446203	Not	ot	CO	2016	0	0	0	0	0	0	0	0	0	0	0	0	
12446294	m	md	JP	2016	0	1	0	0	1	0	0	0	0	0	0	0	71
12446296	m	md	JP	2016	0	1	0	0	1	0	0	0	0	0	0	0	77
12446990	m	md	IT	2016	0	0	0	0	0	0	0	0	0	0	0	0	81
12447959	f	md	CO	2016	0	0	0	0	0	0	0	0	0	0	0	0	43
12448147	f	md	Not re	2016	1	0	0	0	0	0	0	0	0	0	0	0	63
12448436	f	ot	US	2016	0	1	0	0	1	0	0	0	0	0	0	0	77
12448438	m	ot	JP	2016	0	1	0	0	1	0	0	0	0	0	0	0	44
12448520	m	cn	JP	2016	0	1	0	0	1	0	0	0	0	0	0	0	67
12449323	f	cn	FR	2016	0	0	0	0	0	0	0	0	0	0	0	0	32
12449331	f	cn	FR	2016	0	0	0	0	0	0	0	0	0	0	0	0	79
12450330	f	ot	US	2016	0	0	0	0	0	0	0	0	0	0	0	0	86
12450469	f	ph	IL	2016	0	0	0	0	0	0	0	0	0	0	0	0	
12450563	m	ot	US	2016	0	0	0	0	0	0	0	0	0	0	0	0	35
12450656	m	ot	JP	2016	0	1	0	0	1	0	0	0	0	0	0	1	65
12450687	m	cn	JP	2016	0	1	0	0	1	0	0	0	0	0	0	0	71
12450689	m	md	JP	2016	0	1	0	0	1	0	0	0	0	0	0	0	45
12450699	m	ot	BE	2016	0	1	0	0	1	0	0	0	0	0	0	0	58
12450707	m	cn	PT	2016	0	1	0	0	1	0	0	0	0	0	0	0	65
12450708	f	ph	US	2016	0	1	0	0	1	0	0	0	0	0	0	0	

12450968	Not	ot	GR	2016	0	0	0	0	0	0	0	0	0	0	0	0	
12450970	Not	ot	GR	2016	0	0	0	0	0	0	0	0	0	0	0	0	
12450971	f	ot	GR	2016	0	0	0	0	0	0	0	0	0	0	0	0	
12451234	Not	cn	PE	2016	1	0	0	0	0	0	0	0	0	0	0	0	
12451236	Not	ot	PE	2016	1	0	0	0	0	0	0	0	0	0	0	0	
12451677	f	md	Not re	2016	1	0	0	0	0	0	0	0	0	0	0	0	68
12453359	f	md	BR	2016	0	0	0	0	0	0	0	0	0	0	0	0	52
12453723	f	ot	BR	2016	0	1	0	0	1	0	0	0	0	0	0	0	72
12453935	f	md	JP	2016	1	0	0	0	0	0	0	0	0	0	0	0	77
12453975	m	cn	JP	2016	0	0	0	0	0	0	1	0	1	0	0	1	67
12454069	Not	ot	ES	2016	0	0	0	0	0	0	0	0	0	0	0	0	
12454076	Not	ot	ES	2016	0	0	0	0	0	0	0	0	0	0	0	0	
12454082	Not	ot	ES	2016	0	0	0	0	0	0	0	0	0	0	0	0	
12454111	Not	ot	ES	2016	0	0	0	0	0	0	0	0	0	0	0	0	
12454407	Not	ot	ES	2016	0	0	0	0	0	0	0	0	0	0	0	0	
12454506	Not	ot	ES	2016	0	0	0	0	0	0	0	0	0	0	0	0	
12454520	Not	ot	ES	2016	0	0	0	0	0	0	0	0	0	0	0	0	
12454521	Not	ot	ES	2016	0	0	0	0	0	0	0	0	0	0	0	0	
12454553	Not	ot	ES	2016	0	0	0	0	0	0	0	0	0	0	0	0	
12454554	Not	ot	ES	2016	0	0	0	0	0	0	0	0	0	0	0	0	
12454729	m	ot	IT	2016	0	1	0	0	1	0	0	0	0	0	0	0	63
12455241	Not	ot	ES	2016	0	0	0	0	0	0	0	0	0	0	0	0	
12455246	Not	ot	ES	2016	0	0	0	0	0	0	0	0	0	0	0	0	
12455635	m	md	DE	2016	0	1	0	0	0	1	0	0	0	0	0	0	69
12455683	m	ph	US	2016	0	0	0	0	0	0	0	0	0	0	0	0	60
12455728	m	md	DE	2016	0	0	0	0	0	0	0	0	0	0	0	0	57
12455729	m	md	GB	2016	0	0	0	0	0	0	0	0	0	0	0	0	55
12456056	m	md	ES	2016	0	1	0	0	0	1	0	0	0	0	0	0	70
12456142	m	ot	US	2016	0	0	0	0	0	0	0	0	0	0	0	0	83
12456153	m	cn	GB	2016	1	0	0	0	0	0	0	0	0	0	0	0	46
12456719	m	md	AU	2016	0	1	0	0	0	1	0	0	0	0	0	0	68
12456935	m	ot	CN	2016	0	1	0	0	1	0	0	0	0	0	0	0	64
12457143	f	md	US	2016	0	0	0	0	0	0	0	0	0	0	0	0	64
12457417	Not	md	IT	2016	0	0	0	0	0	0	0	0	0	0	0	0	

12457484	f	md	Not re	2016	1	0	0	0	0	0	0	0	0	0	0	0	81
12459226	m	md	JP	2016	0	1	0	0	1	0	0	0	0	0	0	0	85
12459391	f	ot	JP	2016	0	1	0	0	1	0	0	0	0	0	0	0	69
12459427	f	ph	US	2016	1	0	0	0	0	0	0	0	0	0	0	0	78
12459548	m	md	JP	2016	1	0	0	0	0	0	0	0	0	0	0	1	64
12460127	m	cn	IN	2016	0	0	0	0	0	0	0	0	0	0	0	0	66
12460161	m	cn	IN	2016	0	0	0	0	0	0	0	0	0	0	0	0	47
12460193	m	cn	Not re	2016	0	0	0	0	0	0	0	0	0	0	0	0	
12460479	m	md	JP	2016	0	1	0	0	1	0	0	0	0	0	0	0	47
12460483	m	md	JP	2016	0	1	0	0	1	0	0	0	0	0	0	0	56
12460484	f	md	JP	2016	0	1	0	0	1	0	0	0	0	0	0	0	62
12460659	Not	md	US	2016	0	1	0	0	1	0	0	0	0	0	0	0	
12460727	m	ot	JP	2016	0	0	0	0	0	0	0	0	0	0	0	0	68
12461023	Not	md	Not re	2016	1	0	0	0	0	0	0	0	0	0	0	0	
12461581	m	ot	PT	2016	0	1	0	0	1	0	0	0	0	0	0	0	
12461917	m	md	JP	2016	0	1	0	0	1	0	0	0	0	0	0	0	69
12462416	f	Not repo	US	2016	1	0	0	0	0	0	0	0	0	0	0	0	57.3
12462423	f	md	US	2016	1	0	0	0	0	0	0	0	0	0	0	0	60
12462632	f	ph	AU	2016	0	1	0	0	1	0	0	0	0	0	0	0	66
12463240	m	ot	KR	2016	0	1	0	0	1	0	0	0	0	0	0	0	76
12463554	m	cn	GB	2016	1	0	0	0	0	0	0	0	0	0	0	0	63
12463795	f	md	CN	2016	1	0	0	0	0	0	0	0	0	0	0	0	40
12463823	f	ph	US	2016	0	1	0	0	1	0	0	0	0	0	0	0	
12464028	m	ot	US	2016	0	1	0	0	1	0	0	0	0	0	0	0	
12464823	m	cn	US	2016	0	1	0	0	1	0	0	0	0	0	0	0	
12464942	m	md	US	2016	0	0	0	0	0	0	0	0	0	0	0	0	64
12465110	m	cn	GB	2016	1	0	0	0	0	0	0	0	0	0	0	0	
12465115	m	cn	GB	2016	1	0	0	0	0	0	0	0	0	0	0	0	
12465226	m	cn	US	2016	0	0	0	0	0	0	0	0	0	0	0	0	
12465332	m	ot	JP	2016	0	1	0	0	1	0	0	0	0	0	0	0	65
12465421	m	md	JP	2016	0	1	0	0	1	0	0	0	0	0	0	1	53
12465992	f	cn	JP	2016	0	1	0	0	1	0	0	0	0	0	0	0	83
12466023	f	md	GB	2016	0	1	0	0	1	0	0	0	0	0	0	0	
12466024	f	ot	PL	2016	0	1	0	0	1	0	0	0	0	0	0	0	51

12466425	f	ot	FR	2016	1	0	0	0	0	0	0	0	0	0	0	0	61.8
12466598	m	cn	JP	2016	0	1	0	0	1	0	0	0	0	0	0	1	66
12466878	m	cn	JP	2016	0	1	0	0	1	0	0	0	0	0	0	1	64
12467625	m	md	TR	2016	0	1	0	0	1	0	0	0	0	0	0	0	66
12468366	m	md	US	2016	1	0	0	0	0	0	0	0	0	0	0	0	80.5
12469511	m	cn	US	2016	0	1	0	0	1	0	0	0	0	0	0	0	
12469678	m	ot	GB	2016	0	1	0	0	1	0	0	0	0	0	0	0	75
12469684	f	md	CA	2016	0	1	0	0	1	0	0	0	0	0	0	0	
12469701	m	cn	US	2016	0	1	0	0	1	0	0	0	0	0	0	0	
12469729	f	md	FR	2016	1	0	0	0	0	0	0	0	0	0	0	0	78.4
12469807	f	md	GR	2016	1	0	0	0	0	0	0	0	0	0	0	0	82
12469870	m	md	IL	2016	0	1	0	0	0	1	0	0	0	0	0	0	64
12470317	f	cn	JP	2016	0	1	0	0	1	0	0	0	0	0	0	0	60
12470477	f	md	US	2016	0	0	0	0	0	0	0	0	0	0	0	0	87
12470481	f	md	JP	2016	1	0	0	0	0	0	0	0	0	0	0	0	
12471215	f	cn	US	2016	0	1	0	0	1	0	0	0	0	0	0	0	75
12471721	f	md	IT	2016	0	0	0	0	0	0	0	0	0	0	0	0	71
12471726	f	md	NL	2016	0	0	0	0	0	0	0	0	0	0	0	0	
12471882	m	md	NL	2016	0	0	0	0	0	0	0	0	0	0	0	0	
12472417	m	md	JP	2016	1	0	0	0	0	0	0	0	0	0	0	0	76
12472508	f	md	AU	2016	0	1	0	0	1	0	0	0	0	0	0	0	
12473169	f	ot	IT	2016	0	0	0	0	0	0	0	0	0	0	0	0	71
12473316	m	md	US	2016	1	0	0	0	0	0	0	0	0	0	0	0	54
12473616	m	ph	JP	2016	1	0	0	0	0	0	0	0	0	0	0	0	76
12474064	Not	cn	US	2016	1	0	0	0	0	0	0	0	0	0	0	0	
12474296	Not	md	FR	2016	0	1	0	0	1	0	0	0	0	0	0	0	
12474929	f	cn	JP	2016	0	1	0	0	1	0	0	0	0	0	0	0	67
12475389	f	cn	GB	2016	1	0	0	0	0	0	0	0	0	0	0	0	
12475799	m	md	TR	2016	0	1	0	0	1	0	0	0	0	0	0	0	69
12476004	m	ot	CN	2016	0	0	0	0	0	0	0	0	0	0	0	0	65
12476144	m	ph	US	2016	0	0	0	0	0	0	0	0	0	0	0	0	
12476728	f	cn	GB	2016	1	0	0	0	0	0	0	0	0	0	0	0	
12476836	f	cn	IT	2016	0	0	0	0	0	0	0	0	0	0	0	0	71
12476874	Not	ph	US	2016	0	1	0	0	1	0	0	0	0	0	0	0	

12477178	f	md	GB	2016	0	0	0	0	0	0	0	0	0	0	0	0	53
12478019	f	cn	US	2016	0	1	0	0	1	0	0	0	0	0	0	0	
12478027	m	ot	ES	2016	0	1	0	0	1	0	0	0	0	0	0	0	
12478077	m	md	JP	2016	0	1	0	0	1	0	0	0	0	0	0	0	64
12478088	Not	md	JP	2016	0	0	0	0	0	0	0	0	0	0	0	0	
12478104	f	ot	PL	2016	0	1	0	0	1	0	0	0	0	0	0	0	59
12478132	m	ot	US	2016	0	1	0	0	1	0	0	0	0	0	0	0	85
12478181	f	ph	US	2016	0	1	0	0	1	0	0	0	0	0	0	1	
12478236	m	md	US	2016	0	1	1	0	0	0	0	0	0	0	0	0	85
12478497	f	cn	US	2016	1	0	0	0	0	0	0	0	0	0	0	0	
12478808	f	ot	JP	2016	0	1	0	0	1	0	0	0	0	0	0	0	58
12480728	m	ot	TR	2016	0	0	0	0	0	0	0	0	0	0	0	0	60
12481036	m	md	EG	2016	0	0	0	0	0	0	0	0	0	0	0	0	
12481090	m	md	JP	2016	0	1	0	0	1	0	0	0	0	0	0	0	74
12481488	f	md	RO	2016	0	1	0	0	0	1	0	0	0	0	0	0	58
12481757	f	ot	IT	2016	0	0	0	0	0	0	0	0	0	0	0	0	71
12482662	m	md	JP	2016	0	1	0	0	1	0	0	0	0	0	0	0	72
12483127	m	md	NZ	2016	0	1	0	0	0	1	0	0	0	0	0	0	65
12483442	f	md	US	2016	0	1	0	0	1	0	0	0	0	0	0	0	59
12483470	m	md	JP	2016	0	1	0	0	1	0	0	0	0	0	0	0	54
12484379	Not	cn	GB	2016	1	0	0	0	0	0	0	0	0	0	0	1	
12485709	m	md	JP	2016	0	1	0	0	0	1	0	0	0	0	0	0	62
12485937	f	md	US	2016	0	0	0	0	0	0	0	0	0	0	0	0	
12485944	f	ot	GB	2016	0	0	0	0	0	0	0	0	0	0	0	0	53
12486185	m	ot	IT	2016	0	1	0	0	1	0	0	0	0	0	0	0	66
12487447	m	ph	US	2016	0	1	0	0	1	0	0	0	0	0	0	0	70
12487472	m	md	TR	2016	0	0	0	0	0	0	0	0	0	0	0	0	60
12487787	Not	md	US	2016	0	0	0	0	0	0	0	0	0	0	0	0	
12487812	f	md	GB	2016	0	0	0	0	0	0	0	0	0	0	0	0	53
12488032	m	cn	JP	2016	0	1	0	0	1	0	0	0	0	0	0	0	67
12488083	f	md	US	2016	0	1	0	0	1	0	0	0	0	0	0	0	
12488616	f	md	Not re	2016	1	0	0	0	0	0	0	0	0	0	0	0	71
12488653	m	cn	US	2016	0	1	0	0	1	0	0	0	0	0	0	0	
12489009	f	ot	US	2016	0	1	0	0	1	0	0	0	0	0	0	0	

12491365	m	md	US	2016	0	0	0	0	0	0	0	0	0	0	0	0	64
12491412	f	md	IT	2016	0	0	0	0	0	0	0	0	0	0	0	0	71
12491903	m	ot	BE	2016	0	1	0	0	1	0	0	0	0	0	0	0	40
12492215	f	md	GB	2016	0	0	0	0	0	0	0	0	0	0	0	0	53
12493130	f	cn	US	2016	0	0	0	0	0	0	0	0	0	0	0	0	
12493525	f	cn	GB	2016	0	0	0	0	0	0	0	0	0	0	0	0	53
12494010	m	md	FR	2016	1	0	0	0	0	0	0	0	0	0	0	0	
12494297	Not	cn	Not re	2016	0	0	0	0	0	0	0	0	0	0	0	0	
12494808	m	md	TR	2016	0	1	0	0	1	0	0	0	0	0	0	0	58
12494836	Not	Not repo	DE	2016	0	1	0	0	1	0	0	0	0	0	0	0	
12494939	Not	Not repo	DE	2016	0	0	0	0	0	0	0	0	0	0	0	0	
12494942	Not	Not repo	DE	2016	0	0	0	0	0	0	0	0	0	0	0	0	
12495023	m	cn	JP	2016	0	1	0	0	1	0	0	0	0	0	0	0	65
12495118	f	md	GB	2016	0	0	0	0	0	0	0	0	0	0	0	0	53
12495458	m	ot	DE	2016	0	0	0	0	0	0	0	0	0	0	0	0	71
12496003	Not	cn	GB	2016	1	0	0	0	0	0	0	0	0	0	0	0	
12496040	f	md	GB	2016	0	0	0	0	0	0	0	0	0	0	0	0	53
12496052	f	md	US	2016	1	0	0	0	0	0	0	0	0	0	0	1	
12496530	f	ot	US	2016	0	1	0	0	1	0	0	0	0	0	0	0	55
12496624	m	ot	JP	2016	0	0	0	0	0	0	0	0	0	0	0	0	65
12496637	f	md	GB	2016	0	0	0	0	0	0	0	0	0	0	0	0	53
12496919	m	md	Not re	2016	0	0	0	0	0	0	0	0	0	0	0	0	59
12496948	f	md	TR	2016	0	1	0	0	1	0	0	0	0	0	0	0	50
12497002	f	md	US	2016	0	1	1	0	0	0	0	0	0	0	0	0	
12497181	f	md	US	2016	1	0	0	0	0	0	0	0	0	0	0	0	71
12497599	f	cn	US	2016	0	0	0	0	0	0	1	0	1	0	0	0	65
12497664	m	md	ES	2016	0	1	0	0	1	0	0	0	0	0	0	0	66
12497667	f	md	TR	2016	0	1	0	0	1	0	0	0	0	0	0	0	
12497676	m	ot	GB	2016	0	1	0	0	1	0	0	0	0	0	0	1	63
12499197	f	ot	GB	2016	0	0	0	0	0	0	0	0	0	0	0	0	53
12501196	f	md	JP	2016	0	0	0	0	0	0	0	0	0	0	0	0	81
12501208	f	md	Not re	2016	1	0	0	0	0	0	0	0	0	0	0	0	62
12501589	m	md	PT	2016	0	1	0	0	1	0	0	0	0	0	0	0	74
12502634	f	md	US	2016	0	0	0	0	0	0	0	0	0	0	0	0	70

12502655	f	ot	US	2016	0	1	0	0	1	0	0	0	0	0	0	0	55
12502921	m	md	UA	2016	0	1	0	0	0	1	0	0	0	0	0	0	49
12503171	Not	md	JP	2016	0	1	0	0	1	0	0	0	0	0	0	0	
12503608	Not	cn	GB	2016	1	0	0	0	0	0	0	0	0	0	0	0	
12503739	m	cn	JP	2016	0	0	0	0	0	0	0	0	0	0	0	0	75
12503904	f	md	FR	2016	1	0	0	0	0	0	0	0	0	0	0	0	65.7
12504354	Not	md	US	2016	0	1	0	0	1	0	0	0	0	0	0	0	
12504355	f	cn	GB	2016	1	0	0	0	0	0	0	0	0	0	0	0	
12505296	m	cn	US	2016	0	1	1	0	0	0	0	0	0	0	0	0	
12506783	f	ot	US	2016	0	0	0	0	0	0	0	0	0	0	0	0	67
12506852	m	md	JP	2016	0	1	0	0	0	1	0	0	0	0	0	0	71
12507206	m	cn	JP	2016	0	1	0	0	1	0	0	0	0	0	0	0	80
12507272	f	md	TW	2016	0	0	0	0	0	0	0	0	0	0	0	0	77
12507311	Not	ot	SI	2016	1	0	0	0	0	0	0	0	0	0	0	0	
12507543	f	cn	FR	2016	0	0	0	0	0	0	0	0	0	0	0	0	62
12508776	m	md	BR	2016	0	1	0	0	0	1	0	0	0	0	0	0	63
12509169	m	ot	US	2016	0	1	0	0	1	0	0	0	0	0	0	0	62
12511391	Not	ph	US	2016	0	0	0	0	0	0	0	0	0	0	0	0	
12511641	m	cn	JP	2016	0	1	0	0	1	0	0	0	0	0	0	1	67
12515624	m	md	JP	2016	1	0	0	0	0	0	0	0	0	0	0	0	70
12516902	m	md	BE	2016	0	1	0	0	1	0	0	0	0	0	0	0	65
12516996	f	ot	US	2016	0	1	0	0	1	0	0	0	0	0	0	0	75
12517027	f	md	ES	2016	1	0	0	0	0	0	0	0	0	0	0	0	82.7
12517634	f	md	JP	2016	0	0	0	0	0	0	0	0	0	0	0	0	
12517871	Not	ot	CN	2016	0	0	0	0	0	0	0	0	0	0	0	0	
12517913	f	md	JP	2016	0	1	0	0	1	0	0	0	0	0	0	0	67
12518085	m	cn	US	2016	0	1	0	0	1	0	0	0	0	0	0	0	78
12518129	m	ot	US	2016	0	1	0	0	1	0	0	0	0	0	0	1	61
12518412	m	md	ES	2016	0	0	0	0	0	0	0	0	0	0	0	0	71
12518419	f	cn	AR	2016	0	0	0	0	0	0	0	0	0	0	0	0	71
12518712	f	ot	GB	2016	0	1	0	0	1	0	0	0	0	0	0	0	64
12518721	m	ot	US	2016	0	1	0	0	1	0	0	0	0	0	0	0	56
12518723	m	ot	US	2016	0	1	0	0	1	0	0	0	0	0	0	0	42
12519164	m	cn	MY	2016	0	1	1	0	0	0	0	0	0	0	0	0	54

7992883	Not	ot	US	2016	0	0	0	0	0	0	0	0	0	0	0	0	
8280967	m	cn	GB	2016	1	0	0	0	0	0	0	0	0	0	0	0	
8983727	f	cn	US	2016	1	0	0	0	0	0	0	0	0	0	0	0	
9064694	m	md	TR	2016	0	0	0	0	0	0	0	0	0	0	0	0	55
9453940	m	md	JP	2016	0	0	0	0	0	0	0	0	0	0	0	0	60
9487114	m	md	FR	2016	0	0	0	0	0	0	0	0	0	0	0	0	83
9507951	f	md	JP	2016	0	0	0	0	0	0	0	0	0	0	0	0	55
9681999	f	md	Not re	2016	1	0	0	0	0	0	0	0	0	0	0	0	81
9783020	m	cn	GB	2016	1	0	0	0	0	0	0	0	0	0	0	0	72
9807874	m	cn	GB	2016	1	0	0	0	0	0	0	0	0	0	0	0	76
9863286	f	md	US	2016	0	0	0	0	0	0	0	0	0	0	0	0	49
10062493	m	ot	CN	2016	0	0	0	0	0	0	0	0	0	0	0	0	52
10333562	m	md	DE	2016	0	0	0	0	0	0	0	0	0	0	0	0	64
10370957	f	cn	IE	2016	0	0	0	0	0	0	0	0	0	0	0	0	37
10426745	f	cn	GB	2016	1	0	0	0	0	0	0	0	0	0	0	0	
10436451	m	md	JP	2016	0	0	0	0	0	0	0	0	0	0	0	0	55
10518247	m	cn	IE	2016	0	0	0	0	0	0	0	0	0	0	0	0	70
10525950	f	md	FR	2016	0	0	0	0	0	0	0	0	0	0	0	0	65
10538910	f	cn	GB	2016	1	0	0	0	0	0	0	0	0	0	0	0	71
10595203	m	cn	TH	2016	1	0	0	0	0	0	0	0	0	0	0	0	
10638854	m	cn	GB	2016	1	0	0	0	0	0	0	0	0	0	0	0	
10652509	f	md	FR	2016	0	0	0	0	0	0	0	0	0	0	0	0	59
11929773	f	md	US	2016	1	0	0	0	0	0	0	0	0	0	0	0	87
11947749	f	md	Not re	2016	1	0	0	0	0	0	0	0	0	0	0	0	64
12057630	f	cn	JP	2016	0	1	0	0	1	0	0	0	0	0	0	0	68
12073992	f	md	Not re	2016	1	0	0	0	0	0	0	0	0	0	0	0	
12187197	m	md	Not re	2016	0	0	0	0	0	0	0	0	0	0	0	0	76
12193035	m	md	JP	2016	0	0	0	0	0	0	0	0	0	0	0	0	50
12209758	m	cn	JP	2016	0	1	0	0	1	0	0	0	0	0	0	0	78
12211258	f	md	Not re	2016	1	0	0	0	0	0	0	0	0	0	0	0	86
12213606	f	md	US	2016	0	0	0	0	0	0	0	0	0	0	0	0	55
12226355	m	md	US	2016	1	0	0	0	0	0	0	0	0	0	0	0	86
12232056	f	md	JP	2016	1	0	0	0	0	0	0	0	0	0	0	0	78
12236946	m	cn	JP	2016	0	1	0	0	1	0	0	0	0	0	0	0	83

12237744	m	md	JP	2016	0	1	0	0	1	0	0	0	0	0	0	0	82
12246968	m	md	JP	2016	1	0	0	0	0	0	0	0	0	0	0	0	85
12249212	m	cn	JP	2016	0	1	0	0	1	0	0	0	0	0	0	0	57
12249313	m	md	CL	2016	0	0	0	0	0	0	0	0	0	0	0	0	64
12253108	m	cn	JP	2016	0	1	0	0	1	0	0	0	0	0	0	1	55
12253751	m	ot	JP	2016	0	1	0	0	1	0	0	0	0	0	0	0	60
12254000	f	md	CN	2016	1	0	0	0	0	0	0	0	0	0	0	0	75.6
12254799	f	md	JP	2016	1	0	0	0	0	0	0	0	0	0	0	0	67
12254935	f	md	JP	2016	0	0	0	0	0	0	0	0	0	0	0	0	59
12254941	f	ot	FR	2016	0	0	0	0	0	0	0	0	0	0	0	0	60
12257452	m	ot	US	2016	0	1	0	0	1	0	0	0	0	0	0	1	
12257550	f	md	ES	2016	0	0	0	0	0	0	0	0	0	0	0	0	50
12265105	f	cn	FR	2016	0	0	0	0	0	0	0	0	0	0	0	0	60
12265473	m	ot	US	2016	0	1	0	0	0	1	0	0	0	0	0	0	69
12267570	f	ph	FR	2016	1	0	0	0	0	0	0	0	0	0	0	0	84.3
12271486	f	ot	FR	2016	0	0	0	0	0	0	0	0	0	0	0	0	60
12276973	m	cn	JP	2016	0	0	0	0	0	0	0	0	0	0	0	0	74
12280383	f	cn	IE	2016	0	0	0	0	0	0	0	0	0	0	0	0	56
12281239	m	cn	JP	2016	0	1	0	0	1	0	0	0	0	0	0	0	35
12294728	m	cn	JP	2016	0	1	0	0	1	0	0	0	0	0	0	0	74
12300884	f	md	BE	2016	0	1	0	0	1	0	0	0	0	0	0	0	54
12302787	m	md	JP	2016	0	1	0	0	1	0	0	0	0	0	0	0	56
12305514	m	ot	US	2016	0	1	0	0	1	0	0	0	0	0	0	0	67
12308656	m	md	DE	2016	0	1	0	0	1	0	0	0	0	0	0	0	
12310110	m	cn	JP	2016	0	1	0	0	1	0	0	0	0	0	0	0	80
12312575	m	cn	JP	2016	0	1	0	0	1	0	0	0	0	0	0	0	60
12315935	f	ot	JP	2016	0	1	0	0	1	0	0	0	0	0	0	1	63
12316020	m	md	JP	2016	0	1	0	0	1	0	0	0	0	0	0	0	73
12316051	m	md	JP	2016	0	0	0	0	0	0	1	0	1	0	0	1	75
12316231	m	ot	PL	2016	0	1	0	0	1	0	0	0	0	0	0	0	65
12316331	m	ot	GB	2016	0	1	0	0	1	0	0	0	0	0	0	0	69
12317459	m	cn	JP	2016	0	1	0	0	1	0	0	0	0	0	0	0	74
12323233	f	ot	JP	2016	0	1	0	0	1	0	0	0	0	0	0	0	52
12325342	m	ot	ES	2016	0	0	0	0	0	0	0	0	0	0	0	0	64

12326191	f	ot	JP	2016	0	1	0	0	1	0	0	0	0	0	0	0	74
12328973	Not	cn	GB	2016	1	0	0	0	0	0	0	0	0	0	0	0	
12329369	m	cn	US	2016	0	1	0	0	1	0	0	0	0	0	0	0	60
12329541	f	ot	DE	2016	0	1	0	0	1	0	0	0	0	0	0	0	63
12335326	f	Not repo	US	2016	1	0	0	0	0	0	0	0	0	0	0	0	79.4
12335628	m	cn	DE	2016	0	1	0	0	1	0	0	0	0	0	0	0	55
12336895	f	ph	AU	2016	0	1	0	0	1	0	0	0	0	0	0	0	56
12338285	m	cn	DE	2016	0	0	0	0	0	0	0	0	0	0	0	0	80
12339204	m	md	JP	2016	1	0	0	0	0	0	0	0	0	0	0	0	78
12343005	f	md	CN	2016	1	0	0	0	0	0	0	0	0	0	0	0	45.6
12347917	m	md	DE	2016	0	1	0	0	1	0	0	0	0	0	0	0	62
12354540	f	ot	IT	2016	0	0	0	0	0	0	0	0	0	0	0	0	82
12355443	m	ot	ES	2016	0	1	0	0	1	0	0	0	0	0	0	0	62
12367388	f	md	CA	2016	0	0	0	0	0	0	0	0	0	0	0	0	51
12369260	m	ot	IT	2016	0	0	0	0	0	0	0	0	0	0	0	0	76
12370238	f	md	JP	2016	0	0	0	0	0	0	1	0	1	0	0	0	46
12370348	f	ot	US	2016	0	1	1	0	0	0	0	0	0	0	0	0	64
12376418	m	cn	JP	2016	0	0	0	0	0	0	1	0	1	0	0	1	74
12379612	f	md	Not re	2016	1	0	0	0	0	0	0	0	0	0	0	0	71
12386136	m	md	DE	2016	0	1	0	0	1	0	0	0	0	0	0	0	61
12386185	f	cn	US	2016	1	0	0	0	0	0	0	0	0	0	0	0	59.9
12386881	f	md	CN	2016	1	0	0	0	0	0	0	0	0	0	0	0	71.4
12387618	f	cn	JP	2016	0	1	0	0	1	0	0	0	0	0	0	0	76
12390745	Not	cn	JP	2016	0	1	0	0	1	0	0	0	0	0	0	0	63
12391304	m	cn	JP	2016	0	1	0	0	1	0	0	0	0	0	0	0	66
12391309	m	ph	JP	2016	0	1	0	0	1	0	0	0	0	0	0	0	79
12395682	m	cn	IT	2016	0	1	0	0	1	0	0	0	0	0	0	1	79
12395987	f	md	ZA	2016	0	1	0	0	1	0	0	0	0	0	0	0	59
12398425	f	md	JP	2016	0	0	0	0	0	0	0	0	0	0	0	0	47
12399928	m	cn	IT	2016	0	1	0	0	1	0	0	0	0	0	0	0	64
12400462	m	cn	JP	2016	0	1	0	0	1	0	0	0	0	0	0	0	72
12401263	f	cn	GB	2016	1	0	0	0	0	0	0	0	0	0	0	0	73
12403280	m	ot	GR	2016	0	1	0	0	1	0	0	0	0	0	0	1	66
12403509	m	cn	IT	2016	0	1	0	0	1	0	0	0	0	0	0	0	69

12405857	f	md	AU	2016	0	1	0	0	0	1	0	0	0	0	0	0	64
12408651	m	md	ES	2016	0	1	0	0	0	1	0	0	0	0	0	0	62
12408786	f	md	JP	2016	1	0	0	0	0	0	0	0	0	0	0	1	38
12409116	m	cn	JP	2016	0	1	0	0	1	0	0	0	0	0	0	0	75
12409261	f	cn	DE	2016	0	1	0	0	1	0	0	0	0	0	0	0	63
12411669	f	md	Not re	2016	1	0	0	0	0	0	0	0	0	0	0	0	67
12411795	m	ot	IL	2016	1	0	0	0	0	0	0	0	0	0	0	0	85
12411947	m	ph	US	2016	0	0	0	0	0	0	0	0	0	0	0	0	68
12412947	f	md	JP	2016	1	0	0	0	0	0	0	0	0	0	0	0	80
12413498	m	ot	JP	2016	0	1	0	0	1	0	0	0	0	0	0	0	60
12414977	m	md	IT	2016	0	1	0	0	0	1	0	0	0	0	0	0	70
12415745	m	md	TW	2016	0	1	0	0	0	1	0	0	0	0	0	0	45
12418124	m	md	JP	2016	0	1	0	0	1	0	0	0	0	0	0	0	67
12418750	f	ot	ES	2016	0	1	0	0	1	0	0	0	0	0	0	0	51
12420707	m	md	FR	2016	0	0	0	0	0	0	0	0	0	0	0	0	67
12422089	f	md	US	2016	0	1	0	0	0	1	0	0	0	0	0	0	79
12422458	f	md	US	2016	0	1	0	0	0	1	0	0	0	0	0	0	74
12423270	m	md	US	2016	0	1	0	0	0	1	0	0	0	0	0	0	78
12425690	m	ot	CA	2016	0	1	0	0	1	0	0	0	0	0	0	0	60
12426673	f	md	JP	2016	1	0	0	0	0	0	0	0	0	0	0	0	49
12428052	f	md	US	2016	0	1	0	0	0	1	0	0	0	0	0	0	69
12431099	m	md	US	2016	0	1	0	0	0	1	0	0	0	0	0	0	65
12431313	m	md	JP	2016	0	1	0	0	1	0	0	0	0	0	0	0	67
12432307	m	md	JP	2016	0	1	0	0	0	1	0	0	0	0	0	0	68
12433529	m	cn	JP	2016	0	1	0	0	1	0	0	0	0	0	0	0	63
12434058	m	cn	JP	2016	0	1	0	0	1	0	0	0	0	0	0	1	71
12434794	m	md	Not re	2016	0	0	0	0	0	0	0	0	0	0	0	0	51
12436499	f	md	JP	2016	0	1	0	0	1	0	0	0	0	0	0	0	71
12436507	m	md	BE	2016	0	1	0	0	0	1	0	0	0	0	0	1	68
12436562	f	md	JP	2016	0	1	0	0	1	0	0	0	0	0	0	0	67
12437246	m	cn	JP	2016	0	1	0	0	1	0	0	0	0	0	0	0	71
12438029	f	cn	IE	2016	0	0	0	0	0	0	0	0	0	0	0	0	50
12438193	m	ot	ES	2016	0	0	0	0	0	0	0	0	0	0	0	0	62
12446154	f	md	CZ	2016	0	1	0	0	1	0	0	0	0	0	0	0	

12448445	m	md	GB	2016	0	0	0	0	0	0	0	0	0	0	0	0	73
12448533	m	md	JP	2016	0	1	0	0	1	0	0	0	0	0	0	0	67
12449027	f	ot	US	2016	0	1	0	0	1	0	0	0	0	0	0	0	72
12449288	f	md	FR	2016	1	0	0	0	0	0	0	0	0	0	0	0	54.1
12449505	f	cn	JP	2016	0	1	0	0	1	0	0	0	0	0	0	0	69
12450698	m	md	FR	2016	0	1	0	0	1	0	0	0	0	0	0	0	57
12451035	f	ot	DE	2016	0	1	0	0	1	0	0	0	0	0	0	0	29
12452632	f	cn	IT	2016	0	1	0	0	1	0	0	0	0	0	0	0	65
12453973	m	cn	JP	2016	0	1	0	0	1	0	0	0	0	0	0	0	55
12454510	f	cn	JP	2016	0	1	0	0	1	0	0	0	0	0	0	0	61
12454519	m	ot	PT	2016	0	1	0	0	1	0	0	0	0	0	0	0	55
12454709	f	cn	JP	2016	0	1	0	0	1	0	0	0	0	0	0	0	67
12456744	m	cn	US	2016	0	1	0	0	1	0	0	0	0	0	0	0	
12462531	m	md	US	2016	0	1	1	0	0	0	0	0	0	0	0	0	76
12463349	f	ot	CA	2016	0	1	0	0	1	0	0	0	0	0	0	0	63
12465408	f	cn	IT	2016	0	1	0	0	1	0	0	0	0	0	0	0	75
12467958	f	md	DE	2016	0	0	0	0	0	0	0	0	0	0	0	0	71
12468524	f	md	Not re	2016	0	0	0	0	0	0	1	0	1	0	0	0	75
12469009	m	cn	JP	2016	0	1	0	0	1	0	0	0	0	0	0	1	67
12469679	m	ph	JP	2016	0	1	0	0	1	0	0	0	0	0	0	0	68
12469709	f	ot	US	2016	0	1	0	0	1	0	0	0	0	0	0	0	61
12469805	m	md	JP	2016	0	0	0	0	0	0	0	0	0	0	0	0	70
12470406	m	md	TR	2016	0	1	0	0	0	1	0	0	0	0	0	0	76
12470937	m	md	FR	2016	0	0	0	0	0	0	0	0	0	0	0	0	59
12471080	m	md	FR	2016	0	1	0	0	0	1	0	0	0	0	0	1	64
12473086	f	md	JP	2016	0	0	0	0	0	0	0	0	0	0	0	0	74
12473097	m	md	ES	2016	0	0	0	0	0	0	0	0	0	0	0	0	49
12473276	f	ot	US	2016	0	0	0	0	0	0	0	0	0	0	0	0	74
12474866	m	ot	JP	2016	0	1	0	0	1	0	0	0	0	0	0	0	81
12475898	f	md	IT	2016	0	0	0	0	0	0	0	0	0	0	0	0	71
12476378	m	ot	KR	2016	0	0	0	0	0	0	0	0	0	0	0	0	62
12476685	f	md	GB	2016	0	1	0	0	1	0	0	0	0	0	0	0	71
12477381	f	md	KR	2016	0	0	0	0	0	0	0	0	0	0	0	0	56
12477524	m	md	AU	2016	0	0	0	0	0	0	0	0	0	0	0	0	60

12478075	m	ot	JP	2016	0	1	0	0	1	0	0	0	0	0	0	0	71
12478393	f	Not repo	US	2016	1	0	0	0	0	0	0	0	0	0	0	0	
12479757	f	ph	US	2016	0	0	0	0	0	0	0	0	0	0	0	0	45
12482742	m	ot	IN	2016	0	0	0	0	0	0	0	0	0	0	0	0	40
12483121	f	md	IT	2016	0	1	0	0	0	1	0	0	0	0	0	0	65
12483384	f	md	JP	2016	0	0	0	0	0	0	1	0	1	0	0	1	71
12483417	m	md	JP	2016	0	1	0	0	1	0	0	0	0	0	0	0	75
12483482	m	md	JP	2016	0	1	0	0	1	0	0	0	0	0	0	0	48
12484362	m	ot	JP	2016	0	1	0	0	1	0	0	0	0	0	0	0	73
12486363	f	ot	JP	2016	0	1	0	0	1	0	0	0	0	0	0	1	68
12487108	m	cn	JP	2016	0	1	0	0	1	0	0	0	0	0	0	1	68
12487155	f	md	KR	2016	1	0	0	0	0	0	0	0	0	0	0	0	69
12488026	m	cn	GB	2016	1	0	0	0	0	0	0	0	0	0	0	0	
12488036	m	md	JP	2016	0	1	0	0	1	0	0	0	0	0	0	0	65
12488834	f	md	TR	2016	0	0	0	0	0	0	0	0	0	0	0	0	56
12489010	f	md	JP	2016	0	1	0	0	1	0	0	0	0	0	0	0	70
12489504	m	md	TR	2016	0	1	0	0	1	0	0	0	0	0	0	0	
12490828	f	md	JP	2016	1	0	0	0	0	0	0	0	0	0	0	0	81
12492371	f	md	JP	2016	1	0	0	0	0	0	0	0	0	0	0	0	65
12492986	m	ot	Not re	2016	0	0	0	0	0	0	0	0	0	0	0	0	61
12493097	f	md	JP	2016	1	0	0	0	0	0	0	0	0	0	0	0	65
12494197	m	md	JP	2016	0	1	0	0	1	0	0	0	0	0	0	0	72
12494198	m	md	JP	2016	0	1	0	0	1	0	0	0	0	0	0	0	65
12494721	m	md	JP	2016	0	1	0	0	1	0	0	0	0	0	0	0	71
12494810	m	md	TR	2016	0	1	0	0	1	0	0	0	0	0	0	0	76
12495853	f	md	BN	2016	0	0	0	0	0	0	0	0	0	0	0	0	
12495874	m	ph	US	2016	0	1	1	0	0	0	0	0	0	0	0	0	63
12496102	f	md	NL	2016	0	1	0	0	1	0	0	0	0	0	0	0	60
12496272	m	md	TR	2016	0	1	0	0	1	0	0	0	0	0	0	0	64
12496864	Not	md	JP	2016	0	1	0	0	1	0	0	0	0	0	0	0	65
12497075	m	md	JP	2016	1	0	0	0	0	0	0	0	0	0	0	0	74
12497612	m	md	JP	2016	0	1	0	0	1	0	0	0	0	0	0	0	75
12498891	f	md	CA	2016	0	1	0	0	0	1	0	0	0	0	0	0	51
12498902	m	cn	JP	2016	0	1	0	0	1	0	0	0	0	0	0	0	67

12499550	m	md	AU	2016	0	0	0	0	0	0	0	0	0	0	0	0	70
12501236	m	cn	IT	2016	0	1	0	0	1	0	0	0	0	0	0	0	65
12501986	f	md	DE	2016	0	1	0	0	1	0	0	0	0	0	0	0	
12502084	m	md	US	2016	0	0	0	0	0	0	0	0	0	0	0	0	
12502504	f	md	US	2016	0	1	0	0	0	1	0	0	0	0	0	0	65
12502943	m	md	TR	2016	0	1	0	0	1	0	0	0	0	0	0	0	61
12507216	f	ot	US	2016	0	1	0	0	1	0	0	0	0	0	0	0	71
12507310	m	md	JP	2016	0	1	0	0	1	0	0	0	0	0	0	0	69
12507402	m	md	JP	2016	0	1	0	0	1	0	0	0	0	0	0	0	61
12507498	f	md	JP	2016	1	0	0	0	0	0	0	0	0	0	0	0	69
12507928	m	ot	CN	2016	0	1	0	0	1	0	0	0	0	0	0	0	78
12509629	f	md	Not re	2016	1	0	0	0	0	0	0	0	0	0	0	0	56
12510213	m	md	US	2016	0	1	0	0	1	0	0	0	0	0	0	0	63
12510403	f	md	FR	2016	1	0	0	0	0	0	0	0	0	0	0	0	
12511525	m	md	US	2016	0	1	0	0	0	1	0	0	0	0	0	0	61
12517304	f	cn	GB	2016	1	0	0	0	0	0	0	0	0	0	0	0	69
12517462	f	cn	CN	2016	0	0	0	0	0	0	0	0	0	0	0	0	
12518120	m	ot	JP	2016	0	1	0	0	1	0	0	0	0	0	0	0	73
12518152	m	md	GB	2016	0	1	1	0	0	0	0	0	0	0	0	0	68
12519849	m	md	IT	2016	0	0	0	0	0	0	0	0	0	0	0	0	48
12519854	f	md	JP	2016	0	1	0	0	0	1	0	0	0	0	0	0	63
12520524	m	cn	DE	2016	0	1	0	0	1	0	0	0	0	0	0	1	62
12520696	f	ph	US	2016	0	1	0	0	1	0	0	0	0	0	0	0	53
12520746	f	md	GR	2016	0	1	0	0	0	1	0	0	0	0	0	0	62
12520766	f	md	US	2016	0	0	0	0	0	0	1	0	0	0	1	0	83
12520846	m	md	US	2016	0	0	0	0	0	0	0	0	0	0	0	0	50
12521479	m	md	TR	2016	0	1	0	0	1	0	0	0	0	0	0	0	45
12521495	m	md	IT	2016	0	1	0	0	0	1	0	0	0	0	0	0	58
12521872	m	ot	UA	2016	0	0	0	0	0	0	0	0	0	0	0	0	63
12522013	m	cn	IT	2016	0	1	0	0	1	0	0	0	0	0	0	0	70
12522032	m	cn	JP	2016	0	1	0	0	1	0	0	0	0	0	0	0	
12522219	f	cn	US	2016	0	1	0	0	1	0	0	0	0	0	0	1	
12522223	m	cn	US	2016	0	1	0	0	1	0	0	0	0	0	0	0	
12522225	f	ot	US	2016	0	1	0	0	1	0	0	0	0	0	0	0	83

12522734	f	ot	GB	2016	0	1	0	0	1	0	0	0	0	0	0	1	84
12522840	m	cn	JP	2016	0	1	0	0	1	0	0	0	0	0	0	0	78
12523031	f	md	JP	2016	1	0	0	0	0	0	0	0	0	0	0	0	78
12523076	m	ot	US	2016	0	0	0	0	0	0	1	0	1	0	0	0	79
12523188	Not	md	FR	2016	0	1	0	0	1	0	0	0	0	0	0	0	
12523692	Not	ot	BR	2016	0	0	0	0	0	0	0	0	0	0	0	0	
12523754	Not	ot	BR	2016	0	0	0	0	0	0	0	0	0	0	0	0	
12523824	Not	ot	BR	2016	0	0	0	0	0	0	0	0	0	0	0	0	
12523828	Not	ot	BR	2016	0	0	0	0	0	0	0	0	0	0	0	0	
12523841	Not	ot	BR	2016	0	0	0	0	0	0	0	0	0	0	0	0	
12523847	Not	ot	BR	2016	0	0	0	0	0	0	0	0	0	0	0	0	
12524659	m	cn	JP	2016	0	1	0	0	1	0	0	0	0	0	0	0	46
12524864	f	cn	GB	2016	0	0	0	0	0	0	0	0	0	0	0	0	
12524989	m	ot	BR	2016	0	1	0	0	1	0	0	0	0	0	0	0	74
12525083	m	md	JP	2016	0	1	0	0	1	0	0	0	0	0	0	0	73
12525205	Not	md	FR	2016	0	1	0	0	1	0	0	0	0	0	0	0	
12525237	f	ot	US	2016	0	0	0	0	0	0	0	0	0	0	0	0	70
12525802	f	cn	US	2016	1	0	0	0	0	0	0	0	0	0	0	0	
12525821	f	md	JP	2016	0	0	0	0	0	0	1	0	1	0	0	1	82
12525848	f	md	JP	2016	0	1	0	0	1	0	0	0	0	0	0	0	90
12525895	m	cn	JP	2016	0	1	0	0	1	0	0	0	0	0	0	0	48
12526018	Not	md	JP	2016	0	1	0	0	1	0	0	0	0	0	0	0	68
12526836	m	ph	Not re	2016	0	0	0	0	0	0	0	0	0	0	0	0	57
12527216	m	md	RO	2016	0	0	0	0	0	0	0	0	0	0	0	0	
12527233	m	cn	BR	2016	0	0	0	0	0	0	0	0	0	0	0	0	
12527382	m	cn	TH	2016	0	0	0	0	0	0	0	0	0	0	0	0	72
12527463	m	ot	KR	2016	0	0	0	0	0	0	0	0	0	0	0	0	68
12527930	f	md	AU	2016	0	1	0	0	1	0	0	0	0	0	0	0	67
12528000	m	md	PL	2016	0	0	0	0	0	0	0	0	0	0	0	0	65
12528288	m	md	DE	2016	0	1	0	0	1	0	0	0	0	0	0	0	66
12528365	m	md	FR	2016	1	0	0	0	0	0	0	0	0	0	0	0	52
12528372	f	md	Not re	2016	0	0	0	0	0	0	0	0	0	0	0	0	68
12528534	m	ot	US	2016	0	1	0	0	1	0	0	0	0	0	0	0	70
12528559	m	md	GR	2016	0	0	0	0	0	0	0	0	0	0	0	0	70

12528767	Not	ot	JP	2016	0	0	0	0	0	0	0	0	0	0	0	0	
12528969	m	md	JP	2016	0	1	0	0	1	0	0	0	0	0	0	0	69
12529098	m	md	US	2016	1	0	0	0	0	0	0	0	0	0	0	0	60
12529292	m	cn	US	2016	0	1	1	0	0	0	0	0	0	0	0	0	
12529497	Not	ot	DE	2016	0	0	0	0	0	0	0	0	0	0	0	0	
12529502	Not	ot	DE	2016	0	0	0	0	0	0	0	0	0	0	0	0	
12529628	Not	ot	JP	2016	0	0	0	0	0	0	0	0	0	0	0	0	
12529629	Not	ot	JP	2016	0	0	0	0	0	0	0	0	0	0	0	0	
12529639	Not	ot	JP	2016	0	0	0	0	0	0	0	0	0	0	0	0	
12529655	Not	ot	JP	2016	0	0	0	0	0	0	0	0	0	0	0	0	
12529659	Not	ot	JP	2016	0	0	0	0	0	0	0	0	0	0	0	0	
12529660	Not	ot	JP	2016	0	0	0	0	0	0	0	0	0	0	0	0	
12529665	Not	ot	JP	2016	0	0	0	0	0	0	0	0	0	0	0	0	
12529667	Not	ot	JP	2016	0	0	0	0	0	0	0	0	0	0	0	0	
12529673	Not	ot	JP	2016	0	0	0	0	0	0	0	0	0	0	0	0	
12529676	Not	ot	JP	2016	0	0	0	0	0	0	0	0	0	0	0	0	
12529788	Not	ot	DE	2016	0	0	0	0	0	0	0	0	0	0	0	0	
12529810	Not	ot	DE	2016	0	0	0	0	0	0	0	0	0	0	0	0	
12529821	f	cn	US	2016	0	1	0	0	1	0	0	0	0	0	0	0	
12530064	Not	ot	BR	2016	0	0	0	0	0	0	0	0	0	0	0	0	
12530768	f	md	TH	2016	0	0	0	0	0	0	0	0	0	0	0	0	65
12531035	m	md	HU	2016	0	0	0	0	0	0	0	0	0	0	0	0	55
12531603	Not	ot	JP	2016	0	0	0	0	0	0	0	0	0	0	0	0	
12531694	m	md	US	2016	0	1	0	0	1	0	0	0	0	0	0	0	88
12531839	m	ot	TR	2016	0	0	0	0	0	0	0	0	0	0	0	0	60
12534850	m	md	DE	2016	0	1	0	0	1	0	0	0	0	0	0	0	69
12534853	m	ph	US	2016	0	1	0	0	1	0	0	0	0	0	0	0	
12534974	f	md	ES	2016	0	1	0	0	0	1	0	0	0	0	0	0	58
12535114	f	md	JP	2016	0	1	0	0	1	0	0	0	0	0	0	0	68
12535200	m	md	JP	2016	0	1	0	0	1	0	0	0	0	0	0	0	65
12535201	f	ph	CA	2016	0	1	0	0	1	0	0	0	0	0	0	0	72
12535538	m	md	CH	2016	0	0	0	0	0	0	0	0	0	0	0	0	66
12535713	Not	md	JP	2016	1	0	0	0	0	0	0	0	0	0	0	0	
12535714	f	md	FR	2016	1	0	0	0	0	0	0	0	0	0	0	0	

12535729	Not	md	JP	2016	1	0	0	0	0	0	0	0	0	0	0	0	
12535774	Not	md	JP	2016	1	0	0	0	0	0	0	0	0	0	0	0	
12536091	Not	ph	JP	2016	0	1	0	0	1	0	0	0	0	0	0	0	
12536280	m	cn	JP	2016	0	0	0	0	0	0	0	0	0	0	0	0	75
12536371	m	md	ES	2016	0	1	0	0	0	1	0	0	0	0	0	0	61
12537436	m	cn	GB	2016	0	1	0	0	1	0	0	0	0	0	0	0	69
12538555	m	md	ES	2016	0	1	0	0	0	1	0	0	0	0	0	0	77
12538574	Not	ot	US	2016	0	0	0	0	0	0	0	0	0	0	0	0	
12538580	Not	ot	US	2016	1	0	0	0	0	0	0	0	0	0	0	0	
12538584	Not	ot	US	2016	1	0	0	0	0	0	0	0	0	0	0	0	
12538585	Not	ot	US	2016	1	0	0	0	0	0	0	0	0	0	0	1	
12538588	Not	ot	US	2016	1	0	0	0	0	0	0	0	0	0	0	1	
12538590	Not	ot	US	2016	1	0	0	0	0	0	0	0	0	0	0	0	
12538597	Not	ot	US	2016	1	0	0	0	0	0	0	0	0	0	0	0	
12538626	f	cn	GB	2016	0	1	0	0	1	0	0	0	0	0	0	0	74
12538643	Not	ot	FR	2016	0	0	0	0	0	0	0	0	0	0	0	0	
12538658	Not	ot	FR	2016	0	0	0	0	0	0	0	0	0	0	0	0	
12538660	Not	ot	US	2016	0	0	0	0	0	0	0	0	0	0	0	0	
12538661	Not	ot	US	2016	0	0	0	0	0	0	0	0	0	0	0	0	
12538664	Not	ot	US	2016	0	0	0	0	0	0	0	0	0	0	0	0	
12538665	Not	ot	US	2016	1	0	0	0	0	0	0	0	0	0	0	0	
12538670	Not	ot	FR	2016	0	0	0	0	0	0	0	0	0	0	0	0	
12538671	Not	ot	FR	2016	0	0	0	0	0	0	0	0	0	0	0	0	
12538672	Not	ot	FR	2016	0	0	0	0	0	0	0	0	0	0	0	0	
12538674	Not	ot	FR	2016	0	0	0	0	0	0	0	0	0	0	0	0	
12538678	Not	ot	FR	2016	0	0	0	0	0	0	0	0	0	0	0	0	
12538688	Not	ot	FR	2016	0	0	0	0	0	0	0	0	0	0	0	0	
12538689	Not	ot	US	2016	0	0	0	0	0	0	0	0	0	0	0	0	
12538698	Not	ot	US	2016	0	0	0	0	0	0	0	0	0	0	0	0	
12538699	Not	ot	US	2016	0	0	0	0	0	0	0	0	0	0	0	0	
12538701	Not	ot	US	2016	0	0	0	0	0	0	0	0	0	0	0	0	
12538702	Not	ot	US	2016	0	0	0	0	0	0	0	0	0	0	0	0	
12538703	Not	ot	US	2016	0	0	0	0	0	0	0	0	0	0	0	0	
12539023	m	ot	CN	2016	0	0	0	0	0	0	0	0	0	0	0	0	76

12539204	Not	ot	US	2016	0	0	0	0	0	0	0	0	0	0	0	0	
12539220	Not	ot	US	2016	0	0	0	0	0	0	0	0	0	0	0	0	
12539270	Not	ot	US	2016	0	0	0	0	0	0	0	0	0	0	0	0	
12539421	Not	ot	US	2016	0	0	0	0	0	0	0	0	0	0	0	0	
12540058	Not	ot	US	2016	0	0	0	0	0	0	0	0	0	0	0	0	
12540062	Not	ot	US	2016	0	0	0	0	0	0	0	0	0	0	0	0	
12540076	f	md	JP	2016	0	1	0	0	1	0	0	0	0	0	0	0	67
12540202	f	md	AT	2016	1	0	0	0	0	0	0	0	0	0	0	0	67
12540744	m	cn	NL	2016	0	0	0	0	0	0	0	0	0	0	0	0	59
12541201	f	md	IT	2016	0	0	0	0	0	0	0	0	0	0	0	0	46
12541296	m	md	Not re	2016	0	0	0	0	0	0	0	0	0	0	0	0	63
12541685	m	md	PH	2016	1	0	0	0	0	0	0	0	0	0	0	0	65
12541866	f	md	Not re	2016	1	0	0	0	0	0	0	0	0	0	0	0	62
12542263	f	md	FR	2016	0	1	0	0	1	0	0	0	0	0	0	0	60
12542288	Not	ph	AU	2016	0	1	0	0	1	0	0	0	0	0	0	0	45
12542493	m	ot	JP	2016	0	0	0	0	0	0	0	0	0	0	0	0	72
12542497	Not	ot	US	2016	0	0	0	0	0	0	0	0	0	0	0	0	
12542640	Not	ot	US	2016	0	0	0	0	0	0	0	0	0	0	0	0	
12542644	Not	ot	US	2016	0	0	0	0	0	0	0	0	0	0	0	0	
12542796	m	md	JP	2016	0	1	0	0	1	0	0	0	0	0	0	0	68
12543179	m	md	JP	2016	1	0	0	0	0	0	0	0	0	0	0	0	73
12543224	m	ot	FR	2016	0	1	0	0	1	0	0	0	0	0	0	0	44
12543651	f	ot	US	2016	0	0	0	0	0	0	0	0	0	0	0	0	48
12543888	m	md	FR	2016	0	1	0	0	0	1	0	0	0	0	0	0	49
12544825	f	cn	US	2016	0	1	1	0	0	0	0	0	0	0	0	0	65
12545358	m	ot	US	2016	0	1	0	0	1	0	0	0	0	0	0	0	
12546435	f	md	PL	2016	0	0	0	0	0	0	0	0	0	0	0	0	80
12546556	f	ot	IT	2016	0	1	0	0	1	0	0	0	0	0	0	0	59
12546581	m	cn	JP	2016	0	1	0	0	1	0	0	0	0	0	0	0	76
12546586	m	md	JP	2016	0	1	0	0	1	0	0	0	0	0	0	0	75
12547024	m	md	JP	2016	0	1	0	0	1	0	0	0	0	0	0	0	68
12547027	m	cn	JP	2016	0	1	0	0	1	0	0	0	0	0	0	0	57
12547049	m	cn	US	2016	0	1	0	0	1	0	0	0	0	0	0	1	
12547126	m	cn	BR	2016	0	1	0	0	1	0	0	0	0	0	0	0	56

12547127	m	cn	JP	2016	0	1	0	0	1	0	0	0	0	0	0	0	66
12547712	f	md	CH	2016	0	1	0	0	1	0	0	0	0	0	0	0	
12547858	f	md	JP	2016	1	0	0	0	0	0	0	0	0	0	0	0	70
12548071	Not	ot	JP	2016	0	0	0	0	0	0	0	0	0	0	0	0	
12548357	m	cn	CN	2016	0	0	0	0	0	0	0	0	0	0	0	0	
12548758	Not	ot	JP	2016	0	0	0	0	0	0	0	0	0	0	0	0	
12548884	f	cn	GB	2016	1	0	0	0	0	0	0	0	0	0	0	0	52
12548885	m	cn	GB	2016	1	0	0	0	0	0	0	0	0	0	0	0	73
12549324	f	md	JP	2016	1	0	0	0	0	0	0	0	0	0	0	0	85.7
12549769	m	cn	JP	2016	0	1	0	0	1	0	0	0	0	0	0	1	71
12549912	m	md	JP	2016	1	0	0	0	0	0	0	0	0	0	0	0	66
12549917	f	md	MO	2016	1	0	0	0	0	0	0	0	0	0	0	0	64
12549949	f	md	US	2016	0	0	0	0	0	0	0	0	0	0	0	0	53
12550073	Not	md	CH	2016	1	0	0	0	0	0	0	0	0	0	0	0	
12550632	f	md	AT	2016	1	0	0	0	0	0	0	0	0	0	0	0	67
12550677	f	cn	JP	2016	0	1	0	0	1	0	0	0	0	0	0	0	56
12550706	m	ot	FR	2016	0	1	0	0	1	0	0	0	0	0	0	0	73
12550728	m	ot	IT	2016	0	1	0	0	1	0	0	0	0	0	0	0	56
12550987	Not	ot	GR	2016	0	0	0	0	0	0	0	0	0	0	0	0	
12550995	m	md	ES	2016	0	1	0	0	0	1	0	0	0	0	0	0	72
12551215	Not	ot	JP	2016	0	0	0	0	0	0	0	0	0	0	0	0	
12551218	Not	ot	JP	2016	0	0	0	0	0	0	0	0	0	0	0	0	
12551224	Not	ot	JP	2016	0	0	0	0	0	0	0	0	0	0	0	0	
12551225	Not	ot	JP	2016	0	0	0	0	0	0	0	0	0	0	0	0	
12551530	Not	ot	US	2016	0	0	0	0	0	0	0	0	0	0	0	0	
12551533	Not	ot	US	2016	0	0	0	0	0	0	0	0	0	0	0	0	
12551542	Not	ot	US	2016	0	0	0	0	0	0	0	0	0	0	0	0	
12551543	Not	ot	US	2016	0	0	0	0	0	0	0	0	0	0	0	0	
12551549	Not	ot	US	2016	0	0	0	0	0	0	0	0	0	0	0	0	
12551565	Not	ot	US	2016	0	0	0	0	0	0	0	0	0	0	0	0	
12551566	Not	ot	US	2016	0	0	0	0	0	0	0	0	0	0	0	0	
12551660	f	md	JP	2016	0	1	0	0	1	0	0	0	0	0	0	0	57
12552094	Not	ot	US	2016	0	0	0	0	0	0	0	0	0	0	0	0	
12552190	Not	ot	US	2016	0	0	0	0	0	0	0	0	0	0	0	0	

12552251	Not	ot	US	2016	0	0	0	0	0	0	0	0	0	0	0	0	
12552263	Not	ot	US	2016	0	0	0	0	0	0	0	0	0	0	0	0	
12552370	Not	ot	US	2016	0	0	0	0	0	0	0	0	0	0	0	0	
12552392	m	ot	IT	2016	1	0	0	0	0	0	0	0	0	0	0	0	66
12552613	f	md	FR	2016	1	0	0	0	0	0	0	0	0	0	0	0	61
12552686	f	md	BE	2016	0	0	0	0	0	0	0	0	0	0	0	0	84
12552826	Not	Not repo	CN	2016	0	0	0	0	0	0	0	0	0	0	0	0	
12553121	m	md	US	2016	0	1	0	0	0	1	0	0	0	0	0	0	68
12553193	f	md	JP	2016	1	0	0	0	0	0	0	0	0	0	0	0	79
12553228	f	md	FR	2016	0	1	0	0	0	1	0	0	0	0	0	0	60
12553846	f	md	TR	2016	0	1	0	0	1	0	0	0	0	0	0	0	72
12554655	f	md	AU	2016	1	0	0	0	0	0	0	0	0	0	0	0	65
12554753	f	md	KR	2016	1	0	0	0	0	0	0	0	0	0	0	0	71.2
12555205	m	cn	US	2016	0	1	0	0	1	0	0	0	0	0	0	0	
12555522	f	cn	US	2016	0	1	0	0	1	0	0	0	0	0	0	0	
12555801	Not	md	AU	2016	0	1	0	0	1	0	0	0	0	0	0	0	73
12556037	f	cn	JP	2016	0	1	0	0	1	0	0	0	0	0	0	0	61
12556291	Not	cn	IT	2016	0	1	0	0	1	0	0	0	0	0	0	0	61
12556300	f	md	JP	2016	0	1	0	0	1	0	0	0	0	0	0	0	42
12556303	m	ot	KR	2016	0	1	0	0	1	0	0	0	0	0	0	0	55
12556822	f	cn	GB	2016	1	0	0	0	0	0	0	0	0	0	0	0	62
12556919	m	md	JP	2016	0	0	0	0	0	0	0	0	0	0	0	0	56
12557274	Not	ot	AU	2016	1	0	0	0	0	0	0	0	0	0	0	1	
12557572	f	md	Not re	2016	1	0	0	0	0	0	0	0	0	0	0	0	76
12557843	m	md	JP	2016	0	1	0	0	1	0	0	0	0	0	0	1	61
12558115	m	ot	TW	2016	0	0	0	0	0	0	1	0	1	0	0	0	63
12558159	m	md	KR	2016	1	0	0	0	0	0	0	0	0	0	0	0	69
12558287	m	md	FR	2016	0	0	0	0	0	0	0	0	0	0	0	0	59
12558574	f	cn	CH	2016	0	1	0	0	1	0	0	0	0	0	0	0	76
12558629	f	md	CH	2016	0	1	0	0	1	0	0	0	0	0	0	0	64
12558694	m	cn	JP	2016	0	1	0	0	1	0	0	0	0	0	0	0	64
12558892	f	cn	JP	2016	0	1	0	0	1	0	0	0	0	0	0	0	52
12559016	m	ot	US	2016	0	1	0	0	1	0	0	0	0	0	0	0	56
12559642	Not	Not repo	DE	2016	0	0	0	0	0	0	0	0	0	0	0	0	

12560700	m	md	US	2016	0	1	0	0	0	1	0	0	0	0	0	0	69
12561021	m	ot	US	2016	1	0	0	0	0	0	0	0	0	0	0	0	
12561112	m	ot	CA	2016	0	1	1	0	0	0	0	0	0	0	0	0	62
12561428	m	ot	DE	2016	0	0	0	0	0	0	0	0	0	0	0	0	71
12562242	f	md	AU	2016	0	0	0	0	0	0	0	0	0	0	0	0	
12563061	f	ot	GB	2016	0	1	0	0	1	0	0	0	0	0	0	0	69
12563062	f	md	JP	2016	0	0	0	0	0	0	0	0	0	0	0	0	60
12563449	f	md	KR	2016	1	0	0	0	0	0	0	0	0	0	0	0	47
12563647	f	ph	AU	2016	0	0	0	0	0	0	0	0	0	0	0	0	
12563994	m	md	KR	2016	0	1	0	0	0	1	0	0	0	0	0	0	66
12564483	m	md	DE	2016	0	1	0	0	0	1	0	0	0	0	0	0	51
12564623	f	md	US	2016	0	1	0	0	0	1	0	0	0	0	0	0	67
12565039	f	md	JP	2016	0	1	0	0	0	1	0	0	0	0	0	0	69
12565072	m	cn	GB	2016	1	0	0	0	0	0	0	0	0	0	0	0	78
12565083	m	cn	GB	2016	1	0	0	0	0	0	0	0	0	0	0	0	70
12565350	m	ph	JP	2016	0	1	0	0	1	0	0	0	0	0	0	0	
12565433	m	ot	DE	2016	0	0	0	0	0	0	0	0	0	0	0	0	71
12565502	m	md	IT	2016	0	1	0	0	1	0	0	0	0	0	0	0	74
12565505	Not	cn	AU	2016	0	1	0	0	1	0	0	0	0	0	0	0	
12565676	m	ot	DE	2016	0	0	0	0	0	0	0	0	0	0	0	0	71
12566263	f	cn	GB	2016	1	0	0	0	0	0	0	0	0	0	0	0	72
12566502	m	cn	JP	2016	0	1	0	0	1	0	0	0	0	0	0	0	63
12566614	m	cn	JP	2016	0	1	0	0	1	0	0	0	0	0	0	0	43
12566725	m	cn	IL	2016	1	0	0	0	0	0	0	0	0	0	0	0	64
12568507	f	cn	CN	2016	0	0	0	0	0	0	0	0	0	0	0	0	
12569197	f	ot	US	2016	0	0	0	0	0	0	0	0	0	0	0	0	78
12569282	m	md	RU	2016	0	1	0	0	0	1	0	0	0	0	0	0	54
12569317	m	cn	GB	2016	1	0	0	0	0	0	0	0	0	0	0	0	69
12569320	f	cn	GB	2016	1	0	0	0	0	0	0	0	0	0	0	0	81
12569339	f	md	Not re	2016	1	0	0	0	0	0	0	0	0	0	0	0	76
12569523	m	md	KR	2016	0	1	0	0	0	1	0	0	0	0	0	0	66
12570214	m	ot	DE	2016	0	0	0	0	0	0	0	0	0	0	0	0	71
12570245	m	md	TR	2016	0	1	0	0	1	0	0	0	0	0	0	0	65
12570340	m	md	JP	2016	1	0	0	0	0	0	0	0	0	0	0	0	83

12570357	f	cn	US	2016	0	1	0	0	1	0	0	0	0	0	0	0	
12571675	f	cn	US	2016	0	0	0	0	0	0	0	0	0	0	0	0	63
12572246	f	md	AU	2016	0	0	0	0	0	0	0	0	0	0	0	0	73
12572404	m	ot	US	2016	0	1	0	0	1	0	0	0	0	0	0	0	55
12572413	m	md	JP	2016	0	1	0	0	1	0	0	0	0	0	0	0	57
12572422	m	ot	JP	2016	0	1	0	0	1	0	0	0	0	0	0	0	68
12572424	Not	ph	AU	2016	0	1	0	0	1	0	0	0	0	0	0	0	
12572435	m	ot	CA	2016	0	1	0	0	1	0	0	0	0	0	0	0	75
12572544	m	ot	PL	2016	0	1	0	0	1	0	0	0	0	0	0	0	70
12572699	m	cn	CN	2016	0	0	0	0	0	0	0	0	0	0	0	0	67
12573126	m	md	FR	2016	0	1	0	0	0	1	0	0	0	0	0	0	63
12573142	f	md	JP	2016	0	1	0	0	1	0	0	0	0	0	0	0	72
12573146	m	cn	IT	2016	0	1	0	0	1	0	0	0	0	0	0	0	74
12573204	Not	ot	JP	2016	0	0	0	0	0	0	0	0	0	0	0	0	
12573283	m	md	JP	2016	0	0	0	0	0	0	0	0	0	0	0	0	78
12573343	m	ph	JP	2016	0	1	0	0	1	0	0	0	0	0	0	1	70
12573681	f	cn	Not re	2016	0	0	0	0	0	0	0	0	0	0	0	0	
12573915	f	ot	GB	2016	0	0	0	0	0	0	0	0	0	0	0	0	36
12575593	f	cn	GB	2016	1	0	0	0	0	0	0	0	0	0	0	0	
12576006	f	cn	GB	2016	1	0	0	0	0	0	0	0	0	0	0	0	
12576317	m	md	IT	2016	0	1	0	0	1	0	0	0	0	0	0	0	71
12577811	m	md	FR	2016	0	0	0	0	0	0	0	0	0	0	0	0	
12577951	f	md	JP	2016	0	1	0	0	1	0	0	0	0	0	0	0	80
12578271	f	md	US	2016	0	0	0	0	0	0	0	0	0	0	0	0	
12578383	m	cn	JP	2016	0	1	0	0	1	0	0	0	0	0	0	0	66
12579124	Not	md	JP	2016	0	0	0	0	0	0	0	0	0	0	0	0	
12579312	Not	md	JP	2016	0	1	0	0	1	0	0	0	0	0	0	0	
12579505	f	md	US	2016	0	1	0	0	0	1	0	0	0	0	0	0	75
12579991	m	md	JP	2016	0	1	0	0	1	0	0	0	0	0	0	0	62
12579998	f	ot	KR	2016	0	1	0	0	1	0	0	0	0	0	0	0	45
12580208	f	md	KR	2016	1	0	0	0	0	0	0	0	0	0	0	0	59
12580274	m	cn	US	2016	0	1	0	0	1	0	0	0	0	0	0	0	
12580294	m	cn	CN	2016	1	0	0	0	0	0	0	0	0	0	0	0	55
12580297	f	cn	CN	2016	1	0	0	0	0	0	0	0	0	0	0	0	

12581259	m	cn	IT	2016	0	1	0	0	1	0	0	0	0	0	0	0	54
12581559	m	md	ES	2016	1	0	0	0	0	0	0	0	0	0	0	0	70
12581734	m	md	JP	2016	0	1	0	0	1	0	0	0	0	0	0	0	83
12581752	f	md	JP	2016	0	0	0	0	0	0	0	0	0	0	0	0	29
12581794	f	md	JP	2016	0	1	0	0	1	0	0	0	0	0	0	0	
12581816	m	md	JP	2016	0	1	0	0	1	0	0	0	0	0	0	0	
12582071	f	md	US	2016	0	1	0	0	0	1	0	0	0	0	0	0	61
12582259	m	cn	JP	2016	0	1	0	0	1	0	0	0	0	0	0	0	
12582564	m	ot	Not re	2016	0	0	0	0	0	0	0	0	0	0	0	0	47
12582611	m	cn	TR	2016	0	0	0	0	0	0	0	0	0	0	0	0	60
12582681	f	cn	US	2016	0	0	0	0	0	0	0	0	0	0	0	0	
12582801	f	cn	JP	2016	0	1	0	0	1	0	0	0	0	0	0	0	64
12582804	m	cn	JP	2016	0	1	0	0	1	0	0	0	0	0	0	0	60
12582806	m	ot	US	2016	0	1	0	0	1	0	0	0	0	0	0	0	70
12582853	m	md	JP	2016	0	1	0	0	1	0	0	0	0	0	0	0	74
12583218	m	md	JP	2016	0	1	0	0	1	0	0	0	0	0	0	0	67
12583225	f	md	HK	2016	1	0	0	0	0	0	0	0	0	0	0	0	71.7
12583652	m	ot	US	2016	0	1	0	0	1	0	0	0	0	0	0	0	79
12583768	m	cn	IQ	2016	0	0	0	0	0	0	0	0	0	0	0	0	69
12584124	f	cn	GB	2016	1	0	0	0	0	0	0	0	0	0	0	0	68
12584359	Not	ot	IT	2016	0	0	0	0	0	0	0	0	0	0	0	0	
12584462	m	ot	US	2016	0	1	0	0	1	0	0	0	0	0	0	0	83
12584465	m	ot	US	2016	0	1	0	0	1	0	0	0	0	0	0	0	82
12584550	m	cn	JP	2016	0	1	0	0	1	0	0	0	0	0	0	0	70
12584604	f	ot	US	2016	0	1	1	0	0	0	0	0	0	0	0	0	81
12584862	f	cn	JP	2016	0	1	0	0	1	0	0	0	0	0	0	0	68
12584864	f	ot	DE	2016	0	1	0	0	1	0	0	0	0	0	0	0	63
12585619	f	cn	US	2016	0	1	0	0	1	0	0	0	0	0	0	0	70
12585680	m	cn	US	2016	1	0	0	0	0	0	0	0	0	0	0	0	46
12585716	Not	ot	DE	2016	0	0	0	0	0	0	0	0	0	0	0	0	
12585903	m	ot	GB	2016	0	1	0	0	1	0	0	0	0	0	0	0	61
12587381	m	cn	GB	2016	1	0	0	0	0	0	0	0	0	0	0	0	72
12587631	f	ph	US	2016	0	0	0	0	0	0	0	0	0	0	0	0	39
12589826	m	ot	GB	2016	0	1	0	0	1	0	0	0	0	0	0	0	62

12590165	m	ot	US	2016	0	0	0	0	0	0	0	0	0	0	0	0	80
12590235	m	ot	US	2016	0	0	0	0	0	0	0	0	0	0	0	0	66
12590576	f	md	JP	2016	1	0	0	0	0	0	0	0	0	0	0	0	26
12590657	m	cn	JP	2016	0	1	0	0	1	0	0	0	0	0	0	1	62
12590674	m	cn	US	2016	0	1	0	0	1	0	0	0	0	0	0	0	
12590772	Not	ot	DE	2016	0	0	0	0	0	0	0	0	0	0	0	0	
12590828	f	md	JP	2016	0	1	0	0	1	0	0	0	0	0	0	0	58
12591484	m	ph	JP	2016	0	1	0	0	1	0	0	0	0	0	0	0	67
12591559	f	md	DE	2016	0	1	0	0	1	0	0	0	0	0	0	1	64
12591938	m	ot	PL	2016	0	1	0	0	1	0	0	0	0	0	0	0	62
12592277	m	ot	ES	2016	0	1	0	0	1	0	0	0	0	0	0	0	59
12592434	m	ot	US	2016	0	0	0	0	0	0	0	0	0	0	0	0	
12592583	m	cn	US	2016	0	1	0	0	1	0	0	0	0	0	0	0	
12592677	f	ph	US	2016	0	0	0	0	0	0	0	0	0	0	0	0	64
12592682	m	ot	US	2016	0	0	0	0	0	0	0	0	0	0	0	0	43
12593028	m	cn	GB	2016	0	0	0	0	0	0	0	0	0	0	0	0	
12594240	f	md	DE	2016	1	0	0	0	0	0	0	0	0	0	0	0	
12594245	f	md	GB	2016	1	0	0	0	0	0	0	0	0	0	0	0	49
12594285	f	cn	GB	2016	1	0	0	0	0	0	0	0	0	0	0	0	56
12595992	f	md	CO	2016	0	0	0	0	0	0	0	0	0	0	0	0	46
12596007	m	md	CN	2016	0	1	0	0	0	1	0	0	0	0	0	0	45
12596008	m	md	JP	2016	0	0	0	0	0	0	0	0	0	0	0	0	
12596325	m	cn	MY	2016	0	1	1	0	0	0	0	0	0	0	0	0	
12596560	m	md	Not re	2016	1	0	0	0	0	0	0	0	0	0	0	0	58
12596685	f	ot	US	2016	0	1	0	0	1	0	0	0	0	0	0	0	
12596736	m	ot	JP	2016	0	0	0	0	0	0	0	0	0	0	0	0	67
12596842	f	cn	US	2016	0	1	0	0	1	0	0	0	0	0	0	0	69
12596902	Not	ot	JP	2016	0	0	0	0	0	0	0	0	0	0	0	0	
12597050	m	cn	JP	2016	0	1	0	0	1	0	0	0	0	0	0	1	53
12597053	m	cn	GB	2016	1	0	0	0	0	0	0	0	0	0	0	0	77
12597063	Not	md	JP	2016	0	1	0	0	1	0	0	0	0	0	0	0	
12597084	f	cn	GB	2016	1	0	0	0	0	0	0	0	0	0	0	0	70
12597223	f	md	Not re	2016	1	0	0	0	0	0	0	0	0	0	0	0	60
12597258	m	md	US	2016	0	1	0	0	1	0	0	0	0	0	0	0	

12598109	Not	md	JP	2016	1	0	0	0	0	0	0	0	0	0	0	0	
12598137	Not	md	JP	2016	1	0	0	0	0	0	0	0	0	0	0	0	
12598370	f	ph	JP	2016	1	0	0	0	0	0	0	0	0	0	0	0	63
12598885	f	md	CN	2016	1	0	0	0	0	0	0	0	0	0	0	0	60.3
12598956	f	cn	AR	2016	0	0	0	0	0	0	0	0	0	0	0	0	71
12599039	f	md	Not re	2016	1	0	0	0	0	0	0	0	0	0	0	0	54
12599060	f	md	JP	2016	1	0	0	0	0	0	0	0	0	0	0	0	52
12599280	f	Not repo	US	2016	1	0	0	0	0	0	0	0	0	0	0	0	
12599361	f	md	DE	2016	0	0	0	0	0	0	0	0	0	0	0	0	52
12599857	f	cn	DE	2016	0	0	0	0	0	0	0	0	0	0	0	0	66
12600108	f	cn	CA	2016	0	1	1	0	0	0	0	0	0	0	0	0	49
12600206	m	ot	US	2016	0	1	0	0	1	0	0	0	0	0	0	0	
12600265	m	md	PL	2016	0	0	0	0	0	0	0	0	0	0	0	0	69
12600465	m	ot	JP	2016	0	1	0	0	1	0	0	0	0	0	0	0	62
12600510	m	md	KR	2016	1	0	0	0	0	0	0	0	0	0	0	0	45.9
12600907	Not	ot	US	2016	0	0	0	0	0	0	0	0	0	0	0	0	
12600967	f	ot	DE	2016	0	1	0	0	1	0	0	0	0	0	0	0	75
12601033	Not	md	US	2016	0	1	0	0	1	0	0	0	0	0	0	0	
12601167	Not	md	JP	2016	0	1	0	0	1	0	0	0	0	0	0	0	40
12601260	Not	md	CA	2016	1	0	0	0	0	0	0	0	0	0	0	0	
12601266	m	cn	JP	2016	0	1	0	0	1	0	0	0	0	0	0	0	65
12601386	m	md	JP	2016	0	1	0	0	1	0	0	0	0	0	0	0	68
12601688	f	md	IT	2016	0	1	0	0	1	0	0	0	0	0	0	0	62
12601789	f	cn	US	2016	0	0	0	0	0	0	0	0	0	0	0	0	60
12601929	m	cn	CN	2016	0	0	0	0	0	0	0	0	0	0	0	0	
12602116	m	ph	US	2016	0	0	0	0	0	0	0	0	0	0	0	0	30
12602711	f	ph	JP	2016	1	0	0	0	0	0	0	0	0	0	0	0	87
12602869	Not	md	GB	2016	1	0	0	0	0	0	0	0	0	0	0	0	62
12602931	m	md	AU	2016	0	1	0	0	0	1	0	0	0	0	0	0	72
12602945	f	cn	US	2016	0	0	0	0	0	0	0	0	0	0	0	0	50
12603189	m	cn	US	2016	0	0	0	0	0	0	0	0	0	0	0	0	
12603259	m	md	JP	2016	0	1	0	0	0	1	0	0	0	0	0	0	60
12603541	f	md	GB	2016	0	1	1	0	0	0	0	0	0	0	0	0	67
12603616	f	cn	US	2016	0	1	0	0	1	0	0	0	0	0	0	0	

12603682	m	md	GR	2016	0	0	0	0	0	0	0	0	0	0	0	0	57
12604583	m	cn	GB	2016	1	0	0	0	0	0	0	0	0	0	0	0	79
12605124	m	md	Not re	2016	1	0	0	0	0	0	0	0	0	0	0	0	45
12605273	f	ot	NZ	2016	0	1	0	0	1	0	0	0	0	0	0	0	58
12605346	m	md	JP	2016	0	1	0	0	0	1	0	0	0	0	0	0	69
12605481	f	md	CN	2016	0	0	0	0	0	0	0	0	0	0	0	0	
12605503	m	cn	JP	2016	0	1	0	0	1	0	0	0	0	0	0	0	63
12605506	m	md	JP	2016	0	1	0	0	1	0	0	0	0	0	0	0	67
12605573	m	cn	IT	2016	0	1	0	0	1	0	0	0	0	0	0	0	72
12605586	m	md	FR	2016	0	0	0	0	0	0	0	0	0	0	0	0	
12605636	f	ot	JP	2016	0	1	0	0	1	0	0	0	0	0	0	1	70
12605662	f	md	JP	2016	0	1	0	0	0	1	0	0	0	0	0	0	68
12605751	m	ot	US	2016	0	1	0	0	1	0	0	0	0	0	0	0	54
12605754	m	cn	IT	2016	0	1	0	0	1	0	0	0	0	0	0	0	51
12606049	f	md	US	2016	0	0	0	0	0	0	0	0	0	0	0	0	70
12606627	Not	ot	US	2016	0	0	0	0	0	0	0	0	0	0	0	0	
12606642	Not	md	US	2016	0	0	0	0	0	0	0	0	0	0	0	0	
12606819	m	cn	US	2016	0	1	1	0	0	0	0	0	0	0	0	0	68
12606879	m	md	AU	2016	0	0	0	0	0	0	0	0	0	0	0	0	58
12606972	m	cn	CA	2016	0	1	0	0	1	0	0	0	0	0	0	0	
12607131	m	cn	GB	2016	1	0	0	0	0	0	0	0	0	0	0	0	72
12607143	m	cn	GB	2016	1	0	0	0	0	0	0	0	0	0	0	0	79
12607165	m	cn	GB	2016	1	0	0	0	0	0	0	0	0	0	0	0	82
12607461	m	md	JP	2016	0	1	0	0	1	0	0	0	0	0	0	0	63
12607494	f	ot	BR	2016	0	1	0	0	1	0	0	0	0	0	0	0	80
12607769	m	cn	JP	2016	0	1	0	0	1	0	0	0	0	0	0	0	60
12607845	m	ot	US	2016	0	1	0	0	1	0	0	0	0	0	0	0	64
12608033	m	cn	GB	2016	1	0	0	0	0	0	0	0	0	0	0	0	77
12608037	m	cn	GB	2016	1	0	0	0	0	0	0	0	0	0	0	0	80
12608201	m	ot	JP	2016	0	1	0	0	1	0	0	0	0	0	0	0	73
12608486	m	cn	JP	2016	0	1	0	0	1	0	0	0	0	0	0	0	66
12608560	m	md	JP	2016	0	1	0	0	1	0	0	0	0	0	0	1	81
12608825	f	md	BR	2016	1	0	0	0	0	0	0	0	0	0	0	0	56.5
12608994	m	ot	US	2016	0	0	0	0	0	0	0	0	0	0	0	0	72

12609492	f	ot	US	2016	0	1	0	0	1	0	0	0	0	0	0	0	71
12609529	f	md	JP	2016	0	0	0	0	0	0	0	0	0	0	0	0	77
12609618	m	cn	US	2016	0	1	0	0	1	0	0	0	0	0	0	0	52
12609640	f	md	IE	2016	1	0	0	0	0	0	0	0	0	0	0	0	50
12609655	m	cn	GB	2016	0	0	0	0	0	0	0	0	0	0	0	0	
12609994	m	cn	GB	2016	0	0	0	0	0	0	0	0	0	0	0	0	
12610013	m	md	CN	2016	0	0	0	0	0	0	0	0	0	0	0	0	
12610398	f	cn	GB	2016	1	0	0	0	0	0	0	0	0	0	0	0	74
12610402	m	cn	GB	2016	1	0	0	0	0	0	0	0	0	0	0	0	78
12610403	m	cn	GB	2016	1	0	0	0	0	0	0	0	0	0	0	0	85
12610404	m	cn	GB	2016	1	0	0	0	0	0	0	0	0	0	0	0	84
12610408	m	cn	GB	2016	1	0	0	0	0	0	0	0	0	0	0	0	75
12610413	m	cn	GB	2016	1	0	0	0	0	0	0	0	0	0	0	0	78
12610672	m	cn	GB	2016	1	0	0	0	0	0	0	0	0	0	0	0	73
12610682	f	cn	GB	2016	1	0	0	0	0	0	0	0	0	0	0	0	84
12610683	m	cn	GB	2016	1	0	0	0	0	0	0	0	0	0	0	0	71
12611149	m	ot	US	2016	0	1	0	0	1	0	0	0	0	0	0	0	
12611199	m	cn	GB	2016	1	0	0	0	0	0	0	0	0	0	0	0	77
12611200	m	cn	GB	2016	1	0	0	0	0	0	0	0	0	0	0	0	65
12611203	m	cn	GB	2016	1	0	0	0	0	0	0	0	0	0	0	0	58
12611204	m	cn	GB	2016	1	0	0	0	0	0	0	0	0	0	0	0	72
12611205	f	cn	GB	2016	1	0	0	0	0	0	0	0	0	0	0	0	72
12611208	m	cn	GB	2016	1	0	0	0	0	0	0	0	0	0	0	0	78
12611209	m	cn	GB	2016	1	0	0	0	0	0	0	0	0	0	0	0	80
12611212	f	cn	GB	2016	1	0	0	0	0	0	0	0	0	0	0	0	
12611393	f	ot	US	2016	0	0	0	0	0	0	0	0	0	0	0	0	72
12612154	f	cn	US	2016	0	1	0	0	1	0	0	0	0	0	0	0	75
12612813	f	cn	US	2016	0	0	0	0	0	0	1	0	1	0	0	0	
12613116	m	md	DE	2016	0	1	0	0	1	0	0	0	0	0	0	0	55
12613320	Not	ph	US	2016	0	1	0	0	1	0	0	0	0	0	0	0	
12613439	f	cn	JP	2016	0	1	0	0	1	0	0	0	0	0	0	0	77
12613683	f	md	Not re	2016	1	0	0	0	0	0	0	0	0	0	0	0	58
12613710	m	ot	GB	2016	0	0	0	0	0	0	0	0	0	0	0	0	
12613919	m	md	US	2016	0	0	0	0	0	0	0	0	0	0	0	0	

12614116	f	md	AU	2016	0	1	0	0	1	0	0	0	0	0	0	0	59
12614119	Not	ph	AU	2016	0	1	0	0	1	0	0	0	0	0	0	0	
12615649	m	cn	IL	2016	1	0	0	0	0	0	0	0	0	0	0	0	73
12615686	m	cn	JP	2016	0	1	0	0	1	0	0	0	0	0	0	0	63
12615893	m	ot	IN	2016	0	0	0	0	0	0	0	0	0	0	0	0	57
12616008	m	cn	GB	2016	1	0	0	0	0	0	0	0	0	0	0	0	67
12616009	m	cn	GB	2016	1	0	0	0	0	0	0	0	0	0	0	0	71
12616014	f	cn	GB	2016	1	0	0	0	0	0	0	0	0	0	0	0	60
12616022	m	cn	GB	2016	1	0	0	0	0	0	0	0	0	0	0	0	78
12616024	m	ot	GB	2016	1	0	0	0	0	0	0	0	0	0	0	0	76
12616026	m	ot	GB	2016	1	0	0	0	0	0	0	0	0	0	0	0	80
12616029	m	cn	GB	2016	1	0	0	0	0	0	0	0	0	0	0	0	77
12616030	m	cn	GB	2016	1	0	0	0	0	0	0	0	0	0	0	0	79
12616031	m	cn	GB	2016	1	0	0	0	0	0	0	0	0	0	0	0	81
12616034	f	cn	GB	2016	1	0	0	0	0	0	0	0	0	0	0	0	74
12616097	m	cn	JP	2016	0	0	0	0	0	0	0	0	0	0	0	0	66
12616141	m	ot	US	2016	0	1	0	0	1	0	0	0	0	0	0	0	72
12616174	f	cn	JP	2016	0	1	0	0	1	0	0	0	0	0	0	0	55
12616187	m	md	JP	2016	0	1	0	0	1	0	0	0	0	0	0	0	55
12616251	m	cn	GB	2016	1	0	0	0	0	0	0	0	0	0	0	0	87
12616259	m	cn	GB	2016	1	0	0	0	0	0	0	0	0	0	0	0	79
12616260	m	cn	GB	2016	1	0	0	0	0	0	0	0	0	0	0	0	75
12616747	f	md	NL	2016	1	0	0	0	0	0	0	0	0	0	0	1	58.9
12617034	Not	ph	AU	2016	0	1	0	0	1	0	0	0	0	0	0	0	
12617545	Not	cn	US	2016	1	0	0	0	0	0	0	0	0	0	0	0	
12617935	m	ot	JP	2016	0	1	0	0	0	1	0	0	0	0	0	0	60
12618001	f	cn	GB	2016	1	0	0	0	0	0	0	0	0	0	0	0	66
12618002	m	cn	GB	2016	0	0	0	0	0	0	0	0	0	0	0	0	82
12618003	m	cn	GB	2016	0	0	0	0	0	0	0	0	0	0	0	0	78
12618004	m	cn	GB	2016	0	0	0	0	0	0	0	0	0	0	0	0	73
12618005	m	cn	GB	2016	0	0	0	0	0	0	0	0	0	0	0	0	75
12618006	m	cn	GB	2016	0	0	0	0	0	0	0	0	0	0	0	0	79
12618008	f	cn	GB	2016	0	0	0	0	0	0	0	0	0	0	0	0	76
12618009	m	cn	GB	2016	0	0	0	0	0	0	0	0	0	0	0	0	76

12618010	m	cn	GB	2016	0	0	0	0	0	0	0	0	0	0	0	0	76
12618011	f	cn	GB	2016	0	0	0	0	0	0	0	0	0	0	0	0	80
12618012	m	cn	GB	2016	0	0	0	0	0	0	0	0	0	0	0	0	82
12618168	f	md	JP	2016	0	0	0	0	0	0	1	0	1	0	0	1	64
12618289	f	cn	US	2016	0	1	0	0	1	0	0	0	0	0	0	1	72
12618538	Not	md	JP	2016	0	0	0	0	0	0	0	0	0	0	0	0	
12618664	m	cn	GB	2016	0	0	0	0	0	0	0	0	0	0	0	0	32
12618666	m	cn	GB	2016	1	0	0	0	0	0	0	0	0	0	0	0	70
12618673	m	cn	GB	2016	0	0	0	0	0	0	0	0	0	0	0	0	73
12618675	m	cn	GB	2016	0	0	0	0	0	0	0	0	0	0	0	0	80
12618676	f	cn	GB	2016	1	0	0	0	0	0	0	0	0	0	0	0	83
12618680	f	cn	GB	2016	1	0	0	0	0	0	0	0	0	0	0	0	
12618681	m	cn	GB	2016	0	0	0	0	0	0	0	0	0	0	0	0	83
12618690	f	md	NL	2016	0	1	0	0	0	1	0	0	0	0	0	0	70
12618941	f	md	JP	2016	1	0	0	0	0	0	0	0	0	0	0	0	81
12618968	f	ot	CA	2016	0	1	0	0	1	0	0	0	0	0	0	0	
12619289	f	md	US	2016	1	0	0	0	0	0	0	0	0	0	0	0	65
12619501	m	cn	GB	2016	0	0	0	0	0	0	0	0	0	0	0	0	80
12619502	m	cn	GB	2016	0	0	0	0	0	0	0	0	0	0	0	0	76
12619504	f	cn	GB	2016	1	0	0	0	0	0	0	0	0	0	0	0	77
12619507	m	cn	GB	2016	0	0	0	0	0	0	0	0	0	0	0	0	80
12619508	f	cn	GB	2016	0	0	0	0	0	0	0	0	0	0	0	0	80
12619509	m	cn	GB	2016	1	0	0	0	0	0	0	0	0	0	0	0	69
12619510	f	cn	GB	2016	1	0	0	0	0	0	0	0	0	0	0	0	83
12619512	m	cn	GB	2016	1	0	0	0	0	0	0	0	0	0	0	0	80
12619513	m	cn	GB	2016	1	0	0	0	0	0	0	0	0	0	0	0	78
12619514	m	cn	GB	2016	0	0	0	0	0	0	0	0	0	0	0	0	80
12619518	m	cn	GB	2016	1	0	0	0	0	0	0	0	0	0	0	0	76
12619712	m	ph	US	2016	0	1	0	0	1	0	0	0	0	0	0	0	62
12619720	m	ot	IT	2016	0	1	0	0	1	0	0	0	0	0	0	0	75
12619778	f	md	ID	2016	1	0	0	0	0	0	0	0	0	0	0	0	
12619995	f	ot	US	2016	0	1	0	0	1	0	0	0	0	0	0	0	84
12620164	f	ot	Not re	2016	1	0	0	0	0	0	0	0	0	0	0	0	60
12620195	f	md	KR	2016	0	0	0	0	0	0	0	0	0	0	0	0	47

12620400	Not	ot	JP	2016	0	0	0	0	0	0	0	0	0	0	0	0	
12620411	f	ot	US	2016	0	0	0	0	0	0	0	0	0	0	0	0	
12620436	f	ot	US	2016	0	0	0	0	0	0	0	0	0	0	0	0	66
12620584	f	ot	AU	2016	0	1	0	0	1	0	0	0	0	0	0	0	67
12620895	m	cn	JP	2016	0	1	0	0	1	0	0	0	0	0	0	0	65
12621023	m	md	JP	2016	0	1	0	0	1	0	0	0	0	0	0	0	71
12621027	m	md	JP	2016	0	1	0	0	1	0	0	0	0	0	0	0	78
12621431	f	ot	US	2016	0	1	0	0	1	0	0	0	0	0	0	1	68
12621458	m	ot	US	2016	0	0	0	0	0	0	0	0	0	0	0	0	72
12621625	m	ot	US	2016	0	1	0	0	1	0	0	0	0	0	0	0	74
12621966	m	md	JP	2016	0	1	0	0	1	0	0	0	0	0	0	0	
12622280	Not	md	DE	2016	1	0	0	0	0	0	0	0	0	0	0	0	
12622763	m	cn	GB	2016	1	0	0	0	0	0	0	0	0	0	0	0	75
12623253	m	ot	NZ	2016	0	1	0	0	1	0	0	0	0	0	0	0	
12623281	Not	md	JP	2016	0	0	0	0	0	0	0	0	0	0	0	0	
12623421	m	cn	Not re	2016	0	0	0	0	0	0	0	0	0	0	0	0	53
12623492	m	md	Not re	2016	1	0	0	0	0	0	0	0	0	0	0	0	68
12623533	f	cn	GB	2016	1	0	0	0	0	0	0	0	0	0	0	0	
12624162	f	md	FR	2016	0	1	0	0	1	0	0	0	0	0	0	0	
12624220	m	ot	JP	2016	0	0	0	0	0	0	1	0	1	0	0	0	70
12624592	m	md	JP	2016	0	1	0	0	1	0	0	0	0	0	0	0	65
12624621	m	md	DE	2016	0	1	0	0	0	1	0	0	0	0	0	0	79
12624644	Not	ph	AU	2016	0	1	0	0	1	0	0	0	0	0	0	0	58
12624853	m	md	JP	2016	1	0	0	0	0	0	0	0	0	0	0	1	76
12624992	Not	ph	AU	2016	0	1	0	0	1	0	0	0	0	0	0	0	75
12625029	f	cn	VE	2016	0	1	0	0	1	0	0	0	0	0	0	0	63
12625101	f	ot	NL	2016	0	1	0	0	1	0	0	0	0	0	0	0	50
12625201	f	md	AU	2016	0	1	0	0	1	0	0	0	0	0	0	0	
12625269	f	ot	US	2016	0	1	0	0	1	0	0	0	0	0	0	1	
12625518	Not	cn	VE	2016	0	1	0	0	1	0	0	0	0	0	0	0	
12625530	m	md	JP	2016	0	1	0	0	1	0	0	0	0	0	0	0	68
12625548	m	md	JP	2016	0	1	0	0	1	0	0	0	0	0	0	0	
12625942	m	cn	GB	2016	1	0	0	0	0	0	0	0	0	0	0	0	56
12626317	m	cn	GB	2016	0	0	0	0	0	0	0	0	0	0	0	0	78

12626318	f	cn	GB	2016	0	0	0	0	0	0	0	0	0	0	0	0	58
12626319	m	cn	GB	2016	0	0	0	0	0	0	0	0	0	0	0	0	67
12626320	f	cn	GB	2016	1	0	0	0	0	0	0	0	0	0	0	0	73
12626416	Not	cn	VE	2016	0	1	0	0	1	0	0	0	0	0	0	0	
12626547	m	ot	JP	2016	0	1	0	0	1	0	0	0	0	0	0	0	69
12626782	Not	md	FR	2016	0	1	0	0	1	0	0	0	0	0	0	0	
12626890	f	cn	GB	2016	1	0	0	0	0	0	0	0	0	0	0	0	50
12626891	f	cn	GB	2016	1	0	0	0	0	0	0	0	0	0	0	0	49
12626937	Not	md	FR	2016	0	1	0	0	1	0	0	0	0	0	0	0	
12626949	f	md	Not re	2016	1	0	0	0	0	0	0	0	0	0	0	0	51
12627200	m	cn	GB	2016	1	0	0	0	0	0	0	0	0	0	0	0	56
12627414	m	ot	US	2016	0	0	0	0	0	0	0	0	0	0	0	0	45
12627592	f	ot	US	2016	0	0	0	0	0	0	0	0	0	0	0	0	55
12627729	f	ot	US	2016	0	0	0	0	0	0	0	0	0	0	0	0	56
12628156	m	ot	US	2016	0	1	0	0	1	0	0	0	0	0	0	0	57
12628362	m	md	JP	2016	0	1	0	0	1	0	0	0	0	0	0	1	61
12628715	f	md	TR	2016	0	0	0	0	0	0	0	0	0	0	0	0	
12628727	Not	ph	AU	2016	0	1	0	0	1	0	0	0	0	0	0	0	65
12629176	m	md	GB	2016	1	0	0	0	0	0	0	0	0	0	0	0	62
12629186	m	cn	GB	2016	0	0	0	0	0	0	0	0	0	0	0	0	61
12629188	m	cn	GB	2016	1	0	0	0	0	0	0	0	0	0	0	0	63
12629198	f	cn	GB	2016	0	0	0	0	0	0	0	0	0	0	0	0	69
12629201	m	cn	GB	2016	0	0	0	0	0	0	0	0	0	0	0	0	65
12629408	f	ot	US	2016	0	1	0	0	1	0	0	0	0	0	0	0	61
12629454	Not	md	FR	2016	0	1	0	0	1	0	0	0	0	0	0	0	
12629554	m	cn	JP	2016	0	1	0	0	1	0	0	0	0	0	0	0	74
12629915	m	md	CN	2016	0	0	0	0	0	0	0	0	0	0	0	0	
12629929	m	ot	US	2016	0	0	0	0	0	0	0	0	0	0	0	0	67
12630105	f	ph	AU	2016	0	1	0	0	1	0	0	0	0	0	0	0	56
12630281	m	cn	GB	2016	1	0	0	0	0	0	0	0	0	0	0	0	
12630533	m	md	US	2016	1	0	0	0	0	0	0	0	0	0	0	0	59
12630537	m	cn	DE	2016	1	0	0	0	0	0	0	0	0	0	0	0	51
12630627	m	cn	US	2016	0	0	0	0	0	0	0	0	0	0	0	0	74
12633745	m	ph	US	2016	0	1	0	0	1	0	0	0	0	0	0	0	88

12633761	f	md	ES	2016	0	0	0	0	0	0	0	0	0	0	0	0	45
12633802	Not	ph	AU	2016	0	1	0	0	1	0	0	0	0	0	0	0	
12634091	f	md	US	2016	0	0	0	0	0	0	0	0	0	0	0	0	56
12634300	f	md	JP	2016	1	0	0	0	0	0	0	0	0	0	0	0	52
12634352	m	md	JP	2016	0	0	0	0	0	0	1	0	1	0	0	1	64
12634432	f	ot	GB	2016	0	1	0	0	1	0	0	0	0	0	0	0	69
12634733	f	cn	GB	2016	0	0	0	0	0	0	0	0	0	0	0	0	70
12634734	m	cn	GB	2016	0	0	0	0	0	0	0	0	0	0	0	0	64
12634848	f	ot	GB	2016	1	0	0	0	0	0	0	0	0	0	0	0	
12635211	f	cn	VE	2016	0	1	0	0	1	0	0	0	0	0	0	0	56
12635518	m	md	JP	2016	0	1	0	0	1	0	0	0	0	0	0	0	70
12636042	m	md	US	2016	0	1	0	0	0	1	0	0	0	0	0	0	59
12636110	Not	md	PH	2016	0	0	0	0	0	0	0	0	0	0	0	0	
12636133	f	md	GB	2016	0	1	0	0	1	0	0	0	0	0	0	0	52
12636347	m	md	JP	2016	0	1	0	0	1	0	0	0	0	0	0	0	51
12636650	m	md	JP	2016	0	1	0	0	1	0	0	0	0	0	0	0	56
12636987	m	cn	Not re	2016	0	0	0	0	0	0	0	0	0	0	0	0	40
12637028	f	cn	GB	2016	1	0	0	0	0	0	0	0	0	0	0	0	60
12637030	m	cn	GB	2016	0	0	0	0	0	0	0	0	0	0	0	0	88
12637256	f	cn	GB	2016	0	0	0	0	0	0	0	0	0	0	0	0	60
12637292	f	cn	ES	2016	0	1	0	0	1	0	0	0	0	0	0	0	77
12638529	m	ot	US	2016	0	1	0	0	1	0	0	0	0	0	0	0	61
12638666	m	md	JP	2016	0	0	0	0	0	0	1	0	1	0	0	1	64
12639000	m	ot	BR	2016	0	1	0	0	0	1	0	0	0	0	0	0	68
12639422	f	md	JP	2016	0	1	0	0	1	0	0	0	0	0	0	0	58
12639437	m	md	DE	2016	0	1	0	0	0	1	0	0	0	0	0	0	69
12639645	f	md	BE	2016	0	1	0	0	1	0	0	0	0	0	0	0	69
12639715	f	md	JP	2016	0	1	0	0	1	0	0	0	0	0	0	0	76
12639819	m	cn	GB	2016	1	0	0	0	0	0	0	0	0	0	0	1	86
12640057	m	cn	JP	2016	0	0	0	0	0	0	0	0	0	0	0	0	66
12640186	m	cn	GB	2016	1	0	0	0	0	0	0	0	0	0	0	0	61
12640191	m	cn	GB	2016	1	0	0	0	0	0	0	0	0	0	0	0	76
12640372	m	cn	GB	2016	0	0	0	0	0	0	0	0	0	0	0	0	41
12640374	m	cn	GB	2016	0	0	0	0	0	0	0	0	0	0	0	0	62

12640375	f	ot	US	2016	1	0	0	0	0	0	0	0	0	0	0	0	65
12640627	Not	md	JP	2016	0	0	0	0	0	0	0	0	0	0	0	0	
12640744	f	cn	GB	2016	1	0	0	0	0	0	0	0	0	0	0	0	53
12640758	f	cn	GB	2016	0	0	0	0	0	0	0	0	0	0	0	0	60
12640760	m	cn	GB	2016	0	0	0	0	0	0	0	0	0	0	0	0	70
12640761	f	cn	GB	2016	0	0	0	0	0	0	0	0	0	0	0	0	52
12640763	f	cn	GB	2016	1	0	0	0	0	0	0	0	0	0	0	0	57
12640773	f	cn	GB	2016	0	0	0	0	0	0	0	0	0	0	0	0	65
12640776	m	cn	GB	2016	1	0	0	0	0	0	0	0	0	0	0	0	59
12640802	m	md	IT	2016	0	1	0	0	1	0	0	0	0	0	0	0	64
12641107	f	cn	GB	2016	1	0	0	0	0	0	0	0	0	0	0	0	68
12641108	m	cn	GB	2016	1	0	0	0	0	0	0	0	0	0	0	0	65
12641297	f	ph	US	2016	0	0	0	0	0	0	0	0	0	0	0	0	63
12641432	f	cn	US	2016	0	0	0	0	0	0	0	0	0	0	0	0	
12641497	m	cn	BE	2016	0	0	0	0	0	0	0	0	0	0	0	0	
12641499	Not	cn	JP	2016	0	0	0	0	0	0	0	0	0	0	0	0	65
12641667	m	md	TR	2016	0	0	0	0	0	0	0	0	0	0	0	0	58
12641759	f	cn	GB	2016	1	0	0	0	0	0	0	0	0	0	0	0	68
12641760	f	cn	GB	2016	1	0	0	0	0	0	0	0	0	0	0	0	64
12641942	m	ot	JP	2016	0	1	0	0	1	0	0	0	0	0	0	0	68
12642037	m	md	JP	2016	0	1	0	0	1	0	0	0	0	0	0	0	79
12642038	f	md	JP	2016	0	1	0	0	1	0	0	0	0	0	0	0	63
12642096	m	ot	IT	2016	0	0	0	0	0	0	0	0	0	0	0	0	54
12642286	m	md	JP	2016	0	1	0	0	1	0	0	0	0	0	0	1	68
12642394	f	Not repo	US	2016	0	0	0	0	0	0	1	0	1	0	0	0	72.6
12642403	f	md	BE	2016	0	1	0	0	0	1	0	0	0	0	0	0	67
12642682	m	md	FR	2016	0	0	0	0	0	0	0	0	0	0	0	0	70
12642876	Not	cn	US	2016	0	1	0	0	1	0	0	0	0	0	0	0	
12642877	m	cn	JP	2016	0	1	0	0	1	0	0	0	0	0	0	0	85
12642980	Not	ph	JP	2016	0	1	0	0	1	0	0	0	0	0	0	0	
12643096	m	md	JP	2016	1	0	0	0	0	0	0	0	0	0	0	0	61
12643230	m	md	JP	2016	0	1	0	0	0	1	0	0	0	0	0	0	63
12643282	f	cn	AR	2016	0	0	0	0	0	0	0	0	0	0	0	0	50
12643385	f	md	BR	2016	0	1	0	0	0	1	0	0	0	0	0	1	53

12643415	m	cn	JP	2016	0	1	0	0	1	0	0	0	0	0	0	0	79
12643527	f	md	Not re	2016	1	0	0	0	0	0	0	0	0	0	0	0	86
12643687	m	cn	GB	2016	1	0	0	0	0	0	0	0	0	0	0	0	65
12643690	f	cn	GB	2016	0	0	0	0	0	0	0	0	0	0	0	0	64
12643786	f	cn	IE	2016	0	0	0	0	0	0	0	0	0	0	0	0	75
12643832	Not	md	DE	2016	0	0	0	0	0	0	0	0	0	0	0	0	
12643858	f	cn	IE	2016	0	0	0	0	0	0	0	0	0	0	0	0	55
12643861	f	cn	IE	2016	0	0	0	0	0	0	0	0	0	0	0	0	
12644230	f	md	JP	2016	0	1	0	0	1	0	0	0	0	0	0	0	86
12644314	f	cn	JP	2016	0	1	0	0	1	0	0	0	0	0	0	0	76
12644315	f	ot	US	2016	0	0	0	0	0	0	1	0	1	0	0	0	50
12644431	m	ot	FR	2016	0	0	0	0	0	0	0	0	0	0	0	0	61
12644442	m	md	JP	2016	0	0	0	0	0	0	0	0	0	0	0	0	52
12644653	f	md	US	2016	1	0	0	0	0	0	0	0	0	0	0	0	80
12645247	m	md	US	2016	0	1	0	0	0	1	0	0	0	0	0	0	71
12645253	f	md	UA	2016	0	0	0	0	0	0	0	0	0	0	0	0	63
12645340	m	cn	IT	2016	0	1	0	0	1	0	0	0	0	0	0	0	62
12645473	f	ot	BE	2016	0	1	0	0	1	0	0	0	0	0	0	0	65
12645474	m	ot	FR	2016	0	1	0	0	1	0	0	0	0	0	0	0	80
12646000	f	md	US	2016	1	0	0	0	0	0	0	0	0	0	0	0	
12646301	f	cn	US	2016	0	1	1	0	0	0	0	0	0	0	0	0	
12646353	m	cn	JP	2016	0	1	0	0	1	0	0	0	0	0	0	0	54
12646602	m	cn	IT	2016	0	1	0	0	1	0	0	0	0	0	0	0	70
12647003	m	cn	US	2016	0	1	0	0	1	0	0	0	0	0	0	0	
12647210	Not	md	AT	2016	0	0	0	0	0	0	0	0	0	0	0	0	
12647216	Not	md	DE	2016	0	0	0	0	0	0	0	0	0	0	0	0	
12647522	Not	md	BE	2016	0	0	0	0	0	0	0	0	0	0	0	0	
12647807	m	md	ES	2016	0	1	0	0	0	1	0	0	0	0	0	1	74
12647811	f	md	Not re	2016	1	0	0	0	0	0	0	0	0	0	0	0	54
12647860	m	md	JP	2016	0	1	0	0	1	0	0	0	0	0	0	0	60
12647867	m	md	US	2016	0	0	0	0	0	0	0	0	0	0	0	0	62
12647884	f	md	DE	2016	0	0	0	0	0	0	0	0	0	0	0	0	55
12647918	f	md	JP	2016	0	0	0	0	0	0	0	0	0	0	0	0	58
12648077	Not	md	AT	2016	0	0	0	0	0	0	0	0	0	0	0	0	

12648078	Not	md	AT	2016	0	0	0	0	0	0	0	0	0	0	0	0	
12648079	Not	md	BE	2016	0	0	0	0	0	0	0	0	0	0	0	0	
12648102	Not	md	AT	2016	0	0	0	0	0	0	0	0	0	0	0	0	
12648118	Not	md	IE	2016	0	0	0	0	0	0	0	0	0	0	0	0	
12648127	Not	md	DE	2016	0	0	0	0	0	0	0	0	0	0	0	0	
12648159	Not	md	BE	2016	0	0	0	0	0	0	0	0	0	0	0	0	
12648162	Not	md	IE	2016	0	0	0	0	0	0	0	0	0	0	0	0	
12648166	m	cn	GB	2016	1	0	0	0	0	0	0	0	0	0	0	0	
12648525	m	cn	JP	2016	0	1	0	0	1	0	0	0	0	0	0	0	41
12648624	m	ot	CN	2016	0	1	0	0	1	0	0	0	0	0	0	0	54
12648666	f	md	JP	2016	0	1	0	0	1	0	0	0	0	0	0	0	70
12648850	m	ot	US	2016	0	0	0	0	0	0	0	0	0	0	0	0	
12649162	m	md	AU	2016	0	1	0	0	0	1	0	0	0	0	0	1	67
12649203	f	cn	US	2016	0	1	1	0	0	0	0	0	0	0	0	0	
12649213	f	ot	SE	2016	0	1	0	0	1	0	0	0	0	0	0	0	72
12649330	m	ot	RO	2016	0	1	0	0	1	0	0	0	0	0	0	0	
12649831	m	md	JP	2016	1	0	0	0	0	0	0	0	0	0	0	0	60
12649898	m	md	US	2016	0	1	0	1	0	0	0	0	0	0	0	1	59.8
12649903	Not	md	CH	2016	0	0	0	0	0	0	0	0	0	0	0	0	
12649912	Not	md	ES	2016	0	0	0	0	0	0	0	0	0	0	0	0	
12649914	Not	md	FR	2016	0	0	0	0	0	0	0	0	0	0	0	0	
12649948	Not	md	DE	2016	0	0	0	0	0	0	0	0	0	0	0	0	
12650044	Not	md	AT	2016	0	0	0	0	0	0	0	0	0	0	0	0	
12650051	Not	md	AT	2016	0	0	0	0	0	0	0	0	0	0	0	0	
12650053	Not	md	DE	2016	0	0	0	0	0	0	0	0	0	0	0	0	
12650054	Not	md	DE	2016	0	0	0	0	0	0	0	0	0	0	0	0	
12650072	Not	md	ES	2016	0	0	0	0	0	0	0	0	0	0	0	0	
12650074	Not	md	CH	2016	0	0	0	0	0	0	0	0	0	0	0	0	
12650075	Not	md	BE	2016	0	0	0	0	0	0	0	0	0	0	0	0	
12650076	Not	md	CH	2016	0	0	0	0	0	0	0	0	0	0	0	0	
12650080	Not	md	FR	2016	0	0	0	0	0	0	0	0	0	0	0	0	
12650081	m	md	FR	2016	0	0	0	0	0	0	0	0	0	0	0	0	63
12650082	Not	md	DE	2016	0	0	0	0	0	0	0	0	0	0	0	0	
12650087	m	md	JP	2016	0	1	0	0	1	0	0	0	0	0	0	0	69

12650117	f	cn	DE	2016	0	1	0	0	1	0	0	0	0	0	0	0	65
12650355	Not	md	ES	2016	0	0	0	0	0	0	0	0	0	0	0	0	
12650356	Not	md	ES	2016	0	0	0	0	0	0	0	0	0	0	0	0	
12650357	Not	md	ES	2016	0	0	0	0	0	0	0	0	0	0	0	0	
12650358	Not	md	CH	2016	0	0	0	0	0	0	0	0	0	0	0	0	
12650360	Not	md	ES	2016	0	0	0	0	0	0	0	0	0	0	0	0	
12650361	Not	md	CH	2016	0	0	0	0	0	0	0	0	0	0	0	0	
12650363	Not	md	ES	2016	0	0	0	0	0	0	0	0	0	0	0	0	
12650364	Not	md	CH	2016	0	0	0	0	0	0	0	0	0	0	0	0	
12650365	Not	md	ES	2016	0	0	0	0	0	0	0	0	0	0	0	0	
12650366	Not	md	ES	2016	0	0	0	0	0	0	0	0	0	0	0	0	
12650367	Not	md	ES	2016	0	0	0	0	0	0	0	0	0	0	0	0	
12650368	Not	md	ES	2016	0	0	0	0	0	0	0	0	0	0	0	0	
12650369	Not	md	ES	2016	0	0	0	0	0	0	0	0	0	0	0	0	
12650370	Not	md	ES	2016	0	0	0	0	0	0	0	0	0	0	0	0	
12650372	Not	md	ES	2016	0	0	0	0	0	0	0	0	0	0	0	0	
12650611	m	md	US	2016	0	1	0	0	0	1	0	0	0	0	0	0	71
12651350	m	cn	US	2016	0	0	0	0	0	0	0	0	0	0	0	0	
12651352	f	ot	JP	2016	0	0	0	0	0	0	0	0	0	0	0	0	64
12651365	Not	md	FR	2016	0	0	0	0	0	0	0	0	0	0	0	0	
12651379	Not	md	FR	2016	0	0	0	0	0	0	0	0	0	0	0	0	
12651525	f	cn	US	2016	0	0	0	0	0	0	0	0	0	0	0	0	
12651631	m	cn	US	2016	0	1	0	0	1	0	0	0	0	0	0	0	
12652244	m	ot	GB	2016	0	0	0	0	0	0	0	0	0	0	0	0	72
12652296	m	md	KR	2016	0	1	0	0	0	1	0	0	0	0	0	0	68
12652720	m	ot	ES	2016	0	0	0	0	0	0	0	0	0	0	0	0	79
12652851	Not	md	ES	2016	0	0	0	0	0	0	0	0	0	0	0	0	
12653138	f	cn	US	2016	0	0	0	0	0	0	0	0	0	0	0	0	
12653309	f	ot	US	2016	0	0	0	0	0	0	0	0	0	0	0	0	65
12653687	f	md	JP	2016	0	1	0	0	1	0	0	0	0	0	0	1	65
12653721	Not	md	ES	2016	0	0	0	0	0	0	0	0	0	0	0	0	
12653783	Not	md	CH	2016	0	0	0	0	0	0	0	0	0	0	0	0	
12653798	Not	md	CH	2016	0	0	0	0	0	0	0	0	0	0	0	0	
12653871	Not	md	BE	2016	0	0	0	0	0	0	0	0	0	0	0	0	

12653874	Not	md	ES	2016	0	0	0	0	0	0	0	0	0	0	0	0		
12653880	Not	md	BE	2016	0	0	0	0	0	0	0	0	0	0	0	0		
12654068	Not	md	US	2016	0	1	0	0	1	0	0	0	0	0	0	0		
12654075	Not	md	FR	2016	0	0	0	0	0	0	0	0	0	0	0	0		
12654076	Not	md	IE	2016	0	0	0	0	0	0	0	0	0	0	0	0		
12654086	Not	md	ES	2016	0	0	0	0	0	0	0	0	0	0	0	0		
12654220	m	cn	US	2016	0	0	0	0	0	0	0	0	0	0	0	0	52	
12654444	f	md	JP	2016	0	1	0	0	1	0	0	0	0	0	0	1	70	
12654889	m	cn	US	2016	0	1	0	0	1	0	0	0	0	0	0	0	71	
12654908	m	md	HK	2016	1	0	0	0	0	0	0	0	0	0	0	0	53.1	
12655017	m	Not repo	US	2016	1	0	0	0	0	0	0	0	0	0	0	0	62.5	
12655070	f	cn	IE	2016	0	0	0	0	0	0	0	0	0	0	0	0	56	
12655105	m	ph	US	2016	0	0	0	0	0	0	0	0	0	0	0	0	81	
12655649	m	ot	TW	2016	1	0	0	0	0	0	0	0	0	0	0	0	83	
12656041	f	md	US	2016	0	0	0	0	0	0	0	0	0	0	0	0	50	
12656134	m	cn	GB	2016	1	0	0	0	0	0	0	0	0	0	0	0		
12656140	f	md	JP	2016	0	0	0	0	0	0	0	0	0	0	0	0	68	
12656159	m	ot	US	2016	0	1	0	0	1	0	0	0	0	0	0	0		
12656668	m	ot	TW	2016	1	0	0	0	0	0	0	0	0	0	0	0	84	
12656704	m	ot	US	2016	0	0	0	0	0	0	0	0	0	0	0	0	76	
12656756	f	ot	TW	2016	1	0	0	0	0	0	0	0	0	0	0	0	56	
12656827	Not	md	ES	2016	0	0	0	0	0	0	0	0	0	0	0	0		
12656898	f	md	JP	2016	1	0	0	0	0	0	0	0	0	0	0	1	63	
12657790	Not	md	CH	2016	0	0	0	0	0	0	0	0	0	0	0	0		
12657812	Not	md	CH	2016	0	0	0	0	0	0	0	0	0	0	0	0		
12657826	Not	md	CH	2016	0	0	0	0	0	0	0	0	0	0	0	0		
12657844	Not	md	ES	2016	0	0	0	0	0	0	0	0	0	0	0	0		
12657846	Not	md	ES	2016	0	0	0	0	0	0	0	0	0	0	0	0		
12658104	m	md	US	2016	0	0	0	0	0	0	0	0	0	0	0	0	36	
12658313	f	md	FR	2016	1	0	0	0	0	0	0	0	0	0	0	0	51.3	
12658556	m	ot	US	2016	0	1	0	0	1	0	0	0	0	0	0	0		
12658829	f	ot	KR	2016	0	1	0	0	1	0	0	0	0	0	0	0	45	
12658982	m	cn	CN	2016	1	0	0	0	0	0	0	0	0	0	0	0		
12660131	m	md	KR	2016	1	0	0	0	0	0	0	0	0	0	0	0	70	

12660231	f	ot	US	2016	0	1	0	0	1	0	0	0	0	0	0	0	
12660462	Not	md	JP	2016	0	1	0	0	1	0	0	0	0	0	0	0	70
12660483	f	md	JP	2016	0	0	0	0	0	0	1	0	1	0	0	0	75
12660495	Not	md	JP	2016	0	0	0	0	0	0	0	0	0	0	0	0	
12661719	m	md	JP	2016	0	1	0	0	1	0	0	0	0	0	0	0	64
12662456	m	cn	US	2016	0	1	0	0	1	0	0	0	0	0	0	0	68
12662579	m	ph	US	2016	0	1	0	0	1	0	0	0	0	0	0	0	
12662735	m	md	NL	2016	0	1	0	0	0	1	0	0	0	0	0	0	41
12662907	f	cn	US	2016	0	1	0	0	1	0	0	0	0	0	0	0	85
12662979	f	md	NL	2016	0	1	0	0	1	0	0	0	0	0	0	0	55
12663211	Not	md	ES	2016	0	0	0	0	0	0	0	0	0	0	0	0	
12663858	f	ot	US	2016	0	0	0	0	0	0	0	0	0	0	0	0	66
12664380	m	md	JP	2016	0	1	0	0	1	0	0	0	0	0	0	0	68
12664430	Not	md	FR	2016	0	0	0	0	0	0	0	0	0	0	0	0	
12664548	f	cn	US	2016	0	0	0	0	0	0	0	0	0	0	0	0	37
12664586	Not	md	DE	2016	0	0	0	0	0	0	0	0	0	0	0	0	
12664854	m	md	JP	2016	0	1	0	0	1	0	0	0	0	0	0	0	70
12664989	m	cn	US	2016	0	1	1	0	0	0	0	0	0	0	0	1	72
12665147	f	md	NL	2016	0	1	0	0	1	0	0	0	0	0	0	0	52
12666438	m	ot	CN	2016	0	1	0	0	1	0	0	0	0	0	0	0	78
12666511	m	cn	US	2016	0	0	0	0	0	0	0	0	0	0	0	0	59
12666632	m	cn	US	2016	0	1	0	0	1	0	0	0	0	0	0	0	
12666889	m	ph	JP	2016	0	0	0	0	0	0	0	0	0	0	0	0	58
12667150	f	ph	JP	2016	1	0	0	0	0	0	0	0	0	0	0	0	72
12667203	m	md	US	2016	0	1	0	0	1	0	0	0	0	0	0	0	60
12667795	m	md	AE	2016	0	1	1	0	0	0	0	0	0	0	0	0	34
12668583	Not	md	ES	2016	0	0	0	0	0	0	0	0	0	0	0	0	
12668679	m	ot	AU	2016	0	1	0	0	1	0	0	0	0	0	0	0	75
12668718	m	ot	FR	2016	0	0	0	0	0	0	0	0	0	0	0	0	69
12669174	m	Not repo	US	2016	1	0	0	0	0	0	0	0	0	0	0	0	
12669837	m	md	JP	2016	0	1	0	0	1	0	0	0	0	0	0	1	79
12670452	Not	ot	US	2016	0	1	0	0	1	0	0	0	0	0	0	0	
12670675	Not	ot	NL	2016	0	0	0	0	0	0	0	0	0	0	0	0	
12671039	Not	md	US	2016	0	1	0	0	1	0	0	0	0	0	0	1	

12671079	Not	md	JP	2016	0	1	0	0	1	0	0	0	0	0	0	0	
12671478	f	cn	PL	2016	1	0	0	0	0	0	0	0	0	0	0	0	
12671967	Not	ot	JP	2016	0	0	0	0	0	0	0	0	0	0	0	0	
12671990	Not	ot	JP	2016	0	0	0	0	0	0	0	0	0	0	0	0	
12672167	Not	ot	NL	2016	0	0	0	0	0	0	0	0	0	0	0	0	
12672208	Not	ot	NO	2016	0	0	0	0	0	0	0	0	0	0	0	0	
12672232	Not	ot	NO	2016	0	0	0	0	0	0	0	0	0	0	0	0	
12672237	Not	ot	NO	2016	0	0	0	0	0	0	0	0	0	0	0	0	
12672242	Not	ot	NO	2016	0	0	0	0	0	0	0	0	0	0	0	0	
12673867	m	cn	GB	2016	0	1	0	0	1	0	0	0	0	0	0	0	69
12673986	m	md	ZA	2016	0	0	0	0	0	0	0	0	0	0	0	0	76
12674050	f	md	US	2016	1	0	0	0	0	0	0	0	0	0	0	0	
12674052	m	md	JP	2016	0	1	0	0	1	0	0	0	0	0	0	0	46
12674504	f	cn	US	2016	0	0	0	0	0	0	0	0	0	0	0	0	73
12674506	f	md	CA	2016	0	1	0	0	1	0	0	0	0	0	0	0	90
12674509	m	md	JP	2016	0	1	0	0	1	0	0	0	0	0	0	0	67
12674974	f	md	JP	2016	0	1	0	0	1	0	0	0	0	0	0	0	53
12674979	m	cn	JP	2016	0	0	0	0	0	0	1	0	1	0	0	0	67
12675305	Not	md	JP	2016	0	1	0	0	1	0	0	0	0	0	0	0	
12675310	f	md	JP	2016	1	0	0	0	0	0	0	0	0	0	0	0	87
12675879	f	md	US	2016	0	0	0	0	0	0	0	0	0	0	0	0	
12675910	m	md	FR	2016	0	0	0	0	0	0	0	0	0	0	0	0	63
12675980	f	ot	ES	2016	0	0	0	0	0	0	0	0	0	0	0	0	62
12676055	Not	md	GB	2016	0	0	0	0	0	0	0	0	0	0	0	0	
12676462	f	cn	GB	2016	0	0	0	0	0	0	0	0	0	0	0	0	
12676763	m	ot	MX	2016	0	0	0	0	0	0	0	0	0	0	0	0	70
12676838	m	cn	JP	2016	0	1	0	0	1	0	0	0	0	0	0	0	
12677930	f	cn	JP	2016	0	0	0	0	0	0	1	0	1	0	0	1	41
12678161	f	md	TW	2016	1	0	0	0	0	0	0	0	0	0	0	0	55
12678486	m	Not repo	US	2016	1	0	0	0	0	0	0	0	0	0	0	0	61.8
12678673	f	md	JP	2016	1	0	0	0	0	0	0	0	0	0	0	0	67.9
12678923	f	ot	BR	2016	0	1	0	0	1	0	0	0	0	0	0	0	53
12679087	m	Not repo	US	2016	1	0	0	0	0	0	0	0	0	0	0	0	83.6
12679098	Not	md	JP	2016	0	1	0	0	1	0	0	0	0	0	0	0	

12679179	Not	ph	FR	2016	0	1	0	0	1	0	0	0	0	0	0	0	
12679203	f	cn	AR	2016	0	0	0	0	0	0	0	0	0	0	0	0	48
12679229	m	md	JP	2016	0	1	0	0	1	0	0	0	0	0	0	0	38
12679417	f	cn	US	2016	1	0	0	0	0	0	0	0	0	0	0	0	40
12679894	Not	cn	GB	2016	1	0	0	0	0	0	0	0	0	0	0	0	
12679902	f	cn	GB	2016	1	0	0	0	0	0	0	0	0	0	0	0	67
12680565	Not	ph	FR	2016	1	0	0	0	0	0	0	0	0	0	0	0	
12680572	Not	ph	FR	2016	1	0	0	0	0	0	0	0	0	0	0	0	
12681521	m	cn	TR	2016	0	0	0	0	0	0	0	0	0	0	0	0	60
12681664	m	ot	GB	2016	0	0	0	0	0	0	0	0	0	0	0	0	43
12681833	m	cn	JP	2016	0	1	0	0	1	0	0	0	0	0	0	0	78
12682262	f	ot	FR	2016	1	0	0	0	0	0	0	0	0	0	0	0	56
12682283	m	cn	Not re	2016	0	0	0	0	0	0	0	0	0	0	0	0	74
12682289	m	md	US	2016	0	1	0	0	1	0	0	0	0	0	0	0	
12682442	m	md	GB	2016	0	1	0	0	1	0	0	0	0	0	0	0	
12682447	m	Not repo	US	2016	1	0	0	0	0	0	0	0	0	0	0	0	
12682475	f	md	US	2016	0	0	0	0	0	0	0	0	0	0	0	0	61
12682494	f	cn	AU	2016	0	1	0	0	1	0	0	0	0	0	0	0	
12682497	f	md	US	2016	0	1	0	0	1	0	0	0	0	0	0	0	
12682671	m	md	DE	2016	0	1	0	0	0	1	0	0	0	0	0	0	80
12682730	f	cn	GB	2016	0	1	0	0	1	0	0	0	0	0	0	1	57
12682863	f	ph	JP	2016	0	0	0	0	0	0	0	0	0	0	0	0	47
12683052	m	md	Not re	2016	1	0	0	0	0	0	0	0	0	0	0	0	63
12683181	m	cn	Not re	2016	0	0	0	0	0	0	0	0	0	0	0	0	66
12683545	m	ot	DE	2016	0	0	0	0	0	0	0	0	0	0	0	0	63
12683976	m	md	TW	2016	0	0	0	0	0	0	0	0	0	0	0	0	63
12684364	m	md	DE	2016	0	0	0	0	0	0	0	0	0	0	0	0	70
12684663	m	ot	US	2016	0	0	0	0	0	0	0	0	0	0	0	0	70
12685017	f	ot	US	2016	0	0	0	0	0	0	0	0	0	0	0	0	70
12685048	m	cn	JP	2016	0	1	0	0	1	0	0	0	0	0	0	1	69
12685151	m	md	US	2016	0	1	1	0	0	0	0	0	0	0	0	0	
12685241	f	cn	US	2016	0	0	0	0	0	0	0	0	0	0	0	0	38
12685266	f	ot	FR	2016	1	0	0	0	0	0	0	0	0	0	0	0	82
12686249	m	md	JP	2016	1	0	0	0	0	0	0	0	0	0	0	0	90

12686936	f	ot	CA	2016	0	1	0	0	1	0	0	0	0	0	0	0	64
12687010	m	cn	JP	2016	0	1	0	0	1	0	0	0	0	0	0	0	39
12687017	m	cn	PL	2016	0	1	0	0	1	0	0	0	0	0	0	0	61
12687091	m	ot	US	2016	0	1	0	0	1	0	0	0	0	0	0	1	75
12687151	m	ph	JP	2016	0	1	0	0	1	0	0	0	0	0	0	0	66
12687268	f	cn	US	2016	0	1	0	0	1	0	0	0	0	0	0	0	
12687591	Not	ph	AU	2016	0	1	0	0	1	0	0	0	0	0	0	0	58
12687651	f	cn	US	2016	0	1	0	0	1	0	0	0	0	0	0	0	
12688378	Not	ot	CN	2016	1	0	0	0	0	0	0	0	0	0	0	0	
12688421	f	md	US	2016	1	0	0	0	0	0	0	0	0	0	0	1	65.1
12688553	f	md	Not re	2016	1	0	0	0	0	0	0	0	0	0	0	0	83
12688558	m	cn	JP	2016	0	1	0	0	1	0	0	0	0	0	0	0	64
12688913	Not	ot	JP	2016	0	0	0	0	0	0	0	0	0	0	0	0	
12688984	f	cn	US	2016	0	1	0	0	1	0	0	0	0	0	0	0	85
12689747	Not	ot	IT	2016	0	0	0	0	0	0	0	0	0	0	0	0	
12689821	Not	ot	IT	2016	0	0	0	0	0	0	0	0	0	0	0	0	
12690002	f	md	RU	2016	0	0	0	0	0	0	0	0	0	0	0	0	68
12690019	f	ot	US	2016	1	0	0	0	0	0	0	0	0	0	0	0	64
12690079	f	md	JP	2016	1	0	0	0	0	0	0	0	0	0	0	0	86
12690194	f	md	US	2016	0	1	0	0	0	1	0	0	0	0	0	0	71
12690577	m	cn	GB	2016	1	0	0	0	0	0	0	0	0	0	0	0	
12690588	f	cn	GB	2016	1	0	0	0	0	0	0	0	0	0	0	1	58
12690595	f	md	JP	2016	1	0	0	0	0	0	0	0	0	0	0	0	90
12690931	m	md	Not re	2016	0	0	0	0	0	0	0	0	0	0	0	0	61
12691049	m	md	US	2016	0	1	0	0	1	0	0	0	0	0	0	0	
12691119	m	ot	IT	2016	0	0	0	0	0	0	0	0	0	0	0	0	74
12691149	m	md	ZA	2016	0	1	0	0	1	0	0	0	0	0	0	0	62
12691317	Not	md	JP	2016	0	1	0	0	1	0	0	0	0	0	0	0	
12692771	Not	ot	US	2016	0	0	0	0	0	0	0	0	0	0	0	0	
12692815	m	ot	GR	2016	0	1	0	0	1	0	0	0	0	0	0	1	83
12692917	m	ot	IE	2016	0	1	0	0	1	0	0	0	0	0	0	0	71
12693129	f	cn	CA	2016	0	0	0	0	0	0	0	0	0	0	0	0	43
12693168	m	cn	GB	2016	1	0	0	0	0	0	0	0	0	0	0	0	
12693170	Not	cn	GB	2016	1	0	0	0	0	0	0	0	0	0	0	0	

12693972	f	ot	US	2016	0	0	0	0	0	0	0	0	0	0	0	0	50
12694077	Not	ph	AU	2016	0	1	0	0	1	0	0	0	0	0	0	0	
12695047	Not	ot	GR	2016	0	0	0	0	0	0	0	0	0	0	0	0	
12695048	Not	ot	GR	2016	0	0	0	0	0	0	0	0	0	0	0	0	
12695049	Not	ot	GR	2016	0	0	0	0	0	0	0	0	0	0	0	0	
12695395	m	cn	JP	2016	0	1	0	0	1	0	0	0	0	0	0	0	65
12695466	f	ot	US	2016	1	0	0	0	0	0	0	0	0	0	0	0	
12695468	f	md	ID	2016	1	0	0	0	0	0	0	0	0	0	0	0	69
12695480	f	ph	AU	2016	0	1	0	0	1	0	0	0	0	0	0	0	73
12695692	m	cn	US	2016	0	0	0	0	0	0	0	0	0	0	0	0	
12695986	f	md	JP	2016	0	0	0	0	0	0	0	0	0	0	0	0	53
12696354	m	ot	ES	2016	0	1	0	0	1	0	0	0	0	0	0	0	73
12696624	f	cn	US	2016	0	1	0	0	1	0	0	0	0	0	0	0	
12696802	m	ot	US	2016	0	1	0	0	1	0	0	0	0	0	0	0	77
12697565	m	md	US	2016	1	0	0	0	0	0	0	0	0	0	0	0	62.8
12697619	f	ot	US	2016	1	0	0	0	0	0	0	0	0	0	0	0	74
12697863	m	cn	GB	2016	1	0	0	0	0	0	0	0	0	0	0	0	
12698403	m	md	IE	2016	0	1	0	0	1	0	0	0	0	0	0	0	71
12700107	m	cn	JP	2016	0	1	0	0	1	0	0	0	0	0	0	0	76
12700934	f	md	JP	2016	1	0	0	0	0	0	0	0	0	0	0	0	
12700994	m	md	JP	2016	0	1	0	0	1	0	0	0	0	0	0	0	72
12700995	m	ot	US	2016	0	1	0	0	1	0	0	0	0	0	0	0	76
12701020	f	ph	JP	2016	0	1	0	0	1	0	0	0	0	0	0	0	49
12701176	f	cn	RU	2016	1	0	0	0	0	0	0	0	0	0	0	0	
12702005	m	cn	BG	2016	0	1	0	0	1	0	0	0	0	0	0	0	
12702110	m	cn	BG	2016	0	1	0	0	1	0	0	0	0	0	0	0	
12702368	m	md	US	2016	0	1	0	0	0	1	0	0	0	0	0	1	72
12702730	m	ot	JP	2016	0	0	0	0	0	0	0	0	0	0	0	0	65
12702800	m	ph	JP	2016	0	1	0	0	1	0	0	0	0	0	0	0	69
12703926	f	cn	GB	2016	0	1	0	0	1	0	0	0	0	0	0	0	37
12704263	m	md	JP	2016	0	1	0	0	0	1	0	0	0	0	0	0	72
12704412	f	md	JP	2016	0	1	0	0	1	0	0	0	0	0	0	0	67
12704515	f	ot	FR	2016	1	0	0	0	0	0	0	0	0	0	0	0	56
12704646	m	cn	US	2016	0	1	1	0	0	0	0	0	0	0	0	0	58

12704813	f	ot	US	2016	0	1	0	0	1	0	0	0	0	0	0	0	42
12704859	Not	md	US	2016	0	0	0	0	0	0	0	0	0	0	0	0	
12705028	m	ph	US	2016	0	0	0	0	0	0	0	0	0	0	0	0	58
12705083	m	ph	JP	2016	1	0	0	0	0	0	0	0	0	0	0	1	62
12705490	f	ot	US	2016	0	1	0	0	1	0	0	0	0	0	0	0	82
12705542	m	md	JP	2016	0	1	0	0	1	0	0	0	0	0	0	0	72
12705740	m	cn	JP	2016	0	1	0	0	1	0	0	0	0	0	0	0	79
12706592	Not	ot	FR	2016	0	0	0	0	0	0	0	0	0	0	0	0	
12706995	m	ot	BE	2016	0	0	0	0	0	0	0	0	0	0	0	0	65
12707019	Not	md	JP	2016	0	0	0	0	0	0	0	0	0	0	0	0	
12707156	f	cn	GB	2016	1	0	0	0	0	0	0	0	0	0	0	0	
12707534	m	cn	JP	2016	0	1	0	0	1	0	0	0	0	0	0	0	80
12707906	f	ot	FR	2016	1	0	0	0	0	0	0	0	0	0	0	0	82
12707999	f	md	US	2016	1	0	0	0	0	0	0	0	0	0	0	0	
12708287	m	md	JP	2016	0	1	0	0	1	0	0	0	0	0	0	0	63
12708466	f	Not repo	US	2016	1	0	0	0	0	0	0	0	0	0	0	0	66
12708765	f	md	JP	2016	1	0	0	0	0	0	0	0	0	0	0	0	70
12708843	f	md	BE	2016	0	0	0	0	0	0	0	0	0	0	0	0	52
12709023	m	ot	KR	2016	0	1	0	0	1	0	0	0	0	0	0	0	61
12709414	f	md	JP	2016	0	1	0	0	1	0	0	0	0	0	0	0	69
12709547	m	ph	US	2016	0	1	0	0	1	0	0	0	0	0	0	0	74
12709828	m	cn	KR	2016	0	0	0	0	0	0	0	0	0	0	0	0	52
12710026	f	md	JP	2016	0	0	0	0	0	0	1	0	1	0	0	0	77
12710080	Not	ph	JP	2016	0	1	0	0	1	0	0	0	0	0	0	0	
12710215	Not	md	JP	2016	0	1	0	0	1	0	0	0	0	0	0	0	57
12710471	m	cn	JP	2016	0	1	0	0	1	0	0	0	0	0	0	0	69
12710525	m	md	US	2016	0	1	0	0	1	0	0	0	0	0	0	0	
12710680	m	md	JP	2016	0	1	0	0	1	0	0	0	0	0	0	0	84
12711693	f	ot	GB	2016	0	0	0	0	0	0	0	0	0	0	0	0	
12712328	m	md	JP	2016	0	1	0	0	1	0	0	0	0	0	0	0	73
12712375	Not	ot	US	2016	0	1	1	0	0	0	0	0	0	0	0	0	
12712919	m	md	BR	2016	0	0	0	0	0	0	0	0	0	0	0	0	75
12713140	f	md	JP	2016	1	0	0	0	0	0	0	0	0	0	0	0	72
12713268	m	md	IE	2016	1	0	0	0	0	0	0	0	0	0	0	1	51.3

12713813	f	Not repo	US	2016	1	0	0	0	0	0	0	0	0	0	0	0	
12713979	Not	ot	IT	2016	0	0	0	0	0	0	0	0	0	0	0	0	
12714230	m	cn	US	2016	0	0	0	0	0	0	0	0	0	0	0	0	62
12715024	m	ot	Not re	2016	1	0	0	0	0	0	0	0	0	0	0	0	77
12715028	f	md	Not re	2016	1	0	0	0	0	0	0	0	0	0	0	0	87
12715514	m	cn	US	2016	0	1	0	0	1	0	0	0	0	0	0	0	68
12715729	Not	ot	US	2016	0	0	0	0	0	0	0	0	0	0	0	0	
12715823	m	md	US	2016	0	0	0	0	0	0	0	0	0	0	0	0	69
12716435	f	cn	GB	2016	1	0	0	0	0	0	0	0	0	0	0	1	84
12716746	f	cn	GB	2016	1	0	0	0	0	0	0	0	0	0	0	0	74
12717048	f	cn	GB	2016	1	0	0	0	0	0	0	0	0	0	0	0	72
12717079	f	ot	US	2016	0	0	0	0	0	0	0	0	0	0	0	0	82
12717709	m	cn	GB	2016	1	0	0	0	0	0	0	0	0	0	0	0	
12717941	Not	ot	TR	2016	0	0	0	0	0	0	0	0	0	0	0	0	
12718064	f	ot	AU	2016	0	0	0	0	0	0	0	0	0	0	0	0	71
12718145	f	cn	US	2016	0	1	0	0	1	0	0	0	0	0	0	0	
12718195	m	md	JP	2016	0	1	0	0	1	0	0	0	0	0	0	1	49
12718197	f	ot	US	2016	0	1	0	0	1	0	0	0	0	0	0	0	58
12718948	m	ph	JP	2016	1	0	0	0	0	0	0	0	0	0	0	0	74
12719336	f	cn	JP	2016	0	1	0	0	1	0	0	0	0	0	0	0	64
12719907	m	cn	JP	2016	0	1	0	0	1	0	0	0	0	0	0	0	40
12720081	m	cn	US	2016	0	1	1	0	0	0	0	0	0	0	0	1	
12720436	m	ot	SG	2016	0	1	0	0	0	1	0	0	0	0	0	0	69
12721068	m	md	JP	2016	0	1	0	0	1	0	0	0	0	0	0	0	83
12721141	f	cn	GB	2016	1	0	0	0	0	0	0	0	0	0	0	0	
12721437	m	ot	Not re	2016	1	0	0	0	0	0	0	0	0	0	0	0	77
12721438	f	ot	Not re	2016	1	0	0	0	0	0	0	0	0	0	0	0	70
12721439	f	ot	Not re	2016	1	0	0	0	0	0	0	0	0	0	0	0	81
12721441	m	ot	Not re	2016	1	0	0	0	0	0	0	0	0	0	0	0	50
12721442	m	ot	Not re	2016	1	0	0	0	0	0	0	0	0	0	0	0	66
12721448	f	ot	Not re	2016	1	0	0	0	0	0	0	0	0	0	0	0	49
12721482	m	cn	US	2016	0	1	0	0	1	0	0	0	0	0	0	0	
12721570	f	md	FR	2016	1	0	0	0	0	0	0	0	0	0	0	0	72
12721645	m	ot	Not re	2016	1	0	0	0	0	0	0	0	0	0	0	1	88

12721671	m	md	JP	2016	0	1	0	0	1	0	0	0	0	0	0	0	62
12722251	f	md	JP	2016	0	1	0	0	1	0	0	0	0	0	0	0	61
12722561	f	ot	CO	2016	0	1	0	0	1	0	0	0	0	0	0	0	52
12722562	f	ot	GB	2016	0	1	0	0	1	0	0	0	0	0	0	0	71
12723317	m	cn	PR	2016	0	1	0	0	1	0	0	0	0	0	0	0	85
12723455	f	md	US	2016	0	0	0	0	0	0	0	0	0	0	0	0	66
12724035	m	ot	AU	2016	0	1	0	0	1	0	0	0	0	0	0	0	49
12724726	m	cn	US	2016	0	0	0	0	0	0	0	0	0	0	0	0	58
12724800	f	ot	US	2016	0	1	0	0	1	0	0	0	0	0	0	1	58
12725088	m	md	BE	2016	0	0	0	0	0	0	0	0	0	0	0	0	
12725445	m	md	JP	2016	0	1	0	0	1	0	0	0	0	0	0	1	71
12725622	m	md	Not re	2016	0	0	0	0	0	0	0	0	0	0	0	0	55
12725768	f	cn	US	2016	0	1	0	0	1	0	0	0	0	0	0	0	
12725772	f	ot	US	2016	0	1	0	0	1	0	0	0	0	0	0	0	68
12726879	m	ph	US	2016	0	1	0	0	1	0	0	0	0	0	0	0	
12726881	f	cn	CN	2016	0	0	0	0	0	0	0	0	0	0	0	0	
12726933	m	cn	DE	2016	0	1	0	0	1	0	0	0	0	0	0	1	62
12727104	m	md	CL	2016	0	1	0	0	0	1	0	0	0	0	0	0	71
12727206	f	ot	TW	2016	0	1	0	0	1	0	0	0	0	0	0	0	57
12727289	m	md	JP	2016	0	1	0	0	1	0	0	0	0	0	0	0	69
12727290	m	ot	JP	2016	0	1	0	0	1	0	0	0	0	0	0	0	57
12727479	Not	md	JP	2016	1	0	0	0	0	0	0	0	0	0	0	0	
12727591	m	cn	JP	2016	0	1	0	0	1	0	0	0	0	0	0	1	56
12727727	f	ph	FR	2016	1	0	0	0	0	0	0	0	0	0	0	0	56
12727845	Not	md	FR	2016	0	1	0	0	1	0	0	0	0	0	0	0	
12727921	m	md	JP	2016	0	0	0	0	0	0	1	0	1	0	0	0	84
12728044	f	cn	AR	2016	0	0	0	0	0	0	0	0	0	0	0	0	73
12728051	m	ph	JP	2016	0	0	0	0	0	0	0	0	0	0	0	0	69
12728920	m	md	JP	2016	0	0	0	0	0	0	0	0	0	0	0	0	66
12729233	f	md	Not re	2016	1	0	0	0	0	0	0	0	0	0	0	0	63
12729369	f	ot	FR	2016	1	0	0	0	0	0	0	0	0	0	0	0	56
12729627	m	cn	GB	2016	1	0	0	0	0	0	0	0	0	0	0	0	65
12729629	Not	cn	GB	2016	1	0	0	0	0	0	0	0	0	0	0	0	
12729769	m	ot	FR	2016	0	1	0	0	1	0	0	0	0	0	0	0	63

12729878	m	ot	US	2016	0	1	0	0	1	0	0	0	0	0	0	0	65
12730912	m	md	Not re	2016	1	0	0	0	0	0	0	0	0	0	0	0	47
12731041	Not	ot	IT	2016	0	0	0	0	0	0	0	0	0	0	0	0	
12731216	m	md	DE	2016	0	1	0	0	1	0	0	0	0	0	0	1	74
12731340	m	md	AU	2016	0	1	0	0	1	0	0	0	0	0	0	0	82
12731671	f	md	US	2016	0	1	0	0	1	0	0	0	0	0	0	0	
12732003	f	ph	US	2016	0	0	0	0	0	0	0	0	0	0	0	0	49
12732629	m	ot	KR	2016	0	1	0	0	1	0	0	0	0	0	0	1	76
12732967	m	ot	KR	2016	0	0	0	0	0	0	0	0	0	0	0	0	66
12733900	m	md	JP	2016	1	0	0	0	0	0	0	0	0	0	0	0	70
12735377	m	cn	JP	2016	0	1	0	0	1	0	0	0	0	0	0	0	62
12735564	f	ot	GB	2016	0	1	0	0	1	0	0	0	0	0	0	0	70
12735616	f	md	FR	2016	1	0	0	0	0	0	0	0	0	0	0	0	
12735637	m	cn	US	2016	0	1	0	0	1	0	0	0	0	0	0	0	77
12735993	m	cn	JP	2016	0	1	0	0	1	0	0	0	0	0	0	1	72
12736520	m	ot	FR	2016	0	1	0	0	1	0	0	0	0	0	0	0	69
12736884	m	md	CH	2016	0	0	0	0	0	0	0	0	0	0	0	0	
12737083	f	md	FR	2016	0	1	0	0	0	1	0	0	0	0	0	0	60
12737090	f	md	FR	2016	1	0	0	0	0	0	0	0	0	0	0	0	
12737169	m	md	GB	2016	0	1	0	0	1	0	0	0	0	0	0	0	73
12737534	f	md	AU	2016	0	0	0	0	0	0	0	0	0	0	0	0	52
12737551	f	md	AU	2016	0	0	0	0	0	0	0	0	0	0	0	0	47
12737858	Not	ot	US	2016	0	0	0	0	0	0	0	0	0	0	0	0	
12739669	f	ot	GB	2016	0	1	0	0	1	0	0	0	0	0	0	0	74
12739792	f	cn	US	2016	0	0	0	0	0	0	0	0	0	0	0	0	
12739935	m	ot	JP	2016	0	0	0	0	0	0	0	0	0	0	0	0	59
12740669	f	ot	CA	2016	0	1	0	0	1	0	0	0	0	0	0	0	73
12740904	f	cn	JP	2016	0	1	0	0	1	0	0	0	0	0	0	0	76
12740914	f	ph	US	2016	0	1	0	0	1	0	0	0	0	0	0	0	
12740959	m	md	ES	2016	0	1	0	0	0	1	0	0	0	0	0	1	73
12741284	f	md	BE	2016	0	1	0	0	1	0	0	0	0	0	0	0	60
12741333	f	md	US	2016	0	0	0	0	0	0	0	0	0	0	0	0	59
12741896	m	ph	US	2016	0	1	0	0	1	0	0	0	0	0	0	0	59
12741973	f	cn	US	2016	0	1	0	0	1	0	0	0	0	0	0	0	

12742102	f	cn	JP	2016	0	1	0	0	1	0	0	0	0	0	0	0	58
12742531	m	cn	US	2016	0	0	0	0	0	0	0	0	0	0	0	0	78
12742565	f	cn	GB	2016	1	0	0	0	0	0	0	0	0	0	0	0	68
12742566	Not	cn	GB	2016	1	0	0	0	0	0	0	0	0	0	0	0	
12743204	f	ot	US	2016	0	0	0	0	0	0	0	0	0	0	0	0	60
12743537	m	cn	GB	2016	1	0	0	0	0	0	0	0	0	0	0	0	75
12743539	m	cn	GB	2016	1	0	0	0	0	0	0	0	0	0	0	0	60
12743611	m	md	JP	2016	0	1	0	0	1	0	0	0	0	0	0	0	67
12743665	f	md	KR	2016	1	0	0	0	0	0	0	0	0	0	0	0	56
12743862	f	cn	Not re	2016	0	0	0	0	0	0	0	0	0	0	0	0	
12744718	f	ot	US	2016	0	0	0	0	0	0	0	0	0	0	0	0	
12744972	Not	md	US	2016	0	1	0	0	1	0	0	0	0	0	0	0	
12745019	m	md	JP	2016	0	1	0	0	1	0	0	0	0	0	0	1	57
12745089	f	md	AT	2016	0	1	0	0	1	0	0	0	0	0	0	0	72
12745090	f	cn	JP	2016	0	1	0	0	1	0	0	0	0	0	0	0	64
12745316	m	cn	US	2016	0	1	0	0	1	0	0	0	0	0	0	0	
12745318	f	cn	US	2016	0	1	0	0	1	0	0	0	0	0	0	0	
12746284	f	cn	US	2016	0	0	0	0	0	0	0	0	0	0	0	0	62
12746288	Not	ot	IT	2016	0	0	0	0	0	0	0	0	0	0	0	0	
12746298	Not	ot	IT	2016	0	0	0	0	0	0	0	0	0	0	0	0	
12746409	m	ot	US	2016	0	1	0	0	0	1	0	0	0	0	0	0	70
12746640	Not	md	US	2016	0	1	0	0	1	0	0	0	0	0	0	1	
12748402	f	md	Not re	2016	1	0	0	0	0	0	0	0	0	0	0	0	55
12748731	Not	Not repo	AU	2016	1	0	0	0	0	0	0	0	0	0	0	0	
12748824	Not	cn	GB	2016	1	0	0	0	0	0	0	0	0	0	0	0	
12748932	f	ot	US	2016	0	0	0	0	0	0	0	0	0	0	0	0	66
12749487	f	cn	US	2016	1	0	0	0	0	0	0	0	0	0	0	0	78
12749709	f	cn	US	2016	0	0	0	0	0	0	0	0	0	0	0	0	33
12751923	m	md	Not re	2016	1	0	0	0	0	0	0	0	0	0	0	0	
12752762	f	cn	US	2016	0	1	0	0	1	0	0	0	0	0	0	0	
12752845	m	cn	IT	2016	0	1	0	0	1	0	0	0	0	0	0	0	66
12753284	m	cn	JP	2016	0	1	0	0	1	0	0	0	0	0	0	0	65
12753286	m	md	AU	2016	0	1	0	0	1	0	0	0	0	0	0	0	
12754872	f	cn	US	2016	0	0	0	0	0	0	0	0	0	0	0	0	

12754881	f	md	PH	2016	1	0	0	0	0	0	0	0	0	0	0	0	78
12755104	f	md	Not re	2016	1	0	0	0	0	0	0	0	0	0	0	0	60
12755129	m	md	ES	2016	0	0	0	0	0	0	0	0	0	0	0	0	57
12755350	f	cn	JP	2016	0	1	0	0	1	0	0	0	0	0	0	0	65
12755576	Not	ot	NL	2016	0	1	0	0	1	0	0	0	0	0	0	0	
12755577	f	cn	JP	2016	0	1	0	0	1	0	0	0	0	0	0	0	45
12755582	m	ot	US	2016	0	1	0	0	1	0	0	0	0	0	0	0	74
12755626	f	ph	FR	2016	1	0	0	0	0	0	0	0	0	0	0	0	82
12755892	m	ot	GB	2016	0	1	0	0	1	0	0	0	0	0	0	0	67
12756252	m	ot	US	2016	0	0	0	0	0	0	0	0	0	0	0	0	72
12756270	m	ot	BR	2016	0	1	0	0	1	0	0	0	0	0	0	0	74
12756328	m	md	LB	2016	1	0	0	0	0	0	0	0	0	0	0	0	65
12756523	f	md	ES	2016	0	1	0	0	0	1	0	0	0	0	0	0	49
12757442	f	md	US	2016	0	1	0	0	1	0	0	0	0	0	0	0	
12757738	m	md	AU	2016	0	1	0	0	0	1	0	0	0	0	0	0	65
12757791	m	cn	JP	2016	0	1	0	0	1	0	0	0	0	0	0	1	64
12757804	m	ph	JP	2016	0	1	0	0	1	0	0	0	0	0	0	1	65
12758203	m	md	US	2016	0	0	0	0	0	0	0	0	0	0	0	0	45
12758212	m	md	US	2016	0	0	0	0	0	0	0	0	0	0	0	0	46
12759327	Not	ot	IT	2016	0	0	0	0	0	0	0	0	0	0	0	0	
12759328	f	ph	FR	2016	1	0	0	0	0	0	0	0	0	0	0	0	82
12759402	f	ph	FR	2016	1	0	0	0	0	0	0	0	0	0	0	0	56
12760056	Not	ot	IT	2016	0	0	0	0	0	0	0	0	0	0	0	0	
12760060	f	cn	CA	2016	0	0	0	0	0	0	0	0	0	0	0	0	72
12760064	Not	ot	IT	2016	0	0	0	0	0	0	0	0	0	0	0	0	
12761024	m	md	IT	2016	0	1	0	0	1	0	0	0	0	0	0	1	75
12762029	Not	cn	VE	2016	0	1	0	0	1	0	0	0	0	0	0	0	
12763030	m	md	JP	2016	0	1	0	0	1	0	0	0	0	0	0	0	60
12763419	f	md	JP	2016	0	1	0	0	1	0	0	0	0	0	0	0	62
12763924	m	md	US	2016	0	1	1	0	0	0	0	0	0	0	0	1	78
12764516	f	ot	FR	2016	0	1	0	0	1	0	0	0	0	0	0	0	73
12764546	Not	ot	MX	2016	1	0	0	0	0	0	0	0	0	0	0	1	
12764658	f	md	JP	2016	0	1	0	0	1	0	0	0	0	0	0	0	59
12764889	f	cn	US	2016	0	0	0	0	0	0	0	0	0	0	0	0	

12764961	f	md	NL	2016	0	1	0	0	1	0	0	0	0	0	0	0	60
12765024	m	cn	JP	2016	0	1	0	0	1	0	0	0	0	0	0	0	67
12765265	m	md	US	2016	0	0	0	0	0	0	0	0	0	0	0	0	45
12765285	f	md	US	2016	0	0	0	0	0	0	0	0	0	0	0	0	33
12765298	Not	md	US	2016	0	0	0	0	0	0	0	0	0	0	0	0	
12765907	Not	md	JP	2016	0	0	0	0	0	0	0	0	0	0	0	0	
12766507	m	cn	JP	2016	0	1	0	0	1	0	0	0	0	0	0	0	59
12766619	f	ot	PL	2016	0	0	0	0	0	0	0	0	0	0	0	0	53
12767596	m	cn	CA	2016	0	1	0	0	1	0	0	0	0	0	0	0	65
12767729	f	md	Not re	2016	1	0	0	0	0	0	0	0	0	0	0	0	65
12768407	f	ot	JP	2016	0	1	0	0	1	0	0	0	0	0	0	0	67
12769284	Not	md	BE	2016	1	0	0	0	0	0	0	0	0	0	0	0	
12769422	m	ot	US	2016	1	0	0	0	0	0	0	0	0	0	0	0	76
12769654	m	cn	JP	2016	0	1	0	0	1	0	0	0	0	0	0	0	68
12769955	f	md	JP	2016	0	1	0	0	1	0	0	0	0	0	0	1	73
12770034	f	ph	US	2016	0	0	0	0	0	0	0	0	0	0	0	0	45
12770549	m	cn	Not re	2016	0	0	0	0	0	0	0	0	0	0	0	0	54
12770605	m	md	US	2016	1	0	0	0	0	0	0	0	0	0	0	1	51.8
12770877	m	md	Not re	2016	1	0	0	0	0	0	0	0	0	0	0	0	57
12771714	m	ot	FR	2016	0	0	0	0	0	0	0	0	0	0	0	0	59
12771732	m	cn	GB	2016	1	0	0	0	0	0	0	0	0	0	0	0	
12772295	m	md	JP	2016	0	1	0	0	1	0	0	0	0	0	0	0	73
12772660	f	md	DE	2016	0	0	0	0	0	0	0	0	0	0	0	0	45
12772857	f	md	LB	2016	1	0	0	0	0	0	0	0	0	0	0	0	56
12773450	m	md	GB	2016	0	1	0	0	1	0	0	0	0	0	0	0	73
12773548	m	md	GB	2016	0	1	0	0	0	1	0	0	0	0	0	0	72
12773560	f	ot	IE	2016	0	1	0	0	1	0	0	0	0	0	0	0	61
12773636	f	md	US	2016	0	1	0	0	0	1	0	0	0	0	0	0	66
12773772	m	cn	US	2016	0	1	0	0	1	0	0	0	0	0	0	0	
12773967	f	ot	CA	2016	0	1	0	0	1	0	0	0	0	0	0	0	63
12773969	m	cn	IT	2016	0	1	0	0	1	0	0	0	0	0	0	0	76
12774213	f	cn	US	2016	0	0	0	0	0	0	0	0	0	0	0	0	60
12774275	f	cn	US	2016	0	1	0	0	1	0	0	0	0	0	0	1	
12774680	f	cn	US	2016	0	0	0	0	0	0	0	0	0	0	0	0	59

12775093	m	ot	US	2016	0	0	0	0	0	0	0	0	0	0	0	0	75
12775095	f	md	US	2016	0	0	0	0	0	0	0	0	0	0	0	0	67
12775782	m	cn	US	2016	0	0	0	0	0	0	0	0	0	0	0	0	70
12776094	f	md	JP	2016	1	0	0	0	0	0	0	0	0	0	0	0	74
12776307	f	md	US	2016	1	0	0	0	0	0	0	0	0	0	0	0	
12776347	m	ot	PL	2016	0	1	0	0	1	0	0	0	0	0	0	0	70
12776382	m	cn	US	2016	0	1	0	0	1	0	0	0	0	0	0	0	
12776465	Not	md	JP	2016	0	1	0	0	1	0	0	0	0	0	0	0	82
12776515	m	md	JP	2016	0	1	0	0	1	0	0	0	0	0	0	0	65
12776721	f	cn	JP	2016	0	1	0	0	1	0	0	0	0	0	0	0	81
12776754	m	md	Not re	2016	1	0	0	0	0	0	0	0	0	0	0	0	75
12777241	m	ot	US	2016	0	0	0	0	0	0	0	0	0	0	0	0	
12778195	Not	ph	AU	2016	0	1	0	0	1	0	0	0	0	0	0	0	65
12778351	f	cn	US	2016	0	1	0	0	1	0	0	0	0	0	0	0	
12778434	m	md	JP	2016	1	0	0	0	0	0	0	0	0	0	0	0	
12778576	f	ph	US	2016	0	1	0	0	1	0	0	0	0	0	0	0	
12778606	f	ph	FR	2016	0	1	0	0	1	0	0	0	0	0	0	0	
12779053	f	ot	US	2016	0	1	0	0	1	0	0	0	0	0	0	0	80
12779054	m	ot	US	2016	0	1	0	0	1	0	0	0	0	0	0	0	
12779770	m	ph	US	2016	0	1	0	0	1	0	0	0	0	0	0	0	
12779798	Not	ot	IT	2016	0	0	0	0	0	0	0	0	0	0	0	0	
12779876	f	cn	GB	2016	1	0	0	0	0	0	0	0	0	0	0	0	82
12780094	m	cn	JP	2016	0	1	0	0	1	0	0	0	0	0	0	0	67
12780311	m	ph	US	2016	0	0	0	0	0	0	0	0	0	0	0	0	86
12781165	m	md	JP	2016	0	1	0	0	1	0	0	0	0	0	0	0	49
12781221	m	md	ES	2016	0	1	0	0	1	0	0	0	0	0	0	0	63
12781784	m	md	Not re	2016	0	0	0	0	0	0	0	0	0	0	0	0	73
12781954	m	cn	JP	2016	0	1	0	0	1	0	0	0	0	0	0	0	62
12782004	Not	ot	IT	2016	0	0	0	0	0	0	0	0	0	0	0	0	
12782406	m	md	US	2016	0	0	0	0	0	0	0	0	0	0	0	0	74
12782573	f	md	DE	2016	0	1	0	0	1	0	0	0	0	0	0	0	
12782574	m	md	DE	2016	0	1	0	0	1	0	0	0	0	0	0	0	
12783272	m	cn	JP	2016	0	1	0	0	1	0	0	0	0	0	0	0	71
12783708	m	md	JP	2016	0	1	0	0	1	0	0	0	0	0	0	0	76

12783940	m	cn	IE	2016	0	0	0	0	0	0	0	0	0	0	0	0	54
12783941	Not	cn	IE	2016	0	0	0	0	0	0	0	0	0	0	0	0	
12784413	m	md	CA	2016	0	1	0	0	0	1	0	0	0	0	0	1	72
12784490	f	ot	US	2016	0	1	0	0	1	0	0	0	0	0	0	0	
12785112	f	cn	US	2016	0	1	0	0	1	0	0	0	0	0	0	0	
12785826	m	md	US	2016	0	1	0	0	1	0	0	0	0	0	0	0	
12786011	f	md	US	2016	1	0	0	0	0	0	0	0	0	0	0	0	
12786613	f	ot	Not re	2016	1	0	0	0	0	0	0	0	0	0	0	0	68
12786796	m	md	Not re	2016	1	0	0	0	0	0	0	0	0	0	0	0	
12787400	m	ot	UA	2016	0	0	0	0	0	0	0	0	0	0	0	0	63
12787954	f	md	US	2016	1	0	0	0	0	0	0	0	0	0	0	1	47
12788439	m	cn	JP	2016	0	1	0	0	1	0	0	0	0	0	0	0	47
12788524	f	cn	US	2016	1	0	0	0	0	0	0	0	0	0	0	0	
12789161	m	md	JP	2016	0	1	0	0	1	0	0	0	0	0	0	0	61
12789225	m	cn	JP	2016	0	1	0	0	1	0	0	0	0	0	0	0	67
12790092	f	md	Not re	2016	1	0	0	0	0	0	0	0	0	0	0	0	67
12790161	Not	md	JP	2016	0	1	0	0	1	0	0	0	0	0	0	0	
12790191	f	md	JP	2016	1	0	0	0	0	0	0	0	0	0	0	0	63
12790403	Not	cn	JP	2016	0	1	0	0	1	0	0	0	0	0	0	0	
12790805	m	cn	US	2016	0	1	0	0	1	0	0	0	0	0	0	0	
12791403	m	cn	Not re	2016	0	0	0	0	0	0	0	0	0	0	0	0	21
12791500	f	ot	CA	2016	0	1	0	0	1	0	0	0	0	0	0	0	56
12791574	m	cn	IT	2016	0	1	0	0	1	0	0	0	0	0	0	0	69
12795148	m	md	JP	2016	0	1	0	0	1	0	0	0	0	0	0	0	
12797663	m	ot	US	2016	0	1	1	0	0	0	0	0	0	0	0	0	81
12798723	f	md	BR	2016	0	0	0	0	0	0	0	0	0	0	0	0	32
12799824	m	ot	CN	2016	0	1	0	0	1	0	0	0	0	0	0	0	48
12800093	m	cn	FR	2016	0	0	0	0	0	0	0	0	0	0	0	0	82
7131028	f	md	US	2016	0	0	0	0	0	0	0	0	0	0	0	0	55
8862920	f	md	JP	2016	0	0	0	0	0	0	0	0	0	0	0	0	72
8988873	f	md	JP	2016	0	0	0	0	0	0	0	0	0	0	0	0	66
9475015	f	cn	VE	2016	0	0	0	0	0	0	0	0	0	0	0	0	
9745158	f	md	DE	2016	0	0	0	0	0	0	0	0	0	0	0	0	76
9783031	m	cn	GB	2016	1	0	0	0	0	0	0	0	0	0	0	0	84

9788837	f	cn	GB	2016	1	0	0	0	0	0	0	0	0	0	0	0	61
9800969	m	cn	GB	2016	1	0	0	0	0	0	0	0	0	0	0	0	
9818546	f	md	JP	2016	0	0	0	0	0	0	0	0	0	0	0	0	32
9869780	m	ot	US	2016	1	0	0	0	0	0	0	0	0	0	0	0	
9967698	m	md	DE	2016	0	0	0	0	0	0	0	0	0	0	0	0	49
10065497	f	md	US	2016	0	0	0	0	0	0	0	0	0	0	0	0	65
10088147	m	md	JP	2016	0	0	0	0	0	0	0	0	0	0	0	0	72
10265825	m	md	US	2016	0	0	0	0	0	0	0	0	0	0	0	0	75
10660138	f	md	US	2016	0	0	0	0	0	0	0	0	0	0	0	0	67
12117669	f	md	JP	2016	0	1	0	0	0	1	0	0	0	0	0	0	71
12265304	f	cn	JP	2016	0	1	0	0	1	0	0	0	0	0	0	0	73
12316159	f	cn	JP	2016	0	1	0	0	1	0	0	0	0	0	0	0	58
12316412	m	cn	JP	2016	0	1	0	0	1	0	0	0	0	0	0	1	79
12330644	f	md	FR	2016	0	0	0	0	0	0	0	0	0	0	0	0	62
12337997	m	cn	JP	2016	0	1	0	0	1	0	0	0	0	0	0	0	62
12349083	f	cn	US	2016	1	0	0	0	0	0	0	0	0	0	0	0	85
12370459	m	ot	ES	2016	0	1	0	0	1	0	0	0	0	0	0	0	72
12398366	m	cn	JP	2016	0	1	0	0	1	0	0	0	0	0	0	0	73
12410646	m	md	JP	2016	0	1	0	0	1	0	0	0	0	0	0	0	43
12412458	f	md	JP	2016	1	0	0	0	0	0	0	0	0	0	0	0	44
12413187	f	md	FR	2016	0	0	0	0	0	0	0	0	0	0	0	0	85
12421932	m	md	DE	2016	0	1	0	0	0	1	0	0	0	0	0	0	69
12431153	f	cn	US	2016	1	0	0	0	0	0	0	0	0	0	0	0	76
12454508	m	ot	BR	2016	0	1	0	0	1	0	0	0	0	0	0	0	69
12474185	f	md	JP	2016	0	1	0	0	1	0	0	0	0	0	0	0	82
12474959	m	md	JP	2016	1	0	0	0	0	0	0	0	0	0	0	0	59
12478330	m	md	JP	2016	0	0	0	0	0	0	1	0	1	0	0	1	68
12479655	m	ot	US	2016	0	0	0	0	0	0	0	0	0	0	0	0	76
12483440	m	cn	JP	2016	0	1	0	0	1	0	0	0	0	0	0	0	54
12494226	m	cn	JP	2016	0	1	0	0	1	0	0	0	0	0	0	1	74
12498805	m	cn	JP	2016	0	1	0	0	1	0	0	0	0	0	0	0	51
12504481	m	md	JP	2016	0	0	0	0	0	0	0	0	0	0	0	0	64
12506032	f	md	JP	2016	1	0	0	0	0	0	0	0	0	0	0	0	65
12520448	m	md	JP	2016	0	1	0	0	1	0	0	0	0	0	0	0	67

12521949	f	cn	IQ	2016	0	0	0	0	0	0	0	0	0	0	0	0	
12523875	m	cn	IT	2016	0	1	0	0	1	0	0	0	0	0	0	0	86
12524068	f	md	US	2016	0	1	0	0	0	1	0	0	0	0	0	0	72
12525221	m	ot	JP	2016	0	1	0	0	1	0	0	0	0	0	0	0	42
12527902	m	md	FR	2016	0	0	0	0	0	0	0	0	0	0	0	0	71
12528625	f	md	NL	2016	0	0	0	0	0	0	0	0	0	0	0	0	51
12528746	f	md	FR	2016	0	0	0	0	0	0	0	0	0	0	0	0	63
12529955	m	cn	JP	2016	0	1	0	0	1	0	0	0	0	0	0	0	59
12530330	m	md	US	2016	1	0	0	0	0	0	0	0	0	0	0	0	61
12530741	f	ot	AU	2016	0	0	0	0	0	0	0	0	0	0	0	0	55
12530868	m	ot	LT	2016	0	1	0	0	1	0	0	0	0	0	0	0	26
12531648	m	ot	CA	2016	0	1	0	0	1	0	0	0	0	0	0	0	69
12538368	f	md	KR	2016	1	0	0	0	0	0	0	0	0	0	0	0	54
12541584	m	cn	JP	2016	0	1	0	0	1	0	0	0	0	0	0	1	48
12546636	f	ot	BR	2016	0	1	0	0	1	0	0	0	0	0	0	0	57
12549005	m	md	JP	2016	1	0	0	0	0	0	0	0	0	0	0	0	53
12550155	m	md	JP	2016	0	0	0	0	0	0	1	0	1	0	0	1	62
12550182	f	md	US	2016	0	0	0	0	0	0	0	0	0	0	0	0	61
12551121	f	cn	JP	2016	0	0	0	0	0	0	1	0	1	0	0	0	35
12551158	f	ot	US	2016	0	1	0	0	1	0	0	0	0	0	0	0	
12552423	m	md	JP	2016	0	1	0	0	1	0	0	0	0	0	0	1	71
12552971	m	md	JP	2016	0	0	0	0	0	0	1	0	1	0	0	0	38
12557067	m	cn	JP	2016	0	1	0	0	1	0	0	0	0	0	0	0	63
12558715	m	ot	US	2016	0	1	0	0	1	0	0	0	0	0	0	0	57
12559254	m	ot	Not re	2016	0	0	0	0	0	0	0	0	0	0	0	0	71
12569278	m	ot	GB	2016	0	0	0	0	0	0	0	0	0	0	0	0	
12571712	m	md	JP	2016	0	1	0	0	1	0	0	0	0	0	0	0	61
12572433	f	cn	JP	2016	0	1	0	0	1	0	0	0	0	0	0	0	70
12573241	m	ot	GB	2016	0	0	0	0	0	0	0	0	0	0	0	0	71
12576633	m	md	JP	2016	1	0	0	0	0	0	0	0	0	0	0	0	78
12579565	f	md	JP	2016	1	0	0	0	0	0	0	0	0	0	0	0	81
12581253	f	cn	JP	2016	0	1	0	0	1	0	0	0	0	0	0	0	46
12582371	m	md	JP	2016	0	1	0	0	1	0	0	0	0	0	0	1	68
12583228	m	md	BE	2016	0	1	0	0	1	0	0	0	0	0	0	0	75

12584519	m	cn	JP	2016	0	1	0	0	1	0	0	0	0	0	0	0	76
12584553	m	md	CZ	2016	0	1	0	0	1	0	0	0	0	0	0	0	72
12587804	f	md	FR	2016	0	1	0	0	0	1	0	0	0	0	0	0	50
12588110	f	ot	BR	2016	0	1	0	0	1	0	0	0	0	0	0	0	60
12597600	m	ot	US	2016	0	0	0	0	0	0	0	0	0	0	0	0	74
12599872	m	ot	BE	2016	0	1	0	0	1	0	0	0	0	0	0	0	75
12605109	m	md	JP	2016	0	1	0	0	1	0	0	0	0	0	0	0	56
12607993	f	md	IT	2016	0	0	0	0	0	0	0	0	0	0	0	0	47
12611153	f	ot	CA	2016	0	1	0	0	1	0	0	0	0	0	0	0	55
12612585	m	md	LB	2016	1	0	0	0	0	0	0	0	0	0	0	0	56
12620585	m	md	US	2016	0	1	0	0	1	0	0	0	0	0	0	0	
12621441	m	md	JP	2016	0	1	0	0	1	0	0	0	0	0	0	0	65
12622274	m	md	JP	2016	1	0	0	0	0	0	0	0	0	0	0	0	66
12622453	f	md	JP	2016	0	0	0	0	0	0	0	0	0	0	0	0	59
12622794	f	md	JP	2016	1	0	0	0	0	0	0	0	0	0	0	0	72
12623257	f	ph	DE	2016	0	1	0	0	1	0	0	0	0	0	0	0	61
12628354	m	ph	AU	2016	0	1	0	0	1	0	0	0	0	0	0	0	56
12635712	m	md	JP	2016	0	1	0	0	1	0	0	0	0	0	0	0	47
12636134	m	cn	JP	2016	0	1	0	0	1	0	0	0	0	0	0	1	71
12636485	f	ot	JP	2016	0	1	0	0	1	0	0	0	0	0	0	0	73
12639177	m	md	JP	2016	0	1	0	0	1	0	0	0	0	0	0	0	74
12639606	f	md	JP	2016	1	0	0	0	0	0	0	0	0	0	0	1	80
12640631	m	md	NZ	2016	0	0	0	0	0	0	0	0	0	0	0	0	56
12641629	m	md	JP	2016	0	1	0	0	1	0	0	0	0	0	0	0	59
12643797	f	cn	GB	2016	1	0	0	0	0	0	0	0	0	0	0	0	77
12646557	m	cn	JP	2016	0	1	0	0	1	0	0	0	0	0	0	0	59
12646841	f	md	JP	2016	1	0	0	0	0	0	0	0	0	0	0	1	60
12647220	Not	md	ES	2016	0	0	0	0	0	0	0	0	0	0	0	0	
12647806	f	ot	US	2016	1	0	0	0	0	0	0	0	0	0	0	0	
12649859	f	md	US	2016	0	0	0	0	0	0	0	0	0	0	0	0	
12650099	m	ot	NL	2016	0	1	0	0	1	0	0	0	0	0	0	0	74
12650113	m	cn	IT	2016	0	1	0	0	1	0	0	0	0	0	0	0	86
12650158	Not	md	BE	2016	0	0	0	0	0	0	0	0	0	0	0	0	
12650353	Not	md	ES	2016	0	0	0	0	0	0	0	0	0	0	0	0	

12652439	m	ot	US	2016	0	0	0	0	0	0	0	0	0	0	0	0	56
12653873	Not	md	ES	2016	0	0	0	0	0	0	0	0	0	0	0	0	
12654077	Not	md	AT	2016	0	0	0	0	0	0	0	0	0	0	0	0	
12657517	m	md	JP	2016	1	0	0	0	0	0	0	0	0	0	0	0	53
12663435	f	ot	US	2016	1	0	0	0	0	0	0	0	0	0	0	0	56
12663815	Not	md	ES	2016	0	0	0	0	0	0	0	0	0	0	0	0	
12664420	m	md	JP	2016	0	1	0	0	0	1	0	0	0	0	0	0	64
12667720	m	ot	US	2016	0	0	0	0	0	0	0	0	0	0	0	0	88
12669641	f	ph	CA	2016	0	1	0	0	1	0	0	0	0	0	0	0	62
12671016	f	cn	JP	2016	0	1	0	0	1	0	0	0	0	0	0	0	64
12673959	m	md	TR	2016	0	0	0	0	0	0	0	0	0	0	0	0	59
12674795	f	md	TR	2016	0	0	0	0	0	0	0	0	0	0	0	0	67
12674848	m	md	US	2016	0	0	0	0	0	0	0	0	0	0	0	0	58
12675842	m	cn	JP	2016	0	1	0	0	1	0	0	0	0	0	0	1	69
12675883	m	ot	GR	2016	0	1	0	0	1	0	0	0	0	0	0	0	83
12676369	m	cn	JP	2016	0	1	0	0	1	0	0	0	0	0	0	0	72
12678814	m	cn	JP	2016	0	1	0	0	1	0	0	0	0	0	0	0	69
12679568	f	md	US	2016	0	1	0	0	1	0	0	0	0	0	0	0	61
12681461	m	md	HK	2016	1	0	0	0	0	0	0	0	0	0	0	0	65.7
12681834	m	md	JP	2016	0	1	0	0	1	0	0	0	0	0	0	0	69
12686607	f	md	JP	2016	0	0	0	0	0	0	1	0	1	0	0	0	80
12688356	f	md	JP	2016	1	0	0	0	0	0	0	0	0	0	0	1	78
12688580	m	ot	AU	2016	0	1	0	0	1	0	0	0	0	0	0	0	
12690874	f	cn	FR	2016	0	1	0	0	1	0	0	0	0	0	0	0	71
12691312	m	cn	JP	2016	0	1	0	0	1	0	0	0	0	0	0	0	78
12692526	f	ot	JP	2016	0	1	0	0	1	0	0	0	0	0	0	0	68
12693160	m	cn	GB	2016	1	0	0	0	0	0	0	0	0	0	0	0	75
12694199	m	md	US	2016	0	1	1	0	0	0	0	0	0	0	0	0	
12694600	m	cn	US	2016	0	1	1	0	0	0	0	0	0	0	0	0	
12695967	f	ot	TH	2016	0	0	0	0	0	0	0	0	0	0	0	0	78
12696349	m	cn	AT	2016	0	1	1	0	0	0	0	0	0	0	0	0	46
12699528	m	md	BG	2016	0	1	0	0	1	0	0	0	0	0	0	0	
12699729	f	cn	JP	2016	0	1	0	0	1	0	0	0	0	0	0	0	80
12702096	f	md	BG	2016	0	1	0	0	1	0	0	0	0	0	0	0	

12707225	m	md	JP	2016	0	1	0	0	1	0	0	0	0	0	0	0	63
12707349	f	md	DE	2016	1	0	0	0	0	0	0	0	0	0	0	0	79
12707393	m	cn	US	2016	0	1	1	0	0	0	0	0	0	0	0	0	72
12708059	m	cn	JP	2016	0	1	0	0	1	0	0	0	0	0	0	1	75
12710381	f	cn	JP	2016	0	1	0	0	1	0	0	0	0	0	0	0	68
12710515	m	md	JP	2016	0	1	0	0	1	0	0	0	0	0	0	1	56
12711447	m	md	DE	2016	0	0	0	0	0	0	0	0	0	0	0	0	73
12712099	Not	ot	US	2016	0	0	0	0	0	0	0	0	0	0	0	0	
12712173	f	md	US	2016	1	0	0	0	0	0	0	0	0	0	0	0	62
12713311	f	md	JP	2016	1	0	0	0	0	0	0	0	0	0	0	1	82
12716744	Not	cn	GB	2016	1	0	0	0	0	0	0	0	0	0	0	1	
12716861	m	cn	JP	2016	0	1	0	0	1	0	0	0	0	0	0	0	69
12718075	m	cn	US	2016	0	1	1	0	0	0	0	0	0	0	0	1	54
12718639	f	md	JP	2016	1	0	0	0	0	0	0	0	0	0	0	0	62
12719362	m	md	JP	2016	0	1	0	0	1	0	0	0	0	0	0	0	82
12719521	m	cn	JP	2016	0	1	0	0	1	0	0	0	0	0	0	0	82
12719976	m	md	CN	2016	0	1	0	0	0	1	0	0	0	0	0	0	65
12722264	f	ot	CA	2016	0	1	0	0	1	0	0	0	0	0	0	0	49
12722266	m	md	JP	2016	0	1	0	0	0	1	0	0	0	0	0	0	70
12723072	f	ot	HR	2016	0	1	0	0	1	0	0	0	0	0	0	0	56
12723426	m	cn	GB	2016	0	1	1	0	0	0	0	0	0	0	0	0	72
12724297	m	ot	US	2016	0	1	0	0	1	0	0	0	0	0	0	1	
12725274	f	md	US	2016	1	0	0	0	0	0	0	0	0	0	0	0	72.9
12725765	f	md	JP	2016	0	1	0	0	1	0	0	0	0	0	0	0	54
12725769	Not	cn	IT	2016	0	1	0	0	1	0	0	0	0	0	0	0	48
12727243	m	cn	US	2016	0	1	0	0	1	0	0	0	0	0	0	0	
12727350	m	md	BE	2016	0	1	0	0	1	0	0	0	0	0	0	0	
12729090	m	md	JP	2016	1	0	0	0	0	0	0	0	0	0	0	0	58
12729836	m	md	JP	2016	0	1	0	0	1	0	0	0	0	0	0	0	
12730094	f	md	JP	2016	0	0	0	0	0	0	1	0	1	0	0	1	62
12732632	f	cn	JP	2016	0	1	0	0	1	0	0	0	0	0	0	0	62
12733602	f	md	JP	2016	0	1	0	0	1	0	0	0	0	0	0	0	70
12733811	f	md	CA	2016	0	1	0	0	0	1	0	0	0	0	0	0	58
12733832	f	md	JP	2016	1	0	0	0	0	0	0	0	0	0	0	0	75

12735603	m	ot	BE	2016	0	1	0	0	1	0	0	0	0	0	0	0	65
12736758	m	cn	JP	2016	0	0	0	0	0	0	1	0	1	0	0	0	55
12736894	f	md	JP	2016	0	0	0	0	0	0	0	0	0	0	0	0	
12739098	Not	md	JP	2016	0	0	0	0	0	0	0	0	0	0	0	0	
12740524	m	md	JP	2016	0	1	0	0	1	0	0	0	0	0	0	0	66
12741091	m	cn	JP	2016	0	1	0	0	1	0	0	0	0	0	0	0	65
12741093	m	md	JP	2016	0	1	0	0	1	0	0	0	0	0	0	0	68
12741143	m	cn	JP	2016	0	1	0	0	1	0	0	0	0	0	0	1	64
12742567	m	cn	GB	2016	1	0	0	0	0	0	0	0	0	0	0	0	65
12742613	f	md	JP	2016	1	0	0	0	0	0	0	0	0	0	0	1	57
12744720	m	md	CN	2016	0	1	0	0	0	1	0	0	0	0	0	0	63
12745097	f	cn	US	2016	0	1	0	0	1	0	0	0	0	0	0	0	
12745169	m	ot	JP	2016	0	1	0	0	1	0	0	0	0	0	0	0	75
12746489	m	cn	US	2016	0	1	1	0	0	0	0	0	0	0	0	0	43
12748600	m	ot	IT	2016	0	1	0	0	1	0	0	0	0	0	0	0	69
12749649	m	cn	JP	2016	0	1	0	0	1	0	0	0	0	0	0	0	61
12749652	m	md	JP	2016	0	0	0	0	0	0	1	0	1	0	0	1	62
12756117	f	md	GB	2016	1	0	0	0	0	0	0	0	0	0	0	0	81.5
12758527	m	md	SD	2016	0	0	0	0	0	0	0	0	0	0	0	0	76
12758752	m	ot	US	2016	0	1	0	0	1	0	0	0	0	0	0	0	74
12759490	m	cn	JP	2016	0	1	0	0	1	0	0	0	0	0	0	0	78
12759500	m	cn	JP	2016	0	1	0	0	1	0	0	0	0	0	0	0	79
12759731	f	cn	JP	2016	0	1	0	0	1	0	0	0	0	0	0	0	47
12760572	m	md	JP	2016	1	0	0	0	0	0	0	0	0	0	0	1	72
12760961	f	md	JP	2016	1	0	0	0	0	0	0	0	0	0	0	1	85
12762057	f	ot	DE	2016	1	0	0	0	0	0	0	0	0	0	0	1	73
12762122	m	md	US	2016	0	1	0	0	0	1	0	0	0	0	0	0	68
12764689	m	cn	JP	2016	0	1	0	0	1	0	0	0	0	0	0	0	71
12767351	m	md	JP	2016	1	0	0	0	0	0	0	0	0	0	0	0	68
12767811	f	md	CA	2016	0	1	0	0	0	1	0	0	0	0	0	0	58
12768850	f	md	JP	2016	0	0	0	0	0	0	0	0	0	0	0	0	75
12770332	f	cn	IT	2016	0	1	0	0	1	0	0	0	0	0	0	0	68
12773530	m	md	JP	2016	0	1	0	0	1	0	0	0	0	0	0	0	58
12775140	m	md	JP	2016	1	0	0	0	0	0	0	0	0	0	0	0	63

12775143	m	ot	US	2016	0	1	0	0	1	0	0	0	0	0	0	1	64
12778186	f	cn	IT	2016	0	1	0	0	1	0	0	0	0	0	0	0	60
12778299	m	md	JP	2016	0	1	0	0	1	0	0	0	0	0	0	0	76
12781917	f	md	DE	2016	0	0	0	0	0	0	0	0	0	0	0	0	79
12781926	m	ot	IT	2016	0	1	0	0	1	0	0	0	0	0	0	0	78
12781971	m	ot	US	2016	0	1	0	0	1	0	0	0	0	0	0	0	71
12781972	f	md	JP	2016	0	1	0	0	0	1	0	0	0	0	0	0	63
12782017	f	ot	JP	2016	0	1	0	0	1	0	0	0	0	0	0	0	67
12782569	f	md	US	2016	0	1	0	0	0	1	0	0	0	0	0	0	66
12786788	f	md	DE	2016	1	0	0	0	0	0	0	0	0	0	0	0	89
12786793	m	md	BE	2016	1	0	0	0	0	0	0	0	0	0	0	0	56
12786989	m	ph	JP	2016	1	0	0	0	0	0	0	0	0	0	0	0	50
12787114	f	md	IT	2016	1	0	0	0	0	0	0	0	0	0	0	0	82.8
12788041	f	md	IT	2016	1	0	0	0	0	0	0	0	0	0	0	0	80
12788242	f	md	JP	2016	1	0	0	0	0	0	0	0	0	0	0	0	79
12790160	f	md	JP	2016	0	1	0	0	1	0	0	0	0	0	0	0	55
12790742	m	md	DE	2016	0	1	0	0	0	1	0	0	0	0	0	0	58
12796485	m	ot	KR	2016	0	1	0	0	1	0	0	0	0	0	0	0	61
12796604	m	md	KR	2016	0	0	0	0	0	0	0	0	0	0	0	0	67
12796821	m	ot	US	2016	0	1	0	0	1	0	0	0	0	0	0	0	77
12797584	m	md	JP	2016	0	1	0	0	1	0	0	0	0	0	0	0	57
12798000	m	md	JP	2016	0	1	0	0	1	0	0	0	0	0	0	0	65
12798014	m	ph	JP	2016	0	1	0	0	1	0	0	0	0	0	0	0	76
12798088	f	md	JP	2016	0	0	0	0	0	0	0	0	0	0	0	0	67
12798312	m	md	JP	2016	0	1	0	0	1	0	0	0	0	0	0	0	80
12799886	m	ot	JP	2016	0	1	0	0	1	0	0	0	0	0	0	0	52
12799960	m	ot	ES	2016	0	1	0	0	1	0	0	0	0	0	0	0	56
12799998	m	ot	FR	2016	0	1	0	0	1	0	0	0	0	0	0	0	58
12800095	f	cn	US	2016	0	0	0	0	0	0	0	0	0	0	0	0	
12800230	f	md	FR	2016	1	0	0	0	0	0	0	0	0	0	0	0	
12800290	m	ot	BR	2016	0	1	0	0	1	0	0	0	0	0	0	0	67
12800436	m	md	JP	2016	0	1	0	0	1	0	0	0	0	0	0	0	70
12802463	f	cn	GB	2016	1	0	0	0	0	0	0	0	0	0	0	0	
12802552	m	ot	IT	2016	0	1	0	0	1	0	0	0	0	0	0	0	78

12802553	m	cn	IT	2016	0	1	0	0	1	0	0	0	0	0	0	0	76
12802561	m	cn	IT	2016	0	1	0	0	1	0	0	0	0	0	0	0	53
12802579	f	cn	IT	2016	0	1	0	0	1	0	0	0	0	0	0	0	51
12802602	m	cn	GB	2016	1	0	0	0	0	0	0	0	0	0	0	0	
12803405	m	md	GP	2016	0	1	0	0	1	0	0	0	0	0	0	0	
12803545	m	ph	US	2016	1	0	0	0	0	0	0	0	0	0	0	0	
12803851	f	md	DE	2016	0	0	0	0	0	0	0	0	0	0	0	0	51
12804041	m	md	ES	2016	0	0	0	0	0	0	0	0	0	0	0	0	55
12804051	f	md	DE	2016	1	0	0	0	0	0	0	0	0	0	0	0	82
12804150	m	md	GR	2016	1	0	0	0	0	0	0	0	0	0	0	0	61
12804270	m	cn	US	2016	1	0	0	0	0	0	0	0	0	0	0	0	76
12804308	m	md	US	2016	0	0	0	0	0	0	0	0	0	0	0	0	77
12804551	f	cn	US	2016	1	0	0	0	0	0	0	0	0	0	0	0	65
12804612	m	md	BR	2016	1	0	0	0	0	0	0	0	0	0	0	0	80.7
12804614	m	cn	US	2016	1	0	0	0	0	0	0	0	0	0	0	0	67
12804637	f	md	JP	2016	1	0	0	0	0	0	0	0	0	0	0	1	69
12804965	m	md	JP	2016	0	1	0	0	1	0	0	0	0	0	0	0	66
12805205	m	md	JP	2016	0	1	0	0	1	0	0	0	0	0	0	0	79
12805580	m	ot	US	2016	0	1	0	1	0	0	0	0	0	0	0	0	73
12805955	m	ot	US	2016	0	0	0	0	0	0	0	0	0	0	0	0	
12806165	f	ot	US	2016	0	1	1	0	0	0	0	0	0	0	0	0	58
12807286	m	md	IN	2016	0	0	0	0	0	0	0	0	0	0	0	0	61
12807491	m	md	KR	2016	1	0	0	0	0	0	0	0	0	0	0	0	73
12807512	m	md	JP	2016	1	0	0	0	0	0	0	0	0	0	0	0	68
12807649	f	md	DE	2016	0	1	0	0	1	0	0	0	0	0	0	0	
12807701	m	md	US	2016	0	0	0	0	0	0	0	0	0	0	0	0	66
12808244	m	cn	JP	2016	0	1	0	0	1	0	0	0	0	0	0	0	80
12808402	m	cn	US	2016	1	0	0	0	0	0	0	0	0	0	0	0	70
12808709	Not	md	JP	2016	0	0	0	0	0	0	0	0	0	0	0	0	
12808846	m	md	JP	2016	0	1	0	0	1	0	0	0	0	0	0	0	63
12809045	m	md	US	2016	0	0	0	0	0	0	0	0	0	0	0	0	74
12809222	f	cn	CN	2016	1	0	0	0	0	0	0	0	0	0	0	0	60
12809327	f	ot	US	2016	0	1	0	0	1	0	0	0	0	0	0	0	90
12809360	f	ot	GB	2016	0	1	0	0	1	0	0	0	0	0	0	0	63

12809558	m	md	US	2016	0	0	0	0	0	0	0	0	0	0	0	0	78
12809800	f	cn	JP	2016	0	0	0	0	0	0	1	0	1	0	0	0	59
12809976	f	md	KR	2016	1	0	0	0	0	0	0	0	0	0	0	0	74
12810356	m	md	JP	2016	0	0	0	0	0	0	0	0	0	0	0	0	72
12810447	m	cn	JP	2016	0	0	0	0	0	0	0	0	0	0	0	0	69
12810758	m	md	CN	2016	0	1	0	0	0	1	0	0	0	0	0	0	62
12811005	m	md	SK	2016	0	0	0	0	0	0	0	0	0	0	0	0	61
12812470	Not	md	JP	2016	1	0	0	0	0	0	0	0	0	0	0	0	
12812624	m	md	JP	2016	0	1	0	0	1	0	0	0	0	0	0	0	66
12816938	m	md	JP	2016	1	0	0	0	0	0	0	0	0	0	0	0	75
12816977	f	ph	US	2016	0	1	0	0	1	0	0	0	0	0	0	0	51
12818075	f	md	JP	2016	1	0	0	0	0	0	0	0	0	0	0	0	69
12818080	f	md	TW	2016	1	0	0	0	0	0	0	0	0	0	0	0	50
12818181	m	cn	US	2016	1	0	0	0	0	0	0	0	0	0	0	0	83
12818333	m	md	JP	2016	0	1	0	0	1	0	0	0	0	0	0	0	77
12818522	f	cn	JP	2016	0	1	0	0	1	0	0	0	0	0	0	0	79
12818529	m	md	US	2016	0	1	0	0	1	0	0	0	0	0	0	0	
12818648	m	md	US	2016	0	1	0	0	0	1	0	0	0	0	0	0	60
12819811	f	ot	IT	2016	0	1	0	0	1	0	0	0	0	0	0	0	
12820332	m	md	US	2016	0	0	0	0	0	0	0	0	0	0	0	0	81
12820899	m	md	PE	2016	0	1	1	0	0	0	0	0	0	0	0	1	
12820931	f	md	IN	2016	0	0	0	0	0	0	0	0	0	0	0	0	
12820969	Not	md	JP	2016	0	1	0	0	1	0	0	0	0	0	0	0	
12821096	m	cn	JP	2016	0	1	0	0	1	0	0	0	0	0	0	1	65
12821310	f	md	JP	2016	0	1	0	0	1	0	0	0	0	0	0	0	80
12821484	m	ot	IT	2016	0	1	0	0	1	0	0	0	0	0	0	0	73
12821486	Not	cn	IT	2016	0	1	0	0	1	0	0	0	0	0	0	0	59
12821517	m	cn	US	2016	0	1	0	0	1	0	0	0	0	0	0	0	
12821912	f	md	US	2016	0	1	0	0	1	0	0	0	0	0	0	0	
12821979	f	md	US	2016	0	1	0	0	1	0	0	0	0	0	0	0	
12822000	m	md	US	2016	0	1	0	0	1	0	0	0	0	0	0	1	
12822403	f	md	JP	2016	0	1	0	0	0	1	0	0	0	0	0	0	66
12822999	m	ot	US	2016	0	0	0	0	0	0	0	0	0	0	0	0	64
12823230	Not	ot	US	2016	0	1	0	0	1	0	0	0	0	0	0	0	

12823245	m	cn	JP	2016	0	1	0	0	1	0	0	0	0	0	0	0	72
12823737	m	cn	BE	2016	0	0	0	0	0	0	0	0	0	0	0	0	66
12823924	m	md	JP	2016	0	0	0	0	0	0	0	0	0	0	0	0	72
12824175	m	ot	IT	2016	0	1	0	0	1	0	0	0	0	0	0	0	83
12824347	m	ot	JP	2016	0	1	0	0	1	0	0	0	0	0	0	0	65
12825026	f	ot	IT	2016	0	1	0	0	1	0	0	0	0	0	0	0	76
12825148	f	cn	US	2016	0	0	0	0	0	0	0	0	0	0	0	0	
12825151	f	md	BR	2016	1	0	0	0	0	0	0	0	0	0	0	0	77
12825152	m	cn	US	2016	1	0	0	0	0	0	0	0	0	0	0	0	76
12825279	m	md	KR	2016	1	0	0	0	0	0	0	0	0	0	0	0	80
12826656	f	md	PL	2016	1	0	0	0	0	0	0	0	0	0	0	0	66
12827046	Not	md	MX	2016	1	0	0	0	0	0	0	0	0	0	0	0	
12827555	m	cn	FR	2016	0	0	0	0	0	0	0	0	0	0	0	0	
12827728	f	cn	US	2016	1	0	0	0	0	0	0	0	0	0	0	0	77
12827953	f	md	GB	2016	0	0	0	0	0	0	0	0	0	0	0	0	76
12828147	f	md	DE	2016	1	0	0	0	0	0	0	0	0	0	0	0	66
12828248	m	ot	BR	2016	0	1	0	0	1	0	0	0	0	0	0	0	62
12828250	m	ot	JP	2016	0	1	0	0	1	0	0	0	0	0	0	0	56
12828315	Not	md	JP	2016	0	1	0	0	1	0	0	0	0	0	0	1	60
12828347	m	md	US	2016	0	1	0	0	1	0	0	0	0	0	0	0	
12828367	f	md	JP	2016	1	0	0	0	0	0	0	0	0	0	0	0	84
12828406	f	cn	US	2016	1	0	0	0	0	0	0	0	0	0	0	0	58
12830157	f	cn	US	2016	1	0	0	0	0	0	0	0	0	0	0	0	
12830623	Not	md	JP	2016	0	0	0	0	0	0	0	0	0	0	0	0	
12830630	f	ot	IT	2016	0	1	0	0	1	0	0	0	0	0	0	0	64
12830632	m	ot	IT	2016	0	1	0	0	1	0	0	0	0	0	0	0	71
12830891	f	cn	US	2016	1	0	0	0	0	0	0	0	0	0	0	0	73
12831172	f	md	CH	2016	1	0	0	0	0	0	0	0	0	0	0	0	
12831339	f	cn	US	2016	1	0	0	0	0	0	0	0	0	0	0	0	
12831353	m	cn	US	2016	1	0	0	0	0	0	0	0	0	0	0	0	
12831488	m	md	BE	2016	0	0	0	0	0	0	0	0	0	0	0	0	67
12831630	f	cn	CN	2016	0	0	0	0	0	0	0	0	0	0	0	0	
12831779	m	ot	IT	2016	0	1	0	0	1	0	0	0	0	0	0	0	62
12831797	f	md	SE	2016	0	1	0	0	1	0	0	0	0	0	0	0	72

12832081	f	md	JP	2016	0	1	0	0	1	0	0	0	0	0	0	0	65
12832392	m	ot	IT	2016	0	1	0	0	1	0	0	0	0	0	0	1	67
12833461	Not	md	JP	2016	0	1	0	0	1	0	0	0	0	0	0	1	60
12834075	Not	ph	US	2016	0	1	0	0	1	0	0	0	0	0	0	0	
12834254	f	md	CN	2016	1	0	0	0	0	0	0	0	0	0	0	0	60
12834478	m	md	PL	2016	0	0	0	0	0	0	0	0	0	0	0	0	62
12834586	f	md	GB	2016	0	0	0	0	0	0	0	0	0	0	0	0	31
12834948	m	md	KR	2016	1	0	0	0	0	0	0	0	0	0	0	0	53
12835364	m	ot	KR	2016	0	1	0	0	1	0	0	0	0	0	0	0	70
12835387	m	md	JP	2016	0	1	0	0	1	0	0	0	0	0	0	1	72
12835394	m	ot	JP	2016	0	1	0	0	1	0	0	0	0	0	0	0	69
12835395	f	ot	US	2016	0	1	0	0	1	0	0	0	0	0	0	0	68
12835401	m	cn	JP	2016	0	1	0	0	1	0	0	0	0	0	0	1	63
12835402	f	cn	JP	2016	0	1	0	0	1	0	0	0	0	0	0	0	46
12835528	f	cn	US	2016	0	1	0	0	1	0	0	0	0	0	0	0	
12835561	m	md	US	2016	0	1	0	0	0	1	0	0	0	0	0	0	67
12835581	f	md	IT	2016	0	1	0	0	1	0	0	0	0	0	0	0	53
12835910	f	md	JP	2016	1	0	0	0	0	0	0	0	0	0	0	0	68
12836445	m	md	KR	2016	1	0	0	0	0	0	0	0	0	0	0	0	72
12836647	m	md	IT	2016	1	0	0	0	0	0	0	0	0	0	0	0	73
12836671	f	cn	US	2016	0	0	0	0	0	0	0	0	0	0	0	0	74
12837138	m	cn	KR	2016	1	0	0	0	0	0	0	0	0	0	0	0	63
12837326	m	md	DE	2016	1	0	0	0	0	0	0	0	0	0	0	0	74
12837403	m	md	US	2016	1	0	0	0	0	0	0	0	0	0	0	0	83
12837416	f	md	DE	2016	1	0	0	0	0	0	0	0	0	0	0	0	53
12837440	f	cn	US	2016	1	0	0	0	0	0	0	0	0	0	0	0	69
12837453	f	md	JP	2016	1	0	0	0	0	0	0	0	0	0	0	1	64
12837702	Not	md	AU	2016	0	1	0	0	1	0	0	0	0	0	0	0	64
12837782	m	cn	JP	2016	0	1	0	0	1	0	0	0	0	0	0	0	62
12837874	m	ph	US	2016	0	1	0	0	1	0	0	0	0	0	0	0	83
12837952	f	md	FR	2016	1	0	0	0	0	0	0	0	0	0	0	0	
12838184	f	md	US	2016	0	0	0	0	0	0	0	0	0	0	0	0	58
12838529	m	cn	US	2016	0	1	0	0	1	0	0	0	0	0	0	0	
12838600	f	cn	JP	2016	0	1	0	0	1	0	0	0	0	0	0	1	81

12839029	m	ot	GR	2016	0	1	0	0	1	0	0	0	0	0	0	0	79
12839599	f	md	JP	2016	0	0	0	0	0	0	0	0	0	0	0	0	76.8
12839799	m	md	JP	2016	0	1	0	0	1	0	0	0	0	0	0	0	76
12839890	m	md	IN	2016	0	0	0	0	0	0	0	0	0	0	0	0	71
12840233	m	md	DE	2016	0	1	0	0	1	0	0	0	0	0	0	0	
12840333	m	cn	FR	2016	0	0	0	0	0	0	0	0	0	0	0	0	61
12840337	m	md	JP	2016	0	0	0	0	0	0	0	0	0	0	0	0	54
12840444	m	cn	IT	2016	0	1	0	0	1	0	0	0	0	0	0	0	66
12840445	m	cn	JP	2016	0	1	0	0	1	0	0	0	0	0	0	0	73
12840692	f	ot	IN	2016	0	0	0	0	0	0	0	0	0	0	0	0	68
12840835	m	md	JP	2016	0	0	0	0	0	0	0	0	0	0	0	0	71
12841142	Not	ot	JP	2016	0	0	0	0	0	0	0	0	0	0	0	0	
12841223	f	md	US	2016	0	0	0	0	0	0	0	0	0	0	0	0	60
12841242	m	ot	US	2016	1	0	0	0	0	0	0	0	0	0	0	0	
12841662	m	md	AU	2016	0	1	0	0	1	0	0	0	0	0	0	0	56
12841838	m	md	KR	2016	1	0	0	0	0	0	0	0	0	0	0	0	58
12841875	f	md	NO	2016	0	0	0	0	0	0	0	0	0	0	0	0	70
12841974	f	md	HU	2016	1	0	0	0	0	0	0	0	0	0	0	0	51
12842157	Not	md	AU	2016	0	1	0	0	1	0	0	0	0	0	0	0	70
12842269	Not	cn	IT	2016	0	1	0	0	1	0	0	0	0	0	0	0	
12842448	f	ot	IT	2016	0	1	0	0	1	0	0	0	0	0	0	0	56
12842452	f	md	FR	2016	1	0	0	0	0	0	0	0	0	0	0	0	77.1
12842543	f	ph	US	2016	0	1	0	0	1	0	0	0	0	0	0	0	76
12842557	m	cn	GB	2016	0	1	0	0	1	0	0	0	0	0	0	0	
12842567	m	ot	KR	2016	0	1	0	0	1	0	0	0	0	0	0	0	66
12842632	m	ot	IT	2016	0	1	0	0	1	0	0	0	0	0	0	0	48
12842710	m	md	US	2016	0	0	0	0	0	0	0	0	0	0	0	0	
12843004	f	md	KR	2016	1	0	0	0	0	0	0	0	0	0	0	0	81
12843131	m	md	LB	2016	0	0	0	0	0	0	0	0	0	0	0	0	48
12843647	f	md	JP	2016	1	0	0	0	0	0	0	0	0	0	0	0	76
12843897	f	ph	JP	2016	0	1	0	0	1	0	0	0	0	0	0	0	54
12844157	m	md	DK	2016	1	0	0	0	0	0	0	0	0	0	0	0	92
12844968	f	md	US	2016	1	0	0	0	0	0	0	0	0	0	0	0	62
12845038	f	md	KR	2016	1	0	0	0	0	0	0	0	0	0	0	0	54

12845092	f	ot	US	2016	0	0	0	0	0	0	0	0	0	0	0	0	67
12845111	Not	md	JP	2016	0	1	0	0	1	0	0	0	0	0	0	0	
12845374	m	md	US	2016	0	0	0	0	0	0	0	0	0	0	0	0	63
12845733	m	md	KR	2016	1	0	0	0	0	0	0	0	0	0	0	0	61
12845920	f	cn	JP	2016	0	1	0	0	1	0	0	0	0	0	0	1	35
12846264	m	cn	IT	2016	0	1	0	0	1	0	0	0	0	0	0	0	44
12846360	Not	md	IT	2016	0	1	0	0	1	0	0	0	0	0	0	0	
12846406	f	ot	IT	2016	0	1	0	0	1	0	0	0	0	0	0	0	54
12846412	f	md	JP	2016	1	0	0	0	0	0	0	0	0	0	0	0	77
12846551	m	cn	JP	2016	0	1	0	0	1	0	0	0	0	0	0	1	62
12846563	Not	ph	US	2016	0	1	0	0	1	0	0	0	0	0	0	0	
12847473	m	md	BE	2016	0	1	0	0	1	0	0	0	0	0	0	0	52
12847474	f	cn	IT	2016	0	1	0	0	1	0	0	0	0	0	0	0	83
12847668	f	md	JP	2016	1	0	0	0	0	0	0	0	0	0	0	0	82
12848879	m	md	IT	2016	1	0	0	0	0	0	0	0	0	0	0	0	66
12848965	m	md	CN	2016	1	0	0	0	0	0	0	0	0	0	0	0	65
12849107	Not	md	JP	2016	0	1	0	0	1	0	0	0	0	0	0	0	
12849113	m	cn	IT	2016	0	1	0	0	1	0	0	0	0	0	0	0	67
12849114	m	md	RO	2016	1	0	0	0	0	0	0	0	0	0	0	0	62
12849134	f	ot	US	2016	0	0	0	0	0	0	0	0	0	0	0	0	79
12849291	m	md	JP	2016	0	1	0	0	1	0	0	0	0	0	0	0	53
12850070	f	md	US	2016	0	0	0	0	0	0	0	0	0	0	0	0	64
12850209	m	ph	US	2016	1	0	0	0	0	0	0	0	0	0	0	0	66
12850254	m	md	SG	2016	1	0	0	0	0	0	0	0	0	0	0	0	61
12850480	f	md	JP	2016	1	0	0	0	0	0	0	0	0	0	0	0	56
12850504	f	ot	NL	2016	0	1	0	0	1	0	0	0	0	0	0	0	58
12850567	m	md	US	2016	1	0	0	0	0	0	0	0	0	0	0	0	84
12850619	m	ot	IT	2016	0	1	0	0	1	0	0	0	0	0	0	0	62
12850673	m	md	KR	2016	1	0	0	0	0	0	0	0	0	0	0	0	55
12850853	f	cn	US	2016	1	0	0	0	0	0	0	0	0	0	0	0	67
12851123	m	md	IT	2016	0	1	0	0	1	0	0	0	0	0	0	0	
12851226	m	md	JP	2016	0	1	0	0	1	0	0	0	0	0	0	0	66
12851245	m	md	JP	2016	0	1	0	0	1	0	0	0	0	0	0	0	54
12851279	Not	md	CH	2016	0	0	0	0	0	0	0	0	0	0	0	0	

12851287	Not	md	CH	2016	0	0	0	0	0	0	0	0	0	0	0	0	
12851318	f	cn	US	2016	1	0	0	0	0	0	0	0	0	0	0	0	53
12851673	f	ph	US	2016	1	0	0	0	0	0	0	0	0	0	0	0	60
12851678	m	md	DE	2016	0	1	0	0	1	0	0	0	0	0	0	1	67
12851695	m	cn	DE	2016	0	1	0	0	1	0	0	0	0	0	0	0	
12852383	f	cn	US	2016	1	0	0	0	0	0	0	0	0	0	0	0	68
12852440	m	cn	IT	2016	0	1	0	0	1	0	0	0	0	0	0	0	63
12852549	f	cn	GB	2016	1	0	0	0	0	0	0	0	0	0	0	0	
12852717	m	cn	JP	2016	0	1	0	0	1	0	0	0	0	0	0	0	
12852841	f	md	CA	2016	0	1	0	0	1	0	0	0	0	0	0	0	35
12852897	f	md	IE	2016	0	1	0	0	1	0	0	0	0	0	0	0	79
12853260	m	ot	IL	2016	0	0	0	0	0	0	0	0	0	0	0	0	78
12853471	f	cn	IT	2016	0	1	0	0	1	0	0	0	0	0	0	0	75
12853607	Not	md	HK	2016	0	1	0	0	1	0	0	0	0	0	0	0	
12853948	m	md	JP	2016	0	1	0	0	1	0	0	0	0	0	0	0	71
12854028	m	ot	JP	2016	0	1	0	0	0	1	0	0	0	0	0	0	70
12854395	f	ph	US	2016	0	0	0	0	0	0	0	0	0	0	0	0	
12854798	f	ot	US	2016	0	1	0	0	1	0	0	0	0	0	0	0	
12854878	f	md	TW	2016	1	0	0	0	0	0	0	0	0	0	0	0	41
12854961	Not	md	US	2016	1	0	0	0	0	0	0	0	0	0	0	0	
12855383	f	md	DE	2016	1	0	0	0	0	0	0	0	0	0	0	0	68
12855417	f	md	KR	2016	1	0	0	0	0	0	0	0	0	0	0	0	73
12855463	m	cn	US	2016	0	0	0	0	0	0	0	0	0	0	0	0	76
12855582	f	cn	US	2016	1	0	0	0	0	0	0	0	0	0	0	0	67
12855592	Not	md	IT	2016	0	1	0	0	1	0	0	0	0	0	0	0	
12855626	f	cn	US	2016	1	0	0	0	0	0	0	0	0	0	0	0	77
12855705	f	ot	GB	2016	0	1	0	0	1	0	0	0	0	0	0	0	70
12855892	m	cn	US	2016	0	1	0	0	1	0	0	0	0	0	0	0	
12855948	m	ot	DE	2016	0	1	0	0	1	0	0	0	0	0	0	0	63
12856411	Not	cn	IL	2016	0	1	1	0	0	0	0	0	0	0	0	0	
12856553	m	ot	IT	2016	0	1	0	0	1	0	0	0	0	0	0	0	54
12856568	m	md	AU	2016	0	1	0	0	1	0	0	0	0	0	0	0	
12856594	f	md	FR	2016	1	0	0	0	0	0	0	0	0	0	0	0	
12857124	m	cn	JP	2016	0	1	0	0	1	0	0	0	0	0	0	0	69

12857405	m	ot	DE	2016	0	0	0	0	0	0	0	0	0	0	0	0	67
12857666	m	cn	IL	2016	1	0	0	0	0	0	0	0	0	0	0	0	
12858230	f	cn	US	2016	0	1	0	0	1	0	0	0	0	0	0	0	
12858461	f	ot	US	2016	0	1	0	0	1	0	0	0	0	0	0	0	
12858571	m	cn	US	2016	1	0	0	0	0	0	0	0	0	0	0	0	82
12858597	m	cn	US	2016	1	0	0	0	0	0	0	0	0	0	0	0	65
12859108	m	cn	US	2016	1	0	0	0	0	0	0	0	0	0	0	0	60
12859247	f	cn	JP	2016	0	1	0	0	1	0	0	0	0	0	0	1	55
12859276	f	md	KR	2016	1	0	0	0	0	0	0	0	0	0	0	0	70
12859286	m	md	KR	2016	1	0	0	0	0	0	0	0	0	0	0	0	72
12859287	m	md	TR	2016	0	0	0	0	0	0	0	0	0	0	0	0	51
12859386	Not	md	AU	2016	0	1	0	0	1	0	0	0	0	0	0	0	
12859394	m	cn	IT	2016	0	1	0	0	1	0	0	0	0	0	0	0	72
12859424	f	md	KR	2016	1	0	0	0	0	0	0	0	0	0	0	0	71
12859425	f	cn	US	2016	1	0	0	0	0	0	0	0	0	0	0	0	70
12859676	m	md	DE	2016	1	0	0	0	0	0	0	0	0	0	0	0	58
12860054	m	cn	GB	2016	1	0	0	0	0	0	0	0	0	0	0	0	
12860401	m	cn	IT	2016	0	1	0	0	1	0	0	0	0	0	0	0	74
12860502	f	md	BE	2016	0	1	0	0	0	1	0	0	0	0	0	0	79
12860797	m	cn	US	2016	0	1	1	0	0	0	0	0	0	0	0	0	58
12861316	m	ot	IT	2016	0	0	0	0	0	0	0	0	0	0	0	0	40
12861349	m	md	IT	2016	0	1	0	0	1	0	0	0	0	0	0	0	
12861648	m	md	US	2016	0	0	0	0	0	0	0	0	0	0	0	0	74
12861650	m	md	AU	2016	0	1	0	0	1	0	0	0	0	0	0	0	78
12861661	m	ot	IT	2016	0	1	0	0	1	0	0	0	0	0	0	0	66
12861776	Not	md	AT	2016	0	0	0	0	0	0	0	0	0	0	0	0	
12861822	Not	md	DE	2016	0	0	0	0	0	0	0	0	0	0	0	0	
12861833	Not	md	ES	2016	0	0	0	0	0	0	0	0	0	0	0	0	
12862011	m	md	DE	2016	0	1	0	0	0	1	0	0	0	0	0	0	51
12862522	f	ot	DE	2016	0	0	0	0	0	0	0	0	0	0	0	0	79
12862572	m	ot	CZ	2016	0	1	0	0	1	0	0	0	0	0	0	0	80
12863645	m	md	JP	2016	0	1	0	0	1	0	0	0	0	0	0	0	69
12864463	f	ot	PT	2016	0	1	0	0	1	0	0	0	0	0	0	0	76
12864492	f	md	FR	2016	0	0	0	0	0	0	0	0	0	0	0	0	

12864649	m	ot	US	2016	0	1	0	0	1	0	0	0	0	0	0	0		
12864652	m	ot	IT	2016	0	1	0	0	1	0	0	0	0	0	0	0	76
12864658	m	cn	JP	2016	0	1	0	0	1	0	0	0	0	0	0	0	74
12864687	m	cn	US	2016	0	1	0	0	1	0	0	0	0	0	0	0	83
12864773	m	cn	JP	2016	0	1	0	0	1	0	0	0	0	0	0	0	
12865031	m	cn	JP	2016	0	1	0	0	1	0	0	0	0	0	0	0	55
12865038	Not	ot	US	2016	0	1	0	0	1	0	0	0	0	0	0	0	
12865077	Not	ot	CN	2016	0	0	0	0	0	0	0	0	0	0	0	0	
12865519	m	cn	JP	2016	0	1	0	0	1	0	0	0	0	0	0	0	63
12865677	m	md	DE	2016	0	0	0	0	0	0	0	0	0	0	0	0	
12867387	m	cn	US	2016	1	0	0	0	0	0	0	0	0	0	0	0	81
12868139	f	ot	US	2016	0	0	0	0	0	0	0	0	0	0	0	0	75
12868774	Not	md	JP	2016	0	0	0	0	0	0	0	0	0	0	0	0	
12868873	m	cn	Not re	2016	0	1	0	0	1	0	0	0	0	0	0	0	
12870118	m	ph	JP	2016	0	0	0	0	0	0	0	0	0	0	0	0	67
12870166	f	ph	AU	2016	0	0	0	0	0	0	0	0	0	0	0	0	84
12870240	f	cn	AR	2016	0	0	0	0	0	0	0	0	0	0	0	0	72
12870248	m	ot	US	2016	0	0	0	0	0	0	0	0	0	0	0	0	75
12870388	f	cn	GB	2016	1	0	0	0	0	0	0	0	0	0	0	0	
12871230	m	cn	DE	2016	0	1	0	0	1	0	0	0	0	0	0	0	72
12871276	f	md	US	2016	0	1	0	0	1	0	0	0	0	0	0	0	60
12871807	m	md	DE	2016	0	1	0	0	1	0	0	0	0	0	0	0	51
12872021	m	md	JP	2016	1	0	0	0	0	0	0	0	0	0	0	0	65.8
12872060	f	md	CA	2016	0	0	0	0	0	0	0	0	0	0	0	0	
12872552	f	ph	FR	2016	0	1	0	0	1	0	0	0	0	0	0	0	67
12872793	m	md	DE	2016	0	1	0	0	1	0	0	0	0	0	0	0	
12872960	f	cn	JP	2016	0	1	0	0	1	0	0	0	0	0	0	0	58
12873347	m	ot	KR	2016	0	1	0	0	1	0	0	0	0	0	0	0	73
12873486	f	md	JP	2016	1	0	0	0	0	0	0	0	0	0	0	0	83
12873998	Not	ot	CN	2016	0	0	0	0	0	0	0	0	0	0	0	0	
12874212	m	md	IT	2016	1	0	0	0	0	0	0	0	0	0	0	0	62.8
12874483	m	ot	FR	2016	0	1	0	0	1	0	0	0	0	0	0	0	68
12875092	Not	md	JP	2016	0	0	0	0	0	0	0	0	0	0	0	0	
12875098	Not	md	JP	2016	0	0	0	0	0	0	0	0	0	0	0	0	

12875298	f	ot	TR	2016	0	1	0	0	1	0	0	0	0	0	0	0	
12875333	Not	md	JP	2016	0	1	0	0	1	0	0	0	0	0	0	0	
12875444	f	cn	AR	2016	0	0	0	0	0	0	0	0	0	0	0	0	66
12875464	f	md	US	2016	0	1	0	0	0	1	0	0	0	0	0	0	59
12875478	m	cn	GB	2016	0	1	1	0	0	0	0	0	0	0	0	0	67
12875523	f	cn	JP	2016	0	1	0	0	1	0	0	0	0	0	0	0	74
12875598	m	md	US	2016	0	1	1	0	0	0	0	0	0	0	0	0	76
12876175	Not	md	JP	2016	0	0	0	0	0	0	0	0	0	0	0	0	
12876637	m	cn	GB	2016	1	0	0	0	0	0	0	0	0	0	0	0	
12877176	f	ph	JP	2016	1	0	0	0	0	0	0	0	0	0	0	0	86
12877731	m	ph	JP	2016	0	1	0	0	1	0	0	0	0	0	0	0	57
12877922	m	cn	US	2016	0	1	1	0	0	0	0	0	0	0	0	0	36
12878521	Not	md	JP	2016	0	0	0	0	0	0	0	0	0	0	0	0	
12878723	m	cn	US	2016	0	1	1	0	0	0	0	0	0	0	0	0	
12879116	m	md	JP	2016	0	0	0	0	0	0	0	0	0	0	0	0	64
12879219	Not	md	JP	2016	0	0	0	0	0	0	0	0	0	0	0	0	
12879408	Not	md	JP	2016	0	0	0	0	0	0	0	0	0	0	0	0	
12879420	f	md	JP	2016	0	0	0	0	0	0	1	0	1	0	0	0	81
12879459	Not	md	JP	2016	0	0	0	0	0	0	0	0	0	0	0	0	
12880353	f	cn	US	2016	1	0	0	0	0	0	0	0	0	0	0	0	54
12880565	f	cn	TH	2016	0	0	0	0	0	0	0	0	0	0	0	0	43
12880822	f	cn	CA	2016	0	1	0	0	1	0	0	0	0	0	0	0	69
12882010	m	cn	JP	2016	0	1	0	0	1	0	0	0	0	0	0	0	72
12882119	m	cn	GB	2016	1	0	0	0	0	0	0	0	0	0	0	0	
12882235	f	cn	JP	2016	0	1	0	0	1	0	0	0	0	0	0	0	79
12882334	m	cn	US	2016	0	0	0	0	0	0	0	0	0	0	0	0	
12882604	Not	ph	AU	2016	0	1	0	0	1	0	0	0	0	0	0	0	75
12882606	Not	ph	AU	2016	0	1	0	0	1	0	0	0	0	0	0	0	66
12882770	f	cn	US	2016	0	0	0	0	0	0	0	0	0	0	0	0	
12883046	m	ot	IT	2016	0	1	0	0	1	0	0	0	0	0	0	0	68
12883081	f	ot	IT	2016	0	1	0	0	1	0	0	0	0	0	0	0	69
12883097	m	ot	IT	2016	0	1	0	0	1	0	0	0	0	0	0	0	71
12883098	f	ot	IT	2016	0	1	0	0	1	0	0	0	0	0	0	0	68
12883099	f	md	IT	2016	0	1	0	0	1	0	0	0	0	0	0	0	61

12883106	m	cn	IT	2016	0	1	0	0	1	0	0	0	0	0	0	0	73
12883107	m	md	JP	2016	0	1	0	0	1	0	0	0	0	0	0	1	67
12883242	m	md	US	2016	0	1	0	0	0	1	0	0	0	0	0	0	61
12883310	m	cn	IT	2016	0	1	0	0	1	0	0	0	0	0	0	0	61
12883415	m	ot	US	2016	0	1	0	0	0	1	0	0	0	0	0	0	74
12883556	f	md	KR	2016	1	0	0	0	0	0	0	0	0	0	0	0	49
12883570	m	md	JP	2016	1	0	0	0	0	0	0	0	0	0	0	0	80
12883791	m	ot	CA	2016	0	1	0	0	1	0	0	0	0	0	0	0	
12883946	f	cn	US	2016	0	1	0	0	1	0	0	0	0	0	0	0	75
12884438	f	cn	JP	2016	0	1	0	0	1	0	0	0	0	0	0	0	
12884460	f	cn	GB	2016	1	0	0	0	0	0	0	0	0	0	0	0	89
12884600	Not	md	JP	2016	0	0	0	0	0	0	0	0	0	0	0	0	
12884645	Not	md	JP	2016	0	0	0	0	0	0	0	0	0	0	0	0	
12885275	f	md	TH	2016	0	0	0	0	0	0	0	0	0	0	0	0	46
12885663	Not	md	JP	2016	0	0	0	0	0	0	0	0	0	0	0	0	
12885814	f	ot	IT	2016	0	1	0	0	1	0	0	0	0	0	0	0	73
12885820	f	md	SA	2016	0	1	0	0	1	0	0	0	0	0	0	0	
12886181	m	ot	IT	2016	0	1	0	0	1	0	0	0	0	0	0	0	78
12886189	m	cn	JP	2016	0	1	0	0	1	0	0	0	0	0	0	0	57
12886266	f	md	US	2016	0	1	0	0	1	0	0	0	0	0	0	1	
12886438	m	Not repo	US	2016	1	0	0	0	0	0	0	0	0	0	0	0	64.3
12886583	m	cn	JP	2016	0	1	0	0	1	0	0	0	0	0	0	0	76
12886590	f	cn	IT	2016	0	1	0	0	1	0	0	0	0	0	0	0	63
12887159	f	ot	US	2016	0	1	0	0	1	0	0	0	0	0	0	0	
12887164	m	cn	US	2016	0	1	0	0	1	0	0	0	0	0	0	0	
12887171	f	ot	US	2016	0	1	0	0	1	0	0	0	0	0	0	0	
12887680	Not	md	JP	2016	0	0	0	0	0	0	0	0	0	0	0	0	
12887701	Not	md	JP	2016	0	0	0	0	0	0	0	0	0	0	0	0	
12887782	Not	md	JP	2016	0	0	0	0	0	0	0	0	0	0	0	0	
12887806	Not	md	JP	2016	0	0	0	0	0	0	0	0	0	0	0	0	
12887837	m	md	JP	2016	0	1	0	0	1	0	0	0	0	0	0	0	73
12887944	m	md	FR	2016	0	1	0	0	0	1	0	0	0	0	0	0	63
12887951	Not	ph	US	2016	0	1	0	0	1	0	0	0	0	0	0	0	
12888023	f	md	AU	2016	0	1	0	0	0	1	0	0	0	0	0	0	39

12888057	Not	md	JP	2016	0	0	0	0	0	0	0	0	0	0	0	0	
12888117	m	ot	US	2016	0	1	0	0	1	0	0	0	0	0	0	0	
12888471	m	cn	GB	2016	1	0	0	0	0	0	0	0	0	0	0	0	56
12888606	f	cn	JP	2016	0	1	0	0	1	0	0	0	0	0	0	0	74
12888731	m	md	JP	2016	0	0	0	0	0	0	0	0	0	0	0	0	30
12888733	m	md	JP	2016	0	0	0	0	0	0	0	0	0	0	0	0	
12888823	f	cn	US	2016	1	0	0	0	0	0	0	0	0	0	0	0	54
12888964	m	md	DK	2016	0	0	0	0	0	0	0	0	0	0	0	0	60
12888975	f	md	PL	2016	0	0	0	0	0	0	0	0	0	0	0	0	63
12888984	m	md	US	2016	0	1	1	0	0	0	0	0	0	0	0	0	83
12889046	Not	md	US	2016	1	0	0	0	0	0	0	0	0	0	0	0	
12889177	f	md	JP	2016	0	1	0	0	1	0	0	0	0	0	0	0	65
12889327	f	cn	DE	2016	0	0	0	0	0	0	0	0	0	0	0	0	
12889406	f	ot	US	2016	1	0	0	0	0	0	0	0	0	0	0	0	60.9
12889589	m	md	JP	2016	0	1	0	0	1	0	0	0	0	0	0	1	47
12889590	m	md	JP	2016	0	1	0	0	1	0	0	0	0	0	0	0	67
12889759	f	md	DE	2016	0	1	0	0	0	1	0	0	0	0	0	0	80
12890268	Not	md	JP	2016	0	0	0	0	0	0	0	0	0	0	0	0	
12890325	m	cn	JP	2016	0	1	0	0	1	0	0	0	0	0	0	0	68
12891272	Not	md	JP	2016	0	0	0	0	0	0	0	0	0	0	0	0	
12891302	m	cn	JP	2016	1	0	0	0	0	0	0	0	0	0	0	1	70
12891434	m	md	JP	2016	0	1	0	0	1	0	0	0	0	0	0	0	69
12891517	m	ot	CA	2016	0	1	0	0	1	0	0	0	0	0	0	0	77
12891628	m	md	JP	2016	0	1	0	0	1	0	0	0	0	0	0	1	71
12892046	f	md	US	2016	1	0	0	0	0	0	0	0	0	0	0	0	
12892080	m	ot	IT	2016	0	1	0	0	1	0	0	0	0	0	0	0	77
12892136	f	cn	EG	2016	0	1	1	0	0	0	0	0	0	0	0	0	69
12892334	f	md	JP	2016	0	0	0	0	0	0	0	0	0	0	0	0	
12892379	m	md	JP	2016	0	1	0	0	1	0	0	0	0	0	0	0	72
12892828	m	md	SG	2016	0	0	0	0	0	0	0	0	0	0	0	0	68
12892851	m	cn	JP	2016	0	1	0	0	1	0	0	0	0	0	0	0	68
12892854	m	ot	IT	2016	0	1	0	0	1	0	0	0	0	0	0	0	40
12892984	m	cn	GB	2016	1	0	0	0	0	0	0	0	0	0	0	0	
12893040	f	cn	IT	2016	0	1	0	0	1	0	0	0	0	0	0	0	64

12893468	Not	ph	US	2016	0	1	0	0	1	0	0	0	0	0	0	0		
12893566	m	md	JP	2016	0	1	0	0	1	0	0	0	0	0	0	0	56	
12893911	f	cn	Not re	2016	0	0	0	0	0	0	0	0	0	0	0	0		
12894150	m	cn	JP	2016	0	1	0	0	1	0	0	0	0	0	0	0	82	
12894312	f	md	JP	2016	0	1	0	0	1	0	0	0	0	0	0	0	68	
12894535	f	ph	JP	2016	1	0	0	0	0	0	0	0	0	0	0	0	90	
12894852	f	md	KR	2016	1	0	0	0	0	0	0	0	0	0	0	0	66	
12894855	m	ot	KR	2016	0	1	0	0	1	0	0	0	0	0	0	0	70	
12895191	m	cn	US	2016	0	1	0	0	1	0	0	0	0	0	0	0	50	
12895303	f	md	JP	2016	1	0	0	0	0	0	0	0	0	0	0	1	72	
12895329	f	md	JP	2016	1	0	0	0	0	0	0	0	0	0	0	0	81	
12895656	m	md	JP	2016	1	0	0	0	0	0	0	0	0	0	0	0	70	
12896524	f	md	JP	2016	0	0	0	0	0	0	0	0	0	0	0	0		
12896633	m	ot	US	2016	0	1	1	0	0	0	0	0	0	0	0	0	80	
12896717	m	cn	GB	2016	1	0	0	0	0	0	0	0	0	0	0	0	49	
12896753	f	cn	GB	2016	1	0	0	0	0	0	0	0	0	0	0	0	44	
12896890	f	ot	US	2016	0	0	0	0	0	0	0	0	0	0	0	0	40	
12896898	f	ot	US	2016	0	0	0	0	0	0	0	0	0	0	0	0	40	
12896924	f	cn	US	2016	0	0	0	0	0	0	0	0	0	0	0	0		
12897667	f	cn	GB	2016	1	0	0	0	0	0	0	0	0	0	0	0	70	
12898115	f	cn	JP	2016	0	1	1	0	0	0	0	0	0	0	0	0	68	
12898310	f	md	ES	2016	0	1	0	0	0	1	0	0	0	0	0	0	61	
12898318	m	cn	US	2016	0	0	0	0	0	0	0	0	0	0	0	0		
12898552	f	md	JP	2016	1	0	0	0	0	0	0	0	0	0	0	0	73	
12898738	f	md	US	2016	0	1	0	0	0	1	0	0	0	0	0	0	70	
12898851	m	md	JP	2016	1	0	0	0	0	0	0	0	0	0	0	1	87.5	
12898902	m	md	KR	2016	1	0	0	0	0	0	0	0	0	0	0	0	59.8	
12898978	m	md	JP	2016	0	1	0	0	1	0	0	0	0	0	0	0	70	
12899251	Not	ot	US	2016	0	1	0	0	1	0	0	0	0	0	0	0		
12899398	f	cn	GB	2016	1	0	0	0	0	0	0	0	0	0	0	0	49	
12899497	m	cn	US	2016	0	0	0	0	0	0	0	0	0	0	0	0		
12899937	f	cn	US	2016	0	0	0	0	0	0	0	0	0	0	0	0	58	
12899946	m	cn	JP	2016	0	0	0	0	0	0	0	0	0	0	0	0	39	
12900308	f	md	JP	2016	0	0	0	0	0	0	0	0	0	0	0	0	70	

12900786	f	md	JP	2016	0	0	0	0	0	0	0	0	0	0	0	0	40
12900903	f	ot	DE	2016	0	1	0	0	0	1	0	0	0	0	0	0	77
12901038	m	md	JP	2016	0	1	0	0	1	0	0	0	0	0	0	0	77
12901086	f	cn	US	2016	0	1	0	0	1	0	0	0	0	0	0	0	78
12901137	m	ot	PL	2016	0	1	0	0	1	0	0	0	0	0	0	0	66
12901442	f	ph	AU	2016	0	1	0	0	1	0	0	0	0	0	0	0	
12901466	f	ot	IT	2016	0	1	0	0	1	0	0	0	0	0	0	0	70
12901540	f	md	GB	2016	0	1	0	0	1	0	0	0	0	0	0	0	71
12901840	Not	ot	US	2016	0	0	0	0	0	0	0	0	0	0	0	0	
12902014	f	md	CN	2016	0	0	0	0	0	0	0	0	0	0	0	0	52
12903304	m	cn	JP	2016	0	1	0	0	1	0	0	0	0	0	0	0	74
12903456	Not	ph	AU	2016	0	1	0	0	1	0	0	0	0	0	0	0	57
12903461	f	md	BE	2016	0	1	0	0	1	0	0	0	0	0	0	0	80
12903464	Not	ph	AU	2016	0	1	0	0	1	0	0	0	0	0	0	0	
12903530	f	md	US	2016	0	1	0	0	1	0	0	0	0	0	0	0	60
12903659	f	md	DE	2016	0	0	0	0	0	0	0	0	0	0	0	0	35
12903927	Not	md	US	2016	1	0	0	0	0	0	0	0	0	0	0	0	
12904442	m	ot	IN	2016	0	0	0	0	0	0	0	0	0	0	0	0	53
12905338	f	cn	JP	2016	0	1	0	0	1	0	0	0	0	0	0	0	83
12905386	f	cn	IT	2016	0	1	0	0	1	0	0	0	0	0	0	0	63
12905433	m	cn	IT	2016	0	1	0	0	1	0	0	0	0	0	0	0	66
12905477	m	cn	JP	2016	0	1	0	0	1	0	0	0	0	0	0	0	63
12905478	f	cn	IT	2016	0	1	0	0	1	0	0	0	0	0	0	0	65
12905678	m	md	JP	2016	0	1	0	0	1	0	0	0	0	0	0	0	80
12905878	f	cn	US	2016	0	0	0	0	0	0	0	0	0	0	0	0	
12905882	Not	ot	JP	2016	0	0	0	0	0	0	0	0	0	0	0	0	
12905911	Not	ot	JP	2016	0	0	0	0	0	0	0	0	0	0	0	0	
12905920	Not	ot	JP	2016	0	0	0	0	0	0	0	0	0	0	0	0	
12905943	Not	ot	JP	2016	0	0	0	0	0	0	0	0	0	0	0	0	
12906277	f	ot	US	2016	0	0	0	0	0	0	0	0	0	0	0	0	
12906298	f	md	KR	2016	0	0	0	0	0	0	0	0	0	0	0	0	
12906533	m	md	JP	2016	0	1	0	0	1	0	0	0	0	0	0	0	52
12906784	m	ph	JP	2016	0	1	0	0	1	0	0	0	0	0	0	1	74
12907142	m	ph	US	2016	0	1	1	0	0	0	0	0	0	0	0	0	66

12907315	m	cn	JP	2016	0	1	0	0	1	0	0	0	0	0	0	0	72
12907458	f	cn	Not re	2016	0	0	0	0	0	0	0	0	0	0	0	0	65
12907562	m	md	JP	2016	0	1	0	0	1	0	0	0	0	0	0	0	73
12907633	f	ot	IN	2016	0	0	0	0	0	0	0	0	0	0	0	0	60
12907664	f	cn	GB	2016	1	0	0	0	0	0	0	0	0	0	0	0	70
12907671	f	cn	GB	2016	1	0	0	0	0	0	0	0	0	0	0	0	26
12908348	m	cn	JP	2016	0	1	0	0	1	0	0	0	0	0	0	0	68
12908361	m	md	JP	2016	0	1	0	0	1	0	0	0	0	0	0	0	63
12908389	f	cn	IT	2016	0	1	0	0	1	0	0	0	0	0	0	0	52
12908424	f	md	TR	2016	0	0	0	0	0	0	0	0	0	0	0	0	65
12908429	m	cn	JP	2016	0	1	0	0	1	0	0	0	0	0	0	0	74
12908452	f	md	FR	2016	1	0	0	0	0	0	0	0	0	0	0	0	49
12908532	f	ph	CA	2016	0	1	0	0	1	0	0	0	0	0	0	0	
12908644	m	cn	DE	2016	0	1	1	0	0	0	0	0	0	0	0	0	73
12908759	m	md	CN	2016	1	0	0	0	0	0	0	0	0	0	0	0	50
12908791	m	cn	US	2016	1	0	0	0	0	0	0	0	0	0	0	0	85
12908958	m	cn	JP	2016	0	1	0	0	1	0	0	0	0	0	0	0	61
12909192	Not	cn	IT	2016	0	1	0	0	1	0	0	0	0	0	0	0	71
12909283	f	ot	IT	2016	0	1	0	0	1	0	0	0	0	0	0	0	
12909374	Not	cn	IT	2016	0	1	0	0	1	0	0	0	0	0	0	0	
12909418	f	ph	AU	2016	0	1	0	0	1	0	0	0	0	0	0	0	
12909477	f	cn	JP	2016	0	1	0	0	1	0	0	0	0	0	0	1	54
12909936	m	ot	IT	2016	0	1	0	0	1	0	0	0	0	0	0	0	61
12911471	m	ph	AU	2016	0	1	0	0	1	0	0	0	0	0	0	0	
12911924	m	ot	CA	2016	0	1	0	0	1	0	0	0	0	0	0	0	66
12912266	f	md	BG	2016	1	0	0	0	0	0	0	0	0	0	0	0	
12912287	m	md	JP	2016	0	1	0	0	1	0	0	0	0	0	0	0	63
12912440	m	md	US	2016	0	1	0	0	1	0	0	0	0	0	0	0	73
12912579	m	cn	GB	2016	1	0	0	0	0	0	0	0	0	0	0	0	79
12912699	m	md	JP	2016	0	1	0	0	1	0	0	0	0	0	0	0	73
12912702	m	cn	JP	2016	0	0	0	0	0	0	1	0	1	0	0	1	64
12912710	f	cn	JP	2016	0	1	0	0	1	0	0	0	0	0	0	1	74
12912736	f	cn	IT	2016	0	1	0	0	1	0	0	0	0	0	0	0	59
12913214	Not	ot	GR	2016	0	0	0	0	0	0	0	0	0	0	0	0	

12913282	Not	ot	CN	2016	1	0	0	0	0	0	0	0	0	0	0	0	
12913644	f	ot	US	2016	0	1	0	0	1	0	0	0	0	0	0	0	74
12913675	m	md	BE	2016	0	1	0	0	1	0	0	0	0	0	0	0	60
12913680	f	ph	FR	2016	0	1	0	0	1	0	0	0	0	0	0	0	67
12913928	m	cn	CA	2016	0	1	0	0	1	0	0	0	0	0	0	0	72
12914180	m	md	JP	2016	1	0	0	0	0	0	0	0	0	0	0	0	66
12914184	m	md	JP	2016	1	0	0	0	0	0	0	0	0	0	0	0	70.8
12916310	m	cn	US	2016	0	1	1	0	0	0	0	0	0	0	0	0	58
12916502	m	ot	RU	2016	0	1	0	0	1	0	0	0	0	0	0	0	64
12917313	m	cn	BE	2016	0	1	0	0	1	0	0	0	0	0	0	0	56
12918110	f	cn	US	2016	0	1	0	0	1	0	0	0	0	0	0	0	
12918143	m	ot	TW	2016	0	0	0	0	0	0	0	0	0	0	0	0	63
12918150	f	ot	TW	2016	1	0	0	0	0	0	0	0	0	0	0	0	55
12919833	f	cn	JP	2016	0	1	0	0	1	0	0	0	0	0	0	0	27
12920305	m	md	IL	2016	1	0	0	0	0	0	0	0	0	0	0	0	88
12920843	m	md	PL	2016	0	0	0	0	0	0	0	0	0	0	0	0	70
12921130	m	md	KR	2016	0	0	0	0	0	0	0	0	0	0	0	0	68
12921332	m	md	JP	2016	0	1	0	0	1	0	0	0	0	0	0	0	53
12921514	f	ot	US	2016	0	1	0	0	1	0	0	0	0	0	0	0	
12921783	f	cn	US	2016	1	0	0	0	0	0	0	0	0	0	0	0	68
12921799	m	ot	US	2016	0	1	0	0	1	0	0	0	0	0	0	0	
12921964	Not	cn	US	2016	0	1	0	0	1	0	0	0	0	0	0	0	
12922111	Not	cn	US	2016	0	1	0	0	1	0	0	0	0	0	0	0	
12922180	f	md	CH	2016	0	0	0	0	0	0	0	0	0	0	0	0	56
12922359	f	md	US	2016	0	1	1	0	0	0	0	0	0	0	0	0	57
12922421	m	cn	US	2016	0	1	0	0	1	0	0	0	0	0	0	0	
12922587	Not	ph	AU	2016	0	1	0	0	1	0	0	0	0	0	0	0	
12922588	Not	ph	AU	2016	0	1	0	0	1	0	0	0	0	0	0	0	72
12922628	f	md	JP	2016	0	0	0	0	0	0	0	0	0	0	0	0	48
12923054	f	cn	JP	2016	0	1	0	0	1	0	0	0	0	0	0	0	46
12923392	f	md	AT	2016	0	1	0	0	1	0	0	0	0	0	0	0	69
12923907	Not	ph	AU	2016	0	1	0	0	1	0	0	0	0	0	0	0	76
12923929	Not	md	JP	2016	0	0	0	0	0	0	0	0	0	0	0	0	60
12924851	m	cn	JP	2016	0	1	0	0	1	0	0	0	0	0	0	0	57

12925082	f	md	JP	2016	0	0	0	0	0	0	1	0	1	0	0	1	68
12925086	Not	md	PE	2016	1	0	0	0	0	0	0	0	0	0	0	0	
12926035	Not	md	GR	2016	0	0	0	0	0	0	0	0	0	0	0	0	
12926596	Not	ph	AU	2016	0	1	0	0	1	0	0	0	0	0	0	0	90
12926628	f	cn	GB	2016	1	0	0	0	0	0	0	0	0	0	0	0	26
12926726	Not	md	JP	2016	0	1	0	0	1	0	0	0	0	0	0	0	
12926908	f	md	US	2016	1	0	0	0	0	0	0	0	0	0	0	0	
12927189	f	md	FR	2016	1	0	0	0	0	0	0	0	0	0	0	0	69.6
12927220	f	md	JP	2016	0	0	0	0	0	0	0	0	0	0	0	0	42
12927651	m	cn	JP	2016	0	1	0	0	1	0	0	0	0	0	0	0	67
12927679	f	md	JP	2016	1	0	0	0	0	0	0	0	0	0	0	0	81
12927870	f	md	IT	2016	1	0	0	0	0	0	0	0	0	0	0	0	71
12927944	m	ot	BE	2016	0	0	0	0	0	0	0	0	0	0	0	0	41
12928152	m	ph	AU	2016	0	1	0	0	1	0	0	0	0	0	0	0	72
12928161	Not	ph	AU	2016	0	1	0	0	1	0	0	0	0	0	0	0	74
12928177	m	ot	KR	2016	0	1	0	0	1	0	0	0	0	0	0	0	63
12928238	m	md	US	2016	0	1	0	0	0	1	0	0	0	0	0	0	67
12928681	m	md	KR	2016	0	1	0	0	0	1	0	0	0	0	0	0	51
12928697	Not	md	JP	2016	0	1	0	0	1	0	0	0	0	0	0	1	
12928751	f	cn	GB	2016	1	0	0	0	0	0	0	0	0	0	0	0	70
12929321	f	md	TH	2016	1	0	0	0	0	0	0	0	0	0	0	0	65.7
12930037	Not	cn	US	2016	0	0	0	0	0	0	0	0	0	0	0	0	
12930165	m	ot	ES	2016	0	0	0	0	0	0	0	0	0	0	0	0	79
12931838	f	md	JP	2016	1	0	0	0	0	0	0	0	0	0	0	0	72
12931919	m	cn	JP	2016	0	1	0	0	1	0	0	0	0	0	0	0	70
12931958	m	md	JP	2016	0	1	0	0	1	0	0	0	0	0	0	0	75
12932181	Not	md	US	2016	0	1	0	0	1	0	0	0	0	0	0	0	
12932377	m	md	CL	2016	0	1	0	0	0	1	0	0	0	0	0	0	76
12932562	Not	md	AU	2016	0	1	0	0	1	0	0	0	0	0	0	0	55
12932687	f	md	US	2016	0	1	1	0	0	0	0	0	0	0	0	0	59
12932734	Not	cn	GB	2016	1	0	0	0	0	0	0	0	0	0	0	1	
12932736	Not	cn	GB	2016	1	0	0	0	0	0	0	0	0	0	0	1	
12932737	f	cn	GB	2016	1	0	0	0	0	0	0	0	0	0	0	1	84
12932751	m	cn	GB	2016	1	0	0	0	0	0	0	0	0	0	0	0	

12933317	Not	md	AU	2016	0	1	0	0	1	0	0	0	0	0	0	0	73
12933695	m	ot	US	2016	0	1	0	0	1	0	0	0	0	0	0	0	57
12933748	m	ot	CA	2016	0	1	0	0	1	0	0	0	0	0	0	0	67
12934572	m	cn	JP	2016	0	0	0	0	0	0	1	0	1	0	0	0	38
12934631	m	cn	US	2016	1	0	0	0	0	0	0	0	0	0	0	0	61
12934733	f	ot	IT	2016	0	1	0	0	1	0	0	0	0	0	0	0	62
12934835	m	ot	DE	2016	0	1	0	0	1	0	0	0	0	0	0	0	22
12934840	Not	md	US	2016	0	1	0	0	1	0	0	0	0	0	0	0	
12934871	f	ot	IT	2016	0	1	0	0	1	0	0	0	0	0	0	0	71
12934883	f	ot	IT	2016	0	1	0	0	1	0	0	0	0	0	0	0	76
12934886	f	md	AT	2016	0	0	0	0	0	0	0	0	0	0	0	0	62
12934905	m	ot	IT	2016	0	1	0	0	1	0	0	0	0	0	0	0	68
12935028	Not	md	AU	2016	0	1	0	0	1	0	0	0	0	0	0	0	70
12935124	Not	md	AU	2016	0	1	0	0	1	0	0	0	0	0	0	0	65
12935125	f	cn	JP	2016	0	1	0	0	1	0	0	0	0	0	0	0	68
12935593	m	cn	JP	2016	0	1	0	0	1	0	0	0	0	0	0	0	79
12936445	f	md	FR	2016	1	0	0	0	0	0	0	0	0	0	0	0	67.8
12936540	m	cn	GB	2016	1	0	0	0	0	0	0	0	0	0	0	0	
12937004	f	ot	IT	2016	0	1	0	0	1	0	0	0	0	0	0	0	65
12937501	f	ph	JP	2016	0	1	0	0	1	0	0	0	0	0	0	0	56
12937510	f	md	BE	2016	0	1	0	0	1	0	0	0	0	0	0	0	55
12937720	Not	ot	IT	2016	0	1	0	0	1	0	0	0	0	0	0	0	75
12938997	m	md	JP	2016	0	1	0	0	1	0	0	0	0	0	0	0	66
12939021	f	ot	US	2016	0	1	0	0	1	0	0	0	0	0	0	0	66
12939049	Not	md	US	2016	0	1	0	0	1	0	0	0	0	0	0	0	
12939238	m	md	SG	2016	0	1	0	0	1	0	0	0	0	0	0	0	45
12939857	m	md	DE	2016	0	0	0	0	0	0	0	0	0	0	0	0	
12941171	m	ot	KR	2016	0	1	0	0	1	0	0	0	0	0	0	0	56
12941410	m	ot	NL	2016	1	0	0	0	0	0	0	0	0	0	0	1	69.9
12941901	m	ot	TW	2016	1	0	0	0	0	0	0	0	0	0	0	0	87
12941970	m	md	JP	2016	1	0	0	0	0	0	0	0	0	0	0	1	69
12942162	f	ot	CN	2016	1	0	0	0	0	0	0	0	0	0	0	0	41
12942659	f	md	JP	2016	0	1	0	0	1	0	0	0	0	0	0	0	73
12942673	f	md	CN	2016	0	0	0	0	0	0	0	0	0	0	0	0	40

12943044	f	cn	Not re	2016	0	0	0	0	0	0	0	0	0	0	0	0	43
12943422	m	cn	US	2016	0	0	0	0	0	0	0	0	0	0	0	0	
12944416	m	md	TW	2016	0	1	0	0	1	0	0	0	0	0	0	0	45
12944536	f	cn	US	2016	0	1	1	0	0	0	0	0	0	0	0	1	60
12944711	f	cn	US	2016	1	0	0	0	0	0	0	0	0	0	0	0	69
12944867	m	ot	US	2016	0	1	0	0	1	0	0	0	0	0	0	0	69
12944958	f	cn	JP	2016	0	1	0	0	1	0	0	0	0	0	0	0	64
12945370	m	cn	US	2016	0	1	1	0	0	0	0	0	0	0	0	0	77
12945604	m	ot	US	2016	0	1	0	0	1	0	0	0	0	0	0	1	
12945913	f	md	IE	2016	1	0	0	0	0	0	0	0	0	0	0	0	74.3
12945928	m	md	CH	2016	1	0	0	0	0	0	0	0	0	0	0	0	
12945947	m	md	JP	2016	1	0	0	0	0	0	0	0	0	0	0	1	76
12945974	m	cn	CA	2016	0	1	0	0	1	0	0	0	0	0	0	0	67
12946040	f	md	JP	2016	1	0	0	0	0	0	0	0	0	0	0	0	51
12946324	f	ph	US	2016	1	0	0	0	0	0	0	0	0	0	0	0	70
12946372	Not	ot	GR	2016	0	0	0	0	0	0	0	0	0	0	0	0	
12946377	Not	ot	GR	2016	0	0	0	0	0	0	0	0	0	0	0	0	
12946424	Not	ot	GR	2016	0	0	0	0	0	0	0	0	0	0	0	0	
12946429	Not	ot	GR	2016	0	0	0	0	0	0	0	0	0	0	0	0	
12946451	Not	ot	GR	2016	0	0	0	0	0	0	0	0	0	0	0	0	
12946465	m	ot	IT	2016	0	1	0	0	1	0	0	0	0	0	0	0	83
12946478	m	md	JP	2016	0	1	0	0	1	0	0	0	0	0	0	0	70
12946480	m	cn	JP	2016	0	1	0	0	1	0	0	0	0	0	0	0	78
12946555	m	ot	US	2016	0	0	0	0	0	0	0	0	0	0	0	0	
12946640	m	ot	CN	2016	0	1	0	0	1	0	0	0	0	0	0	0	52
12946678	Not	ot	GR	2016	0	0	0	0	0	0	0	0	0	0	0	0	
12946797	f	ot	US	2016	0	1	0	0	1	0	0	0	0	0	0	0	
12946906	m	ot	US	2016	0	0	0	0	0	0	0	0	0	0	0	0	
12947151	m	ot	US	2016	1	0	0	0	0	0	0	0	0	0	0	0	66
12947155	f	ot	US	2016	1	0	0	0	0	0	0	0	0	0	0	0	
12947157	Not	Not repo	US	2016	1	0	0	0	0	0	0	0	0	0	0	0	
12947161	f	ot	US	2016	1	0	0	0	0	0	0	0	0	0	0	0	
12947165	f	ph	US	2016	1	0	0	0	0	0	0	0	0	0	0	0	65.7
12947167	f	cn	US	2016	1	0	0	0	0	0	0	0	0	0	0	0	74.9

12947175	m	md	HU	2016	0	1	0	0	1	0	0	0	0	0	0	0	54
12947185	m	ot	IT	2016	0	1	0	0	1	0	0	0	0	0	0	0	74
12947190	f	ot	IT	2016	0	1	0	0	1	0	0	0	0	0	0	0	68
12947218	m	ot	US	2016	0	1	0	0	1	0	0	0	0	0	0	1	
12947477	m	ot	US	2016	0	1	0	0	1	0	0	0	0	0	0	0	73
12947615	f	cn	US	2016	0	1	0	0	1	0	0	0	0	0	0	0	79
12947740	m	md	JP	2016	0	1	0	0	1	0	0	0	0	0	0	0	67
12948267	f	cn	US	2016	0	1	0	0	1	0	0	0	0	0	0	0	70
12948481	Not	ot	GR	2016	0	0	0	0	0	0	0	0	0	0	0	0	
12948510	Not	ot	GR	2016	0	0	0	0	0	0	0	0	0	0	0	0	
12948563	Not	ot	GR	2016	0	0	0	0	0	0	0	0	0	0	0	0	
12948570	Not	ot	GR	2016	0	0	0	0	0	0	0	0	0	0	0	0	
12948583	Not	ot	GR	2016	0	0	0	0	0	0	0	0	0	0	0	0	
12948586	f	md	JP	2016	0	1	0	0	1	0	0	0	0	0	0	0	77
12948587	m	md	JP	2016	0	1	0	0	1	0	0	0	0	0	0	0	71
12948648	Not	ot	GR	2016	0	0	0	0	0	0	0	0	0	0	0	0	
12948655	Not	ot	GR	2016	0	0	0	0	0	0	0	0	0	0	0	0	
12948658	Not	ot	GR	2016	0	0	0	0	0	0	0	0	0	0	0	0	
12948664	Not	ot	GR	2016	0	0	0	0	0	0	0	0	0	0	0	0	
12948699	Not	ot	GR	2016	0	0	0	0	0	0	0	0	0	0	0	0	
12948700	Not	ot	GR	2016	0	0	0	0	0	0	0	0	0	0	0	0	
12948928	m	md	JP	2016	0	1	0	0	1	0	0	0	0	0	0	0	65
12949208	m	md	JP	2016	0	1	0	0	1	0	0	0	0	0	0	1	67
12949213	f	md	JP	2016	0	1	0	0	1	0	0	0	0	0	0	0	69
12949215	f	md	JP	2016	0	1	0	0	1	0	0	0	0	0	0	0	68
12949221	f	md	JP	2016	1	0	0	0	0	0	0	0	0	0	0	0	67
12949513	f	ot	JP	2016	0	1	0	0	1	0	0	0	0	0	0	0	77
12949682	f	cn	US	2016	0	0	0	0	0	0	0	0	0	0	0	0	61
12949711	f	md	JP	2016	1	0	0	0	0	0	0	0	0	0	0	1	72
12949802	m	ph	US	2016	1	0	0	0	0	0	0	0	0	0	0	0	62
12949817	f	ot	US	2016	1	0	0	0	0	0	0	0	0	0	0	0	68
12950029	m	ph	US	2016	0	1	0	0	1	0	0	0	0	0	0	0	67
12950326	f	md	NL	2016	0	1	0	0	1	0	0	0	0	0	0	0	
12950450	m	cn	US	2016	0	0	0	0	0	0	0	0	0	0	0	0	70

12950657	m	md	IL	2016	1	0	0	0	0	0	0	0	0	0	0	0	75
12950862	m	md	TW	2016	1	0	0	0	0	0	0	0	0	0	0	0	77.6
12951012	m	md	JP	2016	0	1	0	0	1	0	0	0	0	0	0	0	65
12951124	m	md	JP	2016	1	0	0	0	0	0	0	0	0	0	0	0	74
12951208	m	md	AR	2016	0	0	0	0	0	0	0	0	0	0	0	0	52
12951318	Not	ph	AU	2016	0	1	0	0	1	0	0	0	0	0	0	0	73
12951323	m	md	FR	2016	0	1	0	0	1	0	0	0	0	0	0	0	
12951367	Not	ot	GR	2016	0	0	0	0	0	0	0	0	0	0	0	0	
12951624	Not	ot	GR	2016	0	0	0	0	0	0	0	0	0	0	0	0	
12951796	f	md	JP	2016	0	1	0	0	1	0	0	0	0	0	0	0	60
12951810	Not	ph	AU	2016	0	1	0	0	1	0	0	0	0	0	0	0	76
12952165	Not	ph	AU	2016	0	1	0	0	1	0	0	0	0	0	0	0	62
12952277	f	md	IT	2016	0	1	0	0	1	0	0	0	0	0	0	0	61
12952798	Not	ot	GR	2016	0	0	0	0	0	0	0	0	0	0	0	0	
12952887	Not	ot	GR	2016	0	0	0	0	0	0	0	0	0	0	0	0	
12952968	f	cn	CA	2016	0	1	0	0	1	0	0	0	0	0	0	0	
12952969	m	cn	CA	2016	0	1	0	0	1	0	0	0	0	0	0	0	
12953014	m	ot	DE	2016	0	1	0	0	1	0	0	0	0	0	0	0	77
12953018	f	ot	IT	2016	0	1	0	0	1	0	0	0	0	0	0	0	
12953103	Not	ph	JP	2016	1	0	0	0	0	0	0	0	0	0	0	0	
12953176	m	cn	CA	2016	0	1	0	0	1	0	0	0	0	0	0	0	
12953338	Not	ph	AU	2016	0	1	0	0	1	0	0	0	0	0	0	0	56
12953340	m	cn	IT	2016	0	1	0	0	1	0	0	0	0	0	0	0	67
12953482	f	ot	IT	2016	0	1	0	0	1	0	0	0	0	0	0	0	73
12953483	f	cn	CN	2016	1	0	0	0	0	0	0	0	0	0	0	0	65
12953736	m	cn	CA	2016	0	1	0	0	1	0	0	0	0	0	0	0	
12953817	m	cn	US	2016	0	1	0	0	1	0	0	0	0	0	0	0	79
12953820	m	md	BE	2016	0	1	0	0	1	0	0	0	0	0	0	0	58
12953857	f	md	FR	2016	0	1	0	0	0	1	0	0	0	0	0	0	61
12954053	m	cn	CA	2016	0	1	0	0	1	0	0	0	0	0	0	0	
12954152	m	cn	CA	2016	0	1	0	0	1	0	0	0	0	0	0	0	
12954185	m	cn	MY	2016	0	1	1	0	0	0	0	0	0	0	0	0	54
12954521	Not	md	GR	2016	1	0	0	0	0	0	0	0	0	0	0	0	
12954522	Not	md	GR	2016	1	0	0	0	0	0	0	0	0	0	0	0	

12954524	Not	md	GR	2016	1	0	0	0	0	0	0	0	0	0	0	0	
12954534	Not	md	GR	2016	1	0	0	0	0	0	0	0	0	0	0	0	
12954540	Not	md	GR	2016	1	0	0	0	0	0	0	0	0	0	0	0	
12954542	Not	md	GR	2016	1	0	0	0	0	0	0	0	0	0	0	0	
12954544	f	ot	US	2016	0	1	0	0	1	0	0	0	0	0	0	0	52
12954548	Not	md	GR	2016	1	0	0	0	0	0	0	0	0	0	0	0	
12954551	Not	md	GR	2016	1	0	0	0	0	0	0	0	0	0	0	0	
12954577	Not	md	GR	2016	1	0	0	0	0	0	0	0	0	0	0	0	
12954578	Not	md	GR	2016	1	0	0	0	0	0	0	0	0	0	0	0	
12954580	Not	md	GR	2016	1	0	0	0	0	0	0	0	0	0	0	0	
12954590	Not	md	GR	2016	1	0	0	0	0	0	0	0	0	0	0	0	
12954591	Not	md	GR	2016	1	0	0	0	0	0	0	0	0	0	0	0	
12954592	Not	md	GR	2016	1	0	0	0	0	0	0	0	0	0	0	0	
12954593	Not	md	GR	2016	1	0	0	0	0	0	0	0	0	0	0	0	
12954595	Not	md	GR	2016	1	0	0	0	0	0	0	0	0	0	0	0	
12954709	m	cn	CA	2016	0	1	0	0	1	0	0	0	0	0	0	0	
12954734	m	cn	CA	2016	0	1	0	0	1	0	0	0	0	0	0	0	
12954868	m	md	NL	2016	0	0	0	0	0	0	0	0	0	0	0	0	66
12954892	m	cn	CA	2016	0	1	0	0	1	0	0	0	0	0	0	0	
12955499	m	ot	CA	2016	0	1	0	0	1	0	0	0	0	0	0	0	
12955624	m	cn	CA	2016	0	1	0	0	1	0	0	0	0	0	0	0	
12955893	m	ot	US	2016	0	1	0	0	1	0	0	0	0	0	0	1	43
12955958	m	md	DE	2016	0	0	0	0	0	0	0	0	0	0	0	0	70
12955967	m	md	JP	2016	0	1	0	0	1	0	0	0	0	0	0	0	78
12955968	m	ot	CZ	2016	0	1	0	0	1	0	0	0	0	0	0	0	
12955974	f	ot	CZ	2016	0	1	0	0	1	0	0	0	0	0	0	0	
12955975	m	ot	CZ	2016	0	1	0	0	1	0	0	0	0	0	0	1	
12956559	m	cn	IT	2016	0	1	0	0	1	0	0	0	0	0	0	0	67
12956606	m	md	DE	2016	0	0	0	0	0	0	0	0	0	0	0	0	47
12956839	f	md	PE	2016	1	0	0	0	0	0	0	0	0	0	0	0	60
12956840	m	Not repo	US	2016	1	0	0	0	0	0	0	0	0	0	0	0	
12957036	m	cn	CA	2016	0	1	0	0	1	0	0	0	0	0	0	0	
12957206	m	cn	CA	2016	0	1	0	0	1	0	0	0	0	0	0	0	
12957541	f	md	FR	2016	0	1	0	0	1	0	0	0	0	0	0	0	70

12957544	m	ot	IT	2016	0	1	0	0	1	0	0	0	0	0	0	0	77
12957549	f	cn	US	2016	0	1	0	0	1	0	0	0	0	0	0	0	57
12957592	f	ot	IT	2016	0	1	0	0	1	0	0	0	0	0	0	0	60
12957828	m	ot	US	2016	0	1	0	0	1	0	0	0	0	0	0	0	
12958063	f	Not repo	US	2016	1	0	0	0	0	0	0	0	0	0	0	1	56.7
12958108	m	md	JP	2016	0	1	0	0	0	1	0	0	0	0	0	0	60
12958149	f	md	DE	2016	0	0	0	0	0	0	0	0	0	0	0	0	61
12958388	f	ot	GB	2016	0	1	0	0	1	0	0	0	0	0	0	0	
12958506	m	md	JP	2016	0	1	0	0	1	0	0	0	0	0	0	0	69
12958628	m	ot	US	2016	0	1	0	0	1	0	0	0	0	0	0	0	
12958954	f	cn	US	2016	0	0	0	0	0	0	0	0	0	0	0	0	
12958976	m	cn	JP	2016	0	1	0	0	1	0	0	0	0	0	0	1	72
12959403	m	ot	US	2016	0	1	0	0	1	0	0	0	0	0	0	0	
12959404	m	cn	Not re	2016	0	0	0	0	0	0	0	0	0	0	0	0	
12959643	f	md	CZ	2016	0	1	0	0	1	0	0	0	0	0	0	0	
12960985	m	cn	Not re	2016	0	0	0	0	0	0	0	0	0	0	0	0	
12961089	m	md	BR	2016	0	0	0	0	0	0	0	0	0	0	0	0	76
12961323	m	md	TR	2016	0	0	0	0	0	0	0	0	0	0	0	0	62
12961424	m	ot	JP	2016	0	1	0	0	1	0	0	0	0	0	0	1	73
12961568	Not	ot	AU	2016	1	0	0	0	0	0	0	0	0	0	0	0	
12961629	Not	ot	AU	2016	1	0	0	0	0	0	0	0	0	0	0	0	
12961630	m	cn	JP	2016	0	1	0	0	1	0	0	0	0	0	0	1	75
12962026	f	cn	US	2016	0	0	0	0	0	0	0	0	0	0	0	0	74
12962132	m	ot	MX	2016	0	1	0	0	1	0	0	0	0	0	0	0	67
12962641	m	md	ES	2016	1	0	0	0	0	0	0	0	0	0	0	0	57.3
12962895	f	md	JP	2016	0	1	0	0	1	0	0	0	0	0	0	0	66
12963041	f	md	JP	2016	0	1	0	0	1	0	0	0	0	0	0	0	77
12963049	f	cn	US	2016	1	0	0	0	0	0	0	0	0	0	0	0	
12963084	f	md	JP	2016	0	1	0	0	1	0	0	0	0	0	0	0	75
12963097	f	cn	DE	2016	0	1	1	0	0	0	0	0	0	0	0	1	
12963181	m	md	DE	2016	0	1	0	0	0	1	0	0	0	0	0	0	59
12963199	f	cn	ES	2016	0	1	0	0	1	0	0	0	0	0	0	0	43
12963222	m	ot	HR	2016	0	1	0	0	1	0	0	0	0	0	0	0	68
12964021	m	md	SI	2016	0	0	0	0	0	0	0	0	0	0	0	0	56

12964028	Not	ot	IE	2016	0	0	0	0	0	0	0	0	0	0	0	0	59
12964125	f	cn	GB	2016	1	0	0	0	0	0	0	0	0	0	0	0	68
12964635	m	md	JP	2016	0	1	0	0	1	0	0	0	0	0	0	0	74
12964698	Not	md	AU	2016	0	1	0	0	1	0	0	0	0	0	0	0	
12964699	m	md	JP	2016	0	1	0	0	1	0	0	0	0	0	0	0	75
12964705	f	md	GB	2016	0	0	0	0	0	0	0	0	0	0	0	0	80
12965021	f	cn	CH	2016	0	0	0	0	0	0	0	0	0	0	0	0	47
12965040	m	ot	JP	2016	0	0	0	0	0	0	0	0	0	0	0	0	66
12965111	m	cn	JP	2016	0	1	0	0	1	0	0	0	0	0	0	0	73
12965112	m	md	JP	2016	0	1	0	0	1	0	0	0	0	0	0	0	55
12965199	m	ot	IT	2016	0	1	0	0	1	0	0	0	0	0	0	0	73
12965354	f	cn	CA	2016	0	0	0	0	0	0	0	0	0	0	0	0	
12965355	f	cn	US	2016	0	0	0	0	0	0	0	0	0	0	0	0	
12965384	f	ot	CZ	2016	0	0	0	0	0	0	0	0	0	0	0	0	46
12965829	f	md	CN	2016	1	0	0	0	0	0	0	0	0	0	0	1	62.4
12965984	m	ot	MX	2016	0	1	0	0	1	0	0	0	0	0	0	0	80
12966226	f	ot	US	2016	0	1	0	0	1	0	0	0	0	0	0	0	86
12966261	f	md	US	2016	0	0	0	0	0	0	0	0	0	0	0	0	77
12966463	m	cn	US	2016	0	1	1	0	0	0	0	0	0	0	0	0	
12966537	f	ot	CH	2016	0	0	0	0	0	0	0	0	0	0	0	0	69
12967014	m	cn	IT	2016	0	1	0	0	1	0	0	0	0	0	0	0	54
12967249	m	ot	IT	2016	0	1	0	0	1	0	0	0	0	0	0	0	70
12967263	m	md	CN	2016	0	1	0	0	0	1	0	0	0	0	0	0	61
12967392	f	md	FR	2016	1	0	0	0	0	0	0	0	0	0	0	0	
12967703	m	ot	NL	2016	0	0	0	0	0	0	0	0	0	0	0	0	
12968144	m	cn	GB	2016	1	0	0	0	0	0	0	0	0	0	0	0	62
12968147	f	cn	GB	2016	1	0	0	0	0	0	0	0	0	0	0	0	65
12968148	f	cn	GB	2016	1	0	0	0	0	0	0	0	0	0	0	0	56
12968217	Not	md	US	2016	0	1	0	0	1	0	0	0	0	0	0	0	
12968895	Not	md	DE	2016	0	1	0	0	1	0	0	0	0	0	0	0	
12969133	f	cn	GB	2016	1	0	0	0	0	0	0	0	0	0	0	0	
12969159	m	ot	JP	2016	0	0	0	0	0	0	0	0	0	0	0	0	48
12969334	f	cn	GB	2016	1	0	0	0	0	0	0	0	0	0	0	0	64
12969840	m	md	JP	2016	1	0	0	0	0	0	0	0	0	0	0	0	88

12970185	f	ot	US	2016	0	1	0	0	1	0	0	0	0	0	0	0	65
12970389	Not	cn	JP	2016	0	1	0	0	1	0	0	0	0	0	0	0	
12970408	m	cn	IT	2016	0	1	0	0	1	0	0	0	0	0	0	0	65
12970409	m	cn	IT	2016	0	1	0	0	1	0	0	0	0	0	0	0	82
12970433	f	md	US	2016	0	1	0	0	0	1	0	0	0	0	0	0	79
12970499	f	ot	US	2016	0	1	0	0	0	1	0	0	0	0	0	0	79
12970568	Not	cn	JP	2016	0	0	0	0	0	0	0	0	0	0	0	0	
12970595	m	ot	JP	2016	0	0	0	0	0	0	0	0	0	0	0	0	77
12970877	m	md	JP	2016	1	0	0	0	0	0	0	0	0	0	0	1	81.8
12971735	m	md	JP	2016	0	1	0	0	1	0	0	0	0	0	0	0	60
12972045	m	cn	JP	2016	0	1	0	0	1	0	0	0	0	0	0	0	54
12972711	Not	md	JP	2016	0	0	0	0	0	0	0	0	0	0	0	0	
12972818	Not	ot	IT	2016	0	1	0	0	1	0	0	0	0	0	0	0	
12973195	m	ot	IT	2016	0	1	0	0	1	0	0	0	0	0	0	0	66
12973292	f	cn	US	2016	0	1	0	0	1	0	0	0	0	0	0	0	
12973497	m	cn	IN	2016	0	1	0	0	1	0	0	0	0	0	0	0	61
12973824	m	cn	US	2016	0	1	0	0	1	0	0	0	0	0	0	0	
12973835	m	ot	IT	2016	0	1	0	0	1	0	0	0	0	0	0	0	
12974429	f	ot	IT	2016	0	1	0	0	1	0	0	0	0	0	0	0	49
12974706	m	ph	JP	2016	0	1	0	0	1	0	0	0	0	0	0	0	56
12974882	f	md	NL	2016	0	0	0	0	0	0	0	0	0	0	0	0	58.6
12975166	f	md	NL	2016	0	0	0	0	0	0	0	0	0	0	0	0	58.6
12975778	f	ot	US	2016	0	1	0	0	1	0	0	0	0	0	0	0	58
12975932	m	md	JP	2016	1	0	0	0	0	0	0	0	0	0	0	1	56
12975982	m	Not repo	US	2016	1	0	0	0	0	0	0	0	0	0	0	0	
12976074	f	md	JP	2016	1	0	0	0	0	0	0	0	0	0	0	0	71
12976075	m	md	JP	2016	1	0	0	0	0	0	0	0	0	0	0	0	72
12976188	f	ot	US	2016	0	1	0	0	1	0	0	0	0	0	0	0	69
12976725	m	ot	DE	2016	0	0	0	0	0	0	0	0	0	0	0	0	53
12976727	f	cn	Not re	2016	0	0	0	0	0	0	0	0	0	0	0	0	57
12977177	f	md	JP	2016	1	0	0	0	0	0	0	0	0	0	0	0	51
12977778	m	cn	JP	2016	0	1	0	0	1	0	0	0	0	0	0	1	68
12978230	Not	md	US	2016	0	0	0	0	0	0	0	0	0	0	0	0	
12978241	Not	md	US	2016	0	0	0	0	0	0	0	0	0	0	0	0	

12978243	Not	md	US	2016	0	0	0	0	0	0	0	0	0	0	0	0	
12978262	Not	md	US	2016	0	0	0	0	0	0	0	0	0	0	0	0	
12978631	Not	md	BE	2016	0	0	0	0	0	0	0	0	0	0	0	0	
12978823	m	md	JP	2016	0	1	0	0	1	0	0	0	0	0	0	0	63
12978970	f	ot	ES	2016	0	1	0	0	1	0	0	0	0	0	0	0	63
12978989	Not	md	US	2016	0	0	0	0	0	0	0	0	0	0	0	0	
12979271	f	md	KR	2016	1	0	0	0	0	0	0	0	0	0	0	0	76
12979582	f	md	DE	2016	0	0	0	0	0	0	0	0	0	0	0	0	74
12979763	m	cn	JP	2016	0	1	0	0	1	0	0	0	0	0	0	0	58
12980768	m	md	US	2016	0	1	0	1	0	0	0	0	0	0	0	0	64
12981013	m	md	IL	2016	1	0	0	0	0	0	0	0	0	0	0	0	
12981204	f	cn	US	2016	1	0	0	0	0	0	0	0	0	0	0	0	
12981253	f	cn	CA	2016	0	1	0	0	1	0	0	0	0	0	0	0	
12981254	f	cn	CA	2016	0	1	0	0	1	0	0	0	0	0	0	0	
12981255	m	cn	CA	2016	0	1	0	0	1	0	0	0	0	0	0	0	
12981257	f	ot	CA	2016	0	1	0	0	1	0	0	0	0	0	0	0	
12981258	m	ot	CA	2016	0	1	0	0	1	0	0	0	0	0	0	0	
12981259	m	cn	CA	2016	0	1	0	0	1	0	0	0	0	0	0	0	
12981264	f	cn	CA	2016	0	1	0	0	1	0	0	0	0	0	0	0	
12981265	m	cn	CA	2016	0	1	0	0	1	0	0	0	0	0	0	0	
12981412	f	cn	CA	2016	0	1	0	0	1	0	0	0	0	0	0	0	
12981413	m	cn	CA	2016	0	1	0	0	1	0	0	0	0	0	0	0	
12981449	m	cn	CA	2016	0	1	0	0	1	0	0	0	0	0	0	0	
12981473	m	ot	CA	2016	0	1	0	0	1	0	0	0	0	0	0	0	
12981477	f	cn	CA	2016	0	1	0	0	1	0	0	0	0	0	0	0	
12981484	m	cn	CA	2016	0	1	0	0	1	0	0	0	0	0	0	0	
12981766	Not	md	DE	2016	0	1	0	0	1	0	0	0	0	0	0	0	
12981839	Not	md	DE	2016	0	1	0	0	1	0	0	0	0	0	0	0	
12981840	Not	md	DE	2016	0	1	0	0	1	0	0	0	0	0	0	0	
12981842	Not	md	DE	2016	0	1	0	0	1	0	0	0	0	0	0	0	
12981929	m	md	AU	2016	0	1	0	0	1	0	0	0	0	0	0	0	67
12981968	m	cn	CA	2016	0	1	0	0	1	0	0	0	0	0	0	0	
12982122	m	md	BE	2016	0	0	0	0	0	0	0	0	0	0	0	0	
12982145	Not	ph	JP	2016	0	1	0	0	1	0	0	0	0	0	0	0	

12982248	f	md	US	2016	0	0	0	0	0	0	0	0	0	0	0	0	45
12982838	m	cn	JP	2016	0	1	0	0	1	0	0	0	0	0	0	0	70
12982887	f	cn	US	2016	0	1	0	0	1	0	0	0	0	0	0	0	
12982900	m	cn	CA	2016	0	1	0	0	1	0	0	0	0	0	0	0	
12982920	Not	ph	AU	2016	0	1	0	0	1	0	0	0	0	0	0	0	
12983216	Not	ph	AU	2016	0	1	0	0	1	0	0	0	0	0	0	0	70
12983520	f	md	AU	2016	0	1	0	0	1	0	0	0	0	0	0	0	
12983522	f	ot	CA	2016	0	1	0	0	1	0	0	0	0	0	0	0	
12983531	f	cn	US	2016	0	1	0	0	1	0	0	0	0	0	0	0	
12983628	m	md	DE	2016	0	0	0	0	0	0	0	0	0	0	0	0	66
12983933	m	cn	IT	2016	0	1	0	0	1	0	0	0	0	0	0	0	
12984018	f	cn	CN	2016	1	0	0	0	0	0	0	0	0	0	0	0	80
12984022	m	cn	DE	2016	0	1	1	0	0	0	0	0	0	0	0	0	54
12984038	m	ot	DE	2016	0	0	0	0	0	0	0	0	0	0	0	0	53
12984229	m	ot	US	2016	0	0	0	0	0	0	0	0	0	0	0	0	55
12984341	f	ot	CA	2016	0	1	0	0	1	0	0	0	0	0	0	0	
12984465	f	md	DE	2016	0	1	0	0	0	1	0	0	0	0	0	0	61
12984697	f	md	JP	2016	1	0	0	0	0	0	0	0	0	0	0	1	74
12985167	f	ph	JP	2016	1	0	0	0	0	0	0	0	0	0	0	0	67
12985279	f	cn	US	2016	1	0	0	0	0	0	0	0	0	0	0	0	
12985334	Not	md	DE	2016	0	1	0	0	1	0	0	0	0	0	0	0	
12985348	f	cn	JP	2016	0	0	0	0	0	0	1	0	1	0	0	0	84
12985629	m	md	JP	2016	0	1	0	0	1	0	0	0	0	0	0	0	76
12985682	f	md	JP	2016	1	0	0	0	0	0	0	0	0	0	0	0	71
12986180	f	cn	CA	2016	0	1	0	0	1	0	0	0	0	0	0	0	
12986194	f	ot	CA	2016	0	1	0	0	1	0	0	0	0	0	0	0	74
12986200	m	ot	US	2016	0	1	0	0	1	0	0	0	0	0	0	0	
12986271	f	ot	CA	2016	0	1	0	0	1	0	0	0	0	0	0	0	
12986285	f	ot	NL	2016	0	0	0	0	0	0	0	0	0	0	0	0	76
12986474	f	cn	CA	2016	0	1	0	0	1	0	0	0	0	0	0	0	
12986746	m	cn	CA	2016	0	1	0	0	1	0	0	0	0	0	0	0	
12987084	m	cn	CA	2016	0	1	0	0	1	0	0	0	0	0	0	0	
12987345	f	cn	CA	2016	0	1	0	0	1	0	0	0	0	0	0	0	47
12987350	f	md	JP	2016	1	0	0	0	0	0	0	0	0	0	0	0	66

12987355	Not	ot	ES	2016	0	1	0	0	1	0	0	0	0	0	0	1	
12987707	f	md	JP	2016	1	0	0	0	0	0	0	0	0	0	0	0	64
12987964	f	Not repo	US	2016	0	0	0	0	0	0	0	0	0	0	0	0	63
12988029	m	cn	GB	2016	1	0	0	0	0	0	0	0	0	0	0	0	
12988069	Not	ot	CN	2016	0	0	0	0	0	0	0	0	0	0	0	0	
12988103	f	cn	US	2016	1	0	0	0	0	0	0	0	0	0	0	0	70
12988406	f	cn	US	2016	0	1	1	0	0	0	0	0	0	0	0	0	69
12988765	f	md	JP	2016	1	0	0	0	0	0	0	0	0	0	0	1	86
12988839	m	cn	JP	2016	0	1	0	0	1	0	0	0	0	0	0	1	55
12989654	f	ot	DE	2016	0	1	0	0	1	0	0	0	0	0	0	0	66
12989960	m	md	JP	2016	0	1	0	0	1	0	0	0	0	0	0	0	77
12990618	Not	ot	PL	2016	1	0	0	0	0	0	0	0	0	0	0	0	
12990648	m	ot	US	2016	0	1	0	0	1	0	0	0	0	0	0	0	
12990873	f	md	JP	2016	0	1	0	0	1	0	0	0	0	0	0	0	62
12990875	f	md	JP	2016	0	1	0	0	1	0	0	0	0	0	0	0	54
12991667	m	md	US	2016	0	0	0	0	0	0	0	0	0	0	0	0	80
12991733	Not	md	US	2016	0	0	0	0	0	0	0	0	0	0	0	0	
12992054	m	md	IE	2016	0	0	0	0	0	0	0	0	0	0	0	0	65
12992351	m	ot	CA	2016	0	1	0	0	1	0	0	0	0	0	0	0	
12992385	f	cn	CA	2016	0	1	0	0	1	0	0	0	0	0	0	0	
12992388	m	cn	CA	2016	0	1	0	0	1	0	0	0	0	0	0	0	
12992389	f	cn	CA	2016	0	1	0	0	1	0	0	0	0	0	0	0	
12992391	f	cn	CA	2016	0	1	0	0	1	0	0	0	0	0	0	0	
12992422	m	cn	JP	2016	0	1	0	0	1	0	0	0	0	0	0	0	69
12992428	f	md	HU	2016	0	0	0	0	0	0	0	0	0	0	0	0	
12992494	m	cn	CA	2016	0	1	0	0	1	0	0	0	0	0	0	0	
12992587	f	md	DE	2016	0	1	0	0	1	0	0	0	0	0	0	0	53
12992637	m	cn	CA	2016	0	1	0	0	1	0	0	0	0	0	0	0	
12992939	f	cn	CA	2016	0	1	0	0	1	0	0	0	0	0	0	0	
12993148	m	cn	CA	2016	0	1	0	0	1	0	0	0	0	0	0	0	
12993150	m	cn	CA	2016	0	1	0	0	1	0	0	0	0	0	0	0	
12993180	m	ot	CA	2016	0	1	0	0	1	0	0	0	0	0	0	0	
12993181	f	cn	CA	2016	0	1	0	0	1	0	0	0	0	0	0	0	
12994378	m	md	DE	2016	0	1	0	0	0	1	0	0	0	0	0	0	

12994693	f	ot	CA	2016	0	1	0	0	1	0	0	0	0	0	0	0	
12995027	Not	md	JP	2016	0	1	0	0	1	0	0	0	0	0	0	0	
12995373	f	cn	IT	2016	0	1	0	0	1	0	0	0	0	0	0	0	77
12995376	m	cn	JP	2016	0	1	0	0	1	0	0	0	0	0	0	0	71
12995378	m	cn	JP	2016	0	1	0	0	1	0	0	0	0	0	0	0	66
12995381	f	cn	IT	2016	0	1	0	0	1	0	0	0	0	0	0	0	59
12995401	Not	md	FR	2016	0	1	0	0	1	0	0	0	0	0	0	0	
12995425	Not	cn	US	2016	0	0	0	0	0	0	0	0	0	0	0	0	
12995658	m	cn	DE	2016	0	0	0	0	0	0	0	0	0	0	0	0	
12995688	f	md	JP	2016	0	1	0	0	1	0	0	0	0	0	0	0	49
12995690	m	ot	BR	2016	0	1	0	0	1	0	0	0	0	0	0	0	34
12995693	f	md	JP	2016	0	1	0	0	1	0	0	0	0	0	0	0	60
12995733	m	ot	ES	2016	0	1	0	0	0	1	0	0	0	0	0	0	57
12995751	m	cn	CA	2016	0	1	0	0	1	0	0	0	0	0	0	0	
12995752	f	ot	CA	2016	0	1	0	0	1	0	0	0	0	0	0	0	69
12995783	m	ot	FR	2016	0	1	0	0	1	0	0	0	0	0	0	0	65
12995819	m	ph	JP	2016	1	0	0	0	0	0	0	0	0	0	0	0	82.1
12995821	m	ot	AU	2016	0	1	0	0	1	0	0	0	0	0	0	0	80
12995832	m	ot	BR	2016	0	1	0	0	1	0	0	0	0	0	0	0	69
12995949	m	ot	JP	2016	0	1	0	0	1	0	0	0	0	0	0	0	65
12995957	f	ot	BR	2016	0	1	0	0	1	0	0	0	0	0	0	0	38
12996238	m	md	TW	2016	1	0	0	0	0	0	0	0	0	0	0	0	67.2
12996777	m	md	FR	2016	0	0	0	0	0	0	0	0	0	0	0	0	67
12996795	f	cn	CA	2016	0	1	0	0	1	0	0	0	0	0	0	0	
12996831	f	cn	CA	2016	0	1	0	0	1	0	0	0	0	0	0	0	
12996938	m	md	BE	2016	0	1	0	0	1	0	0	0	0	0	0	0	62
12996949	m	ot	CA	2016	0	1	0	0	1	0	0	0	0	0	0	0	
12997339	f	cn	CA	2016	0	1	0	0	1	0	0	0	0	0	0	0	
12997358	f	md	JP	2016	0	0	0	0	0	0	0	0	0	0	0	0	90
12997466	m	cn	JP	2016	0	1	0	0	1	0	0	0	0	0	0	0	76
12997794	f	cn	GB	2016	1	0	0	0	0	0	0	0	0	0	0	0	60
12997798	f	cn	GB	2016	1	0	0	0	0	0	0	0	0	0	0	0	
12997876	f	cn	CA	2016	0	1	0	0	1	0	0	0	0	0	0	0	
12998020	m	cn	GB	2016	1	0	0	0	0	0	0	0	0	0	0	0	62

12998273	f	ot	IT	2016	0	1	0	0	1	0	0	0	0	0	0	0	52
12998274	m	cn	IT	2016	0	1	0	0	1	0	0	0	0	0	0	0	64
12998276	f	md	CA	2016	0	1	0	0	1	0	0	0	0	0	0	0	58
12998613	m	ot	US	2016	0	1	0	0	1	0	0	0	0	0	0	1	68
12999416	f	md	US	2016	1	0	0	0	0	0	0	0	0	0	0	0	
13000374	f	cn	CA	2016	0	1	0	0	1	0	0	0	0	0	0	0	
13000421	m	cn	CA	2016	0	1	0	0	1	0	0	0	0	0	0	0	
13000465	f	cn	CA	2016	0	1	0	0	1	0	0	0	0	0	0	0	
13000524	f	cn	CA	2016	0	1	0	0	1	0	0	0	0	0	0	0	
13000595	m	md	JP	2016	0	0	0	0	0	0	0	0	0	0	0	0	77
13000821	m	cn	JP	2016	0	1	0	0	1	0	0	0	0	0	0	1	73
13001155	m	cn	US	2016	1	0	0	0	0	0	0	0	0	0	0	1	44
13001260	f	md	US	2016	0	0	0	0	0	0	0	0	0	0	0	0	37
13002018	m	cn	CA	2016	0	1	0	0	1	0	0	0	0	0	0	0	
13002267	m	cn	CA	2016	0	1	0	0	1	0	0	0	0	0	0	0	49
13002548	f	cn	CA	2016	0	1	0	0	1	0	0	0	0	0	0	0	
13002995	m	ot	US	2016	0	0	0	0	0	0	0	0	0	0	0	0	68
13003057	Not	ot	DE	2016	0	0	0	0	0	0	0	0	0	0	0	0	
13004196	f	md	JP	2016	1	0	0	0	0	0	0	0	0	0	0	0	68
13004218	f	md	JP	2016	1	0	0	0	0	0	0	0	0	0	0	0	77
13004248	m	md	CN	2016	1	0	0	0	0	0	0	0	0	0	0	0	64
13004639	Not	md	US	2016	0	1	0	0	1	0	0	0	0	0	0	0	
13004799	m	md	DE	2016	0	0	0	0	0	0	0	0	0	0	0	0	66
13004850	m	cn	IT	2016	0	1	0	0	1	0	0	0	0	0	0	0	65
13004949	m	cn	CA	2016	0	1	0	0	1	0	0	0	0	0	0	0	
13004955	f	md	JP	2016	0	1	0	0	1	0	0	0	0	0	0	0	69
13005425	m	md	TH	2016	1	0	0	0	0	0	0	0	0	0	0	0	
13005426	f	cn	GB	2016	1	0	0	0	0	0	0	0	0	0	0	0	69
13005448	m	cn	FR	2016	0	0	0	0	0	0	0	0	0	0	0	0	70
13005462	f	cn	GB	2016	1	0	0	0	0	0	0	0	0	0	0	0	75
13005644	f	cn	US	2016	1	0	0	0	0	0	0	0	0	0	0	0	80
13005693	f	md	US	2016	0	1	1	0	0	0	0	0	0	0	0	0	79
13005823	f	md	NL	2016	0	0	0	0	0	0	0	0	0	0	0	0	65.2
13006472	f	ot	KR	2016	0	0	0	0	0	0	0	0	0	0	0	0	36

13006646	m	cn	JP	2016	0	0	0	0	0	0	0	0	0	0	0	0	73
13006766	f	cn	CA	2016	0	1	0	0	1	0	0	0	0	0	0	0	
13006807	m	ot	US	2016	1	0	0	0	0	0	0	0	0	0	0	0	79
13007190	m	md	JP	2016	0	1	0	0	1	0	0	0	0	0	0	0	63
13007835	f	ot	US	2016	1	0	0	0	0	0	0	0	0	0	0	1	
13007916	f	md	GR	2016	1	0	0	0	0	0	0	0	0	0	0	0	65
13007948	m	md	JP	2016	0	1	0	0	1	0	0	0	0	0	0	0	70
13007953	f	ot	CA	2016	0	1	0	0	1	0	0	0	0	0	0	0	67
13008270	m	cn	CA	2016	0	1	0	0	1	0	0	0	0	0	0	0	
13008360	m	cn	IT	2016	0	1	0	0	1	0	0	0	0	0	0	1	80
13008422	f	cn	CA	2016	0	1	0	0	1	0	0	0	0	0	0	0	
13008544	m	ot	DE	2016	0	0	0	0	0	0	0	0	0	0	0	0	53
13008772	m	cn	CA	2016	0	1	0	0	1	0	0	0	0	0	0	0	61
13009171	m	md	DE	2016	0	0	0	0	0	0	0	0	0	0	0	0	66
13009230	m	ot	DE	2016	0	0	0	0	0	0	0	0	0	0	0	0	53
13009263	Not	ot	CH	2016	0	0	0	0	0	0	0	0	0	0	0	0	
13009685	m	cn	JP	2016	0	0	0	0	0	0	0	0	0	0	0	0	85
13009686	f	cn	JP	2016	0	0	0	0	0	0	0	0	0	0	0	0	75
13009695	Not	cn	GB	2016	1	0	0	0	0	0	0	0	0	0	0	0	
13009719	f	cn	GB	2016	1	0	0	0	0	0	0	0	0	0	0	0	53
13009798	f	md	DE	2016	0	1	0	0	1	0	0	0	0	0	0	0	75
13009840	m	md	FR	2016	0	1	0	0	1	0	0	0	0	0	0	0	
13009869	f	cn	CN	2016	1	0	0	0	0	0	0	0	0	0	0	0	
13010030	m	md	JP	2016	0	1	0	0	1	0	0	0	0	0	0	0	
13010038	f	cn	CA	2016	0	1	0	0	1	0	0	0	0	0	0	0	
13010152	Not	ot	CH	2016	0	1	0	0	1	0	0	0	0	0	0	0	
13010301	m	cn	BR	2016	1	0	0	0	0	0	0	0	0	0	0	0	80
13010358	f	ot	US	2016	0	1	0	0	1	0	0	0	0	0	0	0	62
13011095	f	cn	US	2016	0	0	0	0	0	0	0	0	0	0	0	0	
13011189	m	cn	CA	2016	0	1	0	0	1	0	0	0	0	0	0	0	78
13011249	f	md	US	2016	1	0	0	0	0	0	0	0	0	0	0	0	
13011286	f	md	JP	2016	1	0	0	0	0	0	0	0	0	0	0	0	69
13012206	m	cn	GB	2016	1	0	0	0	0	0	0	0	0	0	0	0	
13012599	m	cn	CA	2016	0	1	0	0	1	0	0	0	0	0	0	0	83

13012601	f	cn	CA	2016	0	1	0	0	1	0	0	0	0	0	0	0	
13012666	f	cn	CA	2016	0	1	0	0	1	0	0	0	0	0	0	0	
13012814	m	cn	JP	2016	0	0	0	0	0	0	0	0	0	0	0	0	71
13013599	f	ot	FR	2016	0	1	0	0	1	0	0	0	0	0	0	0	63
13013612	f	ot	US	2016	0	1	0	0	1	0	0	0	0	0	0	0	71
13013614	f	cn	CA	2016	0	1	0	0	1	0	0	0	0	0	0	0	
13013673	f	cn	CA	2016	0	1	0	0	1	0	0	0	0	0	0	0	77
13013685	f	cn	CA	2016	0	1	0	0	1	0	0	0	0	0	0	0	
13013700	m	md	JP	2016	1	0	0	0	0	0	0	0	0	0	0	0	
13013717	m	md	JP	2016	0	1	0	0	1	0	0	0	0	0	0	0	62
13013722	f	cn	CA	2016	0	1	0	0	1	0	0	0	0	0	0	0	
13013760	m	cn	CA	2016	0	1	0	0	1	0	0	0	0	0	0	0	
13014063	f	md	JP	2016	1	0	0	0	0	0	0	0	0	0	0	1	76
13014394	m	cn	JP	2016	0	1	0	0	1	0	0	0	0	0	0	0	72
13014396	m	cn	JP	2016	0	1	0	0	1	0	0	0	0	0	0	1	65
13014482	f	md	JP	2016	1	0	0	0	0	0	0	0	0	0	0	0	69
13014547	f	ot	BE	2016	0	0	0	0	0	0	0	0	0	0	0	0	86
13014686	f	md	JP	2016	1	0	0	0	0	0	0	0	0	0	0	0	
13014731	m	md	JP	2016	0	1	0	0	1	0	0	0	0	0	0	0	70
13014756	f	md	DE	2016	0	1	0	0	1	0	0	0	0	0	0	0	48
13014925	m	md	AT	2016	1	0	0	0	0	0	0	0	0	0	0	0	76
13015189	f	cn	US	2016	0	1	0	0	1	0	0	0	0	0	0	0	
13015199	Not	ph	AU	2016	0	1	0	0	1	0	0	0	0	0	0	0	78
13015212	m	ot	GB	2016	0	1	0	0	1	0	0	0	0	0	0	0	52
13015218	m	md	JP	2016	0	1	0	0	1	0	0	0	0	0	0	0	69
13015221	m	cn	CA	2016	0	1	0	0	1	0	0	0	0	0	0	0	
13015235	m	ot	US	2016	0	1	0	0	1	0	0	0	0	0	0	0	
13015239	Not	ph	AU	2016	0	1	0	0	1	0	0	0	0	0	0	0	70
13015298	Not	cn	US	2016	0	1	1	0	0	0	0	0	0	0	0	0	
13015594	m	md	US	2016	0	1	1	0	0	0	0	0	0	0	0	0	72
13015824	m	ot	CA	2016	0	1	0	0	1	0	0	0	0	0	0	0	70
13016668	m	cn	CA	2016	0	1	0	0	1	0	0	0	0	0	0	0	63
13016672	f	cn	CA	2016	0	1	0	0	1	0	0	0	0	0	0	0	
13016733	m	md	JP	2016	0	0	0	0	0	0	0	0	0	0	0	0	65

13016937	m	ph	US	2016	0	0	0	0	0	0	0	0	0	0	0	0	58
13016986	f	cn	Not re	2016	0	0	0	0	0	0	0	0	0	0	0	0	58
13017006	f	ot	CA	2016	0	1	0	0	1	0	0	0	0	0	0	0	
13018394	f	ph	JP	2016	0	0	0	0	0	0	1	0	1	0	0	1	78
13018464	m	ot	HU	2016	0	1	0	0	1	0	0	0	0	0	0	1	
13020331	f	cn	US	2016	0	1	1	0	0	0	0	0	0	0	0	0	
13020688	f	ot	ES	2016	0	1	0	0	1	0	0	0	0	0	0	0	63
13020689	f	ot	US	2016	0	1	0	0	1	0	0	0	0	0	0	0	56
13021059	m	md	JP	2016	0	0	0	0	0	0	1	0	1	0	0	0	76
13021359	f	cn	US	2016	0	1	1	0	0	0	0	0	0	0	0	0	
13021434	f	cn	US	2016	0	1	1	0	0	0	0	0	0	0	0	0	
13021561	f	md	JP	2016	0	0	0	0	0	0	0	0	0	0	0	0	84
13021854	m	ot	CA	2016	0	1	0	0	1	0	0	0	0	0	0	0	
13021855	Not	cn	JP	2016	0	1	0	0	1	0	0	0	0	0	0	1	65
13022389	m	md	JP	2016	0	0	0	0	0	0	0	0	0	0	0	0	
13022719	m	cn	CA	2016	0	1	0	0	1	0	0	0	0	0	0	0	55
13022736	m	cn	CA	2016	0	1	0	0	1	0	0	0	0	0	0	0	
13023444	m	cn	US	2016	0	1	1	0	0	0	0	0	0	0	0	0	
13023477	f	cn	CA	2016	0	1	0	0	1	0	0	0	0	0	0	0	70
13023480	m	cn	CA	2016	0	1	0	0	1	0	0	0	0	0	0	0	60
13023592	f	md	ES	2016	1	0	0	0	0	0	0	0	0	0	0	0	76
13023853	f	cn	CA	2016	0	1	0	0	1	0	0	0	0	0	0	0	56
13024559	Not	md	JP	2016	1	0	0	0	0	0	0	0	0	0	0	0	
13024564	f	ot	ES	2016	0	0	0	0	0	0	0	0	0	0	0	0	58
13025375	f	md	IE	2016	1	0	0	0	0	0	0	0	0	0	0	0	68.3
13025382	f	cn	US	2016	0	1	1	0	0	0	0	0	0	0	0	0	
13026247	m	md	JP	2016	1	0	0	0	0	0	0	0	0	0	0	0	73
13026431	m	ot	IT	2016	0	1	0	0	1	0	0	0	0	0	0	1	78
13026703	m	cn	CA	2016	0	1	0	0	1	0	0	0	0	0	0	0	
13026824	f	cn	CA	2016	0	1	0	0	1	0	0	0	0	0	0	0	60
13026844	f	cn	CA	2016	0	1	0	0	1	0	0	0	0	0	0	0	63
13026992	m	cn	JP	2016	0	1	0	0	1	0	0	0	0	0	0	0	73
13027049	Not	ot	US	2016	0	1	0	0	1	0	0	0	0	0	0	0	
13027416	m	md	JP	2016	0	1	0	0	1	0	0	0	0	0	0	1	74

13027479	f	cn	CA	2016	0	1	0	0	1	0	0	0	0	0	0	0	
13028199	f	cn	CA	2016	0	1	0	0	1	0	0	0	0	0	0	0	
13028354	f	ot	CA	2016	0	1	0	0	1	0	0	0	0	0	0	0	
13028589	f	md	DE	2016	0	1	0	0	0	1	0	0	0	0	0	0	60
13028747	f	Not repo	US	2016	1	0	0	0	0	0	0	0	0	0	0	0	83
13028818	m	md	HR	2016	1	0	0	0	0	0	0	0	0	0	0	0	61
13028911	f	md	US	2016	0	1	0	0	1	0	0	0	0	0	0	0	71
13029137	f	cn	CA	2016	0	1	0	0	1	0	0	0	0	0	0	0	
13029374	m	ot	IT	2016	0	1	0	0	1	0	0	0	0	0	0	0	66
13029381	m	cn	CA	2016	0	1	0	0	1	0	0	0	0	0	0	0	
13029999	m	ph	US	2016	0	0	0	0	0	0	0	0	0	0	0	0	74
13030254	m	ph	JP	2016	0	1	0	0	1	0	0	0	0	0	0	0	
13030708	f	md	DE	2016	0	0	0	0	0	0	0	0	0	0	0	0	67
13030720	f	ot	DE	2016	0	0	0	0	0	0	0	0	0	0	0	0	76
13030784	Not	cn	US	2016	1	0	0	0	0	0	0	0	0	0	0	0	
13031109	f	ot	AU	2016	0	0	0	0	0	0	0	0	0	0	0	0	37
13031131	m	md	DE	2016	0	0	0	0	0	0	0	0	0	0	0	0	60
13031584	m	ot	IT	2016	0	1	0	0	1	0	0	0	0	0	0	0	55
13031591	f	md	ES	2016	0	0	0	0	0	0	0	0	0	0	0	0	55
13031594	Not	ot	CN	2016	0	0	0	0	0	0	0	0	0	0	0	0	
13032076	f	cn	GB	2016	1	0	0	0	0	0	0	0	0	0	0	0	69
13032124	f	cn	US	2016	0	1	0	0	1	0	0	0	0	0	0	0	
13032411	f	md	US	2016	0	1	0	0	0	1	0	0	0	0	0	0	55
13032533	Not	md	JP	2016	0	0	0	0	0	0	0	0	0	0	0	0	
13032571	Not	ot	JP	2016	0	0	0	0	0	0	0	0	0	0	0	0	
13032984	Not	cn	GB	2016	0	1	0	0	1	0	0	0	0	0	0	0	
13033059	Not	md	TH	2016	0	1	1	0	0	0	0	0	0	0	0	0	
13033220	Not	md	JP	2016	0	1	0	0	1	0	0	0	0	0	0	0	
13033359	m	md	JP	2016	1	0	0	0	0	0	0	0	0	0	0	0	73
13033381	m	ot	US	2016	0	1	0	0	1	0	0	0	0	0	0	1	65
13033549	m	cn	CA	2016	0	1	0	0	1	0	0	0	0	0	0	0	
13033725	m	md	IT	2016	0	1	0	0	1	0	0	0	0	0	0	0	65
13033846	m	md	JP	2016	0	1	0	0	1	0	0	0	0	0	0	1	44
13034092	f	cn	US	2016	0	1	0	0	1	0	0	0	0	0	0	0	

13034165	m	md	IN	2016	0	1	0	0	1	0	0	0	0	0	0	0	76
13034679	f	cn	US	2016	0	1	1	0	0	0	0	0	0	0	0	0	
13034983	m	cn	US	2016	0	1	1	0	0	0	0	0	0	0	0	0	71
13034999	m	ot	US	2016	0	0	0	0	0	0	0	0	0	0	0	0	66
13035072	m	cn	JP	2016	0	1	0	0	1	0	0	0	0	0	0	0	67
13035334	m	cn	US	2016	0	1	0	0	1	0	0	0	0	0	0	0	65
13035531	f	cn	US	2016	1	0	0	0	0	0	0	0	0	0	0	0	52
13035533	f	cn	US	2016	1	0	0	0	0	0	0	0	0	0	0	0	66
13035534	f	cn	US	2016	1	0	0	0	0	0	0	0	0	0	0	0	57
13035987	f	cn	US	2016	0	0	0	0	0	0	0	0	0	0	0	0	
13036162	m	md	JP	2016	0	0	0	0	0	0	0	0	0	0	0	0	74
13036807	m	cn	GB	2016	0	0	0	0	0	0	1	0	1	0	0	0	76
13036882	m	md	IN	2016	0	1	0	0	1	0	0	0	0	0	0	0	
13037032	Not	ph	AU	2016	0	1	0	0	1	0	0	0	0	0	0	0	
13037052	f	md	CA	2016	0	1	0	0	1	0	0	0	0	0	0	0	56
13037054	m	md	BE	2016	0	1	0	0	1	0	0	0	0	0	0	0	
13037171	m	ot	JP	2016	0	0	0	0	0	0	0	0	0	0	0	0	66
13037233	m	ot	DE	2016	0	1	0	1	0	0	0	0	0	0	0	0	72
13037482	Not	ot	JP	2016	0	0	0	0	0	0	0	0	0	0	0	0	
13037617	m	md	IT	2016	0	1	0	0	1	0	0	0	0	0	0	0	66
13037740	m	md	JP	2016	1	0	0	0	0	0	0	0	0	0	0	0	89
13037921	Not	ph	AU	2016	0	1	0	0	1	0	0	0	0	0	0	0	
13037928	Not	md	JP	2016	0	1	0	0	1	0	0	0	0	0	0	0	70
13037949	m	cn	CA	2016	0	1	0	0	1	0	0	0	0	0	0	1	69
13038480	m	cn	CA	2016	0	1	0	0	1	0	0	0	0	0	0	0	
13038482	m	cn	CA	2016	0	1	0	0	1	0	0	0	0	0	0	0	
13038483	f	cn	CA	2016	0	1	0	0	1	0	0	0	0	0	0	0	
13038498	m	cn	IT	2016	0	1	0	0	1	0	0	0	0	0	0	0	65
13038501	f	cn	US	2016	0	1	0	0	1	0	0	0	0	0	0	0	
13038604	m	cn	CA	2016	0	1	0	0	1	0	0	0	0	0	0	0	
13038606	Not	ph	AU	2016	0	1	0	0	1	0	0	0	0	0	0	0	
13038756	m	cn	JP	2016	0	1	0	0	1	0	0	0	0	0	0	1	54
13039904	m	cn	CA	2016	0	1	0	0	1	0	0	0	0	0	0	0	
13039905	m	cn	CA	2016	0	1	0	0	1	0	0	0	0	0	0	0	

13039937	m	cn	US	2016	0	1	1	0	0	0	0	0	0	0	0	0	
13040356	f	cn	CA	2016	0	1	0	0	1	0	0	0	0	0	0	0	
13042417	m	cn	CA	2016	0	1	0	0	1	0	0	0	0	0	0	0	
13042463	Not	ot	US	2016	0	1	0	0	1	0	0	0	0	0	0	1	
13042538	m	cn	CA	2016	0	1	0	0	1	0	0	0	0	0	0	0	
13043048	f	ot	US	2016	0	1	0	0	1	0	0	0	0	0	0	0	71
13043498	f	cn	CA	2016	0	1	0	0	1	0	0	0	0	0	0	0	
13043689	f	ot	US	2016	0	1	0	0	1	0	0	0	0	0	0	0	
13045046	Not	md	FR	2016	0	1	0	0	1	0	0	0	0	0	0	0	61
13045296	f	md	JP	2016	1	0	0	0	0	0	0	0	0	0	0	0	72
13045345	Not	md	TW	2016	0	1	0	0	1	0	0	0	0	0	0	0	
13046102	m	cn	US	2016	0	0	0	0	0	0	0	0	0	0	0	0	78
13047186	f	cn	CA	2016	0	1	0	0	1	0	0	0	0	0	0	0	
13047301	f	cn	CA	2016	0	1	0	0	1	0	0	0	0	0	0	0	
13047315	m	cn	CA	2016	0	1	0	0	1	0	0	0	0	0	0	0	
13047556	f	md	JP	2016	0	1	0	0	1	0	0	0	0	0	0	0	70
13047561	m	cn	GB	2016	0	1	0	0	1	0	0	0	0	0	0	0	62
13048140	Not	md	IT	2016	0	1	0	0	1	0	0	0	0	0	0	0	
13048414	f	cn	US	2016	0	1	0	0	1	0	0	0	0	0	0	1	
13048446	f	cn	US	2016	0	1	0	0	1	0	0	0	0	0	0	1	
13048904	f	ot	IT	2016	0	1	0	0	1	0	0	0	0	0	0	0	61
13049027	Not	md	AU	2016	0	1	0	0	1	0	0	0	0	0	0	0	73
13049096	m	md	IT	2016	0	1	0	0	1	0	0	0	0	0	0	0	66
13049150	m	cn	CH	2016	0	1	0	0	1	0	0	0	0	0	0	0	62
13049750	m	ot	IT	2016	0	1	0	0	1	0	0	0	0	0	0	0	75
13049790	m	cn	CA	2016	0	1	0	0	1	0	0	0	0	0	0	0	
13049987	f	md	FR	2016	0	0	0	0	0	0	0	0	0	0	0	0	
13050472	f	cn	GB	2016	0	0	0	0	0	0	0	0	0	0	0	0	80
13051908	f	cn	CA	2016	0	1	0	0	1	0	0	0	0	0	0	0	
13052080	m	md	CN	2016	1	0	0	0	0	0	0	0	0	0	0	0	61.9
13052865	m	cn	JP	2016	0	1	0	0	1	0	0	0	0	0	0	0	74
13052932	f	ot	IE	2016	0	1	0	0	1	0	0	0	0	0	0	0	68
13052988	m	md	JP	2016	0	1	0	0	1	0	0	0	0	0	0	0	47
13053015	m	ot	IT	2016	0	1	0	0	1	0	0	0	0	0	0	0	70

13053200	m	cn	JP	2016	0	1	0	0	1	0	0	0	0	0	0	1	80
13053519	f	md	BR	2016	1	0	0	0	0	0	0	0	0	0	0	0	
13053784	f	cn	CA	2016	0	0	0	0	0	0	0	0	0	0	0	0	
13053787	m	md	JP	2016	0	1	0	0	1	0	0	0	0	0	0	0	68
13054188	f	cn	CA	2016	0	1	0	0	1	0	0	0	0	0	0	0	
13054190	f	cn	CA	2016	0	1	0	0	1	0	0	0	0	0	0	0	
13054407	f	cn	US	2016	0	0	0	0	0	0	0	0	0	0	0	0	86
13054507	m	cn	US	2016	0	0	0	0	0	0	0	0	0	0	0	0	70
13054665	m	cn	US	2016	0	1	1	0	0	0	0	0	0	0	0	1	68
13054711	f	cn	CA	2016	0	1	0	0	1	0	0	0	0	0	0	0	
13055577	f	cn	JP	2016	0	1	0	0	1	0	0	0	0	0	0	0	70
13055976	f	cn	US	2016	0	0	0	0	0	0	0	0	0	0	0	0	
13057089	f	md	US	2016	1	0	0	0	0	0	0	0	0	0	0	0	
13057126	f	md	AU	2016	1	0	0	0	0	0	0	0	0	0	0	0	49.9
13057438	m	md	JP	2016	0	0	0	0	0	0	1	0	1	0	0	0	66
13057535	f	ot	CZ	2016	0	1	0	0	1	0	0	0	0	0	0	0	72
13057541	m	ot	IT	2016	0	1	0	0	1	0	0	0	0	0	0	0	55
13057808	Not	ph	GB	2016	0	1	0	0	1	0	0	0	0	0	0	0	50
13057854	m	cn	CA	2016	0	1	0	0	1	0	0	0	0	0	0	0	
13058784	m	ot	IT	2016	0	1	0	0	1	0	0	0	0	0	0	0	78
13058786	m	ot	BE	2016	0	1	0	0	1	0	0	0	0	0	0	0	73
13058986	m	cn	IT	2016	0	1	0	0	0	1	0	0	0	0	0	0	67
13059443	m	cn	GB	2016	1	0	0	0	0	0	0	0	0	0	0	0	68
13059779	m	cn	GB	2016	1	0	0	0	0	0	0	0	0	0	0	0	
13059864	f	cn	US	2016	0	0	0	0	0	0	0	0	0	0	0	0	35
13060082	Not	cn	JP	2016	0	0	0	0	0	0	0	0	0	0	0	0	
13060085	f	cn	US	2016	0	0	0	0	0	0	0	0	0	0	0	0	70
13060086	Not	cn	JP	2016	0	0	0	0	0	0	0	0	0	0	0	0	
13060090	m	ot	ES	2016	0	1	0	0	1	0	0	0	0	0	0	1	78
13060093	m	md	JP	2016	0	1	0	0	1	0	0	0	0	0	0	1	80
13060094	f	ot	IT	2016	0	1	0	0	1	0	0	0	0	0	0	0	51
13060120	m	cn	CA	2016	0	1	0	0	1	0	0	0	0	0	0	0	
13060136	m	cn	US	2016	0	1	0	0	1	0	0	0	0	0	0	0	
13060158	f	ot	US	2016	0	1	0	0	1	0	0	0	0	0	0	0	

13060205	m	md	JP	2016	0	1	0	0	1	0	0	0	0	0	0	0	70
13060214	Not	md	JP	2016	0	1	0	0	1	0	0	0	0	0	0	0	
13060219	f	ot	IT	2016	0	1	0	0	1	0	0	0	0	0	0	0	74
13060221	f	cn	CA	2016	0	1	0	0	1	0	0	0	0	0	0	0	
13060225	f	cn	CA	2016	0	1	0	0	1	0	0	0	0	0	0	0	
13061238	f	md	TR	2016	0	1	0	0	1	0	0	0	0	0	0	0	47
13061307	f	md	JP	2016	0	0	0	0	0	0	0	0	0	0	0	0	39
13061374	Not	md	JP	2016	0	1	0	0	1	0	0	0	0	0	0	0	65
13062518	f	Not repo	US	2016	1	0	0	0	0	0	0	0	0	0	0	0	76
13062579	f	cn	US	2016	1	0	0	0	0	0	0	0	0	0	0	0	88
13063393	f	cn	CA	2016	0	1	0	0	1	0	0	0	0	0	0	0	
13064086	f	ot	JP	2016	1	0	0	0	0	0	0	0	0	0	0	0	73
13064128	f	md	AT	2016	0	0	0	0	0	0	0	0	0	0	0	0	60
13064379	m	cn	CA	2016	0	1	0	0	1	0	0	0	0	0	0	0	
13064550	m	cn	GB	2016	1	0	0	0	0	0	0	0	0	0	0	1	67
13064782	m	cn	IN	2016	0	1	0	0	1	0	0	0	0	0	0	0	
13065301	f	cn	JP	2016	0	0	0	0	0	0	1	0	1	0	0	1	62
13065609	m	md	US	2016	0	1	1	0	0	0	0	0	0	0	0	0	49
13066406	m	cn	JP	2016	0	1	0	0	1	0	0	0	0	0	0	0	85
13066917	f	cn	FR	2016	0	1	0	0	1	0	0	0	0	0	0	0	45
13066936	f	md	JP	2016	1	0	0	0	0	0	0	0	0	0	0	0	55
13067109	f	md	ES	2016	0	1	0	0	1	0	0	0	0	0	0	0	61
13067141	Not	ph	JP	2016	0	1	0	0	1	0	0	0	0	0	0	0	58
13067226	Not	md	GR	2016	0	0	0	0	0	0	0	0	0	0	0	0	
13067357	m	md	JP	2016	0	0	0	0	0	0	0	0	0	0	0	0	63
13067375	m	md	KR	2016	0	1	0	0	0	1	0	0	0	0	0	0	70
13067656	Not	ot	US	2016	0	1	0	0	1	0	0	0	0	0	0	1	
13067671	Not	ot	US	2016	0	1	0	0	1	0	0	0	0	0	0	0	
13067680	Not	ot	US	2016	0	1	0	0	1	0	0	0	0	0	0	0	
13067684	Not	ot	US	2016	0	1	0	0	1	0	0	0	0	0	0	0	
13067690	Not	ot	US	2016	0	1	0	0	1	0	0	0	0	0	0	0	
13067693	Not	ot	US	2016	0	1	0	0	1	0	0	0	0	0	0	0	
13067709	Not	ot	US	2016	0	1	0	0	1	0	0	0	0	0	0	0	
13067716	Not	ot	US	2016	0	1	0	0	1	0	0	0	0	0	0	0	

13067756	f	ot	KR	2016	0	1	0	0	1	0	0	0	0	0	0	0	43
13067768	Not	ot	US	2016	0	1	0	0	1	0	0	0	0	0	0	0	
13067777	Not	ot	US	2016	0	1	0	0	1	0	0	0	0	0	0	0	
13067791	Not	ot	US	2016	0	1	0	0	1	0	0	0	0	0	0	0	
13067798	Not	ot	US	2016	0	1	0	0	1	0	0	0	0	0	0	0	
13067809	Not	ot	US	2016	0	1	0	0	1	0	0	0	0	0	0	0	
13068273	f	md	DE	2016	1	0	0	0	0	0	0	0	0	0	0	0	78
13068713	m	ot	JP	2016	0	1	0	0	1	0	0	0	0	0	0	0	49
13068770	m	cn	CA	2016	0	1	0	0	1	0	0	0	0	0	0	0	
13069681	m	ph	AU	2016	0	1	0	0	1	0	0	0	0	0	0	0	72
13070092	m	md	ES	2016	0	0	0	0	0	0	0	0	0	0	0	0	77
13070296	f	ot	US	2016	0	1	0	0	1	0	0	0	0	0	0	0	54
13070312	m	md	JP	2016	0	1	0	0	1	0	0	0	0	0	0	0	73
13070657	m	md	CL	2016	0	1	0	0	0	1	0	0	0	0	0	0	56
13070679	f	md	JP	2016	0	1	0	0	1	0	0	0	0	0	0	1	75
13070760	f	md	CA	2016	0	1	0	0	0	1	0	0	0	0	0	0	71
13070832	f	md	JP	2016	0	1	0	0	1	0	0	0	0	0	0	0	71
13070849	f	md	JP	2016	1	0	0	0	0	0	0	0	0	0	0	0	62
13071035	m	md	JP	2016	0	1	0	0	1	0	0	0	0	0	0	0	52
13071057	f	cn	JP	2016	0	0	0	0	0	0	1	0	1	0	0	0	77
13071092	m	md	CN	2016	0	0	0	0	0	0	0	0	0	0	0	0	
13071093	m	md	IT	2016	0	1	0	0	0	1	0	0	0	0	0	0	66
13071112	m	md	US	2016	0	0	0	0	0	0	1	0	0	0	1	0	63
13071196	f	md	US	2016	0	1	1	0	0	0	0	0	0	0	0	0	53
13072342	m	md	JP	2016	0	1	0	0	1	0	0	0	0	0	0	0	67
13072393	Not	ot	CN	2016	0	0	0	0	0	0	0	0	0	0	0	0	
13072406	Not	ot	CN	2016	0	0	0	0	0	0	0	0	0	0	0	0	
13072471	f	md	JP	2016	1	0	0	0	0	0	0	0	0	0	0	0	81
13072742	f	md	US	2016	0	0	0	0	0	0	0	0	0	0	0	0	81
13073203	f	cn	JP	2016	0	1	0	0	1	0	0	0	0	0	0	0	65
13073545	m	ot	RU	2016	0	1	0	0	1	0	0	0	0	0	0	0	45
13073641	f	ot	GB	2016	0	0	0	0	0	0	0	0	0	0	0	0	58
13073825	f	ot	FR	2016	0	1	0	0	0	1	0	0	0	0	0	0	50
13074037	Not	md	JP	2016	0	1	0	0	1	0	0	0	0	0	0	1	71

13074179	m	md	JP	2016	1	0	0	0	0	0	0	0	0	0	0	1	68
13074198	m	cn	US	2016	0	1	0	0	1	0	0	0	0	0	0	0	
13074212	f	cn	CA	2016	0	1	0	0	1	0	0	0	0	0	0	0	
13074380	m	cn	TR	2016	0	1	0	0	1	0	0	0	0	0	0	0	74
13074528	m	md	US	2016	0	1	0	0	0	1	0	0	0	0	0	0	23
13074632	m	ot	IT	2016	0	1	0	0	1	0	0	0	0	0	0	0	78
13074648	Not	ot	EG	2016	0	0	0	0	0	0	0	0	0	0	0	0	
13074698	Not	ot	EG	2016	0	0	0	0	0	0	0	0	0	0	0	0	
13074734	Not	ot	EG	2016	0	0	0	0	0	0	0	0	0	0	0	0	
13074750	Not	ot	EG	2016	0	0	0	0	0	0	0	0	0	0	0	0	
13074786	m	ot	JP	2016	0	0	0	0	0	0	0	0	0	0	0	0	83
13074998	f	md	JP	2016	1	0	0	0	0	0	0	0	0	0	0	0	100
13075017	f	md	CY	2016	1	0	0	0	0	0	0	0	0	0	0	0	72
13075164	f	cn	US	2016	1	0	0	0	0	0	0	0	0	0	0	0	
13075217	f	cn	GB	2016	1	0	0	0	0	0	0	0	0	0	0	0	
13075947	Not	md	JP	2016	0	1	0	0	1	0	0	0	0	0	0	0	60
13075998	Not	cn	US	2016	0	1	0	0	1	0	0	0	0	0	0	0	
13076730	m	ot	IT	2016	0	1	0	0	1	0	0	0	0	0	0	0	64
13076731	m	ot	IT	2016	0	1	0	0	1	0	0	0	0	0	0	0	
13077039	f	ph	JP	2016	0	0	0	0	0	0	1	0	1	0	0	0	84
13077070	m	md	SG	2016	0	1	0	0	1	0	0	0	0	0	0	0	66
13077124	m	md	FR	2016	0	0	0	0	0	0	0	0	0	0	0	0	65
13077311	m	md	JP	2016	1	0	0	0	0	0	0	0	0	0	0	1	68
7853959	m	cn	DE	2016	0	0	0	0	0	0	0	0	0	0	0	0	62
7966397	f	md	US	2016	1	0	0	0	0	0	0	0	0	0	0	0	85
7973075	f	md	US	2016	1	0	0	0	0	0	0	0	0	0	0	0	65
8535116	m	md	JP	2016	0	0	0	0	0	0	0	0	0	0	0	0	64
8956262	f	cn	GB	2016	1	0	0	0	0	0	0	0	0	0	0	0	79
8971226	m	md	US	2016	1	0	0	0	0	0	0	0	0	0	0	0	66
9369548	f	cn	US	2016	0	0	0	0	0	0	0	0	0	0	0	0	33
9401446	Not	ot	JP	2016	0	0	0	0	0	0	0	0	0	0	0	0	
9706214	m	ot	DE	2016	0	0	0	0	0	0	0	0	0	0	0	0	73
9822295	Not	Not repo	Not re	2016	1	0	0	0	0	0	0	0	0	0	0	0	
9917729	f	md	US	2016	0	0	0	0	0	0	0	0	0	0	0	0	51

12240250	f	md	US	2017	0	0	0	0	0	0	0	0	0	0	0	0	61
12249704	m	ot	JP	2017	0	1	0	0	1	0	0	0	0	0	0	1	58
12431907	f	md	US	2017	0	1	0	0	0	1	0	0	0	0	0	0	73
12434064	m	md	FR	2017	0	1	0	0	0	1	0	0	0	0	0	0	60
12460514	m	cn	JP	2017	0	1	0	0	1	0	0	0	0	0	0	1	58
12492191	m	ot	TW	2017	0	0	0	0	0	0	0	0	0	0	0	0	74
12525086	m	md	ES	2017	0	1	0	0	0	1	0	0	0	0	0	0	61
12529863	m	cn	JP	2017	0	1	0	0	1	0	0	0	0	0	0	0	72
12569757	m	md	DE	2017	0	1	0	0	0	1	0	0	0	0	0	0	79
12575278	m	md	JP	2017	1	0	0	0	0	0	0	0	0	0	0	0	65
12580165	m	md	FR	2017	0	1	0	0	0	1	0	0	0	0	0	0	78
12583975	m	ot	GB	2017	0	0	0	0	0	0	0	0	0	0	0	0	43
12596601	m	md	JP	2017	0	1	0	0	0	1	0	0	0	0	0	0	70
12607196	m	ot	US	2017	0	0	0	0	0	0	0	0	0	0	0	0	77
12614849	m	md	NL	2017	0	1	0	0	0	1	0	0	0	0	0	0	73
12615547	f	md	CA	2017	0	1	0	0	1	0	0	0	0	0	0	0	65
12624501	m	md	CA	2017	0	1	0	0	0	1	0	0	0	0	0	0	78
12634970	f	md	JP	2017	0	1	0	0	1	0	0	0	0	0	0	0	80
12650050	Not	md	BE	2017	0	0	0	0	0	0	0	0	0	0	0	0	
12650055	Not	md	IE	2017	0	0	0	0	0	0	0	0	0	0	0	0	
12650071	Not	md	IE	2017	0	0	0	0	0	0	0	0	0	0	0	0	
12650154	Not	md	IE	2017	0	0	0	0	0	0	0	0	0	0	0	0	
12650157	Not	md	FR	2017	0	0	0	0	0	0	0	0	0	0	0	0	
12650362	Not	md	ES	2017	0	0	0	0	0	0	0	0	0	0	0	0	
12651706	m	ot	DE	2017	0	1	0	0	0	1	0	0	0	0	0	0	76
12651882	f	md	JP	2017	0	1	0	0	0	1	0	0	0	0	0	0	55
12652846	Not	md	ES	2017	0	0	0	0	0	0	0	0	0	0	0	0	
12654137	f	md	JP	2017	0	0	0	0	0	0	1	0	1	0	0	0	77
12656139	m	md	JP	2017	0	0	0	0	0	0	0	0	0	0	0	0	67
12656620	Not	md	BE	2017	0	0	0	0	0	0	0	0	0	0	0	0	
12669607	f	md	KR	2017	1	0	0	0	0	0	0	0	0	0	0	0	52.3
12673256	m	md	JP	2017	0	1	0	0	0	1	0	0	0	0	0	1	64
12676608	m	ph	US	2017	0	0	0	0	0	0	0	0	0	0	0	0	62
12677960	f	ot	US	2017	0	1	0	0	0	1	0	0	0	0	0	0	69

12682871	f	ot	US	2017	0	0	0	0	0	0	1	0	1	0	0	0	90
12699581	m	md	AU	2017	0	0	0	0	0	0	0	0	0	0	0	0	55
12721151	f	cn	GB	2017	1	0	0	0	0	0	0	0	0	0	0	0	73
12739668	f	ot	GB	2017	0	1	0	0	1	0	0	0	0	0	0	0	75
12745342	m	ph	NL	2017	0	0	0	0	0	0	0	0	0	0	0	0	63
12747455	m	md	JP	2017	0	0	0	0	0	0	0	0	0	0	0	0	70
12774035	f	md	US	2017	0	1	0	0	0	1	0	0	0	0	0	0	64
12774574	f	md	GB	2017	0	0	0	0	0	0	0	0	0	0	0	0	54
12778496	m	md	US	2017	0	1	0	0	0	1	0	0	0	0	0	0	68
12779327	m	md	BR	2017	0	0	0	0	0	0	0	0	0	0	0	0	63
12781047	m	cn	JP	2017	0	1	0	0	1	0	0	0	0	0	0	1	71
12800108	m	cn	US	2017	1	0	0	0	0	0	0	0	0	0	0	1	51.8
12803703	m	ot	DE	2017	0	1	0	0	0	1	0	0	0	0	0	0	65
12805126	f	md	ES	2017	0	1	0	0	0	1	0	0	0	0	0	0	67
12805160	f	ot	BE	2017	0	1	0	0	1	0	0	0	0	0	0	0	62
12807330	m	cn	JP	2017	0	1	0	0	1	0	0	0	0	0	0	1	68
12809068	m	md	CN	2017	1	0	0	0	0	0	0	0	0	0	0	0	58
12809343	f	ot	US	2017	0	0	0	0	0	0	0	0	0	0	0	0	70
12809501	m	md	CN	2017	0	1	0	0	0	1	0	0	0	0	0	0	40
12811901	m	md	AU	2017	0	1	0	0	0	1	0	0	0	0	0	0	69
12815271	m	cn	CA	2017	0	1	0	0	1	0	0	0	0	0	0	0	68
12823362	m	md	JP	2017	0	0	0	0	0	0	0	0	0	0	0	0	75
12825369	f	cn	US	2017	0	1	1	0	0	0	0	0	0	0	0	0	76
12830642	m	cn	JP	2017	0	1	0	0	1	0	0	0	0	0	0	0	63
12835332	f	ot	US	2017	0	0	0	0	0	0	1	0	1	0	0	0	
12838321	m	ot	ES	2017	0	1	0	0	1	0	0	0	0	0	0	0	66
12839613	f	md	US	2017	0	1	1	0	0	0	0	0	0	0	0	1	64
12839905	f	md	IT	2017	0	0	0	0	0	0	0	0	0	0	0	0	49
12842026	f	cn	US	2017	1	0	0	0	0	0	0	0	0	0	0	0	
12842686	m	ot	GR	2017	0	1	0	0	1	0	0	0	0	0	0	0	60
12844605	f	md	JP	2017	1	0	0	0	0	0	0	0	0	0	0	0	63
12847466	m	md	ES	2017	0	1	0	0	0	1	0	0	0	0	0	0	75
12849130	m	md	HK	2017	1	0	0	0	0	0	0	0	0	0	0	0	65
12849288	f	md	CA	2017	0	1	0	0	1	0	0	0	0	0	0	0	73

12851102	m	md	JP	2017	0	1	0	0	0	1	0	0	0	0	0	0	72
12851684	m	md	DE	2017	0	0	0	0	0	0	0	0	0	0	0	0	80
12852590	m	ot	CN	2017	0	1	0	0	1	0	0	0	0	0	0	0	71
12852805	f	md	US	2017	0	1	1	0	0	0	0	0	0	0	0	0	58
12852820	m	ot	US	2017	0	0	0	0	0	0	1	0	1	0	0	0	69
12856504	m	ot	DE	2017	0	1	0	0	0	1	0	0	0	0	0	0	69
12857728	f	cn	GB	2017	1	0	0	0	0	0	0	0	0	0	0	0	
12858469	f	md	JP	2017	0	1	0	0	0	1	0	0	0	0	0	0	55
12858596	m	ot	JP	2017	0	1	0	0	1	0	0	0	0	0	0	0	79
12861831	Not	md	ES	2017	0	0	0	0	0	0	0	0	0	0	0	0	
12865704	m	md	US	2017	0	0	0	0	0	0	0	0	0	0	0	0	63
12866740	f	md	JP	2017	1	0	0	0	0	0	0	0	0	0	0	0	59
12873390	f	md	JP	2017	1	0	0	0	0	0	0	0	0	0	0	0	70
12875470	f	cn	JP	2017	0	1	0	0	1	0	0	0	0	0	0	0	67
12880337	f	cn	GB	2017	1	0	0	0	0	0	0	0	0	0	0	0	68
12883222	f	md	US	2017	0	1	0	0	0	1	0	0	0	0	0	0	47
12883311	m	md	JP	2017	0	1	0	0	1	0	0	0	0	0	0	1	67
12883388	f	md	US	2017	0	1	0	0	0	1	0	0	0	0	0	0	66
12886132	m	md	JP	2017	0	1	0	0	1	0	0	0	0	0	0	0	52
12888976	m	md	AU	2017	0	0	0	0	0	0	0	0	0	0	0	0	73
12890324	m	md	JP	2017	0	1	0	0	1	0	0	0	0	0	0	0	73
12891285	f	md	US	2017	0	0	0	0	0	0	0	0	0	0	0	0	57
12893277	f	md	JP	2017	1	0	0	0	0	0	0	0	0	0	0	0	87
12894289	m	ot	DE	2017	0	0	0	0	0	0	0	0	0	0	0	0	80
12894809	f	md	CA	2017	0	1	0	0	1	0	0	0	0	0	0	0	61
12899955	f	cn	JP	2017	0	1	0	0	1	0	0	0	0	0	0	0	51
12901543	m	cn	JP	2017	0	1	0	0	1	0	0	0	0	0	0	0	72
12902598	m	md	US	2017	0	0	0	0	0	0	0	0	0	0	0	0	75
12908520	m	cn	JP	2017	0	1	0	0	1	0	0	0	0	0	0	0	58
12912327	m	cn	JP	2017	0	1	0	0	1	0	0	0	0	0	0	0	76
12913183	m	md	CH	2017	0	1	0	0	1	0	0	0	0	0	0	0	66
12913485	m	md	MX	2017	0	1	0	0	0	1	0	0	0	0	0	0	63
12913507	f	ot	US	2017	0	1	0	0	1	0	0	0	0	0	0	1	
12913760	m	ot	US	2017	0	1	0	0	1	0	0	0	0	0	0	0	61

12913929	m	md	US	2017	0	1	0	0	1	0	0	0	0	0	0	0	
12914289	f	md	JP	2017	0	0	0	0	0	0	1	0	1	0	0	0	59
12917292	f	cn	IT	2017	0	1	0	0	1	0	0	0	0	0	0	0	74
12918661	f	cn	JP	2017	0	1	0	0	1	0	0	0	0	0	0	0	83
12921638	m	cn	JP	2017	0	0	0	0	0	0	1	0	1	0	0	0	76
12922001	f	ot	KR	2017	0	0	0	0	0	0	0	0	0	0	0	0	51
12922282	m	md	JP	2017	0	1	0	0	1	0	0	0	0	0	0	0	74
12922422	m	md	US	2017	0	1	0	0	1	0	0	0	0	0	0	0	72
12924748	f	ph	JP	2017	0	0	0	0	0	0	0	0	0	0	0	0	30
12927859	m	md	BE	2017	0	1	0	0	0	1	0	0	0	0	0	1	65
12931798	m	cn	JP	2017	0	1	0	0	1	0	0	0	0	0	0	0	64
12934847	m	ot	FR	2017	0	1	0	0	1	0	0	0	0	0	0	0	69
12935694	m	md	IT	2017	0	1	0	0	0	1	0	0	0	0	0	0	67
12936019	f	md	US	2017	0	1	0	0	0	1	0	0	0	0	0	0	75
12936453	m	md	JP	2017	0	1	0	0	0	1	0	0	0	0	0	0	55
12937713	m	ph	US	2017	0	1	0	0	1	0	0	0	0	0	0	0	53
12937718	m	md	JP	2017	0	1	0	0	1	0	0	0	0	0	0	0	63
12938835	m	cn	JP	2017	0	1	0	0	1	0	0	0	0	0	0	1	71
12939963	m	md	FR	2017	0	1	0	0	0	1	0	0	0	0	0	0	52
12940089	m	md	JP	2017	0	1	0	0	1	0	0	0	0	0	0	0	60
12940226	m	ot	US	2017	0	1	0	0	1	0	0	0	0	0	0	0	66
12941293	m	md	JP	2017	0	1	0	0	1	0	0	0	0	0	0	0	79
12942775	m	cn	IT	2017	0	1	0	0	1	0	0	0	0	0	0	0	67
12942864	m	md	IT	2017	0	0	0	0	0	0	0	0	0	0	0	0	68
12944420	m	ot	US	2017	0	1	0	0	1	0	0	0	0	0	0	0	55
12946204	m	cn	US	2017	0	1	1	0	0	0	0	0	0	0	0	0	75
12954200	f	cn	IT	2017	0	1	0	0	1	0	0	0	0	0	0	0	70
12954201	f	cn	IT	2017	0	1	0	0	1	0	0	0	0	0	0	0	79
12955500	m	cn	CA	2017	0	1	0	0	1	0	0	0	0	0	0	0	
12956024	f	md	JP	2017	0	0	0	0	0	0	0	0	0	0	0	0	
12956627	m	cn	US	2017	0	0	0	0	0	0	0	0	0	0	0	0	48
12957378	Not	md	US	2017	0	0	0	0	0	0	0	0	0	0	0	0	
12957578	Not	ot	US	2017	0	0	0	0	0	0	0	0	0	0	0	0	
12957583	m	ot	DE	2017	0	1	0	0	1	0	0	0	0	0	0	0	58

12957919	f	cn	BR	2017	1	0	0	0	0	0	0	0	0	0	0	0	63
12958099	f	md	AT	2017	0	1	0	0	0	1	0	0	0	0	0	0	50
12958554	m	md	JP	2017	0	1	0	0	0	1	0	0	0	0	0	0	66
12959031	f	ot	DE	2017	0	1	0	0	1	0	0	0	0	0	0	1	76
12961097	Not	ot	US	2017	0	1	1	0	0	0	0	0	0	0	0	0	
12962731	f	cn	US	2017	0	1	0	0	1	0	0	0	0	0	0	0	
12964483	m	ot	US	2017	0	0	0	0	0	0	0	0	0	0	0	0	69
12967425	m	md	ES	2017	0	1	0	0	1	0	0	0	0	0	0	1	81
12967426	m	cn	IT	2017	0	1	0	0	1	0	0	0	0	0	0	0	57
12970184	f	md	JP	2017	1	0	0	0	0	0	0	0	0	0	0	0	42
12970459	m	md	AR	2017	0	1	0	0	1	0	0	0	0	0	0	0	53
12970462	f	cn	IT	2017	0	1	0	0	1	0	0	0	0	0	0	0	74
12972094	m	md	ES	2017	0	1	0	0	0	1	0	0	0	0	0	0	79
12972693	m	md	KR	2017	1	0	0	0	0	0	0	0	0	0	0	0	75
12973793	m	cn	JP	2017	0	1	0	0	1	0	0	0	0	0	0	0	31
12973886	m	md	KR	2017	1	0	0	0	0	0	0	0	0	0	0	0	75
12974280	m	md	JP	2017	1	0	0	0	0	0	0	0	0	0	0	0	68
12974574	m	md	JP	2017	0	1	0	0	1	0	0	0	0	0	0	0	75
12975655	m	ot	FR	2017	0	1	0	0	1	0	0	0	0	0	0	0	58
12975986	f	md	JP	2017	1	0	0	0	0	0	0	0	0	0	0	0	69
12977188	m	cn	CA	2017	0	1	0	0	1	0	0	0	0	0	0	0	56
12977381	f	md	US	2017	0	1	0	0	0	1	0	0	0	0	0	0	65
12977808	f	md	NL	2017	0	0	0	0	0	0	0	0	0	0	0	0	73
12980166	m	md	RO	2017	0	0	0	0	0	0	0	0	0	0	0	0	61
12980350	f	cn	KR	2017	1	0	0	0	0	0	0	0	0	0	0	0	74
12981226	m	md	CA	2017	0	1	0	0	1	0	0	0	0	0	0	0	
12981319	m	ot	CA	2017	0	1	0	0	1	0	0	0	0	0	0	0	74
12981410	m	ot	CA	2017	0	1	0	0	1	0	0	0	0	0	0	0	
12983419	f	md	JP	2017	1	0	0	0	0	0	0	0	0	0	0	0	66
12984677	m	ot	ES	2017	0	1	0	0	0	1	0	0	0	0	0	0	70
12985546	m	md	BE	2017	0	1	0	0	1	0	0	0	0	0	0	0	73
12985757	m	ot	CA	2017	0	1	0	0	1	0	0	0	0	0	0	0	
12986015	f	cn	CA	2017	0	1	0	0	1	0	0	0	0	0	0	0	
12986146	m	cn	CA	2017	0	1	0	0	1	0	0	0	0	0	0	0	

12986479	m	cn	CA	2017	0	1	0	0	1	0	0	0	0	0	0	0	71
12987353	f	md	CA	2017	0	1	0	0	1	0	0	0	0	0	0	0	
12987354	m	cn	CA	2017	0	1	0	0	1	0	0	0	0	0	0	0	81
12989787	f	md	CA	2017	0	1	0	0	1	0	0	0	0	0	0	0	
12990889	m	cn	US	2017	0	1	1	0	0	0	0	0	0	0	0	0	80
12990998	m	cn	CA	2017	0	1	0	0	1	0	0	0	0	0	0	0	
12991821	m	md	US	2017	0	0	0	0	0	0	0	0	0	0	0	0	65
12992760	m	md	DE	2017	0	1	0	0	0	1	0	0	0	0	0	0	61
12992860	m	md	ES	2017	0	1	0	0	0	1	0	0	0	0	0	0	57
12994697	f	md	CA	2017	0	1	0	0	1	0	0	0	0	0	0	0	
12996011	f	ot	US	2017	0	1	0	0	1	0	0	0	0	0	0	1	75
12996863	f	cn	CA	2017	0	1	0	0	1	0	0	0	0	0	0	0	
12996950	f	cn	CA	2017	0	1	0	0	1	0	0	0	0	0	0	0	
12996951	f	ot	CA	2017	0	1	0	0	1	0	0	0	0	0	0	0	
12997231	f	ot	KR	2017	0	1	0	0	1	0	0	0	0	0	0	0	67
12998894	f	md	JP	2017	1	0	0	0	0	0	0	0	0	0	0	1	71
13000304	m	md	SI	2017	0	0	0	0	0	0	0	0	0	0	0	0	67
13001907	m	cn	US	2017	0	1	1	0	0	0	0	0	0	0	0	0	52
13002258	f	cn	CA	2017	0	1	0	0	1	0	0	0	0	0	0	0	68
13003706	m	cn	US	2017	0	1	1	0	0	0	0	0	0	0	0	0	42
13004238	m	md	FR	2017	0	0	0	0	0	0	0	0	0	0	0	0	68
13004383	Not	md	US	2017	0	0	0	0	0	0	0	0	0	0	0	0	
13004543	m	md	NL	2017	0	1	0	0	0	1	0	0	0	0	0	0	77
13004635	f	cn	US	2017	1	0	0	0	0	0	0	0	0	0	0	0	
13004957	f	ot	JP	2017	0	1	0	0	1	0	0	0	0	0	0	0	64
13006665	f	ph	JP	2017	1	0	0	0	0	0	0	0	0	0	0	0	59
13008266	f	md	JP	2017	0	1	0	0	1	0	0	0	0	0	0	0	75
13008332	f	md	DE	2017	0	1	0	0	0	1	0	0	0	0	0	0	60
13008513	m	ot	CA	2017	0	1	0	0	1	0	0	0	0	0	0	0	
13010273	f	ot	DE	2017	0	1	0	0	0	1	0	0	0	0	0	0	63
13010439	m	cn	US	2017	0	1	0	0	1	0	0	0	0	0	0	0	
13011104	f	md	IT	2017	0	0	0	0	0	0	0	0	0	0	0	0	62
13011180	f	md	CN	2017	1	0	0	0	0	0	0	0	0	0	0	0	67.9
13011420	f	md	CL	2017	0	1	0	0	0	1	0	0	0	0	0	0	41

13012458	f	cn	GB	2017	1	0	0	0	0	0	0	0	0	0	0	0	89
13013550	f	cn	GB	2017	1	0	0	0	0	0	0	0	0	0	0	0	55
13013671	m	md	JP	2017	0	1	0	0	1	0	0	0	0	0	0	0	63
13014273	f	md	DE	2017	0	1	0	0	1	0	0	0	0	0	0	0	72
13014400	m	ot	CA	2017	0	1	0	0	1	0	0	0	0	0	0	0	
13014501	m	md	JP	2017	0	1	0	0	1	0	0	0	0	0	0	0	56
13014757	m	md	JP	2017	0	1	0	0	1	0	0	0	0	0	0	0	69
13015059	f	md	US	2017	0	1	1	0	0	0	0	0	0	0	0	0	75
13015211	m	ot	CA	2017	0	1	0	0	1	0	0	0	0	0	0	0	75
13015217	f	cn	JP	2017	0	1	0	0	1	0	0	0	0	0	0	0	70
13015806	m	md	CA	2017	0	1	0	0	0	1	0	0	0	0	0	0	65
13016808	m	cn	IT	2017	0	1	0	0	1	0	0	0	0	0	0	0	50
13018224	f	md	CA	2017	0	1	0	0	1	0	0	0	0	0	0	0	
13020854	f	cn	US	2017	0	1	1	0	0	0	0	0	0	0	0	1	65
13021215	m	ot	US	2017	0	1	1	0	0	0	0	0	0	0	0	0	
13022473	f	md	US	2017	0	1	1	0	0	0	0	0	0	0	0	0	77
13023741	f	cn	CA	2017	0	1	0	0	1	0	0	0	0	0	0	0	
13024019	m	ot	US	2017	0	1	1	0	0	0	0	0	0	0	0	0	82
13024193	f	ot	ES	2017	0	1	0	0	0	1	0	0	0	0	0	0	66
13024958	f	md	US	2017	0	1	1	0	0	0	0	0	0	0	0	0	61
13025067	f	md	JP	2017	0	1	0	0	1	0	0	0	0	0	0	1	84
13025130	m	ot	CA	2017	0	1	0	0	1	0	0	0	0	0	0	0	63
13025314	f	ot	CA	2017	0	1	0	0	1	0	0	0	0	0	0	0	
13025861	f	md	JP	2017	1	0	0	0	0	0	0	0	0	0	0	0	80
13026854	f	cn	CA	2017	0	1	0	0	1	0	0	0	0	0	0	0	
13027998	f	ph	US	2017	0	1	1	0	0	0	0	0	0	0	0	0	
13029382	m	cn	US	2017	0	1	0	0	1	0	0	0	0	0	0	0	
13029771	m	md	JP	2017	0	1	0	0	0	1	0	0	0	0	0	0	72
13030214	m	md	CA	2017	0	1	0	0	1	0	0	0	0	0	0	0	63
13030535	m	md	QA	2017	0	0	0	0	0	0	0	0	0	0	0	0	60
13033812	m	md	US	2017	0	1	1	0	0	0	0	0	0	0	0	0	60
13034670	m	md	HK	2017	1	0	0	0	0	0	0	0	0	0	0	1	65
13034820	m	md	ES	2017	0	0	0	0	0	0	0	0	0	0	0	0	51
13034908	f	md	CA	2017	0	1	0	0	1	0	0	0	0	0	0	0	

13035050	m	md	CA	2017	0	1	1	0	0	0	0	0	0	0	0	0	69
13037477	f	md	US	2017	1	0	0	0	0	0	0	0	0	0	0	1	63
13037930	m	md	JP	2017	0	1	0	0	1	0	0	0	0	0	0	1	54
13037950	f	ot	US	2017	0	1	0	0	1	0	0	0	0	0	0	1	
13037961	m	cn	US	2017	0	1	0	0	1	0	0	0	0	0	0	0	81
13039048	f	md	DE	2017	0	0	0	0	0	0	0	0	0	0	0	0	
13040771	m	md	KR	2017	0	1	0	0	0	1	0	0	0	0	0	0	61
13043417	f	md	US	2017	0	1	1	0	0	0	0	0	0	0	0	0	67
13043493	m	md	CN	2017	0	1	0	0	0	1	0	0	0	0	0	0	46
13044487	m	md	CZ	2017	0	1	0	0	1	0	0	0	0	0	0	1	61
13045337	m	md	CA	2017	0	1	0	0	1	0	0	0	0	0	0	0	58
13045880	m	cn	US	2017	0	1	1	0	0	0	0	0	0	0	0	0	54
13046186	m	md	US	2017	0	0	0	0	0	0	0	0	0	0	0	0	66
13046337	f	md	IT	2017	0	0	0	0	0	0	0	0	0	0	0	0	68
13046414	f	md	IT	2017	0	0	0	0	0	0	0	0	0	0	0	0	37
13047024	m	md	JP	2017	0	1	0	0	0	1	0	0	0	0	0	0	55
13047563	m	md	JP	2017	0	1	0	0	1	0	0	0	0	0	0	0	57
13048677	f	cn	GB	2017	1	0	0	0	0	0	0	0	0	0	0	0	72
13049634	f	cn	LB	2017	0	0	0	0	0	0	0	0	0	0	0	0	75
13051180	f	cn	GB	2017	1	0	0	0	0	0	0	0	0	0	0	0	62
13051396	f	cn	US	2017	1	0	0	0	0	0	0	0	0	0	0	1	72
13052462	f	md	TH	2017	1	0	0	0	0	0	0	0	0	0	0	0	78
13052473	m	ot	EG	2017	0	1	0	0	1	0	0	0	0	0	0	0	
13052869	Not	md	CH	2017	0	1	0	0	1	0	0	0	0	0	0	0	
13053170	m	ot	GR	2017	0	1	0	0	1	0	0	0	0	0	0	0	51
13053184	m	md	CN	2017	0	1	0	0	0	1	0	0	0	0	0	0	65
13053226	m	md	DE	2017	0	1	0	0	0	1	0	0	0	0	0	0	66
13053740	m	md	CZ	2017	0	1	0	0	1	0	0	0	0	0	0	0	76
13054137	f	md	FR	2017	0	1	0	0	1	0	0	0	0	0	0	0	
13054233	m	ot	CN	2017	0	0	0	0	0	0	0	0	0	0	0	0	75
13054264	f	md	KR	2017	0	0	0	0	0	0	0	0	0	0	0	0	60
13055564	m	md	JP	2017	0	1	0	0	1	0	0	0	0	0	0	0	69
13055838	f	cn	US	2017	1	0	0	0	0	0	0	0	0	0	0	1	62
13056486	m	ot	AR	2017	0	1	0	0	1	0	0	0	0	0	0	0	80

13056487	f	cn	US	2017	0	1	0	0	1	0	0	0	0	0	0	0	77
13056489	m	ot	FR	2017	0	1	0	0	1	0	0	0	0	0	0	1	68
13056973	f	md	US	2017	0	1	1	0	0	0	0	0	0	0	0	0	72
13057083	m	ot	US	2017	0	0	0	0	0	0	0	0	0	0	0	0	75
13057231	m	cn	JP	2017	0	1	0	0	1	0	0	0	0	0	0	0	63
13057400	m	md	KR	2017	1	0	0	0	0	0	0	0	0	0	0	0	64.2
13057627	f	md	JP	2017	0	1	0	0	0	1	0	0	0	0	0	1	71
13058800	m	md	ES	2017	0	1	0	0	0	1	0	0	0	0	0	0	74
13060213	m	md	JP	2017	0	1	0	0	1	0	0	0	0	0	0	0	73
13060236	f	cn	US	2017	0	1	0	0	1	0	0	0	0	0	0	0	
13060677	Not	cn	US	2017	0	1	1	0	0	0	0	0	0	0	0	0	
13060692	m	md	JP	2017	1	0	0	0	0	0	0	0	0	0	0	1	87.1
13062028	m	md	HU	2017	0	1	0	0	1	0	0	0	0	0	0	0	68
13062241	Not	cn	US	2017	0	1	1	0	0	0	0	0	0	0	0	0	
13062407	m	md	FR	2017	0	1	0	0	1	0	0	0	0	0	0	0	
13062472	m	md	US	2017	0	1	1	0	0	0	0	0	0	0	0	0	80
13063052	m	md	JP	2017	0	1	0	0	1	0	0	0	0	0	0	0	69
13063474	f	md	JP	2017	0	0	0	0	0	0	0	0	0	0	0	0	73
13063628	f	cn	US	2017	0	1	1	0	0	0	0	0	0	0	0	0	
13065363	m	cn	US	2017	0	1	0	0	1	0	0	0	0	0	0	0	
13065398	m	md	PL	2017	0	0	0	0	0	0	0	0	0	0	0	0	73
13065500	m	cn	JP	2017	0	1	0	0	1	0	0	0	0	0	0	0	73
13066655	f	md	DE	2017	0	1	0	0	0	1	0	0	0	0	0	0	60
13066691	m	md	DE	2017	0	1	0	0	0	1	0	0	0	0	0	0	61
13066874	f	ph	US	2017	0	1	0	0	0	1	0	0	0	0	0	0	
13068631	m	cn	HK	2017	0	0	0	0	0	0	0	0	0	0	0	0	
13068725	f	md	DK	2017	0	0	0	0	0	0	0	0	0	0	0	0	80
13068862	m	ot	ES	2017	0	0	0	0	0	0	0	0	0	0	0	0	78
13069827	m	ot	US	2017	0	0	0	0	0	0	0	0	0	0	0	0	76
13070119	m	md	IL	2017	0	0	0	0	0	0	0	0	0	0	0	0	60
13070194	f	md	CA	2017	1	0	0	0	0	0	0	0	0	0	0	0	61
13070639	m	md	JP	2017	0	1	0	0	0	1	0	0	0	0	0	1	70
13071788	f	md	JP	2017	1	0	0	0	0	0	0	0	0	0	0	0	84
13072435	m	md	ES	2017	0	1	0	0	0	1	0	0	0	0	0	0	56

13072483	m	md	PL	2017	0	0	0	0	0	0	0	0	0	0	0	0	76
13073140	m	ph	JP	2017	0	1	0	0	1	0	0	0	0	0	0	0	81
13074159	m	ot	US	2017	0	1	0	0	1	0	0	0	0	0	0	0	61
13074323	m	md	IT	2017	0	1	0	0	1	0	0	0	0	0	0	0	
13075136	f	md	GB	2017	0	0	0	0	0	0	0	0	0	0	0	0	68
13075478	m	ot	IN	2017	0	0	0	0	0	0	0	0	0	0	0	0	54
13075544	m	ot	JP	2017	0	0	0	0	0	0	0	0	0	0	0	0	69
13076114	m	md	IT	2017	0	0	0	0	0	0	0	0	0	0	0	0	37
13076733	m	cn	US	2017	0	1	0	0	1	0	0	0	0	0	0	0	
13076791	m	md	DE	2017	0	0	0	0	0	0	0	0	0	0	0	0	68
13077777	m	md	JP	2017	0	1	0	0	1	0	0	0	0	0	0	0	64
13077828	m	md	IL	2017	0	0	0	0	0	0	0	0	0	0	0	0	31
13077911	m	md	US	2017	0	1	0	0	1	0	0	0	0	0	0	0	61
13078417	f	cn	US	2017	1	0	0	0	0	0	0	0	0	0	0	0	45.8
13078466	m	ot	JP	2017	0	0	0	0	0	0	0	0	0	0	0	0	77
13078623	f	cn	US	2017	0	1	0	0	1	0	0	0	0	0	0	0	
13078675	m	ot	CA	2017	0	1	0	0	1	0	0	0	0	0	0	0	
13078950	m	md	FR	2017	0	0	0	0	0	0	0	0	0	0	0	0	63
13079024	f	cn	US	2017	1	0	0	0	0	0	0	0	0	0	0	0	65
13079040	m	cn	US	2017	0	1	0	0	1	0	0	0	0	0	0	0	
13079066	m	md	JP	2017	0	1	0	0	1	0	0	0	0	0	0	0	80
13079262	f	md	ES	2017	0	1	0	0	0	1	0	0	0	0	0	0	65
13079480	Not	ph	AU	2017	0	1	0	0	1	0	0	0	0	0	0	0	71
13079481	f	cn	JP	2017	0	1	0	0	1	0	0	0	0	0	0	0	61
13079487	m	md	TR	2017	0	1	0	0	1	0	0	0	0	0	0	0	64
13079705	Not	ph	AU	2017	0	1	0	0	1	0	0	0	0	0	0	0	88
13079802	m	cn	US	2017	0	1	0	0	1	0	0	0	0	0	0	0	
13079951	m	cn	GB	2017	1	0	0	0	0	0	0	0	0	0	0	0	65
13080124	f	md	KR	2017	1	0	0	0	0	0	0	0	0	0	0	1	63.8
13080161	m	md	BR	2017	0	0	0	0	0	0	0	0	0	0	0	0	76
13080201	m	md	JP	2017	0	0	0	0	0	0	0	0	0	0	0	0	35
13080202	f	md	JP	2017	0	0	0	0	0	0	0	0	0	0	0	0	58
13080527	f	cn	GB	2017	1	0	0	0	0	0	0	0	0	0	0	0	62
13080806	f	md	DE	2017	0	0	0	0	0	0	0	0	0	0	0	0	80

13081578	m	md	DE	2017	0	0	0	0	0	0	0	0	0	0	0	0	63
13081639	f	md	JP	2017	1	0	0	0	0	0	0	0	0	0	0	0	75
13082024	Not	ot	US	2017	0	0	0	0	0	0	0	0	0	0	0	0	
13082027	Not	ot	US	2017	0	0	0	0	0	0	0	0	0	0	0	0	
13082066	Not	ot	US	2017	0	0	0	0	0	0	0	0	0	0	0	0	
13082133	m	ot	ES	2017	0	1	0	0	0	1	0	0	0	0	0	0	70
13082273	Not	ot	US	2017	0	0	0	0	0	0	0	0	0	0	0	0	
13082291	Not	ot	FR	2017	0	0	0	0	0	0	0	0	0	0	0	0	
13082530	Not	Not repo	CN	2017	1	0	0	0	0	0	0	0	0	0	0	0	
13082779	f	cn	US	2017	1	0	0	0	0	0	0	0	0	0	0	0	90
13082931	m	cn	US	2017	0	1	0	0	1	0	0	0	0	0	0	0	
13082958	m	md	US	2017	0	1	0	0	1	0	0	0	0	0	0	0	
13082959	m	cn	US	2017	0	1	0	0	1	0	0	0	0	0	0	0	
13083264	m	md	JP	2017	0	1	0	0	1	0	0	0	0	0	0	0	78
13083267	m	md	JP	2017	0	1	0	0	1	0	0	0	0	0	0	0	74
13083275	Not	ot	US	2017	0	0	0	0	0	0	0	0	0	0	0	0	
13083374	Not	md	BR	2017	0	0	0	0	0	0	0	0	0	0	0	0	
13083514	m	ot	TR	2017	0	1	0	0	1	0	0	0	0	0	0	0	56
13083919	m	md	CN	2017	0	1	0	0	0	1	0	0	0	0	0	0	66
13083981	m	ot	US	2017	0	1	0	0	1	0	0	0	0	0	0	0	
13083982	f	md	IT	2017	0	1	0	0	1	0	0	0	0	0	0	0	66
13084078	f	cn	CN	2017	1	0	0	0	0	0	0	0	0	0	0	0	70
13084259	f	cn	GB	2017	1	0	0	0	0	0	0	0	0	0	0	0	65
13084425	m	md	ES	2017	0	1	0	0	0	1	0	0	0	0	0	0	69
13084500	m	cn	GB	2017	1	0	0	0	0	0	0	0	0	0	0	0	49
13084566	m	md	US	2017	0	1	1	0	0	0	0	0	0	0	0	0	83
13084569	m	md	KR	2017	0	1	0	0	1	0	0	0	0	0	0	0	54
13084678	Not	md	AU	2017	0	1	0	0	1	0	0	0	0	0	0	0	
13085576	f	ph	JP	2017	1	0	0	0	0	0	0	0	0	0	0	0	59
13085702	m	md	SG	2017	0	0	0	0	0	0	0	0	0	0	0	0	52
13085954	m	ot	US	2017	0	0	0	0	0	0	0	0	0	0	0	0	83
13085955	Not	ot	US	2017	0	0	0	0	0	0	0	0	0	0	0	0	
13086372	f	ot	IL	2017	0	1	0	0	1	0	0	0	0	0	0	0	80
13086524	m	md	ES	2017	0	1	0	0	0	1	0	0	0	0	0	0	51

13086608	m	cn	US	2017	0	1	0	0	1	0	0	0	0	0	0	0	68
13086795	m	md	IL	2017	0	0	0	0	0	0	0	0	0	0	0	0	58
13087036	m	cn	US	2017	0	1	0	0	1	0	0	0	0	0	0	0	55
13087089	m	md	JP	2017	0	1	0	0	1	0	0	0	0	0	0	0	67
13087332	m	md	US	2017	0	1	0	0	1	0	0	0	0	0	0	0	
13087435	m	ot	US	2017	0	1	0	0	1	0	0	0	0	0	0	0	53
13087446	m	ot	JP	2017	0	1	0	0	1	0	0	0	0	0	0	0	46
13087541	m	md	NL	2017	0	1	0	0	0	1	0	0	0	0	0	0	44
13087552	Not	ot	ES	2017	0	0	0	0	0	0	0	0	0	0	0	0	
13088325	f	Not repo	US	2017	1	0	0	0	0	0	0	0	0	0	0	0	72.3
13088419	f	cn	AR	2017	0	0	0	0	0	0	0	0	0	0	0	0	72
13088702	f	Not repo	US	2017	1	0	0	0	0	0	0	0	0	0	0	0	72.9
13088912	f	md	JP	2017	1	0	0	0	0	0	0	0	0	0	0	0	68
13089204	m	md	AU	2017	0	1	0	0	1	0	0	0	0	0	0	0	67
13089310	m	md	JP	2017	1	0	0	0	0	0	0	0	0	0	0	1	80
13089318	Not	ot	JP	2017	0	0	0	0	0	0	0	0	0	0	0	0	
13089319	Not	ot	JP	2017	0	0	0	0	0	0	0	0	0	0	0	0	
13089349	f	cn	US	2017	1	0	0	0	0	0	0	0	0	0	0	0	53
13089634	f	cn	GB	2017	1	0	0	0	0	0	0	0	0	0	0	0	79
13089649	f	cn	GB	2017	1	0	0	0	0	0	0	0	0	0	0	0	37
13089830	f	md	JP	2017	1	0	0	0	0	0	0	0	0	0	0	0	65
13090165	f	md	IT	2017	0	0	0	0	0	0	0	0	0	0	0	0	55
13090214	f	ot	GB	2017	0	1	0	1	0	0	0	0	0	0	0	0	48
13091418	m	ot	IE	2017	0	1	0	0	1	0	0	0	0	0	0	0	68
13091513	m	cn	DE	2017	0	1	0	0	1	0	0	0	0	0	0	0	77
13092065	f	md	CN	2017	0	1	0	0	0	1	0	0	0	0	0	0	48
13092266	m	ot	FR	2017	0	1	0	0	1	0	0	0	0	0	0	0	73
13093502	m	md	PT	2017	0	1	0	0	1	0	0	0	0	0	0	0	37
13093849	m	md	GR	2017	0	0	0	0	0	0	0	0	0	0	0	0	
13093959	m	ot	ES	2017	0	1	0	0	0	1	0	0	0	0	0	0	62
13094199	Not	ot	AU	2017	0	0	0	0	0	0	0	0	0	0	0	0	
13094220	Not	ot	AU	2017	0	0	0	0	0	0	0	0	0	0	0	0	
13094221	Not	ot	AU	2017	0	0	0	0	0	0	0	0	0	0	0	0	
13094355	f	md	US	2017	0	0	0	0	0	0	0	0	0	0	0	0	43

13094716	f	md	US	2017	0	1	0	0	1	0	0	0	0	0	0	0	
13094779	Not	ot	US	2017	0	0	0	0	0	0	0	0	0	0	0	0	
13095153	Not	md	CH	2017	0	0	0	0	0	0	0	0	0	0	0	0	
13095659	m	cn	FR	2017	0	1	0	0	1	0	0	0	0	0	0	0	60
13095678	f	cn	CA	2017	0	1	0	0	1	0	0	0	0	0	0	0	
13095747	m	md	JP	2017	0	1	0	0	1	0	0	0	0	0	0	0	70
13096473	m	cn	US	2017	0	1	0	0	1	0	0	0	0	0	0	0	
13097063	m	md	IL	2017	0	1	0	0	0	1	0	0	0	0	0	0	67
13097160	m	cn	JP	2017	0	1	0	0	1	0	0	0	0	0	0	0	70
13097787	Not	ot	US	2017	0	0	0	0	0	0	0	0	0	0	0	0	
13097796	Not	ot	US	2017	0	0	0	0	0	0	0	0	0	0	0	0	
13097804	m	md	GB	2017	0	0	0	0	0	0	0	0	0	0	0	0	59
13098090	m	md	GB	2017	0	1	0	0	0	1	0	0	0	0	0	0	50
13098217	f	cn	US	2017	0	1	0	0	1	0	0	0	0	0	0	0	
13098687	f	md	JP	2017	0	1	0	0	1	0	0	0	0	0	0	0	80
13098755	m	cn	US	2017	0	0	0	0	0	0	0	0	0	0	0	0	
13098846	m	md	UA	2017	0	0	0	0	0	0	0	0	0	0	0	0	38
13098904	m	cn	JP	2017	0	1	0	0	1	0	0	0	0	0	0	0	66
13099539	m	cn	US	2017	0	1	0	0	1	0	0	0	0	0	0	0	
13099550	f	md	CN	2017	1	0	0	0	0	0	0	0	0	0	0	0	54
13099670	m	cn	US	2017	0	1	0	0	1	0	0	0	0	0	0	0	
13099943	f	ot	US	2017	0	1	1	0	0	0	0	0	0	0	0	0	68
13099972	Not	md	DE	2017	0	0	0	0	0	0	0	0	0	0	0	0	
13099974	Not	md	DE	2017	0	0	0	0	0	0	0	0	0	0	0	0	
13099982	Not	md	IE	2017	0	0	0	0	0	0	0	0	0	0	0	0	
13100299	Not	md	IE	2017	0	0	0	0	0	0	0	0	0	0	0	0	
13100353	m	md	GR	2017	0	0	0	0	0	0	0	0	0	0	0	0	
13100354	m	md	GR	2017	0	0	0	0	0	0	0	0	0	0	0	0	
13100356	m	md	GR	2017	0	0	0	0	0	0	0	0	0	0	0	0	
13100583	f	ot	US	2017	0	1	0	0	1	0	0	0	0	0	0	0	71
13100661	Not	ot	TR	2017	0	0	0	0	0	0	0	0	0	0	0	0	
13100671	Not	ot	TR	2017	0	0	0	0	0	0	0	0	0	0	0	0	
13100673	Not	ot	US	2017	0	0	0	0	0	0	0	0	0	0	0	0	
13100675	Not	ot	TR	2017	0	0	0	0	0	0	0	0	0	0	0	0	

13100955	Not	md	HU	2017	0	1	1	0	0	0	0	0	0	0	0	0	
13101069	m	cn	US	2017	0	1	1	0	0	0	0	0	0	0	0	0	79
13101182	m	cn	US	2017	0	1	1	0	0	0	0	0	0	0	0	0	63
13101302	f	cn	US	2017	0	1	1	0	0	0	0	0	0	0	0	0	
13101384	Not	md	US	2017	0	0	0	0	0	0	0	0	0	0	0	0	
13101675	f	md	US	2017	0	1	0	0	1	0	0	0	0	0	0	0	69
13101917	Not	md	US	2017	0	0	0	0	0	0	0	0	0	0	0	0	
13101934	Not	md	US	2017	0	0	0	0	0	0	0	0	0	0	0	0	
13101995	f	cn	US	2017	0	1	0	0	1	0	0	0	0	0	0	0	79
13102014	m	cn	US	2017	0	1	0	0	1	0	0	0	0	0	0	0	76
13102058	f	md	TH	2017	1	0	0	0	0	0	0	0	0	0	0	0	54
13102237	Not	md	US	2017	0	0	0	0	0	0	0	0	0	0	0	0	
13102247	Not	md	US	2017	0	0	0	0	0	0	0	0	0	0	0	0	
13102374	Not	ot	US	2017	0	0	0	0	0	0	0	0	0	0	0	0	
13102511	m	md	JP	2017	1	0	0	0	0	0	0	0	0	0	0	0	68
13102695	f	cn	US	2017	1	0	0	0	0	0	0	0	0	0	0	0	68
13102800	Not	ot	US	2017	0	0	0	0	0	0	0	0	0	0	0	0	
13102854	m	ph	ES	2017	0	0	0	0	0	0	0	0	0	0	0	0	
13103061	m	ot	CN	2017	0	1	0	0	1	0	0	0	0	0	0	1	72
13103378	Not	md	ES	2017	0	0	0	0	0	0	0	0	0	0	0	0	
13103379	Not	md	IE	2017	0	0	0	0	0	0	0	0	0	0	0	0	
13103476	Not	md	ES	2017	0	0	0	0	0	0	0	0	0	0	0	0	
13103526	f	Not repo	US	2017	1	0	0	0	0	0	0	0	0	0	0	0	
13103721	m	cn	JP	2017	0	1	0	0	1	0	0	0	0	0	0	0	72
13104268	m	md	NL	2017	0	0	0	0	0	0	0	0	0	0	0	0	72
13104451	m	ot	IT	2017	0	1	0	0	1	0	0	0	0	0	0	0	79
13104509	m	ph	US	2017	0	1	1	0	0	0	0	0	0	0	0	0	62
13104632	Not	ot	JP	2017	0	0	0	0	0	0	0	0	0	0	0	0	
13104654	Not	ot	JP	2017	0	0	0	0	0	0	0	0	0	0	0	0	
13104665	Not	ot	JP	2017	0	0	0	0	0	0	0	0	0	0	0	0	
13104666	Not	ot	JP	2017	0	0	0	0	0	0	0	0	0	0	0	0	
13105213	m	ot	US	2017	0	1	0	0	1	0	0	0	0	0	0	0	
13105261	f	cn	CA	2017	0	1	0	0	1	0	0	0	0	0	0	0	56
13105278	m	md	FR	2017	0	1	0	0	0	1	0	0	0	0	0	0	70

13105285	Not	ot	US	2017	0	0	0	0	0	0	0	0	0	0	0	0	
13105288	m	md	US	2017	0	1	0	0	0	1	0	0	0	0	0	0	70
13105307	m	cn	GB	2017	0	0	0	0	0	0	0	0	0	0	0	0	59
13105316	Not	md	US	2017	0	0	0	0	0	0	0	0	0	0	0	0	
13105354	Not	md	US	2017	0	0	0	0	0	0	0	0	0	0	0	0	
13105396	Not	cn	US	2017	0	1	1	0	0	0	0	0	0	0	0	1	
13106231	m	cn	IT	2017	0	1	0	0	1	0	0	0	0	0	0	0	78
13107584	f	cn	US	2017	0	1	0	0	1	0	0	0	0	0	0	0	70
13108393	f	cn	GB	2017	1	0	0	0	0	0	0	0	0	0	0	0	
13108454	m	md	JP	2017	0	1	0	0	1	0	0	0	0	0	0	1	70
13108621	m	cn	US	2017	0	1	1	0	0	0	0	0	0	0	0	0	
13108681	f	ot	US	2017	0	1	0	0	1	0	0	0	0	0	0	1	83
13109313	m	cn	US	2017	0	1	0	0	1	0	0	0	0	0	0	0	
13109333	Not	ot	JP	2017	0	0	0	0	0	0	0	0	0	0	0	0	
13109554	m	ph	JP	2017	0	1	0	0	1	0	0	0	0	0	0	0	61
13109613	m	ph	US	2017	0	0	0	0	0	0	0	0	0	0	0	0	69
13109697	m	ot	GB	2017	0	0	0	0	0	0	0	0	0	0	0	0	59
13111102	m	ph	US	2017	0	1	1	0	0	0	0	0	0	0	0	0	
13111219	f	ot	NL	2017	0	0	0	0	0	0	0	0	0	0	0	0	
13111379	m	cn	US	2017	0	1	0	0	1	0	0	0	0	0	0	0	
13111553	Not	ot	GB	2017	0	0	0	0	0	0	0	0	0	0	0	0	
13112146	f	md	ES	2017	1	0	0	0	0	0	0	0	0	0	0	0	58
13112192	m	cn	US	2017	0	1	0	0	1	0	0	0	0	0	0	0	
13112640	m	md	JP	2017	0	1	0	0	1	0	0	0	0	0	0	0	75
13112700	f	md	CA	2017	0	1	0	0	1	0	0	0	0	0	0	0	
13112755	f	md	JP	2017	1	0	0	0	0	0	0	0	0	0	0	0	78
13112805	f	cn	GB	2017	1	0	0	0	0	0	0	0	0	0	0	0	
13113076	m	ot	GB	2017	0	0	0	0	0	0	0	0	0	0	0	0	59
13113312	Not	ot	US	2017	0	0	0	0	0	0	0	0	0	0	0	0	
13113313	Not	ot	US	2017	0	0	0	0	0	0	0	0	0	0	0	0	
13113395	m	md	JP	2017	0	1	0	0	1	0	0	0	0	0	0	0	61
13113464	f	md	CH	2017	0	0	0	0	0	0	0	0	0	0	0	0	80
13113564	Not	ot	US	2017	0	0	0	0	0	0	0	0	0	0	0	0	
13113591	f	md	JP	2017	1	0	0	0	0	0	0	0	0	0	0	0	58

13114081	Not	ot	US	2017	0	0	0	0	0	0	0	0	0	0	0	0	
13114336	f	md	JP	2017	1	0	0	0	0	0	0	0	0	0	0	0	69
13114411	f	ot	CA	2017	0	1	0	0	0	1	0	0	0	0	0	0	66
13114424	m	cn	US	2017	0	0	0	0	0	0	0	0	0	0	0	0	77
13114493	m	md	TR	2017	0	0	0	0	0	0	0	0	0	0	0	0	80
13114495	m	md	CZ	2017	0	0	0	0	0	0	0	0	0	0	0	0	66
13114550	m	ot	ZA	2017	0	1	0	0	1	0	0	0	0	0	0	0	68
13114855	Not	md	US	2017	0	0	0	0	0	0	0	0	0	0	0	0	
13115886	m	cn	IT	2017	0	1	0	0	1	0	0	0	0	0	0	0	77
13116141	Not	ot	GB	2017	0	0	0	0	0	0	0	0	0	0	0	0	
13116179	m	md	CN	2017	1	0	0	0	0	0	0	0	0	0	0	0	56
13116240	m	md	BE	2017	0	0	0	0	0	0	0	0	0	0	0	0	65
13116529	m	md	GB	2017	0	0	0	0	0	0	0	0	0	0	0	0	59
13116552	f	md	JP	2017	1	0	0	0	0	0	0	0	0	0	0	1	52
13116590	m	ot	DE	2017	0	1	0	0	1	0	0	0	0	0	0	0	79
13116788	f	md	US	2017	1	0	0	0	0	0	0	0	0	0	0	0	
13116967	m	md	AU	2017	0	1	0	0	0	1	0	0	0	0	0	0	62
13117266	Not	cn	GB	2017	1	0	0	0	0	0	0	0	0	0	0	1	
13117292	f	md	CA	2017	0	1	0	0	1	0	0	0	0	0	0	0	
13117311	f	md	JP	2017	1	0	0	0	0	0	0	0	0	0	0	1	94
13117435	m	ot	JP	2017	0	0	0	0	0	0	0	0	0	0	0	0	70
13117436	m	ot	JP	2017	0	0	0	0	0	0	0	0	0	0	0	0	75
13117464	f	md	JP	2017	1	0	0	0	0	0	0	0	0	0	0	0	77.4
13117509	m	ot	JP	2017	0	0	0	0	0	0	0	0	0	0	0	0	70
13117523	f	md	CL	2017	0	1	0	0	0	1	0	0	0	0	0	0	63
13118456	m	ot	JP	2017	0	0	0	0	0	0	0	0	0	0	0	0	83
13118474	m	ot	JP	2017	0	0	0	0	0	0	0	0	0	0	0	0	75
13118481	f	md	KR	2017	0	0	0	0	0	0	0	0	0	0	0	0	69.6
13119960	f	md	CA	2017	0	1	0	0	1	0	0	0	0	0	0	0	63
13119994	f	md	JP	2017	1	0	0	0	0	0	0	0	0	0	0	1	46
13120129	m	ph	GB	2017	0	0	0	0	0	0	0	0	0	0	0	0	59
13120136	f	cn	US	2017	0	0	0	0	0	0	0	0	0	0	0	0	38
13120651	Not	md	JP	2017	1	0	0	0	0	0	0	0	0	0	0	0	
13120666	m	md	JP	2017	1	0	0	0	0	0	0	0	0	0	0	1	

13121065	m	md	GB	2017	0	0	0	0	0	0	0	0	0	0	0	0	59
13122100	f	md	CA	2017	0	1	0	0	1	0	0	0	0	0	0	0	53
13122334	Not	ot	IL	2017	1	0	0	0	0	0	0	0	0	0	0	0	
13122470	f	md	DE	2017	0	0	0	0	0	0	0	0	0	0	0	0	35
13122649	m	cn	JP	2017	0	1	0	0	1	0	0	0	0	0	0	0	61
13122901	Not	md	US	2017	0	0	0	0	0	0	0	0	0	0	0	0	
13122941	m	cn	GB	2017	1	0	0	0	0	0	0	0	0	0	0	0	85
13123445	m	ot	US	2017	0	1	0	0	1	0	0	0	0	0	0	0	76
13123742	m	md	DE	2017	0	1	0	0	1	0	0	0	0	0	0	0	
13123996	m	md	US	2017	0	1	0	0	0	1	0	0	0	0	0	0	68
13124227	m	ot	GB	2017	0	0	0	0	0	0	0	0	0	0	0	0	59
13124248	m	Not repo	US	2017	1	0	0	0	0	0	0	0	0	0	0	0	62.6
13124725	f	md	JP	2017	0	1	0	0	1	0	0	0	0	0	0	0	71
13124764	m	ot	FR	2017	0	1	0	0	1	0	0	0	0	0	0	0	64
13124983	m	ot	US	2017	0	0	0	0	0	0	0	0	0	0	0	0	69
13125018	f	md	CN	2017	0	1	0	0	0	1	0	0	0	0	0	0	69
13125042	f	ph	FR	2017	1	0	0	0	0	0	0	0	0	0	0	0	51.7
13125157	f	md	AU	2017	0	0	0	0	0	0	0	0	0	0	0	0	72
13125208	Not	md	AU	2017	0	1	0	0	1	0	0	0	0	0	0	0	47
13125658	f	cn	US	2017	0	0	0	0	0	0	0	0	0	0	0	0	
13125732	f	md	JP	2017	1	0	0	0	0	0	0	0	0	0	0	0	85
13125838	f	ot	CA	2017	0	0	0	0	0	0	0	0	0	0	0	0	45
13125869	m	cn	JP	2017	0	1	0	0	1	0	0	0	0	0	0	1	67
13126048	m	ph	JP	2017	0	1	0	0	1	0	0	0	0	0	0	0	60
13126357	f	md	ES	2017	0	1	0	0	0	1	0	0	0	0	0	0	72
13126364	f	cn	Not re	2017	0	0	0	0	0	0	0	0	0	0	0	0	
13126582	m	md	KR	2017	0	0	0	0	0	0	0	0	0	0	0	0	70
13127771	f	cn	GB	2017	1	0	0	0	0	0	0	0	0	0	0	1	44
13127969	f	ph	JP	2017	0	0	0	0	0	0	0	0	0	0	0	0	53
13128692	Not	ot	AU	2017	0	1	0	0	1	0	0	0	0	0	0	0	67
13129086	m	cn	JP	2017	0	1	0	0	1	0	0	0	0	0	0	1	57
13129112	f	md	PH	2017	0	1	1	0	0	0	0	0	0	0	0	0	70
13129150	f	ot	JP	2017	0	0	0	0	0	0	0	0	0	0	0	0	68
13129153	m	ot	JP	2017	0	0	0	0	0	0	0	0	0	0	0	0	52

13129406	m	md	JP	2017	0	1	0	0	1	0	0	0	0	0	0	0	72
13129557	f	Not repo	US	2017	1	0	0	0	0	0	0	0	0	0	0	0	
13129658	m	md	JP	2017	0	1	0	0	1	0	0	0	0	0	0	1	64
13129740	m	ph	US	2017	0	0	0	0	0	0	0	0	0	0	0	0	59
13129749	m	ot	IT	2017	0	1	0	0	1	0	0	0	0	0	0	0	70
13130185	m	cn	JP	2017	0	1	0	0	1	0	0	0	0	0	0	0	87
13130377	m	cn	US	2017	1	0	0	0	0	0	0	0	0	0	0	0	
13130651	f	md	US	2017	0	0	0	0	0	0	0	0	0	0	0	0	68
13130668	Not	ot	CH	2017	0	0	0	0	0	0	0	0	0	0	0	0	
13130964	m	cn	US	2017	0	1	0	0	1	0	0	0	0	0	0	0	
13131110	f	md	JP	2017	0	0	0	0	0	0	0	0	0	0	0	0	52
13131149	m	md	SK	2017	0	1	0	0	0	1	0	0	0	0	0	0	69
13131556	Not	md	JP	2017	0	1	0	0	1	0	0	0	0	0	0	0	
13131825	f	md	TH	2017	0	0	0	0	0	0	0	0	0	0	0	0	70
13132072	f	cn	US	2017	1	0	0	0	0	0	0	0	0	0	0	0	65
13132215	f	cn	CN	2017	0	0	0	0	0	0	0	0	0	0	0	0	47
13132332	f	md	JP	2017	0	1	0	0	1	0	0	0	0	0	0	0	72
13132662	m	md	IT	2017	0	0	0	0	0	0	0	0	0	0	0	0	62
13133001	f	ot	CA	2017	0	1	0	0	1	0	0	0	0	0	0	0	50
13134425	m	md	JP	2017	0	1	0	0	1	0	0	0	0	0	0	0	44
13134436	m	md	JP	2017	0	0	0	0	0	0	0	0	0	0	0	0	69
13134572	f	md	JP	2017	0	0	0	0	0	0	0	0	0	0	0	0	74
13134662	Not	md	JP	2017	0	1	0	0	1	0	0	0	0	0	0	0	
13134710	m	md	JP	2017	0	1	0	0	1	0	0	0	0	0	0	1	72
13135006	f	md	TR	2017	0	1	0	0	1	0	0	0	0	0	0	0	68
13135371	m	cn	JP	2017	0	1	0	0	1	0	0	0	0	0	0	0	57
13135397	m	md	JP	2017	0	1	0	0	1	0	0	0	0	0	0	0	67
13135482	m	md	ES	2017	0	1	0	0	0	1	0	0	0	0	0	0	64
13135506	m	ot	US	2017	0	1	0	0	1	0	0	0	0	0	0	0	
13135677	f	ph	US	2017	0	0	0	0	0	0	0	0	0	0	0	0	51
13135722	m	md	KR	2017	0	1	0	0	0	1	0	0	0	0	0	0	62
13135914	m	md	JP	2017	0	1	0	0	1	0	0	0	0	0	0	0	74
13135931	m	md	BE	2017	0	1	0	0	1	0	0	0	0	0	0	0	
13135968	m	md	MX	2017	0	0	0	0	0	0	0	0	0	0	0	0	73

13135993	m	ot	US	2017	0	1	0	0	1	0	0	0	0	0	0	0	
13136751	m	md	KR	2017	0	0	0	0	0	0	0	0	0	0	0	0	72
13137068	m	md	ES	2017	0	1	0	0	0	1	0	0	0	0	0	0	63
13137097	m	md	JP	2017	0	1	0	0	1	0	0	0	0	0	0	1	66
13137519	m	cn	US	2017	0	1	0	0	1	0	0	0	0	0	0	0	
13137752	m	md	JP	2017	0	0	0	0	0	0	0	0	0	0	0	0	62
13137893	m	md	DE	2017	0	1	0	0	1	0	0	0	0	0	0	0	58
13138586	Not	md	JP	2017	0	0	0	0	0	0	0	0	0	0	0	0	
13139144	f	md	JP	2017	0	1	0	0	1	0	0	0	0	0	0	0	59
13139154	f	ot	IT	2017	0	1	0	0	1	0	0	0	0	0	0	0	74
13139157	m	md	DE	2017	0	1	0	0	1	0	0	0	0	0	0	0	84
13139220	f	md	JP	2017	0	0	0	0	0	0	0	0	0	0	0	0	42
13139241	m	cn	US	2017	0	1	1	0	0	0	0	0	0	0	0	0	65
13139248	f	cn	JP	2017	0	1	0	0	1	0	0	0	0	0	0	0	64
13139251	f	cn	GB	2017	0	0	0	0	0	0	0	0	0	0	0	0	67
13139702	m	Not repo	US	2017	1	0	0	0	0	0	0	0	0	0	0	0	80
13139742	f	md	US	2017	1	0	0	0	0	0	0	0	0	0	0	0	53
13139879	f	md	JP	2017	1	0	0	0	0	0	0	0	0	0	0	0	82
13141225	f	ot	AT	2017	0	1	0	0	1	0	0	0	0	0	0	0	64
13141673	f	ph	JP	2017	0	1	0	0	1	0	0	0	0	0	0	0	74
13142348	m	md	JP	2017	0	1	0	0	1	0	0	0	0	0	0	0	66
13142892	f	md	JP	2017	1	0	0	0	0	0	0	0	0	0	0	0	59
13142977	m	md	BE	2017	0	0	0	0	0	0	0	0	0	0	0	0	73
13143374	m	md	AT	2017	0	0	0	0	0	0	0	0	0	0	0	0	81
13143548	m	ot	KR	2017	0	1	0	0	1	0	0	0	0	0	0	0	68
13143647	m	ot	HR	2017	0	1	0	0	1	0	0	0	0	0	0	0	65
13143872	f	cn	TW	2017	1	0	0	0	0	0	0	0	0	0	0	0	68
13144047	m	md	DE	2017	0	1	0	0	1	0	0	0	0	0	0	0	
13144224	m	cn	GB	2017	1	0	0	0	0	0	0	0	0	0	0	0	71
13144346	m	ot	US	2017	0	1	0	0	1	0	0	0	0	0	0	0	68
13144382	m	cn	US	2017	0	1	1	0	0	0	0	0	0	0	0	0	71
13144845	f	md	JP	2017	0	0	0	0	0	0	0	0	0	0	0	0	72
13145084	Not	md	US	2017	0	1	1	0	0	0	0	0	0	0	0	0	
13145458	f	md	US	2017	0	1	0	0	0	1	0	0	0	0	0	0	71

13145910	m	cn	US	2017	1	0	0	0	0	0	0	0	0	0	0	0	41
13146170	f	ot	JP	2017	0	0	0	0	0	0	0	0	0	0	0	0	68
13146444	m	cn	JP	2017	0	1	0	0	1	0	0	0	0	0	0	0	68
13147185	f	md	US	2017	1	0	0	0	0	0	0	0	0	0	0	0	
13147441	m	ot	ES	2017	0	1	0	0	1	0	0	0	0	0	0	0	51
13147737	f	md	AU	2017	0	0	0	0	0	0	0	0	0	0	0	0	75
13147768	f	cn	CN	2017	1	0	0	0	0	0	0	0	0	0	0	0	
13147770	m	cn	GB	2017	1	0	0	0	0	0	0	0	0	0	0	0	74
13147773	m	cn	GB	2017	1	0	0	0	0	0	0	0	0	0	0	0	73
13148092	f	md	GB	2017	0	0	0	0	0	0	0	0	0	0	0	0	61
13148395	m	md	US	2017	0	1	1	0	0	0	0	0	0	0	0	0	62
13148556	m	cn	JP	2017	0	1	0	0	1	0	0	0	0	0	0	0	78
13148561	m	md	JP	2017	0	1	0	0	1	0	0	0	0	0	0	0	67
13149388	m	md	JP	2017	0	0	0	0	0	0	0	0	0	0	0	0	72
13150249	m	md	CN	2017	1	0	0	0	0	0	0	0	0	0	0	0	65.8
13151593	f	Not repo	US	2017	1	0	0	0	0	0	0	0	0	0	0	0	62.8
13151669	m	md	TW	2017	1	0	0	0	0	0	0	0	0	0	0	0	55.8
13151971	f	md	FR	2017	0	1	0	0	0	1	0	0	0	0	0	0	65
13152463	m	cn	US	2017	0	1	1	0	0	0	0	0	0	0	0	0	
13152666	m	md	JP	2017	0	1	0	0	1	0	0	0	0	0	0	0	78
13152790	f	cn	US	2017	0	1	0	0	1	0	0	0	0	0	0	1	65
13152901	f	ot	US	2017	0	1	0	0	1	0	0	0	0	0	0	0	
13152949	Not	cn	US	2017	0	1	0	0	1	0	0	0	0	0	0	0	65
13153068	f	md	JP	2017	1	0	0	0	0	0	0	0	0	0	0	0	81
13153147	f	md	BG	2017	0	1	0	0	1	0	0	0	0	0	0	0	
13155042	f	ph	GB	2017	0	0	0	0	0	0	0	0	0	0	0	0	51
13155082	m	cn	JP	2017	0	0	0	0	0	0	0	0	0	0	0	0	67
13155518	m	cn	US	2017	1	0	0	0	0	0	0	0	0	0	0	0	73
13155898	Not	md	US	2017	0	0	0	0	0	0	0	0	0	0	0	0	
13156109	m	md	HU	2017	0	0	0	0	0	0	0	0	0	0	0	0	67
13156117	m	md	ES	2017	0	0	0	0	0	0	0	0	0	0	0	0	48
13156130	m	md	DE	2017	0	0	0	0	0	0	0	0	0	0	0	0	75
13156294	f	ot	US	2017	0	0	0	0	0	0	0	0	0	0	0	0	65
13156818	m	ot	JP	2017	0	1	0	0	1	0	0	0	0	0	0	0	67

13156898	m	ot	BG	2017	0	1	0	0	1	0	0	0	0	0	0	0	67
13156926	f	ot	US	2017	0	1	0	0	1	0	0	0	0	0	0	0	34
13157056	f	ot	US	2017	0	1	0	0	1	0	0	0	0	0	0	0	74
13157058	f	ph	US	2017	0	1	0	0	1	0	0	0	0	0	0	0	
13157104	m	ot	HR	2017	0	1	0	0	1	0	0	0	0	0	0	0	56
13157120	Not	cn	US	2017	0	1	1	0	0	0	0	0	0	0	0	0	
13157230	f	md	US	2017	0	1	1	0	0	0	0	0	0	0	0	0	46
13157242	f	md	GB	2017	0	0	0	0	0	0	0	0	0	0	0	0	58
13157247	m	ot	HR	2017	0	1	0	0	1	0	0	0	0	0	0	0	
13157356	Not	md	CH	2017	0	0	0	0	0	0	0	0	0	0	0	0	
13157687	m	md	FR	2017	0	1	0	0	0	1	0	0	0	0	0	0	71
13157848	Not	md	AU	2017	0	1	0	0	1	0	0	0	0	0	0	0	
13157857	Not	md	CH	2017	0	0	0	0	0	0	0	0	0	0	0	0	
13158076	Not	md	CH	2017	0	0	0	0	0	0	0	0	0	0	0	0	
13158079	Not	md	CH	2017	0	0	0	0	0	0	0	0	0	0	0	0	
13158221	m	md	US	2017	0	1	1	0	0	0	0	0	0	0	0	0	
13158297	m	ph	US	2017	0	0	0	0	0	0	0	0	0	0	0	0	58
13158366	m	cn	US	2017	0	0	0	0	0	0	0	0	0	0	0	0	67
13158414	f	cn	US	2017	1	0	0	0	0	0	0	0	0	0	0	1	54.4
13158519	f	ph	AU	2017	0	1	0	0	1	0	0	0	0	0	0	1	76
13158602	m	ot	KR	2017	0	0	0	0	0	0	0	0	0	0	0	0	43
13158657	m	cn	JP	2017	0	0	0	0	0	0	0	0	0	0	0	0	67
13158776	f	cn	US	2017	0	1	1	0	0	0	0	0	0	0	0	0	69
13159189	Not	ph	US	2017	0	1	0	0	1	0	0	0	0	0	0	0	72
13159265	m	ot	ES	2017	0	0	0	0	0	0	0	0	0	0	0	0	32
13159495	m	ph	JP	2017	0	1	0	0	1	0	0	0	0	0	0	0	72
13159573	m	ot	US	2017	0	1	0	0	1	0	0	0	0	0	0	0	
13159948	f	cn	CA	2017	0	1	0	0	1	0	0	0	0	0	0	0	
13160058	m	cn	IT	2017	0	1	0	0	1	0	0	0	0	0	0	0	51
13160060	m	md	CH	2017	0	1	0	0	1	0	0	0	0	0	0	0	
13160067	f	cn	CA	2017	0	1	0	0	1	0	0	0	0	0	0	0	
13160243	m	md	JP	2017	0	1	0	0	1	0	0	0	0	0	0	0	64
13160244	f	md	AU	2017	0	1	0	0	1	0	0	0	0	0	0	0	58
13160800	m	cn	JP	2017	0	1	0	0	1	0	0	0	0	0	0	1	80

13161802	m	cn	JP	2017	0	1	0	0	1	0	0	0	0	0	0	1	70
13162033	f	md	US	2017	0	0	0	0	0	0	0	0	0	0	0	0	64
13162429	m	md	CN	2017	0	0	0	0	0	0	0	0	0	0	0	0	61
13162473	f	md	JP	2017	0	1	0	0	1	0	0	0	0	0	0	0	67
13162719	m	cn	US	2017	0	1	0	0	1	0	0	0	0	0	0	0	58
13163260	m	md	US	2017	0	0	0	0	0	0	0	0	0	0	0	0	43
13163303	m	cn	CN	2017	1	0	0	0	0	0	0	0	0	0	0	0	64
13163445	f	md	JP	2017	1	0	0	0	0	0	0	0	0	0	0	1	72
13163660	f	ot	US	2017	0	1	0	0	1	0	0	0	0	0	0	0	
13164158	m	cn	JP	2017	0	1	0	0	1	0	0	0	0	0	0	0	79
13164392	f	md	AU	2017	0	0	0	0	0	0	0	0	0	0	0	0	
13164905	f	md	US	2017	0	1	0	0	0	1	0	0	0	0	0	0	71
13165013	m	cn	US	2017	0	1	1	0	0	0	0	0	0	0	0	0	38
13165347	m	ot	NL	2017	0	1	0	0	1	0	0	0	0	0	0	0	74
13165723	m	ot	JP	2017	0	1	0	0	1	0	0	0	0	0	0	0	60
13165817	m	md	JP	2017	0	1	0	0	1	0	0	0	0	0	0	1	72
13165869	Not	ph	AU	2017	0	1	0	0	1	0	0	0	0	0	0	0	71
13165984	f	md	JP	2017	0	0	0	0	0	0	0	0	0	0	0	0	
13166084	f	md	HU	2017	0	0	0	0	0	0	0	0	0	0	0	0	67
13166274	f	cn	US	2017	0	0	0	0	0	0	0	0	0	0	0	0	59
13166346	m	md	JP	2017	1	0	0	0	0	0	0	0	0	0	0	0	90
13166822	f	cn	GB	2017	1	0	0	0	0	0	0	0	0	0	0	0	85
13167420	m	md	JP	2017	0	1	0	0	1	0	0	0	0	0	0	0	83
13167784	f	md	US	2017	0	0	0	0	0	0	0	0	0	0	0	0	75
13168940	m	cn	JP	2017	0	1	0	0	1	0	0	0	0	0	0	0	71
13169175	m	md	US	2017	0	1	0	0	1	0	0	0	0	0	0	0	
13169177	m	cn	US	2017	0	1	0	0	1	0	0	0	0	0	0	0	
13169733	m	cn	FR	2017	0	1	0	0	1	0	0	0	0	0	0	0	50
13170153	Not	ot	US	2017	1	0	0	0	0	0	0	0	0	0	0	0	
13170284	f	cn	US	2017	0	1	0	0	1	0	0	0	0	0	0	0	
13170305	f	md	FR	2017	0	0	0	0	0	0	0	0	0	0	0	0	28
13170452	f	md	JP	2017	0	1	0	0	1	0	0	0	0	0	0	0	83
13170522	f	md	JP	2017	1	0	0	0	0	0	0	0	0	0	0	0	74
13171025	m	ot	GB	2017	0	0	0	0	0	0	0	0	0	0	0	0	75

13172097	m	cn	US	2017	1	0	0	0	0	0	0	0	0	0	0	0	
13172738	m	cn	FR	2017	0	0	0	0	0	0	0	0	0	0	0	0	
13172775	m	md	JP	2017	1	0	0	0	0	0	0	0	0	0	0	1	81
13174088	f	cn	JP	2017	1	0	0	0	0	0	0	0	0	0	0	0	58
13174309	Not	ph	AU	2017	0	1	0	0	1	0	0	0	0	0	0	0	62
13174331	m	md	JP	2017	0	1	0	0	1	0	0	0	0	0	0	0	85
13175310	m	cn	JP	2017	0	1	0	0	1	0	0	0	0	0	0	0	72
13175617	f	ot	US	2017	0	1	1	0	0	0	0	0	0	0	0	1	56
13176468	Not	ot	US	2017	1	0	0	0	0	0	0	0	0	0	0	0	
13176478	Not	ot	US	2017	1	0	0	0	0	0	0	0	0	0	0	0	
13176479	Not	ot	US	2017	1	0	0	0	0	0	0	0	0	0	0	0	
13176480	Not	ot	US	2017	1	0	0	0	0	0	0	0	0	0	0	0	
13176805	m	ot	GB	2017	0	0	0	0	0	0	0	0	0	0	0	0	63
13177847	m	ph	JP	2017	0	1	0	0	1	0	0	0	0	0	0	0	62
13178156	f	md	US	2017	0	0	0	0	0	0	0	0	0	0	0	0	45
13178421	m	md	JP	2017	0	1	0	0	0	1	0	0	0	0	0	1	72
13178709	m	md	JP	2017	0	0	0	0	0	0	0	0	0	0	0	0	61
13178971	f	md	US	2017	0	0	0	0	0	0	0	0	0	0	0	0	47
13179208	m	cn	US	2017	0	0	0	0	0	0	0	0	0	0	0	0	54
13179264	f	md	KR	2017	1	0	0	0	0	0	0	0	0	0	0	0	48.7
13179355	f	md	US	2017	0	0	0	0	0	0	0	0	0	0	0	0	38
13179359	f	md	JP	2017	0	0	0	0	0	0	0	0	0	0	0	0	46
13179531	f	cn	CA	2017	0	1	0	0	1	0	0	0	0	0	0	0	56
13180022	f	md	TR	2017	0	0	0	0	0	0	0	0	0	0	0	0	61
13183554	m	ph	JP	2017	0	1	0	0	1	0	0	0	0	0	0	0	57
13183564	m	ph	JP	2017	0	1	0	0	1	0	0	0	0	0	0	0	64
13183589	m	ot	JP	2017	0	0	0	0	0	0	0	0	0	0	0	0	67
13183670	f	ot	AT	2017	0	1	0	0	1	0	0	0	0	0	0	0	64
13183704	f	md	JP	2017	1	0	0	0	0	0	0	0	0	0	0	0	74
13184175	f	md	ES	2017	0	1	0	0	0	1	0	0	0	0	0	0	62
13184248	m	md	JP	2017	1	0	0	0	0	0	0	0	0	0	0	0	70
13184344	Not	md	JP	2017	0	1	0	0	1	0	0	0	0	0	0	0	
13184521	m	ot	ES	2017	0	1	0	0	0	1	0	0	0	0	0	0	64
13184566	f	Not repo	US	2017	1	0	0	0	0	0	0	0	0	0	0	0	82.7

13184649	m	md	JP	2017	0	0	0	0	0	0	0	0	0	0	0	0	79
13185254	Not	md	JP	2017	0	1	0	0	1	0	0	0	0	0	0	0	
13185488	m	ph	JP	2017	0	1	0	0	1	0	0	0	0	0	0	1	77
13185572	m	cn	US	2017	0	1	1	0	0	0	0	0	0	0	0	1	77
13185960	m	md	ID	2017	1	0	0	0	0	0	0	0	0	0	0	0	60
13186081	m	cn	CA	2017	0	1	0	0	1	0	0	0	0	0	0	0	62
13186262	m	ot	DE	2017	0	1	0	0	1	0	0	0	0	0	0	0	65
13186580	f	cn	KR	2017	1	0	0	0	0	0	0	0	0	0	0	0	56
13186701	f	cn	US	2017	1	0	0	0	0	0	0	0	0	0	0	0	78
13186932	m	cn	US	2017	0	1	0	0	1	0	0	0	0	0	0	0	61
13187212	f	ph	US	2017	0	1	0	0	1	0	0	0	0	0	0	0	
13187323	m	md	JP	2017	0	1	0	0	1	0	0	0	0	0	0	0	67
13187443	f	cn	US	2017	0	0	0	0	0	0	0	0	0	0	0	0	60
13187916	m	ot	US	2017	0	1	0	0	1	0	0	0	0	0	0	0	70
13188149	f	cn	Not re	2017	0	0	0	0	0	0	0	0	0	0	0	0	59
13188783	f	ot	FR	2017	0	1	0	0	0	1	0	0	0	0	0	0	76
13189615	m	cn	JP	2017	0	1	0	0	1	0	0	0	0	0	0	0	39
13189750	f	cn	AR	2017	0	0	0	0	0	0	0	0	0	0	0	0	63
13189891	m	ot	US	2017	0	0	0	0	0	0	0	0	0	0	0	0	66
13190059	f	md	CN	2017	0	0	0	0	0	0	0	0	0	0	0	0	64
13190966	m	md	JP	2017	0	1	0	0	1	0	0	0	0	0	0	0	51
13191048	m	cn	JP	2017	0	1	0	0	1	0	0	0	0	0	0	0	58
13191217	m	md	ES	2017	0	0	0	0	0	0	0	0	0	0	0	0	48
13191219	f	cn	US	2017	0	1	1	0	0	0	0	0	0	0	0	0	62
13191274	Not	cn	US	2017	0	1	0	0	1	0	0	0	0	0	0	0	
13191422	f	md	DE	2017	0	1	0	0	0	1	0	0	0	0	0	0	67
13191746	f	md	JP	2017	1	0	0	0	0	0	0	0	0	0	0	0	83.9
13191866	m	md	JP	2017	0	1	0	0	0	1	0	0	0	0	0	1	72
13191996	Not	md	US	2017	0	0	0	0	0	0	0	0	0	0	0	0	
13192216	f	md	US	2017	1	0	0	0	0	0	0	0	0	0	0	0	
13192503	m	md	US	2017	0	1	1	0	0	0	0	0	0	0	0	0	65
13192804	m	md	CN	2017	1	0	0	0	0	0	0	0	0	0	0	0	64.3
13193090	f	md	JP	2017	1	0	0	0	0	0	0	0	0	0	0	0	65
13193267	m	md	BR	2017	0	0	0	0	0	0	0	0	0	0	0	0	76

13193362	m	md	JP	2017	0	1	0	0	1	0	0	0	0	0	0	0	64
13193385	m	md	JP	2017	0	1	0	0	1	0	0	0	0	0	0	0	66
13193396	f	md	HU	2017	0	1	0	0	1	0	0	0	0	0	0	0	61
13193409	f	ot	GB	2017	0	1	0	0	1	0	0	0	0	0	0	0	68
13193544	f	md	LB	2017	0	1	0	0	1	0	0	0	0	0	0	0	
13193680	m	cn	JP	2017	0	1	0	0	1	0	0	0	0	0	0	0	66
13193800	f	md	US	2017	0	0	0	0	0	0	0	0	0	0	0	0	64
13194149	f	md	JP	2017	0	1	0	0	1	0	0	0	0	0	0	0	53
13194150	m	md	TR	2017	0	1	0	0	1	0	0	0	0	0	0	0	58
13194180	f	cn	US	2017	1	0	0	0	0	0	0	0	0	0	0	0	55
13194184	m	cn	JP	2017	0	1	0	0	1	0	0	0	0	0	0	0	
13194600	m	md	JP	2017	0	1	0	0	1	0	0	0	0	0	0	1	69
13194961	m	md	DE	2017	0	0	0	0	0	0	0	0	0	0	0	0	55
13195177	f	cn	US	2017	0	1	0	0	1	0	0	0	0	0	0	0	
13195288	m	md	CN	2017	0	1	0	0	0	1	0	0	0	0	0	0	55
13195444	f	md	JP	2017	0	1	0	0	1	0	0	0	0	0	0	0	52
13195461	f	md	US	2017	1	0	0	0	0	0	0	0	0	0	0	0	42
13195633	f	md	JP	2017	0	1	0	0	0	1	0	0	0	0	0	0	51
13195696	m	md	JP	2017	0	0	0	0	0	0	0	0	0	0	0	0	75
13196014	m	cn	FR	2017	0	1	0	0	1	0	0	0	0	0	0	0	70
13196015	Not	cn	IT	2017	0	1	0	0	1	0	0	0	0	0	0	0	
13196031	m	cn	US	2017	0	1	0	0	1	0	0	0	0	0	0	0	
13196057	f	cn	US	2017	0	0	0	0	0	0	0	0	0	0	0	0	
13196181	f	cn	GB	2017	1	0	0	0	0	0	0	0	0	0	0	0	51
13196422	m	cn	US	2017	0	0	0	0	0	0	0	0	0	0	0	0	77
13196496	f	md	JP	2017	0	1	0	0	1	0	0	0	0	0	0	0	64
13196908	m	md	JP	2017	0	1	0	0	1	0	0	0	0	0	0	0	50
13197514	f	ot	US	2017	1	0	0	0	0	0	0	0	0	0	0	0	63
13197846	m	ot	US	2017	0	1	1	0	0	0	0	0	0	0	0	0	76
13199393	f	cn	GB	2017	1	0	0	0	0	0	0	0	0	0	0	0	73
13199766	f	md	DE	2017	0	1	0	0	0	1	0	0	0	0	0	0	66
13200120	f	ot	US	2017	0	1	1	0	0	0	0	0	0	0	0	0	65
13200209	f	cn	US	2017	1	0	0	0	0	0	0	0	0	0	0	0	
13200607	f	md	ES	2017	1	0	0	0	0	0	0	0	0	0	0	0	75.2

13200742	f	md	CA	2017	0	0	0	0	0	0	0	0	0	0	0	0	66
13200946	m	ot	US	2017	0	1	0	0	1	0	0	0	0	0	0	0	
13201871	f	ot	US	2017	0	1	0	0	1	0	0	0	0	0	0	0	
13201988	f	ot	PL	2017	0	1	0	0	1	0	0	0	0	0	0	0	48
13202022	f	md	JP	2017	1	0	0	0	0	0	0	0	0	0	0	0	73
13202239	f	md	US	2017	0	0	0	0	0	0	0	0	0	0	0	0	39
13202302	m	md	US	2017	0	0	0	0	0	0	0	0	0	0	0	0	70
13202356	f	md	US	2017	0	0	0	0	0	0	0	0	0	0	0	0	78
13202667	Not	ot	US	2017	1	0	0	0	0	0	0	0	0	0	0	0	
13202814	m	cn	US	2017	0	1	1	0	0	0	0	0	0	0	0	0	
13202827	Not	ot	US	2017	0	0	0	0	0	0	0	0	0	0	0	0	
13202836	Not	ot	JP	2017	0	0	0	0	0	0	0	0	0	0	0	0	
13202866	Not	ot	JP	2017	0	0	0	0	0	0	0	0	0	0	0	0	
13202895	Not	ot	JP	2017	0	0	0	0	0	0	0	0	0	0	0	0	
13202900	Not	ot	JP	2017	0	0	0	0	0	0	0	0	0	0	0	0	
13202913	Not	ot	US	2017	0	0	0	0	0	0	0	0	0	0	0	0	
13202923	Not	ot	US	2017	0	0	0	0	0	0	0	0	0	0	0	0	
13202936	Not	ot	US	2017	0	0	0	0	0	0	0	0	0	0	0	0	
13202943	Not	ot	US	2017	0	0	0	0	0	0	0	0	0	0	0	0	
13202945	Not	ot	US	2017	0	0	0	0	0	0	0	0	0	0	0	0	
13202952	Not	ot	US	2017	0	0	0	0	0	0	0	0	0	0	0	0	
13202958	Not	ot	US	2017	0	0	0	0	0	0	0	0	0	0	0	0	
13202965	Not	ot	US	2017	0	0	0	0	0	0	0	0	0	0	0	0	
13203011	Not	ot	US	2017	0	0	0	0	0	0	0	0	0	0	0	0	
13203013	Not	ot	US	2017	0	0	0	0	0	0	0	0	0	0	0	0	
13203016	Not	ot	US	2017	0	0	0	0	0	0	0	0	0	0	0	0	
13203034	Not	ot	US	2017	0	0	0	0	0	0	0	0	0	0	0	0	
13203236	Not	ot	US	2017	0	0	0	0	0	0	0	0	0	0	0	0	
13203242	Not	ot	US	2017	0	0	0	0	0	0	0	0	0	0	0	0	
13204452	m	md	US	2017	0	1	0	0	0	1	0	0	0	0	0	0	63
13204505	f	md	NL	2017	0	0	0	0	0	0	0	0	0	0	0	0	89
13205193	m	cn	FR	2017	0	1	0	0	0	1	0	0	0	0	0	0	71
13205427	f	md	CA	2017	1	0	0	0	0	0	0	0	0	0	0	0	82.1
13205913	m	ot	US	2017	0	1	0	0	1	0	0	0	0	0	0	0	48

13207924	m	ot	US	2017	0	0	0	0	0	0	0	0	0	0	0	0	60
13208878	Not	md	JP	2017	0	1	0	0	1	0	0	0	0	0	0	0	
13209981	Not	ph	JP	2017	0	0	0	0	0	0	0	0	0	0	0	0	
13211815	f	md	JP	2017	0	0	0	0	0	0	1	0	1	0	0	0	45
13212161	f	cn	CN	2017	1	0	0	0	0	0	0	0	0	0	0	0	70
13212307	Not	md	CA	2017	1	0	0	0	0	0	0	0	0	0	0	0	
13213150	m	md	US	2017	0	1	0	0	1	0	0	0	0	0	0	0	80
13213335	f	ot	TR	2017	0	0	0	0	0	0	0	0	0	0	0	0	46
13213353	m	ot	ES	2017	0	1	0	0	1	0	0	0	0	0	0	0	67
13213566	m	cn	GB	2017	1	0	0	0	0	0	0	0	0	0	0	0	
13213748	m	md	ES	2017	0	1	0	0	0	1	0	0	0	0	0	0	64
13213843	m	cn	JP	2017	0	1	0	0	1	0	0	0	0	0	0	1	58
13213968	Not	md	US	2017	0	1	0	0	1	0	0	0	0	0	0	0	
13214005	m	md	AU	2017	1	0	0	0	0	0	0	0	0	0	0	0	
13214914	m	md	JP	2017	0	1	0	0	1	0	0	0	0	0	0	0	59
13215154	f	md	JP	2017	0	1	0	0	1	0	0	0	0	0	0	0	67
13215156	m	md	JP	2017	0	1	0	0	1	0	0	0	0	0	0	0	68
13215158	m	ot	AR	2017	0	1	0	0	1	0	0	0	0	0	0	0	59
13216070	f	cn	US	2017	0	1	0	0	1	0	0	0	0	0	0	0	61
13216161	m	md	JP	2017	0	1	0	0	1	0	0	0	0	0	0	1	66
13216238	Not	ot	US	2017	0	0	0	0	0	0	0	0	0	0	0	0	
13216426	m	md	IN	2017	0	1	0	0	1	0	0	0	0	0	0	0	
13216818	Not	ot	US	2017	0	0	0	0	0	0	0	0	0	0	0	0	
13219702	f	md	JP	2017	1	0	0	0	0	0	0	0	0	0	0	0	74
13219928	m	md	ES	2017	0	0	0	0	0	0	0	0	0	0	0	0	73
13220149	f	ot	DE	2017	0	1	0	0	1	0	0	0	0	0	0	0	58
13220424	m	md	JP	2017	0	0	0	0	0	0	0	0	0	0	0	0	88
13221884	m	ph	US	2017	0	1	0	0	1	0	0	0	0	0	0	0	
13222358	m	ot	DE	2017	0	0	0	0	0	0	0	0	0	0	0	0	70
13225134	f	md	US	2017	0	1	0	0	1	0	0	0	0	0	0	0	
13225234	f	md	US	2017	0	1	0	0	0	1	0	0	0	0	0	0	61
13226374	f	cn	AR	2017	0	0	0	0	0	0	0	0	0	0	0	0	60
13226688	m	md	JP	2017	0	1	0	0	0	1	0	0	0	0	0	0	55
13226928	f	md	US	2017	0	1	0	0	0	1	0	0	0	0	0	0	76

13226939	f	ot	US	2017	0	1	1	0	0	0	0	0	0	0	0	0	52
13226974	Not	md	JP	2017	0	1	0	0	1	0	0	0	0	0	0	0	
13227521	m	ph	GB	2017	1	0	0	0	0	0	0	0	0	0	0	0	54
13227871	f	cn	US	2017	0	1	1	0	0	0	0	0	0	0	0	0	70
13228387	f	ph	US	2017	0	0	0	0	0	0	0	0	0	0	0	0	
13228434	Not	md	US	2017	0	0	0	0	0	0	0	0	0	0	0	0	
13228916	m	md	KR	2017	0	0	0	0	0	0	0	0	0	0	0	0	70
13229401	m	md	JP	2017	0	1	0	0	1	0	0	0	0	0	0	0	68
13229505	m	ph	GB	2017	0	1	1	0	0	0	0	0	0	0	0	0	56
13229603	f	cn	US	2017	0	0	0	0	0	0	0	0	0	0	0	0	
13229701	m	cn	US	2017	0	1	0	0	1	0	0	0	0	0	0	0	
13230064	Not	ph	US	2017	0	1	0	0	1	0	0	0	0	0	0	0	
13230172	m	md	DE	2017	0	0	0	0	0	0	0	0	0	0	0	0	47
13230243	m	ot	JP	2017	1	0	0	0	0	0	0	0	0	0	0	0	71
13230494	f	md	JP	2017	1	0	0	0	0	0	0	0	0	0	0	0	67
13230702	m	cn	JP	2017	0	1	0	0	1	0	0	0	0	0	0	0	71
13230891	m	md	PT	2017	0	0	0	0	0	0	0	0	0	0	0	0	66
13231343	m	md	JP	2017	0	1	0	0	1	0	0	0	0	0	0	0	80
13231899	m	md	JP	2017	1	0	0	0	0	0	0	0	0	0	0	0	87
13232151	m	ot	US	2017	0	1	0	0	1	0	0	0	0	0	0	0	
13232279	f	ot	IT	2017	0	1	0	0	1	0	0	0	0	0	0	0	50
13232793	f	md	GR	2017	0	0	0	0	0	0	0	0	0	0	0	0	
13232794	m	md	GR	2017	0	0	0	0	0	0	0	0	0	0	0	0	
13232959	m	cn	JP	2017	0	1	0	0	1	0	0	0	0	0	0	0	77
13233154	f	md	FR	2017	0	1	0	0	0	1	0	0	0	0	0	0	61
13234346	m	ph	JP	2017	0	1	0	0	1	0	0	0	0	0	0	0	62
13234407	f	ot	JP	2017	0	1	0	0	1	0	0	0	0	0	0	0	72
13234916	f	md	JP	2017	0	0	0	0	0	0	0	0	0	0	0	0	72
13234924	f	cn	US	2017	0	0	0	0	0	0	0	0	0	0	0	0	69
13235042	f	md	US	2017	0	0	0	0	0	0	0	0	0	0	0	0	78
13236230	m	ot	US	2017	0	0	0	0	0	0	0	0	0	0	0	0	
13236496	f	md	CN	2017	1	0	0	0	0	0	0	0	0	0	0	0	63
13236512	f	md	IT	2017	1	0	0	0	0	0	0	0	0	0	0	0	61.8
13236740	f	ot	DE	2017	0	0	0	0	0	0	0	0	0	0	0	0	66

13237045	m	md	US	2017	0	1	0	0	1	0	0	0	0	0	0	0	
13237046	f	cn	JP	2017	0	1	0	0	1	0	0	0	0	0	0	0	
13237592	f	cn	US	2017	1	0	0	0	0	0	0	0	0	0	0	0	57
13237603	m	ot	US	2017	0	1	0	0	1	0	0	0	0	0	0	0	82
13237706	Not	ot	CH	2017	0	1	0	0	1	0	0	0	0	0	0	0	
13237721	m	ot	US	2017	0	1	0	0	1	0	0	0	0	0	0	0	
13237928	f	ph	US	2017	0	0	0	0	0	0	0	0	0	0	0	0	50
13237968	m	ph	US	2017	0	0	0	0	0	0	0	0	0	0	0	0	59
13238012	f	md	EG	2017	0	1	0	0	1	0	0	0	0	0	0	0	70
13238057	Not	md	IT	2017	0	1	0	0	1	0	0	0	0	0	0	0	
13238218	m	md	JP	2017	0	1	0	0	0	1	0	0	0	0	0	0	77
13238280	m	cn	US	2017	0	1	0	0	1	0	0	0	0	0	0	0	
13238282	m	md	CA	2017	0	1	0	0	1	0	0	0	0	0	0	0	66
13238461	m	md	JP	2017	0	1	0	0	1	0	0	0	0	0	0	0	67
13238697	m	ot	DE	2017	0	1	0	0	1	0	0	0	0	0	0	0	74
13238700	f	md	IN	2017	0	0	0	0	0	0	0	0	0	0	0	0	40
13238735	m	ot	US	2017	0	0	0	0	0	0	0	0	0	0	0	0	68
13238949	m	cn	JP	2017	0	1	0	0	1	0	0	0	0	0	0	0	70
13239349	m	cn	US	2017	0	1	0	0	1	0	0	0	0	0	0	0	
13239379	m	cn	JP	2017	0	1	0	0	1	0	0	0	0	0	0	0	78
13239530	f	cn	GB	2017	1	0	0	0	0	0	0	0	0	0	0	0	66
13239997	f	md	US	2017	0	0	0	0	0	0	0	0	0	0	0	0	
13240582	f	md	JP	2017	1	0	0	0	0	0	0	0	0	0	0	0	77
13240734	f	md	FR	2017	1	0	0	0	0	0	0	0	0	0	0	0	
13240809	f	md	PT	2017	1	0	0	0	0	0	0	0	0	0	0	0	32.1
13241126	f	md	JP	2017	1	0	0	0	0	0	0	0	0	0	0	0	75
13241171	m	md	CZ	2017	0	0	0	0	0	0	0	0	0	0	0	0	62
13242305	Not	md	JP	2017	0	1	0	0	1	0	0	0	0	0	0	0	62
13242320	m	md	BG	2017	1	0	0	0	0	0	0	0	0	0	0	0	57
13242321	f	cn	GB	2017	1	0	0	0	0	0	0	0	0	0	0	0	
13242538	m	md	US	2017	0	1	1	0	0	0	0	0	0	0	0	1	68
13242626	f	md	BR	2017	0	0	0	0	0	0	0	0	0	0	0	0	72
13242692	f	ot	US	2017	0	1	1	0	0	0	0	0	0	0	0	0	73
13242716	m	md	JP	2017	1	0	0	0	0	0	0	0	0	0	0	0	90

13242724	f	md	CN	2017	1	0	0	0	0	0	0	0	0	0	0	0	37
13243140	Not	md	JP	2017	0	1	0	0	1	0	0	0	0	0	0	0	
13243463	m	ot	CN	2017	0	0	0	0	0	0	0	0	0	0	0	0	64
13243933	f	md	JP	2017	1	0	0	0	0	0	0	0	0	0	0	0	64
13244165	f	cn	GB	2017	1	0	0	0	0	0	0	0	0	0	0	0	80
13244439	f	cn	GB	2017	0	1	0	0	1	0	0	0	0	0	0	0	
13244441	f	cn	JP	2017	0	1	0	0	1	0	0	0	0	0	0	0	72
13244444	f	cn	US	2017	0	1	0	0	1	0	0	0	0	0	0	0	
13244543	Not	ot	JP	2017	0	0	0	0	0	0	0	0	0	0	0	0	
13244819	m	md	AU	2017	0	0	0	0	0	0	0	0	0	0	0	0	59
13244822	Not	ot	JP	2017	0	0	0	0	0	0	0	0	0	0	0	0	
13245374	m	cn	JP	2017	0	0	0	0	0	0	0	0	0	0	0	0	73
13245561	m	md	JP	2017	1	0	0	0	0	0	0	0	0	0	0	0	65
13246046	f	cn	US	2017	0	1	0	0	1	0	0	0	0	0	0	0	50
13246172	f	md	US	2017	0	1	0	0	0	1	0	0	0	0	0	0	56
13246194	m	cn	JP	2017	0	1	0	0	1	0	0	0	0	0	0	0	72
13246555	m	md	FR	2017	0	1	0	0	1	0	0	0	0	0	0	0	70
13246559	Not	md	IT	2017	0	1	0	0	1	0	0	0	0	0	0	0	
13247039	m	ot	DE	2017	0	1	0	0	1	0	0	0	0	0	0	0	67
13247049	m	md	JP	2017	0	1	0	0	1	0	0	0	0	0	0	0	76
13247080	m	md	DE	2017	0	1	0	0	1	0	0	0	0	0	0	0	60
13247106	f	ot	CH	2017	0	1	0	0	1	0	0	0	0	0	0	0	60
13247120	m	cn	US	2017	0	0	0	0	0	0	0	0	0	0	0	0	74
13247138	m	cn	GB	2017	1	0	0	0	0	0	0	0	0	0	0	0	
13247393	m	cn	JP	2017	0	1	0	0	1	0	0	0	0	0	0	1	65
13247669	m	cn	US	2017	0	1	0	0	1	0	0	0	0	0	0	0	
13247739	Not	ot	DE	2017	0	0	0	0	0	0	0	0	0	0	0	0	
13248103	m	ot	IL	2017	0	1	0	0	1	0	0	0	0	0	0	0	62
13250663	Not	md	US	2017	0	0	0	0	0	0	0	0	0	0	0	0	
13250749	m	md	JP	2017	0	0	0	0	0	0	0	0	0	0	0	0	80
13250798	f	cn	Not re	2017	0	1	0	0	1	0	0	0	0	0	0	0	41
13250838	m	cn	US	2017	0	1	0	0	1	0	0	0	0	0	0	0	
13251039	m	cn	JP	2017	0	1	0	0	1	0	0	0	0	0	0	0	71
13251418	f	cn	GB	2017	1	0	0	0	0	0	0	0	0	0	0	0	62

13251592	m	cn	US	2017	0	0	0	0	0	0	0	0	0	0	0	0	53
13251673	Not	cn	JP	2017	0	0	0	0	0	0	0	0	0	0	0	0	
13251678	f	cn	VE	2017	0	0	0	0	0	0	0	0	0	0	0	0	49
13251944	f	md	US	2017	0	1	0	0	0	1	0	0	0	0	0	0	73
13252465	Not	md	JP	2017	0	1	0	0	1	0	0	0	0	0	0	1	
13253067	m	md	JP	2017	0	0	0	0	0	0	0	0	0	0	0	0	
13253610	Not	md	US	2017	0	1	1	0	0	0	0	0	0	0	0	0	
13254014	f	ot	GB	2017	0	1	0	0	1	0	0	0	0	0	0	0	74
13254031	f	md	JP	2017	0	0	0	0	0	0	0	0	0	0	0	0	82
13254242	Not	md	US	2017	0	0	0	0	0	0	0	0	0	0	0	0	
13254398	m	md	AT	2017	0	1	1	0	0	0	0	0	0	0	0	1	58
13254523	m	cn	JP	2017	0	1	0	0	1	0	0	0	0	0	0	0	75
13254526	m	cn	JP	2017	0	1	0	0	1	0	0	0	0	0	0	0	71
13254550	m	md	US	2017	0	0	0	0	0	0	0	0	0	0	0	0	82
13254715	f	cn	US	2017	0	0	0	0	0	0	0	0	0	0	0	0	60
13254716	f	cn	US	2017	0	0	0	0	0	0	0	0	0	0	0	0	67
13255063	m	cn	US	2017	0	1	1	0	0	0	0	0	0	0	0	0	63
13255102	Not	md	US	2017	0	0	0	0	0	0	0	0	0	0	0	0	
13255600	Not	md	US	2017	0	0	0	0	0	0	0	0	0	0	0	0	
13256921	m	cn	JP	2017	0	1	0	0	1	0	0	0	0	0	0	0	77
13257044	Not	ot	US	2017	0	0	0	0	0	0	0	0	0	0	0	0	
13257135	Not	ot	US	2017	0	0	0	0	0	0	0	0	0	0	0	0	
13257228	Not	ot	US	2017	0	0	0	0	0	0	0	0	0	0	0	0	
13257275	m	cn	JP	2017	0	1	0	0	1	0	0	0	0	0	0	0	57
13257287	m	md	CZ	2017	0	0	0	0	0	0	0	0	0	0	0	0	62
13258262	Not	md	US	2017	0	0	0	0	0	0	0	0	0	0	0	0	
13258284	m	md	US	2017	0	0	0	0	0	0	0	0	0	0	0	0	70
13258343	Not	md	US	2017	0	0	0	0	0	0	0	0	0	0	0	0	
13258402	m	md	US	2017	0	0	0	0	0	0	0	0	0	0	0	0	47
13258435	Not	md	US	2017	0	0	0	0	0	0	0	0	0	0	0	0	
13258501	Not	md	US	2017	0	0	0	0	0	0	0	0	0	0	0	0	
13258503	m	cn	IT	2017	0	1	0	0	1	0	0	0	0	0	0	0	74
13258521	m	md	JP	2017	0	1	0	0	1	0	0	0	0	0	0	0	74
13258522	m	md	CA	2017	0	1	0	0	1	0	0	0	0	0	0	1	60

13258700	Not	md	US	2017	0	0	0	0	0	0	0	0	0	0	0	0	
13258971	Not	md	US	2017	0	0	0	0	0	0	0	0	0	0	0	0	
13259074	Not	md	US	2017	0	0	0	0	0	0	0	0	0	0	0	0	
13259122	Not	md	US	2017	0	0	0	0	0	0	0	0	0	0	0	0	
13259123	f	md	JP	2017	1	0	0	0	0	0	0	0	0	0	0	0	80
13259549	f	cn	CN	2017	1	0	0	0	0	0	0	0	0	0	0	0	
13259706	Not	cn	DE	2017	0	1	1	0	0	0	0	0	0	0	0	0	
13259728	f	md	PL	2017	0	0	0	0	0	0	0	0	0	0	0	0	47
13259743	f	md	AU	2017	0	0	0	0	0	0	0	0	0	0	0	0	73
13259827	f	md	KW	2017	0	0	0	0	0	0	0	0	0	0	0	0	67
13259916	f	ot	FI	2017	0	1	1	0	0	0	0	0	0	0	0	1	
13260607	f	md	US	2017	0	1	1	0	0	0	0	0	0	0	0	0	
13260764	m	md	JP	2017	0	1	0	0	1	0	0	0	0	0	0	0	74
13261012	m	ot	FR	2017	0	0	0	0	0	0	0	0	0	0	0	0	63
13261126	m	md	JP	2017	1	0	0	0	0	0	0	0	0	0	0	0	75
13261161	m	ot	GB	2017	0	1	1	0	0	0	0	0	0	0	0	0	56
13261233	f	md	JP	2017	1	0	0	0	0	0	0	0	0	0	0	0	75
13261744	f	cn	US	2017	0	1	1	0	0	0	0	0	0	0	0	0	51
13261765	m	cn	US	2017	0	0	0	0	0	0	0	0	0	0	0	0	67
13261825	f	md	ES	2017	0	1	0	0	0	1	0	0	0	0	0	0	53
13261994	m	ot	US	2017	0	1	0	0	1	0	0	0	0	0	0	0	79
13262144	m	md	JP	2017	1	0	0	0	0	0	0	0	0	0	0	0	
13262675	m	cn	JP	2017	0	1	0	0	1	0	0	0	0	0	0	0	58
13263243	f	md	JP	2017	0	0	0	0	0	0	0	0	0	0	0	0	68
13263249	f	cn	US	2017	0	1	0	0	1	0	0	0	0	0	0	0	
13263404	m	md	JP	2017	0	1	0	0	1	0	0	0	0	0	0	0	60
13264128	f	cn	JP	2017	0	0	0	0	0	0	0	0	0	0	0	0	45
13264176	f	cn	CA	2017	0	1	0	0	1	0	0	0	0	0	0	0	56
13264219	m	md	JP	2017	0	0	0	0	0	0	0	0	0	0	0	0	82
13264731	m	md	JP	2017	1	0	0	0	0	0	0	0	0	0	0	0	67
13265497	m	ot	FR	2017	0	0	0	0	0	0	0	0	0	0	0	0	63
13265539	m	md	JP	2017	0	1	0	0	1	0	0	0	0	0	0	1	63
13265838	f	md	IT	2017	0	0	0	0	0	0	0	0	0	0	0	0	58
13266517	m	md	US	2017	0	1	0	0	1	0	0	0	0	0	0	0	65

13266838	f	cn	JP	2017	0	1	0	0	1	0	0	0	0	0	0	1	79
13267185	f	cn	US	2017	0	1	0	0	1	0	0	0	0	0	0	0	
13267743	f	ot	US	2017	0	1	0	0	1	0	0	0	0	0	0	0	80
13268198	m	md	SA	2017	0	0	0	0	0	0	0	0	0	0	0	0	47
13268757	m	cn	US	2017	0	0	0	0	0	0	0	0	0	0	0	0	84
13268774	f	cn	US	2017	0	0	0	0	0	0	0	0	0	0	0	0	57
13269585	f	md	JP	2017	0	1	0	0	1	0	0	0	0	0	0	0	75
13269642	f	md	US	2017	0	1	0	0	0	1	0	0	0	0	0	0	86
13270116	m	md	JP	2017	0	1	0	0	0	1	0	0	0	0	0	0	59
13270608	f	cn	US	2017	0	1	1	0	0	0	0	0	0	0	0	0	55
13270645	f	Not repo	AU	2017	1	0	0	0	0	0	0	0	0	0	0	0	
13270888	f	cn	US	2017	0	1	0	0	1	0	0	0	0	0	0	0	56
13271177	Not	md	US	2017	0	0	0	0	0	0	0	0	0	0	0	0	
13271286	Not	md	JP	2017	0	1	0	0	1	0	0	0	0	0	0	0	
13271676	f	md	US	2017	0	1	0	0	1	0	0	0	0	0	0	0	
13272296	m	md	FR	2017	1	0	0	0	0	0	0	0	0	0	0	0	
13272968	f	md	DE	2017	0	0	0	0	0	0	0	0	0	0	0	0	41
13273032	f	cn	GB	2017	1	0	0	0	0	0	0	0	0	0	0	0	74
13273333	f	md	US	2017	0	1	1	0	0	0	0	0	0	0	0	0	
13274042	f	cn	JP	2017	0	1	0	0	1	0	0	0	0	0	0	0	72
13274645	m	ot	CH	2017	0	1	0	0	1	0	0	0	0	0	0	0	67
13274679	f	ot	US	2017	0	1	0	0	1	0	0	0	0	0	0	0	70
13275365	m	ot	IT	2017	0	1	0	0	1	0	0	0	0	0	0	0	69
13275430	m	ot	US	2017	0	1	0	0	1	0	0	0	0	0	0	0	
13275957	f	Not repo	US	2017	1	0	0	0	0	0	0	0	0	0	0	0	73.4
13276121	f	md	JP	2017	0	1	0	0	1	0	0	0	0	0	0	0	80
13276430	m	md	IN	2017	0	0	0	0	0	0	0	0	0	0	0	0	65
13276596	f	cn	GB	2017	1	0	0	0	0	0	0	0	0	0	0	0	
13276629	m	md	KR	2017	1	0	0	0	0	0	0	0	0	0	0	0	63
13276641	m	md	KR	2017	0	0	0	0	0	0	0	0	0	0	0	0	62
13276694	m	cn	JP	2017	0	0	0	0	0	0	0	0	0	0	0	0	87
13276792	m	cn	GB	2017	1	0	0	0	0	0	0	0	0	0	0	0	50
13277432	f	md	ES	2017	1	0	0	0	0	0	0	0	0	0	0	0	67.6
13277611	f	md	KR	2017	1	0	0	0	0	0	0	0	0	0	0	0	64.8

13277726	m	md	JP	2017	0	1	0	0	1	0	0	0	0	0	0	0	76
13277790	f	ot	US	2017	0	1	0	0	1	0	0	0	0	0	0	0	55
13277802	m	ot	GB	2017	0	1	0	0	1	0	0	0	0	0	0	0	64
13277998	m	Not repo	US	2017	1	0	0	0	0	0	0	0	0	0	0	0	57.5
13278187	m	ot	TW	2017	0	1	0	0	1	0	0	0	0	0	0	0	57
13278434	Not	md	US	2017	0	1	1	0	0	0	0	0	0	0	0	0	
13278681	Not	ot	AU	2017	1	0	0	0	0	0	0	0	0	0	0	0	
13278706	m	ot	IT	2017	0	0	0	0	0	0	0	0	0	0	0	0	
13280416	m	md	JP	2017	0	1	0	0	1	0	0	0	0	0	0	0	66
13280457	Not	md	US	2017	0	1	0	0	1	0	0	0	0	0	0	0	
13280768	f	Not repo	AU	2017	1	0	0	0	0	0	0	0	0	0	0	0	69.3
13281796	Not	ot	US	2017	0	0	0	0	0	0	0	0	0	0	0	0	
13282387	m	cn	GB	2017	0	1	1	0	0	0	0	0	0	0	0	0	65
13282432	f	md	US	2017	0	0	0	0	0	0	0	0	0	0	0	0	38
13282525	m	cn	CN	2017	1	0	0	0	0	0	0	0	0	0	0	0	69
13283795	f	Not repo	US	2017	1	0	0	0	0	0	0	0	0	0	0	0	73.6
13283852	f	md	JP	2017	0	1	0	0	1	0	0	0	0	0	0	0	56
13284392	f	ot	JP	2017	0	0	0	0	0	0	0	0	0	0	0	0	66
13284518	m	cn	GB	2017	1	0	0	0	0	0	0	0	0	0	0	0	70
13284521	Not	ph	JP	2017	0	1	0	0	1	0	0	0	0	0	0	0	
13284547	m	ot	DE	2017	0	0	0	0	0	0	0	0	0	0	0	0	66
13284786	f	ot	IT	2017	0	1	0	0	1	0	0	0	0	0	0	0	63
13285252	f	md	US	2017	0	0	0	0	0	0	0	0	0	0	0	0	65
13285330	m	md	US	2017	0	0	0	0	0	0	0	0	0	0	0	0	
13285380	m	md	NO	2017	0	0	0	0	0	0	0	0	0	0	0	0	51
13285598	f	cn	US	2017	0	1	0	0	1	0	0	0	0	0	0	0	
13285868	f	ot	US	2017	0	0	0	0	0	0	0	0	0	0	0	0	57
13287115	Not	ot	US	2017	0	1	0	0	1	0	0	0	0	0	0	0	
13287465	f	md	US	2017	0	1	0	0	0	1	0	0	0	0	0	0	82
13287691	m	md	JP	2017	0	1	0	0	1	0	0	0	0	0	0	0	81
13287768	f	cn	US	2017	1	0	0	0	0	0	0	0	0	0	0	1	
13288134	f	md	JP	2017	1	0	0	0	0	0	0	0	0	0	0	0	52
13288195	f	cn	US	2017	0	0	0	0	0	0	0	0	0	0	0	0	
13288440	m	ot	GB	2017	0	0	0	0	0	0	0	0	0	0	0	0	72

13288494	f	md	DE	2017	0	1	0	0	1	0	0	0	0	0	0	0	56
13288668	m	cn	GB	2017	0	0	0	0	0	0	0	0	0	0	0	0	77
13289250	m	md	US	2017	0	0	0	0	0	0	0	0	0	0	0	0	62
13289322	m	ph	JP	2017	0	1	0	0	1	0	0	0	0	0	0	0	59
13289697	Not	ot	GR	2017	0	0	0	0	0	0	0	0	0	0	0	0	
13289703	Not	ot	GR	2017	0	0	0	0	0	0	0	0	0	0	0	0	
13289708	Not	ot	GR	2017	0	0	0	0	0	0	0	0	0	0	0	0	
13289713	m	cn	US	2017	0	0	0	0	0	0	0	0	0	0	0	0	63
13289910	m	md	JP	2017	0	1	0	0	1	0	0	0	0	0	0	0	80
13289917	m	md	JP	2017	1	0	0	0	0	0	0	0	0	0	0	0	66
13290096	m	md	JP	2017	0	1	0	0	1	0	0	0	0	0	0	1	70
13290274	m	md	TH	2017	0	0	0	0	0	0	0	0	0	0	0	0	52
13290349	Not	ph	JP	2017	0	1	0	0	1	0	0	0	0	0	0	0	
13290497	m	cn	JP	2017	0	1	0	0	1	0	0	0	0	0	0	0	48
13290679	f	md	JP	2017	1	0	0	0	0	0	0	0	0	0	0	0	63
13290968	m	ot	DE	2017	0	1	0	0	1	0	0	0	0	0	0	0	65
13291029	f	cn	US	2017	0	1	0	0	1	0	0	0	0	0	0	0	
13292048	f	md	US	2017	0	1	0	0	1	0	0	0	0	0	0	0	
13292636	f	md	CN	2017	1	0	0	0	0	0	0	0	0	0	0	0	52
13292706	m	md	MY	2017	1	0	0	0	0	0	0	0	0	0	0	0	59
13294008	m	md	FR	2017	0	0	0	0	0	0	0	0	0	0	0	0	76
13294189	Not	ot	US	2017	0	1	0	0	1	0	0	0	0	0	0	0	
13294334	f	ph	NL	2017	0	0	0	0	0	0	0	0	0	0	0	0	53
13294697	m	ot	US	2017	0	0	0	0	0	0	0	0	0	0	0	0	
13294701	m	cn	US	2017	0	1	0	0	1	0	0	0	0	0	0	0	
13295024	f	ot	CH	2017	1	0	0	0	0	0	0	0	0	0	0	0	70
13295499	m	md	PE	2017	0	1	0	0	0	1	0	0	0	0	0	1	47
13295658	f	md	JP	2017	0	1	0	0	1	0	0	0	0	0	0	0	63
13295981	f	md	US	2017	1	0	0	0	0	0	0	0	0	0	0	0	51
13295999	f	cn	US	2017	1	0	0	0	0	0	0	0	0	0	0	0	55
13296187	f	ph	NL	2017	0	0	0	0	0	0	0	0	0	0	0	0	53
13296752	m	cn	TW	2017	1	0	0	0	0	0	0	0	0	0	0	0	50
13296838	m	ot	DE	2017	0	1	0	0	1	0	0	0	0	0	0	0	67
13296867	m	cn	CN	2017	0	0	0	0	0	0	0	0	0	0	0	0	

13296974	f	md	ES	2017	0	0	0	0	0	0	0	0	0	0	0	0	51
13297269	m	cn	US	2017	0	1	1	0	0	0	0	0	0	0	0	0	81
13297799	Not	md	US	2017	0	1	0	0	1	0	0	0	0	0	0	0	
13297800	Not	md	US	2017	0	1	0	0	1	0	0	0	0	0	0	0	
13298644	f	ot	US	2017	0	1	0	0	1	0	0	0	0	0	0	0	
13298872	f	md	HU	2017	0	1	0	0	1	0	0	0	0	0	0	0	50
13299218	m	md	DE	2017	0	0	0	0	0	0	0	0	0	0	0	0	83
13299596	m	md	JP	2017	1	0	0	0	0	0	0	0	0	0	0	0	56
13299729	m	cn	KR	2017	1	0	0	0	0	0	0	0	0	0	0	0	79
13300151	m	cn	US	2017	1	0	0	0	0	0	0	0	0	0	0	0	
13300456	f	cn	JP	2017	0	1	0	0	1	0	0	0	0	0	0	0	66
13300565	f	ot	US	2017	1	0	0	0	0	0	0	0	0	0	0	0	
13300607	f	cn	JP	2017	0	1	0	0	1	0	0	0	0	0	0	0	79
13300664	m	md	US	2017	0	1	0	0	1	0	0	0	0	0	0	1	
13300829	m	md	US	2017	0	1	1	0	0	0	0	0	0	0	0	0	68
13301083	Not	ot	KR	2017	0	0	0	0	0	0	0	0	0	0	0	0	
13301820	m	md	IN	2017	0	1	0	0	1	0	0	0	0	0	0	0	55
13301851	Not	md	FR	2017	0	0	0	0	0	0	0	0	0	0	0	0	
13302033	Not	ot	US	2017	1	0	0	0	0	0	0	0	0	0	0	0	
13302382	f	md	PT	2017	0	1	1	0	0	0	0	0	0	0	0	0	75
13302681	f	md	JP	2017	0	0	0	0	0	0	0	0	0	0	0	0	
13303167	m	Not repo	JP	2017	0	0	0	0	0	0	0	0	0	0	0	0	83
13303195	Not	ot	FR	2017	0	1	0	0	1	0	0	0	0	0	0	0	
13303198	m	md	RU	2017	0	1	0	0	0	1	0	0	0	0	0	0	75
13303211	f	cn	US	2017	0	0	0	0	0	0	0	0	0	0	0	0	
13303648	f	Not repo	JP	2017	0	0	0	0	0	0	0	0	0	0	0	0	67
13304001	m	md	JP	2017	0	1	0	0	1	0	0	0	0	0	0	0	76
13304153	Not	md	IT	2017	0	0	0	0	0	0	0	0	0	0	0	0	
13304324	Not	ot	KR	2017	0	0	0	0	0	0	0	0	0	0	0	0	
13304346	Not	ot	KR	2017	0	0	0	0	0	0	0	0	0	0	0	0	
13304349	Not	ot	KR	2017	0	0	0	0	0	0	0	0	0	0	0	0	
13304363	Not	ot	KR	2017	0	0	0	0	0	0	0	0	0	0	0	0	
13304369	Not	ot	KR	2017	0	0	0	0	0	0	0	0	0	0	0	0	
13304370	m	md	JP	2017	0	1	0	0	1	0	0	0	0	0	0	0	63

13304379	Not	ot	KR	2017	0	0	0	0	0	0	0	0	0	0	0	0	
13304410	m	cn	JP	2017	0	1	0	0	1	0	0	0	0	0	0	0	68
13304416	Not	ot	KR	2017	0	0	0	0	0	0	0	0	0	0	0	0	
13304428	Not	ot	KR	2017	0	0	0	0	0	0	0	0	0	0	0	0	
13304432	Not	ot	KR	2017	0	0	0	0	0	0	0	0	0	0	0	0	
13304435	Not	ot	KR	2017	0	0	0	0	0	0	0	0	0	0	0	0	
13304436	Not	ot	KR	2017	0	0	0	0	0	0	0	0	0	0	0	0	
13304456	Not	ot	KR	2017	0	0	0	0	0	0	0	0	0	0	0	0	
13304459	m	ot	FR	2017	0	0	0	0	0	0	0	0	0	0	0	0	63
13304464	Not	ot	KR	2017	0	0	0	0	0	0	0	0	0	0	0	0	
13304469	Not	ot	KR	2017	0	0	0	0	0	0	0	0	0	0	0	0	
13304487	Not	ot	KR	2017	0	0	0	0	0	0	0	0	0	0	0	0	
13304504	Not	ot	KR	2017	0	0	0	0	0	0	0	0	0	0	0	0	
13304555	f	md	JP	2017	0	1	0	0	1	0	0	0	0	0	0	0	72
13304609	Not	ot	KR	2017	0	0	0	0	0	0	0	0	0	0	0	0	
13304750	Not	ot	KR	2017	0	0	0	0	0	0	0	0	0	0	0	0	
13304758	f	cn	US	2017	0	0	0	0	0	0	0	0	0	0	0	0	45
13304774	Not	ot	KR	2017	0	0	0	0	0	0	0	0	0	0	0	0	
13304780	Not	ot	KR	2017	0	0	0	0	0	0	0	0	0	0	0	0	
13304807	Not	ot	KR	2017	0	0	0	0	0	0	0	0	0	0	0	0	
13305371	f	cn	JP	2017	0	1	0	0	1	0	0	0	0	0	0	0	63
13305838	m	md	HU	2017	0	0	0	0	0	0	0	0	0	0	0	0	61
13305847	f	md	AU	2017	0	0	0	0	0	0	0	0	0	0	0	0	60
13306981	f	ot	DE	2017	0	1	0	0	1	0	0	0	0	0	0	0	64
13307085	f	md	US	2017	1	0	0	0	0	0	0	0	0	0	0	0	
13307115	m	md	DE	2017	0	0	0	0	0	0	0	0	0	0	0	0	66
13307127	f	md	JP	2017	0	1	0	0	1	0	0	0	0	0	0	0	69
13307147	m	md	JP	2017	1	0	0	0	0	0	0	0	0	0	0	0	80
13307462	m	md	JP	2017	1	0	0	0	0	0	0	0	0	0	0	0	69.9
13307484	Not	md	FR	2017	0	1	0	0	1	0	0	0	0	0	0	0	
13307513	Not	cn	US	2017	0	1	1	0	0	0	0	0	0	0	0	0	
13307657	m	md	GB	2017	0	0	0	0	0	0	0	0	0	0	0	0	62
13312638	m	md	AT	2017	0	0	0	0	0	0	1	0	1	0	0	0	73
13313021	m	md	CN	2017	1	0	0	0	0	0	0	0	0	0	0	0	72

13313333	f	ot	JP	2017	0	1	0	0	1	0	0	0	0	0	0	0	79
13313451	m	md	JP	2017	0	1	0	0	1	0	0	0	0	0	0	1	63
13313834	f	md	JP	2017	1	0	0	0	0	0	0	0	0	0	0	0	73
13314233	m	md	US	2017	0	1	1	0	0	0	0	0	0	0	0	0	
13314543	f	ph	DE	2017	0	0	0	0	0	0	0	0	0	0	0	0	
13314921	m	ot	GB	2017	1	0	0	0	0	0	0	0	0	0	0	0	88
13315253	f	md	BE	2017	1	0	0	0	0	0	0	0	0	0	0	0	69.1
13315291	f	md	CZ	2017	0	1	0	0	1	0	0	0	0	0	0	1	46
13316382	f	ot	US	2017	1	0	0	0	0	0	0	0	0	0	0	0	38
13316413	f	cn	CA	2017	0	1	1	0	0	0	0	0	0	0	0	0	65
13316775	m	cn	GR	2017	0	1	1	0	0	0	0	0	0	0	0	0	48
13316980	m	cn	JP	2017	0	0	0	0	0	0	0	0	0	0	0	0	79
13317996	Not	md	US	2017	0	0	0	0	0	0	0	0	0	0	0	0	
13319154	m	md	NL	2017	0	0	0	0	0	0	0	0	0	0	0	0	76
13319286	Not	md	JP	2017	0	0	0	0	0	0	0	0	0	0	0	0	
13319370	f	ph	JP	2017	1	0	0	0	0	0	0	0	0	0	0	0	53
13319717	f	md	FR	2017	0	1	0	0	1	0	0	0	0	0	0	0	
13320039	m	cn	JP	2017	0	0	0	0	0	0	0	0	0	0	0	0	65
13320044	m	cn	FR	2017	0	0	0	0	0	0	0	0	0	0	0	0	70
13320173	m	md	HU	2017	0	1	0	0	1	0	0	0	0	0	0	1	58
13320175	Not	md	JP	2017	0	1	0	0	1	0	0	0	0	0	0	0	
13320268	Not	cn	US	2017	1	0	0	0	0	0	0	0	0	0	0	0	
13321073	m	cn	US	2017	0	1	1	0	0	0	0	0	0	0	0	0	55
13321533	Not	ph	JP	2017	1	0	0	0	0	0	0	0	0	0	0	0	
13321830	m	md	SG	2017	0	0	0	0	0	0	0	0	0	0	0	0	79
13322067	m	cn	US	2017	0	1	1	0	0	0	0	0	0	0	0	0	55
13322158	m	ot	US	2017	0	1	0	0	1	0	0	0	0	0	0	0	58
13322266	f	cn	US	2017	0	0	0	0	0	0	0	0	0	0	0	0	73
13322752	Not	md	IN	2017	0	0	0	0	0	0	0	0	0	0	0	0	
13324110	m	cn	GB	2017	1	0	0	0	0	0	0	0	0	0	0	0	
13324211	m	ot	JP	2017	0	1	0	0	1	0	0	0	0	0	0	0	73
13324601	Not	md	JP	2017	0	1	0	0	1	0	0	0	0	0	0	0	
13324605	f	md	JP	2017	0	1	0	0	1	0	0	0	0	0	0	0	65
13325545	m	cn	US	2017	0	0	0	0	0	0	0	0	0	0	0	0	64

13325706	m	md	KR	2017	1	0	0	0	0	0	0	0	0	0	0	1	60
13325970	m	ot	KR	2017	0	1	0	0	1	0	0	0	0	0	0	0	50
13326004	m	cn	US	2017	0	0	0	0	0	0	0	0	0	0	0	0	53
13327009	Not	md	TW	2017	1	0	0	0	0	0	0	0	0	0	0	0	
13327064	m	cn	US	2017	1	0	0	0	0	0	0	0	0	0	0	0	45
13327084	m	md	IT	2017	0	1	0	0	1	0	0	0	0	0	0	0	
13327114	Not	md	IT	2017	1	0	0	0	0	0	0	0	0	0	0	0	61
13328257	m	ot	US	2017	0	1	0	0	1	0	0	0	0	0	0	0	58
13328839	m	ot	ES	2017	0	1	0	0	1	0	0	0	0	0	0	0	60
13328923	m	cn	US	2017	0	0	0	0	0	0	0	0	0	0	0	0	56
13329037	f	ot	JP	2017	0	0	0	0	0	0	0	0	0	0	0	0	41
13329720	m	cn	US	2017	0	0	0	0	0	0	0	0	0	0	0	0	62
13329944	f	md	US	2017	0	1	1	0	0	0	0	0	0	0	0	0	61
13330048	f	md	US	2017	0	0	0	0	0	0	0	0	0	0	0	0	45
13330375	f	cn	US	2017	0	1	0	0	1	0	0	0	0	0	0	0	
13330630	f	ot	JP	2017	0	1	0	0	1	0	0	0	0	0	0	0	68
13332483	m	md	HU	2017	0	1	0	0	1	0	0	0	0	0	0	0	65
13332518	m	md	JP	2017	1	0	0	0	0	0	0	0	0	0	0	0	72
13332757	f	ot	US	2017	0	0	0	0	0	0	0	0	0	0	0	0	
13332901	m	Not repo	US	2017	1	0	0	0	0	0	0	0	0	0	0	0	66.2
13333037	f	cn	US	2017	0	0	0	0	0	0	0	0	0	0	0	0	69
13333407	m	cn	US	2017	0	0	0	0	0	0	0	0	0	0	0	0	55
13333669	f	ot	US	2017	0	1	1	0	0	0	0	0	0	0	0	0	50
13333757	m	md	US	2017	0	0	0	0	0	0	0	0	0	0	0	0	63
13333863	Not	Not repo	Not re	2017	1	0	0	0	0	0	0	0	0	0	0	0	
13334031	f	cn	US	2017	0	1	1	0	0	0	0	0	0	0	0	1	
13334352	m	ot	US	2017	0	1	0	0	1	0	0	0	0	0	0	0	
13334661	f	cn	CN	2017	1	0	0	0	0	0	0	0	0	0	0	0	
13334717	f	cn	US	2017	0	1	1	0	0	0	0	0	0	0	0	0	70
13334812	f	cn	CZ	2017	1	0	0	0	0	0	0	0	0	0	0	0	
13335355	m	md	JP	2017	0	1	0	0	1	0	0	0	0	0	0	0	64
13335578	m	md	JP	2017	0	1	0	0	1	0	0	0	0	0	0	1	59
13335921	Not	ph	JP	2017	0	1	0	0	1	0	0	0	0	0	0	0	
13336099	m	ot	AT	2017	0	1	0	0	1	0	0	0	0	0	0	0	65

13336101	m	cn	JP	2017	0	1	0	0	1	0	0	0	0	0	0	0	69
13336163	Not	ot	JP	2017	0	0	0	0	0	0	0	0	0	0	0	0	
13336349	m	md	DE	2017	0	1	0	0	0	1	0	0	0	0	0	0	55
13336363	f	ot	US	2017	0	1	0	0	1	0	0	0	0	0	0	0	
13336415	f	cn	DK	2017	0	0	0	0	0	0	0	0	0	0	0	0	
13337125	f	md	JP	2017	1	0	0	0	0	0	0	0	0	0	0	0	76
13337204	m	md	JP	2017	1	0	0	0	0	0	0	0	0	0	0	0	72
13337250	m	md	FR	2017	0	0	0	0	0	0	0	0	0	0	0	0	
13337329	m	md	JP	2017	0	1	0	0	1	0	0	0	0	0	0	0	65
13337405	m	ot	US	2017	0	1	0	0	1	0	0	0	0	0	0	0	71
13338821	Not	ph	US	2017	0	0	0	0	0	0	0	0	0	0	0	0	
13338822	Not	md	US	2017	0	0	0	0	0	0	0	0	0	0	0	0	
13338824	Not	md	US	2017	0	0	0	0	0	0	0	0	0	0	0	0	
13338881	f	cn	US	2017	0	1	1	0	0	0	0	0	0	0	0	0	64
13338970	m	md	FR	2017	0	1	0	0	1	0	0	0	0	0	0	0	66
13339290	Not	md	US	2017	0	1	0	0	1	0	0	0	0	0	0	0	
13339300	m	ot	JP	2017	0	0	0	0	0	0	0	0	0	0	0	0	65
13339397	Not	ot	US	2017	0	1	0	0	1	0	0	0	0	0	0	0	
13339555	f	md	JP	2017	0	1	0	0	1	0	0	0	0	0	0	0	70
13339637	m	ot	JP	2017	0	1	0	0	1	0	0	0	0	0	0	0	78
13339648	f	cn	US	2017	0	0	0	0	0	0	0	0	0	0	0	0	
13339651	m	md	JP	2017	0	1	0	0	1	0	0	0	0	0	0	0	66
13339881	f	md	US	2017	0	1	0	0	1	0	0	0	0	0	0	0	82
13340159	m	cn	US	2017	0	1	0	0	1	0	0	0	0	0	0	0	
13340479	Not	ot	US	2017	0	0	0	0	0	0	0	0	0	0	0	0	
13340550	Not	md	SK	2017	0	0	0	0	0	0	0	0	0	0	0	0	
13340620	f	md	CN	2017	0	1	0	0	0	1	0	0	0	0	0	0	58
13340686	f	ph	NL	2017	0	0	0	0	0	0	0	0	0	0	0	0	53
13340933	m	md	JP	2017	0	1	0	0	1	0	0	0	0	0	0	1	79
13341583	Not	md	US	2017	0	1	0	0	0	1	0	0	0	0	0	0	
13341775	m	md	JP	2017	0	1	0	0	1	0	0	0	0	0	0	1	86
13341782	f	ph	JP	2017	0	1	0	0	1	0	0	0	0	0	0	0	53
13342105	m	md	DE	2017	0	1	0	0	1	0	0	0	0	0	0	1	
13342292	m	md	JP	2017	1	0	0	0	0	0	0	0	0	0	0	0	58

13342708	f	md	KR	2017	1	0	0	0	0	0	0	0	0	0	0	0	64.4
13343003	m	cn	US	2017	0	0	0	0	0	0	0	0	0	0	0	0	55
13343067	f	cn	GB	2017	1	0	0	0	0	0	0	0	0	0	0	1	77
13343434	Not	ot	US	2017	0	0	0	0	0	0	0	0	0	0	0	0	74
13343448	f	ot	US	2017	0	0	0	0	0	0	0	0	0	0	0	0	47
13343449	m	ot	US	2017	0	0	0	0	0	0	0	0	0	0	0	0	62
13343544	m	md	JP	2017	0	1	0	0	1	0	0	0	0	0	0	1	61
13343723	f	ot	US	2017	0	0	0	0	0	0	0	0	0	0	0	0	66
13343728	f	md	US	2017	0	0	0	0	0	0	0	0	0	0	0	0	76
13343893	m	cn	US	2017	0	1	0	0	1	0	0	0	0	0	0	0	55
13344249	f	cn	ES	2017	0	1	0	0	1	0	0	0	0	0	0	0	62
13344626	m	ph	JP	2017	1	0	0	0	0	0	0	0	0	0	0	0	64
13344631	f	md	AU	2017	0	1	0	0	1	0	0	0	0	0	0	0	
13345986	m	cn	US	2017	0	1	0	0	0	1	0	0	0	0	0	0	
13347017	Not	md	US	2017	0	1	0	0	1	0	0	0	0	0	0	0	
13347291	m	md	JP	2017	1	0	0	0	0	0	0	0	0	0	0	0	80
13347559	f	ot	CA	2017	0	1	0	0	1	0	0	0	0	0	0	0	58
13347839	f	md	DE	2017	0	1	0	0	1	0	0	0	0	0	0	0	65
13347944	m	md	TR	2017	0	1	0	0	1	0	0	0	0	0	0	0	56
13347955	m	ot	TW	2017	0	1	0	0	1	0	0	0	0	0	0	0	59
13348018	m	ot	TW	2017	0	1	0	0	1	0	0	0	0	0	0	0	54
13348066	m	md	US	2017	1	0	0	0	0	0	0	0	0	0	0	0	
13348172	m	md	TR	2017	0	1	0	0	1	0	0	0	0	0	0	0	66
13348649	m	ph	JP	2017	0	1	0	0	1	0	0	0	0	0	0	0	69
13348726	m	ot	IT	2017	0	1	0	0	1	0	0	0	0	0	0	0	68
13348791	m	cn	US	2017	1	0	0	0	0	0	0	0	0	0	0	0	77
13349214	f	cn	US	2017	0	0	0	0	0	0	0	0	0	0	0	0	
13350413	Not	Not repo	US	2017	1	0	0	0	0	0	0	0	0	0	0	0	
13350471	m	cn	US	2017	1	0	0	0	0	0	0	0	0	0	0	0	80
13350656	m	cn	GB	2017	0	1	1	0	0	0	0	0	0	0	0	1	
13350794	m	cn	JP	2017	0	0	0	0	0	0	0	0	0	0	0	0	75
13350953	f	cn	US	2017	0	0	0	0	0	0	0	0	0	0	0	0	65
13351023	f	md	US	2017	0	0	0	0	0	0	0	0	0	0	0	0	54
13351750	f	cn	US	2017	0	0	0	0	0	0	0	0	0	0	0	0	65

13352391	m	cn	US	2017	0	0	0	0	0	0	0	0	0	0	0	0	61
13352570	m	md	IN	2017	0	0	0	0	0	0	0	0	0	0	0	0	74
13352643	m	cn	US	2017	1	0	0	0	0	0	0	0	0	0	0	0	75
13352745	m	md	US	2017	0	0	0	0	0	0	0	0	0	0	0	0	66
13352750	f	md	US	2017	0	0	0	0	0	0	0	0	0	0	0	0	55
13353610	f	md	JP	2017	1	0	0	0	0	0	0	0	0	0	0	0	70.8
13353968	m	md	CN	2017	0	1	0	0	0	1	0	0	0	0	0	0	58
13354721	m	cn	JP	2017	0	0	0	0	0	0	0	0	0	0	0	0	68
13354722	f	cn	US	2017	0	0	0	0	0	0	0	0	0	0	0	0	76
13355011	m	ot	IT	2017	0	1	0	0	1	0	0	0	0	0	0	0	76
13355381	m	cn	US	2017	0	0	0	0	0	0	0	0	0	0	0	0	72
13355477	m	cn	US	2017	0	1	1	0	0	0	0	0	0	0	0	0	
13355534	m	ot	US	2017	0	1	0	0	1	0	0	0	0	0	0	0	
13355545	f	ot	CA	2017	0	1	0	0	1	0	0	0	0	0	0	0	
13356108	m	md	GB	2017	0	0	0	0	0	0	0	0	0	0	0	0	
13356149	f	cn	TH	2017	0	0	0	0	0	0	0	0	0	0	0	0	64
13356368	m	md	JP	2017	0	1	0	0	1	0	0	0	0	0	0	0	73
13356503	f	ot	PL	2017	0	1	0	0	1	0	0	0	0	0	0	0	52
13356949	m	ot	BR	2017	0	1	0	0	1	0	0	0	0	0	0	0	62
13357099	Not	md	NL	2017	0	0	0	0	0	0	0	0	0	0	0	0	49
13357167	m	md	JP	2017	0	1	0	0	1	0	0	0	0	0	0	0	71
13357245	m	md	JP	2017	0	1	0	0	1	0	0	0	0	0	0	0	68
13357417	m	cn	IT	2017	0	1	0	0	1	0	0	0	0	0	0	0	69
13357504	f	md	JP	2017	1	0	0	0	0	0	0	0	0	0	0	0	89
13358535	m	ot	US	2017	0	1	1	0	0	0	0	0	0	0	0	0	81
13358604	m	md	JP	2017	0	0	0	0	0	0	0	0	0	0	0	0	71
13358795	m	cn	TH	2017	0	0	0	0	0	0	0	0	0	0	0	0	74
13359220	m	md	KR	2017	1	0	0	0	0	0	0	0	0	0	0	0	78.4
13359829	f	ot	US	2017	1	0	0	0	0	0	0	0	0	0	0	0	
13360172	m	cn	JP	2017	0	1	0	0	1	0	0	0	0	0	0	0	64
13360212	m	cn	US	2017	0	0	0	0	0	0	0	0	0	0	0	0	72
13360387	m	cn	JP	2017	0	1	0	0	1	0	0	0	0	0	0	0	55
13360479	m	md	US	2017	0	1	0	0	0	1	0	0	0	0	0	0	61
13360646	m	md	GB	2017	0	1	1	0	0	0	0	0	0	0	0	0	84

13360681	m	md	GR	2017	0	0	0	0	0	0	0	0	0	0	0	0	44
13360771	f	cn	US	2017	1	0	0	0	0	0	0	0	0	0	0	0	
13361098	m	ph	DE	2017	1	0	0	0	0	0	0	0	0	0	0	0	67
13361287	f	cn	AU	2017	0	0	0	0	0	0	0	0	0	0	0	0	
13361312	f	ph	JP	2017	0	1	0	0	1	0	0	0	0	0	0	0	74
13361382	m	ot	CA	2017	0	1	0	0	1	0	0	0	0	0	0	0	72
13361724	m	md	JP	2017	0	0	0	0	0	0	0	0	0	0	0	0	72
13361729	m	Not repo	US	2017	1	0	0	0	0	0	0	0	0	0	0	0	71.9
13361912	f	md	Not re	2017	0	0	0	0	0	0	0	0	0	0	0	0	55
13362094	m	md	EG	2017	0	1	0	0	1	0	0	0	0	0	0	0	52
13362400	m	cn	US	2017	0	1	0	0	1	0	0	0	0	0	0	0	
13362546	f	cn	US	2017	0	1	0	0	1	0	0	0	0	0	0	1	
13363138	m	md	DE	2017	0	0	0	0	0	0	0	0	0	0	0	0	
13363460	f	md	JP	2017	1	0	0	0	0	0	0	0	0	0	0	0	92
13363843	m	ot	US	2017	0	0	0	0	0	0	0	0	0	0	0	0	72
13364980	f	cn	US	2017	0	0	0	0	0	0	0	0	0	0	0	0	47
13366233	f	md	DE	2017	0	0	0	0	0	0	0	0	0	0	0	0	
13366309	f	cn	US	2017	0	0	0	0	0	0	0	0	0	0	0	0	47
13366328	f	md	CA	2017	0	1	0	0	1	0	0	0	0	0	0	0	
13366858	Not	md	BE	2017	0	1	0	0	1	0	0	0	0	0	0	1	
13367182	m	md	JP	2017	0	1	0	0	1	0	0	0	0	0	0	0	60
13367298	Not	ot	US	2017	0	1	0	0	1	0	0	0	0	0	0	0	
13367304	Not	ot	US	2017	0	1	0	0	1	0	0	0	0	0	0	0	
13367307	Not	ot	US	2017	0	1	0	0	1	0	0	0	0	0	0	0	
13367332	m	md	JP	2017	0	1	0	0	1	0	0	0	0	0	0	0	69
13367397	m	md	JP	2017	1	0	0	0	0	0	0	0	0	0	0	0	67
13367495	Not	ot	JP	2017	0	0	0	0	0	0	0	0	0	0	0	0	
13367530	Not	ot	JP	2017	0	0	0	0	0	0	0	0	0	0	0	0	
13367689	m	ot	JP	2017	0	0	0	0	0	0	0	0	0	0	0	0	73
13367700	Not	cn	US	2017	0	1	1	0	0	0	0	0	0	0	0	0	
13367760	m	md	JP	2017	0	1	0	0	1	0	0	0	0	0	0	0	63
13367888	m	ot	US	2017	0	0	0	0	0	0	0	0	0	0	0	0	73
13367988	m	md	US	2017	0	1	0	0	0	1	0	0	0	0	0	0	61
13368330	f	md	JP	2017	0	0	0	0	0	0	0	0	0	0	0	0	

13368463	f	ph	JP	2017	1	0	0	0	0	0	0	0	0	0	0	0	60
13368646	Not	ot	US	2017	0	0	0	0	0	0	0	0	0	0	0	0	
13370690	Not	cn	US	2017	0	1	0	0	1	0	0	0	0	0	0	0	
13370733	Not	cn	DE	2017	1	0	0	0	0	0	0	0	0	0	0	0	
13370993	f	md	US	2017	0	1	0	0	1	0	0	0	0	0	0	0	
13371006	f	md	JP	2017	1	0	0	0	0	0	0	0	0	0	0	0	66
13371056	m	md	JP	2017	0	1	0	0	1	0	0	0	0	0	0	0	70
13371094	m	md	JP	2017	0	1	0	0	1	0	0	0	0	0	0	0	67
13371137	m	ph	JP	2017	0	1	0	0	1	0	0	0	0	0	0	0	67
13371227	Not	cn	US	2017	0	1	0	0	1	0	0	0	0	0	0	0	
13371228	Not	cn	US	2017	0	1	0	0	1	0	0	0	0	0	0	0	
13371230	Not	cn	US	2017	0	1	0	0	1	0	0	0	0	0	0	0	
13371231	Not	cn	US	2017	0	1	0	0	1	0	0	0	0	0	0	0	
13371232	Not	cn	US	2017	0	1	0	0	1	0	0	0	0	0	0	0	
13371348	m	md	IT	2017	0	0	0	0	0	0	0	0	0	0	0	0	69
13371355	m	ph	AU	2017	0	1	0	0	1	0	0	0	0	0	0	0	
13371566	f	md	HU	2017	0	1	0	0	1	0	0	0	0	0	0	1	68
13371568	m	cn	US	2017	0	1	0	0	1	0	0	0	0	0	0	1	
13372582	f	md	FR	2017	0	1	0	0	0	1	0	0	0	0	0	0	66
13372661	f	md	JP	2017	1	0	0	0	0	0	0	0	0	0	0	0	74
13372685	Not	ot	US	2017	1	0	0	0	0	0	0	0	0	0	0	1	
13372736	m	md	RU	2017	1	0	0	0	0	0	0	0	0	0	0	0	55
13372886	f	cn	US	2017	0	1	1	0	0	0	0	0	0	0	0	0	58
13373291	m	md	DE	2017	0	0	0	0	0	0	0	0	0	0	0	0	66
13373744	f	md	JP	2017	1	0	0	0	0	0	0	0	0	0	0	0	78
13374276	m	md	CN	2017	1	0	0	0	0	0	0	0	0	0	0	0	87.6
13375352	Not	ph	AU	2017	0	1	0	0	1	0	0	0	0	0	0	0	68
13375364	f	ot	US	2017	0	1	0	0	1	0	0	0	0	0	0	0	
13375488	f	md	JP	2017	1	0	0	0	0	0	0	0	0	0	0	0	67
13375556	Not	ot	CN	2017	1	0	0	0	0	0	0	0	0	0	0	0	
13375560	m	md	JP	2017	1	0	0	0	0	0	0	0	0	0	0	0	67
13375667	f	md	JP	2017	1	0	0	0	0	0	0	0	0	0	0	0	71
13375962	m	md	US	2017	0	1	0	0	0	1	0	0	0	0	0	0	74
13376006	Not	ph	US	2017	0	0	0	0	0	0	0	0	0	0	0	0	74

13376573	f	md	JP	2017	1	0	0	0	0	0	0	0	0	0	0	0	73
13376574	f	md	JP	2017	1	0	0	0	0	0	0	0	0	0	0	0	75
13376645	m	ph	US	2017	0	0	0	0	0	0	0	0	0	0	0	0	77
13377784	m	ot	IT	2017	0	1	0	0	1	0	0	0	0	0	0	0	69
13377978	m	ot	US	2017	0	1	0	0	1	0	0	0	0	0	0	0	81
13377980	m	md	JP	2017	0	1	0	0	1	0	0	0	0	0	0	0	70
13378114	m	md	JP	2017	0	1	0	0	1	0	0	0	0	0	0	0	69
13378204	f	ph	AU	2017	0	1	0	0	1	0	0	0	0	0	0	0	
13378283	Not	ot	FR	2017	0	0	0	0	0	0	0	0	0	0	0	0	
13378330	m	cn	JP	2017	0	1	0	0	1	0	0	0	0	0	0	0	62
13379605	f	cn	US	2017	0	0	0	0	0	0	0	0	0	0	0	0	42
13380521	m	ph	JP	2017	1	0	0	0	0	0	0	0	0	0	0	0	61
13381341	Not	md	CN	2017	0	0	0	0	0	0	0	0	0	0	0	0	
13382032	m	ot	US	2017	0	0	0	0	0	0	0	0	0	0	0	0	
13382107	m	ot	KR	2017	0	0	0	0	0	0	0	0	0	0	0	0	73
13382207	f	md	ES	2017	0	0	0	0	0	0	0	0	0	0	0	0	66
13382211	m	md	IN	2017	0	0	0	0	0	0	0	0	0	0	0	0	41
13382679	f	ot	US	2017	0	0	0	0	0	0	0	0	0	0	0	0	
13382857	f	Not repo	US	2017	1	0	0	0	0	0	0	0	0	0	0	0	74.1
13383167	f	cn	BR	2017	0	0	0	0	0	0	0	0	0	0	0	0	81
13383355	Not	ot	JP	2017	0	0	0	0	0	0	0	0	0	0	0	0	
13383357	Not	ot	JP	2017	0	0	0	0	0	0	0	0	0	0	0	0	
13383363	Not	ot	JP	2017	0	0	0	0	0	0	0	0	0	0	0	0	
13383365	Not	ot	JP	2017	0	0	0	0	0	0	0	0	0	0	0	0	
13383369	Not	ot	JP	2017	0	0	0	0	0	0	0	0	0	0	0	0	
13383374	Not	ot	JP	2017	0	0	0	0	0	0	0	0	0	0	0	0	
13383486	m	cn	TW	2017	0	1	1	0	0	0	0	0	0	0	0	1	50
13383490	f	md	JP	2017	1	0	0	0	0	0	0	0	0	0	0	0	87
13383561	f	cn	US	2017	0	1	0	0	1	0	0	0	0	0	0	0	
13383746	m	cn	US	2017	0	0	0	0	0	0	0	0	0	0	0	0	
13383786	m	cn	JP	2017	0	1	0	0	1	0	0	0	0	0	0	0	63
13383787	m	cn	KR	2017	1	0	0	0	0	0	0	0	0	0	0	0	59
13383790	m	md	JP	2017	0	1	0	0	1	0	0	0	0	0	0	0	80
13384010	f	md	JP	2017	0	1	0	0	1	0	0	0	0	0	0	0	69

13384126	m	ot	GB	2017	1	0	0	0	0	0	0	0	0	0	0	0	68
13385447	Not	cn	GB	2017	1	0	0	0	0	0	0	0	0	0	0	0	
13385510	m	ph	US	2017	0	0	0	0	0	0	0	0	0	0	0	0	55
13385699	f	ph	CA	2017	0	0	0	0	0	0	0	0	0	0	0	0	
13386021	m	cn	JP	2017	0	1	0	0	1	0	0	0	0	0	0	1	74
13386289	f	md	US	2017	0	0	0	0	0	0	0	0	0	0	0	0	69
13386452	m	Not repo	US	2017	1	0	0	0	0	0	0	0	0	0	0	0	60.7
13386516	m	ot	GB	2017	0	0	0	0	0	0	0	0	0	0	0	0	72
13386583	f	md	JP	2017	1	0	0	0	0	0	0	0	0	0	0	0	76
13387216	m	cn	GB	2017	1	0	0	0	0	0	0	0	0	0	0	0	61
13387854	Not	ot	FR	2017	0	0	0	0	0	0	0	0	0	0	0	0	
13387857	Not	ot	FR	2017	0	0	0	0	0	0	0	0	0	0	0	0	
13387866	Not	ot	FR	2017	0	0	0	0	0	0	0	0	0	0	0	0	
13388123	f	Not repo	US	2017	1	0	0	0	0	0	0	0	0	0	0	0	55.3
13388135	m	Not repo	US	2017	1	0	0	0	0	0	0	0	0	0	0	0	
13388136	f	Not repo	US	2017	1	0	0	0	0	0	0	0	0	0	0	0	60.3
13388199	m	md	JP	2017	0	1	1	0	0	0	0	0	0	0	0	1	80
13388203	f	Not repo	US	2017	1	0	0	0	0	0	0	0	0	0	0	0	
13388345	f	ot	IT	2017	0	1	0	0	1	0	0	0	0	0	0	0	78
13388677	Not	ph	AU	2017	0	1	0	0	1	0	0	0	0	0	0	0	69
13389325	m	cn	ES	2017	0	0	0	0	0	0	0	0	0	0	0	0	72
13389591	Not	ot	GR	2017	0	0	0	0	0	0	0	0	0	0	0	0	
13389646	m	md	JP	2017	0	1	0	0	1	0	0	0	0	0	0	0	47
13389799	Not	md	AU	2017	0	1	0	0	1	0	0	0	0	0	0	0	63
13389826	m	cn	US	2017	0	1	1	0	0	0	0	0	0	0	0	0	
13390069	m	cn	GB	2017	1	0	0	0	0	0	0	0	0	0	0	0	59
13390074	m	cn	GB	2017	1	0	0	0	0	0	0	0	0	0	0	0	67
13390076	f	cn	GB	2017	1	0	0	0	0	0	0	0	0	0	0	0	51
13390077	f	cn	GB	2017	1	0	0	0	0	0	0	0	0	0	0	0	66
13390171	m	cn	GB	2017	1	0	0	0	0	0	0	0	0	0	0	0	58
13390299	Not	md	US	2017	0	0	0	0	0	0	0	0	0	0	0	0	
13390497	Not	ot	JP	2017	0	0	0	0	0	0	0	0	0	0	0	0	
13390502	m	ot	JP	2017	0	1	0	0	1	0	0	0	0	0	0	0	66
13390519	f	cn	GB	2017	1	0	0	0	0	0	0	0	0	0	0	0	85

13390593	m	cn	GB	2017	1	0	0	0	0	0	0	0	0	0	0	0	60
13390594	f	cn	GB	2017	1	0	0	0	0	0	0	0	0	0	0	0	73
13390595	f	cn	GB	2017	1	0	0	0	0	0	0	0	0	0	0	0	74
13390597	f	cn	GB	2017	1	0	0	0	0	0	0	0	0	0	0	0	72
13390604	f	cn	GB	2017	1	0	0	0	0	0	0	0	0	0	0	0	66
13390840	f	cn	GB	2017	1	0	0	0	0	0	0	0	0	0	0	0	76
13390843	f	cn	GB	2017	1	0	0	0	0	0	0	0	0	0	0	0	78
13390903	f	cn	GB	2017	1	0	0	0	0	0	0	0	0	0	0	0	84
13390905	m	md	JP	2017	0	1	0	0	1	0	0	0	0	0	0	0	65
13391723	f	md	AU	2017	0	0	0	0	0	0	0	0	0	0	0	0	72
13392147	m	cn	GB	2017	1	0	0	0	0	0	0	0	0	0	0	0	41
13392155	f	md	CH	2017	0	0	0	0	0	0	0	0	0	0	0	0	
13392798	f	cn	JP	2017	0	1	0	0	1	0	0	0	0	0	0	1	80
13392801	m	ot	IT	2017	0	1	0	0	1	0	0	0	0	0	0	0	66
13393673	m	ph	JP	2017	0	1	0	0	1	0	0	0	0	0	0	0	71
5846197	m	ot	JP	2017	0	0	0	0	0	0	0	0	0	0	0	0	70
6597498	m	cn	JP	2017	0	0	0	0	0	0	0	0	0	0	0	0	82
7010679	m	cn	JP	2017	0	0	0	0	0	0	0	0	0	0	0	0	63
8837118	m	cn	JP	2017	0	0	0	0	0	0	0	0	0	0	0	0	67
9258534	f	ot	CA	2017	0	0	0	0	0	0	0	0	0	0	0	0	66
9332937	f	md	DE	2017	1	0	0	0	0	0	0	0	0	0	0	0	77
9410298	m	ph	US	2017	0	0	0	0	0	0	0	0	0	0	0	0	58
9791977	f	cn	GB	2017	1	0	0	0	0	0	0	0	0	0	0	0	74
10142178	Not	cn	GB	2017	1	0	0	0	0	0	0	0	0	0	0	0	
12667990	m	md	BE	2017	0	0	0	0	0	0	0	0	0	0	0	0	42
12682842	m	cn	JP	2017	0	1	0	0	1	0	0	0	0	0	0	0	71
12716598	m	md	CN	2017	0	1	0	0	0	1	0	0	0	0	0	0	57
12838603	m	md	DE	2017	0	1	0	0	0	1	0	0	0	0	0	0	68
12854450	f	md	US	2017	0	0	0	0	0	0	0	0	0	0	0	0	71
12870408	m	md	JP	2017	0	0	0	0	0	0	1	0	1	0	0	0	76
12884233	f	md	DE	2017	0	1	0	0	0	1	0	0	0	0	0	0	77
12887626	m	ot	US	2017	0	1	1	0	0	0	0	0	0	0	0	0	76
12909613	m	ot	US	2017	0	1	1	0	0	0	0	0	0	0	0	0	65
12924886	f	cn	US	2017	1	0	0	0	0	0	0	0	0	0	0	0	86

12933524	m	ot	JP	2017	0	1	0	0	1	0	0	0	0	0	0	0	65
12949519	f	cn	US	2017	1	0	0	0	0	0	0	0	0	0	0	0	
12972030	m	cn	JP	2017	0	1	0	0	1	0	0	0	0	0	0	0	84
12983568	m	ot	NL	2017	0	1	0	0	1	0	0	0	0	0	0	1	56
12985797	m	cn	US	2017	0	1	1	0	0	0	0	0	0	0	0	0	77
13013702	f	cn	US	2017	1	0	0	0	0	0	0	0	0	0	0	0	69
13015689	m	cn	JP	2017	0	1	0	0	1	0	0	0	0	0	0	0	68
13018220	m	ot	JP	2017	0	1	0	0	1	0	0	0	0	0	0	0	70
13018338	m	md	JP	2017	0	1	0	0	1	0	0	0	0	0	0	0	73
13021351	f	md	NL	2017	0	1	0	0	0	1	0	0	0	0	0	0	61
13037002	m	md	TR	2017	0	0	0	0	0	0	0	0	0	0	0	0	77
13038650	f	md	JP	2017	1	0	0	0	0	0	0	0	0	0	0	0	84
13040613	f	ot	US	2017	0	1	0	0	1	0	0	0	0	0	0	0	69
13046146	m	ot	US	2017	0	1	1	0	0	0	0	0	0	0	0	0	74
13067272	m	md	ES	2017	0	1	0	0	0	1	0	0	0	0	0	0	61
13068851	f	md	JP	2017	0	1	0	0	1	0	0	0	0	0	0	0	54
13069473	m	ot	IN	2017	0	0	0	0	0	0	0	0	0	0	0	0	32
13072490	f	cn	KR	2017	1	0	0	0	0	0	0	0	0	0	0	0	78
13083117	f	md	JP	2017	0	1	0	0	1	0	0	0	0	0	0	0	72
13084720	m	md	US	2017	0	1	1	0	0	0	0	0	0	0	0	0	65
13086826	m	md	JP	2017	0	1	0	0	1	0	0	0	0	0	0	0	68
13090208	m	ot	ES	2017	0	0	0	0	0	0	0	0	0	0	0	0	71
13093129	m	ot	US	2017	0	0	0	0	0	0	0	0	0	0	0	0	87
13100232	m	ot	CA	2017	0	1	0	0	1	0	0	0	0	0	0	0	
13104356	m	cn	JP	2017	0	1	0	0	1	0	0	0	0	0	0	1	70
13106187	m	md	US	2017	0	1	1	0	0	0	0	0	0	0	0	0	84
13109568	m	cn	US	2017	0	1	1	0	0	0	0	0	0	0	0	0	66
13112669	f	cn	JP	2017	0	1	0	0	1	0	0	0	0	0	0	0	63
13114127	m	md	US	2017	0	0	0	0	0	0	0	0	0	0	0	0	76
13117314	f	md	JP	2017	1	0	0	0	0	0	0	0	0	0	0	0	79
13118747	m	cn	JP	2017	0	1	0	0	1	0	0	0	0	0	0	1	68
13120696	m	md	US	2017	0	1	0	0	0	1	0	0	0	0	0	0	75
13130186	f	md	CA	2017	0	1	0	0	1	0	0	0	0	0	0	0	
13131829	f	cn	US	2017	0	1	1	0	0	0	0	0	0	0	0	0	81

13132241	m	md	JP	2017	0	1	0	0	1	0	0	0	0	0	0	0	40
13134098	m	ot	US	2017	0	1	0	0	0	1	0	0	0	0	0	0	75
13134279	f	cn	JP	2017	0	1	0	0	1	0	0	0	0	0	0	0	53
13135879	f	md	JP	2017	0	1	0	0	1	0	0	0	0	0	0	0	68
13136041	m	ot	IT	2017	0	1	0	0	1	0	0	0	0	0	0	0	47
13137033	m	md	IT	2017	0	0	0	0	0	0	0	0	0	0	0	0	70
13144264	m	md	JP	2017	1	0	0	0	0	0	0	0	0	0	0	0	73
13144451	m	cn	JP	2017	0	1	0	0	1	0	0	0	0	0	0	0	57
13148133	f	md	JP	2017	0	0	0	0	0	0	0	0	0	0	0	0	75
13153045	f	cn	US	2017	0	0	0	0	0	0	0	0	0	0	0	0	54
13153458	m	ph	JP	2017	0	0	0	0	0	0	0	0	0	0	0	0	49
13157048	m	ot	IT	2017	0	1	0	0	1	0	0	0	0	0	0	0	69
13157171	f	ot	US	2017	0	1	1	0	0	0	0	0	0	0	0	0	62
13159145	f	ot	IT	2017	0	1	0	0	1	0	0	0	0	0	0	0	65
13159878	m	md	JP	2017	0	1	0	0	1	0	0	0	0	0	0	0	78
13160809	f	md	US	2017	0	1	0	0	0	1	0	0	0	0	0	0	75
13161060	m	md	UA	2017	0	0	0	0	0	0	0	0	0	0	0	0	64
13167454	f	cn	US	2017	0	0	0	0	0	0	0	0	0	0	0	0	68
13176757	m	md	US	2017	0	1	0	0	0	1	0	0	0	0	0	0	72
13177785	f	ot	CA	2017	0	1	0	0	1	0	0	0	0	0	0	0	56
13179412	m	ot	IT	2017	0	1	0	0	0	1	0	0	0	0	0	0	64
13182341	f	cn	BR	2017	1	0	0	0	0	0	0	0	0	0	0	0	
13183793	m	md	JP	2017	0	1	0	0	0	1	0	0	0	0	0	0	53
13186942	m	md	IL	2017	0	1	0	0	0	1	0	0	0	0	0	0	71
13186948	m	ot	FR	2017	0	1	0	0	1	0	0	0	0	0	0	0	61
13187931	m	cn	JP	2017	0	1	0	0	1	0	0	0	0	0	0	0	52
13189238	m	cn	US	2017	0	1	1	0	0	0	0	0	0	0	0	0	
13190463	m	md	US	2017	0	1	1	0	0	0	0	0	0	0	0	0	73
13190605	f	cn	US	2017	0	1	1	0	0	0	0	0	0	0	0	0	70
13191615	m	md	JP	2017	0	1	0	0	0	1	0	0	0	0	0	0	53
13192900	m	md	TR	2017	0	0	0	0	0	0	0	0	0	0	0	0	64
13193289	f	md	US	2017	0	1	1	0	0	0	0	0	0	0	0	0	75
13193723	f	Not repo	US	2017	1	0	0	0	0	0	0	0	0	0	0	0	
13194601	m	md	JP	2017	0	1	0	0	1	0	0	0	0	0	0	0	78

13195850	f	cn	Not re	2017	0	0	0	0	0	0	0	0	0	0	0	0	
13204896	m	cn	US	2017	0	0	0	0	0	0	0	0	0	0	0	0	74
13205726	f	md	JP	2017	0	1	0	0	1	0	0	0	0	0	0	0	71
13209405	f	md	FR	2017	0	1	0	0	0	1	0	0	0	0	0	0	77
13213154	m	md	FR	2017	0	1	0	0	1	0	0	0	0	0	0	0	
13213857	f	cn	US	2017	1	0	0	0	0	0	0	0	0	0	0	0	76
13214251	f	md	US	2017	0	1	0	0	0	1	0	0	0	0	0	1	58
13216186	m	ot	RO	2017	0	1	0	0	1	0	0	0	0	0	0	1	64
13219291	m	cn	US	2017	0	1	1	0	0	0	0	0	0	0	0	0	
13224236	m	md	JP	2017	1	0	0	0	0	0	0	0	0	0	0	1	100
13229242	m	md	KR	2017	0	1	0	0	0	1	0	0	0	0	0	0	62
13229667	Not	ot	GR	2017	0	1	0	0	1	0	0	0	0	0	0	0	55
13237717	m	cn	JP	2017	0	1	0	0	1	0	0	0	0	0	0	0	65
13239347	m	md	US	2017	0	1	1	0	0	0	0	0	0	0	0	0	70
13245787	f	md	US	2017	0	1	0	0	0	1	0	0	0	0	0	0	69
13246949	m	md	US	2017	0	1	0	0	0	1	0	0	0	0	0	0	54
13252323	m	md	US	2017	0	0	0	0	0	0	1	1	0	0	0	0	75
13252898	m	cn	KR	2017	0	0	0	0	0	0	0	0	0	0	0	0	
13254767	m	ot	IT	2017	0	1	0	0	1	0	0	0	0	0	0	0	83
13255994	m	md	GB	2017	0	1	0	0	0	1	0	0	0	0	0	1	50
13256422	f	Not repo	US	2017	0	0	0	0	0	0	0	0	0	0	0	0	
13256962	f	md	FR	2017	0	0	0	0	0	0	0	0	0	0	0	0	50
13257713	m	md	US	2017	0	1	1	0	0	0	0	0	0	0	0	0	94
13258462	f	ot	DE	2017	0	1	0	0	1	0	0	0	0	0	0	0	55
13261530	m	cn	US	2017	0	1	0	0	1	0	0	0	0	0	0	0	66
13263690	f	md	US	2017	0	0	0	0	0	0	0	0	0	0	0	0	55
13263794	f	md	US	2017	0	0	0	0	0	0	0	0	0	0	0	0	55
13264618	f	md	GB	2017	0	1	0	0	1	0	0	0	0	0	0	0	
13264621	Not	ot	GB	2017	0	1	0	0	1	0	0	0	0	0	0	0	
13264708	m	cn	US	2017	0	1	1	0	0	0	0	0	0	0	0	0	68
13265453	f	md	KR	2017	1	0	0	0	0	0	0	0	0	0	0	0	41.2
13265476	m	md	TR	2017	0	0	0	0	0	0	0	0	0	0	0	0	62
13267804	m	ot	US	2017	0	1	0	0	1	0	0	0	0	0	0	0	74
13269401	Not	ot	IT	2017	0	0	0	0	0	0	0	0	0	0	0	0	

13269412	Not	ot	IT	2017	0	0	0	0	0	0	0	0	0	0	0	0	
13269413	Not	ot	IT	2017	0	0	0	0	0	0	0	0	0	0	0	0	
13271360	f	ot	US	2017	0	1	0	0	1	0	0	0	0	0	0	0	48
13273661	m	md	FR	2017	0	1	0	1	0	0	0	0	0	0	0	0	58
13274703	m	ot	CA	2017	0	1	0	0	0	1	0	0	0	0	0	0	64
13275654	f	md	US	2017	1	0	0	0	0	0	0	0	0	0	0	0	62
13276357	f	ot	US	2017	0	0	0	0	0	0	0	0	0	0	0	0	69
13276445	f	md	IT	2017	1	0	0	0	0	0	0	0	0	0	0	0	53.5
13277920	f	md	JP	2017	1	0	0	0	0	0	0	0	0	0	0	0	91.7
13278548	m	md	US	2017	0	1	1	0	0	0	0	0	0	0	0	1	70
13284401	m	md	TR	2017	0	0	0	0	0	0	0	0	0	0	0	0	62
13284934	m	md	CN	2017	1	0	0	0	0	0	0	0	0	0	0	0	53
13289098	f	md	CH	2017	1	0	0	0	0	0	0	0	0	0	0	0	79
13289326	f	md	JP	2017	0	1	0	0	1	0	0	0	0	0	0	1	69
13290020	m	md	US	2017	0	1	1	0	0	0	0	0	0	0	0	0	56
13292090	f	md	AU	2017	1	0	0	0	0	0	0	0	0	0	0	0	82.4
13292618	f	md	SE	2017	0	0	0	0	0	0	0	0	0	0	0	0	70
13294694	m	md	JP	2017	1	0	0	0	0	0	0	0	0	0	0	0	61
13295810	f	cn	JP	2017	0	1	0	0	1	0	0	0	0	0	0	0	79
13298798	f	cn	US	2017	0	1	1	0	0	0	0	0	0	0	0	0	74
13299536	m	md	US	2017	0	1	1	0	0	0	0	0	0	0	0	0	57
13300358	m	cn	JP	2017	0	1	0	0	1	0	0	0	0	0	0	0	66
13301468	m	ot	DE	2017	0	0	0	0	0	0	0	0	0	0	0	0	67
13302473	f	md	JP	2017	0	1	0	0	1	0	0	0	0	0	0	0	74
13303170	m	cn	JP	2017	0	1	0	0	1	0	0	0	0	0	0	0	66
13303695	m	cn	JP	2017	0	0	0	0	0	0	0	0	0	0	0	0	53
13305372	f	md	US	2017	0	1	1	0	0	0	0	0	0	0	0	0	
13306508	m	ph	JP	2017	0	1	0	0	1	0	0	0	0	0	0	1	69
13307029	f	md	JP	2017	1	0	0	0	0	0	0	0	0	0	0	0	65
13307580	Not	cn	US	2017	0	1	1	0	0	0	0	0	0	0	0	0	
13309266	m	md	US	2017	0	1	1	0	0	0	0	0	0	0	0	0	55
13309293	f	md	ES	2017	0	0	0	0	0	0	0	0	0	0	0	0	53
13314468	m	md	JP	2017	1	0	0	0	0	0	0	0	0	0	0	0	70
13314593	f	md	JP	2017	1	0	0	0	0	0	0	0	0	0	0	0	44

13314676	f	cn	US	2017	1	0	0	0	0	0	0	0	0	0	0	0	52
13315191	m	md	US	2017	0	1	1	0	0	0	0	0	0	0	0	0	51
13315948	f	ot	DE	2017	0	1	0	0	1	0	0	0	0	0	0	1	66
13316596	m	md	CN	2017	0	1	0	0	0	1	0	0	0	0	0	0	69
13319893	f	md	US	2017	0	1	0	0	0	1	0	0	0	0	0	0	74
13320244	f	md	JP	2017	0	0	0	0	0	0	1	0	1	0	0	0	45
13324180	f	cn	US	2017	1	0	0	0	0	0	0	0	0	0	0	0	68
13324763	f	md	CH	2017	0	1	0	0	1	0	0	0	0	0	0	0	68
13330516	f	md	CA	2017	0	1	0	0	1	0	0	0	0	0	0	0	
13330799	f	cn	JP	2017	0	1	0	0	1	0	0	0	0	0	0	0	56
13331108	f	md	US	2017	0	1	1	0	0	0	0	0	0	0	0	0	72
13332585	m	ot	ES	2017	0	0	0	0	0	0	0	0	0	0	0	0	68
13332605	f	cn	JP	2017	0	1	0	0	0	1	0	0	0	0	0	1	55
13333012	m	cn	US	2017	0	1	0	0	1	0	0	0	0	0	0	0	75
13333433	Not	cn	JP	2017	0	0	0	0	0	0	0	0	0	0	0	0	
13333708	m	md	US	2017	0	1	1	0	0	0	0	0	0	0	0	0	85
13335504	f	md	DE	2017	0	1	0	0	0	1	0	0	0	0	0	0	59
13335826	m	ot	US	2017	0	1	0	0	1	0	0	0	0	0	0	0	52
13335903	m	ot	GB	2017	0	1	0	0	1	0	0	0	0	0	0	0	75
13336015	f	md	JP	2017	0	1	0	0	1	0	0	0	0	0	0	0	75
13336102	m	cn	JP	2017	0	1	0	0	1	0	0	0	0	0	0	0	71
13336535	m	md	ES	2017	0	1	0	0	0	1	0	0	0	0	0	0	64
13337400	f	md	JP	2017	1	0	0	0	0	0	0	0	0	0	0	0	88
13337689	f	md	JP	2017	1	0	0	0	0	0	0	0	0	0	0	0	62
13339061	f	cn	US	2017	1	0	0	0	0	0	0	0	0	0	0	0	
13340527	m	md	DE	2017	0	1	0	0	1	0	0	0	0	0	0	0	57
13343967	f	cn	US	2017	1	0	0	0	0	0	0	0	0	0	0	1	60
13345854	m	ot	US	2017	0	0	0	0	0	0	0	0	0	0	0	0	73
13347697	f	cn	US	2017	0	1	1	0	0	0	0	0	0	0	0	0	64
13349079	m	ph	US	2017	0	1	1	0	0	0	0	0	0	0	0	0	73
13351499	m	md	IT	2017	1	0	0	0	0	0	0	0	0	0	0	0	74.9
13352394	m	ot	US	2017	0	1	0	0	1	0	0	0	0	0	0	0	65
13353190	m	cn	JP	2017	0	1	1	0	0	0	0	0	0	0	0	0	73
13354144	f	cn	JP	2017	1	0	0	0	0	0	0	0	0	0	0	0	60

13357221	m	md	US	2017	0	1	1	0	0	0	0	0	0	0	0	0	57
13357405	m	cn	DE	2017	0	1	1	0	0	0	0	0	0	0	0	0	60
13358133	m	md	BE	2017	0	1	0	0	0	1	0	0	0	0	0	0	68
13359766	m	ph	JP	2017	1	0	0	0	0	0	0	0	0	0	0	0	74
13359836	m	cn	JP	2017	0	1	0	0	1	0	0	0	0	0	0	0	42
13361630	m	ot	IT	2017	0	1	0	0	1	0	0	0	0	0	0	0	79
13362596	m	md	US	2017	0	0	0	0	0	0	0	0	0	0	0	0	
13363464	m	cn	GB	2017	1	0	0	0	0	0	0	0	0	0	0	0	64
13365892	f	md	US	2017	0	0	0	0	0	0	0	0	0	0	0	0	
13366807	m	md	JP	2017	1	0	0	0	0	0	0	0	0	0	0	0	76
13369359	f	md	KR	2017	0	0	0	0	0	0	0	0	0	0	0	0	65
13369814	m	md	TR	2017	0	0	0	0	0	0	0	0	0	0	0	0	64
13371501	f	ot	ES	2017	0	1	0	0	1	0	0	0	0	0	0	0	73
13371503	f	ph	CA	2017	0	1	0	0	1	0	0	0	0	0	0	0	62
13371562	m	ot	US	2017	0	1	0	0	1	0	0	0	0	0	0	0	77
13372058	f	md	DE	2017	0	0	0	0	0	0	0	0	0	0	0	0	66
13372164	m	md	US	2017	0	1	1	0	0	0	0	0	0	0	0	0	
13372749	m	md	RU	2017	1	0	0	0	0	0	0	0	0	0	0	0	65
13373245	f	md	US	2017	0	1	1	0	0	0	0	0	0	0	0	0	62
13373279	f	cn	JP	2017	0	0	0	0	0	0	0	0	0	0	0	0	85
13374190	m	md	HK	2017	0	1	1	0	0	0	0	0	0	0	0	1	66
13375583	m	cn	JP	2017	0	1	0	0	1	0	0	0	0	0	0	1	54
13375985	f	ot	US	2017	0	1	1	0	0	0	0	0	0	0	0	0	
13376229	m	ph	AU	2017	0	1	0	0	1	0	0	0	0	0	0	0	
13376816	f	md	IT	2017	1	0	0	0	0	0	0	0	0	0	0	0	73
13377025	m	md	US	2017	0	1	0	0	0	1	0	0	0	0	0	0	49
13377577	m	md	US	2017	0	1	1	0	0	0	0	0	0	0	0	1	41
13377925	f	md	FR	2017	0	1	0	0	1	0	0	0	0	0	0	0	66
13379884	m	cn	JP	2017	0	0	0	0	0	0	0	0	0	0	0	0	66
13380288	m	md	JP	2017	0	1	0	0	1	0	0	0	0	0	0	0	74
13380498	f	md	JP	2017	1	0	0	0	0	0	0	0	0	0	0	0	68
13380505	m	md	AT	2017	0	1	0	0	0	1	0	0	0	0	0	0	74
13380891	m	ot	GB	2017	0	1	0	0	1	0	0	0	0	0	0	0	70
13383115	m	cn	JP	2017	0	1	1	0	0	0	0	0	0	0	0	0	75

13383669	f	md	JP	2017	1	0	0	0	0	0	0	0	0	0	0	0	80
13383823	f	md	TH	2017	0	0	0	0	0	0	0	0	0	0	0	0	70
13383881	f	md	US	2017	0	0	0	0	0	0	0	0	0	0	0	0	80
13385626	m	md	JP	2017	0	1	0	0	1	0	0	0	0	0	0	0	79
13386043	m	md	CN	2017	0	1	0	0	0	1	0	0	0	0	0	0	70
13386907	f	md	FR	2017	0	1	0	0	0	1	0	0	0	0	0	0	63
13386986	m	md	JP	2017	0	1	0	0	1	0	0	0	0	0	0	0	70
13387102	m	ph	CH	2017	0	0	0	0	0	0	0	0	0	0	0	0	48
13387561	m	ot	US	2017	0	1	1	0	0	0	0	0	0	0	0	1	78
13387581	m	ot	DE	2017	0	1	1	0	0	0	0	0	0	0	0	0	51
13387726	m	md	US	2017	0	1	1	0	0	0	0	0	0	0	0	0	
13388627	f	cn	US	2017	0	1	1	0	0	0	0	0	0	0	0	0	56
13388761	m	md	JP	2017	0	1	0	0	1	0	0	0	0	0	0	0	66
13389175	m	md	JP	2017	1	0	0	0	0	0	0	0	0	0	0	0	66
13389313	f	md	KR	2017	1	0	0	0	0	0	0	0	0	0	0	0	45.6
13389651	m	ph	JP	2017	0	1	0	0	1	0	0	0	0	0	0	0	73
13389848	m	md	FR	2017	0	1	0	0	0	1	0	0	0	0	0	0	67
13389906	m	md	JP	2017	0	1	0	0	1	0	0	0	0	0	0	0	78
13391303	m	cn	JP	2017	0	1	1	0	0	0	0	0	0	0	0	1	76
13391409	f	cn	US	2017	1	0	0	0	0	0	0	0	0	0	0	0	79
13391984	f	md	FR	2017	1	0	0	0	0	0	0	0	0	0	0	0	63
13392171	m	ph	JP	2017	0	1	0	0	1	0	0	0	0	0	0	1	73
13393142	f	md	TH	2017	0	0	0	0	0	0	0	0	0	0	0	0	65
13393363	f	cn	JP	2017	0	0	0	0	0	0	0	0	0	0	0	0	81
13394418	Not	ot	JP	2017	0	0	0	0	0	0	0	0	0	0	0	0	
13394537	Not	md	US	2017	0	0	0	0	0	0	0	0	0	0	0	0	
13394555	m	ot	AU	2017	0	1	0	0	1	0	0	0	0	0	0	0	60
13394556	f	md	DE	2017	0	1	0	0	1	0	0	0	0	0	0	0	
13394705	m	cn	JP	2017	0	1	1	0	0	0	0	0	0	0	0	1	64
13394853	m	md	IT	2017	0	0	0	0	0	0	0	0	0	0	0	0	
13394919	Not	ot	JP	2017	0	0	0	0	0	0	0	0	0	0	0	0	
13395151	Not	ot	JP	2017	0	0	0	0	0	0	0	0	0	0	0	0	
13395170	Not	ot	JP	2017	0	0	0	0	0	0	0	0	0	0	0	0	
13396512	m	cn	JP	2017	0	1	1	0	0	0	0	0	0	0	0	0	57

13396729	f	cn	GB	2017	1	0	0	0	0	0	0	0	0	0	0	0	
13396739	f	cn	GB	2017	0	0	0	0	0	0	0	0	0	0	0	0	69
13396766	m	md	JP	2017	0	1	0	0	1	0	0	0	0	0	0	0	68
13397226	Not	cn	GB	2017	1	0	0	0	0	0	0	0	0	0	0	0	
13397348	m	ot	US	2017	0	1	0	0	1	0	0	0	0	0	0	0	78
13397418	f	md	JP	2017	1	0	0	0	0	0	0	0	0	0	0	0	72
13397510	m	cn	US	2017	0	1	0	0	1	0	0	0	0	0	0	1	
13398247	f	md	PH	2017	0	1	1	0	0	0	0	0	0	0	0	0	51
13399113	m	ot	IT	2017	0	1	0	0	1	0	0	0	0	0	0	0	60
13399259	m	md	JP	2017	0	1	0	0	1	0	0	0	0	0	0	0	80
13399308	m	cn	US	2017	1	0	0	0	0	0	0	0	0	0	0	0	69
13399492	Not	md	US	2017	0	0	0	0	0	0	0	0	0	0	0	0	
13399717	Not	ot	US	2017	0	1	1	0	0	0	0	0	0	0	0	0	
13399762	m	cn	US	2017	0	1	1	0	0	0	0	0	0	0	0	0	50
13400577	f	md	US	2017	0	1	1	0	0	0	0	0	0	0	0	0	
13400679	f	md	JP	2017	1	0	0	0	0	0	0	0	0	0	0	0	47
13400719	m	ph	JP	2017	0	0	0	0	0	0	0	0	0	0	0	0	66
13400813	f	md	JP	2017	0	0	0	0	0	0	0	0	0	0	0	0	73
13401100	Not	ot	CN	2017	0	0	0	0	0	0	0	0	0	0	0	0	
13401363	m	ot	FR	2017	0	0	0	0	0	0	0	0	0	0	0	0	63
13401471	m	cn	IT	2017	0	1	0	0	1	0	0	0	0	0	0	1	65
13401735	m	ph	JP	2017	0	1	0	0	1	0	0	0	0	0	0	0	80
13401845	m	md	JP	2017	0	1	0	0	1	0	0	0	0	0	0	0	44
13401884	Not	cn	US	2017	0	0	0	0	0	0	0	0	0	0	0	0	
13402089	f	md	US	2017	0	1	1	0	0	0	0	0	0	0	0	0	75
13402556	m	md	GB	2017	0	1	0	0	1	0	0	0	0	0	0	0	57
13402566	m	ot	US	2017	0	1	0	0	1	0	0	0	0	0	0	0	61
13402567	f	cn	US	2017	0	0	0	0	0	0	1	0	1	0	0	0	
13402799	f	md	US	2017	0	0	0	0	0	0	0	0	0	0	0	0	70
13402803	m	md	US	2017	0	0	0	0	0	0	0	0	0	0	0	0	
13402886	f	md	US	2017	0	0	0	0	0	0	0	0	0	0	0	0	40
13402892	f	md	US	2017	0	0	0	0	0	0	0	0	0	0	0	0	50
13402905	m	md	US	2017	0	0	0	0	0	0	0	0	0	0	0	0	
13402908	f	md	US	2017	0	0	0	0	0	0	0	0	0	0	0	0	60

13402911	Not	md	US	2017	0	0	0	0	0	0	0	0	0	0	0	0	
13402919	m	ot	US	2017	0	0	0	0	0	0	0	0	0	0	0	0	58
13402940	m	md	US	2017	0	0	0	0	0	0	0	0	0	0	0	0	
13402945	m	md	US	2017	0	0	0	0	0	0	0	0	0	0	0	0	78
13402971	f	ot	US	2017	0	0	0	0	0	0	0	0	0	0	0	0	40
13402975	m	ot	US	2017	0	0	0	0	0	0	0	0	0	0	0	0	80
13403031	m	md	US	2017	0	0	0	0	0	0	0	0	0	0	0	0	72
13403084	Not	md	US	2017	0	0	0	0	0	0	0	0	0	0	0	0	
13403243	f	md	US	2017	0	0	0	0	0	0	0	0	0	0	0	0	
13403439	m	Not repo	US	2017	1	0	0	0	0	0	0	0	0	0	0	0	56.8
13403590	m	ot	JP	2017	0	1	0	0	1	0	0	0	0	0	0	0	78
13403615	f	cn	US	2017	0	1	1	0	0	0	0	0	0	0	0	0	58
13403908	f	md	NO	2017	0	1	0	0	1	0	0	0	0	0	0	0	66
13404511	m	md	IT	2017	1	0	0	0	0	0	0	0	0	0	0	0	65
13404783	m	md	JP	2017	0	1	0	0	1	0	0	0	0	0	0	0	72
13404864	f	md	US	2017	0	1	1	0	0	0	0	0	0	0	0	0	80
13405085	f	ot	CO	2017	0	1	0	0	1	0	0	0	0	0	0	0	65
13406149	Not	ot	US	2017	0	0	0	0	0	0	0	0	0	0	0	0	
13406152	Not	cn	DE	2017	0	0	0	0	0	0	0	0	0	0	0	0	
13406160	Not	ot	US	2017	0	0	0	0	0	0	0	0	0	0	0	0	
13406162	Not	cn	DE	2017	0	0	0	0	0	0	0	0	0	0	0	0	
13406174	Not	cn	DE	2017	0	0	0	0	0	0	0	0	0	0	0	0	
13406420	m	md	IT	2017	0	1	0	0	0	1	0	0	0	0	0	0	64
13406844	Not	cn	JP	2017	0	0	0	0	0	0	0	0	0	0	0	0	
13406988	f	md	US	2017	0	1	0	0	1	0	0	0	0	0	0	0	
13407226	m	md	ES	2017	0	1	0	0	0	1	0	0	0	0	0	0	64
13407249	Not	ot	US	2017	0	0	0	0	0	0	0	0	0	0	0	0	
13407250	Not	ot	US	2017	0	0	0	0	0	0	0	0	0	0	0	0	
13407251	Not	ot	US	2017	0	0	0	0	0	0	0	0	0	0	0	0	
13407254	Not	ot	US	2017	0	0	0	0	0	0	0	0	0	0	0	0	
13407258	Not	ot	US	2017	0	0	0	0	0	0	0	0	0	0	0	0	
13407482	m	md	NL	2017	0	0	0	0	0	0	0	0	0	0	0	0	74
13407697	f	md	JP	2017	1	0	0	0	0	0	0	0	0	0	0	1	86
13408205	f	md	DE	2017	0	1	0	0	1	0	0	0	0	0	0	0	

13409982	f	ot	JP	2017	0	1	0	0	1	0	0	0	0	0	0	0	50
13413298	m	md	US	2017	0	1	1	0	0	0	0	0	0	0	0	0	
13413316	m	ot	ES	2017	0	0	0	0	0	0	0	0	0	0	0	0	66
13413672	m	ot	US	2017	0	0	0	0	0	0	0	0	0	0	0	0	69
13414404	m	cn	US	2017	0	1	1	0	0	0	0	0	0	0	0	0	65
13414528	f	md	KR	2017	1	0	0	0	0	0	0	0	0	0	0	0	56
13414740	m	cn	US	2017	0	0	0	0	0	0	0	0	0	0	0	0	59
13415606	m	ot	FR	2017	0	0	0	0	0	0	0	0	0	0	0	0	63
13415991	m	md	FR	2017	0	0	0	0	0	0	0	0	0	0	0	0	35
13416123	m	cn	JP	2017	0	1	0	0	1	0	0	0	0	0	0	0	75
13416215	m	ot	ES	2017	0	0	0	0	0	0	0	0	0	0	0	0	68
13416275	f	md	FR	2017	1	0	0	0	0	0	0	0	0	0	0	0	70.6
13416419	m	ot	US	2017	0	1	1	0	0	0	0	0	0	0	0	0	81
13416955	m	ot	US	2017	0	1	0	0	1	0	0	0	0	0	0	0	61
13417006	m	cn	US	2017	0	1	1	0	0	0	0	0	0	0	0	0	
13417018	m	md	IT	2017	1	0	0	0	0	0	0	0	0	0	0	0	78.3
13417523	Not	md	DE	2017	0	0	0	0	0	0	0	0	0	0	0	0	
13417526	m	ot	US	2017	0	0	0	0	0	0	0	0	0	0	0	0	44
13417543	f	cn	US	2017	1	0	0	0	0	0	0	0	0	0	0	0	51
13417568	m	md	FR	2017	0	1	0	0	0	1	0	0	0	0	0	0	60
13417682	m	md	JP	2017	0	1	0	0	1	0	0	0	0	0	0	0	76
13417732	f	md	JP	2017	0	0	0	0	0	0	0	0	0	0	0	0	66
13417803	m	cn	JP	2017	0	1	0	0	1	0	0	0	0	0	0	0	53
13417805	m	cn	JP	2017	0	1	0	0	1	0	0	0	0	0	0	1	79
13418079	m	md	PH	2017	0	1	1	0	0	0	0	0	0	0	0	0	70
13418139	m	cn	JP	2017	0	1	0	0	1	0	0	0	0	0	0	0	79
13418591	f	md	JP	2017	0	0	0	0	0	0	0	0	0	0	0	0	67
13418914	m	cn	DK	2017	0	1	0	0	1	0	0	0	0	0	0	0	78
13419044	m	cn	JP	2017	0	1	0	0	1	0	0	0	0	0	0	0	59
13419078	m	md	US	2017	0	1	0	0	0	1	0	0	0	0	0	0	80
13419164	m	ot	US	2017	0	1	0	0	1	0	0	0	0	0	0	0	
13419211	f	cn	US	2017	0	1	1	0	0	0	0	0	0	0	0	0	
13419248	m	md	DE	2017	0	1	0	0	1	0	0	0	0	0	0	0	77
13419293	m	md	JP	2017	0	0	0	0	0	0	0	0	0	0	0	0	67

13419439	m	ot	GB	2017	0	0	0	0	0	0	0	0	0	0	0	0	63
13419488	m	cn	JP	2017	0	1	0	0	1	0	0	0	0	0	0	0	66
13420173	Not	md	BE	2017	0	1	0	0	1	0	0	0	0	0	0	0	77
13420851	Not	ot	US	2017	0	0	0	0	0	0	0	0	0	0	0	0	
13420852	Not	ot	US	2017	0	0	0	0	0	0	0	0	0	0	0	0	
13420853	Not	ot	US	2017	0	0	0	0	0	0	0	0	0	0	0	0	
13420854	Not	ot	US	2017	0	0	0	0	0	0	0	0	0	0	0	0	
13420855	Not	ot	US	2017	0	0	0	0	0	0	0	0	0	0	0	0	
13420857	Not	ot	US	2017	0	0	0	0	0	0	0	0	0	0	0	0	
13420860	Not	ot	US	2017	0	0	0	0	0	0	0	0	0	0	0	0	
13421722	f	cn	CA	2017	0	1	0	0	1	0	0	0	0	0	0	0	
13421727	m	ot	DE	2017	0	1	0	0	1	0	0	0	0	0	0	0	81
13421743	m	md	JP	2017	0	0	0	0	0	0	0	0	0	0	0	0	63
13422235	m	cn	JP	2017	0	1	1	0	0	0	0	0	0	0	0	1	40
13422922	m	cn	JP	2017	0	1	1	0	0	0	0	0	0	0	0	0	82
13423169	f	md	US	2017	0	1	1	0	0	0	0	0	0	0	0	0	60
13423744	f	md	US	2017	0	1	1	0	0	0	0	0	0	0	0	0	
13423766	f	md	ES	2017	1	0	0	0	0	0	0	0	0	0	0	0	80
13423969	m	md	CL	2017	1	0	0	0	0	0	0	0	0	0	0	0	77
13424040	m	md	CN	2017	1	0	0	0	0	0	0	0	0	0	0	0	55.4
13424539	m	cn	JP	2017	0	1	0	0	1	0	0	0	0	0	0	0	73
13424610	m	md	CA	2017	0	0	0	0	0	0	0	0	0	0	0	0	75
13424654	Not	ot	US	2017	0	1	1	0	0	0	0	0	0	0	0	0	
13424724	m	md	US	2017	0	1	1	0	0	0	0	0	0	0	0	0	
13424985	m	md	JP	2017	0	1	0	0	1	0	0	0	0	0	0	0	79
13425076	m	ot	DE	2017	0	1	0	0	1	0	0	0	0	0	0	0	62
13425159	m	md	CN	2017	0	1	0	0	0	1	0	0	0	0	0	0	48
13425383	m	md	CN	2017	0	0	0	0	0	0	0	0	0	0	0	0	
13425894	m	md	JP	2017	0	1	0	0	1	0	0	0	0	0	0	0	56
13425901	m	ph	US	2017	0	1	0	0	1	0	0	0	0	0	0	1	59
13426480	m	md	JP	2017	0	1	0	0	1	0	0	0	0	0	0	0	71
13426481	m	md	JP	2017	0	1	0	0	1	0	0	0	0	0	0	1	78
13426550	f	md	CN	2017	1	0	0	0	0	0	0	0	0	0	0	0	65.4
13426587	Not	cn	US	2017	0	1	0	0	1	0	0	0	0	0	0	1	

13426594	f	md	JP	2017	0	0	0	0	0	0	0	0	0	0	0	0	
13427024	Not	cn	US	2017	0	1	0	0	1	0	0	0	0	0	0	0	
13427619	m	md	JP	2017	0	1	0	0	1	0	0	0	0	0	0	0	70
13427678	f	ot	US	2017	0	1	0	0	1	0	0	0	0	0	0	0	74
13428200	f	md	BE	2017	0	1	0	0	1	0	0	0	0	0	0	0	57
13428366	f	ot	US	2017	0	1	1	0	0	0	0	0	0	0	0	0	
13428631	Not	ot	AU	2017	0	1	0	0	1	0	0	0	0	0	0	0	61
13428677	Not	cn	US	2017	0	1	0	0	1	0	0	0	0	0	0	1	
13428728	m	md	IT	2017	0	0	0	0	0	0	0	0	0	0	0	0	70
13429095	m	ot	US	2017	0	0	0	0	0	0	0	0	0	0	0	0	
13429198	m	cn	Not re	2017	0	0	0	0	0	0	0	0	0	0	0	0	90
13429211	Not	md	AU	2017	0	1	0	0	1	0	0	0	0	0	0	0	69
13429644	Not	cn	US	2017	0	1	0	0	1	0	0	0	0	0	0	1	
13429755	m	md	BE	2017	0	1	0	0	1	0	0	0	0	0	0	0	
13430707	Not	cn	IT	2017	0	1	0	0	1	0	0	0	0	0	0	0	
13431750	m	ot	JP	2017	0	1	0	0	1	0	0	0	0	0	0	0	67
13432369	f	md	IN	2017	0	0	0	0	0	0	0	0	0	0	0	0	60
13433022	f	md	JP	2017	1	0	0	0	0	0	0	0	0	0	0	0	86
13433425	f	md	JP	2017	1	0	0	0	0	0	0	0	0	0	0	0	37.7
13433458	m	cn	US	2017	0	1	0	0	1	0	0	0	0	0	0	1	
13433542	Not	ot	US	2017	0	1	0	0	1	0	0	0	0	0	0	1	
13433963	m	cn	US	2017	0	1	0	0	1	0	0	0	0	0	0	0	
13433964	m	cn	US	2017	0	1	0	0	1	0	0	0	0	0	0	0	
13434443	m	md	JP	2017	0	1	0	0	1	0	0	0	0	0	0	0	72
13434699	m	ot	JP	2017	0	1	0	0	1	0	0	0	0	0	0	0	65
13435311	Not	cn	US	2017	0	1	0	0	1	0	0	0	0	0	0	0	
13435323	Not	cn	US	2017	0	1	0	0	1	0	0	0	0	0	0	1	
13436802	Not	cn	US	2017	0	1	0	0	1	0	0	0	0	0	0	0	
13437250	Not	cn	GB	2017	1	0	0	0	0	0	0	0	0	0	0	1	
13437280	f	cn	GB	2017	1	0	0	0	0	0	0	0	0	0	0	1	64
13438749	f	md	GB	2017	1	0	0	0	0	0	0	0	0	0	0	0	82
13438860	m	ph	GB	2017	1	0	0	0	0	0	0	0	0	0	0	0	61
13439263	m	md	SA	2017	1	0	0	0	0	0	0	0	0	0	0	0	
13439553	m	md	DE	2017	0	0	0	0	0	0	0	0	0	0	0	0	43

13439601	f	ot	CZ	2017	0	0	0	0	0	0	0	0	0	0	0	0	72
13439928	m	cn	US	2017	0	0	0	0	0	0	0	0	0	0	0	0	43
13440360	f	md	DE	2017	0	1	0	0	1	0	0	0	0	0	0	0	46
13440635	f	md	JP	2017	1	0	0	0	0	0	0	0	0	0	0	0	55
13440745	m	ot	US	2017	1	0	0	0	0	0	0	0	0	0	0	0	59.3
13441040	m	md	KR	2017	0	0	0	0	0	0	0	0	0	0	0	0	59
13441311	f	ot	DE	2017	0	1	0	0	1	0	0	0	0	0	0	0	78
13441339	m	ph	US	2017	0	1	0	0	1	0	0	0	0	0	0	1	
13441648	m	md	GB	2017	0	1	0	0	0	1	0	0	0	0	0	0	73
13441831	f	ot	US	2017	1	0	0	0	0	0	0	0	0	0	0	0	65
13442023	m	md	JP	2017	0	1	0	0	1	0	0	0	0	0	0	0	54
13442085	m	md	FR	2017	0	1	0	0	1	0	0	0	0	0	0	0	
13442267	f	md	US	2017	0	1	0	0	0	1	0	0	0	0	0	0	60
13442499	f	md	JP	2017	1	0	0	0	0	0	0	0	0	0	0	0	63.1
13442509	f	md	CA	2017	0	0	0	0	0	0	0	0	0	0	0	0	
13442938	m	cn	JP	2017	0	1	0	0	1	0	0	0	0	0	0	0	69
13443867	f	cn	JP	2017	0	1	0	0	1	0	0	0	0	0	0	0	80
13444574	f	md	JP	2017	1	0	0	0	0	0	0	0	0	0	0	0	79
13444575	f	md	JP	2017	1	0	0	0	0	0	0	0	0	0	0	0	69.6
13444760	f	md	DE	2017	0	1	0	0	1	0	0	0	0	0	0	0	78
13444869	f	md	FR	2017	1	0	0	0	0	0	0	0	0	0	0	0	
13444882	m	md	GB	2017	0	0	0	0	0	0	0	0	0	0	0	0	65
13445106	m	cn	DE	2017	0	1	0	0	1	0	0	0	0	0	0	1	72
13445183	m	md	GB	2017	0	1	0	1	0	0	0	0	0	0	0	0	76
13445630	m	ph	US	2017	0	1	0	0	1	0	0	0	0	0	0	0	
13446068	Not	ot	IT	2017	0	0	0	0	0	0	0	0	0	0	0	0	
13446189	m	md	NL	2017	0	1	0	0	0	1	0	0	0	0	0	0	53
13446601	f	ot	DE	2017	0	1	0	0	1	0	0	0	0	0	0	0	61
13449217	f	md	JP	2017	1	0	0	0	0	0	0	0	0	0	0	0	85
13449385	f	md	ZA	2017	0	1	0	0	1	0	0	0	0	0	0	0	82
13449501	f	md	JP	2017	0	0	0	0	0	0	0	0	0	0	0	0	45
13449536	m	md	US	2017	0	1	0	0	1	0	0	0	0	0	0	0	
13449537	Not	cn	US	2017	0	1	0	0	1	0	0	0	0	0	0	0	
13450241	m	ot	US	2017	1	0	0	0	0	0	0	0	0	0	0	0	60

13450339	m	cn	US	2017	0	1	0	0	1	0	0	0	0	0	0	0	
13450487	m	md	US	2017	0	0	0	0	0	0	0	0	0	0	0	0	59
13450562	f	md	JP	2017	0	0	0	0	0	0	0	0	0	0	0	0	60
13450688	f	md	CN	2017	0	1	0	0	0	1	0	0	0	0	0	0	59
13450935	m	md	DE	2017	0	1	0	0	0	1	0	0	0	0	0	0	56
13452124	m	cn	US	2017	0	0	0	0	0	0	0	0	0	0	0	0	
13452140	Not	ot	CN	2017	0	0	0	0	0	0	0	0	0	0	0	0	
13452292	f	ot	PL	2017	0	0	0	0	0	0	0	0	0	0	0	0	59
13452344	m	ot	US	2017	0	0	0	0	0	0	0	0	0	0	0	0	66
13452856	m	ot	US	2017	0	0	0	0	0	0	0	0	0	0	0	0	66
13452858	f	ot	US	2017	0	0	0	0	0	0	0	0	0	0	0	0	55
13452903	m	ph	JP	2017	0	0	0	0	0	0	0	0	0	0	0	0	89
13453029	f	md	HU	2017	0	1	0	0	1	0	0	0	0	0	0	0	65
13453102	f	ot	US	2017	0	1	0	0	1	0	0	0	0	0	0	0	
13453111	f	md	JP	2017	1	0	0	0	0	0	0	0	0	0	0	0	62
13453122	f	md	JP	2017	1	0	0	0	0	0	0	0	0	0	0	0	75
13453132	m	md	JP	2017	1	0	0	0	0	0	0	0	0	0	0	0	77
13453154	m	md	JP	2017	1	0	0	0	0	0	0	0	0	0	0	0	60
13453181	f	md	JP	2017	1	0	0	0	0	0	0	0	0	0	0	0	55
13453205	f	md	JP	2017	1	0	0	0	0	0	0	0	0	0	0	0	63
13453212	f	md	JP	2017	1	0	0	0	0	0	0	0	0	0	0	0	52
13453346	f	md	JP	2017	1	0	0	0	0	0	0	0	0	0	0	0	76
13454039	m	Not repo	IT	2017	1	0	0	0	0	0	0	0	0	0	0	0	60
13454621	f	md	US	2017	0	1	0	1	0	0	0	0	0	0	0	0	74
13455221	f	ot	JP	2017	1	0	0	0	0	0	0	0	0	0	0	0	79
13455317	f	ph	US	2017	0	0	0	0	0	0	0	0	0	0	0	0	50
13455388	m	md	JP	2017	1	0	0	0	0	0	0	0	0	0	0	0	56
13455487	f	md	JP	2017	1	0	0	0	0	0	0	0	0	0	0	0	66
13455557	m	md	JP	2017	0	1	1	0	0	0	0	0	0	0	0	0	71
13455762	f	md	JP	2017	1	0	0	0	0	0	0	0	0	0	0	0	77
13455765	m	md	PH	2017	1	0	0	0	0	0	0	0	0	0	0	0	75
13455910	m	ph	US	2017	0	1	1	0	0	0	0	0	0	0	0	0	55
13456004	f	ot	US	2017	0	0	0	0	0	0	0	0	0	0	0	0	26
13456138	f	md	JP	2017	1	0	0	0	0	0	0	0	0	0	0	0	83

13456293	m	Not repo	GB	2017	1	0	0	0	0	0	0	0	0	0	0	0	74
13456355	f	md	JP	2017	1	0	0	0	0	0	0	0	0	0	0	0	67
13456359	f	md	KR	2017	1	0	0	0	0	0	0	0	0	0	0	0	75
13456369	m	md	JP	2017	1	0	0	0	0	0	0	0	0	0	0	1	89
13456566	Not	cn	BE	2017	0	0	0	0	0	0	0	0	0	0	0	0	
13457019	m	md	TH	2017	1	0	0	0	0	0	0	0	0	0	0	0	66
13457169	m	md	JP	2017	0	1	0	0	1	0	0	0	0	0	0	0	57
13457208	m	ph	JP	2017	0	1	0	0	1	0	0	0	0	0	0	0	70
13457437	f	md	JP	2017	1	0	0	0	0	0	0	0	0	0	0	0	65
13457480	m	cn	JP	2017	0	1	1	0	0	0	0	0	0	0	0	0	69
13457486	m	md	CN	2017	0	0	0	0	0	0	0	0	0	0	0	0	65
13457505	f	md	JP	2017	1	0	0	0	0	0	0	0	0	0	0	0	61
13457517	f	md	JP	2017	1	0	0	0	0	0	0	0	0	0	0	0	83
13457521	f	cn	FR	2017	0	1	0	0	0	1	0	0	0	0	0	0	62
13457611	f	md	JP	2017	1	0	0	0	0	0	0	0	0	0	0	0	77
13457624	m	md	JP	2017	1	0	0	0	0	0	0	0	0	0	0	0	57
13457656	m	md	JP	2017	1	0	0	0	0	0	0	0	0	0	0	0	73
13457754	f	md	JP	2017	1	0	0	0	0	0	0	0	0	0	0	0	59
13457755	m	cn	US	2017	0	0	0	0	0	0	0	0	0	0	0	0	58
13457921	m	md	US	2017	0	1	0	0	1	0	0	0	0	0	0	0	
13457938	f	ot	US	2017	0	1	1	0	0	0	0	0	0	0	0	1	84
13457959	f	md	JP	2017	1	0	0	0	0	0	0	0	0	0	0	0	73
13457981	m	md	IN	2017	0	1	0	0	1	0	0	0	0	0	0	0	
13457996	m	md	JP	2017	1	0	0	0	0	0	0	0	0	0	0	0	87
13458003	f	md	JP	2017	1	0	0	0	0	0	0	0	0	0	0	0	60
13458084	f	md	JP	2017	0	1	0	0	0	1	0	0	0	0	0	0	45
13458269	m	md	JP	2017	0	0	0	0	0	0	1	0	1	0	0	0	72
13458318	m	ot	US	2017	0	0	0	0	0	0	0	0	0	0	0	0	
13458422	f	md	JP	2017	1	0	0	0	0	0	0	0	0	0	0	0	71
13458434	m	md	JP	2017	1	0	0	0	0	0	0	0	0	0	0	0	65
13458496	m	ot	JP	2017	0	0	0	0	0	0	1	0	1	0	0	0	73
13458515	m	md	JP	2017	1	0	0	0	0	0	0	0	0	0	0	1	82.9
13458587	f	md	JP	2017	1	0	0	0	0	0	0	0	0	0	0	0	60
13458920	f	md	JP	2017	1	0	0	0	0	0	0	0	0	0	0	0	53

13459042	f	md	JP	2017	1	0	0	0	0	0	0	0	0	0	0	0	75
13459163	m	md	JP	2017	0	0	0	0	0	0	0	0	0	0	0	0	67
13459241	m	md	CN	2017	0	0	0	0	0	0	0	0	0	0	0	0	58
13459262	Not	md	BR	2017	0	1	0	0	1	0	0	0	0	0	0	0	
13459289	m	ot	DE	2017	0	0	0	0	0	0	0	0	0	0	0	0	57
13460120	f	md	DE	2017	0	1	0	0	1	0	0	0	0	0	0	0	
13460922	m	md	CN	2017	0	0	0	0	0	0	0	0	0	0	0	0	42
13460954	Not	ot	US	2017	0	1	0	0	1	0	0	0	0	0	0	0	
13461272	f	md	CN	2017	0	0	0	0	0	0	0	0	0	0	0	0	59
13462022	m	md	JP	2017	0	1	0	0	1	0	0	0	0	0	0	0	55
13462229	Not	ot	US	2017	0	0	0	0	0	0	0	0	0	0	0	0	
13462743	f	md	FR	2017	1	0	0	0	0	0	0	0	0	0	0	0	66.5
13463173	m	md	JP	2017	1	0	0	0	0	0	0	0	0	0	0	1	80
13464936	m	cn	DE	2017	0	1	0	0	1	0	0	0	0	0	0	0	49
13465139	m	ot	DE	2017	0	1	0	0	1	0	0	0	0	0	0	0	68
13465664	f	md	CN	2017	0	1	0	0	0	1	0	0	0	0	0	0	52
13465750	m	md	CN	2017	0	1	0	0	0	1	0	0	0	0	0	0	65
13465844	f	ot	CA	2017	0	1	0	0	1	0	0	0	0	0	0	0	60
13466081	m	ph	US	2017	0	1	0	0	1	0	0	0	0	0	0	0	70
13466188	f	md	US	2017	0	1	0	0	1	0	0	0	0	0	0	0	70
13466278	m	md	ES	2017	0	0	0	0	0	0	0	0	0	0	0	0	86.4
13466335	f	cn	US	2017	0	0	0	0	0	0	0	0	0	0	0	0	
13466869	m	md	JP	2017	0	0	0	0	0	0	0	0	0	0	0	0	67
13466992	m	md	JP	2017	0	1	0	0	0	1	0	0	0	0	0	0	72
13467110	Not	md	US	2017	0	0	0	0	0	0	0	0	0	0	0	0	74
13467277	f	md	DE	2017	0	1	0	0	1	0	0	0	0	0	0	0	
13467455	f	md	IN	2017	0	0	0	0	0	0	0	0	0	0	0	0	65
13467476	f	md	CN	2017	0	1	0	0	0	1	0	0	0	0	0	0	42
13467899	m	md	DE	2017	0	1	0	0	1	0	0	0	0	0	0	0	57
13467930	m	cn	GB	2017	1	0	0	0	0	0	0	0	0	0	0	0	60
13468261	f	md	GB	2017	1	0	0	0	0	0	0	0	0	0	0	0	
13468493	f	Not repo	IT	2017	1	0	0	0	0	0	0	0	0	0	0	0	65
13468557	f	Not repo	GB	2017	1	0	0	0	0	0	0	0	0	0	0	0	75
13468562	f	cn	AR	2017	0	0	0	0	0	0	0	0	0	0	0	0	21

13468801	m	md	JP	2017	1	0	0	0	0	0	0	0	0	0	0	0	72
13468980	f	cn	JP	2017	0	0	0	0	0	0	0	0	0	0	0	0	90
13469393	m	cn	US	2017	1	0	0	0	0	0	0	0	0	0	0	0	79
13469693	f	md	US	2017	0	1	0	0	0	1	0	0	0	0	0	0	61
13470398	m	ot	US	2017	0	1	0	0	1	0	0	0	0	0	0	0	
13470490	Not	ot	GB	2017	0	0	0	0	0	0	0	0	0	0	0	0	
13470646	m	md	AU	2017	0	1	0	0	0	1	0	0	0	0	0	0	71
13470826	f	md	DE	2017	0	1	0	0	1	0	0	0	0	0	0	0	57
13470854	m	md	JP	2017	0	1	0	0	1	0	0	0	0	0	0	0	79
13471000	m	md	JP	2017	0	1	1	0	0	0	0	0	0	0	0	0	66
13471734	f	ot	US	2017	0	0	0	0	0	0	0	0	0	0	0	0	51
13472052	m	cn	JP	2017	0	0	0	0	0	0	0	0	0	0	0	0	65
13472573	m	ph	JP	2017	0	0	0	0	0	0	0	0	0	0	0	0	75
13472635	m	cn	JP	2017	0	0	0	0	0	0	0	0	0	0	0	0	75
13472753	m	cn	JP	2017	0	1	1	0	0	0	0	0	0	0	0	0	69
13472795	f	cn	JP	2017	0	0	0	0	0	0	0	0	0	0	0	0	
13473329	m	cn	JP	2017	0	0	0	0	0	0	0	0	0	0	0	0	76
13473570	m	cn	US	2017	0	1	0	0	1	0	0	0	0	0	0	0	
13473734	f	md	CN	2017	1	0	0	0	0	0	0	0	0	0	0	0	64
13474077	m	ot	US	2017	0	1	1	0	0	0	0	0	0	0	0	0	67
13474123	m	md	US	2017	0	1	0	0	0	1	0	0	0	0	0	0	74
13475960	m	md	JP	2017	0	0	0	0	0	0	0	0	0	0	0	0	57
13476135	m	md	JP	2017	0	1	0	0	0	1	0	0	0	0	0	0	72
13476359	Not	cn	GB	2017	1	0	0	0	0	0	0	0	0	0	0	0	
13476507	f	md	HU	2017	0	0	0	0	0	0	0	0	0	0	0	0	73
13477062	Not	cn	JP	2017	0	0	0	0	0	0	0	0	0	0	0	0	
13477557	f	cn	GB	2017	1	0	0	0	0	0	0	0	0	0	0	0	80
13477622	Not	cn	GB	2017	1	0	0	0	0	0	0	0	0	0	0	0	
13477723	f	ph	TH	2017	0	0	0	0	0	0	0	0	0	0	0	0	49
13477871	f	md	PL	2017	0	0	0	0	0	0	0	0	0	0	0	0	60
13478146	f	Not repo	Not re	2017	1	0	0	0	0	0	0	0	0	0	0	0	71
13478698	m	cn	US	2017	1	0	0	0	0	0	0	0	0	0	0	0	68
13479542	m	md	GB	2017	0	0	0	0	0	0	0	0	0	0	0	0	
13481452	f	md	KR	2017	1	0	0	0	0	0	0	0	0	0	0	0	75

13481659	f	ot	GR	2017	0	0	0	0	0	0	0	0	0	0	0	0	59
13481695	m	md	IT	2017	0	0	0	0	0	0	0	0	0	0	0	0	58
13481844	m	md	JP	2017	0	1	0	0	1	0	0	0	0	0	0	0	70
13482536	Not	Not repo	GB	2017	1	0	0	0	0	0	0	0	0	0	0	0	
13482751	m	cn	US	2017	0	1	1	0	0	0	0	0	0	0	0	1	
13483635	f	md	JP	2017	0	0	0	0	0	0	0	0	0	0	0	0	55
13483967	f	ot	US	2017	0	0	0	0	0	0	0	0	0	0	0	0	
13484349	m	cn	US	2017	0	1	1	0	0	0	0	0	0	0	0	0	65
13484400	m	ot	US	2017	1	0	0	0	0	0	0	0	0	0	0	0	60
13484535	Not	ot	US	2017	1	0	0	0	0	0	0	0	0	0	0	0	87
13484820	m	md	JP	2017	0	1	0	0	1	0	0	0	0	0	0	1	54
13484973	f	md	US	2017	0	0	0	0	0	0	0	0	0	0	0	0	
13485336	f	md	JP	2017	1	0	0	0	0	0	0	0	0	0	0	0	
13485337	f	md	FR	2017	0	1	0	0	0	1	0	0	0	0	0	0	63
13485375	m	md	US	2017	0	1	0	0	1	0	0	0	0	0	0	0	90
13486533	m	md	CN	2017	1	0	0	0	0	0	0	0	0	0	0	0	61.3
13486589	f	Not repo	GB	2017	1	0	0	0	0	0	0	0	0	0	0	0	64
13486602	m	ph	JP	2017	0	1	0	0	1	0	0	0	0	0	0	0	76
13486652	m	md	DE	2017	0	0	0	0	0	0	0	0	0	0	0	0	70
13486937	m	cn	US	2017	0	1	0	0	1	0	0	0	0	0	0	0	
13487005	f	md	CN	2017	0	0	0	0	0	0	0	0	0	0	0	0	48
13487066	f	ph	JP	2017	0	1	0	0	1	0	0	0	0	0	0	0	
13487779	f	md	CA	2017	0	1	0	0	1	0	0	0	0	0	0	0	55
13488536	m	ot	JP	2017	0	1	0	0	1	0	0	0	0	0	0	0	46
13488792	m	cn	US	2017	0	1	1	0	0	0	0	0	0	0	0	1	59
13489288	m	md	JP	2017	0	1	0	0	1	0	0	0	0	0	0	0	70
13489475	f	md	AT	2017	0	1	1	0	0	0	0	0	0	0	0	0	
13489612	f	md	JP	2017	0	1	1	0	0	0	0	0	0	0	0	0	52
13490056	m	Not repo	GB	2017	1	0	0	0	0	0	0	0	0	0	0	0	54
13490285	m	md	JP	2017	0	1	0	0	1	0	0	0	0	0	0	0	62
13490400	m	md	FI	2017	0	0	0	0	0	0	0	0	0	0	0	0	60
13490477	m	ot	US	2017	0	1	0	0	1	0	0	0	0	0	0	0	
13490799	f	Not repo	GB	2017	1	0	0	0	0	0	0	0	0	0	0	0	84
13490980	Not	md	VN	2017	0	0	0	0	0	0	0	0	0	0	0	0	

13491065	f	ot	DE	2017	0	1	0	0	1	0	0	0	0	0	0	0	61
13491350	m	Not repo	GB	2017	1	0	0	0	0	0	0	0	0	0	0	0	74
13491829	f	cn	Not re	2017	0	0	0	0	0	0	0	0	0	0	0	0	
13492122	m	cn	US	2017	0	0	0	0	0	0	0	0	0	0	0	0	
13493239	m	ot	JP	2017	0	1	0	0	1	0	0	0	0	0	0	1	69
13493969	m	cn	GB	2017	0	1	1	0	0	0	0	0	0	0	0	0	
13494210	f	Not repo	GB	2017	1	0	0	0	0	0	0	0	0	0	0	0	77
13494360	Not	Not repo	GB	2017	1	0	0	0	0	0	0	0	0	0	0	0	
13495115	f	md	JP	2017	0	0	0	0	0	0	0	0	0	0	0	0	67
13495511	Not	cn	IN	2017	0	0	0	0	0	0	0	0	0	0	0	0	
13495529	Not	md	IN	2017	0	0	0	0	0	0	0	0	0	0	0	0	
13495538	Not	cn	IN	2017	0	0	0	0	0	0	0	0	0	0	0	0	
13495715	Not	md	US	2017	0	0	0	0	0	0	0	0	0	0	0	0	
13495717	Not	md	US	2017	0	0	0	0	0	0	0	0	0	0	0	0	
13496894	m	md	FR	2017	1	0	0	0	0	0	0	0	0	0	0	0	67.3
13497124	f	cn	GB	2017	1	0	0	0	0	0	0	0	0	0	0	0	48
13497258	m	cn	GB	2017	1	0	0	0	0	0	0	0	0	0	0	0	61
13497388	Not	md	TW	2017	1	0	0	0	0	0	0	0	0	0	0	0	
13497423	f	md	JP	2017	0	0	0	0	0	0	0	0	0	0	0	0	80
13497658	m	cn	US	2017	0	0	0	0	0	0	0	0	0	0	0	0	68
13497944	Not	ot	NO	2017	0	1	0	0	1	0	0	0	0	0	0	0	
13497974	m	ot	US	2017	0	1	0	0	1	0	0	0	0	0	0	0	72
13498016	Not	ot	ES	2017	0	0	0	0	0	0	0	0	0	0	0	0	
13498030	m	ot	IT	2017	0	1	0	0	1	0	0	0	0	0	0	0	70
13498036	m	ot	DE	2017	0	1	0	0	1	0	0	0	0	0	0	0	47
13498078	m	ot	US	2017	0	1	0	0	1	0	0	0	0	0	0	0	79
13498108	m	ot	DE	2017	0	1	0	0	1	0	0	0	0	0	0	0	64
13498109	f	ph	CA	2017	0	1	0	0	1	0	0	0	0	0	0	0	
13498130	f	md	US	2017	0	1	0	0	1	0	0	0	0	0	0	0	
13498142	f	ot	JP	2017	0	1	0	0	1	0	0	0	0	0	0	0	60
13498377	f	Not repo	GB	2017	1	0	0	0	0	0	0	0	0	0	0	0	74
13498887	m	Not repo	JP	2017	0	0	0	0	0	0	0	0	0	0	0	0	82
13498894	m	Not repo	JP	2017	0	0	0	0	0	0	0	0	0	0	0	0	68
13499009	m	cn	GB	2017	0	1	1	0	0	0	0	0	0	0	0	0	

13499617	m	md	JP	2017	0	0	0	0	0	0	0	0	0	0	0	0	
13500184	m	ot	US	2017	1	0	0	0	0	0	0	0	0	0	0	0	
13500775	m	cn	US	2017	0	0	0	0	0	0	0	0	0	0	0	0	61
13500777	m	md	US	2017	0	0	0	0	0	0	0	0	0	0	0	0	
13500778	f	ph	US	2017	0	0	0	0	0	0	0	0	0	0	0	0	
13500779	f	cn	US	2017	0	0	0	0	0	0	0	0	0	0	0	0	
13500783	f	ot	US	2017	0	0	0	0	0	0	0	0	0	0	0	0	38
13501430	f	ph	US	2017	0	0	0	0	0	0	0	0	0	0	0	0	
13501723	Not	Not repo	GB	2017	1	0	0	0	0	0	0	0	0	0	0	0	59
13501830	Not	cn	US	2017	0	1	0	0	1	0	0	0	0	0	0	1	
13501843	Not	md	JP	2017	0	0	0	0	0	0	0	0	0	0	0	0	
13501910	m	cn	JP	2017	0	0	0	0	0	0	1	0	1	0	0	0	78
13502423	Not	ot	JP	2017	1	0	0	0	0	0	0	0	0	0	0	0	
13502489	m	md	BE	2017	0	1	0	0	1	0	0	0	0	0	0	0	60
13502758	Not	cn	GB	2017	1	0	0	0	0	0	0	0	0	0	0	0	
13502762	f	cn	CN	2017	1	0	0	0	0	0	0	0	0	0	0	0	57
13502978	f	md	JP	2017	0	1	0	0	1	0	0	0	0	0	0	0	68
13503496	m	md	JP	2017	0	1	0	0	1	0	0	0	0	0	0	0	74
13503903	f	md	CN	2017	1	0	0	0	0	0	0	0	0	0	0	0	52
13504023	m	cn	JP	2017	0	0	0	0	0	0	0	0	0	0	0	0	64
13504404	m	md	JP	2017	0	1	0	0	1	0	0	0	0	0	0	0	69
13504655	m	Not repo	GB	2017	1	0	0	0	0	0	0	0	0	0	0	0	76
13504942	Not	cn	US	2017	0	1	0	0	1	0	0	0	0	0	0	1	
13504943	Not	cn	US	2017	0	1	0	0	1	0	0	0	0	0	0	1	
13505415	f	ot	NL	2017	0	1	0	0	1	0	0	0	0	0	0	0	65
13505456	m	ot	BE	2017	0	1	0	0	1	0	0	0	0	0	0	0	69
13505595	m	md	JP	2017	1	0	0	0	0	0	0	0	0	0	0	0	68
13505745	f	md	JP	2017	1	0	0	0	0	0	0	0	0	0	0	0	76
13505867	m	ot	US	2017	0	0	0	0	0	0	0	0	0	0	0	0	67
13505877	m	md	US	2017	0	0	0	0	0	0	0	0	0	0	0	0	
13505889	m	ot	US	2017	0	0	0	0	0	0	0	0	0	0	0	0	
13505894	f	cn	US	2017	0	0	0	0	0	0	0	0	0	0	0	0	
13505904	m	md	US	2017	0	0	0	0	0	0	0	0	0	0	0	0	68
13505910	m	ot	US	2017	0	0	0	0	0	0	0	0	0	0	0	0	

13505926	f	ot	US	2017	0	0	0	0	0	0	0	0	0	0	0	0	66
13505956	m	ph	US	2017	0	0	0	0	0	0	0	0	0	0	0	0	
13505962	m	md	US	2017	0	0	0	0	0	0	0	0	0	0	0	0	
13505978	f	ph	US	2017	0	0	0	0	0	0	0	0	0	0	0	0	81
13505991	m	md	US	2017	0	0	0	0	0	0	0	0	0	0	0	0	
13505995	m	cn	US	2017	0	0	0	0	0	0	0	0	0	0	0	0	
13506090	m	ot	US	2017	0	0	0	0	0	0	0	0	0	0	0	0	
13506098	m	md	US	2017	0	0	0	0	0	0	0	0	0	0	0	0	68
13506103	m	ot	US	2017	0	0	0	0	0	0	0	0	0	0	0	0	59
13506116	f	cn	US	2017	0	0	0	0	0	0	0	0	0	0	0	0	
13506152	m	cn	US	2017	0	0	0	0	0	0	0	0	0	0	0	0	
13506158	Not	cn	US	2017	0	0	0	0	0	0	0	0	0	0	0	0	
13506188	f	cn	US	2017	0	0	0	0	0	0	0	0	0	0	0	0	
13506202	m	ot	US	2017	0	0	0	0	0	0	0	0	0	0	0	0	57
13506212	f	md	US	2017	0	0	0	0	0	0	0	0	0	0	0	0	72
13506222	m	md	US	2017	0	0	0	0	0	0	0	0	0	0	0	0	
13506238	f	md	US	2017	0	0	0	0	0	0	0	0	0	0	0	0	54
13506240	m	md	US	2017	0	0	0	0	0	0	0	0	0	0	0	0	
13506253	f	md	US	2017	0	0	0	0	0	0	0	0	0	0	0	0	
13506256	f	md	US	2017	0	0	0	0	0	0	0	0	0	0	0	0	70
13506303	Not	ot	US	2017	0	0	0	0	0	0	0	0	0	0	0	0	
13506306	m	md	US	2017	0	0	0	0	0	0	0	0	0	0	0	0	
13506317	m	ph	US	2017	0	0	0	0	0	0	0	0	0	0	0	0	
13506331	m	ot	US	2017	0	0	0	0	0	0	0	0	0	0	0	0	74
13506334	m	ph	US	2017	0	0	0	0	0	0	0	0	0	0	0	0	79
13506345	f	ph	US	2017	0	0	0	0	0	0	0	0	0	0	0	0	
13506363	f	cn	US	2017	1	0	0	0	0	0	0	0	0	0	0	0	
13506388	m	md	US	2017	0	0	0	0	0	0	0	0	0	0	0	0	85
13506407	m	ot	US	2017	0	0	0	0	0	0	0	0	0	0	0	0	
13506409	f	md	US	2017	0	0	0	0	0	0	0	0	0	0	0	0	
13506415	Not	ot	US	2017	0	0	0	0	0	0	0	0	0	0	0	0	
13506420	f	ot	US	2017	0	0	0	0	0	0	0	0	0	0	0	0	
13506444	Not	md	US	2017	0	0	0	0	0	0	0	0	0	0	0	0	
13506449	m	md	US	2017	0	0	0	0	0	0	0	0	0	0	0	0	63

13506453	Not	ot	US	2017	0	0	0	0	0	0	0	0	0	0	0	0	
13506456	m	cn	US	2017	0	0	0	0	0	0	0	0	0	0	0	0	
13506457	m	cn	US	2017	0	0	0	0	0	0	0	0	0	0	0	0	
13506474	m	cn	US	2017	0	0	0	0	0	0	0	0	0	0	0	0	
13506503	m	ot	US	2017	0	0	0	0	0	0	0	0	0	0	0	0	
13506506	f	ot	US	2017	0	0	0	0	0	0	0	0	0	0	0	0	68
13506520	m	md	US	2017	0	0	0	0	0	0	0	0	0	0	0	0	
13506550	f	md	US	2017	0	0	0	0	0	0	0	0	0	0	0	0	
13506556	f	md	US	2017	0	0	0	0	0	0	0	0	0	0	0	0	56
13506562	f	ot	US	2017	0	0	0	0	0	0	0	0	0	0	0	0	52
13506605	f	md	US	2017	0	0	0	0	0	0	0	0	0	0	0	0	
13506638	f	md	US	2017	0	0	0	0	0	0	0	0	0	0	0	0	
13506645	f	md	US	2017	0	0	0	0	0	0	0	0	0	0	0	0	63
13506700	f	md	US	2017	0	0	0	0	0	0	0	0	0	0	0	0	
13506970	m	md	JP	2017	0	0	0	0	0	0	0	0	0	0	0	0	79
13507044	m	md	US	2017	0	1	0	0	0	1	0	0	0	0	0	0	58
13507503	f	ot	FR	2017	0	1	0	0	1	0	0	0	0	0	0	0	59
13508058	m	ot	IT	2017	0	0	0	0	0	0	0	0	0	0	0	0	68
13508064	f	cn	GB	2017	1	0	0	0	0	0	0	0	0	0	0	0	68
13508098	m	md	JP	2017	0	1	0	0	1	0	0	0	0	0	0	0	46
13508228	m	md	JP	2017	1	0	0	0	0	0	0	0	0	0	0	0	82
13508271	m	md	US	2017	1	0	0	0	0	0	0	0	0	0	0	0	68
13508568	m	Not repo	GB	2017	1	0	0	0	0	0	0	0	0	0	0	0	76
13510082	f	md	BE	2017	0	0	0	0	0	0	0	0	0	0	0	0	55
13510288	m	Not repo	GB	2017	1	0	0	0	0	0	0	0	0	0	0	0	60
13510661	f	md	JP	2017	1	0	0	0	0	0	0	0	0	0	0	0	70
13511165	m	ot	DE	2017	0	1	0	0	1	0	0	0	0	0	0	0	68
13511217	m	cn	US	2017	0	1	0	0	1	0	0	0	0	0	0	0	62
13511630	Not	ot	GR	2017	0	0	0	0	0	0	0	0	0	0	0	0	
13511711	m	Not repo	IT	2017	1	0	0	0	0	0	0	0	0	0	0	0	
13512332	Not	ot	JP	2017	0	0	0	0	0	0	0	0	0	0	0	0	
13512377	m	md	US	2017	1	0	0	0	0	0	0	0	0	0	0	0	71
13512493	m	cn	JP	2017	0	1	0	0	1	0	0	0	0	0	0	0	72
13512937	m	md	JP	2017	1	0	0	0	0	0	0	0	0	0	0	0	68

13513458	f	cn	US	2017	0	1	1	0	0	0	0	0	0	0	0	0	54
13513545	m	md	JP	2017	0	1	0	0	1	0	0	0	0	0	0	0	75
13513663	m	md	RU	2017	1	0	0	0	0	0	0	0	0	0	0	0	66
13514033	m	md	US	2017	0	0	0	0	0	0	0	0	0	0	0	0	
13514341	m	md	TH	2017	0	1	0	0	0	1	0	0	0	0	0	0	75
13514343	m	md	DE	2017	0	1	0	0	1	0	0	0	0	0	0	0	83
13514511	m	md	JP	2017	0	1	0	0	1	0	0	0	0	0	0	0	61
13514512	f	ph	JP	2017	0	1	0	0	1	0	0	0	0	0	0	0	69
13514716	m	cn	US	2017	1	0	0	0	0	0	0	0	0	0	0	0	80
13515028	Not	cn	CN	2017	1	0	0	0	0	0	0	0	0	0	0	0	
13515048	f	md	CN	2017	1	0	0	0	0	0	0	0	0	0	0	0	64.8
13515283	m	md	JP	2017	0	0	0	0	0	0	0	0	0	0	0	0	70
13515443	m	cn	US	2017	0	0	0	0	0	0	0	0	0	0	0	0	75
13515512	m	ot	JP	2017	0	1	0	0	1	0	0	0	0	0	0	0	80
13515781	m	cn	JP	2017	0	0	0	0	0	0	0	0	0	0	0	0	68
13515810	m	md	SI	2017	0	1	1	0	0	0	0	0	0	0	0	0	
13515981	m	ot	JP	2017	0	1	0	0	1	0	0	0	0	0	0	0	83
13516182	m	md	JP	2017	0	0	0	0	0	0	0	0	0	0	0	0	52
13516261	f	md	US	2017	1	0	0	0	0	0	0	0	0	0	0	0	
13516665	f	cn	Not re	2017	0	0	0	0	0	0	0	0	0	0	0	0	43
13516844	m	md	JP	2017	0	1	0	0	1	0	0	0	0	0	0	0	62
13516925	m	md	US	2017	0	0	0	0	0	0	0	0	0	0	0	0	
13517131	m	ot	JP	2017	0	1	0	0	1	0	0	0	0	0	0	0	69
13517659	m	md	HK	2017	0	0	0	0	0	0	0	0	0	0	0	0	36
13517773	m	ph	US	2017	0	1	1	0	0	0	0	0	0	0	0	1	51
13517900	Not	md	JP	2017	0	1	0	0	1	0	0	0	0	0	0	0	
13518611	m	ot	JP	2017	0	1	0	0	1	0	0	0	0	0	0	1	66
13518640	f	cn	GB	2017	1	0	0	0	0	0	0	0	0	0	0	0	74
13518916	f	md	TW	2017	0	1	0	1	0	0	0	0	0	0	0	0	32.9
13519203	m	Not repo	GB	2017	1	0	0	0	0	0	0	0	0	0	0	0	78
13519751	m	cn	US	2017	0	1	0	0	1	0	0	0	0	0	0	0	
13519951	m	ot	US	2017	0	1	0	0	1	0	0	0	0	0	0	0	60
13520595	Not	ph	AU	2017	0	1	0	0	1	0	0	0	0	0	0	0	96
13520698	m	md	FR	2017	0	0	0	0	0	0	0	0	0	0	0	0	

13521231	f	ot	FR	2017	0	0	0	0	0	0	0	0	0	0	0	0	59
13521741	m	md	DE	2017	0	0	0	0	0	0	0	0	0	0	0	0	44
13521867	m	md	US	2017	0	1	0	1	0	0	0	0	0	0	0	0	57
13521907	Not	ot	CN	2017	0	0	0	0	0	0	0	0	0	0	0	0	
13522740	f	cn	US	2017	1	0	0	0	0	0	0	0	0	0	0	0	
13524136	m	ot	GR	2017	0	1	0	0	1	0	0	0	0	0	0	1	66
13524323	f	Not repo	Not re	2017	1	0	0	0	0	0	0	0	0	0	0	0	71
13524568	m	md	JP	2017	0	1	0	0	1	0	0	0	0	0	0	0	81
13524600	m	md	US	2017	0	0	0	0	0	0	0	0	0	0	0	0	
13524607	m	md	US	2017	0	0	0	0	0	0	0	0	0	0	0	0	56
13524627	m	cn	US	2017	0	0	0	0	0	0	0	0	0	0	0	0	
13524664	f	md	US	2017	1	0	0	0	0	0	0	0	0	0	0	0	56
13524716	m	ph	US	2017	0	1	0	0	1	0	0	0	0	0	0	0	
13524802	f	md	US	2017	0	0	0	0	0	0	0	0	0	0	0	0	
13524812	m	ph	US	2017	0	0	0	0	0	0	0	0	0	0	0	0	66
13524836	m	cn	US	2017	0	0	0	0	0	0	0	0	0	0	0	0	
13524850	Not	ot	US	2017	0	0	0	0	0	0	0	0	0	0	0	0	
13524933	m	md	US	2017	0	0	0	0	0	0	0	0	0	0	0	0	
13525021	m	md	US	2017	0	0	0	0	0	0	0	0	0	0	0	0	56
13525023	f	ot	US	2017	0	0	0	0	0	0	0	0	0	0	0	0	
13525026	m	md	US	2017	0	0	0	0	0	0	0	0	0	0	0	0	
13525040	Not	ot	US	2017	0	0	0	0	0	0	0	0	0	0	0	0	
13525045	Not	md	US	2017	0	0	0	0	0	0	0	0	0	0	0	0	
13525057	m	md	US	2017	0	0	0	0	0	0	0	0	0	0	0	0	67
13525065	m	md	US	2017	0	0	0	0	0	0	0	0	0	0	0	0	
13525068	m	cn	US	2017	1	0	0	0	0	0	0	0	0	0	0	0	
13525080	Not	ot	US	2017	0	0	0	0	0	0	0	0	0	0	0	0	
13525081	f	md	US	2017	0	0	0	0	0	0	0	0	0	0	0	0	34
13525094	m	cn	US	2017	0	0	0	0	0	0	0	0	0	0	0	0	
13525124	f	ph	US	2017	0	0	0	0	0	0	0	0	0	0	0	0	63
13525128	Not	ot	US	2017	0	0	0	0	0	0	0	0	0	0	0	0	
13525144	m	cn	US	2017	1	0	0	0	0	0	0	0	0	0	0	0	
13525155	Not	ot	US	2017	0	0	0	0	0	0	0	0	0	0	0	0	
13525193	f	md	US	2017	0	0	0	0	0	0	0	0	0	0	0	0	50

13525220	Not	md	US	2017	0	0	0	0	0	0	0	0	0	0	0	0	
13525254	m	ot	US	2017	0	0	0	0	0	0	0	0	0	0	0	0	84
13525279	Not	md	US	2017	0	0	0	0	0	0	0	0	0	0	0	0	
13525289	Not	ot	US	2017	0	0	0	0	0	0	0	0	0	0	0	0	
13525307	m	ot	US	2017	0	0	0	0	0	0	0	0	0	0	0	0	62
13525342	f	md	US	2017	0	0	0	0	0	0	0	0	0	0	0	0	
13525345	Not	ot	US	2017	0	0	0	0	0	0	0	0	0	0	0	0	
13525411	m	ph	US	2017	0	0	0	0	0	0	0	0	0	0	0	0	
13525667	m	cn	US	2017	1	0	0	0	0	0	0	0	0	0	0	0	
13525929	f	md	CA	2017	0	1	1	0	0	0	0	0	0	0	0	0	65
13526397	m	md	CN	2017	0	0	0	0	0	0	0	0	0	0	0	0	67
13526920	f	ot	HU	2017	0	1	0	0	1	0	0	0	0	0	0	0	57
13526976	f	ot	KR	2017	0	0	0	0	0	0	0	0	0	0	0	0	54
13526997	Not	md	US	2017	1	0	0	0	0	0	0	0	0	0	0	0	
13527040	m	md	JP	2017	0	1	1	0	0	0	0	0	0	0	0	0	80
13527188	m	md	GB	2017	0	0	0	0	0	0	0	0	0	0	0	0	88
13527712	f	md	CH	2017	0	1	0	0	1	0	0	0	0	0	0	0	60
13528263	f	cn	AR	2017	0	0	0	0	0	0	0	0	0	0	0	0	76
13528339	Not	md	US	2017	1	0	0	0	0	0	0	0	0	0	0	0	
13528455	m	ot	US	2017	0	1	0	0	1	0	0	0	0	0	0	0	79
13528667	m	md	US	2017	0	1	0	0	1	0	0	0	0	0	0	1	
13528848	f	cn	US	2017	0	1	1	0	0	0	0	0	0	0	0	0	70
13530874	m	cn	US	2017	0	1	0	0	1	0	0	0	0	0	0	0	51
13531253	f	cn	US	2017	0	0	0	0	0	0	0	0	0	0	0	0	48
13531416	f	cn	US	2017	0	0	0	0	0	0	0	0	0	0	0	0	69
13531685	m	md	TR	2017	0	0	0	0	0	0	0	0	0	0	0	0	47
13532363	m	cn	US	2017	0	1	0	0	1	0	0	0	0	0	0	0	64
13532428	f	md	US	2017	0	0	0	0	0	0	0	0	0	0	0	0	80
13532500	m	md	HR	2017	0	0	0	0	0	0	0	0	0	0	0	0	70
13532511	f	md	JP	2017	0	0	0	0	0	0	0	0	0	0	0	0	31
13532604	f	md	US	2017	0	0	0	0	0	0	0	0	0	0	0	0	71
13532954	m	cn	JP	2017	0	1	0	0	1	0	0	0	0	0	0	0	67
13533074	m	md	US	2017	0	0	0	0	0	0	1	0	0	1	0	0	73.9
13533211	f	md	JP	2017	0	0	0	0	0	0	0	0	0	0	0	0	71

13533374	m	md	US	2017	0	0	0	0	0	0	0	0	0	0	0	0	75
13533459	f	ot	US	2017	0	1	0	0	1	0	0	0	0	0	0	0	64
13533472	m	md	IT	2017	0	1	0	0	1	0	0	0	0	0	0	0	70
13533487	m	md	FR	2017	0	1	0	0	1	0	0	0	0	0	0	0	61
13534317	m	md	DK	2017	0	1	1	0	0	0	0	0	0	0	0	1	57
13535099	m	md	DE	2017	0	0	0	0	0	0	0	0	0	0	0	0	61
13535356	m	md	US	2017	0	1	0	0	0	1	0	0	0	0	0	0	63
13535626	f	md	CN	2017	0	1	0	0	0	1	0	0	0	0	0	0	67
13535700	m	ot	US	2017	0	1	0	0	1	0	0	0	0	0	0	0	54
13536006	m	md	US	2017	1	0	0	0	0	0	0	0	0	0	0	0	65
13536055	m	md	US	2017	1	0	0	0	0	0	0	0	0	0	0	0	79
13536056	m	md	US	2017	1	0	0	0	0	0	0	0	0	0	0	0	57
13536621	m	md	JP	2017	0	1	0	0	1	0	0	0	0	0	0	0	74
13536794	m	ot	DE	2017	0	0	0	0	0	0	0	0	0	0	0	0	72
13537096	f	cn	US	2017	0	1	0	0	1	0	0	0	0	0	0	0	
13537110	f	cn	GB	2017	1	0	0	0	0	0	0	0	0	0	0	0	70
13537113	f	md	CN	2017	1	0	0	0	0	0	0	0	0	0	0	0	70.7
13537834	m	md	EG	2017	0	1	0	0	1	0	0	0	0	0	0	0	67
13538166	m	ot	US	2017	0	1	1	0	0	0	0	0	0	0	0	0	41
13538246	m	cn	JP	2017	0	1	0	0	1	0	0	0	0	0	0	1	81
13538378	m	md	ES	2017	0	1	1	0	0	0	0	0	0	0	0	0	67
13539065	f	ph	JP	2017	0	0	0	0	0	0	0	0	0	0	0	0	
13539219	f	md	FR	2017	0	1	0	0	0	1	0	0	0	0	0	0	62
13539498	f	md	JP	2017	0	0	0	0	0	0	0	0	0	0	0	0	74
13540303	f	cn	JP	2017	0	0	0	0	0	0	0	0	0	0	0	0	
13540340	m	cn	US	2017	0	1	1	0	0	0	0	0	0	0	0	0	
13541273	f	ot	US	2017	0	1	0	0	1	0	0	0	0	0	0	0	54
13541710	Not	cn	US	2017	0	0	0	0	0	0	0	0	0	0	0	0	
13541904	m	ot	JP	2017	0	1	0	0	1	0	0	0	0	0	0	0	75
13541912	m	ot	JP	2017	0	0	0	0	0	0	0	0	0	0	0	0	60
13541954	m	md	JP	2017	0	1	0	0	1	0	0	0	0	0	0	1	75
13542155	Not	md	US	2017	0	0	0	0	0	0	0	0	0	0	0	0	
13542188	f	md	US	2017	0	0	0	0	0	0	0	0	0	0	0	0	67
13542232	f	ot	US	2017	0	0	0	0	0	0	0	0	0	0	0	0	

13542270	Not	md	US	2017	0	0	0	0	0	0	0	0	0	0	0	0	
13542271	f	ph	US	2017	0	0	0	0	0	0	0	0	0	0	0	0	
13542300	Not	md	US	2017	0	0	0	0	0	0	0	0	0	0	0	0	
13542303	m	md	US	2017	0	0	0	0	0	0	0	0	0	0	0	0	
13542304	m	md	US	2017	0	0	0	0	0	0	0	0	0	0	0	0	55
13542336	f	cn	US	2017	0	0	0	0	0	0	0	0	0	0	0	0	
13542366	f	ot	US	2017	0	0	0	0	0	0	0	0	0	0	0	0	
13542387	Not	md	US	2017	0	0	0	0	0	0	0	0	0	0	0	0	
13542405	f	cn	US	2017	0	0	0	0	0	0	0	0	0	0	0	0	70
13542410	f	md	US	2017	0	0	0	0	0	0	0	0	0	0	0	0	
13542418	f	cn	US	2017	1	0	0	0	0	0	0	0	0	0	0	0	
13542423	f	md	US	2017	0	0	0	0	0	0	0	0	0	0	0	0	
13542455	m	md	US	2017	0	0	0	0	0	0	0	0	0	0	0	0	
13542480	m	md	US	2017	0	0	0	0	0	0	0	0	0	0	0	0	
13542527	m	md	US	2017	0	0	0	0	0	0	0	0	0	0	0	0	61
13542539	Not	ph	US	2017	0	0	0	0	0	0	0	0	0	0	0	0	
13542563	f	md	US	2017	0	0	0	0	0	0	0	0	0	0	0	0	
13542566	m	ot	US	2017	0	0	0	0	0	0	0	0	0	0	0	0	
13542567	Not	md	US	2017	0	0	0	0	0	0	0	0	0	0	0	0	
13542575	m	md	US	2017	0	0	0	0	0	0	0	0	0	0	0	0	
13542582	m	cn	US	2017	1	0	0	0	0	0	0	0	0	0	0	0	56
13542583	f	cn	US	2017	0	0	0	0	0	0	0	0	0	0	0	0	
13542585	m	md	US	2017	0	0	0	0	0	0	0	0	0	0	0	0	48
13542621	m	ot	US	2017	0	0	0	0	0	0	0	0	0	0	0	0	
13542624	m	md	US	2017	0	0	0	0	0	0	0	0	0	0	0	0	45
13542673	m	ot	US	2017	0	0	0	0	0	0	0	0	0	0	0	0	70
13542686	m	ot	US	2017	0	0	0	0	0	0	0	0	0	0	0	0	
13542691	m	md	US	2017	0	0	0	0	0	0	0	0	0	0	0	0	
13542709	f	ot	US	2017	0	0	0	0	0	0	0	0	0	0	0	0	42
13542743	Not	md	US	2017	0	0	0	0	0	0	0	0	0	0	0	0	
13542873	m	md	JP	2017	1	0	0	0	0	0	0	0	0	0	0	0	67
13543718	m	md	JP	2017	0	1	0	0	1	0	0	0	0	0	0	0	73
13543735	Not	ot	US	2017	0	1	1	0	0	0	0	0	0	0	0	0	
13543903	m	cn	JP	2017	0	0	0	0	0	0	0	0	0	0	0	0	60

13544091	m	md	GB	2017	0	1	0	0	0	1	0	0	0	0	0	0	61
13544338	m	Not repo	IT	2017	1	0	0	0	0	0	0	0	0	0	0	0	68
13544431	m	md	JP	2017	0	0	0	0	0	0	0	0	0	0	0	0	51
13544501	f	md	US	2017	1	0	0	0	0	0	0	0	0	0	0	0	
13544631	f	ot	DE	2017	0	1	0	0	1	0	0	0	0	0	0	0	64
13544661	m	md	US	2017	0	1	0	0	1	0	0	0	0	0	0	0	
13544747	m	cn	JP	2017	0	1	1	0	0	0	0	0	0	0	0	0	80
13544860	Not	md	TR	2017	0	0	0	0	0	0	0	0	0	0	0	0	
13544865	m	cn	JP	2017	0	0	0	0	0	0	0	0	0	0	0	0	71
13545084	Not	ot	IT	2017	1	0	0	0	0	0	0	0	0	0	0	0	
13545477	Not	md	US	2017	0	0	0	0	0	0	0	0	0	0	0	0	
13545490	Not	md	US	2017	0	0	0	0	0	0	0	0	0	0	0	0	
13545847	m	ot	NL	2017	0	0	0	0	0	0	0	0	0	0	0	0	64
13546671	m	cn	US	2017	0	0	0	0	0	0	0	0	0	0	0	0	37
13546680	m	cn	US	2017	0	0	0	0	0	0	0	0	0	0	0	0	67
13546906	Not	md	US	2017	0	0	0	0	0	0	0	0	0	0	0	0	
13546985	Not	ph	US	2017	0	1	0	0	1	0	0	0	0	0	0	0	
13546995	m	cn	US	2017	0	0	0	0	0	0	0	0	0	0	0	0	70
13547014	f	cn	JP	2017	0	0	0	0	0	0	1	0	1	0	0	1	68
13547074	m	md	JP	2017	0	1	0	0	1	0	0	0	0	0	0	0	75
13547097	f	ot	ES	2017	0	1	0	0	1	0	0	0	0	0	0	1	
13547464	f	cn	US	2017	0	0	0	0	0	0	0	0	0	0	0	0	
13547987	m	cn	US	2017	0	1	0	0	1	0	0	0	0	0	0	0	
13548757	m	md	IL	2017	0	1	0	0	1	0	0	0	0	0	0	0	60
13548793	m	cn	US	2017	0	1	1	0	0	0	0	0	0	0	0	1	
13549449	f	cn	US	2017	0	1	0	0	1	0	0	0	0	0	0	0	
13549539	f	cn	GB	2017	1	0	0	0	0	0	0	0	0	0	0	0	61
13549583	m	md	FR	2017	0	0	0	0	0	0	0	0	0	0	0	0	58
13549856	f	cn	JP	2017	0	1	1	0	0	0	0	0	0	0	0	0	69
13550293	Not	md	PE	2017	0	1	0	0	1	0	0	0	0	0	0	0	
13550724	f	md	JP	2017	1	0	0	0	0	0	0	0	0	0	0	0	63
13550742	f	md	JP	2017	1	0	0	0	0	0	0	0	0	0	0	0	44
13550857	Not	cn	IN	2017	0	1	0	0	1	0	0	0	0	0	0	0	
13550934	m	cn	US	2017	0	0	0	0	0	0	0	0	0	0	0	0	62

13551071	m	cn	US	2017	0	0	0	0	0	0	0	0	0	0	0	0	64
13551268	m	md	US	2017	0	1	1	0	0	0	0	0	0	0	0	0	56
13551559	m	cn	GB	2017	1	0	0	0	0	0	0	0	0	0	0	0	76
13551728	m	md	JP	2017	0	1	0	0	1	0	0	0	0	0	0	0	67
13552288	m	Not repo	US	2017	1	0	0	0	0	0	0	0	0	0	0	1	
13552549	Not	md	AU	2017	0	1	0	0	1	0	0	0	0	0	0	0	
13552633	m	md	JP	2017	1	0	0	0	0	0	0	0	0	0	0	0	60
13552770	m	cn	JP	2017	0	0	0	0	0	0	1	0	1	0	0	1	67
13552847	m	md	AU	2017	1	0	0	0	0	0	0	0	0	0	0	0	62
13553204	m	ph	JP	2017	0	1	0	0	1	0	0	0	0	0	0	0	67
13553206	m	md	JP	2017	0	1	0	0	1	0	0	0	0	0	0	0	73
13553216	m	md	CZ	2017	0	0	0	0	0	0	0	0	0	0	0	0	57
13553482	m	cn	GB	2017	1	0	0	0	0	0	0	0	0	0	0	0	70
13553783	m	cn	JP	2017	0	0	0	0	0	0	0	0	0	0	0	0	68
13554560	f	ot	US	2017	1	0	0	0	0	0	0	0	0	0	0	0	86.1
13554588	f	ph	JP	2017	0	0	0	0	0	0	0	0	0	0	0	0	79
13554652	f	md	JP	2017	0	1	0	0	1	0	0	0	0	0	0	1	78
13557117	m	md	GB	2017	0	0	0	0	0	0	0	0	0	0	0	0	57
13557844	m	cn	GB	2017	1	0	0	0	0	0	0	0	0	0	0	0	62
13558351	f	cn	US	2017	0	1	0	0	1	0	0	0	0	0	0	0	59
13558406	Not	md	JP	2017	0	0	0	0	0	0	0	0	0	0	0	0	71
13559117	m	ot	DE	2017	0	1	0	0	1	0	0	0	0	0	0	0	62
13559121	m	md	JP	2017	0	0	0	0	0	0	0	0	0	0	0	0	55
13559148	f	cn	US	2017	0	1	0	0	1	0	0	0	0	0	0	0	
13559975	Not	ot	JP	2017	0	0	0	0	0	0	0	0	0	0	0	0	
13560075	f	md	JP	2017	0	1	0	0	1	0	0	0	0	0	0	0	65
13560298	f	ot	BR	2017	1	0	0	0	0	0	0	0	0	0	0	0	53
13560431	m	md	JP	2017	0	0	0	0	0	0	0	0	0	0	0	0	
13561144	m	ot	JP	2017	0	1	0	0	1	0	0	0	0	0	0	0	64
13561145	f	md	JP	2017	0	1	0	0	1	0	0	0	0	0	0	0	82
13561409	m	ot	BE	2017	0	1	0	0	1	0	0	0	0	0	0	0	62
13561655	m	cn	US	2017	0	1	1	0	0	0	0	0	0	0	0	0	
13562123	f	md	JP	2017	1	0	0	0	0	0	0	0	0	0	0	0	85
13562622	Not	md	FI	2017	0	0	0	0	0	0	0	0	0	0	0	0	

13562634	f	md	JP	2017	0	0	0	0	0	0	0	0	0	0	0	0	84
13562756	m	md	CN	2017	1	0	0	0	0	0	0	0	0	0	0	0	
13563086	m	ph	US	2017	0	0	0	0	0	0	0	0	0	0	0	0	64
13563157	f	cn	US	2017	0	1	0	0	1	0	0	0	0	0	0	0	
13563169	m	ot	DE	2017	0	1	0	0	0	1	0	0	0	0	0	0	55
13563287	f	md	CL	2017	0	0	0	0	0	0	0	0	0	0	0	0	64
13563496	f	cn	US	2017	0	0	0	0	0	0	0	0	0	0	0	0	67
13563511	f	md	CN	2017	1	0	0	0	0	0	0	0	0	0	0	0	
13563632	f	md	GB	2017	0	0	0	0	0	0	0	0	0	0	0	0	69
13563681	f	cn	US	2017	1	0	0	0	0	0	0	0	0	0	0	0	76
13563753	m	md	KR	2017	1	0	0	0	0	0	0	0	0	0	0	0	71
13564452	m	md	JP	2017	0	0	0	0	0	0	0	0	0	0	0	0	74
13564483	f	cn	JP	2017	0	0	0	0	0	0	0	0	0	0	0	0	79
13564536	Not	Not repo	Not re	2017	1	0	0	0	0	0	0	0	0	0	0	0	
13564669	Not	Not repo	Not re	2017	1	0	0	0	0	0	0	0	0	0	0	0	
13564670	Not	Not repo	Not re	2017	1	0	0	0	0	0	0	0	0	0	0	0	
13564675	Not	Not repo	Not re	2017	1	0	0	0	0	0	0	0	0	0	0	0	
13564676	Not	Not repo	Not re	2017	1	0	0	0	0	0	0	0	0	0	0	0	
13565134	m	md	JP	2017	0	1	0	0	1	0	0	0	0	0	0	0	68
13565257	m	cn	US	2017	0	1	0	0	1	0	0	0	0	0	0	0	58
13565440	f	ot	CA	2017	0	1	0	0	1	0	0	0	0	0	0	0	68
13566908	f	md	US	2017	0	1	0	0	0	1	0	0	0	0	0	0	70
13567196	f	md	JP	2017	1	0	0	0	0	0	0	0	0	0	0	0	88
13567668	Not	md	BN	2017	0	0	0	0	0	0	0	0	0	0	0	0	
13567747	Not	ot	FR	2017	1	0	0	0	0	0	0	0	0	0	0	0	
13567829	Not	ot	FR	2017	1	0	0	0	0	0	0	0	0	0	0	0	
13568194	Not	cn	GB	2017	1	0	0	0	0	0	0	0	0	0	0	0	
13568197	f	ot	US	2017	0	1	1	0	0	0	0	0	0	0	0	0	75
13568797	m	md	IT	2017	0	1	0	0	1	0	0	0	0	0	0	0	
13568920	m	cn	DE	2017	0	1	1	0	0	0	0	0	0	0	0	0	
13568978	m	cn	US	2017	1	0	0	0	0	0	0	0	0	0	0	0	
13569337	m	md	CN	2017	0	0	0	0	0	0	0	0	0	0	0	0	61
13569513	f	cn	US	2017	0	0	0	0	0	0	0	0	0	0	0	0	63
13569530	m	cn	US	2017	0	0	0	0	0	0	0	0	0	0	0	0	82

13569686	f	cn	GB	2017	0	1	1	0	0	0	0	0	0	0	0	0	
13569726	Not	md	HK	2017	0	1	0	0	1	0	0	0	0	0	0	0	
13569757	Not	ph	AU	2017	0	1	0	0	1	0	0	0	0	0	0	0	54
13569798	f	cn	US	2017	0	0	0	0	0	0	0	0	0	0	0	0	86
13570119	m	cn	US	2017	0	0	0	0	0	0	0	0	0	0	0	0	77
13570188	f	cn	US	2017	0	0	0	0	0	0	0	0	0	0	0	0	55
13571748	m	md	GB	2017	0	1	1	0	0	0	0	0	0	0	0	0	62
13571806	Not	cn	US	2017	0	1	1	0	0	0	0	0	0	0	0	0	
13571945	Not	cn	GB	2017	1	0	0	0	0	0	0	0	0	0	0	0	
13571970	m	cn	IN	2017	0	1	0	0	1	0	0	0	0	0	0	0	63
13572023	f	cn	GB	2017	1	0	0	0	0	0	0	0	0	0	0	0	70
13572024	Not	cn	GB	2017	1	0	0	0	0	0	0	0	0	0	0	0	
13572094	m	cn	GB	2017	1	0	0	0	0	0	0	0	0	0	0	0	49
13572101	f	md	CH	2017	0	1	0	1	0	0	0	0	0	0	0	0	74
13572127	m	ot	TW	2017	0	0	0	0	0	0	0	0	0	0	0	0	48
13572313	m	md	JP	2017	0	0	0	0	0	0	0	0	0	0	0	0	66
13572895	m	ph	JP	2017	0	1	1	0	0	0	0	0	0	0	0	0	80
13573016	f	cn	GR	2017	0	1	1	0	0	0	0	0	0	0	0	0	69
13573204	m	md	IT	2017	1	0	0	0	0	0	0	0	0	0	0	0	
13573227	f	md	NL	2017	0	1	0	0	0	1	0	0	0	0	0	0	58
13573386	f	md	AU	2017	0	0	0	0	0	0	0	0	0	0	0	0	57
13573582	f	cn	US	2017	1	0	0	0	0	0	0	0	0	0	0	0	
13573652	f	md	US	2017	1	0	0	0	0	0	0	0	0	0	0	0	
13573665	f	Not repo	US	2017	1	0	0	0	0	0	0	0	0	0	0	1	
13573666	f	Not repo	US	2017	1	0	0	0	0	0	0	0	0	0	0	0	
13573667	Not	md	US	2017	1	0	0	0	0	0	0	0	0	0	0	1	
13573701	m	cn	JP	2017	0	0	0	0	0	0	0	0	0	0	0	0	62
13573754	m	md	JP	2017	1	0	0	0	0	0	0	0	0	0	0	1	75
13573821	f	cn	US	2017	0	0	0	0	0	0	0	0	0	0	0	0	61
13573853	m	cn	GB	2017	1	0	0	0	0	0	0	0	0	0	0	0	
13573981	m	ph	US	2017	0	1	0	0	1	0	0	0	0	0	0	0	
13574035	m	md	JP	2017	0	0	0	0	0	0	0	0	0	0	0	0	75
13574313	m	cn	JP	2017	0	0	0	0	0	0	0	0	0	0	0	0	56
13574682	m	ot	US	2017	0	1	0	0	0	1	0	0	0	0	0	0	62

13574933	f	md	GB	2017	0	0	0	0	0	0	0	0	0	0	0	0	41
13575543	m	md	RU	2017	0	1	0	0	0	1	0	0	0	0	0	0	68
13575808	m	md	CN	2017	1	0	0	0	0	0	0	0	0	0	0	0	53.8
13576312	Not	cn	GB	2017	1	0	0	0	0	0	0	0	0	0	0	0	
13576319	Not	cn	GB	2017	1	0	0	0	0	0	0	0	0	0	0	0	
13576364	m	md	DE	2017	0	1	0	0	1	0	0	0	0	0	0	0	60
13576855	m	cn	JP	2017	0	0	0	0	0	0	0	0	0	0	0	0	71
13576872	m	cn	JP	2017	0	0	0	0	0	0	0	0	0	0	0	0	38
13576940	f	md	US	2017	1	0	0	0	0	0	0	0	0	0	0	0	
13576971	Not	md	US	2017	1	0	0	0	0	0	0	0	0	0	0	0	
13577008	m	ph	US	2017	1	0	0	0	0	0	0	0	0	0	0	0	
13577009	Not	md	US	2017	1	0	0	0	0	0	0	0	0	0	0	0	
13577017	Not	md	US	2017	1	0	0	0	0	0	0	0	0	0	0	0	
13577314	m	cn	US	2017	1	0	0	0	0	0	0	0	0	0	0	0	
13577366	m	md	FR	2017	0	1	0	1	0	0	0	0	0	0	0	0	68.5
13577538	Not	Not repo	Not re	2017	1	0	0	0	0	0	0	0	0	0	0	0	
13578003	m	Not repo	IT	2017	1	0	0	0	0	0	0	0	0	0	0	0	75
13578186	f	cn	US	2017	0	0	0	0	0	0	0	0	0	0	0	0	77
13579367	m	md	JP	2017	0	0	0	0	0	0	0	0	0	0	0	0	
13579401	f	md	CN	2017	1	0	0	0	0	0	0	0	0	0	0	0	73.8
13579482	m	md	US	2017	0	1	0	0	0	1	0	0	0	0	0	0	60
13579571	m	ot	US	2017	0	1	0	0	1	0	0	0	0	0	0	0	
13579880	m	cn	US	2017	0	0	0	0	0	0	0	0	0	0	0	0	54
13579893	m	md	JP	2017	1	0	0	0	0	0	0	0	0	0	0	0	83
13580290	f	md	US	2017	0	1	0	0	0	1	0	0	0	0	0	0	78
13580478	f	md	AU	2017	0	0	0	0	0	0	0	0	0	0	0	0	57
13580624	m	md	EG	2017	0	1	0	0	1	0	0	0	0	0	0	0	56
13580640	f	cn	US	2017	1	0	0	0	0	0	0	0	0	0	0	0	
13580647	Not	md	US	2017	1	0	0	0	0	0	0	0	0	0	0	0	
13581399	Not	ot	CN	2017	0	0	0	0	0	0	0	0	0	0	0	0	
13581527	m	md	ES	2017	0	1	1	0	0	0	0	0	0	0	0	0	47
13581798	m	md	CA	2017	1	0	0	0	0	0	0	0	0	0	0	0	53
13581807	Not	cn	GB	2017	1	0	0	0	0	0	0	0	0	0	0	0	
13581809	Not	Not repo	Not re	2017	1	0	0	0	0	0	0	0	0	0	0	0	

13582037	m	cn	US	2017	0	0	0	0	0	0	0	0	0	0	0	0	49
13582094	f	cn	CA	2017	0	1	1	0	0	0	0	0	0	0	0	1	76
13582141	m	md	DE	2017	0	1	0	0	1	0	0	0	0	0	0	1	
13582325	m	md	JP	2017	0	1	0	0	1	0	0	0	0	0	0	1	68
13582504	f	cn	US	2017	0	0	0	0	0	0	0	0	0	0	0	0	61
13582957	f	ot	FR	2017	0	1	0	0	1	0	0	0	0	0	0	0	68
13583356	f	md	DK	2017	0	1	0	0	1	0	0	0	0	0	0	1	68
13583365	m	cn	JP	2017	0	0	0	0	0	0	0	0	0	0	0	0	72
13583404	m	md	DE	2017	0	1	0	0	1	0	0	0	0	0	0	0	53
13583502	m	ot	US	2017	0	1	0	0	1	0	0	0	0	0	0	0	50
13583597	m	md	US	2017	0	0	0	0	0	0	1	0	0	0	1	0	57
13583607	m	md	FR	2017	0	1	0	0	0	1	0	0	0	0	0	0	51
13583734	m	md	JP	2017	1	0	0	0	0	0	0	0	0	0	0	0	66
13583769	f	ot	US	2017	0	1	0	0	1	0	0	0	0	0	0	0	
13583838	m	ph	US	2017	0	1	0	0	1	0	0	0	0	0	0	0	74
13584386	Not	ot	US	2017	0	0	0	0	0	0	0	0	0	0	0	0	
13584435	m	md	CN	2017	0	1	0	0	0	1	0	0	0	0	0	0	63
13584444	m	md	CN	2017	0	1	0	0	0	1	0	0	0	0	0	0	66
13584961	m	ot	US	2017	0	1	0	0	1	0	0	0	0	0	0	1	67
13585542	m	ot	ES	2017	0	1	0	0	1	0	0	0	0	0	0	0	47
13585788	m	ph	GB	2017	0	0	0	0	0	0	0	0	0	0	0	0	59
13586046	m	md	IL	2017	0	0	0	0	0	0	0	0	0	0	0	0	60
13586242	m	cn	JP	2017	0	0	0	0	0	0	0	0	0	0	0	0	81
13586387	m	cn	GB	2017	1	0	0	0	0	0	0	0	0	0	0	0	
13586492	f	md	CN	2017	1	0	0	0	0	0	0	0	0	0	0	1	71
13587779	Not	ph	AU	2017	0	1	0	0	1	0	0	0	0	0	0	0	62
13588722	Not	md	US	2017	0	1	0	0	1	0	0	0	0	0	0	0	
13588756	f	cn	US	2017	0	0	0	0	0	0	0	0	0	0	0	0	
13588763	m	cn	JP	2017	0	1	1	0	0	0	0	0	0	0	0	0	69
13588972	f	cn	IL	2017	0	1	1	0	0	0	0	0	0	0	0	0	60
13589272	m	cn	JP	2017	0	1	1	0	0	0	0	0	0	0	0	0	72
13589702	m	ot	IT	2017	0	0	0	0	0	0	0	0	0	0	0	0	77
13589764	f	cn	GB	2017	1	0	0	0	0	0	0	0	0	0	0	0	54
13589981	m	md	IT	2017	0	1	0	0	1	0	0	0	0	0	0	0	

13590219	f	cn	US	2017	0	1	0	0	1	0	0	0	0	0	0	0
13590810	f	md	JP	2017	1	0	0	0	0	0	0	0	0	0	0	0	67
13590898	f	cn	US	2017	1	0	0	0	0	0	0	0	0	0	0	0
13590941	m	cn	JP	2017	0	1	1	0	0	0	0	0	0	0	0	0	81
13590960	m	ot	JP	2017	0	1	1	0	0	0	0	0	0	0	0	0	49
13590976	f	ot	US	2017	0	1	0	0	1	0	0	0	0	0	0	0	34
13591253	Not	ph	AU	2017	0	1	0	0	1	0	0	0	0	0	0	0	67
13591255	Not	ph	AU	2017	0	1	0	0	1	0	0	0	0	0	0	0
13591276	f	ph	US	2017	0	0	0	0	0	0	0	0	0	0	0	0	44
13591554	f	cn	US	2017	0	0	0	0	0	0	0	0	0	0	0	0	50
13591949	f	md	JP	2017	0	1	0	0	1	0	0	0	0	0	0	0	68
13591953	Not	ot	IT	2017	0	1	0	0	1	0	0	0	0	0	0	0	69
13592019	m	cn	US	2017	0	0	0	0	0	0	0	0	0	0	0	0	63
13592059	m	md	KR	2017	0	0	0	0	0	0	0	0	0	0	0	0	59
13592318	f	cn	TH	2017	0	0	0	0	0	0	0	0	0	0	0	0	57
13592351	f	ot	JP	2017	0	1	0	0	1	0	0	0	0	0	0	0	72
13592471	m	cn	US	2017	1	0	0	0	0	0	0	0	0	0	0	0	74
13592550	m	md	DE	2017	0	1	0	0	1	0	0	0	0	0	0	0
13592568	f	cn	GB	2017	1	0	0	0	0	0	0	0	0	0	0	0
13592571	m	cn	GB	2017	1	0	0	0	0	0	0	0	0	0	0	0
13592971	m	ph	US	2017	0	0	0	0	0	0	0	0	0	0	0	0	74
13593042	Not	cn	GB	2017	1	0	0	0	0	0	0	0	0	0	0	0
13593046	f	ot	JP	2017	0	1	0	0	1	0	0	0	0	0	0	0	62
13593203	m	ot	US	2017	0	0	0	0	0	0	0	0	0	0	0	0	68
13593566	Not	cn	JP	2017	0	0	0	0	0	0	0	0	0	0	0	0	65
13593622	m	ot	JP	2017	0	0	0	0	0	0	0	0	0	0	0	0	77
13593631	m	ot	JP	2017	0	0	0	0	0	0	0	0	0	0	0	0	80
13594054	f	Not repo	US	2017	1	0	0	0	0	0	0	0	0	0	0	0	78
13594056	m	md	DE	2017	0	1	0	0	1	0	0	0	0	0	0	0
13594463	f	cn	GB	2017	1	0	0	0	0	0	0	0	0	0	0	0
13594615	m	cn	GB	2017	1	0	0	0	0	0	0	0	0	0	0	0
13594616	f	cn	GB	2017	1	0	0	0	0	0	0	0	0	0	0	0	67
13595170	m	md	JP	2017	0	1	0	0	0	1	0	0	0	0	0	1	73
13595354	f	md	JP	2017	1	0	0	0	0	0	0	0	0	0	0	0	74

13596860	f	md	ES	2017	0	1	0	0	1	0	0	0	0	0	0	0	
13597048	Not	md	JP	2017	0	1	0	0	1	0	0	0	0	0	0	0	70
13597118	f	Not repo	AU	2017	1	0	0	0	0	0	0	0	0	0	0	0	
13597249	m	ot	US	2017	0	0	0	0	0	0	0	0	0	0	0	0	74
13597505	m	cn	GB	2017	1	0	0	0	0	0	0	0	0	0	0	0	
13597507	f	cn	CN	2017	1	0	0	0	0	0	0	0	0	0	0	0	
13597790	m	md	IT	2017	0	0	0	0	0	0	0	0	0	0	0	0	51
13598039	f	ot	US	2017	0	1	0	0	1	0	0	0	0	0	0	0	58
13598047	m	cn	US	2017	0	1	0	0	1	0	0	0	0	0	0	0	
13598088	f	md	IT	2017	1	0	0	0	0	0	0	0	0	0	0	0	
13598156	m	md	GB	2017	0	0	0	0	0	0	0	0	0	0	0	0	65
13598208	m	md	GR	2017	1	0	0	0	0	0	0	0	0	0	0	0	80
13598299	f	ot	AU	2017	1	0	0	0	0	0	0	0	0	0	0	1	45.8
13598325	f	cn	GB	2017	1	0	0	0	0	0	0	0	0	0	0	0	
13598433	Not	ot	DE	2017	0	1	0	0	1	0	0	0	0	0	0	0	56
13598852	m	md	NL	2017	0	1	0	1	0	0	0	0	0	0	0	0	65.2
13599514	f	md	US	2017	0	0	0	0	0	0	0	0	0	0	0	0	69
13599557	m	md	US	2017	1	0	0	0	0	0	0	0	0	0	0	0	40
13599694	m	md	JP	2017	0	1	0	0	1	0	0	0	0	0	0	0	75
13599722	f	cn	JP	2017	0	0	0	0	0	0	0	0	0	0	0	0	73
13599896	f	md	IT	2017	1	0	0	0	0	0	0	0	0	0	0	0	
13599958	Not	ot	JP	2017	1	0	0	0	0	0	0	0	0	0	0	1	
13599970	m	md	JP	2017	0	1	1	0	0	0	0	0	0	0	0	0	58
13600553	m	md	GB	2017	0	1	0	1	0	0	0	0	0	0	0	0	76.6
13600760	f	md	DE	2017	0	1	0	1	0	0	0	0	0	0	0	0	53
13600774	f	ot	US	2017	0	1	0	0	1	0	0	0	0	0	0	0	70
13600957	f	md	NO	2017	0	0	0	0	0	0	0	0	0	0	0	0	
13601855	m	cn	US	2017	0	0	0	0	0	0	0	0	0	0	0	0	68
13602261	f	md	JP	2017	0	1	0	0	1	0	0	0	0	0	0	1	68
13602316	m	md	JP	2017	0	1	0	0	1	0	0	0	0	0	0	1	
13602326	f	md	JP	2017	1	0	0	0	0	0	0	0	0	0	0	0	55
13602458	m	ph	JP	2017	0	0	0	0	0	0	0	0	0	0	0	0	70
13602487	m	cn	GB	2017	1	0	0	0	0	0	0	0	0	0	0	0	60
13602528	f	md	AU	2017	0	1	0	0	1	0	0	0	0	0	0	0	83

13602898	f	md	JP	2017	0	0	0	0	0	0	0	0	0	0	0	0	
13602944	Not	ot	US	2017	0	1	0	0	0	1	0	0	0	0	0	1	
13602961	f	md	JP	2017	0	1	0	0	1	0	0	0	0	0	0	0	60
13603106	m	ot	MA	2017	0	0	0	0	0	0	0	0	0	0	0	0	62
13603117	f	md	FR	2017	0	0	0	0	0	0	0	0	0	0	0	0	
13603149	m	md	JP	2017	0	1	0	0	1	0	0	0	0	0	0	1	68
13603164	m	cn	NO	2017	0	1	0	0	1	0	0	0	0	0	0	0	66
13603742	m	cn	JP	2017	0	1	0	0	1	0	0	0	0	0	0	1	71
13603806	f	md	US	2017	0	1	0	0	0	1	0	0	0	0	0	0	77
13603867	f	md	US	2017	0	0	0	0	0	0	0	0	0	0	0	0	
13606025	m	ph	US	2017	0	0	0	0	0	0	0	0	0	0	0	0	72
13607030	f	ot	US	2017	0	1	0	0	1	0	0	0	0	0	0	0	
13607058	f	cn	GB	2017	1	0	0	0	0	0	0	0	0	0	0	0	55
13607059	m	cn	GB	2017	1	0	0	0	0	0	0	0	0	0	0	0	
13607121	f	cn	GB	2017	1	0	0	0	0	0	0	0	0	0	0	0	
13607313	Not	cn	GB	2017	1	0	0	0	0	0	0	0	0	0	0	0	
13607333	m	ot	CA	2017	0	1	0	0	1	0	0	0	0	0	0	0	
13607597	f	md	NL	2017	0	0	0	0	0	0	0	0	0	0	0	0	58
13607703	f	md	IT	2017	1	0	0	0	0	0	0	0	0	0	0	0	57
13607936	m	md	CN	2017	0	1	0	0	0	1	0	0	0	0	0	0	62
13608340	f	cn	US	2017	0	0	0	0	0	0	0	0	0	0	0	0	65
13608479	m	md	US	2017	0	1	0	1	0	0	0	0	0	0	0	1	65.8
13608484	Not	ot	ZA	2017	0	1	0	0	1	0	0	0	0	0	0	0	
13608486	f	md	JP	2017	0	1	0	0	1	0	0	0	0	0	0	0	65
13608570	f	cn	US	2017	0	0	0	0	0	0	0	0	0	0	0	0	72
13608584	m	md	JP	2017	0	1	0	0	1	0	0	0	0	0	0	0	68
13608747	f	ot	US	2017	0	1	0	0	1	0	0	0	0	0	0	0	59
13608749	Not	cn	US	2017	0	1	0	0	1	0	0	0	0	0	0	0	
13609185	f	md	IN	2017	0	1	0	0	1	0	0	0	0	0	0	0	72
13609225	f	cn	US	2017	0	1	0	0	1	0	0	0	0	0	0	0	
13609653	f	md	DE	2017	0	1	1	0	0	0	0	0	0	0	0	0	62
13609937	f	md	IT	2017	0	1	0	0	1	0	0	0	0	0	0	0	
13610872	f	ot	JO	2017	0	0	0	0	0	0	0	0	0	0	0	0	54
13611408	f	cn	JP	2017	0	1	1	0	0	0	0	0	0	0	0	0	

13611560	m	md	IT	2017	0	0	0	0	0	0	0	0	0	0	0	0	84
13612425	f	md	MY	2017	1	0	0	0	0	0	0	0	0	0	0	0	84
13612978	f	cn	GB	2017	1	0	0	0	0	0	0	0	0	0	0	0	
13613515	Not	md	JP	2017	0	0	0	0	0	0	0	0	0	0	0	0	
13613972	f	ot	CA	2017	0	0	0	0	0	0	0	0	0	0	0	0	76
13614228	Not	ot	GB	2017	0	0	0	0	0	0	0	0	0	0	0	0	
13614238	Not	ot	JP	2017	0	0	0	0	0	0	0	0	0	0	0	0	
13614253	Not	ot	JP	2017	0	0	0	0	0	0	0	0	0	0	0	0	
13614256	f	ot	US	2017	0	1	0	0	1	0	0	0	0	0	0	0	73
13614263	Not	ot	JP	2017	0	0	0	0	0	0	0	0	0	0	0	0	
13614621	f	ph	JP	2017	1	0	0	0	0	0	0	0	0	0	0	0	76
13614622	Not	md	JP	2017	1	0	0	0	0	0	0	0	0	0	0	0	
13614674	m	cn	GB	2017	1	0	0	0	0	0	0	0	0	0	0	0	
13614685	m	cn	JP	2017	0	0	0	0	0	0	0	0	0	0	0	0	78
13614726	Not	cn	US	2017	0	1	1	0	0	0	0	0	0	0	0	0	
13614950	m	cn	GB	2017	1	0	0	0	0	0	0	0	0	0	0	0	
13614959	m	cn	GB	2017	1	0	0	0	0	0	0	0	0	0	0	0	
13614998	m	ot	GB	2017	0	0	0	0	0	0	0	0	0	0	0	0	59
13615458	f	md	SA	2017	1	0	0	0	0	0	0	0	0	0	0	0	
13615476	m	md	TR	2017	0	0	0	0	0	0	0	0	0	0	0	0	69
13615698	f	ot	JP	2017	0	1	0	0	1	0	0	0	0	0	0	0	76
13615877	m	md	JP	2017	0	0	0	0	0	0	0	0	0	0	0	0	80
13615921	f	md	US	2017	0	1	1	0	0	0	0	0	0	0	0	0	53
13615938	m	cn	JP	2017	0	1	0	0	1	0	0	0	0	0	0	0	73
13617945	m	ot	JP	2017	1	0	0	0	0	0	0	0	0	0	0	1	82
13617951	m	cn	GB	2017	1	0	0	0	0	0	0	0	0	0	0	0	
13617955	m	cn	GB	2017	1	0	0	0	0	0	0	0	0	0	0	0	
13618188	f	cn	GB	2017	1	0	0	0	0	0	0	0	0	0	0	0	
13618848	m	ot	SG	2017	0	1	0	0	1	0	0	0	0	0	0	0	79
13619465	m	ot	US	2017	0	0	0	0	0	0	0	0	0	0	0	0	84
13619862	f	md	HU	2017	1	0	0	0	0	0	0	0	0	0	0	0	62
13619882	f	md	NO	2017	0	0	0	0	0	0	0	0	0	0	0	0	
13619955	m	Not repo	CA	2017	1	0	0	0	0	0	0	0	0	0	0	0	68
13620055	f	ot	US	2017	1	0	0	0	0	0	0	0	0	0	0	0	76

13620089	m	Not repo	JP	2017	0	0	0	0	0	0	0	0	0	0	0	0	
13620121	Not	md	NO	2017	0	0	0	0	0	0	0	0	0	0	0	0	
13620746	f	md	JP	2017	1	0	0	0	0	0	0	0	0	0	0	0	76
13621077	f	cn	GB	2017	1	0	0	0	0	0	0	0	0	0	0	0	87
13621088	m	cn	JP	2017	0	1	0	0	1	0	0	0	0	0	0	1	74
13621298	Not	cn	US	2017	0	1	1	0	0	0	0	0	0	0	0	0	
13622139	m	cn	GB	2017	1	0	0	0	0	0	0	0	0	0	0	0	62
13622622	m	md	ES	2017	0	1	0	0	1	0	0	0	0	0	0	0	
13622705	m	md	FR	2017	0	0	0	0	0	0	0	0	0	0	0	0	58
13622726	Not	cn	US	2017	0	1	0	0	1	0	0	0	0	0	0	0	
13622792	f	md	JP	2017	1	0	0	0	0	0	0	0	0	0	0	0	56
13622860	m	Not repo	CA	2017	1	0	0	0	0	0	0	0	0	0	0	0	
13622941	f	md	BR	2017	1	0	0	0	0	0	0	0	0	0	0	0	76.5
13623014	m	cn	US	2017	1	0	0	0	0	0	0	0	0	0	0	0	65
13623063	f	cn	US	2017	1	0	0	0	0	0	0	0	0	0	0	0	
13624896	m	cn	US	2017	0	1	0	0	1	0	0	0	0	0	0	0	
13625029	m	md	TH	2017	0	1	0	0	0	1	0	0	0	0	0	0	75
13625247	f	cn	CH	2017	1	0	0	0	0	0	0	0	0	0	0	0	
13625421	f	cn	GB	2017	1	0	0	0	0	0	0	0	0	0	0	0	68
13625628	f	md	JP	2017	1	0	0	0	0	0	0	0	0	0	0	1	80.7
13625777	f	md	NO	2017	0	0	0	0	0	0	0	0	0	0	0	0	
13625852	Not	md	US	2017	1	0	0	0	0	0	0	0	0	0	0	0	
13625854	m	cn	CA	2017	0	1	1	0	0	0	0	0	0	0	0	0	
13625884	f	md	US	2017	1	0	0	0	0	0	0	0	0	0	0	0	
13625895	m	ot	US	2017	1	0	0	0	0	0	0	0	0	0	0	0	69
13625951	f	cn	US	2017	1	0	0	0	0	0	0	0	0	0	0	0	55
13625959	m	md	US	2017	0	0	0	0	0	0	0	0	0	0	0	0	
13625974	f	ot	US	2017	1	0	0	0	0	0	0	0	0	0	0	0	55
13626012	m	ph	US	2017	1	0	0	0	0	0	0	0	0	0	0	0	55
13626022	f	md	US	2017	1	0	0	0	0	0	0	0	0	0	0	0	72
13626027	f	cn	GB	2017	1	0	0	0	0	0	0	0	0	0	0	0	70
13626033	f	ot	US	2017	1	0	0	0	0	0	0	0	0	0	0	0	
13626043	m	cn	US	2017	1	0	0	0	0	0	0	0	0	0	0	0	
13626051	f	ph	US	2017	1	0	0	0	0	0	0	0	0	0	0	0	67

13626119	f	md	US	2017	1	0	0	0	0	0	0	0	0	0	0	1
13626126	f	cn	US	2017	1	0	0	0	0	0	0	0	0	0	0	1
13626138	m	ot	US	2017	1	0	0	0	0	0	0	0	0	0	0	0	74
13626142	f	ph	US	2017	1	0	0	0	0	0	0	0	0	0	0	0	57
13626152	m	md	US	2017	1	0	0	0	0	0	0	0	0	0	0	0	53
13626201	f	md	US	2017	1	0	0	0	0	0	0	0	0	0	0	0	
13626223	m	md	US	2017	1	0	0	0	0	0	0	0	0	0	0	1	
13626230	f	ph	US	2017	1	0	0	0	0	0	0	0	0	0	0	0	58
13626239	m	md	US	2017	1	0	0	0	0	0	0	0	0	0	0	0	
13626243	f	cn	US	2017	1	0	0	0	0	0	0	0	0	0	0	0	69
13626283	f	md	US	2017	1	0	0	0	0	0	0	0	0	0	0	0	64
13626293	f	cn	US	2017	1	0	0	0	0	0	0	0	0	0	0	0	
13626322	m	ot	US	2017	1	0	0	0	0	0	0	0	0	0	0	0	
13626405	f	cn	US	2017	1	0	0	0	0	0	0	0	0	0	0	0	55
13626412	f	cn	US	2017	1	0	0	0	0	0	0	0	0	0	0	0	
13626416	m	md	US	2017	1	0	0	0	0	0	0	0	0	0	0	0	78
13626426	f	md	US	2017	1	0	0	0	0	0	0	0	0	0	0	1	
13626431	m	cn	US	2017	1	0	0	0	0	0	0	0	0	0	0	0	63
13626458	f	cn	US	2017	1	0	0	0	0	0	0	0	0	0	0	0	79
13626465	Not	ot	US	2017	1	0	0	0	0	0	0	0	0	0	0	0	
13626482	f	md	US	2017	1	0	0	0	0	0	0	0	0	0	0	0	
13626503	m	cn	US	2017	1	0	0	0	0	0	0	0	0	0	0	0	56
13626531	f	md	US	2017	1	0	0	0	0	0	0	0	0	0	0	0	
13626618	f	cn	US	2017	1	0	0	0	0	0	0	0	0	0	0	0	61
13626619	f	md	US	2017	1	0	0	0	0	0	0	0	0	0	0	0	70
13626621	Not	cn	US	2017	1	0	0	0	0	0	0	0	0	0	0	0	
13626635	m	cn	US	2017	1	0	0	0	0	0	0	0	0	0	0	0	75
13626639	f	cn	US	2017	1	0	0	0	0	0	0	0	0	0	0	0	60
13626810	m	md	JP	2017	0	0	0	0	0	0	0	0	0	0	0	0	54
13626822	m	cn	GB	2017	1	0	0	0	0	0	0	0	0	0	0	0	91
13626939	f	cn	US	2017	1	0	0	0	0	0	0	0	0	0	0	0	33
13626964	f	cn	US	2017	1	0	0	0	0	0	0	0	0	0	0	0	82
13627138	m	cn	GB	2017	1	0	0	0	0	0	0	0	0	0	0	0	
13627171	f	cn	US	2017	1	0	0	0	0	0	0	0	0	0	0	0	

13627204	f	cn	US	2017	1	0	0	0	0	0	0	0	0	0	0	0	60
13627458	f	md	US	2017	1	0	0	0	0	0	0	0	0	0	0	0	58
13627485	f	cn	US	2017	1	0	0	0	0	0	0	0	0	0	0	0	79
13627508	m	cn	GB	2017	1	0	0	0	0	0	0	0	0	0	0	0	
13627513	f	cn	US	2017	1	0	0	0	0	0	0	0	0	0	0	0	
13627518	f	md	US	2017	1	0	0	0	0	0	0	0	0	0	0	0	71
13627533	m	cn	GB	2017	1	0	0	0	0	0	0	0	0	0	0	0	
13627677	f	cn	US	2017	0	0	0	0	0	0	0	0	0	0	0	0	
13627690	f	ot	US	2017	1	0	0	0	0	0	0	0	0	0	0	0	65
13627695	Not	md	US	2017	1	0	0	0	0	0	0	0	0	0	0	0	
13627706	f	md	US	2017	1	0	0	0	0	0	0	0	0	0	0	0	
13627746	f	cn	GB	2017	1	0	0	0	0	0	0	0	0	0	0	0	
13627770	Not	md	US	2017	1	0	0	0	0	0	0	0	0	0	0	1	
13627775	f	ph	US	2017	1	0	0	0	0	0	0	0	0	0	0	0	67
13627778	f	md	US	2017	1	0	0	0	0	0	0	0	0	0	0	0	
13627849	m	md	US	2017	1	0	0	0	0	0	0	0	0	0	0	0	
13627880	m	md	US	2017	1	0	0	0	0	0	0	0	0	0	0	0	
13627901	f	cn	US	2017	1	0	0	0	0	0	0	0	0	0	0	0	80
13627903	f	cn	US	2017	1	0	0	0	0	0	0	0	0	0	0	0	72
13627927	f	md	US	2017	1	0	0	0	0	0	0	0	0	0	0	1	
13627967	f	ot	US	2017	1	0	0	0	0	0	0	0	0	0	0	0	77
13628045	f	cn	US	2017	1	0	0	0	0	0	0	0	0	0	0	0	60
13628049	f	ot	US	2017	1	0	0	0	0	0	0	0	0	0	0	0	83
13628053	m	cn	US	2017	1	0	0	0	0	0	0	0	0	0	0	0	78
13628096	f	cn	US	2017	1	0	0	0	0	0	0	0	0	0	0	0	
13628136	m	cn	US	2017	1	0	0	0	0	0	0	0	0	0	0	0	
13628145	m	md	US	2017	1	0	0	0	0	0	0	0	0	0	0	0	81
13628179	f	md	US	2017	1	0	0	0	0	0	0	0	0	0	0	1	42
13628189	m	cn	US	2017	1	0	0	0	0	0	0	0	0	0	0	0	59
13628204	m	cn	US	2017	1	0	0	0	0	0	0	0	0	0	0	0	
13628266	f	md	US	2017	1	0	0	0	0	0	0	0	0	0	0	0	
13628272	m	md	US	2017	1	0	0	0	0	0	0	0	0	0	0	1	
13628308	m	ph	US	2017	1	0	0	0	0	0	0	0	0	0	0	0	58
13628340	m	md	US	2017	1	0	0	0	0	0	0	0	0	0	0	0	38

13628358	f	md	US	2017	1	0	0	0	0	0	0	0	0	0	0	0	65
13628364	m	md	US	2017	1	0	0	0	0	0	0	0	0	0	0	0	82
13628386	f	md	NO	2017	0	0	0	0	0	0	0	0	0	0	0	0	63
13628400	m	cn	US	2017	1	0	0	0	0	0	0	0	0	0	0	0	
13628581	f	cn	GB	2017	1	0	0	0	0	0	0	0	0	0	0	0	
13628705	f	cn	US	2017	1	0	0	0	0	0	0	0	0	0	0	0	69
13628793	f	cn	US	2017	1	0	0	0	0	0	0	0	0	0	0	0	79
13628841	Not	cn	JP	2017	0	1	0	0	1	0	0	0	0	0	0	0	
13628901	f	ph	US	2017	1	0	0	0	0	0	0	0	0	0	0	0	83
13628948	f	cn	US	2017	1	0	0	0	0	0	0	0	0	0	0	0	
13628976	m	ot	US	2017	1	0	0	0	0	0	0	0	0	0	0	0	79
13629001	f	cn	US	2017	1	0	0	0	0	0	0	0	0	0	0	0	76
13629075	m	cn	US	2017	1	0	0	0	0	0	0	0	0	0	0	0	79
13629092	f	cn	US	2017	1	0	0	0	0	0	0	0	0	0	0	0	66
13629114	m	cn	US	2017	1	0	0	0	0	0	0	0	0	0	0	0	79
13629120	m	cn	US	2017	1	0	0	0	0	0	0	0	0	0	0	0	81
13629135	m	cn	US	2017	1	0	0	0	0	0	0	0	0	0	0	0	43
13629173	f	ph	US	2017	1	0	0	0	0	0	0	0	0	0	0	0	85
13629214	f	cn	US	2017	1	0	0	0	0	0	0	0	0	0	0	0	73
13629249	f	cn	US	2017	1	0	0	0	0	0	0	0	0	0	0	0	
13629267	f	cn	US	2017	1	0	0	0	0	0	0	0	0	0	0	0	62
13629271	m	cn	US	2017	1	0	0	0	0	0	0	0	0	0	0	0	70
13629288	f	ph	US	2017	1	0	0	0	0	0	0	0	0	0	0	0	72
13629293	m	md	US	2017	1	0	0	0	0	0	0	0	0	0	0	0	81
13629314	m	md	US	2017	1	0	0	0	0	0	0	0	0	0	0	0	
13629316	f	md	US	2017	1	0	0	0	0	0	0	0	0	0	0	0	
13629325	f	cn	US	2017	1	0	0	0	0	0	0	0	0	0	0	0	
13629336	m	cn	US	2017	1	0	0	0	0	0	0	0	0	0	0	0	80
13629344	f	cn	US	2017	1	0	0	0	0	0	0	0	0	0	0	0	65
13629354	Not	ot	CN	2017	0	0	0	0	0	0	0	0	0	0	0	0	
13629372	m	cn	US	2017	1	0	0	0	0	0	0	0	0	0	0	0	55
13629410	f	ot	US	2017	1	0	0	0	0	0	0	0	0	0	0	0	63
13629412	f	md	US	2017	1	0	0	0	0	0	0	0	0	0	0	0	76
13629417	f	ot	US	2017	1	0	0	0	0	0	0	0	0	0	0	0	

13629424	f	cn	US	2017	1	0	0	0	0	0	0	0	0	0	0	0	78
13629443	m	cn	US	2017	1	0	0	0	0	0	0	0	0	0	0	0	
13629473	f	md	US	2017	1	0	0	0	0	0	0	0	0	0	0	0	60
13629482	f	cn	US	2017	1	0	0	0	0	0	0	0	0	0	0	0	65
13629484	m	md	US	2017	1	0	0	0	0	0	0	0	0	0	0	0	66
13629489	f	ot	US	2017	1	0	0	0	0	0	0	0	0	0	0	0	
13629492	f	cn	US	2017	1	0	0	0	0	0	0	0	0	0	0	0	69
13629493	m	md	US	2017	1	0	0	0	0	0	0	0	0	0	0	0	
13629496	f	ot	US	2017	1	0	0	0	0	0	0	0	0	0	0	0	73
13629501	f	cn	US	2017	1	0	0	0	0	0	0	0	0	0	0	0	35
13629504	m	cn	US	2017	1	0	0	0	0	0	0	0	0	0	0	0	87
13629509	f	cn	US	2017	1	0	0	0	0	0	0	0	0	0	0	0	
13629510	f	md	US	2017	1	0	0	0	0	0	0	0	0	0	0	0	75
13629514	m	md	US	2017	1	0	0	0	0	0	0	0	0	0	0	0	74
13629521	f	cn	US	2017	1	0	0	0	0	0	0	0	0	0	0	0	
13629524	f	cn	US	2017	1	0	0	0	0	0	0	0	0	0	0	0	
13629531	f	cn	US	2017	1	0	0	0	0	0	0	0	0	0	0	0	
13629532	f	cn	US	2017	1	0	0	0	0	0	0	0	0	0	0	0	62
13629534	f	md	US	2017	1	0	0	0	0	0	0	0	0	0	0	0	
13629538	Not	ph	US	2017	1	0	0	0	0	0	0	0	0	0	0	0	
13629541	m	cn	US	2017	1	0	0	0	0	0	0	0	0	0	0	0	
13629544	f	cn	US	2017	1	0	0	0	0	0	0	0	0	0	0	0	
13629545	m	cn	US	2017	1	0	0	0	0	0	0	0	0	0	0	0	
13629559	f	cn	US	2017	1	0	0	0	0	0	0	0	0	0	0	0	
13629560	f	ph	US	2017	1	0	0	0	0	0	0	0	0	0	0	0	64
13629567	f	md	US	2017	1	0	0	0	0	0	0	0	0	0	0	0	64
13629568	f	cn	US	2017	1	0	0	0	0	0	0	0	0	0	0	0	
13629572	Not	ot	US	2017	1	0	0	0	0	0	0	0	0	0	0	0	
13629582	m	ot	US	2017	1	0	0	0	0	0	0	0	0	0	0	0	71
13629584	m	ot	US	2017	1	0	0	0	0	0	0	0	0	0	0	0	
13629599	m	cn	US	2017	1	0	0	0	0	0	0	0	0	0	0	0	76
13629607	f	ot	US	2017	1	0	0	0	0	0	0	0	0	0	0	0	
13629633	Not	cn	US	2017	0	0	0	0	0	0	0	0	0	0	0	0	
13629641	f	cn	US	2017	1	0	0	0	0	0	0	0	0	0	0	0	

13629650	Not	ph	US	2017	1	0	0	0	0	0	0	0	0	0	0	0	
13629653	f	md	US	2017	1	0	0	0	0	0	0	0	0	0	0	0	
13629671	Not	md	US	2017	1	0	0	0	0	0	0	0	0	0	0	0	
13629715	f	cn	US	2017	1	0	0	0	0	0	0	0	0	0	0	0	55
13629718	m	ot	US	2017	1	0	0	0	0	0	0	0	0	0	0	0	
13629720	f	cn	GB	2017	1	0	0	0	0	0	0	0	0	0	0	0	
13629755	m	ph	US	2017	1	0	0	0	0	0	0	0	0	0	0	0	
13629757	f	cn	US	2017	1	0	0	0	0	0	0	0	0	0	0	0	48
13629798	f	cn	US	2017	1	0	0	0	0	0	0	0	0	0	0	0	
13629819	f	cn	US	2017	1	0	0	0	0	0	0	0	0	0	0	0	
13629828	m	cn	US	2017	1	0	0	0	0	0	0	0	0	0	0	0	
13629831	f	cn	US	2017	1	0	0	0	0	0	0	0	0	0	0	0	
13629836	f	cn	US	2017	1	0	0	0	0	0	0	0	0	0	0	0	
13629839	f	cn	US	2017	1	0	0	0	0	0	0	0	0	0	0	0	
13629848	f	cn	US	2017	1	0	0	0	0	0	0	0	0	0	0	0	54
13629855	f	ot	US	2017	1	0	0	0	0	0	0	0	0	0	0	0	
13629871	f	cn	US	2017	1	0	0	0	0	0	0	0	0	0	0	0	
13629880	f	ot	US	2017	1	0	0	0	0	0	0	0	0	0	0	0	
13629884	m	ot	US	2017	1	0	0	0	0	0	0	0	0	0	0	0	
13629885	Not	md	US	2017	1	0	0	0	0	0	0	0	0	0	0	0	
13629889	f	cn	US	2017	1	0	0	0	0	0	0	0	0	0	0	0	
13629902	f	cn	US	2017	1	0	0	0	0	0	0	0	0	0	0	0	55
13629906	m	cn	US	2017	1	0	0	0	0	0	0	0	0	0	0	0	60
13629908	f	cn	US	2017	1	0	0	0	0	0	0	0	0	0	0	0	
13629916	m	cn	US	2017	1	0	0	0	0	0	0	0	0	0	0	0	
13629919	m	md	US	2017	1	0	0	0	0	0	0	0	0	0	0	0	
13629922	m	cn	US	2017	1	0	0	0	0	0	0	0	0	0	0	0	
13629935	Not	cn	US	2017	1	0	0	0	0	0	0	0	0	0	0	0	
13629936	f	cn	US	2017	1	0	0	0	0	0	0	0	0	0	0	0	
13629944	f	cn	US	2017	1	0	0	0	0	0	0	0	0	0	0	0	
13629962	m	cn	US	2017	1	0	0	0	0	0	0	0	0	0	0	0	
13629966	m	cn	US	2017	1	0	0	0	0	0	0	0	0	0	0	0	
13629975	m	cn	US	2017	1	0	0	0	0	0	0	0	0	0	0	0	
13629979	f	ph	US	2017	1	0	0	0	0	0	0	0	0	0	0	0	

13629995	f	cn	US	2017	1	0	0	0	0	0	0	0	0	0	0	0	
13629996	m	cn	US	2017	1	0	0	0	0	0	0	0	0	0	0	0	
13630008	f	ot	US	2017	1	0	0	0	0	0	0	0	0	0	0	0	
13630027	f	cn	US	2017	1	0	0	0	0	0	0	0	0	0	0	0	61
13630036	f	md	US	2017	1	0	0	0	0	0	0	0	0	0	0	0	
13630037	m	cn	US	2017	1	0	0	0	0	0	0	0	0	0	0	0	
13630080	f	cn	US	2017	1	0	0	0	0	0	0	0	0	0	0	0	
13630108	f	cn	US	2017	1	0	0	0	0	0	0	0	0	0	0	0	
13630134	f	cn	US	2017	1	0	0	0	0	0	0	0	0	0	0	0	
13630137	m	cn	US	2017	1	0	0	0	0	0	0	0	0	0	0	0	
13630142	f	cn	US	2017	1	0	0	0	0	0	0	0	0	0	0	0	
13630148	f	md	US	2017	1	0	0	0	0	0	0	0	0	0	0	0	
13630151	f	ph	US	2017	1	0	0	0	0	0	0	0	0	0	0	0	
13630164	f	cn	US	2017	1	0	0	0	0	0	0	0	0	0	0	0	
13630168	f	ot	US	2017	1	0	0	0	0	0	0	0	0	0	0	0	
13630172	f	cn	US	2017	1	0	0	0	0	0	0	0	0	0	0	0	
13630175	m	cn	US	2017	1	0	0	0	0	0	0	0	0	0	0	0	
13630213	f	ot	US	2017	1	0	0	0	0	0	0	0	0	0	0	0	
13630222	f	ot	US	2017	1	0	0	0	0	0	0	0	0	0	0	0	
13630247	m	cn	US	2017	1	0	0	0	0	0	0	0	0	0	0	0	
13630263	f	cn	US	2017	1	0	0	0	0	0	0	0	0	0	0	0	66
13630266	f	cn	US	2017	1	0	0	0	0	0	0	0	0	0	0	0	
13630272	m	cn	US	2017	1	0	0	0	0	0	0	0	0	0	0	0	
13630282	f	cn	US	2017	1	0	0	0	0	0	0	0	0	0	0	0	65
13630290	f	cn	US	2017	1	0	0	0	0	0	0	0	0	0	0	0	88
13630292	f	cn	US	2017	1	0	0	0	0	0	0	0	0	0	0	0	
13630296	f	cn	US	2017	1	0	0	0	0	0	0	0	0	0	0	0	
13630329	m	cn	US	2017	1	0	0	0	0	0	0	0	0	0	0	0	
13630368	f	cn	US	2017	1	0	0	0	0	0	0	0	0	0	0	0	74
13630376	f	ot	US	2017	1	0	0	0	0	0	0	0	0	0	0	0	
13630440	Not	cn	US	2017	1	0	0	0	0	0	0	0	0	0	0	0	
13630442	m	cn	US	2017	1	0	0	0	0	0	0	0	0	0	0	0	
13630476	Not	cn	US	2017	1	0	0	0	0	0	0	0	0	0	0	0	
13630481	f	cn	US	2017	1	0	0	0	0	0	0	0	0	0	0	0	

13630496	m	cn	US	2017	1	0	0	0	0	0	0	0	0	0	0	0		
13630514	f	cn	US	2017	1	0	0	0	0	0	0	0	0	0	0	0		
13630553	m	ot	US	2017	1	0	0	0	0	0	0	0	0	0	0	0	74	
13630613	Not	cn	US	2017	1	0	0	0	0	0	0	0	0	0	0	0	85	
13630648	f	md	US	2017	1	0	0	0	0	0	0	0	0	0	0	0		
13630655	f	cn	US	2017	1	0	0	0	0	0	0	0	0	0	0	0		
13630739	f	cn	US	2017	1	0	0	0	0	0	0	0	0	0	0	0	73	
13630770	m	cn	US	2017	1	0	0	0	0	0	0	0	0	0	0	0	46	
13630773	m	cn	US	2017	1	0	0	0	0	0	0	0	0	0	0	0		
13630775	m	cn	US	2017	1	0	0	0	0	0	0	0	0	0	0	0	58	
13630790	f	cn	US	2017	1	0	0	0	0	0	0	0	0	0	0	0		
13630797	f	cn	US	2017	1	0	0	0	0	0	0	0	0	0	0	0		
13630812	m	cn	US	2017	1	0	0	0	0	0	0	0	0	0	0	0		
13630825	f	cn	US	2017	1	0	0	0	0	0	0	0	0	0	0	0		
13630854	f	cn	US	2017	1	0	0	0	0	0	0	0	0	0	0	1		
13630863	f	ot	US	2017	1	0	0	0	0	0	0	0	0	0	0	0		
13630906	m	cn	US	2017	1	0	0	0	0	0	0	0	0	0	0	0		
13630909	m	cn	US	2017	1	0	0	0	0	0	0	0	0	0	0	0		
13630921	m	cn	US	2017	1	0	0	0	0	0	0	0	0	0	0	0		
13630931	Not	ot	US	2017	1	0	0	0	0	0	0	0	0	0	0	0		
13630956	Not	ot	US	2017	1	0	0	0	0	0	0	0	0	0	0	0		
13630999	m	ph	US	2017	1	0	0	0	0	0	0	0	0	0	0	0		
13631025	Not	md	US	2017	1	0	0	0	0	0	0	0	0	0	0	0		
13631032	f	cn	US	2017	1	0	0	0	0	0	0	0	0	0	0	0	82	
13631056	f	cn	US	2017	1	0	0	0	0	0	0	0	0	0	0	0		
13631060	f	md	US	2017	1	0	0	0	0	0	0	0	0	0	0	0		
13631113	f	cn	US	2017	1	0	0	0	0	0	0	0	0	0	0	0		
13631132	f	md	US	2017	1	0	0	0	0	0	0	0	0	0	0	0	45	
13631146	f	ot	US	2017	1	0	0	0	0	0	0	0	0	0	0	0		
13631150	f	md	US	2017	1	0	0	0	0	0	0	0	0	0	0	0		
13631165	m	ot	US	2017	1	0	0	0	0	0	0	0	0	0	0	0		
13631170	f	cn	US	2017	1	0	0	0	0	0	0	0	0	0	0	0		
13631190	f	cn	US	2017	1	0	0	0	0	0	0	0	0	0	0	0		
13631202	m	cn	US	2017	1	0	0	0	0	0	0	0	0	0	0	0	64	

13631213	m	cn	US	2017	1	0	0	0	0	0	0	0	0	0	0	0	
13631215	m	cn	US	2017	1	0	0	0	0	0	0	0	0	0	0	0	
13631223	f	cn	US	2017	1	0	0	0	0	0	0	0	0	0	0	0	67
13631246	f	cn	US	2017	1	0	0	0	0	0	0	0	0	0	0	0	80
13631250	f	cn	US	2017	1	0	0	0	0	0	0	0	0	0	0	0	66
13631251	f	ot	US	2017	1	0	0	0	0	0	0	0	0	0	0	0	
13631258	f	md	US	2017	1	0	0	0	0	0	0	0	0	0	0	0	60
13631271	m	md	US	2017	1	0	0	0	0	0	0	0	0	0	0	0	
13631332	m	md	US	2017	1	0	0	0	0	0	0	0	0	0	0	0	
13631342	m	cn	US	2017	1	0	0	0	0	0	0	0	0	0	0	0	
13631361	f	cn	US	2017	1	0	0	0	0	0	0	0	0	0	0	0	74
13631425	m	md	TR	2017	0	0	0	0	0	0	0	0	0	0	0	0	38
13631426	f	ot	US	2017	1	0	0	0	0	0	0	0	0	0	0	0	
13631442	f	cn	US	2017	1	0	0	0	0	0	0	0	0	0	0	0	
13631449	f	md	US	2017	1	0	0	0	0	0	0	0	0	0	0	0	
13631463	f	md	US	2017	1	0	0	0	0	0	0	0	0	0	0	0	
13631466	f	cn	US	2017	1	0	0	0	0	0	0	0	0	0	0	0	
13631480	m	cn	US	2017	1	0	0	0	0	0	0	0	0	0	0	0	
13631485	f	ot	US	2017	1	0	0	0	0	0	0	0	0	0	0	0	
13631506	f	cn	US	2017	1	0	0	0	0	0	0	0	0	0	0	0	
13631521	m	cn	US	2017	1	0	0	0	0	0	0	0	0	0	0	0	
13631524	f	cn	US	2017	1	0	0	0	0	0	0	0	0	0	0	0	
13631559	f	cn	US	2017	1	0	0	0	0	0	0	0	0	0	0	0	
13631564	Not	md	US	2017	1	0	0	0	0	0	0	0	0	0	0	0	
13631570	f	ot	US	2017	1	0	0	0	0	0	0	0	0	0	0	0	66
13631573	m	cn	US	2017	1	0	0	0	0	0	0	0	0	0	0	0	
13631584	m	cn	US	2017	1	0	0	0	0	0	0	0	0	0	0	0	
13631610	f	md	US	2017	1	0	0	0	0	0	0	0	0	0	0	0	
13631616	f	cn	US	2017	1	0	0	0	0	0	0	0	0	0	0	0	
13631617	m	ot	US	2017	0	1	0	0	1	0	0	0	0	0	0	0	63
13631621	m	cn	US	2017	1	0	0	0	0	0	0	0	0	0	0	0	
13631645	m	cn	US	2017	1	0	0	0	0	0	0	0	0	0	0	0	29
13631684	f	cn	US	2017	1	0	0	0	0	0	0	0	0	0	0	0	
13631721	f	cn	US	2017	1	0	0	0	0	0	0	0	0	0	0	0	81

13631753	m	ot	US	2017	1	0	0	0	0	0	0	0	0	0	0	0	
13631757	m	cn	US	2017	1	0	0	0	0	0	0	0	0	0	0	0	53
13631769	m	cn	US	2017	1	0	0	0	0	0	0	0	0	0	0	0	63
13631775	m	cn	US	2017	1	0	0	0	0	0	0	0	0	0	0	0	
13631778	m	cn	US	2017	1	0	0	0	0	0	0	0	0	0	0	0	
13631801	m	cn	US	2017	1	0	0	0	0	0	0	0	0	0	0	0	68
13631811	f	cn	US	2017	1	0	0	0	0	0	0	0	0	0	0	0	
13631815	f	md	US	2017	1	0	0	0	0	0	0	0	0	0	0	0	63
13631846	Not	ot	US	2017	1	0	0	0	0	0	0	0	0	0	0	0	
13631860	f	cn	US	2017	1	0	0	0	0	0	0	0	0	0	0	0	
13631880	f	cn	US	2017	1	0	0	0	0	0	0	0	0	0	0	0	71
13631889	m	cn	US	2017	1	0	0	0	0	0	0	0	0	0	0	0	67
13631900	m	md	US	2017	1	0	0	0	0	0	0	0	0	0	0	0	
13631910	f	cn	US	2017	1	0	0	0	0	0	0	0	0	0	0	0	
13631916	f	cn	US	2017	1	0	0	0	0	0	0	0	0	0	0	0	86
13631923	Not	md	US	2017	1	0	0	0	0	0	0	0	0	0	0	0	
13631942	f	cn	US	2017	1	0	0	0	0	0	0	0	0	0	0	0	
13631949	m	cn	US	2017	1	0	0	0	0	0	0	0	0	0	0	0	69
13631953	f	md	PL	2017	1	0	0	0	0	0	0	0	0	0	0	0	69
13631956	m	cn	US	2017	1	0	0	0	0	0	0	0	0	0	0	0	
13631972	Not	cn	US	2017	1	0	0	0	0	0	0	0	0	0	0	0	
13632003	f	md	US	2017	1	0	0	0	0	0	0	0	0	0	0	0	
13632021	m	cn	US	2017	1	0	0	0	0	0	0	0	0	0	0	0	
13632023	f	ph	US	2017	1	0	0	0	0	0	0	0	0	0	0	0	
13632033	f	cn	US	2017	1	0	0	0	0	0	0	0	0	0	0	0	
13632131	f	cn	US	2017	1	0	0	0	0	0	0	0	0	0	0	0	
13632152	m	ph	US	2017	1	0	0	0	0	0	0	0	0	0	0	0	70
13632164	m	md	US	2017	1	0	0	0	0	0	0	0	0	0	0	0	
13632169	f	md	US	2017	1	0	0	0	0	0	0	0	0	0	0	0	
13632179	Not	cn	US	2017	1	0	0	0	0	0	0	0	0	0	0	0	
13632194	m	cn	US	2017	1	0	0	0	0	0	0	0	0	0	0	0	41
13632201	m	cn	US	2017	1	0	0	0	0	0	0	0	0	0	0	0	70
13632218	m	cn	US	2017	1	0	0	0	0	0	0	0	0	0	0	0	69
13632219	f	cn	US	2017	1	0	0	0	0	0	0	0	0	0	0	0	66

13632247	Not	ot	US	2017	1	0	0	0	0	0	0	0	0	0	0	0	
13632267	f	ot	US	2017	1	0	0	0	0	0	0	0	0	0	0	0	62
13632328	Not	md	US	2017	1	0	0	0	0	0	0	0	0	0	0	0	
13632338	f	cn	US	2017	1	0	0	0	0	0	0	0	0	0	0	0	79
13632353	f	md	US	2017	1	0	0	0	0	0	0	0	0	0	0	0	86
13632384	m	ph	US	2017	1	0	0	0	0	0	0	0	0	0	0	0	70
13632388	f	cn	US	2017	1	0	0	0	0	0	0	0	0	0	0	0	
13632417	f	md	US	2017	1	0	0	0	0	0	0	0	0	0	0	0	
13632424	m	cn	US	2017	1	0	0	0	0	0	0	0	0	0	0	0	
13632448	m	cn	US	2017	1	0	0	0	0	0	0	0	0	0	0	0	54
13632500	f	cn	US	2017	1	0	0	0	0	0	0	0	0	0	0	0	
13632533	m	md	US	2017	1	0	0	0	0	0	0	0	0	0	0	0	
13632559	f	md	US	2017	1	0	0	0	0	0	0	0	0	0	0	0	
13632570	f	ot	US	2017	1	0	0	0	0	0	0	0	0	0	0	0	
13632582	f	cn	US	2017	1	0	0	0	0	0	0	0	0	0	0	0	
13632600	f	cn	US	2017	1	0	0	0	0	0	0	0	0	0	0	0	81
13632621	m	cn	US	2017	1	0	0	0	0	0	0	0	0	0	0	0	83
13632624	m	md	US	2017	1	0	0	0	0	0	0	0	0	0	0	0	
13632627	Not	md	US	2017	1	0	0	0	0	0	0	0	0	0	0	0	
13632676	f	cn	US	2017	1	0	0	0	0	0	0	0	0	0	0	0	82
13632703	Not	cn	US	2017	1	0	0	0	0	0	0	0	0	0	0	0	
13632716	m	cn	US	2017	1	0	0	0	0	0	0	0	0	0	0	0	
13632774	Not	cn	US	2017	1	0	0	0	0	0	0	0	0	0	0	0	
13632792	Not	cn	US	2017	1	0	0	0	0	0	0	0	0	0	0	0	
13632857	m	cn	US	2017	1	0	0	0	0	0	0	0	0	0	0	0	
13632894	f	cn	US	2017	1	0	0	0	0	0	0	0	0	0	0	0	
13632919	f	cn	US	2017	1	0	0	0	0	0	0	0	0	0	0	0	87
13632936	f	ph	US	2017	1	0	0	0	0	0	0	0	0	0	0	0	
13632983	m	cn	US	2017	1	0	0	0	0	0	0	0	0	0	0	0	
13633024	f	ot	US	2017	1	0	0	0	0	0	0	0	0	0	0	0	
13633054	m	md	US	2017	1	0	0	0	0	0	0	0	0	0	0	0	67
13633066	f	ot	US	2017	1	0	0	0	0	0	0	0	0	0	0	0	
13633080	f	cn	US	2017	1	0	0	0	0	0	0	0	0	0	0	0	
13633121	f	cn	US	2017	1	0	0	0	0	0	0	0	0	0	0	0	34

13633130	f	cn	US	2017	1	0	0	0	0	0	0	0	0	0	0	0	
13633137	m	cn	US	2017	1	0	0	0	0	0	0	0	0	0	0	0	58
13633142	f	cn	US	2017	1	0	0	0	0	0	0	0	0	0	0	0	
13633158	f	cn	US	2017	1	0	0	0	0	0	0	0	0	0	0	0	83
13633173	f	cn	US	2017	1	0	0	0	0	0	0	0	0	0	0	0	
13633190	f	cn	US	2017	0	0	0	0	0	0	0	0	0	0	0	0	
13633217	f	ot	US	2017	1	0	0	0	0	0	0	0	0	0	0	0	
13633219	f	cn	US	2017	1	0	0	0	0	0	0	0	0	0	0	0	
13633231	f	cn	US	2017	1	0	0	0	0	0	0	0	0	0	0	0	41
13633272	Not	md	US	2017	1	0	0	0	0	0	0	0	0	0	0	0	
13633293	f	cn	US	2017	1	0	0	0	0	0	0	0	0	0	0	0	76
13633313	m	ot	US	2017	1	0	0	0	0	0	0	0	0	0	0	0	
13633341	m	ot	US	2017	1	0	0	0	0	0	0	0	0	0	0	0	
13633376	f	ot	US	2017	1	0	0	0	0	0	0	0	0	0	0	0	57
13633387	f	cn	US	2017	1	0	0	0	0	0	0	0	0	0	0	0	
13633403	f	cn	US	2017	1	0	0	0	0	0	0	0	0	0	0	0	
13633475	f	cn	US	2017	1	0	0	0	0	0	0	0	0	0	0	0	
13633504	Not	md	US	2017	1	0	0	0	0	0	0	0	0	0	0	0	
13633558	Not	ot	US	2017	1	0	0	0	0	0	0	0	0	0	0	0	
13633560	Not	md	US	2017	1	0	0	0	0	0	0	0	0	0	0	0	
13633584	f	ot	US	2017	1	0	0	0	0	0	0	0	0	0	0	0	
13633595	m	cn	US	2017	1	0	0	0	0	0	0	0	0	0	0	0	
13633698	f	cn	US	2017	1	0	0	0	0	0	0	0	0	0	0	0	76
13633730	m	cn	US	2017	1	0	0	0	0	0	0	0	0	0	0	0	
13633902	f	cn	US	2017	1	0	0	0	0	0	0	0	0	0	0	0	
13633991	Not	ot	US	2017	1	0	0	0	0	0	0	0	0	0	0	0	
13634007	m	cn	US	2017	1	0	0	0	0	0	0	0	0	0	0	0	63
13634022	m	md	US	2017	1	0	0	0	0	0	0	0	0	0	0	0	
13634047	m	md	US	2017	1	0	0	0	0	0	0	0	0	0	0	0	
13634213	m	ot	US	2017	0	0	0	0	0	0	0	0	0	0	0	0	68
13634237	f	cn	US	2017	1	0	0	0	0	0	0	0	0	0	0	0	
13634246	m	cn	US	2017	1	0	0	0	0	0	0	0	0	0	0	0	63
13634335	f	cn	US	2017	1	0	0	0	0	0	0	0	0	0	0	0	77
13634352	f	md	US	2017	1	0	0	0	0	0	0	0	0	0	0	0	63

13634595	m	cn	US	2017	1	0	0	0	0	0	0	0	0	0	0	0	49
13634686	m	cn	US	2017	1	0	0	0	0	0	0	0	0	0	0	0	70
13634741	f	cn	US	2017	1	0	0	0	0	0	0	0	0	0	0	0	
13634762	f	ot	US	2017	1	0	0	0	0	0	0	0	0	0	0	0	
13634770	f	cn	US	2017	1	0	0	0	0	0	0	0	0	0	0	0	
13634805	f	md	US	2017	1	0	0	0	0	0	0	0	0	0	0	0	
13634810	f	cn	US	2017	1	0	0	0	0	0	0	0	0	0	0	0	
13634843	Not	md	US	2017	1	0	0	0	0	0	0	0	0	0	0	0	
13634858	f	ot	US	2017	1	0	0	0	0	0	0	0	0	0	0	0	72
13635075	f	cn	US	2017	1	0	0	0	0	0	0	0	0	0	0	0	
13635186	f	cn	US	2017	1	0	0	0	0	0	0	0	0	0	0	0	
13635193	f	cn	US	2017	1	0	0	0	0	0	0	0	0	0	0	0	
13635218	f	cn	US	2017	1	0	0	0	0	0	0	0	0	0	0	0	80
13635254	m	cn	US	2017	1	0	0	0	0	0	0	0	0	0	0	0	77
13635311	f	md	US	2017	0	0	0	0	0	0	0	0	0	0	0	0	77
13635363	f	ot	US	2017	1	0	0	0	0	0	0	0	0	0	0	0	
13635371	f	md	US	2017	1	0	0	0	0	0	0	0	0	0	0	0	
13635381	f	cn	US	2017	1	0	0	0	0	0	0	0	0	0	0	0	
13635557	f	md	US	2017	1	0	0	0	0	0	0	0	0	0	0	0	
13635581	m	cn	US	2017	1	0	0	0	0	0	0	0	0	0	0	0	
13635593	f	ot	US	2017	1	0	0	0	0	0	0	0	0	0	0	0	
13635608	m	cn	US	2017	1	0	0	0	0	0	0	0	0	0	0	0	
13635630	m	cn	US	2017	1	0	0	0	0	0	0	0	0	0	0	0	
13635652	f	md	US	2017	1	0	0	0	0	0	0	0	0	0	0	0	
13635715	f	cn	US	2017	1	0	0	0	0	0	0	0	0	0	0	0	
13635717	m	cn	US	2017	1	0	0	0	0	0	0	0	0	0	0	0	
13635718	m	cn	US	2017	1	0	0	0	0	0	0	0	0	0	0	0	
13635723	f	cn	US	2017	1	0	0	0	0	0	0	0	0	0	0	0	
13635725	f	cn	US	2017	1	0	0	0	0	0	0	0	0	0	0	0	
13635744	f	cn	US	2017	1	0	0	0	0	0	0	0	0	0	0	0	75
13635753	f	cn	US	2017	1	0	0	0	0	0	0	0	0	0	0	0	
13635778	f	cn	US	2017	1	0	0	0	0	0	0	0	0	0	0	0	
13635787	Not	md	US	2017	1	0	0	0	0	0	0	0	0	0	0	0	
13635804	f	cn	US	2017	1	0	0	0	0	0	0	0	0	0	0	0	

13635829	m	ot	US	2017	1	0	0	0	0	0	0	0	0	0	0	0	
13635872	f	cn	US	2017	1	0	0	0	0	0	0	0	0	0	0	0	65
13635873	f	ot	US	2017	1	0	0	0	0	0	0	0	0	0	0	0	86
13635890	m	cn	US	2017	1	0	0	0	0	0	0	0	0	0	0	0	
13635931	f	cn	US	2017	1	0	0	0	0	0	0	0	0	0	0	0	74
13635963	m	ph	US	2017	1	0	0	0	0	0	0	0	0	0	0	0	
13635993	f	cn	US	2017	1	0	0	0	0	0	0	0	0	0	0	0	
13636028	f	cn	US	2017	1	0	0	0	0	0	0	0	0	0	0	0	
13636073	m	cn	US	2017	1	0	0	0	0	0	0	0	0	0	0	0	
13636114	m	cn	US	2017	1	0	0	0	0	0	0	0	0	0	0	0	
13636165	Not	ph	US	2017	1	0	0	0	0	0	0	0	0	0	0	0	
13636225	f	cn	US	2017	1	0	0	0	0	0	0	0	0	0	0	0	
13636241	m	cn	US	2017	1	0	0	0	0	0	0	0	0	0	0	0	54
13636296	f	cn	US	2017	1	0	0	0	0	0	0	0	0	0	0	0	67
13636303	f	md	US	2017	1	0	0	0	0	0	0	0	0	0	0	0	
13636309	f	md	US	2017	1	0	0	0	0	0	0	0	0	0	0	0	
13636329	Not	cn	US	2017	1	0	0	0	0	0	0	0	0	0	0	0	
13636352	m	cn	US	2017	1	0	0	0	0	0	0	0	0	0	0	0	64
13636437	m	cn	AE	2017	0	0	0	0	0	0	0	0	0	0	0	0	70
13636439	Not	ph	US	2017	1	0	0	0	0	0	0	0	0	0	0	0	
13636526	m	cn	US	2017	1	0	0	0	0	0	0	0	0	0	0	0	
13636546	m	ph	US	2017	1	0	0	0	0	0	0	0	0	0	0	0	38
13636550	f	ph	US	2017	1	0	0	0	0	0	0	0	0	0	0	0	
13636618	f	cn	US	2017	1	0	0	0	0	0	0	0	0	0	0	0	
13636759	m	ot	US	2017	1	0	0	0	0	0	0	0	0	0	0	0	55
13636760	f	cn	US	2017	1	0	0	0	0	0	0	0	0	0	0	0	
13636800	f	ph	US	2017	1	0	0	0	0	0	0	0	0	0	0	0	
13636816	Not	ot	US	2017	1	0	0	0	0	0	0	0	0	0	0	0	
13636839	m	cn	US	2017	1	0	0	0	0	0	0	0	0	0	0	0	
13636873	m	ot	US	2017	1	0	0	0	0	0	0	0	0	0	0	0	65
13636884	f	ot	US	2017	1	0	0	0	0	0	0	0	0	0	0	0	70
13636911	f	md	US	2017	0	0	0	0	0	0	0	0	0	0	0	0	
13636916	m	md	US	2017	1	0	0	0	0	0	0	0	0	0	0	0	75
13636958	f	cn	US	2017	1	0	0	0	0	0	0	0	0	0	0	0	72

13637251	f	cn	GB	2017	1	0	0	0	0	0	0	0	0	0	0	0	
13637658	f	Not repo	AU	2017	1	0	0	0	0	0	0	0	0	0	0	0	
13637689	f	cn	US	2017	0	0	0	0	0	0	0	0	0	0	0	0	
13637916	m	cn	GB	2017	1	0	0	0	0	0	0	0	0	0	0	0	
13637930	f	md	DE	2017	1	0	0	0	0	0	0	0	0	0	0	0	
13638180	m	md	NL	2017	0	0	0	0	0	0	0	0	0	0	0	0	71
13638293	m	md	JP	2017	0	1	0	1	0	0	0	0	0	0	0	0	69
13638527	Not	cn	JP	2017	0	0	0	0	0	0	0	0	0	0	0	0	
13638750	m	cn	US	2017	0	0	0	0	0	0	0	0	0	0	0	0	55
13638876	f	cn	US	2017	0	1	1	0	0	0	0	0	0	0	0	0	70
13640364	Not	cn	US	2017	0	1	1	0	0	0	0	0	0	0	0	0	
13640493	Not	ot	IT	2017	0	0	0	0	0	0	0	0	0	0	0	0	
13640519	m	md	DE	2017	0	0	0	0	0	0	0	0	0	0	0	0	67
13640556	m	cn	BR	2017	1	0	0	0	0	0	0	0	0	0	0	0	
13640831	f	ot	SG	2017	1	0	0	0	0	0	0	0	0	0	0	0	54
13640916	m	md	JP	2017	0	1	0	0	1	0	0	0	0	0	0	0	61
13640937	f	md	US	2017	0	1	0	0	1	0	0	0	0	0	0	0	
13641122	m	md	US	2017	1	0	0	0	0	0	0	0	0	0	0	0	70
13641330	f	md	JP	2017	0	1	0	0	1	0	0	0	0	0	0	0	74
13641410	f	cn	GB	2017	0	1	1	0	0	0	0	0	0	0	0	0	
13641779	m	cn	GB	2017	1	0	0	0	0	0	0	0	0	0	0	0	
13641783	m	cn	GB	2017	1	0	0	0	0	0	0	0	0	0	0	0	
13642239	m	cn	GB	2017	1	0	0	0	0	0	0	0	0	0	0	0	62
13642313	f	cn	GB	2017	1	0	0	0	0	0	0	0	0	0	0	0	
13642809	m	cn	US	2017	0	1	0	0	1	0	0	0	0	0	0	0	
13643151	f	cn	GB	2017	1	0	0	0	0	0	0	0	0	0	0	0	62
13643171	Not	cn	US	2017	0	1	1	0	0	0	0	0	0	0	0	0	
13643331	f	md	FR	2017	0	1	0	1	0	0	0	0	0	0	0	0	64
13643460	m	md	GB	2017	0	0	0	0	0	0	0	0	0	0	0	0	69
13643585	f	cn	GB	2017	1	0	0	0	0	0	0	0	0	0	0	0	65
13643919	m	ot	CH	2017	0	1	0	0	1	0	0	0	0	0	0	0	65
13645537	f	md	CN	2017	0	0	0	0	0	0	0	0	0	0	0	0	
13645723	f	ph	CA	2017	1	0	0	0	0	0	0	0	0	0	0	0	72
13645753	f	md	IT	2017	0	1	0	0	1	0	0	0	0	0	0	0	

13646117	Not	md	US	2017	0	1	0	0	1	0	0	0	0	0	0	0	
13646373	f	cn	BR	2017	1	0	0	0	0	0	0	0	0	0	0	0	
13646788	f	cn	GB	2017	1	0	0	0	0	0	0	0	0	0	0	0	62
13646799	f	Not repo	SG	2017	1	0	0	0	0	0	0	0	0	0	0	0	52
13646903	f	ot	US	2017	0	1	1	0	0	0	0	0	0	0	0	0	58
13647397	f	cn	US	2017	0	0	0	0	0	0	0	0	0	0	0	0	60
13647409	m	ot	CN	2017	1	0	0	0	0	0	0	0	0	0	0	0	54
13647438	Not	ph	AU	2017	0	1	0	0	1	0	0	0	0	0	0	0	95
13647535	m	cn	GB	2017	0	1	1	0	0	0	0	0	0	0	0	0	70
13647959	m	cn	CA	2017	0	0	0	0	0	0	0	0	0	0	0	0	43
13647984	f	cn	GB	2017	1	0	0	0	0	0	0	0	0	0	0	0	73
13648015	f	cn	GB	2017	1	0	0	0	0	0	0	0	0	0	0	0	70
13648078	m	md	FR	2017	0	1	0	0	1	0	0	0	0	0	0	0	
13648514	m	cn	JP	2017	0	1	1	0	0	0	0	0	0	0	0	1	48
13648535	f	md	JP	2017	1	0	0	0	0	0	0	0	0	0	0	0	73
13648555	m	cn	GB	2017	1	0	0	0	0	0	0	0	0	0	0	0	
13648617	f	cn	GB	2017	1	0	0	0	0	0	0	0	0	0	0	0	
13648635	m	cn	ES	2017	0	0	0	0	0	0	0	0	0	0	0	0	71
13648929	f	ot	BR	2017	0	1	0	0	1	0	0	0	0	0	0	0	73
13648930	m	ot	IE	2017	0	1	0	0	1	0	0	0	0	0	0	0	67
13649366	m	md	GB	2017	0	0	0	0	0	0	0	0	0	0	0	0	65
13649779	Not	ph	AU	2017	0	1	0	0	1	0	0	0	0	0	0	0	72
13649905	m	cn	CA	2017	0	1	0	0	1	0	0	0	0	0	0	0	
13650520	f	ot	US	2017	0	1	0	0	1	0	0	0	0	0	0	1	68
13650793	m	cn	GB	2017	1	0	0	0	0	0	0	0	0	0	0	0	
13650943	f	cn	GB	2017	1	0	0	0	0	0	0	0	0	0	0	0	
13651076	m	cn	GB	2017	1	0	0	0	0	0	0	0	0	0	0	0	
13651196	m	cn	US	2017	0	0	0	0	0	0	0	0	0	0	0	0	
13651321	f	cn	US	2017	0	0	0	0	0	0	0	0	0	0	0	0	67
13651749	Not	md	JP	2017	1	0	0	0	0	0	0	0	0	0	0	0	
13651877	m	cn	GB	2017	1	0	0	0	0	0	0	0	0	0	0	0	
13651973	m	md	JP	2017	0	1	0	0	1	0	0	0	0	0	0	0	77
13652221	f	md	GB	2017	1	0	0	0	0	0	0	0	0	0	0	0	79
13652280	f	cn	GB	2017	1	0	0	0	0	0	0	0	0	0	0	0	

13652355	f	cn	GB	2017	1	0	0	0	0	0	0	0	0	0	0	0	
13653508	f	md	FR	2017	0	1	0	1	0	0	0	0	0	0	0	0	62
13653998	f	ot	JP	2017	0	1	0	0	1	0	0	0	0	0	0	0	56
13654015	m	ot	JP	2017	0	1	0	0	1	0	0	0	0	0	0	0	71
13654892	Not	ph	AU	2017	0	1	0	0	1	0	0	0	0	0	0	0	83
13655136	m	cn	US	2017	0	0	0	0	0	0	0	0	0	0	0	0	63
13655450	m	md	JP	2017	1	0	0	0	0	0	0	0	0	0	0	0	67
13655598	f	cn	JP	2017	0	1	0	0	1	0	0	0	0	0	0	1	60
13656109	Not	ph	AU	2017	0	1	0	0	1	0	0	0	0	0	0	0	76
13656119	m	md	JP	2017	1	0	0	0	0	0	0	0	0	0	0	0	62
13656210	f	md	DE	2017	0	1	0	0	1	0	0	0	0	0	0	0	73
13656606	f	md	JP	2017	0	0	0	0	0	0	0	0	0	0	0	0	62
13656632	m	cn	DE	2017	0	1	1	0	0	0	0	0	0	0	0	0	
13656685	f	md	CN	2017	1	0	0	0	0	0	0	0	0	0	0	0	64.5
13656712	f	cn	DE	2017	0	1	1	0	0	0	0	0	0	0	0	0	70
13657142	Not	ot	US	2017	0	1	0	0	1	0	0	0	0	0	0	0	
13657386	m	md	US	2017	1	0	0	0	0	0	0	0	0	0	0	1	
13657543	m	ot	US	2017	0	1	0	0	1	0	0	0	0	0	0	0	59
13657561	m	cn	US	2017	1	0	0	0	0	0	0	0	0	0	0	0	78
13657631	m	cn	JP	2017	0	0	0	0	0	0	0	0	0	0	0	0	71
13658298	f	md	JP	2017	0	0	0	0	0	0	0	0	0	0	0	0	68
13658610	m	cn	JP	2017	0	1	1	0	0	0	0	0	0	0	0	0	56
13658714	f	md	US	2017	0	1	0	0	1	0	0	0	0	0	0	1	
13658897	m	md	JP	2017	0	1	0	0	1	0	0	0	0	0	0	1	77
13659009	m	cn	GB	2017	1	0	0	0	0	0	0	0	0	0	0	0	77
13659086	f	md	CN	2017	1	0	0	0	0	0	0	0	0	0	0	0	67.8
13659265	f	md	JP	2017	0	1	0	0	1	0	0	0	0	0	0	0	78
13659305	m	cn	JP	2017	0	1	0	0	1	0	0	0	0	0	0	0	74
13659362	f	md	JP	2017	0	0	0	0	0	0	0	0	0	0	0	0	38
13659468	m	Not repo	US	2017	1	0	0	0	0	0	0	0	0	0	0	0	74
13659683	m	ph	US	2017	0	1	1	0	0	0	0	0	0	0	0	0	67
13660214	f	ot	DE	2017	0	1	0	0	1	0	0	0	0	0	0	0	47
13660349	m	cn	GB	2017	1	0	0	0	0	0	0	0	0	0	0	0	
13660364	f	cn	GB	2017	1	0	0	0	0	0	0	0	0	0	0	0	

13660589	f	ph	GB	2017	0	0	0	0	0	0	0	0	0	0	0	0	54
13662140	m	ot	JP	2017	0	1	0	0	1	0	0	0	0	0	0	0	75
13662909	m	ot	JP	2017	0	1	0	0	1	0	0	0	0	0	0	0	69
13663109	Not	md	US	2017	0	1	0	0	1	0	0	0	0	0	0	0	
13663529	m	cn	US	2017	0	0	0	0	0	0	0	0	0	0	0	0	67
13663887	f	ph	JP	2017	1	0	0	0	0	0	0	0	0	0	0	0	86
13664046	Not	ot	AU	2017	0	1	0	0	1	0	0	0	0	0	0	1	80
13664179	m	ot	RU	2017	0	1	0	0	1	0	0	0	0	0	0	0	49
13665072	m	cn	US	2017	0	1	0	0	1	0	0	0	0	0	0	0	59
13665451	m	md	JP	2017	1	0	0	0	0	0	0	0	0	0	0	0	76
13665954	f	md	ES	2017	0	1	0	0	1	0	0	0	0	0	0	0	
13668070	f	md	US	2017	0	0	0	0	0	0	0	0	0	0	0	0	
13668107	m	md	GB	2017	0	1	0	1	0	0	0	0	0	0	0	0	76.3
13668219	m	cn	US	2017	0	1	1	0	0	0	0	0	0	0	0	0	68
13668963	f	md	BE	2017	0	0	0	0	0	0	0	0	0	0	0	0	
13669207	m	cn	US	2017	0	0	0	0	0	0	0	0	0	0	0	0	79
13669470	f	Not repo	CA	2017	0	0	0	0	0	0	1	0	1	0	0	0	
13669877	f	md	US	2017	1	0	0	0	0	0	0	0	0	0	0	0	86
13670140	m	md	JP	2017	0	1	0	0	1	0	0	0	0	0	0	0	61
13670207	m	md	JP	2017	0	0	0	0	0	0	0	0	0	0	0	0	
13670338	m	cn	GB	2017	1	0	0	0	0	0	0	0	0	0	0	0	62
13670551	m	cn	FR	2017	0	1	1	0	0	0	0	0	0	0	0	0	50
13670618	f	ph	GB	2017	0	0	0	0	0	0	0	0	0	0	0	0	54
13671832	m	md	JP	2017	0	1	0	0	1	0	0	0	0	0	0	0	64
13671877	m	md	NL	2017	0	0	0	0	0	0	0	0	0	0	0	0	63
13671936	m	ot	US	2017	0	1	0	0	1	0	0	0	0	0	0	0	62
13672268	m	cn	JP	2017	0	0	0	0	0	0	0	0	0	0	0	0	62
13672488	m	md	FR	2017	0	1	0	0	1	0	0	0	0	0	0	0	
13673593	f	ot	US	2017	0	1	0	0	1	0	0	0	0	0	0	0	74
13675177	f	ot	JP	2017	0	1	0	0	1	0	0	0	0	0	0	1	45
13675226	f	ph	US	2017	0	1	0	0	1	0	0	0	0	0	0	0	
13675514	m	md	DE	2017	0	1	0	0	1	0	0	0	0	0	0	0	
13675537	m	md	FR	2017	1	0	0	0	0	0	0	0	0	0	0	0	38
13675627	m	ot	ES	2017	0	1	0	0	1	0	0	0	0	0	0	0	61

13676281	m	ot	FR	2017	0	0	0	0	0	0	0	0	0	0	0	0	73
13676564	f	cn	JP	2017	0	1	0	0	1	0	0	0	0	0	0	0	65
13677348	m	md	FR	2017	0	0	0	0	0	0	0	0	0	0	0	0	63
13677426	m	md	IL	2017	0	1	0	0	1	0	0	0	0	0	0	0	74
13677524	m	md	IT	2017	0	1	0	0	1	0	0	0	0	0	0	0	70
13677982	m	md	IL	2017	0	1	0	0	1	0	0	0	0	0	0	0	73
13678057	f	md	JP	2017	0	1	0	0	1	0	0	0	0	0	0	0	60
13678505	f	md	JP	2017	1	0	0	0	0	0	0	0	0	0	0	0	70
13678794	Not	cn	US	2017	0	1	0	0	1	0	0	0	0	0	0	0	
13679413	m	md	JP	2017	0	1	0	0	1	0	0	0	0	0	0	0	40
13679709	m	md	JP	2017	0	1	0	0	1	0	0	0	0	0	0	0	
13679747	m	cn	JP	2017	0	1	0	0	1	0	0	0	0	0	0	0	63
13679771	Not	ot	JP	2017	0	0	0	0	0	0	0	0	0	0	0	0	
13679990	f	lw	US	2017	0	0	0	0	0	0	0	0	0	0	0	0	48
13680213	m	ot	CH	2017	0	1	0	0	1	0	0	0	0	0	0	0	61
13680294	m	Not repo	IT	2017	1	0	0	0	0	0	0	0	0	0	0	0	
13680913	f	ot	GB	2017	0	0	0	0	0	0	0	0	0	0	0	0	54
13681086	f	md	JP	2017	0	0	0	0	0	0	0	0	0	0	0	0	70
13681189	f	ot	GB	2017	0	0	0	0	0	0	0	0	0	0	0	0	54
13681539	m	md	US	2017	0	0	0	0	0	0	0	0	0	0	0	0	65
13682601	f	cn	US	2017	0	1	0	0	1	0	0	0	0	0	0	0	
13684536	Not	md	PH	2017	1	0	0	0	0	0	0	0	0	0	0	0	
13684987	m	md	JP	2017	0	0	0	0	0	0	1	0	1	0	0	1	80.3
13685021	Not	ot	JP	2017	0	0	0	0	0	0	0	0	0	0	0	0	
13685060	Not	cn	TW	2017	0	1	1	0	0	0	0	0	0	0	0	1	
13685445	m	cn	GB	2017	1	0	0	0	0	0	0	0	0	0	0	0	76
13685900	Not	ot	JP	2017	0	0	0	0	0	0	0	0	0	0	0	0	
13685985	m	ot	US	2017	0	1	0	0	1	0	0	0	0	0	0	1	
13686568	m	md	KR	2017	0	0	0	0	0	0	0	0	0	0	0	0	
13686636	m	cn	US	2017	0	0	0	0	0	0	0	0	0	0	0	0	57
13686644	f	cn	US	2017	0	0	0	0	0	0	0	0	0	0	0	0	52
13686778	f	ot	GB	2017	0	0	0	0	0	0	0	0	0	0	0	0	54
13687100	m	cn	IL	2017	0	1	1	0	0	0	0	0	0	0	0	0	
13687122	m	ot	GR	2017	0	1	0	0	1	0	0	0	0	0	0	0	64

13687323	f	md	US	2017	1	0	0	0	0	0	0	0	0	0	0	0	50
13687711	f	cn	US	2017	0	0	0	0	0	0	0	0	0	0	0	0	72
13688212	Not	ph	AU	2017	0	1	0	0	1	0	0	0	0	0	0	0	
13688416	Not	ph	AU	2017	0	1	0	0	1	0	0	0	0	0	0	0	
13688534	Not	ph	AU	2017	0	1	0	0	1	0	0	0	0	0	0	0	73
13688535	Not	ph	AU	2017	0	1	0	0	1	0	0	0	0	0	0	0	81
13688662	f	cn	GB	2017	1	0	0	0	0	0	0	0	0	0	0	0	
13688946	f	md	NL	2017	1	0	0	0	0	0	0	0	0	0	0	0	67
13691741	f	ot	FR	2017	0	1	0	0	1	0	0	0	0	0	0	0	66
13691839	m	cn	DE	2017	0	1	1	0	0	0	0	0	0	0	0	0	69
13692339	Not	ot	GB	2017	0	0	0	0	0	0	0	0	0	0	0	0	
13692343	m	ot	JP	2017	0	0	0	0	0	0	0	0	0	0	0	0	68
13692382	m	cn	JP	2017	0	0	0	0	0	0	0	0	0	0	0	0	70
13692475	f	ot	KR	2017	1	0	0	0	0	0	0	0	0	0	0	0	81
13692981	m	md	FR	2017	0	1	0	1	0	0	0	0	0	0	0	0	52
13693289	m	ot	JP	2017	0	1	0	0	1	0	0	0	0	0	0	1	54
13693340	f	ot	DE	2017	0	1	0	0	1	0	0	0	0	0	0	0	63
13694154	m	cn	US	2017	1	0	0	0	0	0	0	0	0	0	0	0	49
13694232	f	cn	GB	2017	1	0	0	0	0	0	0	0	0	0	0	0	71
13694349	m	ot	IT	2017	0	1	0	0	1	0	0	0	0	0	0	0	57
13694425	Not	md	TH	2017	0	0	0	0	0	0	0	0	0	0	0	0	
13695067	m	md	JP	2017	0	1	0	0	1	0	0	0	0	0	0	0	57
13695083	m	md	US	2017	0	1	1	0	0	0	0	0	0	0	0	0	66
13695900	f	ot	US	2017	0	1	0	0	1	0	0	0	0	0	0	0	50
13696380	m	md	US	2017	0	1	0	0	0	1	0	0	0	0	0	0	
13696517	m	cn	US	2017	0	1	0	0	1	0	0	0	0	0	0	0	
13696813	m	ot	US	2017	0	1	0	0	1	0	0	0	0	0	0	0	64
13697435	m	cn	GB	2017	1	0	0	0	0	0	0	0	0	0	0	0	84
13697740	f	cn	US	2017	0	0	0	0	0	0	0	0	0	0	0	0	57
13698137	m	md	US	2017	0	1	0	0	1	0	0	0	0	0	0	1	71
13698171	f	md	US	2017	0	1	0	0	1	0	0	0	0	0	0	0	74
13698427	f	cn	US	2017	0	1	0	0	1	0	0	0	0	0	0	0	
13698627	f	cn	US	2017	0	0	0	0	0	0	0	0	0	0	0	0	68
13698726	m	cn	US	2017	0	0	0	0	0	0	0	0	0	0	0	0	86

13698755	m	md	JP	2017	0	1	0	0	1	0	0	0	0	0	0	0	69
13698852	f	cn	GB	2017	1	0	0	0	0	0	0	0	0	0	0	0	54
13699716	m	cn	IN	2017	0	1	0	0	1	0	0	0	0	0	0	0	54
13699872	m	md	FR	2017	0	1	0	0	1	0	0	0	0	0	0	0	
13699873	m	md	JP	2017	0	1	0	0	1	0	0	0	0	0	0	0	53
13700421	f	ot	US	2017	0	0	0	0	0	0	0	0	0	0	0	0	55
13700423	m	ot	US	2017	0	0	0	0	0	0	0	0	0	0	0	0	66
13701795	Not	md	JP	2017	0	1	0	0	1	0	0	0	0	0	0	0	
13702799	m	cn	US	2017	0	0	0	0	0	0	0	0	0	0	0	0	61
13702930	m	md	JP	2017	0	1	0	0	1	0	0	0	0	0	0	0	78
13703093	Not	ot	BR	2017	0	0	0	0	0	0	0	0	0	0	0	0	
13703344	m	ot	JP	2017	0	1	0	0	1	0	0	0	0	0	0	0	78
13703381	f	cn	GB	2017	1	0	0	0	0	0	0	0	0	0	0	0	
13703941	f	md	US	2017	1	0	0	0	0	0	0	0	0	0	0	0	90
13704228	m	ot	JP	2017	0	1	0	0	1	0	0	0	0	0	0	0	69
13704459	f	ot	DE	2017	0	1	0	0	1	0	0	0	0	0	0	0	65
13704726	Not	cn	US	2017	0	1	0	0	1	0	0	0	0	0	0	0	
13704994	f	ot	US	2017	0	1	0	0	1	0	0	0	0	0	0	0	74
13705270	Not	cn	US	2017	0	1	0	0	1	0	0	0	0	0	0	0	
13705271	Not	cn	US	2017	0	1	0	0	1	0	0	0	0	0	0	0	
13705274	Not	cn	US	2017	0	1	0	0	1	0	0	0	0	0	0	0	
13705289	Not	cn	US	2017	0	1	0	0	1	0	0	0	0	0	0	0	
13706046	m	md	FR	2017	0	1	0	0	0	1	0	0	0	0	0	0	60
13706089	Not	ot	BR	2017	0	0	0	0	0	0	0	0	0	0	0	0	
13706324	Not	ot	BR	2017	0	0	0	0	0	0	0	0	0	0	0	0	
13707445	m	ot	JP	2017	0	1	0	0	1	0	0	0	0	0	0	0	68
13707551	m	md	AT	2017	0	0	0	0	0	0	0	0	0	0	0	0	72
13707563	Not	ot	BR	2017	0	0	0	0	0	0	0	0	0	0	0	0	
13707665	f	ot	DE	2017	0	0	0	0	0	0	0	0	0	0	0	0	63
13708249	m	md	JP	2017	0	1	0	0	1	0	0	0	0	0	0	0	78
7629671	f	md	US	2017	0	0	0	0	0	0	0	0	0	0	0	0	60
8080357	Not	ot	IT	2017	0	0	0	0	0	0	0	0	0	0	0	0	
8084384	Not	ot	IT	2017	0	0	0	0	0	0	0	0	0	0	0	0	
8088204	Not	ot	IT	2017	0	0	0	0	0	0	0	0	0	0	0	0	

8088249	Not	ot	IT	2017	0	0	0	0	0	0	0	0	0	0	0	0	
8122779	f	md	US	2017	0	0	0	0	0	0	0	0	0	0	0	0	32
8122793	m	md	US	2017	0	0	0	0	0	0	0	0	0	0	0	0	79
8160807	f	md	US	2017	0	0	0	0	0	0	0	0	0	0	0	0	43
8298048	m	cn	GB	2017	1	0	0	0	0	0	0	0	0	0	0	0	64
8416994	m	cn	CN	2017	0	0	0	0	0	0	0	0	0	0	0	0	56
8471518	f	cn	GB	2017	1	0	0	0	0	0	0	0	0	0	0	0	83
8632612	m	md	US	2017	0	0	0	0	0	0	0	0	0	0	0	0	76
8775573	m	cn	GB	2017	0	0	0	0	0	0	0	0	0	0	0	0	53
8822685	m	cn	GB	2017	0	0	0	0	0	0	0	0	0	0	0	0	58
10187152	m	md	DE	2017	0	0	0	0	0	0	0	0	0	0	0	0	72
12615978	m	md	HU	2017	0	0	0	0	0	0	0	0	0	0	0	0	67
12623426	f	ot	US	2017	0	1	0	0	1	0	0	0	0	0	0	0	61
12964093	f	cn	FR	2017	0	1	0	0	0	1	0	0	0	0	0	0	61
13008430	m	cn	JP	2017	0	1	0	0	1	0	0	0	0	0	0	1	74
13029326	f	ot	US	2017	0	1	0	0	1	0	0	0	0	0	0	0	77
13034034	m	md	JP	2017	0	1	0	0	0	1	0	0	0	0	0	1	62
13077935	m	md	JP	2017	0	1	0	0	0	1	0	0	0	0	0	0	46
13114115	f	ot	DE	2017	0	1	0	0	0	1	0	0	0	0	0	0	74
13123132	m	ot	BR	2017	0	1	0	0	1	0	0	0	0	0	0	0	58
13135994	m	ot	IT	2017	0	1	0	0	1	0	0	0	0	0	0	0	78
13144744	f	md	JP	2017	1	0	0	0	0	0	0	0	0	0	0	1	62
13150615	m	md	KR	2017	0	0	0	0	0	0	0	0	0	0	0	0	69
13179724	m	cn	JP	2017	0	1	0	0	1	0	0	0	0	0	0	0	77
13181605	f	cn	US	2017	1	0	0	0	0	0	0	0	0	0	0	0	63
13191425	f	md	TW	2017	0	1	0	0	0	1	0	0	0	0	0	0	51
13212659	m	md	JP	2017	0	1	0	0	0	1	0	0	0	0	0	0	66
13219930	f	md	IE	2017	0	0	0	0	0	0	0	0	0	0	0	0	74
13244081	f	md	US	2017	0	0	0	0	0	0	0	0	0	0	0	0	72
13255678	f	md	PL	2017	0	0	0	0	0	0	0	0	0	0	0	0	47
13258868	m	ot	GR	2017	0	1	0	0	1	0	0	0	0	0	0	0	64
13260763	m	ph	JP	2017	0	1	0	0	1	0	0	0	0	0	0	0	60
13265530	m	md	US	2017	0	1	0	0	0	1	0	0	0	0	0	0	54
13276568	m	ph	JP	2017	0	0	0	0	0	0	0	0	0	0	0	0	82

13289383	m	md	JP	2017	0	1	0	0	0	1	0	0	0	0	0	0	75
13298433	f	md	DE	2017	0	1	0	0	0	1	0	0	0	0	0	0	64
13303783	m	md	BE	2017	0	0	0	0	0	0	0	0	0	0	0	0	55
13303965	m	ot	GB	2017	0	1	0	0	1	0	0	0	0	0	0	0	70
13336108	f	ph	JP	2017	0	1	0	0	1	0	0	0	0	0	0	0	45
13338033	Not	cn	GB	2017	1	0	0	0	0	0	0	0	0	0	0	0	61
13339362	f	ot	DE	2017	0	1	0	0	0	1	0	0	0	0	0	0	63
13349866	m	cn	JP	2017	0	1	1	0	0	0	0	0	0	0	0	0	46
13361666	f	md	BR	2017	0	1	0	0	0	1	0	0	0	0	0	0	31
13362042	f	md	JP	2017	0	1	0	0	0	1	0	0	0	0	0	0	54
13374263	m	md	JP	2017	0	1	0	0	0	1	0	0	0	0	0	0	55
13383248	m	md	MY	2017	0	1	0	0	0	1	0	0	0	0	0	0	61
13384886	m	md	JP	2017	0	1	0	0	1	0	0	0	0	0	0	1	70
13394660	m	ot	IT	2017	0	1	0	0	1	0	0	0	0	0	0	0	79
13403909	f	ot	DE	2017	0	1	0	0	1	0	0	0	0	0	0	0	61
13413788	m	cn	JP	2017	0	1	0	0	1	0	0	0	0	0	0	0	72
13417060	f	md	DE	2017	1	0	0	0	0	0	0	0	0	0	0	0	73
13417492	f	md	KR	2017	0	0	0	0	0	0	0	0	0	0	0	0	57
13417808	m	md	JP	2017	0	0	0	0	0	0	0	0	0	0	0	0	66
13417886	f	ot	US	2017	0	1	0	0	1	0	0	0	0	0	0	0	57
13418532	f	ot	JP	2017	0	0	0	0	0	0	0	0	0	0	0	0	67
13420186	f	md	JP	2017	1	0	0	0	0	0	0	0	0	0	0	0	90.2
13420276	m	md	DE	2017	0	0	0	0	0	0	0	0	0	0	0	0	58
13421593	m	ot	GB	2017	0	1	0	0	1	0	0	0	0	0	0	0	59
13421721	f	ot	BR	2017	0	1	0	0	1	0	0	0	0	0	0	0	63
13423364	m	ph	JP	2017	0	1	0	0	1	0	0	0	0	0	0	0	76
13424722	m	ot	IT	2017	0	0	0	0	0	0	0	0	0	0	0	0	72
13424766	f	md	US	2017	0	1	0	0	0	1	0	0	0	0	0	1	64
13426677	m	md	JP	2017	0	1	0	0	0	1	0	0	0	0	0	0	68
13429254	f	cn	US	2017	0	1	0	0	1	0	0	0	0	0	0	0	55
13429903	m	cn	DE	2017	0	1	0	0	1	0	0	0	0	0	0	0	71
13430968	m	ph	FR	2017	0	1	0	0	1	0	0	0	0	0	0	0	59
13433822	f	Not repo	Not re	2017	0	0	0	0	0	0	0	0	0	0	0	0	65
13434746	f	ot	FR	2017	0	1	0	0	1	0	0	0	0	0	0	0	45

13446157	m	cn	JP	2017	0	1	1	0	0	0	0	0	0	0	0	1	84
13446235	f	ot	GB	2017	0	0	0	0	0	0	0	0	0	0	0	0	67
13446524	m	md	US	2017	0	1	0	0	0	1	0	0	0	0	0	0	70
13452417	m	ot	MX	2017	0	1	0	0	1	0	0	0	0	0	0	0	49
13457203	m	ot	KR	2017	0	1	0	0	1	0	0	0	0	0	0	0	47
13458233	m	ot	JP	2017	0	1	0	0	1	0	0	0	0	0	0	0	74
13460998	f	md	CN	2017	1	0	0	0	0	0	0	0	0	0	0	0	70.3
13465254	f	md	JP	2017	0	1	0	0	1	0	0	0	0	0	0	0	52
13474442	m	cn	JP	2017	0	0	0	0	0	0	0	0	0	0	0	0	71
13475713	m	md	DE	2017	0	1	0	0	0	1	0	0	0	0	0	0	81
13477576	f	md	CH	2017	0	0	0	0	0	0	0	0	0	0	0	0	79
13482687	f	md	NL	2017	0	1	0	0	0	1	0	0	0	0	0	0	55
13483563	m	md	JP	2017	0	1	0	0	1	0	0	0	0	0	0	1	69
13483632	f	ot	GR	2017	0	1	0	0	1	0	0	0	0	0	0	0	77
13484145	f	cn	US	2017	1	0	0	0	0	0	0	0	0	0	0	0	82
13484418	m	ot	US	2017	0	1	0	0	1	0	0	0	0	0	0	0	69
13486605	m	ot	JP	2017	0	1	0	0	1	0	0	0	0	0	0	0	66
13490225	f	ot	JP	2017	0	1	0	0	1	0	0	0	0	0	0	0	67
13492431	f	md	DE	2017	1	0	0	0	0	0	0	0	0	0	0	0	67
13495793	m	md	CN	2017	0	1	0	0	0	1	0	0	0	0	0	0	68
13496925	m	cn	JP	2017	0	0	0	0	0	0	0	0	0	0	0	0	68
13498060	m	md	CN	2017	0	1	0	0	0	1	0	0	0	0	0	0	62
13501998	f	cn	JP	2017	0	0	0	0	0	0	1	0	1	0	0	0	53
13502543	m	ot	AU	2017	0	1	0	0	1	0	0	0	0	0	0	0	80
13502652	m	cn	JP	2017	0	1	1	0	0	0	0	0	0	0	0	1	69
13503967	m	cn	US	2017	1	0	0	0	0	0	0	0	0	0	0	0	87
13506969	f	cn	JP	2017	0	1	1	0	0	0	0	0	0	0	0	0	62
13508866	m	md	US	2017	0	1	1	0	0	0	0	0	0	0	0	0	60
13509287	m	md	JP	2017	0	1	1	0	0	0	0	0	0	0	0	0	65
13514801	m	md	FR	2017	0	1	0	1	0	0	0	0	0	0	0	0	41
13517912	f	md	DE	2017	0	0	0	0	0	0	0	0	0	0	0	0	74
13518840	m	md	JP	2017	0	1	0	0	1	0	0	0	0	0	0	1	81
13519986	f	ot	BE	2017	0	1	0	0	1	0	0	0	0	0	0	0	39
13521766	f	md	JP	2017	1	0	0	0	0	0	0	0	0	0	0	1	81

13525510	m	ot	US	2017	0	1	0	0	1	0	0	0	0	0	0	1	64
13525511	m	ot	US	2017	0	1	0	0	1	0	0	0	0	0	0	0	65
13532021	f	cn	JP	2017	0	0	0	0	0	0	0	0	0	0	0	0	
13532482	m	md	BG	2017	0	0	0	0	0	0	0	0	0	0	0	0	60
13534698	m	cn	US	2017	0	1	1	0	0	0	0	0	0	0	0	0	56
13535061	m	ot	US	2017	0	1	0	0	1	0	0	0	0	0	0	0	48
13535573	m	md	CN	2017	0	1	0	0	0	1	0	0	0	0	0	0	51
13535665	m	cn	IN	2017	0	0	0	0	0	0	0	0	0	0	0	0	51
13539110	f	md	JP	2017	0	0	0	0	0	0	0	0	0	0	0	0	47
13540145	m	md	JP	2017	0	1	0	0	1	0	0	0	0	0	0	0	52
13545938	m	ot	JP	2017	0	1	0	0	1	0	0	0	0	0	0	0	81
13546168	f	cn	JP	2017	0	1	1	0	0	0	0	0	0	0	0	0	77
13552370	f	ph	JP	2017	0	0	0	0	0	0	0	0	0	0	0	0	76
13552471	m	ot	DE	2017	0	1	0	0	1	0	0	0	0	0	0	0	51
13552757	m	md	JP	2017	0	1	0	1	0	0	0	0	0	0	0	0	68
13553076	m	md	CA	2017	0	0	0	0	0	0	0	0	0	0	0	0	74
13555201	m	ot	US	2017	0	1	0	0	1	0	0	0	0	0	0	1	58
13558195	f	md	US	2017	1	0	0	0	0	0	0	0	0	0	0	0	75
13565133	m	ph	JP	2017	0	0	0	0	0	0	0	0	0	0	0	0	73
13565421	m	ot	JP	2017	0	1	0	0	1	0	0	0	0	0	0	0	83
13569005	Not	cn	DE	2017	0	1	1	0	0	0	0	0	0	0	0	0	
13569492	m	cn	GR	2017	0	1	1	0	0	0	0	0	0	0	0	0	76
13569519	m	md	JP	2017	0	0	0	0	0	0	0	0	0	0	0	0	75
13570719	m	cn	JP	2017	0	0	0	0	0	0	0	0	0	0	0	0	73
13573150	f	cn	US	2017	0	0	0	0	0	0	0	0	0	0	0	0	
13573912	f	md	BE	2017	0	0	0	0	0	0	0	0	0	0	0	0	58
13576411	f	md	JP	2017	0	1	0	0	1	0	0	0	0	0	0	0	48
13578218	f	cn	GB	2017	1	0	0	0	0	0	0	0	0	0	0	0	66
13578510	f	cn	US	2017	0	1	0	0	1	0	0	0	0	0	0	0	
13578623	m	md	JP	2017	0	0	0	0	0	0	0	0	0	0	0	0	72
13585000	f	md	KR	2017	0	0	0	0	0	0	0	0	0	0	0	0	61
13586038	m	md	DE	2017	0	1	0	0	1	0	0	0	0	0	0	0	
13586748	f	md	DE	2017	0	0	0	0	0	0	0	0	0	0	0	0	66
13587061	f	md	DE	2017	0	1	0	0	1	0	0	0	0	0	0	1	72

13587075	f	md	US	2017	1	0	0	0	0	0	0	0	0	0	0	0	65
13588954	m	cn	JP	2017	0	0	0	0	0	0	0	0	0	0	0	0	78
13590352	m	md	JP	2017	0	1	0	0	1	0	0	0	0	0	0	0	48
13591830	f	md	JP	2017	1	0	0	0	0	0	0	0	0	0	0	0	85
13592159	m	cn	DE	2017	0	1	0	0	1	0	0	0	0	0	0	0	
13592545	f	ot	US	2017	0	1	0	0	1	0	0	0	0	0	0	0	74
13593757	m	md	KR	2017	0	0	0	0	0	0	0	0	0	0	0	0	47
13594617	m	md	CN	2017	1	0	0	0	0	0	0	0	0	0	0	0	75
13597106	f	cn	US	2017	0	1	1	0	0	0	0	0	0	0	0	0	78
13597625	m	cn	US	2017	0	0	0	0	0	0	0	0	0	0	0	0	86
13598288	m	md	JP	2017	0	1	0	0	1	0	0	0	0	0	0	0	75
13600826	f	md	JP	2017	0	1	0	0	1	0	0	0	0	0	0	0	80
13601594	f	ot	FR	2017	0	0	0	0	0	0	0	0	0	0	0	0	59
13601788	m	md	JP	2017	0	1	0	0	0	1	0	0	0	0	0	0	59
13603005	m	ot	AR	2017	0	1	0	0	1	0	0	0	0	0	0	0	62
13604037	f	cn	US	2017	0	1	0	0	1	0	0	0	0	0	0	0	
13608147	m	md	JP	2017	0	1	0	0	1	0	0	0	0	0	0	0	65
13608718	m	md	KR	2017	0	0	0	0	0	0	0	0	0	0	0	0	42
13609122	m	ot	FR	2017	0	1	0	0	1	0	0	0	0	0	0	0	59
13611025	f	cn	DE	2017	0	0	0	0	0	0	0	0	0	0	0	0	62
13611426	m	cn	JP	2017	0	0	0	0	0	0	0	0	0	0	0	0	67
13611631	m	ph	JP	2017	0	1	0	0	1	0	0	0	0	0	0	0	43
13612161	m	md	KR	2017	0	0	0	0	0	0	0	0	0	0	0	0	62
13613044	f	md	US	2017	0	1	0	0	1	0	0	0	0	0	0	0	69
13614515	f	cn	US	2017	1	0	0	0	0	0	0	0	0	0	0	0	71
13615178	f	md	JP	2017	0	0	0	0	0	0	0	0	0	0	0	0	72
13618154	f	md	DE	2017	1	0	0	0	0	0	0	0	0	0	0	0	70
13619617	f	md	JP	2017	0	1	0	1	0	0	0	0	0	0	0	0	69.7
13619927	m	md	JP	2017	0	1	0	1	0	0	0	0	0	0	0	0	57.4
13620433	m	md	KR	2017	0	0	0	0	0	0	0	0	0	0	0	0	59
13620727	f	ph	JP	2017	0	0	0	0	0	0	0	0	0	0	0	0	41
13624230	m	md	IT	2017	0	0	0	0	0	0	0	0	0	0	0	0	59
13629869	f	md	JP	2017	1	0	0	0	0	0	0	0	0	0	0	0	71
13632795	m	md	CN	2017	1	0	0	0	0	0	0	0	0	0	0	0	64

13632882	m	md	JP	2017	1	0	0	0	0	0	0	0	0	0	0	62
13634887	f	cn	US	2017	0	1	0	0	1	0	0	0	0	0	0	0	
13635037	f	cn	US	2017	0	1	0	0	1	0	0	0	0	0	0	0	
13637274	m	md	IT	2017	0	0	0	0	0	0	0	0	0	0	0	0	59
13640144	m	md	US	2017	0	1	0	0	1	0	0	0	0	0	0	0	76
13640212	m	md	JP	2017	0	0	0	0	0	0	0	0	0	0	0	0	57
13641068	f	md	US	2017	0	1	0	0	1	0	0	0	0	0	0	0	72
13643316	m	ot	JP	2017	0	1	0	0	1	0	0	0	0	0	0	0	67
13643602	m	md	JP	2017	0	1	0	0	1	0	0	0	0	0	0	1	61
13645684	m	ot	DE	2017	0	1	0	0	1	0	0	0	0	0	0	0	65
13647075	f	md	IT	2017	1	0	0	0	0	0	0	0	0	0	0	0	67.7
13648438	f	cn	JP	2017	0	0	0	0	0	0	0	0	0	0	0	0	54
13649720	f	ph	US	2017	0	1	0	0	0	1	0	0	0	0	0	0	
13649932	m	ot	US	2017	0	1	0	0	1	0	0	0	0	0	0	0	
13650932	f	Not repo	US	2017	1	0	0	0	0	0	0	0	0	0	0	0	81.5
13654014	m	ot	PL	2017	0	1	0	0	1	0	0	0	0	0	0	0	68
13654027	m	ot	JP	2017	0	1	0	0	1	0	0	0	0	0	0	0	72
13654150	m	ot	JP	2017	0	1	0	0	1	0	0	0	0	0	0	1	76
13654626	f	md	JP	2017	0	0	0	0	0	0	0	0	0	0	0	0	77
13656079	m	md	ES	2017	0	1	0	0	0	1	0	0	0	0	0	1	68
13657457	f	md	JP	2017	0	1	0	0	1	0	0	0	0	0	0	0	78
13658662	m	md	SE	2017	0	1	0	0	1	0	0	0	0	0	0	0	74
13658678	m	md	DE	2017	0	1	0	0	1	0	0	0	0	0	0	0	70
13659267	m	md	JP	2017	0	1	0	0	1	0	0	0	0	0	0	1	82
13660341	f	md	FR	2017	0	0	0	0	0	0	0	0	0	0	0	0	73
13660452	m	md	DE	2017	0	0	0	0	0	0	0	0	0	0	0	0	64
13660530	m	md	JP	2017	1	0	0	0	0	0	0	0	0	0	0	0	71
13660792	f	ot	US	2017	0	0	0	0	0	0	0	0	0	0	0	0	80
13660854	m	md	KR	2017	0	0	0	0	0	0	0	0	0	0	0	0	70
13661911	f	md	JP	2017	0	0	0	0	0	0	0	0	0	0	0	0	71
13662177	m	md	KR	2017	0	0	0	0	0	0	0	0	0	0	0	0	67
13662515	m	cn	JP	2017	0	0	0	0	0	0	0	0	0	0	0	0	66
13662946	f	cn	GB	2017	0	1	1	0	0	0	0	0	0	0	0	0	70
13663065	m	cn	JP	2017	0	1	1	0	0	0	0	0	0	0	0	0	82

13663076	Not	md	JP	2017	1	0	0	0	0	0	0	0	0	0	0	0	
13663196	m	md	FR	2017	1	0	0	0	0	0	0	0	0	0	0	0	51
13663411	m	ot	US	2017	0	0	0	0	0	0	0	0	0	0	0	0	72
13664104	Not	md	JP	2017	1	0	0	0	0	0	0	0	0	0	0	0	
13667403	Not	md	IT	2017	0	1	0	0	1	0	0	0	0	0	0	0	
13667621	m	md	IT	2017	0	1	0	0	1	0	0	0	0	0	0	0	
13668046	m	cn	US	2017	0	0	0	0	0	0	0	0	0	0	0	0	79
13668512	f	md	CN	2017	0	1	0	0	0	1	0	0	0	0	0	0	48
13668780	m	Not repo	US	2017	1	0	0	0	0	0	0	0	0	0	0	0	69
13669044	f	md	JP	2017	1	0	0	0	0	0	0	0	0	0	0	0	71
13669222	f	cn	AR	2017	0	0	0	0	0	0	0	0	0	0	0	0	21
13670025	m	md	JP	2017	0	1	0	0	1	0	0	0	0	0	0	0	61
13671907	f	cn	JP	2017	0	0	0	0	0	0	0	0	0	0	0	0	66
13673354	m	cn	JP	2017	0	1	1	0	0	0	0	0	0	0	0	0	61
13675181	f	md	CA	2017	0	1	0	0	1	0	0	0	0	0	0	0	
13675487	m	md	JP	2017	1	0	0	0	0	0	0	0	0	0	0	0	87
13675527	m	cn	JP	2017	0	1	0	0	1	0	0	0	0	0	0	0	66
13675534	f	md	JP	2017	0	1	0	0	1	0	0	0	0	0	0	0	73
13678448	f	cn	US	2017	0	1	1	0	0	0	0	0	0	0	0	0	63
13679746	m	md	JP	2017	0	1	0	0	1	0	0	0	0	0	0	1	74
13680256	m	md	KR	2017	0	0	0	0	0	0	0	0	0	0	0	0	68
13680298	f	ot	DE	2017	0	1	0	0	1	0	0	0	0	0	0	0	78
13680846	f	md	SG	2017	0	1	0	1	0	0	0	0	0	0	0	0	68.2
13681179	m	md	JP	2017	0	1	0	0	1	0	0	0	0	0	0	0	71
13681400	f	ot	CN	2017	0	1	0	0	1	0	0	0	0	0	0	0	52
13682170	f	md	US	2017	1	0	0	0	0	0	0	0	0	0	0	0	
13682415	m	md	JP	2017	1	0	0	0	0	0	0	0	0	0	0	0	83
13683251	f	ot	US	2017	0	0	0	0	0	0	0	0	0	0	0	0	71
13686731	f	cn	PH	2017	0	0	0	0	0	0	0	0	0	0	0	0	
13686876	f	md	CN	2017	1	0	0	0	0	0	0	0	0	0	0	0	71
13687742	m	ot	DE	2017	0	1	1	0	0	0	0	0	0	0	0	0	68
13688533	m	md	AU	2017	0	1	0	0	1	0	0	0	0	0	0	0	
13688579	f	ot	GB	2017	0	0	0	0	0	0	0	0	0	0	0	0	83
13692241	f	md	IT	2017	1	0	0	0	0	0	0	0	0	0	0	0	

13692253	Not	ph	AU	2017	0	1	0	0	1	0	0	0	0	0	0	0	68
13692451	m	Not repo	AU	2017	1	0	0	0	0	0	0	0	0	0	0	0	58
13692937	f	md	PH	2017	1	0	0	0	0	0	0	0	0	0	0	0	64
13693007	f	ph	JP	2017	1	0	0	0	0	0	0	0	0	0	0	0	92
13693433	Not	cn	JP	2017	0	0	0	0	0	0	0	0	0	0	0	0	75
13694405	f	md	ES	2017	0	0	0	0	0	0	0	0	0	0	0	0	48
13696499	f	ot	DE	2017	0	1	0	0	1	0	0	0	0	0	0	0	53
13696564	f	ot	CZ	2017	0	0	0	0	0	0	0	0	0	0	0	0	81
13696695	m	md	JP	2017	1	0	0	0	0	0	0	0	0	0	0	0	80
13697290	m	cn	DK	2017	0	0	0	0	0	0	0	0	0	0	0	0	
13698428	m	ot	US	2017	0	1	0	0	1	0	0	0	0	0	0	0	75
13698568	f	md	JP	2017	1	0	0	0	0	0	0	0	0	0	0	0	66
13698736	f	cn	US	2017	0	0	0	0	0	0	0	0	0	0	0	0	57
13700357	f	md	FR	2017	0	1	0	0	0	1	0	0	0	0	0	0	73
13700397	f	md	US	2017	1	0	0	0	0	0	0	0	0	0	0	0	56
13700989	f	md	HU	2017	1	0	0	0	0	0	0	0	0	0	0	0	73.3
13702911	m	ot	BE	2017	0	1	0	0	1	0	0	0	0	0	0	0	74
13704091	Not	ot	NL	2017	0	1	0	0	1	0	0	0	0	0	0	0	
13704306	f	md	PT	2017	1	0	0	0	0	0	0	0	0	0	0	0	
13705301	m	cn	US	2017	0	1	0	0	1	0	0	0	0	0	0	0	
13706291	f	md	GB	2017	0	1	0	0	0	1	0	0	0	0	0	0	64
13706930	f	md	JP	2017	1	0	0	0	0	0	0	0	0	0	0	0	72
13707186	m	md	BR	2017	0	0	0	0	0	0	0	0	0	0	0	0	63
13707336	f	ph	DE	2017	0	1	0	0	1	0	0	0	0	0	0	0	
13707422	f	md	IT	2017	1	0	0	0	0	0	0	0	0	0	0	0	
13707607	f	ot	ES	2017	0	0	0	0	0	0	0	0	0	0	0	0	62
13707830	f	md	SK	2017	0	0	0	0	0	0	0	0	0	0	0	0	
13707954	f	md	AU	2017	0	0	0	0	0	0	0	0	0	0	0	0	57
13709678	m	ph	JP	2017	0	0	0	0	0	0	0	0	0	0	0	0	61
13710144	m	md	US	2017	0	0	0	0	0	0	0	0	0	0	0	0	75
13710864	f	Not repo	US	2017	1	0	0	0	0	0	0	0	0	0	0	0	59
13711008	f	cn	GB	2017	1	0	0	0	0	0	0	0	0	0	0	0	
13711220	Not	ot	JP	2017	1	0	0	0	0	0	0	0	0	0	0	0	
13711277	Not	ot	JP	2017	0	0	0	0	0	0	0	0	0	0	0	0	

13711278	Not	ot	JP	2017	1	0	0	0	0	0	0	0	0	0	0	0	
13711289	Not	md	AU	2017	0	1	0	0	1	0	0	0	0	0	0	0	33
13711384	f	ot	FR	2017	0	1	0	0	1	0	0	0	0	0	0	0	73
13711572	m	md	JP	2017	0	1	0	0	1	0	0	0	0	0	0	0	80
13711830	m	md	IT	2017	0	1	0	0	1	0	0	0	0	0	0	0	76
13712650	m	md	JP	2017	0	1	0	0	1	0	0	0	0	0	0	1	73
13712716	m	ot	US	2017	0	1	0	0	1	0	0	0	0	0	0	0	82
13712790	m	md	KR	2017	1	0	0	0	0	0	0	0	0	0	0	0	84
13713273	m	cn	IN	2017	0	1	0	0	1	0	0	0	0	0	0	0	54
13713387	m	md	IN	2017	0	1	0	0	1	0	0	0	0	0	0	0	58
13713388	f	md	IN	2017	0	1	0	0	1	0	0	0	0	0	0	0	42
13713458	f	ot	DE	2017	0	1	0	0	1	0	0	0	0	0	0	0	76
13713579	f	md	JP	2017	1	0	0	0	0	0	0	0	0	0	0	0	71
13713646	m	cn	JP	2017	0	1	0	0	1	0	0	0	0	0	0	0	
13713660	m	md	CN	2017	1	0	0	0	0	0	0	0	0	0	0	0	55
13713664	m	md	JP	2017	1	0	0	0	0	0	0	0	0	0	0	0	69
13713754	m	ot	JP	2017	0	1	0	0	1	0	0	0	0	0	0	1	70
13713818	Not	md	JP	2017	0	0	0	0	0	0	0	0	0	0	0	0	70
13713904	f	ot	JP	2017	0	1	0	0	1	0	0	0	0	0	0	0	72
13713948	f	md	JP	2017	1	0	0	0	0	0	0	0	0	0	0	0	68
13714256	m	md	JP	2017	1	0	0	0	0	0	0	0	0	0	0	0	80
13714329	m	ot	IT	2017	0	1	0	0	1	0	0	0	0	0	0	0	77
13714580	f	cn	US	2017	1	0	0	0	0	0	0	0	0	0	0	0	63
13715481	f	ot	US	2017	0	0	0	0	0	0	0	0	0	0	0	0	63
13715688	m	ph	US	2017	0	0	0	0	0	0	0	0	0	0	0	0	78
13715760	f	ph	JP	2017	0	1	0	0	1	0	0	0	0	0	0	0	70
13715911	m	md	JP	2017	0	1	0	0	1	0	0	0	0	0	0	0	79
13715924	m	ot	US	2017	0	1	0	0	1	0	0	0	0	0	0	0	
13716047	f	md	JP	2017	1	0	0	0	0	0	0	0	0	0	0	0	68
13716197	Not	md	US	2017	0	1	0	0	1	0	0	0	0	0	0	1	
13716200	Not	md	US	2017	0	1	0	0	1	0	0	0	0	0	0	0	
13716291	Not	ot	CH	2017	0	0	0	0	0	0	0	0	0	0	0	0	
13716351	f	cn	GB	2017	1	0	0	0	0	0	0	0	0	0	0	0	76
13716370	Not	md	PT	2017	0	1	0	0	1	0	0	0	0	0	0	0	

13716410	f	ot	US	2017	0	0	0	0	0	0	0	0	0	0	0	0	
13716626	f	md	JP	2017	0	0	0	0	0	0	0	0	0	0	0	0	82
13717007	f	md	JP	2017	1	0	0	0	0	0	0	0	0	0	0	0	67
13717110	m	ot	DE	2017	0	1	0	0	1	0	0	0	0	0	0	0	71
13717964	f	md	JP	2017	0	1	0	0	1	0	0	0	0	0	0	0	50
13718100	Not	md	US	2017	0	1	0	0	1	0	0	0	0	0	0	0	
13718223	m	md	CN	2017	0	0	0	0	0	0	0	0	0	0	0	0	64.6
13718375	f	cn	JP	2017	1	0	0	0	0	0	0	0	0	0	0	0	
13718603	m	ot	ES	2017	0	0	0	0	0	0	0	0	0	0	0	0	74
13719455	f	ot	AU	2017	0	1	0	0	1	0	0	0	0	0	0	0	59
13719539	m	md	US	2017	0	1	0	0	0	1	0	0	0	0	0	0	
13719542	f	md	US	2017	0	1	0	0	0	1	0	0	0	0	0	0	71
13719655	Not	ot	US	2017	0	1	0	0	0	1	0	0	0	0	0	1	
13719766	Not	cn	JP	2017	0	0	0	0	0	0	0	0	0	0	0	0	
13719905	f	cn	US	2017	0	1	0	0	1	0	0	0	0	0	0	0	
13719943	f	md	KR	2017	1	0	0	0	0	0	0	0	0	0	0	1	56
13719973	m	ph	FR	2017	0	1	0	0	1	0	0	0	0	0	0	0	79
13720052	f	ot	US	2017	0	1	0	0	1	0	0	0	0	0	0	0	66
13720156	m	md	CA	2017	1	0	0	0	0	0	0	0	0	0	0	0	65
13720335	m	md	JP	2017	0	1	0	0	1	0	0	0	0	0	0	0	87
13720443	f	ot	JP	2017	0	1	0	0	1	0	0	0	0	0	0	0	76
13720445	f	ot	NL	2017	0	1	0	0	1	0	0	0	0	0	0	0	61
13720508	f	md	US	2017	0	1	0	0	1	0	0	0	0	0	0	0	70
13721306	f	cn	US	2017	1	0	0	0	0	0	0	0	0	0	0	0	81
13721352	f	cn	US	2017	0	0	0	0	0	0	0	0	0	0	0	0	
13721514	f	md	JP	2017	1	0	0	0	0	0	0	0	0	0	0	0	80
13721706	f	md	TR	2017	0	0	0	0	0	0	0	0	0	0	0	0	57
13721714	m	ot	US	2017	0	0	0	0	0	0	0	0	0	0	0	0	60
13721876	f	md	JP	2017	1	0	0	0	0	0	0	0	0	0	0	0	60
13722340	m	md	TH	2017	1	0	0	0	0	0	0	0	0	0	0	0	
13722494	f	cn	GB	2017	1	0	0	0	0	0	0	0	0	0	0	0	
13723110	f	cn	GB	2017	1	0	0	0	0	0	0	0	0	0	0	0	63
13723130	f	md	CN	2017	1	0	0	0	0	0	0	0	0	0	0	0	61.3
13724545	f	cn	IN	2017	0	0	0	0	0	0	0	0	0	0	0	0	54

13724615	f	md	TH	2017	0	0	0	0	0	0	0	0	0	0	0	0	55
13724659	f	md	KR	2017	1	0	0	0	0	0	0	0	0	0	0	0	66
13724690	m	md	JP	2017	0	0	0	0	0	0	0	0	0	0	0	0	43
13724726	Not	md	US	2017	0	1	0	0	1	0	0	0	0	0	0	0	
13725241	Not	ph	AU	2017	0	1	0	0	1	0	0	0	0	0	0	0	
13726226	m	cn	AT	2017	0	0	0	0	0	0	0	0	0	0	0	0	76
13726340	Not	cn	US	2017	0	1	1	0	0	0	0	0	0	0	0	0	
13726732	m	md	JP	2017	0	0	0	0	0	0	0	0	0	0	0	0	76
13726989	m	ot	US	2017	0	0	0	0	0	0	0	0	0	0	0	0	51
13728208	f	md	IT	2017	1	0	0	0	0	0	0	0	0	0	0	0	
13730495	m	cn	US	2017	1	0	0	0	0	0	0	0	0	0	0	0	
13730498	f	cn	GB	2017	1	0	0	0	0	0	0	0	0	0	0	0	
13731492	Not	cn	JP	2017	0	0	0	0	0	0	0	0	0	0	0	0	
13731530	m	cn	US	2017	0	1	1	0	0	0	0	0	0	0	0	0	
13731729	f	ot	JP	2017	0	1	0	0	1	0	0	0	0	0	0	0	81
13731734	m	ot	JP	2017	0	1	0	0	1	0	0	0	0	0	0	0	62
13733538	m	ot	JP	2017	0	1	0	0	1	0	0	0	0	0	0	0	41
13733648	f	cn	US	2017	0	0	0	0	0	0	0	0	0	0	0	0	62
13733730	Not	cn	US	2017	0	1	0	0	1	0	0	0	0	0	0	0	
13733975	Not	cn	TW	2017	0	1	1	0	0	0	0	0	0	0	0	1	
13734166	Not	cn	TW	2017	0	1	1	0	0	0	0	0	0	0	0	0	
13734207	f	md	US	2017	0	1	1	0	0	0	0	0	0	0	0	0	
13734268	m	cn	JP	2017	0	0	0	0	0	0	0	0	0	0	0	0	
13734619	f	md	JP	2017	1	0	0	0	0	0	0	0	0	0	0	0	84
13734814	m	md	JP	2017	0	0	0	0	0	0	0	0	0	0	0	0	65
13735451	m	cn	US	2017	0	0	0	0	0	0	0	0	0	0	0	0	66
13736209	f	cn	FR	2017	0	0	0	0	0	0	0	0	0	0	0	0	
13736234	f	ot	DE	2017	0	0	0	0	0	0	0	0	0	0	0	0	65
13736314	m	md	JP	2017	1	0	0	0	0	0	0	0	0	0	0	1	80.4
13736330	f	ph	DE	2017	0	0	0	0	0	0	0	0	0	0	0	0	82
13736453	m	cn	US	2017	0	1	1	0	0	0	0	0	0	0	0	0	76
13736472	m	ot	IT	2017	0	0	0	0	0	0	0	0	0	0	0	0	69
13736725	m	md	FR	2017	0	1	0	0	0	1	0	0	0	0	0	0	78
13736957	m	ot	CA	2017	0	0	0	0	0	0	0	0	0	0	0	0	44

13736985	m	md	JP	2017	0	0	0	0	0	0	0	0	0	0	0	0	69
13738046	m	ot	US	2017	0	1	0	0	1	0	0	0	0	0	0	0	71
13738086	m	md	JP	2017	0	0	0	0	0	0	0	0	0	0	0	0	78
13738122	m	cn	US	2017	1	0	0	0	0	0	0	0	0	0	0	1	62
13738283	m	ot	JP	2017	0	1	0	0	1	0	0	0	0	0	0	0	50
13738375	m	ot	JP	2017	0	1	0	0	1	0	0	0	0	0	0	0	59
13738806	Not	ph	AU	2017	0	1	0	0	1	0	0	0	0	0	0	0	76
13738840	f	md	TH	2017	0	0	0	0	0	0	0	0	0	0	0	0	
13739227	f	md	DE	2017	0	1	0	0	1	0	0	0	0	0	0	0	71
13739348	f	ot	FR	2017	0	1	0	0	1	0	0	0	0	0	0	0	62
13739522	m	cn	GB	2017	1	0	0	0	0	0	0	0	0	0	0	0	74
13739607	f	cn	FR	2017	0	1	1	0	0	0	0	0	0	0	0	0	
13739954	m	md	JP	2017	1	0	0	0	0	0	0	0	0	0	0	0	44
13740027	f	ot	JP	2017	0	1	0	0	1	0	0	0	0	0	0	0	60
13740060	m	cn	US	2017	0	1	1	0	0	0	0	0	0	0	0	0	
13740154	f	Not repo	US	2017	1	0	0	0	0	0	0	0	0	0	0	0	64.9
13740213	f	md	CH	2017	0	1	0	0	1	0	0	0	0	0	0	1	63
13740265	f	md	US	2017	0	1	0	0	0	1	0	0	0	0	0	0	
13740370	f	ot	US	2017	0	1	1	0	0	0	0	0	0	0	0	0	73
13740750	m	cn	US	2017	0	0	0	0	0	0	0	0	0	0	0	0	61
13740786	m	md	JP	2017	0	1	0	0	1	0	0	0	0	0	0	1	68
13740820	f	ot	US	2017	0	0	0	0	0	0	0	0	0	0	0	0	70
13740903	m	md	JP	2017	0	1	0	0	1	0	0	0	0	0	0	0	50
13741104	m	md	JP	2017	0	1	0	0	1	0	0	0	0	0	0	0	82
13741289	m	ot	JP	2017	0	1	0	0	1	0	0	0	0	0	0	0	47
13741499	f	md	ES	2017	0	1	0	0	0	1	0	0	0	0	0	0	74
13742360	f	cn	CN	2017	1	0	0	0	0	0	0	0	0	0	0	0	82
13742419	Not	ph	AU	2017	0	1	0	0	1	0	0	0	0	0	0	0	52
13742631	f	md	JP	2017	1	0	0	0	0	0	0	0	0	0	0	0	73
13742896	m	md	US	2017	0	1	0	1	0	0	0	0	0	0	0	0	76.6
13743103	f	md	JP	2017	0	1	0	0	1	0	0	0	0	0	0	0	42
13743210	m	ot	JP	2017	0	1	0	0	1	0	0	0	0	0	0	0	52
13743213	m	ot	JP	2017	0	1	0	0	1	0	0	0	0	0	0	0	75
13743230	m	ot	JP	2017	0	1	0	0	1	0	0	0	0	0	0	0	60

13743278	m	ot	JP	2017	0	1	0	0	1	0	0	0	0	0	0	0	70
13743434	f	md	JP	2017	0	0	0	0	0	0	0	0	0	0	0	0	71
13743587	f	ot	CA	2017	0	1	0	0	1	0	0	0	0	0	0	0	66
13743869	m	ot	JP	2017	0	1	0	0	1	0	0	0	0	0	0	1	68
13743912	m	ot	JP	2017	0	1	0	0	1	0	0	0	0	0	0	0	70
13744033	m	ot	JP	2017	0	1	0	0	1	0	0	0	0	0	0	0	77
13744274	m	md	JP	2017	0	1	0	0	1	0	0	0	0	0	0	0	76
13744350	f	ot	JP	2017	0	0	0	0	0	0	0	0	0	0	0	0	67
13744484	m	ot	JP	2017	0	1	0	0	1	0	0	0	0	0	0	0	68
13745039	m	ot	JP	2017	0	1	0	0	1	0	0	0	0	0	0	0	60
13745294	m	cn	TW	2017	0	1	1	0	0	0	0	0	0	0	0	0	
13745540	Not	ph	AU	2017	0	1	0	0	1	0	0	0	0	0	0	0	
13745589	m	md	FR	2017	1	0	0	0	0	0	0	0	0	0	0	0	
13745985	m	md	US	2017	0	0	0	0	0	0	0	0	0	0	0	0	57
13746237	m	md	PT	2017	0	0	0	0	0	0	0	0	0	0	0	0	65
13746366	m	md	JP	2017	0	0	0	0	0	0	0	0	0	0	0	0	64
13746390	m	Not repo	US	2017	1	0	0	0	0	0	0	0	0	0	0	0	
13746431	Not	cn	GB	2017	1	0	0	0	0	0	0	0	0	0	0	0	
13746539	f	md	JP	2017	0	0	0	0	0	0	0	0	0	0	0	0	67
13746584	m	cn	TW	2017	0	0	0	0	0	0	0	0	0	0	0	0	73
13746589	m	cn	TW	2017	0	0	0	0	0	0	0	0	0	0	0	0	56
13746644	f	ot	US	2017	0	1	0	0	1	0	0	0	0	0	0	0	82
13746877	m	cn	US	2017	1	0	0	0	0	0	0	0	0	0	0	0	
13746880	f	cn	GB	2017	1	0	0	0	0	0	0	0	0	0	0	0	71
13747037	m	md	FR	2017	0	0	0	0	0	0	0	0	0	0	0	0	61
13747150	m	md	US	2017	0	1	0	0	1	0	0	0	0	0	0	0	
13747523	m	cn	US	2017	0	0	0	0	0	0	0	0	0	0	0	0	60
13747774	m	md	HU	2017	0	1	0	0	1	0	0	0	0	0	0	0	69
13748182	m	md	US	2017	0	1	0	0	1	0	0	0	0	0	0	0	57
13748447	m	ot	GR	2017	0	1	0	0	1	0	0	0	0	0	0	0	72
13748823	m	md	FR	2017	0	1	0	0	0	1	0	0	0	0	0	0	62
13748896	m	cn	GB	2017	1	0	0	0	0	0	0	0	0	0	0	0	46
13748897	m	Not repo	US	2017	1	0	0	0	0	0	0	0	0	0	0	0	69.3
13749141	m	md	KR	2017	0	1	0	0	0	1	0	0	0	0	0	0	36

13749203	Not	cn	US	2017	0	1	0	0	1	0	0	0	0	0	0	0	
13749618	f	ot	US	2017	0	1	0	0	1	0	0	0	0	0	0	0	68
13750893	Not	ph	AU	2017	0	1	0	0	1	0	0	0	0	0	0	0	74
13751104	f	md	FR	2017	0	1	0	0	1	0	0	0	0	0	0	0	71
13751581	f	md	CN	2017	1	0	0	0	0	0	0	0	0	0	0	0	75.1
13751661	f	ot	JP	2017	0	0	0	0	0	0	0	0	0	0	0	0	67
13752047	m	md	DE	2017	0	0	0	0	0	0	0	0	0	0	0	0	60
13752219	f	md	CN	2017	1	0	0	0	0	0	0	0	0	0	0	0	62.3
13752500	f	ot	US	2017	0	0	0	0	0	0	0	0	0	0	0	0	
13752918	m	md	JP	2017	0	1	0	0	1	0	0	0	0	0	0	1	71
13753385	f	cn	US	2017	0	1	0	0	1	0	0	0	0	0	0	0	
13753406	m	md	FR	2017	0	1	0	0	1	0	0	0	0	0	0	0	
13753604	f	cn	US	2017	0	1	0	0	1	0	0	0	0	0	0	0	60
13753701	f	md	DE	2017	1	0	0	0	0	0	0	0	0	0	0	0	50
13753861	f	md	KR	2017	0	0	0	0	0	0	0	0	0	0	0	0	56
13753977	m	cn	US	2017	0	1	0	0	1	0	0	0	0	0	0	0	50
13754197	f	md	US	2017	0	1	1	0	0	0	0	0	0	0	0	0	
13754284	m	Not repo	Not re	2017	1	0	0	0	0	0	0	0	0	0	0	0	67
13754335	f	ot	AU	2017	1	0	0	0	0	0	0	0	0	0	0	0	48
13754556	f	md	PT	2017	0	0	0	0	0	0	0	0	0	0	0	0	82
13754658	m	md	JP	2017	0	1	0	0	1	0	0	0	0	0	0	0	73
13754752	Not	cn	JP	2017	0	1	1	0	0	0	0	0	0	0	0	0	
13754822	m	cn	JP	2017	0	0	0	0	0	0	0	0	0	0	0	0	
13754889	m	cn	GB	2017	1	0	0	0	0	0	0	0	0	0	0	0	74
13754894	f	ph	GB	2017	0	1	0	0	1	0	0	0	0	0	0	0	69
13754935	Not	ph	AU	2017	0	1	0	0	1	0	0	0	0	0	0	0	43
13754966	f	md	US	2017	0	1	0	0	1	0	0	0	0	0	0	0	
13755544	f	cn	US	2017	0	0	0	0	0	0	0	0	0	0	0	0	67
13755557	m	md	KR	2017	0	0	0	0	0	0	0	0	0	0	0	0	55
13755616	m	ot	JP	2017	0	1	0	0	1	0	0	0	0	0	0	0	77
13755885	m	md	US	2017	0	1	0	0	1	0	0	0	0	0	0	0	
13756293	Not	md	ES	2017	0	1	0	0	1	0	0	0	0	0	0	0	
13756587	f	cn	CA	2017	0	0	0	0	0	0	0	0	0	0	0	0	
13756975	m	cn	US	2017	0	0	0	0	0	0	0	0	0	0	0	0	69

13756987	m	md	US	2017	0	0	0	0	0	0	0	0	0	0	0	0	85
13757020	m	cn	US	2017	0	1	1	0	0	0	0	0	0	0	0	0	
13757572	f	md	NL	2017	0	0	0	0	0	0	0	0	0	0	0	0	65
13757592	Not	ot	IT	2017	0	0	0	0	0	0	0	0	0	0	0	0	
13757609	Not	ot	IT	2017	0	0	0	0	0	0	0	0	0	0	0	0	
13757615	Not	ot	IT	2017	0	0	0	0	0	0	0	0	0	0	0	0	
13757628	Not	ot	IT	2017	0	0	0	0	0	0	0	0	0	0	0	0	
13757752	m	ot	US	2017	0	1	0	0	1	0	0	0	0	0	0	0	
13757968	m	cn	TW	2017	0	1	1	0	0	0	0	0	0	0	0	0	
13758317	f	ot	US	2017	0	0	0	0	0	0	0	0	0	0	0	0	64
13758856	f	ot	US	2017	0	1	0	0	1	0	0	0	0	0	0	0	75
13758904	m	md	JP	2017	1	0	0	0	0	0	0	0	0	0	0	1	86.4
13758942	m	cn	FR	2017	0	0	0	0	0	0	0	0	0	0	0	0	65
13759016	Not	md	KR	2017	0	1	1	0	0	0	0	0	0	0	0	0	
13759028	m	md	JP	2017	0	1	0	0	1	0	0	0	0	0	0	0	70
13759029	m	md	AU	2017	0	1	0	0	1	0	0	0	0	0	0	1	44
13759051	m	md	CN	2017	1	0	0	0	0	0	0	0	0	0	0	0	71.8
13759121	m	md	ES	2017	0	1	0	1	0	0	0	0	0	0	0	0	71.4
13759185	f	cn	GB	2017	1	0	0	0	0	0	0	0	0	0	0	0	68
13759702	f	md	IT	2017	0	1	0	0	1	0	0	0	0	0	0	0	
13760046	m	md	JP	2017	0	1	0	0	1	0	0	0	0	0	0	0	67
13760053	m	ot	DE	2017	0	0	0	0	0	0	0	0	0	0	0	0	69
13760689	m	cn	US	2017	0	1	0	0	1	0	0	0	0	0	0	0	
13761652	m	md	NL	2017	0	0	0	0	0	0	0	0	0	0	0	0	58
13761794	Not	ph	JP	2017	1	0	0	0	0	0	0	0	0	0	0	0	80
13761893	m	cn	JP	2017	0	0	0	0	0	0	0	0	0	0	0	0	63
13762959	m	cn	US	2017	0	0	0	0	0	0	0	0	0	0	0	0	46
13763223	f	Not repo	US	2017	1	0	0	0	0	0	0	0	0	0	0	0	68.4
13763254	f	md	US	2017	0	0	0	0	0	0	0	0	0	0	0	0	58
13763751	f	cn	JP	2017	0	0	0	0	0	0	0	0	0	0	0	0	55
13764566	m	cn	JP	2017	0	1	0	0	1	0	0	0	0	0	0	1	
13765041	m	ot	JP	2017	0	0	0	0	0	0	0	0	0	0	0	0	74
13765082	Not	md	JP	2017	0	1	0	0	1	0	0	0	0	0	0	0	
13765087	f	ot	US	2017	0	1	0	0	1	0	0	0	0	0	0	0	55

13765393	m	ot	DE	2017	0	1	0	0	1	0	0	0	0	0	0	0	79
13765424	m	md	US	2017	0	1	1	0	0	0	0	0	0	0	0	0	46
13765515	Not	md	AT	2017	0	1	1	0	0	0	0	0	0	0	0	0	
13765999	f	ot	US	2017	0	1	0	0	1	0	0	0	0	0	0	0	74
13766218	f	md	JP	2017	1	0	0	0	0	0	0	0	0	0	0	0	70
13766322	m	md	JP	2017	0	1	0	0	1	0	0	0	0	0	0	0	68
13766459	f	ot	US	2017	0	1	0	0	1	0	0	0	0	0	0	0	55
13766611	f	ot	JP	2017	1	0	0	0	0	0	0	0	0	0	0	0	74
13766872	f	md	US	2017	0	1	1	0	0	0	0	0	0	0	0	0	56
13767243	f	md	JP	2017	1	0	0	0	0	0	0	0	0	0	0	0	82
13767287	f	ot	US	2017	0	1	0	0	1	0	0	0	0	0	0	0	70
13767928	m	md	US	2017	0	1	1	0	0	0	0	0	0	0	0	0	70
13768152	f	ot	DE	2017	0	0	0	0	0	0	0	0	0	0	0	0	68
13768278	f	md	US	2017	0	1	0	0	0	1	0	0	0	0	0	0	50
13769205	m	ot	ES	2017	0	1	0	0	1	0	0	0	0	0	0	0	67
13769788	f	cn	US	2017	0	1	1	0	0	0	0	0	0	0	0	0	57
13771141	m	cn	JP	2017	0	0	0	0	0	0	0	0	0	0	0	0	71
13771489	m	md	JP	2017	0	1	0	0	1	0	0	0	0	0	0	0	60
13771839	f	md	JP	2017	1	0	0	0	0	0	0	0	0	0	0	1	90
13771927	f	cn	GB	2017	1	0	0	0	0	0	0	0	0	0	0	0	82
13772015	f	md	BR	2017	0	0	0	0	0	0	0	0	0	0	0	0	44
13772230	f	ot	US	2017	0	1	0	0	1	0	0	0	0	0	0	0	
13773062	f	ot	DE	2017	0	1	0	0	1	0	0	0	0	0	0	0	61
13773075	f	md	JP	2017	0	0	0	0	0	0	0	0	0	0	0	0	69
13773660	m	cn	US	2017	0	0	0	0	0	0	0	0	0	0	0	0	47
13773915	m	md	JP	2017	0	1	0	0	1	0	0	0	0	0	0	0	71
13773922	m	cn	JP	2017	0	1	1	0	0	0	0	0	0	0	0	1	69
13774084	f	ot	JP	2017	0	1	0	0	1	0	0	0	0	0	0	0	60
13774746	f	ot	US	2017	0	1	0	0	1	0	0	0	0	0	0	0	
13775012	Not	md	JP	2017	0	1	0	0	1	0	0	0	0	0	0	0	
13775025	Not	ph	AU	2017	0	1	0	0	1	0	0	0	0	0	0	0	57
13775071	Not	cn	TW	2017	0	0	0	0	0	0	0	0	0	0	0	0	
13775273	m	md	CN	2017	1	0	0	0	0	0	0	0	0	0	0	0	56.5
13775276	f	md	DE	2017	0	1	0	1	0	0	0	0	0	0	0	0	59

13775326	m	cn	GB	2017	0	0	0	0	0	0	0	0	0	0	0	0	80
13775388	f	cn	US	2017	0	0	0	0	0	0	0	0	0	0	0	0	56
13775458	f	cn	US	2017	0	1	1	0	0	0	0	0	0	0	0	0	
13775487	m	md	JP	2017	0	1	0	0	1	0	0	0	0	0	0	0	55
13775648	m	md	JP	2017	0	1	0	0	1	0	0	0	0	0	0	0	64
13775810	Not	cn	GB	2017	1	0	0	0	0	0	0	0	0	0	0	0	
13775853	m	ot	US	2017	0	1	0	0	1	0	0	0	0	0	0	0	39
13775959	Not	md	JP	2017	0	1	0	0	1	0	0	0	0	0	0	0	
13776032	m	ot	US	2017	0	1	0	0	1	0	0	0	0	0	0	0	66
13776211	f	cn	GB	2017	1	0	0	0	0	0	0	0	0	0	0	0	78
13776304	m	md	JP	2017	0	1	0	0	1	0	0	0	0	0	0	0	67
13776719	f	ot	JP	2017	0	1	1	0	0	0	0	0	0	0	0	0	76
13776770	m	md	CN	2017	1	0	0	0	0	0	0	0	0	0	0	0	59
13777180	m	cn	JP	2017	0	1	1	0	0	0	0	0	0	0	0	0	73
13777412	m	ot	GB	2017	0	1	0	0	1	0	0	0	0	0	0	0	69
13777497	m	cn	US	2017	0	1	0	0	1	0	0	0	0	0	0	0	
13777754	f	cn	JP	2017	0	0	0	0	0	0	0	0	0	0	0	0	63
13777819	f	md	JP	2017	0	1	0	0	1	0	0	0	0	0	0	0	54
13777958	f	cn	US	2017	0	1	0	0	1	0	0	0	0	0	0	0	
13778251	f	ot	DE	2017	0	1	0	0	1	0	0	0	0	0	0	0	65
13778265	m	ot	US	2017	0	1	0	0	1	0	0	0	0	0	0	0	67
13778494	m	ot	JP	2017	0	1	0	0	1	0	0	0	0	0	0	0	67
13778663	f	Not repo	US	2017	1	0	0	0	0	0	0	0	0	0	0	0	
13778717	m	cn	US	2017	0	0	0	0	0	0	0	0	0	0	0	0	81
13779301	m	md	BE	2017	0	1	0	0	1	0	0	0	0	0	0	0	77
13779324	f	md	JP	2017	1	0	0	0	0	0	0	0	0	0	0	1	63.9
13779500	m	md	NL	2017	0	0	0	0	0	0	0	0	0	0	0	0	63
13779841	m	cn	TH	2017	0	1	1	0	0	0	0	0	0	0	0	0	61
13780100	f	md	US	2017	0	1	1	0	0	0	0	0	0	0	0	0	56
13780120	m	md	JP	2017	0	1	0	0	1	0	0	0	0	0	0	0	74
13780388	Not	cn	US	2017	0	1	1	0	0	0	0	0	0	0	0	0	
13781203	Not	md	KR	2017	0	1	1	0	0	0	0	0	0	0	0	0	
13781447	m	md	AU	2017	0	1	0	0	0	1	0	0	0	0	0	0	68
13781512	m	md	IT	2017	1	0	0	0	0	0	0	0	0	0	0	0	71

13781627	m	ot	GR	2017	0	1	0	0	1	0	0	0	0	0	0	0	79
13781738	m	ot	AU	2017	0	1	0	0	1	0	0	0	0	0	0	0	74
13782220	m	cn	JP	2017	0	1	0	0	1	0	0	0	0	0	0	0	55
13782482	m	cn	JP	2017	0	1	1	0	0	0	0	0	0	0	0	0	69
13782493	Not	ot	US	2017	0	1	1	0	0	0	0	0	0	0	0	0	61
13783063	Not	cn	GB	2017	1	0	0	0	0	0	0	0	0	0	0	0	
13783664	m	md	KR	2017	0	0	0	0	0	0	0	0	0	0	0	0	41
13784070	f	md	US	2017	0	1	1	0	0	0	0	0	0	0	0	0	
13784909	f	md	JP	2017	0	1	0	0	1	0	0	0	0	0	0	1	76
13785395	m	cn	IN	2017	0	1	0	0	1	0	0	0	0	0	0	0	46
13785481	m	cn	IN	2017	0	1	0	0	1	0	0	0	0	0	0	0	20
13786059	m	md	DK	2017	0	1	1	0	0	0	0	0	0	0	0	0	67
13786582	Not	cn	US	2017	0	1	1	0	0	0	0	0	0	0	0	1	
13788091	f	cn	US	2017	0	1	0	0	1	0	0	0	0	0	0	0	
13788106	f	cn	JP	2017	0	0	0	0	0	0	0	0	0	0	0	0	75
13789891	m	md	US	2017	1	0	0	0	0	0	0	0	0	0	0	0	
13790234	m	cn	US	2017	0	0	0	0	0	0	0	0	0	0	0	0	84
13790804	m	cn	US	2017	0	1	1	0	0	0	0	0	0	0	0	0	81
13790875	Not	md	US	2017	1	0	0	0	0	0	0	0	0	0	0	0	
13790944	f	md	US	2017	0	1	1	0	0	0	0	0	0	0	0	0	60
13791117	f	ot	JP	2017	0	1	0	0	1	0	0	0	0	0	0	0	73
13791186	m	ot	JP	2017	0	1	0	0	1	0	0	0	0	0	0	0	81
13791619	m	md	CN	2017	1	0	0	0	0	0	0	0	0	0	0	0	57
13792446	f	cn	JP	2017	0	1	1	0	0	0	0	0	0	0	0	0	75
13792450	Not	cn	GB	2017	0	1	1	0	0	0	0	0	0	0	0	0	
13792535	m	cn	JP	2017	0	1	1	0	0	0	0	0	0	0	0	0	55
13792596	Not	ot	JP	2017	0	0	0	0	0	0	0	0	0	0	0	0	
13792855	m	md	JP	2017	0	1	1	0	0	0	0	0	0	0	0	0	69
13792882	m	cn	JP	2017	0	0	0	0	0	0	0	0	0	0	0	0	65
13793031	f	md	JP	2017	0	0	0	0	0	0	1	0	1	0	0	0	80
13793166	m	md	JP	2017	1	0	0	0	0	0	0	0	0	0	0	0	86
13793594	f	md	ES	2017	0	1	0	0	0	1	0	0	0	0	0	0	63
13793993	m	cn	US	2017	1	0	0	0	0	0	0	0	0	0	0	0	82
13794054	m	md	JP	2017	0	0	0	0	0	0	0	0	0	0	0	0	70

13794179	m	md	DE	2017	0	1	0	0	1	0	0	0	0	0	0	1	
13794425	m	md	IN	2017	0	1	0	0	1	0	0	0	0	0	0	0	58
13794512	m	md	ES	2017	0	0	0	0	0	0	0	0	0	0	0	0	70
13794539	m	cn	JP	2017	0	1	1	0	0	0	0	0	0	0	0	1	68
13794702	f	cn	JP	2017	0	1	1	0	0	0	0	0	0	0	0	0	78
13794982	Not	md	AU	2017	1	0	0	0	0	0	0	0	0	0	0	0	
13794991	f	ph	JP	2017	0	1	0	0	1	0	0	0	0	0	0	0	73
13794993	m	cn	IN	2017	0	1	0	0	1	0	0	0	0	0	0	0	62
13795072	m	md	DE	2017	0	0	0	0	0	0	0	0	0	0	0	0	79
13795958	m	md	IN	2017	0	1	0	0	1	0	0	0	0	0	0	0	46
13796149	m	md	IN	2017	0	1	0	0	1	0	0	0	0	0	0	0	75
13796163	f	cn	IN	2017	0	1	0	0	1	0	0	0	0	0	0	0	68
13796209	m	md	IN	2017	0	1	0	0	1	0	0	0	0	0	0	0	48
13796907	m	cn	IN	2017	0	1	0	0	1	0	0	0	0	0	0	0	80
13797022	m	cn	IN	2017	0	1	0	0	1	0	0	0	0	0	0	0	59
13797024	m	md	IN	2017	0	1	0	0	1	0	0	0	0	0	0	0	77
13797327	f	md	IN	2017	0	1	0	0	1	0	0	0	0	0	0	0	61
13797336	m	ot	US	2017	0	1	0	0	1	0	0	0	0	0	0	0	63
13797341	m	ot	MA	2017	0	0	0	0	0	0	0	0	0	0	0	0	62
13797360	f	md	US	2017	0	1	0	0	0	1	0	0	0	0	0	0	
13797547	f	ot	US	2017	0	0	0	0	0	0	0	0	0	0	0	0	
13797720	m	cn	JP	2017	0	1	1	0	0	0	0	0	0	0	0	0	75
13797765	Not	ot	CO	2017	0	1	1	0	0	0	0	0	0	0	0	0	
13797964	m	md	IN	2017	0	1	0	0	1	0	0	0	0	0	0	0	55
13797965	m	cn	IN	2017	0	1	0	0	1	0	0	0	0	0	0	0	53
13797966	f	cn	IN	2017	0	1	0	0	1	0	0	0	0	0	0	0	47
13797967	m	cn	IN	2017	0	1	0	0	1	0	0	0	0	0	0	0	54
13798277	f	md	CN	2017	1	0	0	0	0	0	0	0	0	0	0	0	67.7
13798284	f	md	IN	2017	0	1	0	0	1	0	0	0	0	0	0	0	60
13798289	m	md	IN	2017	0	1	0	0	1	0	0	0	0	0	0	0	38
13798614	f	ph	US	2017	0	1	0	0	1	0	0	0	0	0	0	0	
13798623	f	md	CN	2017	1	0	0	0	0	0	0	0	0	0	0	0	49.3
13800316	m	cn	GB	2017	1	0	0	0	0	0	0	0	0	0	0	0	
13800434	m	ot	JP	2017	0	1	0	0	1	0	0	0	0	0	0	0	77

13800803	Not	md	JP	2017	1	0	0	0	0	0	0	0	0	0	0	0	
13800860	Not	cn	GR	2017	0	1	0	0	1	0	0	0	0	0	0	0	
13800908	m	ot	GB	2017	0	0	0	0	0	0	0	0	0	0	0	0	56
13800956	f	cn	US	2017	0	1	1	0	0	0	0	0	0	0	0	0	
13801399	f	md	BE	2017	0	0	0	0	0	0	0	0	0	0	0	0	
13801722	f	md	US	2017	0	1	0	0	1	0	0	0	0	0	0	0	73
13801938	m	cn	JP	2017	0	1	1	0	0	0	0	0	0	0	0	0	68
13801959	f	md	CN	2017	1	0	0	0	0	0	0	0	0	0	0	0	50
13802022	f	md	GR	2017	1	0	0	0	0	0	0	0	0	0	0	0	78
13802074	Not	cn	AU	2017	0	1	0	0	1	0	0	0	0	0	0	0	78
13802172	m	ot	PL	2017	0	1	0	0	1	0	0	0	0	0	0	0	74
13802173	m	ot	US	2017	0	1	0	0	1	0	0	0	0	0	0	0	68
13802368	Not	cn	US	2017	0	0	0	0	0	0	0	0	0	0	0	0	
13802537	m	md	IN	2017	0	1	0	0	1	0	0	0	0	0	0	0	57
13802641	m	cn	JP	2017	0	1	1	0	0	0	0	0	0	0	0	1	
13802783	m	cn	IN	2017	0	1	0	0	1	0	0	0	0	0	0	0	71
13802785	m	md	EG	2017	0	1	0	0	1	0	0	0	0	0	0	0	65
13802900	Not	cn	GR	2017	0	1	0	0	1	0	0	0	0	0	0	0	
13802947	f	cn	IN	2017	0	1	0	0	1	0	0	0	0	0	0	0	63
13802951	f	md	IN	2017	0	1	0	0	1	0	0	0	0	0	0	0	71
13803435	f	md	US	2017	0	1	1	0	0	0	0	0	0	0	0	0	71
13803452	Not	cn	US	2017	0	1	0	0	1	0	0	0	0	0	0	0	
13803589	m	ot	US	2017	0	1	1	0	0	0	0	0	0	0	0	0	61
13804067	m	md	DE	2017	0	1	0	0	1	0	0	0	0	0	0	0	
13804186	f	md	GB	2017	0	1	0	0	0	1	0	0	0	0	0	0	62
13804360	m	ot	US	2017	0	0	0	0	0	0	0	0	0	0	0	0	
13805855	m	Not repo	CA	2017	1	0	0	0	0	0	0	0	0	0	0	0	52
13805908	Not	cn	US	2017	0	1	0	0	1	0	0	0	0	0	0	0	
13807552	f	md	KR	2017	1	0	0	0	0	0	0	0	0	0	0	0	72
13807583	f	md	JP	2017	1	0	0	0	0	0	0	0	0	0	0	1	86
13807933	f	Not repo	US	2017	1	0	0	0	0	0	0	0	0	0	0	0	62.2
13808281	m	ot	GB	2017	0	0	0	0	0	0	0	0	0	0	0	0	70
13810430	m	cn	IN	2017	0	1	0	0	1	0	0	0	0	0	0	0	38
13810433	m	md	IN	2017	0	1	0	0	1	0	0	0	0	0	0	0	51

13810434	m	md	IN	2017	0	1	0	0	1	0	0	0	0	0	0	0	59
13810459	f	md	IN	2017	0	1	0	0	1	0	0	0	0	0	0	1	71
13812756	m	md	JP	2017	0	1	0	0	1	0	0	0	0	0	0	1	74
13812780	f	cn	IN	2017	0	1	0	0	1	0	0	0	0	0	0	0	60
13813017	m	ot	US	2017	0	1	0	0	1	0	0	0	0	0	0	0	75
13813126	m	md	US	2017	0	1	0	0	1	0	0	0	0	0	0	0	
13813128	m	cn	IN	2017	0	1	0	0	1	0	0	0	0	0	0	0	40
13813134	m	cn	IN	2017	0	1	0	0	1	0	0	0	0	0	0	0	82
13814642	m	ot	DE	2017	0	1	0	0	1	0	0	0	0	0	0	0	80
13815042	f	md	AR	2017	0	0	0	0	0	0	0	0	0	0	0	0	48
13815345	f	cn	AR	2017	0	0	0	0	0	0	0	0	0	0	0	0	53
13816041	f	cn	US	2017	0	1	0	0	1	0	0	0	0	0	0	1	
13817114	m	cn	IN	2017	0	1	0	0	1	0	0	0	0	0	0	0	67
13817447	f	md	SE	2017	0	0	0	0	0	0	0	0	0	0	0	0	48
13817462	f	md	JP	2017	0	0	0	0	0	0	0	0	0	0	0	0	69
13818558	f	cn	IN	2017	0	1	0	0	1	0	0	0	0	0	0	0	57
13818563	f	cn	IN	2017	0	1	0	0	1	0	0	0	0	0	0	0	47
13819049	m	ot	US	2017	0	1	0	0	1	0	0	0	0	0	0	0	60
13819065	m	md	JP	2017	0	1	0	0	1	0	0	0	0	0	0	0	61
13819213	f	ot	DE	2017	0	0	0	0	0	0	0	0	0	0	0	0	77
13819365	m	cn	JP	2017	0	0	0	0	0	0	0	0	0	0	0	0	39
13820221	f	md	SK	2017	0	0	0	0	0	0	0	0	0	0	0	0	67
13820241	m	cn	IN	2017	0	1	0	0	1	0	0	0	0	0	0	0	73
13820525	m	md	CN	2017	0	0	0	0	0	0	0	0	0	0	0	0	
13820604	f	ot	ES	2017	1	0	0	0	0	0	0	0	0	0	0	0	78
13820618	f	md	TH	2017	0	0	0	0	0	0	0	0	0	0	0	0	55
13820705	f	md	AT	2017	0	1	0	0	0	1	0	0	0	0	0	0	
13821163	m	md	IN	2017	0	1	0	0	1	0	0	0	0	0	0	0	67
13821491	m	cn	DE	2017	0	1	1	0	0	0	0	0	0	0	0	0	61
13821498	m	cn	IL	2017	0	1	1	0	0	0	0	0	0	0	0	1	
13821510	f	cn	IL	2017	0	1	1	0	0	0	0	0	0	0	0	1	
13822506	f	md	CN	2017	1	0	0	0	0	0	0	0	0	0	0	0	55
13822710	m	md	DE	2017	0	1	0	0	1	0	0	0	0	0	0	0	62
13822713	m	md	JP	2017	0	1	0	0	1	0	0	0	0	0	0	0	52

13823145	f	cn	CA	2017	1	0	0	0	0	0	0	0	0	0	0	0	80.8
13823276	m	cn	US	2017	0	1	0	0	1	0	0	0	0	0	0	0	
13823298	f	md	JP	2017	0	0	0	0	0	0	0	0	0	0	0	0	
13823347	f	cn	IN	2017	0	1	0	0	1	0	0	0	0	0	0	0	38
13823349	m	cn	IN	2017	0	1	0	0	1	0	0	0	0	0	0	0	57
13823421	m	cn	IN	2017	0	1	0	0	1	0	0	0	0	0	0	0	51
13823558	m	md	US	2017	0	1	0	0	1	0	0	0	0	0	0	0	65
13823605	f	md	JP	2017	1	0	0	0	0	0	0	0	0	0	0	0	74
13824068	m	cn	US	2017	0	0	0	0	0	0	0	0	0	0	0	0	73
13824084	m	cn	Not re	2017	0	0	0	0	0	0	0	0	0	0	0	0	32
13824143	m	ot	MX	2017	0	1	0	0	1	0	0	0	0	0	0	0	49
13824382	f	md	JP	2017	1	0	0	0	0	0	0	0	0	0	0	0	56
13824417	m	cn	US	2017	0	0	0	0	0	0	0	0	0	0	0	0	59
13824466	m	md	SK	2017	1	0	0	0	0	0	0	0	0	0	0	0	59
13824795	m	cn	JP	2017	0	0	0	0	0	0	0	0	0	0	0	0	66
13824822	m	md	JP	2017	0	1	0	0	1	0	0	0	0	0	0	0	65
13825697	f	ot	NL	2017	0	1	0	0	1	0	0	0	0	0	0	0	68
13826267	f	md	KR	2017	1	0	0	0	0	0	0	0	0	0	0	0	82
13826274	f	cn	US	2017	0	1	0	0	1	0	0	0	0	0	0	0	
13827474	Not	md	JP	2017	1	0	0	0	0	0	0	0	0	0	0	0	
13827688	f	cn	IT	2017	1	0	0	0	0	0	0	0	0	0	0	0	66.6
13827833	m	cn	US	2017	0	1	0	0	1	0	0	0	0	0	0	0	
13828730	f	ot	US	2017	0	1	0	0	1	0	0	0	0	0	0	0	75
13828959	m	md	JP	2017	0	1	0	0	1	0	0	0	0	0	0	0	57
13829071	m	ph	US	2017	0	0	0	0	0	0	0	0	0	0	0	0	81
13829162	m	md	JP	2017	0	0	0	0	0	0	0	0	0	0	0	0	60
13829329	m	cn	JP	2017	0	1	1	0	0	0	0	0	0	0	0	0	72
13831506	f	md	US	2017	0	0	0	0	0	0	0	0	0	0	0	0	63
13831533	m	md	JP	2017	1	0	0	0	0	0	0	0	0	0	0	0	68
13831535	f	md	JP	2017	1	0	0	0	0	0	0	0	0	0	0	0	57
13831542	f	md	JP	2017	1	0	0	0	0	0	0	0	0	0	0	0	63
13831600	m	md	JP	2017	1	0	0	0	0	0	0	0	0	0	0	0	51
13831625	f	md	JP	2017	1	0	0	0	0	0	0	0	0	0	0	0	65
13831626	m	md	JP	2017	1	0	0	0	0	0	0	0	0	0	0	0	74

13832974	m	ph	FR	2017	0	0	0	0	0	0	0	0	0	0	0	0	69
13833152	f	md	US	2017	0	1	0	0	1	0	0	0	0	0	0	0	90
13833617	Not	cn	JP	2017	0	1	1	0	0	0	0	0	0	0	0	0	76
13833652	m	Not repo	MY	2017	1	0	0	0	0	0	0	0	0	0	0	0	85.3
13833693	Not	ot	US	2017	0	1	0	0	0	1	0	0	0	0	0	0	
13833706	Not	ot	US	2017	0	1	0	0	0	1	0	0	0	0	0	0	
13833713	Not	ot	US	2017	0	1	0	0	0	1	0	0	0	0	0	0	
13833838	m	ot	JP	2017	0	0	0	0	0	0	0	0	0	0	0	0	62
13833913	f	ot	GB	2017	0	1	0	0	1	0	0	0	0	0	0	0	77
13834012	Not	md	MX	2017	0	1	1	0	0	0	0	0	0	0	0	0	
13834142	m	cn	US	2017	0	1	1	0	0	0	0	0	0	0	0	0	50
13835112	f	cn	JP	2017	0	1	1	0	0	0	0	0	0	0	0	0	67
13835178	f	md	JP	2017	1	0	0	0	0	0	0	0	0	0	0	0	79
13835372	f	md	US	2017	0	1	0	0	1	0	0	0	0	0	0	0	57
13835719	f	Not repo	CA	2017	1	0	0	0	0	0	0	0	0	0	0	0	75.5
13835935	f	cn	JP	2017	0	0	0	0	0	0	0	0	0	0	0	0	80
13836394	m	ot	BG	2017	0	0	0	0	0	0	0	0	0	0	0	0	62
13836495	f	md	JP	2017	0	1	0	0	1	0	0	0	0	0	0	0	69
13836545	m	md	JP	2017	0	1	0	0	1	0	0	0	0	0	0	0	70
13837525	f	md	CN	2017	1	0	0	0	0	0	0	0	0	0	0	0	65
13837607	Not	ot	JP	2017	1	0	0	0	0	0	0	0	0	0	0	0	
13838003	m	ot	US	2017	0	1	0	0	1	0	0	0	0	0	0	0	61
13838025	Not	ot	CZ	2017	0	0	0	0	0	0	0	0	0	0	0	0	
13838033	Not	ot	CZ	2017	0	0	0	0	0	0	0	0	0	0	0	0	
13838113	Not	ot	CZ	2017	0	0	0	0	0	0	0	0	0	0	0	0	
13838115	Not	ot	CZ	2017	0	0	0	0	0	0	0	0	0	0	0	0	
13838116	Not	ot	CZ	2017	0	0	0	0	0	0	0	0	0	0	0	0	
13838120	Not	ot	CZ	2017	0	0	0	0	0	0	0	0	0	0	0	0	
13838121	Not	ot	CZ	2017	0	0	0	0	0	0	0	0	0	0	0	0	
13838122	Not	ot	CZ	2017	0	0	0	0	0	0	0	0	0	0	0	0	
13838271	m	ph	JP	2017	0	1	0	0	1	0	0	0	0	0	0	0	70
13838290	f	md	CZ	2017	1	0	0	0	0	0	0	0	0	0	0	0	81
13838543	m	ph	US	2017	0	1	0	0	1	0	0	0	0	0	0	0	55
13839258	m	cn	JP	2017	0	0	0	0	0	0	0	0	0	0	0	0	

13839366	m	ph	US	2017	0	0	0	0	0	0	0	0	0	0	0	0	71
13839798	Not	ot	JP	2017	0	0	0	0	0	0	0	0	0	0	0	0	
13839811	f	cn	GB	2017	1	0	0	0	0	0	0	0	0	0	0	0	53
13840369	m	cn	GB	2017	1	0	0	0	0	0	0	0	0	0	0	0	77
13840434	f	md	CH	2017	0	0	0	0	0	0	0	0	0	0	0	0	
13841215	m	ot	CH	2017	0	1	0	0	1	0	0	0	0	0	0	0	70
13841339	f	cn	CN	2017	1	0	0	0	0	0	0	0	0	0	0	0	70
13842243	m	cn	US	2017	0	1	0	0	1	0	0	0	0	0	0	0	
13842387	f	md	JP	2017	1	0	0	0	0	0	0	0	0	0	0	0	78
13842731	m	md	JP	2017	0	1	0	0	1	0	0	0	0	0	0	0	82
13842827	f	md	GB	2017	0	1	1	0	0	0	0	0	0	0	0	0	74
13842892	Not	cn	JP	2017	0	1	1	0	0	0	0	0	0	0	0	0	
13843169	m	ot	US	2017	0	1	0	0	1	0	0	0	0	0	0	0	89
13843578	m	md	DE	2017	0	0	0	0	0	0	0	0	0	0	0	0	65
13843742	m	cn	US	2017	1	0	0	0	0	0	0	0	0	0	0	0	82
13843880	m	cn	JP	2017	0	1	1	0	0	0	0	0	0	0	0	0	37
13844111	m	md	JP	2017	0	1	1	0	0	0	0	0	0	0	0	0	55
13844154	m	md	JP	2017	0	1	1	0	0	0	0	0	0	0	0	0	69
13844292	f	cn	JP	2017	0	1	1	0	0	0	0	0	0	0	0	0	
13844525	f	ot	US	2017	0	1	0	0	1	0	0	0	0	0	0	0	81
13844531	m	cn	JP	2017	0	1	1	0	0	0	0	0	0	0	0	0	66
13844568	f	md	FR	2017	1	0	0	0	0	0	0	0	0	0	0	0	76.9
13847033	m	ot	DE	2017	0	1	0	0	1	0	0	0	0	0	0	0	57
13847066	f	ot	DE	2017	0	1	0	0	1	0	0	0	0	0	0	0	53
13847549	f	md	US	2017	1	0	0	0	0	0	0	0	0	0	0	0	64.2
13848526	f	cn	US	2017	0	1	1	0	0	0	0	0	0	0	0	0	60
13848534	m	cn	JP	2017	0	1	1	0	0	0	0	0	0	0	0	0	
13848698	f	Not repo	US	2017	1	0	0	0	0	0	0	0	0	0	0	0	
13848761	m	md	JP	2017	0	1	0	0	1	0	0	0	0	0	0	0	76
13848774	f	ot	PL	2017	0	1	0	0	1	0	0	0	0	0	0	0	66
13848787	m	cn	JP	2017	0	1	1	0	0	0	0	0	0	0	0	0	
13848814	m	md	KR	2017	0	1	0	0	0	1	0	0	0	0	0	0	63
13849353	m	md	JP	2017	0	0	0	0	0	0	0	0	0	0	0	0	80
13849833	f	md	JP	2017	0	0	0	0	0	0	0	0	0	0	0	0	82

13850979	Not	md	TW	2017	0	0	0	0	0	0	0	0	0	0	0	0	
13851090	m	cn	JP	2017	0	1	1	0	0	0	0	0	0	0	0	0	61
13851119	m	cn	GB	2017	1	0	0	0	0	0	0	0	0	0	0	0	
13851121	f	cn	GB	2017	1	0	0	0	0	0	0	0	0	0	0	0	74
13851166	m	cn	JP	2017	0	1	1	0	0	0	0	0	0	0	0	0	75
13851405	f	cn	JP	2017	0	1	1	0	0	0	0	0	0	0	0	0	74
13851585	f	md	CN	2017	0	0	0	0	0	0	0	0	0	0	0	0	56
13851804	m	md	JP	2017	0	1	0	0	1	0	0	0	0	0	0	0	71
13852152	m	cn	US	2017	0	0	0	0	0	0	0	0	0	0	0	0	84
13853189	m	md	US	2017	0	1	1	0	0	0	0	0	0	0	0	0	85
13853444	f	cn	JP	2017	0	1	1	0	0	0	0	0	0	0	0	0	64
13854033	m	md	ES	2017	0	1	0	0	1	0	0	0	0	0	0	0	65
13854068	m	cn	US	2017	0	1	0	0	1	0	0	0	0	0	0	0	
13854597	m	md	JP	2017	0	1	0	0	1	0	0	0	0	0	0	0	71
13855438	m	ot	JP	2017	0	1	0	0	1	0	0	0	0	0	0	0	76
13855725	f	md	DE	2017	0	1	0	0	1	0	0	0	0	0	0	0	58
13855727	m	md	JP	2017	0	1	0	0	1	0	0	0	0	0	0	0	75
13855762	f	md	TH	2017	0	0	0	0	0	0	0	0	0	0	0	0	
13856073	m	ph	US	2017	1	0	0	0	0	0	0	0	0	0	0	0	62
13857292	m	cn	GR	2017	0	1	1	0	0	0	0	0	0	0	0	0	66
13857311	m	cn	IN	2017	0	1	0	0	1	0	0	0	0	0	0	0	
13857613	m	cn	US	2017	1	0	0	0	0	0	0	0	0	0	0	0	
13857807	m	ot	IT	2017	0	0	0	0	0	0	0	0	0	0	0	0	65
13857923	m	cn	JP	2017	0	1	1	0	0	0	0	0	0	0	0	0	76
13858143	f	md	IL	2017	0	1	1	0	0	0	0	0	0	0	0	0	
13858158	m	cn	US	2017	0	0	0	0	0	0	0	0	0	0	0	0	73
13858263	Not	ot	DE	2017	0	0	0	0	0	0	0	0	0	0	0	0	
13858510	f	cn	US	2017	0	0	0	0	0	0	0	0	0	0	0	0	70
13858862	m	cn	JP	2017	0	0	0	0	0	0	0	0	0	0	0	0	79
13859909	f	ot	CA	2017	0	0	0	0	0	0	0	0	0	0	0	0	
13859927	f	cn	US	2017	0	0	0	0	0	0	0	0	0	0	0	0	
13859961	f	cn	US	2017	0	0	0	0	0	0	0	0	0	0	0	0	
13860191	f	cn	JP	2017	0	0	0	0	0	0	0	0	0	0	0	0	83
13860822	m	ot	TW	2017	0	0	0	0	0	0	0	0	0	0	0	0	56

13860943	m	md	CN	2017	1	0	0	0	0	0	0	0	0	0	0	0	69.7
13861191	f	md	JP	2017	1	0	0	0	0	0	0	0	0	0	0	0	68
13861489	f	md	DE	2017	0	1	0	0	1	0	0	0	0	0	0	0	79
13861506	m	ot	DE	2017	0	1	0	0	1	0	0	0	0	0	0	0	82
13861507	m	md	BE	2017	0	1	0	0	1	0	0	0	0	0	0	0	54
13861520	f	md	DE	2017	0	1	0	0	1	0	0	0	0	0	0	1	
13861529	m	md	JP	2017	0	1	0	0	1	0	0	0	0	0	0	0	72
13861538	f	cn	US	2017	0	1	0	0	1	0	0	0	0	0	0	0	
13861554	m	md	NL	2017	0	1	0	0	1	0	0	0	0	0	0	0	51
13861726	Not	md	AT	2017	0	0	0	0	0	0	0	0	0	0	0	0	57
13862102	m	md	BE	2017	0	0	0	0	0	0	0	0	0	0	0	0	56
13862613	m	cn	US	2017	1	0	0	0	0	0	0	0	0	0	0	0	74
13862864	m	md	SK	2017	0	1	0	0	0	1	0	0	0	0	0	0	41
13862888	m	ot	JP	2017	0	1	0	0	1	0	0	0	0	0	0	0	67
13863628	m	cn	GR	2017	0	1	1	0	0	0	0	0	0	0	0	1	50
13863853	f	cn	US	2017	0	1	1	0	0	0	0	0	0	0	0	1	
13864042	m	md	DE	2017	0	0	0	0	0	0	0	0	0	0	0	0	60
13864607	m	md	CO	2017	0	0	0	0	0	0	0	0	0	0	0	0	81
13864751	m	cn	US	2017	0	1	1	0	0	0	0	0	0	0	0	0	
13864783	m	cn	US	2017	0	1	0	0	1	0	0	0	0	0	0	0	
13865431	f	md	US	2017	0	0	0	0	0	0	0	0	0	0	0	0	77
13865457	f	cn	GB	2017	1	0	0	0	0	0	0	0	0	0	0	0	73
13865707	f	md	GR	2017	1	0	0	0	0	0	0	0	0	0	0	0	
13866996	m	md	BR	2017	0	0	0	0	0	0	0	0	0	0	0	0	77
13868586	Not	cn	GR	2017	0	1	0	0	1	0	0	0	0	0	0	0	
13868607	f	cn	US	2017	0	1	0	0	1	0	0	0	0	0	0	0	
13869535	m	ot	ES	2017	0	1	0	0	0	1	0	0	0	0	0	0	62
13869545	m	md	JP	2017	0	0	0	0	0	0	0	0	0	0	0	0	82
13869613	f	ph	US	2017	1	0	0	0	0	0	0	0	0	0	0	0	
13870086	f	cn	US	2017	1	0	0	0	0	0	0	0	0	0	0	0	71
13870440	m	md	ES	2017	0	0	0	0	0	0	0	0	0	0	0	0	54
13870610	f	ot	KR	2017	0	0	0	0	0	0	0	0	0	0	0	0	57
13871149	m	cn	DE	2017	0	1	0	0	1	0	0	0	0	0	0	0	76
13871201	m	cn	US	2017	0	1	1	0	0	0	0	0	0	0	0	0	

13871781	Not	cn	JP	2017	0	1	1	0	0	0	0	0	0	0	0	0	
13871903	m	md	JP	2017	1	0	0	0	0	0	0	0	0	0	0	0	51
13871964	m	md	CN	2017	1	0	0	0	0	0	0	0	0	0	0	0	77.4
13872677	f	md	JP	2017	1	0	0	0	0	0	0	0	0	0	0	0	83
13873050	m	md	US	2017	0	1	0	0	0	1	0	0	0	0	0	0	70
13873053	m	md	HU	2017	0	1	0	0	1	0	0	0	0	0	0	0	60
13873194	f	ot	GB	2017	0	0	0	0	0	0	0	0	0	0	0	0	49
13873611	m	ot	US	2017	0	1	0	0	0	1	0	0	0	0	0	0	67
13873876	f	ot	IT	2017	0	1	0	0	1	0	0	0	0	0	0	0	48
13874042	f	cn	US	2017	0	0	0	0	0	0	0	0	0	0	0	0	70
13875211	m	cn	US	2017	0	1	0	0	1	0	0	0	0	0	0	0	92
13875541	m	md	JP	2017	0	1	0	0	1	0	0	0	0	0	0	0	40
13875547	m	md	CH	2017	0	1	0	0	1	0	0	0	0	0	0	0	
13875690	f	ot	HU	2017	0	1	0	0	1	0	0	0	0	0	0	0	61
13875850	m	md	JP	2017	1	0	0	0	0	0	0	0	0	0	0	0	61
13875971	m	md	JP	2017	0	1	0	0	1	0	0	0	0	0	0	0	80
13876355	f	ot	US	2017	0	1	1	0	0	0	0	0	0	0	0	0	66
13876686	m	ot	US	2017	0	1	0	0	1	0	0	0	0	0	0	0	71
13876892	f	cn	US	2017	0	1	0	0	1	0	0	0	0	0	0	0	79
13877331	m	md	TH	2017	0	0	0	0	0	0	0	0	0	0	0	0	50
13877498	f	md	US	2017	0	1	0	0	1	0	0	0	0	0	0	0	
13877717	f	Not repo	JP	2017	0	0	0	0	0	0	0	0	0	0	0	0	49
13878002	f	cn	US	2017	1	0	0	0	0	0	0	0	0	0	0	0	
13878160	f	md	RU	2017	1	0	0	0	0	0	0	0	0	0	0	0	50
13878354	f	cn	US	2017	0	0	0	0	0	0	0	0	0	0	0	0	
13878623	f	cn	US	2017	0	0	0	0	0	0	0	0	0	0	0	0	60
13879035	f	md	KR	2017	1	0	0	0	0	0	0	0	0	0	0	0	58
13880202	m	md	JP	2017	0	1	0	0	1	0	0	0	0	0	0	0	65
13880414	Not	ot	US	2017	0	1	1	0	0	0	0	0	0	0	0	0	
13880918	Not	md	JP	2017	1	0	0	0	0	0	0	0	0	0	0	0	
13880931	f	ot	US	2017	0	1	0	0	0	1	0	0	0	0	0	0	55
13880976	f	md	TR	2017	0	1	0	0	1	0	0	0	0	0	0	0	47
13881700	f	ph	FR	2017	1	0	0	0	0	0	0	0	0	0	0	0	82
13881901	m	md	TR	2017	0	1	0	0	1	0	0	0	0	0	0	0	50

13881998	f	cn	US	2017	0	1	0	0	1	0	0	0	0	0	0	0	
13882121	m	cn	US	2017	0	0	0	0	0	0	0	0	0	0	0	0	
13882465	m	Not repo	US	2017	1	0	0	0	0	0	0	0	0	0	0	0	75.4
13882506	f	ph	FR	2017	1	0	0	0	0	0	0	0	0	0	0	0	56
13882611	m	cn	US	2017	0	1	0	0	1	0	0	0	0	0	0	0	
13883361	f	ph	GB	2017	1	0	0	0	0	0	0	0	0	0	0	0	64
13883932	f	ot	US	2017	0	1	1	0	0	0	0	0	0	0	0	0	72
13883975	m	md	JP	2017	0	0	0	0	0	0	0	0	0	0	0	0	63
13884471	m	md	JP	2017	0	1	0	0	0	1	0	0	0	0	0	0	66
13884544	m	md	CN	2017	0	1	0	0	0	1	0	0	0	0	0	0	65
13884938	f	md	TR	2017	0	0	0	0	0	0	0	0	0	0	0	0	70
13885629	m	md	CH	2017	0	0	0	0	0	0	0	0	0	0	0	0	77
13885633	m	md	MY	2017	1	0	0	0	0	0	0	0	0	0	0	0	
13885765	Not	Not repo	Not re	2017	1	0	0	0	0	0	0	0	0	0	0	0	
13885771	Not	cn	GB	2017	1	0	0	0	0	0	0	0	0	0	0	0	
13886248	m	cn	US	2017	0	1	0	0	1	0	0	0	0	0	0	0	
13886677	Not	ot	US	2017	0	0	0	0	0	0	0	0	0	0	0	0	
13887767	f	md	US	2017	0	1	0	0	1	0	0	0	0	0	0	0	
13888094	f	ot	US	2017	0	0	0	0	0	0	0	0	0	0	0	0	69
13888244	m	md	IL	2017	0	1	0	0	1	0	0	0	0	0	0	0	68
13888282	m	ot	AU	2017	0	1	0	0	1	0	0	0	0	0	0	0	64
13888390	m	md	JP	2017	1	0	0	0	0	0	0	0	0	0	0	0	70
13888526	f	ot	US	2017	0	0	0	0	0	0	0	0	0	0	0	0	
13889033	m	cn	JP	2017	0	1	0	0	1	0	0	0	0	0	0	0	63
13890267	m	ot	ES	2017	0	1	0	0	1	0	0	0	0	0	0	0	64
13890515	m	ph	US	2017	0	1	1	0	0	0	0	0	0	0	0	0	
13890622	f	cn	JP	2017	0	1	1	0	0	0	0	0	0	0	0	0	84
13890789	m	ot	US	2017	0	1	0	0	0	1	0	0	0	0	0	0	
13891151	m	cn	JP	2017	0	1	1	0	0	0	0	0	0	0	0	0	81
13891234	f	cn	GB	2017	1	0	0	0	0	0	0	0	0	0	0	0	77
13891514	m	cn	JP	2017	0	1	1	0	0	0	0	0	0	0	0	0	64
13891550	m	cn	JP	2017	0	0	0	0	0	0	0	0	0	0	0	0	70
13892376	f	cn	US	2017	1	0	0	0	0	0	0	0	0	0	0	0	69
13892595	m	md	CN	2017	1	0	0	0	0	0	0	0	0	0	0	0	78.7

13892893	m	cn	GB	2017	1	0	0	0	0	0	0	0	0	0	0	0	
13893584	Not	ot	US	2017	0	0	0	0	0	0	0	0	0	0	0	0	
13893999	m	md	JP	2017	0	1	0	0	1	0	0	0	0	0	0	0	73
13894156	f	cn	US	2017	0	0	0	0	0	0	0	0	0	0	0	0	51
13894861	m	ot	US	2017	0	1	0	0	1	0	0	0	0	0	0	0	
13895010	f	ot	US	2017	1	0	0	0	0	0	0	0	0	0	0	0	49
13895769	m	ot	GB	2017	0	1	0	0	1	0	0	0	0	0	0	0	42
13895920	m	md	IT	2017	0	1	0	0	1	0	0	0	0	0	0	0	76
13896102	f	cn	US	2017	0	1	1	0	0	0	0	0	0	0	0	0	
13896490	f	ph	US	2017	0	1	1	0	0	0	0	0	0	0	0	0	55
13897013	f	cn	DE	2017	0	1	1	0	0	0	0	0	0	0	0	0	
13897167	m	ot	DE	2017	0	0	0	0	0	0	0	0	0	0	0	0	65
13897392	m	cn	US	2017	0	1	0	0	1	0	0	0	0	0	0	0	69
13897396	f	md	JP	2017	1	0	0	0	0	0	0	0	0	0	0	0	65
13897636	f	cn	IT	2017	0	1	0	0	1	0	0	0	0	0	0	0	
13897792	f	md	JP	2017	0	1	0	0	1	0	0	0	0	0	0	0	70
13897839	m	ot	GR	2017	0	1	0	0	1	0	0	0	0	0	0	0	72
13897984	f	ot	DE	2017	0	1	0	0	1	0	0	0	0	0	0	0	44
13898376	m	md	JP	2017	0	1	0	0	1	0	0	0	0	0	0	0	58
13898458	m	ot	US	2017	0	1	0	0	1	0	0	0	0	0	0	1	65
13898525	m	ot	US	2017	0	1	1	0	0	0	0	0	0	0	0	0	67
13898592	m	cn	US	2017	0	0	0	0	0	0	0	0	0	0	0	0	62
13899258	f	cn	CA	2017	0	0	0	0	0	0	0	0	0	0	0	0	73
13899284	Not	ot	TW	2017	0	0	0	0	0	0	0	0	0	0	0	0	
13899285	Not	ot	TW	2017	0	0	0	0	0	0	0	0	0	0	0	0	
13899754	m	md	BE	2017	0	1	1	0	0	0	0	0	0	0	0	0	
13900156	m	cn	GB	2017	1	0	0	0	0	0	0	0	0	0	0	0	70
13900303	m	ot	JP	2017	0	1	0	0	1	0	0	0	0	0	0	1	80
13900540	m	ot	CN	2017	0	0	0	0	0	0	0	0	0	0	0	0	44
13900760	m	ot	US	2017	0	1	1	0	0	0	0	0	0	0	0	0	
13900987	m	md	IN	2017	0	1	0	0	1	0	0	0	0	0	0	0	29
13901050	f	md	JP	2017	1	0	0	0	0	0	0	0	0	0	0	0	78
13901167	f	cn	US	2017	0	1	0	0	1	0	0	0	0	0	0	0	
13901481	Not	cn	JP	2017	0	0	0	0	0	0	0	0	0	0	0	0	

13901971	f	md	US	2017	0	0	0	0	0	0	0	0	0	0	0	0	
13902031	m	md	RU	2017	0	0	0	0	0	0	0	0	0	0	0	0	
13902604	Not	ot	TW	2017	0	0	0	0	0	0	0	0	0	0	0	0	
13903626	Not	cn	US	2017	0	1	1	0	0	0	0	0	0	0	0	0	
13903876	f	md	JP	2017	0	1	0	0	1	0	0	0	0	0	0	0	62
13904124	m	ot	US	2017	0	0	0	0	0	0	0	0	0	0	0	0	
13904357	f	md	AT	2017	0	1	1	0	0	0	0	0	0	0	0	0	61
13904794	f	md	CN	2017	1	0	0	0	0	0	0	0	0	0	0	0	69
13905338	m	cn	JP	2017	0	0	0	0	0	0	0	0	0	0	0	0	73
13905355	m	cn	JP	2017	0	1	1	0	0	0	0	0	0	0	0	0	67
13905627	m	md	JP	2017	1	0	0	0	0	0	0	0	0	0	0	1	54.7
13905649	m	Not repo	MY	2017	1	0	0	0	0	0	0	0	0	0	0	0	73.3
13905670	m	ot	US	2017	0	1	0	0	1	0	0	0	0	0	0	0	
13905975	f	cn	GB	2017	1	0	0	0	0	0	0	0	0	0	0	0	67
13906028	Not	cn	TH	2017	0	1	1	0	0	0	0	0	0	0	0	0	
13906406	f	ot	US	2017	0	0	0	0	0	0	0	0	0	0	0	0	81
13906903	m	ot	US	2017	0	0	0	0	0	0	0	0	0	0	0	0	
13908208	m	cn	US	2017	0	1	1	0	0	0	0	0	0	0	0	0	70
13908282	f	md	US	2017	0	0	0	0	0	0	1	1	0	0	0	0	69
13908789	m	cn	Not re	2017	0	0	0	0	0	0	0	0	0	0	0	0	
13908852	Not	cn	JP	2017	0	1	1	0	0	0	0	0	0	0	0	0	
13909145	m	cn	JP	2017	0	0	0	0	0	0	0	0	0	0	0	0	59
13909472	f	md	JP	2017	0	1	0	0	1	0	0	0	0	0	0	0	39
13909844	Not	ot	US	2017	0	0	0	0	0	0	0	0	0	0	0	0	
13909857	Not	ot	US	2017	0	0	0	0	0	0	0	0	0	0	0	0	
13909861	Not	ot	US	2017	0	0	0	0	0	0	0	0	0	0	0	0	
13909875	Not	ot	US	2017	0	0	0	0	0	0	0	0	0	0	0	0	
13909876	Not	ot	US	2017	0	0	0	0	0	0	0	0	0	0	0	0	
13909897	Not	ot	US	2017	0	0	0	0	0	0	0	0	0	0	0	0	
13909898	Not	ot	US	2017	0	0	0	0	0	0	0	0	0	0	0	0	
13909935	Not	ot	US	2017	0	0	0	0	0	0	0	0	0	0	0	0	
13909963	Not	ot	US	2017	0	0	0	0	0	0	0	0	0	0	0	0	
13909970	Not	ot	US	2017	0	0	0	0	0	0	0	0	0	0	0	0	
13909971	Not	ot	US	2017	0	0	0	0	0	0	0	0	0	0	0	0	

13909972	Not	ot	US	2017	0	0	0	0	0	0	0	0	0	0	0	0	
13910036	Not	ot	US	2017	0	0	0	0	0	0	0	0	0	0	0	0	
13910040	Not	ot	US	2017	0	0	0	0	0	0	0	0	0	0	0	0	
13910680	m	ot	JP	2017	0	0	0	0	0	0	0	0	0	0	0	0	74
13910687	m	ot	JP	2017	0	0	0	0	0	0	0	0	0	0	0	0	71
13910692	m	ot	JP	2017	0	0	0	0	0	0	0	0	0	0	0	0	59
13911007	m	cn	JP	2017	0	0	0	0	0	0	0	0	0	0	0	0	70
13911073	m	cn	US	2017	0	0	0	0	0	0	0	0	0	0	0	0	73
13911146	f	cn	GB	2017	1	0	0	0	0	0	0	0	0	0	0	0	
13912484	m	md	NZ	2017	0	0	0	0	0	0	0	0	0	0	0	0	69
13912580	f	md	TH	2017	0	0	0	0	0	0	0	0	0	0	0	0	77
13915093	m	md	IN	2017	0	1	0	0	1	0	0	0	0	0	0	0	73
13915233	m	md	JP	2017	1	0	0	0	0	0	0	0	0	0	0	0	57
13915243	Not	cn	GB	2017	1	0	0	0	0	0	0	0	0	0	0	0	
13915319	f	cn	GB	2017	1	0	0	0	0	0	0	0	0	0	0	0	
13915409	m	cn	JP	2017	0	1	1	0	0	0	0	0	0	0	0	0	65
13915880	m	md	US	2017	0	1	0	0	0	1	0	0	0	0	0	0	73
13915886	m	cn	JP	2017	0	1	1	0	0	0	0	0	0	0	0	0	76
13916071	Not	cn	JP	2017	0	1	1	0	0	0	0	0	0	0	0	1	67
13916178	m	cn	JP	2017	0	1	1	0	0	0	0	0	0	0	0	0	73
13916446	m	cn	GB	2017	1	0	0	0	0	0	0	0	0	0	0	0	
13916536	f	md	FR	2017	0	0	0	0	0	0	0	0	0	0	0	0	
13916589	m	md	JP	2017	1	0	0	0	0	0	0	0	0	0	0	0	77
13917378	f	md	JP	2017	0	1	0	0	1	0	0	0	0	0	0	0	67
13917741	m	ot	US	2017	0	1	0	0	1	0	0	0	0	0	0	0	
13919316	f	md	JP	2017	1	0	0	0	0	0	0	0	0	0	0	0	69
13919497	m	md	JP	2017	0	1	0	0	1	0	0	0	0	0	0	0	61
13919768	f	md	JP	2017	0	1	0	0	1	0	0	0	0	0	0	0	39
13919855	m	ot	JP	2017	0	1	0	0	1	0	0	0	0	0	0	0	62
13919967	m	md	US	2017	0	0	0	0	0	0	0	0	0	0	0	0	58
13920312	m	ot	US	2017	0	0	0	0	0	0	0	0	0	0	0	0	68
13920880	m	md	PE	2017	1	0	0	0	0	0	0	0	0	0	0	0	79
13920949	f	md	US	2017	0	1	1	0	0	0	0	0	0	0	0	0	69
13921104	Not	md	JP	2017	0	0	0	0	0	0	0	0	0	0	0	0	

13921389	f	md	JP	2017	1	0	0	0	0	0	0	0	0	0	0	0	88
13922306	Not	ot	IT	2017	0	0	0	0	0	0	0	0	0	0	0	0	
13922493	Not	ot	JP	2017	0	0	0	0	0	0	0	0	0	0	0	0	
13922583	Not	cn	JP	2017	0	0	0	0	0	0	0	0	0	0	0	0	65
13922757	m	cn	US	2017	1	0	0	0	0	0	0	0	0	0	0	0	55
13922860	m	cn	US	2017	0	1	1	0	0	0	0	0	0	0	0	0	
13923072	Not	md	JP	2017	0	1	0	0	1	0	0	0	0	0	0	0	
13924192	f	md	US	2017	1	0	0	0	0	0	0	0	0	0	0	0	60
13924350	Not	ot	US	2017	0	0	0	0	0	0	0	0	0	0	0	0	
13924542	m	ot	US	2017	0	1	1	0	0	0	0	0	0	0	0	0	63
13924594	m	md	US	2017	0	1	1	0	0	0	0	0	0	0	0	0	
13924694	f	md	CN	2017	1	0	0	0	0	0	0	0	0	0	0	0	70
13925738	f	md	CN	2017	1	0	0	0	0	0	0	0	0	0	0	0	50.4
13926036	f	ph	US	2017	0	1	0	0	1	0	0	0	0	0	0	0	60
13926841	f	md	TH	2017	1	0	0	0	0	0	0	0	0	0	0	0	69
13926847	f	cn	US	2017	0	0	0	0	0	0	0	0	0	0	0	0	60
13926864	m	cn	US	2017	0	1	0	0	1	0	0	0	0	0	0	0	
13926881	m	md	JP	2017	0	1	0	0	1	0	0	0	0	0	0	0	71
13926913	f	md	CN	2017	1	0	0	0	0	0	0	0	0	0	0	0	73
13927205	f	ot	CA	2017	0	1	1	0	0	0	0	0	0	0	0	0	55
13927263	f	md	CH	2017	0	0	0	0	0	0	0	0	0	0	0	0	68
13927297	f	md	US	2017	0	1	1	0	0	0	0	0	0	0	0	0	67
13927371	f	md	DK	2017	0	0	0	0	0	0	0	0	0	0	0	0	61
13928348	f	ph	GB	2017	0	1	1	0	0	0	0	0	0	0	0	1	57
13928717	Not	ot	US	2017	0	1	0	0	1	0	0	0	0	0	0	0	
13928751	Not	ot	US	2017	0	1	0	0	1	0	0	0	0	0	0	0	
13929025	m	md	GR	2017	0	1	0	0	1	0	0	0	0	0	0	0	
13929119	m	md	IN	2017	0	1	0	0	1	0	0	0	0	0	0	0	47
13929475	f	md	US	2017	1	0	0	0	0	0	0	0	0	0	0	0	60
13929694	f	cn	US	2017	0	1	0	0	1	0	0	0	0	0	0	0	
13930212	m	ph	JP	2017	0	1	0	0	1	0	0	0	0	0	0	0	37
13930519	m	md	JP	2017	0	1	0	0	1	0	0	0	0	0	0	0	51
13930762	m	cn	GR	2017	0	1	0	0	1	0	0	0	0	0	0	0	55
13931461	Not	md	JP	2017	0	0	0	0	0	0	0	0	0	0	0	0	

13931474	m	ph	JP	2017	0	1	0	0	1	0	0	0	0	0	0	0	51
13931781	m	md	JP	2017	0	0	0	0	0	0	0	0	0	0	0	0	70
13932516	m	ot	DE	2017	0	1	0	0	1	0	0	0	0	0	0	0	77
13932847	m	ot	US	2017	0	1	0	0	1	0	0	0	0	0	0	0	68
13932889	Not	md	DE	2017	0	0	0	0	0	0	0	0	0	0	0	0	
13933110	f	md	US	2017	0	0	0	0	0	0	0	0	0	0	0	0	
13933295	f	md	CN	2017	1	0	0	0	0	0	0	0	0	0	0	0	70.3
13933388	f	md	TW	2017	0	0	0	0	0	0	0	0	0	0	0	0	
13933455	m	md	IT	2017	0	0	0	0	0	0	0	0	0	0	0	0	40
13933457	m	md	IT	2017	0	0	0	0	0	0	0	0	0	0	0	0	73
13933570	m	md	US	2017	0	1	0	0	1	0	0	0	0	0	0	1	
13934324	Not	cn	US	2017	0	1	1	0	0	0	0	0	0	0	0	0	
13934361	m	cn	JP	2017	0	0	0	0	0	0	0	0	0	0	0	0	73
13934540	m	ot	JP	2017	0	1	0	0	1	0	0	0	0	0	0	0	66
13934556	m	ot	US	2017	0	1	0	0	1	0	0	0	0	0	0	0	53
13934672	f	md	DE	2017	1	0	0	0	0	0	0	0	0	0	0	0	
13935281	Not	md	JP	2017	1	0	0	0	0	0	0	0	0	0	0	0	
13935557	m	ph	US	2017	0	1	0	0	1	0	0	0	0	0	0	0	
13935877	f	md	JP	2017	1	0	0	0	0	0	0	0	0	0	0	0	63
13935959	m	ot	US	2017	0	1	0	0	1	0	0	0	0	0	0	0	66
13936179	f	cn	GB	2017	1	0	0	0	0	0	0	0	0	0	0	0	76
13936540	m	cn	JP	2017	0	1	1	0	0	0	0	0	0	0	0	0	75
13937126	Not	md	KR	2017	1	0	0	0	0	0	0	0	0	0	0	0	70.3
13937543	Not	cn	FR	2017	0	1	1	0	0	0	0	0	0	0	0	0	
13937815	f	md	ID	2017	0	0	0	0	0	0	0	0	0	0	0	0	
13938088	f	ot	US	2017	0	0	0	0	0	0	0	0	0	0	0	0	
13938249	m	ot	US	2017	0	1	0	0	1	0	0	0	0	0	0	0	78
13938361	m	ot	US	2017	0	1	1	0	0	0	0	0	0	0	0	0	
13938486	f	cn	US	2017	1	0	0	0	0	0	0	0	0	0	0	1	65.8
13938598	Not	cn	GR	2017	0	1	0	0	1	0	0	0	0	0	0	0	
13938600	Not	cn	GR	2017	0	1	0	0	1	0	0	0	0	0	0	0	
13938603	Not	cn	GR	2017	0	1	0	0	1	0	0	0	0	0	0	0	
13938605	Not	cn	GR	2017	0	1	0	0	1	0	0	0	0	0	0	0	
13938765	f	md	ID	2017	1	0	0	0	0	0	0	0	0	0	0	0	

13938874	m	cn	JP	2017	0	1	1	0	0	0	0	0	0	0	0	0	67
13939183	m	md	ID	2017	1	0	0	0	0	0	0	0	0	0	0	0	
13939684	m	md	US	2017	0	1	1	0	0	0	0	0	0	0	0	0	75
13940048	m	md	JP	2017	0	1	1	0	0	0	0	0	0	0	0	0	76
13940101	f	Not repo	US	2017	1	0	0	0	0	0	0	0	0	0	0	0	59.1
13940298	Not	ot	AU	2017	0	0	0	0	0	0	0	0	0	0	0	0	
13940357	f	md	KR	2017	1	0	0	0	0	0	0	0	0	0	0	0	62
13940426	Not	ot	AU	2017	0	0	0	0	0	0	0	0	0	0	0	0	
13940654	m	ot	DE	2017	0	1	0	0	1	0	0	0	0	0	0	0	56
13940941	Not	md	US	2017	0	0	0	0	0	0	0	0	0	0	0	0	
13940948	f	md	SI	2017	0	0	0	0	0	0	0	0	0	0	0	0	62
13941551	m	cn	JP	2017	0	1	1	0	0	0	0	0	0	0	0	0	70
13942148	f	ot	IT	2017	0	1	0	0	1	0	0	0	0	0	0	0	57
13942459	m	ot	CL	2017	0	1	0	0	1	0	0	0	0	0	0	0	65
13943341	f	ot	CH	2017	0	1	0	0	1	0	0	0	0	0	0	0	75
13943758	Not	cn	US	2017	0	1	1	0	0	0	0	0	0	0	0	0	
13945287	m	ot	US	2017	0	0	0	0	0	0	0	0	0	0	0	0	74
13945472	f	md	JP	2017	1	0	0	0	0	0	0	0	0	0	0	0	63
13945965	m	cn	US	2017	0	1	1	0	0	0	0	0	0	0	0	0	
13946303	f	md	US	2017	0	0	0	0	0	0	0	0	0	0	0	0	55
13946531	f	md	JP	2017	0	0	0	0	0	0	0	0	0	0	0	0	69
13946746	Not	md	FR	2017	1	0	0	0	0	0	0	0	0	0	0	0	
13947091	f	cn	GB	2017	1	0	0	0	0	0	0	0	0	0	0	0	62
13947179	m	ot	GR	2017	0	1	0	0	1	0	0	0	0	0	0	1	69
13947206	m	cn	JP	2017	0	1	1	0	0	0	0	0	0	0	0	1	73
13947248	m	ot	TR	2017	0	1	0	0	1	0	0	0	0	0	0	0	51
13947751	f	cn	US	2017	0	1	0	0	1	0	0	0	0	0	0	0	
13947816	m	md	AU	2017	1	0	0	0	0	0	0	0	0	0	0	0	76.2
13947889	f	md	FR	2017	1	0	0	0	0	0	0	0	0	0	0	0	45
13949126	f	cn	GB	2017	1	0	0	0	0	0	0	0	0	0	0	0	69
13949166	f	cn	GB	2017	1	0	0	0	0	0	0	0	0	0	0	0	63
13949940	m	cn	GB	2017	1	0	0	0	0	0	0	0	0	0	0	0	
13950122	m	md	JP	2017	0	1	0	0	1	0	0	0	0	0	0	0	62
13950633	m	ot	TR	2017	0	1	0	0	1	0	0	0	0	0	0	0	65

13951665	Not	md	DE	2017	0	1	0	0	1	0	0	0	0	0	0	0	67
13951700	m	cn	US	2017	0	1	1	0	0	0	0	0	0	0	0	0	
13952073	f	md	JP	2017	0	1	1	0	0	0	0	0	0	0	0	0	61
13952682	Not	md	CN	2017	0	0	0	0	0	0	0	0	0	0	0	0	
13953123	m	ph	GR	2017	0	1	0	0	1	0	0	0	0	0	0	0	67
13953147	m	ot	DE	2017	0	1	0	0	1	0	0	0	0	0	0	0	67
13953735	m	ph	JP	2017	0	0	0	0	0	0	0	0	0	0	0	0	
13953994	m	ot	US	2017	0	1	0	0	1	0	0	0	0	0	0	0	67
13954370	f	md	US	2017	0	0	0	0	0	0	0	0	0	0	0	0	
13954582	m	cn	JP	2017	0	1	1	0	0	0	0	0	0	0	0	0	59
13955478	m	md	JP	2017	0	1	0	0	1	0	0	0	0	0	0	0	60
13956287	m	ot	CA	2017	0	1	0	0	1	0	0	0	0	0	0	0	67
13956481	Not	ot	NL	2017	1	0	0	0	0	0	0	0	0	0	0	0	
13956512	Not	ot	NL	2017	1	0	0	0	0	0	0	0	0	0	0	0	
13956515	Not	ot	NL	2017	1	0	0	0	0	0	0	0	0	0	0	0	
13956537	Not	ot	NL	2017	1	0	0	0	0	0	0	0	0	0	0	0	
13956539	Not	ot	NL	2017	1	0	0	0	0	0	0	0	0	0	0	0	
13956541	Not	ot	NL	2017	1	0	0	0	0	0	0	0	0	0	0	0	
13957067	f	md	SK	2017	0	0	0	0	0	0	0	0	0	0	0	0	
13957086	m	ot	US	2017	0	1	0	0	1	0	0	0	0	0	0	0	90
13957217	m	md	DE	2017	0	1	0	0	1	0	0	0	0	0	0	0	
13958280	m	md	US	2017	0	1	0	0	0	1	0	0	0	0	0	0	68
13958281	f	cn	US	2017	0	1	0	0	0	1	0	0	0	0	0	0	75
13958291	m	md	US	2017	0	1	0	0	0	1	0	0	0	0	0	0	68
13958293	f	md	US	2017	0	1	0	0	0	1	0	0	0	0	0	0	73
13958294	m	ph	US	2017	0	1	0	0	0	1	0	0	0	0	0	0	
13958364	f	ot	US	2017	0	1	0	0	0	1	0	0	0	0	0	0	62
13958618	f	ph	US	2017	0	1	0	0	0	1	0	0	0	0	0	0	52
13958635	m	ot	JP	2017	0	1	0	0	1	0	0	0	0	0	0	0	73
13958790	f	ot	US	2017	0	1	0	0	1	0	0	0	0	0	0	0	82
13959425	Not	ot	NL	2017	1	0	0	0	0	0	0	0	0	0	0	0	
13959805	f	Not repo	MY	2017	1	0	0	0	0	0	0	0	0	0	0	0	71.8
13960202	m	ot	US	2017	0	1	0	1	0	0	0	0	0	0	0	0	73
13960315	m	cn	US	2017	1	0	0	0	0	0	0	0	0	0	0	0	78

13960904	f	cn	GB	2017	1	0	0	0	0	0	0	0	0	0	0	0	68
13961178	m	md	PH	2017	1	0	0	0	0	0	0	0	0	0	0	0	66
13961341	f	ot	US	2017	0	1	0	0	1	0	0	0	0	0	0	0	81
13962284	m	ph	JP	2017	0	1	0	0	1	0	0	0	0	0	0	0	64
13963765	m	md	JP	2017	0	1	0	0	1	0	0	0	0	0	0	0	68
13963959	f	md	JP	2017	1	0	0	0	0	0	0	0	0	0	0	0	71
13965134	m	ot	CL	2017	1	0	0	0	0	0	0	0	0	0	0	0	65
13965212	f	md	JP	2017	1	0	0	0	0	0	0	0	0	0	0	0	88
13966440	f	cn	US	2017	0	1	0	0	1	0	0	0	0	0	0	0	
13967184	Not	cn	SG	2017	0	1	1	0	0	0	0	0	0	0	0	0	
13967819	m	ot	JP	2017	0	0	0	0	0	0	0	0	0	0	0	0	82
13969614	f	md	CA	2017	0	1	0	0	1	0	0	0	0	0	0	0	71
13970943	f	md	JP	2017	0	0	0	0	0	0	0	0	0	0	0	0	
13971657	f	Not repo	IT	2017	1	0	0	0	0	0	0	0	0	0	0	0	
13972784	m	md	JP	2017	0	1	0	0	1	0	0	0	0	0	0	0	40
13973008	f	md	US	2017	1	0	0	0	0	0	0	0	0	0	0	0	78.4
13973130	m	md	JP	2017	0	1	0	0	0	1	0	0	0	0	0	0	51
13973688	f	ot	US	2017	0	1	0	0	1	0	0	0	0	0	0	1	79
13973897	Not	ot	JP	2017	0	0	0	0	0	0	0	0	0	0	0	0	
13974483	m	md	TR	2017	0	1	0	0	0	1	0	0	0	0	0	0	53
13974828	f	md	TW	2017	1	0	0	0	0	0	0	0	0	0	0	0	59.2
13975160	Not	ot	AR	2017	0	1	0	0	1	0	0	0	0	0	0	0	
13975194	m	md	IL	2017	0	1	0	0	1	0	0	0	0	0	0	0	70
13975229	m	md	JP	2017	0	0	0	0	0	0	0	0	0	0	0	0	
13975807	Not	md	FR	2017	1	0	0	0	0	0	0	0	0	0	0	0	
13975864	m	md	FR	2017	0	0	0	0	0	0	0	0	0	0	0	0	
13975894	f	md	US	2017	0	0	0	0	0	0	0	0	0	0	0	0	
13977135	m	cn	PE	2017	0	1	1	0	0	0	0	0	0	0	0	0	76
13977380	f	md	ID	2017	1	0	0	0	0	0	0	0	0	0	0	0	62
13977471	m	md	ID	2017	1	0	0	0	0	0	0	0	0	0	0	0	65
13977473	f	md	US	2017	1	0	0	0	0	0	0	0	0	0	0	0	76
13977969	m	cn	FR	2017	0	1	1	0	0	0	0	0	0	0	0	0	53
13978025	m	ot	JP	2017	0	1	0	0	1	0	0	0	0	0	0	0	62
13978888	Not	md	US	2017	0	1	0	0	1	0	0	0	0	0	0	0	

13980346	m	cn	US	2017	0	0	0	0	0	0	0	0	0	0	0	0	74
13980672	Not	cn	AU	2017	0	1	1	0	0	0	0	0	0	0	0	0	94
13980852	f	cn	JP	2017	0	1	1	0	0	0	0	0	0	0	0	0	84
13981194	f	cn	JP	2017	0	1	1	0	0	0	0	0	0	0	0	0	76
13981212	m	ot	GB	2017	0	0	0	0	0	0	0	0	0	0	0	0	82
13982051	m	md	IT	2017	0	1	0	0	1	0	0	0	0	0	0	0	
13982377	Not	cn	JP	2017	0	0	0	0	0	0	0	0	0	0	0	0	
13982458	m	ot	JP	2017	0	1	0	0	1	0	0	0	0	0	0	0	67
13982463	Not	md	JP	2017	0	1	0	0	1	0	0	0	0	0	0	0	
13982464	f	ot	JP	2017	0	1	0	0	1	0	0	0	0	0	0	0	73
13983102	m	ot	US	2017	0	1	0	1	0	0	0	0	0	0	0	0	73
13983566	m	cn	GB	2017	1	0	0	0	0	0	0	0	0	0	0	0	47
13983780	m	md	US	2017	0	1	0	0	1	0	0	0	0	0	0	0	
13983901	m	md	JP	2017	0	1	1	0	0	0	0	0	0	0	0	0	67
13983961	f	cn	US	2017	0	1	0	0	1	0	0	0	0	0	0	0	
13985046	f	md	US	2017	1	0	0	0	0	0	0	0	0	0	0	1	70
13986657	m	md	CA	2017	0	1	0	0	1	0	0	0	0	0	0	1	60
13986818	f	md	US	2017	0	0	0	0	0	0	0	0	0	0	0	0	
13986938	f	cn	GB	2017	1	0	0	0	0	0	0	0	0	0	0	0	71
13987060	m	cn	GB	2017	1	0	0	0	0	0	0	0	0	0	0	0	82
13987497	m	cn	SE	2017	0	1	1	0	0	0	0	0	0	0	0	0	
13987665	f	cn	GB	2017	1	0	0	0	0	0	0	0	0	0	0	0	71
13987666	f	cn	GB	2017	1	0	0	0	0	0	0	0	0	0	0	0	75
13987716	f	cn	GB	2017	1	0	0	0	0	0	0	0	0	0	0	0	75
13987743	f	cn	GB	2017	1	0	0	0	0	0	0	0	0	0	0	0	75
13987744	f	cn	GB	2017	1	0	0	0	0	0	0	0	0	0	0	0	74
13987753	f	cn	GB	2017	1	0	0	0	0	0	0	0	0	0	0	0	75
13988151	m	cn	US	2017	1	0	0	0	0	0	0	0	0	0	0	0	47
13989029	m	cn	US	2017	0	1	0	0	1	0	0	0	0	0	0	0	
13989522	m	cn	JP	2017	0	1	1	0	0	0	0	0	0	0	0	0	68
13989933	m	ot	US	2017	0	1	0	0	1	0	0	0	0	0	0	0	
13990352	m	md	KR	2017	0	1	0	1	0	0	0	0	0	0	0	0	74.6
13990470	m	cn	US	2017	0	1	0	0	1	0	0	0	0	0	0	0	
13990594	f	cn	US	2017	1	0	0	0	0	0	0	0	0	0	0	0	85

13990669	m	ot	US	2017	1	0	0	0	0	0	0	0	0	0	0	0	59
13991033	m	cn	JP	2017	0	1	1	0	0	0	0	0	0	0	0	0	65
13991364	m	cn	GB	2017	1	0	0	0	0	0	0	0	0	0	0	0	82
13991475	m	cn	GB	2017	1	0	0	0	0	0	0	0	0	0	0	0	
13992547	m	ph	GR	2017	0	1	0	0	1	0	0	0	0	0	0	0	68
13992959	m	cn	GB	2017	1	0	0	0	0	0	0	0	0	0	0	0	
13993118	m	md	JP	2017	1	0	0	0	0	0	0	0	0	0	0	0	80
13994436	m	ot	US	2017	0	1	0	0	1	0	0	0	0	0	0	0	71
13995105	Not	cn	US	2017	0	1	1	0	0	0	0	0	0	0	0	0	
13996220	m	ot	BE	2017	0	1	0	0	1	0	0	0	0	0	0	0	63
13996331	f	md	JP	2017	1	0	0	0	0	0	0	0	0	0	0	0	86.4
13997123	f	Not repo	MY	2017	1	0	0	0	0	0	0	0	0	0	0	0	56.2
13997262	Not	cn	JP	2017	0	0	0	0	0	0	0	0	0	0	0	0	65
13997689	m	cn	AT	2017	0	1	1	0	0	0	0	0	0	0	0	0	48
13998181	f	md	JP	2017	0	1	0	0	1	0	0	0	0	0	0	0	73
13998623	m	md	FI	2017	0	1	0	0	1	0	0	0	0	0	0	0	76
13999279	f	cn	US	2017	1	0	0	0	0	0	0	0	0	0	0	0	
13999373	m	cn	JP	2017	0	0	0	0	0	0	0	0	0	0	0	0	66
14000772	Not	ph	JP	2017	1	0	0	0	0	0	0	0	0	0	0	0	
14001770	Not	ph	JP	2017	0	0	0	0	0	0	0	0	0	0	0	0	
14001825	m	cn	US	2017	0	1	1	0	0	0	0	0	0	0	0	0	
14003056	m	ot	KR	2017	0	1	0	0	1	0	0	0	0	0	0	0	78
14003115	Not	ot	CN	2017	1	0	0	0	0	0	0	0	0	0	0	1	
14003177	f	cn	US	2017	0	1	1	0	0	0	0	0	0	0	0	0	
14003548	m	md	US	2017	0	1	0	0	1	0	0	0	0	0	0	0	75
14004486	m	md	FR	2017	0	1	0	0	0	1	0	0	0	0	0	0	60
14004588	m	md	EC	2017	1	0	0	0	0	0	0	0	0	0	0	0	39
14005291	m	Not repo	CA	2017	1	0	0	0	0	0	0	0	0	0	0	0	72
14006344	m	cn	GR	2017	0	1	1	0	0	0	0	0	0	0	0	1	
14007589	f	ot	DE	2017	0	1	0	0	1	0	0	0	0	0	0	0	62
14007769	m	md	JP	2017	0	1	0	0	1	0	0	0	0	0	0	0	76
14009324	f	cn	CO	2017	0	1	1	0	0	0	0	0	0	0	0	0	
14010140	m	cn	GR	2017	0	1	1	0	0	0	0	0	0	0	0	0	85
14010179	m	md	JP	2017	0	1	0	0	1	0	0	0	0	0	0	0	

14010557	f	md	US	2017	1	0	0	0	0	0	0	0	0	0	0	0	60
14010774	m	md	FR	2017	0	1	0	0	1	0	0	0	0	0	0	0	59
14010823	Not	md	US	2017	0	1	0	0	1	0	0	0	0	0	0	0	
14010841	Not	md	US	2017	0	1	0	0	1	0	0	0	0	0	0	0	
14010858	f	ot	US	2017	0	1	0	0	1	0	0	0	0	0	0	0	66
14011157	f	ot	JP	2017	1	0	0	0	0	0	0	0	0	0	0	0	68
14011193	Not	md	JP	2017	0	1	0	0	1	0	0	0	0	0	0	0	67
14011400	m	ph	US	2017	0	0	0	0	0	0	0	0	0	0	0	0	56
14011581	m	ot	JP	2017	0	1	0	0	1	0	0	0	0	0	0	0	72
14011721	Not	cn	GB	2017	1	0	0	0	0	0	0	0	0	0	0	1	
14011745	f	md	JP	2017	1	0	0	0	0	0	0	0	0	0	0	0	
14012565	f	md	JP	2017	0	1	0	0	1	0	0	0	0	0	0	0	72
14012860	m	ot	US	2017	1	0	0	0	0	0	0	0	0	0	0	0	70
14013182	f	cn	US	2017	0	1	0	0	1	0	0	0	0	0	0	0	
14014089	m	md	JP	2017	0	1	0	0	1	0	0	0	0	0	0	0	69
14014107	f	cn	GB	2017	0	1	1	0	0	0	0	0	0	0	0	0	
14014203	m	cn	RO	2017	0	0	0	0	0	0	0	0	0	0	0	0	
14014231	f	md	IT	2017	0	1	0	0	1	0	0	0	0	0	0	0	75
14014441	m	md	PH	2017	0	1	1	0	0	0	0	0	0	0	0	0	
14014839	f	cn	JP	2017	0	1	1	0	0	0	0	0	0	0	0	0	63
14016096	m	cn	GB	2017	1	0	0	0	0	0	0	0	0	0	0	0	68
14016097	Not	cn	GB	2017	1	0	0	0	0	0	0	0	0	0	0	0	
14016667	f	ot	US	2017	0	0	0	0	0	0	0	0	0	0	0	0	29
14017024	f	md	AU	2017	1	0	0	0	0	0	0	0	0	0	0	0	
14017726	f	md	RO	2017	1	0	0	0	0	0	0	0	0	0	0	0	
14018038	m	ot	AU	2017	0	1	0	0	1	0	0	0	0	0	0	0	45
14018133	Not	md	US	2017	0	0	0	0	0	0	0	0	0	0	0	0	
14018482	m	ot	JP	2017	0	1	0	0	1	0	0	0	0	0	0	0	74
14019674	f	md	PL	2017	0	0	0	0	0	0	0	0	0	0	0	0	46
14020350	Not	cn	JP	2017	0	1	1	0	0	0	0	0	0	0	0	0	70
14021402	f	ot	FR	2017	0	1	0	0	1	0	0	0	0	0	0	0	68
14022213	m	cn	DE	2017	0	1	1	0	0	0	0	0	0	0	0	0	45
14023737	f	ot	US	2017	0	0	0	0	0	0	0	0	0	0	0	0	
14024412	m	cn	IN	2017	0	1	0	0	1	0	0	0	0	0	0	0	66

14025044	f	cn	US	2017	0	0	0	0	0	0	0	0	0	0	0	0	
14025876	f	ot	US	2017	0	0	0	0	0	0	0	0	0	0	0	0	
14026212	f	ot	FR	2017	1	0	0	0	0	0	0	0	0	0	0	0	50
14026566	f	md	JP	2017	1	0	0	0	0	0	0	0	0	0	0	0	68.3
14026637	m	md	JP	2017	0	1	0	0	1	0	0	0	0	0	0	0	67
14026783	f	ph	US	2017	0	0	0	0	0	0	0	0	0	0	0	0	
14027599	f	cn	US	2017	0	0	0	0	0	0	0	0	0	0	0	0	
14028254	m	ot	US	2017	0	1	1	0	0	0	0	0	0	0	0	0	70
4000005	Not	cn	US	2017	0	0	0	0	0	0	0	0	0	0	0	0	
5766134	m	cn	JP	2017	0	0	0	0	0	0	0	0	0	0	0	0	69
5972553	Not	cn	JP	2017	0	0	0	0	0	0	0	0	0	0	0	0	67
6075878	m	ot	JP	2017	0	0	0	0	0	0	0	0	0	0	0	0	67
6125012	m	cn	JP	2017	0	0	0	0	0	0	0	0	0	0	0	0	66
6147481	m	cn	JP	2017	0	0	0	0	0	0	0	0	0	0	0	0	41
6579097	m	cn	JP	2017	0	0	0	0	0	0	0	0	0	0	0	0	63
6620274	f	cn	JP	2017	0	0	0	0	0	0	0	0	0	0	0	0	88
6975260	m	cn	JP	2017	0	0	0	0	0	0	0	0	0	0	0	0	68
10512717	m	ot	US	2017	0	0	0	0	0	0	0	0	0	0	0	0	71
10592746	m	md	JP	2017	0	0	0	0	0	0	0	0	0	0	0	0	72
12937365	f	md	NL	2017	0	1	0	0	0	1	0	0	0	0	0	0	47
13247935	m	cn	DE	2017	0	1	0	0	1	0	0	0	0	0	0	0	66
13259508	m	ot	JP	2017	0	1	0	0	1	0	0	0	0	0	0	0	79
13273730	m	md	US	2017	0	1	0	0	0	1	0	0	0	0	0	0	75
13294420	m	ot	US	2017	0	1	0	0	0	1	0	0	0	0	0	0	75
13296925	f	md	CH	2017	0	1	0	0	0	1	0	0	0	0	0	0	73
13330816	m	cn	US	2017	0	1	1	0	0	0	0	0	0	0	0	0	80
13342818	f	ot	US	2017	0	1	0	0	1	0	0	0	0	0	0	0	69
13363317	m	md	ES	2017	0	1	0	0	0	1	0	0	0	0	0	0	64
13378205	m	md	TW	2017	0	1	0	0	0	1	0	0	0	0	0	0	64
13394734	m	md	ES	2017	0	1	0	0	0	1	0	0	0	0	0	0	64
13418156	m	ot	JP	2017	0	1	0	0	1	0	0	0	0	0	0	0	60
13436554	m	cn	US	2017	0	1	0	0	1	0	0	0	0	0	0	0	
13449431	m	Not repo	JP	2017	0	0	0	0	0	0	0	0	0	0	0	0	66
13475927	m	md	JP	2017	0	0	0	0	0	0	0	0	0	0	0	0	77

13499025	m	md	JP	2017	0	0	0	0	0	0	0	0	0	0	0	0	71
13508010	m	md	CN	2017	0	1	0	0	0	1	0	0	0	0	0	0	71
13512085	f	md	DE	2017	0	0	0	0	0	0	0	0	0	0	0	0	59
13513678	m	md	JP	2017	1	0	0	0	0	0	0	0	0	0	0	0	80.8
13531037	f	md	ES	2017	0	1	0	0	0	1	0	0	0	0	0	1	57
13554682	f	md	JP	2017	0	1	0	0	1	0	0	0	0	0	0	0	81
13567603	f	Not repo	JP	2017	0	0	0	0	0	0	0	0	0	0	0	0	75
13569920	m	md	JP	2017	0	1	0	0	1	0	0	0	0	0	0	0	58
13571439	f	cn	US	2017	0	0	0	0	0	0	0	0	0	0	0	0	64
13582797	m	md	JP	2017	0	1	0	0	1	0	0	0	0	0	0	0	67
13587115	m	ph	JP	2017	0	1	0	0	1	0	0	0	0	0	0	0	73
13592428	f	md	JP	2017	0	1	0	0	1	0	0	0	0	0	0	0	
13596460	f	cn	US	2017	0	1	0	0	1	0	0	0	0	0	0	0	78
13611609	m	ot	US	2017	0	0	0	0	0	0	1	0	1	0	0	0	67
13615262	m	md	DE	2017	0	1	0	0	0	1	0	0	0	0	0	0	64
13618125	m	md	JP	2017	0	1	0	0	1	0	0	0	0	0	0	0	73
13632797	m	md	JP	2017	0	1	0	0	1	0	0	0	0	0	0	0	49
13641428	f	ot	JP	2017	0	1	0	0	1	0	0	0	0	0	0	0	72
13643306	m	md	JP	2017	0	1	0	0	1	0	0	0	0	0	0	0	56
13654640	f	ot	JP	2017	0	0	0	0	0	0	1	0	1	0	0	0	77
13655421	m	md	JP	2017	0	1	0	0	1	0	0	0	0	0	0	0	74
13658866	m	md	JP	2017	0	1	0	0	1	0	0	0	0	0	0	0	72
13665792	m	ot	DE	2017	0	1	0	0	1	0	0	0	0	0	0	0	73
13668522	m	md	JP	2017	0	1	0	0	1	0	0	0	0	0	0	0	69
13673240	m	cn	JP	2017	0	0	0	0	0	0	0	0	0	0	0	0	70
13680730	m	ot	JP	2017	0	1	0	0	1	0	0	0	0	0	0	0	66
13681826	m	md	CN	2017	0	1	0	0	0	1	0	0	0	0	0	0	68
13684452	f	ot	JP	2017	0	1	0	0	1	0	0	0	0	0	0	0	63
13684909	m	md	JP	2017	1	0	0	0	0	0	0	0	0	0	0	0	44
13686703	m	md	SG	2017	1	0	0	0	0	0	0	0	0	0	0	0	59
13692387	f	md	US	2017	0	1	0	0	0	1	0	0	0	0	0	0	49
13700398	m	ot	GR	2017	0	1	0	0	1	0	0	0	0	0	0	0	81
13703009	f	md	JP	2017	1	0	0	0	0	0	0	0	0	0	0	0	80.8
13708579	f	md	US	2017	1	0	0	0	0	0	0	0	0	0	0	0	77

13708971	f	md	JP	2017	0	0	0	0	0	0	0	0	0	0	0	0	70
13709318	m	md	CN	2017	0	1	0	0	0	1	0	0	0	0	0	0	46
13711088	m	md	JP	2017	0	1	0	0	1	0	0	0	0	0	0	1	79
13715308	m	ot	FR	2017	0	1	0	0	1	0	0	0	0	0	0	0	52
13717009	m	md	GB	2017	0	0	0	0	0	0	0	0	0	0	0	0	80
13721020	f	ot	US	2017	0	1	0	0	1	0	0	0	0	0	0	0	65
13721719	f	cn	US	2017	0	0	0	0	0	0	0	0	0	0	0	0	92
13726303	m	md	JP	2017	0	1	0	0	1	0	0	0	0	0	0	0	73
13726311	f	cn	JP	2017	0	1	0	0	1	0	0	0	0	0	0	1	70
13727302	f	md	IT	2017	0	1	0	0	1	0	0	0	0	0	0	0	66
13731207	m	md	JP	2017	0	1	0	0	1	0	0	0	0	0	0	0	79
13731728	m	ot	JP	2017	0	1	0	0	1	0	0	0	0	0	0	1	64
13733618	m	ot	US	2017	0	1	1	0	0	0	0	0	0	0	0	0	68
13733646	m	md	JP	2017	0	0	0	0	0	0	0	0	0	0	0	0	82
13740887	f	ot	JP	2017	0	1	0	0	1	0	0	0	0	0	0	0	85
13743373	f	md	JP	2017	0	1	0	0	1	0	0	0	0	0	0	0	64
13756395	m	cn	US	2017	1	0	0	0	0	0	0	0	0	0	0	0	63
13761945	m	ph	JP	2017	0	1	1	0	0	0	0	0	0	0	0	0	86
13762322	f	md	JP	2017	0	1	0	0	0	1	0	0	0	0	0	0	45
13763606	m	ot	CA	2017	0	1	0	0	1	0	0	0	0	0	0	0	76
13763635	m	md	JP	2017	0	1	1	0	0	0	0	0	0	0	0	0	55
13767032	m	cn	JP	2017	0	1	1	0	0	0	0	0	0	0	0	1	62
13767615	m	cn	JP	2017	0	1	1	0	0	0	0	0	0	0	0	1	70
13774032	f	ot	US	2017	0	0	0	0	0	0	0	0	0	0	0	0	78
13776035	m	md	US	2017	0	1	0	0	0	1	0	0	0	0	0	0	62
13779287	m	md	JP	2017	0	1	0	0	1	0	0	0	0	0	0	1	64
13779915	f	md	JP	2017	0	0	0	0	0	0	0	0	0	0	0	0	57
13780651	f	ph	US	2017	0	0	0	0	0	0	0	0	0	0	0	0	37
13791363	m	cn	JP	2017	0	1	1	0	0	0	0	0	0	0	0	0	69
13796319	m	ot	JP	2017	0	1	0	0	1	0	0	0	0	0	0	1	71
13797940	m	md	US	2017	0	1	0	0	0	1	0	0	0	0	0	0	66
13798253	m	md	IL	2017	0	0	0	0	0	0	0	0	0	0	0	0	60
13805450	m	md	AU	2017	0	1	0	0	1	0	0	0	0	0	0	0	67
13807527	f	cn	JP	2017	0	1	1	0	0	0	0	0	0	0	0	0	62

13807886	m	md	CN	2017	0	1	0	1	0	0	0	0	0	0	0	0	68.4
13810431	m	md	US	2017	0	1	0	0	1	0	0	0	0	0	0	0	65
13814740	m	ot	JP	2017	0	1	0	0	1	0	0	0	0	0	0	0	69
13819350	m	cn	JP	2017	0	0	0	0	0	0	0	0	0	0	0	0	60
13821460	m	cn	JP	2017	0	1	1	0	0	0	0	0	0	0	0	0	53
13822103	m	ph	JP	2017	0	1	1	0	0	0	0	0	0	0	0	1	60
13822218	f	md	DE	2017	0	1	0	0	0	1	0	0	0	0	0	0	63
13822616	f	md	JP	2017	0	1	0	0	1	0	0	0	0	0	0	0	57
13824481	m	md	FR	2017	0	1	0	0	0	1	0	0	0	0	0	0	65
13828158	Not	cn	US	2017	0	1	0	0	1	0	0	0	0	0	0	0	54
13835171	f	cn	DE	2017	0	1	1	0	0	0	0	0	0	0	0	0	55
13840430	f	md	US	2017	0	0	0	0	0	0	0	0	0	0	0	0	66
13843910	m	ph	JP	2017	0	1	1	0	0	0	0	0	0	0	0	1	72
13844162	f	ot	US	2017	0	0	0	0	0	0	0	0	0	0	0	0	68
13844925	f	md	FR	2017	0	1	0	0	0	1	0	0	0	0	0	0	53
13850645	f	ot	US	2017	0	1	1	0	0	0	0	0	0	0	0	0	69
13857591	f	md	CA	2017	0	0	0	0	0	0	0	0	0	0	0	0	
13858770	m	cn	JP	2017	0	1	1	0	0	0	0	0	0	0	0	0	47
13861008	m	md	CN	2017	1	0	0	0	0	0	0	0	0	0	0	0	65.7
13861184	f	md	JP	2017	1	0	0	0	0	0	0	0	0	0	0	1	73.3
13868551	m	md	JP	2017	0	1	0	0	1	0	0	0	0	0	0	0	48
13868956	f	md	JP	2017	0	0	0	0	0	0	0	0	0	0	0	0	78
13869806	m	cn	JP	2017	0	0	0	0	0	0	0	0	0	0	0	0	75
13873319	f	ot	GR	2017	0	1	0	0	1	0	0	0	0	0	0	0	73
13875682	m	md	JP	2017	0	1	0	0	1	0	0	0	0	0	0	0	79
13875795	m	md	JP	2017	1	0	0	0	0	0	0	0	0	0	0	0	61
13877386	m	cn	JP	2017	0	1	1	0	0	0	0	0	0	0	0	0	64
13879955	f	md	US	2017	0	1	0	0	0	1	0	0	0	0	0	0	69
13880429	f	md	JP	2017	0	1	0	0	1	0	0	0	0	0	0	0	68
13880526	f	ot	US	2017	0	1	0	0	1	0	0	0	0	0	0	0	49
13884037	f	ot	US	2017	0	1	0	0	1	0	0	0	0	0	0	0	64
13891349	m	md	CN	2017	0	1	0	0	0	1	0	0	0	0	0	0	63
13896773	f	cn	US	2017	0	1	1	0	0	0	0	0	0	0	0	0	
13903038	m	ot	DE	2017	0	1	0	0	1	0	0	0	0	0	0	0	73

13904375	f	md	CN	2017	1	0	0	0	0	0	0	0	0	0	0	0	66.9
13904399	m	ot	JP	2017	0	1	1	0	0	0	0	0	0	0	0	0	86
13905340	m	cn	JP	2017	0	1	1	0	0	0	0	0	0	0	0	0	53
13905641	f	md	JP	2017	1	0	0	0	0	0	0	0	0	0	0	1	76.2
13906785	f	md	AU	2017	0	0	0	0	0	0	0	0	0	0	0	0	70
13913045	m	md	CN	2017	1	0	0	0	0	0	0	0	0	0	0	0	54.3
13916645	f	ot	DE	2017	0	0	0	0	0	0	0	0	0	0	0	0	62
13919081	f	md	JP	2017	0	1	0	0	1	0	0	0	0	0	0	0	38
13920903	f	md	JP	2017	0	0	0	0	0	0	0	0	0	0	0	0	55
13926102	f	md	JP	2017	1	0	0	0	0	0	0	0	0	0	0	0	66
13926672	m	md	ES	2017	0	1	0	0	0	1	0	0	0	0	0	0	71
13927723	m	md	JP	2017	0	1	0	0	1	0	0	0	0	0	0	0	68
13928319	m	md	JP	2017	1	0	0	0	0	0	0	0	0	0	0	0	
13930007	m	cn	JP	2017	0	0	0	0	0	0	0	0	0	0	0	0	81
13930084	f	md	GB	2017	0	0	0	0	0	0	0	0	0	0	0	0	73
13931440	m	md	IN	2017	0	0	0	0	0	0	0	0	0	0	0	0	57
13931839	f	ot	US	2017	0	1	0	0	1	0	0	0	0	0	0	0	74
13932532	m	ph	JP	2017	0	0	0	0	0	0	0	0	0	0	0	0	80
13933375	m	md	JP	2017	0	1	0	0	1	0	0	0	0	0	0	0	67
13937949	m	md	JP	2017	0	0	0	0	0	0	0	0	0	0	0	0	57
13938047	f	ot	US	2017	0	1	0	0	1	0	0	0	0	0	0	0	
13938544	m	md	JP	2017	0	1	1	0	0	0	0	0	0	0	0	1	54
13941301	m	Not repo	US	2017	1	0	0	0	0	0	0	0	0	0	0	0	57
13942594	m	ot	US	2017	0	1	0	0	1	0	0	0	0	0	0	0	78
13943694	m	ot	JP	2017	0	1	1	0	0	0	0	0	0	0	0	1	64
13944922	f	md	JP	2017	1	0	0	0	0	0	0	0	0	0	0	0	70
13945687	f	md	JP	2017	1	0	0	0	0	0	0	0	0	0	0	0	63
13947548	m	ot	JP	2017	0	1	0	0	1	0	0	0	0	0	0	0	69
13948104	f	md	JP	2017	0	0	0	0	0	0	0	0	0	0	0	0	40
13948388	f	cn	GB	2017	1	0	0	0	0	0	0	0	0	0	0	0	61
13948725	f	ot	NL	2017	0	1	0	0	1	0	0	0	0	0	0	1	55
13948810	m	md	CN	2017	0	1	0	0	0	1	0	0	0	0	0	0	54
13949348	m	md	FR	2017	0	1	0	1	0	0	0	0	0	0	0	0	69
13951407	f	md	JP	2017	0	1	0	1	0	0	0	0	0	0	0	0	62.7

13952078	m	cn	JP	2017	0	1	1	0	0	0	0	0	0	0	0	0	80
13954412	f	cn	GB	2017	1	0	0	0	0	0	0	0	0	0	0	0	
13958995	f	md	FR	2017	0	0	0	0	0	0	0	0	0	0	0	0	52
13961344	f	ot	DE	2017	0	1	0	0	1	0	0	0	0	0	0	0	61
13962563	m	cn	DE	2017	1	0	0	0	0	0	0	0	0	0	0	0	
13963175	f	ot	CA	2017	0	1	0	0	1	0	0	0	0	0	0	0	63
13963245	f	md	US	2017	0	0	0	0	0	0	0	0	0	0	0	0	58
13964428	f	ot	DE	2017	0	0	0	0	0	0	0	0	0	0	0	0	62
13965350	f	md	GB	2017	0	1	0	0	1	0	0	0	0	0	0	0	59
13966473	m	cn	US	2017	0	1	1	0	0	0	0	0	0	0	0	0	83
13968639	m	md	DE	2017	0	0	0	0	0	0	0	0	0	0	0	0	80
13973227	Not	ph	JP	2017	0	1	1	0	0	0	0	0	0	0	0	0	
13974680	Not	cn	SG	2017	0	1	1	0	0	0	0	0	0	0	0	0	
13974878	m	md	AU	2017	1	0	0	0	0	0	0	0	0	0	0	0	58.3
13975168	f	md	JP	2017	0	1	0	0	1	0	0	0	0	0	0	0	53
13975383	m	md	CN	2017	1	0	0	0	0	0	0	0	0	0	0	0	56
13976792	m	ot	US	2017	0	1	0	0	1	0	0	0	0	0	0	0	61
13977439	f	md	JP	2017	1	0	0	0	0	0	0	0	0	0	0	0	75
13977461	m	ot	CN	2017	0	1	0	0	1	0	0	0	0	0	0	1	59
13977469	f	md	CZ	2017	1	0	0	0	0	0	0	0	0	0	0	0	67
13978014	Not	ot	JP	2017	0	0	0	0	0	0	0	0	0	0	0	0	
13980395	f	md	JP	2017	0	0	0	0	0	0	0	0	0	0	0	0	48
13983321	m	md	FR	2017	0	1	0	1	0	0	0	0	0	0	0	0	51
13985619	Not	ph	JP	2017	1	0	0	0	0	0	0	0	0	0	0	0	
13986503	m	cn	GB	2017	1	0	0	0	0	0	0	0	0	0	0	0	67
13987024	f	ot	US	2017	1	0	0	0	0	0	0	0	0	0	0	0	78
13987192	f	ph	JP	2017	0	0	0	0	0	0	0	0	0	0	0	0	70
13987584	f	md	FR	2017	0	1	0	0	1	0	0	0	0	0	0	0	47
13989664	f	md	CN	2017	1	0	0	0	0	0	0	0	0	0	0	0	61.8
13990254	f	ot	GR	2017	0	1	0	0	1	0	0	0	0	0	0	0	75
13994092	m	md	US	2017	0	1	1	0	0	0	0	0	0	0	0	0	
13994300	m	ot	JP	2017	0	1	0	0	1	0	0	0	0	0	0	0	53
13996990	f	cn	JP	2017	0	1	1	0	0	0	0	0	0	0	0	0	76
13998451	Not	cn	US	2017	0	1	1	0	0	0	0	0	0	0	0	0	

13999507	m	cn	JP	2017	0	1	1	0	0	0	0	0	0	0	0	0	70
13999874	m	cn	BR	2017	1	0	0	0	0	0	0	0	0	0	0	0	74
14001047	f	md	JP	2017	1	0	0	0	0	0	0	0	0	0	0	0	64
14003029	m	md	CN	2017	0	1	0	0	0	1	0	0	0	0	0	0	57
14004515	f	cn	GB	2017	1	0	0	0	0	0	0	0	0	0	0	0	
14006077	m	md	JP	2017	0	0	0	0	0	0	0	0	0	0	0	0	43
14006397	m	md	JP	2017	0	1	0	0	1	0	0	0	0	0	0	0	67
14006614	m	md	IN	2017	0	1	0	0	1	0	0	0	0	0	0	0	78
14007798	m	md	JP	2017	0	1	0	0	1	0	0	0	0	0	0	0	66
14007870	m	ot	DE	2017	0	1	0	0	1	0	0	0	0	0	0	0	74
14008098	m	md	JP	2017	0	1	0	0	1	0	0	0	0	0	0	0	88
14008753	f	md	Not re	2017	0	1	0	0	1	0	0	0	0	0	0	0	
14009242	f	md	JP	2017	1	0	0	0	0	0	0	0	0	0	0	0	82
14010292	m	md	CN	2017	0	1	0	0	0	1	0	0	0	0	0	0	47
14010368	f	md	JP	2017	0	0	0	0	0	0	0	0	0	0	0	0	70
14010387	m	cn	US	2017	1	0	0	0	0	0	0	0	0	0	0	0	
14011351	m	md	ES	2017	0	1	0	0	0	1	0	0	0	0	0	0	68
14012084	f	md	JP	2017	0	0	0	0	0	0	0	0	0	0	0	0	72
14012181	f	md	GB	2017	0	0	0	0	0	0	0	0	0	0	0	0	59
14013181	m	ot	JP	2017	0	1	0	0	1	0	0	0	0	0	0	0	71
14013633	f	md	NL	2017	0	0	0	0	0	0	0	0	0	0	0	0	49
14014415	m	md	DE	2017	0	1	0	0	0	1	0	0	0	0	0	0	65
14015496	f	Not repo	CA	2017	1	0	0	0	0	0	0	0	0	0	0	0	52
14016156	f	md	JP	2017	1	0	0	0	0	0	0	0	0	0	0	0	67.6
14016872	f	md	JP	2017	1	0	0	0	0	0	0	0	0	0	0	1	77
14017800	m	md	SK	2017	0	0	0	0	0	0	0	0	0	0	0	0	65
14018279	m	ot	DE	2017	0	1	0	0	1	0	0	0	0	0	0	0	79
14018480	m	ot	DE	2017	0	1	0	0	1	0	0	0	0	0	0	0	64
14018630	m	md	IN	2017	0	1	0	0	1	0	0	0	0	0	0	0	79
14019354	m	ph	US	2017	0	1	0	0	1	0	0	0	0	0	0	0	73
14019720	m	cn	BE	2017	0	1	1	0	0	0	0	0	0	0	0	0	67
14020306	f	cn	US	2017	0	1	1	0	0	0	0	0	0	0	0	0	68
14022732	f	md	IT	2017	0	1	0	0	1	0	0	0	0	0	0	0	
14024266	m	ot	CH	2017	0	1	0	0	1	0	0	0	0	0	0	0	67

14025364	m	ot	GB	2017	0	1	0	0	1	0	0	0	0	0	0	0	52
14025372	f	ot	FR	2017	0	1	0	0	1	0	0	0	0	0	0	1	71
14026121	f	md	JP	2017	0	0	0	0	0	0	0	0	0	0	0	0	57
14026329	m	cn	US	2017	1	0	0	0	0	0	0	0	0	0	0	0	84
14026558	f	ph	US	2017	0	1	0	0	1	0	0	0	0	0	0	0	59
14027002	f	ot	PL	2017	0	0	0	0	0	0	0	0	0	0	0	0	51
14027530	f	ot	US	2017	0	1	1	0	0	0	0	0	0	0	0	0	73
14028087	Not	cn	US	2017	0	1	1	0	0	0	0	0	0	0	0	0	
14028137	m	md	TW	2017	1	0	0	0	0	0	0	0	0	0	0	0	60
14029971	Not	ot	CL	2017	0	1	0	0	1	0	0	0	0	0	0	0	
14030294	m	md	US	2017	0	0	0	0	0	0	0	0	0	0	0	0	61
14030687	m	md	US	2017	0	0	0	0	0	0	0	0	0	0	0	0	52
14031024	f	md	US	2017	0	0	0	0	0	0	0	0	0	0	0	0	71
14031763	f	md	US	2017	0	0	0	0	0	0	0	0	0	0	0	0	61
14031804	f	md	TW	2017	1	0	0	0	0	0	0	0	0	0	0	0	60
14031828	m	md	JP	2017	1	0	0	0	0	0	0	0	0	0	0	0	80
14032180	m	cn	FR	2017	0	1	1	0	0	0	0	0	0	0	0	0	
14032413	f	cn	US	2017	0	0	0	0	0	0	0	0	0	0	0	0	68
14033427	f	ot	JP	2017	0	1	0	0	1	0	0	0	0	0	0	0	70
14033713	f	ot	JP	2017	0	1	0	0	1	0	0	0	0	0	0	0	66
14033758	m	md	JP	2017	1	0	0	0	0	0	0	0	0	0	0	0	80
14034415	m	md	JP	2017	1	0	0	0	0	0	0	0	0	0	0	0	78
14034461	m	md	TW	2017	1	0	0	0	0	0	0	0	0	0	0	0	70
14034896	m	md	TW	2017	1	0	0	0	0	0	0	0	0	0	0	0	50
14035022	m	cn	US	2017	0	0	0	0	0	0	0	0	0	0	0	0	56
14035024	m	cn	RO	2017	0	0	0	0	0	0	0	0	0	0	0	0	65
14035285	Not	cn	JP	2017	0	1	1	0	0	0	0	0	0	0	0	0	
14035437	m	md	JP	2017	0	0	0	0	0	0	0	0	0	0	0	0	51
14035481	m	md	JP	2017	0	0	0	0	0	0	0	0	0	0	0	0	44
14036526	f	cn	US	2017	0	0	0	0	0	0	0	0	0	0	0	0	
14036699	m	md	TW	2017	1	0	0	0	0	0	0	0	0	0	0	0	70
14037297	f	md	US	2017	1	0	0	0	0	0	0	0	0	0	0	0	84
14037856	f	md	CN	2017	1	0	0	0	0	0	0	0	0	0	0	0	76.4
14037965	f	md	JP	2017	1	0	0	0	0	0	0	0	0	0	0	0	70

14038664	m	md	ES	2017	0	0	0	0	0	0	0	0	0	0	0	0	69
14038670	f	md	AU	2017	0	0	0	0	0	0	0	0	0	0	0	0	75
14039358	f	md	CN	2017	1	0	0	0	0	0	0	0	0	0	0	0	50.2
14039586	m	ot	CL	2017	0	1	0	0	1	0	0	0	0	0	0	0	65
14039623	f	ot	JP	2017	0	1	0	0	1	0	0	0	0	0	0	0	68
14041012	m	md	US	2017	0	1	0	0	1	0	0	0	0	0	0	0	70
14041637	m	md	JP	2017	0	1	0	0	1	0	0	0	0	0	0	0	74
14042670	m	md	MT	2017	0	1	0	0	1	0	0	0	0	0	0	0	64
14044066	m	md	JP	2017	0	1	0	0	1	0	0	0	0	0	0	0	63
14044286	m	md	JP	2017	0	1	0	0	1	0	0	0	0	0	0	0	74
14044301	m	ot	FR	2017	0	0	0	0	0	0	0	0	0	0	0	0	
14044894	f	md	JP	2017	1	0	0	0	0	0	0	0	0	0	0	0	80
14045037	m	cn	RU	2017	0	1	1	0	0	0	0	0	0	0	0	0	
14045105	m	cn	JP	2017	0	0	0	0	0	0	0	0	0	0	0	0	58
14045220	f	cn	US	2017	0	1	0	0	1	0	0	0	0	0	0	0	
14045816	m	md	JP	2017	0	1	0	0	1	0	0	0	0	0	0	1	69
14046229	Not	ot	US	2017	0	0	0	0	0	0	0	0	0	0	0	0	
14046323	f	ot	US	2017	1	0	0	0	0	0	0	0	0	0	0	0	80
14046554	m	md	JP	2017	0	1	0	0	1	0	0	0	0	0	0	1	68
14046801	f	ot	US	2017	0	1	0	0	1	0	0	0	0	0	0	0	68
14046998	m	md	JP	2017	1	0	0	0	0	0	0	0	0	0	0	1	82.5
14047153	m	md	US	2017	0	1	0	0	1	0	0	0	0	0	0	0	70
14051588	f	md	AU	2017	1	0	0	0	0	0	0	0	0	0	0	0	65.7
14052059	Not	md	JP	2017	1	0	0	0	0	0	0	0	0	0	0	0	79
14052184	Not	ot	GB	2017	0	0	0	0	0	0	0	0	0	0	0	0	63
14052187	f	md	AU	2017	0	0	0	0	0	0	0	0	0	0	0	0	70
14053305	f	cn	US	2017	0	1	1	0	0	0	0	0	0	0	0	0	62
14054100	m	md	JP	2017	1	0	0	0	0	0	0	0	0	0	0	0	47
14054446	m	ot	US	2017	0	1	0	0	1	0	0	0	0	0	0	0	69
14054978	m	ot	TW	2017	0	1	0	0	1	0	0	0	0	0	0	0	65
14055072	Not	md	KW	2017	0	1	0	0	0	1	0	0	0	0	0	0	
14055519	m	Not repo	CA	2017	1	0	0	0	0	0	0	0	0	0	0	0	
14055567	f	md	JP	2017	1	0	0	0	0	0	0	0	0	0	0	0	75.6
14055784	m	cn	US	2017	0	1	1	0	0	0	0	0	0	0	0	0	

14055814	m	cn	US	2017	1	0	0	0	0	0	0	0	0	0	0	0	79
14055871	m	md	DE	2017	0	1	0	0	1	0	0	0	0	0	0	0	58
14056237	f	cn	GB	2017	1	0	0	0	0	0	0	0	0	0	0	0	61
14056262	m	md	CN	2017	1	0	0	0	0	0	0	0	0	0	0	0	55.2
14056840	m	md	JP	2017	0	1	0	1	0	0	0	0	0	0	0	0	66
14057012	m	md	CZ	2017	1	0	0	0	0	0	0	0	0	0	0	0	58
14057045	f	md	CN	2017	1	0	0	0	0	0	0	0	0	0	0	0	45.9
14057047	m	md	US	2017	1	0	0	0	0	0	0	0	0	0	0	0	70
14057221	m	ot	DE	2017	0	1	0	0	1	0	0	0	0	0	0	0	67
14057564	m	ot	KR	2017	0	0	0	0	0	0	0	0	0	0	0	0	63
14058124	m	md	NO	2017	0	1	0	0	1	0	0	0	0	0	0	0	70
14060497	f	md	CZ	2017	0	0	0	0	0	0	0	0	0	0	0	0	51
14060968	f	cn	US	2017	1	0	0	0	0	0	0	0	0	0	0	0	58
14061182	f	md	TW	2017	1	0	0	0	0	0	0	0	0	0	0	0	48
14062156	Not	md	US	2017	0	0	0	0	0	0	0	0	0	0	0	0	
14062591	f	cn	US	2017	1	0	0	0	0	0	0	0	0	0	0	0	68
14062675	f	md	DK	2017	0	1	1	0	0	0	0	0	0	0	0	0	69
14063005	Not	md	IT	2017	1	0	0	0	0	0	0	0	0	0	0	0	
14063406	f	cn	GB	2017	1	0	0	0	0	0	0	0	0	0	0	0	65
14064039	m	md	JP	2017	0	0	0	0	0	0	0	0	0	0	0	0	71
14064301	m	md	IT	2017	0	0	0	0	0	0	0	0	0	0	0	0	40
14065438	m	cn	GB	2017	1	0	0	0	0	0	0	0	0	0	0	0	77
14065722	f	cn	GB	2017	1	0	0	0	0	0	0	0	0	0	0	0	62
14066181	f	ph	IT	2017	0	1	0	0	1	0	0	0	0	0	0	0	68
14066272	m	ph	US	2017	0	1	0	0	1	0	0	0	0	0	0	0	
14066893	Not	ot	GB	2017	0	1	0	0	1	0	0	0	0	0	0	0	
14066977	m	md	AE	2017	0	1	0	0	0	1	0	0	0	0	0	0	52
14067216	m	ot	US	2017	0	1	0	0	0	1	0	0	0	0	0	0	
14067300	f	md	JP	2017	0	1	0	0	1	0	0	0	0	0	0	0	66
14067336	m	ph	US	2017	0	0	0	0	0	0	0	0	0	0	0	0	80
14068962	f	md	JP	2017	1	0	0	0	0	0	0	0	0	0	0	0	80
14069545	m	md	JP	2017	0	0	0	0	0	0	0	0	0	0	0	0	69
14069547	m	cn	JP	2017	0	1	1	0	0	0	0	0	0	0	0	0	69
14069729	m	md	JP	2017	0	1	1	0	0	0	0	0	0	0	0	0	80

14069822	m	md	IT	2017	0	0	0	0	0	0	0	0	0	0	0	0	39
14070027	m	cn	TW	2017	0	0	0	0	0	0	0	0	0	0	0	0	64
14070100	m	md	US	2017	0	1	0	0	1	0	0	0	0	0	0	0	
14070197	Not	cn	JP	2017	0	0	0	0	0	0	0	0	0	0	0	0	
14070333	f	cn	US	2017	1	0	0	0	0	0	0	0	0	0	0	0	75
14070391	m	ot	JP	2017	0	0	0	0	0	0	0	0	0	0	0	0	
14070433	m	md	US	2017	0	1	0	0	1	0	0	0	0	0	0	0	60
14070569	Not	cn	US	2017	0	1	0	0	1	0	0	0	0	0	0	0	
14070572	m	cn	US	2017	0	1	0	0	1	0	0	0	0	0	0	0	
14070578	m	md	CN	2017	0	0	0	0	0	0	0	0	0	0	0	0	55.4
14070626	f	md	JP	2017	0	0	0	0	0	0	0	0	0	0	0	0	88
14072458	m	md	IT	2017	0	1	0	0	1	0	0	0	0	0	0	0	67
14072521	f	ot	GB	2017	0	0	0	0	0	0	0	0	0	0	0	0	70
14072868	m	ph	JP	2017	0	0	0	0	0	0	0	0	0	0	0	0	77
14072960	m	md	JP	2017	0	0	0	0	0	0	0	0	0	0	0	0	67
14074501	m	ph	JP	2017	0	1	0	0	1	0	0	0	0	0	0	0	71
14075037	m	cn	JP	2017	0	1	1	0	0	0	0	0	0	0	0	0	54
14075809	m	ot	ES	2017	0	1	0	0	1	0	0	0	0	0	0	0	68
14076207	f	ot	JP	2017	0	1	0	0	1	0	0	0	0	0	0	0	79
14077464	f	md	CZ	2017	1	0	0	0	0	0	0	0	0	0	0	0	76
14077498	m	md	JP	2017	0	0	0	0	0	0	0	0	0	0	0	0	53
14077553	m	cn	HK	2017	0	0	0	0	0	0	0	0	0	0	0	0	41
14077654	f	md	US	2017	0	1	0	0	0	1	0	0	0	0	0	0	65
14077723	m	ot	SG	2017	0	1	0	0	1	0	0	0	0	0	0	0	69
14078047	f	md	AU	2017	1	0	0	0	0	0	0	0	0	0	0	0	51
14078806	m	cn	GB	2017	1	0	0	0	0	0	0	0	0	0	0	0	73
14079671	f	ot	ES	2017	0	1	0	0	1	0	0	0	0	0	0	1	71
14079708	f	ph	GB	2017	0	0	0	0	0	0	0	0	0	0	0	0	57
14081038	f	md	US	2017	0	0	0	0	0	0	0	0	0	0	0	0	82
14081656	m	cn	JP	2017	0	1	0	0	1	0	0	0	0	0	0	0	41
14082922	m	cn	US	2017	0	1	0	0	1	0	0	0	0	0	0	0	70
14083067	f	cn	FR	2017	0	1	1	0	0	0	0	0	0	0	0	0	
14083272	Not	cn	US	2017	0	1	0	0	1	0	0	0	0	0	0	0	
14083881	f	md	FR	2017	0	0	0	0	0	0	0	0	0	0	0	0	

14084164	m	ot	CN	2017	0	1	0	0	1	0	0	0	0	0	0	0	76
14084311	m	ot	US	2017	0	1	0	0	1	0	0	0	0	0	0	0	75
14084848	f	md	JP	2017	1	0	0	0	0	0	0	0	0	0	0	0	75
14085234	f	ot	US	2017	0	1	1	0	0	0	0	0	0	0	0	0	32
14085560	m	cn	GB	2017	1	0	0	0	0	0	0	0	0	0	0	0	73
14085982	m	ot	NO	2017	0	1	0	0	1	0	0	0	0	0	0	0	74
14086502	m	md	KR	2017	0	1	0	1	0	0	0	0	0	0	0	0	76
14088817	m	md	JP	2017	0	1	0	0	1	0	0	0	0	0	0	0	
14089018	f	ot	JP	2017	0	1	0	0	1	0	0	0	0	0	0	0	76
14089119	f	md	GR	2017	0	1	0	0	1	0	0	0	0	0	0	0	69
14089179	m	ot	ES	2017	0	1	0	0	1	0	0	0	0	0	0	0	71
14089246	f	ot	ZA	2017	0	1	0	0	1	0	0	0	0	0	0	0	68
14089285	f	ot	US	2017	0	1	0	0	1	0	0	0	0	0	0	0	89
14089421	m	ot	JP	2017	0	1	0	0	1	0	0	0	0	0	0	0	
14090488	f	md	DE	2017	0	1	0	0	1	0	0	0	0	0	0	0	
14090490	m	ot	JP	2017	0	1	0	0	1	0	0	0	0	0	0	0	60
14090492	Not	cn	GR	2017	0	1	0	0	1	0	0	0	0	0	0	0	
14090495	m	ot	JP	2017	0	1	0	0	1	0	0	0	0	0	0	0	52
14090511	m	ot	JP	2017	0	1	0	0	1	0	0	0	0	0	0	0	50
14090519	f	md	US	2017	0	0	0	0	0	0	0	0	0	0	0	0	
14090536	m	md	FR	2017	0	1	0	0	1	0	0	0	0	0	0	0	55
14090855	m	ot	JP	2017	0	1	0	0	1	0	0	0	0	0	0	0	
14090859	m	ot	JP	2017	0	1	0	0	1	0	0	0	0	0	0	0	
14090863	m	ot	JP	2017	0	1	0	0	1	0	0	0	0	0	0	0	
14090945	m	cn	US	2017	0	1	0	0	1	0	0	0	0	0	0	0	
14091320	m	md	DE	2017	0	1	0	0	1	0	0	0	0	0	0	0	
14091526	f	ph	US	2017	0	1	0	0	1	0	0	0	0	0	0	0	
14091733	f	ot	US	2017	0	0	0	0	0	0	0	0	0	0	0	0	32
14092296	Not	ot	US	2017	0	0	0	0	0	0	0	0	0	0	0	0	
14092297	Not	ot	US	2017	0	0	0	0	0	0	0	0	0	0	0	0	
14092298	Not	ot	US	2017	0	0	0	0	0	0	0	0	0	0	0	0	
14092309	Not	ot	US	2017	0	0	0	0	0	0	0	0	0	0	0	0	
14092312	Not	ot	US	2017	0	0	0	0	0	0	0	0	0	0	0	0	
14092313	Not	ot	US	2017	0	0	0	0	0	0	0	0	0	0	0	0	

14092314	Not	ot	US	2017	0	0	0	0	0	0	0	0	0	0	0	0	
14092638	m	cn	US	2017	0	1	0	0	1	0	0	0	0	0	0	0	78
14093178	m	md	CH	2017	0	0	0	0	0	0	0	0	0	0	0	0	57
14094295	f	cn	JP	2017	0	1	1	0	0	0	0	0	0	0	0	0	49
14094314	m	cn	JP	2017	0	1	1	0	0	0	0	0	0	0	0	0	77
14094316	m	cn	JP	2017	0	0	0	0	0	0	0	0	0	0	0	0	58
14095512	m	cn	FR	2017	0	1	1	0	0	0	0	0	0	0	0	0	54
14095519	Not	ot	ES	2017	0	0	0	0	0	0	0	0	0	0	0	0	
14095799	m	cn	JP	2017	0	0	0	0	0	0	0	0	0	0	0	0	52
14095912	m	md	JP	2017	0	0	0	0	0	0	0	0	0	0	0	0	41
14096047	f	md	CZ	2017	1	0	0	0	0	0	0	0	0	0	0	0	57
14096059	f	cn	JP	2017	0	0	0	0	0	0	0	0	0	0	0	0	57
14096959	f	md	CZ	2017	1	0	0	0	0	0	0	0	0	0	0	0	73
14098077	f	md	CZ	2017	1	0	0	0	0	0	0	0	0	0	0	0	47
14098492	f	ot	US	2017	0	0	0	0	0	0	0	0	0	0	0	0	74
14098498	f	cn	US	2017	1	0	0	0	0	0	0	0	0	0	0	0	67
14098678	m	ph	JP	2017	0	1	0	0	1	0	0	0	0	0	0	0	65
14099260	m	cn	JP	2017	0	1	1	0	0	0	0	0	0	0	0	1	51
14099568	f	cn	US	2017	0	1	1	0	0	0	0	0	0	0	0	0	82
14099798	f	md	FR	2017	1	0	0	0	0	0	0	0	0	0	0	0	77
14100021	f	md	US	2017	0	0	0	0	0	0	0	0	0	0	0	0	
14100041	f	md	US	2017	0	0	0	0	0	0	0	0	0	0	0	0	73
14100404	m	ot	ES	2017	0	1	0	0	1	0	0	0	0	0	0	0	69
14100405	m	ot	FR	2017	0	1	0	0	1	0	0	0	0	0	0	0	75
14100745	f	md	JP	2017	0	0	0	0	0	0	0	0	0	0	0	0	
14101071	m	ot	JP	2017	0	0	0	0	0	0	0	0	0	0	0	0	70
14101076	m	md	CN	2017	1	0	0	0	0	0	0	0	0	0	0	0	55.3
14101142	m	md	JP	2017	0	1	0	0	1	0	0	0	0	0	0	1	65
14101176	f	cn	US	2017	0	1	0	0	1	0	0	0	0	0	0	0	65
14101241	f	cn	US	2017	1	0	0	0	0	0	0	0	0	0	0	0	76
14101325	m	cn	JP	2017	0	1	1	0	0	0	0	0	0	0	0	0	79
14101349	m	md	JP	2017	1	0	0	0	0	0	0	0	0	0	0	0	63
14101400	m	ot	GB	2017	0	0	0	0	0	0	0	0	0	0	0	0	68
14101540	m	ph	US	2017	0	1	1	0	0	0	0	0	0	0	0	0	62

14101722	m	cn	US	2017	0	1	1	0	0	0	0	0	0	0	0	0	
14101903	f	md	JP	2017	0	1	0	0	1	0	0	0	0	0	0	0	63
14103908	m	cn	US	2017	1	0	0	0	0	0	0	0	0	0	0	0	64
14103979	f	ot	US	2017	0	1	0	0	1	0	0	0	0	0	0	0	73
14104890	f	ot	US	2017	0	1	0	0	1	0	0	0	0	0	0	0	70
14105136	Not	ph	GB	2017	0	0	0	0	0	0	0	0	0	0	0	0	
14105339	f	md	DE	2017	0	0	0	0	0	0	0	0	0	0	0	0	66
14105570	m	md	BE	2017	0	0	0	0	0	0	0	0	0	0	0	0	70
14105667	m	cn	JP	2017	0	1	1	0	0	0	0	0	0	0	0	0	62
14106261	m	ot	JP	2017	0	1	0	0	1	0	0	0	0	0	0	0	69
14106273	m	ot	FR	2017	0	1	0	0	1	0	0	0	0	0	0	0	78
14106359	f	md	FR	2017	0	1	0	0	1	0	0	0	0	0	0	0	76
14107241	m	ot	AR	2017	0	1	0	0	1	0	0	0	0	0	0	1	55
14107675	m	cn	JP	2017	0	0	0	0	0	0	0	0	0	0	0	0	75
14107751	m	ot	US	2017	0	0	0	0	0	0	0	0	0	0	0	0	
14108068	f	cn	US	2017	0	1	1	0	0	0	0	0	0	0	0	0	84
14108845	m	md	US	2017	0	0	0	0	0	0	0	0	0	0	0	0	
14110439	f	cn	GB	2017	1	0	0	0	0	0	0	0	0	0	0	0	
14110511	m	ph	US	2017	1	0	0	0	0	0	0	0	0	0	0	0	86
14110826	m	md	JP	2017	1	0	0	0	0	0	0	0	0	0	0	0	55
14111245	f	md	JP	2017	1	0	0	0	0	0	0	0	0	0	0	1	
14111551	f	md	FR	2017	1	0	0	0	0	0	0	0	0	0	0	1	
14111587	Not	ot	CA	2017	0	0	0	0	0	0	0	0	0	0	0	0	
14111588	Not	ot	CA	2017	0	0	0	0	0	0	0	0	0	0	0	0	
14111622	m	ot	NO	2017	0	0	0	0	0	0	0	0	0	0	0	0	42
14112011	m	cn	JP	2017	0	1	1	0	0	0	0	0	0	0	0	0	77
14113404	m	md	KR	2017	0	1	0	0	1	0	0	0	0	0	0	1	
14113612	m	md	FR	2017	0	1	0	0	1	0	0	0	0	0	0	0	
14113804	f	ot	US	2017	0	1	0	0	1	0	0	0	0	0	0	0	63
14114219	f	cn	JP	2017	0	1	1	0	0	0	0	0	0	0	0	0	74
14114613	m	ot	DE	2017	0	1	0	0	1	0	0	0	0	0	0	0	63
14115182	f	md	CN	2017	1	0	0	0	0	0	0	0	0	0	0	1	61
14115625	Not	ot	TW	2017	0	0	0	0	0	0	0	0	0	0	0	0	
14115715	m	cn	US	2017	0	1	1	0	0	0	0	0	0	0	0	0	

14115833	f	md	CA	2017	1	0	0	0	0	0	0	0	0	0	0	0	
14116016	f	md	JP	2017	0	1	1	0	0	0	0	0	0	0	0	0	70
14117064	m	cn	US	2017	1	0	0	0	0	0	0	0	0	0	0	0	91
14117770	f	md	KR	2017	0	0	0	0	0	0	0	0	0	0	0	0	74
14117825	m	cn	JP	2017	0	1	1	0	0	0	0	0	0	0	0	0	90
14118087	m	md	CN	2017	1	0	0	0	0	0	0	0	0	0	0	0	67.9
14118095	f	md	US	2017	0	0	0	0	0	0	0	0	0	0	0	0	
14118724	m	cn	JP	2017	0	1	1	0	0	0	0	0	0	0	0	0	66
14118999	m	ot	JP	2017	0	1	0	0	1	0	0	0	0	0	0	0	66
14119089	f	md	JP	2017	0	0	0	0	0	0	0	0	0	0	0	0	90
14119251	m	cn	US	2017	0	0	0	0	0	0	0	0	0	0	0	0	
14119610	m	cn	JP	2017	0	0	0	0	0	0	0	0	0	0	0	0	55
14119895	Not	md	IT	2017	1	0	0	0	0	0	0	0	0	0	0	0	
14119988	m	md	JP	2017	0	0	0	0	0	0	1	0	1	0	0	0	65
14120157	f	md	IT	2017	1	0	0	0	0	0	0	0	0	0	0	0	77
14120210	m	ot	JP	2017	0	1	0	0	1	0	0	0	0	0	0	0	65
14120398	f	cn	GB	2017	1	0	0	0	0	0	0	0	0	0	0	0	
14120482	m	ot	DK	2017	1	0	0	0	0	0	0	0	0	0	0	0	49
14120685	m	cn	JP	2017	0	1	1	0	0	0	0	0	0	0	0	0	70
14120887	m	md	JP	2017	1	0	0	0	0	0	0	0	0	0	0	0	85.8
14121931	m	ot	PL	2017	0	1	0	0	1	0	0	0	0	0	0	0	51
14121963	m	cn	Not re	2017	0	0	0	0	0	0	0	0	0	0	0	0	60
14122439	m	md	DE	2017	1	0	0	0	0	0	0	0	0	0	0	0	57
14122915	m	md	JP	2017	1	0	0	0	0	0	0	0	0	0	0	0	74.7
14123363	f	md	US	2017	0	1	0	0	0	1	0	0	0	0	0	0	
14123471	f	md	FR	2017	1	0	0	0	0	0	0	0	0	0	0	0	
14123504	m	md	DE	2017	0	0	0	0	0	0	0	0	0	0	0	0	47
14124011	Not	md	US	2017	0	0	0	0	0	0	0	0	0	0	0	0	
14124115	m	md	JP	2017	0	1	0	0	1	0	0	0	0	0	0	0	51
14124483	m	md	NZ	2017	0	0	0	0	0	0	0	0	0	0	0	0	58
14124651	Not	md	PT	2017	1	0	0	0	0	0	0	0	0	0	0	0	
14124665	m	md	FR	2017	0	1	0	0	1	0	0	0	0	0	0	0	56
14125005	f	md	US	2017	0	1	1	0	0	0	0	0	0	0	0	0	73
14125051	Not	md	US	2017	0	0	0	0	0	0	0	0	0	0	0	0	

14125204	f	md	KR	2017	1	0	0	0	0	0	0	0	0	0	0	0	55
14125312	Not	md	US	2017	0	0	0	0	0	0	0	0	0	0	0	0	
14125329	Not	md	US	2017	0	0	0	0	0	0	0	0	0	0	0	0	
14125331	Not	md	US	2017	0	0	0	0	0	0	0	0	0	0	0	0	
14125340	m	cn	DE	2017	0	0	0	0	0	0	0	0	0	0	0	0	
14125392	Not	md	US	2017	0	0	0	0	0	0	0	0	0	0	0	0	
14125602	f	md	BE	2017	1	0	0	0	0	0	0	0	0	0	0	0	66
14125821	Not	cn	Not re	2017	0	0	0	0	0	0	0	0	0	0	0	0	
14125851	m	ot	US	2017	0	1	0	0	1	0	0	0	0	0	0	1	80
14125871	f	Not repo	US	2017	1	0	0	0	0	0	0	0	0	0	0	1	85.5
14126039	Not	md	US	2017	0	0	0	0	0	0	0	0	0	0	0	0	
14126169	f	md	US	2017	0	1	1	0	0	0	0	0	0	0	0	0	66
14126174	Not	md	US	2017	0	0	0	0	0	0	0	0	0	0	0	0	
14126602	f	md	US	2017	0	1	0	0	1	0	0	0	0	0	0	0	70
14127297	f	ot	GB	2017	0	0	0	0	0	0	0	0	0	0	0	0	82
14127517	f	md	TW	2017	0	1	0	0	0	1	0	0	0	0	0	0	32
14128327	m	cn	JP	2017	0	1	1	0	0	0	0	0	0	0	0	0	73
14128680	m	md	DE	2017	0	0	0	0	0	0	0	0	0	0	0	0	47
14128752	m	md	JP	2017	0	1	0	0	1	0	0	0	0	0	0	0	79
14128794	Not	ot	US	2017	0	0	0	0	0	0	0	0	0	0	0	0	
14128812	m	cn	US	2017	0	1	0	0	1	0	0	0	0	0	0	0	
14128837	f	ot	US	2017	0	0	0	0	0	0	0	0	0	0	0	0	
14128911	m	ot	US	2017	0	1	0	0	1	0	0	0	0	0	0	0	69
14132010	Not	md	CA	2017	0	0	0	0	0	0	0	0	0	0	0	0	
14132094	f	md	JP	2017	0	0	0	0	0	0	0	0	0	0	0	0	86
14132245	m	md	JP	2017	0	1	0	0	1	0	0	0	0	0	0	0	63
14132983	f	md	DE	2017	0	0	0	0	0	0	0	0	0	0	0	0	
14133084	Not	ph	FR	2017	0	1	0	0	1	0	0	0	0	0	0	0	
14133309	f	ot	US	2017	0	0	0	0	0	0	0	0	0	0	0	0	
14133399	Not	ph	US	2017	0	0	0	0	0	0	0	0	0	0	0	0	
14133497	m	md	FR	2017	0	1	0	0	1	0	0	0	0	0	0	0	
14137163	f	md	SK	2017	0	0	0	0	0	0	0	0	0	0	0	0	
14137611	Not	md	CA	2017	0	0	0	0	0	0	0	0	0	0	0	0	
14137746	f	md	JP	2017	1	0	0	0	0	0	0	0	0	0	0	0	78.1

14137773	f	cn	DE	2017	0	1	1	0	0	0	0	0	0	0	0	0	
14137855	f	md	JP	2017	1	0	0	0	0	0	0	0	0	0	0	0	85.8
14138180	f	md	MY	2017	1	0	0	0	0	0	0	0	0	0	0	0	45
14138202	f	cn	US	2017	0	0	0	0	0	0	0	0	0	0	0	0	43
14138206	m	cn	US	2017	0	0	0	0	0	0	0	0	0	0	0	0	
14138207	f	md	MY	2017	1	0	0	0	0	0	0	0	0	0	0	0	58
14138216	m	ot	US	2017	0	0	0	0	0	0	0	0	0	0	0	0	62
14138232	f	md	MY	2017	1	0	0	0	0	0	0	0	0	0	0	0	39
14138243	m	md	MY	2017	1	0	0	0	0	0	0	0	0	0	0	0	65
14138847	m	md	FR	2017	0	1	0	0	1	0	0	0	0	0	0	0	81
14138877	f	ot	RO	2017	0	1	0	0	1	0	0	0	0	0	0	0	60
14138905	m	cn	US	2017	0	1	1	0	0	0	0	0	0	0	0	0	
14138928	f	ot	FR	2017	0	1	0	0	1	0	0	0	0	0	0	0	70
14139073	f	md	MY	2017	1	0	0	0	0	0	0	0	0	0	0	0	72
14139083	f	md	MY	2017	1	0	0	0	0	0	0	0	0	0	0	0	67
14139085	f	md	MY	2017	1	0	0	0	0	0	0	0	0	0	0	0	75
14139151	f	md	AE	2017	0	1	0	0	0	1	0	0	0	0	0	0	
14139162	m	cn	CA	2017	0	1	1	0	0	0	0	0	0	0	0	0	49
14139269	m	cn	US	2017	0	1	1	0	0	0	0	0	0	0	0	1	
14139329	m	cn	US	2017	0	1	1	0	0	0	0	0	0	0	0	1	
14140134	m	ot	KR	2017	0	1	0	0	1	0	0	0	0	0	0	0	83
14140184	m	ot	JP	2017	0	1	1	0	0	0	0	0	0	0	0	0	72
14140892	m	cn	NL	2017	0	1	0	0	1	0	0	0	0	0	0	0	64
14141011	m	md	CN	2017	1	0	0	0	0	0	0	0	0	0	0	0	65.6
14141133	m	ot	JP	2017	0	0	0	0	0	0	0	0	0	0	0	0	55
14141297	f	ot	US	2017	0	0	0	0	0	0	0	0	0	0	0	0	
14141455	m	md	JP	2017	0	1	0	0	1	0	0	0	0	0	0	1	70
14141793	f	md	JP	2017	0	0	0	0	0	0	0	0	0	0	0	0	72
14142302	m	cn	JP	2017	0	1	1	0	0	0	0	0	0	0	0	1	76
14142330	f	ot	GB	2017	0	1	0	0	1	0	0	0	0	0	0	0	57
14143127	m	ot	US	2017	0	1	0	0	1	0	0	0	0	0	0	0	68
14143214	m	ot	JP	2017	0	1	0	0	1	0	0	0	0	0	0	0	67
14143309	f	md	CA	2017	1	0	0	0	0	0	0	0	0	0	0	0	62
14143711	Not	ot	TW	2017	0	0	0	0	0	0	0	0	0	0	0	0	

14144070	f	md	JP	2017	0	1	0	0	1	0	0	0	0	0	0	0	70
14144277	m	ot	BE	2017	0	1	0	0	1	0	0	0	0	0	0	0	58
14144425	m	md	JP	2017	0	1	0	0	1	0	0	0	0	0	0	0	78
14144473	Not	cn	US	2017	0	1	0	0	1	0	0	0	0	0	0	0	
14144525	f	cn	US	2017	0	1	0	0	1	0	0	0	0	0	0	0	64
14145092	m	ph	JP	2017	0	1	1	0	0	0	0	0	0	0	0	0	79
14145384	m	ot	DE	2017	0	1	0	0	1	0	0	0	0	0	0	0	66
14146194	m	md	JP	2017	0	1	0	0	1	0	0	0	0	0	0	0	72
14146294	f	md	JP	2017	1	0	0	0	0	0	0	0	0	0	0	0	68
14146347	f	cn	GB	2017	1	0	0	0	0	0	0	0	0	0	0	0	75
14146350	f	cn	GB	2017	1	0	0	0	0	0	0	0	0	0	0	0	
14146413	f	md	PE	2017	0	0	0	0	0	0	0	0	0	0	0	0	
14146891	f	md	JP	2017	1	0	0	0	0	0	0	0	0	0	0	0	83
14147024	f	md	AT	2017	0	1	0	0	1	0	0	0	0	0	0	0	
14148123	m	ot	FR	2017	0	1	0	0	1	0	0	0	0	0	0	0	59
14148176	Not	md	ID	2017	1	0	0	0	0	0	0	0	0	0	0	0	
14148178	m	ot	FR	2017	0	1	0	0	1	0	0	0	0	0	0	0	70
14148493	m	ot	DE	2017	0	0	0	0	0	0	0	0	0	0	0	0	47
14148722	m	lw	US	2017	0	1	0	0	1	0	0	0	0	0	0	0	77
14148934	f	ph	US	2017	1	0	0	0	0	0	0	0	0	0	0	0	80
14149506	Not	ot	JP	2017	0	0	0	0	0	0	0	0	0	0	0	0	
14149513	f	ph	US	2017	0	1	0	0	1	0	0	0	0	0	0	0	73
14151320	m	cn	CA	2017	0	1	0	0	1	0	0	0	0	0	0	0	57
14151662	Not	Not repo	Not re	2017	1	0	0	0	0	0	0	0	0	0	0	0	
14152149	m	md	DE	2017	0	0	0	0	0	0	0	0	0	0	0	0	48
14152307	f	md	JP	2017	0	1	0	0	1	0	0	0	0	0	0	0	71
14152661	f	md	JP	2017	0	0	0	0	0	0	0	0	0	0	0	0	77
14153488	f	md	JP	2017	1	0	0	0	0	0	0	0	0	0	0	0	76
14153878	m	cn	US	2017	0	1	0	0	1	0	0	0	0	0	0	0	65
14153912	m	md	JP	2017	0	1	0	0	1	0	0	0	0	0	0	1	82
14154254	f	cn	DE	2017	1	0	0	0	0	0	0	0	0	0	0	0	53
14154603	f	md	JP	2017	0	1	0	0	1	0	0	0	0	0	0	1	75
14154657	f	cn	JP	2017	0	1	0	0	1	0	0	0	0	0	0	0	67
14154854	m	md	JP	2017	0	1	0	0	1	0	0	0	0	0	0	0	67

14154868	f	ot	US	2017	0	1	1	0	0	0	0	0	0	0	0	1	
14154890	m	md	US	2017	0	1	0	0	0	1	0	0	0	0	0	1	
14154918	m	ot	US	2017	0	1	1	0	0	0	0	0	0	0	0	0	
14155173	Not	ph	JP	2017	0	0	0	0	0	0	0	0	0	0	0	0	77
14155310	f	md	CH	2017	0	0	0	0	0	0	0	0	0	0	0	0	52
14155381	m	md	IN	2017	0	1	0	0	1	0	0	0	0	0	0	0	62
14155395	Not	md	US	2017	0	1	0	0	1	0	0	0	0	0	0	0	
14155514	m	cn	JP	2017	0	1	1	0	0	0	0	0	0	0	0	0	64
14156097	m	cn	JP	2017	0	1	1	0	0	0	0	0	0	0	0	0	76
14156488	m	md	JP	2017	1	0	0	0	0	0	0	0	0	0	0	0	50
14156587	m	ot	DE	2017	0	1	0	0	1	0	0	0	0	0	0	0	49
14156650	f	cn	US	2017	0	1	0	0	1	0	0	0	0	0	0	0	
14156738	f	md	JP	2017	1	0	0	0	0	0	0	0	0	0	0	0	70.8
14156805	m	md	CN	2017	1	0	0	0	0	0	0	0	0	0	0	0	71
14157107	m	cn	JP	2017	0	1	1	0	0	0	0	0	0	0	0	0	70
14157609	f	ph	US	2017	1	0	0	0	0	0	0	0	0	0	0	0	67
14157981	m	ot	DE	2017	0	0	0	0	0	0	0	0	0	0	0	0	60
14158007	f	cn	US	2017	0	1	0	0	1	0	0	0	0	0	0	0	
14158140	f	md	US	2017	0	0	0	0	0	0	0	0	0	0	0	0	66
14158824	m	md	RU	2017	0	1	0	1	0	0	0	0	0	0	0	0	57
14159230	f	md	TW	2017	1	0	0	0	0	0	0	0	0	0	0	0	80
14159297	m	ot	US	2017	0	1	0	0	1	0	0	0	0	0	0	0	50
14159647	m	md	DE	2017	1	0	0	0	0	0	0	0	0	0	0	0	48
14160089	m	md	JP	2017	0	1	0	0	0	1	0	0	0	0	0	0	63
14160129	f	md	US	2017	0	0	0	0	0	0	0	0	0	0	0	0	92
14160195	f	md	TH	2017	1	0	0	0	0	0	0	0	0	0	0	0	68
14161117	m	ph	JP	2017	0	1	1	0	0	0	0	0	0	0	0	0	
14161408	Not	cn	GB	2017	1	0	0	0	0	0	0	0	0	0	0	0	
14161824	f	cn	JP	2017	0	1	1	0	0	0	0	0	0	0	0	0	65
14162129	m	md	TW	2017	1	0	0	0	0	0	0	0	0	0	0	0	70
14162635	f	md	TW	2017	1	0	0	0	0	0	0	0	0	0	0	0	60
14162640	f	md	TW	2017	1	0	0	0	0	0	0	0	0	0	0	0	60
14162788	f	cn	CA	2017	1	0	0	0	0	0	0	0	0	0	0	0	55
14163084	Not	cn	GB	2017	1	0	0	0	0	0	0	0	0	0	0	0	

14163226	f	md	TW	2017	1	0	0	0	0	0	0	0	0	0	0	0	70
14163340	f	md	TW	2017	1	0	0	0	0	0	0	0	0	0	0	0	50
14163353	f	md	TW	2017	1	0	0	0	0	0	0	0	0	0	0	0	80
14163361	f	md	TW	2017	1	0	0	0	0	0	0	0	0	0	0	0	70
14163421	f	md	TW	2017	1	0	0	0	0	0	0	0	0	0	0	0	60
14163448	m	md	TW	2017	1	0	0	0	0	0	0	0	0	0	0	0	70
14163450	f	md	TW	2017	1	0	0	0	0	0	0	0	0	0	0	0	60
14163593	f	md	TW	2017	1	0	0	0	0	0	0	0	0	0	0	0	70
14163595	f	md	TW	2017	1	0	0	0	0	0	0	0	0	0	0	0	70
14163893	Not	md	DE	2017	0	1	0	0	1	0	0	0	0	0	0	0	69
14163966	m	ot	PE	2017	0	1	0	0	1	0	0	0	0	0	0	0	78
14164239	f	cn	US	2017	0	1	1	0	0	0	0	0	0	0	0	0	
14164838	Not	cn	GB	2017	1	0	0	0	0	0	0	0	0	0	0	0	
14164839	Not	cn	GB	2017	1	0	0	0	0	0	0	0	0	0	0	0	
14165142	m	md	JP	2017	0	0	0	0	0	0	0	0	0	0	0	0	43
14165191	m	cn	JP	2017	0	1	1	0	0	0	0	0	0	0	0	0	69
14165224	f	ot	US	2017	1	0	0	0	0	0	0	0	0	0	0	0	
14165451	m	ot	JP	2017	0	1	0	0	1	0	0	0	0	0	0	1	56
14165987	Not	cn	GB	2017	1	0	0	0	0	0	0	0	0	0	0	0	
14166262	m	md	JP	2017	0	1	0	0	1	0	0	0	0	0	0	0	63
14166524	f	md	CH	2017	0	0	0	0	0	0	0	0	0	0	0	0	
14167051	f	md	JP	2017	1	0	0	0	0	0	0	0	0	0	0	0	78
14167989	f	cn	US	2017	1	0	0	0	0	0	0	0	0	0	0	0	61.7
14169101	f	cn	US	2017	1	0	0	0	0	0	0	0	0	0	0	0	59
14169172	f	cn	VE	2017	0	1	0	0	1	0	0	0	0	0	0	0	
14169611	m	md	CH	2017	0	0	0	0	0	0	0	0	0	0	0	0	68
14169899	m	ot	US	2017	0	1	0	0	1	0	0	0	0	0	0	0	
14170089	f	cn	AR	2017	0	0	0	0	0	0	0	0	0	0	0	0	68
14170730	m	ph	US	2017	1	0	0	0	0	0	0	0	0	0	0	0	67
14171085	f	cn	JP	2017	0	0	0	0	0	0	0	0	0	0	0	0	61
14171333	Not	md	JP	2017	1	0	0	0	0	0	0	0	0	0	0	1	
14171906	m	md	DE	2017	1	0	0	0	0	0	0	0	0	0	0	0	43
14172465	m	md	JP	2017	0	1	0	0	1	0	0	0	0	0	0	0	
14173333	m	md	FR	2017	0	1	0	1	0	0	0	0	0	0	0	0	69

14173775	f	md	JP	2017	0	0	0	0	0	0	0	0	0	0	0	0	73
14174637	f	md	CN	2017	1	0	0	0	0	0	0	0	0	0	0	0	71.1
14174686	m	md	US	2017	1	0	0	0	0	0	0	0	0	0	0	0	55
14174738	f	ot	BE	2017	0	0	0	0	0	0	0	0	0	0	0	0	42
14174925	Not	md	CN	2017	1	0	0	0	0	0	0	0	0	0	0	0	
14174928	f	md	DE	2017	1	0	0	0	0	0	0	0	0	0	0	0	43
14174958	Not	md	CN	2017	1	0	0	0	0	0	0	0	0	0	0	0	
14175154	f	md	CN	2017	1	0	0	0	0	0	0	0	0	0	0	0	70
14175207	m	md	US	2017	0	0	0	0	0	0	0	0	0	0	0	0	
14176086	f	md	DE	2017	1	0	0	0	0	0	0	0	0	0	0	0	78
14176359	f	cn	GB	2017	0	1	1	0	0	0	0	0	0	0	0	0	72
14176581	m	ot	SG	2017	0	1	0	0	1	0	0	0	0	0	0	0	69
14176713	m	md	IN	2017	0	1	0	0	1	0	0	0	0	0	0	0	68
14176879	f	md	FR	2017	1	0	0	0	0	0	0	0	0	0	0	0	45
14176991	Not	md	GR	2017	0	1	0	0	1	0	0	0	0	0	0	0	
14177031	f	ot	DE	2017	0	1	0	0	1	0	0	0	0	0	0	0	53
14177451	f	md	US	2017	0	1	1	0	0	0	0	0	0	0	0	0	62
14177535	f	md	JP	2017	1	0	0	0	0	0	0	0	0	0	0	0	74
14177839	Not	ot	US	2017	0	0	0	0	0	0	0	0	0	0	0	0	
14178045	f	md	CN	2017	0	1	0	1	0	0	0	0	0	0	0	0	62
14179104	m	md	JP	2017	0	1	1	0	0	0	0	0	0	0	0	0	47
14179280	f	ph	US	2017	0	1	0	0	1	0	0	0	0	0	0	0	47
14179394	f	ot	US	2017	0	0	0	0	0	0	0	0	0	0	0	0	64
14180566	m	ot	JP	2017	0	1	0	0	1	0	0	0	0	0	0	0	78
14180699	f	md	JP	2017	0	1	0	0	1	0	0	0	0	0	0	0	72
14181625	f	cn	US	2017	1	0	0	0	0	0	0	0	0	0	0	0	66
14182021	Not	ot	US	2017	0	0	0	0	0	0	0	0	0	0	0	0	
14182045	Not	ot	US	2017	0	0	0	0	0	0	0	0	0	0	0	0	
14182046	Not	ot	US	2017	0	0	0	0	0	0	0	0	0	0	0	0	
14182219	Not	ot	US	2017	0	0	0	0	0	0	0	0	0	0	0	0	
14182239	Not	ot	US	2017	0	0	0	0	0	0	0	0	0	0	0	0	
14182249	Not	ot	US	2017	0	0	0	0	0	0	0	0	0	0	0	0	
14182343	Not	ot	US	2017	0	0	0	0	0	0	0	0	0	0	0	0	
14182363	f	md	CA	2017	0	1	1	0	0	0	0	0	0	0	0	0	

14183320	Not	ph	DE	2017	0	1	1	0	0	0	0	0	0	0	0	0	
14183321	Not	ph	DE	2017	0	1	1	0	0	0	0	0	0	0	0	0	
14183322	Not	ph	DE	2017	0	1	1	0	0	0	0	0	0	0	0	0	
14183460	m	md	IN	2017	0	1	0	0	1	0	0	0	0	0	0	0	73
14183769	m	ot	US	2017	0	1	1	0	0	0	0	0	0	0	0	0	
14183859	m	md	UA	2017	0	0	0	0	0	0	0	0	0	0	0	0	47
14184029	m	md	IT	2017	1	0	0	0	0	0	0	0	0	0	0	0	48.3
14184195	f	cn	GB	2017	1	0	0	0	0	0	0	0	0	0	0	0	69
14184352	Not	md	JP	2017	0	0	0	0	0	0	0	0	0	0	0	0	
14184370	m	ph	US	2017	0	0	0	0	0	0	0	0	0	0	0	0	55
14184684	m	md	US	2017	1	0	0	0	0	0	0	0	0	0	0	0	76
14184776	m	cn	US	2017	0	0	0	0	0	0	0	0	0	0	0	0	
14184950	f	md	FR	2017	0	0	0	0	0	0	0	0	0	0	0	0	85
14185153	m	md	US	2017	0	0	0	0	0	0	0	0	0	0	0	0	
14185541	m	cn	GB	2017	1	0	0	0	0	0	0	0	0	0	0	0	66
14185728	m	cn	US	2017	0	1	1	0	0	0	0	0	0	0	0	0	78
14185825	Not	ot	US	2017	0	0	0	0	0	0	0	0	0	0	0	0	
14186239	Not	md	US	2017	0	0	0	0	0	0	0	0	0	0	0	0	
14186241	Not	md	US	2017	0	0	0	0	0	0	0	0	0	0	0	0	
14186243	Not	md	US	2017	0	0	0	0	0	0	0	0	0	0	0	0	
14186249	Not	md	US	2017	0	0	0	0	0	0	0	0	0	0	0	0	
14186283	Not	md	US	2017	0	0	0	0	0	0	0	0	0	0	0	0	
14186680	Not	md	US	2017	0	0	0	0	0	0	0	0	0	0	0	0	
14186761	Not	md	US	2017	0	0	0	0	0	0	0	0	0	0	0	0	
14186763	Not	ph	JP	2017	1	0	0	0	0	0	0	0	0	0	0	0	
14186877	f	md	ES	2017	1	0	0	0	0	0	0	0	0	0	0	1	54
14187065	m	ph	US	2017	0	0	0	0	0	0	0	0	0	0	0	0	
14187130	m	cn	BR	2017	1	0	0	0	0	0	0	0	0	0	0	0	80
14187248	Not	md	US	2017	0	0	0	0	0	0	0	0	0	0	0	0	
14187249	Not	md	US	2017	0	0	0	0	0	0	0	0	0	0	0	0	
14187273	Not	md	US	2017	0	0	0	0	0	0	0	0	0	0	0	0	
14187275	Not	md	US	2017	0	0	0	0	0	0	0	0	0	0	0	0	
14187276	Not	md	US	2017	0	0	0	0	0	0	0	0	0	0	0	0	
14187354	Not	md	US	2017	0	0	0	0	0	0	0	0	0	0	0	0	

14187362	Not	md	US	2017	0	0	0	0	0	0	0	0	0	0	0	0	
14187366	Not	md	US	2017	0	0	0	0	0	0	0	0	0	0	0	0	
14187372	Not	md	US	2017	0	0	0	0	0	0	0	0	0	0	0	0	
14187421	Not	md	US	2017	0	0	0	0	0	0	0	0	0	0	0	0	
14187466	Not	md	US	2017	0	0	0	0	0	0	0	0	0	0	0	0	
14187537	Not	md	US	2017	0	0	0	0	0	0	0	0	0	0	0	0	
14187576	Not	md	US	2017	0	0	0	0	0	0	0	0	0	0	0	0	
14187618	Not	md	US	2017	0	0	0	0	0	0	0	0	0	0	0	0	
14187621	Not	md	US	2017	0	0	0	0	0	0	0	0	0	0	0	0	
14187663	Not	md	US	2017	0	0	0	0	0	0	0	0	0	0	0	0	
14187682	f	md	JP	2017	0	0	0	0	0	0	0	0	0	0	0	0	80
14187702	f	md	JP	2017	1	0	0	0	0	0	0	0	0	0	0	0	82.4
14187941	Not	cn	US	2017	0	1	1	0	0	0	0	0	0	0	0	0	
14189214	m	cn	US	2017	0	1	1	0	0	0	0	0	0	0	0	0	
14189227	f	md	FR	2017	0	1	0	1	0	0	0	0	0	0	0	1	47.5
14189263	m	cn	JP	2017	0	1	1	0	0	0	0	0	0	0	0	0	48
14189458	f	md	CH	2017	0	0	0	0	0	0	0	0	0	0	0	0	70
14189784	Not	md	JP	2017	1	0	0	0	0	0	0	0	0	0	0	0	
14189975	Not	md	JP	2017	0	0	0	0	0	0	0	0	0	0	0	0	
14189980	m	cn	JP	2017	0	1	1	0	0	0	0	0	0	0	0	0	84
14190223	f	md	CN	2017	1	0	0	0	0	0	0	0	0	0	0	0	
14190348	f	md	JP	2017	1	0	0	0	0	0	0	0	0	0	0	0	73
14190498	f	md	JP	2017	1	0	0	0	0	0	0	0	0	0	0	0	74
14191114	m	md	DE	2017	0	0	0	0	0	0	0	0	0	0	0	0	68
14191848	Not	md	JP	2017	1	0	0	0	0	0	0	0	0	0	0	0	
14192213	f	ot	US	2017	0	0	0	0	0	0	0	0	0	0	0	0	51
14192342	f	cn	CN	2017	1	0	0	0	0	0	0	0	0	0	0	0	82
14192896	f	ot	US	2017	0	1	0	0	1	0	0	0	0	0	0	0	71
14193092	m	md	DE	2017	0	0	0	0	0	0	0	0	0	0	0	0	64
14193103	m	md	DE	2017	0	0	0	0	0	0	0	0	0	0	0	0	71
14193104	m	md	CN	2017	0	0	0	0	0	0	0	0	0	0	0	0	63
14193375	m	ot	JP	2017	0	1	0	0	1	0	0	0	0	0	0	0	75
14193855	f	md	CN	2017	1	0	0	0	0	0	0	0	0	0	0	0	66
14193987	m	md	IN	2017	0	1	0	0	1	0	0	0	0	0	0	0	31

14194006	m	ot	KR	2017	0	0	0	0	0	0	0	0	0	0	0	0	63
14194754	Not	md	JP	2017	0	0	0	0	0	0	0	0	0	0	0	0	
14194981	f	md	IT	2017	0	1	0	0	1	0	0	0	0	0	0	0	
14195065	m	md	SK	2017	1	0	0	0	0	0	0	0	0	0	0	0	
14195067	f	md	SK	2017	1	0	0	0	0	0	0	0	0	0	0	0	
14195102	m	md	SK	2017	1	0	0	0	0	0	0	0	0	0	0	0	
14195107	f	md	NZ	2017	0	1	0	1	0	0	0	0	0	0	0	0	64
14195396	f	cn	IN	2017	0	0	0	0	0	0	0	0	0	0	0	0	45
14195681	f	ot	ES	2017	0	1	0	0	1	0	0	0	0	0	0	0	75
14195682	m	ot	JP	2017	0	1	0	0	1	0	0	0	0	0	0	0	73
14196582	f	md	DE	2017	0	0	0	0	0	0	0	0	0	0	0	0	31
14197031	f	md	DE	2017	0	0	0	0	0	0	0	0	0	0	0	0	55
14197034	m	md	DE	2017	1	0	0	0	0	0	0	0	0	0	0	0	62
14197598	Not	md	DE	2017	0	0	0	0	0	0	0	0	0	0	0	0	
14197602	m	md	DE	2017	0	0	0	0	0	0	0	0	0	0	0	0	62
14197826	f	cn	TW	2017	1	0	0	0	0	0	0	0	0	0	0	0	67
14198900	f	ph	US	2017	0	0	0	0	0	0	0	0	0	0	0	0	66
14199629	f	Not repo	KR	2017	1	0	0	0	0	0	0	0	0	0	0	0	66
14199914	f	cn	GB	2017	1	0	0	0	0	0	0	0	0	0	0	0	54
14200178	Not	ot	JP	2017	1	0	0	0	0	0	0	0	0	0	0	0	
14200438	f	md	MY	2017	0	1	0	0	0	1	0	0	0	0	0	0	67
14200725	m	cn	JP	2017	0	1	1	0	0	0	0	0	0	0	0	0	68
14201895	f	ot	JP	2017	0	1	0	0	1	0	0	0	0	0	0	0	61
14202223	f	cn	JP	2017	0	1	1	0	0	0	0	0	0	0	0	0	60
14202467	f	ot	CL	2017	0	1	0	0	1	0	0	0	0	0	0	0	66
14203313	f	md	DE	2017	1	0	0	0	0	0	0	0	0	0	0	0	50
14203552	m	ot	US	2017	0	1	0	0	1	0	0	0	0	0	0	0	61
14203569	f	md	US	2017	0	0	0	0	0	0	0	0	0	0	0	0	83
14203928	m	md	EG	2017	1	0	0	0	0	0	0	0	0	0	0	0	66
14204125	m	md	CH	2017	0	0	0	0	0	0	0	0	0	0	0	0	56
14204284	f	md	IT	2017	0	1	0	0	1	0	0	0	0	0	0	0	
14204997	f	cn	JP	2017	1	0	0	0	0	0	0	0	0	0	0	0	
14205060	f	md	GB	2017	0	0	0	0	0	0	0	0	0	0	0	0	63
14205108	Not	cn	GB	2017	1	0	0	0	0	0	0	0	0	0	0	0	

14205162	Not	md	BE	2017	0	1	0	0	1	0	0	0	0	0	0	0	
14205399	m	cn	JP	2017	0	1	1	0	0	0	0	0	0	0	0	0	60
14205459	m	cn	FR	2017	0	1	1	0	0	0	0	0	0	0	0	0	
14205485	m	md	FR	2017	0	1	0	0	0	1	0	0	0	0	0	0	66
14205490	m	cn	JP	2017	0	1	1	0	0	0	0	0	0	0	0	0	70
14205633	m	md	JP	2017	0	1	0	0	1	0	0	0	0	0	0	0	67
14205815	m	cn	JP	2017	0	1	1	0	0	0	0	0	0	0	0	0	69
14206074	m	md	DE	2017	0	0	0	0	0	0	0	0	0	0	0	0	75
14206213	m	md	DE	2017	0	0	0	0	0	0	1	0	0	0	1	1	46
14206392	f	cn	US	2017	0	1	1	0	0	0	0	0	0	0	0	0	
14206511	Not	ot	US	2017	1	0	0	0	0	0	0	0	0	0	0	1	
14206720	f	cn	GB	2017	0	1	1	0	0	0	0	0	0	0	0	0	54
14208032	m	md	JP	2017	0	0	0	0	0	0	0	0	0	0	0	0	72
14208087	f	cn	SE	2017	0	1	1	0	0	0	0	0	0	0	0	0	74
14208092	f	md	DE	2017	0	1	0	0	1	0	0	0	0	0	0	0	66
14209907	f	ot	US	2017	0	1	0	0	1	0	0	0	0	0	0	0	52
14210196	m	md	ES	2017	0	0	0	0	0	0	0	0	0	0	0	0	70
14210593	m	cn	JP	2017	0	0	0	0	0	0	0	0	0	0	0	0	68
14210674	m	ph	JP	2017	0	1	0	0	1	0	0	0	0	0	0	0	68
14210731	f	cn	JP	2017	0	1	1	0	0	0	0	0	0	0	0	0	66
14211213	f	ot	JP	2017	0	1	0	0	1	0	0	0	0	0	0	0	74
14211276	f	md	US	2017	0	0	0	0	0	0	0	0	0	0	0	0	
14212366	f	md	DE	2017	0	0	0	0	0	0	0	0	0	0	0	0	55
14212420	m	ph	US	2017	0	1	1	0	0	0	0	0	0	0	0	0	79
14212579	f	md	BE	2017	0	0	0	0	0	0	0	0	0	0	0	0	
14212838	f	cn	JP	2017	0	1	1	0	0	0	0	0	0	0	0	0	75
14213694	m	cn	JP	2017	0	1	1	0	0	0	0	0	0	0	0	0	68
14213996	m	cn	JP	2017	0	1	1	0	0	0	0	0	0	0	0	0	81
14214152	m	ot	BE	2017	0	0	0	0	0	0	0	0	0	0	0	0	63
14215544	Not	md	US	2017	1	0	0	0	0	0	0	0	0	0	0	0	
14215549	m	ot	BR	2017	1	0	0	0	0	0	0	0	0	0	0	0	
14215551	f	md	JP	2017	1	0	0	0	0	0	0	0	0	0	0	0	75
14215594	f	cn	JP	2017	1	0	0	0	0	0	0	0	0	0	0	0	
14216402	m	md	JP	2017	1	0	0	0	0	0	0	0	0	0	0	0	79

14216410	f	md	JP	2017	1	0	0	0	0	0	0	0	0	0	0	0	74
14216577	m	ph	JP	2017	0	1	0	0	1	0	0	0	0	0	0	0	78
14216701	f	cn	US	2017	0	1	1	0	0	0	0	0	0	0	0	0	
14216885	m	md	KR	2017	1	0	0	0	0	0	0	0	0	0	0	0	74.4
14216892	m	ot	US	2017	0	0	0	0	0	0	0	0	0	0	0	0	
14217055	m	md	JP	2017	1	0	0	0	0	0	0	0	0	0	0	0	71
14217240	f	md	JP	2017	0	1	0	0	1	0	0	0	0	0	0	0	63
14217257	f	ot	SG	2017	0	1	0	0	1	0	0	0	0	0	0	0	79
14217379	m	ot	JP	2017	0	1	0	0	1	0	0	0	0	0	0	0	80
14217875	f	md	AT	2017	1	0	0	0	0	0	0	0	0	0	0	0	62
14218416	f	md	JP	2017	1	0	0	0	0	0	0	0	0	0	0	0	80
14219606	m	md	IT	2017	1	0	0	0	0	0	0	0	0	0	0	0	67.7
14219789	f	md	KR	2017	1	0	0	0	0	0	0	0	0	0	0	0	28.7
14220671	f	cn	US	2017	0	1	1	0	0	0	0	0	0	0	0	0	
14220803	f	md	JP	2017	1	0	0	0	0	0	0	0	0	0	0	0	75
14220861	f	md	US	2017	1	0	0	0	0	0	0	0	0	0	0	0	
14221061	m	cn	JP	2017	0	1	1	0	0	0	0	0	0	0	0	0	82
14221418	m	cn	JP	2017	0	1	0	0	1	0	0	0	0	0	0	1	71
14221461	m	md	JP	2017	0	0	0	0	0	0	0	0	0	0	0	0	68
14221846	m	md	SG	2017	1	0	0	0	0	0	0	0	0	0	0	0	48
14221946	m	ot	US	2017	0	1	0	0	0	1	0	0	0	0	0	0	
14223336	f	md	PT	2017	0	0	0	0	0	0	1	0	1	0	0	0	71
14223636	f	md	US	2017	0	0	0	0	0	0	0	0	0	0	0	0	
14223639	f	md	JP	2017	0	0	0	0	0	0	0	0	0	0	0	0	67
14223669	f	md	DE	2017	1	0	0	0	0	0	0	0	0	0	0	0	48
14223770	m	md	FR	2017	0	1	0	0	1	0	0	0	0	0	0	0	
14223929	m	cn	FR	2017	0	1	1	0	0	0	0	0	0	0	0	1	65
14224250	Not	ot	US	2017	0	0	0	0	0	0	0	0	0	0	0	0	
14224251	Not	ot	US	2017	0	0	0	0	0	0	0	0	0	0	0	0	
14224252	Not	ot	US	2017	0	0	0	0	0	0	0	0	0	0	0	0	
14224253	Not	ot	US	2017	0	0	0	0	0	0	0	0	0	0	0	0	
14224254	Not	ot	US	2017	0	0	0	0	0	0	0	0	0	0	0	0	
14224255	Not	ot	US	2017	0	0	0	0	0	0	0	0	0	0	0	0	
14224256	Not	ot	US	2017	0	0	0	0	0	0	0	0	0	0	0	0	

14224257	Not	ot	US	2017	0	0	0	0	0	0	0	0	0	0	0	0	
14224258	Not	ot	US	2017	0	0	0	0	0	0	0	0	0	0	0	0	
14224259	Not	ot	US	2017	0	0	0	0	0	0	0	0	0	0	0	0	
14224260	Not	ot	US	2017	0	0	0	0	0	0	0	0	0	0	0	0	
14224261	Not	ot	US	2017	0	0	0	0	0	0	0	0	0	0	0	0	
14224262	Not	ot	US	2017	0	0	0	0	0	0	0	0	0	0	0	0	
14224263	Not	ot	US	2017	0	0	0	0	0	0	0	0	0	0	0	0	
14224264	Not	ot	US	2017	0	0	0	0	0	0	0	0	0	0	0	0	
14224265	Not	ot	Not re	2017	0	0	0	0	0	0	0	0	0	0	0	0	
14224266	Not	ot	US	2017	0	0	0	0	0	0	0	0	0	0	0	0	
14224319	m	ot	GB	2017	0	1	0	0	1	0	0	0	0	0	0	0	72
14224528	f	md	US	2017	1	0	0	0	0	0	0	0	0	0	0	0	
14225526	m	md	AT	2017	0	0	0	0	0	0	0	0	0	0	0	0	62
14225620	f	cn	US	2017	0	1	0	0	1	0	0	0	0	0	0	0	
14226173	m	ph	US	2017	1	0	0	0	0	0	0	0	0	0	0	0	
14226388	Not	ot	US	2017	0	0	0	0	0	0	0	0	0	0	0	0	
14226389	Not	ot	US	2017	0	0	0	0	0	0	0	0	0	0	0	0	
14226390	Not	md	US	2017	0	0	0	0	0	0	0	0	0	0	0	0	
14226455	m	md	JP	2017	0	1	0	0	1	0	0	0	0	0	0	0	73
14226679	f	ot	US	2017	0	0	0	0	0	0	0	0	0	0	0	0	
14227384	f	md	US	2017	1	0	0	0	0	0	0	0	0	0	0	0	66
14227398	f	md	US	2017	1	0	0	0	0	0	0	0	0	0	0	0	70
14227399	f	md	US	2017	1	0	0	0	0	0	0	0	0	0	0	0	64
14227400	f	md	US	2017	1	0	0	0	0	0	0	0	0	0	0	0	
14227401	f	ot	JP	2017	1	0	0	0	0	0	0	0	0	0	0	0	59
14227485	m	cn	US	2017	0	1	1	0	0	0	0	0	0	0	0	0	50
14228209	m	ot	US	2017	0	1	0	0	1	0	0	0	0	0	0	0	67
14228706	m	md	PT	2017	0	0	0	0	0	0	0	0	0	0	0	0	51
14228767	m	cn	GB	2017	0	1	1	0	0	0	0	0	0	0	0	0	61
14228785	m	cn	CN	2017	0	0	0	0	0	0	0	0	0	0	0	0	76
14228838	f	md	NL	2017	1	0	0	0	0	0	0	0	0	0	0	0	
14228966	Not	md	CO	2017	0	0	0	0	0	0	0	0	0	0	0	0	
14229102	m	cn	US	2017	0	0	0	0	0	0	0	0	0	0	0	0	
14229110	f	md	DE	2017	1	0	0	0	0	0	0	0	0	0	0	0	66

14229648	m	cn	GB	2017	1	0	0	0	0	0	0	0	0	0	0	0	71
14229736	m	ot	US	2017	0	0	0	0	0	0	0	0	0	0	0	0	70
14229814	m	cn	JP	2017	0	1	1	0	0	0	0	0	0	0	0	0	65
14229989	f	cn	GB	2017	1	0	0	0	0	0	0	0	0	0	0	0	
14230151	f	md	JP	2017	0	0	0	0	0	0	0	0	0	0	0	0	80
14230317	f	md	JP	2017	1	0	0	0	0	0	0	0	0	0	0	0	83
14231611	m	cn	GB	2017	1	0	0	0	0	0	0	0	0	0	0	0	48
14231941	m	md	AU	2017	1	0	0	0	0	0	0	0	0	0	0	0	58
14232322	f	cn	GB	2017	1	0	0	0	0	0	0	0	0	0	0	0	
14234123	m	cn	NL	2017	0	1	1	0	0	0	0	0	0	0	0	0	72
14234602	Not	ot	US	2017	0	0	0	0	0	0	0	0	0	0	0	0	
14234608	Not	ot	Not re	2017	0	0	0	0	0	0	0	0	0	0	0	0	
14234814	m	md	KR	2017	0	0	0	0	0	0	0	0	0	0	0	0	72
14234991	Not	ot	US	2017	0	0	0	0	0	0	0	0	0	0	0	0	
14234992	Not	ot	US	2017	0	0	0	0	0	0	0	0	0	0	0	0	
14235006	Not	ot	US	2017	0	0	0	0	0	0	0	0	0	0	0	0	
14235024	f	md	CN	2017	1	0	0	0	0	0	0	0	0	0	0	0	44
14235125	f	md	GB	2017	1	0	0	0	0	0	0	0	0	0	0	0	53
14235153	f	md	CN	2017	1	0	0	0	0	0	0	0	0	0	0	0	61.9
14235644	m	md	US	2017	1	0	0	0	0	0	0	0	0	0	0	0	52
14235779	m	cn	JP	2017	0	1	1	0	0	0	0	0	0	0	0	0	60
14236074	Not	ot	US	2017	0	1	0	0	1	0	0	0	0	0	0	0	
14236340	f	cn	US	2017	0	0	0	0	0	0	0	0	0	0	0	0	
14236348	m	md	IT	2017	0	0	0	0	0	0	0	0	0	0	0	0	68
14236813	f	cn	GB	2017	1	0	0	0	0	0	0	0	0	0	0	1	
14236988	m	cn	GR	2017	0	1	1	0	0	0	0	0	0	0	0	1	71
14237711	f	cn	CA	2017	0	1	0	0	1	0	0	0	0	0	0	0	68
14237817	f	ot	US	2017	0	0	0	0	0	0	1	0	1	0	0	0	79
14237917	f	cn	DE	2017	0	1	1	0	0	0	0	0	0	0	0	0	
14238015	m	md	FR	2017	0	1	0	0	1	0	0	0	0	0	0	0	
14238897	m	cn	DE	2017	0	1	1	0	0	0	0	0	0	0	0	1	74
14239090	f	md	JP	2017	0	0	0	0	0	0	0	0	0	0	0	0	80
14239851	f	md	DE	2017	0	1	0	1	0	0	0	0	0	0	0	0	53
14239886	f	md	JP	2017	1	0	0	0	0	0	0	0	0	0	0	0	86

14240000	m	ot	US	2017	0	1	0	0	1	0	0	0	0	0	0	0	58
14240005	f	md	JP	2017	1	0	0	0	0	0	0	0	0	0	0	0	75
14240061	f	md	JP	2017	1	0	0	0	0	0	0	0	0	0	0	0	77
14240242	Not	md	US	2017	0	0	0	0	0	0	0	0	0	0	0	0	
14240424	m	ot	PL	2017	0	1	0	0	1	0	0	0	0	0	0	0	63
14240698	m	cn	JP	2017	0	0	0	0	0	0	0	0	0	0	0	0	75
14241114	m	md	AT	2017	0	1	0	0	1	0	0	0	0	0	0	0	
14241166	m	ot	DE	2017	0	1	0	0	1	0	0	0	0	0	0	0	61
14241296	m	md	FR	2017	0	1	0	0	1	0	0	0	0	0	0	0	
14241594	m	cn	CN	2017	1	0	0	0	0	0	0	0	0	0	0	0	80
14241610	m	md	US	2017	0	1	0	0	0	1	0	0	0	0	0	0	73
14241627	f	md	TW	2017	0	0	0	0	0	0	0	0	0	0	0	0	
14241697	m	md	US	2017	0	1	0	0	0	1	0	0	0	0	0	0	61
14242007	m	cn	PL	2017	1	0	0	0	0	0	0	0	0	0	0	0	63
14242297	m	md	US	2017	0	0	0	0	0	0	0	0	0	0	0	0	
14242466	f	ot	US	2017	0	0	0	0	0	0	0	0	0	0	0	0	
14242571	m	ot	JP	2017	0	1	0	0	1	0	0	0	0	0	0	0	75
14242681	f	ot	US	2017	0	0	0	0	0	0	0	0	0	0	0	0	74
14243150	f	ot	DE	2017	0	0	0	0	0	0	0	0	0	0	0	0	68
14243242	f	md	US	2017	0	0	0	0	0	0	0	0	0	0	0	0	
14243352	m	ot	DE	2017	0	0	0	0	0	0	0	0	0	0	0	0	75
14243425	f	md	ES	2017	0	1	0	0	0	1	0	0	0	0	0	0	62
14243887	m	cn	JP	2017	0	1	1	0	0	0	0	0	0	0	0	0	83
14244155	m	md	US	2017	1	0	0	0	0	0	0	0	0	0	0	0	65
14244327	f	md	IT	2017	0	0	0	0	0	0	0	0	0	0	0	0	52
14245132	f	md	IN	2017	0	1	0	0	1	0	0	0	0	0	0	0	53
14245133	f	md	NL	2017	0	0	0	0	0	0	0	0	0	0	0	0	52
14245270	f	cn	US	2017	0	1	1	0	0	0	0	0	0	0	0	0	69
14245780	f	md	IN	2017	0	1	0	0	1	0	0	0	0	0	0	0	54
14246768	f	cn	US	2017	0	0	0	0	0	0	0	0	0	0	0	0	
14246823	m	cn	US	2017	0	0	0	0	0	0	0	0	0	0	0	0	
14247105	m	cn	US	2017	0	0	0	0	0	0	0	0	0	0	0	0	
14247889	f	md	CN	2017	1	0	0	0	0	0	0	0	0	0	0	0	68
14248521	m	cn	JP	2017	0	1	1	0	0	0	0	0	0	0	0	0	63

14248732	f	cn	US	2017	0	1	1	0	0	0	0	0	0	0	0	0	
14248802	Not	md	JP	2017	0	0	0	0	0	0	0	0	0	0	0	0	
14250297	m	md	JP	2017	1	0	0	0	0	0	0	0	0	0	0	0	69
14250576	Not	ot	AU	2017	0	1	0	0	1	0	0	0	0	0	0	0	
14250790	f	cn	US	2017	0	0	0	0	0	0	0	0	0	0	0	0	
14250867	m	cn	US	2017	0	0	0	0	0	0	0	0	0	0	0	0	
14250884	Not	ot	FR	2017	0	0	0	0	0	0	0	0	0	0	0	0	
14251718	f	ot	US	2017	0	1	1	0	0	0	0	0	0	0	0	0	53
14252650	f	md	US	2017	1	0	0	0	0	0	0	0	0	0	0	0	71
14253050	Not	md	KR	2017	1	0	0	0	0	0	0	0	0	0	0	1	
14253054	m	md	DE	2017	1	0	0	0	0	0	0	0	0	0	0	0	66
14253160	f	md	US	2017	1	0	0	0	0	0	0	0	0	0	0	0	
14253184	f	ot	BE	2017	0	0	0	0	0	0	0	0	0	0	0	0	32
14253237	m	cn	JP	2017	0	1	1	0	0	0	0	0	0	0	0	0	60
14253424	m	ot	US	2017	0	0	0	0	0	0	0	0	0	0	0	0	57
14253624	m	ot	US	2017	0	0	0	0	0	0	0	0	0	0	0	0	72
14253646	Not	ot	MA	2017	0	0	0	0	0	0	0	0	0	0	0	0	
14254520	m	cn	US	2017	0	0	0	0	0	0	0	0	0	0	0	0	
14254635	f	cn	US	2017	0	0	0	0	0	0	0	0	0	0	0	0	
14254755	f	ot	US	2017	0	0	0	0	0	0	0	0	0	0	0	0	
14254842	m	md	IT	2017	0	1	0	0	1	0	0	0	0	0	0	0	
14254950	m	ot	BE	2017	0	0	0	0	0	0	0	0	0	0	0	0	75
14254971	m	md	US	2017	0	0	0	0	0	0	0	0	0	0	0	0	65
14255028	m	Not repo	JP	2017	0	0	0	0	0	0	0	0	0	0	0	0	62
14255265	m	cn	JP	2017	0	1	1	0	0	0	0	0	0	0	0	0	65
14255302	m	md	JP	2017	0	1	1	0	0	0	0	0	0	0	0	0	
14255510	m	md	US	2017	0	1	1	0	0	0	0	0	0	0	0	0	65
14255542	m	md	JP	2017	1	0	0	0	0	0	0	0	0	0	0	0	72
14255785	m	cn	CN	2017	1	0	0	0	0	0	0	0	0	0	0	0	80
14256068	m	md	JP	2017	1	0	0	0	0	0	0	0	0	0	0	0	64
14256681	m	ot	JP	2017	0	1	0	0	1	0	0	0	0	0	0	0	67
14256819	f	cn	JP	2017	0	1	1	0	0	0	0	0	0	0	0	0	69
14256939	m	ot	US	2017	0	1	0	0	1	0	0	0	0	0	0	0	49
14256959	f	ph	US	2017	0	1	1	0	0	0	0	0	0	0	0	0	67

14256961	m	cn	DE	2017	0	1	0	0	1	0	0	0	0	0	0	0	69
14256965	m	md	DE	2017	0	0	0	0	0	0	1	0	1	0	0	0	67
14257364	m	md	KR	2017	1	0	0	0	0	0	0	0	0	0	0	0	54.3
14257390	Not	md	CH	2017	0	0	0	0	0	0	0	0	0	0	0	0	
14258191	m	cn	CN	2017	0	0	0	0	0	0	0	0	0	0	0	0	65
14258501	f	cn	US	2017	0	0	0	0	0	0	0	0	0	0	0	0	
14258712	m	cn	US	2017	0	1	0	0	1	0	0	0	0	0	0	1	73
14258736	f	cn	US	2017	0	0	0	0	0	0	0	0	0	0	0	0	
14260263	f	md	JP	2017	1	0	0	0	0	0	0	0	0	0	0	0	87
14260387	f	ot	IT	2017	0	0	0	0	0	0	0	0	0	0	0	0	50
14260570	m	md	AT	2017	1	0	0	0	0	0	0	0	0	0	0	0	47
14260806	m	cn	US	2017	0	1	1	0	0	0	0	0	0	0	0	1	
14260905	m	ot	US	2017	0	1	0	0	1	0	0	0	0	0	0	0	83
14261006	m	ot	US	2017	0	1	0	0	1	0	0	0	0	0	0	1	70
14261012	f	md	KR	2017	1	0	0	0	0	0	0	0	0	0	0	0	86
14261180	m	cn	US	2017	0	0	0	0	0	0	0	0	0	0	0	0	
14261488	f	md	US	2017	0	1	0	0	0	1	0	0	0	0	0	0	
14261489	f	md	UA	2017	0	1	0	0	0	1	0	0	0	0	0	0	40
14261610	Not	ot	JP	2017	0	0	0	0	0	0	0	0	0	0	0	0	
14261943	f	md	CN	2017	1	0	0	0	0	0	0	0	0	0	0	0	62
14262248	Not	ot	JP	2017	0	0	0	0	0	0	0	0	0	0	0	0	
14262254	Not	ot	JP	2017	0	0	0	0	0	0	0	0	0	0	0	0	
14262548	m	cn	SE	2017	0	1	1	0	0	0	0	0	0	0	0	0	
14262591	f	cn	US	2017	0	0	0	0	0	0	0	0	0	0	0	0	
14262772	m	ot	NL	2017	0	1	0	0	1	0	0	0	0	0	0	0	60
14262805	Not	ot	JP	2017	0	0	0	0	0	0	0	0	0	0	0	0	
14262809	Not	ot	JP	2017	0	0	0	0	0	0	0	0	0	0	0	0	
14262812	Not	ot	JP	2017	0	0	0	0	0	0	0	0	0	0	0	0	
14262895	m	ot	BE	2017	0	0	0	0	0	0	0	0	0	0	0	0	62
14263094	m	md	PT	2017	0	1	1	0	0	0	0	0	0	0	0	0	
14263175	m	md	DE	2017	0	0	0	0	0	0	0	0	0	0	0	0	56
14263416	m	ot	DE	2017	0	0	0	0	0	0	0	0	0	0	0	0	60
14263681	f	cn	US	2017	0	1	1	0	0	0	0	0	0	0	0	0	
14263819	Not	ot	JP	2017	0	0	0	0	0	0	0	0	0	0	0	0	

14263820	Not	ot	JP	2017	0	0	0	0	0	0	0	0	0	0	0	0	
14263864	f	md	CH	2017	0	0	0	0	0	0	0	0	0	0	0	0	69
14265405	Not	md	US	2017	0	0	0	0	0	0	0	0	0	0	0	0	
14265424	Not	ot	PK	2017	0	0	0	0	0	0	0	0	0	0	0	0	
14265432	Not	ot	PK	2017	0	0	0	0	0	0	0	0	0	0	0	0	
14265437	Not	ot	PK	2017	0	0	0	0	0	0	0	0	0	0	0	0	
14265452	Not	md	US	2017	0	0	0	0	0	0	0	0	0	0	0	0	
14265914	Not	ot	JP	2017	0	0	0	0	0	0	0	0	0	0	0	0	
14265915	Not	ot	JP	2017	0	0	0	0	0	0	0	0	0	0	0	0	
14265916	Not	ot	JP	2017	0	0	0	0	0	0	0	0	0	0	0	0	
14265917	Not	ot	JP	2017	0	0	0	0	0	0	0	0	0	0	0	0	
14265918	Not	ot	JP	2017	0	0	0	0	0	0	0	0	0	0	0	0	
14265919	Not	ot	JP	2017	0	0	0	0	0	0	0	0	0	0	0	0	
14265920	Not	ot	JP	2017	0	0	0	0	0	0	0	0	0	0	0	0	
14265921	Not	ot	JP	2017	0	0	0	0	0	0	0	0	0	0	0	0	
14265922	Not	ot	JP	2017	0	0	0	0	0	0	0	0	0	0	0	0	
14265924	Not	ot	JP	2017	0	0	0	0	0	0	0	0	0	0	0	0	
14265927	Not	ot	JP	2017	0	0	0	0	0	0	0	0	0	0	0	0	
14265928	Not	ot	JP	2017	0	0	0	0	0	0	0	0	0	0	0	0	
14265930	Not	ot	JP	2017	0	0	0	0	0	0	0	0	0	0	0	0	
14265931	Not	ot	JP	2017	0	0	0	0	0	0	0	0	0	0	0	0	
14265939	Not	ot	JP	2017	0	0	0	0	0	0	0	0	0	0	0	0	
14265941	Not	ot	JP	2017	0	0	0	0	0	0	0	0	0	0	0	0	
14265950	m	md	JP	2017	0	1	0	0	1	0	0	0	0	0	0	1	76
14265983	Not	ot	JP	2017	0	0	0	0	0	0	0	0	0	0	0	0	
14266002	f	md	DE	2017	0	1	0	0	1	0	0	0	0	0	0	0	64
14266021	Not	ot	JP	2017	0	0	0	0	0	0	0	0	0	0	0	0	
14266186	m	md	JP	2017	1	0	0	0	0	0	0	0	0	0	0	0	78
14266658	m	md	NL	2017	0	1	0	0	1	0	0	0	0	0	0	0	68
14266797	m	cn	US	2017	0	1	0	0	1	0	0	0	0	0	0	0	57
14266946	m	md	JP	2017	0	1	0	0	1	0	0	0	0	0	0	0	53
14267127	m	cn	US	2017	0	0	0	0	0	0	0	0	0	0	0	0	
14267291	f	cn	US	2017	0	0	0	0	0	0	0	0	0	0	0	0	
14267868	m	md	TR	2017	0	0	0	0	0	0	0	0	0	0	0	0	64

14268065	f	ot	US	2017	0	0	0	0	0	0	0	0	0	0	0	0	
14268217	m	cn	FR	2017	0	1	1	0	0	0	0	0	0	0	0	0	71
14268504	f	md	US	2017	1	0	0	0	0	0	0	0	0	0	0	0	
14269064	f	md	US	2017	0	1	0	0	0	1	0	0	0	0	0	0	68
14269227	f	cn	BE	2017	0	0	0	0	0	0	0	0	0	0	0	0	
14270114	Not	md	US	2017	0	0	0	0	0	0	0	0	0	0	0	0	
14270189	Not	md	US	2017	0	0	0	0	0	0	0	0	0	0	0	0	
14270415	Not	md	US	2017	0	0	0	0	0	0	0	0	0	0	0	0	
14271469	f	ot	FR	2017	0	1	0	0	1	0	0	0	0	0	0	0	59
14271563	f	md	CZ	2017	1	0	0	0	0	0	0	0	0	0	0	0	80
14271618	Not	md	US	2017	0	0	0	0	0	0	0	0	0	0	0	0	
14272373	Not	md	US	2017	0	0	0	0	0	0	0	0	0	0	0	0	
14272775	m	cn	US	2017	1	0	0	0	0	0	0	0	0	0	0	0	57
14273448	Not	ot	JP	2017	0	0	0	0	0	0	0	0	0	0	0	0	
14273811	m	cn	US	2017	1	0	0	0	0	0	0	0	0	0	0	0	54
14274822	m	cn	FR	2017	0	1	0	0	1	0	0	0	0	0	0	0	
14274824	m	ot	FR	2017	0	1	0	0	1	0	0	0	0	0	0	0	
14274939	Not	ot	US	2017	0	0	0	0	0	0	0	0	0	0	0	0	
14276004	f	cn	GB	2017	1	0	0	0	0	0	0	0	0	0	0	0	46
14276205	f	md	US	2017	0	0	0	0	0	0	0	0	0	0	0	0	38
14276313	Not	ot	ES	2017	0	0	0	0	0	0	0	0	0	0	0	0	
14276349	Not	ot	ES	2017	0	0	0	0	0	0	0	0	0	0	0	0	
14276352	Not	ot	ES	2017	0	0	0	0	0	0	0	0	0	0	0	0	
14276554	f	cn	US	2017	0	1	0	0	1	0	0	0	0	0	0	0	
14276720	f	md	IT	2017	0	0	0	0	0	0	0	0	0	0	0	0	52
14277045	Not	ot	PK	2017	0	0	0	0	0	0	0	0	0	0	0	0	
14278612	m	md	DE	2017	0	0	0	0	0	0	0	0	0	0	0	0	68
14278704	f	md	DE	2017	1	0	0	0	0	0	0	0	0	0	0	1	
14278931	m	ot	JP	2017	0	1	0	0	1	0	0	0	0	0	0	0	68
14279095	Not	md	JP	2017	0	1	0	0	1	0	0	0	0	0	0	0	
14279120	Not	md	JP	2017	0	1	0	0	1	0	0	0	0	0	0	0	
14279170	m	md	CA	2017	1	0	0	0	0	0	0	0	0	0	0	0	
14279773	m	md	LU	2017	0	1	0	0	1	0	0	0	0	0	0	0	80
14280007	m	md	JP	2017	0	1	0	0	1	0	0	0	0	0	0	0	90

14280009	m	md	JP	2017	0	1	0	0	1	0	0	0	0	0	0	0	56
14280381	m	md	AU	2017	0	1	0	0	1	0	0	0	0	0	0	0	
14280472	m	md	FR	2017	0	1	0	0	0	1	0	0	0	0	0	0	68
14280672	f	ot	PL	2017	0	1	0	0	1	0	0	0	0	0	0	0	68
14280712	Not	md	US	2017	0	1	0	0	1	0	0	0	0	0	0	0	
14280713	f	md	JP	2017	0	1	0	0	1	0	0	0	0	0	0	0	61
14280853	f	ot	US	2017	0	1	0	0	1	0	0	0	0	0	0	0	60
14280854	m	ot	US	2017	0	1	0	0	1	0	0	0	0	0	0	0	50
14281069	m	cn	JP	2017	0	1	1	0	0	0	0	0	0	0	0	0	70
14281096	f	cn	JP	2017	0	1	1	0	0	0	0	0	0	0	0	0	70
14281173	m	md	PL	2017	1	0	0	0	0	0	0	0	0	0	0	0	68
14281275	m	cn	GB	2017	1	0	0	0	0	0	0	0	0	0	0	0	
14282008	f	cn	JP	2017	0	1	1	0	0	0	0	0	0	0	0	0	63
14282093	m	md	JP	2017	0	0	0	0	0	0	0	0	0	0	0	0	64
14282127	f	md	ES	2017	1	0	0	0	0	0	0	0	0	0	0	0	78
14282285	m	Not repo	US	2017	1	0	0	0	0	0	0	0	0	0	0	0	57.9
14282395	f	cn	JP	2017	1	0	0	0	0	0	0	0	0	0	0	0	
14282531	f	ph	CA	2017	1	0	0	0	0	0	0	0	0	0	0	0	
14282804	m	cn	JP	2017	0	0	0	0	0	0	0	0	0	0	0	0	
14282973	m	ot	DE	2017	0	0	0	0	0	0	0	0	0	0	0	0	75
14283033	f	md	DE	2017	1	0	0	0	0	0	0	0	0	0	0	0	66
14283357	f	ot	US	2017	0	1	0	0	1	0	0	0	0	0	0	0	74
14283647	m	cn	FR	2017	0	1	1	0	0	0	0	0	0	0	0	0	60
14283936	f	cn	GB	2017	1	0	0	0	0	0	0	0	0	0	0	0	63
14284106	m	md	JP	2017	0	1	0	0	1	0	0	0	0	0	0	1	77
14284343	f	cn	GB	2017	1	0	0	0	0	0	0	0	0	0	0	0	58
14284625	m	ot	FR	2017	0	1	0	0	1	0	0	0	0	0	0	0	
14284902	f	cn	GB	2017	1	0	0	0	0	0	0	0	0	0	0	0	83
14285037	m	ph	US	2017	1	0	0	0	0	0	0	0	0	0	0	0	58
14285042	f	cn	US	2017	1	0	0	0	0	0	0	0	0	0	0	0	52
14285045	f	cn	US	2017	1	0	0	0	0	0	0	0	0	0	0	0	52
14285048	f	cn	US	2017	1	0	0	0	0	0	0	0	0	0	0	0	
14285066	f	cn	GB	2017	1	0	0	0	0	0	0	0	0	0	0	0	
14285072	Not	cn	GB	2017	1	0	0	0	0	0	0	0	0	0	0	0	

14285094	m	md	JP	2017	0	1	0	0	1	0	0	0	0	0	0	0	50
14285109	Not	cn	GB	2017	1	0	0	0	0	0	0	0	0	0	0	1	
14285178	m	ot	JP	2017	0	1	0	0	1	0	0	0	0	0	0	0	73
14285237	f	cn	GB	2017	1	0	0	0	0	0	0	0	0	0	0	0	
14285270	f	md	DE	2017	0	0	0	0	0	0	0	0	0	0	0	0	54
14285293	f	cn	GB	2017	1	0	0	0	0	0	0	0	0	0	0	0	53
14285583	m	md	JP	2017	0	1	0	0	1	0	0	0	0	0	0	0	59
14285599	f	cn	GB	2017	1	0	0	0	0	0	0	0	0	0	0	0	
14285625	f	cn	GB	2017	1	0	0	0	0	0	0	0	0	0	0	0	57
14285636	m	ot	JP	2017	0	0	0	0	0	0	0	0	0	0	0	0	67
14285692	f	ph	US	2017	1	0	0	0	0	0	0	0	0	0	0	0	89
14285763	f	cn	GB	2017	1	0	0	0	0	0	0	0	0	0	0	0	
14285831	m	cn	GB	2017	1	0	0	0	0	0	0	0	0	0	0	0	58
14285858	Not	md	TW	2017	0	0	0	0	0	0	0	0	0	0	0	0	
14285897	m	cn	GB	2017	1	0	0	0	0	0	0	0	0	0	0	0	
14285976	f	cn	GB	2017	1	0	0	0	0	0	0	0	0	0	0	0	81
14285979	f	cn	GB	2017	1	0	0	0	0	0	0	0	0	0	0	0	90
14285980	m	cn	GB	2017	1	0	0	0	0	0	0	0	0	0	0	0	
14286013	f	cn	US	2017	0	0	0	0	0	0	0	0	0	0	0	0	66
14286224	Not	cn	US	2017	1	0	0	0	0	0	0	0	0	0	0	0	
14286492	Not	cn	GB	2017	1	0	0	0	0	0	0	0	0	0	0	0	
14286577	m	cn	GB	2017	1	0	0	0	0	0	0	0	0	0	0	0	
14286610	m	cn	GB	2017	1	0	0	0	0	0	0	0	0	0	0	0	86
14286636	m	cn	GB	2017	1	0	0	0	0	0	0	0	0	0	0	0	
14286665	f	cn	GB	2017	1	0	0	0	0	0	0	0	0	0	0	0	71
14286680	f	cn	GB	2017	1	0	0	0	0	0	0	0	0	0	0	0	
14286682	m	cn	GB	2017	1	0	0	0	0	0	0	0	0	0	0	0	
14286685	m	cn	GB	2017	1	0	0	0	0	0	0	0	0	0	0	0	
14286769	f	cn	GB	2017	1	0	0	0	0	0	0	0	0	0	0	0	
14286779	f	cn	GB	2017	1	0	0	0	0	0	0	0	0	0	0	0	
14286794	f	cn	GB	2017	1	0	0	0	0	0	0	0	0	0	0	0	
14286877	m	cn	GB	2017	1	0	0	0	0	0	0	0	0	0	0	0	
14286938	m	cn	GB	2017	1	0	0	0	0	0	0	0	0	0	0	0	
14286962	Not	cn	GB	2017	1	0	0	0	0	0	0	0	0	0	0	0	

14286969	f	ph	US	2017	1	0	0	0	0	0	0	0	0	0	0	0	77
14287053	f	cn	GB	2017	1	0	0	0	0	0	0	0	0	0	0	0	
14287065	f	cn	GB	2017	1	0	0	0	0	0	0	0	0	0	0	0	
14287125	f	cn	GB	2017	1	0	0	0	0	0	0	0	0	0	0	0	73
14287147	f	cn	GB	2017	1	0	0	0	0	0	0	0	0	0	0	0	59
14287352	f	cn	GB	2017	1	0	0	0	0	0	0	0	0	0	0	0	
14287513	f	cn	US	2017	1	0	0	0	0	0	0	0	0	0	0	0	77
14287586	f	cn	GB	2017	1	0	0	0	0	0	0	0	0	0	0	0	61
14288462	f	cn	US	2017	0	1	1	0	0	0	0	0	0	0	0	0	
14289484	f	md	ID	2017	1	0	0	0	0	0	0	0	0	0	0	0	59
14289616	Not	ot	US	2017	0	0	0	0	0	0	0	0	0	0	0	0	
14289730	m	ph	JP	2017	0	1	0	0	1	0	0	0	0	0	0	0	72
14289850	m	cn	DE	2017	0	0	0	0	0	0	0	0	0	0	0	0	60
14289883	m	md	JP	2017	0	1	0	0	1	0	0	0	0	0	0	0	69
14290240	f	ot	IN	2017	0	0	0	0	0	0	0	0	0	0	0	0	41
14290294	f	cn	GB	2017	1	0	0	0	0	0	0	0	0	0	0	0	59
14290410	m	Not repo	CA	2017	1	0	0	0	0	0	0	0	0	0	0	0	
14290995	m	md	DE	2017	0	0	0	0	0	0	0	0	0	0	0	0	64
14291362	m	cn	JP	2017	0	1	1	0	0	0	0	0	0	0	0	0	87
14291495	m	md	IL	2017	0	1	0	0	0	1	0	0	0	0	0	0	79
14291682	m	cn	JP	2017	0	1	1	0	0	0	0	0	0	0	0	0	67
14291780	m	cn	FR	2017	0	0	0	0	0	0	0	0	0	0	0	0	65
14292085	f	ot	DE	2017	0	0	0	0	0	0	0	0	0	0	0	0	68
14292732	m	md	TW	2017	1	0	0	0	0	0	0	0	0	0	0	0	74
14293616	f	cn	IN	2017	0	0	0	0	0	0	0	0	0	0	0	0	68
14293866	Not	ot	GB	2017	0	1	0	0	1	0	0	0	0	0	0	0	
14293927	f	ot	US	2017	0	1	1	0	0	0	0	0	0	0	0	0	45
14293989	m	ot	US	2017	0	1	0	0	1	0	0	0	0	0	0	0	
14294002	Not	md	US	2017	0	0	0	0	0	0	0	0	0	0	0	0	
14294272	m	md	US	2017	0	1	0	0	1	0	0	0	0	0	0	0	
14294338	m	md	KW	2017	0	1	0	0	1	0	0	0	0	0	0	0	
14294451	m	cn	US	2017	1	0	0	0	0	0	0	0	0	0	0	0	75
14294614	f	md	CA	2017	0	1	1	0	0	0	0	0	0	0	0	0	
14295145	Not	ot	JP	2017	0	0	0	0	0	0	0	0	0	0	0	0	

14295224	f	ph	GB	2017	0	0	0	0	0	0	0	0	0	0	0	0	51
14295975	m	cn	JP	2017	0	1	1	0	0	0	0	0	0	0	0	0	66
14296863	m	md	DE	2017	0	1	0	0	1	0	0	0	0	0	0	0	
14297077	m	ph	JP	2017	0	1	1	0	0	0	0	0	0	0	0	0	66
14298056	m	cn	FR	2017	0	1	1	0	0	0	0	0	0	0	0	0	87
14299356	Not	ot	GB	2017	0	0	0	0	0	0	0	0	0	0	0	0	
14299618	m	md	JP	2017	0	1	0	0	1	0	0	0	0	0	0	0	80
14299722	Not	ot	US	2017	0	0	0	0	0	0	0	0	0	0	0	0	
14299824	Not	ot	GB	2017	0	0	0	0	0	0	0	0	0	0	0	0	
14299835	m	ph	JP	2017	0	1	1	0	0	0	0	0	0	0	0	0	84
14299851	f	cn	US	2017	1	0	0	0	0	0	0	0	0	0	0	0	
14300292	m	ot	US	2017	0	0	0	0	0	0	0	0	0	0	0	0	
14300400	m	md	JP	2017	0	1	0	0	1	0	0	0	0	0	0	0	80
14300567	f	md	JP	2017	1	0	0	0	0	0	0	0	0	0	0	0	70
14301122	f	md	US	2017	1	0	0	0	0	0	0	0	0	0	0	0	
14301781	m	md	US	2017	0	1	0	0	0	1	0	0	0	0	0	0	61
14302137	m	md	JP	2017	0	1	0	0	1	0	0	0	0	0	0	0	80
14302949	f	md	JP	2017	1	0	0	0	0	0	0	0	0	0	0	0	73
14303022	m	md	JP	2017	0	1	0	0	1	0	0	0	0	0	0	1	49
14303085	m	md	FR	2017	0	0	0	0	0	0	0	0	0	0	0	0	65
14303097	m	ot	DE	2017	0	0	0	0	0	0	0	0	0	0	0	0	72
14306415	m	ot	IN	2017	0	0	0	0	0	0	0	0	0	0	0	0	
14306775	f	cn	US	2017	1	0	0	0	0	0	0	0	0	0	0	0	57
14306839	f	md	GR	2017	0	1	0	1	0	0	0	0	0	0	0	0	64
14307286	f	ot	IN	2017	0	0	0	0	0	0	0	0	0	0	0	0	60
14307893	m	cn	JP	2017	0	0	0	0	0	0	0	0	0	0	0	0	66
14307989	m	ot	US	2017	0	1	0	0	1	0	0	0	0	0	0	0	
14308345	m	cn	JP	2017	0	1	1	0	0	0	0	0	0	0	0	1	70
14308448	f	md	CZ	2017	1	0	0	0	0	0	0	0	0	0	0	0	75
14308891	m	ph	US	2017	0	0	0	0	0	0	0	0	0	0	0	0	54
14309864	m	md	DE	2017	0	0	0	0	0	0	0	0	0	0	0	0	76
14310431	f	cn	US	2017	1	0	0	0	0	0	0	0	0	0	0	0	72
14310621	m	cn	JP	2017	0	1	0	0	1	0	0	0	0	0	0	0	80
14310903	m	cn	IL	2017	0	1	1	0	0	0	0	0	0	0	0	0	53

14311954	m	cn	US	2017	0	1	0	0	1	0	0	0	0	0	0	0	
14311955	m	md	JP	2017	0	1	0	0	1	0	0	0	0	0	0	0	67
14311956	f	md	BR	2017	0	1	0	0	1	0	0	0	0	0	0	0	
14311969	m	cn	IN	2017	0	1	0	0	1	0	0	0	0	0	0	0	56
14312098	m	cn	US	2017	0	1	0	0	1	0	0	0	0	0	0	0	
14312314	m	cn	US	2017	0	1	0	0	1	0	0	0	0	0	0	0	
14312901	f	Not repo	MY	2017	1	0	0	0	0	0	0	0	0	0	0	0	79.7
14312948	m	ot	IT	2017	0	1	0	0	1	0	0	0	0	0	0	0	72
14312951	Not	ot	GB	2017	0	0	0	0	0	0	0	0	0	0	0	0	
14313183	f	md	DE	2017	1	0	0	0	0	0	0	0	0	0	0	0	66
14314344	Not	ot	GB	2017	0	0	0	0	0	0	0	0	0	0	0	0	
14315281	m	md	LB	2017	0	1	1	0	0	0	0	0	0	0	0	0	74
14316276	f	md	JP	2017	0	1	0	0	1	0	0	0	0	0	0	0	84
14316533	f	md	CN	2017	1	0	0	0	0	0	0	0	0	0	0	0	49.8
14316928	f	cn	US	2017	0	0	0	0	0	0	1	0	1	0	0	0	
14317015	f	ph	NL	2017	0	1	1	0	0	0	0	0	0	0	0	0	56
14317106	m	md	JP	2017	1	0	0	0	0	0	0	0	0	0	0	0	56
14317274	m	ot	DE	2017	0	0	0	0	0	0	0	0	0	0	0	0	64
14317298	m	ot	DE	2017	0	0	0	0	0	0	0	0	0	0	0	0	64
14317321	m	cn	GB	2017	1	0	0	0	0	0	0	0	0	0	0	0	66
14317778	m	ot	US	2017	0	1	1	0	0	0	0	0	0	0	0	0	54
14317821	f	cn	GB	2017	1	0	0	0	0	0	0	0	0	0	0	0	67
14317852	f	cn	GB	2017	1	0	0	0	0	0	0	0	0	0	0	0	43
14317871	m	cn	IT	2017	0	0	0	0	0	0	0	0	0	0	0	0	70
14317919	m	cn	GB	2017	1	0	0	0	0	0	0	0	0	0	0	0	61
14317923	f	cn	JP	2017	0	1	1	0	0	0	0	0	0	0	0	0	47
14318018	m	ot	DE	2017	0	0	0	0	0	0	0	0	0	0	0	0	70
14318023	m	ot	DE	2017	0	0	0	0	0	0	0	0	0	0	0	0	56
14318158	f	md	US	2017	1	0	0	0	0	0	0	0	0	0	0	0	
14318326	f	cn	GB	2017	1	0	0	0	0	0	0	0	0	0	0	0	57
14318760	f	md	DE	2017	0	0	0	0	0	0	0	0	0	0	0	0	54
14319861	m	cn	KR	2017	0	1	1	0	0	0	0	0	0	0	0	0	
14320734	Not	ot	US	2017	0	0	0	0	0	0	0	0	0	0	0	0	
14320735	Not	ot	US	2017	0	0	0	0	0	0	0	0	0	0	0	0	

14320736	Not	ot	US	2017	0	0	0	0	0	0	0	0	0	0	0	0	
14320745	Not	ot	US	2017	0	0	0	0	0	0	0	0	0	0	0	0	
14320972	Not	ot	JP	2017	0	0	0	0	0	0	0	0	0	0	0	0	
14321142	Not	ot	IT	2017	0	0	0	0	0	0	0	0	0	0	0	0	
14322005	f	md	SK	2017	0	0	0	0	0	0	0	0	0	0	0	0	
14322016	m	md	SK	2017	0	0	0	0	0	0	0	0	0	0	0	0	
14322364	m	md	DE	2017	0	0	0	0	0	0	0	0	0	0	0	0	75
14322497	Not	ot	GB	2017	1	0	0	0	0	0	0	0	0	0	0	0	
14322498	Not	ot	GB	2017	1	0	0	0	0	0	0	0	0	0	0	0	
14322764	m	md	JP	2017	0	0	0	0	0	0	0	0	0	0	0	0	75
14323452	m	cn	US	2017	0	1	0	0	1	0	0	0	0	0	0	0	
14323526	m	ot	LB	2017	0	1	1	0	0	0	0	0	0	0	0	0	74
14323761	m	cn	CA	2017	0	0	0	0	0	0	0	0	0	0	0	0	
14324355	f	cn	US	2017	0	1	0	0	1	0	0	0	0	0	0	0	
14324703	f	ot	JP	2017	0	1	0	0	1	0	0	0	0	0	0	0	65
14325123	f	cn	US	2017	0	1	0	0	1	0	0	0	0	0	0	0	
14325176	f	Not repo	US	2017	1	0	0	0	0	0	0	0	0	0	0	0	86
14325182	m	ph	JP	2017	1	0	0	0	0	0	0	0	0	0	0	0	89
14325251	m	md	JP	2017	1	0	0	0	0	0	0	0	0	0	0	0	76
14325429	m	md	JP	2017	0	1	0	0	1	0	0	0	0	0	0	1	55
14325932	m	cn	JP	2017	0	1	1	0	0	0	0	0	0	0	0	1	74
14326012	Not	md	JP	2017	0	0	0	0	0	0	0	0	0	0	0	0	
14326064	m	md	JP	2017	0	1	0	0	1	0	0	0	0	0	0	0	66
14326088	m	cn	US	2017	0	1	0	0	1	0	0	0	0	0	0	0	73
14326317	Not	cn	US	2017	0	1	0	0	1	0	0	0	0	0	0	0	
14326586	m	md	JP	2017	0	1	0	0	1	0	0	0	0	0	0	0	65
14326808	m	ot	DE	2017	0	0	0	0	0	0	0	0	0	0	0	0	56
14326895	m	ot	DE	2017	0	0	0	0	0	0	0	0	0	0	0	0	70
14327030	m	md	KR	2017	0	1	0	0	1	0	0	0	0	0	0	0	65
14327123	f	ot	DE	2017	0	0	0	0	0	0	0	0	0	0	0	0	68
14327276	m	ph	US	2017	0	1	1	0	0	0	0	0	0	0	0	0	76
14327445	f	md	JP	2017	0	0	0	0	0	0	0	0	0	0	0	0	76
14327616	m	md	DE	2017	0	0	0	0	0	0	0	0	0	0	0	0	76
14327696	m	cn	JP	2017	0	1	1	0	0	0	0	0	0	0	0	0	78

14328052	m	cn	JP	2017	0	1	1	0	0	0	0	0	0	0	0	0	
14329053	m	cn	JP	2017	0	1	1	0	0	0	0	0	0	0	0	0	66
14329707	m	cn	JP	2017	0	1	1	0	0	0	0	0	0	0	0	0	75
14329801	m	ot	DE	2017	0	0	0	0	0	0	0	0	0	0	0	0	64
14329892	m	md	JP	2017	0	1	0	0	1	0	0	0	0	0	0	1	69
14329977	m	ot	KR	2017	0	1	0	1	0	0	0	0	0	0	0	0	85
14329982	m	ph	JP	2017	0	1	0	0	1	0	0	0	0	0	0	1	61
14330035	m	cn	US	2017	0	0	0	0	0	0	0	0	0	0	0	0	
14330055	m	md	GB	2017	0	1	0	0	1	0	0	0	0	0	0	0	80
14331231	Not	ot	IT	2017	0	0	0	0	0	0	0	0	0	0	0	0	
14331290	m	md	JP	2017	1	0	0	0	0	0	0	0	0	0	0	0	80
14331703	f	cn	GB	2017	1	0	0	0	0	0	0	0	0	0	0	0	68
14331704	m	cn	GB	2017	1	0	0	0	0	0	0	0	0	0	0	0	69
14331791	m	cn	GB	2017	1	0	0	0	0	0	0	0	0	0	0	0	61
14332219	m	cn	GB	2017	1	0	0	0	0	0	0	0	0	0	0	0	49
14332480	f	cn	US	2017	1	0	0	0	0	0	0	0	0	0	0	0	73
14332762	m	cn	JP	2017	0	0	0	0	0	0	0	0	0	0	0	0	75
14333058	m	cn	KR	2017	0	1	1	0	0	0	0	0	0	0	0	0	
14333754	m	cn	GB	2017	1	0	0	0	0	0	0	0	0	0	0	0	34
14333943	f	cn	GB	2017	1	0	0	0	0	0	0	0	0	0	0	0	71
14333955	m	cn	GB	2017	1	0	0	0	0	0	0	0	0	0	0	0	60
14334220	Not	md	JP	2017	0	1	0	0	1	0	0	0	0	0	0	0	
14335529	m	cn	US	2017	0	1	0	0	1	0	0	0	0	0	0	0	54
14335594	m	ot	GB	2017	0	0	0	0	0	0	0	0	0	0	0	0	74
14335626	m	ot	GB	2017	0	0	0	0	0	0	0	0	0	0	0	0	78
14336302	m	ot	GB	2017	0	0	0	0	0	0	0	0	0	0	0	0	80
14336504	m	cn	US	2017	0	1	1	0	0	0	0	0	0	0	0	1	
14336575	f	cn	GB	2017	1	0	0	0	0	0	0	0	0	0	0	0	52
14336581	m	cn	GB	2017	1	0	0	0	0	0	0	0	0	0	0	0	61
14337424	f	md	DE	2017	0	0	0	0	0	0	0	0	0	0	0	0	67
14337458	m	ot	GB	2017	0	1	1	0	0	0	0	0	0	0	0	0	74
14337591	f	cn	GB	2017	1	0	0	0	0	0	0	0	0	0	0	0	72
14337593	m	md	JP	2017	0	1	0	0	1	0	0	0	0	0	0	0	79
14337604	m	cn	GB	2017	1	0	0	0	0	0	0	0	0	0	0	0	56

14337605	m	cn	GB	2017	1	0	0	0	0	0	0	0	0	0	0	0	55
14337668	Not	ot	GB	2017	1	0	0	0	0	0	0	0	0	0	0	0	
14337705	m	ot	US	2017	0	1	0	0	1	0	0	0	0	0	0	0	64
14337773	f	ot	US	2017	0	1	0	0	1	0	0	0	0	0	0	0	68
14337860	m	md	CZ	2017	0	0	0	0	0	0	0	0	0	0	0	0	55
14337933	m	md	CZ	2017	1	0	0	0	0	0	0	0	0	0	0	0	56
14337934	m	ot	US	2017	0	1	0	0	1	0	0	0	0	0	0	0	55
14337958	f	md	JP	2017	1	0	0	0	0	0	0	0	0	0	0	1	73.4
14338227	f	cn	GB	2017	1	0	0	0	0	0	0	0	0	0	0	0	
14339192	m	md	JP	2017	0	1	0	0	1	0	0	0	0	0	0	0	81
14339253	m	md	JP	2017	0	1	0	0	1	0	0	0	0	0	0	1	67
14340025	m	md	DE	2017	0	0	0	0	0	0	0	0	0	0	0	0	76
14340046	m	ph	JP	2017	0	1	0	0	1	0	0	0	0	0	0	0	76
14340091	Not	md	TW	2017	0	0	0	0	0	0	0	0	0	0	0	0	
6932224	m	cn	JP	2017	0	0	0	0	0	0	0	0	0	0	0	0	70
8130498	f	cn	GB	2017	1	0	0	0	0	0	0	0	0	0	0	0	57
8200983	m	ot	US	2017	0	0	0	0	0	0	0	0	0	0	0	0	67
8217049	m	cn	GB	2017	1	0	0	0	0	0	0	0	0	0	0	0	63
8766486	m	ot	US	2017	0	0	0	0	0	0	0	0	0	0	0	0	67
8962265	Not	Not repo	Not re	2017	1	0	0	0	0	0	0	0	0	0	0	0	
9192794	m	ot	DE	2017	0	0	0	0	0	0	0	0	0	0	0	0	58
9292007	m	cn	US	2017	0	0	0	0	0	0	0	0	0	0	0	0	58
9743212	f	ot	US	2017	0	0	0	0	0	0	0	0	0	0	0	0	51
10011758	m	cn	KR	2018	0	0	0	0	0	0	0	0	0	0	0	0	65
10074027	m	cn	US	2018	0	0	0	0	0	0	0	0	0	0	0	0	69
10080037	m	cn	GB	2018	0	0	0	0	0	0	0	0	0	0	0	0	53
10086717	m	cn	NL	2018	0	0	0	0	0	0	0	0	0	0	0	0	72
10231998	m	cn	AU	2018	0	0	0	0	0	0	0	0	0	0	0	0	66
10374095	m	cn	JP	2018	0	0	0	0	0	0	0	0	0	0	0	0	72
10512713	m	cn	AU	2018	0	0	0	0	0	0	0	0	0	0	0	0	66
10609997	m	cn	US	2018	0	0	0	0	0	0	0	0	0	0	0	0	62
10615619	Not	ot	NO	2018	0	0	0	0	0	0	0	0	0	0	0	0	
13095842	f	md	US	2018	0	1	0	0	0	1	0	0	0	0	0	0	68
13266831	m	md	IL	2018	0	1	0	0	0	1	0	0	0	0	0	0	78

13290615	f	md	ES	2018	0	1	0	0	0	1	0	0	0	0	0	0	67
13414110	m	cn	JP	2018	0	1	1	0	0	0	0	0	0	0	0	1	78
13416742	m	ot	DE	2018	0	1	0	0	0	1	0	0	0	0	0	0	67
13460289	f	md	JP	2018	1	0	0	0	0	0	0	0	0	0	0	0	77
13505186	f	md	ES	2018	0	1	0	0	0	1	0	0	0	0	0	0	81
13521451	m	md	IT	2018	0	1	0	0	0	1	0	0	0	0	0	0	58
13563347	m	md	JP	2018	0	1	0	0	0	1	0	0	0	0	0	0	37
13584459	f	md	GB	2018	0	1	0	0	0	1	0	0	0	0	0	0	62
13584493	m	ot	DE	2018	0	1	0	0	1	0	0	0	0	0	0	0	
13601174	m	cn	JP	2018	0	1	1	0	0	0	0	0	0	0	0	0	55
13634174	m	md	ES	2018	0	1	0	0	0	1	0	0	0	0	0	0	52
13659127	f	md	JP	2018	0	1	1	0	0	0	0	0	0	0	0	0	
13676083	m	ot	CH	2018	0	1	0	0	1	0	0	0	0	0	0	0	79
13689462	m	cn	JP	2018	0	1	1	0	0	0	0	0	0	0	0	0	51
13692860	m	md	US	2018	0	1	0	0	0	1	0	0	0	0	0	0	61
13710371	m	md	BR	2018	0	1	0	0	0	1	0	0	0	0	0	0	76
13713916	m	md	JP	2018	0	1	0	0	1	0	0	0	0	0	0	1	68
13716369	m	ot	CH	2018	0	1	0	0	1	0	0	0	0	0	0	0	79
13740576	f	md	DE	2018	0	1	0	0	0	1	0	0	0	0	0	0	74
13742731	f	md	JP	2018	0	0	0	0	0	0	0	0	0	0	0	0	67
13748740	m	ot	JP	2018	0	1	0	0	1	0	0	0	0	0	0	0	70
13763091	m	md	JP	2018	1	0	0	0	0	0	0	0	0	0	0	0	68
13775525	m	ot	US	2018	0	1	0	0	1	0	0	0	0	0	0	0	61
13777419	m	cn	JP	2018	0	1	1	0	0	0	0	0	0	0	0	0	63
13779304	m	ot	JP	2018	0	1	0	0	1	0	0	0	0	0	0	0	65
13803500	m	ot	DE	2018	0	1	1	0	0	0	0	0	0	0	0	0	65
13817648	f	cn	FR	2018	0	0	0	0	0	0	0	0	0	0	0	0	62
13823119	m	ot	JP	2018	0	1	0	0	1	0	0	0	0	0	0	1	59
13836532	m	cn	US	2018	0	1	0	0	1	0	0	0	0	0	0	1	
13843952	m	cn	JP	2018	0	1	1	0	0	0	0	0	0	0	0	0	67
13854647	m	md	JP	2018	0	1	0	0	1	0	0	0	0	0	0	0	69
13857306	m	ot	CN	2018	0	1	0	0	1	0	0	0	0	0	0	0	44
13884372	m	ot	DE	2018	0	1	0	0	1	0	0	0	0	0	0	0	76
13910251	f	cn	JP	2018	0	1	1	0	0	0	0	0	0	0	0	1	71

13915844	f	md	JP	2018	1	0	0	0	0	0	0	0	0	0	0	1	71
13923157	m	md	JP	2018	0	1	0	0	1	0	0	0	0	0	0	1	71
13928835	m	ot	US	2018	0	0	0	0	0	0	0	0	0	0	0	0	67
13932261	m	md	JP	2018	0	1	0	0	0	1	0	0	0	0	0	0	65
13937575	m	ot	JP	2018	0	1	0	0	1	0	0	0	0	0	0	0	75
13961728	f	cn	US	2018	1	0	0	0	0	0	0	0	0	0	0	0	75
13978494	f	ot	JP	2018	0	1	0	0	1	0	0	0	0	0	0	0	77
13982098	m	md	JP	2018	0	1	0	0	0	1	0	0	0	0	0	0	76
13985425	f	md	US	2018	0	1	0	0	1	0	0	0	0	0	0	1	61
13985808	f	cn	US	2018	0	0	0	0	0	0	0	0	0	0	0	0	87
14013266	m	md	JP	2018	0	0	0	0	0	0	0	0	0	0	0	0	65
14014311	f	md	PT	2018	1	0	0	0	0	0	0	0	0	0	0	0	70
14015245	m	md	CN	2018	1	0	0	0	0	0	0	0	0	0	0	0	62.1
14015350	m	ot	US	2018	0	1	0	0	1	0	0	0	0	0	0	0	64
14023282	m	md	JP	2018	1	0	0	0	0	0	0	0	0	0	0	1	80.2
14024114	f	ot	DE	2018	0	1	0	0	1	0	0	0	0	0	0	0	75
14026903	m	ot	DE	2018	0	1	0	0	1	0	0	0	0	0	0	0	55
14031349	f	ph	JP	2018	0	0	0	0	0	0	0	0	0	0	0	0	59
14031657	f	md	JP	2018	1	0	0	0	0	0	0	0	0	0	0	0	83
14033598	m	md	JP	2018	0	1	0	0	1	0	0	0	0	0	0	0	65
14034529	Not	cn	GB	2018	1	0	0	0	0	0	0	0	0	0	0	0	
14035278	m	md	US	2018	0	0	0	0	0	0	0	0	0	0	0	0	65
14037194	m	ot	JP	2018	0	1	0	0	1	0	0	0	0	0	0	0	76
14039584	m	md	JP	2018	0	1	0	0	1	0	0	0	0	0	0	0	55
14044787	m	md	DE	2018	0	1	0	0	0	1	0	0	0	0	0	0	71
14046478	f	ot	NL	2018	0	0	0	0	0	0	0	0	0	0	0	0	48
14047148	m	cn	JP	2018	0	1	1	0	0	0	0	0	0	0	0	0	
14048188	m	md	FR	2018	0	0	0	0	0	0	0	0	0	0	0	0	75
14050314	m	ot	US	2018	0	1	0	0	1	0	0	0	0	0	0	0	52
14055686	m	ot	DE	2018	0	1	0	0	1	0	0	0	0	0	0	0	63
14056706	m	cn	JP	2018	0	1	0	0	1	0	0	0	0	0	0	0	65
14057274	m	ot	US	2018	0	1	0	0	1	0	0	0	0	0	0	0	60
14065548	f	md	JP	2018	1	0	0	0	0	0	0	0	0	0	0	0	84
14066169	m	md	MY	2018	1	0	0	0	0	0	0	0	0	0	0	0	43

14067586	m	md	IT	2018	0	1	0	0	1	0	0	0	0	0	0	0	61
14068063	f	ot	DE	2018	0	1	0	0	1	0	0	0	0	0	0	0	66
14068958	m	md	JP	2018	1	0	0	0	0	0	0	0	0	0	0	1	80
14070204	m	md	JP	2018	0	1	1	0	0	0	0	0	0	0	0	0	79
14071889	m	ot	JP	2018	0	1	0	0	1	0	0	0	0	0	0	1	50
14073371	m	md	CN	2018	0	1	0	0	0	1	0	0	0	0	0	0	57
14074400	f	cn	IT	2018	0	0	0	0	0	0	0	0	0	0	0	0	58
14074777	m	md	FR	2018	0	1	0	0	1	0	0	0	0	0	0	0	53
14074857	f	cn	GB	2018	1	0	0	0	0	0	0	0	0	0	0	0	75
14075097	m	ph	US	2018	0	0	0	0	0	0	0	0	0	0	0	0	58
14075870	f	md	JP	2018	1	0	0	0	0	0	0	0	0	0	0	0	79
14077372	m	ot	JP	2018	0	1	0	0	1	0	0	0	0	0	0	0	76
14079014	Not	cn	GB	2018	1	0	0	0	0	0	0	0	0	0	0	0	
14079177	f	md	LU	2018	0	0	0	0	0	0	0	0	0	0	0	0	34
14085171	m	cn	US	2018	1	0	0	0	0	0	0	0	0	0	0	0	51.8
14090853	Not	ot	JP	2018	0	1	0	0	1	0	0	0	0	0	0	0	
14097456	f	ot	RU	2018	0	1	0	0	1	0	0	0	0	0	0	0	61
14098234	m	ot	US	2018	0	1	0	0	1	0	0	0	0	0	0	0	72
14098407	f	md	CA	2018	0	0	0	0	0	0	0	0	0	0	0	0	
14099237	f	ot	DE	2018	0	1	0	0	1	0	0	0	0	0	0	0	47
14099585	m	ot	US	2018	0	0	0	0	0	0	0	0	0	0	0	0	72
14100838	f	cn	JP	2018	0	0	0	0	0	0	1	1	0	0	0	0	74
14102125	m	md	JP	2018	1	0	0	0	0	0	0	0	0	0	0	0	53
14104026	f	ot	JP	2018	0	0	0	0	0	0	0	0	0	0	0	0	79
14104846	m	md	JP	2018	0	1	0	0	1	0	0	0	0	0	0	0	68
14105653	m	ot	JP	2018	0	1	0	0	1	0	0	0	0	0	0	0	64
14110876	m	md	US	2018	0	1	0	0	0	1	0	0	0	0	0	1	58
14112134	m	md	JP	2018	0	0	0	0	0	0	0	0	0	0	0	0	75
14114781	m	md	FR	2018	0	1	0	0	1	0	0	0	0	0	0	0	63
14115689	Not	Not repo	Not re	2018	1	0	0	0	0	0	0	0	0	0	0	0	
14119207	m	ot	JP	2018	0	1	0	0	1	0	0	0	0	0	0	1	55
14120297	f	ph	JP	2018	0	0	0	0	0	0	0	0	0	0	0	0	65
14121311	f	md	DE	2018	1	0	0	0	0	0	0	0	0	0	0	0	53
14122674	f	ot	DE	2018	0	1	0	0	1	0	0	0	0	0	0	0	75

14123161	m	md	JP	2018	0	1	0	0	1	0	0	0	0	0	0	1	49
14124387	f	lw	US	2018	0	0	0	0	0	0	0	0	0	0	0	0	69
14126247	m	md	LT	2018	0	0	0	0	0	0	0	0	0	0	0	0	47
14128517	m	ot	DE	2018	0	1	0	0	1	0	0	0	0	0	0	0	77
14128956	m	ot	FR	2018	0	1	0	0	1	0	0	0	0	0	0	0	57
14129370	f	ot	US	2018	0	0	0	0	0	0	0	0	0	0	0	0	91
14132872	m	cn	JP	2018	0	1	1	0	0	0	0	0	0	0	0	0	43
14137131	f	md	JP	2018	0	0	0	0	0	0	0	0	0	0	0	0	50
14138056	m	md	LT	2018	0	0	0	0	0	0	0	0	0	0	0	0	72
14138878	f	ot	GB	2018	0	1	0	0	1	0	0	0	0	0	0	0	60
14139347	m	cn	JP	2018	0	1	1	0	0	0	0	0	0	0	0	0	72
14140844	m	md	FR	2018	0	1	0	0	0	1	0	0	0	0	0	0	69
14141825	f	ot	US	2018	0	1	0	0	1	0	0	0	0	0	0	0	64
14143176	m	ot	JP	2018	0	1	0	0	1	0	0	0	0	0	0	0	63
14143423	m	md	JP	2018	0	1	0	0	1	0	0	0	0	0	0	1	47
14143559	m	md	JP	2018	0	1	0	0	1	0	0	0	0	0	0	0	69
14144947	f	md	US	2018	0	1	0	0	0	1	0	0	0	0	0	0	55
14147438	m	ph	JP	2018	0	1	1	0	0	0	0	0	0	0	0	0	60
14148841	m	md	Not re	2018	0	0	0	0	0	0	0	0	0	0	0	0	
14150149	m	ot	IT	2018	0	1	0	0	1	0	0	0	0	0	0	0	72
14150368	f	md	JP	2018	0	1	1	0	0	0	0	0	0	0	0	1	77
14151140	m	md	PT	2018	1	0	0	0	0	0	0	0	0	0	0	0	64
14151305	m	md	DE	2018	0	0	0	0	0	0	0	0	0	0	0	0	47
14151655	m	md	DE	2018	0	0	0	0	0	0	0	0	0	0	0	0	64
14152827	f	md	JP	2018	1	0	0	0	0	0	0	0	0	0	0	0	72
14154505	f	md	US	2018	0	1	0	0	0	1	0	0	0	0	0	0	55
14154982	m	ot	AR	2018	0	1	0	0	1	0	0	0	0	0	0	1	65
14155435	m	md	TR	2018	0	1	0	0	0	1	0	0	0	0	0	0	64
14155728	m	ot	PT	2018	1	0	0	0	0	0	0	0	0	0	0	0	64
14155979	f	cn	JP	2018	0	1	1	0	0	0	0	0	0	0	0	0	60
14157518	f	ot	ES	2018	0	0	0	0	0	0	0	0	0	0	0	0	66
14157597	m	ot	NO	2018	0	1	1	0	0	0	0	0	0	0	0	0	67
14158239	m	cn	JP	2018	0	0	0	0	0	0	0	0	0	0	0	0	70
14158260	f	md	JP	2018	1	0	0	0	0	0	0	0	0	0	0	0	75

14159863	m	md	IL	2018	0	0	0	0	0	0	0	0	0	0	0	0	61
14160319	f	ot	NL	2018	0	1	0	0	0	1	0	0	0	0	0	0	58
14163741	m	ot	JP	2018	0	1	0	0	1	0	0	0	0	0	0	0	70
14164435	m	md	JP	2018	1	0	0	0	0	0	0	0	0	0	0	0	80
14165046	m	md	CN	2018	0	0	0	0	0	0	0	0	0	0	0	0	75
14165963	f	ot	RO	2018	0	1	0	0	1	0	0	0	0	0	0	0	59
14166955	m	ot	US	2018	0	0	0	0	0	0	0	0	0	0	0	0	73
14173278	m	md	JP	2018	0	1	0	0	1	0	0	0	0	0	0	1	74
14173916	f	cn	JP	2018	0	1	1	0	0	0	0	0	0	0	0	1	78
14174099	f	ph	JP	2018	1	0	0	0	0	0	0	0	0	0	0	0	84
14176458	f	ot	DE	2018	0	1	0	0	1	0	0	0	0	0	0	0	55
14176979	f	md	JP	2018	1	0	0	0	0	0	0	0	0	0	0	0	71
14179038	m	ph	FR	2018	0	0	0	0	0	0	0	0	0	0	0	0	49
14181553	m	md	JP	2018	0	1	0	0	1	0	0	0	0	0	0	0	68
14181586	m	md	JP	2018	0	1	0	0	1	0	0	0	0	0	0	0	65
14182221	m	ot	IE	2018	0	1	0	0	1	0	0	0	0	0	0	0	68
14183023	m	cn	JP	2018	0	1	1	0	0	0	0	0	0	0	0	0	60
14183558	m	md	US	2018	0	1	0	0	0	1	0	0	0	0	0	1	71
14184649	m	md	VN	2018	0	1	0	1	0	0	0	0	0	0	0	0	61.7
14185888	f	md	JP	2018	1	0	0	0	0	0	0	0	0	0	0	0	73
14186059	m	cn	JP	2018	0	1	1	0	0	0	0	0	0	0	0	1	67
14187060	f	ph	US	2018	0	0	0	0	0	0	0	0	0	0	0	0	69
14187268	Not	ot	US	2018	0	0	0	0	0	0	0	0	0	0	0	0	
14190308	m	cn	AT	2018	0	0	0	0	0	0	0	0	0	0	0	0	
14193512	m	cn	DE	2018	0	1	1	0	0	0	0	0	0	0	0	0	
14193740	m	ot	CL	2018	0	1	0	0	1	0	0	0	0	0	0	0	68
14194582	m	cn	US	2018	1	0	0	0	0	0	0	0	0	0	0	1	
14195464	m	md	JP	2018	0	1	0	0	1	0	0	0	0	0	0	0	73
14196988	f	md	DE	2018	1	0	0	0	0	0	0	0	0	0	0	0	35
14197674	m	md	JP	2018	0	1	0	0	1	0	0	0	0	0	0	1	59
14197876	f	md	JP	2018	0	1	0	0	1	0	0	0	0	0	0	0	65
14197990	f	md	JP	2018	0	1	0	0	1	0	0	0	0	0	0	1	65
14198084	m	md	JP	2018	0	1	0	0	1	0	0	0	0	0	0	0	71
14200283	f	md	US	2018	0	1	0	0	0	1	0	0	0	0	0	0	63

14202678	f	md	JP	2018	0	1	0	0	1	0	0	0	0	0	0	1	80
14203564	m	cn	US	2018	1	0	0	0	0	0	0	0	0	0	0	0	
14204242	m	md	DE	2018	0	0	0	0	0	0	0	0	0	0	0	0	75
14204484	m	md	JP	2018	0	1	0	0	1	0	0	0	0	0	0	0	68
14206829	f	ot	CA	2018	0	1	0	0	1	0	0	0	0	0	0	0	63
14206998	m	ot	US	2018	0	1	0	0	1	0	0	0	0	0	0	0	
14208409	m	cn	JP	2018	0	1	1	0	0	0	0	0	0	0	0	0	78
14212291	m	cn	JP	2018	0	1	1	0	0	0	0	0	0	0	0	0	66
14213388	m	Not repo	JP	2018	0	0	0	0	0	0	0	0	0	0	0	0	34
14213447	f	cn	US	2018	0	1	1	0	0	0	0	0	0	0	0	0	70
14214064	f	md	MX	2018	0	1	1	0	0	0	0	0	0	0	0	0	56
14215572	f	md	JP	2018	1	0	0	0	0	0	0	0	0	0	0	0	67
14216422	m	md	GB	2018	0	1	0	0	1	0	0	0	0	0	0	0	81
14217349	m	cn	CN	2018	0	0	0	0	0	0	0	0	0	0	0	0	
14217793	m	md	JP	2018	0	1	0	0	1	0	0	0	0	0	0	0	87
14218315	f	ot	US	2018	0	0	0	0	0	0	0	0	0	0	0	0	48
14218540	m	ot	JP	2018	0	1	0	0	1	0	0	0	0	0	0	0	50
14219687	f	md	DE	2018	1	0	0	0	0	0	0	0	0	0	0	0	69
14220771	f	md	US	2018	1	0	0	0	0	0	0	0	0	0	0	0	70
14221705	m	cn	US	2018	0	0	0	0	0	0	0	0	0	0	0	0	84
14221978	m	md	JP	2018	0	0	0	0	0	0	0	0	0	0	0	0	
14225861	m	ot	JP	2018	0	1	0	0	1	0	0	0	0	0	0	0	64
14226899	f	cn	FR	2018	0	1	1	0	0	0	0	0	0	0	0	0	68
14228883	m	md	AT	2018	1	0	0	0	0	0	0	0	0	0	0	0	
14229387	Not	md	IT	2018	1	0	0	0	0	0	0	0	0	0	0	0	
14230711	f	cn	GB	2018	0	0	0	0	0	0	0	0	0	0	0	0	69
14231875	f	cn	GR	2018	0	0	0	0	0	0	0	0	0	0	0	0	54
14233107	f	cn	US	2018	0	1	0	0	1	0	0	0	0	0	0	0	
14233320	f	md	PT	2018	1	0	0	0	0	0	0	0	0	0	0	0	70
14233448	m	ot	JP	2018	0	1	0	0	1	0	0	0	0	0	0	0	53
14233737	m	md	JP	2018	0	1	0	0	1	0	0	0	0	0	0	0	51
14235865	m	md	JP	2018	0	1	0	0	1	0	0	0	0	0	0	0	69
14237477	Not	md	US	2018	0	1	0	0	1	0	0	0	0	0	0	0	
14237913	m	ot	US	2018	0	1	0	0	1	0	0	0	0	0	0	0	71

14237990	f	md	JP	2018	0	1	0	0	1	0	0	0	0	0	0	0	72
14239159	m	md	JP	2018	1	0	0	0	0	0	0	0	0	0	0	0	81.7
14239382	m	md	JP	2018	0	1	0	0	1	0	0	0	0	0	0	0	74
14240486	m	md	JP	2018	0	1	0	0	1	0	0	0	0	0	0	0	76
14241217	f	cn	US	2018	0	1	0	0	1	0	0	0	0	0	0	0	
14241226	f	ot	US	2018	0	1	0	0	1	0	0	0	0	0	0	0	70
14241807	m	md	JP	2018	0	0	0	0	0	0	0	0	0	0	0	0	75
14242207	m	md	JP	2018	0	1	0	0	1	0	0	0	0	0	0	0	68
14243359	m	md	BR	2018	0	1	0	0	0	1	0	0	0	0	0	0	73
14243853	m	md	DE	2018	0	0	0	0	0	0	0	0	0	0	0	0	68
14244436	f	md	FR	2018	1	0	0	0	0	0	0	0	0	0	0	0	50
14244627	m	ot	BR	2018	0	1	0	0	1	0	0	0	0	0	0	0	68
14244866	m	md	JP	2018	0	1	0	0	1	0	0	0	0	0	0	1	67
14247202	m	ot	JP	2018	0	1	0	0	1	0	0	0	0	0	0	0	64
14248606	m	md	JP	2018	1	0	0	0	0	0	0	0	0	0	0	1	78
14248679	m	md	JP	2018	0	1	0	0	1	0	0	0	0	0	0	0	68
14249270	m	cn	JP	2018	0	1	0	0	1	0	0	0	0	0	0	0	50
14249891	m	md	JP	2018	0	1	0	0	1	0	0	0	0	0	0	0	79
14250005	f	ot	BR	2018	0	1	0	0	1	0	0	0	0	0	0	0	61
14251264	m	ot	JP	2018	0	1	0	0	1	0	0	0	0	0	0	0	71
14251824	m	md	HU	2018	0	1	0	0	1	0	0	0	0	0	0	0	74
14252018	m	md	JP	2018	0	1	0	0	1	0	0	0	0	0	0	1	65
14252201	m	md	JP	2018	0	1	0	0	1	0	0	0	0	0	0	1	80
14252478	m	ot	US	2018	0	1	0	0	1	0	0	0	0	0	0	0	55
14252851	m	ot	JP	2018	0	1	0	0	1	0	0	0	0	0	0	1	61
14253448	f	md	JP	2018	0	0	0	0	0	0	0	0	0	0	0	0	73
14255303	m	md	GB	2018	0	1	0	0	1	0	0	0	0	0	0	0	80
14255446	m	md	JP	2018	0	1	0	0	1	0	0	0	0	0	0	0	73
14256070	m	md	CN	2018	1	0	0	0	0	0	0	0	0	0	0	0	39.9
14256847	m	ot	GB	2018	0	1	0	0	1	0	0	0	0	0	0	0	78
14256864	m	ot	BE	2018	0	1	0	0	1	0	0	0	0	0	0	0	69
14257002	f	ot	US	2018	0	1	1	0	0	0	0	0	0	0	0	0	53
14258762	m	md	JP	2018	1	0	0	0	0	0	0	0	0	0	0	0	74
14258807	m	ot	CZ	2018	0	1	0	0	1	0	0	0	0	0	0	1	55

14260900	m	md	JP	2018	0	1	0	0	1	0	0	0	0	0	0	0	51
14261358	f	ph	US	2018	0	1	0	0	1	0	0	0	0	0	0	0	
14261692	m	cn	JP	2018	0	1	1	0	0	0	0	0	0	0	0	0	
14261802	m	cn	US	2018	0	1	1	0	0	0	0	0	0	0	0	0	
14261942	f	md	CN	2018	1	0	0	0	0	0	0	0	0	0	0	0	70
14262924	m	ot	US	2018	0	1	1	0	0	0	0	0	0	0	0	0	76
14263075	m	Not repo	JP	2018	0	0	0	0	0	0	0	0	0	0	0	0	80
14263223	m	md	PL	2018	0	0	0	0	0	0	0	0	0	0	0	0	74
14263622	m	md	KR	2018	0	0	0	0	0	0	0	0	0	0	0	0	69
14263638	f	md	JP	2018	1	0	0	0	0	0	0	0	0	0	0	0	67
14264069	m	md	JP	2018	0	1	0	0	1	0	0	0	0	0	0	0	77
14264837	m	md	DE	2018	0	0	0	0	0	0	0	0	0	0	0	0	47
14265312	m	cn	US	2018	0	1	0	0	1	0	0	0	0	0	0	0	
14265949	m	md	JP	2018	0	1	0	0	1	0	0	0	0	0	0	1	67
14266060	Not	ot	CN	2018	0	1	0	0	1	0	0	0	0	0	0	0	
14266090	f	ot	CL	2018	0	1	0	0	1	0	0	0	0	0	0	0	75
14271982	f	cn	GB	2018	1	0	0	0	0	0	0	0	0	0	0	0	66
14272543	f	md	JP	2018	0	0	0	0	0	0	0	0	0	0	0	0	60
14276465	f	cn	Not re	2018	0	0	0	0	0	0	0	0	0	0	0	0	72
14277227	f	md	JP	2018	0	1	1	0	0	0	0	0	0	0	0	0	64
14277463	m	md	CN	2018	1	0	0	0	0	0	0	0	0	0	0	0	54.7
14278946	f	md	JP	2018	0	1	0	0	1	0	0	0	0	0	0	0	58
14283107	m	ot	US	2018	0	0	0	0	0	0	0	0	0	0	0	0	84
14283563	m	cn	US	2018	1	0	0	0	0	0	0	0	0	0	0	0	82
14283577	m	ot	DE	2018	0	0	0	0	0	0	0	0	0	0	0	0	81
14284246	m	cn	JP	2018	0	1	1	0	0	0	0	0	0	0	0	0	79
14286292	f	md	KR	2018	1	0	0	0	0	0	0	0	0	0	0	0	70.1
14287319	f	ot	JP	2018	0	1	0	0	1	0	0	0	0	0	0	0	73
14288200	f	ot	DE	2018	0	0	0	0	0	0	0	0	0	0	0	0	68
14288460	m	md	JP	2018	0	1	0	0	1	0	0	0	0	0	0	0	69
14288889	m	md	US	2018	0	1	0	1	0	0	0	0	0	0	0	0	70
14289192	Not	cn	JP	2018	0	0	0	0	0	0	0	0	0	0	0	0	
14290041	m	ot	US	2018	0	1	0	0	1	0	0	0	0	0	0	0	50
14290162	f	cn	JP	2018	0	1	1	0	0	0	0	0	0	0	0	0	74

14290259	m	md	DE	2018	0	0	0	0	0	0	0	0	0	0	0	0	64
14290917	f	cn	GB	2018	1	0	0	0	0	0	0	0	0	0	0	0	83
14290996	m	md	DE	2018	0	0	0	0	0	0	0	0	0	0	0	0	64
14291172	f	md	GB	2018	0	1	0	0	0	1	0	0	0	0	0	0	71
14291607	m	cn	US	2018	0	1	1	0	0	0	0	0	0	0	0	0	70
14292272	m	md	NL	2018	0	0	0	0	0	0	0	0	0	0	0	0	77.6
14292344	f	ph	JP	2018	0	0	0	0	0	0	0	0	0	0	0	0	65
14292561	f	ot	US	2018	0	0	0	0	0	0	0	0	0	0	0	0	74
14292592	f	ph	JP	2018	0	1	1	0	0	0	0	0	0	0	0	0	67
14292641	f	cn	US	2018	1	0	0	0	0	0	0	0	0	0	0	0	
14292714	f	md	US	2018	0	1	0	0	0	1	0	0	0	0	0	0	77
14292877	f	ot	JP	2018	0	1	0	0	1	0	0	0	0	0	0	0	57
14294858	f	cn	US	2018	0	1	1	0	0	0	0	0	0	0	0	0	
14295956	m	md	TW	2018	0	0	0	0	0	0	0	0	0	0	0	0	59
14296598	m	cn	JP	2018	0	1	1	0	0	0	0	0	0	0	0	0	54
14297740	f	ot	DE	2018	0	0	0	0	0	0	0	0	0	0	0	0	66
14297791	m	md	JP	2018	1	0	0	0	0	0	0	0	0	0	0	1	64
14298591	m	ot	IT	2018	0	0	0	0	0	0	0	0	0	0	0	0	70
14298592	f	ot	IT	2018	0	0	0	0	0	0	0	0	0	0	0	0	50
14299942	m	cn	GR	2018	0	0	0	0	0	0	0	0	0	0	0	0	
14300710	f	ot	IT	2018	0	0	0	0	0	0	0	0	0	0	0	0	55
14301677	m	ph	JP	2018	1	0	0	0	0	0	0	0	0	0	0	0	66
14302869	m	ph	JP	2018	0	1	1	0	0	0	0	0	0	0	0	0	53
14305685	m	cn	JP	2018	0	1	1	0	0	0	0	0	0	0	0	0	60
14306380	m	md	JP	2018	0	1	0	0	1	0	0	0	0	0	0	1	66
14306781	f	cn	US	2018	1	0	0	0	0	0	0	0	0	0	0	0	
14308390	m	md	KR	2018	1	0	0	0	0	0	0	0	0	0	0	0	75
14308470	f	md	JP	2018	0	0	0	0	0	0	0	0	0	0	0	0	51
14309272	m	md	JP	2018	0	1	0	0	1	0	0	0	0	0	0	0	60
14311108	m	md	JP	2018	0	1	0	1	0	0	0	0	0	0	0	1	84
14311126	m	ph	JP	2018	0	0	0	0	0	0	0	0	0	0	0	0	80
14312101	m	md	JP	2018	0	1	0	0	1	0	0	0	0	0	0	0	75
14312529	m	ot	RU	2018	0	1	0	0	1	0	0	0	0	0	0	0	62
14313140	f	ot	US	2018	0	0	0	0	0	0	0	0	0	0	0	0	52

14313738	f	md	TN	2018	0	0	0	0	0	0	0	0	0	0	0	0	63
14314340	Not	ot	GB	2018	0	0	0	0	0	0	0	0	0	0	0	0	
14314932	m	cn	GR	2018	0	0	0	0	0	0	0	0	0	0	0	0	
14314978	f	ot	PL	2018	0	1	0	0	1	0	0	0	0	0	0	0	68
14315256	m	ot	BR	2018	0	1	0	0	1	0	0	0	0	0	0	0	81
14315298	m	cn	US	2018	0	1	1	0	0	0	0	0	0	0	0	0	
14315815	f	md	CN	2018	1	0	0	0	0	0	0	0	0	0	0	0	60
14316033	f	Not repo	US	2018	1	0	0	0	0	0	0	0	0	0	0	0	
14316196	m	ot	KR	2018	0	1	0	0	1	0	0	0	0	0	0	0	58
14316646	m	md	RU	2018	0	1	0	0	0	1	0	0	0	0	0	0	52
14316973	f	cn	GB	2018	1	0	0	0	0	0	0	0	0	0	0	0	66
14317241	Not	ph	JP	2018	1	0	0	0	0	0	0	0	0	0	0	0	
14317429	m	md	US	2018	0	1	0	0	0	1	0	0	0	0	0	0	70
14318054	m	ph	FR	2018	0	1	1	0	0	0	0	0	0	0	0	0	73
14320126	f	md	CN	2018	0	0	0	0	0	0	0	0	0	0	0	0	70
14322116	m	md	PL	2018	0	1	0	0	0	1	0	0	0	0	0	0	59
14322242	f	md	JP	2018	0	0	0	0	0	0	0	0	0	0	0	0	74
14322475	m	cn	BR	2018	1	0	0	0	0	0	0	0	0	0	0	0	50
14322533	m	md	GB	2018	0	1	0	0	1	0	0	0	0	0	0	0	81
14322700	m	cn	US	2018	1	0	0	0	0	0	0	0	0	0	0	0	84
14323546	m	ot	KR	2018	0	1	0	0	1	0	0	0	0	0	0	0	61
14324226	Not	md	JP	2018	0	0	0	0	0	0	0	0	0	0	0	0	83
14324274	m	cn	GB	2018	1	0	0	0	0	0	0	0	0	0	0	0	54
14324656	f	md	GB	2018	1	0	0	0	0	0	0	0	0	0	0	0	69
14326508	f	md	CA	2018	0	0	0	0	0	0	0	0	0	0	0	0	
14326735	f	md	JP	2018	0	1	0	0	1	0	0	0	0	0	0	0	57
14327506	m	cn	ES	2018	0	1	1	0	0	0	0	0	0	0	0	0	
14327910	m	ot	DE	2018	0	0	0	0	0	0	0	0	0	0	0	0	77
14327932	m	ot	CZ	2018	0	1	0	0	1	0	0	0	0	0	0	0	71
14328163	m	cn	JP	2018	0	1	1	0	0	0	0	0	0	0	0	0	71
14328851	m	cn	GR	2018	0	0	0	0	0	0	0	0	0	0	0	0	
14329377	m	md	AU	2018	0	0	0	0	0	0	0	0	0	0	0	0	76
14329432	m	cn	GB	2018	0	1	1	0	0	0	0	0	0	0	0	0	57
14329823	m	ot	JP	2018	0	1	0	0	1	0	0	0	0	0	0	0	62

14329874	m	md	JP	2018	0	1	0	0	1	0	0	0	0	0	0	0	53
14329929	f	cn	US	2018	0	1	0	0	1	0	0	0	0	0	0	0	
14330207	f	ot	DE	2018	0	0	0	0	0	0	0	0	0	0	0	0	75
14330584	m	ot	GB	2018	0	0	0	0	0	0	0	0	0	0	0	0	74
14331212	m	md	JP	2018	0	1	0	0	1	0	0	0	0	0	0	0	61
14331350	f	ot	US	2018	0	1	0	0	1	0	0	0	0	0	0	0	70
14331540	Not	cn	PT	2018	0	1	0	0	1	0	0	0	0	0	0	0	78
14331541	m	ot	JP	2018	0	1	0	0	1	0	0	0	0	0	0	0	63
14332049	m	ph	JP	2018	1	0	0	0	0	0	0	0	0	0	0	0	82
14332275	m	md	US	2018	0	0	0	0	0	0	0	0	0	0	0	0	80
14332545	f	cn	US	2018	0	1	1	0	0	0	0	0	0	0	0	1	56
14333405	f	md	JP	2018	1	0	0	0	0	0	0	0	0	0	0	1	78
14333870	m	md	IN	2018	0	1	0	0	1	0	0	0	0	0	0	0	73
14334829	f	Not repo	US	2018	1	0	0	0	0	0	0	0	0	0	0	0	
14334931	f	md	US	2018	0	1	0	0	0	1	0	0	0	0	0	0	74
14335161	m	ot	GR	2018	0	1	0	0	1	0	0	0	0	0	0	0	72
14335336	m	md	JP	2018	1	0	0	0	0	0	0	0	0	0	0	0	69
14335392	m	md	JP	2018	0	1	0	0	1	0	0	0	0	0	0	0	67
14336354	m	md	JP	2018	1	0	0	0	0	0	0	0	0	0	0	0	70
14336582	m	cn	GB	2018	1	0	0	0	0	0	0	0	0	0	0	0	52
14337841	f	md	DE	2018	0	0	0	0	0	0	0	0	0	0	0	0	58
14340121	m	cn	GR	2018	0	0	0	0	0	0	0	0	0	0	0	0	
14340245	m	md	DE	2018	0	0	0	0	0	0	0	0	0	0	0	0	64
14340360	f	ph	IE	2018	0	1	0	0	0	1	0	0	0	0	0	0	61
14340493	f	cn	GB	2018	1	0	0	0	0	0	0	0	0	0	0	0	73
14340514	m	cn	GB	2018	1	0	0	0	0	0	0	0	0	0	0	0	
14340515	f	cn	GB	2018	1	0	0	0	0	0	0	0	0	0	0	0	67
14340612	Not	ot	GB	2018	1	0	0	0	0	0	0	0	0	0	0	0	
14340615	Not	ot	GB	2018	1	0	0	0	0	0	0	0	0	0	0	0	
14340638	Not	md	CN	2018	1	0	0	0	0	0	0	0	0	0	0	0	
14341150	m	md	US	2018	0	1	0	0	0	1	0	0	0	0	0	0	
14341326	m	md	HK	2018	0	1	0	0	0	1	0	0	0	0	0	0	67
14341459	m	cn	GB	2018	1	0	0	0	0	0	0	0	0	0	0	0	70
14341780	f	md	DE	2018	0	1	0	0	0	1	0	0	0	0	0	0	65

14341988	m	ot	CA	2018	1	0	0	0	0	0	0	0	0	0	0	0	
14342382	m	cn	IN	2018	0	1	0	0	1	0	0	0	0	0	0	0	63
14342933	m	md	TR	2018	0	0	0	0	0	0	0	0	0	0	0	0	36
14342968	m	md	TR	2018	0	0	0	0	0	0	0	0	0	0	0	0	59
14342981	m	md	TR	2018	0	0	0	0	0	0	0	0	0	0	0	0	44
14343058	m	md	IE	2018	0	0	0	0	0	0	0	0	0	0	0	0	65
14343088	m	ot	DE	2018	0	0	0	0	0	0	0	0	0	0	0	0	56
14343092	m	ot	DE	2018	0	0	0	0	0	0	0	0	0	0	0	0	70
14343743	f	md	DE	2018	0	0	0	0	0	0	0	0	0	0	0	0	74
14343912	f	md	FR	2018	0	0	0	0	0	0	0	0	0	0	0	0	61
14343966	Not	cn	US	2018	1	0	0	0	0	0	0	0	0	0	0	0	
14344540	m	ot	DE	2018	0	0	0	0	0	0	0	0	0	0	0	0	56
14344751	m	md	DE	2018	0	0	0	0	0	0	0	0	0	0	0	0	78
14344859	m	md	JP	2018	0	1	0	1	0	0	0	0	0	0	0	0	58.9
14345123	m	md	ES	2018	0	1	0	0	0	1	0	0	0	0	0	0	56
14345231	f	ot	CA	2018	0	1	0	0	1	0	0	0	0	0	0	0	52
14345614	f	ot	US	2018	0	0	0	0	0	0	0	0	0	0	0	0	
14346109	f	md	JP	2018	1	0	0	0	0	0	0	0	0	0	0	0	75
14346415	m	ot	DE	2018	0	0	0	0	0	0	0	0	0	0	0	0	57
14346987	f	md	US	2018	0	1	1	0	0	0	0	0	0	0	0	0	63
14347585	m	md	JP	2018	0	1	0	0	1	0	0	0	0	0	0	1	78
14347970	f	cn	DE	2018	1	0	0	0	0	0	0	0	0	0	0	0	68
14348854	m	cn	US	2018	0	1	0	0	1	0	0	0	0	0	0	0	
14349001	f	md	DE	2018	0	0	0	0	0	0	0	0	0	0	0	0	74
14349076	f	ot	US	2018	0	1	0	0	1	0	0	0	0	0	0	0	57
14349090	f	md	US	2018	0	1	0	0	0	1	0	0	0	0	0	0	63
14349588	m	md	US	2018	0	1	0	0	0	1	0	0	0	0	0	0	65
14349616	m	cn	GB	2018	1	0	0	0	0	0	0	0	0	0	0	0	65
14349754	f	cn	IL	2018	0	1	1	0	0	0	0	0	0	0	0	0	58
14349780	f	cn	Not re	2018	0	0	0	0	0	0	0	0	0	0	0	0	
14349827	m	cn	Not re	2018	0	0	0	0	0	0	0	0	0	0	0	0	71
14350205	f	cn	DE	2018	0	0	0	0	0	0	0	0	0	0	0	0	56
14350656	f	md	FR	2018	0	1	1	0	0	0	0	0	0	0	0	0	72
14350980	m	cn	JP	2018	0	1	1	0	0	0	0	0	0	0	0	0	70

14350983	Not	cn	IL	2018	0	1	0	0	1	0	0	0	0	0	0	0	
14351058	f	md	DE	2018	0	0	0	0	0	0	0	0	0	0	0	0	74
14351577	m	md	TR	2018	0	0	0	0	0	0	0	0	0	0	0	0	63
14351676	m	md	CN	2018	0	1	0	1	0	0	0	0	0	0	0	0	49.4
14351939	f	md	FR	2018	1	0	0	0	0	0	0	0	0	0	0	0	86
14352674	m	ot	US	2018	0	0	0	0	0	0	0	0	0	0	0	0	73
14353417	f	md	US	2018	0	0	0	0	0	0	0	0	0	0	0	0	
14353459	m	md	CN	2018	0	0	0	0	0	0	0	0	0	0	0	0	66
14353604	m	ot	BE	2018	0	1	0	0	1	0	0	0	0	0	0	1	63
14353789	Not	cn	US	2018	0	1	0	0	1	0	0	0	0	0	0	0	74
14353980	m	md	US	2018	0	1	0	0	0	1	0	0	0	0	0	0	72
14354134	f	ph	JP	2018	1	0	0	0	0	0	0	0	0	0	0	0	87
14354346	f	ot	BE	2018	0	1	0	0	1	0	0	0	0	0	0	0	70
14354437	m	cn	IL	2018	0	1	0	0	1	0	0	0	0	0	0	0	75
14354560	m	md	DE	2018	0	0	0	0	0	0	0	0	0	0	0	0	68
14354838	m	cn	US	2018	0	1	1	0	0	0	0	0	0	0	0	0	73
14355169	f	md	DE	2018	0	0	0	0	0	0	0	0	0	0	0	0	57
14355191	f	md	KR	2018	1	0	0	0	0	0	0	0	0	0	0	0	70.9
14355661	f	ot	US	2018	0	0	0	0	0	0	0	0	0	0	0	0	60
14356004	Not	cn	GB	2018	1	0	0	0	0	0	0	0	0	0	0	0	
14356978	m	md	BE	2018	0	0	0	0	0	0	0	0	0	0	0	0	
14357398	f	ot	RO	2018	0	1	0	0	1	0	0	0	0	0	0	0	79
14357603	f	md	DE	2018	0	1	1	0	0	0	0	0	0	0	0	0	70
14361573	m	cn	DE	2018	0	1	1	0	0	0	0	0	0	0	0	0	61
14361589	f	md	US	2018	1	0	0	0	0	0	0	0	0	0	0	0	68
14361875	Not	md	IN	2018	0	0	0	0	0	0	0	0	0	0	0	0	
14361876	Not	md	IN	2018	0	0	0	0	0	0	0	0	0	0	0	0	
14361878	Not	md	IN	2018	0	0	0	0	0	0	0	0	0	0	0	0	
14361879	Not	md	IN	2018	0	0	0	0	0	0	0	0	0	0	0	0	
14361886	m	md	JP	2018	0	1	1	0	0	0	0	0	0	0	0	0	76
14362067	m	cn	GR	2018	0	0	0	0	0	0	0	0	0	0	0	0	
14362277	m	ot	DE	2018	0	0	0	0	0	0	0	0	0	0	0	0	70
14362625	m	ot	DE	2018	0	1	0	0	1	0	0	0	0	0	0	0	76
14362668	f	ot	GB	2018	0	1	0	1	0	0	0	0	0	0	0	0	58

14363790	f	md	CZ	2018	1	0	0	0	0	0	0	0	0	0	0	0	81
14363954	f	ot	PL	2018	0	1	0	0	1	0	0	0	0	0	0	0	42
14364364	m	ot	JP	2018	0	1	0	0	1	0	0	0	0	0	0	0	62
14365041	f	cn	US	2018	0	1	0	0	0	1	0	0	0	0	0	0	
14365055	Not	md	US	2018	0	1	0	0	0	1	0	0	0	0	0	0	
14365065	f	ot	US	2018	0	1	0	0	0	1	0	0	0	0	0	0	
14365092	f	cn	US	2018	0	1	0	0	0	1	0	0	0	0	0	0	76
14365111	Not	md	US	2018	0	1	0	0	0	1	0	0	0	0	0	0	
14365132	f	md	US	2018	0	1	0	0	0	1	0	0	0	0	0	0	70
14365146	m	ot	US	2018	0	1	0	0	0	1	0	0	0	0	0	1	
14365156	f	ot	US	2018	0	1	0	0	0	1	0	0	0	0	0	0	73
14365185	m	cn	US	2018	0	1	0	0	0	1	0	0	0	0	0	0	60
14366051	Not	ot	JP	2018	0	0	0	0	0	0	0	0	0	0	0	0	
14366067	f	ph	JP	2018	1	0	0	0	0	0	0	0	0	0	0	0	64
14366289	m	cn	US	2018	0	1	1	0	0	0	0	0	0	0	0	0	
14366615	f	md	GB	2018	0	0	0	0	0	0	0	0	0	0	0	0	49
14367598	m	ot	FR	2018	0	1	0	0	1	0	0	0	0	0	0	0	79
14368003	f	cn	GB	2018	1	0	0	0	0	0	0	0	0	0	0	0	59
14368504	m	ot	US	2018	0	1	0	0	1	0	0	0	0	0	0	0	70
14369176	f	Not repo	US	2018	1	0	0	0	0	0	0	0	0	0	0	0	58.1
14369397	Not	ot	JP	2018	1	0	0	0	0	0	0	0	0	0	0	0	
14369410	f	md	DE	2018	1	0	0	0	0	0	0	0	0	0	0	0	79
14369431	f	md	JP	2018	1	0	0	0	0	0	0	0	0	0	0	0	81.8
14369487	Not	ot	US	2018	0	0	0	0	0	0	0	0	0	0	0	0	
14369584	Not	md	US	2018	0	1	0	0	0	1	0	0	0	0	0	0	
14369720	m	cn	IL	2018	0	1	1	0	0	0	0	0	0	0	0	0	53
14369855	m	ot	TR	2018	0	1	0	0	1	0	0	0	0	0	0	0	52
14369957	f	cn	JP	2018	1	0	0	0	0	0	0	0	0	0	0	0	
14370378	m	cn	JP	2018	0	1	1	0	0	0	0	0	0	0	0	1	62
14370582	m	md	JP	2018	0	0	0	0	0	0	0	0	0	0	0	0	81
14370662	m	ot	US	2018	0	0	0	0	0	0	0	0	0	0	0	0	
14371267	f	md	US	2018	0	1	0	1	0	0	0	0	0	0	0	1	
14371487	m	md	JP	2018	1	0	0	0	0	0	0	0	0	0	0	0	69
14371936	m	ot	IT	2018	0	0	0	0	0	0	0	0	0	0	0	0	

14371980	m	md	US	2018	1	0	0	0	0	0	0	0	0	0	0	0	86
14372187	f	md	FR	2018	1	0	0	0	0	0	0	0	0	0	0	1	86
14372255	m	ot	EG	2018	0	0	0	0	0	0	0	0	0	0	0	0	57
14372959	m	md	KR	2018	0	1	0	0	1	0	0	0	0	0	0	0	71
14373548	f	md	NL	2018	0	0	0	0	0	0	0	0	0	0	0	0	65
14374038	Not	ot	US	2018	0	0	0	0	0	0	0	0	0	0	0	0	
14374039	Not	ot	US	2018	0	0	0	0	0	0	0	0	0	0	0	0	
14374090	Not	ot	US	2018	0	0	0	0	0	0	0	0	0	0	0	0	
14374091	Not	ot	US	2018	0	0	0	0	0	0	0	0	0	0	0	0	
14374094	Not	ot	US	2018	0	0	0	0	0	0	0	0	0	0	0	0	
14374164	f	cn	FR	2018	0	1	1	0	0	0	0	0	0	0	0	0	71
14374251	m	md	JP	2018	0	1	0	0	1	0	0	0	0	0	0	1	84
14374484	Not	ot	IN	2018	0	0	0	0	0	0	0	0	0	0	0	0	
14374870	Not	ot	US	2018	0	0	0	0	0	0	0	0	0	0	0	0	
14374871	m	ot	DE	2018	0	1	0	0	1	0	0	0	0	0	0	0	61
14374872	Not	ot	US	2018	0	0	0	0	0	0	0	0	0	0	0	0	
14374875	Not	ot	US	2018	0	0	0	0	0	0	0	0	0	0	0	0	
14375266	m	cn	Not re	2018	0	0	0	0	0	0	0	0	0	0	0	0	56
14375455	m	md	US	2018	0	1	1	0	0	0	0	0	0	0	0	0	70
14375456	m	cn	JP	2018	0	1	1	0	0	0	0	0	0	0	0	0	62
14375728	f	cn	US	2018	0	1	1	0	0	0	0	0	0	0	0	0	
14375908	f	md	JP	2018	0	0	0	0	0	0	0	0	0	0	0	0	45
14375999	m	cn	JP	2018	0	1	0	0	1	0	0	0	0	0	0	0	59
14377428	f	cn	BR	2018	1	0	0	0	0	0	0	0	0	0	0	0	76
14377618	f	ot	US	2018	0	0	0	0	0	0	0	0	0	0	0	0	69
14377725	m	cn	AT	2018	0	1	1	0	0	0	0	0	0	0	0	0	51
14377820	m	ot	JP	2018	0	1	0	0	1	0	0	0	0	0	0	1	76
14378018	m	md	US	2018	0	0	0	0	0	0	0	0	0	0	0	0	68
14378122	f	cn	US	2018	0	1	1	0	0	0	0	0	0	0	0	0	
14378207	f	md	TW	2018	1	0	0	0	0	0	0	0	0	0	0	0	75
14378437	m	md	JP	2018	0	1	0	0	1	0	0	0	0	0	0	0	81
14378439	m	md	DE	2018	0	0	0	0	0	0	0	0	0	0	0	0	73
14378598	f	md	BE	2018	0	1	0	0	1	0	0	0	0	0	0	0	59
14378766	Not	ot	US	2018	0	0	0	0	0	0	0	0	0	0	0	0	

14378768	Not	ot	US	2018	0	0	0	0	0	0	0	0	0	0	0	0	
14378769	Not	ot	US	2018	0	0	0	0	0	0	0	0	0	0	0	0	
14378770	Not	ot	US	2018	0	0	0	0	0	0	0	0	0	0	0	0	
14378961	m	md	JP	2018	0	1	0	0	1	0	0	0	0	0	0	0	48
14379062	f	cn	US	2018	0	1	1	0	0	0	0	0	0	0	0	0	
14379160	m	md	DE	2018	0	0	0	0	0	0	0	0	0	0	0	0	82
14379306	m	md	JP	2018	0	1	0	0	1	0	0	0	0	0	0	1	61
14379318	Not	ot	US	2018	0	0	0	0	0	0	0	0	0	0	0	0	
14379319	Not	ot	US	2018	0	0	0	0	0	0	0	0	0	0	0	0	
14379335	Not	ot	US	2018	0	0	0	0	0	0	0	0	0	0	0	0	
14379336	Not	ot	US	2018	0	0	0	0	0	0	0	0	0	0	0	0	
14379529	f	cn	US	2018	0	1	0	0	1	0	0	0	0	0	0	0	
14379580	Not	ot	GB	2018	0	0	0	0	0	0	0	0	0	0	0	0	
14379687	Not	ot	US	2018	0	0	0	0	0	0	0	0	0	0	0	0	
14379689	Not	ot	US	2018	0	0	0	0	0	0	0	0	0	0	0	0	
14379738	Not	ot	US	2018	0	0	0	0	0	0	0	0	0	0	0	0	
14379752	Not	ot	US	2018	0	0	0	0	0	0	0	0	0	0	0	0	
14379797	f	ot	US	2018	0	1	0	0	1	0	0	0	0	0	0	0	36
14380513	m	md	US	2018	0	0	0	0	0	0	0	0	0	0	0	0	60
14380677	f	cn	GB	2018	1	0	0	0	0	0	0	0	0	0	0	0	51
14381316	Not	ot	JP	2018	1	0	0	0	0	0	0	0	0	0	0	0	
14381363	Not	ot	JP	2018	1	0	0	0	0	0	0	0	0	0	0	0	
14381388	m	cn	US	2018	0	1	1	0	0	0	0	0	0	0	0	0	
14381397	Not	ot	JP	2018	1	0	0	0	0	0	0	0	0	0	0	0	
14382473	f	ph	US	2018	0	0	0	0	0	0	0	0	0	0	0	0	60
14382953	f	md	DE	2018	0	0	0	0	0	0	0	0	0	0	0	0	45
14382989	f	md	KR	2018	0	1	0	0	1	0	0	0	0	0	0	0	64
14383082	m	cn	CN	2018	0	0	0	0	0	0	0	0	0	0	0	0	64
14383347	m	cn	JP	2018	0	1	1	0	0	0	0	0	0	0	0	0	63
14383445	m	md	JP	2018	0	0	0	0	0	0	0	0	0	0	0	0	
14383739	m	cn	GR	2018	0	1	1	0	0	0	0	0	0	0	0	0	59
14384509	m	cn	IL	2018	0	1	1	0	0	0	0	0	0	0	0	0	
14384613	f	cn	US	2018	1	0	0	0	0	0	0	0	0	0	0	0	80
14384659	m	cn	KR	2018	0	1	1	0	0	0	0	0	0	0	0	0	

14384752	f	md	FR	2018	0	1	1	0	0	0	0	0	0	0	0	0	59
14384914	m	md	US	2018	0	0	0	0	0	0	0	0	0	0	0	0	
14385722	f	md	US	2018	0	0	0	0	0	0	0	0	0	0	0	0	76
14385848	m	md	JP	2018	0	1	0	0	1	0	0	0	0	0	0	0	31
14386022	m	cn	US	2018	0	1	1	0	0	0	0	0	0	0	0	0	
14386513	f	cn	US	2018	0	0	0	0	0	0	0	0	0	0	0	0	49
14386637	m	ot	US	2018	0	0	0	0	0	0	0	0	0	0	0	0	
14387249	m	cn	IL	2018	0	1	1	0	0	0	0	0	0	0	0	0	
14387292	m	md	US	2018	0	0	0	0	0	0	0	0	0	0	0	0	
14389692	f	cn	US	2018	1	0	0	0	0	0	0	0	0	0	0	0	62
14389890	m	md	US	2018	0	1	0	1	0	0	0	0	0	0	0	0	62.9
14390647	Not	ot	JP	2018	0	0	0	0	0	0	0	0	0	0	0	0	
14390650	Not	ot	US	2018	0	0	0	0	0	0	0	0	0	0	0	0	
14390934	Not	ot	US	2018	0	0	0	0	0	0	0	0	0	0	0	0	
14391265	m	cn	FR	2018	0	1	0	0	1	0	0	0	0	0	0	0	71
14391414	m	md	JP	2018	0	1	0	0	1	0	0	0	0	0	0	1	65
14391486	f	md	FR	2018	1	0	0	0	0	0	0	0	0	0	0	0	89.4
14391555	m	ot	IN	2018	0	0	0	0	0	0	0	0	0	0	0	0	46
14391582	f	ot	IN	2018	0	0	0	0	0	0	0	0	0	0	0	0	56
14391607	m	ot	CN	2018	0	1	0	0	1	0	0	0	0	0	0	0	56
14391636	m	cn	GR	2018	0	0	0	0	0	0	0	0	0	0	0	0	
14392282	m	ot	CA	2018	0	1	0	0	1	0	0	0	0	0	0	0	68
14392401	f	ph	JP	2018	0	0	0	0	0	0	0	0	0	0	0	0	44
14392437	m	md	MX	2018	0	0	0	0	0	0	0	0	0	0	0	0	56
14392473	f	cn	US	2018	0	0	0	0	0	0	0	0	0	0	0	0	65
14392637	m	cn	JP	2018	0	0	0	0	0	0	0	0	0	0	0	0	42
14392646	m	ot	GB	2018	0	1	0	0	1	0	0	0	0	0	0	0	80
14392801	m	cn	GR	2018	0	1	1	0	0	0	0	0	0	0	0	0	65
14393426	m	md	IE	2018	0	0	0	0	0	0	0	0	0	0	0	0	76
14393564	f	ph	US	2018	0	1	1	0	0	0	0	0	0	0	0	0	57
14395104	f	cn	DE	2018	0	1	1	0	0	0	0	0	0	0	0	0	60
14395131	m	md	JP	2018	0	1	0	0	1	0	0	0	0	0	0	0	70
14399242	f	ot	DE	2018	0	1	0	0	1	0	0	0	0	0	0	0	80
14400369	m	cn	JP	2018	0	1	1	0	0	0	0	0	0	0	0	0	80

14400567	m	md	JP	2018	0	1	0	0	1	0	0	0	0	0	0	0	70
14401574	f	Not repo	US	2018	1	0	0	0	0	0	0	0	0	0	0	0	87.4
14402084	f	cn	ES	2018	0	1	1	0	0	0	0	0	0	0	0	0	62
14402086	m	ot	CH	2018	0	1	1	0	0	0	0	0	0	0	0	1	71
14402141	m	cn	GB	2018	1	0	0	0	0	0	0	0	0	0	0	0	64
14402252	f	md	NL	2018	0	0	0	0	0	0	0	0	0	0	0	0	76
14402257	f	cn	US	2018	0	1	1	0	0	0	0	0	0	0	0	0	
14402548	f	md	DE	2018	0	0	0	0	0	0	0	0	0	0	0	0	67
14402923	m	ot	DE	2018	0	1	0	0	1	0	0	0	0	0	0	0	74
14402994	f	md	DE	2018	0	0	0	0	0	0	0	0	0	0	0	0	74
14403101	f	cn	CN	2018	1	0	0	0	0	0	0	0	0	0	0	0	86
14404707	m	ot	US	2018	0	1	0	0	1	0	0	0	0	0	0	0	
14405245	m	ot	DE	2018	0	1	0	0	1	0	0	0	0	0	0	0	73
14405268	f	ot	US	2018	0	1	0	0	1	0	0	0	0	0	0	0	
14405383	m	cn	CN	2018	0	0	0	0	0	0	0	0	0	0	0	0	
14405404	m	ot	JP	2018	0	1	0	0	1	0	0	0	0	0	0	0	65
14405507	m	cn	JP	2018	0	1	1	0	0	0	0	0	0	0	0	0	82
14405577	Not	md	US	2018	0	0	0	0	0	0	0	0	0	0	0	0	
14405837	f	Not repo	MY	2018	1	0	0	0	0	0	0	0	0	0	0	0	69.3
14405861	m	cn	US	2018	0	1	0	0	1	0	0	0	0	0	0	0	64
14405921	m	ot	ES	2018	0	1	0	0	1	0	0	0	0	0	0	0	61
14406268	f	cn	JP	2018	0	1	1	0	0	0	0	0	0	0	0	0	60
14406440	m	ot	DE	2018	0	1	0	0	1	0	0	0	0	0	0	1	85
14406686	m	ot	JP	2018	0	1	1	0	0	0	0	0	0	0	0	1	86
14408918	m	ot	US	2018	0	1	0	0	1	0	0	0	0	0	0	0	80
14408988	f	cn	FR	2018	0	1	1	0	0	0	0	0	0	0	0	0	83
14409048	m	cn	JP	2018	0	1	1	0	0	0	0	0	0	0	0	0	66
14409272	m	cn	CN	2018	1	0	0	0	0	0	0	0	0	0	0	0	
14409487	f	md	CN	2018	1	0	0	0	0	0	0	0	0	0	0	0	62
14409579	Not	md	JP	2018	1	0	0	0	0	0	0	0	0	0	0	0	
14410288	f	md	IN	2018	0	1	0	0	1	0	0	0	0	0	0	0	67
14410317	f	md	IN	2018	0	1	0	0	1	0	0	0	0	0	0	0	73
14410699	m	md	ES	2018	0	0	0	0	0	0	0	0	0	0	0	0	55
14411135	f	ot	US	2018	0	1	1	0	0	0	0	0	0	0	0	0	70

14411357	f	md	JP	2018	1	0	0	0	0	0	0	0	0	0	0	0	55.8
14411528	f	cn	GB	2018	1	0	0	0	0	0	0	0	0	0	0	0	
14412268	f	md	CN	2018	1	0	0	0	0	0	0	0	0	0	0	0	50.8
14412391	m	md	IE	2018	0	0	0	0	0	0	0	0	0	0	0	0	65
14412673	m	ot	DE	2018	0	0	0	0	0	0	0	0	0	0	0	0	74
14412719	Not	md	JP	2018	1	0	0	0	0	0	0	0	0	0	0	0	90
14413302	Not	ot	US	2018	0	0	0	0	0	0	0	0	0	0	0	0	
14413306	Not	ot	US	2018	0	0	0	0	0	0	0	0	0	0	0	0	
14413308	Not	ot	US	2018	0	1	1	0	0	0	0	0	0	0	0	0	
14413377	Not	ot	US	2018	0	0	0	0	0	0	0	0	0	0	0	0	
14413556	m	ot	CA	2018	0	1	0	0	1	0	0	0	0	0	0	0	62
14413651	m	md	US	2018	0	1	1	0	0	0	0	0	0	0	0	0	56
14413744	m	md	DE	2018	0	0	0	0	0	0	0	0	0	0	0	0	71
14413749	m	cn	PH	2018	0	1	1	0	0	0	0	0	0	0	0	0	77
14415285	m	ot	JP	2018	0	0	0	0	0	0	0	0	0	0	0	0	62
14415431	m	ot	IT	2018	0	0	0	0	0	0	0	0	0	0	0	0	70
14416114	f	ot	JP	2018	0	0	0	0	0	0	0	0	0	0	0	0	70
14416593	m	md	ES	2018	0	1	0	0	0	1	0	0	0	0	0	0	64
14416721	f	cn	GB	2018	1	0	0	0	0	0	0	0	0	0	0	0	76
14416875	f	md	CH	2018	1	0	0	0	0	0	0	0	0	0	0	0	
14417459	m	md	TR	2018	0	0	0	0	0	0	0	0	0	0	0	0	57
14417916	Not	cn	GB	2018	1	0	0	0	0	0	0	0	0	0	0	0	
14418947	Not	cn	GB	2018	1	0	0	0	0	0	0	0	0	0	0	0	
14419111	m	md	DE	2018	1	0	0	0	0	0	0	0	0	0	0	0	81
14419435	Not	cn	GB	2018	1	0	0	0	0	0	0	0	0	0	0	0	
14419436	Not	cn	GB	2018	1	0	0	0	0	0	0	0	0	0	0	0	
14419481	f	cn	GB	2018	1	0	0	0	0	0	0	0	0	0	0	0	
14420086	Not	ot	JP	2018	1	0	0	0	0	0	0	0	0	0	0	0	
14420856	m	md	CZ	2018	1	0	0	0	0	0	0	0	0	0	0	0	67
14422560	m	md	GB	2018	0	1	0	0	1	0	0	0	0	0	0	0	65
14422616	m	md	JP	2018	0	1	0	0	1	0	0	0	0	0	0	0	75
14423470	m	ot	DE	2018	0	0	0	0	0	0	0	0	0	0	0	0	74
14423655	m	ot	US	2018	0	1	1	0	0	0	0	0	0	0	0	0	78
14424045	f	ot	DE	2018	0	0	0	0	0	0	0	0	0	0	0	0	67

14424205	f	md	US	2018	1	0	0	0	0	0	0	0	0	0	0	0	52
14424211	m	ot	US	2018	0	1	1	0	0	0	0	0	0	0	0	0	
14425211	m	md	ES	2018	0	1	0	0	0	1	0	0	0	0	0	0	71
14425219	f	md	JP	2018	0	1	0	0	1	0	0	0	0	0	0	0	64
14425534	f	ot	JP	2018	1	0	0	0	0	0	0	0	0	0	0	1	88
14426067	m	ot	FR	2018	0	1	0	0	1	0	0	0	0	0	0	0	58
14426129	m	md	IT	2018	0	0	0	0	0	0	0	0	0	0	0	0	70
14426166	f	md	JP	2018	0	1	0	0	1	0	0	0	0	0	0	0	48
14426534	m	md	FR	2018	0	0	0	0	0	0	0	0	0	0	0	0	65
14430385	m	ot	ES	2018	0	1	0	0	1	0	0	0	0	0	0	0	64
14430800	Not	ot	CN	2018	0	0	0	0	0	0	0	0	0	0	0	0	
14430932	Not	ot	US	2018	0	0	0	0	0	0	0	0	0	0	0	0	
14431344	m	cn	US	2018	0	0	0	0	0	0	0	0	0	0	0	0	68
14431513	f	cn	US	2018	0	1	1	0	0	0	0	0	0	0	0	0	
14431615	m	ot	GB	2018	0	1	0	0	1	0	0	0	0	0	0	0	67
14432185	m	cn	GB	2018	1	0	0	0	0	0	0	0	0	0	0	0	78
14432220	f	cn	US	2018	0	1	1	0	0	0	0	0	0	0	0	0	
14432806	m	md	CN	2018	0	1	0	1	0	0	0	0	0	0	0	0	62.1
14433129	m	md	JP	2018	0	1	0	0	1	0	0	0	0	0	0	0	70
14433136	m	md	TR	2018	0	0	0	0	0	0	0	0	0	0	0	0	60
14433208	f	cn	GB	2018	1	0	0	0	0	0	0	0	0	0	0	0	52
14433371	f	ph	US	2018	1	0	0	0	0	0	0	0	0	0	0	0	71
14433855	m	md	SK	2018	0	0	0	0	0	0	0	0	0	0	0	0	63
14434325	f	md	DE	2018	0	0	0	0	0	0	0	0	0	0	0	0	58
14434626	m	ot	JP	2018	0	1	0	0	1	0	0	0	0	0	0	0	65
14434660	m	ot	GB	2018	0	1	0	0	1	0	0	0	0	0	0	0	77
14434666	m	md	JP	2018	0	1	0	0	1	0	0	0	0	0	0	0	75
14435001	f	ot	DE	2018	0	1	0	0	1	0	0	0	0	0	0	0	75
14435307	m	md	US	2018	0	1	0	0	1	0	0	0	0	0	0	0	65
14435698	m	md	JP	2018	0	1	0	0	1	0	0	0	0	0	0	0	74
14436081	m	cn	US	2018	0	0	0	0	0	0	0	0	0	0	0	0	55
14436875	f	cn	ES	2018	0	1	1	0	0	0	0	0	0	0	0	0	76
14437071	m	cn	US	2018	0	1	0	0	1	0	0	0	0	0	0	0	69
14437074	f	cn	US	2018	0	1	1	0	0	0	0	0	0	0	0	0	

14437077	m	cn	JP	2018	0	1	1	0	0	0	0	0	0	0	0	0	60
14437671	Not	md	JP	2018	0	0	0	0	0	0	0	0	0	0	0	0	81
14438196	f	md	IT	2018	1	0	0	0	0	0	0	0	0	0	0	0	76.7
14438422	f	cn	JP	2018	0	1	1	0	0	0	0	0	0	0	0	0	80
14438499	f	cn	GB	2018	1	0	0	0	0	0	0	0	0	0	0	0	
14438638	f	md	DE	2018	0	0	0	0	0	0	0	0	0	0	0	0	67
14438667	m	cn	GB	2018	1	0	0	0	0	0	0	0	0	0	0	0	
14438816	f	ot	US	2018	0	1	1	0	0	0	0	0	0	0	0	0	88
14439045	m	md	US	2018	0	1	0	0	1	0	0	0	0	0	0	0	74
14439716	m	ot	IT	2018	0	0	0	0	0	0	0	0	0	0	0	0	70
14440875	m	md	JP	2018	0	1	0	0	1	0	0	0	0	0	0	1	46
14441235	Not	md	TR	2018	0	0	0	0	0	0	0	0	0	0	0	0	
14441300	f	ot	JP	2018	0	0	0	0	0	0	0	0	0	0	0	0	75
14441375	f	ot	US	2018	1	0	0	0	0	0	0	0	0	0	0	0	70.2
14441531	Not	md	TH	2018	1	0	0	0	0	0	0	0	0	0	0	0	72.6
14444576	Not	md	DE	2018	0	0	0	0	0	0	0	0	0	0	0	0	
14444606	f	md	TH	2018	1	0	0	0	0	0	0	0	0	0	0	0	44
14444620	Not	ph	JP	2018	0	0	0	0	0	0	0	0	0	0	0	0	
14444643	m	md	JP	2018	1	0	0	0	0	0	0	0	0	0	0	0	64
14445280	m	cn	IN	2018	0	1	0	0	1	0	0	0	0	0	0	0	50
14445308	f	cn	IN	2018	0	1	0	0	1	0	0	0	0	0	0	0	62
14445363	Not	md	DE	2018	0	0	0	0	0	0	0	0	0	0	0	0	
14445608	f	md	JP	2018	1	0	0	0	0	0	0	0	0	0	0	0	77
14445768	m	ot	JP	2018	0	1	0	0	1	0	0	0	0	0	0	0	52
14445769	f	ot	NL	2018	0	0	0	0	0	0	0	0	0	0	0	0	48
14446064	m	md	JP	2018	0	1	0	1	0	0	0	0	0	0	0	0	76
14446071	f	ot	DE	2018	0	0	0	0	0	0	0	0	0	0	0	0	57
14446236	m	md	JP	2018	0	1	0	0	1	0	0	0	0	0	0	0	69
14446297	m	md	DE	2018	0	0	0	0	0	0	0	0	0	0	0	0	59
14446560	f	Not repo	US	2018	0	1	0	1	0	0	0	0	0	0	0	0	71.1
14446856	f	ot	DE	2018	1	0	0	0	0	0	0	0	0	0	0	0	57
14447088	f	cn	AR	2018	0	0	0	0	0	0	0	0	0	0	0	0	68
14447887	f	md	DE	2018	0	0	0	0	0	0	0	0	0	0	0	0	67
14448230	m	cn	US	2018	1	0	0	0	0	0	0	0	0	0	0	0	66

14449063	f	md	US	2018	0	1	1	0	0	0	0	0	0	0	0	0	70
14449483	f	cn	US	2018	0	1	0	0	1	0	0	0	0	0	0	0	64
14449540	f	ot	DE	2018	0	1	0	0	1	0	0	0	0	0	0	0	79
14449544	m	ot	DE	2018	0	1	0	0	1	0	0	0	0	0	0	0	77
14450200	f	md	DE	2018	0	1	0	0	0	1	0	0	0	0	0	1	61
14450305	f	ot	JP	2018	0	1	0	0	1	0	0	0	0	0	0	1	75
14450324	Not	ot	US	2018	0	1	1	0	0	0	0	0	0	0	0	0	
14450329	Not	ot	US	2018	0	1	1	0	0	0	0	0	0	0	0	0	
14450767	Not	md	JP	2018	0	0	0	0	0	0	0	0	0	0	0	0	80
14450797	Not	ot	US	2018	0	0	0	0	0	0	0	0	0	0	0	0	
14450801	Not	ot	US	2018	0	0	0	0	0	0	0	0	0	0	0	0	
14450890	Not	md	JP	2018	0	0	0	0	0	0	0	0	0	0	0	0	75
14451319	Not	md	JP	2018	0	0	0	0	0	0	0	0	0	0	0	0	80
14451627	Not	md	US	2018	0	0	0	0	0	0	0	0	0	0	0	0	
14452174	m	md	US	2018	0	0	0	0	0	0	0	0	0	0	0	0	
14452280	f	md	JP	2018	0	0	0	0	0	0	0	0	0	0	0	0	45
14452354	Not	md	JP	2018	0	0	0	0	0	0	0	0	0	0	0	0	80
14452755	Not	md	JP	2018	0	0	0	0	0	0	0	0	0	0	0	0	80
14452862	m	cn	JP	2018	0	1	1	0	0	0	0	0	0	0	0	1	55
14453085	m	md	JP	2018	0	1	0	0	0	1	0	0	0	0	0	0	61
14453148	f	ot	DE	2018	0	1	0	0	0	1	0	0	0	0	0	0	66
14453536	Not	cn	GB	2018	1	0	0	0	0	0	0	0	0	0	0	0	
14453894	m	md	JP	2018	0	1	0	1	0	0	0	0	0	0	0	0	65
14454083	f	md	JP	2018	0	1	0	0	1	0	0	0	0	0	0	0	61
14454406	m	ot	DE	2018	0	0	0	0	0	0	0	0	0	0	0	0	57
14454705	f	ot	US	2018	0	1	1	0	0	0	0	0	0	0	0	0	
14455055	f	md	FR	2018	1	0	0	0	0	0	0	0	0	0	0	0	49
14455286	f	ot	US	2018	0	1	0	0	1	0	0	0	0	0	0	0	65
14455315	m	ot	US	2018	0	1	0	0	1	0	0	0	0	0	0	0	68
14455915	m	md	JP	2018	0	0	0	0	0	0	0	0	0	0	0	0	80
14455958	Not	md	JP	2018	0	0	0	0	0	0	0	0	0	0	0	0	76
14456006	Not	md	JP	2018	0	0	0	0	0	0	0	0	0	0	0	0	76
14456036	Not	md	JP	2018	0	0	0	0	0	0	0	0	0	0	0	0	84
14456242	Not	ot	US	2018	0	0	0	0	0	0	0	0	0	0	0	0	

14456243	Not	ot	US	2018	0	0	0	0	0	0	0	0	0	0	0	0	
14456244	Not	ot	US	2018	0	0	0	0	0	0	0	0	0	0	0	0	
14456246	Not	md	JP	2018	0	0	0	0	0	0	0	0	0	0	0	0	78
14456271	f	md	JP	2018	0	1	0	0	1	0	0	0	0	0	0	0	57
14456384	m	md	HU	2018	0	0	0	0	0	0	0	0	0	0	0	0	53
14456421	m	md	JP	2018	0	0	0	0	0	0	0	0	0	0	0	0	87
14456448	Not	md	JP	2018	0	0	0	0	0	0	0	0	0	0	0	0	77
14456465	Not	md	JP	2018	0	0	0	0	0	0	0	0	0	0	0	0	78
14456528	f	md	JP	2018	0	1	0	0	1	0	0	0	0	0	0	0	62
14456534	m	md	JP	2018	1	0	0	0	0	0	0	0	0	0	0	1	78
14456567	Not	ot	US	2018	0	0	0	0	0	0	0	0	0	0	0	0	
14456733	Not	ot	US	2018	0	0	0	0	0	0	0	0	0	0	0	0	
14456734	Not	ot	US	2018	0	0	0	0	0	0	0	0	0	0	0	0	
14456735	Not	ot	US	2018	0	0	0	0	0	0	0	0	0	0	0	0	
14456736	Not	ot	US	2018	0	0	0	0	0	0	0	0	0	0	0	0	
14456750	Not	ot	US	2018	0	0	0	0	0	0	0	0	0	0	0	0	
14457034	m	md	JP	2018	0	0	0	0	0	0	0	0	0	0	0	0	81
14457109	f	md	JP	2018	0	0	0	0	0	0	0	0	0	0	0	0	78
14457370	f	md	JP	2018	0	0	0	0	0	0	0	0	0	0	0	0	76
14457371	Not	md	JP	2018	0	0	0	0	0	0	0	0	0	0	0	0	81
14457514	f	md	JP	2018	0	0	0	0	0	0	0	0	0	0	0	0	77
14457720	Not	md	JP	2018	1	0	0	0	0	0	0	0	0	0	0	0	90
14457792	m	md	JP	2018	0	0	0	0	0	0	0	0	0	0	0	0	82
14457793	m	md	JP	2018	0	0	0	0	0	0	0	0	0	0	0	0	78
14457803	m	ot	US	2018	0	1	0	0	1	0	0	0	0	0	0	1	43
14457816	m	md	JP	2018	0	0	0	0	0	0	0	0	0	0	0	0	79
14457931	f	cn	JP	2018	0	1	1	0	0	0	0	0	0	0	0	0	69
14458434	m	Not repo	KR	2018	1	0	0	0	0	0	0	0	0	0	0	0	60
14458685	m	md	JP	2018	0	1	0	0	1	0	0	0	0	0	0	0	76
14458859	m	cn	US	2018	0	0	0	0	0	0	1	1	0	0	0	0	55
14459729	f	cn	US	2018	1	0	0	0	0	0	0	0	0	0	0	1	55.9
14460472	f	md	TR	2018	0	0	0	0	0	0	0	0	0	0	0	0	56
14460590	m	cn	US	2018	0	1	1	0	0	0	0	0	0	0	0	0	
14461335	m	ot	DE	2018	0	0	0	0	0	0	0	0	0	0	0	0	68

14462066	f	ot	US	2018	0	0	0	0	0	0	0	0	0	0	0	0	67
14462251	f	cn	US	2018	0	1	1	0	0	0	0	0	0	0	0	0	77
14462990	f	md	AT	2018	0	0	0	0	0	0	0	0	0	0	0	0	55
14463318	f	cn	US	2018	0	1	1	0	0	0	0	0	0	0	0	0	
14465486	Not	ot	US	2018	0	0	0	0	0	0	0	0	0	0	0	0	
14465487	Not	ot	US	2018	0	0	0	0	0	0	0	0	0	0	0	0	
14465488	Not	ot	US	2018	0	0	0	0	0	0	0	0	0	0	0	0	
14465490	Not	ot	US	2018	0	0	0	0	0	0	0	0	0	0	0	0	
14465491	Not	ot	US	2018	0	0	0	0	0	0	0	0	0	0	0	0	
14465492	Not	ot	US	2018	0	0	0	0	0	0	0	0	0	0	0	0	
14465493	Not	ot	US	2018	0	0	0	0	0	0	0	0	0	0	0	0	
14465497	Not	ot	US	2018	0	0	0	0	0	0	0	0	0	0	0	0	
14465498	Not	ot	US	2018	0	0	0	0	0	0	0	0	0	0	0	0	
14465564	Not	ot	US	2018	0	0	0	0	0	0	0	0	0	0	0	0	
14465565	Not	ot	US	2018	0	0	0	0	0	0	0	0	0	0	0	0	
14465568	m	md	AU	2018	0	1	0	1	0	0	0	0	0	0	0	1	55
14465598	f	md	JP	2018	1	0	0	0	0	0	0	0	0	0	0	1	86.9
14465603	Not	ot	US	2018	0	0	0	0	0	0	0	0	0	0	0	0	
14465604	Not	ot	US	2018	0	0	0	0	0	0	0	0	0	0	0	0	
14465605	Not	ot	US	2018	0	0	0	0	0	0	0	0	0	0	0	0	
14465606	Not	ot	US	2018	0	0	0	0	0	0	0	0	0	0	0	0	
14465631	Not	ot	US	2018	0	0	0	0	0	0	0	0	0	0	0	0	
14465632	Not	ot	US	2018	0	0	0	0	0	0	0	0	0	0	0	0	
14465633	Not	ot	US	2018	0	0	0	0	0	0	0	0	0	0	0	0	
14465634	Not	ot	US	2018	0	0	0	0	0	0	0	0	0	0	0	0	
14465663	Not	ot	US	2018	0	0	0	0	0	0	0	0	0	0	0	0	
14465664	Not	ot	US	2018	0	0	0	0	0	0	0	0	0	0	0	0	
14465665	Not	ot	US	2018	0	0	0	0	0	0	0	0	0	0	0	0	
14465666	Not	ot	US	2018	0	0	0	0	0	0	0	0	0	0	0	0	
14465667	Not	ot	US	2018	0	0	0	0	0	0	0	0	0	0	0	0	
14465668	Not	ot	US	2018	0	0	0	0	0	0	0	0	0	0	0	0	
14465671	Not	ot	US	2018	0	0	0	0	0	0	0	0	0	0	0	0	
14465672	Not	ot	US	2018	0	0	0	0	0	0	0	0	0	0	0	0	
14465673	Not	ot	US	2018	0	0	0	0	0	0	0	0	0	0	0	0	

14465677	Not	ot	US	2018	0	0	0	0	0	0	0	0	0	0	0	0	
14465678	Not	ot	US	2018	0	0	0	0	0	0	0	0	0	0	0	0	
14465679	Not	ot	US	2018	0	0	0	0	0	0	0	0	0	0	0	0	
14465680	Not	ot	US	2018	0	0	0	0	0	0	0	0	0	0	0	0	
14465681	Not	ot	US	2018	0	0	0	0	0	0	0	0	0	0	0	0	
14466072	m	ot	US	2018	0	0	0	0	0	0	0	0	0	0	0	0	77
14466358	Not	ot	US	2018	0	0	0	0	0	0	0	0	0	0	0	0	
14466362	Not	ot	US	2018	0	0	0	0	0	0	0	0	0	0	0	0	
14466449	Not	ot	US	2018	0	0	0	0	0	0	0	0	0	0	0	0	
14466452	m	md	JP	2018	0	1	0	0	1	0	0	0	0	0	0	0	67
14467582	Not	ot	US	2018	0	0	0	0	0	0	0	0	0	0	0	0	
14467590	Not	ot	US	2018	0	0	0	0	0	0	0	0	0	0	0	0	
14467591	Not	ot	US	2018	0	0	0	0	0	0	0	0	0	0	0	0	
14467598	m	md	JP	2018	0	1	0	0	1	0	0	0	0	0	0	0	59
14467783	f	cn	GB	2018	1	0	0	0	0	0	0	0	0	0	0	0	74
14468034	Not	ot	US	2018	0	0	0	0	0	0	0	0	0	0	0	0	
14469369	m	ot	JP	2018	1	0	0	0	0	0	0	0	0	0	0	0	74
14469728	Not	md	JP	2018	1	0	0	0	0	0	0	0	0	0	0	1	
14470459	m	ot	JP	2018	0	0	0	0	0	0	0	0	0	0	0	0	66
14470509	f	ph	US	2018	0	0	0	0	0	0	1	1	0	0	0	0	70
14472481	Not	ot	US	2018	0	0	0	0	0	0	0	0	0	0	0	0	
14472482	Not	ot	US	2018	0	0	0	0	0	0	0	0	0	0	0	0	
14472502	Not	ot	US	2018	0	0	0	0	0	0	0	0	0	0	0	0	
14472503	Not	ot	US	2018	0	0	0	0	0	0	0	0	0	0	0	0	
14472504	Not	ot	US	2018	0	0	0	0	0	0	0	0	0	0	0	0	
14472507	Not	ot	US	2018	0	0	0	0	0	0	0	0	0	0	0	0	
14472508	Not	ot	US	2018	0	0	0	0	0	0	0	0	0	0	0	0	
14472517	Not	ot	US	2018	0	0	0	0	0	0	0	0	0	0	0	0	
14472656	m	ot	GR	2018	0	1	0	0	1	0	0	0	0	0	0	0	48
14472864	f	md	CN	2018	1	0	0	0	0	0	0	0	0	0	0	0	64.7
14472991	m	cn	US	2018	0	1	1	0	0	0	0	0	0	0	0	0	
14473163	Not	ot	JP	2018	0	0	0	0	0	0	0	0	0	0	0	0	
14473404	m	ot	JP	2018	0	1	0	0	1	0	0	0	0	0	0	0	67
14473554	m	md	JP	2018	0	1	0	0	1	0	0	0	0	0	0	0	60

14473905	m	md	JP	2018	0	0	0	0	0	0	0	0	0	0	0	0	
14473928	f	md	JP	2018	1	0	0	0	0	0	0	0	0	0	0	0	68
14473940	m	Not repo	US	2018	1	0	0	0	0	0	0	0	0	0	0	0	
14474025	m	ot	IL	2018	0	1	0	0	0	1	0	0	0	0	0	0	80
14474036	m	ot	IL	2018	0	1	0	0	0	1	0	0	0	0	0	0	
14474384	m	ph	US	2018	0	1	0	0	1	0	0	0	0	0	0	0	61
14474925	m	cn	JP	2018	0	1	1	0	0	0	0	0	0	0	0	0	79
14474963	m	cn	IN	2018	0	0	0	0	0	0	0	0	0	0	0	0	52
14475409	f	md	JP	2018	1	0	0	0	0	0	0	0	0	0	0	0	84
14475418	m	md	JP	2018	1	0	0	0	0	0	0	0	0	0	0	1	82.1
14475690	f	cn	JP	2018	0	1	1	0	0	0	0	0	0	0	0	0	85
14476143	f	md	DE	2018	0	0	0	0	0	0	0	0	0	0	0	0	74
14477255	f	md	CH	2018	0	0	0	0	0	0	0	0	0	0	0	0	58
14478052	m	md	JP	2018	0	1	0	0	1	0	0	0	0	0	0	0	75
14478215	Not	ot	JP	2018	0	0	0	0	0	0	0	0	0	0	0	0	
14479060	m	ph	US	2018	0	1	0	1	0	0	0	0	0	0	0	0	
14479073	f	md	DE	2018	0	1	0	0	1	0	0	0	0	0	0	0	
14479113	f	md	DE	2018	0	0	0	0	0	0	0	0	0	0	0	0	65
14479265	m	md	JP	2018	1	0	0	0	0	0	0	0	0	0	0	0	74
14479334	m	md	CZ	2018	1	0	0	0	0	0	0	0	0	0	0	0	
14479393	f	md	US	2018	1	0	0	0	0	0	0	0	0	0	0	0	73
14479395	f	cn	US	2018	1	0	0	0	0	0	0	0	0	0	0	0	
14479471	f	Not repo	MY	2018	1	0	0	0	0	0	0	0	0	0	0	0	66.6
14479913	m	md	US	2018	0	1	0	1	0	0	0	0	0	0	0	0	82.5
14479971	m	cn	IN	2018	0	1	1	0	0	0	0	0	0	0	0	0	
14480093	m	md	JP	2018	1	0	0	0	0	0	0	0	0	0	0	0	71.4
14480147	m	ot	US	2018	0	0	0	0	0	0	0	0	0	0	0	0	65
14480321	m	ot	DE	2018	0	0	0	0	0	0	0	0	0	0	0	0	67
14480394	f	md	JP	2018	0	1	0	0	1	0	0	0	0	0	0	0	66
14480395	m	md	JP	2018	0	1	0	0	1	0	0	0	0	0	0	0	63
14480628	m	cn	JP	2018	0	1	1	0	0	0	0	0	0	0	0	0	70
14480874	f	md	AU	2018	0	1	0	0	1	0	0	0	0	0	0	0	67
14481062	m	md	NZ	2018	0	0	0	0	0	0	0	0	0	0	0	0	62
14481173	f	md	US	2018	0	1	0	1	0	0	0	0	0	0	0	0	

14481337	m	cn	JP	2018	0	0	0	0	0	0	0	0	0	0	0	0	75
14481361	m	cn	JP	2018	0	0	0	0	0	0	0	0	0	0	0	0	65
14481782	m	md	JP	2018	0	1	0	0	1	0	0	0	0	0	0	0	70
14481839	f	cn	US	2018	1	0	0	0	0	0	0	0	0	0	0	0	63
14482019	f	md	SK	2018	1	0	0	0	0	0	0	0	0	0	0	0	
14482718	f	md	DE	2018	0	0	0	0	0	0	0	0	0	0	0	0	57
14482785	m	cn	US	2018	0	1	0	0	1	0	0	0	0	0	0	0	
14482971	m	md	DE	2018	0	1	0	0	1	0	0	0	0	0	0	0	81
14482988	m	md	JP	2018	0	1	0	0	1	0	0	0	0	0	0	0	75
14483062	f	md	JP	2018	0	1	0	0	1	0	0	0	0	0	0	0	69
14483193	f	md	DE	2018	0	1	0	0	1	0	0	0	0	0	0	0	
14483514	f	cn	US	2018	1	0	0	0	0	0	0	0	0	0	0	0	73
14483540	m	ot	FR	2018	0	0	0	0	0	0	0	0	0	0	0	0	58
14483615	f	ot	BE	2018	0	1	0	0	1	0	0	0	0	0	0	0	70
14483850	f	md	DE	2018	0	0	0	0	0	0	0	0	0	0	0	0	
14484054	f	cn	CA	2018	0	0	0	0	0	0	0	0	0	0	0	0	
14484583	Not	md	US	2018	0	0	0	0	0	0	0	0	0	0	0	0	
14484634	m	ot	DE	2018	0	1	0	0	1	0	0	0	0	0	0	0	79
14484891	f	md	AU	2018	0	0	0	0	0	0	0	0	0	0	0	0	67
14485017	f	ot	US	2018	0	0	0	0	0	0	0	0	0	0	0	0	63
14486066	Not	ot	US	2018	0	0	0	0	0	0	0	0	0	0	0	0	
14487521	m	md	US	2018	1	0	0	0	0	0	0	0	0	0	0	0	64
14487527	f	cn	US	2018	1	0	0	0	0	0	0	0	0	0	0	1	
14487553	m	md	ID	2018	1	0	0	0	0	0	0	0	0	0	0	0	50
14487708	f	md	IT	2018	0	1	0	1	0	0	0	0	0	0	0	0	78
14487838	f	md	DE	2018	0	0	0	0	0	0	0	0	0	0	0	0	56
14488086	f	ot	CA	2018	0	0	0	0	0	0	0	0	0	0	0	0	65
14488166	f	cn	JP	2018	0	1	1	0	0	0	0	0	0	0	0	0	
14488232	f	ot	NL	2018	0	0	0	0	0	0	0	0	0	0	0	0	48
14488580	f	cn	JP	2018	0	1	1	0	0	0	0	0	0	0	0	0	69
14488727	f	cn	JP	2018	0	1	1	0	0	0	0	0	0	0	0	0	86
14488997	m	md	US	2018	0	0	0	0	0	0	0	0	0	0	0	0	66
14489159	m	md	US	2018	0	0	0	0	0	0	0	0	0	0	0	0	72
14489229	f	md	US	2018	0	0	0	0	0	0	0	0	0	0	0	0	54

14489678	f	ot	US	2018	0	0	0	0	0	0	0	0	0	0	0	0	59
14489921	f	cn	US	2018	0	0	0	0	0	0	0	0	0	0	0	0	
14489931	f	md	DE	2018	0	1	0	0	0	1	0	0	0	0	0	0	58
14489958	m	md	JP	2018	0	1	0	0	1	0	0	0	0	0	0	0	74
14490201	f	md	CN	2018	0	1	0	0	0	1	0	0	0	0	0	0	69
14490221	m	md	JP	2018	1	0	0	0	0	0	0	0	0	0	0	0	69
14490619	m	md	BE	2018	0	1	1	0	0	0	0	0	0	0	0	0	
14490776	m	cn	GB	2018	1	0	0	0	0	0	0	0	0	0	0	0	64
14490852	m	md	CN	2018	0	1	0	0	0	1	0	0	0	0	0	0	63
14491348	f	md	JP	2018	1	0	0	0	0	0	0	0	0	0	0	0	65
14491451	m	md	IN	2018	0	0	0	0	0	0	0	0	0	0	0	0	62
14491492	m	md	JP	2018	0	1	0	0	1	0	0	0	0	0	0	0	60
14491604	m	md	JP	2018	0	0	0	0	0	0	0	0	0	0	0	0	77
14491615	Not	md	JP	2018	1	0	0	0	0	0	0	0	0	0	0	0	80
14491681	m	ot	DE	2018	0	0	0	0	0	0	0	0	0	0	0	0	78
14491695	f	ot	US	2018	0	1	1	0	0	0	0	0	0	0	0	0	71
14491945	f	md	PL	2018	0	0	0	0	0	0	0	0	0	0	0	0	
14492269	m	ot	JP	2018	0	1	0	0	1	0	0	0	0	0	0	0	65
14492487	m	md	KR	2018	1	0	0	0	0	0	0	0	0	0	0	0	64
14492540	f	md	CN	2018	1	0	0	0	0	0	0	0	0	0	0	0	61
14493125	m	md	FR	2018	0	0	0	0	0	0	0	0	0	0	0	0	
14493423	m	md	FR	2018	0	1	0	1	0	0	0	0	0	0	0	0	60.9
14493509	f	md	US	2018	0	0	0	0	0	0	0	0	0	0	0	0	66
14495006	f	cn	GR	2018	0	0	0	0	0	0	0	0	0	0	0	0	54
14495020	f	md	US	2018	0	1	1	0	0	0	0	0	0	0	0	0	62
14495057	m	md	TR	2018	0	0	0	0	0	0	0	0	0	0	0	0	57
14495821	m	cn	GR	2018	0	1	1	0	0	0	0	0	0	0	0	0	77
14496048	m	md	AT	2018	0	0	0	0	0	0	0	0	0	0	0	0	
14496226	m	ot	JP	2018	0	0	0	0	0	0	0	0	0	0	0	0	66
14496468	m	ot	US	2018	0	1	0	0	1	0	0	0	0	0	0	0	60
14496776	m	md	US	2018	0	0	0	0	0	0	0	0	0	0	0	0	77
14497033	m	md	JP	2018	0	1	0	0	1	0	0	0	0	0	0	0	54
14497084	m	md	KR	2018	1	0	0	0	0	0	0	0	0	0	0	0	60
14497269	f	md	JP	2018	0	1	0	0	1	0	0	0	0	0	0	0	70

14497270	m	ot	JP	2018	0	1	0	0	1	0	0	0	0	0	0	0	68
14498112	m	cn	IT	2018	0	1	1	0	0	0	0	0	0	0	0	0	
14498223	m	md	SK	2018	0	0	0	0	0	0	0	0	0	0	0	0	59
14498581	f	md	DE	2018	1	0	0	0	0	0	0	0	0	0	0	0	65
14498805	m	cn	JP	2018	0	1	1	0	0	0	0	0	0	0	0	1	68
14499797	m	cn	JP	2018	0	1	0	0	1	0	0	0	0	0	0	0	
14499937	f	ot	BE	2018	0	1	0	0	1	0	0	0	0	0	0	0	56
14500249	f	cn	JP	2018	0	1	1	0	0	0	0	0	0	0	0	0	75
14500338	f	md	US	2018	0	0	0	0	0	0	0	0	0	0	0	0	71
14501087	m	cn	US	2018	0	0	0	0	0	0	0	0	0	0	0	0	
14501330	m	ot	JP	2018	0	1	0	0	1	0	0	0	0	0	0	0	57
14502152	m	md	US	2018	1	0	0	0	0	0	0	0	0	0	0	0	25
14502415	Not	ot	JP	2018	0	0	0	0	0	0	0	0	0	0	0	0	
14502468	f	md	JP	2018	0	1	0	0	1	0	0	0	0	0	0	0	64
14502520	f	md	US	2018	1	0	0	0	0	0	0	0	0	0	0	0	90
14502648	Not	md	US	2018	1	0	0	0	0	0	0	0	0	0	0	0	
14502679	m	md	JP	2018	0	1	0	0	1	0	0	0	0	0	0	0	58
14502704	m	ot	IN	2018	1	0	0	0	0	0	0	0	0	0	0	0	
14502755	Not	md	US	2018	0	1	0	1	0	0	0	0	0	0	0	1	83
14502959	m	cn	ES	2018	0	1	1	0	0	0	0	0	0	0	0	0	
14505230	Not	ot	US	2018	0	1	1	0	0	0	0	0	0	0	0	0	
14505232	m	cn	US	2018	1	0	0	0	0	0	0	0	0	0	0	0	57
14505247	f	cn	US	2018	0	1	0	0	1	0	0	0	0	0	0	0	
14505649	m	md	CO	2018	0	0	0	0	0	0	0	0	0	0	0	0	
14505992	m	md	US	2018	1	0	0	0	0	0	0	0	0	0	0	0	81.7
14506095	m	ph	US	2018	1	0	0	0	0	0	0	0	0	0	0	0	73
14506907	f	cn	BE	2018	0	1	1	0	0	0	0	0	0	0	0	0	52
14507334	m	ot	US	2018	0	1	0	0	1	0	0	0	0	0	0	0	66
14507846	Not	md	US	2018	1	0	0	0	0	0	0	0	0	0	0	0	
14507872	m	ot	DE	2018	0	1	0	0	1	0	0	0	0	0	0	1	85
14508430	m	md	JP	2018	0	0	0	0	0	0	0	0	0	0	0	0	
14508496	m	md	CN	2018	1	0	0	0	0	0	0	0	0	0	0	0	79
14508846	f	cn	JP	2018	0	0	0	0	0	0	0	0	0	0	0	0	68
14509132	m	cn	IN	2018	0	0	0	0	0	0	0	0	0	0	0	0	38

14509338	m	md	LB	2018	0	1	0	0	1	0	0	0	0	0	0	0	65
14510251	Not	md	US	2018	1	0	0	0	0	0	0	0	0	0	0	0	
14510605	f	md	JP	2018	1	0	0	0	0	0	0	0	0	0	0	0	75
14510668	m	md	JP	2018	0	1	1	0	0	0	0	0	0	0	0	1	62
14510970	m	ot	BE	2018	0	1	0	0	1	0	0	0	0	0	0	1	69
14511025	f	cn	US	2018	1	0	0	0	0	0	0	0	0	0	0	0	68
14511444	m	ot	FR	2018	0	1	0	0	1	0	0	0	0	0	0	0	69
14512121	f	md	JP	2018	0	1	1	0	0	0	0	0	0	0	0	0	81
14512175	m	cn	DE	2018	0	0	0	0	0	0	0	0	0	0	0	0	63
14512259	m	md	JP	2018	0	1	0	0	1	0	0	0	0	0	0	1	65
14514693	m	cn	JP	2018	0	1	1	0	0	0	0	0	0	0	0	0	75
14514748	m	md	DE	2018	0	1	0	0	1	0	0	0	0	0	0	0	59
14515122	m	ph	US	2018	0	1	0	0	1	0	0	0	0	0	0	0	
14515742	f	md	US	2018	1	0	0	0	0	0	0	0	0	0	0	0	
14515746	f	md	US	2018	0	1	0	1	0	0	0	0	0	0	0	0	
14516206	f	md	DK	2018	0	1	1	0	0	0	0	0	0	0	0	0	75
14516530	m	cn	ES	2018	0	1	1	0	0	0	0	0	0	0	0	0	67
14516681	m	cn	US	2018	0	1	1	0	0	0	0	0	0	0	0	0	60
14517106	f	cn	US	2018	0	1	0	0	1	0	0	0	0	0	0	0	56
14517209	m	md	US	2018	0	1	0	0	0	1	0	0	0	0	0	0	76
14517383	m	md	US	2018	0	1	0	0	0	1	0	0	0	0	0	0	71
14517738	f	ph	US	2018	0	1	1	0	0	0	0	0	0	0	0	1	
14517766	f	md	US	2018	1	0	0	0	0	0	0	0	0	0	0	0	64.8
14517820	m	cn	US	2018	0	1	1	0	0	0	0	0	0	0	0	0	
14517911	Not	ot	US	2018	0	0	0	0	0	0	0	0	0	0	0	0	
14518016	m	cn	JP	2018	0	1	1	0	0	0	0	0	0	0	0	0	90
14518110	m	md	CR	2018	0	1	0	0	0	1	0	0	0	0	0	0	63
14518370	f	md	ES	2018	0	1	0	1	0	0	0	0	0	0	0	0	63
14518691	m	md	KR	2018	1	0	0	0	0	0	0	0	0	0	0	0	59
14519082	f	md	US	2018	0	1	1	0	0	0	0	0	0	0	0	0	
14519122	m	cn	JP	2018	0	1	0	0	1	0	0	0	0	0	0	0	72
14519206	m	ot	DE	2018	0	0	0	0	0	0	0	0	0	0	0	0	60
14519273	m	md	DE	2018	0	0	0	0	0	0	0	0	0	0	0	0	65
14519431	m	cn	AT	2018	0	1	1	0	0	0	0	0	0	0	0	1	51

14519840	m	ot	US	2018	0	0	0	0	0	0	1	0	1	0	0	0	55
14519920	m	ot	GR	2018	0	0	0	0	0	0	0	0	0	0	0	0	
14520170	m	md	DE	2018	0	1	0	0	1	0	0	0	0	0	0	0	75
14520625	f	md	US	2018	0	1	1	0	0	0	0	0	0	0	0	0	79
14521024	m	ot	JP	2018	0	0	0	0	0	0	0	0	0	0	0	0	69
14521707	f	ot	US	2018	0	0	0	0	0	0	0	0	0	0	0	0	
14522679	f	md	TH	2018	0	0	0	0	0	0	0	0	0	0	0	0	62
14523401	Not	ot	IN	2018	0	0	0	0	0	0	0	0	0	0	0	0	
14523770	f	cn	US	2018	0	1	1	0	0	0	0	0	0	0	0	0	
14524259	m	md	CN	2018	0	1	0	1	0	0	0	0	0	0	0	0	63.1
14524263	m	ot	US	2018	0	0	0	0	0	0	0	0	0	0	0	0	70
14524551	f	ot	DE	2018	0	0	0	0	0	0	0	0	0	0	0	0	78
14524600	f	md	US	2018	0	1	0	0	0	1	0	0	0	0	0	0	55
14524976	f	cn	GB	2018	1	0	0	0	0	0	0	0	0	0	0	0	74
14525108	f	md	BE	2018	0	0	0	0	0	0	0	0	0	0	0	0	62
14525123	Not	ot	US	2018	0	0	0	0	0	0	0	0	0	0	0	0	
14525622	m	md	DE	2018	0	0	0	0	0	0	0	0	0	0	0	0	68
14526315	f	cn	GB	2018	1	0	0	0	0	0	0	0	0	0	0	0	
14526460	f	Not repo	US	2018	1	0	0	0	0	0	0	0	0	0	0	1	86
14526695	f	ph	JP	2018	1	0	0	0	0	0	0	0	0	0	0	0	75
14527178	m	md	FR	2018	0	1	0	0	1	0	0	0	0	0	0	0	82
14527235	f	cn	US	2018	0	1	1	0	0	0	0	0	0	0	0	0	
14527741	m	ot	US	2018	0	1	0	0	1	0	0	0	0	0	0	0	72
14528457	f	ot	DE	2018	0	1	0	0	1	0	0	0	0	0	0	0	53
14528836	f	md	JP	2018	1	0	0	0	0	0	0	0	0	0	0	0	77
14529349	m	cn	JP	2018	0	0	0	0	0	0	0	0	0	0	0	0	71
14529359	Not	md	US	2018	0	0	0	0	0	0	0	0	0	0	0	0	
14530041	f	cn	GB	2018	1	0	0	0	0	0	0	0	0	0	0	0	
14530203	m	md	DE	2018	0	0	0	0	0	0	0	0	0	0	0	0	68
14530292	f	cn	DE	2018	0	1	1	0	0	0	0	0	0	0	0	0	62
14530906	f	ot	US	2018	0	1	1	0	0	0	0	0	0	0	0	0	52
14530927	m	ot	JP	2018	0	1	0	0	1	0	0	0	0	0	0	0	73
14531360	m	md	IT	2018	0	1	0	1	0	0	0	0	0	0	0	0	73
14531373	m	ph	US	2018	0	1	0	0	1	0	0	0	0	0	0	0	47

14531439	m	md	GR	2018	1	0	0	0	0	0	0	0	0	0	0	1	55
14531837	m	md	JP	2018	0	1	0	0	1	0	0	0	0	0	0	0	56
14532127	f	md	US	2018	0	1	1	0	0	0	0	0	0	0	0	0	78
14532764	m	ot	JP	2018	0	0	0	0	0	0	0	0	0	0	0	0	80
14533215	m	Not repo	CA	2018	1	0	0	0	0	0	0	0	0	0	0	0	52.8
14533479	f	md	JP	2018	0	1	0	0	1	0	0	0	0	0	0	0	63
14534149	f	md	AU	2018	1	0	0	0	0	0	0	0	0	0	0	0	57.5
14534156	f	ot	DE	2018	0	1	0	0	1	0	0	0	0	0	0	0	64
14534728	m	Not repo	US	2018	1	0	0	0	0	0	0	0	0	0	0	0	
14535529	m	ph	US	2018	0	0	0	0	0	0	0	0	0	0	0	0	75
14535690	m	ph	US	2018	0	1	1	0	0	0	0	0	0	0	0	0	67
14535897	m	ot	DE	2018	0	1	0	0	1	0	0	0	0	0	0	0	68
14536166	m	md	US	2018	0	1	0	1	0	0	0	0	0	0	0	0	63.5
14536177	m	cn	GB	2018	1	0	0	0	0	0	0	0	0	0	0	0	85
14536314	f	Not repo	AU	2018	1	0	0	0	0	0	0	0	0	0	0	0	52
14536442	m	md	JP	2018	0	1	0	0	1	0	0	0	0	0	0	0	65
14536475	m	ot	DE	2018	0	1	0	0	1	0	0	0	0	0	0	0	47
14536521	m	md	ID	2018	1	0	0	0	0	0	0	0	0	0	0	0	
14536941	f	ot	FR	2018	0	0	0	0	0	0	0	0	0	0	0	0	63
14537429	Not	ot	US	2018	0	1	1	0	0	0	0	0	0	0	0	0	
14537538	f	cn	US	2018	1	0	0	0	0	0	0	0	0	0	0	0	70
14537658	f	md	US	2018	0	1	0	1	0	0	0	0	0	0	0	0	74.9
14538022	f	cn	IN	2018	0	1	0	0	1	0	0	0	0	0	0	0	58
14538063	m	md	IN	2018	0	1	0	0	1	0	0	0	0	0	0	0	78
14538088	f	ot	GB	2018	0	1	0	0	1	0	0	0	0	0	0	0	59
14538118	m	md	JP	2018	0	1	0	0	1	0	0	0	0	0	0	0	67
14538181	f	md	JP	2018	1	0	0	0	0	0	0	0	0	0	0	1	69.2
14538266	m	md	KR	2018	1	0	0	0	0	0	0	0	0	0	0	0	49
14538285	f	md	JP	2018	1	0	0	0	0	0	0	0	0	0	0	0	
14538308	f	ot	US	2018	0	1	0	0	1	0	0	0	0	0	0	0	
14538381	m	cn	MX	2018	0	1	1	0	0	0	0	0	0	0	0	0	
14538382	f	md	CA	2018	0	1	0	1	0	0	0	0	0	0	0	0	53
14538426	f	Not repo	AU	2018	1	0	0	0	0	0	0	0	0	0	0	0	81.9
14539225	f	md	US	2018	0	1	0	0	1	0	0	0	0	0	0	0	

14539488	f	md	FR	2018	0	0	0	0	0	0	0	0	0	0	0	0	69
14539598	f	md	FI	2018	0	1	0	0	1	0	0	0	0	0	0	0	50
14539996	f	cn	US	2018	0	1	0	0	1	0	0	0	0	0	0	0	
14539997	f	ot	AR	2018	0	1	0	0	1	0	0	0	0	0	0	0	61
14540006	f	cn	GB	2018	1	0	0	0	0	0	0	0	0	0	0	0	
14540042	m	md	DE	2018	0	0	0	0	0	0	0	0	0	0	0	0	68
14540083	f	md	DE	2018	1	0	0	0	0	0	0	0	0	0	0	0	67
14540124	Not	md	JP	2018	0	1	1	0	0	0	0	0	0	0	0	0	80
14540417	m	cn	GB	2018	1	0	0	0	0	0	0	0	0	0	0	0	78
14541777	f	md	DE	2018	0	1	0	0	1	0	0	0	0	0	0	1	55
14542389	f	md	AU	2018	1	0	0	0	0	0	0	0	0	0	0	0	56.7
14542928	Not	ot	AU	2018	0	0	0	0	0	0	0	0	0	0	0	0	
14543027	f	md	DE	2018	0	0	0	0	0	0	0	0	0	0	0	0	67
14543162	f	md	US	2018	0	0	0	0	0	0	0	0	0	0	0	0	
14543366	f	md	ID	2018	1	0	0	0	0	0	0	0	0	0	0	0	
14543373	f	ot	DE	2018	0	1	0	0	1	0	0	0	0	0	0	0	64
14543587	m	ot	ES	2018	0	1	0	0	1	0	0	0	0	0	0	0	56
14544149	Not	ot	AU	2018	0	0	0	0	0	0	0	0	0	0	0	0	
14545319	m	md	BR	2018	0	0	0	0	0	0	0	0	0	0	0	0	35
14545360	m	ot	US	2018	0	1	0	0	1	0	0	0	0	0	0	0	68
14545400	f	ot	US	2018	1	0	0	0	0	0	0	0	0	0	0	0	85
14545441	m	ph	JP	2018	0	1	1	0	0	0	0	0	0	0	0	0	80
14545815	m	ph	US	2018	0	1	0	1	0	0	0	0	0	0	0	0	81.4
14545900	m	cn	JP	2018	0	1	0	0	1	0	0	0	0	0	0	0	
14545910	m	md	BE	2018	0	1	0	0	1	0	0	0	0	0	0	0	70
14545963	m	md	LB	2018	0	1	0	0	1	0	0	0	0	0	0	0	
14546000	m	md	BE	2018	0	0	0	0	0	0	0	0	0	0	0	0	65
14546346	Not	md	RO	2018	0	1	0	0	1	0	0	0	0	0	0	0	
14546435	m	cn	FR	2018	0	1	1	0	0	0	0	0	0	0	0	0	
14546726	f	cn	JP	2018	0	0	0	0	0	0	0	0	0	0	0	0	62
14546778	Not	Not repo	Not re	2018	1	0	0	0	0	0	0	0	0	0	0	0	
14546846	m	ot	US	2018	0	0	0	0	0	0	0	0	0	0	0	0	52
14547273	f	cn	US	2018	1	0	0	0	0	0	0	0	0	0	0	0	69
14547470	f	cn	JP	2018	0	1	1	0	0	0	0	0	0	0	0	0	55

14547971	f	Not repo	CH	2018	0	1	0	1	0	0	0	0	0	0	0	1	74.9
14548267	m	cn	GB	2018	0	1	1	0	0	0	0	0	0	0	0	1	
14548994	m	md	GB	2018	1	0	0	0	0	0	0	0	0	0	0	0	70
14550716	m	ot	ES	2018	0	1	0	0	1	0	0	0	0	0	0	0	69
14550768	f	md	JP	2018	0	0	0	0	0	0	0	0	0	0	0	0	
14550808	m	md	JP	2018	0	1	0	0	1	0	0	0	0	0	0	1	76
14551252	m	cn	US	2018	0	1	0	0	1	0	0	0	0	0	0	0	
14551290	Not	md	US	2018	0	1	0	0	1	0	0	0	0	0	0	0	
14551326	f	md	DE	2018	0	1	0	0	1	0	0	0	0	0	0	0	48
14551640	m	md	HK	2018	0	0	0	0	0	0	0	0	0	0	0	0	
14551679	m	md	JP	2018	0	1	0	0	1	0	0	0	0	0	0	1	71
14551730	f	ot	ES	2018	0	1	0	0	1	0	0	0	0	0	0	0	62
14551758	m	ot	JP	2018	0	1	0	0	1	0	0	0	0	0	0	0	77
14551808	f	ot	JP	2018	0	1	0	0	1	0	0	0	0	0	0	0	67
14551810	Not	md	BE	2018	0	1	0	0	1	0	0	0	0	0	0	0	
14551833	Not	ot	US	2018	0	0	0	0	0	0	0	0	0	0	0	0	
14552055	m	cn	IN	2018	0	1	0	0	1	0	0	0	0	0	0	0	66
14553212	m	md	US	2018	0	1	1	0	0	0	0	0	0	0	0	1	68
14553402	m	ot	US	2018	0	1	0	0	1	0	0	0	0	0	0	0	71
14553452	m	cn	JP	2018	0	1	1	0	0	0	0	0	0	0	0	0	73
14553612	m	cn	US	2018	0	1	1	0	0	0	0	0	0	0	0	0	
14553864	m	ot	FR	2018	0	1	0	0	1	0	0	0	0	0	0	0	70
14554037	f	ot	US	2018	0	0	0	0	0	0	0	0	0	0	0	0	61
14554371	Not	cn	US	2018	0	1	1	0	0	0	0	0	0	0	0	0	
14554417	f	cn	US	2018	1	0	0	0	0	0	0	0	0	0	0	0	
14554464	m	md	JP	2018	0	1	0	0	1	0	0	0	0	0	0	0	70
14555094	m	ot	CN	2018	0	1	0	0	1	0	0	0	0	0	0	0	61
14555100	f	md	DE	2018	0	0	0	0	0	0	0	0	0	0	0	0	57
14555885	m	ot	DE	2018	0	1	0	0	1	0	0	0	0	0	0	0	59
14556080	m	md	PL	2018	0	0	0	0	0	0	0	0	0	0	0	0	71
14556112	Not	ot	IT	2018	0	0	0	0	0	0	0	0	0	0	0	0	
14556129	Not	ot	IT	2018	0	0	0	0	0	0	0	0	0	0	0	0	
14556135	Not	ot	IT	2018	0	0	0	0	0	0	0	0	0	0	0	0	
14556161	m	md	US	2018	0	1	1	0	0	0	0	0	0	0	0	0	

14556239	m	cn	FR	2018	0	1	1	0	0	0	0	0	0	0	0	0	64
14556248	f	md	JP	2018	0	1	0	0	1	0	0	0	0	0	0	0	61
14556274	f	ot	JP	2018	0	1	0	0	1	0	0	0	0	0	0	0	70
14556419	Not	ot	IT	2018	0	0	0	0	0	0	0	0	0	0	0	0	
14556519	m	cn	FR	2018	0	1	1	0	0	0	0	0	0	0	0	0	
14556583	m	cn	CN	2018	0	0	0	0	0	0	0	0	0	0	0	0	
14556624	f	md	US	2018	0	0	0	0	0	0	0	0	0	0	0	0	62
14556627	f	md	US	2018	0	1	0	0	0	1	0	0	0	0	0	0	62
14556846	m	cn	FR	2018	0	1	1	0	0	0	0	0	0	0	0	0	
14557173	m	cn	FR	2018	0	1	1	0	0	0	0	0	0	0	0	0	
14557214	m	cn	DE	2018	0	1	1	0	0	0	0	0	0	0	0	0	
14557693	m	ot	US	2018	0	1	0	0	1	0	0	0	0	0	0	0	69
14557873	f	cn	FR	2018	0	1	1	0	0	0	0	0	0	0	0	0	85
14558063	Not	md	JP	2018	0	0	0	0	0	0	0	0	0	0	0	0	
14558297	Not	ot	IN	2018	0	0	0	0	0	0	0	0	0	0	0	0	
14558408	f	md	IL	2018	0	0	0	0	0	0	0	0	0	0	0	0	76
14558560	f	Not repo	US	2018	0	0	0	0	0	0	0	0	0	0	0	0	86.8
14559699	m	md	ES	2018	0	0	0	0	0	0	0	0	0	0	0	0	71
14559822	m	ot	CN	2018	0	1	0	0	1	0	0	0	0	0	0	0	63
14560874	Not	md	DE	2018	1	0	0	0	0	0	0	0	0	0	0	0	
14561050	m	ot	JP	2018	0	1	0	0	1	0	0	0	0	0	0	0	74
14561128	m	ot	JP	2018	0	0	0	0	0	0	0	0	0	0	0	0	80
14561163	f	cn	NL	2018	1	0	0	0	0	0	0	0	0	0	0	0	
14561546	f	md	JP	2018	1	0	0	0	0	0	0	0	0	0	0	0	62
14561701	m	Not repo	CA	2018	1	0	0	0	0	0	0	0	0	0	0	0	
14561734	m	ot	IN	2018	0	0	0	0	0	0	0	0	0	0	0	0	55
14561746	f	cn	Not re	2018	0	0	0	0	0	0	0	0	0	0	0	0	49
14561841	f	ot	US	2018	0	0	0	0	0	0	0	0	0	0	0	0	61
14562298	m	Not repo	ID	2018	1	0	0	0	0	0	0	0	0	0	0	0	
14562335	Not	ot	IT	2018	0	0	0	0	0	0	0	0	0	0	0	0	
14562705	f	ot	US	2018	0	1	0	0	1	0	0	0	0	0	0	0	57
14563227	m	md	US	2018	0	1	0	1	0	0	0	0	0	0	0	0	63.5
14563262	f	md	DE	2018	0	1	0	0	1	0	0	0	0	0	0	1	55
14563668	f	md	JP	2018	0	1	0	0	1	0	0	0	0	0	0	0	64

14563690	f	md	DE	2018	0	0	0	0	0	0	0	0	0	0	0	0	58
14563948	m	md	US	2018	0	1	1	0	0	0	0	0	0	0	0	0	83
14563949	m	md	US	2018	0	1	1	0	0	0	0	0	0	0	0	0	76
14564085	m	cn	DE	2018	0	1	1	0	0	0	0	0	0	0	0	0	
14564207	m	ot	JP	2018	0	0	0	0	0	0	0	0	0	0	0	0	73
14564223	m	md	LT	2018	0	0	0	0	0	0	0	0	0	0	0	0	68
14564866	f	md	BE	2018	0	1	1	0	0	0	0	0	0	0	0	0	
14565017	f	md	CN	2018	0	1	0	0	0	1	0	0	0	0	0	0	74
14565047	f	cn	US	2018	0	1	0	0	1	0	0	0	0	0	0	0	60
14565090	m	md	US	2018	0	1	0	1	0	0	0	0	0	0	0	0	
14565417	f	md	IT	2018	0	1	0	0	1	0	0	0	0	0	0	0	
14565469	Not	md	DE	2018	0	1	0	0	1	0	0	0	0	0	0	0	
14565545	m	cn	GB	2018	0	1	1	0	0	0	0	0	0	0	0	0	
14565551	m	md	BE	2018	0	1	1	0	0	0	0	0	0	0	0	0	67
14565709	f	ot	US	2018	0	0	0	0	0	0	0	0	0	0	0	0	
14565735	f	md	JP	2018	1	0	0	0	0	0	0	0	0	0	0	1	79
14565776	m	md	JP	2018	0	1	0	0	1	0	0	0	0	0	0	0	
14566193	m	md	JP	2018	0	0	0	0	0	0	0	0	0	0	0	0	74
14566371	m	md	JP	2018	0	1	0	0	1	0	0	0	0	0	0	0	75
14566564	m	md	GR	2018	0	0	0	0	0	0	0	0	0	0	0	0	57
14566687	m	ot	ES	2018	0	1	0	0	1	0	0	0	0	0	0	0	58
14566719	m	md	CA	2018	0	0	0	0	0	0	0	0	0	0	0	0	62
14567103	m	md	JP	2018	0	1	1	0	0	0	0	0	0	0	0	0	67
14567493	f	md	US	2018	0	1	0	0	1	0	0	0	0	0	0	0	
14567648	f	cn	ID	2018	1	0	0	0	0	0	0	0	0	0	0	0	55
14567856	m	md	US	2018	0	1	0	1	0	0	0	0	0	0	0	0	76
14568176	f	cn	GB	2018	1	0	0	0	0	0	0	0	0	0	0	0	69
14568383	f	md	GB	2018	0	0	0	0	0	0	0	0	0	0	0	0	
14568493	m	cn	GB	2018	1	0	0	0	0	0	0	0	0	0	0	0	59
14568497	f	md	US	2018	0	0	0	0	0	0	0	0	0	0	0	0	
14568610	f	ot	IT	2018	0	1	0	0	1	0	0	0	0	0	0	1	74
14568777	m	ot	US	2018	0	1	0	0	1	0	0	0	0	0	0	0	59
14568858	m	cn	CN	2018	1	0	0	0	0	0	0	0	0	0	0	0	67
14568993	m	md	KR	2018	1	0	0	0	0	0	0	0	0	0	0	0	49

14569029	m	md	Not re	2018	1	0	0	0	0	0	0	0	0	0	0	0	87
14569294	m	ot	ES	2018	0	1	0	0	1	0	0	0	0	0	0	0	70
14569475	m	md	US	2018	0	1	0	0	0	1	0	0	0	0	0	0	67
14569514	f	md	JP	2018	0	0	0	0	0	0	0	0	0	0	0	0	32
14569546	f	md	IL	2018	1	0	0	0	0	0	0	0	0	0	0	0	83
14569751	m	cn	GB	2018	0	1	1	0	0	0	0	0	0	0	0	1	74
14570095	f	cn	US	2018	0	1	1	0	0	0	0	0	0	0	0	0	67
14570106	m	cn	BE	2018	0	1	1	0	0	0	0	0	0	0	0	0	
14570107	m	md	BE	2018	0	1	1	0	0	0	0	0	0	0	0	1	71
14570685	Not	ot	NZ	2018	0	0	0	0	0	0	0	0	0	0	0	0	
14570692	Not	ot	DE	2018	0	0	0	0	0	0	0	0	0	0	0	0	
14570694	Not	ot	NZ	2018	0	0	0	0	0	0	0	0	0	0	0	0	
14570697	Not	ot	DE	2018	0	0	0	0	0	0	0	0	0	0	0	0	
14570702	Not	ot	DE	2018	0	1	1	0	0	0	0	0	0	0	0	0	
14570706	Not	ot	DE	2018	0	0	0	0	0	0	0	0	0	0	0	0	
14570707	Not	ot	NZ	2018	0	0	0	0	0	0	0	0	0	0	0	0	
14570708	Not	ot	NZ	2018	0	0	0	0	0	0	0	0	0	0	0	0	
14570709	Not	ot	NZ	2018	0	0	0	0	0	0	0	0	0	0	0	0	
14570710	Not	ot	NZ	2018	0	0	0	0	0	0	0	0	0	0	0	0	
14570711	Not	ot	NZ	2018	0	0	0	0	0	0	0	0	0	0	0	0	
14570712	Not	ot	DE	2018	0	0	0	0	0	0	0	0	0	0	0	0	
14570764	Not	ot	NZ	2018	0	0	0	0	0	0	0	0	0	0	0	0	
14570772	Not	ot	NZ	2018	0	0	0	0	0	0	0	0	0	0	0	0	
14570773	Not	ot	NZ	2018	0	0	0	0	0	0	0	0	0	0	0	0	
14570774	Not	ot	NZ	2018	0	0	0	0	0	0	0	0	0	0	0	0	
14570780	Not	ot	NZ	2018	0	0	0	0	0	0	0	0	0	0	0	0	
14570781	Not	ot	NZ	2018	0	0	0	0	0	0	0	0	0	0	0	0	
14570782	Not	ot	NZ	2018	0	0	0	0	0	0	0	0	0	0	0	0	
14570788	Not	ot	NZ	2018	0	0	0	0	0	0	0	0	0	0	0	0	
14570789	Not	ot	NZ	2018	0	0	0	0	0	0	0	0	0	0	0	0	
14570790	Not	ot	NZ	2018	0	0	0	0	0	0	0	0	0	0	0	0	
14570795	Not	ot	NZ	2018	0	0	0	0	0	0	0	0	0	0	0	0	
14570797	Not	ot	NZ	2018	0	0	0	0	0	0	0	0	0	0	0	0	
14570806	Not	ot	NZ	2018	0	0	0	0	0	0	0	0	0	0	0	0	

14570807	Not	ot	NZ	2018	0	0	0	0	0	0	0	0	0	0	0	0	
14570811	Not	ot	NZ	2018	0	0	0	0	0	0	0	0	0	0	0	0	
14570812	Not	ot	NZ	2018	0	0	0	0	0	0	0	0	0	0	0	0	
14570972	Not	ot	DE	2018	0	0	0	0	0	0	0	0	0	0	0	0	
14570973	Not	ot	DE	2018	0	0	0	0	0	0	0	0	0	0	0	0	
14571074	m	md	JP	2018	0	1	0	0	1	0	0	0	0	0	0	0	67
14571215	Not	ot	NZ	2018	0	0	0	0	0	0	0	0	0	0	0	0	
14571217	Not	ot	NZ	2018	0	0	0	0	0	0	0	0	0	0	0	0	
14571222	Not	ot	NZ	2018	0	0	0	0	0	0	0	0	0	0	0	0	
14571224	Not	ot	NZ	2018	0	0	0	0	0	0	0	0	0	0	0	0	
14571417	m	cn	GB	2018	1	0	0	0	0	0	0	0	0	0	0	0	64
14572326	f	cn	GB	2018	1	0	0	0	0	0	0	0	0	0	0	0	83
14572328	f	cn	US	2018	0	0	0	0	0	0	0	0	0	0	0	0	
14572387	m	ph	JP	2018	0	1	0	0	1	0	0	0	0	0	0	0	73
14572527	m	md	US	2018	0	1	0	1	0	0	0	0	0	0	0	0	66
14572608	f	md	JP	2018	0	1	0	0	1	0	0	0	0	0	0	0	42
14572734	f	md	DE	2018	1	0	0	0	0	0	0	0	0	0	0	0	38
14573023	f	ot	US	2018	0	0	0	0	0	0	0	0	0	0	0	0	
14573523	m	ot	JP	2018	0	0	0	0	0	0	0	0	0	0	0	0	80
14573546	m	md	KR	2018	0	0	0	0	0	0	0	0	0	0	0	0	59
14573554	m	md	JP	2018	0	1	1	0	0	0	0	0	0	0	0	0	68
14573767	f	md	JP	2018	0	0	0	0	0	0	0	0	0	0	0	0	71
14573818	f	md	NL	2018	0	0	0	0	0	0	0	0	0	0	0	0	70
14573946	f	ot	DE	2018	0	1	0	0	1	0	0	0	0	0	0	0	75
14574039	m	md	US	2018	0	1	0	1	0	0	0	0	0	0	0	0	66
14574321	m	md	JP	2018	0	1	0	0	1	0	0	0	0	0	0	0	76
14575631	Not	ot	NZ	2018	0	0	0	0	0	0	0	0	0	0	0	0	
14575742	Not	ot	DE	2018	0	1	1	0	0	0	0	0	0	0	0	0	
14575758	f	ot	US	2018	0	1	0	0	1	0	0	0	0	0	0	0	69
14575779	m	md	ES	2018	0	0	0	0	0	0	0	0	0	0	0	0	62
14575931	f	ot	BG	2018	0	0	0	0	0	0	0	0	0	0	0	0	55
14575985	m	cn	KR	2018	0	1	1	0	0	0	0	0	0	0	0	0	
14575994	m	ph	US	2018	0	0	0	0	0	0	0	0	0	0	0	0	70
14576009	Not	ot	DE	2018	0	1	1	0	0	0	0	0	0	0	0	0	

14576011	Not	ot	DE	2018	0	0	0	0	0	0	0	0	0	0	0	0		
14576012	Not	ot	DE	2018	0	0	0	0	0	0	0	0	0	0	0	0		
14576061	Not	ot	NZ	2018	0	0	0	0	0	0	0	0	0	0	0	0		
14576063	Not	ot	NZ	2018	0	0	0	0	0	0	0	0	0	0	0	0		
14576066	Not	ot	NZ	2018	0	0	0	0	0	0	0	0	0	0	0	0		
14576068	f	md	DE	2018	0	1	1	0	0	0	0	0	0	0	0	0		81
14576071	Not	ot	NZ	2018	0	0	0	0	0	0	0	0	0	0	0	0		
14576072	Not	ot	NZ	2018	0	0	0	0	0	0	0	0	0	0	0	0		
14576223	m	ot	FR	2018	0	1	0	0	1	0	0	0	0	0	0	0		63
14576355	m	md	BE	2018	0	1	0	0	1	0	0	0	0	0	0	0		71
14576422	Not	ot	NZ	2018	0	0	0	0	0	0	0	0	0	0	0	0		
14576616	m	cn	US	2018	1	0	0	0	0	0	0	0	0	0	0	0		
14576651	Not	cn	GB	2018	1	0	0	0	0	0	0	0	0	0	0	0		
14576682	f	ph	JP	2018	1	0	0	0	0	0	0	0	0	0	0	0		85
14577027	m	md	KR	2018	0	0	0	0	0	0	0	0	0	0	0	0		59
14577148	m	cn	JP	2018	0	1	1	0	0	0	0	0	0	0	0	0		
14577630	m	ot	KR	2018	0	1	0	0	1	0	0	0	0	0	0	0		72
14578277	f	md	KR	2018	1	0	0	0	0	0	0	0	0	0	0	0		63
14578575	f	ot	DE	2018	0	0	0	0	0	0	0	0	0	0	0	0		57
14579002	f	md	JP	2018	0	0	0	0	0	0	0	0	0	0	0	0		53
14579157	m	ot	DE	2018	0	0	0	0	0	0	0	0	0	0	0	0		59
14580164	f	md	US	2018	0	1	0	0	1	0	0	0	0	0	0	0		63
14580225	f	ot	CZ	2018	0	0	0	0	0	0	0	0	0	0	0	0		57
14580625	m	ot	US	2018	0	1	1	0	0	0	0	0	0	0	0	0		79
14580632	f	md	US	2018	0	1	1	0	0	0	0	0	0	0	0	0		79
14581085	m	md	CN	2018	0	1	0	0	0	1	0	0	0	0	0	0		69
14581143	m	md	CH	2018	0	0	0	0	0	0	0	0	0	0	0	0		
14581416	f	md	JP	2018	1	0	0	0	0	0	0	0	0	0	0	0		81.8
14581855	f	md	DE	2018	0	1	0	0	1	0	0	0	0	0	0	0		67
14582466	f	ot	DE	2018	0	0	0	0	0	0	0	0	0	0	0	0		74
14582726	Not	ot	JP	2018	0	0	0	0	0	0	0	0	0	0	0	0		
14583142	Not	cn	BR	2018	1	0	0	0	0	0	0	0	0	0	0	0		
14583293	f	md	GB	2018	0	1	1	0	0	0	0	0	0	0	0	0		75
14584112	f	ot	FR	2018	0	1	0	0	1	0	0	0	0	0	0	0		63

14584218	f	md	US	2018	0	1	1	0	0	0	0	0	0	0	0	0	75
14584702	f	cn	DE	2018	0	1	0	0	1	0	0	0	0	0	0	0	62
14584752	Not	md	JP	2018	0	0	0	0	0	0	0	0	0	0	0	0	
14584876	f	cn	JP	2018	0	1	1	0	0	0	0	0	0	0	0	0	74
14584991	m	md	CN	2018	0	1	0	1	0	0	0	0	0	0	0	0	51
14585052	f	md	US	2018	1	0	0	0	0	0	0	0	0	0	0	0	
14585110	f	md	US	2018	0	1	0	0	1	0	0	0	0	0	0	0	
14585252	m	ot	RO	2018	0	0	0	0	0	0	0	0	0	0	0	0	70
14585314	m	md	JP	2018	0	1	0	0	1	0	0	0	0	0	0	0	65
14585918	m	cn	US	2018	0	1	1	0	0	0	0	0	0	0	0	0	80
14586030	m	ot	DE	2018	0	0	0	0	0	0	0	0	0	0	0	0	67
14586319	f	md	JP	2018	1	0	0	0	0	0	0	0	0	0	0	0	73
14586434	Not	md	CN	2018	1	0	0	0	0	0	0	0	0	0	0	0	
14586516	f	md	DE	2018	0	1	0	0	1	0	0	0	0	0	0	1	55
14586966	m	md	DE	2018	0	0	0	0	0	0	0	0	0	0	0	0	58
14587419	f	md	JP	2018	1	0	0	0	0	0	0	0	0	0	0	1	85
14587426	f	cn	CN	2018	1	0	0	0	0	0	0	0	0	0	0	0	93.1
14587430	Not	md	FR	2018	0	0	0	0	0	0	0	0	0	0	0	0	67
14587438	f	cn	US	2018	1	0	0	0	0	0	0	0	0	0	0	0	66
14587470	f	ph	JP	2018	0	0	0	0	0	0	0	0	0	0	0	0	55
14587626	m	md	JP	2018	0	1	0	0	1	0	0	0	0	0	0	0	73
14588207	m	md	US	2018	0	1	0	0	0	1	0	0	0	0	0	0	54
14588537	m	cn	JP	2018	0	1	0	0	1	0	0	0	0	0	0	0	67
14588581	f	ot	US	2018	0	1	1	0	0	0	0	0	0	0	0	0	74
14589062	f	ot	US	2018	0	1	0	0	1	0	0	0	0	0	0	0	53
14589392	Not	md	JP	2018	0	0	0	0	0	0	0	0	0	0	0	0	
14589547	m	md	RO	2018	0	1	0	0	1	0	0	0	0	0	0	0	
14589561	m	md	JP	2018	0	1	0	0	1	0	0	0	0	0	0	0	46
14589642	Not	ot	GB	2018	0	1	1	0	0	0	0	0	0	0	0	0	
14590054	m	cn	JP	2018	0	1	0	0	1	0	0	0	0	0	0	0	73
14590186	m	md	IL	2018	0	1	0	1	0	0	0	0	0	0	0	0	57
14590529	m	ph	US	2018	0	0	0	0	0	0	0	0	0	0	0	0	65
14590613	m	cn	JP	2018	0	1	0	0	1	0	0	0	0	0	0	1	68
14591457	m	ot	JP	2018	0	0	0	0	0	0	0	0	0	0	0	0	80

14591499	f	md	DE	2018	0	0	0	0	0	0	0	0	0	0	0	0	70
14591902	Not	ot	JP	2018	0	0	0	0	0	0	0	0	0	0	0	0	
14592503	f	md	JP	2018	1	0	0	0	0	0	0	0	0	0	0	1	81.6
14592696	f	cn	JP	2018	0	1	1	0	0	0	0	0	0	0	0	0	78
14592827	m	ot	JP	2018	0	1	0	0	1	0	0	0	0	0	0	1	72
14593094	f	md	NZ	2018	0	1	0	1	0	0	0	0	0	0	0	0	62
14593549	m	cn	JP	2018	0	1	1	0	0	0	0	0	0	0	0	0	68
14594514	f	ot	GB	2018	0	1	0	0	1	0	0	0	0	0	0	0	72
14595545	f	md	US	2018	0	1	0	0	1	0	0	0	0	0	0	0	
14595705	Not	ot	JP	2018	1	0	0	0	0	0	0	0	0	0	0	0	
14595716	m	md	JP	2018	0	1	0	0	1	0	0	0	0	0	0	0	73
14595725	m	cn	DE	2018	1	0	0	0	0	0	0	0	0	0	0	0	
14596459	m	md	TH	2018	0	0	0	0	0	0	0	0	0	0	0	0	87
14597057	m	ot	IT	2018	0	1	0	0	1	0	0	0	0	0	0	1	68
14597081	m	md	JP	2018	0	1	0	0	1	0	0	0	0	0	0	0	67
14597223	f	ot	AU	2018	0	1	0	0	1	0	0	0	0	0	0	0	62
14597234	f	cn	DE	2018	0	0	0	0	0	0	1	1	0	0	0	0	66
14597258	f	md	JP	2018	0	0	0	0	0	0	1	0	1	0	0	0	60
14597503	m	md	JP	2018	0	1	0	0	1	0	0	0	0	0	0	0	67
14597553	f	md	BR	2018	0	1	0	0	0	1	0	0	0	0	0	0	61
14597753	f	md	BE	2018	0	0	0	0	0	0	0	0	0	0	0	0	78
14597777	m	cn	JP	2018	0	1	0	0	1	0	0	0	0	0	0	0	
14598549	m	md	GB	2018	0	0	0	0	0	0	0	0	0	0	0	0	
14598581	f	ph	JP	2018	0	1	1	0	0	0	0	0	0	0	0	0	80
14598633	Not	cn	JP	2018	0	0	0	0	0	0	0	0	0	0	0	0	75
14598669	f	cn	AR	2018	0	0	0	0	0	0	0	0	0	0	0	0	70
14598854	f	ot	US	2018	0	1	1	0	0	0	0	0	0	0	0	0	52
14598932	m	cn	GB	2018	1	0	0	0	0	0	0	0	0	0	0	0	85
14599278	m	cn	JP	2018	0	1	1	0	0	0	0	0	0	0	0	0	68
14599342	m	ot	JP	2018	0	1	0	0	1	0	0	0	0	0	0	0	70
14599352	m	cn	US	2018	1	0	0	0	0	0	0	0	0	0	0	0	
14599554	m	cn	IL	2018	0	1	1	0	0	0	0	0	0	0	0	0	77
14599605	f	md	TR	2018	0	0	0	0	0	0	0	0	0	0	0	0	
14599620	m	cn	GB	2018	1	0	0	0	0	0	0	0	0	0	0	0	50

14599789	f	md	DE	2018	0	0	0	0	0	0	0	0	0	0	0	0	55
14600207	f	ph	JP	2018	1	0	0	0	0	0	0	0	0	0	0	1	60
14600487	f	md	DE	2018	0	0	0	0	0	0	0	0	0	0	0	0	63
14601263	f	ph	CA	2018	0	1	0	0	1	0	0	0	0	0	0	0	62
14601573	m	cn	UA	2018	0	1	1	0	0	0	0	0	0	0	0	0	60
14601683	Not	md	IT	2018	1	0	0	0	0	0	0	0	0	0	0	0	
14601792	m	md	JP	2018	0	0	0	0	0	0	0	0	0	0	0	0	60
14601859	Not	ot	JP	2018	0	0	0	0	0	0	0	0	0	0	0	0	
14601867	Not	ot	JP	2018	0	0	0	0	0	0	0	0	0	0	0	0	
14602032	Not	ot	JP	2018	0	0	0	0	0	0	0	0	0	0	0	0	
14602078	f	md	JP	2018	0	1	0	0	1	0	0	0	0	0	0	0	57
14602268	Not	ot	JP	2018	0	0	0	0	0	0	0	0	0	0	0	0	
14602271	Not	ot	JP	2018	0	0	0	0	0	0	0	0	0	0	0	0	
14602611	f	md	JP	2018	0	1	0	0	1	0	0	0	0	0	0	1	61
14602710	m	cn	GB	2018	1	0	0	0	0	0	0	0	0	0	0	0	74
14603061	f	md	KR	2018	0	1	0	0	1	0	0	0	0	0	0	0	74
14603401	m	md	GR	2018	0	0	0	0	0	0	0	0	0	0	0	0	52
14603524	f	ph	CA	2018	0	1	0	0	1	0	0	0	0	0	0	0	66
14604078	m	md	JP	2018	1	0	0	0	0	0	0	0	0	0	0	0	70
14604130	f	md	DE	2018	0	0	0	0	0	0	0	0	0	0	0	0	70
14604189	m	md	CN	2018	1	0	0	0	0	0	0	0	0	0	0	0	74.9
14604575	f	md	CN	2018	1	0	0	0	0	0	0	0	0	0	0	0	61.8
14604576	f	md	JP	2018	1	0	0	0	0	0	0	0	0	0	0	0	75
14604588	f	ph	JP	2018	1	0	0	0	0	0	0	0	0	0	0	0	84.8
14604676	f	cn	GB	2018	1	0	0	0	0	0	0	0	0	0	0	0	
14604751	m	cn	JP	2018	0	1	1	0	0	0	0	0	0	0	0	0	64
14605094	Not	ot	JP	2018	0	0	0	0	0	0	0	0	0	0	0	0	
14605151	f	cn	DE	2018	0	1	1	0	0	0	0	0	0	0	0	0	77
14605213	m	ot	ES	2018	0	1	0	0	1	0	0	0	0	0	0	0	56
14605228	f	md	NL	2018	0	0	0	0	0	0	0	0	0	0	0	0	62
14605330	Not	ot	JP	2018	0	0	0	0	0	0	0	0	0	0	0	0	
14605340	Not	ot	JP	2018	0	0	0	0	0	0	0	0	0	0	0	0	
14605347	m	md	US	2018	0	1	1	0	0	0	0	0	0	0	0	0	49
14605348	m	md	JP	2018	0	1	0	0	1	0	0	0	0	0	0	1	56

14605354	m	ot	AR	2018	0	1	0	0	1	0	0	0	0	0	0	0	63
14606033	Not	ot	JP	2018	0	0	0	0	0	0	0	0	0	0	0	0	
14606046	Not	ot	JP	2018	0	0	0	0	0	0	0	0	0	0	0	0	
14606119	Not	md	DE	2018	0	1	0	0	1	0	0	0	0	0	0	0	
14606155	m	ot	JP	2018	0	1	0	0	1	0	0	0	0	0	0	0	72
14606215	m	ph	DE	2018	0	1	0	0	1	0	0	0	0	0	0	0	62
14606290	m	ot	US	2018	0	1	0	0	1	0	0	0	0	0	0	0	57
14606901	Not	md	JP	2018	0	1	0	0	1	0	0	0	0	0	0	0	
14608100	f	md	JP	2018	0	1	0	0	1	0	0	0	0	0	0	0	80
14608101	m	cn	JP	2018	0	1	1	0	0	0	0	0	0	0	0	0	57
14608274	f	md	US	2018	0	1	0	0	1	0	0	0	0	0	0	0	64
14608386	m	md	JP	2018	0	1	0	0	1	0	0	0	0	0	0	0	72
14608510	f	md	JP	2018	1	0	0	0	0	0	0	0	0	0	0	0	80
14609092	f	md	JP	2018	1	0	0	0	0	0	0	0	0	0	0	0	70
14609804	f	md	IT	2018	1	0	0	0	0	0	0	0	0	0	0	0	72
14609904	f	ot	CL	2018	0	1	0	0	1	0	0	0	0	0	0	0	76
14609905	m	md	JP	2018	0	1	0	0	1	0	0	0	0	0	0	0	61
14610444	Not	md	US	2018	0	1	0	0	1	0	0	0	0	0	0	0	
14610480	Not	ot	DE	2018	0	1	0	0	1	0	0	0	0	0	0	0	
14611127	m	md	UA	2018	0	1	0	0	0	1	0	0	0	0	0	0	71
14611187	f	cn	US	2018	0	1	0	0	1	0	0	0	0	0	0	0	
14611291	f	ot	DE	2018	0	1	0	0	1	0	0	0	0	0	0	0	
14611376	m	cn	IN	2018	0	1	0	0	0	1	0	0	0	0	0	0	44
14611575	Not	md	US	2018	0	1	0	0	1	0	0	0	0	0	0	0	
14611910	m	cn	US	2018	0	1	0	0	1	0	0	0	0	0	0	0	
14612740	m	ot	DE	2018	0	0	0	0	0	0	0	0	0	0	0	0	78
14613242	m	md	JP	2018	0	0	0	0	0	0	0	0	0	0	0	0	80
14613390	f	md	DE	2018	0	0	0	0	0	0	0	0	0	0	0	0	74
14613716	m	md	JP	2018	0	1	1	0	0	0	0	0	0	0	0	0	68
14613866	f	md	JP	2018	1	0	0	0	0	0	0	0	0	0	0	0	82
14614338	f	ot	US	2018	0	1	0	0	1	0	0	0	0	0	0	0	
14614339	m	md	JP	2018	0	1	0	0	1	0	0	0	0	0	0	0	66
14614656	m	md	JP	2018	0	1	0	0	1	0	0	0	0	0	0	0	63
14615374	m	md	JP	2018	1	0	0	0	0	0	0	0	0	0	0	0	85

14615428	m	cn	DE	2018	0	0	0	0	0	0	0	0	0	0	0	0	
14615597	f	md	US	2018	0	1	0	1	0	0	0	0	0	0	0	0	
14615700	m	md	US	2018	0	1	0	1	0	0	0	0	0	0	0	0	45
14615817	m	md	JP	2018	0	1	0	0	1	0	0	0	0	0	0	0	70
14615837	m	md	JP	2018	0	0	0	0	0	0	0	0	0	0	0	0	70
14615861	m	ot	JP	2018	0	1	0	0	1	0	0	0	0	0	0	0	58
14616073	m	cn	JP	2018	0	0	0	0	0	0	0	0	0	0	0	0	61
14616193	m	md	ES	2018	0	1	0	0	0	1	0	0	0	0	0	0	65
14616287	f	cn	US	2018	0	1	0	0	0	1	0	0	0	0	0	0	50
14616547	Not	ot	US	2018	0	1	0	0	0	1	0	0	0	0	0	0	
14616582	Not	ot	US	2018	0	1	0	0	0	1	0	0	0	0	0	0	
14616600	m	md	JP	2018	0	1	0	0	1	0	0	0	0	0	0	0	68
14616913	Not	ot	US	2018	0	1	0	0	0	1	0	0	0	0	0	0	
14617038	f	md	US	2018	0	1	0	0	0	1	0	0	0	0	0	0	36
14617125	f	ph	JP	2018	0	0	0	0	0	0	0	0	0	0	0	0	45
14617359	f	ot	DE	2018	0	0	0	0	0	0	0	0	0	0	0	0	65
14617573	f	cn	US	2018	0	1	0	0	1	0	0	0	0	0	0	0	60
14617750	f	ot	US	2018	0	1	0	0	1	0	0	0	0	0	0	0	68
14617980	m	cn	US	2018	1	0	0	0	0	0	0	0	0	0	0	0	65
14618966	m	md	US	2018	0	1	0	1	0	0	0	0	0	0	0	0	81
14618967	Not	md	CH	2018	0	1	0	1	0	0	0	0	0	0	0	0	
14619172	f	ph	JP	2018	1	0	0	0	0	0	0	0	0	0	0	0	76.1
14619520	f	md	CN	2018	1	0	0	0	0	0	0	0	0	0	0	0	63
14619573	f	md	DE	2018	0	1	0	0	1	0	0	0	0	0	0	1	55
14619874	m	Not repo	US	2018	1	0	0	0	0	0	0	0	0	0	0	0	
14620092	f	cn	US	2018	1	0	0	0	0	0	0	0	0	0	0	0	
14620165	f	md	CH	2018	1	0	0	0	0	0	0	0	0	0	0	0	
14620660	Not	md	PT	2018	1	0	0	0	0	0	0	0	0	0	0	0	
14620831	Not	md	JP	2018	1	0	0	0	0	0	0	0	0	0	0	0	
14620920	m	md	DE	2018	0	0	0	0	0	0	0	0	0	0	0	0	57
14620924	m	md	JP	2018	0	1	0	0	1	0	0	0	0	0	0	0	65
14621314	m	md	AR	2018	0	1	0	0	0	1	0	0	0	0	0	1	57
14621351	f	cn	US	2018	0	1	0	0	1	0	0	0	0	0	0	0	
14621627	f	ot	AT	2018	0	1	0	0	1	0	0	0	0	0	0	0	67

14622332	f	cn	US	2018	0	1	1	0	0	0	0	0	0	0	0	0	
14623131	Not	md	JP	2018	0	0	0	0	0	0	0	0	0	0	0	0	
14623146	m	ot	AU	2018	0	1	1	0	0	0	0	0	0	0	0	0	
14623155	m	md	US	2018	0	1	0	1	0	0	0	0	0	0	0	0	
14623310	m	md	PH	2018	0	1	1	0	0	0	0	0	0	0	0	0	56
14623743	m	cn	JP	2018	0	0	0	0	0	0	0	0	0	0	0	0	80
14624358	m	ot	RO	2018	0	1	0	0	1	0	0	0	0	0	0	0	62
14624435	m	md	JP	2018	0	1	0	0	1	0	0	0	0	0	0	0	68
14624640	m	md	JP	2018	0	1	0	0	1	0	0	0	0	0	0	0	74
14624977	m	md	JP	2018	0	1	0	0	1	0	0	0	0	0	0	0	52
14625039	m	md	JP	2018	0	1	1	0	0	0	0	0	0	0	0	0	79
14626019	m	ot	ES	2018	0	1	0	0	1	0	0	0	0	0	0	0	63
14626180	f	cn	GB	2018	0	1	1	0	0	0	0	0	0	0	0	0	71
14626838	m	ph	US	2018	0	0	0	0	0	0	0	0	0	0	0	0	67
14627165	m	md	JP	2018	0	1	0	0	1	0	0	0	0	0	0	0	68
14627260	m	md	JP	2018	0	1	0	0	1	0	0	0	0	0	0	0	57
14627284	m	md	BR	2018	0	0	0	0	0	0	0	0	0	0	0	0	82
14627400	m	cn	JP	2018	0	1	1	0	0	0	0	0	0	0	0	0	83
14627409	m	cn	JP	2018	0	1	1	0	0	0	0	0	0	0	0	0	90
14628539	m	cn	CN	2018	1	0	0	0	0	0	0	0	0	0	0	0	64
14629300	m	ot	JP	2018	0	1	0	0	1	0	0	0	0	0	0	1	68
14629550	m	ph	US	2018	0	0	0	0	0	0	0	0	0	0	0	0	65
14629569	m	ot	HU	2018	0	1	0	0	1	0	0	0	0	0	0	0	76
14629808	m	ot	SK	2018	0	0	0	0	0	0	0	0	0	0	0	0	63
14629909	f	md	CN	2018	1	0	0	0	0	0	0	0	0	0	0	0	46
14630101	m	ot	DE	2018	0	0	0	0	0	0	0	0	0	0	0	0	80
14630376	Not	cn	SE	2018	0	1	1	0	0	0	0	0	0	0	0	0	
14630395	f	md	JP	2018	0	0	0	0	0	0	0	0	0	0	0	0	75
14630801	m	md	US	2018	1	0	0	0	0	0	0	0	0	0	0	0	38.5
14631165	f	cn	US	2018	0	0	0	0	0	0	0	0	0	0	0	0	68
14631195	Not	md	JP	2018	0	1	0	0	1	0	0	0	0	0	0	0	62
14631972	f	Not repo	AU	2018	1	0	0	0	0	0	0	0	0	0	0	0	
14632227	m	ph	FR	2018	0	1	0	1	0	0	0	0	0	0	0	0	
14632316	f	md	JP	2018	0	1	0	0	1	0	0	0	0	0	0	0	69

14632317	m	md	JP	2018	0	1	0	0	1	0	0	0	0	0	0	1	75
14632487	m	ot	JP	2018	0	1	0	1	0	0	0	0	0	0	0	0	73
14632496	f	cn	US	2018	0	0	0	0	0	0	0	0	0	0	0	0	39
14632499	Not	cn	US	2018	0	0	0	0	0	0	0	0	0	0	0	0	
14632500	f	cn	US	2018	0	0	0	0	0	0	0	0	0	0	0	0	61
14632505	f	md	US	2018	0	1	0	0	0	1	0	0	0	0	0	1	
14632514	f	ph	US	2018	0	0	0	0	0	0	0	0	0	0	0	0	
14632520	f	md	US	2018	0	0	0	0	0	0	0	0	0	0	0	0	
14632529	f	cn	US	2018	0	0	0	0	0	0	0	0	0	0	0	0	
14632537	m	md	US	2018	0	0	0	0	0	0	0	0	0	0	0	0	
14632562	Not	ot	US	2018	0	1	0	0	0	1	0	0	0	0	0	0	
14633097	m	md	JP	2018	0	1	0	0	1	0	0	0	0	0	0	0	61
14633664	f	cn	US	2018	0	1	1	0	0	0	0	0	0	0	0	0	71
14633719	f	cn	US	2018	0	1	0	0	1	0	0	0	0	0	0	0	
14633890	Not	ot	US	2018	0	0	0	0	0	0	0	0	0	0	0	0	
14633915	f	ot	JP	2018	0	1	0	0	1	0	0	0	0	0	0	0	63
14634172	Not	ot	IT	2018	0	0	0	0	0	0	0	0	0	0	0	0	
14634264	f	ot	TW	2018	0	1	0	0	0	1	0	0	0	0	0	0	
14635351	m	cn	JP	2018	0	0	0	0	0	0	0	0	0	0	0	0	80
14635352	f	ot	US	2018	0	1	0	0	1	0	0	0	0	0	0	0	79
14635499	m	md	US	2018	0	1	0	0	0	1	0	0	0	0	0	0	89
14635838	m	md	JP	2018	1	0	0	0	0	0	0	0	0	0	0	0	90
14636832	f	md	AU	2018	1	0	0	0	0	0	0	0	0	0	0	0	84.5
14637544	m	md	IE	2018	0	0	0	0	0	0	0	0	0	0	0	0	60
14637649	f	md	CZ	2018	1	0	0	0	0	0	0	0	0	0	0	0	68
14638550	m	cn	JP	2018	0	1	0	0	1	0	0	0	0	0	0	0	74
14638612	f	cn	US	2018	1	0	0	0	0	0	0	0	0	0	0	0	
14639012	m	cn	US	2018	0	1	0	0	1	0	0	0	0	0	0	0	
14639074	f	cn	US	2018	1	0	0	0	0	0	0	0	0	0	0	0	79
14639082	m	md	JP	2018	0	1	0	0	1	0	0	0	0	0	0	0	68
14639402	f	ph	AU	2018	1	0	0	0	0	0	0	0	0	0	0	1	51.4
14639403	f	cn	US	2018	1	0	0	0	0	0	0	0	0	0	0	0	85.1
14639514	f	md	JP	2018	0	1	0	0	1	0	0	0	0	0	0	0	73
14639623	m	md	JP	2018	0	1	0	0	1	0	0	0	0	0	0	0	75

14639698	f	cn	US	2018	0	1	0	0	1	0	0	0	0	0	0	0	
14639770	f	md	AT	2018	0	0	0	0	0	0	0	0	0	0	0	0	
14640057	m	md	CN	2018	0	1	0	0	0	1	0	0	0	0	0	0	46
14640282	m	md	DE	2018	0	0	0	0	0	0	0	0	0	0	0	0	65
14640746	f	md	JP	2018	1	0	0	0	0	0	0	0	0	0	0	0	85
14640880	m	md	US	2018	0	1	1	0	0	0	0	0	0	0	0	0	72
14640941	f	ot	DE	2018	0	0	0	0	0	0	0	0	0	0	0	0	64
14641357	m	md	JP	2018	0	1	0	0	1	0	0	0	0	0	0	0	55
14641359	m	md	JP	2018	0	1	0	0	1	0	0	0	0	0	0	0	77
14641751	m	cn	GB	2018	1	0	0	0	0	0	0	0	0	0	0	0	
14641915	f	md	IT	2018	1	0	0	0	0	0	0	0	0	0	0	0	
14643100	f	md	US	2018	0	1	0	0	1	0	0	0	0	0	0	0	60
14645580	m	cn	US	2018	0	0	0	0	0	0	0	0	0	0	0	0	
14645711	m	md	FR	2018	0	1	0	0	1	0	0	0	0	0	0	0	
14645968	m	cn	US	2018	0	1	1	0	0	0	0	0	0	0	0	0	79
14646131	Not	ot	ES	2018	0	0	0	0	0	0	0	0	0	0	0	0	
14646136	f	ot	BE	2018	0	1	0	0	1	0	0	0	0	0	0	0	70
14646402	f	md	JP	2018	0	1	0	0	1	0	0	0	0	0	0	0	55
14646442	m	md	GR	2018	1	0	0	0	0	0	0	0	0	0	0	0	58
14646480	f	cn	US	2018	0	1	0	0	1	0	0	0	0	0	0	0	53
14646625	m	ot	DE	2018	0	0	0	0	0	0	0	0	0	0	0	0	63
14646837	f	md	RU	2018	0	1	0	0	0	1	0	0	0	0	0	0	63
14647595	f	ot	SE	2018	0	0	0	0	0	0	0	0	0	0	0	0	42
14647749	f	md	FR	2018	1	0	0	0	0	0	0	0	0	0	0	0	
14647783	m	md	DE	2018	0	1	0	0	1	0	0	0	0	0	0	0	61
14647874	f	ot	US	2018	0	1	0	0	1	0	0	0	0	0	0	1	75
14648063	f	cn	IT	2018	0	0	0	0	0	0	0	0	0	0	0	0	
14648717	m	ot	BR	2018	0	1	0	0	1	0	0	0	0	0	0	0	57
14649305	Not	cn	CN	2018	1	0	0	0	0	0	0	0	0	0	0	0	51
14649369	m	md	DE	2018	0	0	0	0	0	0	0	0	0	0	0	0	65
14649393	m	ot	JP	2018	0	1	0	0	1	0	0	0	0	0	0	0	68
14649805	f	ot	US	2018	0	1	0	0	1	0	0	0	0	0	0	1	51
14649953	m	md	US	2018	0	1	0	1	0	0	0	0	0	0	0	0	63.6
14650130	f	ph	US	2018	0	0	0	0	0	0	0	0	0	0	0	0	

14650574	f	md	US	2018	0	1	0	0	1	0	0	0	0	0	0	0	56
14650734	Not	ot	GB	2018	0	0	0	0	0	0	0	0	0	0	0	0	
14651687	m	ot	US	2018	0	1	1	0	0	0	0	0	0	0	0	0	80
14651955	m	cn	US	2018	1	0	0	0	0	0	0	0	0	0	0	0	
14652132	f	md	US	2018	0	1	1	0	0	0	0	0	0	0	0	0	
14652423	m	cn	JP	2018	0	1	1	0	0	0	0	0	0	0	0	0	80
14652428	m	cn	US	2018	0	1	0	0	1	0	0	0	0	0	0	0	
14652484	f	cn	US	2018	0	1	0	0	1	0	0	0	0	0	0	0	85
14652615	m	md	JP	2018	1	0	0	0	0	0	0	0	0	0	0	0	61
14652701	f	cn	US	2018	0	0	0	0	0	0	0	0	0	0	0	0	53
14652978	f	md	JP	2018	1	0	0	0	0	0	0	0	0	0	0	0	75
14652995	m	md	US	2018	0	0	0	0	0	0	0	0	0	0	0	0	79
14653012	f	cn	JP	2018	0	1	1	0	0	0	0	0	0	0	0	0	54
14653160	f	ot	GB	2018	0	1	0	0	1	0	0	0	0	0	0	0	65
14653187	m	md	AT	2018	0	1	0	0	0	1	0	0	0	0	0	0	63
14653327	f	md	TH	2018	1	0	0	0	0	0	0	0	0	0	0	0	58
14653331	m	md	JP	2018	1	0	0	0	0	0	0	0	0	0	0	0	62
14653365	m	md	JP	2018	0	1	0	0	1	0	0	0	0	0	0	0	80
14654778	f	md	CN	2018	1	0	0	0	0	0	0	0	0	0	0	0	
14655243	f	ot	CA	2018	0	0	0	0	0	0	0	0	0	0	0	0	
14655674	f	ot	BE	2018	0	0	0	0	0	0	0	0	0	0	0	0	72
14656295	f	cn	TW	2018	1	0	0	0	0	0	0	0	0	0	0	0	54
14657060	m	md	GR	2018	0	0	0	0	0	0	0	0	0	0	0	0	71
14657706	f	md	JP	2018	1	0	0	0	0	0	0	0	0	0	0	0	64
14657949	f	md	JP	2018	0	1	0	0	1	0	0	0	0	0	0	0	80
14658179	f	md	JP	2018	0	0	0	0	0	0	0	0	0	0	0	0	43
14658201	m	cn	JP	2018	0	0	0	0	0	0	0	0	0	0	0	0	64
14658208	m	md	JP	2018	0	1	0	0	1	0	0	0	0	0	0	0	72
14658239	m	md	JP	2018	0	1	0	0	1	0	0	0	0	0	0	0	69
14658322	m	md	JP	2018	0	1	0	0	1	0	0	0	0	0	0	0	76
14658385	Not	md	NL	2018	0	0	0	0	0	0	0	0	0	0	0	0	
14658623	f	md	JP	2018	1	0	0	0	0	0	0	0	0	0	0	0	45
14658638	Not	cn	GB	2018	1	0	0	0	0	0	0	0	0	0	0	0	
14658857	m	md	JP	2018	1	0	0	0	0	0	0	0	0	0	0	0	63

14658990	f	cn	ID	2018	1	0	0	0	0	0	0	0	0	0	0	0	
14659248	f	md	US	2018	0	1	1	0	0	0	0	0	0	0	0	0	75
14659310	m	md	CN	2018	0	1	0	0	0	1	0	0	0	0	0	0	62
14659330	f	ot	SE	2018	0	0	0	0	0	0	0	0	0	0	0	0	42
14659404	m	Not repo	SG	2018	1	0	0	0	0	0	0	0	0	0	0	0	68.7
14659406	f	md	JP	2018	1	0	0	0	0	0	0	0	0	0	0	1	73
14659475	m	md	AU	2018	0	1	0	1	0	0	0	0	0	0	0	0	75
14659720	f	cn	IT	2018	1	0	0	0	0	0	0	0	0	0	0	0	72
14660105	m	cn	US	2018	0	0	0	0	0	0	0	0	0	0	0	0	65
14660243	m	md	US	2018	0	1	1	0	0	0	0	0	0	0	0	0	
14660308	f	cn	AR	2018	0	0	0	0	0	0	0	0	0	0	0	0	69
14660545	f	cn	GB	2018	1	0	0	0	0	0	0	0	0	0	0	0	73
14660695	m	md	US	2018	1	0	0	0	0	0	0	0	0	0	0	0	54
14661276	f	md	AT	2018	0	1	1	0	0	0	0	0	0	0	0	0	
14661337	m	ot	US	2018	0	1	0	0	1	0	0	0	0	0	0	0	55
14661384	f	cn	US	2018	0	1	0	0	1	0	0	0	0	0	0	0	
14661407	f	ot	NZ	2018	0	0	0	0	0	0	0	0	0	0	0	0	42
14661457	f	cn	US	2018	0	1	0	0	1	0	0	0	0	0	0	0	
14661942	m	md	DE	2018	1	0	0	0	0	0	0	0	0	0	0	0	
14662032	m	md	IT	2018	0	1	0	0	1	0	0	0	0	0	0	1	70
14662299	m	cn	US	2018	0	1	0	0	1	0	0	0	0	0	0	0	
14662376	f	ot	DE	2018	0	1	0	0	1	0	0	0	0	0	0	0	52
14662423	f	ot	DE	2018	0	1	0	0	1	0	0	0	0	0	0	0	65
14662456	f	md	DE	2018	0	1	0	0	1	0	0	0	0	0	0	1	55
14662654	f	cn	US	2018	0	0	0	0	0	0	0	0	0	0	0	0	
14662967	f	cn	US	2018	1	0	0	0	0	0	0	0	0	0	0	0	70
14663117	Not	md	IT	2018	1	0	0	0	0	0	0	0	0	0	0	0	
14663471	Not	ot	FR	2018	0	0	0	0	0	0	0	0	0	0	0	0	
14663476	Not	ot	FR	2018	0	0	0	0	0	0	0	0	0	0	0	0	
14663591	f	md	CA	2018	0	0	0	0	0	0	0	0	0	0	0	0	
14663644	Not	ot	FR	2018	0	0	0	0	0	0	0	0	0	0	0	0	
14664099	f	md	JP	2018	1	0	0	0	0	0	0	0	0	0	0	0	66
14664133	m	md	US	2018	0	1	1	0	0	0	0	0	0	0	0	0	68
14664205	f	ot	JP	2018	1	0	0	0	0	0	0	0	0	0	0	0	64

14665141	m	ot	US	2018	0	0	0	0	0	0	0	0	0	0	0	0	68
14665475	f	md	BG	2018	1	0	0	0	0	0	0	0	0	0	0	0	57
14665643	f	md	SI	2018	1	0	0	0	0	0	0	0	0	0	0	0	
14665904	Not	ot	FR	2018	0	0	0	0	0	0	0	0	0	0	0	0	
14666286	f	md	JP	2018	0	1	0	0	1	0	0	0	0	0	0	0	30
14666469	m	md	US	2018	1	0	0	0	0	0	0	0	0	0	0	1	64
14666580	m	ot	JP	2018	0	1	0	0	1	0	0	0	0	0	0	0	65
14666736	Not	md	JP	2018	0	1	0	0	1	0	0	0	0	0	0	0	
14666868	f	cn	US	2018	0	1	1	0	0	0	0	0	0	0	0	0	75
14667225	f	ot	CH	2018	0	1	0	0	1	0	0	0	0	0	0	0	76
14667505	f	md	US	2018	0	0	0	0	0	0	1	0	0	0	1	0	50
14667536	m	ot	IT	2018	0	0	0	0	0	0	0	0	0	0	0	0	41
14667543	m	cn	US	2018	0	1	0	0	1	0	0	0	0	0	0	0	
14667975	m	md	JP	2018	0	1	0	0	1	0	0	0	0	0	0	0	62
14668790	f	cn	GB	2018	1	0	0	0	0	0	0	0	0	0	0	0	60
14668791	f	cn	GB	2018	1	0	0	0	0	0	0	0	0	0	0	0	60
14668821	f	ph	GB	2018	1	0	0	0	0	0	0	0	0	0	0	0	58
14669035	f	md	JP	2018	1	0	0	0	0	0	0	0	0	0	0	0	80
14669292	f	ot	US	2018	0	1	0	0	1	0	0	0	0	0	0	0	50
14669307	m	ot	US	2018	0	1	0	0	1	0	0	0	0	0	0	0	83
14669332	f	ot	US	2018	0	1	1	0	0	0	0	0	0	0	0	0	52
14669698	m	cn	US	2018	0	0	0	0	0	0	0	0	0	0	0	0	64
14670200	m	cn	JP	2018	0	1	1	0	0	0	0	0	0	0	0	0	57
14670536	m	md	JP	2018	0	1	0	0	1	0	0	0	0	0	0	0	75
14670553	m	ot	DE	2018	0	0	0	0	0	0	0	0	0	0	0	0	80
14672040	m	md	US	2018	0	0	0	0	0	0	0	0	0	0	0	0	81
14672326	Not	ot	JP	2018	1	0	0	0	0	0	0	0	0	0	0	1	
14673002	f	md	JP	2018	0	1	0	0	1	0	0	0	0	0	0	0	67
14673004	m	md	BR	2018	0	1	0	0	1	0	0	0	0	0	0	0	
14673045	m	md	DE	2018	0	0	0	0	0	0	0	0	0	0	0	0	68
14673305	m	md	US	2018	0	1	0	0	0	1	0	0	0	0	0	0	66
14673387	f	cn	GB	2018	1	0	0	0	0	0	0	0	0	0	0	0	45
14673534	Not	ot	AU	2018	0	1	0	0	1	0	0	0	0	0	0	0	
14673535	Not	ot	AU	2018	0	1	0	0	1	0	0	0	0	0	0	0	

14673536	Not	ot	AU	2018	0	1	0	0	1	0	0	0	0	0	0	0	
14673573	f	md	IT	2018	1	0	0	0	0	0	0	0	0	0	0	0	72
14673686	Not	ot	TW	2018	0	1	0	0	1	0	0	0	0	0	0	0	
14674381	m	cn	GB	2018	1	0	0	0	0	0	0	0	0	0	0	0	63
14674671	f	cn	GB	2018	1	0	0	0	0	0	0	0	0	0	0	0	
14674806	f	ot	US	2018	0	0	0	0	0	0	0	0	0	0	0	0	68
14675078	f	cn	GB	2018	1	0	0	0	0	0	0	0	0	0	0	0	65
14675377	m	md	JP	2018	0	1	0	0	1	0	0	0	0	0	0	0	60
14676038	f	cn	US	2018	1	0	0	0	0	0	0	0	0	0	0	0	
14676305	m	ot	DE	2018	0	1	0	0	1	0	0	0	0	0	0	0	66
14676403	m	md	JP	2018	0	1	0	0	1	0	0	0	0	0	0	0	58
14676426	m	ot	FR	2018	0	1	0	0	1	0	0	0	0	0	0	0	63
14676482	m	ot	US	2018	0	1	0	0	1	0	0	0	0	0	0	0	
14676485	m	ot	US	2018	0	1	0	0	1	0	0	0	0	0	0	0	
14676638	m	cn	CN	2018	0	0	0	0	0	0	0	0	0	0	0	0	
14676727	m	md	JP	2018	0	1	0	0	1	0	0	0	0	0	0	0	60
14676739	m	ot	JP	2018	0	1	0	0	1	0	0	0	0	0	0	0	67
14676765	m	cn	CA	2018	0	1	0	0	1	0	0	0	0	0	0	0	
14678213	Not	ot	FR	2018	0	0	0	0	0	0	0	0	0	0	0	0	
14678768	m	md	JP	2018	0	1	0	0	1	0	0	0	0	0	0	0	75
14679000	m	cn	JP	2018	0	0	0	0	0	0	0	0	0	0	0	0	88
14679078	m	ot	DE	2018	1	0	0	0	0	0	0	0	0	0	0	0	74
14679716	m	ot	US	2018	0	1	0	0	1	0	0	0	0	0	0	0	59
14680696	f	ot	SE	2018	0	0	0	0	0	0	0	0	0	0	0	0	42
14681513	m	cn	LB	2018	1	0	0	0	0	0	0	0	0	0	0	1	
14682534	m	ot	JP	2018	0	1	0	0	1	0	0	0	0	0	0	0	67
14682796	m	md	US	2018	0	1	1	0	0	0	0	0	0	0	0	0	48
14683281	m	Not repo	Not re	2018	0	0	0	0	0	0	0	0	0	0	0	0	60
14683509	m	md	FR	2018	0	0	0	0	0	0	0	0	0	0	0	0	
14684388	Not	ot	US	2018	0	0	0	0	0	0	0	0	0	0	0	0	
14684517	m	ot	JP	2018	0	1	0	0	1	0	0	0	0	0	0	0	67
14684714	f	md	US	2018	0	0	0	0	0	0	0	0	0	0	0	0	68
14686187	m	ot	JP	2018	0	1	0	0	1	0	0	0	0	0	0	0	66
14686255	f	ot	HK	2018	0	1	0	0	1	0	0	0	0	0	0	0	48

14686939	f	ph	US	2018	1	0	0	0	0	0	0	0	0	0	0	56
14687076	f	md	DE	2018	0	1	0	0	1	0	0	0	0	0	0	60
14687175	m	ot	US	2018	1	0	0	0	0	0	0	0	0	0	0	60
14687672	m	md	DE	2018	0	0	0	0	0	0	0	0	0	0	0	59
14687677	f	md	US	2018	1	0	0	0	0	0	0	0	0	0	0	
14687905	f	md	CN	2018	1	0	0	0	0	0	0	0	0	0	0	64.5
14688279	m	ot	IN	2018	1	0	0	0	0	0	0	0	0	0	0	46
14688291	f	cn	US	2018	1	0	0	0	0	0	0	0	0	0	0	89
14688323	m	cn	GB	2018	1	0	0	0	0	0	0	0	0	0	0	60
14688433	m	ot	GB	2018	0	0	0	0	0	0	0	0	0	0	0	76
14688440	f	md	JP	2018	0	0	0	0	0	0	0	0	0	0	0	38
14688621	Not	ot	CN	2018	0	0	0	0	0	0	0	0	0	0	0	
14689285	f	cn	GB	2018	1	0	0	0	0	0	0	0	0	0	0	72
14689668	m	md	JP	2018	0	1	0	0	1	0	0	0	0	0	0	67
14689671	f	ph	US	2018	0	1	0	1	0	0	0	0	0	0	0	50
14690233	f	cn	US	2018	0	0	0	0	0	0	0	0	0	0	0	
14690692	m	cn	IE	2018	0	0	0	0	0	0	0	0	0	0	0	65
14690792	f	ot	US	2018	0	1	0	0	1	0	0	0	0	0	0	46
14691231	m	cn	US	2018	1	0	0	0	0	0	0	0	0	0	0	
14692155	m	md	JP	2018	0	1	0	0	1	0	0	0	0	0	1	76
14692158	Not	ot	US	2018	0	1	0	0	1	0	0	0	0	0	0	
14692160	m	ot	JP	2018	0	0	0	0	0	0	0	0	0	0	0	73
14692224	m	md	JP	2018	0	0	0	0	0	0	0	0	0	0	0	64
14692251	f	ot	IT	2018	0	1	0	0	1	0	0	0	0	0	0	56
14692380	f	ph	US	2018	0	1	0	1	0	0	0	0	0	0	0	75.8
14692572	f	ot	IT	2018	0	1	0	0	1	0	0	0	0	0	0	80
14692614	Not	md	IT	2018	1	0	0	0	0	0	0	0	0	0	0	
14692659	m	ot	IT	2018	0	1	0	0	1	0	0	0	0	0	0	79
14692926	f	md	JP	2018	1	0	0	0	0	0	0	0	0	0	0	69
14693064	m	md	AT	2018	0	0	0	0	0	0	0	0	0	0	0	
14693233	f	md	JP	2018	1	0	0	0	0	0	0	0	0	0	0	58
14693405	m	md	CN	2018	0	0	0	0	0	0	0	0	0	0	0	60
14694058	m	cn	GB	2018	1	0	0	0	0	0	0	0	0	0	0	80
14694297	m	cn	HU	2018	0	0	0	0	0	0	0	0	0	0	0	75

14694300	m	md	LU	2018	0	1	0	0	1	0	0	0	0	0	0	0	73
14694371	m	cn	IE	2018	0	0	0	0	0	0	0	0	0	0	0	0	59
14694630	m	md	HU	2018	0	1	0	0	1	0	0	0	0	0	0	0	
14694716	m	md	IT	2018	0	1	0	0	1	0	0	0	0	0	0	0	
14694862	m	md	IT	2018	0	1	0	0	1	0	0	0	0	0	0	0	78
14695107	Not	ot	US	2018	0	0	0	0	0	0	0	0	0	0	0	0	
14695199	m	md	JP	2018	0	1	0	0	1	0	0	0	0	0	0	0	70
14695403	f	ot	US	2018	0	0	0	0	0	0	0	0	0	0	0	0	68
14695451	m	md	JP	2018	0	1	0	0	1	0	0	0	0	0	0	0	90
14695511	Not	ot	AU	2018	0	0	0	0	0	0	0	0	0	0	0	0	
14695633	m	cn	ES	2018	0	0	0	0	0	0	0	0	0	0	0	0	68
14695753	f	md	IT	2018	1	0	0	0	0	0	0	0	0	0	0	0	72
14696377	m	ot	JP	2018	0	0	0	0	0	0	0	0	0	0	0	0	64
14696378	m	cn	IE	2018	0	0	0	0	0	0	0	0	0	0	0	0	59
14696379	f	cn	US	2018	0	0	0	0	0	0	0	0	0	0	0	0	68
14697981	f	cn	IE	2018	0	0	0	0	0	0	0	0	0	0	0	0	49
14698029	m	ot	JP	2018	0	0	0	0	0	0	0	0	0	0	0	0	73
14698047	m	cn	DE	2018	0	0	0	0	0	0	0	0	0	0	0	0	59
14698249	m	md	US	2018	0	1	0	1	0	0	0	0	0	0	0	0	
14698298	m	md	JP	2018	0	1	0	0	1	0	0	0	0	0	0	0	66
14698646	m	ot	JP	2018	0	0	0	0	0	0	0	0	0	0	0	0	72
14699020	f	md	JP	2018	0	1	0	0	1	0	0	0	0	0	0	0	60
14699042	Not	md	AT	2018	0	0	0	0	0	0	0	0	0	0	0	0	
14699256	f	cn	US	2018	0	1	0	0	1	0	0	0	0	0	0	0	69
14699496	m	ph	US	2018	1	0	0	0	0	0	0	0	0	0	0	0	70
14699559	Not	cn	GB	2018	1	0	0	0	0	0	0	0	0	0	0	0	
14699962	m	cn	US	2018	0	1	1	0	0	0	0	0	0	0	0	0	
14701644	f	cn	CN	2018	0	0	0	0	0	0	0	0	0	0	0	0	
14701673	m	ph	GB	2018	0	1	1	0	0	0	0	0	0	0	0	0	80
14701799	f	md	JP	2018	0	0	0	0	0	0	0	0	0	0	0	0	
14719835	f	cn	US	2018	1	0	0	0	0	0	0	0	0	0	0	0	55
7301929	m	ph	IN	2018	0	0	0	0	0	0	0	0	0	0	0	0	70
8167164	m	cn	GB	2018	1	0	0	0	0	0	0	0	0	0	0	0	39
8318635	m	cn	GB	2018	1	0	0	0	0	0	0	0	0	0	0	0	71

8345516	m	cn	GB	2018	1	0	0	0	0	0	0	0	0	0	0	0	58
8559464	m	cn	IE	2018	0	0	0	0	0	0	0	0	0	0	0	0	55
8651151	f	cn	IE	2018	0	0	0	0	0	0	0	0	0	0	0	0	67
8731639	m	cn	GB	2018	1	0	0	0	0	0	0	0	0	0	0	0	61
8966910	m	cn	IE	2018	0	0	0	0	0	0	0	0	0	0	0	0	66
9033492	m	cn	DE	2018	0	0	0	0	0	0	0	0	0	0	0	0	68
9046569	m	ot	DE	2018	0	0	0	0	0	0	0	0	0	0	0	0	70
9087851	f	cn	GB	2018	1	0	0	0	0	0	0	0	0	0	0	0	65
9232175	m	ot	US	2018	0	0	0	0	0	0	0	0	0	0	0	0	56
9287275	f	cn	IE	2018	0	0	0	0	0	0	0	0	0	0	0	0	67
9332862	f	cn	US	2018	0	0	0	0	0	0	0	0	0	0	0	0	53
9358029	m	cn	GB	2018	1	0	0	0	0	0	0	0	0	0	0	0	61
9361682	m	cn	GB	2018	1	0	0	0	0	0	0	0	0	0	0	0	59
9421810	m	cn	IE	2018	0	0	0	0	0	0	0	0	0	0	0	0	57
9493886	m	md	JP	2018	0	0	0	0	0	0	0	0	0	0	0	0	75
9668610	m	cn	IE	2018	0	0	0	0	0	0	0	0	0	0	0	0	74
9680333	m	cn	TW	2018	0	0	0	0	0	0	0	0	0	0	0	0	
9689819	m	cn	JP	2018	0	0	0	0	0	0	0	0	0	0	0	0	67
9806330	f	cn	BR	2018	0	0	0	0	0	0	0	0	0	0	0	0	66
9812188	m	cn	DK	2018	0	0	0	0	0	0	0	0	0	0	0	0	72
9835677	m	cn	IE	2018	0	0	0	0	0	0	0	0	0	0	0	0	59
9878201	m	md	JP	2018	0	0	0	0	0	0	0	0	0	0	0	0	68
9918176	m	cn	IE	2018	0	0	0	0	0	0	0	0	0	0	0	0	60
9956365	m	cn	IE	2018	0	0	0	0	0	0	0	0	0	0	0	0	66
10033724	m	md	JP	2018	0	0	0	0	0	0	0	0	0	0	0	0	85
10233574	m	cn	AU	2018	0	0	0	0	0	0	0	0	0	0	0	0	62
10686312	f	md	ES	2018	1	0	0	0	0	0	0	0	0	0	0	0	84.7
13363819	m	ot	JP	2018	0	1	0	0	1	0	0	0	0	0	0	0	67
13518745	m	ot	DE	2018	0	1	0	0	1	0	0	0	0	0	0	0	85
13655077	m	ph	JP	2018	0	1	1	0	0	0	0	0	0	0	0	0	64
13754654	f	cn	JP	2018	0	1	1	0	0	0	0	0	0	0	0	1	66
13772629	m	ot	JP	2018	0	1	0	0	1	0	0	0	0	0	0	0	68
13840246	m	ot	JP	2018	0	1	0	0	1	0	0	0	0	0	0	0	72
13891886	m	cn	JP	2018	0	1	1	0	0	0	0	0	0	0	0	0	78

13930046	f	cn	JP	2018	0	0	0	0	0	0	1	1	0	0	0	0	72
13943184	f	md	JP	2018	0	1	0	0	1	0	0	0	0	0	0	0	82
13947881	m	md	US	2018	0	1	0	0	0	1	0	0	0	0	0	0	73
13970482	m	ot	DE	2018	0	1	0	0	1	0	0	0	0	0	0	0	66
13995805	m	cn	JP	2018	0	1	1	0	0	0	0	0	0	0	0	0	64
13997970	f	ot	DE	2018	0	1	0	0	1	0	0	0	0	0	0	0	79
14007868	f	md	JP	2018	0	1	0	0	1	0	0	0	0	0	0	0	68
14033639	m	ot	DE	2018	0	1	0	0	1	0	0	0	0	0	0	0	75
14046263	f	ot	CA	2018	0	0	0	0	0	0	0	0	0	0	0	0	
14090864	m	cn	US	2018	0	1	0	0	1	0	0	0	0	0	0	0	
14092850	m	ot	JP	2018	0	1	0	0	1	0	0	0	0	0	0	0	64
14113872	m	md	JP	2018	0	1	0	0	1	0	0	0	0	0	0	0	58
14116753	m	md	JP	2018	0	1	0	0	1	0	0	0	0	0	0	0	74
14122933	m	ot	JP	2018	0	1	0	0	1	0	0	0	0	0	0	0	68
14132414	m	md	JP	2018	0	1	0	0	1	0	0	0	0	0	0	0	74
14133352	m	md	JP	2018	0	1	0	0	1	0	0	0	0	0	0	0	70
14151165	Not	ot	DE	2018	0	0	0	0	0	0	0	0	0	0	0	0	
14193087	m	cn	US	2018	0	1	1	0	0	0	0	0	0	0	0	1	
14195558	f	ot	DE	2018	0	1	0	0	1	0	0	0	0	0	0	1	51
14207062	m	md	JP	2018	0	1	1	0	0	0	0	0	0	0	0	0	82
14221001	f	md	JP	2018	1	0	0	0	0	0	0	0	0	0	0	0	72.8
14236709	m	md	JP	2018	0	0	0	0	0	0	0	0	0	0	0	0	49
14241830	f	md	JP	2018	0	1	0	0	1	0	0	0	0	0	0	0	44
14241903	m	md	IE	2018	0	1	0	0	0	1	0	0	0	0	0	0	61
14241925	f	ot	DE	2018	0	1	0	0	1	0	0	0	0	0	0	0	53
14249198	m	md	JP	2018	0	1	0	0	1	0	0	0	0	0	0	0	68
14253849	m	md	JP	2018	0	1	0	0	1	0	0	0	0	0	0	0	69
14255101	m	md	JP	2018	0	1	0	0	1	0	0	0	0	0	0	0	75
14261571	m	md	DE	2018	0	0	0	0	0	0	0	0	0	0	0	0	64
14263158	f	cn	US	2018	1	0	0	0	0	0	0	0	0	0	0	0	
14264068	f	md	JP	2018	0	1	0	0	1	0	0	0	0	0	0	0	68
14266842	m	md	JP	2018	0	1	0	0	1	0	0	0	0	0	0	0	52
14268555	m	md	JP	2018	0	1	0	0	1	0	0	0	0	0	0	0	74
14272639	m	md	FR	2018	0	1	0	0	1	0	0	0	0	0	0	0	67

14279156	f	md	JP	2018	0	1	0	0	1	0	0	0	0	0	0	0	67
14279746	m	md	JP	2018	0	1	0	0	1	0	0	0	0	0	0	0	60
14280227	m	ot	JP	2018	0	1	0	0	1	0	0	0	0	0	0	0	79
14287301	f	ot	JP	2018	0	1	0	0	1	0	0	0	0	0	0	0	66
14287339	m	ot	DE	2018	0	1	0	0	1	0	0	0	0	0	0	0	56
14289467	f	md	JP	2018	0	1	0	0	1	0	0	0	0	0	0	0	57
14289830	m	ot	JP	2018	0	1	0	0	1	0	0	0	0	0	0	1	61
14290229	m	md	JP	2018	0	0	0	0	0	0	0	0	0	0	0	0	
14293951	m	md	JP	2018	0	1	0	0	1	0	0	0	0	0	0	0	79
14297529	m	md	JP	2018	0	1	0	0	1	0	0	0	0	0	0	0	72
14298037	f	md	JP	2018	0	1	1	0	0	0	0	0	0	0	0	0	63
14298245	m	md	DE	2018	0	0	0	0	0	0	0	0	0	0	0	0	76
14300349	m	ot	US	2018	0	1	0	0	1	0	0	0	0	0	0	0	70
14308726	f	md	JP	2018	0	1	0	0	1	0	0	0	0	0	0	0	70
14310658	m	md	CH	2018	0	1	0	0	1	0	0	0	0	0	0	0	53
14310815	m	ot	DE	2018	0	1	0	0	1	0	0	0	0	0	0	0	67
14312402	m	md	JP	2018	0	1	0	0	1	0	0	0	0	0	0	1	66
14322541	m	md	JP	2018	0	1	0	0	1	0	0	0	0	0	0	0	78
14324669	f	ot	US	2018	0	1	0	0	1	0	0	0	0	0	0	0	68
14325216	m	md	JP	2018	0	1	0	0	1	0	0	0	0	0	0	0	51
14326531	m	md	JP	2018	0	1	0	0	1	0	0	0	0	0	0	1	65
14326870	f	ot	IT	2018	0	1	0	0	1	0	0	0	0	0	0	0	53
14327227	m	cn	US	2018	0	1	1	0	0	0	0	0	0	0	0	0	80
14327230	m	md	CN	2018	0	1	0	1	0	0	0	0	0	0	0	0	73
14328866	m	ot	DE	2018	0	0	0	0	0	0	0	0	0	0	0	0	77
14329333	f	md	JP	2018	1	0	0	0	0	0	0	0	0	0	0	0	75
14329654	f	md	US	2018	0	1	0	1	0	0	0	0	0	0	0	0	73
14331472	m	md	JP	2018	0	1	0	0	1	0	0	0	0	0	0	0	75
14332695	f	ot	BE	2018	0	0	0	0	0	0	0	0	0	0	0	0	59
14335103	f	ph	JP	2018	0	1	0	0	1	0	0	0	0	0	0	0	56
14335355	m	md	JP	2018	0	1	0	0	1	0	0	0	0	0	0	0	71
14336660	f	md	JP	2018	0	1	0	0	1	0	0	0	0	0	0	0	78
14337623	m	md	JP	2018	0	1	0	0	1	0	0	0	0	0	0	0	78
14338918	m	md	JP	2018	0	1	0	0	1	0	0	0	0	0	0	0	64

14340248	m	md	DE	2018	0	0	0	0	0	0	0	0	0	0	0	0	76
14346552	m	md	TW	2018	0	1	0	1	0	0	0	0	0	0	0	0	66.9
14347468	f	md	JP	2018	0	1	0	0	1	0	0	0	0	0	0	0	69
14349333	m	cn	US	2018	0	1	0	0	1	0	0	0	0	0	0	0	
14350067	m	md	JP	2018	0	1	0	0	1	0	0	0	0	0	0	0	58
14350116	m	md	JP	2018	0	1	0	0	1	0	0	0	0	0	0	0	79
14350419	m	cn	US	2018	0	1	0	0	1	0	0	0	0	0	0	0	
14351707	m	md	JP	2018	0	1	0	0	1	0	0	0	0	0	0	0	71
14351872	m	md	JP	2018	0	1	0	0	1	0	0	0	0	0	0	0	60
14353728	f	cn	US	2018	0	1	0	0	1	0	0	0	0	0	0	0	
14353751	m	cn	US	2018	0	1	0	0	1	0	0	0	0	0	0	0	82
14354753	m	md	JP	2018	0	1	0	0	1	0	0	0	0	0	0	0	66
14356018	f	md	PL	2018	0	1	0	0	0	1	0	0	0	0	0	0	38
14360500	m	cn	US	2018	0	0	0	0	0	0	1	0	1	0	0	0	
14363740	m	ot	US	2018	0	1	0	0	1	0	0	0	0	0	0	0	
14364128	f	md	SK	2018	0	0	0	0	0	0	0	0	0	0	0	0	56
14372163	m	md	US	2018	0	1	0	0	0	1	0	0	0	0	0	0	67
14372546	m	cn	US	2018	0	1	0	0	1	0	0	0	0	0	0	0	
14373549	m	md	DE	2018	0	0	0	0	0	0	0	0	0	0	0	0	63
14373712	f	cn	US	2018	0	1	0	0	1	0	0	0	0	0	0	0	
14374488	f	cn	US	2018	0	1	0	0	1	0	0	0	0	0	0	0	55
14374539	f	md	JP	2018	0	1	0	0	1	0	0	0	0	0	0	0	55
14375141	f	md	JP	2018	0	1	0	0	1	0	0	0	0	0	0	0	70
14375628	m	md	JP	2018	0	1	0	0	1	0	0	0	0	0	0	0	59
14375957	m	cn	US	2018	0	1	1	0	0	0	0	0	0	0	0	0	71
14376311	m	md	JP	2018	0	1	0	0	1	0	0	0	0	0	0	1	68
14377571	m	md	JP	2018	0	1	0	0	1	0	0	0	0	0	0	0	75
14378019	m	ot	DE	2018	0	0	0	0	0	0	0	0	0	0	0	0	77
14378973	m	ot	JP	2018	0	1	0	0	1	0	0	0	0	0	0	0	68
14381039	m	md	JP	2018	0	1	0	0	1	0	0	0	0	0	0	1	64
14381394	m	Not repo	JP	2018	0	0	0	0	0	0	0	0	0	0	0	0	66
14384706	f	md	JP	2018	1	0	0	0	0	0	0	0	0	0	0	0	45
14390129	m	ot	US	2018	0	1	0	0	1	0	0	0	0	0	0	0	
14390138	f	md	JP	2018	1	0	0	0	0	0	0	0	0	0	0	0	78.1

14391299	m	md	JP	2018	0	1	0	0	1	0	0	0	0	0	0	1	84
14393209	m	md	JP	2018	0	1	0	0	1	0	0	0	0	0	0	0	79
14398183	m	md	JP	2018	0	1	0	0	1	0	0	0	0	0	0	1	76
14401764	m	md	CZ	2018	0	1	0	0	1	0	0	0	0	0	0	0	67
14402966	m	md	DE	2018	0	1	1	0	0	0	0	0	0	0	0	0	60
14403345	m	md	JP	2018	0	1	0	0	1	0	0	0	0	0	0	0	71
14403568	m	cn	JP	2018	0	1	1	0	0	0	0	0	0	0	0	1	90
14403657	f	md	DE	2018	0	1	0	0	1	0	0	0	0	0	0	0	65
14404024	m	md	JP	2018	0	0	0	0	0	0	0	0	0	0	0	0	47
14406635	f	ot	DE	2018	0	0	0	0	0	0	0	0	0	0	0	0	45
14413681	f	ot	DE	2018	0	0	0	0	0	0	0	0	0	0	0	0	75
14414342	m	md	US	2018	0	1	0	0	0	1	0	0	0	0	0	0	59
14414588	m	cn	JP	2018	0	1	1	0	0	0	0	0	0	0	0	0	81
14416496	m	cn	JP	2018	0	1	0	0	1	0	0	0	0	0	0	0	76
14418032	f	md	JP	2018	0	1	0	0	1	0	0	0	0	0	0	0	82
14419245	m	cn	GB	2018	1	0	0	0	0	0	0	0	0	0	0	0	50
14419539	f	ot	DE	2018	0	0	0	0	0	0	0	0	0	0	0	0	57
14422036	m	ot	DE	2018	0	1	0	0	1	0	0	0	0	0	0	0	61
14422726	m	md	TW	2018	0	1	0	1	0	0	0	0	0	0	0	0	67
14424009	m	md	US	2018	0	1	0	0	0	1	0	0	0	0	0	0	70
14424377	m	md	JP	2018	0	1	0	0	1	0	0	0	0	0	0	0	69
14424974	m	md	FR	2018	0	0	0	0	0	0	0	0	0	0	0	0	57
14425264	f	md	DE	2018	0	0	0	0	0	0	0	0	0	0	0	0	75
14431700	f	md	JP	2018	1	0	0	0	0	0	0	0	0	0	0	0	85
14432275	m	md	JP	2018	0	1	0	0	1	0	0	0	0	0	0	0	74
14433010	f	ot	MX	2018	0	1	0	0	1	0	0	0	0	0	0	0	82
14436739	m	md	ES	2018	0	1	0	0	0	1	0	0	0	0	0	0	65
14438148	m	md	BE	2018	0	1	0	1	0	0	0	0	0	0	0	0	
14438826	m	md	IT	2018	1	0	0	0	0	0	0	0	0	0	0	0	71.5
14438831	m	ph	JP	2018	1	0	0	0	0	0	0	0	0	0	0	0	61
14444323	m	md	JP	2018	0	1	0	0	1	0	0	0	0	0	0	0	74
14444325	m	md	JP	2018	0	1	0	0	1	0	0	0	0	0	0	0	71
14445464	m	Not repo	Not re	2018	0	0	0	0	0	0	0	0	0	0	0	0	76
14448091	f	Not repo	JP	2018	0	0	0	0	0	0	0	0	0	0	0	0	43

14448977	m	cn	JP	2018	0	1	0	0	1	0	0	0	0	0	0	0	76
14449494	m	md	JP	2018	0	1	0	0	1	0	0	0	0	0	0	0	65
14454094	m	md	JP	2018	0	1	0	0	1	0	0	0	0	0	0	0	67
14455848	m	ot	JP	2018	0	1	0	0	1	0	0	0	0	0	0	0	72
14467917	f	cn	US	2018	0	0	0	0	0	0	0	0	0	0	0	0	74
14468565	f	Not repo	JP	2018	0	0	0	0	0	0	0	0	0	0	0	0	73
14468567	f	Not repo	JP	2018	0	0	0	0	0	0	0	0	0	0	0	0	64
14472216	m	md	FR	2018	0	1	0	1	0	0	0	0	0	0	0	0	63.3
14474938	m	cn	DE	2018	0	0	0	0	0	0	0	0	0	0	0	0	71
14479056	f	md	BE	2018	0	0	0	0	0	0	0	0	0	0	0	0	75
14480200	f	cn	DE	2018	0	1	0	0	1	0	0	0	0	0	0	0	65
14483737	m	md	CA	2018	0	1	0	0	0	1	0	0	0	0	0	0	54
14484710	Not	ot	ES	2018	0	0	0	0	0	0	0	0	0	0	0	0	70
14484787	m	ot	US	2018	0	1	0	0	1	0	0	0	0	0	0	0	48
14485282	f	cn	US	2018	0	0	0	0	0	0	0	0	0	0	0	0	74
14491210	m	md	JP	2018	1	0	0	0	0	0	0	0	0	0	0	1	84.7
14491305	m	ot	DE	2018	0	0	0	0	0	0	0	0	0	0	0	0	76
14491335	f	md	DE	2018	0	0	0	0	0	0	0	0	0	0	0	0	57
14491776	m	md	CH	2018	0	0	0	0	0	0	0	0	0	0	0	0	66
14493582	f	cn	US	2018	0	0	0	0	0	0	0	0	0	0	0	0	80
14496595	f	md	US	2018	0	1	0	0	0	1	0	0	0	0	0	0	69
14496958	f	md	JP	2018	0	1	0	0	1	0	0	0	0	0	0	0	53
14497172	m	md	US	2018	0	0	0	0	0	0	0	0	0	0	0	0	59
14497854	m	cn	RO	2018	0	0	0	0	0	0	0	0	0	0	0	0	
14498863	m	md	JP	2018	0	1	0	0	1	0	0	0	0	0	0	0	71
14505517	f	ot	JP	2018	0	1	0	0	1	0	0	0	0	0	0	0	80
14507548	f	md	KR	2018	1	0	0	0	0	0	0	0	0	0	0	0	58
14508436	m	cn	FR	2018	0	1	0	0	1	0	0	0	0	0	0	0	
14511950	f	md	JP	2018	1	0	0	0	0	0	0	0	0	0	0	0	83
14512503	m	cn	JP	2018	0	1	1	0	0	0	0	0	0	0	0	1	71
14516557	m	ot	JP	2018	0	1	0	0	1	0	0	0	0	0	0	0	81
14516587	m	md	JP	2018	0	1	0	0	1	0	0	0	0	0	0	0	66
14516634	m	cn	JP	2018	0	1	1	0	0	0	0	0	0	0	0	0	70
14516710	f	cn	US	2018	0	1	0	0	1	0	0	0	0	0	0	0	57

14516837	f	md	BE	2018	1	0	0	0	0	0	0	0	0	0	0	0	64.1
14524718	m	cn	US	2018	1	0	0	0	0	0	0	0	0	0	0	0	
14524897	m	md	DE	2018	0	0	0	0	0	0	0	0	0	0	0	0	82
14526329	f	ph	US	2018	0	1	1	0	0	0	0	0	0	0	0	0	
14527503	m	cn	NL	2018	0	1	1	0	0	0	0	0	0	0	0	0	
14528691	m	md	JP	2018	0	1	0	0	1	0	0	0	0	0	0	0	67
14530873	Not	md	JP	2018	0	0	0	0	0	0	0	0	0	0	0	0	
14531085	m	ot	JP	2018	0	1	0	0	1	0	0	0	0	0	0	0	66
14531192	f	md	JP	2018	1	0	0	0	0	0	0	0	0	0	0	0	59
14531297	f	Not repo	US	2018	1	0	0	0	0	0	0	0	0	0	0	0	84.7
14533475	f	md	DE	2018	0	0	0	0	0	0	0	0	0	0	0	0	56
14534476	Not	ph	JP	2018	1	0	0	0	0	0	0	0	0	0	0	0	
14534900	m	md	DE	2018	0	0	0	0	0	0	0	0	0	0	0	0	82
14534969	f	md	DE	2018	1	0	0	0	0	0	0	0	0	0	0	0	38
14534999	f	ot	DE	2018	0	0	0	0	0	0	0	0	0	0	0	0	57
14537824	Not	ph	US	2018	0	1	0	0	1	0	0	0	0	0	0	0	
14538037	f	md	JP	2018	0	1	0	0	1	0	0	0	0	0	0	0	49
14538284	f	cn	US	2018	1	0	0	0	0	0	0	0	0	0	0	0	57.8
14538606	f	Not repo	US	2018	1	0	0	0	0	0	0	0	0	0	0	0	
14538625	m	cn	IT	2018	0	0	0	0	0	0	0	0	0	0	0	0	50
14538776	m	ot	US	2018	0	1	0	0	1	0	0	0	0	0	0	0	72
14538924	f	ot	US	2018	0	1	0	0	1	0	0	0	0	0	0	0	74
14539638	f	md	US	2018	0	1	0	1	0	0	0	0	0	0	0	0	59.7
14541810	m	md	IE	2018	0	0	0	0	0	0	0	0	0	0	0	0	60
14542936	m	ot	JP	2018	0	1	0	0	1	0	0	0	0	0	0	0	80
14546633	m	ot	DE	2018	0	1	0	0	1	0	0	0	0	0	0	0	62
14547564	m	cn	US	2018	0	1	1	0	0	0	0	0	0	0	0	0	
14552521	m	ot	JP	2018	0	1	0	0	1	0	0	0	0	0	0	0	74
14552823	f	ot	DE	2018	0	0	0	0	0	0	0	0	0	0	0	0	69
14552846	Not	Not repo	Not re	2018	1	0	0	0	0	0	0	0	0	0	0	1	
14553193	f	ot	JP	2018	0	1	1	0	0	0	0	0	0	0	0	0	68
14555891	f	md	JP	2018	0	1	0	0	1	0	0	0	0	0	0	0	74
14555902	m	cn	JP	2018	0	1	1	0	0	0	0	0	0	0	0	0	71
14555986	m	ot	DE	2018	0	1	0	0	1	0	0	0	0	0	0	0	69

14556434	m	cn	JP	2018	0	1	1	0	0	0	0	0	0	0	0	0	69
14559417	m	cn	DE	2018	0	0	0	0	0	0	0	0	0	0	0	0	63
14561104	f	ot	FR	2018	0	1	0	0	1	0	0	0	0	0	0	0	62
14565733	f	md	JP	2018	1	0	0	0	0	0	0	0	0	0	0	0	68
14566163	m	md	JP	2018	0	1	1	0	0	0	0	0	0	0	0	1	70
14566261	f	md	US	2018	0	0	0	0	0	0	0	0	0	0	0	0	63
14567160	m	md	JP	2018	1	0	0	0	0	0	0	0	0	0	0	0	58.8
14567319	m	md	FR	2018	0	0	0	0	0	0	0	0	0	0	0	0	53
14567496	m	ot	ES	2018	1	0	0	0	0	0	0	0	0	0	0	0	64
14568034	m	md	CZ	2018	1	0	0	0	0	0	0	0	0	0	0	0	54
14568127	m	md	DE	2018	0	0	0	0	0	0	0	0	0	0	0	0	81
14568306	m	ot	DE	2018	0	1	0	0	1	0	0	0	0	0	0	0	80
14569485	f	md	US	2018	0	1	0	0	0	1	0	0	0	0	0	0	56
14570971	m	ot	JP	2018	0	1	0	0	1	0	0	0	0	0	0	1	74
14571459	m	ot	US	2018	0	0	0	0	0	0	0	0	0	0	0	0	82
14574823	m	md	BR	2018	0	1	0	0	0	1	0	0	0	0	0	0	74
14575856	f	md	AU	2018	0	1	0	0	0	1	0	0	0	0	0	0	
14576830	m	ot	US	2018	0	0	0	0	0	0	0	0	0	0	0	0	67
14579309	m	ot	ES	2018	0	1	0	0	1	0	0	0	0	0	0	0	61
14579907	m	ot	DE	2018	0	1	0	0	1	0	0	0	0	0	0	0	67
14580263	m	md	JP	2018	0	1	0	0	1	0	0	0	0	0	0	0	68
14580431	m	md	IT	2018	0	1	0	0	1	0	0	0	0	0	0	0	58
14581488	m	ot	TW	2018	0	1	0	1	0	0	0	0	0	0	0	0	68
14582599	f	md	DE	2018	0	0	0	0	0	0	0	0	0	0	0	0	57
14583500	m	ot	DE	2018	0	0	0	0	0	0	0	0	0	0	0	0	71
14584165	m	md	US	2018	1	0	0	0	0	0	0	0	0	0	0	1	93.2
14584842	m	md	JP	2018	0	1	0	0	1	0	0	0	0	0	0	0	67
14585410	f	md	ES	2018	0	1	0	0	0	1	0	0	0	0	0	0	60
14586437	f	ph	JP	2018	0	1	1	0	0	0	0	0	0	0	0	0	74
14587547	m	md	JP	2018	1	0	0	0	0	0	0	0	0	0	0	0	81
14587808	m	ph	JP	2018	1	0	0	0	0	0	0	0	0	0	0	1	75
14588811	f	ot	HU	2018	0	1	0	0	1	0	0	0	0	0	0	0	49
14590052	m	md	GB	2018	1	0	0	0	0	0	0	0	0	0	0	0	70
14590517	f	md	JP	2018	0	0	0	0	0	0	1	0	1	0	0	0	78

14591508	f	md	US	2018	0	1	0	1	0	0	0	0	0	0	0	0	59.9
14592742	m	md	JP	2018	0	1	1	0	0	0	0	0	0	0	0	0	72
14592927	f	ot	DK	2018	1	0	0	0	0	0	0	0	0	0	0	0	53
14593448	m	md	JP	2018	0	1	0	0	1	0	0	0	0	0	0	0	75
14593753	m	md	BE	2018	0	0	0	0	0	0	0	0	0	0	0	0	53
14597482	m	md	KR	2018	0	1	0	0	0	1	0	0	0	0	0	0	61
14598242	m	md	DE	2018	0	0	0	0	0	0	0	0	0	0	0	0	59
14599343	m	ph	JP	2018	0	1	0	0	1	0	0	0	0	0	0	0	82
14599436	m	md	JP	2018	0	1	0	0	1	0	0	0	0	0	0	0	70
14599939	m	md	US	2018	0	0	0	0	0	0	0	0	0	0	0	0	72
14600700	m	cn	DE	2018	0	0	0	0	0	0	0	0	0	0	0	0	59
14601799	Not	ph	JP	2018	1	0	0	0	0	0	0	0	0	0	0	0	
14602891	f	md	US	2018	0	0	0	0	0	0	0	0	0	0	0	0	65
14604254	m	md	JP	2018	0	1	0	0	1	0	0	0	0	0	0	0	61
14604267	m	cn	US	2018	0	1	1	0	0	0	0	0	0	0	0	0	78
14604502	f	md	JP	2018	1	0	0	0	0	0	0	0	0	0	0	0	67
14605263	f	ot	FR	2018	0	1	0	0	1	0	0	0	0	0	0	0	56
14607922	m	md	JP	2018	0	1	0	0	1	0	0	0	0	0	0	0	62
14608163	m	ot	US	2018	0	1	0	0	1	0	0	0	0	0	0	0	72
14608275	m	md	JP	2018	0	1	0	0	1	0	0	0	0	0	0	0	74
14608277	f	ph	US	2018	0	1	0	0	1	0	0	0	0	0	0	0	
14608289	m	ot	DE	2018	0	0	0	0	0	0	0	0	0	0	0	0	67
14610205	f	md	JP	2018	0	1	0	0	1	0	0	0	0	0	0	0	73
14611441	m	md	LU	2018	0	1	1	0	0	0	0	0	0	0	0	0	66
14611481	m	ot	IT	2018	0	1	0	0	1	0	0	0	0	0	0	0	50
14611635	f	md	DE	2018	0	0	0	0	0	0	0	0	0	0	0	0	67
14612049	m	md	JP	2018	0	1	0	0	1	0	0	0	0	0	0	0	74
14615524	m	md	BE	2018	0	1	1	0	0	0	0	0	0	0	0	0	65
14617144	f	md	TR	2018	0	0	0	0	0	0	0	0	0	0	0	0	64
14617173	f	md	JP	2018	0	1	0	0	1	0	0	0	0	0	0	0	67
14617408	m	md	HU	2018	0	0	0	0	0	0	0	0	0	0	0	0	81
14617609	f	md	JP	2018	1	0	0	0	0	0	0	0	0	0	0	0	56
14618555	m	cn	JP	2018	0	1	1	0	0	0	0	0	0	0	0	0	65
14619744	m	cn	FR	2018	0	1	0	0	1	0	0	0	0	0	0	0	79

14620115	Not	ot	NL	2018	0	0	0	0	0	0	0	0	0	0	0	0	71
14620819	f	ot	DE	2018	0	1	0	0	1	0	0	0	0	0	0	0	57
14621986	f	md	US	2018	0	1	0	0	1	0	0	0	0	0	0	0	72
14623542	f	md	GB	2018	0	1	1	0	0	0	0	0	0	0	0	0	78
14626181	m	md	AU	2018	0	1	0	0	0	1	0	0	0	0	0	0	70
14626970	f	md	JP	2018	0	0	0	0	0	0	0	0	0	0	0	0	74
14627081	m	ot	US	2018	0	1	0	0	1	0	0	0	0	0	0	0	78
14627480	Not	md	US	2018	0	0	0	0	0	0	0	0	0	0	0	0	
14627488	Not	md	US	2018	0	0	0	0	0	0	0	0	0	0	0	0	
14627494	Not	md	US	2018	0	0	0	0	0	0	0	0	0	0	0	0	
14629127	m	md	JP	2018	0	1	0	0	1	0	0	0	0	0	0	0	66
14630407	m	ot	JP	2018	0	1	0	0	1	0	0	0	0	0	0	0	76
14634400	m	md	JP	2018	1	0	0	0	0	0	0	0	0	0	0	0	59
14634982	m	md	KR	2018	0	0	0	0	0	0	0	0	0	0	0	0	57
14635473	m	cn	GB	2018	1	0	0	0	0	0	0	0	0	0	0	0	59
14636475	f	cn	US	2018	0	0	0	0	0	0	0	0	0	0	0	0	
14639278	m	cn	US	2018	0	1	0	0	1	0	0	0	0	0	0	0	
14640183	m	cn	US	2018	0	1	0	0	1	0	0	0	0	0	0	0	
14641733	m	md	DE	2018	0	0	0	0	0	0	0	0	0	0	0	0	68
14641965	m	ot	DE	2018	0	1	0	0	1	0	0	0	0	0	0	0	65
14642459	f	Not repo	US	2018	1	0	0	0	0	0	0	0	0	0	0	0	89
14643521	m	md	JP	2018	0	1	0	0	1	0	0	0	0	0	0	0	75
14643756	m	md	JP	2018	0	0	0	0	0	0	0	0	0	0	0	0	78
14645553	m	cn	JP	2018	0	1	1	0	0	0	0	0	0	0	0	0	80
14646070	m	cn	JP	2018	0	1	1	0	0	0	0	0	0	0	0	1	68
14646107	f	md	JP	2018	0	1	1	0	0	0	0	0	0	0	0	0	73
14646259	f	ot	KR	2018	0	1	0	0	1	0	0	0	0	0	0	0	59
14648251	f	md	DE	2018	0	0	0	0	0	0	0	0	0	0	0	0	48
14649338	m	md	JP	2018	0	1	1	0	0	0	0	0	0	0	0	1	61
14650262	f	md	US	2018	0	0	0	0	0	0	0	0	0	0	0	0	
14650811	f	cn	DE	2018	0	1	0	0	1	0	0	0	0	0	0	0	65
14651080	m	md	DE	2018	1	0	0	0	0	0	0	0	0	0	0	0	54
14651223	f	md	KR	2018	1	0	0	0	0	0	0	0	0	0	0	0	55
14652167	m	ot	DE	2018	0	0	0	0	0	0	0	0	0	0	0	0	76

14652258	f	ot	DE	2018	0	0	0	0	0	0	0	0	0	0	0	0	78
14653288	m	md	FR	2018	0	1	0	0	1	0	0	0	0	0	0	0	
14653354	Not	md	SE	2018	0	0	0	0	0	0	0	0	0	0	0	0	
14655099	m	md	JP	2018	0	1	0	0	1	0	0	0	0	0	0	0	72
14655821	m	md	FR	2018	0	0	0	0	0	0	0	0	0	0	0	0	56
14656621	m	ot	JP	2018	0	1	0	0	1	0	0	0	0	0	0	0	50
14659136	m	ot	JP	2018	0	1	0	0	1	0	0	0	0	0	0	0	64
14659265	m	ot	JP	2018	0	1	0	0	1	0	0	0	0	0	0	0	80
14661204	m	ot	JP	2018	0	1	0	0	1	0	0	0	0	0	0	0	74
14661232	f	md	CA	2018	0	0	0	0	0	0	0	0	0	0	0	0	
14662686	m	md	JP	2018	0	1	0	0	1	0	0	0	0	0	0	0	82
14662740	m	cn	JP	2018	0	1	1	0	0	0	0	0	0	0	0	0	40
14662993	m	md	JP	2018	0	1	0	0	1	0	0	0	0	0	0	0	70
14663156	m	md	DE	2018	0	0	0	0	0	0	0	0	0	0	0	0	60
14663912	m	md	LV	2018	0	1	0	0	0	1	0	0	0	0	0	0	66
14664556	m	md	GB	2018	0	1	1	0	0	0	0	0	0	0	0	0	74
14664985	f	md	DE	2018	1	0	0	0	0	0	0	0	0	0	0	0	78
14665276	m	ot	DE	2018	0	1	0	0	1	0	0	0	0	0	0	0	54
14665707	f	md	SE	2018	0	0	0	0	0	0	0	0	0	0	0	0	53
14666156	m	md	DE	2018	1	0	0	0	0	0	0	0	0	0	0	0	48
14666980	f	ph	GB	2018	0	0	0	0	0	0	0	0	0	0	0	0	43
14668504	m	cn	GB	2018	1	0	0	0	0	0	0	0	0	0	0	0	66
14668829	f	ot	US	2018	0	1	1	0	0	0	0	0	0	0	0	0	81
14669547	f	md	JP	2018	1	0	0	0	0	0	0	0	0	0	0	0	64.1
14670268	f	md	US	2018	0	1	1	0	0	0	0	0	0	0	0	0	63
14671809	f	md	IT	2018	1	0	0	0	0	0	0	0	0	0	0	0	72
14672087	m	cn	US	2018	0	0	0	0	0	0	0	0	0	0	0	0	71
14672963	f	md	IT	2018	1	0	0	0	0	0	0	0	0	0	0	0	72
14673256	m	ot	JP	2018	1	0	0	0	0	0	0	0	0	0	0	0	75
14673466	m	ph	BR	2018	0	1	0	0	0	1	0	0	0	0	0	0	61
14673672	f	md	US	2018	0	1	1	0	0	0	0	0	0	0	0	0	74
14673700	m	md	US	2018	0	1	0	1	0	0	0	0	0	0	0	0	64
14675339	m	md	DE	2018	0	0	0	0	0	0	0	0	0	0	0	0	68
14676601	m	md	FR	2018	0	1	0	0	1	0	0	0	0	0	0	0	62

14676671	m	md	JP	2018	0	1	0	0	1	0	0	0	0	0	0	0	75
14677041	m	ot	BE	2018	0	1	0	0	1	0	0	0	0	0	0	1	70
14678560	Not	ot	US	2018	0	0	0	0	0	0	0	0	0	0	0	0	
14678642	m	md	JP	2018	0	1	0	0	1	0	0	0	0	0	0	0	74
14679667	f	ot	ES	2018	0	1	0	0	1	0	0	0	0	0	0	0	53
14680459	f	md	JP	2018	0	1	0	0	1	0	0	0	0	0	0	0	
14681489	m	ot	JP	2018	0	1	0	0	1	0	0	0	0	0	0	1	66
14682609	f	md	ES	2018	0	1	0	0	0	1	0	0	0	0	0	0	52
14683443	m	md	BR	2018	0	1	0	0	0	1	0	0	0	0	0	0	61
14684291	f	ph	JP	2018	1	0	0	0	0	0	0	0	0	0	0	0	58.8
14684705	f	ot	CA	2018	0	0	0	0	0	0	0	0	0	0	0	0	53
14685113	m	md	TR	2018	0	0	0	0	0	0	0	0	0	0	0	0	60
14685436	m	cn	US	2018	0	1	1	0	0	0	0	0	0	0	0	1	
14685586	f	cn	GB	2018	1	0	0	0	0	0	0	0	0	0	0	0	
14687490	f	md	JP	2018	0	0	0	0	0	0	0	0	0	0	0	0	48
14687861	m	md	TR	2018	0	1	0	0	0	1	0	0	0	0	0	0	54
14688220	f	cn	GB	2018	1	0	0	0	0	0	0	0	0	0	0	0	
14688618	m	md	FR	2018	0	0	0	0	0	0	0	0	0	0	0	0	78
14688660	f	cn	GR	2018	0	0	0	0	0	0	0	0	0	0	0	0	
14689410	f	md	JP	2018	0	1	0	0	1	0	0	0	0	0	0	0	76
14690504	m	md	DE	2018	0	0	0	0	0	0	0	0	0	0	0	0	51
14690839	m	ot	US	2018	0	1	0	0	1	0	0	0	0	0	0	0	
14691960	m	md	JP	2018	0	1	0	0	1	0	0	0	0	0	0	0	68
14692871	m	ot	DE	2018	0	0	0	0	0	0	0	0	0	0	0	0	83
14693050	f	Not repo	JP	2018	0	0	0	0	0	0	0	0	0	0	0	0	66
14693467	m	md	ES	2018	0	1	0	0	0	1	0	0	0	0	0	0	58
14693545	m	cn	JP	2018	0	1	1	0	0	0	0	0	0	0	0	1	68
14693703	f	md	JP	2018	0	0	0	0	0	0	0	0	0	0	0	0	72
14693766	f	ot	AT	2018	0	1	0	0	1	0	0	0	0	0	0	0	67
14695238	m	md	JP	2018	1	0	0	0	0	0	0	0	0	0	0	1	66
14698642	m	md	TW	2018	0	1	0	0	0	1	0	0	0	0	0	0	50
14698836	m	ot	JP	2018	0	0	0	0	0	0	0	0	0	0	0	0	69
14699123	m	ot	DE	2018	0	0	0	0	0	0	0	0	0	0	0	0	78
14699878	f	md	DE	2018	1	0	0	0	0	0	0	0	0	0	0	0	

14700015	f	ot	US	2018	0	0	0	0	0	0	0	0	0	0	0	0	91
14700073	m	md	ES	2018	0	0	0	0	0	0	0	0	0	0	0	0	75
14702208	m	ot	JP	2018	0	1	0	0	1	0	0	0	0	0	0	0	71
14702573	Not	ot	JP	2018	0	0	0	0	0	0	0	0	0	0	0	0	
14702634	m	md	SE	2018	0	0	0	0	0	0	0	0	0	0	0	0	
14703373	m	md	CH	2018	0	1	0	0	0	1	0	0	0	0	0	0	
14703660	f	cn	GB	2018	1	0	0	0	0	0	0	0	0	0	0	0	46
14704431	m	md	JP	2018	0	1	0	0	1	0	0	0	0	0	0	1	77
14704858	Not	ot	FR	2018	0	0	0	0	0	0	0	0	0	0	0	0	
14705204	f	cn	US	2018	0	1	0	0	1	0	0	0	0	0	0	0	50
14706143	f	cn	IE	2018	0	1	1	0	0	0	0	0	0	0	0	0	52
14706162	m	md	CN	2018	0	1	0	1	0	0	0	0	0	0	0	0	72.5
14706405	f	ot	JP	2018	0	1	0	0	1	0	0	0	0	0	0	0	73
14706570	f	ot	MX	2018	0	1	0	0	1	0	0	0	0	0	0	0	79
14706587	f	md	JP	2018	0	1	0	0	1	0	0	0	0	0	0	0	73
14706860	m	cn	JP	2018	0	1	1	0	0	0	0	0	0	0	0	0	86
14707003	m	md	JP	2018	0	0	0	0	0	0	0	0	0	0	0	0	74
14707038	m	md	JP	2018	0	0	0	0	0	0	0	0	0	0	0	0	42
14707136	f	md	JP	2018	0	0	0	0	0	0	0	0	0	0	0	0	64
14707267	f	md	SE	2018	0	0	0	0	0	0	0	0	0	0	0	0	42
14707324	m	md	JP	2018	0	1	0	0	1	0	0	0	0	0	0	0	72
14707406	f	md	US	2018	0	1	0	0	1	0	0	0	0	0	0	0	78
14707560	m	md	JP	2018	0	0	0	0	0	0	0	0	0	0	0	0	74
14707740	Not	ot	FR	2018	0	0	0	0	0	0	0	0	0	0	0	0	
14707846	f	md	DE	2018	0	0	0	0	0	0	0	0	0	0	0	0	70
14708171	f	md	JP	2018	0	0	0	0	0	0	0	0	0	0	0	0	71
14708198	f	md	US	2018	0	1	0	0	0	1	0	0	0	0	0	1	64
14708207	f	md	CA	2018	0	0	0	0	0	0	0	0	0	0	0	0	86
14708293	f	md	US	2018	0	1	1	0	0	0	0	0	0	0	0	0	72
14708299	m	ot	DE	2018	0	0	0	0	0	0	0	0	0	0	0	0	73
14708432	f	md	JP	2018	0	0	0	0	0	0	0	0	0	0	0	0	40
14708437	m	md	DE	2018	0	1	0	0	0	1	0	0	0	0	0	0	55
14708584	Not	ot	ES	2018	0	1	1	0	0	0	0	0	0	0	0	0	
14708587	Not	ot	ES	2018	0	0	0	0	0	0	0	0	0	0	0	0	

14708756	f	md	US	2018	0	1	1	0	0	0	0	0	0	0	0	0	
14708884	f	cn	US	2018	1	0	0	0	0	0	0	0	0	0	0	0	
14710004	m	md	DE	2018	0	0	0	0	0	0	0	0	0	0	0	0	56
14710111	m	md	JP	2018	1	0	0	0	0	0	0	0	0	0	0	0	59.9
14710575	f	cn	US	2018	0	0	0	0	0	0	0	0	0	0	0	0	41
14711241	m	md	JP	2018	0	0	0	0	0	0	0	0	0	0	0	0	74
14711391	m	ot	JP	2018	0	0	0	0	0	0	0	0	0	0	0	0	76
14711605	m	ot	DE	2018	0	1	0	0	1	0	0	0	0	0	0	0	81
14711748	m	md	US	2018	0	0	0	0	0	0	0	0	0	0	0	0	33
14711826	f	ot	JP	2018	0	1	1	0	0	0	0	0	0	0	0	0	82
14711868	m	ot	IL	2018	0	1	0	0	1	0	0	0	0	0	0	0	48
14711965	f	md	AU	2018	0	0	0	0	0	0	0	0	0	0	0	0	
14712262	f	cn	AU	2018	0	1	0	0	1	0	0	0	0	0	0	0	
14712299	m	md	JP	2018	0	0	0	0	0	0	0	0	0	0	0	0	61
14712384	m	md	JP	2018	0	0	0	0	0	0	0	0	0	0	0	0	73
14712521	m	ot	DE	2018	0	1	0	0	1	0	0	0	0	0	0	0	82
14712554	m	md	JP	2018	0	0	0	0	0	0	0	0	0	0	0	0	67
14712604	f	md	DE	2018	0	1	0	0	1	0	0	0	0	0	0	0	53
14712652	f	md	US	2018	0	1	1	0	0	0	0	0	0	0	0	0	82
14712679	m	ph	JP	2018	0	1	0	0	1	0	0	0	0	0	0	0	65
14712683	f	md	US	2018	0	1	0	0	1	0	0	0	0	0	0	0	54
14712980	f	cn	IT	2018	0	1	1	0	0	0	0	0	0	0	0	0	70
14713319	Not	ph	US	2018	0	1	0	0	1	0	0	0	0	0	0	0	
14713350	f	md	IL	2018	0	1	0	0	0	1	0	0	0	0	0	0	68
14713481	m	md	JP	2018	0	0	0	0	0	0	0	0	0	0	0	0	70
14713686	m	md	JP	2018	1	0	0	0	0	0	0	0	0	0	0	0	72
14713768	m	md	IT	2018	0	0	0	0	0	0	0	0	0	0	0	0	65
14713873	m	md	DE	2018	0	0	0	0	0	0	0	0	0	0	0	0	
14714016	m	md	JP	2018	0	0	0	0	0	0	0	0	0	0	0	0	74
14715310	f	ot	TW	2018	0	1	0	1	0	0	0	0	0	0	0	0	55
14715346	m	md	SG	2018	0	0	0	0	0	0	0	0	0	0	0	0	54
14715367	m	ot	GR	2018	0	0	0	0	0	0	0	0	0	0	0	0	71
14715462	m	md	JP	2018	1	0	0	0	0	0	0	0	0	0	0	0	60
14715880	f	md	JP	2018	1	0	0	0	0	0	0	0	0	0	0	0	84

14715886	m	cn	JP	2018	1	0	0	0	0	0	0	0	0	0	0	0	
14716464	f	md	FR	2018	0	0	0	0	0	0	0	0	0	0	0	0	62
14716885	Not	md	KW	2018	0	0	0	0	0	0	0	0	0	0	0	0	
14716990	m	ot	US	2018	0	1	0	0	1	0	0	0	0	0	0	0	50
14718603	f	ot	DE	2018	0	1	0	0	1	0	0	0	0	0	0	0	52
14718649	f	md	JP	2018	0	0	0	0	0	0	0	0	0	0	0	0	74
14718660	m	md	JP	2018	0	0	0	0	0	0	0	0	0	0	0	0	60
14718680	m	md	JP	2018	0	0	0	0	0	0	0	0	0	0	0	0	64
14718687	f	cn	US	2018	0	1	1	0	0	0	0	0	0	0	0	1	
14718724	m	cn	US	2018	1	0	0	0	0	0	0	0	0	0	0	0	
14718889	f	cn	US	2018	1	0	0	0	0	0	0	0	0	0	0	0	71
14719244	m	md	US	2018	0	1	0	0	1	0	0	0	0	0	0	0	51
14719342	m	ot	GR	2018	0	0	0	0	0	0	0	0	0	0	0	0	71
14719343	m	md	DE	2018	0	0	0	0	0	0	0	0	0	0	0	0	75
14719416	f	ot	DE	2018	0	0	0	0	0	0	0	0	0	0	0	0	68
14719463	Not	md	IT	2018	0	1	0	0	1	0	0	0	0	0	0	0	
14720079	m	cn	JP	2018	0	1	1	0	0	0	0	0	0	0	0	0	68
14720470	m	md	JP	2018	1	0	0	0	0	0	0	0	0	0	0	0	70
14720528	m	md	JP	2018	0	0	0	0	0	0	0	0	0	0	0	0	65
14720570	m	md	JP	2018	0	0	0	0	0	0	0	0	0	0	0	0	73
14720729	m	ot	DE	2018	0	1	0	0	1	0	0	0	0	0	0	0	66
14720844	m	md	JP	2018	0	0	0	0	0	0	0	0	0	0	0	0	70
14721075	f	md	JP	2018	0	0	0	0	0	0	0	0	0	0	0	0	72
14722065	m	md	CH	2018	0	0	0	0	0	0	0	0	0	0	0	0	
14723434	m	md	CN	2018	1	0	0	0	0	0	0	0	0	0	0	0	76.5
14723520	m	md	JP	2018	1	0	0	0	0	0	0	0	0	0	0	0	48
14725344	m	cn	US	2018	0	1	0	0	1	0	0	0	0	0	0	0	
14725415	m	cn	US	2018	1	0	0	0	0	0	0	0	0	0	0	0	78
14725445	m	md	ES	2018	0	0	0	0	0	0	0	0	0	0	0	0	56
14725485	m	md	DE	2018	1	0	0	0	0	0	0	0	0	0	0	0	50
14725644	f	md	JP	2018	0	0	0	0	0	0	0	0	0	0	0	0	60
14725912	Not	ot	JP	2018	0	1	0	0	1	0	0	0	0	0	0	0	
14725971	m	md	US	2018	0	1	0	1	0	0	0	0	0	0	0	0	
14726000	Not	ot	US	2018	0	0	0	0	0	0	0	0	0	0	0	0	

14726075	m	md	US	2018	1	0	0	0	0	0	0	0	0	0	0	0	59
14726381	Not	ot	FR	2018	0	0	0	0	0	0	0	0	0	0	0	0	
14726419	m	md	JP	2018	0	0	0	0	0	0	0	0	0	0	0	0	67
14726562	f	cn	GB	2018	1	0	0	0	0	0	0	0	0	0	0	0	55
14726648	m	md	JP	2018	0	0	0	0	0	0	0	0	0	0	0	0	67
14726984	m	md	JP	2018	0	0	0	0	0	0	0	0	0	0	0	0	68
14727089	m	md	JP	2018	0	1	0	0	0	1	0	0	0	0	0	0	76
14727090	f	md	JP	2018	1	0	0	0	0	0	0	0	0	0	0	0	68.5
14727123	Not	ot	JP	2018	0	1	0	0	1	0	0	0	0	0	0	0	70
14727196	m	Not repo	US	2018	1	0	0	0	0	0	0	0	0	0	0	0	
14727613	m	cn	US	2018	0	1	1	0	0	0	0	0	0	0	0	0	72
14727620	f	ot	JP	2018	0	0	0	0	0	0	0	0	0	0	0	0	80
14727744	m	md	JP	2018	0	0	0	0	0	0	0	0	0	0	0	0	42
14727892	m	md	JP	2018	0	0	0	0	0	0	0	0	0	0	0	0	66
14728103	Not	md	US	2018	1	0	0	0	0	0	0	0	0	0	0	1	
14728436	m	md	RO	2018	0	0	0	0	0	0	0	0	0	0	0	0	
14728446	m	md	US	2018	0	1	1	0	0	0	0	0	0	0	0	0	67
14728696	m	md	DE	2018	0	0	0	0	0	0	0	0	0	0	0	0	81
14728742	f	ot	US	2018	0	1	0	0	1	0	0	0	0	0	0	0	55
14729194	m	ot	JP	2018	0	1	0	0	1	0	0	0	0	0	0	0	67
14729608	m	ot	IT	2018	0	1	0	0	1	0	0	0	0	0	0	0	77
14730419	m	ot	JP	2018	0	1	0	0	1	0	0	0	0	0	0	0	56
14730636	f	cn	TR	2018	0	0	0	0	0	0	0	0	0	0	0	0	
14731220	m	md	BE	2018	0	1	0	0	1	0	0	0	0	0	0	0	
14731310	m	md	ES	2018	0	1	0	0	0	1	0	0	0	0	0	0	76
14731479	f	ph	JP	2018	0	1	0	0	1	0	0	0	0	0	0	0	72
14731674	m	ot	US	2018	0	0	0	0	0	0	0	0	0	0	0	0	61
14731693	m	ot	IN	2018	0	0	0	0	0	0	0	0	0	0	0	0	
14731806	m	cn	CN	2018	0	0	0	0	0	0	0	0	0	0	0	0	
14731807	m	cn	FR	2018	0	0	0	0	0	0	0	0	0	0	0	0	51
14731882	m	md	BR	2018	0	1	0	0	0	1	0	0	0	0	0	0	79
14732048	f	md	JP	2018	1	0	0	0	0	0	0	0	0	0	0	0	70
14732267	m	md	JP	2018	0	0	0	0	0	0	0	0	0	0	0	0	71
14732306	f	md	JP	2018	0	0	0	0	0	0	0	0	0	0	0	0	72

14732318	m	md	ES	2018	0	1	1	0	0	0	0	0	0	0	0	1	67
14732344	m	md	JP	2018	0	0	0	0	0	0	0	0	0	0	0	0	62
14732430	Not	md	JP	2018	0	1	0	0	1	0	0	0	0	0	0	0	
14732576	f	md	JP	2018	0	0	0	0	0	0	0	0	0	0	0	0	63
14732640	m	md	JP	2018	0	0	0	0	0	0	0	0	0	0	0	0	70
14732649	Not	md	CH	2018	0	1	0	0	0	1	0	0	0	0	0	0	
14732773	m	md	JP	2018	0	1	0	0	1	0	0	0	0	0	0	0	69
14732786	f	md	US	2018	0	0	0	0	0	0	0	0	0	0	0	0	59
14733287	m	md	JP	2018	0	0	0	0	0	0	0	0	0	0	0	0	54
14733361	Not	ph	JP	2018	0	1	0	0	1	0	0	0	0	0	0	0	
14733405	Not	md	CH	2018	0	1	0	0	0	1	0	0	0	0	0	0	
14733521	Not	md	CH	2018	0	0	0	0	0	0	0	0	0	0	0	0	
14733523	m	md	JP	2018	0	0	0	0	0	0	0	0	0	0	0	0	65
14733537	Not	md	CH	2018	0	1	0	0	0	1	0	0	0	0	0	0	
14733657	f	cn	JP	2018	0	1	1	0	0	0	0	0	0	0	0	0	57
14733965	m	md	JP	2018	0	0	0	0	0	0	0	0	0	0	0	0	69
14734141	Not	md	CH	2018	0	1	0	0	0	1	0	0	0	0	0	0	
14734153	Not	md	CH	2018	0	1	0	0	0	1	0	0	0	0	0	0	
14735129	f	md	JP	2018	0	0	0	0	0	0	0	0	0	0	0	0	31
14735148	f	cn	KR	2018	0	0	0	0	0	0	0	0	0	0	0	0	
14735160	f	md	DE	2018	0	0	0	0	0	0	0	0	0	0	0	0	61
14735161	f	md	DE	2018	0	0	0	0	0	0	0	0	0	0	0	0	63
14735401	Not	cn	JP	2018	0	1	1	0	0	0	0	0	0	0	0	0	74
14735588	m	md	CA	2018	0	1	1	0	0	0	0	0	0	0	0	0	63
14735601	f	md	DE	2018	0	0	0	0	0	0	0	0	0	0	0	0	70
14735630	f	md	US	2018	0	1	1	0	0	0	0	0	0	0	0	0	70
14737859	m	cn	JP	2018	0	1	1	0	0	0	0	0	0	0	0	0	87
14737908	Not	cn	JP	2018	0	1	1	0	0	0	0	0	0	0	0	0	66
14737940	m	md	JP	2018	0	0	0	0	0	0	0	0	0	0	0	0	60
14738396	m	ot	DE	2018	0	1	1	0	0	0	0	0	0	0	0	0	63
14738786	m	md	JP	2018	0	1	1	0	0	0	0	0	0	0	0	1	74
14739067	f	md	UY	2018	0	1	0	0	0	1	0	0	0	0	0	0	64
14739100	m	md	US	2018	0	1	0	0	0	1	0	0	0	0	0	0	74
14739468	m	cn	US	2018	0	0	0	0	0	0	0	0	0	0	0	0	76

14739564	Not	md	CH	2018	0	1	0	0	0	1	0	0	0	0	0	0		
14739658	f	md	ID	2018	1	0	0	0	0	0	0	0	0	0	0	0	75	
14740416	m	md	JP	2018	0	0	0	0	0	0	0	0	0	0	0	0	70	
14740754	m	ot	TR	2018	0	0	0	0	0	0	0	0	0	0	0	0	49	
14741210	m	cn	JP	2018	0	0	0	0	0	0	0	0	0	0	0	0	75	
14741484	m	cn	US	2018	1	0	0	0	0	0	0	0	0	0	0	0		
14741519	m	cn	JP	2018	0	1	1	0	0	0	0	0	0	0	0	0	44	
14741806	m	cn	TH	2018	0	1	1	0	0	0	0	0	0	0	0	0	63	
14741873	m	cn	VN	2018	0	1	1	0	0	0	0	0	0	0	0	0		
14742223	f	ot	US	2018	0	1	0	1	0	0	0	0	0	0	0	0	70.8	
14742253	m	cn	FR	2018	0	1	1	0	0	0	0	0	0	0	0	0		
14742485	m	md	JP	2018	0	0	0	0	0	0	0	0	0	0	0	0	53	
14742606	f	md	SE	2018	0	0	0	0	0	0	0	0	0	0	0	0	42	
14742748	f	md	JP	2018	0	1	0	0	1	0	0	0	0	0	0	0	80	
14743775	m	md	JP	2018	0	1	0	0	1	0	0	0	0	0	0	1	68	
14743778	m	md	JP	2018	0	1	0	0	1	0	0	0	0	0	0	1	48	
14744372	f	md	US	2018	1	0	0	0	0	0	0	0	0	0	0	0		
14744472	f	ot	DE	2018	0	1	0	0	1	0	0	0	0	0	0	0	74	
14744552	Not	ot	US	2018	1	0	0	0	0	0	0	0	0	0	0	0		
14744732	f	md	US	2018	0	1	0	0	0	1	0	0	0	0	0	0	64	
14744910	f	md	JP	2018	0	1	0	0	0	1	0	0	0	0	0	0	74	
14744925	m	md	JP	2018	1	0	0	0	0	0	0	0	0	0	0	0	67	
14745162	m	md	ID	2018	1	0	0	0	0	0	0	0	0	0	0	0		
14745356	m	md	ES	2018	0	1	0	0	0	1	0	0	0	0	0	0	70	
14745358	Not	md	JP	2018	1	0	0	0	0	0	0	0	0	0	0	0		
14745474	m	md	JP	2018	0	0	0	0	0	0	0	0	0	0	0	0	73	
14746445	f	md	JP	2018	0	0	0	0	0	0	0	0	0	0	0	0	63	
14746958	m	md	FR	2018	0	1	0	0	1	0	0	0	0	0	0	0	63	
14748262	m	ot	AU	2018	0	1	0	0	1	0	0	0	0	0	0	0	77	
14749801	f	md	CZ	2018	1	0	0	0	0	0	0	0	0	0	0	0	79	
14749824	f	md	JP	2018	0	0	0	0	0	0	0	0	0	0	0	0	63	
14750350	m	md	JP	2018	0	0	0	0	0	0	0	0	0	0	0	0	54	
14750778	m	md	JP	2018	0	0	0	0	0	0	0	0	0	0	0	0	63	
14750892	m	md	IN	2018	0	1	0	0	1	0	0	0	0	0	0	0	27	

14750894	m	cn	JP	2018	0	1	0	0	1	0	0	0	0	0	0	0	
14750936	Not	cn	US	2018	0	1	0	0	1	0	0	0	0	0	0	0	
14751078	m	md	DK	2018	0	1	0	0	0	1	0	0	0	0	0	0	65
14751104	f	md	JP	2018	1	0	0	0	0	0	0	0	0	0	0	0	68
14751125	m	md	JP	2018	0	0	0	0	0	0	0	0	0	0	0	0	69
14751337	m	md	JP	2018	0	0	0	0	0	0	0	0	0	0	0	0	66
14751518	f	ot	DE	2018	0	1	0	0	1	0	0	0	0	0	0	0	60
14751702	f	ot	NL	2018	0	0	0	0	0	0	0	0	0	0	0	0	62
14752104	Not	ot	US	2018	0	1	0	0	1	0	0	0	0	0	0	0	
14752120	m	cn	GB	2018	1	0	0	0	0	0	0	0	0	0	0	0	70
14752139	m	cn	GB	2018	1	0	0	0	0	0	0	0	0	0	0	0	
14752448	f	md	US	2018	0	1	0	0	0	1	0	0	0	0	0	1	67
14752633	f	cn	GB	2018	1	0	0	0	0	0	0	0	0	0	0	0	
14753128	m	md	US	2018	0	1	1	0	0	0	0	0	0	0	0	0	
14753171	f	md	DE	2018	0	0	0	0	0	0	0	0	0	0	0	0	53
14753630	f	md	JP	2018	1	0	0	0	0	0	0	0	0	0	0	0	67
14753691	m	ot	FR	2018	0	1	0	1	0	0	0	0	0	0	0	0	39
14753768	f	md	AT	2018	1	0	0	0	0	0	0	0	0	0	0	0	
14754216	f	ot	US	2018	0	0	0	0	0	0	0	0	0	0	0	0	44
14754266	m	md	DE	2018	1	0	0	0	0	0	0	0	0	0	0	0	78
14754649	f	cn	US	2018	1	0	0	0	0	0	0	0	0	0	0	0	
14754847	f	ot	DE	2018	0	1	0	0	1	0	0	0	0	0	0	0	85
14754848	m	md	JP	2018	0	1	0	0	1	0	0	0	0	0	0	0	70
14755105	f	md	US	2018	0	1	0	0	1	0	0	0	0	0	0	0	66
14755143	f	md	IT	2018	0	1	0	0	1	0	0	0	0	0	0	0	60
14756339	m	md	SK	2018	0	1	1	0	0	0	0	0	0	0	0	0	
14756484	m	cn	GB	2018	1	0	0	0	0	0	0	0	0	0	0	0	34
14757242	m	cn	US	2018	0	1	1	0	0	0	0	0	0	0	0	0	
14757479	f	ot	GB	2018	1	0	0	0	0	0	0	0	0	0	0	0	
14757578	Not	ot	JP	2018	1	0	0	0	0	0	0	0	0	0	0	0	
14757594	m	ot	PE	2018	0	1	0	0	1	0	0	0	0	0	0	0	74
14757832	f	md	CZ	2018	1	0	0	0	0	0	0	0	0	0	0	0	70
14757891	m	md	DE	2018	1	0	0	0	0	0	0	0	0	0	0	0	73
14757892	m	md	DE	2018	1	0	0	0	0	0	0	0	0	0	0	0	71

14757914	m	md	DE	2018	1	0	0	0	0	0	0	0	0	0	0	0	61
14757981	m	ot	CA	2018	0	1	0	0	1	0	0	0	0	0	0	0	62
14758583	m	ot	DE	2018	0	1	0	0	1	0	0	0	0	0	0	0	67
14758614	m	md	AU	2018	0	0	0	0	0	0	0	0	0	0	0	0	79
14759404	m	ot	JP	2018	0	0	0	0	0	0	0	0	0	0	0	0	75
14759680	m	ot	JP	2018	0	0	0	0	0	0	0	0	0	0	0	0	69
14759911	m	ot	FR	2018	0	1	0	1	0	0	0	0	0	0	0	0	39
14759913	f	cn	US	2018	0	1	1	0	0	0	0	0	0	0	0	0	
14760015	f	md	CZ	2018	1	0	0	0	0	0	0	0	0	0	0	0	77
14760073	Not	md	JP	2018	1	0	0	0	0	0	0	0	0	0	0	0	
14760158	m	ot	CH	2018	0	1	0	0	1	0	0	0	0	0	0	1	64
14760404	f	cn	CN	2018	0	0	0	0	0	0	0	0	0	0	0	0	71
14761394	f	cn	CA	2018	0	0	0	0	0	0	0	0	0	0	0	0	
14761429	Not	md	US	2018	1	0	0	0	0	0	0	0	0	0	0	0	
14761998	m	md	SG	2018	0	1	0	0	0	1	0	0	0	0	0	0	
14762046	m	ot	JP	2018	0	1	0	0	1	0	0	0	0	0	0	0	70
14762047	m	md	US	2018	0	1	0	0	1	0	0	0	0	0	0	0	72
14762218	f	md	US	2018	0	1	1	0	0	0	0	0	0	0	0	0	63
14762586	m	md	CZ	2018	1	0	0	0	0	0	0	0	0	0	0	0	58
14762884	f	cn	US	2018	0	0	0	0	0	0	0	0	0	0	0	0	76
14762920	m	ot	GB	2018	0	0	0	0	0	0	0	0	0	0	0	0	51
14763139	m	md	GB	2018	1	0	0	0	0	0	0	0	0	0	0	0	69
14763194	m	cn	JP	2018	0	1	1	0	0	0	0	0	0	0	0	0	88
14763270	Not	ot	US	2018	0	1	0	0	1	0	0	0	0	0	0	0	
14763393	f	ot	US	2018	0	0	0	0	0	0	0	0	0	0	0	0	
14763556	m	cn	AR	2018	0	1	1	0	0	0	0	0	0	0	0	0	63
14763707	m	cn	JP	2018	0	1	1	0	0	0	0	0	0	0	0	0	65
14763798	f	md	US	2018	0	0	0	0	0	0	0	0	0	0	0	0	44
14763992	Not	md	HK	2018	0	1	0	0	0	1	0	0	0	0	0	0	
14764171	m	cn	DE	2018	1	0	0	0	0	0	0	0	0	0	0	0	
14764909	m	md	GB	2018	0	0	0	0	0	0	0	0	0	0	0	0	76
14765766	Not	ot	JP	2018	0	1	0	0	1	0	0	0	0	0	0	0	
14765780	f	cn	US	2018	0	1	1	0	0	0	0	0	0	0	0	0	
14765870	m	md	JP	2018	0	1	0	0	1	0	0	0	0	0	0	0	74

14766327	f	ot	US	2018	0	1	0	0	1	0	0	0	0	0	0	0	68
14766676	m	ot	JM	2018	0	0	0	0	0	0	0	0	0	0	0	0	69
14767059	m	ot	BE	2018	0	1	0	0	1	0	0	0	0	0	0	0	53
14767379	m	md	FR	2018	0	1	0	0	1	0	0	0	0	0	0	0	61
14767601	m	cn	US	2018	0	1	0	0	1	0	0	0	0	0	0	0	
14767604	Not	cn	JP	2018	0	1	1	0	0	0	0	0	0	0	0	0	73
14767905	m	cn	US	2018	1	0	0	0	0	0	0	0	0	0	0	0	
14767985	f	md	JP	2018	0	0	0	0	0	0	0	0	0	0	0	0	80
14767996	m	md	AT	2018	0	1	0	1	0	0	0	0	0	0	0	1	56.6
14768064	m	md	JP	2018	0	1	0	0	1	0	0	0	0	0	0	1	
14768301	m	md	JP	2018	1	0	0	0	0	0	0	0	0	0	0	0	67
14768542	m	cn	DE	2018	0	1	0	0	1	0	0	0	0	0	0	0	71
14768646	f	cn	GB	2018	1	0	0	0	0	0	0	0	0	0	0	0	90
14768791	m	ot	ES	2018	1	0	0	0	0	0	0	0	0	0	0	0	64
14768847	m	md	DE	2018	0	1	0	0	0	1	0	0	0	0	0	0	69
14768871	f	md	US	2018	0	0	0	0	0	0	0	0	0	0	0	0	80
14769000	m	ph	GB	2018	0	1	1	0	0	0	0	0	0	0	0	0	87
14769046	m	cn	US	2018	0	1	0	0	1	0	0	0	0	0	0	0	
14769079	f	md	US	2018	0	1	1	0	0	0	0	0	0	0	0	0	47
14769271	m	ot	RO	2018	0	0	0	0	0	0	0	0	0	0	0	0	66
14769550	m	md	JP	2018	0	1	0	0	1	0	0	0	0	0	0	0	63
14769954	m	ot	JP	2018	0	1	0	0	1	0	0	0	0	0	0	0	71
14770030	Not	md	IE	2018	0	0	0	0	0	0	0	0	0	0	0	0	
14770357	m	md	LB	2018	0	1	0	0	1	0	0	0	0	0	0	0	
14770571	m	cn	JP	2018	0	1	1	0	0	0	0	0	0	0	0	0	89
14771878	f	md	DE	2018	1	0	0	0	0	0	0	0	0	0	0	0	44
14772003	f	md	US	2018	1	0	0	0	0	0	0	0	0	0	0	0	
14772065	m	md	US	2018	0	1	0	1	0	0	0	0	0	0	0	1	
14772367	m	Not repo	IT	2018	1	0	0	0	0	0	0	0	0	0	0	0	
14772468	Not	md	GR	2018	0	0	0	0	0	0	0	0	0	0	0	0	
14772677	f	cn	US	2018	0	1	1	0	0	0	0	0	0	0	0	0	40
14772972	f	md	IE	2018	0	1	0	1	0	0	0	0	0	0	0	0	61.8
14772984	m	md	CN	2018	0	1	0	0	0	1	0	0	0	0	0	0	47
14772990	m	ot	DE	2018	0	1	0	0	1	0	0	0	0	0	0	0	56

14773032	m	ot	DE	2018	0	1	0	0	1	0	0	0	0	0	0	0	63
14773240	m	md	GB	2018	0	1	0	0	1	0	0	0	0	0	0	0	80
14773435	Not	ot	FR	2018	0	0	0	0	0	0	0	0	0	0	0	0	
14773695	m	md	BR	2018	0	1	0	0	0	1	0	0	0	0	0	0	66
14773847	m	md	JP	2018	1	0	0	0	0	0	0	0	0	0	0	0	80
14773926	m	md	US	2018	0	1	1	0	0	0	0	0	0	0	0	0	
14774000	m	md	DE	2018	0	0	0	0	0	0	0	0	0	0	0	0	
14774028	m	cn	JP	2018	0	1	1	0	0	0	0	0	0	0	0	0	67
14774432	m	md	JP	2018	0	1	0	0	1	0	0	0	0	0	0	1	62
14774485	f	cn	GB	2018	0	1	1	0	0	0	0	0	0	0	0	0	73
14774957	f	md	ES	2018	1	0	0	0	0	0	0	0	0	0	0	0	
14775119	m	md	JP	2018	0	0	0	0	0	0	0	0	0	0	0	0	
14775120	m	md	JP	2018	0	0	0	0	0	0	0	0	0	0	0	0	
14775121	m	md	JP	2018	0	0	0	0	0	0	0	0	0	0	0	0	
14775409	f	md	JP	2018	1	0	0	0	0	0	0	0	0	0	0	1	73
14775453	m	md	RO	2018	0	0	0	0	0	0	0	0	0	0	0	0	66
14775510	f	md	GB	2018	0	0	0	0	0	0	0	0	0	0	0	0	78
14775871	f	cn	US	2018	0	0	0	0	0	0	0	0	0	0	0	0	
14775952	f	Not repo	US	2018	1	0	0	0	0	0	0	0	0	0	0	0	64.6
14775958	f	md	SI	2018	1	0	0	0	0	0	0	0	0	0	0	0	55
14775997	m	cn	US	2018	1	0	0	0	0	0	0	0	0	0	0	0	77
14775999	m	md	JP	2018	1	0	0	0	0	0	0	0	0	0	0	1	73.1
14776052	f	md	JP	2018	0	0	0	0	0	0	0	0	0	0	0	0	32
14776102	f	cn	US	2018	0	1	1	0	0	0	0	0	0	0	0	0	72
14776614	m	cn	US	2018	0	1	0	0	1	0	0	0	0	0	0	0	60
14776635	m	cn	IT	2018	0	1	1	0	0	0	0	0	0	0	0	0	77
14776831	f	ph	IT	2018	0	1	0	0	1	0	0	0	0	0	0	0	65
14777004	Not	cn	US	2018	0	1	1	0	0	0	0	0	0	0	0	0	
14777128	m	md	FR	2018	0	1	0	0	1	0	0	0	0	0	0	0	68
14777191	m	ot	US	2018	0	1	0	0	1	0	0	0	0	0	0	0	68
14777219	f	md	DE	2018	1	0	0	0	0	0	0	0	0	0	0	0	66
14777234	m	ot	JP	2018	0	1	0	0	1	0	0	0	0	0	0	0	50
14777235	m	ph	JP	2018	1	0	0	0	0	0	0	0	0	0	0	0	45
14777277	m	cn	JP	2018	0	1	1	0	0	0	0	0	0	0	0	1	78

14777412	m	md	US	2018	1	0	0	0	0	0	0	0	0	0	0	0	75
14777584	m	ot	DE	2018	0	0	0	0	0	0	0	0	0	0	0	0	67
14777780	m	cn	IN	2018	0	1	0	0	0	1	0	0	0	0	0	0	65
14778735	m	cn	GB	2018	1	0	0	0	0	0	0	0	0	0	0	0	55
14778925	m	ot	FR	2018	0	1	0	1	0	0	0	0	0	0	0	0	39
14778964	m	md	JP	2018	0	0	0	0	0	0	0	0	0	0	0	0	
14778965	m	md	JP	2018	0	0	0	0	0	0	0	0	0	0	0	0	
14778969	m	md	JP	2018	0	0	0	0	0	0	0	0	0	0	0	0	
14778970	m	md	JP	2018	0	0	0	0	0	0	0	0	0	0	0	0	
14778971	m	md	JP	2018	0	0	0	0	0	0	0	0	0	0	0	0	
14778972	m	md	JP	2018	0	0	0	0	0	0	0	0	0	0	0	0	
14778980	f	md	JP	2018	0	0	0	0	0	0	0	0	0	0	0	0	
14779642	f	md	VN	2018	1	0	0	0	0	0	0	0	0	0	0	0	45
14779730	f	md	DE	2018	1	0	0	0	0	0	0	0	0	0	0	0	69
14780171	m	ot	RO	2018	0	0	0	0	0	0	0	0	0	0	0	0	66
14780251	m	md	DE	2018	0	1	0	0	1	0	0	0	0	0	0	0	
14781468	m	md	JP	2018	0	1	0	0	1	0	0	0	0	0	0	1	75
14781513	m	ot	US	2018	0	1	0	0	1	0	0	0	0	0	0	0	70
14781515	f	cn	US	2018	1	0	0	0	0	0	0	0	0	0	0	0	
14782044	m	ph	JP	2018	1	0	0	0	0	0	0	0	0	0	0	0	80
14782153	m	md	US	2018	0	1	0	1	0	0	0	0	0	0	0	0	73
14782520	f	md	MY	2018	0	0	0	0	0	0	1	0	0	0	1	0	59
14782612	Not	ot	JP	2018	1	0	0	0	0	0	0	0	0	0	0	0	
14782790	m	md	DE	2018	0	0	0	0	0	0	0	0	0	0	0	0	51
14782900	Not	md	AT	2018	0	0	0	0	0	0	0	0	0	0	0	0	
14783000	m	md	JP	2018	0	0	0	0	0	0	0	0	0	0	0	0	60
14783339	f	cn	US	2018	1	0	0	0	0	0	0	0	0	0	0	0	55
14783509	m	md	US	2018	0	1	1	0	0	0	0	0	0	0	0	0	
14783626	m	cn	JP	2018	0	1	1	0	0	0	0	0	0	0	0	1	72
14784455	f	cn	GB	2018	1	0	0	0	0	0	0	0	0	0	0	0	66
14784457	f	cn	GB	2018	1	0	0	0	0	0	0	0	0	0	0	0	65
14784570	m	md	US	2018	0	1	0	1	0	0	0	0	0	0	0	0	
14784602	f	cn	US	2018	1	0	0	0	0	0	0	0	0	0	0	0	77
14784732	Not	md	CL	2018	0	0	0	0	0	0	0	0	0	0	0	0	

14784930	m	ot	GB	2018	0	0	0	0	0	0	0	0	0	0	0	0	63
14785165	f	md	ES	2018	1	0	0	0	0	0	0	0	0	0	0	0	73.9
14785298	f	md	JP	2018	1	0	0	0	0	0	0	0	0	0	0	0	45
14785666	m	ot	US	2018	0	1	0	1	0	0	0	0	0	0	0	0	60
14785798	f	md	KR	2018	1	0	0	0	0	0	0	0	0	0	0	0	58.6
14785816	f	cn	US	2018	0	0	0	0	0	0	0	0	0	0	0	0	
14786113	f	ot	FR	2018	0	1	0	0	1	0	0	0	0	0	0	0	69
14788084	m	cn	US	2018	0	1	0	0	1	0	0	0	0	0	0	0	
14788118	Not	ot	CN	2018	0	0	0	0	0	0	0	0	0	0	0	0	
14788139	m	ot	FR	2018	0	1	0	0	1	0	0	0	0	0	0	0	60
14788450	m	ot	ES	2018	0	1	0	0	1	0	0	0	0	0	0	0	72
14788515	m	Not repo	US	2018	1	0	0	0	0	0	0	0	0	0	0	0	79.4
14788569	m	ot	ES	2018	0	1	0	0	1	0	0	0	0	0	0	0	72
14788679	f	ot	JP	2018	0	1	0	0	1	0	0	0	0	0	0	0	72
14788695	m	md	US	2018	0	1	0	0	1	0	0	0	0	0	0	0	
14788840	f	md	JP	2018	0	1	1	0	0	0	0	0	0	0	0	0	75
14788953	m	ot	JP	2018	1	0	0	0	0	0	0	0	0	0	0	0	76
14788954	f	ot	JP	2018	1	0	0	0	0	0	0	0	0	0	0	0	67
14789505	m	md	DE	2018	0	0	0	0	0	0	0	0	0	0	0	0	63
14789519	m	ot	DE	2018	0	0	0	0	0	0	0	0	0	0	0	0	80
14789717	f	cn	AU	2018	0	1	0	0	1	0	0	0	0	0	0	0	
14789957	m	cn	JP	2018	0	1	1	0	0	0	0	0	0	0	0	1	75
14790097	Not	ot	HU	2018	0	0	0	0	0	0	0	0	0	0	0	0	
14790455	m	ot	DE	2018	0	0	0	0	0	0	0	0	0	0	0	0	73
14790965	m	md	US	2018	0	1	1	0	0	0	0	0	0	0	0	0	
14794195	f	ot	DE	2018	0	1	0	0	1	0	0	0	0	0	0	1	55
14794344	m	ot	US	2018	0	1	0	0	1	0	0	0	0	0	0	0	74
14794403	m	md	GB	2018	0	0	0	0	0	0	0	0	0	0	0	0	65
14794695	m	md	US	2018	0	0	0	0	0	0	0	0	0	0	0	0	56
14794785	Not	cn	GB	2018	1	0	0	0	0	0	0	0	0	0	0	0	
14794789	m	ot	GB	2018	1	0	0	0	0	0	0	0	0	0	0	0	66
14794925	m	ot	DE	2018	0	0	0	0	0	0	0	0	0	0	0	0	47
14794988	m	md	JP	2018	0	1	0	0	1	0	0	0	0	0	0	0	82
14795466	m	cn	JP	2018	0	1	1	0	0	0	0	0	0	0	0	0	77

14795560	f	ot	JP	2018	0	1	0	0	1	0	0	0	0	0	0	0	71
14797090	m	md	US	2018	0	1	0	0	1	0	0	0	0	0	0	0	70
14797652	f	md	JP	2018	1	0	0	0	0	0	0	0	0	0	0	0	81
14797702	f	md	JP	2018	0	1	0	0	1	0	0	0	0	0	0	0	71
14797787	Not	ot	US	2018	0	1	0	0	1	0	0	0	0	0	0	0	
14797977	m	cn	US	2018	1	0	0	0	0	0	0	0	0	0	0	0	88
14798276	Not	cn	GB	2018	1	0	0	0	0	0	0	0	0	0	0	0	
14798591	m	cn	JP	2018	0	1	1	0	0	0	0	0	0	0	0	0	83
14798687	m	Not repo	Not re	2018	0	0	0	0	0	0	0	0	0	0	0	0	58
14798708	f	md	US	2018	0	1	1	0	0	0	0	0	0	0	0	0	66
14798767	f	cn	JP	2018	0	0	0	0	0	0	0	0	0	0	0	0	80
14798811	f	md	GB	2018	0	1	0	0	1	0	0	0	0	0	0	0	62
14798906	m	md	JP	2018	1	0	0	0	0	0	0	0	0	0	0	0	55.6
14799299	m	md	JP	2018	1	0	0	0	0	0	0	0	0	0	0	0	60
14799717	m	Not repo	CA	2018	1	0	0	0	0	0	0	0	0	0	0	1	68
14799729	m	ot	JP	2018	0	1	0	0	1	0	0	0	0	0	0	0	67
14800530	Not	ot	US	2018	1	0	0	0	0	0	0	0	0	0	0	0	
14800534	Not	ot	US	2018	1	0	0	0	0	0	0	0	0	0	0	0	
14800863	f	ot	US	2018	0	1	0	0	1	0	0	0	0	0	0	0	58
14800902	m	md	KR	2018	1	0	0	0	0	0	0	0	0	0	0	0	72
14800953	m	md	CN	2018	0	1	0	1	0	0	0	0	0	0	0	0	57.4
14801179	m	md	US	2018	1	0	0	0	0	0	0	0	0	0	0	0	69.8
14801180	f	md	CA	2018	0	1	0	1	0	0	0	0	0	0	0	0	76
14801210	f	cn	TW	2018	0	0	0	0	0	0	0	0	0	0	0	0	56
14801407	m	md	CZ	2018	1	0	0	0	0	0	0	0	0	0	0	0	52
14802469	m	md	JP	2018	0	1	0	0	1	0	0	0	0	0	0	0	65
14802777	f	md	IE	2018	0	0	0	0	0	0	0	0	0	0	0	0	52
14802813	f	md	FR	2018	0	0	0	0	0	0	0	0	0	0	0	0	62
14802883	m	ot	DE	2018	0	1	0	0	1	0	0	0	0	0	0	0	64
14802903	f	md	US	2018	0	0	0	0	0	0	0	0	0	0	0	0	
14803068	f	md	SG	2018	0	0	0	0	0	0	0	0	0	0	0	0	70
14803271	f	ot	US	2018	0	1	0	0	1	0	0	0	0	0	0	0	62
14803445	f	cn	TH	2018	0	1	1	0	0	0	0	0	0	0	0	0	
14803680	m	ot	US	2018	0	0	0	0	0	0	0	0	0	0	0	0	77

14803768	f	ot	DE	2018	0	1	0	0	1	0	0	0	0	0	0	0	65
14803780	m	md	MY	2018	0	1	0	0	0	1	0	0	0	0	0	0	61
14803821	f	md	JP	2018	0	1	0	0	1	0	0	0	0	0	0	0	70
14803843	f	cn	JP	2018	0	1	1	0	0	0	0	0	0	0	0	0	
14803851	f	cn	US	2018	1	0	0	0	0	0	0	0	0	0	0	0	67
14804270	Not	ph	JP	2018	0	1	0	0	1	0	0	0	0	0	0	0	
14804289	f	md	JP	2018	0	1	0	0	1	0	0	0	0	0	0	0	65
14804398	f	cn	GB	2018	0	0	0	0	0	0	0	0	0	0	0	0	71
14804402	f	md	DE	2018	1	0	0	0	0	0	0	0	0	0	0	0	76
14804410	m	ph	JP	2018	0	1	1	0	0	0	0	0	0	0	0	0	73
14804625	Not	md	JP	2018	1	0	0	0	0	0	0	0	0	0	0	0	
14804724	f	cn	US	2018	0	1	0	0	1	0	0	0	0	0	0	0	
14805062	m	ot	NL	2018	0	0	0	0	0	0	0	0	0	0	0	0	64
14805314	Not	md	JP	2018	1	0	0	0	0	0	0	0	0	0	0	0	72
14806030	m	cn	DE	2018	0	1	1	0	0	0	0	0	0	0	0	0	67
14806303	m	cn	JP	2018	0	1	1	0	0	0	0	0	0	0	0	0	62
14806518	f	Not repo	US	2018	1	0	0	0	0	0	0	0	0	0	0	0	74.2
14807013	m	cn	GB	2018	1	0	0	0	0	0	0	0	0	0	0	0	
14807503	m	cn	JP	2018	0	1	1	0	0	0	0	0	0	0	0	0	80
14808881	m	cn	TH	2018	0	1	1	0	0	0	0	0	0	0	0	0	62
14812677	m	ot	CN	2018	0	1	0	0	1	0	0	0	0	0	0	0	65
14812756	f	md	ID	2018	1	0	0	0	0	0	0	0	0	0	0	0	60
14812923	m	ot	DE	2018	0	0	0	0	0	0	0	0	0	0	0	0	60
14813302	m	md	JP	2018	0	1	0	0	1	0	0	0	0	0	0	0	76
14813414	f	md	DE	2018	1	0	0	0	0	0	0	0	0	0	0	0	67
14813546	f	md	CZ	2018	1	0	0	0	0	0	0	0	0	0	0	0	46
14814022	m	ot	DE	2018	0	0	0	0	0	0	0	0	0	0	0	0	56
14814363	f	cn	US	2018	0	0	0	0	0	0	0	0	0	0	0	0	
14814371	f	md	DE	2018	0	0	0	0	0	0	0	0	0	0	0	0	58
14814414	m	Not repo	US	2018	1	0	0	0	0	0	0	0	0	0	0	0	64.9
14814542	f	cn	US	2018	0	0	0	0	0	0	0	0	0	0	0	0	
14814594	m	md	DE	2018	0	0	0	0	0	0	0	0	0	0	0	0	73
14814607	f	md	JP	2018	1	0	0	0	0	0	0	0	0	0	0	0	65
14814831	f	md	ID	2018	1	0	0	0	0	0	0	0	0	0	0	0	60

14814896	Not	ot	FR	2018	0	0	0	0	0	0	0	0	0	0	0	0	
14815037	m	cn	JP	2018	0	1	1	0	0	0	0	0	0	0	0	0	71
14815113	m	md	DE	2018	0	0	0	0	0	0	0	0	0	0	0	0	47
14815304	Not	ot	FR	2018	0	0	0	0	0	0	0	0	0	0	0	0	
14815551	m	md	JP	2018	1	0	0	0	0	0	0	0	0	0	0	1	62
14815567	f	md	JP	2018	1	0	0	0	0	0	0	0	0	0	0	0	68
14815994	f	cn	GB	2018	1	0	0	0	0	0	0	0	0	0	0	0	71
14816143	f	ot	DE	2018	0	0	0	0	0	0	0	0	0	0	0	0	70
14816388	m	md	FR	2018	0	1	0	1	0	0	0	0	0	0	0	0	67.5
14816506	f	ot	JP	2018	0	0	0	0	0	0	0	0	0	0	0	0	32
14816843	f	md	DE	2018	0	1	0	0	1	0	0	0	0	0	0	1	72
14817019	f	cn	US	2018	0	0	0	0	0	0	0	0	0	0	0	0	
14817050	Not	md	JP	2018	0	0	0	0	0	0	0	0	0	0	0	0	
14817693	f	md	SE	2018	0	0	0	0	0	0	0	0	0	0	0	0	42
14817709	m	md	ID	2018	1	0	0	0	0	0	0	0	0	0	0	0	70
14817710	Not	md	MY	2018	1	0	0	0	0	0	0	0	0	0	0	0	
14818005	Not	md	MY	2018	1	0	0	0	0	0	0	0	0	0	0	0	
14818156	f	md	JP	2018	0	0	0	0	0	0	0	0	0	0	0	0	65
14818227	f	ot	US	2018	0	1	0	0	1	0	0	0	0	0	0	0	66
14818317	f	ot	SG	2018	0	1	0	0	1	0	0	0	0	0	0	0	71
14818350	f	ot	JP	2018	0	1	0	0	1	0	0	0	0	0	0	0	72
14818382	f	md	DE	2018	0	1	0	0	1	0	0	0	0	0	0	0	
14818518	m	ot	US	2018	1	0	0	0	0	0	0	0	0	0	0	0	
14818682	m	cn	US	2018	0	0	0	0	0	0	0	0	0	0	0	0	62
14819357	f	cn	GB	2018	1	0	0	0	0	0	0	0	0	0	0	0	
14820208	m	ot	US	2018	1	0	0	0	0	0	0	0	0	0	0	0	57
14820325	Not	ot	US	2018	0	0	0	0	0	0	0	0	0	0	0	0	
14820364	Not	ot	FR	2018	0	0	0	0	0	0	0	0	0	0	0	0	
14820370	f	md	SK	2018	1	0	0	0	0	0	0	0	0	0	0	0	64
14820469	Not	md	MY	2018	1	0	0	0	0	0	0	0	0	0	0	0	
14820470	Not	md	MY	2018	1	0	0	0	0	0	0	0	0	0	0	0	
14820519	Not	md	MY	2018	1	0	0	0	0	0	0	0	0	0	0	0	
14820535	Not	md	MY	2018	1	0	0	0	0	0	0	0	0	0	0	0	
14820536	Not	md	MY	2018	1	0	0	0	0	0	0	0	0	0	0	0	

14820537	Not	md	MY	2018	1	0	0	0	0	0	0	0	0	0	0	0	
14820539	Not	md	MY	2018	1	0	0	0	0	0	0	0	0	0	0	0	
14820830	m	cn	US	2018	0	1	0	0	1	0	0	0	0	0	0	0	
14820946	m	md	DE	2018	0	0	0	0	0	0	0	0	0	0	0	0	54
14821094	m	cn	US	2018	0	1	0	0	1	0	0	0	0	0	0	0	59
14821131	f	md	US	2018	0	1	0	1	0	0	0	0	0	0	0	0	
14821132	Not	ot	US	2018	0	1	0	1	0	0	0	0	0	0	0	0	
14821141	Not	md	US	2018	0	1	0	1	0	0	0	0	0	0	0	0	
14821940	m	ot	FR	2018	0	0	0	0	0	0	0	0	0	0	0	0	59
14822288	Not	ot	CA	2018	0	0	0	0	0	0	0	0	0	0	0	0	
14822515	f	md	JP	2018	1	0	0	0	0	0	0	0	0	0	0	0	75
14822697	f	cn	Not re	2018	0	0	0	0	0	0	0	0	0	0	0	0	
14822765	f	ot	US	2018	0	0	0	0	0	0	0	0	0	0	0	0	65
14822965	f	ot	JP	2018	0	0	0	0	0	0	0	0	0	0	0	0	79
14823446	Not	md	MY	2018	1	0	0	0	0	0	0	0	0	0	0	0	
14823755	m	ot	FR	2018	0	1	0	1	0	0	0	0	0	0	0	0	39
14824163	m	md	US	2018	0	1	1	0	0	0	0	0	0	0	0	0	76
14825173	f	Not repo	Not re	2018	0	0	0	0	0	0	0	0	0	0	0	0	56
14825177	m	md	DE	2018	0	0	0	0	0	0	0	0	0	0	0	0	80
14825440	f	cn	US	2018	0	1	1	0	0	0	0	0	0	0	0	0	
14825643	Not	ot	LB	2018	0	0	0	0	0	0	0	0	0	0	0	0	
14825752	m	md	CN	2018	0	1	0	0	0	1	0	0	0	0	0	0	65
14825864	m	ot	US	2018	0	0	0	0	0	0	0	0	0	0	0	0	56
14826187	f	cn	US	2018	0	1	0	0	1	0	0	0	0	0	0	0	
14826206	Not	md	PL	2018	1	0	0	0	0	0	0	0	0	0	0	0	64
14826262	m	md	JP	2018	0	1	0	0	1	0	0	0	0	0	0	0	70
14826310	f	md	HU	2018	0	1	0	0	0	1	0	0	0	0	0	0	70
14826463	m	cn	JP	2018	0	1	1	0	0	0	0	0	0	0	0	0	60
14826464	f	cn	JP	2018	0	1	1	0	0	0	0	0	0	0	0	1	71
14828485	m	ot	PL	2018	0	0	0	0	0	0	0	0	0	0	0	0	57
14828779	Not	cn	US	2018	1	0	0	0	0	0	0	0	0	0	0	0	
14828912	f	cn	CA	2018	1	0	0	0	0	0	0	0	0	0	0	0	
14829269	f	ot	DE	2018	0	1	0	0	1	0	0	0	0	0	0	0	76
14829276	f	md	DE	2018	0	0	0	0	0	0	0	0	0	0	0	0	61

14829695	f	md	DE	2018	0	0	0	0	0	0	0	0	0	0	0	0	
14829732	m	md	JP	2018	0	1	0	1	0	0	0	0	0	0	0	0	74
14829815	f	ot	BR	2018	0	0	0	0	0	0	0	0	0	0	0	0	41
14829817	m	ot	JP	2018	0	1	0	0	1	0	0	0	0	0	0	0	74
14829956	m	ot	US	2018	0	1	0	0	1	0	0	0	0	0	0	0	59
14830128	m	md	DE	2018	1	0	0	0	0	0	0	0	0	0	0	0	60
14831026	m	ot	DE	2018	0	1	0	0	1	0	0	0	0	0	0	0	65
14831054	f	ot	FR	2018	0	0	0	0	0	0	0	0	0	0	0	0	49
14831190	Not	md	MY	2018	1	0	0	0	0	0	0	0	0	0	0	0	
14831253	m	md	US	2018	0	1	0	0	0	1	0	0	0	0	0	0	62
14831340	f	md	US	2018	0	1	0	0	0	1	0	0	0	0	0	0	
14831571	Not	md	US	2018	1	0	0	0	0	0	0	0	0	0	0	0	
14831964	f	cn	TH	2018	0	1	1	0	0	0	0	0	0	0	0	0	42
14832044	m	md	AU	2018	0	1	0	0	0	1	0	0	0	0	0	0	80
14832046	m	ph	JP	2018	0	0	0	0	0	0	0	0	0	0	0	0	
14832058	m	md	JP	2018	0	0	0	0	0	0	0	0	0	0	0	0	75
14832115	m	md	GB	2018	0	1	0	0	0	1	0	0	0	0	0	1	51
14832854	m	ot	US	2018	0	0	0	0	0	0	0	0	0	0	0	0	61
14833590	m	md	US	2018	0	0	0	0	0	0	0	0	0	0	0	0	75
14833595	m	cn	US	2018	0	0	0	0	0	0	0	0	0	0	0	0	58
14833597	m	cn	US	2018	0	0	0	0	0	0	0	0	0	0	0	0	
14833675	f	ph	US	2018	0	0	0	0	0	0	0	0	0	0	0	0	
14833688	f	md	US	2018	0	0	0	0	0	0	0	0	0	0	0	0	
14833708	m	cn	US	2018	0	0	0	0	0	0	0	0	0	0	0	0	
14833804	m	ot	US	2018	0	0	0	0	0	0	0	0	0	0	0	0	
14833813	m	cn	US	2018	0	0	0	0	0	0	0	0	0	0	0	0	59
14833842	f	cn	US	2018	1	0	0	0	0	0	0	0	0	0	0	0	76
14833852	m	cn	US	2018	0	0	0	0	0	0	0	0	0	0	0	0	
14833885	f	ot	US	2018	0	0	0	0	0	0	0	0	0	0	0	0	50
14833917	m	ot	US	2018	0	0	0	0	0	0	0	0	0	0	0	0	
14833954	m	ot	US	2018	0	0	0	0	0	0	0	0	0	0	0	0	50
14833979	Not	md	US	2018	0	0	0	0	0	0	0	0	0	0	0	0	
14834011	Not	cn	US	2018	0	0	0	0	0	0	0	0	0	0	0	0	
14834022	f	cn	US	2018	1	0	0	0	0	0	0	0	0	0	0	0	

14834060	f	md	US	2018	0	0	0	0	0	0	0	0	0	0	0	0	
14834064	f	ot	US	2018	0	0	0	0	0	0	0	0	0	0	0	0	70
14834098	m	ot	US	2018	0	0	0	0	0	0	0	0	0	0	0	0	
14834115	f	cn	US	2018	0	0	0	0	0	0	0	0	0	0	0	0	
14834362	m	cn	DE	2018	0	1	1	0	0	0	0	0	0	0	0	0	
14835235	Not	md	MY	2018	1	0	0	0	0	0	0	0	0	0	0	0	
14835246	Not	md	MY	2018	1	0	0	0	0	0	0	0	0	0	0	0	
14835388	Not	md	MY	2018	1	0	0	0	0	0	0	0	0	0	0	0	
14835409	m	ph	BR	2018	0	1	0	0	0	1	0	0	0	0	0	0	73
14835425	Not	md	MY	2018	1	0	0	0	0	0	0	0	0	0	0	0	
14835655	Not	md	MY	2018	1	0	0	0	0	0	0	0	0	0	0	0	
14835732	m	ph	CH	2018	0	0	0	0	0	0	0	0	0	0	0	0	42
14835813	Not	md	MY	2018	1	0	0	0	0	0	0	0	0	0	0	0	
14835911	m	md	JP	2018	1	0	0	0	0	0	0	0	0	0	0	0	84.2
14835920	m	md	JP	2018	1	0	0	0	0	0	0	0	0	0	0	1	70
14835926	Not	md	MY	2018	1	0	0	0	0	0	0	0	0	0	0	0	
14835929	Not	md	MY	2018	1	0	0	0	0	0	0	0	0	0	0	0	
14836003	f	md	AT	2018	0	1	0	0	0	1	0	0	0	0	0	0	
14836238	Not	md	MY	2018	1	0	0	0	0	0	0	0	0	0	0	0	
14836242	m	md	SG	2018	0	1	0	0	0	1	0	0	0	0	0	1	79
14836267	Not	md	MY	2018	1	0	0	0	0	0	0	0	0	0	0	0	
14836268	Not	md	MY	2018	1	0	0	0	0	0	0	0	0	0	0	0	
14836360	f	md	FR	2018	0	1	0	0	0	1	0	0	0	0	0	0	47
14836435	m	ot	JP	2018	0	1	1	0	0	0	0	0	0	0	0	1	64
14836455	f	cn	US	2018	0	1	0	0	1	0	0	0	0	0	0	0	
14836655	Not	md	MY	2018	1	0	0	0	0	0	0	0	0	0	0	0	
14836685	Not	md	MY	2018	1	0	0	0	0	0	0	0	0	0	0	0	
14836731	Not	md	MY	2018	1	0	0	0	0	0	0	0	0	0	0	0	
14836799	f	md	CA	2018	0	0	0	0	0	0	0	0	0	0	0	0	
14837040	m	cn	US	2018	0	1	1	0	0	0	0	0	0	0	0	0	73
14837097	f	md	US	2018	0	1	0	1	0	0	0	0	0	0	0	0	
14838889	m	md	JP	2018	0	0	0	0	0	0	0	0	0	0	0	0	39
14838993	Not	ot	US	2018	0	1	0	0	1	0	0	0	0	0	0	0	
14839536	f	md	ID	2018	1	0	0	0	0	0	0	0	0	0	0	0	

14839686	f	ot	US	2018	1	0	0	0	0	0	0	0	0	0	0	0	
14839892	f	md	DE	2018	0	1	0	0	1	0	0	0	0	0	0	0	66
14839924	f	cn	US	2018	1	0	0	0	0	0	0	0	0	0	0	0	72
14839940	Not	md	MY	2018	1	0	0	0	0	0	0	0	0	0	0	0	
14839950	Not	md	MY	2018	1	0	0	0	0	0	0	0	0	0	0	0	
14839994	m	cn	US	2018	0	1	0	0	1	0	0	0	0	0	0	0	
14840074	f	md	JP	2018	0	1	0	0	1	0	0	0	0	0	0	0	46
14840112	m	md	DE	2018	0	0	0	0	0	0	0	0	0	0	0	0	64
14840154	m	md	US	2018	0	0	0	0	0	0	0	0	0	0	0	0	83
14840215	f	md	JP	2018	1	0	0	0	0	0	0	0	0	0	0	0	67
14840243	m	ot	FR	2018	0	1	0	1	0	0	0	0	0	0	0	0	39
14840459	m	md	DE	2018	0	0	0	0	0	0	0	0	0	0	0	0	58
14840568	f	md	US	2018	0	1	0	0	0	1	0	0	0	0	0	0	65
14840710	f	ph	JP	2018	1	0	0	0	0	0	0	0	0	0	0	0	82
14840754	f	cn	US	2018	0	0	0	0	0	0	0	0	0	0	0	0	
14841254	m	md	US	2018	0	1	0	1	0	0	0	0	0	0	0	1	
14841375	f	cn	US	2018	0	0	0	0	0	0	0	0	0	0	0	0	72
14841507	m	md	IL	2018	0	1	0	0	0	1	0	0	0	0	0	0	
14842009	m	md	BR	2018	0	1	0	1	0	0	0	0	0	0	0	0	62.2
14842746	m	ot	DE	2018	0	0	0	0	0	0	0	0	0	0	0	0	65
14842793	f	ot	BE	2018	0	0	0	0	0	0	0	0	0	0	0	0	59
14843001	m	md	CA	2018	1	0	0	0	0	0	0	0	0	0	0	0	65
14843144	f	ot	BE	2018	0	0	0	0	0	0	0	0	0	0	0	0	
14843411	Not	ot	CA	2018	0	0	0	0	0	0	0	0	0	0	0	0	
14843922	f	md	JP	2018	0	0	0	0	0	0	0	0	0	0	0	0	
14844457	m	ot	JP	2018	0	1	0	0	1	0	0	0	0	0	0	0	54
14845100	m	md	BE	2018	0	0	0	0	0	0	0	0	0	0	0	0	42
14845407	m	md	CN	2018	0	1	0	1	0	0	0	0	0	0	0	0	69.8
14845581	Not	md	JP	2018	1	0	0	0	0	0	0	0	0	0	0	0	
14845809	m	md	US	2018	1	0	0	0	0	0	0	0	0	0	0	0	65
14846001	m	cn	GB	2018	1	0	0	0	0	0	0	0	0	0	0	0	67
14846423	Not	md	TW	2018	1	0	0	0	0	0	0	0	0	0	0	0	
14846610	m	md	KR	2018	0	0	0	0	0	0	0	0	0	0	0	0	68
14846678	Not	ot	AU	2018	0	1	0	0	1	0	0	0	0	0	0	0	

14847230	m	md	DE	2018	0	0	0	0	0	0	0	0	0	0	0	0	
14847253	f	cn	JP	2018	0	1	1	0	0	0	0	0	0	0	0	0	52
14848425	m	cn	BE	2018	0	0	0	0	0	0	0	0	0	0	0	0	42
14848454	m	md	CN	2018	1	0	0	0	0	0	0	0	0	0	0	0	62.7
14848492	m	ot	DE	2018	0	1	0	0	1	0	0	0	0	0	0	0	81
14848534	m	ot	US	2018	1	0	0	0	0	0	0	0	0	0	0	0	
14848717	f	Not repo	TW	2018	1	0	0	0	0	0	0	0	0	0	0	0	80
14848828	m	md	DE	2018	0	1	0	1	0	0	0	0	0	0	0	0	67.6
14849240	m	md	VN	2018	1	0	0	0	0	0	0	0	0	0	0	0	70
14849325	f	md	US	2018	1	0	0	0	0	0	0	0	0	0	0	0	70
14850144	f	ot	DK	2018	1	0	0	0	0	0	0	0	0	0	0	0	53
14850625	f	md	US	2018	0	1	0	1	0	0	0	0	0	0	0	1	
14850628	m	md	JP	2018	0	1	0	0	1	0	0	0	0	0	0	0	65
14851098	f	md	FR	2018	0	1	0	0	1	0	0	0	0	0	0	0	
14851287	f	md	NL	2018	0	1	1	0	0	0	0	0	0	0	0	0	62
14851409	m	md	US	2018	0	1	1	0	0	0	0	0	0	0	0	0	52
14851414	m	md	US	2018	0	1	1	0	0	0	0	0	0	0	0	0	51
14851451	m	md	US	2018	0	1	1	0	0	0	0	0	0	0	0	0	59
14852013	Not	cn	GB	2018	1	0	0	0	0	0	0	0	0	0	0	0	
14852016	m	md	IN	2018	0	1	0	0	0	1	0	0	0	0	0	0	55
14853618	m	ot	CN	2018	0	0	0	0	0	0	0	0	0	0	0	0	76
14854107	f	md	JP	2018	0	0	0	0	0	0	0	0	0	0	0	0	75
14854642	m	ot	DE	2018	0	0	0	0	0	0	0	0	0	0	0	0	80
14854718	f	ot	CA	2018	0	1	0	1	0	0	0	0	0	0	0	0	75
14854728	m	cn	US	2018	0	1	1	0	0	0	0	0	0	0	0	0	
14855842	f	md	IT	2018	0	0	0	0	0	0	0	0	0	0	0	0	47
14856069	f	md	US	2018	0	1	1	0	0	0	0	0	0	0	0	0	73
14857115	f	cn	US	2018	0	1	1	0	0	0	0	0	0	0	0	1	56
14857933	m	md	IL	2018	0	1	0	0	1	0	0	0	0	0	0	0	62
14857982	m	ot	US	2018	0	1	0	0	1	0	0	0	0	0	0	0	75
14858162	m	md	ES	2018	1	0	0	0	0	0	0	0	0	0	0	0	73
14858382	m	ot	JP	2018	0	1	0	0	1	0	0	0	0	0	0	0	73
14859563	f	md	US	2018	0	0	0	0	0	0	0	0	0	0	0	0	65
14860402	f	cn	US	2018	0	1	1	0	0	0	0	0	0	0	0	0	

14861035	m	ot	DE	2018	0	0	0	0	0	0	0	0	0	0	0	0	58
14864751	m	cn	US	2018	0	1	1	0	0	0	0	0	0	0	0	0	
14865979	f	ot	NL	2018	0	1	0	0	1	0	0	0	0	0	0	0	65
14867583	m	md	AU	2018	0	1	0	1	0	0	0	0	0	0	0	0	71.3
14867594	f	ph	JP	2018	1	0	0	0	0	0	0	0	0	0	0	1	77
14867723	Not	md	FR	2018	0	1	0	0	1	0	0	0	0	0	0	0	
14867730	Not	cn	US	2018	0	1	1	0	0	0	0	0	0	0	0	0	
14868254	m	cn	NZ	2018	0	1	0	0	1	0	0	0	0	0	0	0	74
14868274	f	cn	US	2018	0	1	0	0	1	0	0	0	0	0	0	0	
14868427	f	ot	JP	2018	0	1	0	0	1	0	0	0	0	0	0	0	79
14868541	Not	md	IL	2018	0	0	0	0	0	0	0	0	0	0	0	0	
14869494	m	cn	IT	2018	0	1	1	0	0	0	0	0	0	0	0	1	70
14869619	m	ot	US	2018	1	0	0	0	0	0	0	0	0	0	0	0	57
14870024	f	cn	GB	2018	1	0	0	0	0	0	0	0	0	0	0	0	
14870430	m	md	FR	2018	0	1	0	1	0	0	0	0	0	0	0	0	39
14870443	f	cn	US	2018	1	0	0	0	0	0	0	0	0	0	0	0	63
14870472	m	md	JP	2018	0	0	0	0	0	0	0	0	0	0	0	0	62
14871315	f	md	US	2018	1	0	0	0	0	0	0	0	0	0	0	0	62
14872348	m	ot	AU	2018	0	1	0	0	1	0	0	0	0	0	0	0	81
14872822	f	md	JP	2018	1	0	0	0	0	0	0	0	0	0	0	0	47
14872933	f	ph	CA	2018	0	0	0	0	0	0	0	0	0	0	0	0	
14873880	m	md	IL	2018	0	1	0	0	0	1	0	0	0	0	0	1	35
14874273	f	ot	JP	2018	0	0	0	0	0	0	0	0	0	0	0	0	32
14874636	f	cn	GB	2018	1	0	0	0	0	0	0	0	0	0	0	0	73
14874684	f	ot	JP	2018	0	0	0	0	0	0	0	0	0	0	0	0	32
14875572	m	ot	DE	2018	0	0	0	0	0	0	0	0	0	0	0	0	71
14876088	m	cn	GR	2018	0	1	1	0	0	0	0	0	0	0	0	0	63
14876299	m	md	FR	2018	0	0	0	0	0	0	0	0	0	0	0	0	63
14876934	f	ot	Not re	2018	1	0	0	0	0	0	0	0	0	0	0	0	53
14877104	f	cn	AR	2018	0	0	0	0	0	0	0	0	0	0	0	0	33
14878261	m	md	US	2018	0	1	0	0	1	0	0	0	0	0	0	1	
14878743	f	md	CA	2018	0	1	1	0	0	0	0	0	0	0	0	0	
14878779	f	cn	KR	2018	1	0	0	0	0	0	0	0	0	0	0	0	67
14879002	f	cn	IE	2018	0	0	0	0	0	0	1	1	0	0	0	1	56

14879160	f	md	JP	2018	0	1	0	0	1	0	0	0	0	0	0	1	55
14879223	Not	ot	US	2018	0	0	0	0	0	0	0	0	0	0	0	0	
14879609	m	md	DE	2018	0	0	0	0	0	0	0	0	0	0	0	0	
14880062	m	md	KR	2018	0	1	0	1	0	0	0	0	0	0	0	0	65
14880729	m	cn	US	2018	0	1	1	0	0	0	0	0	0	0	0	0	
14881270	f	ot	DE	2018	0	0	0	0	0	0	0	0	0	0	0	0	64
14881895	m	md	FR	2018	0	1	0	1	0	0	0	0	0	0	0	0	67.5
14881983	f	ph	CA	2018	0	1	0	0	1	0	0	0	0	0	0	0	76
14881984	Not	ot	DE	2018	0	1	0	0	1	0	0	0	0	0	0	0	66
14882139	f	md	US	2018	1	0	0	0	0	0	0	0	0	0	0	1	83
14882647	m	md	US	2018	0	1	1	0	0	0	0	0	0	0	0	1	55
14882688	m	cn	US	2018	1	0	0	0	0	0	0	0	0	0	0	0	65
14883188	f	md	ES	2018	1	0	0	0	0	0	0	0	0	0	0	0	81
14883498	m	cn	US	2018	0	1	1	0	0	0	0	0	0	0	0	0	56
14884235	f	cn	US	2018	0	1	1	0	0	0	0	0	0	0	0	0	
14884949	Not	ph	JP	2018	0	1	1	0	0	0	0	0	0	0	0	0	
14884994	m	ot	US	2018	0	1	0	0	1	0	0	0	0	0	0	0	48
14885169	m	cn	JP	2018	0	1	1	0	0	0	0	0	0	0	0	1	69
14885777	Not	md	US	2018	0	1	0	0	0	1	0	0	0	0	0	0	
14885847	f	ph	GB	2018	0	0	0	0	0	0	0	0	0	0	0	0	49
14885873	f	md	BE	2018	0	1	0	0	0	1	0	0	0	0	0	0	65
14887777	m	ot	CN	2018	0	1	1	0	0	0	0	0	0	0	0	1	52
14887872	f	md	US	2018	0	1	0	0	0	1	0	0	0	0	0	1	64
14889356	f	cn	JP	2018	0	1	1	0	0	0	0	0	0	0	0	0	80
14889407	f	md	BE	2018	0	0	0	0	0	0	0	0	0	0	0	0	49
14889541	f	md	US	2018	0	1	0	1	0	0	0	0	0	0	0	0	60
14889551	f	cn	US	2018	0	1	1	0	0	0	0	0	0	0	0	0	
14889698	m	cn	US	2018	0	1	1	0	0	0	0	0	0	0	0	0	55
14890058	m	md	JP	2018	0	1	0	0	1	0	0	0	0	0	0	0	74
14890147	m	ot	JP	2018	0	1	0	0	1	0	0	0	0	0	0	0	54
14890148	m	ot	BE	2018	0	1	0	0	1	0	0	0	0	0	0	0	70
14890416	m	cn	US	2018	0	1	1	0	0	0	0	0	0	0	0	0	84
14891019	m	ot	AU	2018	0	1	0	0	1	0	0	0	0	0	0	0	67
14891186	m	md	JP	2018	0	1	0	0	1	0	0	0	0	0	0	1	77

14891277	f	md	JP	2018	1	0	0	0	0	0	0	0	0	0	0	0	44
14892136	f	md	MY	2018	0	1	0	0	0	1	0	0	0	0	0	0	59
14892478	m	Not repo	Not re	2018	0	1	0	1	0	0	0	0	0	0	0	0	39
14892964	f	md	JP	2018	0	0	0	0	0	0	0	0	0	0	0	0	83
14893188	f	ph	DE	2018	1	0	0	0	0	0	0	0	0	0	0	0	78
14893248	f	md	SK	2018	1	0	0	0	0	0	0	0	0	0	0	0	70
14893657	f	md	US	2018	0	1	0	0	0	1	0	0	0	0	0	0	
14893723	m	md	JP	2018	0	1	0	0	1	0	0	0	0	0	0	1	71
14893793	m	md	JP	2018	0	1	0	0	1	0	0	0	0	0	0	0	66
14894043	f	cn	US	2018	0	1	1	0	0	0	0	0	0	0	0	0	79
14894072	m	md	SK	2018	1	0	0	0	0	0	0	0	0	0	0	0	
14894277	m	md	JP	2018	0	1	0	0	1	0	0	0	0	0	0	1	50
14894481	f	Not repo	US	2018	1	0	0	0	0	0	0	0	0	0	0	0	50
14894504	m	md	JP	2018	0	1	0	0	1	0	0	0	0	0	0	0	58
14894662	m	md	GR	2018	0	0	0	0	0	0	0	0	0	0	0	0	48
14894800	m	cn	US	2018	0	1	0	1	0	0	0	0	0	0	0	1	65
14894965	m	cn	US	2018	0	1	0	0	1	0	0	0	0	0	0	0	
14895101	m	ot	BR	2018	0	1	0	0	0	1	0	0	0	0	0	1	54
14895629	f	md	EG	2018	0	1	0	0	1	0	0	0	0	0	0	0	55
14895753	m	md	CN	2018	0	1	0	0	0	1	0	0	0	0	0	1	58
14895883	f	ph	CA	2018	0	1	1	0	0	0	0	0	0	0	0	1	63
14896132	f	md	IT	2018	0	0	0	0	0	0	0	0	0	0	0	0	42
14896239	f	md	JP	2018	1	0	0	0	0	0	0	0	0	0	0	0	47
14896421	Not	md	US	2018	0	1	0	1	0	0	0	0	0	0	0	1	
14896487	m	md	BR	2018	0	1	0	1	0	0	0	0	0	0	0	0	67
14896864	f	md	JP	2018	1	0	0	0	0	0	0	0	0	0	0	0	73
14897218	f	md	PH	2018	0	1	1	0	0	0	0	0	0	0	0	0	70
14897359	m	ot	FR	2018	0	1	0	0	1	0	0	0	0	0	0	0	53
14897552	m	md	BE	2018	0	1	0	1	0	0	0	0	0	0	0	1	60.5
14897717	f	md	US	2018	0	1	0	1	0	0	0	0	0	0	0	1	
14898538	f	ot	JP	2018	0	1	1	0	0	0	0	0	0	0	0	0	
14898690	f	md	JP	2018	0	1	0	0	1	0	0	0	0	0	0	0	52
14899064	m	md	FR	2018	0	1	0	0	0	1	0	0	0	0	0	0	67
14899109	f	ph	DE	2018	0	1	0	0	1	0	0	0	0	0	0	0	64

14899153	f	md	JP	2018	0	1	0	0	1	0	0	0	0	0	0	0	42
14899204	m	cn	US	2018	1	0	0	0	0	0	0	0	0	0	0	0	
14899243	f	md	US	2018	0	1	0	0	0	1	0	0	0	0	0	0	77
14899471	m	ot	FR	2018	0	1	0	0	1	0	0	0	0	0	0	0	85
14899540	m	md	IT	2018	0	1	0	0	1	0	0	0	0	0	0	0	
14899668	m	ot	US	2018	0	0	0	0	0	0	0	0	0	0	0	0	
14899674	f	md	IT	2018	0	0	0	0	0	0	0	0	0	0	0	0	
14900016	m	cn	JP	2018	0	1	1	0	0	0	0	0	0	0	0	0	72
14900046	m	cn	DE	2018	0	1	1	0	0	0	0	0	0	0	0	0	86
14900356	f	cn	US	2018	0	1	0	0	1	0	0	0	0	0	0	0	
14900374	m	md	AU	2018	0	1	0	1	0	0	0	0	0	0	0	0	58.4
14900405	m	md	JP	2018	0	0	0	0	0	0	0	0	0	0	0	0	81
14900433	f	cn	US	2018	0	1	1	0	0	0	0	0	0	0	0	0	
14900802	f	md	JP	2018	1	0	0	0	0	0	0	0	0	0	0	0	60
14901169	f	md	JP	2018	0	1	0	0	1	0	0	0	0	0	0	0	76
14901256	f	md	RO	2018	0	0	0	0	0	0	0	0	0	0	0	0	50
14901446	f	ot	ES	2018	0	0	0	0	0	0	0	0	0	0	0	0	61
14901954	f	ot	US	2018	0	0	0	0	0	0	0	0	0	0	0	0	55
14902213	f	md	EG	2018	1	0	0	0	0	0	0	0	0	0	0	0	50
14902256	m	md	JP	2018	1	0	0	0	0	0	0	0	0	0	0	0	66
14902366	f	ot	JP	2018	1	0	0	0	0	0	0	0	0	0	0	0	62
14903477	m	ot	US	2018	0	0	0	0	0	0	0	0	0	0	0	0	65
14903754	m	ot	AR	2018	0	1	0	0	1	0	0	0	0	0	0	0	58
14903913	f	ot	US	2018	0	1	1	0	0	0	0	0	0	0	0	0	52
14904206	m	ot	JP	2018	0	1	0	0	1	0	0	0	0	0	0	0	71
14904224	f	cn	CA	2018	0	1	1	0	0	0	0	0	0	0	0	0	82
14904272	m	md	KR	2018	1	0	0	0	0	0	0	0	0	0	0	0	60
14906945	m	md	DE	2018	0	0	0	0	0	0	0	0	0	0	0	0	65
14906966	m	md	JP	2018	0	1	0	0	1	0	0	0	0	0	0	1	79
14906983	m	md	DE	2018	0	0	0	0	0	0	0	0	0	0	0	0	56
14907131	f	md	PL	2018	0	0	0	0	0	0	0	0	0	0	0	0	
14907291	f	ot	DE	2018	0	1	0	0	1	0	0	0	0	0	0	0	65
14908085	f	cn	US	2018	0	1	1	0	0	0	0	0	0	0	0	0	80
14908122	m	md	JP	2018	0	1	1	0	0	0	0	0	0	0	0	1	51

14908135	m	md	CN	2018	0	1	0	1	0	0	0	0	0	0	0	0	69.9
14908162	m	ot	MX	2018	0	0	0	0	0	0	0	0	0	0	0	0	66
14908882	f	cn	US	2018	1	0	0	0	0	0	0	0	0	0	0	0	65
14909098	m	md	US	2018	0	1	1	0	0	0	0	0	0	0	0	0	77
14910010	m	md	JP	2018	1	0	0	0	0	0	0	0	0	0	0	0	68
14910147	f	ot	CZ	2018	0	0	0	0	0	0	0	0	0	0	0	0	57
14910432	m	md	JP	2018	0	1	0	0	1	0	0	0	0	0	0	0	56
14910527	m	ot	GB	2018	0	0	0	0	0	0	0	0	0	0	0	0	42
14910529	m	md	JP	2018	0	1	0	0	1	0	0	0	0	0	0	0	77
14910717	f	ot	CN	2018	0	1	0	0	1	0	0	0	0	0	0	0	61
14911881	f	md	JP	2018	1	0	0	0	0	0	0	0	0	0	0	0	41
14911952	m	ph	ID	2018	1	0	0	0	0	0	0	0	0	0	0	0	
14912233	m	Not repo	TW	2018	1	0	0	0	0	0	0	0	0	0	0	0	49
14912426	m	md	TW	2018	0	1	0	1	0	0	0	0	0	0	0	0	74
14913572	Not	ot	BD	2018	0	0	0	0	0	0	0	0	0	0	0	0	
14913575	m	cn	LB	2018	0	0	0	0	0	0	0	0	0	0	0	0	70
14913672	Not	ot	BD	2018	0	0	0	0	0	0	0	0	0	0	0	0	
14913676	Not	ot	BD	2018	0	0	0	0	0	0	0	0	0	0	0	0	
14913958	m	md	CN	2018	0	1	0	0	0	1	0	0	0	0	0	0	67
14915311	f	ot	DE	2018	0	0	0	0	0	0	0	0	0	0	0	0	48
14915568	m	cn	US	2018	1	0	0	0	0	0	0	0	0	0	0	0	85
14915684	f	ot	DE	2018	0	0	0	0	0	0	0	0	0	0	0	0	60
14916178	f	cn	US	2018	1	0	0	0	0	0	0	0	0	0	0	0	73
14916575	f	md	US	2018	1	0	0	0	0	0	0	0	0	0	0	1	69
14917024	f	ot	SG	2018	0	1	0	0	1	0	0	0	0	0	0	0	52
14917085	m	md	LT	2018	0	0	0	0	0	0	0	0	0	0	0	0	60
14917390	m	ot	JP	2018	0	1	0	0	1	0	0	0	0	0	0	1	55
14917414	m	md	JP	2018	0	1	0	0	1	0	0	0	0	0	0	0	
14917521	m	md	JP	2018	0	0	0	0	0	0	0	0	0	0	0	0	70
14917696	m	ph	JP	2018	0	1	0	0	1	0	0	0	0	0	0	0	82
14917751	m	md	JP	2018	0	0	0	0	0	0	0	0	0	0	0	0	73
14917770	m	cn	GB	2018	0	1	1	0	0	0	0	0	0	0	0	0	88
14917810	m	md	JP	2018	0	1	0	0	1	0	0	0	0	0	0	0	75
14917855	m	md	JP	2018	0	1	0	0	1	0	0	0	0	0	0	1	67

14917890	f	md	GB	2018	0	1	0	0	0	1	0	0	0	0	0	0	73
14918108	f	cn	US	2018	1	0	0	0	0	0	0	0	0	0	0	0	
14918249	Not	ot	JP	2018	0	0	0	0	0	0	0	0	0	0	0	0	
14918373	m	ot	IN	2018	0	0	0	0	0	0	0	0	0	0	0	0	72
14918460	f	cn	GB	2018	1	0	0	0	0	0	0	0	0	0	0	0	
14918466	Not	ot	JP	2018	0	0	0	0	0	0	0	0	0	0	0	0	
14918468	Not	ot	JP	2018	0	0	0	0	0	0	0	0	0	0	0	0	
14918589	f	md	JP	2018	0	1	0	0	1	0	0	0	0	0	0	0	68
14918609	m	md	JP	2018	0	0	0	0	0	0	0	0	0	0	0	0	74
14918648	Not	ot	BD	2018	0	0	0	0	0	0	0	0	0	0	0	0	
14920157	m	md	CO	2018	0	0	0	0	0	0	0	0	0	0	0	0	
14920321	f	md	US	2018	1	0	0	0	0	0	0	0	0	0	0	0	80.8
14920649	f	cn	US	2018	1	0	0	0	0	0	0	0	0	0	0	0	
14921240	m	md	KR	2018	1	0	0	0	0	0	0	0	0	0	0	0	83
14921280	m	ot	FR	2018	0	1	0	0	1	0	0	0	0	0	0	1	79
14921314	m	ot	US	2018	0	1	0	0	1	0	0	0	0	0	0	0	65
14921350	m	ot	ES	2018	0	1	0	0	1	0	0	0	0	0	0	0	62
14921462	f	cn	AU	2018	0	1	0	0	1	0	0	0	0	0	0	0	59
14921506	f	ot	FR	2018	0	1	0	0	1	0	0	0	0	0	0	0	59
14921531	m	ot	FR	2018	0	1	0	0	1	0	0	0	0	0	0	0	61
14921532	f	ot	FR	2018	0	1	0	0	1	0	0	0	0	0	0	0	65
14921604	f	md	JP	2018	0	0	0	0	0	0	0	0	0	0	0	0	80
14921712	f	cn	US	2018	0	1	0	0	1	0	0	0	0	0	0	0	
14921735	m	md	US	2018	0	1	0	1	0	0	0	0	0	0	0	0	
14922797	m	md	DE	2018	0	0	0	0	0	0	0	0	0	0	0	0	59
14922853	m	ot	DE	2018	0	0	0	0	0	0	0	0	0	0	0	0	65
14922867	m	cn	GB	2018	1	0	0	0	0	0	0	0	0	0	0	0	84
14923670	m	md	JP	2018	1	0	0	0	0	0	0	0	0	0	0	0	64.6
14923673	m	md	US	2018	0	1	0	1	0	0	0	0	0	0	0	1	
14924013	m	ot	FR	2018	0	1	0	0	1	0	0	0	0	0	0	0	62
14924127	m	cn	JP	2018	0	1	1	0	0	0	0	0	0	0	0	0	77
14924322	f	md	EG	2018	0	1	0	0	1	0	0	0	0	0	0	0	62
14924651	f	ot	DE	2018	0	0	0	0	0	0	0	0	0	0	0	0	61
14924658	f	ot	DE	2018	0	1	0	0	1	0	0	0	0	0	0	0	52

14924770	m	md	HU	2018	0	1	0	0	1	0	0	0	0	0	0	0	63
14925067	m	md	CH	2018	0	1	0	1	0	0	0	0	0	0	0	0	67.2
14925216	f	md	US	2018	1	0	0	0	0	0	0	0	0	0	0	0	60
14925220	f	cn	US	2018	1	0	0	0	0	0	0	0	0	0	0	0	65
14925221	Not	md	US	2018	1	0	0	0	0	0	0	0	0	0	0	0	
14925251	m	ot	FR	2018	0	1	0	0	1	0	0	0	0	0	0	0	61
14925554	m	ot	JP	2018	0	1	0	0	1	0	0	0	0	0	0	0	55
14925615	Not	ot	US	2018	0	1	0	0	1	0	0	0	0	0	0	0	
14926597	m	ot	BE	2018	0	1	0	0	1	0	0	0	0	0	0	0	71
14926703	m	md	JP	2018	0	1	0	0	1	0	0	0	0	0	0	0	70
14926777	m	md	JP	2018	0	1	0	0	1	0	0	0	0	0	0	0	73
14926839	f	ot	DE	2018	0	1	0	0	1	0	0	0	0	0	0	0	61
14927009	f	ph	JP	2018	1	0	0	0	0	0	0	0	0	0	0	0	79
14927361	Not	ot	JP	2018	0	0	0	0	0	0	0	0	0	0	0	0	
14927617	f	ph	JP	2018	1	0	0	0	0	0	0	0	0	0	0	0	75
14927785	m	cn	CA	2018	0	0	0	0	0	0	0	0	0	0	0	0	48
14927796	m	ot	ES	2018	0	1	0	0	1	0	0	0	0	0	0	0	64
14928164	f	cn	GB	2018	1	0	0	0	0	0	0	0	0	0	0	0	37
14928881	f	md	JP	2018	1	0	0	0	0	0	0	0	0	0	0	1	84
14929104	m	cn	US	2018	0	0	0	0	0	0	0	0	0	0	0	0	
14929267	m	cn	JP	2018	0	1	1	0	0	0	0	0	0	0	0	0	
14929318	Not	ot	GB	2018	0	1	1	0	0	0	0	0	0	0	0	0	
14929430	f	ot	US	2018	1	0	0	0	0	0	0	0	0	0	0	0	
14929462	f	ot	CO	2018	0	0	0	0	0	0	0	0	0	0	0	0	
14929489	m	ot	GB	2018	0	0	0	0	0	0	0	0	0	0	0	0	82
14929522	m	cn	US	2018	0	1	1	0	0	0	0	0	0	0	0	0	
14929952	Not	md	DE	2018	0	1	1	0	0	0	0	0	0	0	0	0	
14929981	Not	ot	NL	2018	0	0	0	0	0	0	0	0	0	0	0	0	
14929982	Not	ot	NL	2018	0	0	0	0	0	0	0	0	0	0	0	0	
14929983	Not	ot	NL	2018	0	0	0	0	0	0	0	0	0	0	0	0	
14929984	Not	ot	NL	2018	0	0	0	0	0	0	0	0	0	0	0	0	
14929985	Not	ot	NL	2018	0	0	0	0	0	0	0	0	0	0	0	0	
14929986	Not	ot	NL	2018	0	0	0	0	0	0	0	0	0	0	0	0	
14929987	Not	ot	NL	2018	0	0	0	0	0	0	0	0	0	0	0	0	

14929988	Not	ot	NL	2018	0	0	0	0	0	0	0	0	0	0	0	0	
14929989	Not	ot	NL	2018	0	0	0	0	0	0	0	0	0	0	0	0	
14929990	Not	ot	NL	2018	0	0	0	0	0	0	0	0	0	0	0	0	
14929991	Not	ot	NL	2018	0	0	0	0	0	0	0	0	0	0	0	0	
14929992	Not	ot	NL	2018	0	0	0	0	0	0	0	0	0	0	0	0	
14929993	Not	ot	NL	2018	0	0	0	0	0	0	0	0	0	0	0	0	
14929994	Not	ot	NL	2018	0	0	0	0	0	0	0	0	0	0	0	0	
14929995	Not	ot	NL	2018	0	0	0	0	0	0	0	0	0	0	0	0	
14929996	Not	ot	NL	2018	0	0	0	0	0	0	0	0	0	0	0	0	
14929997	Not	ot	NL	2018	0	0	0	0	0	0	0	0	0	0	0	0	
14929998	Not	ot	NL	2018	0	0	0	0	0	0	0	0	0	0	0	0	
14929999	Not	ot	NL	2018	0	0	0	0	0	0	0	0	0	0	0	0	
14930006	Not	ot	NL	2018	0	0	0	0	0	0	0	0	0	0	0	0	
14930014	Not	ot	NL	2018	0	0	0	0	0	0	0	0	0	0	0	0	
14930015	Not	ot	NL	2018	0	0	0	0	0	0	0	0	0	0	0	0	
14930016	Not	ot	NL	2018	0	0	0	0	0	0	0	0	0	0	0	0	
14930017	Not	ot	NL	2018	0	0	0	0	0	0	0	0	0	0	0	0	
14930161	m	cn	JP	2018	0	0	0	0	0	0	0	0	0	0	0	0	49
14930358	m	md	JP	2018	0	1	1	0	0	0	0	0	0	0	0	1	49
14930492	f	ot	US	2018	0	0	0	0	0	0	0	0	0	0	0	0	
14930609	m	md	ES	2018	0	1	0	0	1	0	0	0	0	0	0	0	65
14930653	m	md	EG	2018	0	1	0	0	1	0	0	0	0	0	0	0	65
14931029	m	md	DE	2018	0	0	0	0	0	0	0	0	0	0	0	0	
14931113	f	md	US	2018	0	1	0	0	0	1	0	0	0	0	0	1	
14931351	m	cn	JP	2018	0	1	1	0	0	0	0	0	0	0	0	0	53
14931476	f	cn	US	2018	0	1	1	0	0	0	0	0	0	0	0	0	
14931903	m	ot	JP	2018	0	1	0	0	1	0	0	0	0	0	0	0	70
14933145	f	cn	US	2018	1	0	0	0	0	0	0	0	0	0	0	0	81
14933477	m	cn	GB	2018	1	0	0	0	0	0	0	0	0	0	0	0	
14933506	f	md	JP	2018	1	0	0	0	0	0	0	0	0	0	0	0	92
14933635	f	md	DE	2018	0	0	0	0	0	0	0	0	0	0	0	0	
14933844	f	md	BR	2018	0	1	0	0	0	1	0	0	0	0	0	0	57
14934236	f	md	JP	2018	1	0	0	0	0	0	0	0	0	0	0	0	80
14934442	m	ot	US	2018	0	1	0	0	1	0	0	0	0	0	0	0	66

14934469	f	md	DE	2018	0	0	0	0	0	0	0	0	0	0	0	0	61
14934626	f	md	US	2018	0	0	0	0	0	0	0	0	0	0	0	0	73
14934953	f	md	US	2018	1	0	0	0	0	0	0	0	0	0	0	0	
14935022	Not	md	CN	2018	1	0	0	0	0	0	0	0	0	0	0	0	61
14935114	m	ot	DE	2018	0	1	0	0	1	0	0	0	0	0	0	0	74
14935134	m	md	DE	2018	1	0	0	0	0	0	0	0	0	0	0	0	70
14935256	f	ot	GB	2018	0	1	0	0	1	0	0	0	0	0	0	0	81
14935309	f	ot	US	2018	0	1	0	0	1	0	0	0	0	0	0	0	64
14935342	m	md	JP	2018	0	0	0	0	0	0	0	0	0	0	0	0	90
14935378	m	ot	US	2018	0	1	1	0	0	0	0	0	0	0	0	0	69
14935490	Not	md	TH	2018	0	1	1	0	0	0	0	0	0	0	0	0	
14936283	m	md	US	2018	0	1	0	1	0	0	0	0	0	0	0	0	
14936297	f	ph	US	2018	0	1	0	1	0	0	0	0	0	0	0	0	68.7
14936341	Not	md	JP	2018	0	1	0	0	1	0	0	0	0	0	0	0	
14936481	m	cn	CN	2018	0	0	0	0	0	0	0	0	0	0	0	0	59
14936508	f	md	US	2018	1	0	0	0	0	0	0	0	0	0	0	0	
14936783	f	cn	US	2018	0	0	0	0	0	0	0	0	0	0	0	0	
14936997	m	cn	JP	2018	0	1	1	0	0	0	0	0	0	0	0	1	78
14937242	Not	ot	CN	2018	0	1	0	0	0	1	0	0	0	0	0	1	
14937317	m	ot	DE	2018	0	0	0	0	0	0	0	0	0	0	0	0	67
14937643	m	ot	US	2018	0	1	0	0	1	0	0	0	0	0	0	0	49
14937869	m	md	CN	2018	1	0	0	0	0	0	0	0	0	0	0	0	57
14937881	Not	cn	JP	2018	0	0	0	0	0	0	0	0	0	0	0	0	
14937958	f	ot	CA	2018	0	1	1	0	0	0	0	0	0	0	0	0	69
14937970	m	md	FR	2018	0	1	0	0	1	0	0	0	0	0	0	0	70
14938253	m	cn	JP	2018	0	1	1	0	0	0	0	0	0	0	0	0	75
14938363	m	ot	US	2018	1	0	0	0	0	0	0	0	0	0	0	0	
14938389	f	md	JP	2018	0	0	0	0	0	0	0	0	0	0	0	0	70
14938396	m	md	BR	2018	0	1	0	1	0	0	0	0	0	0	0	0	72
14938605	m	md	ES	2018	0	1	0	0	0	1	0	0	0	0	0	0	84
14938698	f	cn	GB	2018	1	0	0	0	0	0	0	0	0	0	0	0	
14939267	m	md	US	2018	0	1	1	0	0	0	0	0	0	0	0	0	76
14939623	m	ot	DE	2018	0	1	0	0	1	0	0	0	0	0	0	0	70
14940053	f	ot	KR	2018	0	0	0	0	0	0	0	0	0	0	0	0	57

14940451	f	md	IN	2018	0	0	0	0	0	0	0	0	0	0	0	0	54
14940625	f	md	DE	2018	0	0	0	0	0	0	0	0	0	0	0	0	66
14940646	f	md	JP	2018	1	0	0	0	0	0	0	0	0	0	0	0	90
14941056	m	cn	TH	2018	0	1	1	0	0	0	0	0	0	0	0	0	58
14942001	m	ot	DE	2018	0	0	0	0	0	0	0	0	0	0	0	0	60
14942040	f	ot	GB	2018	0	1	1	0	0	0	0	0	0	0	0	0	68
14942129	m	ot	JP	2018	0	1	0	0	1	0	0	0	0	0	0	0	63
14942222	m	ot	DE	2018	0	1	0	0	1	0	0	0	0	0	0	0	70
14942536	m	md	JP	2018	0	1	0	0	0	1	0	0	0	0	0	0	50
14942555	f	cn	GB	2018	1	0	0	0	0	0	0	0	0	0	0	0	70
14943045	f	cn	US	2018	0	1	0	1	0	0	0	0	0	0	0	0	
14943163	f	ot	JP	2018	0	1	0	0	1	0	0	0	0	0	0	1	77
14943255	m	cn	US	2018	0	1	1	0	0	0	0	0	0	0	0	0	
14943438	f	ot	JP	2018	0	1	0	0	1	0	0	0	0	0	0	0	68
14943615	m	cn	JP	2018	0	1	1	0	0	0	0	0	0	0	0	0	64
14943919	m	md	US	2018	1	0	0	0	0	0	0	0	0	0	0	0	
14944169	f	ot	IT	2018	1	0	0	0	0	0	0	0	0	0	0	0	72
14944561	m	md	PL	2018	0	1	0	0	0	1	0	0	0	0	0	0	64
14944669	m	cn	HU	2018	0	1	0	0	1	0	0	0	0	0	0	1	62
14944795	m	md	JP	2018	0	1	0	0	1	0	0	0	0	0	0	0	80
14944827	m	md	DE	2018	0	0	0	0	0	0	0	0	0	0	0	0	47
14945527	f	cn	US	2018	0	1	1	0	0	0	0	0	0	0	0	0	
14945903	m	md	JP	2018	0	0	0	0	0	0	0	0	0	0	0	0	60
14946087	f	cn	IL	2018	0	1	1	0	0	0	0	0	0	0	0	0	
14946839	m	cn	US	2018	0	1	1	0	0	0	0	0	0	0	0	0	
14946902	f	md	RO	2018	0	0	0	0	0	0	0	0	0	0	0	0	54
14946910	f	cn	US	2018	0	1	1	0	0	0	0	0	0	0	0	0	43
14947038	f	ot	US	2018	0	1	1	0	0	0	0	0	0	0	0	1	
14947201	f	cn	US	2018	0	1	1	0	0	0	0	0	0	0	0	1	60
14947300	f	md	FR	2018	1	0	0	0	0	0	0	0	0	0	0	0	
14947757	m	cn	US	2018	0	1	1	0	0	0	0	0	0	0	0	0	
14947992	f	md	US	2018	0	1	0	1	0	0	0	0	0	0	0	0	76
14948388	f	ot	US	2018	0	0	0	0	0	0	0	0	0	0	0	0	76
14948659	m	ot	US	2018	0	1	0	0	1	0	0	0	0	0	0	0	65

14949277	f	ot	US	2018	0	1	1	0	0	0	0	0	0	0	0	0	
14949534	f	md	FR	2018	1	0	0	0	0	0	0	0	0	0	0	0	68
14949668	m	md	JP	2018	0	1	0	0	1	0	0	0	0	0	0	0	76
14949980	m	md	JP	2018	0	1	0	0	1	0	0	0	0	0	0	0	49
14950106	m	md	JP	2018	0	1	0	0	1	0	0	0	0	0	0	0	50
14950381	m	md	DE	2018	0	0	0	0	0	0	0	0	0	0	0	0	81
14950394	m	md	JP	2018	0	1	0	0	1	0	0	0	0	0	0	1	55
14950527	Not	ot	JP	2018	0	1	0	0	1	0	0	0	0	0	0	0	
14950557	m	ot	DE	2018	0	0	0	0	0	0	0	0	0	0	0	0	58
14950576	Not	ot	IT	2018	0	1	0	0	1	0	0	0	0	0	0	0	
14950618	f	md	JP	2018	0	1	0	0	1	0	0	0	0	0	0	0	64
14950619	Not	ot	JP	2018	0	1	0	0	1	0	0	0	0	0	0	0	
14950623	Not	ot	JP	2018	0	1	0	0	1	0	0	0	0	0	0	0	
14950648	Not	ot	JP	2018	0	1	0	0	1	0	0	0	0	0	0	0	
14950675	f	ot	AT	2018	0	1	0	0	1	0	0	0	0	0	0	0	67
14950787	Not	ot	JP	2018	0	1	0	0	1	0	0	0	0	0	0	0	
14950796	m	md	JP	2018	0	1	0	0	1	0	0	0	0	0	0	0	82
14951076	m	md	AU	2018	0	1	0	0	0	1	0	0	0	0	0	0	74
14951207	f	md	JP	2018	0	0	0	0	0	0	0	0	0	0	0	0	80
14951233	Not	ot	CN	2018	0	0	0	0	0	0	0	0	0	0	0	0	
14951565	m	cn	US	2018	0	1	1	0	0	0	0	0	0	0	0	0	64
14951651	m	md	JP	2018	0	1	0	0	1	0	0	0	0	0	0	0	53
14951751	f	cn	JP	2018	0	1	1	0	0	0	0	0	0	0	0	0	84
14951754	m	cn	SI	2018	0	1	1	0	0	0	0	0	0	0	0	0	72
14952163	Not	md	IE	2018	0	0	0	0	0	0	0	0	0	0	0	0	
14952783	m	md	JP	2018	0	1	0	0	1	0	0	0	0	0	0	0	50
14952951	m	cn	US	2018	0	1	1	0	0	0	0	0	0	0	0	0	
14953552	m	md	JP	2018	0	1	0	0	1	0	0	0	0	0	0	0	67
14953808	f	md	SG	2018	1	0	0	0	0	0	0	0	0	0	0	0	65
14954934	m	ot	JP	2018	0	1	0	0	1	0	0	0	0	0	0	0	68
14955038	m	md	JP	2018	0	1	0	0	1	0	0	0	0	0	0	0	65
14955183	m	md	JP	2018	0	1	0	0	1	0	0	0	0	0	0	0	73
14955318	Not	md	JP	2018	0	1	0	0	1	0	0	0	0	0	0	0	
14955667	f	ot	DE	2018	0	1	0	0	1	0	0	0	0	0	0	0	56

14955677	m	md	JP	2018	0	1	0	0	0	1	0	0	0	0	0	0	59
14955699	f	ot	GB	2018	0	1	0	0	1	0	0	0	0	0	0	0	83
14955837	m	md	US	2018	0	0	0	0	0	0	0	0	0	0	0	0	
14955863	m	ot	DE	2018	0	0	0	0	0	0	0	0	0	0	0	0	70
14956868	f	cn	GB	2018	1	0	0	0	0	0	0	0	0	0	0	0	78
14956990	m	cn	JP	2018	0	1	1	0	0	0	0	0	0	0	0	0	73
14957375	m	cn	JP	2018	0	1	1	0	0	0	0	0	0	0	0	0	69
14958165	m	ot	DE	2018	0	0	0	0	0	0	0	0	0	0	0	0	78
14958890	m	ot	US	2018	0	0	0	0	0	0	0	0	0	0	0	0	
14959287	m	ot	ES	2018	0	1	0	0	1	0	0	0	0	0	0	0	63
14959288	m	md	JP	2018	0	1	0	0	1	0	0	0	0	0	0	0	76
14959696	Not	ot	IT	2018	0	1	0	0	1	0	0	0	0	0	0	0	
14959865	m	ot	CN	2018	0	1	0	0	1	0	0	0	0	0	0	0	59
14960066	m	cn	US	2018	0	1	1	0	0	0	0	0	0	0	0	0	74
14961000	Not	md	AU	2018	0	1	0	0	1	0	0	0	0	0	0	0	
14961452	m	md	IT	2018	0	1	0	0	1	0	0	0	0	0	0	0	68
14961459	m	md	JP	2018	0	0	0	0	0	0	0	0	0	0	0	0	80
14961877	m	md	DE	2018	0	1	0	0	1	0	0	0	0	0	0	0	71
14962066	Not	md	US	2018	0	0	0	0	0	0	0	0	0	0	0	0	
14962109	f	md	JP	2018	0	1	1	0	0	0	0	0	0	0	0	0	48
14962384	m	cn	TW	2018	1	0	0	0	0	0	0	0	0	0	0	0	70
14962386	m	md	CH	2018	0	1	0	1	0	0	0	0	0	0	0	0	62.6
14962873	m	ot	DE	2018	0	0	0	0	0	0	0	0	0	0	0	0	83
14963036	f	md	DE	2018	0	0	0	0	0	0	0	0	0	0	0	0	66
14963550	m	cn	GB	2018	1	0	0	0	0	0	0	0	0	0	0	0	71
14963576	m	md	SK	2018	0	0	0	0	0	0	0	0	0	0	0	0	84
14964253	m	ot	IN	2018	0	0	0	0	0	0	0	0	0	0	0	0	75
14964361	f	md	IT	2018	1	0	0	0	0	0	0	0	0	0	0	0	72
14964557	m	md	JP	2018	0	1	0	1	0	0	0	0	0	0	0	0	71.8
14964586	m	ot	JP	2018	0	0	0	0	0	0	0	0	0	0	0	0	79
14964665	m	md	IT	2018	1	0	0	0	0	0	0	0	0	0	0	0	68
14964753	f	md	ID	2018	1	0	0	0	0	0	0	0	0	0	0	0	68
14964841	m	ot	DE	2018	0	0	0	0	0	0	0	0	0	0	0	0	73
14965209	f	md	US	2018	0	0	0	0	0	0	0	0	0	0	0	0	65

14966510	m	cn	IL	2018	0	1	1	0	0	0	0	0	0	0	0	0	58
14966519	f	cn	US	2018	1	0	0	0	0	0	0	0	0	0	0	0	70
14966920	m	md	US	2018	0	1	1	0	0	0	0	0	0	0	0	0	
14966971	m	cn	JP	2018	0	0	0	0	0	0	0	0	0	0	0	0	57
14966973	m	md	JP	2018	0	1	0	0	1	0	0	0	0	0	0	1	71
14967626	m	md	AT	2018	0	0	0	0	0	0	0	0	0	0	0	0	
14968062	m	cn	JP	2018	0	1	1	0	0	0	0	0	0	0	0	0	78
14968397	f	cn	GB	2018	1	0	0	0	0	0	0	0	0	0	0	0	
14969102	f	md	JP	2018	0	0	0	0	0	0	0	0	0	0	0	0	69
14969136	f	md	US	2018	0	1	0	1	0	0	0	0	0	0	0	0	
14969547	f	cn	US	2018	0	1	0	0	1	0	0	0	0	0	0	0	57
14969770	f	md	ES	2018	0	0	0	0	0	0	0	0	0	0	0	0	76.1
14970921	f	ph	JP	2018	0	1	0	0	1	0	0	0	0	0	0	0	70
14971063	f	md	JP	2018	0	1	0	0	1	0	0	0	0	0	0	0	65
14971310	m	ot	SE	2018	0	1	1	0	0	0	0	0	0	0	0	0	
14971923	m	md	DE	2018	0	1	0	0	1	0	0	0	0	0	0	0	62
14971990	m	cn	GR	2018	0	0	0	0	0	0	0	0	0	0	0	0	
14972032	m	md	JP	2018	0	1	0	0	1	0	0	0	0	0	0	1	83
14972078	m	md	JP	2018	0	1	0	0	0	1	0	0	0	0	0	0	80
14972544	m	md	JP	2018	0	1	0	0	1	0	0	0	0	0	0	0	63
14972853	m	cn	US	2018	0	0	0	0	0	0	0	0	0	0	0	0	
14972938	f	cn	CN	2018	1	0	0	0	0	0	0	0	0	0	0	0	70
14973294	f	cn	Not re	2018	0	0	0	0	0	0	0	0	0	0	0	0	
14973363	f	ot	SE	2018	0	0	0	0	0	0	0	0	0	0	0	0	42
14973625	f	cn	GB	2018	1	0	0	0	0	0	0	0	0	0	0	0	90
14973671	f	cn	JP	2018	0	0	0	0	0	0	0	0	0	0	0	0	80
14973801	m	cn	US	2018	0	1	1	0	0	0	0	0	0	0	0	0	60
14974358	m	cn	JP	2018	0	1	1	0	0	0	0	0	0	0	0	1	68
14974388	Not	md	US	2018	0	0	0	0	0	0	0	0	0	0	0	0	
14974667	m	md	AU	2018	0	0	0	0	0	0	0	0	0	0	0	0	67
14974758	f	ot	CA	2018	0	1	1	0	0	0	0	0	0	0	0	1	63
14975107	m	md	JP	2018	0	0	0	0	0	0	0	0	0	0	0	0	80
14975204	m	md	JP	2018	0	1	0	0	1	0	0	0	0	0	0	0	81
14976389	f	md	LB	2018	0	1	1	0	0	0	0	0	0	0	0	0	40

14976420	f	md	BE	2018	0	0	0	0	0	0	0	0	0	0	0	0	
14976827	m	md	IT	2018	1	0	0	0	0	0	0	0	0	0	0	0	68
14977071	m	md	EG	2018	0	1	0	0	1	0	0	0	0	0	0	0	60
14977340	f	md	JP	2018	0	0	0	0	0	0	1	0	1	0	0	0	57
14977366	m	md	JP	2018	0	1	0	0	1	0	0	0	0	0	0	0	75
14977531	m	md	GB	2018	0	0	0	0	0	0	0	0	0	0	0	0	
14977613	m	md	GB	2018	0	1	0	1	0	0	0	0	0	0	0	0	73.5
14977664	m	md	JP	2018	0	1	0	0	0	1	0	0	0	0	0	0	66
14978034	f	md	US	2018	0	1	0	0	1	0	0	0	0	0	0	0	70
14978220	m	cn	JP	2018	0	1	1	0	0	0	0	0	0	0	0	0	71
14978318	f	ot	SE	2018	0	0	0	0	0	0	0	0	0	0	0	0	42
14978544	f	md	US	2018	1	0	0	0	0	0	0	0	0	0	0	0	
14978595	m	md	JP	2018	0	1	0	0	1	0	0	0	0	0	0	0	77
14978621	m	ot	DE	2018	0	0	0	0	0	0	0	0	0	0	0	0	66
14978752	f	md	JP	2018	0	1	1	0	0	0	0	0	0	0	0	1	85
14978856	f	Not repo	KR	2018	1	0	0	0	0	0	0	0	0	0	0	0	70
14978960	m	md	JP	2018	0	1	0	0	1	0	0	0	0	0	0	0	49
14979910	m	cn	JP	2018	0	1	0	0	1	0	0	0	0	0	0	0	63
14980249	m	md	LB	2018	1	0	0	0	0	0	0	0	0	0	0	0	80
14980484	m	ot	GR	2018	0	1	0	0	1	0	0	0	0	0	0	0	67
14980573	f	md	DE	2018	0	0	0	0	0	0	0	0	0	0	0	0	
14980924	f	cn	US	2018	0	1	0	0	1	0	0	0	0	0	0	0	
14981340	m	cn	US	2018	0	1	0	0	1	0	0	0	0	0	0	0	
14981508	m	md	DE	2018	0	1	0	0	1	0	0	0	0	0	0	0	
14981599	f	md	CZ	2018	1	0	0	0	0	0	0	0	0	0	0	0	72
14981758	m	ot	DE	2018	0	0	0	0	0	0	0	0	0	0	0	0	77
14981886	m	ph	JP	2018	1	0	0	0	0	0	0	0	0	0	0	0	81
14981959	f	md	US	2018	0	1	0	0	0	1	0	0	0	0	0	0	60
14982065	m	cn	JP	2018	0	1	1	0	0	0	0	0	0	0	0	0	
14982105	m	md	FR	2018	0	1	0	1	0	0	0	0	0	0	0	0	57.8
14982261	m	md	GE	2018	0	1	0	0	0	1	0	0	0	0	0	0	79
14982347	f	md	US	2018	0	1	0	0	1	0	0	0	0	0	0	0	
14982512	m	md	US	2018	0	1	0	0	1	0	0	0	0	0	0	0	
14983342	m	cn	GB	2018	1	0	0	0	0	0	0	0	0	0	0	0	76

14983371	f	md	GR	2018	0	0	0	0	0	0	0	0	0	0	0	0	49
14984677	f	ot	CN	2018	0	0	0	0	0	0	0	0	0	0	0	0	63
14984695	m	md	FR	2018	0	1	0	0	0	1	0	0	0	0	0	0	61
14984733	m	ot	HU	2018	0	1	0	0	1	0	0	0	0	0	0	0	58
14985056	f	ot	GB	2018	0	1	0	0	0	1	0	0	0	0	0	0	
14985139	m	cn	JP	2018	0	0	0	0	0	0	0	0	0	0	0	0	79
14985687	m	cn	DE	2018	0	1	0	0	1	0	0	0	0	0	0	0	70
14985741	m	ot	SK	2018	0	0	0	0	0	0	0	0	0	0	0	0	63
14985886	f	md	ES	2018	0	0	0	0	0	0	0	0	0	0	0	0	56
14986120	m	ot	CN	2018	0	1	0	0	1	0	0	0	0	0	0	1	53
14986139	f	ot	FR	2018	0	1	0	0	1	0	0	0	0	0	0	0	69
14986616	Not	md	JP	2018	0	1	0	0	1	0	0	0	0	0	0	0	70
14986643	m	ot	GB	2018	0	0	0	0	0	0	0	0	0	0	0	0	78
14986663	f	md	JP	2018	0	1	0	0	1	0	0	0	0	0	0	0	70
14986708	m	md	US	2018	1	0	0	0	0	0	0	0	0	0	0	0	81
14986923	f	ot	US	2018	0	1	0	0	1	0	0	0	0	0	0	0	
14987328	m	cn	US	2018	0	1	0	0	1	0	0	0	0	0	0	0	
14987374	m	ph	US	2018	0	0	0	0	0	0	0	0	0	0	0	0	84
14987629	f	ot	FR	2018	0	0	0	0	0	0	0	0	0	0	0	0	66
14987702	f	md	US	2018	1	0	0	0	0	0	0	0	0	0	0	0	59.5
14987715	f	Not repo	JP	2018	0	0	0	0	0	0	0	0	0	0	0	0	85
14988673	f	cn	BR	2018	1	0	0	0	0	0	0	0	0	0	0	0	
14988680	m	md	JP	2018	0	1	0	0	0	1	0	0	0	0	0	0	53
14988802	m	md	ES	2018	1	0	0	0	0	0	0	0	0	0	0	0	64
14989177	m	cn	BR	2018	0	1	1	0	0	0	0	0	0	0	0	0	
14989207	m	cn	JP	2018	0	1	1	0	0	0	0	0	0	0	0	0	70
14989249	f	cn	JP	2018	0	0	0	0	0	0	0	0	0	0	0	0	90
14989451	f	md	DE	2018	1	0	0	0	0	0	0	0	0	0	0	0	
14989749	m	Not repo	JP	2018	0	0	0	0	0	0	0	0	0	0	0	0	77
14990776	m	cn	BR	2018	0	1	0	1	0	0	0	0	0	0	0	0	62.3
14990780	m	md	KR	2018	1	0	0	0	0	0	0	0	0	0	0	1	72.6
14990908	f	md	ES	2018	1	0	0	0	0	0	0	0	0	0	0	1	70.8
14991214	m	cn	JP	2018	0	1	1	0	0	0	0	0	0	0	0	0	83
14991735	f	ot	FR	2018	0	1	0	0	1	0	0	0	0	0	0	0	36

14992183	f	md	US	2018	1	0	0	0	0	0	0	0	0	0	0	0	82
14992594	Not	ot	CN	2018	0	0	0	0	0	0	0	0	0	0	0	0	
14993594	Not	ot	CN	2018	0	0	0	0	0	0	0	0	0	0	0	0	
14993669	f	md	DE	2018	1	0	0	0	0	0	0	0	0	0	0	0	
14993746	f	md	JP	2018	0	0	0	0	0	0	1	0	1	0	0	0	77
14993768	f	ot	JP	2018	0	1	0	0	1	0	0	0	0	0	0	0	77
14993774	f	md	DE	2018	0	1	0	0	0	1	0	0	0	0	0	0	61
14994146	f	cn	US	2018	0	1	0	0	1	0	0	0	0	0	0	0	
14994204	m	ot	JP	2018	0	1	0	0	1	0	0	0	0	0	0	0	52
14994367	m	md	JP	2018	0	1	0	0	0	1	0	0	0	0	0	1	75
14994511	f	ot	US	2018	0	1	0	0	1	0	0	0	0	0	0	1	48
14994939	f	md	IN	2018	0	0	0	0	0	0	0	0	0	0	0	0	58
14995346	m	ot	US	2018	0	1	0	0	1	0	0	0	0	0	0	0	54
14995485	m	md	JP	2018	0	1	0	0	0	1	0	0	0	0	0	0	74
14996195	m	ot	DE	2018	0	1	0	0	1	0	0	0	0	0	0	0	62
14996357	f	md	KR	2018	0	1	0	0	0	1	0	0	0	0	0	0	60
14997219	m	md	ID	2018	1	0	0	0	0	0	0	0	0	0	0	0	68
14997274	m	md	AT	2018	0	0	0	0	0	0	0	0	0	0	0	0	57
14997397	m	md	IN	2018	0	1	0	0	1	0	0	0	0	0	0	0	51
14998130	f	cn	US	2018	1	0	0	0	0	0	0	0	0	0	0	0	
14998372	m	ot	JP	2018	0	1	0	0	1	0	0	0	0	0	0	0	76
14998463	m	ot	DE	2018	0	1	0	0	1	0	0	0	0	0	0	0	72
14998569	f	md	JP	2018	0	1	0	0	1	0	0	0	0	0	0	0	75
14998598	f	cn	US	2018	1	0	0	0	0	0	0	0	0	0	0	0	72
14998614	f	md	US	2018	0	1	0	0	1	0	0	0	0	0	0	0	72
14998620	f	md	JP	2018	0	1	0	0	1	0	0	0	0	0	0	0	67
14998766	m	ph	JP	2018	0	1	0	0	1	0	0	0	0	0	0	0	47
14998811	m	md	SE	2018	0	0	0	0	0	0	0	0	0	0	0	0	77
14999208	Not	md	US	2018	1	0	0	0	0	0	0	0	0	0	0	0	
14999258	m	md	PL	2018	0	1	0	0	0	1	0	0	0	0	0	0	62
14999493	f	md	DE	2018	0	0	0	0	0	0	0	0	0	0	0	0	45
14999918	f	md	NL	2018	0	0	0	0	0	0	0	0	0	0	0	0	76
15000001	m	md	JP	2018	0	1	1	0	0	0	0	0	0	0	0	0	70
15000372	Not	md	JP	2018	0	0	0	0	0	0	0	0	0	0	0	0	80

15000492	m	cn	US	2018	0	0	0	0	0	0	0	0	0	0	0	0	
15000577	m	md	US	2018	0	1	0	1	0	0	0	0	0	0	0	1	
15000933	m	cn	US	2018	1	0	0	0	0	0	0	0	0	0	0	0	69
15001788	Not	md	US	2018	1	0	0	0	0	0	0	0	0	0	0	0	
15001886	f	ot	DE	2018	0	1	0	0	1	0	0	0	0	0	0	0	74
15002253	m	cn	JP	2018	0	1	1	0	0	0	0	0	0	0	0	0	70
15003010	Not	md	US	2018	0	1	0	1	0	0	0	0	0	0	0	0	
15003531	f	md	SK	2018	1	0	0	0	0	0	0	0	0	0	0	0	
15003532	m	cn	US	2018	1	0	0	0	0	0	0	0	0	0	0	0	77
15003560	m	ot	ES	2018	0	1	0	0	1	0	0	0	0	0	0	0	60
15003619	f	md	US	2018	0	1	0	1	0	0	0	0	0	0	0	0	60
15003863	f	cn	JP	2018	0	0	0	0	0	0	0	0	0	0	0	0	70
15003983	m	ph	FR	2018	0	0	0	0	0	0	0	0	0	0	0	0	57
15004026	m	cn	GB	2018	1	0	0	0	0	0	0	0	0	0	0	0	63
15004526	f	ph	US	2018	1	0	0	0	0	0	0	0	0	0	0	0	35
15004542	f	ot	DE	2018	0	1	0	0	1	0	0	0	0	0	0	0	61
15004633	m	cn	JP	2018	0	1	1	0	0	0	0	0	0	0	0	0	86
15005582	f	cn	LB	2018	0	0	0	0	0	0	0	0	0	0	0	0	54
15006353	m	cn	JP	2018	0	1	1	0	0	0	0	0	0	0	0	1	60
15007337	m	ot	US	2018	0	1	0	0	1	0	0	0	0	0	0	0	78
15007339	Not	cn	US	2018	1	0	0	0	0	0	0	0	0	0	0	0	
15007466	m	md	CN	2018	0	1	0	1	0	0	0	0	0	0	0	0	51.2
15007714	m	Not repo	US	2018	1	0	0	0	0	0	0	0	0	0	0	0	66.8
15007833	m	cn	JP	2018	0	1	1	0	0	0	0	0	0	0	0	0	74
15008479	f	cn	US	2018	1	0	0	0	0	0	0	0	0	0	0	0	77
15008790	m	md	JP	2018	0	1	0	0	1	0	0	0	0	0	0	0	60
15008802	f	ot	US	2018	0	1	0	0	1	0	0	0	0	0	0	0	57
15009333	f	cn	GB	2018	0	1	0	0	1	0	0	0	0	0	0	0	60
15009698	f	cn	US	2018	1	0	0	0	0	0	0	0	0	0	0	0	62.8
15009701	f	ot	IT	2018	0	1	0	0	1	0	0	0	0	0	0	0	42
15010356	m	cn	JP	2018	0	1	1	0	0	0	0	0	0	0	0	0	80
15010462	m	cn	JP	2018	0	1	1	0	0	0	0	0	0	0	0	0	
15010781	f	ot	JP	2018	0	1	1	0	0	0	0	0	0	0	0	1	84
15010913	m	cn	ES	2018	0	1	1	0	0	0	0	0	0	0	0	0	71

15010918	f	ot	DE	2018	0	0	0	0	0	0	0	0	0	0	0	0	56
15010950	m	cn	MX	2018	0	1	1	0	0	0	0	0	0	0	0	0	
15010971	f	md	JP	2018	0	0	0	0	0	0	0	0	0	0	0	0	80
15011026	m	cn	JP	2018	0	1	1	0	0	0	0	0	0	0	0	0	82
15011484	m	cn	US	2018	1	0	0	0	0	0	0	0	0	0	0	0	71
15011854	m	ot	US	2018	0	1	0	0	1	0	0	0	0	0	0	0	59
15012615	f	md	JP	2018	0	1	0	0	1	0	0	0	0	0	0	0	71
15012994	m	md	GB	2018	0	0	0	0	0	0	0	0	0	0	0	0	63
15013009	m	ot	DE	2018	0	1	0	0	1	0	0	0	0	0	0	0	64
15013158	Not	ot	FR	2018	0	0	0	0	0	0	0	0	0	0	0	0	
15013481	f	md	AU	2018	0	1	1	0	0	0	0	0	0	0	0	0	55
15013973	f	cn	US	2018	0	1	0	0	1	0	0	0	0	0	0	0	
15014053	f	ot	FR	2018	0	1	0	0	1	0	0	0	0	0	0	0	86
15014108	f	md	BE	2018	0	1	0	0	1	0	0	0	0	0	0	1	58
15014174	m	md	HR	2018	0	1	1	0	0	0	0	0	0	0	0	0	
15014222	f	md	JP	2018	0	1	0	0	1	0	0	0	0	0	0	0	76
15014394	f	cn	US	2018	1	0	0	0	0	0	0	0	0	0	0	0	60
15014445	f	cn	JP	2018	0	1	1	0	0	0	0	0	0	0	0	0	80
15014460	m	md	CZ	2018	1	0	0	0	0	0	0	0	0	0	0	0	70
15014546	m	cn	US	2018	0	1	0	0	1	0	0	0	0	0	0	0	
15014553	f	ot	NL	2018	0	0	0	0	0	0	0	0	0	0	0	0	67
15014730	Not	md	JP	2018	0	1	0	0	1	0	0	0	0	0	0	0	76
15014739	m	md	JP	2018	0	1	0	0	0	1	0	0	0	0	0	0	70
15015116	f	ot	BR	2018	1	0	0	0	0	0	0	0	0	0	0	0	50
15015217	Not	ot	TR	2018	0	0	0	0	0	0	0	0	0	0	0	0	
15015727	f	md	PT	2018	1	0	0	0	0	0	0	0	0	0	0	0	
15015777	f	cn	US	2018	0	0	0	0	0	0	0	0	0	0	0	0	56
15016253	f	Not repo	US	2018	1	0	0	0	0	0	0	0	0	0	0	0	69.7
15016276	f	md	JP	2018	0	0	0	0	0	0	0	0	0	0	0	0	83
15016843	f	cn	US	2018	0	1	1	0	0	0	0	0	0	0	0	1	
15016964	Not	cn	GB	2018	1	0	0	0	0	0	0	0	0	0	0	0	
15017345	m	ot	ES	2018	0	1	0	0	1	0	0	0	0	0	0	0	74
15017464	m	ot	CH	2018	0	1	0	0	1	0	0	0	0	0	0	0	56
15017848	Not	md	JP	2018	0	0	0	0	0	0	0	0	0	0	0	0	

15018267	m	ot	DE	2018	0	0	0	0	0	0	0	0	0	0	0	0	60
15018275	m	md	CA	2018	0	0	0	0	0	0	0	0	0	0	0	0	52
15018637	m	cn	CN	2018	0	0	0	0	0	0	0	0	0	0	0	0	
15018652	m	md	HR	2018	0	1	1	0	0	0	0	0	0	0	0	0	
15018945	m	cn	JP	2018	0	1	1	0	0	0	0	0	0	0	0	0	81
15019408	m	ot	DE	2018	0	0	0	0	0	0	0	0	0	0	0	0	67
15019460	Not	md	JP	2018	0	1	0	0	1	0	0	0	0	0	0	0	
15020223	m	cn	JP	2018	0	1	1	0	0	0	0	0	0	0	0	0	75
15020303	m	md	JP	2018	0	1	0	0	1	0	0	0	0	0	0	1	64
15020528	f	md	AT	2018	1	0	0	0	0	0	0	0	0	0	0	0	63
15020852	m	md	PT	2018	0	0	0	0	0	0	0	0	0	0	0	0	78
15021084	Not	md	FR	2018	0	1	0	0	1	0	0	0	0	0	0	0	
15021578	m	cn	KR	2018	0	1	1	0	0	0	0	0	0	0	0	0	
15022036	m	cn	GB	2018	1	0	0	0	0	0	0	0	0	0	0	0	71
15022753	m	cn	JP	2018	0	1	1	0	0	0	0	0	0	0	0	0	50
15022803	m	cn	US	2018	0	1	1	0	0	0	0	0	0	0	0	0	
15023812	m	ot	FR	2018	0	1	0	0	1	0	0	0	0	0	0	0	56
15023851	m	cn	TW	2018	0	0	0	0	0	0	0	0	0	0	0	0	45
15026593	f	md	GB	2018	0	1	1	0	0	0	0	0	0	0	0	0	65
15026683	Not	cn	US	2018	0	1	1	0	0	0	0	0	0	0	0	0	
15026747	m	md	JP	2018	0	1	0	0	1	0	0	0	0	0	0	1	80
15026804	m	ot	FR	2018	0	1	0	0	1	0	0	0	0	0	0	0	
15026848	m	cn	HU	2018	0	1	0	0	1	0	0	0	0	0	0	0	66
15026902	f	ot	US	2018	0	1	0	0	1	0	0	0	0	0	0	0	79
15026914	f	cn	US	2018	1	0	0	0	0	0	0	0	0	0	0	0	
15027243	m	md	DE	2018	0	0	0	0	0	0	0	0	0	0	0	0	65
15027311	f	cn	US	2018	0	1	1	0	0	0	0	0	0	0	0	0	
15027474	f	md	JP	2018	0	1	0	0	0	1	0	0	0	0	0	0	74
15027513	m	md	JP	2018	0	1	0	0	1	0	0	0	0	0	0	0	62
15027945	f	cn	US	2018	1	0	0	0	0	0	0	0	0	0	0	0	65
15027982	f	md	DE	2018	0	0	0	0	0	0	0	0	0	0	0	0	
15028064	m	md	DE	2018	0	0	0	0	0	0	0	0	0	0	0	0	
15028183	m	ot	DE	2018	0	0	0	0	0	0	0	0	0	0	0	0	51
15028193	m	ot	DE	2018	0	0	0	0	0	0	0	0	0	0	0	0	76

15028339	Not	md	AU	2018	0	0	0	0	0	0	0	0	0	0	0	0	55
15028707	f	cn	US	2018	1	0	0	0	0	0	0	0	0	0	0	0	76
15029115	m	cn	TH	2018	0	0	0	0	0	0	0	0	0	0	0	0	59
15029279	Not	md	JP	2018	1	0	0	0	0	0	0	0	0	0	0	0	
15029333	f	cn	GB	2018	1	0	0	0	0	0	0	0	0	0	0	0	
15030367	Not	cn	US	2018	0	1	1	0	0	0	0	0	0	0	0	1	
15030368	f	cn	US	2018	0	1	1	0	0	0	0	0	0	0	0	0	80
15030369	m	ot	GT	2018	0	1	1	0	0	0	0	0	0	0	0	0	
15032093	m	ot	US	2018	1	0	0	0	0	0	0	0	0	0	0	0	59
15032227	m	cn	US	2018	0	1	1	0	0	0	0	0	0	0	0	0	54
15032286	f	cn	US	2018	0	1	1	0	0	0	0	0	0	0	0	0	
15033801	m	md	JP	2018	0	1	0	0	1	0	0	0	0	0	0	0	80
15033862	m	md	JP	2018	0	1	0	0	1	0	0	0	0	0	0	0	71
15034369	f	ph	CA	2018	1	0	0	0	0	0	0	0	0	0	0	0	64
15034499	f	md	US	2018	1	0	0	0	0	0	0	0	0	0	0	0	78
15034570	f	md	JP	2018	1	0	0	0	0	0	0	0	0	0	0	0	100
15034617	Not	md	JP	2018	0	1	0	0	1	0	0	0	0	0	0	0	
15034706	m	cn	US	2018	0	1	0	0	1	0	0	0	0	0	0	0	
15035188	m	ot	JP	2018	0	1	1	0	0	0	0	0	0	0	0	1	74
15035331	m	md	KR	2018	0	1	0	0	1	0	0	0	0	0	0	0	
15035507	m	md	DE	2018	0	0	0	0	0	0	0	0	0	0	0	0	74
15035832	m	cn	IN	2018	0	0	0	0	0	0	0	0	0	0	0	0	40
15036346	m	cn	US	2018	0	0	0	0	0	0	0	0	0	0	0	0	54
15036435	Not	md	JP	2018	0	1	0	0	1	0	0	0	0	0	0	0	
15036499	m	cn	JP	2018	0	0	0	0	0	0	0	0	0	0	0	0	71
15036922	m	cn	GB	2018	1	0	0	0	0	0	0	0	0	0	0	0	52
15037210	m	md	DE	2018	0	0	0	0	0	0	0	0	0	0	0	0	54
15037388	m	cn	MX	2018	0	1	1	0	0	0	0	0	0	0	0	1	
15037476	m	ot	JP	2018	0	1	0	1	0	0	0	0	0	0	0	0	70
15037661	m	cn	BG	2018	1	0	0	0	0	0	0	0	0	0	0	0	52
15037680	f	cn	DE	2018	0	0	0	0	0	0	0	0	0	0	0	0	57
15038276	f	md	US	2018	0	1	1	0	0	0	0	0	0	0	0	0	
15040788	m	cn	JP	2018	0	0	0	0	0	0	0	0	0	0	0	0	75
15040851	Not	cn	US	2018	0	1	0	0	1	0	0	0	0	0	0	0	

15041342	m	ot	US	2018	0	1	0	0	1	0	0	0	0	0	0	0	61
15041424	f	cn	US	2018	1	0	0	0	0	0	0	0	0	0	0	0	67
15041439	m	ot	NL	2018	0	0	0	0	0	0	0	0	0	0	0	0	64
15041641	f	md	KR	2018	0	1	0	0	0	1	0	0	0	0	0	0	54
15041708	f	md	US	2018	0	1	0	1	0	0	0	0	0	0	0	0	
15041727	m	ph	JP	2018	0	1	0	0	1	0	0	0	0	0	0	0	54
15041857	f	cn	US	2018	1	0	0	0	0	0	0	0	0	0	0	0	85
15041888	m	cn	GB	2018	1	0	0	0	0	0	0	0	0	0	0	0	70
15042306	m	ot	DE	2018	0	0	0	0	0	0	0	0	0	0	0	0	77
15042470	m	md	DE	2018	0	1	1	0	0	0	0	0	0	0	0	0	
15042576	Not	ot	US	2018	0	0	0	0	0	0	0	0	0	0	0	0	
15042642	m	cn	JP	2018	1	0	0	0	0	0	0	0	0	0	0	0	
15042871	m	cn	US	2018	0	1	1	0	0	0	0	0	0	0	0	0	
15042994	m	md	GR	2018	0	0	0	0	0	0	0	0	0	0	0	0	71
15043279	f	ot	KR	2018	0	0	0	0	0	0	0	0	0	0	0	0	57
15043339	f	ot	NL	2018	0	0	0	0	0	0	0	0	0	0	0	0	67
15043402	m	cn	US	2018	1	0	0	0	0	0	0	0	0	0	0	1	51
15043530	f	cn	US	2018	1	0	0	0	0	0	0	0	0	0	0	0	64
15043859	f	md	JP	2018	0	0	0	0	0	0	0	0	0	0	0	0	47
15043902	m	md	US	2018	0	1	0	1	0	0	0	0	0	0	0	1	
15043914	f	ot	DE	2018	0	1	0	0	1	0	0	0	0	0	0	0	
15044020	f	ph	US	2018	0	0	0	0	0	0	0	0	0	0	0	0	63
15044167	m	md	JP	2018	0	0	0	0	0	0	0	0	0	0	0	0	73
15044640	m	ot	DE	2018	0	1	0	0	1	0	0	0	0	0	0	0	70
15044643	m	ot	DE	2018	0	0	0	0	0	0	0	0	0	0	0	0	47
15044730	f	md	JP	2018	1	0	0	0	0	0	0	0	0	0	0	0	68
15046102	m	md	US	2018	0	1	0	1	0	0	0	0	0	0	0	0	60
15046477	m	ot	DE	2018	0	1	0	0	1	0	0	0	0	0	0	0	66
15047846	m	ot	FR	2018	0	1	0	0	1	0	0	0	0	0	0	0	64
15047990	f	cn	EC	2018	0	0	0	0	0	0	0	0	0	0	0	0	
15048189	f	md	IT	2018	1	0	0	0	0	0	0	0	0	0	0	0	44
15048214	f	ot	US	2018	0	1	0	0	1	0	0	0	0	0	0	0	59
15048265	m	md	JP	2018	0	1	0	0	1	0	0	0	0	0	0	0	63
15048281	m	md	JP	2018	0	1	0	0	1	0	0	0	0	0	0	0	48

15048380	f	md	JP	2018	0	1	0	0	1	0	0	0	0	0	0	0	65
15048496	f	md	FR	2018	0	1	0	0	1	0	0	0	0	0	0	0	70
15048514	m	ot	DE	2018	0	0	0	0	0	0	0	0	0	0	0	0	60
15048701	m	md	IT	2018	1	0	0	0	0	0	0	0	0	0	0	0	74
15049006	f	ot	IT	2018	0	0	0	0	0	0	0	0	0	0	0	0	50
15049356	Not	ot	DE	2018	0	1	0	0	1	0	0	0	0	0	0	0	
15049820	m	ot	FR	2018	0	0	0	0	0	0	0	0	0	0	0	0	63
15049880	Not	ph	JP	2018	1	0	0	0	0	0	0	0	0	0	0	0	
15049923	f	cn	JP	2018	0	1	1	0	0	0	0	0	0	0	0	0	79
15050134	f	md	PE	2018	1	0	0	0	0	0	0	0	0	0	0	0	64
15050308	m	md	FR	2018	0	1	0	0	1	0	0	0	0	0	0	0	64
15050390	f	cn	Not re	2018	0	0	0	0	0	0	0	0	0	0	0	0	
15050424	Not	md	CL	2018	0	0	0	0	0	0	0	0	0	0	0	0	
15051529	f	md	US	2018	1	0	0	0	0	0	0	0	0	0	0	0	82
15051729	Not	ot	FR	2018	0	0	0	0	0	0	0	0	0	0	0	0	
15053144	m	md	US	2018	0	1	0	1	0	0	0	0	0	0	0	1	70
15053166	m	Not repo	Not re	2018	0	1	0	0	1	0	0	0	0	0	0	0	63
15053393	f	cn	US	2018	0	1	0	0	1	0	0	0	0	0	0	0	59
15054101	m	cn	US	2018	0	1	0	0	1	0	0	0	0	0	0	0	66
15055252	f	cn	US	2018	0	1	0	0	1	0	0	0	0	0	0	0	58
15057068	m	cn	US	2018	0	1	1	0	0	0	0	0	0	0	0	0	82
15057413	f	ot	DE	2018	0	0	0	0	0	0	0	0	0	0	0	0	75
15057523	m	ot	DE	2018	0	0	0	0	0	0	0	0	0	0	0	0	74
15057938	Not	md	US	2018	0	0	0	0	0	0	0	0	0	0	0	0	
15058031	f	md	US	2018	0	1	1	0	0	0	0	0	0	0	0	0	79
15058155	Not	ot	JP	2018	0	0	0	0	0	0	0	0	0	0	0	0	
15058227	m	md	DE	2018	0	1	1	0	0	0	0	0	0	0	0	0	65
15058313	m	md	JP	2018	1	0	0	0	0	0	0	0	0	0	0	0	73.3
15058348	m	cn	US	2018	1	0	0	0	0	0	0	0	0	0	0	0	70
15058436	m	ot	US	2018	0	1	0	0	1	0	0	0	0	0	0	0	75
15058515	m	ot	DE	2018	0	0	0	0	0	0	0	0	0	0	0	0	73
15058520	m	md	DE	2018	0	0	0	0	0	0	0	0	0	0	0	0	81
15058576	m	cn	JP	2018	0	1	1	0	0	0	0	0	0	0	0	0	
15058634	f	cn	US	2018	0	1	1	0	0	0	0	0	0	0	0	0	76

15059736	f	md	US	2018	0	0	0	0	0	0	0	0	0	0	0	0	65
15060184	m	md	IT	2018	0	0	0	0	0	0	0	0	0	0	0	0	
15060540	Not	ot	GB	2018	0	1	1	0	0	0	0	0	0	0	0	0	
15061099	Not	ot	US	2018	0	1	0	0	0	1	0	0	0	0	0	0	
15061315	f	cn	US	2018	0	1	0	0	0	1	0	0	0	0	0	0	53
15061773	Not	ot	JP	2018	0	0	0	0	0	0	0	0	0	0	0	0	
15061775	m	md	GT	2018	0	1	0	0	0	1	0	0	0	0	0	0	72
15061946	f	ot	BE	2018	0	1	0	0	1	0	0	0	0	0	0	0	70
15064596	m	md	IL	2018	0	1	0	0	1	0	0	0	0	0	0	0	69
15064731	m	ot	JP	2018	0	1	0	0	1	0	0	0	0	0	0	0	88
15064751	m	ot	DE	2018	0	1	0	0	1	0	0	0	0	0	0	0	63
15064868	f	md	US	2018	0	1	0	0	1	0	0	0	0	0	0	0	64
15064944	m	md	JP	2018	0	1	0	0	1	0	0	0	0	0	0	0	53
15065013	f	ot	JP	2018	0	1	0	0	1	0	0	0	0	0	0	0	90
15065043	m	md	AT	2018	0	0	0	0	0	0	0	0	0	0	0	0	
15065052	f	md	JP	2018	0	0	0	0	0	0	0	0	0	0	0	0	68
15066345	m	md	FR	2018	0	0	0	0	0	0	0	0	0	0	0	0	54
15066349	f	cn	US	2018	0	1	0	1	0	0	0	0	0	0	0	1	57.4
15066485	f	md	DK	2018	0	1	1	0	0	0	0	0	0	0	0	0	75
15066722	m	ot	DE	2018	0	0	0	0	0	0	0	0	0	0	0	0	58
15066953	m	md	DE	2018	0	0	0	0	0	0	0	0	0	0	0	0	47
15066991	f	md	BE	2018	0	1	1	0	0	0	0	0	0	0	0	0	58
15069322	m	md	US	2018	0	0	0	0	0	0	0	0	0	0	0	0	
15069646	m	md	US	2018	0	0	0	0	0	0	0	0	0	0	0	0	
15069764	m	md	US	2018	0	0	0	0	0	0	0	0	0	0	0	0	57
15069873	m	cn	US	2018	0	0	0	0	0	0	0	0	0	0	0	0	71
15072884	m	ot	US	2018	0	1	0	0	1	0	0	0	0	0	0	0	50
15072984	m	cn	JP	2018	0	1	1	0	0	0	0	0	0	0	0	0	70
15073164	f	md	US	2018	0	1	0	0	0	1	0	0	0	0	0	0	69
15073309	m	ot	JP	2018	1	0	0	0	0	0	0	0	0	0	0	0	45
15073408	f	md	US	2018	0	0	0	0	0	0	0	0	0	0	0	0	79
15073682	f	md	GB	2018	0	1	0	0	0	1	0	0	0	0	0	1	71
15073920	m	ot	DE	2018	0	0	0	0	0	0	0	0	0	0	0	0	66
15074228	Not	md	JP	2018	1	0	0	0	0	0	0	0	0	0	0	0	80

15074273	Not	cn	GR	2018	0	0	0	0	0	0	0	0	0	0	0	0	49
15074426	f	cn	GB	2018	1	0	0	0	0	0	0	0	0	0	0	0	
15075767	f	cn	US	2018	0	0	0	0	0	0	0	0	0	0	0	0	56
15075988	m	md	US	2018	0	1	0	0	0	1	0	0	0	0	0	0	67
15076855	f	ot	US	2018	0	1	0	0	1	0	0	0	0	0	0	0	57
15076939	f	md	JP	2018	1	0	0	0	0	0	0	0	0	0	0	0	80
15077381	m	md	ES	2018	0	1	0	1	0	0	0	0	0	0	0	0	70
15079638	f	cn	JP	2018	0	1	1	0	0	0	0	0	0	0	0	0	66
15080237	m	ot	KR	2018	0	1	0	0	1	0	0	0	0	0	0	0	64
15080528	f	md	TW	2018	1	0	0	0	0	0	0	0	0	0	0	0	67
15081477	f	ot	DE	2018	0	0	0	0	0	0	0	0	0	0	0	0	56
15081565	f	md	JP	2018	1	0	0	0	0	0	0	0	0	0	0	0	67
15083246	m	cn	JP	2018	0	1	0	0	1	0	0	0	0	0	0	1	76
15083445	f	cn	GB	2018	0	0	0	0	0	0	0	0	0	0	0	0	82
15083674	f	md	JP	2018	0	1	0	0	1	0	0	0	0	0	0	0	
15084415	f	ot	DE	2018	0	1	0	0	1	0	0	0	0	0	0	0	58
15084868	m	ot	DE	2018	0	1	0	0	1	0	0	0	0	0	0	0	67
15084875	m	ot	US	2018	0	1	0	0	1	0	0	0	0	0	0	0	69
15084919	Not	md	US	2018	0	1	0	1	0	0	0	0	0	0	0	1	
15085379	m	ot	US	2018	0	1	1	0	0	0	0	0	0	0	0	0	
15087483	m	md	IT	2018	0	1	0	0	1	0	0	0	0	0	0	0	
15087690	f	md	TW	2018	1	0	0	0	0	0	0	0	0	0	0	0	74
15088004	f	ot	DE	2018	0	0	0	0	0	0	0	0	0	0	0	0	57
15088351	m	md	JP	2018	0	1	0	0	1	0	0	0	0	0	0	0	76
15088503	f	cn	US	2018	1	0	0	0	0	0	0	0	0	0	0	0	59.8
15088526	f	ot	US	2018	0	1	0	0	1	0	0	0	0	0	0	0	
15088561	f	md	TW	2018	1	0	0	0	0	0	0	0	0	0	0	0	46
15089257	m	md	US	2018	0	1	0	0	0	1	0	0	0	0	0	0	
15089491	f	cn	US	2018	0	0	0	0	0	0	0	0	0	0	0	0	42
15089887	m	md	US	2018	0	1	0	1	0	0	0	0	0	0	0	0	
15090127	m	md	AR	2018	0	1	1	0	0	0	0	0	0	0	0	0	57
15090248	Not	md	JP	2018	1	0	0	0	0	0	0	0	0	0	0	0	
15090564	m	lw	US	2018	0	0	0	0	0	0	0	0	0	0	0	0	39
15091669	m	cn	JP	2018	0	1	1	0	0	0	0	0	0	0	0	0	64

15092864	m	ot	ES	2018	0	1	0	0	1	0	0	0	0	0	0	72
15092914	m	ot	DE	2018	0	1	0	0	1	0	0	0	0	0	0	69
15092943	m	ot	CN	2018	0	1	0	0	1	0	0	0	0	0	0	62
15093176	f	md	US	2018	0	1	0	1	0	0	0	0	0	0	0	36
15093460	m	cn	US	2018	1	0	0	0	0	0	0	0	0	0	0	53
15093876	f	md	FR	2018	0	1	0	0	1	0	0	0	0	0	0	36
15094118	f	md	PL	2018	0	1	0	0	0	1	0	0	0	0	0	49
7371877	m	md	JP	2018	0	0	0	0	0	0	0	0	0	0	0	62
12992933	m	md	JP	2018	0	1	0	0	0	1	0	0	0	0	0	71
13747151	m	ot	JP	2018	0	1	0	0	1	0	0	0	0	0	0	60
13845198	f	md	CL	2018	0	1	0	0	0	1	0	0	0	0	0	62
13891395	m	md	ES	2018	0	1	0	0	0	1	0	0	0	0	0	72
14187388	f	md	JP	2018	1	0	0	0	0	0	0	0	0	0	0	63.7
14274278	m	md	JP	2018	0	1	0	0	1	0	0	0	0	0	0	69
14327244	m	md	JP	2018	0	1	0	0	1	0	0	0	0	0	0	78
14327451	f	md	JP	2018	0	1	0	0	1	0	0	0	0	0	0	54
14334855	m	md	JP	2018	0	1	0	0	1	0	0	0	0	0	0	68
14373904	m	md	JP	2018	0	1	0	0	1	0	0	0	0	0	0	74
14374490	m	md	JP	2018	0	1	0	0	1	0	0	0	0	0	0	67
14379717	m	ot	FR	2018	0	1	0	0	1	0	0	0	0	0	1	58
14390337	f	md	JP	2018	0	1	0	0	0	1	0	0	0	0	0	64
14405386	f	cn	DE	2018	0	1	0	0	1	0	0	0	0	0	0	65
14456563	m	md	JP	2018	0	1	0	0	1	0	0	0	0	0	0	76
14508932	m	cn	DE	2018	0	1	0	0	1	0	0	0	0	0	0	67
14554710	f	ot	US	2018	0	1	0	0	1	0	0	0	0	0	0	90
14564247	m	ot	DE	2018	0	1	0	0	1	0	0	0	0	0	0	61
14592638	Not	ot	FR	2018	0	0	0	0	0	0	0	0	0	0	0	
14621171	f	ot	JP	2018	0	1	0	0	1	0	0	0	0	0	0	63
14625148	m	md	ES	2018	0	1	0	0	0	1	0	0	0	0	0	61
14668092	m	md	JP	2018	0	1	0	0	1	0	0	0	0	0	0	64
14671800	m	md	ES	2018	0	1	0	0	0	1	0	0	0	0	0	58
14678367	m	ot	JP	2018	0	1	0	0	1	0	0	0	0	0	0	70
14686514	m	md	Not re	2018	0	1	0	1	0	0	0	0	0	0	0	74
14711165	m	md	JP	2018	0	0	0	0	0	0	0	0	0	0	0	87

14713486	m	md	JP	2018	0	1	0	0	1	0	0	0	0	0	0	0	64
14713504	f	md	RU	2018	0	1	0	1	0	0	0	0	0	0	0	0	51.3
14716262	m	md	EG	2018	0	0	0	0	0	0	0	0	0	0	0	0	55
14719692	m	md	JP	2018	0	1	0	0	1	0	0	0	0	0	0	0	73
14730281	m	ot	US	2018	0	0	0	0	0	0	0	0	0	0	0	0	76
14732241	m	md	JP	2018	0	1	0	0	1	0	0	0	0	0	0	0	76
14741956	m	md	TR	2018	0	0	0	0	0	0	0	0	0	0	0	0	76
14744834	m	ot	DE	2018	0	1	0	0	1	0	0	0	0	0	0	0	77
14754556	m	md	JP	2018	1	0	0	0	0	0	0	0	0	0	0	0	51
14775243	f	ot	DE	2018	0	1	0	0	1	0	0	0	0	0	0	0	63
14778838	m	ot	TR	2018	0	1	1	0	0	0	0	0	0	0	0	0	57
14779049	f	ot	SE	2018	0	0	0	0	0	0	0	0	0	0	0	0	42
14779224	m	md	AT	2018	0	0	0	0	0	0	0	0	0	0	0	0	57
14781788	f	md	JP	2018	0	1	0	0	1	0	0	0	0	0	0	0	71
14790449	m	cn	JP	2018	0	1	1	0	0	0	0	0	0	0	0	0	81
14803581	f	cn	US	2018	1	0	0	0	0	0	0	0	0	0	0	0	
14812684	m	ot	DE	2018	0	1	0	0	1	0	0	0	0	0	0	0	71
14817696	m	ot	GB	2018	0	0	0	0	0	0	0	0	0	0	0	0	39
14822890	m	cn	JP	2018	0	1	1	0	0	0	0	0	0	0	0	0	75
14827703	f	ph	US	2018	0	1	1	0	0	0	0	0	0	0	0	0	57
14835359	m	md	CH	2018	0	1	0	0	1	0	0	0	0	0	0	0	58
14836457	m	ph	JP	2018	0	1	0	0	1	0	0	0	0	0	0	0	69
14838970	m	md	DE	2018	0	0	0	0	0	0	0	0	0	0	0	0	51
14848009	f	ot	DE	2018	0	1	0	0	1	0	0	0	0	0	0	0	67
14848562	m	ot	BG	2018	0	0	0	0	0	0	0	0	0	0	0	0	62
14861086	f	ot	DE	2018	0	1	0	0	1	0	0	0	0	0	0	0	70
14899362	m	md	JP	2018	0	1	0	0	1	0	0	0	0	0	0	0	60
14901057	m	md	FR	2018	0	1	0	0	1	0	0	0	0	0	0	0	64
14901583	m	md	DE	2018	0	0	0	0	0	0	0	0	0	0	0	0	63
14906513	f	md	JP	2018	0	0	0	0	0	0	0	0	0	0	0	0	
14907724	f	cn	US	2018	0	1	1	0	0	0	0	0	0	0	0	0	56
14907931	m	ot	US	2018	0	1	0	0	1	0	0	0	0	0	0	1	57
14907932	m	ot	JP	2018	0	1	0	0	1	0	0	0	0	0	0	0	73
14908049	m	ot	DE	2018	0	1	0	0	1	0	0	0	0	0	0	0	74

14911265	f	ot	FR	2018	0	1	0	0	1	0	0	0	0	0	0	0	65
14911824	m	cn	US	2018	1	0	0	0	0	0	0	0	0	0	0	0	80
14925589	f	ot	GB	2018	0	1	0	0	1	0	0	0	0	0	0	0	65
14926452	m	ot	DE	2018	0	1	0	0	1	0	0	0	0	0	0	0	62
14944739	f	md	JP	2018	0	1	0	0	1	0	0	0	0	0	0	0	57
14945431	f	md	MX	2018	0	1	0	0	0	1	0	0	0	0	0	0	81
14950400	f	md	JP	2018	0	1	0	0	1	0	0	0	0	0	0	0	46
14953142	m	ot	DE	2018	0	0	0	0	0	0	0	0	0	0	0	0	64
14957809	f	md	JP	2018	1	0	0	0	0	0	0	0	0	0	0	0	79
14961386	m	md	US	2018	0	1	0	0	0	1	0	0	0	0	0	0	62
14963144	m	md	FR	2018	0	1	0	0	0	1	0	0	0	0	0	0	67
14967704	m	md	JP	2018	0	1	0	0	0	1	0	0	0	0	0	0	64
14969902	m	md	JP	2018	0	1	0	0	1	0	0	0	0	0	0	0	58
14970808	m	ot	FR	2018	0	1	0	0	1	0	0	0	0	0	0	0	64
14978125	f	cn	US	2018	1	0	0	0	0	0	0	0	0	0	0	0	82
14982839	f	ot	NL	2018	0	0	0	0	0	0	0	0	0	0	0	0	
14984556	f	md	JP	2018	0	1	0	0	1	0	0	0	0	0	0	0	71
14986447	m	ot	FR	2018	0	1	0	0	1	0	0	0	0	0	0	0	52
14990779	m	md	TW	2018	0	1	0	1	0	0	0	0	0	0	0	0	69.5
14991571	m	md	PL	2018	0	0	0	0	0	0	0	0	0	0	0	0	62
14994216	f	md	JP	2018	0	1	0	0	1	0	0	0	0	0	0	0	42
14996584	f	ot	JP	2018	0	1	0	0	1	0	0	0	0	0	0	0	71
14997334	f	ph	US	2018	1	0	0	0	0	0	0	0	0	0	0	0	55
14999038	f	md	JP	2018	0	0	0	0	0	0	0	0	0	0	0	0	62
14999192	m	md	CN	2018	0	1	0	0	0	1	0	0	0	0	0	0	56
14999281	m	md	JP	2018	0	1	0	0	0	1	0	0	0	0	0	1	70
14999705	f	md	JP	2018	1	0	0	0	0	0	0	0	0	0	0	0	66
14999855	m	md	ES	2018	0	1	0	0	0	1	0	0	0	0	0	0	69
15002984	m	md	US	2018	0	1	0	0	1	0	0	0	0	0	0	0	
15003401	f	ph	US	2018	1	0	0	0	0	0	0	0	0	0	0	0	30
15004878	f	md	JP	2018	1	0	0	0	0	0	0	0	0	0	0	0	81.1
15005884	f	md	JP	2018	0	1	0	0	0	1	0	0	0	0	0	0	65
15009404	m	md	JP	2018	0	1	0	0	1	0	0	0	0	0	0	0	76
15011064	f	md	GB	2018	0	1	0	0	0	1	0	0	0	0	0	0	62

15014914	f	md	KR	2018	1	0	0	0	0	0	0	0	0	0	0	0	76
15016027	m	ot	DE	2018	0	0	0	0	0	0	0	0	0	0	0	0	65
15016649	f	ot	JP	2018	1	0	0	0	0	0	0	0	0	0	0	0	69
15018265	m	ot	GB	2018	0	1	1	0	0	0	0	0	0	0	0	0	58
15019610	f	md	KR	2018	1	0	0	0	0	0	0	0	0	0	0	0	84
15019683	m	ot	RO	2018	0	1	0	0	1	0	0	0	0	0	0	0	69
15019941	m	md	KR	2018	1	0	0	0	0	0	0	0	0	0	0	0	69
15020558	f	md	ES	2018	0	1	0	0	0	1	0	0	0	0	0	0	64
15027619	f	cn	US	2018	1	0	0	0	0	0	0	0	0	0	0	0	
15029146	m	md	ES	2018	0	1	0	0	0	1	0	0	0	0	0	0	69
15034029	m	ot	TW	2018	0	1	0	0	1	0	0	0	0	0	0	0	76
15035418	m	md	JP	2018	1	0	0	0	0	0	0	0	0	0	0	0	79
15037444	f	md	PH	2018	1	0	0	0	0	0	0	0	0	0	0	0	59
15037918	m	md	JP	2018	1	0	0	0	0	0	0	0	0	0	0	0	74.6
15040900	m	md	ES	2018	0	0	0	0	0	0	0	0	0	0	0	0	66
15043295	m	md	JP	2018	0	1	0	0	0	1	0	0	0	0	0	0	70
15045527	m	md	HU	2018	0	0	0	0	0	0	0	0	0	0	0	0	65
15047905	f	ot	GB	2018	0	1	0	0	1	0	0	0	0	0	0	1	83
15048243	m	md	ES	2018	0	0	0	0	0	0	0	0	0	0	0	0	65
15049869	m	md	DE	2018	0	1	0	1	0	0	0	0	0	0	0	0	60
15056545	f	md	DK	2018	0	0	0	0	0	0	0	0	0	0	0	0	34
15057434	m	cn	JP	2018	0	1	1	0	0	0	0	0	0	0	0	0	71
15070153	f	md	JP	2018	0	0	0	0	0	0	0	0	0	0	0	0	73
15072607	m	cn	JP	2018	0	1	1	0	0	0	0	0	0	0	0	0	51
15074130	m	md	JP	2018	0	1	0	1	0	0	0	0	0	0	0	0	74
15075582	f	md	JP	2018	0	0	0	0	0	0	0	0	0	0	0	0	70
15077055	m	ot	DE	2018	0	1	0	0	1	0	0	0	0	0	0	0	79
15077398	f	ot	US	2018	0	0	0	0	0	0	0	0	0	0	0	0	66
15080340	m	ot	FR	2018	0	1	0	1	0	0	0	0	0	0	0	0	39
15080996	m	md	GB	2018	0	1	0	0	0	1	0	0	0	0	0	0	60
15082796	m	md	JP	2018	0	1	0	0	0	1	0	0	0	0	0	0	60
15088504	m	md	FR	2018	0	1	0	1	0	0	0	0	0	0	0	0	69.3
15089468	Not	ph	JP	2018	1	0	0	0	0	0	0	0	0	0	0	0	
15089751	f	md	JP	2018	0	1	0	0	0	1	0	0	0	0	0	0	84

15089819	f	md	CN	2018	0	1	0	0	0	1	0	0	0	0	0	0	66
15089946	m	md	AT	2018	0	0	0	0	0	0	0	0	0	0	0	0	30
15092220	f	md	CN	2018	1	0	0	0	0	0	0	0	0	0	0	0	69.2
15093621	f	md	JP	2018	0	0	0	0	0	0	0	0	0	0	0	0	43
15095193	Not	md	JP	2018	0	0	0	0	0	0	0	0	0	0	0	0	
15095225	f	md	JP	2018	0	1	0	0	1	0	0	0	0	0	0	1	62
15095325	m	ph	JP	2018	0	1	0	0	0	1	0	0	0	0	0	0	70
15095658	m	ot	DE	2018	0	0	0	0	0	0	0	0	0	0	0	0	72
15095659	m	md	JP	2018	0	1	0	0	0	1	0	0	0	0	0	0	64
15095887	m	ot	FR	2018	0	1	0	1	0	0	0	0	0	0	0	0	39
15095959	m	md	DE	2018	0	1	0	0	1	0	0	0	0	0	0	1	72
15096289	m	ph	US	2018	0	0	0	0	0	0	0	0	0	0	0	0	66
15096477	m	ot	FR	2018	0	1	0	0	1	0	0	0	0	0	0	0	63
15096591	f	md	JP	2018	0	0	0	0	0	0	0	0	0	0	0	0	79
15096626	m	md	US	2018	0	1	0	1	0	0	0	0	0	0	0	0	67
15096693	f	md	CH	2018	0	1	0	0	0	1	0	0	0	0	0	0	
15096828	f	Not repo	US	2018	1	0	0	0	0	0	0	0	0	0	0	0	54.6
15096890	Not	md	DE	2018	0	0	0	0	0	0	0	0	0	0	0	0	65
15097448	m	ot	US	2018	0	1	1	0	0	0	0	0	0	0	0	0	
15097554	m	ot	US	2018	0	1	1	0	0	0	0	0	0	0	0	0	81
15097609	m	ot	DE	2018	0	1	0	0	1	0	0	0	0	0	0	0	53
15098212	f	ot	JP	2018	0	1	0	0	1	0	0	0	0	0	0	0	60
15098265	m	md	JP	2018	0	1	0	0	1	0	0	0	0	0	0	0	53
15099128	Not	ot	GB	2018	0	0	0	0	0	0	0	0	0	0	0	0	
15099370	m	cn	KR	2018	1	0	0	0	0	0	0	0	0	0	0	0	42
15100229	f	md	DE	2018	0	1	1	0	0	0	0	0	0	0	0	1	64
15100402	f	md	AT	2018	1	0	0	0	0	0	0	0	0	0	0	0	52
15100616	f	md	HR	2018	1	0	0	0	0	0	0	0	0	0	0	0	77
15100630	m	cn	JP	2018	0	1	1	0	0	0	0	0	0	0	0	0	81
15100659	m	ot	DE	2018	0	1	0	0	1	0	0	0	0	0	0	1	59
15100742	f	md	JP	2018	1	0	0	0	0	0	0	0	0	0	0	0	74
15100871	f	md	DE	2018	1	0	0	0	0	0	0	0	0	0	0	0	
15100894	f	cn	US	2018	1	0	0	0	0	0	0	0	0	0	0	0	86
15101584	m	ph	FR	2018	0	1	1	0	0	0	0	0	0	0	0	0	87

15101796	m	md	JP	2018	0	0	0	0	0	0	0	0	0	0	0	0	66
15102997	f	md	DE	2018	1	0	0	0	0	0	0	0	0	0	0	0	
15103619	m	md	PL	2018	0	1	0	0	0	1	0	0	0	0	0	0	81
15104270	m	ot	DE	2018	0	0	0	0	0	0	0	0	0	0	0	0	66
15104750	f	cn	US	2018	1	0	0	0	0	0	0	0	0	0	0	0	71
15105043	m	ot	JP	2018	0	0	0	0	0	0	0	0	0	0	0	0	77
15105398	f	ot	CA	2018	0	1	0	0	1	0	0	0	0	0	0	0	76
15105440	m	md	AU	2018	0	1	0	0	1	0	0	0	0	0	0	0	58
15105508	f	ot	US	2018	0	0	0	0	0	0	0	0	0	0	0	0	48
15105512	f	md	JP	2018	0	1	0	0	1	0	0	0	0	0	0	0	72
15105567	m	md	DE	2018	0	0	0	0	0	0	0	0	0	0	0	0	65
15105666	m	md	JP	2018	0	1	0	0	1	0	0	0	0	0	0	0	79
15105673	f	md	JP	2018	0	0	0	0	0	0	0	0	0	0	0	0	61
15105765	m	md	JP	2018	0	1	0	0	1	0	0	0	0	0	0	0	66
15105894	f	md	JP	2018	0	1	0	0	1	0	0	0	0	0	0	0	77
15106048	m	md	CN	2018	1	0	0	0	0	0	0	0	0	0	0	0	66.2
15106259	m	md	KR	2018	1	0	0	0	0	0	0	0	0	0	0	0	61
15106413	m	ot	IT	2018	0	0	0	0	0	0	0	0	0	0	0	0	65
15106914	m	ot	US	2018	0	1	0	0	1	0	0	0	0	0	0	1	83
15107068	f	md	JP	2018	1	0	0	0	0	0	0	0	0	0	0	0	73
15107568	m	ot	FR	2018	0	1	0	0	1	0	0	0	0	0	0	0	76
15107837	m	md	IT	2018	0	0	0	0	0	0	0	0	0	0	0	0	
15107934	m	cn	FR	2018	0	1	1	0	0	0	0	0	0	0	0	0	70
15107973	f	cn	GB	2018	0	1	1	0	0	0	0	0	0	0	0	0	61
15107976	m	ot	JP	2018	0	1	0	0	1	0	0	0	0	0	0	0	65
15108136	m	md	DE	2018	0	0	0	0	0	0	0	0	0	0	0	0	65
15108238	m	md	NL	2018	0	1	0	0	1	0	0	0	0	0	0	0	66
15108241	f	ot	FR	2018	1	0	0	0	0	0	0	0	0	0	0	0	68
15108854	f	ot	DE	2018	0	1	0	0	1	0	0	0	0	0	0	0	53
15109221	m	md	JP	2018	1	0	0	0	0	0	0	0	0	0	0	0	74
15109548	f	md	GB	2018	0	1	0	0	0	1	0	0	0	0	0	0	49
15109645	m	md	JP	2018	0	1	0	0	0	1	0	0	0	0	0	0	68
15109939	m	md	US	2018	1	0	0	0	0	0	0	0	0	0	0	0	
15110110	Not	md	JP	2018	1	0	0	0	0	0	0	0	0	0	0	0	43

15110253	m	ot	DE	2018	0	0	0	0	0	0	0	0	0	0	0	0	70
15110326	f	md	ZA	2018	0	0	0	0	0	0	0	0	0	0	0	0	
15110750	m	md	JP	2018	0	1	0	0	0	1	0	0	0	0	0	0	57
15111669	f	md	AT	2018	0	0	0	0	0	0	0	0	0	0	0	0	59
15112211	Not	ot	US	2018	0	1	0	0	1	0	0	0	0	0	0	0	
15112223	f	cn	CN	2018	1	0	0	0	0	0	0	0	0	0	0	0	
15112551	m	ot	JP	2018	0	1	0	0	1	0	0	0	0	0	0	0	73
15112892	m	md	JP	2018	0	1	0	0	1	0	0	0	0	0	0	1	60
15113229	f	ot	US	2018	0	1	0	0	1	0	0	0	0	0	0	0	65
15113327	m	md	CN	2018	0	1	0	0	0	1	0	0	0	0	0	0	77
15113558	f	ot	US	2018	0	1	0	0	1	0	0	0	0	0	0	0	58
15113568	m	ot	DE	2018	0	0	0	0	0	0	0	0	0	0	0	0	
15113667	Not	md	CN	2018	0	0	0	0	0	0	0	0	0	0	0	0	
15113676	f	ot	AU	2018	0	1	0	0	1	0	0	0	0	0	0	1	71
15113678	m	ot	US	2018	0	1	0	0	1	0	0	0	0	0	0	0	71
15113848	Not	md	JP	2018	0	1	0	0	1	0	0	0	0	0	0	0	
15113930	f	md	DE	2018	0	0	0	0	0	0	0	0	0	0	0	0	
15113933	m	ot	DE	2018	0	1	0	0	1	0	0	0	0	0	0	0	70
15113946	f	Not repo	US	2018	1	0	0	0	0	0	0	0	0	0	0	0	63.9
15114412	f	md	JP	2018	0	0	0	0	0	0	0	0	0	0	0	0	70
15114581	m	ot	JP	2018	0	1	1	0	0	0	0	0	0	0	0	1	79
15114597	m	md	DE	2018	0	0	0	0	0	0	0	0	0	0	0	0	64
15114600	f	cn	GB	2018	1	0	0	0	0	0	0	0	0	0	0	0	
15114843	m	ot	DE	2018	0	1	0	0	1	0	0	0	0	0	0	0	59
15115023	m	ot	IE	2018	0	1	0	0	1	0	0	0	0	0	0	0	65
15115035	m	cn	JP	2018	0	1	1	0	0	0	0	0	0	0	0	0	78
15115108	f	md	FR	2018	0	0	0	0	0	0	0	0	0	0	0	0	76
15115245	f	ph	JP	2018	0	0	0	0	0	0	0	0	0	0	0	0	75
15115327	m	ot	US	2018	0	1	0	0	1	0	0	0	0	0	0	0	74
15115353	m	md	JP	2018	1	0	0	0	0	0	0	0	0	0	0	0	70
15115530	m	md	JP	2018	0	0	0	0	0	0	0	0	0	0	0	0	51
15116300	m	cn	DE	2018	0	0	0	0	0	0	0	0	0	0	0	0	
15116386	f	cn	IE	2018	0	1	1	0	0	0	0	0	0	0	0	0	57
15116468	f	ot	US	2018	0	0	0	0	0	0	0	0	0	0	0	0	12

15116617	m	md	NL	2018	0	1	0	1	0	0	0	0	0	0	0	0	76.7
15117049	m	md	DE	2018	0	1	0	1	0	0	0	0	0	0	0	0	76.8
15117308	f	ot	PL	2018	0	1	0	0	1	0	0	0	0	0	0	0	75
15117463	m	cn	JP	2018	0	1	1	0	0	0	0	0	0	0	0	1	86
15117522	m	md	TW	2018	0	0	0	0	0	0	0	0	0	0	0	0	
15117570	f	cn	BG	2018	0	1	1	0	0	0	0	0	0	0	0	0	76
15118490	f	md	JP	2018	0	1	0	0	1	0	0	0	0	0	0	1	70
15118743	f	md	BE	2018	1	0	0	0	0	0	0	0	0	0	0	0	84
15118798	f	md	JP	2018	0	1	0	0	0	1	0	0	0	0	0	0	83
15119086	m	cn	SE	2018	0	1	1	0	0	0	0	0	0	0	0	0	
15119111	m	md	JP	2018	0	1	0	0	1	0	0	0	0	0	0	0	63
15119118	m	cn	US	2018	0	1	1	0	0	0	0	0	0	0	0	0	
15119348	Not	md	JP	2018	0	1	0	0	1	0	0	0	0	0	0	0	80
15120470	m	ot	FR	2018	0	1	0	0	1	0	0	0	0	0	0	0	56
15120613	m	ot	GB	2018	0	0	0	0	0	0	0	0	0	0	0	0	74
15120662	f	md	DE	2018	0	1	1	0	0	0	0	0	0	0	0	0	55
15121203	m	cn	DE	2018	0	1	1	0	0	0	0	0	0	0	0	0	
15121224	m	ot	JP	2018	0	1	0	0	1	0	0	0	0	0	0	0	76
15121457	f	Not repo	CA	2018	1	0	0	0	0	0	0	0	0	0	0	0	60.6
15121492	m	ot	DE	2018	0	0	0	0	0	0	0	0	0	0	0	0	66
15121918	m	ot	JP	2018	0	1	0	0	1	0	0	0	0	0	0	0	75
15122059	Not	cn	FR	2018	0	0	0	0	0	0	0	0	0	0	0	0	
15122101	m	ot	JP	2018	0	1	0	0	1	0	0	0	0	0	0	0	71
15122762	f	cn	GR	2018	0	0	0	0	0	0	0	0	0	0	0	0	49
15123408	f	md	BR	2018	0	1	0	0	1	0	0	0	0	0	0	0	66
15123423	m	ot	FR	2018	0	1	0	0	1	0	0	0	0	0	0	0	
15123424	m	ph	JP	2018	0	1	0	0	1	0	0	0	0	0	0	0	67
15123513	f	cn	US	2018	1	0	0	0	0	0	0	0	0	0	0	0	71
15123789	m	ot	JP	2018	0	1	0	0	1	0	0	0	0	0	0	0	41
15124020	m	md	ES	2018	0	1	0	0	0	1	0	0	0	0	0	0	47
15124052	f	cn	US	2018	0	1	1	0	0	0	0	0	0	0	0	0	81
15124111	m	ot	JP	2018	0	1	1	0	0	0	0	0	0	0	0	0	81
15124218	f	md	IT	2018	0	1	0	0	1	0	0	0	0	0	0	0	48
15124567	f	cn	US	2018	0	1	1	0	0	0	0	0	0	0	0	0	71

15124742	m	md	JP	2018	0	1	0	0	0	1	0	0	0	0	0	0	45
15125015	m	cn	JP	2018	0	1	1	0	0	0	0	0	0	0	0	1	70
15125030	f	cn	JP	2018	0	1	1	0	0	0	0	0	0	0	0	0	60
15125236	m	ot	JP	2018	0	1	0	0	1	0	0	0	0	0	0	0	60
15125333	f	md	DE	2018	0	0	0	0	0	0	0	0	0	0	0	0	71
15125406	m	md	US	2018	0	1	0	0	0	1	0	0	0	0	0	0	70
15125575	m	cn	JP	2018	0	1	1	0	0	0	0	0	0	0	0	1	71
15125675	m	cn	JP	2018	0	0	0	0	0	0	0	0	0	0	0	0	77
15125796	f	md	FR	2018	0	0	0	0	0	0	0	0	0	0	0	0	40
15126037	m	md	Not re	2018	0	0	0	0	0	0	0	0	0	0	0	0	52
15126694	f	ot	DE	2018	0	0	0	0	0	0	0	0	0	0	0	0	48
15127141	f	md	KR	2018	1	0	0	0	0	0	0	0	0	0	0	0	75.5
15127242	m	ot	DE	2018	0	0	0	0	0	0	0	0	0	0	0	0	72
15128339	f	md	JP	2018	0	1	0	0	0	1	0	0	0	0	0	0	50
15128393	m	md	JP	2018	0	1	0	0	1	0	0	0	0	0	0	1	64
15128437	f	cn	DE	2018	0	1	1	0	0	0	0	0	0	0	0	0	
15128991	f	cn	FR	2018	0	1	1	0	0	0	0	0	0	0	0	0	67
15129486	m	md	GB	2018	0	1	0	0	0	1	0	0	0	0	0	0	51
15129874	m	cn	JP	2018	0	1	1	0	0	0	0	0	0	0	0	0	66
15130689	f	ot	AU	2018	0	1	0	0	1	0	0	0	0	0	0	0	73
15130727	m	ot	KR	2018	0	1	0	0	1	0	0	0	0	0	0	0	66
15131005	m	ph	US	2018	0	0	0	0	0	0	0	0	0	0	0	0	84
15131285	f	cn	PL	2018	0	0	0	0	0	0	0	0	0	0	0	0	56
15131322	m	ph	JP	2018	0	1	0	0	1	0	0	0	0	0	0	0	69
15131624	m	ot	US	2018	0	0	0	0	0	0	0	0	0	0	0	0	
15131632	m	md	DE	2018	0	0	0	0	0	0	0	0	0	0	0	0	78
15132180	f	md	DE	2018	1	0	0	0	0	0	0	0	0	0	0	0	72
15132489	m	md	IN	2018	0	1	0	0	1	0	0	0	0	0	0	0	72
15132589	m	md	GB	2018	0	1	0	0	0	1	0	0	0	0	0	0	84
15132762	m	md	JP	2018	0	1	0	0	0	1	0	0	0	0	0	0	46
15132827	f	ot	DE	2018	0	0	0	0	0	0	0	0	0	0	0	0	57
15132833	f	md	DE	2018	0	0	0	0	0	0	0	0	0	0	0	0	48
15132952	m	md	JP	2018	0	1	0	0	1	0	0	0	0	0	0	0	
15133002	m	cn	IN	2018	0	1	0	0	1	0	0	0	0	0	0	0	62

15133089	f	md	KR	2018	0	1	0	0	1	0	0	0	0	0	0	0	68
15133202	m	cn	JP	2018	0	1	1	0	0	0	0	0	0	0	0	1	74
15133319	m	cn	JP	2018	0	0	0	0	0	0	0	0	0	0	0	0	61
15133347	m	md	US	2018	0	1	0	1	0	0	0	0	0	0	0	0	73
15133485	f	cn	US	2018	0	0	0	0	0	0	0	0	0	0	0	0	
15134006	f	md	JP	2018	0	0	0	0	0	0	0	0	0	0	0	0	68
15134254	Not	md	JP	2018	0	0	0	0	0	0	0	0	0	0	0	0	
15134917	m	md	JP	2018	0	1	0	0	0	1	0	0	0	0	0	0	45
15134953	f	md	FR	2018	0	1	0	0	0	1	0	0	0	0	0	0	50
15134960	Not	md	JP	2018	0	1	0	0	0	1	0	0	0	0	0	0	
15134973	f	cn	JP	2018	0	1	1	0	0	0	0	0	0	0	0	0	75
15135483	m	md	CA	2018	1	0	0	0	0	0	0	0	0	0	0	0	66.3
15136091	f	md	US	2018	0	1	1	0	0	0	0	0	0	0	0	0	60
15136139	f	md	TH	2018	0	0	0	0	0	0	0	0	0	0	0	0	49
15136205	m	ph	US	2018	0	1	1	0	0	0	0	0	0	0	0	0	71
15136222	m	md	CN	2018	0	1	0	1	0	0	0	0	0	0	0	0	71.1
15136349	f	md	NL	2018	0	1	0	1	0	0	0	0	0	0	0	0	61
15137926	f	ot	FR	2018	0	1	0	0	1	0	0	0	0	0	0	0	68
15137948	m	ot	CN	2018	0	1	0	0	1	0	0	0	0	0	0	0	65
15138126	f	md	NL	2018	0	0	0	0	0	0	0	0	0	0	0	0	69
15138898	m	ot	US	2018	0	1	0	1	0	0	0	0	0	0	0	0	
15138935	f	md	DE	2018	0	0	0	0	0	0	0	0	0	0	0	0	58
15139110	f	md	DE	2018	0	0	0	0	0	0	0	0	0	0	0	0	
15139234	f	cn	US	2018	0	0	0	0	0	0	0	0	0	0	0	0	75
15139615	m	ot	CA	2018	0	1	0	0	1	0	0	0	0	0	0	0	
15140205	m	cn	JP	2018	0	0	0	0	0	0	0	0	0	0	0	0	73
15140302	m	cn	FR	2018	0	1	1	0	0	0	0	0	0	0	0	0	
15140337	f	ot	DE	2018	0	0	0	0	0	0	0	0	0	0	0	0	48
15140434	m	ot	DE	2018	0	0	0	0	0	0	0	0	0	0	0	0	66
15140930	f	md	US	2018	1	0	0	0	0	0	0	0	0	0	0	0	82
15141482	f	ot	US	2018	0	1	0	0	1	0	0	0	0	0	0	0	80
15143030	f	ot	US	2018	0	1	0	0	1	0	0	0	0	0	0	0	83
15143184	m	ot	BE	2018	0	0	0	0	0	0	0	0	0	0	0	0	42
15143408	m	md	US	2018	1	0	0	0	0	0	0	0	0	0	0	0	

15143862	m	ot	US	2018	0	1	0	0	1	0	0	0	0	0	0	0	64
15144096	m	md	JP	2018	0	1	0	1	0	0	0	0	0	0	0	0	55.4
15144491	m	md	DE	2018	0	0	0	0	0	0	0	0	0	0	0	0	56
15144496	m	md	AU	2018	0	0	0	0	0	0	0	0	0	0	0	0	
15144512	m	ot	US	2018	0	1	0	0	1	0	0	0	0	0	0	0	80
15144653	f	cn	DE	2018	0	1	1	0	0	0	0	0	0	0	0	0	62
15144852	m	ot	GB	2018	1	0	0	0	0	0	0	0	0	0	0	0	57
15144856	f	ot	GB	2018	1	0	0	0	0	0	0	0	0	0	0	0	64
15145451	m	cn	JP	2018	0	1	1	0	0	0	0	0	0	0	0	0	59
15146977	f	md	CZ	2018	0	0	0	0	0	0	0	0	0	0	0	0	47
15147118	m	md	JP	2018	0	1	0	0	1	0	0	0	0	0	0	0	75
15147240	m	ot	DE	2018	0	0	0	0	0	0	0	0	0	0	0	0	65
15147366	m	ot	JP	2018	0	1	0	0	1	0	0	0	0	0	0	0	64
15147443	m	md	JP	2018	0	1	0	0	1	0	0	0	0	0	0	1	63
15147538	m	cn	JP	2018	0	1	0	0	1	0	0	0	0	0	0	0	66
15147632	f	cn	US	2018	0	0	0	0	0	0	0	0	0	0	0	0	
15147836	f	md	JP	2018	0	1	0	0	0	1	0	0	0	0	0	1	64
15148012	f	md	PT	2018	0	1	0	0	0	1	0	0	0	0	0	0	70
15148228	f	md	BE	2018	0	1	0	0	0	1	0	0	0	0	0	0	72
15148258	m	md	DE	2018	0	1	1	0	0	0	0	0	0	0	0	0	57
15148558	Not	Not repo	US	2018	0	1	0	1	0	0	0	0	0	0	0	0	
15148704	f	ot	US	2018	0	0	0	0	0	0	0	0	0	0	0	0	66
15149159	f	cn	US	2018	1	0	0	0	0	0	0	0	0	0	0	0	69
15150082	f	md	JP	2018	1	0	0	0	0	0	0	0	0	0	0	0	69
15150329	m	md	JP	2018	1	0	0	0	0	0	0	0	0	0	0	1	77
15150687	m	md	AT	2018	0	1	0	0	1	0	0	0	0	0	0	1	79
15151320	m	md	IL	2018	0	0	0	0	0	0	0	0	0	0	0	0	55
15151338	m	ot	US	2018	0	1	0	0	1	0	0	0	0	0	0	0	
15151730	f	ot	AU	2018	0	1	0	0	1	0	0	0	0	0	0	0	30
15151754	f	cn	US	2018	0	1	1	0	0	0	0	0	0	0	0	0	
15151930	m	cn	US	2018	0	1	0	0	1	0	0	0	0	0	0	0	62
15152069	f	cn	US	2018	1	0	0	0	0	0	0	0	0	0	0	0	84
15152492	m	ot	US	2018	0	1	0	0	1	0	0	0	0	0	0	0	84
15152528	f	cn	US	2018	1	0	0	0	0	0	0	0	0	0	0	0	

15152663	m	md	DE	2018	0	0	0	0	0	0	0	0	0	0	0	0	74
15152695	m	md	US	2018	0	1	0	1	0	0	0	0	0	0	0	1	
15152852	m	ot	IT	2018	0	1	0	0	1	0	0	0	0	0	0	0	79
15153532	f	ot	CH	2018	0	1	0	0	1	0	0	0	0	0	0	0	77
15153582	m	ot	FR	2018	0	1	0	1	0	0	0	0	0	0	0	0	39
15153849	m	md	CH	2018	0	1	0	0	1	0	0	0	0	0	0	0	57
15153963	m	md	JP	2018	1	0	0	0	0	0	0	0	0	0	0	0	
15154247	m	cn	DE	2018	0	1	0	0	1	0	0	0	0	0	0	0	61
15154464	m	ot	GB	2018	1	0	0	0	0	0	0	0	0	0	0	0	66
15154487	Not	cn	JP	2018	0	0	0	0	0	0	0	0	0	0	0	0	
15154616	m	md	VN	2018	1	0	0	0	0	0	0	0	0	0	0	1	35
15154617	m	ph	FR	2018	1	0	0	0	0	0	0	0	0	0	0	0	73
15154646	m	cn	JP	2018	0	1	1	0	0	0	0	0	0	0	0	0	69
15154678	f	ot	US	2018	0	1	1	0	0	0	0	0	0	0	0	0	60
15154680	m	md	FR	2018	0	0	0	0	0	0	0	0	0	0	0	0	63
15154948	m	md	JP	2018	0	1	1	0	0	0	0	0	0	0	0	0	40
15155649	m	ot	JP	2018	0	0	0	0	0	0	0	0	0	0	0	0	69
15156202	f	ot	US	2018	1	0	0	0	0	0	0	0	0	0	0	1	58
15156304	f	ot	AT	2018	0	1	0	0	0	1	0	0	0	0	0	0	
15157132	f	md	US	2018	0	1	0	0	0	1	0	0	0	0	0	0	60
15157209	m	ot	JP	2018	0	1	0	0	1	0	0	0	0	0	0	0	75
15157307	f	ph	FR	2018	0	1	1	0	0	0	0	0	0	0	0	0	66
15157381	m	md	JP	2018	0	1	0	0	1	0	0	0	0	0	0	0	62
15157430	f	cn	RS	2018	0	1	1	0	0	0	0	0	0	0	0	0	40
15157528	f	ot	US	2018	1	0	0	0	0	0	0	0	0	0	0	0	72
15157553	m	md	EG	2018	0	0	0	0	0	0	0	0	0	0	0	0	64
15157577	m	md	JP	2018	0	1	0	0	1	0	0	0	0	0	0	0	71
15157908	m	md	FR	2018	0	1	0	0	1	0	0	0	0	0	0	0	66
15158394	f	cn	US	2018	0	1	0	0	1	0	0	0	0	0	0	0	60
15158448	m	ot	CZ	2018	0	1	0	0	1	0	0	0	0	0	0	0	70
15158780	m	ph	US	2018	0	1	0	0	1	0	0	0	0	0	0	0	68
15158950	m	md	JP	2018	0	1	0	0	0	1	0	0	0	0	0	1	74
15158967	m	ot	DE	2018	0	0	0	0	0	0	0	0	0	0	0	0	84
15159113	Not	ph	JP	2018	1	0	0	0	0	0	0	0	0	0	0	0	

15159749	m	md	DE	2018	0	0	0	0	0	0	0	0	0	0	0	0	64
15159926	f	ot	PL	2018	0	1	0	0	1	0	0	0	0	0	0	0	57
15159933	m	cn	JP	2018	0	1	1	0	0	0	0	0	0	0	0	0	80
15160012	f	md	JP	2018	1	0	0	0	0	0	0	0	0	0	0	0	76
15160148	f	cn	JP	2018	0	0	0	0	0	0	0	0	0	0	0	0	74
15160201	m	cn	US	2018	0	0	0	0	0	0	0	0	0	0	0	0	75
15160596	m	md	PT	2018	1	0	0	0	0	0	0	0	0	0	0	0	
15160668	m	ot	BE	2018	0	0	0	0	0	0	0	0	0	0	0	0	73
15163373	m	cn	JP	2018	0	0	0	0	0	0	0	0	0	0	0	0	76
15163382	m	md	JP	2018	0	0	0	0	0	0	0	0	0	0	0	0	80
15163508	Not	md	ES	2018	0	0	0	0	0	0	0	0	0	0	0	0	
15163551	m	ot	JP	2018	0	1	0	0	1	0	0	0	0	0	0	0	76
15163563	Not	md	CN	2018	0	0	0	0	0	0	0	0	0	0	0	0	
15163735	m	cn	CN	2018	0	0	0	0	0	0	0	0	0	0	0	0	
15164004	m	md	JP	2018	1	0	0	0	0	0	0	0	0	0	0	0	80
15164049	f	md	IT	2018	0	0	0	0	0	0	0	0	0	0	0	0	62
15164546	m	ot	GR	2018	0	1	0	0	1	0	0	0	0	0	0	0	67
15164852	m	ot	US	2018	0	1	0	0	1	0	0	0	0	0	0	0	
15165003	f	ot	JP	2018	0	0	0	0	0	0	0	0	0	0	0	0	78
15165777	m	ph	JP	2018	1	0	0	0	0	0	0	0	0	0	0	0	49
15165836	m	md	DK	2018	0	1	1	0	0	0	0	0	0	0	0	1	63
15167239	m	ot	US	2018	0	1	0	0	1	0	0	0	0	0	0	0	68
15168232	m	ot	CN	2018	0	1	0	0	1	0	0	0	0	0	0	0	68
15168611	f	md	JP	2018	0	1	0	0	1	0	0	0	0	0	0	0	67
15168627	Not	ot	CN	2018	0	0	0	0	0	0	0	0	0	0	0	0	
15168642	m	cn	JP	2018	0	1	1	0	0	0	0	0	0	0	0	0	70
15168707	m	md	US	2018	0	1	0	0	1	0	0	0	0	0	0	0	
15169234	m	ot	DE	2018	0	0	0	0	0	0	0	0	0	0	0	0	60
15169340	f	md	BR	2018	0	1	0	0	0	1	0	0	0	0	0	0	
15169387	m	md	DE	2018	0	0	0	0	0	0	0	0	0	0	0	0	49
15169395	m	md	DE	2018	0	0	0	0	0	0	0	0	0	0	0	0	49
15169478	Not	md	ES	2018	1	0	0	0	0	0	0	0	0	0	0	0	
15169718	m	md	BR	2018	0	1	0	0	0	1	0	0	0	0	0	0	77
15170204	f	ot	DE	2018	0	1	0	0	1	0	0	0	0	0	0	0	49

15170490	m	cn	JP	2018	0	0	0	0	0	0	0	0	0	0	0	0	65
15170715	f	md	DE	2018	0	1	0	1	0	0	0	0	0	0	0	0	54
15170864	m	md	JP	2018	0	1	0	0	1	0	0	0	0	0	0	1	71
15171018	f	Not repo	US	2018	1	0	0	0	0	0	0	0	0	0	0	0	67.8
15171317	m	ot	JP	2018	0	0	0	0	0	0	0	0	0	0	0	0	76
15171495	m	cn	JP	2018	0	1	1	0	0	0	0	0	0	0	0	0	76
15172502	f	cn	US	2018	0	1	0	0	1	0	0	0	0	0	0	0	
15172638	f	md	US	2018	0	1	0	1	0	0	0	0	0	0	0	0	60
15172760	Not	ot	US	2018	0	0	0	0	0	0	0	0	0	0	0	0	
15173040	f	md	JP	2018	0	1	0	0	1	0	0	0	0	0	0	0	81
15173250	m	ot	DE	2018	0	0	0	0	0	0	0	0	0	0	0	0	65
15173282	m	ot	JP	2018	0	1	0	0	1	0	0	0	0	0	0	0	62
15173369	f	ot	ES	2018	0	1	0	0	1	0	0	0	0	0	0	0	61
15173432	f	md	FR	2018	1	0	0	0	0	0	0	0	0	0	0	0	69.1
15177890	m	cn	JP	2018	0	1	1	0	0	0	0	0	0	0	0	0	66
15179146	Not	ot	US	2018	0	0	0	0	0	0	0	0	0	0	0	0	
15179651	f	md	DE	2018	1	0	0	0	0	0	0	0	0	0	0	0	48
15179667	f	ot	PE	2018	0	1	0	0	1	0	0	0	0	0	0	0	49
15179701	f	cn	US	2018	0	1	1	0	0	0	0	0	0	0	0	0	74
15179821	m	ot	US	2018	0	1	1	0	0	0	0	0	0	0	0	0	82
15180633	m	md	JP	2018	0	0	0	0	0	0	0	0	0	0	0	0	51
15180789	Not	md	JP	2018	0	0	0	0	0	0	0	0	0	0	0	0	
15181094	f	cn	CA	2018	0	0	0	0	0	0	0	0	0	0	0	0	71
15181166	f	cn	US	2018	0	0	0	0	0	0	0	0	0	0	0	0	52
15182672	f	cn	JP	2018	0	1	1	0	0	0	0	0	0	0	0	0	80
15182758	f	cn	GB	2018	1	0	0	0	0	0	0	0	0	0	0	0	79
15182803	m	ot	US	2018	0	0	0	0	0	0	0	0	0	0	0	0	
15182836	m	ph	BR	2018	0	1	0	0	0	1	0	0	0	0	0	0	62
15183613	Not	md	US	2018	0	1	0	1	0	0	0	0	0	0	0	0	
15183866	m	ot	CN	2018	0	1	0	0	1	0	0	0	0	0	0	0	75
15185624	f	cn	US	2018	1	0	0	0	0	0	0	0	0	0	0	0	66
15186923	Not	md	US	2018	0	1	1	0	0	0	0	0	0	0	0	0	
15187088	f	cn	US	2018	0	1	0	0	1	0	0	0	0	0	0	0	
15187861	m	md	HU	2018	0	0	0	0	0	0	0	0	0	0	0	0	

15187864	m	md	DE	2018	0	1	0	0	1	0	0	0	0	0	0	0	74
15187865	f	md	JP	2018	0	1	0	0	1	0	0	0	0	0	0	0	67
15188306	m	md	ES	2018	0	1	0	1	0	0	0	0	0	0	0	0	53.6
15188311	m	ot	JP	2018	0	0	0	0	0	0	0	0	0	0	0	0	76
15188323	m	md	EG	2018	0	1	0	0	1	0	0	0	0	0	0	0	60
15188335	m	ot	US	2018	0	1	1	0	0	0	0	0	0	0	0	0	
15188591	f	md	US	2018	0	0	0	0	0	0	0	0	0	0	0	0	
15188680	m	md	JP	2018	0	1	0	0	0	1	0	0	0	0	0	0	78
15188841	m	cn	US	2018	0	0	0	0	0	0	0	0	0	0	0	0	
15188932	f	cn	US	2018	0	1	0	0	1	0	0	0	0	0	0	0	
15189749	f	md	NL	2018	0	0	0	0	0	0	0	0	0	0	0	0	69
15189762	m	md	SK	2018	0	0	0	0	0	0	0	0	0	0	0	0	36
15189773	m	ot	DE	2018	0	0	0	0	0	0	0	0	0	0	0	0	65
15189799	f	md	DE	2018	0	0	0	0	0	0	0	0	0	0	0	0	
15189817	f	cn	GB	2018	1	0	0	0	0	0	0	0	0	0	0	0	68
15189934	m	ot	DE	2018	0	0	0	0	0	0	0	0	0	0	0	0	60
15189937	f	ot	CH	2018	0	1	0	0	1	0	0	0	0	0	0	0	57
15190013	m	md	JP	2018	0	0	0	0	0	0	0	0	0	0	0	0	
15190128	m	ot	IT	2018	0	1	0	0	1	0	0	0	0	0	0	0	73
15190735	f	cn	JP	2018	0	1	1	0	0	0	0	0	0	0	0	0	75
15190791	m	ot	BE	2018	0	0	0	0	0	0	0	0	0	0	0	0	42
15191517	f	ot	US	2018	0	1	0	0	1	0	0	0	0	0	0	0	52
15192346	f	ot	US	2018	0	1	0	0	1	0	0	0	0	0	0	0	60
15192476	m	md	JP	2018	0	1	0	0	1	0	0	0	0	0	0	0	68
15192618	f	md	JP	2018	0	1	0	0	0	1	0	0	0	0	0	0	87
15192624	m	md	JP	2018	0	1	0	0	1	0	0	0	0	0	0	0	79
15193313	f	md	JP	2018	0	0	0	0	0	0	0	0	0	0	0	0	71
15193947	f	md	FR	2018	0	0	0	0	0	0	0	0	0	0	0	0	52
15195722	m	md	GR	2018	0	0	0	0	0	0	0	0	0	0	0	0	66
15196457	m	ot	JP	2018	0	1	0	0	1	0	0	0	0	0	0	0	78
15196862	f	ph	US	2018	0	1	1	0	0	0	0	0	0	0	0	0	82
15196877	f	Not repo	US	2018	1	0	0	0	0	0	0	0	0	0	0	0	
15198410	m	ot	US	2018	0	1	0	0	1	0	0	0	0	0	0	0	69
15198443	Not	ot	GB	2018	0	0	0	0	0	0	0	0	0	0	0	0	

15200113	m	ot	DE	2018	0	1	0	0	1	0	0	0	0	0	0	0	79
15200341	m	md	JP	2018	0	1	0	0	0	1	0	0	0	0	0	0	68
15200390	f	ot	CN	2018	1	0	0	0	0	0	0	0	0	0	0	0	58
15200407	m	ot	CN	2018	1	0	0	0	0	0	0	0	0	0	0	0	57
15200411	f	cn	AU	2018	0	1	0	0	1	0	0	0	0	0	0	0	
15200451	f	ot	PE	2018	0	1	0	0	1	0	0	0	0	0	0	0	49
15200452	m	md	DE	2018	0	0	0	0	0	0	0	0	0	0	0	0	
15200533	m	ot	CN	2018	1	0	0	0	0	0	0	0	0	0	0	0	60
15200539	f	ot	CN	2018	1	0	0	0	0	0	0	0	0	0	0	0	50
15200697	m	md	JP	2018	0	1	0	0	1	0	0	0	0	0	0	0	61
15200864	f	ot	CN	2018	1	0	0	0	0	0	0	0	0	0	0	0	73
15201270	f	cn	CN	2018	1	0	0	0	0	0	0	0	0	0	0	0	86
15201438	f	ph	US	2018	0	1	1	0	0	0	0	0	0	0	0	0	
15201451	m	ot	DE	2018	0	0	0	0	0	0	0	0	0	0	0	0	57
15201529	f	md	JP	2018	0	0	0	0	0	0	0	0	0	0	0	0	60
15201533	f	md	JP	2018	1	0	0	0	0	0	0	0	0	0	0	1	68.4
15201673	m	md	DK	2018	0	1	0	0	0	1	0	0	0	0	0	0	73
15201751	m	ph	FR	2018	1	0	0	0	0	0	0	0	0	0	0	0	80
15202012	m	md	CN	2018	0	1	0	1	0	0	0	0	0	0	0	0	67.3
15202222	m	md	CN	2018	0	1	0	1	0	0	0	0	0	0	0	0	61.3
15202409	f	ot	KR	2018	1	0	0	0	0	0	0	0	0	0	0	0	66
15202705	m	ot	KR	2018	1	0	0	0	0	0	0	0	0	0	0	0	53
15203053	m	cn	US	2018	0	0	0	0	0	0	0	0	0	0	0	0	
15203115	Not	md	US	2018	0	1	0	1	0	0	0	0	0	0	0	1	
15203288	m	md	JP	2018	0	0	0	0	0	0	0	0	0	0	0	0	
15203447	m	ot	DE	2018	0	0	0	0	0	0	0	0	0	0	0	0	
15204525	m	ot	FR	2018	0	1	0	0	1	0	0	0	0	0	0	0	59
15204669	f	ot	US	2018	0	1	0	0	1	0	0	0	0	0	0	0	
15204709	Not	md	JP	2018	1	0	0	0	0	0	0	0	0	0	0	0	
15205769	Not	md	DE	2018	0	0	0	0	0	0	0	0	0	0	0	0	
15205922	m	md	JP	2018	0	1	0	0	1	0	0	0	0	0	0	0	80
15205929	m	md	CZ	2018	1	0	0	0	0	0	0	0	0	0	0	0	69
15205948	f	md	CZ	2018	1	0	0	0	0	0	0	0	0	0	0	0	71
15206080	m	cn	JP	2018	0	1	1	0	0	0	0	0	0	0	0	0	74

15206091	m	md	JP	2018	0	1	0	0	1	0	0	0	0	0	0	1	66
15206848	m	ot	GB	2018	0	0	0	0	0	0	0	0	0	0	0	0	49
15207110	f	md	JP	2018	1	0	0	0	0	0	0	0	0	0	0	0	80.3
15207204	m	md	CN	2018	0	1	0	0	0	1	0	0	0	0	0	0	61
15207256	Not	md	JP	2018	0	0	0	0	0	0	0	0	0	0	0	0	
15208232	m	md	IE	2018	0	1	0	0	0	1	0	0	0	0	0	0	65
15208583	m	ot	CN	2018	0	1	0	0	1	0	0	0	0	0	0	0	52
15209128	Not	md	JP	2018	0	1	1	0	0	0	0	0	0	0	0	1	
15209466	m	ot	DE	2018	0	0	0	0	0	0	0	0	0	0	0	0	54
15209725	m	md	DE	2018	0	0	0	0	0	0	0	0	0	0	0	0	80
15209755	f	ph	US	2018	0	1	0	1	0	0	0	0	0	0	0	1	74
15209776	f	ot	DE	2018	0	1	0	0	1	0	0	0	0	0	0	0	65
15210168	m	cn	JP	2018	0	1	1	0	0	0	0	0	0	0	0	1	72
15210507	m	md	JP	2018	0	1	0	0	1	0	0	0	0	0	0	1	68
15210524	m	cn	DE	2018	0	0	0	0	0	0	0	0	0	0	0	0	
15212009	m	md	HK	2018	0	0	0	0	0	0	0	0	0	0	0	0	60
15213689	m	md	SG	2018	0	1	0	0	0	1	0	0	0	0	0	1	79
15214119	m	md	US	2018	0	1	0	1	0	0	0	0	0	0	0	0	
15214835	f	cn	GB	2018	1	0	0	0	0	0	0	0	0	0	0	0	
15214975	f	cn	JP	2018	0	0	0	0	0	0	0	0	0	0	0	0	65
15215373	f	Not repo	Not re	2018	0	0	0	0	0	0	0	0	0	0	0	0	48
15215606	m	md	DE	2018	0	0	0	0	0	0	0	0	0	0	0	0	61
15216002	f	cn	GB	2018	1	0	0	0	0	0	0	0	0	0	0	0	72
15216040	f	Not repo	US	2018	0	1	1	0	0	0	0	0	0	0	0	1	61
15217070	f	ot	DE	2018	0	0	0	0	0	0	0	0	0	0	0	0	7
15217638	m	md	JP	2018	0	0	0	0	0	0	0	0	0	0	0	0	46
15218104	Not	ot	US	2018	0	1	0	0	1	0	0	0	0	0	0	0	
15218190	m	md	JP	2018	0	1	0	0	1	0	0	0	0	0	0	0	51
15218478	f	md	US	2018	0	1	1	0	0	0	0	0	0	0	0	0	61
15218553	Not	md	JP	2018	0	1	1	0	0	0	0	0	0	0	0	0	
15218581	m	md	FR	2018	0	1	0	0	1	0	0	0	0	0	0	0	65
15218769	m	cn	US	2018	0	1	1	0	0	0	0	0	0	0	0	0	
15218867	f	md	JP	2018	0	1	0	0	1	0	0	0	0	0	0	0	64
15219199	Not	md	JP	2018	0	1	1	0	0	0	0	0	0	0	0	0	

15219505	m	md	CN	2018	0	1	0	0	0	1	0	0	0	0	0	0	74
15219526	f	ot	SG	2018	0	1	0	0	1	0	0	0	0	0	0	0	58
15219550	f	md	CH	2018	0	1	0	1	0	0	0	0	0	0	0	0	58
15219670	f	cn	US	2018	0	0	0	0	0	0	0	0	0	0	0	0	53
15220471	Not	md	JP	2018	0	0	0	0	0	0	0	0	0	0	0	0	
15220591	f	ot	TR	2018	0	1	0	0	1	0	0	0	0	0	0	0	72
15220629	f	md	DK	2018	0	1	0	0	0	1	0	0	0	0	0	0	63
15221454	Not	cn	JP	2018	0	0	0	0	0	0	0	0	0	0	0	0	
15221680	m	ot	US	2018	0	1	0	1	0	0	0	0	0	0	0	0	60
15221809	m	ot	US	2018	1	0	0	0	0	0	0	0	0	0	0	1	77
15222006	m	md	US	2018	0	1	0	0	0	1	0	0	0	0	0	0	
15222010	f	cn	US	2018	0	1	1	0	0	0	0	0	0	0	0	0	91
15222052	m	ot	HU	2018	0	0	0	0	0	0	0	0	0	0	0	0	62
15222332	m	ot	GB	2018	0	1	1	0	0	0	0	0	0	0	0	0	
15222685	Not	ot	CN	2018	0	0	0	0	0	0	0	0	0	0	0	0	
15222839	f	ot	IE	2018	0	1	0	0	1	0	0	0	0	0	0	0	55
15222914	f	md	PL	2018	0	0	0	0	0	0	0	0	0	0	0	0	59
15223215	Not	ot	CN	2018	0	0	0	0	0	0	0	0	0	0	0	0	
15223350	f	md	KR	2018	1	0	0	0	0	0	0	0	0	0	0	0	63
15223432	Not	ot	CN	2018	0	0	0	0	0	0	0	0	0	0	0	0	
15223434	Not	ot	CN	2018	0	0	0	0	0	0	0	0	0	0	0	0	
15223519	m	cn	US	2018	0	0	0	0	0	0	0	0	0	0	0	0	62
15224986	m	md	US	2018	0	0	0	0	0	0	0	0	0	0	0	0	
15225016	f	md	JP	2018	1	0	0	0	0	0	0	0	0	0	0	0	61
15225181	f	md	JP	2018	0	1	1	0	0	0	0	0	0	0	0	0	70
15225324	f	cn	GB	2018	1	0	0	0	0	0	0	0	0	0	0	0	64
15225398	f	ot	US	2018	0	1	0	0	1	0	0	0	0	0	0	0	66
15225431	f	cn	US	2018	0	1	0	0	1	0	0	0	0	0	0	0	
15225945	m	md	JP	2018	1	0	0	0	0	0	0	0	0	0	0	1	66
15226123	f	cn	GB	2018	1	0	0	0	0	0	0	0	0	0	0	0	71
15226428	m	ot	US	2018	0	1	0	0	1	0	0	0	0	0	0	0	65
15226963	m	md	EG	2018	0	1	0	0	1	0	0	0	0	0	0	0	58
15227243	m	md	JP	2018	0	1	0	0	1	0	0	0	0	0	0	0	70
15227334	f	md	JP	2018	0	1	0	0	0	1	0	0	0	0	0	0	67

15227419	m	md	US	2018	0	1	0	1	0	0	0	0	0	0	0	1	
15227468	m	ot	CN	2018	0	1	0	0	1	0	0	0	0	0	0	0	70
15227989	m	cn	JP	2018	0	1	1	0	0	0	0	0	0	0	0	0	74
15228240	m	ot	BE	2018	0	1	0	0	1	0	0	0	0	0	0	0	66
15228254	f	Not repo	US	2018	1	0	0	0	0	0	0	0	0	0	0	0	45.6
15228285	f	md	US	2018	0	1	1	0	0	0	0	0	0	0	0	0	60
15228356	m	ot	ES	2018	0	1	0	0	1	0	0	0	0	0	0	0	77
15228550	f	ot	PE	2018	0	1	0	0	1	0	0	0	0	0	0	0	49
15229808	Not	md	JP	2018	0	1	1	0	0	0	0	0	0	0	0	0	
15229876	f	ot	KR	2018	1	0	0	0	0	0	0	0	0	0	0	0	66
15229888	m	ot	KR	2018	1	0	0	0	0	0	0	0	0	0	0	0	53
15230139	m	cn	US	2018	0	1	0	0	1	0	0	0	0	0	0	0	
15230385	m	md	FR	2018	0	1	0	1	0	0	0	0	0	0	0	0	65
15230478	m	md	FR	2018	0	1	0	0	1	0	0	0	0	0	0	0	77
15230525	m	md	US	2018	0	0	0	0	0	0	0	0	0	0	0	0	
15230535	m	md	JP	2018	0	0	0	0	0	0	0	0	0	0	0	0	82
15230907	m	md	ES	2018	0	1	0	0	0	1	0	0	0	0	0	0	67
15231112	f	md	KR	2018	1	0	0	0	0	0	0	0	0	0	0	0	63
15231318	f	md	JP	2018	0	0	0	0	0	0	0	0	0	0	0	0	84
15231370	f	ot	DE	2018	0	0	0	0	0	0	0	0	0	0	0	0	
15231468	f	cn	US	2018	0	0	0	0	0	0	0	0	0	0	0	0	68
15231511	m	md	CA	2018	0	0	0	0	0	0	0	0	0	0	0	0	53
15231778	f	md	ES	2018	0	1	0	0	0	1	0	0	0	0	0	0	68
15231925	Not	ot	CN	2018	0	0	0	0	0	0	0	0	0	0	0	0	
15232040	m	md	RU	2018	0	1	0	1	0	0	0	0	0	0	0	0	56.3
15232137	Not	md	DE	2018	0	1	0	0	0	1	0	0	0	0	0	0	73
15232290	m	ot	DE	2018	0	0	0	0	0	0	0	0	0	0	0	0	66
15232708	f	ot	PL	2018	0	1	0	0	1	0	0	0	0	0	0	0	
15233001	m	md	DE	2018	0	0	0	0	0	0	0	0	0	0	0	0	73
15233285	f	md	US	2018	1	0	0	0	0	0	0	0	0	0	0	0	
15233420	m	ot	DE	2018	0	0	0	0	0	0	0	0	0	0	0	0	77
15233586	m	cn	JP	2018	0	0	0	0	0	0	0	0	0	0	0	0	53
15233657	m	md	US	2018	0	0	0	0	0	0	0	0	0	0	0	0	78
15233757	m	ot	US	2018	0	1	0	0	1	0	0	0	0	0	0	0	79

15234127	m	cn	KR	2018	1	0	0	0	0	0	0	0	0	0	0	0	71
15234162	m	md	JP	2018	0	1	0	0	1	0	0	0	0	0	0	0	63
15234243	m	md	DE	2018	0	0	0	0	0	0	0	0	0	0	0	0	63
15234550	f	cn	GB	2018	0	1	0	0	1	0	0	0	0	0	0	0	76
15234863	m	ot	ES	2018	0	1	0	0	1	0	0	0	0	0	0	0	65
15234977	f	cn	US	2018	1	0	0	0	0	0	0	0	0	0	0	0	72
15235045	f	ot	TW	2018	0	1	0	0	1	0	0	0	0	0	0	0	69
15235464	f	md	DE	2018	0	0	0	0	0	0	0	0	0	0	0	0	56
15235502	f	md	US	2018	1	0	0	0	0	0	0	0	0	0	0	0	90
15235803	m	md	DE	2018	0	0	0	0	0	0	0	0	0	0	0	0	76
15237205	m	md	JP	2018	1	0	0	0	0	0	0	0	0	0	0	0	70
15237547	m	md	JP	2018	0	1	0	0	0	1	0	0	0	0	0	0	
15237850	m	md	JP	2018	0	0	0	0	0	0	0	0	0	0	0	0	87
15237983	f	ot	US	2018	0	0	0	0	0	0	0	0	0	0	0	0	40
15238170	m	Not repo	JP	2018	0	0	0	0	0	0	0	0	0	0	0	0	75
15238403	m	ot	CN	2018	0	1	0	0	1	0	0	0	0	0	0	0	65
15239239	f	md	BE	2018	0	1	0	1	0	0	0	0	0	0	0	1	70.8
15239271	f	ot	DE	2018	0	0	0	0	0	0	0	0	0	0	0	0	78
15239434	f	cn	GB	2018	1	0	0	0	0	0	0	0	0	0	0	0	
15239471	m	md	CN	2018	1	0	0	0	0	0	0	0	0	0	0	0	80
15239809	m	md	GB	2018	0	0	0	0	0	0	0	0	0	0	0	0	51
15240413	f	cn	KR	2018	0	1	1	0	0	0	0	0	0	0	0	0	
15240478	f	md	DE	2018	0	0	0	0	0	0	0	0	0	0	0	0	74
15240641	m	ot	US	2018	0	1	0	1	0	0	0	0	0	0	0	0	39
15241070	m	ph	US	2018	0	1	1	0	0	0	0	0	0	0	0	0	55
15241560	m	ot	DE	2018	0	0	0	0	0	0	0	0	0	0	0	0	66
15241587	m	ot	DE	2018	0	0	0	0	0	0	0	0	0	0	0	0	
15241594	m	ot	DE	2018	0	0	0	0	0	0	0	0	0	0	0	0	72
15241640	f	ph	CA	2018	0	0	0	0	0	0	0	0	0	0	0	0	72
15241661	f	md	JP	2018	0	0	0	0	0	0	0	0	0	0	0	0	65
15242140	m	cn	JP	2018	0	1	1	0	0	0	0	0	0	0	0	0	84
15242246	m	cn	US	2018	0	1	1	0	0	0	0	0	0	0	0	0	53
15242294	m	ph	JP	2018	0	0	0	0	0	0	0	0	0	0	0	0	74
15242466	m	md	GB	2018	0	0	0	0	0	0	0	0	0	0	0	0	76

15242600	m	md	HU	2018	0	0	0	0	0	0	0	0	0	0	0	0	74
15242609	m	md	IT	2018	0	0	0	0	0	0	0	0	0	0	0	0	50
15242717	Not	ot	US	2018	0	0	0	0	0	0	0	0	0	0	0	0	
15244649	f	md	JP	2018	0	1	0	0	1	0	0	0	0	0	0	1	64
15245382	m	md	DE	2018	0	0	0	0	0	0	0	0	0	0	0	0	63
15245545	f	ot	DE	2018	0	0	0	0	0	0	0	0	0	0	0	0	57
15245623	f	ot	DE	2018	0	0	0	0	0	0	0	0	0	0	0	0	48
15245774	f	md	DE	2018	0	0	0	0	0	0	0	0	0	0	0	0	56
15245875	m	md	DE	2018	0	0	0	0	0	0	0	0	0	0	0	0	57
15245878	f	ot	DE	2018	0	0	0	0	0	0	0	0	0	0	0	0	69
15246016	f	ot	TH	2018	0	1	0	0	1	0	0	0	0	0	0	0	57
15247185	f	ot	FR	2018	0	0	0	0	0	0	0	0	0	0	0	0	76
15247290	f	md	JP	2018	0	1	0	0	1	0	0	0	0	0	0	0	67
15247291	Not	ot	US	2018	0	1	0	0	1	0	0	0	0	0	0	0	
15247376	m	md	JP	2018	0	1	0	0	0	1	0	0	0	0	0	0	62
15247739	f	cn	US	2018	0	1	0	1	0	0	0	0	0	0	0	0	73.7
15248066	f	ot	DE	2018	0	1	0	0	1	0	0	0	0	0	0	1	73
15248239	m	cn	JP	2018	0	0	0	0	0	0	0	0	0	0	0	0	62
15248254	f	cn	US	2018	0	0	0	0	0	0	0	0	0	0	0	0	47
15248560	m	md	JP	2018	0	1	0	0	0	1	0	0	0	0	0	0	80
15248604	m	md	JP	2018	0	1	0	0	1	0	0	0	0	0	0	0	73
15249573	m	ph	US	2018	0	1	1	0	0	0	0	0	0	0	0	1	82
15249707	f	md	TR	2018	1	0	0	0	0	0	0	0	0	0	0	0	59
15249751	f	cn	GB	2018	1	0	0	0	0	0	0	0	0	0	0	0	
15249796	f	cn	US	2018	0	0	0	0	0	0	0	0	0	0	0	0	69
15250065	f	cn	IL	2018	0	0	0	0	0	0	0	0	0	0	0	0	38
15250107	m	md	CA	2018	1	0	0	0	0	0	0	0	0	0	0	0	50
15250400	f	md	US	2018	0	1	0	0	0	1	0	0	0	0	0	0	78
15250454	m	md	DE	2018	0	0	0	0	0	0	0	0	0	0	0	0	74
15250837	m	cn	CA	2018	0	0	0	0	0	0	0	0	0	0	0	0	63
15251042	m	md	US	2018	1	0	0	0	0	0	0	0	0	0	0	1	77
15251195	m	ot	CN	2018	0	1	0	0	1	0	0	0	0	0	0	0	53
15251313	m	md	GB	2018	0	1	0	1	0	0	0	0	0	0	0	0	60.5
15251980	f	cn	US	2018	0	1	0	0	1	0	0	0	0	0	0	0	60

15252422	m	ot	GB	2018	0	1	0	0	1	0	0	0	0	0	0	0	
15253192	m	md	ES	2018	0	1	1	0	0	0	0	0	0	0	0	0	
15253526	f	md	EC	2018	0	1	0	0	0	1	0	0	0	0	0	0	53
15253655	f	cn	IN	2018	0	1	0	0	0	1	0	0	0	0	0	0	83
15253968	m	md	DE	2018	0	0	0	0	0	0	0	0	0	0	0	0	58
15254010	m	ot	US	2018	0	1	0	0	1	0	0	0	0	0	0	0	68
15254023	f	cn	US	2018	1	0	0	0	0	0	0	0	0	0	0	0	73
15254207	m	md	JP	2018	0	1	0	0	1	0	0	0	0	0	0	0	83
15254530	f	cn	US	2018	1	0	0	0	0	0	0	0	0	0	0	0	69
15255160	m	md	US	2018	0	1	0	1	0	0	0	0	0	0	0	1	
15255382	m	md	JP	2018	0	1	0	0	1	0	0	0	0	0	0	0	77
15255510	f	cn	AR	2018	0	0	0	0	0	0	0	0	0	0	0	0	33
15255595	f	md	FR	2018	0	0	0	0	0	0	0	0	0	0	0	0	67
15255767	m	cn	JP	2018	0	1	0	0	1	0	0	0	0	0	0	0	
15256049	m	md	EG	2018	0	1	0	0	0	1	0	0	0	0	0	0	
15256067	m	md	KR	2018	0	1	0	0	0	1	0	0	0	0	0	1	61
15256599	f	md	SG	2018	0	1	0	0	0	1	0	0	0	0	0	0	
15256813	f	ot	CA	2018	0	1	1	0	0	0	0	0	0	0	0	1	58
15256974	Not	cn	US	2018	0	0	0	0	0	0	0	0	0	0	0	0	
15257738	Not	md	AT	2018	0	0	0	0	0	0	0	0	0	0	0	0	
15257855	f	md	US	2018	0	0	0	0	0	0	1	0	1	0	0	0	80
15257919	Not	md	US	2018	1	0	0	0	0	0	0	0	0	0	0	0	
15257921	m	cn	JP	2018	0	1	1	0	0	0	0	0	0	0	0	0	67
15257972	f	md	CH	2018	0	1	0	1	0	0	0	0	0	0	0	0	58
15258124	f	ot	JP	2018	0	0	0	0	0	0	0	0	0	0	0	0	62
15258152	m	cn	JP	2018	0	0	0	0	0	0	0	0	0	0	0	0	56
15258540	m	md	JP	2018	0	1	0	0	1	0	0	0	0	0	0	0	76
15258546	f	cn	US	2018	1	0	0	0	0	0	0	0	0	0	0	0	57
15258899	m	ot	US	2018	1	0	0	0	0	0	0	0	0	0	0	1	77
15258986	m	cn	CN	2018	0	0	0	0	0	0	0	0	0	0	0	0	
15259271	f	cn	KR	2018	0	1	1	0	0	0	0	0	0	0	0	0	
15259727	f	md	FR	2018	0	0	0	0	0	0	0	0	0	0	0	0	74
15259751	f	md	JP	2018	0	1	0	0	0	1	0	0	0	0	0	0	71
15260165	m	md	JP	2018	0	0	0	0	0	0	0	0	0	0	0	0	50

15260729	f	ot	CA	2018	0	1	0	0	1	0	0	0	0	0	0	0	73
15260857	f	ot	PL	2018	0	1	0	0	1	0	0	0	0	0	0	0	71
15261279	m	ot	CN	2018	0	1	0	0	1	0	0	0	0	0	0	0	65
15261566	f	md	MX	2018	0	1	0	1	0	0	0	0	0	0	0	0	69
15261622	f	md	US	2018	1	0	0	0	0	0	0	0	0	0	0	1	62
15262168	m	cn	US	2018	0	1	0	0	1	0	0	0	0	0	0	0	59
15262297	f	cn	US	2018	0	0	0	0	0	0	0	0	0	0	0	0	63
15262622	f	md	DE	2018	1	0	0	0	0	0	0	0	0	0	0	0	38
15262758	m	md	IT	2018	0	1	0	0	1	0	0	0	0	0	0	0	64
15262915	f	cn	IN	2018	0	1	0	0	1	0	0	0	0	0	0	0	51
15263001	m	md	IL	2018	0	0	0	0	0	0	0	0	0	0	0	0	
15263032	m	md	JP	2018	0	1	0	0	1	0	0	0	0	0	0	0	81
15263155	m	md	CH	2018	0	1	0	0	1	0	0	0	0	0	0	0	65
15263201	f	ot	NL	2018	0	0	0	0	0	0	0	0	0	0	0	0	62
15263396	m	md	IL	2018	0	0	0	0	0	0	0	0	0	0	0	0	51
15263814	f	cn	US	2018	0	0	0	0	0	0	0	0	0	0	0	0	72
15263818	m	md	SE	2018	0	1	1	0	0	0	0	0	0	0	0	0	
15263837	m	cn	US	2018	0	1	0	0	1	0	0	0	0	0	0	0	
15264122	m	md	JP	2018	1	0	0	0	0	0	0	0	0	0	0	1	76.7
15264137	f	Not repo	US	2018	0	1	0	1	0	0	0	0	0	0	0	0	53
15264213	f	md	DE	2018	0	1	0	0	1	0	0	0	0	0	0	1	55
15264773	m	md	JP	2018	0	0	0	0	0	0	0	0	0	0	0	0	72
15264833	f	md	JP	2018	0	1	0	0	0	1	0	0	0	0	0	0	55
15265438	f	ot	US	2018	0	0	0	0	0	0	0	0	0	0	0	0	73
15265493	m	md	IN	2018	0	1	0	0	1	0	0	0	0	0	0	0	72
15265691	m	ot	DE	2018	0	0	0	0	0	0	0	0	0	0	0	0	
15265889	m	ot	CA	2018	0	1	0	0	1	0	0	0	0	0	0	0	65
15266056	f	cn	US	2018	0	1	0	0	1	0	0	0	0	0	0	0	59
15266181	f	cn	US	2018	0	0	0	0	0	0	0	0	0	0	0	0	61
15266323	m	md	EG	2018	0	1	0	0	1	0	0	0	0	0	0	0	64
15266541	m	ot	DE	2018	0	0	0	0	0	0	0	0	0	0	0	0	49
15266883	m	ot	DE	2018	0	0	0	0	0	0	0	0	0	0	0	0	61
15268094	m	md	IE	2018	0	0	0	0	0	0	0	0	0	0	0	0	60
15268297	m	cn	US	2018	0	0	0	0	0	0	0	0	0	0	0	0	75

15268298	f	cn	US	2018	0	0	0	0	0	0	0	0	0	0	0	0	66
15268563	m	ot	DE	2018	0	0	0	0	0	0	0	0	0	0	0	0	74
15268619	f	cn	DE	2018	0	1	1	0	0	0	0	0	0	0	0	0	60
15269123	m	md	DE	2018	0	0	0	0	0	0	0	0	0	0	0	0	62
15269177	m	md	DE	2018	0	0	0	0	0	0	0	0	0	0	0	0	
15269411	f	ot	DE	2018	0	0	0	0	0	0	0	0	0	0	0	0	65
15269685	m	md	AR	2018	0	1	0	0	0	1	0	0	0	0	0	0	68
15269867	f	md	US	2018	0	0	0	0	0	0	0	0	0	0	0	0	77
15270429	m	cn	CA	2018	0	0	0	0	0	0	0	0	0	0	0	0	
15270529	m	md	FR	2018	0	0	0	0	0	0	0	0	0	0	0	0	64
15270571	m	cn	CA	2018	0	0	0	0	0	0	0	0	0	0	0	0	
15270573	f	ot	FR	2018	0	0	0	0	0	0	0	0	0	0	0	0	74
15270594	m	md	JP	2018	0	1	0	1	0	0	0	0	0	0	0	0	71
15271279	m	Not repo	JP	2018	0	0	0	0	0	0	0	0	0	0	0	0	65
15271297	m	md	IN	2018	0	0	0	0	0	0	0	0	0	0	0	0	40
15271788	f	cn	US	2018	1	0	0	0	0	0	0	0	0	0	0	0	83
15272018	m	ot	DE	2018	0	1	0	0	1	0	0	0	0	0	0	0	58
15272037	f	ot	JP	2018	0	0	0	0	0	0	0	0	0	0	0	0	60
15272068	m	md	US	2018	0	1	0	0	1	0	0	0	0	0	0	0	73
15272217	f	cn	US	2018	0	1	0	0	1	0	0	0	0	0	0	0	
15272505	f	cn	US	2018	1	0	0	0	0	0	0	0	0	0	0	0	61
15272668	m	md	US	2018	0	1	0	1	0	0	0	0	0	0	0	0	52
15273769	Not	md	US	2018	0	1	0	1	0	0	0	0	0	0	0	0	
15274057	m	md	GB	2018	0	0	0	0	0	0	0	0	0	0	0	0	65
15274077	m	md	DE	2018	0	1	0	0	1	0	0	0	0	0	0	0	85
15274088	f	cn	US	2018	0	0	0	0	0	0	0	0	0	0	0	0	61
15274136	m	cn	JP	2018	0	1	1	0	0	0	0	0	0	0	0	0	74
15274137	m	md	PT	2018	0	0	0	0	0	0	0	0	0	0	0	0	
15274150	m	cn	US	2018	0	1	1	0	0	0	0	0	0	0	0	0	79
15274170	m	md	CN	2018	0	0	0	0	0	0	0	0	0	0	0	0	59
15274203	m	cn	JP	2018	0	1	1	0	0	0	0	0	0	0	0	0	68
15274375	m	ot	JP	2018	0	1	0	0	1	0	0	0	0	0	0	0	59
15274442	m	md	DE	2018	0	1	0	0	1	0	0	0	0	0	0	0	72
15274530	m	ot	FR	2018	0	1	0	0	1	0	0	0	0	0	0	0	

15274573	f	ph	US	2018	0	1	1	0	0	0	0	0	0	0	0	0	
15274647	f	md	IE	2018	1	0	0	0	0	0	0	0	0	0	0	0	53
15274683	m	md	ES	2018	0	1	0	0	1	0	0	0	0	0	0	0	76
15274727	f	md	CN	2018	1	0	0	0	0	0	0	0	0	0	0	0	54.8
15274968	m	md	BR	2018	0	1	0	0	1	0	0	0	0	0	0	0	
15275119	m	md	CN	2018	0	1	0	0	0	1	0	0	0	0	0	0	48
15275977	f	ot	DE	2018	0	1	0	0	1	0	0	0	0	0	0	0	77
15276355	m	cn	US	2018	0	1	1	0	0	0	0	0	0	0	0	0	
15276370	m	md	EG	2018	0	1	0	0	0	1	0	0	0	0	0	0	
15276399	f	md	US	2018	1	0	0	0	0	0	0	0	0	0	0	0	
15276855	m	ot	CN	2018	0	1	0	0	1	0	0	0	0	0	0	0	64
15277018	m	ot	CN	2018	0	1	0	0	1	0	0	0	0	0	0	0	55
15277243	m	md	US	2018	1	0	0	0	0	0	0	0	0	0	0	1	93
15277576	f	cn	US	2018	0	0	0	0	0	0	0	0	0	0	0	0	64
15277604	f	Not repo	ID	2018	1	0	0	0	0	0	0	0	0	0	0	0	
15277738	f	md	JP	2018	1	0	0	0	0	0	0	0	0	0	0	0	85
15277870	f	cn	US	2018	0	1	1	0	0	0	0	0	0	0	0	0	
15278458	m	md	US	2018	0	1	1	0	0	0	0	0	0	0	0	0	
15278486	m	md	CN	2018	0	1	0	1	0	0	0	0	0	0	0	0	71.5
15278491	m	ot	JP	2018	0	1	0	0	1	0	0	0	0	0	0	0	70
15278712	m	md	JP	2018	0	1	0	0	1	0	0	0	0	0	0	0	55
15278834	f	cn	JP	2018	0	1	1	0	0	0	0	0	0	0	0	0	79
15278962	m	md	HK	2018	0	1	0	0	0	1	0	0	0	0	0	0	31
15279097	m	md	DE	2018	0	0	0	0	0	0	0	0	0	0	0	0	58
15279245	f	md	IL	2018	0	1	0	0	1	0	0	0	0	0	0	0	63
15279368	m	md	FR	2018	0	0	0	0	0	0	0	0	0	0	0	0	64
15279478	m	md	IL	2018	0	1	0	0	1	0	0	0	0	0	0	0	67
15280111	Not	ph	JP	2018	1	0	0	0	0	0	0	0	0	0	0	0	
15280661	m	cn	US	2018	0	0	0	0	0	0	0	0	0	0	0	0	
15280727	m	ot	DE	2018	0	0	0	0	0	0	0	0	0	0	0	0	84
15280830	f	cn	US	2018	0	0	0	0	0	0	0	0	0	0	0	0	66
15281344	m	ot	JP	2018	0	0	0	0	0	0	0	0	0	0	0	0	66
15281347	m	ot	JP	2018	0	0	0	0	0	0	0	0	0	0	0	0	70
15281350	m	ot	JP	2018	0	0	0	0	0	0	0	0	0	0	0	0	62

15282100	m	cn	US	2018	0	1	1	0	0	0	0	0	0	0	0	1	
15282501	f	md	US	2018	0	1	0	0	1	0	0	0	0	0	0	0	72
15283078	m	cn	DE	2018	0	1	1	0	0	0	0	0	0	0	0	0	
15283154	f	cn	BR	2018	0	1	0	1	0	0	0	0	0	0	0	0	70
15283276	f	md	CA	2018	0	1	0	0	0	1	0	0	0	0	0	0	83
15283288	f	cn	US	2018	0	0	0	0	0	0	0	0	0	0	0	0	63
15283318	m	md	KR	2018	1	0	0	0	0	0	0	0	0	0	0	0	59
15283403	m	cn	GB	2018	0	1	1	0	0	0	0	0	0	0	0	0	75
15283404	m	md	ES	2018	0	0	0	0	0	0	0	0	0	0	0	0	65
15283839	f	ot	NL	2018	0	0	0	0	0	0	0	0	0	0	0	0	69
15283846	f	md	US	2018	0	1	0	1	0	0	0	0	0	0	0	0	
15284014	m	md	JP	2018	0	0	0	0	0	0	0	0	0	0	0	0	
15284027	m	md	JP	2018	0	0	0	0	0	0	0	0	0	0	0	0	75
15284744	m	cn	US	2018	0	0	0	0	0	0	0	0	0	0	0	0	65
15284877	m	cn	US	2018	0	0	0	0	0	0	0	0	0	0	0	0	75
15284991	f	md	GB	2018	0	1	0	0	0	1	0	0	0	0	0	0	60
15285324	f	md	TW	2018	0	0	0	0	0	0	0	0	0	0	0	0	62
15285443	m	ot	TH	2018	0	1	0	0	1	0	0	0	0	0	0	0	59
15285487	f	md	US	2018	0	1	0	0	1	0	0	0	0	0	0	0	72
15285529	m	md	BR	2018	0	0	0	0	0	0	0	0	0	0	0	0	55
15285534	f	cn	US	2018	0	0	0	0	0	0	0	0	0	0	0	0	
15285535	m	cn	US	2018	0	1	1	0	0	0	0	0	0	0	0	0	
15285565	f	md	FR	2018	0	1	0	1	0	0	0	0	0	0	0	0	46
15285758	m	ph	US	2018	0	0	0	0	0	0	0	0	0	0	0	0	53
15285895	f	md	JP	2018	1	0	0	0	0	0	0	0	0	0	0	1	59
15285949	f	md	JP	2018	1	0	0	0	0	0	0	0	0	0	0	0	48.2
15286004	m	md	CN	2018	0	1	0	1	0	0	0	0	0	0	0	0	65.6
15286147	f	ot	US	2018	0	1	0	0	1	0	0	0	0	0	0	0	56
15286411	m	ot	US	2018	0	1	0	0	1	0	0	0	0	0	0	0	76
15286467	m	ot	DE	2018	0	0	0	0	0	0	0	0	0	0	0	0	
15286787	m	ot	JP	2018	0	1	0	0	1	0	0	0	0	0	0	1	64
15287043	m	cn	US	2018	1	0	0	0	0	0	0	0	0	0	0	0	76
15287245	Not	ph	US	2018	0	1	0	0	1	0	0	0	0	0	0	0	
15287394	m	ot	GB	2018	0	1	1	0	0	0	0	0	0	0	0	1	75

15287456	f	md	TH	2018	1	0	0	0	0	0	0	0	0	0	0	0	66.5
15288164	m	md	JP	2018	0	1	0	0	0	1	0	0	0	0	0	0	60
15288294	f	md	DE	2018	0	1	0	0	0	1	0	0	0	0	0	0	64
15288412	m	md	JP	2018	0	1	0	0	0	1	0	0	0	0	0	0	69
15288960	m	md	IL	2018	0	0	0	0	0	0	0	0	0	0	0	0	
15289712	f	cn	US	2018	0	1	1	0	0	0	0	0	0	0	0	0	
15290305	m	ot	NL	2018	0	0	0	0	0	0	0	0	0	0	0	0	68
15290329	m	md	US	2018	0	1	0	1	0	0	0	0	0	0	0	1	
15290359	m	ot	CN	2018	0	1	0	0	1	0	0	0	0	0	0	0	67
15290473	m	md	DE	2018	1	0	0	0	0	0	0	0	0	0	0	0	53
15290568	m	ph	US	2018	1	0	0	0	0	0	0	0	0	0	0	0	
15290755	f	md	CN	2018	1	0	0	0	0	0	0	0	0	0	0	0	68.4
15291085	f	md	CN	2018	0	1	0	1	0	0	0	0	0	0	0	0	21
15291678	f	md	US	2018	0	0	0	0	0	0	0	0	0	0	0	0	59
15291773	f	cn	CH	2018	0	1	1	0	0	0	0	0	0	0	0	0	49
15291899	f	cn	US	2018	0	0	0	0	0	0	0	0	0	0	0	0	84
15293006	m	md	JP	2018	0	0	0	0	0	0	0	0	0	0	0	0	80
15293245	f	md	TH	2018	0	1	0	0	0	1	0	0	0	0	0	0	52
15293325	m	ot	US	2018	0	1	0	0	1	0	0	0	0	0	0	0	74
15293442	f	cn	US	2018	0	1	1	0	0	0	0	0	0	0	0	0	
15295088	f	cn	US	2018	0	0	0	0	0	0	0	0	0	0	0	0	
15295826	f	md	JP	2018	1	0	0	0	0	0	0	0	0	0	0	0	80
15296209	f	ot	FR	2018	0	1	0	0	1	0	0	0	0	0	0	0	60
15296264	f	md	US	2018	0	1	1	0	0	0	0	0	0	0	0	0	49
15296481	m	cn	JP	2018	0	1	1	0	0	0	0	0	0	0	0	0	68
15296982	m	md	JP	2018	0	1	0	0	1	0	0	0	0	0	0	0	69
15297320	m	ot	DE	2018	0	0	0	0	0	0	0	0	0	0	0	0	64
15297486	f	Not repo	KR	2018	1	0	0	0	0	0	0	0	0	0	0	0	45
15297494	f	md	DE	2018	0	1	0	0	1	0	0	0	0	0	0	0	50
15297595	m	cn	US	2018	0	0	0	0	0	0	0	0	0	0	0	0	60
15297678	Not	cn	ID	2018	0	0	0	0	0	0	0	0	0	0	0	0	
15298045	m	ot	JP	2018	0	1	0	1	0	0	0	0	0	0	0	0	69
15298165	m	md	DE	2018	0	0	0	0	0	0	0	0	0	0	0	0	51
15298263	Not	ot	US	2018	0	0	0	0	0	0	0	0	0	0	0	0	

15298848	m	md	IL	2018	0	1	0	0	0	1	0	0	0	0	0	0	60
15298862	f	md	JP	2018	0	0	0	0	0	0	0	0	0	0	0	0	65
15299259	f	ot	DE	2018	0	0	0	0	0	0	0	0	0	0	0	0	66
15299322	f	md	IT	2018	0	0	0	0	0	0	0	0	0	0	0	0	44
15299458	Not	cn	US	2018	0	1	1	0	0	0	0	0	0	0	0	0	71
15299986	m	md	DE	2018	0	1	1	0	0	0	0	0	0	0	0	1	62
15300005	f	cn	US	2018	0	1	0	0	1	0	0	0	0	0	0	0	57
15300713	f	cn	US	2018	0	0	0	0	0	0	0	0	0	0	0	0	57
15301266	m	md	KR	2018	1	0	0	0	0	0	0	0	0	0	0	0	76
15302059	m	cn	TW	2018	0	0	0	0	0	0	0	0	0	0	0	0	69
15302114	m	cn	CN	2018	0	0	0	0	0	0	0	0	0	0	0	0	71
15302198	m	ot	US	2018	0	1	0	0	1	0	0	0	0	0	0	0	73
15302310	f	md	FR	2018	0	1	0	1	0	0	0	0	0	0	0	0	58
15302373	m	md	SG	2018	0	1	0	1	0	0	0	0	0	0	0	0	79.7
15302855	m	ot	JP	2018	0	0	0	0	0	0	0	0	0	0	0	0	65
15302858	m	ot	JP	2018	0	0	0	0	0	0	0	0	0	0	0	0	67
15302871	m	ot	JP	2018	0	0	0	0	0	0	0	0	0	0	0	0	68
15302876	m	ot	JP	2018	0	0	0	0	0	0	0	0	0	0	0	0	60
15305385	Not	md	CN	2018	0	0	0	0	0	0	0	0	0	0	0	0	
15306002	f	cn	JP	2018	0	1	1	0	0	0	0	0	0	0	0	0	81
15306208	Not	ot	CN	2018	0	0	0	0	0	0	0	0	0	0	0	0	
15306339	f	ph	US	2018	0	1	0	0	1	0	0	0	0	0	0	1	82
15306721	Not	ot	JP	2018	0	0	0	0	0	0	0	0	0	0	0	0	
15306971	m	md	DE	2018	0	0	0	0	0	0	0	0	0	0	0	0	78
15307081	m	cn	US	2018	0	0	0	0	0	0	0	0	0	0	0	0	77
15307371	m	ot	JP	2018	0	1	0	0	1	0	0	0	0	0	0	0	74
15307378	f	md	KR	2018	1	0	0	0	0	0	0	0	0	0	0	0	57
15308107	m	cn	US	2018	0	0	0	0	0	0	0	0	0	0	0	0	67
15308231	m	md	US	2018	0	1	0	1	0	0	0	0	0	0	0	0	
15308691	f	md	US	2018	0	1	1	0	0	0	0	0	0	0	0	0	83
15308997	m	ot	JP	2018	0	0	0	0	0	0	0	0	0	0	0	0	62
15308998	m	ot	JP	2018	0	0	0	0	0	0	0	0	0	0	0	0	66
15308999	m	ot	JP	2018	0	0	0	0	0	0	0	0	0	0	0	0	70
15309461	m	cn	US	2018	0	1	1	0	0	0	0	0	0	0	0	0	

15310470	Not	ph	JP	2018	1	0	0	0	0	0	0	0	0	0	0	0	
15310612	Not	ot	US	2018	0	0	0	0	0	0	0	0	0	0	0	0	
15310627	m	md	IN	2018	0	0	0	0	0	0	0	0	0	0	0	0	42
15310760	f	md	JP	2018	0	1	0	0	1	0	0	0	0	0	0	0	70
15311047	f	md	IN	2018	0	1	0	0	1	0	0	0	0	0	0	0	66
15311072	f	md	JP	2018	1	0	0	0	0	0	0	0	0	0	0	0	52
15311110	m	cn	JP	2018	0	1	1	0	0	0	0	0	0	0	0	0	74
15311709	f	md	US	2018	0	1	1	0	0	0	0	0	0	0	0	0	
15311900	m	cn	US	2018	0	0	0	0	0	0	0	0	0	0	0	0	52
15312042	f	ot	US	2018	0	1	0	0	1	0	0	0	0	0	0	0	
15312227	f	md	US	2018	0	1	0	1	0	0	0	0	0	0	0	0	
15313162	f	md	DE	2018	1	0	0	0	0	0	0	0	0	0	0	0	54
15313214	m	cn	JP	2018	0	1	1	0	0	0	0	0	0	0	0	0	73
15313285	f	cn	US	2018	0	0	0	0	0	0	0	0	0	0	0	0	56
15313476	m	md	US	2018	0	1	0	1	0	0	0	0	0	0	0	1	
15313578	f	md	DK	2018	0	1	1	0	0	0	0	0	0	0	0	0	58
15314101	m	ot	NL	2018	0	0	0	0	0	0	0	0	0	0	0	0	63
15314109	f	md	US	2018	1	0	0	0	0	0	0	0	0	0	0	0	
15314118	m	md	BR	2018	0	1	0	1	0	0	0	0	0	0	0	1	68.2
15314348	f	md	CH	2018	0	1	0	0	1	0	0	0	0	0	0	0	
15314696	m	ot	CN	2018	1	0	0	0	0	0	0	0	0	0	0	0	36
15314855	m	md	DE	2018	0	1	0	0	1	0	0	0	0	0	0	1	70
15315418	m	cn	US	2018	0	1	1	0	0	0	0	0	0	0	0	0	
15316316	f	cn	US	2018	0	1	1	0	0	0	0	0	0	0	0	0	
15316625	f	cn	GB	2018	0	1	1	0	0	0	0	0	0	0	0	0	51
15316831	m	md	US	2018	0	0	0	0	0	0	0	0	0	0	0	0	
15317377	m	md	FR	2018	0	1	0	1	0	0	0	0	0	0	0	0	64
15317821	m	md	AU	2018	0	0	0	0	0	0	0	0	0	0	0	0	72
15317826	m	md	US	2018	0	1	1	0	0	0	0	0	0	0	0	0	74
15318066	f	cn	US	2018	0	1	1	0	0	0	0	0	0	0	0	0	
15318269	f	cn	US	2018	0	1	1	0	0	0	0	0	0	0	0	0	
15318311	m	cn	US	2018	0	0	0	0	0	0	0	0	0	0	0	0	50
15318993	f	ph	US	2018	1	0	0	0	0	0	0	0	0	0	0	0	74
15319194	f	md	SI	2018	0	1	1	0	0	0	0	0	0	0	0	0	75

15319290	m	ot	US	2018	0	1	0	0	1	0	0	0	0	0	0	0	63
15319566	f	md	GB	2018	0	0	0	0	0	0	0	0	0	0	0	0	81
15319931	f	md	US	2018	0	0	0	0	0	0	0	0	0	0	0	0	64
15320483	m	ot	JP	2018	0	0	0	0	0	0	0	0	0	0	0	0	67
15320484	m	ot	JP	2018	0	0	0	0	0	0	0	0	0	0	0	0	65
15320487	m	md	JP	2018	0	1	0	0	0	1	0	0	0	0	0	0	63
15320507	m	ot	JP	2018	0	0	0	0	0	0	0	0	0	0	0	0	68
15320508	m	ot	JP	2018	0	0	0	0	0	0	0	0	0	0	0	0	60
15320515	f	ot	JP	2018	0	1	0	0	1	0	0	0	0	0	0	0	76
15321545	m	ot	FR	2018	0	1	1	0	0	0	0	0	0	0	0	0	73
15322312	m	ot	JP	2018	0	0	0	0	0	0	0	0	0	0	0	0	65
15322313	m	ot	JP	2018	0	0	0	0	0	0	0	0	0	0	0	0	60
15322318	m	ot	JP	2018	0	0	0	0	0	0	0	0	0	0	0	0	67
15322319	m	ot	JP	2018	0	0	0	0	0	0	0	0	0	0	0	0	68
15322508	f	ot	JP	2018	0	0	0	0	0	0	0	0	0	0	0	0	74
15322515	m	Not repo	JP	2018	0	0	0	0	0	0	0	0	0	0	0	0	78
15322640	f	md	US	2018	0	0	0	0	0	0	0	0	0	0	0	0	59
15322994	f	md	SK	2018	0	0	0	0	0	0	0	0	0	0	0	0	64
15323014	f	md	SK	2018	0	0	0	0	0	0	0	0	0	0	0	0	68
15323151	m	cn	US	2018	0	1	1	0	0	0	0	0	0	0	0	0	62
15323363	m	cn	JP	2018	0	1	1	0	0	0	0	0	0	0	0	0	
15323410	m	ot	CN	2018	0	0	0	0	0	0	0	0	0	0	0	0	60
15323721	m	md	JP	2018	0	1	0	0	1	0	0	0	0	0	0	0	67
15324092	m	md	JP	2018	0	1	0	0	0	1	0	0	0	0	0	0	
15324149	f	md	JP	2018	0	1	0	0	1	0	0	0	0	0	0	0	80
15324329	m	md	LB	2018	0	1	0	0	1	0	0	0	0	0	0	0	
15324380	f	ot	US	2018	0	1	0	0	1	0	0	0	0	0	0	1	33
15324555	f	cn	US	2018	0	0	0	0	0	0	0	0	0	0	0	0	73
15324832	f	ot	US	2018	0	1	0	0	1	0	0	0	0	0	0	0	53
15325053	m	cn	US	2018	0	1	0	0	1	0	0	0	0	0	0	0	
15325317	m	md	JP	2018	1	0	0	0	0	0	0	0	0	0	0	0	86
15325529	f	md	DE	2018	0	0	0	0	0	0	0	0	0	0	0	0	66
15325761	m	md	JP	2018	0	1	0	1	0	0	0	0	0	0	0	0	52
15325860	f	md	PT	2018	0	0	0	0	0	0	0	0	0	0	0	0	30

15325976	m	cn	JP	2018	0	1	1	0	0	0	0	0	0	0	0	0		66
15326056	m	ph	US	2018	0	1	0	1	0	0	0	0	0	0	0	0		74
15326101	f	md	IN	2018	0	0	0	0	0	0	0	0	0	0	0	0		
15326109	m	ph	US	2018	0	1	0	1	0	0	0	0	0	0	0	1		74
15326132	m	md	TW	2018	0	0	0	0	0	0	0	0	0	0	0	0		55
15326590	f	cn	JP	2018	0	1	1	0	0	0	0	0	0	0	0	0		72
15326768	f	md	DE	2018	0	1	1	0	0	0	0	0	0	0	0	0		62
15326825	m	md	JP	2018	0	1	0	1	0	0	0	0	0	0	0	1		74
15326854	f	cn	US	2018	0	1	1	0	0	0	0	0	0	0	0	1		68
15326996	Not	md	US	2018	0	1	0	1	0	0	0	0	0	0	0	1		
15327720	m	cn	US	2018	0	1	1	0	0	0	0	0	0	0	0	0		
15327783	f	md	US	2018	0	0	0	0	0	0	0	0	0	0	0	0		89
15327792	m	ph	US	2018	0	0	0	0	0	0	0	0	0	0	0	0		67
15327795	f	cn	US	2018	0	0	0	0	0	0	0	0	0	0	0	0		
15327808	m	md	US	2018	0	1	0	0	0	1	0	0	0	0	0	0		58
15327813	m	cn	US	2018	0	0	0	0	0	0	0	0	0	0	0	0		49
15327831	f	md	US	2018	0	1	0	0	0	1	0	0	0	0	0	0		
15327838	m	md	US	2018	0	1	0	0	0	1	0	0	0	0	0	1		
15327840	m	md	US	2018	0	1	0	0	0	1	0	0	0	0	0	0		
15327842	f	cn	US	2018	0	1	0	0	0	1	0	0	0	0	0	0		
15327845	Not	md	US	2018	0	1	0	0	0	1	0	0	0	0	0	0		
15327862	f	cn	US	2018	0	0	0	0	0	0	0	0	0	0	0	0		
15327864	f	cn	US	2018	0	1	0	0	0	1	0	0	0	0	0	0		73
15327895	f	ot	US	2018	0	0	0	0	0	0	0	0	0	0	0	0		58
15327898	m	cn	US	2018	0	0	0	0	0	0	0	0	0	0	0	0		
15327933	f	cn	US	2018	0	0	0	0	0	0	0	0	0	0	0	0		
15328773	f	ph	FR	2018	0	1	0	1	0	0	0	0	0	0	0	0		64.1
15328804	f	md	JP	2018	1	0	0	0	0	0	0	0	0	0	0	0		84.4
15328806	m	md	JP	2018	0	1	0	0	0	1	0	0	0	0	0	0		65
15328828	f	ot	US	2018	0	1	0	0	1	0	0	0	0	0	0	0		71
15329081	m	ot	JP	2018	0	0	0	0	0	0	0	0	0	0	0	0		65
15329082	m	ot	JP	2018	0	0	0	0	0	0	0	0	0	0	0	0		67
15329085	m	ot	JP	2018	0	0	0	0	0	0	0	0	0	0	0	0		68
15329099	m	ot	JP	2018	0	0	0	0	0	0	0	0	0	0	0	0		60

15329220	f	ot	FR	2018	0	1	0	0	1	0	0	0	0	0	0	0	69
15329230	f	md	JP	2018	0	1	0	0	1	0	0	0	0	0	0	0	70
15329359	f	cn	US	2018	0	1	1	0	0	0	0	0	0	0	0	0	
15329670	m	md	US	2018	0	0	0	0	0	0	0	0	0	0	0	0	45
15329706	m	md	IE	2018	0	1	0	0	0	1	0	0	0	0	0	0	47
15330126	f	cn	US	2018	1	0	0	0	0	0	0	0	0	0	0	0	
15330592	Not	ot	JP	2018	0	0	0	0	0	0	0	0	0	0	0	0	
15330791	m	md	DE	2018	0	0	0	0	0	0	0	0	0	0	0	0	
15331343	f	ot	US	2018	0	1	0	0	1	0	0	0	0	0	0	0	78
15331476	Not	md	AT	2018	0	0	0	0	0	0	0	0	0	0	0	0	
15331805	m	cn	JP	2018	0	1	1	0	0	0	0	0	0	0	0	1	77
15331910	m	md	US	2018	0	1	0	0	0	1	0	0	0	0	0	0	70
15332040	m	md	JP	2018	0	0	0	0	0	0	0	0	0	0	0	0	50
15332271	f	cn	JP	2018	0	1	1	0	0	0	0	0	0	0	0	0	84
15332345	m	md	JP	2018	0	1	0	0	1	0	0	0	0	0	0	0	72
15332564	f	cn	US	2018	0	1	0	0	1	0	0	0	0	0	0	0	
15332657	f	md	JP	2018	0	0	0	0	0	0	0	0	0	0	0	0	80
15332975	m	ot	JP	2018	0	0	0	0	0	0	0	0	0	0	0	0	73
15333145	f	cn	US	2018	0	1	1	0	0	0	0	0	0	0	0	0	
15333611	m	cn	US	2018	1	0	0	0	0	0	0	0	0	0	0	0	75
15333627	m	ot	DE	2018	0	0	0	0	0	0	0	0	0	0	0	0	
15334852	f	ph	GB	2018	0	1	1	0	0	0	0	0	0	0	0	0	51
15335123	f	md	JP	2018	0	1	0	1	0	0	0	0	0	0	0	0	72
15335190	f	md	FR	2018	0	1	0	1	0	0	0	0	0	0	0	0	68.3
15335473	f	md	US	2018	0	1	1	0	0	0	0	0	0	0	0	0	61
15335499	f	Not repo	CA	2018	1	0	0	0	0	0	0	0	0	0	0	0	
15335582	m	ot	CN	2018	0	1	0	0	1	0	0	0	0	0	0	0	61
15335613	f	ot	CO	2018	0	0	0	0	0	0	0	0	0	0	0	0	76
15335710	f	md	JP	2018	0	0	0	0	0	0	0	0	0	0	0	0	
15335711	m	Not repo	JP	2018	0	0	0	0	0	0	0	0	0	0	0	0	33
15335913	f	ot	FR	2018	0	0	0	0	0	0	0	0	0	0	0	0	74
15335939	m	cn	JP	2018	0	1	1	0	0	0	0	0	0	0	0	0	68
15336658	m	ot	US	2018	1	0	0	0	0	0	0	0	0	0	0	0	55
15336803	m	ot	JP	2018	0	1	0	0	1	0	0	0	0	0	0	0	64

15336992	m	ot	JP	2018	0	0	0	0	0	0	0	0	0	0	0	0	66
15337021	m	ot	JP	2018	0	0	0	0	0	0	0	0	0	0	0	0	70
15337022	m	ot	JP	2018	0	0	0	0	0	0	0	0	0	0	0	0	62
15337194	m	cn	US	2018	0	1	1	0	0	0	0	0	0	0	0	0	
15337331	f	md	PL	2018	0	1	0	0	0	1	0	0	0	0	0	0	74
15337417	f	cn	US	2018	1	0	0	0	0	0	0	0	0	0	0	0	67
15337429	m	cn	KR	2018	0	1	1	0	0	0	0	0	0	0	0	0	
15337893	f	cn	US	2018	0	0	0	0	0	0	0	0	0	0	0	0	
15338380	m	md	JP	2018	0	1	0	0	1	0	0	0	0	0	0	0	52
15338446	Not	md	GR	2018	0	1	0	0	1	0	0	0	0	0	0	0	
15338887	m	md	NO	2018	0	1	0	1	0	0	0	0	0	0	0	0	
15339638	f	md	TR	2018	0	0	0	0	0	0	0	0	0	0	0	0	51
15340007	f	ot	US	2018	0	1	0	0	1	0	0	0	0	0	0	0	69
15340063	m	md	KR	2018	1	0	0	0	0	0	0	0	0	0	0	0	55.9
15340272	m	ot	CN	2018	0	1	0	0	1	0	0	0	0	0	0	1	68
15340362	f	ot	DE	2018	0	1	0	0	1	0	0	0	0	0	0	0	61
15340668	m	md	JP	2018	0	1	1	0	0	0	0	0	0	0	0	0	67
15341070	m	cn	US	2018	0	0	0	0	0	0	0	0	0	0	0	0	
15342022	f	md	US	2018	0	1	1	0	0	0	0	0	0	0	0	0	
15342165	m	md	TH	2018	0	0	0	0	0	0	0	0	0	0	0	0	67
15343132	m	ot	JP	2018	0	1	0	0	1	0	0	0	0	0	0	0	58
15343195	Not	md	CN	2018	0	0	0	0	0	0	0	0	0	0	0	0	
15343559	m	ot	US	2018	0	1	0	0	1	0	0	0	0	0	0	0	73
15343790	m	cn	US	2018	0	1	1	0	0	0	0	0	0	0	0	0	
15344366	m	cn	MX	2018	0	1	1	0	0	0	0	0	0	0	0	0	
15344615	f	md	NL	2018	0	1	0	0	1	0	0	0	0	0	0	1	72
15344628	m	cn	US	2018	1	0	0	0	0	0	0	0	0	0	0	0	
15344630	f	cn	US	2018	1	0	0	0	0	0	0	0	0	0	0	0	61.5
15344797	Not	Not repo	JP	2018	0	0	0	0	0	0	0	0	0	0	0	0	
15344837	f	ph	GB	2018	0	0	0	0	0	0	0	0	0	0	0	0	51
15345134	m	ot	DE	2018	0	0	0	0	0	0	0	0	0	0	0	0	8
15345172	f	md	FR	2018	0	0	0	0	0	0	0	0	0	0	0	0	59
15345211	m	md	IN	2018	0	0	0	0	0	0	0	0	0	0	0	0	
15345542	m	md	JP	2018	0	1	0	0	0	1	0	0	0	0	0	0	65

15345564	m	md	AU	2018	0	1	0	0	0	1	0	0	0	0	0	0	78
15345691	f	md	US	2018	0	1	1	0	0	0	0	0	0	0	0	0	
15345713	m	cn	JP	2018	0	1	1	0	0	0	0	0	0	0	0	0	73
15346009	f	md	DK	2018	0	1	1	0	0	0	0	0	0	0	0	0	56
15346097	m	ot	JP	2018	0	1	0	0	1	0	0	0	0	0	0	1	64
15346371	m	md	CN	2018	0	1	0	0	0	1	0	0	0	0	0	0	62
15346847	m	ot	AT	2018	0	0	0	0	0	0	0	0	0	0	0	0	72
15346875	m	ot	FR	2018	0	0	0	0	0	0	0	0	0	0	0	0	66
15347402	m	cn	US	2018	0	1	1	0	0	0	0	0	0	0	0	0	
15347502	m	cn	JP	2018	0	1	1	0	0	0	0	0	0	0	0	0	65
15348002	m	ot	IT	2018	0	1	0	0	1	0	0	0	0	0	0	0	50
15348080	f	md	JP	2018	0	0	0	0	0	0	0	0	0	0	0	0	
15348112	f	md	US	2018	0	1	0	1	0	0	0	0	0	0	0	1	
15348968	m	ot	JP	2018	0	1	0	0	1	0	0	0	0	0	0	0	69
15348988	f	md	FR	2018	0	0	0	0	0	0	0	0	0	0	0	0	60
15349213	m	md	US	2018	0	1	1	0	0	0	0	0	0	0	0	0	
15349239	f	md	US	2018	1	0	0	0	0	0	0	0	0	0	0	0	
15349526	f	ot	HU	2018	0	1	0	0	1	0	0	0	0	0	0	0	68
15349697	f	cn	US	2018	0	0	0	0	0	0	0	0	0	0	0	0	
15349869	m	ot	GB	2018	0	0	0	0	0	0	0	0	0	0	0	0	57
15349980	m	Not repo	JP	2018	0	0	0	0	0	0	0	0	0	0	0	0	64
15350237	m	ot	NL	2018	0	0	0	0	0	0	0	0	0	0	0	0	63
15350600	m	md	GB	2018	0	0	0	0	0	0	0	0	0	0	0	0	57
15350619	m	ot	FR	2018	0	1	0	0	1	0	0	0	0	0	0	0	68
15350956	f	Not repo	US	2018	0	1	0	1	0	0	0	0	0	0	0	0	85
15351010	Not	md	AU	2018	0	1	0	0	1	0	0	0	0	0	0	0	
15351467	f	md	BE	2018	0	1	1	0	0	0	0	0	0	0	0	0	66
15351919	m	md	JP	2018	1	0	0	0	0	0	0	0	0	0	0	0	73
15352162	f	cn	FR	2018	0	0	0	0	0	0	0	0	0	0	0	0	69
15352163	f	cn	FR	2018	0	0	0	0	0	0	0	0	0	0	0	0	50
15352529	m	ot	DE	2018	0	0	0	0	0	0	0	0	0	0	0	0	
15352536	f	ot	DE	2018	0	0	0	0	0	0	0	0	0	0	0	0	66
15352961	m	md	JP	2018	0	1	0	1	0	0	0	0	0	0	0	0	81
15353907	f	md	US	2018	0	1	0	1	0	0	0	0	0	0	0	1	67

15354193	f	ot	GB	2018	0	0	0	0	0	0	0	0	0	0	0	0	81
15354238	m	md	JP	2018	0	1	0	0	1	0	0	0	0	0	0	1	71
15354402	m	ot	FR	2018	0	1	0	0	1	0	0	0	0	0	0	0	53
15354520	Not	md	JP	2018	0	1	0	0	0	1	0	0	0	0	0	1	80
15354727	m	ot	US	2018	0	1	1	0	0	0	0	0	0	0	0	0	30
15355005	m	md	JP	2018	0	0	0	0	0	0	0	0	0	0	0	0	
15355327	m	md	MY	2018	0	1	0	0	0	1	0	0	0	0	0	0	59
15355411	m	ot	US	2018	0	1	0	0	1	0	0	0	0	0	0	0	
15355515	m	md	JP	2018	0	1	0	0	0	1	0	0	0	0	0	0	70
15355790	f	md	JP	2018	1	0	0	0	0	0	0	0	0	0	0	0	88
15356174	f	md	IN	2018	1	0	0	0	0	0	0	0	0	0	0	0	78
15356353	m	md	JP	2018	1	0	0	0	0	0	0	0	0	0	0	0	76
15356497	f	md	JP	2018	1	0	0	0	0	0	0	0	0	0	0	0	60
15356888	m	md	JP	2018	1	0	0	0	0	0	0	0	0	0	0	0	77
15356923	m	md	DE	2018	0	0	0	0	0	0	0	0	0	0	0	0	81
15357087	f	md	JP	2018	0	0	0	0	0	0	0	0	0	0	0	0	44
15357402	m	md	JP	2018	1	0	0	0	0	0	0	0	0	0	0	1	70
15357438	m	ot	JP	2018	1	0	0	0	0	0	0	0	0	0	0	0	79
15357488	f	ot	RO	2018	0	1	0	0	1	0	0	0	0	0	0	0	
15357641	f	ph	US	2018	0	0	0	0	0	0	0	0	0	0	0	0	68
15357738	f	md	DE	2018	1	0	0	0	0	0	0	0	0	0	0	0	64
15357771	m	md	CA	2018	0	0	0	0	0	0	0	0	0	0	0	0	55
15357876	f	md	JP	2018	1	0	0	0	0	0	0	0	0	0	0	1	84.5
15357915	m	ot	ES	2018	0	1	0	0	1	0	0	0	0	0	0	0	76
15357963	m	ot	DE	2018	0	0	0	0	0	0	0	0	0	0	0	0	78
15358048	f	ot	JP	2018	0	0	0	0	0	0	0	0	0	0	0	0	44
15358127	f	ph	US	2018	0	0	0	0	0	0	0	0	0	0	0	0	68
15358466	f	cn	DE	2018	0	1	1	0	0	0	0	0	0	0	0	0	
15359028	f	md	BE	2018	1	0	0	0	0	0	0	0	0	0	0	0	67
15359048	m	ot	RO	2018	0	1	0	0	1	0	0	0	0	0	0	0	60
15359700	m	md	JP	2018	0	1	1	0	0	0	0	0	0	0	0	1	56
15359905	m	md	TH	2018	0	1	0	1	0	0	0	0	0	0	0	0	62.2
15360549	m	md	DE	2018	0	1	0	0	1	0	0	0	0	0	0	0	64
15360919	Not	md	US	2018	0	1	0	0	1	0	0	0	0	0	0	0	

15361101	m	cn	GB	2018	1	0	0	0	0	0	0	0	0	0	0	0	
15361109	m	md	BR	2018	0	1	0	0	0	1	0	0	0	0	0	0	61
15361237	f	ot	FR	2018	0	0	0	0	0	0	0	0	0	0	0	0	77
15361549	m	md	ES	2018	1	0	0	0	0	0	0	0	0	0	0	0	59
15361665	m	ot	DE	2018	0	0	0	0	0	0	0	0	0	0	0	0	78
15361676	m	ot	US	2018	0	1	1	0	0	0	0	0	0	0	0	0	58
15361706	m	md	EG	2018	0	1	0	0	0	1	0	0	0	0	0	0	55
15362056	Not	ph	JP	2018	1	0	0	0	0	0	0	0	0	0	0	0	
15362283	f	ph	CO	2018	0	0	0	0	0	0	0	0	0	0	0	0	
15362356	m	ot	US	2018	0	1	0	0	1	0	0	0	0	0	0	0	78
15362389	m	ot	FR	2018	0	1	0	0	1	0	0	0	0	0	0	0	79
15362391	m	cn	FR	2018	0	0	0	0	0	0	0	0	0	0	0	0	59
15362420	m	ot	US	2018	0	0	0	0	0	0	0	0	0	0	0	0	61
15362551	f	ot	CL	2018	0	1	0	0	1	0	0	0	0	0	0	0	76
15363177	f	cn	IN	2018	0	0	0	0	0	0	0	0	0	0	0	0	65
15363209	m	cn	JP	2018	0	0	0	0	0	0	0	0	0	0	0	0	67
15363507	m	md	DE	2018	0	0	0	0	0	0	0	0	0	0	0	0	
15364683	m	md	JP	2018	0	1	0	0	1	0	0	0	0	0	0	0	62
15364698	m	md	DE	2018	0	0	0	0	0	0	0	0	0	0	0	0	70
15364722	m	md	IL	2018	0	0	0	0	0	0	0	0	0	0	0	0	75
15364896	m	ot	FR	2018	0	1	0	0	1	0	0	0	0	0	0	0	
15365226	m	md	DE	2018	0	0	0	0	0	0	0	0	0	0	0	0	
15365581	f	cn	US	2018	0	1	1	0	0	0	0	0	0	0	0	0	87
15365751	m	ot	FR	2018	0	0	0	0	0	0	0	0	0	0	0	0	66
15365965	m	ot	GR	2018	0	1	0	0	1	0	0	0	0	0	0	0	62
15365991	f	md	DE	2018	0	1	0	1	0	0	0	0	0	0	0	0	65
15366112	m	ot	FR	2018	0	1	0	0	1	0	0	0	0	0	0	0	70
15366706	m	ph	US	2018	0	1	1	0	0	0	0	0	0	0	0	0	55
15366812	f	cn	US	2018	1	0	0	0	0	0	0	0	0	0	0	0	80
15366845	m	md	ES	2018	0	1	0	0	1	0	0	0	0	0	0	0	76
15367078	m	ph	CA	2018	0	1	0	0	1	0	0	0	0	0	0	1	67
15367381	m	md	FR	2018	0	1	0	0	0	1	0	0	0	0	0	0	64
15367500	m	md	IN	2018	0	0	0	0	0	0	0	0	0	0	0	0	56
15367705	f	ot	FR	2018	0	0	0	0	0	0	0	0	0	0	0	0	85

15367727	m	ot	FR	2018	0	0	0	0	0	0	0	0	0	0	0	0	59
15367817	m	cn	US	2018	0	0	0	0	0	0	0	0	0	0	0	0	52
15368021	Not	ph	JP	2018	0	1	0	0	1	0	0	0	0	0	0	0	
15368481	m	ot	CN	2018	0	1	0	0	1	0	0	0	0	0	0	0	65
15368497	m	md	GB	2018	0	0	0	0	0	0	0	0	0	0	0	0	57
15368838	f	ot	US	2018	0	1	0	0	1	0	0	0	0	0	0	0	57
15370010	f	ph	FR	2018	0	0	0	0	0	0	0	0	0	0	0	0	45
15370015	f	md	FR	2018	0	0	0	0	0	0	0	0	0	0	0	0	61
15370096	Not	md	JP	2018	1	0	0	0	0	0	0	0	0	0	0	0	
15370101	f	md	JP	2018	1	0	0	0	0	0	0	0	0	0	0	0	84.4
15370217	f	cn	US	2018	1	0	0	0	0	0	0	0	0	0	0	0	
15370252	m	ot	JP	2018	0	1	1	0	0	0	0	0	0	0	0	0	69
15370603	f	cn	FR	2018	0	1	1	0	0	0	0	0	0	0	0	0	
15370709	Not	md	JP	2018	0	1	0	0	1	0	0	0	0	0	0	0	
15370877	f	cn	FR	2018	0	0	0	0	0	0	0	0	0	0	0	0	77
15370970	Not	md	US	2018	0	1	0	1	0	0	0	0	0	0	0	1	
15371342	f	ot	DE	2018	0	0	0	0	0	0	0	0	0	0	0	0	75
15371599	m	md	JP	2018	0	1	0	0	1	0	0	0	0	0	0	0	75
15371618	m	ot	FR	2018	0	0	0	0	0	0	0	0	0	0	0	0	66
15371621	m	ot	FR	2018	0	0	0	0	0	0	0	0	0	0	0	0	76
15371697	f	Not repo	ID	2018	1	0	0	0	0	0	0	0	0	0	0	0	
15371877	m	ot	DE	2018	0	1	0	0	1	0	0	0	0	0	0	0	60
15371927	f	ot	DE	2018	0	1	0	0	1	0	0	0	0	0	0	0	58
15371928	m	ot	DE	2018	0	1	0	0	1	0	0	0	0	0	0	0	51
15371929	m	ot	DE	2018	0	1	0	0	1	0	0	0	0	0	0	0	65
15372104	m	cn	JP	2018	0	1	1	0	0	0	0	0	0	0	0	1	72
15372349	m	cn	US	2018	0	1	1	0	0	0	0	0	0	0	0	0	65
15372451	f	cn	MX	2018	0	1	1	0	0	0	0	0	0	0	0	1	68
15372675	Not	md	US	2018	0	1	0	0	1	0	0	0	0	0	0	0	
15372714	Not	ot	US	2018	0	1	0	0	0	1	0	0	0	0	0	0	
15372917	m	md	JP	2018	0	1	0	0	1	0	0	0	0	0	0	0	67
15373112	Not	md	US	2018	0	1	0	0	1	0	0	0	0	0	0	1	
15373298	m	md	US	2018	0	1	0	1	0	0	0	0	0	0	0	0	
15373339	f	md	DE	2018	0	1	0	0	0	1	0	0	0	0	0	0	61

15374303	m	ph	US	2018	0	1	0	0	1	0	0	0	0	0	0	0	
15374390	m	md	JP	2018	0	0	0	0	0	0	0	0	0	0	0	0	80
15374565	m	cn	JP	2018	0	0	0	0	0	0	0	0	0	0	0	0	75
15374613	m	md	GB	2018	0	0	0	0	0	0	0	0	0	0	0	0	57
15374712	Not	ot	JP	2018	0	1	0	0	1	0	0	0	0	0	0	0	
15374751	Not	md	US	2018	0	1	0	0	1	0	0	0	0	0	0	0	
15374753	Not	md	US	2018	0	1	0	0	1	0	0	0	0	0	0	0	
15374754	m	md	JP	2018	0	1	0	0	1	0	0	0	0	0	0	0	73
15374908	Not	md	US	2018	0	1	0	0	1	0	0	0	0	0	0	0	
15374925	m	ot	JP	2018	0	1	0	1	0	0	0	0	0	0	0	0	69
15375092	m	ot	RO	2018	0	1	0	0	1	0	0	0	0	0	0	0	
15375093	m	md	JP	2018	0	1	0	0	1	0	0	0	0	0	0	0	66
15375096	m	ot	CN	2018	0	0	0	0	0	0	0	0	0	0	0	0	48
15375295	f	md	EG	2018	0	0	0	0	0	0	0	0	0	0	0	0	43
15375476	f	md	US	2018	0	1	0	1	0	0	0	0	0	0	0	0	
15375587	m	md	JP	2018	1	0	0	0	0	0	0	0	0	0	0	1	74
15375772	m	md	SG	2018	0	1	0	1	0	0	0	0	0	0	0	0	59.6
15375912	m	md	JP	2018	0	1	0	0	1	0	0	0	0	0	0	0	68
15376239	m	ot	CN	2018	0	0	0	0	0	0	0	0	0	0	0	0	49
15376373	m	ot	CN	2018	0	1	0	0	1	0	0	0	0	0	0	1	62
15376410	f	md	DE	2018	0	0	0	0	0	0	0	0	0	0	0	0	58
15376479	f	cn	CH	2018	0	0	0	0	0	0	0	0	0	0	0	0	
15377084	m	cn	JP	2018	0	1	1	0	0	0	0	0	0	0	0	0	74
15377935	Not	ph	JP	2018	0	1	0	0	1	0	0	0	0	0	0	0	
15377993	f	md	JP	2018	0	1	0	0	1	0	0	0	0	0	0	0	70
15378020	m	md	JP	2018	1	0	0	0	0	0	0	0	0	0	0	0	75
15378104	m	md	GB	2018	0	0	0	0	0	0	0	0	0	0	0	0	64
15378391	m	cn	IT	2018	0	1	1	0	0	0	0	0	0	0	0	0	61
15380881	f	ot	CN	2018	0	1	0	0	1	0	0	0	0	0	0	1	54
15382533	m	ot	BE	2018	0	1	0	0	1	0	0	0	0	0	0	0	65
15383822	m	ot	CN	2018	0	1	0	0	1	0	0	0	0	0	0	0	59
15384065	m	md	IN	2018	0	1	0	0	0	1	0	0	0	0	0	0	45
15384180	m	md	PH	2018	0	1	0	0	0	1	0	0	0	0	0	1	44
15384247	m	ot	FR	2018	0	1	0	0	1	0	0	0	0	0	0	0	75

15384395	m	md	JP	2018	0	1	0	0	1	0	0	0	0	0	0	1	64
15384442	f	cn	DE	2018	0	1	1	0	0	0	0	0	0	0	0	0	
15384622	f	cn	US	2018	1	0	0	0	0	0	0	0	0	0	0	0	
15384780	f	md	HK	2018	0	0	0	0	0	0	0	0	0	0	0	0	51
15384800	f	cn	JP	2018	0	1	1	0	0	0	0	0	0	0	0	0	75
15384853	f	cn	US	2018	0	1	0	0	1	0	0	0	0	0	0	0	
15385104	f	cn	GB	2018	0	0	0	0	0	0	0	0	0	0	0	0	80
15385448	f	cn	US	2018	0	0	0	0	0	0	0	0	0	0	0	0	74
15385725	m	cn	JP	2018	0	0	0	0	0	0	0	0	0	0	0	0	70
15386156	m	ph	FR	2018	0	1	0	1	0	0	0	0	0	0	0	0	59.9
15386218	m	ot	TW	2018	0	1	0	0	1	0	0	0	0	0	0	0	61
15386548	m	md	JP	2018	1	0	0	0	0	0	0	0	0	0	0	1	78.9
15386635	m	ot	CN	2018	0	1	0	0	1	0	0	0	0	0	0	0	53
15386735	m	md	JP	2018	0	1	0	0	1	0	0	0	0	0	0	0	57
15386904	m	cn	ID	2018	1	0	0	0	0	0	0	0	0	0	0	0	
15386911	f	cn	US	2018	0	0	0	0	0	0	0	0	0	0	0	0	53
15387325	f	cn	US	2018	1	0	0	0	0	0	0	0	0	0	0	0	76
15387634	m	cn	JP	2018	0	1	1	0	0	0	0	0	0	0	0	0	56
15387872	f	cn	US	2018	0	0	0	0	0	0	0	0	0	0	0	0	
15389030	m	md	US	2018	0	1	1	0	0	0	0	0	0	0	0	0	73
15389109	f	md	UA	2018	0	1	0	0	0	1	0	0	0	0	0	0	
15389661	m	cn	US	2018	0	1	1	0	0	0	0	0	0	0	0	0	
15390009	m	md	DE	2018	0	0	0	0	0	0	0	0	0	0	0	0	72
15390017	m	cn	US	2018	0	1	0	1	0	0	0	0	0	0	0	1	
15390137	f	ot	TW	2018	0	1	0	0	1	0	0	0	0	0	0	0	60
15390209	m	md	IN	2018	0	0	0	0	0	0	0	0	0	0	0	0	47
15390218	m	cn	IT	2018	0	1	1	0	0	0	0	0	0	0	0	0	76
15390228	f	md	IN	2018	0	0	0	0	0	0	0	0	0	0	0	0	53
15390249	f	cn	IT	2018	0	1	1	0	0	0	0	0	0	0	0	0	84
15390624	f	md	PL	2018	0	1	0	0	0	1	0	0	0	0	0	0	69
15392019	m	ph	US	2018	0	1	0	0	0	1	0	0	0	0	0	0	75
15392935	m	ot	JP	2018	0	1	0	0	1	0	0	0	0	0	0	0	69
15392969	f	md	CN	2018	0	1	0	1	0	0	0	0	0	0	0	0	51.9
15393907	Not	md	US	2018	1	0	0	0	0	0	0	0	0	0	0	0	

15394052	f	ot	FR	2018	0	1	0	0	1	0	0	0	0	0	0	0	54
15394137	m	md	US	2018	0	1	0	0	1	0	0	0	0	0	0	0	75
15394273	m	cn	JP	2018	0	1	0	0	1	0	0	0	0	0	0	1	83
15394316	m	ph	JP	2018	0	1	0	0	1	0	0	0	0	0	0	0	69
15394491	m	md	JP	2018	0	1	0	0	1	0	0	0	0	0	0	0	74
15394565	f	md	TH	2018	0	0	0	0	0	0	0	0	0	0	0	0	
15394775	f	ot	US	2018	0	0	0	0	0	0	0	0	0	0	0	0	44
15394841	m	md	BE	2018	0	1	0	0	0	1	0	0	0	0	0	0	55
15394985	m	cn	ES	2018	0	1	1	0	0	0	0	0	0	0	0	0	80
15395003	m	ph	FR	2018	0	1	0	1	0	0	0	0	0	0	0	0	66.8
15395273	f	cn	GB	2018	1	0	0	0	0	0	0	0	0	0	0	0	90
15395811	Not	ot	JP	2018	1	0	0	0	0	0	0	0	0	0	0	0	
15396079	m	md	JP	2018	1	0	0	0	0	0	0	0	0	0	0	0	79
15396713	m	md	GB	2018	0	1	1	0	0	0	0	0	0	0	0	0	85
15397209	m	ot	FR	2018	0	1	0	0	1	0	0	0	0	0	0	0	65
15397237	m	md	CN	2018	0	1	0	0	0	1	0	0	0	0	0	0	75
15397295	f	cn	PL	2018	0	1	1	0	0	0	0	0	0	0	0	0	
15397350	m	ot	DE	2018	0	0	0	0	0	0	0	0	0	0	0	0	77
15397965	m	md	FR	2018	0	1	0	1	0	0	0	0	0	0	0	0	64
15398077	f	md	DE	2018	0	0	0	0	0	0	0	0	0	0	0	0	44
15398092	m	cn	US	2018	0	1	1	0	0	0	0	0	0	0	0	0	
15398382	m	ot	US	2018	0	1	0	0	1	0	0	0	0	0	0	0	
15398554	m	Not repo	JP	2018	0	0	0	0	0	0	0	0	0	0	0	0	
15399649	Not	md	JP	2018	0	1	0	1	0	0	0	0	0	0	0	1	55
15399665	m	cn	JP	2018	0	1	1	0	0	0	0	0	0	0	0	1	80
15399746	m	cn	JP	2018	0	1	1	0	0	0	0	0	0	0	0	0	67
15399808	m	ot	DE	2018	0	1	0	0	1	0	0	0	0	0	0	0	65
15400298	f	ot	AR	2018	0	1	0	0	1	0	0	0	0	0	0	0	60
15400458	m	md	LB	2018	0	1	0	0	1	0	0	0	0	0	0	0	80
15400459	m	md	LB	2018	0	1	0	0	1	0	0	0	0	0	0	0	
15400910	m	md	US	2018	0	1	0	0	0	1	0	0	0	0	0	0	60
15401645	f	ph	US	2018	1	0	0	0	0	0	0	0	0	0	0	1	75
15401657	m	md	BE	2018	0	0	0	0	0	0	0	0	0	0	0	0	
15401947	Not	cn	US	2018	0	0	0	0	0	0	0	0	0	0	0	0	

15401996	m	md	US	2018	0	1	0	1	0	0	0	0	0	0	0	1	
15402099	m	md	EE	2018	0	1	1	0	0	0	0	0	0	0	0	0	57
15402907	m	cn	JP	2018	0	0	0	0	0	0	0	0	0	0	0	0	
15403260	m	ot	CN	2018	0	1	0	0	1	0	0	0	0	0	0	0	60
15403644	m	ot	FR	2018	0	0	0	0	0	0	0	0	0	0	0	0	59
15403690	f	md	FR	2018	0	0	0	0	0	0	0	0	0	0	0	0	71
15403780	m	md	GB	2018	0	1	0	1	0	0	0	0	0	0	0	0	68.4
15403873	m	cn	US	2018	0	1	0	0	1	0	0	0	0	0	0	0	69
15403875	m	cn	JP	2018	0	1	1	0	0	0	0	0	0	0	0	0	
15404168	m	Not repo	JP	2018	0	0	0	0	0	0	0	0	0	0	0	0	
15404357	m	ot	CN	2018	1	0	0	0	0	0	0	0	0	0	0	0	
15404599	m	cn	US	2018	0	1	1	0	0	0	0	0	0	0	0	0	
15404692	f	ot	US	2018	0	0	0	0	0	0	0	0	0	0	0	0	
15404710	Not	md	NL	2018	0	1	0	0	1	0	0	0	0	0	0	0	
15404792	m	md	FR	2018	0	0	0	0	0	0	0	0	0	0	0	0	51
15405507	m	md	JP	2018	0	1	0	0	0	1	0	0	0	0	0	0	68
15405527	Not	md	GB	2018	0	1	0	0	1	0	0	0	0	0	0	0	
15405615	m	ot	JP	2018	0	1	0	0	1	0	0	0	0	0	0	0	80
15405724	Not	md	GB	2018	0	1	0	0	1	0	0	0	0	0	0	0	
15405760	f	md	CA	2018	0	1	0	0	0	1	0	0	0	0	0	0	69
15405913	m	md	CN	2018	0	1	0	1	0	0	0	0	0	0	0	0	58.5
15406002	f	ot	US	2018	0	0	0	0	0	0	0	0	0	0	0	0	58
15406399	f	md	AT	2018	0	1	0	0	0	1	0	0	0	0	0	0	74
15406421	Not	md	US	2018	1	0	0	0	0	0	0	0	0	0	0	0	
15407170	m	cn	US	2018	0	1	0	0	1	0	0	0	0	0	0	0	
15407377	f	cn	US	2018	0	0	0	0	0	0	0	0	0	0	0	0	78
15408357	m	md	JP	2018	0	1	0	1	0	0	0	0	0	0	0	1	62
15408636	m	cn	IT	2018	0	1	0	0	1	0	0	0	0	0	0	0	64
15408638	f	cn	US	2018	0	0	0	0	0	0	0	0	0	0	0	0	58
15408639	m	cn	FR	2018	0	0	0	0	0	0	0	0	0	0	0	0	75
15408766	f	md	JP	2018	1	0	0	0	0	0	0	0	0	0	0	0	75.1
15408804	f	md	CH	2018	0	0	0	0	0	0	0	0	0	0	0	0	49
15408840	Not	md	JP	2018	1	0	0	0	0	0	0	0	0	0	0	1	
15408899	m	md	CN	2018	0	1	0	1	0	0	0	0	0	0	0	0	65

15408957	m	md	JP	2018	0	1	0	1	0	0	0	0	0	0	0	1	71.8
15409865	f	ot	US	2018	0	1	0	0	1	0	0	0	0	0	0	0	61
15410096	m	md	ID	2018	1	0	0	0	0	0	0	0	0	0	0	0	52
15410117	Not	ph	US	2018	0	1	0	0	1	0	0	0	0	0	0	0	
15410181	m	md	SG	2018	0	0	0	0	0	0	1	0	0	0	1	0	
15410442	m	md	JP	2018	1	0	0	0	0	0	0	0	0	0	0	0	74
15410583	m	ph	US	2018	0	1	0	0	1	0	0	0	0	0	0	0	65
15410646	m	ot	JP	2018	0	1	0	0	1	0	0	0	0	0	0	0	64
15410782	f	cn	US	2018	0	1	1	0	0	0	0	0	0	0	0	0	86
15410913	m	md	JP	2018	1	0	0	0	0	0	0	0	0	0	0	0	77
15411016	f	cn	US	2018	1	0	0	0	0	0	0	0	0	0	0	0	87
15412227	m	cn	JP	2018	0	1	1	0	0	0	0	0	0	0	0	0	81
15412585	f	cn	US	2018	0	0	0	0	0	0	0	0	0	0	0	0	53
15412675	m	ot	DE	2018	0	0	0	0	0	0	0	0	0	0	0	0	56
15412698	Not	ot	DE	2018	0	0	0	0	0	0	0	0	0	0	0	0	76
15412765	f	cn	Not re	2018	0	0	0	0	0	0	0	0	0	0	0	0	55
15413071	f	cn	US	2018	0	0	0	0	0	0	0	0	0	0	0	0	67
15413253	m	ot	DE	2018	0	1	0	0	1	0	0	0	0	0	0	0	82
15413527	m	ot	DE	2018	0	0	0	0	0	0	0	0	0	0	0	0	77
15413766	m	cn	US	2018	0	0	0	0	0	0	0	0	0	0	0	0	62
15413952	Not	cn	US	2018	0	0	0	0	0	0	0	0	0	0	0	0	
15413965	f	md	US	2018	1	0	0	0	0	0	0	0	0	0	0	0	40
15414096	m	cn	JP	2018	0	1	1	0	0	0	0	0	0	0	0	1	
15414290	f	md	JP	2018	1	0	0	0	0	0	0	0	0	0	0	1	79
15414500	m	ot	US	2018	1	0	0	0	0	0	0	0	0	0	0	0	
15414822	m	ph	US	2018	0	1	0	1	0	0	0	0	0	0	0	0	53.1
15415503	f	ph	US	2018	0	1	0	0	1	0	0	0	0	0	0	0	60
15416504	f	cn	US	2018	0	1	0	0	1	0	0	0	0	0	0	0	
15416570	f	cn	TW	2018	0	1	0	0	0	1	0	0	0	0	0	0	
15416649	m	cn	US	2018	0	1	1	0	0	0	0	0	0	0	0	0	
15416734	f	md	IE	2018	0	0	0	0	0	0	0	0	0	0	0	0	76
15416871	m	cn	JP	2018	0	1	1	0	0	0	0	0	0	0	0	1	58
15417326	f	ph	US	2018	0	1	0	0	1	0	0	0	0	0	0	0	
15417848	m	cn	US	2018	0	1	1	0	0	0	0	0	0	0	0	0	

15417849	f	cn	PL	2018	0	1	1	0	0	0	0	0	0	0	0	0	
15419017	Not	cn	JP	2018	0	0	0	0	0	0	0	0	0	0	0	0	70
15419154	m	cn	GB	2018	1	0	0	0	0	0	0	0	0	0	0	0	
15419313	f	cn	US	2018	0	0	0	0	0	0	0	0	0	0	0	0	64
15419759	m	cn	IE	2018	0	1	1	0	0	0	0	0	0	0	0	0	63
15419808	m	ot	DE	2018	0	0	0	0	0	0	0	0	0	0	0	0	81
15420064	m	md	US	2018	0	1	0	1	0	0	0	0	0	0	0	0	54
15421077	m	ot	ES	2018	0	1	0	0	1	0	0	0	0	0	0	0	62
15422300	m	md	US	2018	0	1	0	0	1	0	0	0	0	0	0	0	
15422606	m	md	US	2018	0	1	1	0	0	0	0	0	0	0	0	0	83
15423037	f	cn	ID	2018	1	0	0	0	0	0	0	0	0	0	0	0	73.7
15423582	f	ot	US	2018	0	1	0	0	1	0	0	0	0	0	0	0	51
15423983	m	cn	US	2018	0	1	1	0	0	0	0	0	0	0	0	0	72
15424118	m	cn	CA	2018	0	0	0	0	0	0	0	0	0	0	0	0	58
15424430	m	cn	GB	2018	1	0	0	0	0	0	0	0	0	0	0	0	69
15425675	m	cn	MX	2018	0	1	1	0	0	0	0	0	0	0	0	0	76
15425715	m	md	DE	2018	0	0	0	0	0	0	0	0	0	0	0	0	81
15425980	m	ot	DE	2018	0	0	0	0	0	0	0	0	0	0	0	0	72
15426114	m	cn	DE	2018	0	0	0	0	0	0	0	0	0	0	0	0	71
15426293	m	md	JP	2018	0	0	0	0	0	0	0	0	0	0	0	0	69
15426313	m	md	TR	2018	0	0	0	0	0	0	0	0	0	0	0	0	65
15426735	f	md	SI	2018	0	1	0	1	0	0	0	0	0	0	0	0	
15426957	m	cn	US	2018	0	0	0	0	0	0	0	0	0	0	0	0	53
15427119	m	ot	JP	2018	0	1	0	0	1	0	0	0	0	0	0	0	65
15427817	m	ot	NL	2018	0	1	0	0	1	0	0	0	0	0	0	0	64
15428337	m	ph	FR	2018	0	1	0	1	0	0	0	0	0	0	0	0	76.1
15429090	m	md	KR	2018	1	0	0	0	0	0	0	0	0	0	0	0	87
15429712	m	cn	JP	2018	0	1	1	0	0	0	0	0	0	0	0	0	77
15429899	Not	ot	DK	2018	0	1	0	0	1	0	0	0	0	0	0	0	
15429993	m	ot	DK	2018	0	1	0	0	1	0	0	0	0	0	0	0	56
15430139	f	md	JP	2018	1	0	0	0	0	0	0	0	0	0	0	0	70
15430426	m	cn	US	2018	0	1	1	0	0	0	0	0	0	0	0	0	
15430748	m	cn	JP	2018	0	1	0	0	1	0	0	0	0	0	0	0	68
15430812	f	md	US	2018	0	0	0	0	0	0	0	0	0	0	0	0	45

15430814	f	cn	US	2018	1	0	0	0	0	0	0	0	0	0	0	0	68
15430865	Not	ot	CN	2018	0	0	0	0	0	0	0	0	0	0	0	0	
15431177	m	md	TR	2018	0	0	0	0	0	0	0	0	0	0	0	0	51
15431281	f	ot	JP	2018	0	1	0	0	1	0	0	0	0	0	0	0	69
15431313	f	md	TR	2018	0	0	0	0	0	0	0	0	0	0	0	0	54
15431348	f	md	TR	2018	0	0	0	0	0	0	0	0	0	0	0	0	70
15431377	m	md	TR	2018	0	0	0	0	0	0	0	0	0	0	0	0	56
15431466	m	md	TR	2018	0	0	0	0	0	0	0	0	0	0	0	0	66
15431832	m	cn	US	2018	0	0	0	0	0	0	0	0	0	0	0	0	67
15432236	m	cn	PL	2018	0	1	1	0	0	0	0	0	0	0	0	0	
15432259	m	md	US	2018	0	0	0	0	0	0	0	0	0	0	0	0	
15432453	f	md	JP	2018	0	1	0	0	1	0	0	0	0	0	0	0	80
15432464	m	md	US	2018	0	1	0	0	1	0	0	0	0	0	0	0	
15432835	f	md	RO	2018	0	1	0	0	1	0	0	0	0	0	0	0	
15433248	m	ot	US	2018	0	1	0	0	1	0	0	0	0	0	0	0	
15433407	m	cn	ID	2018	1	0	0	0	0	0	0	0	0	0	0	0	38
15433416	f	md	AU	2018	0	0	0	0	0	0	0	0	0	0	0	0	
15433494	f	md	GB	2018	0	1	0	0	0	1	0	0	0	0	0	0	60
15433499	f	md	AU	2018	0	1	0	0	0	1	0	0	0	0	0	0	61
15433563	f	ph	US	2018	0	1	0	0	0	1	0	0	0	0	0	0	
15433769	f	md	JP	2018	0	0	0	0	0	0	0	0	0	0	0	0	74
15434469	m	md	JP	2018	0	0	0	0	0	0	0	0	0	0	0	0	48
15434891	f	ph	US	2018	1	0	0	0	0	0	0	0	0	0	0	0	63
15434898	m	ot	CN	2018	0	1	0	0	1	0	0	0	0	0	0	1	55
15435130	m	md	JP	2018	0	1	0	0	0	1	0	0	0	0	0	0	73
15435585	m	ot	DE	2018	0	1	0	0	1	0	0	0	0	0	0	0	
15435934	m	md	JP	2018	0	1	0	0	1	0	0	0	0	0	0	0	53
15435988	m	ot	JP	2018	0	1	0	0	1	0	0	0	0	0	0	1	79
15436578	m	ot	US	2018	0	0	0	0	0	0	0	0	0	0	0	0	70
15437393	f	ot	US	2018	0	0	0	0	0	0	0	0	0	0	0	0	63
15437764	m	md	JP	2018	0	1	0	0	1	0	0	0	0	0	0	1	65
15438749	f	cn	IN	2018	0	1	0	0	1	0	0	0	0	0	0	0	
15438758	f	Not repo	ID	2018	1	0	0	0	0	0	0	0	0	0	0	0	
15438768	m	ot	JP	2018	0	1	0	0	1	0	0	0	0	0	0	0	70

15438847	m	ot	AU	2018	0	0	0	0	0	0	0	0	0	0	0	0	50
15438917	f	cn	US	2018	0	1	1	0	0	0	0	0	0	0	0	0	84
15438960	f	md	JP	2018	1	0	0	0	0	0	0	0	0	0	0	0	54
15439364	Not	md	JP	2018	1	0	0	0	0	0	0	0	0	0	0	0	
15439530	m	md	JP	2018	0	0	0	0	0	0	0	0	0	0	0	0	67
15439564	f	cn	US	2018	0	1	1	0	0	0	0	0	0	0	0	0	
15439599	m	md	JP	2018	1	0	0	0	0	0	0	0	0	0	0	0	80
15439704	m	ph	JP	2018	0	1	1	0	0	0	0	0	0	0	0	0	77
15439709	m	ot	US	2018	0	1	0	0	1	0	0	0	0	0	0	0	79
15439765	f	ot	CA	2018	1	0	0	0	0	0	0	0	0	0	0	0	60
15439896	f	ot	US	2018	1	0	0	0	0	0	0	0	0	0	0	0	
15439922	m	cn	JP	2018	0	1	1	0	0	0	0	0	0	0	0	0	80
15439955	m	md	ES	2018	0	1	0	0	0	1	0	0	0	0	0	1	64
15440004	m	cn	CN	2018	0	1	0	0	1	0	0	0	0	0	0	0	74
15440009	f	md	JP	2018	0	1	0	0	0	1	0	0	0	0	0	0	80
15440062	f	ot	US	2018	0	1	0	0	1	0	0	0	0	0	0	0	79
15440188	m	ot	DE	2018	0	1	0	0	1	0	0	0	0	0	0	0	85
15440498	m	Not repo	JP	2018	0	0	0	0	0	0	0	0	0	0	0	0	41
15440699	m	ot	ES	2018	0	1	0	0	1	0	0	0	0	0	0	0	64
15440865	f	ph	US	2018	0	0	0	0	0	0	0	0	0	0	0	0	92
15441083	m	md	IN	2018	0	0	0	0	0	0	0	0	0	0	0	0	56
15442900	m	ot	IT	2018	0	0	0	0	0	0	0	0	0	0	0	0	72
10417696	f	md	AT	2018	0	0	0	0	0	0	0	0	0	0	0	0	51
14618876	m	md	JP	2018	0	1	0	0	1	0	0	0	0	0	0	0	67
14626552	m	ot	DE	2018	0	1	0	0	1	0	0	0	0	0	0	0	63
14971866	m	md	JP	2018	0	1	0	0	1	0	0	0	0	0	0	0	45
15003871	m	md	JP	2018	0	1	0	0	1	0	0	0	0	0	0	0	61
15095766	m	md	IT	2018	0	1	0	0	0	1	0	0	0	0	0	0	59
15107967	f	md	JP	2018	0	1	1	0	0	0	0	0	0	0	0	0	78
15108382	m	cn	JP	2018	0	1	1	0	0	0	0	0	0	0	0	0	70
15122658	m	md	CO	2018	0	0	0	0	0	0	0	0	0	0	0	0	
15131118	f	Not repo	US	2018	1	0	0	0	0	0	0	0	0	0	0	0	
15131606	f	ot	JP	2018	0	1	0	0	1	0	0	0	0	0	0	0	66
15135450	m	md	JP	2018	0	1	0	0	1	0	0	0	0	0	0	0	75

15150533	f	md	JP	2018	1	0	0	0	0	0	0	0	0	0	0	0	80
15172560	m	cn	IN	2018	0	1	0	0	1	0	0	0	0	0	0	0	34
15180977	m	ot	JP	2018	0	1	0	0	1	0	0	0	0	0	0	1	47
15188094	m	ot	JP	2018	0	1	0	0	1	0	0	0	0	0	0	0	70
15190851	f	ot	US	2018	0	1	0	0	1	0	0	0	0	0	0	0	61
15194590	f	ph	JP	2018	1	0	0	0	0	0	0	0	0	0	0	1	85.3
15196536	m	md	JP	2018	0	1	0	0	1	0	0	0	0	0	0	0	80
15200085	m	ot	JP	2018	0	1	0	0	1	0	0	0	0	0	0	0	83
15200355	Not	md	BR	2018	0	1	0	0	1	0	0	0	0	0	0	0	
15202206	Not	ot	JP	2018	0	1	0	0	1	0	0	0	0	0	0	0	63
15204799	m	md	JP	2018	0	1	0	0	1	0	0	0	0	0	0	1	67
15206428	m	ot	DE	2018	0	0	0	0	0	0	0	0	0	0	0	0	64
15207276	m	md	JP	2018	0	1	0	0	1	0	0	0	0	0	0	1	64
15209902	f	ot	CZ	2018	0	0	0	0	0	0	0	0	0	0	0	0	57
15214123	m	md	US	2018	0	1	0	1	0	0	0	0	0	0	0	0	
15218113	m	md	JP	2018	0	1	0	0	1	0	0	0	0	0	0	0	74
15218171	m	md	JP	2018	0	1	0	0	1	0	0	0	0	0	0	0	72
15218460	f	cn	JP	2018	0	1	1	0	0	0	0	0	0	0	0	0	77
15219128	f	md	DE	2018	0	0	0	0	0	0	0	0	0	0	0	0	77
15224995	f	Not repo	US	2018	1	0	0	0	0	0	0	0	0	0	0	0	77.6
15225217	m	md	JP	2018	0	0	0	0	0	0	0	0	0	0	0	0	44
15227758	f	md	JP	2018	1	0	0	0	0	0	0	0	0	0	0	0	73
15227818	m	md	JP	2018	0	1	1	0	0	0	0	0	0	0	0	0	77
15231980	m	md	ES	2018	0	1	0	0	0	1	0	0	0	0	0	0	65
15235623	f	ot	DE	2018	0	0	0	0	0	0	0	0	0	0	0	0	59
15238867	f	cn	US	2018	0	1	1	0	0	0	0	0	0	0	0	1	72
15246782	f	md	US	2018	0	1	0	0	1	0	0	0	0	0	0	0	72
15258415	m	ot	DE	2018	0	1	0	0	1	0	0	0	0	0	0	0	56
15260972	f	ot	US	2018	0	1	0	1	0	0	0	0	0	0	0	0	76
15264361	m	md	CA	2018	0	1	0	0	0	1	0	0	0	0	0	1	82
15264625	f	md	JP	2018	0	0	0	0	0	0	0	0	0	0	0	0	72
15268943	m	ot	IL	2018	0	1	1	0	0	0	0	0	0	0	0	0	82
15277744	f	md	JP	2018	1	0	0	0	0	0	0	0	0	0	0	0	60
15282795	f	md	FR	2018	0	0	0	0	0	0	0	0	0	0	0	0	74

15282887	m	md	DE	2018	1	0	0	0	0	0	0	0	0	0	0	0	53
15286181	f	ot	IN	2018	0	0	0	0	0	0	0	0	0	0	0	0	54
15286783	m	md	DE	2018	0	1	1	0	0	0	0	0	0	0	0	0	66
15286811	m	md	KR	2018	0	1	0	0	1	0	0	0	0	0	0	0	64
15289035	m	md	JP	2018	0	1	0	0	1	0	0	0	0	0	0	0	67
15296909	f	ot	DE	2018	0	0	0	0	0	0	0	0	0	0	0	0	66
15298903	f	md	JP	2018	0	1	0	0	0	1	0	0	0	0	0	0	63
15306305	f	md	US	2018	0	1	1	0	0	0	0	0	0	0	0	0	49
15308270	m	md	FR	2018	0	1	0	1	0	0	0	0	0	0	0	0	58
15308876	m	cn	DE	2018	0	1	1	0	0	0	0	0	0	0	0	0	
15312152	f	ot	DE	2018	0	1	0	0	1	0	0	0	0	0	0	0	74
15319677	m	md	ES	2018	0	1	0	0	1	0	0	0	0	0	0	0	76
15323265	f	md	DE	2018	0	0	0	0	0	0	0	0	0	0	0	0	59
15334953	f	md	AT	2018	0	0	0	0	0	0	0	0	0	0	0	0	70
15336797	m	md	JP	2018	0	0	0	0	0	0	0	0	0	0	0	0	
15340024	m	cn	JP	2018	0	1	1	0	0	0	0	0	0	0	0	0	38
15340113	m	ot	CN	2018	0	1	0	0	1	0	0	0	0	0	0	0	63
15340595	f	md	US	2018	0	0	0	0	0	0	0	0	0	0	0	0	41
15340718	m	ot	CN	2018	0	1	0	0	1	0	0	0	0	0	0	0	60
15344726	m	ot	DE	2018	0	0	0	0	0	0	0	0	0	0	0	0	61
15346180	m	md	JP	2018	0	1	0	0	1	0	0	0	0	0	0	0	75
15350811	f	Not repo	Not re	2018	0	1	0	0	1	0	0	0	0	0	0	0	50
15353055	Not	ot	DE	2018	0	0	0	0	0	0	0	0	0	0	0	0	76
15354363	f	ot	AU	2018	1	0	0	0	0	0	0	0	0	0	0	0	46
15354527	f	ot	TW	2018	0	1	0	0	1	0	0	0	0	0	0	0	60
15357269	m	cn	US	2018	0	1	1	0	0	0	0	0	0	0	0	0	67
15368406	f	md	JP	2018	0	0	0	0	0	0	0	0	0	0	0	0	
15372939	f	md	DE	2018	0	1	0	0	1	0	0	0	0	0	0	0	69
15373565	m	ot	FR	2018	0	0	0	0	0	0	0	0	0	0	0	0	59
15373577	f	ot	FR	2018	0	0	0	0	0	0	0	0	0	0	0	0	77
15373638	m	ot	FR	2018	0	0	0	0	0	0	0	0	0	0	0	0	76
15374393	m	ot	DE	2018	0	0	0	0	0	0	0	0	0	0	0	0	8
15374486	m	md	BG	2018	0	1	0	1	0	0	0	0	0	0	0	0	52
15375066	m	md	JP	2018	0	1	0	1	0	0	0	0	0	0	0	1	67

15375821	m	md	CN	2018	0	1	0	1	0	0	0	0	0	0	0	0	73.3
15384089	m	ot	FR	2018	0	0	0	0	0	0	0	0	0	0	0	0	66
15384110	f	ot	FR	2018	0	0	0	0	0	0	0	0	0	0	0	0	50
15385502	m	md	JP	2018	1	0	0	0	0	0	0	0	0	0	0	0	79
15387175	f	md	JP	2018	0	0	0	0	0	0	0	0	0	0	0	0	82
15390299	m	md	JP	2018	0	0	0	0	0	0	0	0	0	0	0	0	
15394724	Not	md	NL	2018	0	1	0	0	1	0	0	0	0	0	0	0	
15405231	m	md	PL	2018	0	1	0	0	0	1	0	0	0	0	0	0	68
15408500	m	md	JP	2018	0	1	0	1	0	0	0	0	0	0	0	0	77
15409749	f	ot	US	2018	0	1	0	0	1	0	0	0	0	0	0	0	70
15409768	m	md	JP	2018	1	0	0	0	0	0	0	0	0	0	0	0	75
15409795	f	cn	US	2018	0	1	1	0	0	0	0	0	0	0	0	0	
15410466	m	ot	CA	2018	0	1	0	0	1	0	0	0	0	0	0	0	78
15410531	f	cn	JP	2018	0	1	1	0	0	0	0	0	0	0	0	0	74
15412128	f	md	JP	2018	0	0	0	0	0	0	0	0	0	0	0	0	44
15413072	f	md	HU	2018	0	0	0	0	0	0	0	0	0	0	0	0	62
15413234	f	md	DE	2018	0	0	0	0	0	0	0	0	0	0	0	0	67
15414723	m	md	CN	2018	0	0	0	0	0	0	0	0	0	0	0	0	66
15414971	f	ot	RO	2018	0	1	0	0	1	0	0	0	0	0	0	0	62
15415311	f	ot	US	2018	0	1	1	0	0	0	0	0	0	0	0	1	72
15416315	f	ot	NL	2018	0	1	0	0	1	0	0	0	0	0	0	0	70
15416972	m	ot	NL	2018	0	1	0	0	1	0	0	0	0	0	0	0	95
15417383	f	md	NZ	2018	0	0	0	0	0	0	0	0	0	0	0	0	64
15419830	f	ot	DE	2018	0	0	0	0	0	0	0	0	0	0	0	0	48
15422957	m	Not repo	US	2018	1	0	0	0	0	0	0	0	0	0	0	0	74.5
15428580	m	ph	US	2018	0	0	0	0	0	0	0	0	0	0	0	0	
15429556	f	cn	US	2018	0	1	0	0	1	0	0	0	0	0	0	0	
15432231	m	ot	US	2018	0	1	0	0	1	0	0	0	0	0	0	0	69
15432735	f	md	DE	2018	1	0	0	0	0	0	0	0	0	0	0	0	84
15437737	f	ot	FR	2018	0	1	0	0	1	0	0	0	0	0	0	0	72
15438801	f	ot	US	2018	0	1	0	0	1	0	0	0	0	0	0	0	55
15438879	m	md	FR	2018	0	0	0	0	0	0	0	0	0	0	0	0	81
15438950	m	ot	DE	2018	0	1	0	0	1	0	0	0	0	0	0	0	74
15438961	m	cn	EC	2018	0	0	0	0	0	0	0	0	0	0	0	0	

15439636	Not	md	JP	2018	1	0	0	0	0	0	0	0	0	0	0	0	
15440174	m	cn	IE	2018	0	1	1	0	0	0	0	0	0	0	0	0	63
15440519	m	md	JP	2018	0	0	0	0	0	0	0	0	0	0	0	0	
15448479	m	md	KR	2018	1	0	0	0	0	0	0	0	0	0	0	0	74
15449319	f	cn	AU	2018	0	1	1	0	0	0	0	0	0	0	0	1	26
15449620	m	ot	JP	2018	0	1	0	0	1	0	0	0	0	0	0	0	56
15449632	Not	md	US	2018	0	1	0	1	0	0	0	0	0	0	0	1	
15449650	f	ot	DE	2018	0	1	0	0	1	0	0	0	0	0	0	0	48
15450950	m	md	IN	2018	0	0	0	0	0	0	0	0	0	0	0	0	73
15451540	f	cn	US	2018	0	1	1	0	0	0	0	0	0	0	0	0	
15451958	f	ot	GB	2018	1	0	0	0	0	0	0	0	0	0	0	0	27
15452864	f	cn	CA	2018	1	0	0	0	0	0	0	0	0	0	0	0	
15452866	Not	md	BE	2018	0	0	0	0	0	0	0	0	0	0	0	0	
15453085	f	cn	IE	2018	0	0	0	0	0	0	0	0	0	0	0	0	58
15453864	f	md	DE	2018	0	0	0	0	0	0	0	0	0	0	0	0	57
15453867	f	md	DE	2018	0	0	0	0	0	0	0	0	0	0	0	0	67
15454031	m	md	DE	2018	0	1	0	0	1	0	0	0	0	0	0	0	73
15454780	f	cn	GB	2018	1	0	0	0	0	0	0	0	0	0	0	1	59
15454889	f	md	JP	2018	0	1	0	0	1	0	0	0	0	0	0	0	52
15454913	f	cn	US	2018	0	0	0	0	0	0	0	0	0	0	0	0	
15455058	Not	ot	US	2018	0	1	0	1	0	0	0	0	0	0	0	1	
15456277	m	cn	JP	2018	0	1	1	0	0	0	0	0	0	0	0	0	72
15456331	m	Not repo	JP	2018	0	0	0	0	0	0	0	0	0	0	0	0	55
15456417	m	md	DE	2018	0	0	0	0	0	0	0	0	0	0	0	0	58
15456437	m	ot	IN	2018	1	0	0	0	0	0	0	0	0	0	0	0	51
15456494	m	cn	JP	2018	0	1	1	0	0	0	0	0	0	0	0	1	69
15456954	m	cn	JP	2018	0	1	1	0	0	0	0	0	0	0	0	0	60
15457027	m	cn	US	2018	0	0	0	0	0	0	0	0	0	0	0	0	57
15457334	m	ot	US	2018	0	1	0	0	1	0	0	0	0	0	0	0	78
15457387	m	md	US	2018	0	1	0	1	0	0	0	0	0	0	0	0	
15457485	m	cn	US	2018	1	0	0	0	0	0	0	0	0	0	0	0	74
15457561	f	md	JP	2018	0	1	0	0	0	1	0	0	0	0	0	0	43
15457941	m	ot	JP	2018	0	1	0	0	1	0	0	0	0	0	0	0	73
15457942	m	ot	JP	2018	0	1	0	0	1	0	0	0	0	0	0	0	69

15458135	Not	md	JP	2018	0	1	0	0	1	0	0	0	0	0	0	0	
15458514	m	md	JP	2018	0	1	0	0	1	0	0	0	0	0	0	0	78
15458680	Not	cn	JP	2018	0	1	1	0	0	0	0	0	0	0	0	0	
15458695	m	cn	US	2018	0	1	0	0	1	0	0	0	0	0	0	0	
15458731	f	ot	US	2018	0	1	0	1	0	0	0	0	0	0	0	0	76
15458964	f	ot	CZ	2018	0	0	0	0	0	0	0	0	0	0	0	0	24
15459067	m	Not repo	JP	2018	0	0	0	0	0	0	0	0	0	0	0	0	50
15459388	m	md	JP	2018	0	1	0	1	0	0	0	0	0	0	0	0	60
15459892	m	md	AR	2018	0	1	0	0	0	1	0	0	0	0	0	0	54
15460151	f	ot	DE	2018	0	1	0	0	1	0	0	0	0	0	0	0	61
15460554	f	ot	IT	2018	0	0	0	0	0	0	0	0	0	0	0	0	50
15460709	m	ot	DE	2018	0	0	0	0	0	0	0	0	0	0	0	0	80
15461028	f	cn	NP	2018	0	0	0	0	0	0	0	0	0	0	0	0	34
15461195	f	ot	US	2018	0	0	0	0	0	0	1	0	1	0	0	0	66
15461418	m	md	JP	2018	0	1	0	1	0	0	0	0	0	0	0	1	69
15462295	Not	md	US	2018	0	1	0	1	0	0	0	0	0	0	0	0	
15462591	f	md	JP	2018	0	1	0	0	0	1	0	0	0	0	0	0	64
15462719	m	ot	JP	2018	0	1	0	0	1	0	0	0	0	0	0	0	69
15462855	m	ph	JP	2018	0	1	0	0	1	0	0	0	0	0	0	0	79
15463531	m	md	US	2018	1	0	0	0	0	0	0	0	0	0	0	0	
15464094	m	md	JP	2018	1	0	0	0	0	0	0	0	0	0	0	0	
15464299	m	md	DE	2018	0	0	0	0	0	0	0	0	0	0	0	0	51
15464366	Not	md	JP	2018	1	0	0	0	0	0	0	0	0	0	0	0	70
15464427	m	md	US	2018	1	0	0	0	0	0	0	0	0	0	0	0	
15464497	m	md	JP	2018	0	1	0	1	0	0	0	0	0	0	0	0	71.5
15464662	f	Not repo	MY	2018	1	0	0	0	0	0	0	0	0	0	0	0	67
15465026	f	ot	US	2018	0	1	0	0	1	0	0	0	0	0	0	0	76
15465227	f	ot	US	2018	0	1	0	0	1	0	0	0	0	0	0	0	
15465236	m	md	JP	2018	0	0	0	0	0	0	0	0	0	0	0	0	61
15465690	m	md	VN	2018	1	0	0	0	0	0	0	0	0	0	0	0	60
15466493	m	ot	US	2018	0	1	0	0	1	0	0	0	0	0	0	0	65
15466999	m	md	US	2018	0	1	0	1	0	0	0	0	0	0	0	0	
15467091	m	ot	US	2018	0	1	0	0	0	1	0	0	0	0	0	0	
15467254	m	ot	DE	2018	0	1	0	0	1	0	0	0	0	0	0	0	63

15467338	m	md	US	2018	0	1	0	1	0	0	0	0	0	0	0	0	
15467442	Not	ot	JP	2018	0	0	0	0	0	0	0	0	0	0	0	0	
15467856	m	md	DE	2018	0	1	0	0	1	0	0	0	0	0	0	0	85
15467861	m	md	NL	2018	0	1	0	0	0	1	0	0	0	0	0	0	
15467874	m	md	JP	2018	0	1	0	0	0	1	0	0	0	0	0	0	50
15468025	f	md	IN	2018	0	1	0	0	0	1	0	0	0	0	0	0	50
15468078	f	cn	ID	2018	1	0	0	0	0	0	0	0	0	0	0	0	
15468088	f	md	JP	2018	1	0	0	0	0	0	0	0	0	0	0	0	67.7
15468210	m	cn	US	2018	0	0	0	0	0	0	0	0	0	0	0	0	78
15468343	m	md	JP	2018	1	0	0	0	0	0	0	0	0	0	0	0	77
15468555	m	cn	IN	2018	0	0	0	0	0	0	0	0	0	0	0	0	50
15468646	m	md	DE	2018	0	0	0	0	0	0	0	0	0	0	0	0	72
15468707	m	md	JP	2018	1	0	0	0	0	0	0	0	0	0	0	0	76.4
15468714	f	ph	US	2018	0	1	0	1	0	0	0	0	0	0	0	0	82.3
15468817	m	md	JP	2018	0	1	0	0	0	1	0	0	0	0	0	0	70
15469170	Not	ot	FR	2018	0	1	0	0	1	0	0	0	0	0	0	0	
15469171	Not	ot	FR	2018	0	1	0	0	1	0	0	0	0	0	0	0	
15469186	Not	ot	FR	2018	0	1	0	0	1	0	0	0	0	0	0	0	
15469311	m	cn	BR	2018	0	0	0	0	0	0	0	0	0	0	0	0	70
15469394	m	cn	JP	2018	0	1	1	0	0	0	0	0	0	0	0	0	60
15469742	m	md	JP	2018	1	0	0	0	0	0	0	0	0	0	0	0	
15469813	m	ot	DE	2018	0	0	0	0	0	0	0	0	0	0	0	0	70
15469846	f	md	DE	2018	0	0	0	0	0	0	0	0	0	0	0	0	67
15469857	f	md	SG	2018	0	0	0	0	0	0	0	0	0	0	0	0	77
15469915	m	md	US	2018	0	0	0	0	0	0	0	0	0	0	0	0	
15469939	Not	md	JP	2018	1	0	0	0	0	0	0	0	0	0	0	0	
15469965	f	ot	DE	2018	0	1	0	0	1	0	0	0	0	0	0	0	61
15470007	f	cn	ID	2018	1	0	0	0	0	0	0	0	0	0	0	0	45
15470148	Not	md	US	2018	1	0	0	0	0	0	0	0	0	0	0	0	
15470206	f	ph	FR	2018	0	1	0	1	0	0	0	0	0	0	0	0	62.8
15470219	f	cn	US	2018	0	1	1	0	0	0	0	0	0	0	0	0	37
15470226	f	cn	AR	2018	0	1	1	0	0	0	0	0	0	0	0	0	54
15470569	m	md	KR	2018	0	1	0	0	1	0	0	0	0	0	0	0	63
15470652	f	cn	US	2018	0	0	0	0	0	0	0	0	0	0	0	0	81

15470950	f	cn	US	2018	0	1	1	0	0	0	0	0	0	0	0	0	
15470999	m	md	DE	2018	0	1	0	0	1	0	0	0	0	0	0	0	85
15472307	m	ph	GB	2018	0	0	0	0	0	0	0	0	0	0	0	0	48
15472556	f	ot	JP	2018	0	1	0	0	1	0	0	0	0	0	0	0	70
15472960	m	md	US	2018	0	1	0	0	1	0	0	0	0	0	0	0	64
15473050	f	cn	US	2018	0	1	0	0	1	0	0	0	0	0	0	0	
15473335	f	cn	US	2018	0	0	0	0	0	0	0	0	0	0	0	0	77
15473365	m	cn	AR	2018	0	1	1	0	0	0	0	0	0	0	0	1	50
15473525	m	ot	BE	2018	0	1	0	0	1	0	0	0	0	0	0	0	58
15473559	m	md	MY	2018	0	1	0	0	0	1	0	0	0	0	0	0	77
15473654	f	cn	US	2018	0	0	0	0	0	0	0	0	0	0	0	0	
15473812	m	md	JP	2018	0	0	0	0	0	0	0	0	0	0	0	0	82
15474514	m	md	CN	2018	1	0	0	0	0	0	0	0	0	0	0	0	60.8
15474515	f	cn	JP	2018	1	0	0	0	0	0	0	0	0	0	0	0	
15474516	f	cn	US	2018	0	1	1	0	0	0	0	0	0	0	0	0	
15474572	f	ot	JP	2018	0	0	0	0	0	0	0	0	0	0	0	0	48
15474747	f	cn	US	2018	1	0	0	0	0	0	0	0	0	0	0	0	86
15475226	f	cn	US	2018	0	0	0	0	0	0	0	0	0	0	0	0	57
15475476	f	cn	US	2018	0	1	1	0	0	0	0	0	0	0	0	0	85
15475512	f	ot	CA	2018	0	1	0	0	1	0	0	0	0	0	0	0	76
15475585	f	cn	US	2018	1	0	0	0	0	0	0	0	0	0	0	0	
15475850	f	ot	GB	2018	1	0	0	0	0	0	0	0	0	0	0	0	27
15475959	m	ot	DE	2018	0	0	0	0	0	0	0	0	0	0	0	0	58
15476174	m	cn	CA	2018	0	1	1	0	0	0	0	0	0	0	0	0	76
15476316	m	Not repo	JP	2018	0	0	0	0	0	0	0	0	0	0	0	0	56
15476850	m	md	BR	2018	0	1	0	1	0	0	0	0	0	0	0	0	61.9
15476862	m	cn	GB	2018	1	0	0	0	0	0	0	0	0	0	0	0	79
15476876	m	md	JP	2018	0	1	0	0	0	1	0	0	0	0	0	0	67
15476968	m	md	JP	2018	1	0	0	0	0	0	0	0	0	0	0	0	67
15477009	m	md	JP	2018	0	0	0	0	0	0	0	0	0	0	0	0	77
15477484	f	md	JP	2018	1	0	0	0	0	0	0	0	0	0	0	0	58
15477529	f	md	PE	2018	0	1	0	1	0	0	0	0	0	0	0	0	54
15477661	f	md	JP	2018	1	0	0	0	0	0	0	0	0	0	0	0	44
15478326	f	md	JP	2018	0	1	0	0	1	0	0	0	0	0	0	0	60

15478558	m	cn	IN	2018	0	1	0	0	1	0	0	0	0	0	0	0	
15478665	m	md	DE	2018	0	1	0	0	1	0	0	0	0	0	0	0	85
15479291	m	ot	FR	2018	0	1	0	0	1	0	0	0	0	0	0	0	70
15479349	m	md	DE	2018	0	0	0	0	0	0	0	0	0	0	0	0	68
15479389	f	cn	US	2018	0	0	0	0	0	0	0	0	0	0	0	0	
15479462	f	cn	US	2018	0	0	0	0	0	0	0	0	0	0	0	0	76
15479482	Not	cn	JP	2018	0	1	1	0	0	0	0	0	0	0	0	0	65
15480288	f	cn	US	2018	0	0	0	0	0	0	0	0	0	0	0	0	74
15480911	f	md	FR	2018	0	0	0	0	0	0	0	0	0	0	0	0	69
15480966	m	md	GR	2018	0	1	0	0	0	1	0	0	0	0	0	0	66
15481356	m	cn	JP	2018	0	1	1	0	0	0	0	0	0	0	0	1	76
15481478	f	md	SE	2018	0	1	0	1	0	0	0	0	0	0	0	0	
15481657	f	md	DK	2018	0	0	0	0	0	0	0	0	0	0	0	0	67
15481799	m	ot	DE	2018	0	1	0	0	1	0	0	0	0	0	0	0	66
15481840	f	ot	US	2018	0	0	0	0	0	0	0	0	0	0	0	0	
15482071	m	cn	US	2018	0	1	1	0	0	0	0	0	0	0	0	0	
15482243	m	ot	US	2018	0	1	0	0	1	0	0	0	0	0	0	0	60
15482665	m	md	CN	2018	0	1	0	1	0	0	0	0	0	0	0	0	59.4
15482686	m	cn	JP	2018	0	1	1	0	0	0	0	0	0	0	0	0	42
15482802	m	md	JP	2018	0	1	0	0	1	0	0	0	0	0	0	0	67
15483170	f	cn	US	2018	0	0	0	0	0	0	0	0	0	0	0	0	61
15483196	m	cn	US	2018	0	0	0	0	0	0	0	0	0	0	0	0	87
15483735	f	md	JP	2018	0	1	0	1	0	0	0	0	0	0	0	1	66.2
15483778	f	md	ES	2018	0	0	0	0	0	0	0	0	0	0	0	0	83
15483836	m	md	US	2018	0	1	0	1	0	0	0	0	0	0	0	1	
15483842	m	ot	JP	2018	0	0	0	0	0	0	0	0	0	0	0	0	72
15483907	f	md	JP	2018	1	0	0	0	0	0	0	0	0	0	0	0	77
15484403	m	ot	DE	2018	0	1	0	0	1	0	0	0	0	0	0	0	69
15484552	f	md	ES	2018	0	1	1	0	0	0	0	0	0	0	0	0	
15484970	m	cn	US	2018	0	0	0	0	0	0	0	0	0	0	0	0	
15484976	f	cn	US	2018	0	0	0	0	0	0	0	0	0	0	0	0	
15484982	f	md	US	2018	0	0	0	0	0	0	0	0	0	0	0	0	49
15485154	f	cn	US	2018	0	0	0	0	0	0	0	0	0	0	0	0	
15485168	f	cn	US	2018	0	0	0	0	0	0	0	0	0	0	0	0	

15485194	f	ot	US	2018	0	0	0	0	0	0	0	0	0	0	0	0	
15485205	f	ph	US	2018	0	0	0	0	0	0	0	0	0	0	0	0	
15485222	Not	ph	US	2018	0	0	0	0	0	0	0	0	0	0	0	0	
15485240	Not	md	US	2018	0	0	0	0	0	0	0	0	0	0	0	0	
15485252	f	ot	US	2018	0	0	0	0	0	0	0	0	0	0	0	0	66
15485319	m	md	US	2018	0	0	0	0	0	0	0	0	0	0	0	0	
15485330	m	cn	US	2018	0	0	0	0	0	0	0	0	0	0	0	0	
15485349	m	md	US	2018	0	0	0	0	0	0	0	0	0	0	0	0	
15485354	f	cn	US	2018	0	0	0	0	0	0	0	0	0	0	0	0	66
15485366	f	cn	US	2018	0	0	0	0	0	0	0	0	0	0	0	0	
15485747	f	md	JP	2018	1	0	0	0	0	0	0	0	0	0	0	0	67.1
15485793	m	md	BG	2018	0	1	0	1	0	0	0	0	0	0	0	0	57.8
15485847	m	md	JP	2018	0	0	0	0	0	0	0	0	0	0	0	0	100
15486066	f	md	US	2018	0	1	0	0	0	1	0	0	0	0	0	0	37
15486201	m	cn	JP	2018	0	1	1	0	0	0	0	0	0	0	0	0	59
15486573	f	md	KR	2018	1	0	0	0	0	0	0	0	0	0	0	0	77
15486774	m	ot	US	2018	0	1	0	0	1	0	0	0	0	0	0	0	
15486792	m	md	JP	2018	0	1	0	0	0	1	0	0	0	0	0	0	
15486804	f	md	JP	2018	1	0	0	0	0	0	0	0	0	0	0	0	75
15486833	m	cn	US	2018	0	1	1	0	0	0	0	0	0	0	0	0	66
15487307	m	md	JP	2018	0	1	0	0	0	1	0	0	0	0	0	0	
15487354	f	md	KR	2018	1	0	0	0	0	0	0	0	0	0	0	0	67
15487537	Not	ot	AU	2018	0	0	0	0	0	0	0	0	0	0	0	0	
15487544	Not	ot	AU	2018	0	0	0	0	0	0	0	0	0	0	0	0	
15487679	f	cn	GB	2018	1	0	0	0	0	0	0	0	0	0	0	0	78
15487768	m	cn	JP	2018	0	1	1	0	0	0	0	0	0	0	0	0	47
15488024	f	ot	IT	2018	1	0	0	0	0	0	0	0	0	0	0	0	50
15488247	m	cn	GB	2018	1	0	0	0	0	0	0	0	0	0	0	0	70
15488299	m	ot	US	2018	0	0	0	0	0	0	0	0	0	0	0	0	
15488857	f	md	JP	2018	1	0	0	0	0	0	0	0	0	0	0	1	56
15488939	m	ph	FR	2018	0	1	0	1	0	0	0	0	0	0	0	0	61.1
15489024	m	md	JP	2018	1	0	0	0	0	0	0	0	0	0	0	0	68
15489165	f	md	US	2018	1	0	0	0	0	0	0	0	0	0	0	0	
15489398	m	md	JP	2018	0	1	0	1	0	0	0	0	0	0	0	1	75

15489986	f	md	US	2018	0	0	0	0	0	0	0	0	0	0	0	0	65
15490131	f	ot	IT	2018	0	1	0	0	1	0	0	0	0	0	0	0	60
15490268	m	md	DE	2018	0	0	0	0	0	0	0	0	0	0	0	0	80
15490793	f	md	JP	2018	1	0	0	0	0	0	0	0	0	0	0	0	69
15491046	f	md	JP	2018	0	0	0	0	0	0	0	0	0	0	0	0	
15491123	m	md	CN	2018	0	1	0	0	0	1	0	0	0	0	0	0	79
15491320	m	ot	JP	2018	0	1	0	0	1	0	0	0	0	0	0	0	60
15491360	m	ot	JP	2018	0	0	0	0	0	0	0	0	0	0	0	0	80
15491620	m	md	JP	2018	0	1	0	0	0	1	0	0	0	0	0	0	68
15492156	m	md	FR	2018	0	1	0	1	0	0	0	0	0	0	0	0	65.8
15492584	f	md	JP	2018	0	0	0	0	0	0	0	0	0	0	0	0	76.5
15492832	m	md	PL	2018	1	0	0	0	0	0	0	0	0	0	0	0	52
15492908	m	cn	CN	2018	1	0	0	0	0	0	0	0	0	0	0	0	65
15492953	Not	md	CH	2018	0	0	0	0	0	0	0	0	0	0	0	0	
15493461	m	cn	US	2018	0	1	1	0	0	0	0	0	0	0	0	0	68
15493570	f	ot	JP	2018	1	0	0	0	0	0	0	0	0	0	0	0	60
15493584	m	ot	JP	2018	0	0	0	0	0	0	0	0	0	0	0	0	
15494168	m	cn	US	2018	0	0	0	0	0	0	0	0	0	0	0	0	69
15494227	Not	md	TR	2018	0	0	0	0	0	0	0	0	0	0	0	0	
15494366	f	cn	US	2018	0	0	0	0	0	0	0	0	0	0	0	0	55
15494426	f	md	US	2018	0	1	1	0	0	0	0	0	0	0	0	0	
15494430	f	ot	US	2018	0	1	0	0	1	0	0	0	0	0	0	0	67
15494520	f	md	JP	2018	1	0	0	0	0	0	0	0	0	0	0	0	80.4
15494807	m	md	JP	2018	0	1	0	0	1	0	0	0	0	0	0	0	70
15495025	f	ot	US	2018	1	0	0	0	0	0	0	0	0	0	0	0	44
15495204	f	ot	CZ	2018	0	0	0	0	0	0	0	0	0	0	0	0	23
15496299	m	cn	US	2018	0	1	0	0	1	0	0	0	0	0	0	0	
15496307	m	md	JP	2018	0	0	0	0	0	0	0	0	0	0	0	0	76
15497061	m	md	JP	2018	0	1	0	0	0	1	0	0	0	0	0	0	54
15497153	m	cn	US	2018	0	1	1	0	0	0	0	0	0	0	0	0	
15498099	m	cn	US	2018	0	0	0	0	0	0	0	0	0	0	0	0	62
15498322	m	ot	GB	2018	1	0	0	0	0	0	0	0	0	0	0	0	80
15498386	m	ot	US	2018	0	1	0	1	0	0	0	0	0	0	0	0	64
15498636	m	cn	JP	2018	0	1	1	0	0	0	0	0	0	0	0	0	77

15498899	f	md	JP	2018	1	0	0	0	0	0	0	0	0	0	0	0	41
15498929	f	cn	GB	2018	1	0	0	0	0	0	0	0	0	0	0	0	60
15499499	m	cn	JP	2018	0	1	1	0	0	0	0	0	0	0	0	0	74
15499860	m	ot	JP	2018	0	0	0	0	0	0	0	0	0	0	0	0	76
15500841	m	cn	DE	2018	0	0	0	0	0	0	0	0	0	0	0	0	
15500991	f	md	SE	2018	0	1	0	0	1	0	0	0	0	0	0	0	74
15504224	m	md	JP	2018	0	1	0	0	1	0	0	0	0	0	0	0	78
15504426	f	cn	GB	2018	1	0	0	0	0	0	0	0	0	0	0	0	
15505218	m	md	NO	2018	0	1	0	1	0	0	0	0	0	0	0	0	70
15505334	m	md	DK	2018	0	1	1	0	0	0	0	0	0	0	0	0	65
15506058	m	cn	JP	2018	0	1	1	0	0	0	0	0	0	0	0	1	83
15506075	Not	md	BE	2018	0	0	0	0	0	0	0	0	0	0	0	0	
15506154	m	md	CN	2018	1	0	0	0	0	0	0	0	0	0	0	0	45
15506549	f	ot	GB	2018	0	1	0	0	1	0	0	0	0	0	0	0	88
15506767	m	ot	JP	2018	0	0	0	0	0	0	0	0	0	0	0	0	76
15506934	m	cn	JP	2018	0	1	1	0	0	0	0	0	0	0	0	0	71
15507518	m	md	CH	2018	0	1	0	0	0	1	0	0	0	0	0	0	
15507576	f	md	JP	2018	0	0	0	0	0	0	0	0	0	0	0	0	63
15507583	f	md	IT	2018	0	0	0	0	0	0	0	0	0	0	0	0	
15507886	f	md	CA	2018	0	1	0	1	0	0	0	0	0	0	0	0	
15508105	m	ot	JP	2018	0	1	0	0	1	0	0	0	0	0	0	0	56
15508193	m	ot	US	2018	0	1	0	0	1	0	0	0	0	0	0	0	56
15508195	m	cn	TH	2018	0	1	0	0	1	0	0	0	0	0	0	0	58
15508324	m	cn	US	2018	0	0	0	0	0	0	0	0	0	0	0	0	52
15508343	m	ot	US	2018	0	1	1	0	0	0	0	0	0	0	0	0	65
15510804	m	md	US	2018	0	0	0	0	0	0	0	0	0	0	0	0	49
15514768	f	ot	JP	2018	0	1	0	0	1	0	0	0	0	0	0	0	61
15514796	m	cn	US	2018	1	0	0	0	0	0	0	0	0	0	0	0	
15515085	f	md	US	2018	0	1	1	0	0	0	0	0	0	0	0	0	
15515087	f	md	JP	2018	0	1	0	0	1	0	0	0	0	0	0	0	81
15515925	f	cn	US	2018	0	1	1	0	0	0	0	0	0	0	0	0	
15516192	m	md	DE	2018	0	0	0	0	0	0	0	0	0	0	0	0	42
15516339	f	ot	JP	2018	0	1	0	0	1	0	0	0	0	0	0	0	60
15517350	f	ot	US	2018	0	1	0	0	1	0	0	0	0	0	0	0	74

15517429	m	md	JP	2018	0	1	0	0	1	0	0	0	0	0	0	0	80
15517718	m	md	FR	2018	0	1	0	0	1	0	0	0	0	0	0	0	64
15518020	m	ot	FR	2018	0	1	0	0	1	0	0	0	0	0	0	0	79
15518057	m	cn	CL	2018	0	1	1	0	0	0	0	0	0	0	0	0	70
15518169	Not	md	JP	2018	0	0	0	0	0	0	0	0	0	0	0	0	
15518179	m	cn	US	2018	0	0	0	0	0	0	0	0	0	0	0	0	52
15518214	m	md	TN	2018	0	0	0	0	0	0	0	0	0	0	0	0	41
15518305	Not	Not repo	ID	2018	1	0	0	0	0	0	0	0	0	0	0	0	
15518539	m	md	JP	2018	0	1	0	1	0	0	0	0	0	0	0	1	82
15518545	m	md	JP	2018	1	0	0	0	0	0	0	0	0	0	0	0	75
15518546	m	ph	FR	2018	0	1	0	1	0	0	0	0	0	0	0	0	61.4
15520093	m	ot	US	2018	0	0	0	0	0	0	0	0	0	0	0	0	61
15522983	m	md	JP	2018	0	1	0	1	0	0	0	0	0	0	0	0	78.4
15523024	f	ot	US	2018	0	1	0	1	0	0	0	0	0	0	0	0	
15523086	f	ot	US	2018	0	1	0	0	1	0	0	0	0	0	0	0	80
15523179	Not	md	JP	2018	0	1	0	0	0	1	0	0	0	0	0	0	66
15523248	m	ot	JP	2018	0	1	0	0	1	0	0	0	0	0	0	0	
15523423	m	Not repo	JP	2018	0	0	0	0	0	0	0	0	0	0	0	0	61
15523441	m	ot	US	2018	0	1	0	0	1	0	0	0	0	0	0	1	68
15523602	m	md	JP	2018	0	1	0	0	1	0	0	0	0	0	0	1	63
15523784	m	md	US	2018	0	1	0	1	0	0	0	0	0	0	0	0	75.8
15523803	m	ot	JP	2018	0	1	0	1	0	0	0	0	0	0	0	0	65
15523805	f	ot	US	2018	0	1	0	0	1	0	0	0	0	0	0	0	44
15523873	Not	md	JP	2018	1	0	0	0	0	0	0	0	0	0	0	1	
15523963	m	md	JP	2018	0	1	0	1	0	0	0	0	0	0	0	1	79.2
15524141	m	cn	JP	2018	0	1	0	0	1	0	0	0	0	0	0	0	50
15524147	Not	md	JP	2018	0	1	0	0	1	0	0	0	0	0	0	0	
15524158	f	md	JP	2018	1	0	0	0	0	0	0	0	0	0	0	0	79
15524516	f	ph	FR	2018	0	1	0	1	0	0	0	0	0	0	0	1	70.2
15524665	Not	md	JP	2018	1	0	0	0	0	0	0	0	0	0	0	1	
15524731	f	md	JP	2018	1	0	0	0	0	0	0	0	0	0	0	0	70
15524890	m	md	JP	2018	0	1	0	0	1	0	0	0	0	0	0	0	71
15525297	f	ph	FR	2018	0	1	0	1	0	0	0	0	0	0	0	1	65.6
15525443	m	md	JP	2018	0	1	0	1	0	0	0	0	0	0	0	1	70.2

15525628	m	md	JP	2018	0	1	0	1	0	0	0	0	0	0	0	1	67
15525630	m	md	JP	2018	1	0	0	0	0	0	0	0	0	0	0	0	75.8
15525681	m	md	ES	2018	0	1	0	0	0	1	0	0	0	0	0	0	70
15525851	Not	ot	US	2018	0	1	0	0	1	0	0	0	0	0	0	0	
15525926	Not	ot	US	2018	0	1	0	0	1	0	0	0	0	0	0	0	
15525928	Not	ot	US	2018	0	1	0	0	1	0	0	0	0	0	0	0	
15525930	Not	ot	US	2018	0	1	0	0	1	0	0	0	0	0	0	0	
15525932	Not	ot	US	2018	0	1	0	0	1	0	0	0	0	0	0	0	
15525992	Not	ot	US	2018	0	1	0	0	1	0	0	0	0	0	0	0	
15526014	f	md	IT	2018	1	0	0	0	0	0	0	0	0	0	0	0	64
15526085	m	md	JP	2018	1	0	0	0	0	0	0	0	0	0	0	1	85.1
15526158	f	cn	CA	2018	0	0	0	0	0	0	0	0	0	0	0	0	47
15526204	f	md	JP	2018	0	0	0	0	0	0	0	0	0	0	0	0	45
15526224	f	cn	IN	2018	0	1	0	0	1	0	0	0	0	0	0	0	
15526248	m	cn	JP	2018	0	0	0	0	0	0	0	0	0	0	0	0	75
15527447	m	md	SK	2018	1	0	0	0	0	0	0	0	0	0	0	0	75
15528037	f	md	JP	2018	0	1	0	0	0	1	0	0	0	0	0	0	64
15528803	f	md	FR	2018	0	1	0	0	0	1	0	0	0	0	0	0	63
15528815	f	md	FR	2018	0	1	0	0	0	1	0	0	0	0	0	0	63
15529984	f	ot	US	2018	0	1	1	0	0	0	0	0	0	0	0	0	63
15530401	m	ot	CN	2018	0	1	0	0	1	0	0	0	0	0	0	0	57
15530534	f	ot	CA	2018	0	0	0	0	0	0	0	0	0	0	0	0	58
15530570	m	ot	BR	2018	0	1	0	0	1	0	0	0	0	0	0	0	62
15530817	m	md	FR	2018	0	1	0	0	1	0	0	0	0	0	0	0	66
15530878	m	md	KR	2018	0	1	0	0	1	0	0	0	0	0	0	0	68
15531063	f	md	JP	2018	0	1	0	0	0	1	0	0	0	0	0	0	70
15531130	m	cn	JP	2018	0	1	1	0	0	0	0	0	0	0	0	1	66
15531189	m	md	JP	2018	0	1	0	0	1	0	0	0	0	0	0	0	55
15531379	f	md	US	2018	0	1	1	0	0	0	0	0	0	0	0	0	64
15531536	f	cn	CN	2018	0	0	0	0	0	0	0	0	0	0	0	0	
15531782	m	cn	CN	2018	0	0	0	0	0	0	0	0	0	0	0	0	
15531832	m	md	JP	2018	0	1	1	0	0	0	0	0	0	0	0	1	43
15531864	f	md	US	2018	0	1	1	0	0	0	0	0	0	0	0	0	79
15531900	m	md	JP	2018	0	1	1	0	0	0	0	0	0	0	0	1	80

15532133	Not	ot	NL	2018	1	0	0	0	0	0	0	0	0	0	0	0	
15532282	m	md	DE	2018	0	1	1	0	0	0	0	0	0	0	0	0	86
15532662	m	md	NL	2018	0	1	0	0	1	0	0	0	0	0	0	0	67
15532921	m	md	JP	2018	0	1	0	0	1	0	0	0	0	0	0	0	68
15533214	m	cn	PL	2018	0	1	1	0	0	0	0	0	0	0	0	0	60
15533549	m	md	JP	2018	0	1	0	1	0	0	0	0	0	0	0	1	80
15533707	f	ph	FR	2018	0	0	0	0	0	0	0	0	0	0	0	0	76
15533734	f	md	RO	2018	1	0	0	0	0	0	0	0	0	0	0	0	
15533735	f	md	JP	2018	1	0	0	0	0	0	0	0	0	0	0	0	
15533946	m	md	JP	2018	1	0	0	0	0	0	0	0	0	0	0	0	73
15534009	f	md	IL	2018	0	0	0	0	0	0	0	0	0	0	0	0	88
15534173	m	cn	ES	2018	0	1	1	0	0	0	0	0	0	0	0	1	59
15534327	m	md	DE	2018	0	0	0	0	0	0	0	0	0	0	0	0	68
15534557	m	md	JP	2018	0	1	0	0	1	0	0	0	0	0	0	1	65
15534572	m	md	AT	2018	0	1	0	0	1	0	0	0	0	0	0	0	
15534671	f	cn	US	2018	1	0	0	0	0	0	0	0	0	0	0	0	76
15534964	m	ph	US	2018	0	1	0	0	1	0	0	0	0	0	0	0	
15535178	m	md	DE	2018	0	1	0	0	0	1	0	0	0	0	0	0	
15535656	Not	md	JP	2018	1	0	0	0	0	0	0	0	0	0	0	0	
15535943	f	cn	US	2018	1	0	0	0	0	0	0	0	0	0	0	0	77
15536092	f	ot	AR	2018	0	1	0	0	1	0	0	0	0	0	0	0	75
15536302	m	md	JP	2018	0	0	0	0	0	0	0	0	0	0	0	0	70
15536590	m	md	JP	2018	0	1	0	1	0	0	0	0	0	0	0	0	79.4
15537016	f	md	CN	2018	0	1	0	1	0	0	0	0	0	0	0	0	44.5
15537048	f	cn	FR	2018	0	0	0	0	0	0	0	0	0	0	0	0	29
15537079	f	md	FR	2018	0	1	0	0	0	1	0	0	0	0	0	0	63
15537325	m	md	DE	2018	0	1	0	0	1	0	0	0	0	0	0	0	63
15537641	Not	md	JP	2018	1	0	0	0	0	0	0	0	0	0	0	0	
15538599	m	ot	PL	2018	0	1	0	0	1	0	0	0	0	0	0	0	79
15538793	m	ot	CN	2018	0	0	0	0	0	0	0	0	0	0	0	0	60
15538879	Not	cn	IT	2018	0	0	0	0	0	0	0	0	0	0	0	0	
15539178	m	ot	CN	2018	0	1	0	0	1	0	0	0	0	0	0	0	71
15539266	f	cn	US	2018	0	1	0	0	1	0	0	0	0	0	0	0	
15539945	f	md	AU	2018	0	0	0	0	0	0	0	0	0	0	0	0	

15540010	m	md	IL	2018	0	0	0	0	0	0	0	0	0	0	0	0	69
15540026	m	ot	JP	2018	0	1	0	0	1	0	0	0	0	0	0	0	73
15540081	f	cn	US	2018	0	0	0	0	0	0	0	0	0	0	0	0	49
15540185	f	ot	CN	2018	1	0	0	0	0	0	0	0	0	0	0	0	62
15540186	m	ot	CN	2018	1	0	0	0	0	0	0	0	0	0	0	0	66
15540222	m	ot	CN	2018	1	0	0	0	0	0	0	0	0	0	0	0	85
15540312	f	ot	CN	2018	1	0	0	0	0	0	0	0	0	0	0	0	79
15540313	f	ot	CN	2018	1	0	0	0	0	0	0	0	0	0	0	0	89
15540412	f	md	US	2018	0	1	0	0	0	1	0	0	0	0	0	0	78
15541128	m	Not repo	CA	2018	1	0	0	0	0	0	0	0	0	0	0	0	
15541598	m	ot	US	2018	0	1	0	0	1	0	0	0	0	0	0	0	55
15542096	f	md	CA	2018	0	1	0	0	0	1	0	0	0	0	0	0	72
15542144	f	ot	CN	2018	1	0	0	0	0	0	0	0	0	0	0	0	66
15542220	m	ot	US	2018	0	1	0	1	0	0	0	0	0	0	0	0	65
15542343	f	ot	CN	2018	1	0	0	0	0	0	0	0	0	0	0	0	75
15542412	m	ot	CN	2018	1	0	0	0	0	0	0	0	0	0	0	0	56
15542421	m	ot	CN	2018	1	0	0	0	0	0	0	0	0	0	0	0	51
15542696	m	md	DE	2018	1	0	0	0	0	0	0	0	0	0	0	0	
15542826	m	ot	US	2018	0	1	0	0	1	0	0	0	0	0	0	0	64
15542901	m	cn	US	2018	1	0	0	0	0	0	0	0	0	0	0	0	66.3
15542906	m	md	US	2018	0	1	0	1	0	0	0	0	0	0	0	0	
15542908	m	md	US	2018	0	1	0	1	0	0	0	0	0	0	0	0	
15542909	m	md	US	2018	0	1	0	1	0	0	0	0	0	0	0	0	
15542910	Not	md	US	2018	0	1	0	1	0	0	0	0	0	0	0	0	
15542911	f	Not repo	US	2018	0	1	0	1	0	0	0	0	0	0	0	0	
15544123	m	ot	US	2018	0	1	0	0	1	0	0	0	0	0	0	0	56
15544415	m	md	FR	2018	0	1	0	0	1	0	0	0	0	0	0	0	
15544480	m	md	JP	2018	0	1	0	0	0	1	0	0	0	0	0	0	76
15544745	m	ot	US	2018	0	1	0	0	1	0	0	0	0	0	0	0	55
15545136	m	md	JP	2018	0	1	0	0	1	0	0	0	0	0	0	0	63
15545489	m	md	JP	2018	0	1	0	1	0	0	0	0	0	0	0	0	63
15545923	f	cn	US	2018	1	0	0	0	0	0	0	0	0	0	0	0	
15546921	f	md	GB	2018	0	0	0	0	0	0	0	0	0	0	0	0	32
15547061	m	ph	JP	2018	1	0	0	0	0	0	0	0	0	0	0	0	76

15547175	m	md	JP	2018	0	0	0	0	0	0	1	0	0	1	0	1	79
15547211	f	cn	US	2018	0	0	0	0	0	0	0	0	0	0	0	0	63
15547297	f	Not repo	ID	2018	1	0	0	0	0	0	0	0	0	0	0	0	
15547495	m	md	JP	2018	1	0	0	0	0	0	0	0	0	0	0	0	72.6
15547506	m	md	US	2018	1	0	0	0	0	0	0	0	0	0	0	0	
15547797	f	cn	US	2018	0	0	0	0	0	0	0	0	0	0	0	0	70
15548007	f	md	DE	2018	0	0	0	0	0	0	0	0	0	0	0	0	73
15548217	f	cn	DE	2018	0	1	1	0	0	0	0	0	0	0	0	0	53
15548325	f	ot	ZA	2018	0	1	0	0	1	0	0	0	0	0	0	0	73
15548649	m	md	JP	2018	0	1	0	0	0	1	0	0	0	0	0	0	74
15548894	m	Not repo	CZ	2018	0	1	0	1	0	0	0	0	0	0	0	1	67.3
15549196	Not	md	CN	2018	0	0	0	0	0	0	0	0	0	0	0	0	
15549544	Not	md	CA	2018	0	1	0	0	1	0	0	0	0	0	0	0	
15549556	m	cn	US	2018	0	0	0	0	0	0	0	0	0	0	0	0	74
15549784	f	md	JP	2018	0	0	0	0	0	0	0	0	0	0	0	0	64
15549788	f	md	IN	2018	0	0	0	0	0	0	0	0	0	0	0	0	66
15549948	m	md	US	2018	0	1	0	1	0	0	0	0	0	0	0	0	
15550093	Not	md	CA	2018	0	1	0	0	1	0	0	0	0	0	0	0	
15550094	Not	md	CA	2018	0	1	0	0	1	0	0	0	0	0	0	0	
15550403	f	cn	GB	2018	1	0	0	0	0	0	0	0	0	0	0	0	55
15550428	m	ot	CN	2018	0	0	0	0	0	0	0	0	0	0	0	0	55
15550880	Not	ot	IT	2018	0	1	0	0	1	0	0	0	0	0	0	0	
15551278	m	md	JP	2018	0	1	0	1	0	0	0	0	0	0	0	0	77.5
15551888	m	md	JP	2018	1	0	0	0	0	0	0	0	0	0	0	0	90
15552062	f	ph	JP	2018	1	0	0	0	0	0	0	0	0	0	0	0	65.6
15552337	m	ot	ES	2018	0	1	0	0	1	0	0	0	0	0	0	0	58
15552566	m	md	US	2018	0	1	0	0	0	1	0	0	0	0	0	0	74
15552610	m	cn	JP	2018	0	0	0	0	0	0	0	0	0	0	0	0	75
15552744	f	cn	DE	2018	0	0	0	0	0	0	0	0	0	0	0	0	66
15552893	m	ot	DE	2018	0	1	0	0	1	0	0	0	0	0	0	0	61
15553078	m	ot	CN	2018	0	1	0	0	1	0	0	0	0	0	0	0	61
15553443	m	md	ES	2018	0	1	0	0	1	0	0	0	0	0	0	0	
15553444	m	ot	FR	2018	0	1	0	0	1	0	0	0	0	0	0	0	63
15553578	f	md	US	2018	1	0	0	0	0	0	0	0	0	0	0	0	76

15553617	m	ot	GB	2018	0	1	0	0	1	0	0	0	0	0	0	0	80
15553883	m	md	US	2018	0	1	0	0	0	1	0	0	0	0	0	0	69
15554009	f	cn	US	2018	1	0	0	0	0	0	0	0	0	0	0	0	86
15554486	m	ot	JP	2018	0	1	0	1	0	0	0	0	0	0	0	0	79
15554706	f	md	US	2018	1	0	0	0	0	0	0	0	0	0	0	0	
15554797	f	ot	DE	2018	0	1	0	0	1	0	0	0	0	0	0	0	79
15554914	m	ot	US	2018	0	1	0	1	0	0	0	0	0	0	0	0	
15555080	m	ot	US	2018	0	1	0	0	1	0	0	0	0	0	0	0	85
15555243	f	ph	CA	2018	0	1	0	0	1	0	0	0	0	0	0	0	
15555433	Not	md	JP	2018	0	1	0	1	0	0	0	0	0	0	0	1	
15555599	m	md	JP	2018	0	1	0	0	0	1	0	0	0	0	0	0	58
15555797	f	cn	US	2018	0	1	1	0	0	0	0	0	0	0	0	0	63
15555962	m	ph	JP	2018	1	0	0	0	0	0	0	0	0	0	0	0	79
15556285	f	cn	US	2018	1	0	0	0	0	0	0	0	0	0	0	0	29
15556762	m	ot	DE	2018	0	1	0	0	1	0	0	0	0	0	0	0	70
15556772	m	ot	CL	2018	0	1	0	0	1	0	0	0	0	0	0	0	55
15556779	f	cn	IL	2018	0	0	0	0	0	0	0	0	0	0	0	0	
15556853	m	md	JP	2018	1	0	0	0	0	0	0	0	0	0	0	0	75.5
15556933	f	md	JP	2018	1	0	0	0	0	0	0	0	0	0	0	0	75
15556973	Not	ot	PL	2018	0	0	0	0	0	0	0	0	0	0	0	0	
15557019	m	md	PL	2018	0	0	0	0	0	0	0	0	0	0	0	0	69
15557020	f	md	JP	2018	1	0	0	0	0	0	0	0	0	0	0	0	68
15557131	m	cn	US	2018	0	1	1	0	0	0	0	0	0	0	0	0	
15557926	m	cn	JP	2018	0	1	1	0	0	0	0	0	0	0	0	0	62
15558028	f	md	US	2018	0	1	1	0	0	0	0	0	0	0	0	0	80
15558049	f	cn	US	2018	0	1	1	0	0	0	0	0	0	0	0	1	
15558385	Not	ot	CA	2018	0	0	0	0	0	0	0	0	0	0	0	0	
15558454	m	md	DE	2018	0	1	0	0	1	0	0	0	0	0	0	0	63
15559128	m	cn	IL	2018	0	0	0	0	0	0	0	0	0	0	0	0	
15559135	f	ot	ES	2018	0	1	0	0	1	0	0	0	0	0	0	0	73
15559204	m	ot	KR	2018	0	1	0	0	1	0	0	0	0	0	0	0	66
15559267	Not	ot	CH	2018	0	1	0	0	1	0	0	0	0	0	0	0	
15560007	m	Not repo	JP	2018	0	0	0	0	0	0	0	0	0	0	0	0	44
15560206	m	cn	US	2018	0	1	1	0	0	0	0	0	0	0	0	0	

15562092	f	cn	PL	2018	0	1	1	0	0	0	0	0	0	0	0	0	59
15563162	m	cn	NL	2018	0	0	0	0	0	0	0	0	0	0	0	0	44
15563358	m	cn	CN	2018	0	1	0	0	1	0	0	0	0	0	0	0	61
15563461	m	md	US	2018	0	1	0	1	0	0	0	0	0	0	0	0	
15564021	m	md	JP	2018	0	1	0	0	1	0	0	0	0	0	0	0	76
15564320	m	md	FR	2018	0	0	0	0	0	0	0	0	0	0	0	0	52
15564826	m	md	US	2018	0	1	0	0	0	1	0	0	0	0	0	0	74
15565382	f	md	JP	2018	0	0	0	0	0	0	0	0	0	0	0	0	70
15565508	f	ot	DE	2018	0	0	0	0	0	0	0	0	0	0	0	0	70
15565801	m	cn	US	2018	1	0	0	0	0	0	0	0	0	0	0	0	73
15566039	m	md	JP	2018	1	0	0	0	0	0	0	0	0	0	0	0	73
15566051	m	ot	JP	2018	0	1	0	0	1	0	0	0	0	0	0	0	69
15566210	Not	ot	JP	2018	0	0	0	0	0	0	0	0	0	0	0	0	
15566257	Not	ot	JP	2018	0	0	0	0	0	0	0	0	0	0	0	0	
15566824	m	md	JP	2018	0	1	1	0	0	0	0	0	0	0	0	0	80
15567063	m	Not repo	JP	2018	0	0	0	0	0	0	0	0	0	0	0	0	62
15567281	f	md	DE	2018	1	0	0	0	0	0	0	0	0	0	0	0	53
15567287	m	md	JP	2018	0	1	0	1	0	0	0	0	0	0	0	0	78
15567358	m	md	JP	2018	0	1	0	0	0	1	0	0	0	0	0	1	76
15567406	m	md	FR	2018	0	1	0	1	0	0	0	0	0	0	0	0	70
15567486	f	md	JP	2018	1	0	0	0	0	0	0	0	0	0	0	0	80
15567546	m	cn	JP	2018	0	1	1	0	0	0	0	0	0	0	0	0	66
15567716	m	cn	US	2018	0	1	1	0	0	0	0	0	0	0	0	0	74
15567852	m	cn	Not re	2018	0	0	0	0	0	0	0	0	0	0	0	0	
15567868	m	cn	US	2018	1	0	0	0	0	0	0	0	0	0	0	0	64
15567900	Not	md	MY	2018	0	0	0	0	0	0	0	0	0	0	0	0	
15567973	m	ot	JP	2018	0	1	0	0	1	0	0	0	0	0	0	1	57
15568003	m	md	JP	2018	0	1	1	0	0	0	0	0	0	0	0	0	64
15568136	f	md	JP	2018	1	0	0	0	0	0	0	0	0	0	0	0	55.2
15568207	f	md	JP	2018	0	1	0	0	1	0	0	0	0	0	0	0	74
15568359	f	cn	US	2018	0	0	0	0	0	0	0	0	0	0	0	0	83
15568399	m	cn	GB	2018	0	1	1	0	0	0	0	0	0	0	0	0	
15568850	f	md	JP	2018	0	1	0	0	1	0	0	0	0	0	0	0	52
15568867	m	md	US	2018	0	1	0	1	0	0	0	0	0	0	0	0	

15568920	m	md	JP	2018	0	1	0	0	0	1	0	0	0	0	0	1	73
15569044	Not	md	US	2018	0	1	0	0	0	1	0	0	0	0	0	0	
15569198	m	md	JP	2018	0	1	0	0	0	1	0	0	0	0	0	0	69
15569866	f	md	AU	2018	0	1	0	0	0	1	0	0	0	0	0	0	
15569944	m	ot	US	2018	0	0	0	0	0	0	0	0	0	0	0	0	
15570116	f	md	FR	2018	0	0	0	0	0	0	0	0	0	0	0	0	61
15570377	m	md	FR	2018	0	1	0	0	0	1	0	0	0	0	0	0	70
15570695	f	ot	US	2018	0	1	0	0	1	0	0	0	0	0	0	1	58
15571047	m	md	JP	2018	0	1	0	1	0	0	0	0	0	0	0	1	62
15571200	m	cn	JP	2018	0	1	1	0	0	0	0	0	0	0	0	0	74
15571220	m	cn	JP	2018	0	1	1	0	0	0	0	0	0	0	0	0	73
15571356	Not	md	AU	2018	0	0	0	0	0	0	0	0	0	0	0	0	
15571456	f	ot	CN	2018	0	1	0	0	1	0	0	0	0	0	0	0	59
15571508	m	ot	CN	2018	0	1	0	0	1	0	0	0	0	0	0	0	73
15571729	m	md	JP	2018	0	0	0	0	0	0	0	0	0	0	0	0	66
15571816	m	md	US	2018	0	1	0	1	0	0	0	0	0	0	0	0	58.6
15572410	Not	ot	US	2018	0	0	0	0	0	0	0	0	0	0	0	0	
15572412	Not	ot	US	2018	0	0	0	0	0	0	0	0	0	0	0	0	
15572413	Not	ot	US	2018	0	0	0	0	0	0	0	0	0	0	0	0	
15572414	Not	ot	US	2018	0	0	0	0	0	0	0	0	0	0	0	0	
15572566	Not	ot	US	2018	0	0	0	0	0	0	0	0	0	0	0	0	
15572567	Not	ot	US	2018	0	0	0	0	0	0	0	0	0	0	0	0	
15572636	f	md	US	2018	0	1	1	0	0	0	0	0	0	0	0	1	63
15572907	m	ot	US	2018	0	1	0	0	1	0	0	0	0	0	0	0	51
15573160	m	md	FR	2018	0	1	0	0	0	1	0	0	0	0	0	0	70
15573196	m	ot	US	2018	0	1	0	0	1	0	0	0	0	0	0	0	68
15573477	f	md	CH	2018	0	1	0	0	0	1	0	0	0	0	0	0	
15573696	f	md	JP	2018	1	0	0	0	0	0	0	0	0	0	0	0	
15573847	m	md	SG	2018	0	1	0	0	0	1	0	0	0	0	0	0	66
15574181	f	cn	GB	2018	1	0	0	0	0	0	0	0	0	0	0	0	42
15574847	m	cn	US	2018	0	1	1	0	0	0	0	0	0	0	0	0	72
15575110	f	ot	JP	2018	0	1	0	0	1	0	0	0	0	0	0	0	69
15575163	m	ot	JP	2018	0	0	0	0	0	0	0	0	0	0	0	0	
15575169	m	ot	JP	2018	0	0	0	0	0	0	0	0	0	0	0	0	

15575170	m	ot	JP	2018	0	0	0	0	0	0	0	0	0	0	0	0	
15575171	m	ot	JP	2018	0	0	0	0	0	0	0	0	0	0	0	0	
15575434	m	md	JP	2018	0	1	0	0	0	1	0	0	0	0	0	0	64
15575443	m	ot	DE	2018	0	1	0	0	0	1	0	0	0	0	0	0	
15575474	m	ot	CN	2018	0	0	0	0	0	0	0	0	0	0	0	0	75
15575596	m	md	MY	2018	0	1	0	1	0	0	0	0	0	0	0	0	59
15575867	f	md	NL	2018	0	1	0	1	0	0	0	0	0	0	0	1	57.9
15576068	f	md	JP	2018	1	0	0	0	0	0	0	0	0	0	0	1	80
15576090	f	cn	US	2018	1	0	0	0	0	0	0	0	0	0	0	0	62
15576116	m	md	CN	2018	0	1	0	1	0	0	0	0	0	0	0	0	63.7
15576128	m	md	JP	2018	1	0	0	0	0	0	0	0	0	0	0	0	76
15576775	f	md	EG	2018	0	0	0	0	0	0	0	0	0	0	0	0	67
15577213	m	ot	US	2018	0	0	0	0	0	0	0	0	0	0	0	0	66
15577333	Not	ot	JP	2018	0	1	0	0	1	0	0	0	0	0	0	0	
15577334	Not	ot	JP	2018	0	1	0	0	1	0	0	0	0	0	0	0	
15578137	m	md	BR	2018	0	1	0	0	0	1	0	0	0	0	0	0	72
15578206	m	cn	US	2018	0	0	0	0	0	0	0	0	0	0	0	0	57
15578239	f	cn	GB	2018	1	0	0	0	0	0	0	0	0	0	0	0	66
15578320	m	md	JP	2018	0	0	0	0	0	0	0	0	0	0	0	0	75
15578609	m	cn	US	2018	0	0	0	0	0	0	0	0	0	0	0	0	78
15578625	m	md	CN	2018	0	0	0	0	0	0	0	0	0	0	0	0	66
15578675	f	md	JP	2018	0	1	0	0	1	0	0	0	0	0	0	0	31
15578846	f	md	JP	2018	0	1	0	0	1	0	0	0	0	0	0	0	70
15579030	m	md	US	2018	0	1	1	0	0	0	0	0	0	0	0	1	
15579547	m	ot	IT	2018	0	1	0	0	1	0	0	0	0	0	0	0	60
15580212	m	cn	US	2018	0	0	0	0	0	0	0	0	0	0	0	0	56
15580431	m	ot	DE	2018	0	0	0	0	0	0	0	0	0	0	0	0	80
15580440	Not	md	US	2018	0	1	0	1	0	0	0	0	0	0	0	1	
15580447	m	ot	GB	2018	0	1	0	0	0	1	0	0	0	0	0	0	
15580451	f	ot	GB	2018	0	0	0	0	0	0	0	0	0	0	0	0	59
15580529	f	cn	US	2018	0	0	0	0	0	0	0	0	0	0	0	0	60
15580643	m	ph	JP	2018	1	0	0	0	0	0	0	0	0	0	0	0	65
15581046	f	md	IN	2018	1	0	0	0	0	0	0	0	0	0	0	1	
15581066	f	ot	TW	2018	1	0	0	0	0	0	0	0	0	0	0	0	63

15582067	f	md	DE	2018	0	1	0	0	1	0	0	0	0	0	0	0	69
15583015	m	cn	US	2018	1	0	0	0	0	0	0	0	0	0	0	0	68
15583418	m	cn	US	2018	0	0	0	0	0	0	0	0	0	0	0	0	77
15584087	m	md	US	2018	0	1	1	0	0	0	0	0	0	0	0	0	70
15584691	f	ot	US	2018	1	0	0	0	0	0	0	0	0	0	0	1	73
15584892	m	md	JP	2018	1	0	0	0	0	0	0	0	0	0	0	0	67
15585057	m	ot	IT	2018	0	1	0	0	1	0	0	0	0	0	0	0	63
15585058	m	ot	CN	2018	0	1	0	0	1	0	0	0	0	0	0	0	61
15585125	f	cn	US	2018	0	0	0	0	0	0	0	0	0	0	0	0	
15585414	f	md	JP	2018	0	0	0	0	0	0	1	0	1	0	0	0	53
15585672	f	md	US	2018	0	1	0	0	1	0	0	0	0	0	0	0	
15585760	m	cn	TH	2018	0	0	0	0	0	0	0	0	0	0	0	0	46
15589119	f	md	US	2018	0	0	0	0	0	0	0	0	0	0	0	0	72
15589291	f	cn	US	2018	0	1	0	1	0	0	0	0	0	0	0	0	78
15589416	m	md	JP	2018	0	1	0	0	0	1	0	0	0	0	0	0	78
15589697	m	cn	US	2018	0	1	1	0	0	0	0	0	0	0	0	1	
15589714	Not	md	CN	2018	1	0	0	0	0	0	0	0	0	0	0	0	
15589783	m	cn	US	2018	0	1	1	0	0	0	0	0	0	0	0	0	65
15590033	m	cn	US	2018	0	0	0	0	0	0	0	0	0	0	0	0	64
15590268	m	md	JP	2018	1	0	0	0	0	0	0	0	0	0	0	0	65.9
15590304	Not	ot	FR	2018	0	0	0	0	0	0	0	0	0	0	0	0	
15590557	m	md	US	2018	0	0	0	0	0	0	0	0	0	0	0	0	42
15590904	f	md	FR	2018	1	0	0	0	0	0	0	0	0	0	0	0	
15591246	m	md	US	2018	0	1	0	1	0	0	0	0	0	0	0	1	
15591733	m	md	US	2018	0	0	0	0	0	0	0	0	0	0	0	0	45
15592121	m	cn	DE	2018	0	1	1	0	0	0	0	0	0	0	0	1	
15592432	f	cn	US	2018	0	1	1	0	0	0	0	0	0	0	0	0	
15592463	m	md	DE	2018	0	1	0	0	0	1	0	0	0	0	0	0	
15592857	m	cn	US	2018	0	0	0	0	0	0	0	0	0	0	0	0	61
15593346	f	md	JP	2018	1	0	0	0	0	0	0	0	0	0	0	0	70
15593370	f	md	JP	2018	0	0	0	0	0	0	0	0	0	0	0	0	47
15594316	m	cn	US	2018	0	0	0	0	0	0	0	0	0	0	0	0	76
15595046	m	md	DE	2018	0	1	1	0	0	0	0	0	0	0	0	0	74
15595065	f	ot	DE	2018	0	0	0	0	0	0	0	0	0	0	0	0	73

15595159	m	ot	FR	2018	0	1	0	0	1	0	0	0	0	0	0	0	67
15595321	m	cn	DE	2018	1	0	0	0	0	0	0	0	0	0	0	0	69
15595450	f	md	JP	2018	1	0	0	0	0	0	0	0	0	0	0	0	59
15595625	m	ph	JP	2018	0	0	0	0	0	0	0	0	0	0	0	0	67
15595640	m	cn	US	2018	0	1	1	0	0	0	0	0	0	0	0	1	
15595663	f	md	US	2018	0	0	0	0	0	0	0	0	0	0	0	0	
15595672	m	md	ES	2018	0	0	0	0	0	0	0	0	0	0	0	0	63
15595950	f	cn	GB	2018	1	0	0	0	0	0	0	0	0	0	0	0	
15596930	Not	md	JP	2018	0	1	0	1	0	0	0	0	0	0	0	1	
15597200	m	md	JP	2018	0	0	0	0	0	0	0	0	0	0	0	0	
15597426	f	cn	US	2018	0	0	0	0	0	0	0	0	0	0	0	0	60
15597444	m	md	US	2018	0	1	0	0	1	0	0	0	0	0	0	0	48
15597953	f	md	JP	2018	1	0	0	0	0	0	0	0	0	0	0	0	80
15597996	Not	ot	JP	2018	1	0	0	0	0	0	0	0	0	0	0	0	
15598151	m	md	US	2018	1	0	0	0	0	0	0	0	0	0	0	0	
15598425	f	md	US	2018	1	0	0	0	0	0	0	0	0	0	0	0	
15598458	f	md	US	2018	1	0	0	0	0	0	0	0	0	0	0	0	81
15598469	m	md	US	2018	1	0	0	0	0	0	0	0	0	0	0	0	
15598493	Not	md	US	2018	1	0	0	0	0	0	0	0	0	0	0	0	
15598496	f	md	US	2018	1	0	0	0	0	0	0	0	0	0	0	0	
15598497	f	cn	US	2018	1	0	0	0	0	0	0	0	0	0	0	0	71
15598515	f	md	US	2018	1	0	0	0	0	0	0	0	0	0	0	0	
15598722	m	md	JP	2018	1	0	0	0	0	0	0	0	0	0	0	0	80
15598858	f	md	NO	2018	1	0	0	0	0	0	0	0	0	0	0	0	62
15598990	f	ot	US	2018	0	0	0	0	0	0	0	0	0	0	0	0	
15599060	Not	ot	JP	2018	1	0	0	0	0	0	0	0	0	0	0	0	
15599115	Not	ot	JP	2018	1	0	0	0	0	0	0	0	0	0	0	0	
15599116	Not	ot	JP	2018	1	0	0	0	0	0	0	0	0	0	0	0	
15599143	f	md	JP	2018	1	0	0	0	0	0	0	0	0	0	0	0	50
15599347	m	cn	JP	2018	0	1	1	0	0	0	0	0	0	0	0	0	59
15599371	m	md	IT	2018	0	0	0	0	0	0	0	0	0	0	0	0	61
15599485	m	ot	ES	2018	0	1	0	0	1	0	0	0	0	0	0	0	53
15599559	m	ot	DE	2018	0	0	0	0	0	0	0	0	0	0	0	0	77
15599666	m	cn	US	2018	0	1	1	0	0	0	0	0	0	0	0	0	

15599723	m	md	JP	2018	0	1	0	0	1	0	0	0	0	0	0	0	77
15599813	m	md	JP	2018	1	0	0	0	0	0	0	0	0	0	0	0	90
15599869	Not	md	US	2018	0	1	1	0	0	0	0	0	0	0	0	1	
15599902	m	md	SK	2018	0	0	0	0	0	0	0	0	0	0	0	0	64
15600034	f	ot	DE	2018	0	1	0	0	1	0	0	0	0	0	0	0	79
15600254	f	md	NL	2018	0	0	0	0	0	0	0	0	0	0	0	0	70
15600495	Not	ot	US	2018	0	0	0	0	0	0	1	1	0	0	0	0	
15600566	f	md	IE	2018	0	1	0	0	0	1	0	0	0	0	0	0	58
15600577	m	cn	US	2018	0	0	0	0	0	0	0	0	0	0	0	0	62
15600842	m	md	CN	2018	0	1	0	0	0	1	0	0	0	0	0	0	70
15600900	f	cn	US	2018	0	1	1	0	0	0	0	0	0	0	0	0	
15600984	m	md	KR	2018	0	1	0	0	1	0	0	0	0	0	0	1	69
15601162	m	cn	FR	2018	0	1	0	0	1	0	0	0	0	0	0	0	73
15601268	m	cn	US	2018	0	0	0	0	0	0	0	0	0	0	0	0	58
15601464	Not	md	MY	2018	1	0	0	0	0	0	0	0	0	0	0	0	
15601581	f	ot	CZ	2018	0	0	0	0	0	0	0	0	0	0	0	0	71
15601644	m	md	JP	2018	0	0	0	0	0	0	0	0	0	0	0	0	68
15601684	m	cn	US	2018	0	0	0	0	0	0	0	0	0	0	0	0	65
15602011	m	md	US	2018	0	1	1	0	0	0	0	0	0	0	0	0	69
15602015	f	cn	CN	2018	1	0	0	0	0	0	0	0	0	0	0	0	72
15602488	m	cn	TH	2018	0	0	0	0	0	0	0	0	0	0	0	0	67
15602764	m	md	JP	2018	1	0	0	0	0	0	0	0	0	0	0	0	70
15602766	m	md	PL	2018	0	0	0	0	0	0	0	0	0	0	0	0	63
15602839	f	md	ID	2018	1	0	0	0	0	0	0	0	0	0	0	0	
15602954	m	ph	JP	2018	1	0	0	0	0	0	0	0	0	0	0	0	82
15603215	m	md	JP	2018	1	0	0	0	0	0	0	0	0	0	0	0	66.5
15603468	f	ot	BR	2018	0	1	0	0	1	0	0	0	0	0	0	0	45
15603648	Not	ot	GB	2018	0	0	0	0	0	0	0	0	0	0	0	0	
15603649	Not	ot	TW	2018	0	0	0	0	0	0	0	0	0	0	0	0	
15603650	Not	ot	DK	2018	0	0	0	0	0	0	0	0	0	0	0	0	
15603663	m	ot	CN	2018	0	0	0	0	0	0	0	0	0	0	0	0	65
15603764	m	cn	GB	2018	1	0	0	0	0	0	0	0	0	0	0	0	41
15603925	m	cn	JP	2018	0	1	1	0	0	0	0	0	0	0	0	0	67
15603949	m	ot	BR	2018	0	1	0	0	1	0	0	0	0	0	0	0	68

15604342	m	cn	US	2018	1	0	0	0	0	0	0	0	0	0	0	0	67
15604953	f	cn	US	2018	0	0	0	0	0	0	0	0	0	0	0	0	70
15605131	f	md	US	2018	0	1	0	0	0	1	0	0	0	0	0	0	58
15605749	f	md	JP	2018	1	0	0	0	0	0	0	0	0	0	0	0	
15605774	m	cn	JP	2018	1	0	0	0	0	0	0	0	0	0	0	0	
15605941	m	md	JP	2018	0	1	0	0	1	0	0	0	0	0	0	0	75
15607052	m	cn	US	2018	1	0	0	0	0	0	0	0	0	0	0	0	79
15607386	m	md	JP	2018	0	1	1	0	0	0	0	0	0	0	0	0	69
15607731	m	md	CN	2018	1	0	0	0	0	0	0	0	0	0	0	0	68.6
15607770	f	md	NL	2018	0	0	0	0	0	0	0	0	0	0	0	0	49
15608031	m	md	JP	2018	1	0	0	0	0	0	0	0	0	0	0	0	71.5
15608607	f	md	CZ	2018	0	0	0	0	0	0	0	0	0	0	0	0	24
15609020	m	md	SK	2018	1	0	0	0	0	0	0	0	0	0	0	0	64
15609050	f	md	US	2018	1	0	0	0	0	0	0	0	0	0	0	0	
15609183	m	cn	Not re	2018	0	0	0	0	0	0	0	0	0	0	0	0	66
15609651	m	ot	JP	2018	0	1	0	0	1	0	0	0	0	0	0	0	
15609673	m	md	IL	2018	0	1	0	0	1	0	0	0	0	0	0	0	70
15609912	Not	ot	IT	2018	0	0	0	0	0	0	0	0	0	0	0	0	
15609955	f	md	JP	2018	0	1	0	0	0	1	0	0	0	0	0	1	73
15609989	Not	md	US	2018	0	1	0	1	0	0	0	0	0	0	0	1	
15610017	Not	ot	CN	2018	0	0	0	0	0	0	0	0	0	0	0	0	
15610349	f	md	DE	2018	0	1	1	0	0	0	0	0	0	0	0	0	67
15611117	Not	ot	CN	2018	0	0	0	0	0	0	0	0	0	0	0	0	
15611397	m	md	JP	2018	0	1	0	0	0	1	0	0	0	0	0	0	50
15611487	f	ot	IT	2018	0	1	0	0	1	0	0	0	0	0	0	0	62
15611684	Not	md	CN	2018	0	0	0	0	0	0	0	0	0	0	0	0	
15611762	m	md	SK	2018	0	0	0	0	0	0	1	0	0	0	1	0	66
15612184	m	md	DE	2018	0	1	0	0	0	1	0	0	0	0	0	0	56
15612215	m	md	JP	2018	0	1	0	1	0	0	0	0	0	0	0	1	41
15612360	m	md	US	2018	0	1	0	1	0	0	0	0	0	0	0	0	
15612407	Not	ph	JP	2018	1	0	0	0	0	0	0	0	0	0	0	0	
15612750	m	md	DE	2018	0	0	0	0	0	0	0	0	0	0	0	0	62
15612765	m	md	DE	2018	0	0	0	0	0	0	0	0	0	0	0	0	62
15612806	f	md	DE	2018	0	0	0	0	0	0	0	0	0	0	0	0	55

15612807	m	md	DE	2018	0	0	0	0	0	0	0	0	0	0	0	0	53
15612809	m	md	DE	2018	0	0	0	0	0	0	0	0	0	0	0	0	56
15612810	m	md	DE	2018	0	0	0	0	0	0	0	0	0	0	0	0	71
15612815	f	md	DE	2018	0	0	0	0	0	0	0	0	0	0	0	0	55
15612817	f	md	DE	2018	0	0	0	0	0	0	0	0	0	0	0	0	42
15613166	m	Not repo	CA	2018	0	1	0	1	0	0	0	0	0	0	0	0	76.6
15613167	f	md	JP	2018	1	0	0	0	0	0	0	0	0	0	0	0	82
15613291	m	md	JP	2018	0	1	0	1	0	0	0	0	0	0	0	1	63.7
15613349	m	md	US	2018	0	0	0	0	0	0	0	0	0	0	0	0	69
15613380	f	md	JP	2018	0	0	0	0	0	0	0	0	0	0	0	0	80
15613395	m	cn	JP	2018	0	1	1	0	0	0	0	0	0	0	0	0	72
15613399	f	cn	ID	2018	1	0	0	0	0	0	0	0	0	0	0	0	
15613475	m	ot	TR	2018	0	1	0	0	1	0	0	0	0	0	0	0	73
15613604	m	ot	JP	2018	0	1	0	0	1	0	0	0	0	0	0	0	80
15613654	f	md	DE	2018	0	0	0	0	0	0	0	0	0	0	0	0	59
15613678	m	ot	US	2018	0	0	0	0	0	0	0	0	0	0	0	0	
15614321	m	ot	FR	2018	0	1	0	0	1	0	0	0	0	0	0	0	71
15614656	m	md	BR	2018	0	1	0	0	1	0	0	0	0	0	0	0	
15614689	Not	md	CH	2018	0	1	0	1	0	0	0	0	0	0	0	0	73
15614766	f	md	FR	2018	0	0	0	0	0	0	0	0	0	0	0	0	
15614811	m	ot	JP	2018	0	1	0	0	1	0	0	0	0	0	0	0	71
15614867	m	md	KR	2018	0	1	0	1	0	0	0	0	0	0	0	0	60.6
15614879	m	cn	JP	2018	0	1	1	0	0	0	0	0	0	0	0	0	80
15614885	Not	ot	US	2018	0	1	0	0	1	0	0	0	0	0	0	0	
15615013	f	cn	US	2018	0	1	1	0	0	0	0	0	0	0	0	0	52
15615469	m	md	JP	2018	1	0	0	0	0	0	0	0	0	0	0	0	71
15615554	f	cn	DE	2018	0	1	1	0	0	0	0	0	0	0	0	1	
15615561	m	ph	JP	2018	1	0	0	0	0	0	0	0	0	0	0	0	
15615837	f	ot	US	2018	0	1	0	0	1	0	0	0	0	0	0	0	61
15615899	f	ot	US	2018	0	1	0	0	1	0	0	0	0	0	0	0	76
15615910	m	ot	DE	2018	0	0	0	0	0	0	0	0	0	0	0	0	
15615985	m	md	BR	2018	0	1	0	0	0	1	0	0	0	0	0	0	72
15616167	m	md	GB	2018	0	1	0	1	0	0	0	0	0	0	0	0	57.5
15616172	f	ot	JP	2018	0	1	0	0	1	0	0	0	0	0	0	0	38

15616259	f	md	MY	2018	0	1	0	0	0	1	0	0	0	0	0	0	73
15616628	f	md	JP	2018	0	1	0	1	0	0	0	0	0	0	0	1	74.1
15616663	m	cn	US	2018	0	0	0	0	0	0	0	0	0	0	0	0	48
15616767	m	md	PT	2018	0	1	0	0	1	0	0	0	0	0	0	0	71
15616934	Not	ot	US	2018	0	1	1	0	0	0	0	0	0	0	0	0	
15616997	m	cn	US	2018	0	1	1	0	0	0	0	0	0	0	0	0	
15617123	Not	md	RU	2018	0	0	0	0	0	0	0	0	0	0	0	0	
15617405	Not	md	US	2018	0	1	0	1	0	0	0	0	0	0	0	0	
15617462	f	ot	US	2018	0	0	0	0	0	0	0	0	0	0	0	0	
15617545	m	md	US	2018	0	1	0	1	0	0	0	0	0	0	0	1	
15617664	f	ot	CZ	2018	0	0	0	0	0	0	0	0	0	0	0	0	24
15617678	m	md	DE	2018	0	0	0	0	0	0	0	0	0	0	0	0	63
15617866	f	md	US	2018	1	0	0	0	0	0	0	0	0	0	0	0	
15617985	m	md	DE	2018	0	0	0	0	0	0	0	0	0	0	0	0	37
15618201	f	md	DE	2018	0	1	1	0	0	0	0	0	0	0	0	0	67
15618202	m	ot	BR	2018	0	1	0	0	1	0	0	0	0	0	0	0	75
15618427	m	cn	IL	2018	0	0	0	0	0	0	0	0	0	0	0	0	
15618530	m	cn	US	2018	1	0	0	0	0	0	0	0	0	0	0	0	
15618844	f	ot	DE	2018	0	0	0	0	0	0	0	0	0	0	0	0	57
15618847	Not	md	DE	2018	0	0	0	0	0	0	0	0	0	0	0	0	
15618919	f	ot	AU	2018	0	1	0	0	1	0	0	0	0	0	0	0	61
15619081	f	md	JP	2018	0	1	0	0	1	0	0	0	0	0	0	0	64
15619362	m	cn	DE	2018	0	1	1	0	0	0	0	0	0	0	0	0	
15620193	m	cn	US	2018	0	0	0	0	0	0	0	0	0	0	0	0	53
15620242	m	cn	US	2018	0	0	0	0	0	0	0	0	0	0	0	0	65
15620424	m	md	HU	2018	0	0	0	0	0	0	0	0	0	0	0	0	
15620702	f	ot	JP	2018	0	0	0	0	0	0	0	0	0	0	0	0	67
15621366	m	md	BR	2018	0	1	0	0	1	0	0	0	0	0	0	0	
15621571	m	ot	JP	2018	0	1	1	0	0	0	0	0	0	0	0	0	73
15621707	m	ot	BG	2018	0	1	0	1	0	0	0	0	0	0	0	0	52
15621906	m	md	DK	2018	0	1	1	0	0	0	0	0	0	0	0	0	75
15621970	f	md	SG	2018	0	0	0	0	0	0	0	0	0	0	0	0	39
15622270	m	ot	GB	2018	0	0	0	0	0	0	0	0	0	0	0	0	70
15622480	Not	md	AT	2018	1	0	0	0	0	0	0	0	0	0	0	0	

15622488	f	md	JP	2018	1	0	0	0	0	0	0	0	0	0	0	0	80
15622503	f	md	US	2018	1	0	0	0	0	0	0	0	0	0	0	0	70
15622619	m	md	JP	2018	0	1	0	1	0	0	0	0	0	0	0	0	62.6
15622712	Not	md	AT	2018	1	0	0	0	0	0	0	0	0	0	0	0	
15622716	m	md	VN	2018	0	1	0	1	0	0	0	0	0	0	0	0	58.7
15622820	f	md	US	2018	0	1	0	1	0	0	0	0	0	0	0	0	
15622856	m	ot	US	2018	0	0	0	0	0	0	0	0	0	0	0	0	67
15623222	m	cn	JP	2018	0	1	1	0	0	0	0	0	0	0	0	0	67
15623244	m	cn	US	2018	0	0	0	0	0	0	0	0	0	0	0	0	74
15623277	m	ot	CN	2018	0	1	0	0	1	0	0	0	0	0	0	1	59
15623282	f	cn	CA	2018	0	0	0	0	0	0	0	0	0	0	0	0	32
15623765	f	md	DE	2018	0	1	0	0	1	0	0	0	0	0	0	0	78
15623817	m	md	JP	2018	0	1	0	1	0	0	0	0	0	0	0	0	66
15623979	f	cn	US	2018	0	0	0	0	0	0	0	0	0	0	0	0	72
15624364	f	md	NL	2018	0	0	0	0	0	0	0	0	0	0	0	0	49
15624731	m	md	JP	2018	1	0	0	0	0	0	0	0	0	0	0	0	78
15625297	m	ot	JP	2018	1	0	0	0	0	0	0	0	0	0	0	0	72
15625413	f	md	KR	2018	1	0	0	0	0	0	0	0	0	0	0	0	78.6
15625474	m	md	JP	2018	0	1	0	1	0	0	0	0	0	0	0	1	70
15625513	Not	cn	JP	2018	0	0	0	0	0	0	0	0	0	0	0	0	60
15625517	Not	ot	NL	2018	0	0	0	0	0	0	0	0	0	0	0	0	
15625523	Not	ot	NL	2018	0	0	0	0	0	0	0	0	0	0	0	0	
15625524	Not	ot	NL	2018	0	0	0	0	0	0	0	0	0	0	0	0	
15625526	Not	ot	NL	2018	0	0	0	0	0	0	0	0	0	0	0	0	
15625708	f	md	IN	2018	0	0	0	0	0	0	0	0	0	0	0	0	63
15626062	f	md	US	2018	0	1	0	1	0	0	0	0	0	0	0	1	
15626198	m	md	JP	2018	0	1	0	0	1	0	0	0	0	0	0	0	65
15626702	m	ot	CN	2018	0	0	0	0	0	0	0	0	0	0	0	0	70
15626866	m	md	DE	2018	0	0	0	0	0	0	0	0	0	0	0	0	64
15627039	Not	ph	JP	2018	1	0	0	0	0	0	0	0	0	0	0	0	
15627334	f	cn	DE	2018	0	1	1	0	0	0	0	0	0	0	0	0	65
15627724	m	md	ES	2018	0	0	0	0	0	0	0	0	0	0	0	0	80
15627983	f	md	DE	2018	0	0	0	0	0	0	0	0	0	0	0	0	55
15628206	f	md	US	2018	0	1	0	1	0	0	0	0	0	0	0	1	

15628257	m	ot	DE	2018	0	0	0	0	0	0	0	0	0	0	0	0	63
15628271	m	ot	DE	2018	0	0	0	0	0	0	0	0	0	0	0	0	37
15628297	f	Not repo	JP	2018	0	0	0	0	0	0	0	0	0	0	0	0	51
15628552	m	cn	DE	2018	0	1	1	0	0	0	0	0	0	0	0	0	75
15628590	Not	md	JP	2018	0	1	1	0	0	0	0	0	0	0	0	1	
15628683	Not	md	JP	2018	1	0	0	0	0	0	0	0	0	0	0	1	
15629017	f	ot	US	2018	0	1	0	0	1	0	0	0	0	0	0	0	71
15629474	Not	md	CN	2018	0	0	0	0	0	0	0	0	0	0	0	0	
15629542	Not	md	CN	2018	0	0	0	0	0	0	0	0	0	0	0	0	
15629856	m	md	DE	2018	0	0	0	0	0	0	0	0	0	0	0	0	72
15630074	Not	md	CN	2018	0	0	0	0	0	0	0	0	0	0	0	0	
15630224	f	cn	Not re	2018	0	0	0	0	0	0	0	0	0	0	0	0	45
15630288	f	cn	AU	2018	0	1	1	0	0	0	0	0	0	0	0	0	
15630364	f	md	US	2018	1	0	0	0	0	0	0	0	0	0	0	0	
15630398	m	md	JP	2018	0	1	0	0	0	1	0	0	0	0	0	0	85
15631048	f	cn	US	2018	0	0	0	0	0	0	0	0	0	0	0	0	56
15631111	m	md	JP	2018	1	0	0	0	0	0	0	0	0	0	0	0	65
15631153	Not	ot	US	2018	0	1	1	0	0	0	0	0	0	0	0	0	
15631774	f	cn	AU	2018	0	0	0	0	0	0	0	0	0	0	0	0	62
15632143	f	ph	GB	2018	0	0	0	0	0	0	0	0	0	0	0	0	60
15632866	f	cn	DE	2018	0	1	1	0	0	0	0	0	0	0	0	1	64
15633348	f	md	FR	2018	0	0	0	0	0	0	0	0	0	0	0	0	66
15633359	m	cn	DE	2018	0	1	1	0	0	0	0	0	0	0	0	0	
15633876	m	cn	JP	2018	0	1	1	0	0	0	0	0	0	0	0	0	78
15633954	f	ot	US	2018	0	1	0	1	0	0	0	0	0	0	0	0	
15634406	Not	md	US	2018	0	0	0	0	0	0	0	0	0	0	0	0	54
15635127	m	md	DE	2018	0	1	0	0	1	0	0	0	0	0	0	0	62
15635329	f	md	JP	2018	1	0	0	0	0	0	0	0	0	0	0	1	79.4
15635439	m	ot	DE	2018	0	1	0	0	1	0	0	0	0	0	0	0	75
15635445	m	md	JP	2018	0	1	0	0	0	1	0	0	0	0	0	0	72
15635772	m	ot	FR	2018	0	1	0	0	1	0	0	0	0	0	0	0	64
15636214	f	md	ES	2018	0	1	0	0	0	1	0	0	0	0	0	0	54
15636316	Not	ph	US	2018	0	1	0	0	1	0	0	0	0	0	0	0	
15636581	f	md	DE	2018	0	1	1	0	0	0	0	0	0	0	0	0	54

15637030	f	ot	FR	2018	0	1	0	0	1	0	0	0	0	0	0	0	56
15637048	f	md	JP	2018	1	0	0	0	0	0	0	0	0	0	0	0	83
15637278	m	md	JO	2018	0	1	1	0	0	0	0	0	0	0	0	1	
15639543	m	ot	US	2018	0	1	0	0	1	0	0	0	0	0	0	0	59
15639808	m	md	DE	2018	0	1	0	0	1	0	0	0	0	0	0	0	64
15639941	f	md	JP	2018	0	1	1	0	0	0	0	0	0	0	0	0	70
15640048	m	cn	JP	2018	0	0	0	0	0	0	0	0	0	0	0	0	75
15640053	m	cn	AR	2018	0	0	0	0	0	0	0	0	0	0	0	0	65
15640953	f	ot	ZA	2018	0	1	0	0	1	0	0	0	0	0	0	0	73
15641008	f	ot	ES	2018	0	1	0	0	1	0	0	0	0	0	0	0	60
15641199	m	ot	JP	2018	0	1	0	0	1	0	0	0	0	0	0	0	78
15641225	m	ot	US	2018	0	1	0	0	1	0	0	0	0	0	0	1	63
15642102	f	cn	US	2018	1	0	0	0	0	0	0	0	0	0	0	0	
15642123	m	cn	US	2018	1	0	0	0	0	0	0	0	0	0	0	0	
15642247	f	md	US	2018	0	1	1	0	0	0	0	0	0	0	0	0	
15642452	Not	md	IT	2018	1	0	0	0	0	0	0	0	0	0	0	0	
15642514	m	md	JP	2018	0	1	0	1	0	0	0	0	0	0	0	1	76
15642598	f	cn	US	2018	1	0	0	0	0	0	0	0	0	0	0	0	
15642683	f	cn	US	2018	1	0	0	0	0	0	0	0	0	0	0	0	76
15642783	f	md	IT	2018	0	0	0	0	0	0	0	0	0	0	0	0	41
15642919	m	md	JP	2018	0	1	0	0	0	1	0	0	0	0	0	0	68
15642951	m	md	JP	2018	0	1	0	1	0	0	0	0	0	0	0	1	45
15643274	f	ot	JP	2018	0	0	0	0	0	0	0	0	0	0	0	0	83
15643546	Not	md	US	2018	0	1	0	0	0	1	0	0	0	0	0	0	80
15644438	Not	ot	CN	2018	1	0	0	0	0	0	0	0	0	0	0	0	
15644688	f	md	US	2018	0	1	0	1	0	0	0	0	0	0	0	0	
15644697	m	md	FR	2018	0	0	0	0	0	0	0	0	0	0	0	0	74
15644747	m	ot	US	2018	0	1	0	0	1	0	0	0	0	0	0	0	47
15644839	f	ot	US	2018	1	0	0	0	0	0	0	0	0	0	0	0	79
15645228	f	ot	CN	2018	0	1	0	0	1	0	0	0	0	0	0	0	63
15645552	f	ot	CZ	2018	0	0	0	0	0	0	0	0	0	0	0	0	57
15646366	f	ph	JP	2018	1	0	0	0	0	0	0	0	0	0	0	0	70
15646451	m	md	JP	2018	0	1	0	0	0	1	0	0	0	0	0	0	72
15646507	m	md	JP	2018	0	1	0	0	1	0	0	0	0	0	0	0	74

15646619	m	Not repo	US	2018	1	0	0	0	0	0	0	0	0	0	0	0	74.1
15646742	f	ot	US	2018	0	1	0	0	1	0	0	0	0	0	0	0	
15646989	f	md	JP	2018	1	0	0	0	0	0	0	0	0	0	0	0	84
15647012	m	md	US	2018	0	1	0	0	0	1	0	0	0	0	0	0	
15647508	m	ot	DE	2018	0	1	0	0	1	0	0	0	0	0	0	0	64
15647740	m	md	NO	2018	0	1	0	0	0	1	0	0	0	0	0	0	42
15647993	m	cn	JP	2018	0	1	1	0	0	0	0	0	0	0	0	1	74
15648033	m	md	CN	2018	0	1	0	0	1	0	0	0	0	0	0	0	69
15648039	m	cn	JP	2018	0	0	0	0	0	0	0	0	0	0	0	0	75
15648247	f	md	JP	2018	0	1	0	0	1	0	0	0	0	0	0	1	84
15648569	m	cn	DE	2018	0	1	1	0	0	0	0	0	0	0	0	0	62
15648730	f	cn	DE	2018	0	1	1	0	0	0	0	0	0	0	0	0	53
15649486	m	cn	US	2018	0	1	1	0	0	0	0	0	0	0	0	1	
15649696	m	md	AU	2018	0	1	0	0	0	1	0	0	0	0	0	0	68
15649956	m	ot	FR	2018	0	1	0	0	1	0	0	0	0	0	0	0	79
15650024	f	md	MY	2018	0	1	0	0	0	1	0	0	0	0	0	1	
15650272	f	cn	DE	2018	1	0	0	0	0	0	0	0	0	0	0	0	
15650325	m	md	JP	2018	0	1	0	0	1	0	0	0	0	0	0	0	72
15650372	m	ot	US	2018	0	1	0	0	1	0	0	0	0	0	0	0	66
15650575	m	md	AU	2018	0	1	0	0	0	1	0	0	0	0	0	0	
15651116	m	cn	US	2018	0	1	1	0	0	0	0	0	0	0	0	0	79
15651519	m	ot	CA	2018	0	1	1	0	0	0	0	0	0	0	0	0	
15651524	f	cn	US	2018	0	1	0	0	1	0	0	0	0	0	0	0	78
15651650	m	md	JP	2018	0	1	0	1	0	0	0	0	0	0	0	1	83.5
15651780	m	ot	JP	2018	1	0	0	0	0	0	0	0	0	0	0	0	
15651814	m	cn	Not re	2018	0	0	0	0	0	0	0	0	0	0	0	0	51
15651817	Not	md	JP	2018	1	0	0	0	0	0	0	0	0	0	0	0	80
15652001	m	cn	JP	2018	0	1	1	0	0	0	0	0	0	0	0	0	75
15652044	f	md	FR	2018	1	0	0	0	0	0	0	0	0	0	0	0	75
15652046	f	md	JP	2018	1	0	0	0	0	0	0	0	0	0	0	0	72
15652134	m	md	JP	2018	0	1	0	1	0	0	0	0	0	0	0	1	69
15652150	f	cn	DE	2018	0	1	1	0	0	0	0	0	0	0	0	0	65
15652166	Not	md	JP	2018	1	0	0	0	0	0	0	0	0	0	0	1	90
15652167	m	md	DE	2018	1	0	0	0	0	0	0	0	0	0	0	1	79

15652240	m	md	TR	2018	0	1	0	0	1	0	0	0	0	0	0	0	46
15652738	Not	md	JP	2018	0	1	1	0	0	0	0	0	0	0	0	0	
15652889	m	ot	AT	2018	0	0	0	0	0	0	0	0	0	0	0	0	54
15653628	m	cn	JP	2018	0	1	1	0	0	0	0	0	0	0	0	0	76
15654486	f	md	BE	2018	0	1	0	0	1	0	0	0	0	0	0	0	57
15654564	f	cn	GB	2018	0	1	1	0	0	0	0	0	0	0	0	0	
15654608	Not	md	BE	2018	0	1	0	0	1	0	0	0	0	0	0	0	69
15654865	m	cn	US	2018	0	1	1	0	0	0	0	0	0	0	0	0	
15654986	f	md	CZ	2018	1	0	0	0	0	0	0	0	0	0	0	0	67
15655324	m	md	JP	2018	1	0	0	0	0	0	0	0	0	0	0	0	80
15655325	m	ph	US	2018	0	1	0	1	0	0	0	0	0	0	0	0	88
15655358	m	md	JP	2018	1	0	0	0	0	0	0	0	0	0	0	0	58
15655871	m	cn	DE	2018	0	1	1	0	0	0	0	0	0	0	0	1	72
15656138	m	md	TW	2018	0	0	0	0	0	0	0	0	0	0	0	0	69
15656804	Not	cn	ID	2018	0	0	0	0	0	0	0	0	0	0	0	0	
15657023	f	cn	GB	2018	1	0	0	0	0	0	0	0	0	0	0	0	65
15657029	f	cn	GB	2018	1	0	0	0	0	0	0	0	0	0	0	0	73
15657858	m	md	JP	2018	0	0	0	0	0	0	0	0	0	0	0	0	
15657861	m	md	JP	2018	0	0	0	0	0	0	0	0	0	0	0	0	
15658002	m	ot	ES	2018	0	1	0	0	1	0	0	0	0	0	0	0	70
15658646	f	md	JP	2018	0	0	0	0	0	0	0	0	0	0	0	0	70
15658878	Not	md	JP	2018	0	1	1	0	0	0	0	0	0	0	0	1	
15658880	m	md	FR	2018	0	0	0	0	0	0	1	0	1	0	0	0	59
15659130	m	md	JP	2018	0	0	0	0	0	0	0	0	0	0	0	0	
15659292	m	ot	IL	2018	0	1	1	0	0	0	0	0	0	0	0	0	76
15659298	Not	md	JP	2018	0	1	1	0	0	0	0	0	0	0	0	0	
15660188	m	cn	DE	2018	0	1	1	0	0	0	0	0	0	0	0	1	66
15660508	m	md	AU	2018	0	0	0	0	0	0	0	0	0	0	0	0	55
15660576	f	md	JP	2018	0	0	0	0	0	0	0	0	0	0	0	0	90
15660603	f	cn	DE	2018	0	1	1	0	0	0	0	0	0	0	0	1	64
15660658	m	ph	US	2018	0	1	0	1	0	0	0	0	0	0	0	0	66
15660698	m	ot	GB	2018	0	0	0	0	0	0	0	0	0	0	0	0	51
15660853	f	cn	GB	2018	1	0	0	0	0	0	0	0	0	0	0	0	81
15660906	Not	md	JP	2018	0	1	1	0	0	0	0	0	0	0	0	0	

15660950	Not	md	GB	2018	0	0	0	0	0	0	0	0	0	0	0	0	
15661335	f	ph	US	2018	0	1	0	1	0	0	0	0	0	0	0	0	76.1
15661370	Not	ot	VN	2018	0	0	0	0	0	0	0	0	0	0	0	0	
15661546	m	md	SE	2018	0	0	0	0	0	0	0	0	0	0	0	0	64
15661577	f	cn	AU	2018	0	1	0	1	0	0	0	0	0	0	0	0	80
15661627	m	md	FR	2018	0	0	0	0	0	0	0	0	0	0	0	0	74
15661637	m	md	FR	2018	0	0	0	0	0	0	0	0	0	0	0	0	
15661753	m	md	DE	2018	0	0	0	0	0	0	0	0	0	0	0	0	50
15661759	f	md	JP	2018	0	0	0	0	0	0	0	0	0	0	0	0	60
15661971	m	cn	JP	2018	0	1	1	0	0	0	0	0	0	0	0	1	65
15662055	m	cn	Not re	2018	0	0	0	0	0	0	0	0	0	0	0	0	
15662310	m	md	DE	2018	0	0	0	0	0	0	0	0	0	0	0	0	53
15662312	m	md	JP	2018	0	0	0	0	0	0	0	0	0	0	0	0	
15662484	f	md	FR	2018	0	1	0	0	1	0	0	0	0	0	0	0	74
15663325	f	md	US	2018	0	1	0	0	1	0	0	0	0	0	0	1	75
15663475	m	ph	JP	2018	0	1	0	0	1	0	0	0	0	0	0	0	72
15663620	f	cn	GB	2018	1	0	0	0	0	0	0	0	0	0	0	0	
15663917	Not	md	CN	2018	1	0	0	0	0	0	0	0	0	0	0	0	
15664051	m	ot	US	2018	0	1	0	0	0	1	0	0	0	0	0	0	
15664066	f	cn	US	2018	0	0	0	0	0	0	0	0	0	0	0	0	70
15664150	f	md	HU	2018	0	0	0	0	0	0	0	0	0	0	0	0	60
15664159	f	cn	GB	2018	1	0	0	0	0	0	0	0	0	0	0	0	70
15664275	m	ph	US	2018	0	1	0	0	1	0	0	0	0	0	0	0	68
15664304	m	ph	US	2018	0	1	0	0	1	0	0	0	0	0	0	0	68
15664332	f	cn	NZ	2018	0	0	0	0	0	0	0	0	0	0	0	0	
15664509	f	ot	JP	2018	1	0	0	0	0	0	0	0	0	0	0	0	64
15664780	m	md	FR	2018	0	0	0	0	0	0	0	0	0	0	0	0	69
15664889	f	ot	JP	2018	1	0	0	0	0	0	0	0	0	0	0	0	48
15664890	f	ot	JP	2018	1	0	0	0	0	0	0	0	0	0	0	0	51
15665207	f	ot	JP	2018	1	0	0	0	0	0	0	0	0	0	0	0	43
15665380	m	md	SE	2018	0	0	0	0	0	0	0	0	0	0	0	0	64
15665610	m	ot	GR	2018	0	1	1	0	0	0	0	0	0	0	0	0	
15665742	Not	ot	VN	2018	0	0	0	0	0	0	0	0	0	0	0	0	
15665757	f	ot	IT	2018	0	0	0	0	0	0	0	0	0	0	0	0	62

15665796	m	md	CN	2018	0	0	0	0	0	0	0	0	0	0	0	0	51
15665817	f	cn	GB	2018	1	0	0	0	0	0	0	0	0	0	0	1	59
15666062	f	ot	JP	2018	1	0	0	0	0	0	0	0	0	0	0	0	61
15666151	m	Not repo	JP	2018	0	0	0	0	0	0	0	0	0	0	0	0	76
15666269	f	ot	JP	2018	1	0	0	0	0	0	0	0	0	0	0	0	74
15666352	f	ot	JP	2018	1	0	0	0	0	0	0	0	0	0	0	0	79
15666460	Not	ot	US	2018	0	1	0	1	0	0	0	0	0	0	0	0	
15667415	f	ot	PL	2018	0	0	0	0	0	0	0	0	0	0	0	0	59
15667458	m	ot	CN	2018	0	1	0	0	1	0	0	0	0	0	0	0	68
15667656	m	ot	CZ	2018	0	0	0	0	0	0	0	0	0	0	0	0	68
15667669	m	ot	DE	2018	0	1	1	0	0	0	0	0	0	0	0	0	75
15667704	m	cn	IN	2018	0	0	0	0	0	0	0	0	0	0	0	0	59
15668109	m	ot	DE	2018	0	1	0	0	1	0	0	0	0	0	0	0	75
15668376	f	cn	US	2018	0	1	0	0	1	0	0	0	0	0	0	0	
15668410	Not	md	JP	2018	0	1	1	0	0	0	0	0	0	0	0	0	
15668557	f	ot	FR	2018	0	1	0	0	1	0	0	0	0	0	0	0	67
15668655	m	cn	US	2018	0	1	0	0	1	0	0	0	0	0	0	0	
15668789	f	cn	US	2018	0	1	1	0	0	0	0	0	0	0	0	0	73
15668888	Not	md	JP	2018	0	1	1	0	0	0	0	0	0	0	0	1	
15669116	m	ot	GB	2018	0	0	0	0	0	0	0	0	0	0	0	0	
15669140	f	ot	US	2018	0	1	0	0	1	0	0	0	0	0	0	0	64
15669182	m	md	JP	2018	0	1	1	0	0	0	0	0	0	0	0	0	59
15669274	m	md	JP	2018	0	1	0	0	0	1	0	0	0	0	0	0	
15669525	m	md	IN	2018	0	1	0	0	1	0	0	0	0	0	0	0	65
15669560	m	md	DE	2018	0	0	0	0	0	0	0	0	0	0	0	0	56
15670508	m	md	JP	2018	1	0	0	0	0	0	0	0	0	0	0	1	74.8
15671663	m	ot	GB	2018	0	1	1	0	0	0	0	0	0	0	0	0	75
15671754	m	ot	CN	2018	0	1	0	0	1	0	0	0	0	0	0	0	72
15671887	m	ot	GB	2018	0	0	0	0	0	0	0	0	0	0	0	0	74
15672646	f	ot	US	2018	0	1	1	0	0	0	0	0	0	0	0	0	74
15673092	f	cn	ID	2018	1	0	0	0	0	0	0	0	0	0	0	0	
15673171	m	cn	JP	2018	0	1	1	0	0	0	0	0	0	0	0	0	70
15673273	m	ot	DE	2018	0	0	0	0	0	0	0	0	0	0	0	0	59
15673702	m	cn	US	2018	1	0	0	0	0	0	0	0	0	0	0	0	

15673935	m	md	EG	2018	0	1	0	0	1	0	0	0	0	0	0	0	52
15673949	m	md	JP	2018	0	1	0	1	0	0	0	0	0	0	0	1	66.9
15674086	m	md	DE	2018	0	1	0	0	1	0	0	0	0	0	0	0	65
15674155	m	cn	US	2018	1	0	0	0	0	0	0	0	0	0	0	0	74
15674205	m	ot	JP	2018	0	1	0	0	1	0	0	0	0	0	0	0	86
15674441	m	md	US	2018	0	1	0	0	1	0	0	0	0	0	0	0	60
15674824	m	ot	JP	2018	0	1	0	0	0	1	0	0	0	0	0	0	68
15674897	Not	md	JP	2018	0	1	1	0	0	0	0	0	0	0	0	1	
15675577	Not	cn	JP	2018	0	1	1	0	0	0	0	0	0	0	0	1	
15675882	Not	ot	US	2018	0	0	0	0	0	0	0	0	0	0	0	0	
15675931	f	md	PE	2018	0	1	0	1	0	0	0	0	0	0	0	0	55
15676191	f	cn	JP	2018	0	1	1	0	0	0	0	0	0	0	0	0	80
15676969	m	cn	DE	2018	0	1	0	0	1	0	0	0	0	0	0	0	61
15676986	Not	md	US	2018	0	1	0	1	0	0	0	0	0	0	0	0	
15677194	m	ph	JP	2018	0	1	0	0	1	0	0	0	0	0	0	0	77
15677712	f	cn	JP	2018	0	0	0	0	0	0	0	0	0	0	0	0	48
15677720	f	cn	AT	2018	0	0	0	0	0	0	0	0	0	0	0	0	
15677843	m	ot	US	2018	0	0	0	0	0	0	0	0	0	0	0	0	52
15678441	m	md	NO	2018	0	1	1	0	0	0	0	0	0	0	0	0	64
15678623	m	md	JP	2018	0	1	0	0	1	0	0	0	0	0	0	0	64
15678757	m	cn	US	2018	0	1	0	0	1	0	0	0	0	0	0	1	66
15679426	f	ot	CA	2018	0	1	0	1	0	0	0	0	0	0	0	0	75
15679472	m	ot	FR	2018	0	1	0	0	1	0	0	0	0	0	0	0	73
15679723	f	ph	US	2018	1	0	0	0	0	0	0	0	0	0	0	0	67
15679798	m	ot	GB	2018	0	0	0	0	0	0	1	0	1	0	0	0	59
15679859	f	ot	AR	2018	0	1	0	0	1	0	0	0	0	0	0	0	62
15680024	m	ot	US	2018	0	1	0	1	0	0	0	0	0	0	0	0	
15680279	f	ot	IT	2018	0	0	0	0	0	0	0	0	0	0	0	0	62
15680452	f	md	CA	2018	0	1	0	1	0	0	0	0	0	0	0	0	
15680492	m	cn	JP	2018	0	1	1	0	0	0	0	0	0	0	0	0	64
15680625	f	md	BR	2018	0	0	0	0	0	0	0	0	0	0	0	0	57
15680723	m	cn	JP	2018	0	1	1	0	0	0	0	0	0	0	0	0	80
15681368	m	cn	FR	2018	0	0	0	0	0	0	0	0	0	0	0	0	61
15681733	f	md	FR	2018	0	0	0	0	0	0	0	0	0	0	0	0	94

15681952	m	md	IT	2018	0	0	0	0	0	0	0	0	0	0	0	0	64
15682054	m	md	JP	2018	0	1	0	1	0	0	0	0	0	0	0	0	60
15682324	m	cn	US	2018	0	1	1	0	0	0	0	0	0	0	0	1	81
15682478	m	md	CA	2018	0	1	1	0	0	0	0	0	0	0	0	0	64
15682737	f	cn	IN	2018	0	1	0	0	1	0	0	0	0	0	0	0	
15682966	f	cn	AU	2018	0	1	1	0	0	0	0	0	0	0	0	0	78
15683560	f	ph	CA	2018	0	1	1	0	0	0	0	0	0	0	0	1	63
15683614	Not	md	BE	2018	0	0	0	0	0	0	0	0	0	0	0	0	
15683782	m	ot	JP	2018	0	0	0	0	0	0	1	0	0	0	1	1	79
15683790	f	md	HR	2018	1	0	0	0	0	0	0	0	0	0	0	0	83
15683902	m	cn	JP	2018	0	1	1	0	0	0	0	0	0	0	0	0	74
15684196	Not	cn	CA	2018	0	1	1	0	0	0	0	0	0	0	0	0	
15684225	f	ot	US	2018	0	1	0	0	1	0	0	0	0	0	0	0	
15684231	f	md	JP	2018	0	1	0	0	0	1	0	0	0	0	0	0	74
15684316	m	md	JP	2018	0	1	0	1	0	0	0	0	0	0	0	1	77
15684349	m	md	US	2018	0	1	0	1	0	0	0	0	0	0	0	1	
15684740	m	md	JP	2018	0	1	0	1	0	0	0	0	0	0	0	1	80
15684750	f	md	JP	2018	0	1	0	0	1	0	0	0	0	0	0	1	66
15685047	m	ot	US	2018	0	1	0	0	1	0	0	0	0	0	0	1	75
15685178	f	cn	IN	2018	0	1	0	0	1	0	0	0	0	0	0	0	66
15685257	m	ot	DE	2018	0	0	0	0	0	0	0	0	0	0	0	0	8
15685579	Not	cn	CA	2018	0	1	1	0	0	0	0	0	0	0	0	0	
15685640	m	cn	IN	2018	0	1	0	0	1	0	0	0	0	0	0	0	
15686351	m	cn	JP	2018	0	1	1	0	0	0	0	0	0	0	0	0	62
15686829	m	md	FR	2018	0	0	0	0	0	0	0	0	0	0	0	0	51
15687569	m	ot	RO	2018	0	1	0	0	1	0	0	0	0	0	0	0	62
15688265	m	cn	DE	2018	0	1	1	0	0	0	0	0	0	0	0	0	
15688290	f	ot	FR	2018	0	1	0	0	1	0	0	0	0	0	0	0	48
15688363	m	md	HK	2018	0	1	1	0	0	0	0	0	0	0	0	1	
15688693	m	ot	US	2018	0	1	0	0	1	0	0	0	0	0	0	0	
15688695	m	ot	US	2018	0	1	0	0	1	0	0	0	0	0	0	0	
15688793	m	md	IT	2018	0	0	0	0	0	0	0	0	0	0	0	0	47
15688876	f	md	CN	2018	0	0	0	0	0	0	0	0	0	0	0	0	66
15688881	m	md	JP	2018	0	1	0	1	0	0	0	0	0	0	0	0	70

15688903	f	md	FR	2018	0	0	0	0	0	0	0	0	0	0	0	0	81
15688996	m	cn	JP	2018	0	1	1	0	0	0	0	0	0	0	0	1	75
15689463	f	ot	DE	2018	0	1	1	0	0	0	0	0	0	0	0	0	53
15690026	m	cn	DE	2018	0	1	1	0	0	0	0	0	0	0	0	0	75
15690182	f	md	JP	2018	1	0	0	0	0	0	0	0	0	0	0	0	90
15690408	f	md	IT	2018	0	0	0	0	0	0	0	0	0	0	0	0	74
15691149	f	md	JP	2018	0	1	0	1	0	0	0	0	0	0	0	1	50
15691653	f	ot	IT	2018	0	1	0	0	1	0	0	0	0	0	0	0	78
15691738	m	cn	US	2018	0	1	1	0	0	0	0	0	0	0	0	0	63
15692357	Not	md	JP	2018	0	1	0	0	1	0	0	0	0	0	0	0	
15692378	f	cn	US	2018	0	1	1	0	0	0	0	0	0	0	0	0	
15692413	Not	ot	AU	2018	0	1	0	0	1	0	0	0	0	0	0	0	
15692414	Not	ot	AU	2018	0	1	0	0	1	0	0	0	0	0	0	0	
15692617	Not	ot	AU	2018	0	1	0	0	1	0	0	0	0	0	0	0	
15692647	f	cn	CA	2018	0	0	0	0	0	0	0	0	0	0	0	0	
15692879	m	ot	JP	2018	0	1	1	0	0	0	0	0	0	0	0	1	71
15693007	m	md	JP	2018	0	1	0	0	0	1	0	0	0	0	0	0	60
15693112	f	ot	JP	2018	0	1	0	1	0	0	0	0	0	0	0	1	70
15693241	m	md	US	2018	0	0	0	0	0	0	0	0	0	0	0	0	
15693448	f	md	JP	2018	0	0	0	0	0	0	0	0	0	0	0	0	76
15693465	m	md	DE	2018	0	0	0	0	0	0	0	0	0	0	0	0	68
15693669	m	md	US	2018	1	0	0	0	0	0	0	0	0	0	0	0	
15694340	m	md	HR	2018	0	0	0	0	0	0	0	0	0	0	0	0	50
15694709	Not	md	JP	2018	0	1	0	0	1	0	0	0	0	0	0	0	
15694915	Not	ot	AU	2018	0	1	0	0	1	0	0	0	0	0	0	0	
15695327	m	cn	US	2018	0	0	0	0	0	0	0	0	0	0	0	0	
15695569	f	cn	US	2018	0	1	1	0	0	0	0	0	0	0	0	0	80
15695587	m	md	PT	2018	1	0	0	0	0	0	0	0	0	0	0	0	
15695593	m	md	JP	2018	0	1	0	1	0	0	0	0	0	0	0	1	67.5
15695727	f	md	JP	2018	0	0	0	0	0	0	0	0	0	0	0	0	66
15695847	m	md	JP	2018	0	1	0	1	0	0	0	0	0	0	0	1	60
15696992	m	md	JP	2018	0	1	0	1	0	0	0	0	0	0	0	1	64
15697010	m	md	US	2018	0	1	0	1	0	0	0	0	0	0	0	0	65
15697488	f	md	PA	2018	0	1	0	0	0	1	0	0	0	0	0	0	

15697738	m	md	JP	2018	0	1	0	1	0	0	0	0	0	0	0	0	80
15698794	f	cn	US	2018	0	1	1	0	0	0	0	0	0	0	0	0	
15699049	m	md	KR	2018	0	1	0	0	0	1	0	0	0	0	0	1	72
15699194	m	cn	PH	2018	0	1	1	0	0	0	0	0	0	0	0	0	62
15699284	m	ot	CN	2018	0	1	0	0	1	0	0	0	0	0	0	0	62
15699417	m	md	JP	2018	0	1	0	0	1	0	0	0	0	0	0	0	65
15699756	m	ph	FR	2018	0	0	0	0	0	0	0	0	0	0	0	0	87
15699941	f	md	CZ	2018	0	0	0	0	0	0	0	0	0	0	0	0	21
15700007	m	md	JP	2018	0	1	0	1	0	0	0	0	0	0	0	1	78.9
15700052	m	cn	US	2018	0	1	1	0	0	0	0	0	0	0	0	0	
15700233	m	ot	CY	2018	1	0	0	0	0	0	0	0	0	0	0	0	58
15700368	f	ph	JP	2018	1	0	0	0	0	0	0	0	0	0	0	0	69
15700486	m	md	NO	2018	0	0	0	0	0	0	0	0	0	0	0	0	52
15700514	f	md	JP	2018	1	0	0	0	0	0	0	0	0	0	0	0	64
15700674	m	md	DE	2018	0	0	0	0	0	0	0	0	0	0	0	0	68
15701601	m	md	CN	2018	0	0	0	0	0	0	0	0	0	0	0	0	58
15701604	f	md	CN	2018	0	0	0	0	0	0	0	0	0	0	0	0	55
15701606	m	md	CN	2018	0	0	0	0	0	0	0	0	0	0	0	0	65
15702023	m	cn	US	2018	0	1	1	0	0	0	0	0	0	0	0	0	58
15702210	Not	md	JP	2018	0	1	0	0	1	0	0	0	0	0	0	0	
15702287	Not	md	JP	2018	0	1	0	0	1	0	0	0	0	0	0	0	
15702440	m	ot	CH	2018	0	1	0	0	1	0	0	0	0	0	0	0	69
15702474	m	md	DE	2018	0	0	0	0	0	0	0	0	0	0	0	0	68
15702820	m	md	JP	2018	0	1	0	0	0	1	0	0	0	0	0	0	73
15703134	Not	md	CN	2018	0	0	0	0	0	0	0	0	0	0	0	0	
15703607	m	cn	US	2018	0	0	0	0	0	0	0	0	0	0	0	0	60
15704163	Not	md	JP	2018	1	0	0	0	0	0	0	0	0	0	0	0	
15704316	Not	md	JP	2018	0	1	0	0	1	0	0	0	0	0	0	0	
15704346	f	md	JP	2018	1	0	0	0	0	0	0	0	0	0	0	1	81
15704454	m	md	DK	2018	0	0	0	0	0	0	0	0	0	0	0	0	69
15705188	m	md	JP	2018	0	0	0	0	0	0	0	0	0	0	0	0	70
15705367	m	cn	JP	2018	0	1	1	0	0	0	0	0	0	0	0	1	70
15705649	f	md	US	2018	0	0	0	0	0	0	0	0	0	0	0	0	68
15706311	m	cn	JP	2018	0	1	1	0	0	0	0	0	0	0	0	1	75

15706585	m	ot	GB	2018	0	0	0	0	0	0	0	0	0	0	0	0	71
15707617	m	cn	IL	2018	0	1	1	0	0	0	0	0	0	0	0	0	86
15707934	m	md	FR	2018	1	0	0	0	0	0	0	0	0	0	0	0	
15708183	m	md	CN	2018	0	0	0	0	0	0	0	0	0	0	0	0	54
15708668	m	md	JP	2018	0	1	0	1	0	0	0	0	0	0	0	1	69
15708830	f	md	JP	2018	0	0	0	0	0	0	0	0	0	0	0	0	57
15709028	m	ot	US	2018	0	1	0	0	1	0	0	0	0	0	0	0	75
15709811	f	md	KR	2018	1	0	0	0	0	0	0	0	0	0	0	0	64.6
15709818	m	ot	JP	2018	0	1	0	1	0	0	0	0	0	0	0	0	70
15710262	m	md	JP	2018	1	0	0	0	0	0	0	0	0	0	0	1	65
15710773	m	ot	CN	2018	0	1	0	0	1	0	0	0	0	0	0	0	46
15711101	m	ph	BR	2018	1	0	0	0	0	0	0	0	0	0	0	0	53
15711247	f	cn	US	2018	0	1	0	0	1	0	0	0	0	0	0	1	
15711393	m	md	US	2018	1	0	0	0	0	0	0	0	0	0	0	0	
15711701	m	md	JP	2018	0	1	0	0	1	0	0	0	0	0	0	0	83
15711772	f	md	US	2018	0	0	0	0	0	0	0	0	0	0	0	0	59
15711871	m	md	JP	2018	0	1	0	0	1	0	0	0	0	0	0	0	78
15712048	f	cn	US	2018	0	1	1	0	0	0	0	0	0	0	0	0	71
15712574	m	md	JP	2018	0	1	0	0	0	1	0	0	0	0	0	0	67
15712649	m	md	JP	2018	0	1	0	0	0	1	0	0	0	0	0	0	76
15713041	f	md	JP	2018	0	1	0	1	0	0	0	0	0	0	0	1	80
15713322	f	ot	US	2018	0	0	0	0	0	0	0	0	0	0	0	0	
15713380	m	ph	US	2018	0	0	0	0	0	0	0	0	0	0	0	0	67
15713704	m	md	FR	2018	0	1	1	0	0	0	0	0	0	0	0	0	65
15713747	m	md	CA	2018	0	1	0	0	0	1	0	0	0	0	0	0	
15714530	f	md	US	2018	0	1	0	1	0	0	0	0	0	0	0	1	71
15714532	m	md	JP	2018	0	1	0	1	0	0	0	0	0	0	0	1	80
15715178	m	md	BE	2018	0	0	0	0	0	0	0	0	0	0	0	0	73
15715337	m	md	JP	2018	0	1	0	0	1	0	0	0	0	0	0	0	73
15715434	m	cn	JP	2018	0	1	1	0	0	0	0	0	0	0	0	0	80
15715474	m	ot	US	2018	0	0	0	0	0	0	0	0	0	0	0	0	46
15715502	f	ot	US	2018	0	1	0	0	1	0	0	0	0	0	0	0	84
15715521	f	md	DE	2018	0	1	1	0	0	0	0	0	0	0	0	0	69
15715527	Not	ot	GB	2018	0	0	0	0	0	0	0	0	0	0	0	0	

15715556	Not	md	US	2018	0	0	0	0	0	0	0	0	0	0	0	0	
15715583	f	md	SI	2018	0	1	1	0	0	0	0	0	0	0	0	0	67
15715864	m	ot	ES	2018	0	0	0	0	0	0	0	0	0	0	0	0	74
15717399	f	ot	JP	2018	0	1	0	1	0	0	0	0	0	0	0	1	73
15717466	f	cn	US	2018	1	0	0	0	0	0	0	0	0	0	0	0	
15717492	f	cn	GB	2018	1	0	0	0	0	0	0	0	0	0	0	0	
15717498	f	cn	GB	2018	1	0	0	0	0	0	0	0	0	0	0	0	
15717524	m	cn	GB	2018	1	0	0	0	0	0	0	0	0	0	0	0	82
15717532	m	cn	GB	2018	1	0	0	0	0	0	0	0	0	0	0	0	
15717571	m	cn	GB	2018	1	0	0	0	0	0	0	0	0	0	0	0	
15717592	m	cn	GB	2018	1	0	0	0	0	0	0	0	0	0	0	0	
15717597	f	md	US	2018	1	0	0	0	0	0	0	0	0	0	0	0	58
15717655	m	cn	GB	2018	1	0	0	0	0	0	0	0	0	0	0	0	
15717667	f	cn	GB	2018	0	0	0	0	0	0	1	1	0	0	0	0	87
15717693	m	cn	GB	2018	1	0	0	0	0	0	0	0	0	0	0	0	
15717709	f	cn	GB	2018	1	0	0	0	0	0	0	0	0	0	0	0	72
15717713	m	cn	GB	2018	1	0	0	0	0	0	0	0	0	0	0	0	61
15717869	f	cn	GB	2018	1	0	0	0	0	0	0	0	0	0	0	0	
15718036	m	cn	DE	2018	0	1	1	0	0	0	0	0	0	0	0	0	
15718050	m	cn	GB	2018	1	0	0	0	0	0	0	0	0	0	0	0	
15718069	m	cn	GB	2018	1	0	0	0	0	0	0	0	0	0	0	0	
15718070	m	cn	GB	2018	1	0	0	0	0	0	0	0	0	0	0	0	61
15718095	f	cn	GB	2018	1	0	0	0	0	0	0	0	0	0	0	0	
15718109	m	cn	GB	2018	1	0	0	0	0	0	0	0	0	0	0	0	
15718147	f	cn	GB	2018	1	0	0	0	0	0	0	0	0	0	0	0	
15718157	m	cn	GB	2018	1	0	0	0	0	0	0	0	0	0	0	0	
15718170	f	cn	GB	2018	1	0	0	0	0	0	0	0	0	0	0	0	92
15718194	m	cn	GB	2018	1	0	0	0	0	0	0	0	0	0	0	0	
15718246	f	cn	GB	2018	1	0	0	0	0	0	0	0	0	0	0	0	
15718263	Not	cn	GB	2018	1	0	0	0	0	0	0	0	0	0	0	0	
15718294	m	cn	GB	2018	1	0	0	0	0	0	0	0	0	0	0	0	66
15718321	f	cn	GB	2018	1	0	0	0	0	0	0	0	0	0	0	0	86
15718322	m	cn	GB	2018	1	0	0	0	0	0	0	0	0	0	0	0	
15718344	f	cn	GB	2018	1	0	0	0	0	0	0	0	0	0	0	0	

15718345	f	cn	GB	2018	1	0	0	0	0	0	0	0	0	0	0	0	68
15718376	f	cn	GB	2018	1	0	0	0	0	0	0	0	0	0	0	0	
15718381	f	cn	GB	2018	1	0	0	0	0	0	0	0	0	0	0	0	
15718396	f	cn	GB	2018	1	0	0	0	0	0	0	0	0	0	0	0	
15718494	f	cn	GB	2018	1	0	0	0	0	0	0	0	0	0	0	0	83
15718534	m	cn	GB	2018	1	0	0	0	0	0	0	0	0	0	0	0	
15718559	f	cn	GB	2018	1	0	0	0	0	0	0	0	0	0	0	0	
15718572	m	cn	GB	2018	1	0	0	0	0	0	0	0	0	0	0	0	
15719169	m	md	JP	2018	0	1	0	0	1	0	0	0	0	0	0	0	58
15719445	m	ot	NL	2018	0	0	0	0	0	0	0	0	0	0	0	0	71
15719801	f	md	US	2018	0	1	0	1	0	0	0	0	0	0	0	0	
15720313	f	ot	RO	2018	0	1	0	0	1	0	0	0	0	0	0	0	74
15720633	f	md	US	2018	1	0	0	0	0	0	0	0	0	0	0	0	60
15720641	Not	cn	JP	2018	0	1	1	0	0	0	0	0	0	0	0	0	70
15720803	f	md	ES	2018	0	1	0	0	1	0	0	0	0	0	0	0	78
15720850	m	md	IN	2018	0	1	0	0	1	0	0	0	0	0	0	0	
15721163	m	md	JP	2018	0	0	0	0	0	0	0	0	0	0	0	0	51
15721688	m	ot	ES	2018	0	1	0	0	1	0	0	0	0	0	0	0	65
15721694	m	md	CN	2018	0	0	0	0	0	0	0	0	0	0	0	0	48
15721733	m	md	CN	2018	0	0	0	0	0	0	0	0	0	0	0	0	71
15721916	m	cn	CN	2018	0	1	0	0	1	0	0	0	0	0	0	0	72
15722030	m	md	CN	2018	0	0	0	0	0	0	0	0	0	0	0	0	
15722045	m	md	AU	2018	0	0	0	0	0	0	1	0	0	0	1	0	
15722139	Not	md	MY	2018	0	1	0	0	0	1	0	0	0	0	0	0	
15722143	Not	cn	SG	2018	1	0	0	0	0	0	0	0	0	0	0	0	
15722586	m	md	HU	2018	0	0	0	0	0	0	0	0	0	0	0	0	69
15722752	m	ot	JP	2018	0	1	1	0	0	0	0	0	0	0	0	0	76
15722816	f	md	NL	2018	0	0	0	0	0	0	0	0	0	0	0	0	78
15722939	f	cn	US	2018	1	0	0	0	0	0	0	0	0	0	0	0	
15723483	m	ot	CN	2018	0	1	0	0	1	0	0	0	0	0	0	0	49
15723616	m	ph	JP	2018	1	0	0	0	0	0	0	0	0	0	0	0	80
15723678	Not	md	JP	2018	0	1	0	0	1	0	0	0	0	0	0	1	
15723857	f	ot	JP	2018	0	1	0	0	1	0	0	0	0	0	0	1	72
15723968	m	md	FR	2018	0	0	0	0	0	0	0	0	0	0	0	0	48

15723972	m	md	JP	2018	0	1	0	1	0	0	0	0	0	0	0	1	71.2
15723975	f	cn	JP	2018	0	1	1	0	0	0	0	0	0	0	0	1	
15724020	m	ot	US	2018	0	1	0	0	1	0	0	0	0	0	0	0	57
15724090	Not	md	JP	2018	1	0	0	0	0	0	0	0	0	0	0	0	
15724137	m	md	US	2018	0	1	0	1	0	0	0	0	0	0	0	0	70
15724402	f	md	US	2018	0	0	0	0	0	0	1	0	0	1	0	0	85.7
15724403	f	ot	GB	2018	0	1	0	1	0	0	0	0	0	0	0	0	64.1
15724717	f	md	US	2018	0	0	0	0	0	0	0	0	0	0	0	0	70
15725279	m	md	JP	2018	0	0	0	0	0	0	0	0	0	0	0	0	66
15725361	f	ph	US	2018	1	0	0	0	0	0	0	0	0	0	0	0	76
15725478	m	md	AT	2018	0	0	0	0	0	0	0	0	0	0	0	0	70
15725566	m	md	AT	2018	0	0	0	0	0	0	0	0	0	0	0	0	68
15726261	m	cn	BE	2018	0	1	1	0	0	0	0	0	0	0	0	0	
15726344	f	md	US	2018	1	0	0	0	0	0	0	0	0	0	0	1	48
15727024	m	md	CN	2018	0	1	0	0	0	1	0	0	0	0	0	0	65
15728487	Not	cn	GB	2018	1	0	0	0	0	0	0	0	0	0	0	0	
15728488	m	md	JP	2018	0	1	0	0	1	0	0	0	0	0	0	0	61
15728683	f	md	JP	2018	0	1	0	1	0	0	0	0	0	0	0	0	80
15729076	m	md	US	2018	0	0	0	0	0	0	0	0	0	0	0	0	74
15729834	f	md	JP	2018	1	0	0	0	0	0	0	0	0	0	0	0	60
15730813	f	md	JP	2018	0	1	0	0	1	0	0	0	0	0	0	1	72
15730886	f	cn	GB	2018	1	0	0	0	0	0	0	0	0	0	0	0	
15731585	m	md	US	2018	0	0	0	0	0	0	0	0	0	0	0	0	73
15731586	Not	ot	GB	2018	0	0	0	0	0	0	0	0	0	0	0	0	
15732855	m	cn	US	2018	0	1	1	0	0	0	0	0	0	0	0	0	
15733024	m	cn	GB	2018	0	0	0	0	0	0	0	0	0	0	0	0	58
15733119	m	ph	IT	2018	0	1	0	0	1	0	0	0	0	0	0	0	79
15733180	m	md	SK	2018	0	1	1	0	0	0	0	0	0	0	0	0	67
15733279	m	md	TR	2018	0	0	0	0	0	0	0	0	0	0	0	0	50
15733474	m	cn	US	2018	1	0	0	0	0	0	0	0	0	0	0	0	
15733744	m	cn	JP	2018	0	1	1	0	0	0	0	0	0	0	0	1	71
15734063	f	md	JP	2018	0	1	0	0	0	1	0	0	0	0	0	0	70
15734171	Not	cn	GB	2018	1	0	0	0	0	0	0	0	0	0	0	0	
15734172	m	cn	GB	2018	1	0	0	0	0	0	0	0	0	0	0	0	

15734173	Not	cn	GB	2018	1	0	0	0	0	0	0	0	0	0	0	0	
15734174	Not	cn	GB	2018	1	0	0	0	0	0	0	0	0	0	0	0	
15734175	Not	cn	GB	2018	1	0	0	0	0	0	0	0	0	0	0	0	
15734323	m	md	JP	2018	0	1	0	1	0	0	0	0	0	0	0	0	70
15734633	m	md	JP	2018	0	0	0	0	0	0	0	0	0	0	0	0	81
15735618	f	ph	JP	2018	0	1	0	0	1	0	0	0	0	0	0	0	
15735923	f	cn	Not re	2018	0	0	0	0	0	0	0	0	0	0	0	0	63
15735925	f	md	IN	2018	0	0	0	0	0	0	0	0	0	0	0	0	
15736090	m	cn	GB	2018	1	0	0	0	0	0	0	0	0	0	0	0	
15736181	m	ot	GB	2018	0	0	0	0	0	0	0	0	0	0	0	0	71
15736432	f	ot	CA	2018	0	1	1	0	0	0	0	0	0	0	0	1	63
15736683	m	cn	IN	2018	0	0	0	0	0	0	0	0	0	0	0	0	68
15736703	m	md	JP	2018	0	0	0	0	0	0	0	0	0	0	0	0	59
15737638	m	md	EG	2018	0	1	0	0	1	0	0	0	0	0	0	0	53
15737914	f	md	PT	2018	0	0	0	0	0	0	0	0	0	0	0	0	
15737989	m	ot	FR	2018	0	1	0	0	1	0	0	0	0	0	0	0	64
15738811	m	md	GB	2018	0	1	1	0	0	0	0	0	0	0	0	0	43
15738834	m	ph	JP	2018	0	0	0	0	0	0	0	0	0	0	0	0	43
15739016	f	ph	CA	2018	0	0	0	0	0	0	0	0	0	0	0	0	
15739113	m	ot	DE	2018	0	0	0	0	0	0	0	0	0	0	0	0	62
15739305	Not	cn	JP	2018	0	1	1	0	0	0	0	0	0	0	0	0	80
15739713	Not	ot	US	2018	0	1	1	0	0	0	0	0	0	0	0	0	
15740192	f	ph	JP	2018	1	0	0	0	0	0	0	0	0	0	0	0	85
15740370	f	md	US	2018	0	1	0	1	0	0	0	0	0	0	0	1	
15740583	f	md	JP	2018	1	0	0	0	0	0	0	0	0	0	0	0	72
15740834	m	md	JP	2018	0	1	0	1	0	0	0	0	0	0	0	1	66
15740839	Not	ot	AU	2018	0	0	0	0	0	0	0	0	0	0	0	0	
15740951	m	md	JP	2018	0	1	0	1	0	0	0	0	0	0	0	0	75
15741327	m	cn	LK	2018	0	1	1	0	0	0	0	0	0	0	0	1	69
15741638	f	md	FR	2018	0	0	0	0	0	0	0	0	0	0	0	0	80
15741893	f	ot	US	2018	0	1	1	0	0	0	0	0	0	0	0	0	73
15741959	Not	cn	JP	2018	0	0	0	0	0	0	0	0	0	0	0	0	
15741972	Not	cn	JP	2018	0	0	0	0	0	0	0	0	0	0	0	0	
15741975	Not	cn	JP	2018	0	0	0	0	0	0	0	0	0	0	0	0	

15741983	Not	cn	JP	2018	0	0	0	0	0	0	0	0	0	0	0	0	
15741989	Not	cn	JP	2018	0	0	0	0	0	0	0	0	0	0	0	0	
15741990	Not	cn	JP	2018	0	0	0	0	0	0	0	0	0	0	0	0	
15741991	Not	cn	JP	2018	0	0	0	0	0	0	0	0	0	0	0	0	
15741992	m	ph	CA	2018	0	0	0	0	0	0	0	0	0	0	0	0	57
15742112	m	md	AU	2018	0	1	1	0	0	0	0	0	0	0	0	0	78
15742190	f	ot	US	2018	0	0	0	0	0	0	0	0	0	0	0	0	86
15742712	m	ot	DE	2018	0	1	0	0	1	0	0	0	0	0	0	0	61
15743046	m	ot	FR	2018	0	1	0	0	1	0	0	0	0	0	0	0	43
15743196	Not	ot	CN	2018	0	0	0	0	0	0	0	0	0	0	0	0	
15743830	f	cn	GR	2018	0	0	0	0	0	0	0	0	0	0	0	0	54
15744186	f	md	HR	2018	1	0	0	0	0	0	0	0	0	0	0	0	78
15744201	m	md	US	2018	0	1	0	0	0	1	0	0	0	0	0	0	78
15744605	f	md	JP	2018	0	0	0	0	0	0	0	0	0	0	0	0	70
15744796	m	md	JP	2018	0	1	0	1	0	0	0	0	0	0	0	1	70
15745163	Not	md	AU	2018	0	0	0	0	0	0	0	0	0	0	0	0	30
15745623	m	md	JP	2018	0	1	0	1	0	0	0	0	0	0	0	0	74
15745624	f	md	JP	2018	1	0	0	0	0	0	0	0	0	0	0	0	70
15745869	m	cn	Not re	2018	0	0	0	0	0	0	0	0	0	0	0	0	73
15745889	f	ot	GB	2018	0	0	0	0	0	0	0	0	0	0	0	0	62
15745972	m	ot	GB	2018	0	1	0	0	0	1	0	0	0	0	0	0	
15746149	f	ot	GB	2018	0	0	0	0	0	0	0	0	0	0	0	0	64
15746836	f	ot	DE	2018	0	1	1	0	0	0	0	0	0	0	0	0	69
15746848	f	ot	DE	2018	0	1	1	0	0	0	0	0	0	0	0	1	72
15747446	f	cn	US	2018	1	0	0	0	0	0	0	0	0	0	0	0	69
15747535	m	cn	LB	2018	0	1	0	0	1	0	0	0	0	0	0	0	73
15747561	m	md	FR	2018	0	1	1	0	0	0	0	0	0	0	0	0	59
15747894	m	cn	CN	2018	0	0	0	0	0	0	0	0	0	0	0	0	
15748573	m	ot	CN	2018	1	0	0	0	0	0	0	0	0	0	0	0	
15749083	f	cn	US	2018	0	1	0	1	0	0	0	0	0	0	0	1	70
15749363	f	cn	US	2018	0	0	0	0	0	0	0	0	0	0	0	0	
15749609	f	ot	CN	2018	1	0	0	0	0	0	0	0	0	0	0	0	
15749923	f	ot	CN	2018	1	0	0	0	0	0	0	0	0	0	0	0	
15749961	f	ph	JP	2018	0	0	0	0	0	0	0	0	0	0	0	0	70

15750074	m	ot	CN	2018	1	0	0	0	0	0	0	0	0	0	0	0	
15750076	m	md	US	2018	1	0	0	0	0	0	0	0	0	0	0	0	
15750143	f	ot	CN	2018	1	0	0	0	0	0	0	0	0	0	0	0	
15750292	Not	md	US	2018	0	1	0	0	1	0	0	0	0	0	0	0	
15750314	f	ot	CN	2018	1	0	0	0	0	0	0	0	0	0	0	0	
15750315	f	ot	CN	2018	1	0	0	0	0	0	0	0	0	0	0	0	
15750521	m	md	CZ	2018	0	1	0	0	1	0	0	0	0	0	0	0	66
15750788	Not	md	JP	2018	1	0	0	0	0	0	0	0	0	0	0	0	
15750859	f	ot	US	2018	0	1	0	0	1	0	0	0	0	0	0	0	70
15751302	f	md	AT	2018	1	0	0	0	0	0	0	0	0	0	0	0	51
15751353	m	ot	US	2018	0	1	0	0	1	0	0	0	0	0	0	0	
15751660	m	cn	JP	2018	0	0	0	0	0	0	0	0	0	0	0	0	56
15751913	m	ot	NL	2018	0	1	0	0	1	0	0	0	0	0	0	0	69
15752052	Not	md	JP	2018	1	0	0	0	0	0	0	0	0	0	0	0	
15752119	m	md	JP	2018	1	0	0	0	0	0	0	0	0	0	0	0	61
15752210	f	cn	GB	2018	0	0	0	0	0	0	0	0	0	0	0	0	62
15752258	m	ot	CN	2018	1	0	0	0	0	0	0	0	0	0	0	0	
15752273	f	ot	IN	2018	0	1	0	0	1	0	0	0	0	0	0	0	57
15752303	m	cn	JP	2018	1	0	0	0	0	0	0	0	0	0	0	0	54
15752304	m	ot	CN	2018	1	0	0	0	0	0	0	0	0	0	0	0	
15752305	m	ot	CN	2018	1	0	0	0	0	0	0	0	0	0	0	0	
15752313	m	ot	CN	2018	1	0	0	0	0	0	0	0	0	0	0	0	
15752391	f	ot	AR	2018	0	1	0	0	1	0	0	0	0	0	0	0	74
15752392	m	md	US	2018	0	1	0	1	0	0	0	0	0	0	0	0	71
15752405	f	cn	US	2018	0	0	0	0	0	0	0	0	0	0	0	0	
15752730	m	ot	IT	2018	0	1	0	0	1	0	0	0	0	0	0	0	46
15753104	m	cn	GT	2018	0	1	1	0	0	0	0	0	0	0	0	0	
15753172	Not	md	CN	2018	0	0	0	0	0	0	0	0	0	0	0	0	
15753236	Not	md	GB	2018	0	0	0	0	0	0	0	0	0	0	0	0	59
15753269	Not	ot	IL	2018	0	1	1	0	0	0	0	0	0	0	0	0	
15753336	Not	cn	GB	2018	0	0	0	0	0	0	0	0	0	0	0	0	69
15753352	m	cn	JP	2018	0	1	1	0	0	0	0	0	0	0	0	1	63
15753527	m	md	NL	2018	0	0	0	0	0	0	0	0	0	0	0	0	63
15753559	Not	cn	GB	2018	0	0	0	0	0	0	0	0	0	0	0	0	

15753786	m	cn	US	2018	0	1	1	0	0	0	0	0	0	0	0	0	
15753936	m	cn	JP	2018	0	1	1	0	0	0	0	0	0	0	0	1	55
15754273	f	md	JP	2018	1	0	0	0	0	0	0	0	0	0	0	0	76
15754349	m	cn	US	2018	0	1	0	0	1	0	0	0	0	0	0	0	85
15754949	Not	md	CN	2018	0	0	0	0	0	0	0	0	0	0	0	0	
15755014	f	md	US	2018	0	1	0	0	1	0	0	0	0	0	0	0	68
15755175	Not	md	CN	2018	0	0	0	0	0	0	0	0	0	0	0	0	
15755247	f	ph	US	2018	0	0	0	0	0	0	0	0	0	0	0	0	53
15755295	m	ot	FR	2018	0	1	0	0	1	0	0	0	0	0	0	0	72
15755493	m	ot	CN	2018	0	0	0	0	0	0	0	0	0	0	0	0	42
15755520	f	cn	US	2018	0	1	0	0	1	0	0	0	0	0	0	0	
15755561	m	md	JP	2018	0	0	0	0	0	0	0	0	0	0	0	0	
15756036	f	md	AU	2018	0	0	0	0	0	0	0	0	0	0	0	0	
15756138	m	ph	JP	2018	0	0	0	0	0	0	0	0	0	0	0	0	70
15756396	m	md	JP	2018	0	1	0	1	0	0	0	0	0	0	0	1	74
15756584	f	md	GB	2018	0	0	0	0	0	0	0	0	0	0	0	0	62
15756646	f	md	GB	2018	0	0	0	0	0	0	0	0	0	0	0	0	78
15756768	f	ot	TW	2018	1	0	0	0	0	0	0	0	0	0	0	0	66
15756815	f	Not repo	AU	2018	1	0	0	0	0	0	0	0	0	0	0	0	67
15756879	f	ot	TW	2018	1	0	0	0	0	0	0	0	0	0	0	0	64
15756908	f	ot	TW	2018	1	0	0	0	0	0	0	0	0	0	0	0	61
15756952	m	ot	TW	2018	1	0	0	0	0	0	0	0	0	0	0	0	85
15756980	f	ph	CH	2018	0	1	1	0	0	0	0	0	0	0	0	0	65
15757088	m	Not repo	JP	2018	1	0	0	0	0	0	0	0	0	0	0	0	54
15757094	Not	ot	IT	2018	0	0	0	0	0	0	0	0	0	0	0	0	
15757143	m	md	JP	2018	0	1	0	1	0	0	0	0	0	0	0	1	82.6
15757549	m	cn	DE	2018	0	1	1	0	0	0	0	0	0	0	0	1	76
15758158	f	md	JP	2018	0	1	0	1	0	0	0	0	0	0	0	1	72
15758179	Not	md	JP	2018	1	0	0	0	0	0	0	0	0	0	0	0	
15758211	m	md	AR	2018	0	1	0	0	0	1	0	0	0	0	0	0	
15758230	m	md	JP	2018	0	1	0	0	0	1	0	0	0	0	0	0	70
15758683	m	ot	DE	2018	0	0	0	0	0	0	0	0	0	0	0	0	52
15758691	m	ot	DE	2018	0	1	1	0	0	0	0	0	0	0	0	0	69
15758956	f	md	US	2018	0	1	1	0	0	0	0	0	0	0	0	0	

15759300	m	ph	US	2018	0	1	1	0	0	0	0	0	0	0	0	0	65
15759314	m	md	JP	2018	1	0	0	0	0	0	0	0	0	0	0	0	80
15759334	Not	md	CN	2018	0	0	0	0	0	0	0	0	0	0	0	0	
15759548	f	ot	US	2018	0	1	0	0	1	0	0	0	0	0	0	0	75
15759571	m	ot	US	2018	0	1	0	0	1	0	0	0	0	0	0	0	
15759572	Not	md	JP	2018	0	1	0	0	1	0	0	0	0	0	0	0	
15760222	m	md	HR	2018	0	0	0	0	0	0	0	0	0	0	0	0	50
15761218	Not	ot	GB	2018	0	0	0	0	0	0	0	0	0	0	0	0	
15761744	m	cn	JP	2018	0	1	1	0	0	0	0	0	0	0	0	0	79
15761785	m	ot	PL	2018	0	0	0	0	0	0	0	0	0	0	0	0	68
15762144	m	ot	CN	2018	0	0	0	0	0	0	0	0	0	0	0	0	53
15762257	m	cn	JP	2018	0	1	1	0	0	0	0	0	0	0	0	0	54
15762268	m	ot	RO	2018	0	0	0	0	0	0	0	0	0	0	0	0	66
15762300	m	cn	GB	2018	1	0	0	0	0	0	0	0	0	0	0	0	
15762462	m	ot	US	2018	0	1	0	0	1	0	0	0	0	0	0	0	69
15762570	f	cn	GB	2018	0	0	0	0	0	0	0	0	0	0	0	0	69
15762820	Not	ot	GB	2018	0	0	0	0	0	0	0	0	0	0	0	0	
15762833	Not	ot	IT	2018	0	0	0	0	0	0	0	0	0	0	0	0	
15762835	Not	ot	IT	2018	0	0	0	0	0	0	0	0	0	0	0	0	
15763286	Not	md	AT	2018	1	0	0	0	0	0	0	0	0	0	0	0	63
15763397	f	cn	CH	2018	1	0	0	0	0	0	0	0	0	0	0	0	
15763658	m	md	JP	2018	0	1	0	0	1	0	0	0	0	0	0	0	61
15763752	Not	md	JP	2018	0	1	0	0	1	0	0	0	0	0	0	0	
15764016	Not	md	JP	2018	0	1	0	1	0	0	0	0	0	0	0	0	69
15764137	m	ot	JP	2018	0	1	0	1	0	0	0	0	0	0	0	0	60.9
15764722	m	cn	JP	2018	0	1	1	0	0	0	0	0	0	0	0	0	76
15766343	f	md	US	2018	0	0	0	0	0	0	0	0	0	0	0	0	80
15766373	Not	cn	JP	2018	0	1	1	0	0	0	0	0	0	0	0	1	80
15766596	m	cn	SA	2018	0	1	1	0	0	0	0	0	0	0	0	0	
15766766	f	md	JP	2018	0	1	0	1	0	0	0	0	0	0	0	1	55
15766957	m	md	JP	2018	0	0	0	0	0	0	1	0	0	1	0	0	70
15767062	m	cn	PL	2018	0	0	0	0	0	0	0	0	0	0	0	0	68
15767142	f	md	JP	2018	1	0	0	0	0	0	0	0	0	0	0	0	84
15767253	m	cn	IN	2018	0	0	0	0	0	0	0	0	0	0	0	0	60

15767304	f	Not repo	CN	2018	1	0	0	0	0	0	0	0	0	0	0	0	60
15767440	m	md	JP	2018	0	1	0	1	0	0	0	0	0	0	0	1	70
15767441	f	ot	JP	2018	0	1	0	1	0	0	0	0	0	0	0	1	68
15767501	f	md	IT	2018	0	0	0	0	0	0	0	0	0	0	0	0	
15767558	Not	ot	US	2018	1	0	0	0	0	0	0	0	0	0	0	0	
15767563	m	md	HU	2018	0	1	0	0	1	0	0	0	0	0	0	0	78
15767643	f	md	DE	2018	1	0	0	0	0	0	0	0	0	0	0	0	68
15767651	f	cn	CN	2018	1	0	0	0	0	0	0	0	0	0	0	0	63
15767703	m	cn	IL	2018	0	1	1	0	0	0	0	0	0	0	0	0	55
15768078	m	ot	FR	2018	0	1	0	0	1	0	0	0	0	0	0	0	68
15768443	Not	ot	US	2018	0	1	0	0	1	0	0	0	0	0	0	0	
15768449	Not	ot	US	2018	0	1	0	0	1	0	0	0	0	0	0	0	
15768460	Not	ot	US	2018	0	1	0	0	1	0	0	0	0	0	0	0	
15768514	Not	ot	US	2018	0	1	0	0	1	0	0	0	0	0	0	0	
15768551	m	ot	JP	2018	0	1	0	0	1	0	0	0	0	0	0	0	65
15769629	m	md	JP	2018	0	1	0	0	0	1	0	0	0	0	0	0	70
15770206	m	md	US	2018	0	0	0	0	0	0	0	0	0	0	0	0	
15770351	m	md	JP	2018	0	1	0	0	0	1	0	0	0	0	0	0	71
15770956	f	ot	US	2018	0	0	0	0	0	0	0	0	0	0	0	0	
15771026	m	md	JP	2018	0	1	0	1	0	0	0	0	0	0	0	1	58
15771306	m	cn	JP	2018	0	1	1	0	0	0	0	0	0	0	0	0	67
15771681	f	ot	US	2018	0	0	0	0	0	0	0	0	0	0	0	0	79
15771738	f	cn	US	2018	0	1	0	0	1	0	0	0	0	0	0	0	
15771822	f	md	JP	2018	0	0	0	0	0	0	0	0	0	0	0	0	54
15772206	m	ot	CN	2018	0	1	0	0	1	0	0	0	0	0	0	0	61
15772507	m	cn	JP	2018	0	1	1	0	0	0	0	0	0	0	0	0	76
15772680	f	md	JP	2018	0	1	0	1	0	0	0	0	0	0	0	1	68.9
15772749	f	ot	GB	2018	0	1	1	0	0	0	0	0	0	0	0	1	72
15772953	f	ot	CH	2018	1	0	0	0	0	0	0	0	0	0	0	0	59
15772955	f	md	JP	2018	1	0	0	0	0	0	0	0	0	0	0	0	50
15772965	m	ot	JP	2018	0	1	0	0	1	0	0	0	0	0	0	0	53
15773067	m	ot	BR	2018	0	1	0	0	1	0	0	0	0	0	0	0	49
15774030	f	cn	US	2018	1	0	0	0	0	0	0	0	0	0	0	0	
15774721	m	md	JP	2018	0	0	0	0	0	0	0	0	0	0	0	0	43

15774873	m	cn	IN	2018	0	1	0	0	1	0	0	0	0	0	0	0	
15774985	Not	ot	US	2018	0	1	0	0	1	0	0	0	0	0	0	0	
15775211	f	cn	DE	2018	0	1	0	0	1	0	0	0	0	0	0	0	65
15775256	m	cn	DE	2018	0	0	0	0	0	0	0	0	0	0	0	0	71
15775597	f	md	US	2018	1	0	0	0	0	0	0	0	0	0	0	0	70
15775811	m	ot	IL	2018	0	1	0	0	1	0	0	0	0	0	0	0	61
15775815	m	ot	IL	2018	0	1	0	0	1	0	0	0	0	0	0	0	74
15775838	m	ot	IL	2018	0	1	0	0	1	0	0	0	0	0	0	0	72
15775841	m	ot	IL	2018	0	1	0	0	1	0	0	0	0	0	0	0	72
15775852	f	ot	IL	2018	0	1	0	0	1	0	0	0	0	0	0	0	80
15776544	f	md	JP	2018	1	0	0	0	0	0	0	0	0	0	0	0	72
15776936	m	ph	JP	2018	0	1	1	0	0	0	0	0	0	0	0	0	
15777073	f	cn	JP	2018	0	1	1	0	0	0	0	0	0	0	0	1	68
15777099	m	md	US	2018	0	1	1	0	0	0	0	0	0	0	0	0	66
15777388	m	md	GB	2018	0	1	0	0	0	1	0	0	0	0	0	0	65
15777780	m	cn	JP	2018	0	1	1	0	0	0	0	0	0	0	0	0	55
15778138	m	md	FR	2018	0	0	0	0	0	0	0	0	0	0	0	0	76
15778186	m	md	US	2018	0	1	0	0	1	0	0	0	0	0	0	0	
15778217	f	ot	US	2018	1	0	0	0	0	0	0	0	0	0	0	0	62
15778406	f	cn	JP	2018	0	1	1	0	0	0	0	0	0	0	0	0	62
15778434	f	cn	CA	2018	0	0	0	0	0	0	0	0	0	0	0	0	73
15786935	m	ph	US	2018	0	0	0	0	0	0	0	0	0	0	0	0	63
15790727	f	ot	US	2018	1	0	0	0	0	0	0	0	0	0	0	0	69
6735619	f	cn	GB	2018	0	0	0	0	0	0	0	0	0	0	0	0	63
13076303	f	md	NL	2019	0	1	0	0	0	1	0	0	0	0	0	0	75
14951173	m	md	PL	2019	0	1	0	0	0	1	0	0	0	0	0	0	51
15324337	f	md	NL	2019	0	1	0	0	0	1	0	0	0	0	0	0	70
15337758	f	md	SK	2019	0	1	0	0	0	1	0	0	0	0	0	0	69
15354477	f	ot	US	2019	0	1	0	0	1	0	0	0	0	0	0	0	
15458852	f	ot	US	2019	0	1	0	0	1	0	0	0	0	0	0	0	69
15459779	m	ot	JP	2019	0	1	0	0	1	0	0	0	0	0	0	1	62
15468181	f	md	JP	2019	0	1	0	0	0	1	0	0	0	0	0	0	63
15469835	f	md	JP	2019	1	0	0	0	0	0	0	0	0	0	0	0	66.1
15481781	m	md	US	2019	0	1	0	0	0	1	0	0	0	0	0	0	76

15483494	m	cn	JP	2019	0	0	0	0	0	0	0	0	0	0	0	0	79
15483963	m	ot	ES	2019	0	1	0	1	0	0	0	0	0	0	0	0	73
15486352	m	md	FR	2019	0	0	0	0	0	0	0	0	0	0	0	0	68
15487245	m	cn	JP	2019	0	1	1	0	0	0	0	0	0	0	0	0	72
15490531	m	ot	IN	2019	0	0	0	0	0	0	0	0	0	0	0	0	36
15496027	m	ot	CN	2019	0	1	0	0	1	0	0	0	0	0	0	0	56
15496849	m	cn	JP	2019	0	1	1	0	0	0	0	0	0	0	0	1	62
15497561	f	ot	US	2019	0	1	0	0	1	0	0	0	0	0	0	0	
15498262	f	md	CA	2019	0	0	0	0	0	0	0	0	0	0	0	0	58
15507831	m	cn	US	2019	0	1	0	0	1	0	0	0	0	0	0	0	
15517317	m	md	US	2019	1	0	0	0	0	0	0	0	0	0	0	0	66
15524725	m	md	FR	2019	0	0	0	0	0	0	0	0	0	0	0	0	72
15530248	m	ot	PL	2019	0	1	0	0	1	0	0	0	0	0	0	0	64
15532611	m	cn	US	2019	1	0	0	0	0	0	0	0	0	0	0	0	73
15533793	m	ot	CN	2019	0	1	0	0	1	0	0	0	0	0	0	0	81
15534033	f	md	JP	2019	1	0	0	0	0	0	0	0	0	0	0	1	83
15537058	f	cn	PH	2019	0	0	0	0	0	0	0	0	0	0	0	0	
15542973	m	md	GB	2019	0	1	0	0	0	1	0	0	0	0	0	0	71
15546723	f	cn	US	2019	0	1	0	0	1	0	0	0	0	0	0	0	70
15549063	m	cn	CN	2019	0	1	0	0	1	0	0	0	0	0	0	0	78
15557844	m	cn	JP	2019	0	1	1	0	0	0	0	0	0	0	0	0	57
15557884	m	md	JP	2019	0	1	1	0	0	0	0	0	0	0	0	1	68
15557937	m	md	US	2019	0	1	1	0	0	0	0	0	0	0	0	0	51
15560154	m	md	US	2019	0	1	1	0	0	0	0	0	0	0	0	0	
15563290	f	md	CN	2019	0	0	0	0	0	0	0	0	0	0	0	0	43
15564087	m	ot	US	2019	0	1	0	0	1	0	0	0	0	0	0	0	53
15566321	m	ot	US	2019	0	0	0	0	0	0	0	0	0	0	0	0	72
15568215	Not	md	NL	2019	0	1	0	0	1	0	0	0	0	0	0	0	
15569116	m	md	JP	2019	0	1	0	0	1	0	0	0	0	0	0	1	68
15569362	m	ot	JP	2019	0	1	0	0	1	0	0	0	0	0	0	0	68
15569923	f	md	JP	2019	0	1	0	0	1	0	0	0	0	0	0	0	48
15571252	Not	md	JP	2019	0	1	0	0	1	0	0	0	0	0	0	0	
15571617	m	ph	JP	2019	0	1	0	0	1	0	0	0	0	0	0	0	76
15571815	f	ph	JP	2019	1	0	0	0	0	0	0	0	0	0	0	0	78

15573202	m	md	US	2019	0	1	0	0	1	0	0	0	0	0	0	0	76
15574739	m	md	JP	2019	0	1	0	0	1	0	0	0	0	0	0	1	65
15577719	f	cn	US	2019	0	1	0	0	1	0	0	0	0	0	0	0	
15578286	f	md	US	2019	0	1	0	0	1	0	0	0	0	0	0	0	
15578624	f	ot	JP	2019	0	1	0	0	1	0	0	0	0	0	0	0	
15578887	Not	ot	JP	2019	0	1	0	0	1	0	0	0	0	0	0	0	
15583422	m	md	FR	2019	0	0	0	0	0	0	0	0	0	0	0	0	48
15588795	f	md	DE	2019	1	0	0	0	0	0	0	0	0	0	0	0	68
15588810	m	cn	MY	2019	1	0	0	0	0	0	0	0	0	0	0	0	40
15591979	f	md	FR	2019	1	0	0	0	0	0	0	0	0	0	0	0	79
15595925	m	ot	JP	2019	0	1	0	0	1	0	0	0	0	0	0	0	
15596853	f	ph	US	2019	0	1	0	0	1	0	0	0	0	0	0	0	53
15596947	f	md	JP	2019	1	0	0	0	0	0	0	0	0	0	0	0	73.1
15597988	m	ot	JP	2019	0	1	0	0	1	0	0	0	0	0	0	1	62
15600772	m	ot	JP	2019	0	1	0	0	1	0	0	0	0	0	0	0	69
15600901	m	cn	US	2019	0	1	0	0	1	0	0	0	0	0	0	0	
15601242	m	md	KR	2019	0	1	0	0	1	0	0	0	0	0	0	1	75
15601587	m	ot	CZ	2019	0	0	0	0	0	0	0	0	0	0	0	0	36
15601668	m	ot	DE	2019	0	1	0	0	1	0	0	0	0	0	0	1	63
15601773	f	md	JP	2019	1	0	0	0	0	0	0	0	0	0	0	1	63
15602371	m	md	CZ	2019	0	0	0	0	0	0	0	0	0	0	0	0	36
15603078	m	md	MX	2019	0	0	0	0	0	0	0	0	0	0	0	0	58
15604884	f	md	FR	2019	1	0	0	0	0	0	0	0	0	0	0	0	69
15605001	m	cn	US	2019	0	1	0	0	1	0	0	0	0	0	0	0	57
15605074	m	cn	US	2019	0	1	0	0	1	0	0	0	0	0	0	0	
15606629	m	md	NL	2019	0	1	0	0	1	0	0	0	0	0	0	1	71
15609645	m	md	JP	2019	0	1	0	0	1	0	0	0	0	0	0	0	78
15609659	m	md	DE	2019	0	1	0	0	1	0	0	0	0	0	0	1	87
15611867	m	ph	JP	2019	0	1	0	0	0	1	0	0	0	0	0	1	79
15614317	m	md	DE	2019	0	1	1	0	0	0	0	0	0	0	0	0	76
15614819	m	ph	JP	2019	0	1	0	0	1	0	0	0	0	0	0	0	79
15615209	f	ot	JP	2019	0	1	0	0	1	0	0	0	0	0	0	0	70
15615898	m	md	CN	2019	0	1	0	0	0	1	0	0	0	0	0	0	80
15616050	m	cn	US	2019	0	1	0	0	1	0	0	0	0	0	0	0	

15618966	f	cn	US	2019	0	1	0	0	1	0	0	0	0	0	0	0	
15619616	f	md	DK	2019	0	0	0	0	0	0	0	0	0	0	0	0	35
15622368	m	cn	JP	2019	0	1	1	0	0	0	0	0	0	0	0	0	71
15623716	m	cn	US	2019	0	1	0	0	1	0	0	0	0	0	0	0	76
15623870	m	cn	LB	2019	0	1	0	0	1	0	0	0	0	0	0	0	73
15624430	m	ot	AU	2019	0	1	0	0	1	0	0	0	0	0	0	0	59
15626135	f	md	JP	2019	1	0	0	0	0	0	0	0	0	0	0	0	75.7
15627213	m	md	JP	2019	0	1	0	1	0	0	0	0	0	0	0	1	59
15627941	f	md	FR	2019	0	0	0	0	0	0	0	0	0	0	0	0	53
15630952	f	ph	FR	2019	0	0	0	0	0	0	0	0	0	0	0	0	59
15631386	m	cn	JP	2019	0	1	1	0	0	0	0	0	0	0	0	0	63
15635233	f	cn	CA	2019	0	1	1	0	0	0	0	0	0	0	0	0	41
15640444	Not	md	JP	2019	0	1	0	0	1	0	0	0	0	0	0	0	
15641385	m	ot	JP	2019	0	1	0	0	1	0	0	0	0	0	0	0	52
15641772	m	md	JP	2019	0	1	0	0	1	0	0	0	0	0	0	0	70
15641947	f	md	JP	2019	0	1	0	0	1	0	0	0	0	0	0	0	74
15642774	m	md	IN	2019	0	1	0	0	1	0	0	0	0	0	0	0	
15643579	f	ot	US	2019	0	0	0	0	0	0	0	0	0	0	0	0	68
15643608	m	md	JP	2019	0	0	0	0	0	0	0	0	0	0	0	0	34
15645017	f	ot	US	2019	0	0	0	0	0	0	0	0	0	0	0	0	77
15646084	Not	md	JP	2019	0	1	0	0	1	0	0	0	0	0	0	0	
15646230	Not	md	JP	2019	0	1	0	0	1	0	0	0	0	0	0	0	
15646472	f	md	JP	2019	0	1	0	0	1	0	0	0	0	0	0	0	70
15647938	m	md	JP	2019	0	1	0	0	1	0	0	0	0	0	0	0	74
15648181	m	md	JP	2019	0	1	0	0	0	1	0	0	0	0	0	0	85
15649553	f	cn	US	2019	0	0	0	0	0	0	0	0	0	0	0	0	
15650225	m	md	KR	2019	0	1	0	0	1	0	0	0	0	0	0	0	60
15650226	Not	ph	JP	2019	0	1	0	0	1	0	0	0	0	0	0	0	
15654173	f	md	US	2019	0	1	0	0	1	0	0	0	0	0	0	0	
15655068	m	md	TW	2019	0	1	0	0	0	1	0	0	0	0	0	0	42
15656774	m	cn	US	2019	1	0	0	0	0	0	0	0	0	0	0	0	
15657079	Not	md	IT	2019	0	0	0	0	0	0	0	0	0	0	0	0	
15658884	f	ph	FR	2019	0	1	0	0	1	0	0	0	0	0	0	0	46
15659952	m	ot	FR	2019	0	1	0	0	1	0	0	0	0	0	0	0	61

15660596	m	md	JP	2019	1	0	0	0	0	0	0	0	0	0	0	0	73.8
15660661	f	md	JP	2019	0	1	0	1	0	0	0	0	0	0	0	0	80
15661934	m	md	AT	2019	0	0	0	0	0	0	0	0	0	0	0	0	54
15663271	m	md	JP	2019	0	1	0	0	1	0	0	0	0	0	0	0	72
15663923	m	md	JP	2019	0	1	0	0	1	0	0	0	0	0	0	1	59
15666173	m	Not repo	JP	2019	0	0	0	0	0	0	0	0	0	0	0	0	57
15668034	m	cn	JP	2019	0	1	1	0	0	0	0	0	0	0	0	0	78
15669052	f	ph	JP	2019	0	1	0	0	0	1	0	0	0	0	0	0	70
15669957	f	md	CA	2019	0	1	1	0	0	0	0	0	0	0	0	0	
15670609	f	cn	JP	2019	0	1	1	0	0	0	0	0	0	0	0	0	61
15673240	f	md	FR	2019	0	0	0	0	0	0	0	0	0	0	0	0	56
15674818	m	md	JP	2019	0	1	0	0	0	1	0	0	0	0	0	0	82
15675543	Not	md	JP	2019	0	1	0	1	0	0	0	0	0	0	0	1	
15676771	f	cn	US	2019	1	0	0	0	0	0	0	0	0	0	0	0	73
15676773	m	ot	JP	2019	0	1	0	1	0	0	0	0	0	0	0	1	55.4
15676784	m	ot	NL	2019	0	1	0	0	1	0	0	0	0	0	0	0	74
15677405	f	md	HK	2019	0	0	0	0	0	0	0	0	0	0	0	0	60
15679207	f	cn	US	2019	0	1	1	0	0	0	0	0	0	0	0	0	
15680254	m	cn	DE	2019	0	1	1	0	0	0	0	0	0	0	0	0	
15681837	f	md	JP	2019	0	1	0	0	1	0	0	0	0	0	0	0	62
15682099	m	cn	DE	2019	0	1	1	0	0	0	0	0	0	0	0	0	
15683021	m	md	AT	2019	0	0	0	0	0	0	0	0	0	0	0	0	54
15684258	m	md	CN	2019	0	1	0	0	0	1	0	0	0	0	0	0	58
15685645	f	md	JP	2019	1	0	0	0	0	0	0	0	0	0	0	1	80.1
15686746	m	ot	US	2019	0	1	0	0	1	0	0	0	0	0	0	0	66
15686763	m	cn	VN	2019	0	1	1	0	0	0	0	0	0	0	0	0	
15688595	m	ot	DE	2019	0	0	0	0	0	0	0	0	0	0	0	0	60
15688723	m	md	DE	2019	0	0	0	0	0	0	0	0	0	0	0	0	65
15688833	m	md	CN	2019	0	1	0	0	0	1	0	0	0	0	0	0	63
15688946	f	ot	US	2019	1	0	0	0	0	0	0	0	0	0	0	0	
15689699	Not	ph	JP	2019	1	0	0	0	0	0	0	0	0	0	0	0	
15690363	m	md	JP	2019	0	1	0	1	0	0	0	0	0	0	0	1	72
15690999	m	md	IT	2019	0	1	1	0	0	0	0	0	0	0	0	0	69
15691144	m	md	JP	2019	0	1	0	1	0	0	0	0	0	0	0	1	71

15693173	f	cn	JP	2019	0	1	1	0	0	0	0	0	0	0	0	0	51
15694398	Not	ot	FR	2019	0	1	1	0	0	0	0	0	0	0	0	0	
15695559	f	cn	CH	2019	0	1	1	0	0	0	0	0	0	0	0	0	64
15697193	f	md	JP	2019	0	1	0	0	1	0	0	0	0	0	0	0	69
15698716	m	md	JP	2019	0	1	0	0	1	0	0	0	0	0	0	0	90
15699243	f	md	JP	2019	0	1	0	0	0	1	0	0	0	0	0	0	70
15699563	m	cn	US	2019	0	1	0	0	1	0	0	0	0	0	0	0	
15700232	f	md	JP	2019	1	0	0	0	0	0	0	0	0	0	0	1	81
15702209	Not	md	JP	2019	0	1	0	0	1	0	0	0	0	0	0	0	
15702216	Not	md	JP	2019	0	1	0	0	1	0	0	0	0	0	0	0	
15702675	m	md	JP	2019	0	1	0	0	1	0	0	0	0	0	0	0	67
15702771	m	ot	DE	2019	0	1	0	0	1	0	0	0	0	0	0	0	57
15703373	m	md	JP	2019	0	1	0	0	0	1	0	0	0	0	0	0	70
15704690	m	Not repo	JP	2019	0	0	0	0	0	0	0	0	0	0	0	0	72
15705419	m	md	JP	2019	0	1	0	0	1	0	0	0	0	0	0	0	75
15705514	m	md	DE	2019	0	1	0	0	1	0	0	0	0	0	0	0	
15707594	m	ot	US	2019	0	1	0	0	1	0	0	0	0	0	0	0	69
15709817	f	md	JP	2019	0	1	0	1	0	0	0	0	0	0	0	1	61.4
15710482	m	md	DE	2019	0	0	0	0	0	0	0	0	0	0	0	0	57
15711097	m	md	VN	2019	0	1	0	1	0	0	0	0	0	0	0	0	71.4
15711913	m	ot	FR	2019	0	1	0	0	1	0	0	0	0	0	0	0	63
15712472	m	cn	JP	2019	0	1	1	0	0	0	0	0	0	0	0	0	71
15715480	m	ot	DE	2019	0	0	0	0	0	0	0	0	0	0	0	0	64
15716911	Not	md	JP	2019	1	0	0	0	0	0	0	0	0	0	0	0	90
15719803	f	Not repo	TW	2019	0	1	0	1	0	0	0	0	0	0	0	1	
15720566	m	md	DE	2019	0	1	0	0	1	0	0	0	0	0	0	0	53
15721138	m	ot	FR	2019	0	1	0	0	1	0	0	0	0	0	0	0	63
15725148	m	md	CN	2019	0	0	0	0	0	0	0	0	0	0	0	0	52
15728726	m	cn	JP	2019	0	1	1	0	0	0	0	0	0	0	0	1	70
15728867	m	ph	JP	2019	0	1	0	0	1	0	0	0	0	0	0	0	79
15729340	f	cn	CH	2019	0	1	1	0	0	0	0	0	0	0	0	0	
15730572	m	ot	US	2019	0	0	0	0	0	0	0	0	0	0	0	0	79
15731729	f	cn	IN	2019	0	0	0	0	0	0	0	0	0	0	0	0	61
15731818	f	ot	US	2019	0	0	0	0	0	0	0	0	0	0	0	0	35

15732580	m	md	JP	2019	1	0	0	0	0	0	0	0	0	0	0	1	82.8
15733252	f	md	JP	2019	0	0	0	0	0	0	0	0	0	0	0	0	49
15734236	m	md	JP	2019	1	0	0	0	0	0	0	0	0	0	0	0	80
15734365	f	cn	JP	2019	0	1	1	0	0	0	0	0	0	0	0	0	80
15735607	m	md	JP	2019	0	1	0	1	0	0	0	0	0	0	0	1	77
15735861	f	ot	CZ	2019	0	0	0	0	0	0	0	0	0	0	0	0	82
15738749	f	md	DE	2019	0	0	0	0	0	0	0	0	0	0	0	0	66
15739088	m	cn	JP	2019	0	1	1	0	0	0	0	0	0	0	0	0	67
15739608	f	md	GB	2019	1	0	0	0	0	0	0	0	0	0	0	0	53
15742437	f	ot	PL	2019	0	1	0	0	1	0	0	0	0	0	0	0	59
15742947	f	md	TW	2019	0	0	0	0	0	0	0	0	0	0	0	0	66
15743037	m	md	TH	2019	0	1	0	0	1	0	0	0	0	0	0	0	49
15745150	Not	md	IN	2019	0	0	0	0	0	0	0	0	0	0	0	0	
15745243	m	md	PL	2019	0	1	0	0	0	1	0	0	0	0	0	0	57
15745859	f	ot	US	2019	0	0	0	0	0	0	0	0	0	0	0	0	70
15747739	m	md	PL	2019	0	0	0	0	0	0	0	0	0	0	0	0	68
15748650	Not	ot	CZ	2019	0	0	0	0	0	0	0	0	0	0	0	0	
15748983	m	md	CN	2019	0	1	0	0	0	1	0	0	0	0	0	0	54
15751484	m	cn	JP	2019	0	1	1	0	0	0	0	0	0	0	0	0	65
15752876	Not	md	US	2019	0	1	1	0	0	0	0	0	0	0	0	0	
15752936	m	ot	AR	2019	0	1	0	0	1	0	0	0	0	0	0	0	60
15756389	f	md	IT	2019	0	1	0	0	1	0	0	0	0	0	0	0	77
15756981	m	md	BE	2019	1	0	0	0	0	0	0	0	0	0	0	0	80.1
15756983	f	md	JP	2019	1	0	0	0	0	0	0	0	0	0	0	0	54
15757087	f	Not repo	US	2019	1	0	0	0	0	0	0	0	0	0	0	0	67
15757529	m	md	JP	2019	0	1	0	1	0	0	0	0	0	0	0	1	72.6
15757533	m	md	JP	2019	0	1	0	1	0	0	0	0	0	0	0	0	73
15757926	m	ot	DE	2019	0	0	0	0	0	0	0	0	0	0	0	0	82
15757932	m	cn	DE	2019	0	0	0	0	0	0	0	0	0	0	0	0	63
15758136	f	md	US	2019	0	0	0	0	0	0	0	0	0	0	0	0	78
15758210	m	md	JP	2019	1	0	0	0	0	0	0	0	0	0	0	1	91
15758217	m	md	JP	2019	1	0	0	0	0	0	0	0	0	0	0	1	78
15759176	f	ot	FR	2019	0	1	0	0	1	0	0	0	0	0	0	0	
15759250	m	ph	JP	2019	0	0	0	0	0	0	0	0	0	0	0	0	78

15759594	f	md	NL	2019	0	0	0	0	0	0	0	0	0	0	0	0	52
15759675	m	md	JP	2019	0	1	0	1	0	0	0	0	0	0	0	1	80
15759781	m	Not repo	GR	2019	1	0	0	0	0	0	0	0	0	0	0	0	
15760443	f	md	CZ	2019	0	0	0	0	0	0	0	0	0	0	0	0	82
15761262	m	md	JP	2019	0	1	0	1	0	0	0	0	0	0	0	1	77
15762685	m	cn	JP	2019	0	1	1	0	0	0	0	0	0	0	0	0	44
15764546	m	md	DE	2019	0	1	0	0	1	0	0	0	0	0	0	0	56
15764856	f	md	DE	2019	0	0	0	0	0	0	0	0	0	0	0	0	
15765183	m	md	JP	2019	0	1	0	0	1	0	0	0	0	0	0	1	76
15767435	m	md	JP	2019	0	1	0	1	0	0	0	0	0	0	0	0	79
15767544	f	md	DE	2019	1	0	0	0	0	0	0	0	0	0	0	0	74.3
15767833	f	md	JP	2019	0	1	0	1	0	0	0	0	0	0	0	1	66.3
15768003	m	md	PL	2019	0	1	0	0	1	0	0	0	0	0	0	0	72
15768243	m	md	FR	2019	0	0	0	0	0	0	0	0	0	0	0	0	63
15768913	f	ot	PL	2019	0	1	0	0	1	0	0	0	0	0	0	0	51
15769099	m	ot	US	2019	0	1	0	0	1	0	0	0	0	0	0	0	62
15769628	m	md	JP	2019	0	0	0	0	0	0	0	0	0	0	0	0	
15769766	f	ph	JP	2019	1	0	0	0	0	0	0	0	0	0	0	0	
15772368	m	md	JP	2019	0	1	0	1	0	0	0	0	0	0	0	0	
15772770	m	md	JP	2019	0	1	0	1	0	0	0	0	0	0	0	1	65
15774334	m	ot	DE	2019	0	0	0	0	0	0	0	0	0	0	0	0	54
15774720	m	Not repo	JP	2019	0	0	0	0	0	0	0	0	0	0	0	0	80
15775034	m	md	JP	2019	0	1	0	0	1	0	0	0	0	0	0	0	65
15776283	Not	md	FR	2019	0	0	0	0	0	0	0	0	0	0	0	0	
15776915	f	cn	JP	2019	0	1	1	0	0	0	0	0	0	0	0	1	60
15778130	m	cn	JP	2019	0	1	1	0	0	0	0	0	0	0	0	1	70
15778916	f	md	DE	2019	0	0	0	0	0	0	0	0	0	0	0	0	61
15779477	f	md	CN	2019	1	0	0	0	0	0	0	0	0	0	0	0	50
15779923	f	md	CN	2019	0	0	0	0	0	0	0	0	0	0	0	0	62
15780199	f	md	US	2019	0	1	0	0	0	1	0	0	0	0	0	1	85
15780355	m	ot	DE	2019	0	0	0	0	0	0	0	0	0	0	0	0	81
15780430	m	cn	TH	2019	0	1	0	0	1	0	0	0	0	0	0	0	56
15780435	m	md	JP	2019	0	1	0	1	0	0	0	0	0	0	0	0	57
15780474	m	md	JP	2019	1	0	0	0	0	0	0	0	0	0	0	0	70

15780476	f	md	JP	2019	0	1	0	1	0	0	0	0	0	0	0	1	85.2
15780595	m	ot	JP	2019	1	0	0	0	0	0	0	0	0	0	0	0	60.5
15780601	m	ot	AT	2019	0	0	0	0	0	0	0	0	0	0	0	0	57
15780726	f	ot	JP	2019	0	1	0	0	1	0	0	0	0	0	0	0	57
15780727	f	cn	JP	2019	0	1	1	0	0	0	0	0	0	0	0	0	60
15780730	f	cn	GB	2019	1	0	0	0	0	0	0	0	0	0	0	0	69
15780735	f	cn	US	2019	1	0	0	0	0	0	0	0	0	0	0	0	
15780762	m	md	JP	2019	0	1	0	0	0	1	0	0	0	0	0	0	76
15780799	f	md	FR	2019	1	0	0	0	0	0	0	0	0	0	0	0	70.5
15781278	m	ot	FR	2019	0	1	0	0	1	0	0	0	0	0	0	0	70
15781384	f	cn	US	2019	0	0	0	0	0	0	0	0	0	0	0	0	69
15781775	m	md	JP	2019	1	0	0	0	0	0	0	0	0	0	0	0	70
15781782	m	md	DK	2019	0	0	0	0	0	0	0	0	0	0	0	0	52
15781884	m	cn	IN	2019	0	1	0	0	1	0	0	0	0	0	0	0	
15782779	f	cn	DE	2019	0	1	1	0	0	0	0	0	0	0	0	1	72
15782831	f	ot	JP	2019	1	0	0	0	0	0	0	0	0	0	0	0	80
15783608	m	md	US	2019	0	1	0	0	0	1	0	0	0	0	0	0	66
15783894	f	md	JP	2019	0	1	0	0	1	0	0	0	0	0	0	0	72
15784019	m	ot	DE	2019	0	1	0	0	1	0	0	0	0	0	0	0	67
15784152	m	md	Not re	2019	0	0	0	0	0	0	0	0	0	0	0	0	57
15784173	m	ot	FR	2019	0	1	0	0	1	0	0	0	0	0	0	0	74
15784251	m	md	JP	2019	0	1	0	0	0	1	0	0	0	0	0	0	73
15784416	m	ot	DE	2019	0	0	0	0	0	0	0	0	0	0	0	0	58
15784806	f	cn	IN	2019	0	1	0	0	1	0	0	0	0	0	0	0	
15784935	m	md	JP	2019	1	0	0	0	0	0	0	0	0	0	0	1	72
15785281	f	md	JP	2019	0	1	0	1	0	0	0	0	0	0	0	1	76
15785398	m	ph	US	2019	0	1	1	0	0	0	0	0	0	0	0	0	61
15785851	m	ot	US	2019	0	1	0	0	1	0	0	0	0	0	0	0	60
15785864	Not	ot	JP	2019	0	0	0	0	0	0	0	0	0	0	0	0	
15785867	f	md	JP	2019	0	0	0	0	0	0	0	0	0	0	0	0	84
15785907	m	md	JP	2019	1	0	0	0	0	0	0	0	0	0	0	0	69.7
15786537	m	cn	GB	2019	1	0	0	0	0	0	0	0	0	0	0	1	65
15786723	m	md	US	2019	0	1	0	0	1	0	0	0	0	0	0	0	
15787023	m	md	US	2019	0	0	0	0	0	0	0	0	0	0	0	0	74

15787217	f	ot	CN	2019	0	1	0	0	1	0	0	0	0	0	0	0	49
15787260	f	ot	PL	2019	0	1	0	0	1	0	0	0	0	0	0	0	61
15787452	m	ot	US	2019	0	1	0	0	1	0	0	0	0	0	0	0	69
15787526	m	ot	US	2019	0	1	0	0	1	0	0	0	0	0	0	0	58
15787573	f	cn	KR	2019	0	1	1	0	0	0	0	0	0	0	0	0	
15787771	m	ph	AU	2019	0	1	0	0	0	1	0	0	0	0	0	0	61
15787844	m	cn	US	2019	0	1	0	0	1	0	0	0	0	0	0	0	
15787860	m	md	DE	2019	0	1	0	0	1	0	0	0	0	0	0	0	69
15787911	m	ot	DE	2019	0	1	0	0	1	0	0	0	0	0	0	0	58
15787941	Not	md	US	2019	0	1	0	0	1	0	0	0	0	0	0	0	
15787942	Not	md	US	2019	0	1	0	0	1	0	0	0	0	0	0	0	
15787943	Not	md	US	2019	0	1	0	0	1	0	0	0	0	0	0	0	
15787944	Not	md	US	2019	0	1	0	0	1	0	0	0	0	0	0	0	
15787950	Not	md	US	2019	0	1	0	0	1	0	0	0	0	0	0	0	
15787951	Not	md	US	2019	0	1	0	0	1	0	0	0	0	0	0	0	
15787959	Not	md	US	2019	0	1	0	0	1	0	0	0	0	0	0	0	
15787963	Not	md	US	2019	0	1	0	0	1	0	0	0	0	0	0	0	
15787964	Not	md	US	2019	0	1	0	0	1	0	0	0	0	0	0	0	
15787965	Not	md	US	2019	0	1	0	0	1	0	0	0	0	0	0	0	
15787972	Not	md	US	2019	0	1	0	0	1	0	0	0	0	0	0	0	
15787973	Not	md	US	2019	0	1	0	0	1	0	0	0	0	0	0	0	
15787974	Not	md	US	2019	0	1	0	0	1	0	0	0	0	0	0	0	
15787975	Not	md	US	2019	0	1	0	0	1	0	0	0	0	0	0	0	
15787976	Not	md	US	2019	0	1	0	0	1	0	0	0	0	0	0	0	
15787977	Not	md	US	2019	0	1	0	0	1	0	0	0	0	0	0	0	
15787984	Not	md	US	2019	0	1	0	0	1	0	0	0	0	0	0	0	
15787985	Not	md	US	2019	0	1	0	0	1	0	0	0	0	0	0	0	
15787986	Not	md	US	2019	0	1	0	0	1	0	0	0	0	0	0	0	
15787997	Not	md	US	2019	0	1	0	0	1	0	0	0	0	0	0	0	
15787998	Not	md	US	2019	0	1	0	0	1	0	0	0	0	0	0	0	
15787999	Not	md	US	2019	0	1	0	0	1	0	0	0	0	0	0	0	
15788005	Not	md	US	2019	0	1	0	0	1	0	0	0	0	0	0	0	
15788006	Not	md	US	2019	0	1	0	0	1	0	0	0	0	0	0	0	
15788007	Not	md	US	2019	0	1	0	0	1	0	0	0	0	0	0	0	

15788017	Not	md	US	2019	0	1	0	0	1	0	0	0	0	0	0	0	
15788018	Not	md	US	2019	0	1	0	0	1	0	0	0	0	0	0	0	
15788019	Not	md	US	2019	0	1	0	0	1	0	0	0	0	0	0	0	
15788023	Not	md	US	2019	0	1	0	0	1	0	0	0	0	0	0	0	
15788024	Not	md	US	2019	0	1	0	0	1	0	0	0	0	0	0	0	
15788035	Not	md	US	2019	0	1	0	0	1	0	0	0	0	0	0	0	
15788231	m	md	IE	2019	0	1	0	0	1	0	0	0	0	0	0	0	
15788525	m	md	TR	2019	0	1	0	0	0	1	0	0	0	0	0	1	34
15788701	f	md	CN	2019	0	1	0	0	0	1	0	0	0	0	0	0	49
15789455	m	md	US	2019	1	0	0	0	0	0	0	0	0	0	0	0	
15789724	m	ph	US	2019	0	1	0	1	0	0	0	0	0	0	0	0	74
15789811	m	md	TW	2019	0	0	0	0	0	0	0	0	0	0	0	0	69
15789819	m	md	SG	2019	0	0	0	0	0	0	0	0	0	0	0	0	57
15790753	m	ot	JP	2019	0	1	0	0	1	0	0	0	0	0	0	0	69
15791184	f	md	FR	2019	1	0	0	0	0	0	0	0	0	0	0	0	57
15792090	m	ot	CN	2019	0	1	0	0	1	0	0	0	0	0	0	0	62
15792744	Not	cn	IN	2019	0	0	0	0	0	0	0	0	0	0	0	0	25
15792991	m	md	CN	2019	0	1	0	0	0	1	0	0	0	0	0	0	59
15793038	Not	md	JP	2019	0	0	0	0	0	0	0	0	0	0	0	0	
15793132	f	md	JP	2019	0	1	0	0	0	1	0	0	0	0	0	0	74
15793447	m	cn	US	2019	0	1	1	0	0	0	0	0	0	0	0	0	72
15793697	m	ot	NO	2019	0	0	0	0	0	0	0	0	0	0	0	0	76
15793969	m	md	AU	2019	0	0	0	0	0	0	0	0	0	0	0	0	77
15794267	m	md	TW	2019	0	0	0	0	0	0	0	0	0	0	0	0	69
15794524	f	ot	US	2019	0	0	0	0	0	0	0	0	0	0	0	0	59
15794542	m	ot	US	2019	0	0	0	0	0	0	0	0	0	0	0	0	72
15794553	f	ph	CA	2019	0	0	0	0	0	0	0	0	0	0	0	0	64
15794626	m	ot	FR	2019	0	1	0	0	1	0	0	0	0	0	0	0	68
15794695	m	md	TH	2019	0	1	0	1	0	0	0	0	0	0	0	0	71.5
15794751	f	md	CA	2019	0	0	0	0	0	0	0	0	0	0	0	0	32
15795435	f	ot	CN	2019	0	0	0	0	0	0	0	0	0	0	0	0	55
15796060	f	cn	US	2019	0	1	0	0	1	0	0	0	0	0	0	0	
15796081	f	md	CH	2019	1	0	0	0	0	0	0	0	0	0	0	0	61
15796175	f	cn	US	2019	0	1	0	0	1	0	0	0	0	0	0	0	

15796332	m	ot	KR	2019	0	1	0	0	1	0	0	0	0	0	0	0	72
15796386	m	cn	TH	2019	0	1	0	0	1	0	0	0	0	0	0	0	73
15796395	f	cn	US	2019	0	1	0	0	1	0	0	0	0	0	0	0	79
15796683	m	md	FR	2019	0	0	0	0	0	0	0	0	0	0	0	0	
15797612	m	md	NO	2019	0	1	0	0	0	1	0	0	0	0	0	0	62
15797902	f	ot	DE	2019	1	0	0	0	0	0	0	0	0	0	0	0	47
15798399	m	md	KR	2019	0	0	0	0	0	0	0	0	0	0	0	0	58
15799537	m	ot	DE	2019	0	0	0	0	0	0	0	0	0	0	0	0	67
15799862	f	md	JP	2019	1	0	0	0	0	0	0	0	0	0	0	0	74
15799865	f	md	JP	2019	1	0	0	0	0	0	0	0	0	0	0	0	74
15799868	f	md	JP	2019	1	0	0	0	0	0	0	0	0	0	0	0	74
15799917	f	cn	US	2019	1	0	0	0	0	0	0	0	0	0	0	0	
15800209	f	ot	US	2019	0	1	0	0	1	0	0	0	0	0	0	0	74
15800368	m	ot	DE	2019	0	0	0	0	0	0	0	0	0	0	0	0	70
15800844	f	md	JP	2019	1	0	0	0	0	0	0	0	0	0	0	0	65
15800963	f	ph	IT	2019	0	1	0	0	1	0	0	0	0	0	0	0	81
15800992	f	md	JP	2019	1	0	0	0	0	0	0	0	0	0	0	0	89
15801028	m	md	JP	2019	1	0	0	0	0	0	0	0	0	0	0	1	62
15801167	f	md	JP	2019	1	0	0	0	0	0	0	0	0	0	0	0	73
15801168	m	md	JP	2019	1	0	0	0	0	0	0	0	0	0	0	1	67
15801185	m	md	JP	2019	1	0	0	0	0	0	0	0	0	0	0	0	55
15801218	f	md	RS	2019	0	1	1	0	0	0	0	0	0	0	0	0	62
15801268	m	md	JP	2019	1	0	0	0	0	0	0	0	0	0	0	0	76
15801270	f	md	JP	2019	1	0	0	0	0	0	0	0	0	0	0	0	55
15801292	f	md	JP	2019	1	0	0	0	0	0	0	0	0	0	0	0	79
15801317	f	md	JP	2019	1	0	0	0	0	0	0	0	0	0	0	0	72
15801352	m	md	JP	2019	0	1	0	0	0	1	0	0	0	0	0	0	66
15801815	m	ot	NL	2019	0	1	0	0	1	0	0	0	0	0	0	0	64
15801816	m	cn	DE	2019	0	0	0	0	0	0	0	0	0	0	0	0	53
15801885	f	md	JP	2019	1	0	0	0	0	0	0	0	0	0	0	0	76
15802770	f	md	ES	2019	0	0	0	0	0	0	0	0	0	0	0	0	72
15803068	m	md	US	2019	0	1	0	1	0	0	0	0	0	0	0	0	60
15803090	f	md	JP	2019	1	0	0	0	0	0	0	0	0	0	0	0	74
15803109	m	ot	US	2019	0	1	0	0	1	0	0	0	0	0	0	0	

15803270	m	cn	US	2019	1	0	0	0	0	0	0	0	0	0	0	0	79
15803487	m	md	JP	2019	1	0	0	0	0	0	0	0	0	0	0	0	68
15803488	f	md	JP	2019	1	0	0	0	0	0	0	0	0	0	0	0	65
15803490	m	md	JP	2019	1	0	0	0	0	0	0	0	0	0	0	0	71
15803739	m	md	JP	2019	1	0	0	0	0	0	0	0	0	0	0	0	74
15803982	f	md	JP	2019	1	0	0	0	0	0	0	0	0	0	0	0	68
15804034	m	ot	DE	2019	0	0	0	0	0	0	0	0	0	0	0	0	80
15804213	f	md	JP	2019	1	0	0	0	0	0	0	0	0	0	0	0	50
15804214	f	md	JP	2019	1	0	0	0	0	0	0	0	0	0	0	0	73
15804541	m	ot	DE	2019	0	0	0	0	0	0	0	0	0	0	0	0	64
15804903	m	ot	JP	2019	0	0	0	0	0	0	0	0	0	0	0	0	73
15805499	f	ph	US	2019	0	1	0	0	1	0	0	0	0	0	0	0	
15805941	m	md	JP	2019	0	1	0	0	0	1	0	0	0	0	0	0	75
15806162	f	md	DE	2019	1	0	0	0	0	0	0	0	0	0	0	0	78
15806565	f	cn	US	2019	1	0	0	0	0	0	0	0	0	0	0	0	88.7
15806595	m	cn	US	2019	0	1	0	0	1	0	0	0	0	0	0	0	79
15807246	m	ot	GB	2019	0	0	0	0	0	0	0	0	0	0	0	0	71
15807823	f	md	KR	2019	0	0	0	0	0	0	0	0	0	0	0	0	57
15808364	f	md	NL	2019	0	0	0	0	0	0	0	0	0	0	0	0	78
15808476	m	md	CN	2019	0	0	0	0	0	0	0	0	0	0	0	0	57
15808611	m	cn	JP	2019	0	1	1	0	0	0	0	0	0	0	0	1	72
15808873	m	md	JP	2019	0	1	1	0	0	0	0	0	0	0	0	0	63
15809213	Not	ot	US	2019	0	1	0	1	0	0	0	0	0	0	0	0	
15809319	Not	md	US	2019	0	0	0	0	0	0	0	0	0	0	0	0	
15810288	f	ot	FR	2019	0	1	0	0	1	0	0	0	0	0	0	0	60
15810698	f	ph	JP	2019	0	0	0	0	0	0	0	0	0	0	0	0	80
15811247	Not	ot	CN	2019	1	0	0	0	0	0	0	0	0	0	0	0	
15811415	f	md	CH	2019	1	0	0	0	0	0	0	0	0	0	0	0	82
15812012	f	ot	AU	2019	1	0	0	0	0	0	0	0	0	0	0	0	
15812362	m	md	JP	2019	0	1	0	0	1	0	0	0	0	0	0	0	81
15812735	Not	md	US	2019	0	0	0	0	0	0	0	0	0	0	0	0	
15812923	f	ot	AU	2019	1	0	0	0	0	0	0	0	0	0	0	0	71.8
15813535	Not	md	US	2019	0	0	0	0	0	0	0	0	0	0	0	0	
15813555	Not	md	US	2019	0	0	0	0	0	0	0	0	0	0	0	0	

15813578	Not	md	US	2019	0	0	0	0	0	0	0	0	0	0	0	0	
15813581	m	ot	FR	2019	0	0	0	0	0	0	0	0	0	0	0	0	69
15813642	f	ot	JP	2019	1	0	0	0	0	0	0	0	0	0	0	0	67
15813800	m	cn	GB	2019	1	0	0	0	0	0	0	0	0	0	0	0	62
15813990	m	md	JP	2019	0	1	0	1	0	0	0	0	0	0	0	1	74.9
15814243	m	md	BR	2019	0	0	0	0	0	0	0	0	0	0	0	0	65
15814482	m	ot	DE	2019	0	0	0	0	0	0	0	0	0	0	0	0	66
15814629	f	ph	GB	2019	1	0	0	0	0	0	0	0	0	0	0	0	71
15815570	m	ph	JP	2019	0	0	0	0	0	0	0	0	0	0	0	0	
15818750	f	cn	IL	2019	0	1	1	0	0	0	0	0	0	0	0	0	
15819209	m	cn	US	2019	0	1	0	0	0	1	0	0	0	0	0	0	65
15819483	m	ot	US	2019	0	1	0	0	1	0	0	0	0	0	0	0	59
15819490	Not	md	JP	2019	0	1	0	0	1	0	0	0	0	0	0	0	
15819816	m	md	JP	2019	0	1	0	0	1	0	0	0	0	0	0	0	72
15819852	Not	cn	SG	2019	0	0	0	0	0	0	0	0	0	0	0	0	
15819868	m	md	JP	2019	0	1	0	0	1	0	0	0	0	0	0	0	67
15820137	Not	ot	CN	2019	1	0	0	0	0	0	0	0	0	0	0	0	
15820139	m	cn	TW	2019	0	1	0	0	0	1	0	0	0	0	0	0	
15820217	f	cn	JP	2019	0	1	1	0	0	0	0	0	0	0	0	0	74
15820226	m	ot	NL	2019	0	0	0	0	0	0	0	0	0	0	0	0	63
15820231	m	ot	NL	2019	0	0	0	0	0	0	0	0	0	0	0	0	65
15820351	m	md	DE	2019	0	1	0	0	0	1	0	0	0	0	0	0	67
15820824	f	ot	US	2019	1	0	0	0	0	0	0	0	0	0	0	0	69
15821456	f	cn	DE	2019	0	1	1	0	0	0	0	0	0	0	0	0	66
15821607	m	ot	CN	2019	0	0	0	0	0	0	0	0	0	0	0	0	62
15821778	f	cn	US	2019	1	0	0	0	0	0	0	0	0	0	0	0	
15821843	m	cn	FR	2019	0	1	1	0	0	0	0	0	0	0	0	0	67
15822651	m	ot	JP	2019	1	0	0	0	0	0	0	0	0	0	0	0	61
15822868	m	md	CN	2019	0	0	0	0	0	0	0	0	0	0	0	0	68
15822924	f	cn	US	2019	0	1	1	0	0	0	0	0	0	0	0	0	73
15823304	f	cn	KR	2019	0	0	0	0	0	0	0	0	0	0	0	0	71
15823926	f	ot	NL	2019	0	0	0	0	0	0	0	0	0	0	0	0	52
15824071	m	cn	DE	2019	0	1	1	0	0	0	0	0	0	0	0	0	
15824617	f	md	CZ	2019	1	0	0	0	0	0	0	0	0	0	0	0	55

15824621	m	cn	DE	2019	0	1	1	0	0	0	0	0	0	0	0	0	
15824771	m	ph	US	2019	0	1	0	0	1	0	0	0	0	0	0	0	
15824871	m	md	JP	2019	0	1	0	0	1	0	0	0	0	0	0	0	58
15825148	f	md	FR	2019	0	0	0	0	0	0	0	0	0	0	0	0	
15825272	f	cn	US	2019	0	0	0	0	0	0	0	0	0	0	0	0	
15825274	f	md	CN	2019	0	1	0	0	0	1	0	0	0	0	0	0	65
15825298	m	md	DE	2019	1	0	0	0	0	0	0	0	0	0	0	0	36
15825321	m	md	AU	2019	0	1	0	0	0	1	0	0	0	0	0	0	77
15825410	m	ot	JP	2019	0	0	0	0	0	0	0	0	0	0	0	0	70
15825445	m	md	FR	2019	0	0	0	0	0	0	0	0	0	0	0	0	70
15825511	f	md	DE	2019	0	0	0	0	0	0	0	0	0	0	0	0	48
15825697	m	md	IT	2019	1	0	0	0	0	0	0	0	0	0	0	0	70
15825859	m	md	SE	2019	0	0	0	0	0	0	0	0	0	0	0	0	71
15825878	f	cn	NL	2019	0	0	0	0	0	0	0	0	0	0	0	0	52
15825972	Not	md	JP	2019	0	1	0	0	1	0	0	0	0	0	0	0	
15826265	f	cn	JP	2019	0	1	0	0	1	0	0	0	0	0	0	0	71
15826285	m	ph	JP	2019	1	0	0	0	0	0	0	0	0	0	0	0	71
15826896	Not	ph	CN	2019	1	0	0	0	0	0	0	0	0	0	0	0	
15827821	m	ph	US	2019	0	1	1	0	0	0	0	0	0	0	0	0	
15828826	f	ot	DE	2019	0	0	0	0	0	0	0	0	0	0	0	0	59
15830554	f	cn	JP	2019	0	1	1	0	0	0	0	0	0	0	0	0	76
15830563	f	ot	JP	2019	0	1	1	0	0	0	0	0	0	0	0	0	72
15830694	m	md	JP	2019	0	1	0	0	0	1	0	0	0	0	0	0	74
15830777	f	md	DE	2019	0	1	0	0	1	0	0	0	0	0	0	0	65
15831060	Not	md	US	2019	0	0	0	0	0	0	0	0	0	0	0	0	
15831182	m	md	FR	2019	0	1	0	0	1	0	0	0	0	0	0	0	74
15831521	m	md	JP	2019	0	1	0	0	0	1	0	0	0	0	0	0	71
15831707	f	md	FR	2019	1	0	0	0	0	0	0	0	0	0	0	0	70
15831763	m	md	JP	2019	0	1	0	0	0	1	0	0	0	0	0	0	82
15832026	m	md	JP	2019	0	1	0	0	1	0	0	0	0	0	0	0	64
15832033	f	cn	NL	2019	1	0	0	0	0	0	0	0	0	0	0	0	37
15832167	m	md	JP	2019	0	0	0	0	0	0	0	0	0	0	0	0	73
15832197	Not	ot	CN	2019	1	0	0	0	0	0	0	0	0	0	0	0	
15832245	f	md	JP	2019	0	0	0	0	0	0	0	0	0	0	0	0	70

15832438	f	ph	SI	2019	1	0	0	0	0	0	0	0	0	0	0	0	60
15832993	m	cn	GR	2019	0	1	1	0	0	0	0	0	0	0	0	0	
15833447	f	ph	FR	2019	0	0	0	0	0	0	0	0	0	0	0	0	55
15833677	f	cn	JP	2019	0	1	1	0	0	0	0	0	0	0	0	1	75
15833739	f	md	US	2019	0	0	0	0	0	0	0	0	0	0	0	0	65
15834097	m	md	JP	2019	1	0	0	0	0	0	0	0	0	0	0	1	81
15834100	f	md	JP	2019	1	0	0	0	0	0	0	0	0	0	0	0	72
15834496	m	ot	JP	2019	0	1	0	0	1	0	0	0	0	0	0	0	68
15834566	m	cn	US	2019	1	0	0	0	0	0	0	0	0	0	0	0	67
15835617	m	md	KR	2019	0	1	0	0	1	0	0	0	0	0	0	0	65
15835934	Not	ot	US	2019	0	1	0	0	1	0	0	0	0	0	0	0	
15836021	f	md	CA	2019	0	1	0	0	0	1	0	0	0	0	0	0	61
15836309	m	md	DE	2019	0	1	0	0	1	0	0	0	0	0	0	0	58
15836826	m	md	US	2019	0	1	0	1	0	0	0	0	0	0	0	0	
15836928	m	md	KR	2019	0	1	0	0	0	1	0	0	0	0	0	0	65
15837675	f	md	JP	2019	1	0	0	0	0	0	0	0	0	0	0	1	80.4
15838342	m	cn	KR	2019	0	1	1	0	0	0	0	0	0	0	0	0	
15838609	m	ot	AU	2019	1	0	0	0	0	0	0	0	0	0	0	0	69.8
15838618	m	cn	JP	2019	1	0	0	0	0	0	0	0	0	0	0	0	
15838675	m	md	JP	2019	0	1	0	1	0	0	0	0	0	0	0	0	61
15839148	m	ot	CA	2019	0	1	0	0	1	0	0	0	0	0	0	0	65
15839313	m	ot	GB	2019	0	0	0	0	0	0	0	0	0	0	0	0	74
15839952	m	ot	US	2019	0	1	0	0	1	0	0	0	0	0	0	0	67
15840420	f	ph	JP	2019	1	0	0	0	0	0	0	0	0	0	0	0	80
15840526	Not	md	US	2019	0	0	0	0	0	0	0	0	0	0	0	0	
15840557	m	md	EG	2019	0	1	0	0	0	1	0	0	0	0	0	0	
15840944	f	md	JP	2019	1	0	0	0	0	0	0	0	0	0	0	0	66
15841031	m	md	PT	2019	0	0	0	0	0	0	0	0	0	0	0	0	58
15841114	m	md	IE	2019	0	1	0	0	0	1	0	0	0	0	0	0	56
15841121	f	md	EG	2019	0	1	0	0	0	1	0	0	0	0	0	0	
15841205	f	md	JP	2019	1	0	0	0	0	0	0	0	0	0	0	0	79.8
15841347	f	ot	IT	2019	1	0	0	0	0	0	0	0	0	0	0	0	71
15841589	m	ph	FR	2019	1	0	0	0	0	0	0	0	0	0	0	0	79
15842029	m	Not repo	CN	2019	1	0	0	0	0	0	0	0	0	0	0	0	

15842370	f	ot	CN	2019	0	1	1	0	0	0	0	0	0	0	0	0	49
15842555	m	md	JP	2019	0	1	0	1	0	0	0	0	0	0	0	0	66
15842640	f	md	IT	2019	1	0	0	0	0	0	0	0	0	0	0	0	80
15842641	f	md	IT	2019	1	0	0	0	0	0	0	0	0	0	0	0	73
15842811	f	md	IT	2019	1	0	0	0	0	0	0	0	0	0	0	0	47
15842840	m	md	CH	2019	0	0	0	0	0	0	0	0	0	0	0	0	83
15843208	f	ot	US	2019	0	0	0	0	0	0	0	0	0	0	0	0	81
15843234	m	md	JP	2019	0	0	0	0	0	0	0	0	0	0	0	0	66
15843403	m	ot	JP	2019	0	1	0	1	0	0	0	0	0	0	0	1	67.2
15843410	Not	Not repo	TW	2019	0	1	0	1	0	0	0	0	0	0	0	1	
15843746	m	ph	FR	2019	0	1	0	1	0	0	0	0	0	0	0	0	53
15843784	m	md	JP	2019	1	0	0	0	0	0	0	0	0	0	0	0	74
15844103	f	md	US	2019	0	1	1	0	0	0	0	0	0	0	0	0	85
15844122	f	md	DK	2019	0	1	1	0	0	0	0	0	0	0	0	0	56
15844352	f	Not repo	Not re	2019	0	0	0	0	0	0	0	0	0	0	0	0	59
15844765	m	md	NL	2019	0	0	0	0	0	0	0	0	0	0	0	0	63
15844887	Not	md	US	2019	1	0	0	0	0	0	0	0	0	0	0	0	
15845263	f	ot	GB	2019	1	0	0	0	0	0	0	0	0	0	0	0	59
15845414	m	md	JP	2019	0	0	0	0	0	0	0	0	0	0	0	0	72
15845438	f	md	JP	2019	0	0	0	0	0	0	0	0	0	0	0	0	54
15845793	m	md	NL	2019	0	0	0	0	0	0	0	0	0	0	0	0	65
15846477	f	md	JP	2019	0	1	0	0	0	1	0	0	0	0	0	0	72
15846503	m	ot	LB	2019	0	1	0	0	1	0	0	0	0	0	0	0	
15847132	Not	md	US	2019	0	0	0	0	0	0	0	0	0	0	0	0	
15847161	f	ot	CA	2019	0	1	0	0	1	0	0	0	0	0	0	0	78
15847375	m	ot	DE	2019	0	1	0	0	1	0	0	0	0	0	0	0	60
15847751	m	md	ES	2019	0	1	0	0	0	1	0	0	0	0	0	0	65
15848569	m	md	JP	2019	0	0	0	0	0	0	0	0	0	0	0	0	65
15848969	m	cn	US	2019	0	0	0	0	0	0	0	0	0	0	0	0	76
15849114	f	md	US	2019	1	0	0	0	0	0	0	0	0	0	0	0	
15849192	f	cn	DE	2019	0	1	1	0	0	0	0	0	0	0	0	0	52
15849313	m	ot	JP	2019	0	1	0	0	1	0	0	0	0	0	0	0	
15849380	m	md	AT	2019	0	0	0	0	0	0	0	0	0	0	0	0	68
15849427	m	ot	JP	2019	0	1	0	0	1	0	0	0	0	0	0	0	

15849563	m	md	JP	2019	0	1	0	0	1	0	0	0	0	0	0	1	74
15849602	Not	ot	JP	2019	0	0	0	0	0	0	0	0	0	0	0	0	
15849677	m	md	IL	2019	1	0	0	0	0	0	0	0	0	0	0	0	45
15850218	f	lw	US	2019	0	0	0	0	0	0	0	0	0	0	0	0	60
15850305	f	ot	NL	2019	1	0	0	0	0	0	0	0	0	0	0	0	79
15850871	m	ot	JP	2019	0	1	0	0	1	0	0	0	0	0	0	0	
15851041	m	md	JP	2019	0	1	0	0	1	0	0	0	0	0	0	1	67
15851134	m	ot	JP	2019	0	1	0	0	1	0	0	0	0	0	0	0	
15851150	m	md	IE	2019	0	1	0	0	0	1	0	0	0	0	0	0	62
15851153	m	ot	JP	2019	0	1	0	0	1	0	0	0	0	0	0	0	
15851209	m	ot	JP	2019	0	1	0	0	1	0	0	0	0	0	0	0	
15851379	m	ot	TW	2019	0	1	0	0	0	1	0	0	0	0	0	0	66
15851408	m	cn	US	2019	1	0	0	0	0	0	0	0	0	0	0	0	56
15851423	m	ot	FR	2019	0	1	0	0	1	0	0	0	0	0	0	0	53
15851478	m	ot	JP	2019	0	1	0	0	1	0	0	0	0	0	0	0	
15851505	m	ot	JP	2019	0	1	0	0	1	0	0	0	0	0	0	0	
15851644	m	ot	DE	2019	0	0	0	0	0	0	0	0	0	0	0	0	58
15851683	f	ot	SE	2019	0	0	0	0	0	0	0	0	0	0	0	0	42
15851706	m	ph	JP	2019	0	1	0	0	1	0	0	0	0	0	0	1	58
15851716	Not	md	JP	2019	1	0	0	0	0	0	0	0	0	0	0	1	61
15851853	m	cn	JP	2019	0	1	1	0	0	0	0	0	0	0	0	0	74
15852589	f	md	GB	2019	1	0	0	0	0	0	0	0	0	0	0	0	66
15852707	m	Not repo	JP	2019	0	0	0	0	0	0	0	0	0	0	0	0	39
15853976	f	md	US	2019	0	0	0	0	0	0	0	0	0	0	0	0	
15854128	f	cn	US	2019	1	0	0	0	0	0	0	0	0	0	0	0	64
15854174	m	md	CN	2019	0	1	0	1	0	0	0	0	0	0	0	0	57.6
15854420	m	md	DE	2019	1	0	0	0	0	0	0	0	0	0	0	0	
15854977	f	ot	NL	2019	1	0	0	0	0	0	0	0	0	0	0	0	67
15854978	f	ot	NL	2019	1	0	0	0	0	0	0	0	0	0	0	0	62
15854988	m	ot	NL	2019	1	0	0	0	0	0	0	0	0	0	0	0	70
15855004	m	ot	GB	2019	0	0	0	0	0	0	0	0	0	0	0	0	80
15855956	m	ot	PT	2019	0	1	0	0	1	0	0	0	0	0	0	0	69
15856579	f	md	JP	2019	0	1	0	0	0	1	0	0	0	0	0	0	62
15856580	m	md	HU	2019	0	0	0	0	0	0	0	0	0	0	0	0	

15856656	m	ot	JP	2019	0	1	0	0	1	0	0	0	0	0	0	0	83
15856909	Not	ot	US	2019	0	0	0	0	0	0	0	0	0	0	0	0	
15857202	m	md	CN	2019	0	0	0	0	0	0	0	0	0	0	0	0	69
15857231	m	ot	IT	2019	0	0	0	0	0	0	0	0	0	0	0	0	80
15857237	f	cn	GR	2019	0	0	0	0	0	0	0	0	0	0	0	0	49
15857403	f	md	IN	2019	0	0	0	0	0	0	0	0	0	0	0	0	47
15857559	f	ot	NL	2019	1	0	0	0	0	0	0	0	0	0	0	0	37
15857570	f	ot	NL	2019	1	0	0	0	0	0	0	0	0	0	0	0	66
15857757	m	Not repo	ID	2019	1	0	0	0	0	0	0	0	0	0	0	0	
15857951	f	md	JP	2019	1	0	0	0	0	0	0	0	0	0	0	1	74.7
15858019	m	md	JP	2019	1	0	0	0	0	0	0	0	0	0	0	1	77
15858324	m	md	CN	2019	0	0	0	0	0	0	0	0	0	0	0	0	55
15858805	f	ot	NL	2019	1	0	0	0	0	0	0	0	0	0	0	0	72
15859561	f	md	JP	2019	0	0	0	0	0	0	0	0	0	0	0	0	75
15859704	f	ot	US	2019	0	0	0	0	0	0	0	0	0	0	0	0	70
15859869	f	cn	US	2019	0	1	1	0	0	0	0	0	0	0	0	0	
15860009	m	ot	JP	2019	0	0	0	0	0	0	0	0	0	0	0	0	49
15860147	f	md	FR	2019	0	0	0	0	0	0	0	0	0	0	0	0	69
15860155	m	md	FR	2019	0	0	0	0	0	0	0	0	0	0	0	0	67
15860250	f	md	FR	2019	0	0	0	0	0	0	0	0	0	0	0	0	66
15860378	m	ot	US	2019	0	1	0	0	1	0	0	0	0	0	0	0	59
15860469	m	ot	MX	2019	0	1	0	0	1	0	0	0	0	0	0	0	66
15860717	f	md	FR	2019	0	1	0	0	1	0	0	0	0	0	0	0	54
15861206	f	cn	US	2019	0	1	0	1	0	0	0	0	0	0	0	0	73.1
15861381	m	cn	JP	2019	0	1	1	0	0	0	0	0	0	0	0	0	70
15862064	f	md	ES	2019	1	0	0	0	0	0	0	0	0	0	0	0	72
15862898	f	md	JP	2019	1	0	0	0	0	0	0	0	0	0	0	0	83
15862916	m	md	DE	2019	0	0	0	0	0	0	0	0	0	0	0	0	59
15863004	f	cn	CA	2019	0	0	0	0	0	0	0	0	0	0	0	0	42
15863178	m	md	US	2019	0	1	0	1	0	0	0	0	0	0	0	0	85
15863523	m	md	JP	2019	0	1	0	1	0	0	0	0	0	0	0	1	71.2
15864087	f	ot	US	2019	1	0	0	0	0	0	0	0	0	0	0	1	
15864162	f	md	CZ	2019	0	0	0	0	0	0	0	0	0	0	0	0	82
15864241	m	ph	US	2019	0	0	0	0	0	0	0	0	0	0	0	0	58

15864408	m	ot	IL	2019	0	0	0	0	0	0	0	0	0	0	0	0	77
15864528	m	cn	JP	2019	0	1	0	0	1	0	0	0	0	0	0	0	59
15864531	f	cn	DE	2019	0	1	1	0	0	0	0	0	0	0	0	0	66
15864741	f	ot	IN	2019	0	0	0	0	0	0	0	0	0	0	0	0	57
15864744	m	cn	DE	2019	0	1	1	0	0	0	0	0	0	0	0	0	
15864916	m	cn	JP	2019	0	1	1	0	0	0	0	0	0	0	0	0	72
15865243	m	ot	CN	2019	0	1	0	0	1	0	0	0	0	0	0	0	67
15865266	f	ot	PL	2019	0	1	0	0	1	0	0	0	0	0	0	0	74
15865562	m	md	JP	2019	0	1	0	0	0	1	0	0	0	0	0	0	60
15865744	f	md	JP	2019	1	0	0	0	0	0	0	0	0	0	0	1	76
15865748	m	md	JP	2019	0	1	0	0	1	0	0	0	0	0	0	0	83
15865981	m	cn	JP	2019	0	1	1	0	0	0	0	0	0	0	0	0	66
15866052	m	cn	JP	2019	0	1	1	0	0	0	0	0	0	0	0	0	72
15866429	f	ot	US	2019	0	1	0	0	1	0	0	0	0	0	0	0	46
15866963	m	ot	DE	2019	0	0	0	0	0	0	0	0	0	0	0	0	77
15867041	m	cn	SE	2019	0	1	1	0	0	0	0	0	0	0	0	0	
15867074	f	ot	FR	2019	0	1	0	0	1	0	0	0	0	0	0	0	65
15867151	m	ot	FR	2019	0	1	0	0	1	0	0	0	0	0	0	0	61
15867176	m	ot	FR	2019	0	1	0	0	1	0	0	0	0	0	0	0	54
15867254	f	ot	FR	2019	0	1	0	0	1	0	0	0	0	0	0	0	59
15867314	f	cn	JP	2019	1	0	0	0	0	0	0	0	0	0	0	0	75
15867329	f	md	JP	2019	1	0	0	0	0	0	0	0	0	0	0	0	72
15867376	f	ot	NL	2019	1	0	0	0	0	0	0	0	0	0	0	0	72
15867493	m	cn	CA	2019	0	0	0	0	0	0	0	0	0	0	0	0	66
15867611	m	ph	JP	2019	0	0	0	0	0	0	0	0	0	0	0	0	67
15867631	m	md	JP	2019	1	0	0	0	0	0	0	0	0	0	0	0	50
15867802	m	md	BE	2019	0	1	1	0	0	0	0	0	0	0	0	0	77
15867877	Not	ot	US	2019	0	0	0	0	0	0	0	0	0	0	0	0	
15868177	f	md	US	2019	0	1	0	1	0	0	0	0	0	0	0	0	
15868253	m	ot	FR	2019	0	1	0	0	1	0	0	0	0	0	0	0	62
15868270	m	cn	KR	2019	0	0	0	0	0	0	1	1	0	0	0	0	
15868340	f	md	PL	2019	1	0	0	0	0	0	0	0	0	0	0	0	60
15868361	Not	md	JP	2019	1	0	0	0	0	0	0	0	0	0	0	0	93
15868465	f	md	JP	2019	0	1	0	1	0	0	0	0	0	0	0	1	64

15868496	m	ot	FR	2019	0	1	0	0	1	0	0	0	0	0	0	0	61
15868620	m	ph	CN	2019	1	0	0	0	0	0	0	0	0	0	0	0	67
15868674	f	ot	NL	2019	1	0	0	0	0	0	0	0	0	0	0	0	66
15868757	Not	ot	US	2019	0	0	0	0	0	0	0	0	0	0	0	0	
15868793	Not	ot	US	2019	0	1	1	0	0	0	0	0	0	0	0	0	
15868800	f	md	CN	2019	0	0	0	0	0	0	0	0	0	0	0	0	79
15868978	m	Not repo	US	2019	1	0	0	0	0	0	0	0	0	0	0	0	90
15868984	m	ot	NL	2019	1	0	0	0	0	0	0	0	0	0	0	0	70
15869385	m	ot	US	2019	0	1	0	0	1	0	0	0	0	0	0	0	66
15869567	m	ot	NL	2019	0	0	0	0	0	0	0	0	0	0	0	0	71
15869580	f	ot	DE	2019	0	1	1	0	0	0	0	0	0	0	0	0	66
15869582	m	md	JP	2019	0	1	1	0	0	0	0	0	0	0	0	0	65
15871154	f	md	FR	2019	0	0	0	0	0	0	0	0	0	0	0	0	56
15872239	Not	md	US	2019	0	0	0	0	0	0	0	0	0	0	0	0	
15872292	Not	md	US	2019	0	0	0	0	0	0	0	0	0	0	0	0	
15875871	m	ot	DE	2019	0	0	0	0	0	0	0	0	0	0	0	0	49
15876989	m	cn	JP	2019	0	1	1	0	0	0	0	0	0	0	0	0	61
15877442	f	md	JP	2019	0	1	0	0	1	0	0	0	0	0	0	0	60
15877831	m	cn	DE	2019	0	1	1	0	0	0	0	0	0	0	0	0	
15877953	m	ot	FR	2019	0	1	0	0	1	0	0	0	0	0	0	0	71
15878406	m	ot	US	2019	0	1	0	0	1	0	0	0	0	0	0	0	
15878420	m	md	IN	2019	0	1	0	0	1	0	0	0	0	0	0	0	73
15878852	Not	md	JP	2019	1	0	0	0	0	0	0	0	0	0	0	1	
15878863	m	ot	US	2019	0	0	0	0	0	0	0	0	0	0	0	0	72
15879103	m	cn	US	2019	0	1	1	0	0	0	0	0	0	0	0	0	70
15879134	m	cn	GB	2019	0	0	0	0	0	0	0	0	0	0	0	0	77
15879709	f	Not repo	JP	2019	0	0	0	0	0	0	0	0	0	0	0	0	74
15879823	f	md	CZ	2019	0	0	0	0	0	0	0	0	0	0	0	0	47
15880233	m	md	JP	2019	1	0	0	0	0	0	0	0	0	0	0	0	82.8
15880696	m	ph	US	2019	0	1	0	1	0	0	0	0	0	0	0	0	66
15880943	m	cn	CA	2019	0	1	1	0	0	0	0	0	0	0	0	0	72
15880958	f	md	JP	2019	1	0	0	0	0	0	0	0	0	0	0	1	65.3
15881008	m	md	US	2019	0	1	0	1	0	0	0	0	0	0	0	0	57
15881009	Not	md	US	2019	0	1	0	1	0	0	0	0	0	0	0	0	

15881080	m	md	US	2019	0	1	0	1	0	0	0	0	0	0	0	0	
15881114	m	cn	JP	2019	0	1	1	0	0	0	0	0	0	0	0	0	72
15881299	m	md	JP	2019	0	1	0	0	1	0	0	0	0	0	0	0	64
15881307	m	ot	FR	2019	0	1	0	0	1	0	0	0	0	0	0	1	79
15881648	m	md	JP	2019	0	0	0	0	0	0	0	0	0	0	0	0	
15881876	f	md	PL	2019	0	1	1	0	0	0	0	0	0	0	0	0	70
15881880	f	cn	JP	2019	0	1	1	0	0	0	0	0	0	0	0	0	76
15881934	f	ot	JP	2019	0	1	1	0	0	0	0	0	0	0	0	0	77
15881951	f	cn	JP	2019	0	1	1	0	0	0	0	0	0	0	0	1	56
15882099	m	cn	JP	2019	0	1	1	0	0	0	0	0	0	0	0	0	65
15882405	m	md	US	2019	1	0	0	0	0	0	0	0	0	0	0	1	
15882526	m	md	FR	2019	0	0	0	0	0	0	0	0	0	0	0	0	77
15882769	f	md	US	2019	0	1	1	0	0	0	0	0	0	0	0	0	
15882888	f	ph	US	2019	0	1	1	0	0	0	0	0	0	0	0	0	
15882948	f	cn	GB	2019	1	0	0	0	0	0	0	0	0	0	0	0	
15883130	f	ot	NL	2019	1	0	0	0	0	0	0	0	0	0	0	0	67
15883177	m	ph	JP	2019	0	1	0	0	0	1	0	0	0	0	0	0	71
15883464	m	ot	ES	2019	0	1	0	0	1	0	0	0	0	0	0	0	59
15883470	f	md	JP	2019	0	1	0	0	0	1	0	0	0	0	0	1	68
15884184	f	ot	NL	2019	1	0	0	0	0	0	0	0	0	0	0	0	79
15884485	m	ot	PL	2019	0	1	0	0	1	0	0	0	0	0	0	0	62
15884510	f	ot	US	2019	0	0	0	0	0	0	0	0	0	0	0	0	
15884731	f	ot	NL	2019	1	0	0	0	0	0	0	0	0	0	0	0	62
15884823	f	md	JP	2019	1	0	0	0	0	0	0	0	0	0	0	0	71
15885043	f	md	JP	2019	0	1	0	1	0	0	0	0	0	0	0	1	71.4
15885093	f	ot	US	2019	0	1	0	0	1	0	0	0	0	0	0	0	
15885108	m	md	JP	2019	0	1	0	1	0	0	0	0	0	0	0	1	70
15885118	Not	md	US	2019	0	1	0	1	0	0	0	0	0	0	0	0	
15885298	m	md	FR	2019	0	0	0	0	0	0	0	0	0	0	0	0	77
15885308	m	md	ES	2019	0	1	0	0	0	1	0	0	0	0	0	0	72
15885345	f	ot	NL	2019	1	0	0	0	0	0	0	0	0	0	0	0	60
15885530	m	cn	JP	2019	0	0	0	0	0	0	0	0	0	0	0	0	64
15885554	m	md	ES	2019	0	0	0	0	0	0	0	0	0	0	0	0	42
15885684	f	md	KR	2019	1	0	0	0	0	0	0	0	0	0	0	0	74

15885687	m	md	ES	2019	0	1	0	1	0	0	0	0	0	0	0	0	
15885719	m	cn	JP	2019	0	1	1	0	0	0	0	0	0	0	0	1	70
15885980	m	md	JP	2019	0	0	0	0	0	0	0	0	0	0	0	0	69
15886108	m	ot	DE	2019	0	0	0	0	0	0	0	0	0	0	0	0	76
15886117	f	cn	DE	2019	0	0	0	0	0	0	0	0	0	0	0	0	
15886209	m	cn	US	2019	0	1	1	0	0	0	0	0	0	0	0	0	
15886222	Not	cn	DE	2019	0	0	0	0	0	0	0	0	0	0	0	0	
15886621	m	md	JP	2019	0	1	0	1	0	0	0	0	0	0	0	0	
15887049	m	cn	DE	2019	0	1	1	0	0	0	0	0	0	0	0	0	75
15887055	f	cn	DE	2019	0	1	1	0	0	0	0	0	0	0	0	0	68
15887124	f	cn	US	2019	0	1	1	0	0	0	0	0	0	0	0	0	
15887154	f	ot	GB	2019	1	0	0	0	0	0	0	0	0	0	0	0	69
15887189	f	md	US	2019	0	0	0	0	0	0	0	0	0	0	0	0	75
15887250	m	cn	US	2019	0	1	1	0	0	0	0	0	0	0	0	0	
15887346	m	md	JP	2019	0	1	0	0	0	1	0	0	0	0	0	0	77
15887446	f	Not repo	PT	2019	1	0	0	0	0	0	0	0	0	0	0	0	63.6
15887506	f	ot	DE	2019	0	0	0	0	0	0	0	0	0	0	0	0	48
15887693	m	cn	US	2019	0	1	0	0	1	0	0	0	0	0	0	0	74
15887695	f	md	IL	2019	0	1	0	0	1	0	0	0	0	0	0	0	51
15887803	m	md	DE	2019	0	0	0	0	0	0	0	0	0	0	0	0	49
15887893	f	md	CN	2019	0	0	0	0	0	0	0	0	0	0	0	0	77
15887912	m	cn	DE	2019	0	1	1	0	0	0	0	0	0	0	0	1	
15888181	m	md	FR	2019	0	1	0	0	1	0	0	0	0	0	0	0	67
15888288	m	md	JP	2019	0	1	0	1	0	0	0	0	0	0	0	1	80
15888320	m	md	JP	2019	0	0	0	0	0	0	0	0	0	0	0	0	68
15888456	Not	md	US	2019	0	1	0	1	0	0	0	0	0	0	0	1	
15888488	f	md	FR	2019	0	1	0	0	0	1	0	0	0	0	0	0	52
15888624	m	md	JP	2019	0	1	0	1	0	0	0	0	0	0	0	0	72.4
15888837	Not	md	JP	2019	1	0	0	0	0	0	0	0	0	0	0	0	100
15889156	m	md	JP	2019	0	1	0	1	0	0	0	0	0	0	0	1	76.5
15889201	m	md	DE	2019	0	0	0	0	0	0	0	0	0	0	0	0	65
15889367	f	md	JP	2019	0	1	0	1	0	0	0	0	0	0	0	1	80
15889447	m	md	JP	2019	0	1	0	1	0	0	0	0	0	0	0	1	73.6
15889703	Not	cn	JP	2019	0	1	1	0	0	0	0	0	0	0	0	1	

15889751	m	ot	NL	2019	0	0	0	0	0	0	0	0	0	0	0	0	71
15889850	Not	md	PL	2019	0	0	0	0	0	0	0	0	0	0	0	0	
15889903	m	cn	JP	2019	0	0	0	0	0	0	0	0	0	0	0	0	71
15889911	f	ot	US	2019	1	0	0	0	0	0	0	0	0	0	0	0	69
15890589	f	md	PL	2019	0	0	0	0	0	0	0	0	0	0	0	0	
15890859	Not	md	JP	2019	1	0	0	0	0	0	0	0	0	0	0	1	
15890936	m	md	JP	2019	0	0	0	0	0	0	0	0	0	0	0	0	19
15890981	m	md	JP	2019	0	1	0	1	0	0	0	0	0	0	0	0	63
15891050	f	cn	CA	2019	0	0	0	0	0	0	0	0	0	0	0	0	41
15891076	m	md	DE	2019	0	1	1	0	0	0	0	0	0	0	0	0	66
15891653	m	ot	US	2019	0	1	0	0	1	0	0	0	0	0	0	0	74
15892269	m	md	JP	2019	0	1	0	1	0	0	0	0	0	0	0	1	71
15892598	m	ph	US	2019	0	0	0	0	0	0	0	0	0	0	0	0	48
15892725	m	md	US	2019	0	0	0	0	0	0	0	0	0	0	0	0	52
15892738	f	cn	US	2019	0	0	0	0	0	0	0	0	0	0	0	0	62
15893298	f	cn	DE	2019	0	1	1	0	0	0	0	0	0	0	0	0	
15893351	m	cn	GR	2019	0	1	1	0	0	0	0	0	0	0	0	0	85
15893642	f	ot	JP	2019	0	0	0	0	0	0	0	0	0	0	0	0	59
15893697	Not	ot	NL	2019	0	1	0	0	1	0	0	0	0	0	0	0	
15893917	m	ph	US	2019	1	0	0	0	0	0	0	0	0	0	0	0	73
15894061	Not	ot	US	2019	0	1	0	0	1	0	0	0	0	0	0	0	
15894063	f	ot	PL	2019	0	1	0	0	1	0	0	0	0	0	0	0	71
15894088	Not	ot	US	2019	0	1	0	0	1	0	0	0	0	0	0	0	
15894090	Not	ot	US	2019	0	1	0	0	1	0	0	0	0	0	0	0	
15894122	Not	ot	US	2019	0	1	0	0	1	0	0	0	0	0	0	0	
15894294	f	ot	NL	2019	1	0	0	0	0	0	0	0	0	0	0	0	37
15894412	m	md	DE	2019	0	1	1	0	0	0	0	0	0	0	0	0	66
15894531	f	md	DE	2019	0	0	0	0	0	0	0	0	0	0	0	0	48
15894897	m	cn	GB	2019	1	0	0	0	0	0	0	0	0	0	0	0	50
15894985	f	md	JP	2019	1	0	0	0	0	0	0	0	0	0	0	0	75
15895569	m	md	US	2019	0	0	0	0	0	0	0	0	0	0	0	0	55
15895788	m	md	JP	2019	1	0	0	0	0	0	0	0	0	0	0	0	76
15896288	Not	md	US	2019	0	0	0	0	0	0	0	0	0	0	0	0	
15896364	m	ot	US	2019	0	1	1	0	0	0	0	0	0	0	0	1	80

15896374	f	md	GB	2019	1	0	0	0	0	0	0	0	0	0	0	0	
15896604	f	cn	CA	2019	0	0	0	0	0	0	0	0	0	0	0	0	57
15896843	f	md	JP	2019	0	0	0	0	0	0	0	0	0	0	0	0	38.6
15896898	m	ot	PL	2019	0	1	1	0	0	0	0	0	0	0	0	0	64
15897004	f	cn	US	2019	0	1	1	0	0	0	0	0	0	0	0	0	
15897038	Not	ot	US	2019	0	0	0	0	0	0	0	0	0	0	0	0	
15897044	m	md	AU	2019	0	0	0	0	0	0	0	0	0	0	0	0	78
15897207	m	ot	ES	2019	0	1	0	0	1	0	0	0	0	0	0	0	54
15897218	m	md	US	2019	0	1	0	1	0	0	0	0	0	0	0	0	62
15897220	m	ot	JP	2019	0	1	0	0	1	0	0	0	0	0	0	0	69
15897267	Not	ph	LU	2019	0	0	0	0	0	0	0	0	0	0	0	0	65
15897274	Not	ph	LU	2019	0	0	0	0	0	0	0	0	0	0	0	0	63
15897366	f	ot	NL	2019	1	0	0	0	0	0	0	0	0	0	0	0	60
15897672	m	cn	US	2019	0	1	0	0	1	0	0	0	0	0	0	0	54
15897685	m	ot	CN	2019	0	0	0	0	0	0	0	0	0	0	0	0	50
15897951	f	ot	US	2019	0	1	0	0	1	0	0	0	0	0	0	0	80
15898290	m	md	IE	2019	0	1	0	0	0	1	0	0	0	0	0	0	68
15898457	m	md	DE	2019	0	0	0	0	0	0	0	0	0	0	0	0	80
15898466	m	md	ES	2019	0	0	0	0	0	0	0	0	0	0	0	0	42
15898669	m	md	NL	2019	0	0	0	0	0	0	0	0	0	0	0	0	44
15898770	m	md	JP	2019	0	1	0	1	0	0	0	0	0	0	0	1	80
15899174	Not	ot	IT	2019	0	1	0	0	1	0	0	0	0	0	0	0	
15899238	m	ot	DE	2019	0	0	0	0	0	0	0	0	0	0	0	0	72
15899378	m	cn	US	2019	0	0	0	0	0	0	0	0	0	0	0	0	63
15899391	Not	cn	LB	2019	1	0	0	0	0	0	0	0	0	0	0	0	
15899474	m	md	FR	2019	0	0	0	0	0	0	0	0	0	0	0	0	77
15899638	m	md	JP	2019	0	1	0	1	0	0	0	0	0	0	0	1	50
15899644	f	md	US	2019	1	0	0	0	0	0	0	0	0	0	0	0	
15899678	m	ph	US	2019	0	0	0	0	0	0	0	0	0	0	0	0	44
15899845	f	lw	US	2019	0	0	0	0	0	0	0	0	0	0	0	0	69
15900036	f	md	US	2019	0	1	0	1	0	0	0	0	0	0	0	1	
15900708	m	cn	JP	2019	0	1	1	0	0	0	0	0	0	0	0	0	68
15900713	m	md	JP	2019	0	1	0	1	0	0	0	0	0	0	0	1	67
15900905	m	md	JP	2019	0	1	0	1	0	0	0	0	0	0	0	1	63

15900921	m	md	JP	2019	0	1	0	0	1	0	0	0	0	0	0	0	68
15900964	m	md	JP	2019	1	0	0	0	0	0	0	0	0	0	0	0	71.5
15901026	m	md	JP	2019	0	1	0	1	0	0	0	0	0	0	0	1	70
15901027	Not	md	JP	2019	1	0	0	0	0	0	0	0	0	0	0	0	
15901134	f	md	US	2019	0	1	0	1	0	0	0	0	0	0	0	1	70
15901177	f	ot	JP	2019	1	0	0	0	0	0	0	0	0	0	0	0	80
15901214	f	cn	AR	2019	0	0	0	0	0	0	0	0	0	0	0	0	45
15901519	f	md	JP	2019	1	0	0	0	0	0	0	0	0	0	0	0	73
15901538	m	ot	FR	2019	0	1	0	0	1	0	0	0	0	0	0	0	64
15901624	m	ph	US	2019	0	1	1	0	0	0	0	0	0	0	0	0	56
15901854	m	cn	JP	2019	0	0	0	0	0	0	0	0	0	0	0	0	66
15902095	f	md	NL	2019	0	0	0	0	0	0	0	0	0	0	0	0	75
15904299	m	md	JP	2019	0	1	0	0	0	1	0	0	0	0	0	0	80
15905024	m	cn	SG	2019	0	1	0	0	0	1	0	0	0	0	0	0	
15905169	m	md	IT	2019	0	1	1	0	0	0	0	0	0	0	0	0	80
15905397	f	md	US	2019	0	0	0	0	0	0	0	0	0	0	0	0	59
15905822	f	ot	US	2019	0	0	0	0	0	0	0	0	0	0	0	0	77
15906174	Not	cn	US	2019	0	0	0	0	0	0	0	0	0	0	0	0	
15906212	f	md	FR	2019	0	0	0	0	0	0	0	0	0	0	0	0	52
15906702	f	ot	US	2019	0	0	0	0	0	0	0	0	0	0	0	0	77
15907705	f	ot	GB	2019	0	1	1	0	0	0	0	0	0	0	0	0	66
15907871	f	md	JP	2019	1	0	0	0	0	0	0	0	0	0	0	1	76
15907940	m	md	NZ	2019	0	0	0	0	0	0	0	0	0	0	0	0	68
15908008	Not	md	US	2019	0	1	0	1	0	0	0	0	0	0	0	1	
15913197	f	cn	JP	2019	0	1	1	0	0	0	0	0	0	0	0	0	64
15913215	m	ot	CN	2019	0	1	0	0	1	0	0	0	0	0	0	1	64
15913304	m	cn	Not re	2019	0	0	0	0	0	0	0	0	0	0	0	0	43
15916055	m	ot	US	2019	0	0	0	0	0	0	0	0	0	0	0	0	61
15916237	f	Not repo	IT	2019	1	0	0	0	0	0	0	0	0	0	0	0	82
15916263	f	ot	US	2019	0	0	0	0	0	0	0	0	0	0	0	0	77
15916789	Not	ot	CN	2019	0	0	0	0	0	0	0	0	0	0	0	0	
15916843	m	md	TW	2019	0	1	0	0	1	0	0	0	0	0	0	0	41
15917008	m	ot	PL	2019	1	0	0	0	0	0	0	0	0	0	0	0	
15917291	m	md	BE	2019	0	1	1	0	0	0	0	0	0	0	0	0	

15917444	f	md	JP	2019	0	1	0	1	0	0	0	0	0	0	0	1	75
15917575	f	md	JP	2019	0	1	0	1	0	0	0	0	0	0	0	0	75
15918436	m	cn	Not re	2019	0	0	0	0	0	0	0	0	0	0	0	0	37
15918462	m	cn	DE	2019	0	1	1	0	0	0	0	0	0	0	0	0	75
15918705	f	ot	FR	2019	0	0	0	0	0	0	0	0	0	0	0	0	76
15919350	f	md	JP	2019	0	1	1	0	0	0	0	0	0	0	0	0	66
15919633	m	md	JP	2019	0	1	0	1	0	0	0	0	0	0	0	1	61
15919640	f	md	JP	2019	1	0	0	0	0	0	0	0	0	0	0	1	80.3
15919832	f	md	CN	2019	0	0	0	0	0	0	0	0	0	0	0	0	44
15920205	m	md	TH	2019	0	1	1	0	0	0	0	0	0	0	0	0	75
15920425	m	cn	KE	2019	0	0	0	0	0	0	0	0	0	0	0	0	40
15920434	Not	md	JP	2019	0	0	0	0	0	0	0	0	0	0	0	0	
15920468	m	md	JP	2019	0	1	0	1	0	0	0	0	0	0	0	1	70
15920551	Not	md	JP	2019	0	1	0	1	0	0	0	0	0	0	0	1	
15920629	m	cn	Not re	2019	0	0	0	0	0	0	0	0	0	0	0	0	66
15920633	f	cn	Not re	2019	0	0	0	0	0	0	0	0	0	0	0	0	
15920691	Not	md	US	2019	0	1	0	1	0	0	0	0	0	0	0	0	
15920692	m	md	JP	2019	0	1	0	1	0	0	0	0	0	0	0	1	33
15920693	Not	md	US	2019	0	1	0	1	0	0	0	0	0	0	0	1	
15920931	m	cn	JP	2019	0	1	1	0	0	0	0	0	0	0	0	0	58
15920968	f	ph	US	2019	0	0	0	0	0	0	0	0	0	0	0	0	66
15921267	m	cn	JP	2019	0	1	1	0	0	0	0	0	0	0	0	0	76
15921952	m	cn	GB	2019	0	1	1	0	0	0	0	0	0	0	0	1	75
15923178	m	md	JP	2019	0	1	0	0	0	1	0	0	0	0	0	0	70
15924197	Not	ot	CN	2019	0	0	0	0	0	0	0	0	0	0	0	0	
15926686	m	ot	JP	2019	0	1	0	0	1	0	0	0	0	0	0	0	70
15926698	f	cn	US	2019	0	1	1	0	0	0	0	0	0	0	0	0	
15928267	m	cn	JP	2019	0	1	1	0	0	0	0	0	0	0	0	0	65
15928481	m	md	JP	2019	0	1	0	1	0	0	0	0	0	0	0	1	
15928890	f	md	CH	2019	0	1	0	1	0	0	0	0	0	0	0	0	76
15928936	f	cn	PL	2019	0	1	1	0	0	0	0	0	0	0	0	0	73
15929144	m	cn	FR	2019	0	0	0	0	0	0	0	0	0	0	0	0	62
15929359	f	ot	DE	2019	0	1	1	0	0	0	0	0	0	0	0	0	
15929522	m	cn	CA	2019	0	0	0	0	0	0	0	0	0	0	0	0	54

15929564	m	ot	GB	2019	0	1	1	0	0	0	0	0	0	0	0	0	75
15930455	m	cn	CN	2019	0	1	0	0	1	0	0	0	0	0	0	0	73
15930693	f	md	PL	2019	0	1	0	0	0	1	0	0	0	0	0	0	61
15930921	f	md	CN	2019	0	0	0	0	0	0	0	0	0	0	0	0	39
15931529	f	ot	GB	2019	1	0	0	0	0	0	0	0	0	0	0	0	37
15931564	f	ot	NL	2019	1	0	0	0	0	0	0	0	0	0	0	0	66
15932027	m	ot	DE	2019	0	1	0	0	1	0	0	0	0	0	0	0	68
15932053	m	cn	CA	2019	0	1	0	0	0	1	0	0	0	0	0	0	
15932072	f	ot	NL	2019	1	0	0	0	0	0	0	0	0	0	0	0	72
15932227	f	md	US	2019	0	0	0	0	0	0	0	0	0	0	0	0	
15932503	f	cn	JP	2019	0	0	0	0	0	0	0	0	0	0	0	0	65
15932588	f	cn	US	2019	1	0	0	0	0	0	0	0	0	0	0	0	87
15932871	m	md	BE	2019	0	1	1	0	0	0	0	0	0	0	0	0	
15932897	f	ot	NL	2019	1	0	0	0	0	0	0	0	0	0	0	0	62
15932900	f	ot	NL	2019	1	0	0	0	0	0	0	0	0	0	0	0	37
15932903	f	md	MQ	2019	0	0	0	0	0	0	0	0	0	0	0	0	59
15933425	Not	ot	PL	2019	0	0	0	0	0	0	0	0	0	0	0	0	
15933622	Not	md	US	2019	1	0	0	0	0	0	0	0	0	0	0	0	
15933677	m	cn	US	2019	0	1	0	0	1	0	0	0	0	0	0	0	
15933901	Not	cn	JP	2019	1	0	0	0	0	0	0	0	0	0	0	0	
15934329	f	cn	US	2019	0	1	0	0	1	0	0	0	0	0	0	0	
15934473	m	ph	JP	2019	0	1	0	0	1	0	0	0	0	0	0	1	65
15934489	f	ph	JP	2019	1	0	0	0	0	0	0	0	0	0	0	0	90
15934491	m	cn	KR	2019	1	0	0	0	0	0	0	0	0	0	0	0	52
15934565	m	ot	CN	2019	0	1	0	0	1	0	0	0	0	0	0	0	72
15934636	m	ot	NL	2019	1	0	0	0	0	0	0	0	0	0	0	0	70
15934645	m	cn	JP	2019	0	1	1	0	0	0	0	0	0	0	0	0	70
15934703	f	ot	CN	2019	0	1	0	0	1	0	0	0	0	0	0	1	74
15934792	f	cn	DE	2019	0	1	1	0	0	0	0	0	0	0	0	0	68
15934796	f	cn	DE	2019	0	1	1	0	0	0	0	0	0	0	0	0	
15934800	m	cn	JP	2019	0	1	1	0	0	0	0	0	0	0	0	0	62
15935100	m	cn	IN	2019	0	1	0	0	1	0	0	0	0	0	0	0	
15935171	f	ot	DE	2019	0	1	1	0	0	0	0	0	0	0	0	0	68
15935199	f	md	JP	2019	0	0	0	0	0	0	0	0	0	0	0	0	65

15935311	f	md	CA	2019	0	0	0	0	0	0	0	0	0	0	0	0	74
15935701	m	md	KR	2019	0	1	0	0	1	0	0	0	0	0	0	0	61
15935719	f	cn	US	2019	0	1	0	0	1	0	0	0	0	0	0	0	
15935925	m	cn	US	2019	0	1	0	0	1	0	0	0	0	0	0	0	
15936172	Not	ot	US	2019	1	0	0	0	0	0	0	0	0	0	0	0	
15936203	f	cn	US	2019	0	1	1	0	0	0	0	0	0	0	0	0	
15936289	m	md	JP	2019	0	1	0	0	0	1	0	0	0	0	0	0	81
15936600	m	ph	US	2019	0	1	0	1	0	0	0	0	0	0	0	1	72
15936793	Not	ot	US	2019	1	0	0	0	0	0	0	0	0	0	0	0	
15937005	m	md	BE	2019	0	1	0	0	0	1	0	0	0	0	0	0	66
15937153	f	md	JP	2019	0	0	0	0	0	0	0	0	0	0	0	0	76
15937273	f	cn	US	2019	1	0	0	0	0	0	0	0	0	0	0	0	63.7
15937547	m	cn	JP	2019	0	1	1	0	0	0	0	0	0	0	0	0	74
15938478	f	md	US	2019	0	1	0	1	0	0	0	0	0	0	0	0	
15939454	m	md	KR	2019	0	1	0	0	1	0	0	0	0	0	0	0	79
15940136	f	ot	US	2019	0	1	0	1	0	0	0	0	0	0	0	0	66.8
15940311	m	cn	KR	2019	0	1	1	0	0	0	0	0	0	0	0	0	56
15940594	m	cn	JP	2019	0	1	1	0	0	0	0	0	0	0	0	1	82
15940766	m	ot	DE	2019	0	1	0	0	1	0	0	0	0	0	0	0	68
15940807	m	md	JP	2019	1	0	0	0	0	0	0	0	0	0	0	1	72
15940928	m	cn	JP	2019	0	1	1	0	0	0	0	0	0	0	0	0	71
15940969	m	cn	KW	2019	1	0	0	0	0	0	0	0	0	0	0	0	63.7
15941036	f	ot	BR	2019	0	1	0	0	1	0	0	0	0	0	0	0	73
15941084	f	md	US	2019	0	1	1	0	0	0	0	0	0	0	0	0	
15941339	m	cn	JP	2019	0	1	1	0	0	0	0	0	0	0	0	0	72
15941697	m	md	JP	2019	0	1	0	0	0	1	0	0	0	0	0	0	60
15941891	m	ot	FR	2019	0	1	0	0	1	0	0	0	0	0	0	0	70
15942263	Not	ot	NL	2019	0	1	0	0	1	0	0	0	0	0	0	0	
15942512	f	ot	US	2019	1	0	0	0	0	0	0	0	0	0	0	0	69
15942904	m	md	JP	2019	0	1	1	0	0	0	0	0	0	0	0	0	76
15943098	f	md	IE	2019	0	1	1	0	0	0	0	0	0	0	0	1	66
15943998	m	md	JO	2019	0	1	0	0	0	1	0	0	0	0	0	1	33
15944054	m	md	JP	2019	0	1	0	0	0	1	0	0	0	0	0	0	66
15944651	f	cn	JP	2019	0	1	1	0	0	0	0	0	0	0	0	0	62

15945506	f	cn	US	2019	0	1	0	0	1	0	0	0	0	0	0	0	
15945561	m	cn	DE	2019	0	1	1	0	0	0	0	0	0	0	0	0	61
15945883	f	ot	US	2019	0	1	0	0	1	0	0	0	0	0	0	0	
15945895	m	md	JP	2019	0	1	0	0	1	0	0	0	0	0	0	0	52
15945919	f	cn	US	2019	0	1	0	0	1	0	0	0	0	0	0	0	
15945967	m	ot	US	2019	0	1	0	0	1	0	0	0	0	0	0	0	
15946122	m	md	JP	2019	0	1	0	1	0	0	0	0	0	0	0	1	78
15946216	m	cn	US	2019	0	1	1	0	0	0	0	0	0	0	0	0	
15946481	m	ot	JP	2019	0	1	0	0	1	0	0	0	0	0	0	0	74
15947540	Not	md	AU	2019	1	0	0	0	0	0	0	0	0	0	0	0	
15947822	Not	ot	IT	2019	0	1	0	0	1	0	0	0	0	0	0	0	
15948114	Not	ot	US	2019	1	0	0	0	0	0	0	0	0	0	0	0	
15948770	m	cn	US	2019	1	0	0	0	0	0	0	0	0	0	0	0	80
15948797	m	md	GB	2019	0	0	0	0	0	0	0	0	0	0	0	0	71
15949052	m	cn	KR	2019	0	1	1	0	0	0	0	0	0	0	0	0	68
15949778	f	ot	ES	2019	0	1	0	0	1	0	0	0	0	0	0	1	78
15949873	m	md	DE	2019	0	1	0	0	0	1	0	0	0	0	0	0	58
15949988	m	md	IN	2019	0	0	0	0	0	0	0	0	0	0	0	0	65
15950584	m	md	JP	2019	1	0	0	0	0	0	0	0	0	0	0	0	80
15950602	m	md	DE	2019	0	1	0	0	0	1	0	0	0	0	0	0	69
15950610	f	md	JP	2019	0	1	0	1	0	0	0	0	0	0	0	0	70
15950638	m	md	JP	2019	0	1	0	0	1	0	0	0	0	0	0	0	60
15950658	m	md	JP	2019	0	1	0	0	1	0	0	0	0	0	0	0	80
15950661	m	md	JP	2019	0	1	0	0	1	0	0	0	0	0	0	0	70
15951002	f	md	FR	2019	1	0	0	0	0	0	0	0	0	0	0	0	75
15951084	Not	md	JP	2019	0	1	0	0	1	0	0	0	0	0	0	0	
15951128	m	md	JP	2019	1	0	0	0	0	0	0	0	0	0	0	0	58
15951320	Not	cn	US	2019	0	0	0	0	0	0	0	0	0	0	0	0	
15951495	m	md	JP	2019	1	0	0	0	0	0	0	0	0	0	0	0	80
15952019	m	cn	JP	2019	0	1	1	0	0	0	0	0	0	0	0	0	77
15952132	m	md	JP	2019	0	1	0	0	1	0	0	0	0	0	0	0	70
15952193	f	cn	US	2019	0	1	1	0	0	0	0	0	0	0	0	0	58
15952506	m	md	GR	2019	0	0	0	0	0	0	0	0	0	0	0	0	74
15952733	m	ot	ES	2019	0	1	0	0	1	0	0	0	0	0	0	0	59

15952774	f	ot	US	2019	0	1	0	0	1	0	0	0	0	0	0	0	57
15953093	f	ot	JP	2019	0	1	0	0	1	0	0	0	0	0	0	1	68
15953244	f	md	NL	2019	0	0	0	0	0	0	0	0	0	0	0	0	82
15953404	Not	ot	US	2019	1	0	0	0	0	0	0	0	0	0	0	0	
15953564	f	md	JP	2019	0	1	0	1	0	0	0	0	0	0	0	1	73
15953873	m	ot	GB	2019	1	0	0	0	0	0	0	0	0	0	0	0	70
15953952	m	md	FR	2019	0	0	0	0	0	0	0	0	0	0	0	0	64
15954343	f	md	US	2019	0	1	1	0	0	0	0	0	0	0	0	0	
15954429	m	cn	US	2019	0	1	1	0	0	0	0	0	0	0	0	0	61
15955007	Not	ph	JP	2019	0	0	0	0	0	0	0	0	0	0	0	0	
15955465	m	md	CH	2019	0	0	0	0	0	0	0	0	0	0	0	0	69
15955469	f	ot	DE	2019	0	1	0	1	0	0	0	0	0	0	0	0	
15956191	m	cn	US	2019	0	1	0	0	1	0	0	0	0	0	0	0	76
15956252	m	ot	DE	2019	0	1	1	0	0	0	0	0	0	0	0	0	62
15956460	Not	md	JP	2019	1	0	0	0	0	0	0	0	0	0	0	0	
15956483	m	ot	US	2019	0	1	1	0	0	0	0	0	0	0	0	0	
15956771	f	md	JP	2019	1	0	0	0	0	0	0	0	0	0	0	1	90
15956981	f	md	JP	2019	1	0	0	0	0	0	0	0	0	0	0	0	80
15956984	m	ph	JP	2019	1	0	0	0	0	0	0	0	0	0	0	0	51
15957061	m	cn	JP	2019	0	1	1	0	0	0	0	0	0	0	0	1	70
15957217	m	ot	US	2019	0	1	0	0	1	0	0	0	0	0	0	0	41
15957455	m	md	JP	2019	1	0	0	0	0	0	0	0	0	0	0	0	77.2
15957544	f	md	FR	2019	0	1	0	1	0	0	0	0	0	0	0	0	
15957556	f	cn	US	2019	0	1	0	1	0	0	0	0	0	0	0	1	87
15957646	f	ph	US	2019	1	0	0	0	0	0	0	0	0	0	0	0	66
15958148	f	cn	CA	2019	0	0	0	0	0	0	0	0	0	0	0	0	45
15958282	f	cn	JP	2019	0	1	1	0	0	0	0	0	0	0	0	0	47
15958455	m	md	JP	2019	0	1	0	1	0	0	0	0	0	0	0	1	65
15958482	m	ot	US	2019	0	1	0	0	1	0	0	0	0	0	0	0	62
15958826	m	ph	JP	2019	1	0	0	0	0	0	0	0	0	0	0	0	84
15959043	f	md	SE	2019	0	1	0	0	1	0	0	0	0	0	0	0	66
15959046	m	md	AU	2019	0	1	0	0	0	1	0	0	0	0	0	0	75
15959277	f	md	DE	2019	0	1	0	0	1	0	0	0	0	0	0	0	62
15959328	f	ot	DE	2019	0	1	0	0	1	0	0	0	0	0	0	0	79

15959391	m	md	IL	2019	0	0	0	0	0	0	0	0	0	0	0	0	71
15959548	m	ot	FR	2019	0	1	0	0	1	0	0	0	0	0	0	0	64
15959794	f	md	US	2019	0	0	0	0	0	0	0	0	0	0	0	0	
15959807	f	cn	US	2019	0	0	0	0	0	0	0	0	0	0	0	0	49
15959816	f	ot	US	2019	0	0	0	0	0	0	0	0	0	0	0	0	65
15959819	m	cn	US	2019	0	0	0	0	0	0	0	0	0	0	0	0	
15959820	m	cn	US	2019	0	0	0	0	0	0	0	0	0	0	0	0	
15959824	Not	ph	US	2019	0	0	0	0	0	0	0	0	0	0	0	0	
15959825	f	md	US	2019	0	0	0	0	0	0	0	0	0	0	0	0	42
15959855	f	cn	US	2019	0	0	0	0	0	0	0	0	0	0	0	0	
15959859	f	cn	US	2019	0	0	0	0	0	0	0	0	0	0	0	0	
15959879	m	md	US	2019	0	0	0	0	0	0	0	0	0	0	0	0	
15960299	m	ot	US	2019	0	0	0	0	0	0	0	0	0	0	0	0	
15960440	f	cn	US	2019	0	0	0	0	0	0	0	0	0	0	0	0	39
15960763	m	md	JP	2019	0	0	0	0	0	0	0	0	0	0	0	0	73
15961271	m	cn	JP	2019	0	1	1	0	0	0	0	0	0	0	0	0	71
15961375	m	md	JP	2019	0	1	0	0	0	1	0	0	0	0	0	0	70
15961651	f	ot	US	2019	0	0	0	0	0	0	0	0	0	0	0	0	40
15961746	m	cn	FR	2019	0	0	0	0	0	0	0	0	0	0	0	0	62
15961879	f	md	US	2019	0	0	0	0	0	0	0	0	0	0	0	0	75
15961894	m	ot	US	2019	0	1	0	0	1	0	0	0	0	0	0	0	55
15962526	m	ot	DE	2019	0	0	0	0	0	0	0	0	0	0	0	0	77
15962861	f	ot	US	2019	0	0	0	0	0	0	0	0	0	0	0	0	53
15962891	f	md	JP	2019	1	0	0	0	0	0	0	0	0	0	0	0	87
15963349	m	md	JP	2019	1	0	0	0	0	0	0	0	0	0	0	1	79
15963540	f	md	DE	2019	1	0	0	0	0	0	0	0	0	0	0	0	
15963550	f	cn	JP	2019	0	1	1	0	0	0	0	0	0	0	0	0	69
15963668	m	md	NL	2019	0	1	1	0	0	0	0	0	0	0	0	0	74
15964600	m	ph	GB	2019	0	0	0	0	0	0	0	0	0	0	0	0	74
15964657	m	md	CN	2019	0	1	0	0	0	1	0	0	0	0	0	0	62
15965033	m	cn	JP	2019	0	1	1	0	0	0	0	0	0	0	0	0	62
15965089	m	cn	JP	2019	0	1	1	0	0	0	0	0	0	0	0	0	52
15965112	f	md	US	2019	0	1	1	0	0	0	0	0	0	0	0	0	84
15965456	m	ph	GB	2019	0	1	1	0	0	0	0	0	0	0	0	0	68

15965633	f	md	JP	2019	1	0	0	0	0	0	0	0	0	0	0	1	49
15966280	m	ph	JP	2019	0	1	0	0	0	1	0	0	0	0	0	0	
15966870	f	ot	GB	2019	0	1	1	0	0	0	0	0	0	0	0	0	
15966891	m	md	JP	2019	0	1	0	1	0	0	0	0	0	0	0	1	72
15967786	f	ot	GB	2019	1	0	0	0	0	0	0	0	0	0	0	0	75
15967844	f	md	AU	2019	1	0	0	0	0	0	0	0	0	0	0	0	70
15967922	f	md	JP	2019	1	0	0	0	0	0	0	0	0	0	0	0	66.5
15968071	m	md	JP	2019	0	1	0	0	0	1	0	0	0	0	0	0	80
15968383	f	ot	JP	2019	0	1	0	0	1	0	0	0	0	0	0	0	74
15968450	Not	md	JP	2019	0	1	0	0	1	0	0	0	0	0	0	0	
15968662	m	Not repo	JP	2019	0	0	0	0	0	0	0	0	0	0	0	0	66
15968779	m	cn	JP	2019	0	1	1	0	0	0	0	0	0	0	0	0	75
15969175	m	cn	JP	2019	0	1	1	0	0	0	0	0	0	0	0	0	70
15969563	m	cn	JP	2019	0	1	1	0	0	0	0	0	0	0	0	0	79
15969948	m	ot	CN	2019	0	0	0	0	0	0	0	0	0	0	0	0	61
15973623	f	cn	US	2019	0	1	1	0	0	0	0	0	0	0	0	0	
15973965	m	cn	US	2019	0	1	0	0	1	0	0	0	0	0	0	0	
15974009	m	md	US	2019	0	0	0	0	0	0	0	0	0	0	0	0	55
15974020	m	md	DE	2019	0	1	0	0	1	0	0	0	0	0	0	0	47
15974421	m	md	PH	2019	0	1	0	0	0	1	0	0	0	0	0	0	70
15974536	f	md	US	2019	0	0	0	0	0	0	0	0	0	0	0	0	59
15974582	f	ot	DE	2019	0	1	0	0	1	0	0	0	0	0	0	0	59
15974747	m	cn	JP	2019	0	1	1	0	0	0	0	0	0	0	0	0	77
15974879	f	cn	JP	2019	0	1	1	0	0	0	0	0	0	0	0	0	62
15974932	m	cn	JP	2019	0	1	1	0	0	0	0	0	0	0	0	0	68
15974945	m	ph	JP	2019	0	1	0	0	0	1	0	0	0	0	0	0	50
15975357	Not	md	JP	2019	0	1	0	0	0	1	0	0	0	0	0	0	63
15975443	f	ot	US	2019	0	0	0	0	0	0	0	0	0	0	0	0	64
15975746	m	cn	JP	2019	0	1	1	0	0	0	0	0	0	0	0	0	74
15975778	m	cn	JP	2019	0	1	1	0	0	0	0	0	0	0	0	0	82
15976116	f	md	AR	2019	0	1	1	0	0	0	0	0	0	0	0	0	
15977212	f	md	IT	2019	0	0	0	0	0	0	0	0	0	0	0	0	61
15977288	Not	ot	CA	2019	0	0	0	0	0	0	0	0	0	0	0	0	
15977375	m	md	JP	2019	0	1	0	1	0	0	0	0	0	0	0	1	80

15977427	m	cn	IL	2019	0	1	1	0	0	0	0	0	0	0	0	0	75
15977638	m	Not repo	JP	2019	0	0	0	0	0	0	0	0	0	0	0	0	71
15977680	Not	cn	JP	2019	0	1	1	0	0	0	0	0	0	0	0	0	55
15977877	m	cn	JP	2019	0	1	1	0	0	0	0	0	0	0	0	0	59
15977881	f	cn	IN	2019	0	1	0	0	1	0	0	0	0	0	0	0	
15978400	m	md	JP	2019	0	1	0	0	0	1	0	0	0	0	0	0	77
15978482	m	md	JP	2019	0	1	1	0	0	0	0	0	0	0	0	0	68
15978927	Not	cn	US	2019	0	1	0	0	1	0	0	0	0	0	0	0	
15979061	m	cn	US	2019	0	1	0	1	0	0	0	0	0	0	0	0	73
15979346	f	md	JP	2019	0	1	0	0	0	1	0	0	0	0	0	0	70
15979753	f	ot	US	2019	0	1	0	0	1	0	0	0	0	0	0	0	75
15979892	m	ph	US	2019	0	1	1	0	0	0	0	0	0	0	0	0	71
15980317	Not	ot	CA	2019	0	0	0	0	0	0	0	0	0	0	0	0	
15980770	Not	ot	CA	2019	0	0	0	0	0	0	0	0	0	0	0	0	
15980827	m	ot	US	2019	0	1	1	0	0	0	0	0	0	0	0	0	58
15981063	m	cn	US	2019	0	1	1	0	0	0	0	0	0	0	0	0	
15981182	f	md	US	2019	0	1	0	0	1	0	0	0	0	0	0	0	70
15981826	f	md	GB	2019	1	0	0	0	0	0	0	0	0	0	0	0	63
15981838	Not	Not repo	ID	2019	1	0	0	0	0	0	0	0	0	0	0	0	
15982201	f	md	IT	2019	1	0	0	0	0	0	0	0	0	0	0	1	79
15982232	f	ot	CA	2019	0	0	0	0	0	0	0	0	0	0	0	0	56
15982613	f	ot	CA	2019	0	1	0	1	0	0	0	0	0	0	0	0	75
15982614	m	cn	BR	2019	1	0	0	0	0	0	0	0	0	0	0	0	
15982651	m	md	KR	2019	1	0	0	0	0	0	0	0	0	0	0	0	62
15982704	m	cn	JP	2019	0	1	1	0	0	0	0	0	0	0	0	0	53
15982778	m	md	BE	2019	0	1	1	0	0	0	0	0	0	0	0	0	50
15983020	f	cn	CA	2019	0	0	0	0	0	0	0	0	0	0	0	0	48
15983573	f	cn	JP	2019	0	1	1	0	0	0	0	0	0	0	0	1	70
15983680	m	md	JP	2019	0	1	0	0	0	1	0	0	0	0	0	0	59
15983729	m	md	JP	2019	0	1	0	0	1	0	0	0	0	0	0	0	84
15984001	m	cn	KR	2019	0	1	1	0	0	0	0	0	0	0	0	0	
15984268	m	md	JP	2019	0	1	0	0	1	0	0	0	0	0	0	0	79
15984298	f	md	US	2019	0	1	0	0	0	1	0	0	0	0	0	0	62
15984426	m	md	JP	2019	0	1	0	1	0	0	0	0	0	0	0	0	48

15984533	m	ot	US	2019	0	0	0	0	0	0	0	0	0	0	0	0	
15984756	m	md	US	2019	0	0	0	0	0	0	0	0	0	0	0	0	74
15985002	m	md	US	2019	0	0	0	0	0	0	0	0	0	0	0	0	56
15985222	m	md	JP	2019	0	1	0	0	0	1	0	0	0	0	0	0	48
15985273	Not	ot	GB	2019	0	0	0	0	0	0	0	0	0	0	0	0	
15985348	Not	md	IN	2019	0	1	0	0	0	1	0	0	0	0	0	0	
15985403	Not	ot	GB	2019	0	0	0	0	0	0	0	0	0	0	0	0	
15985487	Not	ot	GB	2019	0	0	0	0	0	0	0	0	0	0	0	0	
15985657	Not	ot	GB	2019	0	0	0	0	0	0	0	0	0	0	0	0	
15985846	f	md	DE	2019	0	0	0	0	0	0	0	0	0	0	0	0	81
15985990	Not	ot	GB	2019	0	0	0	0	0	0	0	0	0	0	0	0	
15986047	Not	ot	GB	2019	0	0	0	0	0	0	0	0	0	0	0	0	
15986262	m	cn	GB	2019	0	1	1	0	0	0	0	0	0	0	0	0	
15986946	m	cn	JP	2019	0	1	1	0	0	0	0	0	0	0	0	0	63
15987269	m	cn	US	2019	0	1	1	0	0	0	0	0	0	0	0	0	
15987446	m	ot	DE	2019	0	1	1	0	0	0	0	0	0	0	0	0	61
15987577	f	cn	DE	2019	0	1	1	0	0	0	0	0	0	0	0	0	
15987778	f	cn	US	2019	0	0	0	0	0	0	0	0	0	0	0	0	60
15987822	m	cn	US	2019	0	0	0	0	0	0	0	0	0	0	0	0	73
15987895	f	cn	FR	2019	0	0	0	0	0	0	0	0	0	0	0	0	60
15988191	f	md	JP	2019	0	1	0	0	1	0	0	0	0	0	0	0	64
15988202	f	cn	US	2019	0	1	1	0	0	0	0	0	0	0	0	1	46
15988422	m	md	JP	2019	0	1	0	0	0	1	0	0	0	0	0	0	71
15988463	f	cn	US	2019	0	1	0	0	1	0	0	0	0	0	0	0	
15988481	Not	md	US	2019	0	1	0	1	0	0	0	0	0	0	0	0	
15989197	m	md	JP	2019	0	1	0	0	1	0	0	0	0	0	0	1	58
15989708	m	md	JP	2019	0	1	0	0	0	1	0	0	0	0	0	0	60
15989834	m	md	GR	2019	1	0	0	0	0	0	0	0	0	0	0	0	71
15989839	m	cn	JP	2019	0	1	1	0	0	0	0	0	0	0	0	0	84
15990087	m	ot	CA	2019	0	0	0	0	0	0	0	0	0	0	0	0	75
15990178	f	md	JP	2019	0	0	0	0	0	0	0	0	0	0	0	0	62
15990466	Not	md	BE	2019	0	1	1	0	0	0	0	0	0	0	0	1	64
15990888	m	ot	JP	2019	0	1	1	0	0	0	0	0	0	0	0	0	78
15991313	m	md	ES	2019	0	0	0	0	0	0	0	0	0	0	0	0	73

15991329	m	ot	CN	2019	0	1	0	0	1	0	0	0	0	0	0	0	65
15991400	f	md	GB	2019	0	0	0	0	0	0	0	0	0	0	0	0	30
15991823	f	cn	JP	2019	0	1	1	0	0	0	0	0	0	0	0	0	50
15991887	f	cn	AR	2019	1	0	0	0	0	0	0	0	0	0	0	0	60
15992030	f	md	US	2019	1	0	0	0	0	0	0	0	0	0	0	0	
15992198	m	cn	JP	2019	0	1	1	0	0	0	0	0	0	0	0	0	
15993007	f	cn	US	2019	0	1	0	0	1	0	0	0	0	0	0	0	70
15993235	f	cn	US	2019	0	0	0	0	0	0	0	0	0	0	0	0	
15993294	f	ot	FR	2019	0	0	0	0	0	0	0	0	0	0	0	0	59
15993413	f	md	US	2019	0	0	0	0	0	0	0	0	0	0	0	0	53
15993553	f	ot	KR	2019	0	1	0	0	1	0	0	0	0	0	0	0	65
15993798	m	ph	FR	2019	0	0	0	0	0	0	0	0	0	0	0	0	63
15993855	m	ot	NL	2019	0	0	0	0	0	0	0	0	0	0	0	0	71
15994065	m	md	JP	2019	0	1	0	0	0	1	0	0	0	0	0	0	60
15994579	f	cn	BR	2019	1	0	0	0	0	0	0	0	0	0	0	0	60
15994737	f	md	DE	2019	0	0	0	0	0	0	0	0	0	0	0	0	
15995087	f	md	JP	2019	0	1	0	0	0	1	0	0	0	0	0	0	70
15995141	f	md	BE	2019	0	1	1	0	0	0	0	0	0	0	0	0	49
15995197	f	Not repo	ID	2019	1	0	0	0	0	0	0	0	0	0	0	0	
15995209	Not	md	US	2019	0	1	0	1	0	0	0	0	0	0	0	0	
15995283	f	md	JP	2019	0	1	0	1	0	0	0	0	0	0	0	1	77.5
15995451	m	cn	KR	2019	0	1	1	0	0	0	0	0	0	0	0	0	
15995508	f	md	US	2019	1	0	0	0	0	0	0	0	0	0	0	0	
15995567	m	md	JP	2019	1	0	0	0	0	0	0	0	0	0	0	0	69
15995615	m	cn	RO	2019	0	0	0	0	0	0	0	0	0	0	0	0	
15995654	Not	md	DE	2019	0	1	0	1	0	0	0	0	0	0	0	0	
15996410	m	md	US	2019	0	1	0	1	0	0	0	0	0	0	0	0	75
15996586	m	md	JP	2019	0	1	0	1	0	0	0	0	0	0	0	1	
15996589	m	md	JP	2019	0	1	0	1	0	0	0	0	0	0	0	1	80
15996612	f	ot	FR	2019	0	1	0	0	1	0	0	0	0	0	0	0	68
15996823	m	cn	JP	2019	0	1	1	0	0	0	0	0	0	0	0	0	77
15996910	m	cn	JP	2019	0	1	1	0	0	0	0	0	0	0	0	0	60
15997012	m	ph	JP	2019	0	0	0	0	0	0	0	0	0	0	0	0	80
15997208	f	md	JP	2019	1	0	0	0	0	0	0	0	0	0	0	0	52

15997245	m	md	JP	2019	1	0	0	0	0	0	0	0	0	0	0	0	76
15997431	f	ot	RU	2019	0	1	0	0	1	0	0	0	0	0	0	0	61
15997767	m	ot	DE	2019	0	0	0	0	0	0	0	0	0	0	0	0	65
15997869	m	ot	BR	2019	0	1	0	0	1	0	0	0	0	0	0	0	65
15997999	m	md	CH	2019	0	0	0	0	0	0	0	0	0	0	0	0	74
15998062	Not	ot	US	2019	0	1	0	0	1	0	0	0	0	0	0	0	
15998255	m	md	AE	2019	0	1	0	0	0	1	0	0	0	0	0	0	74
15999059	m	cn	JP	2019	0	1	1	0	0	0	0	0	0	0	0	0	70
15999095	Not	ot	GB	2019	0	0	0	0	0	0	0	0	0	0	0	0	
15999140	f	cn	US	2019	0	1	1	0	0	0	0	0	0	0	0	0	60
16000179	m	cn	GB	2019	1	0	0	0	0	0	0	0	0	0	0	0	65
16000266	m	cn	KR	2019	0	1	1	0	0	0	0	0	0	0	0	0	
16000358	f	cn	AR	2019	0	0	0	0	0	0	0	0	0	0	0	0	54
16000386	m	md	DE	2019	0	1	0	0	1	0	0	0	0	0	0	0	69
16000603	m	cn	JP	2019	0	1	1	0	0	0	0	0	0	0	0	0	71
16000922	m	md	LB	2019	1	0	0	0	0	0	0	0	0	0	0	0	49
16002031	m	cn	JP	2019	0	1	1	0	0	0	0	0	0	0	0	0	60
16002306	f	cn	CA	2019	0	0	0	0	0	0	0	0	0	0	0	0	51
16003390	Not	md	US	2019	0	1	0	1	0	0	0	0	0	0	0	0	
16003933	m	cn	US	2019	0	1	0	0	1	0	0	0	0	0	0	0	51
16003966	f	md	ID	2019	0	1	1	0	0	0	0	0	0	0	0	0	
16004212	m	md	UA	2019	0	1	0	0	0	1	0	0	0	0	0	0	59
16004942	f	md	JP	2019	0	1	0	0	0	1	0	0	0	0	0	0	80
16004953	m	md	JP	2019	0	0	0	0	0	0	0	0	0	0	0	0	36
16005066	f	md	KR	2019	0	0	0	0	0	0	0	0	0	0	0	0	57
16005455	m	md	KR	2019	0	1	0	0	1	0	0	0	0	0	0	0	56
16005822	Not	md	US	2019	0	1	0	0	1	0	0	0	0	0	0	0	
16005823	f	ot	GB	2019	0	1	0	0	1	0	0	0	0	0	0	0	88
16005867	Not	md	US	2019	0	1	0	0	1	0	0	0	0	0	0	0	
16005875	f	md	DE	2019	0	1	1	0	0	0	0	0	0	0	0	0	70
16005879	Not	md	US	2019	0	1	0	0	1	0	0	0	0	0	0	0	
16006384	m	cn	JP	2019	0	1	1	0	0	0	0	0	0	0	0	0	72
16006462	m	ot	US	2019	0	1	1	0	0	0	0	0	0	0	0	0	58
16006957	m	md	DE	2019	0	1	1	0	0	0	0	0	0	0	0	0	

16006976	Not	md	JP	2019	0	1	0	1	0	0	0	0	0	0	0	1	
16007180	f	md	FR	2019	1	0	0	0	0	0	0	0	0	0	0	0	
16007269	m	cn	AT	2019	0	1	1	0	0	0	0	0	0	0	0	0	
16007299	m	cn	US	2019	0	0	0	0	0	0	0	0	0	0	0	0	
16007307	m	cn	TH	2019	0	0	0	0	0	0	0	0	0	0	0	0	70
16007325	f	cn	MA	2019	0	1	1	0	0	0	0	0	0	0	0	1	64
16007665	m	cn	JP	2019	0	1	1	0	0	0	0	0	0	0	0	0	86
16008004	f	md	DE	2019	0	0	0	0	0	0	0	0	0	0	0	0	56
16008150	f	ot	US	2019	0	1	0	0	1	0	0	0	0	0	0	0	
16008339	m	cn	JP	2019	0	1	1	0	0	0	0	0	0	0	0	1	
16008416	f	md	JP	2019	0	0	0	0	0	0	1	0	1	0	0	0	81
16008460	f	md	DE	2019	0	1	0	0	1	0	0	0	0	0	0	1	66
16008465	f	md	DK	2019	0	1	1	0	0	0	0	0	0	0	0	0	66
16008645	m	md	DE	2019	0	1	1	0	0	0	0	0	0	0	0	0	70
16008662	Not	ot	LB	2019	0	1	0	0	1	0	0	0	0	0	0	0	
16008679	f	ot	IN	2019	0	1	0	0	1	0	0	0	0	0	0	0	57
16008686	m	cn	JP	2019	0	1	1	0	0	0	0	0	0	0	0	0	55
16008918	Not	md	MY	2019	0	1	0	0	0	1	0	0	0	0	0	1	
16008929	m	cn	JP	2019	0	1	1	0	0	0	0	0	0	0	0	0	69
16009019	f	md	DK	2019	0	1	1	0	0	0	0	0	0	0	0	1	71
16009597	f	ot	MY	2019	0	1	1	0	0	0	0	0	0	0	0	0	83
16009776	Not	md	JP	2019	0	1	0	0	1	0	0	0	0	0	0	0	
16009903	m	md	JP	2019	0	1	0	0	0	1	0	0	0	0	0	0	60
16009938	m	md	JP	2019	0	0	0	0	0	0	0	0	0	0	0	0	70
16010014	m	ot	US	2019	0	1	0	0	1	0	0	0	0	0	0	0	70
16010396	m	md	JP	2019	0	1	0	0	1	0	0	0	0	0	0	0	65
16010875	m	cn	US	2019	0	1	1	0	0	0	0	0	0	0	0	0	
16011018	f	ot	GB	2019	0	0	0	0	0	0	1	0	1	0	0	0	66
16011086	m	cn	JP	2019	0	1	1	0	0	0	0	0	0	0	0	0	74
16011142	f	cn	US	2019	0	0	0	0	0	0	0	0	0	0	0	0	
16011570	f	ot	US	2019	1	0	0	0	0	0	0	0	0	0	0	0	69
16011906	f	cn	US	2019	1	0	0	0	0	0	0	0	0	0	0	0	
16012032	f	ot	DE	2019	0	1	1	0	0	0	0	0	0	0	0	0	70
16012107	m	cn	JP	2019	0	1	1	0	0	0	0	0	0	0	0	0	72

16012218	f	md	DE	2019	1	0	0	0	0	0	0	0	0	0	0	1	70
16012696	f	ph	JP	2019	1	0	0	0	0	0	0	0	0	0	0	0	86
16012950	f	md	CA	2019	0	0	0	0	0	0	0	0	0	0	0	0	77
16013496	m	cn	JP	2019	0	1	1	0	0	0	0	0	0	0	0	0	65
16013873	f	cn	KR	2019	0	0	0	0	0	0	0	0	0	0	0	0	
16014011	Not	ot	GB	2019	0	0	0	0	0	0	0	0	0	0	0	0	
16014073	f	md	CA	2019	0	1	0	0	1	0	0	0	0	0	0	0	
16014121	f	ot	US	2019	1	0	0	0	0	0	0	0	0	0	0	0	87
16014230	m	cn	JP	2019	0	1	1	0	0	0	0	0	0	0	0	0	61
16014571	f	cn	DE	2019	0	1	1	0	0	0	0	0	0	0	0	0	68
16014798	m	ot	ES	2019	0	1	0	0	1	0	0	0	0	0	0	0	60
16015166	m	ot	DE	2019	0	1	0	0	1	0	0	0	0	0	0	0	68
16015509	m	md	JP	2019	0	1	0	0	0	1	0	0	0	0	0	0	60
16015582	f	cn	TW	2019	0	1	0	0	0	1	0	0	0	0	0	0	
16015767	f	md	DE	2019	1	0	0	0	0	0	0	0	0	0	0	0	
16015899	m	md	JP	2019	0	1	0	0	0	1	0	0	0	0	0	0	58
16015901	m	md	JP	2019	0	1	0	0	0	1	0	0	0	0	0	0	79
16015927	f	md	DE	2019	0	1	0	0	1	0	0	0	0	0	0	0	50
16016198	m	md	JP	2019	0	1	0	0	0	1	0	0	0	0	0	0	68
16016261	m	md	FR	2019	0	1	0	0	1	0	0	0	0	0	0	0	50
16016708	f	ot	US	2019	0	0	0	0	0	0	0	0	0	0	0	0	
16018064	Not	md	IE	2019	0	0	0	0	0	0	0	0	0	0	0	0	
16018109	m	ph	JP	2019	0	0	0	0	0	0	1	0	1	0	0	0	74
16018202	m	cn	JP	2019	0	1	1	0	0	0	0	0	0	0	0	0	58
16018370	m	ot	US	2019	0	1	0	0	1	0	0	0	0	0	0	0	84
16018463	m	md	JP	2019	0	1	0	1	0	0	0	0	0	0	0	1	81
16018995	f	md	JP	2019	1	0	0	0	0	0	0	0	0	0	0	1	71
16019135	m	ot	GB	2019	0	0	0	0	0	0	0	0	0	0	0	0	
16019539	m	md	US	2019	0	1	1	0	0	0	0	0	0	0	0	0	
16020033	f	md	JP	2019	1	0	0	0	0	0	0	0	0	0	0	0	82
16020462	f	cn	JP	2019	0	1	1	0	0	0	0	0	0	0	0	0	65
16020719	m	cn	CN	2019	0	1	0	0	1	0	0	0	0	0	0	0	68
16020805	m	md	JP	2019	0	1	0	0	0	1	0	0	0	0	0	0	66
16021316	f	ot	FR	2019	0	1	0	0	1	0	0	0	0	0	0	0	76

16021454	Not	md	US	2019	0	1	0	1	0	0	0	0	0	0	0	0	56
16021609	m	md	CZ	2019	0	1	0	0	1	0	0	0	0	0	0	0	77
16021660	m	cn	JP	2019	0	1	1	0	0	0	0	0	0	0	0	0	73
16021733	f	ph	GB	2019	0	1	1	0	0	0	0	0	0	0	0	0	51
16022115	m	cn	JP	2019	0	0	0	0	0	0	0	0	0	0	0	0	59
16022202	f	ot	US	2019	1	0	0	0	0	0	0	0	0	0	0	0	75
16022897	f	cn	US	2019	0	1	0	1	0	0	0	0	0	0	0	0	
16022971	f	md	JP	2019	1	0	0	0	0	0	0	0	0	0	0	0	80
16023098	m	ot	US	2019	0	1	0	1	0	0	0	0	0	0	0	0	
16023148	f	md	JP	2019	1	0	0	0	0	0	0	0	0	0	0	0	65
16023293	m	md	JP	2019	1	0	0	0	0	0	0	0	0	0	0	0	80
16023627	m	md	JP	2019	0	1	0	1	0	0	0	0	0	0	0	1	69
16023641	m	md	JP	2019	0	1	0	1	0	0	0	0	0	0	0	0	50
16023665	m	cn	JP	2019	0	1	1	0	0	0	0	0	0	0	0	0	72
16023770	m	cn	JP	2019	0	1	1	0	0	0	0	0	0	0	0	1	74
16023896	m	cn	JP	2019	0	1	1	0	0	0	0	0	0	0	0	0	62
16024191	m	md	JP	2019	1	0	0	0	0	0	0	0	0	0	0	0	61
16024347	f	Not repo	ID	2019	1	0	0	0	0	0	0	0	0	0	0	0	
16024655	m	md	ES	2019	0	1	0	0	1	0	0	0	0	0	0	0	76
16024665	m	cn	JP	2019	0	1	1	0	0	0	0	0	0	0	0	0	80
16025026	f	cn	US	2019	0	0	0	0	0	0	0	0	0	0	0	0	55
16025271	m	ot	GB	2019	0	0	0	0	0	0	0	0	0	0	0	0	
16025593	Not	md	JP	2019	0	0	0	0	0	0	0	0	0	0	0	0	
16026659	Not	md	CN	2019	1	0	0	0	0	0	0	0	0	0	0	0	
16026664	m	md	JP	2019	1	0	0	0	0	0	0	0	0	0	0	0	55
16027516	m	md	JP	2019	0	1	0	0	1	0	0	0	0	0	0	0	63
16028085	m	md	JP	2019	0	1	1	0	0	0	0	0	0	0	0	0	77
16028382	m	md	JP	2019	0	0	0	0	0	0	0	0	0	0	0	0	70
16028781	f	md	JP	2019	1	0	0	0	0	0	0	0	0	0	0	0	84
16031354	m	md	JP	2019	1	0	0	0	0	0	0	0	0	0	0	1	72
16031464	m	ot	US	2019	0	1	1	0	0	0	0	0	0	0	0	0	58
16031897	m	cn	JP	2019	0	1	1	0	0	0	0	0	0	0	0	1	
16032401	f	md	SA	2019	1	0	0	0	0	0	0	0	0	0	0	0	
16032470	m	ot	JP	2019	0	1	0	0	1	0	0	0	0	0	0	0	64

16032625	Not	ph	JP	2019	0	1	0	0	1	0	0	0	0	0	0	0	
16033332	Not	md	AU	2019	1	0	0	0	0	0	0	0	0	0	0	0	
16033433	m	ot	CN	2019	0	1	0	0	1	0	0	0	0	0	0	0	66
16033754	f	cn	JP	2019	1	0	0	0	0	0	0	0	0	0	0	0	62
16033760	f	md	BE	2019	0	1	1	0	0	0	0	0	0	0	0	1	69
16035432	m	md	EG	2019	0	1	0	0	1	0	0	0	0	0	0	0	71
16035833	m	md	JP	2019	1	0	0	0	0	0	0	0	0	0	0	1	89
16036021	f	md	US	2019	1	0	0	0	0	0	0	0	0	0	0	0	40
16036121	m	Not repo	AU	2019	1	0	0	0	0	0	0	0	0	0	0	0	
16036997	m	md	JP	2019	0	1	0	1	0	0	0	0	0	0	0	1	60
16037002	f	lw	US	2019	0	0	0	0	0	0	0	0	0	0	0	0	55
16037228	m	Not repo	US	2019	1	0	0	0	0	0	0	0	0	0	0	0	72.2
16037339	f	md	PL	2019	0	0	0	0	0	0	0	0	0	0	0	0	75
16037479	m	md	US	2019	0	1	0	1	0	0	0	0	0	0	0	1	
16038155	f	md	JP	2019	0	1	0	1	0	0	0	0	0	0	0	1	69
16038418	m	ot	NL	2019	0	1	0	0	1	0	0	0	0	0	0	0	85
16038478	m	ot	DE	2019	0	1	0	0	1	0	0	0	0	0	0	0	75
16039015	m	md	JP	2019	0	1	0	1	0	0	0	0	0	0	0	1	77
16039068	f	ot	ES	2019	0	1	0	0	1	0	0	0	0	0	0	0	47
16039070	m	ot	DE	2019	0	1	0	0	1	0	0	0	0	0	0	0	65
16039092	f	md	FR	2019	0	0	0	0	0	0	0	0	0	0	0	0	
16039097	Not	md	JP	2019	0	0	0	0	0	0	0	0	0	0	0	0	87
16039133	f	ot	US	2019	0	1	0	0	1	0	0	0	0	0	0	0	43
16039135	f	ot	US	2019	0	1	0	0	1	0	0	0	0	0	0	0	63
16039300	m	ph	JP	2019	0	1	0	0	0	1	0	0	0	0	0	0	64
16039412	Not	md	JP	2019	0	1	0	0	0	1	0	0	0	0	0	0	70
16039480	f	md	DE	2019	0	1	0	0	0	1	0	0	0	0	0	1	54
16039554	m	ot	FR	2019	0	1	0	0	1	0	0	0	0	0	0	0	86
16039555	m	md	JP	2019	0	1	0	0	0	1	0	0	0	0	0	1	80
16039816	Not	ot	US	2019	0	1	0	0	1	0	0	0	0	0	0	0	58
16039880	f	md	JP	2019	0	1	0	1	0	0	0	0	0	0	0	1	80
16039900	Not	md	JP	2019	1	0	0	0	0	0	0	0	0	0	0	0	
16040037	m	md	US	2019	1	0	0	0	0	0	0	0	0	0	0	1	
16040168	m	cn	US	2019	0	1	1	0	0	0	0	0	0	0	0	0	

16040301	f	md	US	2019	0	1	0	1	0	0	0	0	0	0	0	0	
16040808	Not	cn	KR	2019	1	0	0	0	0	0	0	0	0	0	0	0	
16041349	Not	cn	KR	2019	1	0	0	0	0	0	0	0	0	0	0	0	
16041550	f	ot	DE	2019	1	0	0	0	0	0	0	0	0	0	0	0	27
16042574	Not	md	LB	2019	0	1	0	0	1	0	0	0	0	0	0	0	
16042871	f	cn	IN	2019	0	1	0	0	1	0	0	0	0	0	0	0	54
16042944	Not	md	JP	2019	0	1	0	1	0	0	0	0	0	0	0	1	
16043202	f	md	JP	2019	1	0	0	0	0	0	0	0	0	0	0	0	74.8
16043335	m	cn	JP	2019	0	1	1	0	0	0	0	0	0	0	0	0	89
16043592	m	cn	JP	2019	0	1	1	0	0	0	0	0	0	0	0	0	54
16044204	m	ot	CN	2019	0	1	0	0	1	0	0	0	0	0	0	0	69
16044207	m	cn	BY	2019	0	0	0	0	0	0	0	0	0	0	0	0	61
16044363	m	ot	BE	2019	0	1	1	0	0	0	0	0	0	0	0	0	77
16044500	Not	ot	US	2019	0	1	0	0	1	0	0	0	0	0	0	0	
16044501	Not	ot	US	2019	0	1	0	0	1	0	0	0	0	0	0	0	
16044502	Not	ot	US	2019	0	1	0	0	1	0	0	0	0	0	0	0	
16044503	Not	ot	US	2019	0	1	0	0	1	0	0	0	0	0	0	0	
16044504	Not	ot	US	2019	0	1	0	0	1	0	0	0	0	0	0	0	
16044505	Not	ot	US	2019	0	1	0	0	1	0	0	0	0	0	0	0	
16044508	Not	ot	US	2019	0	1	0	0	1	0	0	0	0	0	0	0	
16044509	Not	ot	US	2019	0	1	0	0	1	0	0	0	0	0	0	0	
16044510	Not	ot	US	2019	0	1	0	0	1	0	0	0	0	0	0	0	
16044511	Not	ot	US	2019	0	1	0	0	1	0	0	0	0	0	0	0	
16044534	Not	ot	US	2019	0	1	0	0	1	0	0	0	0	0	0	0	
16044595	m	cn	JP	2019	0	1	1	0	0	0	0	0	0	0	0	1	67
16044640	m	cn	JP	2019	0	1	1	0	0	0	0	0	0	0	0	0	80
16044771	m	cn	JP	2019	0	1	1	0	0	0	0	0	0	0	0	0	72
16044987	m	md	JP	2019	0	0	0	0	0	0	0	0	0	0	0	0	60
16045122	m	md	CN	2019	0	1	0	0	0	1	0	0	0	0	0	0	60
16045123	f	md	JP	2019	0	0	0	0	0	0	0	0	0	0	0	0	66
16045192	Not	ot	US	2019	0	1	0	0	1	0	0	0	0	0	0	0	
16045204	Not	ot	US	2019	0	1	0	0	1	0	0	0	0	0	0	0	
16045230	Not	ot	US	2019	0	1	0	0	1	0	0	0	0	0	0	0	
16045240	Not	ot	US	2019	0	1	0	0	1	0	0	0	0	0	0	0	

16045394	f	ot	US	2019	0	1	1	0	0	0	0	0	0	0	0	0	56
16045432	Not	ot	US	2019	0	1	0	0	1	0	0	0	0	0	0	0	
16045466	f	md	US	2019	1	0	0	0	0	0	0	0	0	0	0	1	
16045619	Not	ot	US	2019	0	1	0	0	1	0	0	0	0	0	0	0	
16045795	Not	cn	KR	2019	1	0	0	0	0	0	0	0	0	0	0	0	
16045805	Not	cn	KR	2019	1	0	0	0	0	0	0	0	0	0	0	0	
16045836	f	md	DE	2019	0	0	0	0	0	0	0	0	0	0	0	0	
16046052	Not	md	JP	2019	1	0	0	0	0	0	0	0	0	0	0	0	
16046360	Not	md	JP	2019	0	1	0	1	0	0	0	0	0	0	0	1	
16047048	f	md	US	2019	0	1	1	0	0	0	0	0	0	0	0	0	62
16047081	Not	ot	US	2019	0	1	0	0	1	0	0	0	0	0	0	0	
16047434	f	Not repo	AU	2019	1	0	0	0	0	0	0	0	0	0	0	0	74
16047448	f	ot	CA	2019	0	1	0	1	0	0	0	0	0	0	0	0	77
16047654	f	ot	FR	2019	0	1	0	0	1	0	0	0	0	0	0	0	63
16047980	Not	cn	JP	2019	0	1	1	0	0	0	0	0	0	0	0	1	
16048960	f	ot	DE	2019	0	0	0	0	0	0	0	0	0	0	0	0	56
16049314	Not	ot	MA	2019	0	0	0	0	0	0	0	0	0	0	0	0	
16049440	m	cn	JP	2019	0	1	1	0	0	0	0	0	0	0	0	0	
16049474	m	md	MY	2019	1	0	0	0	0	0	0	0	0	0	0	0	47
16049593	f	md	MY	2019	1	0	0	0	0	0	0	0	0	0	0	0	34
16049601	m	cn	DE	2019	0	1	1	0	0	0	0	0	0	0	0	0	70
16049602	m	cn	BE	2019	0	1	1	0	0	0	0	0	0	0	0	0	77
16049885	f	md	MY	2019	1	0	0	0	0	0	0	0	0	0	0	0	64
16049894	f	md	MY	2019	1	0	0	0	0	0	0	0	0	0	0	0	57
16049898	m	md	MY	2019	1	0	0	0	0	0	0	0	0	0	0	0	66
16050368	m	md	JP	2019	1	0	0	0	0	0	0	0	0	0	0	1	59
16050950	m	cn	GB	2019	1	0	0	0	0	0	0	0	0	0	0	0	38
16051036	f	md	JP	2019	1	0	0	0	0	0	0	0	0	0	0	1	70
16051099	m	cn	Not re	2019	0	0	0	0	0	0	0	0	0	0	0	0	61
16051698	f	md	JP	2019	0	1	0	1	0	0	0	0	0	0	0	1	60
16051975	m	md	MY	2019	1	0	0	0	0	0	0	0	0	0	0	0	57
16052339	m	cn	JP	2019	0	1	1	0	0	0	0	0	0	0	0	0	74
16052341	m	cn	JP	2019	0	1	1	0	0	0	0	0	0	0	0	0	71
16052347	m	cn	JP	2019	0	1	1	0	0	0	0	0	0	0	0	0	60

16052364	m	cn	DE	2019	0	1	1	0	0	0	0	0	0	0	0	0	
16052441	f	ph	US	2019	0	0	0	0	0	0	1	1	0	0	0	0	54
16052549	f	md	US	2019	0	1	0	1	0	0	0	0	0	0	0	0	60
16052550	f	md	GB	2019	1	0	0	0	0	0	0	0	0	0	0	0	
16052558	f	md	JP	2019	1	0	0	0	0	0	0	0	0	0	0	0	
16052731	m	cn	JP	2019	0	1	1	0	0	0	0	0	0	0	0	0	72
16052812	m	md	JP	2019	0	1	0	1	0	0	0	0	0	0	0	0	68
16052898	Not	md	JP	2019	1	0	0	0	0	0	0	0	0	0	0	1	80
16052901	m	md	JP	2019	1	0	0	0	0	0	0	0	0	0	0	0	80
16052921	m	cn	JP	2019	0	1	1	0	0	0	0	0	0	0	0	0	55
16052933	f	md	ES	2019	1	0	0	0	0	0	0	0	0	0	0	0	65
16053026	m	md	JP	2019	0	1	0	1	0	0	0	0	0	0	0	1	73.4
16053115	f	md	JP	2019	1	0	0	0	0	0	0	0	0	0	0	0	
16053116	m	md	DE	2019	0	1	0	1	0	0	0	0	0	0	0	1	54
16053126	m	cn	JP	2019	0	1	1	0	0	0	0	0	0	0	0	1	64
16053143	Not	cn	JP	2019	0	1	1	0	0	0	0	0	0	0	0	1	
16053249	m	md	JP	2019	1	0	0	0	0	0	0	0	0	0	0	1	68.8
16053362	m	md	HR	2019	0	1	1	0	0	0	0	0	0	0	0	0	58
16053569	f	cn	JP	2019	0	0	0	0	0	0	0	0	0	0	0	0	70
16054023	f	md	FR	2019	0	0	0	0	0	0	0	0	0	0	0	0	36
16054296	m	md	CN	2019	0	0	0	0	0	0	0	0	0	0	0	0	59
16054353	f	cn	CN	2019	0	0	0	0	0	0	0	0	0	0	0	0	55
16054579	m	cn	Not re	2019	0	0	0	0	0	0	0	0	0	0	0	0	53
16054709	m	cn	Not re	2019	0	0	0	0	0	0	0	0	0	0	0	0	44
16055203	f	cn	US	2019	0	1	1	0	0	0	0	0	0	0	0	0	72
16056058	m	md	JP	2019	0	0	0	0	0	0	0	0	0	0	0	0	58
16056065	m	md	US	2019	0	1	0	0	0	1	0	0	0	0	0	0	60
16056137	m	md	JP	2019	0	1	0	0	0	1	0	0	0	0	0	0	69
16056435	m	md	JP	2019	0	1	0	0	0	1	0	0	0	0	0	1	69
16056809	f	md	JP	2019	0	0	0	0	0	0	0	0	0	0	0	0	
16057460	f	md	US	2019	1	0	0	0	0	0	0	0	0	0	0	1	
16058357	m	cn	JP	2019	0	1	1	0	0	0	0	0	0	0	0	0	72
16058578	m	ot	IT	2019	1	0	0	0	0	0	0	0	0	0	0	0	68
16059293	f	ot	FR	2019	0	0	0	0	0	0	0	0	0	0	0	0	85

16059295	m	ot	FR	2019	0	0	0	0	0	0	0	0	0	0	0	0	66
16059296	f	ot	FR	2019	0	0	0	0	0	0	0	0	0	0	0	0	69
16059297	m	ot	FR	2019	0	0	0	0	0	0	0	0	0	0	0	0	59
16059303	f	ot	FR	2019	0	0	0	0	0	0	0	0	0	0	0	0	77
16059347	m	ot	FR	2019	0	0	0	0	0	0	0	0	0	0	0	0	76
16059349	f	ot	FR	2019	0	0	0	0	0	0	0	0	0	0	0	0	50
16059541	m	cn	GB	2019	0	1	1	0	0	0	0	0	0	0	0	0	
16059650	m	ot	ES	2019	0	1	0	0	1	0	0	0	0	0	0	0	61
16060148	m	md	DE	2019	0	1	0	0	1	0	0	0	0	0	0	0	54
16060164	m	cn	JP	2019	0	1	1	0	0	0	0	0	0	0	0	0	90
16060181	m	cn	JP	2019	0	1	1	0	0	0	0	0	0	0	0	0	90
16060740	f	ot	US	2019	0	1	0	0	1	0	0	0	0	0	0	0	
16061013	f	md	US	2019	0	0	0	0	0	0	0	0	0	0	0	0	62
16061019	m	md	US	2019	0	0	0	0	0	0	0	0	0	0	0	0	55
16061067	m	ot	NL	2019	0	1	0	0	0	1	0	0	0	0	0	0	49
16061253	m	md	US	2019	0	1	0	0	0	1	0	0	0	0	0	0	72
16061294	f	cn	US	2019	0	0	0	0	0	0	0	0	0	0	0	0	
16061397	m	md	US	2019	0	1	0	0	0	1	0	0	0	0	0	0	
16061428	f	ot	US	2019	0	1	0	0	0	1	0	0	0	0	0	0	
16061454	m	cn	US	2019	0	1	0	0	0	1	0	0	0	0	0	0	56
16061459	m	md	US	2019	0	0	0	0	0	0	0	0	0	0	0	0	76
16061883	Not	md	US	2019	1	0	0	0	0	0	0	0	0	0	0	0	
16061905	Not	md	US	2019	1	0	0	0	0	0	0	0	0	0	0	0	
16061916	m	ot	DE	2019	0	1	0	0	1	0	0	0	0	0	0	0	55
16061966	m	cn	JP	2019	0	1	1	0	0	0	0	0	0	0	0	0	69
16062032	Not	md	US	2019	0	1	0	1	0	0	0	0	0	0	0	1	
16062153	m	md	US	2019	0	1	0	1	0	0	0	0	0	0	0	0	55
16062157	m	ot	US	2019	0	1	1	0	0	0	0	0	0	0	0	0	58
16062569	f	md	JP	2019	1	0	0	0	0	0	0	0	0	0	0	0	76
16062736	Not	md	JP	2019	0	0	0	0	0	0	0	0	0	0	0	0	70
16063249	m	cn	JP	2019	0	1	1	0	0	0	0	0	0	0	0	0	68
16063268	m	ot	ES	2019	0	0	0	0	0	0	0	0	0	0	0	0	70
16063332	f	md	JP	2019	0	1	0	1	0	0	0	0	0	0	0	1	69
16063646	m	md	JP	2019	1	0	0	0	0	0	0	0	0	0	0	1	83

16063671	f	ot	NL	2019	0	0	0	0	0	0	0	0	0	0	0	0	78
16063874	f	cn	GR	2019	0	1	1	0	0	0	0	0	0	0	0	0	50
16064893	m	cn	DE	2019	0	1	1	0	0	0	0	0	0	0	0	0	67
16065102	m	md	JP	2019	0	1	0	0	0	1	0	0	0	0	0	0	80
16065845	f	md	JP	2019	0	1	0	0	1	0	0	0	0	0	0	1	68
16066202	f	md	US	2019	0	1	0	1	0	0	0	0	0	0	0	0	54
16066243	f	md	US	2019	0	1	0	0	0	1	0	0	0	0	0	0	72
16066345	m	md	JP	2019	0	1	0	0	1	0	0	0	0	0	0	0	69
16067298	f	md	KR	2019	1	0	0	0	0	0	0	0	0	0	0	0	64.9
16067529	Not	md	CN	2019	1	0	0	0	0	0	0	0	0	0	0	0	
16067656	f	md	US	2019	1	0	0	0	0	0	0	0	0	0	0	0	65.3
16067934	f	md	JP	2019	1	0	0	0	0	0	0	0	0	0	0	0	78
16067935	Not	md	JP	2019	0	1	0	1	0	0	0	0	0	0	0	0	75
16068566	f	cn	US	2019	0	0	0	0	0	0	0	0	0	0	0	0	68
16069137	m	md	JP	2019	0	1	0	0	1	0	0	0	0	0	0	1	75
16069530	m	md	JP	2019	0	0	0	0	0	0	0	0	0	0	0	0	70
16069655	m	ph	JP	2019	0	1	0	0	1	0	0	0	0	0	0	1	61
16069699	m	cn	JP	2019	0	1	1	0	0	0	0	0	0	0	0	1	66
16069735	f	cn	US	2019	0	1	1	0	0	0	0	0	0	0	0	0	
16069756	f	cn	DE	2019	0	1	1	0	0	0	0	0	0	0	0	0	62
16069958	m	ot	GB	2019	0	1	0	0	0	1	0	0	0	0	0	0	47
16070342	m	cn	JP	2019	0	1	1	0	0	0	0	0	0	0	0	0	71
16070362	m	cn	US	2019	0	1	1	0	0	0	0	0	0	0	0	0	47
16070382	f	md	US	2019	0	1	1	0	0	0	0	0	0	0	0	0	
16070425	f	md	US	2019	0	1	1	0	0	0	0	0	0	0	0	0	66
16070939	f	ot	PL	2019	0	0	0	0	0	0	0	0	0	0	0	0	75
16071326	f	ot	US	2019	0	0	0	0	0	0	0	0	0	0	0	0	74
16071899	Not	md	US	2019	0	1	0	1	0	0	0	0	0	0	0	0	
16071921	m	ot	RS	2019	0	1	1	0	0	0	0	0	0	0	0	0	
16072304	Not	md	ES	2019	1	0	0	0	0	0	0	0	0	0	0	0	
16072801	f	ot	JP	2019	1	0	0	0	0	0	0	0	0	0	0	1	80
16072856	m	cn	US	2019	1	0	0	0	0	0	0	0	0	0	0	0	81
16073154	f	cn	US	2019	0	1	1	0	0	0	0	0	0	0	0	0	
16073166	m	md	JP	2019	0	1	0	1	0	0	0	0	0	0	0	1	72

16073507	m	md	JP	2019	0	1	0	0	1	0	0	0	0	0	0	1	74
16073523	m	md	JP	2019	0	1	0	0	1	0	0	0	0	0	0	0	78
16073524	m	ot	US	2019	0	1	0	0	1	0	0	0	0	0	0	0	72
16073592	Not	cn	BE	2019	0	1	1	0	0	0	0	0	0	0	0	0	64
16074065	m	md	JP	2019	1	0	0	0	0	0	0	0	0	0	0	0	66
16074077	m	md	JP	2019	1	0	0	0	0	0	0	0	0	0	0	0	70.1
16074078	f	md	JP	2019	1	0	0	0	0	0	0	0	0	0	0	0	78
16074118	m	ph	JP	2019	0	0	0	0	0	0	0	0	0	0	0	0	90
16074135	m	md	JP	2019	1	0	0	0	0	0	0	0	0	0	0	0	70
16074163	Not	md	US	2019	0	0	0	0	0	0	0	0	0	0	0	0	
16074225	f	ot	FR	2019	0	1	0	0	1	0	0	0	0	0	0	0	53
16074370	Not	md	KR	2019	0	0	0	0	0	0	0	0	0	0	0	0	
16074602	f	ot	US	2019	1	0	0	0	0	0	0	0	0	0	0	1	70
16074604	m	ot	US	2019	0	1	0	0	1	0	0	0	0	0	0	0	72
16074608	m	ot	DE	2019	0	1	0	0	1	0	0	0	0	0	0	0	77
16074649	f	ot	AU	2019	0	1	0	0	1	0	0	0	0	0	0	0	61
16074968	f	md	JP	2019	0	1	0	0	0	1	0	0	0	0	0	0	57
16075403	f	md	JP	2019	0	1	0	0	0	1	0	0	0	0	0	0	70
16075586	m	md	CH	2019	1	0	0	0	0	0	0	0	0	0	0	0	53
16075787	m	cn	JP	2019	0	1	1	0	0	0	0	0	0	0	0	0	78
16076595	f	cn	Not re	2019	0	0	0	0	0	0	0	0	0	0	0	0	38
16077202	m	ot	US	2019	0	1	0	0	1	0	0	0	0	0	0	0	
16077365	f	ph	JP	2019	1	0	0	0	0	0	0	0	0	0	0	0	81
16077460	f	md	US	2019	0	1	1	0	0	0	0	0	0	0	0	1	83
16078064	m	md	JP	2019	0	1	0	1	0	0	0	0	0	0	0	1	79.2
16078191	m	cn	US	2019	0	1	1	0	0	0	0	0	0	0	0	0	94
16078433	m	md	FR	2019	0	1	0	1	0	0	0	0	0	0	0	1	66
16078719	f	cn	US	2019	1	0	0	0	0	0	0	0	0	0	0	0	
16078927	m	ot	US	2019	0	1	0	0	1	0	0	0	0	0	0	0	74
16079081	m	ot	DE	2019	0	0	0	0	0	0	0	0	0	0	0	0	75
16079087	f	md	JP	2019	1	0	0	0	0	0	0	0	0	0	0	1	73.8
16079194	f	md	US	2019	1	0	0	0	0	0	0	0	0	0	0	0	
16079393	m	ph	IT	2019	1	0	0	0	0	0	0	0	0	0	0	0	77
16079478	f	md	DE	2019	0	1	1	0	0	0	0	0	0	0	0	0	55

16079479	m	md	JP	2019	0	1	1	0	0	0	0	0	0	0	0	1	70
16079513	f	md	JP	2019	0	1	0	1	0	0	0	0	0	0	0	1	80
16079668	f	md	NL	2019	1	0	0	0	0	0	0	0	0	0	0	0	53
16079771	f	cn	CN	2019	1	0	0	0	0	0	0	0	0	0	0	0	54
16079780	f	md	HU	2019	1	0	0	0	0	0	0	0	0	0	0	0	
16079943	m	md	BE	2019	0	1	1	0	0	0	0	0	0	0	0	0	77
16080041	m	ot	FR	2019	0	1	0	0	1	0	0	0	0	0	0	0	73
16080676	m	md	JP	2019	0	0	0	0	0	0	0	0	0	0	0	0	
16080734	m	md	HU	2019	0	1	0	0	0	1	0	0	0	0	0	0	67
16080932	f	md	FR	2019	0	0	0	0	0	0	0	0	0	0	0	0	61
16081214	f	cn	US	2019	0	0	0	0	0	0	0	0	0	0	0	0	75
16081927	f	md	PT	2019	0	0	0	0	0	0	0	0	0	0	0	0	40
16082292	Not	md	CZ	2019	1	0	0	0	0	0	0	0	0	0	0	0	
16082742	Not	md	NL	2019	0	1	0	0	0	1	0	0	0	0	0	0	
16083137	m	md	JP	2019	0	0	0	0	0	0	0	0	0	0	0	0	74
16083471	m	md	BE	2019	0	1	1	0	0	0	0	0	0	0	0	0	48
16083849	m	md	FR	2019	0	0	0	0	0	0	0	0	0	0	0	0	58
16084466	f	md	FR	2019	0	0	0	0	0	0	0	0	0	0	0	0	
16084870	m	ph	FR	2019	0	0	0	0	0	0	0	0	0	0	0	0	54
16085293	m	cn	JP	2019	0	1	1	0	0	0	0	0	0	0	0	0	
16085438	m	cn	NL	2019	0	1	1	0	0	0	0	0	0	0	0	0	56
16085756	m	md	DE	2019	0	1	0	0	1	0	0	0	0	0	0	0	
16085900	m	Not repo	ID	2019	1	0	0	0	0	0	0	0	0	0	0	0	77
16086330	m	cn	JP	2019	0	0	0	0	0	0	0	0	0	0	0	0	65
16086333	f	cn	US	2019	0	1	0	0	1	0	0	0	0	0	0	0	
16087407	m	md	IT	2019	0	1	0	0	1	0	0	0	0	0	0	0	70
16087578	f	cn	JP	2019	0	1	1	0	0	0	0	0	0	0	0	0	59
16087668	f	ot	CN	2019	0	1	0	0	1	0	0	0	0	0	0	0	75
16087906	f	md	US	2019	1	0	0	0	0	0	0	0	0	0	0	0	
16087926	m	md	BE	2019	0	1	1	0	0	0	0	0	0	0	0	0	66
16087938	m	cn	JP	2019	0	1	1	0	0	0	0	0	0	0	0	0	61
16088027	f	cn	JP	2019	0	1	1	0	0	0	0	0	0	0	0	0	70
16088034	Not	md	JP	2019	1	0	0	0	0	0	0	0	0	0	0	0	
16088081	f	md	NL	2019	0	0	0	0	0	0	0	0	0	0	0	0	49

16088335	m	ot	FR	2019	0	1	0	0	1	0	0	0	0	0	0	0	80
16088666	f	md	DE	2019	0	1	1	0	0	0	0	0	0	0	0	0	61
16088956	m	md	US	2019	0	1	1	0	0	0	0	0	0	0	0	0	
16089101	m	md	FR	2019	0	1	0	0	1	0	0	0	0	0	0	0	43
16089137	m	cn	JP	2019	0	1	1	0	0	0	0	0	0	0	0	0	70
16089742	m	ot	US	2019	0	0	0	0	0	0	0	0	0	0	0	0	43
16090152	f	ot	US	2019	1	0	0	0	0	0	0	0	0	0	0	0	
16090860	f	ot	GB	2019	0	0	0	0	0	0	0	0	0	0	0	0	56
16091061	f	cn	DE	2019	0	1	1	0	0	0	0	0	0	0	0	0	62
16091249	f	cn	BR	2019	0	0	0	0	0	0	0	0	0	0	0	0	
16091258	f	cn	IL	2019	0	0	0	0	0	0	0	0	0	0	0	0	39
16091485	m	md	US	2019	0	1	0	1	0	0	0	0	0	0	0	1	
16091607	f	cn	US	2019	0	1	1	0	0	0	0	0	0	0	0	0	
16091687	m	cn	GR	2019	0	1	1	0	0	0	0	0	0	0	0	0	70
16091716	m	Not repo	CN	2019	1	0	0	0	0	0	0	0	0	0	0	0	58
16091980	m	md	US	2019	0	1	1	0	0	0	0	0	0	0	0	0	74
16091983	m	ot	AR	2019	0	1	0	0	1	0	0	0	0	0	0	0	69
16092007	m	md	US	2019	0	1	0	0	1	0	0	0	0	0	0	0	64
16092325	m	cn	JP	2019	0	1	1	0	0	0	0	0	0	0	0	0	
16092335	m	cn	JP	2019	0	1	1	0	0	0	0	0	0	0	0	0	60
16092512	Not	md	HK	2019	0	0	0	0	0	0	0	0	0	0	0	0	
16093054	f	ot	DE	2019	1	0	0	0	0	0	0	0	0	0	0	0	53
16093164	m	md	JP	2019	0	1	0	0	0	1	0	0	0	0	0	0	90
16093541	f	cn	JP	2019	0	1	1	0	0	0	0	0	0	0	0	0	61
16093620	f	cn	US	2019	1	0	0	0	0	0	0	0	0	0	0	1	74
16093649	f	md	CZ	2019	1	0	0	0	0	0	0	0	0	0	0	0	65
16093683	Not	ot	CN	2019	0	0	0	0	0	0	0	0	0	0	0	0	
16094072	f	md	CZ	2019	0	0	0	0	0	0	0	0	0	0	0	0	24
16094083	m	md	AT	2019	0	0	0	0	0	0	0	0	0	0	0	0	54
16094969	f	cn	US	2019	0	1	0	0	1	0	0	0	0	0	0	0	64
16095133	Not	ph	JP	2019	1	0	0	0	0	0	0	0	0	0	0	0	
16095824	m	ot	IT	2019	0	1	0	0	1	0	0	0	0	0	0	0	74
16095982	m	md	JP	2019	0	1	0	1	0	0	0	0	0	0	0	1	80
16096173	f	ot	JP	2019	0	0	0	0	0	0	0	0	0	0	0	0	71

16096446	m	md	JP	2019	0	1	0	0	1	0	0	0	0	0	0	0	54
16096881	f	ot	US	2019	0	1	0	0	1	0	0	0	0	0	0	0	61
16096884	f	ot	US	2019	0	1	0	0	1	0	0	0	0	0	0	0	72
16097468	m	md	JP	2019	1	0	0	0	0	0	0	0	0	0	0	1	74
16097470	f	md	US	2019	1	0	0	0	0	0	0	0	0	0	0	0	
16097752	m	cn	JP	2019	0	0	0	0	0	0	0	0	0	0	0	0	78
16098004	m	md	DE	2019	0	0	0	0	0	0	0	0	0	0	0	0	
16098314	f	ot	CA	2019	0	1	0	0	1	0	0	0	0	0	0	0	61
16098522	f	md	FR	2019	0	1	0	0	0	1	0	0	0	0	0	0	56
16098556	m	cn	JP	2019	0	1	1	0	0	0	0	0	0	0	0	0	79
16099134	m	cn	JP	2019	0	1	1	0	0	0	0	0	0	0	0	0	80
16099206	f	cn	US	2019	1	0	0	0	0	0	0	0	0	0	0	0	63
16099654	f	cn	DE	2019	0	1	1	0	0	0	0	0	0	0	0	0	55
16100145	Not	md	US	2019	0	1	0	1	0	0	0	0	0	0	0	0	
16100150	f	ot	FR	2019	0	0	0	0	0	0	0	0	0	0	0	0	54
16101188	Not	md	MY	2019	0	1	0	1	0	0	0	0	0	0	0	0	
16101263	m	md	JP	2019	0	1	0	1	0	0	0	0	0	0	0	1	64
16101271	Not	ph	JP	2019	0	1	0	1	0	0	0	0	0	0	0	0	60
16101586	m	md	IN	2019	0	0	0	0	0	0	0	0	0	0	0	0	62
16101594	m	md	DE	2019	0	0	0	0	0	0	0	0	0	0	0	0	67
16103029	f	ot	US	2019	0	0	0	0	0	0	0	0	0	0	0	0	74
16103095	f	md	US	2019	0	1	0	0	0	1	0	0	0	0	0	0	
16103237	f	cn	GB	2019	1	0	0	0	0	0	0	0	0	0	0	0	69
16103441	m	md	JP	2019	0	1	0	0	1	0	0	0	0	0	0	0	100
16103468	m	ot	CN	2019	0	1	0	0	1	0	0	0	0	0	0	0	65
16104402	m	md	US	2019	0	0	0	0	0	0	0	0	0	0	0	0	
16104940	m	md	SK	2019	0	1	1	0	0	0	0	0	0	0	0	0	73
16105113	m	md	ES	2019	0	1	0	0	0	1	0	0	0	0	0	0	61
16105242	Not	md	US	2019	0	0	0	0	0	0	0	0	0	0	0	0	
16105243	m	cn	GR	2019	0	1	1	0	0	0	0	0	0	0	0	0	60
16105258	m	md	JP	2019	0	1	0	1	0	0	0	0	0	0	0	0	61
16105388	Not	ot	US	2019	1	0	0	0	0	0	0	0	0	0	0	0	75
16105854	f	cn	DE	2019	0	1	1	0	0	0	0	0	0	0	0	0	
16105896	f	md	JP	2019	0	0	0	0	0	0	0	0	0	0	0	0	76

16106236	f	ot	JP	2019	1	0	0	0	0	0	0	0	0	0	0	0	72
16106492	f	md	GB	2019	0	0	0	0	0	0	0	0	0	0	0	0	81
16106495	m	md	DE	2019	0	0	0	0	0	0	0	0	0	0	0	0	66
16106591	f	cn	JP	2019	0	0	0	0	0	0	0	0	0	0	0	0	76
16106594	m	cn	JP	2019	0	1	1	0	0	0	0	0	0	0	0	0	77
16106757	m	cn	FR	2019	0	1	0	0	1	0	0	0	0	0	0	0	43
16107074	m	md	FR	2019	0	0	0	0	0	0	0	0	0	0	0	0	81
16107079	f	md	JP	2019	1	0	0	0	0	0	0	0	0	0	0	1	79
16107172	f	md	JP	2019	1	0	0	0	0	0	0	0	0	0	0	0	79.2
16107173	m	md	JP	2019	1	0	0	0	0	0	0	0	0	0	0	0	70
16107284	m	cn	CA	2019	0	0	0	0	0	0	0	0	0	0	0	0	49
16107361	m	cn	JP	2019	0	1	1	0	0	0	0	0	0	0	0	0	72
16107377	f	md	GR	2019	0	0	0	0	0	0	0	0	0	0	0	0	73
16107616	f	md	JP	2019	1	0	0	0	0	0	0	0	0	0	0	0	75
16107714	m	md	DE	2019	0	0	0	0	0	0	0	0	0	0	0	0	75
16107743	m	Not repo	US	2019	0	1	0	1	0	0	0	0	0	0	0	0	73
16107786	m	ot	CH	2019	0	1	0	0	1	0	0	0	0	0	0	0	73
16108162	m	ot	US	2019	0	1	0	0	1	0	0	0	0	0	0	0	70
16108179	f	md	JP	2019	0	1	0	0	1	0	0	0	0	0	0	0	51
16108180	m	ot	CN	2019	0	1	0	0	1	0	0	0	0	0	0	0	62
16108189	m	ot	RO	2019	0	1	0	0	1	0	0	0	0	0	0	0	74
16108238	f	md	GB	2019	0	0	0	0	0	0	0	0	0	0	0	0	63
16108276	m	md	CZ	2019	0	1	0	0	1	0	0	0	0	0	0	0	
16108323	m	cn	DE	2019	0	1	1	0	0	0	0	0	0	0	0	0	74
16109493	m	md	DE	2019	0	0	0	0	0	0	0	0	0	0	0	0	69
16109802	Not	ot	NL	2019	0	0	0	0	0	0	0	0	0	0	0	0	
16110266	f	md	IT	2019	0	1	0	0	1	0	0	0	0	0	0	0	69
16110611	m	ot	DE	2019	0	1	0	0	1	0	0	0	0	0	0	1	71
16111259	f	md	IL	2019	0	1	0	0	0	1	0	0	0	0	0	0	69
16111505	Not	md	HR	2019	0	1	1	0	0	0	0	0	0	0	0	0	
16111943	f	md	US	2019	1	0	0	0	0	0	0	0	0	0	0	0	54
16112620	m	cn	US	2019	0	0	0	0	0	0	0	0	0	0	0	0	79
16112763	m	md	NL	2019	0	0	0	0	0	0	0	0	0	0	0	0	72
16113172	Not	md	BR	2019	0	0	0	0	0	0	0	0	0	0	0	0	

16113179	f	md	CO	2019	0	0	0	0	0	0	0	0	0	0	0	0	33
16113724	m	ph	FR	2019	0	0	0	0	0	0	0	0	0	0	0	0	71
16113973	Not	cn	JP	2019	0	1	1	0	0	0	0	0	0	0	0	0	70
16114970	m	ph	JP	2019	0	1	0	0	1	0	0	0	0	0	0	0	67
16115293	m	ot	CN	2019	0	1	0	0	1	0	0	0	0	0	0	0	75
16115419	m	ot	DE	2019	0	1	1	0	0	0	0	0	0	0	0	1	65
16115967	m	ot	US	2019	1	0	0	0	0	0	0	0	0	0	0	0	71
16116811	m	md	JP	2019	0	1	0	1	0	0	0	0	0	0	0	1	73
16116818	m	ot	FR	2019	0	0	0	0	0	0	0	0	0	0	0	0	63
16117108	m	md	JP	2019	0	1	0	0	0	1	0	0	0	0	0	0	90
16117271	m	ot	US	2019	1	0	0	0	0	0	0	0	0	0	0	0	83
16117628	f	ot	US	2019	0	1	0	1	0	0	0	0	0	0	0	0	79
16117702	Not	md	HR	2019	0	1	1	0	0	0	0	0	0	0	0	0	
16117821	m	cn	US	2019	0	1	0	0	0	1	0	0	0	0	0	1	66
16117929	m	md	DE	2019	0	0	0	0	0	0	0	0	0	0	0	0	75
16117965	Not	md	JP	2019	1	0	0	0	0	0	0	0	0	0	0	0	70
16118037	f	md	JP	2019	1	0	0	0	0	0	0	0	0	0	0	1	89
16118306	f	md	JP	2019	1	0	0	0	0	0	0	0	0	0	0	0	70
16118315	m	md	IN	2019	0	0	0	0	0	0	0	0	0	0	0	0	37
16118494	f	md	IT	2019	1	0	0	0	0	0	0	0	0	0	0	0	
16119271	m	cn	CA	2019	0	0	0	0	0	0	0	0	0	0	0	0	59
16119729	f	md	US	2019	0	1	1	0	0	0	0	0	0	0	0	0	
16119755	m	cn	JP	2019	0	1	1	0	0	0	0	0	0	0	0	0	
16119819	m	md	JP	2019	0	1	0	1	0	0	0	0	0	0	0	1	73
16120004	m	cn	JP	2019	0	1	1	0	0	0	0	0	0	0	0	0	63
16120023	m	cn	IN	2019	0	0	0	0	0	0	0	0	0	0	0	0	67
16120043	f	ot	JP	2019	0	1	0	0	1	0	0	0	0	0	0	0	74
16120659	Not	md	HR	2019	0	1	1	0	0	0	0	0	0	0	0	0	
16120693	Not	md	HR	2019	0	1	1	0	0	0	0	0	0	0	0	0	
16120744	Not	md	HR	2019	0	1	1	0	0	0	0	0	0	0	0	0	
16120792	Not	md	HR	2019	0	1	1	0	0	0	0	0	0	0	0	0	
16120817	Not	md	HR	2019	0	1	1	0	0	0	0	0	0	0	0	0	
16120944	Not	ot	US	2019	0	1	0	0	1	0	0	0	0	0	0	0	
16121087	m	md	JP	2019	0	1	0	0	0	1	0	0	0	0	0	1	73

16121153	m	cn	JP	2019	0	1	1	0	0	0	0	0	0	0	0	0	90
16121555	Not	md	JP	2019	0	1	0	0	1	0	0	0	0	0	0	0	
16121918	m	ot	JP	2019	0	1	0	1	0	0	0	0	0	0	0	1	58.3
16122185	Not	md	JP	2019	0	1	1	0	0	0	0	0	0	0	0	1	
16122468	Not	md	HR	2019	0	1	1	0	0	0	0	0	0	0	0	0	
16122616	Not	md	US	2019	1	0	0	0	0	0	0	0	0	0	0	0	
16122745	m	ot	ES	2019	0	0	0	0	0	0	0	0	0	0	0	0	
16123082	Not	md	HR	2019	0	1	1	0	0	0	0	0	0	0	0	0	
16123375	m	md	JP	2019	0	1	1	0	0	0	0	0	0	0	0	0	78
16123640	Not	md	JP	2019	0	1	0	1	0	0	0	0	0	0	0	1	
16123644	m	ot	CA	2019	0	0	0	0	0	0	0	0	0	0	0	0	
16124226	f	md	US	2019	1	0	0	0	0	0	0	0	0	0	0	0	66
16124227	m	md	US	2019	1	0	0	0	0	0	0	0	0	0	0	0	
16124609	m	cn	JP	2019	0	1	1	0	0	0	0	0	0	0	0	0	62
16124736	Not	cn	US	2019	0	0	0	0	0	0	0	0	0	0	0	0	
16124784	m	md	JP	2019	0	1	0	1	0	0	0	0	0	0	0	0	80
16124795	m	cn	US	2019	0	1	0	1	0	0	0	0	0	0	0	0	68.5
16124965	m	ot	JP	2019	0	1	0	0	1	0	0	0	0	0	0	0	64
16124991	f	md	JP	2019	0	1	0	0	1	0	0	0	0	0	0	1	66
16125196	f	ot	US	2019	0	1	0	0	1	0	0	0	0	0	0	0	71
16125227	Not	md	JP	2019	0	0	0	0	0	0	0	0	0	0	0	0	
16125273	m	ot	US	2019	0	1	0	0	1	0	0	0	0	0	0	0	73
16125770	Not	ph	FR	2019	0	0	0	0	0	0	0	0	0	0	0	0	
16125966	f	ot	FR	2019	0	1	0	0	1	0	0	0	0	0	0	0	65
16126066	m	ph	FR	2019	0	0	0	0	0	0	0	0	0	0	0	0	71
16126468	m	md	JP	2019	0	1	0	0	0	1	0	0	0	0	0	0	61
16126474	m	md	JP	2019	0	1	0	0	0	1	0	0	0	0	0	0	72
16127194	Not	md	HR	2019	0	1	1	0	0	0	0	0	0	0	0	0	68
16127352	Not	md	HR	2019	0	1	1	0	0	0	0	0	0	0	0	0	
16127680	Not	md	HR	2019	0	1	1	0	0	0	0	0	0	0	0	0	66
16127935	m	cn	JP	2019	0	1	1	0	0	0	0	0	0	0	0	0	56
16128489	f	md	US	2019	0	1	0	1	0	0	0	0	0	0	0	0	58
16129107	Not	md	JP	2019	0	1	0	1	0	0	0	0	0	0	0	1	
16129723	Not	md	JP	2019	1	0	0	0	0	0	0	0	0	0	0	0	

16129780	Not	md	JP	2019	0	1	0	1	0	0	0	0	0	0	0	1	
16130151	f	md	JP	2019	0	0	0	0	0	0	0	0	0	0	0	0	65
16130152	f	md	JP	2019	1	0	0	0	0	0	0	0	0	0	0	0	86
16130788	f	md	US	2019	0	1	0	1	0	0	0	0	0	0	0	0	100
16131852	Not	md	US	2019	0	0	0	0	0	0	0	0	0	0	0	0	
16131910	f	md	US	2019	1	0	0	0	0	0	0	0	0	0	0	0	65
16132218	Not	cn	US	2019	0	0	0	0	0	0	0	0	0	0	0	0	
16132734	m	cn	CN	2019	1	0	0	0	0	0	0	0	0	0	0	0	
16133177	m	md	JP	2019	0	1	0	0	0	1	0	0	0	0	0	0	79
16133192	m	md	HR	2019	0	1	1	0	0	0	0	0	0	0	0	0	
16133834	m	md	JP	2019	0	1	0	0	0	1	0	0	0	0	0	0	84
16133893	m	md	US	2019	0	1	0	0	0	1	0	0	0	0	0	1	65
16134057	m	ot	CA	2019	1	0	0	0	0	0	0	0	0	0	0	0	48
16134340	m	md	GB	2019	0	1	1	0	0	0	0	0	0	0	0	0	
16134733	m	cn	IN	2019	0	0	0	0	0	0	0	0	0	0	0	0	67
16134752	Not	md	HR	2019	0	1	1	0	0	0	0	0	0	0	0	0	
16135150	f	md	DE	2019	0	0	0	0	0	0	0	0	0	0	0	0	81
16135771	m	cn	JP	2019	0	1	1	0	0	0	0	0	0	0	0	0	90
16135804	f	md	JP	2019	0	0	0	0	0	0	0	0	0	0	0	0	75
16135815	f	cn	JP	2019	0	1	1	0	0	0	0	0	0	0	0	0	66
16135892	f	md	JP	2019	1	0	0	0	0	0	0	0	0	0	0	1	68
16136035	m	md	US	2019	0	0	0	0	0	0	1	1	0	0	0	0	78
16136609	m	md	JP	2019	0	1	1	0	0	0	0	0	0	0	0	0	73
16136681	m	md	IN	2019	0	0	0	0	0	0	0	0	0	0	0	0	57
16136718	m	cn	JP	2019	0	1	1	0	0	0	0	0	0	0	0	0	70
16136722	f	md	JP	2019	0	1	1	0	0	0	0	0	0	0	0	0	77
16136983	m	ot	IT	2019	0	1	0	0	1	0	0	0	0	0	0	0	
16137451	Not	ot	NL	2019	0	0	0	0	0	0	0	0	0	0	0	0	
16137470	f	md	PT	2019	1	0	0	0	0	0	0	0	0	0	0	0	90
16137949	Not	ot	NL	2019	0	0	0	0	0	0	0	0	0	0	0	0	
16138499	f	md	IL	2019	0	0	0	0	0	0	0	0	0	0	0	0	75
16139401	f	ot	DE	2019	0	0	0	0	0	0	0	0	0	0	0	0	60
6461423	m	cn	FR	2019	0	0	0	0	0	0	0	0	0	0	0	0	67
6471287	m	cn	FR	2019	0	0	0	0	0	0	0	0	0	0	0	0	67

13656142	f	md	AR	2019	0	1	0	0	0	1	0	0	0	0	0	0	73
14598826	m	ot	DE	2019	0	1	0	0	1	0	0	0	0	0	0	0	69
14784746	m	ot	FR	2019	0	1	1	0	0	0	0	0	0	0	0	0	67
15133686	m	ot	CN	2019	0	1	0	0	1	0	0	0	0	0	0	0	71
15479073	m	md	GB	2019	0	0	0	0	0	0	0	0	0	0	0	0	76
15539648	m	md	JP	2019	0	1	0	0	0	1	0	0	0	0	0	0	73
15580644	m	md	JP	2019	0	1	0	1	0	0	0	0	0	0	0	1	67.3
15645003	m	md	CY	2019	0	0	0	0	0	0	0	0	0	0	0	0	74
15648706	m	md	JP	2019	0	1	0	1	0	0	0	0	0	0	0	1	76
15670131	f	md	DK	2019	0	1	0	0	0	1	0	0	0	0	0	1	68
15681501	m	md	CN	2019	0	1	0	0	0	1	0	0	0	0	0	0	54
15717449	f	cn	JP	2019	0	1	1	0	0	0	0	0	0	0	0	0	65
15722833	f	md	CN	2019	0	0	0	0	0	0	0	0	0	0	0	0	79
15740519	f	md	JP	2019	0	1	0	1	0	0	0	0	0	0	0	1	80
15743153	f	md	JP	2019	0	1	0	0	0	1	0	0	0	0	0	0	76
15744270	m	md	JP	2019	1	0	0	0	0	0	0	0	0	0	0	0	76.9
15750748	Not	md	KR	2019	0	0	0	0	0	0	0	0	0	0	0	0	
15754903	m	md	DE	2019	0	1	0	0	1	0	0	0	0	0	0	0	58
15766964	f	ot	DE	2019	0	0	0	0	0	0	0	0	0	0	0	0	65
15779121	f	md	JP	2019	1	0	0	0	0	0	0	0	0	0	0	0	74.1
15779772	m	cn	DE	2019	0	1	0	0	1	0	0	0	0	0	0	0	64
15786875	f	cn	US	2019	0	1	0	0	1	0	0	0	0	0	0	0	62
15791301	f	cn	US	2019	0	1	1	0	0	0	0	0	0	0	0	0	70
15796050	m	ot	ES	2019	0	1	0	0	1	0	0	0	0	0	0	0	56
15801224	f	cn	US	2019	0	1	0	0	1	0	0	0	0	0	0	0	
15803087	m	ph	US	2019	0	1	0	0	1	0	0	0	0	0	0	1	53
15803111	f	cn	US	2019	0	1	0	0	1	0	0	0	0	0	0	0	71
15805213	m	ot	US	2019	0	1	0	0	1	0	0	0	0	0	0	0	75
15811435	m	md	MA	2019	0	1	0	0	0	1	0	0	0	0	0	0	51
15812461	m	cn	JP	2019	0	1	1	0	0	0	0	0	0	0	0	0	63
15813276	f	ot	DE	2019	0	1	0	0	1	0	0	0	0	0	0	0	64
15818730	m	cn	US	2019	0	1	0	0	1	0	0	0	0	0	0	0	
15821611	m	cn	DE	2019	0	0	0	0	0	0	0	0	0	0	0	0	
15822979	f	md	JP	2019	0	1	0	1	0	0	0	0	0	0	0	1	77.3

15832067	m	ot	AU	2019	0	1	0	0	1	0	0	0	0	0	0	0	67
15832182	m	md	JP	2019	0	1	0	0	1	0	0	0	0	0	0	0	81
15834162	m	md	JP	2019	0	1	0	0	1	0	0	0	0	0	0	0	70
15836500	m	cn	US	2019	0	1	0	0	1	0	0	0	0	0	0	0	72
15838545	m	md	JP	2019	0	1	0	1	0	0	0	0	0	0	0	1	80.5
15840525	m	md	JP	2019	0	1	0	0	1	0	0	0	0	0	0	0	72
15840906	m	md	JP	2019	1	0	0	0	0	0	0	0	0	0	0	0	70
15844402	m	md	JP	2019	0	1	1	0	0	0	0	0	0	0	0	0	65
15844513	m	cn	US	2019	0	1	0	0	1	0	0	0	0	0	0	1	
15850025	f	cn	US	2019	0	0	0	0	0	0	0	0	0	0	0	0	59
15850906	f	md	KR	2019	0	1	0	0	1	0	0	0	0	0	0	0	51
15851133	f	ot	US	2019	0	1	0	0	1	0	0	0	0	0	0	0	71
15851959	m	ph	JP	2019	0	0	0	0	0	0	1	0	1	0	0	0	74
15852519	m	ot	US	2019	0	1	0	0	1	0	0	0	0	0	0	0	73
15859288	m	md	JP	2019	0	1	0	0	1	0	0	0	0	0	0	1	68
15859977	m	md	DE	2019	0	1	1	0	0	0	0	0	0	0	0	0	66
15863510	m	cn	US	2019	0	0	0	0	0	0	0	0	0	0	0	0	
15863522	m	md	JP	2019	0	1	0	1	0	0	0	0	0	0	0	1	75
15865595	f	cn	US	2019	0	1	0	0	1	0	0	0	0	0	0	0	82
15865883	m	md	JP	2019	1	0	0	0	0	0	0	0	0	0	0	0	82.6
15866220	f	cn	US	2019	0	0	0	0	0	0	0	0	0	0	0	0	67
15866887	f	cn	US	2019	0	1	1	0	0	0	0	0	0	0	0	0	
15868830	m	md	IN	2019	0	1	0	1	0	0	0	0	0	0	0	0	53.9
15876999	f	cn	US	2019	0	0	0	0	0	0	0	0	0	0	0	0	57
15877461	m	ot	FR	2019	0	1	0	0	1	0	0	0	0	0	0	0	60
15878320	f	cn	US	2019	0	0	0	0	0	0	0	0	0	0	0	0	57
15879882	f	cn	US	2019	0	1	0	1	0	0	0	0	0	0	0	0	73
15880533	m	ot	FR	2019	0	1	0	0	1	0	0	0	0	0	0	0	66
15881640	f	md	JP	2019	0	0	0	0	0	0	0	0	0	0	0	0	31
15882901	f	cn	DE	2019	0	1	1	0	0	0	0	0	0	0	0	0	70
15882947	m	md	FI	2019	0	1	0	0	0	1	0	0	0	0	0	0	
15883915	m	md	JP	2019	0	1	0	0	1	0	0	0	0	0	0	0	64
15884111	m	md	JP	2019	1	0	0	0	0	0	0	0	0	0	0	1	67
15887291	m	md	US	2019	0	1	0	0	0	1	0	0	0	0	0	0	55

15894391	m	ot	GB	2019	0	0	0	0	0	0	0	0	0	0	0	0	36
15902729	f	md	JP	2019	0	1	0	0	0	1	0	0	0	0	0	0	63
15917113	f	md	TH	2019	0	1	0	0	1	0	0	0	0	0	0	0	66
15917356	m	cn	JP	2019	0	1	1	0	0	0	0	0	0	0	0	0	62
15918977	m	ot	FR	2019	0	1	0	0	1	0	0	0	0	0	0	0	65
15920746	m	cn	JP	2019	0	1	1	0	0	0	0	0	0	0	0	1	64
15921108	f	cn	JP	2019	0	1	1	0	0	0	0	0	0	0	0	0	74
15928999	f	md	FR	2019	0	0	0	0	0	0	0	0	0	0	0	0	74
15929886	m	md	JP	2019	0	1	0	1	0	0	0	0	0	0	0	1	74.3
15936338	m	md	KR	2019	0	1	0	0	1	0	0	0	0	0	0	0	72
15937406	f	md	JP	2019	1	0	0	0	0	0	0	0	0	0	0	0	70.7
15944040	m	md	KR	2019	0	1	0	0	1	0	0	0	0	0	0	0	71
15949255	f	cn	GB	2019	0	1	1	0	0	0	0	0	0	0	0	0	74
15950945	m	md	DE	2019	0	0	0	0	0	0	0	0	0	0	0	0	65
15958244	m	cn	JP	2019	0	1	1	0	0	0	0	0	0	0	0	0	74
15958527	m	ot	US	2019	0	1	1	0	0	0	0	0	0	0	0	0	
15959657	m	md	PT	2019	0	1	0	0	1	0	0	0	0	0	0	0	80
15962862	m	md	CN	2019	0	0	0	0	0	0	0	0	0	0	0	0	60
15962888	m	md	JP	2019	0	1	0	1	0	0	0	0	0	0	0	1	70
15963307	m	md	JP	2019	1	0	0	0	0	0	0	0	0	0	0	0	78.8
15963316	m	ot	GB	2019	0	1	0	0	1	0	0	0	0	0	0	0	75
15965417	f	md	IT	2019	0	1	0	0	0	1	0	0	0	0	0	0	63
15967812	m	ot	JP	2019	0	1	1	0	0	0	0	0	0	0	0	1	68
15967819	m	ot	KR	2019	0	0	0	0	0	0	0	0	0	0	0	0	58
15968596	f	md	CH	2019	0	0	0	0	0	0	0	0	0	0	0	0	56
15974570	m	md	JP	2019	0	1	0	0	0	1	0	0	0	0	0	0	70
15974609	f	ot	DE	2019	0	1	0	0	1	0	0	0	0	0	0	0	75
15977549	m	cn	JP	2019	0	1	1	0	0	0	0	0	0	0	0	1	75
15978233	m	md	JP	2019	0	1	0	0	1	0	0	0	0	0	0	0	61
15978770	m	md	PT	2019	0	1	1	0	0	0	0	0	0	0	0	0	53
15980040	m	ot	DE	2019	0	1	0	0	1	0	0	0	0	0	0	0	70
15980425	f	ot	CA	2019	0	0	0	0	0	0	0	0	0	0	0	0	56
15981380	m	Not repo	JP	2019	0	0	0	0	0	0	0	0	0	0	0	0	62
15984550	f	md	JP	2019	1	0	0	0	0	0	0	0	0	0	0	0	87

15987078	m	md	JP	2019	0	0	0	0	0	0	0	0	0	0	0	0	60
15991595	f	md	JP	2019	1	0	0	0	0	0	0	0	0	0	0	0	45.4
15991655	m	ot	DE	2019	0	1	0	0	1	0	0	0	0	0	0	0	66
15996575	m	md	JP	2019	0	1	0	1	0	0	0	0	0	0	0	0	65.3
15996782	f	Not repo	JP	2019	0	0	0	0	0	0	0	0	0	0	0	0	64
16001069	f	md	JP	2019	0	0	0	0	0	0	0	0	0	0	0	0	83
16001417	f	md	US	2019	0	1	1	0	0	0	0	0	0	0	0	0	
16001916	m	cn	JP	2019	0	1	1	0	0	0	0	0	0	0	0	0	74
16001974	m	cn	JP	2019	0	1	1	0	0	0	0	0	0	0	0	1	74
16003899	m	md	ES	2019	0	1	1	0	0	0	0	0	0	0	0	1	60
16005500	m	md	JP	2019	0	1	0	1	0	0	0	0	0	0	0	1	76.3
16005874	f	ot	IT	2019	0	1	1	0	0	0	0	0	0	0	0	0	60
16007258	m	cn	JP	2019	0	1	1	0	0	0	0	0	0	0	0	0	77
16013472	m	md	JP	2019	1	0	0	0	0	0	0	0	0	0	0	1	66.6
16013994	f	md	JP	2019	1	0	0	0	0	0	0	0	0	0	0	0	67.1
16014214	f	ot	JP	2019	1	0	0	0	0	0	0	0	0	0	0	0	78
16015043	m	cn	JP	2019	0	1	1	0	0	0	0	0	0	0	0	1	83
16015355	m	md	ES	2019	0	1	0	0	0	1	0	0	0	0	0	0	63
16017270	m	cn	JP	2019	0	1	1	0	0	0	0	0	0	0	0	0	67
16021098	m	ot	DE	2019	0	1	1	0	0	0	0	0	0	0	0	0	70
16022660	m	md	AT	2019	0	0	0	0	0	0	0	0	0	0	0	0	84
16023541	m	Not repo	JP	2019	0	0	0	0	0	0	0	0	0	0	0	0	63
16027368	m	cn	GR	2019	0	1	1	0	0	0	0	0	0	0	0	0	
16027568	f	md	JP	2019	0	1	0	0	1	0	0	0	0	0	0	0	76
16028499	Not	md	JP	2019	0	1	0	0	1	0	0	0	0	0	0	0	
16029871	m	md	JP	2019	0	1	0	0	1	0	0	0	0	0	0	0	71
16030520	m	md	IT	2019	0	1	0	0	1	0	0	0	0	0	0	0	93
16031177	m	md	JP	2019	1	0	0	0	0	0	0	0	0	0	0	0	82
16033720	f	md	JP	2019	0	0	0	0	0	0	1	0	0	0	1	0	51
16038263	m	cn	JP	2019	0	1	1	0	0	0	0	0	0	0	0	0	68
16038399	m	cn	JP	2019	0	1	1	0	0	0	0	0	0	0	0	0	76
16038719	m	cn	JP	2019	0	1	1	0	0	0	0	0	0	0	0	0	80
16039328	m	ph	JP	2019	0	1	0	0	1	0	0	0	0	0	0	0	70
16039333	f	ph	JP	2019	0	1	0	0	1	0	0	0	0	0	0	0	37

16044565	f	cn	JP	2019	0	1	1	0	0	0	0	0	0	0	0	62
16044580	f	cn	JP	2019	0	1	1	0	0	0	0	0	0	0	0	66
16045996	m	md	JP	2019	0	1	0	1	0	0	0	0	0	0	0	1	76
16047711	f	md	JP	2019	0	1	1	0	0	0	0	0	0	0	0	62
16048698	m	md	JP	2019	1	0	0	0	0	0	0	0	0	0	0	1	73.7
16050786	f	md	CH	2019	0	0	0	0	0	0	0	0	0	0	0	0	
16051226	m	md	JP	2019	0	1	0	1	0	0	0	0	0	0	0	1	77
16052366	m	cn	JP	2019	0	1	1	0	0	0	0	0	0	0	0	0	59
16052987	f	ot	HU	2019	0	0	0	0	0	0	0	0	0	0	0	0	54
16053111	f	Not repo	CN	2019	1	0	0	0	0	0	0	0	0	0	0	0	66
16055880	m	md	JP	2019	0	1	0	0	0	1	0	0	0	0	0	0	70
16056629	f	md	HU	2019	0	0	0	0	0	0	0	0	0	0	0	0	54
16057970	m	cn	JP	2019	0	1	1	0	0	0	0	0	0	0	0	0	51
16058037	m	cn	JP	2019	0	1	1	0	0	0	0	0	0	0	0	0	82
16063859	m	md	JP	2019	0	1	0	0	1	0	0	0	0	0	0	0	65
16068645	f	cn	US	2019	1	0	0	0	0	0	0	0	0	0	0	0	
16068723	f	md	JP	2019	1	0	0	0	0	0	0	0	0	0	0	1	66
16069136	f	ot	ES	2019	0	1	0	0	1	0	0	0	0	0	0	0	76
16069828	m	ot	JP	2019	0	1	1	0	0	0	0	0	0	0	0	0	76
16070012	m	cn	JP	2019	0	1	1	0	0	0	0	0	0	0	0	0	67
16070656	m	cn	JP	2019	0	1	1	0	0	0	0	0	0	0	0	1	66
16071050	m	ot	JP	2019	0	1	1	0	0	0	0	0	0	0	0	1	61
16071802	m	cn	JP	2019	0	1	1	0	0	0	0	0	0	0	0	0	64
16072712	m	md	JP	2019	0	1	1	0	0	0	0	0	0	0	0	0	
16072996	f	md	JP	2019	1	0	0	0	0	0	0	0	0	0	0	0	70
16073071	m	md	IE	2019	0	0	0	0	0	0	0	0	0	0	0	0	57
16075860	m	cn	JP	2019	0	1	1	0	0	0	0	0	0	0	0	0	64
16076624	f	md	JP	2019	0	0	0	0	0	0	0	0	0	0	0	0	47
16077627	m	ot	JP	2019	0	1	0	0	1	0	0	0	0	0	0	0	78
16079797	m	cn	JP	2019	0	1	1	0	0	0	0	0	0	0	0	0	53
16080047	f	ot	CA	2019	0	1	0	0	1	0	0	0	0	0	0	0	76
16080236	m	md	JP	2019	0	1	0	1	0	0	0	0	0	0	0	0	65
16080801	m	md	JP	2019	0	1	0	0	0	1	0	0	0	0	0	0	75
16085359	m	ot	BR	2019	0	1	0	0	1	0	0	0	0	0	0	0	77

16086601	Not	cn	US	2019	0	0	0	0	0	0	0	0	0	0	0	0	
16087370	m	md	CZ	2019	1	0	0	0	0	0	0	0	0	0	0	0	33
16087373	f	md	JP	2019	0	1	0	0	0	1	0	0	0	0	0	1	65
16089201	m	md	CZ	2019	1	0	0	0	0	0	0	0	0	0	0	0	65
16089782	m	ot	TW	2019	0	1	0	1	0	0	0	0	0	0	0	0	58.1
16092390	m	cn	JP	2019	0	1	1	0	0	0	0	0	0	0	0	0	80
16092911	Not	ot	GB	2019	0	0	0	0	0	0	0	0	0	0	0	0	
16093403	f	md	JP	2019	0	1	0	1	0	0	0	0	0	0	0	1	67.4
16093983	f	md	US	2019	0	1	0	0	0	1	0	0	0	0	0	0	72
16095469	Not	ot	DE	2019	0	1	1	0	0	0	0	0	0	0	0	0	49
16096875	f	ot	US	2019	0	1	0	0	1	0	0	0	0	0	0	0	63
16097454	m	md	JP	2019	0	1	1	0	0	0	0	0	0	0	0	0	76
16097787	f	md	JP	2019	1	0	0	0	0	0	0	0	0	0	0	0	53
16097963	m	ot	JP	2019	0	1	1	0	0	0	0	0	0	0	0	0	68
16097970	f	md	DE	2019	0	0	0	0	0	0	0	0	0	0	0	0	
16099050	m	md	JP	2019	0	1	0	0	0	1	0	0	0	0	0	0	51
16099814	f	md	JP	2019	1	0	0	0	0	0	0	0	0	0	0	0	85
16099980	m	md	DE	2019	0	0	0	0	0	0	0	0	0	0	0	0	69
16102945	m	ot	NL	2019	0	1	0	0	1	0	0	0	0	0	0	0	73
16103272	f	ot	FR	2019	0	1	0	0	1	0	0	0	0	0	0	0	57
16104015	f	md	JP	2019	1	0	0	0	0	0	0	0	0	0	0	1	78.3
16106150	m	md	JP	2019	0	1	0	1	0	0	0	0	0	0	0	1	73
16106784	f	md	JP	2019	1	0	0	0	0	0	0	0	0	0	0	0	74.1
16107081	f	Not repo	US	2019	1	0	0	0	0	0	0	0	0	0	0	1	64
16107370	f	md	NO	2019	0	1	0	1	0	0	0	0	0	0	0	0	
16107780	f	ot	US	2019	0	0	0	0	0	0	0	0	0	0	0	0	58
16108183	f	md	FR	2019	0	1	0	0	1	0	0	0	0	0	0	0	66
16108192	m	ot	US	2019	0	1	0	0	1	0	0	0	0	0	0	1	71
16108283	m	md	DE	2019	0	1	0	0	1	0	0	0	0	0	0	0	53
16108801	m	Not repo	JP	2019	0	0	0	0	0	0	0	0	0	0	0	0	66
16111081	m	ph	US	2019	0	0	0	0	0	0	0	0	0	0	0	0	58
16115225	m	md	JP	2019	0	0	0	0	0	0	0	0	0	0	0	0	39
16116540	f	md	DE	2019	0	1	0	0	0	1	0	0	0	0	0	0	83
16117730	f	Not repo	ID	2019	1	0	0	0	0	0	0	0	0	0	0	0	

16118188	m	cn	JP	2019	0	1	1	0	0	0	0	0	0	0	0	0	67
16119091	m	md	JP	2019	0	1	0	1	0	0	0	0	0	0	0	0	65
16120481	m	ot	FR	2019	0	1	0	0	1	0	0	0	0	0	0	0	72
16121233	m	cn	JP	2019	0	0	0	0	0	0	0	0	0	0	0	0	66
16121840	f	ph	US	2019	0	0	0	0	0	0	0	0	0	0	0	0	66
16123261	m	md	JP	2019	0	1	1	0	0	0	0	0	0	0	0	0	71
16123461	m	md	JP	2019	0	1	0	1	0	0	0	0	0	0	0	1	70
16123569	Not	md	ES	2019	0	0	0	0	0	0	0	0	0	0	0	0	61
16124964	m	ot	ES	2019	0	1	0	0	1	0	0	0	0	0	0	0	60
16126480	m	md	FR	2019	0	1	0	0	0	1	0	0	0	0	0	0	69
16126709	f	cn	JP	2019	0	1	1	0	0	0	0	0	0	0	0	0	74
16127854	m	ot	JP	2019	0	1	1	0	0	0	0	0	0	0	0	0	77
16127855	f	cn	JP	2019	0	1	1	0	0	0	0	0	0	0	0	0	61
16129457	m	md	JP	2019	0	1	0	1	0	0	0	0	0	0	0	1	75
16129555	m	cn	JP	2019	0	1	1	0	0	0	0	0	0	0	0	0	64
16131124	m	ot	JP	2019	0	1	0	0	1	0	0	0	0	0	0	0	67
16132724	f	Not repo	ID	2019	1	0	0	0	0	0	0	0	0	0	0	0	50
16134278	m	cn	JP	2019	0	1	1	0	0	0	0	0	0	0	0	0	78
16136599	m	ot	JP	2019	0	1	1	0	0	0	0	0	0	0	0	0	73
16136617	m	cn	JP	2019	0	1	1	0	0	0	0	0	0	0	0	0	76
16136724	m	ot	JP	2019	0	1	1	0	0	0	0	0	0	0	0	0	83
16136726	m	ot	JP	2019	0	1	1	0	0	0	0	0	0	0	0	0	78
16137058	Not	md	HR	2019	0	1	1	0	0	0	0	0	0	0	0	0	
16137180	Not	md	HR	2019	0	1	1	0	0	0	0	0	0	0	0	0	
16137369	m	md	JP	2019	0	0	0	0	0	0	0	0	0	0	0	0	80
16137573	Not	md	HR	2019	0	1	1	0	0	0	0	0	0	0	0	0	
16137642	f	md	PL	2019	0	1	0	0	0	1	0	0	0	0	0	0	59
16137773	f	md	ES	2019	0	1	0	0	0	1	0	0	0	0	0	0	63
16137864	f	cn	US	2019	0	1	1	0	0	0	0	0	0	0	0	0	
16138063	Not	md	GR	2019	0	0	0	0	0	0	0	0	0	0	0	0	
16138518	f	md	DE	2019	0	0	0	0	0	0	0	0	0	0	0	0	60
16138768	f	md	BE	2019	0	1	0	0	0	1	0	0	0	0	0	1	70
16139406	Not	md	FR	2019	0	0	0	0	0	0	0	0	0	0	0	0	
16140925	Not	ot	JP	2019	0	0	0	0	0	0	0	0	0	0	0	0	

16141395	f	lw	US	2019	0	0	0	0	0	0	0	0	0	0	0	0	64
16143230	f	cn	CA	2019	0	1	1	0	0	0	0	0	0	0	0	0	77
16143383	f	cn	AR	2019	0	0	0	0	0	0	0	0	0	0	0	0	32
16143632	f	cn	US	2019	0	1	1	0	0	0	0	0	0	0	0	0	61
16144451	m	ot	BR	2019	0	1	0	0	1	0	0	0	0	0	0	0	78
16145032	f	cn	US	2019	0	1	1	0	0	0	0	0	0	0	0	0	
16145546	f	md	CA	2019	0	1	0	0	0	1	0	0	0	0	0	0	76
16145603	f	md	DE	2019	0	1	0	0	1	0	0	0	0	0	0	0	66
16145615	m	md	JP	2019	0	1	0	0	0	1	0	0	0	0	0	0	75
16145637	m	ot	AR	2019	0	1	0	0	1	0	0	0	0	0	0	0	64
16145916	f	md	JP	2019	1	0	0	0	0	0	0	0	0	0	0	1	75.3
16145956	m	md	US	2019	0	0	0	0	0	0	0	0	0	0	0	0	42
16146026	m	md	JP	2019	0	1	0	1	0	0	0	0	0	0	0	1	50
16146575	m	cn	HK	2019	0	1	1	0	0	0	0	0	0	0	0	0	
16146672	m	md	JP	2019	0	1	0	0	0	1	0	0	0	0	0	0	69
16148191	f	md	IT	2019	0	1	0	0	1	0	0	0	0	0	0	0	66
16148242	f	md	MY	2019	0	0	0	0	0	0	0	0	0	0	0	0	
16148316	f	cn	IN	2019	0	0	0	0	0	0	0	0	0	0	0	0	43
16148782	Not	md	US	2019	0	0	0	0	0	0	0	0	0	0	0	0	
16149288	f	Not repo	CN	2019	1	0	0	0	0	0	0	0	0	0	0	0	
16149292	m	md	JP	2019	0	1	0	1	0	0	0	0	0	0	0	1	81.4
16149774	m	cn	JP	2019	0	1	1	0	0	0	0	0	0	0	0	0	80
16149782	f	md	JP	2019	1	0	0	0	0	0	0	0	0	0	0	1	61.1
16149835	m	md	JP	2019	0	1	0	1	0	0	0	0	0	0	0	1	70.3
16149862	f	md	JP	2019	0	1	0	0	0	1	0	0	0	0	0	0	80
16149870	m	cn	US	2019	0	1	1	0	0	0	0	0	0	0	0	0	
16149927	f	md	DE	2019	1	0	0	0	0	0	0	0	0	0	0	0	
16149985	f	cn	GB	2019	1	0	0	0	0	0	0	0	0	0	0	0	66
16150006	m	md	JP	2019	0	1	1	0	0	0	0	0	0	0	0	0	78
16150024	f	md	CH	2019	0	0	0	0	0	0	0	0	0	0	0	0	61
16150185	f	Not repo	AU	2019	1	0	0	0	0	0	0	0	0	0	0	0	
16150279	m	ph	JP	2019	0	1	1	0	0	0	0	0	0	0	0	0	69
16150434	f	md	JP	2019	1	0	0	0	0	0	0	0	0	0	0	0	64
16150655	m	ot	ES	2019	0	1	0	0	1	0	0	0	0	0	0	0	62

16151075	m	md	GB	2019	0	1	0	0	1	0	0	0	0	0	0	0	74
16151763	Not	ot	US	2019	0	0	0	0	0	0	0	0	0	0	0	0	72
16151777	m	ot	JP	2019	0	0	0	0	0	0	0	0	0	0	0	0	70
16151890	Not	Not repo	IT	2019	1	0	0	0	0	0	0	0	0	0	0	0	
16151898	m	md	FR	2019	0	1	0	1	0	0	0	0	0	0	0	0	61
16151985	m	md	FI	2019	1	0	0	0	0	0	0	0	0	0	0	0	63
16152073	f	md	IN	2019	0	0	0	0	0	0	0	0	0	0	0	0	70
16152197	m	md	JP	2019	0	1	0	1	0	0	0	0	0	0	0	1	67
16152207	m	ot	FR	2019	0	1	0	0	1	0	0	0	0	0	0	0	71
16152210	f	md	JP	2019	1	0	0	0	0	0	0	0	0	0	0	0	59
16152285	f	md	ES	2019	0	1	0	0	0	1	0	0	0	0	0	0	68
16152901	m	md	RU	2019	0	1	0	1	0	0	0	0	0	0	0	0	67
16153103	m	md	JP	2019	0	0	0	0	0	0	0	0	0	0	0	0	70
16153612	m	md	CN	2019	0	1	0	0	0	1	0	0	0	0	0	0	63
16153630	m	cn	JP	2019	0	1	1	0	0	0	0	0	0	0	0	0	59
16153878	f	md	IN	2019	0	0	0	0	0	0	0	0	0	0	0	0	42
16154042	f	md	DE	2019	1	0	0	0	0	0	0	0	0	0	0	0	73
16154338	m	md	US	2019	1	0	0	0	0	0	0	0	0	0	0	0	
16154415	f	md	DE	2019	0	0	0	0	0	0	0	0	0	0	0	0	60
16154640	m	ot	DE	2019	0	1	0	0	1	0	0	0	0	0	0	0	
16154661	m	cn	CY	2019	0	0	0	0	0	0	0	0	0	0	0	0	74
16154665	m	cn	JP	2019	0	0	0	0	0	0	0	0	0	0	0	0	59
16154680	m	md	DE	2019	0	0	0	0	0	0	0	0	0	0	0	0	45
16154847	m	cn	JP	2019	0	1	1	0	0	0	0	0	0	0	0	0	80
16155059	m	ot	DE	2019	0	1	0	0	1	0	0	0	0	0	0	0	61
16155067	f	md	FI	2019	1	0	0	0	0	0	0	0	0	0	0	0	48
16155124	f	md	US	2019	0	0	0	0	0	0	0	0	0	0	0	0	63
16155191	m	ot	FR	2019	0	1	0	0	1	0	0	0	0	0	0	1	55
16155654	m	ot	DE	2019	0	1	0	0	1	0	0	0	0	0	0	0	69
16155730	m	md	BE	2019	0	0	0	0	0	0	0	0	0	0	0	0	42
16155778	m	md	JP	2019	0	1	0	0	0	1	0	0	0	0	0	0	67
16155836	m	ph	JP	2019	0	1	0	0	1	0	0	0	0	0	0	0	72
16155918	Not	md	JP	2019	0	1	0	0	1	0	0	0	0	0	0	0	
16155990	f	md	JP	2019	0	1	1	0	0	0	0	0	0	0	0	0	90

16156145	m	cn	US	2019	1	0	0	0	0	0	0	0	0	0	0	0	
16156482	f	md	CH	2019	0	1	0	0	0	1	0	0	0	0	0	0	
16156612	f	md	JP	2019	0	1	0	0	0	1	0	0	0	0	0	0	75
16156630	m	md	JP	2019	0	1	0	0	0	1	0	0	0	0	0	1	70
16156719	m	md	MY	2019	0	1	0	0	0	1	0	0	0	0	0	0	
16156930	m	md	US	2019	0	1	1	0	0	0	0	0	0	0	0	0	38
16156931	f	md	US	2019	0	1	1	0	0	0	0	0	0	0	0	0	
16156943	m	md	JP	2019	0	1	0	0	0	1	0	0	0	0	0	0	53
16157052	m	md	FR	2019	0	1	0	0	0	1	0	0	0	0	0	0	64
16157352	m	cn	DE	2019	0	1	1	0	0	0	0	0	0	0	0	1	74
16157370	f	md	JP	2019	1	0	0	0	0	0	0	0	0	0	0	0	69.5
16157749	f	ot	US	2019	0	1	1	0	0	0	0	0	0	0	0	0	72
16157869	m	md	TR	2019	0	0	0	0	0	0	0	0	0	0	0	0	65
16159051	m	md	US	2019	0	1	0	1	0	0	0	0	0	0	0	1	
16159152	m	md	JP	2019	0	0	0	0	0	0	0	0	0	0	0	0	67
16159238	m	md	TR	2019	0	0	0	0	0	0	0	0	0	0	0	0	70
16159283	m	md	ID	2019	1	0	0	0	0	0	0	0	0	0	0	0	55
16159744	f	md	US	2019	0	0	0	0	0	0	0	0	0	0	0	0	62
16159997	m	cn	HR	2019	0	1	1	0	0	0	0	0	0	0	0	0	
16160135	f	cn	US	2019	0	0	0	0	0	0	0	0	0	0	0	0	57
16160347	f	cn	DE	2019	0	1	1	0	0	0	0	0	0	0	0	0	62
16160351	f	ph	GB	2019	1	0	0	0	0	0	0	0	0	0	0	0	71
16160413	f	md	BE	2019	0	1	1	0	0	0	0	0	0	0	0	0	
16160909	m	cn	CN	2019	0	0	0	0	0	0	0	0	0	0	0	0	59
16162031	f	md	IT	2019	0	1	0	0	0	1	0	0	0	0	0	0	74
16162408	m	cn	JP	2019	0	1	1	0	0	0	0	0	0	0	0	0	80
16162600	m	md	AU	2019	0	1	0	0	0	1	0	0	0	0	0	0	71
16162759	f	md	DE	2019	0	0	0	0	0	0	0	0	0	0	0	0	60
16162842	m	md	JP	2019	0	1	0	1	0	0	0	0	0	0	0	1	71
16162947	f	md	JP	2019	1	0	0	0	0	0	0	0	0	0	0	0	50
16162970	f	md	JP	2019	1	0	0	0	0	0	0	0	0	0	0	0	90
16163028	m	ph	JP	2019	1	0	0	0	0	0	0	0	0	0	0	0	78
16163029	m	md	JP	2019	1	0	0	0	0	0	0	0	0	0	0	0	70
16163098	f	cn	JP	2019	0	1	1	0	0	0	0	0	0	0	0	0	69

16163150	f	md	JP	2019	0	0	0	0	0	0	0	0	0	0	0	0	61
16163259	f	ot	SE	2019	0	0	0	0	0	0	0	0	0	0	0	0	42
16163324	m	cn	JP	2019	0	1	1	0	0	0	0	0	0	0	0	1	68
16163666	f	md	TW	2019	0	0	0	0	0	0	0	0	0	0	0	0	77
16163688	m	md	FR	2019	0	1	0	1	0	0	0	0	0	0	0	0	
16163736	f	md	DE	2019	0	0	0	0	0	0	0	0	0	0	0	0	60
16164318	f	Not repo	GR	2019	0	1	0	1	0	0	0	0	0	0	0	0	75
16164335	m	cn	JP	2019	0	1	1	0	0	0	0	0	0	0	0	0	81
16164773	m	cn	JP	2019	0	1	1	0	0	0	0	0	0	0	0	0	76
16164795	f	md	RO	2019	0	0	0	0	0	0	0	0	0	0	0	0	
16165025	f	md	JP	2019	1	0	0	0	0	0	0	0	0	0	0	0	87
16165457	m	ot	FR	2019	0	1	0	0	1	0	0	0	0	0	0	0	64
16165465	m	md	JP	2019	0	1	1	0	0	0	0	0	0	0	0	0	70
16165713	f	cn	US	2019	0	1	0	0	1	0	0	0	0	0	0	0	53
16165912	f	cn	CN	2019	0	1	0	0	1	0	0	0	0	0	0	0	78
16166068	f	cn	US	2019	1	0	0	0	0	0	0	0	0	0	0	0	
16166365	m	md	AT	2019	0	0	0	0	0	0	0	0	0	0	0	0	72
16167077	Not	md	GR	2019	0	0	0	0	0	0	0	0	0	0	0	0	
16167087	Not	ot	CN	2019	0	0	0	0	0	0	0	0	0	0	0	0	
16167127	f	md	JP	2019	0	0	0	0	0	0	0	0	0	0	0	0	90
16167356	f	ot	JP	2019	0	1	0	0	1	0	0	0	0	0	0	0	78
16167358	m	md	US	2019	0	1	0	0	0	1	0	0	0	0	0	0	69
16167379	m	ot	NL	2019	0	0	0	0	0	0	0	0	0	0	0	0	42
16167662	m	ot	JP	2019	0	1	1	0	0	0	0	0	0	0	0	1	77
16167690	m	md	US	2019	0	1	0	0	0	1	0	0	0	0	0	0	62
16167730	f	md	JP	2019	0	1	0	0	0	1	0	0	0	0	0	0	70
16167761	f	md	JP	2019	0	1	0	0	0	1	0	0	0	0	0	1	70
16168250	m	md	KW	2019	1	0	0	0	0	0	0	0	0	0	0	0	64.7
16168273	f	cn	GB	2019	1	0	0	0	0	0	0	0	0	0	0	0	79
16168312	m	md	IN	2019	0	0	0	0	0	0	0	0	0	0	0	0	41
16168455	f	Not repo	US	2019	1	0	0	0	0	0	0	0	0	0	0	1	73
16168457	f	Not repo	US	2019	1	0	0	0	0	0	0	0	0	0	0	1	70
16168575	f	ph	US	2019	1	0	0	0	0	0	0	0	0	0	0	1	62
16168964	m	md	JP	2019	0	0	0	0	0	0	0	0	0	0	0	0	75

16169738	m	cn	JP	2019	0	1	1	0	0	0	0	0	0	0	0	0	70
16170075	m	ot	NL	2019	0	1	1	0	0	0	0	0	0	0	0	0	78
16170085	m	ot	ES	2019	0	0	0	0	0	0	0	0	0	0	0	0	
16170229	m	ot	JP	2019	0	1	0	0	1	0	0	0	0	0	0	0	72
16170704	Not	ot	RS	2019	0	0	0	0	0	0	0	0	0	0	0	0	
16170705	Not	ot	RS	2019	0	0	0	0	0	0	0	0	0	0	0	0	
16170706	Not	ot	RS	2019	0	0	0	0	0	0	0	0	0	0	0	0	
16170707	Not	ot	RS	2019	0	0	0	0	0	0	0	0	0	0	0	0	
16170708	Not	ot	RS	2019	0	0	0	0	0	0	0	0	0	0	0	0	
16170723	Not	ot	RS	2019	0	0	0	0	0	0	0	0	0	0	0	0	
16171198	m	cn	KR	2019	0	0	0	0	0	0	0	0	0	0	0	0	43
16171384	m	md	JP	2019	0	1	0	0	1	0	0	0	0	0	0	0	65
16171570	f	cn	US	2019	0	1	0	0	1	0	0	0	0	0	0	0	
16171799	f	ot	IE	2019	0	1	0	0	1	0	0	0	0	0	0	0	63
16172164	m	md	JP	2019	0	1	0	0	0	1	0	0	0	0	0	0	67
16172440	Not	md	IT	2019	0	0	0	0	0	0	0	0	0	0	0	0	
16172550	f	md	KR	2019	0	0	0	0	0	0	0	0	0	0	0	0	56
16173234	m	cn	GB	2019	0	1	1	0	0	0	0	0	0	0	0	0	
16173455	f	cn	KR	2019	0	0	0	0	0	0	0	0	0	0	0	0	56
16173841	m	md	CN	2019	0	1	0	0	0	1	0	0	0	0	0	0	73
16173889	f	ot	GB	2019	0	1	1	0	0	0	0	0	0	0	0	0	75
16174003	f	md	DE	2019	0	1	1	0	0	0	0	0	0	0	0	0	60
16174026	f	cn	JP	2019	1	0	0	0	0	0	0	0	0	0	0	0	76
16174106	f	cn	IL	2019	1	0	0	0	0	0	0	0	0	0	0	0	52
16174781	f	md	KR	2019	0	0	0	0	0	0	0	0	0	0	0	0	52
16174881	m	md	IT	2019	1	0	0	0	0	0	0	0	0	0	0	0	
16175067	m	cn	DE	2019	1	0	0	0	0	0	0	0	0	0	0	0	58
16175215	f	Not repo	US	2019	1	0	0	0	0	0	0	0	0	0	0	0	
16175250	m	md	JP	2019	1	0	0	0	0	0	0	0	0	0	0	0	83
16175568	m	cn	JP	2019	0	1	1	0	0	0	0	0	0	0	0	0	
16175808	f	md	KR	2019	0	0	0	0	0	0	0	0	0	0	0	0	72
16176015	f	ot	GB	2019	1	0	0	0	0	0	0	0	0	0	0	0	32
16176103	m	md	JP	2019	0	1	0	1	0	0	0	0	0	0	0	0	69
16176725	f	cn	KR	2019	0	0	0	0	0	0	0	0	0	0	0	0	48

16176771	m	cn	JP	2019	0	1	1	0	0	0	0	0	0	0	0	1	57
16176804	m	ot	US	2019	0	1	0	0	1	0	0	0	0	0	0	0	82
16176981	m	md	JP	2019	0	1	0	1	0	0	0	0	0	0	0	1	65
16177364	m	md	KR	2019	0	1	0	0	1	0	0	0	0	0	0	0	73
16177688	Not	md	FR	2019	0	0	0	0	0	0	0	0	0	0	0	0	
16177738	m	cn	JP	2019	0	1	1	0	0	0	0	0	0	0	0	1	74
16177840	f	ot	CA	2019	0	1	0	0	1	0	0	0	0	0	0	0	78
16178062	f	md	JP	2019	1	0	0	0	0	0	0	0	0	0	0	0	79
16178247	m	ot	DE	2019	0	1	0	0	1	0	0	0	0	0	0	0	63
16178411	Not	md	IT	2019	0	0	0	0	0	0	0	0	0	0	0	0	
16178439	f	md	DE	2019	0	1	1	0	0	0	0	0	0	0	0	0	86
16178647	m	ph	US	2019	0	1	0	1	0	0	0	0	0	0	0	0	73
16178902	f	md	JP	2019	0	1	1	0	0	0	0	0	0	0	0	0	53
16178930	f	ot	IT	2019	0	1	1	0	0	0	0	0	0	0	0	0	55
16179057	m	cn	IN	2019	0	1	0	0	1	0	0	0	0	0	0	0	60
16179058	Not	md	DE	2019	1	0	0	0	0	0	0	0	0	0	0	0	65
16179093	m	ot	ES	2019	0	1	0	0	1	0	0	0	0	0	0	0	68
16179731	f	md	IT	2019	0	0	0	0	0	0	0	0	0	0	0	0	
16179782	m	ot	US	2019	1	0	0	0	0	0	0	0	0	0	0	0	83
16179899	Not	cn	JP	2019	0	1	1	0	0	0	0	0	0	0	0	0	80
16179955	m	md	JP	2019	0	1	0	1	0	0	0	0	0	0	0	1	70
16179978	f	md	JP	2019	1	0	0	0	0	0	0	0	0	0	0	0	71.2
16180137	f	md	US	2019	1	0	0	0	0	0	0	0	0	0	0	0	85
16180312	f	cn	KW	2019	1	0	0	0	0	0	0	0	0	0	0	0	55.9
16180646	f	cn	US	2019	0	0	0	0	0	0	0	0	0	0	0	0	78
16180682	m	md	JP	2019	0	1	0	1	0	0	0	0	0	0	0	0	70
16181198	m	cn	DE	2019	0	0	0	0	0	0	0	0	0	0	0	0	58
16181204	m	cn	DE	2019	0	1	1	0	0	0	0	0	0	0	0	1	
16181211	Not	cn	JP	2019	0	0	0	0	0	0	0	0	0	0	0	0	69
16181646	f	cn	US	2019	1	0	0	0	0	0	0	0	0	0	0	0	
16181779	m	ph	BR	2019	0	0	0	0	0	0	0	0	0	0	0	0	83
16181879	m	cn	DE	2019	0	1	1	0	0	0	0	0	0	0	0	1	64
16181883	m	ot	JP	2019	0	1	0	1	0	0	0	0	0	0	0	1	70
16182555	f	cn	NL	2019	0	1	1	0	0	0	0	0	0	0	0	0	

16182568	m	md	JP	2019	0	1	0	0	0	1	0	0	0	0	0	1	79
16182938	m	md	JP	2019	0	1	0	1	0	0	0	0	0	0	0	1	62
16182951	f	cn	US	2019	0	1	1	0	0	0	0	0	0	0	0	0	65
16183124	f	md	KR	2019	1	0	0	0	0	0	0	0	0	0	0	0	46
16183387	m	cn	DE	2019	0	1	1	0	0	0	0	0	0	0	0	1	74
16183665	m	ph	AU	2019	0	1	0	0	0	1	0	0	0	0	0	1	
16183705	f	cn	GB	2019	1	0	0	0	0	0	0	0	0	0	0	0	
16183954	m	md	JP	2019	0	1	0	0	0	1	0	0	0	0	0	0	70
16184874	Not	cn	US	2019	0	0	0	0	0	0	0	0	0	0	0	0	
16185075	m	md	JP	2019	0	1	0	1	0	0	0	0	0	0	0	0	76
16186729	m	ot	FR	2019	0	1	0	0	1	0	0	0	0	0	0	0	51
16186754	m	md	BE	2019	0	1	1	0	0	0	0	0	0	0	0	0	
16186999	Not	md	JP	2019	0	1	0	0	1	0	0	0	0	0	0	0	
16187062	f	md	CA	2019	0	0	0	0	0	0	0	0	0	0	0	0	75
16187096	m	ot	KR	2019	0	1	0	0	1	0	0	0	0	0	0	0	54
16187137	m	md	FR	2019	0	0	0	0	0	0	0	0	0	0	0	0	69
16187341	f	md	JP	2019	0	0	0	0	0	0	0	0	0	0	0	0	79
16187757	f	Not repo	RU	2019	0	1	0	1	0	0	0	0	0	0	0	0	45
16187759	m	ph	JP	2019	1	0	0	0	0	0	0	0	0	0	0	0	68
16187784	m	md	JP	2019	0	0	0	0	0	0	0	0	0	0	0	0	61
16188093	Not	md	CN	2019	0	0	0	0	0	0	0	0	0	0	0	0	
16188164	m	cn	JP	2019	0	1	1	0	0	0	0	0	0	0	0	0	71
16188318	Not	md	BE	2019	0	1	0	0	0	1	0	0	0	0	0	0	49
16188671	m	cn	JP	2019	0	1	1	0	0	0	0	0	0	0	0	0	70
16188966	f	cn	US	2019	1	0	0	0	0	0	0	0	0	0	0	0	52
16189247	Not	md	CN	2019	0	0	0	0	0	0	0	0	0	0	0	0	
16189689	m	cn	JP	2019	0	1	1	0	0	0	0	0	0	0	0	0	66
16190237	m	md	JP	2019	0	1	0	1	0	0	0	0	0	0	0	0	62
16190541	m	md	FR	2019	0	0	0	0	0	0	0	0	0	0	0	0	69
16190619	Not	md	JP	2019	1	0	0	0	0	0	0	0	0	0	0	0	
16190742	f	cn	JP	2019	0	1	1	0	0	0	0	0	0	0	0	0	36
16190816	m	md	JP	2019	0	1	0	1	0	0	0	0	0	0	0	1	65
16191015	m	ot	NL	2019	0	0	0	0	0	0	0	0	0	0	0	0	58
16191175	m	cn	JP	2019	0	1	1	0	0	0	0	0	0	0	0	0	73

16191250	f	md	NL	2019	1	0	0	0	0	0	0	0	0	0	0	0	56
16191334	m	Not repo	RU	2019	0	1	0	1	0	0	0	0	0	0	0	0	28.3
16191361	m	ph	US	2019	0	1	1	0	0	0	0	0	0	0	0	0	63
16191364	m	md	FR	2019	0	0	0	0	0	0	0	0	0	0	0	0	69
16191597	f	cn	IN	2019	0	0	0	0	0	0	0	0	0	0	0	0	68
16192021	f	md	US	2019	1	0	0	0	0	0	0	0	0	0	0	0	77
16192051	m	ot	JP	2019	0	1	0	1	0	0	0	0	0	0	0	0	66
16192062	m	md	JP	2019	0	1	0	1	0	0	0	0	0	0	0	0	80
16192163	m	cn	IT	2019	1	0	0	0	0	0	0	0	0	0	0	0	62
16192170	f	md	JP	2019	0	1	0	1	0	0	0	0	0	0	0	0	74
16192210	m	md	US	2019	1	0	0	0	0	0	0	0	0	0	0	1	80
16192923	m	md	TN	2019	0	1	0	0	0	1	0	0	0	0	0	0	53
16192936	m	cn	US	2019	0	1	1	0	0	0	0	0	0	0	0	0	62
16192967	m	md	US	2019	0	0	0	0	0	0	0	0	0	0	0	0	70
16193111	m	md	FR	2019	0	0	0	0	0	0	0	0	0	0	0	0	69
16194856	f	ot	TW	2019	0	1	0	0	1	0	0	0	0	0	0	1	59
16194911	f	ot	FR	2019	0	1	0	0	1	0	0	0	0	0	0	1	58
16194930	m	md	BE	2019	0	1	0	0	1	0	0	0	0	0	0	0	
16194982	f	md	CN	2019	0	1	0	0	1	0	0	0	0	0	0	0	73
16195008	m	cn	JP	2019	0	1	1	0	0	0	0	0	0	0	0	0	80
16195048	m	ot	FR	2019	0	1	0	0	1	0	0	0	0	0	0	0	68
16195329	m	cn	JP	2019	0	1	1	0	0	0	0	0	0	0	0	0	64
16195398	m	cn	US	2019	0	1	0	0	1	0	0	0	0	0	0	0	
16195439	f	md	JP	2019	1	0	0	0	0	0	0	0	0	0	0	0	
16195681	m	cn	AU	2019	0	1	1	0	0	0	0	0	0	0	0	0	65
16195742	f	ot	JP	2019	0	0	0	0	0	0	0	0	0	0	0	0	73
16195881	m	md	DE	2019	0	0	0	0	0	0	0	0	0	0	0	0	58
16196123	f	cn	GB	2019	1	0	0	0	0	0	0	0	0	0	0	0	
16196196	f	md	CN	2019	0	1	0	0	1	0	0	0	0	0	0	0	75
16196201	m	md	FR	2019	0	1	0	0	1	0	0	0	0	0	0	0	
16197322	f	md	DE	2019	0	1	0	0	0	1	0	0	0	0	0	0	53
16197331	m	cn	JP	2019	0	0	0	0	0	0	0	0	0	0	0	0	60
16197357	m	md	JP	2019	0	1	0	0	0	1	0	0	0	0	0	1	69
16197358	f	md	JP	2019	0	0	0	0	0	0	0	0	0	0	0	0	75

16197562	m	cn	DE	2019	0	1	1	0	0	0	0	0	0	0	0	0	50
16198078	m	md	DE	2019	0	0	0	0	0	0	0	0	0	0	0	0	82
16198154	m	md	AU	2019	0	0	0	0	0	0	0	0	0	0	0	0	50
16198257	f	cn	JP	2019	0	1	1	0	0	0	0	0	0	0	0	0	80
16198272	Not	cn	KR	2019	0	0	0	0	0	0	0	0	0	0	0	0	
16198497	Not	md	TW	2019	0	0	0	0	0	0	0	0	0	0	0	0	
16198529	m	md	US	2019	0	1	0	1	0	0	0	0	0	0	0	1	
16198793	f	cn	FR	2019	0	1	1	0	0	0	0	0	0	0	0	0	85
16198818	m	cn	JP	2019	0	1	1	0	0	0	0	0	0	0	0	0	72
16198826	m	ot	JP	2019	0	1	1	0	0	0	0	0	0	0	0	0	70
16199157	f	md	IT	2019	1	0	0	0	0	0	0	0	0	0	0	0	
16199289	m	md	US	2019	0	1	0	1	0	0	0	0	0	0	0	0	
16199298	Not	md	US	2019	0	1	0	1	0	0	0	0	0	0	0	0	
16199331	m	cn	JP	2019	0	1	1	0	0	0	0	0	0	0	0	0	
16199425	m	ot	DE	2019	0	1	0	0	1	0	0	0	0	0	0	0	60
16199783	f	md	US	2019	0	1	1	0	0	0	0	0	0	0	0	0	
16199799	f	cn	CA	2019	0	0	0	0	0	0	0	0	0	0	0	0	75
16199872	m	ot	DE	2019	0	0	0	0	0	0	0	0	0	0	0	0	6
16199906	m	cn	AU	2019	0	1	1	0	0	0	0	0	0	0	0	0	68
16199981	Not	ot	US	2019	0	1	0	0	1	0	0	0	0	0	0	0	
16200724	m	md	JP	2019	0	1	0	0	0	1	0	0	0	0	0	0	80
16201184	m	cn	JP	2019	0	1	1	0	0	0	0	0	0	0	0	0	70
16201774	m	md	JP	2019	0	1	1	0	0	0	0	0	0	0	0	0	71
16201840	m	ph	US	2019	0	1	1	0	0	0	0	0	0	0	0	0	
16202019	m	cn	JP	2019	0	1	1	0	0	0	0	0	0	0	0	0	
16202369	m	md	JP	2019	0	1	0	1	0	0	0	0	0	0	0	1	50
16203234	m	md	JP	2019	0	1	0	1	0	0	0	0	0	0	0	1	71
16204172	m	cn	IN	2019	0	1	0	0	1	0	0	0	0	0	0	0	55
16204278	Not	md	US	2019	0	1	0	1	0	0	0	0	0	0	0	1	
16204321	Not	md	JP	2019	0	1	0	0	0	1	0	0	0	0	0	0	
16204322	m	md	JP	2019	0	0	0	0	0	0	0	0	0	0	0	0	
16204330	f	cn	GB	2019	1	0	0	0	0	0	0	0	0	0	0	0	51
16204357	m	md	JP	2019	1	0	0	0	0	0	0	0	0	0	0	0	83.4
16204366	m	md	JP	2019	0	1	0	1	0	0	0	0	0	0	0	1	78

16204692	f	cn	Not re	2019	0	0	0	0	0	0	0	0	0	0	0	0	
16205001	m	cn	KR	2019	0	0	0	0	0	0	0	0	0	0	0	0	41
16205277	m	md	JP	2019	0	0	0	0	0	0	0	0	0	0	0	0	80
16205396	m	md	CO	2019	1	0	0	0	0	0	0	0	0	0	0	0	
16205678	Not	md	US	2019	0	1	0	1	0	0	0	0	0	0	0	1	
16205818	f	ot	NZ	2019	0	0	0	0	0	0	0	0	0	0	0	0	42
16206103	m	md	US	2019	0	1	0	1	0	0	0	0	0	0	0	1	62
16206118	m	md	JP	2019	0	1	0	0	0	1	0	0	0	0	0	0	60
16206431	f	ot	US	2019	0	1	0	0	1	0	0	0	0	0	0	0	68
16206591	f	md	CR	2019	0	1	0	0	0	1	0	0	0	0	0	0	70
16206735	f	ot	US	2019	0	1	0	0	1	0	0	0	0	0	0	0	66
16206830	m	ot	FR	2019	0	1	0	0	1	0	0	0	0	0	0	0	58
16206918	f	cn	GB	2019	1	0	0	0	0	0	0	0	0	0	0	0	
16207130	m	md	FR	2019	0	1	0	1	0	0	0	0	0	0	0	0	55
16207236	m	cn	KR	2019	0	1	1	0	0	0	0	0	0	0	0	0	
16207314	m	md	JP	2019	0	1	1	0	0	0	0	0	0	0	0	0	
16207318	m	md	JP	2019	0	1	0	1	0	0	0	0	0	0	0	1	83
16207373	m	cn	JP	2019	0	1	1	0	0	0	0	0	0	0	0	0	61
16207546	m	md	JP	2019	0	1	0	1	0	0	0	0	0	0	0	1	65.2
16207852	m	cn	JP	2019	0	1	1	0	0	0	0	0	0	0	0	1	69
16207988	m	cn	JP	2019	0	1	1	0	0	0	0	0	0	0	0	0	
16208354	m	md	JP	2019	0	0	0	0	0	0	0	0	0	0	0	0	
16208507	m	ot	US	2019	0	0	0	0	0	0	0	0	0	0	0	0	
16208556	Not	md	JP	2019	0	0	0	0	0	0	0	0	0	0	0	0	
16208649	m	ot	NL	2019	0	1	1	0	0	0	0	0	0	0	0	1	74
16208852	m	Not repo	US	2019	1	0	0	0	0	0	0	0	0	0	0	0	
16209197	m	ot	JP	2019	0	1	0	0	1	0	0	0	0	0	0	0	60
16209281	m	cn	JP	2019	0	1	1	0	0	0	0	0	0	0	0	0	67
16209321	Not	md	US	2019	0	1	0	1	0	0	0	0	0	0	0	1	
16209377	m	cn	US	2019	0	0	0	0	0	0	0	0	0	0	0	0	75
16209424	m	cn	JP	2019	0	1	1	0	0	0	0	0	0	0	0	0	38
16209525	f	md	JP	2019	0	0	0	0	0	0	0	0	0	0	0	0	75
16209601	m	md	JP	2019	0	0	0	0	0	0	0	0	0	0	0	0	63
16209922	m	cn	IN	2019	0	0	0	0	0	0	0	0	0	0	0	0	

16210008	m	md	HR	2019	0	1	1	0	0	0	0	0	0	0	0	0	63
16210084	m	md	JP	2019	0	1	0	1	0	0	0	0	0	0	0	0	48
16210264	m	ot	NL	2019	0	0	0	0	0	0	0	0	0	0	0	0	58
16210721	m	md	JP	2019	0	1	0	0	0	1	0	0	0	0	0	0	72
16210755	m	md	FR	2019	0	1	0	0	0	1	0	0	0	0	0	0	64
16210761	m	md	PL	2019	0	1	0	0	0	1	0	0	0	0	0	0	65
16210981	Not	ot	JP	2019	0	0	0	0	0	0	0	0	0	0	0	0	
16211290	Not	ph	CA	2019	0	1	1	0	0	0	0	0	0	0	0	1	
16211619	Not	md	JP	2019	0	1	0	0	0	1	0	0	0	0	0	0	
16211675	m	md	JP	2019	0	1	0	0	0	1	0	0	0	0	0	0	77
16211762	Not	ph	CA	2019	0	1	1	0	0	0	0	0	0	0	0	1	
16211764	Not	ph	CA	2019	0	1	1	0	0	0	0	0	0	0	0	1	
16211878	m	md	KR	2019	0	1	0	0	0	1	0	0	0	0	0	0	62
16211927	m	md	FR	2019	0	0	0	0	0	0	1	0	0	1	0	1	80
16211963	Not	md	IT	2019	1	0	0	0	0	0	0	0	0	0	0	0	
16212127	Not	md	IT	2019	1	0	0	0	0	0	0	0	0	0	0	0	
16212751	m	cn	JP	2019	0	1	1	0	0	0	0	0	0	0	0	0	75
16213528	m	cn	GB	2019	1	0	0	0	0	0	0	0	0	0	0	0	67
16213593	f	ot	FR	2019	0	1	0	0	1	0	0	0	0	0	0	0	66
16213664	m	md	JP	2019	0	1	0	1	0	0	0	0	0	0	0	0	84
16213719	f	ot	NL	2019	0	0	0	0	0	0	0	0	0	0	0	0	78
16213836	f	cn	GB	2019	1	0	0	0	0	0	0	0	0	0	0	0	
16214177	m	md	US	2019	0	0	0	0	0	0	0	0	0	0	0	0	54
16214256	f	cn	IL	2019	0	0	0	0	0	0	0	0	0	0	0	0	70
16214348	f	md	SK	2019	0	0	0	0	0	0	0	0	0	0	0	0	76
16214472	f	ot	US	2019	0	1	0	0	1	0	0	0	0	0	0	0	73
16214659	m	cn	AR	2019	0	1	1	0	0	0	0	0	0	0	0	0	43
16215127	f	ot	DE	2019	0	1	0	0	1	0	0	0	0	0	0	0	68
16215182	f	cn	CN	2019	0	1	0	0	1	0	0	0	0	0	0	0	78
16215188	m	ot	FR	2019	0	1	0	0	1	0	0	0	0	0	0	0	65
16215404	m	md	JP	2019	0	1	0	0	1	0	0	0	0	0	0	0	63
16215622	f	cn	DE	2019	0	1	1	0	0	0	0	0	0	0	0	0	71
16216165	m	md	JP	2019	1	0	0	0	0	0	0	0	0	0	0	0	62
16216626	m	ot	NL	2019	0	0	0	0	0	0	0	0	0	0	0	0	75

16216858	m	ph	JP	2019	0	0	0	0	0	0	0	0	0	0	0	0	
16217213	m	ot	US	2019	0	1	0	0	1	0	0	0	0	0	0	0	69
16218202	f	ot	US	2019	0	1	0	0	1	0	0	0	0	0	0	0	58
16218225	f	cn	GB	2019	1	0	0	0	0	0	0	0	0	0	0	0	
16218471	Not	Not repo	IT	2019	1	0	0	0	0	0	0	0	0	0	0	0	
16218472	f	ph	US	2019	1	0	0	0	0	0	0	0	0	0	0	0	51
16218617	Not	md	JP	2019	0	1	0	1	0	0	0	0	0	0	0	0	
16218943	m	md	JP	2019	0	1	0	1	0	0	0	0	0	0	0	0	57
16218981	f	ot	NL	2019	0	1	1	0	0	0	0	0	0	0	0	0	
16218982	f	ot	FR	2019	0	1	0	0	1	0	0	0	0	0	0	0	57
16219007	m	ot	IT	2019	0	1	0	0	1	0	0	0	0	0	0	0	67
16219011	f	md	FR	2019	1	0	0	0	0	0	0	0	0	0	0	0	
16220055	m	md	JP	2019	0	1	1	0	0	0	0	0	0	0	0	1	68
16220060	m	cn	JP	2019	0	1	1	0	0	0	0	0	0	0	0	0	75
16220197	f	ot	NL	2019	0	1	1	0	0	0	0	0	0	0	0	0	65
16220204	f	ot	DE	2019	0	1	1	0	0	0	0	0	0	0	0	0	60
16220887	m	ot	DE	2019	0	1	1	0	0	0	0	0	0	0	0	0	71
16220982	f	ot	CA	2019	0	1	0	0	1	0	0	0	0	0	0	0	59
16221563	f	ot	FR	2019	0	1	0	0	1	0	0	0	0	0	0	0	53
16221632	m	cn	IL	2019	0	1	1	0	0	0	0	0	0	0	0	0	
16221649	Not	cn	NL	2019	0	1	1	0	0	0	0	0	0	0	0	0	
16221697	m	cn	US	2019	0	1	0	0	1	0	0	0	0	0	0	0	57
16222052	m	ph	JP	2019	0	1	0	0	1	0	0	0	0	0	0	0	80
16222431	Not	ot	CN	2019	0	0	0	0	0	0	1	1	0	0	0	0	
16222491	f	ot	US	2019	1	0	0	0	0	0	0	0	0	0	0	0	65
16222529	m	ot	FR	2019	0	1	0	0	1	0	0	0	0	0	0	0	71
16222788	m	cn	US	2019	1	0	0	0	0	0	0	0	0	0	0	0	
16223164	m	cn	JP	2019	0	1	1	0	0	0	0	0	0	0	0	0	76
16223338	m	md	US	2019	1	0	0	0	0	0	0	0	0	0	0	1	80
16223396	m	cn	JP	2019	0	1	1	0	0	0	0	0	0	0	0	0	55
16223415	f	cn	JP	2019	0	1	1	0	0	0	0	0	0	0	0	0	67
16223596	f	ot	TW	2019	1	0	0	0	0	0	0	0	0	0	0	0	56
16223883	m	md	ES	2019	0	0	0	0	0	0	0	0	0	0	0	0	70
16224371	m	ot	US	2019	0	1	0	0	1	0	0	0	0	0	0	0	

16224814	f	md	JP	2019	1	0	0	0	0	0	0	0	0	0	0	0	75
16225437	m	cn	JP	2019	0	1	1	0	0	0	0	0	0	0	0	0	60
16225480	f	cn	KR	2019	0	0	0	0	0	0	0	0	0	0	0	0	
16225485	f	cn	DE	2019	0	1	1	0	0	0	0	0	0	0	0	0	60
16225554	m	ph	JP	2019	0	1	1	0	0	0	0	0	0	0	0	0	74
16225625	f	cn	US	2019	0	1	1	0	0	0	0	0	0	0	0	0	
16225638	m	cn	GR	2019	0	1	1	0	0	0	0	0	0	0	0	1	65
16225696	m	ot	IT	2019	0	0	0	0	0	0	0	0	0	0	0	0	
16225878	Not	md	JP	2019	0	1	0	0	1	0	0	0	0	0	0	0	
16226213	f	ot	JP	2019	0	1	0	0	1	0	0	0	0	0	0	0	62
16226294	m	md	US	2019	1	0	0	0	0	0	0	0	0	0	0	0	
16227279	f	ot	FR	2019	0	1	0	0	1	0	0	0	0	0	0	0	62
16227610	m	cn	JP	2019	0	1	1	0	0	0	0	0	0	0	0	1	68
16227614	m	md	JP	2019	1	0	0	0	0	0	0	0	0	0	0	0	83
16227657	m	md	FR	2019	0	1	0	0	0	1	0	0	0	0	0	1	66
16227781	m	ot	DE	2019	0	0	0	0	0	0	0	0	0	0	0	0	68
16227819	m	md	JP	2019	0	1	0	1	0	0	0	0	0	0	0	0	49.4
16228178	m	md	PL	2019	1	0	0	0	0	0	0	0	0	0	0	0	67.2
16228317	m	md	US	2019	0	1	1	0	0	0	0	0	0	0	0	0	68
16228365	m	md	AU	2019	0	0	0	0	0	0	0	0	0	0	0	0	
16229499	m	md	JP	2019	0	1	0	1	0	0	0	0	0	0	0	1	69.2
16229571	f	md	JP	2019	1	0	0	0	0	0	0	0	0	0	0	1	79
16229724	m	md	JP	2019	0	1	0	0	1	0	0	0	0	0	0	0	60
16229759	f	cn	NL	2019	0	1	1	0	0	0	0	0	0	0	0	0	65
16229807	m	cn	JP	2019	0	1	1	0	0	0	0	0	0	0	0	0	74
16229829	m	md	JP	2019	0	1	0	1	0	0	0	0	0	0	0	1	75
16230286	f	md	JP	2019	1	0	0	0	0	0	0	0	0	0	0	0	76
16230393	f	md	JP	2019	1	0	0	0	0	0	0	0	0	0	0	0	73.1
16230398	m	ot	JP	2019	0	1	0	1	0	0	0	0	0	0	0	1	76
16230587	m	md	JP	2019	0	0	0	0	0	0	0	0	0	0	0	0	80
16230638	m	md	GR	2019	0	0	0	0	0	0	0	0	0	0	0	0	56
16230676	m	cn	JP	2019	0	1	1	0	0	0	0	0	0	0	0	0	84
16230704	m	cn	JP	2019	0	1	1	0	0	0	0	0	0	0	0	0	77
16230734	m	ot	BE	2019	0	1	1	0	0	0	0	0	0	0	0	0	70

16230762	f	md	US	2019	0	0	0	0	0	0	0	0	0	0	0	0	80
16230848	f	ot	TW	2019	0	1	0	0	1	0	0	0	0	0	0	0	59
16230979	m	md	US	2019	0	0	0	0	0	0	0	0	0	0	0	0	38
16231376	m	cn	JP	2019	0	1	1	0	0	0	0	0	0	0	0	1	81
16231637	Not	ot	US	2019	0	1	0	0	1	0	0	0	0	0	0	0	
16231863	f	md	JP	2019	1	0	0	0	0	0	0	0	0	0	0	0	76
16232157	f	md	US	2019	1	0	0	0	0	0	0	0	0	0	0	0	
16232211	f	cn	KR	2019	0	1	1	0	0	0	0	0	0	0	0	0	
16232430	m	md	JP	2019	0	1	0	0	0	1	0	0	0	0	0	0	70
16232646	f	ot	CA	2019	0	1	1	0	0	0	0	0	0	0	0	1	63
16232770	f	ot	CA	2019	0	1	1	0	0	0	0	0	0	0	0	0	60
16233396	Not	md	IT	2019	1	0	0	0	0	0	0	0	0	0	0	0	
16233590	m	md	JP	2019	1	0	0	0	0	0	0	0	0	0	0	0	67
16234008	m	md	JP	2019	0	1	0	1	0	0	0	0	0	0	0	1	47
16234310	m	ot	FR	2019	0	1	0	0	1	0	0	0	0	0	0	0	43
16234315	m	md	FR	2019	0	0	0	0	0	0	0	0	0	0	0	0	74
16234766	m	md	US	2019	0	1	0	1	0	0	0	0	0	0	0	0	
16234954	m	cn	JP	2019	0	1	1	0	0	0	0	0	0	0	0	1	77
16235073	f	ot	CA	2019	0	1	1	0	0	0	0	0	0	0	0	1	63
16235153	f	cn	CN	2019	1	0	0	0	0	0	0	0	0	0	0	0	
16235259	f	cn	US	2019	0	0	0	0	0	0	0	0	0	0	0	0	72
16235522	f	cn	US	2019	0	1	1	0	0	0	0	0	0	0	0	0	
16235679	m	cn	US	2019	0	1	0	0	1	0	0	0	0	0	0	0	
16235777	f	ot	DE	2019	0	1	1	0	0	0	0	0	0	0	0	0	48
16235928	m	cn	JP	2019	0	1	1	0	0	0	0	0	0	0	0	0	60
16236115	m	md	AT	2019	0	1	0	0	1	0	0	0	0	0	0	0	80
16236382	f	md	JP	2019	0	1	1	0	0	0	0	0	0	0	0	0	52
16236421	m	cn	JP	2019	0	1	1	0	0	0	0	0	0	0	0	0	60
16236443	m	md	FR	2019	0	0	0	0	0	0	0	0	0	0	0	0	43
16237346	m	md	JP	2019	0	1	0	0	1	0	0	0	0	0	0	0	75
16237372	f	cn	Not re	2019	0	0	0	0	0	0	0	0	0	0	0	0	
16237756	f	ot	US	2019	0	1	1	0	0	0	0	0	0	0	0	0	
16237766	Not	md	JP	2019	1	0	0	0	0	0	0	0	0	0	0	0	
16237768	m	md	US	2019	1	0	0	0	0	0	0	0	0	0	0	1	80

16237852	f	cn	JP	2019	0	1	1	0	0	0	0	0	0	0	0	0	80
16238127	m	md	JP	2019	0	1	1	0	0	0	0	0	0	0	0	0	
16238516	m	md	FR	2019	1	0	0	0	0	0	0	0	0	0	0	0	65
16238519	Not	md	JP	2019	0	1	0	1	0	0	0	0	0	0	0	0	
16238529	f	cn	NL	2019	0	1	1	0	0	0	0	0	0	0	0	0	68
16238699	m	md	PL	2019	0	0	0	0	0	0	0	0	0	0	0	0	68
16238810	m	ot	US	2019	1	0	0	0	0	0	0	0	0	0	0	0	80
16239256	Not	cn	CA	2019	0	0	0	0	0	0	0	0	0	0	0	0	
16239258	Not	cn	CA	2019	0	0	0	0	0	0	0	0	0	0	0	0	
16239380	m	cn	US	2019	0	1	1	0	0	0	0	0	0	0	0	0	73
16239528	f	md	JP	2019	0	0	0	0	0	0	0	0	0	0	0	0	75
16239585	m	ot	KR	2019	0	0	0	0	0	0	0	0	0	0	0	0	71
16239908	m	md	IN	2019	0	0	0	0	0	0	0	0	0	0	0	0	61
16240233	m	cn	CN	2019	1	0	0	0	0	0	0	0	0	0	0	0	
16240353	f	ot	CZ	2019	0	0	0	0	0	0	0	0	0	0	0	0	47
16240504	f	ot	NL	2019	0	1	1	0	0	0	0	0	0	0	0	0	60
16240507	f	ot	NL	2019	0	1	1	0	0	0	0	0	0	0	0	0	
16240721	f	md	JP	2019	0	1	1	0	0	0	0	0	0	0	0	0	77
16241361	Not	cn	NL	2019	0	1	1	0	0	0	0	0	0	0	0	0	
16241549	f	md	US	2019	0	0	0	0	0	0	0	0	0	0	0	0	85
16241550	f	ot	US	2019	1	0	0	0	0	0	0	0	0	0	0	0	69
16241612	m	md	MA	2019	0	1	0	0	0	1	0	0	0	0	0	0	
16241845	m	md	JP	2019	0	1	0	1	0	0	0	0	0	0	0	1	65
16241860	m	md	US	2019	0	1	0	0	0	1	0	0	0	0	0	0	60
16242000	m	cn	JP	2019	0	1	1	0	0	0	0	0	0	0	0	0	59
16242138	m	cn	JP	2019	0	0	0	0	0	0	0	0	0	0	0	0	69
16242243	m	md	JP	2019	0	1	0	0	0	1	0	0	0	0	0	0	72
16242713	m	cn	BG	2019	0	1	1	0	0	0	0	0	0	0	0	0	74
16242795	m	md	US	2019	1	0	0	0	0	0	0	0	0	0	0	1	80
16242918	m	md	CR	2019	0	0	0	0	0	0	0	0	0	0	0	0	37
16242967	m	md	US	2019	0	1	0	0	0	1	0	0	0	0	0	0	65
16243236	f	ph	JP	2019	1	0	0	0	0	0	0	0	0	0	0	0	77
16243422	m	md	JP	2019	0	1	0	1	0	0	0	0	0	0	0	1	76
16243948	f	ot	US	2019	0	1	0	0	1	0	0	0	0	0	0	0	

16244120	f	ot	DE	2019	0	0	0	0	0	0	0	0	0	0	0	0	58
16244153	m	md	JP	2019	1	0	0	0	0	0	0	0	0	0	0	0	85.1
16244393	m	md	CZ	2019	0	0	0	0	0	0	0	0	0	0	0	0	68
16244562	f	md	US	2019	0	1	0	0	0	1	0	0	0	0	0	0	66
16244792	f	md	US	2019	0	1	0	0	1	0	0	0	0	0	0	0	71
16244851	f	cn	IN	2019	0	1	0	0	1	0	0	0	0	0	0	0	34
16244967	f	cn	US	2019	1	0	0	0	0	0	0	0	0	0	0	0	55
16244973	m	md	JP	2019	0	1	0	1	0	0	0	0	0	0	0	1	66
16244975	m	md	JP	2019	0	1	0	1	0	0	0	0	0	0	0	0	65
16245050	m	md	US	2019	0	1	0	1	0	0	0	0	0	0	0	0	78
16245129	Not	md	JP	2019	1	0	0	0	0	0	0	0	0	0	0	0	
16245252	m	md	JP	2019	0	1	1	0	0	0	0	0	0	0	0	0	70
16245272	m	cn	JP	2019	0	1	1	0	0	0	0	0	0	0	0	0	70
16245410	Not	md	JP	2019	1	0	0	0	0	0	0	0	0	0	0	0	66
16245452	m	md	JP	2019	0	1	1	0	0	0	0	0	0	0	0	0	76
16245758	f	md	JP	2019	1	0	0	0	0	0	0	0	0	0	0	0	86
16245791	m	cn	JP	2019	0	1	1	0	0	0	0	0	0	0	0	0	65
16245882	m	cn	JP	2019	0	1	1	0	0	0	0	0	0	0	0	0	84
16245955	m	ot	US	2019	0	1	0	0	1	0	0	0	0	0	0	0	43
16245971	m	cn	JP	2019	0	1	1	0	0	0	0	0	0	0	0	0	76
16245972	f	cn	US	2019	0	1	1	0	0	0	0	0	0	0	0	0	60
16245974	f	ot	US	2019	0	1	0	0	1	0	0	0	0	0	0	0	60
16246189	m	md	JP	2019	1	0	0	0	0	0	0	0	0	0	0	1	84.8
16246808	m	cn	RU	2019	0	1	0	0	0	1	0	0	0	0	0	0	65
16246816	f	ot	PL	2019	0	0	0	0	0	0	0	0	0	0	0	0	75
16246865	Not	ot	JP	2019	0	0	0	0	0	0	0	0	0	0	0	0	
16247077	m	md	JP	2019	0	0	0	0	0	0	0	0	0	0	0	0	62
16247150	f	cn	US	2019	0	1	1	0	0	0	0	0	0	0	0	0	
16248292	m	ot	JP	2019	0	1	0	0	1	0	0	0	0	0	0	0	66
16248486	m	ot	JP	2019	0	1	0	0	1	0	0	0	0	0	0	0	73
16248775	m	md	HK	2019	0	1	0	0	0	1	0	0	0	0	0	0	54
16248929	m	md	DE	2019	0	0	0	0	0	0	0	0	0	0	0	0	69
16249064	f	ot	DE	2019	0	1	0	0	1	0	0	0	0	0	0	0	58
16249210	f	ph	JP	2019	0	1	0	0	0	1	0	0	0	0	0	0	70

16249283	Not	cn	CN	2019	0	1	0	0	1	0	0	0	0	0	0	0	
16249317	m	md	DE	2019	0	0	0	0	0	0	0	0	0	0	0	0	75
16249370	m	ph	FR	2019	0	0	0	0	0	0	0	0	0	0	0	0	71
16249527	m	md	HK	2019	0	1	0	0	0	1	0	0	0	0	0	0	55
16249938	f	ot	US	2019	0	1	0	0	1	0	0	0	0	0	0	0	
16250907	Not	md	JP	2019	1	0	0	0	0	0	0	0	0	0	0	0	88
16251308	m	cn	JP	2019	0	1	1	0	0	0	0	0	0	0	0	0	79
16251498	f	cn	US	2019	0	0	0	0	0	0	0	0	0	0	0	0	66
16251592	f	md	FR	2019	0	0	0	0	0	0	0	0	0	0	0	0	54
16251754	m	ot	JP	2019	0	1	1	0	0	0	0	0	0	0	0	0	41
16251785	m	cn	JP	2019	0	1	1	0	0	0	0	0	0	0	0	0	74
16251839	Not	md	US	2019	0	1	0	1	0	0	0	0	0	0	0	0	
16251847	Not	cn	US	2019	0	1	1	0	0	0	0	0	0	0	0	0	
16251886	f	md	JP	2019	1	0	0	0	0	0	0	0	0	0	0	0	69
16252271	f	md	IT	2019	1	0	0	0	0	0	0	0	0	0	0	0	
16252352	m	ot	US	2019	0	0	0	0	0	0	0	0	0	0	0	0	23
16252408	m	md	IE	2019	0	1	0	0	1	0	0	0	0	0	0	0	
16252646	m	md	JP	2019	0	1	0	0	1	0	0	0	0	0	0	0	76
16252647	m	md	JP	2019	0	1	0	0	1	0	0	0	0	0	0	0	69
16252764	m	cn	JP	2019	0	1	1	0	0	0	0	0	0	0	0	1	59
16252795	m	ot	JP	2019	0	1	0	0	1	0	0	0	0	0	0	0	79
16252798	Not	md	DK	2019	0	0	0	0	0	0	0	0	0	0	0	0	
16252841	m	md	BG	2019	0	1	1	0	0	0	0	0	0	0	0	0	57
16252924	m	cn	JP	2019	0	1	1	0	0	0	0	0	0	0	0	0	74
16252956	m	md	US	2019	0	1	1	0	0	0	0	0	0	0	0	0	
16253755	m	md	JP	2019	0	1	0	0	1	0	0	0	0	0	0	0	82
16253765	m	md	DE	2019	0	1	0	0	1	0	0	0	0	0	0	0	72
16253906	m	md	FR	2019	0	1	0	0	1	0	0	0	0	0	0	0	86
16254007	m	cn	US	2019	0	1	0	0	1	0	0	0	0	0	0	0	
16254099	m	cn	JP	2019	0	1	1	0	0	0	0	0	0	0	0	1	61
16254254	Not	md	IT	2019	0	0	0	0	0	0	0	0	0	0	0	0	
16254536	m	ot	NL	2019	0	0	0	0	0	0	0	0	0	0	0	0	75
16254584	f	cn	US	2019	0	1	1	0	0	0	0	0	0	0	0	0	54
16254627	m	md	US	2019	1	0	0	0	0	0	0	0	0	0	0	0	42

16254733	m	md	KR	2019	1	0	0	0	0	0	0	0	0	0	0	0	79
16255148	m	ot	ES	2019	0	1	0	0	1	0	0	0	0	0	0	0	61
16255344	m	md	JP	2019	1	0	0	0	0	0	0	0	0	0	0	0	75.3
16255438	f	md	US	2019	1	0	0	0	0	0	0	0	0	0	0	0	65
16255725	f	md	JP	2019	1	0	0	0	0	0	0	0	0	0	0	0	79.5
16256173	m	cn	JP	2019	0	1	1	0	0	0	0	0	0	0	0	0	73
16256200	m	md	JP	2019	0	1	0	1	0	0	0	0	0	0	0	1	79
16256372	f	md	US	2019	0	0	0	0	0	0	0	0	0	0	0	0	73
16256661	m	cn	JP	2019	0	1	1	0	0	0	0	0	0	0	0	0	66
16256729	m	md	US	2019	0	0	0	0	0	0	0	0	0	0	0	0	
16256941	m	md	DE	2019	0	0	0	0	0	0	0	0	0	0	0	0	63
16257052	Not	md	DE	2019	0	0	0	0	0	0	0	0	0	0	0	0	64
16257086	f	cn	US	2019	0	1	1	0	0	0	0	0	0	0	0	0	75
16257149	m	md	CH	2019	0	1	0	0	0	1	0	0	0	0	0	0	73
16257383	m	md	JP	2019	0	1	0	1	0	0	0	0	0	0	0	1	70
16257571	f	cn	NL	2019	0	1	1	0	0	0	0	0	0	0	0	0	
16257779	f	md	CH	2019	0	1	1	0	0	0	0	0	0	0	0	0	66
16257803	m	md	CH	2019	0	0	0	0	0	0	0	0	0	0	0	0	61
16257974	m	ot	US	2019	0	1	0	0	1	0	0	0	0	0	0	0	76
16258052	Not	md	PL	2019	0	0	0	0	0	0	0	0	0	0	0	0	
16258056	m	ph	JP	2019	0	1	0	1	0	0	0	0	0	0	0	1	69.7
16258198	m	cn	JP	2019	0	1	1	0	0	0	0	0	0	0	0	0	45
16258456	Not	ot	JP	2019	0	0	0	0	0	0	0	0	0	0	0	0	
16258588	f	ot	CA	2019	0	1	0	0	1	0	0	0	0	0	0	0	49
16258912	f	md	JP	2019	0	1	0	0	0	1	0	0	0	0	0	0	47
16259096	Not	ot	US	2019	0	1	0	0	1	0	0	0	0	0	0	0	
16259198	m	ot	FR	2019	0	1	0	0	1	0	0	0	0	0	0	0	64
16259372	m	md	BE	2019	0	0	0	0	0	0	0	0	0	0	0	0	42
16260383	f	ot	DE	2019	0	0	0	0	0	0	0	0	0	0	0	0	81
16260387	m	ot	DE	2019	0	0	0	0	0	0	0	0	0	0	0	0	64
16260559	m	md	JP	2019	1	0	0	0	0	0	0	0	0	0	0	1	80
16262317	f	ot	BE	2019	0	1	0	0	1	0	0	0	0	0	0	0	76
16262596	f	md	KR	2019	0	0	0	0	0	0	0	0	0	0	0	0	74
16262621	f	md	AT	2019	0	0	0	0	0	0	0	0	0	0	0	0	62

16263015	m	ot	US	2019	0	1	0	0	1	0	0	0	0	0	0	0	87
16263212	m	md	JP	2019	0	1	0	0	0	1	0	0	0	0	0	0	58
16263229	f	md	DE	2019	0	0	0	0	0	0	0	0	0	0	0	0	60
16263258	m	cn	JP	2019	0	1	1	0	0	0	0	0	0	0	0	0	62
16264114	f	ph	US	2019	1	0	0	0	0	0	0	0	0	0	0	0	
16264280	m	cn	GR	2019	0	0	0	0	0	0	0	0	0	0	0	0	56
16264361	f	md	JP	2019	1	0	0	0	0	0	0	0	0	0	0	1	82
16264712	m	md	JP	2019	0	1	0	1	0	0	0	0	0	0	0	1	75
16264876	m	cn	CA	2019	0	1	0	0	0	1	0	0	0	0	0	0	73
16265591	f	md	JP	2019	0	1	0	1	0	0	0	0	0	0	0	1	76
16265723	m	cn	JP	2019	0	1	1	0	0	0	0	0	0	0	0	0	80
16265754	Not	md	JP	2019	0	1	0	1	0	0	0	0	0	0	0	0	
16265755	f	md	JP	2019	1	0	0	0	0	0	0	0	0	0	0	0	73
16265899	m	md	JP	2019	1	0	0	0	0	0	0	0	0	0	0	0	47
16266332	m	ot	CH	2019	1	0	0	0	0	0	0	0	0	0	0	0	73.3
16266850	m	md	KR	2019	0	0	0	0	0	0	0	0	0	0	0	0	59
16267077	m	ot	US	2019	0	1	0	0	1	0	0	0	0	0	0	0	60
16267157	m	cn	IL	2019	0	0	0	0	0	0	0	0	0	0	0	0	55
16267170	m	ot	PL	2019	0	1	0	0	1	0	0	0	0	0	0	0	46
16267263	m	md	BR	2019	0	0	0	0	0	0	0	0	0	0	0	0	64
16267726	f	md	JP	2019	0	1	0	1	0	0	0	0	0	0	0	1	60.5
16267774	m	md	US	2019	0	1	0	1	0	0	0	0	0	0	0	1	
16268329	m	md	JP	2019	0	1	0	0	0	1	0	0	0	0	0	0	66
16268352	m	cn	MY	2019	0	1	1	0	0	0	0	0	0	0	0	0	67
16268924	f	cn	JP	2019	0	1	1	0	0	0	0	0	0	0	0	1	67
16269164	Not	ot	US	2019	0	1	0	0	1	0	0	0	0	0	0	0	
16269444	m	ph	FR	2019	0	1	1	0	0	0	0	0	0	0	0	0	63
16269486	f	md	JP	2019	0	1	0	1	0	0	0	0	0	0	0	1	79
16269895	m	md	CN	2019	0	1	0	0	0	1	0	0	0	0	0	0	65
16270674	m	ot	ES	2019	0	1	0	0	1	0	0	0	0	0	0	0	59
16271208	m	md	ID	2019	1	0	0	0	0	0	0	0	0	0	0	1	
16271238	m	ot	KR	2019	0	0	0	0	0	0	0	0	0	0	0	0	68
16271422	m	md	JP	2019	0	1	0	1	0	0	0	0	0	0	0	0	64
16271490	f	md	JP	2019	0	1	0	1	0	0	0	0	0	0	0	1	83

16271591	f	md	SK	2019	1	0	0	0	0	0	0	0	0	0	0	0	76
16271634	f	md	JP	2019	1	0	0	0	0	0	0	0	0	0	0	0	76
16272054	f	cn	US	2019	0	1	1	0	0	0	0	0	0	0	0	1	84
16272123	Not	md	TT	2019	0	1	0	0	0	1	0	0	0	0	0	0	
16272142	m	md	JP	2019	1	0	0	0	0	0	0	0	0	0	0	0	75
16272149	m	md	JP	2019	0	1	0	1	0	0	0	0	0	0	0	1	78
16272417	m	md	NL	2019	0	1	0	1	0	0	0	0	0	0	0	1	72.3
16272453	f	ot	FR	2019	0	1	0	0	1	0	0	0	0	0	0	0	64
16272551	m	md	JP	2019	0	1	0	0	0	1	0	0	0	0	0	0	70
16273254	m	md	FR	2019	0	1	0	0	1	0	0	0	0	0	0	0	51
16273660	m	md	ES	2019	0	1	0	0	0	1	0	0	0	0	0	1	84
16273984	m	cn	US	2019	1	0	0	0	0	0	0	0	0	0	0	0	68
16274183	m	md	DE	2019	0	1	0	0	0	1	0	0	0	0	0	0	69
16274245	m	ot	DE	2019	0	1	1	0	0	0	0	0	0	0	0	1	65
16274334	m	cn	US	2019	0	1	0	0	1	0	0	0	0	0	0	0	
16275127	f	md	IN	2019	0	0	0	0	0	0	0	0	0	0	0	0	50
16275306	m	ot	FR	2019	0	0	0	0	0	0	0	0	0	0	0	0	58
16275903	f	cn	Not re	2019	0	0	0	0	0	0	0	0	0	0	0	0	
16276326	f	cn	CA	2019	0	0	0	0	0	0	0	0	0	0	0	0	57
16276331	f	cn	CA	2019	0	0	0	0	0	0	0	0	0	0	0	0	75
16276475	f	cn	US	2019	0	1	1	0	0	0	0	0	0	0	0	1	46
16276714	f	md	NL	2019	0	0	0	0	0	0	0	0	0	0	0	0	71
16277010	m	ot	NL	2019	0	0	0	0	0	0	0	0	0	0	0	0	70
16277525	f	ot	FR	2019	0	1	0	0	1	0	0	0	0	0	0	0	63
16277954	f	md	IT	2019	1	0	0	0	0	0	0	0	0	0	0	0	
16278015	f	md	DE	2019	0	0	0	0	0	0	0	0	0	0	0	0	58
16278416	f	md	GB	2019	0	0	0	0	0	0	0	0	0	0	0	0	
16279106	f	ot	PR	2019	0	0	0	0	0	0	0	0	0	0	0	0	
16279480	m	md	JP	2019	0	1	1	0	0	0	0	0	0	0	0	0	40
16279730	f	md	US	2019	0	1	0	1	0	0	0	0	0	0	0	0	69
16279943	m	cn	IL	2019	0	1	1	0	0	0	0	0	0	0	0	0	62
16280053	m	md	JP	2019	0	1	0	0	0	1	0	0	0	0	0	0	71
16280697	m	md	DE	2019	0	1	1	0	0	0	0	0	0	0	0	0	
16280855	m	md	VN	2019	0	1	0	0	0	1	0	0	0	0	0	0	59

16280947	m	ot	US	2019	0	1	1	0	0	0	0	0	0	0	0	0	52
16281078	m	md	IT	2019	0	1	0	0	0	1	0	0	0	0	0	0	83
16281079	m	md	IT	2019	0	1	0	0	0	1	0	0	0	0	0	0	60
16282101	Not	ph	JP	2019	0	0	0	0	0	0	0	0	0	0	0	0	65.5
16283465	f	cn	CA	2019	0	0	0	0	0	0	0	0	0	0	0	0	45
16284084	Not	ot	US	2019	0	1	0	0	1	0	0	0	0	0	0	0	
16284295	m	ot	US	2019	0	1	0	0	1	0	0	0	0	0	0	0	
16284447	f	cn	US	2019	1	0	0	0	0	0	0	0	0	0	0	0	57
16285901	f	md	DE	2019	0	1	0	0	0	1	0	0	0	0	0	0	68
16285962	Not	ph	US	2019	1	0	0	0	0	0	0	0	0	0	0	0	
16286441	m	cn	DE	2019	0	1	1	0	0	0	0	0	0	0	0	0	74
16286650	f	cn	CN	2019	1	0	0	0	0	0	0	0	0	0	0	0	63.9
16286816	f	md	ID	2019	1	0	0	0	0	0	0	0	0	0	0	0	
16286910	Not	md	IT	2019	0	1	0	1	0	0	0	0	0	0	0	0	
16287113	m	md	JP	2019	0	1	0	0	0	1	0	0	0	0	0	0	74
16287209	f	cn	IL	2019	0	1	1	0	0	0	0	0	0	0	0	0	82
16287482	m	cn	US	2019	0	1	0	0	1	0	0	0	0	0	0	0	
16287490	m	cn	US	2019	0	1	0	0	1	0	0	0	0	0	0	0	74
16287638	m	cn	CN	2019	1	0	0	0	0	0	0	0	0	0	0	0	77.5
16287715	m	cn	CN	2019	0	1	0	0	1	0	0	0	0	0	0	0	50
16287912	f	cn	IT	2019	0	1	1	0	0	0	0	0	0	0	0	0	79
16288261	m	ot	AR	2019	0	1	0	0	1	0	0	0	0	0	0	0	70
16288457	f	md	SE	2019	0	0	0	0	0	0	0	0	0	0	0	0	74
16288569	f	cn	TH	2019	0	1	0	0	1	0	0	0	0	0	0	0	63
16288995	f	md	US	2019	0	1	0	0	0	1	0	0	0	0	0	0	63
16288996	m	cn	KR	2019	0	1	1	0	0	0	0	0	0	0	0	0	
16289043	m	md	CN	2019	0	1	0	0	1	0	0	0	0	0	0	0	
16289218	m	cn	CA	2019	0	1	0	0	1	0	0	0	0	0	0	0	
16289497	f	cn	KR	2019	1	0	0	0	0	0	0	0	0	0	0	0	68
16289541	m	ph	US	2019	0	0	0	0	0	0	0	0	0	0	0	0	73
16289639	Not	md	DE	2019	1	0	0	0	0	0	0	0	0	0	0	0	
16289800	f	ot	FR	2019	0	1	0	0	1	0	0	0	0	0	0	0	67
16289985	m	md	JP	2019	0	1	0	0	0	1	0	0	0	0	0	0	52
16290384	m	md	TW	2019	0	1	0	1	0	0	0	0	0	0	0	0	58

16290470	m	md	US	2019	1	0	0	0	0	0	0	0	0	0	0	0	
16290481	m	md	BE	2019	0	1	1	0	0	0	0	0	0	0	0	0	70
16290507	f	md	JP	2019	0	0	0	0	0	0	0	0	0	0	0	0	71
16290707	m	md	DE	2019	0	0	0	0	0	0	0	0	0	0	0	0	82
16291651	m	cn	US	2019	0	1	1	0	0	0	0	0	0	0	0	1	49
16291805	m	md	BR	2019	0	1	0	0	0	1	0	0	0	0	0	0	64
16292317	m	cn	JP	2019	0	1	1	0	0	0	0	0	0	0	0	0	66
16293680	m	cn	US	2019	0	1	1	0	0	0	0	0	0	0	0	0	70
16294467	m	ot	ES	2019	0	1	0	0	1	0	0	0	0	0	0	0	66
16294819	f	ot	US	2019	0	1	0	0	1	0	0	0	0	0	0	0	60
16297127	f	md	TW	2019	0	1	0	1	0	0	0	0	0	0	0	0	51
16297475	f	ph	US	2019	0	0	0	0	0	0	0	0	0	0	0	0	63
16297542	f	md	HK	2019	0	1	0	0	0	1	0	0	0	0	0	0	70
16297721	f	ot	US	2019	0	1	0	0	1	0	0	0	0	0	0	0	72
16297733	Not	ot	US	2019	0	1	0	0	1	0	0	0	0	0	0	0	
16298215	m	ot	US	2019	0	1	0	0	1	0	0	0	0	0	0	0	60
16298258	f	md	JP	2019	0	1	0	0	1	0	0	0	0	0	0	0	80
16298269	m	md	FR	2019	0	0	0	0	0	0	0	0	0	0	0	0	69
16298321	Not	md	US	2019	1	0	0	0	0	0	0	0	0	0	0	0	
16298415	f	md	GB	2019	1	0	0	0	0	0	0	0	0	0	0	0	79.2
16298425	f	cn	US	2019	1	0	0	0	0	0	0	0	0	0	0	0	60
16298876	f	cn	KR	2019	0	1	1	0	0	0	0	0	0	0	0	0	
16299431	m	cn	CN	2019	1	0	0	0	0	0	0	0	0	0	0	0	53
16299508	f	cn	CA	2019	0	1	0	1	0	0	0	0	0	0	0	1	56
16299759	m	ot	CA	2019	0	1	1	0	0	0	0	0	0	0	0	0	
16299875	f	cn	CA	2019	0	1	0	0	1	0	0	0	0	0	0	0	72
16300156	Not	md	US	2019	0	0	0	0	0	0	1	1	0	0	0	0	
16300196	f	cn	US	2019	0	1	1	0	0	0	0	0	0	0	0	0	87
16300267	f	cn	CN	2019	1	0	0	0	0	0	0	0	0	0	0	0	
16300994	f	md	HK	2019	0	1	0	0	0	1	0	0	0	0	0	0	65
16301395	m	ot	US	2019	0	1	0	0	1	0	0	0	0	0	0	0	
16301565	m	md	BE	2019	0	1	0	0	0	1	0	0	0	0	0	0	74
16301986	f	md	CA	2019	0	0	0	0	0	0	0	0	0	0	0	0	
16302024	f	md	JP	2019	0	0	0	0	0	0	0	0	0	0	0	0	87

16302795	m	md	DE	2019	0	1	0	0	0	1	0	0	0	0	0	0	79
16303329	f	md	HK	2019	0	1	0	0	0	1	0	0	0	0	0	0	59
16303531	Not	md	CN	2019	0	0	0	0	0	0	0	0	0	0	0	0	
16304581	m	md	ES	2019	0	1	0	1	0	0	0	0	0	0	0	0	66
16304614	f	ot	DE	2019	0	0	0	0	0	0	0	0	0	0	0	0	
16304634	m	cn	US	2019	0	1	0	0	1	0	0	0	0	0	0	0	48
16304660	m	ot	FR	2019	0	1	0	0	1	0	0	0	0	0	0	0	70
16305016	Not	ot	GB	2019	0	0	0	0	0	0	0	0	0	0	0	0	
16305360	f	ot	TW	2019	0	1	0	0	1	0	0	0	0	0	0	0	66
16305488	f	md	JP	2019	1	0	0	0	0	0	0	0	0	0	0	0	65
16305533	m	ot	US	2019	0	1	0	1	0	0	0	0	0	0	0	0	55
16305545	f	cn	FR	2019	0	1	1	0	0	0	0	0	0	0	0	0	
16305703	m	cn	JP	2019	0	0	0	0	0	0	0	0	0	0	0	0	58
16306604	f	cn	US	2019	0	1	1	0	0	0	0	0	0	0	0	0	
16306800	m	cn	JP	2019	0	1	1	0	0	0	0	0	0	0	0	0	67
16306873	m	ot	DE	2019	0	1	1	0	0	0	0	0	0	0	0	0	
16307011	m	cn	IT	2019	0	1	1	0	0	0	0	0	0	0	0	0	78
16307392	m	md	JP	2019	0	1	0	0	0	1	0	0	0	0	0	0	72
16307590	f	cn	US	2019	0	1	1	0	0	0	0	0	0	0	0	0	
16307746	f	md	HK	2019	0	1	0	0	0	1	0	0	0	0	0	0	66
16307770	f	md	US	2019	0	1	0	1	0	0	0	0	0	0	0	1	
16307858	m	cn	JP	2019	0	1	1	0	0	0	0	0	0	0	0	0	71
16308011	m	ot	FR	2019	0	1	0	0	1	0	0	0	0	0	0	0	56
16308227	m	cn	KR	2019	0	1	1	0	0	0	0	0	0	0	0	0	
16308233	f	cn	JP	2019	0	1	1	0	0	0	0	0	0	0	0	0	74
16308446	m	md	JP	2019	0	1	0	0	0	1	0	0	0	0	0	0	85
16308544	f	md	IL	2019	1	0	0	0	0	0	0	0	0	0	0	0	64
16308737	m	cn	FR	2019	0	1	1	0	0	0	0	0	0	0	0	0	70
16309176	f	cn	AR	2019	0	0	0	0	0	0	0	0	0	0	0	0	40
16310175	m	cn	JP	2019	0	1	1	0	0	0	0	0	0	0	0	0	64
16310652	m	ot	JP	2019	1	0	0	0	0	0	0	0	0	0	0	0	49
16311110	m	md	IN	2019	0	0	0	0	0	0	0	0	0	0	0	0	74
16311163	f	Not repo	US	2019	1	0	0	0	0	0	0	0	0	0	0	0	
16311468	m	cn	JP	2019	0	1	1	0	0	0	0	0	0	0	0	0	69

16311501	m	cn	DE	2019	0	1	1	0	0	0	0	0	0	0	0	0	
16311733	Not	md	PL	2019	0	0	0	0	0	0	0	0	0	0	0	0	
16311792	m	ot	KR	2019	0	0	0	0	0	0	0	0	0	0	0	0	61
16311807	m	ot	DE	2019	0	0	0	0	0	0	0	0	0	0	0	0	56
16311912	m	ot	US	2019	0	1	1	0	0	0	0	0	0	0	0	0	79
16312193	m	md	JP	2019	0	1	0	0	1	0	0	0	0	0	0	0	82
16312258	m	md	JP	2019	1	0	0	0	0	0	0	0	0	0	0	0	73
16312272	f	md	JP	2019	0	1	0	0	0	1	0	0	0	0	0	0	81
16312795	f	md	JP	2019	0	1	0	0	0	1	0	0	0	0	0	0	71
16312801	f	cn	GR	2019	0	1	1	0	0	0	0	0	0	0	0	0	
16312876	Not	ot	US	2019	1	0	0	0	0	0	0	0	0	0	0	0	
16312942	f	md	US	2019	1	0	0	0	0	0	0	0	0	0	0	0	
16313441	m	md	HK	2019	0	1	0	0	0	1	0	0	0	0	0	0	64
16313508	m	md	JP	2019	0	1	1	0	0	0	0	0	0	0	0	1	66
16313633	f	md	JP	2019	1	0	0	0	0	0	0	0	0	0	0	0	71
16313894	f	ot	US	2019	0	0	0	0	0	0	0	0	0	0	0	0	69
16314074	m	ot	US	2019	0	1	0	0	1	0	0	0	0	0	0	0	
16314137	m	md	US	2019	0	0	0	0	0	0	0	0	0	0	0	0	66
16314203	m	cn	JP	2019	0	1	1	0	0	0	0	0	0	0	0	0	83
16314433	m	ot	KR	2019	0	0	0	0	0	0	0	0	0	0	0	0	40
16314500	Not	ot	JP	2019	1	0	0	0	0	0	0	0	0	0	0	0	
16315021	f	ph	JP	2019	0	0	0	0	0	0	0	0	0	0	0	0	72
16315058	f	md	JP	2019	1	0	0	0	0	0	0	0	0	0	0	0	88
16315060	f	ot	US	2019	0	1	0	1	0	0	0	0	0	0	0	0	68.7
16315355	m	cn	FR	2019	0	1	1	0	0	0	0	0	0	0	0	0	70
16315640	m	ot	CN	2019	1	0	0	0	0	0	0	0	0	0	0	0	63
16315896	m	md	IN	2019	0	0	0	0	0	0	0	0	0	0	0	0	75
16316098	m	cn	DE	2019	0	1	1	0	0	0	0	0	0	0	0	0	74
16316384	f	cn	BE	2019	0	1	1	0	0	0	0	0	0	0	0	1	77
16316504	m	md	KR	2019	0	1	1	0	0	0	0	0	0	0	0	0	75
16316526	m	cn	IL	2019	0	1	1	0	0	0	0	0	0	0	0	0	70
16316531	f	md	CH	2019	0	1	1	0	0	0	0	0	0	0	0	0	58
16316567	m	cn	US	2019	0	1	1	0	0	0	0	0	0	0	0	0	57
16316633	m	ot	DE	2019	0	1	0	0	1	0	0	0	0	0	0	0	59

16317263	Not	md	US	2019	0	1	0	1	0	0	0	0	0	0	0	0	
16317455	m	md	AR	2019	0	1	0	0	0	1	0	0	0	0	0	0	68
16317491	m	ot	FR	2019	0	1	0	0	1	0	0	0	0	0	0	0	63
16317562	m	md	US	2019	0	1	0	1	0	0	0	0	0	0	0	0	
16317625	m	md	MX	2019	0	1	0	0	0	1	0	0	0	0	0	0	
16317683	m	md	JP	2019	0	1	0	0	1	0	0	0	0	0	0	1	71
16317750	m	md	JP	2019	0	1	0	0	0	1	0	0	0	0	0	0	74
16317755	Not	cn	JP	2019	0	1	1	0	0	0	0	0	0	0	0	0	74
16317785	m	cn	JP	2019	0	1	1	0	0	0	0	0	0	0	0	0	60
16317851	Not	ot	US	2019	0	1	0	0	1	0	0	0	0	0	0	0	
16317919	m	md	JP	2019	0	1	0	0	1	0	0	0	0	0	0	0	64
16318117	m	ot	FR	2019	0	1	0	1	0	0	0	0	0	0	0	0	
16318366	m	ot	DE	2019	0	1	0	0	1	0	0	0	0	0	0	0	73
16318368	m	cn	CA	2019	0	1	0	0	1	0	0	0	0	0	0	0	
16318376	m	cn	US	2019	0	0	0	0	0	0	0	0	0	0	0	0	
16319451	f	cn	US	2019	0	0	0	0	0	0	0	0	0	0	0	0	32
16320173	Not	md	JP	2019	0	1	0	1	0	0	0	0	0	0	0	0	
16320417	f	md	JP	2019	1	0	0	0	0	0	0	0	0	0	0	0	81
16321170	Not	md	JP	2019	0	1	0	0	1	0	0	0	0	0	0	0	
16321923	m	md	JP	2019	0	1	0	1	0	0	0	0	0	0	0	0	66
16321967	f	md	FR	2019	1	0	0	0	0	0	0	0	0	0	0	0	
16322440	m	cn	JP	2019	0	1	1	0	0	0	0	0	0	0	0	0	
16323035	Not	ot	JP	2019	0	0	0	0	0	0	0	0	0	0	0	0	
16323036	Not	ot	JP	2019	0	0	0	0	0	0	0	0	0	0	0	0	
16323077	Not	ot	JP	2019	0	0	0	0	0	0	0	0	0	0	0	0	
16323118	Not	ot	JP	2019	0	0	0	0	0	0	0	0	0	0	0	0	
16323122	Not	ot	JP	2019	0	0	0	0	0	0	0	0	0	0	0	0	
16323125	Not	ot	JP	2019	0	0	0	0	0	0	0	0	0	0	0	0	
16323126	Not	ot	JP	2019	0	0	0	0	0	0	0	0	0	0	0	0	
16323130	Not	ot	JP	2019	0	0	0	0	0	0	0	0	0	0	0	0	
16323131	Not	ot	JP	2019	0	0	0	0	0	0	0	0	0	0	0	0	
16323373	Not	ot	JP	2019	0	0	0	0	0	0	0	0	0	0	0	0	
16323407	f	cn	GB	2019	1	0	0	0	0	0	0	0	0	0	0	0	78
16323561	Not	ot	US	2019	0	1	0	0	1	0	0	0	0	0	0	0	

16323621	m	md	US	2019	0	1	1	0	0	0	0	0	0	0	0	0	
16323670	Not	ot	JP	2019	0	0	0	0	0	0	0	0	0	0	0	0	
16323717	m	cn	JP	2019	0	1	1	0	0	0	0	0	0	0	0	1	66
16323903	f	md	PL	2019	0	1	1	0	0	0	0	0	0	0	0	0	63
16324023	Not	ph	JP	2019	1	0	0	0	0	0	0	0	0	0	0	0	
16324037	m	md	US	2019	0	1	1	0	0	0	0	0	0	0	0	0	74
16324080	m	md	JP	2019	1	0	0	0	0	0	0	0	0	0	0	0	72
16324114	f	md	JP	2019	0	1	0	0	0	1	0	0	0	0	0	1	75
16324405	f	cn	JP	2019	0	1	1	0	0	0	0	0	0	0	0	0	78
16324974	m	Not repo	US	2019	0	1	0	1	0	0	0	0	0	0	0	0	45
16325216	m	cn	JP	2019	0	1	1	0	0	0	0	0	0	0	0	0	85
16325399	f	md	CO	2019	0	0	0	0	0	0	0	0	0	0	0	0	60
16325563	f	cn	US	2019	0	1	1	0	0	0	0	0	0	0	0	0	
16325611	f	md	AT	2019	0	0	0	0	0	0	0	0	0	0	0	0	69
16325689	f	cn	CA	2019	0	1	0	0	1	0	0	0	0	0	0	0	61
16325906	f	md	US	2019	0	1	0	1	0	0	0	0	0	0	0	0	86
16326435	m	ot	US	2019	0	1	0	0	1	0	0	0	0	0	0	0	
16326447	Not	md	US	2019	0	1	0	0	1	0	0	0	0	0	0	0	
16326707	f	ot	SG	2019	0	1	0	0	1	0	0	0	0	0	0	0	66
16326710	m	md	JP	2019	0	1	0	1	0	0	0	0	0	0	0	0	73
16326715	m	md	JP	2019	0	1	0	1	0	0	0	0	0	0	0	1	60
16326727	m	cn	JP	2019	0	0	0	0	0	0	0	0	0	0	0	0	71
16326788	m	md	JP	2019	1	0	0	0	0	0	0	0	0	0	0	0	75
16327017	f	md	JP	2019	1	0	0	0	0	0	0	0	0	0	0	1	76.5
16327229	Not	md	JP	2019	0	0	0	0	0	0	0	0	0	0	0	0	
16327317	m	md	BE	2019	0	1	1	0	0	0	0	0	0	0	0	0	68
16327351	f	ph	US	2019	0	0	0	0	0	0	0	0	0	0	0	0	60
16327490	Not	md	BE	2019	0	1	0	0	1	0	0	0	0	0	0	0	
16327620	m	ot	US	2019	0	1	0	0	1	0	0	0	0	0	0	0	58
16327797	f	md	VN	2019	0	1	0	0	0	1	0	0	0	0	0	0	47
16328646	f	cn	US	2019	0	0	0	0	0	0	0	0	0	0	0	0	73
16329318	m	md	JP	2019	0	1	0	0	1	0	0	0	0	0	0	0	74
16329367	f	md	DE	2019	0	1	0	0	0	1	0	0	0	0	0	0	79
16329464	m	cn	JP	2019	0	1	1	0	0	0	0	0	0	0	0	0	69

16329548	m	cn	JP	2019	0	1	1	0	0	0	0	0	0	0	0	0	78
16329644	m	md	JP	2019	0	1	0	0	0	1	0	0	0	0	0	0	46
16329667	m	cn	IL	2019	0	1	1	0	0	0	0	0	0	0	0	0	50
16329795	m	md	GB	2019	0	1	1	0	0	0	0	0	0	0	0	0	
16329865	m	md	BR	2019	0	1	0	0	0	1	0	0	0	0	0	0	
16330481	Not	md	DE	2019	0	0	0	0	0	0	0	0	0	0	0	0	67
16330997	Not	md	DE	2019	0	1	1	0	0	0	0	0	0	0	0	0	
16332901	f	ph	JP	2019	0	1	0	0	1	0	0	0	0	0	0	1	70
16332943	m	cn	BE	2019	0	1	1	0	0	0	0	0	0	0	0	0	75
16333455	m	cn	JP	2019	0	1	1	0	0	0	0	0	0	0	0	0	79
16333595	f	ot	FR	2019	0	1	0	1	0	0	0	0	0	0	0	0	47
16334265	f	cn	JP	2019	0	0	0	0	0	0	0	0	0	0	0	0	68
16335093	f	cn	GB	2019	0	1	1	0	0	0	0	0	0	0	0	0	
16335133	f	md	MY	2019	0	1	0	0	0	1	0	0	0	0	0	0	
16335653	m	md	DE	2019	0	0	0	0	0	0	0	0	0	0	0	0	58
16335947	m	cn	JP	2019	0	1	1	0	0	0	0	0	0	0	0	0	70
16335982	m	md	JP	2019	0	1	0	1	0	0	0	0	0	0	0	0	58
16335987	m	cn	JP	2019	0	1	1	0	0	0	0	0	0	0	0	0	71
16336067	Not	md	JP	2019	0	1	0	0	0	1	0	0	0	0	0	0	76
16336415	m	cn	GB	2019	0	1	1	0	0	0	0	0	0	0	0	0	
16336662	f	ph	JP	2019	1	0	0	0	0	0	0	0	0	0	0	0	89
16336934	f	cn	BE	2019	0	1	1	0	0	0	0	0	0	0	0	0	82
16336941	Not	md	JP	2019	1	0	0	0	0	0	0	0	0	0	0	0	
16337052	f	md	JP	2019	0	1	0	0	0	1	0	0	0	0	0	0	70
16337288	f	md	AU	2019	0	1	0	0	0	1	0	0	0	0	0	0	
16337695	f	cn	JP	2019	0	1	1	0	0	0	0	0	0	0	0	0	71
16337730	m	ot	IT	2019	0	1	0	0	1	0	0	0	0	0	0	0	50
16337731	f	ot	NL	2019	0	1	0	0	1	0	0	0	0	0	0	0	69
16337734	m	ot	ES	2019	0	1	0	0	1	0	0	0	0	0	0	0	80
16337738	f	ot	IT	2019	0	1	0	0	1	0	0	0	0	0	0	0	51
16337803	Not	md	JP	2019	0	1	0	0	1	0	0	0	0	0	0	0	
16337892	Not	ot	US	2019	0	1	0	0	1	0	0	0	0	0	0	0	
16338012	m	md	JP	2019	0	1	0	1	0	0	0	0	0	0	0	0	67
16338059	m	md	CN	2019	0	0	0	0	0	0	0	0	0	0	0	0	51

16338622	m	md	JP	2019	1	0	0	0	0	0	0	0	0	0	0	0	70
16338786	m	cn	JP	2019	0	1	1	0	0	0	0	0	0	0	0	0	77
16338826	f	ot	US	2019	1	0	0	0	0	0	0	0	0	0	0	0	73
16338835	f	md	DE	2019	0	1	0	1	0	0	0	0	0	0	0	0	71.8
16339100	Not	md	US	2019	0	1	1	0	0	0	0	0	0	0	0	0	
16340004	f	md	FR	2019	0	1	0	1	0	0	0	0	0	0	0	0	
16340037	m	cn	JP	2019	0	1	1	0	0	0	0	0	0	0	0	0	60
16340056	m	cn	US	2019	0	1	1	0	0	0	0	0	0	0	0	0	
16340398	m	ot	KR	2019	0	0	0	0	0	0	0	0	0	0	0	0	64
16340594	f	md	JP	2019	1	0	0	0	0	0	0	0	0	0	0	0	
16340628	m	md	JP	2019	1	0	0	0	0	0	0	0	0	0	0	0	84
16340947	f	ot	US	2019	0	1	0	0	1	0	0	0	0	0	0	0	49
16340977	m	md	CA	2019	1	0	0	0	0	0	0	0	0	0	0	0	69
16341374	m	md	JP	2019	0	1	0	0	1	0	0	0	0	0	0	1	65
16341447	m	md	CY	2019	0	0	0	0	0	0	0	0	0	0	0	0	74
16341524	f	Not repo	US	2019	1	0	0	0	0	0	0	0	0	0	0	0	69
16341718	f	md	ES	2019	0	1	0	0	0	1	0	0	0	0	0	0	55
16341825	m	ph	US	2019	1	0	0	0	0	0	0	0	0	0	0	0	69
16341970	m	ot	US	2019	0	1	0	0	1	0	0	0	0	0	0	0	
16342223	f	Not repo	US	2019	1	0	0	0	0	0	0	0	0	0	0	0	69
16343080	m	cn	Not re	2019	0	0	0	0	0	0	0	0	0	0	0	0	70
16343379	m	md	JP	2019	0	1	0	1	0	0	0	0	0	0	0	0	80
16343432	f	Not repo	IT	2019	1	0	0	0	0	0	0	0	0	0	0	1	72.9
16343563	m	md	IN	2019	0	1	0	0	1	0	0	0	0	0	0	0	70
16343920	f	md	FR	2019	0	0	0	0	0	0	0	0	0	0	0	0	67
16343937	f	cn	CN	2019	1	0	0	0	0	0	0	0	0	0	0	0	71
16344023	m	md	DE	2019	0	1	1	0	0	0	0	0	0	0	0	0	
16344044	Not	md	KR	2019	0	0	0	0	0	0	0	0	0	0	0	0	
16344180	m	cn	JP	2019	0	1	1	0	0	0	0	0	0	0	0	0	70
16344275	f	cn	GB	2019	1	0	0	0	0	0	0	0	0	0	0	0	76
16344408	m	cn	CN	2019	0	0	0	0	0	0	0	0	0	0	0	0	62
16344591	Not	md	JP	2019	1	0	0	0	0	0	0	0	0	0	0	0	
16345034	m	cn	JP	2019	0	1	1	0	0	0	0	0	0	0	0	0	80
16345584	m	md	JP	2019	0	1	0	0	0	1	0	0	0	0	0	0	74

16345633	m	md	JP	2019	0	1	0	0	0	1	0	0	0	0	0	0	70
16345772	f	Not repo	IT	2019	1	0	0	0	0	0	0	0	0	0	0	0	
16345873	m	md	US	2019	1	0	0	0	0	0	0	0	0	0	0	1	
16346262	f	ph	GB	2019	1	0	0	0	0	0	0	0	0	0	0	0	57
16346303	f	cn	US	2019	0	0	0	0	0	0	0	0	0	0	0	0	
16346404	m	ph	JP	2019	1	0	0	0	0	0	0	0	0	0	0	0	82
16346411	m	md	JP	2019	0	1	0	1	0	0	0	0	0	0	0	1	67
16346458	f	md	VN	2019	0	1	0	0	0	1	0	0	0	0	0	1	52
16346499	f	ot	GB	2019	0	1	0	0	1	0	0	0	0	0	0	0	88
16346543	f	md	HR	2019	0	1	1	0	0	0	0	0	0	0	0	0	
16346561	m	cn	JP	2019	0	1	1	0	0	0	0	0	0	0	0	0	78
16346577	Not	ot	IL	2019	0	0	0	0	0	0	0	0	0	0	0	0	
16346892	f	md	JP	2019	1	0	0	0	0	0	0	0	0	0	0	0	82.9
16346968	m	md	MX	2019	0	1	0	0	0	1	0	0	0	0	0	0	
16347093	m	Not repo	ID	2019	1	0	0	0	0	0	0	0	0	0	0	0	
16347097	m	md	JP	2019	0	1	0	1	0	0	0	0	0	0	0	1	76
16347227	f	md	JP	2019	1	0	0	0	0	0	0	0	0	0	0	0	81.5
16347281	m	md	ES	2019	0	1	0	0	0	1	0	0	0	0	0	0	75
16347552	f	ot	FR	2019	1	0	0	0	0	0	0	0	0	0	0	0	48
16347671	m	md	JP	2019	0	1	0	1	0	0	0	0	0	0	0	1	80
16347678	m	md	ES	2019	0	1	0	0	0	1	0	0	0	0	0	0	66
16348345	f	md	GB	2019	1	0	0	0	0	0	0	0	0	0	0	0	75
16348640	f	cn	DE	2019	0	1	1	0	0	0	0	0	0	0	0	0	37
16348771	m	md	JP	2019	0	0	0	0	0	0	0	0	0	0	0	0	65
16348844	m	md	FR	2019	0	1	0	1	0	0	0	0	0	0	0	0	
16348845	m	cn	GR	2019	0	0	0	0	0	0	0	0	0	0	0	0	62
16348878	f	md	ES	2019	0	0	0	0	0	0	0	0	0	0	0	0	73
16349040	f	cn	GB	2019	1	0	0	0	0	0	0	0	0	0	0	0	60
16349161	m	md	JP	2019	0	1	0	1	0	0	0	0	0	0	0	1	
16349410	f	md	DE	2019	1	0	0	0	0	0	0	0	0	0	0	0	45
16349415	f	cn	US	2019	0	0	0	0	0	0	0	0	0	0	0	0	
16349557	m	ot	NL	2019	0	1	1	0	0	0	0	0	0	0	0	0	
16349697	m	cn	US	2019	0	1	1	0	0	0	0	0	0	0	0	0	
16349743	m	cn	US	2019	0	0	0	0	0	0	0	0	0	0	0	0	

16349853	m	md	US	2019	0	0	0	0	0	0	0	0	0	0	0	0	41
16349946	m	cn	DE	2019	0	0	0	0	0	0	0	0	0	0	0	0	65
16349993	f	ot	DE	2019	0	0	0	0	0	0	0	0	0	0	0	0	64
16350550	m	md	JP	2019	0	1	0	0	0	1	0	0	0	0	0	0	62
16350631	Not	md	JP	2019	1	0	0	0	0	0	0	0	0	0	0	0	
16350694	f	cn	US	2019	1	0	0	0	0	0	0	0	0	0	0	0	72
16350757	m	md	IT	2019	0	0	0	0	0	0	0	0	0	0	0	0	
16350802	m	md	JP	2019	0	1	0	0	0	1	0	0	0	0	0	0	70
16350835	m	ot	US	2019	0	0	0	0	0	0	0	0	0	0	0	0	70
16350961	m	md	JP	2019	0	1	1	0	0	0	0	0	0	0	0	0	83
16351104	m	md	JP	2019	0	1	0	0	1	0	0	0	0	0	0	0	45
16351274	Not	ot	US	2019	0	1	1	0	0	0	0	0	0	0	0	0	
16351534	m	md	JP	2019	0	1	0	1	0	0	0	0	0	0	0	1	80
16353195	m	cn	JP	2019	0	0	0	0	0	0	0	0	0	0	0	0	65
16353199	Not	cn	JP	2019	0	0	0	0	0	0	0	0	0	0	0	0	
16353231	m	cn	KR	2019	0	0	0	0	0	0	0	0	0	0	0	0	67
16353351	m	ot	NL	2019	0	1	1	0	0	0	0	0	0	0	0	0	74
16353844	m	md	US	2019	0	1	1	0	0	0	0	0	0	0	0	0	64
16353896	f	ot	IN	2019	1	0	0	0	0	0	0	0	0	0	0	0	
16354489	m	md	JP	2019	0	1	0	1	0	0	0	0	0	0	0	1	84
16354592	m	ot	CN	2019	0	1	0	0	1	0	0	0	0	0	0	0	66
16354633	m	Not repo	US	2019	1	0	0	0	0	0	0	0	0	0	0	0	65.5
16354859	m	ot	CN	2019	0	1	0	0	1	0	0	0	0	0	0	0	54
16354930	f	Not repo	CA	2019	0	1	0	1	0	0	0	0	0	0	0	0	77
16354992	m	cn	MX	2019	0	1	1	0	0	0	0	0	0	0	0	0	83
16355099	m	cn	US	2019	0	1	1	0	0	0	0	0	0	0	0	0	
16355346	Not	cn	JP	2019	0	1	1	0	0	0	0	0	0	0	0	0	70
16355743	m	cn	JP	2019	0	1	1	0	0	0	0	0	0	0	0	0	70
16355868	m	cn	CN	2019	0	1	0	0	1	0	0	0	0	0	0	0	53
16356159	f	ot	US	2019	0	1	0	0	1	0	0	0	0	0	0	0	
16356251	f	md	KR	2019	1	0	0	0	0	0	0	0	0	0	0	0	75
16356278	m	ot	CN	2019	0	1	0	0	1	0	0	0	0	0	0	0	76
16356431	f	ot	US	2019	0	1	0	0	1	0	0	0	0	0	0	0	75
16356452	f	md	RS	2019	0	1	0	0	0	1	0	0	0	0	0	0	62

16356606	m	ot	DE	2019	0	1	0	0	1	0	0	0	0	0	0	0	72
16356640	f	cn	JP	2019	0	1	1	0	0	0	0	0	0	0	0	0	83
16356783	m	cn	JP	2019	0	1	1	0	0	0	0	0	0	0	0	1	76
16356789	m	md	DE	2019	0	1	0	0	1	0	0	0	0	0	0	0	68
16357336	m	md	CN	2019	0	1	0	0	0	1	0	0	0	0	0	1	59
16357475	m	cn	JP	2019	0	1	1	0	0	0	0	0	0	0	0	0	66
16357503	Not	ot	DE	2019	1	0	0	0	0	0	0	0	0	0	0	0	
16357524	Not	ot	DE	2019	1	0	0	0	0	0	0	0	0	0	0	0	
16357612	Not	ot	CN	2019	1	0	0	0	0	0	0	0	0	0	0	0	
16357613	Not	ot	CN	2019	1	0	0	0	0	0	0	0	0	0	0	0	
16357620	m	cn	JP	2019	0	1	1	0	0	0	0	0	0	0	0	0	67
16357828	f	md	JP	2019	0	1	0	1	0	0	0	0	0	0	0	1	65
16357971	m	md	IT	2019	0	1	0	0	1	0	0	0	0	0	0	0	75
16358187	f	md	JP	2019	1	0	0	0	0	0	0	0	0	0	0	1	74
16358268	m	md	JP	2019	0	1	0	1	0	0	0	0	0	0	0	0	60
16358269	m	cn	IN	2019	1	0	0	0	0	0	0	0	0	0	0	0	64
16358275	m	md	DE	2019	0	1	1	0	0	0	0	0	0	0	0	0	62
16358386	Not	md	JP	2019	1	0	0	0	0	0	0	0	0	0	0	0	
16358563	f	cn	US	2019	1	0	0	0	0	0	0	0	0	0	0	0	
16358592	f	cn	JP	2019	0	1	1	0	0	0	0	0	0	0	0	0	58
16358595	m	cn	JP	2019	0	1	1	0	0	0	0	0	0	0	0	0	72
16358599	Not	md	ES	2019	0	0	0	0	0	0	0	0	0	0	0	0	61
16358628	m	md	JP	2019	0	1	1	0	0	0	0	0	0	0	0	0	75
16358861	m	cn	CA	2019	1	0	0	0	0	0	0	0	0	0	0	0	66.1
16358945	m	cn	JP	2019	0	1	1	0	0	0	0	0	0	0	0	0	72
16359420	f	cn	US	2019	1	0	0	0	0	0	0	0	0	0	0	1	
16359585	Not	md	US	2019	0	1	0	0	0	1	0	0	0	0	0	0	
16359636	m	md	JP	2019	0	1	0	0	0	1	0	0	0	0	0	0	77
16359656	Not	md	HK	2019	0	1	0	0	0	1	0	0	0	0	0	0	
16360408	m	ot	GB	2019	0	0	0	0	0	0	0	0	0	0	0	0	72
16360548	m	md	JP	2019	0	1	1	0	0	0	0	0	0	0	0	0	73
16360738	f	md	US	2019	0	0	0	0	0	0	0	0	0	0	0	0	79
16360921	m	md	FR	2019	0	0	0	0	0	0	0	0	0	0	0	0	
16360994	m	md	JP	2019	0	1	0	1	0	0	0	0	0	0	0	1	66

16361110	f	cn	KR	2019	0	0	0	0	0	0	0	0	0	0	0	0	60
16361282	m	cn	JP	2019	0	1	1	0	0	0	0	0	0	0	0	0	79
16361836	f	cn	US	2019	0	0	0	0	0	0	0	0	0	0	0	0	
16361900	m	md	JP	2019	1	0	0	0	0	0	0	0	0	0	0	0	78
16361939	m	md	RU	2019	0	1	0	0	0	1	0	0	0	0	0	0	46
16362027	Not	md	US	2019	0	0	0	0	0	0	0	0	0	0	0	0	
16362617	m	md	IE	2019	0	1	0	0	1	0	0	0	0	0	0	0	65
16362877	m	md	JP	2019	0	1	0	1	0	0	0	0	0	0	0	1	75
16363058	m	cn	JP	2019	0	1	1	0	0	0	0	0	0	0	0	0	69
16363073	f	md	JP	2019	1	0	0	0	0	0	0	0	0	0	0	0	50
16363074	m	cn	JP	2019	0	1	1	0	0	0	0	0	0	0	0	0	68
16363478	m	md	JP	2019	0	1	0	1	0	0	0	0	0	0	0	1	66
16363714	f	md	JP	2019	0	0	0	0	0	0	0	0	0	0	0	0	75
16363760	Not	md	US	2019	0	1	1	0	0	0	0	0	0	0	0	0	
16363802	m	ot	US	2019	0	1	0	0	1	0	0	0	0	0	0	1	62
16363943	m	md	DE	2019	1	0	0	0	0	0	0	0	0	0	0	0	71
16364264	m	md	JP	2019	0	1	1	0	0	0	0	0	0	0	0	0	70
16364470	m	cn	KR	2019	0	0	0	0	0	0	0	0	0	0	0	0	42
16364856	Not	ot	US	2019	0	1	0	0	1	0	0	0	0	0	0	0	
16365129	m	ot	KR	2019	0	1	0	0	1	0	0	0	0	0	0	0	68
16365166	m	cn	JP	2019	0	1	1	0	0	0	0	0	0	0	0	0	61
16365202	f	md	UA	2019	0	1	0	0	0	1	0	0	0	0	0	0	
16365204	m	cn	IT	2019	0	1	1	0	0	0	0	0	0	0	0	0	
16365260	f	md	IL	2019	1	0	0	0	0	0	0	0	0	0	0	0	58
16365349	f	ot	US	2019	0	1	0	0	1	0	0	0	0	0	0	0	
16365399	m	md	JP	2019	0	1	0	1	0	0	0	0	0	0	0	0	69.8
16365672	f	ph	ES	2019	1	0	0	0	0	0	0	0	0	0	0	0	63
16365766	f	cn	JP	2019	0	1	1	0	0	0	0	0	0	0	0	0	69
16366046	m	cn	JP	2019	0	1	1	0	0	0	0	0	0	0	0	0	68
16366062	m	md	JP	2019	1	0	0	0	0	0	0	0	0	0	0	0	70
16366199	Not	md	AU	2019	0	1	0	0	0	1	0	0	0	0	0	0	
16366228	m	md	NL	2019	0	0	0	0	0	0	0	0	0	0	0	0	
16366517	m	ph	JP	2019	0	1	0	0	1	0	0	0	0	0	0	0	76
16367221	m	md	RO	2019	1	0	0	0	0	0	0	0	0	0	0	0	60

16367795	f	md	IN	2019	0	0	0	0	0	0	0	0	0	0	0	0	50
16368014	f	ph	JP	2019	1	0	0	0	0	0	0	0	0	0	0	0	
16368117	Not	ot	US	2019	0	1	0	0	1	0	0	0	0	0	0	1	72
16368302	m	cn	JP	2019	0	1	1	0	0	0	0	0	0	0	0	0	65
16368350	m	md	JP	2019	0	1	0	0	1	0	0	0	0	0	0	0	58
16368624	m	cn	JP	2019	1	0	0	0	0	0	0	0	0	0	0	0	
16368749	m	cn	JP	2019	0	0	0	0	0	0	0	0	0	0	0	0	70
16368878	Not	md	US	2019	0	0	0	0	0	0	0	0	0	0	0	0	
16369207	m	md	ES	2019	0	1	0	0	0	1	0	0	0	0	0	0	67
16369230	f	cn	CA	2019	0	0	0	0	0	0	0	0	0	0	0	0	77
16369268	m	ot	CA	2019	0	1	0	0	1	0	0	0	0	0	0	0	62
16369490	f	md	HU	2019	0	1	0	0	1	0	0	0	0	0	0	1	66
16369532	m	md	ES	2019	0	1	0	0	0	1	0	0	0	0	0	0	50
16369540	m	md	ES	2019	0	1	0	0	0	1	0	0	0	0	0	0	69
16369661	f	cn	JP	2019	0	1	0	0	1	0	0	0	0	0	0	0	75
16369689	m	md	VN	2019	0	1	0	0	0	1	0	0	0	0	0	0	
16369691	f	md	AT	2019	0	0	0	0	0	0	0	0	0	0	0	0	65
16369903	m	cn	IN	2019	0	1	0	0	1	0	0	0	0	0	0	0	62
16369923	m	md	JP	2019	0	1	1	0	0	0	0	0	0	0	0	0	80
16370067	m	md	RS	2019	1	0	0	0	0	0	0	0	0	0	0	0	80
16370120	m	ot	US	2019	0	1	0	0	1	0	0	0	0	0	0	0	48
16370298	Not	md	JP	2019	0	1	0	0	0	1	0	0	0	0	0	0	76
16371049	f	Not repo	US	2019	1	0	0	0	0	0	0	0	0	0	0	1	70
16371681	f	md	AT	2019	0	0	0	0	0	0	0	0	0	0	0	0	58
16371721	m	cn	JP	2019	0	1	1	0	0	0	0	0	0	0	0	1	71
16372033	m	md	FI	2019	0	1	0	0	1	0	0	0	0	0	0	0	61
16372034	m	ot	FR	2019	0	1	0	0	1	0	0	0	0	0	0	0	72
16372328	m	cn	JP	2019	0	0	0	0	0	0	0	0	0	0	0	0	66
16372638	m	md	JP	2019	0	1	1	0	0	0	0	0	0	0	0	0	70
16372701	m	md	NL	2019	0	1	0	0	1	0	0	0	0	0	0	0	74
16372746	f	md	DE	2019	1	0	0	0	0	0	0	0	0	0	0	0	61
16372895	Not	ot	DE	2019	0	1	1	0	0	0	0	0	0	0	0	0	
16373274	f	md	US	2019	0	1	0	1	0	0	0	0	0	0	0	0	76
16373279	m	cn	JP	2019	0	0	0	0	0	0	0	0	0	0	0	0	

16373931	m	cn	DE	2019	0	1	1	0	0	0	0	0	0	0	0	0	49
16374588	m	ot	JP	2019	0	1	0	0	1	0	0	0	0	0	0	0	59
16374589	m	ot	FR	2019	0	1	0	0	1	0	0	0	0	0	0	0	46
16374802	m	Not repo	US	2019	1	0	0	0	0	0	0	0	0	0	0	0	
16375102	f	md	ES	2019	0	1	0	0	1	0	0	0	0	0	0	0	69
16375284	m	ot	CN	2019	1	0	0	0	0	0	0	0	0	0	0	0	54
16375338	m	md	JP	2019	0	1	0	0	0	1	0	0	0	0	0	0	44
16375383	m	md	RS	2019	1	0	0	0	0	0	0	0	0	0	0	0	
16375423	m	md	JP	2019	0	1	0	0	1	0	0	0	0	0	0	1	44
16375481	m	md	SK	2019	1	0	0	0	0	0	0	0	0	0	0	0	84
16375628	m	md	NL	2019	0	1	0	0	1	0	0	0	0	0	0	0	78
16375802	m	cn	US	2019	1	0	0	0	0	0	0	0	0	0	0	0	
16375964	m	cn	JP	2019	0	1	1	0	0	0	0	0	0	0	0	0	44
16376014	m	ot	TW	2019	0	0	0	0	0	0	0	0	0	0	0	0	
16376027	Not	ot	CN	2019	1	0	0	0	0	0	0	0	0	0	0	0	
16376199	m	ot	US	2019	1	0	0	0	0	0	0	0	0	0	0	0	
16376321	Not	ot	CN	2019	1	0	0	0	0	0	0	0	0	0	0	0	
16376569	f	md	JP	2019	1	0	0	0	0	0	0	0	0	0	0	0	60
16376645	f	md	JP	2019	0	1	0	1	0	0	0	0	0	0	0	0	61
16376879	f	cn	JP	2019	1	0	0	0	0	0	0	0	0	0	0	0	
16377452	m	md	JP	2019	0	1	0	1	0	0	0	0	0	0	0	1	73
16377464	m	md	JP	2019	0	1	0	1	0	0	0	0	0	0	0	1	65
16377498	Not	ot	CN	2019	1	0	0	0	0	0	0	0	0	0	0	0	
16377638	f	ot	CN	2019	1	0	0	0	0	0	0	0	0	0	0	0	44
16377640	Not	ot	CN	2019	1	0	0	0	0	0	0	0	0	0	0	0	
16377643	m	ot	CN	2019	1	0	0	0	0	0	0	0	0	0	0	0	51
16377644	f	ot	CN	2019	1	0	0	0	0	0	0	0	0	0	0	0	73
16377777	f	ot	GB	2019	0	1	1	0	0	0	0	0	0	0	0	0	
16377990	m	md	JP	2019	0	1	0	1	0	0	0	0	0	0	0	1	78.2
16378189	m	ot	CN	2019	1	0	0	0	0	0	0	0	0	0	0	0	59
16378231	m	cn	JP	2019	0	1	1	0	0	0	0	0	0	0	0	0	77
16378285	m	ot	AT	2019	0	1	0	0	1	0	0	0	0	0	0	0	79
16378511	f	md	JP	2019	1	0	0	0	0	0	0	0	0	0	0	0	86
16378728	f	md	JP	2019	1	0	0	0	0	0	0	0	0	0	0	0	74.7

16378769	m	ot	JP	2019	0	1	0	1	0	0	0	0	0	0	0	1	69
16378789	f	cn	JP	2019	0	1	1	0	0	0	0	0	0	0	0	0	60
16378802	m	ot	US	2019	0	1	0	0	1	0	0	0	0	0	0	0	
16378822	f	md	US	2019	0	1	0	0	0	1	0	0	0	0	0	0	55
16379190	Not	ot	CN	2019	1	0	0	0	0	0	0	0	0	0	0	0	
16379193	f	cn	JP	2019	0	1	1	0	0	0	0	0	0	0	0	0	50
16379604	m	cn	CA	2019	0	0	0	0	0	0	0	0	0	0	0	0	39
16379786	Not	ot	FR	2019	0	0	0	0	0	0	0	0	0	0	0	0	
16379804	f	cn	DE	2019	0	1	1	0	0	0	0	0	0	0	0	0	
16380086	f	cn	JP	2019	0	1	1	0	0	0	0	0	0	0	0	0	90
16380360	f	md	KR	2019	1	0	0	0	0	0	0	0	0	0	0	0	70
16380536	f	md	MX	2019	0	1	0	0	0	1	0	0	0	0	0	0	
16380617	Not	ot	IT	2019	0	0	0	0	0	0	0	0	0	0	0	0	
16380796	m	ot	FR	2019	0	1	0	0	1	0	0	0	0	0	0	0	71
16380798	m	ot	US	2019	0	1	0	0	1	0	0	0	0	0	0	0	
16380822	m	ot	US	2019	0	1	0	0	1	0	0	0	0	0	0	0	62
16381259	f	ot	FR	2019	0	1	0	0	1	0	0	0	0	0	0	0	61
16381633	m	md	FR	2019	0	1	0	0	1	0	0	0	0	0	0	0	53
16381785	f	ot	FR	2019	0	1	0	0	1	0	0	0	0	0	0	0	51
16381804	f	md	JP	2019	1	0	0	0	0	0	0	0	0	0	0	0	70
16381838	f	cn	Not re	2019	0	0	0	0	0	0	0	0	0	0	0	0	
16382171	Not	cn	GB	2019	1	0	0	0	0	0	0	0	0	0	0	0	49
16382488	m	cn	JP	2019	0	1	1	0	0	0	0	0	0	0	0	0	65
16382612	m	md	JP	2019	0	1	1	0	0	0	0	0	0	0	0	1	
16382675	Not	md	RS	2019	1	0	0	0	0	0	0	0	0	0	0	0	
16382681	m	ot	FR	2019	0	1	0	0	1	0	0	0	0	0	0	0	62
16382732	m	md	EG	2019	0	1	0	0	1	0	0	0	0	0	0	0	62
16382763	Not	md	IN	2019	0	0	0	0	0	0	0	0	0	0	0	0	
16383084	m	ot	BE	2019	0	1	0	0	0	1	0	0	0	0	0	0	70
16383115	m	cn	DE	2019	0	0	0	0	0	0	0	0	0	0	0	0	55
16383154	Not	md	NL	2019	0	1	1	0	0	0	0	0	0	0	0	0	66
16383619	f	ph	US	2019	0	1	1	0	0	0	0	0	0	0	0	0	67
16384176	m	md	JP	2019	0	1	0	1	0	0	0	0	0	0	0	0	65
16384776	m	cn	US	2019	0	1	1	0	0	0	0	0	0	0	0	1	61

16384814	m	md	US	2019	0	1	0	0	1	0	0	0	0	0	0	0	48
16384815	m	md	US	2019	0	0	0	0	0	0	0	0	0	0	0	0	78
16384886	f	cn	DE	2019	0	1	1	0	0	0	0	0	0	0	0	0	37
16384890	m	cn	GB	2019	0	1	1	0	0	0	0	0	0	0	0	0	
16384976	m	cn	DE	2019	0	0	0	0	0	0	0	0	0	0	0	0	
16385105	f	md	BE	2019	1	0	0	0	0	0	0	0	0	0	0	0	72.3
16385343	m	md	JP	2019	0	1	1	0	0	0	0	0	0	0	0	1	66
16385375	f	md	DE	2019	0	0	0	0	0	0	1	1	0	0	0	0	
16385400	m	ot	DE	2019	0	0	0	0	0	0	0	0	0	0	0	0	67
16386013	Not	md	GB	2019	0	0	0	0	0	0	0	0	0	0	0	0	
16386387	f	cn	JP	2019	0	1	1	0	0	0	0	0	0	0	0	0	73
16386665	m	cn	VN	2019	0	1	1	0	0	0	0	0	0	0	0	0	
16387044	m	md	DE	2019	0	1	0	0	0	1	0	0	0	0	0	0	74
16387207	m	md	JP	2019	0	1	0	1	0	0	0	0	0	0	0	1	
16387230	f	md	JP	2019	0	1	0	0	1	0	0	0	0	0	0	0	60
16387247	f	ot	US	2019	0	1	1	0	0	0	0	0	0	0	0	0	68
16387316	m	cn	JP	2019	0	1	1	0	0	0	0	0	0	0	0	0	77
16387458	m	cn	BE	2019	0	1	1	0	0	0	0	0	0	0	0	0	67
16387470	f	md	KR	2019	0	1	0	0	1	0	0	0	0	0	0	0	73
16387485	m	ot	KR	2019	0	1	0	0	1	0	0	0	0	0	0	0	
16387666	m	cn	US	2019	0	0	0	0	0	0	0	0	0	0	0	0	
16387750	Not	cn	GB	2019	1	0	0	0	0	0	0	0	0	0	0	0	
16387776	m	ot	JP	2019	0	0	0	0	0	0	0	0	0	0	0	0	80
16387782	f	cn	JP	2019	0	1	1	0	0	0	0	0	0	0	0	0	66
16387798	f	md	FR	2019	0	1	0	0	1	0	0	0	0	0	0	0	57
16387840	f	ph	FR	2019	1	0	0	0	0	0	0	0	0	0	0	0	71
16387854	m	md	JP	2019	0	1	0	0	0	1	0	0	0	0	0	0	62
16388260	m	cn	CN	2019	0	1	0	0	1	0	0	0	0	0	0	0	
16388404	Not	md	JP	2019	1	0	0	0	0	0	0	0	0	0	0	0	
16388854	m	cn	CN	2019	0	1	0	0	1	0	0	0	0	0	0	0	
16388857	m	md	JP	2019	0	1	0	0	1	0	0	0	0	0	0	0	80
16389075	f	ot	JP	2019	1	0	0	0	0	0	0	0	0	0	0	1	83
16389313	f	md	US	2019	0	1	0	0	1	0	0	0	0	0	0	0	74
16389937	m	md	JP	2019	0	1	0	1	0	0	0	0	0	0	0	1	61

16390008	m	Not repo	CN	2019	1	0	0	0	0	0	0	0	0	0	0	0	79
16390296	f	ph	GB	2019	0	0	0	0	0	0	0	0	0	0	0	0	
16390423	f	cn	MY	2019	0	0	0	0	0	0	0	0	0	0	0	0	60
16391195	f	ot	GB	2019	0	0	0	0	0	0	0	0	0	0	0	0	54
16391349	m	md	JP	2019	0	1	0	1	0	0	0	0	0	0	0	1	65
16391630	m	cn	KW	2019	1	0	0	0	0	0	0	0	0	0	0	0	44.5
16391956	m	cn	JP	2019	0	1	1	0	0	0	0	0	0	0	0	0	60
16392002	m	cn	JP	2019	0	1	1	0	0	0	0	0	0	0	0	0	70
16392561	Not	ot	JP	2019	0	1	0	0	1	0	0	0	0	0	0	0	
16392567	f	cn	CN	2019	0	1	0	0	1	0	0	0	0	0	0	0	40
16392578	f	cn	US	2019	1	0	0	0	0	0	0	0	0	0	0	0	
16392591	m	cn	US	2019	1	0	0	0	0	0	0	0	0	0	0	0	72
16392614	Not	md	IT	2019	1	0	0	0	0	0	0	0	0	0	0	0	
16392748	Not	ot	JP	2019	0	0	0	0	0	0	0	0	0	0	0	0	
16392894	Not	md	BE	2019	1	0	0	0	0	0	0	0	0	0	0	0	
16392896	m	cn	JP	2019	0	1	1	0	0	0	0	0	0	0	0	0	71
16393206	m	cn	JP	2019	0	1	1	0	0	0	0	0	0	0	0	0	72
16393445	f	md	US	2019	0	1	0	1	0	0	0	0	0	0	0	0	63
16393766	m	cn	JP	2019	0	1	1	0	0	0	0	0	0	0	0	0	66
16394123	f	cn	JP	2019	0	1	1	0	0	0	0	0	0	0	0	0	68
16394214	m	ot	JP	2019	0	1	1	0	0	0	0	0	0	0	0	0	70
16394255	Not	md	US	2019	0	1	0	0	0	1	0	0	0	0	0	0	
16394670	Not	md	US	2019	0	1	0	0	0	1	0	0	0	0	0	0	
16395174	f	md	JP	2019	1	0	0	0	0	0	0	0	0	0	0	0	60
16395291	m	cn	JP	2019	0	1	1	0	0	0	0	0	0	0	0	0	71
16395329	m	cn	KR	2019	0	1	1	0	0	0	0	0	0	0	0	0	
16395455	f	Not repo	AU	2019	0	1	0	1	0	0	0	0	0	0	0	0	60.5
16395456	f	md	JP	2019	0	1	0	1	0	0	0	0	0	0	0	0	71.2
16395621	Not	cn	US	2019	0	0	0	0	0	0	0	0	0	0	0	0	
16395759	Not	md	US	2019	0	1	0	0	0	1	0	0	0	0	0	0	
16395764	Not	md	US	2019	0	1	0	0	0	1	0	0	0	0	0	1	
16395768	Not	md	US	2019	0	1	0	0	0	1	0	0	0	0	0	1	
16395772	Not	md	US	2019	0	1	0	0	0	1	0	0	0	0	0	0	
16395773	Not	md	US	2019	0	1	0	0	0	1	0	0	0	0	0	0	

16395775	Not	md	US	2019	0	1	0	0	0	1	0	0	0	0	0	0	
16395798	Not	md	US	2019	0	0	0	0	0	0	0	0	0	0	0	0	
16395930	m	cn	US	2019	0	0	0	0	0	0	0	0	0	0	0	0	67
16396139	m	cn	JP	2019	0	1	1	0	0	0	0	0	0	0	0	0	54
16396284	m	cn	JP	2019	0	1	1	0	0	0	0	0	0	0	0	0	72
16396711	Not	md	JP	2019	0	1	0	0	1	0	0	0	0	0	0	0	
16396930	m	md	JP	2019	0	1	1	0	0	0	0	0	0	0	0	0	58
16396954	f	cn	DE	2019	0	0	0	0	0	0	1	1	0	0	0	0	
16397720	m	md	JP	2019	0	0	0	0	0	0	0	0	0	0	0	0	70
16397824	f	md	JP	2019	1	0	0	0	0	0	0	0	0	0	0	0	60.1
16398452	f	ph	FR	2019	0	0	0	0	0	0	0	0	0	0	0	0	36
16398471	f	md	JP	2019	1	0	0	0	0	0	0	0	0	0	0	0	76
16399041	f	md	PL	2019	1	0	0	0	0	0	0	0	0	0	0	0	
16399160	m	md	JP	2019	0	1	0	0	1	0	0	0	0	0	0	1	74
16399493	m	cn	JP	2019	0	1	1	0	0	0	0	0	0	0	0	0	68
16399958	Not	ot	NL	2019	0	1	1	0	0	0	0	0	0	0	0	0	
16400429	Not	md	DE	2019	0	1	1	0	0	0	0	0	0	0	0	0	
16400455	m	md	JP	2019	0	1	0	1	0	0	0	0	0	0	0	0	78
16400460	m	md	FR	2019	0	0	0	0	0	0	0	0	0	0	0	0	52.5
16400694	m	md	JP	2019	0	1	0	0	1	0	0	0	0	0	0	0	
16400732	f	cn	DE	2019	0	1	1	0	0	0	0	0	0	0	0	0	
16400749	f	cn	JP	2019	0	1	1	0	0	0	0	0	0	0	0	0	66
16401584	m	cn	AU	2019	1	0	0	0	0	0	0	0	0	0	0	0	
16402088	f	md	JP	2019	0	1	0	0	0	1	0	0	0	0	0	0	72
16402167	f	md	JP	2019	0	1	0	0	0	1	0	0	0	0	0	1	70
16403499	m	cn	JP	2019	0	1	1	0	0	0	0	0	0	0	0	0	59
16403653	m	cn	CN	2019	0	1	0	0	1	0	0	0	0	0	0	0	56
16403688	f	md	JP	2019	0	1	0	0	0	1	0	0	0	0	0	1	80
16403790	m	md	CN	2019	0	1	0	0	0	1	0	0	0	0	0	0	73
16403923	Not	ot	US	2019	0	1	0	0	1	0	0	0	0	0	0	0	
16404146	m	ot	JP	2019	0	1	0	1	0	0	0	0	0	0	0	1	74
16404517	Not	md	US	2019	1	0	0	0	0	0	0	0	0	0	0	1	
16404787	m	cn	JP	2019	0	1	1	0	0	0	0	0	0	0	0	0	65
16404954	f	md	JP	2019	0	1	0	1	0	0	0	0	0	0	0	1	77

16405023	Not	md	IT	2019	0	1	0	1	0	0	0	0	0	0	0	0	
16405035	m	md	GR	2019	0	1	0	1	0	0	0	0	0	0	0	0	65
16405079	f	md	JP	2019	1	0	0	0	0	0	0	0	0	0	0	0	69
16405142	m	md	GB	2019	0	0	0	0	0	0	0	0	0	0	0	0	66
16405413	m	ot	JP	2019	0	1	0	1	0	0	0	0	0	0	0	0	70
16405457	m	cn	JP	2019	0	1	1	0	0	0	0	0	0	0	0	0	78
16405919	f	md	CN	2019	0	1	0	0	1	0	0	0	0	0	0	0	
16405981	f	ot	US	2019	0	1	0	0	1	0	0	0	0	0	0	0	49
16406321	m	Not repo	CN	2019	1	0	0	0	0	0	0	0	0	0	0	0	75
16406504	Not	cn	JP	2019	0	1	1	0	0	0	0	0	0	0	0	0	80
16406669	m	ot	PL	2019	0	1	0	0	1	0	0	0	0	0	0	0	62
16407232	m	cn	CN	2019	0	0	0	0	0	0	0	0	0	0	0	0	65
16407466	m	ot	DE	2019	0	0	0	0	0	0	0	0	0	0	0	0	77
16408087	f	md	FR	2019	0	1	0	0	0	1	0	0	0	0	0	0	
16408292	f	cn	KR	2019	0	1	1	0	0	0	0	0	0	0	0	0	
16408718	f	cn	JP	2019	0	1	1	0	0	0	0	0	0	0	0	0	62
16409024	m	md	AT	2019	0	1	0	0	1	0	0	0	0	0	0	0	79
16409025	m	md	AT	2019	0	1	0	0	1	0	0	0	0	0	0	0	53
16409712	m	md	AT	2019	0	1	0	0	1	0	0	0	0	0	0	0	79
16410080	m	cn	JP	2019	0	1	1	0	0	0	0	0	0	0	0	0	65
16410277	m	cn	CN	2019	0	1	0	0	1	0	0	0	0	0	0	0	
16410323	m	ot	MX	2019	0	0	0	0	0	0	0	0	0	0	0	0	60
16410589	m	ot	US	2019	0	1	1	0	0	0	0	0	0	0	0	0	72
16410783	m	ot	KR	2019	0	1	0	0	1	0	0	0	0	0	0	0	68
16410842	m	cn	JP	2019	0	1	1	0	0	0	0	0	0	0	0	1	69
16411048	Not	ot	JP	2019	0	1	0	1	0	0	0	0	0	0	0	1	73
16411352	f	cn	US	2019	1	0	0	0	0	0	0	0	0	0	0	0	59
16412010	m	cn	JP	2019	0	1	1	0	0	0	0	0	0	0	0	0	73
16412398	Not	ot	PL	2019	0	1	0	0	1	0	0	0	0	0	0	0	
16412518	f	cn	HU	2019	0	1	1	0	0	0	0	0	0	0	0	0	68
16412618	f	md	JP	2019	0	1	0	0	0	1	0	0	0	0	0	0	62
16412632	m	cn	JP	2019	0	1	0	0	1	0	0	0	0	0	0	1	66
16412683	m	cn	JP	2019	0	1	1	0	0	0	0	0	0	0	0	0	64
16412769	m	md	FR	2019	0	1	0	0	0	1	0	0	0	0	0	0	77

16412800	f	cn	US	2019	0	1	1	0	0	0	0	0	0	0	0	0	
16412851	m	ph	JP	2019	0	1	0	0	1	0	0	0	0	0	0	1	59
16413051	m	cn	CN	2019	0	1	0	0	1	0	0	0	0	0	0	0	64
16413222	m	md	JP	2019	0	1	0	0	1	0	0	0	0	0	0	0	70
16413582	Not	cn	JP	2019	0	1	1	0	0	0	0	0	0	0	0	0	76
16413632	m	cn	CN	2019	0	0	0	0	0	0	0	0	0	0	0	0	
16413839	f	cn	Not re	2019	0	0	0	0	0	0	0	0	0	0	0	0	
16413856	m	md	BE	2019	0	1	0	0	0	1	0	0	0	0	0	0	70
16414324	Not	ot	CN	2019	1	0	0	0	0	0	0	0	0	0	0	0	
16414362	m	cn	US	2019	0	1	1	0	0	0	0	0	0	0	0	0	58
16414411	m	cn	JP	2019	0	1	1	0	0	0	0	0	0	0	0	0	60
16414469	m	md	JP	2019	0	1	0	0	0	1	0	0	0	0	0	0	65
16414499	m	cn	NP	2019	0	0	0	0	0	0	0	0	0	0	0	0	76
16414878	f	md	US	2019	0	1	1	0	0	0	0	0	0	0	0	0	61
16416194	m	md	DE	2019	0	1	0	0	0	1	0	0	0	0	0	0	77
16416443	m	md	JP	2019	0	1	1	0	0	0	0	0	0	0	0	0	71
16416630	f	md	JP	2019	0	0	0	0	0	0	0	0	0	0	0	0	70
16416679	m	cn	JP	2019	0	1	1	0	0	0	0	0	0	0	0	0	
16416796	m	md	MX	2019	0	0	0	0	0	0	0	0	0	0	0	0	51
16417108	m	cn	FR	2019	0	1	1	0	0	0	0	0	0	0	0	0	60
16417360	m	cn	JP	2019	0	1	1	0	0	0	0	0	0	0	0	0	
16417363	m	md	JP	2019	0	1	1	0	0	0	0	0	0	0	0	1	62
16418641	m	cn	JP	2019	0	1	1	0	0	0	0	0	0	0	0	0	
16418714	m	md	FR	2019	0	1	0	0	1	0	0	0	0	0	0	0	86
16418858	m	cn	JP	2019	0	1	1	0	0	0	0	0	0	0	0	1	69
16418999	f	md	JP	2019	1	0	0	0	0	0	0	0	0	0	0	0	
16419155	m	md	US	2019	0	1	0	1	0	0	0	0	0	0	0	0	
16419178	m	cn	US	2019	1	0	0	0	0	0	0	0	0	0	0	0	
16419316	m	ot	DE	2019	0	1	0	0	1	0	0	0	0	0	0	0	68
16419695	Not	md	US	2019	0	0	0	0	0	0	0	0	0	0	0	0	
16419880	m	md	ES	2019	0	1	0	0	0	1	0	0	0	0	0	0	76
16420168	Not	md	GB	2019	0	0	0	0	0	0	0	0	0	0	0	0	
16420512	m	cn	JP	2019	0	1	1	0	0	0	0	0	0	0	0	0	70
16420997	f	ot	GB	2019	0	0	0	0	0	0	0	0	0	0	0	0	87

16420998	f	ot	GB	2019	0	0	0	0	0	0	0	0	0	0	0	0	67
16421107	f	cn	JP	2019	0	1	1	0	0	0	0	0	0	0	0	0	33
16421161	f	cn	US	2019	1	0	0	0	0	0	0	0	0	0	0	0	
16421659	m	ot	FR	2019	0	1	1	0	0	0	0	0	0	0	0	0	62
16422350	m	cn	DE	2019	0	1	1	0	0	0	0	0	0	0	0	0	62
16422593	m	cn	JP	2019	0	1	1	0	0	0	0	0	0	0	0	0	86
16422599	f	ph	GB	2019	0	0	0	0	0	0	0	0	0	0	0	0	54
16423217	m	cn	US	2019	1	0	0	0	0	0	0	0	0	0	0	0	68
16423267	m	ot	JP	2019	0	0	0	0	0	0	0	0	0	0	0	0	72
16423492	m	md	VN	2019	0	0	0	0	0	0	0	0	0	0	0	0	80
16423603	Not	ot	BE	2019	0	0	0	0	0	0	0	0	0	0	0	0	
16423604	Not	ot	BE	2019	0	0	0	0	0	0	0	0	0	0	0	0	
16423606	m	cn	IN	2019	0	1	0	0	1	0	0	0	0	0	0	0	
16423662	f	md	US	2019	0	1	1	0	0	0	0	0	0	0	0	1	
16423688	m	cn	AU	2019	0	1	1	0	0	0	0	0	0	0	0	0	
16423755	m	cn	JP	2019	0	1	1	0	0	0	0	0	0	0	0	0	75
16423879	m	cn	JP	2019	0	1	1	0	0	0	0	0	0	0	0	0	54
16424628	f	ot	DE	2019	0	0	0	0	0	0	0	0	0	0	0	0	65
16424653	f	ot	CN	2019	1	0	0	0	0	0	0	0	0	0	0	0	
16424704	m	cn	GB	2019	1	0	0	0	0	0	0	0	0	0	0	0	
16425263	f	md	JP	2019	0	1	0	0	0	1	0	0	0	0	0	0	70
16425300	f	ph	US	2019	0	1	0	1	0	0	0	0	0	0	0	0	78.4
16425439	Not	md	US	2019	0	0	0	0	0	0	0	0	0	0	0	0	
16426519	f	Not repo	CN	2019	1	0	0	0	0	0	0	0	0	0	0	1	39.8
16427278	m	md	JP	2019	0	1	0	1	0	0	0	0	0	0	0	1	
16427423	Not	ot	CA	2019	0	0	0	0	0	0	0	0	0	0	0	0	
16427564	m	md	JP	2019	0	1	0	1	0	0	0	0	0	0	0	1	78.7
16427590	m	cn	JP	2019	0	1	1	0	0	0	0	0	0	0	0	0	63
16427752	m	md	JP	2019	0	1	0	1	0	0	0	0	0	0	0	1	71
16427965	f	cn	US	2019	0	1	0	0	1	0	0	0	0	0	0	0	
16428159	m	cn	JP	2019	0	1	1	0	0	0	0	0	0	0	0	0	48
16428219	m	cn	JP	2019	0	1	1	0	0	0	0	0	0	0	0	0	78
16428313	Not	md	JP	2019	0	1	0	0	1	0	0	0	0	0	0	0	
16428790	Not	md	JP	2019	0	1	0	0	1	0	0	0	0	0	0	0	

16428896	m	cn	PH	2019	0	1	1	0	0	0	0	0	0	0	0	0	69
16428901	m	md	JP	2019	0	1	0	1	0	0	0	0	0	0	0	0	74
16428974	m	cn	JP	2019	0	1	1	0	0	0	0	0	0	0	0	0	73
16428991	m	cn	JP	2019	0	1	1	0	0	0	0	0	0	0	0	0	79
16429100	m	ot	US	2019	0	1	0	0	1	0	0	0	0	0	0	1	54
16429128	f	ot	CA	2019	0	1	1	0	0	0	0	0	0	0	0	0	60
16429161	m	md	JP	2019	0	1	0	0	0	1	0	0	0	0	0	0	
16429188	m	ot	CA	2019	0	1	1	0	0	0	0	0	0	0	0	0	82
16429280	f	ot	CA	2019	0	1	1	0	0	0	0	0	0	0	0	0	78
16429356	m	cn	CN	2019	0	1	0	0	1	0	0	0	0	0	0	0	69
16429442	m	cn	JP	2019	0	1	1	0	0	0	0	0	0	0	0	1	62
16429481	f	ot	NL	2019	1	0	0	0	0	0	0	0	0	0	0	0	46
16430495	m	cn	CN	2019	0	1	0	0	1	0	0	0	0	0	0	0	69
16430529	Not	ph	JP	2019	0	1	0	0	1	0	0	0	0	0	0	1	
16430541	f	md	US	2019	0	1	0	0	1	0	0	0	0	0	0	0	61
16430623	m	ph	JP	2019	1	0	0	0	0	0	0	0	0	0	0	0	81
16431006	m	ot	DE	2019	0	1	1	0	0	0	0	0	0	0	0	0	62
16431311	m	cn	JP	2019	0	0	0	0	0	0	0	0	0	0	0	0	80
16431646	f	cn	EG	2019	0	1	1	0	0	0	0	0	0	0	0	0	77
16431890	m	cn	AT	2019	0	1	0	0	1	0	0	0	0	0	0	0	79
16432421	m	ot	FR	2019	1	0	0	0	0	0	0	0	0	0	0	0	79
16433425	f	md	JP	2019	0	1	0	1	0	0	0	0	0	0	0	1	
16433892	m	Not repo	AU	2019	0	1	0	1	0	0	0	0	0	0	0	0	77
16433949	f	md	JP	2019	0	1	0	1	0	0	0	0	0	0	0	1	70
16433977	f	md	JP	2019	1	0	0	0	0	0	0	0	0	0	0	1	79.4
16434232	f	cn	US	2019	1	0	0	0	0	0	0	0	0	0	0	0	
16434528	m	md	JP	2019	0	1	0	0	1	0	0	0	0	0	0	1	75
16435142	f	md	JP	2019	0	1	0	1	0	0	0	0	0	0	0	0	68
16435220	m	cn	JP	2019	0	1	1	0	0	0	0	0	0	0	0	0	75
16435875	m	ot	ZA	2019	0	1	0	0	1	0	0	0	0	0	0	0	76
16438059	m	cn	JP	2019	0	1	1	0	0	0	0	0	0	0	0	0	80
16438583	m	cn	JP	2019	0	1	1	0	0	0	0	0	0	0	0	0	68
16438679	m	cn	JP	2019	0	1	1	0	0	0	0	0	0	0	0	0	69
16438980	m	md	JP	2019	0	1	0	0	1	0	0	0	0	0	0	0	65

16439261	f	md	US	2019	0	1	0	1	0	0	0	0	0	0	0	1		
16439432	m	md	DE	2019	0	1	1	0	0	0	0	0	0	0	0	0	69	
16439532	m	cn	JP	2019	0	1	1	0	0	0	0	0	0	0	0	0	62	
16439587	Not	md	JP	2019	1	0	0	0	0	0	0	0	0	0	0	1		
16439736	f	cn	US	2019	1	0	0	0	0	0	0	0	0	0	0	0	81	
16439855	f	md	DE	2019	0	1	1	0	0	0	0	0	0	0	0	1	55	
16439907	m	cn	US	2019	0	1	1	0	0	0	0	0	0	0	0	0		
16440078	Not	md	SG	2019	0	1	0	1	0	0	0	0	0	0	0	0		
16440142	f	cn	US	2019	1	0	0	0	0	0	0	0	0	0	0	0	72	
16440251	f	ot	US	2019	0	1	0	0	1	0	0	0	0	0	0	0	52	
16440319	f	cn	JP	2019	0	1	1	0	0	0	0	0	0	0	0	0	78	
16440846	m	cn	JP	2019	0	1	1	0	0	0	0	0	0	0	0	0	84	
16440855	Not	ot	IT	2019	0	0	0	0	0	0	0	0	0	0	0	0		
16440856	Not	ot	IT	2019	0	0	0	0	0	0	0	0	0	0	0	0		
16440857	Not	ot	IT	2019	0	0	0	0	0	0	0	0	0	0	0	0		
16441004	f	cn	US	2019	0	1	0	0	1	0	0	0	0	0	0	0		
16441206	f	md	GB	2019	0	0	0	0	0	0	0	0	0	0	0	0		
16442832	m	cn	US	2019	1	0	0	0	0	0	0	0	0	0	0	0	57	
16442972	f	ot	DE	2019	0	1	1	0	0	0	0	0	0	0	0	0	68	
16443505	f	cn	GB	2019	1	0	0	0	0	0	0	0	0	0	0	0	76	
16443773	m	md	JP	2019	0	1	0	0	0	1	0	0	0	0	0	0	63	
16443986	f	cn	GB	2019	1	0	0	0	0	0	0	0	0	0	0	0		
16443990	m	cn	GB	2019	1	0	0	0	0	0	0	0	0	0	0	0		
16444017	m	ot	IT	2019	0	1	0	0	1	0	0	0	0	0	0	0		
16444045	m	md	JP	2019	0	1	0	0	1	0	0	0	0	0	0	1	70	
16444119	m	ot	IE	2019	0	1	0	0	0	1	0	0	0	0	0	0		
16444141	m	md	FI	2019	0	1	0	0	0	1	0	0	0	0	0	0	58	
16444209	m	cn	JP	2019	0	1	1	0	0	0	0	0	0	0	0	0	47	
16444319	Not	ph	IT	2019	0	0	0	0	0	0	0	0	0	0	0	0		
16444997	f	cn	JP	2019	0	1	1	0	0	0	0	0	0	0	0	0	63	
16445157	m	cn	JP	2019	0	1	1	0	0	0	0	0	0	0	0	0	65	
16445163	Not	ph	JP	2019	1	0	0	0	0	0	0	0	0	0	0	0		
16445205	f	cn	US	2019	0	0	0	0	0	0	0	0	0	0	0	0		
16445210	f	cn	KR	2019	0	0	0	0	0	0	0	0	0	0	0	0	56	

16445222	Not	md	JP	2019	0	1	1	0	0	0	0	0	0	0	0	0	
16445238	Not	ot	IT	2019	0	0	0	0	0	0	0	0	0	0	0	0	
16445308	m	ot	US	2019	0	0	0	0	0	0	0	0	0	0	0	0	66
16445847	m	md	JP	2019	0	1	0	1	0	0	0	0	0	0	0	0	64
16445894	Not	md	JP	2019	1	0	0	0	0	0	0	0	0	0	0	0	
16445934	m	ot	CN	2019	0	0	0	0	0	0	0	0	0	0	0	0	73
16446114	m	md	IN	2019	0	1	0	1	0	0	0	0	0	0	0	1	66
16446137	f	md	US	2019	0	0	0	0	0	0	0	0	0	0	0	0	56
16446209	Not	md	PL	2019	1	0	0	0	0	0	0	0	0	0	0	0	
16446483	m	cn	JP	2019	0	1	1	0	0	0	0	0	0	0	0	0	78
16446486	m	md	GB	2019	0	1	0	1	0	0	0	0	0	0	0	0	
16446528	m	ot	BE	2019	0	1	0	0	1	0	0	0	0	0	0	0	66
16446628	f	ot	DK	2019	0	0	0	0	0	0	0	0	0	0	0	0	79
16446630	m	ot	DE	2019	0	1	0	0	1	0	0	0	0	0	0	0	
16447214	f	md	JP	2019	0	1	0	0	1	0	0	0	0	0	0	1	71
16447300	m	md	KR	2019	1	0	0	0	0	0	0	0	0	0	0	0	57
16447564	m	md	JP	2019	0	1	0	0	1	0	0	0	0	0	0	1	70
16448589	m	cn	DE	2019	0	1	1	0	0	0	0	0	0	0	0	0	70
16448792	f	cn	JP	2019	0	1	1	0	0	0	0	0	0	0	0	0	75
16448806	m	md	FR	2019	0	1	0	0	0	1	0	0	0	0	0	0	83
16448859	m	md	JP	2019	0	1	0	0	1	0	0	0	0	0	0	1	66
16449053	m	md	JP	2019	0	1	1	0	0	0	0	0	0	0	0	0	
16449093	f	md	CN	2019	0	1	0	0	0	1	0	0	0	0	0	0	56
16449106	m	ph	JP	2019	0	1	0	0	1	0	0	0	0	0	0	0	70
16449397	m	md	JP	2019	0	1	0	0	1	0	0	0	0	0	0	0	80
16449418	m	ot	FR	2019	0	1	0	0	1	0	0	0	0	0	0	0	51
16449700	m	cn	JP	2019	0	1	1	0	0	0	0	0	0	0	0	0	75
16449721	m	md	JP	2019	0	1	1	0	0	0	0	0	0	0	0	0	47
16449810	f	ot	FR	2019	0	1	0	0	1	0	0	0	0	0	0	0	64
16449910	Not	md	JP	2019	0	1	0	1	0	0	0	0	0	0	0	0	
16450515	f	ot	DE	2019	0	1	1	0	0	0	0	0	0	0	0	0	
16450540	f	Not repo	CN	2019	1	0	0	0	0	0	0	0	0	0	0	0	
16450784	m	cn	JP	2019	0	1	1	0	0	0	0	0	0	0	0	0	71
16450847	m	ot	NL	2019	0	0	0	0	0	0	0	0	0	0	0	0	67

16450856	Not	md	CA	2019	0	0	0	0	0	0	0	0	0	0	0	0	
16450905	f	md	JP	2019	0	0	0	0	0	0	0	0	0	0	0	0	69
16450940	f	md	US	2019	0	1	0	1	0	0	0	0	0	0	0	1	
16451158	Not	cn	JP	2019	0	1	1	0	0	0	0	0	0	0	0	0	76
16451340	f	ot	US	2019	0	0	0	0	0	0	0	0	0	0	0	0	61
16451344	Not	md	GB	2019	0	0	0	0	0	0	0	0	0	0	0	0	
16451429	Not	md	GB	2019	0	0	0	0	0	0	0	0	0	0	0	0	
16451866	Not	ph	US	2019	0	0	0	0	0	0	0	0	0	0	0	0	
16452098	f	ot	US	2019	0	1	1	0	0	0	0	0	0	0	0	0	62
16453667	m	ot	DE	2019	0	1	0	0	1	0	0	0	0	0	0	0	41
16453743	m	Not repo	RU	2019	0	1	0	1	0	0	0	0	0	0	0	0	65.3
16453763	m	Not repo	RU	2019	0	1	0	1	0	0	0	0	0	0	0	0	57.5
16453786	m	md	JP	2019	0	1	0	1	0	0	0	0	0	0	0	0	62
16453966	m	md	JP	2019	0	1	1	0	0	0	0	0	0	0	0	0	72
16454027	m	cn	JP	2019	0	1	1	0	0	0	0	0	0	0	0	0	71
16454150	m	cn	JP	2019	0	1	1	0	0	0	0	0	0	0	0	0	71
16454303	m	ot	TN	2019	0	0	0	0	0	0	0	0	0	0	0	0	75
16454396	f	cn	JP	2019	0	1	1	0	0	0	0	0	0	0	0	0	62
16454500	f	cn	JP	2019	0	1	1	0	0	0	0	0	0	0	0	0	75
16454673	m	md	IT	2019	0	1	0	0	1	0	0	0	0	0	0	1	73
16455230	m	cn	JP	2019	0	1	1	0	0	0	0	0	0	0	0	0	49
16455327	m	md	TR	2019	0	0	0	0	0	0	0	0	0	0	0	0	60
16455782	m	Not repo	CN	2019	1	0	0	0	0	0	0	0	0	0	0	0	63
16455997	f	md	JP	2019	0	0	0	0	0	0	0	0	0	0	0	0	69
16456069	f	md	JP	2019	0	0	0	0	0	0	0	0	0	0	0	0	68
16456184	Not	ot	BE	2019	0	0	0	0	0	0	0	0	0	0	0	0	
16456186	Not	ot	BE	2019	0	0	0	0	0	0	0	0	0	0	0	0	
16456498	f	cn	LB	2019	0	0	0	0	0	0	0	0	0	0	0	0	44
16456549	m	ot	DE	2019	0	1	0	0	1	0	0	0	0	0	0	0	71
16457013	Not	md	SG	2019	0	1	0	1	0	0	0	0	0	0	0	0	
16457428	m	cn	GB	2019	1	0	0	0	0	0	0	0	0	0	0	0	75
16457534	Not	md	JP	2019	1	0	0	0	0	0	0	0	0	0	0	0	
16457536	Not	md	JP	2019	1	0	0	0	0	0	0	0	0	0	0	0	
16459151	m	md	NL	2019	0	1	1	0	0	0	0	0	0	0	0	0	62

16460209	m	md	JP	2019	0	1	0	1	0	0	0	0	0	0	0	1	69.1
16460278	m	ot	CN	2019	0	1	0	0	1	0	0	0	0	0	0	0	65
16460783	f	cn	US	2019	1	0	0	0	0	0	0	0	0	0	0	0	76
16461423	f	md	JP	2019	0	1	0	0	1	0	0	0	0	0	0	0	71
16461615	m	cn	CN	2019	0	1	0	0	1	0	0	0	0	0	0	0	52
16461626	m	cn	JP	2019	0	1	1	0	0	0	0	0	0	0	0	0	72
16461942	m	cn	JP	2019	0	1	1	0	0	0	0	0	0	0	0	1	63
16462446	m	md	ES	2019	0	0	0	0	0	0	0	0	0	0	0	0	70
16462566	Not	md	JP	2019	0	1	1	0	0	0	0	0	0	0	0	0	
16462601	f	cn	US	2019	1	0	0	0	0	0	0	0	0	0	0	0	
16462974	f	md	JP	2019	1	0	0	0	0	0	0	0	0	0	0	0	50
16463030	Not	md	JP	2019	1	0	0	0	0	0	0	0	0	0	0	1	
16463068	f	md	JP	2019	0	1	0	0	0	1	0	0	0	0	0	0	68
16463104	m	Not repo	CN	2019	1	0	0	0	0	0	0	0	0	0	0	0	76.4
16463862	f	ph	GB	2019	0	0	0	0	0	0	0	0	0	0	0	0	
16464378	f	ot	TW	2019	0	0	0	0	0	0	0	0	0	0	0	0	57
16464460	m	md	JP	2019	0	1	0	1	0	0	0	0	0	0	0	0	68
16465070	m	cn	JP	2019	0	1	1	0	0	0	0	0	0	0	0	1	75
16465119	f	cn	CN	2019	1	0	0	0	0	0	0	0	0	0	0	0	62
16465120	f	md	JP	2019	1	0	0	0	0	0	0	0	0	0	0	1	71
16465335	f	cn	DE	2019	0	1	1	0	0	0	0	0	0	0	0	0	68
16465693	m	md	JP	2019	0	1	0	1	0	0	0	0	0	0	0	1	80
16465705	m	cn	CN	2019	0	1	0	0	1	0	0	0	0	0	0	0	
16465732	f	md	GB	2019	0	1	0	1	0	0	0	0	0	0	0	0	64.2
16465783	f	cn	JP	2019	0	1	1	0	0	0	0	0	0	0	0	1	81
16465791	m	ph	JP	2019	0	1	1	0	0	0	0	0	0	0	0	0	78
16465838	m	md	NL	2019	0	1	0	0	1	0	0	0	0	0	0	0	70
16466321	m	cn	BE	2019	0	1	1	0	0	0	0	0	0	0	0	0	50
16466343	m	md	JP	2019	0	1	0	0	0	1	0	0	0	0	0	0	61
16466355	f	cn	SG	2019	0	0	0	0	0	0	0	0	0	0	0	0	
16467244	m	md	VN	2019	0	1	0	0	0	1	0	0	0	0	0	0	
16467301	m	cn	CN	2019	0	1	0	0	1	0	0	0	0	0	0	0	
16467347	f	md	JP	2019	0	0	0	0	0	0	0	0	0	0	0	0	74
16467429	m	md	FR	2019	0	1	0	0	0	1	0	0	0	0	0	0	

16467535	m	md	JP	2019	0	1	0	0	0	1	0	0	0	0	0	0	74
16467704	f	md	DE	2019	0	0	0	0	0	0	1	1	0	0	0	0	
16467973	m	md	JP	2019	0	1	0	0	0	1	0	0	0	0	0	0	67
16468386	m	md	JP	2019	0	1	0	0	1	0	0	0	0	0	0	0	80
16468471	f	cn	CN	2019	0	1	0	0	1	0	0	0	0	0	0	0	
16468563	m	cn	AR	2019	0	0	0	0	0	0	0	0	0	0	0	0	54
16469568	m	md	DE	2019	0	0	0	0	0	0	0	0	0	0	0	0	64
16470062	m	md	US	2019	0	1	0	0	1	0	0	0	0	0	0	0	42
16470068	m	md	JP	2019	0	1	0	0	1	0	0	0	0	0	0	0	79
16470211	f	cn	US	2019	0	1	1	0	0	0	0	0	0	0	0	0	
16470673	m	cn	JP	2019	0	1	1	0	0	0	0	0	0	0	0	0	
16470791	f	md	US	2019	1	0	0	0	0	0	0	0	0	0	0	0	44
16471001	Not	md	IT	2019	1	0	0	0	0	0	0	0	0	0	0	0	
16471096	Not	md	IT	2019	1	0	0	0	0	0	0	0	0	0	0	0	
16471120	m	ot	CN	2019	0	0	0	0	0	0	0	0	0	0	0	0	63
16471125	m	md	JP	2019	1	0	0	0	0	0	0	0	0	0	0	0	63
16471209	f	md	CH	2019	0	0	0	0	0	0	0	0	0	0	0	0	
16471258	m	cn	JP	2019	0	1	1	0	0	0	0	0	0	0	0	0	72
16471432	f	ot	DE	2019	1	0	0	0	0	0	0	0	0	0	0	0	59
16471600	m	md	DE	2019	0	0	0	0	0	0	0	0	0	0	0	0	55
16472589	m	ot	FR	2019	0	1	0	0	1	0	0	0	0	0	0	0	45
16472893	f	ot	JP	2019	0	0	0	0	0	0	0	0	0	0	0	0	69
16473476	Not	md	JP	2019	0	1	0	0	1	0	0	0	0	0	0	0	
16473669	m	ph	JP	2019	0	1	0	0	1	0	0	0	0	0	0	0	50
16473789	f	ot	US	2019	0	1	1	0	0	0	0	0	0	0	0	0	62
16473827	m	ot	GB	2019	0	0	0	0	0	0	0	0	0	0	0	0	61
16473845	Not	md	AU	2019	0	1	0	1	0	0	0	0	0	0	0	0	
16473849	f	Not repo	GB	2019	0	1	0	1	0	0	0	0	0	0	0	0	65
16473940	f	md	IT	2019	1	0	0	0	0	0	0	0	0	0	0	0	
16474632	m	cn	JP	2019	0	1	1	0	0	0	0	0	0	0	0	1	74
16475528	f	cn	JP	2019	0	1	1	0	0	0	0	0	0	0	0	1	64
16475763	m	md	JP	2019	0	1	0	1	0	0	0	0	0	0	0	1	76
16475949	m	md	JP	2019	0	1	0	1	0	0	0	0	0	0	0	1	70
16476214	f	md	JP	2019	0	0	0	0	0	0	0	0	0	0	0	0	69

16476272	m	ph	IT	2019	0	0	0	0	0	0	0	0	0	0	0	0	78
16476637	m	cn	JP	2019	0	1	1	0	0	0	0	0	0	0	0	0	70
16476651	m	cn	JP	2019	0	1	1	0	0	0	0	0	0	0	0	0	50
16476661	m	cn	JP	2019	0	1	1	0	0	0	0	0	0	0	0	0	74
16477261	f	md	FR	2019	0	1	0	0	0	1	0	0	0	0	0	0	75
16477440	m	cn	JP	2019	0	1	1	0	0	0	0	0	0	0	0	0	80
16478016	m	cn	BE	2019	0	0	0	0	0	0	0	0	0	0	0	0	
16478262	m	cn	CN	2019	0	1	0	0	1	0	0	0	0	0	0	1	65
16478416	f	md	US	2019	1	0	0	0	0	0	0	0	0	0	0	1	
16479021	f	md	JP	2019	1	0	0	0	0	0	0	0	0	0	0	0	90
16479647	f	md	DK	2019	0	0	0	0	0	0	0	0	0	0	0	0	79
16479895	f	md	NL	2019	0	0	0	0	0	0	0	0	0	0	0	0	79
16480179	m	ot	JP	2019	0	1	0	1	0	0	0	0	0	0	0	1	81
16480273	f	md	JP	2019	1	0	0	0	0	0	0	0	0	0	0	0	75
16480276	m	ph	FR	2019	0	1	0	1	0	0	0	0	0	0	0	0	68.7
16480278	m	md	JP	2019	0	1	0	1	0	0	0	0	0	0	0	0	78
16480287	m	Not repo	CN	2019	1	0	0	0	0	0	0	0	0	0	0	0	58.8
16480511	m	md	JP	2019	0	1	0	0	1	0	0	0	0	0	0	0	66
16480516	m	ot	JP	2019	0	0	0	0	0	0	0	0	0	0	0	0	77
16481333	Not	md	NL	2019	0	0	0	0	0	0	0	0	0	0	0	0	
16481460	Not	md	JP	2019	0	1	0	0	1	0	0	0	0	0	0	0	
16481519	m	md	JP	2019	0	1	0	0	0	1	0	0	0	0	0	0	
16481542	Not	md	JP	2019	1	0	0	0	0	0	0	0	0	0	0	0	
16481925	f	cn	CN	2019	0	1	0	0	1	0	0	0	0	0	0	0	40
16482632	m	ot	US	2019	0	1	0	0	1	0	0	0	0	0	0	1	72
16482791	f	md	BE	2019	0	1	0	0	0	1	0	0	0	0	0	0	62
16482823	m	md	JP	2019	0	1	0	0	1	0	0	0	0	0	0	1	70
16482878	m	ot	RO	2019	0	1	0	0	1	0	0	0	0	0	0	0	61
16482933	m	md	HK	2019	0	1	0	0	0	1	0	0	0	0	0	0	64
16484890	Not	md	JP	2019	0	1	0	1	0	0	0	0	0	0	0	0	
16485287	f	cn	KR	2019	0	1	1	0	0	0	0	0	0	0	0	0	
16485498	f	md	JP	2019	0	1	0	1	0	0	0	0	0	0	0	1	76
16486790	m	cn	US	2019	0	1	1	0	0	0	0	0	0	0	0	0	
16487240	m	cn	JP	2019	0	1	1	0	0	0	0	0	0	0	0	0	79

16487634	m	ot	MX	2019	0	0	0	0	0	0	0	0	0	0	0	0	50
16487980	m	md	FR	2019	0	0	0	0	0	0	0	0	0	0	0	0	77
16488719	m	ot	AU	2019	0	1	0	0	1	0	0	0	0	0	0	0	65
16488792	m	md	US	2019	0	1	0	0	1	0	0	0	0	0	0	0	77
16488937	m	md	US	2019	0	1	1	0	0	0	0	0	0	0	0	0	85
16489316	Not	md	JP	2019	1	0	0	0	0	0	0	0	0	0	0	0	
16489364	m	cn	JP	2019	0	1	1	0	0	0	0	0	0	0	0	0	62
16489495	f	ot	IT	2019	0	0	0	0	0	0	0	0	0	0	0	0	80
16489518	m	md	JP	2019	0	1	1	0	0	0	0	0	0	0	0	0	75
16489695	m	ot	US	2019	0	1	0	0	1	0	0	0	0	0	0	0	
16490251	m	md	JP	2019	0	1	0	0	0	1	0	0	0	0	0	0	47
16490299	m	ot	NL	2019	0	0	0	0	0	0	0	0	0	0	0	0	67
16490449	m	md	FR	2019	0	1	0	0	0	1	0	0	0	0	0	0	66
16490950	f	cn	CN	2019	1	0	0	0	0	0	0	0	0	0	0	0	
16491340	f	Not repo	CN	2019	1	0	0	0	0	0	0	0	0	0	0	0	67
16491440	m	cn	CN	2019	0	1	0	0	1	0	0	0	0	0	0	0	
16491487	f	cn	CN	2019	1	0	0	0	0	0	0	0	0	0	0	0	62.3
16491747	m	cn	JP	2019	0	1	1	0	0	0	0	0	0	0	0	0	69
16492337	Not	md	JP	2019	1	0	0	0	0	0	0	0	0	0	0	0	80
16492840	m	md	PL	2019	0	0	0	0	0	0	0	0	0	0	0	0	
16493308	m	md	FR	2019	0	1	0	0	0	1	0	0	0	0	0	0	65
16494236	f	md	JP	2019	0	1	0	0	0	1	0	0	0	0	0	0	75
16494329	m	md	DE	2019	0	1	1	0	0	0	0	0	0	0	0	0	62
16494345	m	cn	IT	2019	0	1	1	0	0	0	0	0	0	0	0	0	70
16495570	m	cn	JP	2019	0	1	1	0	0	0	0	0	0	0	0	0	71
16496175	m	md	JP	2019	0	1	0	1	0	0	0	0	0	0	0	1	78
16496230	m	cn	JP	2019	0	1	1	0	0	0	0	0	0	0	0	0	70
16496370	f	cn	JP	2019	0	1	1	0	0	0	0	0	0	0	0	0	67
16496539	f	md	US	2019	0	1	0	0	1	0	0	0	0	0	0	0	87
16497015	f	cn	CN	2019	1	0	0	0	0	0	0	0	0	0	0	0	70.2
16497803	f	ot	US	2019	0	1	0	0	1	0	0	0	0	0	0	0	
16498033	m	md	JP	2019	0	1	0	1	0	0	0	0	0	0	0	1	67
16499006	m	cn	CN	2019	1	0	0	0	0	0	0	0	0	0	0	0	60.8
8172250	m	ot	US	2019	0	0	0	0	0	0	0	0	0	0	0	0	30

8229580	f	ot	US	2019	0	0	0	0	0	0	0	0	0	0	0	0	40
8281894	m	ot	US	2019	0	0	0	0	0	0	0	0	0	0	0	0	58
10676505	m	ot	US	2019	0	1	0	0	1	0	0	0	0	0	0	0	74
15463639	m	md	JP	2019	0	1	0	0	0	1	0	0	0	0	0	0	54
15801126	f	md	CO	2019	0	1	0	0	0	1	0	0	0	0	0	0	83
15821066	f	md	DE	2019	0	1	0	0	1	0	0	0	0	0	0	0	64
15887753	m	md	JP	2019	0	1	0	0	1	0	0	0	0	0	0	1	70
16073024	m	ot	SE	2019	0	1	0	0	1	0	0	0	0	0	0	0	86
16075315	m	md	JP	2019	0	1	0	0	1	0	0	0	0	0	0	0	68
16079258	m	md	JP	2019	0	1	0	0	1	0	0	0	0	0	0	0	53
16080131	m	ot	DE	2019	0	1	0	0	1	0	0	0	0	0	0	1	65
16097017	f	md	CH	2019	0	1	1	0	0	0	0	0	0	0	0	0	70
16097678	m	md	JP	2019	0	0	0	0	0	0	1	0	0	1	0	1	58
16114959	f	ot	JP	2019	1	0	0	0	0	0	0	0	0	0	0	0	77
16115803	m	cn	KR	2019	0	1	1	0	0	0	0	0	0	0	0	0	
16150553	m	ot	JP	2019	0	1	0	0	1	0	0	0	0	0	0	1	73
16152202	m	md	JP	2019	0	1	0	1	0	0	0	0	0	0	0	1	79
16152271	m	md	JP	2019	0	1	0	0	0	1	0	0	0	0	0	0	62
16154776	f	md	JP	2019	0	1	1	0	0	0	0	0	0	0	0	0	70
16155494	m	cn	JP	2019	0	1	1	0	0	0	0	0	0	0	0	0	42
16158655	m	ot	KR	2019	0	0	0	0	0	0	0	0	0	0	0	0	
16164380	m	cn	JP	2019	0	1	1	0	0	0	0	0	0	0	0	0	64
16176469	m	ot	JP	2019	0	1	0	1	0	0	0	0	0	0	0	1	75
16176473	f	md	JP	2019	0	1	0	1	0	0	0	0	0	0	0	1	82
16182697	m	cn	JP	2019	0	1	1	0	0	0	0	0	0	0	0	0	77
16182888	f	md	JP	2019	0	1	1	0	0	0	0	0	0	0	0	1	75
16187499	f	md	DE	2019	0	0	0	0	0	0	0	0	0	0	0	0	64
16191855	f	ot	US	2019	0	1	0	0	1	0	0	0	0	0	0	0	62
16192092	m	cn	JP	2019	0	1	1	0	0	0	0	0	0	0	0	0	77
16192943	m	cn	JP	2019	0	1	1	0	0	0	0	0	0	0	0	0	70
16195654	m	md	CN	2019	0	1	0	0	0	1	0	0	0	0	0	0	70
16201673	m	cn	JP	2019	0	1	1	0	0	0	0	0	0	0	0	0	68
16217029	Not	cn	JP	2019	0	1	0	0	1	0	0	0	0	0	0	0	
16219789	f	md	PT	2019	0	1	0	0	1	0	0	0	0	0	0	0	70

16221305	m	md	JP	2019	0	1	0	0	1	0	0	0	0	0	0	1	81
16225561	f	md	JP	2019	0	1	0	0	1	0	0	0	0	0	0	0	74
16225629	m	md	JP	2019	0	1	0	0	1	0	0	0	0	0	0	0	80
16227520	m	ot	JP	2019	0	1	1	0	0	0	0	0	0	0	0	0	72
16228844	m	md	CN	2019	0	1	0	0	0	1	0	0	0	0	0	0	82
16228940	m	md	JP	2019	0	1	0	0	1	0	0	0	0	0	0	1	72
16236882	m	cn	JP	2019	0	1	1	0	0	0	0	0	0	0	0	0	70
16240991	m	cn	JP	2019	0	1	1	0	0	0	0	0	0	0	0	0	84
16241461	f	cn	US	2019	0	1	0	0	1	0	0	0	0	0	0	0	56
16242693	f	cn	CN	2019	0	1	0	0	1	0	0	0	0	0	0	1	42
16242942	f	md	JP	2019	1	0	0	0	0	0	0	0	0	0	0	0	71
16244308	m	md	JP	2019	0	0	0	0	0	0	0	0	0	0	0	0	50
16248561	m	md	EG	2019	0	1	0	0	1	0	0	0	0	0	0	0	65
16251869	m	md	JP	2019	0	1	1	0	0	0	0	0	0	0	0	0	70
16262206	m	md	CN	2019	0	0	0	0	0	0	0	0	0	0	0	0	65
16264785	f	md	JP	2019	1	0	0	0	0	0	0	0	0	0	0	0	79.7
16265037	m	md	JP	2019	0	1	0	0	1	0	0	0	0	0	0	0	56
16265835	m	md	JP	2019	0	1	0	1	0	0	0	0	0	0	0	0	69
16267533	Not	md	US	2019	0	1	0	0	1	0	0	0	0	0	0	0	
16269382	m	cn	FR	2019	0	1	1	0	0	0	0	0	0	0	0	0	60
16271424	m	md	JP	2019	0	1	0	1	0	0	0	0	0	0	0	1	70
16278863	m	cn	JP	2019	0	1	1	0	0	0	0	0	0	0	0	0	60
16286750	m	ot	US	2019	0	0	0	0	0	0	0	0	0	0	0	0	70
16288606	f	md	US	2019	0	0	0	0	0	0	0	0	0	0	0	0	80
16289042	f	cn	LB	2019	0	1	0	0	1	0	0	0	0	0	0	0	72
16298377	m	cn	US	2019	1	0	0	0	0	0	0	0	0	0	0	0	
16306916	f	Not repo	JP	2019	0	0	0	0	0	0	0	0	0	0	0	0	46
16307453	m	ot	FR	2019	0	1	0	0	1	0	0	0	0	0	0	0	43
16312943	m	md	US	2019	0	1	0	0	0	1	0	0	0	0	0	0	69
16313250	f	ot	JP	2019	0	1	0	1	0	0	0	0	0	0	0	1	55
16313436	m	ot	JP	2019	0	1	1	0	0	0	0	0	0	0	0	1	52
16315128	f	md	JP	2019	1	0	0	0	0	0	0	0	0	0	0	0	70.3
16317583	f	md	JP	2019	0	1	1	0	0	0	0	0	0	0	0	0	64
16318081	f	cn	CA	2019	0	1	0	0	1	0	0	0	0	0	0	0	77

16319756	m	cn	JP	2019	0	1	1	0	0	0	0	0	0	0	0	0	78
16319802	f	md	JP	2019	1	0	0	0	0	0	0	0	0	0	0	1	70
16323369	m	md	JP	2019	0	1	1	0	0	0	0	0	0	0	0	0	80
16327269	m	ot	PL	2019	0	0	0	0	0	0	0	0	0	0	0	0	61
16329653	Not	md	CH	2019	0	1	0	0	0	1	0	0	0	0	0	0	
16333821	m	md	JP	2019	1	0	0	0	0	0	0	0	0	0	0	0	60
16333891	m	cn	JP	2019	0	1	1	0	0	0	0	0	0	0	0	1	78
16343062	f	ph	US	2019	0	0	0	0	0	0	0	0	0	0	0	0	79
16343150	m	md	TW	2019	0	0	0	0	0	0	0	0	0	0	0	0	77
16343546	m	md	JP	2019	1	0	0	0	0	0	0	0	0	0	0	0	68.5
16345366	Not	md	JP	2019	0	1	0	0	0	1	0	0	0	0	0	1	60
16347936	f	md	JP	2019	1	0	0	0	0	0	0	0	0	0	0	0	66
16348428	m	md	IT	2019	0	0	0	0	0	0	0	0	0	0	0	0	61
16349803	m	ot	DE	2019	0	1	0	0	1	0	0	0	0	0	0	0	
16353630	m	ph	JP	2019	1	0	0	0	0	0	0	0	0	0	0	1	84
16358445	m	md	JP	2019	0	1	0	1	0	0	0	0	0	0	0	1	66
16358730	Not	ot	DE	2019	0	0	0	0	0	0	0	0	0	0	0	0	67
16359356	m	cn	JP	2019	0	1	1	0	0	0	0	0	0	0	0	1	70
16361654	f	md	JP	2019	0	1	0	1	0	0	0	0	0	0	0	0	59.4
16364080	f	md	AU	2019	0	0	0	0	0	0	0	0	0	0	0	0	78
16364512	m	md	JP	2019	0	1	0	1	0	0	0	0	0	0	0	1	68
16365940	m	cn	JP	2019	0	1	1	0	0	0	0	0	0	0	0	0	70
16368765	f	ot	JP	2019	0	1	1	0	0	0	0	0	0	0	0	0	60
16370157	f	md	JP	2019	1	0	0	0	0	0	0	0	0	0	0	0	67
16376864	m	md	DE	2019	0	1	0	1	0	0	0	0	0	0	0	0	
16382293	m	ot	US	2019	0	1	0	0	1	0	0	0	0	0	0	0	62
16382447	f	md	DE	2019	0	1	0	0	0	1	0	0	0	0	0	0	52
16386948	m	cn	JP	2019	0	1	1	0	0	0	0	0	0	0	0	1	78
16388277	f	ot	US	2019	0	1	0	0	1	0	0	0	0	0	0	0	60
16390741	f	cn	US	2019	0	0	0	0	0	0	0	0	0	0	0	0	74
16392920	m	md	CN	2019	0	1	0	0	1	0	0	0	0	0	0	0	51
16393181	m	md	JP	2019	0	1	0	0	0	1	0	0	0	0	0	0	75
16393350	f	cn	KR	2019	0	0	0	0	0	0	0	0	0	0	0	0	60
16393404	m	ot	JP	2019	0	1	0	1	0	0	0	0	0	0	0	1	66

16393582	m	md	DE	2019	0	0	0	0	0	0	0	0	0	0	0	0	65
16395550	m	ph	JP	2019	0	0	0	0	0	0	0	0	0	0	0	0	75
16397255	m	md	JP	2019	0	1	0	1	0	0	0	0	0	0	0	0	80
16399920	f	md	JP	2019	1	0	0	0	0	0	0	0	0	0	0	0	68.4
16400640	m	ot	US	2019	0	1	0	0	1	0	0	0	0	0	0	0	62
16406378	m	ph	JP	2019	0	1	1	0	0	0	0	0	0	0	0	0	70
16406772	f	ot	ES	2019	0	1	1	0	0	0	0	0	0	0	0	0	61
16406776	m	ot	ES	2019	0	1	1	0	0	0	0	0	0	0	0	0	63
16407472	m	ot	GB	2019	0	1	0	0	0	1	0	0	0	0	0	0	
16413084	f	cn	US	2019	1	0	0	0	0	0	0	0	0	0	0	0	
16413379	m	cn	JP	2019	0	1	1	0	0	0	0	0	0	0	0	0	80
16414358	m	cn	JP	2019	0	1	1	0	0	0	0	0	0	0	0	0	75
16417966	m	cn	JP	2019	0	1	1	0	0	0	0	0	0	0	0	0	44
16419164	f	md	FR	2019	0	0	0	0	0	0	0	0	0	0	0	0	54
16419821	m	md	ES	2019	0	1	0	0	0	1	0	0	0	0	0	0	75
16420102	m	md	JP	2019	0	1	0	1	0	0	0	0	0	0	0	1	71.8
16420906	m	md	RU	2019	0	1	1	0	0	0	0	0	0	0	0	0	56
16421096	f	md	DE	2019	1	0	0	0	0	0	0	0	0	0	0	0	
16421792	m	ot	JP	2019	0	1	1	0	0	0	0	0	0	0	0	1	69
16423931	f	ot	US	2019	0	0	0	0	0	0	1	0	1	0	0	0	72
16425978	m	cn	JP	2019	0	1	1	0	0	0	0	0	0	0	0	0	70
16426913	m	cn	JP	2019	0	1	1	0	0	0	0	0	0	0	0	0	81
16427359	f	cn	JP	2019	0	1	1	0	0	0	0	0	0	0	0	0	55
16428897	m	cn	JP	2019	0	1	1	0	0	0	0	0	0	0	0	1	70
16430679	f	md	FR	2019	0	0	0	0	0	0	0	0	0	0	0	0	75
16430737	f	md	JP	2019	1	0	0	0	0	0	0	0	0	0	0	0	70.7
16430942	m	md	TW	2019	0	1	0	1	0	0	0	0	0	0	0	0	80
16435231	m	md	JP	2019	0	1	1	0	0	0	0	0	0	0	0	1	80
16438779	m	md	TR	2019	0	0	0	0	0	0	0	0	0	0	0	0	52
16442470	m	ph	JP	2019	0	1	1	0	0	0	0	0	0	0	0	0	60
16443548	m	md	JP	2019	0	0	0	0	0	0	0	0	0	0	0	0	65
16445172	m	md	JP	2019	0	1	0	0	1	0	0	0	0	0	0	0	60
16445377	m	ot	DE	2019	0	1	1	0	0	0	0	0	0	0	0	0	70
16445806	f	md	JP	2019	1	0	0	0	0	0	0	0	0	0	0	1	84

16446212	f	md	JP	2019	1	0	0	0	0	0	0	0	0	0	0	0	76
16446229	Not	md	PL	2019	0	0	0	0	0	0	0	0	0	0	0	0	
16448860	Not	md	JP	2019	0	1	1	0	0	0	0	0	0	0	0	0	
16450742	f	md	PL	2019	0	1	0	0	0	1	0	0	0	0	0	0	70
16452448	m	cn	JP	2019	0	1	1	0	0	0	0	0	0	0	0	0	58
16453193	m	cn	DE	2019	0	1	1	0	0	0	0	0	0	0	0	0	70
16457643	m	md	JP	2019	0	1	0	1	0	0	0	0	0	0	0	1	69
16457943	m	md	JP	2019	0	1	0	0	0	1	0	0	0	0	0	0	70
16462879	m	cn	JP	2019	0	0	0	0	0	0	0	0	0	0	0	0	67
16463996	f	ph	US	2019	0	1	1	0	0	0	0	0	0	0	0	0	76
16465247	f	ot	JP	2019	1	0	0	0	0	0	0	0	0	0	0	0	78
16468816	f	md	JP	2019	1	0	0	0	0	0	0	0	0	0	0	0	74
16468836	m	cn	US	2019	0	1	1	0	0	0	0	0	0	0	0	0	62
16469077	m	md	US	2019	0	1	0	0	0	1	0	0	0	0	0	0	71
16469572	m	ot	JP	2019	0	1	1	0	0	0	0	0	0	0	0	0	83
16469737	m	ot	JP	2019	0	1	1	0	0	0	0	0	0	0	0	0	87
16471161	Not	cn	CN	2019	0	0	0	0	0	0	0	0	0	0	0	0	
16471838	m	md	JP	2019	0	1	0	0	0	1	0	0	0	0	0	0	63
16472296	f	ot	CN	2019	0	1	0	0	1	0	0	0	0	0	0	0	56
16472439	f	ot	NL	2019	0	1	0	0	1	0	0	0	0	0	0	0	66
16474624	m	md	JP	2019	0	1	0	0	0	1	0	0	0	0	0	0	72
16475106	f	cn	DE	2019	0	1	1	0	0	0	0	0	0	0	0	0	71
16475751	Not	md	NO	2019	0	0	0	0	0	0	0	0	0	0	0	0	66
16476093	m	md	JP	2019	0	1	0	1	0	0	0	0	0	0	0	1	77.6
16477303	m	md	KR	2019	1	0	0	0	0	0	0	0	0	0	0	0	70
16478102	m	ot	CN	2019	0	1	0	0	1	0	0	0	0	0	0	0	56
16479756	m	md	DE	2019	0	0	0	0	0	0	0	0	0	0	0	0	80
16480238	m	ot	IT	2019	0	0	0	0	0	0	0	0	0	0	0	0	56
16480290	m	Not repo	CN	2019	1	0	0	0	0	0	0	0	0	0	0	0	
16483038	f	md	JP	2019	1	0	0	0	0	0	0	0	0	0	0	0	80
16483040	f	md	JP	2019	1	0	0	0	0	0	0	0	0	0	0	1	71.5
16483306	m	md	HU	2019	0	0	0	0	0	0	0	0	0	0	0	0	48
16485544	m	md	CN	2019	0	0	0	0	0	0	0	0	0	0	0	0	39
16485965	f	Not repo	CN	2019	1	0	0	0	0	0	0	0	0	0	0	0	

16487631	f	md	JP	2019	0	1	1	0	0	0	0	0	0	0	0	0	71
16489310	f	md	JP	2019	1	0	0	0	0	0	0	0	0	0	0	1	77.6
16489896	m	md	KR	2019	1	0	0	0	0	0	0	0	0	0	0	0	104
16490949	m	Not repo	CN	2019	1	0	0	0	0	0	0	0	0	0	0	0	70
16491751	f	Not repo	CN	2019	1	0	0	0	0	0	0	0	0	0	0	0	
16494030	m	Not repo	CN	2019	1	0	0	0	0	0	0	0	0	0	0	0	74
16495641	f	cn	CN	2019	1	0	0	0	0	0	0	0	0	0	0	0	
16495673	Not	ot	JP	2019	0	1	0	0	1	0	0	0	0	0	0	0	
16496324	m	md	KR	2019	1	0	0	0	0	0	0	0	0	0	0	0	77
16497362	m	ph	NL	2019	0	0	0	0	0	0	0	0	0	0	0	0	74
16497465	f	Not repo	ID	2019	1	0	0	0	0	0	0	0	0	0	0	0	
16498044	f	md	KR	2019	0	1	1	0	0	0	0	0	0	0	0	0	
16498212	m	cn	JP	2019	0	1	1	0	0	0	0	0	0	0	0	0	84
16499235	m	cn	CN	2019	0	1	0	0	1	0	0	0	0	0	0	0	
16499411	Not	md	JP	2019	0	1	0	0	0	1	0	0	0	0	0	0	80
16499650	m	cn	JP	2019	0	1	0	0	0	1	0	0	0	0	0	0	80
16499654	f	cn	CN	2019	1	0	0	0	0	0	0	0	0	0	0	0	
16500115	f	cn	Not re	2019	0	0	0	0	0	0	0	0	0	0	0	0	66
16501337	f	md	DK	2019	0	0	0	0	0	0	0	0	0	0	0	0	79
16502711	m	ot	DE	2019	0	0	0	0	0	0	0	0	0	0	0	0	55
16506653	f	cn	CN	2019	1	0	0	0	0	0	0	0	0	0	0	0	74
16506973	Not	ot	US	2019	0	0	0	0	0	0	0	0	0	0	0	0	
16506974	Not	ot	US	2019	0	0	0	0	0	0	0	0	0	0	0	0	
16506975	Not	ot	US	2019	0	0	0	0	0	0	0	0	0	0	0	0	
16506976	m	ot	IT	2019	0	0	0	0	0	0	0	0	0	0	0	0	56
16506981	Not	ot	US	2019	0	0	0	0	0	0	0	0	0	0	0	0	
16507219	m	cn	JP	2019	0	1	1	0	0	0	0	0	0	0	0	0	80
16507635	m	md	JP	2019	0	1	0	0	1	0	0	0	0	0	0	1	69
16507759	f	cn	CN	2019	1	0	0	0	0	0	0	0	0	0	0	0	60
16508191	f	ph	US	2019	0	0	0	0	0	0	0	0	0	0	0	0	
16509565	m	md	JP	2019	0	0	0	0	0	0	0	0	0	0	0	0	70
16509852	m	ph	US	2019	0	0	0	0	0	0	0	0	0	0	0	0	56
16509893	f	md	RS	2019	0	1	1	0	0	0	0	0	0	0	0	0	
16510390	Not	cn	JP	2019	0	1	1	0	0	0	0	0	0	0	0	0	

16510538	m	md	FR	2019	0	1	1	0	0	0	0	0	0	0	0	0	70
16511596	m	ot	CN	2019	1	0	0	0	0	0	0	0	0	0	0	0	65
16512948	f	md	AT	2019	0	1	1	0	0	0	0	0	0	0	0	0	
16513028	m	md	JP	2019	0	1	0	0	1	0	0	0	0	0	0	1	77
16513551	m	ot	US	2019	0	1	0	0	1	0	0	0	0	0	0	0	73
16513893	m	Not repo	CN	2019	1	0	0	0	0	0	0	0	0	0	0	0	
16514252	f	md	TW	2019	0	1	0	0	0	1	0	0	0	0	0	0	
16514389	f	cn	CN	2019	1	0	0	0	0	0	0	0	0	0	0	0	65
16514460	m	Not repo	KR	2019	0	1	0	1	0	0	0	0	0	0	0	0	73
16514508	Not	ot	CN	2019	0	1	0	0	0	1	0	0	0	0	0	0	
16514534	Not	ot	NL	2019	0	1	1	0	0	0	0	0	0	0	0	0	77
16514605	m	ot	US	2019	0	0	0	0	0	0	0	0	0	0	0	0	73
16514900	m	cn	KR	2019	0	0	0	0	0	0	0	0	0	0	0	0	51
16515612	m	md	RS	2019	0	1	1	0	0	0	0	0	0	0	0	0	64
16517540	m	cn	CN	2019	1	0	0	0	0	0	0	0	0	0	0	0	
16518069	m	ot	TR	2019	0	0	0	0	0	0	0	0	0	0	0	0	52
16518131	m	cn	CA	2019	0	0	0	0	0	0	0	0	0	0	0	0	83
16518633	f	cn	CN	2019	1	0	0	0	0	0	0	0	0	0	0	0	
16518975	f	cn	CN	2019	1	0	0	0	0	0	0	0	0	0	0	0	
16519251	m	md	JP	2019	0	1	0	1	0	0	0	0	0	0	0	1	60
16519253	m	md	JP	2019	0	1	0	1	0	0	0	0	0	0	0	0	61
16519275	m	md	JP	2019	1	0	0	0	0	0	0	0	0	0	0	0	88
16519586	Not	md	IT	2019	1	0	0	0	0	0	0	0	0	0	0	0	
16520231	m	md	FR	2019	1	0	0	0	0	0	0	0	0	0	0	0	
16521159	m	md	JP	2019	0	1	0	0	1	0	0	0	0	0	0	0	80
16521567	f	cn	CN	2019	1	0	0	0	0	0	0	0	0	0	0	0	
16522000	Not	md	US	2019	0	0	0	0	0	0	0	0	0	0	0	0	
16522426	m	md	ES	2019	0	0	0	0	0	0	0	0	0	0	0	0	59
16523366	f	md	DK	2019	0	0	0	0	0	0	0	0	0	0	0	0	79
16523379	f	md	JP	2019	1	0	0	0	0	0	0	0	0	0	0	1	81.1
16523380	f	md	JP	2019	0	1	0	1	0	0	0	0	0	0	0	1	78
16524281	f	Not repo	CN	2019	1	0	0	0	0	0	0	0	0	0	0	0	
16524298	f	Not repo	CN	2019	1	0	0	0	0	0	0	0	0	0	0	0	
16525278	m	md	JP	2019	1	0	0	0	0	0	0	0	0	0	0	0	72

16525450	f	cn	CN	2019	1	0	0	0	0	0	0	0	0	0	0	0	
16525509	f	md	JP	2019	1	0	0	0	0	0	0	0	0	0	0	0	70
16525525	m	cn	JP	2019	0	1	1	0	0	0	0	0	0	0	0	0	49
16525548	m	md	RS	2019	0	1	1	0	0	0	0	0	0	0	0	0	69
16525757	Not	md	JP	2019	0	1	0	0	0	1	0	0	0	0	0	0	
16525760	f	md	JP	2019	0	1	0	0	0	1	0	0	0	0	0	0	70
16525915	m	md	RU	2019	0	1	0	0	0	1	0	0	0	0	0	0	60
16526328	m	md	RS	2019	0	1	1	0	0	0	0	0	0	0	0	0	59
16526429	f	ot	FR	2019	0	1	0	0	1	0	0	0	0	0	0	0	55
16526538	f	Not repo	CN	2019	1	0	0	0	0	0	0	0	0	0	0	0	
16526851	m	md	US	2019	0	1	0	0	1	0	0	0	0	0	0	0	71
16526996	m	cn	DK	2019	0	1	1	0	0	0	0	0	0	0	0	0	
16527279	f	md	JP	2019	1	0	0	0	0	0	0	0	0	0	0	0	80.1
16527819	m	cn	IN	2019	0	0	0	0	0	0	0	0	0	0	0	0	62
16527820	m	ot	IT	2019	1	0	0	0	0	0	0	0	0	0	0	0	70
16528155	m	cn	JP	2019	0	1	1	0	0	0	0	0	0	0	0	0	69
16528450	m	cn	CN	2019	1	0	0	0	0	0	0	0	0	0	0	0	50
16528509	m	Not repo	CN	2019	1	0	0	0	0	0	0	0	0	0	0	0	
16528522	f	Not repo	CN	2019	1	0	0	0	0	0	0	0	0	0	0	0	
16528827	m	ot	DE	2019	0	1	0	0	1	0	0	0	0	0	0	0	34
16529011	m	cn	US	2019	0	0	0	0	0	0	0	0	0	0	0	0	72
16529033	m	cn	JP	2019	0	1	1	0	0	0	0	0	0	0	0	0	78
16529098	m	ot	CN	2019	0	1	0	0	1	0	0	0	0	0	0	0	68
16529100	f	cn	US	2019	0	1	1	0	0	0	0	0	0	0	0	0	
16529317	f	Not repo	JP	2019	0	0	0	0	0	0	0	0	0	0	0	0	74
16529454	Not	ph	JP	2019	0	1	0	0	1	0	0	0	0	0	0	0	
16529560	f	ot	JP	2019	0	0	0	0	0	0	0	0	0	0	0	0	78
16529672	f	cn	CN	2019	0	1	0	0	1	0	0	0	0	0	0	0	
16529765	m	ph	AU	2019	0	1	0	0	0	1	0	0	0	0	0	0	
16529944	f	ph	US	2019	0	0	0	0	0	0	0	0	0	0	0	0	40
16530328	m	cn	JP	2019	0	1	1	0	0	0	0	0	0	0	0	0	84
16530331	f	Not repo	CN	2019	1	0	0	0	0	0	0	0	0	0	0	0	48
16530393	m	ot	TH	2019	0	1	0	0	1	0	0	0	0	0	0	0	68
16530543	m	ph	CN	2019	1	0	0	0	0	0	0	0	0	0	0	0	

16531332	m	md	JP	2019	0	1	0	0	0	1	0	0	0	0	0	0	
16531428	f	md	GB	2019	0	0	0	0	0	0	0	0	0	0	0	0	
16531506	f	md	KR	2019	1	0	0	0	0	0	0	0	0	0	0	0	55
16531513	f	ot	US	2019	0	1	0	0	1	0	0	0	0	0	0	0	67
16531639	f	md	JP	2019	1	0	0	0	0	0	0	0	0	0	0	0	43
16531849	m	md	JP	2019	1	0	0	0	0	0	0	0	0	0	0	0	77
16532022	m	ot	IT	2019	1	0	0	0	0	0	0	0	0	0	0	0	70
16532156	f	cn	JP	2019	0	1	1	0	0	0	0	0	0	0	0	1	75
16532411	m	cn	CN	2019	0	1	0	0	1	0	0	0	0	0	0	0	
16532417	f	cn	GB	2019	1	0	0	0	0	0	0	0	0	0	0	0	68
16532468	f	cn	JP	2019	0	1	1	0	0	0	0	0	0	0	0	0	54
16532593	m	ot	JP	2019	0	1	1	0	0	0	0	0	0	0	0	0	79
16532751	f	md	JP	2019	1	0	0	0	0	0	0	0	0	0	0	0	67
16532768	m	cn	JP	2019	0	0	0	0	0	0	0	0	0	0	0	0	72
16532805	f	cn	JP	2019	0	1	1	0	0	0	0	0	0	0	0	0	48
16533494	f	ot	BE	2019	0	1	1	0	0	0	0	0	0	0	0	0	79
16533583	f	md	AT	2019	0	0	0	0	0	0	0	0	0	0	0	0	
16533603	f	md	US	2019	0	1	0	0	1	0	0	0	0	0	0	0	75
16533615	m	md	JP	2019	1	0	0	0	0	0	0	0	0	0	0	0	67.5
16533716	Not	md	DE	2019	0	1	0	1	0	0	0	0	0	0	0	0	
16533739	m	cn	JP	2019	0	1	1	0	0	0	0	0	0	0	0	0	79
16533754	m	cn	JP	2019	0	1	1	0	0	0	0	0	0	0	0	0	64
16533872	m	cn	JP	2019	0	1	1	0	0	0	0	0	0	0	0	0	59
16533906	f	cn	US	2019	1	0	0	0	0	0	0	0	0	0	0	0	
16533938	f	md	JP	2019	0	1	0	0	1	0	0	0	0	0	0	0	62
16533986	m	md	JP	2019	0	1	1	0	0	0	0	0	0	0	0	0	57
16534133	m	md	JP	2019	0	1	0	1	0	0	0	0	0	0	0	1	66.2
16534173	f	cn	JP	2019	0	1	1	0	0	0	0	0	0	0	0	0	53
16534214	m	ot	US	2019	0	1	0	0	1	0	0	0	0	0	0	1	65
16534240	Not	md	DE	2019	0	1	0	1	0	0	0	0	0	0	0	0	
16534472	f	cn	JP	2019	0	1	1	0	0	0	0	0	0	0	0	0	54
16534599	m	cn	JP	2019	0	1	1	0	0	0	0	0	0	0	0	0	78
16534806	m	cn	JP	2019	0	1	1	0	0	0	0	0	0	0	0	0	74
16534898	m	md	US	2019	0	1	1	0	0	0	0	0	0	0	0	0	69

16534955	m	md	JP	2019	0	1	0	0	0	1	0	0	0	0	0	1	61
16535354	m	cn	US	2019	1	0	0	0	0	0	0	0	0	0	0	0	66.5
16535418	m	ot	CN	2019	0	0	0	0	0	0	0	0	0	0	0	0	67
16535781	m	md	JP	2019	0	1	0	0	0	1	0	0	0	0	0	0	70
16535789	m	ot	IT	2019	0	0	0	0	0	0	0	0	0	0	0	0	56
16535902	f	cn	CN	2019	1	0	0	0	0	0	0	0	0	0	0	0	
16535924	m	md	KR	2019	0	1	0	0	1	0	0	0	0	0	0	0	73
16535950	m	cn	JP	2019	0	1	1	0	0	0	0	0	0	0	0	0	74
16536004	m	Not repo	IT	2019	0	1	0	1	0	0	0	0	0	0	0	0	63.7
16536328	m	md	JP	2019	0	1	0	1	0	0	0	0	0	0	0	0	60
16536529	m	ot	US	2019	0	1	0	0	1	0	0	0	0	0	0	0	64
16536534	m	ot	AU	2019	0	1	0	0	1	0	0	0	0	0	0	0	
16536701	m	cn	CN	2019	0	1	0	0	1	0	0	0	0	0	0	0	
16536797	m	ph	JP	2019	0	1	1	0	0	0	0	0	0	0	0	0	75
16536828	m	cn	CN	2019	0	1	0	0	1	0	0	0	0	0	0	0	
16536829	m	cn	CN	2019	0	1	0	0	1	0	0	0	0	0	0	0	60
16536830	m	cn	CN	2019	0	1	0	0	1	0	0	0	0	0	0	0	64
16536873	f	cn	CN	2019	1	0	0	0	0	0	0	0	0	0	0	0	63
16536874	f	cn	CN	2019	1	0	0	0	0	0	0	0	0	0	0	0	55
16536875	m	cn	CN	2019	1	0	0	0	0	0	0	0	0	0	0	0	
16536888	m	cn	CN	2019	1	0	0	0	0	0	0	0	0	0	0	0	62.8
16536966	m	md	DE	2019	0	0	0	0	0	0	0	0	0	0	0	0	8
16537358	m	cn	CN	2019	1	0	0	0	0	0	0	0	0	0	0	0	
16537359	f	cn	CN	2019	1	0	0	0	0	0	0	0	0	0	0	0	47
16537368	m	cn	CN	2019	1	0	0	0	0	0	0	0	0	0	0	0	
16537369	m	cn	CN	2019	1	0	0	0	0	0	0	0	0	0	0	0	
16537370	f	cn	CN	2019	1	0	0	0	0	0	0	0	0	0	0	0	69
16537382	f	cn	CN	2019	1	0	0	0	0	0	0	0	0	0	0	0	65
16537383	f	cn	CN	2019	1	0	0	0	0	0	0	0	0	0	0	0	
16537384	m	cn	CN	2019	1	0	0	0	0	0	0	0	0	0	0	0	
16537420	f	cn	CN	2019	1	0	0	0	0	0	0	0	0	0	0	0	43
16537436	f	cn	CN	2019	1	0	0	0	0	0	0	0	0	0	0	0	60.8
16537438	m	cn	JP	2019	0	1	1	0	0	0	0	0	0	0	0	1	60
16537470	f	cn	CN	2019	1	0	0	0	0	0	0	0	0	0	0	0	

16537609	f	cn	CN	2019	1	0	0	0	0	0	0	0	0	0	0	0	
16537610	f	cn	CN	2019	1	0	0	0	0	0	0	0	0	0	0	0	
16537613	f	cn	CN	2019	1	0	0	0	0	0	0	0	0	0	0	0	
16537614	f	cn	CN	2019	1	0	0	0	0	0	0	0	0	0	0	69
16537615	f	cn	CN	2019	1	0	0	0	0	0	0	0	0	0	0	75.6
16537644	f	cn	CN	2019	1	0	0	0	0	0	0	0	0	0	0	
16537710	f	cn	CN	2019	1	0	0	0	0	0	0	0	0	0	0	
16537747	f	cn	CN	2019	1	0	0	0	0	0	0	0	0	0	0	
16537748	m	cn	CN	2019	1	0	0	0	0	0	0	0	0	0	0	
16537771	f	cn	CN	2019	1	0	0	0	0	0	0	0	0	0	0	
16537797	f	cn	CN	2019	1	0	0	0	0	0	0	0	0	0	0	
16537798	f	cn	CN	2019	1	0	0	0	0	0	0	0	0	0	0	
16537806	f	cn	CN	2019	1	0	0	0	0	0	0	0	0	0	0	
16537807	f	cn	CN	2019	1	0	0	0	0	0	0	0	0	0	0	
16537837	f	cn	CN	2019	1	0	0	0	0	0	0	0	0	0	0	
16537838	f	cn	CN	2019	1	0	0	0	0	0	0	0	0	0	0	64
16537877	f	cn	CN	2019	1	0	0	0	0	0	0	0	0	0	0	
16537879	f	cn	CN	2019	1	0	0	0	0	0	0	0	0	0	0	
16537918	m	ot	IT	2019	1	0	0	0	0	0	0	0	0	0	0	70
16538264	f	cn	CN	2019	1	0	0	0	0	0	0	0	0	0	0	
16538344	f	md	ES	2019	0	1	1	0	0	0	0	0	0	0	0	71
16538426	f	cn	CN	2019	1	0	0	0	0	0	0	0	0	0	0	
16538427	f	cn	CN	2019	1	0	0	0	0	0	0	0	0	0	0	60
16538478	m	md	UA	2019	0	1	0	0	0	1	0	0	0	0	0	0	70
16538491	f	cn	CN	2019	1	0	0	0	0	0	0	0	0	0	0	65.3
16538493	f	cn	CN	2019	1	0	0	0	0	0	0	0	0	0	0	79
16538494	f	cn	CN	2019	1	0	0	0	0	0	0	0	0	0	0	
16538497	f	cn	CN	2019	1	0	0	0	0	0	0	0	0	0	0	79.1
16538520	f	cn	CN	2019	1	0	0	0	0	0	0	0	0	0	0	
16538522	m	cn	CN	2019	1	0	0	0	0	0	0	0	0	0	0	
16538564	m	md	JP	2019	0	1	1	0	0	0	0	0	0	0	0	71
16538566	f	md	GR	2019	0	1	0	0	0	1	0	0	0	0	0	0	
16538584	m	cn	CN	2019	1	0	0	0	0	0	0	0	0	0	0	53.6
16538585	f	cn	CN	2019	1	0	0	0	0	0	0	0	0	0	0	

16538586	f	cn	CN	2019	1	0	0	0	0	0	0	0	0	0	0	0	
16538587	f	cn	CN	2019	1	0	0	0	0	0	0	0	0	0	0	0	
16538594	f	cn	CN	2019	1	0	0	0	0	0	0	0	0	0	0	0	42
16538641	m	cn	CN	2019	1	0	0	0	0	0	0	0	0	0	0	0	
16538642	f	cn	CN	2019	1	0	0	0	0	0	0	0	0	0	0	0	47
16538644	f	cn	CN	2019	1	0	0	0	0	0	0	0	0	0	0	0	
16538648	f	cn	CN	2019	1	0	0	0	0	0	0	0	0	0	0	0	58.6
16538649	m	cn	CN	2019	1	0	0	0	0	0	0	0	0	0	0	0	
16538650	f	cn	CN	2019	1	0	0	0	0	0	0	0	0	0	0	0	60.3
16538651	m	cn	CN	2019	1	0	0	0	0	0	0	0	0	0	0	0	
16538652	m	cn	CN	2019	1	0	0	0	0	0	0	0	0	0	0	0	
16538653	f	cn	CN	2019	1	0	0	0	0	0	0	0	0	0	0	0	
16538654	m	cn	CN	2019	1	0	0	0	0	0	0	0	0	0	0	0	
16538675	Not	md	JP	2019	0	1	0	0	1	0	0	0	0	0	0	0	
16538714	m	ot	JP	2019	0	1	0	0	1	0	0	0	0	0	0	0	75
16539019	m	md	JP	2019	0	1	0	0	0	1	0	0	0	0	0	0	70
16539241	m	md	JP	2019	0	1	0	0	0	1	0	0	0	0	0	0	69
16540123	f	cn	CN	2019	1	0	0	0	0	0	0	0	0	0	0	0	
16540228	m	ot	JP	2019	0	1	0	0	1	0	0	0	0	0	0	0	50
16540411	f	cn	CN	2019	1	0	0	0	0	0	0	0	0	0	0	0	65
16540415	f	cn	CN	2019	1	0	0	0	0	0	0	0	0	0	0	0	83.5
16540418	f	cn	CN	2019	1	0	0	0	0	0	0	0	0	0	0	0	57.5
16540697	m	cn	CN	2019	1	0	0	0	0	0	0	0	0	0	0	0	64.1
16540708	f	cn	CN	2019	1	0	0	0	0	0	0	0	0	0	0	0	35
16541138	f	cn	CN	2019	1	0	0	0	0	0	0	0	0	0	0	0	74
16541194	m	cn	CN	2019	0	1	0	0	1	0	0	0	0	0	0	0	71
16541278	f	cn	CN	2019	1	0	0	0	0	0	0	0	0	0	0	0	
16541279	m	cn	CN	2019	1	0	0	0	0	0	0	0	0	0	0	0	
16541287	f	cn	CN	2019	1	0	0	0	0	0	0	0	0	0	0	0	45
16541288	f	cn	CN	2019	1	0	0	0	0	0	0	0	0	0	0	0	
16541289	f	cn	CN	2019	1	0	0	0	0	0	0	0	0	0	0	0	
16541290	f	cn	CN	2019	1	0	0	0	0	0	0	0	0	0	0	0	
16541291	f	cn	CN	2019	1	0	0	0	0	0	0	0	0	0	0	0	
16541331	f	md	BR	2019	0	1	0	1	0	0	0	0	0	0	0	0	65

16541332	f	cn	CN	2019	1	0	0	0	0	0	0	0	0	0	0	0	64
16541334	m	cn	CN	2019	1	0	0	0	0	0	0	0	0	0	0	0	
16541414	f	cn	CN	2019	1	0	0	0	0	0	0	0	0	0	0	0	
16541416	f	cn	CN	2019	1	0	0	0	0	0	0	0	0	0	0	0	
16541501	f	cn	CN	2019	1	0	0	0	0	0	0	0	0	0	0	0	
16541579	m	cn	CN	2019	1	0	0	0	0	0	0	0	0	0	0	0	57.3
16541580	m	ot	JP	2019	0	1	0	1	0	0	0	0	0	0	0	1	76
16541631	f	cn	CN	2019	1	0	0	0	0	0	0	0	0	0	0	0	45.3
16541776	f	ot	AT	2019	0	1	1	0	0	0	0	0	0	0	0	0	
16542263	m	ot	HU	2019	0	0	0	0	0	0	0	0	0	0	0	0	
16542790	Not	md	CN	2019	1	0	0	0	0	0	0	0	0	0	0	0	
16542909	f	md	JP	2019	1	0	0	0	0	0	0	0	0	0	0	1	84.6
16543008	Not	ot	IT	2019	1	0	0	0	0	0	0	0	0	0	0	0	
16543158	f	cn	US	2019	1	0	0	0	0	0	0	0	0	0	0	0	
16543526	m	md	JP	2019	0	1	0	0	1	0	0	0	0	0	0	0	76
16543602	f	cn	JP	2019	0	1	1	0	0	0	0	0	0	0	0	0	71
16543766	Not	md	JP	2019	0	1	0	0	1	0	0	0	0	0	0	1	
16543910	m	ot	IT	2019	0	0	0	0	0	0	0	0	0	0	0	0	56
16544330	f	ot	JP	2019	0	0	0	0	0	0	0	0	0	0	0	0	69
16544571	m	md	DE	2019	1	0	0	0	0	0	0	0	0	0	0	0	55
16544698	m	ot	JP	2019	0	1	0	1	0	0	0	0	0	0	0	1	64
16545045	f	md	JP	2019	1	0	0	0	0	0	0	0	0	0	0	0	90
16545153	m	md	US	2019	0	1	0	0	1	0	0	0	0	0	0	0	65
16545159	f	md	JP	2019	1	0	0	0	0	0	0	0	0	0	0	0	66.8
16545249	Not	md	DE	2019	0	0	0	0	0	0	0	0	0	0	0	0	
16545373	f	md	JP	2019	1	0	0	0	0	0	0	0	0	0	0	0	78
16545416	m	cn	JP	2019	0	1	1	0	0	0	0	0	0	0	0	1	66
16545946	Not	md	DE	2019	0	0	0	0	0	0	0	0	0	0	0	0	
16546231	m	ph	JP	2019	0	1	0	1	0	0	0	0	0	0	0	0	68
16546920	f	cn	CN	2019	1	0	0	0	0	0	0	0	0	0	0	0	67.9
16546926	f	Not repo	DE	2019	1	0	0	0	0	0	0	0	0	0	0	0	
16546945	m	cn	JP	2019	0	1	1	0	0	0	0	0	0	0	0	0	67
16547071	m	ot	JP	2019	0	1	1	0	0	0	0	0	0	0	0	0	68
16547096	f	md	JP	2019	1	0	0	0	0	0	0	0	0	0	0	1	71

16547471	Not	md	IT	2019	1	0	0	0	0	0	0	0	0	0	0	0	
16548734	m	cn	CN	2019	0	1	0	0	1	0	0	0	0	0	0	0	56
16548794	m	ot	JP	2019	0	1	1	0	0	0	0	0	0	0	0	0	65
16548822	f	md	FR	2019	1	0	0	0	0	0	0	0	0	0	0	0	75
16548888	Not	ot	US	2019	0	0	0	0	0	0	0	0	0	0	0	0	67
16548923	m	md	JP	2019	0	1	0	0	1	0	0	0	0	0	0	0	81
16549127	f	ot	CN	2019	0	1	0	0	1	0	0	0	0	0	0	0	53
16549487	f	md	JP	2019	0	1	0	0	0	1	0	0	0	0	0	0	55
16549869	m	md	DE	2019	0	1	0	0	0	1	0	0	0	0	0	0	64
16549899	m	md	DE	2019	0	0	0	0	0	0	0	0	0	0	0	0	80
16550801	f	ph	FR	2019	0	0	0	0	0	0	0	0	0	0	0	0	76
16550824	f	Not repo	US	2019	1	0	0	0	0	0	0	0	0	0	0	0	69
16551118	m	cn	JP	2019	0	1	1	0	0	0	0	0	0	0	0	0	80
16551310	m	cn	US	2019	0	1	0	0	1	0	0	0	0	0	0	0	
16551322	f	md	JP	2019	1	0	0	0	0	0	0	0	0	0	0	0	66
16551813	m	cn	CN	2019	0	0	0	0	0	0	0	0	0	0	0	0	62
16551822	f	md	JP	2019	0	1	0	0	1	0	0	0	0	0	0	0	46
16552177	m	ot	JP	2019	0	0	0	0	0	0	0	0	0	0	0	0	
16553250	m	cn	JP	2019	0	1	1	0	0	0	0	0	0	0	0	0	74
16553437	m	md	US	2019	0	0	0	0	0	0	0	0	0	0	0	0	69
16553980	f	md	JP	2019	1	0	0	0	0	0	0	0	0	0	0	0	81.9
16554124	m	cn	CN	2019	1	0	0	0	0	0	0	0	0	0	0	0	
16554809	f	md	JP	2019	0	1	0	1	0	0	0	0	0	0	0	0	54.7
16555327	f	cn	JP	2019	0	1	1	0	0	0	0	0	0	0	0	0	68
16555333	f	md	JP	2019	0	1	0	1	0	0	0	0	0	0	0	1	71
16555575	f	ot	CA	2019	0	1	0	1	0	0	0	0	0	0	0	0	76
16555872	f	md	US	2019	0	1	1	0	0	0	0	0	0	0	0	0	73
16555889	m	ot	NL	2019	1	0	0	0	0	0	0	0	0	0	0	0	70
16555919	m	ot	DE	2019	0	0	0	0	0	0	0	0	0	0	0	0	61
16555930	m	ot	FR	2019	0	1	1	0	0	0	0	0	0	0	0	1	
16555934	Not	ot	DE	2019	0	0	0	0	0	0	0	0	0	0	0	0	
16556002	m	ot	NL	2019	0	0	0	0	0	0	0	0	0	0	0	0	71
16556285	f	ot	CA	2019	0	1	0	0	1	0	0	0	0	0	0	0	62
16556308	f	ot	CN	2019	0	1	0	0	1	0	0	0	0	0	0	0	41

16556370	Not	ot	US	2019	0	0	0	0	0	0	0	0	0	0	0	0	
16556661	m	md	KR	2019	0	1	0	0	1	0	0	0	0	0	0	0	73
16556925	f	md	JP	2019	0	1	0	0	0	1	0	0	0	0	0	0	60
16556941	f	ph	CA	2019	0	1	0	0	1	0	0	0	0	0	0	0	66
16557317	m	md	KR	2019	0	0	0	0	0	0	0	0	0	0	0	0	42
16557339	m	md	KR	2019	0	0	0	0	0	0	0	0	0	0	0	0	56
16557840	f	md	IN	2019	0	0	0	0	0	0	0	0	0	0	0	0	63
16557928	m	ph	JP	2019	0	1	0	0	1	0	0	0	0	0	0	0	70
16558009	m	md	JP	2019	1	0	0	0	0	0	0	0	0	0	0	0	80
16558208	m	cn	KR	2019	0	1	1	0	0	0	0	0	0	0	0	0	
16558375	m	ot	FR	2019	0	0	0	0	0	0	0	0	0	0	0	0	59
16558528	Not	ot	US	2019	0	1	0	0	1	0	0	0	0	0	0	0	
16558578	m	md	RU	2019	0	0	0	0	0	0	0	0	0	0	0	0	33
16559310	m	cn	JP	2019	0	1	1	0	0	0	0	0	0	0	0	0	53
16559479	m	cn	IT	2019	0	0	0	0	0	0	0	0	0	0	0	0	56
16559912	f	md	JP	2019	1	0	0	0	0	0	0	0	0	0	0	0	67.1
16560628	f	ot	US	2019	0	0	0	0	0	0	0	0	0	0	0	0	87
16560756	m	ot	JP	2019	0	1	1	0	0	0	0	0	0	0	0	1	69
16561174	f	ot	CA	2019	0	0	0	0	0	0	0	0	0	0	0	0	65
16561958	m	md	FR	2019	0	0	0	0	0	0	0	0	0	0	0	0	72
16561965	m	md	IT	2019	0	0	0	0	0	0	0	0	0	0	0	0	56
16561969	m	md	JP	2019	0	1	0	0	1	0	0	0	0	0	0	0	62
16562117	m	cn	KR	2019	0	0	0	0	0	0	0	0	0	0	0	0	59
16562595	m	cn	JP	2019	0	1	1	0	0	0	0	0	0	0	0	0	72
16562697	m	cn	JP	2019	0	1	1	0	0	0	0	0	0	0	0	1	57
16562734	m	ot	JP	2019	0	1	1	0	0	0	0	0	0	0	0	0	66
16563069	m	cn	US	2019	1	0	0	0	0	0	0	0	0	0	0	0	
16563647	m	ot	PT	2019	0	1	0	0	1	0	0	0	0	0	0	1	74
16563754	m	cn	US	2019	1	0	0	0	0	0	0	0	0	0	0	0	
16563759	m	md	FR	2019	0	0	0	0	0	0	0	0	0	0	0	0	67
16563854	m	cn	JP	2019	0	1	1	0	0	0	0	0	0	0	0	0	84
16563856	f	cn	CN	2019	1	0	0	0	0	0	0	0	0	0	0	0	64
16563959	f	md	RU	2019	0	1	0	0	0	1	0	0	0	0	0	0	60
16564006	m	md	DE	2019	0	1	0	0	1	0	0	0	0	0	0	0	75

16564170	f	ot	US	2019	0	1	0	0	1	0	0	0	0	0	0	1	71
16564177	f	Not repo	CN	2019	1	0	0	0	0	0	0	0	0	0	0	0	
16564360	m	md	JP	2019	0	0	0	0	0	0	0	0	0	0	0	0	68
16564420	f	md	DK	2019	0	0	0	0	0	0	0	0	0	0	0	0	66
16564469	m	md	JP	2019	0	1	0	1	0	0	0	0	0	0	0	1	70
16564519	f	cn	JP	2019	1	0	0	0	0	0	0	0	0	0	0	0	
16564612	Not	ot	IT	2019	0	0	0	0	0	0	0	0	0	0	0	0	
16564632	f	cn	CN	2019	1	0	0	0	0	0	0	0	0	0	0	0	57.2
16564696	Not	cn	IT	2019	0	0	0	0	0	0	0	0	0	0	0	0	
16564883	m	md	JP	2019	0	1	0	1	0	0	0	0	0	0	0	1	66
16564950	m	ph	TH	2019	0	1	0	0	1	0	0	0	0	0	0	0	56
16565464	f	ot	CA	2019	0	1	0	1	0	0	0	0	0	0	0	0	
16565505	f	cn	IQ	2019	0	0	0	0	0	0	0	0	0	0	0	0	60
16565769	Not	md	ES	2019	0	1	1	0	0	0	0	0	0	0	0	0	79
16565835	m	ot	CO	2019	0	0	0	0	0	0	0	0	0	0	0	0	
16566112	f	cn	JP	2019	0	0	0	0	0	0	0	0	0	0	0	0	60
16566489	m	md	KR	2019	0	0	0	0	0	0	0	0	0	0	0	0	69
16566735	f	md	JP	2019	1	0	0	0	0	0	0	0	0	0	0	0	89.2
16566762	f	ot	ES	2019	0	1	1	0	0	0	0	0	0	0	0	0	87
16566772	m	md	JP	2019	0	1	0	0	1	0	0	0	0	0	0	1	69
16566838	m	md	JP	2019	0	1	1	0	0	0	0	0	0	0	0	0	71
16566894	Not	md	DE	2019	0	1	0	1	0	0	0	0	0	0	0	1	
16567553	m	md	JP	2019	0	1	0	1	0	0	0	0	0	0	0	1	79
16567567	f	cn	KR	2019	0	0	0	0	0	0	0	0	0	0	0	0	63
16567701	f	ot	US	2019	0	0	0	0	0	0	0	0	0	0	0	0	
16567704	m	Not repo	IT	2019	0	1	0	1	0	0	0	0	0	0	0	0	58.4
16567865	m	cn	JP	2019	0	1	1	0	0	0	0	0	0	0	0	0	72
16568091	f	cn	US	2019	0	0	0	0	0	0	0	0	0	0	0	0	64
16568144	Not	md	DE	2019	0	1	0	0	1	0	0	0	0	0	0	1	
16568219	m	ot	FR	2019	0	1	0	0	1	0	0	0	0	0	0	0	69
16568253	m	ot	CN	2019	0	1	0	0	1	0	0	0	0	0	0	0	54
16568372	m	cn	JP	2019	0	1	1	0	0	0	0	0	0	0	0	0	84
16568382	m	cn	KR	2019	0	0	0	0	0	0	0	0	0	0	0	0	
16568513	m	ot	CA	2019	1	0	0	0	0	0	0	0	0	0	0	0	48

16569128	m	cn	JP	2019	0	1	1	0	0	0	0	0	0	0	0	0	69
16569388	m	md	TH	2019	0	1	0	0	0	1	0	0	0	0	0	0	54
16569504	f	md	JP	2019	0	1	0	0	1	0	0	0	0	0	0	0	60
16569617	f	ph	FR	2019	0	1	0	0	1	0	0	0	0	0	0	0	69
16569701	Not	md	CN	2019	0	0	0	0	0	0	0	0	0	0	0	0	
16569854	Not	ot	JP	2019	0	0	0	0	0	0	0	0	0	0	0	0	
16570033	m	ot	CN	2019	0	0	0	0	0	0	0	0	0	0	0	0	30
16570220	f	cn	JP	2019	0	1	1	0	0	0	0	0	0	0	0	0	74
16570322	f	md	US	2019	1	0	0	0	0	0	0	0	0	0	0	0	
16570588	Not	ph	JP	2019	0	1	0	0	1	0	0	0	0	0	0	0	
16570682	m	Not repo	CN	2019	1	0	0	0	0	0	0	0	0	0	0	0	
16570945	f	Not repo	CN	2019	1	0	0	0	0	0	0	0	0	0	0	0	
16571376	f	ph	US	2019	0	1	0	0	1	0	0	0	0	0	0	0	64
16571690	m	md	SK	2019	0	0	0	0	0	0	0	0	0	0	0	0	69
16571696	f	md	IN	2019	0	0	0	0	0	0	0	0	0	0	0	0	48
16572417	m	cn	IT	2019	0	0	0	0	0	0	0	0	0	0	0	0	56
16573046	m	cn	JP	2019	0	1	1	0	0	0	0	0	0	0	0	0	75
16573075	f	md	CA	2019	0	1	0	1	0	0	0	0	0	0	0	0	75
16573310	m	Not repo	GR	2019	1	0	0	0	0	0	0	0	0	0	0	0	71
16573312	f	md	IL	2019	0	1	0	1	0	0	0	0	0	0	0	0	56.4
16573414	Not	ph	JP	2019	1	0	0	0	0	0	0	0	0	0	0	0	
16573474	m	md	US	2019	0	1	0	1	0	0	0	0	0	0	0	0	64
16573478	f	md	JP	2019	0	1	0	1	0	0	0	0	0	0	0	1	71.4
16573482	m	ot	US	2019	0	1	0	1	0	0	0	0	0	0	0	0	
16573545	m	ot	JP	2019	0	1	0	1	0	0	0	0	0	0	0	1	65
16573723	f	md	US	2019	0	0	0	0	0	0	0	0	0	0	0	0	73
16574470	Not	cn	JP	2019	0	1	1	0	0	0	0	0	0	0	0	0	
16574752	m	cn	JP	2019	0	1	1	0	0	0	0	0	0	0	0	0	70
16575288	m	cn	CN	2019	0	1	0	0	1	0	0	0	0	0	0	0	
16575459	m	md	JP	2019	0	1	0	0	0	1	0	0	0	0	0	1	70
16575744	m	cn	KR	2019	1	0	0	0	0	0	0	0	0	0	0	0	64
16575769	Not	ot	JP	2019	1	0	0	0	0	0	0	0	0	0	0	0	
16575979	f	Not repo	CN	2019	1	0	0	0	0	0	0	0	0	0	0	0	55.8
16576581	m	md	JP	2019	0	1	0	0	0	1	0	0	0	0	0	0	74

16576653	f	md	DK	2019	0	0	0	0	0	0	0	0	0	0	0	0	66
16576800	m	ot	FR	2019	0	1	0	0	1	0	0	0	0	0	0	0	80
16576883	m	md	JP	2019	0	1	0	0	0	1	0	0	0	0	0	0	70
16577133	m	md	JP	2019	0	1	0	0	1	0	0	0	0	0	0	0	69
16577209	m	md	JP	2019	0	1	0	0	0	1	0	0	0	0	0	0	73
16577268	m	md	ES	2019	0	1	0	0	0	1	0	0	0	0	0	0	74
16577535	m	ot	IT	2019	1	0	0	0	0	0	0	0	0	0	0	0	
16577587	f	ot	GR	2019	0	1	0	0	0	1	0	0	0	0	0	0	
16577611	Not	md	JP	2019	0	1	0	1	0	0	0	0	0	0	0	0	
16577645	m	ot	DE	2019	0	0	0	0	0	0	0	0	0	0	0	0	61
16577684	Not	md	FR	2019	0	1	0	1	0	0	0	0	0	0	0	0	
16577840	m	ot	JP	2019	0	1	0	0	1	0	0	0	0	0	0	0	69
16577841	m	md	JP	2019	0	1	0	0	1	0	0	0	0	0	0	0	79
16578567	f	md	FR	2019	0	0	0	0	0	0	0	0	0	0	0	0	54
16578737	Not	ot	TW	2019	1	0	0	0	0	0	0	0	0	0	0	0	70
16579068	Not	ot	TW	2019	1	0	0	0	0	0	0	0	0	0	0	0	63
16579083	Not	ph	JP	2019	1	0	0	0	0	0	0	0	0	0	0	0	
16579092	Not	ph	JP	2019	1	0	0	0	0	0	0	0	0	0	0	0	
16579446	m	md	JP	2019	0	1	0	1	0	0	0	0	0	0	0	0	73.5
16579536	m	ot	TR	2019	0	0	0	0	0	0	0	0	0	0	0	0	52
16579559	m	cn	ES	2019	0	1	1	0	0	0	0	0	0	0	0	0	36
16580032	m	md	JP	2019	0	1	0	1	0	0	0	0	0	0	0	1	81
16580059	f	cn	PT	2019	0	0	0	0	0	0	0	0	0	0	0	0	
16580148	f	md	JP	2019	0	1	0	1	0	0	0	0	0	0	0	0	67.7
16580322	m	ot	DE	2019	0	1	1	0	0	0	0	0	0	0	0	0	48
16580540	f	cn	PT	2019	0	0	0	0	0	0	0	0	0	0	0	0	
16581647	f	cn	KR	2019	0	0	0	0	0	0	0	0	0	0	0	0	44
16581929	Not	cn	JP	2019	0	1	1	0	0	0	0	0	0	0	0	0	
16582116	m	md	JP	2019	0	1	0	1	0	0	0	0	0	0	0	1	78
16582434	f	md	VN	2019	0	1	0	0	0	1	0	0	0	0	0	0	62
16582617	f	cn	JP	2019	0	1	1	0	0	0	0	0	0	0	0	0	78
16582763	m	md	AU	2019	0	1	0	0	0	1	0	0	0	0	0	0	
16582773	f	cn	AE	2019	0	0	0	0	0	0	0	0	0	0	0	0	69
16582780	m	md	KR	2019	0	1	1	0	0	0	0	0	0	0	0	0	57

16583007	m	md	JP	2019	0	1	0	1	0	0	0	0	0	0	0	1	80
16583427	f	md	JP	2019	0	1	0	1	0	0	0	0	0	0	0	1	56
16584184	f	cn	CN	2019	1	0	0	0	0	0	0	0	0	0	0	0	
16584237	m	ot	JP	2019	0	0	0	0	0	0	0	0	0	0	0	0	68
16584581	f	md	FR	2019	0	0	0	0	0	0	0	0	0	0	0	0	54
16584600	m	md	JP	2019	0	1	1	0	0	0	0	0	0	0	0	1	54
16584767	m	Not repo	ID	2019	1	0	0	0	0	0	0	0	0	0	0	0	67.2
16584852	f	cn	AT	2019	0	1	1	0	0	0	0	0	0	0	0	0	
16585095	f	md	FR	2019	0	0	0	0	0	0	0	0	0	0	0	0	60
16585124	f	Not repo	KR	2019	1	0	0	0	0	0	0	0	0	0	0	0	70
16585592	m	md	JP	2019	0	1	0	1	0	0	0	0	0	0	0	1	66
16585891	m	ot	JP	2019	0	1	1	0	0	0	0	0	0	0	0	0	85
16585980	m	ot	US	2019	0	1	0	0	1	0	0	0	0	0	0	0	67
16586087	f	ph	US	2019	0	0	0	0	0	0	0	0	0	0	0	0	63
16586343	f	ot	US	2019	0	1	0	0	1	0	0	0	0	0	0	0	71
16586426	Not	md	JP	2019	1	0	0	0	0	0	0	0	0	0	0	0	
16586539	f	md	RU	2019	0	0	0	0	0	0	0	0	0	0	0	0	69
16586579	m	md	TH	2019	0	1	0	1	0	0	0	0	0	0	0	0	75.1
16586727	Not	ot	GB	2019	0	0	0	0	0	0	0	0	0	0	0	0	
16586875	m	md	JP	2019	1	0	0	0	0	0	0	0	0	0	0	1	76
16587083	m	cn	FR	2019	0	1	1	0	0	0	0	0	0	0	0	0	
16587897	f	cn	US	2019	0	1	0	0	1	0	0	0	0	0	0	0	91
16588114	Not	md	US	2019	0	0	0	0	0	0	0	0	0	0	0	0	
16588120	Not	md	US	2019	0	0	0	0	0	0	0	0	0	0	0	0	
16588121	Not	md	US	2019	0	0	0	0	0	0	0	0	0	0	0	0	
16588293	f	Not repo	CN	2019	1	0	0	0	0	0	0	0	0	0	0	0	
16588377	m	ot	US	2019	0	1	0	1	0	0	0	0	0	0	0	1	68
16588479	m	md	FR	2019	0	0	0	0	0	0	0	0	0	0	0	0	72
16588530	f	md	US	2019	0	1	1	0	0	0	0	0	0	0	0	1	77
16588656	Not	cn	JP	2019	0	1	1	0	0	0	0	0	0	0	0	0	
16588797	m	md	IL	2019	0	1	0	0	0	1	0	0	0	0	0	0	
16589141	f	cn	US	2019	1	0	0	0	0	0	0	0	0	0	0	0	
16589463	m	md	DE	2019	0	1	0	0	1	0	0	0	0	0	0	1	52
16590320	Not	md	JP	2019	0	1	0	0	0	1	0	0	0	0	0	0	70

16590412	m	md	JP	2019	0	1	0	0	0	1	0	0	0	0	0	0	
16590774	m	md	JP	2019	0	1	0	0	1	0	0	0	0	0	0	0	72
16590934	f	ot	NL	2019	0	0	0	0	0	0	0	0	0	0	0	0	63
16591402	f	md	TH	2019	0	0	0	0	0	0	0	0	0	0	0	0	
16592149	m	ot	NL	2019	0	0	0	0	0	0	0	0	0	0	0	0	72
16592263	m	cn	KR	2019	0	0	0	0	0	0	0	0	0	0	0	0	48
16592286	f	cn	IN	2019	1	0	0	0	0	0	0	0	0	0	0	0	64
16592330	m	md	JP	2019	0	1	0	1	0	0	0	0	0	0	0	1	70
16592583	m	ot	ES	2019	0	1	1	0	0	0	0	0	0	0	0	0	76
16592833	Not	md	US	2019	0	0	0	0	0	0	0	0	0	0	0	0	
16592843	Not	md	US	2019	0	0	0	0	0	0	0	0	0	0	0	0	
16593062	Not	Not repo	IT	2019	1	0	0	0	0	0	0	0	0	0	0	0	73
16593278	m	cn	JP	2019	0	1	1	0	0	0	0	0	0	0	0	0	
16593343	Not	md	DE	2019	1	0	0	0	0	0	0	0	0	0	0	0	
16593344	m	cn	CN	2019	0	1	0	0	1	0	0	0	0	0	0	0	
16593672	m	md	FR	2019	0	1	0	0	0	1	0	0	0	0	0	1	54
16593688	f	md	JP	2019	1	0	0	0	0	0	0	0	0	0	0	0	72.4
16593803	Not	ot	CN	2019	0	0	0	0	0	0	0	0	0	0	0	0	
16593828	Not	ot	CN	2019	0	0	0	0	0	0	0	0	0	0	0	0	
16594034	f	cn	IL	2019	0	0	0	0	0	0	0	0	0	0	0	0	38
16594305	f	ot	US	2019	0	0	0	0	0	0	0	0	0	0	0	0	61
16594572	m	ot	US	2019	0	1	0	0	1	0	0	0	0	0	0	0	59
16594821	f	cn	US	2019	0	1	1	0	0	0	0	0	0	0	0	0	
16595080	f	cn	JP	2019	0	0	0	0	0	0	0	0	0	0	0	0	70
16595110	f	md	GB	2019	0	0	0	0	0	0	0	0	0	0	0	0	
16595242	Not	Not repo	AU	2019	0	1	0	1	0	0	0	0	0	0	0	0	
16595243	f	md	JP	2019	1	0	0	0	0	0	0	0	0	0	0	0	89
16595445	f	ot	NL	2019	1	0	0	0	0	0	0	0	0	0	0	0	70
16595446	m	cn	GB	2019	1	0	0	0	0	0	0	0	0	0	0	0	
16595803	Not	cn	JP	2019	0	1	1	0	0	0	0	0	0	0	0	1	
16595821	m	md	JP	2019	0	1	0	0	0	1	0	0	0	0	0	1	69
16595908	f	cn	CN	2019	0	1	0	0	1	0	0	0	0	0	0	0	83
16595911	Not	md	FR	2019	0	1	0	0	1	0	0	0	0	0	0	0	
16596147	m	cn	US	2019	1	0	0	0	0	0	0	0	0	0	0	0	48

16596350	f	md	JP	2019	1	0	0	0	0	0	0	0	0	0	0	0	71
16596439	m	cn	CN	2019	0	1	0	0	1	0	0	0	0	0	0	0	
16596454	Not	cn	JP	2019	0	1	1	0	0	0	0	0	0	0	0	0	65
16596674	f	ot	CA	2019	0	0	0	0	0	0	0	0	0	0	0	0	63
16596795	m	ot	CA	2019	0	0	0	0	0	0	0	0	0	0	0	0	48
16596971	f	md	AU	2019	0	1	0	0	0	1	0	0	0	0	0	0	
16597016	m	ot	JP	2019	0	1	1	0	0	0	0	0	0	0	0	0	76
16597082	m	md	JP	2019	1	0	0	0	0	0	0	0	0	0	0	0	65
16597141	f	ot	CA	2019	0	0	0	0	0	0	0	0	0	0	0	0	63
16597699	f	ph	JP	2019	1	0	0	0	0	0	0	0	0	0	0	0	79
16597700	f	md	US	2019	0	1	0	1	0	0	0	0	0	0	0	1	
16598175	Not	cn	JP	2019	0	1	1	0	0	0	0	0	0	0	0	0	72
16598358	f	ot	US	2019	0	1	0	0	1	0	0	0	0	0	0	0	46
16598435	f	Not repo	ID	2019	1	0	0	0	0	0	0	0	0	0	0	0	
16598505	f	ph	FR	2019	0	0	0	0	0	0	0	0	0	0	0	0	69
16598655	m	md	KR	2019	0	1	0	0	0	1	0	0	0	0	0	0	67
16599047	f	ot	NL	2019	0	0	0	0	0	0	0	0	0	0	0	0	66
16599068	Not	md	JP	2019	0	1	0	1	0	0	0	0	0	0	0	1	
16599401	f	md	JP	2019	1	0	0	0	0	0	0	0	0	0	0	0	79
16599466	m	ot	DE	2019	0	1	0	0	1	0	0	0	0	0	0	0	56
16599857	m	md	JP	2019	1	0	0	0	0	0	0	0	0	0	0	0	81
16599978	f	cn	JP	2019	0	1	1	0	0	0	0	0	0	0	0	0	
16600021	f	cn	GB	2019	1	0	0	0	0	0	0	0	0	0	0	0	
16600037	f	ot	US	2019	0	1	0	0	1	0	0	0	0	0	0	0	58
16600064	m	md	JP	2019	0	1	1	0	0	0	0	0	0	0	0	0	62
16600082	f	ot	GB	2019	0	0	0	0	0	0	0	0	0	0	0	0	75
16600344	m	cn	CN	2019	0	1	0	0	1	0	0	0	0	0	0	0	
16600360	f	cn	CN	2019	0	1	0	0	1	0	0	0	0	0	0	0	
16600371	Not	md	NL	2019	0	0	0	0	0	0	0	0	0	0	0	0	
16600372	Not	ot	IT	2019	0	0	0	0	0	0	0	0	0	0	0	0	
16600374	m	cn	JP	2019	0	1	1	0	0	0	0	0	0	0	0	0	81
16600408	f	md	BE	2019	0	1	0	0	1	0	0	0	0	0	0	0	71
16600530	m	md	JP	2019	0	0	0	0	0	0	1	0	0	1	0	1	70
16600648	m	cn	JP	2019	0	1	1	0	0	0	0	0	0	0	0	0	69

16601517	m	cn	JP	2019	0	1	1	0	0	0	0	0	0	0	0	0	65
16601570	f	md	JP	2019	1	0	0	0	0	0	0	0	0	0	0	1	75.7
16601777	m	md	IN	2019	0	0	0	0	0	0	0	0	0	0	0	0	54
16601805	m	cn	JP	2019	0	1	1	0	0	0	0	0	0	0	0	1	72
16606652	f	md	JP	2019	0	1	1	0	0	0	0	0	0	0	0	0	82
16606878	m	md	JP	2019	0	1	0	0	0	1	0	0	0	0	0	0	63
16607137	Not	md	JP	2019	0	1	0	0	1	0	0	0	0	0	0	0	
16608076	m	md	JP	2019	1	0	0	0	0	0	0	0	0	0	0	1	81
16608563	m	cn	JP	2019	0	1	1	0	0	0	0	0	0	0	0	0	68
16610029	f	md	CY	2019	0	1	1	0	0	0	0	0	0	0	0	0	
16610623	f	Not repo	JP	2019	0	0	0	0	0	0	0	0	0	0	0	0	77
16610654	Not	md	US	2019	0	0	0	0	0	0	0	0	0	0	0	0	
16611519	Not	ot	JP	2019	0	0	0	0	0	0	0	0	0	0	0	0	
16611668	Not	ot	JP	2019	0	0	0	0	0	0	0	0	0	0	0	0	
16612010	m	cn	JP	2019	0	1	1	0	0	0	0	0	0	0	0	1	66
16612012	m	ot	JP	2019	0	1	1	0	0	0	0	0	0	0	0	0	71
16612041	Not	ot	US	2019	0	1	0	0	0	1	0	0	0	0	0	0	
16612042	Not	ot	US	2019	0	1	0	0	0	1	0	0	0	0	0	0	
16612047	m	ot	US	2019	0	1	0	0	0	1	0	0	0	0	0	1	
16612048	m	md	US	2019	0	1	0	0	0	1	0	0	0	0	0	0	
16612049	f	md	US	2019	0	1	0	0	0	1	0	0	0	0	0	0	
16612051	m	md	US	2019	0	1	0	0	0	1	0	0	0	0	0	0	
16612069	f	ot	US	2019	0	1	0	0	0	1	0	0	0	0	0	0	67
16612070	m	md	US	2019	0	1	0	0	0	1	0	0	0	0	0	0	80
16613411	Not	ph	CA	2019	0	1	1	0	0	0	0	0	0	0	0	1	
16613808	m	cn	IN	2019	0	0	0	0	0	0	0	0	0	0	0	0	
16614011	m	md	JP	2019	0	1	1	0	0	0	0	0	0	0	0	1	66
16614463	f	md	NZ	2019	0	1	0	0	0	1	0	0	0	0	0	0	53
16614481	m	cn	CN	2019	0	1	0	0	1	0	0	0	0	0	0	0	46
16614489	m	ph	JP	2019	0	1	0	0	1	0	0	0	0	0	0	1	75
16614754	m	md	JP	2019	0	1	1	0	0	0	0	0	0	0	0	0	66
16615005	m	md	JP	2019	0	1	1	0	0	0	0	0	0	0	0	0	72
16615282	Not	md	JP	2019	0	1	0	0	1	0	0	0	0	0	0	0	
16616194	f	ot	US	2019	0	1	0	0	1	0	0	0	0	0	0	0	

16616260	Not	ph	CA	2019	0	1	1	0	0	0	0	0	0	0	0	1	
16616287	f	ot	FR	2019	0	0	0	0	0	0	0	0	0	0	0	0	59
16617476	f	ot	US	2019	0	1	0	0	1	0	0	0	0	0	0	0	46
16618290	m	md	NL	2019	0	0	0	0	0	0	0	0	0	0	0	0	73
16618935	m	cn	JP	2019	0	1	1	0	0	0	0	0	0	0	0	0	68
16618953	Not	ot	US	2019	0	0	0	0	0	0	0	0	0	0	0	0	
16619428	f	md	US	2019	0	1	0	0	1	0	0	0	0	0	0	0	61
16619654	f	Not repo	CA	2019	0	1	0	1	0	0	0	0	0	0	0	0	71
16619846	f	md	US	2019	1	0	0	0	0	0	0	0	0	0	0	0	60
16619860	Not	md	DE	2019	0	1	1	0	0	0	0	0	0	0	0	0	80
16620577	f	cn	DK	2019	0	1	1	0	0	0	0	0	0	0	0	1	81
16620691	m	ot	TW	2019	0	1	0	0	1	0	0	0	0	0	0	0	48
16620999	Not	md	PL	2019	0	0	0	0	0	0	0	0	0	0	0	0	
16621593	m	ot	DE	2019	0	1	1	0	0	0	0	0	0	0	0	0	71
16621755	f	md	IT	2019	1	0	0	0	0	0	0	0	0	0	0	0	
16622374	m	cn	JP	2019	0	1	1	0	0	0	0	0	0	0	0	1	77
16623384	m	ot	FR	2019	0	1	0	0	0	1	0	0	0	0	0	1	54
16623660	Not	cn	US	2019	0	1	0	0	1	0	0	0	0	0	0	0	
16623799	m	md	US	2019	0	0	0	0	0	0	0	0	0	0	0	0	64
16623942	m	cn	JP	2019	0	1	1	0	0	0	0	0	0	0	0	0	73
16623962	f	md	DK	2019	0	1	1	0	0	0	0	0	0	0	0	1	81
16624047	f	cn	CA	2019	0	0	0	0	0	0	0	0	0	0	0	0	59
16624081	f	md	AT	2019	0	1	1	0	0	0	0	0	0	0	0	0	
16624088	m	md	FR	2019	0	1	0	1	0	0	0	0	0	0	0	1	72
16624196	m	cn	CN	2019	0	1	0	0	1	0	0	0	0	0	0	0	
16624231	m	md	HU	2019	0	0	0	0	0	0	0	0	0	0	0	0	
16624268	m	md	CN	2019	0	1	0	0	0	1	0	0	0	0	0	0	71
16624503	m	ot	JP	2019	0	0	0	0	0	0	0	0	0	0	0	0	69
16624886	m	cn	JP	2019	0	1	1	0	0	0	0	0	0	0	0	0	61
16625247	m	md	GB	2019	0	0	0	0	0	0	0	0	0	0	0	0	59
16625569	m	ot	JP	2019	0	1	0	0	1	0	0	0	0	0	0	0	69
16625621	f	ot	JP	2019	1	0	0	0	0	0	0	0	0	0	0	0	71
16625874	Not	md	JP	2019	1	0	0	0	0	0	0	0	0	0	0	0	
16625955	f	md	GR	2019	0	1	0	0	0	1	0	0	0	0	0	0	

16626016	f	ot	CA	2019	0	1	0	0	1	0	0	0	0	0	0	0	53
16626130	Not	md	PL	2019	0	0	0	0	0	0	0	0	0	0	0	0	
16626282	m	Not repo	CN	2019	1	0	0	0	0	0	0	0	0	0	0	0	
16626343	Not	ph	JP	2019	0	1	0	1	0	0	0	0	0	0	0	0	
16626646	f	ot	US	2019	0	1	0	1	0	0	0	0	0	0	0	0	49
16626762	f	Not repo	CN	2019	1	0	0	0	0	0	0	0	0	0	0	0	
16627118	m	ot	JP	2019	0	1	0	1	0	0	0	0	0	0	0	1	72
16627146	Not	ot	US	2019	0	1	0	0	1	0	0	0	0	0	0	0	
16627504	f	ot	JP	2019	0	1	0	0	1	0	0	0	0	0	0	0	68
16627978	m	ot	JP	2019	0	1	0	0	1	0	0	0	0	0	0	0	81
16627981	m	cn	JP	2019	0	1	1	0	0	0	0	0	0	0	0	0	72
16628051	Not	ph	CA	2019	0	1	1	0	0	0	0	0	0	0	0	1	
16628142	m	ph	US	2019	0	1	1	0	0	0	0	0	0	0	0	0	72
16628192	m	ot	US	2019	1	0	0	0	0	0	0	0	0	0	0	0	
16628374	Not	md	US	2019	0	0	0	0	0	0	0	0	0	0	0	0	
16628924	m	md	KP	2019	0	0	0	0	0	0	0	0	0	0	0	0	55
16628984	f	cn	JP	2019	0	1	1	0	0	0	0	0	0	0	0	0	70
16629064	f	md	JP	2019	0	1	0	0	1	0	0	0	0	0	0	0	69
16629280	f	ph	US	2019	0	0	0	0	0	0	0	0	0	0	0	0	69
16629618	m	md	JP	2019	0	1	0	1	0	0	0	0	0	0	0	0	72
16629678	m	cn	JP	2019	0	1	1	0	0	0	0	0	0	0	0	1	77
16629859	m	ot	FR	2019	0	1	0	0	1	0	0	0	0	0	0	0	73
16629886	f	ot	FR	2019	0	1	0	0	1	0	0	0	0	0	0	0	57
16629927	f	cn	SE	2019	0	1	0	0	1	0	0	0	0	0	0	0	82
16629971	f	md	DE	2019	0	0	0	0	0	0	0	0	0	0	0	0	
16630058	f	md	JP	2019	1	0	0	0	0	0	0	0	0	0	0	0	78
16630149	f	md	KR	2019	0	0	0	0	0	0	0	0	0	0	0	0	66
16630331	f	cn	TH	2019	0	1	0	0	1	0	0	0	0	0	0	0	85
16630351	m	md	JP	2019	0	0	0	0	0	0	0	0	0	0	0	0	54
16630490	m	ph	US	2019	0	1	1	0	0	0	0	0	0	0	0	0	87
16630607	m	cn	CN	2019	0	1	0	0	1	0	0	0	0	0	0	0	
16630666	f	md	GB	2019	0	0	0	0	0	0	0	0	0	0	0	0	71
16630789	Not	md	JP	2019	0	1	0	0	1	0	0	0	0	0	0	0	
16630799	f	md	JP	2019	0	1	0	0	1	0	0	0	0	0	0	0	79

16630857	Not	md	US	2019	0	0	0	0	0	0	0	0	0	0	0	0	
16630870	f	cn	US	2019	0	0	0	0	0	0	0	0	0	0	0	0	76
16631417	f	md	GR	2019	0	0	0	0	0	0	0	0	0	0	0	0	80
16631494	m	ot	JP	2019	0	1	0	0	1	0	0	0	0	0	0	0	76
16631545	m	md	JP	2019	0	1	0	1	0	0	0	0	0	0	0	1	78
16632639	m	md	FR	2019	0	1	0	0	1	0	0	0	0	0	0	0	86
16633490	f	ot	CA	2019	0	0	0	0	0	0	0	0	0	0	0	0	69
16633612	m	ot	CN	2019	0	1	0	0	1	0	0	0	0	0	0	0	68
16633814	m	cn	JP	2019	0	1	1	0	0	0	0	0	0	0	0	0	83
16633978	m	md	JP	2019	1	0	0	0	0	0	0	0	0	0	0	1	77
16634017	f	Not repo	CN	2019	1	0	0	0	0	0	0	0	0	0	0	0	63
16634018	f	md	JP	2019	1	0	0	0	0	0	0	0	0	0	0	1	84.8
16634022	m	md	JP	2019	0	1	0	1	0	0	0	0	0	0	0	1	77.9
16634044	Not	md	JP	2019	1	0	0	0	0	0	0	0	0	0	0	0	
16634045	f	md	US	2019	0	1	0	1	0	0	0	0	0	0	0	0	61
16634062	f	cn	US	2019	0	1	0	1	0	0	0	0	0	0	0	0	
16634144	m	ot	IE	2019	0	1	0	0	0	1	0	0	0	0	0	0	77
16634243	m	ot	CN	2019	0	1	0	0	1	0	0	0	0	0	0	0	63
16634522	m	md	PL	2019	0	1	0	0	0	1	0	0	0	0	0	0	69
16634841	f	ot	AU	2019	0	1	0	0	0	1	0	0	0	0	0	0	64
16635026	m	md	DK	2019	0	1	1	0	0	0	0	0	0	0	0	0	67
16635060	m	cn	US	2019	0	1	1	0	0	0	0	0	0	0	0	0	72
16635257	f	md	IT	2019	1	0	0	0	0	0	0	0	0	0	0	0	46.9
16635263	f	md	JP	2019	0	0	0	0	0	0	0	0	0	0	0	0	90
16635363	m	ot	NL	2019	1	0	0	0	0	0	0	0	0	0	0	0	70
16635453	f	md	US	2019	1	0	0	0	0	0	0	0	0	0	0	0	
16635488	m	ot	GB	2019	0	1	0	0	1	0	0	0	0	0	0	0	84
16635595	m	md	JP	2019	1	0	0	0	0	0	0	0	0	0	0	0	
16635924	m	md	IN	2019	0	1	0	1	0	0	0	0	0	0	0	0	70
16635949	f	md	US	2019	1	0	0	0	0	0	0	0	0	0	0	1	63
16635953	m	ph	JP	2019	1	0	0	0	0	0	0	0	0	0	0	0	100
16636946	m	ot	JP	2019	0	1	1	0	0	0	0	0	0	0	0	0	71
16636974	f	md	JP	2019	1	0	0	0	0	0	0	0	0	0	0	1	68.6
16637327	m	ot	JP	2019	0	1	1	0	0	0	0	0	0	0	0	0	74

16637904	f	md	BE	2019	1	0	0	0	0	0	0	0	0	0	0	0	82.7
16638004	m	md	JP	2019	1	0	0	0	0	0	0	0	0	0	0	0	50
16638009	f	md	DK	2019	0	1	1	0	0	0	0	0	0	0	0	0	64
16638297	f	md	AU	2019	0	0	0	0	0	0	0	0	0	0	0	0	62
16638420	m	md	JP	2019	1	0	0	0	0	0	0	0	0	0	0	1	66.8
16638430	m	Not repo	CA	2019	0	1	0	1	0	0	0	0	0	0	0	0	42.8
16639149	m	cn	IL	2019	0	1	1	0	0	0	0	0	0	0	0	1	72
16639165	m	ot	JP	2019	0	0	0	0	0	0	0	0	0	0	0	0	75
16639170	f	ot	FR	2019	0	1	0	0	1	0	0	0	0	0	0	0	72
16639340	m	cn	JP	2019	0	1	1	0	0	0	0	0	0	0	0	0	78
16639359	m	ot	CA	2019	0	1	1	0	0	0	0	0	0	0	0	0	
16640083	f	ot	AT	2019	0	1	1	0	0	0	0	0	0	0	0	0	
16640881	m	ot	US	2019	0	1	1	0	0	0	0	0	0	0	0	0	61
16641002	f	md	KR	2019	1	0	0	0	0	0	0	0	0	0	0	0	72
16641003	m	cn	JP	2019	0	1	1	0	0	0	0	0	0	0	0	1	40
16641081	f	ot	KR	2019	0	1	0	0	1	0	0	0	0	0	0	0	60
16641379	f	md	DE	2019	0	1	0	0	0	1	0	0	0	0	0	0	75
16641999	m	cn	JP	2019	0	0	0	0	0	0	0	0	0	0	0	0	70
16642552	m	cn	JP	2019	0	1	1	0	0	0	0	0	0	0	0	0	72
16643441	Not	md	IT	2019	1	0	0	0	0	0	0	0	0	0	0	0	
16644030	f	cn	US	2019	0	1	1	0	0	0	0	0	0	0	0	0	56
16644084	f	cn	CZ	2019	0	1	1	0	0	0	0	0	0	0	0	0	
16644151	f	md	JP	2019	0	0	0	0	0	0	0	0	0	0	0	0	62
16644159	m	md	VN	2019	0	0	0	0	0	0	0	0	0	0	0	0	42
16644464	Not	md	AT	2019	0	0	0	0	0	0	0	0	0	0	0	0	
16644931	m	cn	ES	2019	0	1	1	0	0	0	0	0	0	0	0	0	36
16645076	Not	md	AT	2019	0	0	0	0	0	0	0	0	0	0	0	0	
16645081	Not	md	AT	2019	0	0	0	0	0	0	0	0	0	0	0	0	
16645323	m	cn	Not re	2019	0	0	0	0	0	0	0	0	0	0	0	0	
16645620	m	md	JP	2019	0	1	0	0	1	0	0	0	0	0	0	1	72
16645744	m	md	RS	2019	0	1	1	0	0	0	0	0	0	0	0	0	
16645960	f	md	DE	2019	0	1	0	0	0	1	0	0	0	0	0	0	75
16646074	m	md	AT	2019	0	1	0	0	1	0	0	0	0	0	0	0	79
16646298	f	md	US	2019	0	1	1	0	0	0	0	0	0	0	0	0	63

16646417	f	md	CZ	2019	1	0	0	0	0	0	0	0	0	0	0	0	87
16646596	m	ot	AT	2019	0	1	0	0	1	0	0	0	0	0	0	1	53
16646722	m	cn	US	2019	0	1	1	0	0	0	0	0	0	0	0	0	60
16646740	m	ot	CH	2019	0	1	1	0	0	0	0	0	0	0	0	0	55
16646900	f	md	FR	2019	0	1	1	0	0	0	0	0	0	0	0	0	63
16646979	m	md	NL	2019	0	0	0	0	0	0	0	0	0	0	0	0	70
16647166	m	md	FR	2019	0	1	0	1	0	0	0	0	0	0	0	0	68.2
16647173	m	md	JP	2019	0	1	0	1	0	0	0	0	0	0	0	0	73
16647293	m	md	JP	2019	1	0	0	0	0	0	0	0	0	0	0	0	79
16647787	m	ot	JP	2019	0	1	1	0	0	0	0	0	0	0	0	1	79
16647814	m	cn	JP	2019	0	1	1	0	0	0	0	0	0	0	0	0	73
16648172	f	md	NP	2019	0	0	0	0	0	0	0	0	0	0	0	0	50
16648192	f	ot	GB	2019	0	1	0	0	0	1	0	0	0	0	0	0	56
16648195	m	md	JP	2019	1	0	0	0	0	0	0	0	0	0	0	0	83
16648210	Not	ot	ES	2019	1	0	0	0	0	0	0	0	0	0	0	0	
16648218	Not	ot	ES	2019	1	0	0	0	0	0	0	0	0	0	0	0	
16648219	Not	ot	ES	2019	1	0	0	0	0	0	0	0	0	0	0	0	
16648223	Not	ot	ES	2019	1	0	0	0	0	0	0	0	0	0	0	0	
16648283	m	cn	Not re	2019	0	0	0	0	0	0	0	0	0	0	0	0	83
16648297	Not	ot	ES	2019	1	0	0	0	0	0	0	0	0	0	0	0	
16648298	Not	ot	ES	2019	1	0	0	0	0	0	0	0	0	0	0	0	
16648299	Not	ot	ES	2019	1	0	0	0	0	0	0	0	0	0	0	0	
16648300	Not	ot	ES	2019	1	0	0	0	0	0	0	0	0	0	0	0	
16648348	Not	ot	ES	2019	1	0	0	0	0	0	0	0	0	0	0	0	
16648355	Not	ot	ES	2019	1	0	0	0	0	0	0	0	0	0	0	0	
16648357	f	md	FR	2019	0	1	0	1	0	0	0	0	0	0	0	1	
16648471	Not	ot	ES	2019	1	0	0	0	0	0	0	0	0	0	0	0	
16648478	f	md	US	2019	1	0	0	0	0	0	0	0	0	0	0	1	
16648698	f	md	US	2019	1	0	0	0	0	0	0	0	0	0	0	0	68
16648710	Not	md	US	2019	0	1	0	1	0	0	0	0	0	0	0	1	
16648755	m	cn	JP	2019	0	1	1	0	0	0	0	0	0	0	0	0	69
16648771	f	ot	CA	2019	0	0	0	0	0	0	0	0	0	0	0	0	63
16648784	Not	ot	ES	2019	1	0	0	0	0	0	0	0	0	0	0	0	
16648787	f	md	JP	2019	1	0	0	0	0	0	0	0	0	0	0	0	89

16648796	m	md	MY	2019	0	1	0	1	0	0	0	0	0	0	0	1	
16648809	Not	ot	ES	2019	1	0	0	0	0	0	0	0	0	0	0	0	
16648811	Not	ot	ES	2019	1	0	0	0	0	0	0	0	0	0	0	0	
16648998	Not	ot	ES	2019	1	0	0	0	0	0	0	0	0	0	0	0	
16649061	m	Not repo	JP	2019	0	0	0	0	0	0	0	0	0	0	0	0	
16649081	m	ot	JP	2019	0	1	0	1	0	0	0	0	0	0	0	1	78
16649085	Not	md	US	2019	0	1	0	1	0	0	0	0	0	0	0	0	72
16649221	m	cn	US	2019	0	0	0	0	0	0	0	0	0	0	0	0	
16649233	m	cn	US	2019	0	1	1	0	0	0	0	0	0	0	0	0	63
16649242	Not	cn	GB	2019	1	0	0	0	0	0	0	0	0	0	0	0	
16649550	m	md	KR	2019	1	0	0	0	0	0	0	0	0	0	0	0	84
16649659	f	md	US	2019	0	0	0	0	0	0	0	0	0	0	0	0	72
16650365	m	ot	FR	2019	0	1	0	0	1	0	0	0	0	0	0	0	78
16650368	f	ph	JP	2019	0	1	0	0	1	0	0	0	0	0	0	0	79
16650620	m	Not repo	JP	2019	0	0	0	0	0	0	0	0	0	0	0	0	80
16650763	m	md	JP	2019	0	1	0	0	0	1	0	0	0	0	0	0	74
16650809	Not	md	JP	2019	0	1	0	0	1	0	0	0	0	0	0	0	
16651085	f	md	KR	2019	1	0	0	0	0	0	0	0	0	0	0	0	41
16651385	m	cn	SA	2019	0	1	1	0	0	0	0	0	0	0	0	0	
16651643	m	ot	US	2019	0	0	0	0	0	0	0	0	0	0	0	0	43
16651646	f	ot	SG	2019	0	0	0	0	0	0	0	0	0	0	0	0	63
16651845	f	ph	US	2019	0	0	0	0	0	0	0	0	0	0	0	0	64
16652278	f	md	DE	2019	0	1	1	0	0	0	0	0	0	0	0	0	55
16652491	m	md	JP	2019	0	1	0	1	0	0	0	0	0	0	0	1	81
16652604	f	md	US	2019	0	1	1	0	0	0	0	0	0	0	0	0	
16653101	f	ph	CA	2019	0	1	1	0	0	0	0	0	0	0	0	1	63
16653156	Not	md	US	2019	0	0	0	0	0	0	0	0	0	0	0	0	
16653283	m	md	JP	2019	0	1	0	0	1	0	0	0	0	0	0	0	53
16653378	m	md	JP	2019	0	1	0	1	0	0	0	0	0	0	0	1	76
16654277	m	ot	RO	2019	0	1	0	0	1	0	0	0	0	0	0	0	64
16654405	f	md	AT	2019	0	1	1	0	0	0	0	0	0	0	0	0	
16654814	m	md	JP	2019	0	1	0	0	0	1	0	0	0	0	0	0	70
16654977	m	md	JP	2019	0	1	0	0	0	1	0	0	0	0	0	0	53
16655032	m	md	JP	2019	0	1	0	0	0	1	0	0	0	0	0	0	

16655592	m	md	JP	2019	0	1	1	0	0	0	0	0	0	0	0	0	70
16656087	Not	md	US	2019	0	0	0	0	0	0	0	0	0	0	0	0	
16656088	Not	md	US	2019	0	0	0	0	0	0	0	0	0	0	0	0	
16656115	f	md	JP	2019	0	1	0	1	0	0	0	0	0	0	0	0	70
16656366	m	md	CN	2019	0	0	0	0	0	0	0	0	0	0	0	0	58
16657255	m	cn	US	2019	0	1	1	0	0	0	0	0	0	0	0	0	63
16657397	f	ph	JP	2019	0	1	0	1	0	0	0	0	0	0	0	0	70
16657729	m	cn	JP	2019	0	1	1	0	0	0	0	0	0	0	0	0	67
16657738	m	cn	CA	2019	0	0	0	0	0	0	0	0	0	0	0	0	65
16657821	m	Not repo	EG	2019	1	0	0	0	0	0	0	0	0	0	0	0	
16657903	m	md	JP	2019	0	1	0	1	0	0	0	0	0	0	0	1	60
16658136	m	md	JP	2019	1	0	0	0	0	0	0	0	0	0	0	0	72
16658500	m	cn	JP	2019	0	1	1	0	0	0	0	0	0	0	0	0	75
16658588	m	ot	CN	2019	0	1	0	0	1	0	0	0	0	0	0	0	60
16658777	f	md	US	2019	0	1	1	0	0	0	0	0	0	0	0	0	
16658950	m	Not repo	US	2019	1	0	0	0	0	0	0	0	0	0	0	0	63
16659234	m	md	JP	2019	0	1	0	0	0	1	0	0	0	0	0	0	39
16659401	m	md	RU	2019	0	1	0	0	0	1	0	0	0	0	0	0	65
16659467	m	md	KR	2019	1	0	0	0	0	0	0	0	0	0	0	0	88
16659471	m	cn	JP	2019	0	1	1	0	0	0	0	0	0	0	0	0	74
16659491	m	md	KR	2019	1	0	0	0	0	0	0	0	0	0	0	0	76
16659899	m	cn	CN	2019	0	1	0	0	1	0	0	0	0	0	0	0	72
16659924	m	cn	JP	2019	0	1	1	0	0	0	0	0	0	0	0	0	70
16660188	m	ot	FR	2019	0	1	0	0	1	0	0	0	0	0	0	0	71
16660519	f	md	DE	2019	0	1	0	0	0	1	0	0	0	0	0	0	74
16660759	f	cn	US	2019	0	1	0	1	0	0	0	0	0	0	0	0	
16660884	m	cn	CN	2019	0	1	0	0	1	0	0	0	0	0	0	0	73
16660889	m	cn	JP	2019	0	1	1	0	0	0	0	0	0	0	0	0	75
16661014	f	md	JP	2019	1	0	0	0	0	0	0	0	0	0	0	0	85
16661323	f	cn	US	2019	0	0	0	0	0	0	0	0	0	0	0	0	68
16661391	m	cn	CN	2019	0	1	0	0	1	0	0	0	0	0	0	0	72
16661396	m	cn	JP	2019	0	1	1	0	0	0	0	0	0	0	0	0	
16661480	m	ot	IN	2019	0	0	0	0	0	0	0	0	0	0	0	0	59
16661582	f	cn	US	2019	1	0	0	0	0	0	0	0	0	0	0	0	50.4

16661684	f	md	JP	2019	0	1	0	1	0	0	0	0	0	0	0	1	80
16661687	f	cn	US	2019	1	0	0	0	0	0	0	0	0	0	0	0	66.8
16661759	m	md	JP	2019	0	1	0	1	0	0	0	0	0	0	0	1	57
16661770	m	cn	CN	2019	0	0	0	0	0	0	0	0	0	0	0	0	66
16661815	m	md	CN	2019	0	1	0	0	0	1	0	0	0	0	0	0	71
16662359	m	md	US	2019	0	0	0	0	0	0	0	0	0	0	0	0	67
16662955	m	md	JP	2019	0	1	1	0	0	0	0	0	0	0	0	1	68
16663154	m	ot	JP	2019	0	1	1	0	0	0	0	0	0	0	0	1	40
16663194	m	md	US	2019	0	0	0	0	0	0	0	0	0	0	0	0	67
16663357	f	md	NL	2019	0	0	0	0	0	0	0	0	0	0	0	0	65
16663601	m	md	JP	2019	0	1	1	0	0	0	0	0	0	0	0	1	70
16663737	f	md	JP	2019	1	0	0	0	0	0	0	0	0	0	0	1	78.6
16663738	m	md	JP	2019	0	1	0	1	0	0	0	0	0	0	0	1	67
16663741	f	md	JP	2019	0	1	0	1	0	0	0	0	0	0	0	1	73
16663868	m	md	KR	2019	0	1	1	0	0	0	0	0	0	0	0	0	67
16663958	Not	cn	JP	2019	0	1	1	0	0	0	0	0	0	0	0	0	
16663987	m	cn	JP	2019	0	1	1	0	0	0	0	0	0	0	0	0	70
16664014	f	cn	PL	2019	1	0	0	0	0	0	0	0	0	0	0	0	52
16664127	f	md	IL	2019	1	0	0	0	0	0	0	0	0	0	0	0	44.1
16664206	m	md	JP	2019	0	1	0	1	0	0	0	0	0	0	0	1	68
16664252	m	md	US	2019	0	1	0	1	0	0	0	0	0	0	0	0	81
16664394	m	md	JP	2019	0	1	1	0	0	0	0	0	0	0	0	0	68
16664408	f	md	KR	2019	1	0	0	0	0	0	0	0	0	0	0	0	66
16664686	Not	md	NL	2019	0	0	0	0	0	0	0	0	0	0	0	0	
16664874	f	lw	US	2019	0	0	0	0	0	0	0	0	0	0	0	0	41
16665168	f	lw	US	2019	0	0	0	0	0	0	0	0	0	0	0	0	58
16667243	f	cn	US	2019	1	0	0	0	0	0	0	0	0	0	0	0	
16667340	m	cn	CN	2019	0	1	0	0	1	0	0	0	0	0	0	0	64
16668165	m	ph	US	2019	0	1	0	1	0	0	0	0	0	0	0	1	72
16668220	m	ot	KR	2019	0	0	0	0	0	0	0	0	0	0	0	0	58
16668294	m	md	DE	2019	0	0	0	0	0	0	0	0	0	0	0	0	80
16668353	f	cn	AT	2019	0	1	1	0	0	0	0	0	0	0	0	0	82
16668542	m	md	JP	2019	0	0	0	0	0	0	0	0	0	0	0	0	67
16669073	f	ph	US	2019	0	1	0	0	0	1	0	0	0	0	0	0	63

16669140	m	cn	CN	2019	0	0	0	0	0	0	0	0	0	0	0	0	63
16669280	m	ph	IT	2019	0	1	0	0	1	0	0	0	0	0	0	0	75
16669318	m	cn	JP	2019	0	1	1	0	0	0	0	0	0	0	0	0	69
16669479	f	cn	Not re	2019	0	0	0	0	0	0	0	0	0	0	0	0	73
16670330	m	md	US	2019	0	1	1	0	0	0	0	0	0	0	0	0	62
16671465	m	md	CN	2019	0	1	0	0	0	1	0	0	0	0	0	0	71
16671737	m	md	JP	2019	0	1	0	0	0	1	0	0	0	0	0	0	
16671786	m	md	EG	2019	0	1	0	0	1	0	0	0	0	0	0	0	40
16671908	m	ot	MY	2019	0	0	0	0	0	0	0	0	0	0	0	0	72
16672358	f	md	DK	2019	0	0	0	0	0	0	0	0	0	0	0	0	62
16672554	m	md	BE	2019	0	0	0	0	0	0	0	0	0	0	0	0	59
16672778	m	ot	BE	2019	0	1	1	0	0	0	0	0	0	0	0	0	64
16672846	Not	md	JP	2019	0	1	1	0	0	0	0	0	0	0	0	1	
16672924	f	md	ID	2019	1	0	0	0	0	0	0	0	0	0	0	0	
16673342	Not	ot	IT	2019	1	0	0	0	0	0	0	0	0	0	0	0	
16673912	f	md	JP	2019	1	0	0	0	0	0	0	0	0	0	0	0	83
16673987	f	md	JP	2019	1	0	0	0	0	0	0	0	0	0	0	0	39
16674402	f	md	US	2019	0	1	0	0	0	1	0	0	0	0	0	0	72
16674683	f	ph	US	2019	0	1	1	0	0	0	0	0	0	0	0	0	60
16674720	Not	ph	JP	2019	1	0	0	0	0	0	0	0	0	0	0	0	
16674766	m	md	JP	2019	0	0	0	0	0	0	0	0	0	0	0	0	85
16674919	m	ot	JP	2019	0	0	0	0	0	0	0	0	0	0	0	0	73
16675067	m	md	AU	2019	0	1	1	0	0	0	0	0	0	0	0	0	58
16675152	m	cn	US	2019	0	0	0	0	0	0	0	0	0	0	0	0	
16675255	m	md	JP	2019	0	1	0	0	0	1	0	0	0	0	0	0	60
16675263	f	md	GB	2019	0	1	0	0	0	1	0	0	0	0	0	0	56
16675464	f	md	HU	2019	0	1	1	0	0	0	0	0	0	0	0	0	68
16675765	f	ot	CA	2019	1	0	0	0	0	0	0	0	0	0	0	1	79.1
16675872	m	md	JP	2019	0	1	0	0	0	1	0	0	0	0	0	0	67
16676007	f	md	JP	2019	1	0	0	0	0	0	0	0	0	0	0	0	65
16676085	m	md	JP	2019	0	1	1	0	0	0	0	0	0	0	0	0	57
16676101	f	ph	JP	2019	0	0	0	0	0	0	0	0	0	0	0	0	70
16676106	f	cn	JP	2019	0	1	1	0	0	0	0	0	0	0	0	0	60
16676316	f	ot	JP	2019	0	1	0	1	0	0	0	0	0	0	0	1	69

16676525	f	cn	CN	2019	1	0	0	0	0	0	0	0	0	0	0	0	
16676677	m	md	JP	2019	0	1	0	0	0	1	0	0	0	0	0	0	50
16676799	f	cn	JP	2019	0	0	0	0	0	0	0	0	0	0	0	0	67
16677167	m	cn	JP	2019	0	1	1	0	0	0	0	0	0	0	0	0	83
16677185	m	ot	JP	2019	0	1	0	1	0	0	0	0	0	0	0	1	75
16677233	f	cn	US	2019	0	0	0	0	0	0	1	1	0	0	0	0	63
16677241	f	cn	CN	2019	1	0	0	0	0	0	0	0	0	0	0	0	49
16677380	m	cn	CN	2019	1	0	0	0	0	0	0	0	0	0	0	0	54.6
16677381	f	cn	CN	2019	1	0	0	0	0	0	0	0	0	0	0	0	
16677384	m	cn	CN	2019	1	0	0	0	0	0	0	0	0	0	0	0	
16677450	m	cn	CN	2019	1	0	0	0	0	0	0	0	0	0	0	0	
16677556	m	cn	CN	2019	1	0	0	0	0	0	0	0	0	0	0	0	
16677561	f	cn	CN	2019	1	0	0	0	0	0	0	0	0	0	0	0	66.3
16677644	f	cn	CN	2019	1	0	0	0	0	0	0	0	0	0	0	0	75
16678039	Not	ot	US	2019	0	0	0	0	0	0	0	0	0	0	0	0	
16678049	f	ot	FR	2019	0	1	0	0	1	0	0	0	0	0	0	0	71
16678378	f	cn	CN	2019	1	0	0	0	0	0	0	0	0	0	0	0	
16678522	m	cn	CN	2019	1	0	0	0	0	0	0	0	0	0	0	0	
16678735	f	cn	CN	2019	1	0	0	0	0	0	0	0	0	0	0	0	
16678869	f	cn	CN	2019	1	0	0	0	0	0	0	0	0	0	0	0	
16678870	f	cn	CN	2019	1	0	0	0	0	0	0	0	0	0	0	0	
16679066	m	ph	JP	2019	1	0	0	0	0	0	0	0	0	0	0	0	57
16679074	m	cn	JP	2019	0	1	1	0	0	0	0	0	0	0	0	1	68
16679142	f	cn	CN	2019	1	0	0	0	0	0	0	0	0	0	0	0	
16679264	f	cn	CN	2019	1	0	0	0	0	0	0	0	0	0	0	0	
16679266	f	cn	CN	2019	1	0	0	0	0	0	0	0	0	0	0	0	
16679320	m	ot	KR	2019	0	1	0	0	1	0	0	0	0	0	0	0	51
16679428	f	md	GB	2019	1	0	0	0	0	0	0	0	0	0	0	1	82.1
16679429	f	Not repo	ID	2019	1	0	0	0	0	0	0	0	0	0	0	0	
16679432	m	cn	CN	2019	1	0	0	0	0	0	0	0	0	0	0	0	
16679493	f	cn	CN	2019	1	0	0	0	0	0	0	0	0	0	0	0	62.9
16679630	f	md	JP	2019	1	0	0	0	0	0	0	0	0	0	0	0	70.6
16679669	Not	ot	US	2019	0	1	0	0	1	0	0	0	0	0	0	0	
16679905	m	md	IN	2019	0	1	0	1	0	0	0	0	0	0	0	0	74

16680017	m	cn	JP	2019	0	1	1	0	0	0	0	0	0	0	0	0	78
16680467	m	cn	JP	2019	0	1	1	0	0	0	0	0	0	0	0	0	73
16680470	f	cn	CN	2019	1	0	0	0	0	0	0	0	0	0	0	0	
16680471	f	cn	CN	2019	1	0	0	0	0	0	0	0	0	0	0	0	70
16680472	m	cn	CN	2019	1	0	0	0	0	0	0	0	0	0	0	0	
16680473	f	cn	CN	2019	1	0	0	0	0	0	0	0	0	0	0	0	77.4
16680474	f	cn	CN	2019	1	0	0	0	0	0	0	0	0	0	0	0	
16680475	f	cn	CN	2019	1	0	0	0	0	0	0	0	0	0	0	0	
16680476	m	cn	CN	2019	1	0	0	0	0	0	0	0	0	0	0	0	
16680490	f	Not repo	CN	2019	1	0	0	0	0	0	0	0	0	0	0	0	55.6
16680491	f	cn	CN	2019	1	0	0	0	0	0	0	0	0	0	0	0	64
16680492	f	cn	CN	2019	1	0	0	0	0	0	0	0	0	0	0	0	
16680493	f	Not repo	CN	2019	1	0	0	0	0	0	0	0	0	0	0	0	55
16680504	f	cn	CN	2019	1	0	0	0	0	0	0	0	0	0	0	0	
16680505	f	cn	CN	2019	1	0	0	0	0	0	0	0	0	0	0	0	
16680506	f	cn	CN	2019	1	0	0	0	0	0	0	0	0	0	0	0	50
16680557	m	cn	JP	2019	0	1	1	0	0	0	0	0	0	0	0	0	81
16680769	m	cn	JP	2019	0	1	1	0	0	0	0	0	0	0	0	0	55
16680799	m	cn	CN	2019	1	0	0	0	0	0	0	0	0	0	0	0	
16680800	f	cn	CN	2019	1	0	0	0	0	0	0	0	0	0	0	0	
16680801	m	cn	CN	2019	1	0	0	0	0	0	0	0	0	0	0	0	64
16680827	f	cn	CN	2019	1	0	0	0	0	0	0	0	0	0	0	0	70
16680828	f	cn	CN	2019	1	0	0	0	0	0	0	0	0	0	0	0	60
16680829	f	cn	CN	2019	1	0	0	0	0	0	0	0	0	0	0	0	43.7
16680830	m	cn	CN	2019	1	0	0	0	0	0	0	0	0	0	0	0	
16680831	m	cn	CN	2019	1	0	0	0	0	0	0	0	0	0	0	0	
16680832	f	cn	CN	2019	1	0	0	0	0	0	0	0	0	0	0	0	
16680833	f	cn	CN	2019	1	0	0	0	0	0	0	0	0	0	0	0	70
16680834	f	cn	CN	2019	1	0	0	0	0	0	0	0	0	0	0	0	
16680835	f	cn	CN	2019	1	0	0	0	0	0	0	0	0	0	0	0	
16680836	f	cn	CN	2019	1	0	0	0	0	0	0	0	0	0	0	0	
16680946	m	md	JP	2019	0	1	0	0	1	0	0	0	0	0	0	0	69
16680997	m	md	TR	2019	1	0	0	0	0	0	0	0	0	0	0	0	
16681112	m	md	PH	2019	0	1	0	0	0	1	0	0	0	0	0	0	

16681269	m	ot	JP	2019	0	1	0	0	0	1	0	0	0	0	0	0	60
16681363	f	cn	CN	2019	1	0	0	0	0	0	0	0	0	0	0	0	66.6
16681407	f	cn	CN	2019	1	0	0	0	0	0	0	0	0	0	0	0	
16681463	m	ot	US	2019	0	0	0	0	0	0	0	0	0	0	0	0	54
16681589	f	cn	CN	2019	1	0	0	0	0	0	0	0	0	0	0	0	
16681681	f	ph	US	2019	0	1	0	0	0	1	0	0	0	0	0	0	72
16681888	m	md	FR	2019	0	1	0	0	0	1	0	0	0	0	0	0	56
16681953	f	ot	US	2019	0	1	0	0	1	0	0	0	0	0	0	0	69
16682152	f	cn	CN	2019	1	0	0	0	0	0	0	0	0	0	0	0	
16682154	f	cn	CN	2019	1	0	0	0	0	0	0	0	0	0	0	0	
16682156	f	cn	CN	2019	1	0	0	0	0	0	0	0	0	0	0	0	
16682157	f	cn	CN	2019	1	0	0	0	0	0	0	0	0	0	0	0	55.6
16682159	m	cn	CN	2019	1	0	0	0	0	0	0	0	0	0	0	0	
16682258	f	cn	CN	2019	1	0	0	0	0	0	0	0	0	0	0	0	69
16682259	f	cn	CN	2019	1	0	0	0	0	0	0	0	0	0	0	0	
16682262	f	cn	CN	2019	1	0	0	0	0	0	0	0	0	0	0	0	
16682263	m	cn	CN	2019	1	0	0	0	0	0	0	0	0	0	0	0	
16682264	f	cn	CN	2019	1	0	0	0	0	0	0	0	0	0	0	0	
16682265	f	cn	CN	2019	1	0	0	0	0	0	0	0	0	0	0	0	
16682266	f	cn	CN	2019	1	0	0	0	0	0	0	0	0	0	0	0	
16682349	f	cn	CN	2019	1	0	0	0	0	0	0	0	0	0	0	0	67.8
16682418	Not	md	JP	2019	1	0	0	0	0	0	0	0	0	0	0	0	80
16682544	m	md	JP	2019	0	1	1	0	0	0	0	0	0	0	0	0	63
16682545	m	ot	JP	2019	0	1	1	0	0	0	0	0	0	0	0	0	71
16682571	m	cn	CN	2019	1	0	0	0	0	0	0	0	0	0	0	0	48
16682572	f	cn	CN	2019	1	0	0	0	0	0	0	0	0	0	0	0	
16682573	m	cn	CN	2019	1	0	0	0	0	0	0	0	0	0	0	0	
16682650	Not	ot	IN	2019	0	0	0	0	0	0	0	0	0	0	0	0	
16682688	f	cn	CN	2019	1	0	0	0	0	0	0	0	0	0	0	0	
16682833	f	cn	CN	2019	1	0	0	0	0	0	0	0	0	0	0	0	
16682837	f	cn	CN	2019	1	0	0	0	0	0	0	0	0	0	0	0	54.5
16683072	m	ph	US	2019	0	1	0	0	0	1	0	0	0	0	0	0	65
16683103	m	Not repo	CN	2019	1	0	0	0	0	0	0	0	0	0	0	0	
16683106	f	cn	CN	2019	1	0	0	0	0	0	0	0	0	0	0	0	42.4

16683206	f	cn	US	2019	0	0	0	0	0	0	0	0	0	0	0	0	
16683221	f	cn	US	2019	1	0	0	0	0	0	0	0	0	0	0	0	
16683222	f	cn	CN	2019	1	0	0	0	0	0	0	0	0	0	0	0	
16683247	f	cn	CN	2019	1	0	0	0	0	0	0	0	0	0	0	0	
16683249	m	cn	CN	2019	1	0	0	0	0	0	0	0	0	0	0	0	60.9
16683250	f	cn	CN	2019	1	0	0	0	0	0	0	0	0	0	0	0	72
16683251	f	cn	CN	2019	1	0	0	0	0	0	0	0	0	0	0	0	
16683252	m	cn	CN	2019	1	0	0	0	0	0	0	0	0	0	0	0	51
16683253	m	cn	CN	2019	1	0	0	0	0	0	0	0	0	0	0	0	
16683254	f	cn	CN	2019	1	0	0	0	0	0	0	0	0	0	0	0	79.7
16683255	f	cn	CN	2019	1	0	0	0	0	0	0	0	0	0	0	0	73
16683256	f	cn	CN	2019	1	0	0	0	0	0	0	0	0	0	0	0	
16683257	f	cn	CN	2019	1	0	0	0	0	0	0	0	0	0	0	0	56
16683258	f	cn	CN	2019	1	0	0	0	0	0	0	0	0	0	0	0	
16683311	f	cn	JP	2019	0	0	0	0	0	0	0	0	0	0	0	0	
16683346	f	cn	CN	2019	1	0	0	0	0	0	0	0	0	0	0	0	66
16683348	f	cn	CN	2019	1	0	0	0	0	0	0	0	0	0	0	0	
16683732	f	md	JP	2019	1	0	0	0	0	0	0	0	0	0	0	0	69.2
16683735	f	cn	CN	2019	1	0	0	0	0	0	0	0	0	0	0	0	74
16684074	f	cn	CN	2019	1	0	0	0	0	0	0	0	0	0	0	0	
16684260	m	ot	FR	2019	0	1	0	0	1	0	0	0	0	0	0	0	70
16684340	f	cn	JP	2019	0	1	1	0	0	0	0	0	0	0	0	0	77
16684377	f	cn	CN	2019	1	0	0	0	0	0	0	0	0	0	0	0	89
16684380	m	md	JP	2019	0	1	0	1	0	0	0	0	0	0	0	1	80
16684479	m	Not repo	CN	2019	1	0	0	0	0	0	0	0	0	0	0	0	70.8
16684517	f	md	JP	2019	0	1	0	1	0	0	0	0	0	0	0	1	74.6
16684520	m	cn	CN	2019	1	0	0	0	0	0	0	0	0	0	0	0	
16684521	m	cn	CN	2019	1	0	0	0	0	0	0	0	0	0	0	1	71.1
16684535	m	md	JP	2019	0	1	0	0	1	0	0	0	0	0	0	0	62
16684547	m	md	JP	2019	0	1	1	0	0	0	0	0	0	0	0	1	70
16685106	m	cn	JP	2019	0	1	1	0	0	0	0	0	0	0	0	1	77
16685352	m	ot	NZ	2019	0	1	0	0	1	0	0	0	0	0	0	0	72
16685841	m	ot	JP	2019	0	1	0	0	1	0	0	0	0	0	0	0	66
16685957	Not	ot	FR	2019	0	1	0	0	1	0	0	0	0	0	0	0	

16686063	f	md	RU	2019	0	1	0	0	0	1	0	0	0	0	0	0	55
16686160	m	ph	JP	2019	0	1	1	0	0	0	0	0	0	0	0	0	74
16686235	Not	ph	JP	2019	0	1	1	0	0	0	0	0	0	0	0	1	74
16686483	m	cn	SG	2019	0	1	0	0	0	1	0	0	0	0	0	0	
16686834	f	cn	US	2019	0	1	1	0	0	0	0	0	0	0	0	0	60
16686919	f	cn	US	2019	1	0	0	0	0	0	0	0	0	0	0	0	
16687113	m	cn	JP	2019	0	1	1	0	0	0	0	0	0	0	0	0	78
16687451	f	ot	GB	2019	0	1	1	0	0	0	0	0	0	0	0	1	49
16687470	m	md	JP	2019	1	0	0	0	0	0	0	0	0	0	0	0	81.6
16687700	f	ph	JP	2019	1	0	0	0	0	0	0	0	0	0	0	0	80
16687753	m	cn	CN	2019	0	1	0	0	1	0	0	0	0	0	0	0	
16687798	m	md	JP	2019	0	1	0	1	0	0	0	0	0	0	0	1	80
16688206	m	md	DE	2019	0	1	1	0	0	0	0	0	0	0	0	0	56
16688262	f	cn	US	2019	0	1	0	1	0	0	0	0	0	0	0	0	
16688280	f	cn	US	2019	0	1	1	0	0	0	0	0	0	0	0	0	
16688362	m	ot	DE	2019	0	1	0	0	1	0	0	0	0	0	0	0	65
16688445	m	md	IN	2019	0	1	0	0	0	1	0	0	0	0	0	0	64
16688761	m	md	AT	2019	0	1	1	0	0	0	0	0	0	0	0	0	65
16688970	m	md	JP	2019	0	1	0	0	1	0	0	0	0	0	0	0	80
16689059	m	md	JP	2019	0	1	0	1	0	0	0	0	0	0	0	1	48
16689146	m	md	US	2019	0	1	0	1	0	0	0	0	0	0	0	0	64
16689190	m	md	JP	2019	0	1	1	0	0	0	0	0	0	0	0	1	70
16689261	f	cn	JP	2019	0	1	1	0	0	0	0	0	0	0	0	0	76
16689262	f	Not repo	CN	2019	1	0	0	0	0	0	0	0	0	0	0	0	46
16689280	m	md	JP	2019	0	1	1	0	0	0	0	0	0	0	0	0	79
16689319	f	md	JP	2019	1	0	0	0	0	0	0	0	0	0	0	1	78.9
16689386	f	Not repo	IT	2019	1	0	0	0	0	0	0	0	0	0	0	0	82
16689646	f	md	JP	2019	1	0	0	0	0	0	0	0	0	0	0	0	73
16689740	m	cn	CN	2019	0	1	0	0	1	0	0	0	0	0	0	0	56
16689751	Not	md	JP	2019	0	1	0	0	1	0	0	0	0	0	0	0	
16689889	m	cn	JP	2019	0	1	1	0	0	0	0	0	0	0	0	0	46
16689989	m	ot	CN	2019	0	1	0	0	1	0	0	0	0	0	0	0	68
16690404	f	md	CN	2019	0	1	0	0	0	1	0	0	0	0	0	0	65
16690551	f	cn	CN	2019	0	0	0	0	0	0	0	0	0	0	0	0	74

16690588	m	cn	JP	2019	0	1	1	0	0	0	0	0	0	0	0	0	65
16690619	m	md	JP	2019	0	1	1	0	0	0	0	0	0	0	0	0	65
16690629	m	cn	JP	2019	0	1	1	0	0	0	0	0	0	0	0	0	70
16690766	m	cn	US	2019	0	1	1	0	0	0	0	0	0	0	0	0	
16690819	f	cn	JP	2019	0	1	1	0	0	0	0	0	0	0	0	0	73
16691136	m	md	IL	2019	1	0	0	0	0	0	0	0	0	0	0	0	85
16691410	m	md	JP	2019	0	1	1	0	0	0	0	0	0	0	0	0	74
16691451	m	cn	JP	2019	1	0	0	0	0	0	0	0	0	0	0	0	
16692021	m	ot	JP	2019	0	1	1	0	0	0	0	0	0	0	0	0	71
16692054	m	md	JP	2019	0	1	0	1	0	0	0	0	0	0	0	1	51
16692111	f	md	JP	2019	1	0	0	0	0	0	0	0	0	0	0	1	
16692157	m	md	US	2019	0	0	0	0	0	0	0	0	0	0	0	0	59
16693427	f	cn	US	2019	0	1	1	0	0	0	0	0	0	0	0	0	75
16693534	Not	ot	FR	2019	0	0	0	0	0	0	0	0	0	0	0	0	
16693539	Not	ot	FR	2019	0	0	0	0	0	0	0	0	0	0	0	0	
16694134	Not	ot	SE	2019	0	0	0	0	0	0	0	0	0	0	0	0	
16694254	Not	ot	SE	2019	0	0	0	0	0	0	0	0	0	0	0	0	
16694298	Not	ot	SE	2019	0	0	0	0	0	0	0	0	0	0	0	0	
16694835	f	ot	CN	2019	0	1	0	0	1	0	0	0	0	0	0	0	47
16694868	f	cn	Not re	2019	0	0	0	0	0	0	0	0	0	0	0	0	
16694939	f	ot	CN	2019	0	0	0	0	0	0	0	0	0	0	0	0	63
16695319	f	cn	US	2019	0	1	1	0	0	0	0	0	0	0	0	0	
16695335	f	md	JP	2019	0	1	1	0	0	0	0	0	0	0	0	0	70
16696012	Not	md	NL	2019	0	0	0	0	0	0	0	0	0	0	0	0	
16696186	m	cn	JP	2019	0	1	1	0	0	0	0	0	0	0	0	0	64
16696547	f	md	TW	2019	0	1	1	0	0	0	0	0	0	0	0	0	64
16697056	f	cn	US	2019	0	0	0	0	0	0	0	0	0	0	0	0	
16697068	f	cn	US	2019	0	0	0	0	0	0	0	0	0	0	0	0	
16697376	f	cn	US	2019	0	0	0	0	0	0	0	0	0	0	0	0	
16697520	Not	md	ES	2019	0	1	1	0	0	0	0	0	0	0	0	0	55
16697776	m	Not repo	CN	2019	1	0	0	0	0	0	0	0	0	0	0	0	44
16697785	m	md	JP	2019	0	1	0	1	0	0	0	0	0	0	0	1	70
16697969	m	ot	MX	2019	1	0	0	0	0	0	0	0	0	0	0	0	
16698273	f	md	JP	2019	1	0	0	0	0	0	0	0	0	0	0	1	56.1

16699346	m	cn	JP	2019	0	1	1	0	0	0	0	0	0	0	0	0	79
16699893	f	cn	JP	2019	0	1	1	0	0	0	0	0	0	0	0	0	42
16699945	f	ot	GB	2019	0	0	0	0	0	0	0	0	0	0	0	0	54
16699951	m	cn	JP	2019	0	1	1	0	0	0	0	0	0	0	0	0	66
16700127	m	ot	BE	2019	0	0	0	0	0	0	0	0	0	0	0	0	59
16700172	m	md	JP	2019	0	0	0	0	0	0	0	0	0	0	0	0	59
16700409	m	cn	TR	2019	0	1	1	0	0	0	0	0	0	0	0	0	
16700419	m	md	JP	2019	0	1	1	0	0	0	0	0	0	0	0	0	73
16700624	m	md	JP	2019	0	1	0	0	1	0	0	0	0	0	0	0	55
16700739	f	ot	US	2019	0	1	0	0	1	0	0	0	0	0	0	0	
16700887	m	cn	JP	2019	0	1	1	0	0	0	0	0	0	0	0	1	80
16701018	f	cn	Not re	2019	0	0	0	0	0	0	0	0	0	0	0	0	
16701047	f	md	ID	2019	0	1	0	0	0	1	0	0	0	0	0	0	
16701059	f	md	US	2019	0	0	0	0	0	0	0	0	0	0	0	0	
16701067	m	md	JP	2019	0	1	0	1	0	0	0	0	0	0	0	1	80
16701072	m	md	TH	2019	0	1	0	0	0	1	0	0	0	0	0	0	71
16701119	f	md	DE	2019	0	1	0	0	0	1	0	0	0	0	0	0	63
16701178	m	md	TH	2019	0	1	0	0	0	1	0	0	0	0	0	0	55
16701401	f	cn	AU	2019	0	1	1	0	0	0	0	0	0	0	0	0	69
16701565	m	md	JP	2019	0	1	0	1	0	0	0	0	0	0	0	1	80
16701662	f	md	JP	2019	1	0	0	0	0	0	0	0	0	0	0	0	45
16701777	Not	md	JP	2019	1	0	0	0	0	0	0	0	0	0	0	0	
16701921	m	cn	JP	2019	0	1	1	0	0	0	0	0	0	0	0	0	68
16702286	m	ot	TW	2019	0	1	0	0	1	0	0	0	0	0	0	0	71
16702868	f	cn	JP	2019	0	1	1	0	0	0	0	0	0	0	0	0	54
16703166	Not	md	CN	2019	1	0	0	0	0	0	0	0	0	0	0	0	78.8
16703490	f	cn	DE	2019	0	0	0	0	0	0	0	0	0	0	0	0	
16703521	m	ot	DE	2019	0	1	1	0	0	0	0	0	0	0	0	0	72
16703525	m	ot	AT	2019	0	1	1	0	0	0	0	0	0	0	0	0	65
16703821	f	md	JP	2019	0	1	0	0	1	0	0	0	0	0	0	0	82
16703937	m	md	JP	2019	0	1	0	1	0	0	0	0	0	0	0	1	72.8
16703992	m	cn	JP	2019	0	1	1	0	0	0	0	0	0	0	0	0	50
16704296	m	cn	JP	2019	0	1	1	0	0	0	0	0	0	0	0	0	53
16704873	f	cn	US	2019	0	0	0	0	0	0	0	0	0	0	0	0	50

16704885	m	cn	US	2019	0	1	1	0	0	0	0	0	0	0	0	0	
16704922	m	cn	CA	2019	0	1	1	0	0	0	0	0	0	0	0	0	82
16705174	m	cn	CN	2019	0	0	0	0	0	0	0	0	0	0	0	0	75
16705237	m	cn	JP	2019	0	1	1	0	0	0	0	0	0	0	0	0	72
16705260	m	cn	JP	2019	0	1	1	0	0	0	0	0	0	0	0	0	70
16705308	f	md	JP	2019	0	0	0	0	0	0	0	0	0	0	0	0	87
16705328	Not	md	CN	2019	0	0	0	0	0	0	0	0	0	0	0	0	
16705339	m	cn	US	2019	0	0	0	0	0	0	0	0	0	0	0	0	
16705381	m	md	JP	2019	0	1	1	0	0	0	0	0	0	0	0	0	64
16705387	m	md	JP	2019	0	1	1	0	0	0	0	0	0	0	0	0	59
16705562	f	md	BE	2019	0	1	0	0	0	1	0	0	0	0	0	0	73
16705567	m	cn	CN	2019	1	0	0	0	0	0	0	0	0	0	0	0	
16705676	f	ot	US	2019	0	1	0	0	1	0	0	0	0	0	0	0	52
16706683	m	md	US	2019	0	0	0	0	0	0	0	0	0	0	0	0	53
16706851	m	cn	JP	2019	0	1	1	0	0	0	0	0	0	0	0	0	
16706881	m	md	HU	2019	0	0	0	0	0	0	0	0	0	0	0	0	67
16707002	m	cn	US	2019	0	1	1	0	0	0	0	0	0	0	0	0	
16707644	m	ph	FR	2019	0	0	0	0	0	0	0	0	0	0	0	0	71
16707893	Not	md	UA	2019	0	0	0	0	0	0	0	0	0	0	0	0	
16708004	f	cn	JP	2019	0	1	1	0	0	0	0	0	0	0	0	0	64
16708063	m	ot	CN	2019	0	1	0	0	1	0	0	0	0	0	0	0	73
16708123	m	ot	JP	2019	0	1	1	0	0	0	0	0	0	0	0	1	78
16708292	m	ot	US	2019	0	1	0	0	1	0	0	0	0	0	0	0	
16708294	m	cn	US	2019	0	1	1	0	0	0	0	0	0	0	0	0	80
16708355	m	md	US	2019	0	1	0	1	0	0	0	0	0	0	0	0	69
16708741	f	md	JP	2019	0	0	0	0	0	0	0	0	0	0	0	0	44
16708797	m	cn	US	2019	0	1	1	0	0	0	0	0	0	0	0	0	74
16708921	m	cn	CN	2019	1	0	0	0	0	0	0	0	0	0	0	0	75.5
16709106	m	cn	CN	2019	0	0	0	0	0	0	0	0	0	0	0	0	
16709803	m	md	US	2019	0	1	0	1	0	0	0	0	0	0	0	0	
16709805	Not	ot	IT	2019	0	1	0	1	0	0	0	0	0	0	0	0	43
16709808	f	md	CA	2019	1	0	0	0	0	0	0	0	0	0	0	0	
16709857	f	cn	US	2019	0	0	0	0	0	0	0	0	0	0	0	0	
16710117	m	md	JP	2019	1	0	0	0	0	0	0	0	0	0	0	0	75

16710205	m	cn	JP	2019	0	1	1	0	0	0	0	0	0	0	0	0	73
16710279	m	md	JP	2019	1	0	0	0	0	0	0	0	0	0	0	0	82
16710691	m	ph	FR	2019	0	1	0	1	0	0	0	0	0	0	0	0	63.4
16710952	f	ot	AU	2019	0	0	0	0	0	0	1	0	0	0	1	0	
16712029	f	ot	CN	2019	1	0	0	0	0	0	0	0	0	0	0	0	65
16712034	f	ot	IT	2019	1	0	0	0	0	0	0	0	0	0	0	0	64
16712169	m	ot	AU	2019	0	1	0	0	1	0	0	0	0	0	0	0	74
16712222	f	cn	GB	2019	0	1	1	0	0	0	0	0	0	0	0	1	49
16712273	m	cn	JP	2019	0	1	1	0	0	0	0	0	0	0	0	1	76
16712279	f	cn	DE	2019	0	1	1	0	0	0	0	0	0	0	0	0	56
16712570	m	ot	ME	2019	0	1	1	0	0	0	0	0	0	0	0	1	78
16712608	f	ot	DE	2019	0	1	1	0	0	0	0	0	0	0	0	0	56
16712825	m	cn	CN	2019	0	1	0	0	1	0	0	0	0	0	0	0	
16713074	f	md	DE	2019	0	1	1	0	0	0	0	0	0	0	0	0	56
16713464	m	md	JP	2019	0	1	0	0	0	1	0	0	0	0	0	0	60
16713584	f	md	US	2019	0	1	1	0	0	0	0	0	0	0	0	0	69
16713875	f	cn	US	2019	1	0	0	0	0	0	0	0	0	0	0	0	
16714030	f	md	JP	2019	0	0	0	0	0	0	0	0	0	0	0	0	70
16714189	m	ot	FR	2019	0	1	0	0	1	0	0	0	0	0	0	0	80
16714197	m	md	JP	2019	0	1	0	0	0	1	0	0	0	0	0	0	54
16714198	Not	md	IL	2019	0	0	0	0	0	0	0	0	0	0	0	0	
16714280	m	ot	KR	2019	0	0	0	0	0	0	0	0	0	0	0	0	67
16714608	f	ot	KR	2019	0	0	0	0	0	0	0	0	0	0	0	0	54
16715015	f	md	US	2019	0	1	0	0	0	1	0	0	0	0	0	0	72
16715017	m	md	DE	2019	1	0	0	0	0	0	0	0	0	0	0	0	
16715064	f	cn	US	2019	0	0	0	0	0	0	0	0	0	0	0	0	
16715617	m	cn	JP	2019	0	1	1	0	0	0	0	0	0	0	0	0	75
16715674	m	cn	JP	2019	0	1	1	0	0	0	0	0	0	0	0	0	63
16715808	m	ot	US	2019	0	0	0	0	0	0	0	0	0	0	0	0	69
16716105	m	cn	JP	2019	0	1	1	0	0	0	0	0	0	0	0	0	66
16716200	f	md	TH	2019	0	0	0	0	0	0	0	0	0	0	0	0	34
16716234	f	cn	CA	2019	0	0	0	0	0	0	0	0	0	0	0	0	45
16716314	m	cn	JP	2019	0	1	1	0	0	0	0	0	0	0	0	0	70
16716450	m	cn	JP	2019	0	1	1	0	0	0	0	0	0	0	0	1	

16716506	f	ot	IE	2019	0	1	0	0	1	0	0	0	0	0	0	0	79
16716555	m	cn	US	2019	0	0	0	0	0	0	0	0	0	0	0	0	81
16716752	m	md	GB	2019	0	0	0	0	0	0	0	0	0	0	0	0	71
16716968	f	cn	JP	2019	0	1	1	0	0	0	0	0	0	0	0	1	
16717380	m	md	US	2019	0	1	0	1	0	0	0	0	0	0	0	0	71
16717519	m	ot	GB	2019	0	0	0	0	0	0	0	0	0	0	0	0	65
16717996	f	ot	AT	2019	0	0	0	0	0	0	0	0	0	0	0	0	
16718017	f	md	KR	2019	0	0	0	0	0	0	0	0	0	0	0	0	
16718331	m	md	US	2019	0	0	0	0	0	0	0	0	0	0	0	0	
16718816	m	ot	FR	2019	0	1	0	0	1	0	0	0	0	0	0	0	61
16718840	Not	ot	DE	2019	0	0	0	0	0	0	0	0	0	0	0	0	
16718985	f	Not repo	AU	2019	1	0	0	0	0	0	0	0	0	0	0	0	
16719110	f	md	US	2019	0	1	0	0	1	0	0	0	0	0	0	0	64
16720031	f	cn	UA	2019	0	0	0	0	0	0	0	0	0	0	0	0	
16720179	Not	ot	US	2019	1	0	0	0	0	0	0	0	0	0	0	0	
16720302	f	ph	FR	2019	0	1	0	0	1	0	0	0	0	0	0	0	
16720331	m	ot	DE	2019	0	1	0	0	1	0	0	0	0	0	0	0	72
16720340	f	cn	CA	2019	0	0	0	0	0	0	0	0	0	0	0	0	33
16720398	m	md	BE	2019	0	1	1	0	0	0	0	0	0	0	0	0	61
16720402	Not	ot	US	2019	1	0	0	0	0	0	0	0	0	0	0	0	
16720723	m	cn	JP	2019	0	1	1	0	0	0	0	0	0	0	0	0	78
16720781	Not	md	PL	2019	0	0	0	0	0	0	0	0	0	0	0	0	
16721178	f	cn	IL	2019	0	1	1	0	0	0	0	0	0	0	0	0	
16721375	f	md	JP	2019	0	1	0	1	0	0	0	0	0	0	0	1	50
16721879	f	md	ES	2019	0	0	0	0	0	0	0	0	0	0	0	0	59
16722088	m	ot	ES	2019	0	1	0	0	1	0	0	0	0	0	0	0	76
16722333	m	ot	US	2019	0	1	0	0	1	0	0	0	0	0	0	0	73
16722999	f	ot	US	2019	0	0	0	0	0	0	0	0	0	0	0	0	64
16723519	f	cn	DE	2019	0	1	1	0	0	0	0	0	0	0	0	0	47
16723751	m	ot	FR	2019	0	1	0	0	1	0	0	0	0	0	0	0	52
16724341	f	md	JP	2019	1	0	0	0	0	0	0	0	0	0	0	0	80
16725239	Not	md	JP	2019	1	0	0	0	0	0	0	0	0	0	0	0	
16725289	m	md	DE	2019	0	1	0	0	1	0	0	0	0	0	0	0	57
16725787	m	md	HU	2019	0	0	0	0	0	0	0	0	0	0	0	0	86

16726646	m	cn	US	2019	1	0	0	0	0	0	0	0	0	0	0	0		
16726764	m	md	DE	2019	0	0	0	0	0	0	0	0	0	0	0	0	80
16726923	Not	md	US	2019	1	0	0	0	0	0	0	0	0	0	0	0	
16727077	f	ot	IT	2019	0	1	0	0	1	0	0	0	0	0	0	0	72
16727090	m	ot	IT	2019	0	1	0	0	1	0	0	0	0	0	0	0	70
16727207	m	ot	IT	2019	0	1	0	0	1	0	0	0	0	0	0	0	61
16727780	m	md	JP	2019	0	1	0	0	0	1	0	0	0	0	0	1	75
16728141	m	md	DE	2019	0	1	0	0	0	1	0	0	0	0	0	0	57
16728363	f	cn	US	2019	0	1	1	0	0	0	0	0	0	0	0	0	
16728738	m	cn	CN	2019	0	1	0	0	1	0	0	0	0	0	0	0	
16728757	f	ot	US	2019	0	1	0	0	1	0	0	0	0	0	0	0	72
16728809	f	md	HK	2019	0	1	0	0	0	1	0	0	0	0	0	0	70
16729091	f	ot	AU	2019	0	0	0	0	0	0	1	0	0	0	1	0	
16729840	f	md	US	2019	1	0	0	0	0	0	0	0	0	0	0	0	66
16730125	f	ot	CN	2019	1	0	0	0	0	0	0	0	0	0	0	0	63
16730276	m	ot	AU	2019	1	0	0	0	0	0	0	0	0	0	0	0	41
16730422	f	ot	NL	2019	0	1	1	0	0	0	0	0	0	0	0	0	56
16730490	f	cn	JP	2019	0	1	1	0	0	0	0	0	0	0	0	0	51
16731218	m	cn	US	2019	0	1	1	0	0	0	0	0	0	0	0	0	
16731270	f	ot	IN	2019	1	0	0	0	0	0	0	0	0	0	0	0	65
16731272	f	ot	IN	2019	1	0	0	0	0	0	0	0	0	0	0	0	56
16731481	m	ot	FR	2019	0	1	0	0	1	0	0	0	0	0	0	0	68
16731484	m	md	IL	2019	0	1	1	0	0	0	0	0	0	0	0	0	70
16732176	f	md	GB	2019	1	0	0	0	0	0	0	0	0	0	0	0	65
16732283	m	ot	FR	2019	0	1	0	0	1	0	0	0	0	0	0	0	46
16732494	f	cn	US	2019	0	1	0	1	0	0	0	0	0	0	0	1	
16732584	m	md	JP	2019	0	1	1	0	0	0	0	0	0	0	0	0	81
16732711	f	md	HK	2019	0	1	0	0	0	1	0	0	0	0	0	0	
16732782	f	md	JP	2019	0	0	0	0	0	0	0	0	0	0	0	0	66
16732884	m	md	JP	2019	0	1	0	0	0	1	0	0	0	0	0	1	52
16733150	f	md	DE	2019	0	1	0	0	0	1	0	0	0	0	0	0	65
16733189	f	cn	US	2019	0	0	0	0	0	0	0	0	0	0	0	0	
16733244	f	cn	JP	2019	0	1	1	0	0	0	0	0	0	0	0	0	64
16733394	m	ph	JP	2019	0	0	0	0	0	0	0	0	0	0	0	0	61

16733407	m	md	JP	2019	0	1	0	1	0	0	0	0	0	0	0	1	73
16733416	m	ot	AU	2019	0	1	0	0	1	0	0	0	0	0	0	0	74
16733548	f	cn	Not re	2019	0	0	0	0	0	0	0	0	0	0	0	0	
16733632	m	cn	US	2019	0	1	1	0	0	0	0	0	0	0	0	0	
16733747	f	ot	HU	2019	0	1	0	0	0	1	0	0	0	0	0	0	72
16734209	f	md	IT	2019	0	0	0	0	0	0	0	0	0	0	0	0	61
16734478	m	cn	US	2019	0	1	0	0	1	0	0	0	0	0	0	1	
16734508	m	cn	JP	2019	0	1	1	0	0	0	0	0	0	0	0	0	79
16734563	m	cn	JP	2019	0	0	0	0	0	0	0	0	0	0	0	0	65
16734794	m	ph	JP	2019	1	0	0	0	0	0	0	0	0	0	0	0	81
16734812	m	cn	JP	2019	0	1	1	0	0	0	0	0	0	0	0	0	70
16734868	m	ph	JP	2019	1	0	0	0	0	0	0	0	0	0	0	1	90
16734869	m	ph	JP	2019	1	0	0	0	0	0	0	0	0	0	0	0	59
16734989	f	md	AU	2019	1	0	0	0	0	0	0	0	0	0	0	0	
16735005	m	md	SE	2019	0	0	0	0	0	0	0	0	0	0	0	0	71
16735117	f	ph	HK	2019	0	0	0	0	0	0	0	0	0	0	0	0	56
16735146	m	md	JP	2019	0	1	0	1	0	0	0	0	0	0	0	1	71
16735148	m	md	JP	2019	0	1	0	1	0	0	0	0	0	0	0	1	68
16735332	m	cn	US	2019	0	1	1	0	0	0	0	0	0	0	0	0	71
16735451	m	md	JP	2019	0	0	0	0	0	0	1	0	0	1	0	1	46.3
16735497	m	md	GB	2019	0	1	0	0	0	1	0	0	0	0	0	0	72
16735584	m	md	DE	2019	0	1	1	0	0	0	0	0	0	0	0	0	69
16735604	f	ot	BE	2019	0	1	0	0	0	1	0	0	0	0	0	0	73
16736030	Not	md	JP	2019	1	0	0	0	0	0	0	0	0	0	0	0	
16736035	f	Not repo	CN	2019	1	0	0	0	0	0	0	0	0	0	0	0	67
16736058	m	ot	NL	2019	0	1	1	0	0	0	0	0	0	0	0	0	65
16736642	m	cn	JP	2019	0	1	1	0	0	0	0	0	0	0	0	0	71
16736874	m	cn	CN	2019	0	1	0	0	1	0	0	0	0	0	0	0	
16736881	m	md	JP	2019	0	1	0	1	0	0	0	0	0	0	0	1	80
16736883	m	md	BE	2019	0	1	0	1	0	0	0	0	0	0	0	1	72.9
16737458	f	md	NL	2019	0	0	0	0	0	0	0	0	0	0	0	0	62
16737748	m	md	US	2019	0	1	1	0	0	0	0	0	0	0	0	0	
16737799	Not	md	HK	2019	0	1	0	0	0	1	0	0	0	0	0	0	
16737826	m	md	JP	2019	0	0	0	0	0	0	0	0	0	0	0	0	60

16738528	f	md	JP	2019	0	0	0	0	0	0	0	0	0	0	0	0	85
16739406	m	md	US	2019	0	1	0	0	1	0	0	0	0	0	0	0	77
16739485	f	md	DE	2019	0	1	1	0	0	0	0	0	0	0	0	0	56
16739967	Not	md	AU	2019	0	1	0	0	0	1	0	0	0	0	0	0	
16740140	f	md	US	2019	0	1	1	0	0	0	0	0	0	0	0	0	
16740463	f	cn	JP	2019	0	1	1	0	0	0	0	0	0	0	0	0	47
16740584	Not	ph	US	2019	0	0	0	0	0	0	0	0	0	0	0	0	
16740709	m	cn	JP	2019	0	0	0	0	0	0	0	0	0	0	0	0	73
16741414	Not	md	KR	2019	0	1	1	0	0	0	0	0	0	0	0	0	69
16741615	m	cn	JP	2019	0	1	1	0	0	0	0	0	0	0	0	0	77
16741947	f	ot	US	2019	0	1	0	0	1	0	0	0	0	0	0	0	58
16742050	f	md	US	2019	0	1	0	1	0	0	0	0	0	0	0	1	81
16742294	Not	ot	CH	2019	0	0	0	0	0	0	0	0	0	0	0	0	
16742618	m	cn	JP	2019	0	1	1	0	0	0	0	0	0	0	0	1	73
16742676	m	cn	JP	2019	0	1	1	0	0	0	0	0	0	0	0	1	73
16742744	m	Not repo	CN	2019	1	0	0	0	0	0	0	0	0	0	0	0	
16742842	Not	md	GB	2019	0	1	0	0	0	1	0	0	0	0	0	0	
16742858	Not	md	HR	2019	0	1	1	0	0	0	0	0	0	0	0	0	
16743857	Not	md	HR	2019	0	1	1	0	0	0	0	0	0	0	0	0	57
16744100	m	cn	JP	2019	0	1	1	0	0	0	0	0	0	0	0	0	73
16744309	f	md	JP	2019	0	1	0	1	0	0	0	0	0	0	0	1	
16744651	f	md	HU	2019	0	0	0	0	0	0	0	0	0	0	0	0	51
16744670	Not	md	AR	2019	0	0	0	0	0	0	0	0	0	0	0	0	
16745024	f	ph	US	2019	0	1	1	0	0	0	0	0	0	0	0	0	72
16745671	Not	md	JP	2019	0	1	0	0	1	0	0	0	0	0	0	0	
16745767	Not	md	JP	2019	0	1	0	0	1	0	0	0	0	0	0	0	
16745817	m	ot	US	2019	0	1	0	1	0	0	0	0	0	0	0	0	75.9
16746618	f	ot	DE	2019	0	1	1	0	0	0	0	0	0	0	0	0	47
16746690	f	ot	CN	2019	0	1	0	0	1	0	0	0	0	0	0	0	56
16747022	m	md	JP	2019	0	1	0	1	0	0	0	0	0	0	0	1	80
16747246	f	ot	US	2019	0	1	0	0	1	0	0	0	0	0	0	0	
16747358	f	md	CZ	2019	0	0	0	0	0	0	0	0	0	0	0	0	
16747399	f	md	CZ	2019	0	0	0	0	0	0	0	0	0	0	0	0	78
16747435	Not	md	BR	2019	0	1	0	1	0	0	0	0	0	0	0	0	

16747441	m	cn	ID	2019	1	0	0	0	0	0	0	0	0	0	0	0	
16747625	f	md	DE	2019	0	1	1	0	0	0	0	0	0	0	0	0	56
16747678	m	ot	JP	2019	0	1	0	1	0	0	0	0	0	0	0	1	71
16748085	m	cn	JP	2019	0	1	1	0	0	0	0	0	0	0	0	0	71
16748557	m	md	JP	2019	0	1	0	0	1	0	0	0	0	0	0	0	77
16748610	m	cn	US	2019	0	1	1	0	0	0	0	0	0	0	0	0	
16748758	Not	md	ID	2019	0	1	0	0	0	1	0	0	0	0	0	0	50
16748784	m	md	CN	2019	1	0	0	0	0	0	0	0	0	0	0	0	63
16749206	m	md	JP	2019	0	1	0	1	0	0	0	0	0	0	0	0	46
16749246	m	md	JP	2019	0	1	0	0	1	0	0	0	0	0	0	0	78
16749338	f	cn	IN	2019	0	0	0	0	0	0	0	0	0	0	0	0	70
16749351	f	ot	US	2019	0	1	0	0	1	0	0	0	0	0	0	0	55
16749805	f	cn	US	2019	0	1	1	0	0	0	0	0	0	0	0	0	67
16750287	f	md	AT	2019	0	0	0	0	0	0	0	0	0	0	0	0	82
16750784	m	ot	US	2019	0	1	0	0	1	0	0	0	0	0	0	0	
16750959	f	md	JP	2019	1	0	0	0	0	0	0	0	0	0	0	0	91
16750960	f	cn	CN	2019	1	0	0	0	0	0	0	0	0	0	0	0	66
16751030	f	cn	US	2019	1	0	0	0	0	0	0	0	0	0	0	0	
16751079	f	md	PL	2019	0	1	1	0	0	0	0	0	0	0	0	0	73
16751373	m	md	JP	2019	0	1	0	1	0	0	0	0	0	0	0	1	75
16751447	m	ph	JP	2019	1	0	0	0	0	0	0	0	0	0	0	1	84
16751560	f	cn	US	2019	0	0	0	0	0	0	0	0	0	0	0	0	
16751788	Not	md	JP	2019	0	1	0	1	0	0	0	0	0	0	0	0	77
16752415	m	ot	IN	2019	0	0	0	0	0	0	0	0	0	0	0	0	52
16752489	m	cn	AT	2019	0	1	1	0	0	0	0	0	0	0	0	0	65
16752673	Not	cn	JP	2019	0	1	1	0	0	0	0	0	0	0	0	0	90
16753111	m	cn	JP	2019	0	1	1	0	0	0	0	0	0	0	0	0	71
16753142	f	md	DE	2019	0	1	1	0	0	0	0	0	0	0	0	0	56
16753174	m	cn	DE	2019	0	1	1	0	0	0	0	0	0	0	0	0	59
16753175	m	md	IL	2019	0	1	0	0	0	1	0	0	0	0	0	0	67
16753387	m	md	JP	2019	0	1	0	0	1	0	0	0	0	0	0	0	78
16753650	m	cn	JP	2019	0	1	1	0	0	0	0	0	0	0	0	0	73
16753783	m	md	JP	2019	0	1	0	1	0	0	0	0	0	0	0	0	69
16753793	m	md	JP	2019	0	1	0	1	0	0	0	0	0	0	0	1	66

16753833	f	cn	US	2019	1	0	0	0	0	0	0	0	0	0	0	0	50
16754030	m	cn	DE	2019	0	1	1	0	0	0	0	0	0	0	0	0	65
16755168	f	md	US	2019	0	0	0	0	0	0	0	0	0	0	0	0	63
16755191	Not	ot	IT	2019	1	0	0	0	0	0	0	0	0	0	0	0	
16755801	m	md	US	2019	0	1	0	0	1	0	0	0	0	0	0	0	
16755821	f	cn	JP	2019	0	1	1	0	0	0	0	0	0	0	0	0	80
16756114	f	ot	AR	2019	0	1	0	0	1	0	0	0	0	0	0	0	66
16756258	f	md	DE	2019	0	0	0	0	0	0	0	0	0	0	0	0	80
16756435	f	ot	US	2019	0	1	0	0	1	0	0	0	0	0	0	0	65
16756474	f	md	BR	2019	0	1	0	1	0	0	0	0	0	0	0	0	
16756980	m	cn	DE	2019	0	1	1	0	0	0	0	0	0	0	0	0	68
16756981	f	cn	DE	2019	0	1	1	0	0	0	0	0	0	0	0	0	47
16757101	Not	md	BR	2019	0	0	0	0	0	0	0	0	0	0	0	0	
16757299	m	md	KR	2019	0	1	1	0	0	0	0	0	0	0	0	0	54
16757614	f	md	JP	2019	0	1	0	0	0	1	0	0	0	0	0	0	65
16757723	m	ph	JP	2019	0	1	0	0	0	1	0	0	0	0	0	1	75
16757885	f	cn	DK	2019	0	1	1	0	0	0	0	0	0	0	0	0	62
16757999	m	Not repo	CN	2019	1	0	0	0	0	0	0	0	0	0	0	0	48.4
16758106	Not	ot	JP	2019	0	1	0	0	1	0	0	0	0	0	0	0	
16758186	f	cn	CN	2019	1	0	0	0	0	0	0	0	0	0	0	0	80
16758601	m	Not repo	CN	2019	1	0	0	0	0	0	0	0	0	0	0	0	53
16758657	f	ot	CN	2019	1	0	0	0	0	0	0	0	0	0	0	0	55
16758720	m	md	US	2019	0	1	0	1	0	0	0	0	0	0	0	0	
16758798	m	Not repo	CA	2019	0	1	0	1	0	0	0	0	0	0	0	0	
16759112	f	cn	Not re	2019	0	0	0	0	0	0	0	0	0	0	0	0	83
16759413	f	md	JP	2019	0	1	0	1	0	0	0	0	0	0	0	1	70
16759644	Not	md	BR	2019	0	1	0	1	0	0	0	0	0	0	0	1	
16759918	m	cn	US	2019	0	0	0	0	0	0	0	0	0	0	0	0	
16760143	m	Not repo	ID	2019	1	0	0	0	0	0	0	0	0	0	0	0	
16760338	f	md	DE	2019	0	1	1	0	0	0	0	0	0	0	0	0	75
16760343	m	cn	KR	2019	0	0	0	0	0	0	0	0	0	0	0	0	
16760369	f	md	JP	2019	1	0	0	0	0	0	0	0	0	0	0	1	60.4
16760558	Not	md	JP	2019	0	0	0	0	0	0	0	0	0	0	0	0	
16760562	m	ot	KR	2019	0	1	1	0	0	0	0	0	0	0	0	0	58

16760626	f	md	US	2019	1	0	0	0	0	0	0	0	0	0	0	1	46
16760861	f	md	JP	2019	1	0	0	0	0	0	0	0	0	0	0	0	78
16761030	m	md	JP	2019	0	1	0	1	0	0	0	0	0	0	0	1	78
16761031	f	md	JP	2019	0	1	0	1	0	0	0	0	0	0	0	1	66
16761364	f	Not repo	CN	2019	1	0	0	0	0	0	0	0	0	0	0	0	67
16761414	f	md	JP	2019	0	1	0	1	0	0	0	0	0	0	0	0	72
16761532	m	ot	DE	2019	0	0	0	0	0	0	0	0	0	0	0	0	80
16762230	f	cn	CA	2019	0	1	0	0	1	0	0	0	0	0	0	0	74
16762259	m	cn	IN	2019	0	1	0	0	1	0	0	0	0	0	0	0	
16762267	f	md	BR	2019	0	0	0	0	0	0	0	0	0	0	0	0	
16762779	m	cn	LB	2019	0	1	0	0	1	0	0	0	0	0	0	0	62
16762819	m	cn	JP	2019	0	1	1	0	0	0	0	0	0	0	0	0	70
16762841	f	cn	JP	2019	0	1	1	0	0	0	0	0	0	0	0	0	80
16762844	m	cn	JP	2019	0	1	1	0	0	0	0	0	0	0	0	0	71
16763019	f	cn	CN	2019	0	1	0	0	1	0	0	0	0	0	0	0	
16763025	m	ot	FR	2019	0	1	0	0	1	0	0	0	0	0	0	0	58
16763044	Not	ph	JP	2019	0	1	1	0	0	0	0	0	0	0	0	1	
16763338	f	cn	JP	2019	0	1	1	0	0	0	0	0	0	0	0	0	84
16763348	f	md	US	2019	0	1	0	0	1	0	0	0	0	0	0	0	38
16763438	f	ot	FR	2019	0	1	0	0	1	0	0	0	0	0	0	0	47
16763546	m	md	US	2019	0	1	1	0	0	0	0	0	0	0	0	0	56
16763659	m	cn	DE	2019	0	1	1	0	0	0	0	0	0	0	0	0	66
16763746	f	cn	US	2019	1	0	0	0	0	0	0	0	0	0	0	0	81
16764093	Not	md	JP	2019	0	1	0	1	0	0	0	0	0	0	0	0	
16764459	m	cn	US	2019	0	1	1	0	0	0	0	0	0	0	0	0	55
16764476	f	ot	FR	2019	0	1	0	0	1	0	0	0	0	0	0	0	63
16764477	f	md	DE	2019	0	0	0	0	0	0	0	0	0	0	0	0	55
16764494	m	cn	JP	2019	0	0	0	0	0	0	0	0	0	0	0	0	70
16764512	m	ot	JP	2019	0	1	0	0	1	0	0	0	0	0	0	1	65
16764637	f	md	JP	2019	1	0	0	0	0	0	0	0	0	0	0	1	74
16764903	m	md	JP	2019	0	1	0	0	0	1	0	0	0	0	0	0	70
16764991	m	cn	SG	2019	0	1	0	0	0	1	0	0	0	0	0	0	
16765003	f	md	BE	2019	0	1	0	0	0	1	0	0	0	0	0	0	73
16765700	f	ot	FR	2019	0	1	0	0	1	0	0	0	0	0	0	1	64

16765702	f	cn	JP	2019	0	1	1	0	0	0	0	0	0	0	0	0	
16765752	f	md	US	2019	1	0	0	0	0	0	0	0	0	0	0	0	66
16765826	m	cn	JP	2019	0	1	1	0	0	0	0	0	0	0	0	0	71
16765985	m	cn	US	2019	0	1	1	0	0	0	0	0	0	0	0	0	65
16766056	f	md	JP	2019	0	1	0	1	0	0	0	0	0	0	0	0	60
16766158	f	md	CA	2019	0	0	0	0	0	0	0	0	0	0	0	0	
16766189	Not	cn	JP	2019	0	0	0	0	0	0	0	0	0	0	0	0	65
16766316	m	cn	US	2019	0	1	1	0	0	0	0	0	0	0	0	0	
16766410	m	md	IN	2019	0	0	0	0	0	0	0	0	0	0	0	0	60
16766470	f	ot	US	2019	0	0	0	0	0	0	0	0	0	0	0	0	
16766608	f	Not repo	CN	2019	1	0	0	0	0	0	0	0	0	0	0	0	81
16767618	f	ot	US	2019	0	1	0	0	1	0	0	0	0	0	0	0	69
16767684	m	md	FR	2019	0	0	0	0	0	0	0	0	0	0	0	0	69
16767711	m	md	JP	2019	0	1	0	0	1	0	0	0	0	0	0	0	76
16767774	f	ot	CA	2019	0	0	0	0	0	0	0	0	0	0	0	0	39
16767809	f	md	JP	2019	1	0	0	0	0	0	0	0	0	0	0	0	79
16767928	m	ot	US	2019	0	1	0	0	1	0	0	0	0	0	0	0	66
16768279	f	md	JP	2019	1	0	0	0	0	0	0	0	0	0	0	0	55
16768390	m	Not repo	CN	2019	1	0	0	0	0	0	0	0	0	0	0	0	66
16768519	m	cn	IN	2019	0	0	0	0	0	0	0	0	0	0	0	0	
16768622	m	ot	US	2019	0	1	0	1	0	0	0	0	0	0	0	0	72
16769198	f	cn	JP	2019	0	1	1	0	0	0	0	0	0	0	0	0	60
16769212	f	cn	DE	2019	0	1	1	0	0	0	0	0	0	0	0	0	31
16769249	m	md	US	2019	0	1	1	0	0	0	0	0	0	0	0	0	60
16769408	m	md	CN	2019	0	0	0	0	0	0	0	0	0	0	0	0	54
16769601	m	cn	CN	2019	1	0	0	0	0	0	0	0	0	0	0	0	64
16769619	Not	ot	CN	2019	1	0	0	0	0	0	0	0	0	0	0	0	
16769829	f	md	JP	2019	0	1	0	0	0	1	0	0	0	0	0	0	75
16769885	f	ot	AU	2019	0	1	0	0	0	1	0	0	0	0	0	0	
16769892	Not	md	CN	2019	0	1	0	0	1	0	0	0	0	0	0	0	
16770008	m	md	VN	2019	0	1	0	0	0	1	0	0	0	0	0	0	
16770063	m	ot	JP	2019	0	1	1	0	0	0	0	0	0	0	0	1	82
16770191	f	cn	CN	2019	1	0	0	0	0	0	0	0	0	0	0	0	52
16770299	f	cn	CN	2019	1	0	0	0	0	0	0	0	0	0	0	0	62

16770300	f	Not repo	CN	2019	1	0	0	0	0	0	0	0	0	0	0	0	66
16770442	m	cn	CA	2019	0	1	1	0	0	0	0	0	0	0	0	0	52
16770650	m	md	US	2019	1	0	0	0	0	0	0	0	0	0	0	0	70
16770727	m	Not repo	CN	2019	1	0	0	0	0	0	0	0	0	0	0	0	61
16770736	m	cn	CN	2019	1	0	0	0	0	0	0	0	0	0	0	0	82
16770737	Not	md	IT	2019	1	0	0	0	0	0	0	0	0	0	0	0	
16770839	f	md	CN	2019	0	1	0	0	0	1	0	0	0	0	0	0	54
16771058	m	md	IT	2019	0	1	0	1	0	0	0	0	0	0	0	0	
16771249	m	md	JP	2019	0	1	0	1	0	0	0	0	0	0	0	1	47
16771250	f	md	JP	2019	1	0	0	0	0	0	0	0	0	0	0	0	
16771255	f	cn	JP	2019	0	1	1	0	0	0	0	0	0	0	0	0	74
16771311	m	ot	IN	2019	0	0	0	0	0	0	0	0	0	0	0	0	60
16771680	f	cn	JP	2019	0	1	1	0	0	0	0	0	0	0	0	0	71
16771825	f	Not repo	CN	2019	1	0	0	0	0	0	0	0	0	0	0	0	53
16771834	f	md	NO	2019	0	0	0	0	0	0	0	0	0	0	0	0	56
16772078	f	md	PL	2019	1	0	0	0	0	0	0	0	0	0	0	0	69
16772654	Not	cn	RU	2019	0	0	0	0	0	0	0	0	0	0	0	0	
16772684	m	md	JP	2019	0	1	0	1	0	0	0	0	0	0	0	1	67
16773028	f	Not repo	ID	2019	1	0	0	0	0	0	0	0	0	0	0	0	
16773061	m	ot	DE	2019	0	1	1	0	0	0	0	0	0	0	0	0	53
16773223	m	cn	DE	2019	0	1	1	0	0	0	0	0	0	0	0	0	59
16773920	f	md	US	2019	0	1	0	0	0	1	0	0	0	0	0	0	89
16774287	f	md	JP	2019	0	0	0	0	0	0	0	0	0	0	0	0	90
16774331	f	cn	US	2019	0	1	1	0	0	0	0	0	0	0	0	0	
16774587	m	md	IN	2019	0	0	0	0	0	0	0	0	0	0	0	0	60
16774621	f	ot	US	2019	0	1	0	0	1	0	0	0	0	0	0	0	
16774692	m	ot	US	2019	0	1	0	0	0	1	0	0	0	0	0	0	
16775185	f	cn	SG	2019	0	0	0	0	0	0	0	0	0	0	0	0	73
16775302	Not	ph	JP	2019	1	0	0	0	0	0	0	0	0	0	0	0	
16775394	m	ot	US	2019	0	0	0	0	0	0	0	0	0	0	0	0	70
16775408	f	Not repo	CN	2019	1	0	0	0	0	0	0	0	0	0	0	0	57
16775463	f	Not repo	CN	2019	1	0	0	0	0	0	0	0	0	0	0	0	61
16775528	Not	md	JP	2019	1	0	0	0	0	0	0	0	0	0	0	0	80
16775529	f	cn	CN	2019	1	0	0	0	0	0	0	0	0	0	0	0	70

16775797	m	cn	AT	2019	0	1	1	0	0	0	0	0	0	0	0	0	65
16775938	m	md	DE	2019	0	0	0	0	0	0	0	0	0	0	0	0	80
16776039	m	cn	CN	2019	0	1	0	0	1	0	0	0	0	0	0	0	62
16776226	f	ot	IT	2019	1	0	0	0	0	0	0	0	0	0	0	0	68.3
16776343	Not	ot	CN	2019	0	0	0	0	0	0	0	0	0	0	0	0	
16776562	m	md	JP	2019	0	1	0	1	0	0	0	0	0	0	0	1	90
16776665	f	md	JP	2019	1	0	0	0	0	0	0	0	0	0	0	0	69
16776835	m	md	JP	2019	0	0	0	0	0	0	0	0	0	0	0	0	81
16776858	Not	ph	JP	2019	0	1	0	0	0	1	0	0	0	0	0	0	
16777101	m	md	LB	2019	0	0	0	0	0	0	0	0	0	0	0	0	70
16777129	Not	ot	US	2019	0	0	0	0	0	0	1	0	1	0	0	0	
16777177	Not	ot	US	2019	0	0	0	0	0	0	1	1	0	0	0	0	
16777720	m	cn	JP	2019	0	1	1	0	0	0	0	0	0	0	0	0	58
16777870	f	md	TH	2019	1	0	0	0	0	0	0	0	0	0	0	0	56
16777924	f	ot	NL	2019	0	0	0	0	0	0	0	0	0	0	0	0	68
16778102	f	cn	US	2019	0	1	1	0	0	0	0	0	0	0	0	0	65
16778189	f	cn	CH	2019	1	0	0	0	0	0	0	0	0	0	0	0	71
16778220	f	ot	CL	2019	0	1	0	0	1	0	0	0	0	0	0	1	64
16778723	m	md	US	2019	0	1	0	0	0	1	0	0	0	0	0	0	73
16778730	f	md	TH	2019	0	0	0	0	0	0	0	0	0	0	0	0	38
16778946	Not	md	JP	2019	1	0	0	0	0	0	0	0	0	0	0	1	
16779045	f	cn	CA	2019	0	1	1	0	0	0	0	0	0	0	0	0	73
16779067	f	Not repo	CN	2019	1	0	0	0	0	0	0	0	0	0	0	0	74
16779427	m	cn	CN	2019	1	0	0	0	0	0	0	0	0	0	0	0	82
16779521	m	cn	US	2019	1	0	0	0	0	0	0	0	0	0	0	0	
16779736	f	md	TN	2019	1	0	0	0	0	0	0	0	0	0	0	0	47
16779768	f	cn	CN	2019	1	0	0	0	0	0	0	0	0	0	0	0	64
16780462	m	md	JP	2019	1	0	0	0	0	0	0	0	0	0	0	1	77.1
16780467	Not	md	US	2019	0	1	1	0	0	0	0	0	0	0	0	0	71
16780491	Not	md	CN	2019	0	0	0	0	0	0	0	0	0	0	0	0	
16780540	m	cn	AT	2019	0	1	1	0	0	0	0	0	0	0	0	0	65
16780652	m	cn	CN	2019	1	0	0	0	0	0	0	0	0	0	0	0	65.5
16780763	m	ot	JP	2019	0	1	0	0	1	0	0	0	0	0	0	0	73
16780862	m	ot	JP	2019	0	0	0	0	0	0	0	0	0	0	0	0	70

16780924	f	md	JP	2019	1	0	0	0	0	0	0	0	0	0	0	0	64.8
16781061	f	ot	NL	2019	0	1	1	0	0	0	0	0	0	0	0	0	47
16781368	m	md	JP	2019	0	1	0	1	0	0	0	0	0	0	0	0	71
16781527	f	md	JP	2019	1	0	0	0	0	0	0	0	0	0	0	0	74
16781666	m	cn	US	2019	0	1	0	1	0	0	0	0	0	0	0	0	59.4
16781682	f	Not repo	IT	2019	0	1	0	1	0	0	0	0	0	0	0	0	70.5
16781685	f	Not repo	CN	2019	1	0	0	0	0	0	0	0	0	0	0	0	
16781862	f	cn	US	2019	0	1	0	0	1	0	0	0	0	0	0	0	
16781901	Not	md	DE	2019	0	1	0	0	1	0	0	0	0	0	0	0	
16781991	m	cn	US	2019	1	0	0	0	0	0	0	0	0	0	0	0	
16782412	m	cn	US	2019	1	0	0	0	0	0	0	0	0	0	0	0	
16782442	m	cn	US	2019	1	0	0	0	0	0	0	0	0	0	0	0	
16782482	f	md	DE	2019	0	1	0	1	0	0	0	0	0	0	0	1	57.7
16782560	f	Not repo	CN	2019	1	0	0	0	0	0	0	0	0	0	0	0	66
16782970	m	ot	DE	2019	0	1	0	0	1	0	0	0	0	0	0	0	60
16782989	Not	md	PL	2019	0	0	0	0	0	0	0	0	0	0	0	0	
16783029	f	ot	DE	2019	0	1	1	0	0	0	0	0	0	0	0	0	31
16783041	m	md	GB	2019	0	1	0	0	0	1	0	0	0	0	0	0	80
16783063	Not	md	US	2019	0	0	0	0	0	0	0	0	0	0	0	0	
16783250	m	md	DE	2019	0	1	0	0	1	0	0	0	0	0	0	1	55
16783316	m	cn	CN	2019	0	1	0	0	1	0	0	0	0	0	0	0	
16783318	m	cn	GR	2019	0	1	1	0	0	0	0	0	0	0	0	1	
16783615	m	md	HK	2019	0	0	0	0	0	0	1	0	0	0	1	0	59
16783818	m	md	DE	2019	0	1	1	0	0	0	0	0	0	0	0	0	68
16783828	Not	md	AU	2019	0	1	0	0	0	1	0	0	0	0	0	0	
16783836	f	md	JP	2019	0	1	0	0	0	1	0	0	0	0	0	0	
16784150	m	cn	CN	2019	0	0	0	0	0	0	0	0	0	0	0	0	45
16784254	f	cn	US	2019	0	1	1	0	0	0	0	0	0	0	0	0	60
16784906	f	md	JP	2019	0	1	0	1	0	0	0	0	0	0	0	1	76
16785701	m	Not repo	CN	2019	1	0	0	0	0	0	0	0	0	0	0	0	65
16785703	Not	md	PT	2019	1	0	0	0	0	0	0	0	0	0	0	0	
16786160	f	cn	JP	2019	0	1	1	0	0	0	0	0	0	0	0	0	71
16786216	f	md	US	2019	0	0	0	0	0	0	0	0	0	0	0	0	61
16786226	m	cn	CN	2019	1	0	0	0	0	0	0	0	0	0	0	0	55

16786353	Not	md	IT	2019	0	0	0	0	0	0	0	0	0	0	0	0	74
16786765	f	md	CA	2019	0	0	0	0	0	0	0	0	0	0	0	0	70
16786812	m	Not repo	CN	2019	1	0	0	0	0	0	0	0	0	0	0	0	56
16786815	f	Not repo	CN	2019	1	0	0	0	0	0	0	0	0	0	0	0	
16786817	f	Not repo	CN	2019	1	0	0	0	0	0	0	0	0	0	0	0	62
16786832	m	ot	IN	2019	0	0	0	0	0	0	0	0	0	0	0	0	60
16786919	m	Not repo	CN	2019	1	0	0	0	0	0	0	0	0	0	0	0	64
16786921	m	cn	CN	2019	1	0	0	0	0	0	0	0	0	0	0	0	54
16786929	Not	ot	IT	2019	0	1	0	1	0	0	0	0	0	0	0	0	
16786944	m	cn	US	2019	1	0	0	0	0	0	0	0	0	0	0	0	
16787017	f	Not repo	CN	2019	1	0	0	0	0	0	0	0	0	0	0	0	63
16787355	m	md	TR	2019	0	1	0	1	0	0	0	0	0	0	0	0	72
16787913	m	ot	DE	2019	0	1	1	0	0	0	0	0	0	0	0	0	59
16788040	f	cn	CN	2019	1	0	0	0	0	0	0	0	0	0	0	0	
16788065	Not	md	PT	2019	0	0	0	0	0	0	0	0	0	0	0	0	
16788299	m	md	JP	2019	0	1	0	0	0	1	0	0	0	0	0	1	78
16788368	m	md	JP	2019	0	1	0	0	0	1	0	0	0	0	0	0	76
16788709	m	md	SE	2019	0	1	0	0	0	1	0	0	0	0	0	0	72
16788858	m	ot	TR	2019	0	0	0	0	0	0	0	0	0	0	0	0	52
16789039	f	md	DE	2019	0	0	0	0	0	0	0	0	0	0	0	0	58
16789049	m	ph	JP	2019	0	1	1	0	0	0	0	0	0	0	0	0	69
16789056	f	md	JP	2019	1	0	0	0	0	0	0	0	0	0	0	1	90
16789097	f	md	HR	2019	0	0	0	0	0	0	0	0	0	0	0	0	70
16789346	m	md	US	2019	0	1	0	1	0	0	0	0	0	0	0	0	67.8
16789612	f	cn	CN	2019	1	0	0	0	0	0	0	0	0	0	0	0	65
16789907	m	ot	ES	2019	0	1	0	0	1	0	0	0	0	0	0	0	79
16790157	f	cn	CN	2019	1	0	0	0	0	0	0	0	0	0	0	0	84
16790954	f	cn	CA	2019	0	1	1	0	0	0	0	0	0	0	0	0	77
16790957	f	cn	CN	2019	0	0	0	0	0	0	0	0	0	0	0	0	79
16791082	m	ot	US	2019	0	1	0	1	0	0	0	0	0	0	0	0	67
16791249	Not	md	US	2019	0	0	0	0	0	0	0	0	0	0	0	0	
16791374	m	md	SG	2019	0	0	0	0	0	0	0	0	0	0	0	0	
16791461	Not	md	IN	2019	1	0	0	0	0	0	0	0	0	0	0	0	
16791516	Not	md	CZ	2019	0	0	0	0	0	0	0	0	0	0	0	0	

16791590	m	md	US	2019	0	1	0	1	0	0	0	0	0	0	0	0	71
16791765	m	ot	ES	2019	0	0	0	0	0	0	0	0	0	0	0	0	37
16791794	f	Not repo	CN	2019	1	0	0	0	0	0	0	0	0	0	0	0	66
16791890	m	ot	KR	2019	0	1	0	0	1	0	0	0	0	0	0	0	76
16791933	m	md	CN	2019	0	0	0	0	0	0	0	0	0	0	0	0	66
16792894	m	Not repo	CN	2019	1	0	0	0	0	0	0	0	0	0	0	0	73
16792935	Not	md	JP	2019	1	0	0	0	0	0	0	0	0	0	0	0	
16792936	Not	md	JP	2019	1	0	0	0	0	0	0	0	0	0	0	0	
16792947	m	md	UY	2019	0	1	0	0	0	1	0	0	0	0	0	1	
16792958	f	cn	CN	2019	1	0	0	0	0	0	0	0	0	0	0	0	59
16792959	Not	md	JP	2019	1	0	0	0	0	0	0	0	0	0	0	0	
16792988	m	md	JP	2019	0	1	0	0	0	1	0	0	0	0	0	0	45
16793006	Not	md	JP	2019	1	0	0	0	0	0	0	0	0	0	0	0	
16793048	Not	md	JP	2019	1	0	0	0	0	0	0	0	0	0	0	0	
16793064	Not	md	JP	2019	1	0	0	0	0	0	0	0	0	0	0	0	
16793142	Not	md	JP	2019	1	0	0	0	0	0	0	0	0	0	0	0	
16793228	m	Not repo	CN	2019	1	0	0	0	0	0	0	0	0	0	0	0	63
16793409	f	md	JP	2019	0	1	0	0	0	1	0	0	0	0	0	1	64
16793458	f	cn	JP	2019	0	1	1	0	0	0	0	0	0	0	0	0	61
16793679	m	cn	CN	2019	1	0	0	0	0	0	0	0	0	0	0	0	56
16793795	Not	md	JP	2019	1	0	0	0	0	0	0	0	0	0	0	0	
16793802	f	Not repo	CN	2019	1	0	0	0	0	0	0	0	0	0	0	0	64
16793878	f	Not repo	CN	2019	1	0	0	0	0	0	0	0	0	0	0	0	50
16794024	m	ot	IN	2019	0	0	0	0	0	0	0	0	0	0	0	0	
16794037	Not	md	JP	2019	1	0	0	0	0	0	0	0	0	0	0	0	
16794053	m	cn	US	2019	0	0	0	0	0	0	0	0	0	0	0	0	
16794061	f	cn	US	2019	0	0	0	0	0	0	0	0	0	0	0	0	
16794064	f	md	US	2019	0	0	0	0	0	0	0	0	0	0	0	0	
16794087	m	ph	US	2019	0	0	0	0	0	0	0	0	0	0	0	0	
16794106	f	md	US	2019	0	0	0	0	0	0	0	0	0	0	0	0	
16794259	m	ot	CA	2019	0	1	1	0	0	0	0	0	0	0	0	0	66
16794268	Not	md	IN	2019	0	1	1	0	0	0	0	0	0	0	0	0	63
16794274	f	md	US	2019	0	0	0	0	0	0	0	0	0	0	0	0	
16794276	Not	ot	US	2019	0	0	0	0	0	0	0	0	0	0	0	0	

16794302	m	cn	US	2019	0	0	0	0	0	0	0	0	0	0	0	0	
16794304	f	md	US	2019	0	0	0	0	0	0	0	0	0	0	0	0	
16794312	m	ot	US	2019	0	0	0	0	0	0	0	0	0	0	0	0	60
16794314	m	cn	US	2019	0	0	0	0	0	0	0	0	0	0	0	0	58
16794343	m	Not repo	CN	2019	1	0	0	0	0	0	0	0	0	0	0	0	58
16794345	f	cn	CN	2019	1	0	0	0	0	0	0	0	0	0	0	0	61
16794506	f	cn	CN	2019	1	0	0	0	0	0	0	0	0	0	0	0	55
16794514	f	cn	JP	2019	0	1	1	0	0	0	0	0	0	0	0	0	70
16794541	f	cn	US	2019	0	0	0	0	0	0	0	0	0	0	0	0	38
16794563	m	Not repo	CN	2019	1	0	0	0	0	0	0	0	0	0	0	0	49
16794953	f	ph	JP	2019	1	0	0	0	0	0	0	0	0	0	0	0	81
16795250	m	Not repo	CN	2019	1	0	0	0	0	0	0	0	0	0	0	0	51.6
16795517	f	md	JP	2019	0	1	0	1	0	0	0	0	0	0	0	1	70
16795534	m	ot	TW	2019	0	1	1	0	0	0	0	0	0	0	0	1	72
16795697	m	cn	JP	2019	0	0	0	0	0	0	0	0	0	0	0	0	90
16795715	m	md	DE	2019	0	0	0	0	0	0	0	0	0	0	0	0	67
16795717	f	cn	CN	2019	1	0	0	0	0	0	0	0	0	0	0	0	61
16795750	m	cn	JP	2019	0	1	1	0	0	0	0	0	0	0	0	0	83
16795785	f	cn	US	2019	1	0	0	0	0	0	0	0	0	0	0	0	
16795826	f	cn	CN	2019	1	0	0	0	0	0	0	0	0	0	0	0	71
16795827	f	cn	CN	2019	1	0	0	0	0	0	0	0	0	0	0	0	48
16795899	m	cn	JP	2019	0	1	1	0	0	0	0	0	0	0	0	0	72
16795969	f	Not repo	CN	2019	1	0	0	0	0	0	0	0	0	0	0	0	54
16795974	m	md	CN	2019	1	0	0	0	0	0	0	0	0	0	0	0	
16796280	m	ot	DE	2019	0	1	1	0	0	0	0	0	0	0	0	0	56
16796349	f	cn	JP	2019	0	1	1	0	0	0	0	0	0	0	0	0	65
16796769	Not	md	JP	2019	1	0	0	0	0	0	0	0	0	0	0	0	
16796797	m	cn	JP	2019	0	1	1	0	0	0	0	0	0	0	0	0	73
16796952	f	ot	NL	2019	0	1	0	0	1	0	0	0	0	0	0	0	70
16796967	m	cn	CN	2019	1	0	0	0	0	0	0	0	0	0	0	0	56
16797133	Not	md	JP	2019	1	0	0	0	0	0	0	0	0	0	0	0	
16797264	f	cn	KR	2019	1	0	0	0	0	0	0	0	0	0	0	0	43
16797384	f	md	JP	2019	0	0	0	0	0	0	0	0	0	0	0	0	50
16797705	m	Not repo	CN	2019	1	0	0	0	0	0	0	0	0	0	0	0	72

16797730	f	cn	CN	2019	1	0	0	0	0	0	0	0	0	0	0	0	68
16797736	m	cn	JP	2019	0	1	1	0	0	0	0	0	0	0	0	0	72
16797816	m	cn	CN	2019	1	0	0	0	0	0	0	0	0	0	0	0	62
16797821	Not	ot	US	2019	0	0	0	0	0	0	0	0	0	0	0	0	
16797857	m	ot	FR	2019	0	1	0	0	1	0	0	0	0	0	0	1	60
16797960	m	md	IN	2019	0	0	0	0	0	0	0	0	0	0	0	0	60
16798210	f	cn	CN	2019	1	0	0	0	0	0	0	0	0	0	0	0	60
16798358	f	cn	CN	2019	1	0	0	0	0	0	0	0	0	0	0	0	65
16798587	f	cn	CN	2019	1	0	0	0	0	0	0	0	0	0	0	0	67
16798592	m	cn	CN	2019	1	0	0	0	0	0	0	0	0	0	0	0	64
16798600	f	Not repo	CN	2019	1	0	0	0	0	0	0	0	0	0	0	0	73
16798641	m	md	IN	2019	0	0	0	0	0	0	0	0	0	0	0	0	60
16798787	f	cn	CN	2019	1	0	0	0	0	0	0	0	0	0	0	0	61
16798789	m	cn	CN	2019	1	0	0	0	0	0	0	0	0	0	0	0	54
16798790	m	ph	JP	2019	1	0	0	0	0	0	0	0	0	0	0	0	58
16798951	f	cn	CN	2019	1	0	0	0	0	0	0	0	0	0	0	0	60
16798952	m	cn	CN	2019	1	0	0	0	0	0	0	0	0	0	0	0	57.2
16799190	m	md	BE	2019	0	1	1	0	0	0	0	0	0	0	0	0	70
16799339	m	cn	JP	2019	0	1	1	0	0	0	0	0	0	0	0	0	90
16799768	f	md	HU	2019	0	0	0	0	0	0	0	0	0	0	0	0	
16799785	m	cn	DE	2019	0	1	1	0	0	0	0	0	0	0	0	0	59
16799875	f	cn	CN	2019	1	0	0	0	0	0	0	0	0	0	0	0	53
16799879	m	md	JP	2019	1	0	0	0	0	0	0	0	0	0	0	1	89
16800353	f	Not repo	CN	2019	1	0	0	0	0	0	0	0	0	0	0	0	86
16800776	f	md	JP	2019	1	0	0	0	0	0	0	0	0	0	0	0	52.5
16801366	m	cn	JP	2019	0	1	1	0	0	0	0	0	0	0	0	0	47
16801426	m	md	JP	2019	0	1	0	1	0	0	0	0	0	0	0	1	71
16801473	f	cn	CN	2019	1	0	0	0	0	0	0	0	0	0	0	0	
16801492	m	ot	TW	2019	0	1	1	0	0	0	0	0	0	0	0	1	57
16801578	f	cn	CN	2019	1	0	0	0	0	0	0	0	0	0	0	0	
16801582	f	ot	NL	2019	0	1	1	0	0	0	0	0	0	0	0	0	31
16801617	f	cn	CN	2019	1	0	0	0	0	0	0	0	0	0	0	0	64
16801619	f	cn	CN	2019	1	0	0	0	0	0	0	0	0	0	0	0	71
16801621	m	ph	JP	2019	1	0	0	0	0	0	0	0	0	0	0	0	

16801622	f	Not repo	CN	2019	1	0	0	0	0	0	0	0	0	0	0	0	51
16801764	f	md	HK	2019	0	1	0	0	0	1	0	0	0	0	0	0	75
16801805	f	md	JP	2019	0	0	0	0	0	0	0	0	0	0	0	0	50
16802220	m	md	JP	2019	0	0	0	0	0	0	0	0	0	0	0	0	90
16802337	m	ot	NL	2019	0	1	1	0	0	0	0	0	0	0	0	0	59
16802465	m	cn	SE	2019	0	1	1	0	0	0	0	0	0	0	0	0	
16802584	m	cn	US	2019	0	0	0	0	0	0	0	0	0	0	0	0	
16802624	m	md	JP	2019	0	1	0	0	0	1	0	0	0	0	0	0	67
16802659	m	md	JP	2019	0	1	0	0	0	1	0	0	0	0	0	0	77
16802688	m	md	JP	2019	0	1	0	0	0	1	0	0	0	0	0	0	69
16802702	m	md	JP	2019	0	1	0	0	0	1	0	0	0	0	0	0	71
16802715	m	Not repo	CN	2019	1	0	0	0	0	0	0	0	0	0	0	0	67
16802908	f	cn	CN	2019	0	1	0	0	1	0	0	0	0	0	0	0	
16802936	m	Not repo	CN	2019	1	0	0	0	0	0	0	0	0	0	0	0	61.6
16803054	m	ph	JP	2019	0	1	0	0	1	0	0	0	0	0	0	1	68
16803063	m	md	JP	2019	0	1	0	0	1	0	0	0	0	0	0	1	
16803184	f	ot	CA	2019	0	1	0	0	1	0	0	0	0	0	0	0	73
16803466	m	md	NL	2019	0	1	0	1	0	0	0	0	0	0	0	1	
16803620	f	ot	JP	2019	1	0	0	0	0	0	0	0	0	0	0	0	69
16803641	m	md	JP	2019	0	1	0	0	1	0	0	0	0	0	0	0	
16803668	m	md	JP	2019	1	0	0	0	0	0	0	0	0	0	0	1	87
16804262	Not	ot	US	2019	0	1	0	0	1	0	0	0	0	0	0	0	
16804380	f	ot	US	2019	0	0	0	0	0	0	0	0	0	0	0	0	
16804404	Not	md	US	2019	0	0	0	0	0	0	0	0	0	0	0	0	
16804513	m	cn	JP	2019	0	1	1	0	0	0	0	0	0	0	0	0	65
16804722	m	md	ID	2019	1	0	0	0	0	0	0	0	0	0	0	0	50
16805075	m	Not repo	CN	2019	1	0	0	0	0	0	0	0	0	0	0	1	50.9
16805077	m	Not repo	CN	2019	1	0	0	0	0	0	0	0	0	0	0	0	53
16805325	f	md	JP	2019	1	0	0	0	0	0	0	0	0	0	0	0	84
16805339	f	md	JP	2019	1	0	0	0	0	0	0	0	0	0	0	0	60
16805426	m	Not repo	MY	2019	0	1	0	1	0	0	0	0	0	0	0	0	60
16805429	m	cn	JP	2019	1	0	0	0	0	0	0	0	0	0	0	0	
16805636	f	cn	US	2019	1	0	0	0	0	0	0	0	0	0	0	0	
16805645	f	cn	CN	2019	1	0	0	0	0	0	0	0	0	0	0	0	65

16805809	m	md	IN	2019	0	1	1	0	0	0	0	0	0	0	0	0	62
16806470	Not	md	JP	2019	0	1	0	1	0	0	0	0	0	0	0	1	70
16806472	f	cn	IN	2019	1	0	0	0	0	0	0	0	0	0	0	0	74
16806513	f	cn	CN	2019	1	0	0	0	0	0	0	0	0	0	0	0	70
16807326	Not	md	CN	2019	0	1	0	0	1	0	0	0	0	0	0	0	65
16807519	f	cn	JP	2019	0	1	1	0	0	0	0	0	0	0	0	0	60
16807602	m	md	CN	2019	0	1	0	0	0	1	0	0	0	0	0	0	63
16807881	f	md	JP	2019	0	1	0	0	1	0	0	0	0	0	0	0	74
16807935	f	Not repo	CN	2019	1	0	0	0	0	0	0	0	0	0	0	0	56
16807936	f	Not repo	CN	2019	1	0	0	0	0	0	0	0	0	0	0	0	80
16808161	f	cn	CN	2019	1	0	0	0	0	0	0	0	0	0	0	0	57
16808218	m	md	JP	2019	0	1	1	0	0	0	0	0	0	0	0	0	66
16808657	m	md	JP	2019	0	1	0	0	0	1	0	0	0	0	0	0	73
16808668	f	cn	CN	2019	1	0	0	0	0	0	0	0	0	0	0	0	57
16808826	m	md	JP	2019	0	1	0	0	0	1	0	0	0	0	0	1	90
16809226	f	md	ES	2019	0	1	1	0	0	0	0	0	0	0	0	0	53
16809605	m	Not repo	US	2019	1	0	0	0	0	0	0	0	0	0	0	0	86
16810138	f	cn	NL	2019	0	1	1	0	0	0	0	0	0	0	0	0	59
16810193	Not	ph	US	2019	0	0	0	0	0	0	0	0	0	0	0	0	
16810315	m	md	US	2019	0	0	0	0	0	0	0	0	0	0	0	0	
16811664	Not	md	IT	2019	1	0	0	0	0	0	0	0	0	0	0	0	
16811922	Not	ot	JP	2019	0	0	0	0	0	0	0	0	0	0	0	0	
16811938	m	ot	US	2019	0	0	0	0	0	0	0	0	0	0	0	0	
16812213	Not	ot	ES	2019	0	0	0	0	0	0	0	0	0	0	0	0	
16813254	Not	md	GR	2019	0	0	0	0	0	0	0	0	0	0	0	0	
16813328	Not	ot	IT	2019	1	0	0	0	0	0	0	0	0	0	0	0	
16813329	Not	md	JP	2019	1	0	0	0	0	0	0	0	0	0	0	0	
16813416	m	ot	CA	2019	0	0	0	0	0	0	0	0	0	0	0	0	54
16813436	f	ot	US	2019	0	1	0	0	1	0	0	0	0	0	0	0	71
16813532	m	md	JP	2019	0	1	0	0	0	1	0	0	0	0	0	0	69
16813640	m	md	JP	2019	0	1	0	0	0	1	0	0	0	0	0	0	72
16813813	f	cn	US	2019	0	1	0	0	1	0	0	0	0	0	0	0	
16814369	m	md	GB	2019	0	1	0	0	0	1	0	0	0	0	0	0	67
16814877	f	md	AU	2019	0	1	0	0	0	1	0	0	0	0	0	0	68

16815190	m	md	GB	2019	0	1	0	0	0	1	0	0	0	0	0	0	80
16817035	f	md	JP	2019	0	0	0	0	0	0	0	0	0	0	0	0	72
16817162	m	Not repo	RU	2019	0	1	0	1	0	0	0	0	0	0	0	0	60
16817263	f	ot	US	2019	0	0	0	0	0	0	0	0	0	0	0	0	78
16817520	m	md	JP	2019	0	0	0	0	0	0	0	0	0	0	0	0	72
16818111	m	cn	JP	2019	0	1	1	0	0	0	0	0	0	0	0	0	70
16818648	m	cn	IN	2019	0	1	0	0	1	0	0	0	0	0	0	0	55
16818788	m	md	VN	2019	1	0	0	0	0	0	0	0	0	0	0	0	55
16819131	m	md	JP	2019	1	0	0	0	0	0	0	0	0	0	0	1	71
16819135	m	md	TW	2019	0	1	0	1	0	0	0	0	0	0	0	0	65
16819182	m	ot	JP	2019	0	1	1	0	0	0	0	0	0	0	0	1	64
16819686	m	ot	TW	2019	0	1	0	0	1	0	0	0	0	0	0	0	71
16819697	f	cn	JP	2019	0	1	1	0	0	0	0	0	0	0	0	0	80
16819834	m	ot	SE	2019	0	1	1	0	0	0	0	0	0	0	0	0	
16819956	m	cn	CN	2019	0	0	0	0	0	0	0	0	0	0	0	0	
16820569	m	ph	JP	2019	0	1	0	0	1	0	0	0	0	0	0	0	80
16820613	f	md	MY	2019	1	0	0	0	0	0	0	0	0	0	0	0	72
16820617	m	ot	SE	2019	0	1	0	0	0	1	0	0	0	0	0	0	
16820653	m	md	BY	2019	0	1	0	0	0	1	0	0	0	0	0	0	
16820740	f	md	US	2019	1	0	0	0	0	0	0	0	0	0	0	0	
16820783	f	Not repo	US	2019	1	0	0	0	0	0	0	0	0	0	0	0	70
16820821	m	md	GB	2019	0	1	0	0	0	1	0	0	0	0	0	0	64
16820863	m	ph	JP	2019	0	1	0	0	1	0	0	0	0	0	0	0	80
16821050	Not	ph	JP	2019	1	0	0	0	0	0	0	0	0	0	0	0	
16821224	m	md	JP	2019	0	1	1	0	0	0	0	0	0	0	0	1	77
16821439	m	md	CA	2019	0	0	0	0	0	0	0	0	0	0	0	0	35
16821612	f	ot	JP	2019	0	1	1	0	0	0	0	0	0	0	0	0	65
16821661	m	md	FR	2019	0	1	0	0	0	1	0	0	0	0	0	0	66
16822148	m	md	JP	2019	0	1	0	1	0	0	0	0	0	0	0	1	70
16822154	m	md	US	2019	0	0	0	0	0	0	0	0	0	0	0	0	
16823613	f	md	JP	2019	1	0	0	0	0	0	0	0	0	0	0	1	90
16823635	f	ot	JP	2019	0	1	0	1	0	0	0	0	0	0	0	1	68.7
16823825	f	md	JP	2019	1	0	0	0	0	0	0	0	0	0	0	0	71
16823827	f	Not repo	CN	2019	1	0	0	0	0	0	0	0	0	0	0	0	73

16823884	Not	md	DE	2019	0	1	0	1	0	0	0	0	0	0	0	1	
16823894	Not	md	PH	2019	0	0	0	0	0	0	0	0	0	0	0	0	40
16823979	f	md	FR	2019	0	1	0	1	0	0	0	0	0	0	0	0	76
16824036	m	cn	JP	2019	0	1	1	0	0	0	0	0	0	0	0	0	70
16824383	m	md	JP	2019	0	1	0	0	1	0	0	0	0	0	0	1	71
16824411	m	cn	JP	2019	0	1	1	0	0	0	0	0	0	0	0	1	79
16824684	f	cn	NL	2019	0	1	0	0	1	0	0	0	0	0	0	0	
16824789	f	cn	CN	2019	0	1	0	0	1	0	0	0	0	0	0	0	
16824934	m	md	NL	2019	0	0	0	0	0	0	0	0	0	0	0	0	69
16825260	m	cn	CN	2019	0	1	0	0	1	0	0	0	0	0	0	0	59
16825280	Not	ot	US	2019	0	0	0	0	0	0	1	0	1	0	0	1	
16825473	Not	ot	US	2019	0	0	0	0	0	0	1	0	1	0	0	1	
16825588	m	md	US	2019	0	1	0	1	0	0	0	0	0	0	0	1	
16825695	Not	ph	JP	2019	0	1	0	0	1	0	0	0	0	0	0	0	
16825733	Not	ot	US	2019	0	0	0	0	0	0	1	0	1	0	0	0	
16825773	m	ph	US	2019	0	0	0	0	0	0	0	0	0	0	0	0	67
16825876	m	ph	US	2019	0	0	0	0	0	0	0	0	0	0	0	0	67
16825919	m	cn	JP	2019	0	1	1	0	0	0	0	0	0	0	0	1	81
16826002	f	md	US	2019	0	0	0	0	0	0	0	0	0	0	0	0	53
16826083	f	ot	US	2019	0	1	0	0	1	0	0	0	0	0	0	0	58
16826479	m	cn	CA	2019	0	1	1	0	0	0	0	0	0	0	0	0	75
16826509	Not	md	TW	2019	0	0	0	0	0	0	0	0	0	0	0	0	77
16826529	Not	md	TW	2019	0	0	0	0	0	0	0	0	0	0	0	0	83
16826533	Not	md	TW	2019	0	0	0	0	0	0	0	0	0	0	0	0	
16826675	f	cn	US	2019	0	0	0	0	0	0	0	0	0	0	0	0	
16826749	f	ot	JP	2019	1	0	0	0	0	0	0	0	0	0	0	0	71
16826750	f	ot	JP	2019	1	0	0	0	0	0	0	0	0	0	0	0	64
16826754	Not	md	TW	2019	0	0	0	0	0	0	0	0	0	0	0	0	
16826757	Not	md	TW	2019	0	0	0	0	0	0	0	0	0	0	0	0	
16826762	Not	md	TW	2019	0	0	0	0	0	0	0	0	0	0	0	0	
16826827	m	md	JP	2019	0	1	0	1	0	0	0	0	0	0	0	1	75
16826851	m	ot	CN	2019	0	1	0	0	0	1	0	0	0	0	0	1	57
16826971	f	md	CN	2019	1	0	0	0	0	0	0	0	0	0	0	1	64.7
16827031	Not	md	JP	2019	0	1	0	1	0	0	0	0	0	0	0	0	

16827568	f	ot	JP	2019	0	1	0	1	0	0	0	0	0	0	0	0	69
16827804	f	cn	JP	2019	0	1	1	0	0	0	0	0	0	0	0	0	68
16828018	m	md	JP	2019	0	1	0	0	1	0	0	0	0	0	0	0	62
16828053	m	cn	CN	2019	1	0	0	0	0	0	0	0	0	0	0	0	60
16828258	m	md	US	2019	0	0	0	0	0	0	0	0	0	0	0	0	53
16828429	m	ot	US	2019	0	1	0	0	1	0	0	0	0	0	0	0	75
16828766	m	ot	US	2019	0	1	0	0	1	0	0	0	0	0	0	0	69
16828820	f	Not repo	CA	2019	1	0	0	0	0	0	0	0	0	0	0	0	
16828916	f	Not repo	CA	2019	0	1	0	1	0	0	0	0	0	0	0	0	
16829047	m	cn	SE	2019	0	1	1	0	0	0	0	0	0	0	0	0	
16829689	Not	md	TW	2019	0	0	0	0	0	0	0	0	0	0	0	0	
16829877	Not	md	TW	2019	0	0	0	0	0	0	0	0	0	0	0	0	
16829898	m	cn	Not re	2019	0	0	0	0	0	0	0	0	0	0	0	0	
16830555	f	cn	US	2019	0	1	1	0	0	0	0	0	0	0	0	0	66
16830695	m	cn	CN	2019	0	1	0	0	1	0	0	0	0	0	0	0	78
16830930	m	cn	DE	2019	0	0	0	0	0	0	0	0	0	0	0	0	68
16831205	m	cn	JP	2019	0	1	1	0	0	0	0	0	0	0	0	0	81
16831323	m	ot	DE	2019	0	1	1	0	0	0	0	0	0	0	0	0	
16831324	m	ot	DE	2019	0	1	1	0	0	0	0	0	0	0	0	0	79
16831341	m	cn	CN	2019	0	0	0	0	0	0	0	0	0	0	0	0	73
16831752	f	ot	US	2019	0	1	0	0	1	0	0	0	0	0	0	0	81
16832769	m	md	CN	2019	0	1	0	0	0	1	0	0	0	0	0	0	70
16832796	Not	md	CN	2019	0	0	0	0	0	0	0	0	0	0	0	0	
16832811	m	md	JP	2019	0	1	0	1	0	0	0	0	0	0	0	1	40
16832855	f	ph	JP	2019	1	0	0	0	0	0	0	0	0	0	0	0	
16832899	Not	md	CN	2019	0	0	0	0	0	0	0	0	0	0	0	0	
16833062	f	md	FR	2019	1	0	0	0	0	0	0	0	0	0	0	0	
16833443	f	Not repo	DE	2019	1	0	0	0	0	0	0	0	0	0	0	0	
16833653	f	ph	US	2019	1	0	0	0	0	0	0	0	0	0	0	0	69.8
16833662	m	ot	NL	2019	0	0	0	0	0	0	0	0	0	0	0	0	
16833674	Not	md	US	2019	0	0	0	0	0	0	0	0	0	0	0	0	
16833713	f	md	US	2019	0	0	0	0	0	0	0	0	0	0	0	0	56
16834268	Not	md	TW	2019	0	0	0	0	0	0	0	0	0	0	0	0	
16834517	f	md	US	2019	0	1	0	1	0	0	0	0	0	0	0	1	62

16834679	f	md	JP	2019	0	1	0	1	0	0	0	0	0	0	0	1	74
16834768	Not	md	AU	2019	0	1	0	0	1	0	0	0	0	0	0	0	
16834984	m	cn	GB	2019	0	1	0	0	0	1	0	0	0	0	0	0	67
16835872	m	cn	JP	2019	0	1	1	0	0	0	0	0	0	0	0	0	80
16835913	m	cn	US	2019	0	1	0	0	1	0	0	0	0	0	0	0	
16836028	m	md	JP	2019	0	1	0	0	0	1	0	0	0	0	0	0	60
16836038	f	md	AT	2019	0	1	0	0	0	1	0	0	0	0	0	0	59
16836333	f	cn	US	2019	0	1	1	0	0	0	0	0	0	0	0	0	68
16836588	f	md	CN	2019	0	0	0	0	0	0	0	0	0	0	0	0	55
16836810	m	md	JP	2019	0	1	0	0	0	1	0	0	0	0	0	0	70
16836937	f	md	GB	2019	0	1	1	0	0	0	0	0	0	0	0	0	74
16837572	m	md	RU	2019	0	1	0	0	0	1	0	0	0	0	0	0	65
16837904	f	md	JP	2019	0	1	0	0	0	1	0	0	0	0	0	0	56
16837905	m	cn	AT	2019	0	1	1	0	0	0	0	0	0	0	0	0	65
16837947	m	cn	JP	2019	0	1	1	0	0	0	0	0	0	0	0	0	70
16838144	m	md	JP	2019	0	1	0	0	0	1	0	0	0	0	0	0	73
16838516	f	cn	CN	2019	1	0	0	0	0	0	0	0	0	0	0	0	58
16838696	f	ot	US	2019	0	1	1	0	0	0	0	0	0	0	0	0	52
16838977	m	cn	US	2019	0	0	0	0	0	0	0	0	0	0	0	0	
16839002	m	md	JP	2019	0	1	1	0	0	0	0	0	0	0	0	0	76
16839557	m	cn	US	2019	0	0	0	0	0	0	0	0	0	0	0	0	
16839562	m	cn	US	2019	0	0	0	0	0	0	0	0	0	0	0	0	
16840100	Not	ot	US	2019	0	0	0	0	0	0	0	0	0	0	0	0	
16840308	Not	ot	US	2019	0	0	0	0	0	0	0	0	0	0	0	0	
16840320	m	ot	GB	2019	0	1	0	0	0	1	0	0	0	0	0	0	67
16840594	f	md	JP	2019	0	1	1	0	0	0	0	0	0	0	0	1	69
16840838	f	Not repo	ID	2019	1	0	0	0	0	0	0	0	0	0	0	0	
16841523	m	cn	DE	2019	0	1	1	0	0	0	0	0	0	0	0	0	
16841527	m	cn	SE	2019	0	1	1	0	0	0	0	0	0	0	0	0	
16841556	f	md	US	2019	1	0	0	0	0	0	0	0	0	0	0	1	
16841560	Not	md	GB	2019	0	1	0	1	0	0	0	0	0	0	0	1	
16841670	Not	ot	JP	2019	0	0	0	0	0	0	0	0	0	0	0	0	
16841685	Not	ot	JP	2019	0	0	0	0	0	0	0	0	0	0	0	0	
16841966	Not	md	NL	2019	0	1	1	0	0	0	0	0	0	0	0	0	65

16842066	f	md	BR	2019	1	0	0	0	0	0	0	0	0	0	0	0	
16842070	Not	md	JP	2019	1	0	0	0	0	0	0	0	0	0	0	0	
16842184	m	cn	DE	2019	0	1	1	0	0	0	0	0	0	0	0	0	
16842274	m	cn	JP	2019	0	1	1	0	0	0	0	0	0	0	0	0	65
16842987	m	cn	JP	2019	0	1	1	0	0	0	0	0	0	0	0	0	66
16843479	Not	ot	JP	2019	1	0	0	0	0	0	0	0	0	0	0	1	
16843874	m	md	VN	2019	0	1	0	0	0	1	0	0	0	0	0	0	
16843941	f	ot	LB	2019	0	1	0	0	1	0	0	0	0	0	0	0	
16844490	Not	ot	JP	2019	0	0	0	0	0	0	0	0	0	0	0	0	
16844528	Not	ot	JP	2019	0	0	0	0	0	0	0	0	0	0	0	0	
16844556	Not	ot	TW	2019	0	0	0	0	0	0	0	0	0	0	0	0	
16845808	m	cn	JP	2019	0	1	1	0	0	0	0	0	0	0	0	0	51
16846148	Not	md	JP	2019	0	1	0	0	1	0	0	0	0	0	0	0	
16846546	f	md	US	2019	0	1	0	1	0	0	0	0	0	0	0	0	
16846706	f	md	CZ	2019	0	0	0	0	0	0	0	0	0	0	0	0	
16846822	f	ot	DE	2019	0	1	1	0	0	0	0	0	0	0	0	0	
16846833	m	md	MY	2019	0	1	1	0	0	0	0	0	0	0	0	0	68
16846851	m	md	US	2019	0	1	1	0	0	0	0	0	0	0	0	0	
16847360	m	cn	JP	2019	0	1	1	0	0	0	0	0	0	0	0	0	
16847412	m	md	DE	2019	0	0	0	0	0	0	0	0	0	0	0	0	67
16847616	Not	ot	IT	2019	1	0	0	0	0	0	0	0	0	0	0	0	
16848302	m	md	TW	2019	0	1	0	0	1	0	0	0	0	0	0	0	36
16848381	m	cn	CN	2019	1	0	0	0	0	0	0	0	0	0	0	0	53
16848501	f	ot	CA	2019	0	0	0	0	0	0	0	0	0	0	0	0	63
16848838	f	cn	US	2019	1	0	0	0	0	0	0	0	0	0	0	0	
16849075	Not	md	AU	2019	0	1	0	0	0	1	0	0	0	0	0	1	
16849385	Not	md	SA	2019	0	1	0	0	1	0	0	0	0	0	0	0	
16849387	m	ot	CN	2019	0	1	0	0	1	0	0	0	0	0	0	0	44
16849427	f	md	KR	2019	0	0	0	0	0	0	0	0	0	0	0	0	60
16849525	m	ot	IE	2019	0	1	0	0	0	1	0	0	0	0	0	0	
16849568	Not	ot	IT	2019	0	0	0	0	0	0	1	0	1	0	0	0	
16849918	f	cn	CN	2019	0	1	0	0	1	0	0	0	0	0	0	0	
16850290	Not	ot	IT	2019	1	0	0	0	0	0	0	0	0	0	0	0	
16850310	m	cn	US	2019	0	1	0	0	1	0	0	0	0	0	0	0	

16850396	f	cn	JP	2019	0	1	1	0	0	0	0	0	0	0	0	0	86
16850459	m	md	JP	2019	0	1	0	1	0	0	0	0	0	0	0	0	90
16850460	m	md	JP	2019	0	1	0	1	0	0	0	0	0	0	0	1	80
16850960	f	md	JP	2019	1	0	0	0	0	0	0	0	0	0	0	0	64.3
16851319	m	cn	IN	2019	0	0	0	0	0	0	0	0	0	0	0	0	34
16851602	m	ot	NL	2019	0	1	1	0	0	0	0	0	0	0	0	0	
16851669	Not	md	LB	2019	0	0	0	0	0	0	0	0	0	0	0	0	
16851802	f	Not repo	CN	2019	1	0	0	0	0	0	0	0	0	0	0	0	68
16851843	Not	ot	US	2019	1	0	0	0	0	0	0	0	0	0	0	1	
16851867	m	cn	US	2019	0	0	0	0	0	0	0	0	0	0	0	0	77
16852048	m	Not repo	CN	2019	1	0	0	0	0	0	0	0	0	0	0	0	60
16852097	f	md	DE	2019	0	1	0	1	0	0	0	0	0	0	0	0	45
16852187	m	cn	JP	2019	0	1	1	0	0	0	0	0	0	0	0	0	70
16852733	f	ot	US	2019	0	1	1	0	0	0	0	0	0	0	0	0	
16853227	m	md	BE	2019	0	1	1	0	0	0	0	0	0	0	0	0	72
16853380	m	md	GB	2019	0	1	0	1	0	0	0	0	0	0	0	0	
16853511	f	Not repo	CN	2019	1	0	0	0	0	0	0	0	0	0	0	0	64
16854384	m	md	ES	2019	0	0	0	0	0	0	0	0	0	0	0	0	42
16854725	m	cn	US	2019	0	1	0	0	1	0	0	0	0	0	0	0	77
16854738	Not	md	JP	2019	0	1	0	1	0	0	0	0	0	0	0	1	
16855006	f	ot	US	2019	0	1	0	0	1	0	0	0	0	0	0	0	62
16855247	m	md	JP	2019	0	1	0	0	0	1	0	0	0	0	0	0	66
16855280	f	cn	JP	2019	0	1	1	0	0	0	0	0	0	0	0	0	62
16855961	m	ot	US	2019	0	1	0	0	1	0	0	0	0	0	0	0	57
16856220	m	md	US	2019	0	0	0	0	0	0	0	0	0	0	0	0	
16857466	f	md	JP	2019	1	0	0	0	0	0	0	0	0	0	0	0	50
16857521	m	cn	SE	2019	0	1	1	0	0	0	0	0	0	0	0	0	
16857640	m	cn	US	2019	0	1	0	0	1	0	0	0	0	0	0	0	
16857668	f	Not repo	CN	2019	1	0	0	0	0	0	0	0	0	0	0	0	
16857953	f	md	GB	2019	0	1	0	1	0	0	0	0	0	0	0	0	
16859431	f	ot	CA	2019	0	0	0	0	0	0	0	0	0	0	0	0	63
16859655	m	cn	DE	2019	0	1	1	0	0	0	0	0	0	0	0	0	69
16859657	m	cn	DE	2019	0	1	1	0	0	0	0	0	0	0	0	1	63
16859919	m	cn	JP	2019	0	1	1	0	0	0	0	0	0	0	0	0	72

16860010	Not	ot	TW	2019	1	0	0	0	0	0	0	0	0	0	0	0	
16860011	Not	ot	CA	2019	1	0	0	0	0	0	0	0	0	0	0	0	
16860085	m	Not repo	US	2019	1	0	0	0	0	0	0	0	0	0	0	0	
16860406	m	md	JP	2019	0	1	0	1	0	0	0	0	0	0	0	0	59
16860410	m	ot	JP	2019	0	1	0	1	0	0	0	0	0	0	0	1	61
16860454	m	ot	US	2019	0	1	0	0	1	0	0	0	0	0	0	0	62
16860491	m	md	JP	2019	0	1	1	0	0	0	0	0	0	0	0	0	69
16860493	m	cn	US	2019	0	1	1	0	0	0	0	0	0	0	0	0	
16860503	f	md	IL	2019	0	1	0	1	0	0	0	0	0	0	0	0	45
16860721	m	md	FR	2019	0	1	0	0	0	1	0	0	0	0	0	0	64
16860772	m	cn	DE	2019	0	1	1	0	0	0	0	0	0	0	0	0	57
16860812	f	Not repo	CN	2019	1	0	0	0	0	0	0	0	0	0	0	0	46
16860949	f	ot	US	2019	0	1	0	0	1	0	0	0	0	0	0	0	71
16861132	m	cn	JP	2019	0	1	1	0	0	0	0	0	0	0	0	0	80
16861146	Not	ot	PT	2019	1	0	0	0	0	0	0	0	0	0	0	0	
16861477	Not	ot	NL	2019	1	0	0	0	0	0	0	0	0	0	0	0	
16861523	m	md	JP	2019	0	1	0	1	0	0	0	0	0	0	0	0	70
16861535	Not	ot	IT	2019	1	0	0	0	0	0	0	0	0	0	0	0	
16861617	f	md	SK	2019	1	0	0	0	0	0	0	0	0	0	0	0	53
16861706	m	cn	JP	2019	0	1	1	0	0	0	0	0	0	0	0	0	62
16861938	m	md	JP	2019	0	1	0	1	0	0	0	0	0	0	0	1	74
16861947	m	ph	JP	2019	0	1	0	1	0	0	0	0	0	0	0	0	61
16862214	m	md	JP	2019	0	1	0	1	0	0	0	0	0	0	0	0	62
16862225	Not	ot	KR	2019	1	0	0	0	0	0	0	0	0	0	0	0	
16862349	Not	ot	CN	2019	1	0	0	0	0	0	0	0	0	0	0	0	
16862398	Not	ot	US	2019	1	0	0	0	0	0	0	0	0	0	0	0	
16862523	m	cn	CN	2019	1	0	0	0	0	0	0	0	0	0	0	0	64
16862585	m	md	US	2019	0	1	0	0	0	1	0	0	0	0	0	0	76
16862680	m	ot	CN	2019	1	0	0	0	0	0	0	0	0	0	0	0	48
16862696	Not	ot	US	2019	1	0	0	0	0	0	0	0	0	0	0	0	
16862821	Not	md	ES	2019	0	1	1	0	0	0	0	0	0	0	0	0	79
16862886	f	Not repo	CN	2019	1	0	0	0	0	0	0	0	0	0	0	0	64
16862887	m	ph	JP	2019	0	1	0	1	0	0	0	0	0	0	0	0	70
16862951	m	ot	FR	2019	0	1	0	0	1	0	0	0	0	0	0	0	64

16862982	m	md	JP	2019	0	1	0	1	0	0	0	0	0	0	0	1	80
16863003	f	cn	CN	2019	1	0	0	0	0	0	0	0	0	0	0	0	43
16863127	Not	Not repo	Not re	2019	0	1	1	0	0	0	0	0	0	0	0	0	79
16863167	m	cn	JP	2019	0	1	1	0	0	0	0	0	0	0	0	0	67
16863238	Not	ot	TW	2019	1	0	0	0	0	0	0	0	0	0	0	0	
16863266	m	md	JP	2019	1	0	0	0	0	0	0	0	0	0	0	0	75
16863630	f	md	JP	2019	0	1	0	1	0	0	0	0	0	0	0	1	60
16863651	Not	md	JP	2019	0	0	0	0	0	0	0	0	0	0	0	0	67
16863902	Not	ot	TW	2019	1	0	0	0	0	0	0	0	0	0	0	0	
16863912	Not	ot	US	2019	1	0	0	0	0	0	0	0	0	0	0	0	
16864214	m	md	NL	2019	0	1	1	0	0	0	0	0	0	0	0	0	65
16864293	f	cn	CN	2019	0	1	0	0	1	0	0	0	0	0	0	0	49
16864537	f	cn	CN	2019	0	1	0	0	1	0	0	0	0	0	0	0	41
16864623	f	cn	CN	2019	0	1	0	0	1	0	0	0	0	0	0	0	63
16864676	m	Not repo	US	2019	0	1	0	1	0	0	0	0	0	0	0	1	
16865087	Not	md	ES	2019	0	1	1	0	0	0	0	0	0	0	0	0	79
16865138	m	md	JP	2019	0	1	0	0	0	1	0	0	0	0	0	0	60
16865202	m	cn	CN	2019	0	0	0	0	0	0	0	0	0	0	0	0	67
16865223	m	ot	US	2019	0	1	0	0	1	0	0	0	0	0	0	0	51
16867004	Not	ot	JP	2019	1	0	0	0	0	0	0	0	0	0	0	1	
16867025	m	md	JP	2019	0	1	0	0	0	1	0	0	0	0	0	0	
16868006	m	cn	IN	2019	0	0	0	0	0	0	0	0	0	0	0	0	48
16868200	m	md	NL	2019	0	0	0	0	0	0	0	0	0	0	0	0	69
16868438	f	Not repo	CN	2019	1	0	0	0	0	0	0	0	0	0	0	0	71
16868681	m	md	GB	2019	0	1	0	0	0	1	0	0	0	0	0	0	67
16868817	m	md	CN	2019	0	0	0	0	0	0	0	0	0	0	0	0	56
16868883	m	cn	JP	2019	0	1	1	0	0	0	0	0	0	0	0	0	78
16868933	m	md	KR	2019	0	1	1	0	0	0	0	0	0	0	0	0	67
16869182	m	ot	TW	2019	0	0	0	0	0	0	0	0	0	0	0	0	83
16869234	f	md	US	2019	0	1	1	0	0	0	0	0	0	0	0	0	73
16869848	f	ot	CA	2019	0	1	1	0	0	0	0	0	0	0	0	0	51
16870218	f	cn	CO	2019	0	1	1	0	0	0	0	0	0	0	0	1	
16871124	f	md	SY	2019	1	0	0	0	0	0	0	0	0	0	0	0	
16871152	Not	md	IT	2019	1	0	0	0	0	0	0	0	0	0	0	0	

16871214	f	cn	US	2019	1	0	0	0	0	0	0	0	0	0	0	0	81
16871803	m	ph	US	2019	0	0	0	0	0	0	0	0	0	0	0	0	73
8205904	m	ot	IT	2019	0	0	0	0	0	0	0	0	0	0	0	0	36
16181463	f	md	JP	2019	1	0	0	0	0	0	0	0	0	0	0	1	77.2
16225036	m	cn	JP	2019	0	1	1	0	0	0	0	0	0	0	0	1	73
16272082	m	md	JP	2019	0	1	0	1	0	0	0	0	0	0	0	0	74.6
16365802	f	md	JP	2019	1	0	0	0	0	0	0	0	0	0	0	1	73.5
16524068	m	md	JP	2019	0	1	0	0	1	0	0	0	0	0	0	1	79
16529450	f	ot	JP	2019	0	1	0	0	1	0	0	0	0	0	0	0	72
16556305	f	ot	CA	2019	0	1	0	0	1	0	0	0	0	0	0	0	59
16557064	Not	ot	JP	2019	0	0	0	0	0	0	0	0	0	0	0	0	
16594864	f	ot	AU	2019	0	1	0	0	1	0	0	0	0	0	0	0	69
16603147	m	md	TW	2019	0	1	1	0	0	0	0	0	0	0	0	0	59
16634145	f	ot	KR	2019	0	1	0	0	1	0	0	0	0	0	0	0	79
16642327	Not	md	LT	2019	0	0	0	0	0	0	0	0	0	0	0	0	
16646201	f	cn	US	2019	1	0	0	0	0	0	0	0	0	0	0	1	64
16647341	m	cn	JP	2019	0	1	1	0	0	0	0	0	0	0	0	1	67
16651226	f	md	GB	2019	0	1	0	0	0	1	0	0	0	0	0	0	81
16677103	m	ph	JP	2019	0	1	0	0	1	0	0	0	0	0	0	0	75
16680798	f	cn	CN	2019	1	0	0	0	0	0	0	0	0	0	0	0	
16680841	f	md	US	2019	0	1	0	0	0	1	0	0	0	0	0	0	63
16682153	m	cn	CN	2019	1	0	0	0	0	0	0	0	0	0	0	0	63.4
16684597	f	md	US	2019	0	1	1	0	0	0	0	0	0	0	0	0	50
16684876	m	md	JP	2019	0	1	0	0	1	0	0	0	0	0	0	0	57
16689279	m	ot	JP	2019	0	1	1	0	0	0	0	0	0	0	0	0	56
16689660	m	md	US	2019	0	0	0	0	0	0	0	0	0	0	0	0	73
16696542	m	Not repo	JP	2019	0	1	0	1	0	0	0	0	0	0	0	0	61
16697859	m	md	ES	2019	0	1	1	0	0	0	0	0	0	0	0	0	81
16702855	m	md	JP	2019	0	1	0	1	0	0	0	0	0	0	0	1	69
16709787	m	md	FR	2019	0	1	0	0	1	0	0	0	0	0	0	0	54
16715813	f	cn	US	2019	0	1	1	0	0	0	0	0	0	0	0	0	49
16716248	m	ot	US	2019	0	1	0	1	0	0	0	0	0	0	0	0	
16720058	m	md	ES	2019	0	0	0	0	0	0	0	0	0	0	0	0	73
16723456	m	cn	KR	2019	1	0	0	0	0	0	0	0	0	0	0	0	69

16729968	m	ot	CN	2019	0	0	0	0	0	0	0	0	0	0	0	0	71
16730266	m	ph	US	2019	0	0	0	0	0	0	0	0	0	0	0	0	58
16734221	m	md	JP	2019	0	1	0	1	0	0	0	0	0	0	0	1	79
16735999	m	ph	US	2019	0	0	0	0	0	0	0	0	0	0	0	0	70
16736146	f	cn	US	2019	1	0	0	0	0	0	0	0	0	0	0	0	50
16740645	f	ot	IT	2019	1	0	0	0	0	0	0	0	0	0	0	0	66
16747667	f	cn	JP	2019	0	1	1	0	0	0	0	0	0	0	0	0	68
16747689	m	md	JP	2019	0	1	0	1	0	0	0	0	0	0	0	1	65
16751730	f	md	JP	2019	1	0	0	0	0	0	0	0	0	0	0	0	
16753685	m	cn	JP	2019	0	1	1	0	0	0	0	0	0	0	0	1	70
16756327	Not	Not repo	IT	2019	1	0	0	0	0	0	0	0	0	0	0	0	
16757016	m	md	MY	2019	0	1	1	0	0	0	0	0	0	0	0	0	73
16760008	m	Not repo	JP	2019	0	1	0	1	0	0	0	0	0	0	0	1	68.5
16762272	m	ot	KR	2019	0	1	0	0	1	0	0	0	0	0	0	0	71
16773026	f	md	JP	2019	0	1	0	1	0	0	0	0	0	0	0	1	70.7
16782485	f	md	JP	2019	1	0	0	0	0	0	0	0	0	0	0	1	69.5
16792437	m	cn	CN	2019	0	0	0	0	0	0	0	0	0	0	0	0	70
16793119	Not	md	JP	2019	1	0	0	0	0	0	0	0	0	0	0	0	
16795696	m	md	JP	2019	0	1	1	0	0	0	0	0	0	0	0	0	53
16796968	m	cn	CN	2019	1	0	0	0	0	0	0	0	0	0	0	0	
16797919	m	ph	JP	2019	0	1	1	0	0	0	0	0	0	0	0	1	84
16805638	f	ot	IT	2019	1	0	0	0	0	0	0	0	0	0	0	0	39.5
16816146	m	md	DE	2019	0	1	1	0	0	0	0	0	0	0	0	0	73
16817163	m	md	JP	2019	1	0	0	0	0	0	0	0	0	0	0	0	69
16824056	m	cn	JP	2019	0	0	0	0	0	0	0	0	0	0	0	0	54
16828165	m	md	FR	2019	0	1	0	0	1	0	0	0	0	0	0	0	63
16829637	m	cn	JP	2019	0	1	1	0	0	0	0	0	0	0	0	1	77
16829770	f	ph	US	2019	0	1	1	0	0	0	0	0	0	0	0	0	79
16831367	m	md	JP	2019	0	1	0	1	0	0	0	0	0	0	0	1	46
16831515	m	md	FR	2019	0	1	0	0	0	1	0	0	0	0	0	0	64
16832232	m	ph	US	2019	0	1	0	1	0	0	0	0	0	0	0	0	78
16837578	m	ph	JP	2019	0	1	1	0	0	0	0	0	0	0	0	1	
16838310	m	cn	KR	2019	1	0	0	0	0	0	0	0	0	0	0	0	37
16841557	f	Not repo	CN	2019	1	0	0	0	0	0	0	0	0	0	0	0	61

16844198	m	md	MX	2019	0	1	0	0	0	1	0	0	0	0	0	0	77
16851357	m	cn	JP	2019	0	1	1	0	0	0	0	0	0	0	0	0	80
16854883	f	cn	CN	2019	1	0	0	0	0	0	0	0	0	0	0	0	57
16857770	m	md	JP	2019	0	1	0	1	0	0	0	0	0	0	0	1	58.9
16857998	f	cn	DE	2019	0	0	0	0	0	0	0	0	0	0	0	0	
16859195	m	cn	CN	2019	1	0	0	0	0	0	0	0	0	0	0	0	69
16859460	m	md	CA	2019	0	1	1	0	0	0	0	0	0	0	0	0	81
16860446	m	md	ES	2019	0	1	1	0	0	0	0	0	0	0	0	0	69
16862218	f	cn	CN	2019	1	0	0	0	0	0	0	0	0	0	0	0	46
16862351	f	Not repo	CN	2019	1	0	0	0	0	0	0	0	0	0	0	0	79.9
16863279	f	Not repo	CN	2019	1	0	0	0	0	0	0	0	0	0	0	0	
16863462	m	md	FR	2019	0	1	1	0	0	0	0	0	0	0	0	0	40
16863512	f	cn	CN	2019	1	0	0	0	0	0	0	0	0	0	0	0	42
16863513	f	Not repo	CN	2019	1	0	0	0	0	0	0	0	0	0	0	0	73
16863514	f	Not repo	CN	2019	1	0	0	0	0	0	0	0	0	0	0	0	48.8
16863844	m	md	JP	2019	0	1	0	1	0	0	0	0	0	0	0	1	78
16864247	m	md	FR	2019	0	1	0	0	1	0	0	0	0	0	0	0	64
16867799	m	ot	DE	2019	0	1	0	0	1	0	0	0	0	0	0	0	68
16870041	m	md	CH	2019	0	0	0	0	0	0	0	0	0	0	0	0	67
16870297	m	ot	SG	2019	0	1	1	0	0	0	0	0	0	0	0	0	57
16871267	m	cn	US	2019	1	0	0	0	0	0	0	0	0	0	0	0	69
16871286	m	cn	JP	2019	0	1	1	0	0	0	0	0	0	0	0	0	69
16871369	m	md	ES	2019	0	0	0	0	0	0	0	0	0	0	0	0	42
16871679	f	cn	CN	2019	1	0	0	0	0	0	0	0	0	0	0	0	41
16871953	m	ot	KR	2019	0	1	0	0	1	0	0	0	0	0	0	1	74
16872026	f	Not repo	CN	2019	1	0	0	0	0	0	0	0	0	0	0	0	54
16872088	Not	ot	IT	2019	1	0	0	0	0	0	0	0	0	0	0	0	
16872256	m	md	JP	2019	0	0	0	0	0	0	0	0	0	0	0	0	66
16872701	m	cn	US	2019	1	0	0	0	0	0	0	0	0	0	0	0	
16872742	Not	ot	IT	2019	1	0	0	0	0	0	0	0	0	0	0	0	
16872830	m	ot	JP	2019	0	1	0	1	0	0	0	0	0	0	0	0	65
16872937	m	cn	JP	2019	0	1	1	0	0	0	0	0	0	0	0	0	67
16872944	f	ph	IT	2019	0	0	0	0	0	0	0	0	0	0	0	0	42
16873006	Not	ot	JP	2019	0	0	0	0	0	0	0	0	0	0	0	0	

16873179	f	md	TW	2019	1	0	0	0	0	0	0	0	0	0	0	0	
16873280	m	cn	CN	2019	1	0	0	0	0	0	0	0	0	0	0	0	63
16873289	m	md	JP	2019	1	0	0	0	0	0	0	0	0	0	0	1	76.6
16873383	f	cn	CN	2019	1	0	0	0	0	0	0	0	0	0	0	0	63
16873628	m	md	IN	2019	0	0	0	0	0	0	0	0	0	0	0	0	60
16873721	m	md	JP	2019	0	1	0	1	0	0	0	0	0	0	0	0	70
16873740	m	cn	HU	2019	0	1	1	0	0	0	0	0	0	0	0	0	71
16873791	f	ot	US	2019	0	1	0	0	1	0	0	0	0	0	0	0	60
16873940	m	md	BR	2019	1	0	0	0	0	0	0	0	0	0	0	0	48
16873979	f	Not repo	CN	2019	1	0	0	0	0	0	0	0	0	0	0	0	67
16874059	m	ot	US	2019	0	1	0	0	1	0	0	0	0	0	0	1	66
16874168	f	Not repo	CN	2019	1	0	0	0	0	0	0	0	0	0	0	0	58
16874260	Not	ot	IT	2019	1	0	0	0	0	0	0	0	0	0	0	0	
16874305	m	md	JP	2019	1	0	0	0	0	0	0	0	0	0	0	1	87
16874484	f	cn	CN	2019	1	0	0	0	0	0	0	0	0	0	0	0	
16874561	f	Not repo	CN	2019	1	0	0	0	0	0	0	0	0	0	0	0	71
16874573	m	ot	CN	2019	0	0	0	0	0	0	0	0	0	0	0	0	65
16874613	f	md	US	2019	0	0	0	0	0	0	0	0	0	0	0	0	53
16874681	m	cn	US	2019	0	0	0	0	0	0	0	0	0	0	0	0	
16875215	f	ot	ZA	2019	0	1	0	0	1	0	0	0	0	0	0	0	56
16875251	m	Not repo	CN	2019	1	0	0	0	0	0	0	0	0	0	0	0	77
16875409	m	ph	JP	2019	0	0	0	0	0	0	0	0	0	0	0	0	68
16875519	f	cn	JP	2019	0	1	1	0	0	0	0	0	0	0	0	0	81
16875733	f	ot	IT	2019	1	0	0	0	0	0	0	0	0	0	0	0	
16876550	f	Not repo	CN	2019	1	0	0	0	0	0	0	0	0	0	0	0	50
16876607	f	cn	SE	2019	0	1	1	0	0	0	0	0	0	0	0	0	63
16876655	m	Not repo	US	2019	0	1	0	1	0	0	0	0	0	0	0	1	62.9
16876820	m	cn	DE	2019	0	0	0	0	0	0	0	0	0	0	0	0	69
16876942	f	cn	CN	2019	1	0	0	0	0	0	0	0	0	0	0	0	51
16876943	m	cn	CN	2019	1	0	0	0	0	0	0	0	0	0	0	0	70
16876945	f	cn	CN	2019	1	0	0	0	0	0	0	0	0	0	0	0	48
16877181	m	md	CZ	2019	0	0	0	0	0	0	0	0	0	0	0	0	
16877319	f	Not repo	CN	2019	1	0	0	0	0	0	0	0	0	0	0	0	46
16877438	f	cn	US	2019	0	0	0	0	0	0	0	0	0	0	0	0	82

16877489	m	Not repo	JP	2019	1	0	0	0	0	0	0	0	0	0	0	0	70.6
16877715	m	cn	FR	2019	0	1	1	0	0	0	0	0	0	0	0	0	81
16877733	Not	md	JP	2019	0	1	0	1	0	0	0	0	0	0	0	1	
16877862	f	cn	Not re	2019	0	0	0	0	0	0	0	0	0	0	0	0	
16877934	m	Not repo	CN	2019	1	0	0	0	0	0	0	0	0	0	0	0	68.1
16878399	m	cn	JP	2019	0	1	1	0	0	0	0	0	0	0	0	0	72
16878407	m	md	RU	2019	0	1	1	0	0	0	0	0	0	0	0	0	60
16878533	Not	ot	BE	2019	0	0	0	0	0	0	0	0	0	0	0	0	
16878690	Not	ot	BE	2019	0	0	0	0	0	0	0	0	0	0	0	0	
16878820	f	cn	DE	2019	0	1	1	0	0	0	0	0	0	0	0	0	
16879383	f	ph	JP	2019	0	1	1	0	0	0	0	0	0	0	0	0	74
16879530	f	md	JP	2019	0	0	0	0	0	0	0	0	0	0	0	0	72
16879772	m	md	IT	2019	1	0	0	0	0	0	0	0	0	0	0	0	
16879775	m	md	JP	2019	0	1	0	1	0	0	0	0	0	0	0	1	70
16880490	f	md	US	2019	0	1	0	0	0	1	0	0	0	0	0	0	47
16880740	f	Not repo	CN	2019	1	0	0	0	0	0	0	0	0	0	0	0	49
16880800	m	md	FR	2019	0	1	0	0	1	0	0	0	0	0	0	0	63
16880858	f	ot	NL	2019	0	1	1	0	0	0	0	0	0	0	0	0	
16880920	Not	ph	JP	2019	1	0	0	0	0	0	0	0	0	0	0	0	
16880933	m	md	PL	2019	0	0	0	0	0	0	0	0	0	0	0	0	61
16881246	m	ph	US	2019	0	1	0	0	1	0	0	0	0	0	0	0	73
16881615	Not	ot	US	2019	0	1	0	0	1	0	0	0	0	0	0	0	
16881743	m	md	US	2019	0	1	0	0	1	0	0	0	0	0	0	0	68
16881762	m	md	FR	2019	0	1	0	0	1	0	0	0	0	0	0	0	63
16881845	f	Not repo	CN	2019	1	0	0	0	0	0	0	0	0	0	0	0	75
16881887	f	ot	US	2019	0	1	0	0	1	0	0	0	0	0	0	0	71
16882181	f	cn	AR	2019	0	1	1	0	0	0	0	0	0	0	0	0	61
16882505	m	ot	CA	2019	0	1	0	0	1	0	0	0	0	0	0	0	59
16882536	m	ot	JP	2019	0	1	1	0	0	0	0	0	0	0	0	1	79
16882699	f	md	US	2019	0	1	0	1	0	0	0	0	0	0	0	0	58
16882774	m	md	JP	2019	0	1	0	0	1	0	0	0	0	0	0	0	46
16883341	f	ot	MX	2019	0	1	0	0	1	0	0	0	0	0	0	0	60
16883454	Not	ot	JP	2019	1	0	0	0	0	0	0	0	0	0	0	0	
16883462	m	md	IT	2019	0	1	0	1	0	0	0	0	0	0	0	1	80.4

16883476	m	md	US	2019	0	1	0	0	1	0	0	0	0	0	0	0	68
16883654	f	cn	CN	2019	1	0	0	0	0	0	0	0	0	0	0	0	62
16883736	m	md	CA	2019	0	1	1	0	0	0	0	0	0	0	0	0	68
16883749	m	md	DE	2019	0	1	0	0	1	0	0	0	0	0	0	0	57
16883836	m	cn	JP	2019	0	1	1	0	0	0	0	0	0	0	0	0	63
16883873	f	cn	AR	2019	0	0	0	0	0	0	0	0	0	0	0	0	73
16884244	m	md	JP	2019	0	1	0	0	0	1	0	0	0	0	0	1	80
16884247	Not	cn	JP	2019	0	1	1	0	0	0	0	0	0	0	0	0	90
16884262	f	md	US	2019	0	1	0	1	0	0	0	0	0	0	0	0	70
16884466	f	md	US	2019	0	0	0	0	0	0	0	0	0	0	0	0	39
16884613	f	cn	CN	2019	1	0	0	0	0	0	0	0	0	0	0	0	89
16884698	f	Not repo	CN	2019	1	0	0	0	0	0	0	0	0	0	0	0	56
16884706	m	md	PT	2019	0	0	0	0	0	0	0	0	0	0	0	0	44
16884891	m	md	JP	2019	0	1	0	1	0	0	0	0	0	0	0	1	
16884894	m	md	JP	2019	0	1	0	1	0	0	0	0	0	0	0	1	72
16884899	f	md	JP	2019	1	0	0	0	0	0	0	0	0	0	0	0	79
16884901	f	cn	CN	2019	1	0	0	0	0	0	0	0	0	0	0	0	64
16884914	f	ot	US	2019	0	1	0	0	1	0	0	0	0	0	0	0	54
16884999	f	cn	CN	2019	1	0	0	0	0	0	0	0	0	0	0	0	64
16885004	f	Not repo	CN	2019	1	0	0	0	0	0	0	0	0	0	0	0	58
16885117	m	Not repo	CN	2019	1	0	0	0	0	0	0	0	0	0	0	0	62
16885283	f	cn	US	2019	1	0	0	0	0	0	0	0	0	0	0	0	72
16885364	m	ot	JP	2019	0	1	0	0	1	0	0	0	0	0	0	0	69
16885404	f	Not repo	CN	2019	1	0	0	0	0	0	0	0	0	0	0	0	69
16885689	f	ot	US	2019	0	1	0	0	1	0	0	0	0	0	0	0	61
16885746	m	ph	JP	2019	0	1	0	1	0	0	0	0	0	0	0	1	69
16885848	m	md	JP	2019	1	0	0	0	0	0	0	0	0	0	0	0	42
16885850	f	cn	CN	2019	1	0	0	0	0	0	0	0	0	0	0	0	64.2
16885859	Not	cn	JP	2019	0	1	1	0	0	0	0	0	0	0	0	0	
16885860	f	md	JP	2019	0	0	0	0	0	0	0	0	0	0	0	0	66
16885907	f	md	JP	2019	0	1	0	1	0	0	0	0	0	0	0	1	80
16886300	f	cn	KR	2019	0	0	0	0	0	0	0	0	0	0	0	0	58
16886302	m	Not repo	US	2019	1	0	0	0	0	0	0	0	0	0	0	0	65.2
16886317	m	cn	CN	2019	1	0	0	0	0	0	0	0	0	0	0	0	74

16886433	f	cn	CN	2019	1	0	0	0	0	0	0	0	0	0	0	0	61
16886449	m	md	JP	2019	0	1	1	0	0	0	0	0	0	0	0	0	57
16886453	m	ot	US	2019	0	1	0	0	1	0	0	0	0	0	0	0	72
16886743	f	Not repo	CN	2019	1	0	0	0	0	0	0	0	0	0	0	0	64
16886744	m	cn	CN	2019	1	0	0	0	0	0	0	0	0	0	0	0	62
16886843	m	md	JP	2019	0	1	0	1	0	0	0	0	0	0	0	0	82.6
16886844	f	md	FR	2019	0	1	0	1	0	0	0	0	0	0	0	0	
16887022	m	md	DE	2019	0	0	0	0	0	0	0	0	0	0	0	0	88
16887050	f	cn	CN	2019	1	0	0	0	0	0	0	0	0	0	0	0	65
16887058	f	cn	JP	2019	0	1	1	0	0	0	0	0	0	0	0	0	70
16887146	Not	cn	US	2019	0	1	1	0	0	0	0	0	0	0	0	0	
16887547	f	ot	US	2019	0	1	0	0	1	0	0	0	0	0	0	0	60
16887572	m	cn	CN	2019	0	1	0	0	1	0	0	0	0	0	0	0	65
16887594	m	cn	CN	2019	0	1	0	0	1	0	0	0	0	0	0	0	
16888326	m	cn	CN	2019	0	1	0	0	1	0	0	0	0	0	0	1	56
16888906	m	cn	JP	2019	0	1	1	0	0	0	0	0	0	0	0	0	55
16889238	m	cn	JP	2019	0	1	1	0	0	0	0	0	0	0	0	0	49
16889268	m	md	US	2019	0	1	1	0	0	0	0	0	0	0	0	0	
16889438	f	cn	CN	2019	1	0	0	0	0	0	0	0	0	0	0	0	75
16889450	f	Not repo	CN	2019	1	0	0	0	0	0	0	0	0	0	0	0	65
16889500	f	Not repo	CN	2019	1	0	0	0	0	0	0	0	0	0	0	0	64
16889785	f	md	TW	2019	1	0	0	0	0	0	0	0	0	0	0	0	60
16890184	m	md	IL	2019	0	1	0	0	0	1	0	0	0	0	0	0	68
16890555	m	cn	CN	2019	1	0	0	0	0	0	0	0	0	0	0	0	69
16890739	f	md	JP	2019	0	0	0	0	0	0	1	1	0	0	0	0	82
16890817	m	cn	JP	2019	0	1	1	0	0	0	0	0	0	0	0	0	
16890961	f	cn	JP	2019	0	1	1	0	0	0	0	0	0	0	0	0	70
16890994	m	cn	CN	2019	1	0	0	0	0	0	0	0	0	0	0	0	
16891074	f	Not repo	CN	2019	1	0	0	0	0	0	0	0	0	0	0	0	
16891075	f	Not repo	CN	2019	1	0	0	0	0	0	0	0	0	0	0	0	69
16891529	f	cn	CN	2019	1	0	0	0	0	0	0	0	0	0	0	0	70
16891531	f	cn	CN	2019	1	0	0	0	0	0	0	0	0	0	0	0	57
16891532	f	cn	CN	2019	1	0	0	0	0	0	0	0	0	0	0	0	61.8
16891923	m	ot	NL	2019	0	1	0	0	0	1	0	0	0	0	0	0	67

16892267	f	Not repo	MY	2019	1	0	0	0	0	0	0	0	0	0	0	0	70.8
16892273	f	md	IT	2019	1	0	0	0	0	0	0	0	0	0	0	0	
16892282	m	cn	CN	2019	1	0	0	0	0	0	0	0	0	0	0	0	64
16892467	m	md	JP	2019	0	1	0	1	0	0	0	0	0	0	0	1	72
16892679	m	cn	JP	2019	0	1	1	0	0	0	0	0	0	0	0	0	82
16892941	f	Not repo	CN	2019	1	0	0	0	0	0	0	0	0	0	0	0	59
16892977	m	md	JP	2019	0	1	0	0	0	1	0	0	0	0	0	0	80
16893299	f	ot	JP	2019	0	1	0	1	0	0	0	0	0	0	0	0	71
16893300	f	Not repo	CN	2019	1	0	0	0	0	0	0	0	0	0	0	0	70
16893301	f	cn	CN	2019	1	0	0	0	0	0	0	0	0	0	0	0	79
16893363	f	cn	CN	2019	1	0	0	0	0	0	0	0	0	0	0	0	72
16893517	f	Not repo	CN	2019	1	0	0	0	0	0	0	0	0	0	0	0	55.3
16893543	f	md	ZA	2019	1	0	0	0	0	0	0	0	0	0	0	0	
16893677	m	md	FR	2019	0	1	1	0	0	0	0	0	0	0	0	0	58
16894237	f	cn	CN	2019	1	0	0	0	0	0	0	0	0	0	0	0	65
16894371	m	Not repo	CN	2019	1	0	0	0	0	0	0	0	0	0	0	0	81
16894373	f	md	JP	2019	1	0	0	0	0	0	0	0	0	0	0	1	66.8
16894858	m	cn	US	2019	0	1	1	0	0	0	0	0	0	0	0	0	45
16894920	m	cn	CN	2019	1	0	0	0	0	0	0	0	0	0	0	0	38
16894922	m	Not repo	JP	2019	0	1	0	1	0	0	0	0	0	0	0	1	71.4
16894933	f	cn	CN	2019	1	0	0	0	0	0	0	0	0	0	0	0	59
16895322	f	Not repo	ID	2019	1	0	0	0	0	0	0	0	0	0	0	0	
16895373	m	cn	US	2019	1	0	0	0	0	0	0	0	0	0	0	0	56
16895408	m	Not repo	ID	2019	1	0	0	0	0	0	0	0	0	0	0	0	
16895473	f	cn	CN	2019	1	0	0	0	0	0	0	0	0	0	0	0	76
16895782	f	cn	CN	2019	1	0	0	0	0	0	0	0	0	0	0	0	60
16895847	f	cn	CN	2019	1	0	0	0	0	0	0	0	0	0	0	0	62
16895891	m	md	GB	2019	0	1	1	0	0	0	0	0	0	0	0	0	68
16896287	m	md	GB	2019	0	1	1	0	0	0	0	0	0	0	0	0	67
16896322	m	md	CN	2019	1	0	0	0	0	0	0	0	0	0	0	0	45
16896460	m	md	PT	2019	0	1	0	0	1	0	0	0	0	0	0	0	55
16896468	m	md	HU	2019	0	0	0	0	0	0	0	0	0	0	0	0	
16896614	f	md	CH	2019	0	1	1	0	0	0	0	0	0	0	0	0	67
16897087	m	ot	US	2019	0	1	0	0	1	0	0	0	0	0	0	0	60

16897089	f	cn	JP	2019	0	1	1	0	0	0	0	0	0	0	0	0	78
16897182	Not	md	FR	2019	0	1	0	0	1	0	0	0	0	0	0	0	
16897356	m	ot	PL	2019	0	1	0	0	1	0	0	0	0	0	0	0	79
16897536	f	ot	US	2019	0	1	1	0	0	0	0	0	0	0	0	0	62
16898407	Not	ph	JP	2019	1	0	0	0	0	0	0	0	0	0	0	0	
16898411	m	ot	FR	2019	0	1	0	0	1	0	0	0	0	0	0	0	72
16898476	f	Not repo	CN	2019	1	0	0	0	0	0	0	0	0	0	0	0	45
16898638	m	md	JP	2019	1	0	0	0	0	0	0	0	0	0	0	0	80
16898642	m	md	KR	2019	1	0	0	0	0	0	0	0	0	0	0	0	67.3
16898643	m	cn	CN	2019	1	0	0	0	0	0	0	0	0	0	0	0	73
16898646	m	md	JP	2019	0	1	0	1	0	0	0	0	0	0	0	0	72
16899128	Not	md	US	2019	1	0	0	0	0	0	0	0	0	0	0	0	
16899141	f	ph	US	2019	1	0	0	0	0	0	0	0	0	0	0	0	69
16899415	f	ot	JP	2019	0	1	0	0	1	0	0	0	0	0	0	0	69
16899754	f	md	NL	2019	0	1	0	0	0	1	0	0	0	0	0	0	59
16900100	m	Not repo	CN	2019	1	0	0	0	0	0	0	0	0	0	0	0	
16900260	m	ph	FR	2019	0	1	0	1	0	0	0	0	0	0	0	0	48.5
16900477	f	ot	US	2019	0	1	0	0	1	0	0	0	0	0	0	0	62
16900487	f	md	JP	2019	0	1	0	0	1	0	0	0	0	0	0	0	75
16901085	m	cn	CA	2019	0	1	1	0	0	0	0	0	0	0	0	0	90
16901329	f	md	DE	2019	0	1	0	0	1	0	0	0	0	0	0	0	58
16901565	m	md	JP	2019	0	0	0	0	0	0	0	0	0	0	0	0	48
16901579	m	md	JP	2019	0	1	0	0	0	1	0	0	0	0	0	0	
16901595	m	ot	RS	2019	0	1	0	0	0	1	0	0	0	0	0	0	
16901681	m	md	DE	2019	0	0	0	0	0	0	0	0	0	0	0	0	59
16901735	f	md	PT	2019	0	1	0	0	1	0	0	0	0	0	0	0	44
16902291	f	cn	US	2019	1	0	0	0	0	0	0	0	0	0	0	0	81
16902646	f	ot	US	2019	0	1	0	0	1	0	0	0	0	0	0	0	68
16902657	f	cn	US	2019	1	0	0	0	0	0	0	0	0	0	0	0	71
16902721	f	cn	IN	2019	0	1	0	0	0	1	0	0	0	0	0	0	61
16902918	m	ph	US	2019	0	0	0	0	0	0	0	0	0	0	0	0	69
16902987	f	cn	CN	2019	0	1	0	0	1	0	0	0	0	0	0	0	
16903142	f	md	JP	2019	0	1	1	0	0	0	0	0	0	0	0	0	68
16903407	m	md	US	2019	0	1	1	0	0	0	0	0	0	0	0	0	

16903941	m	ot	CA	2019	0	1	0	0	1	0	0	0	0	0	0	0	82
16904001	f	ot	FR	2019	0	0	0	0	0	0	0	0	0	0	0	0	59
16904571	m	md	CN	2019	0	1	0	0	0	1	0	0	0	0	0	0	66
16904573	f	md	JP	2019	1	0	0	0	0	0	0	0	0	0	0	0	78
16904959	f	cn	JP	2019	0	1	1	0	0	0	0	0	0	0	0	0	75
16904993	m	ot	JP	2019	0	1	0	1	0	0	0	0	0	0	0	0	77.7
16905093	f	ot	IT	2019	1	0	0	0	0	0	0	0	0	0	0	0	
16905665	m	md	JP	2019	0	1	0	0	1	0	0	0	0	0	0	0	69
16905738	m	cn	JP	2019	0	1	1	0	0	0	0	0	0	0	0	0	61
16905898	f	cn	CN	2019	1	0	0	0	0	0	0	0	0	0	0	0	56.3
16906144	m	cn	JP	2019	0	1	1	0	0	0	0	0	0	0	0	0	77
16906508	f	md	JP	2019	1	0	0	0	0	0	0	0	0	0	0	0	80
16906818	m	cn	JP	2019	0	1	1	0	0	0	0	0	0	0	0	1	57
16906977	m	md	BY	2019	0	1	0	0	0	1	0	0	0	0	0	0	
16907380	f	Not repo	JP	2019	1	0	0	0	0	0	0	0	0	0	0	0	85.4
16907586	m	ot	FR	2019	0	1	0	0	1	0	0	0	0	0	0	0	62
16907592	Not	md	JP	2019	0	1	0	0	1	0	0	0	0	0	0	0	
16907746	m	Not repo	JP	2019	1	0	0	0	0	0	0	0	0	0	0	0	77.6
16908178	Not	ot	US	2019	0	0	0	0	0	0	0	0	0	0	0	0	
16908253	m	cn	DE	2019	0	1	1	0	0	0	0	0	0	0	0	0	
16908314	f	md	JP	2019	1	0	0	0	0	0	0	0	0	0	0	0	75
16908413	m	md	JP	2019	0	1	0	1	0	0	0	0	0	0	0	0	76
16908708	f	Not repo	US	2019	1	0	0	0	0	0	0	0	0	0	0	0	69.3
16908717	f	ot	US	2019	0	1	1	0	0	0	0	0	0	0	0	0	45
16908892	m	md	US	2019	0	0	0	0	0	0	0	0	0	0	0	0	60
16909134	m	cn	CN	2019	1	0	0	0	0	0	0	0	0	0	0	0	56
16909141	m	cn	CN	2019	1	0	0	0	0	0	0	0	0	0	0	0	68
16909172	f	ot	FR	2019	0	0	0	0	0	0	0	0	0	0	0	0	59
16909230	m	cn	JP	2019	1	0	0	0	0	0	0	0	0	0	0	0	
16909308	m	cn	US	2019	0	1	1	0	0	0	0	0	0	0	0	0	
16909416	f	cn	CN	2019	1	0	0	0	0	0	0	0	0	0	0	0	
16909558	m	Not repo	CA	2019	0	1	0	1	0	0	0	0	0	0	0	1	
16909560	m	md	JP	2019	0	1	0	1	0	0	0	0	0	0	0	1	79
16909611	f	Not repo	ID	2019	1	0	0	0	0	0	0	0	0	0	0	0	51.9

16909740	f	Not repo	US	2019	1	0	0	0	0	0	0	0	0	0	0	0	86.1
16909775	f	Not repo	CN	2019	1	0	0	0	0	0	0	0	0	0	0	0	46
16909943	f	md	JP	2019	0	1	0	1	0	0	0	0	0	0	0	1	64
16910082	m	cn	JP	2019	0	1	1	0	0	0	0	0	0	0	0	1	67
16910227	m	cn	JP	2019	0	0	0	0	0	0	0	0	0	0	0	0	80
16910276	m	cn	JP	2019	0	1	1	0	0	0	0	0	0	0	0	0	70
16910348	m	ot	CN	2019	0	1	0	0	1	0	0	0	0	0	0	1	63
16910385	f	md	CN	2019	1	0	0	0	0	0	0	0	0	0	0	0	60
16910469	m	ot	US	2019	0	1	0	0	1	0	0	0	0	0	0	0	77
16911024	f	md	CZ	2019	0	0	0	0	0	0	0	0	0	0	0	0	61
16911170	m	ph	GB	2019	0	1	1	0	0	0	0	0	0	0	0	1	77
16911736	m	md	US	2019	0	0	0	0	0	0	0	0	0	0	0	0	73
16912746	m	md	JP	2019	0	1	0	0	0	1	0	0	0	0	0	0	69
16913226	f	ot	US	2019	1	0	0	0	0	0	0	0	0	0	0	0	72
16913229	f	md	PT	2019	0	0	0	0	0	0	0	0	0	0	0	0	71
16913415	f	ot	DE	2019	0	0	0	0	0	0	0	0	0	0	0	0	
16913844	m	cn	JP	2019	0	1	1	0	0	0	0	0	0	0	0	1	71
16914159	f	md	MX	2019	0	1	1	0	0	0	0	0	0	0	0	0	53
16914905	m	cn	CN	2019	1	0	0	0	0	0	0	0	0	0	0	0	50
16915081	f	md	JP	2019	1	0	0	0	0	0	0	0	0	0	0	0	68
16915095	m	cn	US	2019	0	0	0	0	0	0	0	0	0	0	0	0	
16915206	f	md	JP	2019	1	0	0	0	0	0	0	0	0	0	0	1	81.6
16915250	m	Not repo	JP	2019	0	1	0	1	0	0	0	0	0	0	0	1	77
16915266	m	cn	CN	2019	1	0	0	0	0	0	0	0	0	0	0	0	52
16915958	m	cn	JP	2019	0	1	1	0	0	0	0	0	0	0	0	1	81
16916465	m	cn	US	2019	1	0	0	0	0	0	0	0	0	0	0	0	
16916512	Not	Not repo	JP	2019	1	0	0	0	0	0	0	0	0	0	0	0	
16916892	m	md	ES	2019	0	1	0	0	0	1	0	0	0	0	0	0	83
16916990	m	md	US	2019	0	1	0	0	0	1	0	0	0	0	0	0	75
16917138	m	cn	MM	2019	0	1	0	0	1	0	0	0	0	0	0	0	57
16917484	m	ot	CA	2019	0	0	0	0	0	0	0	0	0	0	0	0	75
16917763	Not	ot	CN	2019	1	0	0	0	0	0	0	0	0	0	0	0	
16918472	f	ot	AT	2019	0	1	0	0	0	1	0	0	0	0	0	0	59
16918637	f	ot	IT	2019	0	0	0	0	0	0	0	0	0	0	0	0	73

16918686	f	cn	JP	2019	0	1	1	0	0	0	0	0	0	0	0	0	61
16918700	m	cn	JP	2019	0	1	1	0	0	0	0	0	0	0	0	0	66
16919299	m	cn	JP	2019	0	1	1	0	0	0	0	0	0	0	0	1	77
16919557	f	md	US	2019	0	0	0	0	0	0	0	0	0	0	0	0	60
16919668	f	md	IN	2019	0	1	0	1	0	0	0	0	0	0	0	0	53
16920054	f	cn	US	2019	1	0	0	0	0	0	0	0	0	0	0	0	46.9
16920813	m	cn	JP	2019	0	1	1	0	0	0	0	0	0	0	0	0	48
16920840	m	md	JP	2019	1	0	0	0	0	0	0	0	0	0	0	0	71
16921206	m	cn	KR	2019	0	0	0	0	0	0	0	0	0	0	0	0	
16921699	f	md	DE	2019	0	0	0	0	0	0	0	0	0	0	0	0	
16922299	f	md	FI	2019	0	1	0	0	0	1	0	0	0	0	0	0	
16922351	Not	md	IT	2019	1	0	0	0	0	0	0	0	0	0	0	0	
16922446	m	md	DE	2019	0	1	0	0	0	1	0	0	0	0	0	0	71
16922620	f	md	SG	2019	0	1	0	0	0	1	0	0	0	0	0	0	
16923003	m	md	BO	2019	0	1	0	0	0	1	0	0	0	0	0	0	
16923225	Not	md	JP	2019	1	0	0	0	0	0	0	0	0	0	0	0	
16923307	m	cn	JP	2019	0	1	1	0	0	0	0	0	0	0	0	0	25
16923663	f	md	US	2019	0	1	0	0	1	0	0	0	0	0	0	0	65
16924370	m	cn	JP	2019	0	0	0	0	0	0	0	0	0	0	0	0	80
16924590	m	cn	JP	2019	0	1	1	0	0	0	0	0	0	0	0	0	62
16924736	Not	md	SA	2019	0	0	0	0	0	0	0	0	0	0	0	0	
16925083	f	cn	US	2019	0	0	0	0	0	0	0	0	0	0	0	0	58
16925107	m	md	US	2019	0	1	1	0	0	0	0	0	0	0	0	0	86
16925271	f	ph	US	2019	1	0	0	0	0	0	0	0	0	0	0	0	69
16925554	m	ot	US	2019	0	1	0	0	1	0	0	0	0	0	0	0	64
16925762	m	md	JP	2019	0	1	0	1	0	0	0	0	0	0	0	1	67
16925771	m	ph	JP	2019	0	1	1	0	0	0	0	0	0	0	0	0	78
16925845	m	cn	GB	2019	0	1	1	0	0	0	0	0	0	0	0	0	
16926165	Not	md	JP	2019	0	1	0	1	0	0	0	0	0	0	0	1	70
16926915	f	ot	CH	2019	0	1	1	0	0	0	0	0	0	0	0	0	65
16926974	m	cn	JP	2019	0	1	1	0	0	0	0	0	0	0	0	0	90
16927177	f	cn	GB	2019	0	1	1	0	0	0	0	0	0	0	0	0	50
16927401	f	ot	US	2019	0	1	0	0	1	0	0	0	0	0	0	0	66
16927771	Not	md	BE	2019	0	1	0	0	0	1	0	0	0	0	0	0	

16927936	m	md	JP	2019	0	1	0	0	0	1	0	0	0	0	0	0	66
16928006	m	md	MX	2019	0	1	0	0	0	1	0	0	0	0	0	0	76
16928070	m	md	US	2019	0	1	1	0	0	0	0	0	0	0	0	0	
16928103	m	cn	JP	2019	0	1	1	0	0	0	0	0	0	0	0	0	59
16928153	m	cn	JP	2019	0	1	1	0	0	0	0	0	0	0	0	0	65
16928230	m	md	HK	2019	0	1	0	0	0	1	0	0	0	0	0	0	64
16928415	m	md	JP	2019	0	1	0	0	0	1	0	0	0	0	0	0	52
16928430	f	md	JP	2019	0	1	0	1	0	0	0	0	0	0	0	0	81
16928761	m	md	CL	2019	0	1	0	0	0	1	0	0	0	0	0	0	72
16929229	m	cn	JP	2019	0	1	1	0	0	0	0	0	0	0	0	0	61
16929290	f	md	DK	2019	0	0	0	0	0	0	0	0	0	0	0	0	64
16929291	f	md	DK	2019	0	0	0	0	0	0	0	0	0	0	0	0	81
16929537	f	md	DK	2019	0	0	0	0	0	0	0	0	0	0	0	0	55
16929539	m	md	DK	2019	0	0	0	0	0	0	0	0	0	0	0	0	74
16929771	m	ot	US	2019	0	0	0	0	0	0	0	0	0	0	0	0	90
16929899	m	cn	JP	2019	0	1	1	0	0	0	0	0	0	0	0	1	70
16929987	m	md	TW	2019	0	0	0	0	0	0	0	0	0	0	0	0	78
16929993	m	md	KR	2019	0	0	0	0	0	0	0	0	0	0	0	0	
16930399	f	cn	JP	2019	0	1	1	0	0	0	0	0	0	0	0	0	45
16930646	m	md	US	2019	0	0	0	0	0	0	0	0	0	0	0	0	58
16931050	f	Not repo	US	2019	1	0	0	0	0	0	0	0	0	0	0	0	63
16931243	m	md	LB	2019	1	0	0	0	0	0	0	0	0	0	0	0	84
16931377	m	ot	AU	2019	0	1	0	0	1	0	0	0	0	0	0	0	58
16931421	Not	md	JP	2019	1	0	0	0	0	0	0	0	0	0	0	0	
16931564	f	cn	DE	2019	0	0	0	0	0	0	0	0	0	0	0	0	76
16931636	m	md	JP	2019	1	0	0	0	0	0	0	0	0	0	0	0	74
16932126	f	ph	JP	2019	0	1	0	0	1	0	0	0	0	0	0	0	67
16932273	m	cn	GR	2019	0	1	1	0	0	0	0	0	0	0	0	0	68
16932538	m	md	JP	2019	0	1	0	1	0	0	0	0	0	0	0	0	
16932541	f	md	JP	2019	0	1	0	1	0	0	0	0	0	0	0	1	
16932659	m	ot	US	2019	0	1	0	1	0	0	0	0	0	0	0	0	63
16933299	f	md	JP	2019	0	1	0	1	0	0	0	0	0	0	0	1	65
16933656	Not	ph	JP	2019	1	0	0	0	0	0	0	0	0	0	0	0	
16933736	m	cn	JP	2019	0	1	1	0	0	0	0	0	0	0	0	0	78

16934109	f	md	SG	2019	0	1	0	0	0	1	0	0	0	0	0	0	73
16934139	f	md	DK	2019	0	0	0	0	0	0	0	0	0	0	0	0	74
16934174	m	ot	US	2019	0	1	0	0	1	0	0	0	0	0	0	0	
16934372	m	md	JP	2019	0	1	1	0	0	0	0	0	0	0	0	1	
16934627	f	ot	FR	2019	0	0	0	0	0	0	0	0	0	0	0	0	59
16934738	Not	md	JP	2019	0	1	0	1	0	0	0	0	0	0	0	0	78.9
16935369	m	md	SA	2019	0	1	1	0	0	0	0	0	0	0	0	0	65
16935404	m	md	KR	2019	0	1	0	0	0	1	0	0	0	0	0	0	77
16935664	f	Not repo	RU	2019	1	0	0	0	0	0	0	0	0	0	0	0	52.2
16935828	f	Not repo	US	2019	1	0	0	0	0	0	0	0	0	0	0	0	
16936333	m	md	KR	2019	0	0	0	0	0	0	0	0	0	0	0	0	59
16936369	Not	md	FR	2019	1	0	0	0	0	0	0	0	0	0	0	0	
16936425	f	md	US	2019	0	1	1	0	0	0	0	0	0	0	0	0	53
16936427	m	ot	NL	2019	0	1	1	0	0	0	0	0	0	0	0	0	67
16936713	f	ph	FR	2019	0	0	0	0	0	0	0	0	0	0	0	0	82
16936755	f	cn	DE	2019	0	1	1	0	0	0	0	0	0	0	0	0	73
16936756	m	md	DE	2019	0	1	0	0	0	1	0	0	0	0	0	0	61
16936767	Not	ot	US	2019	0	0	0	0	0	0	0	0	0	0	0	0	
16936852	m	ot	RO	2019	0	1	0	0	1	0	0	0	0	0	0	0	63
16936947	m	md	JP	2019	0	1	0	1	0	0	0	0	0	0	0	1	67
16936956	f	cn	Not re	2019	0	0	0	0	0	0	0	0	0	0	0	0	68
16937146	f	ot	IT	2019	1	0	0	0	0	0	0	0	0	0	0	0	77.3
16937219	f	cn	JP	2019	0	1	1	0	0	0	0	0	0	0	0	0	90
16937298	f	ph	JP	2019	0	1	0	0	1	0	0	0	0	0	0	0	70
16937350	m	md	JP	2019	0	1	0	1	0	0	0	0	0	0	0	0	46
16937359	f	md	JP	2019	0	1	0	1	0	0	0	0	0	0	0	0	65
16937426	Not	ot	IT	2019	1	0	0	0	0	0	0	0	0	0	0	0	
16937552	m	md	JP	2019	0	1	0	1	0	0	0	0	0	0	0	1	70
16937715	Not	md	PT	2019	0	0	0	0	0	0	0	0	0	0	0	0	
16937738	m	md	JP	2019	0	1	1	0	0	0	0	0	0	0	0	0	72
16937745	m	Not repo	JP	2019	0	1	0	1	0	0	0	0	0	0	0	1	47
16937793	f	cn	JP	2019	0	1	1	0	0	0	0	0	0	0	0	0	74
16937865	f	md	JP	2019	0	1	1	0	0	0	0	0	0	0	0	0	64
16937969	m	md	DE	2019	1	0	0	0	0	0	0	0	0	0	0	0	79

16938371	f	md	BA	2019	0	1	1	0	0	0	0	0	0	0	0	0	70
16938429	f	md	US	2019	0	0	0	0	0	0	0	0	0	0	0	0	
16938834	m	md	IT	2019	0	1	1	0	0	0	0	0	0	0	0	0	74
16938838	m	md	ES	2019	0	0	0	0	0	0	0	0	0	0	0	0	68
16938839	Not	ot	DE	2019	0	0	0	0	0	0	0	0	0	0	0	0	61
16939088	f	cn	JP	2019	0	1	1	0	0	0	0	0	0	0	0	1	83
16939165	m	md	FR	2019	0	1	1	0	0	0	0	0	0	0	0	0	58
16939206	f	md	JP	2019	0	1	0	0	0	1	0	0	0	0	0	0	79
16939259	f	md	DE	2019	0	1	1	0	0	0	0	0	0	0	0	0	52
16939332	m	md	RS	2019	0	1	1	0	0	0	0	0	0	0	0	0	67
16939499	f	cn	CN	2019	0	1	0	0	1	0	0	0	0	0	0	0	59
16940197	m	ph	ES	2019	0	1	1	0	0	0	0	0	0	0	0	0	71
16940202	m	md	DK	2019	0	0	0	0	0	0	0	0	0	0	0	0	74
16940267	f	md	PT	2019	0	1	0	0	1	0	0	0	0	0	0	0	44
16940412	f	md	DK	2019	0	0	0	0	0	0	0	0	0	0	0	0	64
16940774	m	ot	US	2019	0	1	0	1	0	0	0	0	0	0	0	0	63
16940828	m	cn	US	2019	1	0	0	0	0	0	0	0	0	0	0	0	
16941206	m	ot	DE	2019	0	1	0	0	1	0	0	0	0	0	0	0	67
16941342	m	Not repo	CN	2019	1	0	0	0	0	0	0	0	0	0	0	0	61
16941434	m	cn	JP	2019	0	1	1	0	0	0	0	0	0	0	0	0	51
16941620	f	md	DK	2019	0	0	0	0	0	0	0	0	0	0	0	0	81
16941632	m	cn	JP	2019	0	1	1	0	0	0	0	0	0	0	0	0	65
16941689	m	cn	JP	2019	0	1	1	0	0	0	0	0	0	0	0	1	80
16941842	m	ot	JP	2019	0	1	0	0	1	0	0	0	0	0	0	0	79
16941873	m	cn	CN	2019	0	1	0	0	1	0	0	0	0	0	0	0	64
16942082	f	cn	US	2019	0	0	0	0	0	0	0	0	0	0	0	0	
16942559	Not	cn	JP	2019	0	1	1	0	0	0	0	0	0	0	0	0	
16943062	f	Not repo	JP	2019	0	0	0	0	0	0	0	0	0	0	0	0	31
16943084	m	ot	US	2019	0	1	0	0	1	0	0	0	0	0	0	0	66
16943183	f	md	JP	2019	0	1	0	0	1	0	0	0	0	0	0	0	66
16943227	m	cn	JP	2019	0	1	1	0	0	0	0	0	0	0	0	0	70
16943250	m	ot	CN	2019	0	0	0	0	0	0	0	0	0	0	0	0	46
16943381	m	md	IT	2019	0	1	0	0	0	1	0	0	0	0	0	0	61
16943508	m	cn	US	2019	0	0	0	0	0	0	0	0	0	0	0	0	68

16943801	m	md	GB	2019	0	1	1	0	0	0	0	0	0	0	0	0	61
16944176	m	cn	CA	2019	0	1	1	0	0	0	0	0	0	0	0	0	
16944286	m	md	JP	2019	0	1	0	0	1	0	0	0	0	0	0	0	71
16944297	m	md	JP	2019	0	1	0	0	1	0	0	0	0	0	0	1	70
16944446	f	cn	Not re	2019	0	0	0	0	0	0	0	0	0	0	0	0	60
16944607	f	md	DK	2019	0	0	0	0	0	0	0	0	0	0	0	0	74
16944627	f	md	GB	2019	0	0	0	0	0	0	0	0	0	0	0	0	64
16944684	m	ot	FR	2019	0	1	0	0	1	0	0	0	0	0	0	0	71
16944967	m	ot	US	2019	0	0	0	0	0	0	0	0	0	0	0	0	56
16945027	m	ot	IT	2019	1	0	0	0	0	0	0	0	0	0	0	0	
16945133	f	md	JP	2019	0	1	0	1	0	0	0	0	0	0	0	0	82.3
16945594	f	md	JP	2019	0	1	0	0	1	0	0	0	0	0	0	0	70
16945669	m	md	FR	2019	0	1	0	1	0	0	0	0	0	0	0	0	
16945910	m	md	NL	2019	0	0	0	0	0	0	0	0	0	0	0	0	55
16946474	f	cn	CN	2019	1	0	0	0	0	0	0	0	0	0	0	0	50
16946475	f	cn	AE	2019	0	1	0	1	0	0	0	0	0	0	0	0	70.8
16946551	f	md	RO	2019	1	0	0	0	0	0	0	0	0	0	0	0	65
16946615	m	ph	DE	2019	0	1	1	0	0	0	0	0	0	0	0	0	
16946862	m	md	US	2019	0	1	0	1	0	0	0	0	0	0	0	0	77
16947826	f	md	CL	2019	0	1	0	0	0	1	0	0	0	0	0	0	61
16947835	f	md	DE	2019	0	0	0	0	0	0	0	0	0	0	0	0	82
16948303	m	cn	JP	2019	0	1	1	0	0	0	0	0	0	0	0	0	80
16948387	m	md	JP	2019	0	1	0	1	0	0	0	0	0	0	0	0	90
16948434	m	cn	CN	2019	0	1	0	0	1	0	0	0	0	0	0	0	65
16949248	m	md	JP	2019	0	1	0	0	0	1	0	0	0	0	0	0	66
16949450	m	md	JP	2019	0	1	1	0	0	0	0	0	0	0	0	0	66
16949575	m	cn	US	2019	0	1	1	0	0	0	0	0	0	0	0	0	
16949690	m	md	RU	2019	0	1	0	0	0	1	0	0	0	0	0	0	58
16950012	m	cn	JP	2019	0	0	0	0	0	0	0	0	0	0	0	0	67
16950335	f	cn	US	2019	0	0	0	0	0	0	0	0	0	0	0	0	
16950347	f	md	HK	2019	0	1	0	0	0	1	0	0	0	0	0	0	54
16950669	f	md	US	2019	0	0	0	0	0	0	0	0	0	0	0	0	73
16951446	m	md	DE	2019	0	0	0	0	0	0	0	0	0	0	0	0	61
16951469	m	cn	US	2019	0	1	1	0	0	0	0	0	0	0	0	0	

16951609	f	md	SE	2019	1	0	0	0	0	0	0	0	0	0	0	0	58
16951746	m	ot	DE	2019	0	1	0	0	1	0	0	0	0	0	0	0	59
16951928	m	md	PL	2019	0	0	0	0	0	0	0	0	0	0	0	0	72
16952157	f	cn	JP	2019	0	1	1	0	0	0	0	0	0	0	0	0	50
16952245	m	md	JP	2019	0	1	1	0	0	0	0	0	0	0	0	0	72
16952686	m	md	FR	2019	0	1	0	0	0	1	0	0	0	0	0	0	61
16952706	f	md	US	2019	1	0	0	0	0	0	0	0	0	0	0	0	
16952800	m	cn	JP	2019	0	1	1	0	0	0	0	0	0	0	0	1	66
16952833	f	md	DK	2019	0	0	0	0	0	0	0	0	0	0	0	0	55
16952978	m	cn	JP	2019	0	1	1	0	0	0	0	0	0	0	0	0	64
16953056	f	cn	JP	2019	0	1	1	0	0	0	0	0	0	0	0	0	65
16953087	m	md	US	2019	0	1	0	1	0	0	0	0	0	0	0	0	79
16953305	m	md	JP	2019	0	1	0	1	0	0	0	0	0	0	0	0	78
16953309	f	Not repo	JP	2019	0	1	0	1	0	0	0	0	0	0	0	1	40
16953341	m	ot	CN	2019	0	1	0	0	1	0	0	0	0	0	0	0	64
16953357	m	md	JP	2019	0	1	0	1	0	0	0	0	0	0	0	1	61
16953358	m	md	JP	2019	0	1	0	1	0	0	0	0	0	0	0	0	63
16953365	m	cn	GB	2019	0	1	1	0	0	0	0	0	0	0	0	0	62
16954174	Not	ot	NL	2019	0	0	0	0	0	0	0	0	0	0	0	0	
16954468	m	ot	KR	2019	0	0	0	0	0	0	0	0	0	0	0	0	52
16954511	Not	Not repo	AU	2019	1	0	0	0	0	0	0	0	0	0	0	0	
16954512	Not	Not repo	AU	2019	1	0	0	0	0	0	0	0	0	0	0	0	
16954636	m	ot	DE	2019	0	1	0	0	1	0	0	0	0	0	0	0	
16954784	f	cn	US	2019	1	0	0	0	0	0	0	0	0	0	0	0	
16955143	m	Not repo	PT	2019	1	0	0	0	0	0	0	0	0	0	0	0	76
16955659	Not	md	US	2019	0	0	0	0	0	0	0	0	0	0	0	0	
16955975	f	ot	FR	2019	0	1	0	0	1	0	0	0	0	0	0	0	58
16956005	Not	md	US	2019	0	0	0	0	0	0	0	0	0	0	0	0	
16956164	m	cn	US	2019	0	0	0	0	0	0	0	0	0	0	0	0	
16956254	m	Not repo	SG	2019	0	1	0	1	0	0	0	0	0	0	0	0	81
16956525	m	ot	RU	2019	1	0	0	0	0	0	0	0	0	0	0	0	
16956539	m	md	CN	2019	0	0	0	0	0	0	0	0	0	0	0	0	71
16956689	f	md	JP	2019	0	1	0	1	0	0	0	0	0	0	0	0	80
16956764	f	cn	JP	2019	0	1	1	0	0	0	0	0	0	0	0	0	75

16956789	m	md	JP	2019	0	1	0	1	0	0	0	0	0	0	0	1	66
16957026	m	md	JP	2019	0	1	0	1	0	0	0	0	0	0	0	1	81
16957034	m	md	JP	2019	1	0	0	0	0	0	0	0	0	0	0	0	90
16957191	f	md	ES	2019	0	1	0	0	0	1	0	0	0	0	0	0	54
16957192	m	md	JP	2019	0	1	0	1	0	0	0	0	0	0	0	1	61
16957387	m	ot	KR	2019	0	0	0	0	0	0	0	0	0	0	0	0	56
16957424	m	md	US	2019	0	1	1	0	0	0	0	0	0	0	0	0	64
16957946	m	cn	CN	2019	0	0	0	0	0	0	0	0	0	0	0	0	51
16958214	f	md	CN	2019	0	0	0	0	0	0	0	0	0	0	0	0	66
16958249	Not	md	US	2019	0	1	0	1	0	0	0	0	0	0	0	1	
16958437	m	md	FR	2019	1	0	0	0	0	0	0	0	0	0	0	0	40
16958856	Not	ot	NL	2019	0	0	0	0	0	0	0	0	0	0	0	0	
16958864	Not	ot	NL	2019	0	0	0	0	0	0	0	0	0	0	0	0	
16959242	Not	md	FR	2019	1	0	0	0	0	0	0	0	0	0	0	0	
16959255	f	Not repo	US	2019	1	0	0	0	0	0	0	0	0	0	0	0	
16959439	m	md	ID	2019	1	0	0	0	0	0	0	0	0	0	0	0	
16959480	f	cn	US	2019	0	0	0	0	0	0	0	0	0	0	0	0	
16959519	m	cn	JP	2019	0	1	1	0	0	0	0	0	0	0	0	0	73
16959703	f	md	ZA	2019	0	0	0	0	0	0	0	0	0	0	0	0	56
16959722	m	ph	JP	2019	0	1	0	0	1	0	0	0	0	0	0	0	78
16960433	m	md	JP	2019	1	0	0	0	0	0	0	0	0	0	0	1	73
16960482	f	md	US	2019	0	0	0	0	0	0	0	0	0	0	0	0	64
16960552	f	Not repo	JP	2019	1	0	0	0	0	0	0	0	0	0	0	0	
16960769	f	ot	US	2019	1	0	0	0	0	0	0	0	0	0	0	0	85
16961035	m	md	FR	2019	0	0	0	0	0	0	0	0	0	0	0	0	55
16961243	f	Not repo	SG	2019	1	0	0	0	0	0	0	0	0	0	0	0	65.3
16961300	m	md	US	2019	0	0	0	0	0	0	0	0	0	0	0	0	53
16961498	Not	md	ES	2019	0	0	0	0	0	0	0	0	0	0	0	0	
16961576	f	md	ID	2019	1	0	0	0	0	0	0	0	0	0	0	0	59
16962152	m	Not repo	US	2019	1	0	0	0	0	0	0	0	0	0	0	0	86
16962376	Not	ot	CN	2019	1	0	0	0	0	0	0	0	0	0	0	0	
16962441	f	cn	IN	2019	0	0	0	0	0	0	0	0	0	0	0	0	51
16962700	m	md	JP	2019	1	0	0	0	0	0	0	0	0	0	0	0	69
16963151	m	Not repo	KR	2019	0	1	0	1	0	0	0	0	0	0	0	0	48.8

16963152	m	md	GB	2019	0	1	0	1	0	0	0	0	0	0	0	0	77
16963287	f	md	US	2019	1	0	0	0	0	0	0	0	0	0	0	0	57
16963635	m	md	JP	2019	0	1	0	0	1	0	0	0	0	0	0	0	65
16963671	f	ot	US	2019	0	1	0	0	1	0	0	0	0	0	0	0	52
16963867	m	Not repo	US	2019	1	0	0	0	0	0	0	0	0	0	0	0	86
16963899	Not	cn	KR	2019	0	0	0	0	0	0	0	0	0	0	0	0	
16964040	Not	ot	JP	2019	0	0	0	0	0	0	0	0	0	0	0	0	
16964041	Not	ot	JP	2019	0	0	0	0	0	0	0	0	0	0	0	0	
16964485	m	cn	US	2019	0	1	1	0	0	0	0	0	0	0	0	0	
16964922	m	ot	IT	2019	1	0	0	0	0	0	0	0	0	0	0	0	80
16965041	m	md	US	2019	0	1	0	1	0	0	0	0	0	0	0	0	63
16965303	Not	cn	JP	2019	0	1	1	0	0	0	0	0	0	0	0	0	
16965312	Not	cn	JP	2019	0	1	1	0	0	0	0	0	0	0	0	0	
16965369	Not	ot	NL	2019	0	0	0	0	0	0	0	0	0	0	0	0	
16965495	m	ph	CA	2019	0	0	0	0	0	0	0	0	0	0	0	0	55
16966444	f	ot	KR	2019	0	0	0	0	0	0	0	0	0	0	0	0	52
16966557	Not	cn	JP	2019	0	1	1	0	0	0	0	0	0	0	0	0	
16966585	m	cn	JP	2019	0	1	1	0	0	0	0	0	0	0	0	0	75
16966587	Not	cn	JP	2019	0	1	1	0	0	0	0	0	0	0	0	0	
16967524	Not	ot	ES	2019	0	0	0	0	0	0	0	0	0	0	0	0	
16967804	m	md	JP	2019	0	1	0	0	1	0	0	0	0	0	0	0	75
16967910	Not	cn	JP	2019	0	1	1	0	0	0	0	0	0	0	0	1	
16967919	f	md	CH	2019	0	1	1	0	0	0	0	0	0	0	0	0	57
16967963	m	cn	DE	2019	0	1	0	0	1	0	0	0	0	0	0	0	
16968043	f	cn	JP	2019	0	1	1	0	0	0	0	0	0	0	0	0	64
16968085	m	ot	FR	2019	0	1	0	0	1	0	0	0	0	0	0	0	71
16968091	m	md	DE	2019	0	0	0	0	0	0	0	0	0	0	0	0	80
16968127	Not	cn	JP	2019	0	1	1	0	0	0	0	0	0	0	0	0	
16968182	Not	cn	JP	2019	0	1	1	0	0	0	0	0	0	0	0	0	
16968185	Not	cn	JP	2019	0	1	1	0	0	0	0	0	0	0	0	0	
16968263	m	cn	JP	2019	0	1	1	0	0	0	0	0	0	0	0	1	80
16968321	m	md	DE	2019	0	1	0	0	0	1	0	0	0	0	0	0	61
16968336	m	md	US	2019	0	1	0	1	0	0	0	0	0	0	0	0	81
16968392	m	md	JP	2019	0	1	0	0	0	1	0	0	0	0	0	0	72

16968448	m	md	JP	2019	0	1	0	0	0	1	0	0	0	0	0	0	70
16968464	Not	cn	US	2019	0	1	1	0	0	0	0	0	0	0	0	0	
16968598	f	ot	FR	2019	0	1	0	0	1	0	0	0	0	0	0	0	73
16968608	f	cn	JP	2019	0	1	1	0	0	0	0	0	0	0	0	0	73
16968619	m	cn	US	2019	1	0	0	0	0	0	0	0	0	0	0	0	
16968700	f	cn	KR	2019	0	0	0	0	0	0	0	0	0	0	0	0	70
16968761	Not	cn	JP	2019	0	1	1	0	0	0	0	0	0	0	0	0	
16968784	Not	cn	JP	2019	0	1	1	0	0	0	0	0	0	0	0	0	
16968832	Not	cn	JP	2019	0	1	1	0	0	0	0	0	0	0	0	0	
16968833	Not	cn	JP	2019	0	1	1	0	0	0	0	0	0	0	0	0	
16968896	m	md	CA	2019	0	1	0	1	0	0	0	0	0	0	0	0	71.1
16968955	Not	cn	JP	2019	0	1	1	0	0	0	0	0	0	0	0	0	
16968956	Not	cn	JP	2019	0	1	1	0	0	0	0	0	0	0	0	0	
16968981	Not	cn	JP	2019	0	1	1	0	0	0	0	0	0	0	0	0	
16968982	Not	cn	JP	2019	0	1	1	0	0	0	0	0	0	0	0	0	
16969005	Not	cn	JP	2019	0	1	1	0	0	0	0	0	0	0	0	0	
16969036	Not	cn	JP	2019	0	1	1	0	0	0	0	0	0	0	0	0	
16969241	m	ot	FR	2019	0	1	0	0	1	0	0	0	0	0	0	0	60
16969368	Not	cn	JP	2019	0	1	1	0	0	0	0	0	0	0	0	0	
16969369	Not	cn	JP	2019	0	1	1	0	0	0	0	0	0	0	0	0	
16969466	Not	cn	JP	2019	0	1	1	0	0	0	0	0	0	0	0	0	
16969699	f	cn	CN	2019	0	0	0	0	0	0	0	0	0	0	0	0	50
16969859	Not	cn	JP	2019	0	1	1	0	0	0	0	0	0	0	0	0	
16969891	Not	cn	JP	2019	0	1	1	0	0	0	0	0	0	0	0	0	
16970799	m	ot	US	2019	0	1	0	1	0	0	0	0	0	0	0	0	63
16971088	m	cn	JP	2019	0	1	1	0	0	0	0	0	0	0	0	0	72
16971291	f	cn	CA	2019	1	0	0	0	0	0	0	0	0	0	0	0	
16971408	f	md	TW	2019	0	0	0	0	0	0	0	0	0	0	0	0	61
16971417	m	Not repo	US	2019	0	1	0	1	0	0	0	0	0	0	0	0	67
16971418	f	md	JP	2019	0	1	0	1	0	0	0	0	0	0	0	0	85
16971433	f	cn	US	2019	0	0	0	0	0	0	0	0	0	0	0	0	77
16971647	Not	ph	JP	2019	0	0	0	0	0	0	0	0	0	0	0	0	
16971648	Not	cn	DE	2019	0	0	0	0	0	0	0	0	0	0	0	0	
16971816	Not	cn	JP	2019	0	1	1	0	0	0	0	0	0	0	0	0	

16971902	m	ot	KR	2019	0	1	0	0	1	0	0	0	0	0	0	0	70
16971907	m	md	IN	2019	0	0	0	0	0	0	0	0	0	0	0	0	64
16972010	m	ot	FR	2019	0	0	0	0	0	0	0	0	0	0	0	0	65
16972013	f	cn	CH	2019	0	1	1	0	0	0	0	0	0	0	0	0	
16972127	m	md	DE	2019	0	1	0	0	1	0	0	0	0	0	0	0	67
16972257	f	cn	JP	2019	0	0	0	0	0	0	0	0	0	0	0	0	77
16972265	m	ot	US	2019	0	0	0	0	0	0	0	0	0	0	0	0	90
16972578	f	cn	CN	2019	0	0	0	0	0	0	0	0	0	0	0	0	65
16973689	m	md	HK	2019	0	1	0	0	0	1	0	0	0	0	0	0	60
16973996	m	cn	TH	2019	0	1	0	0	1	0	0	0	0	0	0	0	66
16974072	m	ot	GB	2019	0	1	1	0	0	0	0	0	0	0	0	0	62
16974429	f	md	KR	2019	1	0	0	0	0	0	0	0	0	0	0	0	75
16974517	m	md	JP	2019	0	1	0	1	0	0	0	0	0	0	0	1	75
16975438	m	ph	US	2019	0	1	1	0	0	0	0	0	0	0	0	1	63
16975723	f	cn	DE	2019	0	1	1	0	0	0	0	0	0	0	0	0	
16975724	m	md	ES	2019	0	0	0	0	0	0	0	0	0	0	0	0	70
16975961	f	md	JP	2019	0	1	1	0	0	0	0	0	0	0	0	1	73
16975974	m	ot	CA	2019	1	0	0	0	0	0	0	0	0	0	0	0	
16976022	f	cn	CN	2019	1	0	0	0	0	0	0	0	0	0	0	0	76.6
16976033	Not	ot	CN	2019	1	0	0	0	0	0	0	0	0	0	0	0	
16976035	m	cn	CA	2019	1	0	0	0	0	0	0	0	0	0	0	0	
16976117	Not	md	CH	2019	0	1	0	0	1	0	0	0	0	0	0	0	
16976662	f	cn	GB	2019	0	1	1	0	0	0	0	0	0	0	0	0	50
16976791	Not	cn	JP	2019	0	1	1	0	0	0	0	0	0	0	0	0	76
16976796	m	md	MY	2019	0	1	1	0	0	0	0	0	0	0	0	0	57
16976912	m	ot	JP	2019	0	1	0	0	1	0	0	0	0	0	0	0	56
16976971	m	ot	ES	2019	0	1	0	0	1	0	0	0	0	0	0	0	48
16977127	f	md	RU	2019	1	0	0	0	0	0	0	0	0	0	0	0	76
16977386	Not	md	JP	2019	0	1	0	0	0	1	0	0	0	0	0	0	
16977651	Not	cn	JP	2019	0	1	1	0	0	0	0	0	0	0	0	0	
16977826	m	ot	US	2019	0	1	0	0	1	0	0	0	0	0	0	0	41
16978055	m	cn	US	2019	0	0	0	0	0	0	0	0	0	0	0	0	
16978139	Not	cn	JP	2019	0	1	1	0	0	0	0	0	0	0	0	0	
16978143	Not	ot	KR	2019	1	0	0	0	0	0	0	0	0	0	0	0	

16978226	f	md	IN	2019	0	1	0	0	0	1	0	0	0	0	0	0		
16978322	Not	cn	JP	2019	0	1	1	0	0	0	0	0	0	0	0	0		
16978901	f	md	PL	2019	0	0	0	0	0	0	0	0	0	0	0	0		
16979174	m	cn	US	2019	0	1	1	0	0	0	0	0	0	0	0	0		68
16979449	Not	md	JP	2019	0	1	0	1	0	0	0	0	0	0	0	1		80
16979557	m	md	JP	2019	1	0	0	0	0	0	0	0	0	0	0	1		73
16979695	Not	ot	KR	2019	1	0	0	0	0	0	0	0	0	0	0	0		
16979802	m	cn	JP	2019	0	1	1	0	0	0	0	0	0	0	0	0		51
16979807	Not	md	IT	2019	1	0	0	0	0	0	0	0	0	0	0	0		
16979814	f	ot	NL	2019	0	1	1	0	0	0	0	0	0	0	0	0		50
16979881	Not	md	CN	2019	0	0	0	0	0	0	0	0	0	0	0	0		
16979887	f	cn	CA	2019	0	1	0	1	0	0	0	0	0	0	0	0		67.2
16979943	m	md	CA	2019	0	0	0	0	0	0	0	0	0	0	0	0		77
16979965	Not	md	CN	2019	0	0	0	0	0	0	0	0	0	0	0	0		
16979992	m	md	GB	2019	0	1	1	0	0	0	0	0	0	0	0	0		
16980197	m	ot	US	2019	0	0	0	0	0	0	0	0	0	0	0	0		72
16980267	Not	ot	KR	2019	1	0	0	0	0	0	0	0	0	0	0	0		
16980273	Not	ot	KR	2019	1	0	0	0	0	0	0	0	0	0	0	1		
16980601	m	Not repo	IT	2019	1	0	0	0	0	0	0	0	0	0	0	0		88
16980829	m	Not repo	US	2019	1	0	0	0	0	0	0	0	0	0	0	0		77.5
16981028	m	md	FR	2019	0	1	1	0	0	0	0	0	0	0	0	0		61
16981221	m	cn	JP	2019	0	1	1	0	0	0	0	0	0	0	0	0		49
16981225	m	cn	RO	2019	0	1	1	0	0	0	0	0	0	0	0	0		
16981251	f	md	US	2019	0	0	0	0	0	0	0	0	0	0	0	0		
16981262	f	ot	CA	2019	1	0	0	0	0	0	0	0	0	0	0	0		
16981267	Not	cn	JP	2019	0	1	1	0	0	0	0	0	0	0	0	0		
16981299	m	md	JP	2019	0	1	0	1	0	0	0	0	0	0	0	0		60
16982048	f	ot	US	2019	0	0	0	0	0	0	0	0	0	0	0	0		72
16982257	m	md	JP	2019	0	1	0	1	0	0	0	0	0	0	0	1		79
16982283	m	md	FR	2019	0	1	0	1	0	0	0	0	0	0	0	0		
16982290	m	md	JP	2019	0	1	0	1	0	0	0	0	0	0	0	1		
16982310	m	md	JP	2019	0	1	0	1	0	0	0	0	0	0	0	1		66
16982464	f	md	JP	2019	1	0	0	0	0	0	0	0	0	0	0	0		67
16982746	m	md	TR	2019	0	0	0	0	0	0	0	0	0	0	0	0		

16983070	m	cn	US	2019	0	1	1	0	0	0	0	0	0	0	0	0	
16983118	m	ot	NL	2019	0	1	1	0	0	0	0	0	0	0	0	0	62
16983198	f	md	JP	2019	0	1	0	1	0	0	0	0	0	0	0	0	61
16983293	m	md	JP	2019	0	1	0	1	0	0	0	0	0	0	0	0	81
16983455	m	cn	AR	2019	0	0	0	0	0	0	0	0	0	0	0	0	55
16983751	m	cn	CN	2019	0	1	0	0	1	0	0	0	0	0	0	0	
16983876	m	Not repo	JP	2019	0	1	0	1	0	0	0	0	0	0	0	0	70
16983880	Not	Not repo	AU	2019	0	1	0	1	0	0	0	0	0	0	0	0	
16983918	m	md	PL	2019	0	1	1	0	0	0	0	0	0	0	0	0	62
16984626	f	md	CA	2019	1	0	0	0	0	0	0	0	0	0	0	0	
16984627	m	md	JP	2019	1	0	0	0	0	0	0	0	0	0	0	0	71.7
16984695	f	md	US	2019	0	1	0	0	1	0	0	0	0	0	0	1	55
16984724	m	ot	JP	2019	0	0	0	0	0	0	0	0	0	0	0	0	80
16984799	f	md	JP	2019	0	1	0	1	0	0	0	0	0	0	0	0	80
16984942	m	md	US	2019	0	1	0	0	1	0	0	0	0	0	0	1	55
16984943	f	md	US	2019	0	1	0	0	1	0	0	0	0	0	0	1	56
16984965	f	md	US	2019	0	1	0	0	1	0	0	0	0	0	0	1	61
16984966	f	md	US	2019	0	1	0	0	1	0	0	0	0	0	0	1	72
16985095	m	md	BG	2019	1	0	0	0	0	0	0	0	0	0	0	0	67
16985212	m	md	JP	2019	0	1	0	1	0	0	0	0	0	0	0	1	68
16985215	f	md	JP	2019	1	0	0	0	0	0	0	0	0	0	0	0	68
16985243	f	md	SE	2019	0	1	0	0	0	1	0	0	0	0	0	0	67
16985257	f	md	US	2019	0	1	0	0	1	0	0	0	0	0	0	0	47
16985372	m	Not repo	JP	2019	0	1	0	1	0	0	0	0	0	0	0	1	
16985405	m	ot	US	2019	0	0	0	0	0	0	0	0	0	0	0	0	65
16985443	m	cn	JP	2019	0	1	1	0	0	0	0	0	0	0	0	0	81
16985534	m	cn	GB	2019	0	1	1	0	0	0	0	0	0	0	0	0	
16985539	f	cn	GB	2019	0	1	1	0	0	0	0	0	0	0	0	0	50
16985780	m	md	US	2019	0	1	0	0	1	0	0	0	0	0	0	0	61
16985813	m	cn	US	2019	1	0	0	0	0	0	0	0	0	0	0	0	
16986128	m	cn	JP	2019	0	1	1	0	0	0	0	0	0	0	0	0	73
16986147	f	ot	BR	2019	0	1	1	0	0	0	0	0	0	0	0	0	65
16986169	m	cn	JP	2019	0	1	1	0	0	0	0	0	0	0	0	0	45
16986180	m	md	TR	2019	0	1	1	0	0	0	0	0	0	0	0	0	71

16986267	m	md	FR	2019	0	0	0	0	0	0	0	0	0	0	0	0	51
16986368	f	ot	US	2019	0	1	0	0	1	0	0	0	0	0	0	0	79
16986825	f	md	FR	2019	0	1	0	0	0	1	0	0	0	0	0	0	57
16987173	Not	md	AU	2019	0	1	0	0	0	1	0	0	0	0	0	0	
16987329	f	ot	US	2019	0	1	0	0	1	0	0	0	0	0	0	0	79
16987355	m	ot	US	2019	0	0	0	0	0	0	0	0	0	0	0	0	90
16987471	f	md	US	2019	1	0	0	0	0	0	0	0	0	0	0	0	81
16987473	f	md	US	2019	1	0	0	0	0	0	0	0	0	0	0	0	72
16987487	m	md	US	2019	1	0	0	0	0	0	0	0	0	0	0	0	67
16987602	m	md	CN	2019	0	0	0	0	0	0	0	0	0	0	0	0	54
16987796	m	md	JP	2019	0	1	0	0	0	1	0	0	0	0	0	0	64
16987968	m	md	TR	2019	0	0	0	0	0	0	0	0	0	0	0	0	
16987969	m	md	TR	2019	0	0	0	0	0	0	0	0	0	0	0	0	
16987974	m	md	TR	2019	0	0	0	0	0	0	0	0	0	0	0	0	
16988086	m	md	JP	2019	0	1	0	0	0	1	0	0	0	0	0	0	71
16988528	m	md	TR	2019	0	0	0	0	0	0	0	0	0	0	0	0	
16988674	f	cn	US	2019	0	0	0	0	0	0	0	0	0	0	0	0	
16988715	m	ot	US	2019	0	1	0	0	1	0	0	0	0	0	0	0	
16989838	m	md	JP	2019	0	1	0	0	0	1	0	0	0	0	0	0	70
16989841	m	md	IT	2019	0	1	1	0	0	0	0	0	0	0	0	0	74
16990238	m	ot	JP	2019	1	0	0	0	0	0	0	0	0	0	0	0	73.3
16990355	f	cn	CN	2019	1	0	0	0	0	0	0	0	0	0	0	0	69
16990359	f	cn	US	2019	1	0	0	0	0	0	0	0	0	0	0	0	
16990570	Not	Not repo	JP	2019	1	0	0	0	0	0	0	0	0	0	0	0	
16990593	Not	ot	US	2019	0	0	0	0	0	0	0	0	0	0	0	0	
16990609	Not	ot	US	2019	0	0	0	0	0	0	0	0	0	0	0	0	
16990666	m	md	KR	2019	0	1	1	0	0	0	0	0	0	0	0	0	71
16990914	m	md	JP	2019	0	1	0	0	1	0	0	0	0	0	0	0	75
16990926	m	ot	FR	2019	0	1	0	0	1	0	0	0	0	0	0	0	70
16991089	m	cn	GB	2019	0	1	1	0	0	0	0	0	0	0	0	0	
16991228	f	md	NL	2019	0	1	1	0	0	0	0	0	0	0	0	0	66
16991336	f	ot	DE	2019	0	1	0	0	1	0	0	0	0	0	0	0	52
16991362	f	md	ES	2019	0	1	1	0	0	0	0	0	0	0	0	0	55
16991520	f	md	JP	2019	0	1	1	0	0	0	0	0	0	0	0	0	60

16991538	m	md	GB	2019	0	1	0	0	0	1	0	0	0	0	0	0	
16991754	f	cn	GT	2019	0	1	1	0	0	0	0	0	0	0	0	0	
16991883	m	md	DE	2019	0	1	0	1	0	0	0	0	0	0	0	0	77
16992603	m	cn	GR	2019	0	1	1	0	0	0	0	0	0	0	0	0	
16992618	m	md	IL	2019	0	1	0	1	0	0	0	0	0	0	0	0	71
16992757	f	cn	US	2019	1	0	0	0	0	0	0	0	0	0	0	0	65
16992826	m	ph	BR	2019	0	1	0	0	0	1	0	0	0	0	0	0	67
16992910	m	ph	US	2019	0	1	0	1	0	0	0	0	0	0	0	0	59
16992927	Not	ot	JP	2019	0	0	0	0	0	0	0	0	0	0	0	0	
16993418	m	cn	JP	2019	0	1	1	0	0	0	0	0	0	0	0	1	72
16993694	m	ot	JP	2019	0	1	1	0	0	0	0	0	0	0	0	0	71
16994058	m	md	DE	2019	0	0	0	0	0	0	0	0	0	0	0	0	61
16994205	f	md	JP	2019	0	0	0	0	0	0	0	0	0	0	0	0	64
16994297	f	cn	Not re	2019	0	0	0	0	0	0	0	0	0	0	0	0	35
16994300	m	cn	Not re	2019	0	0	0	0	0	0	0	0	0	0	0	0	50
16994311	m	cn	US	2019	0	1	1	0	0	0	0	0	0	0	0	0	80
16994325	m	cn	JP	2019	0	1	1	0	0	0	0	0	0	0	0	0	
16994433	m	ot	JP	2019	0	0	0	0	0	0	0	0	0	0	0	0	
16994617	m	cn	US	2019	1	0	0	0	0	0	0	0	0	0	0	0	63
16994778	m	md	GB	2019	0	1	1	0	0	0	0	0	0	0	0	0	74
16994989	f	md	CA	2019	1	0	0	0	0	0	0	0	0	0	0	0	
16995204	m	Not repo	AU	2019	0	1	0	1	0	0	0	0	0	0	0	0	
16995302	f	cn	US	2019	1	0	0	0	0	0	0	0	0	0	0	0	
16995477	m	md	JP	2019	0	1	1	0	0	0	0	0	0	0	0	0	55
16995644	m	md	JP	2019	0	1	1	0	0	0	0	0	0	0	0	0	63
16995691	f	cn	JP	2019	0	1	1	0	0	0	0	0	0	0	0	0	67
16995812	f	md	US	2019	0	1	0	0	1	0	0	0	0	0	0	0	72
16995863	m	cn	JP	2019	0	1	1	0	0	0	0	0	0	0	0	0	78
16995931	f	md	BE	2019	0	0	0	0	0	0	0	0	0	0	0	0	77
16995976	m	md	JP	2019	0	1	0	0	0	1	0	0	0	0	0	0	78
16996185	m	ot	US	2019	0	1	0	0	1	0	0	0	0	0	0	0	83
16996315	m	ot	US	2019	1	0	0	0	0	0	0	0	0	0	0	0	49
16996379	m	cn	US	2019	0	1	1	0	0	0	0	0	0	0	0	0	48
16996414	m	md	JP	2019	0	0	0	0	0	0	0	0	0	0	0	0	60

16996444	Not	ot	ES	2019	1	0	0	0	0	0	0	0	0	0	0	0	
16997072	m	ph	US	2019	0	1	0	0	1	0	0	0	0	0	0	0	69
16997192	m	md	US	2019	0	1	0	1	0	0	0	0	0	0	0	0	69
16997229	m	md	JP	2019	0	1	0	1	0	0	0	0	0	0	0	1	75
16997456	m	md	IT	2019	0	1	1	0	0	0	0	0	0	0	0	0	74
16997502	m	md	FR	2019	0	1	1	0	0	0	0	0	0	0	0	0	61
16997608	f	md	JP	2019	1	0	0	0	0	0	0	0	0	0	0	1	72
16997706	Not	md	JP	2019	0	1	0	1	0	0	0	0	0	0	0	1	
16997728	f	md	KR	2019	0	1	0	0	0	1	0	0	0	0	0	0	74
16997785	Not	ph	JP	2019	0	1	0	0	1	0	0	0	0	0	0	0	
16998129	m	md	JP	2019	0	1	0	0	0	1	0	0	0	0	0	0	
16998387	m	md	JP	2019	0	1	0	0	1	0	0	0	0	0	0	0	39
16998796	f	ph	VN	2019	1	0	0	0	0	0	0	0	0	0	0	0	54
16999525	m	cn	JP	2019	0	1	1	0	0	0	0	0	0	0	0	0	69
16999568	m	cn	JP	2019	0	1	1	0	0	0	0	0	0	0	0	0	74
16999570	m	cn	JP	2019	0	1	1	0	0	0	0	0	0	0	0	0	62
16999610	f	ot	JP	2019	0	1	0	0	1	0	0	0	0	0	0	0	81
16999612	m	ot	JP	2019	0	1	0	0	1	0	0	0	0	0	0	0	55
16999627	f	cn	JP	2019	0	1	1	0	0	0	0	0	0	0	0	0	73
16999648	m	cn	JP	2019	0	1	1	0	0	0	0	0	0	0	0	0	76
16999652	m	ot	JP	2019	0	1	0	0	1	0	0	0	0	0	0	0	75
16999701	m	Not repo	US	2019	0	0	0	0	0	0	0	0	0	0	0	0	42
16999711	f	md	JP	2019	0	1	0	0	1	0	0	0	0	0	0	0	69
16999753	m	cn	IN	2019	0	1	0	0	1	0	0	0	0	0	0	0	
17000136	m	cn	JP	2019	0	1	1	0	0	0	0	0	0	0	0	0	75
17000175	m	cn	JP	2019	0	1	1	0	0	0	0	0	0	0	0	0	55
17000317	m	cn	JP	2019	0	1	1	0	0	0	0	0	0	0	0	0	80
17000333	f	md	IT	2019	1	0	0	0	0	0	0	0	0	0	0	0	
17000563	f	ot	US	2019	0	1	0	0	1	0	0	0	0	0	0	0	68
17000588	f	md	US	2019	0	1	0	0	1	0	0	0	0	0	0	0	65
17000875	f	md	JP	2019	0	1	0	0	1	0	0	0	0	0	0	0	82
17000889	Not	ph	JP	2019	1	0	0	0	0	0	0	0	0	0	0	0	86
17000953	m	md	JP	2019	0	1	0	1	0	0	0	0	0	0	0	1	80
17001044	f	ot	CA	2019	1	0	0	0	0	0	0	0	0	0	0	0	

17001165	f	cn	CN	2019	1	0	0	0	0	0	0	0	0	0	0	0	43
17001446	f	ot	US	2019	0	0	0	0	0	0	0	0	0	0	0	0	64
17001476	m	cn	JP	2019	0	0	0	0	0	0	0	0	0	0	0	0	65
17001494	m	md	JP	2019	0	1	1	0	0	0	0	0	0	0	0	0	70
17001539	m	md	JP	2019	0	1	1	0	0	0	0	0	0	0	0	1	71
17001540	f	cn	CN	2019	0	0	0	0	0	0	0	0	0	0	0	0	37
17001565	f	ot	CN	2019	1	0	0	0	0	0	0	0	0	0	0	0	58
17001763	m	ot	CN	2019	1	0	0	0	0	0	0	0	0	0	0	0	46
17001765	m	ot	CN	2019	1	0	0	0	0	0	0	0	0	0	0	0	68
17001826	f	md	US	2019	1	0	0	0	0	0	0	0	0	0	0	0	67
17001860	f	md	KR	2019	1	0	0	0	0	0	0	0	0	0	0	0	79
17002155	m	md	US	2019	1	0	0	0	0	0	0	0	0	0	0	0	73
17002287	m	ot	JP	2019	0	0	0	0	0	0	0	0	0	0	0	0	65
17002288	m	cn	JP	2019	0	1	1	0	0	0	0	0	0	0	0	0	80
17002293	m	cn	JP	2019	0	1	1	0	0	0	0	0	0	0	0	0	72
17002649	Not	md	ID	2019	0	1	0	0	0	1	0	0	0	0	0	0	
17002650	Not	cn	PH	2019	0	1	0	0	0	1	0	0	0	0	0	0	
17002769	Not	ot	JP	2019	0	1	0	0	1	0	0	0	0	0	0	0	
17003368	m	cn	US	2019	0	1	1	0	0	0	0	0	0	0	0	0	64
17003509	m	md	US	2019	0	1	0	0	1	0	0	0	0	0	0	0	77
17003511	m	md	US	2019	0	1	0	0	1	0	0	0	0	0	0	0	55
17003514	f	md	US	2019	0	1	0	0	1	0	0	0	0	0	0	0	50
17003813	f	ph	JP	2019	1	0	0	0	0	0	0	0	0	0	0	0	82
17003848	m	ot	JP	2019	0	1	1	0	0	0	0	0	0	0	0	0	68
17003927	m	Not repo	JP	2019	0	1	0	1	0	0	0	0	0	0	0	1	70
17004308	f	md	JP	2019	0	1	0	0	1	0	0	0	0	0	0	0	70
17004359	m	cn	CN	2019	0	0	0	0	0	0	0	0	0	0	0	0	44
17004374	f	Not repo	JP	2019	1	0	0	0	0	0	0	0	0	0	0	0	71.2
17004391	f	cn	CN	2019	1	0	0	0	0	0	0	0	0	0	0	0	57
17004456	f	cn	US	2019	0	0	0	0	0	0	0	0	0	0	0	0	75
17004639	m	ph	JP	2019	0	1	0	0	0	1	0	0	0	0	0	0	59
17004808	m	ot	KR	2019	0	1	1	0	0	0	0	0	0	0	0	0	43
17004823	m	Not repo	CN	2019	1	0	0	0	0	0	0	0	0	0	0	0	67
17004920	m	cn	JP	2019	0	1	1	0	0	0	0	0	0	0	0	0	72

17004936	f	cn	JP	2019	0	1	1	0	0	0	0	0	0	0	0	0	77
17004979	f	cn	FR	2019	0	1	1	0	0	0	0	0	0	0	0	0	65
17005079	m	md	RS	2019	0	1	1	0	0	0	0	0	0	0	0	0	56
17005242	f	md	US	2019	1	0	0	0	0	0	0	0	0	0	0	0	80
17005360	m	ot	JP	2019	0	1	0	1	0	0	0	0	0	0	0	1	72
17005361	f	cn	CA	2019	0	1	0	1	0	0	0	0	0	0	0	1	
17005365	f	md	CN	2019	1	0	0	0	0	0	0	0	0	0	0	0	84
17005440	f	md	PT	2019	0	1	0	0	1	0	0	0	0	0	0	0	44
17005468	m	md	US	2019	0	1	1	0	0	0	0	0	0	0	0	0	66
17005609	f	md	JP	2019	1	0	0	0	0	0	0	0	0	0	0	0	80
17005668	m	cn	JP	2019	0	0	0	0	0	0	0	0	0	0	0	0	65
17005707	f	md	DE	2019	1	0	0	0	0	0	0	0	0	0	0	0	80.4
17005812	m	cn	JP	2019	0	1	1	0	0	0	0	0	0	0	0	0	44
17006156	f	ot	IT	2019	1	0	0	0	0	0	0	0	0	0	0	0	46
17006482	m	cn	JP	2019	0	1	1	0	0	0	0	0	0	0	0	0	69
17006740	m	ot	IT	2019	0	1	1	0	0	0	0	0	0	0	0	0	78
17006822	Not	md	US	2019	0	0	0	0	0	0	0	0	0	0	0	0	
17006941	f	md	IT	2019	0	1	0	0	0	1	0	0	0	0	0	0	70
17006980	m	ot	IT	2019	0	1	1	0	0	0	0	0	0	0	0	0	63
17007531	m	md	VN	2019	0	1	0	0	0	1	0	0	0	0	0	0	
17007741	Not	Not repo	US	2019	0	1	0	1	0	0	0	0	0	0	0	1	
17007801	f	md	US	2019	0	1	0	0	1	0	0	0	0	0	0	1	55
17007804	f	md	US	2019	0	1	0	0	1	0	0	0	0	0	0	0	47
17007805	f	md	US	2019	0	1	0	0	1	0	0	0	0	0	0	1	56
17007812	m	md	US	2019	0	1	0	0	1	0	0	0	0	0	0	1	55
17007887	m	ot	US	2019	0	1	1	0	0	0	0	0	0	0	0	1	
17008234	m	md	JP	2019	0	1	1	0	0	0	0	0	0	0	0	1	60
17008325	m	md	JP	2019	0	1	1	0	0	0	0	0	0	0	0	0	64
17008502	m	md	US	2019	0	1	0	0	1	0	0	0	0	0	0	0	61
17008511	m	cn	IN	2019	0	1	0	0	1	0	0	0	0	0	0	0	23
17008625	f	md	US	2019	0	1	0	0	1	0	0	0	0	0	0	1	61
17008854	m	cn	CN	2019	1	0	0	0	0	0	0	0	0	0	0	0	57
17009204	f	md	IT	2019	1	0	0	0	0	0	0	0	0	0	0	0	
17009247	f	md	US	2019	0	1	0	0	1	0	0	0	0	0	0	0	65

17009283	m	cn	CN	2019	1	0	0	0	0	0	0	0	0	0	0	0	66
17009680	m	md	IT	2019	0	1	1	0	0	0	0	0	0	0	0	0	74
17009862	m	cn	JP	2019	0	1	1	0	0	0	0	0	0	0	0	0	62
17010120	m	cn	JP	2019	0	1	1	0	0	0	0	0	0	0	0	0	45
17010368	m	md	CN	2019	0	1	0	0	0	1	0	0	0	0	0	0	69
17010390	m	md	JP	2019	1	0	0	0	0	0	0	0	0	0	0	0	59
17010399	f	cn	CN	2019	1	0	0	0	0	0	0	0	0	0	0	0	
17010453	f	Not repo	IT	2019	1	0	0	0	0	0	0	0	0	0	0	0	79.4
17010502	f	md	US	2019	1	0	0	0	0	0	0	0	0	0	0	0	75
17010503	f	cn	CN	2019	1	0	0	0	0	0	0	0	0	0	0	0	57
17010580	Not	Not repo	AU	2019	0	1	0	1	0	0	0	0	0	0	0	0	61.8
17010728	m	md	JP	2019	0	1	0	1	0	0	0	0	0	0	0	0	80
17010891	f	cn	CN	2019	1	0	0	0	0	0	0	0	0	0	0	0	52
17010912	f	cn	CN	2019	1	0	0	0	0	0	0	0	0	0	0	0	59
17010979	f	cn	CN	2019	1	0	0	0	0	0	0	0	0	0	0	0	59
17011002	f	md	ES	2019	0	1	0	0	0	1	0	0	0	0	0	0	54
17011376	f	Not repo	CN	2019	1	0	0	0	0	0	0	0	0	0	0	0	87
17011471	m	cn	JP	2019	0	1	1	0	0	0	0	0	0	0	0	0	80
17011712	f	md	JP	2019	0	1	0	0	0	1	0	0	0	0	0	1	71
17011807	f	ot	FR	2019	0	1	0	0	1	0	0	0	0	0	0	1	58
17011821	m	ot	JP	2019	0	1	0	0	1	0	0	0	0	0	0	0	57
17011846	Not	md	CN	2019	1	0	0	0	0	0	0	0	0	0	0	0	
17011922	m	md	NL	2019	0	0	0	0	0	0	0	0	0	0	0	0	
17012296	m	ot	JP	2019	0	0	0	0	0	0	0	0	0	0	0	0	
17012403	Not	ph	JP	2019	0	1	1	0	0	0	0	0	0	0	0	1	72
17012452	m	md	RU	2019	0	1	0	0	0	1	0	0	0	0	0	0	67
17012470	Not	ot	JP	2019	1	0	0	0	0	0	0	0	0	0	0	0	
17012810	m	ot	CA	2019	0	0	0	0	0	0	0	0	0	0	0	0	70
17012817	m	ot	CA	2019	0	1	1	0	0	0	0	0	0	0	0	0	
17012877	m	cn	CN	2019	0	1	0	0	1	0	0	0	0	0	0	0	39
17012885	m	cn	CN	2019	0	1	0	0	1	0	0	0	0	0	0	0	71
17012931	m	cn	US	2019	0	1	0	0	1	0	0	0	0	0	0	0	78
17012945	f	ot	LB	2019	0	1	0	0	1	0	0	0	0	0	0	0	
17013329	f	cn	Not re	2019	0	0	0	0	0	0	0	0	0	0	0	0	61

17013902	f	ot	US	2019	0	0	0	0	0	0	0	0	0	0	0	0	59
17014453	f	Not repo	US	2019	1	0	0	0	0	0	0	0	0	0	0	0	91.6
17015029	m	md	JP	2019	0	1	0	0	0	1	0	0	0	0	0	0	71
17015743	f	cn	US	2019	1	0	0	0	0	0	0	0	0	0	0	0	
17015819	Not	md	JP	2019	0	1	0	0	1	0	0	0	0	0	0	0	
17016021	f	md	ES	2019	0	0	0	0	0	0	0	0	0	0	0	0	68
17016272	m	cn	US	2019	0	0	0	0	0	0	0	0	0	0	0	0	67
17016801	f	md	PT	2019	0	1	0	0	1	0	0	0	0	0	0	0	44
17016971	m	cn	JP	2019	0	1	1	0	0	0	0	0	0	0	0	0	58
17017066	f	ph	JP	2019	1	0	0	0	0	0	0	0	0	0	0	1	68
17017188	m	cn	BR	2019	1	0	0	0	0	0	0	0	0	0	0	0	
17017455	m	cn	JP	2019	0	1	1	0	0	0	0	0	0	0	0	0	71
17017469	Not	ot	FR	2019	1	0	0	0	0	0	0	0	0	0	0	1	
17017540	m	ot	US	2019	1	0	0	0	0	0	0	0	0	0	0	0	74.2
17017541	Not	ot	FR	2019	1	0	0	0	0	0	0	0	0	0	0	0	
17018237	f	md	KR	2019	1	0	0	0	0	0	0	0	0	0	0	0	85
17018767	m	cn	IN	2019	0	1	0	0	1	0	0	0	0	0	0	0	
17018775	m	cn	IN	2019	0	1	0	0	1	0	0	0	0	0	0	0	
17018898	m	ot	GB	2019	0	1	0	0	0	1	0	0	0	0	0	0	66
17018977	m	md	CN	2019	0	1	0	0	0	1	0	0	0	0	0	0	70
17019087	m	md	JP	2019	0	1	0	0	0	1	0	0	0	0	0	0	44
17019466	f	md	ES	2019	0	0	0	0	0	0	0	0	0	0	0	0	53
17019497	f	md	KR	2019	1	0	0	0	0	0	0	0	0	0	0	0	77
17019578	f	md	JP	2019	0	1	0	0	1	0	0	0	0	0	0	0	80
17019948	Not	ot	IT	2019	1	0	0	0	0	0	0	0	0	0	0	0	
17020075	f	cn	US	2019	1	0	0	0	0	0	0	0	0	0	0	0	
17020095	f	ot	CN	2019	1	0	0	0	0	0	0	0	0	0	0	0	66
17020264	m	md	BR	2019	1	0	0	0	0	0	0	0	0	0	0	0	48
17020318	f	md	KR	2019	0	0	0	0	0	0	0	0	0	0	0	0	
17020557	f	Not repo	IT	2019	1	0	0	0	0	0	0	0	0	0	0	0	
17020566	m	cn	JP	2019	0	1	1	0	0	0	0	0	0	0	0	1	77
17020732	Not	md	TN	2019	0	0	0	0	0	0	0	0	0	0	0	0	65
17020812	m	md	AT	2019	0	1	1	0	0	0	0	0	0	0	0	0	
17020837	f	Not repo	CN	2019	1	0	0	0	0	0	0	0	0	0	0	0	64

17020917	f	cn	CN	2019	1	0	0	0	0	0	0	0	0	0	0	0	69
17020934	m	ot	BE	2019	0	1	1	0	0	0	0	0	0	0	0	0	57
17021132	m	md	JP	2019	0	1	1	0	0	0	0	0	0	0	0	0	70
17021262	f	cn	US	2019	1	0	0	0	0	0	0	0	0	0	0	0	64.4
17021736	m	cn	US	2019	0	1	0	1	0	0	0	0	0	0	0	1	
17021796	m	md	ID	2019	1	0	0	0	0	0	0	0	0	0	0	0	
17022258	f	md	FR	2019	1	0	0	0	0	0	0	0	0	0	0	0	
17022383	m	cn	JP	2019	0	1	1	0	0	0	0	0	0	0	0	0	72
17022744	m	cn	JP	2019	0	1	1	0	0	0	0	0	0	0	0	0	71
17023059	m	md	JP	2019	0	1	1	0	0	0	0	0	0	0	0	0	47
17023172	m	md	ES	2019	0	1	1	0	0	0	0	0	0	0	0	0	61
17023280	m	cn	JP	2019	0	1	1	0	0	0	0	0	0	0	0	1	81
17023327	f	md	US	2019	0	1	0	1	0	0	0	0	0	0	0	0	
17023383	f	cn	CN	2019	1	0	0	0	0	0	0	0	0	0	0	0	88.9
17023746	f	cn	CN	2019	1	0	0	0	0	0	0	0	0	0	0	0	76
17024279	m	md	FR	2019	0	1	0	0	0	1	0	0	0	0	0	0	78
17024340	f	cn	CN	2019	1	0	0	0	0	0	0	0	0	0	0	0	86
17024552	f	ot	US	2019	0	0	0	0	0	0	0	0	0	0	0	0	64
17024624	f	ot	US	2019	0	0	0	0	0	0	0	0	0	0	0	0	73
17024628	f	md	RO	2019	0	1	0	0	0	1	0	0	0	0	0	0	42
17024727	f	cn	CN	2019	1	0	0	0	0	0	0	0	0	0	0	0	61
17024839	m	cn	CN	2019	1	0	0	0	0	0	0	0	0	0	0	0	77
17024977	f	cn	CN	2019	1	0	0	0	0	0	0	0	0	0	0	0	63
17025040	f	cn	CN	2019	1	0	0	0	0	0	0	0	0	0	0	0	47
17025096	m	cn	CN	2019	1	0	0	0	0	0	0	0	0	0	0	0	56
17025097	m	cn	CN	2019	1	0	0	0	0	0	0	0	0	0	0	0	76
17025445	f	md	US	2019	0	1	0	1	0	0	0	0	0	0	0	0	49
17025795	m	cn	US	2019	0	0	0	0	0	0	0	0	0	0	0	0	
17025948	m	cn	CN	2019	1	0	0	0	0	0	0	0	0	0	0	0	63
17026040	f	cn	JP	2019	0	1	1	0	0	0	0	0	0	0	0	0	72
17026357	f	cn	CN	2019	1	0	0	0	0	0	0	0	0	0	0	0	74
17026359	m	Not repo	CN	2019	1	0	0	0	0	0	0	0	0	0	0	0	70
17026438	f	cn	CN	2019	1	0	0	0	0	0	0	0	0	0	0	0	50
17026879	f	cn	US	2019	1	0	0	0	0	0	0	0	0	0	0	0	76

17026882	m	md	RU	2019	0	1	0	0	0	1	0	0	0	0	0	0	63
17027084	f	cn	CN	2019	1	0	0	0	0	0	0	0	0	0	0	0	49.8
17027104	m	cn	CN	2019	1	0	0	0	0	0	0	0	0	0	0	0	53
17027180	m	cn	GB	2019	0	1	1	0	0	0	0	0	0	0	0	0	74
17027243	f	cn	CN	2019	1	0	0	0	0	0	0	0	0	0	0	0	62
17027274	m	md	US	2019	0	1	0	1	0	0	0	0	0	0	0	0	81
17027771	m	md	JP	2019	0	1	0	0	1	0	0	0	0	0	0	0	60
17027947	m	md	JP	2019	0	0	0	0	0	0	0	0	0	0	0	0	71
17028019	m	cn	JP	2019	0	1	1	0	0	0	0	0	0	0	0	1	66
17028042	m	ot	US	2019	0	0	0	0	0	0	0	0	0	0	0	0	73
17029002	Not	ot	FR	2019	1	0	0	0	0	0	0	0	0	0	0	0	
17029006	Not	ot	FR	2019	1	0	0	0	0	0	0	0	0	0	0	0	
17029220	Not	ot	JP	2019	0	0	0	0	0	0	0	0	0	0	0	0	
17029345	f	md	JP	2019	0	1	0	0	0	1	0	0	0	0	0	0	52
17029480	f	md	JP	2019	0	0	0	0	0	0	0	0	0	0	0	0	90
17029824	m	md	JP	2019	0	0	0	0	0	0	0	0	0	0	0	0	
17029829	m	md	CN	2019	0	0	0	0	0	0	0	0	0	0	0	0	74
17029831	m	md	CN	2019	0	0	0	0	0	0	0	0	0	0	0	0	50
17029967	m	Not repo	JP	2019	1	0	0	0	0	0	0	0	0	0	0	0	70
17030679	m	cn	CN	2019	0	0	0	0	0	0	0	0	0	0	0	0	52
17030698	m	cn	DE	2019	0	1	1	0	0	0	0	0	0	0	0	0	60
17030941	m	ph	FR	2019	0	0	0	0	0	0	0	0	0	0	0	0	
17031273	f	md	JP	2019	1	0	0	0	0	0	0	0	0	0	0	0	77.8
17032241	m	cn	JP	2019	0	1	1	0	0	0	0	0	0	0	0	0	71
17032281	Not	ot	FR	2019	1	0	0	0	0	0	0	0	0	0	0	0	
17032400	m	md	JP	2019	0	1	0	1	0	0	0	0	0	0	0	1	53
17032458	m	md	JP	2019	0	1	1	0	0	0	0	0	0	0	0	0	70
17032482	Not	md	TR	2019	0	0	0	0	0	0	0	0	0	0	0	0	
17032501	m	md	JP	2019	0	1	0	1	0	0	0	0	0	0	0	1	68
17032594	f	md	US	2019	1	0	0	0	0	0	0	0	0	0	0	0	
17032653	f	md	JP	2019	0	1	0	1	0	0	0	0	0	0	0	1	51
17032654	Not	ot	FR	2019	1	0	0	0	0	0	0	0	0	0	0	0	
17032655	Not	ot	FR	2019	1	0	0	0	0	0	0	0	0	0	0	0	
17032656	Not	ot	FR	2019	1	0	0	0	0	0	0	0	0	0	0	0	

17032717	f	cn	CN	2019	1	0	0	0	0	0	0	0	0	0	0	0	62
17032720	Not	md	IT	2019	1	0	0	0	0	0	0	0	0	0	0	0	
17032727	f	Not repo	CN	2019	1	0	0	0	0	0	0	0	0	0	0	0	66
17032821	m	ot	US	2019	0	1	1	0	0	0	0	0	0	0	0	0	68
17033079	m	cn	CN	2019	0	1	0	0	1	0	0	0	0	0	0	0	82
17033092	m	md	DE	2019	0	1	0	0	1	0	0	0	0	0	0	0	54
17033095	Not	ot	PT	2019	0	0	0	0	0	0	0	0	0	0	0	0	
17033515	Not	md	BR	2019	1	0	0	0	0	0	0	0	0	0	0	0	
17033796	m	md	JP	2019	0	1	1	0	0	0	0	0	0	0	0	0	66
17033856	m	ot	CN	2019	0	0	0	0	0	0	0	0	0	0	0	0	59
17034122	m	md	SG	2019	0	0	0	0	0	0	0	0	0	0	0	0	60
17034132	f	cn	GB	2019	0	1	1	0	0	0	0	0	0	0	0	0	60
17034623	m	cn	CA	2019	0	1	0	0	1	0	0	0	0	0	0	0	67
17034810	f	cn	CN	2019	0	1	0	0	1	0	0	0	0	0	0	0	31
17034977	f	md	FR	2019	0	0	0	0	0	0	0	0	0	0	0	0	83
17035196	m	ot	BE	2019	0	1	0	0	1	0	0	0	0	0	0	0	66
17035254	m	md	US	2019	0	1	0	0	1	0	0	0	0	0	0	0	76
17035333	m	md	JP	2019	0	1	1	0	0	0	0	0	0	0	0	0	77
17035457	m	cn	UA	2019	0	1	1	0	0	0	0	0	0	0	0	0	29
17036182	m	cn	CN	2019	1	0	0	0	0	0	0	0	0	0	0	0	64
17036200	f	md	US	2019	0	1	0	0	0	1	0	0	0	0	0	0	74
17036305	f	md	BR	2019	0	0	0	0	0	0	0	0	0	0	0	0	60
17036366	m	md	RU	2019	0	0	0	0	0	0	0	0	0	0	0	0	71
17036391	m	md	DE	2019	0	1	0	0	1	0	0	0	0	0	0	0	80
17036696	f	cn	CN	2019	1	0	0	0	0	0	0	0	0	0	0	0	65
17036745	f	cn	IN	2019	0	0	0	0	0	0	0	0	0	0	0	0	59
17036911	Not	md	JP	2019	1	0	0	0	0	0	0	0	0	0	0	0	
17037196	f	md	US	2019	0	1	0	0	1	0	0	0	0	0	0	1	73
17037556	Not	ot	IT	2019	0	1	0	1	0	0	0	0	0	0	0	0	
17037778	m	md	JP	2019	0	1	0	0	1	0	0	0	0	0	0	0	73
17037926	f	ot	IT	2019	1	0	0	0	0	0	0	0	0	0	0	0	
17037934	m	Not repo	JP	2019	1	0	0	0	0	0	0	0	0	0	0	1	75
17037937	m	ph	JP	2019	1	0	0	0	0	0	0	0	0	0	0	0	73
17037961	m	md	JP	2019	0	1	1	0	0	0	0	0	0	0	0	0	64

17037980	m	md	NL	2019	0	1	0	0	0	1	0	0	0	0	0	0	61
17037993	f	md	CA	2019	0	1	0	1	0	0	0	0	0	0	0	0	
17038081	m	md	PH	2019	0	1	0	1	0	0	0	0	0	0	0	0	67.3
17038185	m	md	IT	2019	0	1	0	1	0	0	0	0	0	0	0	0	
17038306	f	cn	JP	2019	0	1	1	0	0	0	0	0	0	0	0	1	73
17038432	m	Not repo	GB	2019	0	1	0	1	0	0	0	0	0	0	0	0	57
17038434	m	md	NZ	2019	1	0	0	0	0	0	0	0	0	0	0	0	81
17038445	Not	md	IT	2019	1	0	0	0	0	0	0	0	0	0	0	0	
17038565	m	cn	CA	2019	0	0	0	0	0	0	0	0	0	0	0	0	57
17038567	m	md	JP	2019	0	1	0	1	0	0	0	0	0	0	0	0	75.2
17038596	m	cn	JP	2019	0	1	1	0	0	0	0	0	0	0	0	0	65
17038888	m	md	FR	2019	0	1	0	1	0	0	0	0	0	0	0	0	
17038930	m	md	SA	2019	0	1	0	0	1	0	0	0	0	0	0	0	
17039441	m	cn	JP	2019	0	1	1	0	0	0	0	0	0	0	0	0	63
17039506	f	cn	BR	2019	1	0	0	0	0	0	0	0	0	0	0	0	78
17039591	f	cn	CA	2019	0	0	0	0	0	0	0	0	0	0	0	0	54
17039627	f	md	EC	2019	0	1	0	0	0	1	0	0	0	0	0	0	65
17039669	Not	ot	JP	2019	0	0	0	0	0	0	0	0	0	0	0	0	
17039670	Not	ot	JP	2019	0	0	0	0	0	0	0	0	0	0	0	0	
17040580	f	ph	US	2019	1	0	0	0	0	0	0	0	0	0	0	0	69
17041380	f	cn	Not re	2019	0	0	0	0	0	0	0	0	0	0	0	0	
17041501	m	cn	US	2019	0	0	0	0	0	0	0	0	0	0	0	0	67
17042598	m	ph	US	2019	0	0	0	0	0	0	0	0	0	0	0	0	64
17042664	m	ot	JP	2019	0	1	0	0	1	0	0	0	0	0	0	0	57
17042810	m	ot	CA	2019	0	0	0	0	0	0	0	0	0	0	0	0	49
17042839	m	md	JP	2019	0	0	0	0	0	0	0	0	0	0	0	0	68
17043031	m	md	US	2019	0	1	1	0	0	0	0	0	0	0	0	0	59
17043094	Not	ot	DE	2019	0	0	0	0	0	0	0	0	0	0	0	0	
17043646	Not	ph	US	2019	0	1	0	1	0	0	0	0	0	0	0	1	69
17044151	f	Not repo	US	2019	1	0	0	0	0	0	0	0	0	0	0	0	
17044279	m	md	DE	2019	0	1	0	0	1	0	0	0	0	0	0	0	54
17044308	f	ot	CN	2019	0	1	1	0	0	0	0	0	0	0	0	0	44
17044510	f	cn	CN	2019	1	0	0	0	0	0	0	0	0	0	0	0	62
17045178	Not	ot	PT	2019	0	0	0	0	0	0	0	0	0	0	0	0	

17045892	m	ph	JP	2019	0	1	0	0	0	1	0	0	0	0	0	0	55
17045979	m	md	JP	2019	0	1	0	0	0	1	0	0	0	0	0	0	80
17046130	f	md	JP	2019	0	1	0	0	0	1	0	0	0	0	0	0	55
17046421	m	cn	DE	2019	0	1	1	0	0	0	0	0	0	0	0	0	63
17046431	Not	md	JP	2019	0	0	0	0	0	0	0	0	0	0	0	0	70
17046497	Not	md	US	2019	0	1	0	0	1	0	0	0	0	0	0	0	
17046555	m	ot	CA	2019	0	0	0	0	0	0	0	0	0	0	0	0	
17046654	m	md	US	2019	0	1	1	0	0	0	0	0	0	0	0	0	77
17046752	m	cn	US	2019	1	0	0	0	0	0	0	0	0	0	0	0	
17046767	Not	ph	CA	2019	0	1	1	0	0	0	0	0	0	0	0	1	
17046946	m	ot	US	2019	0	0	0	0	0	0	0	0	0	0	0	0	
17047023	m	cn	CN	2019	1	0	0	0	0	0	0	0	0	0	0	0	86
17047301	f	md	IT	2019	0	1	0	0	0	1	0	0	0	0	0	0	70
17048205	Not	md	JP	2019	1	0	0	0	0	0	0	0	0	0	0	0	
17048394	m	md	NL	2019	0	0	0	0	0	0	0	0	0	0	0	0	
17048547	Not	md	US	2019	0	0	0	0	0	0	0	0	0	0	0	0	
17048584	m	ot	KR	2019	0	1	0	0	1	0	0	0	0	0	0	0	74
17048762	m	cn	IN	2019	0	0	0	0	0	0	0	0	0	0	0	0	62
17048821	m	md	JP	2019	0	1	0	1	0	0	0	0	0	0	0	1	58
17048917	m	ot	US	2019	0	1	0	1	0	0	0	0	0	0	0	0	64
17049113	f	cn	JP	2019	0	1	1	0	0	0	0	0	0	0	0	0	74
17049226	Not	md	ES	2019	0	1	0	1	0	0	0	0	0	0	0	0	
17049233	m	md	JP	2019	0	1	0	1	0	0	0	0	0	0	0	1	65.9
17049394	f	Not repo	CN	2019	1	0	0	0	0	0	0	0	0	0	0	0	52
17049398	Not	Not repo	JP	2019	1	0	0	0	0	0	0	0	0	0	0	0	
17049483	m	Not repo	JP	2019	1	0	0	0	0	0	0	0	0	0	0	0	68
17049504	m	md	TH	2019	0	1	1	0	0	0	0	0	0	0	0	0	63
17049528	f	cn	DE	2019	0	1	1	0	0	0	0	0	0	0	0	0	69
17050294	m	cn	JP	2019	0	1	1	0	0	0	0	0	0	0	0	0	80
17050348	f	md	US	2019	0	0	0	0	0	0	0	0	0	0	0	0	52
17050358	m	md	HU	2019	0	0	0	0	0	0	0	0	0	0	0	0	58
17050372	f	ot	NL	2019	0	1	1	0	0	0	0	0	0	0	0	0	55
17050738	m	ot	KR	2019	0	1	0	0	1	0	0	0	0	0	0	0	74
17051119	m	cn	JP	2019	0	1	1	0	0	0	0	0	0	0	0	0	65

17051123	f	Not repo	JP	2019	1	0	0	0	0	0	0	0	0	0	0	0	65
17051127	m	md	TR	2019	0	1	0	0	0	1	0	0	0	0	0	0	53
17051631	Not	ph	CA	2019	0	1	1	0	0	0	0	0	0	0	0	1	
17051744	f	md	GB	2019	1	0	0	0	0	0	0	0	0	0	0	0	
17051745	m	md	JP	2019	0	0	0	0	0	0	0	0	0	0	0	0	75
17051837	m	ot	US	2019	0	1	0	0	1	0	0	0	0	0	0	0	
17052512	f	md	JP	2019	0	0	0	0	0	0	1	0	0	0	1	0	61
17052742	m	md	JP	2019	0	1	0	0	0	1	0	0	0	0	0	0	54
17052831	m	ph	JP	2019	0	0	0	0	0	0	0	0	0	0	0	0	48
17053110	f	ot	US	2019	0	0	0	0	0	0	0	0	0	0	0	0	45
17053437	Not	cn	DE	2019	0	1	0	0	0	1	0	0	0	0	0	0	
17053581	m	ot	JP	2019	0	1	1	0	0	0	0	0	0	0	0	0	72
17053858	m	ot	US	2019	0	1	0	1	0	0	0	0	0	0	0	0	47
17054112	m	md	JP	2019	0	1	0	1	0	0	0	0	0	0	0	1	55
17054201	m	ot	IN	2019	0	1	1	0	0	0	0	0	0	0	0	0	38
17054226	m	cn	US	2019	0	1	0	1	0	0	0	0	0	0	0	0	85.7
17054299	m	md	US	2019	0	1	0	1	0	0	0	0	0	0	0	0	67
17054734	m	ot	NL	2019	0	1	1	0	0	0	0	0	0	0	0	0	74
17054736	Not	ot	NL	2019	0	1	0	0	0	1	0	0	0	0	0	0	
17055217	f	ot	JP	2019	1	0	0	0	0	0	0	0	0	0	0	0	20
17055332	f	ot	CA	2019	0	0	0	0	0	0	0	0	0	0	0	0	
17055457	f	md	US	2019	0	1	0	0	1	0	0	0	0	0	0	0	65
17055619	m	md	DE	2019	0	1	1	0	0	0	0	0	0	0	0	0	
17055906	m	md	JP	2019	0	1	0	0	1	0	0	0	0	0	0	0	71
17055911	m	Not repo	DE	2019	0	1	0	1	0	0	0	0	0	0	0	0	56
17056081	m	md	JP	2019	0	1	0	0	1	0	0	0	0	0	0	0	73
17056168	f	ot	CA	2019	0	0	0	0	0	0	0	0	0	0	0	0	63
17056346	Not	md	JP	2019	0	1	0	1	0	0	0	0	0	0	0	1	
17056496	f	md	US	2019	0	1	1	0	0	0	0	0	0	0	0	0	47
17056550	f	ot	US	2019	1	0	0	0	0	0	0	0	0	0	0	0	55
17057552	f	Not repo	JP	2019	1	0	0	0	0	0	0	0	0	0	0	1	62
17057759	f	ph	US	2019	0	1	0	0	1	0	0	0	0	0	0	0	76
17057885	m	cn	JP	2019	0	1	1	0	0	0	0	0	0	0	0	0	91
17057934	f	md	DE	2019	1	0	0	0	0	0	0	0	0	0	0	0	

17058093	f	cn	CN	2019	1	0	0	0	0	0	0	0	0	0	0	0	72
17059241	f	md	JP	2019	0	1	0	0	1	0	0	0	0	0	0	1	67
17059276	f	cn	JP	2019	0	1	1	0	0	0	0	0	0	0	0	1	
17059340	m	Not repo	JP	2019	1	0	0	0	0	0	0	0	0	0	0	0	
17059862	m	cn	DE	2019	0	1	1	0	0	0	0	0	0	0	0	0	
17060108	f	Not repo	JP	2019	0	1	0	1	0	0	0	0	0	0	0	0	77
17060383	m	cn	Not re	2019	0	0	0	0	0	0	0	0	0	0	0	0	71
17060449	m	md	JP	2019	1	0	0	0	0	0	0	0	0	0	0	0	69
17060579	m	cn	CN	2019	1	0	0	0	0	0	0	0	0	0	0	0	44
17060583	m	md	US	2019	0	0	0	0	0	0	1	0	0	1	0	0	72
17060722	m	md	JP	2019	0	1	0	1	0	0	0	0	0	0	0	1	68
17060726	Not	md	US	2019	1	0	0	0	0	0	0	0	0	0	0	0	
17061295	m	md	CN	2019	0	0	0	0	0	0	0	0	0	0	0	0	56
17061390	m	md	US	2019	0	0	0	0	0	0	0	0	0	0	0	0	56
17061538	f	cn	JP	2019	0	1	1	0	0	0	0	0	0	0	0	0	52
17061763	m	cn	JP	2019	0	1	1	0	0	0	0	0	0	0	0	0	71
17061806	f	md	CN	2019	0	0	0	0	0	0	0	0	0	0	0	0	44
17061966	m	md	JP	2019	0	1	0	1	0	0	0	0	0	0	0	1	80
17062014	f	ot	BE	2019	0	1	0	0	1	0	0	0	0	0	0	0	72
17062069	m	md	JP	2019	0	1	0	0	0	1	0	0	0	0	0	0	71
17062109	Not	md	JP	2019	1	0	0	0	0	0	0	0	0	0	0	0	
17062176	f	md	FR	2019	1	0	0	0	0	0	0	0	0	0	0	0	67
17062195	f	md	FR	2019	0	1	0	0	0	1	0	0	0	0	0	0	57
17062227	m	md	JP	2019	0	1	0	0	0	1	0	0	0	0	0	1	58
17062337	f	md	US	2019	0	0	0	0	0	0	0	0	0	0	0	0	61
17062486	f	ot	FR	2019	0	1	0	0	1	0	0	0	0	0	0	0	82
17062542	m	ot	JP	2019	0	0	0	0	0	0	0	0	0	0	0	0	60
17062738	m	md	JP	2019	0	1	0	0	1	0	0	0	0	0	0	0	79
17063080	m	md	LB	2019	0	1	0	0	1	0	0	0	0	0	0	0	
17063659	Not	md	CN	2019	1	0	0	0	0	0	0	0	0	0	0	0	
17063732	Not	md	CN	2019	1	0	0	0	0	0	0	0	0	0	0	0	
17063831	m	md	CN	2019	1	0	0	0	0	0	0	0	0	0	0	0	73
17063922	Not	md	JP	2019	0	1	0	1	0	0	0	0	0	0	0	1	
17064912	f	ph	JP	2019	1	0	0	0	0	0	0	0	0	0	0	0	82

17065181	Not	Not repo	JP	2019	0	1	0	1	0	0	0	0	0	0	0	1	
17065185	m	md	JP	2019	0	1	0	1	0	0	0	0	0	0	0	0	
17065505	m	md	JP	2019	0	1	0	0	1	0	0	0	0	0	0	0	66
17065701	m	Not repo	JP	2019	1	0	0	0	0	0	0	0	0	0	0	0	
17065890	Not	md	IT	2019	1	0	0	0	0	0	0	0	0	0	0	0	
17065993	m	Not repo	ID	2019	1	0	0	0	0	0	0	0	0	0	0	0	
17066831	m	ot	FR	2019	0	1	0	0	1	0	0	0	0	0	0	0	82
17066909	m	cn	JP	2019	0	1	1	0	0	0	0	0	0	0	0	0	67
17066912	f	Not repo	CN	2019	1	0	0	0	0	0	0	0	0	0	0	0	54
17066978	m	md	US	2019	1	0	0	0	0	0	0	0	0	0	0	0	87
17067043	m	cn	Not re	2019	0	0	0	0	0	0	0	0	0	0	0	0	
17067106	f	md	DE	2019	0	1	0	1	0	0	0	0	0	0	0	0	66
17067512	f	ot	GB	2019	0	1	0	0	1	0	0	0	0	0	0	0	76
17067773	f	cn	JP	2019	0	0	0	0	0	0	0	0	0	0	0	0	80
17067793	m	md	JP	2019	0	1	0	0	1	0	0	0	0	0	0	0	75
17067794	f	md	JP	2019	0	1	0	0	1	0	0	0	0	0	0	0	81
17068043	Not	md	UA	2019	0	0	0	0	0	0	0	0	0	0	0	0	
17068188	Not	md	CN	2019	0	0	0	0	0	0	0	0	0	0	0	0	
17069072	f	cn	US	2019	0	1	0	0	1	0	0	0	0	0	0	0	62
17069223	f	cn	US	2019	0	1	1	0	0	0	0	0	0	0	0	0	70
17070120	m	cn	JP	2019	0	1	1	0	0	0	0	0	0	0	0	0	75
17070253	m	ot	JP	2019	0	0	0	0	0	0	0	0	0	0	0	0	69
17070718	m	md	US	2019	0	0	0	0	0	0	0	0	0	0	0	0	55
17070750	m	cn	JP	2019	0	1	1	0	0	0	0	0	0	0	0	0	82
17070797	m	cn	JP	2019	0	1	1	0	0	0	0	0	0	0	0	0	60
17071062	m	cn	CN	2019	1	0	0	0	0	0	0	0	0	0	0	0	54
17071151	f	Not repo	US	2019	0	0	0	0	0	0	0	0	0	0	0	0	72
17071158	m	md	CN	2019	0	0	0	0	0	0	0	0	0	0	0	0	64
17071296	m	md	JP	2019	0	1	1	0	0	0	0	0	0	0	0	0	75
17071992	m	md	JP	2019	0	1	0	0	1	0	0	0	0	0	0	0	55
17071998	f	md	JP	2019	0	1	0	0	1	0	0	0	0	0	0	0	82
17072040	Not	md	IT	2019	1	0	0	0	0	0	0	0	0	0	0	0	
17072135	m	md	IT	2019	0	1	1	0	0	0	0	0	0	0	0	0	75
17072306	m	md	US	2019	0	1	0	1	0	0	0	0	0	0	0	0	70

17072314	m	md	JP	2019	0	1	0	1	0	0	0	0	0	0	0	0	58
17072330	Not	cn	MX	2019	0	0	0	0	0	0	0	0	0	0	0	0	27
17072335	m	md	JP	2019	0	1	0	0	1	0	0	0	0	0	0	0	48
17072352	f	Not repo	JP	2019	1	0	0	0	0	0	0	0	0	0	0	0	87
17072436	f	md	AU	2019	0	1	0	0	1	0	0	0	0	0	0	0	73
17072614	m	cn	JP	2019	0	1	1	0	0	0	0	0	0	0	0	0	65
17072722	m	md	HU	2019	0	0	0	0	0	0	0	0	0	0	0	0	58
17072759	m	cn	SA	2019	0	1	1	0	0	0	0	0	0	0	0	0	
17072850	f	md	DK	2019	0	1	1	0	0	0	0	0	0	0	0	0	76
17073035	f	cn	US	2019	1	0	0	0	0	0	0	0	0	0	0	0	
17073113	m	md	NL	2019	0	1	1	0	0	0	0	0	0	0	0	0	71
17073159	Not	md	IT	2019	0	1	0	0	0	1	0	0	0	0	0	0	
17073364	m	cn	US	2019	1	0	0	0	0	0	0	0	0	0	0	0	73
17073395	m	md	JP	2019	0	1	0	0	1	0	0	0	0	0	0	0	68
17073399	m	md	HR	2019	0	1	1	0	0	0	0	0	0	0	0	0	66
17073716	f	cn	JP	2019	0	0	0	0	0	0	0	0	0	0	0	0	77
17073900	m	md	US	2019	0	0	0	0	0	0	0	0	0	0	0	0	73
17074184	Not	cn	JP	2019	0	1	1	0	0	0	0	0	0	0	0	0	85
17074227	m	md	JP	2019	0	1	1	0	0	0	0	0	0	0	0	0	70
17074249	m	ot	DE	2019	1	0	0	0	0	0	0	0	0	0	0	0	61
17074550	m	Not repo	ID	2019	1	0	0	0	0	0	0	0	0	0	0	0	
17074717	m	cn	CN	2019	1	0	0	0	0	0	0	0	0	0	0	0	61
17074809	m	md	JP	2019	0	1	1	0	0	0	0	0	0	0	0	0	69
17074954	f	md	JP	2019	1	0	0	0	0	0	0	0	0	0	0	0	90
17075199	m	md	TW	2019	0	1	0	0	0	1	0	0	0	0	0	0	82
17075255	Not	ot	FR	2019	0	1	0	1	0	0	0	0	0	0	0	0	
17075489	m	md	US	2019	0	0	0	0	0	0	0	0	0	0	0	0	
17075736	m	md	GB	2019	0	1	1	0	0	0	0	0	0	0	0	0	74
17076000	f	Not repo	US	2019	0	0	0	0	0	0	0	0	0	0	0	0	55
17076168	f	ph	JP	2019	0	0	0	0	0	0	1	0	0	0	1	0	61
17076230	f	Not repo	KR	2019	0	0	0	0	0	0	0	0	0	0	0	0	
17076232	Not	md	JP	2019	1	0	0	0	0	0	0	0	0	0	0	0	
17076388	m	cn	KR	2019	1	0	0	0	0	0	0	0	0	0	0	0	70
17076401	m	Not repo	AU	2019	0	1	0	1	0	0	0	0	0	0	0	0	

17076465	m	cn	CA	2019	1	0	0	0	0	0	0	0	0	0	0	0	
17076782	Not	md	JP	2019	1	0	0	0	0	0	0	0	0	0	0	0	
17076796	m	cn	IN	2019	0	1	0	0	1	0	0	0	0	0	0	0	
17076953	f	md	JP	2019	1	0	0	0	0	0	0	0	0	0	0	0	85.5
17077030	f	cn	GB	2019	0	1	1	0	0	0	0	0	0	0	0	0	60
17077807	f	cn	Not re	2019	0	0	0	0	0	0	0	0	0	0	0	0	62
17077834	f	ot	US	2019	0	1	0	1	0	0	0	0	0	0	0	0	49
17078050	m	ot	CN	2019	0	1	0	0	1	0	0	0	0	0	0	0	64
17078224	m	ot	ES	2019	0	1	1	0	0	0	0	0	0	0	0	0	67
17078716	f	md	IT	2019	1	0	0	0	0	0	0	0	0	0	0	0	
17078797	m	md	JP	2019	0	0	0	0	0	0	0	0	0	0	0	0	
17078975	m	cn	JP	2019	0	1	1	0	0	0	0	0	0	0	0	0	70
17079005	m	cn	JP	2019	0	1	1	0	0	0	0	0	0	0	0	0	63
17079412	m	cn	US	2019	1	0	0	0	0	0	0	0	0	0	0	0	
17079716	f	md	JP	2019	0	1	0	1	0	0	0	0	0	0	0	1	79
17079948	Not	md	US	2019	0	1	0	1	0	0	0	0	0	0	0	0	
17080090	m	md	JP	2019	0	1	0	0	0	1	0	0	0	0	0	1	80
17080149	m	md	JP	2019	0	1	0	0	0	1	0	0	0	0	0	0	33
17080539	f	md	JP	2019	0	0	0	0	0	0	0	0	0	0	0	0	80
17080794	f	ot	RU	2019	1	0	0	0	0	0	0	0	0	0	0	0	
17080806	f	cn	US	2019	1	0	0	0	0	0	0	0	0	0	0	0	
17081087	m	md	JP	2019	1	0	0	0	0	0	0	0	0	0	0	0	80
17081503	m	cn	IN	2019	0	1	0	0	1	0	0	0	0	0	0	0	64
17082539	f	cn	JP	2019	0	1	1	0	0	0	0	0	0	0	0	0	69
17082697	f	md	JP	2019	1	0	0	0	0	0	0	0	0	0	0	0	80
17082758	m	md	RU	2019	0	1	1	0	0	0	0	0	0	0	0	0	70
17082845	f	ot	AT	2019	0	1	0	0	0	1	0	0	0	0	0	0	59
17082914	m	cn	KR	2019	0	0	0	0	0	0	0	0	0	0	0	0	
17083041	m	cn	CN	2019	1	0	0	0	0	0	0	0	0	0	0	0	51.7
17083082	m	Not repo	JP	2019	1	0	0	0	0	0	0	0	0	0	0	0	60
17083161	f	cn	US	2019	1	0	0	0	0	0	0	0	0	0	0	0	56
17083443	f	ot	NL	2019	0	1	1	0	0	0	0	0	0	0	0	0	60
17083515	m	md	FR	2019	0	1	0	1	0	0	0	0	0	0	0	0	
17083571	Not	Not repo	JP	2019	1	0	0	0	0	0	0	0	0	0	0	0	

17083668	f	md	US	2019	1	0	0	0	0	0	0	0	0	0	0	0	
17083687	m	ot	IT	2019	0	0	0	0	0	0	0	0	0	0	0	0	56
17083759	f	cn	US	2019	1	0	0	0	0	0	0	0	0	0	0	0	83
17083804	m	cn	Not re	2019	0	0	0	0	0	0	0	0	0	0	0	0	51
17083979	m	ot	GB	2019	0	0	0	0	0	0	0	0	0	0	0	0	67
17084000	m	Not repo	SG	2019	1	0	0	0	0	0	0	0	0	0	0	0	67.3
17084197	f	md	MY	2019	0	1	1	0	0	0	0	0	0	0	0	0	39
17084515	m	cn	DE	2019	0	1	1	0	0	0	0	0	0	0	0	0	60
17084525	f	ot	DE	2019	0	1	0	0	1	0	0	0	0	0	0	0	73
17084661	m	cn	JP	2019	0	1	1	0	0	0	0	0	0	0	0	0	
17085239	m	cn	JP	2019	0	0	0	0	0	0	0	0	0	0	0	0	70
17085310	m	md	NL	2019	0	1	1	0	0	0	0	0	0	0	0	0	71
17085407	m	ot	GR	2019	0	1	0	0	1	0	0	0	0	0	0	0	63
17085458	m	md	JP	2019	0	1	1	0	0	0	0	0	0	0	0	0	79
17086612	Not	md	JP	2019	0	1	0	0	0	1	0	0	0	0	0	0	
17086871	m	md	JP	2019	0	1	0	0	0	1	0	0	0	0	0	0	63
17086904	f	cn	JP	2019	0	1	0	0	1	0	0	0	0	0	0	0	
17087215	Not	md	IT	2019	1	0	0	0	0	0	0	0	0	0	0	0	
17088033	f	md	AT	2019	0	0	0	0	0	0	0	0	0	0	0	0	54
17088106	f	md	FR	2019	0	1	0	1	0	0	0	0	0	0	0	0	
17088571	m	ot	DE	2019	0	1	1	0	0	0	0	0	0	0	0	0	69
17088588	m	md	JP	2019	0	1	0	0	1	0	0	0	0	0	0	0	85
17088713	m	md	FR	2019	0	1	0	1	0	0	0	0	0	0	0	0	74
17088715	m	md	FR	2019	0	1	0	1	0	0	0	0	0	0	0	0	
17088913	m	md	FR	2019	0	1	0	1	0	0	0	0	0	0	0	0	
17089010	m	cn	JP	2019	0	0	0	0	0	0	0	0	0	0	0	0	59
17089011	m	cn	JP	2019	0	1	1	0	0	0	0	0	0	0	0	0	66
17089129	f	md	FR	2019	0	1	0	1	0	0	0	0	0	0	0	0	
17089134	m	md	FR	2019	0	1	0	1	0	0	0	0	0	0	0	1	
17089151	m	md	FR	2019	0	1	0	1	0	0	0	0	0	0	0	0	
17089154	m	md	FR	2019	0	1	0	1	0	0	0	0	0	0	0	0	
17089178	m	Not repo	CA	2019	1	0	0	0	0	0	0	0	0	0	0	0	
17089216	m	md	JP	2019	0	1	0	1	0	0	0	0	0	0	0	1	77
17089222	m	Not repo	JP	2019	0	1	0	1	0	0	0	0	0	0	0	1	62

17089240	f	cn	US	2019	0	1	0	0	1	0	0	0	0	0	0	0	75
17089243	f	md	JP	2019	0	1	0	1	0	0	0	0	0	0	0	0	75
17089245	m	md	FR	2019	0	1	0	1	0	0	0	0	0	0	0	0	
17089254	m	cn	CN	2019	0	1	0	0	1	0	0	0	0	0	0	0	88
17089272	Not	md	IT	2019	1	0	0	0	0	0	0	0	0	0	0	0	
17089273	Not	md	IT	2019	1	0	0	0	0	0	0	0	0	0	0	0	
17089531	m	md	JP	2019	0	1	1	0	0	0	0	0	0	0	0	0	70
17089543	f	md	DE	2019	0	1	1	0	0	0	0	0	0	0	0	0	76
17089556	f	cn	JP	2019	0	1	1	0	0	0	0	0	0	0	0	0	68
17089720	f	cn	US	2019	0	0	0	0	0	0	0	0	0	0	0	0	
17089737	f	cn	US	2019	0	1	1	0	0	0	0	0	0	0	0	0	56
17090657	Not	md	JP	2019	0	1	0	0	1	0	0	0	0	0	0	0	
17090987	f	ot	NL	2019	0	1	0	0	1	0	0	0	0	0	0	0	62
17091056	f	md	JP	2019	0	1	0	0	0	1	0	0	0	0	0	0	68
17091101	f	Not repo	US	2019	0	0	0	0	0	0	0	0	0	0	0	0	72
17091258	f	cn	JP	2019	0	1	1	0	0	0	0	0	0	0	0	1	80
17091405	m	md	US	2019	1	0	0	0	0	0	0	0	0	0	0	0	
17091727	m	md	JP	2019	1	0	0	0	0	0	0	0	0	0	0	0	70
17091728	m	md	JP	2019	0	1	0	1	0	0	0	0	0	0	0	1	
17091769	m	md	JP	2019	0	1	0	0	0	1	0	0	0	0	0	0	72
17091929	m	md	DE	2019	0	1	0	0	1	0	0	0	0	0	0	0	62
17091932	f	md	JP	2019	0	1	1	0	0	0	0	0	0	0	0	0	72
17092349	Not	md	FR	2019	0	0	0	0	0	0	0	0	0	0	0	0	
17092752	m	Not repo	JP	2019	0	1	0	1	0	0	0	0	0	0	0	0	76
17092756	f	md	MY	2019	1	0	0	0	0	0	0	0	0	0	0	0	61
17092790	Not	md	CN	2019	1	0	0	0	0	0	0	0	0	0	0	0	
17092792	Not	md	CN	2019	1	0	0	0	0	0	0	0	0	0	0	0	
17092861	Not	md	JP	2019	0	1	0	1	0	0	0	0	0	0	0	1	
17092921	f	Not repo	DK	2019	1	0	0	0	0	0	0	0	0	0	0	1	71
17092930	f	md	JP	2019	1	0	0	0	0	0	0	0	0	0	0	0	82
17093008	f	ph	JP	2019	1	0	0	0	0	0	0	0	0	0	0	0	69
17093049	Not	ph	JP	2019	1	0	0	0	0	0	0	0	0	0	0	0	
17093058	f	ph	CA	2019	1	0	0	0	0	0	0	0	0	0	0	0	
17093114	m	Not repo	JP	2019	0	1	0	1	0	0	0	0	0	0	0	0	70

17093397	m	cn	Not re	2019	0	0	0	0	0	0	0	0	0	0	0	0	48
17093408	f	ot	JP	2019	1	0	0	0	0	0	0	0	0	0	0	0	70
17093410	f	ot	IT	2019	1	0	0	0	0	0	0	0	0	0	0	0	80
17093453	m	cn	US	2019	0	1	1	0	0	0	0	0	0	0	0	0	
17093610	m	md	DE	2019	0	1	0	0	1	0	0	0	0	0	0	0	54
17093615	f	md	IT	2019	1	0	0	0	0	0	0	0	0	0	0	0	
17093654	m	md	JP	2019	0	1	0	1	0	0	0	0	0	0	0	1	63
17093812	f	Not repo	JP	2019	1	0	0	0	0	0	0	0	0	0	0	0	70
17093819	f	ot	DE	2019	0	1	0	0	1	0	0	0	0	0	0	0	71
17093891	m	cn	JP	2019	0	0	0	0	0	0	0	0	0	0	0	0	59
17093957	Not	md	JP	2019	0	1	0	1	0	0	0	0	0	0	0	1	
17094118	m	ot	US	2019	0	1	0	0	1	0	0	0	0	0	0	1	60
17094141	m	ot	JP	2019	0	1	0	0	1	0	0	0	0	0	0	0	62
17094252	m	cn	JP	2019	0	1	1	0	0	0	0	0	0	0	0	0	70
17094261	m	md	JP	2019	0	0	0	0	0	0	0	0	0	0	0	0	64
17094373	m	md	TW	2019	1	0	0	0	0	0	0	0	0	0	0	0	58
17094389	f	Not repo	CA	2019	1	0	0	0	0	0	0	0	0	0	0	0	88.1
17094396	f	Not repo	US	2019	1	0	0	0	0	0	0	0	0	0	0	0	72
17094565	m	cn	JP	2019	0	1	1	0	0	0	0	0	0	0	0	1	75
17094760	f	ot	FI	2019	0	1	0	0	0	1	0	0	0	0	0	0	54
17095040	f	md	TW	2019	1	0	0	0	0	0	0	0	0	0	0	0	73
17095310	m	md	DE	2019	0	1	0	0	1	0	0	0	0	0	0	0	54
17095392	m	ot	DE	2019	0	1	1	0	0	0	0	0	0	0	0	0	67
17095698	m	md	US	2019	0	1	1	0	0	0	0	0	0	0	0	0	55
17095897	m	md	DE	2019	1	0	0	0	0	0	0	0	0	0	0	0	37
17096068	f	md	JP	2019	0	1	0	0	0	1	0	0	0	0	0	0	73
17096119	f	md	DE	2019	1	0	0	0	0	0	0	0	0	0	0	0	38
17096226	m	md	CN	2019	1	0	0	0	0	0	0	0	0	0	0	0	45
17096322	f	md	JP	2019	0	1	1	0	0	0	0	0	0	0	0	0	67
17096407	m	ot	KR	2019	0	0	0	0	0	0	0	0	0	0	0	0	76
17096570	f	md	CZ	2019	1	0	0	0	0	0	0	0	0	0	0	0	47
17096817	m	md	CZ	2019	1	0	0	0	0	0	0	0	0	0	0	0	77
17096928	Not	md	JP	2019	0	1	0	0	0	1	0	0	0	0	0	0	70
17097083	f	md	CZ	2019	1	0	0	0	0	0	0	0	0	0	0	0	76

17097100	f	md	PL	2019	0	1	0	0	0	1	0	0	0	0	0	0	65
17097498	m	md	UA	2019	0	1	1	0	0	0	0	0	0	0	0	0	74
17097530	m	md	PL	2019	0	1	1	0	0	0	0	0	0	0	0	0	62
17098112	m	md	JP	2019	0	1	1	0	0	0	0	0	0	0	0	0	76
17098170	m	ot	AU	2019	0	0	0	0	0	0	0	0	0	0	0	0	75
17098213	f	md	JP	2019	1	0	0	0	0	0	0	0	0	0	0	0	81
17098324	Not	ot	DE	2019	0	1	0	0	1	0	0	0	0	0	0	0	
17098351	m	cn	JP	2019	0	1	1	0	0	0	0	0	0	0	0	1	81
17098472	m	cn	JP	2019	0	1	1	0	0	0	0	0	0	0	0	1	
17098485	f	cn	US	2019	0	0	0	0	0	0	0	0	0	0	0	0	
17098647	m	md	TW	2019	0	0	0	0	0	0	0	0	0	0	0	0	84
17098957	f	md	JP	2019	0	1	1	0	0	0	0	0	0	0	0	0	67
17099062	m	ot	CN	2019	0	0	0	0	0	0	0	0	0	0	0	0	61
17099073	f	md	JP	2019	1	0	0	0	0	0	0	0	0	0	0	0	73
17099117	f	ot	FR	2019	0	1	1	0	0	0	0	0	0	0	0	0	53
17100277	f	Not repo	MY	2019	1	0	0	0	0	0	0	0	0	0	0	0	
17100385	m	md	FR	2019	0	1	0	1	0	0	0	0	0	0	0	0	
17101429	f	cn	KR	2019	0	0	0	0	0	0	0	0	0	0	0	0	
17101526	m	cn	JP	2019	0	1	1	0	0	0	0	0	0	0	0	1	78
17101544	f	cn	JP	2019	0	1	1	0	0	0	0	0	0	0	0	0	67
17101576	m	md	US	2019	0	1	1	0	0	0	0	0	0	0	0	0	
17101608	m	md	JP	2019	0	0	0	0	0	0	0	0	0	0	0	0	70
17101979	m	cn	JP	2019	0	0	0	0	0	0	0	0	0	0	0	0	49
17102039	Not	md	CN	2019	1	0	0	0	0	0	0	0	0	0	0	0	
17102181	f	ot	HU	2019	0	1	0	0	1	0	0	0	0	0	0	0	73
17102983	Not	md	AU	2019	0	1	0	0	0	1	0	0	0	0	0	0	
17103508	f	md	JP	2019	0	1	0	0	1	0	0	0	0	0	0	0	79
17103572	f	ot	RU	2019	1	0	0	0	0	0	0	0	0	0	0	0	60
17103618	m	md	JP	2019	0	1	0	0	1	0	0	0	0	0	0	0	70
17103628	m	md	DE	2019	0	1	0	0	0	1	0	0	0	0	0	0	76
17103717	m	md	CN	2019	0	1	0	0	0	1	0	0	0	0	0	0	69
17103740	m	Not repo	KR	2019	1	0	0	0	0	0	0	0	0	0	0	0	62.4
17103782	f	ph	JP	2019	0	1	0	0	1	0	0	0	0	0	0	0	79
17103856	m	md	PL	2019	0	1	0	0	0	1	0	0	0	0	0	0	63

17103931	f	cn	JP	2019	0	1	1	0	0	0	0	0	0	0	0	1	70
17104011	m	md	JP	2019	0	1	0	0	1	0	0	0	0	0	0	0	63
17104449	f	cn	US	2019	0	1	1	0	0	0	0	0	0	0	0	0	
17104915	f	ot	NL	2019	0	0	0	0	0	0	0	0	0	0	0	0	55
17104989	m	cn	JP	2019	0	1	1	0	0	0	0	0	0	0	0	0	67
17105212	f	md	JP	2019	0	1	1	0	0	0	0	0	0	0	0	0	64
17105442	Not	md	JP	2019	1	0	0	0	0	0	0	0	0	0	0	0	
17105515	f	ot	PL	2019	0	1	1	0	0	0	0	0	0	0	0	0	58
17105516	m	md	NL	2019	0	0	0	0	0	0	0	0	0	0	0	0	50
17105731	f	md	JP	2019	0	1	0	0	1	0	0	0	0	0	0	0	81
17105914	f	md	DE	2019	0	0	0	0	0	0	0	0	0	0	0	0	
17106073	f	md	PH	2019	0	0	0	0	0	0	0	0	0	0	0	0	42
17106123	Not	md	JP	2019	1	0	0	0	0	0	0	0	0	0	0	0	85
17106338	f	Not repo	ID	2019	1	0	0	0	0	0	0	0	0	0	0	0	
17106342	Not	md	IT	2019	1	0	0	0	0	0	0	0	0	0	0	1	
17106504	m	md	DE	2019	1	0	0	0	0	0	0	0	0	0	0	0	66
17106513	f	cn	US	2019	0	0	0	0	0	0	0	0	0	0	0	0	
17106526	m	ot	RU	2019	1	0	0	0	0	0	0	0	0	0	0	0	60
17106527	m	ot	RU	2019	1	0	0	0	0	0	0	0	0	0	0	0	60
17106534	f	md	JP	2019	1	0	0	0	0	0	0	0	0	0	0	0	76
17106860	m	cn	DE	2019	0	0	0	0	0	0	0	0	0	0	0	0	54
17107125	m	Not repo	JP	2019	0	1	0	1	0	0	0	0	0	0	0	0	70
17107143	f	md	GB	2019	0	1	0	1	0	0	0	0	0	0	0	1	71
17107315	Not	ot	US	2019	0	0	0	0	0	0	0	0	0	0	0	0	
17107330	m	md	DE	2019	0	0	0	0	0	0	0	0	0	0	0	0	
17107574	f	md	IT	2019	1	0	0	0	0	0	0	0	0	0	0	0	81.3
17108757	m	md	JP	2019	0	1	1	0	0	0	0	0	0	0	0	0	80
17108863	m	ph	JP	2019	0	1	1	0	0	0	0	0	0	0	0	0	80
17109248	m	ot	FR	2019	0	1	0	0	1	0	0	0	0	0	0	0	60
17110077	Not	cn	US	2019	0	1	0	0	1	0	0	0	0	0	0	0	
17110110	m	md	JP	2019	0	1	1	0	0	0	0	0	0	0	0	0	77
17110238	f	cn	IN	2019	0	0	0	0	0	0	0	0	0	0	0	0	53
17110322	m	md	JP	2019	0	1	0	0	0	1	0	0	0	0	0	0	41
17110447	m	ot	JP	2019	0	1	1	0	0	0	0	0	0	0	0	1	76

17110679	f	cn	DE	2019	0	0	0	0	0	0	0	0	0	0	0	0	76
17110781	Not	md	JP	2019	0	1	0	1	0	0	0	0	0	0	0	0	80
17111677	m	ot	IT	2019	1	0	0	0	0	0	0	0	0	0	0	0	70
17111903	f	md	JP	2019	0	1	1	0	0	0	0	0	0	0	0	0	67
17111919	m	md	US	2019	0	1	0	1	0	0	0	0	0	0	0	1	76
17111972	Not	Not repo	JP	2019	0	1	0	1	0	0	0	0	0	0	0	0	
17112054	m	md	TH	2019	1	0	0	0	0	0	0	0	0	0	0	0	66
17112080	f	md	DE	2019	1	0	0	0	0	0	0	0	0	0	0	0	73
17112098	m	md	JP	2019	0	1	0	0	0	1	0	0	0	0	0	1	62
17112248	f	Not repo	JP	2019	1	0	0	0	0	0	0	0	0	0	0	0	60
17112290	f	ot	AU	2019	0	1	1	0	0	0	0	0	0	0	0	0	60
17112301	f	cn	GB	2019	0	1	1	0	0	0	0	0	0	0	0	0	60
17112617	m	cn	SE	2019	0	1	1	0	0	0	0	0	0	0	0	0	
17112908	m	ot	ES	2019	0	1	1	0	0	0	0	0	0	0	0	0	67
17113555	Not	md	BE	2019	0	0	0	0	0	0	0	0	0	0	0	0	
17113597	m	md	JP	2019	0	1	0	0	0	1	0	0	0	0	0	0	77
17113720	Not	ot	CN	2019	0	0	0	0	0	0	0	0	0	0	0	0	
17113936	m	md	JP	2019	0	1	0	1	0	0	0	0	0	0	0	0	74
17114014	m	ph	JP	2019	0	0	0	0	0	0	0	0	0	0	0	0	63
17114105	Not	md	US	2019	0	1	0	0	0	1	0	0	0	0	0	0	
17114140	Not	md	US	2019	0	0	0	0	0	0	0	0	0	0	0	0	
17114764	f	md	VN	2019	1	0	0	0	0	0	0	0	0	0	0	0	72
17114823	f	md	JP	2019	1	0	0	0	0	0	0	0	0	0	0	0	81
17115390	Not	ot	IT	2019	1	0	0	0	0	0	0	0	0	0	0	0	
17115439	m	md	AT	2019	0	1	1	0	0	0	0	0	0	0	0	0	54
17115491	m	md	JP	2019	0	0	0	0	0	0	0	0	0	0	0	0	
17115498	m	md	JP	2019	0	0	0	0	0	0	0	0	0	0	0	0	
17115839	Not	md	JP	2019	1	0	0	0	0	0	0	0	0	0	0	0	80
17116053	f	md	DE	2019	0	1	1	0	0	0	0	0	0	0	0	0	62
17116068	m	md	FR	2019	0	1	0	1	0	0	0	0	0	0	0	0	61.8
17116072	Not	ot	PH	2019	0	1	1	0	0	0	0	0	0	0	0	0	55
17116088	m	md	TW	2019	0	1	1	0	0	0	0	0	0	0	0	1	69
17116510	f	cn	US	2019	1	0	0	0	0	0	0	0	0	0	0	0	76
17116537	f	cn	US	2019	1	0	0	0	0	0	0	0	0	0	0	0	

17116556	f	cn	US	2019	1	0	0	0	0	0	0	0	0	0	0	0	
17116560	f	cn	US	2019	1	0	0	0	0	0	0	0	0	0	0	0	
17116573	f	cn	US	2019	1	0	0	0	0	0	0	0	0	0	0	0	83
17116665	f	cn	US	2019	1	0	0	0	0	0	0	0	0	0	0	0	
17116716	f	ot	US	2019	1	0	0	0	0	0	0	0	0	0	0	0	92
17116718	m	md	US	2019	1	0	0	0	0	0	0	0	0	0	0	0	
17116725	m	cn	US	2019	1	0	0	0	0	0	0	0	0	0	0	0	
17116728	f	cn	US	2019	1	0	0	0	0	0	0	0	0	0	0	0	60
17116763	m	cn	US	2019	1	0	0	0	0	0	0	0	0	0	0	0	
17117069	m	Not repo	JP	2019	1	0	0	0	0	0	0	0	0	0	0	0	49
17117079	m	ot	IT	2019	1	0	0	0	0	0	0	0	0	0	0	0	82
17117201	m	Not repo	EG	2019	1	0	0	0	0	0	0	0	0	0	0	0	
17117246	f	md	DE	2019	1	0	0	0	0	0	0	0	0	0	0	0	57.1
17117296	Not	md	IT	2019	1	0	0	0	0	0	0	0	0	0	0	0	
17117605	Not	md	JP	2019	0	0	0	0	0	0	0	0	0	0	0	0	66
17117899	m	cn	SA	2019	0	1	1	0	0	0	0	0	0	0	0	0	
17117917	Not	md	RU	2019	0	0	0	0	0	0	0	0	0	0	0	0	
17117920	m	cn	DE	2019	0	1	1	0	0	0	0	0	0	0	0	0	
17118105	Not	cn	JP	2019	0	1	1	0	0	0	0	0	0	0	0	0	75
17118182	f	md	DE	2019	0	0	0	0	0	0	0	0	0	0	0	0	
17118305	f	ot	US	2019	0	0	0	0	0	0	0	0	0	0	0	0	58
17118463	m	cn	JP	2019	0	1	1	0	0	0	0	0	0	0	0	0	54
17118482	m	ot	US	2019	0	1	0	0	1	0	0	0	0	0	0	0	82
17118587	Not	cn	JP	2019	0	1	1	0	0	0	0	0	0	0	0	0	70
17118673	m	cn	JP	2019	0	1	1	0	0	0	0	0	0	0	0	0	84
17118688	m	md	LB	2019	0	1	0	0	1	0	0	0	0	0	0	0	45
17118872	Not	md	US	2019	0	0	0	0	0	0	0	0	0	0	0	0	
17119508	f	ot	FR	2019	0	1	0	0	1	0	0	0	0	0	0	0	76
17119515	m	md	FR	2019	0	1	0	1	0	0	0	0	0	0	0	0	50
17119519	Not	ot	IT	2019	1	0	0	0	0	0	0	0	0	0	0	0	
17120756	f	md	AU	2019	0	1	0	0	0	1	0	0	0	0	0	0	
17120773	m	md	CN	2019	1	0	0	0	0	0	0	0	0	0	0	0	45
17121369	m	cn	CA	2019	1	0	0	0	0	0	0	0	0	0	0	0	
17121475	m	cn	JP	2019	0	1	1	0	0	0	0	0	0	0	0	0	70

17121636	Not	md	US	2019	1	0	0	0	0	0	0	0	0	0	0	0	
17121693	m	md	FR	2019	0	1	0	1	0	0	0	0	0	0	0	0	
17122133	f	md	MY	2019	0	1	0	0	0	1	0	0	0	0	0	1	68
17123150	f	md	DE	2019	0	0	0	0	0	0	0	0	0	0	0	0	70
17123892	m	md	JP	2019	1	0	0	0	0	0	0	0	0	0	0	1	78
17123984	f	md	IT	2019	0	1	0	0	1	0	0	0	0	0	0	0	72
17124006	m	md	JP	2019	0	0	0	0	0	0	0	0	0	0	0	0	70
17124278	m	ot	DE	2019	0	1	0	0	1	0	0	0	0	0	0	0	79
17124364	f	cn	CN	2019	1	0	0	0	0	0	0	0	0	0	0	0	
17124414	Not	md	GB	2019	0	1	0	1	0	0	0	0	0	0	0	0	
17124416	m	ot	NL	2019	0	1	1	0	0	0	0	0	0	0	0	0	63
17125045	m	md	JP	2019	0	0	0	0	0	0	0	0	0	0	0	0	66
17125046	m	cn	JP	2019	0	1	1	0	0	0	0	0	0	0	0	0	59
17125207	m	md	JP	2019	0	1	0	0	0	1	0	0	0	0	0	1	80
17125303	m	md	JP	2019	0	1	0	0	0	1	0	0	0	0	0	0	74
17125358	f	ot	US	2019	0	1	1	0	0	0	0	0	0	0	0	0	62
17125522	f	cn	US	2019	0	1	1	0	0	0	0	0	0	0	0	0	
17125679	m	md	ES	2019	0	1	1	0	0	0	0	0	0	0	0	0	54
17125999	Not	md	GR	2019	0	0	0	0	0	0	0	0	0	0	0	0	
17126058	m	md	IT	2019	0	1	0	0	1	0	0	0	0	0	0	0	70
17126063	m	md	IT	2019	0	1	0	0	1	0	0	0	0	0	0	0	61
17126225	m	ot	FR	2019	0	1	0	0	1	0	0	0	0	0	0	0	73
17126244	f	md	GB	2019	0	1	0	0	0	1	0	0	0	0	0	0	55
17127314	m	md	JP	2019	0	1	0	0	1	0	0	0	0	0	0	1	64
17127383	m	ot	JP	2019	0	1	0	0	0	1	0	0	0	0	0	0	78
17127412	Not	ot	JP	2019	0	0	0	0	0	0	0	0	0	0	0	0	
17127413	Not	ot	JP	2019	0	0	0	0	0	0	0	0	0	0	0	0	
17127414	m	md	PL	2019	0	0	0	0	0	0	0	0	0	0	0	0	69
17127576	f	ot	PL	2019	0	0	0	0	0	0	0	0	0	0	0	0	80
17127775	m	md	JP	2019	0	1	0	0	1	0	0	0	0	0	0	0	46
17128714	f	ph	FR	2019	0	0	0	0	0	0	0	0	0	0	0	0	
17128812	m	md	US	2019	0	0	0	0	0	0	0	0	0	0	0	0	50
17128877	f	cn	CN	2019	1	0	0	0	0	0	0	0	0	0	0	0	57
17129713	m	md	JP	2019	0	1	1	0	0	0	0	0	0	0	0	0	47

17129743	m	ot	PL	2019	0	0	0	0	0	0	0	0	0	0	0	0	70
17129843	Not	md	JP	2019	0	0	0	0	0	0	0	0	0	0	0	0	75
17130072	f	cn	US	2019	1	0	0	0	0	0	0	0	0	0	0	0	
17130158	f	md	FR	2019	0	1	0	1	0	0	0	0	0	0	0	0	
17130237	m	md	FR	2019	0	1	0	1	0	0	0	0	0	0	0	0	
17130238	f	cn	CN	2019	1	0	0	0	0	0	0	0	0	0	0	0	57
17130316	m	md	CA	2019	0	1	0	1	0	0	0	0	0	0	0	0	
17130381	f	md	DE	2019	0	0	0	0	0	0	0	0	0	0	0	0	
17130449	f	Not repo	CN	2019	1	0	0	0	0	0	0	0	0	0	0	0	66
17130760	f	cn	JP	2019	0	1	1	0	0	0	0	0	0	0	0	0	70
17130788	m	md	CZ	2019	0	0	0	0	0	0	0	0	0	0	0	0	70
17131209	Not	md	JP	2019	0	1	1	0	0	0	0	0	0	0	0	0	47
17131288	f	Not repo	CN	2019	1	0	0	0	0	0	0	0	0	0	0	0	55
17131300	m	md	DK	2019	0	0	0	0	0	0	0	0	0	0	0	0	
17132229	m	md	JP	2019	0	1	0	0	0	1	0	0	0	0	0	0	79
17132527	m	md	AU	2019	0	1	0	0	0	1	0	0	0	0	0	0	
17132637	m	md	JP	2019	0	1	0	0	0	1	0	0	0	0	0	0	67
17132841	m	md	PT	2019	0	1	1	0	0	0	0	0	0	0	0	0	
17132854	m	Not repo	CN	2019	1	0	0	0	0	0	0	0	0	0	0	0	78
17132860	Not	ot	JP	2019	1	0	0	0	0	0	0	0	0	0	0	0	
17132896	f	Not repo	CN	2019	1	0	0	0	0	0	0	0	0	0	0	0	79.7
17133029	m	cn	JP	2019	0	0	0	0	0	0	0	0	0	0	0	0	67
17133367	m	md	JP	2019	0	1	0	0	1	0	0	0	0	0	0	0	54
17134501	m	ph	US	2019	0	1	0	0	0	1	0	0	0	0	0	0	64
17134905	f	cn	IN	2019	0	0	0	0	0	0	0	0	0	0	0	0	58
17134961	m	cn	CN	2019	1	0	0	0	0	0	0	0	0	0	0	0	55
17135213	m	md	JP	2019	1	0	0	0	0	0	0	0	0	0	0	1	45
17135407	Not	md	FR	2019	0	1	0	1	0	0	0	0	0	0	0	0	
17135511	m	md	JP	2019	0	1	0	1	0	0	0	0	0	0	0	1	63
17135954	m	Not repo	CN	2019	1	0	0	0	0	0	0	0	0	0	0	0	71
17135978	f	md	FR	2019	0	0	0	0	0	0	0	0	0	0	0	0	69
17136014	m	cn	CN	2019	1	0	0	0	0	0	0	0	0	0	0	0	76
17136107	f	Not repo	CN	2019	1	0	0	0	0	0	0	0	0	0	0	0	
17136284	f	md	JP	2019	0	1	0	1	0	0	0	0	0	0	0	0	74

17136417	f	cn	CN	2019	1	0	0	0	0	0	0	0	0	0	0	0	55
17136424	m	Not repo	IT	2019	1	0	0	0	0	0	0	0	0	0	0	0	85
17136942	m	ot	US	2019	0	1	0	0	1	0	0	0	0	0	0	0	73
17137573	m	md	JP	2019	0	1	0	0	0	1	0	0	0	0	0	0	76
17137579	m	ot	US	2019	0	1	1	0	0	0	0	0	0	0	0	0	69
17137700	f	ot	GB	2019	0	1	0	0	0	1	0	0	0	0	0	0	55
17138008	Not	ph	JP	2019	0	1	0	0	1	0	0	0	0	0	0	0	
17138122	m	md	TW	2019	0	1	0	0	0	1	0	0	0	0	0	0	67
17139102	m	md	JP	2019	0	1	0	0	0	1	0	0	0	0	0	0	80
17139276	f	cn	CN	2019	0	1	0	0	1	0	0	0	0	0	0	0	
17140218	m	md	DE	2019	0	0	0	0	0	0	0	0	0	0	0	0	
17140512	Not	ph	JP	2019	0	1	0	0	1	0	0	0	0	0	0	0	
17140860	f	ot	BE	2019	0	1	1	0	0	0	0	0	0	0	0	0	
17140868	f	md	IL	2019	0	0	0	0	0	0	0	0	0	0	0	0	
17141350	m	ot	FR	2019	0	1	0	0	1	0	0	0	0	0	0	0	73
17141424	m	cn	AR	2019	0	0	0	0	0	0	0	0	0	0	0	0	55
17141748	f	cn	US	2019	1	0	0	0	0	0	0	0	0	0	0	0	75.4
17142083	f	cn	CN	2019	1	0	0	0	0	0	0	0	0	0	0	0	60
17142088	m	cn	CN	2019	1	0	0	0	0	0	0	0	0	0	0	0	68
17142091	f	Not repo	GR	2019	1	0	0	0	0	0	0	0	0	0	0	0	65
17142121	m	ot	US	2019	0	1	0	0	1	0	0	0	0	0	0	0	
17142156	f	cn	CN	2019	1	0	0	0	0	0	0	0	0	0	0	0	57
17142214	m	cn	US	2019	0	1	1	0	0	0	0	0	0	0	0	0	
17142241	m	md	US	2019	0	0	0	0	0	0	0	0	0	0	0	0	
17142364	f	ot	US	2019	0	1	0	0	1	0	0	0	0	0	0	0	65
17142513	f	ot	US	2019	0	0	0	0	0	0	0	0	0	0	0	0	69
17142514	f	md	US	2019	0	0	0	0	0	0	0	0	0	0	0	0	
17142520	m	cn	US	2019	0	0	0	0	0	0	0	0	0	0	0	0	47
17142522	m	cn	US	2019	0	0	0	0	0	0	0	0	0	0	0	0	
17142621	Not	cn	US	2019	0	1	1	0	0	0	0	0	0	0	0	0	
17142810	f	cn	JP	2019	0	1	1	0	0	0	0	0	0	0	0	0	79
17142876	m	ot	DE	2019	0	1	1	0	0	0	0	0	0	0	0	0	56
17143888	Not	ot	IT	2019	0	1	1	0	0	0	0	0	0	0	0	0	64
17144234	m	md	JP	2019	0	1	0	0	1	0	0	0	0	0	0	0	60

17144560	m	md	JP	2019	0	0	0	0	0	0	0	0	0	0	0	0	60
17144809	m	ot	NL	2019	0	1	1	0	0	0	0	0	0	0	0	0	69
17145418	m	md	FR	2019	0	1	0	0	0	1	0	0	0	0	0	0	83
17145486	f	cn	CN	2019	1	0	0	0	0	0	0	0	0	0	0	0	68
17145570	f	Not repo	CN	2019	1	0	0	0	0	0	0	0	0	0	0	0	70
17145585	f	cn	US	2019	0	0	0	0	0	0	0	0	0	0	0	0	59
17145661	m	Not repo	CN	2019	1	0	0	0	0	0	0	0	0	0	0	0	58
17145745	m	md	JP	2019	0	1	0	1	0	0	0	0	0	0	0	1	60
17145793	f	md	JP	2019	1	0	0	0	0	0	0	0	0	0	0	0	68
17145997	m	cn	CN	2019	1	0	0	0	0	0	0	0	0	0	0	0	61
17146028	m	cn	CN	2019	1	0	0	0	0	0	0	0	0	0	0	0	68
17146097	m	md	FR	2019	0	1	0	1	0	0	0	0	0	0	0	0	
17146108	f	cn	CN	2019	1	0	0	0	0	0	0	0	0	0	0	0	55.9
17146205	f	ot	JP	2019	0	1	1	0	0	0	0	0	0	0	0	1	73
17146242	m	md	JP	2019	0	0	0	0	0	0	0	0	0	0	0	0	57
17146349	f	md	JP	2019	1	0	0	0	0	0	0	0	0	0	0	0	52
17146414	m	cn	IL	2019	0	1	1	0	0	0	0	0	0	0	0	0	
17146473	m	Not repo	CN	2019	1	0	0	0	0	0	0	0	0	0	0	0	63.3
17146506	m	cn	US	2019	0	1	0	0	1	0	0	0	0	0	0	0	
17146770	m	md	DE	2019	0	0	0	0	0	0	0	0	0	0	0	0	80
17146791	f	cn	JP	2019	0	1	1	0	0	0	0	0	0	0	0	0	60
17146954	m	cn	DE	2019	0	1	1	0	0	0	0	0	0	0	0	0	57
17147152	m	cn	JP	2019	0	0	0	0	0	0	0	0	0	0	0	0	70
17147198	f	ph	JP	2019	0	1	0	0	1	0	0	0	0	0	0	0	73
17147411	m	md	KR	2019	0	1	1	0	0	0	0	0	0	0	0	0	65
17147499	f	Not repo	CN	2019	1	0	0	0	0	0	0	0	0	0	0	0	64
17147624	f	cn	CA	2019	0	1	0	1	0	0	0	0	0	0	0	0	
17147737	f	ot	US	2019	0	1	1	0	0	0	0	0	0	0	0	0	62
17147792	f	cn	CN	2019	1	0	0	0	0	0	0	0	0	0	0	0	71
17147874	m	md	JP	2019	0	1	1	0	0	0	0	0	0	0	0	0	80
17148672	m	cn	JP	2019	0	1	1	0	0	0	0	0	0	0	0	0	75
17149227	f	cn	US	2019	1	0	0	0	0	0	0	0	0	0	0	0	
17149325	m	cn	FR	2019	0	1	1	0	0	0	0	0	0	0	0	0	58
17149386	Not	ot	US	2019	0	1	0	0	1	0	0	0	0	0	0	0	

17149400	f	Not repo	CN	2019	1	0	0	0	0	0	0	0	0	0	0	0	57
17149457	m	cn	JP	2019	0	1	1	0	0	0	0	0	0	0	0	0	67
17149513	m	md	US	2019	0	1	0	1	0	0	0	0	0	0	0	1	75
17149518	f	cn	CN	2019	1	0	0	0	0	0	0	0	0	0	0	0	56
17149727	Not	ph	VN	2019	1	0	0	0	0	0	0	0	0	0	0	0	
17149788	m	cn	CN	2019	1	0	0	0	0	0	0	0	0	0	0	0	57
17149790	Not	ot	JP	2019	1	0	0	0	0	0	0	0	0	0	0	0	
17149791	Not	ot	JP	2019	1	0	0	0	0	0	0	0	0	0	0	0	
17149876	f	ot	US	2019	1	0	0	0	0	0	0	0	0	0	0	0	88
17150106	f	ot	AT	2019	0	1	0	0	0	1	0	0	0	0	0	0	59
17150190	f	md	HU	2019	0	1	0	0	1	0	0	0	0	0	0	0	58
17150695	f	md	DE	2019	0	1	0	0	0	1	0	0	0	0	0	0	65
17150890	m	cn	JP	2019	1	0	0	0	0	0	0	0	0	0	0	0	
17150982	m	Not repo	JP	2019	1	0	0	0	0	0	0	0	0	0	0	1	85
17151039	f	cn	DE	2019	0	1	1	0	0	0	0	0	0	0	0	1	73
17151175	m	cn	US	2019	1	0	0	0	0	0	0	0	0	0	0	0	72.2
17151964	m	cn	CA	2019	0	1	0	1	0	0	0	0	0	0	0	0	
17151969	f	ot	CA	2019	0	0	0	0	0	0	0	0	0	0	0	0	45
17152273	m	ot	US	2019	1	0	0	0	0	0	0	0	0	0	0	0	
17152288	m	Not repo	JP	2019	1	0	0	0	0	0	0	0	0	0	0	0	70
17152501	m	md	JP	2019	1	0	0	0	0	0	0	0	0	0	0	1	74
17152791	Not	ot	JP	2019	1	0	0	0	0	0	0	0	0	0	0	0	
17152864	f	cn	CN	2019	1	0	0	0	0	0	0	0	0	0	0	0	50
17152937	f	cn	CN	2019	1	0	0	0	0	0	0	0	0	0	0	0	53.3
17152940	f	cn	CN	2019	1	0	0	0	0	0	0	0	0	0	0	0	72
17153052	m	md	JP	2019	0	1	1	0	0	0	0	0	0	0	0	1	66
17153076	m	md	JP	2019	0	1	0	1	0	0	0	0	0	0	0	1	78
17153077	Not	ot	JP	2019	1	0	0	0	0	0	0	0	0	0	0	0	
17153445	f	Not repo	VN	2019	1	0	0	0	0	0	0	0	0	0	0	0	60
17153457	f	cn	CN	2019	1	0	0	0	0	0	0	0	0	0	0	0	87.5
17153713	m	ot	US	2019	0	1	0	0	1	0	0	0	0	0	0	0	57
17153925	f	cn	JP	2019	0	1	1	0	0	0	0	0	0	0	0	0	
17153935	Not	ot	FR	2019	0	1	0	0	1	0	0	0	0	0	0	0	82
17153985	Not	ot	BE	2019	1	0	0	0	0	0	0	0	0	0	0	0	

17153995	m	cn	IL	2019	0	0	0	0	0	0	0	0	0	0	0	0	38
17154100	m	md	ES	2019	0	0	0	0	0	0	0	0	0	0	0	0	49
17154654	Not	ot	JP	2019	0	0	0	0	0	0	0	0	0	0	0	0	
17154714	m	cn	US	2019	0	0	0	0	0	0	0	0	0	0	0	0	
17155511	f	md	DE	2019	0	1	0	0	1	0	0	0	0	0	0	0	58
17155938	m	md	JP	2019	0	1	0	0	0	1	0	0	0	0	0	0	74
17156133	m	md	US	2019	1	0	0	0	0	0	0	0	0	0	0	0	
17156620	m	md	JP	2019	0	1	0	0	0	1	0	0	0	0	0	0	70
17156667	m	md	DE	2019	0	0	0	0	0	0	0	0	0	0	0	0	68
17156964	f	cn	RU	2019	1	0	0	0	0	0	0	0	0	0	0	0	59
17157291	m	md	JP	2019	0	1	0	0	0	1	0	0	0	0	0	1	70
17157318	m	ot	DE	2019	0	1	1	0	0	0	0	0	0	0	0	0	
17157722	m	ot	CN	2019	0	0	0	0	0	0	0	0	0	0	0	0	62
17157819	m	ot	JP	2019	0	1	1	0	0	0	0	0	0	0	0	1	61
17158149	m	md	IL	2019	0	0	0	0	0	0	0	0	0	0	0	0	
17158436	m	md	JP	2019	0	1	1	0	0	0	0	0	0	0	0	0	68
17158838	f	ot	Not re	2019	0	0	0	0	0	0	0	0	0	0	0	0	60
17159242	Not	md	JP	2019	1	0	0	0	0	0	0	0	0	0	0	1	
17159433	f	ot	JP	2019	0	0	0	0	0	0	0	0	0	0	0	0	75
17159548	m	ot	DE	2019	0	1	1	0	0	0	0	0	0	0	0	0	68
17159638	f	ph	FR	2019	0	1	0	0	1	0	0	0	0	0	0	0	
17159665	m	md	RO	2019	1	0	0	0	0	0	0	0	0	0	0	0	80
17159797	Not	md	JP	2019	0	0	0	0	0	0	1	0	1	0	0	0	
17160204	m	md	CN	2019	0	0	0	0	0	0	0	0	0	0	0	0	53
17160335	Not	md	JP	2019	1	0	0	0	0	0	0	0	0	0	0	1	
17160343	Not	md	JP	2019	1	0	0	0	0	0	0	0	0	0	0	0	
17160454	f	cn	BG	2019	0	1	1	0	0	0	0	0	0	0	0	0	49
17160787	m	ot	GB	2019	0	1	1	0	0	0	0	0	0	0	0	0	62
17160833	m	ot	ES	2019	0	0	0	0	0	0	0	0	0	0	0	0	53
17161180	f	ot	DE	2019	0	0	0	0	0	0	0	0	0	0	0	0	
17161195	m	cn	JP	2019	0	1	1	0	0	0	0	0	0	0	0	0	60
17161661	f	cn	FR	2019	0	1	1	0	0	0	0	0	0	0	0	0	61
17162151	f	ot	JP	2019	0	1	0	0	1	0	0	0	0	0	0	0	60
17162214	m	md	JP	2019	0	1	0	0	1	0	0	0	0	0	0	0	80

17162446	f	Not repo	JP	2019	1	0	0	0	0	0	0	0	0	0	0	1	90
17162659	m	md	JP	2019	0	1	1	0	0	0	0	0	0	0	0	0	68
17163413	m	md	JP	2019	0	1	1	0	0	0	0	0	0	0	0	0	66
17163467	f	md	DE	2019	0	1	0	0	1	0	0	0	0	0	0	0	58
17163881	m	md	FR	2019	0	1	0	0	0	1	0	0	0	0	0	0	40
17164176	Not	md	JP	2019	0	1	0	1	0	0	0	0	0	0	0	1	80
17164245	f	cn	US	2019	0	0	0	0	0	0	0	0	0	0	0	0	
17164320	f	cn	US	2019	0	1	1	0	0	0	0	0	0	0	0	0	
17164365	m	md	JP	2019	0	1	1	0	0	0	0	0	0	0	0	0	80
17164808	m	cn	JP	2019	0	0	0	0	0	0	0	0	0	0	0	0	78
17164948	f	cn	IN	2019	0	0	0	0	0	0	0	0	0	0	0	0	62
17165035	m	cn	JP	2019	0	1	1	0	0	0	0	0	0	0	0	0	69
17165393	m	md	JP	2019	0	1	1	0	0	0	0	0	0	0	0	0	92
17165677	f	md	FR	2019	0	0	0	0	0	0	0	0	0	0	0	0	69
17165898	Not	md	IT	2019	1	0	0	0	0	0	0	0	0	0	0	0	
17165907	m	Not repo	CN	2019	1	0	0	0	0	0	0	0	0	0	0	0	74.7
17165962	m	md	VN	2019	1	0	0	0	0	0	0	0	0	0	0	0	
17166179	m	md	JP	2019	0	1	1	0	0	0	0	0	0	0	0	0	47
17166240	m	cn	JP	2019	0	1	1	0	0	0	0	0	0	0	0	0	60
17166551	m	md	JP	2019	0	1	1	0	0	0	0	0	0	0	0	0	66
17166561	f	ot	JP	2019	0	1	1	0	0	0	0	0	0	0	0	0	72
17166977	f	cn	CN	2019	0	1	0	0	1	0	0	0	0	0	0	0	56
17167072	m	md	JP	2019	0	1	1	0	0	0	0	0	0	0	0	0	76
17167329	f	cn	CN	2019	0	1	0	0	1	0	0	0	0	0	0	0	89
17167591	m	cn	CN	2019	0	1	0	0	1	0	0	0	0	0	0	0	65
17167676	Not	md	US	2019	0	0	0	0	0	0	0	0	0	0	0	0	
17167877	m	cn	CN	2019	0	1	0	0	1	0	0	0	0	0	0	0	72
17168115	m	ot	DE	2019	0	1	0	0	1	0	0	0	0	0	0	0	58
17168192	Not	ot	JP	2019	1	0	0	0	0	0	0	0	0	0	0	0	
17168609	f	md	MX	2019	0	1	0	0	0	1	0	0	0	0	0	0	78
17168775	m	cn	JP	2019	0	0	0	0	0	0	0	0	0	0	0	0	80
17168837	m	md	JP	2019	0	1	1	0	0	0	0	0	0	0	0	0	60
17169014	f	md	RU	2019	0	1	0	1	0	0	0	0	0	0	0	0	63.6
17169024	m	cn	CN	2019	1	0	0	0	0	0	0	0	0	0	0	0	

17169417	m	cn	DE	2019	0	1	1	0	0	0	0	0	0	0	0	0	
17169475	m	md	JP	2019	0	0	0	0	0	0	0	0	0	0	0	0	43
17169585	f	ot	GB	2019	0	1	1	0	0	0	0	0	0	0	0	0	50
17169603	f	cn	CN	2019	1	0	0	0	0	0	0	0	0	0	0	0	56
17169724	f	Not repo	JP	2019	0	1	0	1	0	0	0	0	0	0	0	1	58
17169830	f	cn	US	2019	0	0	0	0	0	0	0	0	0	0	0	0	
17170102	m	ot	GB	2019	0	1	1	0	0	0	0	0	0	0	0	0	
17170213	m	ot	ES	2019	0	1	1	0	0	0	0	0	0	0	0	0	75
17170273	m	cn	JP	2019	0	1	1	0	0	0	0	0	0	0	0	1	66
17170281	f	cn	CN	2019	1	0	0	0	0	0	0	0	0	0	0	0	57
17170340	m	md	JP	2019	0	1	1	0	0	0	0	0	0	0	0	0	67
17170394	m	Not repo	JP	2019	0	1	0	1	0	0	0	0	0	0	0	1	80
17170425	m	cn	CN	2019	0	1	0	0	1	0	0	0	0	0	0	0	77
17170448	m	md	JP	2019	0	0	0	0	0	0	0	0	0	0	0	0	71
17170535	m	md	JP	2019	0	1	0	1	0	0	0	0	0	0	0	0	65
17170775	f	cn	IN	2019	0	1	0	0	1	0	0	0	0	0	0	0	
17170780	m	ot	JP	2019	0	0	0	0	0	0	0	0	0	0	0	0	67
17170781	m	ot	JP	2019	0	0	0	0	0	0	0	0	0	0	0	0	68
17170783	m	ot	JP	2019	0	0	0	0	0	0	0	0	0	0	0	0	55
17170784	m	ot	JP	2019	0	0	0	0	0	0	0	0	0	0	0	0	69
17170790	m	ot	JP	2019	0	0	0	0	0	0	0	0	0	0	0	0	65
17170872	f	cn	CN	2019	1	0	0	0	0	0	0	0	0	0	0	0	55
17170873	f	cn	CN	2019	1	0	0	0	0	0	0	0	0	0	0	0	66
17170929	m	ot	JP	2019	0	1	0	1	0	0	0	0	0	0	0	0	65
17170986	m	ot	US	2019	0	1	0	0	1	0	0	0	0	0	0	0	69
17171058	f	Not repo	JP	2019	1	0	0	0	0	0	0	0	0	0	0	0	69
17171544	f	ot	SE	2019	0	0	0	0	0	0	0	0	0	0	0	0	67
17172021	Not	md	IN	2019	0	1	1	0	0	0	0	0	0	0	0	0	64
17172104	f	md	JP	2019	0	1	1	0	0	0	0	0	0	0	0	0	77
17172401	f	ot	US	2019	0	1	1	0	0	0	0	0	0	0	0	0	51
17172654	m	ph	JP	2019	0	1	1	0	0	0	0	0	0	0	0	0	69
17173069	f	ot	DE	2019	0	0	0	0	0	0	0	0	0	0	0	0	57
17173102	f	Not repo	CN	2019	1	0	0	0	0	0	0	0	0	0	0	0	67.8
17173103	f	cn	CN	2019	1	0	0	0	0	0	0	0	0	0	0	0	74.5

17173272	Not	md	JP	2019	1	0	0	0	0	0	0	0	0	0	0	0	80
17173451	m	cn	JP	2019	0	1	1	0	0	0	0	0	0	0	0	0	70
17173776	f	ot	US	2019	0	0	0	0	0	0	0	0	0	0	0	0	
17174017	m	cn	CA	2019	0	1	0	0	0	1	0	0	0	0	0	0	60
17174026	m	md	AT	2019	0	1	1	0	0	0	0	0	0	0	0	0	54
17174186	Not	md	JP	2019	0	1	0	0	1	0	0	0	0	0	0	1	
17174266	m	ot	JP	2019	0	0	0	0	0	0	0	0	0	0	0	0	49
17174484	Not	ot	JP	2019	1	0	0	0	0	0	0	0	0	0	0	0	
17174606	m	cn	JP	2019	0	1	1	0	0	0	0	0	0	0	0	0	
17174668	f	cn	TW	2019	0	1	0	0	0	1	0	0	0	0	0	0	
17174744	m	ot	DE	2019	0	0	0	0	0	0	0	0	0	0	0	0	64
17175101	Not	md	US	2019	0	0	0	0	0	0	0	0	0	0	0	0	
17175591	f	Not repo	CN	2019	1	0	0	0	0	0	0	0	0	0	0	0	59
17175593	f	cn	CN	2019	1	0	0	0	0	0	0	0	0	0	0	0	
17175683	m	md	JP	2019	0	0	0	0	0	0	0	0	0	0	0	0	71
17175771	m	cn	IN	2019	0	1	0	0	1	0	0	0	0	0	0	0	
17176354	f	md	BE	2019	0	1	0	0	0	1	0	0	0	0	0	0	73
17176808	m	ot	JP	2019	0	1	0	1	0	0	0	0	0	0	0	1	74
17176813	m	ot	JP	2019	0	1	0	1	0	0	0	0	0	0	0	1	76
17176822	f	Not repo	CN	2019	1	0	0	0	0	0	0	0	0	0	0	0	76
17176915	m	md	JP	2019	0	1	0	0	0	1	0	0	0	0	0	0	69
17177209	m	ot	CA	2019	0	1	1	0	0	0	0	0	0	0	0	0	68
17177963	Not	ot	JP	2019	1	0	0	0	0	0	0	0	0	0	0	0	
17178037	f	md	ES	2019	0	1	0	0	1	0	0	0	0	0	0	0	59
17178055	Not	ot	JP	2019	1	0	0	0	0	0	0	0	0	0	0	0	
17178192	f	md	BR	2019	0	1	0	0	1	0	0	0	0	0	0	0	
17178714	Not	ot	JP	2019	0	0	0	0	0	0	0	0	0	0	0	0	
17178828	m	Not repo	JP	2019	0	1	0	1	0	0	0	0	0	0	0	1	70
17178966	f	cn	IN	2019	0	1	0	0	1	0	0	0	0	0	0	0	57
17179114	m	md	JP	2019	0	1	1	0	0	0	0	0	0	0	0	0	63
17179277	f	ot	US	2019	0	1	0	0	1	0	0	0	0	0	0	0	83
17179373	m	cn	GR	2019	0	0	0	0	0	0	0	0	0	0	0	0	53
17180089	m	ot	FR	2019	0	1	0	0	0	1	0	0	0	0	0	0	48
17180246	m	Not repo	JP	2019	0	1	0	1	0	0	0	0	0	0	0	0	80

17180581	f	ot	CA	2019	0	1	0	0	0	1	0	0	0	0	0	0	71
17181661	f	ot	AT	2019	0	1	0	0	0	1	0	0	0	0	0	0	59
17182143	f	cn	US	2019	0	0	0	0	0	0	0	0	0	0	0	0	59
17182148	m	ot	JP	2019	0	0	0	0	0	0	0	0	0	0	0	0	55
17182792	f	ot	DE	2019	0	1	0	0	1	0	0	0	0	0	0	0	60
17183223	Not	md	JP	2019	0	1	1	0	0	0	0	0	0	0	0	0	40
17183379	f	md	IT	2019	1	0	0	0	0	0	0	0	0	0	0	0	79.5
17184783	m	md	JP	2019	0	1	1	0	0	0	0	0	0	0	0	0	50
17184834	f	md	JP	2019	0	1	1	0	0	0	0	0	0	0	0	1	75
17185943	m	ot	GB	2019	0	0	0	0	0	0	0	0	0	0	0	0	81
17186075	f	cn	CA	2019	1	0	0	0	0	0	0	0	0	0	0	0	
17186077	m	Not repo	JP	2019	0	1	0	1	0	0	0	0	0	0	0	1	66
17186167	f	Not repo	CN	2019	1	0	0	0	0	0	0	0	0	0	0	0	72
17186207	f	md	GB	2019	0	1	1	0	0	0	0	0	0	0	0	0	74
17186502	f	md	FR	2019	0	0	0	0	0	0	0	0	0	0	0	0	70
17186596	m	cn	US	2019	0	1	0	0	1	0	0	0	0	0	0	0	
17186754	f	Not repo	JP	2019	1	0	0	0	0	0	0	0	0	0	0	1	81
17186755	f	md	ID	2019	1	0	0	0	0	0	0	0	0	0	0	0	
17186770	m	ot	LB	2019	0	0	0	0	0	0	0	0	0	0	0	0	56
17186811	f	md	JP	2019	1	0	0	0	0	0	0	0	0	0	0	0	79
17186822	f	ot	NL	2019	0	0	0	0	0	0	0	0	0	0	0	0	60
17187815	m	md	JP	2019	0	1	1	0	0	0	0	0	0	0	0	0	72
17187982	f	cn	IL	2019	0	0	0	0	0	0	0	0	0	0	0	0	52
17187985	f	md	JP	2019	0	0	0	0	0	0	0	0	0	0	0	0	90
17188008	m	md	DE	2019	0	0	0	0	0	0	0	0	0	0	0	0	68
17188260	m	md	JP	2019	0	0	0	0	0	0	0	0	0	0	0	0	63
17188491	f	md	JP	2019	0	1	1	0	0	0	0	0	0	0	0	0	72
17189275	m	md	JP	2019	0	0	0	0	0	0	0	0	0	0	0	0	60
17189477	m	cn	DE	2019	0	1	1	0	0	0	0	0	0	0	0	0	57
17189839	f	md	JP	2019	0	0	0	0	0	0	0	0	0	0	0	0	75
17189856	Not	md	CN	2019	0	0	0	0	0	0	0	0	0	0	0	0	
17190274	f	md	DE	2019	0	1	0	0	1	0	0	0	0	0	0	0	58
17190364	m	ot	DE	2019	0	0	0	0	0	0	0	0	0	0	0	0	59
17190368	m	ot	US	2019	0	1	0	0	1	0	0	0	0	0	0	0	81

17190383	m	ot	PL	2019	0	0	0	0	0	0	0	0	0	0	0	0	70
17190724	m	md	FR	2019	0	0	0	0	0	0	1	0	1	0	0	0	75
17191622	m	cn	CN	2019	0	1	0	0	1	0	0	0	0	0	0	0	
17191657	m	cn	JP	2019	0	1	1	0	0	0	0	0	0	0	0	0	74
17191842	f	cn	JP	2019	0	1	1	0	0	0	0	0	0	0	0	0	82
17191956	m	cn	US	2019	0	1	1	0	0	0	0	0	0	0	0	1	
17192201	m	ot	PL	2019	0	1	1	0	0	0	0	0	0	0	0	0	75
17192229	Not	ot	RU	2019	0	1	0	1	0	0	0	0	0	0	0	0	
17192493	f	cn	CN	2019	1	0	0	0	0	0	0	0	0	0	0	0	
17192500	m	Not repo	RU	2019	0	1	0	1	0	0	0	0	0	0	0	1	
17192631	f	ot	CA	2019	0	0	0	0	0	0	0	0	0	0	0	0	69
17192899	m	cn	JP	2019	0	1	1	0	0	0	0	0	0	0	0	0	70
17193091	Not	md	SK	2019	0	1	1	0	0	0	0	0	0	0	0	0	45
17193242	m	md	AU	2019	0	1	1	0	0	0	0	0	0	0	0	0	46
17193349	m	cn	US	2019	0	1	1	0	0	0	0	0	0	0	0	0	66
17193476	m	md	KR	2019	0	1	1	0	0	0	0	0	0	0	0	0	69
17193548	m	md	DE	2019	0	0	0	0	0	0	0	0	0	0	0	0	
17193551	m	md	NL	2019	0	1	0	1	0	0	0	0	0	0	0	0	52
17193906	m	cn	SE	2019	0	1	1	0	0	0	0	0	0	0	0	0	60
17194070	Not	md	BE	2019	0	1	0	0	0	1	0	0	0	0	0	0	
17194566	m	md	JP	2019	0	1	0	1	0	0	0	0	0	0	0	0	64
17194579	m	ot	DE	2019	0	1	1	0	0	0	0	0	0	0	0	0	67
17196283	m	md	PT	2019	0	1	1	0	0	0	0	0	0	0	0	0	66
17197180	m	md	FR	2019	0	1	0	1	0	0	0	0	0	0	0	0	
17197238	f	md	JP	2019	1	0	0	0	0	0	0	0	0	0	0	0	84.6
17197436	f	ot	GR	2019	0	0	0	0	0	0	0	0	0	0	0	0	61
17197944	m	md	FR	2019	0	1	0	0	0	1	0	0	0	0	0	0	69
17198118	m	Not repo	JP	2019	1	0	0	0	0	0	0	0	0	0	0	1	70
17198344	m	Not repo	JP	2019	1	0	0	0	0	0	0	0	0	0	0	0	70
17198736	m	Not repo	JP	2019	0	1	0	1	0	0	0	0	0	0	0	1	70
17199093	f	cn	GB	2019	0	0	0	0	0	0	0	0	0	0	0	0	
17199185	f	cn	JP	2019	0	1	1	0	0	0	0	0	0	0	0	0	70
17199301	m	ot	JP	2019	0	1	0	0	1	0	0	0	0	0	0	0	61
17199842	m	md	JP	2019	0	1	0	0	1	0	0	0	0	0	0	0	60

17199853	m	md	JP	2019	0	1	0	0	1	0	0	0	0	0	0	0	75
17199986	m	cn	JP	2019	0	1	1	0	0	0	0	0	0	0	0	0	80
17200435	m	cn	JP	2019	0	1	1	0	0	0	0	0	0	0	0	0	90
17200662	m	cn	DE	2019	0	1	1	0	0	0	0	0	0	0	0	1	63
17200736	m	ot	DE	2019	0	1	1	0	0	0	0	0	0	0	0	0	
17200773	f	ot	FR	2019	0	0	0	0	0	0	0	0	0	0	0	0	55
17201733	f	cn	US	2019	1	0	0	0	0	0	0	0	0	0	0	0	
17201800	f	md	JP	2019	1	0	0	0	0	0	0	0	0	0	0	0	77
17202715	f	md	HU	2019	0	0	0	0	0	0	0	0	0	0	0	0	
17202990	f	cn	JP	2019	0	1	1	0	0	0	0	0	0	0	0	1	70
17203069	m	Not repo	JP	2019	1	0	0	0	0	0	0	0	0	0	0	1	70
17203072	f	Not repo	JP	2019	0	1	0	1	0	0	0	0	0	0	0	0	60
17203238	Not	md	JP	2019	1	0	0	0	0	0	0	0	0	0	0	0	
17203240	f	Not repo	CN	2019	1	0	0	0	0	0	0	0	0	0	0	0	44
17203241	Not	Not repo	JP	2019	1	0	0	0	0	0	0	0	0	0	0	0	90
17203447	Not	md	JP	2019	0	1	0	1	0	0	0	0	0	0	0	1	60
17203522	Not	cn	US	2019	0	0	0	0	0	0	0	0	0	0	0	0	50
17204333	m	ot	FR	2019	0	1	0	0	1	0	0	0	0	0	0	0	77
17204837	m	md	RU	2019	0	1	0	0	0	1	0	0	0	0	0	0	45
17205292	Not	ot	FR	2019	0	0	0	0	0	0	0	0	0	0	0	0	
17205550	m	md	AU	2019	0	1	0	0	0	1	0	0	0	0	0	0	83
17205689	m	cn	JP	2019	0	1	1	0	0	0	0	0	0	0	0	0	60
17206347	f	Not repo	CN	2019	1	0	0	0	0	0	0	0	0	0	0	0	46
17206511	m	ot	DE	2019	0	1	1	0	0	0	0	0	0	0	0	0	54
17206765	f	ot	IL	2019	1	0	0	0	0	0	0	0	0	0	0	0	65
17206825	f	md	FR	2019	0	1	0	1	0	0	0	0	0	0	0	0	
17207547	f	cn	CN	2019	1	0	0	0	0	0	0	0	0	0	0	0	71
17209041	m	ot	JP	2019	0	0	0	0	0	0	0	0	0	0	0	0	61
17209042	m	ot	JP	2019	0	1	1	0	0	0	0	0	0	0	0	0	
17209332	m	md	SI	2019	0	0	0	0	0	0	0	0	0	0	0	0	46
17209464	f	md	JP	2019	1	0	0	0	0	0	0	0	0	0	0	0	
17209834	m	cn	CN	2019	0	1	0	0	1	0	0	0	0	0	0	0	75
17209858	m	ot	IL	2019	1	0	0	0	0	0	0	0	0	0	0	0	63
17209863	m	Not repo	JP	2019	0	1	0	1	0	0	0	0	0	0	0	0	66

17210028	m	Not repo	JP	2019	0	1	0	1	0	0	0	0	0	0	0	1	67.6
17210119	m	cn	US	2019	1	0	0	0	0	0	0	0	0	0	0	0	
17210179	f	ot	NL	2019	0	1	1	0	0	0	0	0	0	0	0	1	73
17210333	m	ot	IL	2019	1	0	0	0	0	0	0	0	0	0	0	0	75
17210338	f	cn	CA	2019	1	0	0	0	0	0	0	0	0	0	0	0	
17211203	m	ot	DE	2019	0	1	0	0	1	0	0	0	0	0	0	0	56
17211748	m	md	KR	2019	0	1	0	0	0	1	0	0	0	0	0	0	77
17212031	f	cn	US	2019	0	1	1	0	0	0	0	0	0	0	0	0	
17212276	m	md	JP	2019	0	1	0	0	1	0	0	0	0	0	0	0	71
17212426	m	cn	JP	2019	0	1	1	0	0	0	0	0	0	0	0	0	90
17213119	Not	ot	KR	2019	0	1	1	0	0	0	0	0	0	0	0	0	70
17213246	m	md	CN	2019	0	1	1	0	0	0	0	0	0	0	0	0	66
17213376	m	md	CA	2019	0	0	0	0	0	0	0	0	0	0	0	0	23
17213435	Not	ot	JP	2019	0	0	0	0	0	0	0	0	0	0	0	0	
17213542	m	ot	CL	2019	0	0	0	0	0	0	0	0	0	0	0	0	70
17214092	Not	ot	DE	2019	0	0	0	0	0	0	0	0	0	0	0	0	
17214278	Not	ot	DE	2019	0	0	0	0	0	0	0	0	0	0	0	0	
17214548	m	md	DK	2019	0	1	1	0	0	0	0	0	0	0	0	0	74
17214841	Not	md	ES	2019	0	1	1	0	0	0	0	0	0	0	0	0	79
17214859	f	ot	US	2019	0	1	0	0	1	0	0	0	0	0	0	0	58
17215248	m	ot	NL	2019	0	0	0	0	0	0	0	0	0	0	0	0	64
17215249	m	ot	NL	2019	0	0	0	0	0	0	0	0	0	0	0	0	
17215973	m	ot	US	2019	0	0	0	0	0	0	0	0	0	0	0	0	67
17216101	m	md	JP	2019	0	1	0	0	1	0	0	0	0	0	0	0	70
17216335	m	md	CN	2019	0	0	0	0	0	0	0	0	0	0	0	0	70
17216699	m	cn	CN	2019	0	1	0	0	1	0	0	0	0	0	0	0	69
17216731	m	cn	IN	2019	0	1	0	0	1	0	0	0	0	0	0	0	
17216899	Not	ph	JP	2019	0	1	0	0	0	1	0	0	0	0	0	0	80
17217169	f	ot	US	2019	0	1	1	0	0	0	0	0	0	0	0	0	62
17217447	f	md	US	2019	1	0	0	0	0	0	0	0	0	0	0	0	53
17218517	f	cn	JP	2019	0	1	1	0	0	0	0	0	0	0	0	0	61
17218683	m	md	JP	2019	0	1	0	0	1	0	0	0	0	0	0	0	77
17219755	m	md	IN	2019	0	1	1	0	0	0	0	0	0	0	0	0	60
17220294	f	Not repo	ID	2019	1	0	0	0	0	0	0	0	0	0	0	0	77.8

17220343	m	cn	JP	2019	0	1	1	0	0	0	0	0	0	0	0	0	80
17220454	f	md	US	2019	0	0	0	0	0	0	0	0	0	0	0	0	
17250768	f	Not repo	US	2019	0	0	0	0	0	0	0	0	0	0	0	0	88
9607254	m	ot	CA	2019	0	0	0	0	0	0	0	0	0	0	0	0	77
10356338	Not	hp	JP	2020	0	0	0	0	0	0	0	0	0	0	0	0	
16536700	m	hp	CN	2020	0	1	0	0	1	0	0	0	0	0	0	0	71
16582867	m	md	DE	2020	0	1	0	0	0	1	0	0	0	0	0	0	72
16794104	m	md	JP	2020	0	1	0	0	0	1	0	0	0	0	0	0	76
16827032	m	ph	JP	2020	0	1	0	1	0	0	0	0	0	0	0	0	76
16850184	m	md	FR	2020	0	1	1	0	0	0	0	0	0	0	0	0	58
16860005	m	md	VN	2020	0	1	0	0	0	1	0	0	0	0	0	0	76
16872879	m	cn	IN	2020	0	0	0	0	0	0	0	0	0	0	0	0	72
16872914	f	hp	CA	2020	0	0	0	0	0	0	0	0	0	0	0	0	67
16874180	m	md	JP	2020	1	0	0	0	0	0	0	0	0	0	0	0	75.1
16876402	f	ph	US	2020	0	1	1	0	0	0	0	0	0	0	0	0	
16876858	f	hp	US	2020	0	1	0	0	1	0	0	0	0	0	0	0	
16880153	m	md	FR	2020	0	1	0	0	0	1	0	0	0	0	0	0	65
16880510	m	cn	JP	2020	0	1	1	0	0	0	0	0	0	0	0	0	70
16880817	m	cn	DE	2020	0	1	1	0	0	0	0	0	0	0	0	0	57
16884095	m	cn	DE	2020	0	1	1	0	0	0	0	0	0	0	0	1	57
16884801	m	md	FR	2020	0	1	1	0	0	0	0	0	0	0	0	0	58
16885308	f	cn	US	2020	0	1	0	0	1	0	0	0	0	0	0	0	
16885838	m	cn	JP	2020	0	1	1	0	0	0	0	0	0	0	0	0	74
16886441	m	Not repo	JP	2020	0	1	0	1	0	0	0	0	0	0	0	1	71.8
16887427	m	md	FR	2020	0	1	0	0	1	0	0	0	0	0	0	0	72
16887584	m	cn	US	2020	0	1	0	0	1	0	0	0	0	0	0	0	82
16899277	f	md	DE	2020	0	0	0	0	0	0	0	0	0	0	0	0	56
16899740	f	md	DE	2020	0	0	0	0	0	0	0	0	0	0	0	0	66
16900526	m	md	DE	2020	0	0	0	0	0	0	0	0	0	0	0	0	62
16901324	m	md	DE	2020	0	0	0	0	0	0	0	0	0	0	0	0	72
16901542	m	md	DE	2020	0	0	0	0	0	0	0	0	0	0	0	0	72
16904333	m	hp	SG	2020	0	1	0	0	1	0	0	0	0	0	0	0	60
16909563	Not	md	FR	2020	0	0	0	0	0	0	0	0	0	0	0	0	
16910970	f	md	DE	2020	0	0	0	0	0	0	0	0	0	0	0	0	67

16911137	m	md	DE	2020	0	0	0	0	0	0	0	0	0	0	0	0	58
16911140	m	md	DE	2020	0	0	0	0	0	0	0	0	0	0	0	0	73
16913301	f	md	CA	2020	0	0	0	0	0	0	0	0	0	0	0	0	65
16915078	m	md	JP	2020	0	1	0	1	0	0	0	0	0	0	0	0	70
16918748	f	cn	US	2020	0	1	1	0	0	0	0	0	0	0	0	0	93
16918801	m	md	JP	2020	1	0	0	0	0	0	0	0	0	0	0	0	67
16925556	m	hp	DE	2020	0	1	0	0	1	0	0	0	0	0	0	0	57
16927058	f	md	JP	2020	0	1	1	0	0	0	0	0	0	0	0	0	64
16932121	m	md	JP	2020	0	1	0	0	1	0	0	0	0	0	0	1	75
16933720	f	md	IT	2020	0	1	0	0	1	0	0	0	0	0	0	0	79
16934744	m	cn	CN	2020	0	1	0	0	1	0	0	0	0	0	0	0	
16935026	m	md	US	2020	0	1	1	0	0	0	0	0	0	0	0	0	66
16939492	f	md	DE	2020	0	1	0	0	1	0	0	0	0	0	0	0	
16939805	f	hp	DE	2020	0	0	0	0	0	0	0	0	0	0	0	0	56
16939843	f	hp	US	2020	0	1	0	0	1	0	0	0	0	0	0	0	64
16946897	f	md	JP	2020	0	1	0	0	1	0	0	0	0	0	0	0	77
16949683	f	cn	IN	2020	0	0	0	0	0	0	0	0	0	0	0	0	67
16955006	m	cn	JP	2020	0	1	1	0	0	0	0	0	0	0	0	0	70
16955173	f	ph	JP	2020	0	0	0	0	0	0	0	0	0	0	0	0	74
16956647	Not	md	JP	2020	1	0	0	0	0	0	0	0	0	0	0	0	
16956865	m	md	JP	2020	0	1	0	1	0	0	0	0	0	0	0	1	77
16957935	m	md	JP	2020	0	1	0	1	0	0	0	0	0	0	0	1	68
16957983	m	cn	JP	2020	0	1	1	0	0	0	0	0	0	0	0	0	70
16959302	f	md	ES	2020	0	1	1	0	0	0	0	0	0	0	0	0	83
16973108	m	hp	JP	2020	0	1	1	0	0	0	0	0	0	0	0	0	79
16981122	m	cn	US	2020	0	1	1	0	0	0	0	0	0	0	0	0	
16982903	Not	md	US	2020	1	0	0	0	0	0	0	0	0	0	0	0	
16982904	Not	Not repo	AU	2020	0	1	0	1	0	0	0	0	0	0	0	0	
16983194	m	hp	CA	2020	0	1	0	1	0	0	0	0	0	0	0	0	73
16984169	f	md	JP	2020	1	0	0	0	0	0	0	0	0	0	0	0	72
16987328	f	md	VN	2020	0	1	1	0	0	0	0	0	0	0	0	1	48
16988704	f	md	JP	2020	0	1	0	0	0	1	0	0	0	0	0	0	71
16989750	m	md	JP	2020	0	0	0	0	0	0	0	0	0	0	0	0	70
16999918	m	md	FR	2020	0	1	0	0	0	1	0	0	0	0	0	1	67

17002683	m	md	DE	2020	0	1	0	0	0	1	0	0	0	0	0	1	80
17004846	f	md	AU	2020	0	0	0	0	0	0	0	0	0	0	0	0	57
17005672	m	cn	JP	2020	0	0	0	0	0	0	0	0	0	0	0	0	61
17005866	f	md	NL	2020	0	0	0	0	0	0	0	0	0	0	0	0	84
17010919	m	md	US	2020	0	1	0	0	1	0	0	0	0	0	0	0	92
17012350	m	hp	US	2020	0	1	0	0	1	0	0	0	0	0	0	0	92
17015800	m	cn	CN	2020	0	1	0	0	1	0	0	0	0	0	0	0	72
17016139	f	md	JP	2020	1	0	0	0	0	0	0	0	0	0	0	1	81
17018090	f	md	CZ	2020	0	1	1	0	0	0	0	0	0	0	0	0	61
17021126	m	md	JP	2020	0	1	0	0	0	1	0	0	0	0	0	1	70
17021968	m	Not repo	JP	2020	0	1	0	1	0	0	0	0	0	0	0	1	80
17024062	m	hp	CN	2020	0	1	0	0	1	0	0	0	0	0	0	0	67
17029104	m	cn	IN	2020	0	1	0	0	0	1	0	0	0	0	0	0	72
17032745	f	md	HU	2020	0	1	1	0	0	0	0	0	0	0	0	0	72
17036076	m	md	DE	2020	0	1	0	0	0	1	0	0	0	0	0	0	65
17036722	f	cn	US	2020	0	0	0	0	0	0	0	0	0	0	0	0	70
17038079	m	md	JP	2020	0	1	0	1	0	0	0	0	0	0	0	1	70
17038183	Not	md	JP	2020	1	0	0	0	0	0	0	0	0	0	0	0	
17044438	m	md	FR	2020	0	1	0	1	0	0	0	0	0	0	0	0	84
17044467	f	cn	US	2020	0	0	0	0	0	0	0	0	0	0	0	0	
17044512	m	md	FR	2020	0	1	0	1	0	0	0	0	0	0	0	0	67.2
17048128	m	md	US	2020	0	0	0	0	0	0	0	0	0	0	0	0	66
17049675	m	md	US	2020	0	0	0	0	0	0	0	0	0	0	0	0	67
17050404	m	cn	JP	2020	0	1	1	0	0	0	0	0	0	0	0	1	91
17050406	Not	md	US	2020	0	0	0	0	0	0	0	0	0	0	0	0	
17051645	m	md	JP	2020	0	1	0	0	0	1	0	0	0	0	0	0	68
17053888	f	hp	AU	2020	0	1	0	0	1	0	0	0	0	0	0	0	83
17054316	m	Not repo	JP	2020	0	1	0	1	0	0	0	0	0	0	0	1	80
17058862	m	Not repo	IT	2020	0	1	0	1	0	0	0	0	0	0	0	1	73
17060182	m	cn	CN	2020	0	1	0	0	1	0	0	0	0	0	0	0	64
17064568	m	md	IN	2020	0	1	1	0	0	0	0	0	0	0	0	0	60
17065186	f	md	JP	2020	1	0	0	0	0	0	0	0	0	0	0	0	70
17068088	m	hp	JP	2020	0	1	1	0	0	0	0	0	0	0	0	0	69
17069899	m	cn	JP	2020	0	1	1	0	0	0	0	0	0	0	0	0	69

17076183	m	md	AR	2020	0	1	1	0	0	0	0	0	0	0	0	0	63
17078983	m	md	JP	2020	0	1	1	0	0	0	0	0	0	0	0	0	77
17083059	f	md	MY	2020	0	1	1	0	0	0	0	0	0	0	0	0	57
17085506	m	md	DE	2020	0	1	0	0	1	0	0	0	0	0	0	0	49
17088644	m	md	IN	2020	0	1	1	0	0	0	0	0	0	0	0	0	67
17092951	m	md	CN	2020	0	0	0	0	0	0	0	0	0	0	0	0	56
17094365	m	md	MY	2020	0	1	1	0	0	0	0	0	0	0	0	0	61
17096459	f	md	DE	2020	1	0	0	0	0	0	0	0	0	0	0	0	64
17098157	m	md	IN	2020	0	1	1	0	0	0	0	0	0	0	0	0	67
17098166	m	md	ES	2020	0	1	1	0	0	0	0	0	0	0	0	0	77
17105121	f	md	GB	2020	0	1	1	0	0	0	0	0	0	0	0	0	74
17106322	m	md	TR	2020	0	1	1	0	0	0	0	0	0	0	0	0	65
17106341	f	hp	IT	2020	1	0	0	0	0	0	0	0	0	0	0	0	
17109283	f	cn	US	2020	1	0	0	0	0	0	0	0	0	0	0	0	68
17113043	m	md	SK	2020	0	1	1	0	0	0	0	0	0	0	0	0	73
17115590	f	Not repo	JP	2020	1	0	0	0	0	0	0	0	0	0	0	0	63.3
17116054	m	hp	GB	2020	0	1	1	0	0	0	0	0	0	0	0	0	68
17116292	f	md	JP	2020	1	0	0	0	0	0	0	0	0	0	0	1	74
17120427	m	Not repo	JP	2020	0	1	0	1	0	0	0	0	0	0	0	0	67.4
17127315	f	md	JP	2020	0	1	0	0	1	0	0	0	0	0	0	0	80
17128653	f	md	DE	2020	0	1	1	0	0	0	0	0	0	0	0	0	76
17128912	f	md	JP	2020	0	1	1	0	0	0	0	0	0	0	0	0	80
17129125	f	md	NO	2020	0	0	0	0	0	0	0	0	0	0	0	0	74
17130352	m	md	JP	2020	0	1	0	0	1	0	0	0	0	0	0	0	62
17130565	f	md	CN	2020	0	1	0	0	0	1	0	0	0	0	0	0	63
17134771	m	md	JP	2020	0	1	1	0	0	0	0	0	0	0	0	0	60
17135612	m	md	SE	2020	0	1	1	0	0	0	0	0	0	0	0	0	75
17147134	m	md	JP	2020	0	0	0	0	0	0	0	0	0	0	0	0	66
17147625	m	Not repo	JP	2020	0	1	0	1	0	0	0	0	0	0	0	0	72.1
17147787	f	ph	CA	2020	0	0	0	0	0	0	0	0	0	0	0	0	52
17151410	m	md	JP	2020	0	1	0	0	0	1	0	0	0	0	0	0	68
17152866	f	md	JP	2020	1	0	0	0	0	0	0	0	0	0	0	0	80.8
17152989	m	cn	GR	2020	0	1	1	0	0	0	0	0	0	0	0	1	75
17153290	m	md	JP	2020	0	0	0	0	0	0	0	0	0	0	0	0	70

17153318	m	hp	US	2020	0	1	0	0	1	0	0	0	0	0	0	1	72
17153916	m	md	IL	2020	0	1	1	0	0	0	0	0	0	0	0	0	70
17154674	f	md	JP	2020	0	1	0	0	0	1	0	0	0	0	0	0	60
17157003	m	hp	DE	2020	0	1	1	0	0	0	0	0	0	0	0	0	67
17159823	f	cn	JP	2020	0	1	1	0	0	0	0	0	0	0	0	0	78
17163215	f	hp	FR	2020	0	1	0	0	1	0	0	0	0	0	0	0	75
17163588	m	md	JP	2020	0	1	0	0	0	1	0	0	0	0	0	0	68
17165546	m	md	TW	2020	0	1	1	0	0	0	0	0	0	0	0	0	65
17166264	Not	md	JP	2020	0	0	0	0	0	0	0	0	0	0	0	0	90
17166649	f	md	DE	2020	0	0	0	0	0	0	0	0	0	0	0	0	73
17167198	m	md	JP	2020	0	1	0	0	1	0	0	0	0	0	0	0	84
17169623	m	md	KR	2020	0	1	0	0	1	0	0	0	0	0	0	0	60
17170111	m	md	KR	2020	0	1	0	0	1	0	0	0	0	0	0	0	62
17170166	f	hp	US	2020	0	1	0	0	1	0	0	0	0	0	0	0	63
17170590	f	md	JP	2020	0	0	0	0	0	0	0	0	0	0	0	0	82
17171988	m	cn	CN	2020	0	0	0	0	0	0	0	0	0	0	0	0	49
17179707	m	hp	NL	2020	0	1	0	0	0	1	0	0	0	0	0	0	119
17180908	m	md	CN	2020	0	0	0	0	0	0	0	0	0	0	0	0	66
17182205	f	md	GB	2020	0	1	1	0	0	0	0	0	0	0	0	0	74
17182285	m	md	CA	2020	0	1	0	0	0	1	0	0	0	0	0	0	62
17182334	f	md	DE	2020	0	1	0	0	0	1	0	0	0	0	0	0	67
17183768	f	cn	PL	2020	0	0	0	0	0	0	0	0	0	0	0	0	80
17183878	m	hp	CN	2020	0	1	0	0	1	0	0	0	0	0	0	0	54
17184621	m	md	IN	2020	0	1	0	1	0	0	0	0	0	0	0	1	60
17186447	m	md	CN	2020	0	0	0	0	0	0	0	0	0	0	0	0	56
17186557	m	hp	CN	2020	0	1	0	0	1	0	0	0	0	0	0	0	76
17186655	m	hp	US	2020	0	1	0	0	1	0	0	0	0	0	0	0	
17186846	m	md	CN	2020	0	0	0	0	0	0	0	0	0	0	0	0	66
17187788	m	cn	IL	2020	0	1	1	0	0	0	0	0	0	0	0	0	75
17188060	f	hp	DE	2020	0	0	0	0	0	0	0	0	0	0	0	0	70
17188983	m	md	JP	2020	0	1	0	0	0	1	0	0	0	0	0	1	78
17190836	f	md	DE	2020	0	1	0	0	0	1	0	0	0	0	0	0	74
17190872	m	md	KR	2020	1	0	0	0	0	0	0	0	0	0	0	0	62
17191314	f	hp	DE	2020	0	1	1	0	0	0	0	0	0	0	0	0	73

17191529	m	md	MY	2020	0	1	1	0	0	0	0	0	0	0	0	0	63
17191672	Not	hp	US	2020	0	1	0	0	1	0	0	0	0	0	0	0	
17192902	m	md	KR	2020	1	0	0	0	0	0	0	0	0	0	0	1	83
17193329	m	md	TW	2020	1	0	0	0	0	0	0	0	0	0	0	0	
17195209	m	md	DE	2020	0	0	0	0	0	0	0	0	0	0	0	0	68
17195218	f	md	DE	2020	0	0	0	0	0	0	0	0	0	0	0	0	52
17197382	f	Not repo	CN	2020	1	0	0	0	0	0	0	0	0	0	0	0	78
17198751	m	Not repo	CN	2020	1	0	0	0	0	0	0	0	0	0	0	0	68
17198981	m	hp	SE	2020	0	1	1	0	0	0	0	0	0	0	0	0	60
17200117	m	md	JP	2020	0	1	0	0	1	0	0	0	0	0	0	0	71
17200951	m	md	CN	2020	0	1	1	0	0	0	0	0	0	0	0	0	70
17201629	f	ph	US	2020	0	0	0	0	0	0	0	0	0	0	0	0	80
17202145	m	md	JP	2020	0	1	1	0	0	0	0	0	0	0	0	0	65
17204320	m	md	JP	2020	0	1	0	0	1	0	0	0	0	0	0	0	76
17204328	m	hp	US	2020	0	1	0	0	1	0	0	0	0	0	0	0	72
17204647	f	cn	CN	2020	0	1	0	0	1	0	0	0	0	0	0	0	
17204844	f	md	JP	2020	0	1	0	0	0	1	0	0	0	0	0	0	71
17207196	m	md	JP	2020	0	1	1	0	0	0	0	0	0	0	0	0	75
17207482	m	md	JP	2020	0	0	0	0	0	0	0	0	0	0	0	0	75
17208079	Not	md	GB	2020	0	1	1	0	0	0	0	0	0	0	0	0	73
17208508	f	cn	CN	2020	1	0	0	0	0	0	0	0	0	0	0	0	59
17208683	f	md	TR	2020	0	1	1	0	0	0	0	0	0	0	0	0	61
17209861	f	Not repo	JP	2020	0	1	0	1	0	0	0	0	0	0	0	0	70
17209862	f	Not repo	JP	2020	0	1	0	1	0	0	0	0	0	0	0	1	78.4
17209967	f	cn	CN	2020	1	0	0	0	0	0	0	0	0	0	0	0	69
17210613	f	md	RU	2020	0	1	1	0	0	0	0	0	0	0	0	0	75
17212468	m	cn	BE	2020	0	1	1	0	0	0	0	0	0	0	0	0	60
17212495	m	md	DE	2020	0	0	0	0	0	0	0	0	0	0	0	0	68
17212722	f	md	CZ	2020	0	0	0	0	0	0	0	0	0	0	0	0	45
17213070	f	md	KR	2020	1	0	0	0	0	0	0	0	0	0	0	0	78
17213274	m	md	RU	2020	0	1	1	0	0	0	0	0	0	0	0	0	64
17214703	m	md	DE	2020	0	0	0	0	0	0	0	0	0	0	0	0	72
17217556	m	cn	CN	2020	0	1	0	0	1	0	0	0	0	0	0	0	60
17218328	m	md	JP	2020	0	1	0	0	0	1	0	0	0	0	0	0	70

17220224	m	cn	CN	2020	1	0	0	0	0	0	0	0	0	0	0	0	73
17220848	m	hp	DE	2020	0	1	0	0	1	0	0	0	0	0	0	0	68
17221623	f	md	JP	2020	0	1	1	0	0	0	0	0	0	0	0	0	75
17222045	m	Not repo	JP	2020	0	1	0	1	0	0	0	0	0	0	0	0	57
17222144	f	md	JP	2020	1	0	0	0	0	0	0	0	0	0	0	0	84.9
17222476	m	cn	JP	2020	0	1	1	0	0	0	0	0	0	0	0	0	57
17222623	f	cn	CN	2020	0	1	0	0	1	0	0	0	0	0	0	0	
17222856	f	md	US	2020	0	0	0	0	0	0	0	0	0	0	0	0	38
17223022	f	cn	KR	2020	1	0	0	0	0	0	0	0	0	0	0	0	71
17223141	f	cn	JP	2020	0	0	0	0	0	0	0	0	0	0	0	0	67
17223184	m	md	JP	2020	0	1	0	0	0	1	0	0	0	0	0	0	
17223305	f	md	DE	2020	0	0	0	0	0	0	0	0	0	0	0	0	61
17223613	m	hp	DE	2020	0	0	0	0	0	0	0	0	0	0	0	0	53
17223705	m	hp	US	2020	0	1	0	1	0	0	0	0	0	0	0	0	71
17224081	f	md	DE	2020	0	0	0	0	0	0	0	0	0	0	0	0	52
17224270	m	cn	US	2020	0	0	0	0	0	0	0	0	0	0	0	0	
17224371	m	md	CN	2020	0	1	1	0	0	0	0	0	0	0	0	0	56
17224480	m	cn	Not re	2020	0	0	0	0	0	0	0	0	0	0	0	0	
17224702	f	md	DE	2020	0	1	0	0	1	0	0	0	0	0	0	0	58
17225107	m	md	FR	2020	0	1	0	0	1	0	0	0	0	0	0	0	63
17225120	f	hp	AT	2020	0	1	0	0	0	1	0	0	0	0	0	0	59
17225132	m	md	FR	2020	0	1	1	0	0	0	0	0	0	0	0	0	58
17225253	m	cn	DE	2020	0	1	0	0	0	1	0	0	0	0	0	0	77
17225388	m	hp	JP	2020	0	1	1	0	0	0	0	0	0	0	0	1	77
17225509	m	hp	RU	2020	0	1	1	0	0	0	0	0	0	0	0	0	64
17226124	f	hp	CN	2020	0	1	0	0	1	0	0	0	0	0	0	0	54
17227000	f	cn	IN	2020	0	1	0	0	1	0	0	0	0	0	0	0	69
17227092	m	md	JP	2020	0	1	0	0	0	1	0	0	0	0	0	0	80
17227099	m	md	JP	2020	0	1	0	0	0	1	0	0	0	0	0	0	70
17227212	m	hp	DE	2020	0	0	0	0	0	0	0	0	0	0	0	0	59
17227215	f	md	CZ	2020	1	0	0	0	0	0	0	0	0	0	0	0	59
17227273	f	cn	CN	2020	0	1	0	0	1	0	0	0	0	0	0	0	
17227278	f	hp	US	2020	0	0	0	0	0	0	0	0	0	0	0	0	66
17227343	m	md	JP	2020	0	1	0	0	0	1	0	0	0	0	0	0	75

17227376	f	Not repo	JP	2020	0	1	0	1	0	0	0	0	0	0	0	1	80
17227534	f	md	CN	2020	0	1	0	0	1	0	0	0	0	0	0	0	38
17228164	m	Not repo	JP	2020	0	1	0	1	0	0	0	0	0	0	0	1	69
17228225	m	hp	DE	2020	0	0	0	0	0	0	0	0	0	0	0	0	58
17228233	f	md	NL	2020	0	0	0	0	0	0	1	0	0	0	1	0	58
17228674	f	cn	TW	2020	0	0	0	0	0	0	0	0	0	0	0	0	72
17228806	m	cn	IN	2020	0	0	0	0	0	0	0	0	0	0	0	0	33
17228815	f	md	IN	2020	0	0	0	0	0	0	0	0	0	0	0	0	39
17228819	m	md	JP	2020	0	1	0	0	0	1	0	0	0	0	0	0	68
17228919	f	Not repo	CN	2020	1	0	0	0	0	0	0	0	0	0	0	0	64
17228923	m	cn	JP	2020	0	1	1	0	0	0	0	0	0	0	0	0	53
17228968	f	md	TH	2020	0	1	0	0	0	1	0	0	0	0	0	0	48
17229107	f	md	JP	2020	0	1	0	0	0	1	0	0	0	0	0	0	67
17229238	m	cn	CN	2020	1	0	0	0	0	0	0	0	0	0	0	0	59
17229247	f	cn	CN	2020	1	0	0	0	0	0	0	0	0	0	0	0	45
17229401	m	Not repo	JP	2020	1	0	0	0	0	0	0	0	0	0	0	0	
17229462	m	cn	TW	2020	0	1	0	0	0	1	0	0	0	0	0	0	69
17229736	m	md	KR	2020	1	0	0	0	0	0	0	0	0	0	0	0	60
17229988	m	cn	CN	2020	1	0	0	0	0	0	0	0	0	0	0	0	77
17230083	f	cn	JP	2020	0	1	1	0	0	0	0	0	0	0	0	0	77
17230164	f	md	US	2020	0	0	0	0	0	0	0	0	0	0	0	0	48
17230271	m	hp	NL	2020	0	1	1	0	0	0	0	0	0	0	0	0	70
17230285	f	Not repo	JP	2020	1	0	0	0	0	0	0	0	0	0	0	0	67
17230489	m	hp	FR	2020	0	1	0	0	1	0	0	0	0	0	0	0	80
17230526	m	hp	JP	2020	0	1	0	1	0	0	0	0	0	0	0	1	62
17230599	m	hp	TR	2020	0	1	1	0	0	0	0	0	0	0	0	0	69
17231404	m	Not repo	ID	2020	1	0	0	0	0	0	0	0	0	0	0	0	
17232559	m	md	MY	2020	0	1	1	0	0	0	0	0	0	0	0	0	63
17232786	f	md	KR	2020	1	0	0	0	0	0	0	0	0	0	0	0	89
17232887	m	hp	CN	2020	0	1	0	0	1	0	0	0	0	0	0	0	36
17233020	m	hp	JP	2020	0	1	1	0	0	0	0	0	0	0	0	1	81
17233065	m	md	DE	2020	0	0	0	0	0	0	0	0	0	0	0	0	59
17233094	m	hp	DE	2020	0	0	0	0	0	0	0	0	0	0	0	0	53
17233257	f	md	DE	2020	0	1	0	0	1	0	0	0	0	0	0	0	58

17233383	m	hp	DE	2020	0	1	1	0	0	0	0	0	0	0	0	0	68
17233540	f	md	DE	2020	1	0	0	0	0	0	0	0	0	0	0	0	59.4
17233704	f	cn	US	2020	0	1	0	0	0	1	0	0	0	0	0	0	89
17233705	m	md	JP	2020	0	1	0	0	0	1	0	0	0	0	0	0	69
17233809	m	hp	DE	2020	0	0	0	0	0	0	0	0	0	0	0	0	72
17233810	f	hp	DE	2020	0	0	0	0	0	0	0	0	0	0	0	0	67
17233881	f	hp	FR	2020	0	1	0	0	1	0	0	0	0	0	0	0	58
17235156	m	hp	CN	2020	0	1	0	0	1	0	0	0	0	0	0	0	48
17235612	m	cn	CN	2020	0	1	0	0	1	0	0	0	0	0	0	0	41
17235680	f	md	DE	2020	0	0	0	0	0	0	0	0	0	0	0	0	71
17235904	f	hp	DE	2020	0	0	0	0	0	0	0	0	0	0	0	0	56
17235905	m	hp	DE	2020	0	0	0	0	0	0	0	0	0	0	0	0	64
17236002	m	md	DE	2020	0	1	1	0	0	0	0	0	0	0	0	0	70
17236283	m	cn	DE	2020	0	1	1	0	0	0	0	0	0	0	0	1	63
17236445	m	hp	AU	2020	0	1	0	0	0	1	0	0	0	0	0	0	66
17236771	m	md	PL	2020	0	0	0	0	0	0	0	0	0	0	0	0	43
17237563	m	cn	IL	2020	0	1	1	0	0	0	0	0	0	0	0	1	
17237627	m	hp	DE	2020	0	1	1	0	0	0	0	0	0	0	0	0	68
17237858	f	cn	US	2020	1	0	0	0	0	0	0	0	0	0	0	0	69
17237907	m	md	SI	2020	0	0	0	0	0	0	0	0	0	0	0	0	
17238479	f	cn	CN	2020	1	0	0	0	0	0	0	0	0	0	0	0	71.4
17238529	m	md	JP	2020	0	1	0	1	0	0	0	0	0	0	0	1	70
17240207	f	cn	PE	2020	1	0	0	0	0	0	0	0	0	0	0	0	
17240814	f	md	NL	2020	0	0	0	0	0	0	0	0	0	0	0	0	63
17241189	m	hp	DE	2020	0	1	1	0	0	0	0	0	0	0	0	0	54
17241699	m	md	US	2020	0	1	0	0	0	1	0	0	0	0	0	0	
17241753	f	cn	CN	2020	1	0	0	0	0	0	0	0	0	0	0	0	61
17241968	f	cn	Not re	2020	0	0	0	0	0	0	0	0	0	0	0	0	
17241976	m	md	GR	2020	0	1	1	0	0	0	0	0	0	0	0	0	71
17242372	m	md	SI	2020	0	0	0	0	0	0	0	0	0	0	0	0	46
17242406	f	Not repo	GB	2020	0	1	1	0	0	0	0	0	0	0	0	0	65
17242408	m	cn	CN	2020	1	0	0	0	0	0	0	0	0	0	0	0	64
17243280	m	Not repo	CN	2020	1	0	0	0	0	0	0	0	0	0	0	0	61
17244091	m	hp	MX	2020	0	1	0	0	1	0	0	0	0	0	0	0	70

17244099	f	md	CO	2020	0	1	1	0	0	0	0	0	0	0	0	0	36
17244152	m	hp	DE	2020	0	1	1	0	0	0	0	0	0	0	0	0	
17244415	f	md	IT	2020	0	1	0	0	0	1	0	0	0	0	0	1	67
17244520	f	cn	BE	2020	0	1	1	0	0	0	0	0	0	0	0	0	66
17245047	Not	md	CN	2020	0	0	0	0	0	0	0	0	0	0	0	0	
17245255	m	cn	CN	2020	0	1	0	0	1	0	0	0	0	0	0	0	
17245408	f	cn	CN	2020	1	0	0	0	0	0	0	0	0	0	0	0	64
17245452	f	cn	CN	2020	1	0	0	0	0	0	0	0	0	0	0	0	77
17245712	f	cn	CN	2020	1	0	0	0	0	0	0	0	0	0	0	0	83
17245751	f	cn	CN	2020	1	0	0	0	0	0	0	0	0	0	0	0	67.9
17245856	f	cn	KR	2020	1	0	0	0	0	0	0	0	0	0	0	0	44
17246054	m	cn	CN	2020	1	0	0	0	0	0	0	0	0	0	0	0	51
17246055	f	Not repo	CN	2020	1	0	0	0	0	0	0	0	0	0	0	0	76
17246368	f	md	CN	2020	0	1	0	0	0	1	0	0	0	0	0	0	65
17246408	f	md	CO	2020	0	1	1	0	0	0	0	0	0	0	0	0	80
17246617	m	cn	CN	2020	1	0	0	0	0	0	0	0	0	0	0	0	59
17246624	f	Not repo	JP	2020	1	0	0	0	0	0	0	0	0	0	0	0	60
17247327	f	cn	IN	2020	0	0	0	0	0	0	0	0	0	0	0	0	42
17247717	f	cn	CN	2020	1	0	0	0	0	0	0	0	0	0	0	0	
17247750	m	hp	US	2020	0	0	0	0	0	0	0	0	0	0	0	0	61
17247777	m	Not repo	CN	2020	1	0	0	0	0	0	0	0	0	0	0	0	59
17247831	f	cn	CN	2020	1	0	0	0	0	0	0	0	0	0	0	0	53
17247988	m	md	CN	2020	0	1	0	0	1	0	0	0	0	0	0	0	51
17248073	f	cn	CN	2020	1	0	0	0	0	0	0	0	0	0	0	0	60
17248212	f	cn	CN	2020	1	0	0	0	0	0	0	0	0	0	0	0	
17248230	f	hp	DE	2020	0	1	1	0	0	0	0	0	0	0	0	0	73
17248254	f	cn	CA	2020	0	0	0	0	0	0	0	0	0	0	0	0	51
17248279	f	cn	CN	2020	1	0	0	0	0	0	0	0	0	0	0	0	77
17248284	f	md	JP	2020	0	1	0	0	1	0	0	0	0	0	0	0	60
17248291	m	cn	CN	2020	1	0	0	0	0	0	0	0	0	0	0	0	65.6
17248293	f	cn	CN	2020	1	0	0	0	0	0	0	0	0	0	0	0	66.6
17248300	m	Not repo	CN	2020	1	0	0	0	0	0	0	0	0	0	0	0	73
17248352	f	cn	CN	2020	1	0	0	0	0	0	0	0	0	0	0	0	75.5
17248364	m	Not repo	CN	2020	1	0	0	0	0	0	0	0	0	0	0	0	64

17249079	f	cn	DE	2020	0	1	1	0	0	0	0	0	0	0	0	0	73
17249282	f	md	GB	2020	0	1	0	0	0	1	0	0	0	0	0	0	69
17249674	Not	md	MA	2020	0	1	0	0	0	1	0	0	0	0	0	0	
17249949	Not	md	DE	2020	0	0	0	0	0	0	0	0	0	0	0	0	72
17250096	f	hp	GB	2020	0	1	1	0	0	0	0	0	0	0	0	0	65
17250221	Not	hp	DE	2020	0	0	0	0	0	0	0	0	0	0	0	0	58
17250380	f	ph	GB	2020	0	1	1	0	0	0	0	0	0	0	0	0	65
17250920	Not	cn	BR	2020	0	1	1	0	0	0	0	0	0	0	0	0	
17251452	f	cn	US	2020	0	0	0	0	0	0	0	0	0	0	0	0	73
17252496	m	cn	CN	2020	1	0	0	0	0	0	0	0	0	0	0	0	67
17252805	m	cn	JP	2020	0	1	1	0	0	0	0	0	0	0	0	0	80
17253042	m	Not repo	US	2020	1	0	0	0	0	0	0	0	0	0	0	0	75
17253115	m	hp	US	2020	1	0	0	0	0	0	0	0	0	0	0	1	
17253150	m	hp	AR	2020	0	1	0	0	1	0	0	0	0	0	0	0	60
17253336	Not	Not repo	JP	2020	1	0	0	0	0	0	0	0	0	0	0	1	
17253655	m	md	CL	2020	0	1	1	0	0	0	0	0	0	0	0	0	82
17253885	f	cn	DE	2020	0	0	0	0	0	0	0	0	0	0	0	0	
17254115	m	hp	DE	2020	0	1	1	0	0	0	0	0	0	0	0	0	69
17254582	m	md	SI	2020	0	0	0	0	0	0	0	0	0	0	0	0	46
17255010	m	hp	DE	2020	0	0	0	0	0	0	0	0	0	0	0	0	53
17255053	f	cn	US	2020	0	0	0	0	0	0	0	0	0	0	0	0	
17255075	m	md	VN	2020	0	1	0	0	0	1	0	0	0	0	0	0	50
17255169	m	md	JP	2020	0	1	0	0	1	0	0	0	0	0	0	0	70
17255338	m	md	JP	2020	0	1	0	0	1	0	0	0	0	0	0	0	87
17255397	m	cn	CA	2020	0	1	1	0	0	0	0	0	0	0	0	0	
17255536	m	cn	IT	2020	0	1	0	0	0	1	0	0	0	0	0	0	56
17255614	m	hp	NL	2020	0	1	1	0	0	0	0	0	0	0	0	0	77
17255708	m	md	JP	2020	0	1	1	0	0	0	0	0	0	0	0	0	80
17255722	f	md	DE	2020	0	1	1	0	0	0	0	0	0	0	0	0	47
17255771	f	cn	CN	2020	0	1	0	0	1	0	0	0	0	0	0	0	63
17256151	m	md	DE	2020	0	1	1	0	0	0	0	0	0	0	0	0	70
17256554	m	hp	DE	2020	0	0	0	0	0	0	0	0	0	0	0	0	64
17256825	m	hp	CN	2020	0	1	0	0	1	0	0	0	0	0	0	0	43
17256852	f	md	JP	2020	0	1	1	0	0	0	0	0	0	0	0	0	70

17257518	f	hp	DE	2020	0	0	0	0	0	0	0	0	0	0	0	0		
17257557	Not	md	JP	2020	0	1	0	0	1	0	0	0	0	0	0	0		
17257749	f	cn	CN	2020	1	0	0	0	0	0	0	0	0	0	0	0	69	
17257892	Not	md	CN	2020	0	0	0	0	0	0	0	0	0	0	0	0		
17257929	m	hp	PL	2020	0	0	0	0	0	0	0	0	0	0	0	0	70	
17258114	m	md	GR	2020	0	1	1	0	0	0	0	0	0	0	0	0	65	
17258278	f	md	PL	2020	0	0	0	0	0	0	0	0	0	0	0	0	81	
17258334	f	md	AU	2020	0	0	0	0	0	0	0	0	0	0	0	0	63	
17258398	m	cn	JP	2020	0	1	1	0	0	0	0	0	0	0	0	0	67	
17258478	m	md	JP	2020	0	1	1	0	0	0	0	0	0	0	0	0	82	
17258663	m	cn	ID	2020	1	0	0	0	0	0	0	0	0	0	0	0		
17258693	f	cn	JP	2020	0	1	1	0	0	0	0	0	0	0	0	0	72	
17258747	m	hp	CN	2020	0	1	0	0	1	0	0	0	0	0	0	1	54	
17258907	m	md	GR	2020	0	1	1	0	0	0	0	0	0	0	0	0	73	
17259420	f	cn	JP	2020	0	0	0	0	0	0	0	0	0	0	0	0	89	
17259476	m	hp	JP	2020	0	1	1	0	0	0	0	0	0	0	0	1	55	
17259521	f	ph	GB	2020	0	1	1	0	0	0	0	0	0	0	0	0	65	
17259867	Not	md	DE	2020	0	0	0	0	0	0	0	0	0	0	0	0	53	
17260038	m	hp	DE	2020	0	0	0	0	0	0	0	0	0	0	0	0		
17260057	Not	md	DE	2020	0	0	0	0	0	0	0	0	0	0	0	0	64	
17260133	m	hp	DE	2020	0	1	1	0	0	0	0	0	0	0	0	0	68	
17260208	m	md	IN	2020	0	1	1	0	0	0	0	0	0	0	0	0	67	
17260439	f	md	IT	2020	0	1	0	0	0	1	0	0	0	0	0	1	67	
17260760	m	hp	TW	2020	0	1	0	0	0	1	0	0	0	0	0	0	62	
17260796	m	cn	HK	2020	0	1	1	0	0	0	0	0	0	0	0	0		
17260883	f	cn	KR	2020	1	0	0	0	0	0	0	0	0	0	0	0	49	
17260904	f	md	DK	2020	0	1	1	0	0	0	0	0	0	0	0	0	70	
17260914	f	cn	IN	2020	0	1	0	0	0	1	0	0	0	0	0	0	57	
17260926	m	md	KR	2020	0	1	0	0	1	0	0	0	0	0	0	0	70	
17261194	m	md	JP	2020	0	1	1	0	0	0	0	0	0	0	0	0		
17261598	f	md	JP	2020	0	1	0	0	0	1	0	0	0	0	0	0	79	
17261660	m	md	DE	2020	0	0	0	0	0	0	0	0	0	0	0	0	72	
17261759	f	md	JP	2020	0	1	0	0	1	0	0	0	0	0	0	0	64	
17261976	m	cn	IN	2020	0	0	0	0	0	0	0	0	0	0	0	0	37	

17262023	m	Not repo	CN	2020	1	0	0	0	0	0	0	0	0	0	0	0	73.4
17262245	f	md	TW	2020	0	1	0	1	0	0	0	0	0	0	0	0	53
17262251	f	md	JP	2020	1	0	0	0	0	0	0	0	0	0	0	0	70
17262541	m	md	JP	2020	0	1	1	0	0	0	0	0	0	0	0	1	78
17262639	m	md	US	2020	0	0	0	0	0	0	0	0	0	0	0	0	79
17262688	m	hp	US	2020	0	1	1	0	0	0	0	0	0	0	0	0	82
17263061	f	hp	JP	2020	0	1	0	0	1	0	0	0	0	0	0	0	79
17263775	m	md	FR	2020	0	1	1	0	0	0	0	0	0	0	0	0	61
17263894	m	md	TW	2020	0	1	0	1	0	0	0	0	0	0	0	0	73
17263899	f	md	US	2020	0	1	0	1	0	0	0	0	0	0	0	1	
17264646	m	md	JP	2020	0	1	0	0	1	0	0	0	0	0	0	0	31
17264669	f	md	MX	2020	0	1	1	0	0	0	0	0	0	0	0	0	74
17264831	m	cn	JP	2020	0	1	1	0	0	0	0	0	0	0	0	0	80
17264868	m	hp	DE	2020	0	1	0	0	1	0	0	0	0	0	0	0	61
17266103	m	md	DE	2020	0	1	0	0	0	1	0	0	0	0	0	0	
17266392	m	md	DE	2020	0	1	1	0	0	0	0	0	0	0	0	0	72
17266947	m	md	US	2020	0	1	0	0	0	1	0	0	0	0	0	0	95
17266979	m	md	AT	2020	0	0	0	0	0	0	0	0	0	0	0	0	71
17267733	f	md	JP	2020	1	0	0	0	0	0	0	0	0	0	0	0	58
17268016	m	md	KR	2020	0	1	0	0	1	0	0	0	0	0	0	1	59
17268027	m	cn	JP	2020	0	1	1	0	0	0	0	0	0	0	0	0	72
17268351	f	cn	CN	2020	1	0	0	0	0	0	0	0	0	0	0	0	63
17268537	m	hp	JP	2020	0	1	1	0	0	0	0	0	0	0	0	0	76
17268559	f	cn	GB	2020	0	1	1	0	0	0	0	0	0	0	0	0	65
17268630	m	md	GB	2020	0	1	1	0	0	0	0	0	0	0	0	0	64
17268733	f	ph	US	2020	1	0	0	0	0	0	0	0	0	0	0	0	51
17268927	m	md	GB	2020	1	0	0	0	0	0	0	0	0	0	0	0	70
17269210	m	md	BE	2020	0	1	1	0	0	0	0	0	0	0	0	0	71
17269347	f	cn	CA	2020	1	0	0	0	0	0	0	0	0	0	0	0	
17269390	Not	md	JP	2020	1	0	0	0	0	0	0	0	0	0	0	1	
17269396	f	Not repo	JP	2020	1	0	0	0	0	0	0	0	0	0	0	1	86
17269455	m	cn	US	2020	0	1	1	0	0	0	0	0	0	0	0	1	
17269481	m	md	ES	2020	0	1	1	0	0	0	0	0	0	0	0	0	76
17269487	Not	md	CH	2020	0	0	0	0	0	0	0	0	0	0	0	0	

17269569	m	md	KR	2020	0	1	0	0	1	0	0	0	0	0	0	0	76
17269762	f	Not repo	MY	2020	1	0	0	0	0	0	0	0	0	0	0	0	61.6
17269956	f	ph	DE	2020	1	0	0	0	0	0	0	0	0	0	0	0	59
17270832	m	hp	JP	2020	0	1	0	0	0	1	0	0	0	0	0	0	57
17270938	f	md	JP	2020	0	1	0	1	0	0	0	0	0	0	0	1	70
17271243	f	md	US	2020	1	0	0	0	0	0	0	0	0	0	0	0	52
17271793	m	md	JP	2020	0	1	0	1	0	0	0	0	0	0	0	0	78
17271892	m	hp	CN	2020	1	0	0	0	0	0	0	0	0	0	0	0	69
17272147	f	md	JP	2020	1	0	0	0	0	0	0	0	0	0	0	1	59
17272224	f	Not repo	CN	2020	1	0	0	0	0	0	0	0	0	0	0	0	77
17272293	f	cn	CN	2020	1	0	0	0	0	0	0	0	0	0	0	0	80
17272387	f	cn	JP	2020	1	0	0	0	0	0	0	0	0	0	0	0	60
17272601	Not	md	JP	2020	0	1	1	0	0	0	0	0	0	0	0	0	
17272946	f	cn	US	2020	0	0	0	0	0	0	0	0	0	0	0	0	68
17273171	m	Not repo	ID	2020	1	0	0	0	0	0	0	0	0	0	0	0	
17274123	Not	md	US	2020	0	1	0	1	0	0	0	0	0	0	0	0	68
17274124	m	md	US	2020	0	1	0	1	0	0	0	0	0	0	0	0	50
17274125	f	ph	US	2020	0	1	0	1	0	0	0	0	0	0	0	0	
17274166	m	cn	US	2020	0	1	1	0	0	0	0	0	0	0	0	0	64
17274192	Not	md	US	2020	0	1	0	1	0	0	0	0	0	0	0	0	
17274415	m	md	IT	2020	0	1	1	0	0	0	0	0	0	0	0	0	74
17275787	Not	md	JP	2020	0	1	1	0	0	0	0	0	0	0	0	0	
17275998	f	cn	DE	2020	0	1	1	0	0	0	0	0	0	0	0	0	73
17276137	m	hp	JP	2020	0	0	0	0	0	0	0	0	0	0	0	0	79
17276413	f	md	US	2020	0	1	0	1	0	0	0	0	0	0	0	0	
17276479	m	hp	JP	2020	0	0	0	0	0	0	0	0	0	0	0	0	51
17276660	f	md	DE	2020	0	0	0	0	0	0	0	0	0	0	0	0	58
17277144	Not	Not repo	JP	2020	1	0	0	0	0	0	0	0	0	0	0	0	80
17277279	f	ph	GB	2020	0	1	1	0	0	0	0	0	0	0	0	0	65
17277473	f	md	JP	2020	0	0	0	0	0	0	0	0	0	0	0	0	40
17277836	Not	Not repo	JP	2020	0	1	0	1	0	0	0	0	0	0	0	0	
17278140	m	cn	KR	2020	0	0	0	0	0	0	0	0	0	0	0	0	44
17278717	f	ph	JP	2020	0	0	0	0	0	0	0	0	0	0	0	0	89
17278733	m	ph	CA	2020	0	1	0	1	0	0	0	0	0	0	0	0	71.2

17278814	f	md	JP	2020	1	0	0	0	0	0	0	0	0	0	0	1	79.5
17278820	f	md	US	2020	0	1	0	1	0	0	0	0	0	0	0	0	
17278901	f	cn	US	2020	0	1	1	0	0	0	0	0	0	0	0	0	
17279195	m	cn	IN	2020	0	1	0	0	1	0	0	0	0	0	0	0	68
17279695	m	md	US	2020	0	1	0	1	0	0	0	0	0	0	0	0	
17279856	f	md	JP	2020	0	1	0	0	0	1	0	0	0	0	0	0	79
17280235	m	ph	JP	2020	0	1	0	0	0	1	0	0	0	0	0	1	69
17280255	f	cn	IN	2020	0	1	0	0	1	0	0	0	0	0	0	0	51
17281035	m	md	VN	2020	1	0	0	0	0	0	0	0	0	0	0	0	33
17281307	f	Not repo	US	2020	1	0	0	0	0	0	0	0	0	0	0	0	72
17281477	m	md	IL	2020	0	1	0	1	0	0	0	0	0	0	0	0	
17282079	m	cn	CA	2020	0	1	0	1	0	0	0	0	0	0	0	0	
17282265	f	Not repo	JP	2020	1	0	0	0	0	0	0	0	0	0	0	0	33
17282298	Not	md	JP	2020	1	0	0	0	0	0	0	0	0	0	0	0	
17282666	m	md	JP	2020	1	0	0	0	0	0	0	0	0	0	0	0	91
17282926	m	Not repo	JP	2020	0	1	0	1	0	0	0	0	0	0	0	0	80
17282931	m	md	US	2020	0	1	0	1	0	0	0	0	0	0	0	0	75
17283047	m	md	KR	2020	0	1	1	0	0	0	0	0	0	0	0	1	64
17283099	m	md	CA	2020	0	0	0	0	0	0	0	0	0	0	0	0	75
17283155	m	Not repo	MY	2020	1	0	0	0	0	0	0	0	0	0	0	0	58.4
17283252	f	cn	US	2020	0	0	0	0	0	0	0	0	0	0	0	0	49
17283362	f	cn	CN	2020	1	0	0	0	0	0	0	0	0	0	0	0	62
17283640	f	md	IT	2020	1	0	0	0	0	0	0	0	0	0	0	0	89
17284295	f	Not repo	CN	2020	1	0	0	0	0	0	0	0	0	0	0	0	85
17284569	m	ph	US	2020	0	1	0	1	0	0	0	0	0	0	0	0	
17284573	m	hp	JP	2020	0	1	0	1	0	0	0	0	0	0	0	1	72
17284619	m	hp	CA	2020	0	1	1	0	0	0	0	0	0	0	0	0	52
17284661	f	md	JP	2020	1	0	0	0	0	0	0	0	0	0	0	0	69.2
17284746	f	cn	US	2020	1	0	0	0	0	0	0	0	0	0	0	0	
17284766	f	cn	US	2020	1	0	0	0	0	0	0	0	0	0	0	0	
17284894	f	Not repo	JP	2020	0	1	0	1	0	0	0	0	0	0	0	0	71
17285086	f	md	US	2020	0	1	1	0	0	0	0	0	0	0	0	0	68
17285097	m	cn	JP	2020	0	0	0	0	0	0	0	0	0	0	0	0	65
17285659	m	md	JP	2020	0	0	0	0	0	0	0	0	0	0	0	0	76

17285685	f	cn	CN	2020	0	0	0	0	0	0	0	0	0	0	0	0	46
17285863	f	md	ES	2020	0	1	0	0	0	1	0	0	0	0	0	0	53
17286220	m	ph	GB	2020	0	1	1	0	0	0	0	0	0	0	0	0	74
17286788	m	md	JP	2020	0	0	0	0	0	0	0	0	0	0	0	0	76
17286916	m	md	KR	2020	0	1	0	0	1	0	0	0	0	0	0	0	71
17286962	m	cn	JP	2020	0	1	1	0	0	0	0	0	0	0	0	0	50
17287233	m	hp	US	2020	0	1	0	0	0	1	0	0	0	0	0	0	55
17287622	f	cn	CN	2020	0	1	0	0	1	0	0	0	0	0	0	0	60
17288513	Not	hp	CA	2020	0	1	1	0	0	0	0	0	0	0	0	0	73
17289113	m	cn	IN	2020	0	0	0	0	0	0	0	0	0	0	0	0	69
17289208	f	md	DE	2020	0	1	1	0	0	0	0	0	0	0	0	0	
17289220	Not	md	US	2020	1	0	0	0	0	0	0	0	0	0	0	0	
17289510	f	Not repo	RO	2020	1	0	0	0	0	0	0	0	0	0	0	0	70
17289521	m	hp	US	2020	0	1	0	0	1	0	0	0	0	0	0	0	73
17289714	m	md	DE	2020	0	1	1	0	0	0	0	0	0	0	0	0	58
17289773	f	md	BY	2020	0	0	0	0	0	0	0	0	0	0	0	0	53
17289786	Not	cn	KR	2020	0	0	0	0	0	0	0	0	0	0	0	0	
17290101	m	cn	JP	2020	0	1	1	0	0	0	0	0	0	0	0	0	66
17290481	f	hp	AU	2020	0	0	0	0	0	0	0	0	0	0	0	0	54
17290696	f	cn	Not re	2020	0	0	0	0	0	0	0	0	0	0	0	0	
17290748	m	md	JP	2020	0	1	0	0	1	0	0	0	0	0	0	0	72
17290750	m	md	JP	2020	0	1	0	0	1	0	0	0	0	0	0	0	76
17290854	m	cn	GR	2020	0	1	1	0	0	0	0	0	0	0	0	0	70
17291459	f	ph	US	2020	0	0	0	0	0	0	0	0	0	0	0	0	63
17291491	m	cn	JP	2020	0	1	1	0	0	0	0	0	0	0	0	0	80
17292223	m	cn	CN	2020	0	1	0	0	1	0	0	0	0	0	0	1	73
17293161	m	md	TH	2020	0	1	1	0	0	0	0	0	0	0	0	0	63
17293214	m	hp	JP	2020	1	0	0	0	0	0	0	0	0	0	0	0	53
17293386	m	cn	CA	2020	1	0	0	0	0	0	0	0	0	0	0	0	51.8
17293640	m	md	JP	2020	0	1	0	0	0	1	0	0	0	0	0	0	80
17294247	m	md	JP	2020	1	0	0	0	0	0	0	0	0	0	0	1	64
17294253	m	Not repo	JP	2020	1	0	0	0	0	0	0	0	0	0	0	1	80
17294307	f	ph	GB	2020	0	1	1	0	0	0	0	0	0	0	0	0	65
17294780	m	cn	JP	2020	0	1	1	0	0	0	0	0	0	0	0	0	81

17295097	m	cn	JP	2020	0	1	0	0	1	0	0	0	0	0	0	0	48
17295274	f	md	DE	2020	0	0	0	0	0	0	0	0	0	0	0	0	
17296097	m	md	VN	2020	1	0	0	0	0	0	0	0	0	0	0	0	
17296305	f	md	JP	2020	0	1	0	0	0	1	0	0	0	0	0	1	77
17296409	f	md	BE	2020	0	1	1	0	0	0	0	0	0	0	0	1	64
17296537	Not	Not repo	JP	2020	1	0	0	0	0	0	0	0	0	0	0	0	77
17296775	m	Not repo	US	2020	1	0	0	0	0	0	0	0	0	0	0	0	73
17296809	m	md	JP	2020	0	1	0	0	1	0	0	0	0	0	0	0	
17296910	m	md	JP	2020	0	1	0	1	0	0	0	0	0	0	0	1	
17297007	m	md	JP	2020	0	1	0	1	0	0	0	0	0	0	0	0	
17297771	m	cn	CN	2020	0	1	0	0	1	0	0	0	0	0	0	0	75
17298060	m	cn	JP	2020	0	1	1	0	0	0	0	0	0	0	0	0	61
17298468	f	hp	RU	2020	1	0	0	0	0	0	0	0	0	0	0	0	
17298471	f	hp	RU	2020	1	0	0	0	0	0	0	0	0	0	0	0	
17298473	m	hp	RU	2020	1	0	0	0	0	0	0	0	0	0	0	0	
17298581	Not	hp	RU	2020	1	0	0	0	0	0	0	0	0	0	0	0	
17298595	m	hp	DE	2020	0	0	0	0	0	0	0	0	0	0	0	0	
17298680	m	md	SG	2020	0	1	1	0	0	0	0	0	0	0	0	0	63
17298999	Not	hp	KR	2020	0	0	0	0	0	0	0	0	0	0	0	0	
17299250	m	hp	RU	2020	1	0	0	0	0	0	0	0	0	0	0	0	
17299417	m	cn	CN	2020	0	1	0	0	1	0	0	0	0	0	0	0	73
17299419	m	cn	CN	2020	0	1	0	0	1	0	0	0	0	0	0	0	75
17299590	f	hp	RU	2020	1	0	0	0	0	0	0	0	0	0	0	0	
17299607	m	hp	RU	2020	1	0	0	0	0	0	0	0	0	0	0	0	
17299608	f	hp	RU	2020	1	0	0	0	0	0	0	0	0	0	0	0	
17299609	f	hp	RU	2020	1	0	0	0	0	0	0	0	0	0	0	0	
17299610	Not	Not repo	JP	2020	1	0	0	0	0	0	0	0	0	0	0	0	
17299611	f	hp	RU	2020	1	0	0	0	0	0	0	0	0	0	0	0	
17300570	m	hp	RU	2020	1	0	0	0	0	0	0	0	0	0	0	0	
17300580	f	hp	RU	2020	1	0	0	0	0	0	0	0	0	0	0	0	
17300998	Not	md	JP	2020	0	1	0	0	0	1	0	0	0	0	0	0	
17301229	m	hp	DE	2020	0	1	0	0	0	1	0	0	0	0	0	0	61
17301400	f	Not repo	JP	2020	1	0	0	0	0	0	0	0	0	0	0	0	72
17301744	m	hp	US	2020	0	0	0	0	0	0	0	0	0	0	0	0	71

17301748	m	md	CN	2020	0	1	1	0	0	0	0	0	0	0	0	1	68
17301812	m	md	HU	2020	0	1	0	1	0	0	0	0	0	0	0	0	74
17301813	Not	md	JP	2020	0	1	0	1	0	0	0	0	0	0	0	0	75
17301868	f	cn	US	2020	1	0	0	0	0	0	0	0	0	0	0	0	
17302151	f	md	KR	2020	0	0	0	0	0	0	0	0	0	0	0	0	43
17302321	m	md	GR	2020	0	1	1	0	0	0	0	0	0	0	0	0	81
17302323	f	Not repo	JP	2020	1	0	0	0	0	0	0	0	0	0	0	0	79
17302412	m	cn	Not re	2020	0	0	0	0	0	0	0	0	0	0	0	0	
17302533	f	Not repo	GB	2020	0	1	0	1	0	0	0	0	0	0	0	0	30
17302547	m	md	KR	2020	0	0	0	0	0	0	0	0	0	0	0	0	44
17302626	m	hp	JP	2020	0	1	1	0	0	0	0	0	0	0	0	0	55
17302627	f	md	PL	2020	1	0	0	0	0	0	0	0	0	0	0	0	
17302764	m	Not repo	JP	2020	0	1	0	1	0	0	0	0	0	0	0	1	81.8
17303018	m	md	US	2020	0	1	1	0	0	0	0	0	0	0	0	0	73
17303490	m	hp	US	2020	0	1	0	0	1	0	0	0	0	0	0	0	74
17304813	f	ph	GB	2020	0	1	1	0	0	0	0	0	0	0	0	0	65
17305200	Not	md	JP	2020	0	1	0	0	1	0	0	0	0	0	0	0	
17305339	f	hp	FR	2020	0	1	0	0	1	0	0	0	0	0	0	0	62
17305435	Not	md	DE	2020	1	0	0	0	0	0	0	0	0	0	0	0	
17305477	f	md	PL	2020	0	1	1	0	0	0	0	0	0	0	0	0	82
17305694	f	md	JP	2020	1	0	0	0	0	0	0	0	0	0	0	0	100
17305819	f	md	DE	2020	0	1	1	0	0	0	0	0	0	0	0	0	79
17305899	f	md	KR	2020	0	0	0	0	0	0	0	0	0	0	0	0	
17306115	Not	hp	NL	2020	0	0	0	0	0	0	0	0	0	0	0	0	
17306397	f	md	FR	2020	0	1	0	0	0	1	0	0	0	0	0	0	71
17306488	f	cn	US	2020	0	0	0	0	0	0	0	0	0	0	0	0	49
17306731	m	cn	CN	2020	0	1	0	0	1	0	0	0	0	0	0	0	63
17306854	f	md	JP	2020	0	0	0	0	0	0	0	0	0	0	0	0	67
17306868	f	cn	DE	2020	0	1	1	0	0	0	0	0	0	0	0	0	73
17306907	Not	md	DE	2020	1	0	0	0	0	0	0	0	0	0	0	0	
17307013	m	hp	BE	2020	0	0	0	0	0	0	0	0	0	0	0	0	64
17307155	f	md	JP	2020	1	0	0	0	0	0	0	0	0	0	0	0	69
17307651	f	md	ES	2020	0	0	0	0	0	0	0	0	0	0	0	0	56
17307733	f	md	JP	2020	0	1	0	0	0	1	0	0	0	0	0	0	68

17307788	m	Not repo	JP	2020	0	1	0	1	0	0	0	0	0	0	0	1	75
17307840	f	md	PL	2020	0	1	0	0	0	1	0	0	0	0	0	0	53
17308227	m	cn	JP	2020	0	1	1	0	0	0	0	0	0	0	0	0	80
17308231	m	cn	JP	2020	0	1	1	0	0	0	0	0	0	0	0	1	68
17308622	f	md	NL	2020	0	0	0	0	0	0	0	0	0	0	0	0	63
17308686	f	cn	CN	2020	1	0	0	0	0	0	0	0	0	0	0	0	59
17309014	m	md	JP	2020	0	1	0	0	1	0	0	0	0	0	0	0	71
17309015	m	md	JP	2020	0	1	0	0	1	0	0	0	0	0	0	0	51
17309107	m	cn	US	2020	0	0	0	0	0	0	0	0	0	0	0	0	77
17309258	m	cn	JP	2020	0	1	1	0	0	0	0	0	0	0	0	0	61
17310188	m	md	NL	2020	0	1	1	0	0	0	0	0	0	0	0	0	65
17310437	m	hp	US	2020	0	1	0	1	0	0	0	0	0	0	0	0	72
17310465	m	cn	ES	2020	0	1	1	0	0	0	0	0	0	0	0	0	
17311571	f	cn	US	2020	0	0	0	0	0	0	0	0	0	0	0	0	
17312629	m	cn	JP	2020	0	1	0	0	0	1	0	0	0	0	0	1	72
17312980	Not	md	CN	2020	0	1	0	0	0	1	0	0	0	0	0	0	
17312989	f	md	GB	2020	0	1	0	0	0	1	0	0	0	0	0	1	59
17313713	f	cn	IL	2020	0	0	0	0	0	0	0	0	0	0	0	0	73
17313978	f	Not repo	JP	2020	0	1	0	1	0	0	0	0	0	0	0	0	
17313996	m	cn	CN	2020	0	1	0	0	1	0	0	0	0	0	0	0	
17314063	m	hp	US	2020	0	0	0	0	0	0	0	0	0	0	0	0	84
17314585	m	md	KR	2020	0	1	1	0	0	0	0	0	0	0	0	0	80
17314728	f	md	JP	2020	0	1	0	0	0	1	0	0	0	0	0	0	68
17315045	m	hp	DE	2020	0	0	0	0	0	0	0	0	0	0	0	0	80
17315245	m	hp	US	2020	0	0	0	0	0	0	0	0	0	0	0	0	54
17315347	m	Not repo	JP	2020	0	1	0	1	0	0	0	0	0	0	0	0	
17315355	m	cn	JP	2020	0	1	1	0	0	0	0	0	0	0	0	0	72
17315361	m	hp	US	2020	0	0	0	0	0	0	0	0	0	0	0	0	51
17315524	m	md	JP	2020	1	0	0	0	0	0	0	0	0	0	0	1	85
17315525	m	md	JP	2020	0	1	0	1	0	0	0	0	0	0	0	1	80
17315526	m	Not repo	ID	2020	1	0	0	0	0	0	0	0	0	0	0	0	
17316018	m	md	JP	2020	0	1	0	0	0	1	0	0	0	0	0	0	66
17316057	m	md	CN	2020	0	0	0	0	0	0	0	0	0	0	0	0	61
17316069	m	Not repo	JP	2020	0	1	0	1	0	0	0	0	0	0	0	0	75

17316077	Not	Not repo	IT	2020	1	0	0	0	0	0	0	0	0	0	0	0	
17316128	Not	md	JP	2020	1	0	0	0	0	0	0	0	0	0	0	0	
17316182	m	cn	CN	2020	0	1	1	0	0	0	0	0	0	0	0	0	70
17316394	m	Not repo	JP	2020	0	1	0	1	0	0	0	0	0	0	0	0	
17316556	f	cn	JP	2020	0	0	0	0	0	0	0	0	0	0	0	0	71
17316724	f	Not repo	JP	2020	1	0	0	0	0	0	0	0	0	0	0	0	80
17316728	m	Not repo	JP	2020	0	1	0	1	0	0	0	0	0	0	0	1	77.3
17316827	m	md	JP	2020	0	1	1	0	0	0	0	0	0	0	0	1	74
17316954	m	Not repo	JP	2020	1	0	0	0	0	0	0	0	0	0	0	0	87
17317148	Not	md	DE	2020	1	0	0	0	0	0	0	0	0	0	0	0	
17317164	f	hp	SK	2020	1	0	0	0	0	0	0	0	0	0	0	0	58
17317201	m	Not repo	JP	2020	1	0	0	0	0	0	0	0	0	0	0	0	68
17318230	f	hp	SK	2020	1	0	0	0	0	0	0	0	0	0	0	0	72
17318234	f	hp	SK	2020	1	0	0	0	0	0	0	0	0	0	0	0	59
17318257	m	hp	SK	2020	1	0	0	0	0	0	0	0	0	0	0	0	64
17318346	m	md	JP	2020	0	1	0	1	0	0	0	0	0	0	0	1	78
17318434	m	md	KR	2020	0	1	1	0	0	0	0	0	0	0	0	0	65
17318438	Not	md	KR	2020	0	1	1	0	0	0	0	0	0	0	0	0	75
17318659	m	hp	JP	2020	0	1	1	0	0	0	0	0	0	0	0	0	74
17318672	m	cn	JP	2020	0	1	1	0	0	0	0	0	0	0	0	0	71
17318744	Not	md	DE	2020	1	0	0	0	0	0	0	0	0	0	0	0	
17319563	f	md	JP	2020	0	1	0	0	0	1	0	0	0	0	0	0	
17319678	f	ph	GB	2020	0	1	1	0	0	0	0	0	0	0	0	0	65
17319684	m	cn	US	2020	0	1	1	0	0	0	0	0	0	0	0	0	72
17319725	m	md	ES	2020	0	0	0	0	0	0	0	0	0	0	0	0	59
17319803	f	md	US	2020	0	1	1	0	0	0	0	0	0	0	0	0	68
17319806	f	md	US	2020	0	0	0	0	0	0	0	0	0	0	0	0	
17322268	m	md	JP	2020	0	1	0	0	0	1	0	0	0	0	0	0	74
17322912	f	md	GB	2020	0	1	0	0	0	1	0	0	0	0	0	0	55
17322947	Not	md	JP	2020	0	1	0	0	1	0	0	0	0	0	0	0	63
17323647	f	hp	AU	2020	1	0	0	0	0	0	0	0	0	0	0	0	67
17323795	f	cn	US	2020	0	1	1	0	0	0	0	0	0	0	0	0	52
17324012	m	cn	JP	2020	0	1	1	0	0	0	0	0	0	0	0	0	70
17324074	f	cn	US	2020	0	0	0	0	0	0	0	0	0	0	0	0	

17324363	f	md	DE	2020	0	0	0	0	0	0	0	0	0	0	0	0	56
17324411	Not	cn	MY	2020	1	0	0	0	0	0	0	0	0	0	0	0	
17324993	m	hp	FR	2020	0	1	0	0	1	0	0	0	0	0	0	0	71
17325373	m	cn	DE	2020	0	1	1	0	0	0	0	0	0	0	0	0	60
17325428	m	cn	US	2020	1	0	0	0	0	0	0	0	0	0	0	0	
17325430	m	cn	BE	2020	0	1	1	0	0	0	0	0	0	0	0	0	
17325788	m	md	NL	2020	0	1	1	0	0	0	0	0	0	0	0	0	71
17326504	m	hp	JP	2020	0	1	1	0	0	0	0	0	0	0	0	0	69
17326896	m	cn	BE	2020	0	1	1	0	0	0	0	0	0	0	0	0	60
17327161	m	Not repo	JP	2020	0	1	0	1	0	0	0	0	0	0	0	0	69
17327208	f	hp	US	2020	1	0	0	0	0	0	0	0	0	0	0	0	
17327375	m	md	JP	2020	1	0	0	0	0	0	0	0	0	0	0	0	80
17327385	m	md	DE	2020	0	0	0	0	0	0	0	0	0	0	0	0	72
17327456	f	Not repo	JP	2020	0	1	0	1	0	0	0	0	0	0	0	0	
17327541	f	md	CA	2020	0	1	0	1	0	0	0	0	0	0	0	0	56
17327629	f	Not repo	US	2020	0	1	0	1	0	0	0	0	0	0	0	1	80
17327634	f	Not repo	RO	2020	1	0	0	0	0	0	0	0	0	0	0	0	75
17327635	m	md	JP	2020	0	1	0	1	0	0	0	0	0	0	0	0	
17327636	m	Not repo	JP	2020	0	1	0	1	0	0	0	0	0	0	0	0	
17327680	m	md	KR	2020	0	0	0	0	0	0	0	0	0	0	0	0	71
17327742	m	Not repo	JP	2020	0	1	0	1	0	0	0	0	0	0	0	1	56.3
17327751	m	Not repo	JP	2020	0	1	0	1	0	0	0	0	0	0	0	0	
17327993	f	md	GR	2020	0	1	1	0	0	0	0	0	0	0	0	0	62
17328040	m	md	HK	2020	0	1	0	0	0	1	0	0	0	0	0	0	59
17328537	m	cn	JP	2020	0	1	1	0	0	0	0	0	0	0	0	0	58
17329867	m	md	US	2020	0	0	0	0	0	0	0	0	0	0	0	0	
17329944	f	md	JP	2020	1	0	0	0	0	0	0	0	0	0	0	0	90
17330303	m	hp	GB	2020	0	1	0	1	0	0	0	0	0	0	0	0	57
17330574	f	cn	GB	2020	0	1	1	0	0	0	0	0	0	0	0	0	60
17330582	f	Not repo	JP	2020	1	0	0	0	0	0	0	0	0	0	0	0	90
17330609	Not	hp	IT	2020	0	0	0	0	0	0	0	0	0	0	0	0	
17330677	m	md	AU	2020	0	1	1	0	0	0	0	0	0	0	0	0	83
17331035	f	md	NL	2020	0	1	1	0	0	0	0	0	0	0	0	0	
17331567	m	cn	JP	2020	0	1	1	0	0	0	0	0	0	0	0	0	69

17331614	f	hp	US	2020	0	1	0	1	0	0	0	0	0	0	0	0	
17331625	Not	md	CN	2020	0	0	0	0	0	0	0	0	0	0	0	0	
17331987	f	Not repo	JP	2020	1	0	0	0	0	0	0	0	0	0	0	0	80
17331990	f	md	US	2020	0	1	0	1	0	0	0	0	0	0	0	0	
17332438	f	md	FR	2020	0	0	0	0	0	0	0	0	0	0	0	0	61
17332781	f	md	US	2020	1	0	0	0	0	0	0	0	0	0	0	0	63.4
17332782	m	md	JP	2020	0	1	0	1	0	0	0	0	0	0	0	0	
17332798	Not	Not repo	JP	2020	1	0	0	0	0	0	0	0	0	0	0	0	
17332799	m	Not repo	JP	2020	1	0	0	0	0	0	0	0	0	0	0	1	75
17333380	m	md	JP	2020	0	1	1	0	0	0	0	0	0	0	0	0	64
17333382	f	cn	JP	2020	0	1	1	0	0	0	0	0	0	0	0	0	80
17333529	m	md	JP	2020	1	0	0	0	0	0	0	0	0	0	0	0	60
17334174	f	Not repo	JP	2020	1	0	0	0	0	0	0	0	0	0	0	0	74.1
17334323	m	md	JP	2020	0	1	0	0	0	1	0	0	0	0	0	1	65
17334847	m	md	JP	2020	0	1	1	0	0	0	0	0	0	0	0	0	60
17334883	m	cn	JP	2020	0	1	1	0	0	0	0	0	0	0	0	0	72
17336787	m	md	TR	2020	0	1	1	0	0	0	0	0	0	0	0	0	41
17336810	Not	hp	FR	2020	0	0	0	0	0	0	0	0	0	0	0	0	
17336950	m	md	JP	2020	0	1	1	0	0	0	0	0	0	0	0	1	89
17337056	f	hp	AT	2020	0	1	1	0	0	0	0	0	0	0	0	0	62
17337213	f	Not repo	JP	2020	1	0	0	0	0	0	0	0	0	0	0	0	68
17337368	m	hp	US	2020	0	1	1	0	0	0	0	0	0	0	0	0	73
17337443	m	md	DE	2020	0	0	0	0	0	0	0	0	0	0	0	0	80
17337503	f	hp	US	2020	0	0	0	0	0	0	0	0	0	0	0	0	
17337715	f	Not repo	JP	2020	0	1	0	1	0	0	0	0	0	0	0	1	50
17338067	f	md	JP	2020	0	0	0	0	0	0	0	0	0	0	0	0	70
17338289	m	md	JP	2020	0	1	0	0	0	1	0	0	0	0	0	0	75
17338500	m	hp	KR	2020	0	0	0	0	0	0	0	0	0	0	0	0	57
17338501	f	hp	KR	2020	0	0	0	0	0	0	0	0	0	0	0	0	69
17338886	Not	Not repo	JP	2020	1	0	0	0	0	0	0	0	0	0	0	0	
17338919	m	hp	NL	2020	0	1	1	0	0	0	0	0	0	0	0	0	77
17339519	Not	md	JP	2020	0	1	0	1	0	0	0	0	0	0	0	0	
17339863	f	md	DE	2020	1	0	0	0	0	0	0	0	0	0	0	0	58
17340332	f	md	UA	2020	0	1	1	0	0	0	0	0	0	0	0	0	69

17342464	m	hp	US	2020	0	1	1	0	0	0	0	0	0	0	0	0	59
17343829	m	md	GB	2020	0	1	0	1	0	0	0	0	0	0	0	0	61
17344979	m	hp	US	2020	0	0	0	0	0	0	0	0	0	0	0	0	56
17345216	Not	Not repo	JP	2020	0	1	0	1	0	0	0	0	0	0	0	1	
17345232	f	Not repo	US	2020	1	0	0	0	0	0	0	0	0	0	0	0	74
17345245	f	md	JP	2020	0	1	0	1	0	0	0	0	0	0	0	1	
17345959	m	md	DE	2020	0	0	0	0	0	0	0	0	0	0	0	0	80
17346614	f	md	FR	2020	0	0	0	0	0	0	0	0	0	0	0	0	59
17347305	f	md	JP	2020	0	0	0	0	0	0	0	0	0	0	0	0	72
17347375	f	cn	US	2020	1	0	0	0	0	0	0	0	0	0	0	0	
17347481	m	md	JP	2020	0	1	0	0	1	0	0	0	0	0	0	0	59
17347627	m	md	FR	2020	0	1	0	0	0	1	0	0	0	0	0	0	
17347637	m	md	FR	2020	0	1	0	0	0	1	0	0	0	0	0	0	
17347773	m	cn	US	2020	0	0	0	0	0	0	0	0	0	0	0	0	
17347813	m	cn	US	2020	0	0	0	0	0	0	0	0	0	0	0	0	
17348650	f	md	US	2020	0	1	0	1	0	0	0	0	0	0	0	0	59
17348668	m	md	JP	2020	1	0	0	0	0	0	0	0	0	0	0	0	90
17348734	m	cn	US	2020	0	0	0	0	0	0	0	0	0	0	0	0	65
17348856	f	md	PL	2020	0	0	0	0	0	0	0	0	0	0	0	0	63
17348914	f	md	NL	2020	0	0	0	0	0	0	1	0	0	0	1	0	58
17349134	m	md	HK	2020	0	1	1	0	0	0	0	0	0	0	0	0	72
17349193	f	md	SK	2020	0	1	1	0	0	0	0	0	0	0	0	0	76
17349199	m	md	JP	2020	1	0	0	0	0	0	0	0	0	0	0	0	80
17349203	f	md	JP	2020	1	0	0	0	0	0	0	0	0	0	0	0	83
17349730	m	hp	CA	2020	0	1	0	0	1	0	0	0	0	0	0	1	60
17349914	f	md	US	2020	0	1	0	0	0	1	0	0	0	0	0	0	
17349981	Not	md	FR	2020	1	0	0	0	0	0	0	0	0	0	0	0	
17350023	m	cn	EG	2020	0	1	1	0	0	0	0	0	0	0	0	0	
17350036	Not	md	FR	2020	1	0	0	0	0	0	0	0	0	0	0	0	
17350461	Not	md	JP	2020	0	1	0	0	0	1	0	0	0	0	0	0	60
17350573	m	cn	JP	2020	0	1	1	0	0	0	0	0	0	0	0	0	64
17350617	Not	md	DE	2020	1	0	0	0	0	0	0	0	0	0	0	0	
17350849	Not	hp	US	2020	0	0	0	0	0	0	0	0	0	0	0	0	
17351524	m	md	DK	2020	0	0	0	0	0	0	0	0	0	0	0	0	

17351728	m	cn	JP	2020	0	1	1	0	0	0	0	0	0	0	0	0	75
17351854	m	md	IN	2020	0	1	0	1	0	0	0	0	0	0	0	0	59
17351962	m	ph	IS	2020	0	1	1	0	0	0	0	0	0	0	0	0	85
17352042	m	cn	FR	2020	0	1	1	0	0	0	0	0	0	0	0	0	
17352124	m	cn	ID	2020	0	1	1	0	0	0	0	0	0	0	0	0	55
17352497	f	md	JP	2020	1	0	0	0	0	0	0	0	0	0	0	0	58
17352599	m	md	JP	2020	0	1	0	0	0	1	0	0	0	0	0	0	75
17352658	f	md	JP	2020	1	0	0	0	0	0	0	0	0	0	0	0	89
17352795	f	md	JP	2020	1	0	0	0	0	0	0	0	0	0	0	0	75
17352983	f	md	JP	2020	1	0	0	0	0	0	0	0	0	0	0	0	68
17352986	m	md	JP	2020	0	1	0	1	0	0	0	0	0	0	0	1	76
17353035	f	md	JP	2020	1	0	0	0	0	0	0	0	0	0	0	0	71
17353167	m	md	JP	2020	1	0	0	0	0	0	0	0	0	0	0	0	78
17353315	f	cn	US	2020	1	0	0	0	0	0	0	0	0	0	0	0	92
17353370	f	md	US	2020	1	0	0	0	0	0	0	0	0	0	0	0	54
17353434	f	md	JP	2020	1	0	0	0	0	0	0	0	0	0	0	0	81
17353452	m	md	US	2020	0	1	0	1	0	0	0	0	0	0	0	1	
17353561	m	Not repo	JP	2020	0	1	0	1	0	0	0	0	0	0	0	0	
17353801	f	Not repo	JP	2020	0	1	0	1	0	0	0	0	0	0	0	0	
17354412	m	Not repo	JP	2020	0	1	0	1	0	0	0	0	0	0	0	0	
17354558	f	md	JP	2020	1	0	0	0	0	0	0	0	0	0	0	0	90
17354559	Not	md	JP	2020	0	1	0	1	0	0	0	0	0	0	0	0	
17354560	Not	md	JP	2020	1	0	0	0	0	0	0	0	0	0	0	0	
17354717	f	Not repo	JP	2020	0	1	0	1	0	0	0	0	0	0	0	0	
17354721	m	Not repo	JP	2020	0	1	0	1	0	0	0	0	0	0	0	0	
17355167	m	md	CN	2020	1	0	0	0	0	0	0	0	0	0	0	0	57
17355327	m	md	JP	2020	0	1	0	1	0	0	0	0	0	0	0	1	74
17355329	Not	Not repo	JP	2020	1	0	0	0	0	0	0	0	0	0	0	0	
17355475	m	hp	IL	2020	1	0	0	0	0	0	0	0	0	0	0	0	63
17355552	m	md	JP	2020	0	1	0	1	0	0	0	0	0	0	0	0	72
17355556	m	md	JP	2020	0	1	0	1	0	0	0	0	0	0	0	1	60
17355557	f	md	JP	2020	0	1	0	1	0	0	0	0	0	0	0	1	69
17355995	m	hp	JP	2020	0	0	0	0	0	0	0	0	0	0	0	0	66
17356002	m	cn	JP	2020	0	1	1	0	0	0	0	0	0	0	0	0	81

17356006	m	hp	US	2020	0	1	0	1	0	0	0	0	0	0	0	0	
17356008	f	md	PL	2020	0	1	1	0	0	0	0	0	0	0	0	0	68
17356009	m	hp	US	2020	0	1	0	1	0	0	0	0	0	0	0	0	
17356090	m	Not repo	JP	2020	0	1	0	1	0	0	0	0	0	0	0	0	70
17356094	m	Not repo	JP	2020	0	1	0	1	0	0	0	0	0	0	0	0	
17356128	f	md	JP	2020	1	0	0	0	0	0	0	0	0	0	0	0	65
17356159	m	hp	DE	2020	0	1	1	0	0	0	0	0	0	0	0	0	75
17356245	f	md	US	2020	0	1	0	1	0	0	0	0	0	0	0	0	
17356246	f	md	US	2020	0	1	0	1	0	0	0	0	0	0	0	0	
17356247	f	md	US	2020	0	1	0	1	0	0	0	0	0	0	0	0	75
17356260	m	md	JP	2020	1	0	0	0	0	0	0	0	0	0	0	0	71
17356277	f	md	US	2020	0	1	0	1	0	0	0	0	0	0	0	0	
17356278	m	md	US	2020	0	1	0	1	0	0	0	0	0	0	0	0	
17356284	f	ph	US	2020	1	0	0	0	0	0	0	0	0	0	0	0	51
17356289	m	Not repo	JP	2020	0	1	0	1	0	0	0	0	0	0	0	0	
17356344	f	md	JP	2020	1	0	0	0	0	0	0	0	0	0	0	1	65
17356345	m	md	JP	2020	0	1	0	1	0	0	0	0	0	0	0	0	
17356561	m	cn	JP	2020	0	1	1	0	0	0	0	0	0	0	0	0	70
17356562	f	hp	US	2020	1	0	0	0	0	0	0	0	0	0	0	0	59
17356626	f	Not repo	CA	2020	0	1	0	1	0	0	0	0	0	0	0	1	42
17356630	m	Not repo	IT	2020	1	0	0	0	0	0	0	0	0	0	0	0	
17356731	m	md	JP	2020	0	1	0	1	0	0	0	0	0	0	0	1	69
17356946	m	md	US	2020	0	1	0	1	0	0	0	0	0	0	0	0	76
17357459	Not	md	US	2020	0	0	0	0	0	0	0	0	0	0	0	0	
17357493	m	md	JP	2020	0	1	1	0	0	0	0	0	0	0	0	0	71
17358420	m	md	JP	2020	0	0	0	0	0	0	0	0	0	0	0	0	70
17358735	m	hp	DE	2020	0	0	0	0	0	0	0	0	0	0	0	0	62
17359115	m	md	DE	2020	0	0	0	0	0	0	0	0	0	0	0	0	
17359197	m	md	UA	2020	0	1	0	0	0	1	0	0	0	0	0	0	55
17359219	f	md	US	2020	0	1	0	1	0	0	0	0	0	0	0	0	70
17359501	f	ph	JP	2020	0	0	0	0	0	0	0	0	0	0	0	0	
17359723	f	md	US	2020	0	1	1	0	0	0	0	0	0	0	0	0	64
17359913	m	md	US	2020	0	0	0	0	0	0	0	0	0	0	0	0	73
17359938	m	hp	US	2020	0	1	0	0	1	0	0	0	0	0	0	1	64

17360117	Not	hp	PT	2020	0	1	0	1	0	0	0	0	0	0	0	0	
17360118	Not	hp	PT	2020	1	0	0	0	0	0	0	0	0	0	0	0	
17360480	m	md	US	2020	0	0	0	0	0	0	0	0	0	0	0	0	73
17360537	f	cn	Not re	2020	0	0	0	0	0	0	0	0	0	0	0	0	
17360647	m	hp	JP	2020	0	0	0	0	0	0	0	0	0	0	0	0	73
17360723	m	cn	BE	2020	0	1	1	0	0	0	0	0	0	0	0	0	71
17360839	m	hp	GB	2020	0	1	1	0	0	0	0	0	0	0	0	0	74
17361137	Not	hp	PT	2020	1	0	0	0	0	0	0	0	0	0	0	0	
17361163	m	cn	GR	2020	0	1	1	0	0	0	0	0	0	0	0	0	70
17361336	f	hp	PT	2020	1	0	0	0	0	0	0	0	0	0	0	0	73
17361537	Not	hp	PT	2020	1	0	0	0	0	0	0	0	0	0	0	0	
17361656	m	cn	FR	2020	0	1	1	0	0	0	0	0	0	0	0	0	66
17361692	m	md	IL	2020	0	1	1	0	0	0	0	0	0	0	0	0	70
17362066	m	md	JP	2020	0	1	0	0	0	1	0	0	0	0	0	0	83
17362254	Not	hp	PT	2020	1	0	0	0	0	0	0	0	0	0	0	0	
17362312	f	md	US	2020	0	1	0	1	0	0	0	0	0	0	0	0	75
17362566	Not	hp	NL	2020	0	0	0	0	0	0	0	0	0	0	0	0	
17362808	m	hp	US	2020	0	1	0	1	0	0	0	0	0	0	0	0	
17362887	f	cn	US	2020	0	1	1	0	0	0	0	0	0	0	0	0	
17363121	m	hp	US	2020	0	0	0	0	0	0	0	0	0	0	0	0	71
17363177	m	hp	DE	2020	0	0	0	0	0	0	0	0	0	0	0	0	52
17363227	f	cn	JP	2020	0	1	1	0	0	0	0	0	0	0	0	0	66
17363259	f	cn	JP	2020	0	1	1	0	0	0	0	0	0	0	0	0	
17363362	Not	md	RU	2020	0	0	0	0	0	0	0	0	0	0	0	0	
17363385	m	cn	JP	2020	0	0	0	0	0	0	0	0	0	0	0	0	68
17363685	f	md	US	2020	0	1	1	0	0	0	0	0	0	0	0	0	70
17363779	m	md	US	2020	0	1	0	1	0	0	0	0	0	0	0	1	55
17364226	m	cn	IN	2020	0	1	0	0	0	1	0	0	0	0	0	0	
17364291	Not	ph	CA	2020	0	1	1	0	0	0	0	0	0	0	0	1	
17364292	m	cn	JP	2020	0	1	1	0	0	0	0	0	0	0	0	1	88
17364309	f	hp	CA	2020	0	1	0	0	1	0	0	0	0	0	0	0	57
17364476	m	cn	JP	2020	0	1	1	0	0	0	0	0	0	0	0	0	62
17364781	m	md	US	2020	0	1	1	0	0	0	0	0	0	0	0	0	
17364792	Not	hp	US	2020	0	1	0	0	1	0	0	0	0	0	0	1	63

17364844	Not	hp	TR	2020	0	0	0	0	0	0	0	0	0	0	0	0	
17365079	m	md	DE	2020	0	1	0	0	0	1	0	0	0	0	0	0	78
17365494	f	cn	US	2020	1	0	0	0	0	0	0	0	0	0	0	0	73
17365565	m	md	US	2020	0	1	0	1	0	0	0	0	0	0	0	1	
17365782	f	Not repo	JP	2020	0	1	0	1	0	0	0	0	0	0	0	1	77
17365920	m	hp	DE	2020	0	1	0	0	0	1	0	0	0	0	0	1	59
17366771	m	cn	FR	2020	0	1	1	0	0	0	0	0	0	0	0	0	
17366823	f	cn	AR	2020	0	0	0	0	0	0	0	0	0	0	0	0	53
17366980	m	md	ES	2020	0	1	1	0	0	0	0	0	0	0	0	0	76
17367006	f	md	JP	2020	0	0	0	0	0	0	0	0	0	0	0	0	73
17367025	f	hp	US	2020	0	1	0	1	0	0	0	0	0	0	0	0	75
17367100	m	Not repo	JP	2020	0	1	0	1	0	0	0	0	0	0	0	1	76
17367269	m	md	JP	2020	0	1	1	0	0	0	0	0	0	0	0	0	60
17367319	f	hp	PL	2020	0	0	0	0	0	0	0	0	0	0	0	0	80
17367740	m	Not repo	JP	2020	1	0	0	0	0	0	0	0	0	0	0	0	77
17367829	m	cn	US	2020	0	1	0	1	0	0	0	0	0	0	0	0	83.1
17367848	f	Not repo	JP	2020	1	0	0	0	0	0	0	0	0	0	0	0	
17367925	m	hp	KR	2020	0	0	0	0	0	0	0	0	0	0	0	0	70
17368136	f	hp	AT	2020	0	1	0	1	0	0	0	0	0	0	0	0	70
17368157	f	hp	FR	2020	0	1	0	0	1	0	0	0	0	0	0	0	78
17368788	f	cn	JP	2020	0	1	1	0	0	0	0	0	0	0	0	0	90
17368863	m	hp	IT	2020	0	1	0	0	1	0	0	0	0	0	0	0	66
17368972	m	cn	JP	2020	0	1	1	0	0	0	0	0	0	0	0	0	69
17369224	m	hp	JP	2020	0	1	0	0	1	0	0	0	0	0	0	1	82
17369595	m	md	PL	2020	0	0	0	0	0	0	0	0	0	0	0	0	65
17369598	m	md	IT	2020	0	1	0	0	0	1	0	0	0	0	0	0	64
17369614	m	cn	US	2020	1	0	0	0	0	0	0	0	0	0	0	0	
17369816	m	cn	TW	2020	0	1	0	0	0	1	0	0	0	0	0	0	
17369817	f	md	IT	2020	0	0	0	0	0	0	0	0	0	0	0	0	72
17369898	m	md	PL	2020	0	1	0	0	0	1	0	0	0	0	0	1	
17370079	m	cn	JP	2020	0	1	1	0	0	0	0	0	0	0	0	0	71
17370264	f	md	PL	2020	0	0	0	0	0	0	0	0	0	0	0	0	63
17370291	m	cn	JP	2020	0	1	1	0	0	0	0	0	0	0	0	1	80
17370534	m	cn	CN	2020	0	0	0	0	0	0	0	0	0	0	0	0	70

17370715	f	cn	FR	2020	0	1	1	0	0	0	0	0	0	0	0	0	61
17370798	m	Not repo	JP	2020	1	0	0	0	0	0	0	0	0	0	0	0	77
17370978	m	cn	FR	2020	0	1	1	0	0	0	0	0	0	0	0	0	58
17371103	f	Not repo	JP	2020	0	1	0	1	0	0	0	0	0	0	0	0	
17371810	m	md	US	2020	0	1	0	1	0	0	0	0	0	0	0	1	
17371813	m	md	CR	2020	1	0	0	0	0	0	0	0	0	0	0	0	86
17371832	m	md	GB	2020	0	1	0	0	0	1	0	0	0	0	0	0	80
17372023	f	md	US	2020	1	0	0	0	0	0	0	0	0	0	0	0	
17372157	f	cn	US	2020	1	0	0	0	0	0	0	0	0	0	0	0	
17372159	f	Not repo	IT	2020	1	0	0	0	0	0	0	0	0	0	0	1	
17372531	m	md	JP	2020	0	1	0	1	0	0	0	0	0	0	0	1	71.4
17372671	m	Not repo	IT	2020	1	0	0	0	0	0	0	0	0	0	0	0	
17372673	f	md	JP	2020	1	0	0	0	0	0	0	0	0	0	0	0	72
17372718	f	cn	DE	2020	0	1	1	0	0	0	0	0	0	0	0	0	52
17372745	m	cn	JP	2020	0	1	1	0	0	0	0	0	0	0	0	0	73
17372997	f	md	JP	2020	0	1	0	1	0	0	0	0	0	0	0	1	71
17373181	m	md	RO	2020	1	0	0	0	0	0	0	0	0	0	0	0	48.6
17373225	f	cn	IT	2020	0	1	1	0	0	0	0	0	0	0	0	0	60
17373452	Not	hp	DE	2020	0	1	0	0	0	1	0	0	0	0	0	0	
17373641	m	md	JP	2020	1	0	0	0	0	0	0	0	0	0	0	0	72
17373778	f	md	GB	2020	0	0	0	0	0	0	0	0	0	0	0	0	57
17373862	m	md	HU	2020	0	1	0	0	0	1	0	0	0	0	0	0	62
17374079	f	cn	Not re	2020	0	0	0	0	0	0	0	0	0	0	0	0	
17374535	m	cn	JP	2020	0	1	1	0	0	0	0	0	0	0	0	0	39
17374539	m	cn	US	2020	0	0	0	0	0	0	0	0	0	0	0	0	
17374768	Not	cn	JP	2020	0	1	1	0	0	0	0	0	0	0	0	0	90
17374907	f	cn	JP	2020	0	1	1	0	0	0	0	0	0	0	0	0	90
17375313	m	Not repo	JP	2020	0	1	0	1	0	0	0	0	0	0	0	0	
17375396	m	hp	US	2020	0	1	0	1	0	0	0	0	0	0	0	0	75
17375685	Not	cn	US	2020	1	0	0	0	0	0	0	0	0	0	0	0	
17375907	Not	hp	JP	2020	0	0	0	0	0	0	0	0	0	0	0	0	
17376260	m	Not repo	JP	2020	0	1	0	1	0	0	0	0	0	0	0	0	
17377446	m	ph	BR	2020	0	0	0	0	0	0	0	0	0	0	0	0	50
17378002	m	cn	JP	2020	0	1	1	0	0	0	0	0	0	0	0	0	

17378349	m	md	NL	2020	0	0	0	0	0	0	0	0	0	0	0	0	54
17378737	f	md	IL	2020	1	0	0	0	0	0	0	0	0	0	0	0	20
17384209	m	md	SA	2020	0	1	0	0	1	0	0	0	0	0	0	0	
17386487	m	hp	JP	2020	0	1	0	1	0	0	0	0	0	0	0	1	65
17386612	m	hp	US	2020	0	1	0	0	1	0	0	0	0	0	0	0	64
17386654	m	cn	CA	2020	0	1	0	1	0	0	0	0	0	0	0	0	65.7
17386677	m	cn	US	2020	1	0	0	0	0	0	0	0	0	0	0	0	62.6
17386858	m	cn	GR	2020	0	1	1	0	0	0	0	0	0	0	0	0	70
17387401	m	md	JP	2020	0	1	1	0	0	0	0	0	0	0	0	1	62
17388335	m	cn	US	2020	1	0	0	0	0	0	0	0	0	0	0	0	
17388427	Not	md	JP	2020	0	1	0	1	0	0	0	0	0	0	0	0	70
17388575	m	cn	JP	2020	0	1	1	0	0	0	0	0	0	0	0	0	76
17388754	Not	md	ES	2020	0	1	1	0	0	0	0	0	0	0	0	0	
17388942	f	md	US	2020	1	0	0	0	0	0	0	0	0	0	0	0	70
17388992	m	hp	CN	2020	0	0	0	0	0	0	0	0	0	0	0	0	74
17389004	Not	md	PH	2020	0	1	0	0	0	1	0	0	0	0	0	0	
17389026	f	cn	CA	2020	0	0	0	0	0	0	0	0	0	0	0	0	51
17389234	m	md	US	2020	1	0	0	0	0	0	0	0	0	0	0	0	
17389412	f	md	TW	2020	0	0	0	0	0	0	0	0	0	0	0	0	69
17389535	f	md	US	2020	1	0	0	0	0	0	0	0	0	0	0	0	
17389536	f	md	JP	2020	1	0	0	0	0	0	0	0	0	0	0	0	79
17389832	f	md	JP	2020	0	1	1	0	0	0	0	0	0	0	0	0	
17390021	f	Not repo	US	2020	1	0	0	0	0	0	0	0	0	0	0	0	80
17390040	m	md	JP	2020	0	1	0	1	0	0	0	0	0	0	0	1	63.6
17390092	f	cn	US	2020	0	1	0	1	0	0	0	0	0	0	0	0	
17390093	f	md	FR	2020	0	0	0	0	0	0	0	0	0	0	0	0	61
17390172	Not	Not repo	JP	2020	0	1	0	1	0	0	0	0	0	0	0	1	
17390174	m	md	GB	2020	0	1	0	1	0	0	0	0	0	0	0	0	
17390202	f	md	US	2020	0	0	0	0	0	0	0	0	0	0	0	0	63
17390211	f	hp	NL	2020	0	1	1	0	0	0	0	0	0	0	0	0	
17390234	m	md	RO	2020	1	0	0	0	0	0	0	0	0	0	0	0	48.6
17390240	f	Not repo	JP	2020	1	0	0	0	0	0	0	0	0	0	0	0	81
17390418	m	cn	BR	2020	0	1	1	0	0	0	0	0	0	0	0	0	
17390502	f	md	JP	2020	0	1	0	0	0	1	0	0	0	0	0	0	64

17390731	m	Not repo	JP	2020	0	1	0	1	0	0	0	0	0	0	0	0	46
17390892	f	Not repo	US	2020	0	1	0	1	0	0	0	0	0	0	0	0	
17390918	m	md	US	2020	0	1	0	1	0	0	0	0	0	0	0	0	71
17391040	f	cn	JP	2020	0	1	1	0	0	0	0	0	0	0	0	0	65
17391128	m	md	GR	2020	0	0	0	0	0	0	0	0	0	0	0	0	70
17391369	Not	cn	JP	2020	0	1	1	0	0	0	0	0	0	0	0	0	80
17391381	Not	md	PH	2020	0	1	0	0	0	1	0	0	0	0	0	0	
17391525	m	hp	AU	2020	0	1	1	0	0	0	0	0	0	0	0	0	75
17391659	m	Not repo	JP	2020	0	1	0	1	0	0	0	0	0	0	0	1	70
17391936	Not	Not repo	JP	2020	0	1	0	1	0	0	0	0	0	0	0	0	80
17392008	m	md	IT	2020	0	1	0	0	1	0	0	0	0	0	0	0	79
17392092	Not	hp	US	2020	0	0	0	0	0	0	0	0	0	0	0	0	
17392149	Not	hp	US	2020	0	0	0	0	0	0	0	0	0	0	0	0	
17392248	f	hp	JP	2020	0	1	0	0	1	0	0	0	0	0	0	0	59
17392451	f	md	FR	2020	1	0	0	0	0	0	0	0	0	0	0	0	
17393009	Not	md	JP	2020	0	1	0	1	0	0	0	0	0	0	0	1	80
17393673	m	hp	DE	2020	0	0	0	0	0	0	0	0	0	0	0	0	58
17393893	m	cn	TW	2020	0	1	0	0	0	1	0	0	0	0	0	0	
17393952	m	hp	FR	2020	0	1	0	0	1	0	0	0	0	0	0	0	74
17393964	m	md	JP	2020	0	1	0	0	1	0	0	0	0	0	0	0	56
17394115	f	md	SA	2020	0	1	0	0	1	0	0	0	0	0	0	0	
17394201	m	cn	JP	2020	0	1	1	0	0	0	0	0	0	0	0	0	72
17394287	m	ph	US	2020	0	1	0	0	1	0	0	0	0	0	0	0	
17394375	m	md	JP	2020	0	1	0	0	0	1	0	0	0	0	0	1	79
17394533	m	md	JP	2020	0	1	0	0	0	1	0	0	0	0	0	0	66
17395706	f	md	Not re	2020	0	0	0	0	0	0	0	0	0	0	0	0	69
17395885	f	md	US	2020	1	0	0	0	0	0	0	0	0	0	0	0	
17395919	f	md	DE	2020	0	1	1	0	0	0	0	0	0	0	0	0	61
17395967	m	md	FR	2020	0	1	1	0	0	0	0	0	0	0	0	0	49
17396068	f	hp	US	2020	1	0	0	0	0	0	0	0	0	0	0	0	
17397099	f	cn	JP	2020	0	0	0	0	0	0	0	0	0	0	0	0	65
17397202	m	Not repo	JP	2020	0	1	0	1	0	0	0	0	0	0	0	0	77
17397203	m	Not repo	MY	2020	0	1	0	1	0	0	0	0	0	0	0	0	84.5
17397561	m	cn	JP	2020	0	1	1	0	0	0	0	0	0	0	0	0	54

17397674	m	Not repo	US	2020	0	1	0	1	0	0	0	0	0	0	0	0	
17398194	f	cn	JP	2020	0	1	1	0	0	0	0	0	0	0	0	0	70
17398216	m	cn	JP	2020	0	1	1	0	0	0	0	0	0	0	0	0	80
17398218	m	md	JP	2020	0	0	0	0	0	0	0	0	0	0	0	0	55
17398286	f	md	CH	2020	0	1	1	0	0	0	0	0	0	0	0	0	76
17398591	m	hp	FR	2020	0	1	0	0	1	0	0	0	0	0	0	0	65
17398592	m	hp	US	2020	0	1	0	0	1	0	0	0	0	0	0	0	76
17399192	m	cn	JP	2020	0	1	1	0	0	0	0	0	0	0	0	1	84
17399603	m	cn	JP	2020	0	1	1	0	0	0	0	0	0	0	0	0	57
17399896	m	md	DE	2020	0	0	0	0	0	0	0	0	0	0	0	0	72
17400788	f	md	VN	2020	0	1	0	0	0	1	0	0	0	0	0	0	
17401359	f	hp	GB	2020	0	1	1	0	0	0	0	0	0	0	0	0	60
17401484	m	hp	DE	2020	0	1	0	0	0	1	0	0	0	0	0	1	53
17401542	f	md	IN	2020	0	1	0	0	1	0	0	0	0	0	0	0	
17401564	m	cn	US	2020	0	1	1	0	0	0	0	0	0	0	0	0	49
17401595	f	md	US	2020	1	0	0	0	0	0	0	0	0	0	0	0	86
17401778	m	hp	PH	2020	0	1	1	0	0	0	0	0	0	0	0	0	66
17402006	Not	hp	CN	2020	1	0	0	0	0	0	0	0	0	0	0	1	
17402039	m	md	FR	2020	0	1	0	0	0	1	0	0	0	0	0	0	78
17402463	f	md	JP	2020	0	0	0	0	0	0	0	0	0	0	0	0	90
17402689	Not	ph	DE	2020	0	0	0	0	0	0	0	0	0	0	0	0	
17403211	f	md	PL	2020	0	0	0	0	0	0	0	0	0	0	0	0	82
17403346	f	hp	JP	2020	0	1	0	0	1	0	0	0	0	0	0	0	81
17403405	Not	Not repo	DE	2020	0	1	0	1	0	0	0	0	0	0	0	0	
17403481	Not	Not repo	DE	2020	0	1	0	1	0	0	0	0	0	0	0	0	
17403540	m	md	US	2020	0	1	0	1	0	0	0	0	0	0	0	0	
17404059	m	md	IN	2020	0	1	0	1	0	0	0	0	0	0	0	0	73
17404447	f	md	DE	2020	1	0	0	0	0	0	0	0	0	0	0	1	64
17404885	m	cn	JP	2020	0	1	1	0	0	0	0	0	0	0	0	0	74
17404903	Not	md	JP	2020	0	1	0	0	0	1	0	0	0	0	0	0	
17405105	m	cn	JP	2020	0	1	1	0	0	0	0	0	0	0	0	0	73
17405439	f	md	US	2020	1	0	0	0	0	0	0	0	0	0	0	0	
17405725	m	Not repo	JP	2020	0	1	0	1	0	0	0	0	0	0	0	0	60
17405737	m	hp	AU	2020	0	1	0	0	1	0	0	0	0	0	0	0	75

17405801	m	hp	JP	2020	0	0	0	0	0	0	0	0	0	0	0	0	73
17405872	m	md	JP	2020	0	1	0	1	0	0	0	0	0	0	0	0	60
17405960	m	Not repo	MY	2020	1	0	0	0	0	0	0	0	0	0	0	0	76.8
17405962	f	Not repo	US	2020	1	0	0	0	0	0	0	0	0	0	0	0	
17406651	m	cn	JP	2020	0	1	1	0	0	0	0	0	0	0	0	0	70
17406738	m	cn	JP	2020	0	1	1	0	0	0	0	0	0	0	0	0	77
17407136	f	cn	RO	2020	0	1	1	0	0	0	0	0	0	0	0	0	41
17407138	m	ph	US	2020	0	1	0	0	1	0	0	0	0	0	0	0	
17407571	f	md	US	2020	1	0	0	0	0	0	0	0	0	0	0	0	67
17407614	m	md	JP	2020	0	0	0	0	0	0	0	0	0	0	0	0	75
17407692	f	md	GR	2020	0	0	0	0	0	0	0	0	0	0	0	0	
17408515	m	md	JP	2020	0	1	0	1	0	0	0	0	0	0	0	1	53
17408595	m	cn	JP	2020	0	1	1	0	0	0	0	0	0	0	0	0	93
17408746	f	hp	GB	2020	0	1	1	0	0	0	0	0	0	0	0	0	57
17408809	m	md	PT	2020	0	1	1	0	0	0	0	0	0	0	0	0	66
17408956	f	md	JP	2020	0	1	0	1	0	0	0	0	0	0	0	0	48
17409551	m	cn	JP	2020	0	1	1	0	0	0	0	0	0	0	0	0	70
17409596	f	Not repo	JP	2020	0	1	0	1	0	0	0	0	0	0	0	1	60
17409649	m	Not repo	JP	2020	0	1	0	1	0	0	0	0	0	0	0	0	60
17409760	f	cn	US	2020	1	0	0	0	0	0	0	0	0	0	0	0	
17410039	m	cn	IN	2020	0	0	0	0	0	0	0	0	0	0	0	0	38
17410043	f	md	GB	2020	0	0	0	0	0	0	0	0	0	0	0	0	63
17410383	m	md	JP	2020	0	1	0	1	0	0	0	0	0	0	0	1	75
17410773	Not	md	FR	2020	0	1	0	0	0	1	0	0	0	0	0	0	
17411541	f	md	RU	2020	1	0	0	0	0	0	0	0	0	0	0	0	58
17411877	m	cn	IN	2020	0	1	0	0	1	0	0	0	0	0	0	0	
17411960	m	md	GB	2020	0	1	0	0	0	1	0	0	0	0	0	0	48
17412017	m	md	NL	2020	0	1	1	0	0	0	0	0	0	0	0	0	75
17412118	f	md	DE	2020	0	0	0	0	0	0	0	0	0	0	0	0	60
17412305	m	md	DE	2020	0	0	0	0	0	0	0	0	0	0	0	0	72
17412564	m	cn	IN	2020	0	1	0	0	1	0	0	0	0	0	0	0	72
17412593	m	md	CN	2020	0	1	0	0	0	1	0	0	0	0	0	0	68
17412942	f	md	DE	2020	0	0	0	0	0	0	0	0	0	0	0	0	60
17412969	f	cn	CN	2020	0	1	0	0	1	0	0	0	0	0	0	0	

17413284	m	ph	CA	2020	0	1	0	0	1	0	0	0	0	0	0	0	66
17413612	m	md	JP	2020	0	1	1	0	0	0	0	0	0	0	0	0	60
17413629	m	hp	DE	2020	0	1	0	0	1	0	0	0	0	0	0	0	54
17413800	m	cn	BR	2020	0	1	1	0	0	0	0	0	0	0	0	0	65
17414364	f	md	TW	2020	0	1	1	0	0	0	0	0	0	0	0	0	47
17414613	f	Not repo	AU	2020	0	1	0	1	0	0	0	0	0	0	0	0	
17414659	m	md	FR	2020	0	1	0	1	0	0	0	0	0	0	0	0	75
17414870	m	cn	US	2020	1	0	0	0	0	0	0	0	0	0	0	0	
17415002	m	cn	FR	2020	0	1	1	0	0	0	0	0	0	0	0	0	
17415033	m	hp	US	2020	0	1	0	0	0	1	0	0	0	0	0	0	85
17415083	m	md	JP	2020	0	0	0	0	0	0	0	0	0	0	0	0	45
17415642	m	ph	FR	2020	0	1	0	1	0	0	0	0	0	0	0	0	58
17416760	f	md	IT	2020	0	1	1	0	0	0	0	0	0	0	0	0	60
17417712	f	md	JP	2020	1	0	0	0	0	0	0	0	0	0	0	0	70
17417749	m	hp	FR	2020	0	1	0	0	1	0	0	0	0	0	0	0	72
17417786	m	md	US	2020	0	1	0	1	0	0	0	0	0	0	0	0	
17418177	m	md	JP	2020	0	1	0	0	0	1	0	0	0	0	0	0	90
17418205	m	Not repo	GB	2020	0	1	0	1	0	0	0	0	0	0	0	0	
17418353	m	hp	FR	2020	0	1	0	0	1	0	0	0	0	0	0	0	71
17418376	m	md	KR	2020	0	1	0	0	1	0	0	0	0	0	0	0	73
17418690	m	md	US	2020	0	1	0	1	0	0	0	0	0	0	0	0	
17418996	m	md	DE	2020	0	1	0	0	1	0	0	0	0	0	0	0	58
17419496	m	ph	US	2020	0	0	0	0	0	0	0	0	0	0	0	0	71
17419555	m	md	DE	2020	0	0	0	0	0	0	0	0	0	0	0	0	72
17419564	f	cn	CA	2020	0	0	0	0	0	0	0	0	0	0	0	0	
17419874	f	cn	CA	2020	0	1	0	1	0	0	0	0	0	0	0	0	
17419897	m	md	CN	2020	0	1	0	0	0	1	0	0	0	0	0	0	63
17420379	m	Not repo	MY	2020	1	0	0	0	0	0	0	0	0	0	0	0	78.7
17420421	f	md	US	2020	1	0	0	0	0	0	0	0	0	0	0	0	66
17420545	Not	hp	JP	2020	0	0	0	0	0	0	0	0	0	0	0	0	
17420558	m	md	JP	2020	0	1	0	0	0	1	0	0	0	0	0	0	68
17420618	m	md	TW	2020	0	1	0	1	0	0	0	0	0	0	0	0	65.4
17420658	m	md	BR	2020	0	1	0	1	0	0	0	0	0	0	0	0	71.7
17420659	m	md	CA	2020	1	0	0	0	0	0	0	0	0	0	0	0	57

17421297	Not	md	US	2020	0	1	0	0	0	1	0	0	0	0	0	0	
17421340	m	cn	FR	2020	0	1	1	0	0	0	0	0	0	0	0	0	66
17421418	m	md	BE	2020	0	0	0	0	0	0	0	0	0	0	0	0	66
17421420	m	md	US	2020	0	1	0	0	0	1	0	0	0	0	0	0	78
17421588	m	md	US	2020	1	0	0	0	0	0	0	0	0	0	0	0	66
17421933	f	md	US	2020	1	0	0	0	0	0	0	0	0	0	0	0	69
17422057	m	md	KR	2020	0	1	0	0	0	1	0	0	0	0	0	0	64
17422430	f	md	CA	2020	0	0	0	0	0	0	0	0	0	0	0	0	44
17422861	Not	md	JP	2020	0	0	0	0	0	0	0	0	0	0	0	0	
17423323	f	md	JP	2020	0	1	1	0	0	0	0	0	0	0	0	0	76
17423769	f	md	MY	2020	0	1	0	0	0	1	0	0	0	0	0	0	55
17424252	Not	md	US	2020	0	1	0	0	1	0	0	0	0	0	0	0	
17424560	Not	md	HU	2020	0	1	0	0	1	0	0	0	0	0	0	0	
17424821	f	md	JP	2020	0	1	0	0	0	1	0	0	0	0	0	0	58
17424967	m	ph	PT	2020	0	1	1	0	0	0	0	0	0	0	0	0	61
17425507	m	md	JP	2020	0	1	0	0	0	1	0	0	0	0	0	0	60
17425510	f	Not repo	JP	2020	1	0	0	0	0	0	0	0	0	0	0	0	77
17425651	m	cn	CN	2020	0	1	0	0	1	0	0	0	0	0	0	0	77
17425653	f	cn	US	2020	0	1	1	0	0	0	0	0	0	0	0	0	87
17425654	m	cn	JP	2020	0	0	0	0	0	0	0	0	0	0	0	0	72
17425695	m	md	JP	2020	0	1	0	0	0	1	0	0	0	0	0	0	70
17425775	f	cn	US	2020	0	0	0	0	0	0	0	0	0	0	0	0	48
17426068	Not	Not repo	DE	2020	0	1	0	1	0	0	0	0	0	0	0	0	
17426218	m	md	DE	2020	0	1	0	0	0	1	0	0	0	0	0	0	70
17426248	Not	hp	GR	2020	0	0	0	0	0	0	0	0	0	0	0	0	51
17426299	m	cn	JP	2020	0	1	1	0	0	0	0	0	0	0	0	0	70
17426313	f	md	JP	2020	0	1	1	0	0	0	0	0	0	0	0	0	68
17426373	f	md	FR	2020	0	0	0	0	0	0	0	0	0	0	0	0	61
17426388	Not	hp	FR	2020	0	0	0	0	0	0	0	0	0	0	0	0	
17426622	f	hp	US	2020	1	0	0	0	0	0	0	0	0	0	0	0	71
17427150	m	md	KR	2020	0	1	0	0	0	1	0	0	0	0	0	0	71
17427162	m	cn	JP	2020	0	1	1	0	0	0	0	0	0	0	0	0	76
17427361	m	cn	JP	2020	0	1	1	0	0	0	0	0	0	0	0	0	46
17427523	Not	Not repo	DE	2020	0	1	0	1	0	0	0	0	0	0	0	0	

17427793	m	Not repo	GB	2020	0	1	0	1	0	0	0	0	0	0	0	1	
17427865	f	md	JP	2020	0	1	0	1	0	0	0	0	0	0	0	1	72
17427989	m	md	FR	2020	0	1	0	1	0	0	0	0	0	0	0	0	
17428251	m	cn	JP	2020	0	1	1	0	0	0	0	0	0	0	0	0	72
17428351	Not	md	HU	2020	0	1	0	0	1	0	0	0	0	0	0	0	
17428557	m	hp	DE	2020	0	1	1	0	0	0	0	0	0	0	0	0	63
17428593	m	hp	US	2020	0	0	0	0	0	0	0	0	0	0	0	0	
17428854	Not	md	FR	2020	0	1	0	0	0	1	0	0	0	0	0	0	
17428958	m	cn	JP	2020	0	1	1	0	0	0	0	0	0	0	0	0	70
17428975	m	cn	JP	2020	0	1	1	0	0	0	0	0	0	0	0	0	73
17429051	m	cn	US	2020	1	0	0	0	0	0	0	0	0	0	0	0	
17429191	m	cn	US	2020	0	1	1	0	0	0	0	0	0	0	0	0	
17429481	m	hp	DE	2020	0	0	0	0	0	0	0	0	0	0	0	0	68
17429548	m	hp	JP	2020	0	1	1	0	0	0	0	0	0	0	0	0	69
17429654	m	cn	MX	2020	0	1	1	0	0	0	0	0	0	0	0	0	70
17430402	m	md	US	2020	0	0	0	0	0	0	0	0	0	0	0	0	55
17430490	m	md	DK	2020	0	0	0	0	0	0	0	0	0	0	0	0	57
17430865	f	md	JP	2020	0	0	0	0	0	0	0	0	0	0	0	0	77
17431000	m	Not repo	JP	2020	0	1	0	1	0	0	0	0	0	0	0	1	80
17431201	Not	Not repo	DE	2020	0	1	0	1	0	0	0	0	0	0	0	0	
17431450	m	cn	BE	2020	0	1	1	0	0	0	0	0	0	0	0	0	60
17431506	m	cn	CA	2020	0	0	0	0	0	0	0	0	0	0	0	0	84
17431507	m	hp	TR	2020	0	1	1	0	0	0	0	0	0	0	0	0	69
17431914	f	cn	JP	2020	0	0	0	0	0	0	0	0	0	0	0	0	71
17432064	m	md	JP	2020	0	1	0	0	1	0	0	0	0	0	0	0	77
17432412	f	cn	JP	2020	0	0	0	0	0	0	0	0	0	0	0	0	83
17432415	f	cn	JP	2020	0	0	0	0	0	0	0	0	0	0	0	0	55
17432534	m	hp	JP	2020	0	0	0	0	0	0	0	0	0	0	0	0	72
17432613	m	md	IN	2020	0	1	1	0	0	0	0	0	0	0	0	0	48
17432702	f	hp	JP	2020	0	1	0	0	0	1	0	0	0	0	0	0	
17432727	f	hp	US	2020	0	1	0	0	1	0	0	0	0	0	0	0	
17432979	f	Not repo	GR	2020	0	1	0	1	0	0	0	0	0	0	0	0	65
17433002	Not	hp	FR	2020	0	0	0	0	0	0	0	0	0	0	0	0	
17433004	m	cn	TH	2020	0	1	0	0	1	0	0	0	0	0	0	0	82

17433006	m	md	CZ	2020	0	1	0	0	1	0	0	0	0	0	0	0	73
17433080	Not	md	JP	2020	0	1	0	0	0	1	0	0	0	0	0	0	
17433378	m	cn	JP	2020	0	1	1	0	0	0	0	0	0	0	0	0	80
17433382	m	Not repo	JP	2020	0	1	0	1	0	0	0	0	0	0	0	0	69
17433415	f	md	CA	2020	1	0	0	0	0	0	0	0	0	0	0	0	
17433424	f	cn	JP	2020	0	1	1	0	0	0	0	0	0	0	0	0	90
17433639	f	md	DE	2020	1	0	0	0	0	0	0	0	0	0	0	0	
17433883	f	md	TH	2020	1	0	0	0	0	0	0	0	0	0	0	0	71
17434166	f	cn	US	2020	0	0	0	0	0	0	0	0	0	0	0	0	35
17434546	f	md	JP	2020	0	1	0	0	0	1	0	0	0	0	0	0	70
17434736	m	md	DE	2020	0	0	0	0	0	0	0	0	0	0	0	0	
17435170	f	cn	MX	2020	0	1	1	0	0	0	0	0	0	0	0	0	67
17435243	m	cn	JP	2020	0	1	1	0	0	0	0	0	0	0	0	0	88
17435277	m	hp	US	2020	0	1	1	0	0	0	0	0	0	0	0	0	
17435344	f	md	DE	2020	0	0	0	0	0	0	0	0	0	0	0	0	60
17435361	Not	md	JP	2020	0	1	0	0	1	0	0	0	0	0	0	0	
17435386	m	md	KR	2020	0	1	0	0	0	1	0	0	0	0	0	0	64
17435785	f	cn	US	2020	1	0	0	0	0	0	0	0	0	0	0	0	
17436524	m	cn	JP	2020	0	1	1	0	0	0	0	0	0	0	0	0	69
17436834	f	Not repo	US	2020	0	1	0	1	0	0	0	0	0	0	0	0	78
17436875	m	cn	CN	2020	0	1	0	0	1	0	0	0	0	0	0	0	57
17436968	f	md	US	2020	1	0	0	0	0	0	0	0	0	0	0	0	70
17436986	Not	md	TR	2020	0	0	0	0	0	0	0	0	0	0	0	0	
17437248	f	cn	KR	2020	1	0	0	0	0	0	0	0	0	0	0	0	65
17437290	m	md	SA	2020	0	1	0	1	0	0	0	0	0	0	0	0	60
17437373	f	md	CL	2020	0	1	1	0	0	0	0	0	0	0	0	0	85
17437377	m	cn	JP	2020	0	1	1	0	0	0	0	0	0	0	0	0	78
17437515	m	hp	US	2020	0	1	0	0	1	0	0	0	0	0	0	0	64
17437589	f	md	US	2020	0	1	1	0	0	0	0	0	0	0	0	0	75
17437680	Not	md	FR	2020	0	0	0	0	0	0	0	0	0	0	0	0	
17437868	f	hp	FR	2020	0	1	0	0	1	0	0	0	0	0	0	0	74
17437882	m	md	US	2020	0	1	1	0	0	0	0	0	0	0	0	0	71
17437887	m	md	JP	2020	0	1	0	0	0	1	0	0	0	0	0	0	70
17438113	m	hp	US	2020	0	0	0	0	0	0	0	0	0	0	0	0	

17438197	Not	ph	JP	2020	0	1	0	0	1	0	0	0	0	0	0	0	
17438236	m	cn	CN	2020	0	1	0	0	1	0	0	0	0	0	0	0	
17438516	f	md	AU	2020	0	0	0	0	0	0	0	0	0	0	0	0	45
17438646	m	cn	US	2020	0	1	0	1	0	0	0	0	0	0	0	0	81.4
17438712	m	Not repo	GB	2020	0	1	0	1	0	0	0	0	0	0	0	0	
17438936	f	md	NL	2020	0	1	1	0	0	0	0	0	0	0	0	0	66
17439117	Not	md	FR	2020	0	1	0	1	0	0	0	0	0	0	0	0	
17439810	Not	md	DE	2020	0	1	0	1	0	0	0	0	0	0	0	0	
17439816	m	md	JP	2020	1	0	0	0	0	0	0	0	0	0	0	0	80
17439905	m	md	JP	2020	1	0	0	0	0	0	0	0	0	0	0	0	79
17439960	f	md	DE	2020	0	1	0	0	0	1	0	0	0	0	0	0	67
17440420	m	ph	JP	2020	1	0	0	0	0	0	0	0	0	0	0	0	80
17440445	Not	Not repo	DE	2020	0	1	0	1	0	0	0	0	0	0	0	0	
17440456	Not	Not repo	DE	2020	0	1	0	1	0	0	0	0	0	0	0	0	
17440540	f	md	US	2020	0	1	0	1	0	0	0	0	0	0	0	0	
17440828	m	hp	JP	2020	0	0	0	0	0	0	0	0	0	0	0	0	69
17440843	f	md	FR	2020	0	1	0	0	1	0	0	0	0	0	0	0	66
17440847	m	hp	NO	2020	0	0	0	0	0	0	0	0	0	0	0	0	66
17440849	m	md	ES	2020	0	1	1	0	0	0	0	0	0	0	0	0	84
17440928	f	md	US	2020	0	1	0	0	1	0	0	0	0	0	0	0	
17441024	m	cn	JP	2020	0	0	0	0	0	0	0	0	0	0	0	0	
17441213	m	cn	JP	2020	0	1	1	0	0	0	0	0	0	0	0	0	80
17441744	Not	Not repo	DE	2020	0	1	0	1	0	0	0	0	0	0	0	0	
17441826	f	md	US	2020	1	0	0	0	0	0	0	0	0	0	0	0	
17441849	f	ph	GB	2020	0	1	1	0	0	0	0	0	0	0	0	0	65
17442151	Not	md	ES	2020	0	0	0	0	0	0	0	0	0	0	0	0	
17442330	m	cn	JP	2020	0	0	0	0	0	0	0	0	0	0	0	0	60
17442425	m	hp	KR	2020	0	1	1	0	0	0	0	0	0	0	0	0	80
17442696	m	hp	IT	2020	0	1	0	0	1	0	0	0	0	0	0	0	62
17443627	m	Not repo	GR	2020	0	1	0	1	0	0	0	0	0	0	0	0	60
17443817	m	cn	JP	2020	0	0	0	0	0	0	0	0	0	0	0	0	75
17444332	f	cn	Not re	2020	0	0	0	0	0	0	0	0	0	0	0	0	
17444407	m	Not repo	JP	2020	0	1	0	1	0	0	0	0	0	0	0	1	70
17444602	Not	Not repo	DE	2020	0	1	0	1	0	0	0	0	0	0	0	0	

17444729	Not	Not repo	DE	2020	0	1	0	1	0	0	0	0	0	0	0	0	
17444994	m	cn	JP	2020	0	1	1	0	0	0	0	0	0	0	0	0	
17445022	m	Not repo	MY	2020	1	0	0	0	0	0	0	0	0	0	0	0	84.4
17445024	m	Not repo	GR	2020	0	1	0	1	0	0	0	0	0	0	0	0	58
17445027	Not	Not repo	DE	2020	0	1	0	1	0	0	0	0	0	0	0	0	
17445093	Not	Not repo	DE	2020	0	1	0	1	0	0	0	0	0	0	0	0	
17445388	Not	Not repo	DE	2020	0	1	0	1	0	0	0	0	0	0	0	0	
17445419	m	md	BE	2020	0	0	0	0	0	0	0	0	0	0	0	0	
17445546	m	Not repo	JP	2020	0	1	0	1	0	0	0	0	0	0	0	0	80
17446039	Not	Not repo	DE	2020	0	1	0	1	0	0	0	0	0	0	0	0	
17446173	m	cn	FR	2020	0	1	1	0	0	0	0	0	0	0	0	1	
17446624	f	hp	US	2020	1	0	0	0	0	0	0	0	0	0	0	0	69
17446671	f	Not repo	CN	2020	1	0	0	0	0	0	0	0	0	0	0	1	55
17446722	f	Not repo	CA	2020	0	1	0	1	0	0	0	0	0	0	0	0	
17446723	f	hp	US	2020	0	1	0	1	0	0	0	0	0	0	0	0	80
17446844	m	md	JP	2020	1	0	0	0	0	0	0	0	0	0	0	0	87
17446846	Not	hp	FR	2020	0	1	0	1	0	0	0	0	0	0	0	0	
17446906	m	Not repo	JP	2020	0	1	0	1	0	0	0	0	0	0	0	0	77
17446908	m	Not repo	GR	2020	0	1	0	1	0	0	0	0	0	0	0	0	77
17446912	f	Not repo	US	2020	1	0	0	0	0	0	0	0	0	0	0	0	91.7
17446978	m	Not repo	US	2020	1	0	0	0	0	0	0	0	0	0	0	0	82.2
17447032	f	md	US	2020	0	1	0	1	0	0	0	0	0	0	0	1	55
17447033	f	hp	US	2020	0	1	0	1	0	0	0	0	0	0	0	0	
17447038	m	md	JP	2020	1	0	0	0	0	0	0	0	0	0	0	0	89
17447156	m	hp	US	2020	0	1	0	0	1	0	0	0	0	0	0	0	73
17447260	m	cn	JP	2020	0	1	1	0	0	0	0	0	0	0	0	0	95
17447388	Not	md	JP	2020	0	0	0	0	0	0	0	0	0	0	0	0	80
17447643	m	hp	CN	2020	0	1	0	0	1	0	0	0	0	0	0	0	62
17447697	m	md	HU	2020	0	1	1	0	0	0	0	0	0	0	0	0	57
17447769	m	md	DE	2020	0	0	0	0	0	0	0	0	0	0	0	0	62
17447788	m	cn	Not re	2020	0	0	0	0	0	0	0	0	0	0	0	0	
17448336	m	cn	JP	2020	0	1	1	0	0	0	0	0	0	0	0	0	90
17448346	m	md	KR	2020	0	1	0	0	1	0	0	0	0	0	0	0	67
17448391	m	md	JP	2020	0	1	0	0	0	1	0	0	0	0	0	0	72

17448393	m	cn	US	2020	1	0	0	0	0	0	0	0	0	0	0	0	75
17448403	m	md	JP	2020	0	1	0	0	0	1	0	0	0	0	0	0	75
17448411	f	cn	JP	2020	0	1	1	0	0	0	0	0	0	0	0	0	59
17448557	f	md	US	2020	0	1	1	0	0	0	0	0	0	0	0	0	51
17449333	Not	cn	JP	2020	0	1	1	0	0	0	0	0	0	0	0	0	80
17449952	m	cn	JP	2020	0	1	1	0	0	0	0	0	0	0	0	0	70
17450727	f	cn	US	2020	0	0	0	0	0	0	0	0	0	0	0	0	73
17451056	m	md	MY	2020	0	1	1	0	0	0	0	0	0	0	0	0	61
17451058	f	Not repo	MY	2020	1	0	0	0	0	0	0	0	0	0	0	0	58.6
17451190	f	cn	US	2020	0	1	1	0	0	0	0	0	0	0	0	0	
17451735	m	cn	JP	2020	0	1	1	0	0	0	0	0	0	0	0	0	80
17451987	m	md	FR	2020	1	0	0	0	0	0	0	0	0	0	0	0	79
17451991	f	md	DE	2020	0	1	1	0	0	0	0	0	0	0	0	0	
17452077	m	md	CN	2020	0	1	0	0	0	1	0	0	0	0	0	0	70
17452140	f	cn	CA	2020	0	0	0	0	0	0	0	0	0	0	0	0	59
17452181	m	hp	IT	2020	0	0	0	0	0	0	0	0	0	0	0	0	47
17452185	m	md	ES	2020	0	1	1	0	0	0	0	0	0	0	0	0	62
17452477	m	cn	JP	2020	0	1	1	0	0	0	0	0	0	0	0	0	65
17452701	Not	hp	FR	2020	0	1	0	0	1	0	0	0	0	0	0	0	
17452702	Not	hp	FR	2020	0	1	0	0	1	0	0	0	0	0	0	0	
17452707	Not	hp	FR	2020	0	1	0	0	1	0	0	0	0	0	0	0	
17452739	Not	hp	FR	2020	0	1	0	0	1	0	0	0	0	0	0	0	
17452761	m	Not repo	JP	2020	1	0	0	0	0	0	0	0	0	0	0	0	70.9
17452824	m	cn	CA	2020	1	0	0	0	0	0	0	0	0	0	0	0	
17453006	m	Not repo	JP	2020	0	1	0	1	0	0	0	0	0	0	0	1	80
17453108	m	cn	JP	2020	0	1	1	0	0	0	0	0	0	0	0	0	61
17453120	m	md	IN	2020	0	1	1	0	0	0	0	0	0	0	0	0	71
17453619	f	hp	US	2020	0	1	0	0	1	0	0	0	0	0	0	0	62
17453638	f	ph	US	2020	0	1	1	0	0	0	0	0	0	0	0	1	
17453709	f	md	AT	2020	1	0	0	0	0	0	0	0	0	0	0	0	52
17453722	f	md	CL	2020	0	1	1	0	0	0	0	0	0	0	0	0	85
17453825	Not	hp	NL	2020	0	0	0	0	0	0	0	0	0	0	0	0	
17454068	m	hp	US	2020	0	1	0	0	1	0	0	0	0	0	0	0	58
17454565	m	hp	IT	2020	0	1	0	0	1	0	0	0	0	0	0	0	65

17454656	Not	md	TR	2020	1	0	0	0	0	0	0	0	0	0	0	0	
17454674	Not	md	TR	2020	1	0	0	0	0	0	0	0	0	0	0	0	
17454675	Not	md	TR	2020	1	0	0	0	0	0	0	0	0	0	0	0	
17454676	Not	md	TR	2020	1	0	0	0	0	0	0	0	0	0	0	0	
17455393	Not	hp	FR	2020	0	1	0	0	1	0	0	0	0	0	0	0	
17455408	Not	hp	FR	2020	0	1	0	0	1	0	0	0	0	0	0	0	
17455702	f	Not repo	JP	2020	0	0	0	0	0	0	1	0	0	1	0	1	60
17455796	f	hp	US	2020	0	1	1	0	0	0	0	0	0	0	0	0	55
17455834	f	md	FR	2020	0	1	0	1	0	0	0	0	0	0	0	0	
17456635	m	Not repo	AU	2020	0	1	0	1	0	0	0	0	0	0	0	0	70
17456645	m	md	JP	2020	0	1	0	1	0	0	0	0	0	0	0	1	62
17456920	Not	Not repo	DE	2020	0	1	0	1	0	0	0	0	0	0	0	0	
17456966	m	md	DE	2020	1	0	0	0	0	0	0	0	0	0	0	0	81
17457265	Not	md	TR	2020	0	0	0	0	0	0	0	0	0	0	0	0	
17457306	m	md	IT	2020	0	1	1	0	0	0	0	0	0	0	0	0	69
17457491	Not	hp	FR	2020	0	1	0	0	1	0	0	0	0	0	0	0	
17457500	m	md	TR	2020	0	1	1	0	0	0	0	0	0	0	0	0	76
17457509	Not	Not repo	DE	2020	0	1	0	1	0	0	0	0	0	0	0	0	
17457517	f	md	US	2020	0	1	0	0	1	0	0	0	0	0	0	0	66
17457589	m	Not repo	GB	2020	0	1	0	1	0	0	0	0	0	0	0	0	
17457684	Not	hp	US	2020	0	1	0	0	1	0	0	0	0	0	0	0	62
17457722	m	Not repo	JP	2020	0	1	0	1	0	0	0	0	0	0	0	1	74
17457761	Not	md	TR	2020	0	0	0	0	0	0	0	0	0	0	0	0	
17457925	m	cn	JP	2020	0	1	1	0	0	0	0	0	0	0	0	0	70
17457972	m	md	US	2020	0	1	0	1	0	0	0	0	0	0	0	0	
17458063	f	ph	JP	2020	1	0	0	0	0	0	0	0	0	0	0	0	74
17458184	m	Not repo	JP	2020	0	1	0	1	0	0	0	0	0	0	0	1	60
17458187	m	Not repo	JP	2020	0	1	0	1	0	0	0	0	0	0	0	0	70
17458194	m	Not repo	JP	2020	0	1	0	1	0	0	0	0	0	0	0	0	70
17458196	m	Not repo	ID	2020	1	0	0	0	0	0	0	0	0	0	0	0	
17458274	Not	Not repo	DE	2020	0	1	0	1	0	0	0	0	0	0	0	0	
17458967	f	hp	JP	2020	0	1	0	0	1	0	0	0	0	0	0	0	73
17459354	Not	Not repo	JP	2020	1	0	0	0	0	0	0	0	0	0	0	0	
17459409	f	md	US	2020	0	1	1	0	0	0	0	0	0	0	0	0	

17459973	m	md	JP	2020	0	1	0	0	0	1	0	0	0	0	0	0	74
17460015	m	hp	AU	2020	0	1	0	0	1	0	0	0	0	0	0	0	69
17460046	f	md	US	2020	0	0	0	0	0	0	0	0	0	0	0	0	
17460052	Not	md	TR	2020	0	0	0	0	0	0	0	0	0	0	0	0	
17460635	Not	hp	FR	2020	0	1	0	0	1	0	0	0	0	0	0	0	
17460772	m	cn	CN	2020	1	0	0	0	0	0	0	0	0	0	0	0	
17460906	Not	md	JP	2020	0	1	1	0	0	0	0	0	0	0	0	0	
17460930	f	ph	FR	2020	0	0	0	0	0	0	0	0	0	0	0	0	55
17461062	f	ph	FR	2020	0	0	0	0	0	0	0	0	0	0	0	0	69
17461238	m	md	JP	2020	0	1	0	0	0	1	0	0	0	0	0	1	66
17461329	f	cn	JP	2020	0	1	1	0	0	0	0	0	0	0	0	0	79
17461378	m	cn	TW	2020	0	1	0	0	0	1	0	0	0	0	0	0	
17461512	f	md	AT	2020	0	0	0	0	0	0	0	0	0	0	0	0	79
17461529	Not	md	BG	2020	1	0	0	0	0	0	0	0	0	0	0	0	
17461671	Not	md	JP	2020	0	1	0	0	1	0	0	0	0	0	0	0	
17462341	m	hp	GR	2020	0	0	0	0	0	0	0	0	0	0	0	0	74
17462519	f	cn	US	2020	1	0	0	0	0	0	0	0	0	0	0	0	53
17462575	f	cn	US	2020	0	1	0	1	0	0	0	0	0	0	0	0	
17462618	m	cn	CN	2020	0	1	0	0	1	0	0	0	0	0	0	0	62
17463216	m	Not repo	US	2020	1	0	0	0	0	0	0	0	0	0	0	0	54.1
17463217	m	Not repo	JP	2020	1	0	0	0	0	0	0	0	0	0	0	0	66
17463266	f	md	JP	2020	0	1	0	0	0	1	0	0	0	0	0	0	38
17463437	m	md	JP	2020	0	1	0	1	0	0	0	0	0	0	0	1	60
17463499	f	md	JP	2020	0	0	0	0	0	0	0	0	0	0	0	0	
17463823	Not	md	TR	2020	0	0	0	0	0	0	0	0	0	0	0	0	
17463858	Not	md	TR	2020	0	0	0	0	0	0	0	0	0	0	0	0	
17463888	Not	md	TR	2020	0	0	0	0	0	0	0	0	0	0	0	0	
17464221	f	ph	CA	2020	0	0	0	0	0	0	0	0	0	0	0	0	62
17464317	Not	md	TR	2020	1	0	0	0	0	0	0	0	0	0	0	0	
17464319	Not	md	TR	2020	1	0	0	0	0	0	0	0	0	0	0	0	
17464321	Not	md	TR	2020	1	0	0	0	0	0	0	0	0	0	0	0	
17464323	Not	md	TR	2020	1	0	0	0	0	0	0	0	0	0	0	0	
17464361	m	md	US	2020	0	0	0	0	0	0	0	0	0	0	0	0	
17464411	m	hp	IN	2020	0	0	0	0	0	0	0	0	0	0	0	0	70

17464561	m	md	IT	2020	0	1	1	0	0	0	0	0	0	0	0	0	69
17464656	m	hp	DE	2020	0	1	1	0	0	0	0	0	0	0	0	0	79
17464733	f	md	US	2020	0	0	0	0	0	0	0	0	0	0	0	0	
17464745	m	cn	US	2020	0	0	0	0	0	0	0	0	0	0	0	0	35
17464767	Not	cn	US	2020	0	0	0	0	0	0	0	0	0	0	0	0	
17464770	m	md	US	2020	0	0	0	0	0	0	0	0	0	0	0	0	
17464787	m	cn	US	2020	0	0	0	0	0	0	0	0	0	0	0	0	
17464802	f	cn	US	2020	0	0	0	0	0	0	0	0	0	0	0	0	
17464803	f	md	US	2020	0	0	0	0	0	0	0	0	0	0	0	0	
17464804	Not	cn	US	2020	0	0	0	0	0	0	0	0	0	0	0	0	
17464808	m	cn	US	2020	0	0	0	0	0	0	0	0	0	0	0	0	
17464809	f	cn	US	2020	0	0	0	0	0	0	0	0	0	0	0	0	
17464811	m	cn	US	2020	0	0	0	0	0	0	0	0	0	0	0	0	
17464846	m	cn	US	2020	0	0	0	0	0	0	0	0	0	0	0	0	58
17464848	m	ph	US	2020	0	0	0	0	0	0	0	0	0	0	0	0	
17464866	f	md	US	2020	0	0	0	0	0	0	0	0	0	0	0	0	75
17464868	m	cn	US	2020	0	0	0	0	0	0	0	0	0	0	0	0	
17464872	Not	cn	US	2020	0	0	0	0	0	0	0	0	0	0	0	0	
17464879	f	ph	US	2020	0	0	0	0	0	0	0	0	0	0	0	0	
17464887	f	cn	US	2020	0	0	0	0	0	0	0	0	0	0	0	0	
17464901	f	cn	US	2020	0	0	0	0	0	0	0	0	0	0	0	0	71
17464941	m	cn	US	2020	0	0	0	0	0	0	0	0	0	0	0	0	
17464947	f	cn	US	2020	0	0	0	0	0	0	0	0	0	0	0	0	
17464950	f	cn	US	2020	0	0	0	0	0	0	0	0	0	0	0	0	
17464963	f	cn	US	2020	0	0	0	0	0	0	0	0	0	0	0	0	
17464986	f	ph	US	2020	0	0	0	0	0	0	0	0	0	0	0	0	
17464987	m	cn	US	2020	0	0	0	0	0	0	0	0	0	0	0	0	52
17464992	f	cn	US	2020	0	0	0	0	0	0	0	0	0	0	0	0	72
17465096	m	md	PL	2020	0	1	0	0	0	1	0	0	0	0	0	0	66
17465172	m	cn	JP	2020	0	1	1	0	0	0	0	0	0	0	0	0	
17465288	f	md	DE	2020	0	1	0	0	1	0	0	0	0	0	0	0	56
17465358	m	cn	US	2020	0	1	0	0	1	0	0	0	0	0	0	0	78
17465636	m	cn	DK	2020	0	1	0	0	0	1	0	0	0	0	0	0	
17465748	m	Not repo	JP	2020	0	1	0	1	0	0	0	0	0	0	0	1	67

17465807	f	md	AT	2020	1	0	0	0	0	0	0	0	0	0	0	0	60
17465914	f	md	TW	2020	0	1	0	0	0	1	0	0	0	0	0	0	56
17465991	f	ph	GB	2020	0	0	0	0	0	0	0	0	0	0	0	0	60
17466176	f	cn	US	2020	1	0	0	0	0	0	0	0	0	0	0	1	80
17466215	f	cn	JP	2020	0	1	1	0	0	0	0	0	0	0	0	0	80
17466256	f	cn	US	2020	1	0	0	0	0	0	0	0	0	0	0	1	80
17466635	f	md	JP	2020	1	0	0	0	0	0	0	0	0	0	0	0	80.6
17467146	m	ph	JP	2020	1	0	0	0	0	0	0	0	0	0	0	0	86
17467195	f	md	RO	2020	0	1	1	0	0	0	0	0	0	0	0	0	41
17467492	m	md	US	2020	0	0	0	0	0	0	0	0	0	0	0	0	
17467750	f	cn	US	2020	0	0	0	0	0	0	0	0	0	0	0	0	76
17467889	Not	hp	US	2020	0	0	0	0	0	0	0	0	0	0	0	0	
17468183	f	Not repo	JP	2020	1	0	0	0	0	0	0	0	0	0	0	1	90
17468185	m	Not repo	JP	2020	0	1	0	1	0	0	0	0	0	0	0	1	80
17468520	f	ph	US	2020	0	0	0	0	0	0	0	0	0	0	0	0	69
17468651	f	hp	JP	2020	0	1	1	0	0	0	0	0	0	0	0	0	66
17469307	m	md	CN	2020	0	0	0	0	0	0	0	0	0	0	0	0	69
17469309	f	Not repo	ID	2020	1	0	0	0	0	0	0	0	0	0	0	0	
17469381	m	hp	JP	2020	0	0	0	0	0	0	1	0	1	0	0	1	59
17469433	m	hp	JP	2020	0	0	0	0	0	0	1	0	1	0	0	0	63
17469586	f	md	JP	2020	0	1	0	1	0	0	0	0	0	0	0	1	72
17469593	m	hp	IN	2020	0	0	0	0	0	0	0	0	0	0	0	0	71
17469684	m	hp	CN	2020	0	1	0	0	1	0	0	0	0	0	0	0	78
17469789	f	cn	JP	2020	0	0	0	0	0	0	0	0	0	0	0	0	76
17469912	f	md	CA	2020	0	0	0	0	0	0	0	0	0	0	0	0	61
17470722	Not	md	US	2020	1	0	0	0	0	0	0	0	0	0	0	0	
17470762	m	md	BE	2020	0	1	1	0	0	0	0	0	0	0	0	0	
17470788	f	md	US	2020	1	0	0	0	0	0	0	0	0	0	0	0	76
17471251	Not	md	JP	2020	0	0	0	0	0	0	0	0	0	0	0	0	80
17471254	m	cn	JP	2020	0	1	1	0	0	0	0	0	0	0	0	0	
17471292	m	md	JP	2020	0	1	0	0	0	1	0	0	0	0	0	0	80
17471723	m	hp	JP	2020	1	0	0	0	0	0	0	0	0	0	0	0	71
17471724	m	Not repo	JP	2020	0	1	0	1	0	0	0	0	0	0	0	0	66.3
17471725	m	hp	JP	2020	0	0	0	0	0	0	1	0	1	0	0	0	75

17471792	m	md	JP	2020	0	0	0	0	0	0	0	0	0	0	0	0	68
17471852	m	md	JP	2020	0	0	0	0	0	0	0	0	0	0	0	0	57
17471853	m	md	JP	2020	0	0	0	0	0	0	0	0	0	0	0	0	72
17471966	Not	md	IN	2020	1	0	0	0	0	0	0	0	0	0	0	1	
17472027	m	hp	US	2020	0	1	0	0	1	0	0	0	0	0	0	0	
17472242	f	ph	GB	2020	0	0	0	0	0	0	0	0	0	0	0	0	60
17472517	m	md	JP	2020	0	0	0	0	0	0	0	0	0	0	0	0	66
17472519	m	md	JP	2020	0	0	0	0	0	0	0	0	0	0	0	0	69
17472563	m	md	JP	2020	0	0	0	0	0	0	0	0	0	0	0	0	65
17472592	m	md	JP	2020	0	0	0	0	0	0	0	0	0	0	0	0	70
17472609	m	md	JP	2020	0	0	0	0	0	0	0	0	0	0	0	0	54
17473060	m	md	JP	2020	0	0	0	0	0	0	0	0	0	0	0	0	57
17473129	f	md	SG	2020	1	0	0	0	0	0	0	0	0	0	0	0	60
17473756	m	cn	US	2020	0	1	0	1	0	0	0	0	0	0	0	0	
17473790	m	hp	LB	2020	0	1	1	0	0	0	0	0	0	0	0	0	58
17474423	Not	Not repo	DE	2020	0	1	0	1	0	0	0	0	0	0	0	0	
17474432	f	Not repo	TW	2020	0	1	0	1	0	0	0	0	0	0	0	0	70
17474444	f	Not repo	ID	2020	1	0	0	0	0	0	0	0	0	0	0	0	
17474468	Not	Not repo	DE	2020	0	1	0	1	0	0	0	0	0	0	0	0	
17474646	m	cn	MY	2020	1	0	0	0	0	0	0	0	0	0	0	0	71
17474664	Not	Not repo	DE	2020	0	1	0	1	0	0	0	0	0	0	0	0	
17474674	m	cn	JP	2020	1	0	0	0	0	0	0	0	0	0	0	0	71
17474878	f	Not repo	JP	2020	1	0	0	0	0	0	0	0	0	0	0	0	74
17474934	f	md	US	2020	1	0	0	0	0	0	0	0	0	0	0	0	
17474963	f	md	PL	2020	0	0	0	0	0	0	0	0	0	0	0	0	
17475602	Not	hp	US	2020	0	0	0	0	0	0	0	0	0	0	0	0	
17476085	m	md	CL	2020	0	1	0	0	0	1	0	0	0	0	0	0	74
17476096	m	Not repo	JP	2020	0	1	0	1	0	0	0	0	0	0	0	1	78
17476099	f	md	JP	2020	1	0	0	0	0	0	0	0	0	0	0	0	67
17476875	m	hp	NO	2020	0	0	0	0	0	0	0	0	0	0	0	0	66
17477627	f	md	TR	2020	0	0	0	0	0	0	0	0	0	0	0	0	62
17477641	f	cn	JP	2020	0	1	1	0	0	0	0	0	0	0	0	0	75
17477736	Not	cn	JP	2020	0	1	1	0	0	0	0	0	0	0	0	0	77
17477799	f	Not repo	JP	2020	1	0	0	0	0	0	0	0	0	0	0	0	66

17477805	m	Not repo	GB	2020	0	1	0	1	0	0	0	0	0	0	0	0	
17478039	f	hp	US	2020	0	1	1	0	0	0	0	0	0	0	0	0	55
17478143	m	md	GR	2020	0	1	1	0	0	0	0	0	0	0	0	0	62
17478146	f	hp	JP	2020	1	0	0	0	0	0	0	0	0	0	0	0	74
17478230	m	md	FR	2020	0	1	0	1	0	0	0	0	0	0	0	0	
17478552	Not	md	JP	2020	0	1	0	0	1	0	0	0	0	0	0	1	
17478586	m	md	GR	2020	0	1	1	0	0	0	0	0	0	0	0	0	81
17478752	f	hp	NL	2020	0	0	0	0	0	0	0	0	0	0	0	0	
17478781	m	cn	JP	2020	0	1	1	0	0	0	0	0	0	0	0	0	66
17478950	m	hp	JP	2020	0	0	0	0	0	0	1	0	1	0	0	1	77
17479395	Not	md	GR	2020	0	0	0	0	0	0	0	0	0	0	0	0	
17479499	Not	hp	US	2020	0	1	0	0	1	0	0	0	0	0	0	0	
17479787	Not	md	US	2020	0	0	0	0	0	0	0	0	0	0	0	0	
17479790	Not	cn	UY	2020	0	1	1	0	0	0	0	0	0	0	0	0	
17480670	m	md	SE	2020	1	0	0	0	0	0	0	0	0	0	0	0	45
17481083	m	cn	GB	2020	0	1	1	0	0	0	0	0	0	0	0	0	55
17481333	m	md	IN	2020	0	1	0	0	1	0	0	0	0	0	0	0	54
17481611	f	md	US	2020	1	0	0	0	0	0	0	0	0	0	0	0	84
17481956	m	cn	JP	2020	0	1	1	0	0	0	0	0	0	0	0	0	56
17482248	m	md	MX	2020	0	1	1	0	0	0	0	0	0	0	0	0	54
17482285	m	cn	DE	2020	0	1	1	0	0	0	0	0	0	0	0	0	67
17482369	f	md	VN	2020	1	0	0	0	0	0	0	0	0	0	0	0	
17482461	f	md	DE	2020	0	1	0	0	0	1	0	0	0	0	0	0	57
17482532	m	md	ES	2020	0	0	0	0	0	0	0	0	0	0	0	0	59
17482557	m	cn	CA	2020	0	0	0	0	0	0	0	0	0	0	0	0	78
17482828	m	cn	NP	2020	0	0	0	0	0	0	0	0	0	0	0	0	66
17483241	f	md	JP	2020	1	0	0	0	0	0	0	0	0	0	0	0	75
17483443	m	ph	US	2020	0	0	0	0	0	0	0	0	0	0	0	0	68
17483509	Not	hp	CN	2020	0	0	0	0	0	0	0	0	0	0	0	0	
17483703	Not	hp	CN	2020	0	0	0	0	0	0	0	0	0	0	0	0	
17483705	Not	hp	CN	2020	0	0	0	0	0	0	0	0	0	0	0	0	
17483710	Not	hp	CN	2020	0	0	0	0	0	0	0	0	0	0	0	0	
17483762	f	md	US	2020	0	1	1	0	0	0	0	0	0	0	0	0	55
17483834	Not	hp	BE	2020	0	0	0	0	0	0	0	0	0	0	0	0	

17483876	m	md	US	2020	1	0	0	0	0	0	0	0	0	0	0	0	70
17484897	Not	md	CA	2020	0	0	0	0	0	0	0	0	0	0	0	0	
17484999	f	cn	US	2020	0	1	0	0	1	0	0	0	0	0	0	0	
17485296	f	md	DE	2020	0	1	1	0	0	0	0	0	0	0	0	0	
17485372	m	hp	TW	2020	0	1	0	0	1	0	0	0	0	0	0	0	72
17485420	m	hp	CN	2020	0	1	0	0	1	0	0	0	0	0	0	0	71
17485454	Not	md	CL	2020	1	0	0	0	0	0	0	0	0	0	0	0	39
17485688	m	md	IT	2020	0	1	1	0	0	0	0	0	0	0	0	0	
17485855	f	md	SK	2020	0	1	0	0	0	1	0	0	0	0	0	0	72
17486189	f	md	US	2020	0	1	1	0	0	0	0	0	0	0	0	0	75
17486239	f	md	US	2020	0	1	1	0	0	0	0	0	0	0	0	0	61
17486399	f	hp	US	2020	0	1	0	0	1	0	0	0	0	0	0	0	73
17486683	m	md	FR	2020	0	1	0	0	1	0	0	0	0	0	0	0	66
17487050	m	md	JP	2020	0	1	0	0	0	1	0	0	0	0	0	0	80
17487106	f	md	JP	2020	0	1	0	0	0	1	0	0	0	0	0	0	66
17487346	m	cn	JP	2020	0	1	1	0	0	0	0	0	0	0	0	0	60
17487897	f	md	US	2020	0	0	0	0	0	0	0	0	0	0	0	0	42
17488246	f	ph	GB	2020	0	0	0	0	0	0	0	0	0	0	0	0	60
17488305	m	ph	US	2020	0	0	0	0	0	0	0	0	0	0	0	0	71
17488574	f	md	IN	2020	0	1	1	0	0	0	0	0	0	0	0	0	37
17488618	f	md	JP	2020	0	1	1	0	0	0	0	0	0	0	0	0	54
17488767	m	hp	US	2020	0	0	0	0	0	0	0	0	0	0	0	0	59
17489237	m	hp	JP	2020	0	1	1	0	0	0	0	0	0	0	0	0	63
17489351	m	cn	JP	2020	0	1	1	0	0	0	0	0	0	0	0	0	74
17489597	m	cn	CA	2020	0	0	0	0	0	0	0	0	0	0	0	0	37
17490639	m	md	DE	2020	0	0	0	0	0	0	0	0	0	0	0	0	79
17490775	m	md	JP	2020	0	1	0	0	1	0	0	0	0	0	0	0	73
17490896	m	md	JP	2020	0	0	0	0	0	0	0	0	0	0	0	0	41
17491011	m	md	BE	2020	0	1	1	0	0	0	0	0	0	0	0	0	71
17491056	m	Not repo	JP	2020	1	0	0	0	0	0	0	0	0	0	0	0	
17491079	m	md	IT	2020	0	0	0	0	0	0	1	0	0	0	1	0	61
17491124	m	hp	SE	2020	0	0	0	0	0	0	0	0	0	0	0	0	57
17492113	m	cn	JP	2020	0	1	1	0	0	0	0	0	0	0	0	0	70
17492813	f	cn	CN	2020	0	0	0	0	0	0	0	0	0	0	0	0	66

17494002	m	hp	AT	2020	0	0	0	0	0	0	0	0	0	0	0	0	72
17495028	m	cn	JP	2020	0	1	1	0	0	0	0	0	0	0	0	0	85
17495208	Not	md	HU	2020	0	0	0	0	0	0	0	0	0	0	0	0	70
17495468	m	hp	DE	2020	0	1	0	0	0	1	0	0	0	0	0	0	69
17496102	f	cn	US	2020	0	1	1	0	0	0	0	0	0	0	0	0	
17496222	m	hp	IN	2020	0	1	1	0	0	0	0	0	0	0	0	0	48
17496261	m	cn	JP	2020	0	1	1	0	0	0	0	0	0	0	0	1	63
17496388	m	cn	CN	2020	0	1	0	0	1	0	0	0	0	0	0	0	70
17497352	f	Not repo	JP	2020	0	1	0	1	0	0	0	0	0	0	0	1	79
17497402	f	cn	US	2020	0	1	1	0	0	0	0	0	0	0	0	1	73
17497490	f	Not repo	JP	2020	0	1	0	1	0	0	0	0	0	0	0	0	63
17497514	f	md	ES	2020	0	0	0	0	0	0	0	0	0	0	0	0	67
17497693	m	md	JP	2020	0	1	0	1	0	0	0	0	0	0	0	1	69
17498020	f	Not repo	CN	2020	1	0	0	0	0	0	0	0	0	0	0	0	75
17498022	f	hp	CN	2020	1	0	0	0	0	0	0	0	0	0	0	0	40
17498502	m	Not repo	LB	2020	1	0	0	0	0	0	0	0	0	0	0	0	
17498672	f	md	DE	2020	0	1	1	0	0	0	0	0	0	0	0	0	66
17498887	m	md	CZ	2020	1	0	0	0	0	0	0	0	0	0	0	0	67
17499065	m	hp	NL	2020	0	0	0	0	0	0	0	0	0	0	0	0	61
17499214	f	hp	US	2020	0	0	0	0	0	0	0	0	0	0	0	0	
17499259	m	md	BE	2020	0	1	1	0	0	0	0	0	0	0	0	0	75
17499968	m	hp	IT	2020	0	1	1	0	0	0	0	0	0	0	0	0	69
17499992	m	hp	US	2020	0	1	1	0	0	0	0	0	0	0	0	0	55
17500168	m	md	KR	2020	0	1	0	0	1	0	0	0	0	0	0	0	69
17500192	f	md	CL	2020	0	1	0	0	0	1	0	0	0	0	0	0	38
17500209	f	md	JP	2020	0	1	0	0	0	1	0	0	0	0	0	1	80
17500405	m	md	JP	2020	0	1	1	0	0	0	0	0	0	0	0	0	68
17500503	Not	md	US	2020	0	1	0	0	1	0	0	0	0	0	0	0	
17500837	f	cn	CN	2020	0	1	0	0	1	0	0	0	0	0	0	0	
17500921	m	Not repo	DK	2020	0	1	0	1	0	0	0	0	0	0	0	1	71
17501171	m	md	JP	2020	0	1	0	1	0	0	0	0	0	0	0	1	84
17501993	m	md	US	2020	1	0	0	0	0	0	0	0	0	0	0	0	70
17502055	f	md	PH	2020	0	1	1	0	0	0	0	0	0	0	0	0	65
17502103	f	md	JP	2020	0	1	0	1	0	0	0	0	0	0	0	1	60

17502216	f	ph	GB	2020	0	0	0	0	0	0	0	0	0	0	0	0	60
17504316	m	hp	US	2020	0	0	0	0	0	0	0	0	0	0	0	0	58
17504746	m	md	US	2020	0	1	0	1	0	0	0	0	0	0	0	0	72
17504751	Not	hp	CA	2020	0	1	1	0	0	0	0	0	0	0	0	0	73
17504826	m	md	KR	2020	1	0	0	0	0	0	0	0	0	0	0	0	70.9
17504849	f	hp	US	2020	0	1	1	0	0	0	0	0	0	0	0	0	67
17504854	m	Not repo	JP	2020	1	0	0	0	0	0	0	0	0	0	0	0	57
17505044	m	hp	US	2020	0	1	0	0	1	0	0	0	0	0	0	0	53
17505504	f	md	US	2020	0	1	1	0	0	0	0	0	0	0	0	0	55
17505802	f	cn	JP	2020	0	1	1	0	0	0	0	0	0	0	0	0	82
17505858	m	Not repo	MY	2020	1	0	0	0	0	0	0	0	0	0	0	0	69.4
17505917	f	md	US	2020	0	1	0	0	0	1	0	0	0	0	0	0	65
17507410	m	md	CN	2020	0	1	0	0	0	1	0	0	0	0	0	0	57
17507517	m	Not repo	JP	2020	1	0	0	0	0	0	0	0	0	0	0	0	70
17507519	m	cn	US	2020	0	1	0	1	0	0	0	0	0	0	0	0	
17507971	m	hp	CZ	2020	0	0	0	0	0	0	0	0	0	0	0	0	
17507994	f	md	AT	2020	0	0	0	0	0	0	0	0	0	0	0	0	
17508610	f	cn	JP	2020	0	1	1	0	0	0	0	0	0	0	0	0	76
17508969	m	md	DE	2020	0	1	1	0	0	0	0	0	0	0	0	0	
17509117	m	hp	NL	2020	1	0	0	0	0	0	0	0	0	0	0	0	80
17509131	f	Not repo	AU	2020	1	0	0	0	0	0	0	0	0	0	0	0	
17509271	m	hp	US	2020	0	1	0	0	1	0	0	0	0	0	0	0	78
17509342	m	cn	US	2020	0	1	0	1	0	0	0	0	0	0	0	0	
17509377	f	cn	US	2020	0	1	0	1	0	0	0	0	0	0	0	0	
17509518	m	Not repo	AU	2020	1	0	0	0	0	0	0	0	0	0	0	0	
17509522	f	md	GB	2020	0	1	0	1	0	0	0	0	0	0	0	1	71
17509667	m	ph	US	2020	0	1	0	0	1	0	0	0	0	0	0	0	86
17509893	Not	ph	JP	2020	0	1	0	1	0	0	0	0	0	0	0	0	
17510008	Not	md	JP	2020	1	0	0	0	0	0	0	0	0	0	0	1	80
17510304	m	md	JP	2020	1	0	0	0	0	0	0	0	0	0	0	0	72
17510628	Not	hp	CN	2020	0	0	0	0	0	0	0	0	0	0	0	0	
17510633	m	md	NL	2020	0	1	0	0	0	1	0	0	0	0	0	0	70
17510660	m	Not repo	AE	2020	0	1	0	1	0	0	0	0	0	0	0	0	
17510680	f	md	JP	2020	1	0	0	0	0	0	0	0	0	0	0	0	81

17510684	m	Not repo	MY	2020	1	0	0	0	0	0	0	0	0	0	0	0	82.3
17511022	m	md	US	2020	0	0	0	0	0	0	0	0	0	0	0	0	78
17511036	m	Not repo	IT	2020	0	1	0	1	0	0	0	0	0	0	0	0	57.1
17511369	f	md	DE	2020	0	0	0	0	0	0	0	0	0	0	0	0	82
17512265	f	md	CZ	2020	0	0	0	0	0	0	0	0	0	0	0	0	81
17512332	m	md	FR	2020	0	1	1	0	0	0	0	0	0	0	0	1	
17512555	f	md	DE	2020	0	0	0	0	0	0	0	0	0	0	0	0	72
17514418	m	cn	CN	2020	0	0	0	0	0	0	0	0	0	0	0	0	
17514449	m	ph	US	2020	0	1	1	0	0	0	0	0	0	0	0	0	73
17514764	f	md	DE	2020	0	1	1	0	0	0	0	0	0	0	0	0	
17514770	m	hp	FR	2020	0	1	0	0	1	0	0	0	0	0	0	0	73
17514778	m	hp	CA	2020	0	1	0	0	0	1	0	0	0	0	0	0	
17514854	m	md	JP	2020	0	1	0	0	0	1	0	0	0	0	0	0	80
17515110	m	Not repo	TW	2020	1	0	0	0	0	0	0	0	0	0	0	0	89
17515334	m	hp	AT	2020	0	0	0	0	0	0	0	0	0	0	0	0	72
17515429	m	hp	AT	2020	0	0	0	0	0	0	0	0	0	0	0	0	72
17515915	m	cn	DE	2020	0	1	1	0	0	0	0	0	0	0	0	0	
17516247	m	cn	NP	2020	0	0	0	0	0	0	0	0	0	0	0	0	56
17516268	m	md	CN	2020	0	0	0	0	0	0	0	0	0	0	0	0	57
17516543	f	cn	CN	2020	1	0	0	0	0	0	0	0	0	0	0	0	72
17516544	f	md	VN	2020	1	0	0	0	0	0	0	0	0	0	0	0	57
17516709	f	cn	CN	2020	0	0	0	0	0	0	0	0	0	0	0	0	
17517174	f	cn	JP	2020	0	1	1	0	0	0	0	0	0	0	0	0	80
17517983	f	hp	GB	2020	0	0	0	0	0	0	0	0	0	0	0	0	67
17518179	Not	md	JP	2020	0	1	0	0	0	1	0	0	0	0	0	0	60
17518209	m	md	KR	2020	0	1	0	0	1	0	0	0	0	0	0	0	74
17518359	m	md	KR	2020	0	1	0	0	1	0	0	0	0	0	0	0	69
17518633	f	hp	US	2020	0	1	0	0	1	0	0	0	0	0	0	0	65
17518993	m	md	KR	2020	0	1	0	0	1	0	0	0	0	0	0	0	68
17519172	m	md	US	2020	0	1	1	0	0	0	0	0	0	0	0	0	59
17519182	m	md	US	2020	0	1	1	0	0	0	0	0	0	0	0	0	66
17519183	m	md	US	2020	0	1	1	0	0	0	0	0	0	0	0	0	64
17519240	m	md	US	2020	0	1	1	0	0	0	0	0	0	0	0	0	57
17519245	m	md	US	2020	0	1	1	0	0	0	0	0	0	0	0	0	58

17519371	f	md	FR	2020	0	0	0	0	0	0	0	0	0	0	0	0	61
17519606	Not	hp	TR	2020	0	0	0	0	0	0	0	0	0	0	0	0	
17519735	m	cn	JP	2020	0	0	0	0	0	0	0	0	0	0	0	0	67
17519806	m	Not repo	JP	2020	0	1	0	1	0	0	0	0	0	0	0	1	80
17520139	m	ph	FR	2020	0	0	0	0	0	0	0	0	0	0	0	0	73
17520243	m	md	PH	2020	0	1	1	0	0	0	0	0	0	0	0	0	58
17520282	m	md	TR	2020	0	1	1	0	0	0	0	0	0	0	0	0	70
17520338	Not	md	IN	2020	0	0	0	0	0	0	0	0	0	0	0	0	
17520500	f	hp	US	2020	0	0	0	0	0	0	0	0	0	0	0	0	
17520764	f	cn	IL	2020	0	1	1	0	0	0	0	0	0	0	0	0	60
17520943	m	md	JP	2020	0	1	0	0	1	0	0	0	0	0	0	0	39
17521181	m	md	JP	2020	0	0	0	0	0	0	0	0	0	0	0	0	41
17521477	Not	cn	IN	2020	0	0	0	0	0	0	0	0	0	0	0	0	31
17521812	f	md	CH	2020	0	0	0	0	0	0	0	0	0	0	0	0	52
17522475	f	md	KR	2020	0	1	0	0	1	0	0	0	0	0	0	0	49
17523047	m	md	KR	2020	0	1	0	0	1	0	0	0	0	0	0	0	79
17523225	m	md	KR	2020	0	1	0	0	1	0	0	0	0	0	0	0	60
17523254	f	md	GB	2020	1	0	0	0	0	0	0	0	0	0	0	0	77
17523444	m	md	KR	2020	0	1	0	0	1	0	0	0	0	0	0	0	77
17523711	m	md	DE	2020	0	1	0	0	0	1	0	0	0	0	0	0	70
17523941	f	md	KR	2020	0	1	0	0	1	0	0	0	0	0	0	0	43
17524074	m	md	KR	2020	0	1	0	0	1	0	0	0	0	0	0	0	61
17524078	f	md	FR	2020	0	0	0	0	0	0	0	0	0	0	0	0	74
17524234	m	md	KR	2020	0	1	0	0	1	0	0	0	0	0	0	0	65
17525602	f	md	DE	2020	0	1	0	0	0	1	0	0	0	0	0	0	79
17525924	f	ph	GB	2020	0	1	1	0	0	0	0	0	0	0	0	0	72
17525936	m	hp	GB	2020	0	1	1	0	0	0	0	0	0	0	0	0	69
17526031	f	cn	JP	2020	0	1	1	0	0	0	0	0	0	0	0	0	80
17526081	Not	md	PT	2020	0	1	1	0	0	0	0	0	0	0	0	0	
17526348	m	cn	FR	2020	0	1	1	0	0	0	0	0	0	0	0	0	
17526465	m	cn	FR	2020	0	1	1	0	0	0	0	0	0	0	0	0	66
17526783	f	cn	US	2020	0	0	0	0	0	0	0	0	0	0	0	0	76
17527025	m	md	KR	2020	0	1	0	0	1	0	0	0	0	0	0	0	71
17527412	f	md	KR	2020	0	1	0	0	1	0	0	0	0	0	0	0	58

17528027	f	Not repo	AU	2020	0	1	0	1	0	0	0	0	0	0	0	1	
17528280	m	hp	CN	2020	0	0	0	0	0	0	0	0	0	0	0	0	72
17528959	f	ph	JP	2020	1	0	0	0	0	0	0	0	0	0	0	0	74
17529070	f	cn	US	2020	0	0	0	0	0	0	0	0	0	0	0	0	
17529094	m	md	US	2020	0	0	0	0	0	0	0	0	0	0	0	0	54
17529095	f	cn	US	2020	0	0	0	0	0	0	0	0	0	0	0	0	
17529096	m	ph	US	2020	0	0	0	0	0	0	0	0	0	0	0	0	70
17529107	m	cn	US	2020	0	0	0	0	0	0	0	0	0	0	0	0	
17529110	f	cn	US	2020	0	0	0	0	0	0	0	0	0	0	0	0	
17529112	f	cn	US	2020	0	0	0	0	0	0	0	0	0	0	0	0	
17529694	f	hp	US	2020	0	1	0	0	1	0	0	0	0	0	0	0	62
17529915	f	md	DE	2020	0	1	0	0	1	0	0	0	0	0	0	0	75
17530263	f	md	LB	2020	1	0	0	0	0	0	0	0	0	0	0	0	
17530419	m	Not repo	US	2020	1	0	0	0	0	0	0	0	0	0	0	0	
17530666	f	hp	US	2020	0	1	0	1	0	0	0	0	0	0	0	0	
17531099	f	hp	JP	2020	1	0	0	0	0	0	0	0	0	0	0	1	78
17531359	f	md	VN	2020	1	0	0	0	0	0	0	0	0	0	0	0	55
17531361	Not	Not repo	AU	2020	1	0	0	0	0	0	0	0	0	0	0	0	
17531362	f	cn	CN	2020	1	0	0	0	0	0	0	0	0	0	0	0	71
17531793	m	Not repo	JP	2020	0	1	0	1	0	0	0	0	0	0	0	1	55.5
17532047	m	cn	KW	2020	1	0	0	0	0	0	0	0	0	0	0	0	
17532380	f	md	IT	2020	0	1	0	0	0	1	0	0	0	0	0	0	42
17533009	f	md	VN	2020	1	0	0	0	0	0	0	0	0	0	0	0	49
17533163	f	md	JP	2020	1	0	0	0	0	0	0	0	0	0	0	0	80
17533370	m	md	DK	2020	0	1	1	0	0	0	0	0	0	0	0	0	72
17534159	f	md	US	2020	0	0	0	0	0	0	0	0	0	0	0	0	
17534183	f	cn	CH	2020	1	0	0	0	0	0	0	0	0	0	0	0	
17534259	m	md	JP	2020	0	1	0	1	0	0	0	0	0	0	0	0	70
17534487	f	Not repo	CN	2020	1	0	0	0	0	0	0	0	0	0	0	0	55
17534570	m	md	IT	2020	0	1	0	0	1	0	0	0	0	0	0	0	70
17535419	f	hp	FR	2020	0	1	0	0	1	0	0	0	0	0	0	0	47
17536016	m	hp	US	2020	0	1	0	0	1	0	0	0	0	0	0	0	77
17536118	m	cn	GB	2020	0	1	1	0	0	0	0	0	0	0	0	0	
17536279	f	Not repo	CN	2020	1	0	0	0	0	0	0	0	0	0	0	0	55

17536351	m	cn	CA	2020	1	0	0	0	0	0	0	0	0	0	0	0	63
17536397	Not	cn	IT	2020	0	0	0	0	0	0	0	0	0	0	0	0	70
17536457	f	md	FR	2020	0	1	0	1	0	0	0	0	0	0	0	0	
17536514	m	cn	CA	2020	1	0	0	0	0	0	0	0	0	0	0	0	48.2
17536926	m	hp	BE	2020	0	1	1	0	0	0	0	0	0	0	0	0	60
17537778	m	ph	JP	2020	0	1	0	1	0	0	0	0	0	0	0	0	70
17538039	m	hp	RU	2020	0	1	0	1	0	0	0	0	0	0	0	0	
17538318	m	md	TR	2020	0	0	0	0	0	0	0	0	0	0	0	0	68
17538892	f	cn	CA	2020	0	1	0	1	0	0	0	0	0	0	0	0	
17539028	f	hp	NL	2020	0	1	1	0	0	0	0	0	0	0	0	0	
17539041	f	hp	US	2020	0	1	1	0	0	0	0	0	0	0	0	0	
17540176	m	ph	JP	2020	1	0	0	0	0	0	0	0	0	0	0	0	
17540272	f	cn	US	2020	1	0	0	0	0	0	0	0	0	0	0	0	58
17540287	f	md	JP	2020	1	0	0	0	0	0	0	0	0	0	0	0	90
17540288	f	cn	CN	2020	1	0	0	0	0	0	0	0	0	0	0	0	53.7
17540316	f	Not repo	CN	2020	1	0	0	0	0	0	0	0	0	0	0	0	61
17540445	f	Not repo	CN	2020	1	0	0	0	0	0	0	0	0	0	0	0	64
17540701	f	cn	JP	2020	0	1	1	0	0	0	0	0	0	0	0	0	85
17540713	f	Not repo	JP	2020	1	0	0	0	0	0	0	0	0	0	0	0	68.9
17540802	m	cn	US	2020	0	1	1	0	0	0	0	0	0	0	0	0	
17540837	m	md	CA	2020	0	0	0	0	0	0	0	0	0	0	0	0	37
17540898	f	cn	CA	2020	0	0	0	0	0	0	0	0	0	0	0	0	62
17540990	m	md	TR	2020	0	1	1	0	0	0	0	0	0	0	0	0	66
17541041	m	md	FR	2020	0	1	0	1	0	0	0	0	0	0	0	0	
17541188	m	cn	JP	2020	0	0	0	0	0	0	0	0	0	0	0	0	60
17541242	m	md	RU	2020	0	1	0	1	0	0	0	0	0	0	0	1	69
17541525	f	Not repo	JP	2020	1	0	0	0	0	0	0	0	0	0	0	1	76
17541741	m	cn	CA	2020	0	0	0	0	0	0	0	0	0	0	0	0	58
17542000	m	hp	JP	2020	0	1	1	0	0	0	0	0	0	0	0	1	80
17542211	f	cn	BA	2020	0	1	1	0	0	0	0	0	0	0	0	0	
17542499	m	md	DK	2020	0	1	1	0	0	0	0	0	0	0	0	0	70
17542929	m	cn	JP	2020	0	1	1	0	0	0	0	0	0	0	0	0	70
17543318	m	cn	KR	2020	1	0	0	0	0	0	0	0	0	0	0	0	53
17543606	m	md	US	2020	0	0	0	0	0	0	0	0	0	0	0	0	83

17543609	m	hp	SE	2020	0	0	0	0	0	0	0	0	0	0	0	0	57
17543740	m	cn	CA	2020	0	1	0	0	0	1	0	0	0	0	0	0	
17544409	m	md	ES	2020	0	1	1	0	0	0	0	0	0	0	0	0	69
17544536	f	md	NL	2020	1	0	0	0	0	0	0	0	0	0	0	0	56
17545146	f	md	NL	2020	1	0	0	0	0	0	0	0	0	0	0	0	57
17545649	Not	Not repo	JP	2020	1	0	0	0	0	0	0	0	0	0	0	1	
17545651	f	cn	CN	2020	1	0	0	0	0	0	0	0	0	0	0	0	65
17545793	m	md	DK	2020	0	1	1	0	0	0	0	0	0	0	0	0	72
17547352	m	md	ES	2020	0	1	0	0	1	0	0	0	0	0	0	0	65
17547369	m	cn	GR	2020	0	1	1	0	0	0	0	0	0	0	0	1	55
17547506	m	hp	US	2020	0	1	0	0	1	0	0	0	0	0	0	0	60
17547570	f	md	SE	2020	0	1	0	0	1	0	0	0	0	0	0	0	73
17547757	m	cn	JP	2020	0	1	1	0	0	0	0	0	0	0	0	0	82
17547766	f	md	JP	2020	0	1	1	0	0	0	0	0	0	0	0	0	71
17547816	m	hp	CN	2020	0	1	0	0	1	0	0	0	0	0	0	0	65
17547864	m	md	US	2020	1	0	0	0	0	0	0	0	0	0	0	0	70
17548468	f	Not repo	US	2020	1	0	0	0	0	0	0	0	0	0	0	0	49
17548860	m	cn	JP	2020	0	1	1	0	0	0	0	0	0	0	0	0	45
17548896	m	md	JP	2020	0	1	0	0	0	1	0	0	0	0	0	0	70
17549272	f	md	DE	2020	0	1	1	0	0	0	0	0	0	0	0	0	
17549810	Not	md	JP	2020	0	0	0	0	0	0	0	0	0	0	0	0	78
17550102	f	cn	US	2020	0	0	0	0	0	0	0	0	0	0	0	0	
17550541	m	cn	JP	2020	0	1	1	0	0	0	0	0	0	0	0	0	59
17550554	m	md	US	2020	0	0	0	0	0	0	0	0	0	0	0	0	
17550781	m	cn	US	2020	1	0	0	0	0	0	0	0	0	0	0	0	
17550890	m	md	JP	2020	0	1	0	1	0	0	0	0	0	0	0	1	68
17550891	m	md	FR	2020	0	1	0	1	0	0	0	0	0	0	0	1	
17550892	Not	Not repo	CN	2020	1	0	0	0	0	0	0	0	0	0	0	0	
17550936	f	cn	US	2020	0	1	0	0	1	0	0	0	0	0	0	0	
17551097	Not	md	US	2020	0	0	0	0	0	0	0	0	0	0	0	0	
17551105	Not	md	US	2020	0	0	0	0	0	0	0	0	0	0	0	0	
17551106	Not	md	US	2020	0	0	0	0	0	0	0	0	0	0	0	0	
17551110	Not	md	US	2020	0	0	0	0	0	0	0	0	0	0	0	0	
17551350	m	hp	JP	2020	0	1	1	0	0	0	0	0	0	0	0	1	56

17551680	m	cn	CN	2020	0	1	0	0	1	0	0	0	0	0	0	0	57
17552060	f	cn	RU	2020	1	0	0	0	0	0	0	0	0	0	0	0	52
17552559	f	hp	CN	2020	1	0	0	0	0	0	0	0	0	0	0	0	66
17552560	m	md	JP	2020	0	1	0	1	0	0	0	0	0	0	0	1	71
17553125	m	hp	JP	2020	0	1	1	0	0	0	0	0	0	0	0	0	68
17553173	f	Not repo	US	2020	0	0	0	0	0	0	0	0	0	0	0	0	53
17553720	Not	md	US	2020	0	0	0	0	0	0	0	0	0	0	0	0	
17554270	m	md	CN	2020	0	1	0	0	0	1	0	0	0	0	0	0	65
17554350	f	hp	AU	2020	0	1	0	1	0	0	0	0	0	0	0	1	78.2
17554793	f	Not repo	NZ	2020	1	0	0	0	0	0	0	0	0	0	0	0	49.6
17554984	Not	hp	CN	2020	1	0	0	0	0	0	0	0	0	0	0	0	
17555344	f	cn	CN	2020	1	0	0	0	0	0	0	0	0	0	0	0	58
17555612	Not	md	US	2020	0	0	0	0	0	0	0	0	0	0	0	0	
17555641	m	md	JP	2020	0	1	0	0	0	1	0	0	0	0	0	0	62
17555858	f	cn	US	2020	0	1	1	0	0	0	0	0	0	0	0	0	62
17555950	f	md	CN	2020	1	0	0	0	0	0	0	0	0	0	0	0	69
17556487	f	hp	JP	2020	0	0	0	0	0	0	0	0	0	0	0	0	53
17556646	m	md	BR	2020	0	1	1	0	0	0	0	0	0	0	0	0	58
17557037	m	cn	JP	2020	0	1	1	0	0	0	0	0	0	0	0	0	77
17557320	f	cn	CN	2020	1	0	0	0	0	0	0	0	0	0	0	0	79
17557322	f	hp	JP	2020	1	0	0	0	0	0	0	0	0	0	0	0	74
17557370	Not	Not repo	BR	2020	1	0	0	0	0	0	0	0	0	0	0	0	
17557579	m	ph	JP	2020	0	1	1	0	0	0	0	0	0	0	0	0	72
17557672	m	Not repo	JP	2020	0	0	0	0	0	0	0	0	0	0	0	0	62
17557923	m	Not repo	CN	2020	1	0	0	0	0	0	0	0	0	0	0	0	77
17558624	m	hp	JP	2020	0	1	1	0	0	0	0	0	0	0	0	0	75
17559127	f	md	JP	2020	0	1	0	0	0	1	0	0	0	0	0	0	77
17559348	f	hp	FR	2020	0	1	0	0	1	0	0	0	0	0	0	0	73
17559838	m	cn	JP	2020	0	0	0	0	0	0	0	0	0	0	0	0	66
17559852	f	hp	US	2020	0	1	0	0	1	0	0	0	0	0	0	0	75
17559872	m	Not repo	CN	2020	1	0	0	0	0	0	0	0	0	0	0	0	64
17559901	m	md	VN	2020	0	1	0	0	0	1	0	0	0	0	0	0	
17560226	m	md	JP	2020	0	0	0	0	0	0	0	0	0	0	0	0	
17560359	f	hp	FI	2020	0	0	0	0	0	0	0	0	0	0	0	0	41

17560427	m	md	ES	2020	0	0	0	0	0	0	0	0	0	0	0	0	39
17560610	m	ph	US	2020	0	0	0	0	0	0	0	0	0	0	0	0	74
17560789	f	cn	US	2020	0	1	1	0	0	0	0	0	0	0	0	0	
17560915	f	hp	CN	2020	0	0	0	0	0	0	0	0	0	0	0	0	62
17561021	m	cn	IN	2020	0	0	0	0	0	0	0	0	0	0	0	0	
17561350	m	hp	DE	2020	0	1	1	0	0	0	0	0	0	0	0	0	68
17561431	m	md	JP	2020	0	1	0	1	0	0	0	0	0	0	0	1	
17561597	m	md	JP	2020	0	1	0	1	0	0	0	0	0	0	0	1	60
17561790	f	md	ES	2020	1	0	0	0	0	0	0	0	0	0	0	0	55
17562510	f	Not repo	CN	2020	1	0	0	0	0	0	0	0	0	0	0	0	67
17562668	f	hp	NL	2020	0	0	0	0	0	0	0	0	0	0	0	0	60
17562957	Not	md	SE	2020	0	1	0	0	0	1	0	0	0	0	0	0	
17562984	m	md	CA	2020	0	0	0	0	0	0	0	0	0	0	0	0	64
17563963	m	md	RO	2020	0	1	0	0	0	1	0	0	0	0	0	0	
17565745	f	md	IT	2020	0	0	0	0	0	0	0	0	0	0	0	0	
17565839	f	md	AT	2020	0	0	0	0	0	0	0	0	0	0	0	0	
17565864	m	ph	US	2020	0	1	1	0	0	0	0	0	0	0	0	1	81
17566230	m	hp	Not re	2020	0	1	1	0	0	0	0	0	0	0	0	0	
17566463	m	md	FR	2020	0	1	0	1	0	0	0	0	0	0	0	0	
17566770	f	hp	Not re	2020	0	0	0	0	0	0	0	0	0	0	0	0	69
17566958	m	md	JP	2020	0	0	0	0	0	0	0	0	0	0	0	0	76
17566968	f	hp	GB	2020	0	1	1	0	0	0	0	0	0	0	0	0	65
17567163	f	md	TR	2020	0	1	1	0	0	0	0	0	0	0	0	0	58
17567653	Not	hp	AU	2020	0	0	0	0	0	0	0	0	0	0	0	0	
17567730	f	Not repo	JP	2020	1	0	0	0	0	0	0	0	0	0	0	0	84
17567852	m	md	FR	2020	0	1	0	1	0	0	0	0	0	0	0	0	62
17567868	m	md	PL	2020	0	0	0	0	0	0	0	0	0	0	0	0	63
17567881	Not	cn	TW	2020	0	1	1	0	0	0	0	0	0	0	0	0	66
17567911	f	cn	CN	2020	1	0	0	0	0	0	0	0	0	0	0	0	
17567956	Not	md	JP	2020	0	1	0	0	1	0	0	0	0	0	0	0	
17567967	Not	md	JP	2020	0	1	0	0	1	0	0	0	0	0	0	0	
17568066	m	hp	JP	2020	1	0	0	0	0	0	0	0	0	0	0	0	56
17568113	m	hp	JP	2020	1	0	0	0	0	0	0	0	0	0	0	0	69
17568122	m	md	JP	2020	0	1	0	1	0	0	0	0	0	0	0	1	69

17568126	m	cn	CN	2020	1	0	0	0	0	0	0	0	0	0	0	0	46
17568854	Not	cn	US	2020	0	1	1	0	0	0	0	0	0	0	0	0	
17569109	f	cn	US	2020	0	0	0	0	0	0	0	0	0	0	0	0	
17569357	m	md	JP	2020	0	1	0	0	1	0	0	0	0	0	0	0	55
17569360	m	cn	DE	2020	0	1	1	0	0	0	0	0	0	0	0	0	
17569710	m	hp	US	2020	0	1	0	0	1	0	0	0	0	0	0	0	
17569866	m	md	JP	2020	0	1	0	0	1	0	0	0	0	0	0	0	69
17570085	f	ph	FR	2020	0	1	1	0	0	0	0	0	0	0	0	0	68
17570089	f	cn	CN	2020	0	1	0	0	1	0	0	0	0	0	0	0	79
17570596	m	hp	SI	2020	0	0	0	0	0	0	0	0	0	0	0	0	46
17570778	f	md	BE	2020	0	1	0	0	1	0	0	0	0	0	0	0	69
17571160	f	hp	CN	2020	1	0	0	0	0	0	0	0	0	0	0	0	71
17571488	m	hp	AU	2020	0	1	0	0	0	1	0	0	0	0	0	0	66
17572089	m	hp	DK	2020	0	0	0	0	0	0	0	0	0	0	0	0	65
17572382	m	hp	IT	2020	0	1	1	0	0	0	0	0	0	0	0	1	41
17572494	m	md	JP	2020	0	1	0	0	1	0	0	0	0	0	0	1	68
17572573	f	ph	GB	2020	0	1	0	1	0	0	0	0	0	0	0	0	71
17572726	m	hp	CN	2020	1	0	0	0	0	0	0	0	0	0	0	0	62
17572728	m	hp	CN	2020	1	0	0	0	0	0	0	0	0	0	0	0	56
17573250	f	hp	US	2020	0	1	0	1	0	0	0	0	0	0	0	0	
17573588	f	hp	US	2020	0	0	0	0	0	0	0	0	0	0	0	0	74
17574779	m	cn	CN	2020	0	1	0	0	1	0	0	0	0	0	0	0	
17575203	m	hp	CA	2020	0	1	1	0	0	0	0	0	0	0	0	0	52
17575865	m	hp	CA	2020	0	0	0	0	0	0	0	0	0	0	0	0	70
17576111	Not	hp	US	2020	0	1	0	0	1	0	0	0	0	0	0	0	
17576386	f	md	ES	2020	0	0	0	0	0	0	0	0	0	0	0	0	52
17576785	m	md	KR	2020	0	1	1	0	0	0	0	0	0	0	0	0	74
17576923	f	md	US	2020	1	0	0	0	0	0	0	0	0	0	0	0	68
17577967	f	md	US	2020	0	0	0	0	0	0	0	0	0	0	0	0	58
17578179	f	cn	US	2020	0	0	0	0	0	0	0	0	0	0	0	0	
17578242	f	hp	US	2020	0	1	1	0	0	0	0	0	0	0	0	0	70
17578257	f	cn	JP	2020	0	1	1	0	0	0	0	0	0	0	0	0	84
17578258	m	hp	SE	2020	0	0	0	0	0	0	0	0	0	0	0	0	57
17578901	f	cn	IL	2020	0	0	0	0	0	0	0	0	0	0	0	0	53

17579450	f	md	NL	2020	0	1	1	0	0	0	0	0	0	0	0	0	74
17579540	m	Not repo	JP	2020	1	0	0	0	0	0	0	0	0	0	0	0	82
17579741	f	md	ES	2020	0	0	0	0	0	0	0	0	0	0	0	0	52
17579782	m	md	DE	2020	0	1	0	0	0	1	0	0	0	0	0	0	80
17579790	m	md	FR	2020	0	1	0	0	0	1	0	0	0	0	0	0	61
17580020	m	cn	DE	2020	0	1	1	0	0	0	0	0	0	0	0	0	
17580240	Not	hp	KR	2020	0	0	0	0	0	0	0	0	0	0	0	0	
17581127	f	md	ES	2020	0	0	0	0	0	0	0	0	0	0	0	0	64
17581192	m	cn	JP	2020	0	1	1	0	0	0	0	0	0	0	0	0	
17581213	m	hp	US	2020	0	0	0	0	0	0	0	0	0	0	0	0	61
17581896	f	Not repo	CN	2020	1	0	0	0	0	0	0	0	0	0	0	0	69
17581958	f	cn	CA	2020	0	1	0	1	0	0	0	0	0	0	0	0	80.9
17583051	m	Not repo	JP	2020	1	0	0	0	0	0	0	0	0	0	0	0	
17583489	f	md	KR	2020	0	1	0	0	0	1	0	0	0	0	0	0	65
17583664	m	hp	AU	2020	0	1	1	0	0	0	0	0	0	0	0	0	78
17584053	m	hp	JP	2020	0	0	0	0	0	0	0	0	0	0	0	0	71
17584570	m	hp	JP	2020	0	1	1	0	0	0	0	0	0	0	0	0	72
17585055	m	hp	CL	2020	0	0	0	0	0	0	0	0	0	0	0	0	66
17585243	m	hp	DE	2020	0	1	1	0	0	0	0	0	0	0	0	0	66
17585886	m	md	JP	2020	0	1	1	0	0	0	0	0	0	0	0	0	71
17585973	m	hp	CN	2020	1	0	0	0	0	0	0	0	0	0	0	0	68
17586733	f	md	JP	2020	0	1	0	1	0	0	0	0	0	0	0	1	71.7
17586769	m	cn	LB	2020	0	1	0	0	1	0	0	0	0	0	0	0	
17586770	f	ph	JP	2020	0	1	0	1	0	0	0	0	0	0	0	1	69
17587111	m	hp	FI	2020	0	1	1	0	0	0	0	0	0	0	0	0	64
17587321	m	hp	CN	2020	1	0	0	0	0	0	0	0	0	0	0	0	63
17587927	Not	cn	US	2020	0	1	1	0	0	0	0	0	0	0	0	0	
17588551	m	hp	CA	2020	0	0	0	0	0	0	0	0	0	0	0	0	
17588623	f	cn	MX	2020	0	0	0	0	0	0	0	0	0	0	0	0	
17590001	m	md	JP	2020	0	0	0	0	0	0	0	0	0	0	0	0	62
17591350	f	md	ES	2020	0	0	0	0	0	0	0	0	0	0	0	0	52
17591487	m	cn	US	2020	0	1	0	1	0	0	0	0	0	0	0	0	58.3
17591585	m	Not repo	JP	2020	0	1	0	1	0	0	0	0	0	0	0	1	70
17591592	m	hp	GB	2020	0	1	1	0	0	0	0	0	0	0	0	0	67

17591595	m	md	IL	2020	0	1	0	1	0	0	0	0	0	0	0	0	80
17592162	m	hp	AU	2020	0	1	0	0	0	1	0	0	0	0	0	0	66
17592989	m	md	AU	2020	0	1	1	0	0	0	0	0	0	0	0	0	49
17593051	f	cn	FR	2020	1	0	0	0	0	0	0	0	0	0	0	0	67
17593320	f	cn	US	2020	1	0	0	0	0	0	0	0	0	0	0	0	
17593428	f	cn	US	2020	1	0	0	0	0	0	0	0	0	0	0	0	
17594212	f	cn	FR	2020	1	0	0	0	0	0	0	0	0	0	0	0	67
17594277	f	md	AT	2020	0	0	0	0	0	0	0	0	0	0	0	0	
17594446	m	md	CL	2020	0	1	1	0	0	0	0	0	0	0	0	0	61
17594579	f	md	US	2020	1	0	0	0	0	0	0	0	0	0	0	1	
17596272	m	cn	CN	2020	0	1	0	0	1	0	0	0	0	0	0	0	58
17596525	m	hp	US	2020	0	1	0	0	1	0	0	0	0	0	0	0	65
17597292	m	hp	FR	2020	0	1	0	0	1	0	0	0	0	0	0	0	60
17597635	f	md	DE	2020	0	1	0	0	0	1	0	0	0	0	0	0	62
17597660	f	cn	Not re	2020	0	0	0	0	0	0	0	0	0	0	0	0	
17597947	m	hp	US	2020	0	0	0	0	0	0	0	0	0	0	0	0	59
17598856	f	md	VN	2020	1	0	0	0	0	0	0	0	0	0	0	0	
17598999	m	hp	US	2020	0	1	0	0	1	0	0	0	0	0	0	0	75
17599193	m	hp	JP	2020	0	0	0	0	0	0	0	0	0	0	0	0	61
17599871	m	cn	JP	2020	0	1	1	0	0	0	0	0	0	0	0	0	83
17600222	m	md	DE	2020	0	1	1	0	0	0	0	0	0	0	0	0	
17600234	m	Not repo	JP	2020	1	0	0	0	0	0	0	0	0	0	0	1	83
17600294	m	cn	AE	2020	1	0	0	0	0	0	0	0	0	0	0	0	56.1
17602995	m	md	JP	2020	0	1	0	0	0	1	0	0	0	0	0	0	60
17604009	f	hp	ES	2020	0	1	0	0	1	0	0	0	0	0	0	0	73
17604257	f	Not repo	TW	2020	1	0	0	0	0	0	0	0	0	0	0	0	56
17605146	f	cn	GB	2020	0	1	1	0	0	0	0	0	0	0	0	0	81
17605151	m	Not repo	AU	2020	1	0	0	0	0	0	0	0	0	0	0	0	
17606851	m	hp	JP	2020	0	1	1	0	0	0	0	0	0	0	0	0	84
17606857	m	hp	JP	2020	0	1	0	1	0	0	0	0	0	0	0	1	74
17607665	m	md	IE	2020	0	0	0	0	0	0	0	0	0	0	0	0	68
17607831	Not	cn	JP	2020	0	1	1	0	0	0	0	0	0	0	0	0	77
17608441	m	cn	IN	2020	0	1	0	0	1	0	0	0	0	0	0	0	
9323966	m	md	NL	2020	0	0	0	0	0	0	0	0	0	0	0	0	43

16979180	f	ph	US	2020	0	0	0	0	0	0	0	0	0	0	0	0	69
17017268	m	md	PL	2020	0	1	1	0	0	0	0	0	0	0	0	0	66
17151690	m	md	CN	2020	0	0	0	0	0	0	0	0	0	0	0	0	53
17179351	m	md	JP	2020	0	0	0	0	0	0	0	0	0	0	0	0	74
17222957	m	hp	DE	2020	0	0	0	0	0	0	0	0	0	0	0	0	60
17228598	m	md	US	2020	0	0	0	0	0	0	0	0	0	0	0	0	
17229204	m	md	JP	2020	0	1	0	0	0	1	0	0	0	0	0	0	68
17230137	m	md	JP	2020	0	1	1	0	0	0	0	0	0	0	0	1	77
17243502	f	cn	CA	2020	0	0	0	0	0	0	0	0	0	0	0	0	54
17248634	f	hp	BE	2020	0	0	0	0	0	0	0	0	0	0	0	0	68
17259288	f	hp	US	2020	0	1	1	0	0	0	0	0	0	0	0	0	
17260058	f	md	DE	2020	0	0	0	0	0	0	0	0	0	0	0	0	67
17269922	m	hp	CN	2020	0	1	0	0	1	0	0	0	0	0	0	0	65
17281830	f	Not repo	JP	2020	1	0	0	0	0	0	0	0	0	0	0	0	80
17302205	m	Not repo	ID	2020	1	0	0	0	0	0	0	0	0	0	0	0	
17306695	f	md	IN	2020	0	1	0	0	1	0	0	0	0	0	0	0	
17307149	f	md	JP	2020	0	1	0	0	1	0	0	0	0	0	0	0	54
17307169	m	md	JP	2020	0	1	0	0	1	0	0	0	0	0	0	0	72
17310252	f	md	JP	2020	0	1	0	0	1	0	0	0	0	0	0	0	76
17313310	m	md	JP	2020	0	1	0	0	1	0	0	0	0	0	0	0	68
17331695	f	md	AU	2020	0	0	0	0	0	0	0	0	0	0	0	0	64
17363080	m	md	MY	2020	0	1	1	0	0	0	0	0	0	0	0	0	61
17367739	m	Not repo	CA	2020	0	1	0	1	0	0	0	0	0	0	0	0	
17370789	m	cn	US	2020	0	1	0	0	1	0	0	0	0	0	0	0	
17414273	f	md	GB	2020	0	1	1	0	0	0	0	0	0	0	0	0	57
17425893	m	cn	EG	2020	0	1	1	0	0	0	0	0	0	0	0	0	57
17432627	f	md	UA	2020	0	1	1	0	0	0	0	0	0	0	0	0	58
17432733	f	md	DE	2020	0	1	1	0	0	0	0	0	0	0	0	0	64
17459014	f	md	JP	2020	0	1	1	0	0	0	0	0	0	0	0	0	82
17467021	m	md	JP	2020	0	1	0	0	1	0	0	0	0	0	0	0	78
17469596	m	md	US	2020	0	1	0	1	0	0	0	0	0	0	0	0	57
17470105	m	md	HU	2020	0	1	1	0	0	0	0	0	0	0	0	0	77
17470254	m	cn	JP	2020	0	1	1	0	0	0	0	0	0	0	0	1	71
17474462	m	md	TR	2020	0	1	1	0	0	0	0	0	0	0	0	0	60

17475492	f	cn	US	2020	1	0	0	0	0	0	0	0	0	0	0	0	59
17498264	m	hp	JP	2020	1	0	0	0	0	0	0	0	0	0	0	0	73
17503354	m	hp	CN	2020	0	1	0	0	1	0	0	0	0	0	0	0	60
17504052	Not	md	MX	2020	0	0	0	0	0	0	0	0	0	0	0	0	
17505929	m	hp	FR	2020	0	1	0	0	1	0	0	0	0	0	0	1	65
17507655	Not	md	JP	2020	0	1	0	0	1	0	0	0	0	0	0	0	
17516755	m	md	JP	2020	0	1	0	0	1	0	0	0	0	0	0	0	59
17520218	m	md	AR	2020	0	1	1	0	0	0	0	0	0	0	0	0	81
17521001	m	hp	US	2020	0	1	1	0	0	0	0	0	0	0	0	0	79
17528863	m	md	ES	2020	0	1	0	0	0	1	0	0	0	0	0	0	84
17529435	f	md	JP	2020	0	1	0	1	0	0	0	0	0	0	0	0	75.9
17536339	m	md	FR	2020	0	1	0	0	0	1	0	0	0	0	0	0	52
17536788	m	cn	CN	2020	0	1	0	0	1	0	0	0	0	0	0	0	
17540158	m	md	JP	2020	0	1	0	1	0	0	0	0	0	0	0	1	63.8
17541106	m	md	FR	2020	0	1	1	0	0	0	0	0	0	0	0	0	60
17541660	m	hp	IT	2020	0	1	0	0	1	0	0	0	0	0	0	0	67
17544070	f	md	TW	2020	0	0	0	0	0	0	0	0	0	0	0	0	
17546450	m	ph	BE	2020	0	1	1	0	0	0	0	0	0	0	0	0	73
17552413	m	Not repo	JP	2020	0	1	0	1	0	0	0	0	0	0	0	1	69.6
17565632	m	md	KR	2020	0	1	0	0	0	1	0	0	0	0	0	0	72
17567562	m	md	CL	2020	0	1	0	0	0	1	0	0	0	0	0	0	68
17568010	f	hp	PT	2020	0	0	0	0	0	0	0	0	0	0	0	0	60
17568572	f	cn	IL	2020	0	0	0	0	0	0	0	0	0	0	0	0	73
17571507	m	hp	CN	2020	0	1	1	0	0	0	0	0	0	0	0	0	65
17574059	m	hp	IT	2020	0	1	0	0	1	0	0	0	0	0	0	0	49
17575159	m	md	US	2020	0	0	0	0	0	0	0	0	0	0	0	0	75
17578055	Not	hp	US	2020	0	1	0	0	1	0	0	0	0	0	0	0	
17580654	m	Not repo	CN	2020	1	0	0	0	0	0	0	0	0	0	0	0	78.8
17582344	m	hp	JP	2020	0	0	0	0	0	0	0	0	0	0	0	0	80
17587949	f	md	RU	2020	0	0	0	0	0	0	0	0	0	0	0	0	60
17588188	f	hp	CN	2020	0	1	0	0	1	0	0	0	0	0	0	0	70
17593019	m	hp	IN	2020	0	1	1	0	0	0	0	0	0	0	0	0	55
17594570	m	hp	DE	2020	0	1	0	0	1	0	0	0	0	0	0	0	61
17597730	m	cn	CN	2020	0	0	0	0	0	0	0	0	0	0	0	0	

17598855	f	md	VN	2020	1	0	0	0	0	0	0	0	0	0	0	0	
17599903	f	ph	JP	2020	1	0	0	0	0	0	0	0	0	0	0	0	81
17600053	m	hp	VN	2020	0	1	1	0	0	0	0	0	0	0	0	1	62
17600237	f	md	JP	2020	1	0	0	0	0	0	0	0	0	0	0	0	76
17601989	f	hp	GR	2020	0	1	1	0	0	0	0	0	0	0	0	0	
17606927	f	hp	CN	2020	0	1	0	0	1	0	0	0	0	0	0	0	63
17608125	f	cn	CN	2020	0	1	0	0	1	0	0	0	0	0	0	0	64
17608728	m	cn	KR	2020	1	0	0	0	0	0	0	0	0	0	0	0	63
17608970	f	cn	AE	2020	1	0	0	0	0	0	0	0	0	0	0	0	30.1
17609056	f	cn	CN	2020	0	1	0	0	1	0	0	0	0	0	0	0	55
17609473	m	hp	JP	2020	0	1	0	0	1	0	0	0	0	0	0	0	71
17609621	m	md	PL	2020	0	0	0	0	0	0	0	0	0	0	0	0	
17610076	f	cn	AR	2020	0	0	0	0	0	0	0	0	0	0	0	0	69
17610588	m	hp	US	2020	0	0	0	0	0	0	0	0	0	0	0	0	69
17611345	m	cn	JP	2020	0	1	1	0	0	0	0	0	0	0	0	0	78
17611375	m	hp	TR	2020	0	0	0	0	0	0	0	0	0	0	0	0	56
17611638	m	Not repo	JP	2020	1	0	0	0	0	0	0	0	0	0	0	0	77
17612034	Not	hp	ES	2020	0	1	0	0	1	0	0	0	0	0	0	0	
17612439	m	md	JP	2020	0	0	0	0	0	0	0	0	0	0	0	0	69
17613165	m	cn	US	2020	0	1	1	0	0	0	0	0	0	0	0	0	77
17613199	m	cn	CN	2020	0	1	0	0	1	0	0	0	0	0	0	0	64
17613883	f	cn	IL	2020	0	0	0	0	0	0	0	0	0	0	0	0	
17614141	m	md	PL	2020	0	0	0	0	0	0	0	0	0	0	0	0	82
17614858	m	hp	US	2020	0	1	0	0	1	0	0	0	0	0	0	0	
17616139	m	md	AT	2020	0	1	1	0	0	0	0	0	0	0	0	0	73
17616224	m	md	JP	2020	0	1	0	1	0	0	0	0	0	0	0	0	67
17616301	m	cn	Not re	2020	0	0	0	0	0	0	0	0	0	0	0	0	
17616518	m	md	US	2020	0	1	0	1	0	0	0	0	0	0	0	1	60
17616520	m	hp	NL	2020	0	1	1	0	0	0	0	0	0	0	0	0	73
17616592	f	hp	US	2020	1	0	0	0	0	0	0	0	0	0	0	0	89
17617745	m	cn	CN	2020	0	1	0	0	1	0	0	0	0	0	0	0	67
17617954	m	cn	NO	2020	0	1	1	0	0	0	0	0	0	0	0	0	42
17618935	f	cn	US	2020	1	0	0	0	0	0	0	0	0	0	0	0	67.3
17619009	m	md	DE	2020	0	0	0	0	0	0	0	0	0	0	0	0	74

17619738	m	md	US	2020	0	1	1	0	0	0	0	0	0	0	0	0		
17619749	f	cn	PH	2020	0	0	0	0	0	0	0	0	0	0	0	0	75
17621133	m	md	FR	2020	0	1	0	0	0	1	0	0	0	0	0	1	68
17621157	f	hp	CN	2020	0	0	0	0	0	0	0	0	0	0	0	0	41
17621259	m	hp	US	2020	0	1	0	0	1	0	0	0	0	0	0	0	
17621261	m	hp	DE	2020	0	1	0	0	1	0	0	0	0	0	0	0	55
17621753	m	Not repo	JP	2020	0	1	0	1	0	0	0	0	0	0	0	1	67
17622139	f	md	JP	2020	1	0	0	0	0	0	0	0	0	0	0	0	80
17622165	f	cn	US	2020	1	0	0	0	0	0	0	0	0	0	0	0	
17622234	f	md	GB	2020	0	1	0	0	0	1	0	0	0	0	0	0	55
17622350	m	md	JP	2020	0	1	0	1	0	0	0	0	0	0	0	1	73
17622550	m	hp	JP	2020	0	1	1	0	0	0	0	0	0	0	0	0	67
17623305	m	hp	TR	2020	0	0	0	0	0	0	0	0	0	0	0	0	66
17623478	m	cn	JP	2020	0	1	1	0	0	0	0	0	0	0	0	0	69
17624280	m	md	JP	2020	0	1	0	1	0	0	0	0	0	0	0	0	69
17625400	Not	md	JP	2020	0	0	0	0	0	0	0	0	0	0	0	0	
17626547	m	cn	GB	2020	0	1	1	0	0	0	0	0	0	0	0	1	61
17627774	f	Not repo	US	2020	0	1	0	1	0	0	0	0	0	0	0	0	72.7
17628404	m	hp	AU	2020	0	1	0	0	0	1	0	0	0	0	0	0	66
17628715	f	Not repo	CA	2020	1	0	0	0	0	0	0	0	0	0	0	0	63.7
17630092	m	md	JP	2020	0	0	0	0	0	0	0	0	0	0	0	0	
17630134	f	md	JP	2020	0	0	0	0	0	0	0	0	0	0	0	0	67
17631249	m	Not repo	JP	2020	1	0	0	0	0	0	0	0	0	0	0	0	82
17631432	f	hp	DE	2020	0	0	0	0	0	0	0	0	0	0	0	0	67
17631582	f	hp	US	2020	1	0	0	0	0	0	0	0	0	0	0	0	89
17631734	f	hp	PH	2020	0	0	0	0	0	0	0	0	0	0	0	0	79
17631913	m	md	JP	2020	0	0	0	0	0	0	0	0	0	0	0	0	68
17632089	f	cn	CA	2020	0	0	0	0	0	0	0	0	0	0	0	0	35
17632332	m	md	UA	2020	0	1	0	1	0	0	0	0	0	0	0	0	66
17633265	m	hp	AR	2020	0	1	0	0	1	0	0	0	0	0	0	0	72
17633344	f	hp	US	2020	0	1	0	0	1	0	0	0	0	0	0	0	85
17633412	m	md	BE	2020	0	1	1	0	0	0	0	0	0	0	0	0	69
17634715	m	md	IT	2020	0	1	0	0	1	0	0	0	0	0	0	0	67
17634887	f	hp	ES	2020	0	1	0	0	1	0	0	0	0	0	0	0	66

17635103	m	md	SG	2020	1	0	0	0	0	0	0	0	0	0	0	0	55
17635188	f	ph	CA	2020	0	1	0	0	1	0	0	0	0	0	0	0	72
17635712	m	hp	NL	2020	0	1	0	0	1	0	0	0	0	0	0	0	62
17635903	f	hp	IT	2020	0	1	0	0	1	0	0	0	0	0	0	0	76
17635968	f	hp	US	2020	0	1	0	0	1	0	0	0	0	0	0	0	62
17636088	f	cn	JP	2020	1	0	0	0	0	0	0	0	0	0	0	0	55
17636150	m	hp	FR	2020	0	1	0	0	1	0	0	0	0	0	0	0	71
17636448	Not	md	JP	2020	0	1	0	0	0	1	0	0	0	0	0	0	70
17637584	m	cn	CN	2020	1	0	0	0	0	0	0	0	0	0	0	0	69
17637598	f	cn	US	2020	1	0	0	0	0	0	0	0	0	0	0	1	
17637938	f	cn	CN	2020	1	0	0	0	0	0	0	0	0	0	0	0	84
17638130	m	md	JP	2020	0	1	0	1	0	0	0	0	0	0	0	0	73
17638311	Not	md	US	2020	0	1	0	0	1	0	0	0	0	0	0	0	
17638381	Not	hp	KR	2020	0	0	0	0	0	0	0	0	0	0	0	0	
17638517	f	hp	US	2020	1	0	0	0	0	0	0	0	0	0	0	0	89
17638539	Not	md	US	2020	0	0	0	0	0	0	0	0	0	0	0	0	
17638542	f	md	US	2020	0	0	0	0	0	0	0	0	0	0	0	0	60
17639069	m	md	US	2020	0	1	0	1	0	0	0	0	0	0	0	0	
17639158	m	md	JP	2020	0	0	0	0	0	0	0	0	0	0	0	0	59
17639189	f	md	US	2020	0	1	1	0	0	0	0	0	0	0	0	1	59
17639208	f	Not repo	CN	2020	1	0	0	0	0	0	0	0	0	0	0	0	58
17639272	m	Not repo	CN	2020	1	0	0	0	0	0	0	0	0	0	0	0	68
17639616	f	md	US	2020	0	1	0	1	0	0	0	0	0	0	0	0	55
17639905	f	cn	US	2020	1	0	0	0	0	0	0	0	0	0	0	0	
17640115	m	hp	CA	2020	0	0	0	0	0	0	0	0	0	0	0	0	
17640823	m	hp	NL	2020	0	1	1	0	0	0	0	0	0	0	0	0	
17640866	m	md	IT	2020	0	0	0	0	0	0	0	0	0	0	0	0	
17640993	m	md	JP	2020	0	1	1	0	0	0	0	0	0	0	0	0	67
17641326	f	cn	US	2020	0	1	1	0	0	0	0	0	0	0	0	0	86
17641374	m	md	CA	2020	0	1	1	0	0	0	0	0	0	0	0	0	63
17641502	f	cn	US	2020	0	1	1	0	0	0	0	0	0	0	0	0	
17641528	f	md	DE	2020	0	1	1	0	0	0	0	0	0	0	0	0	54
17641666	m	cn	JP	2020	0	1	1	0	0	0	0	0	0	0	0	0	61
17641756	m	md	AU	2020	0	0	0	0	0	0	1	0	0	0	1	0	

17642056	m	md	AT	2020	0	1	1	0	0	0	0	0	0	0	0	0	73
17642206	m	cn	CN	2020	1	0	0	0	0	0	0	0	0	0	0	0	73
17642557	f	md	LT	2020	0	1	0	0	0	1	0	0	0	0	0	0	57
17643997	Not	md	CN	2020	0	0	0	0	0	0	0	0	0	0	0	0	
17645158	m	hp	JP	2020	0	0	0	0	0	0	0	0	0	0	0	0	
17645238	m	Not repo	CN	2020	1	0	0	0	0	0	0	0	0	0	0	0	75
17645249	f	Not repo	CN	2020	1	0	0	0	0	0	0	0	0	0	0	0	55
17645731	f	cn	CN	2020	1	0	0	0	0	0	0	0	0	0	0	0	44
17645799	m	cn	CN	2020	1	0	0	0	0	0	0	0	0	0	0	0	54
17645860	m	cn	JP	2020	0	1	1	0	0	0	0	0	0	0	0	0	52
17646274	m	md	DE	2020	0	1	1	0	0	0	0	0	0	0	0	0	85
17646366	f	Not repo	CN	2020	1	0	0	0	0	0	0	0	0	0	0	0	63
17646963	m	cn	US	2020	1	0	0	0	0	0	0	0	0	0	0	0	
17647726	f	Not repo	US	2020	1	0	0	0	0	0	0	0	0	0	0	0	59
17647838	Not	cn	JP	2020	0	1	1	0	0	0	0	0	0	0	0	0	
17648023	f	cn	CN	2020	1	0	0	0	0	0	0	0	0	0	0	0	68
17648752	m	cn	US	2020	0	1	0	0	1	0	0	0	0	0	0	0	71
17648956	m	Not repo	CN	2020	1	0	0	0	0	0	0	0	0	0	0	0	
17649058	m	hp	JP	2020	0	0	0	0	0	0	0	0	0	0	0	0	
17649484	m	hp	US	2020	0	1	1	0	0	0	0	0	0	0	0	0	77
17649598	f	cn	CN	2020	1	0	0	0	0	0	0	0	0	0	0	0	70
17649609	f	md	CN	2020	0	0	0	0	0	0	0	0	0	0	0	0	32
17650348	m	md	US	2020	0	0	0	0	0	0	0	0	0	0	0	0	
17650486	m	md	JP	2020	0	1	0	0	1	0	0	0	0	0	0	0	62
17651385	f	cn	RU	2020	1	0	0	0	0	0	0	0	0	0	0	0	60
17651407	m	hp	US	2020	0	0	0	0	0	0	0	0	0	0	0	0	66
17651476	m	hp	US	2020	0	0	0	0	0	0	0	0	0	0	0	0	66
17651649	m	hp	JP	2020	0	0	0	0	0	0	0	0	0	0	0	0	
17651659	f	cn	US	2020	1	0	0	0	0	0	0	0	0	0	0	0	
17651747	m	cn	US	2020	1	0	0	0	0	0	0	0	0	0	0	0	
17651762	f	Not repo	AU	2020	1	0	0	0	0	0	0	0	0	0	0	0	57
17651837	f	md	DE	2020	0	0	0	0	0	0	0	0	0	0	0	0	67
17652116	m	md	US	2020	0	1	0	0	1	0	0	0	0	0	0	0	71
17652701	m	Not repo	MY	2020	1	0	0	0	0	0	0	0	0	0	0	0	67.9

17652789	f	hp	GB	2020	0	0	0	0	0	0	0	0	0	0	0	0	65
17652795	m	hp	GB	2020	0	0	0	0	0	0	0	0	0	0	0	0	66
17653095	m	hp	BE	2020	0	0	0	0	0	0	0	0	0	0	0	0	69
17653749	f	md	GB	2020	0	1	0	0	0	1	0	0	0	0	0	1	70
17654177	m	hp	IT	2020	1	0	0	0	0	0	0	0	0	0	0	0	64
17654205	f	md	US	2020	0	1	0	0	0	1	0	0	0	0	0	0	84
17654541	m	md	CA	2020	0	1	1	0	0	0	0	0	0	0	0	0	68
17655424	m	Not repo	CN	2020	1	0	0	0	0	0	0	0	0	0	0	0	48
17655777	f	hp	JP	2020	0	0	0	0	0	0	0	0	0	0	0	0	77
17655919	f	Not repo	ID	2020	1	0	0	0	0	0	0	0	0	0	0	0	72
17656722	m	hp	JP	2020	0	1	0	1	0	0	0	0	0	0	0	0	72
17657305	m	md	JP	2020	0	1	0	0	0	1	0	0	0	0	0	0	59
17657967	m	cn	CN	2020	0	0	0	0	0	0	0	0	0	0	0	0	66
17658095	m	hp	JP	2020	0	0	0	0	0	0	0	0	0	0	0	0	61
17658849	m	cn	JP	2020	0	1	1	0	0	0	0	0	0	0	0	0	84
17658894	m	hp	DK	2020	1	0	0	0	0	0	0	0	0	0	0	0	72
17659225	f	hp	NL	2020	0	1	0	0	1	0	0	0	0	0	0	0	51
17659630	m	hp	IT	2020	1	0	0	0	0	0	0	0	0	0	0	0	70
17660601	f	md	US	2020	0	0	0	0	0	0	0	0	0	0	0	0	63
17660936	m	md	KR	2020	0	0	0	0	0	0	0	0	0	0	0	0	72
17661724	m	md	ES	2020	0	0	0	0	0	0	0	0	0	0	0	0	
17661741	m	md	JP	2020	0	0	0	0	0	0	0	0	0	0	0	0	68
17661907	f	ph	US	2020	0	0	0	0	0	0	0	0	0	0	0	0	81
17662131	f	cn	US	2020	1	0	0	0	0	0	0	0	0	0	0	0	
17663048	m	ph	JP	2020	0	1	0	0	0	1	0	0	0	0	0	0	70
17663138	f	hp	US	2020	0	1	0	0	1	0	0	0	0	0	0	0	73
17664429	m	md	US	2020	0	0	0	0	0	0	0	0	0	0	0	0	67
17664598	f	md	RU	2020	0	1	0	0	0	1	0	0	0	0	0	0	42
17665088	f	md	MY	2020	0	0	0	0	0	0	0	0	0	0	0	0	74
17665248	f	Not repo	FR	2020	0	1	0	1	0	0	0	0	0	0	0	0	60
17665604	f	hp	TR	2020	1	0	0	0	0	0	0	0	0	0	0	0	48
17665732	m	cn	CN	2020	1	0	0	0	0	0	0	0	0	0	0	0	69
17666109	f	Not repo	CN	2020	1	0	0	0	0	0	0	0	0	0	0	0	63
17666349	m	Not repo	AU	2020	0	1	0	1	0	0	0	0	0	0	0	1	76.9

17666457	m	cn	JP	2020	0	0	0	0	0	0	0	0	0	0	0	0	64
17666926	f	md	DE	2020	1	0	0	0	0	0	0	0	0	0	0	0	82.9
17667455	f	hp	US	2020	0	0	0	0	0	0	0	0	0	0	0	0	70
17667611	m	cn	US	2020	0	1	1	0	0	0	0	0	0	0	0	0	73
17668167	m	hp	US	2020	0	0	0	0	0	0	0	0	0	0	0	0	72
17668681	m	md	ES	2020	0	1	0	0	0	1	0	0	0	0	0	0	66
17668755	f	hp	US	2020	0	1	1	0	0	0	0	0	0	0	0	0	55
17668847	m	md	KR	2020	0	1	0	0	0	1	0	0	0	0	0	0	74
17669196	Not	cn	JP	2020	0	1	1	0	0	0	0	0	0	0	0	0	62
17669672	f	cn	US	2020	0	1	1	0	0	0	0	0	0	0	0	0	59
17669934	m	hp	BE	2020	0	0	0	0	0	0	0	0	0	0	0	0	79
17670943	f	Not repo	US	2020	1	0	0	0	0	0	0	0	0	0	0	0	
17671068	f	md	DE	2020	1	0	0	0	0	0	0	0	0	0	0	0	73
17671373	m	md	RU	2020	0	1	0	1	0	0	0	0	0	0	0	0	65
17672679	f	cn	CN	2020	1	0	0	0	0	0	0	0	0	0	0	0	62.8
17673957	m	md	JP	2020	0	1	0	0	0	1	0	0	0	0	0	0	47
17674499	m	hp	FR	2020	0	1	0	0	1	0	0	0	0	0	0	0	67
17674742	m	md	FR	2020	0	1	0	0	0	1	0	0	0	0	0	0	69
17674858	m	md	CN	2020	0	1	0	0	0	1	0	0	0	0	0	0	68
17675010	f	cn	IN	2020	0	1	0	0	1	0	0	0	0	0	0	0	68
17675045	f	md	SG	2020	0	0	0	0	0	0	0	0	0	0	0	0	
17675260	f	md	JP	2020	1	0	0	0	0	0	0	0	0	0	0	0	70
17675922	m	md	US	2020	1	0	0	0	0	0	0	0	0	0	0	0	80
17675935	f	Not repo	MY	2020	1	0	0	0	0	0	0	0	0	0	0	0	62.6
17676548	m	hp	US	2020	0	0	0	0	0	0	0	0	0	0	0	0	59
17677106	f	md	FR	2020	0	1	0	0	1	0	0	0	0	0	0	0	71
17677703	m	cn	US	2020	0	1	1	0	0	0	0	0	0	0	0	0	
17678372	f	ph	US	2020	0	0	0	0	0	0	0	0	0	0	0	0	47
17678444	m	md	IT	2020	0	0	0	0	0	0	0	0	0	0	0	0	
17678510	m	md	JP	2020	0	0	0	0	0	0	0	0	0	0	0	0	
17678861	f	cn	HU	2020	1	0	0	0	0	0	0	0	0	0	0	0	
17679073	m	md	JP	2020	0	1	0	0	0	1	0	0	0	0	0	1	58
17679082	m	cn	CA	2020	0	0	0	0	0	0	0	0	0	0	0	0	84
17679564	Not	md	JP	2020	0	1	0	1	0	0	0	0	0	0	0	0	

17679992	m	md	UA	2020	0	1	1	0	0	0	0	0	0	0	0	0	69
17680015	f	hp	JP	2020	0	1	1	0	0	0	0	0	0	0	0	0	76
17680058	m	hp	DK	2020	0	1	1	0	0	0	0	0	0	0	0	0	58
17680060	m	hp	CA	2020	0	0	0	0	0	0	0	0	0	0	0	0	66
17680109	m	md	JP	2020	0	1	0	0	0	1	0	0	0	0	0	1	73
17680195	m	cn	CN	2020	1	0	0	0	0	0	0	0	0	0	0	0	
17680517	m	cn	JP	2020	0	1	1	0	0	0	0	0	0	0	0	0	82
17680631	m	ph	IT	2020	0	0	0	0	0	0	0	0	0	0	0	0	78
17681103	m	md	AT	2020	0	1	1	0	0	0	0	0	0	0	0	0	73
17681129	m	Not repo	ID	2020	1	0	0	0	0	0	0	0	0	0	0	0	
17681136	m	Not repo	ID	2020	1	0	0	0	0	0	0	0	0	0	0	0	
17681408	m	hp	CN	2020	0	1	0	0	1	0	0	0	0	0	0	0	48
17681415	f	hp	US	2020	0	1	0	0	1	0	0	0	0	0	0	0	64
17681841	Not	md	JP	2020	0	1	0	0	0	1	0	0	0	0	0	0	
17681843	Not	md	JP	2020	0	1	0	0	0	1	0	0	0	0	0	0	
17681849	Not	md	JP	2020	0	1	0	0	0	1	0	0	0	0	0	0	
17681859	f	hp	IT	2020	0	1	0	0	1	0	0	0	0	0	0	0	
17681934	m	md	JP	2020	0	1	0	0	0	1	0	0	0	0	0	1	69
17682913	m	hp	GR	2020	0	1	0	0	1	0	0	0	0	0	0	0	71
17682958	f	ph	JP	2020	0	1	0	0	0	1	0	0	0	0	0	0	69
17683607	m	md	JP	2020	0	1	0	0	0	1	0	0	0	0	0	1	68
17683683	m	cn	CN	2020	0	1	0	0	1	0	0	0	0	0	0	0	63
17683784	f	md	CN	2020	0	0	0	0	0	0	0	0	0	0	0	0	48
17684003	f	cn	US	2020	1	0	0	0	0	0	0	0	0	0	0	0	
17684070	f	md	BE	2020	0	0	0	0	0	0	1	1	0	0	0	0	39
17684145	f	md	FR	2020	0	1	0	0	0	1	0	0	0	0	0	0	49
17684340	f	Not repo	ID	2020	1	0	0	0	0	0	0	0	0	0	0	0	67.6
17684835	m	md	US	2020	0	0	0	0	0	0	0	0	0	0	0	0	61
17686501	m	cn	JP	2020	0	1	1	0	0	0	0	0	0	0	0	0	67
17686510	m	cn	CN	2020	0	1	0	0	1	0	0	0	0	0	0	1	
17686544	m	cn	JP	2020	0	1	1	0	0	0	0	0	0	0	0	0	60
17687403	m	md	JP	2020	0	1	0	1	0	0	0	0	0	0	0	0	70
17687466	Not	hp	RU	2020	0	1	0	1	0	0	0	0	0	0	0	0	
17687544	f	md	US	2020	1	0	0	0	0	0	0	0	0	0	0	0	72

17687940	m	hp	FR	2020	0	1	0	0	1	0	0	0	0	0	0	0	76
17687941	m	hp	US	2020	0	1	0	0	1	0	0	0	0	0	0	0	75
17688084	Not	hp	FR	2020	0	0	0	0	0	0	0	0	0	0	0	0	
17688140	Not	hp	FR	2020	0	0	0	0	0	0	0	0	0	0	0	0	
17688181	f	md	JP	2020	0	0	0	0	0	0	0	0	0	0	0	0	60
17688360	m	hp	JP	2020	0	0	0	0	0	0	0	0	0	0	0	0	64
17688383	f	hp	US	2020	0	1	0	0	1	0	0	0	0	0	0	0	64
17688399	m	cn	US	2020	0	1	1	0	0	0	0	0	0	0	0	0	
17688519	m	Not repo	JP	2020	1	0	0	0	0	0	0	0	0	0	0	0	69
17688521	Not	md	JP	2020	0	1	0	1	0	0	0	0	0	0	0	0	
17688864	Not	cn	US	2020	0	0	0	0	0	0	0	0	0	0	0	0	
17688887	m	hp	CA	2020	0	1	1	0	0	0	0	0	0	0	0	0	66
17689124	f	md	US	2020	0	1	0	0	0	1	0	0	0	0	0	0	58
17689284	m	cn	DE	2020	0	1	1	0	0	0	0	0	0	0	0	0	
17689874	Not	md	US	2020	0	0	0	0	0	0	0	0	0	0	0	0	
17689976	m	md	TR	2020	0	1	1	0	0	0	0	0	0	0	0	0	61
17690285	m	md	KR	2020	0	1	0	0	1	0	0	0	0	0	0	0	68
17690320	f	hp	BR	2020	0	1	1	0	0	0	0	0	0	0	0	0	61
17690393	m	md	JP	2020	0	1	0	0	0	1	0	0	0	0	0	0	59
17690414	f	cn	CN	2020	0	1	0	0	1	0	0	0	0	0	0	0	
17690531	m	hp	FR	2020	0	1	1	0	0	0	0	0	0	0	0	0	77
17691094	f	md	BE	2020	0	0	0	0	0	0	1	1	0	0	0	0	38
17691130	m	hp	US	2020	0	1	0	0	1	0	0	0	0	0	0	0	
17691405	f	cn	BR	2020	1	0	0	0	0	0	0	0	0	0	0	0	
17691754	m	md	US	2020	0	0	0	0	0	0	0	0	0	0	0	0	65
17692483	m	ph	US	2020	0	1	0	1	0	0	0	0	0	0	0	0	83
17693175	m	Not repo	JP	2020	0	1	0	1	0	0	0	0	0	0	0	1	69
17693726	f	md	NL	2020	0	0	0	0	0	0	0	0	0	0	0	0	
17693884	Not	Not repo	RU	2020	0	1	0	1	0	0	0	0	0	0	0	0	
17694107	f	md	US	2020	1	0	0	0	0	0	0	0	0	0	0	0	85
17694116	m	Not repo	ID	2020	1	0	0	0	0	0	0	0	0	0	0	0	71.6
17694443	f	Not repo	US	2020	0	1	0	1	0	0	0	0	0	0	0	1	63
17694601	f	cn	KR	2020	0	0	0	0	0	0	0	0	0	0	0	0	60
17695343	m	md	US	2020	1	0	0	0	0	0	0	0	0	0	0	0	90

17696315	m	cn	CN	2020	0	1	0	0	1	0	0	0	0	0	0	0	65
17696572	m	ph	JP	2020	0	1	1	0	0	0	0	0	0	0	0	0	66
17696887	f	cn	CN	2020	0	1	0	0	1	0	0	0	0	0	0	0	64
17696955	m	md	JP	2020	0	1	1	0	0	0	0	0	0	0	0	1	70
17696967	m	md	FR	2020	0	1	0	0	0	1	0	0	0	0	0	0	70
17697041	m	md	US	2020	0	1	1	0	0	0	0	0	0	0	0	0	58
17697163	Not	cn	US	2020	0	1	0	0	0	1	0	0	0	0	0	0	70
17697247	f	cn	KR	2020	1	0	0	0	0	0	0	0	0	0	0	0	46
17697427	Not	hp	NL	2020	0	0	0	0	0	0	0	0	0	0	0	0	
17697439	Not	hp	NL	2020	0	0	0	0	0	0	0	0	0	0	0	0	
17698380	m	cn	JP	2020	0	1	1	0	0	0	0	0	0	0	0	0	
17698758	m	md	PL	2020	0	0	0	0	0	0	0	0	0	0	0	0	66
17699052	f	Not repo	CN	2020	1	0	0	0	0	0	0	0	0	0	0	0	71.7
17699680	m	hp	US	2020	0	0	0	0	0	0	0	0	0	0	0	0	59
17699705	Not	md	KW	2020	0	0	0	0	0	0	0	0	0	0	0	0	
17700383	f	cn	CN	2020	1	0	0	0	0	0	0	0	0	0	0	0	
17700773	m	md	DE	2020	0	1	1	0	0	0	0	0	0	0	0	0	67
17702495	m	md	JP	2020	0	1	0	0	1	0	0	0	0	0	0	0	71
17703137	f	Not repo	AU	2020	1	0	0	0	0	0	0	0	0	0	0	0	74.2
17705313	m	md	JP	2020	0	1	0	1	0	0	0	0	0	0	0	0	56
17705326	f	cn	JP	2020	1	0	0	0	0	0	0	0	0	0	0	0	
17705921	f	Not repo	CN	2020	1	0	0	0	0	0	0	0	0	0	0	0	85
17705935	m	md	BG	2020	0	0	0	0	0	0	0	0	0	0	0	0	40
17706042	Not	hp	RU	2020	0	1	0	1	0	0	0	0	0	0	0	0	
17706202	m	hp	DE	2020	0	0	0	0	0	0	0	0	0	0	0	0	48
17706825	f	cn	CN	2020	0	1	0	0	1	0	0	0	0	0	0	0	75
17707001	Not	md	JP	2020	0	1	0	0	1	0	0	0	0	0	0	0	84
17707051	f	cn	CN	2020	0	1	0	0	1	0	0	0	0	0	0	1	51
17707107	m	md	JP	2020	0	1	0	0	1	0	0	0	0	0	0	0	90
17707693	m	md	US	2020	1	0	0	0	0	0	0	0	0	0	0	0	71
17707976	m	md	JP	2020	0	1	0	0	0	1	0	0	0	0	0	0	91
17708000	Not	hp	JP	2020	0	0	0	0	0	0	0	0	0	0	0	0	
17708108	m	cn	JP	2020	0	1	1	0	0	0	0	0	0	0	0	0	72
17709788	f	hp	US	2020	0	1	0	0	1	0	0	0	0	0	0	0	87

17710065	m	cn	IL	2020	0	0	0	0	0	0	0	0	0	0	0	0	47
17710330	m	cn	CA	2020	0	0	0	0	0	0	0	0	0	0	0	0	53
17710604	m	md	JP	2020	0	0	0	0	0	0	0	0	0	0	0	0	70
17711228	f	md	ES	2020	0	0	0	0	0	0	0	0	0	0	0	0	61
17711421	m	Not repo	MY	2020	1	0	0	0	0	0	0	0	0	0	0	0	68.2
17711879	Not	Not repo	JP	2020	1	0	0	0	0	0	0	0	0	0	0	0	
17713691	f	md	US	2020	0	1	0	1	0	0	0	0	0	0	0	0	
17714043	m	cn	SG	2020	0	1	1	0	0	0	0	0	0	0	0	0	
17714373	m	cn	JP	2020	0	1	1	0	0	0	0	0	0	0	0	0	81
17714595	f	cn	US	2020	0	1	1	0	0	0	0	0	0	0	0	0	84
17714781	m	md	JP	2020	0	1	0	1	0	0	0	0	0	0	0	1	78
17715054	f	cn	US	2020	0	1	0	1	0	0	0	0	0	0	0	0	
17715056	f	cn	US	2020	0	1	0	1	0	0	0	0	0	0	0	0	
17715600	m	hp	KR	2020	0	1	1	0	0	0	0	0	0	0	0	0	45
17715714	f	md	LB	2020	1	0	0	0	0	0	0	0	0	0	0	0	
17715979	f	hp	NL	2020	0	1	0	0	1	0	0	0	0	0	0	0	
17715992	m	hp	CN	2020	0	1	0	0	1	0	0	0	0	0	0	0	57
17716548	m	Not repo	US	2020	1	0	0	0	0	0	0	0	0	0	0	0	
17716671	Not	hp	JP	2020	1	0	0	0	0	0	0	0	0	0	0	0	
17716938	f	md	TW	2020	0	1	0	0	0	1	0	0	0	0	0	0	53
17717511	f	hp	DE	2020	0	0	0	0	0	0	0	0	0	0	0	0	63
17717711	m	Not repo	CN	2020	1	0	0	0	0	0	0	0	0	0	0	0	61
17717740	m	Not repo	JP	2020	1	0	0	0	0	0	0	0	0	0	0	0	51.2
17718084	f	md	US	2020	0	1	0	0	0	1	0	0	0	0	0	0	71
17718378	m	hp	DE	2020	0	1	0	0	0	1	0	0	0	0	0	0	54
17718398	f	md	US	2020	1	0	0	0	0	0	0	0	0	0	0	0	
17718441	m	md	IL	2020	0	1	1	0	0	0	0	0	0	0	0	0	66
17718548	m	md	IT	2020	0	1	1	0	0	0	0	0	0	0	0	0	70
17718841	m	cn	AR	2020	0	0	0	0	0	0	0	0	0	0	0	0	75
17719712	m	hp	DE	2020	0	0	0	0	0	0	0	0	0	0	0	0	72
17719752	m	cn	CA	2020	0	0	0	0	0	0	0	0	0	0	0	0	
17720111	f	ph	US	2020	0	0	0	0	0	0	0	0	0	0	0	0	47
17720324	m	cn	KR	2020	0	1	1	0	0	0	0	0	0	0	0	0	66
17720411	f	hp	US	2020	0	0	0	0	0	0	0	0	0	0	0	0	63

17720902	f	md	US	2020	0	0	0	0	0	0	0	0	0	0	0	0	52
17721061	f	md	US	2020	0	0	0	0	0	0	0	0	0	0	0	0	63
17721203	m	hp	JP	2020	1	0	0	0	0	0	0	0	0	0	0	0	69
17721382	f	hp	JP	2020	1	0	0	0	0	0	0	0	0	0	0	0	71
17721383	m	hp	JP	2020	1	0	0	0	0	0	0	0	0	0	0	0	79
17721395	f	hp	JP	2020	1	0	0	0	0	0	0	0	0	0	0	0	75
17721398	m	hp	JP	2020	1	0	0	0	0	0	0	0	0	0	0	1	81
17721399	f	hp	JP	2020	1	0	0	0	0	0	0	0	0	0	0	0	84
17721400	f	hp	JP	2020	1	0	0	0	0	0	0	0	0	0	0	0	59
17721401	f	hp	JP	2020	1	0	0	0	0	0	0	0	0	0	0	0	69
17721402	m	hp	JP	2020	1	0	0	0	0	0	0	0	0	0	0	0	56
17721403	f	hp	JP	2020	1	0	0	0	0	0	0	0	0	0	0	0	62
17721404	m	hp	JP	2020	1	0	0	0	0	0	0	0	0	0	0	0	63
17721405	f	hp	JP	2020	1	0	0	0	0	0	0	0	0	0	0	0	49
17721496	f	cn	SG	2020	0	0	0	0	0	0	0	0	0	0	0	0	74
17721518	f	hp	JP	2020	0	1	1	0	0	0	0	0	0	0	0	0	70
17721804	m	hp	AU	2020	0	1	1	0	0	0	0	0	0	0	0	0	61
17721925	m	cn	JP	2020	0	1	1	0	0	0	0	0	0	0	0	0	66
17722939	f	md	FR	2020	0	1	0	0	0	1	0	0	0	0	0	0	51
17723313	f	Not repo	PH	2020	1	0	0	0	0	0	0	0	0	0	0	0	57.6
17723412	m	hp	JP	2020	0	1	1	0	0	0	0	0	0	0	0	0	78
17723432	f	Not repo	BE	2020	0	0	0	0	0	0	1	1	0	0	0	0	
17723440	m	hp	FR	2020	0	1	1	0	0	0	0	0	0	0	0	0	79
17723634	m	ph	JP	2020	1	0	0	0	0	0	0	0	0	0	0	1	86.3
17724175	m	hp	JP	2020	0	1	1	0	0	0	0	0	0	0	0	1	69
17724264	m	cn	JP	2020	0	1	1	0	0	0	0	0	0	0	0	0	82
17724337	f	md	DK	2020	0	0	0	0	0	0	0	0	0	0	0	0	33
17725290	m	md	UA	2020	0	1	1	0	0	0	0	0	0	0	0	0	65
17725431	m	md	KR	2020	0	1	1	0	0	0	0	0	0	0	0	0	61
17725768	f	cn	SG	2020	0	1	1	0	0	0	0	0	0	0	0	0	67
17726233	m	md	FR	2020	0	1	0	0	0	1	0	0	0	0	0	0	69
17726312	f	md	JP	2020	1	0	0	0	0	0	0	0	0	0	0	1	64
17726419	m	hp	US	2020	0	1	0	0	1	0	0	0	0	0	0	0	
17726723	f	cn	US	2020	0	1	1	0	0	0	0	0	0	0	0	0	

17727078	m	cn	CN	2020	0	1	0	0	1	0	0	0	0	0	0	0	71
17727270	m	cn	KR	2020	1	0	0	0	0	0	0	0	0	0	0	0	75
17727593	m	md	JP	2020	0	1	0	1	0	0	0	0	0	0	0	0	70
17727905	f	cn	US	2020	0	0	0	0	0	0	0	0	0	0	0	0	60
17728164	f	ph	FR	2020	0	1	0	1	0	0	0	0	0	0	0	0	58
17728178	f	Not repo	CN	2020	1	0	0	0	0	0	0	0	0	0	0	0	79
17728311	f	cn	US	2020	1	0	0	0	0	0	0	0	0	0	0	0	90
17728419	m	md	JP	2020	0	1	0	1	0	0	0	0	0	0	0	1	74
17728521	m	cn	CN	2020	1	0	0	0	0	0	0	0	0	0	0	0	74
17729603	Not	cn	JP	2020	0	1	1	0	0	0	0	0	0	0	0	1	80
17729728	m	hp	IN	2020	0	0	0	0	0	0	0	0	0	0	0	0	
17729837	f	hp	FR	2020	0	0	0	0	0	0	0	0	0	0	0	0	74
17730011	f	md	BE	2020	0	0	0	0	0	0	1	1	0	0	0	0	
17730547	m	md	DE	2020	0	0	0	0	0	0	0	0	0	0	0	0	
17730551	m	cn	JP	2020	0	1	1	0	0	0	0	0	0	0	0	0	68
17730754	m	hp	DE	2020	0	1	0	0	0	1	0	0	0	0	0	0	70
17730954	Not	hp	CA	2020	0	1	0	1	0	0	0	0	0	0	0	0	
17730968	Not	cn	JP	2020	0	0	0	0	0	0	0	0	0	0	0	0	76
17731428	m	cn	JP	2020	0	0	0	0	0	0	0	0	0	0	0	0	69
17731508	f	md	US	2020	0	1	0	0	1	0	0	0	0	0	0	0	62
17731805	Not	cn	JP	2020	0	1	0	1	0	0	0	0	0	0	0	0	
17732578	f	Not repo	CN	2020	1	0	0	0	0	0	0	0	0	0	0	0	71
17733309	f	ph	JP	2020	0	1	0	1	0	0	0	0	0	0	0	0	67
17733765	m	cn	JP	2020	0	1	1	0	0	0	0	0	0	0	0	0	74
17733775	f	md	JP	2020	0	1	0	0	0	1	0	0	0	0	0	0	80
17733958	m	ph	US	2020	0	0	0	0	0	0	0	0	0	0	0	0	64
17734117	m	hp	US	2020	0	0	0	0	0	0	0	0	0	0	0	0	68
17734127	m	cn	US	2020	0	0	0	0	0	0	0	0	0	0	0	0	74
17735083	Not	md	JP	2020	1	0	0	0	0	0	0	0	0	0	0	0	
17735910	m	md	IT	2020	0	1	1	0	0	0	0	0	0	0	0	0	70
17736282	m	ph	JP	2020	0	0	0	0	0	0	0	0	0	0	0	0	83
17736662	m	md	BR	2020	0	1	1	0	0	0	0	0	0	0	0	0	72
17737240	f	cn	IL	2020	0	0	0	0	0	0	0	0	0	0	0	0	69
17737348	f	md	TW	2020	1	0	0	0	0	0	0	0	0	0	0	0	60

17737360	f	hp	NL	2020	0	1	1	0	0	0	0	0	0	0	0	0	30
17739941	f	hp	CA	2020	0	1	1	0	0	0	0	0	0	0	0	0	77
17740113	f	md	CN	2020	1	0	0	0	0	0	0	0	0	0	0	0	75
17740952	f	md	KR	2020	0	0	0	0	0	0	0	0	0	0	0	0	
17741244	f	hp	US	2020	0	1	0	0	1	0	0	0	0	0	0	0	62
17741726	m	hp	ES	2020	0	1	0	0	1	0	0	0	0	0	0	0	62
17741872	m	cn	JP	2020	0	1	1	0	0	0	0	0	0	0	0	0	77
17742168	m	md	JP	2020	0	1	0	0	1	0	0	0	0	0	0	1	65
17742354	m	md	KR	2020	0	1	0	0	0	1	0	0	0	0	0	0	79
17742371	f	hp	CN	2020	1	0	0	0	0	0	0	0	0	0	0	0	
17742809	f	hp	CA	2020	0	0	0	0	0	0	0	0	0	0	0	0	69
17742994	f	hp	IT	2020	0	1	0	0	1	0	0	0	0	0	0	0	
17743484	m	Not repo	CN	2020	1	0	0	0	0	0	0	0	0	0	0	0	78
17743671	f	md	JP	2020	0	1	0	0	0	1	0	0	0	0	0	0	80
17744230	m	hp	ES	2020	0	1	1	0	0	0	0	0	0	0	0	1	65
17744821	m	md	US	2020	0	1	0	0	1	0	0	0	0	0	0	0	53
17745073	m	md	JP	2020	0	1	0	0	0	1	0	0	0	0	0	0	
17745852	f	hp	CA	2020	0	0	0	0	0	0	0	0	0	0	0	0	66
17746227	f	Not repo	MY	2020	1	0	0	0	0	0	0	0	0	0	0	0	83.5
17746353	m	cn	GR	2020	0	1	1	0	0	0	0	0	0	0	0	0	90
17746751	m	hp	JP	2020	0	1	1	0	0	0	0	0	0	0	0	0	75
17747052	m	hp	ES	2020	0	0	0	0	0	0	0	0	0	0	0	0	53
17747315	m	cn	JP	2020	0	1	1	0	0	0	0	0	0	0	0	0	69
17749215	m	cn	US	2020	0	0	0	0	0	0	0	0	0	0	0	0	64
17749406	f	Not repo	DE	2020	1	0	0	0	0	0	0	0	0	0	0	0	
17749912	m	md	FR	2020	0	1	0	1	0	0	0	0	0	0	0	1	
17750498	f	Not repo	IL	2020	1	0	0	0	0	0	0	0	0	0	0	0	69
17750504	Not	Not repo	BR	2020	1	0	0	0	0	0	0	0	0	0	0	0	
17750586	Not	cn	JP	2020	0	1	1	0	0	0	0	0	0	0	0	0	
17750694	m	cn	Not re	2020	0	0	0	0	0	0	0	0	0	0	0	0	70
17750714	f	cn	CN	2020	0	1	0	0	1	0	0	0	0	0	0	1	63
17750779	f	md	US	2020	0	1	0	0	1	0	0	0	0	0	0	0	59
17750780	m	md	US	2020	0	1	0	0	1	0	0	0	0	0	0	0	62
17750783	m	md	US	2020	0	1	0	0	1	0	0	0	0	0	0	0	74

17750784	f	md	US	2020	0	1	0	0	1	0	0	0	0	0	0	0	47
17751048	Not	hp	JP	2020	0	0	0	0	0	0	0	0	0	0	0	0	
17751530	m	cn	PE	2020	0	1	1	0	0	0	0	0	0	0	0	0	39
17751569	f	cn	AR	2020	0	0	0	0	0	0	0	0	0	0	0	0	53
17751649	f	ph	US	2020	0	0	0	0	0	0	0	0	0	0	0	0	71
17752226	m	hp	CN	2020	0	0	0	0	0	0	0	0	0	0	0	0	64
17752362	m	ph	JP	2020	1	0	0	0	0	0	0	0	0	0	0	0	72
17752439	m	cn	GR	2020	0	1	1	0	0	0	0	0	0	0	0	1	
17752513	m	md	US	2020	0	1	1	0	0	0	0	0	0	0	0	0	78
17752540	m	Not repo	CN	2020	1	0	0	0	0	0	0	0	0	0	0	0	66
17753588	m	md	DE	2020	0	1	0	0	0	1	0	0	0	0	0	0	59
17754427	f	ph	US	2020	0	1	1	0	0	0	0	0	0	0	0	0	62
17755525	f	Not repo	US	2020	1	0	0	0	0	0	0	0	0	0	0	0	80.1
17756049	f	hp	GB	2020	0	0	0	0	0	0	0	0	0	0	0	0	78
17756191	m	md	AU	2020	0	1	1	0	0	0	0	0	0	0	0	0	70
17757096	m	md	TW	2020	0	0	0	0	0	0	0	0	0	0	0	0	
17757315	Not	hp	JP	2020	0	0	0	0	0	0	0	0	0	0	0	0	
17758233	f	md	DE	2020	1	0	0	0	0	0	0	0	0	0	0	0	46
17759094	m	md	JP	2020	0	1	0	0	0	1	0	0	0	0	0	0	55
17760055	Not	hp	JP	2020	0	0	0	0	0	0	0	0	0	0	0	0	
17760114	m	Not repo	US	2020	1	0	0	0	0	0	0	0	0	0	0	0	67.3
17760475	Not	md	US	2020	0	0	0	0	0	0	0	0	0	0	0	0	
17760477	Not	md	US	2020	0	0	0	0	0	0	0	0	0	0	0	0	
17760511	Not	md	US	2020	0	0	0	0	0	0	0	0	0	0	0	0	
17760620	Not	md	US	2020	0	1	0	0	0	1	0	0	0	0	0	0	
17760638	Not	md	US	2020	0	1	0	0	0	1	0	0	0	0	0	0	
17760669	Not	md	US	2020	0	1	0	0	0	1	0	0	0	0	0	0	
17761223	m	md	DE	2020	0	1	0	0	0	1	0	0	0	0	0	0	73
17761243	Not	md	US	2020	0	1	0	0	0	1	0	0	0	0	0	0	
17761449	f	md	FR	2020	0	1	0	0	0	1	0	0	0	0	0	0	67
17761553	f	Not repo	MY	2020	1	0	0	0	0	0	0	0	0	0	0	0	82.4
17761748	m	Not repo	IT	2020	1	0	0	0	0	0	0	0	0	0	0	0	
17761753	f	cn	US	2020	1	0	0	0	0	0	0	0	0	0	0	0	90
17761767	f	Not repo	DE	2020	1	0	0	0	0	0	0	0	0	0	0	0	

17761792	Not	md	US	2020	0	1	0	0	0	1	0	0	0	0	0	0	
17761798	Not	md	US	2020	0	1	0	0	0	1	0	0	0	0	0	0	
17761806	Not	md	US	2020	0	1	0	0	0	1	0	0	0	0	0	1	
17761935	Not	md	US	2020	0	1	0	0	0	1	0	0	0	0	0	0	
17761956	Not	md	US	2020	0	1	0	0	0	1	0	0	0	0	0	0	
17761964	Not	md	US	2020	0	1	0	0	0	1	0	0	0	0	0	0	
17761988	Not	md	US	2020	0	1	0	0	0	1	0	0	0	0	0	0	
17762018	Not	md	US	2020	0	1	0	0	0	1	0	0	0	0	0	0	
17762149	f	md	US	2020	1	0	0	0	0	0	0	0	0	0	0	0	65
17762340	f	md	CO	2020	0	1	1	0	0	0	0	0	0	0	0	0	48
17762409	m	hp	NL	2020	0	1	0	1	0	0	0	0	0	0	0	0	52
17762648	f	md	TW	2020	0	1	0	0	0	1	0	0	0	0	0	0	64
17764569	f	cn	US	2020	1	0	0	0	0	0	0	0	0	0	0	0	56.2
17764735	f	hp	CA	2020	0	0	0	0	0	0	0	0	0	0	0	0	39
17765239	f	cn	US	2020	0	0	0	0	0	0	0	0	0	0	0	0	
17765756	f	Not repo	DE	2020	1	0	0	0	0	0	0	0	0	0	0	0	
17765810	f	md	HU	2020	0	0	0	0	0	0	0	0	0	0	0	0	64
17766256	m	md	ES	2020	0	1	1	0	0	0	0	0	0	0	0	0	61
17766417	Not	md	US	2020	0	1	0	0	0	1	0	0	0	0	0	0	
17766553	m	cn	GR	2020	0	1	1	0	0	0	0	0	0	0	0	0	
17766712	f	md	AU	2020	0	1	1	0	0	0	0	0	0	0	0	0	75
17766779	m	hp	CA	2020	0	1	1	0	0	0	0	0	0	0	0	0	66
17767006	f	Not repo	CN	2020	1	0	0	0	0	0	0	0	0	0	0	0	59
17767395	m	Not repo	CN	2020	1	0	0	0	0	0	0	0	0	0	0	0	
17767515	Not	md	VN	2020	1	0	0	0	0	0	0	0	0	0	0	0	
17767635	f	Not repo	US	2020	1	0	0	0	0	0	0	0	0	0	0	0	72.7
17767918	m	hp	TR	2020	0	0	0	0	0	0	0	0	0	0	0	0	66
17768062	m	hp	AT	2020	0	1	1	0	0	0	0	0	0	0	0	0	77
17768435	m	cn	CN	2020	0	1	0	0	1	0	0	0	0	0	0	0	
17768544	m	md	JP	2020	0	1	0	0	1	0	0	0	0	0	0	1	68
17769085	m	hp	US	2020	0	1	1	0	0	0	0	0	0	0	0	0	55
17769361	m	hp	TR	2020	0	1	0	0	1	0	0	0	0	0	0	1	66
17769762	Not	hp	US	2020	0	0	0	0	0	0	0	0	0	0	0	0	
17769985	f	md	US	2020	0	1	0	1	0	0	0	0	0	0	0	0	

17769987	f	md	CN	2020	1	0	0	0	0	0	0	0	0	0	0	0	36
17769989	m	cn	CN	2020	0	1	0	0	1	0	0	0	0	0	0	0	67
17770147	m	md	DE	2020	0	1	0	0	0	1	0	0	0	0	0	0	54
17770304	m	md	US	2020	0	0	0	0	0	0	0	0	0	0	0	0	55
17770417	m	Not repo	CN	2020	1	0	0	0	0	0	0	0	0	0	0	0	47
17770477	f	cn	DE	2020	0	1	1	0	0	0	0	0	0	0	0	0	64
17770492	f	hp	DE	2020	0	1	1	0	0	0	0	0	0	0	0	0	
17770500	m	hp	FR	2020	0	1	0	0	1	0	0	0	0	0	0	0	43
17770524	m	Not repo	PT	2020	0	1	0	1	0	0	0	0	0	0	0	0	60.7
17770805	f	ph	US	2020	1	0	0	0	0	0	0	0	0	0	0	1	51
17770957	f	hp	GB	2020	0	0	0	0	0	0	0	0	0	0	0	0	60
17771223	m	Not repo	CN	2020	1	0	0	0	0	0	0	0	0	0	0	0	81
17772039	f	md	JP	2020	0	1	0	0	0	1	0	0	0	0	0	0	80
17772061	f	md	BR	2020	1	0	0	0	0	0	0	0	0	0	0	0	46
17772359	m	cn	CN	2020	1	0	0	0	0	0	0	0	0	0	0	0	77
17772521	f	cn	CA	2020	1	0	0	0	0	0	0	0	0	0	0	0	
17772640	m	hp	TR	2020	0	1	1	0	0	0	0	0	0	0	0	0	77
17772778	m	cn	CN	2020	0	1	0	0	1	0	0	0	0	0	0	0	52
17773488	f	hp	US	2020	0	1	1	0	0	0	0	0	0	0	0	0	83
17774100	f	cn	CN	2020	1	0	0	0	0	0	0	0	0	0	0	0	75
17774168	f	cn	US	2020	0	1	1	0	0	0	0	0	0	0	0	0	90
17774489	m	md	CZ	2020	0	1	1	0	0	0	0	0	0	0	0	0	67
17775405	f	cn	US	2020	0	1	1	0	0	0	0	0	0	0	0	0	67
17777030	f	cn	CN	2020	1	0	0	0	0	0	0	0	0	0	0	0	69
17777712	m	md	JP	2020	0	1	1	0	0	0	0	0	0	0	0	0	73
17778076	f	Not repo	CN	2020	1	0	0	0	0	0	0	0	0	0	0	0	73
17778898	m	cn	KR	2020	0	0	0	0	0	0	0	0	0	0	0	0	
17778990	f	Not repo	CN	2020	1	0	0	0	0	0	0	0	0	0	0	0	91
17779287	m	md	PL	2020	0	0	0	0	0	0	0	0	0	0	0	0	66
17779509	m	Not repo	CN	2020	1	0	0	0	0	0	0	0	0	0	0	0	60
17779954	f	cn	CN	2020	1	0	0	0	0	0	0	0	0	0	0	0	53
17779960	f	hp	KR	2020	0	0	0	0	0	0	0	0	0	0	0	0	60
17780747	f	hp	NL	2020	0	1	0	0	1	0	0	0	0	0	0	0	
17780911	m	md	FR	2020	0	1	0	0	0	1	0	0	0	0	0	0	69

17781077	f	md	CN	2020	1	0	0	0	0	0	0	0	0	0	0	0	66.6
17781083	f	Not repo	CN	2020	1	0	0	0	0	0	0	0	0	0	0	0	82
17781618	f	Not repo	CN	2020	1	0	0	0	0	0	0	0	0	0	0	0	
17782352	m	cn	IL	2020	0	0	0	0	0	0	0	0	0	0	0	0	65
17782820	f	md	JP	2020	0	1	0	0	0	1	0	0	0	0	0	0	73
17783698	m	hp	FR	2020	0	1	0	0	1	0	0	0	0	0	0	0	85
17783776	m	md	RO	2020	0	0	0	0	0	0	0	0	0	0	0	0	42
17784192	f	lw	US	2020	0	0	0	0	0	0	0	0	0	0	0	0	54
17784768	f	hp	DE	2020	0	1	1	0	0	0	0	0	0	0	0	0	30
17784889	f	hp	Not re	2020	0	0	0	0	0	0	0	0	0	0	0	0	
17785515	m	cn	KR	2020	0	0	0	0	0	0	0	0	0	0	0	0	
17785972	f	cn	KR	2020	0	0	0	0	0	0	0	0	0	0	0	0	
17786377	f	cn	CN	2020	0	1	0	0	1	0	0	0	0	0	0	0	62
17786545	m	hp	US	2020	1	0	0	0	0	0	0	0	0	0	0	0	
17787033	m	cn	JP	2020	0	1	1	0	0	0	0	0	0	0	0	0	73
17787927	f	Not repo	MY	2020	1	0	0	0	0	0	0	0	0	0	0	0	
17788461	f	cn	US	2020	1	0	0	0	0	0	0	0	0	0	0	0	89
17788912	m	cn	US	2020	0	1	1	0	0	0	0	0	0	0	0	0	73
17788919	f	cn	CN	2020	1	0	0	0	0	0	0	0	0	0	0	0	51
17789148	f	md	DE	2020	1	0	0	0	0	0	0	0	0	0	0	0	55.3
17790259	f	cn	CN	2020	1	0	0	0	0	0	0	0	0	0	0	0	63
17790992	f	cn	SG	2020	0	0	0	0	0	0	0	0	0	0	0	0	73
17791184	f	hp	US	2020	0	1	1	0	0	0	0	0	0	0	0	0	81
17791532	m	hp	KR	2020	0	1	1	0	0	0	0	0	0	0	0	0	67
17791670	m	hp	CN	2020	0	0	0	0	0	0	0	0	0	0	0	0	50
17791762	m	hp	LB	2020	0	0	0	0	0	0	0	0	0	0	0	0	56
17791768	f	md	JP	2020	1	0	0	0	0	0	0	0	0	0	0	0	76
17791772	m	hp	JP	2020	0	1	0	1	0	0	0	0	0	0	0	0	65
17791775	f	cn	CN	2020	1	0	0	0	0	0	0	0	0	0	0	0	62
17791942	f	cn	US	2020	0	0	0	0	0	0	0	0	0	0	0	0	
17792304	f	md	BE	2020	0	0	0	0	0	0	0	0	0	0	0	0	74
17793997	f	cn	Not re	2020	0	0	0	0	0	0	0	0	0	0	0	0	55
17794061	m	md	FR	2020	0	1	0	0	0	1	0	0	0	0	0	0	69
17794355	m	md	BE	2020	0	1	0	0	0	1	0	0	0	0	0	0	74

17794427	m	md	HR	2020	1	0	0	0	0	0	0	0	0	0	0	0	70
17794704	m	Not repo	CN	2020	1	0	0	0	0	0	0	0	0	0	0	0	66
17794755	Not	md	JP	2020	1	0	0	0	0	0	0	0	0	0	0	0	
17794766	f	md	JP	2020	1	0	0	0	0	0	0	0	0	0	0	0	60
17794772	m	Not repo	JP	2020	1	0	0	0	0	0	0	0	0	0	0	0	
17794808	m	Not repo	CN	2020	1	0	0	0	0	0	0	0	0	0	0	0	
17795085	f	md	PT	2020	0	0	0	0	0	0	0	0	0	0	0	0	63
17795181	f	md	FI	2020	0	1	0	0	0	1	0	0	0	0	0	1	
17795237	m	md	IL	2020	0	0	0	0	0	0	0	0	0	0	0	0	73
17795324	f	md	IN	2020	1	0	0	0	0	0	0	0	0	0	0	0	51
17795329	m	md	DK	2020	0	1	1	0	0	0	0	0	0	0	0	0	48
17795733	m	hp	DE	2020	0	1	1	0	0	0	0	0	0	0	0	0	54
17796311	Not	cn	AT	2020	0	1	1	0	0	0	0	0	0	0	0	1	
17796585	f	cn	CA	2020	0	1	1	0	0	0	0	0	0	0	0	1	63
17797300	m	hp	US	2020	0	1	1	0	0	0	0	0	0	0	0	0	74
17798182	f	cn	CN	2020	1	0	0	0	0	0	0	0	0	0	0	0	57
17799086	m	Not repo	GR	2020	0	1	0	1	0	0	0	0	0	0	0	0	
17799192	Not	md	JP	2020	0	0	0	0	0	0	0	0	0	0	0	0	75
17799906	m	md	GR	2020	0	1	1	0	0	0	0	0	0	0	0	0	
17799943	m	cn	JP	2020	0	1	1	0	0	0	0	0	0	0	0	0	60
17799944	Not	cn	JP	2020	0	1	1	0	0	0	0	0	0	0	0	0	75
17799974	m	md	JP	2020	0	1	1	0	0	0	0	0	0	0	0	0	64
17800737	m	cn	JP	2020	0	1	1	0	0	0	0	0	0	0	0	0	68
17800882	f	ph	GB	2020	1	0	0	0	0	0	0	0	0	0	0	0	71
17800888	f	md	CN	2020	1	0	0	0	0	0	0	0	0	0	0	0	63
17801061	f	cn	JP	2020	0	1	1	0	0	0	0	0	0	0	0	0	50
17801330	f	md	JP	2020	0	0	0	0	0	0	1	1	0	0	0	0	82
17801346	m	cn	CN	2020	0	1	0	0	1	0	0	0	0	0	0	0	37
17801361	m	md	JP	2020	0	0	0	0	0	0	0	0	0	0	0	0	70
17801634	f	hp	RU	2020	1	0	0	0	0	0	0	0	0	0	0	0	70
17801688	f	md	US	2020	0	1	1	0	0	0	0	0	0	0	0	0	81
17801949	m	hp	FR	2020	0	0	0	0	0	0	0	0	0	0	0	0	51
17802141	m	md	JP	2020	0	1	1	0	0	0	0	0	0	0	0	1	78
17802649	f	hp	US	2020	0	1	1	0	0	0	0	0	0	0	0	0	81

17802902	Not	Not repo	JP	2020	1	0	0	0	0	0	0	0	0	0	0	0	
17802903	f	hp	HK	2020	1	0	0	0	0	0	0	0	0	0	0	0	68
17803093	f	hp	DE	2020	0	1	1	0	0	0	0	0	0	0	0	0	68
17803094	Not	hp	IT	2020	0	0	0	0	0	0	0	0	0	0	0	0	70
17803111	m	cn	US	2020	1	0	0	0	0	0	0	0	0	0	0	0	72.7
17803654	f	md	US	2020	1	0	0	0	0	0	0	0	0	0	0	0	62
17803762	f	cn	CN	2020	0	0	0	0	0	0	0	0	0	0	0	0	52
17803803	f	cn	NP	2020	0	0	0	0	0	0	0	0	0	0	0	0	58
17804006	m	cn	ID	2020	0	1	1	0	0	0	0	0	0	0	0	0	
17804072	Not	hp	JP	2020	1	0	0	0	0	0	0	0	0	0	0	0	
17804517	Not	hp	JP	2020	1	0	0	0	0	0	0	0	0	0	0	0	
17804524	f	Not repo	ID	2020	1	0	0	0	0	0	0	0	0	0	0	0	62.2
17804525	Not	hp	JP	2020	1	0	0	0	0	0	0	0	0	0	0	0	
17804563	m	md	AT	2020	0	1	1	0	0	0	0	0	0	0	0	1	77
17804659	Not	hp	JP	2020	1	0	0	0	0	0	0	0	0	0	0	0	
17804663	Not	hp	JP	2020	1	0	0	0	0	0	0	0	0	0	0	0	
17804724	m	hp	NL	2020	1	0	0	0	0	0	0	0	0	0	0	0	80
17804820	f	md	US	2020	1	0	0	0	0	0	0	0	0	0	0	0	71
17804851	m	md	US	2020	1	0	0	0	0	0	0	0	0	0	0	0	65
17805580	m	hp	NL	2020	0	1	0	0	0	1	0	0	0	0	0	0	59
17805855	Not	cn	JP	2020	0	1	1	0	0	0	0	0	0	0	0	0	56
17806151	f	cn	JP	2020	0	1	1	0	0	0	0	0	0	0	0	0	59
17806372	f	hp	DE	2020	0	1	0	0	0	1	0	0	0	0	0	0	79
17806645	f	cn	US	2020	1	0	0	0	0	0	0	0	0	0	0	0	
17807126	m	cn	CN	2020	1	0	0	0	0	0	0	0	0	0	0	0	59
17807220	m	md	KR	2020	0	1	0	1	0	0	0	0	0	0	0	0	58
17807522	Not	Not repo	JP	2020	0	1	0	1	0	0	0	0	0	0	0	0	
17807780	f	hp	DE	2020	0	0	0	0	0	0	0	0	0	0	0	0	67
17807803	m	md	JP	2020	0	1	0	1	0	0	0	0	0	0	0	1	77
17808131	f	hp	CA	2020	0	0	0	0	0	0	0	0	0	0	0	0	67
17808368	f	md	LB	2020	1	0	0	0	0	0	0	0	0	0	0	0	
17808944	m	hp	CN	2020	0	0	0	0	0	0	0	0	0	0	0	0	63
17809311	m	md	US	2020	0	0	0	0	0	0	0	0	0	0	0	0	66
17809335	m	hp	SE	2020	0	0	0	0	0	0	0	0	0	0	0	0	57

17809690	m	md	IN	2020	0	1	0	1	0	0	0	0	0	0	0	0	73
17809727	m	ph	JP	2020	0	1	0	0	1	0	0	0	0	0	0	0	61
17810065	m	md	CN	2020	0	0	0	0	0	0	0	0	0	0	0	0	52
17810081	m	hp	US	2020	0	1	0	0	1	0	0	0	0	0	0	0	
17810196	Not	md	US	2020	1	0	0	0	0	0	0	0	0	0	0	0	
17810328	Not	cn	JP	2020	0	1	1	0	0	0	0	0	0	0	0	1	72
17810331	f	ph	JP	2020	0	0	0	0	0	0	0	0	0	0	0	0	71
17810675	f	cn	US	2020	1	0	0	0	0	0	0	0	0	0	0	0	
17810803	f	lw	DK	2020	0	0	0	0	0	0	0	0	0	0	0	0	55
17811707	Not	md	BR	2020	0	0	0	0	0	0	0	0	0	0	0	0	
17811936	Not	md	JP	2020	0	1	0	1	0	0	0	0	0	0	0	0	
17811939	m	Not repo	TW	2020	0	1	0	1	0	0	0	0	0	0	0	0	62
17812018	Not	cn	JP	2020	0	1	1	0	0	0	0	0	0	0	0	1	74
17812022	f	Not repo	JP	2020	1	0	0	0	0	0	0	0	0	0	0	0	71
17812023	f	hp	US	2020	1	0	0	0	0	0	0	0	0	0	0	0	
17812037	f	Not repo	CN	2020	1	0	0	0	0	0	0	0	0	0	0	0	65
17812094	m	md	JP	2020	0	1	0	0	0	1	0	0	0	0	0	0	56
17812203	f	md	US	2020	0	0	0	0	0	0	0	0	0	0	0	0	73
17812315	m	md	JP	2020	0	1	1	0	0	0	0	0	0	0	0	0	54
17812352	m	md	US	2020	0	0	0	0	0	0	0	0	0	0	0	0	80
17812381	f	cn	US	2020	0	0	0	0	0	0	0	0	0	0	0	0	65
17812462	m	md	US	2020	0	1	1	0	0	0	0	0	0	0	0	0	74
17813141	m	hp	DE	2020	0	0	0	0	0	0	0	0	0	0	0	0	76
17813168	f	Not repo	RO	2020	1	0	0	0	0	0	0	0	0	0	0	0	65.8
17813601	m	md	GB	2020	0	0	0	0	0	0	0	0	0	0	0	0	55
17813676	f	Not repo	AU	2020	1	0	0	0	0	0	0	0	0	0	0	1	55
17813824	m	ph	CA	2020	1	0	0	0	0	0	0	0	0	0	0	0	62.9
17813941	m	md	IT	2020	0	1	1	0	0	0	0	0	0	0	0	0	55
17814290	f	md	US	2020	0	0	0	0	0	0	0	0	0	0	0	0	52
17814315	m	hp	ES	2020	0	1	0	0	1	0	0	0	0	0	0	0	68
17814416	f	md	KR	2020	0	1	0	0	0	1	0	0	0	0	0	0	68
17814618	m	md	US	2020	0	1	0	1	0	0	0	0	0	0	0	0	60
17814647	Not	hp	JP	2020	1	0	0	0	0	0	0	0	0	0	0	0	
17814648	Not	hp	JP	2020	1	0	0	0	0	0	0	0	0	0	0	0	

17814673	Not	hp	JP	2020	1	0	0	0	0	0	0	0	0	0	0	0	
17814775	m	cn	CN	2020	0	1	0	0	1	0	0	0	0	0	0	0	46
17815279	f	md	US	2020	0	1	1	0	0	0	0	0	0	0	0	0	81
17815542	m	hp	ES	2020	0	0	0	0	0	0	0	0	0	0	0	0	62
17815577	f	cn	DE	2020	0	1	1	0	0	0	0	0	0	0	0	0	75
17815695	m	hp	NL	2020	0	0	0	0	0	0	0	0	0	0	0	0	65
17815801	Not	cn	IN	2020	0	1	1	0	0	0	0	0	0	0	0	0	
17815917	f	Not repo	CN	2020	1	0	0	0	0	0	0	0	0	0	0	0	47
17815951	m	cn	JP	2020	0	1	1	0	0	0	0	0	0	0	0	0	80
17816361	m	ph	JP	2020	0	1	0	1	0	0	0	0	0	0	0	0	70
17816459	m	md	US	2020	0	0	0	0	0	0	0	0	0	0	0	0	30
17816510	m	cn	US	2020	0	0	0	0	0	0	0	0	0	0	0	0	
17816606	f	Not repo	CN	2020	0	1	0	1	0	0	0	0	0	0	0	0	
17817026	Not	hp	JP	2020	1	0	0	0	0	0	0	0	0	0	0	0	
17817027	Not	hp	JP	2020	1	0	0	0	0	0	0	0	0	0	0	0	
17817029	m	cn	JP	2020	0	0	0	0	0	0	0	0	0	0	0	0	60
17817221	f	md	JP	2020	0	0	0	0	0	0	0	0	0	0	0	0	61
17817389	Not	hp	JP	2020	1	0	0	0	0	0	0	0	0	0	0	0	
17817440	Not	hp	JP	2020	1	0	0	0	0	0	0	0	0	0	0	0	
17817441	Not	hp	JP	2020	1	0	0	0	0	0	0	0	0	0	0	0	
17817646	f	cn	FR	2020	0	1	1	0	0	0	0	0	0	0	0	0	61
17817723	m	md	GB	2020	0	0	0	0	0	0	0	0	0	0	0	0	55
17817766	m	hp	US	2020	0	1	0	0	1	0	0	0	0	0	0	0	
17817950	f	Not repo	ID	2020	1	0	0	0	0	0	0	0	0	0	0	0	68.1
17818188	m	Not repo	ID	2020	1	0	0	0	0	0	0	0	0	0	0	0	
17818330	f	cn	US	2020	0	1	0	0	1	0	0	0	0	0	0	0	62
17818331	f	md	US	2020	0	1	0	0	1	0	0	0	0	0	0	0	
17818361	m	md	IT	2020	0	0	0	0	0	0	0	0	0	0	0	0	41
17818470	m	ph	JP	2020	0	1	1	0	0	0	0	0	0	0	0	0	71
17818479	m	cn	IL	2020	0	0	0	0	0	0	0	0	0	0	0	0	
17818672	f	md	JP	2020	0	1	0	0	1	0	0	0	0	0	0	0	
17818676	m	md	US	2020	0	1	1	0	0	0	0	0	0	0	0	0	74
17818865	m	md	KR	2020	0	1	0	0	0	1	0	0	0	0	0	0	69
17819332	f	md	CZ	2020	1	0	0	0	0	0	0	0	0	0	0	0	46

17819342	m	cn	GB	2020	0	1	1	0	0	0	0	0	0	0	0	0	70
17819444	m	hp	US	2020	0	1	1	0	0	0	0	0	0	0	0	0	74
17819770	Not	hp	DE	2020	1	0	0	0	0	0	0	0	0	0	0	0	
17820342	f	md	IT	2020	0	1	0	0	0	1	0	0	0	0	0	1	82
17820527	m	cn	JP	2020	0	1	1	0	0	0	0	0	0	0	0	0	75
17820586	f	hp	DE	2020	0	1	1	0	0	0	0	0	0	0	0	0	30
17820692	m	cn	GR	2020	0	1	1	0	0	0	0	0	0	0	0	0	
17820712	m	hp	US	2020	1	0	0	0	0	0	0	0	0	0	0	0	68
17820781	m	md	GB	2020	0	0	0	0	0	0	0	0	0	0	0	0	55
17820809	Not	hp	CO	2020	1	0	0	0	0	0	0	0	0	0	0	0	
17821220	m	cn	JP	2020	0	0	0	0	0	0	0	0	0	0	0	0	70
17821413	f	hp	US	2020	0	0	0	0	0	0	0	0	0	0	0	0	57
17821419	m	md	IT	2020	0	1	1	0	0	0	0	0	0	0	0	0	71
17821459	m	md	TW	2020	0	0	0	0	0	0	0	0	0	0	0	0	
17821876	Not	hp	DE	2020	1	0	0	0	0	0	0	0	0	0	0	0	
17821877	Not	hp	DE	2020	1	0	0	0	0	0	0	0	0	0	0	0	
17821878	Not	hp	DE	2020	1	0	0	0	0	0	0	0	0	0	0	0	
17821879	Not	hp	DE	2020	1	0	0	0	0	0	0	0	0	0	0	0	
17821880	Not	hp	DE	2020	1	0	0	0	0	0	0	0	0	0	0	0	
17821881	Not	hp	DE	2020	1	0	0	0	0	0	0	0	0	0	0	0	
17821903	Not	hp	DE	2020	1	0	0	0	0	0	0	0	0	0	0	0	
17821904	Not	hp	DE	2020	1	0	0	0	0	0	0	0	0	0	0	0	
17822103	f	hp	JP	2020	0	0	0	0	0	0	0	0	0	0	0	0	
17822104	f	hp	JP	2020	0	0	0	0	0	0	0	0	0	0	0	0	
17822134	Not	hp	DE	2020	1	0	0	0	0	0	0	0	0	0	0	0	
17822198	Not	hp	DE	2020	1	0	0	0	0	0	0	0	0	0	0	0	
17822204	Not	hp	DE	2020	1	0	0	0	0	0	0	0	0	0	0	0	
17822325	Not	hp	DE	2020	1	0	0	0	0	0	0	0	0	0	0	0	
17822375	Not	hp	CN	2020	0	0	0	0	0	0	0	0	0	0	0	0	
17822427	f	cn	CN	2020	0	0	0	0	0	0	0	0	0	0	0	0	73
17822452	Not	hp	DE	2020	1	0	0	0	0	0	0	0	0	0	0	0	
17822454	Not	hp	DE	2020	1	0	0	0	0	0	0	0	0	0	0	0	
17822506	m	ph	JP	2020	0	1	0	0	0	1	0	0	0	0	0	0	80
17822517	Not	hp	DE	2020	1	0	0	0	0	0	0	0	0	0	0	0	

17822633	Not	hp	DE	2020	1	0	0	0	0	0	0	0	0	0	0	0	
17822634	Not	hp	DE	2020	1	0	0	0	0	0	0	0	0	0	0	0	
17822636	Not	Not repo	CN	2020	0	1	0	1	0	0	0	0	0	0	0	0	
17822637	f	cn	CN	2020	1	0	0	0	0	0	0	0	0	0	0	0	60
17822649	f	cn	US	2020	1	0	0	0	0	0	0	0	0	0	0	0	51.6
17822662	Not	hp	CN	2020	0	0	0	0	0	0	0	0	0	0	0	0	
17822796	Not	hp	DE	2020	1	0	0	0	0	0	0	0	0	0	0	0	
17822808	Not	hp	DE	2020	1	0	0	0	0	0	0	0	0	0	0	0	
17822865	Not	hp	DE	2020	1	0	0	0	0	0	0	0	0	0	0	0	
17822925	Not	hp	DE	2020	1	0	0	0	0	0	0	0	0	0	0	0	
17822962	Not	hp	DE	2020	1	0	0	0	0	0	0	0	0	0	0	0	
17823086	Not	hp	DE	2020	1	0	0	0	0	0	0	0	0	0	0	0	
17823153	Not	hp	DE	2020	1	0	0	0	0	0	0	0	0	0	0	0	
17823210	Not	hp	DE	2020	1	0	0	0	0	0	0	0	0	0	0	0	
17823293	Not	hp	DE	2020	1	0	0	0	0	0	0	0	0	0	0	0	
17823309	Not	hp	DE	2020	1	0	0	0	0	0	0	0	0	0	0	0	
17823311	Not	hp	DE	2020	1	0	0	0	0	0	0	0	0	0	0	0	
17823359	m	md	RO	2020	1	0	0	0	0	0	0	0	0	0	0	0	78.2
17823451	Not	hp	DE	2020	1	0	0	0	0	0	0	0	0	0	0	0	
17823452	Not	hp	DE	2020	1	0	0	0	0	0	0	0	0	0	0	0	
17823528	Not	hp	DE	2020	1	0	0	0	0	0	0	0	0	0	0	0	
17823768	Not	hp	DE	2020	1	0	0	0	0	0	0	0	0	0	0	0	
17823769	Not	hp	DE	2020	1	0	0	0	0	0	0	0	0	0	0	0	
17823770	Not	hp	DE	2020	1	0	0	0	0	0	0	0	0	0	0	0	
17823877	m	md	US	2020	0	1	1	0	0	0	0	0	0	0	0	0	58
17824079	m	md	PL	2020	0	1	0	0	0	1	0	0	0	0	0	0	67
17824148	f	md	JP	2020	1	0	0	0	0	0	0	0	0	0	0	0	76.5
17824183	Not	hp	DE	2020	1	0	0	0	0	0	0	0	0	0	0	0	
17824277	Not	hp	DE	2020	1	0	0	0	0	0	0	0	0	0	0	0	
17824371	Not	hp	DE	2020	1	0	0	0	0	0	0	0	0	0	0	0	
17824826	m	hp	CA	2020	0	1	1	0	0	0	0	0	0	0	0	0	66
17824885	Not	hp	DE	2020	1	0	0	0	0	0	0	0	0	0	0	0	
17824961	Not	hp	DE	2020	1	0	0	0	0	0	0	0	0	0	0	0	
17824997	Not	hp	DE	2020	1	0	0	0	0	0	0	0	0	0	0	0	

17824998	Not	hp	DE	2020	1	0	0	0	0	0	0	0	0	0	0	0	
17825227	Not	hp	DE	2020	1	0	0	0	0	0	0	0	0	0	0	0	
17825250	Not	hp	DE	2020	1	0	0	0	0	0	0	0	0	0	0	0	
17825446	Not	hp	DE	2020	1	0	0	0	0	0	0	0	0	0	0	0	
17825490	m	cn	JP	2020	0	0	0	0	0	0	0	0	0	0	0	0	70
17825719	m	ph	GB	2020	0	0	0	0	0	0	0	0	0	0	0	0	59
17825889	Not	Not repo	JP	2020	1	0	0	0	0	0	0	0	0	0	0	0	
17825922	m	cn	CN	2020	0	1	0	1	0	0	0	0	0	0	0	0	54
17826003	Not	hp	DE	2020	1	0	0	0	0	0	0	0	0	0	0	0	
17826004	Not	hp	DE	2020	1	0	0	0	0	0	0	0	0	0	0	0	
17826028	Not	hp	DE	2020	1	0	0	0	0	0	0	0	0	0	0	0	
17826029	Not	hp	DE	2020	1	0	0	0	0	0	0	0	0	0	0	0	
17826148	Not	hp	DE	2020	1	0	0	0	0	0	0	0	0	0	0	0	
17826184	Not	hp	DE	2020	1	0	0	0	0	0	0	0	0	0	0	0	
17826244	m	cn	JO	2020	0	1	0	0	0	1	0	0	0	0	0	0	51
17826448	f	hp	US	2020	0	1	1	0	0	0	0	0	0	0	0	0	81
17826580	m	hp	GB	2020	0	1	1	0	0	0	0	0	0	0	0	0	68
17826666	f	md	DE	2020	0	1	0	0	0	1	0	0	0	0	0	1	67
17826897	f	md	DE	2020	0	0	0	0	0	0	0	0	0	0	0	0	
17826963	m	hp	BR	2020	0	1	1	0	0	0	0	0	0	0	0	1	72
17827038	Not	hp	DE	2020	1	0	0	0	0	0	0	0	0	0	0	0	
17827082	m	md	NO	2020	0	0	0	0	0	0	0	0	0	0	0	0	35
17827152	f	cn	JP	2020	0	1	1	0	0	0	0	0	0	0	0	0	69
17827258	Not	hp	DE	2020	1	0	0	0	0	0	0	0	0	0	0	0	
17827267	f	md	JP	2020	0	1	0	0	0	1	0	0	0	0	0	0	80
17827331	m	ph	JP	2020	0	1	0	0	1	0	0	0	0	0	0	0	89
17827563	m	md	JP	2020	0	1	0	0	0	1	0	0	0	0	0	0	86
17827758	f	Not repo	CA	2020	1	0	0	0	0	0	0	0	0	0	0	0	
17828431	m	md	GB	2020	0	0	0	0	0	0	0	0	0	0	0	0	55
17828566	f	md	US	2020	0	0	0	0	0	0	0	0	0	0	0	0	
17828702	f	hp	US	2020	0	0	0	0	0	0	0	0	0	0	0	0	65
17828741	m	md	JP	2020	0	1	0	0	0	1	0	0	0	0	0	0	70
17828761	Not	md	JP	2020	1	0	0	0	0	0	0	0	0	0	0	0	
17829780	f	cn	US	2020	0	1	1	0	0	0	0	0	0	0	0	0	

17829853	f	hp	TR	2020	0	0	0	0	0	0	0	0	0	0	0	0	68
17829879	Not	hp	DE	2020	1	0	0	0	0	0	0	0	0	0	0	0	
17829883	Not	md	PL	2020	0	0	0	0	0	0	0	0	0	0	0	0	
17829982	Not	hp	DE	2020	1	0	0	0	0	0	0	0	0	0	0	0	
17830118	m	md	DE	2020	1	0	0	0	0	0	0	0	0	0	0	0	57
17830679	f	md	FI	2020	0	1	0	0	0	1	0	0	0	0	0	1	66
17830885	f	md	RS	2020	1	0	0	0	0	0	0	0	0	0	0	0	64
17830971	m	cn	AT	2020	0	1	1	0	0	0	0	0	0	0	0	0	77
17831032	m	md	US	2020	0	0	0	0	0	0	0	0	0	0	0	0	58
17831601	m	Not repo	IT	2020	1	0	0	0	0	0	0	0	0	0	0	0	
17833404	f	md	US	2020	1	0	0	0	0	0	0	0	0	0	0	0	
17833405	f	hp	US	2020	0	1	1	0	0	0	0	0	0	0	0	0	81
17833744	f	md	KR	2020	0	1	0	0	0	1	0	0	0	0	0	0	68
17834091	f	hp	IE	2020	0	0	0	0	0	0	0	0	0	0	0	0	66
17834719	m	hp	US	2020	0	1	1	0	0	0	0	0	0	0	0	0	74
17835431	m	hp	ID	2020	0	0	0	0	0	0	0	0	0	0	0	0	67
17836004	f	hp	NL	2020	0	1	1	0	0	0	0	0	0	0	0	0	75
17836220	f	hp	CA	2020	0	0	0	0	0	0	0	0	0	0	0	0	63
17836276	m	md	FR	2020	0	0	0	0	0	0	0	0	0	0	0	0	47
17836764	m	hp	FR	2020	0	0	0	0	0	0	0	0	0	0	0	0	
17836775	f	hp	IE	2020	0	0	0	0	0	0	0	0	0	0	0	0	66
17837248	m	hp	US	2020	0	1	0	0	1	0	0	0	0	0	0	0	
17837284	Not	hp	CN	2020	0	0	0	0	0	0	0	0	0	0	0	0	
17837913	m	ph	US	2020	0	1	1	0	0	0	0	0	0	0	0	0	59
17838225	m	hp	CN	2020	0	1	0	0	1	0	0	0	0	0	0	1	69
17839351	f	cn	CN	2020	0	1	0	0	1	0	0	0	0	0	0	0	48
17839355	m	cn	DE	2020	0	1	1	0	0	0	0	0	0	0	0	0	69
17839614	m	Not repo	IT	2020	0	1	0	1	0	0	0	0	0	0	0	0	
17839808	m	hp	CA	2020	0	1	1	0	0	0	0	0	0	0	0	0	73
17839996	f	Not repo	SG	2020	1	0	0	0	0	0	0	0	0	0	0	0	
17840323	f	Not repo	CN	2020	1	0	0	0	0	0	0	0	0	0	0	0	47.6
17840529	m	md	US	2020	0	0	0	0	0	0	0	0	0	0	0	0	74
17840696	m	cn	JP	2020	0	1	1	0	0	0	0	0	0	0	0	0	65
17841442	f	ph	JP	2020	1	0	0	0	0	0	0	0	0	0	0	0	

17842473	f	cn	DE	2020	0	1	1	0	0	0	0	0	0	0	0	0	59
17842719	m	md	US	2020	0	0	0	0	0	0	0	0	0	0	0	0	74
17842946	m	hp	FR	2020	0	0	0	0	0	0	0	0	0	0	0	0	
17843099	f	hp	DE	2020	0	1	1	0	0	0	0	0	0	0	0	1	55
17843182	m	md	AR	2020	0	1	0	0	0	1	0	0	0	0	0	0	41
17843290	f	hp	CA	2020	0	0	0	0	0	0	0	0	0	0	0	0	
17843481	m	cn	JP	2020	0	1	1	0	0	0	0	0	0	0	0	0	67
17843684	Not	md	JP	2020	0	1	1	0	0	0	0	0	0	0	0	0	80
17844597	m	md	US	2020	0	1	1	0	0	0	0	0	0	0	0	0	74
17845261	m	hp	Not re	2020	0	1	1	0	0	0	0	0	0	0	0	0	73
17845435	f	cn	SE	2020	1	0	0	0	0	0	0	0	0	0	0	0	
17845732	f	hp	US	2020	0	0	0	0	0	0	0	0	0	0	0	0	54
17846001	f	cn	US	2020	0	1	0	0	1	0	0	0	0	0	0	0	
17846824	f	cn	US	2020	0	0	0	0	0	0	0	0	0	0	0	0	
17846941	m	hp	DE	2020	0	1	1	0	0	0	0	0	0	0	0	0	
17847138	m	md	KR	2020	0	0	0	0	0	0	0	0	0	0	0	0	60
17847357	f	Not repo	CA	2020	1	0	0	0	0	0	0	0	0	0	0	0	
17847377	m	Not repo	TW	2020	0	1	0	1	0	0	0	0	0	0	0	0	84
17847502	f	Not repo	TW	2020	0	1	0	1	0	0	0	0	0	0	0	0	70
17847506	f	cn	US	2020	1	0	0	0	0	0	0	0	0	0	0	0	79.7
17847507	m	Not repo	IT	2020	0	1	0	1	0	0	0	0	0	0	0	0	75.7
17847558	f	md	DE	2020	0	0	0	0	0	0	0	0	0	0	0	0	80
17847602	m	hp	JP	2020	0	1	0	0	1	0	0	0	0	0	0	0	66
17847659	f	cn	CA	2020	0	1	1	0	0	0	0	0	0	0	0	0	72
17847983	f	md	CZ	2020	0	0	0	0	0	0	0	0	0	0	0	0	45
17848134	m	cn	JP	2020	0	1	1	0	0	0	0	0	0	0	0	0	60
17848492	m	md	JP	2020	0	1	0	0	0	1	0	0	0	0	0	0	60
17848856	m	hp	US	2020	0	1	1	0	0	0	0	0	0	0	0	0	74
17849092	f	ph	FR	2020	0	0	0	0	0	0	0	0	0	0	0	0	52
17849847	m	hp	JP	2020	0	1	1	0	0	0	0	0	0	0	0	0	
17850218	m	md	KR	2020	0	0	0	0	0	0	0	0	0	0	0	0	
17850379	m	md	PL	2020	0	0	0	0	0	0	0	0	0	0	0	0	69
17850475	f	cn	JP	2020	1	0	0	0	0	0	0	0	0	0	0	0	
17850529	f	hp	JP	2020	0	1	0	1	0	0	0	0	0	0	0	1	75

17850595	m	hp	CA	2020	0	0	0	0	0	0	0	0	0	0	0	0	59
17851009	m	cn	CA	2020	0	1	0	1	0	0	0	0	0	0	0	0	
17851383	m	Not repo	CN	2020	1	0	0	0	0	0	0	0	0	0	0	0	67
17851815	f	ph	JP	2020	0	1	0	0	1	0	0	0	0	0	0	0	73
17853207	f	cn	Not re	2020	0	0	0	0	0	0	0	0	0	0	0	0	
17853488	f	hp	US	2020	0	1	0	0	1	0	0	0	0	0	0	0	61
17853781	f	hp	FR	2020	0	1	0	0	1	0	0	0	0	0	0	0	71
17853856	m	cn	JP	2020	0	0	0	0	0	0	0	0	0	0	0	0	72
17854005	f	md	TW	2020	0	1	0	0	0	1	0	0	0	0	0	0	53
17854754	m	hp	FR	2020	0	0	0	0	0	0	0	0	0	0	0	0	
17855126	f	md	JP	2020	0	1	0	1	0	0	0	0	0	0	0	1	73
17855127	f	Not repo	RU	2020	1	0	0	0	0	0	0	0	0	0	0	0	60
17855417	m	md	JP	2020	1	0	0	0	0	0	0	0	0	0	0	0	76.3
17855633	f	cn	CN	2020	1	0	0	0	0	0	0	0	0	0	0	0	72
17856218	Not	hp	CN	2020	0	1	0	0	1	0	0	0	0	0	0	0	
17856229	Not	hp	CN	2020	0	1	0	0	1	0	0	0	0	0	0	0	
17856286	Not	hp	CN	2020	0	1	0	0	1	0	0	0	0	0	0	0	
17856300	Not	hp	CN	2020	0	1	0	0	1	0	0	0	0	0	0	0	
17856327	m	hp	JP	2020	0	1	0	1	0	0	0	0	0	0	0	1	60.5
17856328	Not	hp	CN	2020	0	1	0	0	1	0	0	0	0	0	0	0	
17856334	Not	hp	CN	2020	0	1	0	0	1	0	0	0	0	0	0	0	
17856369	Not	hp	CN	2020	0	1	0	0	1	0	0	0	0	0	0	0	
17856400	m	md	ES	2020	0	1	0	0	0	1	0	0	0	0	0	1	77
17856693	m	cn	CA	2020	0	1	0	1	0	0	0	0	0	0	0	0	
17856743	f	cn	US	2020	0	0	0	0	0	0	0	0	0	0	0	0	59
17856800	f	hp	US	2020	0	1	0	0	1	0	0	0	0	0	0	0	65
17856863	f	cn	Not re	2020	0	0	0	0	0	0	0	0	0	0	0	0	55
17857361	m	Not repo	AE	2020	1	0	0	0	0	0	0	0	0	0	0	0	
17857391	m	cn	NL	2020	0	0	0	0	0	0	0	0	0	0	0	0	21
17857477	f	md	DE	2020	0	1	1	0	0	0	0	0	0	0	0	0	78
17857495	m	ph	GR	2020	0	1	0	0	0	1	0	0	0	0	0	0	80
17857755	Not	hp	JP	2020	1	0	0	0	0	0	0	0	0	0	0	0	
17857827	m	hp	US	2020	0	1	0	0	1	0	0	0	0	0	0	0	71
17857945	f	md	BR	2020	0	1	0	1	0	0	0	0	0	0	0	0	

17859903	f	md	IE	2020	0	0	0	0	0	0	0	0	0	0	0	0	66
17860035	f	cn	IN	2020	0	0	0	0	0	0	0	0	0	0	0	0	51
17860085	f	md	JP	2020	1	0	0	0	0	0	0	0	0	0	0	0	75
17860245	m	cn	AE	2020	1	0	0	0	0	0	0	0	0	0	0	0	69
17860853	m	cn	PH	2020	0	0	0	0	0	0	0	0	0	0	0	0	62
17861619	m	cn	Not re	2020	0	0	0	0	0	0	0	0	0	0	0	0	32
17862027	m	hp	US	2020	0	1	0	0	1	0	0	0	0	0	0	0	45
17862224	f	md	ES	2020	0	1	0	1	0	0	0	0	0	0	0	0	73
17863366	f	Not repo	US	2020	1	0	0	0	0	0	0	0	0	0	0	0	64.8
17863367	Not	Not repo	JP	2020	1	0	0	0	0	0	0	0	0	0	0	0	
17864124	f	Not repo	CN	2020	1	0	0	0	0	0	0	0	0	0	0	0	61.4
17864125	m	md	VN	2020	1	0	0	0	0	0	0	0	0	0	0	0	75
17864228	Not	Not repo	JP	2020	0	1	0	1	0	0	0	0	0	0	0	0	
17864294	Not	md	AT	2020	0	1	1	0	0	0	0	0	0	0	0	1	
17864303	m	cn	Not re	2020	0	0	0	0	0	0	0	0	0	0	0	0	32
17864327	m	Not repo	CN	2020	1	0	0	0	0	0	0	0	0	0	0	0	73.6
17864429	Not	md	US	2020	0	0	0	0	0	0	0	0	0	0	0	0	
17864436	m	cn	US	2020	0	0	0	0	0	0	0	0	0	0	0	0	
17864579	m	md	DK	2020	0	0	0	0	0	0	0	0	0	0	0	0	54
17864612	Not	Not repo	JP	2020	1	0	0	0	0	0	0	0	0	0	0	0	
17864631	m	ph	FR	2020	0	0	0	0	0	0	0	0	0	0	0	0	64
17864799	f	cn	AE	2020	1	0	0	0	0	0	0	0	0	0	0	0	52
17864800	m	Not repo	CN	2020	1	0	0	0	0	0	0	0	0	0	0	0	71
17864809	f	Not repo	CN	2020	1	0	0	0	0	0	0	0	0	0	0	0	49
17864810	m	Not repo	JP	2020	0	1	0	1	0	0	0	0	0	0	0	0	45
17864932	f	Not repo	AE	2020	1	0	0	0	0	0	0	0	0	0	0	0	41
17864991	m	cn	AE	2020	1	0	0	0	0	0	0	0	0	0	0	0	52
17865972	f	hp	US	2020	0	0	0	0	0	0	0	0	0	0	0	0	
17865991	m	md	DK	2020	0	0	0	0	0	0	0	0	0	0	0	0	54
17866121	f	cn	LK	2020	0	1	1	0	0	0	0	0	0	0	0	0	74
17866296	m	hp	NL	2020	0	0	0	0	0	0	0	0	0	0	0	0	
17866342	m	md	GR	2020	0	0	0	0	0	0	0	0	0	0	0	0	67
17866517	m	cn	AE	2020	1	0	0	0	0	0	0	0	0	0	0	0	66
17866518	m	Not repo	AE	2020	1	0	0	0	0	0	0	0	0	0	0	0	41

17866519	m	cn	AE	2020	1	0	0	0	0	0	0	0	0	0	0	0	69
17866520	m	cn	AE	2020	1	0	0	0	0	0	0	0	0	0	0	0	48.9
17866789	f	Not repo	CN	2020	1	0	0	0	0	0	0	0	0	0	0	0	68
17866866	Not	hp	FR	2020	0	0	0	0	0	0	0	0	0	0	0	0	
17866929	Not	md	JP	2020	0	0	0	0	0	0	0	0	0	0	0	0	
17866984	m	ph	FR	2020	0	0	0	0	0	0	0	0	0	0	0	0	64
17867028	f	cn	CN	2020	1	0	0	0	0	0	0	0	0	0	0	0	62
17867047	f	Not repo	CN	2020	1	0	0	0	0	0	0	0	0	0	0	0	
17867114	f	md	CN	2020	1	0	0	0	0	0	0	0	0	0	0	1	53
17867176	m	md	KR	2020	0	1	0	0	0	1	0	0	0	0	0	0	71
17867307	f	Not repo	CN	2020	1	0	0	0	0	0	0	0	0	0	0	0	84
17867700	m	md	KR	2020	0	1	0	0	0	1	0	0	0	0	0	0	62
17868006	Not	cn	JP	2020	0	1	1	0	0	0	0	0	0	0	0	0	80
17868353	m	md	TH	2020	0	1	0	0	0	1	0	0	0	0	0	0	71
17868626	m	cn	JP	2020	0	1	1	0	0	0	0	0	0	0	0	0	67
17868766	m	cn	PL	2020	0	0	0	0	0	0	0	0	0	0	0	0	65
17868971	f	cn	DE	2020	0	1	0	0	0	1	0	0	0	0	0	0	53
17869056	m	hp	US	2020	0	1	1	0	0	0	0	0	0	0	0	0	74
17869782	m	hp	FR	2020	0	1	0	0	1	0	0	0	0	0	0	0	67
17869826	Not	md	JP	2020	1	0	0	0	0	0	0	0	0	0	0	0	
17870242	m	md	ES	2020	0	0	0	0	0	0	0	0	0	0	0	0	45
17870731	m	md	JP	2020	0	0	0	0	0	0	0	0	0	0	0	0	58
17870869	f	Not repo	TW	2020	1	0	0	0	0	0	0	0	0	0	0	0	57
17871315	f	cn	Not re	2020	0	0	0	0	0	0	0	0	0	0	0	0	
17871526	f	hp	NL	2020	0	1	0	0	1	0	0	0	0	0	0	0	61
17872210	f	Not repo	CN	2020	1	0	0	0	0	0	0	0	0	0	0	0	74
17872282	f	cn	Not re	2020	0	0	0	0	0	0	0	0	0	0	0	0	
17872810	Not	hp	FR	2020	0	0	0	0	0	0	0	0	0	0	0	0	
17872950	m	md	JP	2020	0	1	0	0	1	0	0	0	0	0	0	1	65
17872979	m	cn	JP	2020	0	1	1	0	0	0	0	0	0	0	0	0	71
17873116	m	cn	JP	2020	0	1	1	0	0	0	0	0	0	0	0	1	80
17873368	m	hp	NL	2020	0	1	0	0	1	0	0	0	0	0	0	0	81
17873536	f	md	JP	2020	0	1	0	0	0	1	0	0	0	0	0	0	83
17873567	f	md	IT	2020	0	1	0	0	0	1	0	0	0	0	0	0	57

17873871	Not	md	CN	2020	0	1	0	0	1	0	0	0	0	0	0	0	
17875207	m	md	AT	2020	0	1	0	0	0	1	0	0	0	0	0	0	80
17875619	f	hp	CN	2020	0	0	0	0	0	0	0	0	0	0	0	0	73
17875834	m	cn	JP	2020	0	1	1	0	0	0	0	0	0	0	0	0	60
17875837	f	Not repo	CN	2020	1	0	0	0	0	0	0	0	0	0	0	0	
17876400	m	hp	JP	2020	0	1	1	0	0	0	0	0	0	0	0	0	73
17876754	Not	md	FR	2020	0	1	0	0	1	0	0	0	0	0	0	0	
17876893	m	md	Not re	2020	0	1	0	0	0	1	0	0	0	0	0	0	66
17877271	f	cn	CA	2020	0	0	0	0	0	0	0	0	0	0	0	0	62
17877896	m	hp	GR	2020	0	1	0	0	1	0	0	0	0	0	0	0	67
17877901	m	hp	GR	2020	0	1	0	0	1	0	0	0	0	0	0	0	77
17877906	m	hp	GR	2020	0	1	0	0	1	0	0	0	0	0	0	0	64
17877911	m	hp	GR	2020	0	1	0	0	1	0	0	0	0	0	0	0	60
17877996	f	cn	US	2020	0	1	1	0	0	0	0	0	0	0	0	0	
17878362	f	md	JP	2020	0	0	0	0	0	0	0	0	0	0	0	0	72
17878498	f	cn	Not re	2020	0	0	0	0	0	0	0	0	0	0	0	0	
17878700	m	md	KR	2020	0	1	0	0	0	1	0	0	0	0	0	0	61
17879404	m	md	DE	2020	0	0	0	0	0	0	0	0	0	0	0	0	
17879705	f	md	JP	2020	0	1	1	0	0	0	0	0	0	0	0	0	76
17879755	f	ph	JP	2020	1	0	0	0	0	0	0	0	0	0	0	0	73
17880059	f	cn	JP	2020	0	1	1	0	0	0	0	0	0	0	0	0	84
17880148	f	cn	CN	2020	0	1	0	0	1	0	0	0	0	0	0	0	
17880267	f	md	FR	2020	1	0	0	0	0	0	0	0	0	0	0	0	78
17880312	m	md	DE	2020	0	1	0	0	0	1	0	0	0	0	0	0	59
17880516	f	cn	US	2020	0	0	0	0	0	0	0	0	0	0	0	0	55
17880907	f	md	GB	2020	0	1	0	0	0	1	0	0	0	0	0	0	55
17881078	m	cn	US	2020	0	0	0	0	0	0	0	0	0	0	0	0	68
17881313	m	ph	IT	2020	0	0	0	0	0	0	0	0	0	0	0	0	22
17881376	f	cn	SE	2020	1	0	0	0	0	0	0	0	0	0	0	0	
17881767	m	cn	MX	2020	0	0	0	0	0	0	0	0	0	0	0	0	
17881769	m	cn	CN	2020	0	0	0	0	0	0	0	0	0	0	0	0	49
17882133	m	hp	Not re	2020	0	0	0	0	0	0	0	0	0	0	0	0	57
17882424	f	hp	US	2020	0	0	0	0	0	0	0	0	0	0	0	0	57
17882463	m	md	JP	2020	0	1	1	0	0	0	0	0	0	0	0	0	80

17882552	f	hp	US	2020	0	0	0	0	0	0	0	0	0	0	0	0	
17882695	m	cn	US	2020	0	1	0	0	1	0	0	0	0	0	0	0	
17882808	m	md	NO	2020	0	1	1	0	0	0	0	0	0	0	0	0	54
17882888	Not	Not repo	JP	2020	1	0	0	0	0	0	0	0	0	0	0	0	
17883187	m	md	JP	2020	0	1	0	0	0	1	0	0	0	0	0	0	70
17883268	f	hp	DE	2020	0	0	0	0	0	0	0	0	0	0	0	0	62
17883769	m	md	JP	2020	0	1	0	0	0	1	0	0	0	0	0	0	72
17883986	Not	hp	CN	2020	0	0	0	0	0	0	0	0	0	0	0	0	
17884690	m	cn	JP	2020	0	1	1	0	0	0	0	0	0	0	0	1	63
17884913	f	hp	DE	2020	0	0	0	0	0	0	0	0	0	0	0	0	60
17885173	m	md	PL	2020	0	0	0	0	0	0	0	0	0	0	0	0	63
17885516	m	md	JP	2020	0	1	0	1	0	0	0	0	0	0	0	0	63
17886650	m	md	LB	2020	1	0	0	0	0	0	0	0	0	0	0	0	
17886807	m	md	LB	2020	0	1	0	0	1	0	0	0	0	0	0	0	
17886880	Not	md	US	2020	0	0	0	0	0	0	0	0	0	0	0	0	
17886955	m	md	US	2020	0	1	1	0	0	0	0	0	0	0	0	0	60
17886961	m	cn	GR	2020	0	1	1	0	0	0	0	0	0	0	0	0	73
17887042	f	hp	CN	2020	1	0	0	0	0	0	0	0	0	0	0	0	56
17887376	f	md	US	2020	0	1	1	0	0	0	0	0	0	0	0	0	
17887541	f	hp	CN	2020	1	0	0	0	0	0	0	0	0	0	0	0	62
17887678	f	Not repo	AU	2020	1	0	0	0	0	0	0	0	0	0	0	0	
17888060	f	hp	US	2020	1	0	0	0	0	0	0	0	0	0	0	0	69.5
17888276	m	hp	US	2020	0	1	0	0	1	0	0	0	0	0	0	0	
17888526	f	Not repo	JP	2020	0	1	0	1	0	0	0	0	0	0	0	1	68
17889671	f	md	JP	2020	0	0	0	0	0	0	1	1	0	0	0	0	77
17889943	m	md	JP	2020	0	1	0	1	0	0	0	0	0	0	0	0	59
17889944	f	cn	CN	2020	1	0	0	0	0	0	0	0	0	0	0	0	54
17890054	f	md	US	2020	0	0	0	0	0	0	0	0	0	0	0	0	72
17890079	Not	md	CO	2020	0	0	0	0	0	0	0	0	0	0	0	0	
17890107	m	hp	IT	2020	0	1	1	0	0	0	0	0	0	0	0	0	79
17890127	m	cn	US	2020	1	0	0	0	0	0	0	0	0	0	0	0	
17890129	f	cn	US	2020	0	1	0	1	0	0	0	0	0	0	0	1	
17890333	f	md	US	2020	0	0	0	0	0	0	0	0	0	0	0	0	72
17890583	f	Not repo	CN	2020	1	0	0	0	0	0	0	0	0	0	0	0	58

17890815	m	cn	US	2020	0	0	0	0	0	0	0	0	0	0	0	0	
17891083	f	md	US	2020	0	0	0	0	0	0	0	0	0	0	0	0	58
17891305	f	hp	US	2020	0	0	0	0	0	0	0	0	0	0	0	0	65
17891329	m	md	CA	2020	0	1	0	1	0	0	0	0	0	0	0	1	
17891425	m	hp	JP	2020	0	1	1	0	0	0	0	0	0	0	0	0	66
17891707	f	md	DE	2020	0	1	1	0	0	0	0	0	0	0	0	0	59
17891897	f	md	US	2020	0	0	0	0	0	0	0	0	0	0	0	0	53
17892278	m	md	GB	2020	0	1	0	1	0	0	0	0	0	0	0	0	
17892368	Not	hp	CN	2020	1	0	0	0	0	0	0	0	0	0	0	0	
17892390	Not	hp	CN	2020	1	0	0	0	0	0	0	0	0	0	0	0	
17892468	m	md	BE	2020	0	1	0	0	0	1	0	0	0	0	0	0	47
17892899	f	md	TW	2020	0	0	0	0	0	0	0	0	0	0	0	0	
17893091	m	cn	US	2020	0	1	0	1	0	0	0	0	0	0	0	0	50
17893733	m	md	PL	2020	0	1	0	0	0	1	0	0	0	0	0	0	69
17894058	f	cn	JP	2020	0	1	1	0	0	0	0	0	0	0	0	0	68
17894568	m	ph	US	2020	0	1	1	0	0	0	0	0	0	0	0	0	70
17894981	m	md	GB	2020	0	1	1	0	0	0	0	0	0	0	0	0	72
17895296	m	md	IT	2020	0	1	1	0	0	0	0	0	0	0	0	0	63
17895536	f	hp	JP	2020	0	0	0	0	0	0	1	1	0	0	0	0	77
17895871	f	md	US	2020	0	0	0	0	0	0	0	0	0	0	0	0	45
17896815	m	md	GB	2020	0	1	1	0	0	0	0	0	0	0	0	0	58
17896858	f	cn	ID	2020	0	1	0	1	0	0	0	0	0	0	0	0	63
17896951	f	Not repo	IL	2020	0	1	0	1	0	0	0	0	0	0	0	0	
17898177	m	hp	JP	2020	0	0	0	0	0	0	0	0	0	0	0	0	52
17898365	f	cn	Not re	2020	0	0	0	0	0	0	0	0	0	0	0	0	
17898967	m	md	CA	2020	0	1	0	1	0	0	0	0	0	0	0	1	80
17899832	m	md	CN	2020	0	1	0	0	1	0	0	0	0	0	0	1	63
17900038	f	md	JP	2020	0	1	1	0	0	0	0	0	0	0	0	0	72
17900215	f	cn	GR	2020	0	1	1	0	0	0	0	0	0	0	0	0	65
17900460	m	md	JP	2020	0	0	0	0	0	0	0	0	0	0	0	0	74
17900608	m	md	IT	2020	0	1	1	0	0	0	0	0	0	0	0	0	71
17900694	m	md	JP	2020	0	1	0	0	1	0	0	0	0	0	0	1	67
17901129	m	Not repo	SG	2020	1	0	0	0	0	0	0	0	0	0	0	0	68.2
17901622	Not	hp	ES	2020	0	0	0	0	0	0	0	0	0	0	0	0	

17901790	Not	cn	JP	2020	0	1	1	0	0	0	0	0	0	0	0	0	90
17901959	m	hp	NL	2020	0	0	0	0	0	0	0	0	0	0	0	0	45
17902125	f	cn	JP	2020	0	1	1	0	0	0	0	0	0	0	0	0	62
17902277	m	ph	JP	2020	0	1	1	0	0	0	0	0	0	0	0	0	72
17904477	f	cn	Not re	2020	0	0	0	0	0	0	0	0	0	0	0	0	
17905824	m	cn	VN	2020	1	0	0	0	0	0	0	0	0	0	0	0	
17906437	m	cn	JP	2020	0	0	0	0	0	0	0	0	0	0	0	0	70
17906912	m	cn	US	2020	0	1	1	0	0	0	0	0	0	0	0	0	49
17906916	f	cn	CA	2020	0	0	0	0	0	0	0	0	0	0	0	0	75
17907249	m	hp	CN	2020	0	0	0	0	0	0	0	0	0	0	0	0	63
17907544	m	md	DE	2020	0	0	0	0	0	0	0	0	0	0	0	0	71
17907561	f	Not repo	AU	2020	1	0	0	0	0	0	0	0	0	0	0	0	
17907669	Not	Not repo	JP	2020	1	0	0	0	0	0	0	0	0	0	0	1	
17907670	m	md	JP	2020	0	1	0	1	0	0	0	0	0	0	0	1	75
17908547	f	md	JP	2020	1	0	0	0	0	0	0	0	0	0	0	0	86
17909223	m	Not repo	JP	2020	0	1	0	1	0	0	0	0	0	0	0	0	80
17909285	m	hp	JP	2020	1	0	0	0	0	0	0	0	0	0	0	0	54
17909311	f	hp	CH	2020	1	0	0	0	0	0	0	0	0	0	0	0	61
17909557	Not	md	IT	2020	0	0	0	0	0	0	0	0	0	0	0	0	
17909802	m	md	CN	2020	1	0	0	0	0	0	0	0	0	0	0	0	42
17909818	m	hp	JP	2020	0	1	0	1	0	0	0	0	0	0	0	1	76
17910043	Not	ph	JP	2020	1	0	0	0	0	0	0	0	0	0	0	0	
17910046	f	md	US	2020	0	0	0	0	0	0	0	0	0	0	0	0	61
17910932	f	md	DE	2020	0	0	0	0	0	0	0	0	0	0	0	0	
17911551	m	hp	IN	2020	0	0	0	0	0	0	0	0	0	0	0	0	63
17911552	m	hp	IN	2020	0	0	0	0	0	0	0	0	0	0	0	0	
17911909	m	hp	PH	2020	0	1	1	0	0	0	0	0	0	0	0	0	55
17911968	m	hp	JP	2020	0	0	0	0	0	0	0	0	0	0	0	0	52
17912178	Not	Not repo	JP	2020	1	0	0	0	0	0	0	0	0	0	0	0	
17912253	m	hp	NL	2020	0	0	0	0	0	0	0	0	0	0	0	0	54
17912344	m	md	JP	2020	0	1	0	1	0	0	0	0	0	0	0	1	70
17912947	f	md	TH	2020	0	0	0	0	0	0	0	0	0	0	0	0	
17913673	f	Not repo	GR	2020	1	0	0	0	0	0	0	0	0	0	0	0	80
17913683	f	cn	US	2020	1	0	0	0	0	0	0	0	0	0	0	0	64

17914083	f	hp	NL	2020	0	1	0	0	1	0	0	0	0	0	0	0	61
17914410	f	md	JP	2020	0	1	0	1	0	0	0	0	0	0	0	1	69
17914898	f	md	US	2020	0	1	0	0	0	1	0	0	0	0	0	0	64
17915104	Not	md	US	2020	1	0	0	0	0	0	0	0	0	0	0	0	
17915462	f	ph	US	2020	1	0	0	0	0	0	0	0	0	0	0	0	
17916059	f	md	IL	2020	0	0	0	0	0	0	0	0	0	0	0	0	64
17916075	m	md	JP	2020	0	0	0	0	0	0	0	0	0	0	0	0	68
17916153	f	md	FR	2020	1	0	0	0	0	0	0	0	0	0	0	0	78
17916231	m	Not repo	JP	2020	0	1	0	1	0	0	0	0	0	0	0	1	60
17916411	f	md	JP	2020	1	0	0	0	0	0	0	0	0	0	0	0	61
17916417	f	md	JP	2020	1	0	0	0	0	0	0	0	0	0	0	0	79
17916433	m	hp	KR	2020	0	1	1	0	0	0	0	0	0	0	0	0	52
17916505	f	cn	JP	2020	0	1	1	0	0	0	0	0	0	0	0	0	60
17916803	m	md	GB	2020	0	1	1	0	0	0	0	0	0	0	0	0	58
17916854	Not	ph	JP	2020	0	1	0	1	0	0	0	0	0	0	0	0	
17917273	m	Not repo	AU	2020	1	0	0	0	0	0	0	0	0	0	0	0	65
17917402	f	cn	Not re	2020	0	0	0	0	0	0	0	0	0	0	0	0	
17917413	f	md	AU	2020	0	1	1	0	0	0	0	0	0	0	0	0	64
17917416	f	md	JP	2020	0	1	1	0	0	0	0	0	0	0	0	0	61
17917437	f	Not repo	JP	2020	1	0	0	0	0	0	0	0	0	0	0	1	79
17918064	m	md	FR	2020	0	1	0	0	0	1	0	0	0	0	0	0	46
17918119	f	cn	JP	2020	0	1	1	0	0	0	0	0	0	0	0	0	66
17918231	f	md	FR	2020	0	1	0	1	0	0	0	0	0	0	0	0	80
17919313	m	md	JP	2020	0	1	1	0	0	0	0	0	0	0	0	0	72
17919337	f	cn	JP	2020	0	1	1	0	0	0	0	0	0	0	0	0	80
17919932	f	cn	CA	2020	1	0	0	0	0	0	0	0	0	0	0	0	
17920365	f	md	CA	2020	0	0	0	0	0	0	0	0	0	0	0	0	
17920698	Not	md	HR	2020	0	1	1	0	0	0	0	0	0	0	0	0	
17920703	m	hp	JP	2020	0	1	1	0	0	0	0	0	0	0	0	0	72
17921101	f	md	BE	2020	0	0	0	0	0	0	0	0	0	0	0	0	
17922667	Not	md	JP	2020	0	1	0	0	0	1	0	0	0	0	0	0	46
17923201	f	cn	DE	2020	0	1	1	0	0	0	0	0	0	0	0	1	55
17923767	m	Not repo	JP	2020	1	0	0	0	0	0	0	0	0	0	0	0	67
17925051	m	hp	FR	2020	0	1	0	0	1	0	0	0	0	0	0	0	60

17925451	m	md	DE	2020	0	0	0	0	0	0	0	0	0	0	0	0	58
17925649	m	hp	DE	2020	0	1	0	0	0	1	0	0	0	0	0	0	70
17926124	f	md	GB	2020	1	0	0	0	0	0	0	0	0	0	0	0	64
17926125	m	Not repo	JP	2020	1	0	0	0	0	0	0	0	0	0	0	0	70
17926152	f	md	DE	2020	0	1	1	0	0	0	0	0	0	0	0	1	55
17926462	m	hp	CN	2020	0	1	0	0	0	1	0	0	0	0	0	0	53
17926847	f	md	US	2020	0	0	0	0	0	0	0	0	0	0	0	0	82
17927504	m	hp	DE	2020	0	0	0	0	0	0	0	0	0	0	0	0	76
17928064	m	md	US	2020	0	1	1	0	0	0	0	0	0	0	0	0	50
17928160	f	Not repo	JP	2020	1	0	0	0	0	0	0	0	0	0	0	0	70
17928261	f	cn	US	2020	0	1	1	0	0	0	0	0	0	0	0	0	
17928330	f	md	CA	2020	1	0	0	0	0	0	0	0	0	0	0	0	
17928708	m	md	AT	2020	1	0	0	0	0	0	0	0	0	0	0	0	80
17929267	m	md	JP	2020	0	1	0	0	0	1	0	0	0	0	0	0	63
17931087	f	md	GB	2020	1	0	0	0	0	0	0	0	0	0	0	0	35
17931251	f	hp	NL	2020	0	1	0	0	1	0	0	0	0	0	0	0	
17931326	f	cn	US	2020	0	0	0	0	0	0	0	0	0	0	0	0	58
17931347	m	Not repo	GB	2020	1	0	0	0	0	0	0	0	0	0	0	0	62
17931575	m	md	JP	2020	0	1	0	0	1	0	0	0	0	0	0	0	77
17933963	m	cn	US	2020	0	1	1	0	0	0	0	0	0	0	0	0	90
17934067	m	Not repo	GR	2020	0	1	0	1	0	0	0	0	0	0	0	0	60
17934073	f	md	JP	2020	0	1	0	0	0	1	0	0	0	0	0	0	
17934176	m	md	JP	2020	0	1	0	0	0	1	0	0	0	0	0	0	
17934321	f	md	DE	2020	0	0	0	0	0	0	0	0	0	0	0	0	65
17934570	f	cn	US	2020	1	0	0	0	0	0	0	0	0	0	0	1	63.3
17934724	m	md	HU	2020	0	1	0	0	1	0	0	0	0	0	0	0	
17935680	f	md	JP	2020	1	0	0	0	0	0	0	0	0	0	0	0	80
17936208	f	md	DE	2020	0	1	1	0	0	0	0	0	0	0	0	0	75
17936327	m	md	JP	2020	0	1	0	0	0	1	0	0	0	0	0	0	72
17936372	f	md	JP	2020	0	1	0	0	0	1	0	0	0	0	0	0	
17936426	Not	Not repo	JP	2020	1	0	0	0	0	0	0	0	0	0	0	0	
17936804	m	cn	IL	2020	0	1	1	0	0	0	0	0	0	0	0	0	
17938250	f	hp	NL	2020	0	1	0	0	1	0	0	0	0	0	0	0	
17938807	Not	md	JP	2020	0	1	0	0	1	0	0	0	0	0	0	1	

17938828	f	hp	RO	2020	0	1	0	0	1	0	0	0	0	0	0	0	63
17938907	f	cn	US	2020	0	1	0	1	0	0	0	0	0	0	0	0	75.6
17938959	f	md	MY	2020	1	0	0	0	0	0	0	0	0	0	0	0	
17939029	m	cn	CN	2020	0	0	0	0	0	0	0	0	0	0	0	0	55
17939346	m	hp	JP	2020	0	0	0	0	0	0	0	0	0	0	0	0	68
17939385	m	cn	US	2020	0	1	1	0	0	0	0	0	0	0	0	0	70
17939438	m	cn	GB	2020	0	1	1	0	0	0	0	0	0	0	0	0	72
17939826	Not	ph	CA	2020	0	1	1	0	0	0	0	0	0	0	0	1	
17940191	m	md	VN	2020	1	0	0	0	0	0	0	0	0	0	0	0	
17940535	f	ph	JP	2020	0	1	1	0	0	0	0	0	0	0	0	0	
17940694	Not	md	DE	2020	0	1	1	0	0	0	0	0	0	0	0	1	
17941468	m	hp	US	2020	0	1	1	0	0	0	0	0	0	0	0	0	77
17941718	f	md	ES	2020	0	0	0	0	0	0	0	0	0	0	0	0	83
17941795	Not	cn	JP	2020	0	0	0	0	0	0	0	0	0	0	0	0	70
17941943	m	cn	JP	2020	0	1	1	0	0	0	0	0	0	0	0	0	70
17942173	f	cn	CA	2020	0	0	0	0	0	0	0	0	0	0	0	0	57
17942272	m	hp	FR	2020	0	1	0	0	0	1	0	0	0	0	0	0	69
17942860	f	hp	DE	2020	0	1	1	0	0	0	0	0	0	0	0	0	30
17942913	m	hp	DE	2020	0	1	1	0	0	0	0	0	0	0	0	0	68
17943589	m	md	JP	2020	0	1	0	1	0	0	0	0	0	0	0	0	64
17943676	m	cn	US	2020	0	1	1	0	0	0	0	0	0	0	0	0	60
17943694	f	cn	JP	2020	0	0	0	0	0	0	0	0	0	0	0	0	60
17943864	m	cn	US	2020	0	1	1	0	0	0	0	0	0	0	0	1	
17943963	f	md	AT	2020	0	0	0	0	0	0	0	0	0	0	0	0	
17944608	m	cn	JP	2020	0	1	1	0	0	0	0	0	0	0	0	0	73
17944878	m	cn	JP	2020	0	0	0	0	0	0	0	0	0	0	0	0	64
17944951	f	cn	DE	2020	0	1	1	0	0	0	0	0	0	0	0	1	55
17945422	m	hp	DE	2020	0	1	0	0	0	1	0	0	0	0	0	0	65
17945538	m	hp	DE	2020	0	1	0	0	0	1	0	0	0	0	0	1	67
17945615	m	hp	GB	2020	0	0	0	0	0	0	0	0	0	0	0	0	55
17946027	f	md	JP	2020	0	1	0	0	0	1	0	0	0	0	0	1	61
17946215	f	md	FR	2020	1	0	0	0	0	0	0	0	0	0	0	0	
17946907	m	hp	US	2020	0	0	0	0	0	0	0	0	0	0	0	0	77
17948446	m	cn	PL	2020	0	0	0	0	0	0	0	0	0	0	0	0	65

17948612	Not	md	UA	2020	0	0	0	0	0	0	0	0	0	0	0	0	
17948658	m	Not repo	RO	2020	0	0	0	0	0	0	0	0	0	0	0	0	
17949369	m	cn	JP	2020	0	1	1	0	0	0	0	0	0	0	0	0	54
17949601	Not	md	JP	2020	0	1	0	0	1	0	0	0	0	0	0	0	
17950157	m	hp	SK	2020	0	1	1	0	0	0	0	0	0	0	0	0	45
17950690	f	md	JP	2020	0	1	0	0	0	1	0	0	0	0	0	0	67
17951329	m	Not repo	ID	2020	1	0	0	0	0	0	0	0	0	0	0	0	
17951697	Not	md	HK	2020	0	1	0	0	0	1	0	0	0	0	0	0	
17951711	m	cn	CN	2020	0	1	0	0	1	0	0	0	0	0	0	0	62
17951924	m	cn	US	2020	0	0	0	0	0	0	0	0	0	0	0	0	81
17951991	f	md	DE	2020	0	1	1	0	0	0	0	0	0	0	0	1	55
17952128	m	md	JP	2020	0	0	0	0	0	0	0	0	0	0	0	0	60
17952182	m	cn	BG	2020	1	0	0	0	0	0	0	0	0	0	0	0	33
17952452	m	cn	RU	2020	0	1	0	0	0	1	0	0	0	0	0	0	60
17952494	m	cn	JP	2020	0	1	1	0	0	0	0	0	0	0	0	0	60
17952521	m	cn	JP	2020	0	1	1	0	0	0	0	0	0	0	0	0	60
17952597	m	hp	DK	2020	0	0	0	0	0	0	0	0	0	0	0	0	38
17952854	m	md	NL	2020	1	0	0	0	0	0	0	0	0	0	0	0	66
17952925	m	md	DE	2020	0	1	1	0	0	0	0	0	0	0	0	0	65
17953280	m	hp	PL	2020	0	0	0	0	0	0	0	0	0	0	0	0	69
17954484	Not	md	JP	2020	0	1	0	0	0	1	0	0	0	0	0	0	
17954724	f	md	FR	2020	1	0	0	0	0	0	0	0	0	0	0	0	50
17955533	f	hp	AT	2020	0	1	1	0	0	0	0	0	0	0	0	0	
17956253	m	md	JP	2020	0	1	0	0	0	1	0	0	0	0	0	1	70
17956386	m	Not repo	JP	2020	0	1	0	1	0	0	0	0	0	0	0	0	71
17956552	m	Not repo	IL	2020	0	1	0	1	0	0	0	0	0	0	0	0	71
17956979	m	md	JP	2020	0	1	0	1	0	0	0	0	0	0	0	0	70
17957384	Not	md	CH	2020	0	0	0	0	0	0	0	0	0	0	0	0	55
17957626	m	hp	JP	2020	0	1	0	1	0	0	0	0	0	0	0	1	68
17958458	f	md	DE	2020	0	1	1	0	0	0	0	0	0	0	0	0	73
17959142	m	md	KR	2020	0	1	0	0	1	0	0	0	0	0	0	0	59
17959499	Not	cn	JP	2020	0	1	1	0	0	0	0	0	0	0	0	0	60
17959806	f	cn	AE	2020	1	0	0	0	0	0	0	0	0	0	0	0	30.4
17960672	f	md	JP	2020	0	0	0	0	0	0	0	0	0	0	0	0	81

17961256	m	md	CN	2020	0	1	0	0	0	1	0	0	0	0	0	0	50
17961731	m	cn	US	2020	0	0	0	0	0	0	0	0	0	0	0	0	86
17962738	f	hp	CA	2020	0	1	0	1	0	0	0	0	0	0	0	1	68.8
17962929	m	md	KR	2020	0	1	0	0	1	0	0	0	0	0	0	0	58
17963792	f	ph	TR	2020	0	0	0	0	0	0	0	0	0	0	0	0	58
17963930	f	hp	CZ	2020	0	0	0	0	0	0	0	0	0	0	0	0	45
17964165	f	md	FR	2020	1	0	0	0	0	0	0	0	0	0	0	0	
17964222	f	Not repo	ID	2020	1	0	0	0	0	0	0	0	0	0	0	0	45.2
17965020	Not	md	NL	2020	0	0	0	0	0	0	0	0	0	0	0	0	
17965038	m	md	IN	2020	0	0	0	0	0	0	0	0	0	0	0	0	73
8966540	f	hp	CA	2020	1	0	0	0	0	0	0	0	0	0	0	0	70
10035487	Not	hp	CA	2020	0	0	0	0	0	0	0	0	0	0	0	0	
10151005	m	cn	GB	2020	1	0	0	0	0	0	0	0	0	0	0	0	69
10921080	f	md	AU	2020	0	0	0	0	0	0	0	0	0	0	0	0	43
11010560	m	md	US	2020	0	1	0	1	0	0	0	0	0	0	0	0	68
11024789	m	md	US	2020	0	0	0	0	0	0	0	0	0	0	0	0	68
11647723	m	md	FR	2020	1	0	0	0	0	0	0	0	0	0	0	0	
11693735	f	md	TW	2020	1	0	0	0	0	0	0	0	0	0	0	0	63.7
11858341	f	md	US	2020	0	0	0	0	0	0	0	0	0	0	0	0	82
11909631	m	md	TW	2020	0	0	0	0	0	0	0	0	0	0	0	0	49
11961914	m	md	DE	2020	0	0	0	0	0	0	0	0	0	0	0	0	64
11976566	f	md	US	2020	0	0	0	0	0	0	0	0	0	0	0	0	34
11982345	m	md	JP	2020	0	0	0	0	0	0	0	0	0	0	0	0	66
12134123	m	md	US	2020	0	0	0	0	0	0	0	0	0	0	0	0	39
12172766	Not	hp	JP	2020	1	0	0	0	0	0	0	0	0	0	0	0	
12173725	f	md	PL	2020	0	0	0	0	0	0	0	0	0	0	0	0	42
12210030	f	md	NL	2020	0	0	0	0	0	0	0	0	0	0	0	0	71
12214474	m	md	FI	2020	0	0	0	0	0	0	0	0	0	0	0	0	63
12262497	m	md	KR	2020	0	0	0	0	0	0	0	0	0	0	0	0	74
12298984	f	md	JP	2020	0	0	0	0	0	0	0	0	0	0	0	0	74
12345699	m	md	US	2020	0	0	0	0	0	0	0	0	0	0	0	0	58
12350079	m	md	JP	2020	0	0	0	0	0	0	0	0	0	0	0	0	65
12411317	f	md	DE	2020	0	0	0	0	0	0	0	0	0	0	0	0	65
12565700	f	md	CA	2020	0	1	0	1	0	0	0	0	0	0	0	0	65

12570560	m	md	DE	2020	0	1	0	1	0	0	0	0	0	0	0	0	53
12579914	f	md	JP	2020	0	0	0	0	0	0	0	0	0	0	0	0	52
12587462	f	md	CA	2020	0	1	0	1	0	0	0	0	0	0	0	0	73
12600993	f	md	JP	2020	0	0	0	0	0	0	0	0	0	0	0	0	61
12693249	m	md	IT	2020	0	0	0	0	0	0	0	0	0	0	0	0	79
12717819	f	md	CA	2020	0	1	0	1	0	0	0	0	0	0	0	0	65
12832963	m	md	TR	2020	0	0	0	0	0	0	0	0	0	0	0	0	60
12912685	f	md	JP	2020	0	1	0	1	0	0	0	0	0	0	0	0	56
12933181	m	md	PL	2020	0	1	0	1	0	0	0	0	0	0	0	0	70
12947795	f	md	IE	2020	0	0	0	0	0	0	0	0	0	0	0	0	36
12955077	f	md	US	2020	0	0	0	0	0	0	0	0	0	0	0	0	63
12959305	f	md	HK	2020	0	0	0	0	0	0	0	0	0	0	0	0	52
12967420	m	md	DE	2020	0	0	0	0	0	0	0	0	0	0	0	0	55
12975929	f	md	TW	2020	1	0	0	0	0	0	0	0	0	0	0	0	77.1
13001796	m	md	CA	2020	0	1	0	1	0	0	0	0	0	0	0	0	76
13001831	f	md	US	2020	0	0	0	0	0	0	0	0	0	0	0	0	70
13020136	m	cn	US	2020	0	0	0	0	0	0	0	0	0	0	0	0	59
13078071	f	md	JP	2020	0	0	0	0	0	0	0	0	0	0	0	0	38
13191741	f	md	CR	2020	0	0	0	0	0	0	0	0	0	0	0	0	49
13195730	m	md	SG	2020	0	0	0	0	0	0	0	0	0	0	0	0	49
13230112	m	md	KR	2020	0	0	0	0	0	0	0	0	0	0	0	0	61
13254704	f	md	TH	2020	0	0	0	0	0	0	0	0	0	0	0	0	47
13254832	m	md	US	2020	0	0	0	0	0	0	0	0	0	0	0	0	52
13259804	m	cn	DE	2020	0	0	0	0	0	0	0	0	0	0	0	0	
13260716	m	md	CA	2020	0	1	0	1	0	0	0	0	0	0	0	0	76
13262655	f	md	TW	2020	1	0	0	0	0	0	0	0	0	0	0	0	77.1
13268944	m	md	RU	2020	0	0	0	0	0	0	0	0	0	0	0	0	54
13280459	f	md	IT	2020	0	0	0	0	0	0	0	0	0	0	0	0	29
13340230	m	md	DE	2020	0	0	0	0	0	0	0	0	0	0	0	0	76
13340615	m	md	JP	2020	0	0	0	0	0	0	0	0	0	0	0	0	68
13344446	f	md	CN	2020	0	0	0	0	0	0	0	0	0	0	0	0	64
13350003	f	md	CA	2020	0	1	0	1	0	0	0	0	0	0	0	0	70
13430173	m	md	AU	2020	0	0	0	0	0	0	0	0	0	0	0	0	67
13441484	f	md	JP	2020	0	0	0	0	0	0	0	0	0	0	0	0	74

13441620	Not	hp	JP	2020	0	0	0	0	0	0	0	0	0	0	0	0	
13450904	f	md	AU	2020	0	0	0	0	0	0	0	0	0	0	0	0	
13515374	f	md	US	2020	0	1	0	1	0	0	0	0	0	0	0	0	65.1
13516460	f	md	CA	2020	0	1	0	1	0	0	0	0	0	0	0	0	70
13516726	Not	md	HK	2020	0	0	0	0	0	0	0	0	0	0	0	0	
13518362	m	md	JP	2020	0	1	0	1	0	0	0	0	0	0	0	0	72.3
13518383	f	md	DE	2020	0	1	0	1	0	0	0	0	0	0	0	0	46
13519111	m	md	IT	2020	0	1	0	1	0	0	0	0	0	0	0	0	74
13519134	f	md	FR	2020	0	1	0	1	0	0	0	0	0	0	0	0	68.9
13523308	m	md	TW	2020	0	1	0	1	0	0	0	0	0	0	0	0	56
13523331	f	md	DE	2020	0	1	0	1	0	0	0	0	0	0	0	0	76.8
13524024	m	md	US	2020	0	1	0	1	0	0	0	0	0	0	0	1	57
13537024	f	md	CL	2020	0	1	0	1	0	0	0	0	0	0	0	0	77.5
13537083	m	md	TR	2020	0	1	0	1	0	0	0	0	0	0	0	1	56.8
13540939	m	md	US	2020	0	1	0	1	0	0	0	0	0	0	0	0	66
13540947	m	md	FR	2020	0	1	0	1	0	0	0	0	0	0	0	0	48
13540958	m	md	TR	2020	0	1	0	1	0	0	0	0	0	0	0	0	73.3
13541171	m	md	IL	2020	0	1	0	1	0	0	0	0	0	0	0	0	65.3
13541433	m	md	FR	2020	0	1	0	1	0	0	0	0	0	0	0	1	57.6
13545128	m	md	ES	2020	0	1	0	1	0	0	0	0	0	0	0	0	49
13546762	m	md	UA	2020	0	1	0	1	0	0	0	0	0	0	0	0	45.8
13549345	m	md	CL	2020	0	1	0	1	0	0	0	0	0	0	0	0	83.7
13551371	m	md	BR	2020	0	1	0	1	0	0	0	0	0	0	0	0	52.7
13552420	m	md	ES	2020	0	1	0	1	0	0	0	0	0	0	0	0	68.6
13552666	m	md	VN	2020	0	1	0	1	0	0	0	0	0	0	0	0	53.1
13552900	m	cn	PH	2020	0	0	0	0	0	0	0	0	0	0	0	0	81
13555073	m	md	AU	2020	0	1	0	1	0	0	0	0	0	0	0	0	79.1
13557680	f	md	CA	2020	0	1	0	1	0	0	0	0	0	0	0	0	74
13558869	f	md	US	2020	0	1	0	1	0	0	0	0	0	0	0	0	69.9
13559448	m	md	PH	2020	0	1	0	1	0	0	0	0	0	0	0	0	49
13560199	m	md	NL	2020	0	1	0	1	0	0	0	0	0	0	0	0	68
13561178	f	md	RU	2020	0	1	0	1	0	0	0	0	0	0	0	0	72.7
13562966	Not	hp	KR	2020	0	0	0	0	0	0	0	0	0	0	0	0	
13569351	f	md	KR	2020	0	1	0	1	0	0	0	0	0	0	0	0	47.6

13570980	f	md	CA	2020	0	1	0	1	0	0	0	0	0	0	0	0	71
13570981	m	md	BE	2020	0	1	0	1	0	0	0	0	0	0	0	0	64
13574134	f	md	RU	2020	0	1	0	1	0	0	0	0	0	0	0	0	66
13577365	m	md	JP	2020	0	1	0	1	0	0	0	0	0	0	0	1	57.4
13577885	f	md	US	2020	0	1	0	1	0	0	0	0	0	0	0	1	59
13577991	m	md	FR	2020	0	1	0	1	0	0	0	0	0	0	0	0	48
13582220	m	md	FR	2020	0	1	0	1	0	0	0	0	0	0	0	0	75.1
13586382	f	md	CN	2020	0	1	0	1	0	0	0	0	0	0	0	0	66
13586457	f	md	GB	2020	0	1	0	1	0	0	0	0	0	0	0	0	50
13586709	m	md	PL	2020	0	1	0	1	0	0	0	0	0	0	0	0	63.7
13591171	f	md	CL	2020	0	1	0	1	0	0	0	0	0	0	0	0	75.7
13591679	m	md	CN	2020	0	0	0	0	0	0	0	0	0	0	0	0	44
13593730	m	md	DE	2020	0	1	0	1	0	0	0	0	0	0	0	0	70.2
13595006	f	md	US	2020	0	1	0	1	0	0	0	0	0	0	0	0	79.6
13595169	m	md	JP	2020	0	1	0	1	0	0	0	0	0	0	0	0	65.9
13598742	f	md	DE	2020	0	1	0	1	0	0	0	0	0	0	0	0	77
13598995	m	md	JP	2020	0	1	0	1	0	0	0	0	0	0	0	0	67.7
13600048	m	md	RU	2020	0	1	0	1	0	0	0	0	0	0	0	0	70.6
13602592	m	md	US	2020	0	1	0	1	0	0	0	0	0	0	0	0	58.6
13602743	m	md	UA	2020	0	1	0	1	0	0	0	0	0	0	0	0	41.1
13612396	m	md	KR	2020	0	1	0	1	0	0	0	0	0	0	0	0	74.8
13614618	m	md	CA	2020	0	1	0	1	0	0	0	0	0	0	0	1	72
13615651	m	md	VN	2020	0	1	0	1	0	0	0	0	0	0	0	0	83.6
13616040	m	md	IT	2020	0	1	0	1	0	0	0	0	0	0	0	0	70
13620500	m	md	FR	2020	0	1	0	1	0	0	0	0	0	0	0	0	54.2
13628597	f	md	NL	2020	0	1	0	1	0	0	0	0	0	0	0	0	67
13634460	m	md	US	2020	0	1	0	1	0	0	0	0	0	0	0	0	67
13638054	m	md	JP	2020	0	1	0	1	0	0	0	0	0	0	0	1	79.7
13643433	m	md	KR	2020	0	1	0	1	0	0	0	0	0	0	0	1	42.1
13643532	m	md	US	2020	0	1	0	1	0	0	0	0	0	0	0	0	68.7
13652367	m	md	BR	2020	0	1	0	1	0	0	0	0	0	0	0	0	77.3
13656211	f	md	PL	2020	0	1	0	1	0	0	0	0	0	0	0	1	62.9
13657213	m	md	ES	2020	0	1	0	1	0	0	0	0	0	0	0	0	53.3
13665648	m	md	US	2020	0	1	0	1	0	0	0	0	0	0	0	0	58.9

13667712	m	md	US	2020	0	1	0	1	0	0	0	0	0	0	0	0	60
13669321	f	md	AU	2020	0	0	0	0	0	0	0	0	0	0	0	0	39
13671867	m	md	US	2020	0	1	0	1	0	0	0	0	0	0	0	1	69.3
13676468	m	md	SG	2020	0	1	0	1	0	0	0	0	0	0	0	0	44.2
13677714	f	md	GB	2020	0	1	0	1	0	0	0	0	0	0	0	0	58.1
13681513	m	md	GB	2020	0	1	0	1	0	0	0	0	0	0	0	0	52.3
13685627	m	md	BG	2020	0	1	0	1	0	0	0	0	0	0	0	0	72
13686812	f	md	CA	2020	0	1	0	1	0	0	0	0	0	0	0	0	48
13690293	m	md	CA	2020	0	1	0	1	0	0	0	0	0	0	0	0	68
13693477	f	md	FR	2020	0	1	0	1	0	0	0	0	0	0	0	0	71
13699109	m	md	FR	2020	0	1	0	1	0	0	0	0	0	0	0	0	72.2
13701816	f	md	CA	2020	0	1	0	1	0	0	0	0	0	0	0	0	48
13711638	f	md	FR	2020	0	1	0	1	0	0	0	0	0	0	0	0	71
13712690	Not	cn	CA	2020	0	1	0	1	0	0	0	0	0	0	0	0	
13721128	f	md	JP	2020	0	1	0	1	0	0	0	0	0	0	0	0	73
13724525	Not	cn	CA	2020	0	1	0	1	0	0	0	0	0	0	0	0	
13730058	Not	cn	CA	2020	0	1	0	1	0	0	0	0	0	0	0	0	
13731632	f	md	FI	2020	0	1	0	1	0	0	0	0	0	0	0	0	76.4
13731668	m	md	MX	2020	0	1	0	1	0	0	0	0	0	0	0	0	70.4
13742833	m	md	JP	2020	0	1	0	1	0	0	0	0	0	0	0	0	73.4
13744488	m	md	ES	2020	0	0	0	0	0	0	0	0	0	0	0	0	83
13744630	f	md	CN	2020	0	0	0	0	0	0	0	0	0	0	0	0	64
13747283	m	md	JP	2020	0	1	0	1	0	0	0	0	0	0	0	1	58.6
13760245	f	md	GB	2020	0	1	0	1	0	0	0	0	0	0	0	0	56.5
13765632	m	md	UA	2020	0	1	0	1	0	0	0	0	0	0	0	0	53.2
13783580	f	md	CA	2020	0	1	0	1	0	0	0	0	0	0	0	0	72
13784711	m	md	CN	2020	0	1	0	1	0	0	0	0	0	0	0	0	67.5
13791485	m	md	KR	2020	0	1	0	1	0	0	0	0	0	0	0	0	81.1
13794717	f	md	JP	2020	0	0	0	0	0	0	0	0	0	0	0	0	60
13801105	m	md	QA	2020	0	1	0	1	0	0	0	0	0	0	0	0	54.4
13801346	m	md	QA	2020	0	1	0	1	0	0	0	0	0	0	0	0	54.4
13803653	f	md	CA	2020	0	1	0	1	0	0	0	0	0	0	0	0	58
13803976	f	md	CN	2020	0	1	0	1	0	0	0	0	0	0	0	0	70.5
13815403	m	md	KR	2020	0	1	0	1	0	0	0	0	0	0	0	0	62

13828189	m	md	UA	2020	0	1	0	1	0	0	0	0	0	0	0	0	60.6
13828261	m	md	ES	2020	0	0	0	0	0	0	0	0	0	0	0	0	71
13838166	f	md	PE	2020	0	1	0	1	0	0	0	0	0	0	0	0	62.3
13841340	f	md	CA	2020	0	1	0	1	0	0	0	0	0	0	0	0	76
13843863	m	md	KR	2020	0	1	0	1	0	0	0	0	0	0	0	0	62
13843927	f	md	GB	2020	0	0	0	0	0	0	0	0	0	0	0	0	55
13849097	f	md	GB	2020	0	1	0	1	0	0	0	0	0	0	0	0	66
13854267	m	md	PT	2020	0	1	0	1	0	0	0	0	0	0	0	0	69.6
13855508	f	md	PH	2020	0	1	0	1	0	0	0	0	0	0	0	0	63.9
13869297	m	md	AU	2020	0	0	0	0	0	0	0	0	0	0	0	0	55
13871166	f	md	PE	2020	0	1	0	1	0	0	0	0	0	0	0	0	62.5
13871771	m	md	HK	2020	0	1	0	1	0	0	0	0	0	0	0	1	48.9
13871867	m	md	GB	2020	0	0	0	0	0	0	0	0	0	0	0	0	51
13872737	f	md	CA	2020	0	1	0	1	0	0	0	0	0	0	0	0	60
13880194	f	md	CA	2020	0	1	0	1	0	0	0	0	0	0	0	0	66
13882645	m	md	GB	2020	0	1	0	1	0	0	0	0	0	0	0	0	51
13885061	m	md	MY	2020	0	1	0	1	0	0	0	0	0	0	0	0	58.5
13887127	f	md	US	2020	0	1	0	1	0	0	0	0	0	0	0	0	64
13897346	m	md	MY	2020	0	1	0	1	0	0	0	0	0	0	0	1	77.4
13901537	f	md	CA	2020	0	1	0	1	0	0	0	0	0	0	0	0	73
13901809	m	md	SE	2020	0	1	0	1	0	0	0	0	0	0	0	0	60.7
13916195	m	md	US	2020	0	1	0	1	0	0	0	0	0	0	0	0	56.2
13929684	f	md	CA	2020	0	1	0	1	0	0	0	0	0	0	0	0	76
13946394	m	md	CN	2020	0	1	0	1	0	0	0	0	0	0	0	0	68.7
13950253	f	md	CA	2020	0	1	0	1	0	0	0	0	0	0	0	0	57
13981756	f	md	CN	2020	0	1	0	1	0	0	0	0	0	0	0	0	70.6
13987464	m	md	KR	2020	0	1	0	1	0	0	0	0	0	0	0	0	63
14010250	m	md	IT	2020	0	0	0	0	0	0	0	0	0	0	0	0	59
14019743	f	md	JP	2020	0	1	0	1	0	0	0	0	0	0	0	0	56.1
14022532	f	md	KR	2020	0	1	0	1	0	0	0	0	0	0	0	1	76
14034338	f	md	CA	2020	0	1	0	1	0	0	0	0	0	0	0	0	65
14035119	m	md	US	2020	0	1	0	1	0	0	0	0	0	0	0	0	56.2
14056825	Not	hp	GB	2020	0	0	0	0	0	0	0	0	0	0	0	0	
14085384	m	md	DE	2020	0	1	0	1	0	0	0	0	0	0	0	0	72.7

14087909	m	md	KR	2020	0	1	0	1	0	0	0	0	0	0	0	0	68.2
14100534	m	md	CA	2020	0	1	0	1	0	0	0	0	0	0	0	0	79
14100845	m	md	KR	2020	0	1	0	1	0	0	0	0	0	0	0	0	81.1
14102488	m	md	RU	2020	0	1	0	1	0	0	0	0	0	0	0	0	66
14111894	f	md	GR	2020	0	1	0	1	0	0	0	0	0	0	0	0	65
14121335	f	md	KR	2020	0	1	0	1	0	0	0	0	0	0	0	0	72.1
14129468	f	md	CA	2020	0	1	0	1	0	0	0	0	0	0	0	0	64
14129784	m	md	RU	2020	0	1	0	1	0	0	0	0	0	0	0	0	58.7
14132241	f	md	KR	2020	0	1	0	1	0	0	0	0	0	0	0	1	76.7
14133102	f	md	UA	2020	0	1	0	1	0	0	0	0	0	0	0	0	70
14133271	m	md	JP	2020	0	1	0	1	0	0	0	0	0	0	0	0	58.6
14142788	f	md	CA	2020	0	1	0	1	0	0	0	0	0	0	0	0	64
14155063	f	md	US	2020	0	1	0	1	0	0	0	0	0	0	0	0	60
14179453	m	md	CA	2020	0	1	0	1	0	0	0	0	0	0	0	0	59
14182076	m	md	IT	2020	0	0	0	0	0	0	0	0	0	0	0	0	24
14197856	m	md	US	2020	0	1	0	1	0	0	0	0	0	0	0	0	69.3
14201748	m	md	UA	2020	0	1	0	1	0	0	0	0	0	0	0	0	64.7
14233020	m	hp	GB	2020	0	0	0	0	0	0	0	0	0	0	0	0	55
14297714	f	md	ZA	2020	0	1	0	1	0	0	0	0	0	0	0	0	67
14302952	m	md	JP	2020	0	1	0	1	0	0	0	0	0	0	0	0	62.1
14323384	f	md	CA	2020	0	1	0	1	0	0	0	0	0	0	0	0	64
14325793	f	cn	PT	2020	0	1	0	1	0	0	0	0	0	0	0	0	58
14329397	f	md	CA	2020	0	0	0	0	0	0	0	0	0	0	0	0	66
14330518	f	md	PT	2020	0	1	0	1	0	0	0	0	0	0	0	0	58
14335911	m	md	BR	2020	0	1	0	1	0	0	0	0	0	0	0	0	68.4
14338498	f	md	PT	2020	0	0	0	0	0	0	0	0	0	0	0	0	58
14343737	m	md	US	2020	0	1	0	1	0	0	0	0	0	0	0	0	66
14344929	m	md	IT	2020	0	0	0	0	0	0	0	0	0	0	0	0	53
14345145	m	md	FR	2020	0	0	0	0	0	0	0	0	0	0	0	0	75
14345252	f	md	CN	2020	0	1	0	1	0	0	0	0	0	0	0	0	35.6
14356597	m	md	IN	2020	0	1	0	1	0	0	0	0	0	0	0	0	55.9
14364380	m	hp	CA	2020	0	1	0	1	0	0	0	0	0	0	0	0	74
14367993	m	md	ZA	2020	0	1	0	1	0	0	0	0	0	0	0	0	54
14370433	m	md	IN	2020	0	1	0	1	0	0	0	0	0	0	0	0	55.8

14370883	m	md	IN	2020	0	1	0	1	0	0	0	0	0	0	0	0	55.8
14371238	m	md	US	2020	0	1	0	1	0	0	0	0	0	0	0	0	56.7
14378715	m	md	US	2020	1	0	0	0	0	0	0	0	0	0	0	0	43.7
14382722	f	md	IT	2020	0	1	0	1	0	0	0	0	0	0	0	0	61
14383324	f	ph	GB	2020	0	0	0	0	0	0	0	0	0	0	0	0	
14384431	m	md	GR	2020	0	1	0	1	0	0	0	0	0	0	0	0	58.7
14384595	m	md	ZA	2020	0	1	0	1	0	0	0	0	0	0	0	0	54
14393522	m	md	KR	2020	0	1	0	1	0	0	0	0	0	0	0	0	78.3
14409265	m	md	KR	2020	0	1	0	1	0	0	0	0	0	0	0	0	69.8
14411888	m	md	CN	2020	0	1	0	1	0	0	0	0	0	0	0	0	64.4
14431049	f	md	BE	2020	0	0	0	0	0	0	0	0	0	0	0	0	
14435370	f	md	DK	2020	0	0	0	0	0	0	0	0	0	0	0	0	70
14445882	f	md	US	2020	0	0	0	0	0	0	1	0	0	1	0	0	63
14453802	f	md	CA	2020	0	1	0	1	0	0	0	0	0	0	0	0	67
14465732	f	md	IT	2020	0	0	0	0	0	0	0	0	0	0	0	0	39
14466281	f	md	US	2020	0	1	0	1	0	0	0	0	0	0	0	0	57.6
14475005	f	md	CA	2020	0	1	0	1	0	0	0	0	0	0	0	0	59
14478535	f	md	ES	2020	0	0	0	0	0	0	0	0	0	0	0	0	53
14508295	f	md	JP	2020	0	0	0	0	0	0	0	0	0	0	0	0	70
14531169	m	md	US	2020	0	0	0	0	0	0	0	0	0	0	0	0	53
14536380	m	md	KR	2020	0	1	0	1	0	0	0	0	0	0	0	0	59
14559643	m	md	FR	2020	0	0	0	0	0	0	0	0	0	0	0	0	43
14560651	m	md	GB	2020	0	0	0	0	0	0	0	0	0	0	0	0	
14561101	m	md	GB	2020	0	1	0	1	0	0	0	0	0	0	0	0	56.5
14570458	m	md	FR	2020	0	0	0	0	0	0	0	0	0	0	0	0	43
14576321	m	md	KR	2020	0	0	0	0	0	0	0	0	0	0	0	0	54
14583011	f	md	IN	2020	0	1	0	1	0	0	0	0	0	0	0	0	59
14587714	f	md	CA	2020	0	1	0	1	0	0	0	0	0	0	0	0	62
14589492	m	md	CN	2020	0	1	0	1	0	0	0	0	0	0	0	0	61.6
14590049	m	md	JP	2020	0	1	0	1	0	0	0	0	0	0	0	0	66.9
14637219	m	md	CA	2020	0	1	0	1	0	0	0	0	0	0	0	0	62
14675554	m	md	UA	2020	0	1	0	1	0	0	0	0	0	0	0	0	71.3
14678871	m	md	RU	2020	0	1	0	1	0	0	0	0	0	0	0	0	68.8
14681190	f	md	CA	2020	0	1	0	1	0	0	0	0	0	0	0	0	69

14700303	m	md	FR	2020	0	0	0	0	0	0	0	0	0	0	0	0	43
14702644	f	md	CA	2020	0	1	0	1	0	0	0	0	0	0	0	0	66
14715557	m	md	CN	2020	0	1	0	1	0	0	0	0	0	0	0	0	65.3
14737322	m	md	CA	2020	0	1	0	1	0	0	0	0	0	0	0	0	56
14753518	m	hp	FR	2020	0	0	0	0	0	0	0	0	0	0	0	0	43
14754500	f	md	CA	2020	0	1	0	1	0	0	0	0	0	0	0	0	69
14766631	m	md	AU	2020	0	1	0	1	0	0	0	0	0	0	0	0	65
14766909	m	md	AU	2020	0	1	0	1	0	0	0	0	0	0	0	0	65
14768199	f	md	FR	2020	0	0	0	0	0	0	0	0	0	0	0	0	78
14772769	f	md	CA	2020	0	1	0	1	0	0	0	0	0	0	0	0	65
14778481	m	md	RU	2020	0	1	0	1	0	0	0	0	0	0	0	0	68.9
14784253	m	md	US	2020	0	1	0	1	0	0	0	0	0	0	0	0	70
14807475	m	md	JP	2020	0	1	0	1	0	0	0	0	0	0	0	0	70.9
14814643	f	md	CN	2020	0	0	0	0	0	0	0	0	0	0	0	0	48
14818278	f	md	PL	2020	0	0	0	0	0	0	0	0	0	0	0	0	70
14822175	f	md	BR	2020	0	1	0	1	0	0	0	0	0	0	0	0	66
14827345	m	md	US	2020	0	1	0	1	0	0	0	0	0	0	0	0	69
14838512	m	md	CA	2020	0	1	0	1	0	0	0	0	0	0	0	0	64
14840641	m	md	FR	2020	0	0	0	0	0	0	0	0	0	0	0	0	43
14842312	f	md	CA	2020	0	1	0	1	0	0	0	0	0	0	0	0	63
14843364	m	md	ZA	2020	0	1	0	1	0	0	0	0	0	0	0	0	68
14848723	m	md	AU	2020	0	1	0	1	0	0	0	0	0	0	0	0	66
14848932	m	md	BR	2020	0	1	0	1	0	0	0	0	0	0	0	0	66.2
14855263	m	md	GB	2020	0	0	0	0	0	0	0	0	0	0	0	0	
14874040	m	md	VN	2020	0	1	0	1	0	0	0	0	0	0	0	0	58.5
14894732	m	md	IL	2020	0	0	0	0	0	0	0	0	0	0	0	0	82
14897556	m	md	AU	2020	0	1	0	1	0	0	0	0	0	0	0	0	72
14906553	m	md	GB	2020	0	0	0	0	0	0	0	0	0	0	0	0	55
14906836	f	md	KR	2020	0	0	0	0	0	0	0	0	0	0	0	0	52
14915282	m	md	CA	2020	0	1	0	1	0	0	0	0	0	0	0	0	54
14917572	m	md	FR	2020	0	0	0	0	0	0	0	0	0	0	0	0	48
14920484	f	md	GB	2020	0	1	0	1	0	0	0	0	0	0	0	0	81.9
14922336	m	md	IT	2020	0	0	0	0	0	0	0	0	0	0	0	0	74
14925956	m	md	ZA	2020	0	1	0	1	0	0	0	0	0	0	0	0	66

14928506	f	md	IT	2020	0	0	0	0	0	0	0	0	0	0	0	0	58
14933273	f	md	GB	2020	0	0	0	0	0	0	0	0	0	0	0	0	54
14933379	f	md	AU	2020	0	0	0	0	0	0	0	0	0	0	0	0	60
14937446	m	md	KR	2020	0	1	0	1	0	0	0	0	0	0	0	0	66
14943780	f	md	AU	2020	0	1	0	1	0	0	0	0	0	0	0	0	55
14943836	f	md	US	2020	0	1	0	1	0	0	0	0	0	0	0	0	55.6
14943847	m	md	CA	2020	0	1	0	1	0	0	0	0	0	0	0	0	74
14953986	m	md	CA	2020	0	1	0	1	0	0	0	0	0	0	0	0	78
14961028	m	md	AU	2020	0	1	0	1	0	0	0	0	0	0	0	0	66
14961973	m	md	AU	2020	0	1	0	1	0	0	0	0	0	0	0	0	50
14970578	m	md	ES	2020	0	1	0	0	1	0	0	0	0	0	0	0	63
14987458	m	md	IT	2020	0	1	0	1	0	0	0	0	0	0	0	0	70
14990842	m	md	IL	2020	0	1	0	1	0	0	0	0	0	0	0	0	65.3
15003943	f	md	AU	2020	0	0	0	0	0	0	0	0	0	0	0	0	
15007644	f	md	AU	2020	0	1	0	1	0	0	0	0	0	0	0	0	55
15009245	m	hp	US	2020	0	0	0	0	0	0	0	0	0	0	0	0	53
15027196	f	md	CN	2020	0	0	0	0	0	0	0	0	0	0	0	0	39
15030376	m	md	CA	2020	0	1	0	1	0	0	0	0	0	0	0	0	61
15041812	m	md	HK	2020	0	0	0	0	0	0	0	0	0	0	0	0	72
15044302	m	md	US	2020	0	1	0	1	0	0	0	0	0	0	0	0	67.8
15048412	m	md	AU	2020	0	0	0	0	0	0	0	0	0	0	0	0	43
15053014	m	md	DE	2020	0	1	0	1	0	0	0	0	0	0	0	0	60
15077203	m	md	CN	2020	0	1	0	1	0	0	0	0	0	0	0	0	55.8
15084555	m	md	JP	2020	0	1	0	1	0	0	0	0	0	0	0	0	74.6
15087616	f	md	CA	2020	0	1	0	1	0	0	0	0	0	0	0	0	72
15094196	Not	md	BR	2020	0	0	0	0	0	0	0	0	0	0	0	0	
15095866	m	md	GB	2020	0	0	0	0	0	0	0	0	0	0	0	0	72
15108620	f	md	CA	2020	0	1	0	1	0	0	0	0	0	0	0	0	62
15111906	m	md	BR	2020	0	1	0	1	0	0	0	0	0	0	0	0	61.2
15113378	m	md	AU	2020	0	0	0	0	0	0	0	0	0	0	0	0	
15115587	f	md	CA	2020	0	1	0	1	0	0	0	0	0	0	0	0	63
15123685	f	md	ES	2020	0	0	0	0	0	0	0	0	0	0	0	0	
15127136	m	md	AU	2020	0	1	0	1	0	0	0	0	0	0	0	0	40
15132319	m	md	AU	2020	0	1	0	1	0	0	0	0	0	0	0	0	72

15136345	f	md	CA	2020	0	1	0	1	0	0	0	0	0	0	0	0	66
15138368	m	md	BR	2020	0	0	0	0	0	0	0	0	0	0	0	0	31
15147894	f	md	KR	2020	0	0	0	0	0	0	0	0	0	0	0	0	70
15149945	m	md	BR	2020	0	1	0	1	0	0	0	0	0	0	0	0	52.4
15150534	m	md	KR	2020	0	1	0	1	0	0	0	0	0	0	0	0	76.6
15159244	f	hp	JP	2020	0	1	0	0	1	0	0	0	0	0	0	0	48
15160194	m	md	US	2020	0	1	0	1	0	0	0	0	0	0	0	0	76.2
15205517	m	md	CA	2020	0	0	0	0	0	0	0	0	0	0	0	0	53
15209802	m	md	JP	2020	0	1	0	1	0	0	0	0	0	0	0	0	77.4
15215962	f	md	CA	2020	0	1	0	1	0	0	0	0	0	0	0	0	59
15246907	f	md	AU	2020	0	1	0	1	0	0	0	0	0	0	0	0	58
15253360	f	md	CA	2020	0	1	0	1	0	0	0	0	0	0	0	0	55
15258479	m	md	DE	2020	0	0	0	0	0	0	0	0	0	0	0	0	50
15290833	m	md	VN	2020	0	1	0	1	0	0	0	0	0	0	0	0	69.6
15302438	m	md	JP	2020	0	1	0	1	0	0	0	0	0	0	0	0	70.3
15306019	m	md	US	2020	0	0	0	0	0	0	0	0	0	0	0	0	47
15312088	m	md	TR	2020	0	1	0	1	0	0	0	0	0	0	0	0	68.3
15320252	m	md	JP	2020	0	1	0	1	0	0	0	0	0	0	0	0	70
15324397	m	md	PT	2020	0	1	0	1	0	0	0	0	0	0	0	0	67.1
15328406	f	md	IT	2020	0	0	0	0	0	0	0	0	0	0	0	0	46
15340385	m	md	CA	2020	0	1	0	1	0	0	0	0	0	0	0	0	72
15341242	m	md	TH	2020	0	0	0	0	0	0	0	0	0	0	0	0	75
15342568	m	md	CA	2020	0	1	0	1	0	0	0	0	0	0	0	0	57
15351793	m	md	PL	2020	0	1	0	1	0	0	0	0	0	0	0	0	59
15375599	m	md	CA	2020	0	1	0	1	0	0	0	0	0	0	0	0	57
15377498	f	md	CA	2020	0	1	0	1	0	0	0	0	0	0	0	0	62
15386547	m	md	TH	2020	0	1	0	1	0	0	0	0	0	0	0	0	65.6
15387225	m	md	AU	2020	0	1	0	1	0	0	0	0	0	0	0	1	72
15397362	m	hp	DE	2020	0	0	0	0	0	0	0	0	0	0	0	0	55
15406551	f	md	ES	2020	0	0	0	0	0	0	0	0	0	0	0	0	63
15418219	m	md	DE	2020	0	0	0	0	0	0	0	0	0	0	0	0	55
15426732	m	md	US	2020	0	1	0	1	0	0	0	0	0	0	0	0	80
15435344	f	md	HU	2020	0	1	0	1	0	0	0	0	0	0	0	0	66
15450528	m	md	TH	2020	0	1	0	1	0	0	0	0	0	0	0	0	57.6

15459393	f	md	US	2020	1	0	0	0	0	0	0	0	0	0	0	0	30
15479538	m	md	CA	2020	0	1	0	1	0	0	0	0	0	0	0	1	64
15481430	m	md	RU	2020	0	1	0	1	0	0	0	0	0	0	0	0	59
15486005	m	md	RU	2020	0	1	0	1	0	0	0	0	0	0	0	0	58.8
15491210	m	md	AT	2020	0	0	0	0	0	0	0	0	0	0	0	0	53
15492658	m	md	TW	2020	0	1	0	1	0	0	0	0	0	0	0	0	51.7
15494365	m	md	JP	2020	0	1	0	1	0	0	0	0	0	0	0	0	77.8
15500977	m	md	JP	2020	0	1	0	1	0	0	0	0	0	0	0	0	74
15506242	m	md	KR	2020	0	1	0	1	0	0	0	0	0	0	0	1	63.3
15508678	m	md	DE	2020	0	0	0	0	0	0	0	0	0	0	0	0	55
15533481	Not	hp	CA	2020	0	0	0	0	0	0	0	0	0	0	0	0	
15534591	m	md	CA	2020	0	1	0	1	0	0	0	0	0	0	0	0	81
15538131	f	md	CA	2020	0	1	0	1	0	0	0	0	0	0	0	0	70
15538353	m	md	CA	2020	0	1	0	1	0	0	0	0	0	0	0	0	64
15542142	m	md	CA	2020	0	1	0	1	0	0	0	0	0	0	0	1	69
15555241	m	md	IT	2020	0	0	0	0	0	0	0	0	0	0	0	0	83
15556325	f	md	CA	2020	0	1	0	1	0	0	0	0	0	0	0	0	61
15565674	m	md	JP	2020	0	1	0	1	0	0	0	0	0	0	0	1	71.2
15576183	m	md	CA	2020	0	1	0	1	0	0	0	0	0	0	0	0	65
15591464	Not	cn	RO	2020	0	0	0	0	0	0	0	0	0	0	0	0	
15603153	f	md	DE	2020	0	1	0	1	0	0	0	0	0	0	0	0	72
15607239	m	md	TH	2020	0	1	0	1	0	0	0	0	0	0	0	0	77.4
15610703	f	md	AU	2020	0	1	0	1	0	0	0	0	0	0	0	0	58
15633537	m	md	CN	2020	0	1	0	1	0	0	0	0	0	0	0	0	66.5
15666104	m	hp	DK	2020	0	0	0	0	0	0	0	0	0	0	0	0	38
15673340	m	hp	DK	2020	0	0	0	0	0	0	0	0	0	0	0	0	38
15693502	f	md	AU	2020	0	1	0	1	0	0	0	0	0	0	0	0	65
15752185	f	md	US	2020	0	1	0	1	0	0	0	0	0	0	0	0	76
15773828	m	md	AR	2020	0	0	0	0	0	0	0	0	0	0	0	0	44
15775174	m	md	CN	2020	0	0	0	0	0	0	0	0	0	0	0	0	67
15792968	Not	md	AU	2020	0	0	0	0	0	0	0	0	0	0	0	0	29
15802901	m	hp	CA	2020	0	0	0	0	0	0	0	0	0	0	0	0	
15847081	m	md	DK	2020	0	0	0	0	0	0	0	0	0	0	0	0	38
15849534	m	cn	DK	2020	0	0	0	0	0	0	0	0	0	0	0	0	38

15896617	f	md	CA	2020	0	1	0	1	0	0	0	0	0	0	0	0	57
15945816	m	md	FR	2020	0	0	0	0	0	0	0	0	0	0	0	0	42
15977167	Not	md	US	2020	0	0	0	0	0	0	0	0	0	0	0	0	
15990339	f	md	US	2020	0	0	0	0	0	0	0	0	0	0	0	0	
16003428	m	md	DE	2020	0	0	0	0	0	0	0	0	0	0	0	0	42
16015277	Not	hp	JP	2020	0	0	0	0	0	0	0	0	0	0	0	0	
16023744	f	md	FR	2020	0	1	0	0	0	1	0	0	0	0	0	0	49
16056447	f	md	US	2020	0	1	0	0	0	1	0	0	0	0	0	0	80
16083913	f	hp	JP	2020	0	0	0	0	0	0	0	0	0	0	0	0	68
16084406	f	md	IT	2020	0	0	0	0	0	0	0	0	0	0	0	0	70
16143571	m	hp	DE	2020	0	0	0	0	0	0	0	0	0	0	0	0	44
16149451	m	cn	DE	2020	0	0	0	0	0	0	0	0	0	0	0	0	42
16157669	f	cn	CA	2020	0	1	1	0	0	0	0	0	0	0	0	0	77
16165026	m	md	NL	2020	0	1	0	1	0	0	0	0	0	0	0	0	68
16207405	Not	hp	IL	2020	0	0	0	0	0	0	0	0	0	0	0	0	
16225053	m	md	JP	2020	0	1	0	1	0	0	0	0	0	0	0	0	74
16244568	f	md	KR	2020	0	0	0	0	0	0	0	0	0	0	0	0	51
16308061	f	md	JP	2020	0	0	0	0	0	0	0	0	0	0	0	0	74
16326712	m	md	CA	2020	0	1	0	1	0	0	0	0	0	0	0	0	62
16350770	m	md	KR	2020	0	1	0	0	1	0	0	0	0	0	0	0	
16359785	m	md	AR	2020	0	0	0	0	0	0	0	0	0	0	0	0	44
16413352	f	md	AU	2020	0	1	0	1	0	0	0	0	0	0	0	0	59
16422235	m	md	CA	2020	0	1	0	1	0	0	0	0	0	0	0	0	77
16430949	f	md	KR	2020	0	0	0	0	0	0	0	0	0	0	0	0	78
16440051	m	Not repo	US	2020	0	0	0	0	0	0	0	0	0	0	0	0	66
16489688	f	md	ES	2020	0	0	0	0	0	0	0	0	0	0	0	0	70
16498305	m	md	KR	2020	0	0	0	0	0	0	0	0	0	0	0	0	73
16500346	m	md	GB	2020	0	0	0	0	0	0	0	0	0	0	0	0	59
16521571	m	md	CA	2020	0	1	0	1	0	0	0	0	0	0	0	0	56
16565023	f	hp	IT	2020	0	0	0	0	0	0	0	0	0	0	0	0	46
16644479	f	md	CA	2020	0	1	0	1	0	0	0	0	0	0	0	0	70
16675065	f	md	GB	2020	0	0	0	0	0	0	0	0	0	0	0	0	45
16696475	f	md	DK	2020	0	0	0	0	0	0	0	0	0	0	0	0	67
16714764	m	md	KR	2020	0	0	0	0	0	0	0	0	0	0	0	0	50

16738530	f	md	CA	2020	0	0	0	0	0	0	0	0	0	0	0	0	
16753032	f	md	GB	2020	0	1	0	1	0	0	0	0	0	0	0	0	81.7
16772957	m	md	AU	2020	0	1	0	1	0	0	0	0	0	0	0	0	46
16788053	m	cn	KR	2020	0	0	0	0	0	0	0	0	0	0	0	0	
16795354	f	cn	CA	2020	0	1	1	0	0	0	0	0	0	0	0	0	77
16811200	Not	hp	ES	2020	0	0	0	0	0	0	0	0	0	0	0	0	
16828636	m	md	AU	2020	0	1	0	1	0	0	0	0	0	0	0	0	46
16868931	f	md	ES	2020	0	1	1	0	0	0	0	0	0	0	0	0	53
16879863	f	md	ES	2020	0	1	1	0	0	0	0	0	0	0	0	0	53
16915619	f	cn	CA	2020	0	1	1	0	0	0	0	0	0	0	0	0	77
16921823	m	md	KR	2020	0	1	1	0	0	0	0	0	0	0	0	0	69
16926546	m	md	KR	2020	0	1	1	0	0	0	0	0	0	0	0	0	70
16926548	m	md	FR	2020	0	1	1	0	0	0	0	0	0	0	0	0	61
16927374	m	md	ES	2020	0	0	0	0	0	0	0	0	0	0	0	0	60
16927702	m	md	CA	2020	0	1	1	0	0	0	0	0	0	0	0	0	66
16932143	f	md	ES	2020	0	1	1	0	0	0	0	0	0	0	0	0	53
16932497	m	md	PL	2020	0	0	0	0	0	0	0	0	0	0	0	0	63
16933844	f	md	DE	2020	0	1	1	0	0	0	0	0	0	0	0	0	55
16934834	m	md	KR	2020	0	1	1	0	0	0	0	0	0	0	0	0	61
16934842	m	md	DE	2020	0	0	0	0	0	0	0	0	0	0	0	0	61
16938835	f	md	DE	2020	0	0	0	0	0	0	0	0	0	0	0	0	51
16942476	m	hp	NL	2020	0	0	0	0	0	0	0	0	0	0	0	0	50
16942477	f	md	IT	2020	0	0	0	0	0	0	0	0	0	0	0	0	56
16962859	f	md	DK	2020	0	0	0	0	0	0	0	0	0	0	0	0	72
16963098	f	md	DK	2020	0	0	0	0	0	0	0	0	0	0	0	0	72
16969032	m	md	RU	2020	0	0	0	0	0	0	0	0	0	0	0	0	34
16977189	m	md	DE	2020	0	0	0	0	0	0	0	0	0	0	0	0	61
16980784	m	hp	CN	2020	0	0	0	0	0	0	0	0	0	0	0	0	72
16985117	Not	md	US	2020	0	0	0	0	0	0	0	0	0	0	0	0	
16993660	f	md	DK	2020	0	0	0	0	0	0	0	0	0	0	0	0	67
17005758	f	md	IL	2020	0	0	0	0	0	0	0	0	0	0	0	0	81
17006288	f	cn	CA	2020	0	1	1	0	0	0	0	0	0	0	0	0	77
17007593	Not	hp	JP	2020	0	0	0	0	0	0	0	0	0	0	0	0	
17007966	f	md	AU	2020	0	1	0	1	0	0	0	0	0	0	0	0	59

17015640	Not	hp	JP	2020	0	0	0	0	0	0	0	0	0	0	0	0	
17022554	f	md	CN	2020	0	0	0	0	0	0	0	0	0	0	0	0	67
17032618	m	md	ES	2020	0	1	1	0	0	0	0	0	0	0	0	0	75
17044506	m	md	CN	2020	0	0	0	0	0	0	0	0	0	0	0	0	64
17049609	m	hp	CN	2020	0	0	0	0	0	0	0	0	0	0	0	0	67
17064574	m	md	IN	2020	0	1	1	0	0	0	0	0	0	0	0	0	60
17067856	m	md	DK	2020	0	1	1	0	0	0	0	0	0	0	0	0	74
17078519	f	md	CA	2020	0	0	0	0	0	0	0	0	0	0	0	0	
17089716	m	cn	CN	2020	0	1	0	0	1	0	0	0	0	0	0	0	65
17111251	m	hp	CN	2020	0	0	0	0	0	0	0	0	0	0	0	0	67
17112915	f	md	TW	2020	0	0	0	0	0	0	0	0	0	0	0	0	57
17113836	m	hp	US	2020	0	1	0	0	1	0	0	0	0	0	0	0	70
17134859	m	md	CN	2020	0	0	0	0	0	0	0	0	0	0	0	0	70
17158540	m	md	BH	2020	0	0	0	0	0	0	0	0	0	0	0	0	
17173060	m	md	BE	2020	0	0	0	0	0	0	0	0	0	0	0	0	67
17174108	f	md	JP	2020	0	1	0	0	0	1	0	0	0	0	0	0	71
17195452	f	md	IT	2020	0	0	0	0	0	0	0	0	0	0	0	0	78
17199861	m	md	BH	2020	0	0	0	0	0	0	0	0	0	0	0	0	
17218060	f	md	CN	2020	0	0	0	0	0	0	0	0	0	0	0	0	51
17241854	f	md	TH	2020	0	0	0	0	0	0	0	0	0	0	0	0	57
17242987	m	hp	CN	2020	0	0	0	0	0	0	0	0	0	0	0	0	64
17243193	m	md	CN	2020	0	0	0	0	0	0	0	0	0	0	0	0	56
17248192	m	md	CN	2020	0	0	0	0	0	0	0	0	0	0	0	0	70
17275597	m	md	KR	2020	0	0	0	0	0	0	0	0	0	0	0	0	72
17277384	f	md	KR	2020	0	0	0	0	0	0	0	0	0	0	0	0	69
17277651	m	md	TR	2020	0	1	1	0	0	0	0	0	0	0	0	0	77
17282845	f	md	IT	2020	0	0	0	0	0	0	0	0	0	0	0	0	78
17285594	m	md	IN	2020	0	1	1	0	0	0	0	0	0	0	0	0	72
17303516	Not	md	FR	2020	0	0	0	0	0	0	0	0	0	0	0	0	
17315113	f	hp	JP	2020	0	1	0	0	1	0	0	0	0	0	0	0	51
17370641	m	md	DE	2020	0	0	0	0	0	0	0	0	0	0	0	0	
17375037	m	md	TR	2020	0	1	1	0	0	0	0	0	0	0	0	1	64
17391982	Not	hp	US	2020	0	0	0	0	0	0	0	0	0	0	0	0	
17392150	Not	hp	US	2020	0	0	0	0	0	0	0	0	0	0	0	0	

17392168	Not	hp	US	2020	0	0	0	0	0	0	0	0	0	0	0	0	
17397584	m	md	CH	2020	0	0	0	0	0	0	0	0	0	0	0	0	57
17400177	m	md	JP	2020	0	1	0	0	0	1	0	0	0	0	0	0	70
17411331	f	cn	JP	2020	0	1	1	0	0	0	0	0	0	0	0	0	72
17421376	m	md	IN	2020	0	1	1	0	0	0	0	0	0	0	0	0	72
17429376	m	md	KR	2020	0	1	1	0	0	0	0	0	0	0	0	0	75
17436835	f	md	CA	2020	0	1	0	1	0	0	0	0	0	0	0	0	64
17438993	f	md	KR	2020	0	0	0	0	0	0	0	0	0	0	0	0	83
17440931	f	hp	CN	2020	0	0	0	0	0	0	0	0	0	0	0	0	43
17447608	m	hp	DK	2020	0	0	0	0	0	0	0	0	0	0	0	0	65
17448426	Not	hp	JP	2020	0	0	0	0	0	0	0	0	0	0	0	0	
17453570	m	md	CN	2020	0	0	0	0	0	0	0	0	0	0	0	0	70
17456084	m	md	JP	2020	0	1	0	0	0	1	0	0	0	0	0	0	62
17464732	f	cn	US	2020	0	0	0	0	0	0	0	0	0	0	0	0	
17464736	f	hp	US	2020	0	0	0	0	0	0	0	0	0	0	0	0	
17464743	m	ph	US	2020	0	0	0	0	0	0	0	0	0	0	0	0	
17464927	f	ph	US	2020	0	0	0	0	0	0	0	0	0	0	0	0	65
17464931	f	md	US	2020	0	0	0	0	0	0	0	0	0	0	0	0	
17483479	f	md	VN	2020	1	0	0	0	0	0	0	0	0	0	0	0	46.6
17483967	f	hp	US	2020	0	0	0	0	0	0	0	0	0	0	0	0	75
17483996	f	hp	US	2020	0	0	0	0	0	0	0	0	0	0	0	0	45
17487630	f	md	CA	2020	0	0	0	0	0	0	0	0	0	0	0	0	
17506574	f	cn	CA	2020	0	1	1	0	0	0	0	0	0	0	0	0	77
17519783	m	md	CN	2020	0	0	0	0	0	0	0	0	0	0	0	0	47
17531846	m	md	TH	2020	0	1	0	1	0	0	0	0	0	0	0	0	59.4
17547799	f	cn	JP	2020	0	1	1	0	0	0	0	0	0	0	0	0	64
17565813	m	md	CN	2020	0	0	0	0	0	0	0	0	0	0	0	0	61
17571474	f	md	BR	2020	0	0	0	0	0	0	0	0	0	0	0	0	61
17607832	m	hp	CN	2020	0	0	0	0	0	0	0	0	0	0	0	0	70
17616945	m	hp	GB	2020	0	1	1	0	0	0	0	0	0	0	0	0	67
17623398	m	md	CN	2020	0	0	0	0	0	0	0	0	0	0	0	0	68
17624421	f	md	AR	2020	0	1	1	0	0	0	0	0	0	0	0	0	53
17624462	f	md	CL	2020	0	1	1	0	0	0	0	0	0	0	0	0	60
17628453	m	md	JP	2020	0	1	0	0	0	1	0	0	0	0	0	0	76

17634438	m	hp	AR	2020	0	1	0	0	1	0	0	0	0	0	0	0	61
17635261	m	md	KR	2020	0	0	0	0	0	0	0	0	0	0	0	0	77
17649968	m	hp	CN	2020	0	1	1	0	0	0	0	0	0	0	0	0	65
17662562	m	hp	IT	2020	0	1	1	0	0	0	0	0	0	0	0	0	41
17669518	f	hp	JP	2020	0	0	0	0	0	0	0	0	0	0	0	0	72
17669702	Not	hp	IN	2020	0	0	0	0	0	0	0	0	0	0	0	0	
17670581	m	md	TH	2020	0	1	1	0	0	0	0	0	0	0	0	0	63
17671343	m	hp	JP	2020	0	0	0	0	0	0	0	0	0	0	0	0	81
17671354	Not	hp	JP	2020	0	0	0	0	0	0	0	0	0	0	0	0	
17671355	f	hp	JP	2020	0	0	0	0	0	0	0	0	0	0	0	0	66
17671377	f	hp	JP	2020	0	0	0	0	0	0	0	0	0	0	0	0	63
17671392	f	hp	JP	2020	0	0	0	0	0	0	0	0	0	0	0	0	70
17691180	f	md	JP	2020	0	1	0	0	0	1	0	0	0	0	0	0	80
17693486	m	hp	IN	2020	0	1	1	0	0	0	0	0	0	0	0	0	55
17693883	f	md	JP	2020	0	0	0	0	0	0	1	0	0	1	0	1	66
17703579	f	cn	DE	2020	0	1	1	0	0	0	0	0	0	0	0	1	55
17705033	f	md	KR	2020	0	0	0	0	0	0	0	0	0	0	0	0	66
17710503	m	md	JP	2020	0	1	0	0	0	1	0	0	0	0	0	0	49
17715449	m	hp	DE	2020	0	0	0	0	0	0	0	0	0	0	0	0	72
17721284	m	cn	JP	2020	0	1	1	0	0	0	0	0	0	0	0	0	69
17729111	f	cn	US	2020	0	0	0	0	0	0	0	0	0	0	0	0	
17730140	m	md	JP	2020	0	0	0	0	0	0	0	0	0	0	0	0	75
17733868	Not	hp	JP	2020	0	0	0	0	0	0	0	0	0	0	0	0	
17734021	Not	hp	JP	2020	0	0	0	0	0	0	0	0	0	0	0	0	
17739947	f	cn	CA	2020	0	1	1	0	0	0	0	0	0	0	0	0	77
17743521	m	hp	AU	2020	0	0	0	0	0	0	0	0	0	0	0	0	
17747732	m	hp	AU	2020	0	1	1	0	0	0	0	0	0	0	0	0	59
17754520	m	hp	IT	2020	0	1	0	0	1	0	0	0	0	0	0	0	76
17754613	f	md	KR	2020	0	0	0	0	0	0	0	0	0	0	0	0	64
17755223	f	cn	AU	2020	0	0	0	0	0	0	0	0	0	0	0	0	62
17755808	f	cn	DE	2020	0	1	0	0	0	1	0	0	0	0	0	0	53
17761275	m	hp	DK	2020	0	0	0	0	0	0	0	0	0	0	0	0	38
17762165	f	md	TW	2020	0	0	0	0	0	0	0	0	0	0	0	0	57
17765406	m	md	JP	2020	0	1	0	0	0	1	0	0	0	0	0	0	79

17769338	m	cn	US	2020	0	1	0	0	1	0	0	0	0	0	0	0	74
17772947	f	md	CA	2020	0	1	1	0	0	0	0	0	0	0	0	0	59
17787707	m	hp	CN	2020	0	0	0	0	0	0	0	0	0	0	0	0	46
17787770	m	md	CN	2020	0	0	0	0	0	0	0	0	0	0	0	0	50
17787836	f	Not repo	CN	2020	1	0	0	0	0	0	0	0	0	0	0	0	58
17796710	m	md	AU	2020	0	0	0	0	0	0	0	0	0	0	0	0	
17797356	f	cn	AU	2020	0	0	0	0	0	0	0	0	0	0	0	0	
17799275	m	md	JP	2020	0	1	0	1	0	0	0	0	0	0	0	0	61
17801488	m	md	JP	2020	0	0	0	0	0	0	0	0	0	0	0	0	76
17803233	m	hp	CN	2020	0	0	0	0	0	0	0	0	0	0	0	0	69
17805837	m	cn	US	2020	0	0	0	0	0	0	0	0	0	0	0	0	
17808465	m	md	GR	2020	0	1	1	0	0	0	0	0	0	0	0	0	72
17812191	m	md	DE	2020	0	0	0	0	0	0	0	0	0	0	0	0	72
17812192	m	md	DE	2020	0	0	0	0	0	0	0	0	0	0	0	0	73
17814962	f	md	CN	2020	0	0	0	0	0	0	0	0	0	0	0	0	54
17816041	f	hp	JP	2020	1	0	0	0	0	0	0	0	0	0	0	0	63
17816825	m	md	KR	2020	0	1	1	0	0	0	0	0	0	0	0	0	77
17826668	m	md	IT	2020	0	1	0	0	0	1	0	0	0	0	0	0	80
17830490	Not	md	IT	2020	0	1	0	0	1	0	0	0	0	0	0	0	
17830600	m	md	ES	2020	0	1	1	0	0	0	0	0	0	0	0	0	75
17831408	m	md	ZA	2020	0	1	1	0	0	0	0	0	0	0	0	0	62
17836427	m	md	TR	2020	0	1	1	0	0	0	0	0	0	0	0	0	60
17839819	f	hp	GB	2020	0	1	1	0	0	0	0	0	0	0	0	0	57
17840913	m	hp	NL	2020	0	0	0	0	0	0	0	0	0	0	0	0	69
17846483	f	hp	US	2020	1	0	0	0	0	0	0	0	0	0	0	0	
17855125	m	md	JP	2020	0	1	0	1	0	0	0	0	0	0	0	1	80
17856114	m	hp	AU	2020	0	1	1	0	0	0	0	0	0	0	0	1	81
17857057	m	hp	DE	2020	0	1	0	0	0	1	0	0	0	0	0	0	57
17864017	f	cn	JP	2020	0	1	1	0	0	0	0	0	0	0	0	0	80
17864299	m	hp	TR	2020	0	1	1	0	0	0	0	0	0	0	0	0	77
17868119	Not	md	GB	2020	0	0	0	0	0	0	0	0	0	0	0	0	
17868202	m	md	JP	2020	0	1	0	0	0	1	0	0	0	0	0	0	78
17870378	m	md	RU	2020	0	1	1	0	0	0	0	0	0	0	0	0	51
17870650	m	md	CN	2020	0	0	0	0	0	0	0	0	0	0	0	0	65

17871695	m	cn	US	2020	0	0	0	0	0	0	0	0	0	0	0	0	86
17871775	Not	hp	US	2020	0	0	0	0	0	0	1	0	0	0	1	1	
17875313	m	hp	CL	2020	0	1	1	0	0	0	0	0	0	0	0	0	28
17880338	Not	md	JP	2020	0	1	0	1	0	0	0	0	0	0	0	0	
17882719	m	md	JP	2020	0	1	0	0	0	1	0	0	0	0	0	0	93
17884703	Not	Not repo	JP	2020	0	1	0	1	0	0	0	0	0	0	0	0	
17887676	m	cn	US	2020	0	1	1	0	0	0	0	0	0	0	0	0	79
17888249	f	Not repo	US	2020	0	1	0	1	0	0	0	0	0	0	0	0	62
17889753	Not	md	JP	2020	0	1	0	1	0	0	0	0	0	0	0	1	
17891085	f	hp	US	2020	0	0	0	0	0	0	0	0	0	0	0	0	72
17900720	m	cn	JP	2020	0	0	0	0	0	0	0	0	0	0	0	0	80
17903287	f	md	VN	2020	1	0	0	0	0	0	0	0	0	0	0	0	70
17903670	Not	md	DE	2020	0	1	1	0	0	0	0	0	0	0	0	1	55
17908525	m	md	UA	2020	0	1	1	0	0	0	0	0	0	0	0	0	69
17909782	m	md	RU	2020	0	1	1	0	0	0	0	0	0	0	0	0	61
17911259	m	hp	DK	2020	0	0	0	0	0	0	0	0	0	0	0	0	38
17916606	Not	md	NL	2020	0	1	1	0	0	0	0	0	0	0	0	1	55
17917931	m	md	HU	2020	0	1	0	0	1	0	0	0	0	0	0	0	
17919169	f	md	TR	2020	0	0	0	0	0	0	0	0	0	0	0	0	38
17921182	m	md	AT	2020	0	1	0	0	1	0	0	0	0	0	0	0	49
17928214	m	ph	JP	2020	0	1	0	0	0	1	0	0	0	0	0	0	70
17929994	f	md	DE	2020	0	0	0	0	0	0	0	0	0	0	0	0	46
17931127	m	cn	JP	2020	0	1	1	0	0	0	0	0	0	0	0	0	73
17935450	f	md	JP	2020	0	1	1	0	0	0	0	0	0	0	0	0	76
17938654	f	hp	DE	2020	0	1	1	0	0	0	0	0	0	0	0	0	76
17938908	f	Not repo	JP	2020	1	0	0	0	0	0	0	0	0	0	0	0	56
17939296	m	cn	US	2020	0	1	1	0	0	0	0	0	0	0	0	0	77
17940933	m	cn	CN	2020	0	1	0	0	1	0	0	0	0	0	0	0	70
17943765	m	md	US	2020	0	1	0	0	0	1	0	0	0	0	0	0	70
17943920	m	md	JP	2020	1	0	0	0	0	0	0	0	0	0	0	0	85
17945143	m	Not repo	CN	2020	1	0	0	0	0	0	0	0	0	0	0	0	81.8
17945324	m	Not repo	CN	2020	0	1	0	1	0	0	0	0	0	0	0	1	78.2
17948524	m	cn	AT	2020	0	1	1	0	0	0	0	0	0	0	0	0	77
17950818	m	md	DE	2020	0	1	1	0	0	0	0	0	0	0	0	0	69

17952573	m	hp	CN	2020	1	0	0	0	0	0	0	0	0	0	0	0	64
17953024	m	md	IT	2020	1	0	0	0	0	0	0	0	0	0	0	0	70
17953408	m	hp	DE	2020	0	1	0	0	0	1	0	0	0	0	0	0	59
17953503	f	md	JP	2020	0	1	0	1	0	0	0	0	0	0	0	0	70
17954756	f	hp	DE	2020	0	1	0	0	1	0	0	0	0	0	0	0	64
17956425	m	hp	AU	2020	0	0	0	0	0	0	0	0	0	0	0	0	66
17956441	f	hp	AU	2020	0	0	0	0	0	0	0	0	0	0	0	0	77
17956775	m	hp	JP	2020	0	0	0	0	0	0	0	0	0	0	0	0	59
17961453	m	cn	CN	2020	0	1	0	0	1	0	0	0	0	0	0	0	58
17962810	f	hp	US	2020	0	0	0	0	0	0	0	0	0	0	0	0	55
17963869	Not	md	NL	2020	0	0	0	0	0	0	0	0	0	0	0	0	
17964854	m	hp	NL	2020	0	0	0	0	0	0	0	0	0	0	0	0	58
17965060	f	cn	CA	2020	1	0	0	0	0	0	0	0	0	0	0	0	64.5
17965708	Not	md	CN	2020	0	0	0	0	0	0	0	0	0	0	0	0	
17965783	f	md	AU	2020	0	0	0	0	0	0	0	0	0	0	0	0	32
17965813	Not	md	JP	2020	0	1	0	1	0	0	0	0	0	0	0	0	
17966152	f	cn	US	2020	0	1	0	0	1	0	0	0	0	0	0	0	
17966413	m	md	CN	2020	0	1	1	0	0	0	0	0	0	0	0	0	
17966591	m	hp	US	2020	0	1	0	0	1	0	0	0	0	0	0	0	71
17966889	m	md	JP	2020	0	0	0	0	0	0	0	0	0	0	0	0	90
17967885	m	cn	US	2020	0	1	0	0	1	0	0	0	0	0	0	0	
17969417	m	cn	CN	2020	0	0	0	0	0	0	0	0	0	0	0	0	78
17969428	f	md	TW	2020	0	0	0	0	0	0	0	0	0	0	0	0	51
17969643	f	cn	DE	2020	0	1	1	0	0	0	0	0	0	0	0	0	71
17969729	m	md	IN	2020	0	1	1	0	0	0	0	0	0	0	0	0	67
17969880	m	Not repo	MY	2020	1	0	0	0	0	0	0	0	0	0	0	0	60.4
17970487	f	cn	US	2020	0	0	0	0	0	0	0	0	0	0	0	0	67
17971205	f	ph	JP	2020	1	0	0	0	0	0	0	0	0	0	0	0	92
17971395	f	md	JP	2020	0	0	0	0	0	0	0	0	0	0	0	0	
17971849	m	cn	JP	2020	0	1	1	0	0	0	0	0	0	0	0	0	71
17971866	f	md	CA	2020	0	0	0	0	0	0	0	0	0	0	0	0	55
17971950	m	Not repo	NZ	2020	0	1	0	1	0	0	0	0	0	0	0	0	
17972123	m	md	US	2020	0	0	0	0	0	0	0	0	0	0	0	0	67
17973344	m	cn	JP	2020	0	1	1	0	0	0	0	0	0	0	0	1	59

17973411	m	md	JP	2020	0	1	1	0	0	0	0	0	0	0	0	1	70
17973757	f	md	FR	2020	0	1	0	1	0	0	0	0	0	0	0	0	73
17973761	f	cn	JP	2020	1	0	0	0	0	0	0	0	0	0	0	0	
17973988	f	cn	AU	2020	1	0	0	0	0	0	0	0	0	0	0	0	50
17974758	f	md	ES	2020	0	1	1	0	0	0	0	0	0	0	0	0	54
17975079	f	cn	AR	2020	0	0	0	0	0	0	0	0	0	0	0	0	73
17975607	f	cn	AR	2020	0	0	0	0	0	0	0	0	0	0	0	0	69
17975796	f	cn	IL	2020	0	0	0	0	0	0	0	0	0	0	0	0	
17976275	m	md	US	2020	0	1	0	0	0	1	0	0	0	0	0	0	71
17976559	f	ph	JP	2020	0	0	0	0	0	0	0	0	0	0	0	0	72
17976890	f	cn	CN	2020	0	1	0	0	1	0	0	0	0	0	0	0	69
17976900	f	cn	CN	2020	0	1	0	1	0	0	0	0	0	0	0	0	61
17976908	Not	hp	US	2020	1	0	0	0	0	0	0	0	0	0	0	0	
17976927	m	md	JP	2020	0	1	0	1	0	0	0	0	0	0	0	1	70
17976948	Not	ph	US	2020	0	1	0	1	0	0	0	0	0	0	0	0	
17977105	f	cn	US	2020	0	0	0	0	0	0	0	0	0	0	0	0	
17977166	f	md	JP	2020	0	0	0	0	0	0	0	0	0	0	0	0	0
17977243	m	cn	CN	2020	1	0	0	0	0	0	0	0	0	0	0	0	56
17977937	m	cn	TW	2020	0	1	0	0	0	1	0	0	0	0	0	0	
17978430	m	md	US	2020	0	1	1	0	0	0	0	0	0	0	0	0	
17978462	m	Not repo	JP	2020	0	1	0	1	0	0	0	0	0	0	0	0	70
17978525	f	cn	US	2020	1	0	0	0	0	0	0	0	0	0	0	0	77.3
17978930	m	Not repo	US	2020	1	0	0	0	0	0	0	0	0	0	0	0	
17979057	m	Not repo	KR	2020	1	0	0	0	0	0	0	0	0	0	0	0	67.6
17979181	f	ph	JP	2020	1	0	0	0	0	0	0	0	0	0	0	0	72
17979686	m	Not repo	IN	2020	0	1	0	1	0	0	0	0	0	0	0	0	48.1
17979905	m	cn	Not re	2020	0	0	0	0	0	0	0	0	0	0	0	0	87
17980007	f	Not repo	ID	2020	1	0	0	0	0	0	0	0	0	0	0	0	
17980019	m	Not repo	JP	2020	0	1	0	1	0	0	0	0	0	0	0	1	67
17980096	Not	md	NL	2020	1	0	0	0	0	0	0	0	0	0	0	0	
17980434	f	md	RS	2020	0	0	0	0	0	0	0	0	0	0	0	0	61
17980540	m	cn	US	2020	0	0	0	0	0	0	0	0	0	0	0	0	67
17980651	f	cn	CN	2020	0	0	0	0	0	0	0	0	0	0	0	0	55
17980843	Not	hp	NL	2020	1	0	0	0	0	0	0	0	0	0	0	0	

17981347	m	cn	CN	2020	0	1	0	0	1	0	0	0	0	0	0	0	59
17982209	m	md	JP	2020	0	0	0	0	0	0	0	0	0	0	0	0	67
17982432	m	md	NL	2020	0	0	0	0	0	0	0	0	0	0	0	0	61
17982472	Not	cn	JP	2020	0	1	1	0	0	0	0	0	0	0	0	0	70
17982634	m	md	JP	2020	0	1	1	0	0	0	0	0	0	0	0	0	78
17983212	Not	md	NO	2020	0	0	0	0	0	0	0	0	0	0	0	0	
17983232	m	hp	SK	2020	0	1	1	0	0	0	0	0	0	0	0	0	56
17983325	f	md	KR	2020	0	0	0	0	0	0	0	0	0	0	0	0	57
17983532	m	hp	MY	2020	0	1	1	0	0	0	0	0	0	0	0	0	68
17983636	m	md	JP	2020	0	1	0	0	0	1	0	0	0	0	0	0	75
17983848	m	md	JP	2020	0	1	0	0	0	1	0	0	0	0	0	0	70
17983901	f	cn	CN	2020	1	0	0	0	0	0	0	0	0	0	0	0	63
17984226	f	cn	CN	2020	1	0	0	0	0	0	0	0	0	0	0	0	56
17984229	f	cn	CN	2020	1	0	0	0	0	0	0	0	0	0	0	0	55
17984231	f	cn	CN	2020	1	0	0	0	0	0	0	0	0	0	0	0	76
17984377	f	cn	AR	2020	0	0	0	0	0	0	0	0	0	0	0	0	68
17985658	m	hp	US	2020	0	1	0	0	1	0	0	0	0	0	0	0	48
17985961	m	cn	CN	2020	0	1	0	0	1	0	0	0	0	0	0	0	
17986025	m	cn	TR	2020	0	1	0	0	1	0	0	0	0	0	0	0	60
17986108	f	md	GB	2020	0	1	0	0	0	1	0	0	0	0	0	0	
17986164	f	Not repo	CA	2020	1	0	0	0	0	0	0	0	0	0	0	0	57.3
17986237	m	cn	JP	2020	0	1	1	0	0	0	0	0	0	0	0	0	77
17986252	m	cn	JP	2020	0	1	1	0	0	0	0	0	0	0	0	0	64
17986636	Not	cn	JP	2020	0	1	1	0	0	0	0	0	0	0	0	0	70
17986830	f	cn	JP	2020	0	1	1	0	0	0	0	0	0	0	0	0	78
17986832	Not	md	JP	2020	0	0	0	0	0	0	0	0	0	0	0	0	70
17986962	m	Not repo	Not re	2020	0	0	0	0	0	0	0	0	0	0	0	0	52
17987086	m	hp	US	2020	0	1	0	0	1	0	0	0	0	0	0	0	68
17987957	f	hp	DE	2020	0	1	1	0	0	0	0	0	0	0	0	0	59
17988157	m	cn	JP	2020	0	1	1	0	0	0	0	0	0	0	0	0	70
17988433	m	hp	CN	2020	1	0	0	0	0	0	0	0	0	0	0	0	85
17988457	m	cn	US	2020	0	1	1	0	0	0	0	0	0	0	0	0	68
17988771	Not	md	JP	2020	0	1	0	1	0	0	0	0	0	0	0	0	
17989033	f	Not repo	JP	2020	0	1	0	1	0	0	0	0	0	0	0	1	69

17989164	m	Not repo	CO	2020	0	0	0	0	0	0	1	0	0	0	1	0	51
17989165	m	Not repo	BG	2020	1	0	0	0	0	0	0	0	0	0	0	0	
17989223	f	md	FR	2020	1	0	0	0	0	0	0	0	0	0	0	0	
17989776	m	md	AR	2020	0	1	1	0	0	0	0	0	0	0	0	0	74
17990539	f	cn	IN	2020	0	0	0	0	0	0	0	0	0	0	0	0	58
17990794	m	hp	DE	2020	0	1	1	0	0	0	0	0	0	0	0	0	69
17991112	f	cn	JP	2020	0	1	1	0	0	0	0	0	0	0	0	0	68
17991141	m	Not repo	RU	2020	1	0	0	0	0	0	0	0	0	0	0	0	46.4
17991360	m	hp	KR	2020	0	1	0	0	1	0	0	0	0	0	0	0	56
17991388	f	cn	Not re	2020	0	0	0	0	0	0	0	0	0	0	0	0	57
17991750	m	md	NL	2020	0	0	0	0	0	0	0	0	0	0	0	0	61
17992026	f	cn	KR	2020	0	1	1	0	0	0	0	0	0	0	0	0	
17992164	m	hp	US	2020	0	1	0	0	1	0	0	0	0	0	0	0	69
17992360	Not	hp	NL	2020	1	0	0	0	0	0	0	0	0	0	0	0	
17994125	f	cn	US	2020	1	0	0	0	0	0	0	0	0	0	0	0	71
17994663	m	md	JP	2020	0	1	0	1	0	0	0	0	0	0	0	1	66
17994669	m	cn	IN	2020	0	0	0	0	0	0	0	0	0	0	0	0	33
17994875	m	md	BR	2020	0	1	0	1	0	0	0	0	0	0	0	1	54
17995189	f	hp	US	2020	0	1	0	0	1	0	0	0	0	0	0	1	55
17995223	m	md	JP	2020	0	1	0	1	0	0	0	0	0	0	0	0	71
17995235	m	md	JP	2020	0	1	0	0	1	0	0	0	0	0	0	0	80
17995272	Not	cn	JP	2020	0	0	0	0	0	0	0	0	0	0	0	0	70
17995764	m	hp	DK	2020	0	0	0	0	0	0	0	0	0	0	0	0	
17995849	f	hp	DE	2020	0	1	1	0	0	0	0	0	0	0	0	0	71
17995869	m	md	NL	2020	0	0	0	0	0	0	0	0	0	0	0	0	61
17996210	f	md	CA	2020	0	1	0	1	0	0	0	0	0	0	0	1	76.7
17996637	m	hp	CN	2020	0	1	0	0	1	0	0	0	0	0	0	0	48
17996845	Not	hp	NL	2020	1	0	0	0	0	0	0	0	0	0	0	0	
17996859	Not	md	NL	2020	1	0	0	0	0	0	0	0	0	0	0	0	
17997144	Not	hp	JP	2020	0	0	0	0	0	0	0	0	0	0	0	0	
17997174	m	hp	FR	2020	0	1	0	0	1	0	0	0	0	0	0	0	62
17997202	f	cn	JP	2020	0	1	1	0	0	0	0	0	0	0	0	0	68
17997333	m	hp	HU	2020	0	0	0	0	0	0	0	0	0	0	0	0	69
17997567	m	hp	PL	2020	0	0	0	0	0	0	0	0	0	0	0	0	63

17997750	m	md	JP	2020	0	1	0	0	0	1	0	0	0	0	0	1	74
17998090	m	cn	US	2020	0	0	0	0	0	0	0	0	0	0	0	0	47
17998382	f	md	JP	2020	0	0	0	0	0	0	0	0	0	0	0	0	80
17998589	f	md	JP	2020	0	1	0	0	0	1	0	0	0	0	0	0	56
17998935	m	hp	US	2020	0	0	0	0	0	0	0	0	0	0	0	0	44
17999474	m	hp	US	2020	1	0	0	0	0	0	0	0	0	0	0	0	72.1
17999717	m	cn	CN	2020	0	1	0	0	1	0	0	0	0	0	0	0	72
17999815	Not	md	CN	2020	1	0	0	0	0	0	0	0	0	0	0	0	
18000039	f	Not repo	JP	2020	0	1	0	1	0	0	0	0	0	0	0	0	78
18000040	f	Not repo	JP	2020	0	1	0	1	0	0	0	0	0	0	0	0	58
18000372	m	cn	JP	2020	0	1	1	0	0	0	0	0	0	0	0	0	75
18000443	f	md	KR	2020	0	0	0	0	0	0	0	0	0	0	0	0	86
18000597	m	ph	CA	2020	0	1	0	0	1	0	0	0	0	0	0	0	66
18000790	Not	Not repo	AU	2020	0	1	0	1	0	0	0	0	0	0	0	0	
18000838	m	md	IN	2020	1	0	0	0	0	0	0	0	0	0	0	0	61
18000873	Not	Not repo	AU	2020	0	1	0	1	0	0	0	0	0	0	0	0	
18000874	Not	Not repo	AU	2020	0	1	0	1	0	0	0	0	0	0	0	0	
18001025	f	md	FR	2020	0	1	0	0	0	1	0	0	0	0	0	0	
18001082	m	cn	AE	2020	0	1	0	1	0	0	0	0	0	0	0	0	51.5
18001799	m	md	AU	2020	0	1	1	0	0	0	0	0	0	0	0	1	70
18001833	m	hp	Not re	2020	0	1	1	0	0	0	0	0	0	0	0	0	72
18002097	m	cn	CN	2020	1	0	0	0	0	0	0	0	0	0	0	0	50
18002193	m	md	RU	2020	0	1	1	0	0	0	0	0	0	0	0	0	68
18002252	m	md	JP	2020	0	1	0	0	0	1	0	0	0	0	0	0	79
18002436	m	cn	AE	2020	0	1	0	1	0	0	0	0	0	0	0	0	39.1
18003781	f	cn	JP	2020	0	1	1	0	0	0	0	0	0	0	0	0	55
18004481	f	hp	FR	2020	0	1	1	0	0	0	0	0	0	0	0	0	49
18004617	f	hp	FR	2020	0	1	1	0	0	0	0	0	0	0	0	0	57
18004711	f	md	FR	2020	0	1	0	0	0	1	0	0	0	0	0	0	51
18005198	m	cn	JP	2020	0	1	1	0	0	0	0	0	0	0	0	0	75
18005573	m	ph	JP	2020	0	0	0	0	0	0	0	0	0	0	0	0	64
18005676	f	cn	JP	2020	0	1	1	0	0	0	0	0	0	0	0	0	74
18005913	f	md	FR	2020	0	0	0	0	0	0	0	0	0	0	0	0	75
18006404	m	ph	US	2020	0	1	1	0	0	0	0	0	0	0	0	0	76

18007108	f	hp	JP	2020	1	0	0	0	0	0	0	0	0	0	0	0	58
18007290	m	md	JP	2020	0	1	0	0	0	1	0	0	0	0	0	0	69
18007534	Not	md	IN	2020	1	0	0	0	0	0	0	0	0	0	0	0	
18007590	f	md	BE	2020	0	0	0	0	0	0	0	0	0	0	0	0	67
18007609	f	cn	CN	2020	1	0	0	0	0	0	0	0	0	0	0	0	62
18007615	m	Not repo	JP	2020	1	0	0	0	0	0	0	0	0	0	0	0	90
18008162	f	cn	CN	2020	1	0	0	0	0	0	0	0	0	0	0	0	57
18009525	f	hp	KR	2020	0	1	0	0	1	0	0	0	0	0	0	0	60
18009577	m	cn	JP	2020	0	1	1	0	0	0	0	0	0	0	0	0	73
18009664	m	cn	JP	2020	0	1	1	0	0	0	0	0	0	0	0	0	80
18009807	m	md	JP	2020	0	1	0	0	0	1	0	0	0	0	0	0	45
18009911	f	hp	CN	2020	1	0	0	0	0	0	0	0	0	0	0	0	32
18010000	m	md	JP	2020	0	0	0	0	0	0	0	0	0	0	0	0	56
18010191	f	cn	JP	2020	0	1	1	0	0	0	0	0	0	0	0	0	78
18010331	m	hp	AU	2020	0	1	0	0	1	0	0	0	0	0	0	0	64
18010477	m	md	TW	2020	1	0	0	0	0	0	0	0	0	0	0	0	59
18010487	m	md	IN	2020	0	0	0	0	0	0	0	0	0	0	0	0	56
18010957	f	cn	Not re	2020	0	0	0	0	0	0	0	0	0	0	0	0	
18011124	f	hp	DE	2020	0	1	1	0	0	0	0	0	0	0	0	0	71
18011330	m	cn	JP	2020	0	1	1	0	0	0	0	0	0	0	0	0	90
18012218	m	cn	CN	2020	0	1	0	0	1	0	0	0	0	0	0	0	
18012292	f	Not repo	TW	2020	0	1	0	1	0	0	0	0	0	0	0	0	81
18012519	m	md	IQ	2020	0	1	1	0	0	0	0	0	0	0	0	0	74
18012759	f	md	VN	2020	1	0	0	0	0	0	0	0	0	0	0	0	58
18012763	f	md	FR	2020	1	0	0	0	0	0	0	0	0	0	0	0	
18013572	m	md	KW	2020	0	0	0	0	0	0	0	0	0	0	0	0	
18014243	m	md	JP	2020	0	1	0	1	0	0	0	0	0	0	0	1	65
18014349	f	cn	TH	2020	0	0	0	0	0	0	0	0	0	0	0	0	63
18014466	m	hp	NL	2020	0	1	0	0	1	0	0	0	0	0	0	0	66
18015607	f	Not repo	MY	2020	1	0	0	0	0	0	0	0	0	0	0	0	65.7
18015686	m	cn	US	2020	0	0	0	0	0	0	0	0	0	0	0	0	
18015793	f	hp	US	2020	1	0	0	0	0	0	0	0	0	0	0	0	40
18015797	m	Not repo	ID	2020	0	1	0	1	0	0	0	0	0	0	0	0	83.3
18016077	m	Not repo	MY	2020	1	0	0	0	0	0	0	0	0	0	0	0	58.2

18016108	m	md	ES	2020	0	1	1	0	0	0	0	0	0	0	0	0		75
18016456	m	hp	FR	2020	0	1	0	0	1	0	0	0	0	0	0	0		50
18016501	f	md	GB	2020	0	1	0	0	0	1	0	0	0	0	0	0		55
18016540	f	hp	US	2020	0	0	0	0	0	0	0	0	0	0	0	0		
18017421	m	hp	MY	2020	0	1	1	0	0	0	0	0	0	0	0	0		64
18017431	f	md	RU	2020	0	0	0	0	0	0	0	0	0	0	0	0		29
18017719	m	md	FR	2020	0	0	0	0	0	0	0	0	0	0	0	0		77
18017876	m	cn	CN	2020	0	1	0	0	1	0	0	0	0	0	0	0		
18019324	m	cn	Not re	2020	0	0	0	0	0	0	0	0	0	0	0	0		
18019522	f	cn	Not re	2020	0	0	0	0	0	0	0	0	0	0	0	0		34
18019734	f	md	CO	2020	0	1	1	0	0	0	0	0	0	0	0	0		49
18019950	Not	md	JP	2020	0	1	0	0	1	0	0	0	0	0	0	0		70
18019981	m	Not repo	JP	2020	1	0	0	0	0	0	0	0	0	0	0	0		76
18020142	f	hp	IN	2020	0	0	0	0	0	0	0	0	0	0	0	0		
18020706	m	hp	JP	2020	0	0	0	0	0	0	0	0	0	0	0	0		58
18021153	m	hp	MY	2020	0	1	1	0	0	0	0	0	0	0	0	1		73
18021334	f	md	JP	2020	1	0	0	0	0	0	0	0	0	0	0	0		
18021678	m	cn	CN	2020	0	1	0	0	1	0	0	0	0	0	0	0		79
18021754	m	md	GB	2020	0	1	1	0	0	0	0	0	0	0	0	0		72
18021786	m	md	FR	2020	0	1	0	0	0	1	0	0	0	0	0	0		64
18022114	m	md	HU	2020	0	0	0	0	0	0	0	0	0	0	0	0		69
18022409	m	md	JP	2020	0	1	0	0	0	1	0	0	0	0	0	0		
18022444	m	md	DE	2020	0	1	0	0	0	1	0	0	0	0	0	0		64
18022557	f	cn	Not re	2020	0	0	0	0	0	0	0	0	0	0	0	0		
18022653	f	md	ES	2020	0	0	0	0	0	0	0	0	0	0	0	0		80
18023294	Not	cn	MX	2020	0	0	0	0	0	0	0	0	0	0	0	0		
18023345	m	hp	US	2020	0	1	0	0	1	0	0	0	0	0	0	0		57
18024215	m	md	JP	2020	0	1	0	0	1	0	0	0	0	0	0	0		63
18024301	f	md	FR	2020	0	1	0	0	0	1	0	0	0	0	0	0		51
18024418	f	cn	MX	2020	0	0	0	0	0	0	0	0	0	0	0	0		
18024600	f	hp	US	2020	0	1	1	0	0	0	0	0	0	0	0	0		
18024623	m	ph	JP	2020	0	1	0	0	1	0	0	0	0	0	0	0		84
18024908	m	hp	ES	2020	0	1	1	0	0	0	0	0	0	0	0	0		66
18025278	f	md	JP	2020	0	0	0	0	0	0	0	0	0	0	0	0		60

18025774	m	md	JP	2020	0	1	0	1	0	0	0	0	0	0	0	1	72
18025776	m	Not repo	JP	2020	0	1	0	1	0	0	0	0	0	0	0	1	55.7
18026177	f	Not repo	SG	2020	1	0	0	0	0	0	0	0	0	0	0	0	65.9
18026329	f	hp	GB	2020	0	1	0	0	0	1	0	0	0	0	0	0	55
18027174	f	hp	US	2020	0	1	0	0	1	0	0	0	0	0	0	0	68
18027380	m	Not repo	MY	2020	0	1	0	1	0	0	0	0	0	0	0	0	60.3
18027924	m	md	JP	2020	1	0	0	0	0	0	0	0	0	0	0	0	70
18028109	m	cn	JP	2020	0	1	1	0	0	0	0	0	0	0	0	0	71
18028625	f	cn	CN	2020	1	0	0	0	0	0	0	0	0	0	0	0	68
18028993	f	Not repo	US	2020	1	0	0	0	0	0	0	0	0	0	0	0	65.5
18029103	f	md	ES	2020	0	1	1	0	0	0	0	0	0	0	0	0	69
18029401	m	cn	JP	2020	0	1	1	0	0	0	0	0	0	0	0	0	71
18029436	Not	md	US	2020	0	0	0	0	0	0	0	0	0	0	0	0	
18029457	f	hp	DE	2020	0	0	0	0	0	0	0	0	0	0	0	0	60
18029657	f	md	CO	2020	0	1	1	0	0	0	0	0	0	0	0	0	80
18029767	m	cn	JP	2020	0	1	1	0	0	0	0	0	0	0	0	1	76
18029916	f	ph	US	2020	0	0	0	0	0	0	0	0	0	0	0	0	
18030093	f	cn	US	2020	1	0	0	0	0	0	0	0	0	0	0	0	64
18030774	f	md	FR	2020	0	1	0	1	0	0	0	0	0	0	0	0	82
18030979	m	md	JP	2020	0	1	0	0	0	1	0	0	0	0	0	0	71
18031049	m	md	JP	2020	0	1	0	0	0	1	0	0	0	0	0	0	80
18031075	f	md	TW	2020	0	0	0	0	0	0	0	0	0	0	0	0	51
18031797	Not	Not repo	TW	2020	0	1	0	1	0	0	0	0	0	0	0	0	
18031799	f	Not repo	JP	2020	1	0	0	0	0	0	0	0	0	0	0	0	83
18031800	Not	Not repo	AU	2020	1	0	0	0	0	0	0	0	0	0	0	0	61.6
18031801	Not	Not repo	TW	2020	0	1	0	1	0	0	0	0	0	0	0	0	
18031915	m	cn	MX	2020	0	0	0	0	0	0	0	0	0	0	0	0	
18032370	f	md	CY	2020	0	0	0	0	0	0	0	0	0	0	0	0	
18033108	m	hp	HU	2020	0	0	0	0	0	0	0	0	0	0	0	0	69
18033251	Not	md	JP	2020	1	0	0	0	0	0	0	0	0	0	0	0	
18033261	f	md	US	2020	0	0	0	0	0	0	0	0	0	0	0	0	91
18033733	m	ph	JP	2020	0	1	1	0	0	0	0	0	0	0	0	0	70
18033893	m	md	JP	2020	1	0	0	0	0	0	0	0	0	0	0	0	49
18034127	m	cn	CN	2020	0	1	0	0	1	0	0	0	0	0	0	0	71

18034582	m	cn	Not re	2020	0	0	0	0	0	0	0	0	0	0	0	0	
18034990	m	Not repo	AU	2020	0	1	0	1	0	0	0	0	0	0	0	0	
18035035	m	cn	CA	2020	0	0	0	0	0	0	0	0	0	0	0	0	55
18035570	Not	md	JP	2020	0	1	0	1	0	0	0	0	0	0	0	0	
18035789	m	cn	JP	2020	0	1	1	0	0	0	0	0	0	0	0	0	74
18036679	m	hp	NL	2020	0	0	0	0	0	0	0	0	0	0	0	0	61
18036894	f	Not repo	JP	2020	0	1	0	1	0	0	0	0	0	0	0	0	60
18036973	f	md	BE	2020	0	0	0	0	0	0	0	0	0	0	0	0	
18037280	f	hp	BR	2020	0	1	1	0	0	0	0	0	0	0	0	0	79
18037301	m	md	CH	2020	0	0	0	0	0	0	0	0	0	0	0	0	
18037514	f	md	IN	2020	0	0	0	0	0	0	0	0	0	0	0	0	43
18037716	m	Not repo	CN	2020	1	0	0	0	0	0	0	0	0	0	0	0	74
18037725	f	cn	US	2020	0	1	0	1	0	0	0	0	0	0	0	1	
18037735	f	ph	JP	2020	0	1	0	1	0	0	0	0	0	0	0	0	78
18037788	m	ph	JP	2020	0	1	1	0	0	0	0	0	0	0	0	0	70
18037986	f	Not repo	US	2020	1	0	0	0	0	0	0	0	0	0	0	0	59.9
18038048	f	cn	JP	2020	0	0	0	0	0	0	0	0	0	0	0	0	75
18038355	Not	cn	JP	2020	0	1	1	0	0	0	0	0	0	0	0	0	50
18038784	f	ph	CA	2020	0	0	0	0	0	0	0	0	0	0	0	0	76
18039361	m	md	JP	2020	0	1	0	0	1	0	0	0	0	0	0	0	63
18039481	m	hp	CA	2020	0	1	1	0	0	0	0	0	0	0	0	0	52
18039981	Not	md	US	2020	0	0	0	0	0	0	0	0	0	0	0	0	
18040731	f	hp	DE	2020	0	1	1	0	0	0	0	0	0	0	0	0	56
18041079	Not	hp	JP	2020	0	0	0	0	0	0	0	0	0	0	0	0	
18041269	Not	hp	JP	2020	0	0	0	0	0	0	0	0	0	0	0	0	
18041360	f	cn	Not re	2020	0	0	0	0	0	0	0	0	0	0	0	0	
18041856	m	hp	FR	2020	0	1	0	0	1	0	0	0	0	0	0	0	50
18042120	Not	hp	CA	2020	0	1	1	0	0	0	0	0	0	0	0	0	73
18042229	m	cn	JP	2020	0	1	1	0	0	0	0	0	0	0	0	0	88
18042569	m	hp	CA	2020	0	1	1	0	0	0	0	0	0	0	0	0	52
18042890	m	hp	JP	2020	0	0	0	0	0	0	0	0	0	0	0	0	75
18042944	m	hp	JP	2020	0	0	0	0	0	0	0	0	0	0	0	0	74
18042977	f	md	PL	2020	0	1	0	0	0	1	0	0	0	0	0	0	65
18043073	Not	hp	CA	2020	0	1	1	0	0	0	0	0	0	0	0	0	73

18044175	f	hp	US	2020	0	1	0	0	1	0	0	0	0	0	0	0	
18044568	m	md	IN	2020	0	0	0	0	0	0	0	0	0	0	0	0	67
18044570	m	md	IN	2020	0	0	0	0	0	0	0	0	0	0	0	0	66
18044595	m	md	IN	2020	0	0	0	0	0	0	0	0	0	0	0	0	73
18045130	m	hp	ES	2020	0	1	1	0	0	0	0	0	0	0	0	1	57
18045701	m	Not repo	US	2020	1	0	0	0	0	0	0	0	0	0	0	0	89
18045724	f	md	ES	2020	0	0	0	0	0	0	0	0	0	0	0	0	69
18045888	f	md	US	2020	0	0	0	0	0	0	0	0	0	0	0	0	
18045997	f	cn	MX	2020	0	0	0	0	0	0	0	0	0	0	0	0	
18046290	m	cn	JP	2020	0	1	1	0	0	0	0	0	0	0	0	0	79
18046901	Not	md	TH	2020	0	1	0	0	0	1	0	0	0	0	0	0	
18046938	Not	hp	US	2020	0	1	0	0	0	1	0	0	0	0	0	0	
18046944	m	cn	US	2020	0	1	0	0	0	1	0	0	0	0	0	0	65
18046947	m	ph	US	2020	0	1	0	0	0	1	0	0	0	0	0	0	74
18046953	m	hp	US	2020	0	1	0	0	0	1	0	0	0	0	0	0	72
18047038	f	md	US	2020	0	1	0	0	0	1	0	0	0	0	0	0	
18047070	m	hp	US	2020	0	1	0	0	0	1	0	0	0	0	0	0	64
18047071	m	md	US	2020	0	1	0	0	0	1	0	0	0	0	0	0	
18047079	m	ph	US	2020	0	1	0	0	0	1	0	0	0	0	0	0	68
18047081	Not	ph	US	2020	0	1	0	0	0	1	0	0	0	0	0	0	
18047088	m	hp	US	2020	0	1	0	0	0	1	0	0	0	0	0	0	72
18047114	f	cn	JP	2020	0	0	0	0	0	0	0	0	0	0	0	0	69
18047212	m	md	JP	2020	1	0	0	0	0	0	0	0	0	0	0	1	69
18047226	m	cn	CN	2020	0	1	0	0	1	0	0	0	0	0	0	0	78
18048952	m	hp	DE	2020	0	0	0	0	0	0	0	0	0	0	0	0	79
18048986	Not	Not repo	JP	2020	0	1	0	1	0	0	0	0	0	0	0	1	70
18049326	f	Not repo	MY	2020	1	0	0	0	0	0	0	0	0	0	0	0	63.5
18049680	Not	md	US	2020	1	0	0	0	0	0	0	0	0	0	0	0	
18049933	m	cn	US	2020	0	1	1	0	0	0	0	0	0	0	0	0	82
18050135	m	md	NZ	2020	0	1	0	0	0	1	0	0	0	0	0	0	65
18050191	f	md	JP	2020	0	1	0	1	0	0	0	0	0	0	0	1	69
18050289	m	md	AT	2020	0	1	1	0	0	0	0	0	0	0	0	0	75
18050396	m	md	JP	2020	0	1	0	1	0	0	0	0	0	0	0	1	70
18050398	f	Not repo	JP	2020	1	0	0	0	0	0	0	0	0	0	0	0	81

18050452	f	md	AU	2020	0	1	1	0	0	0	0	0	0	0	0	1	72
18050558	f	Not repo	CN	2020	1	0	0	0	0	0	0	0	0	0	0	0	66
18050738	m	md	FR	2020	0	1	1	0	0	0	0	0	0	0	0	0	63
18050916	f	cn	Not re	2020	0	0	0	0	0	0	0	0	0	0	0	0	52
18050938	m	Not repo	ID	2020	1	0	0	0	0	0	0	0	0	0	0	0	70
18050955	m	hp	US	2020	0	0	0	0	0	0	0	0	0	0	0	0	
18051018	m	hp	CN	2020	1	0	0	0	0	0	0	0	0	0	0	0	65
18051395	m	hp	DE	2020	0	1	1	0	0	0	0	0	0	0	0	0	67
18051617	f	md	TW	2020	0	0	0	0	0	0	0	0	0	0	0	0	70
18051752	m	hp	IT	2020	0	1	0	0	1	0	0	0	0	0	0	0	53
18051845	f	cn	JP	2020	0	1	1	0	0	0	0	0	0	0	0	0	66
18052106	f	ph	US	2020	0	0	0	0	0	0	0	0	0	0	0	0	55
18052196	f	hp	GB	2020	0	1	0	0	0	1	0	0	0	0	0	0	
18052231	m	hp	KR	2020	0	1	1	0	0	0	0	0	0	0	0	0	61
18053006	f	hp	US	2020	0	1	0	0	1	0	0	0	0	0	0	0	62
18053169	Not	md	JP	2020	0	0	0	0	0	0	0	0	0	0	0	0	
18053863	m	md	JP	2020	0	1	0	1	0	0	0	0	0	0	0	1	74.5
18054065	f	cn	US	2020	0	0	0	0	0	0	0	0	0	0	0	0	
18054246	m	ph	US	2020	0	1	0	0	1	0	0	0	0	0	0	0	60
18054726	m	hp	JP	2020	0	0	0	0	0	0	1	0	0	1	0	1	79
18054765	Not	md	TW	2020	0	0	0	0	0	0	0	0	0	0	0	0	
18055391	m	cn	JP	2020	0	1	1	0	0	0	0	0	0	0	0	0	62
18055622	m	cn	CN	2020	0	0	0	0	0	0	0	0	0	0	0	0	50
18055674	m	cn	JP	2020	0	1	1	0	0	0	0	0	0	0	0	0	68
18055764	f	cn	Not re	2020	0	0	0	0	0	0	0	0	0	0	0	0	
18055999	f	cn	US	2020	1	0	0	0	0	0	0	0	0	0	0	0	
18056527	f	md	BR	2020	0	0	0	0	0	0	0	0	0	0	0	0	
18056737	m	cn	JP	2020	0	1	1	0	0	0	0	0	0	0	0	0	
18056762	m	md	GR	2020	0	1	1	0	0	0	0	0	0	0	0	0	73
18056989	m	ph	JP	2020	0	1	1	0	0	0	0	0	0	0	0	0	70
18057529	m	cn	US	2020	0	0	0	0	0	0	0	0	0	0	0	0	
18057559	f	cn	CA	2020	0	0	0	0	0	0	0	0	0	0	0	0	30
18057571	f	hp	NL	2020	0	1	0	0	1	0	0	0	0	0	0	0	60
18057687	m	hp	TH	2020	0	1	0	0	1	0	0	0	0	0	0	0	65

18057746	f	hp	JP	2020	0	0	0	0	0	0	0	0	0	0	0	0	35
18057952	f	md	ES	2020	0	1	1	0	0	0	0	0	0	0	0	1	73
18058780	m	cn	US	2020	0	1	1	0	0	0	0	0	0	0	0	0	
18059161	f	cn	US	2020	0	1	1	0	0	0	0	0	0	0	0	0	
18059187	m	Not repo	JP	2020	0	1	0	1	0	0	0	0	0	0	0	0	79
18059198	f	ph	US	2020	1	0	0	0	0	0	0	0	0	0	0	0	70.1
18061213	f	md	IN	2020	0	0	0	0	0	0	0	0	0	0	0	0	40
18061566	f	hp	US	2020	0	1	1	0	0	0	0	0	0	0	0	0	55
18061626	m	md	DE	2020	0	0	0	0	0	0	0	0	0	0	0	0	56
18061753	m	cn	JP	2020	0	0	0	0	0	0	0	0	0	0	0	0	65
18062089	m	cn	CN	2020	1	0	0	0	0	0	0	0	0	0	0	0	57
18062779	f	lw	US	2020	0	0	0	0	0	0	0	0	0	0	0	0	40
18063159	m	cn	IL	2020	0	0	0	0	0	0	0	0	0	0	0	0	73
18063219	m	hp	CA	2020	0	1	1	0	0	0	0	0	0	0	0	0	71
18063274	m	md	HU	2020	0	0	0	0	0	0	0	0	0	0	0	0	69
18063443	m	cn	Not re	2020	0	0	0	0	0	0	0	0	0	0	0	0	
18063927	f	hp	CN	2020	1	0	0	0	0	0	0	0	0	0	0	0	54
18065398	m	hp	CN	2020	1	0	0	0	0	0	0	0	0	0	0	0	85
18065647	m	Not repo	TW	2020	0	1	0	1	0	0	0	0	0	0	0	0	67
18065836	m	hp	NL	2020	0	1	1	0	0	0	0	0	0	0	0	0	72
18065875	Not	cn	CL	2020	0	1	1	0	0	0	0	0	0	0	0	0	
18065971	m	hp	US	2020	1	0	0	0	0	0	0	0	0	0	0	0	44
18066072	m	Not repo	MY	2020	1	0	0	0	0	0	0	0	0	0	0	0	
18066156	f	ph	MY	2020	1	0	0	0	0	0	0	0	0	0	0	0	
18066689	f	hp	CN	2020	1	0	0	0	0	0	0	0	0	0	0	0	47.5
18066690	f	Not repo	CN	2020	1	0	0	0	0	0	0	0	0	0	0	0	54.2
18067338	m	ph	JP	2020	0	1	0	0	0	1	0	0	0	0	0	0	70
18067445	f	cn	US	2020	0	0	0	0	0	0	0	0	0	0	0	0	79
18067495	f	cn	CA	2020	1	0	0	0	0	0	0	0	0	0	0	0	
18068293	f	Not repo	ID	2020	1	0	0	0	0	0	0	0	0	0	0	0	50.7
18068542	m	cn	DE	2020	0	1	1	0	0	0	0	0	0	0	0	0	71
18068686	m	Not repo	JP	2020	0	1	0	1	0	0	0	0	0	0	0	0	80
18069021	m	hp	SG	2020	0	0	0	0	0	0	0	0	0	0	0	0	54
18069613	f	cn	JP	2020	0	1	1	0	0	0	0	0	0	0	0	0	47

18069621	m	cn	JP	2020	0	1	1	0	0	0	0	0	0	0	0	0	75
18069969	f	cn	Not re	2020	0	0	0	0	0	0	0	0	0	0	0	0	
18070019	f	cn	US	2020	0	1	1	0	0	0	0	0	0	0	0	0	
18072325	m	md	IQ	2020	1	0	0	0	0	0	0	0	0	0	0	0	
18072364	m	cn	Not re	2020	0	0	0	0	0	0	0	0	0	0	0	0	
18073322	Not	hp	AT	2020	0	0	0	0	0	0	0	0	0	0	0	0	
18073376	f	cn	US	2020	1	0	0	0	0	0	0	0	0	0	0	0	44.1
18073413	m	hp	US	2020	1	0	0	0	0	0	0	0	0	0	0	0	66
18073528	f	Not repo	TW	2020	0	1	0	1	0	0	0	0	0	0	0	0	69
18075957	m	hp	US	2020	0	1	0	0	1	0	0	0	0	0	0	0	59
18076661	f	hp	US	2020	0	1	0	0	0	1	0	0	0	0	0	0	76
18077217	m	cn	IN	2020	0	0	0	0	0	0	0	0	0	0	0	0	47
18077260	m	hp	DE	2020	0	1	1	0	0	0	0	0	0	0	0	0	70
18077372	m	md	JP	2020	0	1	0	0	0	1	0	0	0	0	0	1	69
18077420	m	md	FR	2020	1	0	0	0	0	0	0	0	0	0	0	0	
18077445	m	md	RU	2020	0	0	0	0	0	0	0	0	0	0	0	0	
18077590	f	cn	JP	2020	0	1	1	0	0	0	0	0	0	0	0	0	77
18077599	Not	md	US	2020	0	1	0	1	0	0	0	0	0	0	0	0	
18077740	Not	md	MY	2020	0	1	0	0	0	1	0	0	0	0	0	0	
18077930	m	md	US	2020	0	0	0	0	0	0	0	0	0	0	0	0	
18078349	f	cn	Not re	2020	0	0	0	0	0	0	0	0	0	0	0	0	
18078532	f	md	JP	2020	1	0	0	0	0	0	0	0	0	0	0	0	70
18078668	m	md	JP	2020	0	0	0	0	0	0	0	0	0	0	0	0	
18078708	f	cn	JP	2020	0	1	1	0	0	0	0	0	0	0	0	0	84
18079156	m	md	JP	2020	0	0	0	0	0	0	0	0	0	0	0	0	69
18079467	f	md	JP	2020	0	1	0	1	0	0	0	0	0	0	0	1	65
18079470	m	md	JP	2020	0	1	0	1	0	0	0	0	0	0	0	0	
18079471	Not	md	JP	2020	1	0	0	0	0	0	0	0	0	0	0	0	
18079629	m	ph	JP	2020	1	0	0	0	0	0	0	0	0	0	0	1	75
18080413	m	hp	DE	2020	0	1	1	0	0	0	0	0	0	0	0	0	71
18080431	m	hp	FR	2020	0	1	0	0	1	0	0	0	0	0	0	0	66
18080579	f	md	JP	2020	0	1	0	1	0	0	0	0	0	0	0	0	80
18081093	f	hp	IN	2020	0	0	0	0	0	0	0	0	0	0	0	0	56
18081310	f	cn	CA	2020	1	0	0	0	0	0	0	0	0	0	0	0	52.2

18081454	m	hp	AU	2020	0	1	1	0	0	0	0	0	0	0	0	0	59
18081479	f	md	JP	2020	0	0	0	0	0	0	1	1	0	0	0	0	77
18081652	m	Not repo	JP	2020	0	1	0	1	0	0	0	0	0	0	0	1	69
18081728	f	md	DE	2020	0	1	0	0	0	1	0	0	0	0	0	0	71
18081832	f	cn	JP	2020	0	1	1	0	0	0	0	0	0	0	0	0	69
18082685	m	md	JP	2020	0	1	0	0	0	1	0	0	0	0	0	0	60
18082697	m	hp	JP	2020	0	1	0	0	0	1	0	0	0	0	0	0	70
18082828	m	md	TR	2020	0	0	0	0	0	0	0	0	0	0	0	0	40
18083332	f	md	TW	2020	0	1	0	0	0	1	0	0	0	0	0	0	64
18083374	f	md	DE	2020	0	0	0	0	0	0	0	0	0	0	0	0	
18083557	m	cn	US	2020	0	0	0	0	0	0	0	0	0	0	0	0	
18084000	f	md	AU	2020	0	0	0	0	0	0	0	0	0	0	0	0	
18084380	m	md	JP	2020	0	1	0	1	0	0	0	0	0	0	0	0	80
18086224	f	Not repo	MY	2020	1	0	0	0	0	0	0	0	0	0	0	0	79.6
18086608	m	hp	ES	2020	0	1	1	0	0	0	0	0	0	0	0	0	84
18086916	m	md	DE	2020	0	0	0	0	0	0	0	0	0	0	0	0	54
18086960	f	cn	CA	2020	0	0	0	0	0	0	1	1	0	0	0	0	58
18087113	Not	md	PT	2020	0	0	0	0	0	0	0	0	0	0	0	0	
18087583	m	md	IN	2020	0	1	0	1	0	0	0	0	0	0	0	0	73
18087883	f	cn	CN	2020	0	1	0	0	1	0	0	0	0	0	0	0	57
18088401	f	cn	CN	2020	1	0	0	0	0	0	0	0	0	0	0	0	
18088489	m	Not repo	CN	2020	1	0	0	0	0	0	0	0	0	0	0	0	45.1
18088516	m	md	CO	2020	0	1	1	0	0	0	0	0	0	0	0	0	63
18088551	m	cn	CN	2020	0	1	0	0	1	0	0	0	0	0	0	0	64
18088630	m	Not repo	JP	2020	0	1	0	1	0	0	0	0	0	0	0	0	70
18089018	m	md	AU	2020	0	0	0	0	0	0	0	0	0	0	0	0	
18089041	f	md	HU	2020	0	0	0	0	0	0	0	0	0	0	0	0	53
18089725	f	md	US	2020	0	0	0	0	0	0	0	0	0	0	0	0	77
18089764	f	md	US	2020	0	0	0	0	0	0	0	0	0	0	0	0	65
18090368	m	md	PL	2020	0	1	0	1	0	0	0	0	0	0	0	0	
18090560	f	md	AU	2020	0	1	0	0	1	0	0	0	0	0	0	0	80
18090787	m	cn	CN	2020	0	1	0	0	1	0	0	0	0	0	0	0	58
18093778	m	md	JP	2020	0	1	0	1	0	0	0	0	0	0	0	1	70
18093825	m	md	JP	2020	0	1	0	1	0	0	0	0	0	0	0	0	75

18093826	m	Not repo	JP	2020	1	0	0	0	0	0	0	0	0	0	0	0	70
18093975	m	md	JP	2020	1	0	0	0	0	0	0	0	0	0	0	0	90
18094608	f	cn	DE	2020	0	1	0	0	0	1	0	0	0	0	0	0	53
18094740	m	hp	JP	2020	0	1	1	0	0	0	0	0	0	0	0	0	
18094742	m	hp	DE	2020	0	1	1	0	0	0	0	0	0	0	0	0	67
18094743	m	md	JP	2020	0	1	0	0	0	1	0	0	0	0	0	0	67
18094756	f	md	US	2020	0	0	0	0	0	0	0	0	0	0	0	0	70
18095094	f	md	CA	2020	0	0	0	0	0	0	0	0	0	0	0	0	74
18095124	f	Not repo	US	2020	1	0	0	0	0	0	0	0	0	0	0	0	63
18095198	m	cn	CN	2020	0	1	0	0	1	0	0	0	0	0	0	0	47
18095460	f	Not repo	CN	2020	1	0	0	0	0	0	0	0	0	0	0	0	
18095670	f	Not repo	MY	2020	1	0	0	0	0	0	0	0	0	0	0	0	55.4
18095781	m	cn	IN	2020	0	0	0	0	0	0	0	0	0	0	0	0	68
18096011	f	Not repo	JP	2020	0	1	0	1	0	0	0	0	0	0	0	0	79
18096149	m	md	DE	2020	0	0	0	0	0	0	0	0	0	0	0	0	44
18096220	Not	md	JP	2020	0	0	0	0	0	0	0	0	0	0	0	0	
18096278	f	Not repo	JP	2020	0	1	0	1	0	0	0	0	0	0	0	1	78
18096497	m	cn	Not re	2020	0	0	0	0	0	0	0	0	0	0	0	0	
18096565	m	md	US	2020	0	0	0	0	0	0	0	0	0	0	0	0	70
18096676	f	md	MX	2020	0	1	1	0	0	0	0	0	0	0	0	0	52
18096901	m	cn	CN	2020	0	1	0	0	1	0	0	0	0	0	0	0	
18097350	m	hp	US	2020	0	1	0	0	1	0	0	0	0	0	0	0	61
18097711	Not	hp	SG	2020	0	0	0	0	0	0	0	0	0	0	0	0	
18097857	m	cn	CN	2020	0	1	0	0	1	0	0	0	0	0	0	0	57
18098127	m	hp	MY	2020	0	0	0	0	0	0	0	0	0	0	0	0	64
18098250	Not	hp	US	2020	0	1	0	1	0	0	0	0	0	0	0	1	74
18098255	Not	hp	US	2020	0	1	0	1	0	0	0	0	0	0	0	1	86
18098260	Not	hp	US	2020	0	1	0	1	0	0	0	0	0	0	0	1	72
18098305	f	md	AT	2020	1	0	0	0	0	0	0	0	0	0	0	0	74
18098535	m	md	AT	2020	1	0	0	0	0	0	0	0	0	0	0	0	41
18098661	m	Not repo	JP	2020	0	1	0	1	0	0	0	0	0	0	0	0	81
18098663	f	md	CA	2020	1	0	0	0	0	0	0	0	0	0	0	0	58
18098857	m	md	FR	2020	0	0	0	0	0	0	0	0	0	0	0	0	69
18098905	f	hp	CN	2020	0	0	0	0	0	0	0	0	0	0	0	0	50

18098986	m	md	DE	2020	0	1	0	0	0	1	0	0	0	0	0	0	59
18099092	f	cn	US	2020	1	0	0	0	0	0	0	0	0	0	0	0	78.3
18099195	f	md	US	2020	0	1	0	1	0	0	0	0	0	0	0	0	75.2
18099357	m	md	GB	2020	0	0	0	0	0	0	0	0	0	0	0	0	62
18100039	Not	hp	US	2020	0	1	0	1	0	0	0	0	0	0	0	1	61
18100427	f	hp	IE	2020	0	1	0	0	1	0	0	0	0	0	0	0	55
18100490	m	md	TR	2020	0	1	1	0	0	0	0	0	0	0	0	0	55
18100510	f	cn	US	2020	0	1	1	0	0	0	0	0	0	0	0	0	54
18100965	m	md	NL	2020	0	1	1	0	0	0	0	0	0	0	0	0	49
18101298	Not	md	MY	2020	1	0	0	0	0	0	0	0	0	0	0	0	
18101374	m	md	RO	2020	0	0	0	0	0	0	0	0	0	0	0	0	50
18101615	m	hp	CN	2020	0	0	0	0	0	0	0	0	0	0	0	0	63
18101619	m	cn	Not re	2020	0	0	0	0	0	0	0	0	0	0	0	0	
18101709	f	md	AT	2020	1	0	0	0	0	0	0	0	0	0	0	0	72
18101715	f	md	AT	2020	1	0	0	0	0	0	0	0	0	0	0	0	78
18101739	f	md	AT	2020	1	0	0	0	0	0	0	0	0	0	0	0	74
18101741	f	md	AT	2020	1	0	0	0	0	0	0	0	0	0	0	0	62
18101745	f	md	AT	2020	1	0	0	0	0	0	0	0	0	0	0	0	63
18102860	m	hp	US	2020	0	1	1	0	0	0	0	0	0	0	0	0	
18103199	f	md	JP	2020	0	1	0	1	0	0	0	0	0	0	0	1	79
18103758	m	md	JP	2020	0	1	0	1	0	0	0	0	0	0	0	0	72
18103764	f	md	JP	2020	0	1	0	1	0	0	0	0	0	0	0	1	56
18104068	m	md	GB	2020	0	0	0	0	0	0	0	0	0	0	0	0	66
18105351	f	md	CZ	2020	0	0	0	0	0	0	0	0	0	0	0	0	72
18105372	m	hp	CN	2020	0	1	0	0	1	0	0	0	0	0	0	0	67
18105383	m	cn	US	2020	0	1	1	0	0	0	0	0	0	0	0	0	58
18105613	m	Not repo	US	2020	0	1	0	1	0	0	0	0	0	0	0	0	
18105923	m	Not repo	JP	2020	0	1	0	1	0	0	0	0	0	0	0	1	78
18106299	m	hp	US	2020	1	0	0	0	0	0	0	0	0	0	0	0	66
18106610	m	Not repo	ID	2020	0	1	0	1	0	0	0	0	0	0	0	0	38.9
18106991	f	cn	AU	2020	0	1	1	0	0	0	0	0	0	0	0	0	74
18107222	Not	md	CN	2020	0	0	0	0	0	0	0	0	0	0	0	0	
18107423	f	cn	US	2020	0	1	1	0	0	0	0	0	0	0	0	0	
18107648	f	md	JP	2020	0	0	0	0	0	0	0	0	0	0	0	0	74

18109088	m	Not repo	JP	2020	0	1	0	1	0	0	0	0	0	0	0	1	59
18109089	Not	hp	US	2020	0	1	0	1	0	0	0	0	0	0	0	1	75
18109122	m	cn	BE	2020	0	1	1	0	0	0	0	0	0	0	0	0	
18109123	m	hp	CN	2020	0	1	1	0	0	0	0	0	0	0	0	0	55
18109365	m	cn	JP	2020	0	1	1	0	0	0	0	0	0	0	0	0	85
18109424	f	Not repo	MY	2020	1	0	0	0	0	0	0	0	0	0	0	0	73
18109518	Not	hp	US	2020	0	1	0	1	0	0	0	0	0	0	0	1	80
18109520	Not	hp	US	2020	0	1	0	1	0	0	0	0	0	0	0	1	71
18109524	Not	hp	US	2020	0	1	0	1	0	0	0	0	0	0	0	1	59
18109525	Not	hp	US	2020	0	1	0	1	0	0	0	0	0	0	0	1	56
18109526	Not	hp	US	2020	0	1	0	1	0	0	0	0	0	0	0	1	61
18109529	Not	hp	US	2020	0	1	0	1	0	0	0	0	0	0	0	1	66
18109532	m	Not repo	US	2020	1	0	0	0	0	0	0	0	0	0	0	0	76.6
18109600	f	Not repo	ID	2020	1	0	0	0	0	0	0	0	0	0	0	0	58.1
18109672	f	md	JP	2020	0	1	0	0	0	1	0	0	0	0	0	1	62
18109789	m	hp	US	2020	1	0	0	0	0	0	0	0	0	0	0	0	44
18109839	m	md	JP	2020	0	1	0	1	0	0	0	0	0	0	0	0	74
18109840	m	hp	JP	2020	1	0	0	0	0	0	0	0	0	0	0	0	78
18110091	m	Not repo	Not re	2020	0	1	1	0	0	0	0	0	0	0	0	0	73
18110288	m	hp	MX	2020	0	1	0	1	0	0	0	0	0	0	0	0	69
18110454	m	Not repo	JP	2020	0	1	0	1	0	0	0	0	0	0	0	1	76
18110571	Not	hp	NL	2020	0	0	0	0	0	0	0	0	0	0	0	0	
18111178	Not	hp	PT	2020	0	1	1	0	0	0	0	0	0	0	0	0	
18111678	f	Not repo	JP	2020	1	0	0	0	0	0	0	0	0	0	0	0	70
18111734	f	ph	CA	2020	0	1	0	1	0	0	0	0	0	0	0	0	
18111922	f	md	US	2020	0	1	0	0	1	0	0	0	0	0	0	1	66
18112053	m	cn	CN	2020	0	1	0	0	1	0	0	0	0	0	0	0	
18112182	m	md	RO	2020	0	0	0	0	0	0	0	0	0	0	0	0	50
18112508	m	Not repo	JP	2020	0	1	0	1	0	0	0	0	0	0	0	1	36
18112692	m	md	CN	2020	0	0	0	0	0	0	0	0	0	0	0	0	34
18112758	f	md	FR	2020	0	1	0	0	0	1	0	0	0	0	0	0	51
18112926	m	md	JP	2020	0	1	0	0	0	1	0	0	0	0	0	0	90
18113508	f	Not repo	JP	2020	1	0	0	0	0	0	0	0	0	0	0	0	75.9
18114062	m	md	JP	2020	0	1	0	1	0	0	0	0	0	0	0	1	77.5

18114067	f	md	JP	2020	1	0	0	0	0	0	0	0	0	0	0	0	88
18114303	m	md	JP	2020	0	1	0	1	0	0	0	0	0	0	0	1	70
18114304	f	md	JP	2020	0	1	0	1	0	0	0	0	0	0	0	1	74
18114812	m	hp	CN	2020	0	1	0	0	1	0	0	0	0	0	0	0	69
18115044	Not	md	JP	2020	0	1	0	0	0	1	0	0	0	0	0	0	
18115567	m	md	JP	2020	1	0	0	0	0	0	0	0	0	0	0	0	75
18115679	Not	md	JP	2020	0	1	0	0	1	0	0	0	0	0	0	0	
18115778	m	Not repo	JP	2020	0	1	0	1	0	0	0	0	0	0	0	1	40
18115893	f	hp	JP	2020	1	0	0	0	0	0	0	0	0	0	0	0	57
18116050	m	cn	CN	2020	0	1	0	0	1	0	0	0	0	0	0	0	61
18116305	m	md	JP	2020	0	1	0	0	1	0	0	0	0	0	0	0	71
18117386	m	hp	US	2020	0	1	0	0	1	0	0	0	0	0	0	1	77
18117593	m	hp	US	2020	0	1	0	0	1	0	0	0	0	0	0	1	73
18117836	f	hp	US	2020	0	1	0	0	1	0	0	0	0	0	0	1	77
18118040	f	cn	CN	2020	0	1	0	0	1	0	0	0	0	0	0	0	
18118876	f	cn	CN	2020	0	0	0	0	0	0	0	0	0	0	0	0	35
18118914	Not	md	PL	2020	0	0	0	0	0	0	0	0	0	0	0	0	
18119853	f	cn	CN	2020	0	1	0	0	1	0	0	0	0	0	0	0	71
18119930	m	md	JP	2020	0	1	0	1	0	0	0	0	0	0	0	0	69
18119939	f	Not repo	CN	2020	1	0	0	0	0	0	0	0	0	0	0	0	67
18119996	f	Not repo	JP	2020	0	1	0	1	0	0	0	0	0	0	0	1	76
18120000	m	md	JP	2020	1	0	0	0	0	0	0	0	0	0	0	0	73
18120005	f	md	JP	2020	1	0	0	0	0	0	0	0	0	0	0	0	78
18120046	m	cn	CN	2020	0	1	0	0	1	0	0	0	0	0	0	0	
18120688	m	hp	CL	2020	0	1	0	0	1	0	0	0	0	0	0	0	81
18120825	f	hp	JP	2020	1	0	0	0	0	0	0	0	0	0	0	0	73
18121114	m	hp	KR	2020	0	1	1	0	0	0	0	0	0	0	0	0	77
18121313	m	md	IN	2020	0	1	0	1	0	0	0	0	0	0	0	0	46
18122022	f	cn	CN	2020	0	0	0	0	0	0	0	0	0	0	0	0	
18122045	f	hp	FR	2020	0	0	0	0	0	0	0	0	0	0	0	0	73
18122128	m	hp	FR	2020	0	0	0	0	0	0	0	0	0	0	0	0	63
18122163	f	md	JP	2020	0	1	0	0	0	1	0	0	0	0	0	0	78
18122168	m	ph	JP	2020	0	1	0	0	0	1	0	0	0	0	0	0	90
18123127	Not	md	FR	2020	0	1	0	0	1	0	0	0	0	0	0	0	

18123250	f	md	HU	2020	1	0	0	0	0	0	0	0	0	0	0	0	80
18123433	m	md	US	2020	1	0	0	0	0	0	0	0	0	0	0	0	66
18123434	f	md	US	2020	1	0	0	0	0	0	0	0	0	0	0	0	53
18123505	m	Not repo	Not re	2020	1	0	0	0	0	0	0	0	0	0	0	0	44
18124604	f	md	JP	2020	0	1	0	1	0	0	0	0	0	0	0	1	67
18125320	Not	hp	JP	2020	0	0	0	0	0	0	0	0	0	0	0	0	
18125673	m	hp	US	2020	0	0	0	0	0	0	0	0	0	0	0	0	
18125877	f	Not repo	CN	2020	1	0	0	0	0	0	0	0	0	0	0	0	86
18125885	m	Not repo	JP	2020	0	1	0	1	0	0	0	0	0	0	0	0	64
18125899	m	cn	JP	2020	1	0	0	0	0	0	0	0	0	0	0	0	
18125908	f	cn	US	2020	1	0	0	0	0	0	0	0	0	0	0	0	76
18125997	m	Not repo	US	2020	1	0	0	0	0	0	0	0	0	0	0	0	74.3
18126011	m	Not repo	JP	2020	0	1	0	1	0	0	0	0	0	0	0	1	73
18126582	m	md	AT	2020	1	0	0	0	0	0	0	0	0	0	0	0	51
18126583	f	md	AT	2020	1	0	0	0	0	0	0	0	0	0	0	0	88
18126593	f	md	AT	2020	1	0	0	0	0	0	0	0	0	0	0	0	72
18126875	f	cn	IL	2020	0	0	0	0	0	0	0	0	0	0	0	0	53
18127608	m	md	JP	2020	0	1	0	0	0	1	0	0	0	0	0	0	68
18128334	m	md	SG	2020	0	0	0	0	0	0	0	0	0	0	0	0	
18129415	f	cn	US	2020	1	0	0	0	0	0	0	0	0	0	0	0	
18129493	m	md	CN	2020	0	1	0	0	0	1	0	0	0	0	0	0	69
18129517	f	md	BR	2020	1	0	0	0	0	0	0	0	0	0	0	0	60
18130196	f	cn	CN	2020	0	1	0	0	1	0	0	0	0	0	0	0	
18130798	m	md	JP	2020	0	1	0	1	0	0	0	0	0	0	0	1	67
18131018	f	cn	CN	2020	1	0	0	0	0	0	0	0	0	0	0	0	69
18131085	m	md	CA	2020	0	1	1	0	0	0	0	0	0	0	0	0	60
18131112	f	ph	JP	2020	1	0	0	0	0	0	0	0	0	0	0	1	75.6
18131151	Not	md	JP	2020	0	0	0	0	0	0	0	0	0	0	0	0	
18131618	m	md	JP	2020	0	1	0	0	0	1	0	0	0	0	0	0	80
18132168	f	cn	JP	2020	0	1	1	0	0	0	0	0	0	0	0	0	80
18132824	m	hp	JP	2020	0	1	0	0	1	0	0	0	0	0	0	0	66
18132980	m	cn	JP	2020	0	1	1	0	0	0	0	0	0	0	0	1	70
18133925	m	md	TH	2020	0	0	0	0	0	0	0	0	0	0	0	0	59
18133986	m	md	JP	2020	0	1	0	0	1	0	0	0	0	0	0	0	66

18134353	f	md	GB	2020	0	1	0	0	0	1	0	0	0	0	0	0	71
18134900	Not	md	JP	2020	0	0	0	0	0	0	0	0	0	0	0	0	69
18134972	m	md	IN	2020	0	1	0	1	0	0	0	0	0	0	0	0	64
18135001	f	cn	CA	2020	0	1	1	0	0	0	0	0	0	0	0	0	77
18135124	m	hp	CN	2020	0	0	0	0	0	0	0	0	0	0	0	0	75
18135450	m	cn	CN	2020	0	1	0	0	1	0	0	0	0	0	0	0	54
18136079	Not	md	JP	2020	0	1	0	0	0	1	0	0	0	0	0	0	
18136163	m	hp	TW	2020	0	1	0	1	0	0	0	0	0	0	0	0	57
18136347	f	hp	CN	2020	1	0	0	0	0	0	0	0	0	0	0	1	50
18136392	m	hp	CN	2020	1	0	0	0	0	0	0	0	0	0	0	1	59
18136397	f	hp	CN	2020	1	0	0	0	0	0	0	0	0	0	0	1	39
18136398	f	hp	CN	2020	1	0	0	0	0	0	0	0	0	0	0	1	51
18136400	f	hp	CN	2020	1	0	0	0	0	0	0	0	0	0	0	1	63
18136401	f	hp	CN	2020	1	0	0	0	0	0	0	0	0	0	0	1	52
18136406	m	hp	CN	2020	1	0	0	0	0	0	0	0	0	0	0	1	59
18136474	m	cn	US	2020	0	1	1	0	0	0	0	0	0	0	0	0	68
18136485	f	hp	CN	2020	1	0	0	0	0	0	0	0	0	0	0	1	63
18136487	f	hp	CN	2020	1	0	0	0	0	0	0	0	0	0	0	1	52
18136500	f	hp	CN	2020	1	0	0	0	0	0	0	0	0	0	0	1	69
18136501	f	hp	CN	2020	1	0	0	0	0	0	0	0	0	0	0	1	65
18136551	m	cn	Not re	2020	0	0	0	0	0	0	0	0	0	0	0	0	
18136908	f	md	RU	2020	1	0	0	0	0	0	0	0	0	0	0	0	43
18137267	m	cn	Not re	2020	0	0	0	0	0	0	0	0	0	0	0	0	
18137642	f	md	RU	2020	1	0	0	0	0	0	0	0	0	0	0	0	42
18137856	m	hp	US	2020	0	1	1	0	0	0	0	0	0	0	0	1	61
18137981	m	cn	JP	2020	0	1	1	0	0	0	0	0	0	0	0	0	70
18138083	m	cn	JP	2020	0	0	0	0	0	0	0	0	0	0	0	0	50
18138221	m	cn	JP	2020	0	1	1	0	0	0	0	0	0	0	0	0	75
18138230	f	md	JP	2020	0	1	0	0	0	1	0	0	0	0	0	1	70
18138271	m	cn	JP	2020	0	1	1	0	0	0	0	0	0	0	0	0	
18138750	f	md	PL	2020	0	1	0	1	0	0	0	0	0	0	0	0	
18139449	m	md	JP	2020	0	1	1	0	0	0	0	0	0	0	0	0	73
18139969	m	md	JP	2020	1	0	0	0	0	0	0	0	0	0	0	0	72
18140575	f	md	JP	2020	1	0	0	0	0	0	0	0	0	0	0	0	66

18140720	f	hp	CA	2020	0	1	1	0	0	0	0	0	0	0	0	0	77
18141277	f	cn	Not re	2020	0	0	0	0	0	0	0	0	0	0	0	0	
18141565	f	md	CZ	2020	1	0	0	0	0	0	0	0	0	0	0	0	72
18141575	f	cn	CN	2020	0	1	0	0	1	0	0	0	0	0	0	0	34
18141756	m	hp	CN	2020	0	0	0	0	0	0	0	0	0	0	0	0	65
18142127	m	hp	JP	2020	0	1	0	1	0	0	0	0	0	0	0	1	74
18142402	m	md	JP	2020	0	1	0	0	1	0	0	0	0	0	0	0	83
18142460	f	cn	CN	2020	0	1	0	0	1	0	0	0	0	0	0	0	
18142588	m	md	PL	2020	0	1	1	0	0	0	0	0	0	0	0	0	58
18143098	m	cn	JP	2020	0	1	1	0	0	0	0	0	0	0	0	0	70
18143295	f	cn	US	2020	0	0	0	0	0	0	1	1	0	0	0	0	59
18143676	f	md	US	2020	0	1	0	0	0	1	0	0	0	0	0	0	59
18143816	f	md	TH	2020	0	0	0	0	0	0	0	0	0	0	0	0	47
18144695	m	Not repo	AU	2020	0	1	0	1	0	0	0	0	0	0	0	0	
18145763	f	hp	JP	2020	1	0	0	0	0	0	0	0	0	0	0	0	73.2
18145926	Not	md	JP	2020	0	0	0	0	0	0	0	0	0	0	0	0	
18145935	m	md	JP	2020	0	1	1	0	0	0	0	0	0	0	0	0	81
18146129	f	hp	CZ	2020	0	0	0	0	0	0	0	0	0	0	0	0	72
18146212	m	cn	AR	2020	0	0	0	0	0	0	0	0	0	0	0	0	33
18146435	f	cn	US	2020	1	0	0	0	0	0	0	0	0	0	0	0	62
18147083	m	cn	US	2020	0	1	1	0	0	0	0	0	0	0	0	0	
18147359	m	hp	US	2020	0	0	0	0	0	0	0	0	0	0	0	0	65
18147368	f	hp	US	2020	0	0	0	0	0	0	0	0	0	0	0	0	77
18147370	m	md	JP	2020	0	1	0	1	0	0	0	0	0	0	0	0	64
18147415	f	hp	CA	2020	1	0	0	0	0	0	0	0	0	0	0	0	72
18147519	Not	md	CN	2020	0	0	0	0	0	0	0	0	0	0	0	0	
18147597	m	hp	IN	2020	0	0	0	0	0	0	0	0	0	0	0	0	72
18147821	f	md	US	2020	0	0	0	0	0	0	0	0	0	0	0	0	80
18147883	m	md	CN	2020	0	0	0	0	0	0	0	0	0	0	0	0	70
18148564	m	md	JP	2020	0	1	0	0	1	0	0	0	0	0	0	0	73
18148658	Not	Not repo	AU	2020	1	0	0	0	0	0	0	0	0	0	0	0	
18148905	f	md	US	2020	0	1	0	0	0	1	0	0	0	0	0	0	47
18149210	f	md	CZ	2020	1	0	0	0	0	0	0	0	0	0	0	0	56
18149453	f	md	CZ	2020	0	0	0	0	0	0	0	0	0	0	0	0	72

18150137	f	cn	CN	2020	1	0	0	0	0	0	0	0	0	0	0	0	66
18150166	m	md	TR	2020	0	1	1	0	0	0	0	0	0	0	0	0	55
18150372	m	Not repo	ID	2020	1	0	0	0	0	0	0	0	0	0	0	0	52.5
18150373	m	Not repo	AU	2020	1	0	0	0	0	0	0	0	0	0	0	0	
18150847	f	Not repo	JP	2020	1	0	0	0	0	0	0	0	0	0	0	0	82
18150889	m	md	FR	2020	0	1	0	0	1	0	0	0	0	0	0	0	65
18152035	m	cn	FR	2020	0	1	1	0	0	0	0	0	0	0	0	0	78
18152309	m	Not repo	CN	2020	1	0	0	0	0	0	0	0	0	0	0	0	80
18152375	m	hp	US	2020	0	0	0	0	0	0	0	0	0	0	0	0	69
18152410	m	md	KR	2020	0	1	1	0	0	0	0	0	0	0	0	0	58
18152500	m	Not repo	JP	2020	0	1	0	1	0	0	0	0	0	0	0	1	73
18153159	Not	md	CN	2020	0	0	0	0	0	0	0	0	0	0	0	0	
18153814	m	hp	US	2020	0	1	1	0	0	0	0	0	0	0	0	1	61
18153822	f	hp	DE	2020	0	1	1	0	0	0	0	0	0	0	0	0	60
18153995	f	cn	Not re	2020	0	0	0	0	0	0	0	0	0	0	0	0	
18154021	Not	hp	US	2020	0	0	0	0	0	0	0	0	0	0	0	0	
18154274	f	md	US	2020	0	1	1	0	0	0	0	0	0	0	0	0	
18154276	m	md	CN	2020	0	0	0	0	0	0	0	0	0	0	0	0	33
18154485	m	md	US	2020	0	0	0	0	0	0	0	0	0	0	0	0	69
18156586	f	md	NL	2020	0	0	0	0	0	0	0	0	0	0	0	0	74
18156857	f	md	AT	2020	1	0	0	0	0	0	0	0	0	0	0	0	67
18157747	f	cn	US	2020	0	0	0	0	0	0	0	0	0	0	0	0	85
18158154	m	hp	US	2020	1	0	0	0	0	0	0	0	0	0	0	0	
18158876	f	hp	US	2020	1	0	0	0	0	0	0	0	0	0	0	0	74
18158944	f	md	KR	2020	0	0	0	0	0	0	0	0	0	0	0	0	88
18159402	Not	md	CN	2020	0	0	0	0	0	0	0	0	0	0	0	0	
18159424	f	md	CZ	2020	0	0	0	0	0	0	0	0	0	0	0	0	72
18159469	m	ph	CA	2020	0	1	0	1	0	0	0	0	0	0	0	1	70
18160223	m	hp	IN	2020	0	1	1	0	0	0	0	0	0	0	0	0	48
18160392	f	md	AT	2020	1	0	0	0	0	0	0	0	0	0	0	0	52
18160711	m	cn	US	2020	1	0	0	0	0	0	0	0	0	0	0	0	81
18160872	f	hp	CN	2020	0	0	0	0	0	0	0	0	0	0	0	0	62
18162227	m	cn	JP	2020	0	1	1	0	0	0	0	0	0	0	0	1	90
18162290	m	hp	US	2020	0	0	0	0	0	0	0	0	0	0	0	0	52

18162441	m	hp	MX	2020	0	0	0	0	0	0	0	0	0	0	0	0	69
18162868	f	md	BE	2020	0	1	1	0	0	0	0	0	0	0	0	0	58
18163433	m	cn	PL	2020	0	1	1	0	0	0	0	0	0	0	0	0	71
18163605	m	hp	US	2020	0	1	1	0	0	0	0	0	0	0	0	1	61
18164535	m	md	CH	2020	0	0	0	0	0	0	0	0	0	0	0	0	64
18164920	f	hp	US	2020	0	1	1	0	0	0	0	0	0	0	0	0	57
18165388	m	hp	FR	2020	1	0	0	0	0	0	0	0	0	0	0	0	71
18165631	f	hp	CN	2020	1	0	0	0	0	0	0	0	0	0	0	0	66
18165942	m	hp	US	2020	0	1	1	0	0	0	0	0	0	0	0	0	
18166363	f	hp	US	2020	0	1	1	0	0	0	0	0	0	0	0	0	57
18167931	f	md	JP	2020	1	0	0	0	0	0	0	0	0	0	0	0	78
18168369	m	cn	Not re	2020	0	0	0	0	0	0	0	0	0	0	0	0	
18168868	f	md	US	2020	0	1	1	0	0	0	0	0	0	0	0	0	57
18169216	m	md	CN	2020	0	1	0	0	0	1	0	0	0	0	0	1	66
18170112	m	md	JP	2020	0	1	0	0	0	1	0	0	0	0	0	0	73
18170341	f	cn	Not re	2020	0	0	0	0	0	0	0	0	0	0	0	0	
18170828	Not	md	JP	2020	0	1	0	1	0	0	0	0	0	0	0	0	
18171036	m	Not repo	CN	2020	1	0	0	0	0	0	0	0	0	0	0	0	77
18171290	f	md	US	2020	0	0	0	0	0	0	0	0	0	0	0	0	
18171832	f	cn	US	2020	0	0	0	0	0	0	0	0	0	0	0	0	
18171870	f	Not repo	CN	2020	1	0	0	0	0	0	0	0	0	0	0	0	60
18171991	f	hp	CN	2020	0	0	0	0	0	0	0	0	0	0	0	0	45
18172121	Not	cn	CN	2020	0	0	0	0	0	0	0	0	0	0	0	0	
18172220	m	md	FR	2020	0	1	0	0	1	0	0	0	0	0	0	0	60
18173015	m	md	VN	2020	1	0	0	0	0	0	0	0	0	0	0	0	
18173209	m	md	JP	2020	0	1	0	1	0	0	0	0	0	0	0	1	80
18173357	Not	md	JP	2020	0	1	0	1	0	0	0	0	0	0	0	0	
18173377	f	md	US	2020	0	1	0	0	0	1	0	0	0	0	0	0	50
18173546	f	Not repo	JP	2020	1	0	0	0	0	0	0	0	0	0	0	1	72
18174051	m	cn	IN	2020	0	0	0	0	0	0	0	0	0	0	0	0	72
18174556	m	hp	KR	2020	0	0	0	0	0	0	0	0	0	0	0	0	79
18174936	Not	hp	AU	2020	0	1	0	0	0	1	0	0	0	0	0	0	
18176010	f	hp	IN	2020	0	0	0	0	0	0	0	0	0	0	0	0	
18176194	m	cn	CN	2020	1	0	0	0	0	0	0	0	0	0	0	0	64

18176424	f	hp	FR	2020	0	0	0	0	0	0	0	0	0	0	0	0	73
18176641	m	hp	US	2020	0	1	1	0	0	0	0	0	0	0	0	1	61
18177037	m	hp	US	2020	0	1	0	0	1	0	0	0	0	0	0	0	50
18177395	f	Not repo	CA	2020	1	0	0	0	0	0	0	0	0	0	0	0	39.2
18177704	m	md	FR	2020	0	1	0	0	0	1	0	0	0	0	0	0	76
18177761	Not	hp	AU	2020	0	1	0	0	0	1	0	0	0	0	0	0	
18177802	m	md	AT	2020	0	1	0	0	0	1	0	0	0	0	0	0	88
18178200	m	md	JP	2020	1	0	0	0	0	0	0	0	0	0	0	0	78
18178657	f	md	IL	2020	0	0	0	0	0	0	0	0	0	0	0	0	
18178837	m	Not repo	IN	2020	0	1	0	1	0	0	0	0	0	0	0	0	65
18179051	f	md	KR	2020	1	0	0	0	0	0	0	0	0	0	0	0	
18179365	f	md	RU	2020	1	0	0	0	0	0	0	0	0	0	0	0	35
18179382	m	md	AT	2020	0	1	1	0	0	0	0	0	0	0	0	1	77
18179523	m	ph	JP	2020	0	1	0	0	1	0	0	0	0	0	0	0	71
18180052	f	hp	US	2020	1	0	0	0	0	0	0	0	0	0	0	0	87.5
18180138	Not	md	JP	2020	0	1	0	1	0	0	0	0	0	0	0	0	
18180147	f	md	JP	2020	0	1	1	0	0	0	0	0	0	0	0	0	61
18180339	f	Not repo	ID	2020	1	0	0	0	0	0	0	0	0	0	0	0	
18180343	f	Not repo	AU	2020	1	0	0	0	0	0	0	0	0	0	0	0	91
18180762	f	hp	US	2020	0	1	0	0	1	0	0	0	0	0	0	0	81
18180901	m	md	CZ	2020	1	0	0	0	0	0	0	0	0	0	0	0	70
18181081	f	md	RO	2020	1	0	0	0	0	0	0	0	0	0	0	0	83.5
18181121	f	md	JP	2020	0	1	0	0	0	1	0	0	0	0	0	0	74
18181155	Not	md	JP	2020	0	1	0	1	0	0	0	0	0	0	0	0	
18181222	m	cn	DE	2020	0	1	1	0	0	0	0	0	0	0	0	0	58
18181297	f	hp	US	2020	0	0	0	0	0	0	0	0	0	0	0	0	73
18181440	f	Not repo	US	2020	1	0	0	0	0	0	0	0	0	0	0	0	86.7
18181856	f	ph	PT	2020	0	1	1	0	0	0	0	0	0	0	0	0	66
18181872	f	cn	RU	2020	1	0	0	0	0	0	0	0	0	0	0	0	53
18181941	m	md	JP	2020	0	1	0	1	0	0	0	0	0	0	0	1	75
18182195	m	hp	US	2020	0	1	1	0	0	0	0	0	0	0	0	1	61
18182357	f	Not repo	SG	2020	1	0	0	0	0	0	0	0	0	0	0	0	
18182436	m	hp	KR	2020	0	1	1	0	0	0	0	0	0	0	0	0	79
18182781	f	Not repo	CN	2020	1	0	0	0	0	0	0	0	0	0	0	0	67

18182797	m	ph	FR	2020	0	1	1	0	0	0	0	0	0	0	0	0	52
18182822	m	cn	US	2020	0	1	1	0	0	0	0	0	0	0	0	0	63
18182841	m	cn	Not re	2020	0	0	0	0	0	0	0	0	0	0	0	0	64
18182848	m	Not repo	JP	2020	1	0	0	0	0	0	0	0	0	0	0	1	71
18182856	f	cn	US	2020	1	0	0	0	0	0	0	0	0	0	0	0	
18182992	m	hp	US	2020	0	1	1	0	0	0	0	0	0	0	0	0	
18183412	f	hp	DE	2020	0	1	1	0	0	0	0	0	0	0	0	0	
18183454	f	hp	PT	2020	0	1	1	0	0	0	0	0	0	0	0	1	66
18183931	f	md	AT	2020	1	0	0	0	0	0	0	0	0	0	0	0	62
18184246	f	ph	PT	2020	0	1	1	0	0	0	0	0	0	0	0	1	66
18184659	m	md	CN	2020	0	0	0	0	0	0	0	0	0	0	0	0	65
18186271	f	md	JP	2020	1	0	0	0	0	0	0	0	0	0	0	1	78
18186272	m	md	JP	2020	1	0	0	0	0	0	0	0	0	0	0	0	54
18186365	f	md	JP	2020	1	0	0	0	0	0	0	0	0	0	0	0	77
18186636	f	md	FR	2020	0	1	0	0	0	1	0	0	0	0	0	0	50
18186642	f	md	JP	2020	1	0	0	0	0	0	0	0	0	0	0	0	85
18186643	m	md	JP	2020	1	0	0	0	0	0	0	0	0	0	0	0	35
18186644	m	md	JP	2020	1	0	0	0	0	0	0	0	0	0	0	0	63
18187139	f	md	JP	2020	1	0	0	0	0	0	0	0	0	0	0	0	71
18187492	m	cn	CN	2020	0	1	0	0	1	0	0	0	0	0	0	0	59
18188610	f	md	US	2020	0	1	1	0	0	0	0	0	0	0	0	1	65
18188638	m	hp	US	2020	0	1	0	0	1	0	0	0	0	0	0	1	73
18188808	f	md	NL	2020	0	0	0	0	0	0	0	0	0	0	0	0	74
18188822	f	md	NL	2020	0	0	0	0	0	0	0	0	0	0	0	0	75
18189646	Not	md	US	2020	0	1	0	1	0	0	0	0	0	0	0	1	
18190158	m	md	CN	2020	0	0	0	0	0	0	0	0	0	0	0	0	54
18190969	m	cn	US	2020	0	1	1	0	0	0	0	0	0	0	0	0	
18191029	m	cn	US	2020	0	0	0	0	0	0	0	0	0	0	0	0	
18191044	m	cn	US	2020	0	0	0	0	0	0	0	0	0	0	0	0	
18191053	f	cn	US	2020	0	0	0	0	0	0	0	0	0	0	0	0	
18191063	f	cn	US	2020	0	0	0	0	0	0	0	0	0	0	0	0	
18191102	m	cn	US	2020	0	0	0	0	0	0	0	0	0	0	0	0	58
18191107	m	cn	US	2020	0	0	0	0	0	0	0	0	0	0	0	0	51
18191129	f	cn	US	2020	0	0	0	0	0	0	0	0	0	0	0	0	62

18191135	f	ph	US	2020	0	0	0	0	0	0	0	0	0	0	0	0	88
18191142	m	cn	US	2020	0	0	0	0	0	0	0	0	0	0	0	0	
18191148	f	hp	US	2020	0	0	0	0	0	0	0	0	0	0	0	0	
18191149	f	md	US	2020	0	0	0	0	0	0	0	0	0	0	0	0	74
18191152	f	md	US	2020	0	0	0	0	0	0	0	0	0	0	0	0	58
18191154	f	md	US	2020	0	0	0	0	0	0	0	0	0	0	0	0	62
18191165	m	cn	US	2020	0	0	0	0	0	0	0	0	0	0	0	0	
18191186	Not	cn	US	2020	0	0	0	0	0	0	0	0	0	0	0	0	
18191211	m	ph	US	2020	0	0	0	0	0	0	0	0	0	0	0	0	67
18191217	m	hp	US	2020	0	0	0	0	0	0	0	0	0	0	0	0	
18191220	f	md	US	2020	0	0	0	0	0	0	0	0	0	0	0	0	
18191221	m	cn	US	2020	0	0	0	0	0	0	0	0	0	0	0	0	
18191254	m	cn	US	2020	0	0	0	0	0	0	0	0	0	0	0	0	
18191260	Not	md	US	2020	0	0	0	0	0	0	0	0	0	0	0	0	
18191261	Not	hp	US	2020	0	0	0	0	0	0	0	0	0	0	0	0	
18191275	f	md	JP	2020	1	0	0	0	0	0	0	0	0	0	0	0	64
18191280	f	md	JP	2020	1	0	0	0	0	0	0	0	0	0	0	0	85
18191281	m	md	JP	2020	1	0	0	0	0	0	0	0	0	0	0	0	61
18191286	f	md	JP	2020	1	0	0	0	0	0	0	0	0	0	0	1	81
18191287	f	md	JP	2020	1	0	0	0	0	0	0	0	0	0	0	0	66
18191288	f	md	JP	2020	1	0	0	0	0	0	0	0	0	0	0	0	73
18191289	f	md	JP	2020	1	0	0	0	0	0	0	0	0	0	0	1	73
18191310	f	md	JP	2020	1	0	0	0	0	0	0	0	0	0	0	1	79
18191407	m	md	JP	2020	1	0	0	0	0	0	0	0	0	0	0	0	60
18191431	f	md	JP	2020	1	0	0	0	0	0	0	0	0	0	0	0	69
18191434	f	md	JP	2020	1	0	0	0	0	0	0	0	0	0	0	0	70
18191448	m	md	JP	2020	1	0	0	0	0	0	0	0	0	0	0	0	74
18191453	f	md	JP	2020	1	0	0	0	0	0	0	0	0	0	0	0	72
18191520	f	md	JP	2020	1	0	0	0	0	0	0	0	0	0	0	0	57
18191610	f	md	JP	2020	1	0	0	0	0	0	0	0	0	0	0	0	78
18191796	Not	md	US	2020	0	0	0	0	0	0	0	0	0	0	0	0	70
18191919	f	md	JP	2020	1	0	0	0	0	0	0	0	0	0	0	0	85
18191920	m	md	JP	2020	1	0	0	0	0	0	0	0	0	0	0	0	86
18191921	f	md	JP	2020	1	0	0	0	0	0	0	0	0	0	0	0	68

18191922	f	md	JP	2020	1	0	0	0	0	0	0	0	0	0	0	1	73
18191923	f	md	JP	2020	1	0	0	0	0	0	0	0	0	0	0	0	73
18191925	f	md	JP	2020	1	0	0	0	0	0	0	0	0	0	0	0	74
18191926	f	md	JP	2020	1	0	0	0	0	0	0	0	0	0	0	0	42
18192063	m	md	JP	2020	1	0	0	0	0	0	0	0	0	0	0	0	76
18192066	m	md	JP	2020	1	0	0	0	0	0	0	0	0	0	0	0	76
18192368	m	md	JP	2020	1	0	0	0	0	0	0	0	0	0	0	0	72
18192713	m	cn	JP	2020	0	1	1	0	0	0	0	0	0	0	0	0	
18193255	m	cn	CN	2020	0	1	0	0	1	0	0	0	0	0	0	0	67
18193259	m	cn	CN	2020	0	1	0	0	1	0	0	0	0	0	0	0	
18193388	f	ph	US	2020	0	0	0	0	0	0	0	0	0	0	0	0	55
18193427	m	md	JP	2020	0	1	0	1	0	0	0	0	0	0	0	1	67
18193428	m	md	JP	2020	0	1	0	1	0	0	0	0	0	0	0	1	73.4
18193437	Not	md	JP	2020	0	1	0	1	0	0	0	0	0	0	0	0	
18193438	f	cn	AE	2020	0	1	0	1	0	0	0	0	0	0	0	0	53.8
18193502	f	md	US	2020	0	0	0	0	0	0	0	0	0	0	0	0	73
18193512	f	cn	US	2020	1	0	0	0	0	0	0	0	0	0	0	0	84
18193961	m	hp	FR	2020	0	1	1	0	0	0	0	0	0	0	0	0	52
18194378	f	md	JP	2020	1	0	0	0	0	0	0	0	0	0	0	1	72
18194384	m	md	ES	2020	0	1	1	0	0	0	0	0	0	0	0	0	75
18194451	f	md	JP	2020	1	0	0	0	0	0	0	0	0	0	0	0	81
18194609	m	md	JP	2020	1	0	0	0	0	0	0	0	0	0	0	0	77
18194626	f	md	JP	2020	1	0	0	0	0	0	0	0	0	0	0	0	87
18194789	f	hp	JP	2020	1	0	0	0	0	0	0	0	0	0	0	0	90
18194849	m	md	TH	2020	0	1	0	0	0	1	0	0	0	0	0	0	
18195141	Not	Not repo	JP	2020	0	1	0	1	0	0	0	0	0	0	0	0	
18195205	m	hp	US	2020	0	1	1	0	0	0	0	0	0	0	0	1	61
18195240	m	hp	JP	2020	0	1	0	1	0	0	0	0	0	0	0	0	70
18195338	m	ph	FR	2020	0	1	1	0	0	0	0	0	0	0	0	0	52
18195356	f	md	JP	2020	1	0	0	0	0	0	0	0	0	0	0	0	76
18195535	f	md	JP	2020	1	0	0	0	0	0	0	0	0	0	0	0	67
18195814	f	md	US	2020	1	0	0	0	0	0	0	0	0	0	0	0	53.5
18195986	m	cn	CN	2020	0	1	0	0	1	0	0	0	0	0	0	0	61
18196172	m	md	US	2020	0	0	0	0	0	0	0	0	0	0	0	0	69

18196771	Not	Not repo	AU	2020	0	1	0	1	0	0	0	0	0	0	0	0	
18197813	f	Not repo	CN	2020	1	0	0	0	0	0	0	0	0	0	0	0	80
18197836	m	md	AR	2020	0	0	0	0	0	0	0	0	0	0	0	0	67
18197914	Not	cn	JP	2020	1	0	0	0	0	0	0	0	0	0	0	0	
18198192	m	hp	ES	2020	0	0	0	0	0	0	0	0	0	0	0	0	59
18198271	m	md	JP	2020	0	1	0	0	1	0	0	0	0	0	0	0	86
18198557	m	ph	FR	2020	1	0	0	0	0	0	0	0	0	0	0	0	67
18198803	m	hp	ES	2020	0	1	0	0	0	1	0	0	0	0	0	0	57
18199027	m	md	ES	2020	0	1	1	0	0	0	0	0	0	0	0	0	71
18199196	m	hp	GE	2020	0	0	0	0	0	0	0	0	0	0	0	0	58
18199221	m	md	CA	2020	1	0	0	0	0	0	0	0	0	0	0	0	
18199268	f	Not repo	JP	2020	1	0	0	0	0	0	0	0	0	0	0	0	77
18199305	m	md	US	2020	0	1	1	0	0	0	0	0	0	0	0	0	
18199802	f	cn	CN	2020	0	1	0	0	1	0	0	0	0	0	0	0	
18200204	m	cn	CN	2020	0	1	0	0	1	0	0	0	0	0	0	0	55
18200389	m	md	US	2020	0	0	0	0	0	0	0	0	0	0	0	0	
18200393	Not	md	JP	2020	0	1	0	0	0	1	0	0	0	0	0	0	70
18200401	m	cn	SG	2020	0	0	0	0	0	0	0	0	0	0	0	0	49
18200631	f	md	DE	2020	0	0	0	0	0	0	0	0	0	0	0	0	67
18200661	m	md	US	2020	0	1	1	0	0	0	0	0	0	0	0	1	61
18200837	m	cn	JP	2020	0	1	1	0	0	0	0	0	0	0	0	0	71
18200855	m	hp	US	2020	0	1	0	0	1	0	0	0	0	0	0	0	68
18200917	Not	hp	CA	2020	0	0	0	0	0	0	0	0	0	0	0	0	
18201010	f	Not repo	CN	2020	1	0	0	0	0	0	0	0	0	0	0	0	56
18201172	f	cn	US	2020	1	0	0	0	0	0	0	0	0	0	0	0	86
18201731	m	hp	IL	2020	0	0	0	0	0	0	0	0	0	0	0	0	
18202612	m	hp	CN	2020	0	1	1	0	0	0	0	0	0	0	0	0	35
18202676	f	md	DE	2020	0	1	1	0	0	0	0	0	0	0	0	0	52
18203227	m	cn	CN	2020	0	1	0	0	1	0	0	0	0	0	0	0	62
18203409	m	hp	JP	2020	0	0	0	0	0	0	0	0	0	0	0	0	75
18203546	m	hp	IL	2020	0	0	0	0	0	0	0	0	0	0	0	0	
18203660	m	hp	CN	2020	0	1	0	0	1	0	0	0	0	0	0	0	
18203871	m	hp	DE	2020	0	0	0	0	0	0	0	0	0	0	0	0	
18204241	m	cn	CN	2020	0	1	0	0	1	0	0	0	0	0	0	0	75

18204260	m	cn	CN	2020	0	1	0	0	1	0	0	0	0	0	0	0	66
18204269	f	ph	PT	2020	0	1	1	0	0	0	0	0	0	0	0	1	66
18204274	f	hp	CA	2020	1	0	0	0	0	0	0	0	0	0	0	0	56.5
18204575	m	cn	IN	2020	0	1	0	0	1	0	0	0	0	0	0	0	73
18204685	m	ph	US	2020	0	1	0	1	0	0	0	0	0	0	0	0	66
18204862	m	cn	CN	2020	0	1	0	0	1	0	0	0	0	0	0	0	57
18205040	Not	md	CN	2020	0	0	0	0	0	0	0	0	0	0	0	0	
18205401	m	ph	FR	2020	0	1	1	0	0	0	0	0	0	0	0	0	52
18205523	m	md	FR	2020	0	1	1	0	0	0	0	0	0	0	0	0	62
18205941	m	cn	CN	2020	0	1	0	0	1	0	0	0	0	0	0	0	
18205943	Not	hp	IT	2020	0	0	0	0	0	0	0	0	0	0	0	0	
18205957	Not	hp	DE	2020	0	0	0	0	0	0	0	0	0	0	0	0	74
18205979	f	cn	US	2020	0	1	1	0	0	0	0	0	0	0	0	0	93
18206122	m	cn	IN	2020	0	1	0	0	1	0	0	0	0	0	0	0	74
18206124	m	cn	IN	2020	0	1	0	0	1	0	0	0	0	0	0	0	
18206230	Not	hp	FR	2020	0	0	0	0	0	0	0	0	0	0	0	0	
18206969	m	cn	CN	2020	0	1	0	0	1	0	0	0	0	0	0	0	57
18207022	m	cn	Not re	2020	0	0	0	0	0	0	0	0	0	0	0	0	62
18207050	m	md	JP	2020	0	1	0	1	0	0	0	0	0	0	0	0	50
18207249	f	md	JP	2020	0	0	0	0	0	0	0	0	0	0	0	0	72
18207318	m	md	CN	2020	0	1	1	0	0	0	0	0	0	0	0	0	65
18207404	m	md	JP	2020	0	1	0	1	0	0	0	0	0	0	0	1	70
18207525	f	Not repo	AU	2020	1	0	0	0	0	0	0	0	0	0	0	0	
18207622	m	cn	CN	2020	0	1	0	0	1	0	0	0	0	0	0	0	50
18207838	Not	hp	JP	2020	1	0	0	0	0	0	0	0	0	0	0	0	
18208199	m	cn	CN	2020	0	1	0	0	1	0	0	0	0	0	0	0	78
18208447	m	cn	US	2020	1	0	0	0	0	0	0	0	0	0	0	0	74
18208668	f	hp	US	2020	0	1	0	1	0	0	0	0	0	0	0	1	
18208688	m	cn	CN	2020	0	1	0	0	1	0	0	0	0	0	0	0	
18208927	m	md	FR	2020	0	0	0	0	0	0	0	0	0	0	0	0	75
18208963	m	md	TM	2020	0	1	0	0	0	1	0	0	0	0	0	0	46
18209016	m	cn	CN	2020	0	1	0	0	1	0	0	0	0	0	0	0	66
18209021	m	cn	CN	2020	0	1	0	0	1	0	0	0	0	0	0	0	70
18209208	f	cn	US	2020	0	0	0	0	0	0	0	0	0	0	0	0	

18209418	m	hp	JP	2020	0	1	1	0	0	0	0	0	0	0	0	0	69
18209522	m	hp	FR	2020	0	1	0	0	1	0	0	0	0	0	0	0	69
18209736	m	cn	CN	2020	0	1	0	0	1	0	0	0	0	0	0	0	74
18209737	m	cn	CN	2020	0	1	0	0	1	0	0	0	0	0	0	0	
18210457	m	cn	US	2020	1	0	0	0	0	0	0	0	0	0	0	0	
18210723	f	cn	Not re	2020	0	0	0	0	0	0	0	0	0	0	0	0	44
18211033	Not	md	JP	2020	0	0	0	0	0	0	0	0	0	0	0	0	
18211123	m	ph	JP	2020	0	1	0	0	0	1	0	0	0	0	0	0	66
18211148	Not	md	JP	2020	0	0	0	0	0	0	0	0	0	0	0	0	
18211149	Not	md	JP	2020	0	0	0	0	0	0	0	0	0	0	0	0	
18211188	f	Not repo	MY	2020	1	0	0	0	0	0	0	0	0	0	0	0	83.2
18211311	m	cn	CN	2020	0	1	0	0	1	0	0	0	0	0	0	0	57
18211312	f	hp	US	2020	0	1	0	0	1	0	0	0	0	0	0	0	70
18211666	m	md	JP	2020	0	1	0	0	0	1	0	0	0	0	0	0	80
18211923	Not	md	JP	2020	0	1	0	0	0	1	0	0	0	0	0	0	60
18212174	m	hp	US	2020	0	0	0	0	0	0	0	0	0	0	0	0	58
18212246	m	md	VN	2020	1	0	0	0	0	0	0	0	0	0	0	0	
18212568	f	hp	RU	2020	0	1	1	0	0	0	0	0	0	0	0	0	48
18212726	f	Not repo	JP	2020	1	0	0	0	0	0	0	0	0	0	0	0	80
18212758	f	md	US	2020	0	1	0	0	0	1	0	0	0	0	0	0	61
18212850	f	cn	CN	2020	0	1	0	0	1	0	0	0	0	0	0	0	79
18212856	m	cn	CN	2020	0	1	0	0	1	0	0	0	0	0	0	0	
18213090	m	cn	GT	2020	0	1	1	0	0	0	0	0	0	0	0	0	
18213154	m	Not repo	JP	2020	1	0	0	0	0	0	0	0	0	0	0	0	76
18213511	f	ph	CA	2020	0	0	0	0	0	0	0	0	0	0	0	0	68
18213882	f	md	JP	2020	1	0	0	0	0	0	0	0	0	0	0	0	85
18213883	m	cn	CN	2020	0	1	0	0	1	0	0	0	0	0	0	0	
18213991	m	md	JP	2020	0	1	0	0	0	1	0	0	0	0	0	0	
18214082	f	hp	DE	2020	0	1	1	0	0	0	0	0	0	0	0	0	30
18214261	m	cn	CN	2020	0	1	0	0	1	0	0	0	0	0	0	0	
18214580	f	Not repo	JP	2020	0	1	0	1	0	0	0	0	0	0	0	1	74
18214708	m	hp	CA	2020	0	0	0	0	0	0	0	0	0	0	0	0	58
18214714	m	cn	US	2020	0	0	0	0	0	0	0	0	0	0	0	0	61
18214745	m	Not repo	JP	2020	0	1	0	1	0	0	0	0	0	0	0	1	62

18214960	m	Not repo	JP	2020	0	1	0	1	0	0	0	0	0	0	0	0	76
18215135	m	cn	FR	2020	0	1	1	0	0	0	0	0	0	0	0	0	
18215173	m	cn	JP	2020	0	1	1	0	0	0	0	0	0	0	0	0	64
18215193	m	md	KW	2020	0	0	0	0	0	0	0	0	0	0	0	0	
18215236	f	hp	US	2020	0	0	0	0	0	0	0	0	0	0	0	0	
18215346	m	md	JP	2020	0	1	0	0	0	1	0	0	0	0	0	0	60
18215392	m	cn	CN	2020	0	1	0	0	1	0	0	0	0	0	0	0	64
18215453	Not	md	JP	2020	0	0	0	0	0	0	0	0	0	0	0	0	
18215590	m	cn	JP	2020	0	1	1	0	0	0	0	0	0	0	0	0	67
18215711	Not	md	JP	2020	0	0	0	0	0	0	0	0	0	0	0	0	
18215853	m	md	JP	2020	0	1	0	0	0	1	0	0	0	0	0	0	52
18215876	m	cn	CN	2020	0	1	0	0	1	0	0	0	0	0	0	0	69
18216059	m	md	KR	2020	0	1	0	0	1	0	0	0	0	0	0	0	65
18216321	m	md	FR	2020	1	0	0	0	0	0	0	0	0	0	0	0	71
18216411	Not	md	JP	2020	0	1	0	0	0	1	0	0	0	0	0	0	
18216658	m	md	KR	2020	0	1	0	0	0	1	0	0	0	0	0	0	68
18216898	f	Not repo	MY	2020	1	0	0	0	0	0	0	0	0	0	0	0	
18216903	f	md	JP	2020	1	0	0	0	0	0	0	0	0	0	0	0	78.9
18216997	f	cn	CN	2020	0	1	0	0	1	0	0	0	0	0	0	0	
18217000	f	hp	US	2020	0	0	0	0	0	0	0	0	0	0	0	0	73
18217260	f	md	NL	2020	0	0	0	0	0	0	0	0	0	0	0	0	61
18217378	Not	md	JP	2020	1	0	0	0	0	0	0	0	0	0	0	0	
18217382	m	cn	JP	2020	0	1	1	0	0	0	0	0	0	0	0	0	71
18217467	Not	hp	CN	2020	1	0	0	0	0	0	0	0	0	0	0	0	
18218043	m	cn	CN	2020	0	1	0	0	1	0	0	0	0	0	0	0	
18218066	m	cn	CN	2020	0	1	0	0	1	0	0	0	0	0	0	0	
18218117	m	md	JP	2020	0	1	0	1	0	0	0	0	0	0	0	0	71
18218142	f	md	US	2020	0	0	0	0	0	0	0	0	0	0	0	0	73
18218212	m	cn	AR	2020	0	0	0	0	0	0	0	0	0	0	0	0	72
18218573	m	cn	CN	2020	0	1	0	0	1	0	0	0	0	0	0	0	
18218590	m	ph	HU	2020	0	1	1	0	0	0	0	0	0	0	0	0	64
18218752	m	Not repo	ID	2020	1	0	0	0	0	0	0	0	0	0	0	0	57.4
18218839	m	cn	CN	2020	1	0	0	0	0	0	0	0	0	0	0	0	47
18218898	m	md	JP	2020	0	1	0	0	1	0	0	0	0	0	0	0	72

18218924	m	cn	CN	2020	0	1	0	0	1	0	0	0	0	0	0	0	
18218945	f	md	JP	2020	1	0	0	0	0	0	0	0	0	0	0	0	80.9
18219144	f	hp	US	2020	0	0	0	0	0	0	0	0	0	0	0	0	68
18219213	f	cn	CN	2020	0	1	0	0	1	0	0	0	0	0	0	0	
18219479	m	cn	CN	2020	0	1	0	0	1	0	0	0	0	0	0	0	
18219483	m	cn	CN	2020	0	1	0	0	1	0	0	0	0	0	0	0	
18219490	m	cn	CN	2020	0	1	0	0	1	0	0	0	0	0	0	0	
18219702	m	cn	CN	2020	0	1	0	0	1	0	0	0	0	0	0	0	64
18219894	m	md	ES	2020	0	1	0	0	0	1	0	0	0	0	0	0	57
18219993	Not	Not repo	AU	2020	0	1	0	1	0	0	0	0	0	0	0	0	
18220308	f	md	NL	2020	0	0	0	0	0	0	0	0	0	0	0	0	75
18221652	Not	md	CR	2020	0	0	0	0	0	0	0	0	0	0	0	0	
18221788	Not	hp	CN	2020	1	0	0	0	0	0	0	0	0	0	0	0	
18222320	m	hp	CA	2020	0	0	0	0	0	0	0	0	0	0	0	0	72
18222411	f	hp	JP	2020	1	0	0	0	0	0	0	0	0	0	0	0	52
18222412	f	md	JP	2020	1	0	0	0	0	0	0	0	0	0	0	0	80
18222854	m	Not repo	CN	2020	1	0	0	0	0	0	0	0	0	0	0	0	64.3
18223362	Not	hp	CN	2020	1	0	0	0	0	0	0	0	0	0	0	0	
18223471	m	Not repo	GB	2020	1	0	0	0	0	0	0	0	0	0	0	0	64
18223620	m	md	FR	2020	1	0	0	0	0	0	0	0	0	0	0	0	71
18223682	Not	hp	TW	2020	0	0	0	0	0	0	0	0	0	0	0	0	
18223791	m	hp	DE	2020	0	1	1	0	0	0	0	0	0	0	0	0	68
18224554	m	cn	CN	2020	0	1	0	0	1	0	0	0	0	0	0	0	
18224775	m	cn	CN	2020	0	1	0	0	1	0	0	0	0	0	0	0	
18224933	f	md	NL	2020	0	0	0	0	0	0	0	0	0	0	0	0	76
18224940	Not	md	RU	2020	0	1	0	1	0	0	0	0	0	0	0	0	
18224999	m	md	AR	2020	0	1	0	0	0	1	0	0	0	0	0	0	
18225018	m	cn	US	2020	1	0	0	0	0	0	0	0	0	0	0	0	70
18225059	m	hp	CN	2020	0	1	0	0	1	0	0	0	0	0	0	0	62
18225447	m	hp	LB	2020	0	1	0	0	1	0	0	0	0	0	0	0	
18225656	m	ph	ID	2020	0	1	1	0	0	0	0	0	0	0	0	0	69
18226587	m	hp	FR	2020	0	1	0	0	1	0	0	0	0	0	0	0	73
18227431	f	md	JP	2020	0	1	0	0	0	1	0	0	0	0	0	0	60
18227580	f	md	JP	2020	1	0	0	0	0	0	0	0	0	0	0	0	76

18227690	m	md	JP	2020	0	0	0	0	0	0	0	0	0	0	0	0	70
18227723	m	hp	IE	2020	0	1	0	0	1	0	0	0	0	0	0	1	72
18227768	Not	hp	TW	2020	0	0	0	0	0	0	0	0	0	0	0	0	
18227783	m	cn	CN	2020	0	1	0	0	1	0	0	0	0	0	0	0	55
18227795	f	Not repo	CN	2020	1	0	0	0	0	0	0	0	0	0	0	0	75
18227885	Not	hp	TW	2020	0	0	0	0	0	0	0	0	0	0	0	0	
18227936	Not	hp	TW	2020	0	0	0	0	0	0	0	0	0	0	0	0	
18227973	f	hp	NL	2020	0	1	1	0	0	0	0	0	0	0	0	0	30
18227979	m	md	JP	2020	0	1	0	1	0	0	0	0	0	0	0	0	80
18228077	f	md	JP	2020	0	1	0	1	0	0	0	0	0	0	0	1	65
18228157	Not	Not repo	CN	2020	0	1	0	1	0	0	0	0	0	0	0	0	
18228229	m	Not repo	JP	2020	0	1	0	1	0	0	0	0	0	0	0	1	71
18228266	f	cn	CN	2020	0	1	0	0	1	0	0	0	0	0	0	0	
18228484	m	hp	NL	2020	0	1	1	0	0	0	0	0	0	0	0	0	68
18228724	m	cn	CN	2020	0	1	0	0	1	0	0	0	0	0	0	0	
18228978	f	cn	FR	2020	0	1	1	0	0	0	0	0	0	0	0	0	
18229081	f	cn	CN	2020	0	1	0	0	1	0	0	0	0	0	0	1	
18229117	m	md	KR	2020	0	1	0	0	1	0	0	0	0	0	0	0	
18229149	m	md	US	2020	0	0	0	0	0	0	0	0	0	0	0	0	73
18229229	m	cn	CA	2020	0	0	0	0	0	0	0	0	0	0	0	0	80
18229537	m	md	JP	2020	0	1	0	1	0	0	0	0	0	0	0	0	75
18229640	m	md	JP	2020	0	1	0	1	0	0	0	0	0	0	0	0	70
18229788	f	md	SG	2020	0	1	0	0	0	1	0	0	0	0	0	0	
18229836	m	cn	CN	2020	0	1	0	0	1	0	0	0	0	0	0	0	
18229842	m	cn	CN	2020	0	1	0	0	1	0	0	0	0	0	0	0	57
18229843	m	cn	CN	2020	0	1	0	0	1	0	0	0	0	0	0	0	
18230014	m	md	KR	2020	0	1	0	0	1	0	0	0	0	0	0	0	
18230053	m	cn	CN	2020	0	1	0	0	1	0	0	0	0	0	0	0	
18230141	m	md	TR	2020	0	0	0	0	0	0	0	0	0	0	0	0	55
18230268	m	cn	CN	2020	0	1	0	0	1	0	0	0	0	0	0	0	
18230427	m	md	US	2020	0	1	1	0	0	0	0	0	0	0	0	0	
18230482	m	md	FR	2020	0	1	0	1	0	0	0	0	0	0	0	1	64
18230492	f	hp	US	2020	1	0	0	0	0	0	0	0	0	0	0	0	73
18230543	m	md	FR	2020	1	0	0	0	0	0	0	0	0	0	0	0	71

18230708	m	cn	CN	2020	0	1	0	0	1	0	0	0	0	0	0	0	
18230881	m	cn	Not re	2020	0	0	0	0	0	0	0	0	0	0	0	0	
18231226	f	cn	CN	2020	0	1	0	0	1	0	0	0	0	0	0	0	
18231333	f	Not repo	AU	2020	0	1	0	1	0	0	0	0	0	0	0	1	59
18231482	m	cn	US	2020	1	0	0	0	0	0	0	0	0	0	0	0	50
18231497	f	cn	US	2020	1	0	0	0	0	0	0	0	0	0	0	0	62
18231510	m	hp	FR	2020	0	0	0	0	0	0	0	0	0	0	0	0	67
18231528	m	md	JP	2020	0	1	0	1	0	0	0	0	0	0	0	1	66
18231809	Not	md	US	2020	0	1	0	1	0	0	0	0	0	0	0	1	
18232142	m	md	JP	2020	0	1	0	1	0	0	0	0	0	0	0	0	82
18233004	f	cn	CN	2020	0	1	0	0	1	0	0	0	0	0	0	0	
18233007	m	Not repo	CN	2020	0	1	0	1	0	0	0	0	0	0	0	0	60
18233065	f	cn	Not re	2020	0	0	0	0	0	0	0	0	0	0	0	0	
18233201	Not	ph	JP	2020	1	0	0	0	0	0	0	0	0	0	0	0	
18233363	m	md	JP	2020	1	0	0	0	0	0	0	0	0	0	0	0	68
18233386	f	cn	Not re	2020	0	0	0	0	0	0	0	0	0	0	0	0	
18233573	f	cn	CN	2020	0	1	0	0	1	0	0	0	0	0	0	0	
18233723	m	Not repo	JP	2020	0	1	0	1	0	0	0	0	0	0	0	0	72
18233775	f	cn	US	2020	1	0	0	0	0	0	0	0	0	0	0	1	71
18233958	m	cn	CN	2020	0	1	0	0	1	0	0	0	0	0	0	0	74
18233994	m	cn	CN	2020	0	1	0	0	1	0	0	0	0	0	0	0	72
18234006	m	cn	CN	2020	0	1	0	0	1	0	0	0	0	0	0	0	
18234007	m	cn	CN	2020	0	1	0	0	1	0	0	0	0	0	0	0	
18234008	m	cn	CN	2020	0	1	0	0	1	0	0	0	0	0	0	0	
18234009	m	cn	CN	2020	0	1	0	0	1	0	0	0	0	0	0	0	
18234155	m	Not repo	JP	2020	0	1	0	1	0	0	0	0	0	0	0	1	77
18234271	m	cn	CN	2020	0	1	0	0	1	0	0	0	0	0	0	0	
18234278	m	cn	CN	2020	0	1	0	0	1	0	0	0	0	0	0	0	
18234365	f	md	DE	2020	0	1	1	0	0	0	0	0	0	0	0	0	30
18234436	m	Not repo	JP	2020	0	1	0	1	0	0	0	0	0	0	0	1	69
18234562	m	md	JP	2020	0	1	0	1	0	0	0	0	0	0	0	1	64
18234584	m	cn	CN	2020	0	1	0	0	1	0	0	0	0	0	0	1	
18234664	f	md	JP	2020	0	1	0	1	0	0	0	0	0	0	0	0	65
18234671	m	Not repo	JP	2020	0	1	0	1	0	0	0	0	0	0	0	1	65

18234673	m	md	JP	2020	0	1	0	1	0	0	0	0	0	0	0	0	75
18234675	m	cn	Not re	2020	0	0	0	0	0	0	0	0	0	0	0	0	
18234923	m	md	JP	2020	0	1	0	1	0	0	0	0	0	0	0	0	52
18234928	m	md	JP	2020	0	1	0	1	0	0	0	0	0	0	0	1	65
18235101	f	md	JP	2020	0	1	0	1	0	0	0	0	0	0	0	0	69
18235319	f	cn	Not re	2020	0	0	0	0	0	0	0	0	0	0	0	0	
18235692	m	hp	CA	2020	0	1	1	0	0	0	0	0	0	0	0	1	77
18236145	m	md	DE	2020	0	1	0	0	0	1	0	0	0	0	0	0	68
18236230	f	md	JP	2020	0	1	0	0	0	1	0	0	0	0	0	0	62
18236235	m	md	KR	2020	0	1	0	0	0	1	0	0	0	0	0	0	86
18236857	f	Not repo	Not re	2020	1	0	0	0	0	0	0	0	0	0	0	0	75
18237193	Not	md	US	2020	0	0	0	0	0	0	0	0	0	0	0	0	
18237218	f	md	US	2020	0	0	0	0	0	0	0	0	0	0	0	0	79
18237430	f	md	GB	2020	0	1	0	0	0	1	0	0	0	0	0	0	75
18237752	m	hp	DE	2020	0	1	1	0	0	0	0	0	0	0	0	0	62
18237942	f	ph	CA	2020	0	0	0	0	0	0	0	0	0	0	0	0	68
18237946	f	hp	IN	2020	0	0	0	0	0	0	0	0	0	0	0	0	57
18238598	m	cn	CN	2020	1	0	0	0	0	0	0	0	0	0	0	0	76
18238624	m	cn	JP	2020	0	1	1	0	0	0	0	0	0	0	0	0	90
18238678	f	cn	CN	2020	0	0	0	0	0	0	0	0	0	0	0	0	61
18239007	m	cn	CN	2020	0	1	0	0	1	0	0	0	0	0	0	0	
18239028	f	md	JP	2020	0	1	1	0	0	0	0	0	0	0	0	0	75
18239031	m	cn	CN	2020	0	1	0	0	1	0	0	0	0	0	0	0	
18239045	m	cn	CN	2020	0	1	0	0	1	0	0	0	0	0	0	0	66
18239050	m	md	CR	2020	1	0	0	0	0	0	0	0	0	0	0	0	61
18239067	m	Not repo	ID	2020	1	0	0	0	0	0	0	0	0	0	0	0	88.2
18239146	m	cn	CN	2020	0	1	0	0	1	0	0	0	0	0	0	0	62
18239389	m	md	JP	2020	0	1	0	0	0	1	0	0	0	0	0	0	73
18239428	m	md	JP	2020	0	1	0	0	0	1	0	0	0	0	0	0	77
18239462	m	hp	US	2020	0	1	0	0	1	0	0	0	0	0	0	0	78
18239690	m	md	PT	2020	0	1	1	0	0	0	0	0	0	0	0	0	66
18239732	m	cn	CN	2020	0	1	0	0	1	0	0	0	0	0	0	0	
18239740	m	cn	CN	2020	0	1	0	0	1	0	0	0	0	0	0	0	
18239831	Not	md	ES	2020	0	1	1	0	0	0	0	0	0	0	0	0	

18239860	m	cn	CN	2020	0	1	0	0	1	0	0	0	0	0	0	0	
18240131	m	cn	CN	2020	0	1	0	0	1	0	0	0	0	0	0	0	63
18240311	f	md	JP	2020	0	0	0	0	0	0	0	0	0	0	0	0	70
18240580	f	md	US	2020	0	0	0	0	0	0	0	0	0	0	0	0	
18240581	f	md	US	2020	0	0	0	0	0	0	0	0	0	0	0	0	79
18240608	m	ph	CA	2020	0	0	0	0	0	0	0	0	0	0	0	0	56
18241423	m	cn	CN	2020	0	1	0	0	1	0	0	0	0	0	0	0	
18241426	f	md	US	2020	0	0	0	0	0	0	0	0	0	0	0	0	79
18241474	f	cn	CN	2020	0	1	0	0	1	0	0	0	0	0	0	0	57
18242609	m	cn	CN	2020	0	1	0	0	1	0	0	0	0	0	0	1	46
18242771	f	hp	JP	2020	1	0	0	0	0	0	0	0	0	0	0	0	63
18242854	f	hp	US	2020	0	1	1	0	0	0	0	0	0	0	0	0	76
18242873	f	hp	JP	2020	1	0	0	0	0	0	0	0	0	0	0	0	77
18243020	f	hp	US	2020	0	1	1	0	0	0	0	0	0	0	0	0	58
18243059	m	hp	US	2020	0	1	1	0	0	0	0	0	0	0	0	0	68
18243071	f	ph	CA	2020	0	0	0	0	0	0	0	0	0	0	0	0	68
18243162	m	hp	JP	2020	0	1	0	0	1	0	0	0	0	0	0	0	57
18243226	m	hp	US	2020	0	1	0	1	0	0	0	0	0	0	0	0	61
18243234	f	hp	US	2020	0	1	1	0	0	0	0	0	0	0	0	0	71
18243267	m	hp	US	2020	0	1	1	0	0	0	0	0	0	0	0	0	78
18243335	m	cn	CN	2020	0	1	0	0	1	0	0	0	0	0	0	0	65
18243429	m	cn	CN	2020	0	1	0	0	1	0	0	0	0	0	0	0	
18243592	f	hp	US	2020	0	1	1	0	0	0	0	0	0	0	0	0	64
18243667	f	hp	TR	2020	0	1	1	0	0	0	0	0	0	0	0	0	58
18243805	m	cn	CN	2020	0	1	0	0	1	0	0	0	0	0	0	0	
18243929	m	hp	GB	2020	0	1	0	0	0	1	0	0	0	0	0	0	76
18244006	Not	md	US	2020	0	0	0	0	0	0	0	0	0	0	0	0	
18244017	f	cn	Not re	2020	0	0	0	0	0	0	0	0	0	0	0	0	
18244513	f	cn	IN	2020	0	0	0	0	0	0	0	0	0	0	0	0	61
18244859	m	md	US	2020	0	1	0	0	0	1	0	0	0	0	0	0	53
18245120	f	cn	CA	2020	0	1	1	0	0	0	0	0	0	0	0	0	72
18245492	f	md	RU	2020	0	1	1	0	0	0	0	0	0	0	0	0	74
18245895	f	hp	US	2020	0	1	0	1	0	0	0	0	0	0	0	0	63
18246271	f	cn	US	2020	1	0	0	0	0	0	0	0	0	0	0	0	59

18247395	m	cn	CN	2020	1	0	0	0	0	0	0	0	0	0	0	0	
18247494	f	md	JP	2020	1	0	0	0	0	0	0	0	0	0	0	0	76
18247496	m	md	DE	2020	1	0	0	0	0	0	0	0	0	0	0	0	
18247815	m	md	FR	2020	0	1	1	0	0	0	0	0	0	0	0	0	71
18248513	m	cn	CN	2020	0	1	0	0	1	0	0	0	0	0	0	0	
18248698	m	cn	US	2020	0	1	0	1	0	0	0	0	0	0	0	1	63.8
18249104	m	md	CA	2020	0	1	0	0	0	1	0	0	0	0	0	0	64
18249123	Not	md	JP	2020	0	1	0	0	0	1	0	0	0	0	0	0	72
18249190	m	md	IT	2020	0	0	0	0	0	0	0	0	0	0	0	0	42
18249615	m	ph	JP	2020	0	1	0	0	1	0	0	0	0	0	0	0	67
18249922	f	cn	Not re	2020	0	0	0	0	0	0	0	0	0	0	0	0	69
18250169	Not	md	US	2020	0	0	0	0	0	0	0	0	0	0	0	0	
18250418	f	hp	CN	2020	0	0	0	0	0	0	0	0	0	0	0	0	65
18250912	m	hp	IT	2020	0	1	1	0	0	0	0	0	0	0	0	0	72
18250920	f	cn	CN	2020	0	1	0	0	1	0	0	0	0	0	0	0	
18251166	Not	md	JP	2020	0	1	0	0	0	1	0	0	0	0	0	0	80
18251247	f	md	RU	2020	1	0	0	0	0	0	0	0	0	0	0	0	67
18251602	m	cn	US	2020	0	0	0	0	0	0	0	0	0	0	0	0	
18251917	f	cn	US	2020	0	0	0	0	0	0	0	0	0	0	0	0	
18252724	Not	md	JP	2020	0	1	0	1	0	0	0	0	0	0	0	0	
18253271	f	hp	NL	2020	0	0	0	0	0	0	0	0	0	0	0	0	76
18253948	m	ph	US	2020	0	1	0	1	0	0	0	0	0	0	0	0	74.2
18254008	m	cn	CN	2020	0	1	0	0	1	0	0	0	0	0	0	0	
18254052	f	cn	CN	2020	0	1	0	0	1	0	0	0	0	0	0	0	
18254084	m	cn	CN	2020	0	1	0	0	1	0	0	0	0	0	0	0	66
18254092	m	cn	CN	2020	0	1	0	0	1	0	0	0	0	0	0	0	
18254306	f	md	JP	2020	0	1	0	0	0	1	0	0	0	0	0	0	50
18254779	f	hp	CN	2020	1	0	0	0	0	0	0	0	0	0	0	0	
18254785	m	cn	Not re	2020	0	0	0	0	0	0	0	0	0	0	0	0	
18255075	f	md	US	2020	0	1	1	0	0	0	0	0	0	0	0	0	55
18255163	f	hp	CN	2020	1	0	0	0	0	0	0	0	0	0	0	0	
18255221	Not	hp	JP	2020	1	0	0	0	0	0	0	0	0	0	0	0	
18255415	m	cn	CN	2020	0	1	0	0	1	0	0	0	0	0	0	1	76
18255757	f	cn	CN	2020	0	1	0	0	1	0	0	0	0	0	0	0	64

18255988	m	md	DK	2020	0	0	0	0	0	0	0	0	0	0	0	0	57
18256065	m	md	JP	2020	0	0	0	0	0	0	0	0	0	0	0	0	
18256117	m	cn	CA	2020	1	0	0	0	0	0	0	0	0	0	0	0	
18256214	Not	md	MY	2020	1	0	0	0	0	0	0	0	0	0	0	0	
18256533	m	cn	Not re	2020	0	0	0	0	0	0	0	0	0	0	0	0	
18256543	Not	hp	JP	2020	1	0	0	0	0	0	0	0	0	0	0	0	
18256544	m	hp	CN	2020	1	0	0	0	0	0	0	0	0	0	0	0	
18256545	f	hp	CN	2020	1	0	0	0	0	0	0	0	0	0	0	0	
18256546	Not	hp	JP	2020	1	0	0	0	0	0	0	0	0	0	0	0	
18256627	f	md	CA	2020	1	0	0	0	0	0	0	0	0	0	0	0	
18256802	f	cn	CN	2020	0	1	0	0	1	0	0	0	0	0	0	0	
18257375	Not	md	US	2020	1	0	0	0	0	0	0	0	0	0	0	0	
18257550	f	cn	CA	2020	1	0	0	0	0	0	0	0	0	0	0	0	72
18257714	f	md	BE	2020	0	0	0	0	0	0	0	0	0	0	0	0	76
18257840	f	cn	US	2020	1	0	0	0	0	0	0	0	0	0	0	0	
18258212	m	cn	Not re	2020	0	0	0	0	0	0	0	0	0	0	0	0	
18258602	f	md	NL	2020	0	0	0	0	0	0	0	0	0	0	0	0	75
18258744	m	cn	JP	2020	0	1	1	0	0	0	0	0	0	0	0	1	80
18258749	f	cn	CN	2020	1	0	0	0	0	0	0	0	0	0	0	0	64
18258842	f	cn	IN	2020	0	0	0	0	0	0	0	0	0	0	0	0	
18258990	f	ph	US	2020	0	1	0	0	1	0	0	0	0	0	0	0	73
18259707	Not	md	MY	2020	1	0	0	0	0	0	0	0	0	0	0	0	
18259767	f	md	DE	2020	0	0	0	0	0	0	0	0	0	0	0	0	
18259809	f	cn	Not re	2020	0	0	0	0	0	0	0	0	0	0	0	0	70
18260019	f	md	NL	2020	0	0	0	0	0	0	0	0	0	0	0	0	75
18260299	m	md	JP	2020	0	0	0	0	0	0	0	0	0	0	0	0	
18260300	m	md	FR	2020	0	0	0	0	0	0	0	0	0	0	0	0	78
18260344	m	md	JP	2020	0	1	0	0	0	1	0	0	0	0	0	0	61
18260345	f	md	BE	2020	0	0	0	0	0	0	0	0	0	0	0	0	75
18260394	f	md	FR	2020	0	1	0	0	0	1	0	0	0	0	0	0	61
18260575	f	md	NL	2020	0	0	0	0	0	0	0	0	0	0	0	0	76
18260893	Not	md	Not re	2020	1	0	0	0	0	0	0	0	0	0	0	0	
18260936	m	hp	FR	2020	0	0	0	0	0	0	0	0	0	0	0	0	67
18261195	m	hp	NL	2020	0	1	0	0	1	0	0	0	0	0	0	0	

18261610	f	md	JP	2020	1	0	0	0	0	0	0	0	0	0	0	0	
18263240	m	md	JP	2020	0	0	0	0	0	0	0	0	0	0	0	0	66
18263865	m	cn	JP	2020	0	1	1	0	0	0	0	0	0	0	0	0	
18264128	f	md	US	2020	0	0	0	0	0	0	0	0	0	0	0	0	
18264323	f	Not repo	AU	2020	0	1	0	1	0	0	0	0	0	0	0	0	53
18264679	m	cn	US	2020	1	0	0	0	0	0	0	0	0	0	0	0	
18264888	f	md	FR	2020	0	1	0	0	0	1	0	0	0	0	0	0	50
18266119	m	hp	FR	2020	0	0	0	0	0	0	0	0	0	0	0	0	67
18266412	m	Not repo	CN	2020	1	0	0	0	0	0	0	0	0	0	0	0	73
18266561	f	hp	NL	2020	0	0	0	0	0	0	0	0	0	0	0	0	74
18266636	f	md	JP	2020	0	1	0	0	0	1	0	0	0	0	0	0	60
18268297	m	hp	TH	2020	0	0	0	0	0	0	0	0	0	0	0	0	
18268480	Not	hp	JP	2020	1	0	0	0	0	0	0	0	0	0	0	0	
18269059	m	cn	CN	2020	1	0	0	0	0	0	0	0	0	0	0	0	57
18269841	m	md	IL	2020	0	1	0	0	1	0	0	0	0	0	0	0	66
18270520	m	hp	CA	2020	0	1	0	0	1	0	0	0	0	0	0	0	72
18271122	m	cn	JP	2020	0	1	1	0	0	0	0	0	0	0	0	0	84
18271465	m	md	JP	2020	0	1	0	0	0	1	0	0	0	0	0	0	69
18271466	m	md	JP	2020	0	1	0	0	0	1	0	0	0	0	0	0	69
18271499	f	md	JP	2020	0	1	0	0	0	1	0	0	0	0	0	0	74
18271536	f	md	JP	2020	0	1	0	0	0	1	0	0	0	0	0	0	61
18272497	f	md	JP	2020	0	1	0	0	0	1	0	0	0	0	0	0	68
18272579	m	cn	US	2020	0	1	1	0	0	0	0	0	0	0	0	0	
18273989	m	md	JP	2020	0	1	0	1	0	0	0	0	0	0	0	1	70
18274396	m	md	JP	2020	1	0	0	0	0	0	0	0	0	0	0	0	60
18274490	Not	hp	US	2020	0	0	0	0	0	0	0	0	0	0	0	0	
18274519	m	hp	CN	2020	0	1	0	0	1	0	0	0	0	0	0	0	63
18274926	f	hp	AU	2020	0	0	0	0	0	0	0	0	0	0	0	0	62
18275055	f	cn	CN	2020	1	0	0	0	0	0	0	0	0	0	0	0	71
18275433	m	Not repo	CN	2020	1	0	0	0	0	0	0	0	0	0	0	0	63
18275754	m	cn	JP	2020	0	1	1	0	0	0	0	0	0	0	0	0	77
18275902	m	md	JP	2020	0	1	0	0	0	1	0	0	0	0	0	0	80
18275904	f	md	HK	2020	0	0	0	0	0	0	0	0	0	0	0	0	
18275910	f	md	JP	2020	0	0	0	0	0	0	0	0	0	0	0	0	80

18275914	m	md	JP	2020	0	0	0	0	0	0	0	0	0	0	0	0	70
18275938	m	md	JP	2020	0	1	0	0	0	1	0	0	0	0	0	0	62
18276081	f	cn	CN	2020	1	0	0	0	0	0	0	0	0	0	0	0	71
18277283	m	cn	AR	2020	0	0	0	0	0	0	0	0	0	0	0	0	75
18277379	f	hp	IN	2020	0	0	0	0	0	0	0	0	0	0	0	0	91
18277405	Not	Not repo	JP	2020	0	1	0	1	0	0	0	0	0	0	0	0	
18277578	f	cn	CN	2020	0	0	0	0	0	0	0	0	0	0	0	0	47
18277603	f	hp	US	2020	0	0	0	0	0	0	0	0	0	0	0	0	
18277651	f	Not repo	CN	2020	1	0	0	0	0	0	0	0	0	0	0	0	74
18277906	m	hp	US	2020	0	0	0	0	0	0	0	0	0	0	0	0	
18278321	Not	hp	CN	2020	1	0	0	0	0	0	0	0	0	0	0	0	
18278376	m	Not repo	CN	2020	1	0	0	0	0	0	0	0	0	0	0	0	
18278576	f	Not repo	RU	2020	1	0	0	0	0	0	0	0	0	0	0	0	60.7
18278778	f	md	AT	2020	0	0	0	0	0	0	0	0	0	0	0	0	
18278969	Not	cn	US	2020	1	0	0	0	0	0	0	0	0	0	0	0	
18279045	m	Not repo	JP	2020	0	1	0	1	0	0	0	0	0	0	0	0	75
18279258	m	md	NO	2020	0	0	0	0	0	0	0	0	0	0	0	0	32
18279282	f	cn	Not re	2020	0	0	0	0	0	0	0	0	0	0	0	0	39
18279339	f	Not repo	TW	2020	1	0	0	0	0	0	0	0	0	0	0	0	78
18279472	m	hp	CN	2020	0	1	0	0	1	0	0	0	0	0	0	0	62
18279506	m	Not repo	JP	2020	0	1	0	1	0	0	0	0	0	0	0	1	85
18279657	m	md	IL	2020	0	1	0	0	1	0	0	0	0	0	0	0	
18279879	Not	md	JP	2020	0	0	0	0	0	0	0	0	0	0	0	0	
18280023	m	cn	Not re	2020	0	0	0	0	0	0	0	0	0	0	0	0	
18280089	m	cn	Not re	2020	0	0	0	0	0	0	0	0	0	0	0	0	
18280213	m	md	JP	2020	0	1	0	0	0	1	0	0	0	0	0	0	68
18280328	f	cn	CN	2020	1	0	0	0	0	0	0	0	0	0	0	0	64
18281021	m	md	KR	2020	0	1	0	0	0	1	0	0	0	0	0	0	78
18282298	f	hp	CA	2020	0	0	0	0	0	0	0	0	0	0	0	0	63
18282354	m	Not repo	CN	2020	1	0	0	0	0	0	0	0	0	0	0	0	62
18282486	f	Not repo	US	2020	1	0	0	0	0	0	0	0	0	0	0	0	
18282503	f	Not repo	CN	2020	1	0	0	0	0	0	0	0	0	0	0	0	45
18282504	f	cn	CN	2020	1	0	0	0	0	0	0	0	0	0	0	0	63
18282591	f	Not repo	CN	2020	1	0	0	0	0	0	0	0	0	0	0	0	65

18282595	m	md	US	2020	0	0	0	0	0	0	0	0	0	0	0	0	92
18282703	f	hp	US	2020	0	1	1	0	0	0	0	0	0	0	0	0	
18282722	m	Not repo	CN	2020	1	0	0	0	0	0	0	0	0	0	0	0	56
18282965	m	md	DE	2020	0	0	0	0	0	0	0	0	0	0	0	0	
18283055	f	Not repo	CN	2020	1	0	0	0	0	0	0	0	0	0	0	0	78
18283070	f	hp	US	2020	0	1	0	0	1	0	0	0	0	0	0	0	
18283155	m	Not repo	CA	2020	1	0	0	0	0	0	0	0	0	0	0	0	56
18283285	f	cn	IL	2020	0	0	0	0	0	0	0	0	0	0	0	0	
18283462	f	md	CH	2020	0	0	0	0	0	0	0	0	0	0	0	0	
18283883	Not	cn	US	2020	0	0	0	0	0	0	0	0	0	0	0	0	
18284185	f	Not repo	CN	2020	1	0	0	0	0	0	0	0	0	0	0	0	72
18284420	m	hp	RU	2020	0	1	0	1	0	0	0	0	0	0	0	0	60
18284549	f	Not repo	CN	2020	1	0	0	0	0	0	0	0	0	0	0	0	55
18284837	m	cn	IL	2020	0	0	0	0	0	0	0	0	0	0	0	0	58
18284873	m	cn	CN	2020	1	0	0	0	0	0	0	0	0	0	0	0	63
18284937	f	Not repo	ID	2020	1	0	0	0	0	0	0	0	0	0	0	0	65.1
18285276	f	cn	CN	2020	1	0	0	0	0	0	0	0	0	0	0	0	62
18285353	m	Not repo	IN	2020	0	1	0	1	0	0	0	0	0	0	0	0	65.7
18285456	f	Not repo	MY	2020	1	0	0	0	0	0	0	0	0	0	0	0	56.8
18285559	m	hp	IL	2020	0	0	0	0	0	0	0	0	0	0	0	0	82
18286373	m	hp	DE	2020	0	1	1	0	0	0	0	0	0	0	0	0	62
18286537	f	cn	LB	2020	0	1	0	0	1	0	0	0	0	0	0	0	69
18286577	m	md	JP	2020	0	1	0	0	0	1	0	0	0	0	0	0	70
18286581	f	md	JP	2020	0	1	0	0	0	1	0	0	0	0	0	0	64
18287183	f	md	US	2020	0	1	0	0	0	1	0	0	0	0	0	0	47
18287746	m	cn	Not re	2020	0	0	0	0	0	0	0	0	0	0	0	0	
18288282	f	md	NL	2020	0	0	0	0	0	0	0	0	0	0	0	0	65
18288474	m	hp	US	2020	0	1	1	0	0	0	0	0	0	0	0	1	73
18288603	m	cn	FR	2020	0	1	1	0	0	0	0	0	0	0	0	0	65
18288684	m	md	FR	2020	0	1	0	0	1	0	0	0	0	0	0	0	65
18288875	m	md	FR	2020	0	1	0	1	0	0	0	0	0	0	0	0	
18289325	f	Not repo	ID	2020	1	0	0	0	0	0	0	0	0	0	0	0	
18289329	m	cn	AE	2020	1	0	0	0	0	0	0	0	0	0	0	0	47.7
18289335	f	cn	US	2020	1	0	0	0	0	0	0	0	0	0	0	0	70

18289420	f	Not repo	IL	2020	1	0	0	0	0	0	0	0	0	0	0	0	79
18289925	m	hp	CA	2020	0	1	1	0	0	0	0	0	0	0	0	0	68
18289926	m	md	FR	2020	0	1	0	0	0	1	0	0	0	0	0	0	81
18290830	Not	hp	CN	2020	0	0	0	0	0	0	0	0	0	0	0	0	
18290832	Not	hp	FR	2020	0	0	0	0	0	0	0	0	0	0	0	0	
18290839	Not	hp	CN	2020	0	0	0	0	0	0	0	0	0	0	0	0	
18291220	f	hp	DE	2020	0	1	0	0	0	1	0	0	0	0	0	0	62
18291311	m	md	BR	2020	0	1	0	1	0	0	0	0	0	0	0	0	
18291539	f	cn	US	2020	1	0	0	0	0	0	0	0	0	0	0	0	
18291629	f	hp	GB	2020	0	0	0	0	0	0	1	1	0	0	0	0	72
18291741	f	md	DE	2020	1	0	0	0	0	0	0	0	0	0	0	0	45
18292153	f	md	DK	2020	0	1	1	0	0	0	0	0	0	0	0	1	80
18292351	f	md	JP	2020	0	1	0	0	0	1	0	0	0	0	0	0	
18292899	f	hp	JP	2020	1	0	0	0	0	0	0	0	0	0	0	0	
18293306	m	ph	US	2020	0	0	0	0	0	0	0	0	0	0	0	0	61
18293421	Not	hp	JP	2020	0	0	0	0	0	0	0	0	0	0	0	0	
18294567	f	md	ES	2020	0	1	1	0	0	0	0	0	0	0	0	0	53
18294628	m	cn	Not re	2020	0	0	0	0	0	0	0	0	0	0	0	0	
18294638	m	cn	CA	2020	0	0	0	0	0	0	0	0	0	0	0	0	59
18295386	m	cn	Not re	2020	0	0	0	0	0	0	0	0	0	0	0	0	72
18295543	f	cn	US	2020	0	0	0	0	0	0	0	0	0	0	0	0	
18295615	m	md	JP	2020	0	1	0	1	0	0	0	0	0	0	0	0	79
18295617	m	md	JP	2020	0	0	0	0	0	0	1	0	0	1	0	1	66
18295952	m	md	CN	2020	0	0	0	0	0	0	0	0	0	0	0	0	55
18296011	f	md	RO	2020	0	1	1	0	0	0	0	0	0	0	0	0	66
18296022	f	cn	US	2020	1	0	0	0	0	0	0	0	0	0	0	0	
18296031	m	cn	US	2020	0	0	0	0	0	0	0	0	0	0	0	0	
18296620	f	md	HU	2020	0	0	0	0	0	0	0	0	0	0	0	0	
18296622	Not	md	BR	2020	0	0	0	0	0	0	0	0	0	0	0	0	35
18297404	f	md	BE	2020	0	0	0	0	0	0	0	0	0	0	0	0	76
18298232	f	cn	US	2020	1	0	0	0	0	0	0	0	0	0	0	0	59
18299226	m	cn	CA	2020	0	0	0	0	0	0	0	0	0	0	0	0	65
18299527	m	md	AU	2020	0	0	0	0	0	0	0	0	0	0	0	0	54
18300201	m	hp	CA	2020	0	0	0	0	0	0	0	0	0	0	0	0	70

18300827	m	md	VN	2020	0	1	0	0	0	1	0	0	0	0	0	0	60
18300909	f	md	FR	2020	0	1	0	0	0	1	0	0	0	0	0	0	57
18300913	Not	md	JP	2020	1	0	0	0	0	0	0	0	0	0	0	0	70
18301128	f	Not repo	MY	2020	1	0	0	0	0	0	0	0	0	0	0	0	81.7
18301890	m	cn	CN	2020	0	1	0	0	1	0	0	0	0	0	0	0	60
18302352	f	cn	CN	2020	1	0	0	0	0	0	0	0	0	0	0	0	79
18302497	f	md	DE	2020	0	0	0	0	0	0	0	0	0	0	0	0	55
18302726	m	cn	CN	2020	0	1	0	0	1	0	0	0	0	0	0	0	
18302846	f	md	JP	2020	1	0	0	0	0	0	0	0	0	0	0	0	87
18303151	m	cn	CN	2020	1	0	0	0	0	0	0	0	0	0	0	0	65
18303263	Not	md	US	2020	1	0	0	0	0	0	0	0	0	0	0	0	
18303379	m	Not repo	JP	2020	1	0	0	0	0	0	0	0	0	0	0	1	86
18303926	m	Not repo	CN	2020	1	0	0	0	0	0	0	0	0	0	0	0	52
18304220	f	cn	US	2020	0	1	0	0	1	0	0	0	0	0	0	0	
18304392	m	Not repo	CN	2020	1	0	0	0	0	0	0	0	0	0	0	0	71
18304488	m	cn	CN	2020	0	1	0	0	1	0	0	0	0	0	0	0	
18304658	m	cn	CN	2020	0	1	0	0	1	0	0	0	0	0	0	0	
18304729	f	hp	CA	2020	0	1	1	0	0	0	0	0	0	0	0	0	62
18304814	f	hp	GB	2020	0	1	0	0	1	0	0	0	0	0	0	0	56
18305129	f	cn	Not re	2020	0	0	0	0	0	0	0	0	0	0	0	0	
18305449	m	hp	IN	2020	0	0	0	0	0	0	0	0	0	0	0	0	
18305830	m	ph	US	2020	0	1	0	1	0	0	0	0	0	0	0	0	76
18305887	m	cn	US	2020	0	1	1	0	0	0	0	0	0	0	0	0	
18306061	m	md	JP	2020	0	1	0	0	0	1	0	0	0	0	0	0	60
18306110	f	Not repo	JP	2020	1	0	0	0	0	0	0	0	0	0	0	0	60
18306320	m	md	EG	2020	0	1	0	0	1	0	0	0	0	0	0	0	
18306703	f	cn	AU	2020	0	0	0	0	0	0	0	0	0	0	0	0	58
18306887	f	cn	Not re	2020	0	0	0	0	0	0	0	0	0	0	0	0	
18307070	m	md	JP	2020	1	0	0	0	0	0	0	0	0	0	0	0	83
18308050	Not	cn	Not re	2020	0	0	0	0	0	0	0	0	0	0	0	0	
18308210	Not	ph	US	2020	0	0	0	0	0	0	0	0	0	0	0	0	
18308817	m	md	DE	2020	1	0	0	0	0	0	0	0	0	0	0	0	78
18308822	f	Not repo	MY	2020	1	0	0	0	0	0	0	0	0	0	0	0	81.8
18308830	m	cn	CN	2020	1	0	0	0	0	0	0	0	0	0	0	0	48

18308833	f	md	US	2020	0	1	0	1	0	0	0	0	0	0	0	1	
18308849	f	Not repo	ID	2020	0	1	0	1	0	0	0	0	0	0	0	0	73.1
18309557	f	cn	Not re	2020	0	0	0	0	0	0	0	0	0	0	0	0	
18309758	f	md	CA	2020	0	0	0	0	0	0	0	0	0	0	0	0	31
18310910	m	md	EG	2020	0	1	0	0	1	0	0	0	0	0	0	0	
18311582	Not	hp	US	2020	1	0	0	0	0	0	0	0	0	0	0	0	
18312349	f	md	CA	2020	0	0	0	0	0	0	0	0	0	0	0	0	36
18312364	f	cn	Not re	2020	0	0	0	0	0	0	0	0	0	0	0	0	45
18312577	m	cn	Not re	2020	0	0	0	0	0	0	0	0	0	0	0	0	67
18312797	f	md	HR	2020	1	0	0	0	0	0	0	0	0	0	0	0	
18312885	f	Not repo	MY	2020	1	0	0	0	0	0	0	0	0	0	0	0	41.3
18313104	f	hp	GB	2020	0	1	0	0	0	1	0	0	0	0	0	0	55
18313182	Not	Not repo	JP	2020	1	0	0	0	0	0	0	0	0	0	0	0	
18313363	f	cn	US	2020	1	0	0	0	0	0	0	0	0	0	0	0	71.5
18313372	f	Not repo	ID	2020	1	0	0	0	0	0	0	0	0	0	0	0	
18313377	f	Not repo	JP	2020	1	0	0	0	0	0	0	0	0	0	0	0	76
18313388	m	cn	JP	2020	0	1	1	0	0	0	0	0	0	0	0	0	72
18313495	m	ph	CA	2020	0	0	0	0	0	0	0	0	0	0	0	0	59
18313864	f	cn	US	2020	0	0	0	0	0	0	0	0	0	0	0	0	57
18314047	f	cn	CN	2020	1	0	0	0	0	0	0	0	0	0	0	0	62
18314178	m	md	IN	2020	0	0	0	0	0	0	0	0	0	0	0	0	78
18314242	f	cn	IN	2020	0	1	0	0	1	0	0	0	0	0	0	0	
18314685	m	md	US	2020	0	0	0	0	0	0	0	0	0	0	0	0	56
18314959	m	md	JP	2020	0	1	0	1	0	0	0	0	0	0	0	0	76
18315627	f	cn	Not re	2020	0	0	0	0	0	0	0	0	0	0	0	0	41
18316152	f	md	TW	2020	1	0	0	0	0	0	0	0	0	0	0	0	59
18316170	f	Not repo	ID	2020	1	0	0	0	0	0	0	0	0	0	0	0	73.6
18316224	f	Not repo	MY	2020	1	0	0	0	0	0	0	0	0	0	0	0	74.2
18316240	f	cn	CN	2020	1	0	0	0	0	0	0	0	0	0	0	0	79
18316502	m	md	US	2020	0	0	0	0	0	0	0	0	0	0	0	0	
18318736	f	hp	US	2020	1	0	0	0	0	0	0	0	0	0	0	0	58
18319460	Not	hp	ES	2020	0	0	0	0	0	0	0	0	0	0	0	0	
18319497	f	cn	Not re	2020	0	0	0	0	0	0	0	0	0	0	0	0	68
18319874	m	cn	US	2020	1	0	0	0	0	0	0	0	0	0	0	0	

18320446	f	ph	PT	2020	0	0	0	0	0	0	0	0	0	0	0	0	63
18320810	f	hp	US	2020	0	0	0	0	0	0	0	0	0	0	0	0	77
18321474	f	hp	FR	2020	0	0	0	0	0	0	0	0	0	0	0	0	57
18321738	m	cn	IN	2020	0	0	0	0	0	0	0	0	0	0	0	0	34
18322494	f	Not repo	TW	2020	1	0	0	0	0	0	0	0	0	0	0	0	65
18322551	f	cn	NL	2020	0	1	1	0	0	0	0	0	0	0	0	0	74
18322624	m	hp	JP	2020	1	0	0	0	0	0	0	0	0	0	0	0	68
18322985	f	hp	US	2020	0	1	0	0	1	0	0	0	0	0	0	0	73
18322996	m	cn	LB	2020	0	1	0	0	1	0	0	0	0	0	0	0	75
18323652	f	md	JP	2020	0	0	0	0	0	0	0	0	0	0	0	0	77
18324741	f	md	FR	2020	0	0	0	0	0	0	0	0	0	0	0	0	53
18325078	f	md	JP	2020	0	1	0	0	1	0	0	0	0	0	0	0	80
18325084	m	hp	US	2020	0	1	0	0	1	0	0	0	0	0	0	0	66
18325186	m	md	ES	2020	0	0	0	0	0	0	0	0	0	0	0	0	
18325202	m	md	SE	2020	0	0	0	0	0	0	0	0	0	0	0	0	72
18325483	f	hp	IN	2020	0	0	0	0	0	0	0	0	0	0	0	0	57
18325671	m	cn	Not re	2020	0	0	0	0	0	0	0	0	0	0	0	0	
18326069	m	hp	CN	2020	0	0	0	0	0	0	0	0	0	0	0	0	62
18326193	f	hp	IN	2020	0	0	0	0	0	0	0	0	0	0	0	0	53
18326221	m	Not repo	TW	2020	0	1	0	1	0	0	0	0	0	0	0	0	64
18326570	f	Not repo	TW	2020	0	1	0	1	0	0	0	0	0	0	0	0	64
18326875	m	md	JP	2020	0	1	0	1	0	0	0	0	0	0	0	0	70
18327028	m	Not repo	MY	2020	1	0	0	0	0	0	0	0	0	0	0	0	66.5
18328041	f	Not repo	Not re	2020	0	0	0	0	0	0	1	1	0	0	0	0	72
18328739	f	md	VN	2020	1	0	0	0	0	0	0	0	0	0	0	0	
18330317	f	Not repo	CN	2020	0	1	0	1	0	0	0	0	0	0	0	0	35
18330318	m	md	VN	2020	1	0	0	0	0	0	0	0	0	0	0	0	55
18330359	m	md	JP	2020	0	1	0	0	1	0	0	0	0	0	0	0	70
18330954	m	cn	CN	2020	0	1	0	1	0	0	0	0	0	0	0	0	68
18331158	m	md	FR	2020	0	1	0	1	0	0	0	0	0	0	0	1	67
18331517	Not	md	CN	2020	0	1	0	1	0	0	0	0	0	0	0	0	
18331560	f	md	NL	2020	0	0	0	0	0	0	0	0	0	0	0	0	65
18332526	m	md	FR	2020	0	1	1	0	0	0	0	0	0	0	0	1	83
18333024	Not	md	FR	2020	0	1	0	0	0	1	0	0	0	0	0	0	

18339171	f	ph	US	2020	0	0	0	0	0	0	0	0	0	0	0	0	62
7085278	m	md	US	2020	0	0	0	0	0	0	0	0	0	0	0	0	63
7380338	f	md	US	2020	0	0	0	0	0	0	0	0	0	0	0	0	61.8
8291661	m	hp	US	2020	0	0	0	0	0	0	0	0	0	0	0	0	57
8434798	f	cn	GB	2020	1	0	0	0	0	0	0	0	0	0	0	0	73
8592429	m	md	GB	2020	0	0	0	0	0	0	0	0	0	0	0	0	67.4
8742371	m	hp	US	2020	1	0	0	0	0	0	0	0	0	0	0	0	57
8934852	m	md	DE	2020	1	0	0	0	0	0	0	0	0	0	0	0	74
8942437	f	md	US	2020	1	0	0	0	0	0	0	0	0	0	0	0	61
8942495	m	md	US	2020	1	0	0	0	0	0	0	0	0	0	0	0	64
8987441	m	md	US	2020	1	0	0	0	0	0	0	0	0	0	0	0	65
8997451	f	md	FR	2020	1	0	0	0	0	0	0	0	0	0	0	0	79
9098834	f	md	US	2020	1	0	0	0	0	0	0	0	0	0	0	0	62
9105923	m	md	FR	2020	1	0	0	0	0	0	0	0	0	0	0	0	62
9124508	f	md	US	2020	1	0	0	0	0	0	0	0	0	0	0	0	74
9224747	m	md	GB	2020	1	0	0	0	0	0	0	0	0	0	0	0	63
9242216	m	md	US	2020	1	0	0	0	0	0	0	0	0	0	0	1	59
9245525	m	hp	US	2020	1	0	0	0	0	0	0	0	0	0	0	0	68
9275008	f	md	BE	2020	1	0	0	0	0	0	0	0	0	0	0	0	61
9331780	m	md	IT	2020	1	0	0	0	0	0	0	0	0	0	0	0	43
9436499	m	md	US	2020	0	0	0	0	0	0	0	0	0	0	0	0	67
9472620	m	cn	GB	2020	1	0	0	0	0	0	0	0	0	0	0	0	73
9503026	f	cn	US	2020	1	0	0	0	0	0	0	0	0	0	0	0	
9507532	f	md	PL	2020	1	0	0	0	0	0	0	0	0	0	0	0	63
9518722	m	cn	GB	2020	1	0	0	0	0	0	0	0	0	0	0	0	63
9625792	m	md	AU	2020	1	0	0	0	0	0	0	0	0	0	0	0	69
9671181	Not	hp	JP	2020	1	0	0	0	0	0	0	0	0	0	0	0	
9671193	Not	hp	JP	2020	1	0	0	0	0	0	0	0	0	0	0	0	
9681529	m	md	US	2020	0	0	0	0	0	0	0	0	0	0	0	0	56
9695309	m	md	JP	2020	1	0	0	0	0	0	0	0	0	0	0	0	63
9831957	f	hp	US	2020	0	0	0	0	0	0	0	0	0	0	0	0	56
9913421	f	cn	GB	2020	1	0	0	0	0	0	0	0	0	0	0	0	78
9943169	m	md	IT	2020	0	0	0	0	0	0	0	0	0	0	0	0	61
9943342	f	md	US	2020	0	0	0	0	0	0	0	0	0	0	0	0	55

15413999	f	hp	ES	2020	0	0	0	0	0	0	0	0	0	0	0	0	61
17277619	f	md	TW	2020	0	0	0	0	0	0	0	0	0	0	0	0	80
17550262	m	md	AR	2020	0	1	1	0	0	0	0	0	0	0	0	0	73
17657008	f	md	US	2020	0	0	0	0	0	0	0	0	0	0	0	0	39
17734300	m	hp	CN	2020	0	0	0	0	0	0	0	0	0	0	0	0	45
17741940	m	md	AU	2020	0	1	1	0	0	0	0	0	0	0	0	0	70
17819634	m	cn	IL	2020	0	0	0	0	0	0	0	0	0	0	0	0	85
17830180	f	md	ES	2020	0	1	1	0	0	0	0	0	0	0	0	0	58
17860505	m	md	TR	2020	0	1	1	0	0	0	0	0	0	0	0	0	42
17907905	f	md	KR	2020	1	0	0	0	0	0	0	0	0	0	0	0	62.7
17918056	m	cn	JP	2020	0	1	1	0	0	0	0	0	0	0	0	1	76
17930090	f	md	US	2020	0	0	0	0	0	0	0	0	0	0	0	0	65
17967016	m	hp	FR	2020	0	1	0	0	1	0	0	0	0	0	0	0	71
17978558	m	md	JP	2020	0	1	1	0	0	0	0	0	0	0	0	0	81
17978815	f	cn	US	2020	0	0	0	0	0	0	0	0	0	0	0	0	63
17981538	m	md	JP	2020	0	1	0	0	0	1	0	0	0	0	0	0	71
17989355	m	Not repo	JP	2020	0	1	0	1	0	0	0	0	0	0	0	0	58
17992084	f	cn	CN	2020	0	1	0	0	1	0	0	0	0	0	0	0	72
17999437	f	md	FR	2020	0	0	0	0	0	0	0	0	0	0	0	0	66
18010687	m	hp	IT	2020	0	0	0	0	0	0	0	0	0	0	0	0	73
18011044	m	cn	Not re	2020	0	0	0	0	0	0	0	0	0	0	0	0	
18011188	f	hp	MY	2020	0	1	1	0	0	0	0	0	0	0	0	0	55
18014213	m	md	JP	2020	0	1	0	0	1	0	0	0	0	0	0	0	46
18020694	m	md	JP	2020	0	1	0	0	1	0	0	0	0	0	0	0	52
18021217	f	md	DE	2020	0	0	0	0	0	0	0	0	0	0	0	0	70
18022906	m	hp	DE	2020	0	1	0	0	0	1	0	0	0	0	0	0	59
18023348	m	cn	CN	2020	0	1	0	0	1	0	0	0	0	0	0	1	66
18032368	f	md	TR	2020	0	0	0	0	0	0	0	0	0	0	0	0	72
18032398	f	md	TR	2020	0	0	0	0	0	0	0	0	0	0	0	0	54
18037720	m	Not repo	JP	2020	0	1	0	1	0	0	0	0	0	0	0	1	72
18037838	m	md	ES	2020	0	1	1	0	0	0	0	0	0	0	0	0	71
18040675	m	md	JP	2020	0	1	1	0	0	0	0	0	0	0	0	0	64
18043607	f	md	SE	2020	0	1	0	0	0	1	0	0	0	0	0	0	65
18044253	f	Not repo	JP	2020	1	0	0	0	0	0	0	0	0	0	0	0	85

18045287	m	Not repo	JP	2020	0	1	0	1	0	0	0	0	0	0	0	1	45
18046404	m	cn	JP	2020	0	1	1	0	0	0	0	0	0	0	0	0	69
18049604	m	hp	US	2020	0	1	0	0	1	0	0	0	0	0	0	1	84
18050388	m	hp	JP	2020	1	0	0	0	0	0	0	0	0	0	0	0	76
18052918	f	Not repo	Not re	2020	1	0	0	0	0	0	0	0	0	0	0	0	63
18057343	m	md	JP	2020	0	1	0	0	0	1	0	0	0	0	0	0	65
18057618	f	md	JP	2020	0	1	0	0	0	1	0	0	0	0	0	0	69
18062403	m	cn	CN	2020	0	1	0	0	1	0	0	0	0	0	0	0	59
18065133	m	md	CN	2020	0	0	0	0	0	0	0	0	0	0	0	0	70
18065947	m	md	CN	2020	0	1	1	0	0	0	0	0	0	0	0	0	56
18066133	f	cn	AU	2020	0	1	0	0	1	0	0	0	0	0	0	0	73
18069312	f	cn	Not re	2020	0	0	0	0	0	0	0	0	0	0	0	0	
18082386	m	hp	ES	2020	0	1	1	0	0	0	0	0	0	0	0	0	66
18083119	m	md	DE	2020	0	1	0	0	0	1	0	0	0	0	0	0	55
18084369	m	md	IT	2020	0	1	0	0	1	0	0	0	0	0	0	0	
18089233	m	md	TR	2020	0	1	1	0	0	0	0	0	0	0	0	0	64
18092885	m	md	ES	2020	0	1	1	0	0	0	0	0	0	0	0	0	70
18096936	m	cn	CN	2020	0	1	0	0	1	0	0	0	0	0	0	0	
18100770	m	hp	JP	2020	0	1	0	0	1	0	0	0	0	0	0	0	65
18101460	f	cn	Not re	2020	0	0	0	0	0	0	0	0	0	0	0	0	
18105070	f	cn	US	2020	0	1	0	0	1	0	0	0	0	0	0	0	
18105691	m	md	DE	2020	0	1	1	0	0	0	0	0	0	0	0	0	
18106954	m	hp	DE	2020	0	1	0	0	1	0	0	0	0	0	0	0	70
18111624	m	ph	JP	2020	0	1	0	0	0	1	0	0	0	0	0	0	50
18112302	m	md	JP	2020	0	1	0	0	0	1	0	0	0	0	0	1	72
18114176	f	hp	JP	2020	0	1	0	0	1	0	0	0	0	0	0	0	66
18115899	f	hp	JP	2020	1	0	0	0	0	0	0	0	0	0	0	0	82
18120287	f	md	JP	2020	0	1	0	1	0	0	0	0	0	0	0	1	69
18120910	m	md	JP	2020	1	0	0	0	0	0	0	0	0	0	0	0	91
18121299	m	md	US	2020	1	0	0	0	0	0	0	0	0	0	0	0	81
18122879	m	md	DE	2020	0	0	0	0	0	0	0	0	0	0	0	0	
18124908	f	md	IN	2020	0	1	0	1	0	0	0	0	0	0	0	0	69
18125253	m	md	IL	2020	0	1	1	0	0	0	0	0	0	0	0	0	66
18125478	f	md	ES	2020	0	1	1	0	0	0	0	0	0	0	0	0	72

18125773	m	md	KR	2020	0	1	1	0	0	0	0	0	0	0	0	0	60
18127235	m	md	FR	2020	0	1	0	0	0	1	0	0	0	0	0	0	73
18127236	m	md	ES	2020	0	1	0	0	0	1	0	0	0	0	0	0	56
18127641	m	ph	JP	2020	0	1	0	0	0	1	0	0	0	0	0	0	76
18128540	m	md	CH	2020	0	1	1	0	0	0	0	0	0	0	0	0	69
18131105	m	cn	FR	2020	0	1	1	0	0	0	0	0	0	0	0	0	78
18131482	m	hp	CN	2020	0	1	0	0	1	0	0	0	0	0	0	0	63
18133874	m	md	JP	2020	0	0	0	0	0	0	0	0	0	0	0	0	59
18140366	m	ph	JP	2020	1	0	0	0	0	0	0	0	0	0	0	0	72
18142142	f	md	ES	2020	0	1	1	0	0	0	0	0	0	0	0	0	58
18146513	m	md	US	2020	0	1	1	0	0	0	0	0	0	0	0	0	65
18148069	m	hp	JP	2020	0	1	0	0	1	0	0	0	0	0	0	0	
18148568	m	md	JP	2020	0	1	0	0	1	0	0	0	0	0	0	0	73
18149448	m	cn	IL	2020	0	0	0	0	0	0	0	0	0	0	0	0	37
18151586	f	md	US	2020	0	1	0	0	0	1	0	0	0	0	0	0	59
18157665	f	md	IL	2020	0	0	0	0	0	0	0	0	0	0	0	0	64
18158544	m	hp	DE	2020	0	0	0	0	0	0	0	0	0	0	0	0	
18161292	f	md	FR	2020	0	1	0	0	0	1	0	0	0	0	0	0	71
18164697	f	cn	US	2020	0	0	0	0	0	0	0	0	0	0	0	0	
18170525	m	md	CN	2020	0	1	1	0	0	0	0	0	0	0	0	1	46
18181045	Not	md	PL	2020	0	0	0	0	0	0	0	0	0	0	0	0	
18182687	f	hp	DE	2020	0	1	0	0	0	1	0	0	0	0	0	0	63
18188247	m	Not repo	JP	2020	0	0	0	0	0	0	0	0	0	0	0	0	64
18188868	m	md	DE	2020	0	1	0	0	0	1	0	0	0	0	0	0	68
18196120	m	cn	JP	2020	0	1	1	0	0	0	0	0	0	0	0	0	80
18198222	f	md	DE	2020	0	1	0	0	0	1	0	0	0	0	0	0	66
18205929	m	md	CN	2020	0	1	0	0	0	1	0	0	0	0	0	0	69
18207389	f	md	JP	2020	1	0	0	0	0	0	0	0	0	0	0	0	59
18207820	m	md	DE	2020	0	1	1	0	0	0	0	0	0	0	0	0	77
18210418	f	md	DE	2020	0	1	0	0	0	1	0	0	0	0	0	0	62
18215838	m	md	HK	2020	0	1	0	0	0	1	0	0	0	0	0	0	52
18215960	f	Not repo	NZ	2020	0	1	0	1	0	0	0	0	0	0	0	1	65.3
18216366	f	hp	JP	2020	0	1	0	1	0	0	0	0	0	0	0	0	71
18218563	m	cn	US	2020	0	0	0	0	0	0	0	0	0	0	0	0	

18219875	m	md	IT	2020	0	1	1	0	0	0	0	0	0	0	0	0	63
18223372	m	hp	FR	2020	0	1	0	0	1	0	0	0	0	0	0	0	61
18226245	m	hp	FR	2020	0	1	0	0	1	0	0	0	0	0	0	0	73
18226614	m	md	JP	2020	0	0	0	0	0	0	0	0	0	0	0	0	57
18230699	f	cn	CN	2020	0	1	0	0	1	0	0	0	0	0	0	0	
18231579	f	md	KR	2020	0	1	0	0	0	1	0	0	0	0	0	0	71
18233148	m	cn	JP	2020	0	1	1	0	0	0	0	0	0	0	0	0	72
18233355	f	md	CA	2020	0	0	0	0	0	0	0	0	0	0	0	0	
18236081	m	md	DE	2020	0	0	0	0	0	0	0	0	0	0	0	0	78
18236535	f	cn	Not re	2020	0	0	0	0	0	0	0	0	0	0	0	0	
18238492	f	md	FR	2020	0	0	0	0	0	0	0	0	0	0	0	0	70
18238535	f	hp	US	2020	0	0	0	0	0	0	0	0	0	0	0	0	65
18240215	m	Not repo	Not re	2020	1	0	0	0	0	0	0	0	0	0	0	0	77
18243879	m	cn	Not re	2020	0	0	0	0	0	0	0	0	0	0	0	0	
18247384	f	Not repo	Not re	2020	1	0	0	0	0	0	0	0	0	0	0	1	65
18247633	f	cn	IN	2020	0	0	0	0	0	0	0	0	0	0	0	0	48
18249796	m	Not repo	US	2020	1	0	0	0	0	0	0	0	0	0	0	0	75.9
18250895	m	md	DE	2020	0	0	0	0	0	0	0	0	0	0	0	0	62
18251568	m	hp	BG	2020	0	1	0	0	0	1	0	0	0	0	0	0	68
18252738	f	Not repo	JP	2020	1	0	0	0	0	0	0	0	0	0	0	0	75
18257206	m	md	IL	2020	0	1	1	0	0	0	0	0	0	0	0	0	76
18257272	Not	md	JP	2020	1	0	0	0	0	0	0	0	0	0	0	0	90
18258802	m	hp	US	2020	0	1	0	0	1	0	0	0	0	0	0	0	
18258862	f	md	US	2020	0	0	0	0	0	0	0	0	0	0	0	0	67
18261395	f	md	US	2020	0	1	1	0	0	0	0	0	0	0	0	0	
18263942	m	hp	DE	2020	0	1	0	0	1	0	0	0	0	0	0	0	60
18264079	m	Not repo	Not re	2020	1	0	0	0	0	0	0	0	0	0	0	1	67
18264326	f	Not repo	CN	2020	1	0	0	0	0	0	0	0	0	0	0	0	
18265517	m	md	HR	2020	0	1	1	0	0	0	0	0	0	0	0	0	57
18265579	m	hp	FR	2020	0	1	0	0	1	0	0	0	0	0	0	0	68
18266692	m	hp	DE	2020	0	0	0	0	0	0	0	0	0	0	0	0	78
18267285	m	md	FR	2020	0	1	0	0	0	1	0	0	0	0	0	0	85
18268294	m	md	JP	2020	0	1	1	0	0	0	0	0	0	0	0	0	69
18268835	m	md	US	2020	0	1	1	0	0	0	0	0	0	0	0	0	71

18271606	f	md	CN	2020	0	0	0	0	0	0	0	0	0	0	0	0	35
18272500	f	hp	KZ	2020	0	0	0	0	0	0	0	0	0	0	0	0	
18273491	f	md	NL	2020	0	0	0	0	0	0	0	0	0	0	0	0	76
18277774	f	ph	JP	2020	0	1	0	1	0	0	0	0	0	0	0	1	68
18277808	m	hp	KR	2020	0	0	0	0	0	0	0	0	0	0	0	0	65
18278929	m	hp	DE	2020	0	0	0	0	0	0	0	0	0	0	0	0	62
18282325	m	md	RO	2020	0	1	1	0	0	0	0	0	0	0	0	0	75
18283626	m	hp	CN	2020	0	1	0	0	1	0	0	0	0	0	0	0	72
18283730	m	cn	CN	2020	0	1	0	0	1	0	0	0	0	0	0	0	58
18283974	m	md	EE	2020	0	1	1	0	0	0	0	0	0	0	0	0	55
18284488	f	cn	US	2020	1	0	0	0	0	0	0	0	0	0	0	0	52
18286501	f	cn	JP	2020	0	1	1	0	0	0	0	0	0	0	0	0	67
18286558	f	hp	FI	2020	0	0	0	0	0	0	0	0	0	0	0	0	67
18286585	m	md	JP	2020	0	1	0	0	0	1	0	0	0	0	0	0	60
18287881	m	md	JP	2020	1	0	0	0	0	0	0	0	0	0	0	0	83.5
18288827	f	hp	DE	2020	0	1	0	0	0	1	0	0	0	0	0	0	62
18289931	f	cn	US	2020	0	0	0	0	0	0	0	0	0	0	0	0	
18292275	m	ph	FR	2020	0	0	0	0	0	0	0	0	0	0	0	0	
18295625	f	md	JP	2020	0	0	0	0	0	0	0	0	0	0	0	0	70
18296043	f	cn	Not re	2020	0	0	0	0	0	0	0	0	0	0	0	0	
18296086	m	md	JP	2020	0	1	0	0	0	1	0	0	0	0	0	0	80
18296821	m	md	DE	2020	0	1	1	0	0	0	0	0	0	0	0	0	65
18299137	f	hp	ES	2020	0	0	0	0	0	0	0	0	0	0	0	0	55
18300230	f	md	FR	2020	0	0	0	0	0	0	0	0	0	0	0	0	82
18300795	f	md	JP	2020	0	0	0	0	0	0	0	0	0	0	0	0	80
18300910	f	md	NL	2020	0	1	0	0	0	1	0	0	0	0	0	0	63
18301029	f	md	JP	2020	0	1	0	0	0	1	0	0	0	0	0	0	80
18301750	m	md	CH	2020	0	1	1	0	0	0	0	0	0	0	0	0	69
18302531	m	cn	Not re	2020	0	0	0	0	0	0	0	0	0	0	0	0	
18302654	f	md	GB	2020	0	0	0	0	0	0	0	0	0	0	0	0	71
18302910	m	md	CN	2020	0	1	0	0	0	1	0	0	0	0	0	0	57
18303154	f	md	JP	2020	0	0	0	0	0	0	0	0	0	0	0	0	56
18303476	m	md	GR	2020	0	1	1	0	0	0	0	0	0	0	0	0	79
18305270	m	hp	CN	2020	0	1	0	0	1	0	0	0	0	0	0	0	45

18305925	f	md	JP	2020	0	1	0	0	0	1	0	0	0	0	0	0	74
18308014	f	hp	US	2020	0	1	1	0	0	0	0	0	0	0	0	0	67
18308460	m	cn	Not re	2020	0	0	0	0	0	0	0	0	0	0	0	0	63
18308498	f	md	GB	2020	0	1	0	0	0	1	0	0	0	0	0	0	75
18309539	m	hp	US	2020	0	1	1	0	0	0	0	0	0	0	0	0	77
18309870	f	md	JP	2020	0	0	0	0	0	0	0	0	0	0	0	0	62
18310945	m	md	ES	2020	0	0	0	0	0	0	0	0	0	0	0	0	65
18311477	m	md	CN	2020	0	0	0	0	0	0	0	0	0	0	0	0	61
18313949	m	md	KR	2020	0	1	1	0	0	0	0	0	0	0	0	0	79
18315622	f	md	IN	2020	0	0	0	0	0	0	0	0	0	0	0	0	59
18316156	Not	md	JP	2020	1	0	0	0	0	0	0	0	0	0	0	0	
18316273	f	md	PL	2020	1	0	0	0	0	0	0	0	0	0	0	0	
18316274	f	md	US	2020	1	0	0	0	0	0	0	0	0	0	0	0	87
18316491	m	md	VN	2020	0	1	0	0	0	1	0	0	0	0	0	0	67
18316516	Not	md	JP	2020	0	1	0	0	0	1	0	0	0	0	0	1	58
18318150	f	md	US	2020	0	0	0	0	0	0	0	0	0	0	0	0	
18318753	m	md	VN	2020	0	1	0	0	0	1	0	0	0	0	0	0	58
18318824	f	hp	ES	2020	0	0	0	0	0	0	0	0	0	0	0	0	64
18319430	m	md	MX	2020	0	1	1	0	0	0	0	0	0	0	0	0	62
18320387	m	hp	DE	2020	0	1	0	0	1	0	0	0	0	0	0	0	80
18320456	f	md	TR	2020	0	0	0	0	0	0	0	0	0	0	0	0	64
18321800	f	cn	US	2020	0	0	0	0	0	0	0	0	0	0	0	0	
18321832	m	md	DE	2020	0	1	0	0	0	1	0	0	0	0	0	0	79
18322009	m	md	IT	2020	0	1	1	0	0	0	0	0	0	0	0	0	78
18323639	m	md	JP	2020	0	1	0	0	0	1	0	0	0	0	0	0	80
18323859	m	md	ES	2020	0	0	0	0	0	0	0	0	0	0	0	0	
18323871	f	Not repo	CN	2020	1	0	0	0	0	0	0	0	0	0	0	0	73
18324035	f	hp	CA	2020	1	0	0	0	0	0	0	0	0	0	0	0	72
18325209	m	md	ES	2020	0	0	0	0	0	0	0	0	0	0	0	0	
18325212	m	hp	ES	2020	0	0	0	0	0	0	0	0	0	0	0	0	
18326128	f	hp	NL	2020	0	0	0	0	0	0	1	0	0	0	1	0	65
18326898	m	md	NL	2020	0	1	0	0	0	1	0	0	0	0	0	0	49
18328118	m	cn	IT	2020	0	1	1	0	0	0	0	0	0	0	0	0	82
18328167	m	ph	US	2020	0	1	1	0	0	0	0	0	0	0	0	0	

18328479	m	md	SI	2020	0	0	0	0	0	0	0	0	0	0	0	0	37
18328536	m	cn	ID	2020	1	0	0	0	0	0	0	0	0	0	0	0	70
18330919	f	hp	ES	2020	0	0	0	0	0	0	0	0	0	0	0	0	64
18331940	m	cn	Not re	2020	0	0	0	0	0	0	0	0	0	0	0	0	
18332124	m	md	KR	2020	0	1	0	0	0	1	0	0	0	0	0	0	63
18332464	f	md	FR	2020	0	0	0	0	0	0	0	0	0	0	0	0	53
18332505	f	cn	Not re	2020	0	0	0	0	0	0	0	0	0	0	0	0	
18333022	m	md	FR	2020	0	1	0	0	0	1	0	0	0	0	0	0	74
18333062	f	md	CN	2020	0	1	0	0	0	1	0	0	0	0	0	0	61
18333250	m	cn	CN	2020	0	1	0	0	1	0	0	0	0	0	0	0	
18333263	m	md	IT	2020	1	0	0	0	0	0	0	0	0	0	0	0	76
18333330	f	cn	FR	2020	1	0	0	0	0	0	0	0	0	0	0	0	
18333432	f	md	HR	2020	0	0	0	0	0	0	0	0	0	0	0	0	
18333497	m	hp	RU	2020	0	1	0	1	0	0	0	0	0	0	0	0	70
18333829	m	md	NP	2020	0	0	0	0	0	0	0	0	0	0	0	0	24
18334241	f	hp	US	2020	0	1	0	0	1	0	0	0	0	0	0	0	
18334333	m	Not repo	IN	2020	0	1	0	1	0	0	0	0	0	0	0	0	63.8
18334390	Not	md	US	2020	0	0	0	0	0	0	0	0	0	0	0	0	
18334570	Not	md	US	2020	0	0	0	0	0	0	0	0	0	0	0	0	
18334715	m	md	JP	2020	0	0	0	0	0	0	0	0	0	0	0	0	80
18334985	m	md	IN	2020	0	0	0	0	0	0	0	0	0	0	0	0	74
18334991	f	Not repo	CN	2020	0	1	0	1	0	0	0	0	0	0	0	0	58
18335046	f	md	JP	2020	0	1	0	0	0	1	0	0	0	0	0	0	69
18335627	m	cn	IN	2020	0	1	0	0	1	0	0	0	0	0	0	0	
18335682	f	cn	CA	2020	0	1	1	0	0	0	0	0	0	0	0	0	72
18335762	m	md	TW	2020	0	1	0	0	0	1	0	0	0	0	0	0	
18335851	Not	md	US	2020	0	0	0	0	0	0	0	0	0	0	0	0	
18336035	m	cn	CN	2020	0	1	0	0	1	0	0	0	0	0	0	0	62
18336175	m	hp	NL	2020	0	1	1	0	0	0	0	0	0	0	0	0	82
18336233	Not	hp	KR	2020	0	0	0	0	0	0	0	0	0	0	0	0	
18336845	m	cn	JP	2020	0	1	1	0	0	0	0	0	0	0	0	0	85
18336965	m	Not repo	MY	2020	1	0	0	0	0	0	0	0	0	0	0	0	81.4
18337128	f	md	JP	2020	0	1	0	0	0	1	0	0	0	0	0	0	60
18337130	f	cn	TW	2020	0	0	0	0	0	0	0	0	0	0	0	0	

18337290	m	hp	IT	2020	0	0	0	0	0	0	0	0	0	0	0	0	69
18337430	m	hp	FR	2020	0	0	0	0	0	0	0	0	0	0	0	0	
18337464	f	cn	DE	2020	0	1	1	0	0	0	0	0	0	0	0	0	35
18337505	f	md	ES	2020	0	0	0	0	0	0	0	0	0	0	0	0	64
18338388	m	Not repo	CN	2020	0	1	0	1	0	0	0	0	0	0	0	0	70
18338449	f	md	HU	2020	0	1	1	0	0	0	0	0	0	0	0	0	52
18338515	f	ph	JP	2020	1	0	0	0	0	0	0	0	0	0	0	0	90
18338556	f	cn	US	2020	0	1	0	0	1	0	0	0	0	0	0	0	
18338578	f	Not repo	US	2020	1	0	0	0	0	0	0	0	0	0	0	0	73.3
18338902	m	hp	ES	2020	0	0	0	0	0	0	0	0	0	0	0	0	
18338904	f	ph	JP	2020	1	0	0	0	0	0	0	0	0	0	0	0	73
18338905	Not	md	JP	2020	0	1	0	1	0	0	0	0	0	0	0	0	
18339109	Not	md	NL	2020	0	0	0	0	0	0	1	0	0	0	1	0	51
18339132	m	Not repo	MY	2020	1	0	0	0	0	0	0	0	0	0	0	0	70.6
18339263	Not	md	US	2020	0	0	0	0	0	0	0	0	0	0	0	0	
18339357	f	md	CA	2020	0	0	0	0	0	0	0	0	0	0	0	0	58
18339559	m	hp	LB	2020	0	1	0	0	1	0	0	0	0	0	0	0	60
18340165	f	md	RO	2020	1	0	0	0	0	0	0	0	0	0	0	0	
18340206	f	md	JP	2020	0	1	0	1	0	0	0	0	0	0	0	0	69
18340410	m	cn	US	2020	0	1	0	0	1	0	0	0	0	0	0	0	
18340418	f	cn	CN	2020	1	0	0	0	0	0	0	0	0	0	0	0	83
18340444	Not	hp	CH	2020	0	1	0	1	0	0	0	0	0	0	0	0	
18340494	f	md	DE	2020	0	1	1	0	0	0	0	0	0	0	0	0	
18340609	f	md	JP	2020	0	1	0	0	1	0	0	0	0	0	0	0	78
18340811	m	cn	JP	2020	0	1	1	0	0	0	0	0	0	0	0	0	71
18340846	m	cn	US	2020	0	1	1	0	0	0	0	0	0	0	0	0	
18341119	m	cn	CA	2020	0	1	1	0	0	0	0	0	0	0	0	0	70
18341349	m	hp	IN	2020	0	0	0	0	0	0	0	0	0	0	0	0	
18341797	m	md	CN	2020	0	0	0	0	0	0	0	0	0	0	0	0	50
18341897	m	md	IT	2020	1	0	0	0	0	0	0	0	0	0	0	0	66
18342224	f	md	IT	2020	1	0	0	0	0	0	0	0	0	0	0	0	64
18342243	m	md	IT	2020	1	0	0	0	0	0	0	0	0	0	0	0	78
18342439	f	cn	CN	2020	0	1	0	0	1	0	0	0	0	0	0	1	66
18342458	Not	md	PL	2020	0	0	0	0	0	0	0	0	0	0	0	0	65

18342611	Not	cn	CN	2020	0	1	0	0	1	0	0	0	0	0	0	0	
18342715	m	hp	KR	2020	0	1	1	0	0	0	0	0	0	0	0	0	79
18342937	Not	Not repo	Not re	2020	1	0	0	0	0	0	0	0	0	0	0	0	
18344452	m	cn	Not re	2020	0	0	0	0	0	0	0	0	0	0	0	0	64
18344617	Not	hp	CN	2020	0	0	0	0	0	0	0	0	0	0	0	0	
18344618	Not	hp	CN	2020	0	0	0	0	0	0	0	0	0	0	0	0	
18344620	Not	hp	CN	2020	0	0	0	0	0	0	0	0	0	0	0	0	
18344677	m	md	CH	2020	0	0	0	0	0	0	0	0	0	0	0	0	61
18344935	m	cn	DE	2020	0	1	1	0	0	0	0	0	0	0	0	0	59
18344974	f	cn	US	2020	0	1	1	0	0	0	0	0	0	0	0	0	59
18344985	f	md	JP	2020	1	0	0	0	0	0	0	0	0	0	0	0	86.3
18345492	f	Not repo	JP	2020	1	0	0	0	0	0	0	0	0	0	0	0	79
18345548	f	cn	JP	2020	0	1	1	0	0	0	0	0	0	0	0	0	71
18345673	m	md	TW	2020	1	0	0	0	0	0	0	0	0	0	0	0	95
18345679	m	cn	US	2020	1	0	0	0	0	0	0	0	0	0	0	0	74
18345985	m	cn	JP	2020	0	1	1	0	0	0	0	0	0	0	0	0	56
18346579	f	md	JP	2020	0	0	0	0	0	0	0	0	0	0	0	0	62
18346580	Not	hp	CN	2020	0	0	0	0	0	0	0	0	0	0	0	0	
18346724	m	md	NO	2020	0	0	0	0	0	0	0	0	0	0	0	0	32
18346804	m	cn	JP	2020	0	1	1	0	0	0	0	0	0	0	0	0	56
18346839	m	cn	JP	2020	0	1	1	0	0	0	0	0	0	0	0	0	73
18346901	f	hp	US	2020	0	1	1	0	0	0	0	0	0	0	0	0	57
18347197	f	md	BG	2020	0	1	0	0	0	1	0	0	0	0	0	0	
18347664	m	hp	IT	2020	0	0	0	0	0	0	0	0	0	0	0	0	69
18347809	m	md	JP	2020	0	1	0	0	0	1	0	0	0	0	0	0	76
18348234	f	hp	ES	2020	0	0	0	0	0	0	0	0	0	0	0	0	64
18348446	m	md	DE	2020	0	0	0	0	0	0	0	0	0	0	0	0	
18348831	f	hp	GB	2020	0	1	0	0	0	1	0	0	0	0	0	0	71
18348897	f	cn	Not re	2020	0	0	0	0	0	0	0	0	0	0	0	0	
18349168	m	cn	JP	2020	0	1	1	0	0	0	0	0	0	0	0	1	80
18349519	f	Not repo	MY	2020	1	0	0	0	0	0	0	0	0	0	0	0	81.4
18350043	m	md	ES	2020	0	0	0	0	0	0	0	0	0	0	0	0	71
18350113	f	md	AT	2020	0	1	1	0	0	0	0	0	0	0	0	0	66
18350462	m	hp	BE	2020	0	1	0	0	0	1	0	0	0	0	0	0	73

18350523	m	md	CN	2020	1	0	0	0	0	0	0	0	0	0	0	0	61
18350533	f	hp	US	2020	0	1	0	0	1	0	0	0	0	0	0	0	72
18350668	f	cn	US	2020	1	0	0	0	0	0	0	0	0	0	0	0	66.8
18350931	m	cn	US	2020	1	0	0	0	0	0	0	0	0	0	0	0	53.2
18350932	m	Not repo	MY	2020	1	0	0	0	0	0	0	0	0	0	0	0	55.7
18351042	m	hp	JP	2020	0	1	1	0	0	0	0	0	0	0	0	0	39
18351115	m	cn	IN	2020	0	1	0	0	1	0	0	0	0	0	0	0	68
18351133	m	md	BG	2020	0	1	0	1	0	0	0	0	0	0	0	0	61
18351146	f	cn	Not re	2020	0	0	0	0	0	0	0	0	0	0	0	0	
18351344	m	md	ES	2020	0	0	0	0	0	0	0	0	0	0	0	0	
18351482	f	md	NL	2020	0	0	0	0	0	0	0	0	0	0	0	0	65
18351663	m	cn	JP	2020	0	1	0	1	0	0	0	0	0	0	0	0	85
18351702	f	cn	CN	2020	0	1	0	0	1	0	0	0	0	0	0	0	
18351792	m	cn	Not re	2020	0	0	0	0	0	0	0	0	0	0	0	0	74
18351948	m	cn	JP	2020	0	1	1	0	0	0	0	0	0	0	0	0	76
18352020	f	cn	DE	2020	0	1	1	0	0	0	0	0	0	0	0	0	
18352118	f	md	US	2020	0	0	0	0	0	0	0	0	0	0	0	0	
18352342	f	md	ES	2020	0	0	0	0	0	0	0	0	0	0	0	0	64
18352729	f	md	SK	2020	0	0	0	0	0	0	0	0	0	0	0	0	60
18353210	f	cn	CA	2020	0	1	1	0	0	0	0	0	0	0	0	0	71
18353552	f	ph	JP	2020	0	0	0	0	0	0	0	0	0	0	0	0	
18353698	m	md	US	2020	0	1	1	0	0	0	0	0	0	0	0	0	88
18353767	f	cn	CA	2020	0	0	0	0	0	0	1	1	0	0	0	0	66
18353857	f	md	JP	2020	0	0	0	0	0	0	0	0	0	0	0	0	71
18353858	m	md	JP	2020	0	1	0	0	0	1	0	0	0	0	0	0	80
18354104	f	hp	IT	2020	0	1	1	0	0	0	0	0	0	0	0	0	60
18354384	m	cn	CA	2020	0	1	0	1	0	0	0	0	0	0	0	0	
18354406	m	md	US	2020	0	0	0	0	0	0	0	0	0	0	0	0	59
18354447	m	md	US	2020	0	0	0	0	0	0	0	0	0	0	0	0	71
18354661	f	cn	US	2020	0	0	0	0	0	0	0	0	0	0	0	0	
18355158	m	cn	CN	2020	0	1	0	0	1	0	0	0	0	0	0	1	
18355211	f	md	JP	2020	1	0	0	0	0	0	0	0	0	0	0	0	90
18355525	f	md	US	2020	0	1	1	0	0	0	0	0	0	0	0	0	
18355931	m	md	GR	2020	0	1	1	0	0	0	0	0	0	0	0	1	78

18355932	m	hp	CA	2020	0	0	0	0	0	0	0	0	0	0	0	0	
18355972	f	cn	Not re	2020	0	0	0	0	0	0	0	0	0	0	0	0	41
18355973	m	cn	Not re	2020	0	0	0	0	0	0	0	0	0	0	0	0	
18356043	m	md	DE	2020	0	1	0	1	0	0	0	0	0	0	0	0	66
18356191	f	md	LB	2020	0	1	0	1	0	0	0	0	0	0	0	0	68
18356325	f	cn	US	2020	0	0	0	0	0	0	0	0	0	0	0	0	31
18356533	f	Not repo	JP	2020	1	0	0	0	0	0	0	0	0	0	0	0	82
18357093	m	ph	JP	2020	0	1	0	0	1	0	0	0	0	0	0	0	53
18357578	m	cn	CA	2020	0	1	0	0	0	1	0	0	0	0	0	0	
18357582	f	cn	US	2020	0	1	1	0	0	0	0	0	0	0	0	0	78
18358010	m	hp	AU	2020	0	1	0	0	1	0	0	0	0	0	0	0	79
18358034	f	cn	JP	2020	0	1	1	0	0	0	0	0	0	0	0	0	84
18358169	Not	md	JP	2020	0	1	0	0	0	1	0	0	0	0	0	0	60
18358173	m	md	JP	2020	0	1	0	0	0	1	0	0	0	0	0	0	80
18358181	m	ph	JP	2020	0	1	0	0	0	1	0	0	0	0	0	0	69
18359371	f	hp	CA	2020	0	0	0	0	0	0	0	0	0	0	0	0	63
18359373	f	cn	CA	2020	0	1	1	0	0	0	0	0	0	0	0	0	72
18359977	m	md	AU	2020	0	1	0	0	0	1	0	0	0	0	0	0	74
18359988	m	md	JP	2020	0	0	0	0	0	0	0	0	0	0	0	0	60
18360738	f	md	FR	2020	0	1	0	0	0	1	0	0	0	0	0	0	57
18361298	m	cn	AE	2020	0	1	0	1	0	0	0	0	0	0	0	0	64.1
18361369	m	md	NO	2020	0	0	0	0	0	0	0	0	0	0	0	0	32
18361723	f	cn	CA	2020	0	0	0	0	0	0	0	0	0	0	0	0	66
18361862	m	hp	US	2020	0	1	0	0	1	0	0	0	0	0	0	0	67
18361938	m	cn	CN	2020	0	1	0	0	1	0	0	0	0	0	0	0	76
18361966	m	md	JP	2020	0	1	0	0	0	1	0	0	0	0	0	0	80
18362024	f	md	JP	2020	0	1	0	0	0	1	0	0	0	0	0	0	71
18362165	m	md	KR	2020	0	1	0	0	0	1	0	0	0	0	0	0	67
18362579	m	md	JP	2020	0	1	0	0	1	0	0	0	0	0	0	1	65
18363076	m	md	NL	2020	0	1	0	0	1	0	0	0	0	0	0	0	70
18363132	m	hp	ES	2020	0	0	0	0	0	0	0	0	0	0	0	0	
18363554	m	md	JP	2020	1	0	0	0	0	0	0	0	0	0	0	0	80
18363708	f	md	FR	2020	1	0	0	0	0	0	0	0	0	0	0	0	51
18364032	f	cn	Not re	2020	0	0	0	0	0	0	0	0	0	0	0	0	

18364522	f	Not repo	JP	2020	1	0	0	0	0	0	0	0	0	0	0	0	80
18365343	f	md	HU	2020	0	1	0	0	0	1	0	0	0	0	0	0	36
18365399	f	Not repo	JP	2020	1	0	0	0	0	0	0	0	0	0	0	0	
18365576	m	Not repo	JP	2020	0	1	0	1	0	0	0	0	0	0	0	0	72
18366061	m	cn	US	2020	1	0	0	0	0	0	0	0	0	0	0	0	
18366204	f	ph	JP	2020	1	0	0	0	0	0	0	0	0	0	0	0	72
18366380	m	md	DE	2020	1	0	0	0	0	0	0	0	0	0	0	0	60
18366628	Not	hp	JP	2020	0	1	0	0	0	1	0	0	0	0	0	0	
18366639	Not	hp	JP	2020	0	1	0	0	0	1	0	0	0	0	0	0	
18366685	f	md	AT	2020	0	0	0	0	0	0	0	0	0	0	0	0	30
18366893	f	hp	HR	2020	0	0	0	0	0	0	0	0	0	0	0	0	
18367011	Not	hp	DE	2020	0	1	0	1	0	0	0	0	0	0	0	0	
18367297	m	Not repo	US	2020	1	0	0	0	0	0	0	0	0	0	0	1	79
18367369	f	md	US	2020	1	0	0	0	0	0	0	0	0	0	0	0	70
18367457	Not	hp	DE	2020	0	1	0	1	0	0	0	0	0	0	0	0	
18367464	Not	hp	DE	2020	0	1	0	1	0	0	0	0	0	0	0	0	
18367574	m	cn	US	2020	0	1	1	0	0	0	0	0	0	0	0	1	
18367658	Not	hp	DE	2020	0	1	0	1	0	0	0	0	0	0	0	0	
18368327	m	cn	AE	2020	0	1	0	1	0	0	0	0	0	0	0	0	70.2
18368798	m	cn	US	2020	0	0	0	0	0	0	0	0	0	0	0	0	
18368832	m	md	SI	2020	0	0	0	0	0	0	0	0	0	0	0	0	37
18368897	m	hp	US	2020	1	0	0	0	0	0	0	0	0	0	0	0	74.8
18369440	m	md	NG	2020	0	0	0	0	0	0	0	0	0	0	0	0	
18369449	m	md	JP	2020	0	0	0	0	0	0	0	0	0	0	0	0	
18369467	Not	md	US	2020	0	0	0	0	0	0	0	0	0	0	0	0	
18370723	m	hp	JP	2020	1	0	0	0	0	0	0	0	0	0	0	0	68
18370935	f	hp	US	2020	0	0	0	0	0	0	0	0	0	0	0	0	57
18371348	m	cn	US	2020	0	0	0	0	0	0	0	0	0	0	0	0	
18371395	f	Not repo	FR	2020	1	0	0	0	0	0	0	0	0	0	0	0	51
18371573	Not	cn	TW	2020	0	0	0	0	0	0	0	0	0	0	0	0	
18372186	f	hp	GB	2020	0	1	0	0	0	1	0	0	0	0	0	0	71
18373087	Not	md	AT	2020	1	0	0	0	0	0	0	0	0	0	0	0	78
18373452	f	md	JP	2020	0	0	0	0	0	0	0	0	0	0	0	0	52
18373884	m	md	JP	2020	0	0	0	0	0	0	0	0	0	0	0	0	76

18374095	m	md	JP	2020	0	1	0	1	0	0	0	0	0	0	0	0	64
18374222	Not	ph	JP	2020	0	0	0	0	0	0	0	0	0	0	0	0	70
18374425	f	hp	DE	2020	0	1	0	0	0	1	0	0	0	0	0	0	66
18374755	f	md	JP	2020	0	1	0	0	1	0	0	0	0	0	0	1	71
18375350	f	cn	US	2020	0	0	0	0	0	0	0	0	0	0	0	0	54
18375387	m	cn	JP	2020	0	1	1	0	0	0	0	0	0	0	0	0	88
18375574	m	hp	FR	2020	0	0	0	0	0	0	0	0	0	0	0	0	
18375695	Not	ph	JP	2020	0	0	0	0	0	0	0	0	0	0	0	0	
18376330	m	ph	US	2020	0	1	1	0	0	0	0	0	0	0	0	0	57
18376548	Not	md	US	2020	0	0	0	0	0	0	0	0	0	0	0	0	
18376621	f	Not repo	ID	2020	1	0	0	0	0	0	0	0	0	0	0	0	61.6
18376856	m	cn	JP	2020	0	1	1	0	0	0	0	0	0	0	0	0	52
18377061	m	md	US	2020	0	1	1	0	0	0	0	0	0	0	0	0	
18377635	f	cn	IN	2020	0	0	0	0	0	0	0	0	0	0	0	0	23
18378654	m	md	JP	2020	0	1	0	0	0	1	0	0	0	0	0	0	
18379378	f	hp	US	2020	0	0	0	0	0	0	0	0	0	0	0	0	
18379420	Not	md	NL	2020	0	0	0	0	0	0	1	0	0	0	1	0	51
18379500	Not	hp	JP	2020	0	0	0	0	0	0	0	0	0	0	0	0	
18379648	f	cn	JP	2020	0	1	1	0	0	0	0	0	0	0	0	0	76
18379712	Not	hp	JP	2020	0	1	0	0	0	1	0	0	0	0	0	0	
18380082	m	md	DK	2020	0	1	1	0	0	0	0	0	0	0	0	0	76
18380327	m	cn	JP	2020	0	1	1	0	0	0	0	0	0	0	0	0	80
18380493	Not	md	PL	2020	0	1	0	0	0	1	0	0	0	0	0	0	
18381465	m	Not repo	CN	2020	0	1	0	1	0	0	0	0	0	0	0	0	74
18382017	m	md	JP	2020	1	0	0	0	0	0	0	0	0	0	0	1	90
18382225	f	ph	JP	2020	0	1	1	0	0	0	0	0	0	0	0	0	80
18382291	f	md	US	2020	0	0	0	0	0	0	0	0	0	0	0	0	
18382329	m	hp	US	2020	0	0	0	0	0	0	0	0	0	0	0	0	38
18382425	f	md	VN	2020	1	0	0	0	0	0	0	0	0	0	0	0	68
18382764	f	hp	FR	2020	1	0	0	0	0	0	0	0	0	0	0	0	51
18382779	f	md	FR	2020	0	0	0	0	0	0	0	0	0	0	0	0	53
18383230	m	Not repo	JP	2020	1	0	0	0	0	0	0	0	0	0	0	0	88
18383231	m	Not repo	ID	2020	0	1	0	1	0	0	0	0	0	0	0	0	78.7
18383470	f	cn	CA	2020	1	0	0	0	0	0	0	0	0	0	0	0	

18383472	f	md	FR	2020	1	0	0	0	0	0	0	0	0	0	0	0	60.4
18383568	Not	md	JP	2020	0	0	0	0	0	0	0	0	0	0	0	0	68
18383865	m	hp	US	2020	0	0	0	0	0	0	0	0	0	0	0	0	
18383967	m	md	TR	2020	0	0	0	0	0	0	0	0	0	0	0	0	57
18384060	Not	md	JP	2020	0	1	0	0	1	0	0	0	0	0	0	0	
18384269	m	hp	DE	2020	0	1	1	0	0	0	0	0	0	0	0	0	77
18384600	m	md	US	2020	0	0	0	0	0	0	0	0	0	0	0	0	75
18384906	m	cn	US	2020	0	1	1	0	0	0	0	0	0	0	0	0	
18385057	Not	md	JP	2020	0	0	0	0	0	0	0	0	0	0	0	0	80
18385120	m	md	IL	2020	0	0	0	0	0	0	0	0	0	0	0	0	61
18385650	m	md	FR	2020	0	0	0	0	0	0	0	0	0	0	0	0	65
18385682	f	md	DE	2020	1	0	0	0	0	0	0	0	0	0	0	0	83
18385709	m	md	JP	2020	0	1	0	0	0	1	0	0	0	0	0	0	70
18385808	f	Not repo	BG	2020	1	0	0	0	0	0	0	0	0	0	0	0	
18386136	Not	hp	JP	2020	0	0	0	0	0	0	0	0	0	0	0	0	
18386156	m	ph	FR	2020	0	0	0	0	0	0	0	0	0	0	0	0	79
18386383	m	md	SI	2020	0	0	0	0	0	0	0	0	0	0	0	0	37
18386432	f	ph	US	2020	0	0	0	0	0	0	0	0	0	0	0	0	78
18386799	f	md	JP	2020	0	1	0	0	0	1	0	0	0	0	0	0	69
18386823	f	cn	US	2020	1	0	0	0	0	0	0	0	0	0	0	0	
18387148	f	md	ES	2020	0	0	0	0	0	0	0	0	0	0	0	0	64
18387242	f	hp	DE	2020	0	1	1	0	0	0	0	0	0	0	0	0	
18387249	f	hp	HU	2020	0	1	0	0	0	1	0	0	0	0	0	0	36
18387391	f	ph	US	2020	0	0	0	0	0	0	0	0	0	0	0	0	54
18387536	f	ph	JP	2020	0	0	0	0	0	0	0	0	0	0	0	0	84
18387577	f	cn	Not re	2020	0	0	0	0	0	0	0	0	0	0	0	0	
18387720	m	cn	US	2020	0	0	0	0	0	0	0	0	0	0	0	0	
18388426	m	hp	US	2020	0	1	0	0	1	0	0	0	0	0	0	0	50
18388836	f	cn	US	2020	0	0	0	0	0	0	0	0	0	0	0	0	75
18388878	f	md	DE	2020	0	1	1	0	0	0	0	0	0	0	0	0	
18389050	f	md	US	2020	1	0	0	0	0	0	0	0	0	0	0	0	
18389666	f	md	FR	2020	1	0	0	0	0	0	0	0	0	0	0	0	51
18389965	f	cn	IN	2020	0	0	0	0	0	0	0	0	0	0	0	0	
18390444	f	md	AU	2020	0	0	0	0	0	0	0	0	0	0	0	0	

18390912	m	md	US	2020	0	1	1	0	0	0	0	0	0	0	0	1	73
18391136	f	hp	US	2020	0	1	0	0	1	0	0	0	0	0	0	0	
18391250	m	md	NL	2020	0	1	0	0	0	1	0	0	0	0	0	0	47
18392616	Not	md	JP	2020	1	0	0	0	0	0	0	0	0	0	0	0	
18392757	f	cn	Not re	2020	0	0	0	0	0	0	0	0	0	0	0	0	
18392911	Not	md	US	2020	0	1	1	0	0	0	0	0	0	0	0	0	
18392937	m	ph	JP	2020	0	0	0	0	0	0	0	0	0	0	0	0	64
18393065	f	md	GB	2020	0	1	0	0	0	1	0	0	0	0	0	0	71
18393155	m	md	DE	2020	0	0	0	0	0	0	0	0	0	0	0	0	
18393309	Not	md	JP	2020	1	0	0	0	0	0	0	0	0	0	0	0	
18393462	m	md	JP	2020	0	1	0	1	0	0	0	0	0	0	0	0	69
18393646	m	md	JP	2020	0	1	0	1	0	0	0	0	0	0	0	1	70
18393736	f	md	FR	2020	0	1	0	0	0	1	0	0	0	0	0	0	50
18393835	m	ph	FR	2020	0	0	0	0	0	0	0	0	0	0	0	0	79
18393918	m	Not repo	CN	2020	0	1	0	1	0	0	0	0	0	0	0	0	65
18393976	f	md	FR	2020	0	0	0	0	0	0	0	0	0	0	0	0	53
18393983	f	cn	US	2020	1	0	0	0	0	0	0	0	0	0	0	0	74.5
18393989	f	cn	CN	2020	0	1	0	1	0	0	0	0	0	0	0	0	64
18393998	m	Not repo	MY	2020	1	0	0	0	0	0	0	0	0	0	0	0	55.8
18394021	m	cn	Not re	2020	0	0	0	0	0	0	0	0	0	0	0	0	61
18394249	Not	md	US	2020	0	0	0	0	0	0	0	0	0	0	0	0	
18394368	m	cn	CN	2020	0	1	0	0	1	0	0	0	0	0	0	0	77
18394374	f	md	DE	2020	0	1	0	0	1	0	0	0	0	0	0	0	63
18394383	f	hp	NL	2020	0	1	1	0	0	0	0	0	0	0	0	0	35
18394422	m	hp	SI	2020	0	0	0	0	0	0	0	0	0	0	0	0	37
18394446	f	hp	ES	2020	0	1	1	0	0	0	0	0	0	0	0	0	55
18394612	f	Not repo	CN	2020	0	1	0	1	0	0	0	0	0	0	0	0	50
18394620	m	Not repo	CN	2020	0	1	0	1	0	0	0	0	0	0	0	0	68
18394632	f	Not repo	JP	2020	1	0	0	0	0	0	0	0	0	0	0	0	94
18394689	m	cn	CN	2020	0	1	0	1	0	0	0	0	0	0	0	0	77
18394918	f	hp	BR	2020	0	1	0	0	1	0	0	0	0	0	0	0	60
18395009	f	md	IT	2020	0	1	0	0	0	1	0	0	0	0	0	0	50
18395359	f	cn	Not re	2020	0	0	0	0	0	0	0	0	0	0	0	0	
18395720	f	cn	US	2020	0	1	0	1	0	0	0	0	0	0	0	0	63.7

18396318	f	cn	CN	2020	0	0	0	0	0	0	0	0	0	0	0	0	58
18396324	m	md	CN	2020	0	1	0	0	0	1	0	0	0	0	0	0	41
18396378	f	md	NL	2020	0	1	0	0	0	1	0	0	0	0	0	0	58
18396425	m	cn	JP	2020	0	1	1	0	0	0	0	0	0	0	0	1	75
18397593	m	hp	CN	2020	0	0	0	0	0	0	0	0	0	0	0	0	62
18397719	f	md	FR	2020	0	1	0	0	0	1	0	0	0	0	0	0	50
18397831	Not	md	MY	2020	1	0	0	0	0	0	0	0	0	0	0	0	
18398064	m	ph	US	2020	0	0	0	0	0	0	0	0	0	0	0	0	61
18398501	m	hp	GB	2020	0	1	0	0	0	1	0	0	0	0	0	0	49
18398827	f	Not repo	MY	2020	1	0	0	0	0	0	0	0	0	0	0	0	68.4
18398948	m	cn	JP	2020	0	1	1	0	0	0	0	0	0	0	0	0	80
18399030	m	hp	JP	2020	0	1	0	0	1	0	0	0	0	0	0	0	66
18399135	m	md	CN	2020	0	1	1	0	0	0	0	0	0	0	0	0	61
18399517	m	cn	AE	2020	1	0	0	0	0	0	0	0	0	0	0	0	47.8
18400410	m	md	CN	2020	0	0	0	0	0	0	0	0	0	0	0	0	70
18400425	m	Not repo	CN	2020	0	1	0	1	0	0	0	0	0	0	0	0	
18400506	m	Not repo	MY	2020	1	0	0	0	0	0	0	0	0	0	0	0	67.8
18400769	m	cn	CN	2020	0	1	0	1	0	0	0	0	0	0	0	0	49
18401297	f	md	JP	2020	0	1	0	1	0	0	0	0	0	0	0	0	48
18401681	m	cn	CN	2020	0	1	0	0	1	0	0	0	0	0	0	0	57
18401870	m	cn	US	2020	1	0	0	0	0	0	0	0	0	0	0	1	59.3
18401872	m	Not repo	MY	2020	1	0	0	0	0	0	0	0	0	0	0	0	69
18402049	m	md	MY	2020	1	0	0	0	0	0	0	0	0	0	0	0	
18402582	f	cn	IN	2020	0	0	0	0	0	0	0	0	0	0	0	0	55
18402653	m	cn	JP	2020	0	1	1	0	0	0	0	0	0	0	0	0	90
18402686	m	Not repo	MY	2020	1	0	0	0	0	0	0	0	0	0	0	0	69.5
18402709	Not	md	MY	2020	1	0	0	0	0	0	0	0	0	0	0	0	
18402719	Not	md	JP	2020	0	1	0	1	0	0	0	0	0	0	0	1	
18402733	Not	md	AU	2020	0	0	0	0	0	0	0	0	0	0	0	0	
18403099	f	cn	JP	2020	0	1	1	0	0	0	0	0	0	0	0	0	69
18403235	f	md	JP	2020	0	1	0	0	0	1	0	0	0	0	0	0	72
18403632	f	hp	RO	2020	0	0	0	0	0	0	0	0	0	0	0	0	
18403788	m	md	US	2020	0	0	0	0	0	0	0	0	0	0	0	0	62
18404411	m	md	KR	2020	0	0	0	0	0	0	0	0	0	0	0	0	49

18404577	Not	hp	ES	2020	0	0	0	0	0	0	0	0	0	0	0	0	
18404711	m	cn	US	2020	0	0	0	0	0	0	0	0	0	0	0	0	67
18404898	m	md	JP	2020	0	1	0	1	0	0	0	0	0	0	0	0	71
18405055	m	md	JP	2020	0	1	0	1	0	0	0	0	0	0	0	1	68.9
18405056	Not	Not repo	GB	2020	0	1	0	1	0	0	0	0	0	0	0	1	
18405495	Not	md	JP	2020	0	0	0	0	0	0	0	0	0	0	0	0	
18405845	f	hp	CA	2020	1	0	0	0	0	0	0	0	0	0	0	0	72
18406251	f	md	BE	2020	1	0	0	0	0	0	0	0	0	0	0	0	
18406253	Not	Not repo	JP	2020	1	0	0	0	0	0	0	0	0	0	0	0	
18406364	m	hp	DE	2020	0	1	0	0	0	1	0	0	0	0	0	0	59
18406711	m	md	IL	2020	0	1	1	0	0	0	0	0	0	0	0	0	72
18406877	f	md	SK	2020	0	0	0	0	0	0	0	0	0	0	0	0	58
18407161	f	cn	US	2020	0	0	0	0	0	0	0	0	0	0	0	0	69
18407181	m	md	US	2020	1	0	0	0	0	0	0	0	0	0	0	0	60
18407182	f	Not repo	ID	2020	1	0	0	0	0	0	0	0	0	0	0	0	36.8
18407189	f	Not repo	JP	2020	0	1	0	1	0	0	0	0	0	0	0	1	67
18407194	f	md	JP	2020	1	0	0	0	0	0	0	0	0	0	0	0	90
18407195	f	md	JP	2020	1	0	0	0	0	0	0	0	0	0	0	0	77
18407208	f	Not repo	CN	2020	1	0	0	0	0	0	0	0	0	0	0	0	52
18407211	Not	md	JP	2020	0	0	0	0	0	0	0	0	0	0	0	0	74
18407218	f	md	JP	2020	1	0	0	0	0	0	0	0	0	0	0	0	80
18407275	f	md	JP	2020	0	0	0	0	0	0	0	0	0	0	0	0	52
18407319	m	md	JP	2020	0	1	0	0	0	1	0	0	0	0	0	0	44
18407341	f	cn	US	2020	1	0	0	0	0	0	0	0	0	0	0	0	34.8
18407370	Not	hp	US	2020	0	0	0	0	0	0	0	0	0	0	0	0	
18407651	f	hp	CA	2020	0	0	0	0	0	0	0	0	0	0	0	0	67
18407698	m	md	SK	2020	0	0	0	0	0	0	0	0	0	0	0	0	49
18407819	m	hp	DE	2020	0	1	0	0	0	1	0	0	0	0	0	0	65
18407879	m	md	JP	2020	0	1	0	1	0	0	0	0	0	0	0	0	70
18407950	m	md	US	2020	0	1	0	0	1	0	0	0	0	0	0	0	60
18408034	Not	md	BD	2020	0	0	0	0	0	0	0	0	0	0	0	0	
18408324	Not	hp	KR	2020	0	1	0	0	0	1	0	0	0	0	0	0	
18408995	Not	md	MY	2020	1	0	0	0	0	0	0	0	0	0	0	0	
18409624	Not	md	JP	2020	0	1	0	0	0	1	0	0	0	0	0	0	70

18409649	f	md	JP	2020	0	1	0	0	0	1	0	0	0	0	0	0	77
18409736	f	Not repo	Not re	2020	0	0	0	0	0	0	1	1	0	0	0	0	71
18409760	m	md	JP	2020	0	1	0	0	1	0	0	0	0	0	0	0	59
18409957	Not	hp	CN	2020	0	0	0	0	0	0	0	0	0	0	0	0	
18410162	m	md	CA	2020	0	0	0	0	0	0	0	0	0	0	0	0	61
18410182	f	md	HU	2020	0	0	0	0	0	0	0	0	0	0	0	0	65
18410604	m	hp	NL	2020	0	0	0	0	0	0	0	0	0	0	0	0	79
18411676	f	md	FR	2020	1	0	0	0	0	0	0	0	0	0	0	0	51
18411696	m	cn	CN	2020	0	1	0	0	1	0	0	0	0	0	0	0	76
18411706	m	hp	FR	2020	0	1	0	0	1	0	0	0	0	0	0	0	71
18411768	f	md	HU	2020	0	1	0	0	0	1	0	0	0	0	0	0	36
18412302	f	hp	DE	2020	0	1	0	0	1	0	0	0	0	0	0	0	70
18412785	f	cn	CN	2020	1	0	0	0	0	0	0	0	0	0	0	0	63
18413220	m	Not repo	JP	2020	0	1	0	1	0	0	0	0	0	0	0	0	71
18413248	m	Not repo	ID	2020	1	0	0	0	0	0	0	0	0	0	0	0	90.6
18413254	Not	Not repo	JP	2020	1	0	0	0	0	0	0	0	0	0	0	0	
18413257	Not	md	JP	2020	1	0	0	0	0	0	0	0	0	0	0	0	
18413303	m	md	CR	2020	1	0	0	0	0	0	0	0	0	0	0	0	
18413522	m	hp	CN	2020	0	0	0	0	0	0	0	0	0	0	0	0	52
18413525	m	md	KR	2020	0	1	0	0	0	1	0	0	0	0	0	0	63
18413718	f	Not repo	CN	2020	1	0	0	0	0	0	0	0	0	0	0	0	83
18413921	f	md	FR	2020	1	0	0	0	0	0	0	0	0	0	0	0	
18413922	f	cn	CN	2020	1	0	0	0	0	0	0	0	0	0	0	0	58
18413972	m	cn	JP	2020	0	1	1	0	0	0	0	0	0	0	0	0	78
18414401	m	cn	US	2020	0	1	1	0	0	0	0	0	0	0	0	0	
18414460	m	md	US	2020	0	0	0	0	0	0	0	0	0	0	0	0	62
18415141	m	hp	US	2020	0	0	0	0	0	0	0	0	0	0	0	0	64
18415202	f	md	CN	2020	1	0	0	0	0	0	0	0	0	0	0	0	70.2
18415204	Not	md	JP	2020	0	1	0	1	0	0	0	0	0	0	0	0	
18415209	m	Not repo	CN	2020	1	0	0	0	0	0	0	0	0	0	0	0	74
18415211	f	cn	CN	2020	1	0	0	0	0	0	0	0	0	0	0	0	55
18415236	m	hp	US	2020	0	1	1	0	0	0	0	0	0	0	0	0	71
18415270	m	hp	LU	2020	0	0	0	0	0	0	0	0	0	0	0	0	51
18415325	m	md	US	2020	0	1	1	0	0	0	0	0	0	0	0	0	67

18415338	f	md	JP	2020	0	0	0	0	0	0	0	0	0	0	0	0	85
18415384	f	cn	CN	2020	1	0	0	0	0	0	0	0	0	0	0	0	59
18415515	Not	md	JP	2020	0	1	0	0	0	1	0	0	0	0	0	0	
18415590	f	cn	CN	2020	1	0	0	0	0	0	0	0	0	0	0	0	63
18415591	m	Not repo	KR	2020	0	1	0	1	0	0	0	0	0	0	0	0	62
18415839	f	cn	US	2020	1	0	0	0	0	0	0	0	0	0	0	0	
18416207	m	md	DE	2020	0	1	0	1	0	0	0	0	0	0	0	0	61
18416228	m	hp	US	2020	0	0	0	0	0	0	0	0	0	0	0	0	62
18416274	m	md	FR	2020	0	1	0	1	0	0	0	0	0	0	0	0	
18416276	f	cn	CN	2020	1	0	0	0	0	0	0	0	0	0	0	0	65
18416283	f	Not repo	CN	2020	1	0	0	0	0	0	0	0	0	0	0	0	67
18416285	Not	md	DE	2020	1	0	0	0	0	0	0	0	0	0	0	0	
18416286	f	cn	CN	2020	1	0	0	0	0	0	0	0	0	0	0	0	57
18416349	f	cn	CN	2020	1	0	0	0	0	0	0	0	0	0	0	0	42
18416353	f	Not repo	CN	2020	1	0	0	0	0	0	0	0	0	0	0	0	63
18416358	f	cn	US	2020	1	0	0	0	0	0	0	0	0	0	0	0	87
18416485	f	Not repo	CN	2020	1	0	0	0	0	0	0	0	0	0	0	0	65
18416542	f	ph	US	2020	0	1	0	1	0	0	0	0	0	0	0	0	68.3
18417714	m	hp	TH	2020	0	1	1	0	0	0	0	0	0	0	0	0	70
18417822	m	md	US	2020	0	0	0	0	0	0	0	0	0	0	0	0	62
18417970	m	md	JP	2020	0	1	0	0	1	0	0	0	0	0	0	0	69
18418209	m	md	SG	2020	1	0	0	0	0	0	0	0	0	0	0	0	80
18418395	f	md	CN	2020	1	0	0	0	0	0	0	0	0	0	0	0	56
18418399	f	cn	US	2020	0	0	0	0	0	0	0	0	0	0	0	0	63
18418454	m	md	DE	2020	0	1	0	0	0	1	0	0	0	0	0	0	65
18418595	m	cn	FR	2020	0	1	1	0	0	0	0	0	0	0	0	0	78
18419097	Not	hp	US	2020	0	1	1	0	0	0	0	0	0	0	0	0	74
18419404	m	ph	FR	2020	0	0	0	0	0	0	0	0	0	0	0	0	79
18419474	f	cn	Not re	2020	0	0	0	0	0	0	0	0	0	0	0	0	
18419732	f	md	IL	2020	0	1	0	1	0	0	0	0	0	0	0	0	69
18420007	m	md	IL	2020	0	1	0	0	1	0	0	0	0	0	0	0	86
18420052	m	md	IL	2020	0	1	0	0	1	0	0	0	0	0	0	1	
18420146	m	md	US	2020	0	0	0	0	0	0	0	0	0	0	0	0	62
18420231	f	cn	US	2020	0	0	0	0	0	0	0	0	0	0	0	0	80

18420659	m	md	AU	2020	0	1	1	0	0	0	0	0	0	0	0	0	
18420934	m	cn	CN	2020	1	0	0	0	0	0	0	0	0	0	0	0	53
18421642	f	md	AT	2020	0	1	0	1	0	0	0	0	0	0	0	0	60
18422007	f	Not repo	US	2020	1	0	0	0	0	0	0	0	0	0	0	0	68.7
18422058	f	cn	IN	2020	0	0	0	0	0	0	0	0	0	0	0	0	51
18422408	Not	Not repo	JP	2020	0	1	0	1	0	0	0	0	0	0	0	0	
18422429	m	md	CN	2020	0	1	1	0	0	0	0	0	0	0	0	0	66
18422509	m	md	MY	2020	1	0	0	0	0	0	0	0	0	0	0	0	
18422515	m	Not repo	CA	2020	0	1	0	1	0	0	0	0	0	0	0	1	69.7
18422592	f	hp	US	2020	0	1	1	0	0	0	0	0	0	0	0	0	64
18422598	m	Not repo	CN	2020	1	0	0	0	0	0	0	0	0	0	0	0	50
18422666	f	Not repo	CN	2020	1	0	0	0	0	0	0	0	0	0	0	0	68
18422825	m	cn	JP	2020	0	1	1	0	0	0	0	0	0	0	0	0	48
18423627	m	md	Not re	2020	1	0	0	0	0	0	0	0	0	0	0	0	70
18424300	m	md	DE	2020	0	1	0	0	0	1	0	0	0	0	0	0	71
18424324	f	md	US	2020	0	1	0	0	0	1	0	0	0	0	0	0	58
18424347	f	cn	US	2020	0	1	0	0	0	1	0	0	0	0	0	0	68
18424352	m	md	TR	2020	0	1	0	0	0	1	0	0	0	0	0	0	36
18424803	f	Not repo	Not re	2020	1	0	0	0	0	0	0	0	0	0	0	0	63
18425245	f	cn	US	2020	0	1	1	0	0	0	0	0	0	0	0	0	94
18425373	m	hp	DE	2020	0	1	1	0	0	0	0	0	0	0	0	0	73
18425429	f	md	US	2020	0	1	0	0	0	1	0	0	0	0	0	0	62
18425440	f	md	BE	2020	0	1	1	0	0	0	0	0	0	0	0	0	
18425493	f	md	BR	2020	0	1	0	0	0	1	0	0	0	0	0	0	66
18425659	f	Not repo	Not re	2020	1	0	0	0	0	0	0	0	0	0	0	0	72
18425695	Not	ph	JP	2020	0	0	0	0	0	0	0	0	0	0	0	0	73
18427162	Not	md	JP	2020	0	0	0	0	0	0	0	0	0	0	0	0	
18427278	f	cn	CN	2020	1	0	0	0	0	0	0	0	0	0	0	0	62
18427671	m	ph	FR	2020	0	0	0	0	0	0	0	0	0	0	0	0	79
18427855	m	hp	US	2020	0	1	1	0	0	0	0	0	0	0	0	0	71
18428056	f	hp	US	2020	0	1	0	0	1	0	0	0	0	0	0	0	53
18428458	m	hp	KR	2020	0	1	0	0	1	0	0	0	0	0	0	0	68
18428520	m	hp	NL	2020	0	1	0	0	1	0	0	0	0	0	0	0	68
18428717	f	hp	NL	2020	0	1	0	1	0	0	0	0	0	0	0	1	60

18429042	m	Not repo	CN	2020	0	1	0	1	0	0	0	0	0	0	0	0	64
18429047	f	Not repo	CN	2020	1	0	0	0	0	0	0	0	0	0	0	0	69
18429360	m	hp	TH	2020	0	0	0	0	0	0	0	0	0	0	0	0	
18429441	m	md	AT	2020	0	0	0	0	0	0	0	0	0	0	0	0	74
18429702	f	cn	CN	2020	0	1	0	0	1	0	0	0	0	0	0	0	63
18429727	f	cn	CN	2020	1	0	0	0	0	0	0	0	0	0	0	0	80
18429817	m	hp	PL	2020	0	1	1	0	0	0	0	0	0	0	0	0	65
18430087	m	md	US	2020	0	0	0	0	0	0	0	0	0	0	0	0	62
18430238	f	Not repo	KR	2020	1	0	0	0	0	0	0	0	0	0	0	0	67.7
18430254	f	hp	AU	2020	0	0	0	0	0	0	0	0	0	0	0	0	33
18430325	m	md	JP	2020	0	1	0	0	1	0	0	0	0	0	0	0	72
18430387	Not	md	JP	2020	0	1	0	0	0	1	0	0	0	0	0	0	
18431068	f	Not repo	NZ	2020	1	0	0	0	0	0	0	0	0	0	0	0	
18431161	m	cn	Not re	2020	0	0	0	0	0	0	0	0	0	0	0	0	
18431179	m	hp	DE	2020	0	1	0	0	0	1	0	0	0	0	0	0	64
18431702	m	md	JP	2020	0	1	0	1	0	0	0	0	0	0	0	1	76
18432159	m	Not repo	MY	2020	1	0	0	0	0	0	0	0	0	0	0	0	72.1
18432249	m	Not repo	CN	2020	0	1	0	1	0	0	0	0	0	0	0	0	48
18432251	m	md	JP	2020	0	1	0	1	0	0	0	0	0	0	0	1	77
18432286	f	md	US	2020	0	1	0	0	1	0	0	0	0	0	0	0	
18432893	f	cn	CA	2020	0	1	1	0	0	0	0	0	0	0	0	0	55
18433141	m	cn	JP	2020	0	1	1	0	0	0	0	0	0	0	0	0	69
18433267	m	Not repo	MY	2020	1	0	0	0	0	0	0	0	0	0	0	0	
18433300	m	md	JP	2020	0	1	0	0	1	0	0	0	0	0	0	1	87
18433414	m	hp	DE	2020	0	1	1	0	0	0	0	0	0	0	0	0	
18433795	m	cn	JP	2020	0	1	1	0	0	0	0	0	0	0	0	0	82
18434097	m	cn	CA	2020	0	0	0	0	0	0	0	0	0	0	0	0	
18434235	Not	ph	JP	2020	0	1	0	1	0	0	0	0	0	0	0	0	
18434459	m	cn	CN	2020	0	1	0	0	1	0	0	0	0	0	0	0	69
18434706	m	md	DE	2020	1	0	0	0	0	0	0	0	0	0	0	0	82
18435249	m	hp	CN	2020	0	1	1	0	0	0	0	0	0	0	0	0	69
18435273	f	md	KR	2020	1	0	0	0	0	0	0	0	0	0	0	0	67.7
18435347	f	md	CH	2020	0	0	0	0	0	0	0	0	0	0	0	0	81
18435363	f	md	JP	2020	0	1	0	0	0	1	0	0	0	0	0	0	75

18435374	m	md	US	2020	0	0	0	0	0	0	0	0	0	0	0	0	62
18435800	m	md	MY	2020	0	1	1	0	0	0	0	0	0	0	0	0	69
18436770	m	Not repo	CN	2020	1	0	0	0	0	0	0	0	0	0	0	0	60.7
18436987	m	md	JP	2020	0	1	0	1	0	0	0	0	0	0	0	0	76
18437036	f	cn	CA	2020	0	1	1	0	0	0	0	0	0	0	0	0	81
18437214	f	cn	US	2020	0	1	1	0	0	0	0	0	0	0	0	0	
18437420	f	hp	JP	2020	1	0	0	0	0	0	0	0	0	0	0	0	53
18437421	m	Not repo	KR	2020	1	0	0	0	0	0	0	0	0	0	0	0	74.6
18437749	f	cn	IN	2020	0	0	0	0	0	0	0	0	0	0	0	0	32
18437796	m	Not repo	MY	2020	1	0	0	0	0	0	0	0	0	0	0	0	63.6
18437927	Not	Not repo	AU	2020	0	1	0	1	0	0	0	0	0	0	0	1	
18437931	m	md	US	2020	0	0	0	0	0	0	0	0	0	0	0	0	62
18437934	m	hp	JP	2020	0	1	0	1	0	0	0	0	0	0	0	0	77
18438040	m	Not repo	JP	2020	1	0	0	0	0	0	0	0	0	0	0	0	77
18438127	f	ph	US	2020	0	0	0	0	0	0	0	0	0	0	0	0	54
18438308	f	cn	CN	2020	1	0	0	0	0	0	0	0	0	0	0	0	66
18438310	f	Not repo	CN	2020	1	0	0	0	0	0	0	0	0	0	0	0	68
18438532	m	hp	TH	2020	0	1	1	0	0	0	0	0	0	0	0	0	70
18438575	f	md	KR	2020	0	0	0	0	0	0	0	0	0	0	0	0	
18439178	m	hp	GR	2020	0	1	1	0	0	0	0	0	0	0	0	0	76
18439512	m	md	US	2020	0	1	1	0	0	0	0	0	0	0	0	0	65
18439581	f	cn	Not re	2020	0	0	0	0	0	0	0	0	0	0	0	0	
18439827	Not	hp	IT	2020	0	1	1	0	0	0	0	0	0	0	0	0	
18439938	m	md	JP	2020	0	0	0	0	0	0	0	0	0	0	0	0	73
18440032	f	md	BE	2020	0	0	0	0	0	0	0	0	0	0	0	0	
18440115	f	Not repo	CN	2020	1	0	0	0	0	0	0	0	0	0	0	0	68
18440468	m	md	JP	2020	0	1	1	0	0	0	0	0	0	0	0	1	59
18440599	f	hp	CN	2020	0	0	0	0	0	0	0	0	0	0	0	0	70
18440636	m	md	CL	2020	0	1	1	0	0	0	0	0	0	0	0	1	57
18440748	f	md	CA	2020	0	0	0	0	0	0	0	0	0	0	0	0	
18440759	m	cn	US	2020	0	0	0	0	0	0	0	0	0	0	0	0	61
18440868	m	md	JP	2020	0	1	0	1	0	0	0	0	0	0	0	1	75
18440869	f	Not repo	CN	2020	1	0	0	0	0	0	0	0	0	0	0	0	60
18441117	m	Not repo	GR	2020	0	1	0	1	0	0	0	0	0	0	0	1	73

18441285	f	cn	JP	2020	1	0	0	0	0	0	0	0	0	0	0	0	
18441305	m	md	DE	2020	0	1	0	0	0	1	0	0	0	0	0	0	64
18441595	f	Not repo	CN	2020	1	0	0	0	0	0	0	0	0	0	0	0	57
18442028	f	cn	US	2020	0	1	0	0	1	0	0	0	0	0	0	0	73
18442183	m	Not repo	GR	2020	0	1	0	1	0	0	0	0	0	0	0	0	60
18442266	f	cn	CN	2020	1	0	0	0	0	0	0	0	0	0	0	0	75
18442322	f	Not repo	CN	2020	1	0	0	0	0	0	0	0	0	0	0	0	69
18442323	f	cn	CN	2020	1	0	0	0	0	0	0	0	0	0	0	0	65
18442328	m	Not repo	CN	2020	1	0	0	0	0	0	0	0	0	0	0	0	54
18442403	f	Not repo	CN	2020	1	0	0	0	0	0	0	0	0	0	0	0	68
18442570	m	Not repo	NZ	2020	0	1	0	1	0	0	0	0	0	0	0	0	
18443125	f	md	US	2020	0	0	0	0	0	0	0	0	0	0	0	0	
18443227	m	cn	CN	2020	1	0	0	0	0	0	0	0	0	0	0	0	55
18443247	m	hp	JP	2020	0	1	1	0	0	0	0	0	0	0	0	0	80
18443374	m	md	US	2020	0	0	0	0	0	0	0	0	0	0	0	0	
18443389	m	hp	US	2020	0	1	0	0	1	0	0	0	0	0	0	0	74
18443505	m	md	CA	2020	0	0	0	0	0	0	0	0	0	0	0	0	61
18443668	m	md	US	2020	0	0	0	0	0	0	0	0	0	0	0	0	77
18443833	m	md	CN	2020	0	1	0	0	0	1	0	0	0	0	0	0	65
18443987	f	md	RO	2020	0	0	0	0	0	0	0	0	0	0	0	0	
18444207	Not	md	US	2020	0	0	0	0	0	0	0	0	0	0	0	0	
18444220	f	cn	US	2020	0	0	0	0	0	0	0	0	0	0	0	0	
18444641	m	md	UA	2020	0	1	1	0	0	0	0	0	0	0	0	0	67
18445027	f	Not repo	CN	2020	1	0	0	0	0	0	0	0	0	0	0	0	75.5
18445230	f	cn	CA	2020	0	1	1	0	0	0	0	0	0	0	0	0	71
18445252	m	md	CA	2020	1	0	0	0	0	0	0	0	0	0	0	0	62.9
18445886	f	Not repo	TW	2020	1	0	0	0	0	0	0	0	0	0	0	0	77
18446022	f	cn	CN	2020	0	1	0	0	1	0	0	0	0	0	0	0	
18446510	m	hp	US	2020	1	0	0	0	0	0	0	0	0	0	0	0	
18446801	m	md	JP	2020	0	1	0	0	1	0	0	0	0	0	0	0	59
18447585	f	cn	US	2020	0	0	0	0	0	0	0	0	0	0	0	0	40
18447750	f	cn	CN	2020	1	0	0	0	0	0	0	0	0	0	0	0	70
18447914	f	md	JP	2020	0	1	0	0	0	1	0	0	0	0	0	0	43
18448181	m	md	KR	2020	0	1	0	0	0	1	0	0	0	0	0	0	81

18448224	m	md	FR	2020	0	1	1	0	0	0	0	0	0	0	0	0	78
18448663	m	ph	US	2020	0	0	0	0	0	0	0	0	0	0	0	0	
18448963	m	hp	JP	2020	0	1	1	0	0	0	0	0	0	0	0	0	59
18449262	f	md	FI	2020	0	0	0	0	0	0	0	0	0	0	0	0	82
18449491	f	cn	CN	2020	1	0	0	0	0	0	0	0	0	0	0	0	65
18449641	f	cn	Not re	2020	0	1	1	0	0	0	0	0	0	0	0	0	64
18449808	Not	hp	US	2020	0	1	0	0	1	0	0	0	0	0	0	0	
18449813	m	md	CN	2020	0	1	1	0	0	0	0	0	0	0	0	0	54
18450000	f	md	AU	2020	0	1	0	0	0	1	0	0	0	0	0	0	33
18450197	m	cn	CN	2020	1	0	0	0	0	0	0	0	0	0	0	0	63.8
18450209	m	hp	JP	2020	1	0	0	0	0	0	0	0	0	0	0	0	85
18450211	f	Not repo	CN	2020	1	0	0	0	0	0	0	0	0	0	0	0	67
18450214	m	Not repo	CN	2020	1	0	0	0	0	0	0	0	0	0	0	0	
18450498	f	Not repo	CN	2020	1	0	0	0	0	0	0	0	0	0	0	0	61
18450518	m	Not repo	CN	2020	1	0	0	0	0	0	0	0	0	0	0	0	54.2
18450576	m	Not repo	CN	2020	1	0	0	0	0	0	0	0	0	0	0	0	85
18450607	f	cn	CN	2020	1	0	0	0	0	0	0	0	0	0	0	0	51
18450629	m	md	ES	2020	0	0	0	0	0	0	0	0	0	0	0	0	
18450822	m	md	JP	2020	0	0	0	0	0	0	0	0	0	0	0	0	70
18450891	m	md	IL	2020	0	1	0	0	1	0	0	0	0	0	0	0	62
18450934	m	Not repo	CN	2020	1	0	0	0	0	0	0	0	0	0	0	0	78
18450940	f	cn	CN	2020	1	0	0	0	0	0	0	0	0	0	0	0	51
18451705	m	cn	US	2020	0	0	0	0	0	0	0	0	0	0	0	0	78
18451713	f	cn	CN	2020	0	1	0	0	1	0	0	0	0	0	0	0	65
18451789	Not	Not repo	JP	2020	0	1	0	1	0	0	0	0	0	0	0	0	
18453211	m	hp	FR	2020	0	0	0	0	0	0	0	0	0	0	0	0	57
18453418	Not	md	JP	2020	0	1	0	0	1	0	0	0	0	0	0	0	
18453737	m	cn	CA	2020	0	0	0	0	0	0	0	0	0	0	0	0	46
18453817	m	ph	US	2020	0	1	0	0	1	0	0	0	0	0	0	0	76
18453992	f	cn	CN	2020	1	0	0	0	0	0	0	0	0	0	0	0	72
18454014	Not	Not repo	JP	2020	0	1	0	1	0	0	0	0	0	0	0	1	
18454016	m	hp	DE	2020	0	1	0	0	0	1	0	0	0	0	0	0	71
18454044	m	md	FR	2020	0	1	1	0	0	0	0	0	0	0	0	0	68
18454218	f	Not repo	CN	2020	1	0	0	0	0	0	0	0	0	0	0	0	68

18454227	m	cn	CA	2020	0	0	0	0	0	0	0	0	0	0	0	0	78
18454255	f	Not repo	LB	2020	1	0	0	0	0	0	0	0	0	0	0	0	
18454316	m	hp	JP	2020	0	1	1	0	0	0	0	0	0	0	0	1	59
18455134	Not	md	JP	2020	0	1	0	1	0	0	0	0	0	0	0	0	
18456418	m	cn	JP	2020	0	0	0	0	0	0	0	0	0	0	0	0	60
18457000	m	hp	JP	2020	0	1	0	1	0	0	0	0	0	0	0	1	63
18457045	m	md	DE	2020	0	1	1	0	0	0	0	0	0	0	0	0	49
18457067	Not	md	JP	2020	0	0	0	0	0	0	0	0	0	0	0	0	75
18457154	f	md	IL	2020	0	1	1	0	0	0	0	0	0	0	0	0	
18457195	Not	md	JP	2020	1	0	0	0	0	0	0	0	0	0	0	0	
18457339	m	md	FR	2020	0	1	1	0	0	0	0	0	0	0	0	0	55
18457449	f	cn	US	2020	0	1	1	0	0	0	0	0	0	0	0	0	74
18457584	m	ph	JP	2020	0	0	0	0	0	0	0	0	0	0	0	0	79
18457602	m	hp	KR	2020	0	1	0	0	1	0	0	0	0	0	0	0	73
18459079	m	hp	US	2020	0	0	0	0	0	0	0	0	0	0	0	0	39
18459129	Not	md	PT	2020	0	0	0	0	0	0	0	0	0	0	0	0	
18459298	m	md	US	2020	0	1	1	0	0	0	0	0	0	0	0	0	67
18459618	m	Not repo	JP	2020	1	0	0	0	0	0	0	0	0	0	0	0	77.4
18459708	m	md	US	2020	0	0	0	0	0	0	0	0	0	0	0	0	62
18460000	m	cn	IL	2020	0	1	1	0	0	0	0	0	0	0	0	0	
18460269	f	md	US	2020	0	1	1	0	0	0	0	0	0	0	0	0	73
18460462	m	ph	US	2020	0	1	0	0	0	1	0	0	0	0	0	0	79
18460494	f	ph	JP	2020	0	0	0	0	0	0	0	0	0	0	0	0	58
18460508	f	cn	US	2020	0	0	0	0	0	0	0	0	0	0	0	0	53
18460512	f	cn	US	2020	0	0	0	0	0	0	0	0	0	0	0	0	50
18460514	f	cn	US	2020	0	0	0	0	0	0	0	0	0	0	0	0	52
18460600	f	md	CR	2020	0	0	0	0	0	0	0	0	0	0	0	0	
18460641	m	md	KR	2020	0	1	0	0	0	1	0	0	0	0	0	0	77
18461113	m	md	JP	2020	1	0	0	0	0	0	0	0	0	0	0	1	75
18461145	Not	md	JP	2020	0	1	0	0	0	1	0	0	0	0	0	0	
18461234	f	ph	VN	2020	0	1	0	0	0	1	0	0	0	0	0	0	64
18461304	m	cn	CA	2020	0	0	0	0	0	0	0	0	0	0	0	0	76
18461323	f	cn	US	2020	0	0	0	0	0	0	0	0	0	0	0	0	50
18461715	f	md	CN	2020	0	1	1	0	0	0	0	0	0	0	0	0	56

18461997	f	md	BE	2020	0	0	0	0	0	0	0	0	0	0	0	0	
18462392	f	md	KR	2020	0	0	0	0	0	0	0	0	0	0	0	0	62
18462493	m	cn	CN	2020	0	1	0	0	1	0	0	0	0	0	0	0	55
18462557	f	Not repo	CN	2020	1	0	0	0	0	0	0	0	0	0	0	0	70
18462605	f	md	US	2020	0	1	1	0	0	0	0	0	0	0	0	0	64
18462852	m	cn	Not re	2020	0	0	0	0	0	0	0	0	0	0	0	0	
18463039	f	cn	NL	2020	0	1	1	0	0	0	0	0	0	0	0	0	
18463172	m	cn	CN	2020	1	0	0	0	0	0	0	0	0	0	0	0	85
18463214	Not	md	KR	2020	0	0	0	0	0	0	0	0	0	0	0	0	
18463532	f	md	BE	2020	0	0	0	0	0	0	0	0	0	0	0	0	74
18463641	f	cn	CA	2020	1	0	0	0	0	0	0	0	0	0	0	0	
18463712	f	cn	CA	2020	1	0	0	0	0	0	0	0	0	0	0	0	
18463873	m	cn	CN	2020	1	0	0	0	0	0	0	0	0	0	0	0	65
18464049	Not	hp	JP	2020	0	0	0	0	0	0	0	0	0	0	0	0	
18464260	Not	md	JP	2020	0	1	1	0	0	0	0	0	0	0	0	0	77
18464600	f	cn	IL	2020	0	0	0	0	0	0	0	0	0	0	0	0	69
18464763	m	hp	GR	2020	0	1	1	0	0	0	0	0	0	0	0	0	81
18465067	f	md	US	2020	0	0	0	0	0	0	0	0	0	0	0	0	
18465769	m	md	TR	2020	0	0	0	0	0	0	0	0	0	0	0	0	59
18466304	m	cn	US	2020	0	0	0	0	0	0	0	0	0	0	0	0	53
18466326	f	cn	US	2020	0	0	0	0	0	0	0	0	0	0	0	0	50
18466381	m	md	JP	2020	0	1	0	0	0	1	0	0	0	0	0	0	60
18466427	m	ph	JP	2020	0	1	0	0	0	1	0	0	0	0	0	0	75
18467092	Not	md	JP	2020	1	0	0	0	0	0	0	0	0	0	0	0	
18467416	f	hp	US	2020	1	0	0	0	0	0	0	0	0	0	0	0	73
18468071	m	ph	US	2020	0	1	1	0	0	0	0	0	0	0	0	1	71
18468075	m	Not repo	ID	2020	0	1	0	1	0	0	0	0	0	0	0	0	61.3
18468534	m	cn	US	2020	0	0	0	0	0	0	0	0	0	0	0	0	
18468687	m	md	PH	2020	0	0	0	0	0	0	0	0	0	0	0	0	36
18468691	f	md	KR	2020	0	0	0	0	0	0	0	0	0	0	0	0	56
18468768	m	md	DE	2020	0	1	0	0	0	1	0	0	0	0	0	0	
18468946	f	Not repo	CN	2020	1	0	0	0	0	0	0	0	0	0	0	0	
18468964	m	hp	NL	2020	0	1	0	0	0	1	0	0	0	0	0	0	71
18469110	f	md	FR	2020	1	0	0	0	0	0	0	0	0	0	0	1	60

18469373	f	md	DE	2020	0	0	0	0	0	0	0	0	0	0	0	0	49
18469378	f	hp	JP	2020	0	0	0	0	0	0	0	0	0	0	0	0	66
18469435	m	hp	DE	2020	0	1	1	0	0	0	0	0	0	0	0	0	
18469792	m	md	US	2020	0	0	0	0	0	0	0	0	0	0	0	0	62
18470152	Not	md	JP	2020	0	1	0	0	0	1	0	0	0	0	0	0	70
18471010	Not	Not repo	AU	2020	1	0	0	0	0	0	0	0	0	0	0	0	
18471135	m	md	CN	2020	0	1	0	0	0	1	0	0	0	0	0	0	70
18471149	m	md	GB	2020	0	0	0	0	0	0	0	0	0	0	0	0	57
18471458	m	md	GB	2020	0	1	0	1	0	0	0	0	0	0	0	0	71.6
18471589	f	ph	JP	2020	1	0	0	0	0	0	0	0	0	0	0	0	70
18471634	f	hp	US	2020	1	0	0	0	0	0	0	0	0	0	0	0	
18471649	m	md	DE	2020	0	1	1	0	0	0	0	0	0	0	0	0	55
18471687	f	cn	KW	2020	1	0	0	0	0	0	0	0	0	0	0	0	54.2
18472117	f	Not repo	CN	2020	1	0	0	0	0	0	0	0	0	0	0	0	65
18472525	f	md	US	2020	1	0	0	0	0	0	0	0	0	0	0	0	73
18472739	Not	md	JP	2020	0	0	0	0	0	0	0	0	0	0	0	0	
18473669	m	md	JP	2020	0	1	0	1	0	0	0	0	0	0	0	1	80
18473778	f	cn	CN	2020	1	0	0	0	0	0	0	0	0	0	0	0	70
18473781	Not	md	JP	2020	1	0	0	0	0	0	0	0	0	0	0	0	
18473782	m	cn	CN	2020	1	0	0	0	0	0	0	0	0	0	0	0	66
18473784	f	Not repo	CN	2020	1	0	0	0	0	0	0	0	0	0	0	0	68
18473786	f	Not repo	ID	2020	1	0	0	0	0	0	0	0	0	0	0	0	64.1
18473891	f	Not repo	ID	2020	0	1	0	1	0	0	0	0	0	0	0	0	64.6
18473929	f	hp	NL	2020	0	1	1	0	0	0	0	0	0	0	0	0	
18474052	f	cn	CA	2020	0	0	0	0	0	0	0	0	0	0	0	0	
18474141	f	md	JP	2020	1	0	0	0	0	0	0	0	0	0	0	0	76
18474150	f	Not repo	CN	2020	1	0	0	0	0	0	0	0	0	0	0	0	57.6
18474393	Not	cn	JP	2020	0	1	1	0	0	0	0	0	0	0	0	1	
18474475	f	Not repo	CN	2020	1	0	0	0	0	0	0	0	0	0	0	0	40
18474529	m	Not repo	CN	2020	1	0	0	0	0	0	0	0	0	0	0	0	50
18474851	f	md	BE	2020	0	1	1	0	0	0	0	0	0	0	0	0	
18474890	f	cn	CN	2020	1	0	0	0	0	0	0	0	0	0	0	0	63
18474898	m	hp	JP	2020	1	0	0	0	0	0	0	0	0	0	0	0	63
18475038	m	hp	JP	2020	1	0	0	0	0	0	0	0	0	0	0	0	68

18475040	f	hp	JP	2020	1	0	0	0	0	0	0	0	0	0	0	0	60
18475044	m	hp	JP	2020	1	0	0	0	0	0	0	0	0	0	0	0	56
18475051	f	cn	US	2020	1	0	0	0	0	0	0	0	0	0	0	0	
18475239	m	hp	JP	2020	1	0	0	0	0	0	0	0	0	0	0	0	51
18475240	m	hp	JP	2020	1	0	0	0	0	0	0	0	0	0	0	0	65
18475333	f	Not repo	CN	2020	1	0	0	0	0	0	0	0	0	0	0	0	68
18475474	f	Not repo	IT	2020	1	0	0	0	0	0	0	0	0	0	0	0	
18475492	m	md	IL	2020	0	0	0	0	0	0	0	0	0	0	0	0	65
18476047	Not	Not repo	AU	2020	0	1	0	1	0	0	0	0	0	0	0	0	
18476687	f	cn	Not re	2020	0	0	0	0	0	0	0	0	0	0	0	0	
18476838	m	md	TR	2020	0	0	0	0	0	0	0	0	0	0	0	0	55
18476932	f	hp	SI	2020	0	1	0	0	0	1	0	0	0	0	0	0	60
18476980	f	hp	DE	2020	0	1	1	0	0	0	0	0	0	0	0	0	69
18477802	Not	hp	DE	2020	0	0	0	0	0	0	0	0	0	0	0	0	
18477869	f	Not repo	CN	2020	1	0	0	0	0	0	0	0	0	0	0	0	76
18477901	m	Not repo	ID	2020	1	0	0	0	0	0	0	0	0	0	0	0	64.7
18478003	f	md	KR	2020	0	0	0	0	0	0	0	0	0	0	0	0	61
18478009	f	md	KR	2020	0	0	0	0	0	0	0	0	0	0	0	0	81
18478020	m	md	IL	2020	0	1	0	0	0	1	0	0	0	0	0	0	51
18478078	f	cn	US	2020	1	0	0	0	0	0	0	0	0	0	0	0	
18478570	f	hp	CN	2020	0	1	0	0	1	0	0	0	0	0	0	0	53
18478619	m	md	US	2020	0	1	1	0	0	0	0	0	0	0	0	0	67
18478836	f	hp	US	2020	0	1	0	0	1	0	0	0	0	0	0	0	72
18478891	m	Not repo	ID	2020	1	0	0	0	0	0	0	0	0	0	0	0	66
18479085	m	Not repo	ID	2020	1	0	0	0	0	0	0	0	0	0	0	0	66.9
18479180	m	hp	FR	2020	0	1	1	0	0	0	0	0	0	0	0	0	67
18479544	f	hp	CN	2020	0	1	1	0	0	0	0	0	0	0	0	0	71
18479766	f	md	DE	2020	0	1	1	0	0	0	0	0	0	0	0	0	69
18480043	m	md	US	2020	0	0	0	0	0	0	0	0	0	0	0	0	65
18480228	f	Not repo	CA	2020	1	0	0	0	0	0	0	0	0	0	0	0	
18480358	m	md	IN	2020	0	0	0	0	0	0	0	0	0	0	0	0	71
18480674	m	cn	US	2020	1	0	0	0	0	0	0	0	0	0	0	0	69
18481458	m	md	JP	2020	0	1	0	0	1	0	0	0	0	0	0	0	73
18481900	f	md	US	2020	0	1	0	0	0	1	0	0	0	0	0	0	90

18481950	f	md	GB	2020	0	1	1	0	0	0	0	0	0	0	0	0	63
18481984	m	hp	JP	2020	1	0	0	0	0	0	0	0	0	0	0	0	64
18481985	f	hp	JP	2020	1	0	0	0	0	0	0	0	0	0	0	0	67
18482032	m	cn	Not re	2020	0	0	0	0	0	0	0	0	0	0	0	0	
18484465	m	md	US	2020	0	1	0	1	0	0	0	0	0	0	0	1	82.1
18484748	f	md	KR	2020	0	1	0	0	0	1	0	0	0	0	0	0	62
18484985	m	hp	FR	2020	0	0	0	0	0	0	0	0	0	0	0	0	72
18485053	m	md	FR	2020	0	1	0	1	0	0	0	0	0	0	0	0	80
18485744	f	md	FR	2020	1	0	0	0	0	0	0	0	0	0	0	0	78
18485768	f	cn	US	2020	1	0	0	0	0	0	0	0	0	0	0	0	66
18486208	m	md	AR	2020	0	1	1	0	0	0	0	0	0	0	0	0	60
18486966	f	cn	CN	2020	1	0	0	0	0	0	0	0	0	0	0	0	69
18486967	f	md	PL	2020	0	1	0	1	0	0	0	0	0	0	0	0	
18487010	m	md	CN	2020	0	1	0	1	0	0	0	0	0	0	0	0	61
18487179	m	md	FR	2020	0	1	0	1	0	0	0	0	0	0	0	1	
18487389	m	cn	US	2020	0	1	0	0	1	0	0	0	0	0	0	0	
18487633	m	cn	CN	2020	0	1	0	0	1	0	0	0	0	0	0	0	
18487735	f	md	FR	2020	0	1	0	1	0	0	0	0	0	0	0	0	
18487737	f	cn	US	2020	1	0	0	0	0	0	0	0	0	0	0	0	68
18488689	f	md	US	2020	1	0	0	0	0	0	0	0	0	0	0	0	73
18489075	m	hp	RU	2020	0	1	0	1	0	0	0	0	0	0	0	0	60
18489374	Not	md	JP	2020	0	0	0	0	0	0	0	0	0	0	0	0	48
18489540	f	cn	Not re	2020	0	0	0	0	0	0	0	0	0	0	0	0	
18489615	f	ph	JP	2020	1	0	0	0	0	0	0	0	0	0	0	0	60
18490418	f	ph	US	2020	1	0	0	0	0	0	0	0	0	0	0	0	60
18490749	Not	hp	US	2020	1	0	0	0	0	0	0	0	0	0	0	0	
18490891	Not	hp	IT	2020	0	0	0	0	0	0	0	0	0	0	0	0	
18491287	m	md	JP	2020	0	1	1	0	0	0	0	0	0	0	0	0	73
18491377	f	md	KR	2020	0	1	0	0	1	0	0	0	0	0	0	0	56
18491433	f	hp	NL	2020	0	1	1	0	0	0	0	0	0	0	0	0	69
18491553	m	hp	TW	2020	1	0	0	0	0	0	0	0	0	0	0	0	72
18491743	f	md	DE	2020	0	1	1	0	0	0	0	0	0	0	0	0	54
18491745	f	md	BE	2020	0	1	1	0	0	0	0	0	0	0	0	0	
18491763	m	Not repo	CA	2020	1	0	0	0	0	0	0	0	0	0	0	0	48.5

18492164	f	hp	US	2020	0	1	0	0	1	0	0	0	0	0	0	0	70
18492326	f	md	US	2020	0	0	0	0	0	0	0	0	0	0	0	0	60
18492381	f	ph	JP	2020	0	0	0	0	0	0	0	0	0	0	0	0	80
18492398	m	hp	CN	2020	0	1	0	0	1	0	0	0	0	0	0	0	68
18492862	m	md	GB	2020	1	0	0	0	0	0	0	0	0	0	0	0	61
18493455	m	md	JP	2020	0	1	0	0	0	1	0	0	0	0	0	0	70
18493542	f	md	US	2020	0	1	0	0	0	1	0	0	0	0	0	0	74
18493714	m	cn	CN	2020	0	1	0	0	1	0	0	0	0	0	0	0	81
18493944	f	hp	US	2020	0	1	0	0	1	0	0	0	0	0	0	0	76
18494177	m	hp	NL	2020	0	0	0	0	0	0	0	0	0	0	0	0	
18496967	m	Not repo	MY	2020	0	1	0	1	0	0	0	0	0	0	0	0	68.5
18497154	m	cn	US	2020	1	0	0	0	0	0	0	0	0	0	0	0	62.7
18497304	m	md	US	2020	0	1	0	0	0	1	0	0	0	0	0	0	77
18497485	f	md	US	2020	0	1	0	1	0	0	0	0	0	0	0	0	
18497486	f	md	FR	2020	1	0	0	0	0	0	0	0	0	0	0	0	
18497956	f	hp	CN	2020	0	0	0	0	0	0	0	0	0	0	0	0	77
18498072	m	md	US	2020	0	0	0	0	0	0	0	0	0	0	0	0	
18498262	f	Not repo	AU	2020	1	0	0	0	0	0	0	0	0	0	0	0	
18498397	Not	cn	TW	2020	1	0	0	0	0	0	0	0	0	0	0	0	
18498832	f	cn	CN	2020	0	0	0	0	0	0	0	0	0	0	0	0	
18498971	f	ph	FR	2020	0	0	0	0	0	0	0	0	0	0	0	0	68
18499043	m	md	KR	2020	0	0	0	0	0	0	0	0	0	0	0	0	49
18499085	f	md	US	2020	1	0	0	0	0	0	0	0	0	0	0	0	73
18499872	m	hp	US	2020	0	1	0	0	1	0	0	0	0	0	0	0	
18500021	m	Not repo	CN	2020	1	0	0	0	0	0	0	0	0	0	0	0	69
18500038	f	cn	CA	2020	0	1	1	0	0	0	0	0	0	0	0	0	60
18500198	f	md	JP	2020	0	1	0	0	0	1	0	0	0	0	0	0	60
18500356	f	md	US	2020	0	1	0	0	0	1	0	0	0	0	0	0	76
18500531	f	ph	US	2020	0	1	0	1	0	0	0	0	0	0	0	0	65
18500548	f	md	US	2020	1	0	0	0	0	0	0	0	0	0	0	0	73
18500603	m	cn	US	2020	0	0	0	0	0	0	0	0	0	0	0	0	40
18500853	m	md	JP	2020	0	1	1	0	0	0	0	0	0	0	0	1	70
18500875	m	md	JP	2020	0	0	0	0	0	0	0	0	0	0	0	0	
18501208	m	hp	JP	2020	1	0	0	0	0	0	0	0	0	0	0	0	74

18501487	m	md	CH	2020	0	0	0	0	0	0	0	0	0	0	0	0	56
18501641	Not	ph	US	2020	0	0	0	0	0	0	0	0	0	0	0	0	
18501716	Not	md	JP	2020	0	1	0	1	0	0	0	0	0	0	0	0	
18501898	f	Not repo	MY	2020	1	0	0	0	0	0	0	0	0	0	0	0	70.5
18501899	m	Not repo	TW	2020	0	1	0	1	0	0	0	0	0	0	0	0	57
18501911	f	Not repo	MY	2020	1	0	0	0	0	0	0	0	0	0	0	0	81.1
18501912	m	Not repo	TW	2020	1	0	0	0	0	0	0	0	0	0	0	0	64
18502178	m	cn	GR	2020	0	1	1	0	0	0	0	0	0	0	0	0	
18502540	f	md	US	2020	0	1	1	0	0	0	0	0	0	0	0	0	
18502943	m	cn	CN	2020	0	1	0	0	1	0	0	0	0	0	0	0	
18503122	f	md	DE	2020	0	1	1	0	0	0	0	0	0	0	0	0	69
18503235	f	cn	Not re	2020	0	0	0	0	0	0	0	0	0	0	0	0	
18503637	f	cn	CN	2020	1	0	0	0	0	0	0	0	0	0	0	0	52
18503640	m	Not repo	CN	2020	1	0	0	0	0	0	0	0	0	0	0	0	76.5
18503641	Not	md	JP	2020	1	0	0	0	0	0	0	0	0	0	0	1	
18503645	f	md	JP	2020	1	0	0	0	0	0	0	0	0	0	0	1	69
18503723	f	md	FR	2020	0	1	0	1	0	0	0	0	0	0	0	0	68
18503727	m	md	JP	2020	0	1	1	0	0	0	0	0	0	0	0	0	72
18503758	m	cn	JP	2020	0	1	1	0	0	0	0	0	0	0	0	0	63
18503826	m	cn	US	2020	1	0	0	0	0	0	0	0	0	0	0	0	61.8
18503973	m	cn	DE	2020	0	1	1	0	0	0	0	0	0	0	0	0	
18504111	m	md	ME	2020	0	1	1	0	0	0	0	0	0	0	0	0	
18504528	m	hp	DE	2020	0	1	1	0	0	0	0	0	0	0	0	0	64
18504647	m	md	KR	2020	0	1	0	0	1	0	0	0	0	0	0	1	61
18504754	m	md	DE	2020	0	1	1	0	0	0	0	0	0	0	0	0	49
18504832	m	cn	JP	2020	0	1	1	0	0	0	0	0	0	0	0	1	75
18505111	m	md	KR	2020	0	1	0	0	1	0	0	0	0	0	0	0	48
18505181	m	md	MX	2020	0	0	0	0	0	0	0	0	0	0	0	0	41
18505383	f	md	DE	2020	0	1	1	0	0	0	0	0	0	0	0	0	69
18505407	m	cn	CN	2020	0	1	0	0	1	0	0	0	0	0	0	0	
18505667	f	md	DE	2020	1	0	0	0	0	0	0	0	0	0	0	0	
18505703	Not	cn	JP	2020	0	1	1	0	0	0	0	0	0	0	0	0	60
18505819	m	md	KR	2020	0	1	0	0	1	0	0	0	0	0	0	0	57
18505877	m	cn	CA	2020	1	0	0	0	0	0	0	0	0	0	0	0	

18505989	m	Not repo	Not re	2020	0	0	0	0	0	0	0	0	0	0	0	0	64
18506116	m	md	DE	2020	0	0	0	0	0	0	0	0	0	0	0	0	55
18506161	f	Not repo	JP	2020	1	0	0	0	0	0	0	0	0	0	0	0	68
18506386	m	hp	TH	2020	0	1	1	0	0	0	0	0	0	0	0	0	62
18507386	m	md	CL	2020	0	1	1	0	0	0	0	0	0	0	0	0	61
18507422	f	hp	US	2020	0	1	0	0	1	0	0	0	0	0	0	0	52
18507652	f	Not repo	CA	2020	1	0	0	0	0	0	0	0	0	0	0	0	
18507666	m	md	KR	2020	0	1	0	0	1	0	0	0	0	0	0	0	71
18507671	m	md	KR	2020	0	1	0	0	1	0	0	0	0	0	0	0	48
18507720	f	cn	JP	2020	0	1	1	0	0	0	0	0	0	0	0	0	76
18507746	m	Not repo	CN	2020	1	0	0	0	0	0	0	0	0	0	0	0	58.6
18508391	f	Not repo	AU	2020	1	0	0	0	0	0	0	0	0	0	0	0	63
18508453	f	cn	LB	2020	0	1	0	0	0	1	0	0	0	0	0	0	
18508456	m	cn	US	2020	0	0	0	0	0	0	0	0	0	0	0	0	53
18508461	m	md	US	2020	0	1	1	0	0	0	0	0	0	0	0	0	
18508468	m	ph	US	2020	0	0	0	0	0	0	0	0	0	0	0	0	
18508595	f	md	KR	2020	0	1	0	0	1	0	0	0	0	0	0	1	60
18508967	m	hp	GR	2020	0	1	1	0	0	0	0	0	0	0	0	0	65
18508987	m	cn	JP	2020	0	1	1	0	0	0	0	0	0	0	0	0	80
18508994	f	md	JP	2020	0	1	0	0	0	1	0	0	0	0	0	0	
18509895	m	hp	DE	2020	0	1	0	0	1	0	0	0	0	0	0	0	36
18510027	f	hp	DE	2020	0	1	1	0	0	0	0	0	0	0	0	0	
18510294	f	md	BE	2020	0	1	1	0	0	0	0	0	0	0	0	0	
18510358	f	md	MY	2020	1	0	0	0	0	0	0	0	0	0	0	0	
18510360	Not	md	JP	2020	0	0	0	0	0	0	0	0	0	0	0	0	
18510418	m	cn	DE	2020	0	1	1	0	0	0	0	0	0	0	0	0	
18510472	m	cn	DE	2020	0	1	1	0	0	0	0	0	0	0	0	0	84
18510522	m	cn	PH	2020	0	1	0	0	0	1	0	0	0	0	0	0	
18510882	m	cn	US	2020	0	1	0	0	1	0	0	0	0	0	0	0	74
18510899	f	Not repo	MY	2020	1	0	0	0	0	0	0	0	0	0	0	0	65.7
18510907	f	md	IT	2020	0	1	0	0	0	1	0	0	0	0	0	0	72
18511062	m	md	JP	2020	0	1	1	0	0	0	0	0	0	0	0	0	72
18511489	Not	ph	US	2020	0	0	0	0	0	0	0	0	0	0	0	0	
18511589	Not	md	SG	2020	1	0	0	0	0	0	0	0	0	0	0	0	

18511652	m	hp	DE	2020	0	1	1	0	0	0	0	0	0	0	0	0	49
18511785	Not	md	JP	2020	1	0	0	0	0	0	0	0	0	0	0	0	
18511807	Not	md	JP	2020	0	0	0	0	0	0	0	0	0	0	0	0	
18512813	f	cn	CN	2020	1	0	0	0	0	0	0	0	0	0	0	0	79
18512867	f	cn	US	2020	0	0	0	0	0	0	0	0	0	0	0	0	
18513159	f	cn	US	2020	0	0	0	0	0	0	0	0	0	0	0	0	36
18513164	m	cn	Not re	2020	0	0	0	0	0	0	0	0	0	0	0	0	
18513176	f	md	JP	2020	0	1	0	0	0	1	0	0	0	0	0	1	78
18513209	m	cn	JP	2020	0	1	1	0	0	0	0	0	0	0	0	0	77
18513258	f	md	US	2020	0	1	0	0	1	0	0	0	0	0	0	1	72
18513470	f	md	JP	2020	1	0	0	0	0	0	0	0	0	0	0	1	83.2
18513676	m	md	ES	2020	0	1	0	0	0	1	0	0	0	0	0	0	67
18513923	f	ph	FR	2020	0	0	0	0	0	0	0	0	0	0	0	0	68
18513973	m	md	DE	2020	0	1	1	0	0	0	0	0	0	0	0	0	72
18514190	f	md	US	2020	0	1	1	0	0	0	0	0	0	0	0	0	52
18514223	f	cn	US	2020	1	0	0	0	0	0	0	0	0	0	0	0	
18514345	f	md	US	2020	0	0	0	0	0	0	0	0	0	0	0	0	67
18514400	f	cn	US	2020	0	0	0	0	0	0	0	0	0	0	0	0	
18515157	f	Not repo	AU	2020	0	1	0	1	0	0	0	0	0	0	0	0	
18516062	m	hp	CN	2020	0	1	0	0	1	0	0	0	0	0	0	1	66
18516222	m	hp	JP	2020	0	1	0	1	0	0	0	0	0	0	0	0	53
18516248	Not	cn	US	2020	0	0	0	0	0	0	0	0	0	0	0	0	
18516428	m	md	MY	2020	1	0	0	0	0	0	0	0	0	0	0	0	
18516703	f	md	US	2020	0	1	0	0	0	1	0	0	0	0	0	0	90
18517486	f	cn	CA	2020	0	0	0	0	0	0	0	0	0	0	0	0	76
18517561	m	md	IT	2020	0	1	1	0	0	0	0	0	0	0	0	0	61
18518302	f	hp	KR	2020	0	1	0	0	1	0	0	0	0	0	0	0	65
18518339	m	ph	US	2020	0	1	0	1	0	0	0	0	0	0	0	0	
18518361	m	md	PL	2020	0	0	0	0	0	0	0	0	0	0	0	0	70
18518437	Not	md	DE	2020	0	1	0	0	1	0	0	0	0	0	0	0	36
18518456	f	cn	CA	2020	1	0	0	0	0	0	0	0	0	0	0	0	
18518541	f	Not repo	AU	2020	1	0	0	0	0	0	0	0	0	0	0	0	69
18518542	Not	Not repo	TH	2020	1	0	0	0	0	0	0	0	0	0	0	0	
18518557	f	ph	FR	2020	0	0	0	0	0	0	0	0	0	0	0	0	68

18518772	f	hp	NL	2020	0	0	0	0	0	0	0	0	0	0	0	0	68
18519103	m	hp	NL	2020	0	1	1	0	0	0	0	0	0	0	0	0	49
18519207	f	Not repo	CN	2020	1	0	0	0	0	0	0	0	0	0	0	0	
18519234	m	md	TR	2020	0	0	0	0	0	0	0	0	0	0	0	0	77
18519490	m	hp	JP	2020	0	1	1	0	0	0	0	0	0	0	0	0	66
18519950	m	cn	US	2020	0	1	1	0	0	0	0	0	0	0	0	0	84
18520310	f	cn	US	2020	0	0	0	0	0	0	0	0	0	0	0	0	50
18520314	f	cn	US	2020	0	0	0	0	0	0	0	0	0	0	0	0	52
18520359	f	md	DE	2020	0	1	1	0	0	0	0	0	0	0	0	0	54
18520559	m	md	US	2020	0	1	0	0	0	1	0	0	0	0	0	0	39
18521112	m	md	AU	2020	0	0	0	0	0	0	0	0	0	0	0	0	
18521298	m	md	JP	2020	0	0	0	0	0	0	0	0	0	0	0	0	52
18521311	f	hp	US	2020	0	1	0	0	1	0	0	0	0	0	0	0	79
18521483	f	cn	Not re	2020	0	0	0	0	0	0	0	0	0	0	0	0	
18521515	Not	md	JP	2020	0	0	0	0	0	0	0	0	0	0	0	0	
18521762	Not	hp	DE	2020	0	0	0	0	0	0	0	0	0	0	0	0	
18521844	f	cn	US	2020	0	0	0	0	0	0	0	0	0	0	0	0	50
18521943	m	hp	CN	2020	0	1	1	0	0	0	0	0	0	0	0	0	59
18522490	m	md	FR	2020	0	1	0	1	0	0	0	0	0	0	0	0	62
18522495	f	md	JP	2020	0	1	1	0	0	0	0	0	0	0	0	0	86
18522541	Not	md	JP	2020	0	1	0	1	0	0	0	0	0	0	0	0	
18522544	f	md	JP	2020	1	0	0	0	0	0	0	0	0	0	0	0	80
18523199	m	ph	JP	2020	0	1	0	0	1	0	0	0	0	0	0	0	77
18523264	m	Not repo	AU	2020	0	1	0	1	0	0	0	0	0	0	0	0	
18523278	f	ph	FR	2020	0	0	0	0	0	0	0	0	0	0	0	0	68
18523492	f	hp	BE	2020	0	0	0	0	0	0	0	0	0	0	0	0	
18523878	m	md	IL	2020	0	0	0	0	0	0	0	0	0	0	0	0	71
18524229	m	md	CA	2020	0	0	0	0	0	0	0	0	0	0	0	0	57
18524278	m	hp	US	2020	0	1	0	0	1	0	0	0	0	0	0	0	69
18525251	f	cn	GB	2020	0	1	1	0	0	0	0	0	0	0	0	0	
18525284	m	hp	CN	2020	0	1	1	0	0	0	0	0	0	0	0	0	
18525443	m	md	JP	2020	0	1	0	0	0	1	0	0	0	0	0	0	80
18526216	m	md	JP	2020	1	0	0	0	0	0	0	0	0	0	0	0	72
18526631	f	hp	BE	2020	0	1	1	0	0	0	0	0	0	0	0	0	

18526840	f	md	DE	2020	0	1	1	0	0	0	0	0	0	0	0	0	54
18527140	f	Not repo	MY	2020	1	0	0	0	0	0	0	0	0	0	0	0	81.2
18527203	Not	md	JP	2020	1	0	0	0	0	0	0	0	0	0	0	0	
18527321	Not	md	JP	2020	1	0	0	0	0	0	0	0	0	0	0	0	
18527323	f	md	JP	2020	1	0	0	0	0	0	0	0	0	0	0	0	71
18527338	f	md	DE	2020	0	0	0	0	0	0	0	0	0	0	0	0	65
18527408	m	md	KR	2020	0	1	1	0	0	0	0	0	0	0	0	0	77
18527491	m	cn	IL	2020	0	1	1	0	0	0	0	0	0	0	0	0	75
18527506	f	hp	NL	2020	0	1	1	0	0	0	0	0	0	0	0	0	54
18527595	f	hp	ES	2020	0	0	0	0	0	0	0	0	0	0	0	0	55
18527719	f	cn	US	2020	0	0	0	0	0	0	0	0	0	0	0	0	89
18528004	f	hp	NL	2020	0	1	0	0	1	0	0	0	0	0	0	0	53
18528171	m	hp	DE	2020	0	1	1	0	0	0	0	0	0	0	0	0	54
18528395	Not	md	US	2020	1	0	0	0	0	0	0	0	0	0	0	0	
18528400	Not	cn	US	2020	0	0	0	0	0	0	0	0	0	0	0	0	
18528568	f	cn	IN	2020	0	0	0	0	0	0	0	0	0	0	0	0	70
18528598	m	md	MY	2020	1	0	0	0	0	0	0	0	0	0	0	0	
18528661	f	hp	NL	2020	0	1	0	0	1	0	0	0	0	0	0	1	64
18528958	m	hp	JP	2020	0	1	0	1	0	0	0	0	0	0	0	1	67
18528965	f	md	US	2020	1	0	0	0	0	0	0	0	0	0	0	0	65
18529342	m	hp	US	2020	0	1	0	0	0	1	0	0	0	0	0	0	70
18529387	f	cn	US	2020	0	1	1	0	0	0	0	0	0	0	0	0	81
18530072	m	md	IT	2020	0	1	1	0	0	0	0	0	0	0	0	0	61
18530136	f	md	US	2020	1	0	0	0	0	0	0	0	0	0	0	0	76
18530197	m	cn	US	2020	0	1	1	0	0	0	0	0	0	0	0	0	
18530392	m	cn	Not re	2020	0	0	0	0	0	0	0	0	0	0	0	0	
18530827	f	cn	Not re	2020	0	0	0	0	0	0	0	0	0	0	0	0	
18531444	f	cn	US	2020	0	0	0	0	0	0	0	0	0	0	0	0	54
18531752	m	cn	RS	2020	0	1	0	0	0	1	0	0	0	0	0	0	65
18532038	m	md	JP	2020	0	1	0	0	0	1	0	0	0	0	0	0	82
18532934	m	ph	JP	2020	0	1	1	0	0	0	0	0	0	0	0	0	67
18533116	f	hp	US	2020	0	1	0	0	0	1	0	0	0	0	0	0	79
18533133	m	hp	CN	2020	0	1	1	0	0	0	0	0	0	0	0	0	
18533365	f	hp	US	2020	0	1	0	0	0	1	0	0	0	0	0	0	79

18533398	f	hp	US	2020	0	1	0	0	0	1	0	0	0	0	0	0		78
18533493	f	hp	US	2020	0	1	0	0	0	1	0	0	0	0	0	0		78
18533543	f	ph	FR	2020	0	0	0	0	0	0	0	0	0	0	0	0		68
18533564	m	cn	IL	2020	0	1	1	0	0	0	0	0	0	0	0	0		75
18533724	f	hp	US	2020	0	1	0	0	0	1	0	0	0	0	0	0		79
18533780	f	hp	US	2020	0	1	0	0	0	1	0	0	0	0	0	0		78
18533891	m	hp	JP	2020	0	1	1	0	0	0	0	0	0	0	0	0		81
18533899	m	hp	US	2020	0	1	0	0	1	0	0	0	0	0	0	0		63
18534179	f	md	ES	2020	0	1	0	1	0	0	0	0	0	0	0	0		
18534841	m	cn	CN	2020	0	0	0	0	0	0	0	0	0	0	0	0		47
18534885	m	hp	NL	2020	0	0	0	0	0	0	0	0	0	0	0	0		75
18534996	Not	hp	FR	2020	0	0	0	0	0	0	0	0	0	0	0	0		
18534997	Not	hp	FR	2020	0	0	0	0	0	0	0	0	0	0	0	0		
18535004	Not	hp	FR	2020	0	0	0	0	0	0	0	0	0	0	0	0		
18535366	m	Not repo	ID	2020	0	1	0	1	0	0	0	0	0	0	0	0		57
18535410	f	cn	Not re	2020	0	0	0	0	0	0	0	0	0	0	0	0		
18535867	f	md	IN	2020	0	0	0	0	0	0	0	0	0	0	0	0		78
18535870	m	cn	PL	2020	0	1	1	0	0	0	0	0	0	0	0	0		
18536029	m	md	JP	2020	0	1	0	0	1	0	0	0	0	0	0	0		70
18536201	m	cn	US	2020	0	0	0	0	0	0	0	0	0	0	0	0		76
18536360	f	md	US	2020	0	0	0	0	0	0	0	0	0	0	0	0		
18536380	m	md	US	2020	0	0	0	0	0	0	0	0	0	0	0	0		
18536451	m	Not repo	CN	2020	0	1	0	1	0	0	0	0	0	0	0	0		35
18536870	f	cn	JP	2020	0	1	1	0	0	0	0	0	0	0	0	0		68
18537091	f	hp	CA	2020	0	1	1	0	0	0	0	0	0	0	0	0		
18537364	f	hp	US	2020	0	1	0	1	0	0	0	0	0	0	0	0		55
18537366	m	md	JP	2020	0	1	0	1	0	0	0	0	0	0	0	1		52.7
18538089	f	Not repo	ID	2020	1	0	0	0	0	0	0	0	0	0	0	0		72.3
18538195	f	cn	Not re	2020	0	0	0	0	0	0	0	0	0	0	0	0		
18538562	m	ph	JP	2020	0	1	0	0	0	1	0	0	0	0	0	0		61
18539095	m	cn	CA	2020	0	0	0	0	0	0	0	0	0	0	0	0		75
18539530	m	hp	DE	2020	0	0	0	0	0	0	0	0	0	0	0	0		66
18539571	Not	cn	US	2020	1	0	0	0	0	0	0	0	0	0	0	0		
18539578	f	cn	US	2020	1	0	0	0	0	0	0	0	0	0	0	0		

18539584	f	cn	US	2020	1	0	0	0	0	0	0	0	0	0	0	0	70
18539586	Not	cn	US	2020	1	0	0	0	0	0	0	0	0	0	0	0	
18539591	f	cn	US	2020	1	0	0	0	0	0	0	0	0	0	0	0	69
18539597	f	cn	US	2020	1	0	0	0	0	0	0	0	0	0	0	0	87
18539645	f	cn	US	2020	1	0	0	0	0	0	0	0	0	0	0	0	68
18539747	f	cn	BE	2020	0	1	1	0	0	0	0	0	0	0	0	0	
18539807	f	cn	GB	2020	0	1	1	0	0	0	0	0	0	0	0	0	
18540092	m	Not repo	CN	2020	1	0	0	0	0	0	0	0	0	0	0	1	57
18540344	f	md	BE	2020	0	1	1	0	0	0	0	0	0	0	0	0	
18540823	f	cn	US	2020	1	0	0	0	0	0	0	0	0	0	0	0	67.2
18540834	Not	md	DE	2020	1	0	0	0	0	0	0	0	0	0	0	0	
18541275	f	hp	NL	2020	0	1	1	0	0	0	0	0	0	0	0	0	
18541516	f	hp	CN	2020	0	1	1	0	0	0	0	0	0	0	0	0	56
18541717	Not	hp	FR	2020	0	0	0	0	0	0	0	0	0	0	0	0	
18541846	m	cn	US	2020	0	1	1	0	0	0	0	0	0	0	0	0	76
18542056	m	cn	JP	2020	0	0	0	0	0	0	0	0	0	0	0	0	71
18542164	f	md	US	2020	0	0	0	0	0	0	0	0	0	0	0	0	74
18542184	m	md	US	2020	0	0	0	0	0	0	0	0	0	0	0	0	63
18542328	m	cn	CA	2020	0	1	0	1	0	0	0	0	0	0	0	0	66
18542408	m	md	CA	2020	0	1	1	0	0	0	0	0	0	0	0	0	62
18542432	m	Not repo	MY	2020	1	0	0	0	0	0	0	0	0	0	0	0	65.2
18542433	f	Not repo	MY	2020	1	0	0	0	0	0	0	0	0	0	0	0	67.8
18542436	m	md	JP	2020	1	0	0	0	0	0	0	0	0	0	0	0	77.8
18542447	f	Not repo	CN	2020	1	0	0	0	0	0	0	0	0	0	0	0	64
18542449	f	Not repo	CN	2020	1	0	0	0	0	0	0	0	0	0	0	0	56
18542613	m	cn	JP	2020	0	1	1	0	0	0	0	0	0	0	0	1	70
18542681	m	cn	JP	2020	0	1	1	0	0	0	0	0	0	0	0	0	80
18543014	m	md	US	2020	0	1	1	0	0	0	0	0	0	0	0	0	74
18543019	m	cn	FR	2020	0	1	1	0	0	0	0	0	0	0	0	0	71
18543041	f	cn	FR	2020	0	1	1	0	0	0	0	0	0	0	0	0	59
18543045	m	md	US	2020	0	1	1	0	0	0	0	0	0	0	0	0	79
18543314	f	cn	US	2020	0	0	0	0	0	0	1	0	0	0	1	0	
18543796	f	ph	FR	2020	0	0	0	0	0	0	0	0	0	0	0	0	
18543841	m	md	JP	2020	0	1	0	1	0	0	0	0	0	0	0	0	

18544117	f	md	JP	2020	0	1	0	0	0	1	0	0	0	0	0	0	76
18544155	m	md	JP	2020	0	1	0	0	0	1	0	0	0	0	0	0	40
18544737	f	md	AU	2020	0	1	0	1	0	0	0	0	0	0	0	0	71
18544968	m	md	JP	2020	0	1	0	1	0	0	0	0	0	0	0	0	54
18545143	m	cn	JP	2020	0	1	1	0	0	0	0	0	0	0	0	0	80
18545259	m	hp	CZ	2020	0	0	0	0	0	0	0	0	0	0	0	0	75
18545375	Not	md	FR	2020	0	1	0	1	0	0	0	0	0	0	0	0	
18545823	m	hp	US	2020	0	0	0	0	0	0	0	0	0	0	0	0	72
18545870	f	md	FR	2020	0	0	0	0	0	0	0	0	0	0	0	0	36
18545873	Not	md	TR	2020	0	0	0	0	0	0	0	0	0	0	0	0	
18546379	Not	md	JP	2020	0	1	0	1	0	0	0	0	0	0	0	0	
18546777	f	md	JP	2020	1	0	0	0	0	0	0	0	0	0	0	0	74
18546835	m	hp	IL	2020	0	0	0	0	0	0	0	0	0	0	0	0	73
18546874	m	md	US	2020	0	1	1	0	0	0	0	0	0	0	0	0	52
18546913	m	ph	JP	2020	0	1	0	0	1	0	0	0	0	0	0	0	67
18547010	m	md	IT	2020	0	0	0	0	0	0	0	0	0	0	0	0	70
18547069	f	ph	JP	2020	1	0	0	0	0	0	0	0	0	0	0	0	87
18547072	f	Not repo	JP	2020	0	1	0	1	0	0	0	0	0	0	0	1	67
18547596	m	hp	DE	2020	0	1	1	0	0	0	0	0	0	0	0	0	51
18547621	m	hp	CN	2020	0	1	0	0	1	0	0	0	0	0	0	0	68
18547665	m	cn	CA	2020	0	0	0	0	0	0	0	0	0	0	0	0	70
18547723	Not	md	MY	2020	0	1	0	0	0	1	0	0	0	0	0	0	
18547730	f	hp	JP	2020	0	0	0	0	0	0	0	0	0	0	0	0	85
18547770	m	md	JP	2020	0	1	0	0	0	1	0	0	0	0	0	0	76
18547978	f	hp	US	2020	0	0	0	0	0	0	0	0	0	0	0	0	
18548036	f	md	HU	2020	0	0	0	0	0	0	0	0	0	0	0	0	63
18548062	m	cn	Not re	2020	0	0	0	0	0	0	0	0	0	0	0	0	
18548439	f	cn	Not re	2020	0	0	0	0	0	0	0	0	0	0	0	0	
18548566	m	md	ES	2020	0	1	0	0	0	1	0	0	0	0	0	0	39
18548579	m	hp	CN	2020	0	1	0	0	1	0	0	0	0	0	0	0	58
18548784	f	cn	US	2020	0	1	1	0	0	0	0	0	0	0	0	0	56
18549175	m	md	HU	2020	0	0	0	0	0	0	0	0	0	0	0	0	
18549402	f	cn	CA	2020	0	1	0	1	0	0	0	0	0	0	0	0	74
18549635	Not	cn	JP	2020	0	1	1	0	0	0	0	0	0	0	0	1	80

18549750	Not	md	IT	2020	0	0	0	0	0	0	0	0	0	0	0	0	
18549945	Not	Not repo	JP	2020	1	0	0	0	0	0	0	0	0	0	0	0	
18549946	m	cn	CN	2020	0	1	0	1	0	0	0	0	0	0	0	0	52
18550046	m	hp	FR	2020	0	1	1	0	0	0	0	0	0	0	0	1	66
18550092	m	cn	DE	2020	0	1	1	0	0	0	0	0	0	0	0	0	49
18550221	Not	md	DE	2020	0	0	0	0	0	0	0	0	0	0	0	0	
18550451	m	cn	CN	2020	1	0	0	0	0	0	0	0	0	0	0	0	67
18550580	Not	md	FR	2020	0	1	0	0	1	0	0	0	0	0	0	0	
18550586	Not	md	JP	2020	1	0	0	0	0	0	0	0	0	0	0	0	
18550774	f	cn	DK	2020	0	1	1	0	0	0	0	0	0	0	0	0	79
18550938	f	md	JP	2020	1	0	0	0	0	0	0	0	0	0	0	0	80
18551081	f	hp	FR	2020	0	1	1	0	0	0	0	0	0	0	0	0	36
18551203	m	md	US	2020	0	0	0	0	0	0	0	0	0	0	0	0	
18551254	f	ph	JP	2020	1	0	0	0	0	0	0	0	0	0	0	0	85
18551310	m	md	US	2020	1	0	0	0	0	0	0	0	0	0	0	0	65
18551312	m	md	JP	2020	0	1	0	1	0	0	0	0	0	0	0	1	76
18551767	m	cn	Not re	2020	0	0	0	0	0	0	0	0	0	0	0	0	76
18551861	f	md	BE	2020	0	1	1	0	0	0	0	0	0	0	0	0	64
18551955	m	md	KR	2020	0	1	0	0	0	1	0	0	0	0	0	0	61
18551989	f	cn	SG	2020	0	0	0	0	0	0	0	0	0	0	0	0	62
18552117	m	hp	US	2020	0	0	0	0	0	0	0	0	0	0	0	0	
18552670	m	hp	RO	2020	0	0	0	0	0	0	0	0	0	0	0	0	67
18552737	m	hp	JP	2020	0	1	0	1	0	0	0	0	0	0	0	0	53
18552742	m	md	TH	2020	0	1	0	0	0	1	0	0	0	0	0	0	67
18553139	m	md	JP	2020	0	1	0	0	0	1	0	0	0	0	0	0	80
18553567	f	md	US	2020	0	1	0	0	0	1	0	0	0	0	0	0	71
18553640	m	md	CN	2020	0	1	0	0	1	0	0	0	0	0	0	0	
18553761	f	md	CH	2020	0	0	0	0	0	0	0	0	0	0	0	0	
18554033	f	cn	DE	2020	0	1	0	0	0	1	0	0	0	0	0	0	58
18554034	f	md	AT	2020	0	0	0	0	0	0	0	0	0	0	0	0	33
18554355	m	md	JP	2020	0	1	0	0	1	0	0	0	0	0	0	0	60
18554851	m	cn	DE	2020	0	1	1	0	0	0	0	0	0	0	0	0	64
18555156	m	Not repo	CN	2020	0	1	0	1	0	0	0	0	0	0	0	0	32
18555254	Not	md	JP	2020	0	1	0	1	0	0	0	0	0	0	0	0	

18555265	m	md	JP	2020	0	1	0	1	0	0	0	0	0	0	0	0	72
18555469	f	Not repo	JP	2020	1	0	0	0	0	0	0	0	0	0	0	0	
18555503	f	cn	Not re	2020	0	0	0	0	0	0	0	0	0	0	0	0	58
18555553	m	ph	CA	2020	0	0	0	0	0	0	0	0	0	0	0	0	58
18555585	m	md	US	2020	0	0	0	0	0	0	0	0	0	0	0	0	65.7
18555604	m	md	JP	2020	0	1	0	1	0	0	0	0	0	0	0	1	70
18555670	f	md	CN	2020	1	0	0	0	0	0	0	0	0	0	0	0	59.4
18556615	m	cn	US	2020	0	1	1	0	0	0	0	0	0	0	0	1	
18556940	f	cn	Not re	2020	0	0	0	0	0	0	0	0	0	0	0	0	
18556959	f	cn	Not re	2020	0	0	0	0	0	0	0	0	0	0	0	0	
18557111	f	cn	Not re	2020	0	0	0	0	0	0	0	0	0	0	0	0	
18557151	f	hp	DE	2020	0	1	0	0	0	1	0	0	0	0	0	0	62
18557456	f	cn	SG	2020	0	1	0	0	0	1	0	0	0	0	0	0	64
18557493	f	hp	US	2020	0	1	1	0	0	0	0	0	0	0	0	0	47
18557948	m	md	NL	2020	0	0	0	0	0	0	0	0	0	0	0	0	50
18558512	m	md	FR	2020	0	1	1	0	0	0	0	0	0	0	0	1	66
18558836	Not	md	JP	2020	0	1	0	0	0	1	0	0	0	0	0	0	80
18559344	f	cn	JP	2020	0	0	0	0	0	0	0	0	0	0	0	0	63
18559483	f	md	HK	2020	0	1	1	0	0	0	0	0	0	0	0	0	62
18559740	f	Not repo	MY	2020	1	0	0	0	0	0	0	0	0	0	0	0	55.8
18559797	f	cn	BE	2020	0	1	1	0	0	0	0	0	0	0	0	0	
18559877	m	md	JP	2020	1	0	0	0	0	0	0	0	0	0	0	0	78
18559930	f	md	JP	2020	1	0	0	0	0	0	0	0	0	0	0	0	62
18559933	m	md	JP	2020	1	0	0	0	0	0	0	0	0	0	0	0	49
18560128	m	md	KR	2020	0	1	0	0	1	0	0	0	0	0	0	0	60
18560254	m	md	JP	2020	0	1	0	1	0	0	0	0	0	0	0	0	81
18560349	m	md	JP	2020	0	1	0	1	0	0	0	0	0	0	0	1	77
18560634	f	md	JP	2020	1	0	0	0	0	0	0	0	0	0	0	1	79
18560636	m	Not repo	MY	2020	1	0	0	0	0	0	0	0	0	0	0	0	
18560772	m	cn	US	2020	0	0	0	0	0	0	0	0	0	0	0	0	58
18560793	m	hp	JP	2020	1	0	0	0	0	0	0	0	0	0	0	0	73
18560908	f	Not repo	MY	2020	1	0	0	0	0	0	0	0	0	0	0	0	64.7
18560942	f	cn	US	2020	0	0	0	0	0	0	0	0	0	0	0	0	
18561224	m	cn	US	2020	0	1	1	0	0	0	0	0	0	0	0	0	

18561267	m	md	FR	2020	0	1	1	0	0	0	0	0	0	0	0	1	66
18562168	m	cn	DK	2020	0	1	1	0	0	0	0	0	0	0	0	0	71
18562232	Not	md	CN	2020	1	0	0	0	0	0	0	0	0	0	0	1	
18562268	m	hp	CN	2020	0	1	1	0	0	0	0	0	0	0	0	0	54
18562291	m	hp	JP	2020	0	1	0	1	0	0	0	0	0	0	0	0	54
18562412	f	md	JP	2020	1	0	0	0	0	0	0	0	0	0	0	0	71
18562511	f	ph	JP	2020	0	0	0	0	0	0	0	0	0	0	0	0	67
18562719	f	md	US	2020	0	1	1	0	0	0	0	0	0	0	0	0	69
18563043	m	md	FR	2020	0	1	1	0	0	0	0	0	0	0	0	1	66
18563556	f	cn	TH	2020	0	0	0	0	0	0	0	0	0	0	0	0	57
18563734	f	md	RS	2020	1	0	0	0	0	0	0	0	0	0	0	0	71
18563753	m	md	JP	2020	1	0	0	0	0	0	0	0	0	0	0	0	70
18563754	f	md	JP	2020	1	0	0	0	0	0	0	0	0	0	0	0	72
18563913	m	Not repo	CN	2020	1	0	0	0	0	0	0	0	0	0	0	0	80
18564110	m	Not repo	IN	2020	0	1	0	1	0	0	0	0	0	0	0	0	60.2
18564177	m	md	JP	2020	1	0	0	0	0	0	0	0	0	0	0	0	70
18564289	m	md	JP	2020	0	1	0	1	0	0	0	0	0	0	0	1	60.6
18564379	m	md	JP	2020	1	0	0	0	0	0	0	0	0	0	0	0	77
18564592	f	md	JP	2020	1	0	0	0	0	0	0	0	0	0	0	0	72
18564881	f	md	JP	2020	1	0	0	0	0	0	0	0	0	0	0	0	59
18564884	f	Not repo	MY	2020	1	0	0	0	0	0	0	0	0	0	0	0	84.2
18564906	m	cn	JP	2020	0	1	1	0	0	0	0	0	0	0	0	0	90
18564973	f	md	JP	2020	1	0	0	0	0	0	0	0	0	0	0	0	72
18564975	f	md	JP	2020	1	0	0	0	0	0	0	0	0	0	0	0	69
18564979	m	md	JP	2020	1	0	0	0	0	0	0	0	0	0	0	0	54
18565154	m	md	JP	2020	1	0	0	0	0	0	0	0	0	0	0	0	79
18565155	m	Not repo	CN	2020	1	0	0	0	0	0	0	0	0	0	0	0	73
18565280	m	hp	KR	2020	0	1	1	0	0	0	0	0	0	0	0	0	76
18565295	f	Not repo	CN	2020	1	0	0	0	0	0	0	0	0	0	0	0	48
18565296	m	md	JP	2020	1	0	0	0	0	0	0	0	0	0	0	0	70
18565362	Not	hp	FR	2020	0	0	0	0	0	0	0	0	0	0	0	0	
18565395	f	Not repo	CN	2020	1	0	0	0	0	0	0	0	0	0	0	0	71
18565459	f	md	JP	2020	1	0	0	0	0	0	0	0	0	0	0	0	70
18565461	f	md	JP	2020	1	0	0	0	0	0	0	0	0	0	0	0	72

18565464	f	md	JP	2020	1	0	0	0	0	0	0	0	0	0	0	0	77
18565577	f	md	JP	2020	1	0	0	0	0	0	0	0	0	0	0	0	46
18565580	m	md	JP	2020	1	0	0	0	0	0	0	0	0	0	0	0	85
18565677	f	Not repo	CN	2020	1	0	0	0	0	0	0	0	0	0	0	0	58
18565678	f	Not repo	US	2020	1	0	0	0	0	0	0	0	0	0	0	0	70
18565939	m	cn	CN	2020	1	0	0	0	0	0	0	0	0	0	0	0	
18565947	m	cn	CN	2020	1	0	0	0	0	0	0	0	0	0	0	0	58
18566045	f	Not repo	CN	2020	1	0	0	0	0	0	0	0	0	0	0	0	59
18566053	Not	hp	FR	2020	0	0	0	0	0	0	0	0	0	0	0	0	
18566059	f	md	JP	2020	1	0	0	0	0	0	0	0	0	0	0	0	54
18566128	m	md	ES	2020	0	1	1	0	0	0	0	0	0	0	0	0	61
18566163	m	md	RS	2020	1	0	0	0	0	0	0	0	0	0	0	0	81
18566168	Not	md	JP	2020	0	0	0	0	0	0	0	0	0	0	0	0	
18566199	f	md	JP	2020	0	1	0	1	0	0	0	0	0	0	0	1	62
18566252	m	Not repo	CN	2020	1	0	0	0	0	0	0	0	0	0	0	0	61
18566684	m	Not repo	CN	2020	1	0	0	0	0	0	0	0	0	0	0	0	80
18566733	Not	md	HK	2020	0	0	0	0	0	0	0	0	0	0	0	0	
18566756	m	hp	JP	2020	0	1	0	1	0	0	0	0	0	0	0	1	64
18567145	m	hp	US	2020	0	1	0	1	0	0	0	0	0	0	0	0	75
18567174	m	md	JP	2020	0	1	0	0	0	1	0	0	0	0	0	0	80
18567429	f	cn	US	2020	0	1	1	0	0	0	0	0	0	0	0	0	
18567635	m	md	JP	2020	0	1	0	0	0	1	0	0	0	0	0	0	85
18567658	Not	md	FR	2020	0	1	1	0	0	0	0	0	0	0	0	1	
18567806	f	md	JP	2020	0	1	0	0	0	1	0	0	0	0	0	0	70
18567923	m	md	JP	2020	0	1	0	0	0	1	0	0	0	0	0	0	71
18568055	m	cn	CN	2020	1	0	0	0	0	0	0	0	0	0	0	0	45
18568245	f	md	JP	2020	1	0	0	0	0	0	0	0	0	0	0	1	67
18568265	f	hp	CN	2020	0	0	0	0	0	0	0	0	0	0	0	0	39
18568350	Not	hp	CN	2020	0	0	0	0	0	0	0	0	0	0	0	0	
18568379	m	cn	US	2020	0	0	0	0	0	0	0	0	0	0	0	0	
18568393	f	md	JP	2020	1	0	0	0	0	0	0	0	0	0	0	0	86
18568429	f	cn	CN	2020	1	0	0	0	0	0	0	0	0	0	0	0	57
18568508	Not	hp	TR	2020	1	0	0	0	0	0	0	0	0	0	0	0	
18568630	m	md	JP	2020	1	0	0	0	0	0	0	0	0	0	0	0	36

18568634	m	cn	JP	2020	0	1	1	0	0	0	0	0	0	0	0	0	71
18568814	Not	hp	TR	2020	1	0	0	0	0	0	0	0	0	0	0	0	
18568816	f	Not repo	CN	2020	1	0	0	0	0	0	0	0	0	0	0	0	48
18568818	f	Not repo	CN	2020	1	0	0	0	0	0	0	0	0	0	0	0	50
18568979	m	md	JP	2020	1	0	0	0	0	0	0	0	0	0	0	0	79
18569088	f	hp	JP	2020	1	0	0	0	0	0	0	0	0	0	0	0	81
18569399	f	Not repo	CN	2020	1	0	0	0	0	0	0	0	0	0	0	0	
18569403	f	md	JP	2020	1	0	0	0	0	0	0	0	0	0	0	0	74
18569563	m	md	NL	2020	0	0	0	0	0	0	0	0	0	0	0	0	50
18569579	m	hp	FR	2020	0	1	1	0	0	0	0	0	0	0	0	0	78
18569582	f	md	JP	2020	1	0	0	0	0	0	0	0	0	0	0	0	70
18569583	f	md	JP	2020	1	0	0	0	0	0	0	0	0	0	0	0	71
18569670	m	md	JP	2020	1	0	0	0	0	0	0	0	0	0	0	0	67
18569671	f	md	JP	2020	1	0	0	0	0	0	0	0	0	0	0	0	89
18569823	f	md	JP	2020	1	0	0	0	0	0	0	0	0	0	0	0	80
18569906	m	cn	FR	2020	0	1	1	0	0	0	0	0	0	0	0	0	71
18569922	m	Not repo	CN	2020	1	0	0	0	0	0	0	0	0	0	0	0	71.6
18570123	m	ph	US	2020	0	0	0	0	0	0	0	0	0	0	0	0	67
18570178	m	md	JP	2020	1	0	0	0	0	0	0	0	0	0	0	0	85
18570192	Not	md	DE	2020	0	1	0	1	0	0	0	0	0	0	0	1	
18570212	f	md	US	2020	0	0	0	0	0	0	0	0	0	0	0	0	66
18570493	m	md	JP	2020	0	1	1	0	0	0	0	0	0	0	0	0	58
18570880	f	md	JP	2020	1	0	0	0	0	0	0	0	0	0	0	1	76
18571404	f	cn	CN	2020	1	0	0	0	0	0	0	0	0	0	0	0	53
18571446	f	cn	CN	2020	1	0	0	0	0	0	0	0	0	0	0	0	64
18571448	f	cn	CN	2020	1	0	0	0	0	0	0	0	0	0	0	0	64
18571449	m	Not repo	CN	2020	1	0	0	0	0	0	0	0	0	0	0	0	55
18571455	m	md	JP	2020	0	1	0	1	0	0	0	0	0	0	0	1	81
18571457	m	Not repo	CN	2020	1	0	0	0	0	0	0	0	0	0	0	0	60
18571517	m	md	FR	2020	0	1	1	0	0	0	0	0	0	0	0	1	66
18571588	f	Not repo	CN	2020	1	0	0	0	0	0	0	0	0	0	0	0	72
18571695	f	cn	CN	2020	1	0	0	0	0	0	0	0	0	0	0	0	58
18571772	m	hp	JP	2020	0	1	0	1	0	0	0	0	0	0	0	1	53
18571951	f	md	AR	2020	0	1	1	0	0	0	0	0	0	0	0	0	44

18572085	f	md	JP	2020	1	0	0	0	0	0	0	0	0	0	0	0	76
18572176	Not	md	NL	2020	0	0	0	0	0	0	0	0	0	0	0	0	50
18572303	m	md	JP	2020	0	1	1	0	0	0	0	0	0	0	0	0	78
18572411	f	cn	Not re	2020	0	0	0	0	0	0	0	0	0	0	0	0	52
18572577	f	md	JP	2020	1	0	0	0	0	0	0	0	0	0	0	0	75
18572874	f	md	TN	2020	0	1	0	0	0	1	0	0	0	0	0	0	62
18573192	f	hp	IL	2020	0	1	1	0	0	0	0	0	0	0	0	0	86
18573424	m	md	FR	2020	0	1	1	0	0	0	0	0	0	0	0	1	66
18573586	f	hp	NL	2020	0	1	0	0	1	0	0	0	0	0	0	0	55
18573595	f	hp	US	2020	0	1	0	1	0	0	0	0	0	0	0	0	80.7
18573698	m	cn	US	2020	0	1	0	0	0	1	0	0	0	0	0	0	67
18574583	m	md	JP	2020	0	1	0	0	0	1	0	0	0	0	0	0	70
18574757	f	md	JP	2020	1	0	0	0	0	0	0	0	0	0	0	1	72
18574864	m	hp	DE	2020	0	0	0	0	0	0	0	0	0	0	0	0	84
18574884	m	Not repo	JP	2020	0	1	0	1	0	0	0	0	0	0	0	1	84
18575108	m	hp	FR	2020	0	0	0	0	0	0	0	0	0	0	0	0	71
18575110	f	hp	FR	2020	0	1	0	0	1	0	0	0	0	0	0	0	51
18575112	f	hp	FR	2020	0	1	0	0	1	0	0	0	0	0	0	0	61
18575181	m	Not repo	AU	2020	0	1	0	1	0	0	0	0	0	0	0	0	
18575258	m	cn	AR	2020	0	0	0	0	0	0	0	0	0	0	0	0	48
18575391	f	cn	US	2020	0	0	0	0	0	0	0	0	0	0	0	0	63
18575482	f	hp	BE	2020	0	0	0	0	0	0	0	0	0	0	0	0	76
18575540	m	md	CN	2020	0	0	0	0	0	0	0	0	0	0	0	0	65
18575554	m	cn	CN	2020	0	1	0	0	1	0	0	0	0	0	0	0	60
18575657	Not	hp	HK	2020	0	1	0	0	0	1	0	0	0	0	0	0	
18575670	Not	hp	HK	2020	0	1	0	0	0	1	0	0	0	0	0	0	
18575671	Not	hp	HK	2020	0	1	0	0	0	1	0	0	0	0	0	0	
18575672	Not	hp	HK	2020	0	1	0	0	0	1	0	0	0	0	0	0	
18575676	Not	hp	HK	2020	0	1	0	0	0	1	0	0	0	0	0	0	
18576087	m	md	JP	2020	0	1	0	1	0	0	0	0	0	0	0	0	73
18576152	m	md	JP	2020	0	1	0	1	0	0	0	0	0	0	0	1	79
18576153	f	md	JP	2020	1	0	0	0	0	0	0	0	0	0	0	0	90
18576409	f	cn	US	2020	0	0	0	0	0	0	0	0	0	0	0	0	54
18576412	m	hp	NL	2020	0	1	1	0	0	0	0	0	0	0	0	1	66

18576458	m	md	JP	2020	1	0	0	0	0	0	0	0	0	0	0	0	
18576620	m	hp	VN	2020	0	1	1	0	0	0	0	0	0	0	0	0	
18576637	m	md	DE	2020	0	1	1	0	0	0	0	0	0	0	0	0	78
18576858	m	md	JP	2020	0	1	0	0	0	1	0	0	0	0	0	1	77
18577045	m	cn	JP	2020	0	1	1	0	0	0	0	0	0	0	0	1	65
18577073	m	md	JP	2020	0	1	1	0	0	0	0	0	0	0	0	0	71
18577567	Not	md	JP	2020	0	1	0	0	0	1	0	0	0	0	0	0	
18577895	m	cn	US	2020	0	0	0	0	0	0	0	0	0	0	0	0	66
18577992	m	md	ES	2020	0	1	0	0	0	1	0	0	0	0	0	0	60
18578403	m	hp	CN	2020	0	0	0	0	0	0	0	0	0	0	0	0	73
18578476	f	md	RU	2020	0	1	0	1	0	0	0	0	0	0	0	0	62
18578711	m	Not repo	MY	2020	1	0	0	0	0	0	0	0	0	0	0	0	66
18579078	m	md	FR	2020	0	1	1	0	0	0	0	0	0	0	0	1	66
18579562	m	md	DE	2020	0	0	0	0	0	0	0	0	0	0	0	0	
18579743	f	md	JP	2020	1	0	0	0	0	0	0	0	0	0	0	0	71
18579747	Not	md	JP	2020	0	1	0	1	0	0	0	0	0	0	0	0	
18579925	f	cn	CA	2020	0	0	0	0	0	0	0	0	0	0	0	0	77
18580088	f	md	US	2020	0	1	0	0	1	0	0	0	0	0	0	0	54
18580129	m	md	DE	2020	0	1	0	1	0	0	0	0	0	0	0	1	56
18580436	Not	cn	JP	2020	0	1	1	0	0	0	0	0	0	0	0	0	80
18580486	f	cn	CN	2020	1	0	0	0	0	0	0	0	0	0	0	0	65
18580751	m	hp	JP	2020	1	0	0	0	0	0	0	0	0	0	0	0	71
18580789	f	hp	JP	2020	1	0	0	0	0	0	0	0	0	0	0	0	89
18580790	f	md	DE	2020	0	1	0	1	0	0	0	0	0	0	0	0	55.8
18580853	f	md	US	2020	0	0	0	0	0	0	0	0	0	0	0	0	82
18580936	f	hp	JP	2020	1	0	0	0	0	0	0	0	0	0	0	0	71
18580989	f	hp	JP	2020	1	0	0	0	0	0	0	0	0	0	0	0	58
18581063	m	hp	JP	2020	1	0	0	0	0	0	0	0	0	0	0	0	78
18581064	f	hp	JP	2020	1	0	0	0	0	0	0	0	0	0	0	0	75
18581071	f	cn	US	2020	1	0	0	0	0	0	0	0	0	0	0	0	64.7
18581123	f	hp	JP	2020	1	0	0	0	0	0	0	0	0	0	0	0	68
18581125	f	hp	JP	2020	1	0	0	0	0	0	0	0	0	0	0	0	65
18581215	m	Not repo	JP	2020	0	1	0	1	0	0	0	0	0	0	0	0	75
18581219	m	md	PL	2020	0	1	0	1	0	0	0	0	0	0	0	0	

18581525	f	ph	FR	2020	0	0	0	0	0	0	0	0	0	0	0	0	68
18581552	m	md	UA	2020	0	0	0	0	0	0	0	0	0	0	0	0	
18581606	f	md	DK	2020	0	0	0	0	0	0	0	0	0	0	0	0	57
18581628	m	Not repo	CN	2020	1	0	0	0	0	0	0	0	0	0	0	0	55.5
18581902	f	cn	US	2020	1	0	0	0	0	0	0	0	0	0	0	0	63.9
18582240	m	cn	US	2020	0	0	0	0	0	0	0	0	0	0	0	0	63
18582798	m	cn	Not re	2020	0	0	0	0	0	0	0	0	0	0	0	0	
18583279	f	hp	IE	2020	0	1	0	0	1	0	0	0	0	0	0	0	72
18583281	m	md	SG	2020	1	0	0	0	0	0	0	0	0	0	0	0	54.1
18583424	m	md	JP	2020	0	1	0	0	1	0	0	0	0	0	0	1	68
18583691	m	cn	GB	2020	0	1	1	0	0	0	0	0	0	0	0	1	66
18584020	f	md	JP	2020	0	1	0	0	1	0	0	0	0	0	0	0	76
18584281	m	md	KR	2020	0	1	0	0	1	0	0	0	0	0	0	0	67
18584345	Not	hp	US	2020	0	1	0	0	1	0	0	0	0	0	0	0	
18584842	m	md	JP	2020	0	1	0	0	0	1	0	0	0	0	0	0	
18585009	m	md	JP	2020	0	1	0	0	1	0	0	0	0	0	0	1	68
18585194	m	cn	Not re	2020	0	0	0	0	0	0	0	0	0	0	0	0	68
18585317	f	hp	PH	2020	0	1	1	0	0	0	0	0	0	0	0	0	65
18585322	f	hp	DK	2020	0	1	1	0	0	0	0	0	0	0	0	0	79
18585499	m	Not repo	JP	2020	0	1	0	1	0	0	0	0	0	0	0	1	63
18586383	f	md	DE	2020	0	0	0	0	0	0	0	0	0	0	0	0	
18586501	f	md	JP	2020	0	1	0	0	0	1	0	0	0	0	0	0	72
18587196	m	ph	JP	2020	0	1	1	0	0	0	0	0	0	0	0	0	69
18587376	f	cn	CZ	2020	1	0	0	0	0	0	0	0	0	0	0	0	61
18587468	f	Not repo	ID	2020	1	0	0	0	0	0	0	0	0	0	0	0	
18587472	m	ph	JP	2020	1	0	0	0	0	0	0	0	0	0	0	0	53
18587571	m	cn	JP	2020	0	1	1	0	0	0	0	0	0	0	0	0	70
18587591	f	cn	US	2020	1	0	0	0	0	0	0	0	0	0	0	0	87
18587858	m	md	FR	2020	0	1	1	0	0	0	0	0	0	0	0	1	66
18588470	m	cn	JP	2020	0	1	1	0	0	0	0	0	0	0	0	1	66
18588598	m	cn	CN	2020	0	0	0	0	0	0	0	0	0	0	0	0	62
18588703	m	hp	NL	2020	0	1	1	0	0	0	0	0	0	0	0	1	66
18589121	m	cn	TH	2020	0	1	0	0	0	1	0	0	0	0	0	0	67
18589204	Not	md	JP	2020	0	0	0	0	0	0	0	0	0	0	0	0	

18589587	f	md	DK	2020	1	0	0	0	0	0	0	0	0	0	0	0	62
18590136	f	md	DE	2020	0	0	0	0	0	0	0	0	0	0	0	0	49
18590368	Not	md	JP	2020	1	0	0	0	0	0	0	0	0	0	0	0	
18590385	f	md	BE	2020	0	0	0	0	0	0	0	0	0	0	0	0	
18590512	f	md	BE	2020	0	1	1	0	0	0	0	0	0	0	0	0	
18590536	Not	ph	JP	2020	1	0	0	0	0	0	0	0	0	0	0	0	
18590951	Not	hp	US	2020	0	0	0	0	0	0	0	0	0	0	0	0	
18590982	Not	md	JP	2020	1	0	0	0	0	0	0	0	0	0	0	0	
18590983	f	md	JP	2020	0	1	0	1	0	0	0	0	0	0	0	0	69
18591033	m	cn	HU	2020	0	0	0	0	0	0	0	0	0	0	0	0	77
18591286	f	hp	IN	2020	0	0	0	0	0	0	0	0	0	0	0	0	60
18591826	f	Not repo	TW	2020	1	0	0	0	0	0	0	0	0	0	0	0	63
18592089	m	md	CN	2020	0	1	1	0	0	0	0	0	0	0	0	0	44
18592136	f	Not repo	CN	2020	1	0	0	0	0	0	0	0	0	0	0	0	69
18592143	m	hp	JP	2020	0	1	0	1	0	0	0	0	0	0	0	1	66
18592300	f	cn	HU	2020	1	0	0	0	0	0	0	0	0	0	0	0	68
18592361	m	md	JP	2020	0	1	1	0	0	0	0	0	0	0	0	0	59
18592392	f	Not repo	ID	2020	0	1	0	1	0	0	0	0	0	0	0	0	52.5
18592686	f	Not repo	AU	2020	1	0	0	0	0	0	0	0	0	0	0	0	
18592847	m	cn	JP	2020	0	1	1	0	0	0	0	0	0	0	0	0	
18592939	Not	md	HK	2020	0	0	0	0	0	0	0	0	0	0	0	0	
18592952	Not	md	HK	2020	1	0	0	0	0	0	0	0	0	0	0	0	
18593001	m	hp	ES	2020	0	1	0	0	1	0	0	0	0	0	0	0	70
18593547	f	cn	US	2020	0	0	0	0	0	0	0	0	0	0	0	0	83
18593717	f	md	DE	2020	0	0	0	0	0	0	0	0	0	0	0	0	49
18594186	Not	Not repo	AU	2020	0	1	0	1	0	0	0	0	0	0	0	0	
18594189	m	hp	US	2020	0	1	0	0	1	0	0	0	0	0	0	0	57
18594342	m	md	FR	2020	0	1	0	1	0	0	0	0	0	0	0	0	
18595177	Not	md	JP	2020	0	1	0	1	0	0	0	0	0	0	0	1	
18595345	f	md	BR	2020	0	0	0	0	0	0	0	0	0	0	0	0	60
18595501	m	md	US	2020	0	0	0	0	0	0	0	0	0	0	0	0	
18595710	m	cn	RU	2020	0	1	1	0	0	0	0	0	0	0	0	0	66
18595769	f	md	IL	2020	1	0	0	0	0	0	0	0	0	0	0	0	83
18596202	m	hp	SG	2020	0	1	0	1	0	0	0	0	0	0	0	0	50.7

18596293	f	md	JP	2020	1	0	0	0	0	0	0	0	0	0	0	0	73
18596563	f	cn	CN	2020	1	0	0	0	0	0	0	0	0	0	0	0	73
18596615	m	hp	DE	2020	0	1	1	0	0	0	0	0	0	0	0	0	78
18597067	f	Not repo	CN	2020	1	0	0	0	0	0	0	0	0	0	0	0	56.3
18597080	m	cn	DE	2020	0	1	1	0	0	0	0	0	0	0	0	0	73
18597140	f	cn	Not re	2020	0	0	0	0	0	0	0	0	0	0	0	0	
18597671	m	md	JP	2020	0	1	0	0	0	1	0	0	0	0	0	0	59
18597766	f	md	HK	2020	0	1	0	0	0	1	0	0	0	0	0	0	
18597797	m	hp	CN	2020	0	1	0	0	1	0	0	0	0	0	0	0	64
18597843	f	md	HK	2020	0	1	0	0	0	1	0	0	0	0	0	0	
18598025	m	md	HK	2020	0	1	0	0	0	1	0	0	0	0	0	0	
18598060	f	cn	CN	2020	1	0	0	0	0	0	0	0	0	0	0	0	74
18598135	m	cn	CN	2020	0	1	0	0	1	0	0	0	0	0	0	0	82
18598208	m	hp	US	2020	0	1	1	0	0	0	0	0	0	0	0	0	89
18598366	f	cn	Not re	2020	0	0	0	0	0	0	0	0	0	0	0	0	
18598415	f	hp	CN	2020	0	0	0	0	0	0	0	0	0	0	0	0	50
18598623	f	md	JP	2020	0	1	0	0	0	1	0	0	0	0	0	0	74
18598673	f	cn	US	2020	0	1	0	0	1	0	0	0	0	0	0	0	
18598678	m	ph	IE	2020	0	0	0	0	0	0	0	0	0	0	0	0	76
18598806	m	hp	JP	2020	0	0	0	0	0	0	0	0	0	0	0	0	70
18599287	m	md	JP	2020	0	1	0	0	0	1	0	0	0	0	0	1	66
18599548	m	hp	JP	2020	0	1	1	0	0	0	0	0	0	0	0	0	73
18599745	f	md	TW	2020	0	1	0	0	0	1	0	0	0	0	0	0	73
18600485	m	md	CZ	2020	0	0	0	0	0	0	0	0	0	0	0	0	75
18600553	f	hp	US	2020	1	0	0	0	0	0	0	0	0	0	0	0	73
18600872	f	md	JP	2020	1	0	0	0	0	0	0	0	0	0	0	0	72
18600899	m	cn	FR	2020	0	1	1	0	0	0	0	0	0	0	0	0	71
18601305	m	md	JP	2020	0	1	0	0	1	0	0	0	0	0	0	1	79
18601367	f	hp	CN	2020	0	0	0	0	0	0	0	0	0	0	0	0	65
18601403	f	md	CZ	2020	0	0	0	0	0	0	0	0	0	0	0	0	
18601415	f	md	IE	2020	0	0	0	0	0	0	1	0	0	0	1	0	60
18601626	f	md	JP	2020	1	0	0	0	0	0	0	0	0	0	0	0	60
18601635	m	cn	Not re	2020	0	0	0	0	0	0	0	0	0	0	0	0	
18601909	m	md	ES	2020	0	1	1	0	0	0	0	0	0	0	0	0	66

18602087	f	hp	IN	2020	0	0	0	0	0	0	0	0	0	0	0	0	50
18602090	m	md	UA	2020	0	1	1	0	0	0	0	0	0	0	0	0	67
18602224	m	hp	US	2020	0	1	1	0	0	0	0	0	0	0	0	0	66
18602602	f	md	JP	2020	0	1	0	0	1	0	0	0	0	0	0	0	71
18602853	Not	Not repo	JP	2020	1	0	0	0	0	0	0	0	0	0	0	0	
18603058	f	hp	US	2020	0	0	0	0	0	0	0	0	0	0	0	0	68
18603499	f	md	JP	2020	0	1	0	0	1	0	0	0	0	0	0	0	66
18603899	m	md	JP	2020	0	1	0	0	0	1	0	0	0	0	0	0	77
18603931	m	md	JP	2020	0	1	0	0	1	0	0	0	0	0	0	0	64
18605130	m	hp	AR	2020	0	1	0	0	0	1	0	0	0	0	0	0	
18605154	f	md	JP	2020	0	1	0	1	0	0	0	0	0	0	0	1	78
18605165	m	hp	DK	2020	0	1	1	0	0	0	0	0	0	0	0	0	71
18605247	m	md	JP	2020	0	1	0	0	0	1	0	0	0	0	0	0	73
18605339	m	md	JP	2020	0	1	0	0	1	0	0	0	0	0	0	1	70
18605443	m	cn	US	2020	0	0	0	0	0	0	0	0	0	0	0	0	58
18605590	m	md	ES	2020	0	0	0	0	0	0	0	0	0	0	0	0	61
18606075	m	Not repo	TW	2020	1	0	0	0	0	0	0	0	0	0	0	0	64
18606242	f	hp	SG	2020	1	0	0	0	0	0	0	0	0	0	0	0	70.8
18606244	Not	cn	VN	2020	1	0	0	0	0	0	0	0	0	0	0	0	
18606364	f	ph	MY	2020	1	0	0	0	0	0	0	0	0	0	0	0	67.7
18606410	f	md	DK	2020	0	1	1	0	0	0	0	0	0	0	0	0	79
18606715	Not	md	US	2020	0	0	0	0	0	0	0	0	0	0	0	0	
18606778	f	cn	CN	2020	1	0	0	0	0	0	0	0	0	0	0	0	70
18606878	f	hp	US	2020	1	0	0	0	0	0	0	0	0	0	0	0	43
18606962	m	md	US	2020	0	1	0	1	0	0	0	0	0	0	0	0	57.3
18607060	f	cn	Not re	2020	0	0	0	0	0	0	0	0	0	0	0	0	
18607341	Not	ph	TR	2020	0	0	0	0	0	0	0	0	0	0	0	0	
18607568	m	md	DK	2020	0	1	1	0	0	0	0	0	0	0	0	0	71
18607679	f	md	CA	2020	1	0	0	0	0	0	0	0	0	0	0	0	
18607691	m	md	CA	2020	1	0	0	0	0	0	0	0	0	0	0	1	55
18607749	m	Not repo	CN	2020	1	0	0	0	0	0	0	0	0	0	0	0	54
18607905	f	cn	US	2020	0	0	0	0	0	0	0	0	0	0	0	0	
18608407	f	cn	US	2020	0	1	1	0	0	0	0	0	0	0	0	1	79
18608882	Not	md	MY	2020	0	1	0	0	0	1	0	0	0	0	0	0	48

18609202	m	cn	CN	2020	0	1	0	0	1	0	0	0	0	0	0	0	76
18609466	f	hp	FR	2020	0	0	0	0	0	0	0	0	0	0	0	0	61
18609477	m	md	DE	2020	1	0	0	0	0	0	0	0	0	0	0	0	71
18609729	Not	md	FR	2020	0	0	0	0	0	0	0	0	0	0	0	0	
18609795	f	cn	JP	2020	0	1	1	0	0	0	0	0	0	0	0	0	60
18609825	m	cn	JP	2020	0	1	1	0	0	0	0	0	0	0	0	0	75
18609872	m	md	JP	2020	0	1	0	0	0	1	0	0	0	0	0	0	82
18609934	m	cn	JP	2020	0	1	1	0	0	0	0	0	0	0	0	0	70
18609989	m	cn	Not re	2020	0	0	0	0	0	0	0	0	0	0	0	0	
18610443	m	md	CN	2020	0	1	0	0	0	1	0	0	0	0	0	0	75
18610836	m	Not repo	JP	2020	1	0	0	0	0	0	0	0	0	0	0	0	76
18612124	m	Not repo	ID	2020	0	1	0	1	0	0	0	0	0	0	0	0	71.4
18612166	f	Not repo	ID	2020	0	1	0	1	0	0	0	0	0	0	0	0	70.6
18612183	m	md	FR	2020	0	1	1	0	0	0	0	0	0	0	0	1	66
18612359	m	Not repo	ID	2020	0	1	0	1	0	0	0	0	0	0	0	0	57.4
18612365	m	cn	SG	2020	1	0	0	0	0	0	0	0	0	0	0	0	
18612480	Not	hp	IT	2020	0	0	0	0	0	0	0	0	0	0	0	0	
18612685	m	cn	JP	2020	0	0	0	0	0	0	0	0	0	0	0	0	70
18614422	m	md	US	2020	0	0	0	0	0	0	0	0	0	0	0	0	
18614785	m	hp	ES	2020	0	1	0	0	1	0	0	0	0	0	0	0	77
18615112	f	hp	US	2020	0	1	0	0	0	1	0	0	0	0	0	0	65
18615140	m	hp	US	2020	0	1	0	0	0	1	0	0	0	0	0	0	45
18615319	f	hp	SG	2020	1	0	0	0	0	0	0	0	0	0	0	0	69.8
18615414	m	cn	JP	2020	0	0	0	0	0	0	0	0	0	0	0	0	71
18615656	m	md	JP	2020	0	0	0	0	0	0	0	0	0	0	0	0	70
18616649	m	cn	CN	2020	0	1	0	0	1	0	0	0	0	0	0	0	59
18616771	m	hp	US	2020	0	1	0	0	0	1	0	0	0	0	0	0	45
18616835	m	hp	SG	2020	0	1	1	0	0	0	0	0	0	0	0	0	41
18616882	m	md	DE	2020	0	0	0	0	0	0	0	0	0	0	0	0	78
18617055	f	md	FR	2020	0	1	0	0	0	1	0	0	0	0	0	0	70
18617121	m	ph	US	2020	0	1	0	1	0	0	0	0	0	0	0	0	
18617351	Not	Not repo	AU	2020	0	1	0	1	0	0	0	0	0	0	0	0	
18618177	f	cn	US	2020	0	0	0	0	0	0	0	0	0	0	0	0	83
18618202	m	ph	US	2020	0	1	1	0	0	0	0	0	0	0	0	1	72

18618230	Not	md	JP	2020	0	0	0	0	0	0	0	0	0	0	0	0	
18618254	m	md	CA	2020	0	0	0	0	0	0	0	0	0	0	0	0	74
18618666	f	ph	US	2020	0	1	1	0	0	0	0	0	0	0	0	0	77
18619503	f	md	NL	2020	1	0	0	0	0	0	0	0	0	0	0	0	
18619567	f	hp	JP	2020	1	0	0	0	0	0	0	0	0	0	0	0	81
18619795	Not	md	US	2020	0	0	0	0	0	0	0	0	0	0	0	0	
18619839	m	md	ES	2020	0	1	1	0	0	0	0	0	0	0	0	0	66
18620435	f	Not repo	JP	2020	1	0	0	0	0	0	0	0	0	0	0	1	83.1
18620637	m	md	JP	2020	0	1	0	0	0	1	0	0	0	0	0	1	64
18620680	m	hp	FR	2020	0	1	0	0	1	0	0	0	0	0	0	0	68
18620773	f	md	RO	2020	1	0	0	0	0	0	0	0	0	0	0	0	70
18621433	m	hp	DE	2020	0	1	0	0	1	0	0	0	0	0	0	0	73
18621631	f	Not repo	ID	2020	1	0	0	0	0	0	0	0	0	0	0	0	41.8
18621870	m	hp	FR	2020	0	1	0	0	1	0	0	0	0	0	0	0	71
18621872	f	hp	FR	2020	0	1	0	0	1	0	0	0	0	0	0	0	51
18621907	f	hp	FR	2020	0	1	0	0	1	0	0	0	0	0	0	0	61
18622121	f	hp	DE	2020	0	1	1	0	0	0	0	0	0	0	0	0	75
18622538	m	cn	FR	2020	0	1	1	0	0	0	0	0	0	0	0	0	65
18622928	m	hp	AU	2020	0	1	0	0	1	0	0	0	0	0	0	0	69
18623005	m	md	JP	2020	1	0	0	0	0	0	0	0	0	0	0	0	
18623025	Not	md	JP	2020	0	0	0	0	0	0	0	0	0	0	0	0	
18623074	f	md	BG	2020	1	0	0	0	0	0	0	0	0	0	0	0	
18623168	f	hp	NL	2020	0	1	1	0	0	0	0	0	0	0	0	0	79
18623169	m	hp	NL	2020	0	1	1	0	0	0	0	0	0	0	0	0	78
18623650	m	md	US	2020	0	1	1	0	0	0	0	0	0	0	0	0	
18623652	m	md	US	2020	0	0	0	0	0	0	0	0	0	0	0	0	
18623654	m	md	US	2020	0	1	1	0	0	0	0	0	0	0	0	0	
18623864	m	cn	US	2020	0	0	0	0	0	0	0	0	0	0	0	0	
18623956	m	md	TR	2020	0	0	0	0	0	0	0	0	0	0	0	0	60
18624133	m	md	US	2020	0	1	1	0	0	0	0	0	0	0	0	0	
18624145	m	md	US	2020	0	1	1	0	0	0	0	0	0	0	0	0	
18624271	m	hp	IT	2020	0	0	0	0	0	0	0	0	0	0	0	0	77
18624625	f	hp	SG	2020	1	0	0	0	0	0	0	0	0	0	0	0	82.6
18624782	f	cn	TH	2020	0	0	0	0	0	0	0	0	0	0	0	0	

18624796	m	md	CN	2020	0	0	0	0	0	0	0	0	0	0	0	0	
18624891	m	cn	AU	2020	0	1	1	0	0	0	0	0	0	0	0	0	74
18625001	m	md	AR	2020	0	0	0	0	0	0	0	0	0	0	0	0	
18625273	m	cn	FR	2020	0	1	1	0	0	0	0	0	0	0	0	0	65
18625294	m	md	US	2020	0	0	0	0	0	0	0	0	0	0	0	0	
18625297	m	md	US	2020	0	1	1	0	0	0	0	0	0	0	0	0	
18625298	m	md	US	2020	0	1	1	0	0	0	0	0	0	0	0	0	
18625301	m	md	US	2020	0	1	1	0	0	0	0	0	0	0	0	0	
18625303	m	md	US	2020	0	1	1	0	0	0	0	0	0	0	0	0	
18626895	f	md	DE	2020	0	0	0	0	0	0	0	0	0	0	0	0	77
18627141	f	cn	US	2020	1	0	0	0	0	0	0	0	0	0	0	0	62
18627656	f	Not repo	JP	2020	0	1	0	1	0	0	0	0	0	0	0	1	73
18627658	Not	md	JP	2020	1	0	0	0	0	0	0	0	0	0	0	0	100
18627757	f	md	NL	2020	0	0	0	0	0	0	0	0	0	0	0	0	69
18628263	m	hp	CN	2020	0	1	0	0	1	0	0	0	0	0	0	0	64
18628806	m	hp	NL	2020	0	1	1	0	0	0	0	0	0	0	0	0	71
18629142	m	hp	JP	2020	0	1	0	1	0	0	0	0	0	0	0	0	57
18629158	f	Not repo	US	2020	0	0	0	0	0	0	0	0	0	0	0	0	93
18629439	m	hp	FR	2020	0	1	0	0	1	0	0	0	0	0	0	0	71
18629524	f	md	JP	2020	0	1	1	0	0	0	0	0	0	0	0	0	73
18629560	m	hp	AU	2020	0	0	0	0	0	0	0	0	0	0	0	0	69
18629935	f	cn	US	2020	0	1	0	0	1	0	0	0	0	0	0	0	
18630226	f	md	HU	2020	0	0	0	0	0	0	0	0	0	0	0	0	
18630353	Not	hp	US	2020	0	0	0	0	0	0	0	0	0	0	0	0	
18630429	f	md	BE	2020	1	0	0	0	0	0	0	0	0	0	0	1	59
18630524	m	cn	JP	2020	0	1	1	0	0	0	0	0	0	0	0	0	80
18630639	f	md	CN	2020	0	1	0	1	0	0	0	0	0	0	0	0	71
18632522	f	hp	US	2020	0	0	0	0	0	0	0	0	0	0	0	0	
18632563	m	hp	BE	2020	0	1	0	0	0	1	0	0	0	0	0	0	61
18632963	f	Not repo	CA	2020	1	0	0	0	0	0	0	0	0	0	0	0	58.7
18632966	m	md	CN	2020	0	0	0	0	0	0	0	0	0	0	0	0	57
18633025	f	cn	Not re	2020	0	0	0	0	0	0	0	0	0	0	0	0	43
18633504	f	md	DE	2020	0	0	0	0	0	0	0	0	0	0	0	0	49
18634187	Not	md	VN	2020	1	0	0	0	0	0	0	0	0	0	0	0	

18634307	m	cn	IL	2020	0	0	0	0	0	0	0	0	0	0	0	0	52
18634958	m	md	JP	2020	0	1	0	0	1	0	0	0	0	0	0	0	60
18635872	m	md	JP	2020	0	0	0	0	0	0	0	0	0	0	0	0	50
18636042	m	md	AU	2020	0	1	1	0	0	0	0	0	0	0	0	0	61
18636073	m	cn	US	2020	1	0	0	0	0	0	0	0	0	0	0	0	77
18636074	Not	hp	JP	2020	0	0	0	0	0	0	0	0	0	0	0	0	
18637125	Not	md	JP	2020	0	1	0	0	0	1	0	0	0	0	0	0	
18637413	m	md	US	2020	1	0	0	0	0	0	0	0	0	0	0	0	46
18637415	m	md	US	2020	1	0	0	0	0	0	0	0	0	0	0	0	
18637430	m	Not repo	JP	2020	1	0	0	0	0	0	0	0	0	0	0	0	76
18637437	Not	Not repo	JP	2020	1	0	0	0	0	0	0	0	0	0	0	0	
18637572	m	Not repo	CN	2020	1	0	0	0	0	0	0	0	0	0	0	0	46.7
18637597	m	hp	US	2020	0	1	1	0	0	0	0	0	0	0	0	0	77
18637721	f	cn	DK	2020	1	0	0	0	0	0	0	0	0	0	0	0	62
18637773	f	cn	SG	2020	1	0	0	0	0	0	0	0	0	0	0	0	79.4
18637971	f	cn	JP	2020	0	1	1	0	0	0	0	0	0	0	0	0	78
18638062	m	md	JP	2020	0	1	0	0	0	1	0	0	0	0	0	0	62
18638764	m	md	US	2020	0	0	0	0	0	0	0	0	0	0	0	0	40
18639007	m	cn	JP	2020	0	0	0	0	0	0	0	0	0	0	0	0	76
18639041	m	hp	CN	2020	0	1	0	0	1	0	0	0	0	0	0	0	53
18639080	f	md	NL	2020	0	1	1	0	0	0	0	0	0	0	0	0	
18639523	f	hp	CN	2020	1	0	0	0	0	0	0	0	0	0	0	0	50
18640777	f	md	FR	2020	0	0	0	0	0	0	0	0	0	0	0	0	62
18641835	m	hp	GB	2020	0	1	1	0	0	0	0	0	0	0	0	0	56
18642050	f	hp	US	2020	0	1	0	0	0	1	0	0	0	0	0	0	65
18642370	m	md	US	2020	0	0	0	0	0	0	0	0	0	0	0	0	70
18642569	m	md	TW	2020	0	0	0	0	0	0	0	0	0	0	0	0	67
18642896	m	md	JP	2020	0	0	0	0	0	0	0	0	0	0	0	0	78
18643153	f	md	US	2020	1	0	0	0	0	0	0	0	0	0	0	0	73
18643299	Not	md	US	2020	0	0	0	0	0	0	0	0	0	0	0	0	
18643450	m	ph	IE	2020	0	0	0	0	0	0	0	0	0	0	0	0	76
18643652	f	cn	CA	2020	0	0	0	0	0	0	0	0	0	0	0	0	75
18643819	m	md	US	2020	1	0	0	0	0	0	0	0	0	0	0	0	
18643821	m	md	US	2020	1	0	0	0	0	0	0	0	0	0	0	0	

18643822	m	md	US	2020	1	0	0	0	0	0	0	0	0	0	0	0	
18644058	m	Not repo	CN	2020	0	1	0	1	0	0	0	0	0	0	0	0	78
18644413	f	cn	JP	2020	0	0	0	0	0	0	0	0	0	0	0	0	84
18644421	f	md	FR	2020	0	1	0	1	0	0	0	0	0	0	0	1	47
18644568	m	md	RO	2020	0	0	0	0	0	0	0	0	0	0	0	0	
18645033	f	hp	DE	2020	0	0	0	0	0	0	0	0	0	0	0	0	68
18645744	f	cn	CN	2020	0	1	0	0	1	0	0	0	0	0	0	0	
18647000	m	hp	US	2020	0	1	1	0	0	0	0	0	0	0	0	0	77
18648068	m	hp	SG	2020	0	1	0	1	0	0	0	0	0	0	0	0	71.2
18648090	m	cn	DE	2020	0	1	1	0	0	0	0	0	0	0	0	0	54
18649156	Not	md	JP	2020	0	1	0	1	0	0	0	0	0	0	0	0	79
18649192	m	md	FR	2020	0	1	0	0	0	1	0	0	0	0	0	0	53
18650338	m	hp	ES	2020	0	0	0	0	0	0	0	0	0	0	0	0	
18650409	f	hp	ES	2020	0	0	0	0	0	0	0	0	0	0	0	0	64
18650718	f	Not repo	TW	2020	1	0	0	0	0	0	0	0	0	0	0	0	59
18651315	f	md	CN	2020	0	0	0	0	0	0	0	0	0	0	0	0	31
18651389	m	hp	DE	2020	0	1	0	0	1	0	0	0	0	0	0	0	77
18651391	m	md	IT	2020	0	1	0	0	0	1	0	0	0	0	0	0	53
18651601	f	md	NO	2020	0	1	1	0	0	0	0	0	0	0	0	0	68
18651676	Not	cn	US	2020	0	1	0	0	1	0	0	0	0	0	0	0	
18652461	f	cn	JP	2020	0	0	0	0	0	0	0	0	0	0	0	0	90
18653143	f	md	JP	2020	1	0	0	0	0	0	0	0	0	0	0	1	
18654129	f	cn	US	2020	0	0	0	0	0	0	0	0	0	0	0	0	
18654442	f	cn	CA	2020	0	0	0	0	0	0	0	0	0	0	0	0	33
18654734	Not	md	JP	2020	1	0	0	0	0	0	0	0	0	0	0	0	
18654735	f	cn	SG	2020	1	0	0	0	0	0	0	0	0	0	0	0	64.9
18654897	Not	hp	US	2020	1	0	0	0	0	0	0	0	0	0	0	0	
18655018	m	ph	IE	2020	0	0	0	0	0	0	0	0	0	0	0	0	76
18655086	m	md	JP	2020	0	1	0	0	0	1	0	0	0	0	0	0	78
18655125	m	md	US	2020	0	1	0	0	0	1	0	0	0	0	0	0	81
18655190	m	md	PL	2020	0	0	0	0	0	0	0	0	0	0	0	0	
18655420	m	md	FR	2020	1	0	0	0	0	0	0	0	0	0	0	0	66
18655684	f	md	JP	2020	1	0	0	0	0	0	0	0	0	0	0	0	
18655750	f	md	US	2020	0	1	1	0	0	0	0	0	0	0	0	0	54

18655958	f	md	IL	2020	0	0	0	0	0	0	0	0	0	0	0	0	
18656071	m	md	JP	2020	1	0	0	0	0	0	0	0	0	0	0	1	73
18656176	Not	hp	DE	2020	1	0	0	0	0	0	0	0	0	0	0	0	
18656189	Not	md	US	2020	1	0	0	0	0	0	0	0	0	0	0	0	
18656382	f	Not repo	CA	2020	1	0	0	0	0	0	0	0	0	0	0	0	73.2
18657718	Not	ph	US	2020	0	0	0	0	0	0	0	0	0	0	0	0	
18657848	f	cn	ID	2020	1	0	0	0	0	0	0	0	0	0	0	0	65
18657852	f	md	CA	2020	1	0	0	0	0	0	0	0	0	0	0	0	
18657917	f	hp	DE	2020	0	1	0	0	1	0	0	0	0	0	0	1	66
18658033	f	cn	CN	2020	0	1	0	0	1	0	0	0	0	0	0	0	62
18658238	m	md	US	2020	0	1	1	0	0	0	0	0	0	0	0	0	77
18658425	f	md	ES	2020	0	1	0	0	1	0	0	0	0	0	0	0	70
18658878	m	cn	Not re	2020	0	0	0	0	0	0	0	0	0	0	0	0	
18659028	m	cn	IL	2020	0	0	0	0	0	0	0	0	0	0	0	0	
18659171	f	ph	BE	2020	0	1	0	0	0	1	0	0	0	0	0	0	61
18659247	f	hp	JP	2020	0	1	0	0	0	1	0	0	0	0	0	0	63
18659264	m	cn	US	2020	0	1	1	0	0	0	0	0	0	0	0	0	
18659274	f	cn	Not re	2020	0	0	0	0	0	0	0	0	0	0	0	0	52
18660226	m	md	NL	2020	0	0	0	0	0	0	0	0	0	0	0	0	49
18661120	m	md	FR	2020	1	0	0	0	0	0	0	0	0	0	0	0	
18661310	Not	Not repo	JP	2020	0	1	0	1	0	0	0	0	0	0	0	0	
18661436	m	hp	IN	2020	0	0	0	0	0	0	0	0	0	0	0	0	69
18661609	f	md	NL	2020	0	0	0	0	0	0	0	0	0	0	0	0	70
18661755	f	cn	SG	2020	1	0	0	0	0	0	0	0	0	0	0	0	85.9
18662306	f	Not repo	SG	2020	0	1	0	1	0	0	0	0	0	0	0	0	62.6
18662632	f	cn	CA	2020	0	0	0	0	0	0	0	0	0	0	0	0	59
18663333	f	cn	SG	2020	1	0	0	0	0	0	0	0	0	0	0	0	60.5
18663549	m	Not repo	ID	2020	1	0	0	0	0	0	0	0	0	0	0	0	60.8
18663756	m	cn	US	2020	0	1	0	1	0	0	0	0	0	0	0	0	61.3
18664655	Not	Not repo	AU	2020	1	0	0	0	0	0	0	0	0	0	0	0	
18664694	f	hp	IN	2020	0	0	0	0	0	0	0	0	0	0	0	0	68
18664781	m	cn	US	2020	1	0	0	0	0	0	0	0	0	0	0	0	70
18664828	m	md	ES	2020	0	1	0	0	0	1	0	0	0	0	0	0	61
18664834	m	hp	IE	2020	0	0	0	0	0	0	0	0	0	0	0	0	76

18664863	m	Not repo	CN	2020	0	1	0	1	0	0	0	0	0	0	0	0	53
18665369	m	Not repo	JP	2020	0	1	0	1	0	0	0	0	0	0	0	0	80
18665497	m	md	JP	2020	0	1	0	0	0	1	0	0	0	0	0	0	
18665722	f	md	CN	2020	0	0	0	0	0	0	0	0	0	0	0	0	
18665873	m	cn	Not re	2020	0	0	0	0	0	0	0	0	0	0	0	0	
18666242	m	hp	IE	2020	0	0	0	0	0	0	0	0	0	0	0	0	76
18666551	m	cn	Not re	2020	0	0	0	0	0	0	0	0	0	0	0	0	
18666553	m	cn	Not re	2020	0	0	0	0	0	0	0	0	0	0	0	0	
18666666	m	ph	CN	2020	0	1	0	0	1	0	0	0	0	0	0	0	
18666943	m	md	JP	2020	0	1	0	0	1	0	0	0	0	0	0	0	75
18667442	Not	md	JP	2020	0	0	0	0	0	0	0	0	0	0	0	0	40
18667470	m	md	AU	2020	0	1	0	0	0	1	0	0	0	0	0	0	
18667574	m	md	JP	2020	0	1	0	0	1	0	0	0	0	0	0	1	73
18667844	f	md	US	2020	0	0	0	0	0	0	0	0	0	0	0	0	77
18668458	m	hp	RO	2020	1	0	0	0	0	0	0	0	0	0	0	0	
18669703	f	cn	CN	2020	0	1	0	0	1	0	0	0	0	0	0	0	46
18669760	m	md	MY	2020	1	0	0	0	0	0	0	0	0	0	0	0	
18669979	m	ph	JP	2020	0	0	0	0	0	0	0	0	0	0	0	0	73
18670852	m	cn	Not re	2020	0	0	0	0	0	0	0	0	0	0	0	0	
18671344	f	hp	NO	2020	0	1	1	0	0	0	0	0	0	0	0	0	68
18671426	m	cn	CN	2020	0	1	0	1	0	0	0	0	0	0	0	0	83
18671917	f	cn	JP	2020	0	1	1	0	0	0	0	0	0	0	0	0	80
18672141	f	hp	AU	2020	0	0	0	0	0	0	0	0	0	0	0	0	57
18672342	Not	md	JP	2020	0	1	0	0	0	1	0	0	0	0	0	0	64
18672593	m	cn	CN	2020	0	1	0	1	0	0	0	0	0	0	0	0	58
18672832	m	md	BE	2020	0	1	0	0	0	1	0	0	0	0	0	0	61
18672967	m	md	JP	2020	0	1	1	0	0	0	0	0	0	0	0	1	66
18673123	m	md	US	2020	0	1	1	0	0	0	0	0	0	0	0	0	81
18673293	f	md	MY	2020	1	0	0	0	0	0	0	0	0	0	0	0	
18673463	m	cn	JP	2020	0	1	1	0	0	0	0	0	0	0	0	0	77
18673565	m	md	US	2020	0	1	1	0	0	0	0	0	0	0	0	0	58
18673614	m	md	US	2020	0	1	1	0	0	0	0	0	0	0	0	0	76
18673693	Not	ph	JP	2020	0	0	0	0	0	0	0	0	0	0	0	0	60
18673733	m	md	VN	2020	1	0	0	0	0	0	0	0	0	0	0	0	36.6

18674021	f	md	NO	2020	0	1	1	0	0	0	0	0	0	0	0	0	68
18674444	m	cn	CN	2020	0	1	0	0	1	0	0	0	0	0	0	0	53
18675098	f	cn	Not re	2020	0	0	0	0	0	0	0	0	0	0	0	0	65
18675221	m	cn	JP	2020	0	0	0	0	0	0	0	0	0	0	0	0	75
18676412	m	cn	JP	2020	0	0	0	0	0	0	0	0	0	0	0	0	78
18676493	f	cn	Not re	2020	0	0	0	0	0	0	0	0	0	0	0	0	
18676563	Not	md	JP	2020	0	1	1	0	0	0	0	0	0	0	0	0	75
18676591	f	cn	Not re	2020	0	0	0	0	0	0	0	0	0	0	0	0	
18676914	m	cn	FR	2020	0	1	1	0	0	0	0	0	0	0	0	0	65
18677976	f	md	DE	2020	0	0	0	0	0	0	0	0	0	0	0	0	49
18678018	f	hp	US	2020	0	1	0	1	0	0	0	0	0	0	0	0	56
18678275	m	md	US	2020	0	0	0	0	0	0	0	0	0	0	0	0	75
18678571	Not	md	JP	2020	1	0	0	0	0	0	0	0	0	0	0	0	
18678592	m	Not repo	CN	2020	0	1	0	1	0	0	0	0	0	0	0	0	88
18678704	m	hp	US	2020	0	1	0	0	1	0	0	0	0	0	0	0	57
18679266	Not	md	IN	2020	1	0	0	0	0	0	0	0	0	0	0	0	
18679439	m	cn	JP	2020	0	1	1	0	0	0	0	0	0	0	0	0	70
18679547	m	md	CN	2020	0	0	0	0	0	0	0	0	0	0	0	0	45
18679808	m	cn	Not re	2020	0	0	0	0	0	0	0	0	0	0	0	0	74
18680161	m	hp	AU	2020	0	0	0	0	0	0	0	0	0	0	0	0	69
18681646	m	md	US	2020	0	0	0	0	0	0	0	0	0	0	0	0	60
18683210	Not	ph	JP	2020	0	1	0	0	0	1	0	0	0	0	0	0	
18683459	m	ph	JP	2020	0	0	0	0	0	0	0	0	0	0	0	0	76
18683652	m	hp	JP	2020	0	1	0	0	0	1	0	0	0	0	0	0	70
18684030	f	cn	Not re	2020	0	0	0	0	0	0	0	0	0	0	0	0	
18684498	m	md	JP	2020	0	1	0	1	0	0	0	0	0	0	0	0	67
18684500	f	md	US	2020	1	0	0	0	0	0	0	0	0	0	0	0	
18685195	m	md	JP	2020	0	1	0	0	1	0	0	0	0	0	0	0	80
18685973	m	hp	US	2020	0	1	0	0	1	0	0	0	0	0	0	0	70
18686486	f	Not repo	JP	2020	1	0	0	0	0	0	0	0	0	0	0	0	67
18686740	m	md	JP	2020	0	1	0	0	0	1	0	0	0	0	0	0	70
18687972	m	md	JP	2020	0	1	0	1	0	0	0	0	0	0	0	1	68
18688246	f	md	JP	2020	0	1	0	0	0	1	0	0	0	0	0	0	77
18688286	f	md	JP	2020	1	0	0	0	0	0	0	0	0	0	0	0	76

18688449	m	ph	JP	2020	0	1	0	1	0	0	0	0	0	0	0	0	60
18688547	Not	hp	TR	2020	1	0	0	0	0	0	0	0	0	0	0	0	
18688555	Not	hp	US	2020	1	0	0	0	0	0	0	0	0	0	0	0	
18688703	Not	hp	TR	2020	1	0	0	0	0	0	0	0	0	0	0	0	
18688713	Not	hp	TR	2020	1	0	0	0	0	0	0	0	0	0	0	0	
18688914	Not	hp	US	2020	1	0	0	0	0	0	0	0	0	0	0	0	
18688947	f	md	JP	2020	1	0	0	0	0	0	0	0	0	0	0	0	69
18689671	m	hp	SG	2020	1	0	0	0	0	0	0	0	0	0	0	0	86.3
18689805	f	Not repo	ID	2020	1	0	0	0	0	0	0	0	0	0	0	0	82.8
18690180	f	md	NO	2020	0	1	1	0	0	0	0	0	0	0	0	0	68
18690384	f	hp	ES	2020	0	1	1	0	0	0	0	0	0	0	0	0	66
17953386	f	cn	Not re	2021	0	0	0	0	0	0	0	0	0	0	0	0	78
17968222	m	hp	DE	2021	0	1	1	0	0	0	0	0	0	0	0	0	65
18063941	m	cn	Not re	2021	0	0	0	0	0	0	0	0	0	0	0	0	
18126912	m	cn	FR	2021	0	1	1	0	0	0	0	0	0	0	0	0	78
18129557	f	hp	FR	2021	0	0	0	0	0	0	0	0	0	0	0	0	69
18164360	m	md	DE	2021	0	1	1	0	0	0	0	0	0	0	0	0	51
18165590	f	md	JP	2021	0	1	0	0	0	1	0	0	0	0	0	0	67
18188225	m	md	JP	2021	0	1	0	0	1	0	0	0	0	0	0	0	55
18206478	m	md	JP	2021	0	0	0	0	0	0	0	0	0	0	0	0	67
18215230	m	hp	DE	2021	0	1	0	0	0	1	0	0	0	0	0	0	68
18323993	f	cn	US	2021	0	0	0	0	0	0	0	0	0	0	0	0	
18333393	m	cn	IL	2021	0	0	0	0	0	0	0	0	0	0	0	0	85
18336890	Not	md	JP	2021	0	0	0	0	0	0	0	0	0	0	0	0	
18338380	Not	md	JP	2021	0	1	0	1	0	0	0	0	0	0	0	0	
18349342	Not	md	JP	2021	0	1	0	0	1	0	0	0	0	0	0	0	
18350902	m	md	KR	2021	0	1	0	0	1	0	0	0	0	0	0	0	69
18355619	m	md	FR	2021	0	1	1	0	0	0	0	0	0	0	0	0	64
18357186	Not	md	JP	2021	0	1	0	0	1	0	0	0	0	0	0	0	
18372759	m	md	JP	2021	0	1	0	0	1	0	0	0	0	0	0	0	68
18379972	f	hp	US	2021	0	1	0	0	1	0	0	0	0	0	0	0	58
18394909	f	hp	US	2021	0	1	0	0	1	0	0	0	0	0	0	0	68
18427521	f	hp	KR	2021	0	0	0	0	0	0	0	0	0	0	0	0	58
18435047	f	cn	Not re	2021	0	0	0	0	0	0	0	0	0	0	0	0	54

18452072	f	md	US	2021	0	1	1	0	0	0	0	0	0	0	0	0	75
18455835	f	md	CN	2021	0	1	0	0	0	1	0	0	0	0	0	0	47
18461678	m	hp	JP	2021	0	1	0	0	1	0	0	0	0	0	0	0	75
18470736	m	md	FR	2021	0	1	0	0	0	1	0	0	0	0	0	0	47
18484612	f	hp	ES	2021	0	1	1	0	0	0	0	0	0	0	0	0	84
18486018	f	cn	US	2021	0	1	0	0	1	0	0	0	0	0	0	0	70
18504821	m	md	US	2021	0	1	1	0	0	0	0	0	0	0	0	0	67
18527972	m	md	TR	2021	0	1	1	0	0	0	0	0	0	0	0	0	70
18546368	m	hp	CN	2021	0	1	0	0	1	0	0	0	0	0	0	0	64
18547356	m	cn	Not re	2021	0	0	0	0	0	0	0	0	0	0	0	0	
18549880	m	md	DE	2021	0	1	0	0	0	1	0	0	0	0	0	0	68
18550109	f	md	JP	2021	0	0	0	0	0	0	0	0	0	0	0	0	79
18554848	m	hp	TR	2021	0	0	0	0	0	0	0	0	0	0	0	0	71
18555659	m	md	JP	2021	0	1	0	1	0	0	0	0	0	0	0	1	65
18555920	f	cn	US	2021	0	1	1	0	0	0	0	0	0	0	0	0	74
18562741	m	md	US	2021	0	1	1	0	0	0	0	0	0	0	0	0	71
18563071	f	md	ES	2021	0	1	1	0	0	0	0	0	0	0	0	0	60
18564363	f	md	AU	2021	0	1	1	0	0	0	0	0	0	0	0	0	73
18566274	f	md	JP	2021	0	1	0	0	1	0	0	0	0	0	0	0	77
18566329	m	md	TW	2021	0	1	1	0	0	0	0	0	0	0	0	0	71
18567325	f	cn	CN	2021	0	0	0	0	0	0	0	0	0	0	0	0	62
18568289	m	md	JP	2021	0	1	0	0	0	1	0	0	0	0	0	0	51
18569042	m	md	Not re	2021	0	0	0	0	0	0	0	0	0	0	0	0	75
18572078	f	cn	Not re	2021	0	0	0	0	0	0	0	0	0	0	0	0	
18580031	m	cn	FR	2021	0	0	0	0	0	0	0	0	0	0	0	0	65
18580905	m	md	HK	2021	0	0	0	0	0	0	0	0	0	0	0	0	70
18581901	m	md	JP	2021	1	0	0	0	0	0	0	0	0	0	0	0	79
18587734	f	Not repo	CN	2021	1	0	0	0	0	0	0	0	0	0	0	1	65
18588115	m	ph	FR	2021	0	1	1	0	0	0	0	0	0	0	0	0	64
18592421	f	md	HK	2021	0	1	0	0	0	1	0	0	0	0	0	0	67
18592459	m	md	CN	2021	0	1	0	1	0	0	0	0	0	0	0	0	60.6
18592558	m	md	HK	2021	0	1	0	0	0	1	0	0	0	0	0	0	71
18592625	m	md	HK	2021	0	1	0	0	0	1	0	0	0	0	0	0	56
18595397	f	ph	JP	2021	0	1	0	0	1	0	0	0	0	0	0	0	79

18598631	m	cn	JP	2021	0	1	1	0	0	0	0	0	0	0	0	0	65
18599935	f	hp	AU	2021	0	1	0	1	0	0	0	0	0	0	0	1	84
18601508	f	md	UA	2021	0	1	0	0	0	1	0	0	0	0	0	0	71
18602399	m	hp	US	2021	0	1	0	0	1	0	0	0	0	0	0	0	68
18603039	m	cn	HU	2021	0	0	0	0	0	0	0	0	0	0	0	0	77
18606457	m	md	JP	2021	0	1	0	0	1	0	0	0	0	0	0	0	64
18606763	m	hp	CN	2021	0	1	0	0	1	0	0	0	0	0	0	1	78
18606766	f	hp	TW	2021	0	1	1	0	0	0	0	0	0	0	0	0	67
18607630	f	md	US	2021	0	1	0	0	0	1	0	0	0	0	0	0	90
18619496	m	md	CN	2021	0	0	0	0	0	0	0	0	0	0	0	0	58
18620794	f	md	US	2021	0	1	0	0	0	1	0	0	0	0	0	0	90
18624035	m	hp	NL	2021	0	1	1	0	0	0	0	0	0	0	0	0	65
18627614	m	md	DE	2021	0	0	0	0	0	0	0	0	0	0	0	0	55
18628200	f	hp	FR	2021	0	0	0	0	0	0	0	0	0	0	0	0	55
18632304	f	md	TR	2021	0	1	0	0	0	1	0	0	0	0	0	0	60
18643004	f	hp	US	2021	0	1	0	0	1	0	0	0	0	0	0	0	72
18643155	f	ph	US	2021	0	0	0	0	0	0	0	0	0	0	0	0	
18644136	f	md	NL	2021	0	0	0	0	0	0	0	0	0	0	0	0	55
18645447	m	ph	US	2021	0	0	0	0	0	0	0	0	0	0	0	0	66
18652244	m	hp	CN	2021	0	1	0	0	1	0	0	0	0	0	0	0	72
18652593	f	md	DE	2021	1	0	0	0	0	0	0	0	0	0	0	0	
18652822	f	md	DE	2021	1	0	0	0	0	0	0	0	0	0	0	0	
18658875	f	hp	DE	2021	0	1	0	0	1	0	0	0	0	0	0	1	55
18659986	m	cn	CN	2021	1	0	0	0	0	0	0	0	0	0	0	0	57
18665237	f	cn	US	2021	1	0	0	0	0	0	0	0	0	0	0	0	46.9
18665343	f	md	IN	2021	1	0	0	0	0	0	0	0	0	0	0	0	53.4
18665872	m	md	KR	2021	0	1	0	0	0	1	0	0	0	0	0	0	66
18670507	m	md	JP	2021	0	1	0	1	0	0	0	0	0	0	0	0	70.5
18672175	m	md	CN	2021	0	1	0	0	0	1	0	0	0	0	0	0	63
18675612	f	cn	Not re	2021	0	0	0	0	0	0	0	0	0	0	0	0	
18676860	f	md	JP	2021	0	0	0	0	0	0	0	0	0	0	0	0	84
18677664	m	md	CH	2021	0	1	0	0	0	1	0	0	0	0	0	0	67
18687430	f	md	CN	2021	1	0	0	0	0	0	0	0	0	0	0	0	73
18689417	f	hp	ES	2021	0	1	1	0	0	0	0	0	0	0	0	0	71

18691410	m	md	JP	2021	0	1	0	1	0	0	0	0	0	0	0	0	75
18691536	f	ph	FR	2021	1	0	0	0	0	0	0	0	0	0	0	1	76
18691799	m	md	JP	2021	0	1	0	1	0	0	0	0	0	0	0	1	59
18692606	f	hp	IT	2021	0	0	0	0	0	0	0	0	0	0	0	0	83
18693836	Not	md	CN	2021	0	0	0	0	0	0	0	0	0	0	0	0	
18693874	f	hp	CN	2021	0	1	0	1	0	0	0	0	0	0	0	0	82
18693877	m	hp	CN	2021	1	0	0	0	0	0	0	0	0	0	0	0	53
18694851	f	Not repo	JP	2021	1	0	0	0	0	0	0	0	0	0	0	0	
18694854	m	Not repo	CN	2021	0	1	0	1	0	0	0	0	0	0	0	0	80
18694925	f	ph	US	2021	0	1	1	0	0	0	0	0	0	0	0	0	75
18695010	m	cn	CN	2021	0	1	0	0	1	0	0	0	0	0	0	0	
18695290	m	md	CN	2021	0	0	0	0	0	0	0	0	0	0	0	0	70
18695446	f	hp	NL	2021	0	1	1	0	0	0	0	0	0	0	0	0	68
18696042	m	md	ES	2021	0	0	0	0	0	0	0	0	0	0	0	0	65
18696900	m	Not repo	CN	2021	0	1	0	1	0	0	0	0	0	0	0	0	85.7
18697057	f	hp	US	2021	0	1	1	0	0	0	0	0	0	0	0	0	65
18697089	f	md	NO	2021	0	1	1	0	0	0	0	0	0	0	0	0	68
18697169	f	hp	US	2021	0	1	1	0	0	0	0	0	0	0	0	0	64
18697485	Not	cn	US	2021	0	1	0	0	1	0	0	0	0	0	0	0	
18697627	m	md	US	2021	0	1	1	0	0	0	0	0	0	0	0	0	58
18698230	f	md	CL	2021	0	1	1	0	0	0	0	0	0	0	0	0	56
18699143	m	hp	DE	2021	0	1	1	0	0	0	0	0	0	0	0	0	
18700370	f	hp	IT	2021	0	1	1	0	0	0	0	0	0	0	0	0	67
18700548	m	md	CZ	2021	0	0	0	0	0	0	0	0	0	0	0	0	64
18701099	f	md	FR	2021	0	1	0	0	0	1	0	0	0	0	0	0	37
18701959	m	md	DE	2021	0	1	0	0	1	0	0	0	0	0	0	0	71
18702009	m	hp	DE	2021	0	0	0	0	0	0	0	0	0	0	0	0	55
18702236	Not	cn	SG	2021	1	0	0	0	0	0	0	0	0	0	0	0	92
18702393	Not	cn	SG	2021	1	0	0	0	0	0	0	0	0	0	0	0	80.9
18702494	f	md	GB	2021	0	1	0	0	0	1	0	0	0	0	0	0	82
18703382	m	cn	Not re	2021	0	0	0	0	0	0	0	0	0	0	0	0	
18703424	f	md	FR	2021	0	0	0	0	0	0	0	0	0	0	0	0	37
18703832	f	cn	US	2021	0	0	0	0	0	0	0	0	0	0	0	0	65
18703984	m	md	FR	2021	0	1	0	0	0	1	0	0	0	0	0	0	53

18704346	Not	hp	CN	2021	0	1	0	0	1	0	0	0	0	0	0	0	
18704907	m	md	JP	2021	0	1	0	0	0	1	0	0	0	0	0	0	76
18705460	m	hp	DE	2021	0	1	0	0	0	1	0	0	0	0	0	0	79
18705581	f	md	JP	2021	0	0	0	0	0	0	0	0	0	0	0	0	78
18706270	f	hp	NL	2021	0	1	1	0	0	0	0	0	0	0	0	0	62
18706508	m	hp	ES	2021	0	1	1	0	0	0	0	0	0	0	0	0	68
18706546	f	md	US	2021	1	0	0	0	0	0	0	0	0	0	0	0	
18707165	m	cn	US	2021	0	0	0	0	0	0	0	0	0	0	0	0	73
18707461	f	md	US	2021	0	0	0	0	0	0	0	0	0	0	0	0	
18707542	f	cn	Not re	2021	0	0	0	0	0	0	0	0	0	0	0	0	
18708315	m	ph	CA	2021	0	0	0	0	0	0	0	0	0	0	0	0	59
18708478	f	cn	Not re	2021	0	0	0	0	0	0	0	0	0	0	0	0	63
18709113	f	hp	US	2021	0	1	1	0	0	0	0	0	0	0	0	0	73
18709211	Not	hp	JP	2021	0	1	0	0	0	1	0	0	0	0	0	1	
18709921	m	hp	US	2021	0	1	0	0	1	0	0	0	0	0	0	0	41
18710487	m	md	AT	2021	0	1	0	0	0	1	0	0	0	0	0	0	75
18710597	f	hp	US	2021	0	1	1	0	0	0	0	0	0	0	0	0	64
18710910	m	md	FR	2021	0	1	1	0	0	0	0	0	0	0	0	0	61
18710956	m	md	US	2021	0	1	1	0	0	0	0	0	0	0	0	0	58
18712150	m	hp	TW	2021	0	0	0	0	0	0	0	0	0	0	0	0	75
18712389	f	hp	US	2021	0	1	1	0	0	0	0	0	0	0	0	0	64
18712423	f	md	DE	2021	0	0	0	0	0	0	0	0	0	0	0	0	63
18713198	f	lw	DK	2021	0	1	1	0	0	0	0	0	0	0	0	0	73
18713550	m	hp	IL	2021	0	0	0	0	0	0	0	0	0	0	0	0	43
18713951	f	hp	US	2021	0	0	0	0	0	0	0	0	0	0	0	0	72
18714077	m	md	FR	2021	0	1	1	0	0	0	0	0	0	0	0	0	61
18714163	f	hp	US	2021	0	0	0	0	0	0	0	0	0	0	0	0	71
18714544	f	cn	Not re	2021	0	0	0	0	0	0	0	0	0	0	0	0	
18714655	f	md	US	2021	0	0	0	0	0	0	0	0	0	0	0	0	
18715935	m	md	MN	2021	0	0	0	0	0	0	0	0	0	0	0	0	70
18715953	m	hp	DE	2021	0	1	1	0	0	0	0	0	0	0	0	1	64
18715962	f	hp	US	2021	0	1	1	0	0	0	0	0	0	0	0	0	64
18716340	f	Not repo	CN	2021	0	1	0	1	0	0	0	0	0	0	0	0	75
18716522	f	hp	FR	2021	0	1	0	0	1	0	0	0	0	0	0	0	75

18716949	f	Not repo	TW	2021	1	0	0	0	0	0	0	0	0	0	0	0	88
18717468	m	hp	CN	2021	0	1	0	0	1	0	0	0	0	0	0	0	60
18717907	f	md	NL	2021	0	0	0	0	0	0	0	0	0	0	0	0	65
18717940	m	cn	JP	2021	0	1	1	0	0	0	0	0	0	0	0	0	72
18718135	m	md	IN	2021	0	0	0	0	0	0	0	0	0	0	0	0	39
18718147	f	hp	US	2021	1	0	0	0	0	0	0	0	0	0	0	0	67
18718158	m	md	NL	2021	0	1	1	0	0	0	0	0	0	0	0	0	78
18718324	f	md	DE	2021	0	1	0	0	1	0	0	0	0	0	0	0	73
18718663	m	cn	CN	2021	0	1	0	1	0	0	0	0	0	0	0	0	66
18718679	f	Not repo	CN	2021	0	1	0	1	0	0	0	0	0	0	0	0	65
18718684	m	Not repo	CN	2021	0	1	0	1	0	0	0	0	0	0	0	0	74
18718751	f	cn	CA	2021	1	0	0	0	0	0	0	0	0	0	0	0	82.4
18718752	f	md	US	2021	0	1	0	1	0	0	0	0	0	0	0	0	
18719128	m	Not repo	CN	2021	0	1	0	1	0	0	0	0	0	0	0	0	82
18720583	m	Not repo	ID	2021	1	0	0	0	0	0	0	0	0	0	0	0	55.4
18721029	m	Not repo	CN	2021	0	1	0	1	0	0	0	0	0	0	0	0	73
18721164	f	cn	Not re	2021	0	0	0	0	0	0	0	0	0	0	0	0	46
18722591	m	cn	JP	2021	0	1	1	0	0	0	0	0	0	0	0	0	70
18723866	m	Not repo	CN	2021	0	1	0	1	0	0	0	0	0	0	0	0	67
18724051	f	cn	JP	2021	0	1	1	0	0	0	0	0	0	0	0	0	84
18726176	m	md	FR	2021	0	0	0	0	0	0	0	0	0	0	0	0	68
18727089	Not	md	CN	2021	0	0	0	0	0	0	0	0	0	0	0	0	
18727115	m	hp	JP	2021	0	0	0	0	0	0	0	0	0	0	0	0	60
18727530	m	Not repo	CN	2021	0	1	0	1	0	0	0	0	0	0	0	0	58
18727531	m	Not repo	ID	2021	1	0	0	0	0	0	0	0	0	0	0	0	78.7
18728138	f	hp	US	2021	0	1	1	0	0	0	0	0	0	0	0	0	64
18728664	m	cn	JP	2021	0	1	1	0	0	0	0	0	0	0	0	0	75
18728750	Not	ph	CN	2021	0	0	0	0	0	0	0	0	0	0	0	0	
18729549	f	md	JP	2021	1	0	0	0	0	0	0	0	0	0	0	0	
18729642	Not	md	JP	2021	1	0	0	0	0	0	0	0	0	0	0	0	
18729649	m	md	CA	2021	1	0	0	0	0	0	0	0	0	0	0	0	
18729653	f	md	JP	2021	1	0	0	0	0	0	0	0	0	0	0	0	86
18729836	m	md	JP	2021	1	0	0	0	0	0	0	0	0	0	0	0	78
18729918	m	Not repo	CN	2021	0	1	0	1	0	0	0	0	0	0	0	0	58

18729920	m	Not repo	CN	2021	0	1	0	1	0	0	0	0	0	0	0	0	70
18729941	m	md	KR	2021	0	1	0	1	0	0	0	0	0	0	0	0	
18730528	m	hp	IT	2021	0	0	0	0	0	0	0	0	0	0	0	0	59
18730774	f	Not repo	MY	2021	1	0	0	0	0	0	0	0	0	0	0	0	61.2
18731030	m	hp	JP	2021	0	1	0	1	0	0	0	0	0	0	0	0	52
18731228	Not	md	US	2021	0	1	0	1	0	0	0	0	0	0	0	1	
18731239	m	hp	IN	2021	0	0	0	0	0	0	0	0	0	0	0	0	67
18731372	f	cn	TH	2021	0	0	0	0	0	0	0	0	0	0	0	0	
18731396	f	Not repo	AU	2021	1	0	0	0	0	0	0	0	0	0	0	0	73.9
18731721	f	md	JP	2021	0	1	0	1	0	0	0	0	0	0	0	0	72
18731724	f	cn	US	2021	0	1	1	0	0	0	0	0	0	0	0	0	61
18732270	m	md	US	2021	0	1	1	0	0	0	0	0	0	0	0	0	58
18732351	m	ph	CA	2021	0	0	0	0	0	0	0	0	0	0	0	0	59
18732371	m	hp	TR	2021	0	1	1	0	0	0	0	0	0	0	0	0	55
18732634	m	cn	JP	2021	0	1	1	0	0	0	0	0	0	0	0	0	60
18732998	f	cn	Not re	2021	0	0	0	0	0	0	0	0	0	0	0	0	
18733158	f	md	FR	2021	0	0	0	0	0	0	1	0	0	0	1	0	54
18733390	f	md	JP	2021	0	1	0	0	0	1	0	0	0	0	0	0	70
18733521	m	md	JP	2021	0	1	0	0	1	0	0	0	0	0	0	0	57
18733763	f	cn	CN	2021	1	0	0	0	0	0	0	0	0	0	0	0	70.3
18734056	f	md	JP	2021	0	1	0	0	0	1	0	0	0	0	0	0	80
18734125	m	hp	NL	2021	1	0	0	0	0	0	0	0	0	0	0	0	70.1
18734192	m	cn	Not re	2021	0	0	0	0	0	0	0	0	0	0	0	0	62
18734938	Not	Not repo	AU	2021	0	1	0	1	0	0	0	0	0	0	0	0	
18735067	m	cn	JP	2021	0	1	1	0	0	0	0	0	0	0	0	1	85
18735219	Not	md	CN	2021	0	0	0	0	0	0	0	0	0	0	0	0	
18735594	f	Not repo	MY	2021	1	0	0	0	0	0	0	0	0	0	0	0	59.8
18735600	m	hp	NL	2021	0	1	1	0	0	0	0	0	0	0	0	0	61
18735612	f	ph	US	2021	0	0	0	0	0	0	0	0	0	0	0	0	64
18735628	m	hp	US	2021	0	1	0	0	1	0	0	0	0	0	0	0	69
18735975	f	hp	US	2021	0	1	1	0	0	0	0	0	0	0	0	0	64
18736379	f	hp	DE	2021	0	1	0	0	1	0	0	0	0	0	0	0	63
18736945	m	hp	US	2021	0	1	1	0	0	0	0	0	0	0	0	0	69
18737423	m	md	TW	2021	0	1	1	0	0	0	0	0	0	0	0	0	65

18737457	f	md	NO	2021	0	1	1	0	0	0	0	0	0	0	0	0	70
18738032	f	cn	ID	2021	1	0	0	0	0	0	0	0	0	0	0	0	70
18738319	f	md	JP	2021	0	1	0	0	0	1	0	0	0	0	0	1	80
18738424	f	cn	CN	2021	1	0	0	0	0	0	0	0	0	0	0	0	87
18738426	f	md	CN	2021	1	0	0	0	0	0	0	0	0	0	0	0	51
18738430	f	cn	CN	2021	1	0	0	0	0	0	0	0	0	0	0	0	73
18738441	m	Not repo	CN	2021	1	0	0	0	0	0	0	0	0	0	0	0	59
18738562	m	cn	SE	2021	1	0	0	0	0	0	0	0	0	0	0	0	45
18740115	f	md	CN	2021	0	0	0	0	0	0	0	0	0	0	0	0	48
18740116	m	md	CN	2021	0	0	0	0	0	0	0	0	0	0	0	0	65
18740292	m	cn	AR	2021	0	0	0	0	0	0	0	0	0	0	0	0	48
18740397	m	cn	US	2021	1	0	0	0	0	0	0	0	0	0	0	0	70
18740438	f	cn	IN	2021	0	0	0	0	0	0	0	0	0	0	0	0	48
18740845	m	hp	PL	2021	0	0	0	0	0	0	0	0	0	0	0	0	48
18741616	m	md	JP	2021	0	1	0	0	0	1	0	0	0	0	0	0	44
18742044	m	md	BY	2021	0	0	0	0	0	0	0	0	0	0	0	0	27
18742380	f	md	CO	2021	0	0	0	0	0	0	0	0	0	0	0	0	61
18742702	m	ph	CA	2021	0	0	0	0	0	0	0	0	0	0	0	0	59
18743332	Not	ph	FR	2021	0	0	0	0	0	0	0	0	0	0	0	0	
18744062	m	md	JP	2021	0	1	0	0	1	0	0	0	0	0	0	0	55
18744120	m	md	JP	2021	0	1	0	0	1	0	0	0	0	0	0	0	49
18744122	f	md	JP	2021	0	1	0	0	1	0	0	0	0	0	0	0	67
18744183	m	md	JP	2021	0	1	0	0	1	0	0	0	0	0	0	0	70
18744301	m	md	JP	2021	0	1	0	0	0	1	0	0	0	0	0	0	62
18744595	m	md	JP	2021	0	1	0	0	1	0	0	0	0	0	0	0	69
18744696	f	cn	US	2021	1	0	0	0	0	0	0	0	0	0	0	0	72
18744909	m	md	JP	2021	0	1	0	0	0	1	0	0	0	0	0	0	70
18745294	m	hp	IT	2021	0	0	0	0	0	0	0	0	0	0	0	0	
18745394	f	cn	AR	2021	0	0	0	0	0	0	0	0	0	0	0	0	52
18745559	f	Not repo	MY	2021	1	0	0	0	0	0	0	0	0	0	0	0	64.8
18745680	f	Not repo	MY	2021	1	0	0	0	0	0	0	0	0	0	0	0	72.2
18745782	f	md	DE	2021	0	1	0	0	0	1	0	0	0	0	0	0	62
18745855	m	hp	JP	2021	0	1	0	1	0	0	0	0	0	0	0	0	45
18746049	m	hp	HU	2021	0	0	0	0	0	0	0	0	0	0	0	0	70

18746072	m	md	DE	2021	1	0	0	0	0	0	0	0	0	0	0	0		
18746255	f	md	US	2021	1	0	0	0	0	0	0	0	0	0	0	0		
18746256	Not	ph	JP	2021	1	0	0	0	0	0	0	0	0	0	0	0		
18746283	m	md	FR	2021	0	1	1	0	0	0	0	0	0	0	0	0	61	
18746446	m	md	DE	2021	0	0	0	0	0	0	0	0	0	0	0	0	79	
18746754	Not	md	JP	2021	0	1	0	0	1	0	0	0	0	0	0	0		
18746780	m	md	JP	2021	0	1	0	0	0	1	0	0	0	0	0	0	69	
18747123	f	cn	MY	2021	1	0	0	0	0	0	0	0	0	0	0	0		
18747124	f	md	US	2021	1	0	0	0	0	0	0	0	0	0	0	0		
18747247	f	md	IN	2021	1	0	0	0	0	0	0	0	0	0	0	0	53.5	
18747495	f	hp	SG	2021	1	0	0	0	0	0	0	0	0	0	0	0	76	
18747497	m	cn	CN	2021	0	1	0	0	1	0	0	0	0	0	0	0		
18747544	f	cn	CN	2021	1	0	0	0	0	0	0	0	0	0	0	1	61	
18747685	m	md	TR	2021	0	0	0	0	0	0	0	0	0	0	0	0	78	
18747941	f	md	HU	2021	0	0	0	0	0	0	0	0	0	0	0	0	66	
18748051	f	Not repo	CN	2021	1	0	0	0	0	0	0	0	0	0	0	0	63	
18748240	m	md	JP	2021	0	1	0	1	0	0	0	0	0	0	0	0	69	
18748244	m	md	JP	2021	1	0	0	0	0	0	0	0	0	0	0	0	64	
18748290	m	hp	IL	2021	0	0	0	0	0	0	0	0	0	0	0	0	68	
18748299	f	md	ID	2021	1	0	0	0	0	0	0	0	0	0	0	0	75	
18748580	m	md	IN	2021	0	0	0	0	0	0	0	0	0	0	0	0	69	
18748602	f	md	IN	2021	0	0	0	0	0	0	0	0	0	0	0	0	65	
18749183	m	md	FR	2021	0	1	1	0	0	0	0	0	0	0	0	0	61	
18749341	m	cn	Not re	2021	0	0	0	0	0	0	0	0	0	0	0	0		
18749393	f	md	IN	2021	0	0	0	0	0	0	0	0	0	0	0	0	51	
18750241	m	ph	CA	2021	0	0	0	0	0	0	0	0	0	0	0	0	59	
18750505	f	md	US	2021	0	1	1	0	0	0	0	0	0	0	0	0	54	
18750838	m	md	AT	2021	0	0	0	0	0	0	0	0	0	0	0	0	75	
18751426	f	md	FR	2021	0	0	0	0	0	0	0	0	0	0	0	0		
18751684	f	cn	Not re	2021	0	0	0	0	0	0	0	0	0	0	0	0		
18751841	m	md	US	2021	0	0	0	0	0	0	0	0	0	0	0	0		
18751859	m	hp	FR	2021	0	1	0	0	1	0	0	0	0	0	0	0	67	
18752477	m	cn	US	2021	1	0	0	0	0	0	0	0	0	0	0	0	67	
18753072	f	hp	BE	2021	0	0	0	0	0	0	0	0	0	0	0	0	74	

18753099	m	hp	US	2021	0	1	0	0	1	0	0	0	0	0	0	0	74
18753143	m	Not repo	CN	2021	0	1	0	1	0	0	0	0	0	0	0	0	64
18753412	f	cn	Not re	2021	0	0	0	0	0	0	0	0	0	0	0	0	
18753905	f	md	JP	2021	0	0	0	0	0	0	0	0	0	0	0	0	
18754183	m	md	JP	2021	0	1	1	0	0	0	0	0	0	0	0	0	62
18754809	m	md	JP	2021	0	1	0	0	0	1	0	0	0	0	0	0	80
18754902	m	md	JP	2021	0	1	0	0	0	1	0	0	0	0	0	0	71
18755029	f	md	RO	2021	1	0	0	0	0	0	0	0	0	0	0	0	63
18755190	m	hp	GR	2021	0	1	1	0	0	0	0	0	0	0	0	0	70
18755933	Not	hp	US	2021	0	0	0	0	0	0	0	0	0	0	0	0	
18756425	m	cn	MY	2021	0	0	0	0	0	0	0	0	0	0	0	0	64
18756482	m	cn	CN	2021	0	1	0	1	0	0	0	0	0	0	0	0	70
18756614	m	md	FR	2021	0	1	0	0	0	1	0	0	0	0	0	0	53
18756654	m	md	FR	2021	0	1	1	0	0	0	0	0	0	0	0	0	61
18756692	f	Not repo	NZ	2021	1	0	0	0	0	0	0	0	0	0	0	0	
18756971	f	cn	JP	2021	1	0	0	0	0	0	0	0	0	0	0	0	
18757052	f	cn	AR	2021	0	0	0	0	0	0	0	0	0	0	0	0	82
18757632	f	Not repo	MY	2021	1	0	0	0	0	0	0	0	0	0	0	0	80.7
18757901	Not	md	JP	2021	0	0	0	0	0	0	0	0	0	0	0	0	
18758049	m	Not repo	CA	2021	1	0	0	0	0	0	0	0	0	0	0	0	88.1
18758117	f	cn	Not re	2021	0	0	0	0	0	0	0	0	0	0	0	0	
18758328	Not	md	CN	2021	0	0	0	0	0	0	0	0	0	0	0	0	
18758417	m	md	IL	2021	0	0	0	0	0	0	0	0	0	0	0	0	
18758565	f	cn	CN	2021	0	1	0	0	1	0	0	0	0	0	0	0	64
18758848	m	hp	US	2021	0	1	0	1	0	0	0	0	0	0	0	0	
18760402	m	md	CN	2021	0	0	0	0	0	0	0	0	0	0	0	0	66
18760944	m	md	JP	2021	0	0	0	0	0	0	0	0	0	0	0	0	53
18761289	f	cn	US	2021	0	1	0	0	1	0	0	0	0	0	0	0	73
18761321	f	md	JP	2021	0	0	0	0	0	0	0	0	0	0	0	0	80
18761490	m	md	DK	2021	0	0	0	0	0	0	0	0	0	0	0	0	74
18761526	m	hp	FR	2021	0	1	0	0	1	0	0	0	0	0	0	0	66
18761646	m	Not repo	CN	2021	0	1	0	1	0	0	0	0	0	0	0	0	65
18761709	m	md	US	2021	0	1	0	1	0	0	0	0	0	0	0	0	
18761717	m	md	CN	2021	0	0	0	0	0	0	0	0	0	0	0	0	76

18762184	m	hp	CN	2021	0	0	0	0	0	0	0	0	0	0	0	0	70
18762220	m	hp	JP	2021	0	1	1	0	0	0	0	0	0	0	0	0	75
18762423	m	Not repo	CN	2021	0	1	0	1	0	0	0	0	0	0	0	0	73
18762424	m	cn	CN	2021	0	1	0	1	0	0	0	0	0	0	0	0	71
18762434	Not	cn	CN	2021	0	1	0	1	0	0	0	0	0	0	0	0	68
18762678	m	cn	IN	2021	0	0	0	0	0	0	0	0	0	0	0	0	52
18762893	m	Not repo	ID	2021	0	1	0	1	0	0	0	0	0	0	0	0	57.4
18762895	m	md	JP	2021	0	1	0	1	0	0	0	0	0	0	0	1	
18762971	m	Not repo	ID	2021	1	0	0	0	0	0	0	0	0	0	0	0	61.4
18763069	f	md	SK	2021	0	0	0	0	0	0	0	0	0	0	0	0	81
18764164	Not	md	KR	2021	0	0	0	0	0	0	0	0	0	0	0	0	
18764173	Not	hp	KR	2021	0	0	0	0	0	0	0	0	0	0	0	0	
18764188	Not	hp	US	2021	0	0	0	0	0	0	0	0	0	0	0	0	
18764412	m	cn	PL	2021	0	1	0	1	0	0	0	0	0	0	0	0	
18764478	f	cn	US	2021	1	0	0	0	0	0	0	0	0	0	0	0	
18764494	f	hp	US	2021	0	1	1	0	0	0	0	0	0	0	0	0	64
18764668	Not	hp	US	2021	0	0	0	0	0	0	0	0	0	0	0	0	
18764669	Not	hp	US	2021	0	0	0	0	0	0	0	0	0	0	0	0	
18764675	Not	hp	US	2021	0	0	0	0	0	0	0	0	0	0	0	0	
18764681	Not	hp	US	2021	0	0	0	0	0	0	0	0	0	0	0	0	
18764684	Not	hp	US	2021	0	0	0	0	0	0	0	0	0	0	0	0	
18764685	Not	hp	US	2021	0	0	0	0	0	0	0	0	0	0	0	0	
18764691	Not	hp	US	2021	0	0	0	0	0	0	0	0	0	0	0	0	
18764833	f	md	CN	2021	0	1	0	0	0	1	0	0	0	0	0	0	66
18764873	Not	md	JP	2021	1	0	0	0	0	0	0	0	0	0	0	0	
18764874	m	md	MX	2021	0	1	0	1	0	0	0	0	0	0	0	0	82.8
18765137	Not	hp	US	2021	0	1	0	1	0	0	0	0	0	0	0	0	
18765279	m	md	IN	2021	0	0	0	0	0	0	0	0	0	0	0	0	38
18765540	Not	md	NO	2021	0	0	0	0	0	0	0	0	0	0	0	0	46
18765615	Not	hp	US	2021	0	0	0	0	0	0	0	0	0	0	0	0	
18765677	Not	md	US	2021	1	0	0	0	0	0	0	0	0	0	0	1	
18765958	m	md	KR	2021	0	1	0	0	0	1	0	0	0	0	0	1	62
18766049	f	md	FR	2021	0	1	0	0	0	1	0	0	0	0	0	0	37
18766462	m	cn	CN	2021	0	1	0	0	1	0	0	0	0	0	0	0	79

18766652	Not	md	CN	2021	0	0	0	0	0	0	0	0	0	0	0	0		
18766797	Not	md	LU	2021	0	1	0	0	1	0	0	0	0	0	0	0		
18766921	m	ph	JP	2021	0	1	0	0	1	0	0	0	0	0	0	0	70	
18767471	m	md	CA	2021	0	0	0	0	0	0	0	0	0	0	0	0	82	
18767472	f	cn	BR	2021	1	0	0	0	0	0	0	0	0	0	0	0	67	
18767879	m	hp	IT	2021	0	0	0	0	0	0	0	0	0	0	0	0		
18768187	m	md	SK	2021	1	0	0	0	0	0	0	0	0	0	0	0		
18768224	f	cn	AR	2021	0	0	0	0	0	0	0	0	0	0	0	0	67	
18768255	m	Not repo	JP	2021	1	0	0	0	0	0	0	0	0	0	0	1	73.2	
18768384	f	Not repo	CA	2021	1	0	0	0	0	0	0	0	0	0	0	0		
18768390	m	md	JP	2021	0	1	0	1	0	0	0	0	0	0	0	0	72	
18768623	Not	hp	US	2021	0	0	0	0	0	0	0	0	0	0	0	0		
18769207	f	cn	JP	2021	0	1	1	0	0	0	0	0	0	0	0	0	79	
18769775	Not	md	HU	2021	0	0	0	0	0	0	0	0	0	0	0	0	66	
18769847	m	cn	CN	2021	0	0	0	0	0	0	0	0	0	0	0	0	75	
18770120	Not	hp	US	2021	0	1	0	1	0	0	0	0	0	0	0	0		
18770517	m	cn	DE	2021	0	1	1	0	0	0	0	0	0	0	0	0	54	
18770764	m	md	DE	2021	0	1	1	0	0	0	0	0	0	0	0	0	52	
18770813	Not	md	JP	2021	0	1	0	1	0	0	0	0	0	0	0	1		
18770844	Not	md	MY	2021	0	0	0	0	0	0	0	0	0	0	0	0		
18771090	Not	md	GB	2021	0	0	0	0	0	0	0	0	0	0	0	0		
18771721	f	hp	US	2021	0	1	0	0	1	0	0	0	0	0	0	0	74	
18772486	Not	Not repo	AU	2021	1	0	0	0	0	0	0	0	0	0	0	0		
18773084	Not	md	US	2021	0	0	0	0	0	0	0	0	0	0	0	0		
18773711	m	ph	US	2021	0	1	0	0	1	0	0	0	0	0	0	0		
18774230	m	Not repo	MY	2021	1	0	0	0	0	0	0	0	0	0	0	0	65.4	
18774231	Not	md	JP	2021	1	0	0	0	0	0	0	0	0	0	0	0		
18774566	f	Not repo	ID	2021	1	0	0	0	0	0	0	0	0	0	0	0		
18774569	Not	md	JP	2021	1	0	0	0	0	0	0	0	0	0	0	0		
18774575	f	Not repo	JP	2021	1	0	0	0	0	0	0	0	0	0	0	0	67	
18774583	f	md	FR	2021	1	0	0	0	0	0	0	0	0	0	0	0	60	
18774700	m	Not repo	ID	2021	1	0	0	0	0	0	0	0	0	0	0	0	78.5	
18774737	m	cn	BG	2021	0	1	1	0	0	0	0	0	0	0	0	0	65	
18774762	m	hp	US	2021	0	1	0	1	0	0	0	0	0	0	0	0	65	

18774902	m	md	US	2021	0	0	0	0	0	0	0	0	0	0	0	0	65
18775005	m	md	CN	2021	0	0	0	0	0	0	0	0	0	0	0	0	62
18775021	m	md	US	2021	0	0	0	0	0	0	0	0	0	0	0	0	50
18775038	Not	md	US	2021	0	1	1	0	0	0	0	0	0	0	0	0	
18775066	Not	md	JP	2021	1	0	0	0	0	0	0	0	0	0	0	0	
18775113	m	cn	FR	2021	0	1	1	0	0	0	0	0	0	0	0	0	65
18775237	m	hp	CA	2021	0	0	0	0	0	0	0	0	0	0	0	0	59
18775349	m	md	JP	2021	0	1	0	0	0	1	0	0	0	0	0	0	58
18775350	m	md	RO	2021	0	0	0	0	0	0	0	0	0	0	0	0	
18775805	m	md	KR	2021	0	0	0	0	0	0	0	0	0	0	0	0	75
18776022	m	Not repo	CN	2021	0	1	0	1	0	0	0	0	0	0	0	0	67.4
18776175	f	hp	JP	2021	1	0	0	0	0	0	0	0	0	0	0	0	70
18776246	f	hp	US	2021	1	0	0	0	0	0	0	0	0	0	0	0	56
18776564	m	ph	US	2021	0	0	0	0	0	0	0	0	0	0	0	0	
18776610	m	hp	RO	2021	0	1	0	0	1	0	0	0	0	0	0	0	73
18776694	f	cn	CN	2021	1	0	0	0	0	0	0	0	0	0	0	0	76
18776769	f	hp	US	2021	0	0	0	0	0	0	0	0	0	0	0	0	76
18776903	f	hp	US	2021	0	1	1	0	0	0	0	0	0	0	0	0	64
18777083	f	cn	CN	2021	0	1	0	0	1	0	0	0	0	0	0	0	66
18777690	m	md	US	2021	0	1	0	1	0	0	0	0	0	0	0	0	
18778025	m	cn	JP	2021	0	1	1	0	0	0	0	0	0	0	0	0	59
18779357	m	md	SK	2021	1	0	0	0	0	0	0	0	0	0	0	0	
18780360	f	cn	Not re	2021	0	0	0	0	0	0	0	0	0	0	0	0	60
18781473	f	md	JP	2021	0	1	0	0	0	1	0	0	0	0	0	0	68
18782953	m	hp	US	2021	0	1	0	1	0	0	0	0	0	0	0	0	65
18783437	m	Not repo	MY	2021	1	0	0	0	0	0	0	0	0	0	0	0	89.6
18783719	m	cn	FR	2021	0	1	1	0	0	0	0	0	0	0	0	0	65
18784868	m	hp	DE	2021	0	1	0	0	0	1	0	0	0	0	0	0	54
18784985	f	Not repo	MY	2021	1	0	0	0	0	0	0	0	0	0	0	0	52.9
18785099	m	md	US	2021	0	1	0	1	0	0	0	0	0	0	0	0	
18785268	Not	md	BE	2021	0	1	0	0	0	1	0	0	0	0	0	0	
18785809	f	md	ID	2021	0	1	1	0	0	0	0	0	0	0	0	0	
18785839	f	hp	PL	2021	0	0	0	0	0	0	0	0	0	0	0	0	54
18785952	m	Not repo	ID	2021	1	0	0	0	0	0	0	0	0	0	0	0	49.1

18786021	Not	cn	JP	2021	0	1	1	0	0	0	0	0	0	0	0	0	55
18786111	m	Not repo	ID	2021	1	0	0	0	0	0	0	0	0	0	0	0	59
18786298	m	cn	US	2021	0	1	0	0	1	0	0	0	0	0	0	0	
18786557	Not	Not repo	JP	2021	0	1	0	1	0	0	0	0	0	0	0	1	
18786650	m	md	IL	2021	0	0	0	0	0	0	0	0	0	0	0	0	58
18786665	f	Not repo	AU	2021	1	0	0	0	0	0	0	0	0	0	0	0	
18786751	m	md	US	2021	0	1	0	1	0	0	0	0	0	0	0	0	
18786879	f	md	JP	2021	1	0	0	0	0	0	0	0	0	0	0	0	66
18787192	f	hp	JP	2021	0	1	1	0	0	0	0	0	0	0	0	0	69
18787298	m	cn	Not re	2021	0	0	0	0	0	0	0	0	0	0	0	0	
18788659	f	hp	US	2021	1	0	0	0	0	0	0	0	0	0	0	0	64
18788702	m	md	RO	2021	0	1	0	1	0	0	0	0	0	0	0	0	
18789094	f	ph	PT	2021	0	1	0	0	0	1	0	0	0	0	0	0	68
18790010	f	cn	CH	2021	0	1	1	0	0	0	0	0	0	0	0	0	
18790963	Not	Not repo	ID	2021	1	0	0	0	0	0	0	0	0	0	0	0	
18791172	f	hp	US	2021	0	1	1	0	0	0	0	0	0	0	0	0	56
18791475	f	ph	JP	2021	1	0	0	0	0	0	0	0	0	0	0	0	86
18792076	m	md	JP	2021	0	0	0	0	0	0	0	0	0	0	0	0	79
18792347	m	cn	CN	2021	0	1	0	0	1	0	0	0	0	0	0	0	55
18792561	Not	hp	JP	2021	0	1	0	0	0	1	0	0	0	0	0	1	
18792695	m	md	JP	2021	0	1	0	0	0	1	0	0	0	0	0	0	70
18793120	m	cn	NL	2021	0	1	0	1	0	0	0	0	0	0	0	0	
18793442	Not	cn	JP	2021	1	0	0	0	0	0	0	0	0	0	0	0	
18793476	f	cn	CN	2021	0	1	0	0	1	0	0	0	0	0	0	0	81
18793483	m	md	SE	2021	0	0	0	0	0	0	0	0	0	0	0	0	70
18793556	f	md	FR	2021	0	1	0	0	0	1	0	0	0	0	0	0	37
18793589	m	cn	IT	2021	0	1	1	0	0	0	0	0	0	0	0	0	54
18793779	m	md	JP	2021	0	1	0	1	0	0	0	0	0	0	0	1	
18794015	m	md	IN	2021	0	1	0	0	0	1	0	0	0	0	0	0	72
18794671	m	hp	AU	2021	0	1	1	0	0	0	0	0	0	0	0	0	62
18794871	m	hp	CN	2021	0	1	0	0	1	0	0	0	0	0	0	0	66
18795029	m	cn	US	2021	0	1	1	0	0	0	0	0	0	0	0	0	77
18795088	m	md	CN	2021	0	1	0	0	0	1	0	0	0	0	0	0	81
18795289	f	Not repo	TW	2021	1	0	0	0	0	0	0	0	0	0	0	0	65

18795629	f	cn	US	2021	0	1	1	0	0	0	0	0	0	0	0	0	66
18795988	m	hp	US	2021	0	1	0	0	1	0	0	0	0	0	0	1	68
18796571	m	hp	US	2021	0	1	0	0	1	0	0	0	0	0	0	0	66
18796630	m	hp	FR	2021	0	0	0	0	0	0	0	0	0	0	0	0	59
18796830	m	md	MY	2021	1	0	0	0	0	0	0	0	0	0	0	0	
18797086	Not	Not repo	ID	2021	1	0	0	0	0	0	0	0	0	0	0	0	
18797118	m	md	US	2021	0	1	0	1	0	0	0	0	0	0	0	0	
18797143	f	cn	US	2021	1	0	0	0	0	0	0	0	0	0	0	0	75.8
18797271	f	Not repo	US	2021	1	0	0	0	0	0	0	0	0	0	0	0	80.2
18797343	m	cn	TW	2021	0	0	0	0	0	0	0	0	0	0	0	0	
18797467	m	md	AT	2021	0	0	0	0	0	0	0	0	0	0	0	0	56
18798248	m	cn	JP	2021	0	1	1	0	0	0	0	0	0	0	0	0	80
18798413	m	md	IT	2021	0	0	0	0	0	0	0	0	0	0	0	0	
18798498	f	cn	US	2021	1	0	0	0	0	0	0	0	0	0	0	0	
18798620	m	md	JP	2021	0	1	0	0	0	1	0	0	0	0	0	0	71
18799194	m	Not repo	Not re	2021	0	1	0	1	0	0	0	0	0	0	0	0	
18799196	m	cn	JP	2021	0	1	1	0	0	0	0	0	0	0	0	0	73
18799256	m	Not repo	CN	2021	1	0	0	0	0	0	0	0	0	0	0	0	55
18799782	f	ph	US	2021	0	1	1	0	0	0	0	0	0	0	0	0	62
18799816	f	md	US	2021	1	0	0	0	0	0	0	0	0	0	0	0	74
18800163	m	md	AU	2021	0	0	0	0	0	0	0	0	0	0	0	0	66
18800306	f	md	AU	2021	0	0	0	0	0	0	0	0	0	0	0	0	77
18801380	f	cn	BE	2021	0	1	1	0	0	0	0	0	0	0	0	0	
18801419	m	Not repo	CN	2021	0	1	0	1	0	0	0	0	0	0	0	1	63
18801524	f	hp	JP	2021	0	1	1	0	0	0	0	0	0	0	0	0	69
18802100	f	Not repo	ID	2021	0	1	0	1	0	0	0	0	0	0	0	0	71.2
18802674	f	cn	RO	2021	0	1	1	0	0	0	0	0	0	0	0	0	
18802875	f	md	JP	2021	0	1	0	0	0	1	0	0	0	0	0	0	80
18803369	m	cn	US	2021	0	0	0	0	0	0	0	0	0	0	0	0	71
18803804	m	md	CN	2021	0	1	1	0	0	0	0	0	0	0	0	1	66
18805762	m	hp	US	2021	0	0	0	0	0	0	0	0	0	0	0	0	66
18806083	f	md	JP	2021	0	0	0	0	0	0	0	0	0	0	0	0	80
18806758	m	Not repo	JP	2021	0	1	0	1	0	0	0	0	0	0	0	1	67
18809487	m	cn	JP	2021	0	1	1	0	0	0	0	0	0	0	0	0	70

18810031	f	hp	DE	2021	0	1	0	0	1	0	0	0	0	0	0	0	78
18810096	m	hp	IN	2021	1	0	0	0	0	0	0	0	0	0	0	0	53
18810099	m	md	US	2021	0	1	1	0	0	0	0	0	0	0	0	0	66
18810214	f	hp	DE	2021	0	0	0	0	0	0	0	0	0	0	0	0	63
18810248	f	md	JP	2021	0	1	0	1	0	0	0	0	0	0	0	1	77
18810681	f	md	HU	2021	1	0	0	0	0	0	0	0	0	0	0	0	80
18810780	m	Not repo	GR	2021	0	1	0	1	0	0	0	0	0	0	0	0	
18810796	f	ph	JP	2021	0	1	1	0	0	0	0	0	0	0	0	0	82
18810829	f	md	AT	2021	0	1	0	0	0	1	0	0	0	0	0	0	76
18810845	m	cn	IT	2021	0	1	1	0	0	0	0	0	0	0	0	0	54
18810853	f	ph	CH	2021	1	0	0	0	0	0	0	0	0	0	0	0	
18810876	m	Not repo	ID	2021	1	0	0	0	0	0	0	0	0	0	0	0	63.8
18810970	m	cn	JP	2021	0	1	1	0	0	0	0	0	0	0	0	0	66
18811000	f	md	FR	2021	0	0	0	0	0	0	0	0	0	0	0	0	
18811240	m	md	DE	2021	0	1	0	0	1	0	0	0	0	0	0	0	
18811517	m	cn	FR	2021	0	1	1	0	0	0	0	0	0	0	0	0	68
18811732	m	md	JP	2021	0	1	1	0	0	0	0	0	0	0	0	0	73
18811749	f	hp	JP	2021	0	1	0	0	0	1	0	0	0	0	0	0	72
18811750	f	md	AT	2021	1	0	0	0	0	0	0	0	0	0	0	0	45
18811819	m	cn	JP	2021	0	0	0	0	0	0	0	0	0	0	0	0	57
18811884	f	cn	Not re	2021	0	0	0	0	0	0	0	0	0	0	0	0	
18811995	m	md	CN	2021	0	1	1	0	0	0	0	0	0	0	0	0	69
18812035	m	hp	JP	2021	0	1	0	0	1	0	0	0	0	0	0	0	69
18812400	f	ph	US	2021	0	1	0	1	0	0	0	0	0	0	0	0	
18812571	m	cn	Not re	2021	0	0	0	0	0	0	0	0	0	0	0	0	79
18812729	m	hp	US	2021	1	0	0	0	0	0	0	0	0	0	0	0	64
18813572	f	md	TW	2021	0	1	0	0	1	0	0	0	0	0	0	0	46
18814222	f	cn	US	2021	1	0	0	0	0	0	0	0	0	0	0	0	74.1
18814388	m	hp	JP	2021	0	1	1	0	0	0	0	0	0	0	0	0	46
18816247	f	md	JP	2021	1	0	0	0	0	0	0	0	0	0	0	0	66
18817343	m	md	IL	2021	0	1	1	0	0	0	0	0	0	0	0	0	56
18818247	f	hp	RO	2021	1	0	0	0	0	0	0	0	0	0	0	0	63
18818956	m	Not repo	Not re	2021	0	1	1	0	0	0	0	0	0	0	0	0	69
18819063	m	md	JP	2021	0	1	0	0	1	0	0	0	0	0	0	0	68

18819245	m	ph	US	2021	0	0	0	0	0	0	0	0	0	0	0	0	64
18819261	m	hp	CN	2021	1	0	0	0	0	0	0	0	0	0	0	1	60
18819420	m	hp	AU	2021	0	1	1	0	0	0	0	0	0	0	0	0	62
18820039	m	hp	DE	2021	0	1	1	0	0	0	0	0	0	0	0	0	65
18820588	m	Not repo	MY	2021	1	0	0	0	0	0	0	0	0	0	0	0	79.9
18820661	f	Not repo	ID	2021	1	0	0	0	0	0	0	0	0	0	0	0	79.4
18820672	m	Not repo	GR	2021	0	1	0	1	0	0	0	0	0	0	0	0	
18820696	m	cn	Not re	2021	0	0	0	0	0	0	0	0	0	0	0	0	
18820898	Not	md	JP	2021	1	0	0	0	0	0	0	0	0	0	0	0	
18820945	f	hp	US	2021	0	0	0	0	0	0	0	0	0	0	0	0	64
18821426	f	hp	CA	2021	0	0	0	0	0	0	1	1	0	0	0	0	58
18821557	m	md	GB	2021	0	0	0	0	0	0	0	0	0	0	0	0	60
18821747	m	md	JP	2021	1	0	0	0	0	0	0	0	0	0	0	0	73
18821762	f	cn	CN	2021	1	0	0	0	0	0	0	0	0	0	0	0	89
18822068	f	cn	US	2021	0	0	0	0	0	0	0	0	0	0	0	0	57
18822091	f	cn	CN	2021	1	0	0	0	0	0	0	0	0	0	0	0	59.7
18822946	m	hp	US	2021	0	1	0	0	0	1	0	0	0	0	0	0	46
18823043	f	hp	DE	2021	0	0	0	0	0	0	0	0	0	0	0	0	77
18824857	m	md	JP	2021	0	1	0	1	0	0	0	0	0	0	0	0	67
18825191	m	md	CN	2021	0	1	0	0	0	1	0	0	0	0	0	0	67
18825583	m	md	GB	2021	0	0	0	0	0	0	0	0	0	0	0	0	72
18826652	m	hp	RU	2021	0	1	0	0	1	0	0	0	0	0	0	0	63
18826979	m	hp	AU	2021	0	1	1	0	0	0	0	0	0	0	0	0	62
18831997	f	cn	JP	2021	0	1	1	0	0	0	0	0	0	0	0	0	61
18832179	m	md	AR	2021	0	1	0	0	0	1	0	0	0	0	0	0	46
18832843	m	cn	JP	2021	0	1	1	0	0	0	0	0	0	0	0	1	58
18832930	m	ph	JP	2021	0	1	0	0	1	0	0	0	0	0	0	0	70
18833895	m	ph	JP	2021	0	1	0	0	1	0	0	0	0	0	0	1	71
18834012	m	md	HR	2021	0	1	0	0	0	1	0	0	0	0	0	0	72
18834987	m	md	IT	2021	0	1	0	0	1	0	0	0	0	0	0	0	
18836568	m	md	PL	2021	0	0	0	0	0	0	0	0	0	0	0	0	60
18836608	m	cn	NP	2021	0	0	0	0	0	0	0	0	0	0	0	0	76
18836714	m	hp	AU	2021	0	1	1	0	0	0	0	0	0	0	0	0	62
18837820	f	md	US	2021	0	1	0	0	1	0	0	0	0	0	0	0	72

18838525	Not	Not repo	JP	2021	0	1	0	1	0	0	0	0	0	0	0	1	
18839136	f	ph	JP	2021	1	0	0	0	0	0	0	0	0	0	0	0	64
18839167	f	cn	FR	2021	0	1	1	0	0	0	0	0	0	0	0	0	
18839236	f	md	CH	2021	0	0	0	0	0	0	0	0	0	0	0	0	71
18839446	f	md	JP	2021	0	0	0	0	0	0	1	0	0	1	0	1	79
18839568	f	hp	CA	2021	0	0	0	0	0	0	0	0	0	0	0	0	43
18840206	Not	ph	JP	2021	0	1	0	1	0	0	0	0	0	0	0	0	
18840207	m	md	TW	2021	1	0	0	0	0	0	0	0	0	0	0	1	53
18840496	f	Not repo	DO	2021	1	0	0	0	0	0	0	0	0	0	0	0	64.6
18841244	f	md	JP	2021	0	1	0	1	0	0	0	0	0	0	0	1	45
18841443	Not	ph	TH	2021	0	1	0	0	1	0	0	0	0	0	0	0	
18841552	m	md	BG	2021	0	1	0	0	0	1	0	0	0	0	0	0	70
18841654	m	md	BE	2021	0	1	1	0	0	0	0	0	0	0	0	0	68
18841707	Not	md	JP	2021	0	1	0	0	0	1	0	0	0	0	0	0	60
18841732	f	hp	US	2021	0	0	0	0	0	0	0	0	0	0	0	0	
18841830	f	Not repo	KW	2021	1	0	0	0	0	0	0	0	0	0	0	0	43.8
18843066	m	md	FR	2021	0	1	0	1	0	0	0	0	0	0	0	0	
18843206	f	md	CN	2021	0	0	0	0	0	0	0	0	0	0	0	0	40
18843855	m	hp	CA	2021	0	0	0	0	0	0	0	0	0	0	0	0	57
18845152	f	hp	CN	2021	0	0	0	0	0	0	0	0	0	0	0	0	30
18845933	f	cn	US	2021	0	0	0	0	0	0	0	0	0	0	0	0	64
18846463	m	md	JP	2021	1	0	0	0	0	0	0	0	0	0	0	1	80.9
18847710	f	md	JP	2021	0	1	0	1	0	0	0	0	0	0	0	1	69
18847713	m	md	JP	2021	0	1	0	1	0	0	0	0	0	0	0	1	56
18848395	m	md	FR	2021	0	1	1	0	0	0	0	0	0	0	0	0	78
18849094	f	md	CZ	2021	0	0	0	0	0	0	0	0	0	0	0	0	60
18849124	f	hp	DE	2021	0	1	0	0	1	0	0	0	0	0	0	0	76
18850778	f	cn	US	2021	1	0	0	0	0	0	0	0	0	0	0	0	82
18850799	f	hp	CN	2021	1	0	0	0	0	0	0	0	0	0	0	0	46
18850938	f	cn	CN	2021	0	0	0	0	0	0	0	0	0	0	0	0	52
18851288	f	md	TH	2021	0	0	0	0	0	0	0	0	0	0	0	0	58
18851435	Not	ph	US	2021	0	1	0	0	1	0	0	0	0	0	0	0	
18851516	m	Not repo	JP	2021	0	1	0	1	0	0	0	0	0	0	0	1	81
18851727	f	md	PL	2021	0	0	0	0	0	0	0	0	0	0	0	0	

18851795	Not	md	FR	2021	1	0	0	0	0	0	0	0	0	0	0	0	60
18851820	m	hp	BG	2021	0	1	0	0	0	1	0	0	0	0	0	0	70
18852693	m	cn	Not re	2021	0	0	0	0	0	0	0	0	0	0	0	0	62
18852697	f	md	MY	2021	0	0	0	0	0	0	0	0	0	0	0	0	53
18852867	m	md	IS	2021	0	0	0	0	0	0	1	1	0	0	0	0	54
18853096	Not	md	JP	2021	1	0	0	0	0	0	0	0	0	0	0	0	
18853101	f	Not repo	ID	2021	1	0	0	0	0	0	0	0	0	0	0	0	81.6
18853280	m	hp	IN	2021	1	0	0	0	0	0	0	0	0	0	0	0	53
18853325	f	Not repo	CA	2021	1	0	0	0	0	0	0	0	0	0	0	0	
18853416	Not	md	JP	2021	0	1	0	1	0	0	0	0	0	0	0	0	
18853417	Not	Not repo	SG	2021	1	0	0	0	0	0	0	0	0	0	0	0	76
18853418	Not	md	JP	2021	0	1	0	1	0	0	0	0	0	0	0	1	
18853451	m	hp	AU	2021	0	1	1	0	0	0	0	0	0	0	0	0	62
18853531	f	md	JP	2021	1	0	0	0	0	0	0	0	0	0	0	0	66
18853534	f	md	AU	2021	0	1	0	1	0	0	0	0	0	0	0	0	
18853614	m	md	US	2021	0	0	0	0	0	0	0	0	0	0	0	0	82
18853673	m	md	JP	2021	0	1	0	1	0	0	0	0	0	0	0	1	63
18853674	m	md	JP	2021	0	1	0	1	0	0	0	0	0	0	0	1	53
18853679	m	md	CN	2021	0	0	0	0	0	0	0	0	0	0	0	0	69
18853808	Not	md	ES	2021	0	0	0	0	0	0	0	0	0	0	0	0	
18853850	f	Not repo	ID	2021	1	0	0	0	0	0	0	0	0	0	0	0	50.4
18854005	m	md	JP	2021	0	0	0	0	0	0	0	0	0	0	0	0	88
18854375	Not	hp	FR	2021	0	0	0	0	0	0	0	0	0	0	0	0	
18854626	f	Not repo	CN	2021	0	1	0	1	0	0	0	0	0	0	0	0	60
18854685	m	md	KR	2021	0	1	0	0	1	0	0	0	0	0	0	1	54
18854970	m	md	CO	2021	1	0	0	0	0	0	0	0	0	0	0	0	45
18855026	m	cn	IN	2021	0	0	0	0	0	0	0	0	0	0	0	0	43
18855133	f	Not repo	MY	2021	1	0	0	0	0	0	0	0	0	0	0	0	53.7
18855136	m	Not repo	MY	2021	1	0	0	0	0	0	0	0	0	0	0	0	75.2
18855137	f	md	CA	2021	1	0	0	0	0	0	0	0	0	0	0	0	
18855473	m	cn	DE	2021	0	1	1	0	0	0	0	0	0	0	0	0	53
18855661	f	hp	NO	2021	1	0	0	0	0	0	0	0	0	0	0	0	35
18855703	f	md	US	2021	0	1	0	0	1	0	0	0	0	0	0	0	68
18856111	f	md	JP	2021	0	1	1	0	0	0	0	0	0	0	0	0	80

18856219	m	md	FR	2021	0	1	0	0	0	1	0	0	0	0	0	0	55
18856348	Not	hp	US	2021	1	0	0	0	0	0	0	0	0	0	0	0	
18856460	m	md	JP	2021	0	1	0	0	1	0	0	0	0	0	0	0	72
18856778	m	md	JP	2021	0	1	1	0	0	0	0	0	0	0	0	0	46
18857068	m	cn	JP	2021	0	1	1	0	0	0	0	0	0	0	0	0	80
18857838	f	md	GB	2021	0	1	0	0	0	1	0	0	0	0	0	0	54
18860442	f	hp	KR	2021	0	0	0	0	0	0	0	0	0	0	0	0	60
18860579	m	cn	US	2021	1	0	0	0	0	0	0	0	0	0	0	0	66
18860601	Not	md	JP	2021	0	1	0	0	1	0	0	0	0	0	0	0	
18860728	m	cn	US	2021	0	0	0	0	0	0	0	0	0	0	0	0	
18860951	f	md	DE	2021	0	0	0	0	0	0	0	0	0	0	0	0	78
18861053	m	hp	DE	2021	0	1	1	0	0	0	0	0	0	0	0	0	80
18861086	f	md	AU	2021	0	1	1	0	0	0	0	0	0	0	0	0	
18861094	f	hp	DK	2021	0	1	1	0	0	0	0	0	0	0	0	0	62
18861347	m	md	CN	2021	0	1	0	0	0	1	0	0	0	0	0	0	75
18861924	f	hp	SI	2021	0	0	0	0	0	0	0	0	0	0	0	0	70
18862232	m	cn	Not re	2021	0	0	0	0	0	0	0	0	0	0	0	0	
18862801	m	md	JP	2021	0	1	0	1	0	0	0	0	0	0	0	0	67
18863304	m	md	FR	2021	0	1	0	0	0	1	0	0	0	0	0	0	68
18863590	m	cn	Not re	2021	0	0	0	0	0	0	0	0	0	0	0	0	53
18863651	f	md	SK	2021	1	0	0	0	0	0	0	0	0	0	0	0	61
18863669	Not	cn	SG	2021	1	0	0	0	0	0	0	0	0	0	0	0	69.8
18863857	f	md	US	2021	0	1	1	0	0	0	0	0	0	0	0	0	55
18864128	m	md	JP	2021	0	1	0	0	1	0	0	0	0	0	0	0	74
18864293	f	md	CN	2021	0	1	0	1	0	0	0	0	0	0	0	0	64
18864565	f	cn	DK	2021	0	0	0	0	0	0	0	0	0	0	0	0	
18864700	m	hp	DE	2021	0	1	1	0	0	0	0	0	0	0	0	0	55
18864712	m	hp	CA	2021	0	1	0	0	1	0	0	0	0	0	0	0	
18864818	f	cn	Not re	2021	0	0	0	0	0	0	0	0	0	0	0	0	51
18865477	m	Not repo	ID	2021	0	1	0	1	0	0	0	0	0	0	0	0	
18866533	m	md	US	2021	0	1	1	0	0	0	0	0	0	0	0	0	67
18866601	f	cn	CN	2021	0	0	0	0	0	0	0	0	0	0	0	0	72
18867006	m	md	FR	2021	0	1	0	0	0	1	0	0	0	0	0	0	68
18867150	f	hp	SG	2021	1	0	0	0	0	0	0	0	0	0	0	0	84.1

18867386	m	md	JP	2021	0	0	0	0	0	0	0	0	0	0	0	0	64
18867626	f	md	PL	2021	1	0	0	0	0	0	0	0	0	0	0	0	
18868166	m	md	CH	2021	0	1	1	0	0	0	0	0	0	0	0	0	66
18868704	m	hp	SG	2021	1	0	0	0	0	0	0	0	0	0	0	0	75.5
18868864	Not	md	JP	2021	0	0	0	0	0	0	0	0	0	0	0	0	78
18869067	m	cn	SG	2021	1	0	0	0	0	0	0	0	0	0	0	0	81.9
18869478	m	md	JP	2021	0	1	1	0	0	0	0	0	0	0	0	0	76
18869670	m	md	US	2021	0	0	0	0	0	0	0	0	0	0	0	0	
18869810	m	md	IL	2021	0	1	1	0	0	0	0	0	0	0	0	0	66
18870036	m	hp	AU	2021	0	1	1	0	0	0	0	0	0	0	0	0	62
18870082	f	hp	IE	2021	0	1	1	0	0	0	0	0	0	0	0	0	53
18870160	f	hp	SG	2021	1	0	0	0	0	0	0	0	0	0	0	0	69.8
18870362	f	md	ES	2021	0	1	0	0	1	0	0	0	0	0	0	0	
18870472	m	cn	SG	2021	1	0	0	0	0	0	0	0	0	0	0	0	60.2
18870557	m	md	CH	2021	0	1	1	0	0	0	0	0	0	0	0	0	66
18871251	m	md	JP	2021	0	1	0	0	0	1	0	0	0	0	0	0	80
18871418	m	hp	DE	2021	0	1	0	0	0	1	0	0	0	0	0	0	60
18872158	f	ph	NZ	2021	1	0	0	0	0	0	0	0	0	0	0	0	71
18872889	m	md	CN	2021	0	1	1	0	0	0	0	0	0	0	0	0	62
18872911	m	md	FR	2021	0	1	1	0	0	0	0	0	0	0	0	0	49
18873026	m	cn	JP	2021	0	1	1	0	0	0	0	0	0	0	0	0	65
18873116	Not	md	CN	2021	0	0	0	0	0	0	0	0	0	0	0	0	
18873125	m	md	JP	2021	0	1	0	1	0	0	0	0	0	0	0	0	
18873229	f	md	DE	2021	0	0	0	0	0	0	0	0	0	0	0	0	63
18873246	f	ph	JP	2021	1	0	0	0	0	0	0	0	0	0	0	0	80
18873309	Not	md	TR	2021	0	0	0	0	0	0	0	0	0	0	0	0	
18873445	f	cn	TH	2021	0	0	0	0	0	0	0	0	0	0	0	0	
18873675	f	md	JP	2021	0	1	0	0	0	1	0	0	0	0	0	0	68
18874226	m	cn	CN	2021	0	1	0	0	1	0	0	0	0	0	0	0	
18874259	f	cn	US	2021	0	0	0	0	0	0	0	0	0	0	0	0	
18874770	Not	md	US	2021	0	0	0	0	0	0	0	0	0	0	0	0	
18874829	f	hp	CN	2021	1	0	0	0	0	0	0	0	0	0	0	0	65
18874830	f	cn	CN	2021	1	0	0	0	0	0	0	0	0	0	0	0	
18874840	f	hp	CN	2021	1	0	0	0	0	0	0	0	0	0	0	0	70

18874874	f	hp	CN	2021	1	0	0	0	0	0	0	0	0	0	0	0	68
18874992	Not	cn	US	2021	0	0	0	0	0	0	0	0	0	0	0	0	
18875383	f	md	IE	2021	0	0	0	0	0	0	1	0	0	0	1	0	60
18875429	f	md	US	2021	1	0	0	0	0	0	0	0	0	0	0	0	
18875565	f	hp	US	2021	0	0	0	0	0	0	0	0	0	0	0	0	77
18875653	f	md	FR	2021	1	0	0	0	0	0	0	0	0	0	0	1	70.7
18875667	f	md	ES	2021	0	1	0	0	1	0	0	0	0	0	0	0	
18876176	f	md	US	2021	1	0	0	0	0	0	0	0	0	0	0	0	60
18877469	f	md	JP	2021	0	1	0	1	0	0	0	0	0	0	0	1	71
18877789	f	ph	JP	2021	1	0	0	0	0	0	0	0	0	0	0	0	75
18878912	f	md	US	2021	0	0	0	0	0	0	0	0	0	0	0	0	74
18879169	f	Not repo	SG	2021	1	0	0	0	0	0	0	0	0	0	0	0	59.6
18879275	Not	md	US	2021	1	0	0	0	0	0	0	0	0	0	0	0	
18879326	m	hp	US	2021	0	0	0	0	0	0	0	0	0	0	0	0	77
18879597	m	md	CN	2021	0	0	0	0	0	0	0	0	0	0	0	0	59
18880006	f	ph	US	2021	0	0	0	0	0	0	0	0	0	0	0	0	
18880261	f	md	MA	2021	1	0	0	0	0	0	0	0	0	0	0	0	60
18880457	m	hp	FR	2021	0	1	0	0	1	0	0	0	0	0	0	0	75
18881103	m	md	JP	2021	0	1	0	0	1	0	0	0	0	0	0	0	74
18881414	m	md	BE	2021	0	1	0	0	1	0	0	0	0	0	0	0	82
18881425	f	ph	US	2021	0	1	0	1	0	0	0	0	0	0	0	0	
18881483	m	hp	CA	2021	0	1	0	0	1	0	0	0	0	0	0	0	61
18882739	m	md	CH	2021	0	1	1	0	0	0	0	0	0	0	0	0	66
18883092	Not	hp	CN	2021	0	0	0	0	0	0	0	0	0	0	0	0	
18883698	Not	hp	CN	2021	0	0	0	0	0	0	0	0	0	0	0	0	
18884258	m	md	JP	2021	0	1	0	0	1	0	0	0	0	0	0	0	79
18886238	m	cn	CH	2021	0	1	1	0	0	0	0	0	0	0	0	0	63
18886269	m	cn	LB	2021	0	0	0	0	0	0	0	0	0	0	0	0	58
18886391	f	cn	US	2021	1	0	0	0	0	0	0	0	0	0	0	0	
18886413	m	Not repo	AU	2021	0	1	0	1	0	0	0	0	0	0	0	0	60
18886965	f	Not repo	JP	2021	1	0	0	0	0	0	0	0	0	0	0	0	77
18887165	m	hp	US	2021	0	0	0	0	0	0	0	0	0	0	0	0	40
18887189	m	md	US	2021	0	1	0	1	0	0	0	0	0	0	0	1	73.3
18887817	f	hp	CN	2021	1	0	0	0	0	0	0	0	0	0	0	0	46

18888809	m	cn	MY	2021	0	1	1	0	0	0	0	0	0	0	0	0	48
18888890	m	cn	JP	2021	0	1	1	0	0	0	0	0	0	0	0	0	53
18888910	Not	Not repo	JP	2021	1	0	0	0	0	0	0	0	0	0	0	0	
18889023	m	Not repo	AU	2021	0	1	0	1	0	0	0	0	0	0	0	0	
18889051	f	md	US	2021	0	0	0	0	0	0	0	0	0	0	0	0	65
18889056	f	cn	US	2021	0	0	0	0	0	0	0	0	0	0	0	0	
18889061	f	md	US	2021	0	0	0	0	0	0	0	0	0	0	0	0	65
18889084	m	Not repo	ID	2021	1	0	0	0	0	0	0	0	0	0	0	0	
18889150	f	Not repo	ID	2021	1	0	0	0	0	0	0	0	0	0	0	0	66.6
18889163	m	md	JP	2021	0	1	0	1	0	0	0	0	0	0	0	1	72.8
18889242	m	md	AU	2021	1	0	0	0	0	0	0	0	0	0	0	0	
18889523	m	md	TR	2021	0	0	0	0	0	0	0	0	0	0	0	0	48
18889542	f	md	HR	2021	0	1	1	0	0	0	0	0	0	0	0	0	75
18889797	f	hp	DE	2021	0	1	1	0	0	0	0	0	0	0	0	0	70
18890645	f	md	JP	2021	0	1	0	0	0	1	0	0	0	0	0	0	70
18891848	Not	hp	US	2021	0	0	0	0	0	0	0	0	0	0	0	0	
18894418	f	cn	US	2021	0	0	0	0	0	0	0	0	0	0	0	0	
18894470	f	Not repo	MY	2021	1	0	0	0	0	0	0	0	0	0	0	0	61.7
18894531	Not	ph	JP	2021	1	0	0	0	0	0	0	0	0	0	0	0	67
18894537	m	ph	JP	2021	1	0	0	0	0	0	0	0	0	0	0	0	62
18894877	m	md	ID	2021	0	1	0	0	0	1	0	0	0	0	0	0	
18895003	m	cn	FR	2021	0	1	1	0	0	0	0	0	0	0	0	0	49
18895163	f	md	US	2021	0	0	0	0	0	0	0	0	0	0	0	0	
18896142	m	md	JP	2021	0	1	0	0	0	1	0	0	0	0	0	0	
18896288	Not	ph	JP	2021	1	0	0	0	0	0	0	0	0	0	0	0	
18896433	f	md	FR	2021	1	0	0	0	0	0	0	0	0	0	0	0	
18897992	f	md	IE	2021	0	0	0	0	0	0	1	0	0	0	1	0	60
18898149	f	cn	CN	2021	0	0	0	0	0	0	0	0	0	0	0	0	65
18898182	Not	md	JP	2021	0	1	0	0	1	0	0	0	0	0	0	0	
18898772	m	md	JP	2021	0	1	0	1	0	0	0	0	0	0	0	1	60
18898820	Not	md	JP	2021	0	1	0	0	1	0	0	0	0	0	0	0	
18899179	m	md	KR	2021	0	1	0	0	0	1	0	0	0	0	0	0	76
18899240	f	md	RO	2021	0	1	0	1	0	0	0	0	0	0	0	0	64
18899245	m	md	JP	2021	0	1	0	1	0	0	0	0	0	0	0	0	

18899248	f	md	RO	2021	0	1	0	1	0	0	0	0	0	0	0	0	64
18899401	m	md	JP	2021	0	1	0	0	1	0	0	0	0	0	0	1	60
18899665	m	hp	IT	2021	0	0	0	0	0	0	0	0	0	0	0	0	68
18900026	m	hp	NL	2021	0	1	1	0	0	0	0	0	0	0	0	0	61
18900055	m	md	TR	2021	0	1	0	0	0	1	0	0	0	0	0	0	77
18900156	f	Not repo	US	2021	1	0	0	0	0	0	0	0	0	0	0	1	75
18900917	m	md	JP	2021	0	1	0	1	0	0	0	0	0	0	0	0	
18901066	m	md	JP	2021	0	0	0	0	0	0	0	0	0	0	0	0	57
18901481	f	Not repo	CN	2021	0	1	0	1	0	0	0	0	0	0	0	0	59
18901816	f	Not repo	MY	2021	1	0	0	0	0	0	0	0	0	0	0	0	48.8
18901836	m	md	JP	2021	0	1	0	0	0	1	0	0	0	0	0	0	80
18902137	f	hp	DE	2021	0	1	0	1	0	0	0	0	0	0	0	0	61
18902281	m	hp	JP	2021	0	1	1	0	0	0	0	0	0	0	0	0	46
18902466	Not	hp	JP	2021	0	1	1	0	0	0	0	0	0	0	0	0	
18902659	f	cn	US	2021	1	0	0	0	0	0	0	0	0	0	0	0	
18902791	f	hp	US	2021	0	1	1	0	0	0	0	0	0	0	0	0	58
18903179	m	md	JP	2021	0	1	0	0	1	0	0	0	0	0	0	0	64
18903245	m	Not repo	CN	2021	0	1	0	1	0	0	0	0	0	0	0	0	60
18903285	Not	md	JP	2021	0	1	0	0	0	1	0	0	0	0	0	0	
18904181	f	Not repo	BE	2021	1	0	0	0	0	0	0	0	0	0	0	0	
18904340	f	hp	CA	2021	0	1	0	1	0	0	0	0	0	0	0	0	75
18904586	m	md	CN	2021	0	1	0	0	0	1	0	0	0	0	0	0	69
18904777	m	cn	CN	2021	0	0	0	0	0	0	0	0	0	0	0	0	60
18904814	m	md	GB	2021	0	1	0	0	0	1	0	0	0	0	0	0	76
18905086	f	md	US	2021	1	0	0	0	0	0	0	0	0	0	0	0	
18905693	f	md	NO	2021	0	1	1	0	0	0	0	0	0	0	0	0	68
18906061	m	hp	JP	2021	0	1	0	0	1	0	0	0	0	0	0	0	84
18906318	f	md	DK	2021	0	0	0	0	0	0	0	0	0	0	0	0	56
18906580	f	cn	Not re	2021	0	0	0	0	0	0	0	0	0	0	0	0	
18906616	m	md	DE	2021	1	0	0	0	0	0	0	0	0	0	0	0	61
18906631	f	md	JP	2021	0	0	0	0	0	0	0	0	0	0	0	0	61
18906758	m	md	JP	2021	0	0	0	0	0	0	0	0	0	0	0	0	72
18907182	m	hp	CA	2021	0	1	0	0	1	0	0	0	0	0	0	0	53
18907766	m	md	JP	2021	0	1	0	0	1	0	0	0	0	0	0	0	59

18907837	f	ph	CA	2021	0	1	0	0	1	0	0	0	0	0	0	0	61
18908146	f	Not repo	JP	2021	1	0	0	0	0	0	0	0	0	0	0	0	80
18908158	f	md	JP	2021	0	1	0	1	0	0	0	0	0	0	0	1	72.2
18908462	m	cn	BD	2021	0	1	0	0	0	1	0	0	0	0	0	0	68
18908615	m	cn	Not re	2021	0	0	0	0	0	0	0	0	0	0	0	0	50
18908927	f	md	JP	2021	1	0	0	0	0	0	0	0	0	0	0	0	53
18910587	m	hp	DE	2021	0	1	1	0	0	0	0	0	0	0	0	0	73
18910991	Not	ph	JP	2021	1	0	0	0	0	0	0	0	0	0	0	0	
18911178	f	md	JP	2021	1	0	0	0	0	0	0	0	0	0	0	0	73
18911489	f	cn	CN	2021	1	0	0	0	0	0	0	0	0	0	0	0	78.9
18912479	m	Not repo	CN	2021	0	1	0	1	0	0	0	0	0	0	0	0	76.7
18912877	Not	hp	CN	2021	0	0	0	0	0	0	0	0	0	0	0	0	
18912887	m	hp	US	2021	0	1	0	1	0	0	0	0	0	0	0	0	67
18913155	f	ph	JP	2021	1	0	0	0	0	0	0	0	0	0	0	0	77
18913388	Not	md	JP	2021	0	1	0	0	1	0	0	0	0	0	0	0	
18914687	m	hp	AU	2021	0	1	1	0	0	0	0	0	0	0	0	0	62
18915147	m	cn	US	2021	0	0	0	0	0	0	0	0	0	0	0	0	40
18915411	f	md	JP	2021	1	0	0	0	0	0	0	0	0	0	0	0	60
18915446	f	md	JP	2021	1	0	0	0	0	0	0	0	0	0	0	0	55
18915448	f	md	JP	2021	1	0	0	0	0	0	0	0	0	0	0	0	67
18915449	m	md	JP	2021	1	0	0	0	0	0	0	0	0	0	0	0	74
18915699	m	cn	Not re	2021	0	0	0	0	0	0	0	0	0	0	0	0	59
18915702	f	md	JP	2021	1	0	0	0	0	0	0	0	0	0	0	0	65
18915714	f	md	JP	2021	1	0	0	0	0	0	0	0	0	0	0	0	64
18915876	Not	md	US	2021	0	0	0	0	0	0	0	0	0	0	0	0	
18916082	Not	md	US	2021	0	1	0	0	1	0	0	0	0	0	0	0	
18916171	f	cn	AU	2021	0	1	1	0	0	0	0	0	0	0	0	0	
18916306	f	hp	US	2021	0	0	0	0	0	0	0	0	0	0	0	0	71
18916376	f	md	JP	2021	1	0	0	0	0	0	0	0	0	0	0	0	72
18916378	f	md	JP	2021	1	0	0	0	0	0	0	0	0	0	0	0	76
18916524	Not	md	JP	2021	0	0	0	0	0	0	0	0	0	0	0	0	
18916713	m	md	IL	2021	0	1	1	0	0	0	0	0	0	0	0	0	71
18916757	f	md	ES	2021	0	1	0	0	1	0	0	0	0	0	0	0	
18916773	m	cn	IN	2021	0	0	0	0	0	0	0	0	0	0	0	0	63

18916786	f	md	ES	2021	0	1	1	0	0	0	0	0	0	0	0	0	65
18916924	m	ph	JP	2021	0	0	0	0	0	0	1	0	0	1	0	1	71
18917092	m	md	US	2021	0	1	1	0	0	0	0	0	0	0	0	0	70
18917115	Not	Not repo	BR	2021	1	0	0	0	0	0	0	0	0	0	0	0	
18917197	m	md	JP	2021	1	0	0	0	0	0	0	0	0	0	0	0	78
18917383	f	md	CA	2021	0	0	0	0	0	0	0	0	0	0	0	0	35
18917716	f	cn	US	2021	1	0	0	0	0	0	0	0	0	0	0	0	52.7
18917769	f	hp	ES	2021	0	1	1	0	0	0	0	0	0	0	0	0	70
18917828	f	cn	Not re	2021	0	0	0	0	0	0	0	0	0	0	0	0	
18917849	f	hp	DE	2021	0	0	0	0	0	0	0	0	0	0	0	0	63
18918211	m	Not repo	MY	2021	1	0	0	0	0	0	0	0	0	0	0	0	92
18918257	f	md	US	2021	1	0	0	0	0	0	0	0	0	0	0	0	
18918375	f	md	BE	2021	0	1	0	1	0	0	0	0	0	0	0	0	76
18918689	f	md	US	2021	0	1	0	0	0	1	0	0	0	0	0	0	64
18918751	f	ph	JP	2021	0	0	0	0	0	0	0	0	0	0	0	0	60
18918904	Not	md	US	2021	0	0	0	0	0	0	0	0	0	0	0	0	
18919403	f	md	DE	2021	0	0	0	0	0	0	0	0	0	0	0	0	
18919813	f	cn	IN	2021	0	1	0	0	1	0	0	0	0	0	0	0	
18919957	f	Not repo	MY	2021	1	0	0	0	0	0	0	0	0	0	0	0	63.3
18920370	Not	md	US	2021	0	0	0	0	0	0	0	0	0	0	0	0	
18920424	m	hp	ES	2021	0	1	0	0	1	0	0	0	0	0	0	0	75
18920437	m	md	DE	2021	0	0	0	0	0	0	0	0	0	0	0	0	56
18920511	f	md	US	2021	0	0	0	0	0	0	0	0	0	0	0	0	68
18920916	m	md	PT	2021	0	1	1	0	0	0	0	0	0	0	0	0	
18920937	f	cn	US	2021	0	1	1	0	0	0	0	0	0	0	0	0	85
18920948	m	md	JP	2021	0	1	0	0	1	0	0	0	0	0	0	0	72
18921159	m	cn	US	2021	0	1	1	0	0	0	0	0	0	0	0	0	
18921218	m	md	JP	2021	1	0	0	0	0	0	0	0	0	0	0	0	66
18921229	m	md	JP	2021	1	0	0	0	0	0	0	0	0	0	0	0	83
18921248	Not	hp	JP	2021	0	0	0	0	0	0	0	0	0	0	0	0	
18922486	f	Not repo	MY	2021	1	0	0	0	0	0	0	0	0	0	0	0	50.5
18922784	f	md	JP	2021	1	0	0	0	0	0	0	0	0	0	0	0	67
18922785	m	md	JP	2021	1	0	0	0	0	0	0	0	0	0	0	0	61
18922924	m	md	JP	2021	1	0	0	0	0	0	0	0	0	0	0	0	75

18923103	Not	Not repo	AU	2021	0	1	0	1	0	0	0	0	0	0	0	0	
18923435	f	hp	CA	2021	0	1	1	0	0	0	0	0	0	0	0	0	64
18923622	m	cn	FR	2021	0	1	1	0	0	0	0	0	0	0	0	0	49
18924056	f	Not repo	JP	2021	1	0	0	0	0	0	0	0	0	0	0	1	92
18924123	m	md	NO	2021	1	0	0	0	0	0	0	0	0	0	0	0	73.1
18924256	f	cn	NP	2021	0	0	0	0	0	0	0	0	0	0	0	0	35
18924438	m	md	JP	2021	0	1	0	0	1	0	0	0	0	0	0	0	80
18924544	f	Not repo	MY	2021	1	0	0	0	0	0	0	0	0	0	0	0	71.7
18924546	Not	Not repo	AU	2021	0	1	0	1	0	0	0	0	0	0	0	0	
18924575	f	md	TR	2021	0	0	0	0	0	0	0	0	0	0	0	0	37
18925206	m	cn	JP	2021	0	1	1	0	0	0	0	0	0	0	0	0	70
18925260	f	cn	US	2021	0	1	1	0	0	0	0	0	0	0	0	0	60
18925264	m	md	ID	2021	0	1	0	0	0	1	0	0	0	0	0	0	76
18925407	m	Not repo	MY	2021	1	0	0	0	0	0	0	0	0	0	0	0	56.5
18925665	Not	ph	JP	2021	0	1	0	0	1	0	0	0	0	0	0	0	
18925709	Not	ph	JP	2021	1	0	0	0	0	0	0	0	0	0	0	0	
18925719	m	hp	US	2021	0	1	0	0	1	0	0	0	0	0	0	1	85
18926091	m	md	JP	2021	0	1	0	0	1	0	0	0	0	0	0	0	55
18926786	f	md	AT	2021	0	0	0	0	0	0	0	0	0	0	0	0	73
18926798	m	hp	JP	2021	0	0	0	0	0	0	0	0	0	0	0	0	70
18926813	m	cn	Not re	2021	0	0	0	0	0	0	0	0	0	0	0	0	
18926895	m	md	JP	2021	0	1	0	0	1	0	0	0	0	0	0	0	72
18927535	m	md	JP	2021	1	0	0	0	0	0	0	0	0	0	0	0	69
18927848	f	md	JP	2021	0	1	0	1	0	0	0	0	0	0	0	1	76
18927849	f	ph	JP	2021	1	0	0	0	0	0	0	0	0	0	0	0	77
18928232	m	md	GB	2021	0	1	0	0	0	1	0	0	0	0	0	0	76
18928348	m	md	JP	2021	1	0	0	0	0	0	0	0	0	0	0	0	67
18928384	Not	hp	MX	2021	0	0	0	0	0	0	0	0	0	0	0	0	
18928664	m	hp	AU	2021	0	1	1	0	0	0	0	0	0	0	0	0	62
18928796	f	md	US	2021	0	1	0	0	0	1	0	0	0	0	0	0	59
18929029	f	hp	US	2021	0	0	0	0	0	0	0	0	0	0	0	0	
18929215	m	cn	Not re	2021	0	0	0	0	0	0	0	0	0	0	0	0	
18929658	m	cn	Not re	2021	0	0	0	0	0	0	0	0	0	0	0	0	
18929682	f	cn	US	2021	1	0	0	0	0	0	0	0	0	0	0	0	

18929723	m	md	GB	2021	0	1	0	0	0	1	0	0	0	0	0	0	76
18929724	m	md	JP	2021	0	1	0	0	1	0	0	0	0	0	0	0	55
18930139	m	cn	Not re	2021	0	0	0	0	0	0	0	0	0	0	0	0	
18930328	Not	hp	CN	2021	0	0	0	0	0	0	0	0	0	0	0	0	
18930512	f	cn	IN	2021	0	0	0	0	0	0	0	0	0	0	0	0	72
18930518	m	cn	IN	2021	0	0	0	0	0	0	0	0	0	0	0	0	41
18930768	f	Not repo	AU	2021	0	1	0	1	0	0	0	0	0	0	0	0	
18930857	Not	md	JP	2021	1	0	0	0	0	0	0	0	0	0	0	0	
18931004	Not	Not repo	JP	2021	1	0	0	0	0	0	0	0	0	0	0	0	
18931623	f	hp	US	2021	1	0	0	0	0	0	0	0	0	0	0	0	64
18931827	m	md	JP	2021	0	1	0	0	0	1	0	0	0	0	0	0	57
18931908	Not	md	JP	2021	1	0	0	0	0	0	0	0	0	0	0	0	
18932305	m	md	US	2021	0	1	0	0	1	0	0	0	0	0	0	0	
18932843	f	md	CA	2021	1	0	0	0	0	0	0	0	0	0	0	0	58
18932907	m	md	JP	2021	0	1	0	0	0	1	0	0	0	0	0	0	58
18933004	m	cn	JP	2021	0	1	1	0	0	0	0	0	0	0	0	0	70
18933249	m	md	JP	2021	0	1	0	0	0	1	0	0	0	0	0	1	80
18933361	m	md	JP	2021	0	1	0	0	1	0	0	0	0	0	0	0	78
18933451	m	hp	LB	2021	0	1	1	0	0	0	0	0	0	0	0	0	61
18933553	m	md	US	2021	0	1	0	1	0	0	0	0	0	0	0	0	65
18933619	f	cn	CA	2021	1	0	0	0	0	0	0	0	0	0	0	0	72
18935018	Not	ph	JP	2021	0	1	0	0	1	0	0	0	0	0	0	0	
18935041	m	md	PL	2021	0	1	0	0	0	1	0	0	0	0	0	0	54
18935624	Not	md	NL	2021	0	0	0	0	0	0	0	0	0	0	0	0	
18935936	f	md	IT	2021	0	0	0	0	0	0	0	0	0	0	0	0	74
18935996	f	md	JP	2021	0	0	0	0	0	0	0	0	0	0	0	0	
18936044	f	md	AU	2021	0	1	0	0	1	0	0	0	0	0	0	0	79
18936221	m	md	JP	2021	0	1	0	0	0	1	0	0	0	0	0	0	80
18936481	f	cn	Not re	2021	0	0	0	0	0	0	0	0	0	0	0	0	
18936650	m	ph	US	2021	0	1	1	0	0	0	0	0	0	0	0	0	
18936716	m	Not repo	MY	2021	1	0	0	0	0	0	0	0	0	0	0	0	73.4
18937055	m	hp	CA	2021	0	0	0	0	0	0	0	0	0	0	0	0	59
18937193	m	md	JP	2021	0	0	0	0	0	0	0	0	0	0	0	0	65
18938384	f	md	CA	2021	1	0	0	0	0	0	0	0	0	0	0	0	

18939776	m	md	JP	2021	0	1	0	1	0	0	0	0	0	0	0	1	
18940233	m	md	US	2021	0	0	0	0	0	0	0	0	0	0	0	0	68
18940319	m	md	CH	2021	0	1	1	0	0	0	0	0	0	0	0	0	
18940699	m	Not repo	TW	2021	0	1	0	1	0	0	0	0	0	0	0	0	69
18940979	m	cn	CN	2021	0	1	0	1	0	0	0	0	0	0	0	0	46
18941019	f	md	JP	2021	0	1	0	1	0	0	0	0	0	0	0	0	68
18941166	m	ph	US	2021	1	0	0	0	0	0	0	0	0	0	0	0	69
18941228	m	md	JP	2021	0	1	0	0	0	1	0	0	0	0	0	0	68
18941275	Not	ph	JP	2021	0	1	0	0	1	0	0	0	0	0	0	1	
18941334	m	cn	CN	2021	0	1	0	1	0	0	0	0	0	0	0	0	55
18941982	f	md	US	2021	0	1	0	1	0	0	0	0	0	0	0	0	68
18941989	m	hp	CN	2021	0	1	0	0	1	0	0	0	0	0	0	0	68
18942456	m	md	CN	2021	0	0	0	0	0	0	0	0	0	0	0	0	64
18943789	m	md	JP	2021	0	1	0	0	0	1	0	0	0	0	0	0	
18944007	m	cn	Not re	2021	0	0	0	0	0	0	0	0	0	0	0	0	91
18944084	f	Not repo	US	2021	1	0	0	0	0	0	0	0	0	0	0	0	
18944429	m	md	US	2021	0	1	1	0	0	0	0	0	0	0	0	0	65
18944573	Not	hp	JP	2021	0	1	0	0	0	1	0	0	0	0	0	1	
18944574	Not	hp	JP	2021	0	1	0	0	0	1	0	0	0	0	0	1	
18944645	m	md	US	2021	0	1	1	0	0	0	0	0	0	0	0	0	
18944837	f	md	JP	2021	1	0	0	0	0	0	0	0	0	0	0	0	53
18944838	m	md	JP	2021	1	0	0	0	0	0	0	0	0	0	0	0	70
18944843	f	md	JP	2021	1	0	0	0	0	0	0	0	0	0	0	0	63
18945136	f	hp	US	2021	0	0	0	0	0	0	0	0	0	0	0	0	56
18945217	Not	ph	JP	2021	0	1	0	1	0	0	0	0	0	0	0	0	
18945252	Not	hp	JP	2021	0	1	0	0	0	1	0	0	0	0	0	1	
18945293	m	Not repo	CN	2021	0	1	0	1	0	0	0	0	0	0	0	0	68
18945362	Not	md	JP	2021	0	1	0	1	0	0	0	0	0	0	0	1	
18945363	m	md	JP	2021	1	0	0	0	0	0	0	0	0	0	0	0	80
18945542	m	md	IT	2021	0	0	0	0	0	0	0	0	0	0	0	0	79
18945629	Not	ph	JP	2021	0	1	0	1	0	0	0	0	0	0	0	1	
18945687	Not	md	JP	2021	0	0	0	0	0	0	0	0	0	0	0	0	
18945721	m	cn	Not re	2021	0	0	0	0	0	0	0	0	0	0	0	0	
18946267	f	hp	SG	2021	1	0	0	0	0	0	0	0	0	0	0	0	89.3

18946657	f	md	HR	2021	0	1	1	0	0	0	0	0	0	0	0	0	75
18947006	m	md	JP	2021	1	0	0	0	0	0	0	0	0	0	0	0	63
18947268	m	hp	KR	2021	0	1	1	0	0	0	0	0	0	0	0	0	69
18947515	m	hp	IN	2021	0	0	0	0	0	0	0	0	0	0	0	0	35
18948048	f	md	JP	2021	1	0	0	0	0	0	0	0	0	0	0	0	38
18948109	f	hp	JP	2021	1	0	0	0	0	0	0	0	0	0	0	0	38
18948234	f	md	JP	2021	1	0	0	0	0	0	0	0	0	0	0	0	88
18948909	m	hp	DE	2021	0	0	0	0	0	0	0	0	0	0	0	0	56
18950831	Not	md	US	2021	1	0	0	0	0	0	0	0	0	0	0	0	
18951087	m	ph	ES	2021	0	1	1	0	0	0	0	0	0	0	0	0	79
18951472	m	cn	US	2021	0	1	0	0	1	0	0	0	0	0	0	0	
18951655	f	cn	Not re	2021	0	0	0	0	0	0	0	0	0	0	0	0	
18951847	m	cn	CN	2021	0	1	0	0	1	0	0	0	0	0	0	0	58
18951918	m	md	GB	2021	0	1	0	0	0	1	0	0	0	0	0	0	73
18952540	m	hp	DE	2021	0	1	0	0	0	1	0	0	0	0	0	0	69
18952586	Not	hp	JP	2021	0	0	0	0	0	0	0	0	0	0	0	0	
18952587	Not	hp	JP	2021	0	0	0	0	0	0	0	0	0	0	0	0	
18952589	Not	hp	JP	2021	0	0	0	0	0	0	0	0	0	0	0	0	
18952591	Not	hp	JP	2021	0	0	0	0	0	0	0	0	0	0	0	0	
18952913	f	md	JP	2021	1	0	0	0	0	0	0	0	0	0	0	0	72
18952928	m	md	JP	2021	1	0	0	0	0	0	0	0	0	0	0	0	74
18952946	m	md	FR	2021	0	1	0	0	1	0	0	0	0	0	0	1	82
18953115	m	md	SG	2021	0	1	0	0	0	1	0	0	0	0	0	0	70
18953387	f	cn	Not re	2021	0	0	0	0	0	0	0	0	0	0	0	0	
18953555	f	md	ES	2021	0	1	1	0	0	0	0	0	0	0	0	0	65
18954640	m	hp	DE	2021	0	1	1	0	0	0	0	0	0	0	0	0	62
18954652	Not	md	US	2021	0	0	0	0	0	0	0	0	0	0	0	0	91
18954830	f	md	ES	2021	0	1	0	0	1	0	0	0	0	0	0	0	
18954947	m	md	JP	2021	0	1	0	0	0	1	0	0	0	0	0	0	50
18955028	m	cn	CN	2021	0	1	0	1	0	0	0	0	0	0	0	0	70
18955069	f	cn	Not re	2021	0	0	0	0	0	0	0	0	0	0	0	0	
18955085	m	hp	US	2021	0	1	0	1	0	0	0	0	0	0	0	0	
18955340	Not	hp	US	2021	0	0	0	0	0	0	0	0	0	0	0	0	
18955359	m	md	RS	2021	0	1	0	0	0	1	0	0	0	0	0	0	64

18955449	m	Not repo	ID	2021	0	1	0	1	0	0	0	0	0	0	0	0	63.7
18955573	m	cn	NP	2021	0	0	0	0	0	0	0	0	0	0	0	0	77
18955634	f	md	PL	2021	0	0	0	0	0	0	0	0	0	0	0	0	74
18956149	m	hp	CN	2021	0	1	0	0	1	0	0	0	0	0	0	0	63
18956313	m	md	US	2021	0	1	0	0	1	0	0	0	0	0	0	1	60
18956432	m	ph	JP	2021	0	1	0	0	1	0	0	0	0	0	0	1	74
18956546	m	md	JP	2021	0	1	0	0	1	0	0	0	0	0	0	0	71
18957397	f	md	AT	2021	0	0	0	0	0	0	0	0	0	0	0	0	
18957803	f	md	JP	2021	1	0	0	0	0	0	0	0	0	0	0	0	62
18957804	f	cn	CA	2021	0	0	0	0	0	0	0	0	0	0	0	0	39
18957805	f	md	JP	2021	1	0	0	0	0	0	0	0	0	0	0	0	52
18957908	f	md	JP	2021	1	0	0	0	0	0	0	0	0	0	0	0	68
18957909	f	md	JP	2021	1	0	0	0	0	0	0	0	0	0	0	0	73
18957922	m	md	JP	2021	1	0	0	0	0	0	0	0	0	0	0	0	62
18958291	f	md	JP	2021	1	0	0	0	0	0	0	0	0	0	0	0	52
18958338	m	hp	IT	2021	0	0	0	0	0	0	0	0	0	0	0	0	40
18958561	m	ph	CA	2021	0	0	0	0	0	0	0	0	0	0	0	0	62
18958971	Not	ph	JP	2021	1	0	0	0	0	0	0	0	0	0	0	0	
18958983	m	hp	IT	2021	0	1	1	0	0	0	0	0	0	0	0	0	65
18959061	f	md	JP	2021	1	0	0	0	0	0	0	0	0	0	0	0	70
18959066	Not	md	US	2021	0	0	0	0	0	0	0	0	0	0	0	0	
18959100	f	cn	CH	2021	0	0	0	0	0	0	0	0	0	0	0	0	
18959261	Not	md	JP	2021	0	1	0	1	0	0	0	0	0	0	0	1	
18959273	f	Not repo	JP	2021	1	0	0	0	0	0	0	0	0	0	0	0	
18959452	f	Not repo	ID	2021	1	0	0	0	0	0	0	0	0	0	0	0	57.1
18959453	m	hp	US	2021	1	0	0	0	0	0	0	0	0	0	0	0	89
18959743	m	cn	CA	2021	1	0	0	0	0	0	0	0	0	0	0	0	62
18959899	m	md	KR	2021	0	0	0	0	0	0	0	0	0	0	0	0	80
18960037	m	Not repo	JP	2021	0	1	0	1	0	0	0	0	0	0	0	1	70
18960529	m	md	JP	2021	0	1	0	0	0	1	0	0	0	0	0	0	80
18960762	m	md	CN	2021	1	0	0	0	0	0	0	0	0	0	0	0	73.4
18961349	f	cn	TH	2021	0	1	0	0	0	1	0	0	0	0	0	0	
18961532	m	Not repo	JP	2021	0	1	0	1	0	0	0	0	0	0	0	0	80
18961551	Not	Not repo	JP	2021	0	1	0	1	0	0	0	0	0	0	0	1	

18961675	f	hp	GB	2021	0	1	1	0	0	0	0	0	0	0	0	0	72
18962145	Not	md	JP	2021	1	0	0	0	0	0	0	0	0	0	0	0	
18963024	f	hp	FR	2021	0	0	0	0	0	0	0	0	0	0	0	0	73
18963233	Not	cn	US	2021	0	0	0	0	0	0	0	0	0	0	0	0	
18963305	m	cn	US	2021	0	1	0	0	1	0	0	0	0	0	0	0	75
18964441	f	Not repo	PL	2021	1	0	0	0	0	0	0	0	0	0	0	0	
18964850	f	md	FR	2021	1	0	0	0	0	0	0	0	0	0	0	1	
18966279	m	md	CO	2021	0	0	0	0	0	0	0	0	0	0	0	0	53
18966592	m	md	US	2021	0	0	0	0	0	0	0	0	0	0	0	0	70
18966681	m	Not repo	JP	2021	1	0	0	0	0	0	0	0	0	0	0	0	82
18967721	f	md	JP	2021	1	0	0	0	0	0	0	0	0	0	0	0	74
18967722	f	md	JP	2021	1	0	0	0	0	0	0	0	0	0	0	0	65
18967723	f	md	JP	2021	1	0	0	0	0	0	0	0	0	0	0	0	68
18967811	m	hp	DE	2021	0	1	0	0	1	0	0	0	0	0	0	0	76
18967879	f	md	JP	2021	1	0	0	0	0	0	0	0	0	0	0	0	67
18967892	f	md	CH	2021	0	0	0	0	0	0	0	0	0	0	0	0	85
18968030	f	cn	LT	2021	0	1	1	0	0	0	0	0	0	0	0	0	55
18968110	Not	Not repo	JP	2021	1	0	0	0	0	0	0	0	0	0	0	0	
18968153	f	hp	US	2021	1	0	0	0	0	0	0	0	0	0	0	0	64
18968404	m	ph	US	2021	0	1	0	0	1	0	0	0	0	0	0	0	73
18968773	m	cn	Not re	2021	0	0	0	0	0	0	0	0	0	0	0	0	63
18969483	f	Not repo	ID	2021	1	0	0	0	0	0	0	0	0	0	0	0	77.1
18969562	m	cn	SG	2021	0	1	0	1	0	0	0	0	0	0	0	0	66.5
18969645	m	hp	TR	2021	0	1	1	0	0	0	0	0	0	0	0	0	61
18970334	Not	hp	KR	2021	0	1	0	0	1	0	0	0	0	0	0	1	
18971109	Not	hp	KR	2021	0	1	0	0	1	0	0	0	0	0	0	0	
18971209	f	cn	US	2021	0	1	0	0	1	0	0	0	0	0	0	0	
18971234	f	md	JP	2021	1	0	0	0	0	0	0	0	0	0	0	0	76
18971299	m	hp	US	2021	0	1	0	1	0	0	0	0	0	0	0	0	65
18971612	m	cn	US	2021	0	0	0	0	0	0	0	0	0	0	0	0	
18971720	Not	hp	KR	2021	0	1	0	0	1	0	0	0	0	0	0	0	
18971921	m	hp	FR	2021	0	0	0	0	0	0	0	0	0	0	0	0	86
18972123	f	md	DE	2021	0	0	0	0	0	0	0	0	0	0	0	0	
18972896	f	Not repo	DE	2021	0	1	0	1	0	0	0	0	0	0	0	0	65

18973089	m	cn	TW	2021	0	1	0	1	0	0	0	0	0	0	0	0	69
18973134	m	md	TW	2021	0	0	0	0	0	0	0	0	0	0	0	0	
18973147	f	Not repo	ID	2021	1	0	0	0	0	0	0	0	0	0	0	0	58.6
18973287	m	md	CH	2021	0	0	0	0	0	0	0	0	0	0	0	0	76
18973676	f	ph	JP	2021	1	0	0	0	0	0	0	0	0	0	0	0	82
18973684	m	md	JP	2021	0	1	0	1	0	0	0	0	0	0	0	0	70
18973762	m	md	CN	2021	1	0	0	0	0	0	0	0	0	0	0	0	48.8
18973838	f	md	AT	2021	0	0	0	0	0	0	0	0	0	0	0	0	
18973880	m	cn	JP	2021	0	1	1	0	0	0	0	0	0	0	0	1	
18973914	m	md	PH	2021	0	1	1	0	0	0	0	0	0	0	0	1	
18973956	f	hp	US	2021	0	1	0	0	1	0	0	0	0	0	0	0	68
18974277	f	md	ES	2021	0	1	1	0	0	0	0	0	0	0	0	0	65
18974360	m	hp	US	2021	0	1	0	0	1	0	0	0	0	0	0	0	72
18974873	f	cn	US	2021	1	0	0	0	0	0	0	0	0	0	0	0	55
18974945	f	cn	Not re	2021	0	0	0	0	0	0	0	0	0	0	0	0	
18975356	f	cn	US	2021	0	1	0	0	1	0	0	0	0	0	0	0	75
18975420	m	cn	US	2021	0	0	0	0	0	0	0	0	0	0	0	0	
18975449	m	cn	IN	2021	0	1	0	0	1	0	0	0	0	0	0	0	
18975469	m	cn	IN	2021	0	0	0	0	0	0	0	0	0	0	0	0	48
18975823	m	ph	JP	2021	0	1	0	1	0	0	0	0	0	0	0	1	70.4
18975825	m	hp	JP	2021	0	1	0	1	0	0	0	0	0	0	0	0	47
18976090	m	hp	IT	2021	0	1	1	0	0	0	0	0	0	0	0	0	65
18978176	m	md	DE	2021	1	0	0	0	0	0	0	0	0	0	0	0	70
18979200	Not	hp	AT	2021	0	0	0	0	0	0	0	0	0	0	0	0	
18979389	m	cn	US	2021	1	0	0	0	0	0	0	0	0	0	0	0	63
18979641	m	Not repo	JP	2021	0	0	0	0	0	0	0	0	0	0	0	0	67
18980106	f	md	JP	2021	1	0	0	0	0	0	0	0	0	0	0	0	45
18980113	f	md	JP	2021	1	0	0	0	0	0	0	0	0	0	0	0	68
18980114	m	md	JP	2021	1	0	0	0	0	0	0	0	0	0	0	0	76
18980130	f	md	JP	2021	1	0	0	0	0	0	0	0	0	0	0	0	70
18980131	f	md	JP	2021	1	0	0	0	0	0	0	0	0	0	0	0	66
18980334	f	md	JP	2021	1	0	0	0	0	0	0	0	0	0	0	0	64
18980363	m	md	GR	2021	0	1	1	0	0	0	0	0	0	0	0	1	68
18980373	f	md	US	2021	0	0	0	0	0	0	0	0	0	0	0	0	66

18980416	m	md	VN	2021	0	1	0	0	0	1	0	0	0	0	0	0	
18980757	m	md	JP	2021	0	1	1	0	0	0	0	0	0	0	0	0	79
18980789	Not	md	JP	2021	0	0	0	0	0	0	0	0	0	0	0	0	
18981225	m	md	JP	2021	1	0	0	0	0	0	0	0	0	0	0	0	67
18981226	f	md	JP	2021	1	0	0	0	0	0	0	0	0	0	0	0	73
18981636	f	md	JP	2021	1	0	0	0	0	0	0	0	0	0	0	0	65
18981692	m	Not repo	ID	2021	1	0	0	0	0	0	0	0	0	0	0	0	58.5
18981807	Not	md	US	2021	0	0	0	0	0	0	0	0	0	0	0	0	
18981879	m	md	CA	2021	0	0	0	0	0	0	0	0	0	0	0	0	56
18982235	f	md	AT	2021	0	0	0	0	0	0	0	0	0	0	0	0	
18982243	m	cn	CN	2021	0	1	0	1	0	0	0	0	0	0	0	0	59
18982316	m	cn	Not re	2021	0	0	0	0	0	0	0	0	0	0	0	0	78
18982805	f	cn	US	2021	1	0	0	0	0	0	0	0	0	0	0	0	55
18983107	f	cn	US	2021	1	0	0	0	0	0	0	0	0	0	0	0	48.2
18983201	m	md	JP	2021	0	1	0	1	0	0	0	0	0	0	0	0	90
18983606	Not	md	JP	2021	0	1	0	0	0	1	0	0	0	0	0	0	
18984099	f	cn	US	2021	1	0	0	0	0	0	0	0	0	0	0	0	80
18984179	m	md	US	2021	0	1	0	0	0	1	0	0	0	0	0	1	60
18984344	f	md	FR	2021	0	1	0	0	0	1	0	0	0	0	0	0	75
18984509	Not	md	US	2021	0	0	0	0	0	0	0	0	0	0	0	0	
18984545	f	md	FR	2021	0	1	0	0	0	1	0	0	0	0	0	0	74
18984567	m	md	US	2021	0	1	0	0	0	1	0	0	0	0	0	0	73
18985499	f	hp	US	2021	0	0	0	0	0	0	0	0	0	0	0	0	69
18985869	f	cn	US	2021	0	1	1	0	0	0	0	0	0	0	0	0	66
18986015	f	md	JP	2021	1	0	0	0	0	0	0	0	0	0	0	0	48
18986024	f	md	JP	2021	1	0	0	0	0	0	0	0	0	0	0	0	75
18986026	f	md	JP	2021	1	0	0	0	0	0	0	0	0	0	0	0	81
18986163	m	hp	US	2021	0	0	0	0	0	0	0	0	0	0	0	0	62
18986378	m	md	CN	2021	0	0	0	0	0	0	0	0	0	0	0	0	73
18986452	Not	md	AU	2021	0	0	0	0	0	0	0	0	0	0	0	0	
18986484	f	hp	CN	2021	0	1	0	0	1	0	0	0	0	0	0	0	43
18986701	Not	md	JP	2021	0	1	0	1	0	0	0	0	0	0	0	0	
18987608	Not	md	AU	2021	0	0	0	0	0	0	0	0	0	0	0	0	
18987632	m	cn	US	2021	1	0	0	0	0	0	0	0	0	0	0	0	70

18987696	f	cn	US	2021	0	0	0	0	0	0	0	0	0	0	0	0	
18987838	Not	md	AU	2021	0	0	0	0	0	0	0	0	0	0	0	0	
18987998	f	md	JP	2021	1	0	0	0	0	0	0	0	0	0	0	0	59
18988326	Not	md	US	2021	0	0	0	0	0	0	0	0	0	0	0	0	
18988584	f	md	FR	2021	0	1	0	0	0	1	0	0	0	0	0	0	74
18989376	m	cn	JP	2021	0	1	1	0	0	0	0	0	0	0	0	0	
18989474	m	ph	CA	2021	0	0	0	0	0	0	0	0	0	0	0	0	56
18989803	m	md	JP	2021	0	1	0	0	1	0	0	0	0	0	0	0	70
18990202	Not	Not repo	JP	2021	0	1	0	1	0	0	0	0	0	0	0	0	
18990343	f	md	JP	2021	0	1	0	0	1	0	0	0	0	0	0	0	80
18990462	m	md	JP	2021	1	0	0	0	0	0	0	0	0	0	0	0	
18990568	m	cn	JP	2021	0	0	0	0	0	0	0	0	0	0	0	0	78
18990668	f	cn	Not re	2021	0	0	0	0	0	0	0	0	0	0	0	0	
18990736	Not	hp	US	2021	0	0	0	0	0	0	0	0	0	0	0	0	60
18990853	f	md	FR	2021	0	1	0	0	0	1	0	0	0	0	0	0	74
18991106	m	cn	US	2021	0	0	0	0	0	0	0	0	0	0	0	0	40
18991144	f	hp	US	2021	0	1	1	0	0	0	0	0	0	0	0	0	73
18991317	m	hp	US	2021	0	1	0	0	1	0	0	0	0	0	0	0	35
18991398	f	cn	US	2021	0	1	1	0	0	0	0	0	0	0	0	0	59
18991693	m	Not repo	Not re	2021	0	1	0	0	1	0	0	0	0	0	0	0	
18991975	m	hp	GR	2021	0	1	1	0	0	0	0	0	0	0	0	1	68
18992587	Not	md	JP	2021	0	1	0	1	0	0	0	0	0	0	0	0	
18992681	f	Not repo	ID	2021	1	0	0	0	0	0	0	0	0	0	0	0	66.6
18992833	m	cn	CA	2021	0	0	0	0	0	0	0	0	0	0	0	0	61
18993215	m	md	JP	2021	0	1	0	1	0	0	0	0	0	0	0	0	
18993338	Not	md	BR	2021	0	0	0	0	0	0	0	0	0	0	0	0	
18993880	f	hp	FR	2021	0	0	0	0	0	0	0	0	0	0	0	0	71
18994055	m	md	JP	2021	0	1	0	0	0	1	0	0	0	0	0	0	
18994057	f	hp	JP	2021	0	1	0	0	1	0	0	0	0	0	0	0	73
18994642	m	cn	IL	2021	0	0	0	0	0	0	0	0	0	0	0	0	65
18994749	f	md	FR	2021	0	0	0	0	0	0	0	0	0	0	0	0	74
18994981	m	hp	IT	2021	0	1	0	0	1	0	0	0	0	0	0	1	41
18995669	m	md	ES	2021	0	0	0	0	0	0	0	0	0	0	0	0	65
18996310	m	hp	IT	2021	0	0	0	0	0	0	0	0	0	0	0	0	40

18996945	m	hp	US	2021	0	1	1	0	0	0	0	0	0	0	0	0	81
18997024	Not	md	AU	2021	0	0	0	0	0	0	0	0	0	0	0	0	
18997373	Not	Not repo	PL	2021	0	1	0	1	0	0	0	0	0	0	0	0	
18997731	Not	md	US	2021	0	0	0	0	0	0	0	0	0	0	0	0	
18997948	m	cn	CN	2021	0	1	0	0	1	0	0	0	0	0	0	0	61
18998090	Not	md	MX	2021	0	0	0	0	0	0	0	0	0	0	0	0	
18998206	f	cn	IN	2021	0	0	0	0	0	0	0	0	0	0	0	0	60
18998207	f	cn	IN	2021	0	0	0	0	0	0	0	0	0	0	0	0	54
18998274	m	md	JP	2021	0	1	0	0	1	0	0	0	0	0	0	0	60
18998548	m	md	ES	2021	0	1	1	0	0	0	0	0	0	0	0	0	74
18998700	m	md	GR	2021	0	1	1	0	0	0	0	0	0	0	0	0	66
18998723	f	Not repo	HU	2021	1	0	0	0	0	0	0	0	0	0	0	0	
18999399	f	cn	US	2021	0	1	0	1	0	0	0	0	0	0	0	0	
18999552	m	cn	CN	2021	0	1	0	0	1	0	0	0	0	0	0	0	
18999861	f	md	RU	2021	0	0	0	0	0	0	0	0	0	0	0	0	70
19000418	f	md	US	2021	0	1	1	0	0	0	0	0	0	0	0	0	61
19000915	f	md	CA	2021	1	0	0	0	0	0	0	0	0	0	0	0	
19000916	m	Not repo	PL	2021	0	1	0	1	0	0	0	0	0	0	0	0	55
19000989	m	hp	ES	2021	0	1	0	0	1	0	0	0	0	0	0	0	65
19002249	Not	md	AU	2021	0	0	0	0	0	0	0	0	0	0	0	0	
19002367	Not	Not repo	AU	2021	0	1	0	1	0	0	0	0	0	0	0	0	
19002479	Not	md	AU	2021	0	0	0	0	0	0	0	0	0	0	0	0	
19002525	f	hp	GR	2021	0	1	0	0	1	0	0	0	0	0	0	0	68
19002542	f	md	ES	2021	0	1	0	0	0	1	0	0	0	0	0	0	77
19002704	m	md	JP	2021	0	1	0	0	1	0	0	0	0	0	0	0	68
19002751	f	md	JP	2021	0	1	0	1	0	0	0	0	0	0	0	1	83
19002796	f	ph	FR	2021	0	0	0	0	0	0	0	0	0	0	0	0	59
19002835	Not	md	AU	2021	0	0	0	0	0	0	0	0	0	0	0	0	
19002839	f	md	JP	2021	0	1	0	1	0	0	0	0	0	0	0	0	51
19002939	Not	md	AU	2021	0	0	0	0	0	0	0	0	0	0	0	0	
19003057	f	hp	FR	2021	0	1	0	0	1	0	0	0	0	0	0	0	80
19003430	f	md	US	2021	0	0	0	0	0	0	0	0	0	0	0	0	
19003498	m	md	JP	2021	0	1	0	0	1	0	0	0	0	0	0	0	70
19003603	m	hp	JP	2021	0	1	0	0	0	1	0	0	0	0	0	0	59

19003611	m	md	JP	2021	0	1	0	0	1	0	0	0	0	0	0	0	73
19003795	f	cn	US	2021	1	0	0	0	0	0	0	0	0	0	0	0	75
19003797	f	hp	US	2021	1	0	0	0	0	0	0	0	0	0	0	0	63
19004081	f	md	JP	2021	1	0	0	0	0	0	0	0	0	0	0	0	61
19004168	f	md	ES	2021	0	1	0	0	0	1	0	0	0	0	0	0	61
19004357	f	cn	JP	2021	1	0	0	0	0	0	0	0	0	0	0	0	
19004487	m	md	HR	2021	0	0	0	0	0	0	0	0	0	0	0	0	68
19004576	m	md	JP	2021	1	0	0	0	0	0	0	0	0	0	0	0	70
19004700	f	md	IN	2021	1	0	0	0	0	0	0	0	0	0	0	0	59.9
19004767	m	Not repo	US	2021	1	0	0	0	0	0	0	0	0	0	0	0	75.7
19004807	m	md	ID	2021	0	1	0	0	0	1	0	0	0	0	0	0	37
19004874	Not	hp	SG	2021	1	0	0	0	0	0	0	0	0	0	0	0	65.6
19004877	Not	cn	SG	2021	1	0	0	0	0	0	0	0	0	0	0	0	72.7
19005091	f	md	FR	2021	0	1	0	0	0	1	0	0	0	0	0	0	58
19005465	Not	md	VN	2021	0	0	0	0	0	0	0	0	0	0	0	0	
19005482	f	cn	Not re	2021	0	0	0	0	0	0	0	0	0	0	0	0	60
19005527	m	hp	DE	2021	0	1	0	0	0	1	0	0	0	0	0	0	53
19006024	f	cn	TW	2021	0	0	0	0	0	0	0	0	0	0	0	0	
19006410	Not	md	JP	2021	0	1	0	0	0	1	0	0	0	0	0	0	80
19006457	m	md	JP	2021	0	1	0	0	0	1	0	0	0	0	0	0	
19006495	Not	md	JP	2021	0	1	0	0	0	1	0	0	0	0	0	0	80
19007378	f	cn	US	2021	1	0	0	0	0	0	0	0	0	0	0	1	69
19007718	f	cn	JP	2021	0	1	1	0	0	0	0	0	0	0	0	0	70
19007799	m	cn	CN	2021	0	0	0	0	0	0	0	0	0	0	0	0	81
19007951	m	cn	US	2021	0	1	0	0	1	0	0	0	0	0	0	0	
19008477	m	hp	IT	2021	0	0	0	0	0	0	0	0	0	0	0	0	41
19008831	m	hp	IT	2021	0	0	0	0	0	0	0	0	0	0	0	0	41
19009082	f	md	MY	2021	0	1	0	0	0	1	0	0	0	0	0	0	73
19009088	f	hp	IN	2021	0	0	0	0	0	0	0	0	0	0	0	0	72
19009331	f	ph	JP	2021	0	1	1	0	0	0	0	0	0	0	0	0	72
19009714	f	md	ID	2021	0	1	0	0	0	1	0	0	0	0	0	0	
19009913	Not	hp	DE	2021	1	0	0	0	0	0	0	0	0	0	0	0	50
19010475	f	md	JP	2021	1	0	0	0	0	0	0	0	0	0	0	0	66
19010524	m	md	AT	2021	0	0	0	0	0	0	0	0	0	0	0	0	60

19010789	Not	md	PL	2021	0	0	0	0	0	0	0	0	0	0	0	0	
19010880	m	md	IT	2021	0	1	1	0	0	0	0	0	0	0	0	0	65
19011395	m	Not repo	JP	2021	0	1	0	1	0	0	0	0	0	0	0	1	
19011858	m	md	Not re	2021	0	0	0	0	0	0	0	0	0	0	0	0	74
19012098	Not	md	LU	2021	0	1	0	0	1	0	0	0	0	0	0	0	62
19012385	Not	hp	CA	2021	0	1	0	0	1	0	0	0	0	0	0	0	
19012509	m	md	FR	2021	0	0	0	0	0	0	0	0	0	0	0	0	48
19012567	f	hp	US	2021	0	1	1	0	0	0	0	0	0	0	0	0	76
19012603	f	ph	US	2021	0	0	0	0	0	0	0	0	0	0	0	0	73
19012755	m	md	TW	2021	0	0	0	0	0	0	0	0	0	0	0	0	80
19012838	f	Not repo	AU	2021	0	1	0	1	0	0	0	0	0	0	0	0	60
19012847	m	md	JP	2021	1	0	0	0	0	0	0	0	0	0	0	1	43
19012968	f	cn	JP	2021	0	1	1	0	0	0	0	0	0	0	0	0	53
19013250	f	md	HU	2021	0	0	0	0	0	0	0	0	0	0	0	0	66
19014010	m	md	JP	2021	0	1	0	0	1	0	0	0	0	0	0	0	75
19014218	m	md	AT	2021	0	0	0	0	0	0	0	0	0	0	0	0	60
19014788	f	md	JP	2021	0	1	0	0	0	1	0	0	0	0	0	0	70
19014793	f	md	JP	2021	0	1	0	0	1	0	0	0	0	0	0	0	70
19014794	m	md	CN	2021	0	0	0	0	0	0	0	0	0	0	0	0	55
19014801	m	hp	JP	2021	0	1	0	1	0	0	0	0	0	0	0	1	63
19015050	f	cn	IN	2021	0	0	0	0	0	0	0	0	0	0	0	0	47
19015128	f	md	JP	2021	0	1	0	0	0	1	0	0	0	0	0	0	61
19015270	f	ph	CA	2021	0	1	0	0	1	0	0	0	0	0	0	0	67
19015429	m	hp	IT	2021	0	0	0	0	0	0	0	0	0	0	0	0	41
19015622	m	hp	US	2021	0	0	0	0	0	0	0	0	0	0	0	0	
19015830	f	md	CA	2021	0	0	0	0	0	0	0	0	0	0	0	0	86
19015855	Not	md	KR	2021	0	0	0	0	0	0	0	0	0	0	0	0	
19016060	f	md	ES	2021	1	0	0	0	0	0	0	0	0	0	0	1	51
19017428	m	md	ID	2021	0	1	0	0	0	1	0	0	0	0	0	0	37
19017868	m	md	IN	2021	0	0	0	0	0	0	0	0	0	0	0	0	60
19017995	m	cn	Not re	2021	0	0	0	0	0	0	0	0	0	0	0	0	
19018647	m	hp	US	2021	0	0	0	0	0	0	0	0	0	0	0	0	
19019131	m	hp	US	2021	0	0	0	0	0	0	0	0	0	0	0	0	
19019447	m	Not repo	JP	2021	0	1	0	1	0	0	0	0	0	0	0	0	78

19019609	Not	md	JP	2021	0	1	0	0	1	0	0	0	0	0	0	0	
19020287	m	md	JP	2021	0	1	0	0	1	0	0	0	0	0	0	0	71
19020361	m	hp	CN	2021	0	1	0	0	1	0	0	0	0	0	0	0	66
19021343	Not	md	JP	2021	1	0	0	0	0	0	0	0	0	0	0	0	
19021508	Not	md	AU	2021	0	0	0	0	0	0	0	0	0	0	0	0	
19021527	m	md	JP	2021	0	1	0	0	1	0	0	0	0	0	0	0	49
19021826	f	Not repo	JP	2021	0	1	0	1	0	0	0	0	0	0	0	1	71
19022325	m	md	CA	2021	0	0	0	0	0	0	0	0	0	0	0	0	56
19022461	m	ph	CZ	2021	0	0	0	0	0	0	0	0	0	0	0	0	
19023260	f	md	JP	2021	1	0	0	0	0	0	0	0	0	0	0	0	80
19023645	m	md	US	2021	0	0	0	0	0	0	0	0	0	0	0	0	53
19024310	m	cn	CN	2021	0	1	0	0	1	0	0	0	0	0	0	0	
19024475	m	md	DE	2021	0	1	0	0	0	1	0	0	0	0	0	0	58
19024919	f	hp	DE	2021	0	0	0	0	0	0	0	0	0	0	0	0	64
19025217	f	md	JP	2021	1	0	0	0	0	0	0	0	0	0	0	0	70
19025241	f	md	JP	2021	1	0	0	0	0	0	0	0	0	0	0	0	52
19025417	m	md	JP	2021	1	0	0	0	0	0	0	0	0	0	0	0	67
19025509	m	md	JP	2021	1	0	0	0	0	0	0	0	0	0	0	0	64
19025621	m	hp	BR	2021	0	0	0	0	0	0	0	0	0	0	0	0	63
19026030	m	md	JP	2021	0	1	0	0	1	0	0	0	0	0	0	0	80
19026282	m	cn	CA	2021	0	1	0	0	1	0	0	0	0	0	0	0	54
19026389	m	md	IL	2021	0	1	1	0	0	0	0	0	0	0	0	0	59
19026473	f	cn	DE	2021	0	1	1	0	0	0	0	0	0	0	0	0	
19027427	f	ph	US	2021	0	0	0	0	0	0	0	0	0	0	0	0	
19027568	m	Not repo	JP	2021	0	1	0	1	0	0	0	0	0	0	0	1	73
19028134	m	md	JP	2021	0	1	0	0	1	0	0	0	0	0	0	0	68
19028325	m	Not repo	JP	2021	0	1	0	1	0	0	0	0	0	0	0	0	74
19028328	m	Not repo	JP	2021	0	1	0	1	0	0	0	0	0	0	0	1	75
19028468	f	md	IT	2021	0	0	0	0	0	0	0	0	0	0	0	0	
19029056	m	Not repo	JP	2021	0	1	0	1	0	0	0	0	0	0	0	0	54
19029083	f	cn	IN	2021	0	0	0	0	0	0	0	0	0	0	0	0	56
19029723	m	Not repo	JP	2021	0	1	0	1	0	0	0	0	0	0	0	1	76
19029818	m	md	CN	2021	0	0	0	0	0	0	0	0	0	0	0	0	35
19029864	m	Not repo	JP	2021	0	1	0	1	0	0	0	0	0	0	0	1	60

19030003	Not	md	MY	2021	1	0	0	0	0	0	0	0	0	0	0	0	
19030037	m	hp	BR	2021	0	0	0	0	0	0	0	0	0	0	0	0	72
19030038	m	hp	BR	2021	0	0	0	0	0	0	0	0	0	0	0	0	71
19030200	f	md	ES	2021	0	0	0	0	0	0	0	0	0	0	0	0	69
19030431	f	md	JP	2021	0	0	0	0	0	0	0	0	0	0	0	0	28
19030591	m	hp	JP	2021	0	1	0	0	1	0	0	0	0	0	0	0	75
19030754	m	hp	BR	2021	0	0	0	0	0	0	0	0	0	0	0	0	70
19030756	f	hp	BR	2021	0	0	0	0	0	0	0	0	0	0	0	0	61
19031057	f	cn	JP	2021	0	1	1	0	0	0	0	0	0	0	0	0	66
19031088	Not	md	MX	2021	0	0	0	0	0	0	0	0	0	0	0	0	115
19031475	f	md	US	2021	0	1	0	1	0	0	0	0	0	0	0	1	55
19031594	m	md	JP	2021	1	0	0	0	0	0	0	0	0	0	0	0	74
19031704	m	Not repo	JP	2021	1	0	0	0	0	0	0	0	0	0	0	0	72.7
19032063	m	md	JP	2021	0	1	0	1	0	0	0	0	0	0	0	0	71
19032314	m	cn	JP	2021	0	0	0	0	0	0	0	0	0	0	0	0	87
19032516	m	md	JP	2021	1	0	0	0	0	0	0	0	0	0	0	0	58
19033844	m	ph	CA	2021	0	0	0	0	0	0	0	0	0	0	0	0	56
19033917	Not	hp	FR	2021	0	1	0	0	1	0	0	0	0	0	0	0	73
19034534	f	md	US	2021	0	0	0	0	0	0	0	0	0	0	0	0	60
19034749	f	cn	US	2021	0	1	1	0	0	0	0	0	0	0	0	0	55
19034762	Not	md	JP	2021	0	1	0	0	0	1	0	0	0	0	0	0	80
19034993	f	cn	US	2021	1	0	0	0	0	0	0	0	0	0	0	0	59
19035072	f	cn	US	2021	1	0	0	0	0	0	0	0	0	0	0	0	72.3
19035073	m	md	CA	2021	1	0	0	0	0	0	0	0	0	0	0	0	
19036099	f	md	ID	2021	0	1	0	0	0	1	0	0	0	0	0	0	
19036234	m	cn	JP	2021	0	1	1	0	0	0	0	0	0	0	0	0	
19036474	m	md	JP	2021	0	1	0	0	0	1	0	0	0	0	0	0	60
19036921	f	md	RS	2021	0	0	0	0	0	0	0	0	0	0	0	0	58
19037053	m	cn	BE	2021	0	0	0	0	0	0	0	0	0	0	0	0	
19037312	m	md	JP	2021	0	1	0	0	1	0	0	0	0	0	0	0	70
19037443	m	md	JP	2021	0	1	0	0	0	1	0	0	0	0	0	0	60
19037603	m	md	IT	2021	0	1	1	0	0	0	0	0	0	0	0	0	65
19037657	f	cn	CN	2021	0	1	0	0	1	0	0	0	0	0	0	0	
19039216	Not	hp	DE	2021	1	0	0	0	0	0	0	0	0	0	0	0	

19039228	Not	hp	DE	2021	1	0	0	0	0	0	0	0	0	0	0	0	
19039258	Not	hp	DE	2021	1	0	0	0	0	0	0	0	0	0	0	0	
19039365	Not	hp	DE	2021	1	0	0	0	0	0	0	0	0	0	0	0	
19039433	Not	hp	DE	2021	1	0	0	0	0	0	0	0	0	0	0	0	
19039453	Not	hp	DE	2021	1	0	0	0	0	0	0	0	0	0	0	0	
19039494	Not	hp	DE	2021	1	0	0	0	0	0	0	0	0	0	0	0	
19039519	Not	hp	DE	2021	1	0	0	0	0	0	0	0	0	0	0	0	
19039520	Not	hp	DE	2021	1	0	0	0	0	0	0	0	0	0	0	0	
19039521	Not	hp	DE	2021	1	0	0	0	0	0	0	0	0	0	0	0	
19039546	Not	hp	DE	2021	1	0	0	0	0	0	0	0	0	0	0	0	
19039660	Not	hp	DE	2021	1	0	0	0	0	0	0	0	0	0	0	0	
19039661	Not	hp	DE	2021	1	0	0	0	0	0	0	0	0	0	0	0	
19039724	Not	hp	DE	2021	1	0	0	0	0	0	0	0	0	0	0	0	
19039745	m	md	JP	2021	0	1	0	0	1	0	0	0	0	0	0	0	68
19039828	m	cn	TH	2021	0	0	0	0	0	0	0	0	0	0	0	0	
19039835	Not	hp	DE	2021	1	0	0	0	0	0	0	0	0	0	0	0	
19039868	Not	hp	DE	2021	1	0	0	0	0	0	0	0	0	0	0	0	
19039880	Not	hp	DE	2021	1	0	0	0	0	0	0	0	0	0	0	0	
19039881	Not	hp	DE	2021	1	0	0	0	0	0	0	0	0	0	0	0	
19039882	Not	hp	DE	2021	1	0	0	0	0	0	0	0	0	0	0	0	
19039967	Not	hp	DE	2021	1	0	0	0	0	0	0	0	0	0	0	0	
19040030	m	cn	US	2021	0	1	1	0	0	0	0	0	0	0	0	1	
19040075	Not	hp	DE	2021	1	0	0	0	0	0	0	0	0	0	0	0	
19040522	Not	hp	DE	2021	1	0	0	0	0	0	0	0	0	0	0	0	
19040523	Not	hp	DE	2021	1	0	0	0	0	0	0	0	0	0	0	0	
19040585	f	cn	CA	2021	1	0	0	0	0	0	0	0	0	0	0	0	
19040644	m	hp	KR	2021	0	0	0	0	0	0	0	0	0	0	0	0	31
19040673	f	cn	US	2021	1	0	0	0	0	0	0	0	0	0	0	0	71
19040942	f	md	AT	2021	1	0	0	0	0	0	0	0	0	0	0	0	78
19041620	m	Not repo	JP	2021	0	1	0	1	0	0	0	0	0	0	0	1	75
19041788	m	cn	IN	2021	0	1	0	0	1	0	0	0	0	0	0	0	
19042384	f	md	NO	2021	0	1	1	0	0	0	0	0	0	0	0	0	70
19042585	f	hp	US	2021	0	0	0	0	0	0	0	0	0	0	0	0	79
19042931	f	md	IN	2021	0	0	0	0	0	0	0	0	0	0	0	0	64

19042953	f	md	JP	2021	1	0	0	0	0	0	0	0	0	0	0	0	
19042984	f	md	JP	2021	1	0	0	0	0	0	0	0	0	0	0	0	76
19042990	m	md	JP	2021	1	0	0	0	0	0	0	0	0	0	0	0	48
19043186	Not	hp	DE	2021	1	0	0	0	0	0	0	0	0	0	0	0	
19043251	Not	hp	DE	2021	1	0	0	0	0	0	0	0	0	0	0	0	
19043490	m	cn	IN	2021	0	1	0	0	1	0	0	0	0	0	0	0	67
19043578	Not	hp	DE	2021	1	0	0	0	0	0	0	0	0	0	0	0	
19043616	Not	hp	DE	2021	1	0	0	0	0	0	0	0	0	0	0	0	
19043617	Not	hp	DE	2021	1	0	0	0	0	0	0	0	0	0	0	0	
19043621	Not	hp	DE	2021	1	0	0	0	0	0	0	0	0	0	0	0	
19043656	m	cn	IN	2021	0	0	0	0	0	0	0	0	0	0	0	0	63
19043823	Not	hp	DE	2021	1	0	0	0	0	0	0	0	0	0	0	0	
19043985	Not	hp	DE	2021	1	0	0	0	0	0	0	0	0	0	0	0	
19043986	Not	hp	DE	2021	1	0	0	0	0	0	0	0	0	0	0	0	
19043988	Not	hp	DE	2021	1	0	0	0	0	0	0	0	0	0	0	0	
19044242	Not	hp	DE	2021	1	0	0	0	0	0	0	0	0	0	0	0	
19044327	f	md	US	2021	0	0	0	0	0	0	0	0	0	0	0	0	
19045926	m	ph	NL	2021	0	0	0	0	0	0	0	0	0	0	0	0	
19045995	m	md	JP	2021	0	1	0	0	1	0	0	0	0	0	0	0	70
19046033	m	Not repo	CN	2021	1	0	0	0	0	0	0	0	0	0	0	0	67
19046098	m	cn	JP	2021	0	1	1	0	0	0	0	0	0	0	0	0	79
19046104	m	md	JP	2021	0	1	0	1	0	0	0	0	0	0	0	1	65
19046260	m	ph	JP	2021	0	1	0	0	0	1	0	0	0	0	0	0	50
19046897	f	cn	Not re	2021	0	0	0	0	0	0	0	0	0	0	0	0	
19046919	m	cn	US	2021	1	0	0	0	0	0	0	0	0	0	0	0	
19047019	f	cn	US	2021	1	0	0	0	0	0	0	0	0	0	0	0	
19047021	m	md	JP	2021	0	1	0	1	0	0	0	0	0	0	0	0	63
19047046	f	cn	Not re	2021	0	0	0	0	0	0	0	0	0	0	0	0	41
19047286	Not	md	ID	2021	0	0	0	0	0	0	0	0	0	0	0	0	
19047328	m	md	JP	2021	0	1	0	1	0	0	0	0	0	0	0	0	62
19047436	m	Not repo	JP	2021	0	1	0	1	0	0	0	0	0	0	0	0	71
19047467	m	cn	JP	2021	0	1	1	0	0	0	0	0	0	0	0	0	68
19047485	f	cn	US	2021	0	1	0	1	0	0	0	0	0	0	0	1	
19047519	Not	cn	JP	2021	0	1	1	0	0	0	0	0	0	0	0	0	70

19047537	m	hp	CA	2021	0	0	0	0	0	0	0	0	0	0	0	0	56
19047754	m	hp	US	2021	0	0	0	0	0	0	0	0	0	0	0	0	71
19048352	f	md	NO	2021	0	1	1	0	0	0	0	0	0	0	0	0	
19048423	f	md	CA	2021	0	0	0	0	0	0	0	0	0	0	0	0	
19048605	m	cn	Not re	2021	0	0	0	0	0	0	0	0	0	0	0	0	
19049514	m	cn	Not re	2021	0	0	0	0	0	0	0	0	0	0	0	0	79
19049783	f	hp	CA	2021	0	1	1	0	0	0	0	0	0	0	0	0	59
19050097	m	cn	JP	2021	0	1	1	0	0	0	0	0	0	0	0	0	70
19050731	m	Not repo	JP	2021	0	1	0	1	0	0	0	0	0	0	0	0	72
19051677	m	hp	UA	2021	0	0	0	0	0	0	0	0	0	0	0	0	73
19051749	m	md	IL	2021	0	1	1	0	0	0	0	0	0	0	0	1	75
19051893	m	hp	KR	2021	0	0	0	0	0	0	0	0	0	0	0	0	60
19052098	m	md	DE	2021	0	1	0	1	0	0	0	0	0	0	0	0	70
19052112	m	hp	US	2021	0	0	0	0	0	0	0	0	0	0	0	0	63
19052118	m	hp	JP	2021	0	1	0	0	1	0	0	0	0	0	0	0	75
19052146	Not	md	JP	2021	0	0	0	0	0	0	0	0	0	0	0	0	
19052567	m	cn	Not re	2021	0	0	0	0	0	0	0	0	0	0	0	0	
19052791	m	md	US	2021	0	1	0	0	0	1	0	0	0	0	0	0	79
19053062	f	hp	FR	2021	1	0	0	0	0	0	0	0	0	0	0	0	
19053142	Not	md	US	2021	1	0	0	0	0	0	0	0	0	0	0	0	
19053211	m	hp	US	2021	0	0	0	0	0	0	0	0	0	0	0	0	
19053437	m	ph	CA	2021	0	0	0	0	0	0	0	0	0	0	0	0	56
19053458	f	Not repo	MY	2021	1	0	0	0	0	0	0	0	0	0	0	0	52.8
19053460	Not	md	JP	2021	0	1	0	1	0	0	0	0	0	0	0	0	
19053508	m	md	US	2021	0	0	0	0	0	0	0	0	0	0	0	0	
19053641	m	md	JP	2021	0	1	0	0	0	1	0	0	0	0	0	1	74
19054296	f	hp	NL	2021	0	1	1	0	0	0	0	0	0	0	0	0	70
19055394	f	md	JP	2021	1	0	0	0	0	0	0	0	0	0	0	0	73
19056112	f	md	NZ	2021	0	1	1	0	0	0	0	0	0	0	0	0	76
19056116	f	cn	CA	2021	1	0	0	0	0	0	0	0	0	0	0	0	63.3
19056224	m	md	JP	2021	0	1	0	0	1	0	0	0	0	0	0	0	50
19056280	Not	hp	US	2021	0	0	0	0	0	0	0	0	0	0	0	0	
19056498	m	hp	DE	2021	0	1	0	0	0	1	0	0	0	0	0	0	58
19056856	f	md	JP	2021	0	0	0	0	0	0	0	0	0	0	0	0	81

19057045	m	hp	US	2021	0	1	0	0	1	0	0	0	0	0	0	0	61
19057070	f	hp	US	2021	0	1	0	0	1	0	0	0	0	0	0	0	48
19057072	f	hp	US	2021	0	1	0	0	1	0	0	0	0	0	0	0	81
19057093	m	hp	US	2021	0	1	0	0	1	0	0	0	0	0	0	0	68
19057098	m	hp	US	2021	0	1	0	0	1	0	0	0	0	0	0	0	65
19057138	m	hp	US	2021	0	1	0	0	1	0	0	0	0	0	0	0	73
19057166	f	ph	US	2021	0	0	0	0	0	0	0	0	0	0	0	0	63
19057197	f	ph	JP	2021	0	1	0	0	1	0	0	0	0	0	0	0	88
19057358	f	ph	IN	2021	0	0	0	0	0	0	0	0	0	0	0	0	58
19057514	f	cn	Not re	2021	0	0	0	0	0	0	0	0	0	0	0	0	66
19057642	f	cn	US	2021	1	0	0	0	0	0	0	0	0	0	0	0	
19057786	f	hp	US	2021	0	1	0	0	1	0	0	0	0	0	0	0	44
19058044	f	Not repo	US	2021	1	0	0	0	0	0	0	0	0	0	0	0	76.9
19058403	Not	hp	US	2021	0	1	0	1	0	0	0	0	0	0	0	0	
19058428	f	md	JP	2021	0	1	0	1	0	0	0	0	0	0	0	0	71
19058678	m	Not repo	JP	2021	0	1	0	1	0	0	0	0	0	0	0	0	72
19058705	Not	hp	US	2021	0	0	0	0	0	0	0	0	0	0	0	0	
19058764	m	md	FR	2021	0	1	0	1	0	0	0	0	0	0	0	0	81
19058774	m	md	JP	2021	0	1	0	1	0	0	0	0	0	0	0	1	
19059041	m	cn	IN	2021	0	0	0	0	0	0	0	0	0	0	0	0	60
19059183	f	hp	JP	2021	0	1	0	1	0	0	0	0	0	0	0	1	75
19059264	f	hp	TR	2021	0	1	0	0	1	0	0	0	0	0	0	0	72
19059276	f	md	JP	2021	0	1	0	0	0	1	0	0	0	0	0	0	69
19059463	Not	md	US	2021	0	0	0	0	0	0	0	0	0	0	0	0	
19059464	Not	md	US	2021	0	0	0	0	0	0	0	0	0	0	0	0	
19059465	m	md	US	2021	0	0	0	0	0	0	0	0	0	0	0	0	70
19059469	f	cn	US	2021	0	0	0	0	0	0	0	0	0	0	0	0	65
19059474	m	md	ES	2021	0	1	0	0	0	1	0	0	0	0	0	0	78
19059638	m	md	JP	2021	0	1	0	0	1	0	0	0	0	0	0	0	70
19059985	m	md	LB	2021	1	0	0	0	0	0	0	0	0	0	0	0	80
19060213	m	md	BR	2021	0	0	0	0	0	0	0	0	0	0	0	0	70
19060246	m	cn	US	2021	0	0	0	0	0	0	0	0	0	0	0	0	
19060769	f	ph	JP	2021	0	0	0	0	0	0	0	0	0	0	0	0	
19060849	m	md	JP	2021	0	1	0	0	0	1	0	0	0	0	0	0	

19061135	f	md	US	2021	0	1	0	0	1	0	0	0	0	0	0	0	61
19061190	f	md	RO	2021	0	0	0	0	0	0	1	0	0	0	1	0	64
19062504	m	cn	Not re	2021	0	0	0	0	0	0	0	0	0	0	0	0	
19062646	f	cn	US	2021	1	0	0	0	0	0	0	0	0	0	0	0	71
19063288	f	md	US	2021	0	0	0	0	0	0	0	0	0	0	0	0	62
19063382	m	cn	Not re	2021	0	0	0	0	0	0	0	0	0	0	0	0	
19063818	m	cn	Not re	2021	0	0	0	0	0	0	0	0	0	0	0	0	73
19063912	m	Not repo	JP	2021	1	0	0	0	0	0	0	0	0	0	0	0	60
19063917	m	cn	US	2021	1	0	0	0	0	0	0	0	0	0	0	0	63.5
19064104	Not	md	JP	2021	0	1	0	0	0	1	0	0	0	0	0	0	
19064115	Not	Not repo	DE	2021	1	0	0	0	0	0	0	0	0	0	0	0	
19064167	m	md	JP	2021	1	0	0	0	0	0	0	0	0	0	0	0	
19064382	f	md	JP	2021	1	0	0	0	0	0	0	0	0	0	0	0	82
19064443	f	hp	US	2021	0	0	0	0	0	0	0	0	0	0	0	0	62
19064495	m	md	DE	2021	0	0	0	0	0	0	0	0	0	0	0	0	
19064504	Not	hp	DE	2021	1	0	0	0	0	0	0	0	0	0	0	0	
19064511	Not	hp	DE	2021	1	0	0	0	0	0	0	0	0	0	0	0	
19064512	Not	hp	DE	2021	1	0	0	0	0	0	0	0	0	0	0	0	
19064558	Not	hp	DE	2021	1	0	0	0	0	0	0	0	0	0	0	0	
19064560	Not	Not repo	DE	2021	1	0	0	0	0	0	0	0	0	0	0	0	
19064565	Not	Not repo	DE	2021	1	0	0	0	0	0	0	0	0	0	0	0	
19064570	Not	Not repo	DE	2021	1	0	0	0	0	0	0	0	0	0	0	0	
19064842	m	hp	JP	2021	0	1	0	1	0	0	0	0	0	0	0	1	72
19064902	f	cn	CN	2021	0	1	0	0	1	0	0	0	0	0	0	1	65
19064986	Not	hp	DE	2021	1	0	0	0	0	0	0	0	0	0	0	0	
19064989	Not	Not repo	DE	2021	1	0	0	0	0	0	0	0	0	0	0	0	
19064993	Not	hp	DE	2021	1	0	0	0	0	0	0	0	0	0	0	0	
19064995	Not	hp	DE	2021	1	0	0	0	0	0	0	0	0	0	0	0	
19064997	Not	hp	DE	2021	1	0	0	0	0	0	0	0	0	0	0	0	
19065122	m	md	JP	2021	0	1	0	0	0	1	0	0	0	0	0	0	70
19065472	Not	Not repo	DE	2021	1	0	0	0	0	0	0	0	0	0	0	0	
19065943	f	cn	Not re	2021	0	0	0	0	0	0	0	0	0	0	0	0	46
19066196	m	md	US	2021	0	1	0	0	1	0	0	0	0	0	0	1	65
19066207	m	md	IN	2021	0	0	0	0	0	0	0	0	0	0	0	0	63

19066684	f	md	US	2021	0	1	0	0	1	0	0	0	0	0	0	0	67
19066993	m	ph	JP	2021	0	1	1	0	0	0	0	0	0	0	0	0	73
19067181	f	cn	US	2021	1	0	0	0	0	0	0	0	0	0	0	0	84
19068705	m	cn	US	2021	0	1	1	0	0	0	0	0	0	0	0	0	75
19069002	m	md	JP	2021	0	0	0	0	0	0	0	0	0	0	0	0	70
19069118	m	md	JP	2021	0	1	0	0	0	1	0	0	0	0	0	0	
19069130	m	hp	CA	2021	0	1	1	0	0	0	0	0	0	0	0	0	78
19069700	f	cn	Not re	2021	0	0	0	0	0	0	0	0	0	0	0	0	
19069713	f	cn	Not re	2021	0	0	0	0	0	0	0	0	0	0	0	0	
19069730	m	cn	IN	2021	0	1	0	0	1	0	0	0	0	0	0	0	50
19070031	f	hp	FR	2021	0	1	0	0	1	0	0	0	0	0	0	0	84
19070066	f	Not repo	JP	2021	1	0	0	0	0	0	0	0	0	0	0	0	76
19070504	m	cn	JP	2021	0	0	0	0	0	0	0	0	0	0	0	0	72
19071024	m	hp	RU	2021	0	0	0	0	0	0	0	0	0	0	0	0	61
19071152	f	md	US	2021	0	1	0	0	1	0	0	0	0	0	0	0	64
19072102	f	hp	GB	2021	0	1	1	0	0	0	0	0	0	0	0	0	61
19072339	m	Not repo	KW	2021	1	0	0	0	0	0	0	0	0	0	0	0	48
19072573	Not	md	DE	2021	0	0	0	0	0	0	0	0	0	0	0	0	
19072596	f	md	DE	2021	0	0	0	0	0	0	0	0	0	0	0	0	58
19072910	m	hp	DE	2021	0	1	1	0	0	0	0	0	0	0	0	0	52
19072944	f	hp	US	2021	0	1	1	0	0	0	0	0	0	0	0	0	57
19073315	f	hp	CA	2021	0	1	0	0	1	0	0	0	0	0	0	0	73
19073673	m	md	JP	2021	0	1	0	0	0	1	0	0	0	0	0	0	65
19074026	f	cn	US	2021	1	0	0	0	0	0	0	0	0	0	0	0	63
19074130	Not	hp	DE	2021	1	0	0	0	0	0	0	0	0	0	0	0	
19074135	Not	hp	DE	2021	1	0	0	0	0	0	0	0	0	0	0	0	
19074136	Not	hp	DE	2021	1	0	0	0	0	0	0	0	0	0	0	0	
19074158	m	hp	DE	2021	0	1	0	0	1	0	0	0	0	0	0	0	77
19074176	f	md	JP	2021	0	1	0	0	1	0	0	0	0	0	0	0	83
19074314	Not	Not repo	DE	2021	1	0	0	0	0	0	0	0	0	0	0	0	
19074607	Not	hp	DE	2021	1	0	0	0	0	0	0	0	0	0	0	0	
19074987	Not	hp	DE	2021	1	0	0	0	0	0	0	0	0	0	0	0	
19074989	f	Not repo	GB	2021	0	1	0	1	0	0	0	0	0	0	0	1	78
19075084	Not	hp	US	2021	0	0	0	0	0	0	0	0	0	0	0	0	

19075139	Not	hp	DE	2021	1	0	0	0	0	0	0	0	0	0	0	0	
19075142	Not	md	DE	2021	1	0	0	0	0	0	0	0	0	0	0	0	
19075145	Not	hp	DE	2021	1	0	0	0	0	0	0	0	0	0	0	0	
19075147	m	md	JP	2021	1	0	0	0	0	0	0	0	0	0	0	0	60
19075606	f	hp	US	2021	0	0	0	0	0	0	0	0	0	0	0	0	
19075695	m	md	JP	2021	0	1	0	0	1	0	0	0	0	0	0	0	67
19075957	Not	hp	DE	2021	1	0	0	0	0	0	0	0	0	0	0	0	
19075960	Not	hp	DE	2021	1	0	0	0	0	0	0	0	0	0	0	0	
19075963	Not	Not repo	DE	2021	1	0	0	0	0	0	0	0	0	0	0	0	
19075991	Not	ph	JP	2021	1	0	0	0	0	0	0	0	0	0	0	0	
19076744	f	md	FR	2021	0	0	0	0	0	0	0	0	0	0	0	0	72
19077002	m	md	JP	2021	0	1	0	0	0	1	0	0	0	0	0	0	70
19077085	m	md	TW	2021	0	1	0	0	0	1	0	0	0	0	0	0	68
19077097	f	hp	CA	2021	0	1	1	0	0	0	0	0	0	0	0	0	67
19077609	m	md	BR	2021	0	0	0	0	0	0	0	0	0	0	0	0	
19078338	f	hp	CA	2021	0	1	1	0	0	0	0	0	0	0	0	0	70
19078441	f	Not repo	US	2021	0	1	1	0	0	0	0	0	0	0	0	0	82
19078899	Not	ph	JP	2021	1	0	0	0	0	0	0	0	0	0	0	0	
19079489	m	hp	US	2021	1	0	0	0	0	0	0	0	0	0	0	0	71
19079789	m	md	JP	2021	0	1	0	1	0	0	0	0	0	0	0	0	60
19079949	m	md	DE	2021	0	0	0	0	0	0	0	0	0	0	0	0	
19080116	Not	md	CA	2021	0	0	0	0	0	0	0	0	0	0	0	0	
19080600	m	cn	US	2021	0	1	1	0	0	0	0	0	0	0	0	0	
19081114	Not	cn	US	2021	0	1	1	0	0	0	0	0	0	0	0	0	
19081155	f	hp	US	2021	0	1	1	0	0	0	0	0	0	0	0	0	54
19081163	m	hp	US	2021	0	1	1	0	0	0	0	0	0	0	0	0	65
19081623	Not	hp	DE	2021	1	0	0	0	0	0	0	0	0	0	0	0	
18184868	m	md	CN	2021	0	1	0	0	0	1	0	0	0	0	0	0	68
18273118	m	hp	US	2021	0	0	0	0	0	0	0	0	0	0	0	0	
18342090	m	md	CN	2021	0	0	0	0	0	0	0	0	0	0	0	0	66
18344546	f	md	CN	2021	0	1	1	0	0	0	0	0	0	0	0	0	60
18427142	m	md	PL	2021	0	0	0	0	0	0	0	0	0	0	0	0	33
18432634	m	md	CL	2021	0	1	1	0	0	0	0	0	0	0	0	0	57
18506242	m	cn	IL	2021	0	0	0	0	0	0	0	0	0	0	0	0	74

18590604	m	md	JP	2021	0	1	1	0	0	0	0	0	0	0	0	0	76
18608738	m	hp	IN	2021	0	1	1	0	0	0	0	0	0	0	0	1	66
18617376	m	md	JP	2021	0	0	0	0	0	0	0	0	0	0	0	0	74
18636770	m	md	JP	2021	0	1	0	1	0	0	0	0	0	0	0	1	77
18650084	m	hp	CN	2021	0	0	0	0	0	0	0	0	0	0	0	0	48
18675779	m	md	FR	2021	0	1	1	0	0	0	0	0	0	0	0	0	42
18677339	m	md	CN	2021	0	0	0	0	0	0	0	0	0	0	0	0	72
18690716	f	md	IT	2021	0	0	0	0	0	0	0	0	0	0	0	0	36
18692481	f	md	CN	2021	0	1	0	0	1	0	0	0	0	0	0	0	70
18694270	f	md	JP	2021	0	1	0	0	1	0	0	0	0	0	0	0	76
18696854	m	hp	KR	2021	0	1	0	0	1	0	0	0	0	0	0	1	77
18702381	m	md	CN	2021	0	1	1	0	0	0	0	0	0	0	0	0	63
18702492	m	md	DE	2021	0	1	0	0	0	1	0	0	0	0	0	0	79
18707189	f	md	US	2021	0	1	0	0	1	0	0	0	0	0	0	0	
18714060	m	cn	JP	2021	0	1	1	0	0	0	0	0	0	0	0	0	68
18726924	m	cn	CN	2021	0	1	0	0	1	0	0	0	0	0	0	0	76
18745985	m	md	SI	2021	0	0	0	0	0	0	0	0	0	0	0	0	74
18747007	Not	hp	US	2021	0	0	0	0	0	0	0	0	0	0	0	0	
18749335	f	cn	Not re	2021	0	0	0	0	0	0	0	0	0	0	0	0	
18751683	m	md	CN	2021	0	1	0	0	0	1	0	0	0	0	0	0	62
18756452	m	hp	FR	2021	0	1	0	0	1	0	0	0	0	0	0	0	73
18757741	m	md	CN	2021	0	1	1	0	0	0	0	0	0	0	0	0	70
18760915	m	md	ES	2021	0	1	0	0	0	1	0	0	0	0	0	0	73
18767907	m	md	IL	2021	0	1	0	0	0	1	0	0	0	0	0	0	67
18778980	m	md	BG	2021	0	1	0	0	0	1	0	0	0	0	0	0	70
18799916	m	ph	FR	2021	1	0	0	0	0	0	0	0	0	0	0	0	
18801843	m	md	KR	2021	0	1	1	0	0	0	0	0	0	0	0	1	74
18810653	m	hp	IL	2021	0	0	0	0	0	0	0	0	0	0	0	0	71
18811269	f	md	MX	2021	0	1	0	1	0	0	0	0	0	0	0	0	89.8
18819485	m	ph	JP	2021	0	1	0	0	1	0	0	0	0	0	0	0	81
18824054	m	hp	US	2021	0	1	1	0	0	0	0	0	0	0	0	0	74
18841847	m	md	JP	2021	0	1	1	0	0	0	0	0	0	0	0	1	59
18853536	m	Not repo	JP	2021	0	1	0	1	0	0	0	0	0	0	0	0	76
18855786	f	md	JP	2021	0	0	0	0	0	0	0	0	0	0	0	0	70

18862238	m	cn	Not re	2021	0	0	0	0	0	0	0	0	0	0	0	0	
18868235	f	cn	US	2021	0	0	0	0	0	0	0	0	0	0	0	0	48
18870056	f	cn	KR	2021	1	0	0	0	0	0	0	0	0	0	0	0	53
18871510	Not	md	JP	2021	0	1	0	0	0	1	0	0	0	0	0	0	
18873887	m	cn	JP	2021	0	1	1	0	0	0	0	0	0	0	0	0	60
18874316	m	md	AR	2021	0	1	1	0	0	0	0	0	0	0	0	0	54
18883829	m	hp	KR	2021	0	1	1	0	0	0	0	0	0	0	0	0	76
18887632	f	md	JP	2021	0	0	0	0	0	0	0	0	0	0	0	0	82
18888098	f	cn	JP	2021	0	0	0	0	0	0	0	0	0	0	0	0	90
18891098	m	md	DE	2021	0	1	0	0	1	0	0	0	0	0	0	0	54
18900520	f	md	GB	2021	0	1	0	0	0	1	0	0	0	0	0	0	54
18907018	f	md	DE	2021	0	1	1	0	0	0	0	0	0	0	0	0	65
18907401	m	md	JP	2021	0	1	1	0	0	0	0	0	0	0	0	0	68
18907504	m	Not repo	US	2021	1	0	0	0	0	0	0	0	0	0	0	0	75.4
18910872	Not	md	JP	2021	0	0	0	0	0	0	0	0	0	0	0	0	73
18917381	m	hp	BE	2021	0	0	0	0	0	0	0	0	0	0	0	0	50
18918652	f	cn	Not re	2021	0	0	0	0	0	0	0	0	0	0	0	0	
18918727	f	md	AT	2021	0	1	0	0	0	1	0	0	0	0	0	0	52
18922841	m	Not repo	IS	2021	0	0	0	0	0	0	1	1	0	0	0	0	54
18926316	f	cn	CA	2021	0	1	0	0	0	1	0	0	0	0	0	0	
18927551	m	md	JP	2021	0	0	0	0	0	0	0	0	0	0	0	0	79
18928406	m	md	FR	2021	0	0	0	0	0	0	0	0	0	0	0	0	67
18929522	f	md	US	2021	0	0	0	0	0	0	0	0	0	0	0	0	55
18938139	m	md	KR	2021	0	0	0	0	0	0	0	0	0	0	0	0	66
18946272	m	cn	CA	2021	1	0	0	0	0	0	0	0	0	0	0	0	41.3
18966712	f	md	US	2021	0	1	0	0	0	1	0	0	0	0	0	0	79
18967361	f	cn	IL	2021	0	0	0	0	0	0	0	0	0	0	0	0	33
18968369	m	md	DE	2021	0	1	0	0	0	1	0	0	0	0	0	0	53
18971726	m	cn	CN	2021	0	1	0	0	1	0	0	0	0	0	0	0	74
18973698	Not	md	JP	2021	0	0	0	0	0	0	0	0	0	0	0	0	
18975719	f	Not repo	JP	2021	1	0	0	0	0	0	0	0	0	0	0	0	65
18975837	f	md	IT	2021	1	0	0	0	0	0	0	0	0	0	0	0	67
18977276	Not	md	JP	2021	0	0	0	0	0	0	0	0	0	0	0	0	
18986068	f	ph	HU	2021	0	0	0	0	0	0	0	0	0	0	0	0	

18988739	f	cn	Not re	2021	0	0	0	0	0	0	0	0	0	0	0	0	
18989726	m	md	JP	2021	0	1	1	0	0	0	0	0	0	0	0	0	70
18994222	m	hp	US	2021	0	0	0	0	0	0	0	0	0	0	0	0	69
19003074	Not	hp	FR	2021	0	1	0	0	1	0	0	0	0	0	0	0	68
19003771	m	md	RU	2021	0	1	1	0	0	0	0	0	0	0	0	0	64
19004581	m	md	JP	2021	0	1	0	0	0	1	0	0	0	0	0	0	68
19009580	m	md	ES	2021	0	1	1	0	0	0	0	0	0	0	0	0	74
19009884	f	md	JP	2021	1	0	0	0	0	0	0	0	0	0	0	0	63
19011825	f	md	SG	2021	0	0	0	0	0	0	0	0	0	0	0	0	58
19012846	f	md	JP	2021	1	0	0	0	0	0	0	0	0	0	0	0	52
19012939	m	md	DE	2021	0	0	0	0	0	0	0	0	0	0	0	0	59
19013079	f	cn	US	2021	0	1	1	0	0	0	0	0	0	0	0	0	
19018975	m	md	CN	2021	0	1	1	0	0	0	0	0	0	0	0	0	62
19020333	m	md	IL	2021	0	0	0	0	0	0	0	0	0	0	0	0	71
19020515	f	md	US	2021	0	1	0	0	0	1	0	0	0	0	0	0	45
19023024	f	md	CL	2021	0	1	1	0	0	0	0	0	0	0	0	0	58
19023585	m	hp	US	2021	0	1	1	0	0	0	0	0	0	0	0	0	58
19024500	f	md	IT	2021	0	0	0	0	0	0	0	0	0	0	0	0	67
19025119	m	hp	US	2021	0	0	0	0	0	0	0	0	0	0	0	0	74
19025227	f	md	JP	2021	1	0	0	0	0	0	0	0	0	0	0	0	64
19025264	f	md	JP	2021	1	0	0	0	0	0	0	0	0	0	0	0	44
19025464	m	md	JP	2021	1	0	0	0	0	0	0	0	0	0	0	0	78
19027747	m	md	GB	2021	0	1	1	0	0	0	0	0	0	0	0	0	70
19029801	f	md	US	2021	0	1	1	0	0	0	0	0	0	0	0	1	81
19030005	f	md	CN	2021	0	1	0	0	0	1	0	0	0	0	0	0	57
19032099	m	hp	RU	2021	0	0	0	0	0	0	0	0	0	0	0	0	66
19032439	f	md	IL	2021	0	0	0	0	0	0	0	0	0	0	0	0	60
19032657	m	md	ES	2021	0	1	1	0	0	0	0	0	0	0	0	0	73
19034015	f	md	CN	2021	0	0	0	0	0	0	0	0	0	0	0	0	77
19035690	f	cn	Not re	2021	0	0	0	0	0	0	0	0	0	0	0	0	
19035732	f	cn	HR	2021	0	1	1	0	0	0	0	0	0	0	0	1	57
19036208	m	md	JP	2021	0	1	0	0	1	0	0	0	0	0	0	1	70
19037232	m	md	IN	2021	0	0	0	0	0	0	0	0	0	0	0	0	58
19038772	f	md	DE	2021	0	1	1	0	0	0	0	0	0	0	0	0	70

19039043	m	md	GB	2021	0	0	0	0	0	0	0	0	0	0	0	0		66
19041153	f	md	IN	2021	0	0	0	0	0	0	0	0	0	0	0	0		56
19041160	Not	md	JP	2021	0	1	0	0	0	1	0	0	0	0	0	0		74
19041871	m	cn	Not re	2021	0	0	0	0	0	0	0	0	0	0	0	0		
19043234	m	md	ES	2021	0	1	1	0	0	0	0	0	0	0	0	0		54
19046934	m	cn	Not re	2021	0	0	0	0	0	0	0	0	0	0	0	0		
19048796	f	md	BE	2021	1	0	0	0	0	0	0	0	0	0	0	0		65
19050325	f	md	US	2021	0	1	1	0	0	0	0	0	0	0	0	0		66
19050472	m	md	US	2021	0	1	1	0	0	0	0	0	0	0	0	0		79
19050603	f	md	US	2021	0	1	1	0	0	0	0	0	0	0	0	0		59
19050660	f	md	US	2021	0	1	1	0	0	0	0	0	0	0	0	0		64
19050755	f	md	US	2021	0	1	1	0	0	0	0	0	0	0	0	0		68
19050765	m	cn	JP	2021	0	1	1	0	0	0	0	0	0	0	0	0		72
19053288	m	ph	JP	2021	0	0	0	0	0	0	0	0	0	0	0	0		74
19055432	m	md	JP	2021	0	1	0	0	0	1	0	0	0	0	0	0		69
19056853	f	hp	GR	2021	0	1	0	0	1	0	0	0	0	0	0	0		72
19057417	f	md	GR	2021	0	1	0	0	0	1	0	0	0	0	0	0		83
19058958	f	md	DE	2021	0	1	1	0	0	0	0	0	0	0	0	0		84
19061107	Not	md	JP	2021	0	1	0	0	0	1	0	0	0	0	0	0		70
19062311	m	md	BR	2021	0	1	1	0	0	0	0	0	0	0	0	0		72
19063776	f	md	US	2021	0	0	0	0	0	0	0	0	0	0	0	0		65
19063860	m	hp	TR	2021	0	0	0	0	0	0	0	0	0	0	0	0		55
19064043	f	Not repo	ID	2021	1	0	0	0	0	0	0	0	0	0	0	0		69.4
19064575	m	cn	CN	2021	0	1	0	1	0	0	0	0	0	0	0	0		51
19064630	m	hp	BE	2021	0	0	0	0	0	0	0	0	0	0	0	0		74
19065013	f	Not repo	JP	2021	1	0	0	0	0	0	0	0	0	0	0	0		76
19066676	Not	md	JP	2021	0	1	0	0	1	0	0	0	0	0	0	0		90
19067470	m	md	CN	2021	0	0	0	0	0	0	0	0	0	0	0	0		50
19067624	m	Not repo	CN	2021	0	1	0	1	0	0	0	0	0	0	0	0		60
19069259	m	md	FR	2021	0	1	0	0	0	1	0	0	0	0	0	0		39
19069311	m	md	CN	2021	0	1	0	0	0	1	0	0	0	0	0	0		60
19069346	m	hp	NL	2021	0	1	0	0	1	0	0	0	0	0	0	0		66
19070167	m	hp	CN	2021	0	1	0	0	1	0	0	0	0	0	0	0		57
19070861	m	md	JP	2021	0	1	0	0	1	0	0	0	0	0	0	0		59

19072899	m	hp	KR	2021	0	0	0	0	0	0	0	0	0	0	0	0	67
19073058	m	md	JP	2021	0	1	0	0	0	1	0	0	0	0	0	0	76
19073752	m	md	US	2021	0	1	0	0	0	1	0	0	0	0	0	0	75
19076350	m	md	FR	2021	0	1	0	0	0	1	0	0	0	0	0	0	82
19079181	m	md	US	2021	0	1	1	0	0	0	0	0	0	0	0	0	73
19080893	Not	md	JP	2021	0	0	0	0	0	0	0	0	0	0	0	0	74
19081512	m	md	JP	2021	0	0	0	0	0	0	0	0	0	0	0	0	66
19081606	m	hp	IL	2021	0	0	0	0	0	0	0	0	0	0	0	0	63
19081932	m	md	JP	2021	0	0	0	0	0	0	0	0	0	0	0	0	
19082073	m	md	JP	2021	0	1	0	1	0	0	0	0	0	0	0	1	69
19082730	m	hp	CA	2021	0	1	1	0	0	0	0	0	0	0	0	1	62
19083043	m	md	ES	2021	0	1	1	0	0	0	0	0	0	0	0	0	65
19083459	f	Not repo	ID	2021	1	0	0	0	0	0	0	0	0	0	0	0	68.5
19083522	f	hp	DE	2021	0	1	1	0	0	0	0	0	0	0	0	0	80
19083535	Not	Not repo	DE	2021	1	0	0	0	0	0	0	0	0	0	0	0	
19083536	Not	hp	DE	2021	1	0	0	0	0	0	0	0	0	0	0	0	
19083787	m	hp	DE	2021	0	1	1	0	0	0	0	0	0	0	0	0	74
19083945	f	md	PT	2021	0	0	0	0	0	0	0	0	0	0	0	0	59
19084271	f	hp	DE	2021	0	1	1	0	0	0	0	0	0	0	0	0	84
19084337	f	hp	JP	2021	0	1	0	1	0	0	0	0	0	0	0	1	74
19084774	Not	Not repo	DE	2021	1	0	0	0	0	0	0	0	0	0	0	0	
19084832	Not	md	JP	2021	0	1	0	1	0	0	0	0	0	0	0	1	
19085044	Not	hp	JP	2021	0	0	0	0	0	0	0	0	0	0	0	0	
19085193	f	cn	US	2021	0	0	0	0	0	0	0	0	0	0	0	0	88
19085528	m	md	AU	2021	0	1	0	1	0	0	0	0	0	0	0	0	
19085621	m	hp	IT	2021	0	1	0	0	1	0	0	0	0	0	0	1	41
19085810	f	md	JP	2021	0	1	0	1	0	0	0	0	0	0	0	1	70
19086538	m	cn	IN	2021	0	0	0	0	0	0	0	0	0	0	0	0	51
19086657	f	ph	US	2021	0	0	0	0	0	0	0	0	0	0	0	0	59
19086680	f	md	JP	2021	1	0	0	0	0	0	0	0	0	0	0	0	64
19086795	f	md	US	2021	0	0	0	0	0	0	0	0	0	0	0	0	63
19087163	Not	hp	NO	2021	1	0	0	0	0	0	0	0	0	0	0	0	
19087203	f	Not repo	DE	2021	1	0	0	0	0	0	0	0	0	0	0	0	
19087327	m	md	ES	2021	0	0	0	0	0	0	0	0	0	0	0	0	74

19087343	f	md	DE	2021	0	1	1	0	0	0	0	0	0	0	0	0	80
19087651	m	md	CN	2021	0	1	0	0	0	1	0	0	0	0	0	0	59
19088139	f	hp	NO	2021	0	1	1	0	0	0	0	0	0	0	0	0	67
19088434	f	md	JP	2021	0	0	0	0	0	0	0	0	0	0	0	0	72
19088491	Not	hp	NO	2021	1	0	0	0	0	0	0	0	0	0	0	0	
19088699	m	hp	US	2021	0	1	1	0	0	0	0	0	0	0	0	0	58
19089165	f	md	GB	2021	0	0	0	0	0	0	1	0	0	0	1	0	67
19089372	f	hp	US	2021	0	0	0	0	0	0	0	0	0	0	0	0	
19089965	f	hp	IN	2021	0	0	0	0	0	0	0	0	0	0	0	0	65
19090173	Not	md	JP	2021	0	0	0	0	0	0	0	0	0	0	0	0	
19090674	f	md	CA	2021	0	0	0	0	0	0	0	0	0	0	0	0	46
19090912	Not	md	NL	2021	0	0	0	0	0	0	0	0	0	0	0	0	
19090953	m	hp	IL	2021	0	0	0	0	0	0	0	0	0	0	0	0	
19091302	f	md	KR	2021	0	0	0	0	0	0	0	0	0	0	0	0	51
19091495	m	hp	DE	2021	0	1	0	0	1	0	0	0	0	0	0	1	68
19091956	f	ph	US	2021	0	0	0	0	0	0	0	0	0	0	0	0	69
19092198	Not	hp	CN	2021	0	0	0	0	0	0	0	0	0	0	0	0	
19092216	f	cn	MX	2021	0	0	0	0	0	0	0	0	0	0	0	0	
19092620	m	md	ES	2021	0	1	1	0	0	0	0	0	0	0	0	0	78
19092794	f	Not repo	ID	2021	1	0	0	0	0	0	0	0	0	0	0	0	63
19093096	f	hp	RO	2021	1	0	0	0	0	0	0	0	0	0	0	0	78
19093124	m	cn	CN	2021	0	1	0	0	1	0	0	0	0	0	0	0	76
19093404	m	cn	CA	2021	1	0	0	0	0	0	0	0	0	0	0	0	56.8
19093479	m	cn	JP	2021	0	1	1	0	0	0	0	0	0	0	0	0	71
19093542	f	cn	JP	2021	0	1	1	0	0	0	0	0	0	0	0	0	72
19093691	f	cn	Not re	2021	0	0	0	0	0	0	0	0	0	0	0	0	45
19093703	f	md	FR	2021	1	0	0	0	0	0	0	0	0	0	0	0	
19093733	m	md	KR	2021	0	0	0	0	0	0	0	0	0	0	0	0	
19093765	f	md	FR	2021	1	0	0	0	0	0	0	0	0	0	0	0	
19093803	m	cn	CN	2021	0	1	0	0	1	0	0	0	0	0	0	0	72
19093994	f	md	NL	2021	0	1	0	0	0	1	0	0	0	0	0	0	71
19094373	m	md	AR	2021	0	1	1	0	0	0	0	0	0	0	0	0	67
19094421	f	cn	JP	2021	0	1	1	0	0	0	0	0	0	0	0	0	80
19094673	Not	md	MY	2021	1	0	0	0	0	0	0	0	0	0	0	0	

19094824	f	cn	CA	2021	1	0	0	0	0	0	0	0	0	0	0	0	
19094868	m	cn	JP	2021	0	1	1	0	0	0	0	0	0	0	0	0	54
19094965	m	md	US	2021	0	1	1	0	0	0	0	0	0	0	0	1	65
19095004	Not	md	DE	2021	0	1	0	0	0	1	0	0	0	0	0	0	
19095259	m	md	US	2021	1	0	0	0	0	0	0	0	0	0	0	1	80
19095408	f	cn	JP	2021	0	1	1	0	0	0	0	0	0	0	0	0	80
19095415	f	cn	US	2021	0	1	1	0	0	0	0	0	0	0	0	0	
19096288	m	cn	IN	2021	0	0	0	0	0	0	0	0	0	0	0	0	70
19097422	f	cn	US	2021	0	0	0	0	0	0	0	0	0	0	0	0	
19097554	m	cn	JP	2021	0	0	0	0	0	0	0	0	0	0	0	0	61
19097856	f	md	JP	2021	0	1	0	0	1	0	0	0	0	0	0	0	75
19097971	f	cn	CN	2021	0	0	0	0	0	0	0	0	0	0	0	0	62
19097974	f	hp	DE	2021	0	1	1	0	0	0	0	0	0	0	0	0	80
19098293	m	Not repo	JP	2021	0	1	0	1	0	0	0	0	0	0	0	1	70
19098368	m	Not repo	RO	2021	0	1	0	1	0	0	0	0	0	0	0	0	
19098370	m	Not repo	JP	2021	0	1	0	1	0	0	0	0	0	0	0	1	68
19098615	m	md	JP	2021	0	0	0	0	0	0	0	0	0	0	0	0	55
19098661	f	md	TR	2021	0	0	0	0	0	0	0	0	0	0	0	0	60
19098682	f	md	IN	2021	0	0	0	0	0	0	0	0	0	0	0	0	71
19098863	m	cn	JP	2021	0	1	1	0	0	0	0	0	0	0	0	0	
19098906	f	md	US	2021	0	1	0	0	0	1	0	0	0	0	0	0	
19098919	f	cn	SK	2021	0	0	0	0	0	0	0	0	0	0	0	0	
19099035	m	md	CA	2021	0	1	0	0	0	1	0	0	0	0	0	1	71
19099334	f	Not repo	CN	2021	0	1	0	1	0	0	0	0	0	0	0	1	32
19099362	m	md	JP	2021	0	1	0	1	0	0	0	0	0	0	0	0	70
19099491	m	Not repo	JP	2021	0	1	0	1	0	0	0	0	0	0	0	1	
19100131	m	hp	CN	2021	1	0	0	0	0	0	0	0	0	0	0	0	
19100233	m	hp	US	2021	0	1	1	0	0	0	0	0	0	0	0	0	71
19100266	f	hp	US	2021	0	1	1	0	0	0	0	0	0	0	0	0	63
19100280	f	hp	US	2021	0	1	1	0	0	0	0	0	0	0	0	0	70
19100615	f	md	JP	2021	0	1	0	0	0	1	0	0	0	0	0	0	76
19100623	m	cn	JP	2021	0	1	1	0	0	0	0	0	0	0	0	0	70
19100741	Not	hp	CN	2021	1	0	0	0	0	0	0	0	0	0	0	0	
19100859	f	md	JP	2021	0	1	0	0	0	1	0	0	0	0	0	0	70

19101042	f	md	JP	2021	0	0	0	0	0	0	0	0	0	0	0	0	72
19101761	f	hp	IN	2021	0	0	0	0	0	0	0	0	0	0	0	0	
19102076	m	md	US	2021	0	1	1	0	0	0	0	0	0	0	0	0	
19102723	f	hp	DE	2021	0	1	1	0	0	0	0	0	0	0	0	0	84
19103390	m	ph	JP	2021	1	0	0	0	0	0	0	0	0	0	0	0	67
19103550	m	md	KR	2021	0	1	0	0	0	1	0	0	0	0	0	0	66
19104265	f	hp	CN	2021	1	0	0	0	0	0	0	0	0	0	0	0	
19104267	f	hp	DK	2021	1	0	0	0	0	0	0	0	0	0	0	0	57
19104268	f	hp	GB	2021	0	1	0	1	0	0	0	0	0	0	0	0	68
19104512	m	hp	FR	2021	0	1	0	0	1	0	0	0	0	0	0	0	71
19104585	Not	Not repo	JP	2021	1	0	0	0	0	0	0	0	0	0	0	0	
19104846	m	cn	Not re	2021	0	0	0	0	0	0	0	0	0	0	0	0	
19105051	f	Not repo	Not re	2021	1	0	0	0	0	0	0	0	0	0	0	0	71
19105101	m	md	JP	2021	0	1	0	0	1	0	0	0	0	0	0	0	71
19106061	f	ph	FR	2021	0	1	0	1	0	0	0	0	0	0	0	0	
19106521	m	md	JP	2021	0	1	0	0	1	0	0	0	0	0	0	0	80
19108074	f	cn	CN	2021	0	0	0	0	0	0	0	0	0	0	0	0	52
19108429	f	md	US	2021	0	1	1	0	0	0	0	0	0	0	0	0	85
19108947	m	cn	JP	2021	0	1	1	0	0	0	0	0	0	0	0	0	75
19109108	m	md	KR	2021	0	1	0	0	0	1	0	0	0	0	0	0	67
19109182	m	md	JP	2021	1	0	0	0	0	0	0	0	0	0	0	1	77
19109937	Not	hp	US	2021	0	1	0	0	0	1	0	0	0	0	0	1	
19110235	m	md	JP	2021	0	1	0	1	0	0	0	0	0	0	0	1	70
19110434	m	hp	JP	2021	0	1	0	1	0	0	0	0	0	0	0	1	72.4
19110872	m	cn	IN	2021	0	1	0	0	1	0	0	0	0	0	0	0	59
19110888	m	Not repo	MY	2021	1	0	0	0	0	0	0	0	0	0	0	0	77.8
19110910	f	md	CN	2021	0	0	0	0	0	0	0	0	0	0	0	0	62
19112203	f	md	US	2021	0	0	0	0	0	0	0	0	0	0	0	0	79
19112981	f	hp	RO	2021	0	1	0	0	1	0	0	0	0	0	0	0	46
19113043	Not	md	GR	2021	0	0	0	0	0	0	0	0	0	0	0	0	
19113046	Not	md	GR	2021	0	0	0	0	0	0	0	0	0	0	0	0	
19113612	f	md	FR	2021	1	0	0	0	0	0	0	0	0	0	0	0	62
19113905	f	cn	US	2021	1	0	0	0	0	0	0	0	0	0	0	0	84.7
19114371	m	md	US	2021	0	1	1	0	0	0	0	0	0	0	0	0	

19114789	f	md	JP	2021	0	1	0	0	0	1	0	0	0	0	0	0	65
19114829	m	md	GR	2021	0	1	0	0	1	0	0	0	0	0	0	0	62
19114859	f	cn	US	2021	0	1	1	0	0	0	0	0	0	0	0	0	
19114987	m	cn	CN	2021	0	1	0	0	1	0	0	0	0	0	0	0	53
19115622	Not	hp	DE	2021	1	0	0	0	0	0	0	0	0	0	0	0	
19115624	Not	ph	JP	2021	1	0	0	0	0	0	0	0	0	0	0	0	
19116271	m	md	VN	2021	0	0	0	0	0	0	1	0	0	0	1	0	59
19116273	m	md	TW	2021	0	0	0	0	0	0	0	0	0	0	0	0	73
19116366	f	cn	US	2021	0	0	0	0	0	0	0	0	0	0	0	0	30
19116383	m	md	DE	2021	0	1	0	0	0	1	0	0	0	0	0	0	79
19116424	Not	hp	CN	2021	0	0	0	0	0	0	0	0	0	0	0	0	
19116446	f	cn	Not re	2021	0	0	0	0	0	0	0	0	0	0	0	0	50
19116453	m	Not repo	RU	2021	0	1	0	1	0	0	0	0	0	0	0	0	48
19116480	f	cn	US	2021	0	0	0	0	0	0	0	0	0	0	0	0	30
19116623	m	md	CN	2021	0	1	1	0	0	0	0	0	0	0	0	0	57
19116668	m	md	JP	2021	0	0	0	0	0	0	0	0	0	0	0	0	37
19117381	f	cn	VN	2021	1	0	0	0	0	0	0	0	0	0	0	0	75.6
19117507	m	cn	Not re	2021	0	0	0	0	0	0	0	0	0	0	0	0	61
19117570	m	cn	Not re	2021	0	0	0	0	0	0	0	0	0	0	0	0	
19117656	m	hp	CN	2021	0	1	0	0	1	0	0	0	0	0	0	0	68
19117984	m	md	JP	2021	1	0	0	0	0	0	0	0	0	0	0	0	81
19117992	m	hp	CN	2021	0	1	0	0	1	0	0	0	0	0	0	1	59
19118036	m	hp	JP	2021	0	1	0	1	0	0	0	0	0	0	0	1	78
19118180	f	cn	Not re	2021	0	0	0	0	0	0	0	0	0	0	0	0	
19118293	m	md	FR	2021	0	1	0	0	1	0	0	0	0	0	0	0	79
19118500	m	ph	JP	2021	0	1	0	0	1	0	0	0	0	0	0	1	69
19119035	f	md	JP	2021	0	1	0	0	0	1	0	0	0	0	0	0	78
19119631	m	cn	CN	2021	0	1	0	0	1	0	0	0	0	0	0	0	67
19119801	Not	md	NL	2021	0	0	0	0	0	0	0	0	0	0	0	0	
19119926	m	cn	Not re	2021	0	0	0	0	0	0	0	0	0	0	0	0	92
19120055	m	md	JP	2021	0	1	0	0	0	1	0	0	0	0	0	0	66
19122243	Not	hp	JP	2021	0	0	0	0	0	0	0	0	0	0	0	0	
19122250	f	md	BE	2021	0	0	0	0	0	0	0	0	0	0	0	0	
19122276	Not	md	US	2021	0	0	0	0	0	0	0	0	0	0	0	0	

19122304	m	md	JP	2021	0	1	0	0	0	1	0	0	0	0	0	1	80
19122737	f	md	DE	2021	0	0	0	0	0	0	0	0	0	0	0	0	76
19122848	f	cn	JO	2021	1	0	0	0	0	0	0	0	0	0	0	0	83
19122863	Not	md	US	2021	0	0	0	0	0	0	0	0	0	0	0	0	
19123300	m	cn	Not re	2021	0	0	0	0	0	0	0	0	0	0	0	0	41
19123691	m	hp	IL	2021	0	0	0	0	0	0	0	0	0	0	0	0	69
19123767	f	hp	DE	2021	0	1	1	0	0	0	0	0	0	0	0	0	54
19124415	m	hp	NL	2021	0	1	0	0	1	0	0	0	0	0	0	0	58
19124665	f	cn	GB	2021	1	0	0	0	0	0	0	0	0	0	0	0	
19124701	f	cn	JP	2021	0	1	1	0	0	0	0	0	0	0	0	0	71
19124800	f	Not repo	ID	2021	1	0	0	0	0	0	0	0	0	0	0	0	80.8
19124801	m	Not repo	GB	2021	0	1	0	1	0	0	0	0	0	0	0	0	
19124809	m	hp	CA	2021	0	0	0	0	0	0	0	0	0	0	0	0	77
19124840	m	cn	CN	2021	0	1	0	0	1	0	0	0	0	0	0	0	68
19124980	f	Not repo	JP	2021	1	0	0	0	0	0	0	0	0	0	0	0	70
19125017	f	md	JP	2021	0	1	0	0	0	1	0	0	0	0	0	0	90
19125038	m	md	JP	2021	0	1	0	0	0	1	0	0	0	0	0	1	60
19125467	m	md	KR	2021	0	0	0	0	0	0	0	0	0	0	0	0	79
19126251	m	md	JP	2021	0	1	0	1	0	0	0	0	0	0	0	0	77
19126288	m	hp	CN	2021	0	1	1	0	0	0	0	0	0	0	0	0	72
19126297	f	hp	BR	2021	0	0	0	0	0	0	0	0	0	0	0	0	32
19127010	m	hp	US	2021	0	1	0	0	1	0	0	0	0	0	0	0	78
19128405	f	ph	US	2021	0	0	0	0	0	0	0	0	0	0	0	0	
19128631	m	md	JP	2021	0	1	0	0	1	0	0	0	0	0	0	0	71
19129115	m	Not repo	JP	2021	0	1	0	1	0	0	0	0	0	0	0	1	82
19129116	m	Not repo	JP	2021	0	1	0	1	0	0	0	0	0	0	0	1	75
19129238	m	md	JP	2021	0	1	0	1	0	0	0	0	0	0	0	1	65
19129347	m	md	CL	2021	0	1	1	0	0	0	0	0	0	0	0	0	55
19129920	f	Not repo	ID	2021	1	0	0	0	0	0	0	0	0	0	0	0	
19130126	m	hp	CN	2021	0	1	0	0	1	0	0	0	0	0	0	0	48
19130473	m	cn	CN	2021	0	1	0	0	1	0	0	0	0	0	0	0	79
19130659	f	md	JP	2021	0	0	0	0	0	0	0	0	0	0	0	0	37
19130759	m	hp	JP	2021	0	1	0	1	0	0	0	0	0	0	0	1	65
19130974	Not	md	US	2021	0	0	0	0	0	0	0	0	0	0	0	0	

19131331	Not	md	JP	2021	0	1	0	0	1	0	0	0	0	0	0	0	66
19131616	f	ph	JP	2021	1	0	0	0	0	0	0	0	0	0	0	1	
19131623	m	cn	CN	2021	0	1	0	0	1	0	0	0	0	0	0	0	49
19131879	f	cn	JP	2021	0	1	1	0	0	0	0	0	0	0	0	0	70
19133088	m	cn	JP	2021	0	1	1	0	0	0	0	0	0	0	0	0	65
19134152	f	md	US	2021	0	1	1	0	0	0	0	0	0	0	0	0	
19134225	Not	md	IN	2021	0	0	0	0	0	0	0	0	0	0	0	0	
19134900	m	cn	US	2021	0	1	1	0	0	0	0	0	0	0	0	0	
19134964	m	md	US	2021	0	0	0	0	0	0	0	0	0	0	0	0	
19135232	f	md	KR	2021	0	1	0	0	0	1	0	0	0	0	0	1	46
19135246	f	md	CN	2021	0	0	0	0	0	0	0	0	0	0	0	0	63
19136103	m	cn	JP	2021	0	1	1	0	0	0	0	0	0	0	0	0	80
19136140	m	hp	JP	2021	0	1	0	1	0	0	0	0	0	0	0	1	49
19136622	f	hp	US	2021	0	0	0	0	0	0	0	0	0	0	0	0	
19136824	m	md	JP	2021	0	1	0	1	0	0	0	0	0	0	0	0	73
19137968	Not	md	JP	2021	0	1	0	0	1	0	0	0	0	0	0	0	
19138047	f	md	CA	2021	0	0	0	0	0	0	0	0	0	0	0	0	68
19138208	f	md	JP	2021	0	1	0	0	0	1	0	0	0	0	0	0	64
19138508	m	md	IT	2021	0	1	0	0	0	1	0	0	0	0	0	0	73
19138521	m	cn	US	2021	0	1	1	0	0	0	0	0	0	0	0	0	
19138588	f	hp	IN	2021	0	0	0	0	0	0	0	0	0	0	0	0	
19138824	m	md	FR	2021	0	1	0	0	0	1	0	0	0	0	0	0	68
19139097	f	cn	FR	2021	0	0	0	0	0	0	0	0	0	0	0	0	
19139592	f	Not repo	CN	2021	0	1	0	1	0	0	0	0	0	0	0	0	33
19139773	f	md	US	2021	0	1	0	0	1	0	0	0	0	0	0	0	65
19139783	f	cn	Not re	2021	0	0	0	0	0	0	0	0	0	0	0	0	64
19139812	f	md	US	2021	0	0	0	0	0	0	0	0	0	0	0	0	62
19139829	m	md	ES	2021	0	0	0	0	0	0	0	0	0	0	0	0	63
19139852	f	md	US	2021	0	0	0	0	0	0	0	0	0	0	0	0	62
19140041	f	hp	NL	2021	1	0	0	0	0	0	0	0	0	0	0	0	57
19140812	m	md	JP	2021	0	0	0	0	0	0	0	0	0	0	0	0	90
19140916	Not	md	US	2021	0	0	0	0	0	0	0	0	0	0	0	0	
19140937	Not	hp	KR	2021	0	1	0	0	0	1	0	0	0	0	0	0	
19141056	Not	md	JP	2021	0	1	0	0	1	0	0	0	0	0	0	0	

19141157	m	cn	GR	2021	0	1	1	0	0	0	0	0	0	0	0	0	68
19141621	m	hp	IT	2021	0	1	0	0	1	0	0	0	0	0	0	0	78
19141792	f	hp	FR	2021	0	1	0	0	1	0	0	0	0	0	0	0	78
19142227	f	md	FR	2021	1	0	0	0	0	0	0	0	0	0	0	0	38
19142439	m	cn	CN	2021	0	0	0	0	0	0	0	0	0	0	0	0	50
19142473	f	md	US	2021	0	0	0	0	0	0	0	0	0	0	0	0	57
19142681	f	cn	Not re	2021	0	0	0	0	0	0	0	0	0	0	0	0	
19142838	m	md	US	2021	0	0	0	0	0	0	0	0	0	0	0	0	
19143228	f	Not repo	JP	2021	1	0	0	0	0	0	0	0	0	0	0	0	58.3
19143338	Not	md	JP	2021	0	1	0	0	0	1	0	0	0	0	0	0	59
19143772	m	hp	ES	2021	0	0	0	0	0	0	0	0	0	0	0	0	85
19144208	f	md	IL	2021	1	0	0	0	0	0	0	0	0	0	0	0	50
19144368	m	md	DE	2021	0	1	0	1	0	0	0	0	0	0	0	0	73
19145019	m	md	AU	2021	0	1	1	0	0	0	0	0	0	0	0	0	76
19145081	m	md	CN	2021	0	0	0	0	0	0	0	0	0	0	0	0	69
19145199	m	Not repo	ID	2021	0	1	0	1	0	0	0	0	0	0	0	0	67.8
19145604	m	md	IN	2021	0	0	0	0	0	0	0	0	0	0	0	0	69
19145649	Not	md	JP	2021	1	0	0	0	0	0	0	0	0	0	0	0	88
19145819	m	cn	US	2021	1	0	0	0	0	0	0	0	0	0	0	0	
19146139	f	md	PL	2021	0	1	1	0	0	0	0	0	0	0	0	0	67
19146266	f	md	CN	2021	0	1	1	0	0	0	0	0	0	0	0	0	37
19146313	f	md	JP	2021	0	0	0	0	0	0	0	0	0	0	0	0	80
19146410	f	Not repo	MY	2021	1	0	0	0	0	0	0	0	0	0	0	0	65.7
19146451	f	cn	US	2021	0	0	0	0	0	0	0	0	0	0	0	0	
19146583	f	md	CN	2021	0	0	0	0	0	0	0	0	0	0	0	0	67
19146643	f	md	JP	2021	0	0	0	0	0	0	0	0	0	0	0	0	90
19146645	f	cn	US	2021	0	0	0	0	0	0	0	0	0	0	0	0	
19146695	Not	md	JP	2021	0	1	0	0	1	0	0	0	0	0	0	0	
19146966	m	hp	KR	2021	0	1	0	0	1	0	0	0	0	0	0	0	59
19147001	m	hp	KR	2021	0	1	0	0	1	0	0	0	0	0	0	0	58
19147031	m	cn	Not re	2021	0	0	0	0	0	0	0	0	0	0	0	0	28
19147060	Not	hp	CN	2021	0	0	0	0	0	0	0	0	0	0	0	0	
19147781	f	cn	CN	2021	0	1	0	0	1	0	0	0	0	0	0	0	51
19147939	m	md	US	2021	0	1	0	0	0	1	0	0	0	0	0	1	72

19148194	m	md	JP	2021	0	1	0	0	0	1	0	0	0	0	0	0	48
19148205	m	cn	JP	2021	0	1	1	0	0	0	0	0	0	0	0	0	80
19148310	m	cn	TW	2021	0	1	0	0	0	1	0	0	0	0	0	0	
19148324	m	hp	CN	2021	0	0	0	0	0	0	0	0	0	0	0	0	63
19148444	f	cn	CA	2021	1	0	0	0	0	0	0	0	0	0	0	0	
19148558	m	cn	CN	2021	0	1	0	0	1	0	0	0	0	0	0	0	84
19148573	f	Not repo	ID	2021	0	1	0	1	0	0	0	0	0	0	0	0	41.5
19148884	Not	hp	JP	2021	0	1	0	0	0	1	0	0	0	0	0	0	
19149146	f	md	KR	2021	0	1	0	0	0	1	0	0	0	0	0	1	46
19149368	f	hp	US	2021	0	1	0	0	1	0	0	0	0	0	0	0	57
19149765	m	hp	JP	2021	0	1	0	0	0	1	0	0	0	0	0	0	51
19150014	m	hp	DE	2021	0	1	0	0	1	0	0	0	0	0	0	0	58
19150133	m	cn	Not re	2021	0	0	0	0	0	0	0	0	0	0	0	0	
19150661	m	cn	JP	2021	0	1	1	0	0	0	0	0	0	0	0	0	80
19153221	m	md	CN	2021	0	0	0	0	0	0	1	0	0	0	1	0	71
19153272	f	hp	JP	2021	0	0	0	0	0	0	0	0	0	0	0	0	62
19153277	m	hp	JP	2021	0	0	0	0	0	0	0	0	0	0	0	0	67
19153279	f	hp	JP	2021	0	0	0	0	0	0	0	0	0	0	0	0	61
19153540	m	cn	US	2021	0	1	1	0	0	0	0	0	0	0	0	0	
19153769	m	Not repo	ID	2021	1	0	0	0	0	0	0	0	0	0	0	0	68.5
19153895	f	cn	GB	2021	1	0	0	0	0	0	0	0	0	0	0	0	
19154186	m	hp	CN	2021	0	0	0	0	0	0	0	0	0	0	0	0	45
19154612	m	md	US	2021	0	1	0	0	1	0	0	0	0	0	0	0	
19154726	Not	md	JP	2021	0	1	0	0	0	1	0	0	0	0	0	0	
19154732	f	cn	AT	2021	0	1	1	0	0	0	0	0	0	0	0	0	
19155001	m	hp	CN	2021	1	0	0	0	0	0	0	0	0	0	0	0	33
19155697	f	hp	NL	2021	0	0	0	0	0	0	0	0	0	0	0	0	60
19155781	f	Not repo	US	2021	1	0	0	0	0	0	0	0	0	0	0	0	74.3
19156121	m	cn	JP	2021	0	1	1	0	0	0	0	0	0	0	0	0	
19156147	f	Not repo	ID	2021	1	0	0	0	0	0	0	0	0	0	0	0	75
19156164	m	hp	US	2021	0	1	0	0	1	0	0	0	0	0	0	0	64
19156728	f	md	JP	2021	0	0	0	0	0	0	0	0	0	0	0	0	49
19156826	f	Not repo	CA	2021	1	0	0	0	0	0	0	0	0	0	0	0	61.6
19156908	m	hp	GR	2021	0	0	0	0	0	0	0	0	0	0	0	0	76

19157267	f	cn	US	2021	0	0	0	0	0	0	0	0	0	0	0	0	39
19157440	Not	md	JP	2021	0	1	0	0	0	1	0	0	0	0	0	0	56
19157567	m	hp	CA	2021	0	0	0	0	0	0	0	0	0	0	0	0	
19157856	m	cn	Not re	2021	0	0	0	0	0	0	0	0	0	0	0	0	60
19158027	Not	hp	FR	2021	1	0	0	0	0	0	0	0	0	0	0	0	
19158028	Not	hp	FR	2021	1	0	0	0	0	0	0	0	0	0	0	0	
19158029	Not	hp	FR	2021	1	0	0	0	0	0	0	0	0	0	0	0	
19158031	Not	hp	FR	2021	1	0	0	0	0	0	0	0	0	0	0	0	
19158032	Not	hp	FR	2021	1	0	0	0	0	0	0	0	0	0	0	0	
19158037	Not	hp	FR	2021	1	0	0	0	0	0	0	0	0	0	0	0	
19158277	m	md	BR	2021	0	1	1	0	0	0	0	0	0	0	0	0	72
19158290	m	hp	IN	2021	0	0	0	0	0	0	0	0	0	0	0	0	42
19158357	f	md	JP	2021	0	1	0	0	1	0	0	0	0	0	0	0	86
19158358	m	md	JP	2021	0	1	0	0	1	0	0	0	0	0	0	0	55
19158489	m	cn	Not re	2021	0	0	0	0	0	0	0	0	0	0	0	0	60
19159096	m	md	JP	2021	0	0	0	0	0	0	0	0	0	0	0	0	50
19159097	m	md	JP	2021	0	1	0	0	1	0	0	0	0	0	0	0	66
19159345	m	Not repo	CN	2021	0	1	0	1	0	0	0	0	0	0	0	1	68
19159563	m	md	KR	2021	0	0	0	0	0	0	0	0	0	0	0	0	
19159807	m	hp	PL	2021	0	0	0	0	0	0	0	0	0	0	0	0	70
19159859	m	cn	Not re	2021	0	0	0	0	0	0	0	0	0	0	0	0	56
19160263	m	hp	US	2021	0	1	0	0	1	0	0	0	0	0	0	0	49
19160265	m	hp	US	2021	0	1	0	0	1	0	0	0	0	0	0	0	66
19160272	f	Not repo	BR	2021	0	0	0	0	0	0	1	1	0	0	0	0	71
19160441	f	hp	US	2021	0	1	0	0	1	0	0	0	0	0	0	0	68
19160872	m	hp	US	2021	0	1	0	0	1	0	0	0	0	0	0	0	57
19161425	m	md	CN	2021	0	1	0	0	0	1	0	0	0	0	0	0	68
19161862	m	cn	JP	2021	0	1	1	0	0	0	0	0	0	0	0	0	82
19162259	m	md	FR	2021	0	1	0	0	0	1	0	0	0	0	0	0	72
19162593	m	hp	US	2021	0	1	0	0	1	0	0	0	0	0	0	0	64
19163046	m	md	CN	2021	0	1	0	0	1	0	0	0	0	0	0	0	48
19163215	m	hp	CN	2021	0	0	0	0	0	0	0	0	0	0	0	0	51
19163601	Not	hp	FR	2021	1	0	0	0	0	0	0	0	0	0	0	0	
19163602	Not	hp	FR	2021	1	0	0	0	0	0	0	0	0	0	0	0	

19163615	Not	hp	FR	2021	1	0	0	0	0	0	0	0	0	0	0	0	
19163617	Not	hp	FR	2021	1	0	0	0	0	0	0	0	0	0	0	0	
19163622	Not	hp	FR	2021	1	0	0	0	0	0	0	0	0	0	0	0	
19163836	Not	Not repo	ID	2021	1	0	0	0	0	0	0	0	0	0	0	0	
19163997	f	cn	JP	2021	0	1	1	0	0	0	0	0	0	0	0	0	60
19164020	f	cn	Not re	2021	0	0	0	0	0	0	0	0	0	0	0	0	34
19164225	m	md	US	2021	0	0	0	0	0	0	0	0	0	0	0	0	49
19164540	Not	hp	TW	2021	1	0	0	0	0	0	0	0	0	0	0	0	
19164824	Not	md	IT	2021	0	0	0	0	0	0	0	0	0	0	0	0	
19165830	f	Not repo	JP	2021	0	1	0	1	0	0	0	0	0	0	0	0	55
19166233	f	cn	US	2021	0	1	1	0	0	0	0	0	0	0	0	0	
19166442	m	hp	ES	2021	0	1	0	0	1	0	0	0	0	0	0	0	69
19166586	f	cn	CN	2021	0	1	0	0	1	0	0	0	0	0	0	0	69
19166652	m	cn	US	2021	0	1	1	0	0	0	0	0	0	0	0	0	
19166985	m	md	DK	2021	0	1	1	0	0	0	0	0	0	0	0	0	61
19167143	Not	md	US	2021	0	1	0	0	1	0	0	0	0	0	0	0	
19167145	Not	md	JP	2021	0	0	0	0	0	0	0	0	0	0	0	0	
19167190	m	md	JP	2021	0	1	0	0	1	0	0	0	0	0	0	0	80
19167391	f	md	ES	2021	0	1	0	0	0	1	0	0	0	0	0	0	64
19167693	m	cn	Not re	2021	0	0	0	0	0	0	0	0	0	0	0	0	
19167831	f	hp	DK	2021	1	0	0	0	0	0	0	0	0	0	0	0	57
19168471	m	md	US	2021	0	0	0	0	0	0	0	0	0	0	0	0	57
19169112	Not	Not repo	BR	2021	0	1	0	1	0	0	0	0	0	0	0	0	
19169122	Not	md	GB	2021	0	1	0	1	0	0	0	0	0	0	0	0	65
19169237	m	cn	IN	2021	0	0	0	0	0	0	0	0	0	0	0	0	
19170002	m	md	KR	2021	0	0	0	0	0	0	0	0	0	0	0	0	50
19170059	f	hp	PL	2021	1	0	0	0	0	0	0	0	0	0	0	0	
19170096	m	md	CN	2021	0	0	0	0	0	0	0	0	0	0	0	0	62
19170626	m	md	MY	2021	0	1	1	0	0	0	0	0	0	0	0	0	71
19170640	f	hp	GB	2021	0	1	0	0	1	0	0	0	0	0	0	0	55
19170751	m	md	CN	2021	0	1	0	0	1	0	0	0	0	0	0	0	48
19170999	m	Not repo	BR	2021	0	1	0	1	0	0	0	0	0	0	0	1	66
19171524	m	hp	US	2021	0	0	0	0	0	0	0	0	0	0	0	0	87
19172515	f	cn	US	2021	0	0	0	0	0	0	0	0	0	0	0	0	

19172551	m	hp	IN	2021	0	0	0	0	0	0	0	0	0	0	0	0	45
19173302	f	cn	CA	2021	0	0	0	0	0	0	1	1	0	0	0	0	66
19173372	f	Not repo	JP	2021	1	0	0	0	0	0	0	0	0	0	0	0	40
19173381	f	hp	US	2021	0	0	0	0	0	0	0	0	0	0	0	0	60
19173823	m	cn	CN	2021	0	1	0	0	1	0	0	0	0	0	0	0	66
19173846	f	cn	Not re	2021	0	0	0	0	0	0	0	0	0	0	0	0	57
19173904	m	hp	US	2021	0	1	0	0	1	0	0	0	0	0	0	0	72
19174106	f	md	JP	2021	0	1	1	0	0	0	0	0	0	0	0	0	73
19174145	m	md	ES	2021	0	1	0	0	0	1	0	0	0	0	0	0	64
19174296	m	cn	JP	2021	0	1	1	0	0	0	0	0	0	0	0	0	78
19174406	f	md	DK	2021	0	0	0	0	0	0	0	0	0	0	0	0	
19174413	m	md	JP	2021	0	1	1	0	0	0	0	0	0	0	0	0	74
19174569	m	hp	DE	2021	0	0	0	0	0	0	0	0	0	0	0	0	80
19174693	m	Not repo	JP	2021	1	0	0	0	0	0	0	0	0	0	0	0	83
19174694	f	cn	CN	2021	1	0	0	0	0	0	0	0	0	0	0	0	73.1
19174696	f	ph	JP	2021	1	0	0	0	0	0	0	0	0	0	0	0	72
19174697	f	Not repo	MY	2021	1	0	0	0	0	0	0	0	0	0	0	0	70
19175254	Not	md	ES	2021	0	0	0	0	0	0	0	0	0	0	0	0	
19175343	f	cn	CA	2021	1	0	0	0	0	0	0	0	0	0	0	0	
19175470	f	hp	CN	2021	0	0	0	0	0	0	0	0	0	0	0	0	70
19175952	Not	ph	JP	2021	0	1	1	0	0	0	0	0	0	0	0	0	74
19176215	m	md	CH	2021	0	0	0	0	0	0	0	0	0	0	0	0	64
19176263	m	cn	DE	2021	0	1	1	0	0	0	0	0	0	0	0	0	
19176470	m	Not repo	ID	2021	1	0	0	0	0	0	0	0	0	0	0	0	79.6
19176506	m	md	JP	2021	0	0	0	0	0	0	0	0	0	0	0	0	83
19176522	f	md	FR	2021	1	0	0	0	0	0	0	0	0	0	0	0	
19176543	m	cn	DE	2021	0	1	1	0	0	0	0	0	0	0	0	0	71
19176564	m	md	JP	2021	0	1	0	0	0	1	0	0	0	0	0	0	80
19176572	f	md	JP	2021	0	1	0	0	0	1	0	0	0	0	0	0	
19176953	m	cn	Not re	2021	0	0	0	0	0	0	0	0	0	0	0	0	
19177186	f	cn	AR	2021	1	0	0	0	0	0	0	0	0	0	0	0	78
19177284	f	cn	US	2021	1	0	0	0	0	0	0	0	0	0	0	0	
19177828	f	md	GB	2021	1	0	0	0	0	0	0	0	0	0	0	0	72
19177973	m	cn	JP	2021	0	1	1	0	0	0	0	0	0	0	0	0	87

19178769	f	cn	JP	2021	0	1	1	0	0	0	0	0	0	0	0	0	75
19178915	m	cn	CN	2021	0	1	0	0	1	0	0	0	0	0	0	0	66
19179061	m	cn	CN	2021	0	1	0	0	1	0	0	0	0	0	0	0	73
19179310	m	cn	Not re	2021	0	0	0	0	0	0	0	0	0	0	0	0	
19179698	f	ph	JP	2021	1	0	0	0	0	0	0	0	0	0	0	0	
19179808	Not	md	JP	2021	0	1	0	0	1	0	0	0	0	0	0	0	
19180232	m	hp	JP	2021	0	0	0	0	0	0	0	0	0	0	0	0	72
19180384	m	cn	JP	2021	0	1	1	0	0	0	0	0	0	0	0	0	66
19180864	m	hp	IT	2021	0	0	0	0	0	0	0	0	0	0	0	0	71
19180896	m	cn	Not re	2021	0	0	0	0	0	0	0	0	0	0	0	0	57
19181338	m	Not repo	MY	2021	1	0	0	0	0	0	0	0	0	0	0	0	56.8
19181344	f	cn	US	2021	1	0	0	0	0	0	0	0	0	0	0	0	90
19181545	Not	md	JP	2021	0	1	0	0	1	0	0	0	0	0	0	1	
19181604	Not	md	JP	2021	0	1	0	0	1	0	0	0	0	0	0	0	
19181767	Not	md	JP	2021	0	1	0	0	1	0	0	0	0	0	0	1	
19182123	Not	md	JP	2021	0	1	0	0	1	0	0	0	0	0	0	0	
19182468	m	md	FR	2021	0	1	0	1	0	0	0	0	0	0	0	0	60.8
19182522	m	md	US	2021	0	0	0	0	0	0	0	0	0	0	0	0	78
19182850	m	md	JP	2021	0	0	0	0	0	0	0	0	0	0	0	0	83
19182895	m	md	JP	2021	0	1	0	0	0	1	0	0	0	0	0	0	72
19183396	f	cn	CA	2021	1	0	0	0	0	0	0	0	0	0	0	0	
19183742	m	md	ES	2021	1	0	0	0	0	0	0	0	0	0	0	0	84
19184143	m	cn	CA	2021	1	0	0	0	0	0	0	0	0	0	0	0	
19184707	m	hp	IT	2021	0	0	0	0	0	0	0	0	0	0	0	0	69
19184793	m	md	JP	2021	0	1	0	0	1	0	0	0	0	0	0	1	50
19184806	f	cn	Not re	2021	0	0	0	0	0	0	0	0	0	0	0	0	
19185085	f	md	US	2021	0	0	0	0	0	0	0	0	0	0	0	0	83
19185112	m	hp	US	2021	0	1	1	0	0	0	0	0	0	0	0	0	47
19185939	f	md	DE	2021	0	1	0	0	0	1	0	0	0	0	0	0	53
19185987	f	md	IN	2021	0	0	0	0	0	0	0	0	0	0	0	0	29
19186012	f	cn	Not re	2021	0	0	0	0	0	0	0	0	0	0	0	0	42
19186087	m	cn	Not re	2021	0	0	0	0	0	0	0	0	0	0	0	0	73
19186299	f	hp	DE	2021	0	0	0	0	0	0	0	0	0	0	0	0	61
19186439	m	cn	US	2021	0	1	1	0	0	0	0	0	0	0	0	0	82

19186491	m	hp	ES	2021	0	1	0	0	1	0	0	0	0	0	0	0	70
19186882	f	md	CN	2021	0	1	0	0	0	1	0	0	0	0	0	0	68
19186891	Not	md	US	2021	0	1	0	1	0	0	0	0	0	0	0	0	
19187345	m	hp	FR	2021	0	1	0	0	1	0	0	0	0	0	0	0	64
19187502	f	md	TR	2021	0	0	0	0	0	0	0	0	0	0	0	0	64
19187599	f	Not repo	CA	2021	1	0	0	0	0	0	0	0	0	0	0	0	
19187751	Not	cn	US	2021	0	0	0	0	0	0	0	0	0	0	0	0	
19188216	f	cn	BE	2021	0	1	1	0	0	0	0	0	0	0	0	0	
19188218	m	cn	US	2021	0	1	0	1	0	0	0	0	0	0	0	0	73
19188226	f	md	US	2021	0	1	0	1	0	0	0	0	0	0	0	0	
19188231	f	ph	US	2021	0	1	0	1	0	0	0	0	0	0	0	0	
19188277	Not	ph	JP	2021	0	1	0	0	1	0	0	0	0	0	0	0	
19188418	f	md	US	2021	0	0	0	0	0	0	0	0	0	0	0	0	52
19188522	m	md	AR	2021	0	1	0	1	0	0	0	0	0	0	0	0	
19188572	m	hp	CA	2021	0	1	0	0	0	1	0	0	0	0	0	0	
19188582	f	md	JP	2021	0	1	0	0	0	1	0	0	0	0	0	0	90
19188746	m	md	JP	2021	0	1	1	0	0	0	0	0	0	0	0	0	74
19189180	m	hp	JP	2021	0	1	0	1	0	0	0	0	0	0	0	0	69
19189709	f	hp	US	2021	0	0	0	0	0	0	0	0	0	0	0	0	
19189927	f	md	DE	2021	0	1	0	0	1	0	0	0	0	0	0	0	63
19189941	m	md	JP	2021	0	1	0	0	1	0	0	0	0	0	0	0	72
19190032	m	cn	JP	2021	0	1	1	0	0	0	0	0	0	0	0	0	64
19190259	Not	ph	JP	2021	1	0	0	0	0	0	0	0	0	0	0	0	
19190303	Not	md	BR	2021	0	0	0	0	0	0	0	0	0	0	0	0	
19190326	Not	md	JP	2021	0	1	0	0	0	1	0	0	0	0	0	1	60
19190418	m	cn	SG	2021	0	1	0	0	0	1	0	0	0	0	0	0	69
19190708	m	cn	Not re	2021	0	0	0	0	0	0	0	0	0	0	0	0	70
19190772	m	cn	US	2021	0	1	0	1	0	0	0	0	0	0	0	0	
19191046	f	cn	Not re	2021	0	0	0	0	0	0	0	0	0	0	0	0	92
19191541	m	md	US	2021	0	0	0	0	0	0	0	0	0	0	0	0	78
19191762	m	md	US	2021	0	0	0	0	0	0	0	0	0	0	0	0	
19191852	m	hp	US	2021	0	1	0	0	1	0	0	0	0	0	0	0	54
19192347	m	md	IL	2021	0	1	0	1	0	0	0	0	0	0	0	0	59
19193299	m	md	NO	2021	0	1	1	0	0	0	0	0	0	0	0	1	70

19193321	f	md	US	2021	0	0	0	0	0	0	0	0	0	0	0	0	82
19193491	f	Not repo	AU	2021	1	0	0	0	0	0	0	0	0	0	0	0	
19193734	f	hp	RO	2021	1	0	0	0	0	0	0	0	0	0	0	0	78
19194606	m	cn	US	2021	0	1	0	1	0	0	0	0	0	0	0	0	
19194608	Not	Not repo	GB	2021	0	1	0	1	0	0	0	0	0	0	0	0	
19194610	m	hp	PT	2021	1	0	0	0	0	0	0	0	0	0	0	0	70
19195035	m	md	TR	2021	0	0	0	0	0	0	0	0	0	0	0	0	55
19195171	m	ph	PT	2021	0	1	0	0	0	1	0	0	0	0	0	0	66
19195460	f	md	US	2021	0	1	1	0	0	0	0	0	0	0	0	1	59
19195549	m	hp	FR	2021	0	1	0	0	1	0	0	0	0	0	0	0	88
19196467	f	cn	Not re	2021	0	0	0	0	0	0	0	0	0	0	0	0	
19196719	f	md	DE	2021	0	1	0	0	1	0	0	0	0	0	0	0	62
19196781	f	hp	US	2021	0	0	0	0	0	0	0	0	0	0	0	0	
19196832	m	ph	JP	2021	0	0	0	0	0	0	0	0	0	0	0	0	70
19197482	Not	hp	RU	2021	0	1	0	1	0	0	0	0	0	0	0	0	
19197828	m	ph	CA	2021	0	0	0	0	0	0	0	0	0	0	0	0	76
19198097	f	cn	US	2021	0	1	0	1	0	0	0	0	0	0	0	0	43.1
19198208	f	cn	US	2021	0	0	0	0	0	0	0	0	0	0	0	0	
19198361	f	md	DE	2021	0	1	0	0	0	1	0	0	0	0	0	0	65
19198776	m	hp	RU	2021	0	1	0	1	0	0	0	0	0	0	0	0	
19198778	Not	Not repo	VN	2021	1	0	0	0	0	0	0	0	0	0	0	1	
19199573	m	cn	US	2021	0	1	0	1	0	0	0	0	0	0	0	0	83.7
19199694	m	md	JP	2021	0	1	0	0	0	1	0	0	0	0	0	1	80
19199712	Not	md	JP	2021	0	1	0	0	1	0	0	0	0	0	0	0	
19200002	m	md	CA	2021	0	0	0	0	0	0	0	0	0	0	0	0	78
19200361	m	hp	US	2021	0	1	0	1	0	0	0	0	0	0	0	0	
19200365	m	ph	JP	2021	1	0	0	0	0	0	0	0	0	0	0	0	84
19200773	m	cn	Not re	2021	0	0	0	0	0	0	0	0	0	0	0	0	
19200895	m	hp	IT	2021	0	0	0	0	0	0	0	0	0	0	0	0	33
19200930	Not	hp	RU	2021	0	1	0	1	0	0	0	0	0	0	0	0	
19200955	m	cn	US	2021	0	1	1	0	0	0	0	0	0	0	0	0	55
19201176	Not	ph	JP	2021	1	0	0	0	0	0	0	0	0	0	0	0	
19201195	f	hp	CA	2021	0	1	0	1	0	0	0	0	0	0	0	0	75
19201205	Not	hp	US	2021	0	0	0	0	0	0	0	0	0	0	0	0	

19201210	m	cn	KR	2021	0	0	0	0	0	0	0	0	0	0	0	0	73
19201476	m	cn	Not re	2021	0	0	0	0	0	0	0	0	0	0	0	0	
19201822	m	md	JP	2021	0	1	0	0	0	1	0	0	0	0	0	0	
19201823	Not	md	JP	2021	0	1	0	0	0	1	0	0	0	0	0	1	
19201883	f	md	US	2021	0	0	0	0	0	0	0	0	0	0	0	0	51
19202227	f	md	US	2021	0	1	1	0	0	0	0	0	0	0	0	0	53
19202281	f	cn	CN	2021	0	1	0	0	1	0	0	0	0	0	0	0	56
19202318	f	hp	US	2021	0	0	0	0	0	0	0	0	0	0	0	0	56
19202337	m	md	US	2021	0	1	1	0	0	0	0	0	0	0	0	0	
19202355	m	md	CN	2021	0	1	1	0	0	0	0	0	0	0	0	0	67
19202659	m	hp	IN	2021	0	0	0	0	0	0	0	0	0	0	0	0	65
19202669	f	md	IT	2021	0	1	0	0	0	1	0	0	0	0	0	0	59
19202685	m	Not repo	VN	2021	1	0	0	0	0	0	0	0	0	0	0	0	65
19202730	f	hp	IT	2021	0	0	0	0	0	0	0	0	0	0	0	0	31
19202732	f	cn	Not re	2021	0	0	0	0	0	0	0	0	0	0	0	0	
19202763	m	cn	Not re	2021	0	0	0	0	0	0	0	0	0	0	0	0	66
19202902	m	cn	JP	2021	0	1	1	0	0	0	0	0	0	0	0	0	77
19202995	f	hp	JP	2021	0	1	0	1	0	0	0	0	0	0	0	0	77
19203014	m	md	DE	2021	0	0	0	0	0	0	0	0	0	0	0	0	57
19203390	m	md	CO	2021	0	1	1	0	0	0	0	0	0	0	0	0	66
19203729	f	hp	IT	2021	0	0	0	0	0	0	0	0	0	0	0	0	60
19204061	f	md	US	2021	0	0	0	0	0	0	0	0	0	0	0	0	56
19204301	m	md	JP	2021	0	1	0	0	1	0	0	0	0	0	0	1	70
19204554	f	md	CN	2021	1	0	0	0	0	0	0	0	0	0	0	0	64
19204670	m	cn	IN	2021	0	1	0	0	1	0	0	0	0	0	0	0	60
19204988	m	md	CN	2021	0	1	0	0	0	1	0	0	0	0	0	0	68
19205084	m	Not repo	JP	2021	1	0	0	0	0	0	0	0	0	0	0	0	
19205156	m	hp	KR	2021	0	0	0	0	0	0	0	0	0	0	0	0	52
19205248	m	Not repo	AU	2021	0	1	0	1	0	0	0	0	0	0	0	0	
19205987	f	md	US	2021	0	0	0	0	0	0	0	0	0	0	0	0	
19206086	f	hp	IT	2021	0	0	0	0	0	0	0	0	0	0	0	0	31
19206177	f	md	US	2021	0	0	0	0	0	0	0	0	0	0	0	0	
19206608	f	md	JP	2021	0	1	0	0	0	1	0	0	0	0	0	0	49
19206728	m	ph	JP	2021	0	1	0	0	1	0	0	0	0	0	0	0	79

19206774	f	md	CZ	2021	1	0	0	0	0	0	0	0	0	0	0	0	72
19207017	f	md	US	2021	1	0	0	0	0	0	0	0	0	0	0	0	
19207201	m	cn	CN	2021	1	0	0	0	0	0	0	0	0	0	0	0	
19207234	m	hp	JP	2021	0	1	0	1	0	0	0	0	0	0	0	1	68
19207235	m	Not repo	CN	2021	0	1	0	1	0	0	0	0	0	0	0	0	58
19207236	f	cn	CA	2021	1	0	0	0	0	0	0	0	0	0	0	0	
19207239	m	hp	CH	2021	1	0	0	0	0	0	0	0	0	0	0	0	
19207246	Not	Not repo	FR	2021	1	0	0	0	0	0	0	0	0	0	0	0	
19207285	m	hp	IN	2021	0	0	0	0	0	0	0	0	0	0	0	0	52
19207319	Not	md	US	2021	0	0	0	0	0	0	0	0	0	0	0	0	
19207333	f	md	US	2021	0	0	0	0	0	0	0	0	0	0	0	0	53
19207671	Not	md	JP	2021	1	0	0	0	0	0	0	0	0	0	0	0	
19207786	m	Not repo	CA	2021	1	0	0	0	0	0	0	0	0	0	0	0	59.3
19208315	m	Not repo	CN	2021	0	1	0	1	0	0	0	0	0	0	0	0	49
19208316	m	Not repo	CN	2021	0	1	0	1	0	0	0	0	0	0	0	0	63
19208755	m	md	JP	2021	0	1	0	0	1	0	0	0	0	0	0	0	67
19210279	f	cn	FR	2021	0	1	0	0	1	0	0	0	0	0	0	0	68
19210295	f	md	US	2021	0	1	1	0	0	0	0	0	0	0	0	0	75
19210581	f	md	US	2021	0	0	0	0	0	0	0	0	0	0	0	0	60
19210733	m	cn	CN	2021	0	0	0	0	0	0	0	0	0	0	0	0	53
19210836	f	md	US	2021	0	0	0	0	0	0	0	0	0	0	0	0	52
19210886	f	Not repo	AU	2021	1	0	0	0	0	0	0	0	0	0	0	0	66.8
19210900	m	cn	CN	2021	0	1	0	1	0	0	0	0	0	0	0	0	71
19211058	f	ph	US	2021	0	0	0	0	0	0	0	0	0	0	0	0	81
19211178	f	md	US	2021	0	0	0	0	0	0	0	0	0	0	0	0	53
19211205	f	Not repo	CN	2021	0	1	0	1	0	0	0	0	0	0	0	0	49
19211386	m	Not repo	CN	2021	0	1	0	1	0	0	0	0	0	0	0	0	86
19211682	f	md	PT	2021	0	0	0	0	0	0	0	0	0	0	0	0	66
19211781	m	ph	JP	2021	0	1	0	1	0	0	0	0	0	0	0	1	
19211871	m	Not repo	US	2021	1	0	0	0	0	0	0	0	0	0	0	0	
19212228	m	ph	US	2021	0	0	0	0	0	0	0	0	0	0	0	0	65
19212333	m	md	KR	2021	0	1	0	0	0	1	0	0	0	0	0	0	71
19212556	m	md	JP	2021	0	1	0	1	0	0	0	0	0	0	0	1	56
19212619	m	Not repo	CN	2021	0	1	0	1	0	0	0	0	0	0	0	0	78

19212784	m	md	NL	2021	0	0	0	0	0	0	0	0	0	0	0	0	
19212806	f	cn	US	2021	1	0	0	0	0	0	0	0	0	0	0	0	53.1
19212906	Not	hp	ES	2021	0	1	0	0	0	1	0	0	0	0	0	1	
19213617	Not	Not repo	FR	2021	1	0	0	0	0	0	0	0	0	0	0	0	
19213678	Not	md	US	2021	0	0	0	0	0	0	0	0	0	0	0	0	
19213690	Not	ph	JP	2021	1	0	0	0	0	0	0	0	0	0	0	0	
19214991	m	ph	US	2021	0	0	0	0	0	0	0	0	0	0	0	0	
19215918	f	cn	US	2021	0	1	1	0	0	0	0	0	0	0	0	0	
19215951	f	hp	AT	2021	0	1	1	0	0	0	0	0	0	0	0	0	
19216046	m	Not repo	JP	2021	1	0	0	0	0	0	0	0	0	0	0	0	80
19216382	f	hp	CN	2021	0	0	0	0	0	0	0	0	0	0	0	0	47
19216389	Not	Not repo	AU	2021	1	0	0	0	0	0	0	0	0	0	0	0	
19216393	Not	Not repo	AU	2021	0	1	0	1	0	0	0	0	0	0	0	1	
19216497	Not	md	JP	2021	0	1	0	1	0	0	0	0	0	0	0	0	
19216577	f	hp	US	2021	0	1	1	0	0	0	0	0	0	0	0	0	61
19217135	f	Not repo	US	2021	1	0	0	0	0	0	0	0	0	0	0	0	66
19217318	f	md	PL	2021	0	0	0	0	0	0	0	0	0	0	0	0	50
19217497	m	hp	JP	2021	0	1	1	0	0	0	0	0	0	0	0	0	72
19217503	f	md	FR	2021	1	0	0	0	0	0	0	0	0	0	0	0	48
19218036	Not	ph	AU	2021	0	1	0	0	1	0	0	0	0	0	0	0	
19218257	m	hp	GR	2021	0	1	0	0	1	0	0	0	0	0	0	0	62
19218270	m	md	KR	2021	0	0	0	0	0	0	0	0	0	0	0	0	61
19218590	m	md	KR	2021	0	1	0	0	0	1	0	0	0	0	0	0	90
19219067	m	cn	DE	2021	0	0	0	0	0	0	0	0	0	0	0	0	79
19219500	Not	md	HU	2021	0	1	0	0	1	0	0	0	0	0	0	0	
19219919	m	cn	DE	2021	0	1	1	0	0	0	0	0	0	0	0	0	71
19220008	m	md	JP	2021	0	0	0	0	0	0	0	0	0	0	0	0	61
19220171	Not	hp	US	2021	0	0	0	0	0	0	0	0	0	0	0	0	
19220394	m	hp	JP	2021	1	0	0	0	0	0	0	0	0	0	0	0	46
19220767	m	Not repo	HU	2021	1	0	0	0	0	0	0	0	0	0	0	0	
19220978	f	cn	Not re	2021	0	0	0	0	0	0	0	0	0	0	0	0	
19221108	f	hp	US	2021	0	1	0	1	0	0	0	0	0	0	0	0	
19221109	m	Not repo	ID	2021	1	0	0	0	0	0	0	0	0	0	0	0	77.7
19221274	f	hp	US	2021	0	0	0	0	0	0	0	0	0	0	0	0	72

19221399	m	cn	KR	2021	0	0	0	0	0	0	0	0	0	0	0	0	
19221918	m	md	GB	2021	0	1	0	0	0	1	0	0	0	0	0	0	63
19221951	m	Not repo	AU	2021	1	0	0	0	0	0	0	0	0	0	0	0	
19222045	m	md	BY	2021	0	0	0	0	0	0	0	0	0	0	0	0	52
19222054	f	md	FR	2021	0	0	0	0	0	0	0	0	0	0	0	0	58
19222056	m	md	FR	2021	0	0	0	0	0	0	0	0	0	0	0	0	69
19222112	f	hp	KR	2021	0	0	0	0	0	0	0	0	0	0	0	0	48
19222512	Not	ph	JP	2021	0	1	0	0	1	0	0	0	0	0	0	0	
19222646	f	cn	AT	2021	0	1	1	0	0	0	0	0	0	0	0	0	
19223313	Not	hp	US	2021	0	0	0	0	0	0	0	0	0	0	0	0	
19224909	f	md	CA	2021	1	0	0	0	0	0	0	0	0	0	0	0	
19225237	Not	md	JP	2021	0	1	0	1	0	0	0	0	0	0	0	0	
19225251	m	cn	US	2021	0	0	0	0	0	0	0	0	0	0	0	0	81
19225899	f	cn	US	2021	1	0	0	0	0	0	0	0	0	0	0	0	86
19226284	Not	cn	DE	2021	0	1	0	1	0	0	0	0	0	0	0	0	
19226637	m	md	US	2021	0	1	0	0	1	0	0	0	0	0	0	0	77
19226639	f	hp	US	2021	0	1	0	1	0	0	0	0	0	0	0	0	77.9
19226648	m	md	FR	2021	0	1	0	0	0	1	0	0	0	0	0	0	81
19227583	m	cn	US	2021	1	0	0	0	0	0	0	0	0	0	0	0	65.1
19227609	Not	Not repo	PL	2021	0	1	0	1	0	0	0	0	0	0	0	0	
19228081	Not	ph	US	2021	0	0	0	0	0	0	0	0	0	0	0	0	
19228195	m	md	JP	2021	0	1	0	0	1	0	0	0	0	0	0	0	77
19228244	m	md	JP	2021	0	1	0	0	1	0	0	0	0	0	0	0	49
19228757	f	md	GB	2021	0	1	0	0	0	1	0	0	0	0	0	0	65
19228999	f	md	US	2021	0	1	0	0	0	1	0	0	0	0	0	0	76
19229017	f	cn	TH	2021	0	0	0	0	0	0	0	0	0	0	0	0	72
19229049	m	cn	JP	2021	0	1	1	0	0	0	0	0	0	0	0	0	70
19229287	f	md	US	2021	0	0	0	0	0	0	0	0	0	0	0	0	64
19229431	m	md	BR	2021	1	0	0	0	0	0	0	0	0	0	0	0	65
19229815	f	cn	US	2021	1	0	0	0	0	0	0	0	0	0	0	0	83
19229929	f	cn	US	2021	0	1	1	0	0	0	0	0	0	0	0	0	
19230406	m	md	ES	2021	0	1	0	0	0	1	0	0	0	0	0	0	59
19230816	f	cn	DE	2021	0	1	1	0	0	0	0	0	0	0	0	0	
19230882	m	cn	CN	2021	0	1	0	0	1	0	0	0	0	0	0	0	75

19230955	m	md	GB	2021	0	1	0	1	0	0	0	0	0	0	0	1	
19230984	f	md	US	2021	0	1	1	0	0	0	0	0	0	0	0	0	61
19231025	f	cn	Not re	2021	0	0	0	0	0	0	0	0	0	0	0	0	51
19231057	Not	hp	US	2021	0	0	0	0	0	0	0	0	0	0	0	0	
19231118	m	hp	FR	2021	0	1	0	0	1	0	0	0	0	0	0	0	59
19231123	m	md	US	2021	0	0	0	0	0	0	0	0	0	0	0	0	
19231772	f	md	DE	2021	0	1	0	0	0	1	0	0	0	0	0	0	53
19231893	m	cn	IN	2021	0	0	0	0	0	0	0	0	0	0	0	0	62
19232216	f	md	NO	2021	0	1	1	0	0	0	0	0	0	0	0	0	70
19232260	m	cn	US	2021	0	1	0	1	0	0	0	0	0	0	0	1	76.3
19232379	f	Not repo	AU	2021	1	0	0	0	0	0	0	0	0	0	0	0	
19233284	f	md	DE	2021	1	0	0	0	0	0	0	0	0	0	0	0	
19233301	f	md	US	2021	0	1	0	0	0	1	0	0	0	0	0	0	76
19233678	Not	hp	US	2021	0	0	0	0	0	0	0	0	0	0	0	0	
19233898	m	hp	FR	2021	0	1	0	0	1	0	0	0	0	0	0	0	86
19234564	m	ph	TW	2021	0	1	0	0	1	0	0	0	0	0	0	0	64
19234712	m	md	DE	2021	0	1	0	0	0	1	0	0	0	0	0	0	62
19235971	f	cn	US	2021	1	0	0	0	0	0	0	0	0	0	0	0	
19236393	Not	md	US	2021	0	1	0	0	1	0	0	0	0	0	0	0	
19236833	m	md	KR	2021	0	0	0	0	0	0	0	0	0	0	0	0	78
19238082	f	Not repo	AU	2021	0	1	0	1	0	0	0	0	0	0	0	0	
19238491	m	md	DE	2021	0	1	0	0	1	0	0	0	0	0	0	0	70
19238567	Not	md	JP	2021	0	1	0	0	1	0	0	0	0	0	0	1	70
19238807	m	md	DE	2021	0	1	1	0	0	0	0	0	0	0	0	0	71
19239212	f	md	DE	2021	0	1	0	0	0	1	0	0	0	0	0	0	53
19239221	m	cn	JP	2021	0	1	1	0	0	0	0	0	0	0	0	0	82
19239458	m	cn	PL	2021	1	0	0	0	0	0	0	0	0	0	0	0	74
19239583	m	md	DE	2021	0	1	0	1	0	0	0	0	0	0	0	0	75
19239686	m	hp	NL	2021	0	1	1	0	0	0	0	0	0	0	0	0	71
19240577	m	cn	JP	2021	0	1	1	0	0	0	0	0	0	0	0	0	80
19240744	Not	md	US	2021	0	0	0	0	0	0	0	0	0	0	0	0	
19241051	Not	md	JP	2021	0	0	0	0	0	0	0	0	0	0	0	0	
19242081	m	hp	TW	2021	0	0	0	0	0	0	0	0	0	0	0	0	55
19242194	m	hp	ES	2021	0	1	1	0	0	0	0	0	0	0	0	0	71

19242203	m	md	DE	2021	0	0	0	0	0	0	0	0	0	0	0	0	57
19242278	m	cn	CN	2021	0	1	0	0	1	0	0	0	0	0	0	0	62
19242421	f	md	US	2021	0	0	0	0	0	0	0	0	0	0	0	0	
19243048	f	md	US	2021	0	1	1	0	0	0	0	0	0	0	0	0	61
19243193	Not	md	US	2021	0	0	0	0	0	0	0	0	0	0	0	0	
19243392	m	ph	GB	2021	0	1	0	0	0	1	0	0	0	0	0	0	71
19243494	m	md	IN	2021	0	1	0	0	1	0	0	0	0	0	0	0	
19243498	m	md	JP	2021	0	1	0	0	1	0	0	0	0	0	0	0	74
19243589	m	md	AR	2021	0	1	0	0	0	1	0	0	0	0	0	0	
19243659	Not	md	US	2021	1	0	0	0	0	0	0	0	0	0	0	1	
19244234	m	cn	IN	2021	0	1	0	0	1	0	0	0	0	0	0	0	38
19244276	m	md	KR	2021	0	1	0	0	0	1	0	0	0	0	0	0	68
19244359	m	md	NL	2021	0	0	0	0	0	0	0	0	0	0	0	0	80
19244631	f	md	CA	2021	0	1	1	0	0	0	0	0	0	0	0	0	75
19245115	m	md	US	2021	0	0	0	0	0	0	0	0	0	0	0	0	
19245136	m	cn	IN	2021	0	1	0	0	1	0	0	0	0	0	0	0	70
19245209	Not	md	DE	2021	0	1	0	0	0	1	0	0	0	0	0	0	
19245814	m	hp	IT	2021	0	1	1	0	0	0	0	0	0	0	0	0	54
19246184	f	hp	JP	2021	0	1	1	0	0	0	0	0	0	0	0	0	77
19246402	Not	md	JP	2021	0	0	0	0	0	0	0	0	0	0	0	0	
19246505	Not	md	JP	2021	0	0	0	0	0	0	0	0	0	0	0	0	
19246517	f	hp	US	2021	0	0	0	0	0	0	0	0	0	0	0	0	79
19246652	f	Not repo	ID	2021	1	0	0	0	0	0	0	0	0	0	0	0	79.9
19246813	f	hp	FR	2021	0	1	0	0	1	0	0	0	0	0	0	0	66
19247147	m	cn	MY	2021	0	0	0	0	0	0	0	0	0	0	0	0	57
19247740	f	hp	NL	2021	0	0	0	0	0	0	0	0	0	0	0	0	61
19247890	m	hp	DE	2021	0	0	0	0	0	0	0	0	0	0	0	0	60
19249357	m	Not repo	JP	2021	0	1	0	1	0	0	0	0	0	0	0	1	81
19250246	m	hp	AM	2021	0	0	0	0	0	0	0	0	0	0	0	0	61
19250491	f	cn	Not re	2021	0	0	0	0	0	0	0	0	0	0	0	0	57
19250569	f	ph	US	2021	1	0	0	0	0	0	0	0	0	0	0	0	77
19250858	m	md	JP	2021	0	1	0	1	0	0	0	0	0	0	0	0	79
19251004	Not	hp	EG	2021	0	0	0	0	0	0	0	0	0	0	0	0	
19251059	f	hp	US	2021	0	1	1	0	0	0	0	0	0	0	0	0	72

19251203	m	md	CH	2021	0	1	0	0	0	1	0	0	0	0	0	0	72
19251674	f	md	JP	2021	0	1	0	0	1	0	0	0	0	0	0	0	60
19251808	m	hp	US	2021	0	1	1	0	0	0	0	0	0	0	0	0	79
19252151	Not	hp	CN	2021	0	0	0	0	0	0	0	0	0	0	0	0	
19252782	f	md	IN	2021	0	0	0	0	0	0	0	0	0	0	0	0	
19253819	m	md	JP	2021	0	0	0	0	0	0	0	0	0	0	0	0	61
19253829	m	md	US	2021	0	0	0	0	0	0	0	0	0	0	0	0	
19254092	Not	md	BR	2021	0	0	0	0	0	0	0	0	0	0	0	0	70
19254475	f	md	JP	2021	0	1	0	0	0	1	0	0	0	0	0	0	80
19255201	m	hp	US	2021	0	0	0	0	0	0	0	0	0	0	0	0	73
19255991	f	md	US	2021	0	1	1	0	0	0	0	0	0	0	0	0	61
19257000	f	md	JP	2021	0	1	0	0	1	0	0	0	0	0	0	0	76
19257031	m	md	JP	2021	0	1	0	0	1	0	0	0	0	0	0	0	80
19257038	f	cn	US	2021	0	0	0	0	0	0	0	0	0	0	0	0	
19257170	m	ph	JP	2021	0	1	0	0	1	0	0	0	0	0	0	0	67
19257304	f	cn	CN	2021	0	0	0	0	0	0	0	0	0	0	0	0	70
19257310	m	Not repo	CN	2021	0	1	0	1	0	0	0	0	0	0	0	0	49
19257332	f	Not repo	RO	2021	1	0	0	0	0	0	0	0	0	0	0	0	72
19257802	f	hp	DE	2021	0	1	0	0	0	1	0	0	0	0	0	0	53
19257913	m	md	CN	2021	0	1	1	0	0	0	0	0	0	0	0	0	64
19258042	m	md	KR	2021	0	1	1	0	0	0	0	0	0	0	0	0	43
19258504	m	md	CH	2021	0	0	0	0	0	0	0	0	0	0	0	0	77
19258510	m	md	CA	2021	1	0	0	0	0	0	0	0	0	0	0	0	47.9
19258787	f	md	FR	2021	1	0	0	0	0	0	0	0	0	0	0	1	79.1
19258884	Not	Not repo	AU	2021	1	0	0	0	0	0	0	0	0	0	0	0	
19258998	f	Not repo	ID	2021	1	0	0	0	0	0	0	0	0	0	0	0	67.1
19259044	m	md	US	2021	0	0	0	0	0	0	0	0	0	0	0	0	80
19259327	f	md	IT	2021	0	0	0	0	0	0	0	0	0	0	0	0	31
19259345	f	md	JP	2021	1	0	0	0	0	0	0	0	0	0	0	0	78
19259366	f	ph	JP	2021	0	0	0	0	0	0	0	0	0	0	0	0	80
19259390	Not	md	KR	2021	0	1	0	0	0	1	0	0	0	0	0	0	
19259476	f	cn	IN	2021	0	0	0	0	0	0	0	0	0	0	0	0	58
19259969	m	hp	KR	2021	0	0	0	0	0	0	0	0	0	0	0	0	52
19260218	f	md	US	2021	0	0	0	0	0	0	0	0	0	0	0	0	64

19260232	m	md	UA	2021	0	1	1	0	0	0	0	0	0	0	0	0	72
19260628	f	md	DE	2021	1	0	0	0	0	0	0	0	0	0	0	0	73
19261177	f	md	CZ	2021	0	1	0	0	0	1	0	0	0	0	0	0	70
19261432	m	hp	DE	2021	0	0	0	0	0	0	0	0	0	0	0	0	60
19262713	f	hp	US	2021	0	0	0	0	0	0	0	0	0	0	0	0	61
19262719	f	cn	US	2021	0	0	0	0	0	0	0	0	0	0	0	0	
19263284	m	hp	JP	2021	0	1	0	0	0	1	0	0	0	0	0	0	69
19263366	m	hp	ES	2021	0	1	1	0	0	0	0	0	0	0	0	0	71
19263534	f	md	JP	2021	0	1	0	0	1	0	0	0	0	0	0	0	78
19263690	f	ph	US	2021	0	1	1	0	0	0	0	0	0	0	0	0	85
19263744	f	cn	US	2021	0	0	0	0	0	0	0	0	0	0	0	0	
19263956	Not	md	JP	2021	0	1	1	0	0	0	0	0	0	0	0	0	90
19263991	Not	md	JP	2021	0	1	0	0	0	1	0	0	0	0	0	0	
19264198	Not	cn	JP	2021	1	0	0	0	0	0	0	0	0	0	0	0	
19264230	Not	hp	US	2021	0	0	0	0	0	0	0	0	0	0	0	0	
19264778	f	hp	US	2021	0	0	0	0	0	0	0	0	0	0	0	0	
19265238	f	md	IT	2021	0	1	1	0	0	0	0	0	0	0	0	0	
19265759	m	md	KR	2021	0	1	0	0	1	0	0	0	0	0	0	0	90
19265784	m	md	KR	2021	0	1	0	0	0	1	0	0	0	0	0	0	72
19265925	f	hp	DE	2021	0	1	0	0	1	0	0	0	0	0	0	0	72
19265994	m	cn	CN	2021	0	1	0	0	1	0	0	0	0	0	0	0	67
19266032	Not	hp	UA	2021	0	0	0	0	0	0	0	0	0	0	0	0	
19266074	m	cn	US	2021	0	1	1	0	0	0	0	0	0	0	0	0	
19266194	Not	hp	UA	2021	0	0	0	0	0	0	0	0	0	0	0	0	
19266405	f	hp	DE	2021	0	0	0	0	0	0	0	0	0	0	0	0	68
19266435	m	hp	DE	2021	0	0	0	0	0	0	0	0	0	0	0	0	57
19266480	f	hp	IN	2021	0	0	0	0	0	0	0	0	0	0	0	0	43
19266575	f	hp	US	2021	0	1	0	0	1	0	0	0	0	0	0	0	74
19266778	f	cn	US	2021	1	0	0	0	0	0	0	0	0	0	0	0	86
19266986	f	md	PT	2021	0	0	0	0	0	0	0	0	0	0	0	0	66
19267282	Not	hp	JP	2021	1	0	0	0	0	0	0	0	0	0	0	1	
19267333	m	Not repo	CN	2021	0	1	0	1	0	0	0	0	0	0	0	0	72
19267563	m	md	DE	2021	0	0	0	0	0	0	0	0	0	0	0	0	
19267704	m	hp	TW	2021	1	0	0	0	0	0	0	0	0	0	0	0	70

19267896	m	cn	Not re	2021	0	0	0	0	0	0	0	0	0	0	0	0	
19267991	f	md	JP	2021	0	0	0	0	0	0	0	0	0	0	0	0	37
19268038	Not	md	US	2021	0	1	1	0	0	0	0	0	0	0	0	0	
19268065	Not	hp	UA	2021	0	0	0	0	0	0	0	0	0	0	0	0	
19268387	m	hp	US	2021	0	0	0	0	0	0	0	0	0	0	0	0	83
19268722	f	hp	IL	2021	0	0	0	0	0	0	0	0	0	0	0	0	72
19269105	m	md	US	2021	0	0	0	0	0	0	0	0	0	0	0	0	69
19269135	f	hp	US	2021	0	0	0	0	0	0	0	0	0	0	0	0	67
19269181	f	hp	US	2021	0	0	0	0	0	0	0	0	0	0	0	0	
19269204	m	md	NO	2021	0	0	0	0	0	0	0	0	0	0	0	0	45
19269205	f	md	RS	2021	0	0	0	0	0	0	0	0	0	0	0	0	55
19269836	f	hp	JP	2021	0	1	1	0	0	0	0	0	0	0	0	0	77
19269953	Not	md	JP	2021	1	0	0	0	0	0	0	0	0	0	0	0	
19270542	f	cn	Not re	2021	0	0	0	0	0	0	0	0	0	0	0	0	66
19270544	f	hp	US	2021	0	0	0	0	0	0	0	0	0	0	0	0	72
19270637	f	cn	US	2021	0	0	0	0	0	0	0	0	0	0	0	0	
19270886	m	md	JP	2021	0	1	0	0	0	1	0	0	0	0	0	0	66
19270936	m	md	JP	2021	0	1	0	0	1	0	0	0	0	0	0	1	72
19271009	f	Not repo	CN	2021	0	1	0	1	0	0	0	0	0	0	0	0	42
19271178	m	Not repo	RU	2021	0	1	0	1	0	0	0	0	0	0	0	0	
19271190	f	md	GB	2021	0	1	0	0	0	1	0	0	0	0	0	0	44
19271227	m	hp	CN	2021	1	0	0	0	0	0	0	0	0	0	0	0	68
19271426	m	cn	US	2021	0	1	0	0	1	0	0	0	0	0	0	0	63
19271674	f	md	JP	2021	0	1	0	1	0	0	0	0	0	0	0	0	62
19271709	f	md	US	2021	0	0	0	0	0	0	0	0	0	0	0	0	67
19272279	m	md	JP	2021	0	1	0	0	0	1	0	0	0	0	0	0	70
19272369	m	ph	JP	2021	0	1	0	0	1	0	0	0	0	0	0	0	
19272517	m	hp	ES	2021	0	1	1	0	0	0	0	0	0	0	0	0	71
19272613	f	cn	JP	2021	0	1	1	0	0	0	0	0	0	0	0	0	64
19272737	Not	md	JP	2021	0	1	0	0	1	0	0	0	0	0	0	0	70
19272761	f	md	US	2021	0	1	1	0	0	0	0	0	0	0	0	0	56
19273062	m	cn	CN	2021	0	1	0	0	1	0	0	0	0	0	0	1	64
19273392	m	md	PT	2021	0	1	0	0	1	0	0	0	0	0	0	0	45
19273855	f	cn	Not re	2021	0	0	0	0	0	0	0	0	0	0	0	0	64

19273981	Not	hp	JP	2021	0	1	0	0	0	1	0	0	0	0	0	0	
19274185	m	md	JP	2021	0	1	0	0	0	1	0	0	0	0	0	0	80
19274434	m	md	JP	2021	0	1	0	1	0	0	0	0	0	0	0	0	
19274634	f	hp	FR	2021	0	1	0	0	1	0	0	0	0	0	0	0	49
19274656	m	md	JP	2021	0	1	0	0	0	1	0	0	0	0	0	0	80
19274999	m	md	JP	2021	0	1	0	0	1	0	0	0	0	0	0	0	72
19275206	Not	hp	ES	2021	0	1	0	0	0	1	0	0	0	0	0	0	
19275252	Not	md	JP	2021	0	1	0	0	1	0	0	0	0	0	0	0	
19275556	m	hp	US	2021	0	1	1	0	0	0	0	0	0	0	0	0	83
19275576	m	Not repo	JP	2021	0	1	0	1	0	0	0	0	0	0	0	0	
19275736	m	hp	CH	2021	1	0	0	0	0	0	0	0	0	0	0	0	
19275942	f	hp	DE	2021	0	1	1	0	0	0	0	0	0	0	0	0	
19276972	Not	md	JP	2021	0	1	0	0	1	0	0	0	0	0	0	0	
19277184	f	Not repo	AU	2021	0	1	0	1	0	0	0	0	0	0	0	0	
19277279	m	md	JP	2021	0	1	0	0	0	1	0	0	0	0	0	0	
19277835	m	md	JP	2021	0	1	0	0	0	1	0	0	0	0	0	0	
19278175	f	ph	US	2021	0	0	0	0	0	0	0	0	0	0	0	0	54
19278327	Not	md	JP	2021	0	0	0	0	0	0	0	0	0	0	0	0	
19278732	m	md	PT	2021	0	1	0	0	0	1	0	0	0	0	0	0	71
19278734	m	md	CN	2021	0	1	0	0	0	1	0	0	0	0	0	0	71
19279180	m	cn	JP	2021	0	1	1	0	0	0	0	0	0	0	0	0	
19279652	m	md	JP	2021	0	0	0	0	0	0	0	0	0	0	0	0	
19279666	f	md	DE	2021	0	1	1	0	0	0	0	0	0	0	0	0	
19279685	Not	hp	FR	2021	0	0	0	0	0	0	0	0	0	0	0	0	
19279918	m	ph	AU	2021	0	0	0	0	0	0	0	0	0	0	0	0	74
19280170	Not	md	FR	2021	0	0	0	0	0	0	0	0	0	0	0	0	77
19280313	f	md	FR	2021	0	1	0	0	0	1	0	0	0	0	0	0	77
19280314	m	md	FR	2021	0	1	0	0	0	1	0	0	0	0	0	0	85
19280319	m	md	FR	2021	0	1	0	0	0	1	0	0	0	0	0	0	55
19280368	f	md	FR	2021	0	1	0	0	0	1	0	0	0	0	0	0	55
19280544	f	md	FR	2021	0	1	0	0	0	1	0	0	0	0	0	0	50
19281630	m	cn	Not re	2021	0	0	0	0	0	0	0	0	0	0	0	0	49
19281707	m	cn	CN	2021	0	1	0	0	1	0	0	0	0	0	0	0	66
19282378	m	cn	CA	2021	1	0	0	0	0	0	0	0	0	0	0	0	

19282504	Not	hp	CH	2021	1	0	0	0	0	0	0	0	0	0	0	0	
19283023	f	Not repo	JP	2021	1	0	0	0	0	0	0	0	0	0	0	0	70
19283282	f	hp	CN	2021	0	1	0	0	1	0	0	0	0	0	0	1	67
19283543	f	cn	CA	2021	1	0	0	0	0	0	0	0	0	0	0	0	
19283881	Not	md	HU	2021	0	0	0	0	0	0	0	0	0	0	0	0	
19284094	m	md	US	2021	0	1	1	0	0	0	0	0	0	0	0	0	75
19285157	f	cn	IN	2021	0	1	0	0	1	0	0	0	0	0	0	0	81
19285334	Not	ph	JP	2021	0	1	0	1	0	0	0	0	0	0	0	0	
19286105	f	cn	Not re	2021	0	0	0	0	0	0	0	0	0	0	0	0	58
19286431	m	md	DE	2021	0	1	0	0	0	1	0	0	0	0	0	0	53
19286501	Not	hp	US	2021	0	0	0	0	0	0	0	0	0	0	0	0	
19286596	Not	md	JP	2021	0	1	0	1	0	0	0	0	0	0	0	1	
19287190	f	Not repo	ID	2021	1	0	0	0	0	0	0	0	0	0	0	0	77.1
19287202	f	hp	US	2021	0	1	0	0	1	0	0	0	0	0	0	0	71
19287642	m	hp	CA	2021	0	0	0	0	0	0	0	0	0	0	0	0	78
19287872	Not	hp	FR	2021	0	1	0	0	1	0	0	0	0	0	0	0	65
19287902	f	Not repo	ID	2021	1	0	0	0	0	0	0	0	0	0	0	0	59.4
19287919	f	md	BR	2021	0	1	1	0	0	0	0	0	0	0	0	0	63
19288136	m	md	JP	2021	1	0	0	0	0	0	0	0	0	0	0	0	49
19288259	f	Not repo	CA	2021	1	0	0	0	0	0	0	0	0	0	0	0	83.3
19289028	Not	cn	JP	2021	0	1	1	0	0	0	0	0	0	0	0	0	60
19289205	m	md	CN	2021	0	0	0	0	0	0	0	0	0	0	0	0	65
19289244	m	cn	Not re	2021	0	0	0	0	0	0	0	0	0	0	0	0	34
19289433	m	cn	Not re	2021	0	0	0	0	0	0	0	0	0	0	0	0	52
19289681	f	cn	Not re	2021	0	0	0	0	0	0	0	0	0	0	0	0	
19289732	f	cn	US	2021	0	0	0	0	0	0	0	0	0	0	0	0	
19290011	f	cn	Not re	2021	0	0	0	0	0	0	0	0	0	0	0	0	65
19290600	f	cn	Not re	2021	0	0	0	0	0	0	0	0	0	0	0	0	41
19290701	f	ph	AU	2021	0	0	0	0	0	0	0	0	0	0	0	0	60
19290703	m	cn	Not re	2021	0	0	0	0	0	0	0	0	0	0	0	0	84
19291159	f	hp	DE	2021	0	1	0	0	1	0	0	0	0	0	0	1	82
19291530	m	md	JP	2021	0	1	0	0	1	0	0	0	0	0	0	0	70
19291560	Not	md	JP	2021	0	1	0	0	1	0	0	0	0	0	0	0	
19292039	Not	md	JP	2021	0	1	0	0	0	1	0	0	0	0	0	0	67

19292385	f	cn	CN	2021	0	0	0	0	0	0	0	0	0	0	0	0	60
19293041	m	hp	US	2021	0	1	0	0	1	0	0	0	0	0	0	0	57
19293353	f	md	US	2021	0	1	0	0	0	1	0	0	0	0	0	0	87
19294805	f	cn	CN	2021	0	1	0	0	1	0	0	0	0	0	0	0	55
19295029	m	Not repo	RO	2021	1	0	0	0	0	0	0	0	0	0	0	0	
19295643	f	cn	Not re	2021	0	0	0	0	0	0	0	0	0	0	0	0	58
19295747	m	hp	IN	2021	0	0	0	0	0	0	0	0	0	0	0	0	60
19298101	Not	md	JP	2021	0	1	1	0	0	0	0	0	0	0	0	0	
19298122	m	Not repo	CN	2021	0	1	0	1	0	0	0	0	0	0	0	0	68
19298424	m	md	SK	2021	1	0	0	0	0	0	0	0	0	0	0	0	51
19298662	m	md	JP	2021	0	1	0	0	0	1	0	0	0	0	0	0	80
19299062	m	hp	IN	2021	0	0	0	0	0	0	0	0	0	0	0	0	38
19299065	f	hp	US	2021	0	0	0	0	0	0	0	0	0	0	0	0	50
19299297	f	hp	DE	2021	0	0	0	0	0	0	0	0	0	0	0	0	76
19299316	Not	md	JP	2021	1	0	0	0	0	0	0	0	0	0	0	0	
19299432	f	hp	DE	2021	0	0	0	0	0	0	0	0	0	0	0	0	76
19299749	f	hp	NL	2021	0	1	1	0	0	0	0	0	0	0	0	0	
19300077	f	cn	US	2021	0	1	1	0	0	0	0	0	0	0	0	0	67
19300331	m	hp	US	2021	0	0	0	0	0	0	0	0	0	0	0	0	59
19300334	m	hp	US	2021	0	0	0	0	0	0	0	0	0	0	0	0	65
19300655	Not	md	DE	2021	0	1	0	0	0	1	0	0	0	0	0	0	
19300762	m	cn	US	2021	1	0	0	0	0	0	0	0	0	0	0	0	
19301332	f	hp	US	2021	0	0	0	0	0	0	0	0	0	0	0	0	56
19301745	f	hp	DE	2021	0	0	0	0	0	0	0	0	0	0	0	0	76
19302202	m	hp	IT	2021	0	1	1	0	0	0	0	0	0	0	0	0	54
19302280	m	hp	NL	2021	0	1	0	0	0	1	0	0	0	0	0	0	62
19302598	m	cn	JP	2021	0	1	1	0	0	0	0	0	0	0	0	0	80
19302826	Not	md	JP	2021	1	0	0	0	0	0	0	0	0	0	0	0	
19303379	f	Not repo	AT	2021	0	1	0	1	0	0	0	0	0	0	0	0	72
19303487	m	cn	Not re	2021	0	0	0	0	0	0	0	0	0	0	0	0	67
19303629	m	hp	US	2021	0	1	0	0	1	0	0	0	0	0	0	0	62
19303647	m	md	JP	2021	0	1	0	0	1	0	0	0	0	0	0	0	70
19303711	f	ph	IT	2021	0	0	0	0	0	0	0	0	0	0	0	0	72
19303758	m	md	SG	2021	0	1	0	0	0	1	0	0	0	0	0	0	61

19303790	Not	md	JP	2021	0	1	0	0	1	0	0	0	0	0	0	0	
19304027	f	Not repo	AU	2021	1	0	0	0	0	0	0	0	0	0	0	0	
19304067	Not	md	JP	2021	0	1	0	0	1	0	0	0	0	0	0	0	80
19304167	f	Not repo	GB	2021	0	1	0	1	0	0	0	0	0	0	0	0	75
19304404	Not	md	JP	2021	0	0	0	0	0	0	0	0	0	0	0	0	
19304412	Not	md	JP	2021	1	0	0	0	0	0	0	0	0	0	0	0	90
19304416	f	Not repo	ID	2021	1	0	0	0	0	0	0	0	0	0	0	0	86.2
19304822	m	Not repo	JP	2021	1	0	0	0	0	0	0	0	0	0	0	0	81
19305111	m	cn	CN	2021	0	1	0	0	1	0	0	0	0	0	0	0	61
19305147	Not	md	NL	2021	0	1	0	0	1	0	0	0	0	0	0	0	
19305202	f	md	CA	2021	0	0	0	0	0	0	1	1	0	0	0	0	58
19305345	f	md	IN	2021	0	0	0	0	0	0	0	0	0	0	0	0	45
19305404	m	hp	DE	2021	0	1	0	0	1	0	0	0	0	0	0	1	65
19305980	Not	Not repo	JP	2021	1	0	0	0	0	0	0	0	0	0	0	0	
19306128	m	hp	US	2021	0	0	0	0	0	0	0	0	0	0	0	0	64
19306237	m	cn	Not re	2021	0	0	0	0	0	0	0	0	0	0	0	0	53
19307034	f	md	IL	2021	1	0	0	0	0	0	0	0	0	0	0	0	
19307196	m	hp	US	2021	0	0	0	0	0	0	0	0	0	0	0	0	50
19307570	Not	cn	US	2021	0	1	1	0	0	0	0	0	0	0	0	0	
19307805	Not	cn	US	2021	0	1	1	0	0	0	0	0	0	0	0	0	
19307816	Not	cn	US	2021	0	1	1	0	0	0	0	0	0	0	0	0	
19307841	Not	Not repo	AU	2021	1	0	0	0	0	0	0	0	0	0	0	0	
19308088	m	Not repo	CN	2021	0	1	0	1	0	0	0	0	0	0	0	0	58
19308515	Not	Not repo	AU	2021	1	0	0	0	0	0	0	0	0	0	0	0	
19308531	f	md	RU	2021	0	0	0	0	0	0	0	0	0	0	0	0	79
19308586	m	hp	DE	2021	0	1	0	0	1	0	0	0	0	0	0	0	70
19308587	f	hp	DE	2021	0	0	0	0	0	0	0	0	0	0	0	0	76
19308661	m	md	FR	2021	0	1	0	0	0	1	0	0	0	0	0	0	81
19309442	f	hp	CN	2021	1	0	0	0	0	0	0	0	0	0	0	0	76
19309445	Not	Not repo	JP	2021	1	0	0	0	0	0	0	0	0	0	0	0	
19309553	f	hp	DE	2021	0	0	0	0	0	0	0	0	0	0	0	0	69
19309743	m	hp	JP	2021	0	1	1	0	0	0	0	0	0	0	0	0	78
19310140	f	hp	GB	2021	0	1	0	0	0	1	0	0	0	0	0	0	44
19310189	f	hp	IT	2021	1	0	0	0	0	0	0	0	0	0	0	0	60

19310264	f	md	JP	2021	0	1	0	1	0	0	0	0	0	0	0	1	80
19310321	m	md	FR	2021	0	1	0	0	0	1	0	0	0	0	0	0	78
19310513	m	hp	IT	2021	0	0	0	0	0	0	0	0	0	0	0	0	54
19310533	f	cn	US	2021	1	0	0	0	0	0	0	0	0	0	0	0	95.4
19310562	f	md	IT	2021	0	0	0	0	0	0	0	0	0	0	0	0	
19310626	f	md	DE	2021	0	0	0	0	0	0	1	0	0	0	1	0	72
19310666	m	hp	CA	2021	0	1	1	0	0	0	0	0	0	0	0	0	
19311822	m	md	US	2021	0	0	0	0	0	0	0	0	0	0	0	0	53
19312046	Not	hp	UA	2021	0	0	0	0	0	0	0	0	0	0	0	0	
19312113	f	ph	TH	2021	0	0	0	0	0	0	0	0	0	0	0	0	
19312138	f	cn	CN	2021	0	1	0	0	1	0	0	0	0	0	0	0	46
19312647	f	hp	PR	2021	0	1	0	0	1	0	0	0	0	0	0	0	81
19313072	Not	ph	JP	2021	0	1	0	0	1	0	0	0	0	0	0	0	
19315397	Not	ph	JP	2021	1	0	0	0	0	0	0	0	0	0	0	0	
19315812	f	cn	PE	2021	1	0	0	0	0	0	0	0	0	0	0	0	
19316541	f	md	CN	2021	1	0	0	0	0	0	0	0	0	0	0	0	85.9
19318027	f	hp	DE	2021	0	0	0	0	0	0	0	0	0	0	0	0	76
19318717	m	Not repo	RO	2021	1	0	0	0	0	0	0	0	0	0	0	0	
19318869	f	cn	TW	2021	0	0	0	0	0	0	0	0	0	0	0	0	
19318995	f	md	MY	2021	0	0	0	0	0	0	0	0	0	0	0	0	65
19320205	f	md	DE	2021	0	0	0	0	0	0	0	0	0	0	0	0	49
19320575	Not	md	US	2021	0	1	0	0	1	0	0	0	0	0	0	0	
19320621	m	cn	JP	2021	0	1	1	0	0	0	0	0	0	0	0	0	70
19321547	m	cn	AR	2021	0	0	0	0	0	0	0	0	0	0	0	0	48
19321625	f	md	US	2021	0	0	0	0	0	0	0	0	0	0	0	0	
19321852	Not	Not repo	AU	2021	1	0	0	0	0	0	0	0	0	0	0	0	
19322515	m	cn	CN	2021	0	1	0	0	1	0	0	0	0	0	0	0	29
19322993	f	hp	US	2021	0	1	0	0	1	0	0	0	0	0	0	0	
19323458	f	md	US	2021	0	0	0	0	0	0	0	0	0	0	0	0	69
19324923	m	md	PL	2021	0	1	1	0	0	0	0	0	0	0	0	0	81
19325466	m	hp	KR	2021	0	1	1	0	0	0	0	0	0	0	0	0	57
19325950	f	md	GR	2021	0	0	0	0	0	0	0	0	0	0	0	0	47
19326459	f	md	AR	2021	0	1	0	0	0	1	0	0	0	0	0	0	74
19326667	m	Not repo	CN	2021	0	1	0	1	0	0	0	0	0	0	0	0	55

19327318	m	cn	CN	2021	0	1	0	0	1	0	0	0	0	0	0	0	71
19327433	f	md	DE	2021	1	0	0	0	0	0	0	0	0	0	0	0	73
19327604	f	Not repo	MY	2021	1	0	0	0	0	0	0	0	0	0	0	0	79.8
19328301	Not	md	GB	2021	0	0	0	0	0	0	0	0	0	0	0	0	
19328433	Not	cn	JP	2021	0	1	1	0	0	0	0	0	0	0	0	0	80
19328435	f	cn	JP	2021	0	1	1	0	0	0	0	0	0	0	0	0	64
19328765	m	md	US	2021	0	0	0	0	0	0	0	0	0	0	0	0	69
19329051	m	md	GR	2021	0	1	0	0	1	0	0	0	0	0	0	0	
19329891	Not	cn	JP	2021	0	1	1	0	0	0	0	0	0	0	0	0	
19330130	f	hp	US	2021	0	1	1	0	0	0	0	0	0	0	0	0	
19330179	m	md	TH	2021	0	0	0	0	0	0	0	0	0	0	0	0	
19330499	Not	hp	IN	2021	0	1	0	1	0	0	0	0	0	0	0	0	
19330540	m	cn	CN	2021	0	1	0	0	1	0	0	0	0	0	0	0	71
19330646	m	cn	Not re	2021	0	0	0	0	0	0	0	0	0	0	0	0	51
19330903	f	md	US	2021	0	0	0	0	0	0	0	0	0	0	0	0	62
19331530	m	cn	IN	2021	0	0	0	0	0	0	0	0	0	0	0	0	59
19331593	m	cn	Not re	2021	0	0	0	0	0	0	0	0	0	0	0	0	
19331866	f	md	FR	2021	0	0	0	0	0	0	0	0	0	0	0	0	38
19332469	f	hp	CN	2021	0	0	0	0	0	0	0	0	0	0	0	0	62
19333030	f	cn	Not re	2021	0	0	0	0	0	0	0	0	0	0	0	0	68
19349815	f	hp	DE	2021	0	0	0	0	0	0	0	0	0	0	0	0	76
19349845	f	md	DE	2021	0	0	0	0	0	0	0	0	0	0	0	0	69
19349867	f	hp	DE	2021	0	0	0	0	0	0	0	0	0	0	0	0	76
19350597	Not	hp	IT	2021	1	0	0	0	0	0	0	0	0	0	0	0	81
19350947	f	md	KR	2021	0	1	1	0	0	0	0	0	0	0	0	0	52
19351528	m	hp	US	2021	0	1	0	0	1	0	0	0	0	0	0	0	
19351552	m	ph	US	2021	0	1	0	0	1	0	0	0	0	0	0	0	
19351629	m	hp	GB	2021	0	1	1	0	0	0	0	0	0	0	0	0	55
19351672	f	cn	CN	2021	0	1	0	0	1	0	0	0	0	0	0	0	69
19351789	Not	Not repo	JP	2021	1	0	0	0	0	0	0	0	0	0	0	0	
19351995	m	md	RO	2021	0	0	0	0	0	0	0	0	0	0	0	0	57
19352009	m	md	IN	2021	0	0	0	0	0	0	0	0	0	0	0	0	59
19352014	f	md	GB	2021	0	1	0	0	0	1	0	0	0	0	0	0	44
19352128	Not	md	JP	2021	0	1	0	0	1	0	0	0	0	0	0	0	

19352150	m	md	CA	2021	0	1	0	0	1	0	0	0	0	0	0	0	50
19352338	f	Not repo	JP	2021	1	0	0	0	0	0	0	0	0	0	0	0	
19352403	Not	cn	JP	2021	0	1	1	0	0	0	0	0	0	0	0	0	90
19352469	f	cn	JP	2021	0	1	0	1	0	0	0	0	0	0	0	0	
19352478	m	md	IL	2021	0	1	1	0	0	0	0	0	0	0	0	0	78
19352733	m	hp	ES	2021	0	1	0	0	1	0	0	0	0	0	0	0	75
19352793	m	md	JP	2021	0	1	0	0	1	0	0	0	0	0	0	0	79
19352801	m	cn	JP	2021	0	1	1	0	0	0	0	0	0	0	0	1	70
19352872	f	md	JP	2021	0	0	0	0	0	0	1	0	1	0	0	0	79
19353021	f	md	CN	2021	1	0	0	0	0	0	0	0	0	0	0	0	50.2
19353080	f	md	JP	2021	0	0	0	0	0	0	0	0	0	0	0	0	58
19353100	m	cn	JP	2021	0	0	0	0	0	0	0	0	0	0	0	0	55
19354124	Not	md	JP	2021	0	1	0	0	1	0	0	0	0	0	0	0	
19354126	Not	md	JP	2021	0	1	0	0	1	0	0	0	0	0	0	0	
19354622	f	md	GB	2021	0	1	0	0	0	1	0	0	0	0	0	0	44
19354788	f	Not repo	Not re	2021	0	0	0	0	0	0	0	0	0	0	0	0	60
19354829	m	hp	PL	2021	0	0	0	0	0	0	0	0	0	0	0	0	33
19355034	f	md	BG	2021	1	0	0	0	0	0	0	0	0	0	0	0	
19355314	m	cn	CN	2021	0	1	0	0	1	0	0	0	0	0	0	0	72
19355729	m	md	JP	2021	0	1	0	0	0	1	0	0	0	0	0	0	73
19356403	m	cn	PL	2021	0	1	1	0	0	0	0	0	0	0	0	0	
19357386	m	cn	CN	2021	0	0	0	0	0	0	0	0	0	0	0	0	
19357826	m	md	US	2021	0	1	0	0	0	1	0	0	0	0	0	0	69
19358092	f	md	MY	2021	0	1	0	0	0	1	0	0	0	0	0	0	54
19358129	m	hp	JP	2021	1	0	0	0	0	0	0	0	0	0	0	0	87
19358145	f	ph	IT	2021	0	0	0	0	0	0	0	0	0	0	0	0	72
19358211	m	Not repo	ID	2021	1	0	0	0	0	0	0	0	0	0	0	0	73.2
19358461	f	md	US	2021	0	0	0	0	0	0	0	0	0	0	0	0	63
19358484	m	cn	JP	2021	0	1	1	0	0	0	0	0	0	0	0	0	70
19358797	m	md	JP	2021	0	1	0	0	1	0	0	0	0	0	0	1	73
19359651	f	Not repo	JP	2021	1	0	0	0	0	0	0	0	0	0	0	0	
19359702	f	md	US	2021	0	0	0	0	0	0	0	0	0	0	0	0	71
19359784	f	md	IN	2021	0	0	0	0	0	0	0	0	0	0	0	0	47
19359990	m	cn	Not re	2021	0	0	0	0	0	0	0	0	0	0	0	0	

19360004	f	md	US	2021	1	0	0	0	0	0	0	0	0	0	0	0	72
19360008	m	cn	IN	2021	0	0	0	0	0	0	0	0	0	0	0	0	55
19360064	m	Not repo	CN	2021	0	1	0	1	0	0	0	0	0	0	0	0	58
19360213	f	md	GB	2021	0	1	0	0	0	1	0	0	0	0	0	0	44
19360955	f	md	DE	2021	1	0	0	0	0	0	0	0	0	0	0	0	
19361060	f	md	JP	2021	0	1	0	1	0	0	0	0	0	0	0	0	73
19361122	m	md	JP	2021	0	1	0	0	1	0	0	0	0	0	0	0	67
19361182	m	cn	CN	2021	0	1	0	0	1	0	0	0	0	0	0	0	60
19361287	m	cn	IN	2021	0	0	0	0	0	0	0	0	0	0	0	0	67
19361306	m	cn	JP	2021	0	1	1	0	0	0	0	0	0	0	0	0	68
19361416	m	Not repo	US	2021	0	1	0	1	0	0	0	0	0	0	0	0	45
19361613	f	Not repo	JP	2021	0	1	0	1	0	0	0	0	0	0	0	0	
19361657	f	Not repo	JP	2021	1	0	0	0	0	0	0	0	0	0	0	1	83
19361795	m	cn	RU	2021	0	0	0	0	0	0	0	0	0	0	0	0	60
19362704	m	md	AT	2021	0	0	0	0	0	0	0	0	0	0	0	0	
19362927	Not	hp	JP	2021	1	0	0	0	0	0	0	0	0	0	0	0	
19363015	m	md	US	2021	0	0	0	0	0	0	0	0	0	0	0	0	92
19363172	Not	Not repo	JP	2021	0	1	0	1	0	0	0	0	0	0	0	1	
19363414	m	md	ES	2021	0	1	0	0	0	1	0	0	0	0	0	0	76
19364175	m	ph	US	2021	0	1	0	1	0	0	0	0	0	0	0	0	89
19364389	m	cn	CN	2021	0	1	0	0	1	0	0	0	0	0	0	0	62
19364750	m	md	JP	2021	0	1	0	0	0	1	0	0	0	0	0	0	60
19364825	m	Not repo	RO	2021	0	1	0	1	0	0	0	0	0	0	0	0	
19365778	m	Not repo	JP	2021	0	1	0	1	0	0	0	0	0	0	0	0	
19366874	f	md	PL	2021	0	1	0	0	1	0	0	0	0	0	0	0	64
19366924	f	md	JP	2021	0	0	0	0	0	0	0	0	0	0	0	0	90
19366932	m	md	DK	2021	1	0	0	0	0	0	0	0	0	0	0	0	59.2
19367161	f	cn	Not re	2021	0	0	0	0	0	0	0	0	0	0	0	0	59
19367180	m	cn	Not re	2021	0	0	0	0	0	0	0	0	0	0	0	0	
19367496	m	cn	FR	2021	0	1	1	0	0	0	0	0	0	0	0	0	68
19367519	m	Not repo	ID	2021	1	0	0	0	0	0	0	0	0	0	0	0	73.1
19367642	m	cn	BE	2021	0	1	1	0	0	0	0	0	0	0	0	0	
19367712	f	hp	CH	2021	1	0	0	0	0	0	0	0	0	0	0	0	47
19369131	m	Not repo	JP	2021	1	0	0	0	0	0	0	0	0	0	0	0	

19369182	m	cn	Not re	2021	0	0	0	0	0	0	0	0	0	0	0	0	48
19369817	Not	md	US	2021	0	1	0	0	0	1	0	0	0	0	0	0	
19370061	f	md	JP	2021	0	1	0	0	1	0	0	0	0	0	0	0	36
19370894	m	hp	US	2021	0	1	0	1	0	0	0	0	0	0	0	1	82
19371343	f	hp	TW	2021	0	0	0	0	0	0	0	0	0	0	0	0	
19371672	m	md	CA	2021	1	0	0	0	0	0	0	0	0	0	0	0	73.3
19371699	f	md	US	2021	0	0	0	0	0	0	0	0	0	0	0	0	56
19372138	f	md	GB	2021	0	1	0	0	0	1	0	0	0	0	0	0	75
19372224	Not	md	JP	2021	0	1	0	0	1	0	0	0	0	0	0	1	
19372583	f	md	JP	2021	1	0	0	0	0	0	0	0	0	0	0	0	
19372785	f	cn	PE	2021	1	0	0	0	0	0	0	0	0	0	0	0	82
19373168	f	md	BE	2021	1	0	0	0	0	0	0	0	0	0	0	0	
19373255	m	Not repo	CN	2021	0	1	0	1	0	0	0	0	0	0	0	0	69
19373444	m	md	JP	2021	0	1	0	0	1	0	0	0	0	0	0	0	70
19373565	Not	md	JP	2021	0	1	0	0	1	0	0	0	0	0	0	0	
19373668	m	md	FR	2021	0	1	1	0	0	0	0	0	0	0	0	0	71
19374343	m	cn	IL	2021	0	1	1	0	0	0	0	0	0	0	0	0	
19374424	f	cn	JP	2021	0	1	1	0	0	0	0	0	0	0	0	0	77
19375130	f	Not repo	ID	2021	1	0	0	0	0	0	0	0	0	0	0	0	51.3
19375249	f	md	CH	2021	0	0	0	0	0	0	0	0	0	0	0	0	44
19375289	m	md	JP	2021	0	0	0	0	0	0	0	0	0	0	0	0	80
19375293	f	md	JP	2021	0	0	0	0	0	0	0	0	0	0	0	0	57
19375350	f	hp	GB	2021	0	1	0	0	0	1	0	0	0	0	0	0	75
19375391	f	cn	TH	2021	0	0	0	0	0	0	0	0	0	0	0	0	92
19375415	m	md	JP	2021	0	1	0	0	1	0	0	0	0	0	0	0	60
19375428	m	cn	IN	2021	0	1	0	0	1	0	0	0	0	0	0	0	70
19375433	f	md	JP	2021	0	1	0	0	1	0	0	0	0	0	0	0	76
19375502	Not	md	JP	2021	0	1	0	0	1	0	0	0	0	0	0	0	
19375747	m	hp	ES	2021	0	0	0	0	0	0	0	0	0	0	0	0	59
19375927	f	md	HU	2021	0	0	0	0	0	0	0	0	0	0	0	0	66
19376108	f	cn	US	2021	0	1	1	0	0	0	0	0	0	0	0	0	72
19376508	m	cn	IN	2021	0	1	0	0	1	0	0	0	0	0	0	0	70
19376877	f	hp	CN	2021	1	0	0	0	0	0	0	0	0	0	0	0	80
19376906	m	md	US	2021	0	0	0	0	0	0	0	0	0	0	0	0	

19377938	Not	md	JP	2021	0	1	0	0	1	0	0	0	0	0	0	0	
19377961	m	hp	JP	2021	0	1	1	0	0	0	0	0	0	0	0	0	67
19378028	f	hp	US	2021	0	0	0	0	0	0	0	0	0	0	0	0	56
19378832	m	cn	US	2021	0	1	0	1	0	0	0	0	0	0	0	0	64.2
19380025	f	cn	CN	2021	0	1	0	0	1	0	0	0	0	0	0	0	71
19380511	m	md	JP	2021	0	1	0	0	1	0	0	0	0	0	0	0	54
19380742	f	md	US	2021	0	1	0	0	0	1	0	0	0	0	0	0	80
19381418	m	md	TR	2021	0	1	1	0	0	0	0	0	0	0	0	0	61
19381550	m	md	DE	2021	0	0	0	0	0	0	0	0	0	0	0	0	
19381658	m	ph	JP	2021	0	1	0	0	0	1	0	0	0	0	0	0	69
19381668	m	md	AT	2021	1	0	0	0	0	0	0	0	0	0	0	0	
19382027	m	md	ES	2021	0	1	1	0	0	0	0	0	0	0	0	0	74
19382353	f	md	DE	2021	0	1	1	0	0	0	0	0	0	0	0	0	67
19382359	m	md	DE	2021	0	1	1	0	0	0	0	0	0	0	0	0	73
19382427	f	md	JP	2021	0	0	0	0	0	0	0	0	0	0	0	0	68
19382552	f	md	IQ	2021	0	1	0	0	0	1	0	0	0	0	0	0	
19382618	f	md	US	2021	0	1	0	1	0	0	0	0	0	0	0	0	67
19382652	m	ph	TH	2021	0	0	0	0	0	0	0	0	0	0	0	0	
19382931	m	md	BR	2021	0	0	0	0	0	0	0	0	0	0	0	0	60
19383994	f	hp	IN	2021	0	0	0	0	0	0	0	0	0	0	0	0	38
19384638	m	cn	IN	2021	0	1	0	0	1	0	0	0	0	0	0	0	70
19384853	f	md	JP	2021	0	0	0	0	0	0	0	0	0	0	0	0	60
19385319	Not	md	PH	2021	0	1	0	0	0	1	0	0	0	0	0	0	
19387001	m	md	FR	2021	0	0	0	0	0	0	0	0	0	0	0	0	56
19387116	m	md	JP	2021	0	1	0	0	1	0	0	0	0	0	0	0	79
19387314	f	md	JP	2021	0	0	0	0	0	0	0	0	0	0	0	0	62
19388038	m	md	JP	2021	0	1	0	0	1	0	0	0	0	0	0	0	54
19388125	m	md	GB	2021	0	0	0	0	0	0	0	0	0	0	0	0	55
19389163	m	md	ME	2021	0	1	1	0	0	0	0	0	0	0	0	0	56
19389168	f	Not repo	MY	2021	1	0	0	0	0	0	0	0	0	0	0	0	56.1
19389606	f	Not repo	JP	2021	1	0	0	0	0	0	0	0	0	0	0	1	80
19389608	f	md	HU	2021	0	0	0	0	0	0	0	0	0	0	0	0	66
19389623	f	cn	US	2021	1	0	0	0	0	0	0	0	0	0	0	0	66
19389818	f	cn	CN	2021	0	1	0	0	1	0	0	0	0	0	0	0	37

19390141	m	cn	JP	2021	0	1	1	0	0	0	0	0	0	0	0	0	74
19390337	f	cn	US	2021	0	1	1	0	0	0	0	0	0	0	0	0	
19390364	m	cn	CN	2021	0	1	1	0	0	0	0	0	0	0	0	0	56
19390508	f	Not repo	ID	2021	1	0	0	0	0	0	0	0	0	0	0	0	53.7
19390642	m	Not repo	MY	2021	1	0	0	0	0	0	0	0	0	0	0	0	56
19391119	f	Not repo	ID	2021	1	0	0	0	0	0	0	0	0	0	0	0	
19391677	f	ph	TH	2021	0	0	0	0	0	0	0	0	0	0	0	0	44
19392010	m	md	IL	2021	0	1	0	0	0	1	0	0	0	0	0	0	67
19392070	f	cn	CA	2021	1	0	0	0	0	0	0	0	0	0	0	0	
19392125	m	ph	JP	2021	0	0	0	0	0	0	0	0	0	0	0	0	36
19392430	m	cn	US	2021	1	0	0	0	0	0	0	0	0	0	0	1	61
19392569	f	cn	US	2021	0	1	1	0	0	0	0	0	0	0	0	0	
19393317	m	cn	CA	2021	0	0	0	0	0	0	0	0	0	0	0	0	56
19394481	m	md	JP	2021	0	1	0	0	1	0	0	0	0	0	0	1	67
19394490	m	hp	NL	2021	0	1	1	0	0	0	0	0	0	0	0	0	73
19394810	f	Not repo	MY	2021	1	0	0	0	0	0	0	0	0	0	0	0	83.4
19395357	m	md	CH	2021	0	0	0	0	0	0	0	0	0	0	0	0	
19395596	m	md	BG	2021	0	1	0	1	0	0	0	0	0	0	0	0	
19395599	m	md	US	2021	0	0	0	0	0	0	0	0	0	0	0	0	76
19395655	m	md	CA	2021	0	1	0	1	0	0	0	0	0	0	0	0	66
19395674	f	ph	JP	2021	1	0	0	0	0	0	0	0	0	0	0	0	79
19395726	m	Not repo	JP	2021	1	0	0	0	0	0	0	0	0	0	0	0	82
19395751	f	md	US	2021	0	0	0	0	0	0	0	0	0	0	0	0	63
19396023	m	cn	CN	2021	0	1	0	0	1	0	0	0	0	0	0	0	68
19396229	f	cn	IN	2021	0	1	0	0	1	0	0	0	0	0	0	0	73
19396369	m	cn	US	2021	0	1	1	0	0	0	0	0	0	0	0	0	62
19396564	f	md	AT	2021	0	0	0	0	0	0	0	0	0	0	0	0	
19396565	f	cn	US	2021	1	0	0	0	0	0	0	0	0	0	0	0	85
19396645	f	cn	Not re	2021	0	0	0	0	0	0	0	0	0	0	0	0	
19397008	f	md	US	2021	0	0	0	0	0	0	0	0	0	0	0	0	65
19397254	m	cn	Not re	2021	0	0	0	0	0	0	0	0	0	0	0	0	
19397264	m	hp	US	2021	0	1	0	0	1	0	0	0	0	0	0	0	57
19397398	m	md	DE	2021	0	1	1	0	0	0	0	0	0	0	0	0	73
19397450	f	Not repo	JP	2021	1	0	0	0	0	0	0	0	0	0	0	0	89

19399279	f	ph	JP	2021	1	0	0	0	0	0	0	0	0	0	0	0	72
19399337	m	hp	JP	2021	0	1	0	0	1	0	0	0	0	0	0	0	61
19399376	m	md	JP	2021	0	0	0	0	0	0	0	0	0	0	0	0	75
19399461	f	md	JP	2021	1	0	0	0	0	0	0	0	0	0	0	0	78
19399522	f	cn	CN	2021	1	0	0	0	0	0	0	0	0	0	0	0	63
19399716	f	ph	JP	2021	1	0	0	0	0	0	0	0	0	0	0	0	80
19399739	m	ph	JP	2021	0	1	0	0	1	0	0	0	0	0	0	0	72
19399894	f	cn	CA	2021	0	0	0	0	0	0	1	1	0	0	0	0	58
19400104	f	hp	GB	2021	0	0	0	0	0	0	0	0	0	0	0	0	61
19400165	Not	md	BR	2021	0	0	0	0	0	0	0	0	0	0	0	0	
19400364	Not	md	JP	2021	0	1	0	0	0	1	0	0	0	0	0	0	
19400772	m	hp	FR	2021	0	1	0	0	1	0	0	0	0	0	0	0	64
19400859	f	md	DE	2021	0	0	0	0	0	0	0	0	0	0	0	0	
19401068	m	hp	GR	2021	0	1	0	0	1	0	0	0	0	0	0	0	55
19401140	m	md	US	2021	0	1	1	0	0	0	0	0	0	0	0	0	68
19401174	m	md	JP	2021	0	0	0	0	0	0	0	0	0	0	0	0	73
19401505	f	md	RU	2021	0	0	0	0	0	0	0	0	0	0	0	0	37
19401677	f	cn	US	2021	0	0	0	0	0	0	0	0	0	0	0	0	64
19401686	m	cn	JP	2021	0	1	1	0	0	0	0	0	0	0	0	0	74
19401926	m	cn	JP	2021	0	1	1	0	0	0	0	0	0	0	0	0	80
19401986	m	hp	CN	2021	0	0	0	0	0	0	0	0	0	0	0	0	62
19402849	f	cn	CA	2021	0	0	0	0	0	0	1	1	0	0	0	0	58
19402985	Not	hp	CN	2021	1	0	0	0	0	0	0	0	0	0	0	0	
19403064	m	cn	IN	2021	0	0	0	0	0	0	0	0	0	0	0	0	78
19403120	Not	hp	CN	2021	1	0	0	0	0	0	0	0	0	0	0	0	
19403151	Not	hp	CN	2021	1	0	0	0	0	0	0	0	0	0	0	0	
19403152	Not	hp	CN	2021	1	0	0	0	0	0	0	0	0	0	0	0	
19403716	m	md	SG	2021	0	0	0	0	0	0	0	0	0	0	0	0	
19405049	Not	md	JP	2021	1	0	0	0	0	0	0	0	0	0	0	0	
19405052	m	cn	TW	2021	0	1	0	0	0	1	0	0	0	0	0	0	
19405129	m	md	NL	2021	0	0	0	0	0	0	0	0	0	0	0	0	71
19405133	f	hp	DK	2021	1	0	0	0	0	0	0	0	0	0	0	0	57
19405461	m	md	JP	2021	1	0	0	0	0	0	0	0	0	0	0	0	90
19405462	Not	md	JP	2021	0	1	0	1	0	0	0	0	0	0	0	1	

19405463	Not	ph	JP	2021	1	0	0	0	0	0	0	0	0	0	0	0	
19405464	Not	md	JP	2021	0	1	0	1	0	0	0	0	0	0	0	1	
19405798	m	Not repo	CN	2021	0	1	0	1	0	0	0	0	0	0	0	0	57
19405799	Not	md	JP	2021	0	1	0	1	0	0	0	0	0	0	0	1	
19405935	Not	hp	NL	2021	0	1	0	0	1	0	0	0	0	0	0	0	72
19405942	Not	Not repo	SE	2021	1	0	0	0	0	0	0	0	0	0	0	0	
19406237	f	Not repo	MY	2021	1	0	0	0	0	0	0	0	0	0	0	0	50.8
19406240	Not	Not repo	SE	2021	1	0	0	0	0	0	0	0	0	0	0	0	
19406338	Not	Not repo	AU	2021	0	1	0	1	0	0	0	0	0	0	0	0	
19406349	f	md	NL	2021	0	0	0	0	0	0	0	0	0	0	0	0	65
19406650	Not	Not repo	SE	2021	1	0	0	0	0	0	0	0	0	0	0	0	
19406651	Not	md	PH	2021	1	0	0	0	0	0	0	0	0	0	0	0	
19406774	m	cn	JP	2021	0	1	1	0	0	0	0	0	0	0	0	0	74
19406941	m	cn	BD	2021	0	1	0	0	0	1	0	0	0	0	0	0	61
19406985	Not	Not repo	JP	2021	1	0	0	0	0	0	0	0	0	0	0	0	
19407012	f	md	ES	2021	0	0	0	0	0	0	0	0	0	0	0	0	81
19407086	Not	Not repo	JP	2021	1	0	0	0	0	0	0	0	0	0	0	0	
19408015	m	cn	Not re	2021	0	0	0	0	0	0	0	0	0	0	0	0	
19408018	Not	md	MX	2021	0	0	0	0	0	0	0	0	0	0	0	0	
19408317	m	md	JP	2021	0	1	0	0	0	1	0	0	0	0	0	0	71
19408346	m	md	KR	2021	0	1	0	0	0	1	0	0	0	0	0	0	85
19408351	Not	md	JP	2021	0	1	0	0	0	1	0	0	0	0	0	0	
19408730	f	md	IN	2021	0	0	0	0	0	0	0	0	0	0	0	0	65
19408886	m	md	DE	2021	0	1	1	0	0	0	0	0	0	0	0	0	73
19410582	m	md	GB	2021	0	1	0	0	0	1	0	0	0	0	0	0	76
19410827	m	cn	IN	2021	0	1	0	0	1	0	0	0	0	0	0	0	58
19410841	Not	hp	CA	2021	0	0	0	0	0	0	0	0	0	0	0	0	
19410884	f	md	TW	2021	0	1	0	0	0	1	0	0	0	0	0	0	57
19410954	f	cn	JP	2021	0	1	1	0	0	0	0	0	0	0	0	0	
19411736	f	md	NL	2021	0	0	0	0	0	0	0	0	0	0	0	0	74
19412184	f	md	JP	2021	0	1	0	0	1	0	0	0	0	0	0	0	80
19412202	m	cn	CN	2021	0	1	0	0	1	0	0	0	0	0	0	0	55
19412268	m	md	NO	2021	0	1	1	0	0	0	0	0	0	0	0	1	70
19412626	m	hp	CA	2021	0	0	0	0	0	0	0	0	0	0	0	0	69

19412630	f	md	JP	2021	0	0	0	0	0	0	0	0	0	0	0	0	60
19412631	Not	hp	FR	2021	0	0	0	0	0	0	0	0	0	0	0	0	
19412665	m	md	CA	2021	1	0	0	0	0	0	0	0	0	0	0	0	70
19412743	m	hp	JP	2021	0	1	0	0	1	0	0	0	0	0	0	0	68
19412768	m	Not repo	MY	2021	1	0	0	0	0	0	0	0	0	0	0	0	
19412928	Not	md	JP	2021	0	1	0	1	0	0	0	0	0	0	0	0	80
19413365	m	hp	US	2021	0	1	1	0	0	0	0	0	0	0	0	0	86
19413441	m	hp	US	2021	0	1	0	0	1	0	0	0	0	0	0	0	62
19413520	f	md	US	2021	0	0	0	0	0	0	0	0	0	0	0	0	
19413558	m	hp	US	2021	0	1	0	0	1	0	0	0	0	0	0	0	74
19413562	m	md	US	2021	0	1	0	0	1	0	0	0	0	0	0	0	58
19413585	m	hp	NL	2021	0	1	0	0	1	0	0	0	0	0	0	0	69
19413783	m	md	HK	2021	0	0	0	0	0	0	0	0	0	0	0	0	89
19414075	m	md	SK	2021	0	0	0	0	0	0	0	0	0	0	0	0	
19414232	m	md	US	2021	0	1	0	0	0	1	0	0	0	0	0	0	76
19414395	m	md	DE	2021	0	1	0	0	1	0	0	0	0	0	0	0	52
19414634	f	md	JP	2021	0	1	0	1	0	0	0	0	0	0	0	0	77
19414651	m	md	JP	2021	0	1	0	0	1	0	0	0	0	0	0	0	79
19414665	m	hp	JP	2021	0	1	0	0	1	0	0	0	0	0	0	0	71
19414783	m	hp	ES	2021	0	1	1	0	0	0	0	0	0	0	0	0	69
19414891	m	hp	JP	2021	0	1	0	0	1	0	0	0	0	0	0	0	61
19415122	f	hp	US	2021	0	0	0	0	0	0	0	0	0	0	0	0	
19415150	f	cn	Not re	2021	0	0	0	0	0	0	0	0	0	0	0	0	42
19415154	m	hp	FR	2021	0	1	1	0	0	0	0	0	0	0	0	0	68
19415330	m	md	JP	2021	0	0	0	0	0	0	0	0	0	0	0	0	57
19416046	m	md	FR	2021	0	1	1	0	0	0	0	0	0	0	0	0	68
19416548	m	hp	US	2021	0	1	0	0	1	0	0	0	0	0	0	0	
19417520	m	md	JP	2021	1	0	0	0	0	0	0	0	0	0	0	0	61
19417621	m	md	JP	2021	0	1	0	1	0	0	0	0	0	0	0	1	
19417686	f	md	FR	2021	0	0	0	0	0	0	0	0	0	0	0	0	64
19417891	m	Not repo	ID	2021	1	0	0	0	0	0	0	0	0	0	0	0	
19417936	m	hp	CA	2021	0	1	1	0	0	0	0	0	0	0	0	1	77
19419009	m	md	JP	2021	0	1	0	1	0	0	0	0	0	0	0	0	40
19419096	m	Not repo	JP	2021	1	0	0	0	0	0	0	0	0	0	0	0	

19419229	f	cn	JP	2021	0	1	1	0	0	0	0	0	0	0	0	0	80
19419267	f	hp	US	2021	0	0	0	0	0	0	0	0	0	0	0	0	64
19419469	f	Not repo	DE	2021	0	1	0	1	0	0	0	0	0	0	0	0	57
19419501	Not	cn	JP	2021	0	1	1	0	0	0	0	0	0	0	0	0	
19419596	Not	md	BR	2021	0	0	0	0	0	0	0	0	0	0	0	0	
19419604	m	md	JP	2021	0	1	0	0	1	0	0	0	0	0	0	0	80
19419654	m	Not repo	US	2021	1	0	0	0	0	0	0	0	0	0	0	0	
19419672	f	md	DE	2021	0	0	0	0	0	0	0	0	0	0	0	0	56
19419677	f	md	NL	2021	0	1	1	0	0	0	0	0	0	0	0	0	57
19420629	m	cn	FR	2021	0	1	1	0	0	0	0	0	0	0	0	0	68
19420716	m	md	CN	2021	0	0	0	0	0	0	0	0	0	0	0	0	69
19420896	m	cn	DE	2021	0	1	1	0	0	0	0	0	0	0	0	0	48
19421115	m	md	JP	2021	0	0	0	0	0	0	0	0	0	0	0	0	73
19421304	f	hp	ES	2021	0	0	0	0	0	0	0	0	0	0	0	0	65
19421682	m	md	JP	2021	0	1	0	0	0	1	0	0	0	0	0	0	80
19422306	m	hp	CN	2021	1	0	0	0	0	0	0	0	0	0	0	0	45
19423078	f	cn	Not re	2021	0	0	0	0	0	0	0	0	0	0	0	0	54
19423080	m	cn	JP	2021	0	1	1	0	0	0	0	0	0	0	0	0	70
19424103	m	Not repo	LB	2021	0	1	0	1	0	0	0	0	0	0	0	0	48
19425365	f	md	CN	2021	0	1	1	0	0	0	0	0	0	0	0	0	46
19425427	m	cn	Not re	2021	0	0	0	0	0	0	0	0	0	0	0	0	55
19425450	m	cn	JP	2021	0	1	1	0	0	0	0	0	0	0	0	0	71
19425985	f	hp	CN	2021	1	0	0	0	0	0	0	0	0	0	0	0	38
19425986	f	hp	CN	2021	1	0	0	0	0	0	0	0	0	0	0	0	69
19425988	f	hp	CN	2021	1	0	0	0	0	0	0	0	0	0	0	0	72
19425990	Not	Not repo	AU	2021	0	1	0	1	0	0	0	0	0	0	0	0	
19426031	m	md	JP	2021	0	0	0	0	0	0	0	0	0	0	0	0	70
19426076	Not	md	MX	2021	0	1	1	0	0	0	0	0	0	0	0	0	
19426370	m	hp	US	2021	0	0	0	0	0	0	0	0	0	0	0	0	84
19426518	m	md	JP	2021	0	0	0	0	0	0	0	0	0	0	0	0	
19426585	m	md	US	2021	0	1	1	0	0	0	0	0	0	0	0	0	
19426841	f	md	CN	2021	0	0	0	0	0	0	0	0	0	0	0	0	69
19426907	f	hp	US	2021	0	1	0	0	1	0	0	0	0	0	0	0	66
19426929	m	hp	JP	2021	0	1	0	0	1	0	0	0	0	0	0	0	61

19427140	f	md	NZ	2021	0	1	0	0	0	1	0	0	0	0	0	0	56
19427437	f	cn	Not re	2021	0	0	0	0	0	0	0	0	0	0	0	0	
19427457	m	md	US	2021	0	1	1	0	0	0	0	0	0	0	0	0	
19428090	f	Not repo	ID	2021	1	0	0	0	0	0	0	0	0	0	0	0	56.7
19428367	m	hp	CN	2021	1	0	0	0	0	0	0	0	0	0	0	0	58
19428373	f	hp	CN	2021	1	0	0	0	0	0	0	0	0	0	0	0	53
19428374	m	hp	CN	2021	1	0	0	0	0	0	0	0	0	0	0	0	81
19428423	f	Not repo	ID	2021	1	0	0	0	0	0	0	0	0	0	0	0	74.3
19428558	Not	md	JP	2021	0	1	0	0	0	1	0	0	0	0	0	0	70
19429235	m	cn	IN	2021	0	1	0	0	1	0	0	0	0	0	0	0	64
19429275	Not	md	JP	2021	1	0	0	0	0	0	0	0	0	0	0	0	
19429425	Not	hp	CA	2021	0	0	0	0	0	0	0	0	0	0	0	0	
19429449	m	md	DE	2021	0	0	0	0	0	0	0	0	0	0	0	0	60
19429592	m	hp	CN	2021	0	1	1	0	0	0	0	0	0	0	0	0	83
19429921	m	cn	Not re	2021	0	0	0	0	0	0	0	0	0	0	0	0	76
19430044	f	cn	US	2021	0	1	0	0	1	0	0	0	0	0	0	0	74
19430047	m	md	JP	2021	0	1	0	0	0	1	0	0	0	0	0	0	74
19430076	Not	md	JP	2021	1	0	0	0	0	0	0	0	0	0	0	0	78
19430079	f	cn	CN	2021	0	1	0	0	1	0	0	0	0	0	0	0	64
19430085	Not	ph	JP	2021	1	0	0	0	0	0	0	0	0	0	0	0	
19431163	m	md	US	2021	0	0	0	0	0	0	0	0	0	0	0	0	
19432118	m	Not repo	CN	2021	1	0	0	0	0	0	0	0	0	0	0	0	
19432139	m	hp	GB	2021	1	0	0	0	0	0	0	0	0	0	0	1	
19432536	f	md	DE	2021	1	0	0	0	0	0	0	0	0	0	0	0	
19432604	m	md	FR	2021	1	0	0	0	0	0	0	0	0	0	0	0	
19432932	f	cn	NP	2021	0	0	0	0	0	0	0	0	0	0	0	0	56
19432986	m	cn	FR	2021	0	1	1	0	0	0	0	0	0	0	0	0	68
19433198	f	md	AT	2021	0	0	0	0	0	0	0	0	0	0	0	0	56
19433280	m	md	CN	2021	0	1	0	0	0	1	0	0	0	0	0	0	69
19433372	m	cn	CN	2021	0	1	0	0	1	0	0	0	0	0	0	0	66
19433994	m	md	JP	2021	0	1	1	0	0	0	0	0	0	0	0	0	80
19434042	f	md	JP	2021	1	0	0	0	0	0	0	0	0	0	0	0	80
19434565	m	cn	JP	2021	0	1	1	0	0	0	0	0	0	0	0	0	58
19435699	m	hp	IT	2021	1	0	0	0	0	0	0	0	0	0	0	0	74

19435997	f	cn	BE	2021	1	0	0	0	0	0	0	0	0	0	0	0	
19436059	f	Not repo	MY	2021	0	1	0	1	0	0	0	0	0	0	0	0	34
19436172	f	ph	JP	2021	1	0	0	0	0	0	0	0	0	0	0	0	85
19436234	f	hp	CA	2021	0	1	0	1	0	0	0	0	0	0	0	0	75
19436421	m	hp	CN	2021	1	0	0	0	0	0	0	0	0	0	0	0	69
19436556	Not	md	JP	2021	0	1	0	1	0	0	0	0	0	0	0	1	
19436694	m	cn	Not re	2021	0	0	0	0	0	0	0	0	0	0	0	0	
19436721	f	md	DE	2021	0	0	0	0	0	0	0	0	0	0	0	0	56
19436887	f	md	ES	2021	0	0	0	0	0	0	0	0	0	0	0	0	
19438136	m	Not repo	MY	2021	1	0	0	0	0	0	0	0	0	0	0	0	57.3
19438179	f	md	DE	2021	0	1	1	0	0	0	0	0	0	0	0	0	60
19438251	m	Not repo	JP	2021	1	0	0	0	0	0	0	0	0	0	0	0	85
19438284	f	md	FR	2021	0	0	0	0	0	0	0	0	0	0	0	0	
19438721	m	md	JP	2021	0	1	0	0	1	0	0	0	0	0	0	0	70
19438917	f	cn	TW	2021	0	0	0	0	0	0	0	0	0	0	0	0	
19439580	Not	hp	CN	2021	1	0	0	0	0	0	0	0	0	0	0	0	
19439741	m	Not repo	US	2021	0	1	0	1	0	0	0	0	0	0	0	0	59.9
19439746	m	Not repo	US	2021	1	0	0	0	0	0	0	0	0	0	0	0	
19440038	m	md	JP	2021	0	1	0	0	0	1	0	0	0	0	0	0	60
19440048	m	md	JP	2021	0	1	0	0	0	1	0	0	0	0	0	0	
19440053	f	md	JP	2021	0	1	0	0	0	1	0	0	0	0	0	0	70
19440070	m	md	TW	2021	0	1	0	0	0	1	0	0	0	0	0	0	62
19440329	m	cn	JP	2021	0	1	1	0	0	0	0	0	0	0	0	0	72
19440358	m	Not repo	PL	2021	0	1	0	1	0	0	0	0	0	0	0	0	
19440521	f	hp	FR	2021	0	1	0	0	1	0	0	0	0	0	0	0	67
19441459	Not	cn	DE	2021	1	0	0	0	0	0	0	0	0	0	0	0	
19441794	m	cn	CA	2021	0	1	1	0	0	0	0	0	0	0	0	0	
19441985	m	md	NL	2021	0	1	0	0	0	1	0	0	0	0	0	0	65
19442008	m	md	NL	2021	0	1	1	0	0	0	0	0	0	0	0	0	74
19442016	m	md	DE	2021	0	1	0	0	0	1	0	0	0	0	0	0	80
19442024	m	md	DE	2021	0	1	0	0	1	0	0	0	0	0	0	0	58
19442123	Not	Not repo	DE	2021	1	0	0	0	0	0	0	0	0	0	0	0	
19442124	Not	hp	DE	2021	1	0	0	0	0	0	0	0	0	0	0	0	
19442125	Not	hp	DE	2021	1	0	0	0	0	0	0	0	0	0	0	0	

19442938	m	Not repo	KR	2021	1	0	0	0	0	0	0	0	0	0	0	0	65
19442982	f	md	DE	2021	0	1	0	0	1	0	0	0	0	0	0	0	67
19443086	Not	Not repo	JP	2021	1	0	0	0	0	0	0	0	0	0	0	0	
19443311	Not	cn	DE	2021	0	1	1	0	0	0	0	0	0	0	0	1	63
19443431	Not	md	JP	2021	1	0	0	0	0	0	0	0	0	0	0	0	
19443492	f	Not repo	HU	2021	1	0	0	0	0	0	0	0	0	0	0	0	79.8
19443850	m	md	TR	2021	0	1	0	0	0	1	0	0	0	0	0	0	81
19444327	m	Not repo	JP	2021	0	1	0	1	0	0	0	0	0	0	0	0	50
19444467	m	cn	NL	2021	0	1	1	0	0	0	0	0	0	0	0	0	72
19444677	m	md	FR	2021	0	1	1	0	0	0	0	0	0	0	0	0	61
19444901	Not	md	CN	2021	0	0	0	0	0	0	0	0	0	0	0	0	
19444906	m	md	PL	2021	0	0	0	0	0	0	0	0	0	0	0	0	56
19444928	Not	md	CN	2021	0	0	0	0	0	0	0	0	0	0	0	0	
19444938	Not	md	CN	2021	0	0	0	0	0	0	0	0	0	0	0	0	
19444940	Not	md	CN	2021	0	0	0	0	0	0	0	0	0	0	0	0	
19444941	Not	md	CN	2021	0	0	0	0	0	0	0	0	0	0	0	0	
19444947	Not	md	CN	2021	0	0	0	0	0	0	0	0	0	0	0	0	
19444951	Not	md	CN	2021	0	0	0	0	0	0	0	0	0	0	0	0	
19444952	Not	md	CN	2021	0	0	0	0	0	0	0	0	0	0	0	0	
19444953	Not	md	CN	2021	0	0	0	0	0	0	0	0	0	0	0	0	
19444955	Not	md	CN	2021	0	0	0	0	0	0	0	0	0	0	0	0	
19444956	Not	md	CN	2021	0	0	0	0	0	0	0	0	0	0	0	0	
19444958	Not	md	CN	2021	0	0	0	0	0	0	0	0	0	0	0	0	
19445350	m	md	JP	2021	0	1	1	0	0	0	0	0	0	0	0	0	63
19445479	m	md	PL	2021	0	0	0	0	0	0	0	0	0	0	0	0	64
19447397	m	Not repo	JP	2021	0	1	0	1	0	0	0	0	0	0	0	0	79
19447892	f	md	BR	2021	0	0	0	0	0	0	0	0	0	0	0	0	77
19448766	f	md	AU	2021	0	0	0	0	0	0	1	0	0	1	0	0	
19448906	m	cn	JP	2021	0	0	0	0	0	0	0	0	0	0	0	0	76
19448942	m	Not repo	IT	2021	1	0	0	0	0	0	0	0	0	0	0	0	74
19449190	f	hp	CN	2021	0	0	0	0	0	0	0	0	0	0	0	0	46
19449198	f	cn	Not re	2021	0	0	0	0	0	0	0	0	0	0	0	0	
19449348	f	md	CN	2021	1	0	0	0	0	0	0	0	0	0	0	0	82
19449350	f	Not repo	CA	2021	1	0	0	0	0	0	0	0	0	0	0	0	

19449418	m	hp	CN	2021	0	0	0	0	0	0	0	0	0	0	0	0	64
19449853	f	md	AU	2021	0	0	0	0	0	0	1	0	0	1	0	0	
19449963	f	Not repo	GB	2021	1	0	0	0	0	0	0	0	0	0	0	1	85
19450103	f	md	RU	2021	0	1	0	0	0	1	0	0	0	0	0	0	59
19450185	m	cn	DE	2021	0	1	1	0	0	0	0	0	0	0	0	0	67
19450279	Not	Not repo	SE	2021	1	0	0	0	0	0	0	0	0	0	0	0	
19450521	m	Not repo	JP	2021	1	0	0	0	0	0	0	0	0	0	0	0	83
19450565	m	md	JP	2021	1	0	0	0	0	0	0	0	0	0	0	0	89
19450734	Not	hp	JP	2021	1	0	0	0	0	0	0	0	0	0	0	0	
19451240	f	hp	US	2021	0	0	0	0	0	0	0	0	0	0	0	0	71
19451268	m	ph	JP	2021	0	1	0	0	0	1	0	0	0	0	0	1	71
19451321	m	hp	US	2021	0	1	0	0	1	0	0	0	0	0	0	0	72
19451386	m	md	JP	2021	0	1	0	0	1	0	0	0	0	0	0	0	76
19451688	f	hp	US	2021	0	1	0	0	1	0	0	0	0	0	0	0	63
19452248	f	cn	SG	2021	1	0	0	0	0	0	0	0	0	0	0	0	78.8
19452721	f	md	DE	2021	0	0	0	0	0	0	0	0	0	0	0	0	72
19452806	m	md	JP	2021	0	1	0	0	0	1	0	0	0	0	0	0	70
19453014	m	md	GB	2021	0	1	0	0	1	0	0	0	0	0	0	0	69
19453184	f	md	JP	2021	0	0	0	0	0	0	0	0	0	0	0	0	
19453278	Not	Not repo	JP	2021	1	0	0	0	0	0	0	0	0	0	0	0	
19453347	f	md	JP	2021	1	0	0	0	0	0	0	0	0	0	0	0	68
19453726	f	hp	AT	2021	0	0	0	0	0	0	0	0	0	0	0	0	58
19453743	f	md	NL	2021	0	1	0	0	0	1	0	0	0	0	0	0	75
19453790	m	Not repo	CN	2021	0	1	0	1	0	0	0	0	0	0	0	0	58
19453947	m	Not repo	CN	2021	0	1	0	1	0	0	0	0	0	0	0	0	59
19454014	f	md	JP	2021	0	1	0	0	0	1	0	0	0	0	0	0	70
19454198	m	md	MY	2021	1	0	0	0	0	0	0	0	0	0	0	0	78
19454299	m	cn	CA	2021	1	0	0	0	0	0	0	0	0	0	0	0	
19454414	f	Not repo	JP	2021	1	0	0	0	0	0	0	0	0	0	0	0	69
19454525	f	md	JP	2021	0	1	0	0	0	1	0	0	0	0	0	0	69
19454644	f	hp	CN	2021	1	0	0	0	0	0	0	0	0	0	0	0	62
19454956	m	hp	CA	2021	0	1	1	0	0	0	0	0	0	0	0	0	54
19455159	f	Not repo	ID	2021	1	0	0	0	0	0	0	0	0	0	0	0	49.6
19455650	f	md	US	2021	0	1	0	0	0	1	0	0	0	0	0	0	72

19455684	f	md	US	2021	0	0	0	0	0	0	0	0	0	0	0	0	66
19455701	f	hp	US	2021	1	0	0	0	0	0	0	0	0	0	0	0	64
19456114	Not	md	JP	2021	0	1	0	0	1	0	0	0	0	0	0	0	
19456140	f	md	AT	2021	0	1	0	0	0	1	0	0	0	0	0	0	52
19456165	f	md	PT	2021	0	0	0	0	0	0	0	0	0	0	0	0	
19456525	f	hp	US	2021	0	1	0	0	1	0	0	0	0	0	0	0	62
19456584	f	md	NL	2021	0	0	0	0	0	0	0	0	0	0	0	0	74
19456650	f	md	FR	2021	0	0	0	0	0	0	0	0	0	0	0	0	
19456971	f	cn	US	2021	1	0	0	0	0	0	0	0	0	0	0	0	
19457421	m	md	JP	2021	0	1	0	0	0	1	0	0	0	0	0	1	67
19457486	f	cn	Not re	2021	0	0	0	0	0	0	0	0	0	0	0	0	62
19457678	m	hp	US	2021	0	1	1	0	0	0	0	0	0	0	0	0	83
19457684	m	md	DE	2021	0	1	0	0	1	0	0	0	0	0	0	0	52
19457686	m	md	DE	2021	0	1	1	0	0	0	0	0	0	0	0	0	48
19457840	f	md	DE	2021	0	0	0	0	0	0	0	0	0	0	0	0	69
19457936	m	ph	JP	2021	1	0	0	0	0	0	0	0	0	0	0	0	75
19458053	f	cn	US	2021	0	1	1	0	0	0	0	0	0	0	0	0	53
19458226	f	md	JP	2021	0	1	0	0	1	0	0	0	0	0	0	0	59
19458723	f	cn	Not re	2021	0	0	0	0	0	0	0	0	0	0	0	0	63
19458750	f	hp	DE	2021	0	1	0	0	1	0	0	0	0	0	0	0	57
19459301	f	Not repo	DE	2021	1	0	0	0	0	0	0	0	0	0	0	0	49
19459366	m	md	JP	2021	0	0	0	0	0	0	0	0	0	0	0	0	65
19459425	m	cn	JP	2021	0	1	1	0	0	0	0	0	0	0	0	0	
19459478	Not	Not repo	AU	2021	0	1	0	1	0	0	0	0	0	0	0	0	
19459597	f	hp	NL	2021	0	0	0	0	0	0	0	0	0	0	0	0	74
19459625	m	hp	CN	2021	0	1	0	0	1	0	0	0	0	0	0	0	49
19459639	f	cn	CN	2021	0	1	0	0	1	0	0	0	0	0	0	0	54
19459672	m	md	TR	2021	0	0	0	0	0	0	0	0	0	0	0	0	63
19459688	Not	hp	JP	2021	1	0	0	0	0	0	0	0	0	0	0	0	
19459696	Not	hp	JP	2021	1	0	0	0	0	0	0	0	0	0	0	0	
19459903	m	cn	IN	2021	0	1	0	0	1	0	0	0	0	0	0	1	70
19460016	m	md	JP	2021	0	1	0	0	0	1	0	0	0	0	0	1	70
19460100	m	md	DE	2021	0	1	0	0	0	1	0	0	0	0	0	0	55
19460838	f	md	US	2021	1	0	0	0	0	0	0	0	0	0	0	0	66

19460913	f	ph	JP	2021	0	0	0	0	0	0	0	0	0	0	0	0	40
19460931	m	ph	JP	2021	0	0	0	0	0	0	0	0	0	0	0	0	40
19461352	f	hp	FR	2021	0	1	0	0	1	0	0	0	0	0	0	0	78
19461365	Not	md	FR	2021	0	1	0	1	0	0	0	0	0	0	0	1	
19461533	Not	ph	JP	2021	1	0	0	0	0	0	0	0	0	0	0	0	
19462085	m	cn	JP	2021	0	1	1	0	0	0	0	0	0	0	0	0	90
19462534	f	md	CA	2021	1	0	0	0	0	0	0	0	0	0	0	0	73.5
19462632	f	Not repo	ID	2021	1	0	0	0	0	0	0	0	0	0	0	0	65.6
19462702	m	Not repo	IT	2021	0	1	0	1	0	0	0	0	0	0	0	0	78
19462733	f	md	ES	2021	0	0	0	0	0	0	0	0	0	0	0	0	38
19462784	f	md	KR	2021	0	0	0	0	0	0	0	0	0	0	0	0	64
19462818	m	ph	JP	2021	0	1	0	0	1	0	0	0	0	0	0	1	72
19462847	f	cn	CN	2021	0	1	0	0	1	0	0	0	0	0	0	0	65
19463021	Not	md	JP	2021	0	1	0	1	0	0	0	0	0	0	0	0	
19463104	f	md	FR	2021	0	0	0	0	0	0	1	0	0	1	0	0	
19463216	f	cn	US	2021	1	0	0	0	0	0	0	0	0	0	0	0	
19463653	Not	hp	JP	2021	1	0	0	0	0	0	0	0	0	0	0	0	
19463654	Not	hp	JP	2021	1	0	0	0	0	0	0	0	0	0	0	0	
19463676	Not	hp	JP	2021	1	0	0	0	0	0	0	0	0	0	0	0	
19463747	m	cn	JP	2021	0	1	1	0	0	0	0	0	0	0	0	0	67
19464131	m	Not repo	AU	2021	0	1	0	1	0	0	0	0	0	0	0	1	
19464298	f	cn	Not re	2021	0	0	0	0	0	0	0	0	0	0	0	0	
19464318	f	hp	DE	2021	0	1	1	0	0	0	0	0	0	0	0	0	
19464581	f	cn	Not re	2021	0	0	0	0	0	0	0	0	0	0	0	0	55
19464625	m	hp	DE	2021	0	1	1	0	0	0	0	0	0	0	0	0	48
19465480	Not	hp	JP	2021	1	0	0	0	0	0	0	0	0	0	0	0	
19465628	f	cn	Not re	2021	0	0	0	0	0	0	0	0	0	0	0	0	
19465823	m	md	JP	2021	0	1	0	0	1	0	0	0	0	0	0	0	75
19465927	m	cn	IN	2021	0	1	0	0	1	0	0	0	0	0	0	0	78
19466179	f	md	JP	2021	0	1	0	0	0	1	0	0	0	0	0	0	80
19466782	m	md	IL	2021	0	1	0	0	1	0	0	0	0	0	0	0	60
19466975	f	cn	JP	2021	0	1	1	0	0	0	0	0	0	0	0	0	65
19467118	f	cn	Not re	2021	0	0	0	0	0	0	0	0	0	0	0	0	
19467302	Not	hp	JP	2021	1	0	0	0	0	0	0	0	0	0	0	0	

19467713	m	cn	US	2021	0	0	0	0	0	0	0	0	0	0	0	0	74
19468383	m	cn	US	2021	0	0	0	0	0	0	0	0	0	0	0	0	68
19468393	m	ph	JP	2021	0	1	0	0	1	0	0	0	0	0	0	0	73
19468464	m	md	DE	2021	0	1	1	0	0	0	0	0	0	0	0	0	48
19468912	m	Not repo	KR	2021	1	0	0	0	0	0	0	0	0	0	0	0	65
19469002	Not	md	NL	2021	0	0	0	0	0	0	0	0	0	0	0	0	
19469003	Not	md	NL	2021	0	0	0	0	0	0	0	0	0	0	0	0	
19469004	Not	md	NL	2021	0	0	0	0	0	0	0	0	0	0	0	0	
19469005	Not	md	NL	2021	0	0	0	0	0	0	0	0	0	0	0	0	
19469021	Not	md	NL	2021	0	0	0	0	0	0	0	0	0	0	0	0	
19469022	Not	md	NL	2021	0	0	0	0	0	0	0	0	0	0	0	0	
19469023	Not	md	NL	2021	0	0	0	0	0	0	0	0	0	0	0	0	
19469024	Not	md	NL	2021	0	0	0	0	0	0	0	0	0	0	0	0	
19469132	m	ph	US	2021	0	1	0	0	1	0	0	0	0	0	0	0	
19469197	Not	hp	FR	2021	0	1	0	0	1	0	0	0	0	0	0	0	73
19469379	f	md	JP	2021	1	0	0	0	0	0	0	0	0	0	0	0	80
19469392	f	md	BG	2021	0	0	0	0	0	0	0	0	0	0	0	0	51
19469490	f	Not repo	IT	2021	1	0	0	0	0	0	0	0	0	0	0	0	48
19469749	m	hp	CA	2021	0	1	0	0	1	0	0	0	0	0	0	0	67
19470114	m	hp	CA	2021	0	1	0	0	1	0	0	0	0	0	0	0	67
19470251	f	md	US	2021	0	0	0	0	0	0	0	0	0	0	0	0	68
19470354	Not	hp	JP	2021	0	1	0	1	0	0	0	0	0	0	0	0	55
19470401	f	md	IT	2021	0	1	1	0	0	0	0	0	0	0	0	0	68
19470458	Not	md	ES	2021	0	1	1	0	0	0	0	0	0	0	0	0	
19470468	Not	md	ES	2021	0	1	1	0	0	0	0	0	0	0	0	0	
19470511	m	cn	IN	2021	0	1	0	0	1	0	0	0	0	0	0	0	62
19471403	m	md	FR	2021	0	1	0	0	0	1	0	0	0	0	0	1	77
19471706	f	md	US	2021	1	0	0	0	0	0	0	0	0	0	0	0	61
19471985	f	Not repo	JP	2021	1	0	0	0	0	0	0	0	0	0	0	0	67
19472764	m	md	AT	2021	0	0	0	0	0	0	0	0	0	0	0	0	79
19473587	f	md	PA	2021	1	0	0	0	0	0	0	0	0	0	0	0	76.2
19473977	Not	md	MY	2021	1	0	0	0	0	0	0	0	0	0	0	0	
19474281	f	md	BG	2021	0	0	0	0	0	0	0	0	0	0	0	0	51
19474349	f	md	US	2021	0	0	0	0	0	0	0	0	0	0	0	0	83

19474449	f	cn	Not re	2021	0	0	0	0	0	0	0	0	0	0	0	0	40
19474469	f	cn	US	2021	0	1	0	1	0	0	0	0	0	0	0	1	77.2
19474670	m	hp	US	2021	0	0	0	0	0	0	0	0	0	0	0	0	
19475415	f	Not repo	US	2021	1	0	0	0	0	0	0	0	0	0	0	0	
19475783	f	md	JP	2021	0	0	0	0	0	0	0	0	0	0	0	0	76
19476592	m	hp	TW	2021	0	0	0	0	0	0	0	0	0	0	0	0	
19476881	m	hp	NL	2021	0	1	1	0	0	0	0	0	0	0	0	0	48
19476982	m	hp	DE	2021	0	1	0	0	0	1	0	0	0	0	0	0	55
19477000	Not	ph	JP	2021	0	0	0	0	0	0	1	1	0	0	0	0	
19477431	m	cn	US	2021	0	0	0	0	0	0	0	0	0	0	0	0	94
19477611	m	hp	US	2021	0	1	1	0	0	0	0	0	0	0	0	0	71
19477971	m	hp	IN	2021	0	0	0	0	0	0	0	0	0	0	0	0	60
19478544	Not	md	US	2021	0	0	0	0	0	0	0	0	0	0	0	0	
19478663	m	md	DE	2021	0	1	1	0	0	0	0	0	0	0	0	0	48
19478755	f	cn	IN	2021	0	0	0	0	0	0	0	0	0	0	0	0	61
19478829	m	Not repo	ID	2021	1	0	0	0	0	0	0	0	0	0	0	0	84.2
19479203	f	md	DE	2021	1	0	0	0	0	0	0	0	0	0	0	0	61
19479262	m	md	JP	2021	0	1	0	0	1	0	0	0	0	0	0	0	64
19479282	m	hp	NL	2021	0	1	1	0	0	0	0	0	0	0	0	0	72
19481132	f	md	JP	2021	0	1	0	0	0	1	0	0	0	0	0	0	
18618749	m	md	KR	2021	0	1	1	0	0	0	0	0	0	0	0	0	70
18695843	f	md	IT	2021	0	1	1	0	0	0	0	0	0	0	0	0	62
18762146	m	hp	UA	2021	0	0	0	0	0	0	0	0	0	0	0	0	72
18767207	m	md	DE	2021	0	0	0	0	0	0	0	0	0	0	0	0	59
18769799	m	cn	IN	2021	0	0	0	0	0	0	0	0	0	0	0	0	50
18873234	m	md	CN	2021	0	1	1	0	0	0	0	0	0	0	0	0	68
18902530	f	md	US	2021	0	1	0	0	0	1	0	0	0	0	0	0	64
18902629	f	md	JP	2021	0	1	0	0	1	0	0	0	0	0	0	1	63
18916719	f	ph	CA	2021	0	1	0	0	1	0	0	0	0	0	0	0	68
18971564	m	md	FR	2021	0	0	0	0	0	0	0	0	0	0	0	0	67
18991171	m	md	FR	2021	0	0	0	0	0	0	0	0	0	0	0	0	67
19017940	f	hp	DE	2021	0	1	0	0	1	0	0	0	0	0	0	0	75
19029121	f	md	DE	2021	0	1	0	0	1	0	0	0	0	0	0	0	75
19030348	f	md	JP	2021	0	0	0	0	0	0	0	0	0	0	0	0	71

19034101	f	cn	JP	2021	0	1	1	0	0	0	0	0	0	0	0	0	77
19039084	m	md	JP	2021	0	1	0	0	1	0	0	0	0	0	0	0	65
19055727	m	md	IT	2021	0	0	0	0	0	0	0	0	0	0	0	0	71
19071336	m	md	JP	2021	0	1	0	0	1	0	0	0	0	0	0	0	90
19071531	m	md	JP	2021	0	1	0	0	1	0	0	0	0	0	0	0	73
19085453	m	md	JP	2021	0	1	0	0	1	0	0	0	0	0	0	0	53
19091667	f	md	FR	2021	0	1	0	0	0	1	0	0	0	0	0	0	63
19094996	Not	md	FR	2021	0	1	0	0	1	0	0	0	0	0	0	0	
19098807	f	md	JP	2021	0	1	0	0	1	0	0	0	0	0	0	0	61
19100066	f	md	JP	2021	0	1	0	0	1	0	0	0	0	0	0	0	67
19100978	f	md	US	2021	0	1	0	0	0	1	0	0	0	0	0	0	72
19101631	m	md	JP	2021	0	1	0	0	1	0	0	0	0	0	0	1	66
19103420	m	md	NL	2021	0	0	0	0	0	0	0	0	0	0	0	0	64
19103763	f	cn	Not re	2021	0	0	0	0	0	0	0	0	0	0	0	0	59
19104272	m	ph	JP	2021	0	1	0	0	1	0	0	0	0	0	0	0	80
19105289	f	md	JP	2021	0	1	0	0	1	0	0	0	0	0	0	0	36
19106293	m	md	JP	2021	0	1	0	0	1	0	0	0	0	0	0	0	78
19114768	m	cn	US	2021	0	1	0	0	1	0	0	0	0	0	0	0	57
19116038	Not	md	JP	2021	0	1	0	0	1	0	0	0	0	0	0	0	
19118227	m	md	TW	2021	0	1	1	0	0	0	0	0	0	0	0	0	63
19126245	f	md	JP	2021	0	0	0	0	0	0	0	0	0	0	0	0	64
19139792	f	md	GB	2021	0	1	1	0	0	0	0	0	0	0	0	0	67
19141569	m	hp	FR	2021	0	1	0	0	1	0	0	0	0	0	0	0	85
19143503	m	md	JP	2021	0	1	0	0	1	0	0	0	0	0	0	0	70
19147022	f	cn	US	2021	0	1	0	0	1	0	0	0	0	0	0	0	82
19153610	m	md	JP	2021	0	1	1	0	0	0	0	0	0	0	0	0	72
19162545	f	md	DE	2021	0	0	0	0	0	0	0	0	0	0	0	0	61
19163835	m	md	US	2021	0	1	0	0	0	1	0	0	0	0	0	0	77
19164477	m	hp	US	2021	0	0	0	0	0	0	0	0	0	0	0	0	74
19166465	f	md	US	2021	0	0	0	0	0	0	0	0	0	0	0	0	61
19169946	f	hp	US	2021	0	0	0	0	0	0	0	0	0	0	0	0	
19174775	m	md	KR	2021	0	1	0	1	0	0	0	0	0	0	0	0	65.3
19180610	m	md	CN	2021	0	1	0	0	0	1	0	0	0	0	0	0	69
19182782	m	md	CN	2021	0	1	1	0	0	0	0	0	0	0	0	0	70

19183519	f	cn	Not re	2021	0	0	0	0	0	0	0	0	0	0	0	0	55
19184087	f	cn	Not re	2021	0	0	0	0	0	0	0	0	0	0	0	0	54
19186588	f	cn	JP	2021	0	0	0	0	0	0	0	0	0	0	0	0	71
19187290	m	md	IT	2021	0	0	0	0	0	0	0	0	0	0	0	0	64
19188538	m	md	JP	2021	0	0	0	0	0	0	0	0	0	0	0	0	75
19189036	m	md	JP	2021	0	1	0	0	1	0	0	0	0	0	0	0	84
19190992	m	md	GB	2021	0	1	0	0	0	1	0	0	0	0	0	0	82
19194609	Not	md	GB	2021	0	1	0	1	0	0	0	0	0	0	0	0	74
19198839	m	md	JP	2021	0	1	0	1	0	0	0	0	0	0	0	1	64
19201678	f	cn	FR	2021	0	0	0	0	0	0	0	0	0	0	0	0	43
19208307	f	md	GB	2021	0	0	0	0	0	0	1	0	0	0	1	0	67
19208540	m	md	JP	2021	0	1	0	0	1	0	0	0	0	0	0	0	70
19212893	f	md	GB	2021	0	1	1	0	0	0	0	0	0	0	0	0	59
19218223	m	hp	BG	2021	0	0	0	0	0	0	0	0	0	0	0	0	74
19221973	f	md	JP	2021	0	1	1	0	0	0	0	0	0	0	0	0	77
19222958	f	cn	US	2021	0	1	1	0	0	0	0	0	0	0	0	0	66
19224215	m	md	KR	2021	0	0	0	0	0	0	0	0	0	0	0	0	67
19224551	f	md	TR	2021	0	0	0	0	0	0	0	0	0	0	0	0	70
19228882	m	ph	US	2021	0	0	0	0	0	0	0	0	0	0	0	0	77
19241049	m	cn	Not re	2021	0	0	0	0	0	0	0	0	0	0	0	0	
19241224	f	md	US	2021	0	1	0	0	0	1	0	0	0	0	0	1	73
19250256	m	md	IT	2021	0	1	1	0	0	0	0	0	0	0	0	0	54
19257986	m	md	PL	2021	0	1	0	0	0	1	0	0	0	0	0	0	70
19258391	m	md	FR	2021	0	1	0	0	0	1	0	0	0	0	0	0	63
19259347	f	Not repo	JP	2021	1	0	0	0	0	0	0	0	0	0	0	0	78
19262605	f	md	JP	2021	0	1	0	0	1	0	0	0	0	0	0	0	70
19262840	f	md	JP	2021	0	1	0	0	1	0	0	0	0	0	0	0	57
19269146	f	cn	CN	2021	0	1	0	0	1	0	0	0	0	0	0	0	61
19270661	Not	md	PL	2021	0	1	0	0	0	1	0	0	0	0	0	0	
19270941	m	md	JP	2021	0	1	0	1	0	0	0	0	0	0	0	0	77
19276928	f	md	BR	2021	0	1	0	0	0	1	0	0	0	0	0	0	81
19283431	f	md	JP	2021	0	0	0	0	0	0	0	0	0	0	0	0	68
19285464	m	md	CN	2021	0	0	0	0	0	0	0	0	0	0	0	0	68
19289940	f	ph	JP	2021	0	1	1	0	0	0	0	0	0	0	0	0	70

19299395	f	ph	JP	2021	0	0	0	0	0	0	0	0	0	0	0	0	43
19302108	m	md	JP	2021	0	1	0	0	1	0	0	0	0	0	0	1	85
19304443	f	md	CN	2021	0	0	0	0	0	0	0	0	0	0	0	0	53
19305087	m	md	JP	2021	0	1	0	1	0	0	0	0	0	0	0	1	62
19307803	Not	cn	US	2021	0	1	1	0	0	0	0	0	0	0	0	0	
19308926	m	md	JP	2021	0	1	0	0	1	0	0	0	0	0	0	0	71
19313389	m	md	MY	2021	0	0	0	0	0	0	0	0	0	0	0	0	77
19319230	m	md	CN	2021	0	1	0	1	0	0	0	0	0	0	0	0	60.2
19327896	m	cn	SG	2021	0	0	0	0	0	0	0	0	0	0	0	0	
19328738	m	cn	JP	2021	0	1	1	0	0	0	0	0	0	0	0	0	55
19352235	m	cn	JP	2021	0	1	1	0	0	0	0	0	0	0	0	0	
19357723	f	hp	BE	2021	0	0	0	0	0	0	0	0	0	0	0	0	82
19360271	m	md	IT	2021	0	1	0	0	0	1	0	0	0	0	0	0	64
19361149	m	hp	GT	2021	1	0	0	0	0	0	0	0	0	0	0	0	
19362831	f	md	CA	2021	0	1	1	0	0	0	0	0	0	0	0	0	69
19366352	f	md	KR	2021	0	0	0	0	0	0	0	0	0	0	0	0	72
19369606	f	md	US	2021	0	1	0	0	0	1	0	0	0	0	0	0	83
19369801	m	md	GR	2021	0	1	1	0	0	0	0	0	0	0	0	0	54
19370153	f	md	DE	2021	0	1	0	0	1	0	0	0	0	0	0	0	62
19371204	f	md	FR	2021	0	1	0	0	0	1	0	0	0	0	0	0	57
19372106	m	md	JP	2021	0	1	0	0	1	0	0	0	0	0	0	0	61
19372420	m	ph	US	2021	0	1	0	1	0	0	0	0	0	0	0	0	68
19372912	f	md	US	2021	0	1	0	0	0	1	0	0	0	0	0	0	75
19373105	f	md	FR	2021	0	0	0	0	0	0	0	0	0	0	0	0	
19373533	m	hp	DE	2021	0	1	1	0	0	0	0	0	0	0	0	0	
19373641	m	md	IT	2021	0	1	0	0	0	1	0	0	0	0	0	0	76
19374630	m	md	DE	2021	0	1	0	0	1	0	0	0	0	0	0	0	58
19375491	m	md	JP	2021	0	1	0	0	0	1	0	0	0	0	0	0	
19376441	f	cn	JP	2021	0	1	0	1	0	0	0	0	0	0	0	0	77
19377185	f	md	JP	2021	0	0	0	0	0	0	0	0	0	0	0	0	77
19377485	m	Not repo	JP	2021	0	1	0	1	0	0	0	0	0	0	0	1	75.2
19380514	m	cn	CN	2021	0	0	0	0	0	0	0	0	0	0	0	0	65
19381694	m	cn	US	2021	0	1	0	0	1	0	0	0	0	0	0	0	76
19382219	m	md	JP	2021	0	1	0	0	0	1	0	0	0	0	0	0	73

19382818	m	cn	Not re	2021	0	0	0	0	0	0	0	0	0	0	0	0	
19386016	m	md	CN	2021	0	1	1	0	0	0	0	0	0	0	0	0	66
19388075	m	md	DE	2021	0	0	0	0	0	0	0	0	0	0	0	0	70
19388937	m	ph	JP	2021	0	0	0	0	0	0	0	0	0	0	0	0	57
19390062	m	ph	JP	2021	0	1	0	0	1	0	0	0	0	0	0	0	59
19391947	Not	md	MX	2021	0	0	0	0	0	0	0	0	0	0	0	0	
19399372	m	md	IL	2021	0	1	0	1	0	0	0	0	0	0	0	0	
19401707	f	ph	JP	2021	0	0	0	0	0	0	0	0	0	0	0	0	52
19402705	f	md	US	2021	0	1	0	0	0	1	0	0	0	0	0	0	83
19402780	m	md	JP	2021	0	0	0	0	0	0	0	0	0	0	0	0	51
19403348	f	md	US	2021	0	1	0	0	0	1	0	0	0	0	0	0	66
19406795	f	md	US	2021	1	0	0	0	0	0	0	0	0	0	0	0	55
19407147	f	md	CA	2021	1	0	0	0	0	0	0	0	0	0	0	0	77.3
19412200	m	cn	CN	2021	0	1	0	0	1	0	0	0	0	0	0	0	60
19413299	m	md	US	2021	0	1	1	0	0	0	0	0	0	0	0	0	53
19417171	f	hp	US	2021	0	1	1	0	0	0	0	0	0	0	0	0	67
19417175	m	hp	US	2021	0	1	1	0	0	0	0	0	0	0	0	0	77
19417840	f	md	GB	2021	0	1	1	0	0	0	0	0	0	0	0	0	68
19419156	m	md	GB	2021	0	1	0	0	0	1	0	0	0	0	0	0	77
19419678	f	hp	NL	2021	0	0	0	0	0	0	0	0	0	0	0	0	60
19420952	f	cn	CN	2021	0	1	0	0	1	0	0	0	0	0	0	0	67
19421260	f	md	PE	2021	0	1	0	0	0	1	0	0	0	0	0	0	96
19421927	f	md	BR	2021	0	0	0	0	0	0	0	0	0	0	0	0	67
19422158	m	md	JP	2021	0	1	0	0	1	0	0	0	0	0	0	0	72
19422283	f	hp	IT	2021	0	0	0	0	0	0	0	0	0	0	0	0	60
19425479	f	ph	JP	2021	0	0	0	0	0	0	0	0	0	0	0	0	54
19426214	Not	hp	DE	2021	0	1	0	0	1	0	0	0	0	0	0	0	62
19428385	m	md	IT	2021	0	1	0	0	0	1	0	0	0	0	0	0	67
19429154	m	md	JP	2021	0	1	0	0	0	1	0	0	0	0	0	0	66
19429965	m	md	ES	2021	0	1	0	0	0	1	0	0	0	0	0	0	76
19431035	f	md	US	2021	1	0	0	0	0	0	0	0	0	0	0	0	60
19431722	f	md	US	2021	0	0	0	0	0	0	0	0	0	0	0	0	60
19433196	f	hp	KR	2021	0	0	0	0	0	0	0	0	0	0	0	0	54
19433277	f	md	BR	2021	0	0	0	0	0	0	0	0	0	0	0	0	60

19433617	m	cn	Not re	2021	0	0	0	0	0	0	0	0	0	0	0	0	57
19435968	m	hp	GB	2021	0	1	0	0	0	1	0	0	0	0	0	0	76
19436240	f	md	ES	2021	0	0	0	0	0	0	0	0	0	0	0	0	71
19436245	m	md	JP	2021	0	1	0	1	0	0	0	0	0	0	0	1	72
19436565	m	md	KR	2021	1	0	0	0	0	0	0	0	0	0	0	0	65.4
19436895	m	md	FR	2021	0	0	0	0	0	0	0	0	0	0	0	0	73
19437398	f	md	DE	2021	0	0	0	0	0	0	0	0	0	0	0	0	72
19437686	Not	md	PL	2021	0	0	0	0	0	0	0	0	0	0	0	0	
19437758	m	md	TW	2021	0	0	0	0	0	0	0	0	0	0	0	0	
19439636	m	hp	DE	2021	0	1	0	0	1	0	0	0	0	0	0	0	58
19441806	m	ph	JP	2021	0	1	1	0	0	0	0	0	0	0	0	0	65
19441914	m	md	GB	2021	0	1	0	0	0	1	0	0	0	0	0	0	76
19442945	Not	hp	US	2021	0	1	0	1	0	0	0	0	0	0	0	0	65.7
19443523	m	hp	DE	2021	0	1	1	0	0	0	0	0	0	0	0	0	
19443670	m	cn	JP	2021	0	1	1	0	0	0	0	0	0	0	0	0	
19443690	Not	md	IN	2021	0	0	0	0	0	0	0	0	0	0	0	0	
19450423	m	md	JP	2021	0	1	0	0	1	0	0	0	0	0	0	0	66
19453320	f	md	JP	2021	0	1	0	0	0	1	0	0	0	0	0	1	78
19453486	m	md	CN	2021	0	1	1	0	0	0	0	0	0	0	0	0	58
19454180	m	md	US	2021	0	1	1	0	0	0	0	0	0	0	0	0	58
19458589	m	md	FR	2021	0	0	0	0	0	0	0	0	0	0	0	0	
19458822	m	hp	PL	2021	0	0	0	0	0	0	0	0	0	0	0	0	75
19459365	f	md	US	2021	0	1	0	0	0	1	0	0	0	0	0	0	58
19459477	m	hp	CN	2021	0	1	0	0	1	0	0	0	0	0	0	0	56
19459618	m	hp	US	2021	0	1	0	0	1	0	0	0	0	0	0	0	81
19460443	m	md	DE	2021	0	1	1	0	0	0	0	0	0	0	0	0	60
19461808	m	md	IT	2021	0	1	0	0	0	1	0	0	0	0	0	0	67
19462479	m	md	ES	2021	0	1	0	0	0	1	0	0	0	0	0	0	76
19463727	m	hp	JP	2021	0	1	0	1	0	0	0	0	0	0	0	1	68
19465826	Not	md	JP	2021	0	1	0	0	1	0	0	0	0	0	0	0	
19466136	m	md	JP	2021	0	1	0	0	1	0	0	0	0	0	0	1	76
19466914	f	cn	JP	2021	0	1	1	0	0	0	0	0	0	0	0	1	71
19467234	f	cn	JP	2021	0	0	0	0	0	0	0	0	0	0	0	0	80
19467261	m	md	US	2021	0	0	0	0	0	0	0	0	0	0	0	0	74

19468463	f	cn	US	2021	0	1	1	0	0	0	0	0	0	0	0	0	
19469114	f	md	US	2021	0	0	0	0	0	0	0	0	0	0	0	0	74
19469362	f	md	NL	2021	0	0	0	0	0	0	0	0	0	0	0	0	57
19470693	f	md	US	2021	0	0	0	0	0	0	0	0	0	0	0	0	62
19472074	m	md	JP	2021	0	1	0	0	1	0	0	0	0	0	0	0	57
19472128	m	md	JP	2021	0	1	0	0	1	0	0	0	0	0	0	0	67
19472226	Not	hp	JP	2021	0	0	0	0	0	0	0	0	0	0	0	0	
19472303	m	cn	PL	2021	0	1	1	0	0	0	0	0	0	0	0	0	
19472641	f	md	US	2021	0	0	0	0	0	0	0	0	0	0	0	0	76
19472689	f	md	DE	2021	0	1	0	0	1	0	0	0	0	0	0	0	57
19473488	f	md	JP	2021	1	0	0	0	0	0	0	0	0	0	0	0	65
19473868	m	cn	CA	2021	0	0	0	0	0	0	0	0	0	0	0	0	62
19475680	m	md	JP	2021	0	1	0	0	1	0	0	0	0	0	0	0	72
19476016	m	md	JP	2021	0	0	0	0	0	0	0	0	0	0	0	0	80
19476594	f	Not repo	RO	2021	1	0	0	0	0	0	0	0	0	0	0	0	65
19477566	f	hp	US	2021	0	0	0	0	0	0	0	0	0	0	0	0	72
19477848	f	hp	CA	2021	0	1	0	0	1	0	0	0	0	0	0	0	71
19479527	m	md	JP	2021	0	1	0	0	1	0	0	0	0	0	0	0	58
19479929	f	md	JP	2021	0	0	0	0	0	0	0	0	0	0	0	0	49
19480717	m	md	IT	2021	0	1	0	0	0	1	0	0	0	0	0	0	67
19480933	f	ph	JP	2021	0	0	0	0	0	0	0	0	0	0	0	0	60
19480940	m	md	BG	2021	0	0	0	0	0	0	0	0	0	0	0	0	68
19481100	m	md	JP	2021	1	0	0	0	0	0	0	0	0	0	0	0	72
19481215	f	hp	JP	2021	0	1	0	0	0	1	0	0	0	0	0	0	73
19481525	m	md	JP	2021	0	0	0	0	0	0	0	0	0	0	0	0	72
19481642	m	Not repo	PL	2021	0	1	0	1	0	0	0	0	0	0	0	0	
19481689	f	md	IT	2021	0	1	1	0	0	0	0	0	0	0	0	0	68
19482240	m	hp	US	2021	0	1	1	0	0	0	0	0	0	0	0	0	71
19482286	f	md	AT	2021	0	0	0	0	0	0	0	0	0	0	0	0	67
19482465	Not	ph	JP	2021	1	0	0	0	0	0	0	0	0	0	0	0	
19482481	f	md	FR	2021	0	0	0	0	0	0	0	0	0	0	0	0	55
19482634	Not	Not repo	HK	2021	1	0	0	0	0	0	0	0	0	0	0	0	
19482802	f	md	GB	2021	0	0	0	0	0	0	0	0	0	0	0	0	
19482839	m	md	JP	2021	0	1	0	0	1	0	0	0	0	0	0	0	71

19483208	m	hp	US	2021	0	1	1	0	0	0	0	0	0	0	0	0	71
19483221	f	Not repo	MY	2021	1	0	0	0	0	0	0	0	0	0	0	0	75
19483235	m	hp	TW	2021	1	0	0	0	0	0	0	0	0	0	0	0	74
19483305	f	md	JP	2021	0	1	0	0	1	0	0	0	0	0	0	0	60
19483801	f	md	KR	2021	0	0	0	0	0	0	0	0	0	0	0	0	61
19483810	f	md	DE	2021	1	0	0	0	0	0	0	0	0	0	0	0	67
19483850	m	Not repo	FR	2021	0	1	0	1	0	0	0	0	0	0	0	1	76.8
19484261	m	md	KR	2021	0	0	0	0	0	0	0	0	0	0	0	0	48
19484344	f	md	JP	2021	0	1	0	0	1	0	0	0	0	0	0	0	76
19484511	m	md	JP	2021	0	1	0	0	1	0	0	0	0	0	0	0	74
19484520	m	cn	US	2021	0	1	1	0	0	0	0	0	0	0	0	0	68
19484689	m	cn	JP	2021	0	1	1	0	0	0	0	0	0	0	0	0	
19484763	m	cn	US	2021	1	0	0	0	0	0	0	0	0	0	0	0	58.3
19484813	f	ph	MY	2021	1	0	0	0	0	0	0	0	0	0	0	0	65
19485002	m	cn	JP	2021	0	1	1	0	0	0	0	0	0	0	0	1	75
19485123	m	hp	JP	2021	0	1	1	0	0	0	0	0	0	0	0	0	76
19485209	m	cn	CN	2021	0	1	0	0	1	0	0	0	0	0	0	0	58
19485666	f	hp	TR	2021	0	0	0	0	0	0	0	0	0	0	0	0	68
19486300	m	cn	DE	2021	0	1	1	0	0	0	0	0	0	0	0	0	73
19486468	m	Not repo	CN	2021	0	1	0	1	0	0	0	0	0	0	0	0	73
19486497	f	md	DE	2021	0	1	0	0	1	0	0	0	0	0	0	0	63
19486499	m	hp	IE	2021	0	1	0	0	1	0	0	0	0	0	0	0	57
19486669	m	Not repo	JP	2021	1	0	0	0	0	0	0	0	0	0	0	0	74
19486670	m	Not repo	ID	2021	1	0	0	0	0	0	0	0	0	0	0	0	
19486832	m	md	JP	2021	0	1	0	0	1	0	0	0	0	0	0	0	70
19486865	f	md	US	2021	0	0	0	0	0	0	0	0	0	0	0	0	
19486883	m	cn	Not re	2021	0	0	0	0	0	0	0	0	0	0	0	0	
19487051	m	md	JP	2021	0	1	0	0	0	1	0	0	0	0	0	0	70
19487092	f	md	JP	2021	1	0	0	0	0	0	0	0	0	0	0	0	63
19487193	f	Not repo	IT	2021	1	0	0	0	0	0	0	0	0	0	0	0	70
19487260	m	cn	CN	2021	0	1	0	1	0	0	0	0	0	0	0	0	74
19487261	m	ph	JP	2021	1	0	0	0	0	0	0	0	0	0	0	0	81
19487306	f	md	US	2021	0	0	0	0	0	0	0	0	0	0	0	0	
19487406	m	hp	JP	2021	0	1	1	0	0	0	0	0	0	0	0	0	

19487501	m	md	JP	2021	0	0	0	0	0	0	0	0	0	0	0	0	70
19487745	f	cn	Not re	2021	0	0	0	0	0	0	0	0	0	0	0	0	80
19487776	f	md	NL	2021	0	0	0	0	0	0	0	0	0	0	0	0	
19487901	m	Not repo	IT	2021	1	0	0	0	0	0	0	0	0	0	0	0	69
19488087	f	md	FR	2021	0	0	0	0	0	0	0	0	0	0	0	0	49
19488151	m	Not repo	ID	2021	1	0	0	0	0	0	0	0	0	0	0	0	57.5
19488416	f	hp	US	2021	0	0	0	0	0	0	0	0	0	0	0	0	64
19488472	m	md	JP	2021	0	1	0	0	1	0	0	0	0	0	0	0	57
19488665	f	hp	GB	2021	0	0	0	0	0	0	0	0	0	0	0	0	
19488741	m	cn	Not re	2021	0	0	0	0	0	0	0	0	0	0	0	0	70
19488789	m	md	FR	2021	0	0	0	0	0	0	0	0	0	0	0	0	
19488819	f	cn	US	2021	0	1	1	0	0	0	0	0	0	0	0	1	70
19488820	m	md	DE	2021	0	0	0	0	0	0	0	0	0	0	0	0	61
19489258	m	md	IN	2021	0	0	0	0	0	0	0	0	0	0	0	0	47
19489381	m	hp	IN	2021	0	0	0	0	0	0	0	0	0	0	0	0	51
19489454	Not	md	DE	2021	0	1	0	0	1	0	0	0	0	0	0	0	67
19489510	m	cn	Not re	2021	0	0	0	0	0	0	0	0	0	0	0	0	38
19489808	f	hp	JP	2021	0	1	0	0	1	0	0	0	0	0	0	0	50
19489889	m	md	JP	2021	0	1	0	0	1	0	0	0	0	0	0	0	77
19490048	m	hp	NL	2021	0	1	0	0	1	0	0	0	0	0	0	0	86
19490751	m	cn	CN	2021	0	1	0	0	1	0	0	0	0	0	0	0	51
19490782	m	hp	JP	2021	0	1	0	0	1	0	0	0	0	0	0	0	64
19490984	f	md	SI	2021	1	0	0	0	0	0	0	0	0	0	0	0	77
19491007	m	cn	JP	2021	0	1	1	0	0	0	0	0	0	0	0	0	84
19491149	m	hp	US	2021	0	1	0	0	1	0	0	0	0	0	0	0	
19491262	f	Not repo	ID	2021	1	0	0	0	0	0	0	0	0	0	0	0	
19491323	f	Not repo	PL	2021	0	1	0	1	0	0	0	0	0	0	0	1	
19491325	f	cn	US	2021	1	0	0	0	0	0	0	0	0	0	0	0	62.1
19491327	f	md	NL	2021	0	1	0	0	0	1	0	0	0	0	0	0	75
19492913	Not	md	JP	2021	0	1	0	1	0	0	0	0	0	0	0	1	
19493069	f	hp	HK	2021	0	1	1	0	0	0	0	0	0	0	0	0	63
19493562	m	ph	JP	2021	0	1	0	0	1	0	0	0	0	0	0	0	83
19493655	f	md	US	2021	0	1	0	0	0	1	0	0	0	0	0	0	72
19494189	m	md	DE	2021	0	0	0	0	0	0	0	0	0	0	0	0	55

19494260	Not	md	DE	2021	0	1	0	0	1	0	0	0	0	0	0	0	67
19494495	m	hp	US	2021	0	1	0	0	1	0	0	0	0	0	0	0	82
19494559	f	hp	DE	2021	0	1	0	0	1	0	0	0	0	0	0	0	62
19494561	m	hp	BG	2021	0	0	0	0	0	0	0	0	0	0	0	0	73
19494885	f	Not repo	ID	2021	1	0	0	0	0	0	0	0	0	0	0	0	
19494988	m	Not repo	ID	2021	1	0	0	0	0	0	0	0	0	0	0	0	66
19494994	Not	Not repo	SE	2021	1	0	0	0	0	0	0	0	0	0	0	0	
19495156	m	md	FR	2021	0	0	0	0	0	0	0	0	0	0	0	0	43
19495174	m	md	JP	2021	0	1	0	0	0	1	0	0	0	0	0	0	80
19495189	f	md	JP	2021	0	1	0	0	0	1	0	0	0	0	0	1	71
19495528	f	md	SG	2021	0	0	0	0	0	0	0	0	0	0	0	0	67
19495677	m	md	ES	2021	0	0	0	0	0	0	0	0	0	0	0	0	70
19496159	m	cn	Not re	2021	0	0	0	0	0	0	0	0	0	0	0	0	57
19497513	m	hp	DE	2021	0	1	0	0	1	0	0	0	0	0	0	0	58
19497537	m	md	PL	2021	0	0	0	0	0	0	0	0	0	0	0	0	66
19497653	f	hp	CA	2021	0	1	0	0	1	0	0	0	0	0	0	0	62
19497768	f	hp	US	2021	0	1	1	0	0	0	0	0	0	0	0	0	75
19497877	f	cn	US	2021	1	0	0	0	0	0	0	0	0	0	0	0	
19497984	f	cn	Not re	2021	0	1	1	0	0	0	0	0	0	0	0	0	64
19498102	f	md	CA	2021	0	0	0	0	0	0	0	0	0	0	0	0	
19498300	m	ph	JP	2021	0	0	0	0	0	0	0	0	0	0	0	0	81
19498491	m	md	FR	2021	0	1	0	0	0	1	0	0	0	0	0	0	71
19498594	f	cn	JP	2021	0	1	1	0	0	0	0	0	0	0	0	0	90
19498708	m	hp	US	2021	0	1	1	0	0	0	0	0	0	0	0	0	71
19498852	m	Not repo	CN	2021	1	0	0	0	0	0	0	0	0	0	0	0	82
19498884	m	md	US	2021	0	0	0	0	0	0	0	0	0	0	0	0	24
19498901	m	md	NL	2021	0	0	0	0	0	0	0	0	0	0	0	0	75
19499636	f	hp	IT	2021	0	0	0	0	0	0	0	0	0	0	0	0	60
19499701	m	md	JP	2021	1	0	0	0	0	0	0	0	0	0	0	0	90
19499733	f	hp	IT	2021	0	0	0	0	0	0	0	0	0	0	0	0	61
19499780	m	cn	IR	2021	1	0	0	0	0	0	0	0	0	0	0	0	77
19499785	m	hp	NL	2021	0	0	0	0	0	0	0	0	0	0	0	0	59
19499790	f	hp	NL	2021	0	0	0	0	0	0	0	0	0	0	0	0	73
19499842	f	md	JP	2021	0	0	0	0	0	0	0	0	0	0	0	0	60

19500135	m	md	DE	2021	0	0	0	0	0	0	0	0	0	0	0	0	70
19500313	f	md	CN	2021	0	0	0	0	0	0	0	0	0	0	0	0	49
19500387	m	md	US	2021	0	1	1	0	0	0	0	0	0	0	0	0	65
19500442	m	Not repo	JP	2021	0	1	0	1	0	0	0	0	0	0	0	0	66
19500504	f	md	US	2021	1	0	0	0	0	0	0	0	0	0	0	0	63
19500513	m	Not repo	CN	2021	0	1	0	1	0	0	0	0	0	0	0	1	67
19500936	m	cn	CN	2021	0	1	0	0	1	0	0	0	0	0	0	0	52
19501002	f	md	PL	2021	0	1	0	0	0	1	0	0	0	0	0	0	70
19501207	m	cn	JP	2021	0	1	1	0	0	0	0	0	0	0	0	0	72
19501482	m	md	BE	2021	0	1	0	0	0	1	0	0	0	0	0	0	77
19501592	f	cn	Not re	2021	0	0	0	0	0	0	0	0	0	0	0	0	64
19501637	Not	md	JP	2021	0	0	0	0	0	0	0	0	0	0	0	0	50
19502032	f	md	CO	2021	0	1	0	1	0	0	0	0	0	0	0	0	65
19502366	f	md	CA	2021	0	0	0	0	0	0	1	1	0	0	0	0	58
19502485	m	hp	NL	2021	0	1	1	0	0	0	0	0	0	0	0	0	72
19502687	m	hp	US	2021	0	0	0	0	0	0	0	0	0	0	0	0	71
19503101	f	md	TW	2021	0	0	0	0	0	0	0	0	0	0	0	0	73
19503110	f	md	FR	2021	0	0	0	0	0	0	0	0	0	0	0	0	50
19503184	f	md	FR	2021	0	0	0	0	0	0	0	0	0	0	0	0	50
19503185	Not	hp	US	2021	0	0	0	0	0	0	0	0	0	0	0	0	
19503192	Not	ph	JP	2021	1	0	0	0	0	0	0	0	0	0	0	0	
19503207	f	md	JP	2021	0	1	0	0	1	0	0	0	0	0	0	1	66
19503272	m	md	FR	2021	0	0	0	0	0	0	0	0	0	0	0	0	78
19503419	m	hp	US	2021	0	1	1	0	0	0	0	0	0	0	0	0	71
19503492	m	cn	JP	2021	0	1	1	0	0	0	0	0	0	0	0	0	58
19503561	m	md	KR	2021	0	1	0	0	1	0	0	0	0	0	0	0	67
19503599	m	cn	JP	2021	0	1	1	0	0	0	0	0	0	0	0	0	68
19503642	m	cn	US	2021	0	0	0	0	0	0	0	0	0	0	0	0	55
19503658	f	hp	IT	2021	1	0	0	0	0	0	0	0	0	0	0	0	71
19503823	f	md	JP	2021	1	0	0	0	0	0	0	0	0	0	0	0	70
19503868	f	md	JP	2021	0	1	0	0	1	0	0	0	0	0	0	0	80
19503969	f	md	ES	2021	0	0	0	0	0	0	0	0	0	0	0	0	37
19504089	m	md	RU	2021	0	0	0	0	0	0	0	0	0	0	0	0	
19504287	f	md	FR	2021	0	0	0	0	0	0	0	0	0	0	0	0	60

19504358	f	hp	DE	2021	0	1	0	0	1	0	0	0	0	0	0	0	63
19504384	Not	ph	US	2021	0	0	0	0	0	0	0	0	0	0	0	0	
19504402	f	md	KR	2021	0	1	0	0	0	1	0	0	0	0	0	1	48
19504462	Not	md	US	2021	0	0	0	0	0	0	0	0	0	0	0	0	
19504670	m	ph	JP	2021	0	1	0	0	1	0	0	0	0	0	0	0	55
19505050	m	cn	GR	2021	0	1	1	0	0	0	0	0	0	0	0	0	72
19505090	f	md	JP	2021	0	0	0	0	0	0	0	0	0	0	0	0	70
19505161	Not	Not repo	JP	2021	1	0	0	0	0	0	0	0	0	0	0	1	
19506046	Not	hp	FR	2021	0	1	0	0	1	0	0	0	0	0	0	0	67
19506380	m	md	CN	2021	0	1	0	1	0	0	0	0	0	0	0	0	69
19506706	f	Not repo	PL	2021	0	1	0	1	0	0	0	0	0	0	0	0	
19506920	f	md	AU	2021	0	0	0	0	0	0	0	0	0	0	0	0	
19507018	m	cn	CN	2021	0	1	0	1	0	0	0	0	0	0	0	0	65
19507024	f	md	SG	2021	1	0	0	0	0	0	0	0	0	0	0	0	
19507087	m	md	KR	2021	0	1	0	0	0	1	0	0	0	0	0	0	59
19507160	m	md	CN	2021	0	1	1	0	0	0	0	0	0	0	0	0	64
19507523	Not	cn	US	2021	1	0	0	0	0	0	0	0	0	0	0	0	81
19507636	Not	md	IT	2021	0	0	0	0	0	0	0	0	0	0	0	0	56
19507658	m	Not repo	US	2021	0	1	0	1	0	0	0	0	0	0	0	0	
19507815	m	cn	Not re	2021	0	0	0	0	0	0	0	0	0	0	0	0	
19507852	Not	hp	US	2021	0	1	0	0	1	0	0	0	0	0	0	0	
19508064	m	cn	DE	2021	0	1	1	0	0	0	0	0	0	0	0	0	
19508100	f	md	DE	2021	0	1	0	1	0	0	0	0	0	0	0	1	61
19508367	m	cn	JP	2021	0	1	1	0	0	0	0	0	0	0	0	0	80
19508410	Not	md	JP	2021	0	0	0	0	0	0	0	0	0	0	0	0	
19508595	f	md	DE	2021	0	1	1	0	0	0	0	0	0	0	0	0	
19508663	m	md	NL	2021	0	1	1	0	0	0	0	0	0	0	0	0	72
19508887	m	hp	FR	2021	0	0	0	0	0	0	0	0	0	0	0	0	61
19508939	m	cn	Not re	2021	0	0	0	0	0	0	0	0	0	0	0	0	54
19509408	f	md	DE	2021	0	0	0	0	0	0	0	0	0	0	0	0	49
19509473	Not	Not repo	JP	2021	1	0	0	0	0	0	0	0	0	0	0	1	
19509586	m	cn	US	2021	0	1	1	0	0	0	0	0	0	0	0	0	
19509733	f	cn	CN	2021	1	0	0	0	0	0	0	0	0	0	0	0	63
19509737	Not	hp	US	2021	0	1	1	0	0	0	0	0	0	0	0	1	

19509832	f	cn	US	2021	0	1	1	0	0	0	0	0	0	0	0	0	
19510350	f	md	US	2021	0	1	0	0	1	0	0	0	0	0	0	0	66
19510662	Not	cn	SG	2021	1	0	0	0	0	0	0	0	0	0	0	0	68
19510790	f	md	PT	2021	0	1	1	0	0	0	0	0	0	0	0	0	60
19510810	m	md	ES	2021	0	1	0	0	0	1	0	0	0	0	0	0	62
19511238	f	md	ES	2021	0	0	0	0	0	0	0	0	0	0	0	0	76
19511303	m	cn	JP	2021	0	1	1	0	0	0	0	0	0	0	0	0	70
19511398	f	md	JP	2021	1	0	0	0	0	0	0	0	0	0	0	0	80
19511483	m	md	AT	2021	0	1	0	0	0	1	0	0	0	0	0	0	72
19511542	f	md	DE	2021	0	0	0	0	0	0	0	0	0	0	0	0	55
19511706	m	md	DE	2021	0	1	0	0	1	0	0	0	0	0	0	0	58
19512202	Not	md	CN	2021	1	0	0	0	0	0	0	0	0	0	0	0	
19512377	f	hp	AU	2021	0	1	0	0	1	0	0	0	0	0	0	0	75
19512395	m	cn	JP	2021	0	1	1	0	0	0	0	0	0	0	0	0	56
19512480	Not	ph	JP	2021	1	0	0	0	0	0	0	0	0	0	0	0	
19513143	f	cn	US	2021	1	0	0	0	0	0	0	0	0	0	0	0	67.9
19513472	Not	ph	JP	2021	1	0	0	0	0	0	0	0	0	0	0	0	
19513918	f	md	US	2021	0	0	0	0	0	0	0	0	0	0	0	0	72
19514136	f	md	JP	2021	1	0	0	0	0	0	0	0	0	0	0	0	72
19514314	f	hp	US	2021	0	1	0	0	1	0	0	0	0	0	0	0	64
19514525	f	hp	MY	2021	0	0	0	0	0	0	0	0	0	0	0	0	41
19514601	m	hp	KR	2021	0	0	0	0	0	0	0	0	0	0	0	0	73
19514604	m	hp	KR	2021	0	0	0	0	0	0	0	0	0	0	0	0	71
19514617	m	hp	RU	2021	0	1	0	1	0	0	0	0	0	0	0	1	60
19514625	f	hp	DE	2021	0	1	0	0	1	0	0	0	0	0	0	0	80
19514883	f	cn	CA	2021	0	0	0	0	0	0	0	0	0	0	0	0	53
19514950	f	md	CH	2021	0	1	0	0	1	0	0	0	0	0	0	0	70
19515189	m	cn	IN	2021	0	0	0	0	0	0	0	0	0	0	0	0	36
19515417	m	hp	IT	2021	0	1	0	0	0	1	0	0	0	0	0	0	67
19515693	f	cn	Not re	2021	0	0	0	0	0	0	0	0	0	0	0	0	76
19515818	Not	md	US	2021	0	0	0	0	0	0	0	0	0	0	0	0	
19517623	m	md	JP	2021	0	1	0	0	0	1	0	0	0	0	0	0	80
19517649	f	Not repo	IT	2021	0	1	0	1	0	0	0	0	0	0	0	0	58
19517981	Not	md	JP	2021	0	0	0	0	0	0	0	0	0	0	0	0	

19518523	m	cn	JP	2021	0	1	1	0	0	0	0	0	0	0	0	0	70
19518526	f	hp	IN	2021	0	0	0	0	0	0	0	0	0	0	0	0	39
19518528	Not	hp	AT	2021	0	1	1	0	0	0	0	0	0	0	0	0	69
19519069	m	md	JP	2021	0	1	0	0	1	0	0	0	0	0	0	0	65
19519521	m	md	JP	2021	0	1	0	0	1	0	0	0	0	0	0	0	81
19519691	f	md	FR	2021	0	1	0	0	0	1	0	0	0	0	0	0	49
19519900	f	Not repo	MY	2021	1	0	0	0	0	0	0	0	0	0	0	0	
19520061	m	md	JP	2021	1	0	0	0	0	0	0	0	0	0	0	0	90
19520062	f	md	JP	2021	0	1	0	0	0	1	0	0	0	0	0	1	71
19520619	m	md	KR	2021	0	0	0	0	0	0	0	0	0	0	0	0	74
19520645	m	cn	Not re	2021	0	0	0	0	0	0	0	0	0	0	0	0	43
19520647	m	md	JP	2021	0	1	0	0	0	1	0	0	0	0	0	0	73
19520765	Not	md	DE	2021	0	1	0	0	1	0	0	0	0	0	0	0	67
19520769	Not	md	DE	2021	0	1	0	0	1	0	0	0	0	0	0	0	67
19521074	f	md	JP	2021	0	1	0	0	0	1	0	0	0	0	0	0	
19521116	f	hp	DE	2021	0	1	1	0	0	0	0	0	0	0	0	0	65
19521141	m	Not repo	MY	2021	1	0	0	0	0	0	0	0	0	0	0	0	70
19521431	f	cn	JP	2021	0	1	1	0	0	0	0	0	0	0	0	0	70
19521466	m	cn	IN	2021	0	1	0	0	1	0	0	0	0	0	0	0	52
19522040	m	hp	HU	2021	0	0	0	0	0	0	0	0	0	0	0	0	70
19522274	f	md	US	2021	0	1	1	0	0	0	0	0	0	0	0	0	50
19522636	f	md	JP	2021	0	0	0	0	0	0	0	0	0	0	0	0	80
19523819	f	md	DE	2021	0	0	0	0	0	0	0	0	0	0	0	0	
19525163	m	md	JP	2021	0	1	0	0	0	1	0	0	0	0	0	0	73
19526520	Not	md	FR	2021	0	1	0	0	0	1	0	0	0	0	0	0	
19527086	m	md	JP	2021	0	1	0	0	0	1	0	0	0	0	0	0	47
19528163	f	hp	CA	2021	0	1	0	0	0	1	0	0	0	0	0	0	
19528693	f	md	CA	2021	0	0	0	0	0	0	0	0	0	0	0	0	61
19528860	f	cn	IL	2021	0	1	1	0	0	0	0	0	0	0	0	0	
19529129	f	md	CA	2021	1	0	0	0	0	0	0	0	0	0	0	0	
19529334	m	Not repo	ID	2021	1	0	0	0	0	0	0	0	0	0	0	0	63.7
19529991	m	cn	Not re	2021	0	0	0	0	0	0	0	0	0	0	0	0	75
19530026	f	cn	Not re	2021	0	0	0	0	0	0	0	0	0	0	0	0	57
19530061	f	md	JP	2021	0	0	0	0	0	0	0	0	0	0	0	0	72

19530073	m	Not repo	IT	2021	0	1	0	1	0	0	0	0	0	0	0	0	78
19530293	Not	cn	SG	2021	1	0	0	0	0	0	0	0	0	0	0	0	81.8
19530991	m	cn	JP	2021	0	1	1	0	0	0	0	0	0	0	0	0	85
19531222	f	md	DE	2021	0	0	0	0	0	0	0	0	0	0	0	0	61
19533603	f	md	JP	2021	0	0	0	0	0	0	0	0	0	0	0	0	71
19535198	f	md	GB	2021	0	0	0	0	0	0	0	0	0	0	0	0	79
19536701	f	cn	US	2021	0	1	1	0	0	0	0	0	0	0	0	0	74
19537592	Not	md	DE	2021	0	0	0	0	0	0	0	0	0	0	0	0	
19537604	Not	md	DE	2021	0	0	0	0	0	0	0	0	0	0	0	0	
19537607	Not	md	DE	2021	0	0	0	0	0	0	0	0	0	0	0	0	
19537639	Not	md	DE	2021	0	0	0	0	0	0	0	0	0	0	0	0	
19537642	Not	md	DE	2021	0	0	0	0	0	0	0	0	0	0	0	0	
19538031	f	md	US	2021	0	1	1	0	0	0	0	0	0	0	0	0	52
19538901	f	Not repo	IT	2021	1	0	0	0	0	0	0	0	0	0	0	0	71
19540319	m	md	JP	2021	0	1	0	1	0	0	0	0	0	0	0	1	70
19540827	m	md	ES	2021	0	1	0	0	0	1	0	0	0	0	0	0	68
19541253	f	hp	CA	2021	0	1	0	0	1	0	0	0	0	0	0	0	70
19542311	f	md	FR	2021	0	1	0	0	0	1	0	0	0	0	0	0	70
19542517	m	cn	CN	2021	0	1	0	0	1	0	0	0	0	0	0	0	62
19543273	f	ph	JP	2021	1	0	0	0	0	0	0	0	0	0	0	0	83
19543400	f	ph	JP	2021	1	0	0	0	0	0	0	0	0	0	0	0	77
19544123	f	md	GB	2021	0	1	0	0	0	1	0	0	0	0	0	0	75
19544327	m	md	CZ	2021	0	1	1	0	0	0	0	0	0	0	0	0	64
19544511	m	hp	BE	2021	0	0	0	0	0	0	0	0	0	0	0	0	69
19545423	f	hp	FR	2021	1	0	0	0	0	0	0	0	0	0	0	0	65
19546233	f	md	NL	2021	0	1	0	0	0	1	0	0	0	0	0	0	75
19546545	Not	md	JP	2021	0	1	0	0	0	1	0	0	0	0	0	0	80
19546678	Not	md	US	2021	0	0	0	0	0	0	0	0	0	0	0	0	42
19548344	m	Not repo	ID	2021	0	1	0	1	0	0	0	0	0	0	0	0	70.7
19549372	m	Not repo	GB	2021	1	0	0	0	0	0	0	0	0	0	0	0	69
19549829	Not	md	DE	2021	0	0	0	0	0	0	0	0	0	0	0	0	
19551380	m	cn	JP	2021	0	1	1	0	0	0	0	0	0	0	0	0	70
19552126	m	cn	JP	2021	0	1	1	0	0	0	0	0	0	0	0	0	71
19552432	Not	md	US	2021	0	0	0	0	0	0	0	0	0	0	0	0	

19552476	m	md	US	2021	0	0	0	0	0	0	0	0	0	0	0	0	80
19553553	f	cn	Not re	2021	0	0	0	0	0	0	0	0	0	0	0	0	
19554084	f	cn	JP	2021	0	1	1	0	0	0	0	0	0	0	0	0	64
19555643	f	md	JP	2021	0	1	0	1	0	0	0	0	0	0	0	0	74
19556115	m	hp	IL	2021	0	0	0	0	0	0	0	0	0	0	0	0	65
19556435	m	cn	FR	2021	0	1	1	0	0	0	0	0	0	0	0	0	69
19556813	Not	md	IT	2021	0	0	0	0	0	0	0	0	0	0	0	0	
19557349	m	cn	JP	2021	0	1	1	0	0	0	0	0	0	0	0	0	65
19557982	m	cn	FR	2021	0	1	1	0	0	0	0	0	0	0	0	0	76
19559359	m	md	DE	2021	0	1	0	0	1	0	0	0	0	0	0	0	67
19559745	m	md	JP	2021	0	1	0	0	0	1	0	0	0	0	0	0	41
19559942	m	hp	JP	2021	0	1	1	0	0	0	0	0	0	0	0	0	68
19560973	m	cn	CN	2021	0	1	0	0	1	0	0	0	0	0	0	0	59
19561205	m	md	DE	2021	0	0	0	0	0	0	0	0	0	0	0	0	70
19561324	m	cn	US	2021	0	1	1	0	0	0	0	0	0	0	0	0	37
19561445	Not	md	JP	2021	0	1	0	0	0	1	0	0	0	0	0	0	
19561824	m	cn	FR	2021	0	1	1	0	0	0	0	0	0	0	0	0	72
19562058	Not	md	JP	2021	0	1	0	0	0	1	0	0	0	0	0	0	65
19562126	f	Not repo	ID	2021	1	0	0	0	0	0	0	0	0	0	0	0	55.2
19562381	m	md	JP	2021	0	1	0	0	0	1	0	0	0	0	0	0	73
19562509	f	md	DE	2021	1	0	0	0	0	0	0	0	0	0	0	0	61
19562739	f	ph	IS	2021	0	1	1	0	0	0	0	0	0	0	0	1	
19562902	f	hp	US	2021	0	1	0	0	1	0	0	0	0	0	0	0	77
19563181	f	md	GB	2021	0	1	0	0	0	1	0	0	0	0	0	0	71
19563461	f	md	JP	2021	1	0	0	0	0	0	0	0	0	0	0	0	79
19563471	f	Not repo	JP	2021	1	0	0	0	0	0	0	0	0	0	0	0	90
19563473	m	hp	CN	2021	1	0	0	0	0	0	0	0	0	0	0	0	61.4
19563523	f	md	JP	2021	0	0	0	0	0	0	0	0	0	0	0	0	80
19563602	m	cn	DE	2021	0	1	1	0	0	0	0	0	0	0	0	0	55
19563674	f	cn	SG	2021	1	0	0	0	0	0	0	0	0	0	0	0	62.1
19563840	m	cn	US	2021	1	0	0	0	0	0	0	0	0	0	0	0	75
19564736	Not	md	DE	2021	0	1	0	0	0	1	0	0	0	0	0	0	
19564737	m	md	CH	2021	0	1	0	0	0	1	0	0	0	0	0	0	74
19564798	f	cn	US	2021	1	0	0	0	0	0	0	0	0	0	0	0	81.5

19564989	f	md	JP	2021	0	0	0	0	0	0	0	0	0	0	0	0	74
19565180	f	md	CZ	2021	0	1	0	0	1	0	0	0	0	0	0	1	72
19565192	m	hp	CN	2021	0	0	0	0	0	0	0	0	0	0	0	0	72
19565202	m	hp	US	2021	0	1	1	0	0	0	0	0	0	0	0	0	71
19565444	Not	md	JP	2021	0	1	0	0	1	0	0	0	0	0	0	0	
19565517	m	md	JP	2021	0	1	0	0	1	0	0	0	0	0	0	1	60
19565853	Not	md	DE	2021	0	1	0	0	1	0	0	0	0	0	0	0	67
19566053	Not	md	JP	2021	0	1	0	0	0	1	0	0	0	0	0	0	50
19566391	f	cn	Not re	2021	0	0	0	0	0	0	0	0	0	0	0	0	53
19566565	m	cn	CN	2021	0	1	0	0	1	0	0	0	0	0	0	0	70
19566898	m	md	JP	2021	0	0	0	0	0	0	0	0	0	0	0	0	61
19566984	f	hp	AT	2021	0	0	0	0	0	0	0	0	0	0	0	0	64
19566985	m	md	JP	2021	0	1	0	1	0	0	0	0	0	0	0	0	66
19566989	f	cn	US	2021	1	0	0	0	0	0	0	0	0	0	0	0	73.5
19566992	f	md	US	2021	0	1	0	1	0	0	0	0	0	0	0	1	79.8
19567367	f	md	BG	2021	0	1	0	0	0	1	0	0	0	0	0	0	65
19567550	f	md	GB	2021	0	1	1	0	0	0	0	0	0	0	0	0	48
19568398	f	md	US	2021	0	0	0	0	0	0	0	0	0	0	0	0	40
19568626	m	md	IT	2021	0	1	1	0	0	0	0	0	0	0	0	0	78
19568974	m	cn	Not re	2021	0	0	0	0	0	0	0	0	0	0	0	0	64
19569108	f	md	CA	2021	0	1	1	0	0	0	0	0	0	0	0	0	62
19569521	m	hp	FR	2021	0	1	0	0	1	0	0	0	0	0	0	0	70
19569756	m	hp	CN	2021	0	1	1	0	0	0	0	0	0	0	0	0	58
19569787	f	cn	JM	2021	0	1	1	0	0	0	0	0	0	0	0	0	
19569954	m	cn	IN	2021	0	0	0	0	0	0	0	0	0	0	0	0	25
19570372	Not	hp	CN	2021	1	0	0	0	0	0	0	0	0	0	0	0	
19570501	m	cn	FR	2021	0	0	0	0	0	0	0	0	0	0	0	0	44
19570616	f	Not repo	CN	2021	0	1	0	1	0	0	0	0	0	0	0	0	69
19570624	f	md	DE	2021	0	0	0	0	0	0	0	0	0	0	0	0	59
19570694	f	md	CN	2021	0	0	0	0	0	0	0	0	0	0	0	0	63
19570768	m	hp	CZ	2021	0	1	1	0	0	0	0	0	0	0	0	0	59
19570794	m	md	CL	2021	0	1	1	0	0	0	0	0	0	0	0	0	66
19570812	Not	ph	JP	2021	1	0	0	0	0	0	0	0	0	0	0	0	
19570872	m	Not repo	JP	2021	1	0	0	0	0	0	0	0	0	0	0	0	68

19570923	m	md	AT	2021	0	0	0	0	0	0	0	0	0	0	0	0	56
19570987	f	md	ES	2021	0	1	1	0	0	0	0	0	0	0	0	0	71
19570997	Not	hp	US	2021	0	0	0	0	0	0	0	0	0	0	0	0	
19571263	Not	ph	JP	2021	0	1	0	0	1	0	0	0	0	0	0	0	80
19571526	m	md	US	2021	0	0	0	0	0	0	0	0	0	0	0	0	53
19571536	m	Not repo	IT	2021	0	0	0	0	0	0	1	0	0	1	0	0	65
19572148	m	md	JP	2021	0	1	0	0	1	0	0	0	0	0	0	0	70
19572152	f	cn	CH	2021	0	1	1	0	0	0	0	0	0	0	0	0	73
19572632	f	cn	CA	2021	0	0	0	0	0	0	0	0	0	0	0	0	39
19572764	m	md	DE	2021	0	1	1	0	0	0	0	0	0	0	0	0	56
19573196	f	cn	TW	2021	0	0	0	0	0	0	0	0	0	0	0	0	68
19573203	Not	hp	CN	2021	0	0	0	0	0	0	0	0	0	0	0	0	
19573210	Not	md	JP	2021	0	1	0	0	0	1	0	0	0	0	0	0	70
19573220	Not	md	JP	2021	0	1	0	0	0	1	0	0	0	0	0	0	77
19573365	m	md	DE	2021	0	0	0	0	0	0	0	0	0	0	0	0	70
19573366	f	md	DE	2021	0	0	0	0	0	0	0	0	0	0	0	0	38
19574299	m	cn	JP	2021	0	1	1	0	0	0	0	0	0	0	0	0	84
19574341	m	md	JP	2021	0	1	0	0	1	0	0	0	0	0	0	0	70
19574388	m	md	TR	2021	0	1	0	0	0	1	0	0	0	0	0	0	59
19574516	m	cn	JP	2021	0	1	1	0	0	0	0	0	0	0	0	0	71
19574817	m	cn	JP	2021	0	0	0	0	0	0	0	0	0	0	0	0	67
19575398	m	md	JP	2021	0	1	0	0	0	1	0	0	0	0	0	0	71
19575584	f	hp	US	2021	0	1	0	0	1	0	0	0	0	0	0	0	77
19575637	m	md	DE	2021	0	1	1	0	0	0	0	0	0	0	0	0	
19575680	m	cn	Not re	2021	0	0	0	0	0	0	0	0	0	0	0	0	30
19577109	m	md	CN	2021	0	1	1	0	0	0	0	0	0	0	0	0	51
19577647	m	Not repo	MY	2021	0	1	0	1	0	0	0	0	0	0	0	0	72
19578010	f	md	PE	2021	0	0	0	0	0	0	0	0	0	0	0	0	48
19578096	f	md	FR	2021	0	1	0	0	0	1	0	0	0	0	0	0	55
19578128	m	hp	GR	2021	0	1	0	0	1	0	0	0	0	0	0	0	70
19578169	f	md	NP	2021	0	0	0	0	0	0	0	0	0	0	0	0	53
19578227	m	md	JP	2021	0	0	0	0	0	0	0	0	0	0	0	0	69
19578434	Not	md	US	2021	0	0	0	0	0	0	0	0	0	0	0	0	
19579095	m	hp	US	2021	0	1	1	0	0	0	0	0	0	0	0	0	71

19579529	m	md	JP	2021	0	1	0	0	1	0	0	0	0	0	0	0	67
19580446	f	hp	JP	2021	0	1	0	0	1	0	0	0	0	0	0	1	78
19581136	Not	md	JP	2021	0	1	0	0	1	0	0	0	0	0	0	0	
19581731	f	cn	Not re	2021	0	0	0	0	0	0	0	0	0	0	0	0	71
19581831	m	md	CN	2021	1	0	0	0	0	0	0	0	0	0	0	0	49
19582047	m	hp	IT	2021	0	1	0	0	0	1	0	0	0	0	0	0	
19582771	m	md	CN	2021	0	0	0	0	0	0	0	0	0	0	0	0	71
19582926	m	md	JP	2021	1	0	0	0	0	0	0	0	0	0	0	0	
19583060	m	md	HR	2021	0	1	1	0	0	0	0	0	0	0	0	0	59
19583224	m	md	JP	2021	0	1	0	0	1	0	0	0	0	0	0	0	71
19583231	f	md	US	2021	0	0	0	0	0	0	0	0	0	0	0	0	61
19583597	f	ph	JP	2021	0	1	0	0	1	0	0	0	0	0	0	1	81
19583622	f	md	JP	2021	0	1	0	0	0	1	0	0	0	0	0	0	52
19583845	Not	md	CA	2021	0	0	0	0	0	0	0	0	0	0	0	0	
19583938	m	md	ES	2021	0	0	0	0	0	0	0	0	0	0	0	0	83
19583988	m	md	ES	2021	0	1	0	0	0	1	0	0	0	0	0	0	68
19584129	m	Not repo	JP	2021	1	0	0	0	0	0	0	0	0	0	0	1	83
19584220	f	hp	US	2021	0	0	0	0	0	0	0	0	0	0	0	0	67
19584424	Not	hp	SG	2021	1	0	0	0	0	0	0	0	0	0	0	0	57.6
19584547	m	cn	JP	2021	0	1	1	0	0	0	0	0	0	0	0	0	46
19584679	m	md	US	2021	0	0	0	0	0	0	0	0	0	0	0	0	
19584688	f	cn	US	2021	0	1	0	1	0	0	0	0	0	0	0	0	83.6
19584700	f	md	US	2021	0	1	0	0	0	1	0	0	0	0	0	0	71
19584752	f	cn	Not re	2021	0	0	0	0	0	0	0	0	0	0	0	0	
19584757	Not	ph	JP	2021	0	1	0	0	1	0	0	0	0	0	0	0	
19585053	m	hp	TR	2021	0	0	0	0	0	0	0	0	0	0	0	0	74
19585074	f	cn	US	2021	0	1	0	0	0	1	0	0	0	0	0	0	
19585084	m	hp	DE	2021	0	1	1	0	0	0	0	0	0	0	0	0	65
19585088	f	cn	US	2021	0	1	0	0	0	1	0	0	0	0	0	0	
19585106	f	cn	US	2021	0	1	0	0	0	1	0	0	0	0	0	0	
19585299	m	md	JP	2021	0	1	0	0	0	1	0	0	0	0	0	0	60
19585489	m	md	US	2021	0	1	0	0	0	1	0	0	0	0	0	0	
19585509	Not	md	US	2021	0	1	0	0	0	1	0	0	0	0	0	0	
19585520	m	cn	US	2021	0	1	0	0	0	1	0	0	0	0	0	0	

19585525	m	md	US	2021	0	1	0	0	0	1	0	0	0	0	0	0	79
19585536	m	cn	Not re	2021	0	0	0	0	0	0	0	0	0	0	0	0	68
19585572	f	hp	US	2021	0	1	0	0	0	1	0	0	0	0	0	0	52
19585621	f	hp	FR	2021	0	1	0	0	1	0	0	0	0	0	0	0	76
19585658	f	md	JP	2021	0	1	0	0	1	0	0	0	0	0	0	0	66
19585806	f	cn	IN	2021	0	0	0	0	0	0	0	0	0	0	0	0	81
19585859	m	md	CN	2021	0	1	0	0	0	1	0	0	0	0	0	0	63
19586122	Not	md	JP	2021	0	1	0	0	1	0	0	0	0	0	0	0	
19586180	f	cn	US	2021	0	1	0	0	0	1	0	0	0	0	0	0	80
19586355	Not	hp	JP	2021	1	0	0	0	0	0	0	0	0	0	0	0	
19586380	Not	ph	JP	2021	1	0	0	0	0	0	0	0	0	0	0	0	
19586471	m	md	US	2021	0	1	1	0	0	0	0	0	0	0	0	0	62
19586581	Not	md	US	2021	0	0	0	0	0	0	0	0	0	0	0	0	
19586909	m	cn	JP	2021	0	1	1	0	0	0	0	0	0	0	0	0	70
19587538	f	md	RU	2021	0	0	0	0	0	0	0	0	0	0	0	0	82
19587719	m	md	DE	2021	0	1	0	0	0	1	0	0	0	0	0	0	54
19587802	m	md	IT	2021	0	1	1	0	0	0	0	0	0	0	0	0	78
19587847	f	cn	Not re	2021	0	0	0	0	0	0	0	0	0	0	0	0	41
19588382	f	md	US	2021	0	0	0	0	0	0	0	0	0	0	0	0	53
19588466	m	md	JP	2021	0	1	1	0	0	0	0	0	0	0	0	0	79
19588655	f	hp	US	2021	0	1	0	0	1	0	0	0	0	0	0	0	74
19588667	f	Not repo	ID	2021	1	0	0	0	0	0	0	0	0	0	0	0	
19588764	m	md	IQ	2021	0	1	1	0	0	0	0	0	0	0	0	0	80
19588902	f	md	JP	2021	0	0	0	0	0	0	0	0	0	0	0	0	50
19588999	f	md	JP	2021	1	0	0	0	0	0	0	0	0	0	0	0	67
19589007	m	Not repo	CA	2021	1	0	0	0	0	0	0	0	0	0	0	0	68
19589012	Not	md	JP	2021	0	0	0	0	0	0	0	0	0	0	0	0	65
19589090	m	md	ES	2021	0	1	0	0	0	1	0	0	0	0	0	0	62
19589112	f	Not repo	ID	2021	1	0	0	0	0	0	0	0	0	0	0	0	64.8
19589266	Not	cn	JP	2021	0	1	1	0	0	0	0	0	0	0	0	0	70
19589420	m	Not repo	CN	2021	0	1	0	1	0	0	0	0	0	0	0	1	57
19589514	f	md	JP	2021	0	1	0	1	0	0	0	0	0	0	0	0	54
19589523	f	md	DE	2021	1	0	0	0	0	0	0	0	0	0	0	0	
19589646	m	md	TR	2021	0	1	0	0	0	1	0	0	0	0	0	0	67

19590677	f	md	CZ	2021	0	0	0	0	0	0	0	0	0	0	0	0	
19591032	Not	md	US	2021	0	0	0	0	0	0	0	0	0	0	0	0	
19591187	m	md	FR	2021	0	0	0	0	0	0	0	0	0	0	0	0	76
19591227	f	cn	US	2021	1	0	0	0	0	0	0	0	0	0	0	0	
19591410	Not	cn	JP	2021	0	1	1	0	0	0	0	0	0	0	0	0	70
19591428	m	md	US	2021	0	0	0	0	0	0	0	0	0	0	0	0	48
19591449	Not	md	JP	2021	0	1	0	0	1	0	0	0	0	0	0	0	
19591511	m	ph	JP	2021	0	1	0	0	0	1	0	0	0	0	0	0	80
19591910	f	cn	US	2021	1	0	0	0	0	0	0	0	0	0	0	0	
19591964	f	md	BG	2021	1	0	0	0	0	0	0	0	0	0	0	0	
19591966	m	Not repo	IT	2021	0	1	0	1	0	0	0	0	0	0	0	0	70
19593184	Not	hp	DE	2021	0	1	0	0	1	0	0	0	0	0	0	0	67
19593446	m	Not repo	IT	2021	1	0	0	0	0	0	0	0	0	0	0	0	49
19593539	f	md	US	2021	0	1	0	0	1	0	0	0	0	0	0	0	
19593807	m	md	JP	2021	0	1	0	0	0	1	0	0	0	0	0	0	44
19594427	m	md	JP	2021	0	1	0	0	0	1	0	0	0	0	0	0	45
19594633	f	md	JP	2021	0	1	0	0	1	0	0	0	0	0	0	1	51
19594878	m	hp	JP	2021	0	1	1	0	0	0	0	0	0	0	0	0	50
19594900	m	md	DE	2021	0	1	1	0	0	0	0	0	0	0	0	0	
19594966	f	hp	GB	2021	0	1	1	0	0	0	0	0	0	0	0	0	
19594974	f	cn	CA	2021	0	0	0	0	0	0	0	0	0	0	0	0	74
19595034	m	md	JP	2021	0	0	0	0	0	0	0	0	0	0	0	0	59
19595225	m	cn	JP	2021	0	1	1	0	0	0	0	0	0	0	0	0	80
19595379	m	cn	US	2021	0	0	0	0	0	0	0	0	0	0	0	0	
19595747	f	hp	MY	2021	0	0	0	0	0	0	0	0	0	0	0	0	41
19595790	m	cn	JP	2021	0	1	1	0	0	0	0	0	0	0	0	0	
19595942	m	md	JP	2021	0	0	0	0	0	0	0	0	0	0	0	0	79
19596313	m	cn	CN	2021	0	1	0	0	1	0	0	0	0	0	0	0	64
19596750	m	md	KR	2021	0	0	0	0	0	0	0	0	0	0	0	0	60
19596848	m	md	CN	2021	0	0	0	0	0	0	0	0	0	0	0	0	61
19597234	Not	cn	JP	2021	0	1	1	0	0	0	0	0	0	0	0	0	82
19597984	m	Not repo	IT	2021	0	1	0	1	0	0	0	0	0	0	0	0	60
19597988	m	cn	IN	2021	1	0	0	0	0	0	0	0	0	0	0	0	64
19598401	f	md	GB	2021	0	1	0	0	0	1	0	0	0	0	0	0	44

19598510	f	md	DE	2021	0	0	0	0	0	0	0	0	0	0	0	0	55
19598709	m	md	DE	2021	0	1	1	0	0	0	0	0	0	0	0	0	55
19598977	m	hp	US	2021	0	1	0	0	1	0	0	0	0	0	0	0	69
19599313	Not	md	KR	2021	1	0	0	0	0	0	0	0	0	0	0	0	
19599507	m	md	DE	2021	0	1	0	0	1	0	0	0	0	0	0	0	58
19599723	m	ph	US	2021	0	0	0	0	0	0	0	0	0	0	0	0	59
19601412	m	cn	US	2021	0	1	1	0	0	0	0	0	0	0	0	0	61
19602183	m	md	JP	2021	0	1	0	0	0	1	0	0	0	0	0	0	
19602204	Not	md	JP	2021	0	1	0	1	0	0	0	0	0	0	0	0	
19602239	m	md	JP	2021	0	1	0	0	0	1	0	0	0	0	0	0	60
19602387	m	hp	AU	2021	0	1	1	0	0	0	0	0	0	0	0	0	61
19602727	m	md	JP	2021	0	1	1	0	0	0	0	0	0	0	0	0	62
19603009	m	md	DE	2021	0	0	0	0	0	0	0	0	0	0	0	0	54
19603365	m	cn	CN	2021	0	1	0	1	0	0	0	0	0	0	0	1	81
19603470	Not	md	CN	2021	0	1	1	0	0	0	0	0	0	0	0	0	51
19603546	f	Not repo	CN	2021	0	1	0	1	0	0	0	0	0	0	0	0	68
19603555	Not	md	JP	2021	0	1	0	1	0	0	0	0	0	0	0	1	
19603580	m	md	US	2021	0	1	1	0	0	0	0	0	0	0	0	0	71
19603623	f	cn	Not re	2021	0	0	0	0	0	0	0	0	0	0	0	0	66
19604188	f	Not repo	US	2021	1	0	0	0	0	0	0	0	0	0	0	0	60
19604792	Not	md	JP	2021	1	0	0	0	0	0	0	0	0	0	0	0	
19604828	m	md	JP	2021	0	1	0	0	1	0	0	0	0	0	0	0	61
19605083	f	hp	US	2021	0	0	0	0	0	0	0	0	0	0	0	0	
19605084	Not	hp	US	2021	0	0	0	0	0	0	0	0	0	0	0	0	
19605146	f	Not repo	JP	2021	1	0	0	0	0	0	0	0	0	0	0	0	56
19605228	f	cn	CN	2021	1	0	0	0	0	0	0	0	0	0	0	0	63
19605295	f	Not repo	JP	2021	1	0	0	0	0	0	0	0	0	0	0	0	90
19605311	Not	hp	US	2021	0	0	0	0	0	0	0	0	0	0	0	0	
19605359	m	hp	JP	2021	0	1	0	0	1	0	0	0	0	0	0	0	75
19605500	f	md	GB	2021	0	1	0	0	0	1	0	0	0	0	0	0	65
19605529	f	cn	CN	2021	0	1	0	0	1	0	0	0	0	0	0	0	67
19605549	m	md	JP	2021	0	1	0	0	0	1	0	0	0	0	0	0	66
19605571	f	cn	Not re	2021	0	0	0	0	0	0	0	0	0	0	0	0	
19605595	m	ph	FR	2021	0	0	0	0	0	0	0	0	0	0	0	0	57

19605823	m	md	DE	2021	0	1	0	0	1	0	0	0	0	0	0	0	58
19605851	Not	Not repo	JP	2021	1	0	0	0	0	0	0	0	0	0	0	0	
19605969	f	cn	US	2021	0	1	0	0	1	0	0	0	0	0	0	0	62
19605982	m	md	CN	2021	0	1	0	0	0	1	0	0	0	0	0	0	55
19607480	m	hp	JP	2021	0	1	0	0	1	0	0	0	0	0	0	1	68
19607825	m	cn	JP	2021	0	1	1	0	0	0	0	0	0	0	0	1	79
19608153	Not	hp	CN	2021	1	0	0	0	0	0	0	0	0	0	0	0	
19608303	f	hp	NL	2021	0	0	0	0	0	0	0	0	0	0	0	0	69
19608305	f	hp	NL	2021	0	0	0	0	0	0	0	0	0	0	0	0	79
19609144	m	cn	JP	2021	0	1	1	0	0	0	0	0	0	0	0	0	60
19609146	Not	md	JP	2021	0	1	0	1	0	0	0	0	0	0	0	0	
19609597	Not	md	DE	2021	0	1	0	0	1	0	0	0	0	0	0	0	67
19609966	m	cn	CN	2021	0	1	0	0	1	0	0	0	0	0	0	0	
19610101	m	hp	Not re	2021	0	0	0	0	0	0	0	0	0	0	0	0	68
19610288	m	md	DE	2021	0	0	0	0	0	0	0	0	0	0	0	0	
19610530	m	md	JP	2021	0	1	0	0	1	0	0	0	0	0	0	0	56
19610618	m	Not repo	MY	2021	1	0	0	0	0	0	0	0	0	0	0	0	55
19610624	Not	md	JP	2021	0	1	0	0	1	0	0	0	0	0	0	0	
19610771	f	hp	IN	2021	1	0	0	0	0	0	0	0	0	0	0	0	57
19610823	m	cn	DK	2021	0	1	1	0	0	0	0	0	0	0	0	0	71
19610996	m	Not repo	MY	2021	1	0	0	0	0	0	0	0	0	0	0	0	
19611392	Not	md	JP	2021	0	1	0	0	0	1	0	0	0	0	0	0	
19611448	m	md	US	2021	0	0	0	0	0	0	0	0	0	0	0	0	66
19611497	m	md	CN	2021	0	0	0	0	0	0	0	0	0	0	0	0	57
19612036	m	md	FR	2021	0	0	0	0	0	0	0	0	0	0	0	0	81
19612262	m	hp	JP	2021	0	0	0	0	0	0	0	0	0	0	0	0	72
19612989	f	cn	US	2021	0	1	1	0	0	0	0	0	0	0	0	0	
19613099	f	md	US	2021	0	1	1	0	0	0	0	0	0	0	0	0	59
19613928	m	md	RU	2021	0	0	0	0	0	0	0	0	0	0	0	0	71
19613941	f	md	ES	2021	0	0	0	0	0	0	0	0	0	0	0	0	64
19613953	m	hp	IN	2021	1	0	0	0	0	0	0	0	0	0	0	0	70
19614009	m	hp	IN	2021	1	0	0	0	0	0	0	0	0	0	0	0	57
19614169	m	hp	IN	2021	1	0	0	0	0	0	0	0	0	0	0	0	45
19614309	m	md	GR	2021	0	1	1	0	0	0	0	0	0	0	0	0	51

19614662	m	cn	US	2021	1	0	0	0	0	0	0	0	0	0	0	0	78
19614752	m	hp	US	2021	0	1	1	0	0	0	0	0	0	0	0	0	71
19614759	m	md	FR	2021	0	1	0	0	0	1	0	0	0	0	0	0	68
19615487	m	md	FR	2021	0	0	0	0	0	0	0	0	0	0	0	0	50
19615601	m	cn	JP	2021	0	1	1	0	0	0	0	0	0	0	0	0	80
19615758	m	cn	IN	2021	0	0	0	0	0	0	0	0	0	0	0	0	38
19617093	Not	md	JP	2021	0	1	0	0	0	1	0	0	0	0	0	0	
19617686	m	md	CN	2021	0	1	0	0	0	1	0	0	0	0	0	0	71
19617825	m	md	MY	2021	0	0	0	0	0	0	0	0	0	0	0	0	69
19617836	m	cn	US	2021	0	0	0	0	0	0	0	0	0	0	0	0	62
19618105	m	hp	IN	2021	1	0	0	0	0	0	0	0	0	0	0	0	56
19619760	m	cn	CN	2021	0	1	0	0	1	0	0	0	0	0	0	0	72
19619778	Not	hp	PT	2021	1	0	0	0	0	0	0	0	0	0	0	0	
19620303	f	md	DE	2021	0	1	0	0	1	0	0	0	0	0	0	0	57
19620333	f	md	MX	2021	0	1	0	0	0	1	0	0	0	0	0	0	82
19620371	f	cn	SG	2021	0	0	0	0	0	0	0	0	0	0	0	0	
19620707	Not	md	JP	2021	0	1	0	0	1	0	0	0	0	0	0	0	69
19621484	f	cn	US	2021	0	0	0	0	0	0	0	0	0	0	0	0	
19621588	f	hp	IN	2021	0	0	0	0	0	0	0	0	0	0	0	0	74
19621708	f	md	IN	2021	0	0	0	0	0	0	0	0	0	0	0	0	32
19622307	m	md	JP	2021	0	1	0	0	1	0	0	0	0	0	0	0	70
19623544	m	cn	FR	2021	0	0	0	0	0	0	0	0	0	0	0	0	44
19623959	f	cn	Not re	2021	0	0	0	0	0	0	0	0	0	0	0	0	67
19624456	f	cn	CA	2021	1	0	0	0	0	0	0	0	0	0	0	0	
19624474	f	md	FR	2021	0	0	0	0	0	0	0	0	0	0	0	0	48
19624955	f	md	NL	2021	1	0	0	0	0	0	0	0	0	0	0	0	
19625087	m	hp	DE	2021	0	1	0	0	0	1	0	0	0	0	0	0	54
19625098	f	cn	Not re	2021	0	0	0	0	0	0	0	0	0	0	0	0	46
19625134	m	ph	US	2021	0	0	0	0	0	0	0	0	0	0	0	0	
19625141	f	hp	BG	2021	0	1	0	0	0	1	0	0	0	0	0	0	65
19625417	Not	md	US	2021	0	0	0	0	0	0	0	0	0	0	0	0	
19626210	m	hp	NL	2021	0	1	1	0	0	0	0	0	0	0	0	0	72
19626223	m	hp	IT	2021	0	0	0	0	0	0	0	0	0	0	0	0	65
19627147	f	hp	US	2021	1	0	0	0	0	0	0	0	0	0	0	0	65.9

19627307	f	md	DE	2021	1	0	0	0	0	0	0	0	0	0	0	0	78
19627311	m	md	JP	2021	0	1	0	0	1	0	0	0	0	0	0	0	68
19627535	f	md	US	2021	0	0	0	0	0	0	0	0	0	0	0	0	
19627681	m	cn	Not re	2021	0	0	0	0	0	0	0	0	0	0	0	0	54
19627700	m	cn	CN	2021	0	1	0	1	0	0	0	0	0	0	0	0	44
19627761	Not	hp	PT	2021	1	0	0	0	0	0	0	0	0	0	0	0	
19627858	f	md	GB	2021	0	1	0	0	0	1	0	0	0	0	0	0	54
19628072	m	md	US	2021	0	0	0	0	0	0	0	0	0	0	0	0	30
19628073	m	md	FR	2021	0	1	1	0	0	0	0	0	0	0	0	0	69
19628254	f	md	RU	2021	0	1	1	0	0	0	0	0	0	0	0	0	67
19628541	f	md	HU	2021	0	0	0	0	0	0	0	0	0	0	0	0	
19628983	f	md	ES	2021	0	0	0	0	0	0	0	0	0	0	0	0	60
19629648	Not	md	MY	2021	0	1	1	0	0	0	0	0	0	0	0	0	
19629706	f	md	VN	2021	1	0	0	0	0	0	0	0	0	0	0	0	46.6
19630020	m	hp	NL	2021	0	0	0	0	0	0	0	0	0	0	0	0	44
19630171	m	md	JP	2021	0	0	0	0	0	0	0	0	0	0	0	0	59
19630543	Not	hp	NL	2021	0	1	0	0	1	0	0	0	0	0	0	0	67
19630552	f	Not repo	BG	2021	0	1	0	1	0	0	0	0	0	0	0	0	
19630990	m	md	JP	2021	0	1	0	1	0	0	0	0	0	0	0	0	
19632181	m	cn	Not re	2021	0	0	0	0	0	0	0	0	0	0	0	0	67
19632744	m	md	BR	2021	0	0	0	0	0	0	0	0	0	0	0	0	87
19632790	m	md	JP	2021	0	1	0	0	1	0	0	0	0	0	0	0	32
19632931	f	cn	US	2021	1	0	0	0	0	0	0	0	0	0	0	0	76.4
19632957	m	cn	FR	2021	0	1	1	0	0	0	0	0	0	0	0	0	72
19633723	m	md	FR	2021	0	1	0	0	0	1	0	0	0	0	0	0	69
19633787	m	cn	IN	2021	0	0	0	0	0	0	0	0	0	0	0	0	55
19633911	m	cn	SG	2021	1	0	0	0	0	0	0	0	0	0	0	0	70.5
19634353	f	hp	US	2021	0	1	0	0	1	0	0	0	0	0	0	0	77
19634545	f	md	GB	2021	0	1	0	0	0	1	0	0	0	0	0	0	54
19635088	f	md	JP	2021	0	1	0	0	0	1	0	0	0	0	0	0	70
19635253	f	md	FR	2021	0	1	0	0	0	1	0	0	0	0	0	0	55
19635518	Not	hp	SG	2021	1	0	0	0	0	0	0	0	0	0	0	0	65
19635544	m	hp	DE	2021	0	1	0	0	1	0	0	0	0	0	0	0	67
19635771	f	md	JP	2021	0	0	0	0	0	0	0	0	0	0	0	0	50

19636029	f	md	FR	2021	0	1	0	0	0	1	0	0	0	0	0	1	74
19636691	Not	Not repo	SE	2021	1	0	0	0	0	0	0	0	0	0	0	0	
19636822	m	cn	IN	2021	0	0	0	0	0	0	0	0	0	0	0	0	52
19636904	m	cn	CN	2021	0	1	0	1	0	0	0	0	0	0	0	0	66
19637041	Not	cn	SG	2021	1	0	0	0	0	0	0	0	0	0	0	0	76.4
19637053	m	cn	Not re	2021	0	0	0	0	0	0	0	0	0	0	0	0	51
19637102	m	ph	JP	2021	0	1	0	0	1	0	0	0	0	0	0	0	59
19637288	Not	hp	SG	2021	1	0	0	0	0	0	0	0	0	0	0	0	47.7
19637899	Not	hp	SG	2021	1	0	0	0	0	0	0	0	0	0	0	0	79.1
19638672	f	md	NL	2021	0	0	0	0	0	0	0	0	0	0	0	0	57
19639309	m	hp	VN	2021	0	1	0	1	0	0	0	0	0	0	0	0	
19639783	m	cn	Not re	2021	0	0	0	0	0	0	0	0	0	0	0	0	62
19640048	m	md	FR	2021	0	1	1	0	0	0	0	0	0	0	0	0	72
19640552	m	hp	BR	2021	0	0	0	0	0	0	0	0	0	0	0	0	71
19640573	m	cn	JP	2021	0	1	1	0	0	0	0	0	0	0	0	1	70
19640743	m	md	JP	2021	0	1	0	0	1	0	0	0	0	0	0	0	79
19640812	m	hp	BR	2021	0	0	0	0	0	0	0	0	0	0	0	0	63
19640960	m	hp	BR	2021	0	0	0	0	0	0	0	0	0	0	0	0	70
19640988	m	hp	BR	2021	0	0	0	0	0	0	0	0	0	0	0	0	72
19641172	Not	cn	SG	2021	1	0	0	0	0	0	0	0	0	0	0	0	61.8
19641515	Not	md	JP	2021	0	1	0	0	0	1	0	0	0	0	0	1	
19641746	f	md	KR	2021	0	0	0	0	0	0	0	0	0	0	0	0	79
19641772	Not	md	SG	2021	1	0	0	0	0	0	0	0	0	0	0	0	79
19641773	m	Not repo	ID	2021	1	0	0	0	0	0	0	0	0	0	0	0	62.3
19641926	m	hp	US	2021	0	0	0	0	0	0	0	0	0	0	0	0	44
19642651	f	Not repo	MY	2021	1	0	0	0	0	0	0	0	0	0	0	0	73.7
19642859	f	hp	BR	2021	0	0	0	0	0	0	0	0	0	0	0	0	61
19642925	f	hp	US	2021	0	0	0	0	0	0	0	0	0	0	0	0	
19643050	f	md	IL	2021	0	0	0	0	0	0	0	0	0	0	0	0	59
19643074	m	md	IL	2021	0	1	0	1	0	0	0	0	0	0	0	0	74
19643489	f	md	DE	2021	0	0	0	0	0	0	0	0	0	0	0	0	
19644554	f	hp	ES	2021	0	0	0	0	0	0	0	0	0	0	0	0	64
19644571	m	md	US	2021	0	1	1	0	0	0	0	0	0	0	0	0	74
19645267	f	hp	US	2021	0	1	0	0	1	0	0	0	0	0	0	0	81

19645276	f	md	ES	2021	0	1	0	0	0	1	0	0	0	0	0	0	58
19645287	Not	md	IT	2021	0	0	0	0	0	0	0	0	0	0	0	0	
19645652	f	md	KR	2021	0	0	0	0	0	0	0	0	0	0	0	0	60
19645693	f	md	CA	2021	0	1	0	0	0	1	0	0	0	0	0	0	66
19645956	m	md	LB	2021	0	1	0	0	1	0	0	0	0	0	0	0	
19645964	m	md	JP	2021	0	1	0	0	1	0	0	0	0	0	0	0	60
19646354	m	md	FR	2021	0	0	0	0	0	0	0	0	0	0	0	0	44
19646675	m	hp	CN	2021	0	1	1	0	0	0	0	0	0	0	0	0	55
19646899	f	md	KR	2021	0	0	0	0	0	0	0	0	0	0	0	0	
19646915	m	cn	FR	2021	0	1	1	0	0	0	0	0	0	0	0	0	72
19646989	f	cn	CN	2021	0	0	0	0	0	0	0	0	0	0	0	0	64
19647015	m	cn	US	2021	1	0	0	0	0	0	0	0	0	0	0	0	87
19647019	f	cn	US	2021	1	0	0	0	0	0	0	0	0	0	0	0	62.2
19647091	m	hp	US	2021	0	1	1	0	0	0	0	0	0	0	0	0	68
19647195	f	cn	US	2021	0	0	0	0	0	0	0	0	0	0	0	0	
19647637	f	hp	TR	2021	0	0	0	0	0	0	0	0	0	0	0	0	62
19647659	f	cn	IN	2021	0	0	0	0	0	0	0	0	0	0	0	0	58
19647661	m	hp	TR	2021	0	0	0	0	0	0	0	0	0	0	0	0	71
19647730	Not	hp	JP	2021	0	1	0	0	1	0	0	0	0	0	0	0	
19647731	Not	hp	JP	2021	0	1	0	0	1	0	0	0	0	0	0	1	
19647733	Not	hp	JP	2021	0	1	0	0	1	0	0	0	0	0	0	0	
19647734	Not	hp	JP	2021	0	1	0	0	1	0	0	0	0	0	0	0	
19647846	f	md	US	2021	0	0	0	0	0	0	0	0	0	0	0	0	
19647869	m	hp	US	2021	0	1	0	0	1	0	0	0	0	0	0	0	68
19647943	m	md	PL	2021	0	1	1	0	0	0	0	0	0	0	0	0	60
19648060	m	hp	US	2021	1	0	0	0	0	0	0	0	0	0	0	0	
19648688	f	md	FR	2021	0	0	0	0	0	0	0	0	0	0	0	0	56
19648713	f	md	JP	2021	0	1	0	0	0	1	0	0	0	0	0	1	70
19649029	f	cn	US	2021	1	0	0	0	0	0	0	0	0	0	0	0	
19649053	m	md	US	2021	0	1	1	0	0	0	0	0	0	0	0	1	59
19649265	m	cn	CN	2021	0	1	0	0	1	0	0	0	0	0	0	0	64
19649396	m	hp	GB	2021	0	0	0	0	0	0	0	0	0	0	0	0	68
19649832	m	cn	US	2021	0	1	1	0	0	0	0	0	0	0	0	0	
19650295	Not	md	DE	2021	0	1	0	0	1	0	0	0	0	0	0	0	67

19650439	f	md	TW	2021	0	0	0	0	0	0	0	0	0	0	0	0	67
19650507	Not	hp	JP	2021	0	0	0	0	0	0	0	0	0	0	0	0	
19650540	Not	hp	JP	2021	0	1	0	0	1	0	0	0	0	0	0	0	
19650542	Not	hp	JP	2021	0	0	0	0	0	0	0	0	0	0	0	0	
19650544	Not	hp	JP	2021	0	0	0	0	0	0	0	0	0	0	0	0	
19650752	f	md	JP	2021	0	1	0	0	1	0	0	0	0	0	0	0	80
19650847	f	md	CN	2021	0	1	1	0	0	0	0	0	0	0	0	0	67
19651263	m	Not repo	ID	2021	1	0	0	0	0	0	0	0	0	0	0	0	72.5
19651407	f	md	NL	2021	1	0	0	0	0	0	0	0	0	0	0	0	
19651545	f	cn	US	2021	1	0	0	0	0	0	0	0	0	0	0	0	70.1
19651637	m	md	CN	2021	0	0	0	0	0	0	0	0	0	0	0	0	70
19651684	Not	hp	JP	2021	0	0	0	0	0	0	0	0	0	0	0	0	
19652611	f	cn	US	2021	0	0	0	0	0	0	0	0	0	0	0	0	68
19652873	Not	hp	US	2021	1	0	0	0	0	0	0	0	0	0	0	0	49.9
19652959	m	cn	JP	2021	0	1	1	0	0	0	0	0	0	0	0	0	90
19653098	f	md	ES	2021	0	0	0	0	0	0	0	0	0	0	0	0	54
19653227	f	Not repo	US	2021	1	0	0	0	0	0	0	0	0	0	0	0	77
19653232	m	Not repo	ID	2021	1	0	0	0	0	0	0	0	0	0	0	0	61.3
19653240	f	md	GB	2021	0	1	0	0	0	1	0	0	0	0	0	0	71
19653250	m	cn	CN	2021	0	1	0	0	1	0	0	0	0	0	0	0	73
19653289	f	md	CN	2021	0	0	0	0	0	0	0	0	0	0	0	0	65
19653377	f	md	US	2021	0	0	0	0	0	0	0	0	0	0	0	0	
19653487	m	cn	IN	2021	0	0	0	0	0	0	0	0	0	0	0	0	46
19653562	m	cn	FR	2021	0	1	1	0	0	0	0	0	0	0	0	0	49
19653673	Not	md	JP	2021	0	0	0	0	0	0	0	0	0	0	0	0	
19653791	Not	cn	JP	2021	0	1	1	0	0	0	0	0	0	0	0	1	83
19653902	Not	md	US	2021	0	0	0	0	0	0	0	0	0	0	0	0	
19654740	m	cn	JP	2021	0	1	1	0	0	0	0	0	0	0	0	0	50
19654804	m	cn	JP	2021	0	1	1	0	0	0	0	0	0	0	0	0	74
19655296	f	md	CN	2021	1	0	0	0	0	0	0	0	0	0	0	1	48
19655939	Not	hp	US	2021	0	0	0	0	0	0	0	0	0	0	0	0	
19656199	f	md	GB	2021	0	1	0	0	0	1	0	0	0	0	0	0	71
19656241	m	md	PT	2021	0	1	1	0	0	0	0	0	0	0	0	0	61
19656326	m	md	PT	2021	0	1	1	0	0	0	0	0	0	0	0	0	

19656327	Not	hp	GB	2021	0	1	0	0	1	0	0	0	0	0	0	0	
19656377	m	ph	CA	2021	0	0	0	0	0	0	0	0	0	0	0	0	76
19656409	Not	md	JP	2021	1	0	0	0	0	0	0	0	0	0	0	0	
19656457	Not	md	JP	2021	0	1	0	1	0	0	0	0	0	0	0	0	
19656459	Not	Not repo	AU	2021	1	0	0	0	0	0	0	0	0	0	0	0	
19656933	m	cn	US	2021	1	0	0	0	0	0	0	0	0	0	0	0	
19656979	f	md	US	2021	0	0	0	0	0	0	0	0	0	0	0	0	72
19656995	m	md	JP	2021	0	1	0	0	0	1	0	0	0	0	0	0	73
19657094	m	cn	FR	2021	0	0	0	0	0	0	0	0	0	0	0	0	44
19657232	f	md	GB	2021	0	0	0	0	0	0	1	0	0	0	1	0	66
19657306	f	cn	Not re	2021	0	0	0	0	0	0	0	0	0	0	0	0	69
19657580	f	md	US	2021	0	0	0	0	0	0	0	0	0	0	0	0	
19657805	m	md	JP	2021	0	1	0	0	1	0	0	0	0	0	0	0	76
19657830	m	cn	Not re	2021	0	0	0	0	0	0	0	0	0	0	0	0	
19657854	m	md	US	2021	0	0	0	0	0	0	0	0	0	0	0	0	
19657873	f	md	JP	2021	0	1	0	0	0	1	0	0	0	0	0	0	60
19658032	m	md	ES	2021	0	0	0	0	0	0	0	0	0	0	0	0	78
19658037	m	md	JP	2021	0	1	0	0	0	1	0	0	0	0	0	0	76
19658071	Not	ph	JP	2021	0	1	0	0	1	0	0	0	0	0	0	0	
19658414	m	Not repo	CN	2021	0	1	0	1	0	0	0	0	0	0	0	1	58
19658415	Not	cn	SG	2021	1	0	0	0	0	0	0	0	0	0	0	0	
19658517	m	Not repo	US	2021	0	1	0	1	0	0	0	0	0	0	0	0	50
19658539	f	md	JP	2021	0	1	0	0	1	0	0	0	0	0	0	0	80
19658567	m	md	US	2021	0	0	0	0	0	0	0	0	0	0	0	0	
19658774	m	md	CN	2021	0	1	1	0	0	0	0	0	0	0	0	0	51
19659479	m	md	JP	2021	0	1	1	0	0	0	0	0	0	0	0	0	72
19659501	m	cn	JP	2021	0	1	1	0	0	0	0	0	0	0	0	0	60
19660074	Not	hp	CN	2021	0	0	0	0	0	0	0	0	0	0	0	0	
19660318	f	md	GB	2021	0	0	0	0	0	0	0	0	0	0	0	0	66
19660324	Not	cn	US	2021	1	0	0	0	0	0	0	0	0	0	0	0	
19660521	Not	md	JP	2021	1	0	0	0	0	0	0	0	0	0	0	0	
19660920	f	cn	CN	2021	0	1	0	0	1	0	0	0	0	0	0	0	74
19660933	m	md	JP	2021	0	1	0	0	1	0	0	0	0	0	0	0	77
19660943	m	md	JP	2021	0	1	0	1	0	0	0	0	0	0	0	1	75

19661131	m	md	JP	2021	0	1	0	1	0	0	0	0	0	0	0	1	72
19661204	f	md	JP	2021	0	0	0	0	0	0	0	0	0	0	0	0	67
19661239	m	md	JP	2021	0	1	0	0	1	0	0	0	0	0	0	0	70
19661327	m	Not repo	JP	2021	0	1	0	1	0	0	0	0	0	0	0	0	44
19661328	m	hp	RU	2021	0	1	0	1	0	0	0	0	0	0	0	0	50
19661333	m	ph	US	2021	1	0	0	0	0	0	0	0	0	0	0	0	
19661334	f	hp	GB	2021	0	1	1	0	0	0	0	0	0	0	0	0	73
19661364	f	md	BE	2021	0	0	0	0	0	0	0	0	0	0	0	0	70
19661648	f	cn	US	2021	0	0	0	0	0	0	0	0	0	0	0	0	
19661661	m	ph	JP	2021	0	1	0	1	0	0	0	0	0	0	0	0	
19661835	m	md	KR	2021	0	1	0	0	0	1	0	0	0	0	0	0	49
19661864	m	md	GB	2021	0	1	1	0	0	0	0	0	0	0	0	0	67
19661964	f	lw	US	2021	0	0	0	0	0	0	0	0	0	0	0	0	64
19661973	Not	md	JP	2021	0	1	0	1	0	0	0	0	0	0	0	1	
19662200	m	hp	US	2021	0	0	0	0	0	0	0	0	0	0	0	0	86
19662212	Not	Not repo	JP	2021	0	1	0	1	0	0	0	0	0	0	0	1	
19662413	m	cn	JP	2021	0	1	1	0	0	0	0	0	0	0	0	1	77
19662571	Not	md	FR	2021	0	0	0	0	0	0	0	0	0	0	0	0	
19662939	f	md	PT	2021	0	0	0	0	0	0	0	0	0	0	0	0	51
19662955	f	hp	GB	2021	0	0	0	0	0	0	0	0	0	0	0	0	71
19663229	f	md	SK	2021	1	0	0	0	0	0	0	0	0	0	0	0	76
19663273	m	ph	FR	2021	0	0	0	0	0	0	0	0	0	0	0	0	55
19663513	f	cn	US	2021	1	0	0	0	0	0	0	0	0	0	0	0	
19663539	Not	Not repo	SE	2021	1	0	0	0	0	0	0	0	0	0	0	0	
19665146	f	md	JP	2021	0	0	0	0	0	0	0	0	0	0	0	0	76
19665248	f	md	US	2021	0	0	0	0	0	0	0	0	0	0	0	0	
19665683	m	md	CZ	2021	1	0	0	0	0	0	0	0	0	0	0	0	50
19665773	f	cn	DE	2021	0	1	1	0	0	0	0	0	0	0	0	0	73
19666336	m	md	JP	2021	0	1	0	0	1	0	0	0	0	0	0	0	60
19666440	Not	Not repo	JP	2021	0	1	0	1	0	0	0	0	0	0	0	1	
19666553	f	ph	TW	2021	1	0	0	0	0	0	0	0	0	0	0	0	59
19666570	m	cn	Not re	2021	0	0	0	0	0	0	0	0	0	0	0	0	59
19666576	m	md	JP	2021	0	1	0	0	1	0	0	0	0	0	0	0	80
19666788	f	hp	CN	2021	1	0	0	0	0	0	0	0	0	0	0	0	62

19666809	m	hp	BG	2021	0	0	0	0	0	0	0	0	0	0	0	0	73
19666856	f	ph	US	2021	0	0	0	0	0	0	0	0	0	0	0	0	83
19666934	f	md	JP	2021	0	0	0	0	0	0	0	0	0	0	0	0	80
19667086	m	hp	CN	2021	0	0	0	0	0	0	0	0	0	0	0	0	55
19667123	Not	md	JP	2021	0	1	0	1	0	0	0	0	0	0	0	1	
19667329	m	hp	GB	2021	0	0	0	0	0	0	0	0	0	0	0	0	68
19667400	Not	Not repo	NZ	2021	1	0	0	0	0	0	0	0	0	0	0	0	
19667805	m	hp	GB	2021	0	0	0	0	0	0	0	0	0	0	0	0	68
19668358	f	cn	US	2021	1	0	0	0	0	0	0	0	0	0	0	0	64.9
19668468	Not	md	JP	2021	0	1	0	1	0	0	0	0	0	0	0	0	
19668630	m	cn	ID	2021	0	1	1	0	0	0	0	0	0	0	0	0	55
19668654	m	Not repo	JP	2021	0	1	0	1	0	0	0	0	0	0	0	1	76
19668706	f	hp	US	2021	1	0	0	0	0	0	0	0	0	0	0	1	67.7
19668757	m	Not repo	ID	2021	1	0	0	0	0	0	0	0	0	0	0	0	79.7
19668835	Not	Not repo	SE	2021	1	0	0	0	0	0	0	0	0	0	0	0	
19669126	f	hp	FR	2021	0	1	0	0	1	0	0	0	0	0	0	0	
19669173	m	md	KR	2021	0	0	0	0	0	0	0	0	0	0	0	0	78
19669687	m	md	JP	2021	1	0	0	0	0	0	0	0	0	0	0	1	87
19669726	Not	hp	JP	2021	0	0	0	0	0	0	0	0	0	0	0	0	
19670071	m	cn	CN	2021	0	1	0	0	1	0	0	0	0	0	0	0	57
19670072	m	hp	ES	2021	0	1	0	0	1	0	0	0	0	0	0	0	61
19670222	f	Not repo	US	2021	1	0	0	0	0	0	0	0	0	0	0	0	88
19670223	Not	cn	US	2021	1	0	0	0	0	0	0	0	0	0	0	0	
19670446	f	md	CN	2021	0	1	0	0	0	1	0	0	0	0	0	0	58
19670458	f	cn	LB	2021	1	0	0	0	0	0	0	0	0	0	0	0	63
19670464	m	md	AT	2021	0	0	0	0	0	0	0	0	0	0	0	0	65
19670471	f	md	DE	2021	0	0	0	0	0	0	0	0	0	0	0	0	70
19670787	m	Not repo	JP	2021	1	0	0	0	0	0	0	0	0	0	0	0	57
19670807	m	md	US	2021	0	1	1	0	0	0	0	0	0	0	0	0	71
19670846	f	cn	SG	2021	1	0	0	0	0	0	0	0	0	0	0	0	
19671063	m	Not repo	CN	2021	0	1	0	1	0	0	0	0	0	0	0	0	44
19671314	f	md	CA	2021	0	1	0	0	0	1	0	0	0	0	0	0	68
19672437	m	cn	JP	2021	0	1	1	0	0	0	0	0	0	0	0	1	75
19672897	Not	hp	US	2021	0	0	0	0	0	0	0	0	0	0	0	0	

19673216	f	md	GB	2021	0	1	0	0	0	1	0	0	0	0	0	0	71
19673753	f	lw	US	2021	0	0	0	0	0	0	0	0	0	0	0	0	45
19673895	f	lw	US	2021	0	0	0	0	0	0	0	0	0	0	0	0	54
19673933	m	cn	JP	2021	0	1	1	0	0	0	0	0	0	0	0	0	73
19674111	f	md	AT	2021	0	0	0	0	0	0	1	0	0	0	1	0	50
19674193	m	md	CN	2021	0	0	0	0	0	0	0	0	0	0	0	0	63
19674345	m	cn	JP	2021	0	1	1	0	0	0	0	0	0	0	0	0	66
19674556	m	cn	CN	2021	0	1	0	0	1	0	0	0	0	0	0	0	59
19675035	m	hp	GB	2021	0	1	0	0	0	1	0	0	0	0	0	0	42
19675051	f	cn	US	2021	0	0	0	0	0	0	0	0	0	0	0	0	
19675076	f	cn	US	2021	0	0	0	0	0	0	0	0	0	0	0	0	
19675077	Not	hp	US	2021	0	0	0	0	0	0	0	0	0	0	0	0	
19675092	m	ph	US	2021	0	0	0	0	0	0	0	0	0	0	0	0	41
19675221	m	md	NL	2021	0	1	0	0	0	1	0	0	0	0	0	0	58
19675321	m	md	JP	2021	0	1	0	0	1	0	0	0	0	0	0	0	60
19675436	m	hp	GB	2021	0	0	0	0	0	0	0	0	0	0	0	0	73
19676061	f	hp	DE	2021	0	1	1	0	0	0	0	0	0	0	0	0	
19676430	Not	hp	JP	2021	0	0	0	0	0	0	0	0	0	0	0	0	
19677057	m	hp	GB	2021	0	0	0	0	0	0	0	0	0	0	0	0	68
19677334	m	hp	US	2021	0	1	0	0	1	0	0	0	0	0	0	0	77
19677414	m	md	DE	2021	0	0	0	0	0	0	0	0	0	0	0	0	61
19677964	Not	md	GB	2021	0	0	0	0	0	0	0	0	0	0	0	0	
19677978	f	md	GB	2021	0	0	0	0	0	0	0	0	0	0	0	0	51
19677980	f	cn	AR	2021	0	0	0	0	0	0	0	0	0	0	0	0	65
19678004	f	md	FR	2021	0	1	0	0	0	1	0	0	0	0	0	0	76
19678156	m	cn	SG	2021	0	0	0	0	0	0	0	0	0	0	0	0	
19678247	m	Not repo	JP	2021	0	1	0	1	0	0	0	0	0	0	0	1	64
19678413	f	cn	CN	2021	0	1	0	0	1	0	0	0	0	0	0	0	44
19678532	Not	hp	ES	2021	0	0	0	0	0	0	0	0	0	0	0	0	
19678751	m	md	JP	2021	0	1	0	0	0	1	0	0	0	0	0	0	56
19679232	f	md	JP	2021	1	0	0	0	0	0	0	0	0	0	0	0	83
19680138	m	hp	TH	2021	0	0	0	0	0	0	0	0	0	0	0	0	89
19680276	m	md	CA	2021	0	1	0	0	1	0	0	0	0	0	0	0	
19680424	f	cn	US	2021	0	1	0	1	0	0	0	0	0	0	0	0	57

19680711	m	md	CN	2021	0	1	0	1	0	0	0	0	0	0	0	1	56
19680774	f	hp	US	2021	0	0	0	0	0	0	0	0	0	0	0	0	62
19680882	m	cn	IN	2021	0	0	0	0	0	0	0	0	0	0	0	0	72
19681177	Not	ph	JP	2021	0	1	0	1	0	0	0	0	0	0	0	0	
19681199	f	md	CA	2021	0	0	0	0	0	0	0	0	0	0	0	0	84
19681510	f	md	PL	2021	0	1	0	1	0	0	0	0	0	0	0	0	
19681673	Not	md	JP	2021	1	0	0	0	0	0	0	0	0	0	0	0	
19681791	Not	hp	JP	2021	0	0	0	0	0	0	0	0	0	0	0	0	
19681822	m	md	FR	2021	0	1	0	0	0	1	0	0	0	0	0	0	59
19681871	Not	hp	FR	2021	0	0	0	0	0	0	0	0	0	0	0	0	
19682031	f	md	KR	2021	0	0	0	0	0	0	0	0	0	0	0	0	38
19682162	f	md	FR	2021	0	1	1	0	0	0	0	0	0	0	0	0	60
19682198	m	ph	JP	2021	0	0	0	0	0	0	0	0	0	0	0	0	73
19682212	Not	hp	US	2021	0	1	0	0	1	0	0	0	0	0	0	0	
19682223	Not	hp	US	2021	0	1	0	0	1	0	0	0	0	0	0	0	
19682398	f	md	JP	2021	0	1	0	0	0	1	0	0	0	0	0	1	83
19682473	m	md	JP	2021	0	1	0	0	0	1	0	0	0	0	0	1	
19682527	Not	md	JP	2021	0	1	0	0	0	1	0	0	0	0	0	0	80
19682749	f	cn	CA	2021	0	0	0	0	0	0	0	0	0	0	0	0	64
19682839	m	hp	US	2021	0	0	0	0	0	0	0	0	0	0	0	0	
19682901	Not	hp	US	2021	0	1	0	0	1	0	0	0	0	0	0	0	
19682909	Not	hp	US	2021	0	1	0	0	1	0	0	0	0	0	0	0	
19682911	Not	hp	US	2021	0	1	0	0	1	0	0	0	0	0	0	0	
19682920	Not	hp	US	2021	0	1	0	0	1	0	0	0	0	0	0	0	
19682922	m	cn	CN	2021	0	1	0	0	1	0	0	0	0	0	0	0	58
19682961	Not	hp	US	2021	0	1	0	0	1	0	0	0	0	0	0	0	
19683378	m	cn	CN	2021	0	1	0	0	1	0	0	0	0	0	0	0	64
19683687	f	ph	JP	2021	0	0	0	0	0	0	0	0	0	0	0	0	47
19683825	Not	hp	DE	2021	0	0	0	0	0	0	1	0	0	0	1	0	
19683934	m	md	JP	2021	0	1	0	0	1	0	0	0	0	0	0	0	70
19683946	f	ph	JP	2021	0	0	0	0	0	0	0	0	0	0	0	0	80
19684263	m	hp	DE	2021	0	1	0	0	1	0	0	0	0	0	0	1	64
19684458	m	hp	JP	2021	0	1	0	0	1	0	0	0	0	0	0	0	62
19684491	Not	hp	US	2021	0	1	0	0	1	0	0	0	0	0	0	0	

19684493	Not	hp	US	2021	0	1	0	0	1	0	0	0	0	0	0	0	
19684714	m	md	DE	2021	0	0	0	0	0	0	0	0	0	0	0	0	66
19684932	m	md	JP	2021	0	1	0	0	1	0	0	0	0	0	0	0	69
19685219	m	cn	LB	2021	1	0	0	0	0	0	0	0	0	0	0	0	72
19685220	m	Not repo	JP	2021	1	0	0	0	0	0	0	0	0	0	0	0	70
19685251	f	md	BE	2021	0	0	0	0	0	0	0	0	0	0	0	0	73
19685320	m	md	US	2021	0	0	0	0	0	0	0	0	0	0	0	0	64
19685606	m	hp	GB	2021	0	0	0	0	0	0	0	0	0	0	0	0	68
19685688	m	md	JP	2021	0	1	0	0	0	1	0	0	0	0	0	0	69
19685877	f	md	NL	2021	0	0	0	0	0	0	0	0	0	0	0	0	74
19686438	m	md	RU	2021	0	0	0	0	0	0	0	0	0	0	0	0	74
19686695	m	hp	DE	2021	0	0	0	0	0	0	0	0	0	0	0	0	71
19686923	m	Not repo	CN	2021	0	1	0	1	0	0	0	0	0	0	0	0	51
19687179	f	md	CN	2021	0	1	1	0	0	0	0	0	0	0	0	0	67
19687289	m	hp	BE	2021	0	1	0	0	1	0	0	0	0	0	0	0	66
19687454	f	Not repo	LB	2021	0	1	0	1	0	0	0	0	0	0	0	0	50
19687960	f	ph	JP	2021	0	0	0	0	0	0	0	0	0	0	0	0	42
19688954	m	cn	TW	2021	0	0	0	0	0	0	0	0	0	0	0	0	49
19688999	m	hp	IT	2021	0	0	0	0	0	0	0	0	0	0	0	0	58
19689083	f	cn	US	2021	0	0	0	0	0	0	0	0	0	0	0	0	52
19689288	m	md	JP	2021	0	1	0	0	1	0	0	0	0	0	0	1	70
19689341	f	ph	JP	2021	0	0	0	0	0	0	0	0	0	0	0	0	70
19689561	f	hp	GB	2021	0	0	0	0	0	0	0	0	0	0	0	0	78
19690284	m	hp	NL	2021	0	0	0	0	0	0	0	0	0	0	0	0	76
19690445	f	md	US	2021	0	0	0	0	0	0	0	0	0	0	0	0	59
19690471	f	cn	IN	2021	0	0	0	0	0	0	0	0	0	0	0	0	48
19690475	f	md	TR	2021	0	0	0	0	0	0	0	0	0	0	0	0	60
19690477	f	md	FR	2021	0	0	0	0	0	0	0	0	0	0	0	0	45
19690781	f	cn	US	2021	1	0	0	0	0	0	0	0	0	0	0	0	
19691673	m	cn	JP	2021	0	1	1	0	0	0	0	0	0	0	0	0	76
19691780	m	Not repo	CN	2021	0	1	0	1	0	0	0	0	0	0	0	0	63
19691906	Not	hp	US	2021	0	1	0	0	0	1	0	0	0	0	0	0	
19691930	f	md	PL	2021	0	0	0	0	0	0	0	0	0	0	0	0	74
19692007	m	md	JP	2021	0	0	0	0	0	0	0	0	0	0	0	0	79

19692274	m	cn	CN	2021	0	1	0	0	1	0	0	0	0	0	0	0	66
19692275	m	cn	CN	2021	0	1	0	0	1	0	0	0	0	0	0	0	62
19692282	Not	md	PL	2021	0	1	0	0	0	1	0	0	0	0	0	0	
19692526	Not	hp	US	2021	0	1	0	0	0	1	0	0	0	0	0	0	
19692883	m	md	US	2021	0	0	0	0	0	0	0	0	0	0	0	0	
19693023	f	md	US	2021	0	0	0	0	0	0	0	0	0	0	0	0	
19695128	f	cn	US	2021	0	0	0	0	0	0	0	0	0	0	0	0	
19695318	m	md	FR	2021	0	0	0	0	0	0	0	0	0	0	0	0	75
19695335	m	md	CN	2021	0	0	0	0	0	0	0	0	0	0	0	0	57
19695376	m	hp	FR	2021	0	1	0	0	1	0	0	0	0	0	0	0	70
19696759	m	md	IL	2021	0	1	1	0	0	0	0	0	0	0	0	0	76
19696772	f	hp	GB	2021	0	1	1	0	0	0	0	0	0	0	0	0	73
19697141	f	md	JP	2021	0	0	0	0	0	0	0	0	0	0	0	0	72
19697964	m	md	JP	2021	0	1	0	0	0	1	0	0	0	0	0	0	70
19698153	m	Not repo	TW	2021	1	0	0	0	0	0	0	0	0	0	0	0	69.9
19698401	m	cn	JP	2021	0	1	1	0	0	0	0	0	0	0	0	0	75
19699061	Not	md	JP	2021	0	1	0	0	0	1	0	0	0	0	0	0	69
19699791	m	hp	JP	2021	0	1	0	0	1	0	0	0	0	0	0	0	55
19700248	f	md	HR	2021	0	1	1	0	0	0	0	0	0	0	0	0	75
19700499	m	hp	RU	2021	0	1	0	1	0	0	0	0	0	0	0	0	62
19700745	m	md	JP	2021	0	1	0	0	1	0	0	0	0	0	0	0	66
19700906	m	md	JP	2021	0	1	1	0	0	0	0	0	0	0	0	1	76
19700919	m	md	JP	2021	0	1	0	0	0	1	0	0	0	0	0	0	60
19700932	f	md	CN	2021	0	0	0	0	0	0	0	0	0	0	0	0	64
19700942	m	md	TR	2021	0	0	0	0	0	0	0	0	0	0	0	0	67
19701369	f	cn	Not re	2021	0	0	0	0	0	0	0	0	0	0	0	0	57
19701525	m	md	RO	2021	0	1	0	0	1	0	0	0	0	0	0	0	64
19701528	m	cn	CN	2021	0	1	0	0	1	0	0	0	0	0	0	0	58
19701559	Not	hp	US	2021	0	0	0	0	0	0	0	0	0	0	0	0	
19701703	m	md	JP	2021	0	1	0	0	0	1	0	0	0	0	0	1	70
19701729	m	hp	NL	2021	0	1	0	0	1	0	0	0	0	0	0	0	70
19701815	m	md	CN	2021	0	0	0	0	0	0	1	0	0	0	1	0	49
19702216	m	hp	DE	2021	0	0	0	0	0	0	0	0	0	0	0	0	47
19702245	Not	hp	CN	2021	1	0	0	0	0	0	0	0	0	0	0	0	

19702772	f	hp	GB	2021	0	1	0	0	0	1	0	0	0	0	0	0	66
19704037	m	hp	US	2021	0	1	0	0	0	1	0	0	0	0	0	0	69
19704162	m	hp	DE	2021	0	1	0	0	1	0	0	0	0	0	0	0	71
19704252	f	md	PE	2021	0	1	0	0	0	1	0	0	0	0	0	0	79
19704307	m	md	CN	2021	0	0	0	0	0	0	0	0	0	0	0	0	63
19704554	f	md	KR	2021	0	1	0	0	0	1	0	0	0	0	0	0	65
19704983	f	md	GB	2021	0	0	0	0	0	0	0	0	0	0	0	0	66
19705340	f	cn	US	2021	0	1	1	0	0	0	0	0	0	0	0	0	56
19706443	f	hp	JP	2021	1	0	0	0	0	0	0	0	0	0	0	0	93
19706453	m	md	ES	2021	0	1	0	0	0	1	0	0	0	0	0	0	78
19706657	m	md	CN	2021	0	1	1	0	0	0	0	0	0	0	0	0	51
19706766	m	md	DE	2021	0	0	0	0	0	0	0	0	0	0	0	0	66
19707158	f	cn	Not re	2021	0	0	0	0	0	0	0	0	0	0	0	0	63
19707416	m	Not repo	CN	2021	1	0	0	0	0	0	0	0	0	0	0	0	69
19707419	f	cn	US	2021	1	0	0	0	0	0	0	0	0	0	0	0	66.7
19707442	Not	hp	JP	2021	0	0	0	0	0	0	0	0	0	0	0	0	
19707443	Not	md	US	2021	0	0	0	0	0	0	0	0	0	0	0	0	
19707675	f	cn	JP	2021	0	1	1	0	0	0	0	0	0	0	0	0	81
19707859	m	Not repo	RU	2021	0	1	0	1	0	0	0	0	0	0	0	0	
19707865	f	hp	CN	2021	0	0	0	0	0	0	0	0	0	0	0	0	69
19709052	Not	hp	US	2021	0	1	0	0	0	1	0	0	0	0	0	0	
19709064	Not	hp	US	2021	0	1	0	0	0	1	0	0	0	0	0	0	
19709081	f	cn	US	2021	1	0	0	0	0	0	0	0	0	0	0	0	53.4
19709083	Not	hp	US	2021	0	1	0	0	0	1	0	0	0	0	0	0	
19709096	Not	hp	US	2021	0	1	0	0	0	1	0	0	0	0	0	0	
19709105	Not	hp	US	2021	0	1	0	0	0	1	0	0	0	0	0	0	
19709123	m	md	NL	2021	0	1	0	0	0	1	0	0	0	0	0	0	58
19709292	m	md	DE	2021	0	1	0	0	0	1	0	0	0	0	0	0	53
19709366	f	cn	US	2021	1	0	0	0	0	0	0	0	0	0	0	0	75.4
19709891	f	cn	Not re	2021	0	0	0	0	0	0	0	0	0	0	0	0	66
19710002	f	cn	US	2021	0	1	1	0	0	0	0	0	0	0	0	0	
19710070	m	hp	KR	2021	0	0	0	0	0	0	0	0	0	0	0	0	73
19710205	f	md	AT	2021	0	0	0	0	0	0	0	0	0	0	0	0	82
19710555	Not	hp	FR	2021	0	0	0	0	0	0	0	0	0	0	0	0	

19710773	f	hp	IN	2021	0	0	0	0	0	0	0	0	0	0	0	0	49
19710776	m	hp	IN	2021	0	0	0	0	0	0	0	0	0	0	0	0	71
19711182	m	md	KR	2021	0	0	0	0	0	0	0	0	0	0	0	0	
19711358	Not	hp	FR	2021	0	0	0	0	0	0	0	0	0	0	0	0	
19711615	f	Not repo	MY	2021	1	0	0	0	0	0	0	0	0	0	0	0	65.4
19711713	m	cn	US	2021	1	0	0	0	0	0	0	0	0	0	0	0	62
19711820	f	hp	JP	2021	1	0	0	0	0	0	0	0	0	0	0	0	41
19711822	f	hp	JP	2021	1	0	0	0	0	0	0	0	0	0	0	0	59
19711823	m	hp	JP	2021	1	0	0	0	0	0	0	0	0	0	0	0	69
19711824	m	hp	JP	2021	1	0	0	0	0	0	0	0	0	0	0	0	66
19712001	m	md	CA	2021	0	0	0	0	0	0	0	0	0	0	0	0	71
19712088	f	cn	CA	2021	0	0	0	0	0	0	0	0	0	0	0	0	79
19712300	f	cn	US	2021	0	0	0	0	0	0	0	0	0	0	0	0	86
19712495	m	cn	JP	2021	0	1	1	0	0	0	0	0	0	0	0	0	65
19712708	m	hp	RO	2021	0	0	0	0	0	0	0	0	0	0	0	0	70
19713609	m	hp	JP	2021	0	1	0	0	1	0	0	0	0	0	0	0	
19714025	m	md	JP	2021	0	1	0	0	1	0	0	0	0	0	0	1	70
19714185	m	cn	CA	2021	0	1	0	0	1	0	0	0	0	0	0	0	54
19714347	Not	hp	US	2021	0	0	0	0	0	0	0	0	0	0	0	0	
19714433	f	cn	SG	2021	0	1	0	0	0	1	0	0	0	0	0	0	62
19714535	f	md	JP	2021	0	1	0	0	1	0	0	0	0	0	0	0	60
19714787	f	md	GB	2021	0	1	0	0	0	1	0	0	0	0	0	0	66
19714789	f	cn	IN	2021	0	1	0	0	1	0	0	0	0	0	0	0	59
19714965	m	hp	US	2021	0	1	0	0	0	1	0	0	0	0	0	0	
19714986	f	md	IN	2021	0	0	0	0	0	0	0	0	0	0	0	0	48
19715435	f	md	ES	2021	0	0	0	0	0	0	0	0	0	0	0	0	65
19716123	Not	ph	US	2021	0	0	0	0	0	0	0	0	0	0	0	0	
19716125	Not	md	JP	2021	0	1	0	0	0	1	0	0	0	0	0	1	60
19716573	f	md	HK	2021	0	0	0	0	0	0	0	0	0	0	0	0	
19716576	m	cn	Not re	2021	0	0	0	0	0	0	0	0	0	0	0	0	
19716766	m	md	JP	2021	0	1	0	0	1	0	0	0	0	0	0	0	69
19716899	m	cn	IN	2021	0	1	0	0	1	0	0	0	0	0	0	0	68
19717211	m	md	JP	2021	0	1	0	1	0	0	0	0	0	0	0	0	72
19717217	Not	hp	US	2021	0	1	0	0	1	0	0	0	0	0	0	0	

19717223	m	hp	JP	2021	1	0	0	0	0	0	0	0	0	0	0	0	61
19717224	Not	hp	US	2021	0	1	0	0	1	0	0	0	0	0	0	0	
19717232	Not	hp	US	2021	0	1	0	0	1	0	0	0	0	0	0	0	
19717239	Not	hp	US	2021	0	1	0	0	1	0	0	0	0	0	0	0	
19717242	Not	hp	US	2021	0	1	0	0	1	0	0	0	0	0	0	0	
19717246	Not	hp	US	2021	0	1	0	0	1	0	0	0	0	0	0	0	
19717258	Not	hp	US	2021	0	1	0	0	1	0	0	0	0	0	0	0	
19717259	Not	hp	US	2021	0	1	0	0	1	0	0	0	0	0	0	0	
19717271	Not	hp	US	2021	0	1	0	0	1	0	0	0	0	0	0	0	
19717274	Not	hp	US	2021	0	1	0	0	1	0	0	0	0	0	0	0	
19717276	Not	hp	US	2021	0	1	0	0	1	0	0	0	0	0	0	0	
19717282	Not	hp	US	2021	0	1	0	0	1	0	0	0	0	0	0	0	
19717285	Not	hp	US	2021	0	1	0	0	1	0	0	0	0	0	0	0	
19717289	Not	hp	US	2021	0	1	0	0	1	0	0	0	0	0	0	0	
19717300	Not	hp	US	2021	0	1	0	0	1	0	0	0	0	0	0	0	
19717301	Not	hp	US	2021	0	1	0	0	1	0	0	0	0	0	0	0	
19717303	Not	hp	US	2021	0	1	0	0	1	0	0	0	0	0	0	0	
19717675	m	md	JP	2021	1	0	0	0	0	0	0	0	0	0	0	1	30
19717706	Not	hp	US	2021	0	1	0	0	1	0	0	0	0	0	0	0	
19717708	Not	hp	US	2021	0	1	0	0	1	0	0	0	0	0	0	0	
19717718	Not	hp	US	2021	0	1	0	0	1	0	0	0	0	0	0	0	
19717719	Not	hp	US	2021	0	1	0	0	1	0	0	0	0	0	0	0	
19717721	Not	hp	US	2021	0	1	0	0	1	0	0	0	0	0	0	0	
19717774	m	md	DE	2021	0	0	0	0	0	0	0	0	0	0	0	0	61
19717803	f	md	ES	2021	0	1	0	0	0	1	0	0	0	0	0	0	61
19717933	f	Not repo	SG	2021	1	0	0	0	0	0	0	0	0	0	0	0	78.6
19717981	m	md	FR	2021	0	1	0	0	0	1	0	0	0	0	0	0	61
19717986	f	cn	CN	2021	0	0	0	0	0	0	0	0	0	0	0	0	66
19718271	f	hp	UA	2021	0	0	0	0	0	0	0	0	0	0	0	0	
19718579	Not	hp	US	2021	0	1	0	0	1	0	0	0	0	0	0	0	
19718580	Not	hp	US	2021	0	1	0	0	1	0	0	0	0	0	0	0	
19719141	Not	md	JP	2021	0	1	0	0	0	1	0	0	0	0	0	0	
19719398	f	cn	US	2021	0	0	0	0	0	0	0	0	0	0	0	0	63
19719551	f	hp	JP	2021	1	0	0	0	0	0	0	0	0	0	0	0	50

19719554	f	hp	JP	2021	1	0	0	0	0	0	0	0	0	0	0	0	55
19719560	f	cn	US	2021	1	0	0	0	0	0	0	0	0	0	0	0	88
19719679	f	cn	US	2021	1	0	0	0	0	0	0	0	0	0	0	0	
19719764	m	hp	JP	2021	1	0	0	0	0	0	0	0	0	0	0	0	71
19720376	f	cn	CN	2021	0	1	0	0	1	0	0	0	0	0	0	0	64
19720399	f	cn	US	2021	1	0	0	0	0	0	0	0	0	0	0	0	83
19720408	Not	md	JP	2021	1	0	0	0	0	0	0	0	0	0	0	0	
19720521	m	hp	CN	2021	0	1	0	0	1	0	0	0	0	0	0	0	61
19720580	Not	hp	US	2021	0	1	0	0	1	0	0	0	0	0	0	0	
19720581	Not	hp	US	2021	0	1	0	0	1	0	0	0	0	0	0	0	68
19720583	Not	hp	US	2021	0	1	0	0	1	0	0	0	0	0	0	0	
19720584	Not	hp	US	2021	0	1	0	0	1	0	0	0	0	0	0	0	
19720585	Not	hp	US	2021	0	1	0	0	1	0	0	0	0	0	0	0	
19721036	m	hp	DE	2021	0	1	0	0	1	0	0	0	0	0	0	0	59
19721100	m	cn	SG	2021	0	1	0	0	0	1	0	0	0	0	0	0	
19721211	m	md	JP	2021	0	0	0	0	0	0	0	0	0	0	0	0	75
19721507	f	md	JP	2021	0	1	0	1	0	0	0	0	0	0	0	0	
19721758	f	md	GB	2021	0	1	0	0	0	1	0	0	0	0	0	0	66
19721827	Not	hp	US	2021	0	1	0	0	1	0	0	0	0	0	0	0	
19721832	Not	hp	US	2021	0	1	0	0	1	0	0	0	0	0	0	0	
19721841	Not	hp	US	2021	0	1	0	0	1	0	0	0	0	0	0	0	
19722425	f	hp	DE	2021	0	1	1	0	0	0	0	0	0	0	0	0	73
19723310	m	hp	US	2021	0	0	0	0	0	0	0	0	0	0	0	0	
19723397	m	md	JP	2021	0	1	0	0	1	0	0	0	0	0	0	0	78
19723399	m	cn	CN	2021	0	1	0	0	1	0	0	0	0	0	0	0	76
19723881	f	cn	CN	2021	0	0	0	0	0	0	0	0	0	0	0	0	48
19724159	f	md	JP	2021	0	0	0	0	0	0	0	0	0	0	0	0	76
19724303	f	hp	DE	2021	0	1	0	0	1	0	0	0	0	0	0	1	67
19724361	f	md	DE	2021	0	1	1	0	0	0	0	0	0	0	0	0	73
19724557	f	cn	US	2021	0	0	0	0	0	0	0	0	0	0	0	0	82
19724559	m	hp	US	2021	0	0	0	0	0	0	0	0	0	0	0	0	83
19724789	f	md	JP	2021	0	1	0	0	0	1	0	0	0	0	0	0	65
19725075	m	md	CN	2021	0	1	1	0	0	0	0	0	0	0	0	0	68
19725195	f	ph	US	2021	0	0	0	0	0	0	0	0	0	0	0	0	84

19726086	m	hp	JP	2021	0	0	0	0	0	0	0	0	0	0	0	0	74
19726204	Not	hp	US	2021	0	1	0	0	1	0	0	0	0	0	0	0	
19726205	Not	hp	US	2021	0	1	0	0	1	0	0	0	0	0	0	0	
19726206	Not	hp	US	2021	0	1	0	0	1	0	0	0	0	0	0	0	
19726209	Not	hp	US	2021	0	1	0	0	1	0	0	0	0	0	0	0	
19727213	f	hp	DE	2021	0	0	0	0	0	0	0	0	0	0	0	0	61
19727263	m	md	US	2021	0	0	0	0	0	0	0	0	0	0	0	0	
19727277	m	md	FR	2021	0	1	0	0	0	1	0	0	0	0	0	0	69
19727668	f	cn	Not re	2021	0	0	0	0	0	0	0	0	0	0	0	0	62
19727710	m	cn	US	2021	0	1	1	0	0	0	0	0	0	0	0	1	62
19728291	f	md	GB	2021	0	1	1	0	0	0	0	0	0	0	0	0	69
19728371	f	md	AT	2021	0	0	0	0	0	0	0	0	0	0	0	0	69
19728645	f	hp	JP	2021	0	1	1	0	0	0	0	0	0	0	0	0	69
19729301	f	md	TH	2021	0	0	0	0	0	0	0	0	0	0	0	0	65
19729314	f	cn	US	2021	0	0	0	0	0	0	0	0	0	0	0	0	
19729503	f	cn	JP	2021	1	0	0	0	0	0	0	0	0	0	0	0	
19729941	m	md	DE	2021	0	0	0	0	0	0	0	0	0	0	0	0	61
19730119	m	Not repo	ID	2021	1	0	0	0	0	0	0	0	0	0	0	0	
19730248	Not	Not repo	SE	2021	1	0	0	0	0	0	0	0	0	0	0	0	
19730318	m	Not repo	DE	2021	1	0	0	0	0	0	0	0	0	0	0	0	
19730323	m	Not repo	ID	2021	1	0	0	0	0	0	0	0	0	0	0	0	61.3
19730796	Not	Not repo	SE	2021	1	0	0	0	0	0	0	0	0	0	0	0	
19731028	f	Not repo	MY	2021	1	0	0	0	0	0	0	0	0	0	0	0	81
19731218	m	cn	Not re	2021	0	0	0	0	0	0	0	0	0	0	0	0	
19731476	f	cn	US	2021	1	0	0	0	0	0	0	0	0	0	0	0	82.3
19731638	f	md	SE	2021	0	0	0	0	0	0	0	0	0	0	0	0	73
19731885	m	hp	US	2021	0	1	0	0	1	0	0	0	0	0	0	0	50
19732033	m	md	IN	2021	0	0	0	0	0	0	0	0	0	0	0	0	54
19732043	m	md	US	2021	0	0	0	0	0	0	0	0	0	0	0	0	
19733193	m	hp	CN	2021	0	1	0	0	1	0	0	0	0	0	0	0	49
19734138	f	cn	BE	2021	0	0	0	0	0	0	0	0	0	0	0	0	
19734482	f	cn	US	2021	1	0	0	0	0	0	0	0	0	0	0	0	
19734716	Not	Not repo	US	2021	1	0	0	0	0	0	0	0	0	0	0	0	
19734742	f	hp	GB	2021	0	1	0	0	0	1	0	0	0	0	0	0	71

19734941	m	cn	CN	2021	1	0	0	0	0	0	0	0	0	0	0	0	47
19735497	m	md	RU	2021	0	1	1	0	0	0	0	0	0	0	0	0	59
19735523	f	cn	IN	2021	0	1	0	0	1	0	0	0	0	0	0	0	47
19735654	m	cn	US	2021	1	0	0	0	0	0	0	0	0	0	0	0	72
19735947	f	cn	CN	2021	0	0	0	0	0	0	0	0	0	0	0	0	37
19736169	f	hp	FR	2021	0	0	0	0	0	0	0	0	0	0	0	0	70
19736207	m	md	US	2021	0	0	0	0	0	0	0	0	0	0	0	0	
19736218	m	md	FR	2021	0	1	0	0	1	0	0	0	0	0	0	0	87
19736374	m	cn	US	2021	1	0	0	0	0	0	0	0	0	0	0	0	
19736473	m	cn	JP	2021	0	1	1	0	0	0	0	0	0	0	0	0	70
19736495	Not	md	JP	2021	0	1	0	1	0	0	0	0	0	0	0	0	
19736527	Not	md	JP	2021	1	0	0	0	0	0	0	0	0	0	0	0	85
19736606	f	hp	US	2021	0	1	1	0	0	0	0	0	0	0	0	1	68
19736687	m	md	JP	2021	0	1	0	0	0	1	0	0	0	0	0	0	
19737496	f	cn	US	2021	0	0	0	0	0	0	0	0	0	0	0	0	
19737868	f	md	NL	2021	0	0	0	0	0	0	0	0	0	0	0	0	58
19737986	m	cn	Not re	2021	0	0	0	0	0	0	0	0	0	0	0	0	
19738006	m	md	DE	2021	0	1	0	0	0	1	0	0	0	0	0	0	58
19738120	f	md	DE	2021	0	1	0	0	1	0	0	0	0	0	0	0	52
19738237	m	cn	IN	2021	0	1	0	0	1	0	0	0	0	0	0	0	66
19738868	f	md	GB	2021	0	1	0	0	0	1	0	0	0	0	0	0	71
19738957	Not	hp	CN	2021	1	0	0	0	0	0	0	0	0	0	0	0	
19738959	Not	hp	CN	2021	1	0	0	0	0	0	0	0	0	0	0	0	
19739476	f	md	DE	2021	0	0	0	0	0	0	0	0	0	0	0	0	61
19739551	m	md	US	2021	1	0	0	0	0	0	0	0	0	0	0	0	72
19739595	m	md	FR	2021	0	1	1	0	0	0	0	0	0	0	0	0	74
19739810	f	md	PL	2021	1	0	0	0	0	0	0	0	0	0	0	0	62
19740052	m	cn	Not re	2021	0	0	0	0	0	0	0	0	0	0	0	0	
19740111	m	cn	CN	2021	0	1	0	0	1	0	0	0	0	0	0	0	60
19740282	f	md	JP	2021	0	1	0	0	0	1	0	0	0	0	0	0	76
19740308	f	md	NO	2021	0	1	1	0	0	0	0	0	0	0	0	0	70
19740402	f	md	BR	2021	0	1	1	0	0	0	0	0	0	0	0	0	74
19740579	m	md	GB	2021	0	1	0	0	0	1	0	0	0	0	0	0	61
19740677	f	cn	CA	2021	0	0	0	0	0	0	1	1	0	0	0	0	66

19740820	m	cn	DE	2021	0	1	1	0	0	0	0	0	0	0	0	0	
19740888	m	cn	IN	2021	0	1	0	0	1	0	0	0	0	0	0	0	68
19740934	Not	hp	CN	2021	1	0	0	0	0	0	0	0	0	0	0	0	
19741090	Not	hp	CN	2021	1	0	0	0	0	0	0	0	0	0	0	0	
19741094	Not	hp	CN	2021	1	0	0	0	0	0	0	0	0	0	0	0	
19741134	Not	hp	CN	2021	1	0	0	0	0	0	0	0	0	0	0	0	
19741135	Not	hp	CN	2021	1	0	0	0	0	0	0	0	0	0	0	0	
19741136	Not	hp	CN	2021	1	0	0	0	0	0	0	0	0	0	0	0	
19741171	Not	hp	CN	2021	1	0	0	0	0	0	0	0	0	0	0	0	
19741172	Not	hp	CN	2021	1	0	0	0	0	0	0	0	0	0	0	0	
19741173	Not	hp	CN	2021	1	0	0	0	0	0	0	0	0	0	0	0	
19741208	Not	hp	CN	2021	1	0	0	0	0	0	0	0	0	0	0	0	
19741209	Not	hp	CN	2021	1	0	0	0	0	0	0	0	0	0	0	0	
19741260	m	cn	JP	2021	0	1	1	0	0	0	0	0	0	0	0	1	90
19741267	Not	hp	CN	2021	1	0	0	0	0	0	0	0	0	0	0	0	
19741268	Not	hp	CN	2021	1	0	0	0	0	0	0	0	0	0	0	0	
19741269	Not	hp	CN	2021	1	0	0	0	0	0	0	0	0	0	0	0	
19741575	Not	hp	ES	2021	0	0	0	0	0	0	0	0	0	0	0	0	
19741600	f	cn	JP	2021	0	1	1	0	0	0	0	0	0	0	0	0	70
19741882	f	cn	CA	2021	1	0	0	0	0	0	0	0	0	0	0	0	73.1
19741920	Not	hp	CN	2021	1	0	0	0	0	0	0	0	0	0	0	0	
19741929	Not	hp	CN	2021	1	0	0	0	0	0	0	0	0	0	0	0	
19742110	f	md	GB	2021	0	1	0	0	0	1	0	0	0	0	0	0	71
19742127	Not	hp	CN	2021	1	0	0	0	0	0	0	0	0	0	0	0	
19742137	Not	hp	CN	2021	1	0	0	0	0	0	0	0	0	0	0	0	
19742242	Not	hp	CN	2021	1	0	0	0	0	0	0	0	0	0	0	0	
19742319	Not	cn	US	2021	1	0	0	0	0	0	0	0	0	0	0	0	
19742729	f	md	NL	2021	0	0	0	0	0	0	0	0	0	0	0	0	79
19743017	Not	hp	CN	2021	1	0	0	0	0	0	0	0	0	0	0	0	
19743076	m	hp	US	2021	0	0	0	0	0	0	0	0	0	0	0	0	76
19743438	f	Not repo	ID	2021	1	0	0	0	0	0	0	0	0	0	0	0	78.7
19743513	f	Not repo	GB	2021	0	1	0	0	0	1	0	0	0	0	0	0	71
19743699	m	md	JP	2021	0	1	0	0	1	0	0	0	0	0	0	0	71
19743890	Not	md	JP	2021	0	1	0	0	1	0	0	0	0	0	0	0	

19744187	f	cn	US	2021	1	0	0	0	0	0	0	0	0	0	0	0	64
19744970	m	Not repo	ID	2021	1	0	0	0	0	0	0	0	0	0	0	0	
19744972	f	hp	NL	2021	0	1	1	0	0	0	0	0	0	0	0	0	73
19745017	f	md	SE	2021	0	0	0	0	0	0	0	0	0	0	0	0	57
19745468	m	cn	CN	2021	0	1	0	0	1	0	0	0	0	0	0	0	58
19745542	Not	md	US	2021	0	0	0	0	0	0	0	0	0	0	0	0	
19745573	Not	ph	US	2021	0	0	0	0	0	0	0	0	0	0	0	0	
19745581	m	ph	JP	2021	1	0	0	0	0	0	0	0	0	0	0	0	80
19745587	f	md	IT	2021	0	1	0	0	0	1	0	0	0	0	0	0	47
19745640	f	hp	CA	2021	1	0	0	0	0	0	0	0	0	0	0	0	72
19745709	f	md	US	2021	0	0	0	0	0	0	0	0	0	0	0	0	41
19745746	m	Not repo	ID	2021	1	0	0	0	0	0	0	0	0	0	0	0	62.2
19746180	f	hp	BE	2021	0	0	0	0	0	0	0	0	0	0	0	0	72
19746371	m	md	JP	2021	0	1	0	0	1	0	0	0	0	0	0	0	50
19746378	m	hp	ES	2021	0	0	0	0	0	0	0	0	0	0	0	0	60
19746557	m	hp	IL	2021	0	0	0	0	0	0	0	0	0	0	0	0	65
19746609	Not	md	ES	2021	1	0	0	0	0	0	0	0	0	0	0	0	
19746983	f	cn	PK	2021	0	1	0	0	0	1	0	0	0	0	0	0	75
19747188	m	md	JP	2021	0	0	0	0	0	0	0	0	0	0	0	0	77
19747405	m	md	PL	2021	0	0	0	0	0	0	0	0	0	0	0	0	66
19747474	f	hp	NL	2021	0	1	0	0	1	0	0	0	0	0	0	0	84
19748052	m	md	ES	2021	0	1	0	0	0	1	0	0	0	0	0	0	64
19748177	f	md	US	2021	0	1	1	0	0	0	0	0	0	0	0	1	75
19748293	m	hp	IN	2021	0	0	0	0	0	0	0	0	0	0	0	0	45
19748519	m	md	FR	2021	0	1	0	0	0	1	0	0	0	0	0	0	54
19750002	f	md	RU	2021	1	0	0	0	0	0	0	0	0	0	0	0	72
19750029	m	md	IL	2021	0	1	1	0	0	0	0	0	0	0	0	0	77
19750086	m	Not repo	ID	2021	1	0	0	0	0	0	0	0	0	0	0	0	
19750370	f	cn	US	2021	0	1	1	0	0	0	0	0	0	0	0	0	60
19750371	m	ph	US	2021	0	1	1	0	0	0	0	0	0	0	0	0	62
19750382	f	md	JP	2021	1	0	0	0	0	0	0	0	0	0	0	0	67
19750628	f	cn	Not re	2021	0	0	0	0	0	0	0	0	0	0	0	0	
19750772	m	cn	US	2021	0	1	0	1	0	0	0	0	0	0	0	0	
19750942	m	cn	IN	2021	0	0	0	0	0	0	0	0	0	0	0	0	70

19750988	f	hp	DE	2021	0	1	0	0	1	0	0	0	0	0	0	0	65
19751009	Not	hp	US	2021	0	0	0	0	0	0	0	0	0	0	0	0	
19751105	m	md	JP	2021	0	0	0	0	0	0	0	0	0	0	0	0	79
19751178	m	cn	CN	2021	0	0	0	0	0	0	0	0	0	0	0	0	52
19751414	Not	md	JP	2021	1	0	0	0	0	0	0	0	0	0	0	0	
19751768	m	hp	JP	2021	0	1	0	0	1	0	0	0	0	0	0	0	62
19752624	f	md	JP	2021	0	1	0	0	0	1	0	0	0	0	0	0	70
19752629	f	hp	CH	2021	0	1	1	0	0	0	0	0	0	0	0	0	87
19752633	Not	md	JP	2021	0	1	0	0	1	0	0	0	0	0	0	0	80
19752853	f	hp	BG	2021	0	1	0	0	0	1	0	0	0	0	0	0	44
19752927	f	md	FR	2021	0	1	0	0	0	1	0	0	0	0	0	0	71
19752928	Not	hp	US	2021	0	1	1	0	0	0	0	0	0	0	0	0	
19752982	f	md	JP	2021	0	0	0	0	0	0	0	0	0	0	0	0	80
19753003	m	cn	US	2021	0	1	1	0	0	0	0	0	0	0	0	0	
19753052	f	md	AE	2021	0	1	0	0	0	1	0	0	0	0	0	0	52
19753053	m	md	JP	2021	0	1	0	0	0	1	0	0	0	0	0	1	80
19753172	m	md	US	2021	0	1	1	0	0	0	0	0	0	0	0	0	73
19753191	m	cn	CN	2021	0	1	0	0	1	0	0	0	0	0	0	0	66
19753196	m	md	FR	2021	0	1	0	0	0	1	0	0	0	0	0	0	61
19753202	m	ph	JP	2021	0	1	0	0	1	0	0	0	0	0	0	0	80
19753227	m	cn	CN	2021	0	1	0	0	1	0	0	0	0	0	0	0	63
19753253	Not	ph	JP	2021	0	1	0	0	1	0	0	0	0	0	0	0	
19753733	Not	md	JP	2021	0	1	0	0	1	0	0	0	0	0	0	0	
19754448	f	md	JP	2021	0	0	0	0	0	0	0	0	0	0	0	0	
19754872	m	md	JP	2021	0	0	0	0	0	0	0	0	0	0	0	0	70
19754926	f	md	JP	2021	1	0	0	0	0	0	0	0	0	0	0	0	75
19755238	m	md	ES	2021	0	1	1	0	0	0	0	0	0	0	0	0	59
19755381	m	Not repo	CN	2021	0	1	0	1	0	0	0	0	0	0	0	1	62.6
19755398	Not	hp	GB	2021	0	1	0	0	0	1	0	0	0	0	0	0	
19755810	m	cn	JP	2021	0	1	1	0	0	0	0	0	0	0	0	0	70
19755863	f	md	JP	2021	0	0	0	0	0	0	0	0	0	0	0	0	65
19755892	Not	md	US	2021	0	0	0	0	0	0	0	0	0	0	0	0	
19756137	m	cn	JP	2021	0	0	0	0	0	0	0	0	0	0	0	0	73
19756564	m	md	NL	2021	0	0	0	0	0	0	0	0	0	0	0	0	

19756565	f	md	FR	2021	0	0	0	0	0	0	0	0	0	0	0	0	61
19756634	m	md	JP	2021	0	1	0	0	0	1	0	0	0	0	0	0	
19756829	f	cn	DE	2021	0	1	0	0	1	0	0	0	0	0	0	0	65
19757256	m	md	IN	2021	1	0	0	0	0	0	0	0	0	0	0	0	46
19757465	m	hp	US	2021	0	0	0	0	0	0	0	0	0	0	0	0	73
19757481	m	md	IL	2021	0	1	0	1	0	0	0	0	0	0	0	0	71
19757509	f	md	PT	2021	0	1	0	0	0	1	0	0	0	0	0	0	70
19757644	Not	md	JP	2021	0	1	0	0	0	1	0	0	0	0	0	0	70
19758468	m	hp	DE	2021	0	1	0	0	1	0	0	0	0	0	0	0	80
19759171	Not	md	IT	2021	0	1	0	0	0	1	0	0	0	0	0	0	
19760054	m	hp	JP	2021	0	1	0	0	1	0	0	0	0	0	0	0	71
19760656	Not	md	JP	2021	0	1	0	0	0	1	0	0	0	0	0	0	70
19761216	f	md	BG	2021	0	1	0	0	0	1	0	0	0	0	0	0	44
19761373	m	md	JP	2021	0	1	0	0	1	0	0	0	0	0	0	0	80
19761382	f	cn	CN	2021	0	0	0	0	0	0	0	0	0	0	0	0	59
19761967	m	md	TR	2021	0	1	1	0	0	0	0	0	0	0	0	0	56
19762009	f	ph	JP	2021	1	0	0	0	0	0	0	0	0	0	0	0	79
19762081	f	md	JP	2021	0	1	0	0	0	1	0	0	0	0	0	0	76
19762505	m	md	JP	2021	0	1	0	0	1	0	0	0	0	0	0	0	51
19762536	m	md	JP	2021	0	1	0	0	1	0	0	0	0	0	0	0	74
19762652	f	md	TR	2021	0	0	0	0	0	0	0	0	0	0	0	0	83
19763020	f	cn	JP	2021	0	1	1	0	0	0	0	0	0	0	0	0	77
19763174	m	cn	SE	2021	0	1	1	0	0	0	0	0	0	0	0	0	67
19763207	m	cn	SG	2021	1	0	0	0	0	0	0	0	0	0	0	0	56.3
19763236	m	md	NL	2021	0	0	0	0	0	0	0	0	0	0	0	0	66
19763240	m	md	PA	2021	0	1	0	0	0	1	0	0	0	0	0	0	69
19763254	m	md	DE	2021	0	1	0	0	1	0	0	0	0	0	0	0	80
19763338	f	md	NL	2021	0	0	0	0	0	0	0	0	0	0	0	0	66
19763357	m	Not repo	MY	2021	0	1	0	1	0	0	0	0	0	0	0	0	74
19763557	f	md	NL	2021	0	0	0	0	0	0	0	0	0	0	0	0	56
19763854	f	cn	CN	2021	0	0	0	0	0	0	0	0	0	0	0	0	70
19764238	f	md	US	2021	0	1	0	0	1	0	0	0	0	0	0	0	54
19764247	Not	hp	US	2021	1	0	0	0	0	0	0	0	0	0	0	0	
19764248	Not	hp	US	2021	1	0	0	0	0	0	0	0	0	0	0	0	

19764256	Not	hp	US	2021	1	0	0	0	0	0	0	0	0	0	0	0	
19764257	Not	hp	US	2021	1	0	0	0	0	0	0	0	0	0	0	0	
19764638	Not	hp	US	2021	1	0	0	0	0	0	0	0	0	0	0	0	
19764724	m	cn	JP	2021	0	1	1	0	0	0	0	0	0	0	0	0	60
19765034	f	cn	CN	2021	0	0	0	0	0	0	0	0	0	0	0	0	55
19765323	f	cn	IN	2021	0	0	0	0	0	0	0	0	0	0	0	0	60
19765455	f	Not repo	MY	2021	1	0	0	0	0	0	0	0	0	0	0	0	
19765549	Not	hp	US	2021	1	0	0	0	0	0	0	0	0	0	0	0	
19765550	Not	hp	US	2021	1	0	0	0	0	0	0	0	0	0	0	0	
19765551	Not	hp	US	2021	1	0	0	0	0	0	0	0	0	0	0	0	
19765557	Not	hp	US	2021	1	0	0	0	0	0	0	0	0	0	0	0	
19765562	Not	hp	US	2021	1	0	0	0	0	0	0	0	0	0	0	0	
19765563	Not	hp	US	2021	1	0	0	0	0	0	0	0	0	0	0	0	
19765724	Not	hp	US	2021	1	0	0	0	0	0	0	0	0	0	0	0	
19765728	Not	hp	US	2021	1	0	0	0	0	0	0	0	0	0	0	0	
19765729	Not	hp	US	2021	1	0	0	0	0	0	0	0	0	0	0	0	
19765730	Not	hp	US	2021	1	0	0	0	0	0	0	0	0	0	0	0	
19765741	Not	hp	US	2021	1	0	0	0	0	0	0	0	0	0	0	0	
19765847	m	md	US	2021	0	1	1	0	0	0	0	0	0	0	0	0	75
19765853	f	ph	FR	2021	0	0	0	0	0	0	0	0	0	0	0	0	
19765869	Not	md	PT	2021	0	1	0	0	0	1	0	0	0	0	0	0	
19765907	m	md	IL	2021	0	1	1	0	0	0	0	0	0	0	0	0	65
19766281	m	hp	FR	2021	0	1	0	0	1	0	0	0	0	0	0	0	73
19766460	Not	hp	US	2021	1	0	0	0	0	0	0	0	0	0	0	0	
19766461	Not	hp	US	2021	1	0	0	0	0	0	0	0	0	0	0	0	
19766560	Not	hp	US	2021	1	0	0	0	0	0	0	0	0	0	0	0	
19766561	Not	hp	US	2021	1	0	0	0	0	0	0	0	0	0	0	0	
19766568	m	cn	IN	2021	0	0	0	0	0	0	0	0	0	0	0	0	73
19766576	Not	hp	US	2021	1	0	0	0	0	0	0	0	0	0	0	0	
19766577	Not	hp	US	2021	1	0	0	0	0	0	0	0	0	0	0	0	
19766583	f	cn	IN	2021	0	0	0	0	0	0	0	0	0	0	0	0	68
19766656	Not	hp	US	2021	1	0	0	0	0	0	0	0	0	0	0	0	
19766657	Not	hp	US	2021	1	0	0	0	0	0	0	0	0	0	0	0	
19766658	Not	hp	US	2021	1	0	0	0	0	0	0	0	0	0	0	0	

19766660	Not	hp	US	2021	1	0	0	0	0	0	0	0	0	0	0	0	
19766662	Not	hp	US	2021	1	0	0	0	0	0	0	0	0	0	0	0	
19766663	Not	hp	US	2021	1	0	0	0	0	0	0	0	0	0	0	0	
19766882	m	cn	IN	2021	0	0	0	0	0	0	0	0	0	0	0	0	60
19768014	f	md	FR	2021	0	1	1	0	0	0	0	0	0	0	0	0	
19768043	Not	md	KR	2021	1	0	0	0	0	0	0	0	0	0	0	0	
19768054	m	cn	JP	2021	0	1	1	0	0	0	0	0	0	0	0	0	49
19768117	m	Not repo	MY	2021	1	0	0	0	0	0	0	0	0	0	0	0	54.9
19768145	m	md	KR	2021	0	1	0	0	0	1	0	0	0	0	0	0	59
19768304	m	cn	JP	2021	0	1	1	0	0	0	0	0	0	0	0	0	68
19768509	m	ph	US	2021	0	0	0	0	0	0	0	0	0	0	0	0	56
19768709	f	md	US	2021	0	0	0	0	0	0	0	0	0	0	0	0	
19768782	f	Not repo	US	2021	1	0	0	0	0	0	0	0	0	0	0	0	75.3
19768958	m	Not repo	CN	2021	0	1	0	1	0	0	0	0	0	0	0	0	59.2
19769208	m	Not repo	CN	2021	0	1	0	1	0	0	0	0	0	0	0	1	61
19769385	f	hp	DE	2021	0	0	0	0	0	0	0	0	0	0	0	0	75
19769401	m	md	GB	2021	0	1	0	0	0	1	0	0	0	0	0	0	65
19769467	m	md	JP	2021	0	1	0	0	1	0	0	0	0	0	0	1	78
19769519	Not	md	US	2021	0	0	0	0	0	0	0	0	0	0	0	0	80
19769738	f	Not repo	MY	2021	1	0	0	0	0	0	0	0	0	0	0	0	
19770130	m	md	JP	2021	0	1	0	0	1	0	0	0	0	0	0	0	58
19770747	m	hp	NL	2021	0	0	0	0	0	0	0	0	0	0	0	0	56
19770772	f	md	JP	2021	0	1	0	0	0	1	0	0	0	0	0	0	66
19770786	f	md	JP	2021	1	0	0	0	0	0	0	0	0	0	0	0	68.2
19770810	Not	md	JP	2021	0	1	0	0	1	0	0	0	0	0	0	0	
19771261	Not	cn	JP	2021	0	1	1	0	0	0	0	0	0	0	0	0	87
19771518	f	md	HK	2021	1	0	0	0	0	0	0	0	0	0	0	0	55
19771529	Not	Not repo	JP	2021	0	1	0	1	0	0	0	0	0	0	0	0	
19771551	m	hp	IT	2021	0	1	0	0	1	0	0	0	0	0	0	0	52
19771687	m	cn	SG	2021	0	1	0	0	0	1	0	0	0	0	0	0	69
19771776	m	md	JP	2021	0	1	0	0	1	0	0	0	0	0	0	0	75
19771969	f	md	JP	2021	1	0	0	0	0	0	0	0	0	0	0	0	65
19772384	Not	cn	JP	2021	0	1	1	0	0	0	0	0	0	0	0	0	50
19772491	m	md	US	2021	0	1	1	0	0	0	0	0	0	0	0	0	56

19772569	m	cn	JP	2021	0	1	1	0	0	0	0	0	0	0	0	0	62
19772682	m	md	US	2021	0	1	1	0	0	0	0	0	0	0	0	0	57
19772761	f	md	US	2021	0	1	1	0	0	0	0	0	0	0	0	0	61
19773074	f	md	FR	2021	0	0	0	0	0	0	0	0	0	0	0	0	47
19773194	m	Not repo	AU	2021	1	0	0	0	0	0	0	0	0	0	0	0	
19773221	f	md	FR	2021	0	1	0	0	0	1	0	0	0	0	0	0	82
19773564	m	md	JP	2021	0	0	0	0	0	0	0	0	0	0	0	0	55
19773888	f	md	US	2021	0	0	0	0	0	0	1	0	1	0	0	0	44
19774603	f	cn	US	2021	0	1	0	1	0	0	0	0	0	0	0	0	
19774912	f	cn	ID	2021	0	1	1	0	0	0	0	0	0	0	0	0	
19774967	m	Not repo	CA	2021	1	0	0	0	0	0	0	0	0	0	0	0	
19775130	m	md	PL	2021	0	0	0	0	0	0	0	0	0	0	0	0	69
19775428	f	Not repo	MY	2021	1	0	0	0	0	0	0	0	0	0	0	0	70
19775452	f	hp	CA	2021	0	0	0	0	0	0	0	0	0	0	0	0	63
19775612	m	md	AT	2021	0	0	0	0	0	0	0	0	0	0	0	0	67
19775982	f	md	JP	2021	0	1	0	0	0	1	0	0	0	0	0	0	80
19776084	m	cn	JP	2021	1	0	0	0	0	0	0	0	0	0	0	0	86
19777068	Not	md	JP	2021	0	1	0	0	0	1	0	0	0	0	0	0	70
19777743	m	md	FR	2021	0	1	0	0	0	1	0	0	0	0	0	0	84
19778556	Not	cn	JP	2021	0	1	1	0	0	0	0	0	0	0	0	0	80
19778581	m	hp	CN	2021	0	0	0	0	0	0	0	0	0	0	0	0	58
19779265	m	cn	Not re	2021	0	0	0	0	0	0	0	0	0	0	0	0	27
19779318	m	md	PL	2021	0	0	0	0	0	0	0	0	0	0	0	0	66
19779740	m	Not repo	CN	2021	0	1	0	1	0	0	0	0	0	0	0	0	62
19779751	Not	ph	PL	2021	0	0	0	0	0	0	0	0	0	0	0	0	
19780458	m	md	FR	2021	0	1	0	0	0	1	0	0	0	0	0	0	61
19780501	Not	md	JP	2021	0	1	0	1	0	0	0	0	0	0	0	0	
19780509	m	hp	GR	2021	0	1	0	0	1	0	0	0	0	0	0	0	68
19780980	f	hp	US	2021	0	0	0	0	0	0	0	0	0	0	0	0	72
19781057	m	Not repo	CN	2021	0	1	0	1	0	0	0	0	0	0	0	0	75
19781180	m	cn	CN	2021	0	1	0	0	1	0	0	0	0	0	0	0	43
19781267	f	cn	US	2021	1	0	0	0	0	0	0	0	0	0	0	0	64.3
19781518	f	hp	RO	2021	1	0	0	0	0	0	0	0	0	0	0	0	78
19781579	f	md	FR	2021	0	1	0	1	0	0	0	0	0	0	0	1	57

19782216	f	md	GB	2021	0	1	0	0	0	1	0	0	0	0	0	0	71
19782987	f	cn	US	2021	0	1	1	0	0	0	0	0	0	0	0	0	74
19783315	m	md	JP	2021	0	1	1	0	0	0	0	0	0	0	0	0	75
19783393	Not	md	US	2021	0	0	0	0	0	0	0	0	0	0	0	0	
19784116	f	hp	SG	2021	1	0	0	0	0	0	0	0	0	0	0	0	83
19784491	Not	md	US	2021	0	0	0	0	0	0	0	0	0	0	0	0	
19784595	f	md	US	2021	0	0	0	0	0	0	0	0	0	0	0	0	
19785230	f	md	JP	2021	0	1	1	0	0	0	0	0	0	0	0	0	65
19785308	f	hp	DE	2021	0	1	0	0	1	0	0	0	0	0	0	0	70
19785386	m	cn	IN	2021	0	0	0	0	0	0	0	0	0	0	0	0	29
19785452	m	md	US	2021	0	0	0	0	0	0	0	0	0	0	0	0	76
19785663	f	cn	SG	2021	1	0	0	0	0	0	0	0	0	0	0	0	83.6
19785713	m	hp	JP	2021	0	1	1	0	0	0	0	0	0	0	0	0	78
19785792	m	Not repo	CN	2021	0	1	0	1	0	0	0	0	0	0	0	0	61
19785984	f	cn	US	2021	0	0	0	0	0	0	0	0	0	0	0	0	74
19786230	m	md	JP	2021	1	0	0	0	0	0	0	0	0	0	0	0	71
19786231	m	md	JP	2021	1	0	0	0	0	0	0	0	0	0	0	0	57
19786256	Not	md	NL	2021	0	0	0	0	0	0	0	0	0	0	0	0	
19786444	m	cn	CA	2021	0	1	1	0	0	0	0	0	0	0	0	0	
19786882	f	hp	US	2021	0	1	0	1	0	0	0	0	0	0	0	0	59.7
19786931	Not	md	NL	2021	0	0	0	0	0	0	0	0	0	0	0	0	
19786935	f	hp	US	2021	0	0	0	0	0	0	0	0	0	0	0	0	56
19787258	m	hp	MY	2021	0	0	0	0	0	0	0	0	0	0	0	0	53
19787259	f	md	JP	2021	0	0	0	0	0	0	0	0	0	0	0	0	50
19787404	m	md	DE	2021	0	0	0	0	0	0	0	0	0	0	0	0	55
19787853	m	cn	JP	2021	0	1	1	0	0	0	0	0	0	0	0	0	
19787858	m	Not repo	PL	2021	0	1	0	1	0	0	0	0	0	0	0	0	
19787918	m	cn	SG	2021	1	0	0	0	0	0	0	0	0	0	0	0	85.1
19787948	m	ph	CA	2021	0	0	0	0	0	0	0	0	0	0	0	0	52
19788302	f	cn	Not re	2021	0	0	0	0	0	0	0	0	0	0	0	0	57
19788334	Not	md	PL	2021	0	0	0	0	0	0	0	0	0	0	0	0	50
19788734	f	md	FR	2021	0	0	0	0	0	0	0	0	0	0	0	0	
19788984	f	cn	Not re	2021	0	0	0	0	0	0	0	0	0	0	0	0	57
19789133	f	md	DE	2021	0	0	0	0	0	0	0	0	0	0	0	0	61

19789208	m	hp	CA	2021	1	0	0	0	0	0	0	0	0	0	0	0	70
19790019	m	md	JP	2021	0	1	0	0	1	0	0	0	0	0	0	0	73
19790047	m	md	JP	2021	0	1	0	0	1	0	0	0	0	0	0	0	70
19790304	f	md	DE	2021	0	0	0	0	0	0	0	0	0	0	0	0	70
19790430	m	cn	JP	2021	0	1	1	0	0	0	0	0	0	0	0	0	70
19790993	Not	hp	US	2021	0	0	0	0	0	0	0	0	0	0	0	0	
19790995	f	md	JP	2021	0	0	0	0	0	0	0	0	0	0	0	0	
19791063	f	hp	US	2021	0	1	1	0	0	0	0	0	0	0	0	0	60
19792808	f	hp	US	2021	0	0	0	0	0	0	0	0	0	0	0	0	56
19792845	m	cn	IN	2021	0	0	0	0	0	0	0	0	0	0	0	0	31
19792847	m	md	JP	2021	0	0	0	0	0	0	0	0	0	0	0	0	77
19792934	f	cn	IN	2021	0	0	0	0	0	0	0	0	0	0	0	0	49
19792950	m	cn	JP	2021	0	1	1	0	0	0	0	0	0	0	0	0	70
19793189	f	md	JP	2021	0	1	0	0	1	0	0	0	0	0	0	0	69
19793427	f	cn	CA	2021	1	0	0	0	0	0	0	0	0	0	0	0	63.8
19793618	m	hp	ES	2021	0	0	0	0	0	0	0	0	0	0	0	0	61
19793634	f	md	JP	2021	0	1	0	0	0	1	0	0	0	0	0	0	60
19793945	m	cn	CN	2021	0	1	0	0	1	0	0	0	0	0	0	0	49
19794064	f	md	FR	2021	0	0	0	0	0	0	0	0	0	0	0	0	61
19794216	m	md	JP	2021	0	1	0	0	1	0	0	0	0	0	0	0	44
19794281	m	md	AU	2021	0	1	1	0	0	0	0	0	0	0	0	0	50
19794598	f	Not repo	CN	2021	0	1	0	1	0	0	0	0	0	0	0	0	64.8
19794634	f	md	MY	2021	1	0	0	0	0	0	0	0	0	0	0	0	
19794803	m	cn	CN	2021	0	1	0	0	1	0	0	0	0	0	0	0	52
19794960	f	hp	JP	2021	0	1	0	0	1	0	0	0	0	0	0	0	
19794972	f	md	MY	2021	1	0	0	0	0	0	0	0	0	0	0	0	
19795296	m	md	MY	2021	1	0	0	0	0	0	0	0	0	0	0	0	
19795320	f	md	FR	2021	0	0	0	0	0	0	0	0	0	0	0	0	43
19795388	Not	md	US	2021	0	0	0	0	0	0	0	0	0	0	0	0	
19795946	Not	md	US	2021	0	0	0	0	0	0	0	0	0	0	0	0	
19796149	f	Not repo	IN	2021	0	1	0	1	0	0	0	0	0	0	0	0	57
19796889	m	cn	JP	2021	0	1	1	0	0	0	0	0	0	0	0	0	88
19797038	m	md	JP	2021	0	1	0	0	0	1	0	0	0	0	0	0	
19797064	f	md	TW	2021	0	0	0	0	0	0	0	0	0	0	0	0	73

19797287	m	hp	US	2021	0	1	1	0	0	0	0	0	0	0	0	0	71
19797312	m	md	FR	2021	0	1	0	1	0	0	0	0	0	0	0	0	71
19797424	m	md	MY	2021	1	0	0	0	0	0	0	0	0	0	0	0	
19797800	f	cn	Not re	2021	0	0	0	0	0	0	0	0	0	0	0	0	59
19798713	f	Not repo	ID	2021	1	0	0	0	0	0	0	0	0	0	0	0	73.8
19798737	m	cn	IN	2021	0	0	0	0	0	0	0	0	0	0	0	0	48
19798891	f	hp	KR	2021	0	0	0	0	0	0	0	0	0	0	0	0	48
19798904	f	md	JP	2021	1	0	0	0	0	0	0	0	0	0	0	0	90
19799013	Not	ph	JP	2021	0	1	0	0	0	1	0	0	0	0	0	0	
19799257	m	cn	CN	2021	0	1	0	1	0	0	0	0	0	0	0	1	53
19799501	Not	md	NL	2021	0	0	0	0	0	0	0	0	0	0	0	0	
19799687	f	md	JP	2021	0	1	0	0	1	0	0	0	0	0	0	0	58
19800082	m	cn	CN	2021	0	1	0	0	1	0	0	0	0	0	0	0	69
19800325	f	md	GB	2021	0	1	0	0	0	1	0	0	0	0	0	0	71
19800597	Not	md	JP	2021	0	1	0	0	0	1	0	0	0	0	0	0	70
19801221	f	cn	US	2021	0	0	0	0	0	0	0	0	0	0	0	0	73
19801285	f	md	JP	2021	0	1	0	0	0	1	0	0	0	0	0	0	60
19801547	m	md	US	2021	0	0	0	0	0	0	0	0	0	0	0	0	
19801920	m	md	JP	2021	0	1	0	0	0	1	0	0	0	0	0	0	60
19802279	f	Not repo	ID	2021	1	0	0	0	0	0	0	0	0	0	0	0	72.6
19802989	m	hp	US	2021	0	1	0	0	1	0	0	0	0	0	0	0	71
19803433	f	hp	JP	2021	0	1	0	1	0	0	0	0	0	0	0	0	75
19803453	m	md	PL	2021	0	0	0	0	0	0	0	0	0	0	0	0	69
19803921	f	Not repo	CN	2021	0	1	0	1	0	0	0	0	0	0	0	0	43
19803922	m	md	PL	2021	0	0	0	0	0	0	0	0	0	0	0	0	66
19804519	f	Not repo	ID	2021	1	0	0	0	0	0	0	0	0	0	0	0	78.4
19804565	Not	md	TH	2021	0	0	0	0	0	0	0	0	0	0	0	0	
19804721	f	cn	IN	2021	0	0	0	0	0	0	0	0	0	0	0	0	62
19805205	m	md	JP	2021	0	1	0	0	0	1	0	0	0	0	0	1	
19805369	m	hp	JP	2021	1	0	0	0	0	0	0	0	0	0	0	0	73
19805370	f	cn	US	2021	1	0	0	0	0	0	0	0	0	0	0	0	84
19805389	f	md	US	2021	0	0	0	0	0	0	0	0	0	0	0	0	
19806009	m	cn	IN	2021	0	1	0	0	1	0	0	0	0	0	0	0	71
19806451	f	hp	JP	2021	0	1	0	0	1	0	0	0	0	0	0	0	50

19808003	Not	hp	US	2021	1	0	0	0	0	0	0	0	0	0	0	0	
19808309	Not	ph	PT	2021	0	1	0	0	0	1	0	0	0	0	0	0	
19808327	Not	hp	US	2021	0	1	0	0	1	0	0	0	0	0	0	0	
19808478	f	md	CA	2021	0	0	0	0	0	0	0	0	0	0	0	0	68
19808594	m	cn	Not re	2021	0	0	0	0	0	0	0	0	0	0	0	0	49
19808775	m	hp	DE	2021	0	1	1	0	0	0	0	0	0	0	0	0	
19809136	f	Not repo	RO	2021	0	1	0	1	0	0	0	0	0	0	0	0	58
19809356	m	ph	CA	2021	0	0	0	0	0	0	0	0	0	0	0	0	52
19809519	Not	md	US	2021	0	0	0	0	0	0	0	0	0	0	0	0	
19809532	f	cn	US	2021	0	0	0	0	0	0	0	0	0	0	0	0	54
19809542	f	ph	US	2021	0	0	0	0	0	0	0	0	0	0	0	0	
19809556	f	ph	US	2021	0	0	0	0	0	0	0	0	0	0	0	0	
19809559	f	cn	US	2021	0	0	0	0	0	0	0	0	0	0	0	0	
19809585	f	cn	US	2021	0	0	0	0	0	0	0	0	0	0	0	0	
19809594	f	cn	US	2021	0	0	0	0	0	0	0	0	0	0	0	0	53
19809602	f	cn	US	2021	0	0	0	0	0	0	0	0	0	0	0	0	51
19809617	f	ph	US	2021	0	0	0	0	0	0	0	0	0	0	0	0	
19809624	f	cn	US	2021	0	0	0	0	0	0	0	0	0	0	0	0	
19809632	m	cn	US	2021	0	0	0	0	0	0	0	0	0	0	0	0	
19809636	m	cn	US	2021	0	0	0	0	0	0	0	0	0	0	0	0	
19809638	f	md	US	2021	0	0	0	0	0	0	0	0	0	0	0	0	60
19809641	f	cn	US	2021	0	0	0	0	0	0	0	0	0	0	0	0	
19809644	f	cn	US	2021	0	0	0	0	0	0	0	0	0	0	0	0	45
19809649	Not	cn	US	2021	0	0	0	0	0	0	0	0	0	0	0	0	
19809660	m	cn	US	2021	0	0	0	0	0	0	0	0	0	0	0	0	
19809661	f	md	US	2021	0	0	0	0	0	0	0	0	0	0	0	0	62
19809699	f	cn	US	2021	0	0	0	0	0	0	0	0	0	0	0	0	
19809711	f	ph	US	2021	0	0	0	0	0	0	0	0	0	0	0	0	43
19809726	Not	cn	US	2021	0	0	0	0	0	0	0	0	0	0	0	0	
19809739	f	ph	US	2021	0	0	0	0	0	0	0	0	0	0	0	0	75
19809755	f	md	Not re	2021	0	0	0	0	0	0	0	0	0	0	0	0	
19809758	f	cn	US	2021	0	0	0	0	0	0	0	0	0	0	0	0	66
19809759	f	cn	US	2021	0	0	0	0	0	0	0	0	0	0	0	0	46
19809777	f	cn	US	2021	0	0	0	0	0	0	0	0	0	0	0	0	

19809780	m	cn	US	2021	0	0	0	0	0	0	0	0	0	0	0	0	
19809790	f	cn	US	2021	0	0	0	0	0	0	0	0	0	0	0	0	45
19809794	m	cn	US	2021	1	0	0	0	0	0	0	0	0	0	0	0	61.8
19809833	m	hp	US	2021	0	0	0	0	0	0	0	0	0	0	0	0	80
19809835	f	cn	US	2021	0	0	0	0	0	0	0	0	0	0	0	0	
19809842	f	cn	US	2021	0	0	0	0	0	0	0	0	0	0	0	0	
19809852	f	hp	US	2021	0	0	0	0	0	0	0	0	0	0	0	0	66
19809854	m	hp	US	2021	1	0	0	0	0	0	0	0	0	0	0	0	74
19809943	m	md	JP	2021	0	1	0	0	1	0	0	0	0	0	0	0	73
19810270	f	Not repo	CA	2021	1	0	0	0	0	0	0	0	0	0	0	0	
19810636	f	md	RU	2021	0	0	0	0	0	0	0	0	0	0	0	0	67
19810640	m	cn	IN	2021	0	0	0	0	0	0	0	0	0	0	0	0	61
19810717	f	md	DE	2021	0	1	1	0	0	0	0	0	0	0	0	0	73
19811044	f	cn	US	2021	0	1	0	1	0	0	0	0	0	0	0	0	
19811126	m	md	JP	2021	0	1	0	0	1	0	0	0	0	0	0	0	68
19811359	m	cn	IN	2021	0	1	0	0	1	0	0	0	0	0	0	0	71
19811524	m	md	US	2021	0	0	0	0	0	0	0	0	0	0	0	0	66
19811894	m	Not repo	DE	2021	0	1	0	1	0	0	0	0	0	0	0	0	
19812081	m	md	JP	2021	0	1	0	0	1	0	0	0	0	0	0	0	72
19813244	f	ph	JP	2021	0	0	0	0	0	0	0	0	0	0	0	0	70
19813858	m	md	CH	2021	0	1	0	0	0	1	0	0	0	0	0	0	61
19813938	f	md	JP	2021	0	1	0	1	0	0	0	0	0	0	0	0	
19814053	m	md	DE	2021	0	1	0	0	0	1	0	0	0	0	0	0	67
19814318	m	Not repo	MY	2021	1	0	0	0	0	0	0	0	0	0	0	0	
19814517	f	cn	IN	2021	0	1	0	0	1	0	0	0	0	0	0	0	53
19814615	f	md	BE	2021	0	0	0	0	0	0	0	0	0	0	0	0	56
19814683	m	md	IN	2021	0	0	0	0	0	0	0	0	0	0	0	0	68
19815205	f	cn	IN	2021	0	0	0	0	0	0	0	0	0	0	0	0	54
19815244	m	md	JP	2021	1	0	0	0	0	0	0	0	0	0	0	0	83
19815300	m	hp	CA	2021	0	1	0	0	1	0	0	0	0	0	0	0	54
19815395	f	Not repo	CN	2021	0	1	0	1	0	0	0	0	0	0	0	0	65
19815781	m	Not repo	AU	2021	1	0	0	0	0	0	0	0	0	0	0	0	58
19815800	m	cn	IN	2021	0	0	0	0	0	0	0	0	0	0	0	0	51
19815832	m	cn	IN	2021	0	0	0	0	0	0	0	0	0	0	0	0	67

19816312	f	cn	CA	2021	0	0	0	0	0	0	0	0	0	0	0	0	38
19816877	m	hp	DE	2021	0	0	0	0	0	0	0	0	0	0	0	0	60
19817119	m	hp	FR	2021	0	1	0	0	1	0	0	0	0	0	0	0	51
19817128	f	md	DE	2021	1	0	0	0	0	0	0	0	0	0	0	0	65
19817259	m	hp	CN	2021	0	0	0	0	0	0	0	0	0	0	0	0	71
19818110	m	md	JP	2021	0	1	0	0	0	1	0	0	0	0	0	1	70
19818313	m	hp	IN	2021	0	0	0	0	0	0	0	0	0	0	0	0	64
19818417	m	md	FR	2021	0	0	0	0	0	0	0	0	0	0	0	0	66
19818554	f	md	JP	2021	0	0	0	0	0	0	0	0	0	0	0	0	40
19818728	m	md	CA	2021	0	1	0	0	1	0	0	0	0	0	0	0	
19818850	m	cn	CA	2021	0	0	0	0	0	0	0	0	0	0	0	0	71
19818888	m	cn	JP	2021	0	1	1	0	0	0	0	0	0	0	0	0	60
19819338	m	cn	JP	2021	0	1	1	0	0	0	0	0	0	0	0	0	
19819594	Not	cn	JP	2021	0	1	1	0	0	0	0	0	0	0	0	0	
19820729	m	Not repo	MY	2021	1	0	0	0	0	0	0	0	0	0	0	0	67
19821641	m	md	JP	2021	0	1	0	1	0	0	0	0	0	0	0	1	78
19822542	m	md	JP	2021	0	1	0	1	0	0	0	0	0	0	0	1	66
19822766	m	cn	JP	2021	0	1	1	0	0	0	0	0	0	0	0	0	79
19822821	m	cn	IN	2021	0	0	0	0	0	0	0	0	0	0	0	0	50
19822822	m	cn	IN	2021	0	0	0	0	0	0	0	0	0	0	0	0	59
19822829	m	md	JP	2021	0	0	0	0	0	0	0	0	0	0	0	0	74
19822833	f	cn	IN	2021	0	0	0	0	0	0	0	0	0	0	0	0	46
19822923	m	Not repo	MY	2021	1	0	0	0	0	0	0	0	0	0	0	0	25
19823048	f	cn	IN	2021	0	0	0	0	0	0	0	0	0	0	0	0	53
19823063	m	cn	IN	2021	0	0	0	0	0	0	0	0	0	0	0	0	47
19823098	f	Not repo	BG	2021	1	0	0	0	0	0	0	0	0	0	0	0	
19823099	f	Not repo	ID	2021	1	0	0	0	0	0	0	0	0	0	0	0	72
19823159	f	cn	Not re	2021	0	0	0	0	0	0	0	0	0	0	0	0	
19823221	f	Not repo	PL	2021	1	0	0	0	0	0	0	0	0	0	0	0	
19823578	m	md	CH	2021	0	0	0	0	0	0	0	0	0	0	0	0	
19823647	f	hp	GB	2021	0	0	0	0	0	0	0	0	0	0	0	0	55
19823668	f	md	TR	2021	0	0	0	0	0	0	0	0	0	0	0	0	68
19823800	m	md	FR	2021	0	0	0	0	0	0	0	0	0	0	0	0	72
19823978	f	ph	GB	2021	0	1	1	0	0	0	0	0	0	0	0	0	69

19824092	f	Not repo	AU	2021	0	1	0	1	0	0	0	0	0	0	0	0	
19824093	m	md	JP	2021	0	1	0	1	0	0	0	0	0	0	0	1	63
19824146	f	md	DE	2021	0	0	0	0	0	0	0	0	0	0	0	0	77
19824167	f	cn	IN	2021	0	0	0	0	0	0	0	0	0	0	0	0	57
19824178	m	cn	IN	2021	0	0	0	0	0	0	0	0	0	0	0	0	60
19824224	f	cn	IN	2021	0	0	0	0	0	0	0	0	0	0	0	0	47
19824228	f	md	JP	2021	0	1	0	0	1	0	0	0	0	0	0	0	65
19826061	f	hp	JP	2021	0	1	0	1	0	0	0	0	0	0	0	0	75
19826668	f	md	SG	2021	0	1	0	0	0	1	0	0	0	0	0	0	
19826862	f	md	JP	2021	0	1	0	0	1	0	0	0	0	0	0	0	65
19827090	m	Not repo	MY	2021	1	0	0	0	0	0	0	0	0	0	0	0	78.5
19827156	m	hp	MY	2021	1	0	0	0	0	0	0	0	0	0	0	0	63.7
19827164	f	md	SK	2021	1	0	0	0	0	0	0	0	0	0	0	0	77
19827754	f	md	US	2021	0	0	0	0	0	0	0	0	0	0	0	0	63
19827874	f	cn	IN	2021	0	0	0	0	0	0	0	0	0	0	0	0	36
19827885	m	cn	IN	2021	0	0	0	0	0	0	0	0	0	0	0	0	57
19827927	f	cn	IN	2021	0	0	0	0	0	0	0	0	0	0	0	0	38
19827934	Not	md	AU	2021	1	0	0	0	0	0	0	0	0	0	0	0	
19828139	m	ph	JP	2021	1	0	0	0	0	0	0	0	0	0	0	0	77
19828235	m	hp	US	2021	0	1	1	0	0	0	0	0	0	0	0	0	80
19828435	f	md	GB	2021	0	1	0	0	0	1	0	0	0	0	0	0	71
19828725	m	hp	US	2021	0	1	1	0	0	0	0	0	0	0	0	0	80
19828997	f	cn	AT	2021	0	1	1	0	0	0	0	0	0	0	0	0	
19829038	f	md	DE	2021	0	0	0	0	0	0	0	0	0	0	0	0	57
19829439	f	cn	Not re	2021	0	0	0	0	0	0	0	0	0	0	0	0	
19829468	f	cn	Not re	2021	0	0	0	0	0	0	0	0	0	0	0	0	
19829469	m	cn	US	2021	1	0	0	0	0	0	0	0	0	0	0	0	60.7
19829564	m	md	CN	2021	0	1	0	0	0	1	0	0	0	0	0	0	68
19829828	m	hp	FR	2021	0	1	0	0	1	0	0	0	0	0	0	0	79
19830306	f	md	CN	2021	0	0	0	0	0	0	0	0	0	0	0	0	63
19830372	f	ph	US	2021	0	0	0	0	0	0	0	0	0	0	0	0	54
19832312	m	cn	JP	2021	0	1	1	0	0	0	0	0	0	0	0	0	71
19832384	Not	cn	GB	2021	0	1	1	0	0	0	0	0	0	0	0	0	
19833852	Not	md	JP	2021	1	0	0	0	0	0	0	0	0	0	0	0	

19834318	Not	md	JP	2021	0	1	0	0	1	0	0	0	0	0	0	0	
19834320	f	md	DE	2021	0	1	0	0	1	0	0	0	0	0	0	0	62
19834941	f	Not repo	JP	2021	0	1	0	1	0	0	0	0	0	0	0	1	60
19834943	m	md	JP	2021	0	1	0	1	0	0	0	0	0	0	0	0	69
19835034	f	cn	US	2021	0	1	1	0	0	0	0	0	0	0	0	0	
19835140	f	md	JP	2021	0	1	0	0	0	1	0	0	0	0	0	0	62
19835369	f	hp	US	2021	0	0	0	0	0	0	0	0	0	0	0	0	
19835509	Not	cn	US	2021	0	0	0	0	0	0	0	0	0	0	0	0	
19835584	m	ph	CA	2021	0	0	0	0	0	0	0	0	0	0	0	0	52
19835639	m	cn	CN	2021	0	1	0	0	1	0	0	0	0	0	0	0	71
19835675	f	ph	CA	2021	0	0	0	0	0	0	0	0	0	0	0	0	73
19835683	m	cn	IN	2021	0	0	0	0	0	0	0	0	0	0	0	0	50
19835890	m	cn	IN	2021	0	0	0	0	0	0	0	0	0	0	0	0	72
19836936	f	hp	US	2021	0	1	0	0	1	0	0	0	0	0	0	0	67
19837035	f	md	US	2021	0	0	0	0	0	0	0	0	0	0	0	0	53
19837081	f	md	US	2021	0	1	0	0	0	1	0	0	0	0	0	0	29
19837091	Not	md	US	2021	0	0	0	0	0	0	0	0	0	0	0	0	
19837161	m	cn	JP	2021	0	1	1	0	0	0	0	0	0	0	0	1	74
19837528	f	md	FR	2021	0	0	0	0	0	0	0	0	0	0	0	0	60
19837580	m	md	JP	2021	0	1	1	0	0	0	0	0	0	0	0	0	78
19838036	f	cn	IN	2021	0	0	0	0	0	0	0	0	0	0	0	0	70
19840216	Not	hp	SG	2021	1	0	0	0	0	0	0	0	0	0	0	0	68.5
19840418	m	hp	US	2021	0	1	0	0	1	0	0	0	0	0	0	0	66
19840435	m	md	CN	2021	0	1	0	1	0	0	0	0	0	0	0	0	70
19840548	Not	md	US	2021	0	0	0	0	0	0	0	0	0	0	0	0	
19840599	f	md	BR	2021	0	0	0	0	0	0	0	0	0	0	0	0	73
19840610	Not	md	JP	2021	0	1	0	0	1	0	0	0	0	0	0	0	
19840717	Not	md	JP	2021	0	1	0	1	0	0	0	0	0	0	0	0	
19840719	Not	Not repo	AU	2021	0	1	0	1	0	0	0	0	0	0	0	0	
19840884	m	hp	US	2021	0	1	0	0	1	0	0	0	0	0	0	0	83
19840987	Not	Not repo	AU	2021	0	1	0	1	0	0	0	0	0	0	0	0	
19841255	m	cn	SE	2021	0	1	1	0	0	0	0	0	0	0	0	0	67
19841726	m	Not repo	CN	2021	0	1	0	1	0	0	0	0	0	0	0	0	
19841972	f	hp	IT	2021	0	0	0	0	0	0	0	0	0	0	0	0	80

19841974	m	md	DE	2021	1	0	0	0	0	0	0	0	0	0	0	0	62
19842488	m	md	JP	2021	0	1	0	0	0	1	0	0	0	0	0	1	76
19843508	m	md	JP	2021	0	1	0	0	1	0	0	0	0	0	0	0	73
19843557	f	md	JP	2021	0	1	0	0	1	0	0	0	0	0	0	0	70
19843699	Not	hp	US	2021	0	1	0	0	0	1	0	0	0	0	0	0	
19843907	m	md	DE	2021	0	1	0	0	0	1	0	0	0	0	0	0	69
19843970	m	md	CH	2021	0	0	0	0	0	0	0	0	0	0	0	0	74
19844078	m	Not repo	CN	2021	0	1	0	1	0	0	0	0	0	0	0	0	52
19844084	Not	md	BG	2021	0	1	0	1	0	0	0	0	0	0	0	0	
19844235	f	md	GB	2021	0	1	0	0	0	1	0	0	0	0	0	0	63
19844293	Not	Not repo	US	2021	1	0	0	0	0	0	0	0	0	0	0	0	
19844364	m	cn	CN	2021	0	1	0	0	1	0	0	0	0	0	0	0	73
19844715	m	md	JP	2021	1	0	0	0	0	0	0	0	0	0	0	1	82
19844824	m	Not repo	CN	2021	0	1	0	1	0	0	0	0	0	0	0	0	81
19845028	m	hp	CA	2021	0	1	0	0	1	0	0	0	0	0	0	0	75
19845243	m	Not repo	CN	2021	0	1	0	1	0	0	0	0	0	0	0	1	61
19845421	m	md	JP	2021	0	0	0	0	0	0	0	0	0	0	0	0	71
19845735	f	md	JP	2021	0	0	0	0	0	0	0	0	0	0	0	0	67
19845742	f	md	JP	2021	0	0	0	0	0	0	0	0	0	0	0	0	68
19845838	f	md	BE	2021	0	0	0	0	0	0	0	0	0	0	0	0	68
19845846	Not	md	US	2021	0	0	0	0	0	0	0	0	0	0	0	0	
19845903	m	ph	DE	2021	0	1	1	0	0	0	0	0	0	0	0	1	52
19846048	f	md	CN	2021	0	0	0	0	0	0	0	0	0	0	0	0	49
19846743	Not	md	JP	2021	0	1	0	1	0	0	0	0	0	0	0	0	
19846744	f	cn	CN	2021	1	0	0	0	0	0	0	0	0	0	0	0	70.2
19847154	f	md	AU	2021	1	0	0	0	0	0	0	0	0	0	0	0	
19847509	m	cn	TW	2021	0	0	0	0	0	0	0	0	0	0	0	0	63
19847564	f	Not repo	ID	2021	1	0	0	0	0	0	0	0	0	0	0	0	
19847607	Not	md	US	2021	0	0	0	0	0	0	0	0	0	0	0	0	
19847673	f	Not repo	CN	2021	1	0	0	0	0	0	0	0	0	0	0	0	77
19849226	m	md	JP	2021	0	0	0	0	0	0	0	0	0	0	0	0	76
19849259	f	cn	Not re	2021	0	0	0	0	0	0	0	0	0	0	0	0	42
19849382	m	hp	CN	2021	0	1	0	0	1	0	0	0	0	0	0	0	59
19849467	m	hp	ES	2021	1	0	0	0	0	0	0	0	0	0	0	0	84

19849614	f	cn	IN	2021	1	0	0	0	0	0	0	0	0	0	0	0	50
19849620	m	hp	FR	2021	0	1	0	0	1	0	0	0	0	0	0	0	82
19849739	m	md	US	2021	0	0	0	0	0	0	0	0	0	0	0	0	
19849853	Not	md	US	2021	0	0	0	0	0	0	0	0	0	0	0	0	
19849951	f	hp	US	2021	0	1	1	0	0	0	0	0	0	0	0	0	
19849960	m	cn	JP	2021	0	0	0	0	0	0	0	0	0	0	0	0	60
19850088	Not	md	US	2021	0	0	0	0	0	0	0	0	0	0	0	0	
19850179	Not	md	US	2021	0	0	0	0	0	0	0	0	0	0	0	0	
19850202	m	hp	IT	2021	0	1	0	0	1	0	0	0	0	0	0	0	70
19850220	Not	md	US	2021	0	0	0	0	0	0	0	0	0	0	0	0	
19850239	f	md	GB	2021	0	1	0	0	0	1	0	0	0	0	0	0	63
19850249	Not	md	US	2021	0	0	0	0	0	0	0	0	0	0	0	0	
19850260	f	hp	IT	2021	0	1	0	0	1	0	0	0	0	0	0	0	63
19850294	f	cn	JP	2021	0	0	0	0	0	0	0	0	0	0	0	0	66
19850450	Not	md	US	2021	0	0	0	0	0	0	0	0	0	0	0	0	
19850487	Not	md	US	2021	0	0	0	0	0	0	0	0	0	0	0	0	
19850509	Not	md	US	2021	0	0	0	0	0	0	0	0	0	0	0	0	
19853503	m	cn	Not re	2021	0	0	0	0	0	0	0	0	0	0	0	0	47
19853517	f	cn	Not re	2021	0	0	0	0	0	0	0	0	0	0	0	0	78
19853542	f	md	DE	2021	0	0	0	0	0	0	0	0	0	0	0	0	69
19853607	m	md	FR	2021	0	1	0	0	0	1	0	0	0	0	0	0	77
19853868	Not	md	US	2021	0	0	0	0	0	0	0	0	0	0	0	0	
19853889	Not	md	US	2021	0	0	0	0	0	0	0	0	0	0	0	0	
19853955	m	cn	JP	2021	0	1	1	0	0	0	0	0	0	0	0	1	77
19854077	m	md	US	2021	1	0	0	0	0	0	0	0	0	0	0	0	72
19854155	Not	md	US	2021	0	0	0	0	0	0	0	0	0	0	0	0	
19854596	m	cn	CN	2021	0	1	0	0	1	0	0	0	0	0	0	0	76
19854638	m	md	CN	2021	0	1	0	0	0	1	0	0	0	0	0	0	49
19854667	m	hp	JP	2021	0	1	1	0	0	0	0	0	0	0	0	0	66
19854778	Not	md	US	2021	0	0	0	0	0	0	0	0	0	0	0	0	
19855050	f	Not repo	US	2021	1	0	0	0	0	0	0	0	0	0	0	0	67
19855261	Not	hp	JP	2021	0	1	0	0	1	0	0	0	0	0	0	0	
19855474	Not	md	US	2021	0	0	0	0	0	0	0	0	0	0	0	0	
19855559	f	md	JP	2021	0	0	0	0	0	0	0	0	0	0	0	0	

19855566	f	md	DE	2021	0	1	0	0	1	0	0	0	0	0	0	0	62
19855730	m	hp	US	2021	0	1	0	0	1	0	0	0	0	0	0	0	63
19855857	f	Not repo	US	2021	1	0	0	0	0	0	0	0	0	0	0	1	
19855871	Not	hp	JP	2021	0	1	0	0	1	0	0	0	0	0	0	0	
19855996	Not	md	US	2021	0	0	0	0	0	0	0	0	0	0	0	0	
19856005	Not	md	US	2021	0	0	0	0	0	0	0	0	0	0	0	0	
19856368	m	Not repo	CA	2021	1	0	0	0	0	0	0	0	0	0	0	0	
19856519	f	hp	CN	2021	0	1	0	0	1	0	0	0	0	0	0	0	73
19856870	Not	md	JP	2021	0	1	0	0	1	0	0	0	0	0	0	0	
19856871	Not	md	US	2021	0	0	0	0	0	0	0	0	0	0	0	0	
19856898	Not	md	US	2021	0	0	0	0	0	0	0	0	0	0	0	0	
19856989	Not	md	US	2021	0	0	0	0	0	0	0	0	0	0	0	0	
19857013	Not	md	US	2021	0	0	0	0	0	0	0	0	0	0	0	0	
19857084	Not	md	US	2021	0	0	0	0	0	0	0	0	0	0	0	0	
19857097	m	md	JP	2021	0	0	0	0	0	0	0	0	0	0	0	0	70
19857101	Not	md	US	2021	0	0	0	0	0	0	0	0	0	0	0	0	
19857102	Not	md	US	2021	0	0	0	0	0	0	0	0	0	0	0	0	
19857109	f	cn	Not re	2021	0	0	0	0	0	0	0	0	0	0	0	0	
19857165	f	cn	CN	2021	0	1	0	0	1	0	0	0	0	0	0	0	60
19857170	m	cn	IN	2021	0	1	0	0	1	0	0	0	0	0	0	0	64
19857201	Not	md	US	2021	0	0	0	0	0	0	0	0	0	0	0	0	
19857216	f	md	DE	2021	0	0	0	0	0	0	0	0	0	0	0	0	51
19857308	m	ph	JP	2021	0	1	0	0	1	0	0	0	0	0	0	0	73
19857369	Not	md	GB	2021	0	0	0	0	0	0	0	0	0	0	0	0	
19857385	m	cn	IN	2021	0	1	0	0	1	0	0	0	0	0	0	0	63
19857439	Not	md	US	2021	0	0	0	0	0	0	0	0	0	0	0	0	
19857444	Not	md	US	2021	0	0	0	0	0	0	0	0	0	0	0	0	
19857449	Not	md	US	2021	0	0	0	0	0	0	0	0	0	0	0	0	
19857466	Not	md	US	2021	0	0	0	0	0	0	0	0	0	0	0	0	
19857754	m	cn	IN	2021	0	1	0	0	1	0	0	0	0	0	0	0	67
19858096	m	md	JP	2021	0	1	0	0	1	0	0	0	0	0	0	1	73
19858107	m	ph	JP	2021	0	1	0	0	0	1	0	0	0	0	0	0	77
19860034	Not	md	US	2021	0	0	0	0	0	0	0	0	0	0	0	0	
19860038	Not	md	US	2021	0	0	0	0	0	0	0	0	0	0	0	0	

19860359	m	cn	IN	2021	0	1	0	0	1	0	0	0	0	0	0	0	59
19860608	m	cn	IN	2021	0	1	0	0	1	0	0	0	0	0	0	0	65
19860702	Not	md	US	2021	0	0	0	0	0	0	0	0	0	0	0	0	
19860712	m	hp	US	2021	0	0	0	0	0	0	0	0	0	0	0	0	
19860893	m	md	JP	2021	0	1	0	0	0	1	0	0	0	0	0	0	80
19860970	f	hp	CA	2021	0	0	0	0	0	0	0	0	0	0	0	0	
19861318	f	hp	US	2021	0	0	0	0	0	0	0	0	0	0	0	0	70
19861340	Not	md	US	2021	0	0	0	0	0	0	0	0	0	0	0	0	
19861619	Not	cn	Not re	2021	0	0	0	0	0	0	0	0	0	0	0	0	
19861688	m	cn	IN	2021	0	0	0	0	0	0	0	0	0	0	0	0	78
19861832	Not	md	JP	2021	0	0	0	0	0	0	0	0	0	0	0	0	
19861851	m	cn	US	2021	0	1	1	0	0	0	0	0	0	0	0	0	73
19861883	Not	md	JP	2021	0	0	0	0	0	0	0	0	0	0	0	0	
19861897	f	cn	IN	2021	0	0	0	0	0	0	0	0	0	0	0	0	38
19861917	Not	md	JP	2021	0	0	0	0	0	0	0	0	0	0	0	0	
19862060	m	md	CN	2021	0	1	0	1	0	0	0	0	0	0	0	0	76.7
19862095	Not	md	JP	2021	0	0	0	0	0	0	0	0	0	0	0	0	
19862337	m	md	IL	2021	0	1	0	1	0	0	0	0	0	0	0	0	
19862463	Not	hp	US	2021	0	0	0	0	0	0	0	0	0	0	0	0	
19862585	f	md	CN	2021	0	0	0	0	0	0	0	0	0	0	0	0	69
19862588	Not	Not repo	JP	2021	1	0	0	0	0	0	0	0	0	0	0	0	
19862626	m	cn	JP	2021	0	1	1	0	0	0	0	0	0	0	0	0	74
19862731	f	cn	US	2021	0	1	1	0	0	0	0	0	0	0	0	0	81
19862735	f	Not repo	US	2021	0	1	0	1	0	0	0	0	0	0	0	0	
19862777	m	md	JP	2021	0	1	0	0	0	1	0	0	0	0	0	0	72
19862791	f	md	JP	2021	0	1	0	0	0	1	0	0	0	0	0	0	70
19862833	m	hp	MY	2021	0	0	0	0	0	0	0	0	0	0	0	0	74
19862874	Not	md	JP	2021	0	0	0	0	0	0	0	0	0	0	0	0	
19863664	m	md	HR	2021	0	1	1	0	0	0	0	0	0	0	0	0	52
19863886	f	md	US	2021	1	0	0	0	0	0	0	0	0	0	0	1	
19864503	f	md	JP	2021	0	0	0	0	0	0	0	0	0	0	0	0	72
19864514	f	cn	IN	2021	0	0	0	0	0	0	0	0	0	0	0	0	48
19864569	m	md	GB	2021	0	1	0	0	0	1	0	0	0	0	0	0	76
19864636	f	md	US	2021	0	0	0	0	0	0	0	0	0	0	0	0	

19864667	m	Not repo	ID	2021	1	0	0	0	0	0	0	0	0	0	0	0	63.7
19864782	f	cn	IN	2021	0	0	0	0	0	0	0	0	0	0	0	0	69
19864828	f	md	JP	2021	0	0	0	0	0	0	0	0	0	0	0	0	44
19864921	m	md	US	2021	0	0	0	0	0	0	0	0	0	0	0	0	90
19865139	Not	hp	JP	2021	0	0	0	0	0	0	0	0	0	0	0	0	90
19865240	m	hp	ES	2021	0	1	0	0	1	0	0	0	0	0	0	0	74
19865689	f	md	JP	2021	0	1	0	0	0	1	0	0	0	0	0	0	67
19865829	m	md	RO	2021	0	1	1	0	0	0	0	0	0	0	0	0	58
19865842	Not	md	US	2021	0	0	0	0	0	0	0	0	0	0	0	0	
19865850	f	cn	US	2021	1	0	0	0	0	0	0	0	0	0	0	0	71.8
19865880	m	md	JP	2021	0	1	0	0	0	1	0	0	0	0	0	0	73
19865977	Not	md	US	2021	0	0	0	0	0	0	0	0	0	0	0	0	
19865987	Not	md	US	2021	0	0	0	0	0	0	0	0	0	0	0	0	
19866323	f	md	DK	2021	0	0	0	0	0	0	0	0	0	0	0	0	69
19866514	f	md	GB	2021	0	1	0	0	0	1	0	0	0	0	0	0	63
19866518	m	hp	NO	2021	0	0	0	0	0	0	0	0	0	0	0	0	
19867277	f	md	FR	2021	0	0	0	0	0	0	0	0	0	0	0	0	60
19867328	f	cn	CA	2021	0	0	0	0	0	0	0	0	0	0	0	0	59
19867416	f	md	US	2021	0	1	1	0	0	0	0	0	0	0	0	0	
19867472	m	md	JP	2021	0	1	0	0	0	1	0	0	0	0	0	0	80
19867566	Not	md	US	2021	0	0	0	0	0	0	0	0	0	0	0	0	
19867601	m	hp	JP	2021	0	0	0	0	0	0	0	0	0	0	0	0	82
19867664	Not	md	US	2021	0	0	0	0	0	0	0	0	0	0	0	0	
19867937	f	cn	SG	2021	0	0	0	0	0	0	0	0	0	0	0	0	
19868236	m	md	JP	2021	0	0	0	0	0	0	0	0	0	0	0	0	70
19868273	m	md	ID	2021	0	1	1	0	0	0	0	0	0	0	0	0	55
19868343	Not	md	US	2021	0	0	0	0	0	0	0	0	0	0	0	0	
19868489	Not	md	US	2021	0	0	0	0	0	0	0	0	0	0	0	0	
19868582	f	md	JP	2021	0	1	0	0	0	1	0	0	0	0	0	0	60
19868701	f	md	IN	2021	0	0	0	0	0	0	0	0	0	0	0	0	57
19868726	f	cn	JP	2021	0	1	1	0	0	0	0	0	0	0	0	0	65
19868926	f	cn	IN	2021	0	0	0	0	0	0	0	0	0	0	0	0	67
19868952	m	cn	JP	2021	0	1	1	0	0	0	0	0	0	0	0	0	60
19869199	m	cn	JP	2021	0	1	1	0	0	0	0	0	0	0	0	0	80

19869259	f	Not repo	ID	2021	1	0	0	0	0	0	0	0	0	0	0	0	64.3
19869406	m	cn	CN	2021	0	1	0	0	1	0	0	0	0	0	0	0	78
19870533	m	Not repo	JP	2021	0	1	0	1	0	0	0	0	0	0	0	1	70
19871066	Not	hp	NL	2021	0	0	0	0	0	0	0	0	0	0	0	0	
19871353	m	cn	JP	2021	0	1	1	0	0	0	0	0	0	0	0	0	80
19871518	f	md	FR	2021	0	0	0	0	0	0	0	0	0	0	0	0	
19871855	m	md	ES	2021	0	1	0	0	0	1	0	0	0	0	0	0	62
19872632	m	hp	JP	2021	0	1	0	0	1	0	0	0	0	0	0	0	
19872732	m	cn	TH	2021	0	0	0	0	0	0	0	0	0	0	0	0	48
19872954	f	hp	KR	2021	0	0	0	0	0	0	0	0	0	0	0	0	
19872967	m	Not repo	JP	2021	0	1	0	1	0	0	0	0	0	0	0	0	68
19873273	f	md	GB	2021	0	1	0	0	0	1	0	0	0	0	0	0	54
19873701	m	md	IN	2021	0	0	0	0	0	0	0	0	0	0	0	0	35
19873787	f	hp	FR	2021	1	0	0	0	0	0	0	0	0	0	0	0	68
19873831	m	hp	FR	2021	1	0	0	0	0	0	0	0	0	0	0	0	77
19873864	f	hp	FR	2021	1	0	0	0	0	0	0	0	0	0	0	0	71
19874586	f	cn	JP	2021	0	1	1	0	0	0	0	0	0	0	0	1	73
19875212	f	cn	US	2021	1	0	0	0	0	0	0	0	0	0	0	0	
19875241	m	hp	FR	2021	0	1	0	0	1	0	0	0	0	0	0	0	77
19876262	m	Not repo	CN	2021	0	1	0	1	0	0	0	0	0	0	0	1	61
19876653	f	hp	BE	2021	0	0	0	0	0	0	0	0	0	0	0	0	72
19876682	m	cn	US	2021	0	0	0	0	0	0	0	0	0	0	0	0	86
19877337	Not	Not repo	US	2021	1	0	0	0	0	0	0	0	0	0	0	0	
19877356	m	cn	CN	2021	0	1	0	0	1	0	0	0	0	0	0	0	69
19877785	f	cn	Not re	2021	0	0	0	0	0	0	0	0	0	0	0	0	56
19877802	f	md	IL	2021	1	0	0	0	0	0	0	0	0	0	0	0	
19877898	m	Not repo	MY	2021	0	1	0	1	0	0	0	0	0	0	0	0	76
19878002	f	hp	US	2021	0	0	0	0	0	0	0	0	0	0	0	0	55
19878344	m	md	JP	2021	0	1	0	0	1	0	0	0	0	0	0	0	68
19878997	m	cn	CN	2021	0	1	0	0	1	0	0	0	0	0	0	0	77
19879304	f	hp	US	2021	1	0	0	0	0	0	0	0	0	0	0	0	77
19879724	f	md	JP	2021	0	1	0	0	0	1	0	0	0	0	0	1	
19879751	f	ph	JP	2021	1	0	0	0	0	0	0	0	0	0	0	0	85
19879752	f	md	JP	2021	1	0	0	0	0	0	0	0	0	0	0	0	

19880025	f	cn	US	2021	0	0	0	0	0	0	0	0	0	0	0	0	
19880079	Not	md	CA	2021	1	0	0	0	0	0	0	0	0	0	0	0	
19880192	f	cn	JP	2021	0	1	1	0	0	0	0	0	0	0	0	0	83
19880437	Not	hp	US	2021	0	1	0	0	1	0	0	0	0	0	0	1	
19880518	f	Not repo	GR	2021	1	0	0	0	0	0	0	0	0	0	0	0	66
19880609	m	Not repo	MY	2021	1	0	0	0	0	0	0	0	0	0	0	0	
19880805	m	md	US	2021	0	0	0	0	0	0	0	0	0	0	0	0	73
19880881	f	md	FR	2021	0	0	0	0	0	0	0	0	0	0	0	0	
19881126	f	md	JP	2021	0	1	0	0	1	0	0	0	0	0	0	0	73
19881320	f	md	JP	2021	0	1	0	0	0	1	0	0	0	0	0	1	90
19881423	m	md	DE	2021	0	1	0	0	0	1	0	0	0	0	0	0	69
19881537	f	md	CN	2021	0	0	0	0	0	0	0	0	0	0	0	0	61
19882005	m	md	JP	2021	0	1	0	0	1	0	0	0	0	0	0	1	54
19882118	m	md	DE	2021	0	1	0	1	0	0	0	0	0	0	0	0	
19882127	m	cn	US	2021	0	1	1	0	0	0	0	0	0	0	0	0	
19882147	f	md	PL	2021	0	0	0	0	0	0	0	0	0	0	0	0	78
19882184	f	cn	US	2021	1	0	0	0	0	0	0	0	0	0	0	0	
19882271	m	md	NL	2021	0	0	0	0	0	0	0	0	0	0	0	0	70
19882273	m	md	JP	2021	0	0	0	0	0	0	0	0	0	0	0	0	74
19882840	f	md	JP	2021	0	1	0	0	0	1	0	0	0	0	0	0	76
19883396	m	cn	CN	2021	0	1	0	0	1	0	0	0	0	0	0	0	51
19883414	m	md	JP	2021	0	1	0	0	1	0	0	0	0	0	0	0	60
19883663	m	md	JP	2021	0	1	0	0	0	1	0	0	0	0	0	0	62
19883999	f	md	GB	2021	0	1	0	0	0	1	0	0	0	0	0	0	71
19884051	m	cn	JP	2021	0	1	1	0	0	0	0	0	0	0	0	1	74
19885035	f	md	FR	2021	0	1	0	0	0	1	0	0	0	0	0	1	57
19885618	m	cn	IN	2021	0	1	0	0	1	0	0	0	0	0	0	0	70
19885619	f	cn	US	2021	1	0	0	0	0	0	0	0	0	0	0	0	58
19885684	Not	md	JP	2021	0	1	0	0	0	1	0	0	0	0	0	0	70
19885733	m	cn	IN	2021	0	1	0	0	1	0	0	0	0	0	0	0	69
19885752	f	cn	SG	2021	0	0	0	0	0	0	0	0	0	0	0	0	53
19885821	f	cn	MY	2021	1	0	0	0	0	0	0	0	0	0	0	0	
19885822	Not	cn	US	2021	1	0	0	0	0	0	0	0	0	0	0	0	77.7
19886046	m	cn	IN	2021	0	0	0	0	0	0	0	0	0	0	0	0	61

19886169	f	cn	IN	2021	0	0	0	0	0	0	0	0	0	0	0	0	38
19886404	f	cn	IN	2021	0	0	0	0	0	0	0	0	0	0	0	0	47
19886508	Not	hp	US	2021	0	0	0	0	0	0	0	0	0	0	0	0	
19886864	m	cn	FR	2021	0	1	1	0	0	0	0	0	0	0	0	0	
19887200	f	md	FR	2021	0	0	0	0	0	0	0	0	0	0	0	0	67
19887480	Not	hp	ES	2021	0	0	0	0	0	0	0	0	0	0	0	0	
19887638	m	hp	NL	2021	0	0	0	0	0	0	0	0	0	0	0	0	70
19888036	Not	hp	SG	2021	1	0	0	0	0	0	0	0	0	0	0	0	69
19888077	f	cn	Not re	2021	0	0	0	0	0	0	0	0	0	0	0	0	61
19888247	f	cn	SG	2021	1	0	0	0	0	0	0	0	0	0	0	0	75.5
19888272	m	hp	US	2021	0	0	0	0	0	0	0	0	0	0	0	0	
19888910	f	hp	JP	2021	1	0	0	0	0	0	0	0	0	0	0	0	70
19889425	f	cn	US	2021	1	0	0	0	0	0	0	0	0	0	0	1	58
19889557	Not	cn	SG	2021	1	0	0	0	0	0	0	0	0	0	0	0	79.7
19889767	f	md	CN	2021	0	1	0	0	0	1	0	0	0	0	0	0	60
19890004	m	md	DE	2021	0	1	1	0	0	0	0	0	0	0	0	0	39
19890126	f	Not repo	MY	2021	1	0	0	0	0	0	0	0	0	0	0	0	59.6
19890346	f	hp	IT	2021	1	0	0	0	0	0	0	0	0	0	0	0	54
19890355	m	cn	SG	2021	1	0	0	0	0	0	0	0	0	0	0	0	71.4
19890558	Not	cn	SG	2021	1	0	0	0	0	0	0	0	0	0	0	0	75
19890824	Not	md	NL	2021	0	0	0	0	0	0	0	0	0	0	0	0	
19890830	m	hp	CH	2021	0	1	1	0	0	0	0	0	0	0	0	0	58
19891075	f	md	HK	2021	0	0	0	0	0	0	0	0	0	0	0	0	
19891210	f	cn	IN	2021	0	0	0	0	0	0	0	0	0	0	0	0	43
19891346	f	cn	IN	2021	0	0	0	0	0	0	0	0	0	0	0	0	52
19891454	f	md	US	2021	0	0	0	0	0	0	0	0	0	0	0	0	61
19892734	f	md	US	2021	0	0	0	0	0	0	0	0	0	0	0	0	70
19893783	m	md	DE	2021	0	1	1	0	0	0	0	0	0	0	0	0	73
19894098	m	hp	IN	2021	0	0	0	0	0	0	0	0	0	0	0	0	46
19894695	m	md	DE	2021	0	1	0	0	0	1	0	0	0	0	0	0	69
19894811	f	hp	US	2021	0	0	0	0	0	0	0	0	0	0	0	0	75
19895153	f	md	FR	2021	0	1	0	0	0	1	0	0	0	0	0	0	43
19895463	f	cn	CN	2021	0	0	0	0	0	0	0	0	0	0	0	0	71
19895750	f	md	GB	2021	0	1	0	0	0	1	0	0	0	0	0	0	50

19895867	Not	md	NL	2021	0	0	0	0	0	0	0	0	0	0	0	0	
19895979	f	md	GB	2021	0	1	0	0	0	1	0	0	0	0	0	0	
19896061	Not	md	JP	2021	1	0	0	0	0	0	0	0	0	0	0	0	
19896086	f	md	US	2021	0	0	0	0	0	0	0	0	0	0	0	0	53
19896400	f	md	JP	2021	0	0	0	0	0	0	0	0	0	0	0	0	53
19896505	m	md	JP	2021	0	0	0	0	0	0	0	0	0	0	0	0	80
19898702	f	md	CN	2021	0	0	0	0	0	0	0	0	0	0	0	0	64
19899196	m	cn	PK	2021	0	1	0	0	0	1	0	0	0	0	0	0	60
19899249	m	cn	US	2021	0	1	1	0	0	0	0	0	0	0	0	0	76
19899345	f	Not repo	MY	2021	1	0	0	0	0	0	0	0	0	0	0	0	
19899404	m	cn	Not re	2021	0	0	0	0	0	0	0	0	0	0	0	0	72
19899767	m	ph	US	2021	0	0	0	0	0	0	0	0	0	0	0	0	73
19899826	f	cn	US	2021	0	0	0	0	0	0	0	0	0	0	0	0	
19900158	m	hp	NL	2021	0	1	0	0	1	0	0	0	0	0	0	0	63
19900207	f	hp	AU	2021	0	1	1	0	0	0	0	0	0	0	0	0	
19900234	f	md	FR	2021	0	1	0	0	0	1	0	0	0	0	0	0	59
19900393	m	hp	US	2021	0	0	0	0	0	0	0	0	0	0	0	0	71
19900585	m	md	US	2021	0	0	0	0	0	0	0	0	0	0	0	0	72
19900703	m	hp	US	2021	0	1	1	0	0	0	0	0	0	0	0	0	68
19900969	Not	hp	CN	2021	0	0	0	0	0	0	0	0	0	0	0	0	
19901123	m	md	IT	2021	0	0	0	0	0	0	0	0	0	0	0	0	
19901175	f	md	AT	2021	0	0	0	0	0	0	0	0	0	0	0	0	62
19901813	f	ph	JP	2021	0	0	0	0	0	0	0	0	0	0	0	0	90
19901863	m	cn	US	2021	0	0	0	0	0	0	0	0	0	0	0	0	
19902025	m	md	JP	2021	0	1	0	0	1	0	0	0	0	0	0	0	70
19902194	m	md	NL	2021	0	0	0	0	0	0	0	0	0	0	0	0	70
19905093	m	md	JP	2021	0	0	0	0	0	0	0	0	0	0	0	0	88
19905208	m	hp	FR	2021	0	1	0	0	1	0	0	0	0	0	0	0	69
19906260	m	md	FR	2021	0	0	0	0	0	0	0	0	0	0	0	0	76
19906306	f	cn	Not re	2021	0	0	0	0	0	0	0	0	0	0	0	0	48
19906582	m	md	JP	2021	0	1	0	1	0	0	0	0	0	0	0	1	64.2
19906824	f	cn	JP	2021	0	1	1	0	0	0	0	0	0	0	0	0	90
19907383	m	cn	US	2021	0	1	1	0	0	0	0	0	0	0	0	0	75
19907968	m	ph	IS	2021	0	1	0	0	0	1	0	0	0	0	0	0	

19908009	m	md	JP	2021	0	1	0	0	0	1	0	0	0	0	0	1	75
19064628	f	md	CA	2021	0	1	1	0	0	0	0	0	0	0	0	0	62
19115659	m	md	RU	2021	0	1	1	0	0	0	0	0	0	0	0	0	63
19217114	m	md	AT	2021	0	0	0	0	0	0	0	0	0	0	0	0	42
19306318	m	md	DE	2021	0	0	0	0	0	0	0	0	0	0	0	0	89
19308625	f	md	CR	2021	0	0	0	0	0	0	0	0	0	0	0	0	73
19328744	f	md	CN	2021	0	0	0	0	0	0	0	0	0	0	0	0	67
19359734	m	md	JP	2021	0	1	0	0	1	0	0	0	0	0	0	0	61
19391919	m	md	DE	2021	0	1	0	0	1	0	0	0	0	0	0	0	58
19415011	m	ph	JP	2021	0	1	0	0	0	1	0	0	0	0	0	0	57
19436942	f	md	FR	2021	0	0	0	0	0	0	0	0	0	0	0	0	69
19454409	f	md	ES	2021	0	1	0	0	0	1	0	0	0	0	0	0	62
19467881	m	md	TR	2021	0	1	0	0	0	1	0	0	0	0	0	0	59
19472731	m	ph	JP	2021	0	1	0	0	1	0	0	0	0	0	0	0	73
19474304	m	md	JP	2021	0	1	0	0	1	0	0	0	0	0	0	0	74
19487436	m	hp	JP	2021	0	1	0	0	1	0	0	0	0	0	0	0	77
19488895	m	md	FR	2021	0	1	0	0	0	1	0	0	0	0	0	0	74
19495560	f	md	FR	2021	0	1	0	0	1	0	0	0	0	0	0	0	
19495586	f	md	FR	2021	0	1	0	0	1	0	0	0	0	0	0	0	
19499887	m	hp	US	2021	0	1	1	0	0	0	0	0	0	0	0	0	71
19502563	m	md	JP	2021	0	0	0	0	0	0	0	0	0	0	0	0	87
19509375	f	md	JP	2021	0	1	0	0	1	0	0	0	0	0	0	0	71
19515003	m	md	IT	2021	0	0	0	0	0	0	0	0	0	0	0	0	66
19516907	Not	md	JP	2021	0	1	0	0	1	0	0	0	0	0	0	0	
19522145	m	md	US	2021	0	0	0	0	0	0	0	0	0	0	0	0	54
19549373	f	md	JP	2021	1	0	0	0	0	0	0	0	0	0	0	0	68
19567973	f	cn	DE	2021	0	0	0	0	0	0	0	0	0	0	0	0	72
19578177	Not	md	FR	2021	0	1	0	0	0	1	0	0	0	0	0	0	
19581969	Not	md	JP	2021	0	0	0	0	0	0	0	0	0	0	0	0	
19592008	m	md	JP	2021	0	0	0	0	0	0	0	0	0	0	0	0	80
19594173	m	md	US	2021	0	0	0	0	0	0	0	0	0	0	0	0	64
19594524	Not	md	JP	2021	0	1	0	0	0	1	0	0	0	0	0	0	
19596901	m	md	JP	2021	0	1	0	0	1	0	0	0	0	0	0	0	73
19606171	Not	md	JP	2021	0	1	1	0	0	0	0	0	0	0	0	0	80

19624481	m	md	JP	2021	0	1	0	0	1	0	0	0	0	0	0	0	69
19627328	f	md	JP	2021	0	1	0	0	1	0	0	0	0	0	0	1	55
19640786	m	md	JP	2021	0	1	0	0	1	0	0	0	0	0	0	0	61
19641320	f	md	FR	2021	0	1	0	0	0	1	0	0	0	0	0	0	54
19645042	m	md	FR	2021	0	1	0	0	0	1	0	0	0	0	0	0	56
19646412	m	md	KR	2021	0	0	0	0	0	0	0	0	0	0	0	0	70
19646948	m	md	JP	2021	0	1	0	0	0	1	0	0	0	0	0	0	68
19647638	m	hp	CN	2021	0	0	0	0	0	0	0	0	0	0	0	0	59
19649643	f	md	GB	2021	0	0	0	0	0	0	0	0	0	0	0	0	54
19654436	m	md	JP	2021	0	1	1	0	0	0	0	0	0	0	0	0	80
19656548	m	hp	FR	2021	0	1	0	0	1	0	0	0	0	0	0	0	70
19657032	f	cn	CN	2021	0	1	0	0	1	0	0	0	0	0	0	0	72
19670667	m	md	CA	2021	0	0	0	0	0	0	0	0	0	0	0	0	79
19679228	f	hp	US	2021	0	1	0	0	1	0	0	0	0	0	0	0	66
19682539	m	md	JP	2021	0	1	0	0	0	1	0	0	0	0	0	0	
19683857	f	md	JP	2021	0	0	0	0	0	0	0	0	0	0	0	0	61
19705838	m	md	US	2021	0	0	0	0	0	0	0	0	0	0	0	0	64
19707230	m	md	DE	2021	0	1	0	0	1	0	0	0	0	0	0	1	63
19708619	m	md	FR	2021	0	0	0	0	0	0	0	0	0	0	0	0	67
19710774	m	md	JP	2021	0	1	0	0	0	1	0	0	0	0	0	0	56
19712410	f	md	GB	2021	0	1	0	0	0	1	0	0	0	0	0	0	71
19715920	m	hp	DE	2021	0	1	0	0	1	0	0	0	0	0	0	0	80
19719090	m	cn	US	2021	1	0	0	0	0	0	0	0	0	0	0	0	68
19719715	f	cn	GB	2021	1	0	0	0	0	0	0	0	0	0	0	0	
19723385	m	hp	CN	2021	0	0	0	0	0	0	0	0	0	0	0	0	55
19725904	f	hp	NL	2021	0	0	0	0	0	0	0	0	0	0	0	0	59
19726626	m	hp	NL	2021	0	0	0	0	0	0	0	0	0	0	0	0	63
19743428	f	cn	FR	2021	0	0	0	0	0	0	0	0	0	0	0	0	74
19746019	f	md	JP	2021	0	1	0	1	0	0	0	0	0	0	0	1	79
19752994	f	md	JP	2021	0	0	0	0	0	0	0	0	0	0	0	0	60
19756904	f	hp	FR	2021	0	0	0	0	0	0	0	0	0	0	0	0	47
19764953	m	md	DE	2021	0	0	0	0	0	0	0	0	0	0	0	0	60
19765627	m	hp	BE	2021	0	1	1	0	0	0	0	0	0	0	0	0	67
19766607	m	hp	CN	2021	0	0	0	0	0	0	0	0	0	0	0	0	63

19774975	f	md	NZ	2021	0	1	0	0	0	1	0	0	0	0	0	0	57
19775921	f	cn	CN	2021	0	1	0	0	1	0	0	0	0	0	0	0	43
19776839	f	hp	US	2021	0	0	0	0	0	0	0	0	0	0	0	0	55
19778481	m	cn	JP	2021	0	0	0	0	0	0	0	0	0	0	0	0	59
19780274	m	hp	ES	2021	0	1	0	0	1	0	0	0	0	0	0	0	82
19782751	m	hp	GR	2021	0	1	0	0	1	0	0	0	0	0	0	0	79
19785763	f	hp	DE	2021	0	1	0	0	1	0	0	0	0	0	0	0	70
19785931	f	md	US	2021	0	1	1	0	0	0	0	0	0	0	0	0	77
19786639	f	hp	KR	2021	0	0	0	0	0	0	0	0	0	0	0	0	62
19787091	f	md	JP	2021	0	0	0	0	0	0	0	0	0	0	0	0	69
19788233	f	md	JP	2021	0	0	0	0	0	0	0	0	0	0	0	0	71
19795723	m	md	DE	2021	0	0	0	0	0	0	0	0	0	0	0	0	65
19800386	Not	md	FR	2021	0	1	0	0	0	1	0	0	0	0	0	0	
19807938	m	md	PL	2021	0	0	0	0	0	0	0	0	0	0	0	0	40
19808130	m	hp	CN	2021	0	1	0	0	1	0	0	0	0	0	0	0	69
19808383	m	md	ES	2021	0	1	0	0	0	1	0	0	0	0	0	0	76
19810719	f	md	JP	2021	1	0	0	0	0	0	0	0	0	0	0	0	74
19812372	m	md	JP	2021	0	1	1	0	0	0	0	0	0	0	0	0	79
19814545	m	md	CH	2021	0	1	1	0	0	0	0	0	0	0	0	0	63
19814613	f	md	JP	2021	0	0	0	0	0	0	0	0	0	0	0	0	80
19815107	f	Not repo	Not re	2021	0	0	0	0	0	0	0	0	0	0	0	0	66
19815510	f	md	IN	2021	0	0	0	0	0	0	0	0	0	0	0	0	69
19816119	m	hp	US	2021	0	1	0	0	1	0	0	0	0	0	0	0	58
19818077	m	md	JP	2021	0	1	0	0	0	1	0	0	0	0	0	0	80
19822546	m	md	JP	2021	0	1	0	1	0	0	0	0	0	0	0	0	74
19822932	f	md	FR	2021	0	0	0	0	0	0	0	0	0	0	0	0	60
19826456	m	hp	BE	2021	0	1	0	0	1	0	0	0	0	0	0	1	75
19829226	f	md	JP	2021	0	1	0	0	1	0	0	0	0	0	0	0	70
19832035	f	md	JP	2021	0	0	0	0	0	0	0	0	0	0	0	0	72
19832300	m	cn	CN	2021	0	0	0	0	0	0	0	0	0	0	0	0	77
19832337	m	cn	JP	2021	1	0	0	0	0	0	0	0	0	0	0	0	68
19834653	m	cn	US	2021	0	1	1	0	0	0	0	0	0	0	0	0	78
19834760	m	md	DE	2021	0	1	1	0	0	0	0	0	0	0	0	0	
19835739	m	md	BG	2021	0	0	0	0	0	0	0	0	0	0	0	0	74

19836996	f	cn	US	2021	0	0	0	0	0	0	0	0	0	0	0	0	70
19839583	m	md	TR	2021	0	1	0	0	0	1	0	0	0	0	0	1	65
19839858	f	md	JP	2021	1	0	0	0	0	0	0	0	0	0	0	0	70.8
19843480	f	md	US	2021	0	1	1	0	0	0	0	0	0	0	0	0	
19844643	m	md	DE	2021	0	1	0	0	1	0	0	0	0	0	0	0	49
19845126	f	hp	AU	2021	0	1	0	0	1	0	0	0	0	0	0	0	67
19849290	m	md	ES	2021	0	0	0	0	0	0	0	0	0	0	0	0	75
19850297	m	hp	ES	2021	0	1	0	0	1	0	0	0	0	0	0	0	69
19854685	f	cn	CN	2021	0	0	0	0	0	0	0	0	0	0	0	0	58
19854926	m	md	KR	2021	0	0	0	0	0	0	0	0	0	0	0	0	76
19857747	f	md	CH	2021	0	0	0	0	0	0	0	0	0	0	0	0	71
19862064	Not	md	GB	2021	0	0	0	0	0	0	0	0	0	0	0	0	
19864630	m	ph	JP	2021	0	1	1	0	0	0	0	0	0	0	0	0	73
19864826	f	md	JP	2021	0	0	0	0	0	0	0	0	0	0	0	0	45
19866478	m	ph	JP	2021	0	1	0	0	0	1	0	0	0	0	0	0	60
19867298	m	hp	CA	2021	0	0	0	0	0	0	0	0	0	0	0	0	75
19868888	m	md	JP	2021	0	0	0	0	0	0	0	0	0	0	0	0	67
19868980	f	hp	GB	2021	0	1	0	0	1	0	0	0	0	0	0	0	67
19874916	m	md	DE	2021	0	1	0	0	0	1	0	0	0	0	0	0	62
19875314	f	md	DE	2021	0	1	0	0	1	0	0	0	0	0	0	1	82
19875751	f	hp	US	2021	0	1	0	0	1	0	0	0	0	0	0	0	62
19877043	m	md	SI	2021	0	0	0	0	0	0	0	0	0	0	0	0	70
19877867	f	hp	US	2021	0	1	0	0	1	0	0	0	0	0	0	0	76
19878510	f	cn	US	2021	0	1	1	0	0	0	0	0	0	0	0	0	60
19878699	m	md	CN	2021	0	0	0	0	0	0	0	0	0	0	0	0	44
19879416	m	ph	JP	2021	1	0	0	0	0	0	0	0	0	0	0	0	63
19881701	m	md	NL	2021	0	0	0	0	0	0	0	0	0	0	0	0	70
19885848	m	cn	US	2021	0	0	0	0	0	0	1	1	0	0	0	0	79
19886712	m	md	BE	2021	1	0	0	0	0	0	0	0	0	0	0	0	72.6
19888917	m	md	IN	2021	0	0	0	0	0	0	0	0	0	0	0	0	67
19890300	m	hp	CH	2021	0	1	0	0	1	0	0	0	0	0	0	0	72
19890351	Not	md	JP	2021	1	0	0	0	0	0	0	0	0	0	0	0	
19890941	f	md	JP	2021	1	0	0	0	0	0	0	0	0	0	0	0	75
19892594	Not	md	JP	2021	0	1	0	0	1	0	0	0	0	0	0	0	

19893756	m	hp	GR	2021	0	1	0	0	1	0	0	0	0	0	0	0	76
19896536	m	md	JP	2021	0	1	0	0	1	0	0	0	0	0	0	1	59
19899034	m	md	DE	2021	0	0	0	0	0	0	0	0	0	0	0	0	61
19899126	m	md	KR	2021	0	1	0	0	0	1	0	0	0	0	0	0	65
19899187	f	md	JP	2021	1	0	0	0	0	0	0	0	0	0	0	0	87.3
19901364	m	md	KR	2021	0	0	0	0	0	0	0	0	0	0	0	0	63
19902856	f	md	GB	2021	0	0	0	0	0	0	0	0	0	0	0	0	40
19902932	f	md	BE	2021	0	0	0	0	0	0	0	0	0	0	0	0	76
19903359	f	md	GB	2021	0	1	0	0	0	1	0	0	0	0	0	0	50
19903492	m	md	JP	2021	0	1	0	0	1	0	0	0	0	0	0	1	78
19903593	m	cn	CO	2021	0	0	0	0	0	0	0	0	0	0	0	0	
19904069	f	cn	ID	2021	1	0	0	0	0	0	0	0	0	0	0	0	66.6
19904537	f	md	JP	2021	1	0	0	0	0	0	0	0	0	0	0	0	
19904605	f	md	FR	2021	0	0	0	0	0	0	0	0	0	0	0	0	
19904631	m	cn	US	2021	0	0	0	0	0	0	0	0	0	0	0	0	59
19904959	m	md	JP	2021	0	1	0	1	0	0	0	0	0	0	0	0	
19905199	f	md	BG	2021	1	0	0	0	0	0	0	0	0	0	0	0	
19905338	m	cn	CN	2021	0	1	0	1	0	0	0	0	0	0	0	0	46.5
19905352	f	ph	MY	2021	1	0	0	0	0	0	0	0	0	0	0	0	57.8
19906118	Not	md	FR	2021	0	0	0	0	0	0	0	0	0	0	0	0	
19906169	f	md	DE	2021	0	1	0	0	1	0	0	0	0	0	0	0	82
19906670	m	cn	IN	2021	0	0	0	0	0	0	0	0	0	0	0	0	49
19906891	m	md	MY	2021	0	1	1	0	0	0	0	0	0	0	0	0	71
19907259	m	md	IN	2021	0	1	0	0	1	0	0	0	0	0	0	0	47
19907285	m	cn	IN	2021	0	1	0	0	1	0	0	0	0	0	0	0	60
19907311	Not	hp	JP	2021	0	1	1	0	0	0	0	0	0	0	0	0	79
19907345	m	md	BG	2021	0	1	0	1	0	0	0	0	0	0	0	1	
19907793	m	md	JP	2021	0	1	0	0	1	0	0	0	0	0	0	0	80
19907884	f	cn	US	2021	1	0	0	0	0	0	0	0	0	0	0	0	
19908129	f	md	GB	2021	0	0	0	0	0	0	0	0	0	0	0	0	54
19908143	m	md	SI	2021	0	0	0	0	0	0	0	0	0	0	0	0	70
19908146	f	hp	IT	2021	0	0	0	0	0	0	0	0	0	0	0	0	74
19908225	m	md	FR	2021	0	1	0	1	0	0	0	0	0	0	0	0	
19908260	f	md	ES	2021	0	0	0	0	0	0	0	0	0	0	0	0	77

19908424	f	md	JP	2021	0	1	0	0	0	1	0	0	0	0	0	0	
19908464	m	cn	AR	2021	0	0	0	0	0	0	0	0	0	0	0	0	44
19908773	m	md	JP	2021	0	1	0	1	0	0	0	0	0	0	0	1	77
19908775	m	md	US	2021	0	0	0	0	0	0	0	0	0	0	0	0	69
19908823	f	md	US	2021	0	1	0	0	0	1	0	0	0	0	0	0	65
19908960	m	md	IT	2021	0	0	0	0	0	0	0	0	0	0	0	0	56
19909719	m	ph	DE	2021	0	1	1	0	0	0	0	0	0	0	0	0	39
19909923	m	md	DE	2021	0	1	0	0	1	0	0	0	0	0	0	0	58
19910028	f	cn	CN	2021	0	0	0	0	0	0	0	0	0	0	0	0	67
19910093	f	ph	JP	2021	0	0	0	0	0	0	0	0	0	0	0	0	
19910495	m	md	DE	2021	0	1	0	0	1	0	0	0	0	0	0	0	
19910832	m	md	ES	2021	0	1	0	0	0	1	0	0	0	0	0	0	60
19910890	f	ph	JP	2021	0	0	0	0	0	0	0	0	0	0	0	0	74
19911188	f	cn	IN	2021	0	1	0	0	1	0	0	0	0	0	0	0	78
19911347	f	md	JP	2021	0	0	0	0	0	0	0	0	0	0	0	0	85
19911731	m	md	KR	2021	0	0	0	0	0	0	0	0	0	0	0	0	80
19912043	m	md	JP	2021	0	1	0	0	1	0	0	0	0	0	0	0	71
19912292	m	hp	CN	2021	0	1	0	0	1	0	0	0	0	0	0	0	70
19912391	m	md	CN	2021	0	0	0	0	0	0	0	0	0	0	0	0	72
19912474	f	cn	US	2021	0	0	0	0	0	0	0	0	0	0	0	0	75
19912542	m	md	KR	2021	1	0	0	0	0	0	0	0	0	0	0	0	52
19912557	f	md	US	2021	0	0	0	0	0	0	0	0	0	0	0	0	
19912708	m	md	DE	2021	0	1	1	0	0	0	0	0	0	0	0	0	39
19912757	m	md	NL	2021	0	1	0	0	0	1	0	0	0	0	0	0	56
19913242	f	md	DE	2021	0	0	0	0	0	0	0	0	0	0	0	0	58
19913245	f	cn	IL	2021	0	0	0	0	0	0	0	0	0	0	0	0	65
19913258	m	md	CL	2021	0	1	1	0	0	0	0	0	0	0	0	0	55
19913261	m	cn	US	2021	0	1	0	1	0	0	0	0	0	0	0	1	74
19913427	f	md	JP	2021	0	0	0	0	0	0	0	0	0	0	0	0	
19913531	m	md	GB	2021	0	1	0	0	0	1	0	0	0	0	0	0	76
19913906	m	md	KR	2021	0	0	0	0	0	0	0	0	0	0	0	0	
19914114	Not	md	JP	2021	0	1	0	0	0	1	0	0	0	0	0	0	
19915616	f	hp	US	2021	0	1	0	0	1	0	0	0	0	0	0	0	67
19916552	f	md	CO	2021	0	0	0	0	0	0	0	0	0	0	0	0	45

19916767	m	hp	JP	2021	0	1	0	0	1	0	0	0	0	0	0	0	42
19917893	m	hp	US	2021	0	1	1	0	0	0	0	0	0	0	0	0	
19918358	m	md	JP	2021	0	1	0	0	0	1	0	0	0	0	0	0	86
19918363	m	hp	FR	2021	1	0	0	0	0	0	0	0	0	0	0	0	77
19918364	f	hp	FR	2021	1	0	0	0	0	0	0	0	0	0	0	0	68
19918365	f	hp	FR	2021	1	0	0	0	0	0	0	0	0	0	0	0	71
19918401	m	md	AT	2021	0	1	0	0	0	1	0	0	0	0	0	0	66
19918445	m	md	KR	2021	0	0	0	0	0	0	0	0	0	0	0	0	
19918473	f	hp	NL	2021	0	1	0	0	1	0	0	0	0	0	0	0	58
19918474	f	cn	CN	2021	0	0	0	0	0	0	0	0	0	0	0	0	72
19918478	f	hp	US	2021	1	0	0	0	0	0	0	0	0	0	0	0	79
19918640	m	md	GR	2021	0	0	0	0	0	0	0	0	0	0	0	0	67
19918649	f	md	SI	2021	0	1	1	0	0	0	0	0	0	0	0	0	75
19918957	m	md	PL	2021	1	0	0	0	0	0	0	0	0	0	0	0	
19919144	f	md	TH	2021	1	0	0	0	0	0	0	0	0	0	0	0	57
19919293	f	md	JP	2021	0	1	0	0	0	1	0	0	0	0	0	0	61
19919368	m	md	JP	2021	0	1	0	0	1	0	0	0	0	0	0	0	60
19919505	f	cn	US	2021	1	0	0	0	0	0	0	0	0	0	0	0	67
19919858	m	cn	SG	2021	0	1	0	0	0	1	0	0	0	0	0	0	
19920057	m	md	FI	2021	0	1	0	0	1	0	0	0	0	0	0	1	68
19920094	f	cn	DE	2021	0	1	1	0	0	0	0	0	0	0	0	0	77
19920104	f	cn	DE	2021	0	1	0	0	0	1	0	0	0	0	0	0	67
19920342	f	md	JP	2021	0	1	0	1	0	0	0	0	0	0	0	0	71
19920413	f	cn	US	2021	0	1	1	0	0	0	0	0	0	0	0	0	
19920546	m	cn	US	2021	0	1	1	0	0	0	0	0	0	0	0	0	
19920551	m	hp	NL	2021	0	1	0	0	0	1	0	0	0	0	0	0	69
19920911	m	md	JP	2021	0	1	1	0	0	0	0	0	0	0	0	0	64
19920935	m	md	CH	2021	0	1	1	0	0	0	0	0	0	0	0	0	
19921060	m	hp	BE	2021	0	0	0	0	0	0	0	0	0	0	0	0	70
19921197	m	md	RU	2021	0	0	0	0	0	0	0	0	0	0	0	0	30
19921363	m	hp	CN	2021	0	1	0	0	1	0	0	0	0	0	0	1	67
19921465	m	md	KR	2021	0	0	0	0	0	0	0	0	0	0	0	0	76
19921892	f	md	GB	2021	0	1	0	0	0	1	0	0	0	0	0	0	75
19922158	m	hp	FR	2021	0	1	1	0	0	0	0	0	0	0	0	0	64

19922864	m	md	JP	2021	0	1	0	0	1	0	0	0	0	0	0	0	79
19922908	f	ph	MY	2021	1	0	0	0	0	0	0	0	0	0	0	0	49.5
19922971	Not	hp	US	2021	0	0	0	0	0	0	0	0	0	0	0	0	
19923031	m	md	JP	2021	0	0	0	0	0	0	0	0	0	0	0	0	62
19923087	f	hp	FR	2021	1	0	0	0	0	0	0	0	0	0	0	0	71
19923143	f	hp	FR	2021	1	0	0	0	0	0	0	0	0	0	0	0	68
19923160	m	hp	FR	2021	1	0	0	0	0	0	0	0	0	0	0	0	77
19923435	f	md	SI	2021	0	1	1	0	0	0	0	0	0	0	0	0	70
19923550	m	md	JP	2021	0	1	0	0	0	1	0	0	0	0	0	0	
19923552	m	md	IT	2021	0	1	1	0	0	0	0	0	0	0	0	0	64
19923644	f	md	GR	2021	0	1	1	0	0	0	0	0	0	0	0	0	60
19924133	m	md	JP	2021	0	1	0	0	0	1	0	0	0	0	0	1	80
19924138	m	hp	US	2021	0	1	1	0	0	0	0	0	0	0	0	0	68
19924240	m	cn	JP	2021	1	0	0	0	0	0	0	0	0	0	0	0	75
19924293	m	md	FR	2021	0	1	1	0	0	0	0	0	0	0	0	0	68
19924311	m	md	KR	2021	0	0	0	0	0	0	0	0	0	0	0	0	71
19924435	m	md	JP	2021	0	0	0	0	0	0	0	0	0	0	0	0	80
19924506	m	md	KR	2021	0	0	0	0	0	0	0	0	0	0	0	0	76
19924561	m	cn	TH	2021	0	0	0	0	0	0	0	0	0	0	0	0	68
19924814	m	md	TR	2021	0	1	1	0	0	0	0	0	0	0	0	0	62
19924858	m	md	AU	2021	0	0	0	0	0	0	0	0	0	0	0	0	54
19924859	Not	hp	US	2021	0	0	0	0	0	0	0	0	0	0	0	0	
19924879	Not	cn	US	2021	1	0	0	0	0	0	0	0	0	0	0	0	
19924919	m	md	GB	2021	0	0	0	0	0	0	0	0	0	0	0	0	72
19924944	m	cn	GB	2021	1	0	0	0	0	0	0	0	0	0	0	0	52
19925174	f	md	JP	2021	1	0	0	0	0	0	0	0	0	0	0	0	85
19925296	f	md	DE	2021	0	1	1	0	0	0	0	0	0	0	0	0	54
19925596	m	md	JP	2021	1	0	0	0	0	0	0	0	0	0	0	0	77
19925602	Not	md	JP	2021	0	1	0	0	1	0	0	0	0	0	0	0	79
19925696	f	md	FR	2021	1	0	0	0	0	0	0	0	0	0	0	0	68
19925697	f	md	FR	2021	1	0	0	0	0	0	0	0	0	0	0	0	71
19925870	m	md	JP	2021	0	1	1	0	0	0	0	0	0	0	0	0	66
19926240	f	hp	US	2021	0	0	0	0	0	0	0	0	0	0	0	0	55
19926406	f	hp	CN	2021	1	0	0	0	0	0	0	0	0	0	0	0	52

19926495	m	md	JP	2021	0	0	0	0	0	0	0	0	0	0	0	0	88
19926673	f	md	FR	2021	0	1	0	0	0	1	0	0	0	0	0	0	55
19927026	f	hp	US	2021	0	0	0	0	0	0	0	0	0	0	0	0	62
19928040	f	md	FR	2021	0	0	0	0	0	0	0	0	0	0	0	0	
19928073	f	md	CA	2021	0	0	0	0	0	0	0	0	0	0	0	0	
19928102	m	cn	DE	2021	1	0	0	0	0	0	0	0	0	0	0	0	85.8
19928251	m	cn	FR	2021	0	1	1	0	0	0	0	0	0	0	0	0	
19928435	Not	hp	JP	2021	0	0	0	0	0	0	0	0	0	0	0	0	
19928922	m	md	JP	2021	0	0	0	0	0	0	0	0	0	0	0	0	
19928924	m	md	JP	2021	0	1	0	0	1	0	0	0	0	0	0	0	80
19929002	f	cn	CN	2021	0	1	0	1	0	0	0	0	0	0	0	1	69
19929095	f	md	HK	2021	1	0	0	0	0	0	0	0	0	0	0	0	
19929146	f	md	FR	2021	0	0	0	0	0	0	0	0	0	0	0	0	72
19929166	f	md	JP	2021	1	0	0	0	0	0	0	0	0	0	0	0	70
19929511	f	md	JP	2021	0	0	0	0	0	0	0	0	0	0	0	0	79
19929591	Not	hp	CN	2021	0	0	0	0	0	0	0	0	0	0	0	0	
19930236	Not	hp	CN	2021	0	0	0	0	0	0	0	0	0	0	0	0	
19930268	Not	hp	CN	2021	0	0	0	0	0	0	0	0	0	0	0	0	
19930345	f	cn	US	2021	0	0	0	0	0	0	0	0	0	0	0	0	71
19930542	f	md	IE	2021	0	1	0	0	1	0	0	0	0	0	0	0	66
19930673	m	md	JP	2021	0	0	0	0	0	0	0	0	0	0	0	0	
19930682	m	hp	JP	2021	0	1	0	0	1	0	0	0	0	0	0	0	76
19930742	m	md	JP	2021	0	1	0	0	0	1	0	0	0	0	0	0	62
19930800	m	hp	US	2021	0	0	0	0	0	0	0	0	0	0	0	0	
19930803	m	md	IT	2021	0	1	0	0	0	1	0	0	0	0	0	0	67
19930883	m	hp	GR	2021	0	1	1	0	0	0	0	0	0	0	0	0	51
19931000	m	md	FR	2021	0	0	0	0	0	0	0	0	0	0	0	0	74
19931219	Not	hp	CN	2021	0	0	0	0	0	0	0	0	0	0	0	0	
19931373	f	md	JP	2021	0	1	0	0	1	0	0	0	0	0	0	0	31
19931412	f	md	JP	2021	0	1	0	0	1	0	0	0	0	0	0	0	78
19931553	Not	hp	CN	2021	0	0	0	0	0	0	0	0	0	0	0	0	
19931581	Not	hp	CN	2021	0	0	0	0	0	0	0	0	0	0	0	0	
19931617	Not	hp	CN	2021	0	0	0	0	0	0	0	0	0	0	0	0	
19931658	f	cn	GB	2021	0	0	0	0	0	0	0	0	0	0	0	0	67

19932052	Not	ph	MY	2021	1	0	0	0	0	0	0	0	0	0	0	0	
19932230	f	md	JP	2021	1	0	0	0	0	0	0	0	0	0	0	0	64
19932298	f	hp	US	2021	0	0	0	0	0	0	0	0	0	0	0	0	55
19932647	f	cn	CA	2021	1	0	0	0	0	0	0	0	0	0	0	0	
19932738	Not	hp	CN	2021	0	0	0	0	0	0	0	0	0	0	0	0	
19932748	Not	hp	DE	2021	1	0	0	0	0	0	0	0	0	0	0	0	
19932813	f	hp	US	2021	0	1	0	0	1	0	0	0	0	0	0	0	80
19933030	f	md	JP	2021	0	0	0	0	0	0	0	0	0	0	0	0	76
19933511	f	md	US	2021	0	0	0	0	0	0	0	0	0	0	0	0	
19933602	f	cn	PE	2021	1	0	0	0	0	0	0	0	0	0	0	0	69.3
19933606	f	md	AU	2021	1	0	0	0	0	0	0	0	0	0	0	0	49
19933837	f	hp	BE	2021	0	1	0	1	0	0	0	0	0	0	0	0	69
19934226	m	cn	IN	2021	0	0	0	0	0	0	0	0	0	0	0	0	70
19934402	m	md	IT	2021	0	1	0	0	0	1	0	0	0	0	0	0	86
19934429	f	md	IT	2021	1	0	0	0	0	0	0	0	0	0	0	0	
19934445	f	cn	IN	2021	0	1	0	0	1	0	0	0	0	0	0	0	71
19934751	m	md	JP	2021	0	1	0	0	1	0	0	0	0	0	0	0	71
19934766	m	hp	US	2021	0	0	0	0	0	0	0	0	0	0	0	0	59
19935410	m	cn	ID	2021	1	0	0	0	0	0	0	0	0	0	0	0	59.6
19935789	Not	ph	US	2021	0	0	0	0	0	0	0	0	0	0	0	0	50
19935910	Not	md	JP	2021	0	1	0	0	1	0	0	0	0	0	0	0	81
19936513	f	cn	US	2021	0	0	0	0	0	0	0	0	0	0	0	0	
19936722	m	ph	PK	2021	0	1	0	0	0	1	0	0	0	0	0	0	52
19937244	m	md	FR	2021	0	1	1	0	0	0	0	0	0	0	0	0	68
19937431	m	md	JP	2021	0	1	1	0	0	0	0	0	0	0	0	0	72
19937718	f	md	DE	2021	0	0	0	0	0	0	0	0	0	0	0	0	58
19938053	f	hp	US	2021	0	0	0	0	0	0	0	0	0	0	0	0	64
19938221	m	md	JP	2021	0	1	1	0	0	0	0	0	0	0	0	0	80
19938554	m	md	JP	2021	0	1	1	0	0	0	0	0	0	0	0	0	70
19938763	m	md	PE	2021	0	1	0	0	0	1	0	0	0	0	0	0	83
19939076	m	hp	US	2021	0	0	0	0	0	0	0	0	0	0	0	0	65
19939106	f	md	JP	2021	0	1	0	0	0	1	0	0	0	0	0	0	71
19939112	f	md	DE	2021	0	0	0	0	0	0	0	0	0	0	0	0	58
19939176	m	md	IL	2021	0	0	0	0	0	0	0	0	0	0	0	0	65

19939399	m	hp	IL	2021	0	1	0	1	0	0	0	0	0	0	0	0	58
19939504	Not	md	IT	2021	1	0	0	0	0	0	0	0	0	0	0	0	
19940233	f	ph	CA	2021	1	0	0	0	0	0	0	0	0	0	0	0	64.6
19940677	f	md	JP	2021	0	0	0	0	0	0	0	0	0	0	0	0	69
19940853	Not	hp	JP	2021	0	0	0	0	0	0	0	0	0	0	0	0	
19940883	m	hp	US	2021	0	1	1	0	0	0	0	0	0	0	0	1	
19941244	f	md	BE	2021	0	1	1	0	0	0	0	0	0	0	0	0	
19941642	f	md	MY	2021	1	0	0	0	0	0	0	0	0	0	0	0	
19941748	m	md	IT	2021	0	0	0	0	0	0	0	0	0	0	0	0	70
19941848	Not	md	GR	2021	0	1	0	0	0	1	0	0	0	0	0	0	
19941952	f	md	JP	2021	0	0	0	0	0	0	0	0	0	0	0	0	70
19942032	m	md	CN	2021	0	1	0	1	0	0	0	0	0	0	0	0	66
19942120	f	hp	US	2021	1	0	0	0	0	0	0	0	0	0	0	0	48.4
19942359	f	md	CN	2021	0	0	0	0	0	0	0	0	0	0	0	0	70
19942649	Not	md	JP	2021	0	0	0	0	0	0	0	0	0	0	0	0	76
19943562	f	md	FR	2021	1	0	0	0	0	0	0	0	0	0	0	0	71
19943793	f	md	DE	2021	0	1	0	0	1	0	0	0	0	0	0	0	74
19943925	f	hp	US	2021	0	0	0	0	0	0	0	0	0	0	0	0	
19944137	Not	hp	JP	2021	0	0	0	0	0	0	0	0	0	0	0	0	
19944366	f	md	DE	2021	0	0	0	0	0	0	0	0	0	0	0	0	
19944390	f	hp	GB	2021	0	1	0	0	1	0	0	0	0	0	0	0	74
19944411	m	md	GB	2021	0	0	0	0	0	0	0	0	0	0	0	0	59
19944991	Not	md	CN	2021	0	0	0	0	0	0	0	0	0	0	0	0	
19945318	Not	md	JP	2021	0	1	0	0	1	0	0	0	0	0	0	0	
19945391	f	md	JP	2021	0	1	1	0	0	0	0	0	0	0	0	0	
19945425	f	hp	NL	2021	0	1	0	0	1	0	0	0	0	0	0	0	82
19945571	f	md	US	2021	0	0	0	0	0	0	0	0	0	0	0	0	65
19945608	m	md	JP	2021	1	0	0	0	0	0	0	0	0	0	0	1	
19945771	m	md	JP	2021	0	0	0	0	0	0	0	0	0	0	0	0	80
19945851	m	md	JP	2021	0	0	0	0	0	0	0	0	0	0	0	0	
19946755	f	md	DE	2021	0	1	1	0	0	0	0	0	0	0	0	0	66
19947008	m	cn	IN	2021	0	1	0	0	1	0	0	0	0	0	0	0	48
19947047	m	md	DE	2021	0	1	1	0	0	0	0	0	0	0	0	0	71
19947130	f	md	JP	2021	0	1	1	0	0	0	0	0	0	0	0	0	79

19947362	f	md	CH	2021	0	1	0	0	0	1	0	0	0	0	0	0	69
19947364	Not	ph	JP	2021	0	1	0	1	0	0	0	0	0	0	0	0	
19947365	m	md	FR	2021	0	1	0	1	0	0	0	0	0	0	0	0	64
19947414	m	md	NL	2021	0	1	1	0	0	0	0	0	0	0	0	0	72
19948546	m	hp	US	2021	0	0	0	0	0	0	0	0	0	0	0	0	73
19948610	m	hp	MY	2021	1	0	0	0	0	0	0	0	0	0	0	0	
19948765	m	md	JP	2021	0	0	0	0	0	0	0	0	0	0	0	0	50
19948769	f	md	JP	2021	0	0	0	0	0	0	0	0	0	0	0	0	80
19948893	m	md	JP	2021	0	1	1	0	0	0	0	0	0	0	0	0	78
19949268	m	hp	US	2021	0	0	0	0	0	0	0	0	0	0	0	0	59
19949287	m	hp	US	2021	0	0	0	0	0	0	0	0	0	0	0	0	59
19949436	f	md	US	2021	0	1	0	0	0	1	0	0	0	0	0	0	59
19949437	f	md	CU	2021	0	0	0	0	0	0	0	0	0	0	0	0	79
19949586	m	hp	US	2021	0	0	0	0	0	0	0	0	0	0	0	0	73
19949603	f	cn	CA	2021	0	1	1	0	0	0	0	0	0	0	0	0	72
19949613	m	hp	US	2021	0	0	0	0	0	0	0	0	0	0	0	0	73
19949774	m	md	JP	2021	0	1	0	0	1	0	0	0	0	0	0	0	80
19949823	m	ph	US	2021	1	0	0	0	0	0	0	0	0	0	0	1	78
19950033	f	cn	CN	2021	0	0	0	0	0	0	0	0	0	0	0	0	
19950311	m	cn	CN	2021	0	1	0	1	0	0	0	0	0	0	0	0	56
19950344	f	md	JP	2021	0	1	0	0	0	1	0	0	0	0	0	0	62
19950419	m	md	JP	2021	0	1	0	0	1	0	0	0	0	0	0	0	72
19950606	f	md	GR	2021	0	0	0	0	0	0	0	0	0	0	0	0	43
19950831	m	md	FR	2021	0	0	0	0	0	0	0	0	0	0	0	0	
19950839	Not	hp	US	2021	0	0	0	0	0	0	0	0	0	0	0	0	
19950922	f	cn	US	2021	0	0	0	0	0	0	0	0	0	0	0	0	
19950973	m	md	FR	2021	0	0	0	0	0	0	0	0	0	0	0	0	65
19951031	m	hp	US	2021	0	1	1	0	0	0	0	0	0	0	0	0	68
19951232	f	md	US	2021	0	1	0	0	0	1	0	0	0	0	0	0	77
19951237	Not	hp	JP	2021	0	1	1	0	0	0	0	0	0	0	0	1	
19951792	f	cn	IN	2021	0	0	0	0	0	0	0	0	0	0	0	0	65
19951805	f	cn	US	2021	0	0	0	0	0	0	0	0	0	0	0	0	
19952146	m	ph	MY	2021	1	0	0	0	0	0	0	0	0	0	0	0	36.4
19952373	f	md	DE	2021	0	1	0	0	1	0	0	0	0	0	0	0	82

19952470	Not	md	JP	2021	0	1	0	0	1	0	0	0	0	0	0	0	
19952523	f	md	CL	2021	0	1	1	0	0	0	0	0	0	0	0	0	71
19952636	m	md	GR	2021	0	0	0	0	0	0	0	0	0	0	0	0	55
19952910	f	md	DE	2021	1	0	0	0	0	0	0	0	0	0	0	0	
19953473	Not	cn	CA	2021	0	1	1	0	0	0	0	0	0	0	0	0	73
19953641	f	cn	FR	2021	0	0	0	0	0	0	0	0	0	0	0	0	
19953845	m	md	MX	2021	0	1	1	0	0	0	0	0	0	0	0	0	81
19954164	f	md	SA	2021	1	0	0	0	0	0	0	0	0	0	0	0	
19954256	f	md	NL	2021	0	1	0	0	0	1	0	0	0	0	0	0	75
19954369	f	cn	CA	2021	1	0	0	0	0	0	0	0	0	0	0	0	58
19954482	m	md	JP	2021	0	1	0	1	0	0	0	0	0	0	0	1	80
19954573	m	cn	ID	2021	1	0	0	0	0	0	0	0	0	0	0	0	66.2
19954620	f	cn	GB	2021	1	0	0	0	0	0	0	0	0	0	0	0	76
19954677	m	cn	KR	2021	0	0	0	0	0	0	0	0	0	0	0	0	
19954683	f	ph	JP	2021	1	0	0	0	0	0	0	0	0	0	0	0	55
19954695	Not	cn	GB	2021	1	0	0	0	0	0	0	0	0	0	0	0	
19954720	m	hp	US	2021	1	0	0	0	0	0	0	0	0	0	0	0	63
19954821	f	md	GB	2021	0	0	0	0	0	0	0	0	0	0	0	0	
19954893	f	md	JP	2021	0	1	1	0	0	0	0	0	0	0	0	0	51
19955092	Not	md	BE	2021	0	0	0	0	0	0	0	0	0	0	0	0	
19955381	f	md	JP	2021	1	0	0	0	0	0	0	0	0	0	0	0	85
19955649	m	md	BR	2021	0	0	0	0	0	0	0	0	0	0	0	0	72
19955652	f	md	JP	2021	0	1	0	0	0	1	0	0	0	0	0	0	80
19955657	m	md	JP	2021	0	1	0	0	0	1	0	0	0	0	0	0	60
19955732	Not	md	JP	2021	0	0	0	0	0	0	0	0	0	0	0	0	
19955932	m	md	DE	2021	0	0	0	0	0	0	0	0	0	0	0	0	45
19955940	Not	hp	JP	2021	0	1	1	0	0	0	0	0	0	0	0	1	
19956049	f	md	KR	2021	0	1	0	1	0	0	0	0	0	0	0	1	76.3
19956209	m	md	JP	2021	0	1	1	0	0	0	0	0	0	0	0	0	81
19956339	f	md	DE	2021	0	1	0	0	1	0	0	0	0	0	0	0	68
19956550	f	md	CL	2021	0	1	1	0	0	0	0	0	0	0	0	0	68
19956676	f	cn	CA	2021	0	0	0	0	0	0	0	0	0	0	0	0	68
19956699	m	cn	US	2021	1	0	0	0	0	0	0	0	0	0	0	0	71.6
19956747	f	md	CN	2021	0	0	0	0	0	0	0	0	0	0	0	0	74

19956765	m	md	IT	2021	1	0	0	0	0	0	0	0	0	0	0	0	60
19956909	m	hp	US	2021	0	1	1	0	0	0	0	0	0	0	0	0	80
19957292	m	cn	US	2021	1	0	0	0	0	0	0	0	0	0	0	0	68.2
19957401	f	md	NL	2021	0	0	0	0	0	0	0	0	0	0	0	0	66
19957492	f	md	JP	2021	0	1	0	0	1	0	0	0	0	0	0	0	71
19957608	m	cn	CN	2021	0	1	0	0	1	0	0	0	0	0	0	0	58
19957732	m	md	JP	2021	0	0	0	0	0	0	0	0	0	0	0	0	64
19958033	m	cn	CN	2021	0	1	0	1	0	0	0	0	0	0	0	1	60
19958229	Not	hp	FR	2021	0	0	0	0	0	0	0	0	0	0	0	0	
19958270	f	hp	BG	2021	1	0	0	0	0	0	0	0	0	0	0	0	
19958460	f	md	JP	2021	0	0	0	0	0	0	0	0	0	0	0	0	70
19958509	Not	cn	JP	2021	1	0	0	0	0	0	0	0	0	0	0	0	
19958568	m	cn	ID	2021	1	0	0	0	0	0	0	0	0	0	0	0	
19958600	m	md	JP	2021	0	1	0	0	0	1	0	0	0	0	0	0	69
19958680	f	hp	FR	2021	1	0	0	0	0	0	0	0	0	0	0	0	34
19958992	m	md	RU	2021	1	0	0	0	0	0	0	0	0	0	0	0	52
19959122	f	md	CA	2021	0	0	0	0	0	0	0	0	0	0	0	0	68
19959151	Not	ph	JP	2021	1	0	0	0	0	0	0	0	0	0	0	0	
19959536	m	cn	CA	2021	0	0	0	0	0	0	0	0	0	0	0	0	71
19959577	m	md	GB	2021	0	1	0	0	0	1	0	0	0	0	0	0	63
19959621	m	md	AT	2021	0	1	1	0	0	0	0	0	0	0	0	0	62
19960025	m	md	PL	2021	1	0	0	0	0	0	0	0	0	0	0	0	
19960155	m	cn	ID	2021	1	0	0	0	0	0	0	0	0	0	0	0	
19960284	m	ph	US	2021	0	0	0	0	0	0	0	0	0	0	0	0	62
19960478	f	cn	CN	2021	0	1	0	1	0	0	0	0	0	0	0	1	56.8
19960555	f	cn	TH	2021	0	0	0	0	0	0	0	0	0	0	0	0	56
19960574	m	ph	JP	2021	0	1	1	0	0	0	0	0	0	0	0	0	72
19960605	m	md	JP	2021	0	1	0	0	0	1	0	0	0	0	0	0	70
19960606	m	md	JP	2021	0	1	0	0	0	1	0	0	0	0	0	0	58
19960612	m	md	CN	2021	0	1	0	0	0	1	0	0	0	0	0	0	60
19960932	f	cn	US	2021	0	0	0	0	0	0	0	0	0	0	0	0	
19960940	f	md	DE	2021	0	0	0	0	0	0	0	0	0	0	0	0	58
19960956	m	hp	DK	2021	0	0	0	0	0	0	0	0	0	0	0	0	56
19961305	f	md	DE	2021	0	1	1	0	0	0	0	0	0	0	0	0	77

19961566	f	cn	US	2021	0	1	1	0	0	0	0	0	0	0	0	0	
19961593	f	md	TW	2021	0	1	0	0	0	1	0	0	0	0	0	1	
19962069	m	md	AU	2021	0	1	1	0	0	0	0	0	0	0	0	0	57
19962612	f	md	JP	2021	0	1	0	0	0	1	0	0	0	0	0	0	54
19962690	f	hp	DE	2021	0	1	1	0	0	0	0	0	0	0	0	0	63
19962696	m	cn	MY	2021	1	0	0	0	0	0	0	0	0	0	0	0	44.6
19962853	m	md	AT	2021	0	0	0	0	0	0	0	0	0	0	0	0	54
19962862	f	md	JP	2021	0	1	0	0	0	1	0	0	0	0	0	0	74
19963035	f	md	SG	2021	0	0	0	0	0	0	0	0	0	0	0	0	69
19963454	f	hp	GB	2021	0	0	0	0	0	0	0	0	0	0	0	0	63
19963476	m	md	JP	2021	0	1	0	0	0	1	0	0	0	0	0	0	65
19964061	f	cn	SG	2021	1	0	0	0	0	0	0	0	0	0	0	0	73
19964270	f	md	JP	2021	0	1	0	0	0	1	0	0	0	0	0	0	62
19964414	f	md	AU	2021	0	1	0	1	0	0	0	0	0	0	0	0	
19964454	f	md	DE	2021	0	1	0	0	1	0	0	0	0	0	0	0	74
19964506	m	md	JP	2021	0	1	1	0	0	0	0	0	0	0	0	0	74
19964509	f	md	JP	2021	0	1	0	0	0	1	0	0	0	0	0	0	67
19964521	m	md	DE	2021	0	1	0	0	1	0	0	0	0	0	0	0	49
19964536	f	md	JP	2021	0	1	0	1	0	0	0	0	0	0	0	0	79
19964712	Not	md	AT	2021	0	1	1	0	0	0	0	0	0	0	0	0	69
19964770	m	md	JP	2021	0	1	0	0	0	1	0	0	0	0	0	0	73
19964818	f	hp	CA	2021	1	0	0	0	0	0	0	0	0	0	0	0	63
19964882	f	md	JP	2021	0	1	0	0	0	1	0	0	0	0	0	0	70
19965035	f	md	US	2021	0	0	0	0	0	0	0	0	0	0	0	0	
19965121	f	md	JP	2021	0	1	0	0	1	0	0	0	0	0	0	1	77
19965127	f	cn	SG	2021	1	0	0	0	0	0	0	0	0	0	0	0	71.4
19965133	f	hp	ES	2021	0	0	0	0	0	0	0	0	0	0	0	0	67
19965164	m	md	IT	2021	0	0	0	0	0	0	0	0	0	0	0	0	75
19965500	f	cn	US	2021	1	0	0	0	0	0	0	0	0	0	0	0	
19966009	f	md	SE	2021	0	0	0	0	0	0	0	0	0	0	0	0	31
19966118	f	cn	SG	2021	1	0	0	0	0	0	0	0	0	0	0	0	64.4
19966254	f	md	CH	2021	0	1	0	0	0	1	0	0	0	0	0	0	72
19966487	m	ph	JP	2021	0	1	0	0	0	1	0	0	0	0	0	0	60
19966489	m	md	JP	2021	0	1	1	0	0	0	0	0	0	0	0	1	90

19966722	f	md	JP	2021	0	1	0	0	1	0	0	0	0	0	0	0	66
19966750	m	hp	KR	2021	0	0	0	0	0	0	0	0	0	0	0	0	65
19967209	m	hp	US	2021	0	1	1	0	0	0	0	0	0	0	0	0	68
19967662	m	md	US	2021	0	0	0	0	0	0	0	0	0	0	0	0	
19967802	f	md	GB	2021	0	1	0	0	0	1	0	0	0	0	0	0	71
19967913	m	md	JP	2021	0	1	1	0	0	0	0	0	0	0	0	0	80
19968318	f	cn	US	2021	0	0	0	0	0	0	0	0	0	0	0	0	
19968427	f	md	IT	2021	1	0	0	0	0	0	0	0	0	0	0	0	
19968594	m	md	US	2021	0	0	0	0	0	0	0	0	0	0	0	0	36
19968806	f	md	JP	2021	0	1	0	0	0	1	0	0	0	0	0	0	70
19968916	f	hp	US	2021	0	1	0	0	1	0	0	0	0	0	0	0	63
19969081	f	hp	GB	2021	0	0	0	0	0	0	0	0	0	0	0	0	63
19969145	f	cn	US	2021	0	1	0	1	0	0	0	0	0	0	0	1	85
19969259	m	md	FR	2021	0	0	0	0	0	0	0	0	0	0	0	0	62
19969335	f	md	JP	2021	0	1	0	0	0	1	0	0	0	0	0	0	72
19969536	m	hp	NL	2021	0	0	0	0	0	0	0	0	0	0	0	0	61
19969539	m	cn	CN	2021	0	1	0	0	1	0	0	0	0	0	0	0	68
19969579	m	md	DE	2021	0	0	0	0	0	0	0	0	0	0	0	0	61
19969761	f	md	US	2021	0	1	0	1	0	0	0	0	0	0	0	1	61
19969765	Not	md	JP	2021	0	1	1	0	0	0	0	0	0	0	0	0	60
19969827	f	md	US	2021	0	0	0	0	0	0	0	0	0	0	0	0	
19969898	m	md	PA	2021	0	0	0	0	0	0	0	0	0	0	0	0	58
19969930	m	md	SI	2021	0	1	0	1	0	0	0	0	0	0	0	0	58.2
19970063	Not	cn	US	2021	0	0	0	0	0	0	0	0	0	0	0	0	
19970203	m	md	BG	2021	0	1	0	0	0	1	0	0	0	0	0	0	66
19970526	f	cn	CN	2021	0	1	0	0	1	0	0	0	0	0	0	0	56
19970679	m	md	JP	2021	0	1	1	0	0	0	0	0	0	0	0	0	72
19970956	f	md	US	2021	0	0	0	0	0	0	1	0	1	0	0	0	81
19971303	Not	md	FR	2021	1	0	0	0	0	0	0	0	0	0	0	0	
19971517	m	md	FR	2021	0	0	0	0	0	0	0	0	0	0	0	0	51
19971846	f	md	JP	2021	0	0	0	0	0	0	0	0	0	0	0	0	
19971862	m	md	JP	2021	0	0	0	0	0	0	0	0	0	0	0	0	
19972038	f	md	FR	2021	0	1	0	0	0	1	0	0	0	0	0	0	51
19972923	m	md	DE	2021	0	1	0	0	0	1	0	0	0	0	0	0	

19972933	Not	md	JP	2021	0	1	0	0	0	1	0	0	0	0	0	0	
19972946	m	md	JP	2021	0	1	1	0	0	0	0	0	0	0	0	0	85
19973183	m	cn	CN	2021	0	1	0	0	1	0	0	0	0	0	0	0	27
19973649	f	cn	US	2021	1	0	0	0	0	0	0	0	0	0	0	0	
19973813	m	md	JP	2021	0	0	0	0	0	0	0	0	0	0	0	0	43
19973830	f	cn	DE	2021	0	0	0	0	0	0	0	0	0	0	0	0	
19975213	f	md	PL	2021	1	0	0	0	0	0	0	0	0	0	0	0	
19975227	m	md	DE	2021	0	1	1	0	0	0	0	0	0	0	0	0	
19975322	f	md	NO	2021	1	0	0	0	0	0	0	0	0	0	0	0	75
19975468	f	md	FR	2021	0	0	0	0	0	0	0	0	0	0	0	0	56
19975769	f	hp	GB	2021	0	0	0	0	0	0	0	0	0	0	0	0	63
19976014	f	md	NL	2021	0	0	0	0	0	0	0	0	0	0	0	0	44
19976800	f	md	JP	2021	0	0	0	0	0	0	0	0	0	0	0	0	80
19976904	f	md	JP	2021	0	1	0	0	0	1	0	0	0	0	0	0	66
19977709	m	md	DE	2021	0	1	0	0	1	0	0	0	0	0	0	0	
19978824	f	md	ES	2021	0	0	0	0	0	0	0	0	0	0	0	0	56
19978908	m	hp	CH	2021	0	0	0	0	0	0	0	0	0	0	0	0	72
19979038	m	md	US	2021	0	1	0	0	0	1	0	0	0	0	0	0	68
19979040	m	md	RU	2021	0	1	0	0	0	1	0	0	0	0	0	0	74
19979141	f	md	SG	2021	1	0	0	0	0	0	0	0	0	0	0	0	89
19979610	m	md	DE	2021	1	0	0	0	0	0	0	0	0	0	0	0	75
19979616	Not	md	JP	2021	0	1	0	0	0	1	0	0	0	0	0	0	70
19979836	m	md	JP	2021	0	1	0	0	0	1	0	0	0	0	0	1	59
19980046	f	cn	GB	2021	1	0	0	0	0	0	0	0	0	0	0	0	71
19980321	m	md	DE	2021	0	1	0	0	0	1	0	0	0	0	0	0	60
19980912	m	md	AR	2021	0	1	1	0	0	0	0	0	0	0	0	0	69
19981385	m	md	JP	2021	0	1	0	0	1	0	0	0	0	0	0	0	63
19981438	m	hp	JP	2021	0	1	0	0	1	0	0	0	0	0	0	0	76
19981480	f	ph	JP	2021	1	0	0	0	0	0	0	0	0	0	0	0	
19981512	m	hp	PL	2021	0	1	0	0	1	0	0	0	0	0	0	0	65
19981603	f	hp	NL	2021	0	0	0	0	0	0	0	0	0	0	0	0	
19982315	m	md	JP	2021	0	1	1	0	0	0	0	0	0	0	0	1	81
19982506	f	cn	CA	2021	0	1	0	0	1	0	0	0	0	0	0	0	79
19982615	f	md	JP	2021	0	0	0	0	0	0	0	0	0	0	0	0	67

19982636	m	md	US	2021	0	0	0	0	0	0	0	0	0	0	0	0	60
19982653	m	cn	CN	2021	0	1	0	1	0	0	0	0	0	0	0	0	72
19982852	f	hp	US	2021	1	0	0	0	0	0	0	0	0	0	0	0	53.7
19982854	Not	md	CH	2021	0	1	0	1	0	0	0	0	0	0	0	0	79
19983195	m	hp	US	2021	0	1	1	0	0	0	0	0	0	0	0	0	80
19983218	f	md	JP	2021	0	1	1	0	0	0	0	0	0	0	0	0	80
19983285	m	md	BE	2021	0	1	0	1	0	0	0	0	0	0	0	0	72.9
19983934	m	hp	NL	2021	0	1	0	1	0	0	0	0	0	0	0	0	85
19983988	m	md	FR	2021	0	0	0	0	0	0	0	0	0	0	0	0	72
19984087	m	cn	TH	2021	1	0	0	0	0	0	0	0	0	0	0	0	59
19984100	f	hp	US	2021	0	1	0	0	1	0	0	0	0	0	0	0	63
19984423	f	cn	CN	2021	0	0	0	0	0	0	0	0	0	0	0	0	67
19984683	m	md	JP	2021	0	1	1	0	0	0	0	0	0	0	0	0	74
19984714	f	cn	CN	2021	1	0	0	0	0	0	0	0	0	0	0	0	68
19985017	Not	md	IT	2021	1	0	0	0	0	0	0	0	0	0	0	0	
19985195	f	md	CH	2021	0	1	0	1	0	0	0	0	0	0	0	1	73
19985255	m	hp	RO	2021	0	0	0	0	0	0	0	0	0	0	0	0	71
19985267	Not	md	IT	2021	1	0	0	0	0	0	0	0	0	0	0	0	
19985416	m	md	JP	2021	0	1	0	1	0	0	0	0	0	0	0	1	
19985469	f	md	IT	2021	0	0	0	0	0	0	0	0	0	0	0	0	63
19985535	Not	ph	JP	2021	0	0	0	0	0	0	0	0	0	0	0	0	
19985590	Not	md	JP	2021	0	0	0	0	0	0	0	0	0	0	0	0	40
19985718	m	md	CH	2021	0	0	0	0	0	0	0	0	0	0	0	0	75
19985911	Not	md	CH	2021	0	1	0	1	0	0	0	0	0	0	0	0	75
19985913	Not	md	CH	2021	0	1	0	1	0	0	0	0	0	0	0	0	64
19986495	m	md	BG	2021	0	1	0	0	0	1	0	0	0	0	0	0	66
19986666	f	hp	US	2021	1	0	0	0	0	0	0	0	0	0	0	0	58
19986688	f	cn	CN	2021	0	1	0	0	1	0	0	0	0	0	0	0	62
19987616	f	ph	JP	2021	0	0	0	0	0	0	0	0	0	0	0	0	59
19987699	m	md	RU	2021	0	1	0	1	0	0	0	0	0	0	0	0	59.8
19987750	m	ph	US	2021	0	0	0	0	0	0	0	0	0	0	0	0	65
19987788	f	md	FR	2021	0	0	0	0	0	0	0	0	0	0	0	0	67
19988041	Not	md	FR	2021	1	0	0	0	0	0	0	0	0	0	0	0	
19988097	m	md	JP	2021	0	1	0	0	0	1	0	0	0	0	0	0	60

19988129	Not	md	JP	2021	0	1	0	0	0	1	0	0	0	0	0	0	
19988281	m	md	JP	2021	0	1	1	0	0	0	0	0	0	0	0	0	80
19988294	m	md	JP	2021	0	1	1	0	0	0	0	0	0	0	0	0	
19988326	f	cn	KR	2021	0	0	0	0	0	0	0	0	0	0	0	0	
19988363	m	md	JP	2021	0	1	0	0	0	1	0	0	0	0	0	0	80
19989110	f	md	BE	2021	0	1	1	0	0	0	0	0	0	0	0	0	
19989134	m	md	FR	2021	0	1	0	0	1	0	0	0	0	0	0	0	73
19989619	Not	cn	US	2021	0	0	0	0	0	0	0	0	0	0	0	0	
19990063	f	cn	US	2021	0	0	0	0	0	0	0	0	0	0	0	0	
19990065	Not	hp	US	2021	0	0	0	0	0	0	0	0	0	0	0	0	
19990389	m	ph	JP	2021	0	1	0	0	1	0	0	0	0	0	0	1	66
19991342	m	hp	MY	2021	1	0	0	0	0	0	0	0	0	0	0	0	56.9
19991703	m	hp	RO	2021	0	1	0	0	1	0	0	0	0	0	0	0	71
19991968	m	md	MY	2021	1	0	0	0	0	0	0	0	0	0	0	0	
19992316	m	md	JP	2021	0	1	0	0	1	0	0	0	0	0	0	0	60
19992870	f	md	DE	2021	0	0	0	0	0	0	0	0	0	0	0	0	
19993236	m	md	JP	2021	0	0	0	0	0	0	0	0	0	0	0	0	66
19993286	m	md	US	2021	0	1	0	1	0	0	0	0	0	0	0	0	75
19993594	m	md	JP	2021	0	1	0	0	0	1	0	0	0	0	0	0	57
19993787	f	md	FR	2021	0	0	0	0	0	0	0	0	0	0	0	0	69
19993843	m	md	JP	2021	0	1	0	0	1	0	0	0	0	0	0	1	80
19994019	f	hp	DE	2021	0	1	0	0	1	0	0	0	0	0	0	0	62
19994316	m	hp	US	2021	0	1	1	0	0	0	0	0	0	0	0	0	77
19995238	m	hp	NL	2021	0	0	0	0	0	0	0	0	0	0	0	0	45
19995588	f	md	GB	2021	0	0	0	0	0	0	0	0	0	0	0	0	83
19995886	f	md	US	2021	0	0	0	0	0	0	0	0	0	0	0	0	82
19995904	m	md	JP	2021	0	1	1	0	0	0	0	0	0	0	0	0	73
19995941	m	md	ID	2021	0	0	0	0	0	0	0	0	0	0	0	0	
19996184	m	md	DE	2021	0	1	0	0	1	0	0	0	0	0	0	0	
19996386	f	ph	AR	2021	0	0	0	0	0	0	0	0	0	0	0	0	57
19996544	m	cn	ID	2021	1	0	0	0	0	0	0	0	0	0	0	0	61.2
19996671	m	md	JP	2021	0	1	1	0	0	0	0	0	0	0	0	0	77
19996683	f	md	GB	2021	0	1	0	0	0	1	0	0	0	0	0	0	63
19996943	f	md	US	2021	0	0	0	0	0	0	0	0	0	0	0	0	60

19997064	m	md	JP	2021	0	0	0	0	0	0	0	0	0	0	0	0	67
19997087	m	hp	US	2021	0	1	0	0	1	0	0	0	0	0	0	0	71
19997097	f	ph	FR	2021	0	0	0	0	0	0	0	0	0	0	0	0	
19997109	m	cn	IN	2021	0	0	0	0	0	0	0	0	0	0	0	0	47
19997123	m	cn	US	2021	1	0	0	0	0	0	0	0	0	0	0	0	
19997313	m	md	DE	2021	0	1	0	0	1	0	0	0	0	0	0	0	
19997860	m	ph	JP	2021	0	1	1	0	0	0	0	0	0	0	0	0	80
19997970	f	md	FR	2021	1	0	0	0	0	0	0	0	0	0	0	0	
19998093	f	md	US	2021	0	1	0	1	0	0	0	0	0	0	0	0	45
19998301	m	md	JP	2021	0	1	0	0	1	0	0	0	0	0	0	0	79
19998326	f	md	JP	2021	0	1	0	0	1	0	0	0	0	0	0	0	70
19998365	m	md	JP	2021	0	0	0	0	0	0	0	0	0	0	0	0	75
19998608	f	md	PL	2021	0	0	0	0	0	0	0	0	0	0	0	0	74
19999008	f	cn	ID	2021	1	0	0	0	0	0	0	0	0	0	0	0	57.9
19999123	m	cn	CN	2021	0	1	0	0	1	0	0	0	0	0	0	0	54
19999126	m	hp	FR	2021	0	1	0	0	1	0	0	0	0	0	0	0	61
19999605	m	cn	CN	2021	0	1	0	1	0	0	0	0	0	0	0	0	70
19999611	m	hp	RU	2021	0	1	0	1	0	0	0	0	0	0	0	0	65
19999803	m	md	JP	2021	0	1	1	0	0	0	0	0	0	0	0	0	71
19999957	m	md	FR	2021	0	1	0	0	0	1	0	0	0	0	0	1	73
19999970	m	md	JP	2021	0	1	1	0	0	0	0	0	0	0	0	0	70
20000154	f	md	DE	2021	0	0	0	0	0	0	0	0	0	0	0	0	51
20000408	Not	ph	JP	2021	0	0	0	0	0	0	0	0	0	0	0	0	60
20000757	f	hp	US	2021	0	1	0	0	1	0	0	0	0	0	0	0	67
20001322	f	cn	US	2021	0	0	0	0	0	0	0	0	0	0	0	0	
20001529	Not	md	IT	2021	1	0	0	0	0	0	0	0	0	0	0	0	
20001532	m	md	RU	2021	1	0	0	0	0	0	0	0	0	0	0	0	60
20001589	f	cn	JP	2021	1	0	0	0	0	0	0	0	0	0	0	0	
20001772	m	cn	CN	2021	0	1	0	1	0	0	0	0	0	0	0	0	57
20001838	m	md	FR	2021	0	1	1	0	0	0	0	0	0	0	0	0	72
20001839	m	md	FR	2021	0	0	0	0	0	0	0	0	0	0	0	0	44
20002205	m	hp	NL	2021	0	0	0	0	0	0	0	0	0	0	0	0	62
20002270	f	hp	NL	2021	0	0	0	0	0	0	0	0	0	0	0	0	
20002388	m	md	JP	2021	0	1	1	0	0	0	0	0	0	0	0	0	80

20002755	f	cn	ID	2021	1	0	0	0	0	0	0	0	0	0	0	0	
20002928	m	md	CN	2021	0	1	1	0	0	0	0	0	0	0	0	0	57
20002933	f	md	CA	2021	0	0	0	0	0	0	0	0	0	0	0	0	52
20003107	f	cn	US	2021	1	0	0	0	0	0	0	0	0	0	0	1	68.9
20003223	f	hp	ID	2021	1	0	0	0	0	0	0	0	0	0	0	1	69
20003353	m	md	US	2021	0	0	0	0	0	0	0	0	0	0	0	0	78
20003505	m	md	DE	2021	0	0	0	0	0	0	0	0	0	0	0	0	77
20003696	f	hp	DK	2021	1	0	0	0	0	0	0	0	0	0	0	0	57
20003831	f	md	JP	2021	0	0	0	0	0	0	0	0	0	0	0	0	72
20003968	f	md	JP	2021	0	0	0	0	0	0	0	0	0	0	0	0	
20003980	f	hp	FR	2021	0	0	0	0	0	0	0	0	0	0	0	0	78
20004096	m	hp	US	2021	0	0	0	0	0	0	0	0	0	0	0	0	42
20004318	m	cn	CN	2021	0	0	0	0	0	0	0	0	0	0	0	0	
20004342	f	md	DE	2021	0	0	0	0	0	0	0	0	0	0	0	0	63
20004437	m	md	JP	2021	0	1	1	0	0	0	0	0	0	0	0	0	81
20004448	f	md	NL	2021	1	0	0	0	0	0	0	0	0	0	0	0	
20004527	f	md	JP	2021	0	0	0	0	0	0	0	0	0	0	0	0	61
20004539	m	hp	HU	2021	0	1	0	0	1	0	0	0	0	0	0	0	70
20004857	m	md	CA	2021	0	0	0	0	0	0	0	0	0	0	0	0	68
20005498	f	md	DE	2021	0	1	0	0	1	0	0	0	0	0	0	0	67
20005664	f	cn	CN	2021	0	0	0	0	0	0	0	0	0	0	0	0	68
20005817	m	hp	JP	2021	0	1	0	1	0	0	0	0	0	0	0	1	75
20005900	m	md	DE	2021	0	1	0	0	1	0	0	0	0	0	0	0	59
20006188	Not	cn	CN	2021	0	0	0	0	0	0	0	0	0	0	0	0	
20006542	f	cn	GB	2021	1	0	0	0	0	0	0	0	0	0	0	0	67
20006548	m	cn	CN	2021	0	1	0	0	1	0	0	0	0	0	0	0	68
20006588	m	md	ES	2021	0	0	0	0	0	0	0	0	0	0	0	0	50
20007023	Not	cn	CN	2021	0	0	0	0	0	0	0	0	0	0	0	0	
20007208	m	md	CN	2021	0	0	0	0	0	0	0	0	0	0	0	0	69
20007278	f	hp	NL	2021	0	0	0	0	0	0	0	0	0	0	0	0	59
20008259	m	md	DE	2021	0	1	0	0	0	1	0	0	0	0	0	0	69
20008300	Not	cn	US	2021	1	0	0	0	0	0	0	0	0	0	0	0	
20008738	f	md	JP	2021	0	0	0	0	0	0	0	0	0	0	0	0	76
20008838	Not	hp	CN	2021	0	0	0	0	0	0	0	0	0	0	0	0	

20009173	m	md	JP	2021	0	1	0	0	0	1	0	0	0	0	0	0	70
20009284	Not	hp	AU	2021	0	1	0	0	0	1	0	0	0	0	0	0	
20009489	f	md	US	2021	0	0	0	0	0	0	0	0	0	0	0	0	70
20010099	m	md	JP	2021	0	1	0	0	0	1	0	0	0	0	0	0	72
20010281	f	md	JP	2021	1	0	0	0	0	0	0	0	0	0	0	0	90
20010449	f	cn	TH	2021	1	0	0	0	0	0	0	0	0	0	0	0	
20010666	Not	md	FR	2021	1	0	0	0	0	0	0	0	0	0	0	0	
20010833	f	ph	MY	2021	1	0	0	0	0	0	0	0	0	0	0	0	
20010872	m	md	US	2021	0	0	0	0	0	0	0	0	0	0	0	0	
20010880	m	md	DE	2021	0	0	0	0	0	0	0	0	0	0	0	0	70
20010967	m	md	CZ	2021	0	1	0	0	1	0	0	0	0	0	0	0	75
20011413	f	cn	LB	2021	0	1	0	0	1	0	0	0	0	0	0	0	73
20011510	m	md	DE	2021	0	1	0	0	1	0	0	0	0	0	0	1	52
20012302	f	cn	CN	2021	1	0	0	0	0	0	0	0	0	0	0	0	68
20012309	m	cn	MY	2021	1	0	0	0	0	0	0	0	0	0	0	0	78.9
20012636	Not	md	DE	2021	0	1	0	0	1	0	0	0	0	0	0	0	
20012739	f	md	JP	2021	0	0	0	0	0	0	0	0	0	0	0	0	46
20012787	m	cn	KR	2021	0	0	0	0	0	0	0	0	0	0	0	0	78
20012943	m	md	IL	2021	0	0	0	0	0	0	0	0	0	0	0	0	65
20012981	m	md	JP	2021	0	0	0	0	0	0	0	0	0	0	0	0	
20013202	m	ph	PL	2021	0	0	0	0	0	0	0	0	0	0	0	0	70
20013210	m	md	FR	2021	0	1	0	0	0	1	0	0	0	0	0	0	56
20013222	Not	md	US	2021	1	0	0	0	0	0	0	0	0	0	0	0	30
20013238	f	cn	TH	2021	1	0	0	0	0	0	0	0	0	0	0	0	61.3
20013410	m	md	KR	2021	0	1	0	0	0	1	0	0	0	0	0	0	75
20013422	f	cn	US	2021	0	1	1	0	0	0	0	0	0	0	0	0	
20013582	m	hp	US	2021	0	1	1	0	0	0	0	0	0	0	0	0	64
20013614	m	md	JP	2021	0	0	0	0	0	0	0	0	0	0	0	0	90
20013965	m	cn	ID	2021	1	0	0	0	0	0	0	0	0	0	0	0	79.3
20013971	f	md	JP	2021	1	0	0	0	0	0	0	0	0	0	0	0	67
20014173	m	md	CN	2021	1	0	0	0	0	0	0	0	0	0	0	0	48
20015637	f	ph	US	2021	0	1	0	0	1	0	0	0	0	0	0	0	67
20017262	m	md	DE	2021	0	0	0	0	0	0	0	0	0	0	0	0	70
20017320	m	md	DE	2021	0	1	0	0	1	0	0	0	0	0	0	1	52

20017405	m	cn	IN	2021	0	0	0	0	0	0	0	0	0	0	0	0	69
20017549	m	md	JP	2021	0	1	0	0	0	1	0	0	0	0	0	0	80
20018515	f	md	US	2021	0	0	0	0	0	0	0	0	0	0	0	0	66
20019045	m	cn	IN	2021	0	1	0	0	1	0	0	0	0	0	0	0	74
20019806	f	md	JP	2021	1	0	0	0	0	0	0	0	0	0	0	0	74
20019814	Not	hp	CN	2021	0	0	0	0	0	0	0	0	0	0	0	0	
20019854	m	hp	US	2021	0	1	0	0	1	0	0	0	0	0	0	0	
20020449	m	cn	CN	2021	0	1	0	1	0	0	0	0	0	0	0	0	51
20020478	f	md	US	2021	1	0	0	0	0	0	0	0	0	0	0	0	
20020488	f	cn	AR	2021	0	0	0	0	0	0	0	0	0	0	0	0	31
20020810	Not	md	JP	2021	0	0	0	0	0	0	0	0	0	0	0	0	85
20020817	m	md	DE	2021	0	1	0	0	1	0	0	0	0	0	0	0	
20020833	Not	md	HK	2021	0	0	0	0	0	0	0	0	0	0	0	0	
20020919	m	md	NL	2021	0	1	0	0	0	1	0	0	0	0	0	0	58
20020948	m	md	KR	2021	0	1	1	0	0	0	0	0	0	0	0	0	54
20021736	m	md	JP	2021	0	1	0	0	1	0	0	0	0	0	0	0	83
20021817	m	md	JP	2021	0	1	0	0	0	1	0	0	0	0	0	0	80
20022003	m	cn	IN	2021	0	1	0	0	1	0	0	0	0	0	0	0	70
20022278	f	md	DE	2021	0	1	1	0	0	0	0	0	0	0	0	0	63
20022345	m	hp	JP	2021	0	1	0	0	0	1	0	0	0	0	0	0	72
20022358	f	hp	US	2021	0	1	0	0	1	0	0	0	0	0	0	0	64
20022528	m	md	GB	2021	0	0	0	0	0	0	0	0	0	0	0	0	62
20022533	m	md	JP	2021	0	1	0	0	0	1	0	0	0	0	0	0	90
20023007	m	cn	IN	2021	0	1	0	0	1	0	0	0	0	0	0	0	82
20023008	m	hp	JP	2021	0	1	0	0	1	0	0	0	0	0	0	0	65
20023510	f	cn	SG	2021	1	0	0	0	0	0	0	0	0	0	0	0	77.5
20023812	f	cn	SG	2021	1	0	0	0	0	0	0	0	0	0	0	0	86.9
20023908	m	cn	SG	2021	1	0	0	0	0	0	0	0	0	0	0	0	78.7
20024183	f	md	DE	2021	0	1	0	0	1	0	0	0	0	0	0	0	63
20024441	m	md	JP	2021	1	0	0	0	0	0	0	0	0	0	0	0	58
20024462	f	md	JP	2021	0	0	0	0	0	0	0	0	0	0	0	0	60
20024526	f	md	DE	2021	0	1	0	0	1	0	0	0	0	0	0	0	80
20024608	f	cn	US	2021	1	0	0	0	0	0	0	0	0	0	0	0	62.1
20024825	m	md	DE	2021	0	0	0	0	0	0	0	0	0	0	0	0	69

20025001	f	md	RO	2021	1	0	0	0	0	0	0	0	0	0	0	0	
20025013	m	md	JP	2021	0	1	0	0	1	0	0	0	0	0	0	0	73
20025741	f	hp	US	2021	0	1	1	0	0	0	0	0	0	0	0	1	
20025762	f	md	FR	2021	0	0	0	0	0	0	0	0	0	0	0	0	61
20025787	f	cn	DE	2021	0	0	0	0	0	0	0	0	0	0	0	0	72
20026028	m	md	JP	2021	0	0	0	0	0	0	0	0	0	0	0	0	67
20026089	m	md	JP	2021	0	1	0	0	1	0	0	0	0	0	0	0	68
20026185	Not	hp	GB	2021	0	0	0	0	0	0	0	0	0	0	0	0	
20026188	m	md	JP	2021	0	1	0	0	0	1	0	0	0	0	0	0	78
20026455	f	hp	US	2021	0	1	0	0	1	0	0	0	0	0	0	0	76
20026511	m	md	JP	2021	0	1	1	0	0	0	0	0	0	0	0	0	72
20026973	m	md	FR	2021	0	1	0	0	1	0	0	0	0	0	0	0	76
20026975	m	md	JP	2021	0	1	0	0	1	0	0	0	0	0	0	0	82
20027371	m	md	JP	2021	0	1	0	0	0	1	0	0	0	0	0	0	68
20027520	m	hp	KR	2021	1	0	0	0	0	0	0	0	0	0	0	0	52
20027579	m	md	DE	2021	0	0	0	0	0	0	0	0	0	0	0	0	77
20027838	f	cn	CA	2021	0	0	0	0	0	0	0	0	0	0	0	0	60
20027943	m	md	JP	2021	0	0	0	0	0	0	0	0	0	0	0	0	54
20028250	Not	md	JP	2021	0	1	0	0	0	1	0	0	0	0	0	0	
20028447	m	cn	IN	2021	0	0	0	0	0	0	0	0	0	0	0	0	54
20028777	f	hp	US	2021	1	0	0	0	0	0	0	0	0	0	0	0	73
20029019	m	cn	IN	2021	0	0	0	0	0	0	0	0	0	0	0	0	66
20029124	m	md	JP	2021	0	1	0	0	1	0	0	0	0	0	0	0	39
20029226	f	md	JP	2021	0	1	0	0	0	1	0	0	0	0	0	0	60
20029442	f	md	JP	2021	1	0	0	0	0	0	0	0	0	0	0	0	81
20029527	Not	md	JP	2021	0	1	0	0	0	1	0	0	0	0	0	0	
20029723	Not	md	JP	2021	0	1	0	0	0	1	0	0	0	0	0	0	
20029931	f	hp	KR	2021	0	0	0	0	0	0	0	0	0	0	0	0	57
20029965	Not	md	JP	2021	0	1	0	0	0	1	0	0	0	0	0	0	
20029993	Not	md	JP	2021	0	1	0	0	0	1	0	0	0	0	0	0	
20030131	m	md	NL	2021	1	0	0	0	0	0	0	0	0	0	0	1	71
20030235	m	md	CZ	2021	0	1	0	0	1	0	0	0	0	0	0	0	66
20030254	Not	md	JP	2021	0	0	0	0	0	0	1	0	0	0	1	0	69
20030293	m	md	JP	2021	0	1	0	0	1	0	0	0	0	0	0	1	63

20030465	f	cn	QA	2021	0	0	0	0	0	0	0	0	0	0	0	0	
20030560	m	md	DE	2021	0	1	1	0	0	0	0	0	0	0	0	0	68
20030685	m	md	CZ	2021	0	1	0	0	1	0	0	0	0	0	0	0	73
20030811	f	md	NL	2021	0	0	0	0	0	0	0	0	0	0	0	0	83
20030837	Not	md	JP	2021	0	1	0	1	0	0	0	0	0	0	0	0	
20030965	f	hp	DE	2021	0	0	0	0	0	0	0	0	0	0	0	0	
20030974	m	md	AT	2021	0	1	0	0	0	1	0	0	0	0	0	0	70
20030977	Not	md	FR	2021	1	0	0	0	0	0	0	0	0	0	0	0	
20031111	m	md	JP	2021	0	1	0	1	0	0	0	0	0	0	0	1	68.6
20031112	f	md	CZ	2021	0	1	0	0	1	0	0	0	0	0	0	0	60
20032041	Not	md	JP	2021	0	1	1	0	0	0	0	0	0	0	0	0	84
20032187	f	hp	US	2021	0	0	0	0	0	0	0	0	0	0	0	0	57
20032396	f	md	CH	2021	1	0	0	0	0	0	0	0	0	0	0	0	73
20032430	m	md	DE	2021	0	1	0	0	1	0	0	0	0	0	0	0	75
20033133	m	ph	FR	2021	0	0	0	0	0	0	0	0	0	0	0	0	64
20033136	f	md	CH	2021	0	0	0	0	0	0	0	0	0	0	0	0	72
20033288	Not	md	US	2021	0	0	0	0	0	0	0	0	0	0	0	0	
20033478	m	md	CZ	2021	0	1	0	0	1	0	0	0	0	0	0	0	79
20033482	m	md	CZ	2021	0	1	0	0	1	0	0	0	0	0	0	0	78
20033523	m	md	IL	2021	1	0	0	0	0	0	0	0	0	0	0	0	77
20033778	m	hp	US	2021	0	1	1	0	0	0	0	0	0	0	0	0	64
20033796	m	cn	IN	2021	0	0	0	0	0	0	0	0	0	0	0	0	42
20034509	m	md	CN	2021	0	0	0	0	0	0	0	0	0	0	0	0	71
20034524	m	cn	CN	2021	0	1	0	1	0	0	0	0	0	0	0	0	54
20034585	f	md	CZ	2021	0	1	0	0	1	0	0	0	0	0	0	0	69
20034642	m	cn	IN	2021	0	1	0	0	1	0	0	0	0	0	0	0	77
20034696	f	md	DE	2021	0	1	0	0	1	0	0	0	0	0	0	0	68
20034807	m	md	AR	2021	0	1	1	0	0	0	0	0	0	0	0	0	70
20035324	m	hp	ES	2021	0	1	0	0	1	0	0	0	0	0	0	0	59
20035325	m	hp	ES	2021	0	0	0	0	0	0	0	0	0	0	0	0	60
20035929	m	md	DE	2021	0	0	0	0	0	0	0	0	0	0	0	0	70
20036016	m	md	JP	2021	0	0	0	0	0	0	0	0	0	0	0	0	80
20036172	Not	md	JP	2021	0	1	0	0	0	1	0	0	0	0	0	0	59
20036314	f	cn	ID	2021	1	0	0	0	0	0	0	0	0	0	0	0	54.1

20036327	m	md	BE	2021	0	0	0	0	0	0	0	0	0	0	0	0	
20036344	m	md	JP	2021	0	1	0	1	0	0	0	0	0	0	0	1	79
20036421	f	md	JP	2021	0	1	0	0	0	1	0	0	0	0	0	0	51
20036574	f	md	JP	2021	0	1	0	0	0	1	0	0	0	0	0	0	78
20036575	Not	md	JP	2021	0	1	0	0	0	1	0	0	0	0	0	0	
20036627	Not	hp	CN	2021	0	0	0	0	0	0	0	0	0	0	0	0	
20036654	m	hp	CN	2021	0	0	0	0	0	0	0	0	0	0	0	0	61
20037012	m	md	CZ	2021	0	1	0	0	1	0	0	0	0	0	0	0	66
20037135	Not	md	JP	2021	0	1	0	0	0	1	0	0	0	0	0	1	
20037149	Not	md	DE	2021	0	1	1	0	0	0	0	0	0	0	0	1	57
20037418	Not	hp	IT	2021	0	0	0	0	0	0	0	0	0	0	0	0	
20037433	m	md	CZ	2021	0	1	0	0	1	0	0	0	0	0	0	0	58
20037564	Not	hp	CN	2021	0	0	0	0	0	0	0	0	0	0	0	0	
20037608	Not	md	JP	2021	0	1	0	0	0	1	0	0	0	0	0	0	72
20037724	f	cn	JP	2021	1	0	0	0	0	0	0	0	0	0	0	0	
20037901	m	hp	JP	2021	0	1	1	0	0	0	0	0	0	0	0	0	71
20037914	f	hp	JP	2021	0	1	1	0	0	0	0	0	0	0	0	0	77
20037928	m	hp	JP	2021	0	1	1	0	0	0	0	0	0	0	0	0	45
20037938	m	hp	JP	2021	0	1	1	0	0	0	0	0	0	0	0	0	45
20037939	Not	md	JP	2021	0	1	0	0	0	1	0	0	0	0	0	0	
20037978	Not	md	JP	2021	0	1	0	0	0	1	0	0	0	0	0	0	
20038021	Not	md	HK	2021	0	0	0	0	0	0	0	0	0	0	0	0	
20038128	f	cn	IN	2021	0	1	0	0	1	0	0	0	0	0	0	0	35
20038425	m	md	DE	2021	0	1	1	0	0	0	0	0	0	0	0	0	
20038894	f	md	IT	2021	0	0	0	0	0	0	0	0	0	0	0	0	
20038920	m	md	FR	2021	0	0	0	0	0	0	0	0	0	0	0	0	67
20039133	f	md	CZ	2021	0	1	0	0	1	0	0	0	0	0	0	0	72
20039201	Not	cn	US	2021	1	0	0	0	0	0	0	0	0	0	0	0	
20039206	Not	md	US	2021	0	0	0	0	0	0	0	0	0	0	0	0	
20039390	m	md	JP	2021	0	1	1	0	0	0	0	0	0	0	0	0	67
20039530	m	md	DE	2021	0	1	0	0	1	0	0	0	0	0	0	0	68
20039607	Not	md	US	2021	0	1	0	0	0	1	0	0	0	0	0	0	
20039920	m	md	GB	2021	0	1	1	0	0	0	0	0	0	0	0	1	
20040062	m	md	DE	2021	0	0	0	0	0	0	0	0	0	0	0	0	77

20040189	m	md	NL	2021	0	0	0	0	0	0	0	0	0	0	0	0	
20041296	Not	md	JP	2021	0	1	0	0	0	1	0	0	0	0	0	0	83
20041312	f	md	JP	2021	0	1	0	0	0	1	0	0	0	0	0	0	70
20041323	f	cn	IN	2021	0	1	0	0	1	0	0	0	0	0	0	0	72
20041326	m	md	JP	2021	0	1	0	0	0	1	0	0	0	0	0	0	
20041327	Not	md	JP	2021	0	1	0	0	0	1	0	0	0	0	0	0	
20042032	m	hp	US	2021	0	1	0	0	1	0	0	0	0	0	0	0	70
20042063	m	md	US	2021	0	1	1	0	0	0	0	0	0	0	0	0	56
20042160	Not	md	FR	2021	1	0	0	0	0	0	0	0	0	0	0	0	
20042483	m	ph	JP	2021	0	1	0	0	1	0	0	0	0	0	0	0	60
20043089	f	md	CN	2021	0	0	0	0	0	0	0	0	0	0	0	0	62
20043531	f	cn	GB	2021	1	0	0	0	0	0	0	0	0	0	0	0	
20043705	m	cn	US	2021	1	0	0	0	0	0	0	0	0	0	0	0	33
20043706	Not	md	US	2021	0	1	0	1	0	0	0	0	0	0	0	0	
20044044	f	md	DE	2021	0	1	1	0	0	0	0	0	0	0	0	0	61
20044575	f	hp	CN	2021	0	0	0	0	0	0	0	0	0	0	0	0	62
20044659	m	md	JP	2021	0	1	0	0	0	1	0	0	0	0	0	0	70
20044674	f	md	RS	2021	0	1	1	0	0	0	0	0	0	0	0	0	70
20044719	f	md	JP	2021	0	0	0	0	0	0	0	0	0	0	0	0	60
20044720	Not	md	US	2021	0	0	0	0	0	0	0	0	0	0	0	0	
20044724	m	md	JP	2021	0	0	0	0	0	0	0	0	0	0	0	0	80
20044868	f	md	KR	2021	0	0	0	0	0	0	0	0	0	0	0	0	82
20045338	m	md	JP	2021	0	1	1	0	0	0	0	0	0	0	0	0	80
20045364	Not	md	FR	2021	0	1	0	1	0	0	0	0	0	0	0	1	
20045368	Not	md	FR	2021	1	0	0	0	0	0	0	0	0	0	0	0	
20045393	Not	md	FR	2021	0	1	0	1	0	0	0	0	0	0	0	0	
20045568	m	hp	CN	2021	1	0	0	0	0	0	0	0	0	0	0	1	58
20045611	m	md	DE	2021	1	0	0	0	0	0	0	0	0	0	0	0	
20045661	m	md	CZ	2021	0	1	0	0	1	0	0	0	0	0	0	0	77
20045777	f	md	ES	2021	0	0	0	0	0	0	0	0	0	0	0	0	54
20046410	m	cn	CN	2021	0	1	0	0	1	0	0	0	0	0	0	0	56
20046540	f	md	US	2021	1	0	0	0	0	0	0	0	0	0	0	0	
20046794	Not	md	FR	2021	1	0	0	0	0	0	0	0	0	0	0	0	
20047266	m	md	ES	2021	0	1	0	0	0	1	0	0	0	0	0	0	58

20047675	f	md	US	2021	0	1	0	0	1	0	0	0	0	0	0	0	66
20048173	m	hp	CN	2021	0	0	0	0	0	0	0	0	0	0	0	0	68
20048189	f	md	HU	2021	0	0	0	0	0	0	0	0	0	0	0	0	66
20048464	m	cn	ID	2021	1	0	0	0	0	0	0	0	0	0	0	0	
20048465	f	cn	JP	2021	1	0	0	0	0	0	0	0	0	0	0	0	80
20049027	m	md	JP	2021	0	1	0	0	0	1	0	0	0	0	0	0	70
20049068	m	md	JP	2021	0	1	0	0	0	1	0	0	0	0	0	0	79
20049082	f	md	KR	2021	1	0	0	0	0	0	0	0	0	0	0	0	
20049116	m	hp	LB	2021	0	1	0	0	1	0	0	0	0	0	0	0	55
20049151	m	md	JP	2021	0	1	0	0	1	0	0	0	0	0	0	0	73
20049186	f	md	JP	2021	0	0	0	0	0	0	0	0	0	0	0	0	72
20049355	f	md	US	2021	0	0	0	0	0	0	0	0	0	0	0	0	83
20049357	f	md	DE	2021	0	0	0	0	0	0	0	0	0	0	0	0	51
20049773	Not	md	FR	2021	0	1	0	1	0	0	0	0	0	0	0	0	
20049965	f	md	US	2021	0	0	0	0	0	0	0	0	0	0	0	0	
20050312	Not	md	FR	2021	0	1	0	1	0	0	0	0	0	0	0	0	
20050313	Not	md	FR	2021	0	1	0	1	0	0	0	0	0	0	0	0	
20050374	m	md	JP	2021	0	1	1	0	0	0	0	0	0	0	0	0	57
20050454	m	ph	JP	2021	0	1	1	0	0	0	0	0	0	0	0	0	
20050480	f	md	IQ	2021	0	1	1	0	0	0	0	0	0	0	0	0	
20050612	f	md	BE	2021	0	0	0	0	0	0	0	0	0	0	0	0	65
20050770	Not	md	CN	2021	0	0	0	0	0	0	0	0	0	0	0	0	
20051244	m	md	JP	2021	0	1	0	0	0	1	0	0	0	0	0	0	68
20052073	m	md	US	2021	0	1	0	0	0	1	0	0	0	0	0	0	66
20052104	m	md	DE	2021	0	1	0	0	1	0	0	0	0	0	0	0	64
20052108	m	md	NL	2021	0	0	0	0	0	0	0	0	0	0	0	0	76
20052219	f	md	JP	2021	1	0	0	0	0	0	0	0	0	0	0	0	77
20052894	f	md	JP	2021	1	0	0	0	0	0	0	0	0	0	0	1	52
20052926	f	md	DE	2021	0	0	0	0	0	0	0	0	0	0	0	0	61
20053138	m	md	ES	2021	0	1	0	0	0	1	0	0	0	0	0	0	68
20053407	m	md	JP	2021	1	0	0	0	0	0	0	0	0	0	0	0	80
20053522	m	hp	NL	2021	0	1	0	0	1	0	0	0	0	0	0	0	
20053921	m	hp	NL	2021	0	1	0	0	1	0	0	0	0	0	0	1	52
20053929	f	hp	US	2021	0	1	1	0	0	0	0	0	0	0	0	0	

20053941	m	md	JP	2021	0	1	1	0	0	0	0	0	0	0	0	1	86
20054030	f	md	CH	2021	1	0	0	0	0	0	0	0	0	0	0	0	73
20054157	m	cn	CN	2021	0	1	0	0	1	0	0	0	0	0	0	0	61
20054237	Not	md	JP	2021	1	0	0	0	0	0	0	0	0	0	0	0	
20054848	Not	md	FR	2021	0	1	0	1	0	0	0	0	0	0	0	0	
20054878	m	md	DE	2021	0	1	0	0	1	0	0	0	0	0	0	0	62
20055009	Not	ph	FR	2021	0	1	0	1	0	0	0	0	0	0	0	0	
20055100	m	md	DE	2021	0	1	0	0	1	0	0	0	0	0	0	0	60
20055169	m	md	US	2021	0	0	0	0	0	0	0	0	0	0	0	0	78
20055232	f	hp	US	2021	0	0	0	0	0	0	0	0	0	0	0	0	62
20055347	m	md	TH	2021	1	0	0	0	0	0	0	0	0	0	0	0	60
20055409	m	hp	IL	2021	0	1	0	0	0	1	0	0	0	0	0	1	67
20055494	Not	md	FR	2021	0	1	0	1	0	0	0	0	0	0	0	0	
20055495	Not	md	FR	2021	1	0	0	0	0	0	0	0	0	0	0	0	
20056116	m	md	JP	2021	0	1	0	0	1	0	0	0	0	0	0	1	55
20056169	m	md	JP	2021	0	1	0	0	1	0	0	0	0	0	0	1	72
20056424	m	md	JP	2021	0	0	0	0	0	0	0	0	0	0	0	0	70
20056648	m	cn	CA	2021	0	0	0	0	0	0	0	0	0	0	0	0	32
20057011	Not	cn	SG	2021	1	0	0	0	0	0	0	0	0	0	0	0	73.6
20057421	m	hp	US	2021	0	1	0	0	1	0	0	0	0	0	0	0	53
20057506	f	cn	CN	2021	0	1	0	1	0	0	0	0	0	0	0	0	68
20058019	f	cn	GB	2021	1	0	0	0	0	0	0	0	0	0	0	0	90
20058022	m	md	ES	2021	0	1	1	0	0	0	0	0	0	0	0	0	73
20058323	Not	hp	FR	2021	0	0	0	0	0	0	0	0	0	0	0	0	
20058640	m	hp	KR	2021	1	0	0	0	0	0	0	0	0	0	0	0	52
20058911	Not	md	FR	2021	1	0	0	0	0	0	0	0	0	0	0	0	
20058914	Not	md	FR	2021	0	1	0	1	0	0	0	0	0	0	0	0	
20059114	m	cn	US	2021	0	0	0	0	0	0	0	0	0	0	0	0	
20059124	m	md	IT	2021	0	1	0	0	0	1	0	0	0	0	0	0	56
20059366	m	hp	DE	2021	0	0	0	0	0	0	0	0	0	0	0	0	65
20059770	m	cn	GB	2021	0	1	1	0	0	0	0	0	0	0	0	0	
20060141	Not	md	JP	2021	0	1	0	0	1	0	0	0	0	0	0	0	
20060209	Not	md	FR	2021	0	1	0	0	1	0	0	0	0	0	0	0	71
20060310	Not	hp	CN	2021	0	0	0	0	0	0	0	0	0	0	0	0	

20060386	f	md	DE	2021	0	0	0	0	0	0	0	0	0	0	0	0	51
20060746	m	cn	CN	2021	0	1	0	0	1	0	0	0	0	0	0	0	72
20060998	m	md	DE	2021	0	1	1	0	0	0	0	0	0	0	0	0	57
20061272	f	md	CA	2021	1	0	0	0	0	0	0	0	0	0	0	0	71.4
20061287	m	cn	CA	2021	0	0	0	0	0	0	0	0	0	0	0	0	39
20061380	f	md	CU	2021	0	0	0	0	0	0	0	0	0	0	0	0	
20061767	m	hp	CN	2021	1	0	0	0	0	0	0	0	0	0	0	1	57
20061831	m	md	JP	2021	0	1	0	0	1	0	0	0	0	0	0	0	60
20061875	m	md	JP	2021	0	0	0	0	0	0	0	0	0	0	0	0	67
20062058	m	md	JP	2021	0	1	0	0	0	1	0	0	0	0	0	0	77
20062185	f	md	FR	2021	0	1	0	0	1	0	0	0	0	0	0	0	
20062237	m	md	US	2021	0	0	0	0	0	0	0	0	0	0	0	0	
20062320	f	cn	SG	2021	1	0	0	0	0	0	0	0	0	0	0	0	78.6
20062371	f	hp	DE	2021	0	0	0	0	0	0	0	0	0	0	0	0	76
20062393	f	md	CA	2021	1	0	0	0	0	0	0	0	0	0	0	0	58
20062489	f	cn	US	2021	1	0	0	0	0	0	0	0	0	0	0	0	64
20062528	f	cn	TH	2021	1	0	0	0	0	0	0	0	0	0	0	0	62.8
20062530	m	md	JP	2021	0	1	0	1	0	0	0	0	0	0	0	1	
20062531	Not	cn	SG	2021	0	1	0	1	0	0	0	0	0	0	0	0	
20063155	m	hp	CN	2021	0	0	0	0	0	0	0	0	0	0	0	0	38
20063276	Not	md	US	2021	0	0	0	0	0	0	0	0	0	0	0	0	
20063749	f	hp	CN	2021	1	0	0	0	0	0	0	0	0	0	0	0	68
20063767	m	md	FR	2021	0	1	1	0	0	0	0	0	0	0	0	0	75
20063794	f	Not repo	US	2021	0	1	1	0	0	0	0	0	0	0	0	0	81
20063840	Not	md	JP	2021	0	0	0	0	0	0	0	0	0	0	0	0	75
20063863	Not	md	FR	2021	0	1	0	0	1	0	0	0	0	0	0	0	71
20064055	f	cn	SG	2021	1	0	0	0	0	0	0	0	0	0	0	0	85.6
20064691	f	md	US	2021	1	0	0	0	0	0	0	0	0	0	0	0	
20065116	m	md	JP	2021	0	1	0	0	0	1	0	0	0	0	0	0	
20066064	Not	cn	CN	2021	0	1	0	0	1	0	0	0	0	0	0	0	82
20066584	Not	md	CA	2021	0	1	0	0	1	0	0	0	0	0	0	0	
20067112	f	hp	CA	2021	1	0	0	0	0	0	0	0	0	0	0	0	
20067360	m	cn	SG	2021	1	0	0	0	0	0	0	0	0	0	0	0	67.3
20067449	m	md	BG	2021	1	0	0	0	0	0	0	0	0	0	0	0	

20067833	Not	md	JP	2021	0	1	0	1	0	0	0	0	0	0	0	0	
20067909	Not	md	TR	2021	0	0	0	0	0	0	0	0	0	0	0	0	
20068533	Not	cn	US	2021	0	1	0	1	0	0	0	0	0	0	0	0	
20070267	m	cn	ID	2021	1	0	0	0	0	0	0	0	0	0	0	0	78.8
20070387	m	hp	FR	2021	0	1	0	0	1	0	0	0	0	0	0	0	57
20070392	Not	cn	SG	2021	1	0	0	0	0	0	0	0	0	0	0	0	80.1
20071108	f	hp	DE	2021	0	0	0	0	0	0	0	0	0	0	0	0	55
20071436	m	md	DE	2021	0	1	0	0	1	0	0	0	0	0	0	0	65
20071723	Not	md	AM	2021	0	1	0	0	0	1	0	0	0	0	0	0	66
20072391	f	hp	RU	2021	0	0	0	0	0	0	0	0	0	0	0	0	60
20072560	f	ph	CN	2021	0	0	0	0	0	0	0	0	0	0	0	0	55
20072849	m	ph	JP	2021	0	1	1	0	0	0	0	0	0	0	0	0	72
20072898	m	md	JP	2021	0	1	1	0	0	0	0	0	0	0	0	0	71
20073086	m	md	AR	2021	1	0	0	0	0	0	0	0	0	0	0	0	47
20074187	Not	md	CN	2021	0	0	0	0	0	0	0	0	0	0	0	0	
20074310	m	md	JP	2021	0	1	0	1	0	0	0	0	0	0	0	1	73
20074405	m	hp	CN	2021	0	1	0	0	1	0	0	0	0	0	0	0	62
20074454	m	md	BG	2021	0	0	0	0	0	0	0	0	0	0	0	0	44
20074767	m	md	JP	2021	0	1	1	0	0	0	0	0	0	0	0	0	74
20074932	m	hp	US	2021	0	1	1	0	0	0	0	0	0	0	0	0	
20075216	m	md	BG	2021	1	0	0	0	0	0	0	0	0	0	0	0	
20075321	f	md	FR	2021	0	1	1	0	0	0	0	0	0	0	0	0	61
20075482	m	md	CA	2021	1	0	0	0	0	0	0	0	0	0	0	0	
20075559	f	md	JP	2021	0	1	0	1	0	0	0	0	0	0	0	0	
20075735	Not	md	JP	2021	0	1	0	1	0	0	0	0	0	0	0	1	
20076344	Not	cn	SG	2021	1	0	0	0	0	0	0	0	0	0	0	0	57.8
20076435	m	md	TR	2021	0	0	0	0	0	0	0	0	0	0	0	0	80
20076514	m	cn	IN	2021	0	0	0	0	0	0	0	0	0	0	0	0	61
20076563	f	md	JP	2021	1	0	0	0	0	0	0	0	0	0	0	0	
20076747	f	hp	JP	2021	1	0	0	0	0	0	0	0	0	0	0	0	77
20076748	Not	md	JP	2021	0	1	0	1	0	0	0	0	0	0	0	0	
20076864	Not	cn	US	2021	1	0	0	0	0	0	0	0	0	0	0	0	73.5
20077445	m	cn	LB	2021	1	0	0	0	0	0	0	0	0	0	0	0	56
20077446	Not	cn	SG	2021	1	0	0	0	0	0	0	0	0	0	0	0	72

20077519	f	md	BR	2021	0	1	1	0	0	0	0	0	0	0	0	0	63
20077541	m	md	JP	2021	0	1	1	0	0	0	0	0	0	0	0	0	80
20077731	f	cn	US	2021	1	0	0	0	0	0	0	0	0	0	0	0	71
20077732	f	md	US	2021	0	1	1	0	0	0	0	0	0	0	0	0	60
20078149	m	cn	SG	2021	0	1	0	0	0	1	0	0	0	0	0	0	77
20078591	f	md	JP	2021	0	1	0	0	0	1	0	0	0	0	0	0	70
20079055	m	cn	CN	2021	0	1	0	0	1	0	0	0	0	0	0	0	66
20079285	m	md	GB	2021	0	1	0	0	0	1	0	0	0	0	0	0	67
20079343	m	hp	JP	2021	0	1	0	0	1	0	0	0	0	0	0	0	74
20079378	m	md	GB	2021	0	1	0	0	0	1	0	0	0	0	0	0	67
20079734	Not	cn	US	2021	1	0	0	0	0	0	0	0	0	0	0	0	87.7
20079817	Not	hp	AU	2021	0	1	0	0	0	1	0	0	0	0	0	0	
20079849	f	md	DK	2021	1	0	0	0	0	0	0	0	0	0	0	1	63
20079977	m	cn	BY	2021	0	1	0	0	0	1	0	0	0	0	0	0	63
20080176	f	md	DE	2021	0	0	0	0	0	0	0	0	0	0	0	0	64
20080208	Not	hp	CN	2021	0	0	0	0	0	0	0	0	0	0	0	0	
20080703	m	md	JP	2021	0	1	0	1	0	0	0	0	0	0	0	1	69
20080727	f	hp	MY	2021	0	0	0	0	0	0	0	0	0	0	0	0	72
20080956	f	md	US	2021	0	1	0	1	0	0	0	0	0	0	0	0	58.8
20080972	m	md	DE	2021	0	1	1	0	0	0	0	0	0	0	0	0	57
20082293	m	cn	US	2021	1	0	0	0	0	0	0	0	0	0	0	0	58.8
20082369	f	md	PT	2021	0	0	0	0	0	0	0	0	0	0	0	0	70
20082377	Not	cn	HK	2021	0	0	0	0	0	0	0	0	0	0	0	0	73
20082463	m	hp	US	2021	1	0	0	0	0	0	0	0	0	0	0	0	
20082683	m	md	SE	2021	0	1	1	0	0	0	0	0	0	0	0	0	67
20082839	f	md	US	2021	0	0	0	0	0	0	0	0	0	0	0	0	73
20082844	Not	md	US	2021	1	0	0	0	0	0	0	0	0	0	0	0	
20082865	f	md	AT	2021	0	0	0	0	0	0	0	0	0	0	0	0	
20082994	f	ph	US	2021	1	0	0	0	0	0	0	0	0	0	0	0	85.1
20083297	Not	hp	GR	2021	0	0	0	0	0	0	0	0	0	0	0	0	
20083764	f	md	GB	2021	0	1	0	1	0	0	0	0	0	0	0	0	
20083941	m	md	JP	2021	0	1	0	0	1	0	0	0	0	0	0	1	74
20084386	Not	md	SG	2021	0	0	0	0	0	0	0	0	0	0	0	0	
20084752	f	cn	CN	2021	0	0	0	0	0	0	0	0	0	0	0	0	57

20084865	m	hp	ES	2021	0	0	0	0	0	0	0	0	0	0	0	0	61
20085407	m	md	JP	2021	1	0	0	0	0	0	0	0	0	0	0	0	70
20085422	m	cn	US	2021	0	0	0	0	0	0	0	0	0	0	0	0	
20085843	m	hp	ES	2021	0	0	0	0	0	0	0	0	0	0	0	0	61
20085923	m	cn	US	2021	0	0	0	0	0	0	0	0	0	0	0	0	68
20085955	f	md	JP	2021	0	1	0	1	0	0	0	0	0	0	0	1	64
20085956	m	md	US	2021	0	1	0	1	0	0	0	0	0	0	0	1	75
20086120	f	cn	US	2021	0	1	1	0	0	0	0	0	0	0	0	0	
20086183	m	cn	SG	2021	1	0	0	0	0	0	0	0	0	0	0	0	81.9
20086660	m	cn	DE	2021	0	1	1	0	0	0	0	0	0	0	0	0	57
20087132	Not	cn	GB	2021	0	0	0	0	0	0	0	0	0	0	0	0	
20087607	m	cn	IN	2021	0	0	0	0	0	0	0	0	0	0	0	0	69
20088223	f	ph	JP	2021	0	0	0	0	0	0	0	0	0	0	0	0	77
20088277	m	md	DE	2021	0	1	0	0	1	0	0	0	0	0	0	0	74
20088492	Not	md	GB	2021	0	0	0	0	0	0	0	0	0	0	0	0	
20088648	Not	md	US	2021	0	0	0	0	0	0	0	0	0	0	0	0	
20089017	Not	cn	CN	2021	0	0	0	0	0	0	0	0	0	0	0	0	
20089075	m	md	GB	2021	0	1	0	0	0	1	0	0	0	0	0	0	67
20089554	Not	hp	SG	2021	1	0	0	0	0	0	0	0	0	0	0	0	67
20089648	f	ph	CA	2021	0	0	0	0	0	0	0	0	0	0	0	0	73
20089884	m	md	CA	2021	1	0	0	0	0	0	0	0	0	0	0	0	
20089899	f	cn	TW	2021	0	0	0	0	0	0	0	0	0	0	0	0	78
20090197	f	cn	US	2021	0	0	0	0	0	0	0	0	0	0	0	0	
20090203	m	cn	TH	2021	1	0	0	0	0	0	0	0	0	0	0	0	79.8
20090372	f	md	RU	2021	0	0	0	0	0	0	0	0	0	0	0	0	57
20090458	Not	md	JP	2021	0	1	0	1	0	0	0	0	0	0	0	0	
20090467	f	hp	US	2021	0	0	0	0	0	0	0	0	0	0	0	0	
20090547	f	cn	TH	2021	1	0	0	0	0	0	0	0	0	0	0	0	87.1
20090620	f	hp	US	2021	1	0	0	0	0	0	0	0	0	0	0	0	67.9
20090624	f	ph	JP	2021	0	1	0	1	0	0	0	0	0	0	0	0	71
20090808	f	md	BG	2021	1	0	0	0	0	0	0	0	0	0	0	0	
20090907	f	md	US	2021	0	0	0	0	0	0	0	0	0	0	0	0	73
20090924	m	md	BE	2021	0	0	0	0	0	0	0	0	0	0	0	0	78
20091416	m	cn	US	2021	1	0	0	0	0	0	0	0	0	0	0	0	

20091454	f	md	CN	2021	1	0	0	0	0	0	0	0	0	0	0	0	84
20091691	Not	hp	US	2021	1	0	0	0	0	0	0	0	0	0	0	0	68.2
20091753	m	md	IL	2021	0	0	0	0	0	0	0	0	0	0	0	0	70
20091762	f	cn	US	2021	1	0	0	0	0	0	0	0	0	0	0	0	62
20091908	m	md	JP	2021	0	1	0	1	0	0	0	0	0	0	0	1	64
20091945	Not	md	GB	2021	1	0	0	0	0	0	0	0	0	0	0	0	
20091972	m	cn	GB	2021	1	0	0	0	0	0	0	0	0	0	0	0	47
20092261	f	md	DE	2021	0	0	0	0	0	0	0	0	0	0	0	0	72
20092294	Not	md	JP	2021	0	1	1	0	0	0	0	0	0	0	0	0	50
20092341	m	cn	TH	2021	1	0	0	0	0	0	0	0	0	0	0	0	60.4
20092604	m	cn	CN	2021	0	0	0	0	0	0	0	0	0	0	0	0	83
20092607	f	cn	CN	2021	0	0	0	0	0	0	0	0	0	0	0	0	51
20092782	f	md	FR	2021	0	1	0	0	0	1	0	0	0	0	0	0	59
20093148	m	md	JP	2021	0	1	1	0	0	0	0	0	0	0	0	0	80
20093496	f	hp	FR	2021	0	0	0	0	0	0	0	0	0	0	0	0	68
20093648	f	md	BE	2021	0	1	0	0	0	1	0	0	0	0	0	0	73
20094141	m	md	HK	2021	0	0	0	0	0	0	0	0	0	0	0	0	76
20095097	m	cn	US	2021	0	1	1	0	0	0	0	0	0	0	0	0	56
20095960	m	hp	CN	2021	0	1	0	0	1	0	0	0	0	0	0	0	63
20096162	Not	hp	US	2021	1	0	0	0	0	0	0	0	0	0	0	0	
20097123	m	ph	JP	2021	0	1	0	0	1	0	0	0	0	0	0	1	68
20097235	Not	hp	GB	2021	0	0	0	0	0	0	0	0	0	0	0	0	
20098150	m	cn	CN	2021	0	1	0	1	0	0	0	0	0	0	0	0	52
20098214	f	md	JP	2021	0	1	0	0	1	0	0	0	0	0	0	0	74
20098385	f	hp	JP	2021	0	0	0	0	0	0	0	0	0	0	0	0	36
20098489	m	md	NL	2021	0	0	0	0	0	0	0	0	0	0	0	0	70
20098840	m	hp	JP	2021	0	1	0	0	0	1	0	0	0	0	0	0	74
20099081	f	hp	JP	2021	0	1	1	0	0	0	0	0	0	0	0	0	70
20099111	m	cn	IN	2021	0	0	0	0	0	0	0	0	0	0	0	0	78
20099337	f	md	BE	2021	0	0	0	0	0	0	0	0	0	0	0	0	65
20099427	f	hp	US	2021	0	0	0	0	0	0	0	0	0	0	0	0	
20099486	m	cn	US	2021	1	0	0	0	0	0	0	0	0	0	0	0	71
20099561	f	md	FR	2021	0	0	0	0	0	0	0	0	0	0	0	0	52
20099591	f	hp	US	2021	0	0	0	0	0	0	0	0	0	0	0	0	

20099857	f	md	US	2021	0	0	0	0	0	0	0	0	0	0	0	0	
20100021	m	ph	JP	2021	0	1	0	0	1	0	0	0	0	0	0	1	68
20100254	f	cn	GB	2021	1	0	0	0	0	0	0	0	0	0	0	0	59
20100511	m	md	JP	2021	0	1	0	0	0	1	0	0	0	0	0	0	74
20100651	m	md	IT	2021	0	1	1	0	0	0	0	0	0	0	0	0	68
20101056	m	ph	ES	2021	0	1	0	0	0	1	0	0	0	0	0	0	64
20101239	m	cn	IN	2021	0	0	0	0	0	0	0	0	0	0	0	0	55
20101435	Not	md	US	2021	0	0	0	0	0	0	0	0	0	0	0	0	
20101494	m	hp	US	2021	0	0	0	0	0	0	0	0	0	0	0	0	53
20101571	m	md	JP	2021	0	1	0	0	0	1	0	0	0	0	0	0	79
20101854	Not	cn	US	2021	1	0	0	0	0	0	0	0	0	0	0	0	
20102245	f	cn	US	2021	0	0	0	0	0	0	0	0	0	0	0	0	59
20102380	f	cn	CA	2021	1	0	0	0	0	0	0	0	0	0	0	0	
20102477	Not	hp	SG	2021	1	0	0	0	0	0	0	0	0	0	0	0	65
20102485	m	md	JP	2021	0	1	0	0	1	0	0	0	0	0	0	0	90
20102648	m	cn	LB	2021	1	0	0	0	0	0	0	0	0	0	0	0	62
20102656	f	md	KR	2021	0	0	0	0	0	0	0	0	0	0	0	0	69
20103150	Not	md	HU	2021	0	0	0	0	0	0	0	0	0	0	0	0	
20103203	f	md	JP	2021	0	1	0	1	0	0	0	0	0	0	0	0	63
20103251	m	md	JP	2021	0	1	0	1	0	0	0	0	0	0	0	0	70
20103254	f	cn	ID	2021	1	0	0	0	0	0	0	0	0	0	0	0	61.5
20103431	Not	md	JP	2021	1	0	0	0	0	0	0	0	0	0	0	0	
20103640	f	md	JP	2021	1	0	0	0	0	0	0	0	0	0	0	0	75.1
20103706	m	md	DE	2021	0	1	0	0	1	0	0	0	0	0	0	0	74
20103721	f	md	DE	2021	0	1	1	0	0	0	0	0	0	0	0	0	63
20103900	f	cn	US	2021	0	0	0	0	0	0	0	0	0	0	0	0	45
20104528	m	ph	US	2021	0	0	0	0	0	0	0	0	0	0	0	0	80
20104997	m	md	JP	2021	0	0	0	0	0	0	0	0	0	0	0	0	60
20105179	m	md	CN	2021	0	0	0	0	0	0	0	0	0	0	0	0	57
20106766	m	cn	ID	2021	1	0	0	0	0	0	0	0	0	0	0	0	54.8
20107114	Not	hp	SG	2021	1	0	0	0	0	0	0	0	0	0	0	0	59
20107299	m	cn	CN	2021	0	1	0	1	0	0	0	0	0	0	0	1	58
20107592	m	md	JP	2021	0	0	0	0	0	0	0	0	0	0	0	0	66
20108013	m	cn	CN	2021	0	1	0	1	0	0	0	0	0	0	0	0	71

20108280	m	md	CA	2021	0	1	1	0	0	0	0	0	0	0	0	1	68
20108298	f	md	JP	2021	1	0	0	0	0	0	0	0	0	0	0	0	82
20108679	m	md	US	2021	0	0	0	0	0	0	0	0	0	0	0	0	
20109032	f	md	US	2021	0	0	0	0	0	0	0	0	0	0	0	0	59
20109106	f	md	TR	2021	0	0	0	0	0	0	0	0	0	0	0	0	55
20109138	f	cn	TH	2021	1	0	0	0	0	0	0	0	0	0	0	0	75
20109343	m	md	JP	2021	0	1	0	0	0	1	0	0	0	0	0	0	60
20109461	m	hp	AU	2021	1	0	0	0	0	0	0	0	0	0	0	0	
20109506	f	hp	MY	2021	1	0	0	0	0	0	0	0	0	0	0	0	69.9
20109573	m	md	FR	2021	0	1	0	1	0	0	0	0	0	0	0	0	64.1
20109698	m	md	JP	2021	1	0	0	0	0	0	0	0	0	0	0	0	74
20109699	m	cn	CN	2021	0	0	0	0	0	0	0	0	0	0	0	0	59
20109700	f	md	JP	2021	1	0	0	0	0	0	0	0	0	0	0	0	86
20109829	f	cn	US	2021	0	0	0	0	0	0	0	0	0	0	0	0	
20110376	f	md	JP	2021	1	0	0	0	0	0	0	0	0	0	0	0	81
20110627	f	md	US	2021	0	0	0	0	0	0	0	0	0	0	0	0	
20110828	m	cn	GB	2021	1	0	0	0	0	0	0	0	0	0	0	0	
20110957	m	md	JP	2021	0	1	0	0	1	0	0	0	0	0	0	0	66
20111102	m	md	BE	2021	0	0	0	0	0	0	0	0	0	0	0	0	57
20111355	Not	md	FR	2021	0	0	0	0	0	0	0	0	0	0	0	0	
20111356	f	cn	CA	2021	0	1	0	0	1	0	0	0	0	0	0	0	75
20111422	Not	md	JP	2021	0	1	0	0	1	0	0	0	0	0	0	0	74
20111758	f	cn	US	2021	1	0	0	0	0	0	0	0	0	0	0	0	
20111979	m	md	JP	2021	0	1	0	0	0	1	0	0	0	0	0	0	53
20112779	Not	md	JP	2021	1	0	0	0	0	0	0	0	0	0	0	0	
20112839	f	md	PL	2021	0	1	0	1	0	0	0	0	0	0	0	0	63
20113075	f	md	JP	2021	0	1	0	1	0	0	0	0	0	0	0	1	68
20113160	Not	cn	US	2021	1	0	0	0	0	0	0	0	0	0	0	0	86
20113616	m	cn	JP	2021	1	0	0	0	0	0	0	0	0	0	0	0	
20113617	f	md	FR	2021	1	0	0	0	0	0	0	0	0	0	0	0	80
20113716	m	md	BG	2021	0	0	0	0	0	0	0	0	0	0	0	0	71
20113840	m	cn	ID	2021	1	0	0	0	0	0	0	0	0	0	0	0	74.3
20113886	f	md	JP	2021	1	0	0	0	0	0	0	0	0	0	0	0	79
20113921	Not	md	GB	2021	1	0	0	0	0	0	0	0	0	0	0	0	

20113949	m	md	RU	2021	0	1	0	1	0	0	0	0	0	0	0	0	
20113962	f	cn	CN	2021	0	1	0	0	1	0	0	0	0	0	0	0	25
20114037	Not	md	JP	2021	0	1	1	0	0	0	0	0	0	0	0	0	80
20115077	f	md	JP	2021	0	1	1	0	0	0	0	0	0	0	0	0	50
20115094	Not	ph	JP	2021	0	0	0	0	0	0	0	0	0	0	0	0	50
20115135	m	ph	JP	2021	0	0	0	0	0	0	0	0	0	0	0	0	80
20115672	Not	md	CN	2021	0	0	0	0	0	0	0	0	0	0	0	0	
20116367	f	ph	JP	2021	0	0	0	0	0	0	0	0	0	0	0	0	50
20117185	f	md	CN	2021	1	0	0	0	0	0	0	0	0	0	0	0	45.2
20118388	Not	md	FR	2021	0	1	0	1	0	0	0	0	0	0	0	0	64.4
20118768	Not	md	US	2021	0	0	0	0	0	0	0	0	0	0	0	0	
20118903	m	md	JP	2021	0	1	1	0	0	0	0	0	0	0	0	0	70
20118910	Not	md	US	2021	0	0	0	0	0	0	0	0	0	0	0	0	
20119100	f	md	PL	2021	1	0	0	0	0	0	0	0	0	0	0	0	
20119251	f	md	SE	2021	1	0	0	0	0	0	0	0	0	0	0	0	
20119316	f	hp	US	2021	1	0	0	0	0	0	0	0	0	0	0	0	62
20119345	m	md	KR	2021	0	0	0	0	0	0	0	0	0	0	0	0	57
20119836	f	cn	IN	2021	0	0	0	0	0	0	0	0	0	0	0	0	57
20120016	m	md	AU	2021	0	1	1	0	0	0	0	0	0	0	0	0	
20120102	f	hp	RO	2021	1	0	0	0	0	0	0	0	0	0	0	0	
20120132	f	md	JP	2021	1	0	0	0	0	0	0	0	0	0	0	0	57
20120189	m	cn	IN	2021	0	0	0	0	0	0	0	0	0	0	0	0	46
20120224	f	md	IT	2021	0	0	0	0	0	0	0	0	0	0	0	0	54
20120307	m	md	JP	2021	0	1	1	0	0	0	0	0	0	0	0	0	77
20120351	m	md	JP	2021	0	1	1	0	0	0	0	0	0	0	0	0	81
20120925	m	md	JP	2021	0	1	0	0	0	1	0	0	0	0	0	0	70
20120933	f	md	JP	2021	0	1	0	0	0	1	0	0	0	0	0	0	79
20120978	m	hp	US	2021	0	0	0	0	0	0	0	0	0	0	0	0	
20121141	m	hp	AU	2021	0	0	0	0	0	0	0	0	0	0	0	0	74
20122774	m	hp	JP	2021	0	1	0	0	1	0	0	0	0	0	0	0	75
20123423	m	cn	IN	2021	0	1	0	0	1	0	0	0	0	0	0	0	91
20123666	f	md	JP	2021	0	0	0	0	0	0	0	0	0	0	0	0	64
20123707	m	hp	CH	2021	0	0	0	0	0	0	0	0	0	0	0	0	79
20124111	m	hp	US	2021	0	1	1	0	0	0	0	0	0	0	0	0	84

20124575	f	cn	IN	2021	0	0	0	0	0	0	0	0	0	0	0	0	48
20124795	f	md	US	2021	1	0	0	0	0	0	0	0	0	0	0	0	
20125086	f	md	FR	2021	0	0	0	0	0	0	0	0	0	0	0	0	
20125150	f	md	US	2021	0	0	0	0	0	0	0	0	0	0	0	0	76
20125243	f	md	IL	2021	0	0	0	0	0	0	0	0	0	0	0	0	65
20125251	m	md	JP	2021	0	0	0	0	0	0	0	0	0	0	0	0	82
20125541	f	md	CN	2021	0	0	0	0	0	0	0	0	0	0	0	0	65
20125683	f	md	FR	2021	0	1	0	0	0	1	0	0	0	0	0	0	55
20126150	m	hp	NL	2021	0	1	1	0	0	0	0	0	0	0	0	0	57
20126567	f	md	ES	2021	0	0	0	0	0	0	0	0	0	0	0	0	66
20126667	Not	md	DE	2021	0	0	0	0	0	0	0	0	0	0	0	0	72
20127002	m	cn	TH	2021	1	0	0	0	0	0	0	0	0	0	0	0	64
20127597	m	md	MY	2021	1	0	0	0	0	0	0	0	0	0	0	0	
20127852	f	cn	US	2021	0	1	1	0	0	0	0	0	0	0	0	0	61
20127971	f	md	US	2021	0	0	0	0	0	0	0	0	0	0	0	0	
20127976	f	hp	US	2021	0	0	0	0	0	0	0	0	0	0	0	0	66
20128105	m	cn	IN	2021	0	0	0	0	0	0	0	0	0	0	0	0	82
20128191	f	hp	FR	2021	1	0	0	0	0	0	0	0	0	0	0	0	34
20128411	f	md	US	2021	0	0	0	0	0	0	0	0	0	0	0	0	69
20128813	f	md	IT	2021	0	0	0	0	0	0	0	0	0	0	0	0	
20128965	m	md	DE	2021	0	1	1	0	0	0	0	0	0	0	0	0	60
20129286	m	cn	MY	2021	1	0	0	0	0	0	0	0	0	0	0	0	
20130420	m	hp	RO	2021	0	1	0	0	1	0	0	0	0	0	0	0	61
20130533	f	md	JP	2021	0	1	0	0	0	1	0	0	0	0	0	0	72
20130755	f	cn	MY	2021	1	0	0	0	0	0	0	0	0	0	0	0	76.6
20130940	m	md	JP	2021	0	1	1	0	0	0	0	0	0	0	0	0	80
20131195	m	hp	RU	2021	0	1	0	1	0	0	0	0	0	0	0	0	
20131249	m	cn	CN	2021	0	1	0	0	1	0	0	0	0	0	0	0	72
20131494	Not	md	US	2021	0	0	0	0	0	0	0	0	0	0	0	0	
20131529	Not	md	US	2021	0	0	0	0	0	0	0	0	0	0	0	0	
20131636	Not	md	JP	2021	1	0	0	0	0	0	0	0	0	0	0	0	
20131991	f	cn	DE	2021	0	1	0	0	1	0	0	0	0	0	0	0	61
20132047	m	cn	DE	2021	0	1	1	0	0	0	0	0	0	0	0	0	73
20132207	f	md	JP	2021	0	0	0	0	0	0	0	0	0	0	0	0	80

20132301	m	md	GB	2021	0	1	1	0	0	0	0	0	0	0	0	1	
20132457	m	ph	JP	2021	0	1	0	1	0	0	0	0	0	0	0	0	67
20132458	m	md	CN	2021	0	1	0	1	0	0	0	0	0	0	0	0	50.3
20132509	Not	hp	US	2021	0	0	0	0	0	0	0	0	0	0	0	0	
20132598	m	md	BE	2021	0	1	1	0	0	0	0	0	0	0	0	0	70
20132679	m	md	JP	2021	0	1	0	0	1	0	0	0	0	0	0	1	69
20132693	m	md	JP	2021	0	1	0	0	1	0	0	0	0	0	0	0	67
20133338	m	md	NL	2021	0	1	0	0	1	0	0	0	0	0	0	0	62
20133432	f	ph	JP	2021	0	0	0	0	0	0	0	0	0	0	0	0	78
20133460	Not	md	JP	2021	0	1	0	0	0	1	0	0	0	0	0	0	90
20133477	Not	md	JP	2021	0	1	0	0	0	1	0	0	0	0	0	0	80
20133861	m	md	JP	2021	0	1	0	0	0	1	0	0	0	0	0	0	71
20133876	m	md	JP	2021	0	1	0	0	1	0	0	0	0	0	0	0	60
20134041	f	cn	IN	2021	1	0	0	0	0	0	0	0	0	0	0	1	83
20134646	Not	md	JP	2021	1	0	0	0	0	0	0	0	0	0	0	0	
20134990	m	md	IT	2021	0	0	0	0	0	0	0	0	0	0	0	0	71
20135039	m	cn	CN	2021	0	1	0	0	1	0	0	0	0	0	0	1	46
20135246	m	md	BG	2021	1	0	0	0	0	0	0	0	0	0	0	0	59
20135266	f	cn	ZA	2021	0	1	1	0	0	0	0	0	0	0	0	0	75
20135589	m	ph	MY	2021	1	0	0	0	0	0	0	0	0	0	0	0	60.3
20135785	m	hp	NL	2021	0	0	0	0	0	0	0	0	0	0	0	0	83
20135949	m	md	NL	2021	0	1	0	0	1	0	0	0	0	0	0	0	62
20136223	f	ph	FR	2021	0	0	0	0	0	0	0	0	0	0	0	0	
20136291	m	md	CN	2021	1	0	0	0	0	0	0	0	0	0	0	0	
20136425	f	cn	TH	2021	1	0	0	0	0	0	0	0	0	0	0	0	55.2
20136617	f	md	DE	2021	0	1	0	0	1	0	0	0	0	0	0	0	65
20136629	f	md	JP	2021	1	0	0	0	0	0	0	0	0	0	0	0	63.1
20136654	m	cn	JP	2021	0	1	0	0	1	0	0	0	0	0	0	0	
20136833	Not	md	JP	2021	1	0	0	0	0	0	0	0	0	0	0	1	
20137934	m	cn	CN	2021	0	0	0	0	0	0	0	0	0	0	0	0	58
20138010	m	cn	IN	2021	0	1	0	0	0	1	0	0	0	0	0	0	76
20138157	m	md	KR	2021	0	1	0	0	0	1	0	0	0	0	0	0	71
20138381	f	md	JP	2021	0	1	0	0	1	0	0	0	0	0	0	0	73
20138660	m	cn	TH	2021	1	0	0	0	0	0	0	0	0	0	0	0	68.5

20138994	f	md	FR	2021	0	1	0	0	1	0	0	0	0	0	0	0	76
20139055	f	md	DE	2021	0	1	1	0	0	0	0	0	0	0	0	0	63
20139058	f	md	DE	2021	0	1	0	0	1	0	0	0	0	0	0	0	65
20139083	m	cn	SG	2021	0	0	0	0	0	0	0	0	0	0	0	0	
20139816	f	ph	US	2021	1	0	0	0	0	0	0	0	0	0	0	0	87
20140380	m	hp	GB	2021	0	0	0	0	0	0	0	0	0	0	0	0	65
20140385	m	md	CH	2021	0	1	0	0	0	1	0	0	0	0	0	0	70
20140780	m	md	DE	2021	0	1	0	0	1	0	0	0	0	0	0	0	60
20140869	m	md	TW	2021	0	0	0	0	0	0	0	0	0	0	0	0	
20141343	f	cn	TH	2021	1	0	0	0	0	0	0	0	0	0	0	0	
20141891	f	hp	PT	2021	1	0	0	0	0	0	0	0	0	0	0	0	77
20141909	m	md	DE	2021	0	1	0	0	1	0	0	0	0	0	0	1	52
20142077	f	md	DE	2021	0	0	0	0	0	0	0	0	0	0	0	0	
20142815	m	ph	JP	2021	0	0	0	0	0	0	0	0	0	0	0	0	80
20143027	m	md	NL	2021	0	1	0	0	1	0	0	0	0	0	0	0	62
20143149	f	cn	US	2021	1	0	0	0	0	0	0	0	0	0	0	0	82.4
20143305	m	md	JP	2021	0	0	0	0	0	0	0	0	0	0	0	0	80
20143516	m	md	US	2021	0	1	1	0	0	0	0	0	0	0	0	0	73
20143656	m	md	NL	2021	0	1	0	0	1	0	0	0	0	0	0	0	62
20143669	m	md	JP	2021	0	1	1	0	0	0	0	0	0	0	0	0	76
20144146	Not	hp	CN	2021	0	0	0	0	0	0	0	0	0	0	0	0	
20144147	Not	hp	CN	2021	0	0	0	0	0	0	0	0	0	0	0	0	
20144325	m	cn	ID	2021	1	0	0	0	0	0	0	0	0	0	0	0	67.8
20144454	Not	hp	ES	2021	0	0	0	0	0	0	0	0	0	0	0	0	
20144482	Not	md	JP	2021	0	1	1	0	0	0	0	0	0	0	0	0	
20144564	m	cn	CN	2021	0	1	0	1	0	0	0	0	0	0	0	0	74
20144969	Not	ph	US	2021	1	0	0	0	0	0	0	0	0	0	0	0	
20145124	f	md	JP	2021	0	0	0	0	0	0	0	0	0	0	0	0	43
20145158	Not	md	JP	2021	0	1	0	1	0	0	0	0	0	0	0	0	
20145231	m	md	JP	2021	0	0	0	0	0	0	0	0	0	0	0	0	90
20145490	m	md	FR	2021	0	0	0	0	0	0	0	0	0	0	0	0	52
20145510	m	md	TR	2021	0	1	1	0	0	0	0	0	0	0	0	0	73
20145576	f	md	JP	2021	0	1	1	0	0	0	0	0	0	0	0	1	80
20145621	m	cn	JO	2021	0	1	0	0	0	1	0	0	0	0	0	0	70

20147535	Not	md	JP	2021	0	0	0	0	0	0	0	0	0	0	0	0	
20147749	m	md	JP	2021	0	1	0	0	0	1	0	0	0	0	0	0	65
20147760	m	md	JP	2021	0	1	0	0	0	1	0	0	0	0	0	0	63
20147804	m	md	BG	2021	0	0	0	0	0	0	0	0	0	0	0	0	71
20147807	m	cn	IN	2021	0	0	0	0	0	0	0	0	0	0	0	0	39
20147992	Not	cn	CN	2021	0	0	0	0	0	0	0	0	0	0	0	0	
20147993	f	cn	CN	2021	0	0	0	0	0	0	0	0	0	0	0	0	76
20148584	f	ph	PT	2021	0	0	0	0	0	0	0	0	0	0	0	0	71
20148625	m	md	DE	2021	0	1	0	0	1	0	0	0	0	0	0	0	59
20149052	m	md	DE	2021	0	1	1	0	0	0	0	0	0	0	0	0	54
20149570	m	cn	CN	2021	0	1	0	0	1	0	0	0	0	0	0	0	52
20149681	f	ph	JP	2021	0	1	0	0	0	1	0	0	0	0	0	0	
20149740	m	md	FR	2021	0	1	0	0	0	1	0	0	0	0	0	0	59
20149750	m	md	JP	2021	0	1	1	0	0	0	0	0	0	0	0	0	64
20150265	f	ph	JP	2021	0	0	0	0	0	0	0	0	0	0	0	0	62
20150368	f	md	JP	2021	0	1	0	0	0	1	0	0	0	0	0	0	67
20150409	m	md	JP	2021	0	1	1	0	0	0	0	0	0	0	0	0	80
20150548	Not	md	JP	2021	0	1	0	0	0	1	0	0	0	0	0	0	80
20150627	m	ph	JP	2021	0	1	1	0	0	0	0	0	0	0	0	0	80
20150731	Not	hp	CN	2021	0	0	0	0	0	0	0	0	0	0	0	0	
20150773	Not	hp	CN	2021	0	0	0	0	0	0	0	0	0	0	0	0	
20150774	Not	hp	CN	2021	0	0	0	0	0	0	0	0	0	0	0	0	
20150775	Not	hp	CN	2021	0	0	0	0	0	0	0	0	0	0	0	0	
20150812	m	md	IT	2021	0	0	0	0	0	0	0	0	0	0	0	0	78
20150840	m	hp	CN	2021	0	0	0	0	0	0	0	0	0	0	0	0	71
20151406	m	hp	US	2021	0	0	0	0	0	0	0	0	0	0	0	0	
20151549	m	md	CN	2021	0	1	1	0	0	0	0	0	0	0	0	0	57
20151691	Not	hp	US	2021	1	0	0	0	0	0	0	0	0	0	0	0	
20151822	f	cn	ID	2021	1	0	0	0	0	0	0	0	0	0	0	0	76
20151951	m	md	FR	2021	0	0	0	0	0	0	0	0	0	0	0	0	83
20152064	Not	md	FR	2021	0	1	0	0	0	1	0	0	0	0	0	0	
20152299	m	cn	IN	2021	0	0	0	0	0	0	0	0	0	0	0	0	81
20152564	m	md	JP	2021	0	1	0	1	0	0	0	0	0	0	0	1	74
20152699	f	md	AU	2021	1	0	0	0	0	0	0	0	0	0	0	0	

20152833	m	Not repo	US	2021	0	1	1	0	0	0	0	0	0	0	0	0	76
20152973	m	hp	GB	2021	0	0	0	0	0	0	0	0	0	0	0	0	65
20152995	Not	md	IN	2021	1	0	0	0	0	0	0	0	0	0	0	0	
20153075	f	md	FR	2021	0	0	0	0	0	0	0	0	0	0	0	0	74
20153079	Not	md	FR	2021	0	0	0	0	0	0	0	0	0	0	0	0	
20153261	f	md	JP	2021	0	1	1	0	0	0	0	0	0	0	0	0	71
20153407	m	md	JP	2021	0	1	0	0	0	1	0	0	0	0	0	0	75
20153468	m	md	JP	2021	0	1	0	0	1	0	0	0	0	0	0	0	77
20153762	Not	hp	FR	2021	1	0	0	0	0	0	0	0	0	0	0	0	
20153845	m	md	IT	2021	0	1	0	0	0	1	0	0	0	0	0	0	80
20153864	f	hp	US	2021	1	0	0	0	0	0	0	0	0	0	0	0	62
20153874	f	cn	SG	2021	0	1	0	0	0	1	0	0	0	0	0	0	64
20153940	m	cn	IN	2021	0	1	0	0	1	0	0	0	0	0	0	0	70
20153954	m	cn	IN	2021	0	1	0	0	1	0	0	0	0	0	0	0	68
20153966	f	cn	CN	2021	0	1	0	1	0	0	0	0	0	0	0	0	71
20154721	Not	md	JP	2021	1	0	0	0	0	0	0	0	0	0	0	0	
20155325	m	md	JP	2021	0	1	0	1	0	0	0	0	0	0	0	1	69
20155602	m	md	RO	2021	0	1	0	0	1	0	0	0	0	0	0	0	71
20155632	Not	cn	JP	2021	0	0	0	0	0	0	0	0	0	0	0	0	
20155671	m	md	JP	2021	0	1	1	0	0	0	0	0	0	0	0	0	77
20155717	m	cn	TH	2021	1	0	0	0	0	0	0	0	0	0	0	0	61.2
20155946	Not	md	JP	2021	0	1	1	0	0	0	0	0	0	0	0	0	62
20155987	Not	md	FR	2021	0	1	0	0	0	1	0	0	0	0	0	0	
20156040	Not	md	JP	2021	0	1	1	0	0	0	0	0	0	0	0	0	
20156346	m	md	JP	2021	0	1	1	0	0	0	0	0	0	0	0	0	75
20156620	f	ph	IN	2021	0	0	0	0	0	0	0	0	0	0	0	0	72
20156742	Not	md	IN	2021	1	0	0	0	0	0	0	0	0	0	0	0	
20156776	m	md	JP	2021	0	1	1	0	0	0	0	0	0	0	0	0	72
20156821	m	cn	LB	2021	0	1	0	0	1	0	0	0	0	0	0	0	80
20156822	Not	hp	IN	2021	1	0	0	0	0	0	0	0	0	0	0	0	
20156870	m	md	JP	2021	0	1	0	1	0	0	0	0	0	0	0	1	72
20156872	m	cn	CN	2021	0	1	0	1	0	0	0	0	0	0	0	1	66
20157048	m	md	JP	2021	0	1	0	1	0	0	0	0	0	0	0	1	73
20157224	f	md	JP	2021	0	1	0	1	0	0	0	0	0	0	0	0	71

20157238	Not	hp	JP	2021	0	1	0	0	1	0	0	0	0	0	0	0	
20157280	Not	hp	JP	2021	0	1	0	0	1	0	0	0	0	0	0	0	
20157289	f	md	CA	2021	0	1	0	0	1	0	0	0	0	0	0	0	80
20157413	f	md	US	2021	0	1	1	0	0	0	0	0	0	0	0	0	66
20157667	Not	md	US	2021	0	0	0	0	0	0	0	0	0	0	0	0	
20158358	f	hp	US	2021	0	0	0	0	0	0	0	0	0	0	0	0	56
20158456	m	md	JP	2021	0	1	0	0	1	0	0	0	0	0	0	0	59
20158470	m	md	JP	2021	0	1	0	0	1	0	0	0	0	0	0	0	70
20158550	f	md	IL	2021	1	0	0	0	0	0	0	0	0	0	0	0	64
20158641	Not	md	AT	2021	0	0	0	0	0	0	0	0	0	0	0	0	
20158801	m	hp	NL	2021	0	1	0	0	1	0	0	0	0	0	0	0	62
20158811	m	md	JP	2021	0	0	0	0	0	0	0	0	0	0	0	0	20
20159439	m	md	FR	2021	0	0	0	0	0	0	0	0	0	0	0	0	83
20159443	m	md	FR	2021	0	0	0	0	0	0	0	0	0	0	0	0	52
20159880	m	md	DE	2021	0	1	0	0	1	0	0	0	0	0	0	0	74
20159907	Not	md	IN	2021	1	0	0	0	0	0	0	0	0	0	0	0	
20159914	Not	md	DE	2021	0	0	0	0	0	0	0	0	0	0	0	0	
20159997	m	md	GR	2021	0	1	1	0	0	0	0	0	0	0	0	0	56
20160083	f	md	JP	2021	0	1	1	0	0	0	0	0	0	0	0	0	64
20160200	m	cn	CN	2021	0	1	0	0	0	1	0	0	0	0	0	0	
20160304	m	md	JP	2021	0	1	1	0	0	0	0	0	0	0	0	1	57
20160355	f	md	US	2021	0	1	0	0	1	0	0	0	0	0	0	0	82
20160540	f	ph	JP	2021	1	0	0	0	0	0	0	0	0	0	0	0	85
20160542	m	ph	JP	2021	0	1	0	1	0	0	0	0	0	0	0	1	70
20160619	f	md	US	2021	0	1	0	1	0	0	0	0	0	0	0	1	67
20160628	Not	md	JP	2021	0	1	1	0	0	0	0	0	0	0	0	0	
20160833	Not	hp	IN	2021	1	0	0	0	0	0	0	0	0	0	0	0	
20160980	Not	ph	JP	2021	1	0	0	0	0	0	0	0	0	0	0	0	
20160991	m	hp	NL	2021	0	1	0	0	1	0	0	0	0	0	0	0	62
20161482	f	md	JP	2021	0	1	0	0	1	0	0	0	0	0	0	0	55
20161500	f	hp	NL	2021	0	0	0	0	0	0	0	0	0	0	0	0	59
20161524	f	md	JP	2021	0	0	0	0	0	0	0	0	0	0	0	0	53
20161706	m	md	JP	2021	0	1	0	0	1	0	0	0	0	0	0	0	69
20161908	f	ph	NZ	2021	0	0	0	0	0	0	0	0	0	0	0	0	71

20161951	f	md	JP	2021	1	0	0	0	0	0	0	0	0	0	0	0	66
20161952	f	md	JP	2021	0	1	0	1	0	0	0	0	0	0	0	1	68.4
20161998	m	md	JP	2021	0	1	0	1	0	0	0	0	0	0	0	0	60
20162199	Not	hp	HK	2021	0	0	0	0	0	0	0	0	0	0	0	0	
20162254	Not	hp	HK	2021	0	0	0	0	0	0	0	0	0	0	0	0	
20162288	Not	hp	HK	2021	0	0	0	0	0	0	0	0	0	0	0	0	
20162465	m	md	JP	2021	0	0	0	0	0	0	0	0	0	0	0	0	70
20162471	f	md	JP	2021	0	0	0	0	0	0	0	0	0	0	0	0	70
20162516	f	md	JP	2021	0	1	0	0	1	0	0	0	0	0	0	0	73
20163167	m	cn	CN	2021	0	0	0	0	0	0	0	0	0	0	0	0	57
20163458	m	cn	HR	2021	0	0	0	0	0	0	0	0	0	0	0	0	74
20163575	Not	hp	ES	2021	0	0	0	0	0	0	0	0	0	0	0	0	
20163599	f	hp	CN	2021	0	1	1	0	0	0	0	0	0	0	0	0	77
20163611	f	hp	CN	2021	0	1	1	0	0	0	0	0	0	0	0	0	88
20163713	m	hp	CN	2021	0	1	1	0	0	0	0	0	0	0	0	0	83
20163722	m	hp	CN	2021	0	1	1	0	0	0	0	0	0	0	0	0	73
20163991	m	md	GB	2021	0	0	0	0	0	0	0	0	0	0	0	0	64
20164211	m	ph	US	2021	0	0	0	0	0	0	0	0	0	0	0	0	50
20164320	m	md	KR	2021	0	1	0	0	0	1	0	0	0	0	0	0	67
20164453	f	cn	JP	2021	1	0	0	0	0	0	0	0	0	0	0	0	
20164614	f	md	JP	2021	0	1	0	0	1	0	0	0	0	0	0	0	73
20164926	m	md	DE	2021	0	1	0	0	1	0	0	0	0	0	0	1	52
20164928	f	md	IT	2021	0	1	0	0	0	1	0	0	0	0	0	0	56
20164996	m	md	DE	2021	0	1	1	0	0	0	0	0	0	0	0	0	60
20165252	m	md	JP	2021	1	0	0	0	0	0	0	0	0	0	0	0	69
20165324	m	md	JP	2021	0	1	0	0	1	0	0	0	0	0	0	0	68
20165357	Not	md	IN	2021	1	0	0	0	0	0	0	0	0	0	0	0	
20165611	m	cn	CN	2021	0	1	0	1	0	0	0	0	0	0	0	0	55
20165683	m	md	SI	2021	0	0	0	0	0	0	0	0	0	0	0	0	54
20165908	Not	hp	HK	2021	0	0	0	0	0	0	0	0	0	0	0	0	
20165909	Not	hp	HK	2021	0	0	0	0	0	0	0	0	0	0	0	0	
20165910	Not	hp	HK	2021	0	0	0	0	0	0	0	0	0	0	0	0	
20166091	f	hp	US	2021	0	1	0	0	1	0	0	0	0	0	0	0	66
20166286	m	md	JP	2021	0	1	1	0	0	0	0	0	0	0	0	0	70

20166600	Not	hp	HK	2021	0	0	0	0	0	0	0	0	0	0	0	0	
20166601	Not	hp	HK	2021	0	0	0	0	0	0	0	0	0	0	0	0	
20166602	Not	hp	HK	2021	0	0	0	0	0	0	0	0	0	0	0	0	
20166603	Not	hp	HK	2021	0	0	0	0	0	0	0	0	0	0	0	0	
20166604	Not	hp	HK	2021	0	0	0	0	0	0	0	0	0	0	0	0	
20166605	Not	hp	HK	2021	0	0	0	0	0	0	0	0	0	0	0	0	
20166606	Not	hp	HK	2021	0	0	0	0	0	0	0	0	0	0	0	0	
20166652	m	md	JP	2021	0	1	0	0	1	0	0	0	0	0	0	0	75
20166689	Not	hp	HK	2021	0	0	0	0	0	0	0	0	0	0	0	0	
20166913	m	hp	GB	2021	0	0	0	0	0	0	0	0	0	0	0	0	65
20166984	f	md	KR	2021	1	0	0	0	0	0	0	0	0	0	0	0	82.3
20167020	f	hp	JP	2021	0	1	1	0	0	0	0	0	0	0	0	0	70
20167442	m	md	JP	2021	0	1	0	0	1	0	0	0	0	0	0	0	72
20167842	m	md	HU	2021	0	1	0	0	0	1	0	0	0	0	0	0	
20168029	f	md	JP	2021	0	1	0	0	1	0	0	0	0	0	0	0	70
20168519	m	hp	AU	2021	0	1	1	0	0	0	0	0	0	0	0	0	55
20168862	m	md	JP	2021	0	1	0	0	1	0	0	0	0	0	0	1	82
20169114	f	md	JP	2021	0	1	0	0	1	0	0	0	0	0	0	0	70
20169138	Not	hp	CN	2021	0	0	0	0	0	0	0	0	0	0	0	0	
20169149	m	cn	CN	2021	0	1	0	0	1	0	0	0	0	0	0	0	65
20169249	m	md	JP	2021	0	1	1	0	0	0	0	0	0	0	0	0	
20169290	m	cn	ID	2021	1	0	0	0	0	0	0	0	0	0	0	0	59.7
20169385	Not	hp	CN	2021	0	0	0	0	0	0	0	0	0	0	0	0	
20169386	Not	hp	CN	2021	0	0	0	0	0	0	0	0	0	0	0	0	
20169690	m	md	JP	2021	0	1	0	1	0	0	0	0	0	0	0	0	63
20169764	f	md	CN	2021	0	0	0	0	0	0	0	0	0	0	0	0	65
20169770	m	md	JP	2021	0	1	0	1	0	0	0	0	0	0	0	1	66
20169883	m	md	IT	2021	1	0	0	0	0	0	0	0	0	0	0	1	70
20169938	m	md	IT	2021	0	0	0	0	0	0	0	0	0	0	0	0	53
20169956	Not	hp	CN	2021	0	0	0	0	0	0	0	0	0	0	0	0	
20169974	m	md	TR	2021	0	0	0	0	0	0	0	0	0	0	0	0	56
20170003	m	cn	VN	2021	1	0	0	0	0	0	0	0	0	0	0	0	81.8
20170111	m	cn	GB	2021	0	0	0	0	0	0	0	0	0	0	0	0	82
20170136	m	md	JP	2021	0	1	0	1	0	0	0	0	0	0	0	1	67

20170271	m	hp	CA	2021	1	0	0	0	0	0	0	0	0	0	0	0	77.1
20170331	m	cn	US	2021	0	1	1	0	0	0	0	0	0	0	0	0	
20170594	f	Not repo	US	2021	0	0	0	0	0	0	0	0	0	0	0	0	74
20170604	Not	ph	JP	2021	1	0	0	0	0	0	0	0	0	0	0	0	
20170695	f	cn	MY	2021	1	0	0	0	0	0	0	0	0	0	0	0	61.8
20170780	Not	cn	US	2021	1	0	0	0	0	0	0	0	0	0	0	0	60
20170817	f	cn	US	2021	0	1	1	0	0	0	0	0	0	0	0	0	
20170833	m	md	JP	2021	0	1	0	0	1	0	0	0	0	0	0	0	76
20171097	m	hp	CN	2021	0	0	0	0	0	0	0	0	0	0	0	0	54
20171290	m	cn	MY	2021	1	0	0	0	0	0	0	0	0	0	0	0	74.8
20171313	Not	hp	CN	2021	0	0	0	0	0	0	0	0	0	0	0	0	
20171315	Not	hp	CN	2021	0	0	0	0	0	0	0	0	0	0	0	0	
20171375	Not	cn	US	2021	1	0	0	0	0	0	0	0	0	0	0	0	81.8
20171468	Not	cn	SG	2021	1	0	0	0	0	0	0	0	0	0	0	0	51
20171649	f	md	DE	2021	0	1	0	0	1	0	0	0	0	0	0	0	82
20171722	Not	hp	CN	2021	0	0	0	0	0	0	0	0	0	0	0	0	
20171723	Not	hp	CN	2021	0	0	0	0	0	0	0	0	0	0	0	0	
20171724	Not	hp	CN	2021	0	0	0	0	0	0	0	0	0	0	0	0	
20171725	Not	hp	CN	2021	0	0	0	0	0	0	0	0	0	0	0	0	
20171986	m	md	JP	2021	0	1	0	0	0	1	0	0	0	0	0	0	76
20172100	f	md	JP	2021	0	1	0	0	0	1	0	0	0	0	0	0	59
20172107	Not	hp	CN	2021	0	0	0	0	0	0	0	0	0	0	0	0	
20172298	f	cn	DE	2021	0	1	0	0	1	0	0	0	0	0	0	0	65
20172509	Not	md	JP	2021	0	1	0	0	0	1	0	0	0	0	0	0	
20172714	m	hp	US	2021	0	0	0	0	0	0	0	0	0	0	0	0	53
20172728	m	md	JP	2021	0	1	0	0	0	1	0	0	0	0	0	0	60
20172761	Not	md	CN	2021	0	0	0	0	0	0	0	0	0	0	0	0	
20172775	Not	hp	CN	2021	0	0	0	0	0	0	0	0	0	0	0	0	
20172960	m	hp	MA	2021	0	0	0	0	0	0	0	0	0	0	0	0	50
20172968	Not	md	JP	2021	0	1	0	0	0	1	0	0	0	0	0	0	50
20173061	m	md	DE	2021	0	1	0	0	1	0	0	0	0	0	0	0	60
20173192	f	hp	IL	2021	0	0	0	0	0	0	0	0	0	0	0	0	71
20173202	m	cn	US	2021	0	0	0	0	0	0	0	0	0	0	0	0	
20173242	m	cn	US	2021	0	1	1	0	0	0	0	0	0	0	0	0	76

20173265	m	md	DE	2021	0	1	0	0	1	0	0	0	0	0	0	1	52
20173438	m	ph	JP	2021	0	1	1	0	0	0	0	0	0	0	0	0	70
20173524	Not	md	JP	2021	0	1	0	0	1	0	0	0	0	0	0	0	
20173545	f	md	AU	2021	0	1	1	0	0	0	0	0	0	0	0	0	79
20173619	Not	md	JP	2021	0	1	0	0	1	0	0	0	0	0	0	0	
20174329	m	md	CA	2021	0	1	0	0	0	1	0	0	0	0	0	0	63
20174611	f	ph	US	2021	0	0	0	0	0	0	0	0	0	0	0	0	76
20175163	m	cn	GB	2021	1	0	0	0	0	0	0	0	0	0	0	0	67
20175184	m	cn	GB	2021	1	0	0	0	0	0	0	0	0	0	0	0	
20175278	m	md	JP	2021	0	1	0	1	0	0	0	0	0	0	0	1	48
20175293	m	md	JP	2021	0	1	1	0	0	0	0	0	0	0	0	0	78
20175479	m	hp	FR	2021	0	1	0	0	1	0	0	0	0	0	0	0	72
20175681	Not	md	CN	2021	0	0	0	0	0	0	0	0	0	0	0	0	
20175851	m	ph	FR	2021	0	1	1	0	0	0	0	0	0	0	0	0	73
20176282	m	hp	NL	2021	0	1	1	0	0	0	0	0	0	0	0	0	60
20176333	Not	hp	US	2021	0	1	1	0	0	0	0	0	0	0	0	0	
20176454	m	md	US	2021	0	1	0	0	0	1	0	0	0	0	0	0	67
20176706	m	md	JP	2021	0	1	0	0	1	0	0	0	0	0	0	0	77
20176972	m	md	DE	2021	0	1	1	0	0	0	0	0	0	0	0	0	
20176991	Not	hp	IT	2021	0	1	0	1	0	0	0	0	0	0	0	0	81
20177185	f	md	JP	2021	0	1	0	0	1	0	0	0	0	0	0	0	82
20177199	m	md	AU	2021	0	0	0	0	0	0	0	0	0	0	0	0	
20177413	f	ph	US	2021	0	1	1	0	0	0	0	0	0	0	0	0	
20177498	m	md	JP	2021	0	1	0	0	0	1	0	0	0	0	0	0	60
20178718	m	md	JP	2021	0	1	0	0	1	0	0	0	0	0	0	0	77
20179158	f	hp	FR	2021	0	0	0	0	0	0	0	0	0	0	0	0	56
20179603	m	md	JP	2021	0	1	0	0	0	1	0	0	0	0	0	0	70
20179784	f	md	JP	2021	1	0	0	0	0	0	0	0	0	0	0	1	78
20179888	m	cn	IN	2021	0	1	0	0	1	0	0	0	0	0	0	0	60
20180073	m	md	JP	2021	0	1	0	0	0	1	0	0	0	0	0	0	70
20180174	Not	md	JP	2021	0	1	1	0	0	0	0	0	0	0	0	0	70
20180252	m	cn	CN	2021	0	0	0	0	0	0	0	0	0	0	0	0	61
20180839	f	cn	CA	2021	1	0	0	0	0	0	0	0	0	0	0	0	49
20180966	f	md	RS	2021	1	0	0	0	0	0	0	0	0	0	0	0	66

20181013	Not	md	JP	2021	1	0	0	0	0	0	0	0	0	0	0	0	
20181150	Not	md	IN	2021	1	0	0	0	0	0	0	0	0	0	0	0	
20181541	m	cn	CN	2021	0	0	0	0	0	0	0	0	0	0	0	0	73
20181833	m	md	JP	2021	0	1	0	0	1	0	0	0	0	0	0	0	53
20181865	m	md	JP	2021	0	1	0	0	1	0	0	0	0	0	0	0	78
20181895	f	hp	US	2021	0	0	0	0	0	0	0	0	0	0	0	0	67
20182181	Not	cn	JP	2021	0	0	0	0	0	0	0	0	0	0	0	0	75
20182187	m	cn	NL	2021	0	1	0	0	1	0	0	0	0	0	0	0	62
20182351	f	cn	AR	2021	0	0	0	0	0	0	0	0	0	0	0	0	71
20182442	Not	hp	US	2021	0	0	0	0	0	0	0	0	0	0	0	0	
20182590	Not	md	NZ	2021	1	0	0	0	0	0	0	0	0	0	0	0	
20182983	m	cn	US	2021	0	0	0	0	0	0	0	0	0	0	0	0	78
20183058	m	md	JP	2021	0	1	0	0	1	0	0	0	0	0	0	0	69
20183090	f	md	GB	2021	1	0	0	0	0	0	0	0	0	0	0	0	51
20184364	m	ph	JP	2021	0	1	1	0	0	0	0	0	0	0	0	0	73
20184600	m	cn	CN	2021	0	1	0	0	1	0	0	0	0	0	0	0	67
20185704	m	ph	US	2021	0	0	0	0	0	0	0	0	0	0	0	0	74
20185958	m	ph	FR	2021	0	0	0	0	0	0	0	0	0	0	0	0	54
20186031	Not	md	DE	2021	0	0	0	0	0	0	0	0	0	0	0	0	
20186048	f	cn	US	2021	1	0	0	0	0	0	0	0	0	0	0	0	
20186508	m	md	JP	2021	0	0	0	0	0	0	0	0	0	0	0	0	
20186600	Not	cn	GB	2021	1	0	0	0	0	0	0	0	0	0	0	0	71
20186910	m	ph	JP	2021	0	1	0	0	1	0	0	0	0	0	0	0	68
20188556	m	md	JP	2021	0	1	0	0	1	0	0	0	0	0	0	0	74
20188563	m	ph	JP	2021	0	1	0	0	1	0	0	0	0	0	0	0	68
20188751	Not	md	JP	2021	1	0	0	0	0	0	0	0	0	0	0	0	
20188863	f	cn	ID	2021	1	0	0	0	0	0	0	0	0	0	0	0	54.2
20189154	Not	cn	CN	2021	0	0	0	0	0	0	0	0	0	0	0	0	
20189186	m	hp	NL	2021	0	0	0	0	0	0	0	0	0	0	0	0	62
20189261	m	hp	CN	2021	0	1	1	0	0	0	0	0	0	0	0	0	72
20189262	m	md	JP	2021	0	1	1	0	0	0	0	0	0	0	0	0	75
20189376	m	md	JP	2021	0	1	0	0	0	1	0	0	0	0	0	0	60
20189378	m	md	JP	2021	0	1	0	0	0	1	0	0	0	0	0	1	90
20189546	m	md	JP	2021	0	1	0	0	0	1	0	0	0	0	0	0	70

20189697	Not	md	JP	2021	0	1	0	0	1	0	0	0	0	0	0	0	80
20189737	m	md	JP	2021	0	1	1	0	0	0	0	0	0	0	0	0	80
20189918	Not	md	NL	2021	0	0	0	0	0	0	0	0	0	0	0	0	
20190282	m	hp	IL	2021	0	1	1	0	0	0	0	0	0	0	0	0	
20190908	Not	md	JP	2021	0	1	0	1	0	0	0	0	0	0	0	0	
20190945	m	md	BR	2021	0	0	0	0	0	0	0	0	0	0	0	0	74
20191426	m	md	JP	2021	0	1	0	0	1	0	0	0	0	0	0	0	72
20191866	m	cn	CN	2021	0	1	0	0	1	0	0	0	0	0	0	0	56
20191900	m	cn	IN	2021	0	1	0	0	1	0	0	0	0	0	0	0	50
20192067	m	cn	IN	2021	0	1	0	0	1	0	0	0	0	0	0	0	61
20192564	Not	md	US	2021	0	0	0	0	0	0	0	0	0	0	0	0	
20193105	m	hp	US	2021	0	1	0	0	1	0	0	0	0	0	0	0	67
20193133	m	md	JP	2021	0	1	0	1	0	0	0	0	0	0	0	1	80
20193144	m	md	JP	2021	0	1	0	0	0	1	0	0	0	0	0	0	70
20193961	Not	hp	IT	2021	0	1	0	0	0	1	0	0	0	0	0	1	
20194087	m	md	JP	2021	0	1	0	1	0	0	0	0	0	0	0	1	71
20194243	m	md	CZ	2021	0	0	0	0	0	0	0	0	0	0	0	0	63
20194502	m	md	US	2021	0	1	0	1	0	0	0	0	0	0	0	0	58
20194681	f	md	TW	2021	0	0	0	0	0	0	0	0	0	0	0	0	72
20194753	m	ph	JP	2021	0	0	0	0	0	0	0	0	0	0	0	0	
20194772	f	md	GB	2021	0	0	0	0	0	0	0	0	0	0	0	0	76
20194915	m	md	JP	2021	0	1	0	0	0	1	0	0	0	0	0	1	65
20194931	Not	cn	DE	2021	0	1	0	0	1	0	0	0	0	0	0	0	74
20195112	m	hp	CN	2021	0	0	0	0	0	0	0	0	0	0	0	0	71
20195215	f	md	CN	2021	1	0	0	0	0	0	0	0	0	0	0	0	51
20195303	f	md	US	2021	0	0	0	0	0	0	0	0	0	0	0	0	41
20195537	m	hp	NL	2021	0	1	0	0	1	0	0	0	0	0	0	0	68
20195707	f	md	FR	2021	0	1	0	0	0	1	0	0	0	0	0	0	59
20195983	f	cn	US	2021	0	1	0	0	0	1	0	0	0	0	0	1	
20196385	m	md	TW	2021	0	0	0	0	0	0	0	0	0	0	0	0	
20196404	m	hp	JP	2021	0	1	0	0	1	0	0	0	0	0	0	1	69
20196812	m	md	JP	2021	0	0	0	0	0	0	0	0	0	0	0	0	69
20196866	f	md	US	2021	0	1	0	0	1	0	0	0	0	0	0	0	90
20196874	f	md	US	2021	0	1	0	0	0	1	0	0	0	0	0	0	65

20197146	m	md	JP	2021	0	1	0	0	0	1	0	0	0	0	0	0	68
20197701	Not	cn	CN	2021	0	0	0	0	0	0	0	0	0	0	0	0	
20197735	f	md	JP	2021	0	1	0	0	1	0	0	0	0	0	0	0	82
20198865	f	hp	JP	2021	0	0	0	0	0	0	1	0	1	0	0	0	67
20198987	f	cn	CA	2021	1	0	0	0	0	0	0	0	0	0	0	0	69.2
20198992	f	md	CN	2021	1	0	0	0	0	0	0	0	0	0	0	0	65
20199053	Not	md	FR	2021	0	1	0	0	1	0	0	0	0	0	0	0	70
20199159	m	md	DE	2021	0	1	0	0	1	0	0	0	0	0	0	0	68
20199191	Not	md	JP	2021	1	0	0	0	0	0	0	0	0	0	0	0	
20199193	f	ph	JP	2021	1	0	0	0	0	0	0	0	0	0	0	0	
20199355	f	cn	US	2021	1	0	0	0	0	0	0	0	0	0	0	0	66
20199449	Not	md	FR	2021	0	1	0	1	0	0	0	0	0	0	0	0	
20199490	Not	md	AU	2021	0	1	0	0	1	0	0	0	0	0	0	0	
20199592	Not	cn	RO	2021	0	1	0	0	1	0	0	0	0	0	0	0	55
20200802	m	md	CN	2021	0	1	0	1	0	0	0	0	0	0	0	0	67
20201725	f	cn	FR	2021	0	0	0	0	0	0	0	0	0	0	0	0	60
20201996	m	md	CN	2021	0	0	0	0	0	0	0	0	0	0	0	0	48
20202026	m	md	CN	2021	0	0	0	0	0	0	0	0	0	0	0	0	57
20202442	f	md	US	2021	0	1	1	0	0	0	0	0	0	0	0	0	68
20202485	Not	md	JP	2021	0	1	0	0	0	1	0	0	0	0	0	0	70
20203380	m	md	US	2021	0	0	0	0	0	0	0	0	0	0	0	0	70
20203756	f	hp	US	2021	0	1	1	0	0	0	0	0	0	0	0	0	40
20203903	m	md	JP	2021	0	1	0	0	1	0	0	0	0	0	0	0	80
20203980	m	cn	JP	2021	0	1	0	0	1	0	0	0	0	0	0	0	
20204027	m	md	JP	2021	0	1	0	0	0	1	0	0	0	0	0	0	70
20204269	m	ph	US	2021	0	0	0	0	0	0	0	0	0	0	0	0	54
20204277	m	md	NL	2021	0	1	0	0	0	1	0	0	0	0	0	0	75
20204694	f	md	DE	2021	0	1	0	0	1	0	0	0	0	0	0	1	65
20205536	f	md	AU	2021	0	0	0	0	0	0	0	0	0	0	0	0	74
20207282	m	ph	US	2021	0	0	0	0	0	0	0	0	0	0	0	0	71
20207293	Not	md	JP	2021	0	1	0	0	1	0	0	0	0	0	0	0	
20207550	f	md	JP	2021	0	0	0	0	0	0	0	0	0	0	0	0	57
20208468	m	md	JP	2021	0	1	0	0	0	1	0	0	0	0	0	0	73
20208597	m	hp	US	2021	0	0	0	0	0	0	0	0	0	0	0	0	53

20208839	Not	md	IN	2021	1	0	0	0	0	0	0	0	0	0	0	0	
20208936	f	md	JP	2021	0	1	1	0	0	0	0	0	0	0	0	0	78
20209511	f	hp	US	2021	0	1	0	0	1	0	0	0	0	0	0	0	
20209998	m	md	JP	2021	0	1	0	0	1	0	0	0	0	0	0	0	78
20210183	m	hp	NL	2021	0	1	0	0	1	0	0	0	0	0	0	0	75
20211139	m	md	DE	2021	0	1	0	0	1	0	0	0	0	0	0	0	76
20211395	m	cn	US	2021	0	0	0	0	0	0	0	0	0	0	0	0	75
20211396	m	md	JP	2021	1	0	0	0	0	0	0	0	0	0	0	0	77
20211484	f	hp	JP	2021	0	0	0	0	0	0	0	0	0	0	0	0	69
20211597	m	md	JP	2021	0	1	1	0	0	0	0	0	0	0	0	0	63
20211639	f	md	DE	2021	0	1	0	0	1	0	0	0	0	0	0	1	82
20212425	m	md	JP	2021	0	1	1	0	0	0	0	0	0	0	0	0	70
20212600	m	md	FR	2021	0	0	0	0	0	0	0	0	0	0	0	0	52
20212759	m	md	JP	2021	0	1	0	0	1	0	0	0	0	0	0	0	67
20213798	f	md	BR	2021	1	0	0	0	0	0	0	0	0	0	0	0	73
20213932	f	md	US	2021	0	0	0	0	0	0	0	0	0	0	0	0	
20214101	m	cn	US	2021	0	1	1	0	0	0	0	0	0	0	0	0	
20214184	f	hp	US	2021	0	1	1	0	0	0	0	0	0	0	0	0	40
20214660	Not	hp	JP	2021	1	0	0	0	0	0	0	0	0	0	0	0	
20214879	f	hp	LB	2021	0	1	0	0	1	0	0	0	0	0	0	0	56
20215313	Not	md	JP	2021	0	1	1	0	0	0	0	0	0	0	0	0	
20215357	m	md	JP	2021	0	1	0	0	1	0	0	0	0	0	0	0	66
20215833	f	md	JP	2021	0	1	0	0	1	0	0	0	0	0	0	0	60
20215876	Not	md	DE	2021	0	0	0	0	0	0	0	0	0	0	0	0	
20215893	f	md	JP	2021	0	1	0	0	1	0	0	0	0	0	0	0	70
20216009	Not	md	JP	2021	0	1	0	0	1	0	0	0	0	0	0	0	
20216126	f	md	DE	2021	0	1	0	0	1	0	0	0	0	0	0	1	65
20216128	m	md	BE	2021	0	1	0	0	0	1	0	0	0	0	0	0	76
20216174	m	md	IQ	2021	0	1	1	0	0	0	0	0	0	0	0	0	75
20216559	m	cn	TH	2021	1	0	0	0	0	0	0	0	0	0	0	0	67
20216641	m	ph	US	2021	0	1	1	0	0	0	0	0	0	0	0	0	78
20216702	m	md	DE	2021	0	1	1	0	0	0	0	0	0	0	0	1	78
20216759	Not	md	US	2021	0	0	0	0	0	0	0	0	0	0	0	0	
20216785	Not	md	GB	2021	1	0	0	0	0	0	0	0	0	0	0	0	

20217117	m	ph	JP	2021	1	0	0	0	0	0	0	0	0	0	0	0	
20217349	Not	md	AR	2021	0	0	0	0	0	0	0	0	0	0	0	0	
20217558	m	cn	AT	2021	0	1	1	0	0	0	0	0	0	0	0	0	54
20218603	f	hp	US	2021	0	1	1	0	0	0	0	0	0	0	0	0	40
20219103	m	md	JP	2021	0	1	0	0	0	1	0	0	0	0	0	0	51
20219392	f	md	KR	2021	0	0	0	0	0	0	0	0	0	0	0	0	69
20219531	f	md	CN	2021	0	0	0	0	0	0	0	0	0	0	0	0	64
20219602	f	md	NP	2021	0	0	0	0	0	0	0	0	0	0	0	0	46
20219784	f	md	MY	2021	0	0	0	0	0	0	0	0	0	0	0	0	67
20219924	f	cn	CN	2021	0	1	0	0	1	0	0	0	0	0	0	0	70
20220190	f	md	FR	2021	0	1	0	0	1	0	0	0	0	0	0	0	79
20220467	m	md	IL	2021	0	0	0	0	0	0	0	0	0	0	0	0	63
20220885	m	cn	CN	2021	0	1	0	0	1	0	0	0	0	0	0	0	52
20221010	m	md	FR	2021	0	1	0	0	1	0	0	0	0	0	0	0	63
20221034	f	md	CN	2021	0	1	0	0	0	1	0	0	0	0	0	0	63
20221355	m	cn	CN	2021	0	0	0	0	0	0	0	0	0	0	0	0	62
20221820	m	cn	US	2021	0	1	1	0	0	0	0	0	0	0	0	0	
20221970	f	cn	GB	2021	0	0	0	0	0	0	1	0	1	0	0	0	67
20222112	f	md	IT	2021	1	0	0	0	0	0	0	0	0	0	0	0	88
20222187	f	md	IT	2021	1	0	0	0	0	0	0	0	0	0	0	0	88
20222332	f	cn	CN	2021	0	1	0	0	1	0	0	0	0	0	0	0	80
20222591	f	cn	GB	2021	1	0	0	0	0	0	0	0	0	0	0	0	52
20222643	m	md	JP	2021	0	1	0	1	0	0	0	0	0	0	0	1	75
20222718	f	md	NL	2021	0	0	0	0	0	0	0	0	0	0	0	0	71
20222761	m	md	JP	2021	0	0	0	0	0	0	0	0	0	0	0	0	80
20223303	m	md	JP	2021	0	1	1	0	0	0	0	0	0	0	0	0	77
20223348	Not	cn	US	2021	1	0	0	0	0	0	0	0	0	0	0	0	
20223704	m	md	FR	2021	0	1	1	0	0	0	0	0	0	0	0	0	
20223782	m	md	NP	2021	0	0	0	0	0	0	0	0	0	0	0	0	46
20223792	f	md	NP	2021	0	0	0	0	0	0	0	0	0	0	0	0	74
20223843	f	hp	IT	2021	0	1	0	0	1	0	0	0	0	0	0	0	60
20223980	f	md	US	2021	0	0	0	0	0	0	0	0	0	0	0	0	88
20224153	f	md	FR	2021	0	0	0	0	0	0	0	0	0	0	0	0	57
20224304	m	md	CN	2021	0	0	0	0	0	0	0	0	0	0	0	0	63

20225096	m	md	JP	2021	0	1	1	0	0	0	0	0	0	0	0	0	
20225105	m	md	JP	2021	0	1	0	0	1	0	0	0	0	0	0	0	59
20225772	m	cn	CN	2021	0	1	0	0	1	0	0	0	0	0	0	0	72
20225967	Not	cn	US	2021	1	0	0	0	0	0	0	0	0	0	0	0	76.2
20225990	m	md	JP	2021	0	1	1	0	0	0	0	0	0	0	0	0	73
20226267	Not	hp	CN	2021	0	0	0	0	0	0	0	0	0	0	0	0	
20226424	m	md	JP	2021	0	0	0	0	0	0	0	0	0	0	0	0	74
20226551	m	cn	IN	2021	0	0	0	0	0	0	0	0	0	0	0	0	48
20226567	f	cn	IN	2021	0	0	0	0	0	0	0	0	0	0	0	0	74
20226635	Not	hp	CN	2021	0	0	0	0	0	0	0	0	0	0	0	0	
20227122	Not	md	FR	2021	0	1	0	0	1	0	0	0	0	0	0	0	63
20227468	f	md	DE	2021	0	1	1	0	0	0	0	0	0	0	0	0	72
20227844	f	md	JP	2021	0	1	0	0	1	0	0	0	0	0	0	0	80
20227868	m	md	JP	2021	0	1	1	0	0	0	0	0	0	0	0	0	74
20227882	m	md	JP	2021	0	1	1	0	0	0	0	0	0	0	0	0	75
20227937	f	hp	US	2021	0	0	0	0	0	0	0	0	0	0	0	0	70
20227946	f	md	JP	2021	0	1	0	1	0	0	0	0	0	0	0	1	
20227973	m	md	US	2021	0	1	0	0	0	1	0	0	0	0	0	0	70
20227981	f	md	JP	2021	0	1	0	1	0	0	0	0	0	0	0	1	65
20228110	f	cn	MY	2021	1	0	0	0	0	0	0	0	0	0	0	0	86.5
20228129	m	md	FR	2021	0	1	0	0	1	0	0	0	0	0	0	0	48
20228218	f	cn	TH	2021	1	0	0	0	0	0	0	0	0	0	0	0	89.9
20228248	m	md	JP	2021	0	1	0	0	1	0	0	0	0	0	0	1	60
20228288	f	cn	US	2021	0	1	1	0	0	0	0	0	0	0	0	0	
20228301	m	md	JP	2021	0	1	1	0	0	0	0	0	0	0	0	0	79
20228313	f	md	CA	2021	1	0	0	0	0	0	0	0	0	0	0	0	
20228431	m	md	SK	2021	0	0	0	0	0	0	0	0	0	0	0	0	32
20229367	m	md	TW	2021	0	0	0	0	0	0	0	0	0	0	0	0	72
20229429	f	md	DE	2021	0	1	1	0	0	0	0	0	0	0	0	0	75
20229577	f	md	JP	2021	0	1	0	0	0	1	0	0	0	0	0	0	60
20229609	m	md	JP	2021	0	1	0	0	0	1	0	0	0	0	0	0	80
20230832	f	md	JP	2021	0	1	0	0	1	0	0	0	0	0	0	0	47
20231348	f	md	JP	2021	0	1	0	0	1	0	0	0	0	0	0	0	47
20231825	f	cn	CN	2021	0	0	0	0	0	0	0	0	0	0	0	0	70

20231832	m	md	JP	2021	0	0	0	0	0	0	0	0	0	0	0	0	77
20231923	m	md	CA	2021	0	0	0	0	0	0	0	0	0	0	0	0	78
20231925	f	cn	CA	2021	0	1	0	1	0	0	0	0	0	0	0	0	40
20231990	f	cn	ID	2021	1	0	0	0	0	0	0	0	0	0	0	0	
20232262	Not	md	NL	2021	0	1	0	0	0	1	0	0	0	0	0	1	
20232675	m	md	US	2021	1	0	0	0	0	0	0	0	0	0	0	0	
20232927	f	md	LB	2021	0	1	0	0	1	0	0	0	0	0	0	0	82
20233055	m	md	FR	2021	0	0	0	0	0	0	0	0	0	0	0	0	97
20233065	m	md	JP	2021	0	1	0	0	1	0	0	0	0	0	0	0	71
20233118	Not	md	JP	2021	0	1	0	1	0	0	0	0	0	0	0	0	
20233503	Not	md	JP	2021	0	1	0	1	0	0	0	0	0	0	0	1	
20233591	f	cn	CN	2021	0	1	0	0	1	0	0	0	0	0	0	0	76
20233608	f	md	JP	2021	0	1	1	0	0	0	0	0	0	0	0	0	80
20233680	f	hp	BR	2021	0	1	0	0	1	0	0	0	0	0	0	0	
20233804	f	md	FR	2021	0	0	0	0	0	0	0	0	0	0	0	0	
20233860	m	md	JP	2021	0	1	1	0	0	0	0	0	0	0	0	0	57
20233863	m	md	JP	2021	0	1	1	0	0	0	0	0	0	0	0	0	90
20233873	m	ph	JP	2021	0	1	0	0	1	0	0	0	0	0	0	0	59
20234025	f	hp	US	2021	0	0	0	0	0	0	0	0	0	0	0	0	
20234034	m	md	JP	2021	0	1	1	0	0	0	0	0	0	0	0	0	75
20234433	m	hp	GB	2021	0	1	1	0	0	0	0	0	0	0	0	0	69
20235299	m	md	JP	2021	0	1	0	0	0	1	0	0	0	0	0	0	60
20235384	m	md	NL	2021	0	1	0	0	0	1	0	0	0	0	0	0	75
20235517	f	cn	US	2021	1	0	0	0	0	0	0	0	0	0	0	0	65
20235576	m	cn	ID	2021	1	0	0	0	0	0	0	0	0	0	0	0	50.2
20235582	f	md	US	2021	0	0	0	0	0	0	0	0	0	0	0	0	76
20235650	Not	md	NL	2021	0	1	0	0	0	1	0	0	0	0	0	0	
20235721	m	md	JP	2021	0	1	1	0	0	0	0	0	0	0	0	0	78
20235784	m	md	JP	2021	0	1	1	0	0	0	0	0	0	0	0	0	74
20236222	f	ph	CA	2021	0	1	1	0	0	0	0	0	0	0	0	0	72
20236539	m	md	JP	2021	0	1	0	0	1	0	0	0	0	0	0	0	74
20236573	m	ph	JP	2021	0	1	1	0	0	0	0	0	0	0	0	0	81
20236670	m	md	JP	2021	0	1	0	0	1	0	0	0	0	0	0	0	79
20236711	m	md	JP	2021	0	1	0	0	1	0	0	0	0	0	0	0	68

20237170	m	cn	TW	2021	0	1	0	1	0	0	0	0	0	0	0	0	72.8
20237171	f	cn	US	2021	1	0	0	0	0	0	0	0	0	0	0	0	82
20238263	f	md	JP	2021	0	1	0	0	1	0	0	0	0	0	0	0	44
20238937	f	cn	US	2021	1	0	0	0	0	0	0	0	0	0	0	0	86
20239016	f	cn	US	2021	1	0	0	0	0	0	0	0	0	0	0	0	
20239075	f	ph	US	2021	0	0	0	0	0	0	0	0	0	0	0	0	72
20239164	m	md	AT	2021	0	1	1	0	0	0	0	0	0	0	0	0	
20239820	f	hp	US	2021	0	0	0	0	0	0	0	0	0	0	0	0	
20240012	m	cn	CN	2021	0	1	0	0	1	0	0	0	0	0	0	0	75
20240206	f	md	JP	2021	0	1	1	0	0	0	0	0	0	0	0	0	80
20240219	m	cn	IN	2021	0	1	0	0	1	0	0	0	0	0	0	0	74
20240299	Not	md	DE	2021	0	0	0	0	0	0	0	0	0	0	0	0	
20240303	f	md	FR	2021	0	0	0	0	0	0	0	0	0	0	0	0	
20240355	m	md	KR	2021	0	0	0	0	0	0	0	0	0	0	0	0	65
20241711	f	md	DE	2021	0	0	0	0	0	0	0	0	0	0	0	0	
20241754	m	cn	IN	2021	1	0	0	0	0	0	0	0	0	0	0	0	77
20246012	m	md	JP	2021	0	1	1	0	0	0	0	0	0	0	0	0	72
20246903	m	hp	KR	2021	0	0	0	0	0	0	0	0	0	0	0	0	50
20247383	f	md	DE	2021	0	1	0	0	1	0	0	0	0	0	0	1	82
20247960	f	cn	IL	2021	0	0	0	0	0	0	0	0	0	0	0	0	
20249339	f	md	US	2021	0	1	1	0	0	0	0	0	0	0	0	0	82
20249698	f	md	BR	2021	0	0	0	0	0	0	0	0	0	0	0	0	72
20253222	f	md	NL	2021	0	0	0	0	0	0	0	0	0	0	0	0	71
20254810	f	cn	CN	2021	0	1	0	1	0	0	0	0	0	0	0	0	48
20255249	f	hp	US	2021	0	1	1	0	0	0	0	0	0	0	0	0	40
20255371	f	md	US	2021	0	1	1	0	0	0	0	0	0	0	0	0	40
20255382	m	md	JP	2021	0	0	0	0	0	0	0	0	0	0	0	0	70
20255494	m	hp	JP	2021	0	1	1	0	0	0	0	0	0	0	0	0	76
20255677	m	cn	CN	2021	0	0	0	0	0	0	0	0	0	0	0	0	56
20255960	m	cn	DE	2021	0	1	1	0	0	0	0	0	0	0	0	0	64
20256264	Not	md	NL	2021	0	0	0	0	0	0	0	0	0	0	0	0	
20256360	m	cn	CN	2021	0	0	0	0	0	0	0	0	0	0	0	0	61
20257480	f	cn	TH	2021	1	0	0	0	0	0	0	0	0	0	0	0	65
20257493	f	ph	US	2021	0	0	0	0	0	0	0	0	0	0	0	0	77

20257661	f	cn	AM	2021	0	0	0	0	0	0	0	0	0	0	0	0	44
20257679	m	md	KR	2021	0	0	0	0	0	0	0	0	0	0	0	0	59
20258276	f	cn	CN	2021	0	1	0	1	0	0	0	0	0	0	0	1	63
20258514	Not	hp	US	2021	0	0	0	0	0	0	0	0	0	0	0	0	
20258712	Not	md	DE	2021	0	1	0	0	1	0	0	0	0	0	0	0	74
20259313	Not	md	US	2021	0	0	0	0	0	0	0	0	0	0	0	0	
20259344	m	cn	CA	2021	0	0	0	0	0	0	0	0	0	0	0	0	
20262486	m	md	JP	2021	0	1	0	0	1	0	0	0	0	0	0	0	69
20262918	m	md	GB	2021	0	1	1	0	0	0	0	0	0	0	0	1	
20262978	f	cn	ID	2021	1	0	0	0	0	0	0	0	0	0	0	0	62.2
20263374	m	md	FR	2021	0	0	0	0	0	0	0	0	0	0	0	0	65
20263469	f	cn	US	2021	1	0	0	0	0	0	0	0	0	0	0	0	88
20263675	Not	ph	JP	2021	1	0	0	0	0	0	0	0	0	0	0	0	
20263897	m	md	JP	2021	0	1	0	0	1	0	0	0	0	0	0	0	69
20263979	m	ph	CA	2021	0	0	0	0	0	0	0	0	0	0	0	0	59
20264346	f	cn	US	2021	1	0	0	0	0	0	0	0	0	0	0	0	75.5
20264866	f	cn	CN	2021	0	0	0	0	0	0	0	0	0	0	0	0	68
20264983	Not	md	JP	2021	1	0	0	0	0	0	0	0	0	0	0	0	
20265045	f	cn	CN	2021	0	0	0	0	0	0	0	0	0	0	0	0	65
20265654	f	cn	CN	2021	0	0	0	0	0	0	0	0	0	0	0	0	66
20265672	Not	cn	SG	2021	1	0	0	0	0	0	0	0	0	0	0	0	
20266316	Not	hp	IN	2021	1	0	0	0	0	0	0	0	0	0	0	0	
20266921	f	ph	NL	2021	1	0	0	0	0	0	0	0	0	0	0	0	86
20266952	m	cn	MY	2021	1	0	0	0	0	0	0	0	0	0	0	0	
20268552	f	hp	CN	2021	0	0	0	0	0	0	1	1	0	0	0	0	49
20344157	m	hp	JP	2021	0	1	0	0	1	0	0	0	0	0	0	0	64
20419252	f	md	JP	2021	0	1	0	0	1	0	0	0	0	0	0	0	73
20428301	m	md	JP	2021	0	1	0	0	1	0	0	0	0	0	0	0	68
20487178	m	md	JP	2021	0	1	0	0	1	0	0	0	0	0	0	1	64
20529587	m	md	JP	2021	0	1	0	0	1	0	0	0	0	0	0	1	72
19381331	f	md	GB	2022	0	1	0	0	0	1	0	0	0	0	0	0	75
19483068	m	md	JP	2022	0	1	0	0	1	0	0	0	0	0	0	0	45
19484326	m	ph	JP	2022	0	1	0	0	1	0	0	0	0	0	0	0	61
19495011	m	hp	JP	2022	0	1	0	0	1	0	0	0	0	0	0	0	77

19506432	m	md	JP	2022	0	1	0	0	1	0	0	0	0	0	0	1	70
19507337	m	md	DE	2022	0	0	0	0	0	0	0	0	0	0	0	0	59
19512327	m	md	JP	2022	0	1	0	0	1	0	0	0	0	0	0	0	80
19568893	f	md	GB	2022	0	1	0	0	0	1	0	0	0	0	0	0	71
19576728	f	md	GB	2022	0	1	0	0	0	1	0	0	0	0	0	0	71
19596996	m	md	JP	2022	0	1	0	0	1	0	0	0	0	0	0	0	77
19639135	f	md	JP	2022	0	1	0	0	1	0	0	0	0	0	0	0	51
19670554	m	md	JP	2022	0	0	0	0	0	0	0	0	0	0	0	0	87
19721806	f	hp	GB	2022	0	1	0	0	0	1	0	0	0	0	0	0	71
19722378	f	md	GB	2022	0	1	0	0	0	1	0	0	0	0	0	0	71
19727724	m	md	RU	2022	0	0	0	0	0	0	0	0	0	0	0	0	62
19730158	f	md	GB	2022	0	1	0	0	0	1	0	0	0	0	0	0	71
19734548	Not	hp	GB	2022	0	1	0	0	0	1	0	0	0	0	0	0	
19735778	f	md	ES	2022	0	1	0	0	0	1	0	0	0	0	0	0	61
19742202	Not	md	GB	2022	0	1	0	0	0	1	0	0	0	0	0	0	
19749338	Not	md	GB	2022	0	1	0	0	0	1	0	0	0	0	0	0	
19753188	f	md	GB	2022	0	1	0	0	0	1	0	0	0	0	0	0	71
19758048	m	md	CN	2022	0	1	0	1	0	0	0	0	0	0	0	0	64.1
19762113	m	cn	US	2022	0	0	0	0	0	0	0	0	0	0	0	0	
19766101	f	md	GB	2022	0	1	0	0	0	1	0	0	0	0	0	0	71
19773425	f	md	JP	2022	0	1	0	0	1	0	0	0	0	0	0	0	
19776193	f	md	GB	2022	0	1	0	0	0	1	0	0	0	0	0	0	71
19781070	f	cn	IN	2022	0	0	0	0	0	0	0	0	0	0	0	0	63
19787871	m	hp	CN	2022	0	0	0	0	0	0	0	0	0	0	0	0	62
19797131	Not	md	GB	2022	0	1	0	0	0	1	0	0	0	0	0	0	
19803064	m	md	ES	2022	0	0	0	0	0	0	0	0	0	0	0	0	73
19805625	Not	md	KR	2022	0	1	0	0	0	1	0	0	0	0	0	0	
19835973	m	md	IT	2022	0	0	0	0	0	0	0	0	0	0	0	0	75
19849285	m	md	ES	2022	0	0	0	0	0	0	0	0	0	0	0	0	75
19883783	f	md	FR	2022	0	0	0	0	0	0	0	0	0	0	0	0	73
19885589	m	md	DE	2022	0	0	0	0	0	0	0	0	0	0	0	0	48
19888996	f	md	DE	2022	0	1	0	0	1	0	0	0	0	0	0	0	67
19901273	f	md	CN	2022	0	0	0	0	0	0	0	0	0	0	0	0	73
19905013	f	md	GB	2022	0	1	0	0	0	1	0	0	0	0	0	0	50

19906020	f	md	JP	2022	0	1	0	0	1	0	0	0	0	0	0	0	70
19906090	m	md	IT	2022	0	0	0	0	0	0	0	0	0	0	0	0	65
19908845	m	md	GB	2022	0	1	1	0	0	0	0	0	0	0	0	0	75
19911189	Not	md	JP	2022	0	1	0	0	1	0	0	0	0	0	0	0	
19911201	Not	md	JP	2022	0	1	0	0	1	0	0	0	0	0	0	0	
19912407	Not	md	JP	2022	0	1	0	0	1	0	0	0	0	0	0	0	
19913332	Not	md	JP	2022	0	1	0	0	1	0	0	0	0	0	0	0	
19914305	f	md	GB	2022	0	1	0	0	0	1	0	0	0	0	0	0	50
19919367	m	md	JP	2022	0	1	0	0	1	0	0	0	0	0	0	0	60
19920147	m	md	JP	2022	0	1	0	0	1	0	0	0	0	0	0	0	67
19921455	m	md	CN	2022	0	1	0	0	0	1	0	0	0	0	0	0	69
19922587	m	cn	CN	2022	0	1	0	0	1	0	0	0	0	0	0	0	53
19923792	f	md	JP	2022	0	1	0	0	1	0	0	0	0	0	0	0	64
19924708	m	md	JP	2022	0	1	0	0	1	0	0	0	0	0	0	1	73
19927893	f	md	TH	2022	0	1	0	0	1	0	0	0	0	0	0	0	28
19928071	m	md	JP	2022	0	0	0	0	0	0	0	0	0	0	0	0	71
19930851	m	md	JP	2022	0	1	0	0	1	0	0	0	0	0	0	0	79
19931540	m	md	JP	2022	0	1	0	0	1	0	0	0	0	0	0	0	63
19932202	f	md	JP	2022	0	1	0	0	1	0	0	0	0	0	0	0	74
19933778	f	md	JP	2022	0	1	0	0	1	0	0	0	0	0	0	0	64
19933789	f	md	NL	2022	0	0	0	0	0	0	0	0	0	0	0	0	74
19934208	f	md	JP	2022	0	1	0	0	1	0	0	0	0	0	0	0	77
19936863	m	cn	CN	2022	0	0	0	0	0	0	0	0	0	0	0	0	66
19937255	m	md	DE	2022	0	0	0	0	0	0	0	0	0	0	0	0	76
19940078	m	cn	IN	2022	0	0	0	0	0	0	0	0	0	0	0	0	60
19942124	m	md	JP	2022	0	1	0	0	1	0	0	0	0	0	0	0	77
19942126	f	md	JP	2022	0	1	0	0	1	0	0	0	0	0	0	0	66
19942139	f	md	JP	2022	0	1	0	0	1	0	0	0	0	0	0	0	68
19942197	m	hp	JP	2022	0	1	0	0	1	0	0	0	0	0	0	0	74
19943897	m	md	JP	2022	0	1	0	0	1	0	0	0	0	0	0	0	57
19943933	f	cn	US	2022	0	1	1	0	0	0	0	0	0	0	0	0	66
19944853	Not	md	JP	2022	0	1	0	0	1	0	0	0	0	0	0	0	
19947322	f	hp	FR	2022	0	0	0	0	0	0	0	0	0	0	0	0	78
19947642	m	md	JP	2022	0	1	0	0	1	0	0	0	0	0	0	0	75

19947837	Not	md	DE	2022	0	1	0	0	1	0	0	0	0	0	0	0	
19947936	m	md	ES	2022	0	1	1	0	0	0	0	0	0	0	0	0	62
19948214	f	md	JP	2022	0	1	0	0	1	0	0	0	0	0	0	0	76
19948556	m	md	DE	2022	0	1	0	0	1	0	0	0	0	0	0	0	64
19950838	m	md	FR	2022	0	0	0	0	0	0	0	0	0	0	0	0	78
19955526	m	md	JP	2022	0	1	0	0	1	0	0	0	0	0	0	1	57
19956588	f	md	FR	2022	0	1	0	0	0	1	0	0	0	0	0	0	63
19956844	m	md	JP	2022	0	1	0	0	1	0	0	0	0	0	0	0	68
19956998	f	md	GB	2022	0	1	1	0	0	0	0	0	0	0	0	0	61
19957528	m	md	JP	2022	0	1	0	0	1	0	0	0	0	0	0	0	70
19957784	Not	md	US	2022	0	1	0	0	0	1	0	0	0	0	0	0	
19958029	f	md	JP	2022	0	1	0	0	0	1	0	0	0	0	0	0	55
19962702	m	md	JP	2022	0	0	0	0	0	0	0	0	0	0	0	0	80
19964259	f	md	JP	2022	0	1	0	0	1	0	0	0	0	0	0	0	73
19966823	m	md	JP	2022	0	1	0	0	1	0	0	0	0	0	0	1	73
19967861	Not	md	JP	2022	0	1	0	0	1	0	0	0	0	0	0	0	
19968458	m	md	JP	2022	0	1	0	0	1	0	0	0	0	0	0	0	60
19969995	Not	md	GB	2022	0	0	0	0	0	0	0	0	0	0	0	0	
19971866	m	md	JP	2022	0	1	1	0	0	0	0	0	0	0	0	0	60
19972082	m	md	JP	2022	0	1	0	0	1	0	0	0	0	0	0	0	65
19972196	m	md	JP	2022	0	1	0	0	1	0	0	0	0	0	0	0	67
19973205	m	md	JP	2022	0	1	0	0	1	0	0	0	0	0	0	0	85
19973921	m	md	JP	2022	0	1	0	0	1	0	0	0	0	0	0	0	80
19974339	f	hp	FR	2022	0	0	0	0	0	0	0	0	0	0	0	0	78
19975921	f	md	DE	2022	0	0	0	0	0	0	0	0	0	0	0	0	45
19981212	f	md	JP	2022	0	0	0	0	0	0	0	0	0	0	0	0	76
19982673	m	md	JP	2022	0	1	0	0	1	0	0	0	0	0	0	0	
19983022	f	md	GB	2022	0	0	0	0	0	0	0	0	0	0	0	0	71
19983247	m	md	JP	2022	0	1	0	0	1	0	0	0	0	0	0	0	72
19985950	m	md	JP	2022	0	1	0	0	1	0	0	0	0	0	0	0	50
19986025	f	cn	CA	2022	0	1	0	0	1	0	0	0	0	0	0	0	65
19988002	f	md	ES	2022	0	0	0	0	0	0	0	0	0	0	0	0	76
19990109	m	hp	NL	2022	0	1	0	0	1	0	0	0	0	0	0	0	49
19990380	m	md	JP	2022	0	1	1	0	0	0	0	0	0	0	0	1	74

19991202	f	cn	US	2022	0	0	0	0	0	0	0	0	0	0	0	0	
19993261	f	md	DE	2022	0	0	0	0	0	0	0	0	0	0	0	0	48
19998699	Not	md	JP	2022	0	1	0	0	1	0	0	0	0	0	0	0	57
19999155	f	md	FR	2022	0	0	0	0	0	0	0	0	0	0	0	0	68
20000518	m	md	JP	2022	0	1	1	0	0	0	0	0	0	0	0	0	57
20002088	m	md	US	2022	0	1	0	0	0	1	0	0	0	0	0	0	84
20007678	m	md	JP	2022	0	1	0	0	1	0	0	0	0	0	0	0	74
20008354	m	md	JP	2022	0	1	0	0	1	0	0	0	0	0	0	0	85
20009300	m	md	LT	2022	0	1	0	0	0	1	0	0	0	0	0	0	65
20009372	m	md	US	2022	0	0	0	0	0	0	0	0	0	0	0	0	81
20010197	f	cn	IN	2022	0	0	0	0	0	0	0	0	0	0	0	0	55
20010920	f	md	KR	2022	0	0	0	0	0	0	0	0	0	0	0	0	77
20011970	m	md	DE	2022	0	1	0	0	1	0	0	0	0	0	0	0	64
20012965	m	md	US	2022	0	0	0	0	0	0	0	0	0	0	0	0	46
20013891	m	md	FR	2022	0	0	0	0	0	0	0	0	0	0	0	0	78
20013982	m	md	NO	2022	0	0	0	0	0	0	0	0	0	0	0	0	37
20020293	m	ph	JP	2022	0	1	0	0	1	0	0	0	0	0	0	0	68
20020701	m	md	BE	2022	0	0	0	0	0	0	0	0	0	0	0	0	84
20022494	m	md	JP	2022	0	1	0	0	0	1	0	0	0	0	0	1	76
20022641	m	md	JP	2022	0	1	0	0	1	0	0	0	0	0	0	0	62
20023112	f	md	FR	2022	0	0	0	0	0	0	0	0	0	0	0	0	
20023692	m	md	JP	2022	0	1	0	0	1	0	0	0	0	0	0	0	71
20023916	m	md	CN	2022	0	0	0	0	0	0	0	0	0	0	0	0	51
20024325	m	md	JP	2022	0	1	0	0	1	0	0	0	0	0	0	1	63
20024533	m	md	JP	2022	0	1	0	0	0	1	0	0	0	0	0	1	75
20024806	m	md	NO	2022	0	0	0	0	0	0	0	0	0	0	0	0	37
20027449	m	md	JP	2022	0	1	0	0	0	1	0	0	0	0	0	0	63
20027868	m	md	JP	2022	0	1	0	0	1	0	0	0	0	0	0	1	63
20028066	m	md	JP	2022	0	1	0	0	1	0	0	0	0	0	0	0	59
20030026	f	md	GB	2022	0	1	0	0	0	1	0	0	0	0	0	0	
20033476	m	md	JP	2022	0	1	0	0	1	0	0	0	0	0	0	0	73
20033483	m	md	JP	2022	0	1	0	0	1	0	0	0	0	0	0	0	67
20034789	f	md	AR	2022	0	0	0	0	0	0	0	0	0	0	0	0	66
20035326	m	md	JP	2022	0	1	0	0	1	0	0	0	0	0	0	1	49

20036577	m	md	ES	2022	0	1	0	0	0	1	0	0	0	0	0	0	46
20037805	m	md	JP	2022	0	1	0	0	1	0	0	0	0	0	0	0	58
20038159	Not	md	US	2022	0	1	0	0	0	1	0	0	0	0	0	0	
20038758	Not	md	JP	2022	0	1	0	0	1	0	0	0	0	0	0	0	70
20041303	m	md	JP	2022	0	1	0	0	1	0	0	0	0	0	0	0	80
20041310	m	md	JP	2022	0	1	0	0	1	0	0	0	0	0	0	1	77
20041313	m	md	JP	2022	0	1	0	0	0	1	0	0	0	0	0	0	74
20042288	m	hp	JP	2022	0	1	0	0	1	0	0	0	0	0	0	0	49
20045578	m	hp	CN	2022	0	0	0	0	0	0	0	0	0	0	0	0	63
20046461	m	md	FR	2022	0	0	0	0	0	0	0	0	0	0	0	0	81
20048902	m	md	CN	2022	0	1	0	0	0	1	0	0	0	0	0	0	69
20050198	m	md	IQ	2022	0	1	1	0	0	0	0	0	0	0	0	0	
20050460	f	md	JP	2022	0	0	0	0	0	0	0	0	0	0	0	0	60
20052587	f	md	BE	2022	0	0	0	0	0	0	0	0	0	0	0	0	72
20052923	m	md	IQ	2022	0	1	1	0	0	0	0	0	0	0	0	0	
20053499	m	md	IQ	2022	0	1	1	0	0	0	0	0	0	0	0	0	
20053566	m	md	IQ	2022	0	1	1	0	0	0	0	0	0	0	0	0	
20053652	m	md	IQ	2022	0	1	1	0	0	0	0	0	0	0	0	0	
20053935	m	md	IQ	2022	0	1	1	0	0	0	0	0	0	0	0	0	
20054331	Not	hp	IT	2022	0	0	0	0	0	0	0	0	0	0	0	0	
20054849	m	md	IQ	2022	0	1	1	0	0	0	0	0	0	0	0	0	
20055011	f	hp	US	2022	0	1	0	0	1	0	0	0	0	0	0	0	
20055416	m	md	JP	2022	0	1	1	0	0	0	0	0	0	0	0	0	65
20055955	f	md	DE	2022	0	0	0	0	0	0	0	0	0	0	0	0	75
20056404	f	md	JP	2022	0	1	0	0	1	0	0	0	0	0	0	0	50
20057127	f	md	DE	2022	0	0	0	0	0	0	0	0	0	0	0	0	66
20059468	m	md	CN	2022	0	1	1	0	0	0	0	0	0	0	0	0	67
20059493	m	md	IQ	2022	0	1	1	0	0	0	0	0	0	0	0	0	
20059568	f	md	CN	2022	0	1	1	0	0	0	0	0	0	0	0	0	50
20059771	m	md	IQ	2022	0	1	1	0	0	0	0	0	0	0	0	0	
20061054	f	cn	MX	2022	0	1	0	0	0	1	0	0	0	0	0	0	65
20061199	m	md	JP	2022	0	1	0	0	1	0	0	0	0	0	0	0	74
20061915	m	md	JP	2022	0	1	0	1	0	0	0	0	0	0	0	1	72
20064816	m	md	FR	2022	0	0	0	0	0	0	0	0	0	0	0	0	63

20066730	m	md	JP	2022	0	1	0	0	1	0	0	0	0	0	0	0	63
20066827	m	md	JP	2022	0	1	0	0	1	0	0	0	0	0	0	1	72
20069511	f	md	NL	2022	0	0	0	0	0	0	0	0	0	0	0	0	61
20070416	m	md	JP	2022	0	1	0	0	1	0	0	0	0	0	0	1	78
20071217	m	md	JP	2022	0	1	0	0	1	0	0	0	0	0	0	1	75
20071788	m	hp	NL	2022	0	1	0	0	1	0	0	0	0	0	0	0	66
20072587	m	md	CN	2022	0	1	0	0	0	1	0	0	0	0	0	0	68
20072940	m	md	JP	2022	0	1	0	0	1	0	0	0	0	0	0	0	51
20073009	m	md	PA	2022	0	1	0	0	0	1	0	0	0	0	0	0	64
20078623	m	md	JP	2022	0	1	1	0	0	0	0	0	0	0	0	1	
20080285	f	md	US	2022	0	1	0	0	0	1	0	0	0	0	0	0	60
20081585	m	md	JP	2022	1	0	0	0	0	0	0	0	0	0	0	0	
20083309	m	md	JP	2022	0	1	0	0	1	0	0	0	0	0	0	0	37
20086012	m	ph	JP	2022	0	1	1	0	0	0	0	0	0	0	0	0	68
20086202	m	md	JP	2022	0	1	0	0	1	0	0	0	0	0	0	0	56
20086667	m	md	JP	2022	0	1	0	0	1	0	0	0	0	0	0	0	79
20089227	m	md	AR	2022	0	1	0	0	0	1	0	0	0	0	0	0	71
20089718	m	md	JP	2022	0	0	0	0	0	0	0	0	0	0	0	0	64
20089878	f	cn	US	2022	0	0	0	0	0	0	0	0	0	0	0	0	
20090871	f	md	JP	2022	0	0	0	0	0	0	0	0	0	0	0	0	47
20095096	m	md	JP	2022	0	1	1	0	0	0	0	0	0	0	0	1	69
20096671	m	md	FR	2022	0	0	0	0	0	0	0	0	0	0	0	0	80
20097072	m	md	JP	2022	0	1	0	0	1	0	0	0	0	0	0	0	67
20099551	m	md	FR	2022	0	0	0	0	0	0	0	0	0	0	0	0	49
20103338	m	md	DE	2022	0	0	0	0	0	0	0	0	0	0	0	0	56
20103339	m	md	FR	2022	0	0	0	0	0	0	0	0	0	0	0	0	64
20103626	f	md	DE	2022	0	1	1	0	0	0	0	0	0	0	0	0	61
20104550	f	md	JP	2022	0	0	0	0	0	0	0	0	0	0	0	0	60
20109400	m	md	DE	2022	0	0	0	0	0	0	0	0	0	0	0	0	70
20109694	m	md	FR	2022	0	1	1	0	0	0	0	0	0	0	0	0	51
20109788	m	md	DE	2022	0	0	0	0	0	0	0	0	0	0	0	0	54
20109822	f	hp	NL	2022	0	1	0	0	1	0	0	0	0	0	0	0	64
20112770	m	md	CN	2022	0	0	0	0	0	0	0	0	0	0	0	0	56
20115267	m	md	JP	2022	0	1	0	0	1	0	0	0	0	0	0	0	80

20116246	f	md	DE	2022	0	0	0	0	0	0	0	0	0	0	0	0	
20119341	m	hp	SG	2022	0	1	0	0	0	1	0	0	0	0	0	0	79
20119529	m	md	JP	2022	0	1	0	0	1	0	0	0	0	0	0	1	65
20120055	f	ph	JP	2022	1	0	0	0	0	0	0	0	0	0	0	1	65
20120138	m	md	JP	2022	0	1	0	0	1	0	0	0	0	0	0	0	51
20120149	m	md	ES	2022	0	1	1	0	0	0	0	0	0	0	0	1	65
20120407	m	md	JP	2022	0	1	1	0	0	0	0	0	0	0	0	0	79
20125826	m	md	MX	2022	0	1	0	1	0	0	0	0	0	0	0	1	71
20126674	m	md	DE	2022	0	1	0	0	0	1	0	0	0	0	0	0	63
20128092	Not	md	GR	2022	0	0	0	0	0	0	0	0	0	0	0	0	
20128109	f	md	FR	2022	0	0	0	0	0	0	0	0	0	0	0	0	59
20128468	m	md	ES	2022	0	1	0	0	0	1	0	0	0	0	0	0	65
20129272	m	md	FR	2022	0	0	0	0	0	0	0	0	0	0	0	0	74
20130497	f	hp	FR	2022	0	1	0	0	1	0	0	0	0	0	0	0	51
20131050	m	md	ES	2022	0	1	1	0	0	0	0	0	0	0	0	0	71
20131813	m	md	JP	2022	0	1	0	0	1	0	0	0	0	0	0	0	70
20132621	m	hp	CL	2022	0	1	0	0	1	0	0	0	0	0	0	0	77
20133737	f	hp	JP	2022	0	0	0	0	0	0	0	0	0	0	0	0	80
20133965	m	md	JP	2022	0	1	0	0	1	0	0	0	0	0	0	0	71
20134436	m	cn	US	2022	0	0	0	0	0	0	0	0	0	0	0	0	82
20136044	m	md	FR	2022	0	0	0	0	0	0	0	0	0	0	0	0	78
20136350	m	md	CA	2022	0	0	0	0	0	0	0	0	0	0	0	0	67
20136870	m	md	FR	2022	0	0	0	0	0	0	0	0	0	0	0	0	55
20137796	f	md	FR	2022	0	1	0	0	0	1	0	0	0	0	0	0	75
20139071	f	md	CN	2022	0	1	1	0	0	0	0	0	0	0	0	0	51
20144729	m	md	KR	2022	0	1	1	0	0	0	0	0	0	0	0	0	65
20145163	f	hp	JP	2022	0	1	0	0	1	0	0	0	0	0	0	0	77
20146146	m	md	JP	2022	0	1	0	1	0	0	0	0	0	0	0	0	74
20146285	Not	md	JP	2022	0	1	0	0	1	0	0	0	0	0	0	1	
20146529	m	hp	CN	2022	0	0	0	0	0	0	0	0	0	0	0	0	65
20147985	m	md	CN	2022	0	0	0	0	0	0	0	0	0	0	0	0	69
20148063	f	md	CN	2022	0	0	0	0	0	0	0	0	0	0	0	0	74
20149654	f	md	DE	2022	0	1	0	0	1	0	0	0	0	0	0	0	62
20149853	m	md	FR	2022	0	0	0	0	0	0	0	0	0	0	0	0	70

20150006	f	md	JP	2022	0	1	0	0	1	0	0	0	0	0	0	0	81
20150025	m	md	JP	2022	0	1	0	0	1	0	0	0	0	0	0	0	74
20150399	m	hp	US	2022	0	1	0	1	0	0	0	0	0	0	0	0	
20150821	m	md	JP	2022	0	0	0	0	0	0	0	0	0	0	0	0	86
20151263	m	md	DE	2022	0	1	0	0	1	0	0	0	0	0	0	0	61
20151920	m	md	FR	2022	0	1	0	0	1	0	0	0	0	0	0	0	84
20151927	f	md	US	2022	0	1	1	0	0	0	0	0	0	0	0	0	64
20152457	m	md	KR	2022	0	0	0	0	0	0	0	0	0	0	0	0	
20153086	f	md	FR	2022	0	0	0	0	0	0	0	0	0	0	0	0	75
20153088	f	cn	US	2022	0	0	0	0	0	0	1	1	0	0	0	0	
20153214	m	hp	US	2022	0	1	1	0	0	0	0	0	0	0	0	0	59
20154111	f	md	CA	2022	0	1	0	0	1	0	0	0	0	0	0	0	80
20154654	m	md	JP	2022	0	1	0	0	1	0	0	0	0	0	0	0	72
20155247	m	md	CN	2022	0	1	0	1	0	0	0	0	0	0	0	0	69
20156111	f	md	US	2022	0	0	0	0	0	0	0	0	0	0	0	0	76
20156222	m	md	GB	2022	0	1	1	0	0	0	0	0	0	0	0	0	50
20156266	m	cn	TH	2022	1	0	0	0	0	0	0	0	0	0	0	0	65.5
20156349	m	md	US	2022	0	0	0	0	0	0	0	0	0	0	0	0	60
20160337	f	cn	JP	2022	0	0	0	0	0	0	0	0	0	0	0	0	75
20161746	m	md	AU	2022	0	0	0	0	0	0	0	0	0	0	0	0	73
20164744	m	cn	US	2022	0	1	1	0	0	0	0	0	0	0	0	0	70
20165494	m	md	DE	2022	0	0	0	0	0	0	0	0	0	0	0	0	
20165608	f	cn	US	2022	0	1	0	1	0	0	0	0	0	0	0	0	50
20166287	m	md	CN	2022	0	1	0	1	0	0	0	0	0	0	0	0	59.7
20166480	m	md	DE	2022	0	1	0	0	1	0	0	0	0	0	0	0	37
20166562	f	md	DE	2022	0	0	0	0	0	0	0	0	0	0	0	0	64
20166731	m	hp	CN	2022	0	0	0	0	0	0	0	0	0	0	0	0	72
20167474	m	hp	NL	2022	0	1	0	0	1	0	0	0	0	0	0	0	66
20167611	m	md	JP	2022	0	1	0	0	1	0	0	0	0	0	0	0	71
20169855	m	md	FR	2022	0	1	0	0	1	0	0	0	0	0	0	0	63
20171299	m	md	FR	2022	0	0	0	0	0	0	0	0	0	0	0	0	69
20171443	m	md	PL	2022	0	1	0	0	0	1	0	0	0	0	0	0	61
20172763	m	md	JP	2022	0	1	0	0	0	1	0	0	0	0	0	0	71
20172999	m	md	JP	2022	0	0	0	0	0	0	0	0	0	0	0	0	89

20173501	f	md	DE	2022	0	0	0	0	0	0	0	0	0	0	0	0	65
20173697	m	md	JP	2022	0	1	0	0	1	0	0	0	0	0	0	0	69
20174135	f	md	DE	2022	0	0	0	0	0	0	0	0	0	0	0	0	
20176903	m	md	JP	2022	0	1	0	0	1	0	0	0	0	0	0	0	85
20177315	m	cn	US	2022	0	0	0	0	0	0	0	0	0	0	0	0	
20177560	m	md	CN	2022	0	1	1	0	0	0	0	0	0	0	0	0	62
20177913	m	ph	JP	2022	0	1	0	0	1	0	0	0	0	0	0	1	78
20178108	f	cn	US	2022	0	1	1	0	0	0	0	0	0	0	0	0	
20178744	m	md	JP	2022	0	1	0	0	1	0	0	0	0	0	0	0	62
20179001	m	md	JP	2022	0	1	1	0	0	0	0	0	0	0	0	0	71
20179588	m	md	FR	2022	0	1	0	0	1	0	0	0	0	0	0	0	83
20182553	m	md	ES	2022	0	1	0	0	0	1	0	0	0	0	0	0	71
20182765	m	md	JP	2022	0	1	0	0	1	0	0	0	0	0	0	0	78
20184363	f	ph	US	2022	0	1	1	0	0	0	0	0	0	0	0	0	
20186912	m	md	NL	2022	0	0	0	0	0	0	0	0	0	0	0	0	60
20187054	m	md	KR	2022	0	1	1	0	0	0	0	0	0	0	0	0	59
20188685	m	md	JP	2022	0	1	0	0	1	0	0	0	0	0	0	0	58
20189119	m	hp	CA	2022	0	1	0	0	1	0	0	0	0	0	0	0	63
20190098	Not	md	BG	2022	1	0	0	0	0	0	0	0	0	0	0	0	66
20191699	f	md	JP	2022	0	1	0	0	1	0	0	0	0	0	0	0	79
20192746	m	md	JP	2022	0	1	1	0	0	0	0	0	0	0	0	0	77
20193306	m	md	CN	2022	0	0	0	0	0	0	0	0	0	0	0	0	71
20193366	f	md	CN	2022	0	0	0	0	0	0	0	0	0	0	0	0	53
20194088	m	md	JP	2022	0	1	0	1	0	0	0	0	0	0	0	0	71
20195566	m	hp	US	2022	0	0	0	0	0	0	0	0	0	0	0	0	78
20196504	m	hp	US	2022	0	1	0	0	1	0	0	0	0	0	0	0	60
20197141	m	hp	FR	2022	0	1	0	0	1	0	0	0	0	0	0	0	74
20197301	m	md	FR	2022	0	1	0	0	0	1	0	0	0	0	0	0	75
20197411	m	md	JP	2022	0	0	0	0	0	0	0	0	0	0	0	0	80
20198743	f	md	DE	2022	0	1	0	0	1	0	0	0	0	0	0	0	65
20199111	f	md	BE	2022	0	1	0	0	0	1	0	0	0	0	0	0	73
20199901	f	md	CN	2022	0	1	1	0	0	0	0	0	0	0	0	0	57
20200023	f	md	CA	2022	0	1	1	0	0	0	0	0	0	0	0	0	65
20200325	m	md	CA	2022	0	1	1	0	0	0	0	0	0	0	0	0	69

20200699	m	md	CN	2022	0	1	0	0	0	1	0	0	0	0	0	1	58
20201430	f	md	ES	2022	0	0	0	0	0	0	0	0	0	0	0	0	70
20201508	m	md	DE	2022	0	1	0	0	1	0	0	0	0	0	0	0	49
20201800	m	hp	CN	2022	0	0	0	0	0	0	0	0	0	0	0	0	71
20201846	f	md	CN	2022	0	0	0	0	0	0	0	0	0	0	0	0	68
20201856	m	md	JP	2022	0	1	1	0	0	0	0	0	0	0	0	0	60
20202230	m	hp	CN	2022	0	1	0	0	1	0	0	0	0	0	0	0	62
20203171	f	md	JP	2022	0	1	1	0	0	0	0	0	0	0	0	0	82
20204153	m	md	JP	2022	0	1	0	0	0	1	0	0	0	0	0	0	70
20204900	m	md	JP	2022	0	1	1	0	0	0	0	0	0	0	0	1	78
20205162	f	ph	JP	2022	0	1	1	0	0	0	0	0	0	0	0	0	60
20205553	f	md	BG	2022	0	0	0	0	0	0	0	0	0	0	0	0	64
20206705	m	md	AU	2022	0	0	0	0	0	0	0	0	0	0	0	0	75
20206735	m	md	JP	2022	0	1	0	0	1	0	0	0	0	0	0	0	83
20206763	m	md	TR	2022	0	0	0	0	0	0	0	0	0	0	0	0	60
20206984	Not	md	JP	2022	0	1	0	0	0	1	0	0	0	0	0	0	65
20207718	m	md	DE	2022	0	0	0	0	0	0	0	0	0	0	0	0	58
20208482	m	md	JP	2022	0	1	1	0	0	0	0	0	0	0	0	0	72
20209020	m	md	PL	2022	0	0	0	0	0	0	0	0	0	0	0	0	68
20209111	m	hp	CL	2022	0	1	0	0	1	0	0	0	0	0	0	0	67
20209112	f	hp	IE	2022	0	1	0	0	1	0	0	0	0	0	0	0	62
20210192	f	md	JP	2022	0	1	0	0	0	1	0	0	0	0	0	0	72
20210884	m	md	JP	2022	0	0	0	0	0	0	0	0	0	0	0	0	80
20212736	m	md	CL	2022	0	1	1	0	0	0	0	0	0	0	0	0	85
20214323	m	md	US	2022	0	0	0	0	0	0	0	0	0	0	0	0	49
20218698	m	md	JP	2022	0	0	0	0	0	0	0	0	0	0	0	0	80
20219534	m	md	MY	2022	0	0	0	0	0	0	0	0	0	0	0	0	62
20219865	f	hp	US	2022	0	1	1	0	0	0	0	0	0	0	0	0	40
20219901	f	hp	US	2022	0	1	1	0	0	0	0	0	0	0	0	0	
20220246	f	ph	NL	2022	1	0	0	0	0	0	0	0	0	0	0	0	86
20221186	m	md	JP	2022	0	1	0	0	1	0	0	0	0	0	0	0	71
20223743	m	hp	CN	2022	0	0	0	0	0	0	0	0	0	0	0	0	62
20223859	m	md	IT	2022	0	0	0	0	0	0	0	0	0	0	0	0	46
20223937	m	md	JP	2022	0	1	0	1	0	0	0	0	0	0	0	0	60

20224293	m	cn	CN	2022	0	1	0	1	0	0	0	0	0	0	0	1	
20224647	m	hp	AU	2022	0	1	0	0	1	0	0	0	0	0	0	0	74
20224728	f	cn	US	2022	1	0	0	0	0	0	0	0	0	0	0	0	74
20224964	f	md	FI	2022	0	0	0	0	0	0	0	0	0	0	0	0	74
20225259	f	md	US	2022	0	1	0	0	0	1	0	0	0	0	0	0	65
20225426	m	md	JP	2022	0	0	0	0	0	0	0	0	0	0	0	0	80
20226434	f	hp	CA	2022	0	0	0	0	0	0	0	0	0	0	0	0	49
20227657	m	md	US	2022	0	0	0	0	0	0	0	0	0	0	0	0	
20228035	m	md	JP	2022	0	1	0	0	1	0	0	0	0	0	0	0	72
20228120	m	md	DE	2022	0	1	0	0	1	0	0	0	0	0	0	1	52
20228271	Not	md	TR	2022	0	1	0	0	1	0	0	0	0	0	0	0	
20228282	f	md	JP	2022	0	1	0	0	1	0	0	0	0	0	0	0	69
20228390	m	md	FR	2022	0	1	0	0	1	0	0	0	0	0	0	0	78
20229201	m	hp	GB	2022	0	0	0	0	0	0	0	0	0	0	0	0	57
20229243	f	md	IT	2022	0	0	0	0	0	0	0	0	0	0	0	0	72
20229327	m	ph	JP	2022	0	1	0	0	0	1	0	0	0	0	0	0	66
20229608	m	md	JP	2022	0	1	0	0	0	1	0	0	0	0	0	0	75
20230202	f	md	ES	2022	0	0	0	0	0	0	0	0	0	0	0	0	70
20231511	f	md	CZ	2022	0	1	0	0	1	0	0	0	0	0	0	0	76
20232060	m	md	CN	2022	0	0	0	0	0	0	0	0	0	0	0	0	70
20232176	f	md	JP	2022	0	0	0	0	0	0	0	0	0	0	0	0	73
20232203	m	ph	JP	2022	0	1	0	0	1	0	0	0	0	0	0	0	51
20232222	f	md	JP	2022	0	1	0	0	1	0	0	0	0	0	0	0	70
20232647	Not	md	JP	2022	0	0	0	0	0	0	0	0	0	0	0	0	
20232968	Not	md	JP	2022	0	0	0	0	0	0	0	0	0	0	0	0	
20233036	m	md	IT	2022	0	0	0	0	0	0	0	0	0	0	0	0	77
20233056	m	md	GB	2022	0	0	0	0	0	0	0	0	0	0	0	0	64
20233179	m	hp	JP	2022	0	1	0	0	1	0	0	0	0	0	0	1	67
20233578	Not	md	JP	2022	0	1	0	0	1	0	0	0	0	0	0	0	
20233585	m	md	CA	2022	0	0	0	0	0	0	0	0	0	0	0	0	57
20233925	m	ph	JP	2022	0	1	0	0	1	0	0	0	0	0	0	0	59
20237109	f	hp	US	2022	0	1	1	0	0	0	0	0	0	0	0	0	40
20237336	m	cn	GB	2022	1	0	0	0	0	0	0	0	0	0	0	0	70
20239260	m	hp	DE	2022	0	1	0	0	1	0	0	0	0	0	0	0	78

20239399	f	hp	ES	2022	0	1	0	0	1	0	0	0	0	0	0	0	66
20239541	f	cn	ID	2022	1	0	0	0	0	0	0	0	0	0	0	0	
20241060	f	md	US	2022	0	0	0	0	0	0	0	0	0	0	0	0	31
20242100	f	md	RU	2022	0	1	1	0	0	0	0	0	0	0	0	0	70
20245664	f	md	FR	2022	0	0	0	0	0	0	0	0	0	0	0	0	86
20245811	m	md	ES	2022	0	1	0	0	0	1	0	0	0	0	0	0	75
20246183	f	md	JP	2022	0	1	0	0	1	0	0	0	0	0	0	0	67
20246699	f	hp	TH	2022	1	0	0	0	0	0	0	0	0	0	0	0	39.7
20247110	f	md	JP	2022	0	1	0	0	0	1	0	0	0	0	0	0	63
20247341	f	md	PL	2022	0	0	0	0	0	0	0	0	0	0	0	0	
20249289	m	md	JP	2022	0	0	0	0	0	0	0	0	0	0	0	0	70
20252182	f	md	ES	2022	0	1	0	0	0	1	0	0	0	0	0	0	63
20256336	m	ph	JP	2022	0	1	0	1	0	0	0	0	0	0	0	1	60
20257796	f	md	ES	2022	0	1	0	0	0	1	0	0	0	0	0	0	63
20258007	f	md	CN	2022	0	0	0	0	0	0	0	0	0	0	0	0	64
20264607	m	md	KR	2022	1	0	0	0	0	0	0	0	0	0	0	0	77
20268438	m	md	MX	2022	0	0	0	0	0	0	0	0	0	0	0	0	49
20276588	m	md	JP	2022	0	1	0	0	1	0	0	0	0	0	0	1	79
20278013	Not	hp	US	2022	0	1	1	0	0	0	0	0	0	0	0	0	
20278043	f	hp	JP	2022	1	0	0	0	0	0	0	0	0	0	0	0	67
20278089	Not	hp	US	2022	0	1	1	0	0	0	0	0	0	0	0	0	
20278651	m	md	FR	2022	1	0	0	0	0	0	0	0	0	0	0	0	
20278654	m	hp	IN	2022	0	1	0	0	1	0	0	0	0	0	0	0	55
20278920	m	md	US	2022	0	0	0	0	0	0	0	0	0	0	0	0	53
20278941	f	md	CN	2022	0	0	0	0	0	0	0	0	0	0	0	0	64
20279848	m	md	CN	2022	0	0	0	0	0	0	0	0	0	0	0	0	55
20279914	m	md	JP	2022	0	1	0	0	0	1	0	0	0	0	0	0	70
20279972	f	cn	GB	2022	0	0	0	0	0	0	1	1	0	0	0	0	49
20280164	f	md	FR	2022	0	0	0	0	0	0	0	0	0	0	0	0	52
20280187	f	md	DE	2022	0	0	0	0	0	0	0	0	0	0	0	0	74
20280320	m	hp	NL	2022	0	1	0	0	0	1	0	0	0	0	0	0	72
20280494	m	cn	US	2022	1	0	0	0	0	0	0	0	0	0	0	0	91.5
20280575	f	cn	IN	2022	0	0	0	0	0	0	0	0	0	0	0	0	55
20281029	f	cn	CA	2022	1	0	0	0	0	0	0	0	0	0	0	0	

20281494	f	cn	SG	2022	1	0	0	0	0	0	0	0	0	0	0	0	73
20282483	m	ph	US	2022	1	0	0	0	0	0	0	0	0	0	0	0	41
20283253	m	md	JP	2022	0	1	0	0	1	0	0	0	0	0	0	1	65
20286594	m	md	IT	2022	0	0	0	0	0	0	0	0	0	0	0	0	
20288424	m	md	JP	2022	0	0	0	0	0	0	0	0	0	0	0	0	60
20288688	m	cn	CN	2022	0	1	0	0	1	0	0	0	0	0	0	0	76
20288754	m	cn	DE	2022	0	0	0	0	0	0	0	0	0	0	0	0	
20288873	m	cn	IN	2022	0	1	0	0	1	0	0	0	0	0	0	0	50
20289124	m	hp	JP	2022	0	1	0	0	1	0	0	0	0	0	0	0	59
20289211	m	md	CN	2022	0	0	0	0	0	0	0	0	0	0	0	0	70
20289548	m	md	FR	2022	0	1	1	0	0	0	0	0	0	0	0	0	68
20289551	f	cn	IN	2022	0	0	0	0	0	0	0	0	0	0	0	0	77
20289800	f	md	GB	2022	0	1	0	0	0	1	0	0	0	0	0	0	50
20289907	f	cn	IN	2022	0	0	0	0	0	0	0	0	0	0	0	0	55
20290264	Not	cn	US	2022	1	0	0	0	0	0	0	0	0	0	0	0	63
20290377	m	md	CH	2022	0	1	1	0	0	0	0	0	0	0	0	0	64
20291161	Not	cn	US	2022	1	0	0	0	0	0	0	0	0	0	0	0	
20291207	f	md	FR	2022	0	0	0	0	0	0	0	0	0	0	0	0	
20291218	f	cn	US	2022	1	0	0	0	0	0	0	0	0	0	0	0	
20291418	m	cn	IN	2022	0	1	0	0	1	0	0	0	0	0	0	0	52
20292045	f	cn	FR	2022	0	0	0	0	0	0	0	0	0	0	0	0	
20292114	m	hp	BR	2022	0	1	0	0	1	0	0	0	0	0	0	0	71
20292153	f	md	NL	2022	0	0	0	0	0	0	0	0	0	0	0	0	71
20292349	m	md	ES	2022	0	1	0	0	0	1	0	0	0	0	0	0	75
20292548	m	cn	DE	2022	0	1	0	0	1	0	0	0	0	0	0	0	72
20292633	m	md	JP	2022	0	1	0	0	0	1	0	0	0	0	0	0	80
20294586	f	md	JP	2022	1	0	0	0	0	0	0	0	0	0	0	0	60
20296995	f	md	FR	2022	0	1	0	0	1	0	0	0	0	0	0	0	67
20297043	f	md	FR	2022	0	0	0	0	0	0	0	0	0	0	0	0	
20297045	m	md	FR	2022	0	1	0	0	1	0	0	0	0	0	0	0	
20297097	m	hp	JP	2022	0	1	1	0	0	0	0	0	0	0	0	0	76
20297237	m	md	FR	2022	0	1	1	0	0	0	0	0	0	0	0	0	
20297253	m	md	FR	2022	1	0	0	0	0	0	0	0	0	0	0	0	
20298160	f	ph	US	2022	0	1	1	0	0	0	0	0	0	0	0	0	79

20298308	m	md	FR	2022	1	0	0	0	0	0	0	0	0	0	0	0		
20298309	f	cn	US	2022	0	0	0	0	0	0	0	0	0	0	0	0		
20298711	f	hp	TW	2022	1	0	0	0	0	0	0	0	0	0	0	0	53	
20298860	m	hp	TW	2022	0	0	0	0	0	0	0	0	0	0	0	0	63	
20299123	f	md	NL	2022	0	0	0	0	0	0	0	0	0	0	0	0	71	
20299370	f	ph	US	2022	0	0	0	0	0	0	0	0	0	0	0	0	62	
20299701	m	hp	NL	2022	0	0	0	0	0	0	0	0	0	0	0	0		
20299759	f	md	FR	2022	0	0	0	0	0	0	0	0	0	0	0	0	56	
20300289	m	hp	KR	2022	0	0	0	0	0	0	0	0	0	0	0	0	58	
20300367	f	hp	TW	2022	0	0	0	0	0	0	0	0	0	0	0	0	60	
20300595	f	md	AT	2022	0	1	0	1	0	0	0	0	0	0	0	0	81	
20302116	m	hp	US	2022	0	1	0	0	1	0	0	0	0	0	0	0		
20302135	m	md	ES	2022	0	0	0	0	0	0	0	0	0	0	0	0	44	
20302142	m	md	IT	2022	0	0	0	0	0	0	0	0	0	0	0	0	75	
20302580	f	md	FR	2022	0	0	0	0	0	0	0	0	0	0	0	0		
20302769	f	md	FR	2022	0	1	1	0	0	0	0	0	0	0	0	0	64	
20303140	m	cn	IN	2022	0	1	0	0	0	1	0	0	0	0	0	0	26	
20303792	Not	hp	US	2022	0	0	0	0	0	0	0	0	0	0	0	0		
20304206	m	md	DE	2022	0	0	0	0	0	0	0	0	0	0	0	0		
20304263	f	md	DE	2022	0	1	0	0	1	0	0	0	0	0	0	1	65	
20304353	f	cn	DE	2022	1	0	0	0	0	0	0	0	0	0	0	0	61	
20304634	Not	md	JP	2022	0	0	0	0	0	0	0	0	0	0	0	0		
20304635	m	md	JP	2022	0	1	1	0	0	0	0	0	0	0	0	0	76	
20305046	m	hp	JP	2022	0	0	0	0	0	0	0	0	0	0	0	0	69	
20305148	m	md	PL	2022	0	1	0	0	0	1	0	0	0	0	0	0	80	
20305175	m	cn	DE	2022	0	0	0	0	0	0	0	0	0	0	0	0		
20305415	f	cn	IN	2022	0	0	0	0	0	0	0	0	0	0	0	0	39	
20306472	f	md	ES	2022	0	0	0	0	0	0	0	0	0	0	0	0	55	
20306495	f	md	TW	2022	0	0	0	0	0	0	0	0	0	0	0	0	65	
20306502	m	md	JP	2022	0	1	1	0	0	0	0	0	0	0	0	0	58	
20306615	m	md	JP	2022	0	1	0	0	1	0	0	0	0	0	0	0		
20306646	f	hp	US	2022	0	1	1	0	0	0	0	0	0	0	0	0	40	
20306773	f	md	ES	2022	0	0	0	0	0	0	0	0	0	0	0	0	45	
20307109	Not	hp	DE	2022	1	0	0	0	0	0	0	0	0	0	0	0		

20307742	m	md	FR	2022	0	0	0	0	0	0	0	0	0	0	0	0	
20308153	f	ph	NL	2022	1	0	0	0	0	0	0	0	0	0	0	0	86
20308291	m	md	FR	2022	0	1	1	0	0	0	0	0	0	0	0	0	
20308389	Not	md	JP	2022	0	0	0	0	0	0	0	0	0	0	0	0	
20308397	f	hp	US	2022	0	1	1	0	0	0	0	0	0	0	0	0	40
20308432	f	md	FR	2022	0	1	0	0	1	0	0	0	0	0	0	0	70
20308991	Not	hp	US	2022	0	0	0	0	0	0	0	0	0	0	0	0	
20309162	m	md	JP	2022	0	1	0	0	0	1	0	0	0	0	0	0	52
20309371	f	cn	CA	2022	1	0	0	0	0	0	0	0	0	0	0	0	
20309536	m	cn	NL	2022	1	0	0	0	0	0	0	0	0	0	0	0	
20309857	m	hp	NL	2022	0	1	0	0	1	0	0	0	0	0	0	0	78
20310302	f	md	US	2022	0	0	0	0	0	0	0	0	0	0	0	0	
20310345	m	md	CN	2022	0	1	0	1	0	0	0	0	0	0	0	0	70
20310439	Not	md	US	2022	0	0	0	0	0	0	0	0	0	0	0	0	
20310445	m	md	FR	2022	0	1	1	0	0	0	0	0	0	0	0	0	
20310774	m	md	NL	2022	0	1	0	0	0	1	0	0	0	0	0	0	75
20311016	f	md	FR	2022	0	0	0	0	0	0	0	0	0	0	0	0	70
20311142	f	md	CN	2022	0	0	0	0	0	0	0	0	0	0	0	0	52
20311271	f	md	JP	2022	0	0	0	0	0	0	0	0	0	0	0	0	72
20311314	f	md	CN	2022	0	0	0	0	0	0	0	0	0	0	0	0	63
20311493	f	cn	CN	2022	0	0	0	0	0	0	0	0	0	0	0	0	69
20311535	m	cn	IN	2022	0	0	0	0	0	0	0	0	0	0	0	0	70
20312510	f	md	DE	2022	0	1	1	0	0	0	0	0	0	0	0	0	71
20313111	f	md	DE	2022	0	1	0	0	0	1	0	0	0	0	0	0	78
20314502	f	cn	US	2022	0	0	0	0	0	0	0	0	0	0	0	0	
20314599	f	ph	NL	2022	1	0	0	0	0	0	0	0	0	0	0	0	86
20315305	f	hp	US	2022	0	1	1	0	0	0	0	0	0	0	0	0	
20316077	m	md	FR	2022	0	1	1	0	0	0	0	0	0	0	0	0	68
20316235	f	md	FR	2022	0	0	0	0	0	0	0	0	0	0	0	0	
20316331	Not	md	US	2022	0	1	1	0	0	0	0	0	0	0	0	0	
20316469	f	cn	CN	2022	0	0	0	0	0	0	0	0	0	0	0	0	56
20316490	Not	md	US	2022	0	1	1	0	0	0	0	0	0	0	0	0	
20316514	Not	hp	SG	2022	0	1	0	0	0	1	0	0	0	0	0	0	73
20316520	f	md	NL	2022	0	0	0	0	0	0	0	0	0	0	0	0	51

20316651	f	hp	IT	2022	0	1	1	0	0	0	0	0	0	0	0	0	60
20316822	f	ph	NL	2022	1	0	0	0	0	0	0	0	0	0	0	0	86
20316885	m	hp	RO	2022	0	1	1	0	0	0	0	0	0	0	0	0	53
20317052	f	md	PL	2022	0	0	0	0	0	0	0	0	0	0	0	0	75
20317246	Not	hp	JP	2022	0	0	0	0	0	0	0	0	0	0	0	0	
20317377	m	md	DE	2022	0	1	1	0	0	0	0	0	0	0	0	0	82
20317863	f	md	DE	2022	0	0	0	0	0	0	0	0	0	0	0	0	64
20317937	f	md	FR	2022	0	1	1	0	0	0	0	0	0	0	0	0	64
20318132	m	md	US	2022	0	0	0	0	0	0	0	0	0	0	0	0	
20318516	m	md	FR	2022	1	0	0	0	0	0	0	0	0	0	0	0	
20318518	f	md	FR	2022	0	0	0	0	0	0	0	0	0	0	0	0	
20318519	m	md	FR	2022	0	1	0	0	1	0	0	0	0	0	0	0	
20318605	m	md	JP	2022	0	1	0	1	0	0	0	0	0	0	0	1	78
20318630	Not	md	US	2022	0	0	0	0	0	0	0	0	0	0	0	0	
20318642	f	md	FR	2022	0	0	0	0	0	0	0	0	0	0	0	0	
20318645	f	md	FR	2022	0	0	0	0	0	0	0	0	0	0	0	0	
20318666	f	cn	MY	2022	1	0	0	0	0	0	0	0	0	0	0	0	63.6
20318668	f	ph	MY	2022	1	0	0	0	0	0	0	0	0	0	0	0	71.9
20318669	f	md	JP	2022	1	0	0	0	0	0	0	0	0	0	0	0	79
20318932	m	hp	NL	2022	0	1	1	0	0	0	0	0	0	0	0	0	
20319184	m	md	JP	2022	1	0	0	0	0	0	0	0	0	0	0	0	62
20319917	m	cn	CN	2022	0	0	0	0	0	0	0	0	0	0	0	0	59
20319966	m	cn	LB	2022	0	1	0	0	0	1	0	0	0	0	0	0	81
20320645	m	md	FR	2022	0	1	1	0	0	0	0	0	0	0	0	0	77
20320795	Not	md	JP	2022	0	0	0	0	0	0	0	0	0	0	0	0	
20321081	Not	md	US	2022	1	0	0	0	0	0	0	0	0	0	0	0	
20321304	m	hp	JP	2022	0	0	0	0	0	0	0	0	0	0	0	0	75
20321936	m	md	FR	2022	0	0	0	0	0	0	0	0	0	0	0	0	
20322159	f	cn	US	2022	1	0	0	0	0	0	0	0	0	0	0	0	69
20322543	m	ph	US	2022	0	0	0	0	0	0	0	0	0	0	0	0	77
20322598	m	md	FR	2022	0	1	1	0	0	0	0	0	0	0	0	0	
20322604	m	md	FR	2022	0	0	0	0	0	0	0	0	0	0	0	0	
20322621	f	md	JP	2022	1	0	0	0	0	0	0	0	0	0	0	0	61
20322650	f	md	DE	2022	0	0	0	0	0	0	0	0	0	0	0	0	

20323326	m	md	FR	2022	0	1	1	0	0	0	0	0	0	0	0	0	
20323478	m	cn	TW	2022	0	1	0	0	0	1	0	0	0	0	0	1	
20323484	f	md	JP	2022	1	0	0	0	0	0	0	0	0	0	0	1	73.2
20323690	f	md	AU	2022	1	0	0	0	0	0	0	0	0	0	0	0	
20323963	m	cn	JP	2022	0	1	0	0	1	0	0	0	0	0	0	0	76
20324141	f	md	KR	2022	0	0	0	0	0	0	0	0	0	0	0	0	73
20325004	m	hp	CN	2022	0	1	0	0	1	0	0	0	0	0	0	0	75
20325072	f	hp	US	2022	0	1	0	0	1	0	0	0	0	0	0	1	72
20325111	m	hp	TW	2022	0	0	0	0	0	0	0	0	0	0	0	0	63
20325322	m	md	FR	2022	0	0	0	0	0	0	0	0	0	0	0	0	
20325336	m	cn	IN	2022	0	0	0	0	0	0	0	0	0	0	0	0	52
20325552	m	md	JP	2022	0	1	0	0	1	0	0	0	0	0	0	0	55
20325893	f	cn	US	2022	0	1	1	0	0	0	0	0	0	0	0	0	
20326095	m	md	US	2022	1	0	0	0	0	0	0	0	0	0	0	0	51
20326339	m	cn	CN	2022	0	1	0	0	1	0	0	0	0	0	0	0	49
20327271	m	md	FR	2022	0	0	0	0	0	0	0	0	0	0	0	0	
20327541	m	md	DE	2022	0	0	0	0	0	0	0	0	0	0	0	0	
20327608	Not	hp	US	2022	1	0	0	0	0	0	0	0	0	0	0	0	55
20327784	f	md	FR	2022	0	1	1	0	0	0	0	0	0	0	0	0	64
20327905	f	cn	DE	2022	0	1	0	0	1	0	0	0	0	0	0	0	62
20327927	m	md	CN	2022	0	0	0	0	0	0	0	0	0	0	0	0	71
20328345	f	cn	US	2022	0	1	1	0	0	0	0	0	0	0	0	0	
20328360	f	cn	CN	2022	0	1	0	1	0	0	0	0	0	0	0	1	61
20328489	f	md	GB	2022	0	1	0	0	0	1	0	0	0	0	0	0	71
20328560	Not	hp	JP	2022	0	0	0	0	0	0	0	0	0	0	0	0	
20328736	m	cn	US	2022	1	0	0	0	0	0	0	0	0	0	0	0	
20328807	m	ph	FR	2022	0	0	0	0	0	0	0	0	0	0	0	0	
20328859	m	cn	US	2022	0	1	0	1	0	0	0	0	0	0	0	1	80
20329224	m	md	FR	2022	0	1	1	0	0	0	0	0	0	0	0	0	
20329394	f	md	FR	2022	0	0	0	0	0	0	0	0	0	0	0	0	
20329458	m	hp	FR	2022	0	1	0	0	1	0	0	0	0	0	0	0	73
20329662	f	md	NL	2022	0	0	0	0	0	0	0	0	0	0	0	0	58
20329663	f	md	NL	2022	0	0	0	0	0	0	0	0	0	0	0	0	66
20329670	f	md	NL	2022	0	0	0	0	0	0	0	0	0	0	0	0	58

20330138	f	cn	US	2022	0	1	1	0	0	0	0	0	0	0	0	0	
20330244	f	hp	US	2022	0	1	1	0	0	0	0	0	0	0	0	0	40
20330436	f	cn	CN	2022	0	1	0	0	1	0	0	0	0	0	0	0	60
20330745	f	md	JP	2022	0	1	0	0	1	0	0	0	0	0	0	0	69
20331535	m	md	DE	2022	0	1	0	0	1	0	0	0	0	0	0	0	50
20331558	m	md	ES	2022	0	1	0	0	0	1	0	0	0	0	0	0	67
20331583	Not	hp	CN	2022	0	0	0	0	0	0	0	0	0	0	0	0	
20331584	f	ph	FR	2022	0	1	0	0	1	0	0	0	0	0	0	0	68
20332450	Not	md	AU	2022	1	0	0	0	0	0	0	0	0	0	0	0	
20332497	m	md	JP	2022	0	1	0	0	0	1	0	0	0	0	0	0	80
20332565	m	md	DE	2022	0	0	0	0	0	0	0	0	0	0	0	0	60
20332693	f	md	PL	2022	0	0	0	0	0	0	0	0	0	0	0	0	76
20332833	m	cn	CN	2022	0	1	0	1	0	0	0	0	0	0	0	0	71
20332835	m	md	CN	2022	1	0	0	0	0	0	0	0	0	0	0	0	78
20332870	f	hp	US	2022	0	1	0	0	1	0	0	0	0	0	0	0	
20333336	m	md	FR	2022	0	1	1	0	0	0	0	0	0	0	0	0	
20333408	f	md	US	2022	1	0	0	0	0	0	0	0	0	0	0	0	52
20333461	m	hp	CH	2022	0	0	0	0	0	0	0	0	0	0	0	0	73
20333588	m	md	FR	2022	0	0	0	0	0	0	0	0	0	0	0	0	
20333592	m	md	CN	2022	0	1	1	0	0	0	0	0	0	0	0	0	56
20333684	f	md	KR	2022	0	0	0	0	0	0	0	0	0	0	0	0	63
20333688	m	hp	DE	2022	0	1	1	0	0	0	0	0	0	0	0	0	61
20333699	m	hp	JP	2022	0	1	1	0	0	0	0	0	0	0	0	0	76
20333762	f	md	CN	2022	0	0	0	0	0	0	0	0	0	0	0	0	72
20333871	f	md	CA	2022	0	0	0	0	0	0	0	0	0	0	0	0	60
20333945	m	md	US	2022	0	1	1	0	0	0	0	0	0	0	0	0	80
20334223	m	hp	US	2022	0	1	0	1	0	0	0	0	0	0	0	1	70
20334755	Not	cn	US	2022	1	0	0	0	0	0	0	0	0	0	0	0	60.9
20335102	f	md	JP	2022	0	0	0	0	0	0	0	0	0	0	0	0	40
20335357	f	md	JP	2022	1	0	0	0	0	0	0	0	0	0	0	0	79
20335418	m	cn	CN	2022	0	1	0	1	0	0	0	0	0	0	0	0	72
20336043	f	md	FR	2022	0	0	0	0	0	0	0	0	0	0	0	0	56
20336063	m	md	JP	2022	0	1	0	1	0	0	0	0	0	0	0	0	55
20336150	m	cn	US	2022	1	0	0	0	0	0	0	0	0	0	0	0	

20336418	m	md	NL	2022	0	0	0	0	0	0	0	0	0	0	0	0	74
20336646	m	md	NL	2022	0	0	0	0	0	0	0	0	0	0	0	0	
20336766	f	hp	AR	2022	0	1	0	0	1	0	0	0	0	0	0	0	66
20336789	m	md	CN	2022	1	0	0	0	0	0	0	0	0	0	0	0	69
20336791	f	cn	US	2022	1	0	0	0	0	0	0	0	0	0	0	0	
20336849	f	cn	CN	2022	0	1	0	0	1	0	0	0	0	0	0	0	49
20336922	f	md	GB	2022	0	1	0	0	0	1	0	0	0	0	0	0	
20336923	m	md	JP	2022	0	1	1	0	0	0	0	0	0	0	0	0	72
20338529	f	ph	US	2022	1	0	0	0	0	0	0	0	0	0	0	0	78
20338683	m	md	IL	2022	0	1	0	0	0	1	0	0	0	0	0	0	85
20338800	m	md	JP	2022	0	1	0	0	1	0	0	0	0	0	0	0	66
20339068	f	cn	US	2022	0	1	0	0	1	0	0	0	0	0	0	1	63
20339152	m	hp	US	2022	0	1	0	0	1	0	0	0	0	0	0	0	
20339592	m	md	JP	2022	0	1	0	0	1	0	0	0	0	0	0	0	66
20340131	m	md	IL	2022	0	1	0	0	1	0	0	0	0	0	0	0	53
20340173	m	md	FR	2022	0	0	0	0	0	0	0	0	0	0	0	0	52
20340453	m	md	FR	2022	0	0	0	0	0	0	0	0	0	0	0	0	70
20340483	m	md	DE	2022	0	1	0	0	1	0	0	0	0	0	0	0	49
20340634	Not	md	DE	2022	1	0	0	0	0	0	0	0	0	0	0	0	51
20340730	m	md	JP	2022	0	0	0	0	0	0	0	0	0	0	0	0	60
20341063	f	md	DE	2022	0	1	1	0	0	0	0	0	0	0	0	0	
20341350	f	md	GB	2022	0	1	1	0	0	0	0	0	0	0	0	0	73
20341488	m	md	JP	2022	0	1	0	0	1	0	0	0	0	0	0	0	65
20341492	m	cn	IN	2022	0	1	0	0	0	1	0	0	0	0	0	0	47
20341849	m	cn	US	2022	1	0	0	0	0	0	0	0	0	0	0	0	73.7
20342175	m	md	ES	2022	0	1	0	0	0	1	0	0	0	0	0	0	72
20342703	m	md	DE	2022	0	1	0	0	1	0	0	0	0	0	0	0	61
20342811	Not	md	JP	2022	0	1	0	1	0	0	0	0	0	0	0	0	
20342857	m	md	FR	2022	0	1	0	1	0	0	0	0	0	0	0	0	70
20343225	f	md	JP	2022	0	0	0	0	0	0	0	0	0	0	0	0	48
20343386	m	md	JP	2022	0	0	0	0	0	0	0	0	0	0	0	0	64
20344696	Not	md	FR	2022	0	0	0	0	0	0	0	0	0	0	0	0	61
20344714	f	md	CN	2022	0	0	0	0	0	0	0	0	0	0	0	0	
20344813	f	md	JP	2022	0	1	0	0	1	0	0	0	0	0	0	0	59

20344814	m	md	JP	2022	0	1	0	0	1	0	0	0	0	0	0	0	68
20344842	f	md	JP	2022	0	1	0	0	1	0	0	0	0	0	0	0	59
20344883	m	hp	ES	2022	0	1	0	0	1	0	0	0	0	0	0	0	77
20345012	m	ph	JP	2022	0	1	0	0	1	0	0	0	0	0	0	0	60
20345072	m	md	BR	2022	0	0	0	0	0	0	0	0	0	0	0	0	74
20345232	m	cn	DE	2022	0	0	0	0	0	0	0	0	0	0	0	0	63
20345430	m	cn	CA	2022	0	1	0	0	1	0	0	0	0	0	0	0	54
20345501	m	hp	RU	2022	0	1	0	1	0	0	0	0	0	0	0	0	50
20345502	m	hp	RU	2022	0	1	0	1	0	0	0	0	0	0	0	0	65
20346151	Not	md	DE	2022	1	0	0	0	0	0	0	0	0	0	0	0	37
20346261	f	md	DE	2022	0	1	0	0	1	0	0	0	0	0	0	0	58
20346405	Not	md	FR	2022	0	0	0	0	0	0	0	0	0	0	0	0	61
20346560	f	md	FR	2022	0	0	0	0	0	0	0	0	0	0	0	0	78
20346663	f	hp	DE	2022	0	1	1	0	0	0	0	0	0	0	0	0	76
20346980	m	md	JP	2022	0	1	0	1	0	0	0	0	0	0	0	1	81
20347311	m	md	JP	2022	0	1	0	0	1	0	0	0	0	0	0	0	65
20347754	m	hp	CN	2022	0	0	0	0	0	0	0	0	0	0	0	0	59
20347896	m	md	JP	2022	0	1	0	0	1	0	0	0	0	0	0	1	69
20348023	Not	md	DE	2022	1	0	0	0	0	0	0	0	0	0	0	0	61
20348027	m	md	JP	2022	0	1	0	0	1	0	0	0	0	0	0	0	65
20348040	m	cn	IN	2022	0	1	0	0	1	0	0	0	0	0	0	0	62
20348056	m	md	JP	2022	0	1	0	0	1	0	0	0	0	0	0	0	57
20348347	Not	md	IT	2022	0	0	0	0	0	0	0	0	0	0	0	0	
20348349	m	md	JP	2022	0	1	0	0	1	0	0	0	0	0	0	0	67
20349193	f	hp	US	2022	0	1	0	0	1	0	0	0	0	0	0	0	67
20349211	Not	md	US	2022	0	0	0	0	0	0	0	0	0	0	0	0	
20349675	Not	hp	CN	2022	1	0	0	0	0	0	0	0	0	0	0	0	
20349766	m	md	BG	2022	0	0	0	0	0	0	0	0	0	0	0	0	74
20349776	m	cn	CA	2022	0	0	0	0	0	0	0	0	0	0	0	0	64
20349969	f	md	JP	2022	0	1	0	0	1	0	0	0	0	0	0	0	72
20349989	m	md	CN	2022	0	1	1	0	0	0	0	0	0	0	0	0	58
20350162	f	hp	GB	2022	0	1	0	0	0	1	0	0	0	0	0	0	55
20350360	f	cn	TH	2022	1	0	0	0	0	0	0	0	0	0	0	0	83
20350515	m	md	ES	2022	0	0	0	0	0	0	0	0	0	0	0	0	84

20350689	Not	ph	JP	2022	1	0	0	0	0	0	0	0	0	0	0	0	
20350692	m	cn	CN	2022	0	1	0	1	0	0	0	0	0	0	0	0	59
20351336	f	md	DE	2022	0	0	0	0	0	0	0	0	0	0	0	0	
20351411	m	md	CN	2022	0	1	0	0	0	1	0	0	0	0	0	0	60
20351596	m	cn	CN	2022	0	1	0	1	0	0	0	0	0	0	0	0	72
20351828	m	md	FR	2022	1	0	0	0	0	0	0	0	0	0	0	0	77
20351856	m	hp	US	2022	0	0	0	0	0	0	0	0	0	0	0	0	
20353283	f	md	JP	2022	0	0	0	0	0	0	0	0	0	0	0	0	54
20353463	Not	md	JP	2022	0	1	0	0	0	1	0	0	0	0	0	0	
20353773	Not	hp	NL	2022	0	0	0	0	0	0	0	0	0	0	0	0	
20353924	m	md	JP	2022	0	1	0	0	0	1	0	0	0	0	0	0	65
20354382	Not	md	FR	2022	0	0	0	0	0	0	0	0	0	0	0	0	61
20354519	m	hp	US	2022	1	0	0	0	0	0	0	0	0	0	0	0	74
20354545	f	md	GB	2022	0	1	0	0	0	1	0	0	0	0	0	0	55
20354669	m	md	PT	2022	0	0	0	0	0	0	0	0	0	0	0	0	83
20354943	m	hp	DE	2022	0	1	1	0	0	0	0	0	0	0	0	0	40
20355523	f	md	FR	2022	1	0	0	0	0	0	0	0	0	0	0	0	60
20355613	f	cn	JP	2022	0	0	0	0	0	0	0	0	0	0	0	0	61
20355659	m	cn	CA	2022	0	0	0	0	0	0	0	0	0	0	0	0	42
20355858	m	md	JP	2022	0	1	0	1	0	0	0	0	0	0	0	1	67
20355862	f	cn	CN	2022	0	1	0	1	0	0	0	0	0	0	0	0	52
20355978	m	md	JP	2022	0	1	0	0	1	0	0	0	0	0	0	0	64
20356082	Not	md	JP	2022	0	1	1	0	0	0	0	0	0	0	0	1	90
20356100	m	cn	TH	2022	0	1	1	0	0	0	0	0	0	0	0	0	59
20357160	f	md	FR	2022	0	0	0	0	0	0	0	0	0	0	0	0	62
20357164	f	md	JP	2022	1	0	0	0	0	0	0	0	0	0	0	0	69
20357165	m	md	JP	2022	0	1	0	1	0	0	0	0	0	0	0	0	63
20358085	m	hp	CN	2022	0	0	0	0	0	0	0	0	0	0	0	0	55
20358336	m	ph	MY	2022	0	0	0	0	0	0	0	0	0	0	0	0	62
20358618	Not	hp	ES	2022	1	0	0	0	0	0	0	0	0	0	0	0	
20358833	m	ph	US	2022	0	1	1	0	0	0	0	0	0	0	0	0	74
20358852	Not	hp	ES	2022	1	0	0	0	0	0	0	0	0	0	0	0	
20358853	m	md	IT	2022	1	0	0	0	0	0	0	0	0	0	0	0	87
20358865	Not	hp	ES	2022	1	0	0	0	0	0	0	0	0	0	0	0	

20359099	f	cn	TH	2022	1	0	0	0	0	0	0	0	0	0	0	0	50.1
20359145	m	md	JP	2022	0	0	0	0	0	0	0	0	0	0	0	0	50
20360027	f	md	JP	2022	0	1	0	0	1	0	0	0	0	0	0	0	59
20360203	Not	md	JP	2022	0	1	0	0	1	0	0	0	0	0	0	0	
20360390	f	md	FR	2022	1	0	0	0	0	0	0	0	0	0	0	0	
20360448	m	md	JP	2022	0	1	0	0	1	0	0	0	0	0	0	0	73
20360843	m	md	LB	2022	0	1	0	0	1	0	0	0	0	0	0	0	
20360997	f	md	JP	2022	0	0	0	0	0	0	0	0	0	0	0	0	62
20361211	Not	md	JP	2022	1	0	0	0	0	0	0	0	0	0	0	0	
20361311	Not	md	KR	2022	1	0	0	0	0	0	0	0	0	0	0	0	
20361363	m	md	AU	2022	1	0	0	0	0	0	0	0	0	0	0	0	
20361430	m	md	DE	2022	0	0	0	0	0	0	0	0	0	0	0	0	81
20361510	f	md	DE	2022	0	0	0	0	0	0	0	0	0	0	0	0	67
20361529	f	md	DE	2022	0	0	0	0	0	0	0	0	0	0	0	0	67
20361721	m	cn	CN	2022	0	1	0	1	0	0	0	0	0	0	0	0	68
20361724	Not	ph	JP	2022	1	0	0	0	0	0	0	0	0	0	0	0	
20362411	m	md	CN	2022	0	0	0	0	0	0	0	0	0	0	0	0	60
20362536	Not	md	US	2022	0	0	0	0	0	0	0	0	0	0	0	0	
20362631	f	md	DE	2022	0	0	0	0	0	0	0	0	0	0	0	0	71
20362927	f	cn	JP	2022	1	0	0	0	0	0	0	0	0	0	0	0	79
20363112	m	cn	ID	2022	1	0	0	0	0	0	0	0	0	0	0	0	56.6
20363303	m	cn	ID	2022	1	0	0	0	0	0	0	0	0	0	0	0	
20363342	Not	md	JP	2022	0	1	0	0	1	0	0	0	0	0	0	0	
20363411	f	cn	CN	2022	0	1	0	1	0	0	0	0	0	0	0	1	70
20363412	f	hp	US	2022	1	0	0	0	0	0	0	0	0	0	0	0	
20363605	m	hp	CN	2022	0	0	0	0	0	0	0	0	0	0	0	0	50
20363633	f	hp	TW	2022	0	0	0	0	0	0	0	0	0	0	0	0	60
20363689	m	md	TW	2022	0	0	0	0	0	0	0	0	0	0	0	0	67
20363950	m	md	JP	2022	0	1	0	0	1	0	0	0	0	0	0	0	57
20364124	m	hp	CN	2022	0	1	0	0	1	0	0	0	0	0	0	0	72
20364151	m	ph	JP	2022	0	1	1	0	0	0	0	0	0	0	0	0	72
20364193	m	md	JP	2022	0	1	0	0	1	0	0	0	0	0	0	0	71
20364541	f	md	ES	2022	0	1	0	0	1	0	0	0	0	0	0	0	
20364867	m	md	JP	2022	0	1	0	0	0	1	0	0	0	0	0	0	80

20364999	m	md	CN	2022	0	1	0	0	0	1	0	0	0	0	0	0	66
20365147	f	md	FR	2022	1	0	0	0	0	0	0	0	0	0	0	0	76
20365181	f	md	DE	2022	0	0	0	0	0	0	0	0	0	0	0	0	
20365811	f	cn	US	2022	0	0	0	0	0	0	0	0	0	0	0	0	
20365847	m	hp	KR	2022	1	0	0	0	0	0	0	0	0	0	0	0	53
20365850	f	hp	KR	2022	1	0	0	0	0	0	0	0	0	0	0	0	66
20367251	m	md	JP	2022	0	1	0	0	1	0	0	0	0	0	0	0	83
20367258	m	md	FR	2022	0	1	0	0	1	0	0	0	0	0	0	1	78
20367266	m	cn	CN	2022	0	1	0	0	1	0	0	0	0	0	0	1	51
20367658	f	md	DE	2022	0	1	0	0	1	0	0	0	0	0	0	1	82
20368200	m	md	ES	2022	0	1	0	0	0	1	0	0	0	0	0	0	71
20368277	f	md	CL	2022	0	0	0	0	0	0	0	0	0	0	0	0	59
20369422	Not	md	US	2022	0	1	0	0	0	1	0	0	0	0	0	0	
20369726	m	md	AU	2022	0	0	0	0	0	0	1	0	0	0	1	0	79.3
20369787	m	md	JP	2022	0	1	0	0	0	1	0	0	0	0	0	0	70
20369985	f	cn	TH	2022	0	0	0	0	0	0	0	0	0	0	0	0	64
20370100	Not	md	JP	2022	0	0	0	0	0	0	0	0	0	0	0	0	
20370355	m	hp	NL	2022	0	1	0	0	1	0	0	0	0	0	0	0	49
20370638	f	md	US	2022	0	0	0	0	0	0	0	0	0	0	0	0	70
20370944	f	cn	IN	2022	0	0	0	0	0	0	0	0	0	0	0	0	56
20371216	f	cn	CA	2022	0	0	0	0	0	0	0	0	0	0	0	0	33
20371277	m	hp	NL	2022	0	1	0	0	1	0	0	0	0	0	0	0	61
20371337	m	md	CA	2022	0	1	0	1	0	0	0	0	0	0	0	0	
20371385	Not	cn	IN	2022	1	0	0	0	0	0	0	0	0	0	0	0	
20371641	m	ph	JP	2022	0	1	0	0	1	0	0	0	0	0	0	0	83
20371715	f	hp	CN	2022	0	1	1	0	0	0	0	0	0	0	0	0	71
20371968	f	md	HR	2022	0	1	1	0	0	0	0	0	0	0	0	0	77
20372130	f	md	JP	2022	0	0	0	0	0	0	0	0	0	0	0	0	67
20372191	m	md	JP	2022	0	1	0	0	1	0	0	0	0	0	0	0	71
20372775	m	md	JP	2022	0	1	0	0	1	0	0	0	0	0	0	0	71
20372794	f	md	FR	2022	0	0	0	0	0	0	0	0	0	0	0	0	48
20373509	f	hp	US	2022	0	0	0	0	0	0	0	0	0	0	0	0	
20373671	m	md	RU	2022	0	1	0	0	0	1	0	0	0	0	0	0	48
20374255	m	md	JP	2022	0	1	0	0	0	1	0	0	0	0	0	0	61

20374421	f	md	IT	2022	0	0	0	0	0	0	0	0	0	0	0	0	73
20374494	m	md	JP	2022	0	1	0	0	1	0	0	0	0	0	0	0	72
20374552	Not	ph	JP	2022	1	0	0	0	0	0	0	0	0	0	0	0	
20374634	f	hp	MY	2022	1	0	0	0	0	0	0	0	0	0	0	0	
20374635	f	md	JP	2022	1	0	0	0	0	0	0	0	0	0	0	0	
20375014	m	md	JP	2022	0	1	1	0	0	0	0	0	0	0	0	0	75
20375121	f	md	DE	2022	0	1	1	0	0	0	0	0	0	0	0	0	61
20375302	m	md	JP	2022	0	1	0	1	0	0	0	0	0	0	0	0	67
20375312	m	md	IT	2022	0	1	1	0	0	0	0	0	0	0	0	0	59
20375637	f	cn	EG	2022	0	0	0	0	0	0	0	0	0	0	0	0	62
20375715	m	md	RU	2022	0	1	0	0	0	1	0	0	0	0	0	0	76
20375932	m	md	DE	2022	0	0	0	0	0	0	0	0	0	0	0	0	68
20376228	f	md	IN	2022	0	0	0	0	0	0	0	0	0	0	0	0	47
20376265	m	cn	CN	2022	0	1	0	1	0	0	0	0	0	0	0	1	54
20376450	f	ph	TH	2022	1	0	0	0	0	0	0	0	0	0	0	0	79
20376457	m	hp	JP	2022	0	1	0	0	1	0	0	0	0	0	0	1	65
20376554	Not	md	JP	2022	0	1	0	1	0	0	0	0	0	0	0	0	
20376609	m	md	JP	2022	0	1	0	1	0	0	0	0	0	0	0	1	86
20376612	m	md	FR	2022	0	1	0	1	0	0	0	0	0	0	0	0	81
20376946	m	md	DE	2022	0	1	0	0	1	0	0	0	0	0	0	0	56
20377232	m	md	AT	2022	0	0	0	0	0	0	0	0	0	0	0	0	59
20377422	m	md	JP	2022	0	1	0	0	1	0	0	0	0	0	0	0	57
20377457	Not	md	IT	2022	1	0	0	0	0	0	0	0	0	0	0	0	
20377530	f	md	JP	2022	1	0	0	0	0	0	0	0	0	0	0	0	
20377597	f	md	JP	2022	0	0	0	0	0	0	0	0	0	0	0	0	75
20377754	f	ph	JP	2022	1	0	0	0	0	0	0	0	0	0	0	0	104
20377767	m	md	JP	2022	0	1	0	0	0	1	0	0	0	0	0	1	55
20377985	m	md	JP	2022	0	1	1	0	0	0	0	0	0	0	0	0	82
20378116	m	md	US	2022	0	1	0	0	1	0	0	0	0	0	0	0	72
20378210	f	hp	IT	2022	0	0	0	0	0	0	0	0	0	0	0	0	66
20378388	Not	md	DE	2022	0	1	1	0	0	0	0	0	0	0	0	0	
20378609	m	cn	GB	2022	1	0	0	0	0	0	0	0	0	0	0	0	64
20378729	m	cn	US	2022	1	0	0	0	0	0	0	0	0	0	0	0	53.4
20378777	m	cn	CN	2022	0	1	0	1	0	0	0	0	0	0	0	0	59

20378852	f	ph	JP	2022	0	0	0	0	0	0	0	0	0	0	0	0	72
20378882	f	md	JP	2022	0	0	0	0	0	0	0	0	0	0	0	0	
20379035	m	hp	US	2022	0	1	1	0	0	0	0	0	0	0	0	0	61
20379140	m	md	FR	2022	0	1	1	0	0	0	0	0	0	0	0	0	55
20379206	m	cn	CN	2022	0	0	0	0	0	0	0	0	0	0	0	0	
20379404	f	hp	US	2022	0	0	0	0	0	0	0	0	0	0	0	0	
20379478	m	md	DE	2022	0	1	0	0	1	0	0	0	0	0	0	0	72
20379480	m	md	JP	2022	0	1	0	0	0	1	0	0	0	0	0	0	61
20379757	m	cn	VN	2022	0	1	1	0	0	0	0	0	0	0	0	1	59
20379859	f	md	DE	2022	0	1	1	0	0	0	0	0	0	0	0	0	53
20380035	Not	cn	DE	2022	0	1	1	0	0	0	0	0	0	0	0	0	
20380036	f	cn	DE	2022	0	1	1	0	0	0	0	0	0	0	0	0	
20380043	f	hp	GB	2022	0	1	1	0	0	0	0	0	0	0	0	0	78
20380213	m	md	JP	2022	0	1	0	0	1	0	0	0	0	0	0	0	64
20380875	m	md	JP	2022	0	1	0	0	0	1	0	0	0	0	0	0	50
20381320	m	md	JP	2022	0	1	0	0	1	0	0	0	0	0	0	0	70
20381807	m	md	JP	2022	0	0	0	0	0	0	0	0	0	0	0	0	80
20382026	Not	md	JP	2022	0	1	0	0	1	0	0	0	0	0	0	0	
20382598	m	hp	DE	2022	0	1	0	0	1	0	0	0	0	0	0	0	78
20382669	m	hp	DE	2022	0	1	1	0	0	0	0	0	0	0	0	1	45
20382810	m	md	JP	2022	0	1	0	0	1	0	0	0	0	0	0	1	80
20382813	m	md	JP	2022	0	1	0	0	1	0	0	0	0	0	0	1	64
20382982	m	md	IT	2022	0	1	1	0	0	0	0	0	0	0	0	0	78
20382994	f	md	JP	2022	0	1	0	0	1	0	0	0	0	0	0	0	82
20383178	Not	cn	SG	2022	1	0	0	0	0	0	0	0	0	0	0	0	84.3
20383433	f	md	DE	2022	0	0	0	0	0	0	0	0	0	0	0	0	65
20383809	f	cn	JP	2022	1	0	0	0	0	0	0	0	0	0	0	0	
20383851	f	cn	CZ	2022	0	1	0	0	1	0	0	0	0	0	0	0	76
20384011	Not	md	JP	2022	0	1	0	0	0	1	0	0	0	0	0	0	80
20384177	m	md	US	2022	0	0	0	0	0	0	0	0	0	0	0	0	63
20384683	m	cn	CN	2022	0	1	0	0	1	0	0	0	0	0	0	0	76
20384726	f	md	RU	2022	0	0	0	0	0	0	0	0	0	0	0	0	52
20384730	m	md	DE	2022	0	1	0	0	1	0	0	0	0	0	0	0	49
20385188	f	hp	US	2022	0	1	1	0	0	0	0	0	0	0	0	0	69

20385619	m	md	DE	2022	0	1	0	0	1	0	0	0	0	0	0	0	
20385982	m	md	DE	2022	0	0	0	0	0	0	0	0	0	0	0	0	68
20386051	m	md	IN	2022	0	1	1	0	0	0	0	0	0	0	0	0	42
20387147	f	hp	IT	2022	1	0	0	0	0	0	0	0	0	0	0	0	79
20387841	f	md	US	2022	0	0	0	0	0	0	0	0	0	0	0	0	
20389348	m	md	DE	2022	0	0	0	0	0	0	0	0	0	0	0	0	68
20389350	Not	md	US	2022	0	0	0	0	0	0	0	0	0	0	0	0	
20389401	f	hp	IT	2022	0	0	0	0	0	0	0	0	0	0	0	0	52
20390010	m	hp	KR	2022	0	0	0	0	0	0	0	0	0	0	0	0	75
20390058	m	md	US	2022	0	0	0	0	0	0	0	0	0	0	0	0	64
20390244	m	hp	IT	2022	0	0	0	0	0	0	0	0	0	0	0	0	57
20390245	f	hp	IT	2022	0	0	0	0	0	0	0	0	0	0	0	0	46
20390287	m	cn	PK	2022	0	0	0	0	0	0	0	0	0	0	0	0	30
20390447	f	cn	IL	2022	0	1	1	0	0	0	0	0	0	0	0	0	69
20390899	m	md	US	2022	0	0	0	0	0	0	0	0	0	0	0	0	
20390982	m	md	JP	2022	0	1	0	0	0	1	0	0	0	0	0	0	79
20391157	Not	md	JP	2022	1	0	0	0	0	0	0	0	0	0	0	0	
20391170	m	md	JP	2022	0	1	0	0	1	0	0	0	0	0	0	0	63
20391189	m	cn	DE	2022	0	1	1	0	0	0	0	0	0	0	0	0	59
20391378	f	cn	UA	2022	1	0	0	0	0	0	0	0	0	0	0	0	50.7
20391471	m	hp	IT	2022	0	0	0	0	0	0	0	0	0	0	0	0	57
20391518	f	hp	IT	2022	0	0	0	0	0	0	0	0	0	0	0	0	60
20391521	m	hp	IT	2022	0	0	0	0	0	0	0	0	0	0	0	0	68
20391555	m	cn	IN	2022	0	0	0	0	0	0	0	0	0	0	0	0	20
20391568	m	hp	IT	2022	0	0	0	0	0	0	0	0	0	0	0	0	32
20391570	Not	cn	US	2022	1	0	0	0	0	0	0	0	0	0	0	0	71
20391677	Not	hp	US	2022	1	0	0	0	0	0	0	0	0	0	0	0	
20391772	f	hp	IT	2022	0	0	0	0	0	0	0	0	0	0	0	0	36
20391794	f	hp	IT	2022	0	0	0	0	0	0	0	0	0	0	0	0	47
20391822	m	hp	IT	2022	0	0	0	0	0	0	0	0	0	0	0	0	
20391862	m	md	JP	2022	0	0	0	0	0	0	0	0	0	0	0	0	76
20391865	f	hp	IT	2022	0	0	0	0	0	0	0	0	0	0	0	0	49
20392017	m	ph	US	2022	0	1	1	0	0	0	0	0	0	0	0	0	75
20393153	f	md	FR	2022	0	1	0	0	1	0	0	0	0	0	0	0	65

20393245	m	md	JP	2022	0	0	0	0	0	0	0	0	0	0	0	0	58
20393397	Not	md	UA	2022	0	0	0	0	0	0	0	0	0	0	0	0	
20393403	m	md	MY	2022	0	1	1	0	0	0	0	0	0	0	0	0	37
20393420	m	cn	US	2022	0	1	1	0	0	0	0	0	0	0	0	0	
20393442	f	cn	DO	2022	1	0	0	0	0	0	0	0	0	0	0	0	
20393520	m	md	JP	2022	0	1	1	0	0	0	0	0	0	0	0	0	79
20393584	m	ph	NZ	2022	0	0	0	0	0	0	0	0	0	0	0	0	66
20393585	f	hp	IT	2022	0	0	0	0	0	0	0	0	0	0	0	0	48
20393635	m	md	RU	2022	0	1	0	0	0	1	0	0	0	0	0	0	62
20393750	m	ph	US	2022	0	0	0	0	0	0	0	0	0	0	0	0	62
20394011	m	md	JP	2022	0	0	0	0	0	0	0	0	0	0	0	0	80
20394154	m	ph	JP	2022	0	1	0	0	0	1	0	0	0	0	0	0	65
20394363	f	md	CZ	2022	0	0	0	0	0	0	0	0	0	0	0	0	
20394486	f	md	CL	2022	0	1	0	0	0	1	0	0	0	0	0	0	62
20394521	f	cn	CN	2022	0	0	0	0	0	0	0	0	0	0	0	0	64
20394535	f	md	US	2022	0	0	0	0	0	0	0	0	0	0	0	0	
20394540	m	md	DE	2022	0	1	0	0	1	0	0	0	0	0	0	0	44
20394749	Not	ph	US	2022	0	0	0	0	0	0	0	0	0	0	0	0	
20403817	f	cn	US	2022	0	1	0	0	0	1	0	0	0	0	0	0	
20404004	m	cn	CN	2022	0	0	0	0	0	0	0	0	0	0	0	0	59
20404124	f	cn	CN	2022	0	0	0	0	0	0	0	0	0	0	0	0	61
20404202	m	md	RO	2022	0	1	0	1	0	0	0	0	0	0	0	1	62
20404641	f	cn	IL	2022	0	0	0	0	0	0	0	0	0	0	0	0	53
20404646	m	md	RO	2022	0	1	0	1	0	0	0	0	0	0	0	0	62.7
20404662	m	ph	JP	2022	0	1	1	0	0	0	0	0	0	0	0	0	80
20404963	f	hp	US	2022	0	0	0	0	0	0	0	0	0	0	0	0	67
20405315	Not	md	KR	2022	0	1	0	0	0	1	0	0	0	0	0	0	
20405325	f	ph	US	2022	0	0	0	0	0	0	0	0	0	0	0	0	67
20405362	m	md	FR	2022	0	1	0	0	1	0	0	0	0	0	0	0	74
20405455	Not	md	JP	2022	0	1	0	0	1	0	0	0	0	0	0	0	
20405755	Not	md	CH	2022	0	0	0	0	0	0	0	0	0	0	0	0	
20405816	Not	md	CH	2022	0	0	0	0	0	0	0	0	0	0	0	0	
20405817	Not	md	CH	2022	0	0	0	0	0	0	0	0	0	0	0	0	
20405832	m	cn	NL	2022	0	0	0	0	0	0	0	0	0	0	0	0	62

20405837	m	md	FR	2022	0	1	0	0	1	0	0	0	0	0	0	0	76
20405992	m	md	IT	2022	0	0	0	0	0	0	0	0	0	0	0	0	61
20406060	f	md	US	2022	0	0	0	0	0	0	0	0	0	0	0	0	
20406108	Not	md	ES	2022	0	0	0	0	0	0	0	0	0	0	0	0	69
20406125	m	md	MX	2022	0	1	1	0	0	0	0	0	0	0	0	0	76
20406402	m	hp	IT	2022	0	0	0	0	0	0	0	0	0	0	0	0	60
20406606	m	md	FR	2022	0	0	0	0	0	0	0	0	0	0	0	0	66
20406610	Not	hp	JP	2022	0	0	0	0	0	0	0	0	0	0	0	0	50
20406679	m	md	US	2022	0	0	0	0	0	0	0	0	0	0	0	0	
20406680	f	ph	US	2022	0	0	0	0	0	0	0	0	0	0	0	0	
20406701	m	md	BE	2022	0	1	0	0	1	0	0	0	0	0	0	0	63
20406734	f	ph	JP	2022	0	1	1	0	0	0	0	0	0	0	0	0	
20407007	f	md	FR	2022	0	1	0	0	0	1	0	0	0	0	0	0	68
20407052	m	hp	CN	2022	0	0	0	0	0	0	0	0	0	0	0	0	66
20407083	m	hp	US	2022	0	0	0	0	0	0	0	0	0	0	0	0	81
20407175	m	hp	TR	2022	0	0	0	0	0	0	0	0	0	0	0	0	72
20407205	f	cn	CN	2022	0	1	0	0	1	0	0	0	0	0	0	1	69
20407259	f	md	JP	2022	0	1	1	0	0	0	0	0	0	0	0	0	84
20407302	f	md	JP	2022	0	1	0	0	1	0	0	0	0	0	0	0	62
20407341	f	cn	US	2022	0	0	0	0	0	0	0	0	0	0	0	0	
20407344	m	cn	PT	2022	0	1	1	0	0	0	0	0	0	0	0	0	64
20407591	Not	cn	FR	2022	0	1	0	0	1	0	0	0	0	0	0	0	
20407617	m	hp	US	2022	0	0	0	0	0	0	0	0	0	0	0	0	59
20407697	m	md	JP	2022	0	1	0	0	1	0	0	0	0	0	0	0	64
20407955	f	md	DE	2022	0	0	0	0	0	0	0	0	0	0	0	0	58
20408053	f	hp	CN	2022	0	1	0	0	1	0	0	0	0	0	0	0	52
20408132	m	md	JP	2022	0	1	0	0	1	0	0	0	0	0	0	0	74
20408253	m	md	JP	2022	0	1	0	0	1	0	0	0	0	0	0	0	74
20408295	m	md	JP	2022	0	1	0	0	1	0	0	0	0	0	0	1	77
20408325	f	ph	US	2022	0	0	0	0	0	0	0	0	0	0	0	0	72
20408339	m	ph	JP	2022	0	1	0	0	0	1	0	0	0	0	0	0	80
20408557	f	md	JP	2022	0	1	0	0	0	1	0	0	0	0	0	0	65
20408776	f	hp	DE	2022	0	1	1	0	0	0	0	0	0	0	0	0	62
20409040	Not	md	JP	2022	0	0	0	0	0	0	0	0	0	0	0	0	72

20409059	f	md	TH	2022	1	0	0	0	0	0	0	0	0	0	0	0	50
20409160	m	cn	CA	2022	1	0	0	0	0	0	0	0	0	0	0	0	
20410575	f	hp	NL	2022	0	1	0	0	1	0	0	0	0	0	0	0	76
20410707	Not	md	JP	2022	0	1	0	0	1	0	0	0	0	0	0	0	
20411568	m	cn	CN	2022	0	1	0	1	0	0	0	0	0	0	0	1	67
20411795	m	md	DE	2022	0	1	1	0	0	0	0	0	0	0	0	0	62
20411993	f	md	US	2022	0	1	1	0	0	0	0	0	0	0	0	0	57
20412456	m	hp	IT	2022	1	0	0	0	0	0	0	0	0	0	0	0	69
20412723	m	md	FR	2022	0	0	0	0	0	0	0	0	0	0	0	0	73
20412860	m	md	DE	2022	0	1	0	0	1	0	0	0	0	0	0	0	56
20412879	f	md	US	2022	0	1	0	0	0	1	0	0	0	0	0	0	84
20414171	m	md	FR	2022	0	0	0	0	0	0	0	0	0	0	0	0	49
20414331	m	md	US	2022	0	0	0	0	0	0	0	0	0	0	0	0	68
20414339	m	md	JP	2022	0	1	0	0	1	0	0	0	0	0	0	0	80
20414430	f	cn	SG	2022	0	1	0	0	0	1	0	0	0	0	0	0	
20414597	f	cn	US	2022	1	0	0	0	0	0	0	0	0	0	0	0	74
20414627	m	md	JP	2022	0	1	0	0	1	0	0	0	0	0	0	0	73
20414892	m	md	JP	2022	0	1	0	0	0	1	0	0	0	0	0	0	70
20414906	m	md	JP	2022	0	1	0	0	1	0	0	0	0	0	0	0	74
20415032	m	md	JP	2022	0	1	0	0	0	1	0	0	0	0	0	1	80
20415087	m	cn	KR	2022	0	0	0	0	0	0	0	0	0	0	0	0	55
20415123	m	md	US	2022	0	0	0	0	0	0	0	0	0	0	0	0	50
20415268	f	hp	ES	2022	1	0	0	0	0	0	0	0	0	0	0	0	67
20415413	f	md	TR	2022	0	0	0	0	0	0	0	0	0	0	0	0	58
20415500	m	ph	JP	2022	0	1	1	0	0	0	0	0	0	0	0	0	68
20415610	f	md	DE	2022	0	1	1	0	0	0	0	0	0	0	0	0	54
20415660	m	cn	CA	2022	1	0	0	0	0	0	0	0	0	0	0	0	80.9
20416345	f	cn	BR	2022	1	0	0	0	0	0	0	0	0	0	0	0	
20416473	m	hp	JP	2022	0	1	0	1	0	0	0	0	0	0	0	0	60
20416549	m	md	JP	2022	1	0	0	0	0	0	0	0	0	0	0	0	
20416757	f	md	ES	2022	0	0	0	0	0	0	0	0	0	0	0	0	39
20417321	f	md	JP	2022	0	1	1	0	0	0	0	0	0	0	0	0	
20417643	f	md	DE	2022	0	1	1	0	0	0	0	0	0	0	0	0	71
20418074	f	md	DE	2022	0	1	1	0	0	0	0	0	0	0	0	0	54

20418446	f	md	RU	2022	0	0	0	0	0	0	0	0	0	0	0	0	56
20418484	m	md	DK	2022	0	0	0	0	0	0	0	0	0	0	0	0	74
20418507	f	hp	KR	2022	0	0	0	0	0	0	0	0	0	0	0	0	74
20418753	m	md	JP	2022	0	1	0	0	1	0	0	0	0	0	0	0	77
20419184	m	md	CN	2022	0	0	0	0	0	0	0	0	0	0	0	0	
20419251	m	hp	DE	2022	0	1	0	0	1	0	0	0	0	0	0	0	67
20419401	m	md	DE	2022	0	0	0	0	0	0	0	0	0	0	0	0	68
20419655	Not	hp	CN	2022	1	0	0	0	0	0	0	0	0	0	0	0	
20419815	m	ph	JP	2022	0	1	0	0	1	0	0	0	0	0	0	0	66
20419931	f	hp	US	2022	0	0	0	0	0	0	0	0	0	0	0	0	
20420337	m	md	JP	2022	0	1	0	0	1	0	0	0	0	0	0	1	65
20421084	f	cn	US	2022	1	0	0	0	0	0	0	0	0	0	0	0	72
20421154	m	hp	CN	2022	0	0	0	0	0	0	0	0	0	0	0	0	69
20421457	m	hp	IT	2022	0	0	0	0	0	0	1	1	0	0	0	0	61
20421508	m	cn	US	2022	0	0	0	0	0	0	0	0	0	0	0	0	63
20421770	f	md	JP	2022	0	0	0	0	0	0	0	0	0	0	0	0	70
20421830	m	md	IT	2022	0	1	0	0	0	1	0	0	0	0	0	0	62
20422040	m	md	JP	2022	0	1	0	0	1	0	0	0	0	0	0	0	70
20422063	m	md	CH	2022	0	1	0	0	0	1	0	0	0	0	0	0	66
20422139	m	cn	CN	2022	0	1	0	1	0	0	0	0	0	0	0	1	64
20422758	f	ph	FR	2022	0	0	0	0	0	0	0	0	0	0	0	0	67
20422774	f	md	US	2022	0	0	0	0	0	0	0	0	0	0	0	0	
20422796	m	md	CN	2022	0	1	0	1	0	0	0	0	0	0	0	0	68.6
20422996	m	md	JP	2022	0	1	0	0	1	0	0	0	0	0	0	0	78
20423024	Not	md	PL	2022	0	0	0	0	0	0	0	0	0	0	0	0	
20423287	f	cn	TH	2022	1	0	0	0	0	0	0	0	0	0	0	0	69
20423305	Not	md	JP	2022	0	1	0	0	0	1	0	0	0	0	0	0	89
20423382	f	md	FR	2022	0	0	0	0	0	0	0	0	0	0	0	0	76
20423416	f	md	JP	2022	0	1	1	0	0	0	0	0	0	0	0	0	79
20423447	f	md	JP	2022	0	0	0	0	0	0	0	0	0	0	0	0	37
20424474	f	md	IT	2022	0	0	0	0	0	0	0	0	0	0	0	0	53
20424780	f	md	CH	2022	0	0	0	0	0	0	0	0	0	0	0	0	67
20425590	f	md	AU	2022	0	0	0	0	0	0	0	0	0	0	0	0	46
20425687	Not	hp	US	2022	0	0	0	0	0	0	0	0	0	0	0	0	

20425947	m	md	FR	2022	0	1	1	0	0	0	0	0	0	0	0	0	72
20426612	m	hp	CN	2022	0	0	0	0	0	0	0	0	0	0	0	0	39
20427365	f	hp	CN	2022	1	0	0	0	0	0	0	0	0	0	0	0	81
20427805	f	md	DE	2022	0	1	1	0	0	0	0	0	0	0	0	0	54
20427818	m	md	JP	2022	0	1	0	0	1	0	0	0	0	0	0	0	70
20427880	f	hp	CA	2022	0	1	1	0	0	0	0	0	0	0	0	0	64
20428102	m	md	DE	2022	0	1	0	0	1	0	0	0	0	0	0	0	72
20428240	m	md	FR	2022	0	0	0	0	0	0	0	0	0	0	0	0	86
20428274	m	md	JP	2022	0	1	0	0	1	0	0	0	0	0	0	0	63
20428603	f	cn	CN	2022	0	1	0	1	0	0	0	0	0	0	0	0	61
20428650	m	md	JP	2022	0	1	0	0	1	0	0	0	0	0	0	1	59
20428800	f	md	JP	2022	0	1	0	0	0	1	0	0	0	0	0	0	66
20428944	Not	md	US	2022	0	0	0	0	0	0	0	0	0	0	0	0	
20429418	Not	md	JP	2022	0	1	0	0	1	0	0	0	0	0	0	0	62
20429566	m	md	JP	2022	0	1	0	0	1	0	0	0	0	0	0	0	67
20429823	m	md	DE	2022	0	1	0	0	1	0	0	0	0	0	0	0	72
20429865	Not	cn	JP	2022	0	0	0	0	0	0	0	0	0	0	0	0	75
20429876	f	md	JP	2022	0	1	0	0	1	0	0	0	0	0	0	0	66
20429877	Not	md	JP	2022	1	0	0	0	0	0	0	0	0	0	0	0	
20429879	Not	ph	JP	2022	0	1	0	1	0	0	0	0	0	0	0	0	
20429880	Not	md	JP	2022	0	1	0	1	0	0	0	0	0	0	0	0	
20430074	Not	ph	JP	2022	1	0	0	0	0	0	0	0	0	0	0	0	
20430843	m	md	CA	2022	1	0	0	0	0	0	0	0	0	0	0	0	
20431014	m	md	SG	2022	1	0	0	0	0	0	0	0	0	0	0	0	66.9
20431365	Not	hp	JP	2022	0	0	0	0	0	0	0	0	0	0	0	0	
20431392	m	md	FR	2022	0	1	1	0	0	0	0	0	0	0	0	0	59
20431532	f	hp	US	2022	0	0	0	0	0	0	0	0	0	0	0	0	
20431593	f	cn	CA	2022	1	0	0	0	0	0	0	0	0	0	0	0	
20431732	f	cn	US	2022	0	1	0	1	0	0	0	0	0	0	0	0	73.1
20431845	m	md	IT	2022	0	0	0	0	0	0	0	0	0	0	0	0	68
20431848	f	hp	CA	2022	0	1	1	0	0	0	0	0	0	0	0	0	58
20431885	m	cn	PK	2022	0	1	0	0	0	1	0	0	0	0	0	0	46
20431967	m	md	JP	2022	0	0	0	0	0	0	0	0	0	0	0	0	65
20432248	f	md	JP	2022	1	0	0	0	0	0	0	0	0	0	0	0	69

20432348	f	md	ES	2022	0	0	0	0	0	0	0	0	0	0	0	0	66
20432513	f	hp	US	2022	0	0	0	0	0	0	0	0	0	0	0	0	69
20432530	f	cn	US	2022	0	0	0	0	0	0	0	0	0	0	0	0	
20432532	m	md	JP	2022	0	1	1	0	0	0	0	0	0	0	0	0	80
20433546	Not	hp	FR	2022	1	0	0	0	0	0	0	0	0	0	0	0	
20433967	f	cn	IN	2022	0	1	0	0	1	0	0	0	0	0	0	0	66
20434162	m	cn	CN	2022	0	0	0	0	0	0	0	0	0	0	0	0	68
20434740	m	cn	IN	2022	0	0	0	0	0	0	0	0	0	0	0	0	65
20434834	m	cn	IN	2022	0	0	0	0	0	0	0	0	0	0	0	0	72
20434960	f	cn	CA	2022	0	0	0	0	0	0	0	0	0	0	0	0	
20435369	m	md	JP	2022	0	1	0	0	1	0	0	0	0	0	0	0	39
20435389	m	md	JP	2022	0	1	0	0	1	0	0	0	0	0	0	0	66
20435399	f	ph	JP	2022	0	0	0	0	0	0	0	0	0	0	0	0	70
20435766	m	md	FR	2022	0	0	0	0	0	0	0	0	0	0	0	0	
20436443	m	md	JP	2022	0	1	0	0	1	0	0	0	0	0	0	0	50
20436679	m	md	JP	2022	0	1	0	0	1	0	0	0	0	0	0	0	70
20436680	m	cn	CN	2022	0	1	0	0	1	0	0	0	0	0	0	0	66
20437404	f	md	DE	2022	0	1	1	0	0	0	0	0	0	0	0	0	54
20437563	m	md	DE	2022	0	1	0	0	1	0	0	0	0	0	0	0	59
20437817	m	md	US	2022	0	0	0	0	0	0	0	0	0	0	0	0	75
20437846	m	cn	CN	2022	0	1	0	1	0	0	0	0	0	0	0	0	60
20437885	Not	md	IT	2022	1	0	0	0	0	0	0	0	0	0	0	0	
20438074	Not	hp	US	2022	0	0	0	0	0	0	0	0	0	0	0	0	
20438362	m	md	JP	2022	0	0	0	0	0	0	0	0	0	0	0	0	72
20438709	f	Not repo	US	2022	0	0	0	0	0	0	0	0	0	0	0	0	80
20439293	m	md	TR	2022	0	0	0	0	0	0	0	0	0	0	0	0	55
20439323	m	md	TR	2022	0	0	0	0	0	0	0	0	0	0	0	0	61
20439324	f	md	FR	2022	0	0	0	0	0	0	0	0	0	0	0	0	62
20439558	m	cn	IN	2022	0	1	0	1	0	0	0	0	0	0	0	0	75
20439651	m	md	CN	2022	0	0	0	0	0	0	1	1	0	0	0	0	48
20440050	m	md	US	2022	0	0	0	0	0	0	0	0	0	0	0	0	
20440283	f	md	GB	2022	0	0	0	0	0	0	0	0	0	0	0	0	56
20440451	m	hp	US	2022	0	0	0	0	0	0	0	0	0	0	0	0	36
20440616	f	md	FI	2022	0	0	0	0	0	0	0	0	0	0	0	0	74

20440784	m	hp	GB	2022	0	0	0	0	0	0	0	0	0	0	0	0	68
20440894	Not	md	JP	2022	0	1	0	0	1	0	0	0	0	0	0	0	72
20440918	m	md	JP	2022	0	0	0	0	0	0	0	0	0	0	0	0	68
20441133	m	md	JP	2022	0	1	0	0	1	0	0	0	0	0	0	0	76
20441265	m	md	JP	2022	0	1	0	0	1	0	0	0	0	0	0	0	51
20441266	Not	md	JP	2022	0	1	0	0	1	0	0	0	0	0	0	0	
20442074	Not	md	JP	2022	0	1	0	0	1	0	0	0	0	0	0	0	83
20442132	Not	hp	DE	2022	0	1	1	0	0	0	0	0	0	0	0	0	54
20442473	m	hp	IL	2022	0	0	0	0	0	0	0	0	0	0	0	0	64
20442595	f	md	IT	2022	0	0	0	0	0	0	0	0	0	0	0	0	74
20442977	m	md	DE	2022	0	0	0	0	0	0	0	0	0	0	0	0	64
20443191	f	hp	DE	2022	0	1	1	0	0	0	0	0	0	0	0	0	44
20444251	f	md	FR	2022	0	0	0	0	0	0	0	0	0	0	0	0	
20444419	m	md	CN	2022	0	1	0	1	0	0	0	0	0	0	0	0	72
20444624	m	hp	NL	2022	0	1	1	0	0	0	0	0	0	0	0	0	59
20444728	m	ph	JP	2022	0	1	1	0	0	0	0	0	0	0	0	0	64
20444983	m	md	JP	2022	0	1	0	0	1	0	0	0	0	0	0	0	59
20445018	m	cn	CN	2022	0	1	0	0	1	0	0	0	0	0	0	0	54
20445352	Not	md	FR	2022	0	0	0	0	0	0	0	0	0	0	0	0	
20445521	Not	cn	SG	2022	1	0	0	0	0	0	0	0	0	0	0	0	78.6
20445525	f	cn	SG	2022	1	0	0	0	0	0	0	0	0	0	0	0	77
20445559	m	md	JP	2022	0	1	0	0	1	0	0	0	0	0	0	0	
20445591	f	cn	SG	2022	1	0	0	0	0	0	0	0	0	0	0	0	74
20445933	Not	ph	US	2022	0	0	0	0	0	0	0	0	0	0	0	0	
20446119	m	hp	US	2022	0	1	1	0	0	0	0	0	0	0	0	0	
20446361	m	md	DE	2022	0	1	0	0	1	0	0	0	0	0	0	0	59
20446412	m	md	JP	2022	0	1	0	0	1	0	0	0	0	0	0	0	63
20446840	f	hp	US	2022	1	0	0	0	0	0	0	0	0	0	0	0	81
20447084	m	md	JP	2022	0	1	1	0	0	0	0	0	0	0	0	0	68
20447139	m	md	JP	2022	0	1	0	0	1	0	0	0	0	0	0	0	76
20447270	m	md	FR	2022	0	0	0	0	0	0	0	0	0	0	0	0	75
20447271	m	md	IT	2022	0	0	0	0	0	0	0	0	0	0	0	0	71
20447272	f	md	FR	2022	0	0	0	0	0	0	0	0	0	0	0	0	70
20447379	f	md	US	2022	0	0	0	0	0	0	0	0	0	0	0	0	

20447395	f	ph	US	2022	0	0	0	0	0	0	0	0	0	0	0	0	71
20447457	m	md	US	2022	0	0	0	0	0	0	0	0	0	0	0	0	
20447488	f	md	US	2022	0	0	0	0	0	0	0	0	0	0	0	0	26
20447661	f	hp	US	2022	0	0	0	0	0	0	0	0	0	0	0	0	53
20447959	m	md	JP	2022	0	1	1	0	0	0	0	0	0	0	0	0	63
20448335	f	md	BE	2022	0	0	0	0	0	0	0	0	0	0	0	0	58
20448346	f	md	JP	2022	0	1	0	0	1	0	0	0	0	0	0	0	66
20448580	Not	md	US	2022	0	0	0	0	0	0	0	0	0	0	0	0	
20449035	m	md	DE	2022	0	1	0	0	1	0	0	0	0	0	0	0	72
20449860	f	md	JP	2022	0	1	0	0	1	0	0	0	0	0	0	0	78
20450007	Not	cn	SG	2022	1	0	0	0	0	0	0	0	0	0	0	0	67.4
20450198	f	cn	US	2022	0	1	0	1	0	0	0	0	0	0	0	0	
20450201	m	cn	US	2022	0	1	0	1	0	0	0	0	0	0	0	0	66.9
20450401	m	cn	ID	2022	1	0	0	0	0	0	0	0	0	0	0	0	58.6
20450726	Not	md	JP	2022	0	1	1	0	0	0	0	0	0	0	0	0	72
20451027	m	hp	US	2022	0	1	0	0	1	0	0	0	0	0	0	0	76
20451224	f	md	JP	2022	1	0	0	0	0	0	0	0	0	0	0	1	69
20451467	f	hp	IL	2022	0	1	0	0	1	0	0	0	0	0	0	0	69
20451674	Not	md	JP	2022	0	1	1	0	0	0	0	0	0	0	0	0	80
20451701	m	md	CN	2022	0	0	0	0	0	0	0	0	0	0	0	0	64
20451747	f	md	BR	2022	0	0	0	0	0	0	0	0	0	0	0	0	66
20451872	f	md	JP	2022	0	1	0	0	1	0	0	0	0	0	0	0	73
20452008	Not	hp	US	2022	0	0	0	0	0	0	0	0	0	0	0	0	
20452025	f	hp	CA	2022	0	1	0	0	1	0	0	0	0	0	0	0	64
20452165	m	md	JP	2022	0	0	0	0	0	0	0	0	0	0	0	0	71
20452230	m	md	JP	2022	0	1	0	0	1	0	0	0	0	0	0	0	73
20452559	m	md	JP	2022	0	1	0	0	1	0	0	0	0	0	0	0	79
20452578	m	md	JP	2022	0	1	0	0	1	0	0	0	0	0	0	0	69
20452665	f	ph	NL	2022	1	0	0	0	0	0	0	0	0	0	0	0	86
20453077	m	md	DE	2022	0	1	0	0	1	0	0	0	0	0	0	0	74
20453247	Not	md	FR	2022	0	0	0	0	0	0	0	0	0	0	0	0	
20453400	m	md	TR	2022	0	0	0	0	0	0	0	0	0	0	0	0	
20453596	Not	md	JP	2022	0	1	0	0	1	0	0	0	0	0	0	0	
20454071	f	md	JP	2022	0	1	0	0	1	0	0	0	0	0	0	0	71

20454086	m	cn	IN	2022	0	1	0	0	0	1	0	0	0	0	0	0	68
20454719	f	cn	GB	2022	1	0	0	0	0	0	0	0	0	0	0	0	86
20454964	m	md	JP	2022	0	0	0	0	0	0	0	0	0	0	0	0	70
20454982	m	md	JP	2022	0	1	0	0	1	0	0	0	0	0	0	0	48
20455088	Not	md	JP	2022	0	1	0	0	1	0	0	0	0	0	0	0	
20455095	f	hp	JP	2022	0	1	0	0	1	0	0	0	0	0	0	0	65
20455256	m	md	DE	2022	0	0	0	0	0	0	0	0	0	0	0	0	69
20455259	f	hp	NL	2022	0	1	1	0	0	0	0	0	0	0	0	0	54
20455708	f	hp	IL	2022	0	1	1	0	0	0	0	0	0	0	0	0	
20455733	Not	md	FR	2022	0	1	0	0	1	0	0	0	0	0	0	0	71
20455790	m	md	JP	2022	0	1	0	1	0	0	0	0	0	0	0	1	66
20456166	m	hp	JP	2022	0	1	0	0	1	0	0	0	0	0	0	0	75
20456392	f	hp	NL	2022	0	1	1	0	0	0	0	0	0	0	0	0	64
20456493	m	md	FR	2022	0	1	0	0	1	0	0	0	0	0	0	0	69
20457229	m	md	US	2022	0	0	0	0	0	0	0	0	0	0	0	0	35
20457264	m	md	JP	2022	0	1	0	0	0	1	0	0	0	0	0	0	50
20457519	m	md	JP	2022	0	1	0	0	1	0	0	0	0	0	0	0	70
20457527	f	md	JP	2022	0	1	0	0	1	0	0	0	0	0	0	0	
20457587	f	md	JP	2022	0	1	1	0	0	0	0	0	0	0	0	0	75
20457635	m	cn	CN	2022	0	0	0	0	0	0	0	0	0	0	0	0	42
20457888	m	md	JP	2022	0	1	1	0	0	0	0	0	0	0	0	0	
20458449	m	md	JP	2022	0	1	0	0	1	0	0	0	0	0	0	0	58
20458597	Not	md	JP	2022	0	1	0	0	1	0	0	0	0	0	0	0	70
20458899	f	cn	CA	2022	0	0	0	0	0	0	0	0	0	0	0	0	77
20458933	m	hp	FR	2022	0	1	0	0	1	0	0	0	0	0	0	0	65
20459695	m	md	KR	2022	0	1	0	0	0	1	0	0	0	0	0	0	73
20459809	f	md	DE	2022	0	1	0	0	0	1	0	0	0	0	0	0	74
20459834	m	md	JP	2022	0	1	0	0	0	1	0	0	0	0	0	1	82
20459857	m	md	JP	2022	0	0	0	0	0	0	0	0	0	0	0	0	
20459876	Not	md	JP	2022	0	1	0	0	0	1	0	0	0	0	0	0	
20459972	f	md	JP	2022	0	1	0	0	0	1	0	0	0	0	0	0	70
20459990	m	md	JP	2022	0	1	0	0	0	1	0	0	0	0	0	0	67
20460043	m	md	BG	2022	0	0	0	0	0	0	0	0	0	0	0	0	74
20460079	m	md	JP	2022	0	1	0	0	1	0	0	0	0	0	0	1	72

20460471	m	md	US	2022	0	0	0	0	0	0	0	0	0	0	0	0	
20460635	m	cn	DE	2022	0	0	0	0	0	0	0	0	0	0	0	0	
20460819	Not	md	JP	2022	1	0	0	0	0	0	0	0	0	0	0	0	
20460820	m	cn	ID	2022	1	0	0	0	0	0	0	0	0	0	0	0	71.1
20461189	Not	md	DE	2022	0	0	0	0	0	0	0	0	0	0	0	0	72
20461301	Not	cn	SG	2022	1	0	0	0	0	0	0	0	0	0	0	0	53
20461352	Not	md	DE	2022	0	0	0	0	0	0	0	0	0	0	0	0	64
20461457	f	cn	IN	2022	0	1	0	0	1	0	0	0	0	0	0	0	44
20461715	f	hp	CA	2022	0	0	0	0	0	0	0	0	0	0	0	0	82
20461931	m	md	DE	2022	0	0	0	0	0	0	0	0	0	0	0	0	72
20461950	m	ph	MY	2022	1	0	0	0	0	0	0	0	0	0	0	0	
20461955	Not	md	NL	2022	0	0	0	0	0	0	0	0	0	0	0	0	
20462043	m	hp	FR	2022	0	1	0	0	1	0	0	0	0	0	0	0	81
20462118	Not	cn	SG	2022	1	0	0	0	0	0	0	0	0	0	0	0	64
20462587	f	cn	ID	2022	1	0	0	0	0	0	0	0	0	0	0	0	74.2
20462592	m	md	ZA	2022	1	0	0	0	0	0	0	0	0	0	0	0	
20462645	m	md	JP	2022	0	1	1	0	0	0	0	0	0	0	0	0	80
20462976	m	cn	IN	2022	0	1	0	0	1	0	0	0	0	0	0	0	67
20462979	m	hp	US	2022	0	1	0	0	1	0	0	0	0	0	0	0	
20462999	m	cn	IN	2022	0	1	0	0	1	0	0	0	0	0	0	0	81
20463032	f	md	JP	2022	0	1	1	0	0	0	0	0	0	0	0	1	78
20463051	m	md	IN	2022	1	0	0	0	0	0	0	0	0	0	0	0	56
20463176	f	md	ES	2022	0	0	0	0	0	0	0	0	0	0	0	0	37
20465930	f	md	JP	2022	0	0	0	0	0	0	0	0	0	0	0	0	41
20466445	f	hp	FR	2022	0	1	0	0	1	0	0	0	0	0	0	0	58
20466655	m	md	JP	2022	0	0	0	0	0	0	0	0	0	0	0	0	64
20466668	f	md	JP	2022	0	1	0	0	1	0	0	0	0	0	0	0	74
20466866	f	md	DE	2022	0	1	1	0	0	0	0	0	0	0	0	0	54
20467244	m	md	JP	2022	0	0	0	0	0	0	0	0	0	0	0	0	50
20467246	m	cn	KR	2022	0	1	0	0	1	0	0	0	0	0	0	0	77
20467476	m	cn	CA	2022	0	1	0	0	1	0	0	0	0	0	0	0	
20468382	f	md	JP	2022	0	1	0	0	1	0	0	0	0	0	0	0	66
20469296	m	cn	IN	2022	0	1	0	1	0	0	0	0	0	0	0	0	57
20469515	Not	ph	NL	2022	0	0	0	0	0	0	0	0	0	0	0	0	

20469539	m	md	FR	2022	0	1	1	0	0	0	0	0	0	0	0	0	59
20469544	m	md	FR	2022	0	1	1	0	0	0	0	0	0	0	0	0	59
20469687	f	hp	US	2022	1	0	0	0	0	0	0	0	0	0	0	0	
20469784	m	md	JP	2022	0	0	0	0	0	0	0	0	0	0	0	0	80
20469882	f	hp	JP	2022	0	1	0	0	1	0	0	0	0	0	0	0	78
20469923	f	md	JP	2022	0	0	0	0	0	0	0	0	0	0	0	0	79
20470147	m	md	JP	2022	0	1	0	0	0	1	0	0	0	0	0	0	70
20470326	f	hp	JP	2022	0	1	0	0	1	0	0	0	0	0	0	0	78
20470430	f	cn	CA	2022	0	0	0	0	0	0	0	0	0	0	0	0	56
20470431	f	md	KR	2022	0	0	0	0	0	0	0	0	0	0	0	0	70
20470602	m	md	IT	2022	0	0	0	0	0	0	0	0	0	0	0	0	
20470652	f	md	FR	2022	0	0	0	0	0	0	0	0	0	0	0	0	69
20470667	f	md	FR	2022	0	0	0	0	0	0	0	0	0	0	0	0	54
20471004	m	cn	IN	2022	0	1	0	1	0	0	0	0	0	0	0	0	71.8
20471057	Not	cn	SG	2022	1	0	0	0	0	0	0	0	0	0	0	0	91
20471110	m	md	CN	2022	0	1	0	0	0	1	0	0	0	0	0	0	49
20471308	f	md	BR	2022	0	1	0	0	1	0	0	0	0	0	0	0	
20471347	m	md	JP	2022	0	1	0	0	1	0	0	0	0	0	0	0	
20472166	f	hp	CN	2022	0	0	0	0	0	0	0	0	0	0	0	0	39
20473052	m	cn	IL	2022	0	0	0	0	0	0	0	0	0	0	0	0	
20473583	f	md	CA	2022	0	0	0	0	0	0	1	1	0	0	0	0	58
20474092	m	hp	JP	2022	0	1	0	0	0	1	0	0	0	0	0	0	60
20474115	m	hp	DE	2022	0	1	0	0	1	0	0	0	0	0	0	1	63
20474587	f	md	MY	2022	1	0	0	0	0	0	0	0	0	0	0	0	
20474675	m	ph	JP	2022	0	1	1	0	0	0	0	0	0	0	0	0	69
20474913	f	hp	DE	2022	0	1	1	0	0	0	0	0	0	0	0	0	62
20475028	f	md	CA	2022	1	0	0	0	0	0	0	0	0	0	0	0	58
20475092	m	cn	DE	2022	0	0	0	0	0	0	0	0	0	0	0	0	64
20475155	m	md	FR	2022	0	1	1	0	0	0	0	0	0	0	0	0	59
20475214	Not	md	JP	2022	1	0	0	0	0	0	0	0	0	0	0	0	
20475404	m	md	JP	2022	0	1	0	0	1	0	0	0	0	0	0	0	73
20475451	m	md	DE	2022	0	0	0	0	0	0	0	0	0	0	0	0	
20475565	f	cn	US	2022	0	1	1	0	0	0	0	0	0	0	0	0	
20475647	f	md	AR	2022	0	0	0	0	0	0	0	0	0	0	0	0	65

20475882	m	md	JP	2022	0	1	0	0	1	0	0	0	0	0	0	0	71
20476198	Not	hp	CN	2022	1	0	0	0	0	0	0	0	0	0	0	0	84
20476581	m	md	DE	2022	0	0	0	0	0	0	0	0	0	0	0	0	
20476927	m	hp	JP	2022	0	1	1	0	0	0	0	0	0	0	0	0	44
20476994	m	md	IQ	2022	0	1	0	0	0	1	0	0	0	0	0	0	76
20478123	m	md	JP	2022	0	1	0	0	1	0	0	0	0	0	0	0	47
20478187	m	hp	JP	2022	0	1	0	0	1	0	0	0	0	0	0	0	51
20478218	f	hp	GB	2022	0	1	0	0	0	1	0	0	0	0	0	0	75
20478227	Not	md	JP	2022	0	1	0	0	1	0	0	0	0	0	0	0	
20478263	m	md	MY	2022	1	0	0	0	0	0	0	0	0	0	0	0	
20478317	Not	md	JP	2022	0	1	0	0	1	0	0	0	0	0	0	0	
20478560	m	md	CN	2022	0	1	0	1	0	0	0	0	0	0	0	0	67.8
20478759	f	md	MX	2022	0	0	0	0	0	0	0	0	0	0	0	0	38
20478762	Not	md	JP	2022	0	0	0	0	0	0	0	0	0	0	0	0	90
20478795	f	md	TW	2022	0	0	0	0	0	0	0	0	0	0	0	0	46
20479426	m	cn	CA	2022	0	1	0	0	1	0	0	0	0	0	0	0	
20479527	f	hp	PL	2022	0	0	0	0	0	0	0	0	0	0	0	0	54
20479543	f	cn	JP	2022	0	0	0	0	0	0	0	0	0	0	0	0	49
20479554	m	cn	JP	2022	0	0	0	0	0	0	0	0	0	0	0	0	53
20479560	f	md	CA	2022	0	0	0	0	0	0	1	1	0	0	0	0	58
20479567	f	hp	CA	2022	0	0	0	0	0	0	0	0	0	0	0	0	77
20479652	f	md	US	2022	0	1	0	0	0	1	0	0	0	0	0	0	81
20479829	f	ph	US	2022	0	1	1	0	0	0	0	0	0	0	0	0	69
20479883	Not	md	FR	2022	0	1	0	1	0	0	0	0	0	0	0	0	
20480194	f	md	JP	2022	1	0	0	0	0	0	0	0	0	0	0	0	78
20480260	f	cn	TH	2022	1	0	0	0	0	0	0	0	0	0	0	0	71
20480264	f	md	US	2022	1	0	0	0	0	0	0	0	0	0	0	0	
20480377	m	md	GB	2022	0	1	1	0	0	0	0	0	0	0	0	0	73
20480802	m	cn	IN	2022	0	0	0	0	0	0	0	0	0	0	0	0	59
20480973	f	cn	IN	2022	0	0	0	0	0	0	0	0	0	0	0	0	51
20481020	Not	md	TR	2022	0	0	0	0	0	0	0	0	0	0	0	0	
20481055	Not	md	TR	2022	0	0	0	0	0	0	0	0	0	0	0	0	
20481186	m	hp	CN	2022	0	0	0	0	0	0	0	0	0	0	0	0	
20481284	m	cn	IN	2022	0	1	0	0	0	1	0	0	0	0	0	0	

20481550	m	md	FR	2022	0	0	0	0	0	0	0	0	0	0	0	0	62
20481664	m	md	JP	2022	0	1	0	0	1	0	0	0	0	0	0	0	67
20481665	m	hp	US	2022	0	1	0	0	1	0	0	0	0	0	0	0	50
20481799	m	md	JP	2022	0	1	0	0	1	0	0	0	0	0	0	0	79
20481968	m	md	IT	2022	0	0	0	0	0	0	0	0	0	0	0	0	
20482113	m	hp	DE	2022	0	0	0	0	0	0	0	0	0	0	0	0	40
20482154	m	md	JP	2022	0	0	0	0	0	0	0	0	0	0	0	0	79
20482321	m	md	PT	2022	0	0	0	0	0	0	0	0	0	0	0	0	76
20482538	Not	md	JP	2022	0	1	0	0	0	1	0	0	0	0	0	0	70
20482570	m	md	JP	2022	0	1	0	0	0	1	0	0	0	0	0	0	72
20482610	Not	hp	IL	2022	0	1	0	0	1	0	0	0	0	0	0	0	
20483009	f	md	JP	2022	0	1	0	0	1	0	0	0	0	0	0	0	74
20483285	Not	md	JP	2022	0	1	0	0	1	0	0	0	0	0	0	0	
20483303	f	cn	US	2022	1	0	0	0	0	0	0	0	0	0	0	0	66
20483436	m	hp	CN	2022	0	0	0	0	0	0	0	0	0	0	0	0	62
20483543	f	cn	US	2022	0	0	0	0	0	0	0	0	0	0	0	0	
20483833	m	md	JP	2022	0	1	0	0	1	0	0	0	0	0	0	0	65
20484303	f	md	JP	2022	0	1	0	0	1	0	0	0	0	0	0	0	77
20484679	f	md	BE	2022	0	1	0	0	0	1	0	0	0	0	0	0	68
20484694	f	cn	TH	2022	1	0	0	0	0	0	0	0	0	0	0	0	84
20484789	f	md	TW	2022	0	0	0	0	0	0	0	0	0	0	0	0	50
20485084	Not	md	JP	2022	0	1	0	0	0	1	0	0	0	0	0	0	70
20485667	m	md	JP	2022	0	1	0	0	1	0	0	0	0	0	0	0	53
20485725	f	cn	GB	2022	1	0	0	0	0	0	0	0	0	0	0	0	70
20485961	f	md	PL	2022	0	1	0	1	0	0	0	0	0	0	0	0	60
20486531	Not	ph	JP	2022	1	0	0	0	0	0	0	0	0	0	0	0	
20486563	m	md	JP	2022	0	1	0	0	1	0	0	0	0	0	0	0	73
20486570	m	ph	JP	2022	0	1	0	0	1	0	0	0	0	0	0	1	78
20486871	f	md	CN	2022	0	1	1	0	0	0	0	0	0	0	0	0	51
20487076	m	md	JP	2022	0	1	0	0	1	0	0	0	0	0	0	0	65
20487640	m	ph	JP	2022	0	0	0	0	0	0	0	0	0	0	0	0	70
20487757	f	cn	US	2022	1	0	0	0	0	0	0	0	0	0	0	0	51.1
20488223	m	md	JP	2022	0	1	1	0	0	0	0	0	0	0	0	0	70
20488514	f	md	FR	2022	0	0	0	0	0	0	0	0	0	0	0	0	

20489071	f	cn	JP	2022	1	0	0	0	0	0	0	0	0	0	0	0	65
20489256	m	md	JP	2022	0	1	0	0	1	0	0	0	0	0	0	0	74
20489257	m	md	JP	2022	0	1	0	1	0	0	0	0	0	0	0	0	65
20490136	m	md	JP	2022	1	0	0	0	0	0	0	0	0	0	0	0	
20490142	Not	ph	JP	2022	1	0	0	0	0	0	0	0	0	0	0	0	
20490895	f	md	DE	2022	0	0	0	0	0	0	0	0	0	0	0	0	60
20491293	m	md	JP	2022	0	1	0	0	1	0	0	0	0	0	0	0	65
20491295	f	cn	IN	2022	0	0	0	0	0	0	0	0	0	0	0	0	50
20491354	m	cn	IN	2022	0	1	0	0	0	1	0	0	0	0	0	0	
20491368	m	hp	US	2022	0	1	0	0	1	0	0	0	0	0	0	0	72
20491372	m	md	JP	2022	1	0	0	0	0	0	0	0	0	0	0	0	69
20491488	m	md	MX	2022	0	0	0	0	0	0	0	0	0	0	0	0	49
20491514	m	cn	CN	2022	0	1	0	0	1	0	0	0	0	0	0	0	68
20491538	m	md	US	2022	0	1	0	0	0	1	0	0	0	0	0	0	57
20491547	m	md	JP	2022	0	1	1	0	0	0	0	0	0	0	0	0	62
20491671	m	ph	US	2022	0	0	0	0	0	0	0	0	0	0	0	0	
20492086	m	md	CN	2022	0	1	1	0	0	0	0	0	0	0	0	0	59
20492360	m	md	JP	2022	0	1	0	0	0	1	0	0	0	0	0	0	80
20492411	f	hp	CA	2022	0	0	0	0	0	0	0	0	0	0	0	0	77
20492440	f	md	US	2022	0	0	0	0	0	0	0	0	0	0	0	0	69
20492893	m	md	JP	2022	0	0	0	0	0	0	0	0	0	0	0	0	84
20493312	f	cn	PH	2022	1	0	0	0	0	0	0	0	0	0	0	0	72
20493389	m	md	JP	2022	0	1	0	0	1	0	0	0	0	0	0	0	78
20493480	f	md	US	2022	0	0	0	0	0	0	0	0	0	0	0	0	73
20493610	m	md	JP	2022	0	1	0	0	1	0	0	0	0	0	0	0	67
20493625	f	md	IN	2022	0	1	0	0	1	0	0	0	0	0	0	0	42
20493709	Not	md	IT	2022	0	0	0	0	0	0	0	0	0	0	0	0	
20494815	m	md	IT	2022	0	1	1	0	0	0	0	0	0	0	0	0	59
20494980	Not	cn	CN	2022	0	0	0	0	0	0	0	0	0	0	0	0	
20495018	m	ph	CA	2022	0	0	0	0	0	0	0	0	0	0	0	0	52
20495124	m	hp	JP	2022	0	1	0	0	1	0	0	0	0	0	0	0	65
20495125	m	md	JP	2022	0	1	0	0	1	0	0	0	0	0	0	0	76
20495320	f	cn	GB	2022	1	0	0	0	0	0	0	0	0	0	0	0	40
20495351	f	md	CA	2022	0	0	0	0	0	0	0	0	0	0	0	0	54

20495445	f	md	SG	2022	0	0	0	0	0	0	0	0	0	0	0	0	52
20495546	m	md	DE	2022	0	1	0	0	1	0	0	0	0	0	0	0	68
20495784	Not	cn	US	2022	0	0	0	0	0	0	0	0	0	0	0	0	
20496573	f	hp	RO	2022	1	0	0	0	0	0	0	0	0	0	0	0	
20496643	m	md	JP	2022	0	1	1	0	0	0	0	0	0	0	0	0	78
20497159	f	cn	JP	2022	0	1	0	0	1	0	0	0	0	0	0	1	54
20497166	m	cn	CN	2022	0	1	0	1	0	0	0	0	0	0	0	0	70
20498532	m	md	JP	2022	0	1	0	0	1	0	0	0	0	0	0	0	81
20499022	m	md	BG	2022	0	1	0	0	0	1	0	0	0	0	0	0	47
20499035	m	md	CN	2022	0	1	0	0	0	1	0	0	0	0	0	0	51
20499287	Not	md	IT	2022	0	0	0	0	0	0	0	0	0	0	0	0	65
20499919	m	md	CN	2022	0	0	0	0	0	0	0	0	0	0	0	0	68
20500014	m	hp	DE	2022	0	1	1	0	0	0	0	0	0	0	0	0	50
20500120	Not	ph	JP	2022	1	0	0	0	0	0	0	0	0	0	0	0	
20500201	m	md	JP	2022	0	1	0	0	1	0	0	0	0	0	0	0	71
20500258	m	cn	JP	2022	0	0	0	0	0	0	1	1	0	0	0	0	
20500363	f	hp	CA	2022	0	0	0	0	0	0	0	0	0	0	0	0	77
20500423	m	cn	CA	2022	0	0	0	0	0	0	0	0	0	0	0	0	70
20500468	f	hp	AU	2022	0	0	0	0	0	0	0	0	0	0	0	0	51
20500688	m	md	JP	2022	0	1	1	0	0	0	0	0	0	0	0	0	80
20501154	m	hp	CN	2022	0	0	0	0	0	0	0	0	0	0	0	0	59
20501410	f	md	CA	2022	0	0	0	0	0	0	0	0	0	0	0	0	54
20501849	f	md	ES	2022	0	0	0	0	0	0	0	0	0	0	0	0	57
20501873	m	md	JP	2022	0	1	0	0	1	0	0	0	0	0	0	0	64
20502504	m	hp	AU	2022	0	1	1	0	0	0	0	0	0	0	0	1	67
20502520	m	ph	CA	2022	0	1	1	0	0	0	0	0	0	0	0	0	82
20502609	f	md	DE	2022	0	0	0	0	0	0	0	0	0	0	0	0	
20502706	m	md	FR	2022	0	0	0	0	0	0	0	0	0	0	0	0	52
20502718	f	md	JP	2022	0	0	0	0	0	0	0	0	0	0	0	0	68
20502743	Not	hp	JP	2022	0	0	0	0	0	0	0	0	0	0	0	0	
20502970	f	hp	JP	2022	1	0	0	0	0	0	0	0	0	0	0	0	84
20503365	f	cn	DE	2022	0	1	1	0	0	0	0	0	0	0	0	0	60
20503972	f	md	HR	2022	0	1	1	0	0	0	0	0	0	0	0	0	79
20504573	f	hp	US	2022	0	0	0	0	0	0	0	0	0	0	0	0	

20504708	f	md	FR	2022	0	0	0	0	0	0	0	0	0	0	0	0	59
20504735	m	md	FR	2022	0	0	0	0	0	0	0	0	0	0	0	0	61
20504852	f	cn	US	2022	1	0	0	0	0	0	0	0	0	0	0	0	74
20504956	Not	md	JP	2022	0	1	0	0	1	0	0	0	0	0	0	0	
20504984	m	md	JP	2022	0	1	0	0	1	0	0	0	0	0	0	1	75
20505102	m	cn	US	2022	0	1	1	0	0	0	0	0	0	0	0	0	
20505187	f	md	CN	2022	0	1	0	0	0	1	0	0	0	0	0	0	35
20505346	m	ph	JP	2022	0	1	0	0	1	0	0	0	0	0	0	1	61
20505672	m	md	JP	2022	0	1	0	0	1	0	0	0	0	0	0	0	57
20505836	Not	ph	JP	2022	1	0	0	0	0	0	0	0	0	0	0	0	
20505875	f	md	KR	2022	0	0	0	0	0	0	0	0	0	0	0	0	65
20506419	m	md	NZ	2022	0	0	0	0	0	0	0	0	0	0	0	0	66
20506420	m	md	DE	2022	0	0	0	0	0	0	0	0	0	0	0	0	83
20506501	f	cn	ID	2022	1	0	0	0	0	0	0	0	0	0	0	0	61.3
20506623	f	ph	US	2022	0	0	0	0	0	0	0	0	0	0	0	0	64
20506910	f	cn	TW	2022	1	0	0	0	0	0	0	0	0	0	0	0	50
20507364	m	cn	IN	2022	0	1	0	0	0	1	0	0	0	0	0	0	
20507439	f	cn	KR	2022	0	0	0	0	0	0	0	0	0	0	0	0	65
20507720	m	md	BE	2022	0	0	0	0	0	0	1	0	0	1	0	1	72
20508079	f	md	FR	2022	0	0	0	0	0	0	0	0	0	0	0	0	81
20508239	m	md	JP	2022	0	1	0	0	0	1	0	0	0	0	0	0	80
20508256	m	md	JP	2022	0	1	0	1	0	0	0	0	0	0	0	1	65
20508432	f	md	FR	2022	0	0	0	0	0	0	0	0	0	0	0	0	
20508560	m	md	HR	2022	0	1	1	0	0	0	0	0	0	0	0	0	80
20508563	m	md	JP	2022	0	1	1	0	0	0	0	0	0	0	0	0	80
20508718	Not	hp	US	2022	0	0	0	0	0	0	0	0	0	0	0	0	
20508990	f	cn	DE	2022	0	0	0	0	0	0	0	0	0	0	0	0	
20509108	f	cn	CA	2022	0	0	0	0	0	0	1	1	0	0	0	0	58
20509412	f	cn	CA	2022	0	0	0	0	0	0	0	0	0	0	0	0	75
20509584	m	hp	RU	2022	0	1	0	0	1	0	0	0	0	0	0	0	75
20509715	m	md	JP	2022	0	1	0	0	1	0	0	0	0	0	0	0	66
20509904	m	md	IT	2022	0	0	0	0	0	0	0	0	0	0	0	0	73
20510143	f	hp	CA	2022	0	0	0	0	0	0	1	1	0	0	0	0	58
20510445	f	cn	CA	2022	0	0	0	0	0	0	1	1	0	0	0	0	58

20510637	f	md	US	2022	0	1	0	0	0	1	0	0	0	0	0	0	75
20511163	m	hp	ES	2022	0	1	0	0	1	0	0	0	0	0	0	1	71
20511258	m	md	US	2022	0	0	0	0	0	0	0	0	0	0	0	0	65
20511314	f	cn	CA	2022	0	0	0	0	0	0	0	0	0	0	0	0	63
20511624	m	md	US	2022	0	0	0	0	0	0	0	0	0	0	0	0	65
20512174	m	md	JP	2022	0	1	0	1	0	0	0	0	0	0	0	1	74
20512246	Not	ph	JP	2022	1	0	0	0	0	0	0	0	0	0	0	0	
20512305	f	md	US	2022	0	1	0	1	0	0	0	0	0	0	0	0	
20512870	f	md	FR	2022	0	0	0	0	0	0	0	0	0	0	0	0	67
20512877	m	cn	CA	2022	0	1	0	0	1	0	0	0	0	0	0	0	58
20512924	m	md	JP	2022	0	1	0	0	1	0	0	0	0	0	0	0	73
20512934	m	md	JP	2022	1	0	0	0	0	0	0	0	0	0	0	0	73
20513568	m	hp	RU	2022	0	1	0	1	0	0	0	0	0	0	0	0	74
20513950	m	hp	JP	2022	0	1	0	1	0	0	0	0	0	0	0	0	75
20514159	Not	ph	JP	2022	1	0	0	0	0	0	0	0	0	0	0	0	
20514275	f	md	JP	2022	0	1	0	0	1	0	0	0	0	0	0	0	71
20514464	m	md	JP	2022	1	0	0	0	0	0	0	0	0	0	0	0	66
20514653	f	cn	CN	2022	0	0	0	0	0	0	0	0	0	0	0	0	49
20515102	f	md	JP	2022	0	1	0	0	0	1	0	0	0	0	0	0	80
20515161	f	md	JP	2022	0	1	0	1	0	0	0	0	0	0	0	0	70
20515209	Not	ph	JP	2022	1	0	0	0	0	0	0	0	0	0	0	0	
20515259	m	md	TW	2022	0	0	0	0	0	0	0	0	0	0	0	0	69
20515540	m	md	CN	2022	0	0	0	0	0	0	0	0	0	0	0	0	68
20515691	m	cn	CN	2022	0	1	0	0	1	0	0	0	0	0	0	0	69
20516627	m	md	FR	2022	0	0	0	0	0	0	0	0	0	0	0	0	65
20516649	f	md	IT	2022	0	0	0	0	0	0	0	0	0	0	0	0	66
20517027	f	md	US	2022	0	0	0	0	0	0	0	0	0	0	0	0	70
20517058	f	ph	JP	2022	0	1	1	0	0	0	0	0	0	0	0	0	59
20517205	f	hp	AU	2022	0	0	0	0	0	0	0	0	0	0	0	0	
20517359	m	md	JP	2022	0	1	0	0	1	0	0	0	0	0	0	1	58
20517430	m	md	JP	2022	0	0	0	0	0	0	0	0	0	0	0	0	60
20517496	f	hp	JP	2022	1	0	0	0	0	0	0	0	0	0	0	0	84
20517512	m	cn	GB	2022	1	0	0	0	0	0	0	0	0	0	0	0	75
20518147	m	cn	SG	2022	1	0	0	0	0	0	0	0	0	0	0	0	82.3

20518232	f	md	JP	2022	0	1	0	0	0	1	0	0	0	0	0	0	80
20518704	m	md	JP	2022	0	1	0	1	0	0	0	0	0	0	0	1	61
20519069	f	cn	BR	2022	0	0	0	0	0	0	0	0	0	0	0	0	
20519835	m	md	JP	2022	0	1	0	0	0	1	0	0	0	0	0	0	65
20520091	f	hp	US	2022	0	0	0	0	0	0	0	0	0	0	0	0	72
20520108	m	cn	US	2022	0	0	0	0	0	0	0	0	0	0	0	0	
20520372	Not	md	JP	2022	1	0	0	0	0	0	0	0	0	0	0	0	
20520452	m	md	JP	2022	0	1	1	0	0	0	0	0	0	0	0	0	74
20520768	f	cn	SG	2022	1	0	0	0	0	0	0	0	0	0	0	0	89.4
20521291	f	hp	US	2022	0	1	0	1	0	0	0	0	0	0	0	0	
20521569	Not	md	JP	2022	0	1	1	0	0	0	0	0	0	0	0	0	80
20521606	f	md	US	2022	0	0	0	0	0	0	0	0	0	0	0	0	64
20523085	f	cn	IN	2022	0	0	0	0	0	0	0	0	0	0	0	0	75
20523814	m	md	RO	2022	0	0	0	0	0	0	0	0	0	0	0	0	51
20523903	f	md	JP	2022	0	1	0	0	1	0	0	0	0	0	0	0	78
20523998	m	md	PT	2022	0	0	0	0	0	0	0	0	0	0	0	0	72
20524260	m	cn	CN	2022	0	0	0	0	0	0	0	0	0	0	0	0	58
20524666	m	cn	IN	2022	0	0	0	0	0	0	0	0	0	0	0	0	51
20525482	m	md	JP	2022	0	1	0	0	1	0	0	0	0	0	0	0	69
20525703	m	md	JP	2022	0	1	1	0	0	0	0	0	0	0	0	0	68
20525759	m	cn	CA	2022	0	1	0	0	1	0	0	0	0	0	0	0	
20525798	m	cn	CA	2022	0	0	0	0	0	0	0	0	0	0	0	0	71
20525977	f	cn	US	2022	1	0	0	0	0	0	0	0	0	0	0	0	70
20526044	m	md	CN	2022	0	0	0	0	0	0	0	0	0	0	0	0	59
20526292	f	cn	DE	2022	0	0	0	0	0	0	0	0	0	0	0	0	49
20526575	m	md	KR	2022	0	1	1	0	0	0	0	0	0	0	0	0	65
20526654	m	md	JP	2022	0	1	0	0	1	0	0	0	0	0	0	1	72
20526656	f	cn	IT	2022	0	0	0	0	0	0	0	0	0	0	0	0	39
20526982	m	md	FR	2022	0	1	0	0	0	1	0	0	0	0	0	0	48
20527072	m	md	DE	2022	0	0	0	0	0	0	0	0	0	0	0	0	68
20527129	m	md	US	2022	0	0	0	0	0	0	0	0	0	0	0	0	
20527263	m	ph	US	2022	0	0	0	0	0	0	0	0	0	0	0	0	53
20527716	f	md	CN	2022	0	1	0	1	0	0	0	0	0	0	0	1	55
20527849	m	md	JP	2022	0	1	0	0	1	0	0	0	0	0	0	0	51

20528329	f	md	FR	2022	0	0	0	0	0	0	0	0	0	0	0	0	
20528993	f	md	US	2022	0	0	0	0	0	0	0	0	0	0	0	0	
20529030	m	cn	IN	2022	0	0	0	0	0	0	0	0	0	0	0	0	37
20529327	m	md	JP	2022	0	1	0	0	1	0	0	0	0	0	0	0	74
20529462	m	md	JP	2022	0	1	0	0	1	0	0	0	0	0	0	0	70
20529538	m	md	DE	2022	0	0	0	0	0	0	0	0	0	0	0	0	60
20529553	m	md	JP	2022	0	1	0	0	0	1	0	0	0	0	0	0	
20529853	m	md	IT	2022	0	1	1	0	0	0	0	0	0	0	0	0	70
20529886	f	md	JP	2022	0	1	0	0	1	0	0	0	0	0	0	0	66
20529908	m	md	JP	2022	0	1	0	0	1	0	0	0	0	0	0	0	68
20530101	m	md	JP	2022	0	1	0	0	1	0	0	0	0	0	0	0	78
20530423	m	md	JP	2022	0	1	0	0	1	0	0	0	0	0	0	0	49
20531090	m	md	FR	2022	0	1	0	0	0	1	0	0	0	0	0	0	56
20531133	m	cn	IN	2022	0	0	0	0	0	0	0	0	0	0	0	0	51
20531625	m	md	JP	2022	0	1	0	1	0	0	0	0	0	0	0	1	68
20531809	m	md	JP	2022	1	0	0	0	0	0	0	0	0	0	0	0	79
20532359	m	md	JP	2022	0	1	0	0	1	0	0	0	0	0	0	0	77
20532608	Not	md	JP	2022	0	1	0	1	0	0	0	0	0	0	0	1	
20532755	Not	md	JP	2022	0	0	0	0	0	0	0	0	0	0	0	0	
20532932	f	md	CA	2022	1	0	0	0	0	0	0	0	0	0	0	0	
20533279	m	ph	JP	2022	0	1	0	0	1	0	0	0	0	0	0	0	60
20533384	m	md	JP	2022	0	1	1	0	0	0	0	0	0	0	0	0	44
20533748	m	hp	CN	2022	0	1	0	0	1	0	0	0	0	0	0	0	
20533833	m	md	JP	2022	0	1	0	1	0	0	0	0	0	0	0	0	76
20533867	m	md	JP	2022	0	1	0	0	1	0	0	0	0	0	0	0	72
20534095	m	md	US	2022	0	1	0	0	0	1	0	0	0	0	0	0	49
20534133	m	hp	JP	2022	0	1	0	0	1	0	0	0	0	0	0	1	65
20534168	m	md	IT	2022	0	0	0	0	0	0	0	0	0	0	0	0	57
20534358	f	md	AU	2022	0	1	1	0	0	0	0	0	0	0	0	0	57
20534649	Not	md	JP	2022	0	1	0	0	0	1	0	0	0	0	0	0	
20534691	m	cn	SG	2022	0	0	0	0	0	0	0	0	0	0	0	0	47
20534719	f	ph	US	2022	0	0	0	0	0	0	0	0	0	0	0	0	60
20534754	f	md	CN	2022	0	0	0	0	0	0	0	0	0	0	0	0	70
20535129	f	md	KR	2022	0	1	0	0	0	1	0	0	0	0	0	0	65

20535763	f	cn	US	2022	1	0	0	0	0	0	0	0	0	0	0	0	
20535985	m	md	JP	2022	0	1	0	0	1	0	0	0	0	0	0	0	73
20536022	f	md	JP	2022	0	1	0	0	1	0	0	0	0	0	0	0	70
20536244	m	md	JP	2022	0	1	0	0	0	1	0	0	0	0	0	0	77
20536248	m	md	JP	2022	0	1	0	0	0	1	0	0	0	0	0	1	78
20536289	f	md	JP	2022	0	1	0	0	1	0	0	0	0	0	0	0	70
20536442	m	md	JP	2022	0	1	0	0	1	0	0	0	0	0	0	0	80
20536670	m	md	JP	2022	0	0	0	0	0	0	0	0	0	0	0	0	70
20536770	m	md	TW	2022	0	0	0	0	0	0	0	0	0	0	0	0	
20536947	m	md	AT	2022	0	1	0	0	0	1	0	0	0	0	0	0	63
20537107	m	md	JP	2022	0	1	1	0	0	0	0	0	0	0	0	0	75
20537976	f	cn	US	2022	0	1	1	0	0	0	0	0	0	0	0	0	
20537977	m	cn	TW	2022	0	1	0	0	1	0	0	0	0	0	0	0	59
20538212	Not	md	JP	2022	0	0	0	0	0	0	1	0	0	1	0	0	
20538838	f	hp	NL	2022	0	0	0	0	0	0	0	0	0	0	0	0	
20538950	f	md	AU	2022	0	0	0	0	0	0	0	0	0	0	0	0	71
20539175	f	md	FR	2022	0	0	0	0	0	0	0	0	0	0	0	0	68
20539377	f	cn	CA	2022	1	0	0	0	0	0	0	0	0	0	0	0	58
20539472	m	cn	CN	2022	0	1	0	1	0	0	0	0	0	0	0	0	64
20539635	f	md	CN	2022	0	0	0	0	0	0	0	0	0	0	0	0	56
20539725	m	md	IE	2022	1	0	0	0	0	0	0	0	0	0	0	0	82
20540055	m	md	CH	2022	0	1	1	0	0	0	0	0	0	0	0	0	58
20540761	m	md	BR	2022	0	0	0	0	0	0	0	0	0	0	0	0	69
20540826	f	md	FR	2022	0	0	0	0	0	0	0	0	0	0	0	0	65
20541102	f	hp	US	2022	0	0	0	0	0	0	0	0	0	0	0	0	
20541252	f	hp	US	2022	0	0	0	0	0	0	0	0	0	0	0	0	58
20541353	m	hp	US	2022	0	0	0	0	0	0	0	0	0	0	0	0	
20541833	m	cn	IN	2022	0	0	0	0	0	0	0	0	0	0	0	0	44
20542089	f	md	JP	2022	0	0	0	0	0	0	0	0	0	0	0	0	40
20542154	m	md	JP	2022	0	1	0	0	0	1	0	0	0	0	0	0	80
20542228	Not	md	IE	2022	1	0	0	0	0	0	0	0	0	0	0	0	
20542312	m	md	AU	2022	1	0	0	0	0	0	0	0	0	0	0	0	
20542361	f	cn	US	2022	0	0	0	0	0	0	0	0	0	0	0	0	
20542435	f	md	US	2022	0	1	0	0	0	1	0	0	0	0	0	0	57

20543122	m	cn	FR	2022	0	0	0	0	0	0	0	0	0	0	0	0	
20543286	f	hp	JP	2022	0	1	1	0	0	0	0	0	0	0	0	0	80
20543299	m	md	MY	2022	0	0	0	0	0	0	0	0	0	0	0	0	60
20543478	m	md	JP	2022	0	1	0	0	1	0	0	0	0	0	0	0	70
20543480	f	md	PT	2022	0	0	0	0	0	0	0	0	0	0	0	0	64
20543595	f	md	IT	2022	0	0	0	0	0	0	0	0	0	0	0	0	
20543616	m	md	IT	2022	0	0	0	0	0	0	0	0	0	0	0	0	
20543902	m	md	JP	2022	0	1	0	0	0	1	0	0	0	0	0	0	56
20544033	m	md	DE	2022	0	1	0	0	1	0	0	0	0	0	0	0	69
20544192	m	hp	TW	2022	0	1	1	0	0	0	0	0	0	0	0	0	72
20544226	m	md	JP	2022	0	1	0	0	0	1	0	0	0	0	0	0	
20544617	m	md	JP	2022	0	0	0	0	0	0	0	0	0	0	0	0	59
20544749	m	hp	DE	2022	0	0	0	0	0	0	0	0	0	0	0	0	
20544784	f	cn	CA	2022	0	0	0	0	0	0	0	0	0	0	0	0	68
20544786	Not	hp	CA	2022	0	1	1	0	0	0	0	0	0	0	0	0	73
20545660	f	cn	IN	2022	0	0	0	0	0	0	0	0	0	0	0	0	61
20545663	m	md	DE	2022	0	1	0	0	1	0	0	0	0	0	0	0	74
20546324	m	hp	JP	2022	0	0	0	0	0	0	0	0	0	0	0	0	
20546362	f	cn	US	2022	1	0	0	0	0	0	0	0	0	0	0	0	
20546646	f	md	IT	2022	0	0	0	0	0	0	0	0	0	0	0	0	33
20546650	f	md	FR	2022	0	0	0	0	0	0	0	0	0	0	0	0	60
20546824	m	md	JP	2022	0	1	1	0	0	0	0	0	0	0	0	0	78
20546968	m	md	CA	2022	0	0	0	0	0	0	0	0	0	0	0	0	
20547447	m	cn	US	2022	0	1	1	0	0	0	0	0	0	0	0	0	63
20547642	f	hp	JP	2022	1	0	0	0	0	0	0	0	0	0	0	0	84
20547803	f	md	ES	2022	0	0	0	0	0	0	0	0	0	0	0	0	71
20547871	m	cn	GB	2022	1	0	0	0	0	0	0	0	0	0	0	0	71
20548610	m	md	GR	2022	0	1	1	0	0	0	0	0	0	0	0	0	61
20548659	m	hp	DE	2022	0	1	1	0	0	0	0	0	0	0	0	1	50
20548849	m	hp	CA	2022	0	1	1	0	0	0	0	0	0	0	0	0	60
20548906	f	md	US	2022	0	0	0	0	0	0	0	0	0	0	0	0	79
20548961	f	md	JP	2022	1	0	0	0	0	0	0	0	0	0	0	0	92
20549509	m	md	JP	2022	0	1	0	0	1	0	0	0	0	0	0	0	80
20550725	m	md	CH	2022	0	0	0	0	0	0	0	0	0	0	0	0	66

20550864	f	cn	US	2022	0	0	0	0	0	0	0	0	0	0	0	0	
20551332	Not	md	US	2022	0	0	0	0	0	0	0	0	0	0	0	0	
20551391	Not	md	IT	2022	0	0	0	0	0	0	0	0	0	0	0	0	67
20551425	f	md	NO	2022	0	0	0	0	0	0	0	0	0	0	0	0	57
20551466	m	md	JP	2022	0	1	1	0	0	0	0	0	0	0	0	0	44
20551580	m	md	DE	2022	0	1	0	0	1	0	0	0	0	0	0	0	59
20551603	m	md	JP	2022	0	1	0	0	1	0	0	0	0	0	0	0	80
20551768	m	hp	IL	2022	0	0	0	0	0	0	0	0	0	0	0	0	54
20551837	Not	md	JP	2022	0	1	0	0	1	0	0	0	0	0	0	1	71
20552302	f	cn	IN	2022	0	0	0	0	0	0	0	0	0	0	0	0	49
20552308	f	cn	IN	2022	0	0	0	0	0	0	0	0	0	0	0	0	58
20552309	m	cn	IN	2022	0	0	0	0	0	0	0	0	0	0	0	0	
20552732	f	md	DE	2022	0	1	1	0	0	0	0	0	0	0	0	0	54
20552749	m	md	JP	2022	0	1	0	0	1	0	0	0	0	0	0	0	72
20552787	f	md	US	2022	1	0	0	0	0	0	0	0	0	0	0	0	
20552857	f	ph	JP	2022	1	0	0	0	0	0	0	0	0	0	0	0	73
20553155	m	ph	FR	2022	0	1	1	0	0	0	0	0	0	0	0	0	
20553216	f	cn	IN	2022	0	0	0	0	0	0	0	0	0	0	0	0	53
20553217	f	cn	IN	2022	0	0	0	0	0	0	0	0	0	0	0	0	67
20553528	Not	md	US	2022	0	0	0	0	0	0	0	0	0	0	0	0	
20553700	f	ph	MY	2022	1	0	0	0	0	0	0	0	0	0	0	0	65
20553768	m	md	RO	2022	0	0	0	0	0	0	0	0	0	0	0	0	51
20553972	m	md	JP	2022	0	1	0	0	1	0	0	0	0	0	0	0	
20554186	f	hp	US	2022	0	1	1	0	0	0	0	0	0	0	0	0	82
20554263	m	cn	VN	2022	0	1	0	1	0	0	0	0	0	0	0	0	
20554265	m	md	FR	2022	0	0	0	0	0	0	0	0	0	0	0	0	
20554334	m	md	JP	2022	0	1	0	0	1	0	0	0	0	0	0	0	70
20554348	m	cn	VN	2022	0	1	0	1	0	0	0	0	0	0	0	0	
20554459	m	md	DE	2022	0	0	0	0	0	0	0	0	0	0	0	0	78
20554469	Not	md	IT	2022	0	0	0	0	0	0	0	0	0	0	0	0	
20554489	m	md	ES	2022	0	0	0	0	0	0	0	0	0	0	0	0	74
20554696	m	md	CN	2022	0	0	0	0	0	0	0	0	0	0	0	0	69
20554950	m	hp	US	2022	0	1	0	0	1	0	0	0	0	0	0	0	75
20555009	m	md	DE	2022	0	1	1	0	0	0	0	0	0	0	0	0	68

20555033	Not	cn	CN	2022	0	0	0	0	0	0	0	0	0	0	0	0		
20556186	m	md	JP	2022	0	1	0	0	0	1	0	0	0	0	0	0	55	
20556304	m	md	CH	2022	0	1	0	0	0	1	0	0	0	0	0	0	74	
20556831	Not	hp	JP	2022	1	0	0	0	0	0	0	0	0	0	0	0		
20556832	f	md	FR	2022	0	0	0	0	0	0	0	0	0	0	0	0		
20557483	m	hp	GB	2022	0	0	0	0	0	0	1	0	0	0	1	0	42	
20557491	f	md	PE	2022	0	1	0	0	0	1	0	0	0	0	0	0	59	
20557603	f	md	JP	2022	0	1	0	0	0	1	0	0	0	0	0	0	70	
20557621	Not	md	JP	2022	0	0	0	0	0	0	0	0	0	0	0	0	80	
20557665	f	md	FI	2022	0	0	0	0	0	0	0	0	0	0	0	0	81	
20557676	m	cn	CN	2022	0	0	0	0	0	0	0	0	0	0	0	0	45	
20558522	m	cn	CN	2022	0	1	0	0	1	0	0	0	0	0	0	0	82	
20558874	m	cn	IN	2022	0	0	0	0	0	0	0	0	0	0	0	0	70	
20558893	f	ph	NL	2022	1	0	0	0	0	0	0	0	0	0	0	0	86	
20559016	m	md	FR	2022	0	1	1	0	0	0	0	0	0	0	0	0	59	
20559647	f	md	JP	2022	0	0	0	0	0	0	0	0	0	0	0	0	75	
20559756	m	cn	IN	2022	0	0	0	0	0	0	0	0	0	0	0	0	57	
20559889	f	md	DE	2022	0	1	0	0	1	0	0	0	0	0	0	0	83	
20560746	m	hp	JP	2022	0	1	0	0	1	0	0	0	0	0	0	0		
20560945	f	md	BE	2022	0	0	0	0	0	0	0	0	0	0	0	0	77	
20560977	f	md	BR	2022	0	0	0	0	0	0	0	0	0	0	0	0		
20561285	m	md	GB	2022	0	1	0	0	0	1	0	0	0	0	0	0	78	
20561394	m	md	FR	2022	0	0	0	0	0	0	0	0	0	0	0	0		
20561567	m	cn	KR	2022	0	0	0	0	0	0	0	0	0	0	0	0	54	
20562213	f	hp	FR	2022	0	0	0	0	0	0	0	0	0	0	0	0	70	
20562369	m	md	JP	2022	0	1	0	0	1	0	0	0	0	0	0	0	79	
20562503	m	md	JP	2022	0	1	1	0	0	0	0	0	0	0	0	0	70	
20563350	m	hp	JP	2022	1	0	0	0	0	0	0	0	0	0	0	0	71	
20564038	m	md	JP	2022	0	1	0	0	1	0	0	0	0	0	0	0	60	
20564666	f	cn	LB	2022	0	1	0	0	0	1	0	0	0	0	0	0		
20564974	m	ph	FR	2022	0	0	0	0	0	0	0	0	0	0	0	0	77	
20565446	m	md	CN	2022	1	0	0	0	0	0	0	0	0	0	0	0	63	
20565542	Not	ph	JP	2022	1	0	0	0	0	0	0	0	0	0	0	0		
20565560	m	md	JP	2022	0	1	0	0	1	0	0	0	0	0	0	0	74	

20565563	m	md	JP	2022	0	1	0	0	1	0	0	0	0	0	0	0	77
20565570	m	md	JP	2022	0	1	0	0	1	0	0	0	0	0	0	0	80
20565581	m	md	JP	2022	0	1	0	0	1	0	0	0	0	0	0	0	77
20566083	f	hp	JP	2022	0	1	0	0	1	0	0	0	0	0	0	0	65
20566157	m	md	JP	2022	0	1	0	0	1	0	0	0	0	0	0	0	82
20566310	f	md	FR	2022	0	0	0	0	0	0	0	0	0	0	0	0	63
20566389	f	md	JP	2022	1	0	0	0	0	0	0	0	0	0	0	0	
20566777	Not	ph	JP	2022	1	0	0	0	0	0	0	0	0	0	0	0	
20566794	f	md	GR	2022	0	1	1	0	0	0	0	0	0	0	0	0	74
20566853	m	ph	US	2022	0	0	0	0	0	0	0	0	0	0	0	0	47
20566855	f	md	JP	2022	0	1	0	0	1	0	0	0	0	0	0	0	61
20567071	m	md	JP	2022	0	1	0	1	0	0	0	0	0	0	0	1	80
20567363	m	md	FR	2022	0	1	1	0	0	0	0	0	0	0	0	0	63
20567581	m	md	JP	2022	0	1	0	0	1	0	0	0	0	0	0	0	63
20567602	Not	ph	JP	2022	1	0	0	0	0	0	0	0	0	0	0	0	
20567659	f	md	JP	2022	0	1	0	0	1	0	0	0	0	0	0	0	67
20567660	m	md	JP	2022	0	1	0	0	1	0	0	0	0	0	0	0	61
20567690	f	md	JP	2022	0	1	0	0	1	0	0	0	0	0	0	0	
20567735	Not	md	FR	2022	0	0	0	0	0	0	0	0	0	0	0	0	
20567737	Not	md	FR	2022	0	0	0	0	0	0	0	0	0	0	0	0	
20567788	f	cn	US	2022	0	0	0	0	0	0	0	0	0	0	0	0	
20567915	m	md	JP	2022	1	0	0	0	0	0	0	0	0	0	0	1	74
20567925	f	md	JP	2022	1	0	0	0	0	0	0	0	0	0	0	1	
20568099	m	md	DE	2022	0	1	0	0	1	0	0	0	0	0	0	0	70
20568267	m	md	JP	2022	0	1	0	0	1	0	0	0	0	0	0	0	62
20568269	m	md	JP	2022	0	1	0	0	1	0	0	0	0	0	0	1	82
20568271	Not	ph	JP	2022	0	1	0	0	1	0	0	0	0	0	0	0	
20568682	m	cn	RO	2022	0	0	0	0	0	0	0	0	0	0	0	0	51
20568695	m	md	JP	2022	0	1	0	0	1	0	0	0	0	0	0	0	74
20569310	f	md	JP	2022	0	1	0	0	1	0	0	0	0	0	0	0	89
20569524	f	md	DE	2022	0	0	0	0	0	0	0	0	0	0	0	0	68
20569600	m	md	JP	2022	0	1	0	0	1	0	0	0	0	0	0	0	65
20569603	m	md	JP	2022	0	1	0	0	1	0	0	0	0	0	0	0	79
20569831	f	cn	US	2022	0	0	0	0	0	0	0	0	0	0	0	0	

20570158	m	md	FR	2022	0	0	0	0	0	0	0	0	0	0	0	0	
20571037	m	md	GR	2022	0	1	1	0	0	0	0	0	0	0	0	0	55
20571064	f	md	GB	2022	0	1	1	0	0	0	0	0	0	0	0	0	42
20571117	f	cn	TW	2022	0	0	0	0	0	0	0	0	0	0	0	0	69
20571194	f	cn	IN	2022	0	0	0	0	0	0	0	0	0	0	0	0	64
20571327	f	md	JP	2022	0	0	0	0	0	0	0	0	0	0	0	0	82
20571646	m	md	JP	2022	0	1	0	0	1	0	0	0	0	0	0	0	77
20571650	m	md	JP	2022	0	1	0	0	1	0	0	0	0	0	0	0	74
20571652	Not	hp	JP	2022	0	1	0	0	1	0	0	0	0	0	0	0	67
20571656	m	md	JP	2022	0	1	0	0	1	0	0	0	0	0	0	0	81
20571659	m	md	JP	2022	0	1	0	0	1	0	0	0	0	0	0	0	59
20571675	f	md	JP	2022	0	1	0	0	1	0	0	0	0	0	0	1	67
20571783	m	md	DE	2022	0	1	0	0	1	0	0	0	0	0	0	0	66
20572481	m	md	JP	2022	0	1	1	0	0	0	0	0	0	0	0	0	72
20572721	f	hp	IL	2022	1	0	0	0	0	0	0	0	0	0	0	0	74
20572916	f	md	DE	2022	0	0	0	0	0	0	0	0	0	0	0	0	71
20572993	m	md	CN	2022	0	0	0	0	0	0	0	0	0	0	0	0	57
20573054	m	md	CO	2022	1	0	0	0	0	0	0	0	0	0	0	0	71
20573538	f	hp	IL	2022	0	0	0	0	0	0	1	1	0	0	0	0	60
20573540	f	hp	IL	2022	0	0	0	0	0	0	1	0	1	0	0	0	70
20573541	m	md	JP	2022	0	1	0	1	0	0	0	0	0	0	0	1	71
20573593	m	md	CZ	2022	0	1	0	0	1	0	0	0	0	0	0	0	58
20573702	f	hp	IL	2022	1	0	0	0	0	0	0	0	0	0	0	0	66
20573793	Not	md	GB	2022	0	0	0	0	0	0	0	0	0	0	0	0	71
20573977	f	hp	JP	2022	0	1	0	0	1	0	0	0	0	0	0	0	60
20574056	m	hp	CN	2022	0	0	0	0	0	0	0	0	0	0	0	0	68
20574151	f	md	DE	2022	0	1	1	0	0	0	0	0	0	0	0	0	60
20574302	f	md	JP	2022	0	1	0	0	1	0	0	0	0	0	0	0	69
20574493	Not	cn	CN	2022	0	0	0	0	0	0	0	0	0	0	0	0	
20574848	f	ph	US	2022	0	0	0	0	0	0	0	0	0	0	0	0	62
20574849	f	md	JP	2022	0	0	0	0	0	0	0	0	0	0	0	0	53
20574899	f	md	US	2022	0	0	0	0	0	0	0	0	0	0	0	0	73
20575260	m	md	IT	2022	0	1	0	0	1	0	0	0	0	0	0	0	
20575659	m	md	JP	2022	0	1	0	0	1	0	0	0	0	0	0	0	69

20576027	m	md	US	2022	0	0	0	0	0	0	0	0	0	0	0	0	53
20576145	m	hp	US	2022	0	1	1	0	0	0	0	0	0	0	0	0	62
20576236	m	hp	US	2022	0	1	1	0	0	0	0	0	0	0	0	0	81
20576709	Not	md	CO	2022	0	1	0	0	1	0	0	0	0	0	0	0	
20577336	f	cn	GR	2022	0	0	0	0	0	0	0	0	0	0	0	0	64
20577383	f	md	GB	2022	0	1	1	0	0	0	0	0	0	0	0	0	42
20577404	Not	md	US	2022	1	0	0	0	0	0	0	0	0	0	0	0	
20577450	m	md	GB	2022	0	1	0	0	0	1	0	0	0	0	0	0	78
20577482	f	hp	US	2022	0	1	1	0	0	0	0	0	0	0	0	0	84
20577541	f	md	JP	2022	0	1	0	0	1	0	0	0	0	0	0	1	77
20577544	m	md	JP	2022	0	1	0	0	1	0	0	0	0	0	0	0	53
20577546	m	md	JP	2022	0	1	0	0	1	0	0	0	0	0	0	0	64
20577662	f	hp	US	2022	0	1	1	0	0	0	0	0	0	0	0	0	69
20578145	Not	cn	Not re	2022	0	1	0	0	1	0	0	0	0	0	0	0	
20578289	m	md	JP	2022	0	1	0	0	1	0	0	0	0	0	0	0	69
20578566	m	md	JP	2022	0	1	1	0	0	0	0	0	0	0	0	0	62
20578809	m	md	JP	2022	0	1	0	0	1	0	0	0	0	0	0	0	65
20578857	m	md	JP	2022	0	1	0	0	1	0	0	0	0	0	0	0	63
20578928	f	md	GB	2022	0	1	1	0	0	0	0	0	0	0	0	0	42
20579017	m	md	FR	2022	0	0	0	0	0	0	0	0	0	0	0	0	74
20579018	Not	md	FR	2022	0	0	0	0	0	0	0	0	0	0	0	0	
20579063	m	md	AU	2022	0	0	0	0	0	0	0	0	0	0	0	0	76
20579204	m	md	JP	2022	0	1	0	0	1	0	0	0	0	0	0	0	72
20579225	f	cn	CN	2022	0	1	0	0	1	0	0	0	0	0	0	0	74
20579230	m	md	JP	2022	0	1	0	0	1	0	0	0	0	0	0	0	72
20579361	f	md	JP	2022	0	1	1	0	0	0	0	0	0	0	0	1	73
20579474	m	md	GB	2022	0	1	0	0	0	1	0	0	0	0	0	0	78
20579565	f	md	JP	2022	0	1	0	0	1	0	0	0	0	0	0	0	66
20579583	f	md	JP	2022	0	1	0	0	1	0	0	0	0	0	0	0	71
20579899	f	md	ES	2022	0	1	0	0	0	1	0	0	0	0	0	0	69
20580036	Not	cn	CN	2022	0	0	0	0	0	0	0	0	0	0	0	0	
20580069	m	md	JP	2022	0	0	0	0	0	0	0	0	0	0	0	0	76
20580081	Not	md	JP	2022	0	1	0	0	1	0	0	0	0	0	0	0	
20580126	Not	md	JP	2022	1	0	0	0	0	0	0	0	0	0	0	0	

20580158	Not	md	JP	2022	1	0	0	0	0	0	0	0	0	0	0	0	
20580236	Not	md	JP	2022	0	1	0	0	1	0	0	0	0	0	0	0	
20580393	m	md	CN	2022	0	1	0	0	0	1	0	0	0	0	0	0	56
20580513	Not	md	JP	2022	1	0	0	0	0	0	0	0	0	0	0	0	
20580738	m	md	JP	2022	0	1	0	0	1	0	0	0	0	0	0	1	
20580833	Not	md	JP	2022	1	0	0	0	0	0	0	0	0	0	0	0	
20580901	Not	md	JP	2022	1	0	0	0	0	0	0	0	0	0	0	0	
20581086	f	md	JP	2022	0	0	0	0	0	0	0	0	0	0	0	0	80
20581330	m	ph	JP	2022	0	1	0	0	1	0	0	0	0	0	0	0	60
20581476	Not	md	JP	2022	1	0	0	0	0	0	0	0	0	0	0	0	
20581684	m	cn	US	2022	0	1	1	0	0	0	0	0	0	0	0	0	
20581861	m	md	RO	2022	0	1	0	1	0	0	0	0	0	0	0	0	
20581862	f	cn	CN	2022	0	1	0	1	0	0	0	0	0	0	0	0	52
20581999	m	cn	US	2022	1	0	0	0	0	0	0	0	0	0	0	0	
20582112	m	md	JP	2022	0	0	0	0	0	0	0	0	0	0	0	0	73
20582175	f	md	DE	2022	0	1	0	0	1	0	0	0	0	0	0	0	57
20582183	Not	md	JP	2022	0	1	0	0	1	0	0	0	0	0	0	0	
20582305	m	cn	CN	2022	0	0	0	0	0	0	0	0	0	0	0	0	62
20582542	f	md	GB	2022	0	1	1	0	0	0	0	0	0	0	0	0	42
20582899	m	md	JP	2022	1	0	0	0	0	0	0	0	0	0	0	0	
20582914	f	Not repo	Not re	2022	0	0	0	0	0	0	0	0	0	0	0	0	51
20582982	m	md	JP	2022	0	1	0	0	1	0	0	0	0	0	0	0	68
20582999	f	cn	CN	2022	1	0	0	0	0	0	0	0	0	0	0	0	78
20583203	m	md	CA	2022	1	0	0	0	0	0	0	0	0	0	0	0	
20583459	m	md	JP	2022	0	1	0	0	1	0	0	0	0	0	0	0	58
20583471	f	md	RO	2022	1	0	0	0	0	0	0	0	0	0	0	0	
20583494	m	md	JP	2022	0	1	0	0	1	0	0	0	0	0	0	0	72
20583521	f	md	JP	2022	0	1	0	0	1	0	0	0	0	0	0	0	68
20583523	f	md	JP	2022	0	1	0	0	1	0	0	0	0	0	0	0	72
20583565	m	md	SA	2022	0	0	0	0	0	0	0	0	0	0	0	0	64
20584166	m	hp	JP	2022	0	1	0	0	0	1	0	0	0	0	0	0	72
20584180	m	md	JP	2022	0	1	0	0	1	0	0	0	0	0	0	0	53
20584181	m	hp	CH	2022	0	1	0	0	1	0	0	0	0	0	0	0	73
20584372	f	md	KR	2022	0	0	0	0	0	0	0	0	0	0	0	0	79

20584502	f	md	JP	2022	0	0	0	0	0	0	0	0	0	0	0	0	53
20584532	f	md	PT	2022	0	1	1	0	0	0	0	0	0	0	0	0	57
20584554	Not	md	JP	2022	0	1	0	0	1	0	0	0	0	0	0	0	80
20584555	m	md	JP	2022	0	1	0	0	1	0	0	0	0	0	0	0	67
20584595	Not	md	BE	2022	0	1	0	0	1	0	0	0	0	0	0	0	80
20584886	Not	hp	US	2022	0	0	0	0	0	0	0	0	0	0	0	0	
20585014	m	cn	MY	2022	0	0	0	0	0	0	0	0	0	0	0	0	78
20585084	f	md	CA	2022	1	0	0	0	0	0	0	0	0	0	0	0	
20585151	m	md	JP	2022	0	1	0	0	1	0	0	0	0	0	0	0	70
20585153	m	md	JP	2022	0	1	0	0	1	0	0	0	0	0	0	0	71
20585616	m	md	JP	2022	0	1	0	0	1	0	0	0	0	0	0	0	69
20585731	m	md	JP	2022	0	1	0	0	1	0	0	0	0	0	0	0	67
20585791	Not	md	GB	2022	0	0	0	0	0	0	0	0	0	0	0	0	
20585899	Not	hp	US	2022	0	0	0	0	0	0	0	0	0	0	0	0	
20586048	Not	md	GB	2022	0	0	0	0	0	0	0	0	0	0	0	0	77
20586262	f	md	JP	2022	0	1	0	0	1	0	0	0	0	0	0	0	73
20586728	m	md	JP	2022	0	1	0	0	1	0	0	0	0	0	0	0	58
20586749	m	md	JP	2022	0	1	0	0	1	0	0	0	0	0	0	0	80
20586793	Not	md	JP	2022	0	1	0	0	1	0	0	0	0	0	0	1	
20586829	m	cn	CN	2022	0	1	0	0	1	0	0	0	0	0	0	0	71
20586830	m	cn	CN	2022	0	1	0	0	1	0	0	0	0	0	0	0	64
20586854	f	md	JP	2022	0	1	0	0	1	0	0	0	0	0	0	0	
20586882	m	md	JP	2022	0	0	0	0	0	0	0	0	0	0	0	0	50
20586962	m	cn	CN	2022	0	1	0	0	1	0	0	0	0	0	0	0	66
20587187	f	cn	US	2022	1	0	0	0	0	0	0	0	0	0	0	0	
20587268	m	md	JP	2022	0	1	0	0	1	0	0	0	0	0	0	0	70
20587296	m	md	JP	2022	0	1	0	0	1	0	0	0	0	0	0	0	70
20587495	f	ph	US	2022	0	0	0	0	0	0	0	0	0	0	0	0	
20587581	m	md	JP	2022	0	1	0	0	1	0	0	0	0	0	0	0	69
20587740	f	hp	TR	2022	1	0	0	0	0	0	0	0	0	0	0	0	70
20587742	Not	md	JP	2022	0	1	0	0	1	0	0	0	0	0	0	0	
20587764	m	cn	CN	2022	0	1	0	0	1	0	0	0	0	0	0	0	65
20587805	m	md	JP	2022	0	1	0	0	1	0	0	0	0	0	0	0	58
20588716	Not	ph	US	2022	0	1	1	0	0	0	0	0	0	0	0	0	

20588740	f	md	CN	2022	0	0	0	0	0	0	0	0	0	0	0	0	53
20589088	m	md	JP	2022	0	1	0	0	1	0	0	0	0	0	0	0	48
20589164	f	md	GB	2022	0	1	1	0	0	0	0	0	0	0	0	0	42
20589257	m	md	JP	2022	0	1	0	0	1	0	0	0	0	0	0	0	79
20589328	m	md	JP	2022	0	1	0	0	1	0	0	0	0	0	0	0	68
20589472	m	hp	NL	2022	0	0	0	0	0	0	0	0	0	0	0	0	51
20589862	f	md	CH	2022	0	1	1	0	0	0	0	0	0	0	0	0	66
20589894	m	md	DE	2022	0	1	1	0	0	0	0	0	0	0	0	1	64
20590022	m	cn	US	2022	0	1	1	0	0	0	0	0	0	0	0	0	
20590129	m	cn	ID	2022	1	0	0	0	0	0	0	0	0	0	0	0	
20590155	f	md	KR	2022	0	0	0	0	0	0	0	0	0	0	0	0	
20590413	Not	ph	JP	2022	0	1	0	0	1	0	0	0	0	0	0	0	
20590570	m	cn	KR	2022	0	0	0	0	0	0	0	0	0	0	0	0	71
20590627	m	cn	JP	2022	0	0	0	0	0	0	0	0	0	0	0	0	86
20590715	m	md	CA	2022	1	0	0	0	0	0	0	0	0	0	0	0	90
20591004	f	md	CN	2022	0	1	1	0	0	0	0	0	0	0	0	0	72
20591225	m	cn	US	2022	1	0	0	0	0	0	0	0	0	0	0	0	
20591228	f	cn	ID	2022	1	0	0	0	0	0	0	0	0	0	0	0	59.7
20591373	f	cn	CA	2022	1	0	0	0	0	0	0	0	0	0	0	0	75.6
20591375	f	md	JP	2022	1	0	0	0	0	0	0	0	0	0	0	0	63
20591382	m	hp	US	2022	0	0	0	0	0	0	0	0	0	0	0	0	63
20591430	m	md	JP	2022	0	1	0	1	0	0	0	0	0	0	0	1	69
20591518	m	md	CH	2022	0	1	0	0	0	1	0	0	0	0	0	0	69
20591605	m	md	FR	2022	0	0	0	0	0	0	0	0	0	0	0	0	54
20591742	m	ph	JP	2022	0	1	0	0	1	0	0	0	0	0	0	0	70
20591765	m	md	JP	2022	0	1	0	0	1	0	0	0	0	0	0	0	71
20591779	m	cn	IN	2022	0	1	0	0	1	0	0	0	0	0	0	0	48
20591781	m	cn	IN	2022	0	1	0	0	1	0	0	0	0	0	0	0	64
20592096	f	md	GB	2022	0	0	0	0	0	0	0	0	0	0	0	0	68
20592504	m	cn	US	2022	0	0	0	0	0	0	0	0	0	0	0	0	81
20592970	m	md	JP	2022	0	1	0	0	1	0	0	0	0	0	0	0	61
20593108	Not	md	CL	2022	0	1	0	0	0	1	0	0	0	0	0	0	
20593175	m	md	JP	2022	0	1	0	0	1	0	0	0	0	0	0	0	79
20593953	m	md	JP	2022	0	1	0	0	1	0	0	0	0	0	0	0	73

20594027	m	md	JP	2022	0	1	0	0	1	0	0	0	0	0	0	0	73
20594136	Not	md	JP	2022	0	1	1	0	0	0	0	0	0	0	0	0	50
20594217	f	cn	US	2022	1	0	0	0	0	0	0	0	0	0	0	0	67
20594815	m	md	JP	2022	0	1	0	0	1	0	0	0	0	0	0	0	78
20594897	m	md	FR	2022	0	0	0	0	0	0	0	0	0	0	0	0	
20595249	f	md	FR	2022	0	0	0	0	0	0	0	0	0	0	0	0	59
20595251	Not	md	GB	2022	0	0	0	0	0	0	0	0	0	0	0	0	73
20595252	Not	md	GB	2022	0	0	0	0	0	0	0	0	0	0	0	0	64
20595253	m	md	FR	2022	0	0	0	0	0	0	0	0	0	0	0	0	56
20595657	m	md	JP	2022	0	1	0	0	0	1	0	0	0	0	0	0	90
20595772	f	hp	US	2022	0	0	0	0	0	0	0	0	0	0	0	0	74
20595806	Not	md	JP	2022	0	0	0	0	0	0	0	0	0	0	0	0	50
20596076	f	md	CH	2022	0	0	0	0	0	0	0	0	0	0	0	0	
20596780	f	md	US	2022	0	0	0	0	0	0	0	0	0	0	0	0	75
20596835	m	cn	GR	2022	0	1	1	0	0	0	0	0	0	0	0	0	61
20596994	Not	md	JP	2022	0	1	0	0	1	0	0	0	0	0	0	0	
20597175	f	md	JP	2022	0	1	0	1	0	0	0	0	0	0	0	1	56
20597553	Not	md	FR	2022	1	0	0	0	0	0	0	0	0	0	0	0	
20597733	m	md	JP	2022	0	1	0	0	1	0	0	0	0	0	0	0	77
20597806	f	md	JP	2022	1	0	0	0	0	0	0	0	0	0	0	0	81
20597904	m	md	JP	2022	0	1	0	0	1	0	0	0	0	0	0	0	69
20597994	m	cn	US	2022	0	0	0	0	0	0	0	0	0	0	0	0	43
20598203	m	md	JP	2022	0	1	0	0	0	1	0	0	0	0	0	0	70
20598280	f	cn	US	2022	0	0	0	0	0	0	0	0	0	0	0	0	
20598788	m	hp	FR	2022	0	1	0	0	1	0	0	0	0	0	0	0	75
20598937	m	md	NL	2022	0	1	0	0	0	1	0	0	0	0	0	0	76
20599349	f	hp	ES	2022	0	1	0	0	1	0	0	0	0	0	0	0	63
20599368	f	md	DE	2022	0	1	0	0	1	0	0	0	0	0	0	0	85
20599432	m	md	JP	2022	0	1	0	0	0	1	0	0	0	0	0	0	63
20599433	f	md	SG	2022	0	1	0	0	0	1	0	0	0	0	0	0	46
20600288	m	md	JP	2022	0	1	0	0	1	0	0	0	0	0	0	0	73
20600307	m	md	JP	2022	0	1	0	0	1	0	0	0	0	0	0	0	79
20600309	m	md	JP	2022	0	1	0	0	1	0	0	0	0	0	0	1	78
20600739	f	md	US	2022	0	1	0	0	0	1	0	0	0	0	0	0	56

20601033	f	md	CN	2022	1	0	0	0	0	0	0	0	0	0	0	0	48
20601049	f	Not repo	TR	2022	1	0	0	0	0	0	0	0	0	0	0	0	70
20601139	f	md	GB	2022	0	1	0	0	0	1	0	0	0	0	0	0	54
20601211	m	md	US	2022	0	0	0	0	0	0	0	0	0	0	0	0	
20601493	m	hp	NL	2022	0	1	0	0	1	0	0	0	0	0	0	0	74
20601810	f	ph	DE	2022	0	1	1	0	0	0	0	0	0	0	0	0	
20602071	f	md	FR	2022	0	0	0	0	0	0	0	0	0	0	0	0	
20602193	m	md	CA	2022	1	0	0	0	0	0	0	0	0	0	0	0	
20602197	m	ph	JP	2022	0	1	1	0	0	0	0	0	0	0	0	0	76
20602257	m	md	JP	2022	0	1	0	0	0	1	0	0	0	0	0	0	
20602481	f	md	KR	2022	0	0	0	0	0	0	0	0	0	0	0	0	82
20602514	m	cn	CN	2022	0	0	0	0	0	0	0	0	0	0	0	0	70
20602654	f	md	CH	2022	0	0	0	0	0	0	0	0	0	0	0	0	54
20602971	f	md	FR	2022	1	0	0	0	0	0	0	0	0	0	0	0	85
20603093	m	md	JP	2022	0	1	0	0	0	1	0	0	0	0	0	0	70
20603134	f	cn	IN	2022	0	0	0	0	0	0	0	0	0	0	0	0	61
20603208	m	md	US	2022	0	1	0	1	0	0	0	0	0	0	0	0	76
20603289	Not	md	ES	2022	0	0	0	0	0	0	0	0	0	0	0	0	67
20603378	f	md	DK	2022	0	0	0	0	0	0	0	0	0	0	0	0	71
20603402	Not	md	ES	2022	0	0	0	0	0	0	0	0	0	0	0	0	53
20603449	f	Not repo	US	2022	0	0	0	0	0	0	0	0	0	0	0	0	
20603459	Not	cn	US	2022	1	0	0	0	0	0	0	0	0	0	0	0	
20603460	m	md	TR	2022	0	0	0	0	0	0	0	0	0	0	0	0	65
20603533	m	md	JP	2022	0	1	0	0	0	1	0	0	0	0	0	0	
20603617	Not	md	JP	2022	0	0	0	0	0	0	0	0	0	0	0	0	
20603758	Not	md	JP	2022	1	0	0	0	0	0	0	0	0	0	0	0	
20604593	m	cn	CN	2022	0	1	0	1	0	0	0	0	0	0	0	0	66
20604683	f	md	DK	2022	1	0	0	0	0	0	0	0	0	0	0	0	
20604871	Not	md	JP	2022	1	0	0	0	0	0	0	0	0	0	0	0	
20604888	f	md	IT	2022	0	0	0	0	0	0	0	0	0	0	0	0	73
20605203	m	ph	JP	2022	0	0	0	0	0	0	0	0	0	0	0	0	49
20605783	m	md	IT	2022	0	0	0	0	0	0	0	0	0	0	0	0	
20605803	m	md	JP	2022	0	0	0	0	0	0	0	0	0	0	0	0	57
20605836	Not	md	JP	2022	0	1	1	0	0	0	0	0	0	0	0	0	80

20605950	Not	hp	GB	2022	0	1	0	0	0	1	0	0	0	0	0	0	
20606339	f	md	IT	2022	0	0	0	0	0	0	0	0	0	0	0	0	82
20606975	f	cn	US	2022	0	0	0	0	0	0	0	0	0	0	0	0	
20606983	m	cn	IT	2022	0	0	0	0	0	0	0	0	0	0	0	0	
20607026	m	md	JP	2022	0	1	0	0	0	1	0	0	0	0	0	0	45
20607245	Not	ph	JP	2022	1	0	0	0	0	0	0	0	0	0	0	0	
20607334	m	md	FR	2022	0	1	0	0	0	1	0	0	0	0	0	0	48
20607421	Not	ph	TH	2022	0	0	0	0	0	0	0	0	0	0	0	0	
20607435	Not	ph	TH	2022	0	0	0	0	0	0	0	0	0	0	0	0	
20607665	f	md	DE	2022	0	0	0	0	0	0	0	0	0	0	0	0	
20607696	f	cn	JP	2022	1	0	0	0	0	0	0	0	0	0	0	0	
20608227	m	md	TH	2022	0	0	0	0	0	0	0	0	0	0	0	0	57
20608493	m	md	LT	2022	0	1	0	0	0	1	0	0	0	0	0	0	64
20608498	f	md	CH	2022	0	0	0	0	0	0	0	0	0	0	0	0	67
20608563	m	md	JP	2022	0	1	0	1	0	0	0	0	0	0	0	1	66
20608567	Not	cn	SG	2022	1	0	0	0	0	0	0	0	0	0	0	0	
20609073	f	md	BR	2022	0	0	0	0	0	0	0	0	0	0	0	0	76
20609095	f	cn	GB	2022	0	1	1	0	0	0	0	0	0	0	0	0	42
20609102	m	md	JP	2022	1	0	0	0	0	0	0	0	0	0	0	0	
20609186	m	md	KR	2022	0	1	1	0	0	0	0	0	0	0	0	0	60
20609192	f	md	KR	2022	0	1	1	0	0	0	0	0	0	0	0	0	54
20609344	m	md	IT	2022	0	0	0	0	0	0	0	0	0	0	0	0	63
20610321	m	md	JP	2022	0	1	0	1	0	0	0	0	0	0	0	0	
20611213	Not	md	FR	2022	1	0	0	0	0	0	0	0	0	0	0	0	
20611418	m	cn	GB	2022	1	0	0	0	0	0	0	0	0	0	0	0	70
20611681	Not	md	FR	2022	1	0	0	0	0	0	0	0	0	0	0	0	
20611682	Not	md	JP	2022	1	0	0	0	0	0	0	0	0	0	0	0	
20611877	m	cn	CN	2022	0	1	0	1	0	0	0	0	0	0	0	0	63
20612508	f	md	CH	2022	0	0	0	0	0	0	0	0	0	0	0	0	74
20612903	m	md	PA	2022	0	0	0	0	0	0	0	0	0	0	0	0	
20613357	m	md	LT	2022	0	1	0	0	0	1	0	0	0	0	0	0	64
20613486	Not	md	JP	2022	0	0	0	0	0	0	0	0	0	0	0	0	59
20613751	Not	md	JP	2022	0	1	0	0	0	1	0	0	0	0	0	0	80
20613774	f	md	GB	2022	0	1	1	0	0	0	0	0	0	0	0	0	42

20614537	Not	hp	JP	2022	1	0	0	0	0	0	0	0	0	0	0	0	
20614583	f	ph	US	2022	0	0	0	0	0	0	0	0	0	0	0	0	
20614832	Not	cn	CN	2022	0	0	0	0	0	0	0	0	0	0	0	0	
20614919	m	cn	PH	2022	0	1	0	0	0	1	0	0	0	0	0	0	61
20615444	f	md	JP	2022	0	0	0	0	0	0	0	0	0	0	0	0	50
20615568	f	md	CL	2022	0	1	0	0	0	1	0	0	0	0	0	0	38
20615571	m	md	KR	2022	0	0	0	0	0	0	0	0	0	0	0	0	73
20615634	m	hp	TW	2022	0	1	1	0	0	0	0	0	0	0	0	0	72
20616004	m	md	KR	2022	0	0	0	0	0	0	0	0	0	0	0	0	
20616032	m	md	DE	2022	0	1	1	0	0	0	0	0	0	0	0	1	64
20616845	m	md	JP	2022	0	1	1	0	0	0	0	0	0	0	0	0	77
20617061	m	md	JP	2022	0	1	0	0	0	1	0	0	0	0	0	0	70
20617546	Not	md	JP	2022	0	0	0	0	0	0	0	0	0	0	0	0	
20617560	Not	md	JP	2022	0	0	0	0	0	0	0	0	0	0	0	0	
20617658	Not	md	TW	2022	0	0	0	0	0	0	0	0	0	0	0	0	
20617752	f	cn	TH	2022	1	0	0	0	0	0	0	0	0	0	0	0	46.5
20617790	m	md	JP	2022	0	1	0	0	0	1	0	0	0	0	0	0	70
20617991	Not	md	ES	2022	0	0	0	0	0	0	0	0	0	0	0	0	59
20618288	f	md	AR	2022	0	1	0	0	0	1	0	0	0	0	0	0	40
20618464	Not	cn	US	2022	1	0	0	0	0	0	0	0	0	0	0	0	
20618534	m	md	JP	2022	0	1	0	0	1	0	0	0	0	0	0	0	77
20618681	f	md	US	2022	0	0	0	0	0	0	0	0	0	0	0	0	77
20618758	Not	cn	US	2022	0	0	0	0	0	0	0	0	0	0	0	0	
20618840	m	md	IT	2022	0	0	0	0	0	0	0	0	0	0	0	0	71
20620012	f	md	JP	2022	0	0	0	0	0	0	0	0	0	0	0	0	
20620809	m	md	JP	2022	0	1	0	0	1	0	0	0	0	0	0	0	79
20621355	f	hp	AU	2022	0	0	0	0	0	0	0	0	0	0	0	0	78
20621699	Not	md	JP	2022	0	1	0	1	0	0	0	0	0	0	0	0	
20621864	m	hp	CA	2022	0	0	0	0	0	0	0	0	0	0	0	0	59
20622899	m	md	AU	2022	0	0	0	0	0	0	0	0	0	0	0	0	
20623031	m	ph	CA	2022	0	1	0	1	0	0	0	0	0	0	0	0	
20623724	f	md	KR	2022	0	0	0	0	0	0	0	0	0	0	0	0	56
20623750	Not	md	JP	2022	0	1	1	0	0	0	0	0	0	0	0	0	80
20623770	m	md	JP	2022	0	1	1	0	0	0	0	0	0	0	0	0	73

20623946	m	md	JP	2022	0	1	0	0	1	0	0	0	0	0	0	1	
20624036	m	md	JP	2022	0	1	1	0	0	0	0	0	0	0	0	0	71
20624456	f	md	JP	2022	0	1	0	0	1	0	0	0	0	0	0	0	63
20624745	m	md	FR	2022	0	1	0	1	0	0	0	0	0	0	0	0	
20625295	m	md	JP	2022	0	1	0	0	0	1	0	0	0	0	0	0	60
20625836	m	md	FR	2022	0	0	0	0	0	0	0	0	0	0	0	0	
20626353	m	hp	GB	2022	0	1	1	0	0	0	0	0	0	0	0	0	74
20626355	f	hp	GB	2022	0	1	1	0	0	0	0	0	0	0	0	0	74
20626367	f	hp	GB	2022	0	1	1	0	0	0	0	0	0	0	0	0	65
20626657	m	md	US	2022	0	1	0	0	0	1	0	0	0	0	0	0	56
20626914	f	md	DE	2022	0	1	0	0	1	0	0	0	0	0	0	0	53
20626923	m	cn	JP	2022	0	0	0	0	0	0	0	0	0	0	0	0	45
20627373	m	md	KR	2022	0	0	0	0	0	0	0	0	0	0	0	0	
20627481	m	cn	PH	2022	0	1	0	0	0	1	0	0	0	0	0	0	66
20627728	m	md	JP	2022	0	1	0	0	0	1	0	0	0	0	0	0	90
20628095	f	cn	IN	2022	0	0	0	0	0	0	0	0	0	0	0	0	50
20628275	Not	md	US	2022	0	0	0	0	0	0	0	0	0	0	0	0	
20628408	m	md	IE	2022	0	0	0	0	0	0	0	0	0	0	0	0	73
20628506	f	cn	IN	2022	0	0	0	0	0	0	0	0	0	0	0	0	60
20628801	f	cn	US	2022	1	0	0	0	0	0	0	0	0	0	0	0	46
20628840	f	md	NL	2022	0	1	0	1	0	0	0	0	0	0	0	0	53
20628980	f	ph	JP	2022	1	0	0	0	0	0	0	0	0	0	0	0	72
20629094	Not	md	JP	2022	1	0	0	0	0	0	0	0	0	0	0	0	
20629218	f	cn	MY	2022	0	0	0	0	0	0	0	0	0	0	0	0	43
20629408	m	md	JP	2022	0	0	0	0	0	0	0	0	0	0	0	0	
20629593	m	md	JP	2022	0	0	0	0	0	0	0	0	0	0	0	0	73
20629642	m	md	JP	2022	1	0	0	0	0	0	0	0	0	0	0	0	74
20629721	m	hp	RU	2022	0	1	0	1	0	0	0	0	0	0	0	0	
20629796	m	md	CH	2022	0	0	0	0	0	0	0	0	0	0	0	0	68
20630135	m	md	FR	2022	0	0	0	0	0	0	0	0	0	0	0	0	75
20630407	m	cn	CN	2022	0	1	0	1	0	0	0	0	0	0	0	0	59
20630605	m	md	JP	2022	0	0	0	0	0	0	0	0	0	0	0	0	76
20632202	m	cn	IN	2022	0	0	0	0	0	0	0	0	0	0	0	0	71
20632259	m	cn	PK	2022	0	1	0	0	0	1	0	0	0	0	0	0	65

20632640	f	md	DE	2022	0	1	0	0	1	0	0	0	0	0	0	0	85
20633134	m	hp	US	2022	0	0	0	0	0	0	0	0	0	0	0	0	63
20633207	m	md	JP	2022	0	0	0	0	0	0	0	0	0	0	0	0	
20633380	f	hp	US	2022	0	0	0	0	0	0	0	0	0	0	0	0	74
20633481	Not	cn	SG	2022	1	0	0	0	0	0	0	0	0	0	0	0	79.5
20633533	f	md	BR	2022	0	0	0	0	0	0	0	0	0	0	0	0	83
20633556	m	md	CH	2022	0	0	0	0	0	0	0	0	0	0	0	0	57
20633605	m	hp	US	2022	1	0	0	0	0	0	0	0	0	0	0	0	87
20633721	m	cn	TH	2022	1	0	0	0	0	0	0	0	0	0	0	0	90.9
20633900	m	md	JP	2022	0	1	0	0	1	0	0	0	0	0	0	0	75
20633902	f	md	JP	2022	0	1	0	0	1	0	0	0	0	0	0	0	68
20633946	m	md	JP	2022	0	1	0	0	1	0	0	0	0	0	0	0	72
20633989	f	cn	US	2022	1	0	0	0	0	0	0	0	0	0	0	0	52
20634033	m	md	JP	2022	0	1	0	0	1	0	0	0	0	0	0	0	73
20634188	m	md	JP	2022	0	1	0	0	1	0	0	0	0	0	0	0	84
20634491	f	md	DE	2022	0	1	1	0	0	0	0	0	0	0	0	0	70
20634610	Not	md	JP	2022	0	1	1	0	0	0	0	0	0	0	0	0	80
20635201	m	md	JP	2022	0	0	0	0	0	0	0	0	0	0	0	0	
20635344	m	md	DE	2022	0	1	1	0	0	0	0	0	0	0	0	0	
20635511	m	md	JP	2022	0	1	0	0	1	0	0	0	0	0	0	0	75
20636701	f	cn	CN	2022	0	0	0	0	0	0	0	0	0	0	0	0	
20637026	m	md	JP	2022	0	1	1	0	0	0	0	0	0	0	0	0	
20637083	m	md	IL	2022	0	1	1	0	0	0	0	0	0	0	0	0	69
20637152	m	md	CH	2022	0	0	0	0	0	0	0	0	0	0	0	0	70
20637231	Not	md	SG	2022	0	0	0	0	0	0	0	0	0	0	0	0	
20637818	f	hp	CA	2022	1	0	0	0	0	0	0	0	0	0	0	0	
20638005	m	cn	ID	2022	1	0	0	0	0	0	0	0	0	0	0	0	71.7
20638253	m	md	JP	2022	0	1	0	0	1	0	0	0	0	0	0	0	77
20638272	m	md	JP	2022	0	1	0	0	1	0	0	0	0	0	0	0	60
20638297	m	md	BG	2022	1	0	0	0	0	0	0	0	0	0	0	0	67
20638398	m	cn	US	2022	1	0	0	0	0	0	0	0	0	0	0	0	80
20638476	m	md	SI	2022	0	0	0	0	0	0	0	0	0	0	0	0	
20638576	m	md	DE	2022	0	1	0	0	1	0	0	0	0	0	0	0	71
20638609	m	md	JP	2022	0	1	0	0	1	0	0	0	0	0	0	0	73

20638620	m	md	CZ	2022	0	1	0	0	1	0	0	0	0	0	0	0	67
20638778	m	cn	TH	2022	1	0	0	0	0	0	0	0	0	0	0	0	79.3
20638818	m	md	DE	2022	0	1	1	0	0	0	0	0	0	0	0	1	64
20638909	m	md	JP	2022	0	0	0	0	0	0	0	0	0	0	0	0	80
20638921	Not	md	JP	2022	0	1	0	0	0	1	0	0	0	0	0	1	
20639222	Not	md	PL	2022	0	0	0	0	0	0	0	0	0	0	0	0	
20639294	f	md	MK	2022	0	0	0	0	0	0	0	0	0	0	0	0	50
20639297	m	hp	CA	2022	0	0	0	0	0	0	0	0	0	0	0	0	53
20639409	m	md	JP	2022	0	1	0	0	1	0	0	0	0	0	0	0	75
20639499	f	hp	US	2022	0	0	0	0	0	0	0	0	0	0	0	0	41
20640054	f	md	FR	2022	0	0	0	0	0	0	0	0	0	0	0	0	70
20641416	f	md	GB	2022	0	0	0	0	0	0	0	0	0	0	0	0	68
20641656	f	cn	US	2022	0	1	0	0	1	0	0	0	0	0	0	0	57
20641738	m	cn	IN	2022	0	1	0	0	1	0	0	0	0	0	0	0	31
20641861	f	md	VN	2022	0	1	0	0	0	1	0	0	0	0	0	0	68
20642415	Not	md	US	2022	0	0	0	0	0	0	0	0	0	0	0	0	
20642443	m	hp	IN	2022	0	1	0	0	1	0	0	0	0	0	0	0	70
20642512	m	cn	IN	2022	0	1	0	0	1	0	0	0	0	0	0	0	
20643381	m	hp	US	2022	0	1	1	0	0	0	0	0	0	0	0	0	
20643854	f	md	GB	2022	0	0	0	0	0	0	0	0	0	0	0	0	
20643966	m	md	CH	2022	0	0	0	0	0	0	0	0	0	0	0	0	
20643991	f	hp	AT	2022	0	0	0	0	0	0	0	0	0	0	0	0	57
20644339	f	md	CA	2022	1	0	0	0	0	0	0	0	0	0	0	0	
20644363	f	md	IT	2022	0	1	1	0	0	0	0	0	0	0	0	0	66
20644964	f	md	IT	2022	0	0	0	0	0	0	0	0	0	0	0	0	84
20645022	Not	hp	IT	2022	1	0	0	0	0	0	0	0	0	0	0	0	
20645093	m	md	JP	2022	0	1	0	0	1	0	0	0	0	0	0	0	68
20645105	Not	hp	TW	2022	1	0	0	0	0	0	0	0	0	0	0	0	
20645577	Not	cn	CN	2022	0	0	0	0	0	0	0	0	0	0	0	0	
20645682	f	cn	US	2022	1	0	0	0	0	0	0	0	0	0	0	0	74
20646196	m	hp	ES	2022	0	1	0	0	1	0	0	0	0	0	0	0	57
20646976	f	md	FR	2022	0	0	0	0	0	0	0	0	0	0	0	0	52
20647033	Not	md	JP	2022	0	1	0	0	0	1	0	0	0	0	0	0	
20647480	f	md	CN	2022	0	0	0	0	0	0	0	0	0	0	0	0	49

20647485	f	md	US	2022	0	0	0	0	0	0	0	0	0	0	0	0	75
20647492	m	md	AU	2022	1	0	0	0	0	0	0	0	0	0	0	0	
20647575	Not	cn	CN	2022	0	0	0	0	0	0	0	0	0	0	0	0	
20647638	m	md	JP	2022	0	1	0	0	0	1	0	0	0	0	0	0	70
20648123	m	hp	IT	2022	0	1	1	0	0	0	0	0	0	0	0	0	66
20648248	Not	md	ES	2022	0	0	0	0	0	0	0	0	0	0	0	0	64
20648317	m	md	RU	2022	0	0	0	0	0	0	0	0	0	0	0	0	
20648553	m	md	US	2022	0	0	0	0	0	0	0	0	0	0	0	0	
20649040	m	md	JP	2022	0	1	1	0	0	0	0	0	0	0	0	0	73
20649045	f	md	FR	2022	1	0	0	0	0	0	0	0	0	0	0	0	
20649120	f	md	JP	2022	1	0	0	0	0	0	0	0	0	0	0	1	35
20649147	Not	md	US	2022	0	1	0	0	0	1	0	0	0	0	0	0	
20649301	m	cn	IN	2022	0	1	0	0	0	1	0	0	0	0	0	0	
20649332	m	md	JP	2022	0	1	0	0	0	1	0	0	0	0	0	0	63
20649806	m	hp	CN	2022	0	0	0	0	0	0	0	0	0	0	0	0	61
20649821	Not	md	GB	2022	0	0	0	0	0	0	0	0	0	0	0	0	74
20649900	m	md	JP	2022	0	1	0	0	1	0	0	0	0	0	0	0	55
20649987	m	md	US	2022	0	0	0	0	0	0	0	0	0	0	0	0	
20650044	f	hp	PL	2022	0	1	0	1	0	0	0	0	0	0	0	0	60
20650474	m	md	JP	2022	0	1	0	0	1	0	0	0	0	0	0	0	58
20651091	f	md	JP	2022	0	1	0	0	1	0	0	0	0	0	0	0	
20651451	m	hp	JP	2022	0	1	1	0	0	0	0	0	0	0	0	0	45
20652057	f	cn	US	2022	0	0	0	0	0	0	0	0	0	0	0	0	
20652115	m	md	ES	2022	0	1	0	0	0	1	0	0	0	0	0	0	64
20652253	m	ph	JP	2022	0	1	0	0	1	0	0	0	0	0	0	1	69
20652686	Not	md	GB	2022	0	0	0	0	0	0	0	0	0	0	0	0	62
20652872	m	cn	PH	2022	0	1	0	0	0	1	0	0	0	0	0	0	84
20652893	f	hp	US	2022	0	0	0	0	0	0	0	0	0	0	0	0	52
20653179	Not	ph	JP	2022	1	0	0	0	0	0	0	0	0	0	0	0	
20653329	f	md	DE	2022	0	1	1	0	0	0	0	0	0	0	0	0	60
20653917	m	cn	US	2022	0	0	0	0	0	0	0	0	0	0	0	0	
20654111	f	hp	JP	2022	1	0	0	0	0	0	0	0	0	0	0	0	80
20654532	f	md	JP	2022	1	0	0	0	0	0	0	0	0	0	0	0	
20654798	f	md	FR	2022	0	0	0	0	0	0	0	0	0	0	0	0	

20654918	f	hp	CA	2022	1	0	0	0	0	0	0	0	0	0	0	0	77.4
20655291	f	ph	JP	2022	0	0	0	0	0	0	0	0	0	0	0	0	60
20655636	f	md	US	2022	0	0	0	0	0	0	0	0	0	0	0	0	70
20655782	m	cn	US	2022	1	0	0	0	0	0	0	0	0	0	0	0	
20656171	f	md	JP	2022	0	0	0	0	0	0	0	0	0	0	0	0	
20656246	f	md	US	2022	1	0	0	0	0	0	0	0	0	0	0	0	57
20657699	Not	hp	JP	2022	0	1	0	0	1	0	0	0	0	0	0	0	
20657926	Not	ph	US	2022	0	0	0	0	0	0	0	0	0	0	0	0	
20658187	m	hp	IT	2022	0	1	1	0	0	0	0	0	0	0	0	0	68
20658708	m	cn	CN	2022	0	1	0	1	0	0	0	0	0	0	0	0	37.2
20658767	Not	hp	US	2022	0	1	0	1	0	0	0	0	0	0	0	0	
20658889	f	cn	JP	2022	0	0	0	0	0	0	0	0	0	0	0	0	49
20658992	f	cn	ID	2022	1	0	0	0	0	0	0	0	0	0	0	0	50.9
20659113	f	hp	CN	2022	0	0	0	0	0	0	0	0	0	0	0	0	
20659280	m	md	JP	2022	0	1	1	0	0	0	0	0	0	0	0	0	78
20659813	m	md	FR	2022	0	1	0	0	0	1	0	0	0	0	0	0	79
20660005	f	cn	ID	2022	1	0	0	0	0	0	0	0	0	0	0	0	48
20660178	m	md	DE	2022	0	1	0	0	1	0	0	0	0	0	0	0	69
20660240	Not	md	JP	2022	0	1	1	0	0	0	0	0	0	0	0	1	80
20660911	f	hp	MX	2022	0	0	0	0	0	0	0	0	0	0	0	0	69
20660919	m	cn	ID	2022	1	0	0	0	0	0	0	0	0	0	0	0	61.1
20661058	m	md	JP	2022	0	1	0	0	1	0	0	0	0	0	0	0	68
20661074	m	cn	CN	2022	0	1	0	1	0	0	0	0	0	0	0	0	67
20661111	m	md	JP	2022	0	1	0	0	1	0	0	0	0	0	0	0	65
19106562	m	md	JP	2022	0	1	0	0	1	0	0	0	0	0	0	0	79
19413547	f	md	US	2022	0	0	0	0	0	0	0	0	0	0	0	0	64
19588508	m	md	EE	2022	0	1	1	0	0	0	0	0	0	0	0	0	49
19630943	m	md	CN	2022	0	1	1	0	0	0	0	0	0	0	0	1	66
19892172	f	hp	US	2022	0	0	0	0	0	0	0	0	0	0	0	0	66
19905160	f	md	CN	2022	0	1	0	0	0	1	0	0	0	0	0	0	47
19924873	m	md	FR	2022	0	0	0	0	0	0	0	0	0	0	0	0	91
19984982	f	md	JP	2022	0	1	1	0	0	0	0	0	0	0	0	0	60
20000152	m	md	FR	2022	0	0	0	0	0	0	0	0	0	0	0	0	25
20017916	m	md	JP	2022	0	1	0	0	1	0	0	0	0	0	0	0	68

20021765	f	md	US	2022	0	0	0	0	0	0	0	0	0	0	0	0	
20027872	m	md	JP	2022	0	1	0	0	1	0	0	0	0	0	0	0	77
20038805	m	md	IT	2022	0	0	0	0	0	0	0	0	0	0	0	0	72
20044621	m	md	IN	2022	0	0	0	0	0	0	0	0	0	0	0	0	49
20045166	m	md	JP	2022	0	1	0	0	1	0	0	0	0	0	0	0	54
20050922	m	md	CH	2022	0	0	0	0	0	0	0	0	0	0	0	0	65
20077472	m	hp	CN	2022	0	0	0	0	0	0	0	0	0	0	0	0	64
20081080	m	md	IT	2022	0	1	1	0	0	0	0	0	0	0	0	0	66
20084808	m	md	JP	2022	0	1	0	0	0	1	0	0	0	0	0	0	74
20093883	m	md	JP	2022	0	1	0	0	1	0	0	0	0	0	0	0	59
20108692	m	md	RO	2022	0	1	1	0	0	0	0	0	0	0	0	0	70
20126602	f	md	KR	2022	0	0	0	0	0	0	0	0	0	0	0	0	
20128792	f	md	CN	2022	0	0	0	0	0	0	0	0	0	0	0	0	60
20134682	f	ph	FR	2022	0	0	0	0	0	0	0	0	0	0	0	0	
20135342	f	md	JP	2022	0	1	0	0	1	0	0	0	0	0	0	0	65
20136725	f	md	JP	2022	0	0	0	0	0	0	0	0	0	0	0	0	81
20141240	f	hp	FR	2022	0	0	0	0	0	0	0	0	0	0	0	0	78
20146499	m	md	FR	2022	0	1	0	0	0	1	0	0	0	0	0	0	59
20169227	f	md	FR	2022	0	1	0	0	0	1	0	0	0	0	0	0	73
20170204	m	md	JP	2022	0	1	0	0	1	0	0	0	0	0	0	0	41
20182379	f	md	JP	2022	0	1	0	0	1	0	0	0	0	0	0	0	70
20183752	f	md	JP	2022	0	1	0	0	1	0	0	0	0	0	0	0	59
20188873	f	md	JP	2022	1	0	0	0	0	0	0	0	0	0	0	0	71
20208502	f	cn	PH	2022	1	0	0	0	0	0	0	0	0	0	0	0	
20210936	m	md	JP	2022	0	0	0	0	0	0	0	0	0	0	0	0	67
20217493	m	md	JP	2022	0	1	0	0	1	0	0	0	0	0	0	0	67
20228279	m	hp	JP	2022	0	0	0	0	0	0	0	0	0	0	0	0	74
20231968	m	md	KR	2022	0	0	0	0	0	0	0	0	0	0	0	0	
20242820	f	hp	AR	2022	0	1	0	0	1	0	0	0	0	0	0	0	65
20246203	m	md	ES	2022	0	1	0	0	0	1	0	0	0	0	0	0	67
20246270	f	hp	DE	2022	0	1	0	0	1	0	0	0	0	0	0	0	66
20249037	f	md	DE	2022	0	0	0	0	0	0	0	0	0	0	0	0	73
20263528	f	hp	JP	2022	0	0	0	0	0	0	0	0	0	0	0	0	58
20273497	f	md	GB	2022	0	0	0	0	0	0	1	0	0	0	1	0	55

20299486	f	md	JP	2022	0	1	0	0	1	0	0	0	0	0	0	1	74
20301680	f	md	US	2022	0	0	0	0	0	0	0	0	0	0	0	0	65
20307287	f	md	FR	2022	0	0	0	0	0	0	0	0	0	0	0	0	86
20310130	f	md	CL	2022	0	1	1	0	0	0	0	0	0	0	0	1	82
20313118	m	md	CN	2022	0	0	0	0	0	0	0	0	0	0	0	0	57
20317088	m	md	JP	2022	0	1	0	0	1	0	0	0	0	0	0	0	76
20318515	f	md	FR	2022	0	0	0	0	0	0	0	0	0	0	0	0	86
20320956	m	md	GB	2022	0	1	1	0	0	0	0	0	0	0	0	1	
20326015	f	cn	CN	2022	0	0	0	0	0	0	0	0	0	0	0	0	60
20328204	f	ph	JP	2022	0	0	0	0	0	0	0	0	0	0	0	0	66
20342927	m	hp	US	2022	0	1	0	0	1	0	0	0	0	0	0	0	59
20343916	m	md	JP	2022	0	1	0	0	1	0	0	0	0	0	0	1	70
20353622	m	md	JP	2022	0	1	0	0	1	0	0	0	0	0	0	0	78
20363409	m	md	JP	2022	0	1	0	1	0	0	0	0	0	0	0	0	71
20363855	f	md	FR	2022	0	0	0	0	0	0	0	0	0	0	0	0	60
20371795	f	hp	JP	2022	0	1	0	0	1	0	0	0	0	0	0	0	50
20372247	m	md	GB	2022	0	1	1	0	0	0	0	0	0	0	0	1	
20377166	f	md	AR	2022	0	0	0	0	0	0	0	0	0	0	0	0	81
20404174	m	md	JP	2022	0	1	0	0	0	1	0	0	0	0	0	0	63
20415592	m	md	JP	2022	0	0	0	0	0	0	0	0	0	0	0	0	70
20415978	m	md	IT	2022	0	0	0	0	0	0	0	0	0	0	0	0	76
20418379	f	ph	FR	2022	0	0	0	0	0	0	0	0	0	0	0	0	64
20420911	m	md	FR	2022	0	0	0	0	0	0	0	0	0	0	0	0	53
20435121	m	md	FR	2022	0	1	0	0	1	0	0	0	0	0	0	0	67
20435305	m	md	JP	2022	0	1	0	0	1	0	0	0	0	0	0	0	52
20437350	f	md	JP	2022	0	1	1	0	0	0	0	0	0	0	0	0	70
20441754	Not	md	JP	2022	0	1	0	0	1	0	0	0	0	0	0	0	77
20446751	m	hp	JP	2022	0	0	0	0	0	0	0	0	0	0	0	0	67
20450893	f	ph	US	2022	0	0	0	0	0	0	0	0	0	0	0	0	
20451148	m	md	TR	2022	0	0	0	0	0	0	0	0	0	0	0	0	53
20452591	m	md	JP	2022	0	1	0	0	1	0	0	0	0	0	0	0	72
20459864	f	md	PL	2022	0	1	0	0	0	1	0	0	0	0	0	0	76
20461732	f	md	GT	2022	0	1	0	0	0	1	0	0	0	0	0	0	56
20462112	m	hp	BE	2022	0	1	0	0	1	0	0	0	0	0	0	0	57

20466584	f	hp	IT	2022	0	0	0	0	0	0	0	0	0	0	0	0	67
20470055	Not	md	JP	2022	0	1	0	0	0	1	0	0	0	0	0	0	80
20471022	f	md	JP	2022	0	1	0	0	1	0	0	0	0	0	0	0	56
20475995	f	md	JP	2022	1	0	0	0	0	0	0	0	0	0	0	1	76
20476325	f	md	DE	2022	0	1	0	1	0	0	0	0	0	0	0	0	47
20476537	f	md	CL	2022	0	0	0	0	0	0	0	0	0	0	0	0	83
20478113	f	hp	AR	2022	0	0	0	0	0	0	0	0	0	0	0	0	83
20481651	Not	md	JP	2022	0	1	0	0	1	0	0	0	0	0	0	0	50
20483985	f	hp	US	2022	0	1	0	0	0	1	0	0	0	0	0	0	
20485072	f	hp	US	2022	0	0	0	0	0	0	0	0	0	0	0	0	67
20488052	m	hp	FR	2022	0	1	0	0	1	0	0	0	0	0	0	0	60
20488451	m	md	JP	2022	0	1	0	0	1	0	0	0	0	0	0	0	72
20490691	m	md	JP	2022	0	1	0	0	1	0	0	0	0	0	0	1	74
20490897	Not	md	FR	2022	0	0	0	0	0	0	0	0	0	0	0	0	73
20491359	Not	md	JP	2022	0	1	0	0	0	1	0	0	0	0	0	0	72
20496778	f	md	DE	2022	0	0	0	0	0	0	0	0	0	0	0	0	66
20504968	m	md	JP	2022	0	1	0	0	1	0	0	0	0	0	0	0	68
20507423	f	hp	US	2022	0	0	0	0	0	0	0	0	0	0	0	0	62
20507681	m	md	CH	2022	0	0	0	0	0	0	0	0	0	0	0	0	69
20508611	m	md	JP	2022	0	1	0	0	1	0	0	0	0	0	0	0	68
20509341	f	md	JP	2022	0	1	0	0	1	0	0	0	0	0	0	0	59
20509928	f	md	FR	2022	0	0	0	0	0	0	0	0	0	0	0	0	68
20512568	m	md	CN	2022	0	1	1	0	0	0	0	0	0	0	0	0	52
20516189	f	md	IT	2022	0	1	1	0	0	0	0	0	0	0	0	0	61
20517152	f	cn	IN	2022	0	0	0	0	0	0	0	0	0	0	0	0	55
20518246	f	md	JP	2022	0	1	0	1	0	0	0	0	0	0	0	1	74
20520209	f	md	US	2022	0	0	0	0	0	0	0	0	0	0	0	0	72
20521095	m	hp	RO	2022	0	0	0	0	0	0	0	0	0	0	0	0	52
20527921	f	md	CA	2022	1	0	0	0	0	0	0	0	0	0	0	0	52.9
20529947	m	md	JP	2022	0	1	0	0	1	0	0	0	0	0	0	1	72
20530524	m	md	JP	2022	0	1	0	0	1	0	0	0	0	0	0	0	73
20532117	f	md	DE	2022	0	1	1	0	0	0	0	0	0	0	0	0	60
20533555	f	cn	US	2022	0	0	0	0	0	0	0	0	0	0	0	0	
20538097	m	md	JP	2022	0	1	1	0	0	0	0	0	0	0	0	0	77

20538965	m	md	GB	2022	0	1	0	0	0	1	0	0	0	0	0	0	65
20539978	m	md	US	2022	0	0	0	0	0	0	0	0	0	0	0	0	64
20543142	m	hp	FR	2022	0	1	0	0	1	0	0	0	0	0	0	0	74
20544709	f	md	DE	2022	0	1	1	0	0	0	0	0	0	0	0	0	60
20544942	f	md	GB	2022	0	1	1	0	0	0	0	0	0	0	0	0	42
20545864	m	hp	JP	2022	0	0	0	0	0	0	0	0	0	0	0	0	71
20546442	f	md	JP	2022	1	0	0	0	0	0	0	0	0	0	0	0	67
20546578	m	ph	PT	2022	0	1	0	0	0	1	0	0	0	0	0	0	
20547170	f	md	SI	2022	0	0	0	0	0	0	0	0	0	0	0	0	70
20551011	m	ph	PT	2022	0	1	0	0	0	1	0	0	0	0	0	0	
20551238	m	ph	PT	2022	0	1	0	0	0	1	0	0	0	0	0	0	
20551312	f	cn	US	2022	1	0	0	0	0	0	0	0	0	0	0	0	
20551589	m	cn	CN	2022	0	1	0	0	1	0	0	0	0	0	0	0	59
20551836	m	md	JP	2022	0	1	0	0	1	0	0	0	0	0	0	0	52
20551997	m	md	PT	2022	0	1	0	1	0	0	0	0	0	0	0	0	78
20553327	f	hp	HR	2022	0	1	0	0	1	0	0	0	0	0	0	0	63
20553987	f	cn	LB	2022	0	1	0	0	0	1	0	0	0	0	0	0	72
20554611	m	md	SG	2022	1	0	0	0	0	0	0	0	0	0	0	0	75
20556301	m	md	CH	2022	0	1	0	0	0	1	0	0	0	0	0	0	69
20557377	f	md	DE	2022	0	1	1	0	0	0	0	0	0	0	0	0	60
20558046	f	md	DE	2022	0	1	1	0	0	0	0	0	0	0	0	0	60
20558860	f	md	JP	2022	0	1	0	0	1	0	0	0	0	0	0	0	74
20560895	m	md	JP	2022	0	1	0	0	0	1	0	0	0	0	0	0	90
20562648	m	md	JP	2022	0	1	0	0	1	0	0	0	0	0	0	0	72
20562814	m	md	GR	2022	0	0	0	0	0	0	0	0	0	0	0	0	73
20566081	f	md	JP	2022	0	1	0	0	1	0	0	0	0	0	0	0	69
20568726	f	hp	PL	2022	0	0	0	0	0	0	0	0	0	0	0	0	74
20569421	m	md	BE	2022	0	0	0	0	0	0	0	0	0	0	0	0	69
20570021	f	md	JP	2022	0	0	0	0	0	0	1	0	0	0	1	0	80
20571688	m	md	JP	2022	0	1	0	0	1	0	0	0	0	0	0	0	71
20573198	f	md	NL	2022	0	1	1	0	0	0	0	0	0	0	0	0	66
20573342	f	md	FR	2022	1	0	0	0	0	0	0	0	0	0	0	0	71.3
20575392	m	ph	JP	2022	0	0	0	0	0	0	0	0	0	0	0	0	60
20576606	m	md	JP	2022	0	1	0	0	1	0	0	0	0	0	0	0	70

20577548	m	md	JP	2022	0	1	0	0	1	0	0	0	0	0	0	0	72
20577600	f	md	IT	2022	0	1	1	0	0	0	0	0	0	0	0	0	70
20580759	f	md	JP	2022	0	1	0	0	1	0	0	0	0	0	0	0	80
20580841	f	md	JP	2022	0	1	1	0	0	0	0	0	0	0	0	0	73
20581182	f	md	JP	2022	0	1	1	0	0	0	0	0	0	0	0	0	57
20582180	m	md	JP	2022	0	1	0	0	1	0	0	0	0	0	0	1	74
20582344	m	md	JP	2022	0	1	0	1	0	0	0	0	0	0	0	0	
20582850	f	hp	NL	2022	0	1	1	0	0	0	0	0	0	0	0	0	60
20583030	m	md	DE	2022	0	0	0	0	0	0	0	0	0	0	0	0	69
20583519	Not	md	JP	2022	0	1	0	0	1	0	0	0	0	0	0	0	79
20585152	m	md	JP	2022	0	1	0	0	1	0	0	0	0	0	0	0	79
20585321	m	md	JP	2022	0	1	0	0	1	0	0	0	0	0	0	0	70
20586169	m	md	JP	2022	0	1	0	0	1	0	0	0	0	0	0	0	77
20586724	m	md	JP	2022	0	1	0	0	1	0	0	0	0	0	0	0	46
20587873	m	ph	PT	2022	0	1	0	0	0	1	0	0	0	0	0	0	67
20588848	m	md	JP	2022	0	1	0	0	1	0	0	0	0	0	0	0	68
20588875	f	md	DE	2022	0	1	1	0	0	0	0	0	0	0	0	0	60
20589214	m	hp	ES	2022	0	1	0	0	1	0	0	0	0	0	0	0	67
20589717	m	md	JP	2022	0	1	0	0	1	0	0	0	0	0	0	0	78
20590403	m	md	JP	2022	0	1	1	0	0	0	0	0	0	0	0	0	60
20590794	Not	md	AU	2022	0	0	0	0	0	0	0	0	0	0	0	0	
20592494	m	hp	DE	2022	0	1	0	0	1	0	0	0	0	0	0	0	59
20593361	m	md	JP	2022	0	1	0	0	1	0	0	0	0	0	0	1	
20594018	m	md	GB	2022	0	1	0	0	0	1	0	0	0	0	0	0	65
20595818	f	md	FR	2022	0	0	0	0	0	0	0	0	0	0	0	0	58
20596748	m	md	JP	2022	0	1	0	0	1	0	0	0	0	0	0	0	77
20597301	f	md	FR	2022	0	0	0	0	0	0	0	0	0	0	0	0	86
20597682	m	md	GB	2022	0	1	0	0	0	1	0	0	0	0	0	0	65
20598397	m	md	JP	2022	0	1	0	0	1	0	0	0	0	0	0	0	71
20598706	m	hp	JP	2022	0	0	0	0	0	0	0	0	0	0	0	0	63
20598894	m	hp	FR	2022	0	1	0	0	1	0	0	0	0	0	0	0	64
20599016	f	md	JP	2022	0	1	0	0	1	0	0	0	0	0	0	0	62
20600860	f	md	JP	2022	0	1	0	0	0	1	0	0	0	0	0	0	47
20600933	m	md	JP	2022	0	1	0	0	1	0	0	0	0	0	0	1	66

20602260	m	md	ES	2022	0	0	0	0	0	0	0	0	0	0	0	0	69
20603339	m	md	JP	2022	0	0	0	0	0	0	0	0	0	0	0	0	60
20604640	f	md	BE	2022	0	0	0	0	0	0	0	0	0	0	0	0	64
20605007	m	cn	US	2022	0	1	1	0	0	0	0	0	0	0	0	0	
20606494	f	md	NL	2022	0	1	1	0	0	0	0	0	0	0	0	0	66
20606590	m	md	LT	2022	0	1	0	0	0	1	0	0	0	0	0	0	73
20609869	f	md	JP	2022	0	0	0	0	0	0	0	0	0	0	0	0	90
20610056	m	md	JP	2022	0	1	1	0	0	0	0	0	0	0	0	0	75
20611621	m	md	GB	2022	0	1	0	0	0	1	0	0	0	0	0	0	65
20611758	f	md	AU	2022	0	0	0	0	0	0	0	0	0	0	0	0	
20612335	f	hp	US	2022	1	0	0	0	0	0	0	0	0	0	0	0	54.6
20614057	m	hp	CN	2022	1	0	0	0	0	0	0	0	0	0	0	0	69
20618349	m	md	KR	2022	0	0	0	0	0	0	0	0	0	0	0	0	56
20621293	m	cn	PH	2022	0	0	0	0	0	0	0	0	0	0	0	0	39
20622298	f	md	FR	2022	0	0	0	0	0	0	0	0	0	0	0	0	68
20622411	f	md	AU	2022	0	0	0	0	0	0	0	0	0	0	0	0	66
20623195	f	cn	CH	2022	0	0	0	0	0	0	0	0	0	0	0	0	
20623710	m	md	JP	2022	0	1	1	0	0	0	0	0	0	0	0	0	60
20624220	m	md	JP	2022	0	0	0	0	0	0	0	0	0	0	0	0	80
20626075	m	md	FR	2022	0	1	0	0	0	1	0	0	0	0	0	0	51
20626489	f	hp	GB	2022	0	1	1	0	0	0	0	0	0	0	0	0	53
20626917	f	cn	JP	2022	0	0	0	0	0	0	0	0	0	0	0	0	65
20627376	f	md	US	2022	0	0	0	0	0	0	0	0	0	0	0	0	67
20630987	m	md	CN	2022	0	0	0	0	0	0	0	0	0	0	0	0	45
20631600	f	md	US	2022	0	1	1	0	0	0	0	0	0	0	0	0	64
20631659	m	md	PT	2022	0	1	1	0	0	0	0	0	0	0	0	0	62
20631909	m	cn	US	2022	0	1	0	1	0	0	0	0	0	0	0	0	82.3
20632339	m	md	JP	2022	0	1	0	0	0	1	0	0	0	0	0	1	80
20632353	f	cn	US	2022	0	0	0	0	0	0	0	0	0	0	0	0	89
20632731	f	md	AR	2022	0	1	1	0	0	0	0	0	0	0	0	0	54
20632949	m	md	RU	2022	0	0	0	0	0	0	0	0	0	0	0	0	70
20633307	m	md	TR	2022	0	0	0	0	0	0	0	0	0	0	0	0	74
20633452	f	md	US	2022	0	0	0	0	0	0	0	0	0	0	0	0	
20633588	m	md	US	2022	0	0	0	0	0	0	0	0	0	0	0	0	72

20633614	m	md	JP	2022	0	0	0	0	0	0	0	0	0	0	0	0	63
20633693	f	md	RU	2022	0	0	0	0	0	0	0	0	0	0	0	0	65
20634272	f	md	NL	2022	0	1	1	0	0	0	0	0	0	0	0	0	66
20634509	m	md	DE	2022	0	1	0	0	1	0	0	0	0	0	0	0	59
20636726	m	md	MY	2022	0	0	0	0	0	0	0	0	0	0	0	0	39
20636940	m	ph	JP	2022	0	1	0	0	1	0	0	0	0	0	0	0	80
20637980	m	md	JP	2022	0	1	1	0	0	0	0	0	0	0	0	0	45
20638042	m	hp	US	2022	0	0	0	0	0	0	0	0	0	0	0	0	
20638430	f	md	FR	2022	0	0	0	0	0	0	0	0	0	0	0	0	47
20638587	m	md	JP	2022	0	1	0	0	1	0	0	0	0	0	0	1	79
20638605	m	md	GB	2022	0	1	0	1	0	0	0	0	0	0	0	0	53
20640051	f	md	SI	2022	0	0	0	0	0	0	0	0	0	0	0	0	70
20640052	f	md	SI	2022	0	0	0	0	0	0	0	0	0	0	0	0	67
20640786	m	md	JP	2022	0	1	0	0	1	0	0	0	0	0	0	0	65
20641046	m	md	JP	2022	0	1	0	0	1	0	0	0	0	0	0	1	67
20641086	m	hp	SG	2022	0	1	0	0	1	0	0	0	0	0	0	0	
20642598	f	cn	CH	2022	0	0	0	0	0	0	0	0	0	0	0	0	
20642600	m	cn	CH	2022	0	0	0	0	0	0	0	0	0	0	0	0	
20642602	m	cn	CH	2022	0	0	0	0	0	0	0	0	0	0	0	0	
20643456	m	ph	CA	2022	0	1	0	1	0	0	0	0	0	0	0	0	
20644312	m	md	JP	2022	0	1	0	0	1	0	0	0	0	0	0	0	76
20644759	Not	md	BR	2022	0	0	0	0	0	0	0	0	0	0	0	0	
20646133	m	ph	JP	2022	0	1	0	0	1	0	0	0	0	0	0	0	80
20647693	Not	cn	CN	2022	0	0	0	0	0	0	0	0	0	0	0	0	
20649003	f	md	JP	2022	0	1	0	0	0	1	0	0	0	0	0	0	50
20649034	Not	md	JP	2022	0	1	0	0	1	0	0	0	0	0	0	0	
20649525	m	md	NL	2022	0	1	0	0	0	1	0	0	0	0	0	0	76
20652446	m	ph	JP	2022	0	1	0	0	1	0	0	0	0	0	0	0	80
20652907	m	md	AU	2022	0	1	0	0	0	1	0	0	0	0	0	0	
20654276	m	md	DE	2022	0	1	0	0	1	0	0	0	0	0	0	0	73
20654596	m	md	GR	2022	0	0	0	0	0	0	0	0	0	0	0	0	68
20655285	m	hp	BE	2022	0	0	0	0	0	0	0	0	0	0	0	0	73
20655442	m	cn	TW	2022	0	0	0	0	0	0	0	0	0	0	0	0	
20655758	m	md	CN	2022	0	1	1	0	0	0	0	0	0	0	0	0	

20655942	f	md	CH	2022	0	0	0	0	0	0	0	0	0	0	0	0	
20655967	f	cn	TH	2022	1	0	0	0	0	0	0	0	0	0	0	0	
20656288	m	hp	ES	2022	0	1	0	0	1	0	0	0	0	0	0	0	61
20656667	f	hp	DE	2022	0	1	0	0	1	0	0	0	0	0	0	0	62
20657181	m	md	JP	2022	0	1	0	0	1	0	0	0	0	0	0	0	68
20659660	m	md	FR	2022	0	0	0	0	0	0	0	0	0	0	0	0	65
20660411	m	md	IT	2022	0	0	0	0	0	0	0	0	0	0	0	0	60
20661006	m	md	JP	2022	0	1	0	0	1	0	0	0	0	0	0	0	71
20661325	m	md	JP	2022	0	1	0	1	0	0	0	0	0	0	0	0	50
20661792	m	cn	GB	2022	0	0	0	0	0	0	0	0	0	0	0	0	70
20661845	m	md	JP	2022	0	0	0	0	0	0	0	0	0	0	0	0	80
20661846	m	hp	DK	2022	0	0	0	0	0	0	0	0	0	0	0	0	57
20662657	f	md	NL	2022	0	0	0	0	0	0	0	0	0	0	0	0	70
20663040	m	md	FR	2022	0	0	0	0	0	0	0	0	0	0	0	0	
20663156	m	md	FR	2022	0	0	0	0	0	0	0	0	0	0	0	0	
20663217	m	md	AU	2022	1	0	0	0	0	0	0	0	0	0	0	0	
20663219	f	hp	IN	2022	1	0	0	0	0	0	0	0	0	0	0	0	53
20663621	f	hp	AT	2022	0	1	0	0	0	1	0	0	0	0	0	0	49
20663653	m	cn	US	2022	0	0	0	0	0	0	0	0	0	0	0	0	70
20663699	f	cn	CN	2022	0	0	0	0	0	0	0	0	0	0	0	0	53
20663943	f	md	BR	2022	1	0	0	0	0	0	0	0	0	0	0	0	59.7
20664028	Not	md	JP	2022	0	1	0	0	0	1	0	0	0	0	0	0	
20664089	m	md	JP	2022	1	0	0	0	0	0	0	0	0	0	0	0	70
20664243	f	md	JP	2022	0	1	0	1	0	0	0	0	0	0	0	1	60
20664344	f	cn	US	2022	1	0	0	0	0	0	0	0	0	0	0	0	69
20664360	m	md	RO	2022	0	1	0	1	0	0	0	0	0	0	0	0	
20664365	Not	ph	JP	2022	1	0	0	0	0	0	0	0	0	0	0	0	
20664370	m	md	JP	2022	0	1	0	1	0	0	0	0	0	0	0	0	75
20664374	m	cn	US	2022	1	0	0	0	0	0	0	0	0	0	0	0	
20664487	m	md	JP	2022	0	1	0	1	0	0	0	0	0	0	0	0	69
20664532	f	md	US	2022	0	1	0	1	0	0	0	0	0	0	0	0	69
20664549	m	cn	AR	2022	0	0	0	0	0	0	0	0	0	0	0	0	41
20664825	m	cn	CN	2022	0	1	0	1	0	0	0	0	0	0	0	1	72
20664827	m	cn	CN	2022	0	1	0	1	0	0	0	0	0	0	0	0	57

20664893	m	md	DE	2022	0	1	0	0	1	0	0	0	0	0	0	0	60
20665042	m	md	NL	2022	0	0	0	0	0	0	0	0	0	0	0	0	72
20665165	m	hp	JP	2022	0	1	1	0	0	0	0	0	0	0	0	0	69
20665186	f	cn	CA	2022	0	0	0	0	0	0	0	0	0	0	0	0	46
20665197	m	md	JP	2022	1	0	0	0	0	0	0	0	0	0	0	0	72
20665204	Not	md	RU	2022	0	1	0	0	0	1	0	0	0	0	0	0	63
20665368	m	cn	EG	2022	0	1	1	0	0	0	0	0	0	0	0	0	67
20665530	m	md	TR	2022	0	1	0	0	0	1	0	0	0	0	0	0	57
20665644	m	md	AT	2022	0	1	0	0	0	1	0	0	0	0	0	0	
20665917	Not	md	DE	2022	0	1	0	0	1	0	0	0	0	0	0	0	61
20667020	m	cn	US	2022	0	1	0	0	0	1	0	0	0	0	0	0	
20667473	f	md	DE	2022	0	1	0	0	1	0	0	0	0	0	0	0	85
20667507	m	cn	CN	2022	0	0	0	0	0	0	0	0	0	0	0	0	68
20667628	m	cn	CA	2022	0	1	1	0	0	0	0	0	0	0	0	0	
20667749	f	md	SG	2022	0	1	0	0	0	1	0	0	0	0	0	0	68
20667867	f	ph	JP	2022	0	1	0	0	1	0	0	0	0	0	0	0	
20667870	m	md	AU	2022	0	0	0	0	0	0	0	0	0	0	0	0	73
20668059	f	cn	US	2022	0	1	0	0	0	1	0	0	0	0	0	0	63
20668150	f	md	JP	2022	0	1	0	0	0	1	0	0	0	0	0	0	80
20668328	m	cn	CA	2022	1	0	0	0	0	0	0	0	0	0	0	0	66.7
20668360	f	md	IT	2022	0	1	1	0	0	0	0	0	0	0	0	0	61
20668470	f	cn	US	2022	0	1	0	1	0	0	0	0	0	0	0	1	
20668498	f	cn	US	2022	0	0	0	0	0	0	0	0	0	0	0	0	
20668538	f	md	JP	2022	0	0	0	0	0	0	0	0	0	0	0	0	71
20668539	f	md	JP	2022	0	0	0	0	0	0	0	0	0	0	0	0	52
20669223	Not	md	US	2022	0	0	0	0	0	0	0	0	0	0	0	0	
20669268	m	md	MK	2022	0	0	0	0	0	0	0	0	0	0	0	0	65
20669642	Not	cn	US	2022	0	0	0	0	0	0	0	0	0	0	0	0	
20669652	f	md	US	2022	0	0	0	0	0	0	0	0	0	0	0	0	
20669780	Not	cn	US	2022	0	0	0	0	0	0	0	0	0	0	0	0	
20669796	m	md	IL	2022	0	0	0	0	0	0	0	0	0	0	0	0	82
20669994	Not	md	PL	2022	0	1	0	1	0	0	0	0	0	0	0	0	
20670111	Not	md	US	2022	0	0	0	0	0	0	0	0	0	0	0	0	
20670135	m	md	TR	2022	0	0	0	0	0	0	0	0	0	0	0	0	59

20670196	Not	cn	US	2022	0	0	0	0	0	0	0	0	0	0	0	0	
20670374	m	md	FR	2022	0	1	0	0	0	1	0	0	0	0	0	0	67
20670438	f	cn	SG	2022	1	0	0	0	0	0	0	0	0	0	0	0	85.3
20670482	Not	cn	US	2022	0	0	0	0	0	0	0	0	0	0	0	0	
20670493	Not	cn	US	2022	0	0	0	0	0	0	0	0	0	0	0	0	
20670505	Not	cn	US	2022	0	0	0	0	0	0	0	0	0	0	0	0	
20670631	Not	cn	US	2022	0	0	0	0	0	0	0	0	0	0	0	0	
20670650	Not	cn	US	2022	0	0	0	0	0	0	0	0	0	0	0	0	
20670808	Not	cn	US	2022	0	0	0	0	0	0	0	0	0	0	0	0	
20670829	Not	cn	US	2022	0	0	0	0	0	0	0	0	0	0	0	0	
20670841	Not	cn	US	2022	0	0	0	0	0	0	0	0	0	0	0	0	
20670859	Not	cn	US	2022	0	0	0	0	0	0	0	0	0	0	0	0	
20670942	f	ph	JP	2022	0	0	0	0	0	0	0	0	0	0	0	0	
20670985	Not	cn	US	2022	0	0	0	0	0	0	0	0	0	0	0	0	
20671002	Not	cn	US	2022	0	0	0	0	0	0	0	0	0	0	0	0	
20671011	Not	cn	US	2022	0	0	0	0	0	0	0	0	0	0	0	0	
20671015	Not	cn	US	2022	0	0	0	0	0	0	0	0	0	0	0	0	
20671736	f	ph	US	2022	0	0	0	0	0	0	0	0	0	0	0	0	62
20671807	m	md	CZ	2022	0	1	0	0	1	0	0	0	0	0	0	0	73
20671883	f	md	US	2022	0	1	1	0	0	0	0	0	0	0	0	0	61
20672196	m	md	JP	2022	0	1	0	0	1	0	0	0	0	0	0	0	
20672214	m	md	JP	2022	0	1	0	0	1	0	0	0	0	0	0	0	
20672242	m	md	JP	2022	0	1	0	0	1	0	0	0	0	0	0	0	80
20672257	m	cn	CN	2022	0	0	0	0	0	0	0	0	0	0	0	0	58
20672392	m	md	FR	2022	0	1	0	0	0	1	0	0	0	0	0	0	51
20672416	Not	cn	CN	2022	0	0	0	0	0	0	0	0	0	0	0	0	
20672888	Not	cn	US	2022	0	0	0	0	0	0	0	0	0	0	0	0	
20672935	Not	cn	US	2022	0	0	0	0	0	0	0	0	0	0	0	0	
20672937	Not	cn	US	2022	0	0	0	0	0	0	0	0	0	0	0	0	
20672940	Not	cn	US	2022	0	0	0	0	0	0	0	0	0	0	0	0	
20672941	Not	cn	US	2022	0	0	0	0	0	0	0	0	0	0	0	0	
20673026	Not	cn	US	2022	0	0	0	0	0	0	0	0	0	0	0	0	
20673047	m	ph	US	2022	0	0	0	0	0	0	0	0	0	0	0	0	68
20673133	m	md	JP	2022	0	1	0	0	0	1	0	0	0	0	0	0	82

20673159	Not	cn	US	2022	0	0	0	0	0	0	0	0	0	0	0	0	
20673160	Not	cn	US	2022	0	0	0	0	0	0	0	0	0	0	0	0	
20673167	Not	cn	US	2022	0	0	0	0	0	0	0	0	0	0	0	0	
20673292	Not	cn	US	2022	0	0	0	0	0	0	0	0	0	0	0	0	
20673293	Not	cn	US	2022	0	0	0	0	0	0	0	0	0	0	0	0	
20673471	Not	cn	US	2022	0	0	0	0	0	0	0	0	0	0	0	0	
20673473	Not	cn	US	2022	0	0	0	0	0	0	0	0	0	0	0	0	
20673478	Not	cn	US	2022	0	0	0	0	0	0	0	0	0	0	0	0	
20673485	m	md	IL	2022	0	1	1	0	0	0	0	0	0	0	0	0	71
20673563	Not	cn	US	2022	0	0	0	0	0	0	0	0	0	0	0	0	
20673714	Not	cn	US	2022	0	0	0	0	0	0	0	0	0	0	0	0	
20673718	Not	cn	US	2022	0	0	0	0	0	0	0	0	0	0	0	0	
20673719	Not	cn	US	2022	0	0	0	0	0	0	0	0	0	0	0	0	
20673742	Not	cn	US	2022	0	0	0	0	0	0	0	0	0	0	0	0	
20673750	m	hp	IT	2022	0	1	1	0	0	0	0	0	0	0	0	0	68
20673760	Not	ph	CA	2022	0	0	0	0	0	0	0	0	0	0	0	0	
20673908	Not	cn	US	2022	0	0	0	0	0	0	0	0	0	0	0	0	
20674099	m	cn	ID	2022	1	0	0	0	0	0	0	0	0	0	0	0	23.4
20674150	f	md	GB	2022	0	1	1	0	0	0	0	0	0	0	0	0	42
20674165	m	hp	JP	2022	0	0	0	0	0	0	0	0	0	0	0	0	50
20674206	f	md	PL	2022	0	0	0	0	0	0	0	0	0	0	0	0	64
20674299	m	cn	CN	2022	0	1	0	1	0	0	0	0	0	0	0	0	54
20674370	Not	md	PL	2022	0	0	0	0	0	0	0	0	0	0	0	0	65
20674420	Not	cn	US	2022	0	0	0	0	0	0	0	0	0	0	0	0	
20674468	Not	md	US	2022	0	1	0	1	0	0	0	0	0	0	0	0	71.7
20674469	Not	cn	US	2022	0	0	0	0	0	0	0	0	0	0	0	0	
20674485	f	hp	AT	2022	0	1	1	0	0	0	0	0	0	0	0	0	56
20674547	Not	cn	US	2022	0	0	0	0	0	0	0	0	0	0	0	0	
20674556	m	md	JP	2022	0	1	0	0	0	1	0	0	0	0	0	0	60
20674770	m	cn	IN	2022	0	0	0	0	0	0	0	0	0	0	0	0	70
20674786	m	md	CA	2022	0	0	0	0	0	0	0	0	0	0	0	0	72
20674840	f	md	US	2022	0	0	0	0	0	0	0	0	0	0	0	0	35
20675260	Not	cn	US	2022	0	0	0	0	0	0	0	0	0	0	0	0	
20675303	m	md	JP	2022	0	1	0	0	1	0	0	0	0	0	0	0	54

20675333	m	md	FR	2022	0	0	0	0	0	0	0	0	0	0	0	0	53
20675360	Not	cn	US	2022	0	0	0	0	0	0	0	0	0	0	0	0	
20675399	Not	cn	US	2022	0	0	0	0	0	0	0	0	0	0	0	0	
20675409	Not	cn	US	2022	0	0	0	0	0	0	0	0	0	0	0	0	
20675416	m	cn	CA	2022	1	0	0	0	0	0	0	0	0	0	0	0	
20675484	m	md	JP	2022	0	0	0	0	0	0	0	0	0	0	0	0	53
20675536	Not	cn	US	2022	0	0	0	0	0	0	0	0	0	0	0	0	
20675695	Not	cn	US	2022	0	0	0	0	0	0	0	0	0	0	0	0	
20675975	f	md	JP	2022	0	0	0	0	0	0	0	0	0	0	0	0	72
20675977	Not	cn	US	2022	0	0	0	0	0	0	0	0	0	0	0	0	
20675999	Not	cn	US	2022	0	0	0	0	0	0	0	0	0	0	0	0	
20676124	f	md	CN	2022	0	0	0	0	0	0	0	0	0	0	0	0	56
20676455	Not	md	JP	2022	0	1	0	0	1	0	0	0	0	0	0	0	77
20676480	Not	md	JP	2022	0	0	0	0	0	0	0	0	0	0	0	0	
20676712	m	cn	PH	2022	0	1	0	0	0	1	0	0	0	0	0	0	63
20676738	f	md	US	2022	0	0	0	0	0	0	0	0	0	0	0	0	60
20676954	Not	cn	JP	2022	0	0	0	0	0	0	0	0	0	0	0	0	
20677096	m	md	JP	2022	0	1	0	0	1	0	0	0	0	0	0	1	64
20677117	Not	cn	JP	2022	0	0	0	0	0	0	0	0	0	0	0	0	
20677118	Not	cn	JP	2022	0	0	0	0	0	0	0	0	0	0	0	0	
20677120	Not	cn	JP	2022	0	0	0	0	0	0	0	0	0	0	0	0	
20677124	Not	cn	JP	2022	0	0	0	0	0	0	0	0	0	0	0	0	
20677125	Not	cn	JP	2022	0	0	0	0	0	0	0	0	0	0	0	0	
20677290	f	hp	US	2022	0	0	0	0	0	0	0	0	0	0	0	0	69
20677405	m	md	TW	2022	0	0	0	0	0	0	0	0	0	0	0	0	72
20677455	Not	cn	JP	2022	0	0	0	0	0	0	0	0	0	0	0	0	
20677588	f	cn	US	2022	0	0	0	0	0	0	0	0	0	0	0	0	
20677770	m	hp	US	2022	0	0	0	0	0	0	0	0	0	0	0	0	73
20678269	Not	md	US	2022	0	0	0	0	0	0	0	0	0	0	0	0	
20679664	m	md	FR	2022	0	1	0	1	0	0	0	0	0	0	0	0	
20679688	f	md	FR	2022	0	0	0	0	0	0	0	0	0	0	0	0	90
20680029	m	hp	US	2022	0	0	0	0	0	0	0	0	0	0	0	0	68
20680138	f	md	DE	2022	0	0	0	0	0	0	0	0	0	0	0	0	71
20680227	m	md	JP	2022	0	1	0	0	1	0	0	0	0	0	0	1	77

20680418	f	md	JP	2022	0	1	0	1	0	0	0	0	0	0	0	0	71
20680466	Not	cn	US	2022	0	0	0	0	0	0	0	0	0	0	0	0	
20680499	m	hp	US	2022	0	1	0	1	0	0	0	0	0	0	0	0	68
20680523	m	md	JP	2022	0	0	0	0	0	0	0	0	0	0	0	0	75
20680538	m	cn	EG	2022	0	0	0	0	0	0	0	0	0	0	0	0	51
20680545	f	md	CN	2022	0	0	0	0	0	0	0	0	0	0	0	0	62
20680591	Not	md	PL	2022	0	0	0	0	0	0	0	0	0	0	0	0	
20680602	Not	md	PL	2022	0	0	0	0	0	0	0	0	0	0	0	0	
20680795	f	cn	KR	2022	0	0	0	0	0	0	0	0	0	0	0	0	83
20680813	m	cn	TH	2022	0	1	1	0	0	0	0	0	0	0	0	0	61
20680985	f	md	CN	2022	0	0	0	0	0	0	0	0	0	0	0	0	65
20681060	f	md	CN	2022	0	0	0	0	0	0	0	0	0	0	0	0	71
20681438	m	md	DE	2022	0	1	0	0	0	1	0	0	0	0	0	0	
20681587	m	md	JP	2022	0	1	0	0	1	0	0	0	0	0	0	0	54
20681867	m	md	IL	2022	0	1	1	0	0	0	0	0	0	0	0	0	54
20681941	f	hp	AR	2022	0	1	0	0	1	0	0	0	0	0	0	0	66
20682090	f	md	US	2022	0	0	0	0	0	0	0	0	0	0	0	0	52
20682159	f	md	JP	2022	0	1	0	0	1	0	0	0	0	0	0	0	
20682480	Not	md	JP	2022	0	1	0	0	1	0	0	0	0	0	0	0	
20682483	m	hp	ES	2022	1	0	0	0	0	0	0	0	0	0	0	0	84
20682530	m	hp	JP	2022	0	1	1	0	0	0	0	0	0	0	0	0	72
20682615	f	hp	US	2022	0	0	0	0	0	0	0	0	0	0	0	0	52
20682848	m	ph	JP	2022	1	0	0	0	0	0	0	0	0	0	0	0	
20683048	Not	hp	CN	2022	0	0	0	0	0	0	0	0	0	0	0	0	62
20683049	Not	hp	CN	2022	0	1	1	0	0	0	0	0	0	0	0	0	62
20683225	f	md	JP	2022	0	0	0	0	0	0	0	0	0	0	0	0	88
20683474	m	md	IL	2022	0	1	1	0	0	0	0	0	0	0	0	0	76
20683491	f	hp	IL	2022	0	0	0	0	0	0	0	0	0	0	0	0	64
20683683	m	md	KR	2022	0	1	0	0	1	0	0	0	0	0	0	0	83
20683714	m	md	US	2022	0	0	0	0	0	0	0	0	0	0	0	0	
20683804	f	cn	TH	2022	1	0	0	0	0	0	0	0	0	0	0	0	65.7
20683960	m	md	JP	2022	1	0	0	0	0	0	0	0	0	0	0	0	80
20684071	f	cn	TW	2022	0	0	0	0	0	0	0	0	0	0	0	0	41
20684289	f	cn	GB	2022	1	0	0	0	0	0	0	0	0	0	0	0	73

20684359	Not	ph	JP	2022	1	0	0	0	0	0	0	0	0	0	0	0	
20684386	f	md	PT	2022	0	1	1	0	0	0	0	0	0	0	0	0	57
20684840	f	md	JP	2022	0	0	0	0	0	0	0	0	0	0	0	0	58
20684896	f	md	JP	2022	0	1	0	0	0	1	0	0	0	0	0	0	62
20685016	f	md	JP	2022	0	1	0	1	0	0	0	0	0	0	0	1	70
20685157	f	cn	US	2022	0	1	1	0	0	0	0	0	0	0	0	0	62
20685226	m	md	US	2022	0	0	0	0	0	0	0	0	0	0	0	0	78
20685418	f	ph	JP	2022	0	1	0	0	1	0	0	0	0	0	0	0	70
20685487	Not	md	US	2022	1	0	0	0	0	0	0	0	0	0	0	0	71.7
20685535	m	cn	IN	2022	0	0	0	0	0	0	0	0	0	0	0	0	60
20685579	f	hp	US	2022	0	1	1	0	0	0	0	0	0	0	0	0	69
20685904	Not	md	US	2022	0	1	0	0	1	0	0	0	0	0	0	0	74
20686371	m	md	CN	2022	1	0	0	0	0	0	0	0	0	0	0	0	79
20687135	m	cn	CN	2022	0	1	0	0	1	0	0	0	0	0	0	0	67
20687398	m	ph	IE	2022	0	0	0	0	0	0	0	0	0	0	0	0	
20688259	Not	cn	CN	2022	0	0	0	0	0	0	0	0	0	0	0	0	
20688305	m	md	JP	2022	0	1	1	0	0	0	0	0	0	0	0	0	74
20688377	f	hp	US	2022	0	0	0	0	0	0	0	0	0	0	0	0	
20688406	f	md	DE	2022	0	1	0	0	1	0	0	0	0	0	0	0	85
20688554	m	md	ES	2022	0	0	0	0	0	0	0	0	0	0	0	0	60
20688746	Not	ph	CA	2022	0	1	1	0	0	0	0	0	0	0	0	1	
20689304	m	hp	US	2022	0	0	0	0	0	0	0	0	0	0	0	0	
20689651	f	md	JP	2022	0	1	1	0	0	0	0	0	0	0	0	0	78
20689659	m	hp	TH	2022	1	0	0	0	0	0	0	0	0	0	0	0	73
20689947	m	md	GR	2022	0	0	0	0	0	0	0	0	0	0	0	0	
20689977	m	cn	IN	2022	0	0	0	0	0	0	0	0	0	0	0	0	82
20690135	m	ph	JP	2022	0	1	0	1	0	0	0	0	0	0	0	0	59
20690223	f	md	JP	2022	1	0	0	0	0	0	0	0	0	0	0	0	83
20690230	Not	md	PL	2022	0	1	0	1	0	0	0	0	0	0	0	0	
20690244	m	hp	US	2022	1	0	0	0	0	0	0	0	0	0	0	0	50
20690485	Not	md	PL	2022	0	1	0	1	0	0	0	0	0	0	0	1	
20690572	f	md	VN	2022	0	0	0	0	0	0	0	0	0	0	0	0	75
20690795	f	md	RU	2022	0	0	0	0	0	0	0	0	0	0	0	0	71
20690840	m	md	JP	2022	0	1	0	1	0	0	0	0	0	0	0	0	72

20690905	m	hp	US	2022	0	0	0	0	0	0	0	0	0	0	0	0	68
20690930	m	ph	US	2022	0	0	0	0	0	0	0	0	0	0	0	0	48
20690992	m	md	JP	2022	0	1	0	1	0	0	0	0	0	0	0	1	68
20691054	f	cn	VN	2022	1	0	0	0	0	0	0	0	0	0	0	0	
20691260	Not	md	PL	2022	0	1	0	1	0	0	0	0	0	0	0	0	
20691264	f	md	FR	2022	0	0	0	0	0	0	0	0	0	0	0	0	61
20691425	f	md	MY	2022	0	0	0	0	0	0	0	0	0	0	0	0	63
20691437	m	cn	SG	2022	1	0	0	0	0	0	0	0	0	0	0	0	84.4
20691439	Not	cn	US	2022	1	0	0	0	0	0	0	0	0	0	0	0	70.4
20691734	m	hp	KR	2022	0	0	0	0	0	0	0	0	0	0	0	0	66
20691759	f	cn	US	2022	1	0	0	0	0	0	0	0	0	0	0	0	69
20691878	m	md	AT	2022	0	0	0	0	0	0	0	0	0	0	0	0	70
20691898	m	md	JP	2022	0	1	1	0	0	0	0	0	0	0	0	0	76
20691999	f	md	US	2022	0	1	0	0	0	1	0	0	0	0	0	0	64
20692106	m	md	JP	2022	0	1	0	0	0	1	0	0	0	0	0	0	60
20692584	f	hp	DE	2022	0	1	0	0	1	0	0	0	0	0	0	0	68
20692615	f	hp	US	2022	0	0	0	0	0	0	0	0	0	0	0	0	82
20692678	Not	md	JP	2022	1	0	0	0	0	0	0	0	0	0	0	0	
20693076	m	md	JP	2022	0	1	0	0	1	0	0	0	0	0	0	0	71
20693328	f	ph	US	2022	1	0	0	0	0	0	0	0	0	0	0	0	
20693469	f	hp	ES	2022	0	1	0	0	1	0	0	0	0	0	0	0	54
20693685	Not	hp	US	2022	0	0	0	0	0	0	0	0	0	0	0	0	
20693815	m	md	JP	2022	0	1	0	0	1	0	0	0	0	0	0	1	72
20693858	f	md	DE	2022	0	1	0	0	1	0	0	0	0	0	0	0	54
20694003	f	md	SE	2022	0	0	0	0	0	0	0	0	0	0	0	0	
20694010	f	md	CN	2022	0	0	0	0	0	0	0	0	0	0	0	0	38
20694026	m	md	RU	2022	0	1	0	0	0	1	0	0	0	0	0	0	74
20694050	m	md	JP	2022	0	1	0	0	1	0	0	0	0	0	0	0	78
20694341	Not	md	JP	2022	0	1	0	0	1	0	0	0	0	0	0	0	70
20694723	f	cn	SG	2022	1	0	0	0	0	0	0	0	0	0	0	0	63.5
20694803	f	cn	US	2022	1	0	0	0	0	0	0	0	0	0	0	0	70.1
20694811	f	cn	TH	2022	1	0	0	0	0	0	0	0	0	0	0	0	71
20694822	m	md	JP	2022	0	1	0	1	0	0	0	0	0	0	0	0	71
20695004	f	cn	JP	2022	0	0	0	0	0	0	0	0	0	0	0	0	52

20695033	m	md	KR	2022	0	0	0	0	0	0	0	0	0	0	0	0	66
20695173	m	hp	US	2022	0	1	0	1	0	0	0	0	0	0	0	0	68
20695507	m	cn	US	2022	1	0	0	0	0	0	0	0	0	0	0	0	64.9
20695508	m	cn	IN	2022	1	0	0	0	0	0	0	0	0	0	0	0	74
20695924	m	md	JP	2022	0	1	0	0	1	0	0	0	0	0	0	1	57
20696012	f	md	GR	2022	0	0	0	0	0	0	0	0	0	0	0	0	64
20696095	m	hp	US	2022	0	0	0	0	0	0	0	0	0	0	0	0	41
20696203	m	cn	IN	2022	0	0	0	0	0	0	0	0	0	0	0	0	52
20696350	m	hp	JP	2022	0	1	1	0	0	0	0	0	0	0	0	1	72
20696418	m	hp	GB	2022	0	1	0	1	0	0	0	0	0	0	0	0	53
20696731	Not	md	AU	2022	0	0	0	0	0	0	0	0	0	0	0	0	62
20696831	f	cn	GB	2022	1	0	0	0	0	0	0	0	0	0	0	0	
20697113	m	hp	IT	2022	0	1	1	0	0	0	0	0	0	0	0	0	68
20697434	m	cn	CA	2022	0	0	0	0	0	0	0	0	0	0	0	0	63
20697629	m	md	FR	2022	0	1	1	0	0	0	0	0	0	0	0	0	75
20697655	Not	md	JP	2022	0	1	0	0	1	0	0	0	0	0	0	0	
20697930	f	md	NL	2022	0	0	0	0	0	0	0	0	0	0	0	0	70
20698253	f	ph	CA	2022	0	1	1	0	0	0	0	0	0	0	0	0	76
20698435	Not	cn	CN	2022	0	0	0	0	0	0	0	0	0	0	0	0	
20698641	f	hp	US	2022	0	0	0	0	0	0	0	0	0	0	0	0	88
20698695	f	hp	AT	2022	0	1	1	0	0	0	0	0	0	0	0	0	69
20698698	f	hp	AT	2022	0	1	1	0	0	0	0	0	0	0	0	0	57
20698699	m	hp	AT	2022	0	1	0	0	1	0	0	0	0	0	0	0	43
20698717	f	hp	AT	2022	0	1	1	0	0	0	0	0	0	0	0	0	56
20698895	m	hp	CA	2022	0	1	1	0	0	0	0	0	0	0	0	0	82
20698934	f	cn	US	2022	0	1	1	0	0	0	0	0	0	0	0	0	86
20699014	m	md	JP	2022	0	1	1	0	0	0	0	0	0	0	0	0	90
20699487	m	md	JP	2022	0	0	0	0	0	0	0	0	0	0	0	0	49
20700082	m	hp	IT	2022	0	1	1	0	0	0	0	0	0	0	0	0	68
20700233	f	md	JP	2022	0	1	0	0	1	0	0	0	0	0	0	0	58
20700274	m	hp	IT	2022	0	1	1	0	0	0	0	0	0	0	0	0	68
20700306	m	md	LT	2022	0	1	0	0	0	1	0	0	0	0	0	0	73
20700437	f	cn	EG	2022	0	0	0	0	0	0	0	0	0	0	0	0	71
20700796	m	md	DK	2022	0	0	0	0	0	0	0	0	0	0	0	0	57

20701280	m	hp	US	2022	0	0	0	0	0	0	0	0	0	0	0	0	69
20701290	f	md	FR	2022	0	0	0	0	0	0	0	0	0	0	0	0	90
20701709	m	hp	PL	2022	0	1	0	1	0	0	0	0	0	0	0	0	
20701761	m	md	FR	2022	0	0	0	0	0	0	0	0	0	0	0	0	70
20701762	Not	md	IT	2022	0	1	0	0	0	1	0	0	0	0	0	0	
20701790	m	cn	IN	2022	1	0	0	0	0	0	0	0	0	0	0	0	40
20701823	m	cn	AU	2022	0	0	0	0	0	0	0	0	0	0	0	0	78
20702084	Not	md	PL	2022	0	1	0	1	0	0	0	0	0	0	0	0	
20702156	f	md	CA	2022	1	0	0	0	0	0	0	0	0	0	0	0	79.6
20702192	m	cn	AU	2022	0	1	1	0	0	0	0	0	0	0	0	0	55
20702244	f	ph	FR	2022	0	0	0	0	0	0	0	0	0	0	0	0	
20702785	m	ph	JP	2022	0	1	0	0	1	0	0	0	0	0	0	0	80
20702857	m	hp	DE	2022	0	1	0	0	1	0	0	0	0	0	0	0	59
20703208	m	md	FR	2022	0	0	0	0	0	0	0	0	0	0	0	0	67
20703627	m	cn	IN	2022	0	1	0	0	1	0	0	0	0	0	0	0	
20703751	m	hp	IT	2022	0	1	1	0	0	0	0	0	0	0	0	0	68
20704021	m	cn	CN	2022	0	1	0	0	1	0	0	0	0	0	0	0	61
20704151	m	md	JP	2022	0	0	0	0	0	0	0	0	0	0	0	0	88
20704458	m	md	ES	2022	0	1	0	0	0	1	0	0	0	0	0	0	74
20704468	f	md	JP	2022	0	1	0	0	1	0	0	0	0	0	0	1	64
20704656	m	md	US	2022	0	0	0	0	0	0	0	0	0	0	0	0	68
20704781	m	md	DE	2022	0	1	1	0	0	0	0	0	0	0	0	0	60
20704841	Not	hp	JP	2022	0	0	0	0	0	0	0	0	0	0	0	0	
20705081	f	md	FR	2022	0	1	1	0	0	0	0	0	0	0	0	0	
20705129	f	hp	TH	2022	1	0	0	0	0	0	0	0	0	0	0	0	35
20705665	f	hp	CA	2022	0	1	1	0	0	0	0	0	0	0	0	0	
20705763	m	cn	FR	2022	0	1	1	0	0	0	0	0	0	0	0	0	72
20705899	m	md	PL	2022	0	1	0	0	0	1	0	0	0	0	0	0	
20705902	m	md	JP	2022	0	0	0	0	0	0	0	0	0	0	0	0	70
20705929	f	cn	GR	2022	0	0	0	0	0	0	0	0	0	0	0	0	64
20706183	Not	hp	US	2022	0	0	0	0	0	0	0	0	0	0	0	0	
20706348	m	md	TR	2022	0	0	0	0	0	0	0	0	0	0	0	0	73
20706445	Not	md	JP	2022	0	1	0	1	0	0	0	0	0	0	0	0	
20706625	m	md	KR	2022	0	0	0	0	0	0	0	0	0	0	0	0	81

20706662	m	md	JP	2022	0	0	0	0	0	0	0	0	0	0	0	0	56
20706787	m	md	GR	2022	0	0	0	0	0	0	0	0	0	0	0	0	
20706960	f	md	AT	2022	0	0	0	0	0	0	0	0	0	0	0	0	55
20707152	m	md	JP	2022	0	1	1	0	0	0	0	0	0	0	0	0	58
20707289	f	hp	IL	2022	0	0	0	0	0	0	0	0	0	0	0	0	55
20707366	f	md	KR	2022	0	0	0	0	0	0	0	0	0	0	0	0	50
20707550	m	hp	ES	2022	1	0	0	0	0	0	0	0	0	0	0	0	84
20707633	Not	ph	HK	2022	0	1	0	0	0	1	0	0	0	0	0	0	
20707833	f	md	DE	2022	0	1	0	0	1	0	0	0	0	0	0	0	
20707860	f	md	DE	2022	0	1	0	0	1	0	0	0	0	0	0	0	65
20707867	f	md	JP	2022	0	0	0	0	0	0	0	0	0	0	0	0	74
20707942	m	cn	CN	2022	0	1	0	0	1	0	0	0	0	0	0	0	84
20708118	m	md	CY	2022	0	1	0	0	1	0	0	0	0	0	0	0	75
20708822	Not	md	JP	2022	0	1	1	0	0	0	0	0	0	0	0	0	79
20709108	m	hp	US	2022	0	0	0	0	0	0	0	0	0	0	0	0	68
20709324	f	md	BG	2022	0	1	0	0	0	1	0	0	0	0	0	0	56
20709489	f	md	CA	2022	1	0	0	0	0	0	0	0	0	0	0	0	
20709635	m	hp	US	2022	0	0	0	0	0	0	0	0	0	0	0	0	55
20709872	m	cn	AR	2022	0	0	0	0	0	0	0	0	0	0	0	0	41
20709896	m	hp	US	2022	0	1	0	1	0	0	0	0	0	0	0	0	68
20709908	f	md	JP	2022	0	1	1	0	0	0	0	0	0	0	0	0	79
20709959	m	md	TR	2022	0	0	0	0	0	0	0	0	0	0	0	0	42
20709970	m	cn	US	2022	0	1	0	1	0	0	0	0	0	0	0	1	63
20710011	m	cn	CN	2022	0	1	0	1	0	0	0	0	0	0	0	0	64
20710054	f	md	FR	2022	0	0	0	0	0	0	0	0	0	0	0	0	
20710056	m	md	NL	2022	0	1	0	0	0	1	0	0	0	0	0	0	76
20710107	Not	md	US	2022	0	0	0	0	0	0	0	0	0	0	0	0	
20710177	m	md	JP	2022	0	1	0	1	0	0	0	0	0	0	0	1	78
20710376	f	md	DE	2022	0	1	0	0	1	0	0	0	0	0	0	0	85
20710429	f	md	FR	2022	0	0	0	0	0	0	0	0	0	0	0	0	52
20710475	f	md	PL	2022	1	0	0	0	0	0	0	0	0	0	0	0	
20710516	m	hp	KR	2022	0	0	0	0	0	0	0	0	0	0	0	0	70
20710951	f	cn	US	2022	0	0	0	0	0	0	0	0	0	0	0	0	
20711391	f	cn	IN	2022	0	0	0	0	0	0	0	0	0	0	0	0	18

20711458	f	hp	US	2022	0	0	0	0	0	0	0	0	0	0	0	0	61
20711546	m	md	JP	2022	0	1	0	1	0	0	0	0	0	0	0	0	75
20711597	f	cn	NL	2022	0	1	1	0	0	0	0	0	0	0	0	0	66
20711608	m	md	JP	2022	0	1	0	1	0	0	0	0	0	0	0	0	62
20711610	f	md	AU	2022	1	0	0	0	0	0	0	0	0	0	0	0	52
20711724	Not	md	JP	2022	0	1	0	1	0	0	0	0	0	0	0	0	40
20711874	m	md	JP	2022	0	0	0	0	0	0	0	0	0	0	0	0	69
20712105	f	md	IL	2022	0	0	0	0	0	0	0	0	0	0	0	0	63
20712283	f	md	US	2022	1	0	0	0	0	0	0	0	0	0	0	0	58
20712415	m	hp	CN	2022	0	0	0	0	0	0	0	0	0	0	0	0	68
20712420	m	md	JP	2022	0	1	0	0	1	0	0	0	0	0	0	0	68
20712457	Not	cn	SG	2022	1	0	0	0	0	0	0	0	0	0	0	0	69.8
20712508	f	md	AU	2022	1	0	0	0	0	0	0	0	0	0	0	0	
20712601	m	md	JP	2022	0	0	0	0	0	0	0	0	0	0	0	0	60
20712643	m	md	GB	2022	0	1	0	0	0	1	0	0	0	0	0	0	65
20712663	m	md	JP	2022	0	1	0	0	1	0	0	0	0	0	0	0	80
20712784	m	md	JP	2022	0	1	1	0	0	0	0	0	0	0	0	0	90
20712829	Not	cn	SG	2022	1	0	0	0	0	0	0	0	0	0	0	0	72.5
20712877	m	md	CN	2022	0	1	0	1	0	0	0	0	0	0	0	0	72.6
20712899	m	md	JP	2022	0	1	1	0	0	0	0	0	0	0	0	0	69
20713091	m	md	FR	2022	0	1	0	1	0	0	0	0	0	0	0	0	
20713125	m	cn	CN	2022	0	1	0	0	1	0	0	0	0	0	0	0	72
20713176	m	hp	US	2022	1	0	0	0	0	0	0	0	0	0	0	0	
20713178	m	cn	IN	2022	1	0	0	0	0	0	0	0	0	0	0	0	60
20713194	m	md	JP	2022	0	1	0	0	1	0	0	0	0	0	0	0	80
20713204	f	hp	TR	2022	0	0	0	0	0	0	0	0	0	0	0	0	64
20713375	m	md	KR	2022	0	0	0	0	0	0	0	0	0	0	0	0	73
20713734	m	md	CN	2022	0	0	0	0	0	0	0	0	0	0	0	0	73
20714004	Not	cn	SG	2022	1	0	0	0	0	0	0	0	0	0	0	0	78.2
20714012	m	hp	JP	2022	0	1	1	0	0	0	0	0	0	0	0	0	80
20714197	f	hp	SG	2022	1	0	0	0	0	0	0	0	0	0	0	0	
20714299	f	cn	CN	2022	0	1	0	0	1	0	0	0	0	0	0	0	62
20714425	f	md	CZ	2022	0	1	1	0	0	0	0	0	0	0	0	0	
20714561	m	md	BE	2022	0	1	0	0	1	0	0	0	0	0	0	0	80

20714621	m	md	JP	2022	0	1	0	0	1	0	0	0	0	0	0	0	62
20714967	m	ph	JP	2022	1	0	0	0	0	0	0	0	0	0	0	0	
20715227	m	cn	CA	2022	1	0	0	0	0	0	0	0	0	0	0	0	81.3
20715296	m	md	JP	2022	0	1	0	1	0	0	0	0	0	0	0	1	84
20716226	m	hp	US	2022	0	1	0	0	1	0	0	0	0	0	0	0	77
20716558	f	md	JP	2022	0	1	0	0	1	0	0	0	0	0	0	0	74
20717534	f	ph	US	2022	0	0	0	0	0	0	0	0	0	0	0	0	88
20717681	f	cn	IN	2022	0	1	0	0	1	0	0	0	0	0	0	0	57
20717845	f	hp	US	2022	0	0	0	0	0	0	0	0	0	0	0	0	
20718591	f	cn	AR	2022	0	0	0	0	0	0	0	0	0	0	0	0	65
20718777	f	md	JP	2022	0	1	0	0	0	1	0	0	0	0	0	0	79
20718827	f	cn	IT	2022	0	1	1	0	0	0	0	0	0	0	0	0	43
20718829	Not	cn	JP	2022	0	0	0	0	0	0	0	0	0	0	0	0	
20718838	f	cn	US	2022	0	0	0	0	0	0	0	0	0	0	0	0	62
20719221	f	md	JP	2022	0	0	0	0	0	0	0	0	0	0	0	0	79
20719517	f	cn	CA	2022	0	0	0	0	0	0	0	0	0	0	0	0	84
20719547	m	md	BR	2022	0	0	0	0	0	0	0	0	0	0	0	0	75
20719609	f	cn	US	2022	0	0	0	0	0	0	0	0	0	0	0	0	73
20719809	m	hp	JP	2022	0	1	0	0	1	0	0	0	0	0	0	0	74
20719875	Not	hp	SG	2022	1	0	0	0	0	0	0	0	0	0	0	0	67
20720040	f	md	DE	2022	0	0	0	0	0	0	0	0	0	0	0	0	61
20720121	f	md	NO	2022	0	0	0	0	0	0	0	0	0	0	0	0	60
20720922	Not	cn	SG	2022	1	0	0	0	0	0	0	0	0	0	0	0	68.3
20720928	f	md	RU	2022	0	1	0	0	0	1	0	0	0	0	0	0	66
20720953	m	hp	IN	2022	0	1	0	0	1	0	0	0	0	0	0	0	62
20721323	m	ph	US	2022	0	0	0	0	0	0	0	0	0	0	0	0	73
20721582	m	md	US	2022	0	0	0	0	0	0	0	0	0	0	0	0	58
20721650	m	md	JP	2022	0	1	0	0	0	1	0	0	0	0	0	0	65
20721717	Not	ph	US	2022	0	0	0	0	0	0	0	0	0	0	0	0	
20721744	f	md	JP	2022	0	1	0	0	1	0	0	0	0	0	0	0	73
20722313	f	md	CZ	2022	0	1	1	0	0	0	0	0	0	0	0	0	53
20722324	m	md	TW	2022	0	1	0	0	0	1	0	0	0	0	0	1	61
20722328	m	md	JP	2022	0	1	1	0	0	0	0	0	0	0	0	0	68
20722348	m	md	JP	2022	0	0	0	0	0	0	0	0	0	0	0	0	63

20722359	m	cn	IN	2022	0	0	0	0	0	0	0	0	0	0	0	0	46
20722482	m	md	JP	2022	0	1	0	0	1	0	0	0	0	0	0	0	68
20722527	f	ph	JP	2022	0	0	0	0	0	0	0	0	0	0	0	0	70
20722566	m	cn	CN	2022	0	1	0	1	0	0	0	0	0	0	0	1	66
20722617	m	hp	AU	2022	0	1	0	0	1	0	0	0	0	0	0	0	64
20722646	Not	md	JP	2022	0	1	0	0	1	0	0	0	0	0	0	0	
20723313	m	md	JP	2022	0	0	0	0	0	0	0	0	0	0	0	0	70
20723375	f	hp	CN	2022	0	1	1	0	0	0	0	0	0	0	0	0	52
20723479	f	hp	CN	2022	0	1	1	0	0	0	0	0	0	0	0	0	63
20723483	m	hp	CN	2022	0	1	1	0	0	0	0	0	0	0	0	0	52
20723499	f	hp	CN	2022	0	1	1	0	0	0	0	0	0	0	0	0	43
20723500	f	md	US	2022	0	0	0	0	0	0	0	0	0	0	0	0	54
20723503	m	md	IL	2022	0	0	0	0	0	0	0	0	0	0	0	0	78
20723529	m	cn	US	2022	1	0	0	0	0	0	0	0	0	0	0	0	81
20723531	f	md	DE	2022	0	1	1	0	0	0	0	0	0	0	0	0	60
20724222	Not	md	JP	2022	1	0	0	0	0	0	0	0	0	0	0	0	
20724357	m	cn	NP	2022	0	0	0	0	0	0	0	0	0	0	0	0	67
20724703	f	ph	FR	2022	0	0	0	0	0	0	0	0	0	0	0	0	
20724888	f	md	DE	2022	0	1	1	0	0	0	0	0	0	0	0	0	58
20725040	f	hp	CN	2022	0	1	1	0	0	0	0	0	0	0	0	0	
20725070	f	hp	CN	2022	0	1	1	0	0	0	0	0	0	0	0	0	60
20725114	f	hp	CN	2022	0	1	1	0	0	0	0	0	0	0	0	0	68
20725502	f	md	CN	2022	0	0	0	0	0	0	0	0	0	0	0	0	66
20725551	m	md	JP	2022	0	1	0	1	0	0	0	0	0	0	0	1	77
20725553	Not	md	ES	2022	1	0	0	0	0	0	0	0	0	0	0	0	
20725634	f	cn	US	2022	1	0	0	0	0	0	0	0	0	0	0	0	
20725953	m	hp	TW	2022	0	0	0	0	0	0	0	0	0	0	0	0	56
20726097	f	md	JP	2022	1	0	0	0	0	0	0	0	0	0	0	0	89
20726459	m	md	JP	2022	0	1	1	0	0	0	0	0	0	0	0	0	
20727061	m	cn	PH	2022	0	0	0	0	0	0	0	0	0	0	0	0	62
20727068	m	md	US	2022	0	1	0	0	0	1	0	0	0	0	0	0	63
20727081	f	md	US	2022	0	1	0	0	0	1	0	0	0	0	0	0	75
20727315	m	md	FR	2022	0	0	0	0	0	0	0	0	0	0	0	0	65
20727376	Not	md	RU	2022	0	1	0	0	0	1	0	0	0	0	0	0	

20727451	m	hp	US	2022	0	0	0	0	0	0	0	0	0	0	0	0	63
20727540	f	cn	US	2022	1	0	0	0	0	0	0	0	0	0	0	0	
20727715	m	hp	US	2022	0	0	0	0	0	0	0	0	0	0	0	0	40
20727885	f	hp	US	2022	0	1	0	0	1	0	0	0	0	0	0	0	73
20728267	f	hp	TR	2022	1	0	0	0	0	0	0	0	0	0	0	0	70
20728606	Not	md	JP	2022	0	1	0	0	1	0	0	0	0	0	0	0	
20728931	Not	md	CN	2022	0	0	0	0	0	0	0	0	0	0	0	0	
20729336	m	md	CA	2022	0	1	0	0	1	0	0	0	0	0	0	0	
20729436	f	md	JP	2022	0	1	0	0	0	1	0	0	0	0	0	0	
20729603	f	md	US	2022	0	0	0	0	0	0	0	0	0	0	0	0	
20730251	m	cn	AR	2022	0	0	0	0	0	0	0	0	0	0	0	0	74
20730260	m	cn	US	2022	0	0	0	0	0	0	0	0	0	0	0	0	61
20730262	m	cn	IN	2022	0	0	0	0	0	0	0	0	0	0	0	0	55
20730531	f	md	JP	2022	0	1	0	0	0	1	0	0	0	0	0	0	60
20730536	f	md	AU	2022	1	0	0	0	0	0	0	0	0	0	0	0	
20731221	m	hp	US	2022	0	0	0	0	0	0	0	0	0	0	0	0	
20731702	m	md	JP	2022	0	1	0	1	0	0	0	0	0	0	0	0	75
20731783	Not	md	AU	2022	1	0	0	0	0	0	0	0	0	0	0	0	
20731820	f	md	DE	2022	0	0	0	0	0	0	0	0	0	0	0	0	71
20731823	m	md	FR	2022	0	0	0	0	0	0	0	0	0	0	0	0	69
20731979	m	md	JP	2022	0	1	0	0	1	0	0	0	0	0	0	1	75
20732265	Not	md	US	2022	0	0	0	0	0	0	0	0	0	0	0	0	
20732282	m	md	FR	2022	0	1	1	0	0	0	0	0	0	0	0	0	67
20732294	f	hp	NL	2022	0	1	0	0	1	0	0	0	0	0	0	0	85
20732451	m	md	AT	2022	0	1	0	0	0	1	0	0	0	0	0	0	63
20732775	Not	hp	SG	2022	1	0	0	0	0	0	0	0	0	0	0	0	93
20733000	m	md	JP	2022	0	1	0	0	1	0	0	0	0	0	0	1	68
20733106	m	md	JP	2022	0	1	0	0	1	0	0	0	0	0	0	1	78
20733153	Not	hp	FR	2022	0	1	0	0	1	0	0	0	0	0	0	0	63
20733265	m	cn	IN	2022	0	1	0	0	1	0	0	0	0	0	0	0	56
20733351	m	md	JP	2022	0	0	0	0	0	0	0	0	0	0	0	0	78
20733515	m	md	IT	2022	0	0	0	0	0	0	0	0	0	0	0	0	
20733529	m	md	JP	2022	1	0	0	0	0	0	0	0	0	0	0	0	85
20733638	f	cn	US	2022	0	0	0	0	0	0	0	0	0	0	0	0	

20733731	f	md	JP	2022	0	1	0	0	1	0	0	0	0	0	0	0	67
20733993	m	md	JP	2022	0	1	0	0	0	1	0	0	0	0	0	0	68
20734231	f	cn	ID	2022	1	0	0	0	0	0	0	0	0	0	0	0	67.8
20734312	m	cn	CA	2022	0	0	0	0	0	0	0	0	0	0	0	0	71
20734497	m	hp	JP	2022	0	1	0	0	1	0	0	0	0	0	0	0	54
20734633	f	hp	US	2022	0	0	0	0	0	0	0	0	0	0	0	0	
20734741	f	md	DE	2022	0	1	0	0	1	0	0	0	0	0	0	0	65
20734785	m	cn	US	2022	0	0	0	0	0	0	0	0	0	0	0	0	
20735045	m	hp	US	2022	0	0	0	0	0	0	0	0	0	0	0	0	68
20735061	f	cn	DE	2022	0	1	1	0	0	0	0	0	0	0	0	0	60
20735138	f	md	US	2022	0	1	0	1	0	0	0	0	0	0	0	1	
20735184	m	md	DE	2022	0	1	0	0	1	0	0	0	0	0	0	0	60
20735303	f	hp	RU	2022	0	1	0	1	0	0	0	0	0	0	0	0	60
20735330	f	hp	AT	2022	0	0	0	0	0	0	0	0	0	0	0	0	69
20735331	m	md	IT	2022	0	1	1	0	0	0	0	0	0	0	0	0	81
20735433	m	md	KR	2022	0	0	0	0	0	0	0	0	0	0	0	0	75
20735575	f	md	US	2022	0	0	0	0	0	0	0	0	0	0	0	0	63
20735746	f	md	FR	2022	0	0	0	0	0	0	0	0	0	0	0	0	
20735872	f	cn	US	2022	0	0	0	0	0	0	0	0	0	0	0	0	
20735972	f	md	JP	2022	0	0	0	0	0	0	0	0	0	0	0	0	71
20736031	f	md	BR	2022	0	0	0	0	0	0	0	0	0	0	0	0	
20736089	Not	hp	BY	2022	0	1	0	0	0	1	0	0	0	0	0	0	41
20736155	Not	md	JP	2022	0	1	0	1	0	0	0	0	0	0	0	1	
20736532	m	hp	US	2022	0	0	0	0	0	0	0	0	0	0	0	0	56
20736576	m	hp	CN	2022	0	1	0	0	1	0	0	0	0	0	0	0	53
20736898	f	ph	US	2022	0	0	0	0	0	0	0	0	0	0	0	0	37
20736919	f	hp	AT	2022	0	0	0	0	0	0	0	0	0	0	0	0	49
20737023	m	md	JP	2022	0	1	1	0	0	0	0	0	0	0	0	0	80
20737025	f	md	US	2022	0	0	0	0	0	0	0	0	0	0	0	0	
20737211	m	hp	US	2022	0	1	0	1	0	0	0	0	0	0	0	0	68
20737277	Not	md	AU	2022	1	0	0	0	0	0	0	0	0	0	0	0	
20737314	m	cn	CN	2022	0	1	0	1	0	0	0	0	0	0	0	0	66
20737334	m	md	JP	2022	0	1	0	0	1	0	0	0	0	0	0	0	81
20737766	f	cn	CN	2022	0	1	0	0	1	0	0	0	0	0	0	0	65

20738049	m	md	RU	2022	0	0	0	0	0	0	0	0	0	0	0	0	66
20738196	m	md	JP	2022	0	1	0	0	1	0	0	0	0	0	0	0	73
20738341	m	cn	CN	2022	0	0	0	0	0	0	0	0	0	0	0	0	57
20738342	m	md	JP	2022	0	0	0	0	0	0	0	0	0	0	0	0	74
20738384	f	md	RU	2022	0	1	0	0	0	1	0	0	0	0	0	0	51
20738413	f	cn	HK	2022	0	0	0	0	0	0	0	0	0	0	0	0	47
20738444	m	md	TW	2022	0	0	0	0	0	0	0	0	0	0	0	0	
20738506	m	md	JP	2022	0	0	0	0	0	0	0	0	0	0	0	0	70
20738557	m	hp	FR	2022	0	1	1	0	0	0	0	0	0	0	0	0	65
20738717	Not	cn	CN	2022	0	0	0	0	0	0	0	0	0	0	0	0	
20738816	f	hp	JP	2022	0	1	1	0	0	0	0	0	0	0	0	0	77
20738903	f	md	NL	2022	0	0	0	0	0	0	0	0	0	0	0	0	70
20739185	Not	cn	KR	2022	1	0	0	0	0	0	0	0	0	0	0	0	
20739207	m	md	FR	2022	0	1	1	0	0	0	0	0	0	0	0	0	58
20739247	Not	ph	JP	2022	1	0	0	0	0	0	0	0	0	0	0	0	
20739486	f	hp	NL	2022	0	1	1	0	0	0	0	0	0	0	0	0	66
20739591	m	md	JP	2022	0	1	1	0	0	0	0	0	0	0	0	0	58
20739623	m	ph	US	2022	0	0	0	0	0	0	0	0	0	0	0	0	62
20739664	m	md	DE	2022	0	1	0	0	1	0	0	0	0	0	0	0	59
20739737	Not	cn	US	2022	1	0	0	0	0	0	0	0	0	0	0	0	65.3
20739738	Not	cn	SG	2022	1	0	0	0	0	0	0	0	0	0	0	0	66.2
20740702	m	md	AU	2022	0	0	0	0	0	0	0	0	0	0	0	0	63
20740707	m	cn	ID	2022	1	0	0	0	0	0	0	0	0	0	0	0	65.5
20740802	m	ph	AU	2022	0	1	0	0	1	0	0	0	0	0	0	0	65
20740878	f	md	IN	2022	0	0	0	0	0	0	0	0	0	0	0	0	68
20740926	m	md	US	2022	0	0	0	0	0	0	0	0	0	0	0	0	
20741063	m	hp	RU	2022	1	0	0	0	0	0	0	0	0	0	0	0	
20741073	f	md	CH	2022	0	1	0	0	0	1	0	0	0	0	0	0	44
20741258	f	md	CN	2022	0	0	0	0	0	0	0	0	0	0	0	0	52
20741405	f	md	US	2022	0	0	0	0	0	0	0	0	0	0	0	0	40
20741524	m	ph	JP	2022	0	1	1	0	0	0	0	0	0	0	0	0	80
20741700	m	md	JP	2022	0	1	0	0	0	1	0	0	0	0	0	0	70
20742210	f	md	BG	2022	0	1	0	0	0	1	0	0	0	0	0	0	65
20742831	f	md	JP	2022	0	0	0	0	0	0	0	0	0	0	0	0	74

20743075	m	md	US	2022	0	0	0	0	0	0	0	0	0	0	0	0	
20743132	f	md	BR	2022	0	1	0	0	1	0	0	0	0	0	0	0	
20743480	m	md	US	2022	0	0	0	0	0	0	0	0	0	0	0	0	
20743632	m	cn	KR	2022	0	0	0	0	0	0	0	0	0	0	0	0	44
20743670	m	md	US	2022	1	0	0	0	0	0	0	0	0	0	0	0	79
20743679	m	hp	US	2022	0	0	0	0	0	0	0	0	0	0	0	0	68
20743902	m	md	FR	2022	0	0	0	0	0	0	0	0	0	0	0	0	73
20743942	m	md	JP	2022	0	1	0	0	1	0	0	0	0	0	0	0	81
20744027	f	md	JP	2022	0	1	0	0	1	0	0	0	0	0	0	0	63
20744028	f	md	DE	2022	0	1	0	0	1	0	0	0	0	0	0	0	69
20744196	m	cn	GB	2022	1	0	0	0	0	0	0	0	0	0	0	0	
20744343	m	hp	US	2022	0	1	0	0	1	0	0	0	0	0	0	0	68
20744589	m	md	DE	2022	0	1	0	0	1	0	0	0	0	0	0	0	
20744814	m	cn	IN	2022	0	0	0	0	0	0	0	0	0	0	0	0	76
20745037	f	md	US	2022	1	0	0	0	0	0	0	0	0	0	0	0	
20745187	m	hp	US	2022	0	0	0	0	0	0	0	0	0	0	0	0	71
20745193	m	md	DE	2022	0	1	0	0	1	0	0	0	0	0	0	0	69
20745456	f	md	SE	2022	0	0	0	0	0	0	0	0	0	0	0	0	
20745723	f	md	JP	2022	0	1	1	0	0	0	0	0	0	0	0	0	70
20746073	f	md	JP	2022	0	1	0	0	0	1	0	0	0	0	0	0	80
20746095	m	hp	TH	2022	0	0	0	0	0	0	0	0	0	0	0	0	60
20746107	m	md	JP	2022	0	1	0	0	1	0	0	0	0	0	0	0	80
20746419	m	hp	US	2022	0	0	0	0	0	0	0	0	0	0	0	0	68
20746571	f	md	JP	2022	0	1	1	0	0	0	0	0	0	0	0	0	60
20746875	f	md	PL	2022	0	1	0	0	0	1	0	0	0	0	0	0	67
20746920	f	cn	CZ	2022	0	1	1	0	0	0	0	0	0	0	0	0	53
20746922	m	cn	JP	2022	0	0	0	0	0	0	0	0	0	0	0	0	85
20746946	f	md	BE	2022	0	0	0	0	0	0	0	0	0	0	0	0	45
20747765	f	md	CZ	2022	0	1	1	0	0	0	0	0	0	0	0	0	71
20747905	f	cn	CN	2022	0	1	0	0	1	0	0	0	0	0	0	0	56
20748088	m	md	JP	2022	0	0	0	0	0	0	0	0	0	0	0	0	
20748215	m	md	CN	2022	0	0	0	0	0	0	0	0	0	0	0	0	48
20748458	m	hp	US	2022	0	1	0	1	0	0	0	0	0	0	0	0	68
20748637	f	md	US	2022	0	1	0	0	1	0	0	0	0	0	0	0	71

20748954	f	md	FR	2022	0	0	0	0	0	0	0	0	0	0	0	0	69
20749289	m	cn	US	2022	0	0	0	0	0	0	0	0	0	0	0	0	
20749810	m	md	CA	2022	1	0	0	0	0	0	0	0	0	0	0	0	
20749813	f	cn	US	2022	1	0	0	0	0	0	0	0	0	0	0	0	67.6
20749842	f	md	JP	2022	0	1	0	0	1	0	0	0	0	0	0	0	70
20749897	f	cn	US	2022	1	0	0	0	0	0	0	0	0	0	0	0	58.4
20749967	Not	md	JP	2022	0	1	0	1	0	0	0	0	0	0	0	1	
20750543	m	cn	DE	2022	0	0	0	0	0	0	0	0	0	0	0	0	53
20750559	Not	hp	FR	2022	0	0	0	0	0	0	0	0	0	0	0	0	
20750719	f	md	CA	2022	0	0	0	0	0	0	0	0	0	0	0	0	
20750764	m	md	JP	2022	0	1	0	0	1	0	0	0	0	0	0	0	65
20750772	m	md	JP	2022	0	1	0	0	1	0	0	0	0	0	0	0	65
20750870	f	hp	SE	2022	0	0	0	0	0	0	0	0	0	0	0	0	
20751056	f	md	DE	2022	0	1	0	0	1	0	0	0	0	0	0	0	75
20751075	m	cn	US	2022	0	0	0	0	0	0	0	0	0	0	0	0	
20751090	m	md	JP	2022	0	1	0	0	1	0	0	0	0	0	0	0	64
20751095	f	md	HK	2022	0	0	0	0	0	0	0	0	0	0	0	0	
20751307	f	md	NL	2022	0	0	0	0	0	0	0	0	0	0	0	0	70
20751702	m	hp	SE	2022	1	0	0	0	0	0	0	0	0	0	0	0	68
20751999	f	md	CN	2022	0	0	0	0	0	0	0	0	0	0	0	0	50
20752276	m	ph	FR	2022	0	0	0	0	0	0	0	0	0	0	0	0	
20752290	m	md	CN	2022	0	0	0	0	0	0	0	0	0	0	0	0	75
20752306	f	cn	US	2022	0	1	1	0	0	0	0	0	0	0	0	0	
20753258	f	md	PT	2022	0	0	0	0	0	0	0	0	0	0	0	0	82
20754016	f	md	JP	2022	0	1	0	1	0	0	0	0	0	0	0	1	71
20754437	f	hp	SE	2022	0	1	0	0	1	0	0	0	0	0	0	0	68
20754497	m	ph	JP	2022	0	0	0	0	0	0	0	0	0	0	0	0	80
20755000	m	md	JP	2022	0	0	0	0	0	0	0	0	0	0	0	0	73
20755402	Not	md	SG	2022	0	0	0	0	0	0	0	0	0	0	0	0	
20755971	m	cn	CN	2022	0	0	0	0	0	0	0	0	0	0	0	0	68
20756025	Not	md	GB	2022	1	0	0	0	0	0	0	0	0	0	0	0	
20756190	m	hp	JP	2022	0	1	0	0	1	0	0	0	0	0	0	0	71
20756644	Not	cn	SG	2022	1	0	0	0	0	0	0	0	0	0	0	0	80
20757262	m	md	JP	2022	0	1	0	0	1	0	0	0	0	0	0	0	84

20757428	m	cn	CN	2022	0	1	0	1	0	0	0	0	0	0	0	1	
20758470	m	md	US	2022	0	0	0	0	0	0	0	0	0	0	0	0	58
20758530	m	hp	JP	2022	0	1	1	0	0	0	0	0	0	0	0	0	67
20758639	m	cn	US	2022	0	0	0	0	0	0	0	0	0	0	0	0	78
20758686	m	md	FR	2022	0	0	0	0	0	0	0	0	0	0	0	0	
20758812	m	md	FR	2022	0	1	1	0	0	0	0	0	0	0	0	0	
20758909	f	md	CN	2022	0	0	0	0	0	0	0	0	0	0	0	0	56
20759021	f	md	JP	2022	0	1	1	0	0	0	0	0	0	0	0	0	67
20759699	m	cn	TH	2022	0	0	0	0	0	0	0	0	0	0	0	0	71
20759720	f	cn	IN	2022	0	1	0	0	0	1	0	0	0	0	0	0	72
20759831	f	cn	FR	2022	0	0	0	0	0	0	0	0	0	0	0	0	44
20759840	f	md	CZ	2022	0	1	1	0	0	0	0	0	0	0	0	0	
20760105	m	cn	CA	2022	0	0	0	0	0	0	0	0	0	0	0	0	58
20760392	f	cn	US	2022	0	1	0	0	1	0	0	0	0	0	0	0	62
20760415	m	md	JP	2022	0	1	0	0	1	0	0	0	0	0	0	0	70
20760527	m	hp	DE	2022	0	0	0	0	0	0	0	0	0	0	0	0	47
20760668	m	hp	IT	2022	0	1	1	0	0	0	0	0	0	0	0	0	68
20760728	m	Not repo	Not re	2022	0	1	1	0	0	0	0	0	0	0	0	0	57
20760847	Not	hp	AE	2022	0	1	0	0	1	0	0	0	0	0	0	0	
20760958	f	cn	IN	2022	0	0	0	0	0	0	0	0	0	0	0	0	72
20761064	m	cn	AT	2022	0	0	0	0	0	0	0	0	0	0	0	0	50
20761066	f	md	FR	2022	0	0	0	0	0	0	0	0	0	0	0	0	
20761229	m	md	IT	2022	0	1	0	0	1	0	0	0	0	0	0	0	65
20761779	Not	md	JP	2022	0	0	0	0	0	0	0	0	0	0	0	0	70
20761888	m	md	IQ	2022	0	1	0	0	0	1	0	0	0	0	0	0	58
20761960	m	md	FR	2022	0	0	0	0	0	0	0	0	0	0	0	0	
20762229	m	cn	VN	2022	0	0	0	0	0	0	0	0	0	0	0	0	65
20762487	m	md	US	2022	0	0	0	0	0	0	0	0	0	0	0	0	69
20762546	m	hp	IT	2022	0	1	1	0	0	0	0	0	0	0	0	0	68
20762832	m	hp	IT	2022	0	1	1	0	0	0	0	0	0	0	0	0	68
20762867	f	ph	CA	2022	1	0	0	0	0	0	0	0	0	0	0	0	64
20763099	f	cn	US	2022	1	0	0	0	0	0	0	0	0	0	0	0	55
20763180	f	md	NP	2022	0	0	0	0	0	0	0	0	0	0	0	0	62
20763614	m	cn	IN	2022	0	0	0	0	0	0	0	0	0	0	0	0	36

20763697	m	ph	JP	2022	0	1	1	0	0	0	0	0	0	0	0	0	80
20764031	Not	ph	JP	2022	1	0	0	0	0	0	0	0	0	0	0	0	
20764071	m	md	JP	2022	0	1	1	0	0	0	0	0	0	0	0	0	73
20764084	m	md	JP	2022	0	1	0	0	0	1	0	0	0	0	0	0	
20764611	f	md	FR	2022	0	0	0	0	0	0	0	0	0	0	0	0	
20764876	Not	md	US	2022	0	0	0	0	0	0	0	0	0	0	0	0	
20765510	f	hp	US	2022	0	1	0	0	0	1	0	0	0	0	0	0	
20766105	f	hp	RU	2022	0	0	0	0	0	0	0	0	0	0	0	0	78
20766665	m	hp	ES	2022	0	1	0	0	1	0	0	0	0	0	0	0	76
20766835	Not	md	JP	2022	0	1	0	1	0	0	0	0	0	0	0	1	
20767255	f	hp	US	2022	0	1	0	0	1	0	0	0	0	0	0	0	64
20767277	Not	ph	US	2022	0	0	0	0	0	0	0	0	0	0	0	0	
20767523	m	cn	CN	2022	0	1	0	0	1	0	0	0	0	0	0	0	
20767881	m	cn	US	2022	0	0	0	0	0	0	0	0	0	0	0	0	59
20767914	m	md	RU	2022	0	0	0	0	0	0	0	0	0	0	0	0	43
20767960	Not	ph	US	2022	0	0	0	0	0	0	0	0	0	0	0	0	
20768029	m	cn	IN	2022	0	1	0	0	0	1	0	0	0	0	0	0	
20768042	m	ph	US	2022	0	0	0	0	0	0	0	0	0	0	0	0	41
20768055	m	cn	US	2022	0	0	0	0	0	0	0	0	0	0	0	0	
20768305	m	md	JP	2022	0	1	1	0	0	0	0	0	0	0	0	0	78
20768456	f	md	AT	2022	0	1	0	0	0	1	0	0	0	0	0	0	47
20768474	f	cn	CA	2022	1	0	0	0	0	0	0	0	0	0	0	0	
20768675	f	md	JP	2022	0	0	0	0	0	0	0	0	0	0	0	0	40
20768803	f	cn	IN	2022	0	0	0	0	0	0	0	0	0	0	0	0	50
20768862	m	cn	SG	2022	0	1	0	0	0	1	0	0	0	0	0	0	80
20768876	m	md	DK	2022	0	0	0	0	0	0	0	0	0	0	0	0	57
20768944	Not	hp	IT	2022	0	1	1	0	0	0	0	0	0	0	0	0	
20768952	f	cn	GB	2022	0	0	0	0	0	0	1	1	0	0	0	0	58
20768983	m	md	CN	2022	0	1	1	0	0	0	0	0	0	0	0	0	68
20768991	m	cn	CN	2022	0	1	0	1	0	0	0	0	0	0	0	1	47
20768992	f	cn	MY	2022	1	0	0	0	0	0	0	0	0	0	0	0	
20769004	Not	cn	CN	2022	0	0	0	0	0	0	0	0	0	0	0	0	
20769024	f	md	US	2022	0	1	1	0	0	0	0	0	0	0	0	0	60
20769026	Not	md	JP	2022	0	0	0	0	0	0	0	0	0	0	0	0	

20769056	Not	md	JP	2022	0	0	0	0	0	0	0	0	0	0	0	0	
20769075	m	md	JP	2022	0	0	0	0	0	0	0	0	0	0	0	0	72
20769144	m	md	AU	2022	0	1	0	0	0	1	0	0	0	0	0	0	33
20769155	Not	md	US	2022	0	0	0	0	0	0	0	0	0	0	0	0	
20769467	m	md	RU	2022	0	1	1	0	0	0	0	0	0	0	0	0	51
20769553	f	md	JP	2022	0	1	0	0	0	1	0	0	0	0	0	0	80
20769652	m	md	NL	2022	0	1	0	0	0	1	0	0	0	0	0	0	70
20769734	f	md	JP	2022	0	0	0	0	0	0	0	0	0	0	0	0	66
20769770	f	md	DE	2022	0	1	0	0	1	0	0	0	0	0	0	0	69
20769771	m	md	CN	2022	0	1	1	0	0	0	0	0	0	0	0	0	63
20769779	m	md	JP	2022	0	1	1	0	0	0	0	0	0	0	0	0	70
20769860	m	cn	IN	2022	0	0	0	0	0	0	0	0	0	0	0	0	75
20769884	m	hp	US	2022	0	0	0	0	0	0	0	0	0	0	0	0	68
20769888	f	md	JP	2022	0	1	1	0	0	0	0	0	0	0	0	0	74
20769972	f	md	IT	2022	0	1	1	0	0	0	0	0	0	0	0	0	43
20770076	m	md	JP	2022	0	1	0	0	1	0	0	0	0	0	0	0	65
20770380	f	md	JP	2022	0	1	0	0	1	0	0	0	0	0	0	0	44
20770657	f	md	JP	2022	0	0	0	0	0	0	0	0	0	0	0	0	46
20770718	f	cn	US	2022	1	0	0	0	0	0	0	0	0	0	0	0	
20770822	f	md	JP	2022	0	1	0	1	0	0	0	0	0	0	0	0	
20770838	f	md	RU	2022	0	1	1	0	0	0	0	0	0	0	0	0	50
20770898	m	hp	ES	2022	0	1	0	0	1	0	0	0	0	0	0	0	61
20770945	m	hp	FR	2022	0	1	0	0	1	0	0	0	0	0	0	1	66
20771322	Not	cn	CN	2022	0	0	0	0	0	0	0	0	0	0	0	0	
20771417	Not	md	US	2022	0	1	0	1	0	0	0	0	0	0	0	0	
20771433	f	md	JP	2022	1	0	0	0	0	0	0	0	0	0	0	0	68
20771439	m	hp	US	2022	0	1	0	1	0	0	0	0	0	0	0	0	
20771450	m	cn	US	2022	0	1	0	1	0	0	0	0	0	0	0	0	
20771744	f	md	NL	2022	0	0	0	0	0	0	0	0	0	0	0	0	71
20771967	m	cn	IN	2022	0	0	0	0	0	0	0	0	0	0	0	0	68
20772139	m	md	JP	2022	0	1	0	0	1	0	0	0	0	0	0	0	77
20772144	m	md	TW	2022	0	0	0	0	0	0	0	0	0	0	0	0	74
20772790	m	cn	CN	2022	0	1	0	0	1	0	0	0	0	0	0	0	73
20772975	Not	md	JP	2022	0	1	0	0	1	0	0	0	0	0	0	0	

20772995	f	md	US	2022	0	1	0	0	1	0	0	0	0	0	0	0	63
20773074	f	hp	CN	2022	0	0	0	0	0	0	0	0	0	0	0	0	46
20773284	m	md	US	2022	0	1	1	0	0	0	0	0	0	0	0	0	
20773829	f	ph	JP	2022	0	1	0	0	1	0	0	0	0	0	0	0	65
20774001	Not	md	JP	2022	0	0	0	0	0	0	0	0	0	0	0	0	
20774262	m	md	JP	2022	0	1	0	0	1	0	0	0	0	0	0	0	80
20774414	m	md	JP	2022	0	1	0	0	0	1	0	0	0	0	0	0	
20774825	m	hp	JP	2022	0	1	0	0	1	0	0	0	0	0	0	0	54
20774963	Not	cn	JP	2022	0	0	0	0	0	0	0	0	0	0	0	0	
20775016	Not	hp	US	2022	1	0	0	0	0	0	0	0	0	0	0	0	
20775041	f	cn	KR	2022	0	0	0	0	0	0	0	0	0	0	0	0	63
20775644	Not	ph	FR	2022	0	0	0	0	0	0	0	0	0	0	0	0	
20775645	m	ph	FR	2022	0	0	0	0	0	0	0	0	0	0	0	0	
20775691	f	cn	SG	2022	0	1	0	0	0	1	0	0	0	0	0	0	68
20775701	m	md	JP	2022	0	1	0	0	0	1	0	0	0	0	0	0	60
20775819	m	md	JP	2022	0	1	0	0	0	1	0	0	0	0	0	0	70
20775844	m	md	TW	2022	0	0	0	0	0	0	0	0	0	0	0	0	57
20775892	Not	md	NL	2022	0	0	0	0	0	0	0	0	0	0	0	0	
20775936	m	cn	IN	2022	0	0	0	0	0	0	0	0	0	0	0	0	38
20775968	f	cn	US	2022	0	0	0	0	0	0	0	0	0	0	0	0	47
20776204	f	cn	SG	2022	0	1	0	0	0	1	0	0	0	0	0	0	
20776708	m	hp	FR	2022	0	1	0	1	0	0	0	0	0	0	0	0	72
20776741	f	cn	DE	2022	0	0	0	0	0	0	0	0	0	0	0	0	58
20776755	m	hp	JP	2022	0	0	0	0	0	0	0	0	0	0	0	0	64
20777087	m	md	FR	2022	0	0	0	0	0	0	0	0	0	0	0	0	70
20777222	m	md	FR	2022	0	1	1	0	0	0	0	0	0	0	0	0	58
20777739	m	md	CN	2022	1	0	0	0	0	0	0	0	0	0	0	0	73
20778281	Not	md	CH	2022	0	0	0	0	0	0	0	0	0	0	0	0	72
20778309	f	md	US	2022	0	0	0	0	0	0	0	0	0	0	0	0	
20778800	Not	md	AU	2022	0	1	1	0	0	0	0	0	0	0	0	0	
20778802	m	md	CN	2022	0	1	0	0	0	1	0	0	0	0	0	0	36
20778838	m	md	JP	2022	0	1	0	0	0	1	0	0	0	0	0	0	
20778926	m	cn	IN	2022	0	0	0	0	0	0	0	0	0	0	0	0	46
20779145	m	cn	IN	2022	0	0	0	0	0	0	0	0	0	0	0	0	42

20779151	m	cn	IN	2022	0	0	0	0	0	0	0	0	0	0	0	0	48
20779322	Not	md	FR	2022	0	0	0	0	0	0	0	0	0	0	0	0	
20779374	Not	md	RU	2022	0	0	0	0	0	0	0	0	0	0	0	0	
20779396	f	cn	TH	2022	1	0	0	0	0	0	0	0	0	0	0	0	66.9
20779423	m	cn	CN	2022	0	1	0	1	0	0	0	0	0	0	0	0	62
20779440	Not	hp	SG	2022	1	0	0	0	0	0	0	0	0	0	0	0	89.4
20779479	m	md	JP	2022	0	1	1	0	0	0	0	0	0	0	0	0	80
20779511	m	md	BE	2022	0	1	0	1	0	0	0	0	0	0	0	0	66
20779513	m	md	CN	2022	0	1	0	1	0	0	0	0	0	0	0	0	75.5
20779518	Not	ph	JP	2022	1	0	0	0	0	0	0	0	0	0	0	0	
20779523	Not	hp	TH	2022	1	0	0	0	0	0	0	0	0	0	0	0	
20779524	m	cn	KR	2022	0	0	0	0	0	0	0	0	0	0	0	0	56
20779656	f	md	TH	2022	0	1	0	0	0	1	0	0	0	0	0	0	63
20779713	f	md	TW	2022	0	0	0	0	0	0	0	0	0	0	0	0	71
20779832	m	md	FR	2022	0	0	0	0	0	0	0	0	0	0	0	0	
20780147	f	md	GB	2022	0	0	0	0	0	0	0	0	0	0	0	0	53
20780230	m	md	JP	2022	0	0	0	0	0	0	0	0	0	0	0	0	71
20780316	m	md	JP	2022	0	1	0	0	0	1	0	0	0	0	0	0	80
20780348	m	md	JP	2022	0	1	0	0	1	0	0	0	0	0	0	0	66
20780461	m	cn	US	2022	0	1	0	0	1	0	0	0	0	0	0	0	61
20780904	f	ph	JP	2022	0	0	0	0	0	0	0	0	0	0	0	0	90
20781096	m	md	JP	2022	0	1	0	0	0	1	0	0	0	0	0	0	66
20781229	f	md	NZ	2022	1	0	0	0	0	0	0	0	0	0	0	0	
20781947	f	md	CZ	2022	0	1	1	0	0	0	0	0	0	0	0	0	60
20782004	m	hp	CN	2022	0	0	0	0	0	0	0	0	0	0	0	0	71
20782393	m	md	CN	2022	0	0	0	0	0	0	0	0	0	0	0	0	67
20782434	m	md	AU	2022	0	0	0	0	0	0	0	0	0	0	0	0	56
20782628	f	cn	US	2022	0	0	0	0	0	0	0	0	0	0	0	0	
20782633	Not	hp	US	2022	0	0	0	0	0	0	0	0	0	0	0	0	
20782999	m	md	JP	2022	0	1	0	0	0	1	0	0	0	0	0	0	60
20783059	f	md	JP	2022	0	1	0	0	0	1	0	0	0	0	0	0	70
20783153	Not	md	US	2022	0	0	0	0	0	0	0	0	0	0	0	0	
20783173	f	md	AR	2022	0	0	0	0	0	0	0	0	0	0	0	0	
20783199	m	md	JP	2022	0	1	0	0	0	1	0	0	0	0	0	0	70

20783331	f	md	US	2022	0	0	0	0	0	0	0	0	0	0	0	0	68
20783424	f	cn	IT	2022	0	0	0	0	0	0	0	0	0	0	0	0	69
20783491	Not	md	PA	2022	0	0	0	0	0	0	0	0	0	0	0	0	
20783497	m	cn	CH	2022	0	1	1	0	0	0	0	0	0	0	0	0	
20783692	m	md	JP	2022	1	0	0	0	0	0	0	0	0	0	0	0	68
20783818	Not	md	JP	2022	1	0	0	0	0	0	0	0	0	0	0	0	
20783961	m	md	SA	2022	0	1	0	1	0	0	0	0	0	0	0	1	69
20783995	f	md	FR	2022	0	0	0	0	0	0	0	0	0	0	0	0	78
20784059	m	cn	CN	2022	0	1	0	0	1	0	0	0	0	0	0	0	65
20784136	m	md	JP	2022	0	1	0	1	0	0	0	0	0	0	0	0	
20784179	f	cn	CA	2022	0	0	0	0	0	0	0	0	0	0	0	0	
20784288	f	md	FR	2022	0	0	0	0	0	0	0	0	0	0	0	0	62
20784451	m	hp	JP	2022	0	1	0	0	1	0	0	0	0	0	0	0	59
20784551	m	md	DE	2022	0	0	0	0	0	0	0	0	0	0	0	0	64
20784578	m	md	CH	2022	0	1	0	0	0	1	0	0	0	0	0	0	72
20784808	m	md	JP	2022	0	1	0	0	0	1	0	0	0	0	0	0	51
20784811	m	md	MY	2022	0	0	0	0	0	0	0	0	0	0	0	0	40
20784952	f	hp	US	2022	0	1	1	0	0	0	0	0	0	0	0	0	
20785056	m	hp	TW	2022	1	0	0	0	0	0	0	0	0	0	0	0	66
20786619	m	md	JP	2022	0	1	0	0	1	0	0	0	0	0	0	0	56
20787714	f	md	JP	2022	0	0	0	0	0	0	0	0	0	0	0	0	73
20787884	m	cn	IN	2022	0	1	0	0	0	1	0	0	0	0	0	0	68
20787908	f	md	DE	2022	0	0	0	0	0	0	0	0	0	0	0	0	70
20787971	f	md	DE	2022	0	1	0	0	1	0	0	0	0	0	0	0	58
20788082	Not	hp	IT	2022	0	1	0	0	0	1	0	0	0	0	0	0	
20788901	m	md	IL	2022	0	1	0	0	1	0	0	0	0	0	0	0	71
20789052	f	md	AU	2022	1	0	0	0	0	0	0	0	0	0	0	0	50.9
20789292	Not	md	IT	2022	1	0	0	0	0	0	0	0	0	0	0	0	
20789991	Not	md	JP	2022	1	0	0	0	0	0	0	0	0	0	0	0	
20790066	f	cn	JP	2022	1	0	0	0	0	0	0	0	0	0	0	0	
20791000	m	hp	CN	2022	0	0	0	0	0	0	0	0	0	0	0	0	68
20791436	m	md	DE	2022	0	1	0	0	1	0	0	0	0	0	0	0	74
20791642	f	md	FR	2022	0	0	0	0	0	0	0	0	0	0	0	0	76
20791980	f	md	BR	2022	0	0	0	0	0	0	0	0	0	0	0	0	66

20791981	f	md	BR	2022	0	0	0	0	0	0	0	0	0	0	0	0	72
20792242	Not	ph	JP	2022	1	0	0	0	0	0	0	0	0	0	0	0	
20792305	m	hp	JP	2022	0	1	0	0	1	0	0	0	0	0	0	0	80
20792432	m	md	JP	2022	0	1	0	0	1	0	0	0	0	0	0	1	56
20793298	m	md	US	2022	0	1	0	1	0	0	0	0	0	0	0	0	69
20793363	Not	md	JP	2022	0	0	0	0	0	0	0	0	0	0	0	0	80
20793687	m	cn	TW	2022	0	0	0	0	0	0	0	0	0	0	0	0	84
20794502	Not	md	US	2022	0	1	0	0	1	0	0	0	0	0	0	0	
20794927	m	md	JP	2022	0	1	0	0	0	1	0	0	0	0	0	0	66
20795344	m	ph	IT	2022	0	1	0	0	0	1	0	0	0	0	0	0	
20795406	m	md	IT	2022	0	1	1	0	0	0	0	0	0	0	0	0	81
20795417	m	md	JP	2022	0	0	0	0	0	0	0	0	0	0	0	0	80
20795813	m	hp	IL	2022	0	0	0	0	0	0	0	0	0	0	0	0	71
20796265	m	cn	TH	2022	1	0	0	0	0	0	0	0	0	0	0	0	67.9
20796308	Not	md	FR	2022	0	0	0	0	0	0	0	0	0	0	0	0	
20796465	m	md	FR	2022	0	0	0	0	0	0	0	0	0	0	0	0	50
20796502	m	md	FR	2022	0	1	1	0	0	0	0	0	0	0	0	0	
20796560	f	md	FR	2022	0	0	0	0	0	0	0	0	0	0	0	0	
20796580	m	hp	DE	2022	0	1	0	0	1	0	0	0	0	0	0	0	59
20796609	m	cn	US	2022	0	0	0	0	0	0	0	0	0	0	0	0	65
20796680	m	md	NL	2022	0	0	0	0	0	0	0	0	0	0	0	0	74
20796704	m	md	CN	2022	0	0	0	0	0	0	0	0	0	0	0	0	73
20796743	Not	hp	DE	2022	0	1	0	0	0	1	0	0	0	0	0	0	
20796852	m	hp	CN	2022	0	0	0	0	0	0	0	0	0	0	0	0	60
20796871	m	md	IL	2022	0	0	0	0	0	0	0	0	0	0	0	0	68
20797541	m	md	CN	2022	0	0	0	0	0	0	0	0	0	0	0	0	75
20797596	f	cn	US	2022	0	0	0	0	0	0	0	0	0	0	0	0	
20797786	f	cn	CA	2022	1	0	0	0	0	0	0	0	0	0	0	0	45
20798019	m	hp	IN	2022	0	1	0	0	1	0	0	0	0	0	0	0	41
20798121	m	md	US	2022	0	0	0	0	0	0	0	0	0	0	0	0	76
20798154	m	hp	FR	2022	0	1	0	0	1	0	0	0	0	0	0	0	72
20798716	f	hp	TW	2022	1	0	0	0	0	0	0	0	0	0	0	0	68
20799100	m	cn	CN	2022	0	1	0	0	1	0	0	0	0	0	0	0	34
20799469	f	md	BR	2022	0	1	0	0	1	0	0	0	0	0	0	0	

20799508	f	cn	CN	2022	0	1	0	0	1	0	0	0	0	0	0	0	36
20799516	Not	md	DE	2022	0	1	0	0	1	0	0	0	0	0	0	1	60
20800522	f	md	US	2022	0	0	0	0	0	0	0	0	0	0	0	0	77
20800601	m	cn	CN	2022	0	1	0	0	1	0	0	0	0	0	0	0	78
20802695	f	md	JP	2022	0	0	0	0	0	0	0	0	0	0	0	0	73
20802770	f	md	JP	2022	0	1	0	0	1	0	0	0	0	0	0	0	68
20803137	m	md	NL	2022	0	1	0	0	0	1	0	0	0	0	0	0	70
20803453	m	md	BR	2022	1	0	0	0	0	0	0	0	0	0	0	1	71
20803618	f	hp	DK	2022	0	0	0	0	0	0	0	0	0	0	0	0	39
20803636	m	cn	IN	2022	0	0	0	0	0	0	0	0	0	0	0	0	40
20803655	f	md	IT	2022	0	0	0	0	0	0	0	0	0	0	0	0	74
20803911	f	md	KR	2022	0	0	0	0	0	0	0	0	0	0	0	0	60
20803947	m	hp	ES	2022	0	0	0	0	0	0	0	0	0	0	0	0	60
20804118	f	md	JP	2022	0	1	1	0	0	0	0	0	0	0	0	0	89
20804137	f	md	JP	2022	0	1	0	0	1	0	0	0	0	0	0	0	66
20804577	m	md	FR	2022	0	1	1	0	0	0	0	0	0	0	0	0	58
20804616	m	md	FR	2022	0	1	1	0	0	0	0	0	0	0	0	0	58
20804859	f	md	FR	2022	0	0	0	0	0	0	0	0	0	0	0	0	
20804866	m	hp	FR	2022	0	0	0	0	0	0	0	0	0	0	0	0	56
20805192	m	hp	CL	2022	0	0	0	0	0	0	0	0	0	0	0	0	70
20805313	m	md	JP	2022	0	1	0	0	1	0	0	0	0	0	0	0	47
20806763	f	md	IT	2022	0	0	0	0	0	0	0	0	0	0	0	0	
20806795	m	md	FR	2022	0	0	0	0	0	0	0	0	0	0	0	0	69
20806942	m	md	ES	2022	0	0	0	0	0	0	0	0	0	0	0	0	64
20807230	Not	md	IL	2022	0	0	0	0	0	0	0	0	0	0	0	0	
20807418	m	md	FR	2022	0	1	1	0	0	0	0	0	0	0	0	0	
20807473	m	md	LT	2022	0	1	0	0	0	1	0	0	0	0	0	0	73
20807920	m	md	KR	2022	0	1	0	0	1	0	0	0	0	0	0	0	71
20807938	Not	md	BG	2022	1	0	0	0	0	0	0	0	0	0	0	0	
20807939	f	cn	CA	2022	1	0	0	0	0	0	0	0	0	0	0	0	
20807956	m	md	JP	2022	0	1	0	0	0	1	0	0	0	0	0	0	73
20808423	m	md	DE	2022	0	0	0	0	0	0	0	0	0	0	0	0	67
20809019	f	md	US	2022	0	0	0	0	0	0	0	0	0	0	0	0	69
20809278	m	hp	JP	2022	0	1	0	0	1	0	0	0	0	0	0	0	71

20809445	Not	cn	JP	2022	1	0	0	0	0	0	0	0	0	0	0	0	
20809498	Not	md	JP	2022	0	1	0	0	1	0	0	0	0	0	0	0	
20809638	f	cn	US	2022	0	0	0	0	0	0	0	0	0	0	0	0	
20809845	f	md	DE	2022	0	0	0	0	0	0	0	0	0	0	0	0	76
20810060	f	md	TH	2022	1	0	0	0	0	0	0	0	0	0	0	0	51.7
20810211	m	md	CA	2022	0	0	0	0	0	0	0	0	0	0	0	0	66
20810317	f	md	CN	2022	0	0	0	0	0	0	0	0	0	0	0	0	56
20810422	Not	md	BG	2022	1	0	0	0	0	0	0	0	0	0	0	0	
20810582	f	hp	NL	2022	0	1	0	0	0	1	0	0	0	0	0	0	47
20810724	f	md	KR	2022	0	1	0	0	1	0	0	0	0	0	0	1	59
20811045	m	md	IL	2022	0	0	0	0	0	0	0	0	0	0	0	0	72
20811547	f	hp	DE	2022	0	0	0	0	0	0	0	0	0	0	0	0	68
20811742	m	md	KR	2022	0	1	0	0	1	0	0	0	0	0	0	0	61
20812353	f	md	JP	2022	0	1	0	0	1	0	0	0	0	0	0	0	71
20812542	f	cn	US	2022	0	0	0	0	0	0	0	0	0	0	0	0	
20812817	m	hp	US	2022	0	1	1	0	0	0	0	0	0	0	0	0	
20812869	m	cn	SG	2022	0	1	0	0	0	1	0	0	0	0	0	0	69
20813925	Not	md	DE	2022	0	0	0	0	0	0	0	0	0	0	0	0	
20814270	f	md	JP	2022	0	1	0	0	1	0	0	0	0	0	0	0	90
20814523	m	hp	AU	2022	0	0	0	0	0	0	0	0	0	0	0	0	65
20815254	f	md	NL	2022	0	1	0	1	0	0	0	0	0	0	0	0	54
20815365	m	md	FR	2022	0	1	1	0	0	0	0	0	0	0	0	0	58
20815820	m	ph	JP	2022	0	1	1	0	0	0	0	0	0	0	0	0	66
20816135	f	md	AR	2022	0	0	0	0	0	0	0	0	0	0	0	0	
20816205	f	hp	FR	2022	0	0	0	0	0	0	0	0	0	0	0	0	74
20816318	m	md	KR	2022	0	1	0	0	1	0	0	0	0	0	0	0	89
20817886	f	md	ES	2022	0	1	0	0	0	1	0	0	0	0	0	0	73
20818007	m	hp	ES	2022	0	1	0	0	1	0	0	0	0	0	0	0	60
20818842	f	cn	TH	2022	1	0	0	0	0	0	0	0	0	0	0	0	80.2
20819119	f	md	JP	2022	0	0	0	0	0	0	0	0	0	0	0	0	
20819555	m	ph	CA	2022	0	0	0	0	0	0	0	0	0	0	0	0	82
20819705	f	md	BR	2022	0	1	1	0	0	0	0	0	0	0	0	0	63
20819737	m	md	ES	2022	0	1	0	0	1	0	0	0	0	0	0	0	45
20819805	f	md	FR	2022	0	0	0	0	0	0	0	0	0	0	0	0	

20819864	m	md	DE	2022	0	1	0	0	1	0	0	0	0	0	0	0	66
20819904	m	hp	AU	2022	0	0	0	0	0	0	0	0	0	0	0	0	65
20819925	f	md	AT	2022	0	1	0	0	0	1	0	0	0	0	0	0	47
20820558	Not	hp	US	2022	0	0	0	0	0	0	0	0	0	0	0	0	
20821244	f	md	US	2022	0	1	1	0	0	0	0	0	0	0	0	0	
20821778	Not	md	IL	2022	0	0	0	0	0	0	0	0	0	0	0	0	
20822036	f	md	KR	2022	0	0	0	0	0	0	0	0	0	0	0	0	44
20822250	f	cn	BE	2022	0	0	0	0	0	0	0	0	0	0	0	0	
20822290	f	cn	TH	2022	0	0	0	0	0	0	0	0	0	0	0	0	52
20822306	m	cn	US	2022	0	0	0	0	0	0	0	0	0	0	0	0	
20822385	f	hp	CA	2022	0	1	1	0	0	0	0	0	0	0	0	0	58
20822404	f	hp	CA	2022	0	1	1	0	0	0	0	0	0	0	0	0	58
20822719	m	md	IN	2022	0	1	0	0	1	0	0	0	0	0	0	0	52
20822833	f	md	CA	2022	0	0	0	0	0	0	0	0	0	0	0	0	
20822912	m	hp	TH	2022	1	0	0	0	0	0	0	0	0	0	0	0	53.5
20822935	m	cn	TH	2022	1	0	0	0	0	0	0	0	0	0	0	0	68
20823460	m	cn	IN	2022	0	0	0	0	0	0	0	0	0	0	0	0	79
20824542	m	md	NL	2022	0	0	0	0	0	0	0	0	0	0	0	0	70
20824631	m	md	JP	2022	0	0	0	0	0	0	0	0	0	0	0	0	71
20824700	m	md	GR	2022	0	1	0	0	1	0	0	0	0	0	0	0	83
20825053	f	cn	US	2022	1	0	0	0	0	0	0	0	0	0	0	0	52
20825244	f	cn	US	2022	0	1	1	0	0	0	0	0	0	0	0	0	
20825259	f	hp	US	2022	0	0	0	0	0	0	0	0	0	0	0	0	
20825542	m	cn	ID	2022	1	0	0	0	0	0	0	0	0	0	0	0	82.1
20825682	m	cn	TH	2022	1	0	0	0	0	0	0	0	0	0	0	0	68.6
20825825	f	cn	ID	2022	1	0	0	0	0	0	0	0	0	0	0	0	59.2
20825846	m	hp	JP	2022	0	1	1	0	0	0	0	0	0	0	0	0	44
20825860	m	md	LT	2022	0	1	0	0	0	1	0	0	0	0	0	0	73
20825908	f	md	US	2022	0	0	0	0	0	0	0	0	0	0	0	0	61
20826051	f	cn	TH	2022	1	0	0	0	0	0	0	0	0	0	0	0	76
20826217	m	cn	TH	2022	1	0	0	0	0	0	0	0	0	0	0	0	70
20826550	Not	md	ES	2022	1	0	0	0	0	0	0	0	0	0	0	0	
20826830	f	md	AT	2022	0	1	0	0	0	1	0	0	0	0	0	0	47
20826844	f	hp	FR	2022	0	1	0	0	0	1	0	0	0	0	0	0	64

20826912	Not	hp	US	2022	0	0	0	0	0	0	0	0	0	0	0	0	
20827082	m	md	BR	2022	0	0	0	0	0	0	0	0	0	0	0	0	65
20827300	Not	cn	SG	2022	1	0	0	0	0	0	0	0	0	0	0	0	63.4
20827303	m	md	CN	2022	0	1	0	1	0	0	0	0	0	0	0	1	58
20827382	Not	md	IT	2022	1	0	0	0	0	0	0	0	0	0	0	0	
20827440	m	md	CN	2022	0	1	0	1	0	0	0	0	0	0	0	0	
20827444	m	hp	CN	2022	0	0	0	0	0	0	0	0	0	0	0	0	63
20827829	Not	md	US	2022	0	0	0	0	0	0	0	0	0	0	0	0	
20828356	f	hp	TH	2022	1	0	0	0	0	0	0	0	0	0	0	0	69.1
20828357	f	md	GB	2022	1	0	0	0	0	0	0	0	0	0	0	0	
20828654	f	md	FR	2022	0	0	0	0	0	0	0	0	0	0	0	0	
20828678	f	md	JP	2022	0	1	1	0	0	0	0	0	0	0	0	0	63
20828688	f	md	JP	2022	0	0	0	0	0	0	0	0	0	0	0	0	50
20829706	f	md	US	2022	0	1	1	0	0	0	0	0	0	0	0	0	82
20830506	m	md	DE	2022	0	1	0	0	1	0	0	0	0	0	0	0	56
20830541	Not	md	GB	2022	0	0	0	0	0	0	0	0	0	0	0	0	
20830542	Not	md	GB	2022	0	0	0	0	0	0	0	0	0	0	0	0	
20830545	Not	md	GB	2022	0	0	0	0	0	0	0	0	0	0	0	0	
20830556	Not	cn	FR	2022	0	0	0	0	0	0	0	0	0	0	0	0	
20830830	Not	hp	JP	2022	0	1	0	0	1	0	0	0	0	0	0	0	
20830895	m	hp	CN	2022	0	1	0	0	1	0	0	0	0	0	0	0	71
20832194	m	hp	CA	2022	0	1	0	0	1	0	0	0	0	0	0	0	54
20833184	m	md	TH	2022	0	0	0	0	0	0	0	0	0	0	0	0	
20833652	m	cn	TH	2022	1	0	0	0	0	0	0	0	0	0	0	0	60.3
20833851	f	cn	LB	2022	0	0	0	0	0	0	0	0	0	0	0	0	37
20833855	Not	md	DE	2022	0	0	0	0	0	0	0	0	0	0	0	0	
20833858	m	md	NL	2022	0	1	0	0	0	1	0	0	0	0	0	0	70
20834232	f	md	CA	2022	1	0	0	0	0	0	0	0	0	0	0	0	64
20834255	m	md	ES	2022	0	1	0	0	0	1	0	0	0	0	0	1	59
20834487	m	hp	CN	2022	0	0	0	0	0	0	0	0	0	0	0	0	57
20834821	m	hp	IT	2022	1	0	0	0	0	0	0	0	0	0	0	0	69
20834871	Not	md	FR	2022	0	0	0	0	0	0	0	0	0	0	0	0	
20834877	m	md	GB	2022	0	0	0	0	0	0	0	0	0	0	0	0	70
20835796	m	md	CN	2022	0	0	0	0	0	0	0	0	0	0	0	0	60

20835936	m	md	JP	2022	0	0	0	0	0	0	0	0	0	0	0	0	68
20836188	m	md	JP	2022	0	1	0	0	1	0	0	0	0	0	0	1	48
20836606	m	md	JP	2022	0	1	0	0	1	0	0	0	0	0	0	0	70
20836830	m	cn	CA	2022	0	0	0	0	0	0	0	0	0	0	0	0	54
20836995	m	cn	CN	2022	0	1	0	0	1	0	0	0	0	0	0	0	72
20837201	f	md	GR	2022	0	1	1	0	0	0	0	0	0	0	0	0	
20838112	f	hp	GB	2022	0	0	0	0	0	0	0	0	0	0	0	0	
20838590	m	cn	CA	2022	0	0	0	0	0	0	0	0	0	0	0	0	
20839400	f	cn	US	2022	1	0	0	0	0	0	0	0	0	0	0	0	
20839495	f	cn	US	2022	0	0	0	0	0	0	0	0	0	0	0	0	75
20839545	f	md	JP	2022	0	0	0	0	0	0	0	0	0	0	0	0	77
20839570	f	hp	SG	2022	0	1	0	0	0	1	0	0	0	0	0	0	
20839572	m	md	KR	2022	0	1	0	0	0	1	0	0	0	0	0	0	72
20839620	m	md	DE	2022	0	1	0	0	1	0	0	0	0	0	0	0	63
20839703	f	md	DE	2022	0	1	1	0	0	0	0	0	0	0	0	0	47
20839782	f	cn	US	2022	1	0	0	0	0	0	0	0	0	0	0	0	73.3
20839874	m	cn	SE	2022	0	0	0	0	0	0	0	0	0	0	0	0	73
20839942	m	md	FR	2022	0	1	1	0	0	0	0	0	0	0	0	0	
20840135	f	cn	US	2022	1	0	0	0	0	0	0	0	0	0	0	0	67.6
20840404	m	md	TW	2022	0	0	0	0	0	0	0	0	0	0	0	0	61
20840426	m	md	JP	2022	1	0	0	0	0	0	0	0	0	0	0	1	72
20840455	Not	cn	US	2022	0	0	0	0	0	0	0	0	0	0	0	0	
20840482	f	cn	ID	2022	1	0	0	0	0	0	0	0	0	0	0	0	73
20840488	m	md	CN	2022	0	1	0	1	0	0	0	0	0	0	0	0	73.4
20840585	m	md	JP	2022	0	1	0	1	0	0	0	0	0	0	0	1	76
20840587	m	md	NL	2022	0	1	0	1	0	0	0	0	0	0	0	0	61
20840613	f	md	AT	2022	0	1	0	0	0	1	0	0	0	0	0	0	45
20841301	Not	hp	ID	2022	1	0	0	0	0	0	0	0	0	0	0	0	
20841402	m	md	CZ	2022	0	1	0	0	1	0	0	0	0	0	0	0	73
20841423	m	md	JP	2022	0	1	1	0	0	0	0	0	0	0	0	0	62
20841605	m	md	JP	2022	0	0	0	0	0	0	0	0	0	0	0	0	70
20841637	m	md	FR	2022	0	0	0	0	0	0	0	0	0	0	0	0	
20841738	m	md	JP	2022	0	0	0	0	0	0	0	0	0	0	0	0	
20841885	m	ph	JP	2022	0	0	0	0	0	0	0	0	0	0	0	0	80

20841905	m	md	LB	2022	0	1	0	0	1	0	0	0	0	0	0	0	
20842037	m	cn	IN	2022	0	1	0	0	1	0	0	0	0	0	0	0	56
20842318	m	md	DE	2022	0	0	0	0	0	0	0	0	0	0	0	0	70
20842436	m	md	JP	2022	0	1	0	0	1	0	0	0	0	0	0	0	60
20842829	Not	md	CN	2022	0	0	0	0	0	0	0	0	0	0	0	0	
20843942	m	cn	ID	2022	1	0	0	0	0	0	0	0	0	0	0	0	55.2
20843997	f	cn	IN	2022	1	0	0	0	0	0	0	0	0	0	0	0	81
20845377	f	cn	US	2022	0	0	0	0	0	0	0	0	0	0	0	0	
20845454	m	md	JP	2022	0	1	0	0	1	0	0	0	0	0	0	0	81
20845652	f	md	MY	2022	1	0	0	0	0	0	0	0	0	0	0	0	82
20845718	f	md	HR	2022	0	1	1	0	0	0	0	0	0	0	0	1	76
20845759	m	md	DE	2022	0	0	0	0	0	0	0	0	0	0	0	0	57
20845791	Not	md	AR	2022	0	0	0	0	0	0	0	0	0	0	0	0	
20846620	f	md	FR	2022	0	0	0	0	0	0	0	0	0	0	0	0	53
20846686	m	ph	JP	2022	0	1	1	0	0	0	0	0	0	0	0	0	65
20846926	m	cn	CN	2022	0	0	0	0	0	0	0	0	0	0	0	0	53
20847434	m	md	FR	2022	0	1	0	0	0	1	0	0	0	0	0	0	71
20847436	m	md	CN	2022	0	0	0	0	0	0	0	0	0	0	0	0	40
20847505	m	cn	IN	2022	0	1	0	0	1	0	0	0	0	0	0	0	76
20847535	f	md	CN	2022	0	0	0	0	0	0	0	0	0	0	0	0	72
20848108	f	hp	BR	2022	0	1	1	0	0	0	0	0	0	0	0	0	
20848312	f	md	JP	2022	0	1	1	0	0	0	0	0	0	0	0	0	74
20848414	Not	ph	US	2022	0	0	0	0	0	0	0	0	0	0	0	0	
20848613	f	hp	ID	2022	1	0	0	0	0	0	0	0	0	0	0	0	62.3
20848736	f	md	JP	2022	0	0	0	0	0	0	0	0	0	0	0	0	
20848769	f	cn	ID	2022	1	0	0	0	0	0	0	0	0	0	0	0	54.9
20848813	m	md	JP	2022	0	0	0	0	0	0	0	0	0	0	0	0	86
20848819	m	md	US	2022	0	0	0	0	0	0	0	0	0	0	0	0	
20848995	m	md	IL	2022	0	0	0	0	0	0	0	0	0	0	0	0	60
20849072	f	cn	TH	2022	1	0	0	0	0	0	0	0	0	0	0	0	74.4
20849674	f	md	JP	2022	0	0	0	0	0	0	0	0	0	0	0	0	49
20849793	f	hp	SG	2022	0	1	0	0	0	1	0	0	0	0	0	0	46
20850058	Not	cn	CN	2022	0	0	0	0	0	0	0	0	0	0	0	0	
20850074	m	md	AU	2022	0	1	1	0	0	0	0	0	0	0	0	1	71

20850226	m	cn	ID	2022	1	0	0	0	0	0	0	0	0	0	0	0	45.5
20850259	f	hp	US	2022	0	0	0	0	0	0	0	0	0	0	0	0	
20850265	f	cn	GR	2022	0	1	0	0	1	0	0	0	0	0	0	0	74
20850695	m	md	DE	2022	0	1	0	0	1	0	0	0	0	0	0	0	74
20850868	m	md	DE	2022	0	0	0	0	0	0	0	0	0	0	0	0	86
20851197	m	md	NL	2022	0	1	1	0	0	0	0	0	0	0	0	0	70
20851250	m	md	US	2022	0	0	0	0	0	0	0	0	0	0	0	0	67
20851275	m	md	PL	2022	0	1	1	0	0	0	0	0	0	0	0	0	
20851322	Not	ph	JP	2022	0	0	0	0	0	0	0	0	0	0	0	0	
20851617	m	md	KR	2022	0	0	0	0	0	0	0	0	0	0	0	0	75
20851852	f	ph	US	2022	0	0	0	0	0	0	0	0	0	0	0	0	
20851913	f	md	NO	2022	0	1	1	0	0	0	0	0	0	0	0	0	79
20852205	Not	hp	JP	2022	0	0	0	0	0	0	0	0	0	0	0	0	80
20852223	m	md	BR	2022	0	1	1	0	0	0	0	0	0	0	0	0	72
20852352	Not	md	JP	2022	0	0	0	0	0	0	0	0	0	0	0	0	
20852482	Not	md	JP	2022	0	1	0	0	1	0	0	0	0	0	0	0	
20852494	f	md	DE	2022	0	1	1	0	0	0	0	0	0	0	0	0	
20853147	f	hp	US	2022	0	0	0	0	0	0	0	0	0	0	0	0	
20853256	m	md	US	2022	0	0	0	0	0	0	0	0	0	0	0	0	83
20853405	f	hp	NL	2022	0	0	0	0	0	0	0	0	0	0	0	0	70
20853493	f	md	QA	2022	0	0	0	0	0	0	0	0	0	0	0	0	60
20853533	m	cn	MY	2022	0	0	0	0	0	0	0	0	0	0	0	0	69
20853581	f	md	US	2022	0	1	1	0	0	0	0	0	0	0	0	0	57
20853762	Not	md	GB	2022	1	0	0	0	0	0	0	0	0	0	0	0	
20853893	m	md	CN	2022	0	1	0	1	0	0	0	0	0	0	0	1	56
20854113	m	md	IN	2022	0	1	0	0	0	1	0	0	0	0	0	0	55
20854316	f	cn	CN	2022	1	0	0	0	0	0	0	0	0	0	0	0	
20854322	m	hp	US	2022	1	0	0	0	0	0	0	0	0	0	0	0	57
20854373	Not	md	JP	2022	0	1	1	0	0	0	0	0	0	0	0	0	70
20854387	f	md	US	2022	0	0	0	0	0	0	0	0	0	0	0	0	
20854487	m	cn	CA	2022	1	0	0	0	0	0	0	0	0	0	0	0	58.3
20854803	f	md	DE	2022	0	1	1	0	0	0	0	0	0	0	0	1	74
20854890	f	md	FR	2022	1	0	0	0	0	0	0	0	0	0	0	0	
20854897	m	hp	US	2022	0	0	0	0	0	0	0	0	0	0	0	0	67

20855484	f	hp	IE	2022	0	1	0	0	1	0	0	0	0	0	0	0	66
20855719	f	cn	ID	2022	1	0	0	0	0	0	0	0	0	0	0	0	68.8
20855773	f	hp	AR	2022	0	1	0	0	1	0	0	0	0	0	0	0	66
20855929	m	md	JP	2022	0	1	0	1	0	0	0	0	0	0	0	1	69
20855954	m	hp	JP	2022	0	0	0	0	0	0	0	0	0	0	0	0	65
20856036	m	md	JP	2022	0	1	1	0	0	0	0	0	0	0	0	0	80
20856127	f	md	JP	2022	0	1	0	0	1	0	0	0	0	0	0	0	80
20857718	f	md	JP	2022	0	0	0	0	0	0	0	0	0	0	0	0	80
20859877	Not	cn	SG	2022	1	0	0	0	0	0	0	0	0	0	0	0	86.8
20860025	m	md	RU	2022	0	1	0	0	0	1	0	0	0	0	0	0	
20860662	f	md	JP	2022	0	1	0	0	0	1	0	0	0	0	0	0	73
20860733	Not	cn	SG	2022	1	0	0	0	0	0	0	0	0	0	0	0	61.7
20860734	Not	cn	SG	2022	1	0	0	0	0	0	0	0	0	0	0	0	69.6
20860786	f	md	HU	2022	0	0	0	0	0	0	0	0	0	0	0	0	62
20860806	m	md	NL	2022	0	1	0	0	0	1	0	0	0	0	0	0	70
20860821	f	md	CA	2022	0	1	0	1	0	0	0	0	0	0	0	0	75.2
20861107	Not	hp	CN	2022	0	1	0	0	0	1	0	0	0	0	0	0	
20861135	Not	cn	SG	2022	1	0	0	0	0	0	0	0	0	0	0	0	89.3
20861252	f	hp	NL	2022	0	1	1	0	0	0	0	0	0	0	0	0	57
20861260	f	cn	US	2022	0	1	1	0	0	0	0	0	0	0	0	0	67
20861511	m	md	JP	2022	0	1	0	0	1	0	0	0	0	0	0	0	65
20861789	m	hp	BG	2022	0	0	0	0	0	0	0	0	0	0	0	0	68
20861796	f	ph	HR	2022	0	1	1	0	0	0	0	0	0	0	0	0	57
20861831	f	cn	TH	2022	1	0	0	0	0	0	0	0	0	0	0	0	66
20862116	f	cn	TH	2022	0	0	0	0	0	0	0	0	0	0	0	0	26
20862122	f	md	CA	2022	0	0	0	0	0	0	0	0	0	0	0	0	38
20862123	Not	md	JP	2022	0	0	0	0	0	0	0	0	0	0	0	0	84
20862656	Not	ph	JP	2022	0	1	0	1	0	0	0	0	0	0	0	0	
20862658	f	cn	AT	2022	0	0	0	0	0	0	0	0	0	0	0	0	32
20862881	m	ph	JP	2022	0	0	0	0	0	0	0	0	0	0	0	0	87
20862930	f	cn	KR	2022	0	0	0	0	0	0	0	0	0	0	0	0	51
20863028	f	md	IL	2022	0	0	0	0	0	0	0	0	0	0	0	0	79
20863185	m	md	JP	2022	0	1	0	0	1	0	0	0	0	0	0	0	57
20863500	m	md	NP	2022	0	0	0	0	0	0	0	0	0	0	0	0	72

20863585	f	hp	US	2022	0	1	1	0	0	0	0	0	0	0	0	0	88
20863671	f	hp	US	2022	0	0	0	0	0	0	0	0	0	0	0	0	55
20863890	f	md	UA	2022	0	0	0	0	0	0	0	0	0	0	0	0	51
20864090	m	md	JP	2022	0	1	0	0	1	0	0	0	0	0	0	0	72
20864119	m	md	JP	2022	0	0	0	0	0	0	0	0	0	0	0	0	60
20864123	m	hp	RU	2022	0	0	0	0	0	0	0	0	0	0	0	0	62
20864168	m	md	JP	2022	0	1	0	0	1	0	0	0	0	0	0	0	48
20864791	Not	hp	CN	2022	0	1	0	0	0	1	0	0	0	0	0	0	
20864850	Not	md	JP	2022	0	1	0	0	1	0	0	0	0	0	0	0	
20865128	f	cn	CA	2022	0	0	0	0	0	0	0	0	0	0	0	0	71
20865275	m	md	JP	2022	0	1	0	0	0	1	0	0	0	0	0	0	70
20865373	m	md	JP	2022	0	1	0	0	1	0	0	0	0	0	0	0	67
20865502	m	cn	GB	2022	0	0	0	0	0	0	1	1	0	0	0	0	57
20865734	f	md	JP	2022	0	1	0	0	0	1	0	0	0	0	0	0	66
20865965	m	hp	CN	2022	0	1	0	0	1	0	0	0	0	0	0	0	66
20866322	m	cn	CA	2022	1	0	0	0	0	0	0	0	0	0	0	0	
20866638	f	cn	ID	2022	1	0	0	0	0	0	0	0	0	0	0	0	72
20866815	m	md	JP	2022	0	1	0	0	0	1	0	0	0	0	0	0	54
20866984	f	md	SK	2022	0	0	0	0	0	0	0	0	0	0	0	0	72
20867669	m	hp	NL	2022	0	1	0	0	1	0	0	0	0	0	0	0	74
20867807	f	md	ES	2022	0	1	0	0	0	1	0	0	0	0	0	0	51
20867812	m	md	JP	2022	0	1	0	0	0	1	0	0	0	0	0	0	64
20867852	f	cn	CN	2022	0	0	0	0	0	0	0	0	0	0	0	0	53
20867873	m	md	JP	2022	0	1	1	0	0	0	0	0	0	0	0	0	62
20867939	m	hp	US	2022	1	0	0	0	0	0	0	0	0	0	0	0	82
20868233	m	hp	JP	2022	0	1	1	0	0	0	0	0	0	0	0	0	44
20868339	f	md	CL	2022	0	0	0	0	0	0	0	0	0	0	0	0	48
20868393	f	md	FR	2022	0	0	0	0	0	0	0	0	0	0	0	0	44
20868441	f	md	DE	2022	0	1	0	0	1	0	0	0	0	0	0	0	68
20868573	m	md	FR	2022	0	0	0	0	0	0	0	0	0	0	0	0	73
20868626	m	hp	BG	2022	1	0	0	0	0	0	0	0	0	0	0	0	59
20868994	m	md	JP	2022	0	1	0	0	1	0	0	0	0	0	0	0	60
20869072	f	md	BE	2022	0	1	1	0	0	0	0	0	0	0	0	0	
20869160	f	md	US	2022	0	0	0	0	0	0	0	0	0	0	0	0	

20869653	Not	md	JP	2022	0	1	0	0	1	0	0	0	0	0	0	0	
20870697	m	md	AU	2022	0	1	0	1	0	0	0	0	0	0	0	1	70
20870817	f	ph	FR	2022	0	0	0	0	0	0	0	0	0	0	0	0	
20870879	m	md	JP	2022	0	1	1	0	0	0	0	0	0	0	0	1	90
20871494	f	hp	US	2022	0	1	0	0	1	0	0	0	0	0	0	0	63
20871815	f	hp	US	2022	0	1	1	0	0	0	0	0	0	0	0	0	75
20872058	Not	md	CH	2022	0	1	1	0	0	0	0	0	0	0	0	0	
20872542	f	md	PT	2022	0	1	1	0	0	0	0	0	0	0	0	0	57
20872544	Not	cn	BY	2022	0	1	0	0	0	1	0	0	0	0	0	0	57
20872825	f	md	DE	2022	0	1	0	0	1	0	0	0	0	0	0	0	85
20872840	f	md	PT	2022	0	1	1	0	0	0	0	0	0	0	0	0	57
20873139	f	md	FR	2022	0	0	0	0	0	0	0	0	0	0	0	0	
20873149	m	md	DE	2022	0	0	0	0	0	0	0	0	0	0	0	0	72
20873428	m	cn	ID	2022	1	0	0	0	0	0	0	0	0	0	0	0	81.5
20873449	m	md	IT	2022	0	0	0	0	0	0	0	0	0	0	0	0	60
20873580	m	md	JP	2022	0	1	0	0	1	0	0	0	0	0	0	0	72
20873590	f	md	FR	2022	0	0	0	0	0	0	0	0	0	0	0	0	69
20873798	f	hp	RU	2022	0	1	0	0	0	1	0	0	0	0	0	0	70
20873915	m	md	CN	2022	0	1	0	1	0	0	0	0	0	0	0	0	52.1
20874285	f	md	SA	2022	0	0	0	0	0	0	0	0	0	0	0	0	
20874502	f	md	JP	2022	0	0	0	0	0	0	0	0	0	0	0	0	71
20874527	m	hp	DE	2022	0	1	0	0	1	0	0	0	0	0	0	0	58
20874993	f	hp	US	2022	0	0	0	0	0	0	0	0	0	0	0	0	
20875084	m	md	IN	2022	0	1	0	0	1	0	0	0	0	0	0	0	75
20875133	m	md	DE	2022	0	1	0	0	1	0	0	0	0	0	0	1	49
20875483	m	md	AU	2022	0	1	0	1	0	0	0	0	0	0	0	0	60
20875497	f	md	US	2022	0	0	0	0	0	0	0	0	0	0	0	0	
20875532	m	md	JP	2022	0	1	0	0	1	0	0	0	0	0	0	0	80
20875709	m	ph	JP	2022	0	1	1	0	0	0	0	0	0	0	0	0	80
20875870	Not	md	GB	2022	1	0	0	0	0	0	0	0	0	0	0	0	
20875945	m	hp	IT	2022	0	1	0	0	0	1	0	0	0	0	0	0	82
20876341	m	md	JP	2022	0	1	0	0	1	0	0	0	0	0	0	1	37
20876411	Not	hp	CN	2022	1	0	0	0	0	0	0	0	0	0	0	0	
20876505	Not	md	JP	2022	0	1	0	0	1	0	0	0	0	0	0	0	

20876534	Not	hp	CN	2022	1	0	0	0	0	0	0	0	0	0	0	0	
20876595	m	hp	TW	2022	0	0	0	0	0	0	0	0	0	0	0	0	67
20877052	m	md	GB	2022	1	0	0	0	0	0	0	0	0	0	0	0	
20877271	Not	md	JP	2022	0	1	1	0	0	0	0	0	0	0	0	0	
20877785	f	hp	SE	2022	0	1	0	0	1	0	0	0	0	0	0	0	68
20877880	f	hp	US	2022	0	0	0	0	0	0	0	0	0	0	0	0	
20878281	m	cn	CN	2022	0	1	0	1	0	0	0	0	0	0	0	1	62
20878628	Not	hp	CN	2022	1	0	0	0	0	0	0	0	0	0	0	0	
20878630	Not	hp	CN	2022	1	0	0	0	0	0	0	0	0	0	0	0	
20878634	Not	hp	CN	2022	1	0	0	0	0	0	0	0	0	0	0	0	
20878771	Not	hp	CN	2022	1	0	0	0	0	0	0	0	0	0	0	0	
20878772	Not	hp	CN	2022	1	0	0	0	0	0	0	0	0	0	0	0	
20878810	Not	hp	CN	2022	1	0	0	0	0	0	0	0	0	0	0	0	
20879135	Not	ph	JP	2022	0	1	0	0	0	1	0	0	0	0	0	0	70
20879346	f	cn	SE	2022	0	1	0	0	1	0	0	0	0	0	0	0	68
20879543	Not	md	ES	2022	0	0	0	0	0	0	0	0	0	0	0	0	69
20879822	m	hp	RU	2022	0	0	0	0	0	0	0	0	0	0	0	0	62
20880248	Not	hp	CN	2022	1	0	0	0	0	0	0	0	0	0	0	0	
20880253	Not	hp	CN	2022	1	0	0	0	0	0	0	0	0	0	0	0	
20880279	m	cn	SG	2022	0	1	0	0	0	1	0	0	0	0	0	0	70
20880366	m	cn	EG	2022	0	0	0	0	0	0	0	0	0	0	0	0	55
20880580	m	md	DE	2022	0	0	0	0	0	0	0	0	0	0	0	0	61
20881095	m	cn	CN	2022	0	1	0	0	1	0	0	0	0	0	0	0	55
20881491	Not	hp	CN	2022	1	0	0	0	0	0	0	0	0	0	0	0	
20881687	f	md	DE	2022	0	1	1	0	0	0	0	0	0	0	0	0	60
20881799	f	md	US	2022	0	0	0	0	0	0	0	0	0	0	0	0	
20881841	f	md	US	2022	0	1	1	0	0	0	0	0	0	0	0	0	57
20881863	f	md	JP	2022	0	0	0	0	0	0	0	0	0	0	0	0	93
20881924	Not	md	BR	2022	0	1	0	0	1	0	0	0	0	0	0	0	
20881978	f	ph	JP	2022	0	0	0	0	0	0	0	0	0	0	0	0	44
20882105	Not	cn	SG	2022	1	0	0	0	0	0	0	0	0	0	0	0	67
20882434	m	md	JP	2022	0	1	0	0	1	0	0	0	0	0	0	0	70
20882775	Not	md	GB	2022	1	0	0	0	0	0	0	0	0	0	0	0	
20882788	f	md	DE	2022	0	1	0	0	1	0	0	0	0	0	0	0	65

20882851	Not	md	US	2022	0	0	0	0	0	0	0	0	0	0	0	0	
20883194	f	cn	CN	2022	0	1	0	1	0	0	0	0	0	0	0	1	47
20883266	m	md	DE	2022	0	1	0	0	1	0	0	0	0	0	0	0	
20883311	m	cn	US	2022	0	1	1	0	0	0	0	0	0	0	0	0	73
20883423	m	ph	JP	2022	0	1	1	0	0	0	0	0	0	0	0	0	74
20883509	m	md	CN	2022	0	0	0	0	0	0	0	0	0	0	0	0	69
20883675	Not	ph	JP	2022	1	0	0	0	0	0	0	0	0	0	0	0	
20883717	Not	md	JP	2022	0	0	0	0	0	0	0	0	0	0	0	0	
20883790	Not	ph	JP	2022	1	0	0	0	0	0	0	0	0	0	0	0	
20883941	m	cn	ID	2022	1	0	0	0	0	0	0	0	0	0	0	0	76.2
20884111	m	md	JP	2022	0	1	1	0	0	0	0	0	0	0	0	1	80
20884143	m	ph	US	2022	0	0	0	0	0	0	0	0	0	0	0	0	61
20885052	m	hp	US	2022	0	1	1	0	0	0	0	0	0	0	0	0	61
20885079	f	md	DE	2022	1	0	0	0	0	0	0	0	0	0	0	0	78
20885105	m	md	JP	2022	0	1	0	0	1	0	0	0	0	0	0	0	61
20885136	f	md	CA	2022	0	1	1	0	0	0	0	0	0	0	0	0	58
20885286	Not	cn	SG	2022	0	1	0	1	0	0	0	0	0	0	0	0	
20885290	m	cn	ID	2022	1	0	0	0	0	0	0	0	0	0	0	0	
20885518	Not	hp	CN	2022	0	0	0	0	0	0	0	0	0	0	0	0	
20886001	f	cn	FR	2022	0	1	0	0	1	0	0	0	0	0	0	0	63
20886499	f	md	US	2022	0	0	0	0	0	0	0	0	0	0	0	0	88
20886555	m	hp	TW	2022	0	0	0	0	0	0	0	0	0	0	0	0	67
20886747	m	md	JP	2022	0	1	0	0	0	1	0	0	0	0	0	0	80
20887111	f	hp	CA	2022	0	0	0	0	0	0	0	0	0	0	0	0	77
20887451	Not	md	RU	2022	0	1	0	0	0	1	0	0	0	0	0	0	59
20887454	f	md	RU	2022	0	1	0	0	0	1	0	0	0	0	0	0	61
20888040	m	hp	BE	2022	0	0	0	0	0	0	0	0	0	0	0	0	72
20888100	f	cn	US	2022	0	0	0	0	0	0	0	0	0	0	0	0	
20889335	f	md	CZ	2022	0	0	0	0	0	0	0	0	0	0	0	0	63
20889607	f	md	JP	2022	0	1	0	0	1	0	0	0	0	0	0	0	75
20889609	m	cn	CN	2022	0	1	0	0	1	0	0	0	0	0	0	0	66
20889891	f	md	FR	2022	0	0	0	0	0	0	0	0	0	0	0	0	
20890096	Not	md	JP	2022	0	1	0	0	1	0	0	0	0	0	0	0	80
20890100	Not	hp	GB	2022	1	0	0	0	0	0	0	0	0	0	0	0	

20890111	f	md	DE	2022	0	1	0	0	1	0	0	0	0	0	0	0	75
20890150	m	cn	CN	2022	0	1	0	0	1	0	0	0	0	0	0	0	74
20890167	m	md	JP	2022	0	1	1	0	0	0	0	0	0	0	0	0	60
20890170	f	cn	CN	2022	0	1	0	0	1	0	0	0	0	0	0	0	78
20890949	Not	md	JP	2022	0	1	0	0	1	0	0	0	0	0	0	0	
20891075	m	md	TH	2022	0	1	0	1	0	0	0	0	0	0	0	0	69
20891168	f	md	JP	2022	0	0	0	0	0	0	0	0	0	0	0	0	68
20891668	Not	cn	US	2022	0	0	0	0	0	0	0	0	0	0	0	0	
20891750	f	md	MY	2022	1	0	0	0	0	0	0	0	0	0	0	0	
20892017	m	cn	IN	2022	0	0	0	0	0	0	0	0	0	0	0	0	31
20892074	m	md	GR	2022	0	1	1	0	0	0	0	0	0	0	0	0	70
20892522	m	cn	US	2022	0	1	1	0	0	0	0	0	0	0	0	0	
20892590	m	md	JP	2022	0	1	0	0	1	0	0	0	0	0	0	0	73
20892814	f	md	FR	2022	1	0	0	0	0	0	0	0	0	0	0	0	
20892830	m	md	JP	2022	0	1	0	0	1	0	0	0	0	0	0	0	72
20892847	m	cn	IN	2022	0	1	0	0	1	0	0	0	0	0	0	0	54
20893064	m	md	JP	2022	0	1	1	0	0	0	0	0	0	0	0	0	63
20893130	f	md	JP	2022	0	1	0	0	1	0	0	0	0	0	0	0	64
20893185	f	hp	US	2022	0	1	0	0	1	0	0	0	0	0	0	0	73
20893403	m	md	JP	2022	0	1	1	0	0	0	0	0	0	0	0	0	68
20893480	m	md	JP	2022	0	1	1	0	0	0	0	0	0	0	0	0	
20893820	m	hp	JP	2022	0	1	0	0	1	0	0	0	0	0	0	0	69
20893908	m	md	JP	2022	0	1	0	1	0	0	0	0	0	0	0	1	54
20894481	m	md	US	2022	0	0	0	0	0	0	0	0	0	0	0	0	69
20894857	Not	md	IT	2022	0	0	0	0	0	0	0	0	0	0	0	0	54
20895334	f	cn	MY	2022	1	0	0	0	0	0	0	0	0	0	0	0	
20895340	m	md	JP	2022	0	1	0	1	0	0	0	0	0	0	0	0	72
20895383	m	cn	CA	2022	0	1	0	0	1	0	0	0	0	0	0	0	73
20895412	m	md	RU	2022	0	1	0	0	0	1	0	0	0	0	0	1	58
20895454	f	md	US	2022	0	0	0	0	0	0	0	0	0	0	0	0	57
20895500	m	hp	US	2022	0	1	1	0	0	0	0	0	0	0	0	0	59
20895634	m	md	JP	2022	0	1	1	0	0	0	0	0	0	0	0	0	58
20895784	m	md	CN	2022	0	0	0	0	0	0	0	0	0	0	0	0	63
20895882	m	md	JP	2022	0	0	0	0	0	0	0	0	0	0	0	0	

20895987	f	md	DE	2022	0	1	0	0	1	0	0	0	0	0	0	0	
20896005	m	md	AT	2022	0	1	0	0	1	0	0	0	0	0	0	0	81
20896039	m	md	JP	2022	0	1	0	1	0	0	0	0	0	0	0	0	65
20896086	m	md	CN	2022	0	0	0	0	0	0	0	0	0	0	0	0	73
20896308	f	md	US	2022	0	0	0	0	0	0	0	0	0	0	0	0	77
20896352	m	md	PT	2022	0	0	0	0	0	0	0	0	0	0	0	0	
20897126	f	hp	IN	2022	0	1	0	0	1	0	0	0	0	0	0	0	72
20897393	Not	md	US	2022	0	0	0	0	0	0	0	0	0	0	0	0	
20897560	m	md	PL	2022	0	1	0	0	0	1	0	0	0	0	0	0	74
20897785	m	md	JP	2022	0	0	0	0	0	0	0	0	0	0	0	0	38
20898121	f	hp	US	2022	0	1	0	0	1	0	0	0	0	0	0	0	59
20898125	f	md	JP	2022	0	1	0	0	1	0	0	0	0	0	0	0	57
20898194	Not	ph	US	2022	0	0	0	0	0	0	0	0	0	0	0	0	
20898598	f	cn	US	2022	0	0	0	0	0	0	0	0	0	0	0	0	88
20898732	f	md	US	2022	1	0	0	0	0	0	0	0	0	0	0	1	70
20898733	m	md	JP	2022	0	1	1	0	0	0	0	0	0	0	0	0	80
20899078	f	md	CN	2022	0	0	0	0	0	0	0	0	0	0	0	0	72
20899334	m	md	JP	2022	0	1	1	0	0	0	0	0	0	0	0	0	76
20899737	f	md	RS	2022	1	0	0	0	0	0	0	0	0	0	0	0	67
20899817	m	md	RS	2022	1	0	0	0	0	0	0	0	0	0	0	0	91
20900000	f	md	JP	2022	0	0	0	0	0	0	0	0	0	0	0	0	69
20900006	f	md	BG	2022	0	1	0	0	0	1	0	0	0	0	0	0	71
20900046	f	md	RS	2022	1	0	0	0	0	0	0	0	0	0	0	0	71
20900092	m	cn	IN	2022	0	0	0	0	0	0	0	0	0	0	0	0	39
20900163	m	md	DE	2022	0	0	0	0	0	0	0	0	0	0	0	0	61
20900235	f	ph	CA	2022	0	0	0	0	0	0	0	0	0	0	0	0	73
20900329	m	md	GR	2022	0	1	1	0	0	0	0	0	0	0	0	0	80
20900613	m	md	FR	2022	0	0	0	0	0	0	0	0	0	0	0	0	73
20900620	m	md	US	2022	0	1	1	0	0	0	0	0	0	0	0	0	61
20900663	f	cn	US	2022	1	0	0	0	0	0	0	0	0	0	0	0	
20900918	m	md	RS	2022	1	0	0	0	0	0	0	0	0	0	0	0	66
20901085	m	cn	IN	2022	1	0	0	0	0	0	0	0	0	0	0	0	75
20901383	f	cn	TH	2022	1	0	0	0	0	0	0	0	0	0	0	0	71.6
20901388	m	md	JP	2022	0	1	0	0	0	1	0	0	0	0	0	0	60

20901520	m	md	JP	2022	0	1	1	0	0	0	0	0	0	0	0	0	80
20901645	Not	md	RU	2022	0	1	0	0	0	1	0	0	0	0	0	1	
20901649	f	md	RS	2022	1	0	0	0	0	0	0	0	0	0	0	0	48
20901749	m	md	JP	2022	0	1	0	0	0	1	0	0	0	0	0	0	59
20901801	Not	md	AR	2022	0	0	0	0	0	0	0	0	0	0	0	0	
20901803	f	md	RS	2022	1	0	0	0	0	0	0	0	0	0	0	0	82
20901863	f	md	RS	2022	1	0	0	0	0	0	0	0	0	0	0	0	77
20901923	f	ph	FR	2022	0	0	0	0	0	0	0	0	0	0	0	0	
20901982	m	cn	US	2022	1	0	0	0	0	0	0	0	0	0	0	0	
20902589	m	md	KR	2022	0	0	0	0	0	0	0	0	0	0	0	0	79
20902733	f	md	CN	2022	1	0	0	0	0	0	0	0	0	0	0	0	69
20902980	Not	md	OM	2022	0	0	0	0	0	0	0	0	0	0	0	0	
20903147	f	md	BE	2022	0	0	0	0	0	0	0	0	0	0	0	0	54
20903299	m	hp	US	2022	0	0	0	0	0	0	0	0	0	0	0	0	74
20903399	m	md	NZ	2022	1	0	0	0	0	0	0	0	0	0	0	0	56.5
20903466	m	md	NL	2022	0	1	1	0	0	0	0	0	0	0	0	0	70
20903500	m	md	US	2022	0	0	0	0	0	0	0	0	0	0	0	0	
20903593	m	hp	PL	2022	0	1	1	0	0	0	0	0	0	0	0	0	67
20903710	Not	md	JP	2022	1	0	0	0	0	0	0	0	0	0	0	0	
20903828	m	md	CN	2022	0	0	0	0	0	0	0	0	0	0	0	0	65
20904075	f	md	JP	2022	0	1	0	0	1	0	0	0	0	0	0	0	64
20904413	m	cn	US	2022	0	1	1	0	0	0	0	0	0	0	0	0	57
20904550	m	md	US	2022	1	0	0	0	0	0	0	0	0	0	0	0	57
20905681	f	hp	SE	2022	0	1	0	0	1	0	0	0	0	0	0	0	68
20905985	m	md	RU	2022	0	1	0	0	0	1	0	0	0	0	0	1	56
20906572	m	md	PL	2022	0	1	1	0	0	0	0	0	0	0	0	0	68
20906802	m	cn	TH	2022	0	1	0	0	0	1	0	0	0	0	0	0	
20906808	f	hp	TH	2022	0	0	0	0	0	0	0	0	0	0	0	0	61
20906859	m	hp	US	2022	0	1	1	0	0	0	0	0	0	0	0	0	59
20906882	m	cn	IN	2022	0	0	0	0	0	0	0	0	0	0	0	0	32
20906944	f	md	JP	2022	0	0	0	0	0	0	0	0	0	0	0	0	50
20906972	m	md	JP	2022	0	1	0	0	0	1	0	0	0	0	0	0	80
20907031	m	hp	US	2022	0	1	0	0	1	0	0	0	0	0	0	0	68
20907204	f	md	JP	2022	1	0	0	0	0	0	0	0	0	0	0	0	61

20907213	Not	ph	JP	2022	1	0	0	0	0	0	0	0	0	0	0	0	
20907342	m	md	AT	2022	0	1	0	0	1	0	0	0	0	0	0	0	81
20907512	m	cn	HK	2022	0	0	0	0	0	0	0	0	0	0	0	0	51
20907519	f	md	JP	2022	0	1	1	0	0	0	0	0	0	0	0	0	75
20907549	f	md	US	2022	0	1	0	0	1	0	0	0	0	0	0	0	48
20907650	f	hp	TH	2022	0	0	0	0	0	0	0	0	0	0	0	0	61
20907667	f	cn	IN	2022	0	0	0	0	0	0	0	0	0	0	0	0	59
20908015	f	md	JP	2022	0	0	0	0	0	0	0	0	0	0	0	0	80
20908476	f	cn	ID	2022	1	0	0	0	0	0	0	0	0	0	0	0	59.9
20908613	f	ph	Not re	2022	0	0	0	0	0	0	0	0	0	0	0	0	80
20908928	m	md	BE	2022	0	1	0	0	0	1	0	0	0	0	0	0	62
20909011	m	md	JP	2022	0	1	0	0	1	0	0	0	0	0	0	0	59
20910713	f	md	FR	2022	0	1	0	0	0	1	0	0	0	0	0	0	
20910843	f	md	FR	2022	0	0	0	0	0	0	0	0	0	0	0	0	
20910912	f	cn	ID	2022	1	0	0	0	0	0	0	0	0	0	0	0	62.7
20911059	m	hp	US	2022	0	1	0	0	1	0	0	0	0	0	0	0	67
20911176	Not	hp	US	2022	1	0	0	0	0	0	0	0	0	0	0	0	
20911200	m	md	JP	2022	0	1	1	0	0	0	0	0	0	0	0	0	78
20911376	m	md	PL	2022	0	0	0	0	0	0	0	0	0	0	0	0	72
20911589	m	hp	FR	2022	0	1	0	0	1	0	0	0	0	0	0	0	77
20912099	f	md	FR	2022	0	0	0	0	0	0	0	0	0	0	0	0	67
20912151	f	cn	ID	2022	1	0	0	0	0	0	0	0	0	0	0	0	
20912194	Not	md	AR	2022	0	0	0	0	0	0	0	0	0	0	0	0	
20912219	f	cn	ID	2022	1	0	0	0	0	0	0	0	0	0	0	0	
20912300	f	cn	ID	2022	1	0	0	0	0	0	0	0	0	0	0	0	
20912370	f	md	CA	2022	1	0	0	0	0	0	0	0	0	0	0	0	
20912445	m	cn	IN	2022	0	0	0	0	0	0	0	0	0	0	0	0	51
20912490	m	md	CN	2022	0	0	0	0	0	0	0	0	0	0	0	0	48
20912600	m	hp	US	2022	0	1	1	0	0	0	0	0	0	0	0	0	
20912675	Not	hp	CN	2022	1	0	0	0	0	0	0	0	0	0	0	0	
20912719	m	md	DE	2022	0	1	0	0	1	0	0	0	0	0	0	0	64
20912754	f	ph	HR	2022	0	1	1	0	0	0	0	0	0	0	0	0	57
20912767	f	cn	CA	2022	1	0	0	0	0	0	0	0	0	0	0	0	
20912943	m	md	AU	2022	0	0	0	0	0	0	0	0	0	0	0	0	63

20913323	m	md	JP	2022	0	1	1	0	0	0	0	0	0	0	0	0	
20913652	Not	hp	IT	2022	0	0	0	0	0	0	1	1	0	0	0	0	
20913769	m	md	DE	2022	0	1	0	0	1	0	0	0	0	0	0	0	60
20913960	m	cn	IN	2022	0	0	0	0	0	0	0	0	0	0	0	0	
20917100	f	md	DE	2022	0	1	0	0	1	0	0	0	0	0	0	0	81
20917128	m	md	JP	2022	0	1	0	0	1	0	0	0	0	0	0	0	70
20917335	m	hp	BG	2022	0	1	0	0	1	0	0	0	0	0	0	1	
20917336	m	hp	BG	2022	0	1	0	0	1	0	0	0	0	0	0	0	65
20917647	m	md	CZ	2022	0	1	0	0	1	0	0	0	0	0	0	0	73
20917800	f	cn	DE	2022	0	1	1	0	0	0	0	0	0	0	0	0	
20917861	m	hp	TW	2022	0	0	0	0	0	0	0	0	0	0	0	0	70
20918594	m	md	PL	2022	0	0	0	0	0	0	0	0	0	0	0	0	45
20918712	m	hp	JP	2022	0	0	0	0	0	0	0	0	0	0	0	0	66
20918717	Not	cn	HK	2022	0	0	0	0	0	0	0	0	0	0	0	0	
20919311	Not	hp	US	2022	1	0	0	0	0	0	0	0	0	0	0	0	67.5
20919521	Not	md	US	2022	0	0	0	0	0	0	0	0	0	0	0	0	
20920202	Not	md	TW	2022	0	0	0	0	0	0	0	0	0	0	0	0	
20920646	m	md	FR	2022	0	1	0	0	0	1	0	0	0	0	0	0	43
20920808	m	md	JP	2022	0	1	0	1	0	0	0	0	0	0	0	0	
20920952	m	cn	IN	2022	0	0	0	0	0	0	0	0	0	0	0	0	62
20921084	m	md	FR	2022	0	0	0	0	0	0	0	0	0	0	0	0	70
20921125	f	md	CA	2022	1	0	0	0	0	0	0	0	0	0	0	0	59.3
20921300	f	md	JP	2022	0	1	1	0	0	0	0	0	0	0	0	0	82
20921443	f	cn	GB	2022	0	0	0	0	0	0	0	0	0	0	0	0	57
20921571	m	cn	IN	2022	0	1	0	0	0	1	0	0	0	0	0	0	46
20921584	m	ph	JP	2022	0	0	0	0	0	0	0	0	0	0	0	0	58
20921879	f	cn	CA	2022	0	1	0	0	1	0	0	0	0	0	0	0	73
20921902	f	md	JP	2022	0	1	0	0	1	0	0	0	0	0	0	0	85
20921966	m	md	CN	2022	0	0	0	0	0	0	0	0	0	0	0	0	62
20922291	m	md	JP	2022	0	1	1	0	0	0	0	0	0	0	0	0	79
20923026	Not	md	RU	2022	0	1	0	0	0	1	0	0	0	0	0	0	
20923181	m	hp	PT	2022	0	1	1	0	0	0	0	0	0	0	0	0	44
20923243	m	md	CN	2022	0	0	0	0	0	0	0	0	0	0	0	0	68
20923774	m	cn	CN	2022	0	1	0	1	0	0	0	0	0	0	0	0	60

20923968	f	md	TH	2022	0	1	0	1	0	0	0	0	0	0	0	0	80
20923997	f	md	FR	2022	0	0	0	0	0	0	0	0	0	0	0	0	63
20924030	f	md	NZ	2022	1	0	0	0	0	0	0	0	0	0	0	0	
20924230	m	md	CN	2022	0	0	0	0	0	0	0	0	0	0	0	0	61
20925108	m	md	JP	2022	0	1	0	1	0	0	0	0	0	0	0	0	71
20925195	f	md	NP	2022	0	0	0	0	0	0	0	0	0	0	0	0	71
20925278	m	md	CA	2022	0	0	0	0	0	0	0	0	0	0	0	0	
20925289	f	md	JP	2022	1	0	0	0	0	0	0	0	0	0	0	0	69
20925409	m	md	AT	2022	0	1	0	0	0	1	0	0	0	0	0	0	59
20926020	m	md	IT	2022	0	0	0	0	0	0	0	0	0	0	0	0	76
20926398	f	cn	US	2022	1	0	0	0	0	0	0	0	0	0	0	0	71.2
20926437	m	cn	US	2022	0	1	1	0	0	0	0	0	0	0	0	0	
20926477	f	md	FR	2022	0	0	0	0	0	0	0	0	0	0	0	0	
20926492	m	ph	US	2022	0	0	0	0	0	0	0	0	0	0	0	0	71
20926807	m	md	JP	2022	0	1	0	0	1	0	0	0	0	0	0	0	52
20926821	Not	hp	DE	2022	0	1	0	0	1	0	0	0	0	0	0	0	61
20927634	m	ph	PT	2022	0	0	0	0	0	0	0	0	0	0	0	0	65
20928494	f	md	JP	2022	0	1	0	0	1	0	0	0	0	0	0	0	63
20928611	m	md	JP	2022	0	1	0	0	1	0	0	0	0	0	0	0	80
20928772	m	hp	BE	2022	0	1	0	0	1	0	0	0	0	0	0	0	62
20928854	f	md	IL	2022	0	0	0	0	0	0	0	0	0	0	0	0	64
20929305	f	md	GB	2022	0	1	0	0	0	1	0	0	0	0	0	0	65
20929363	f	md	GB	2022	0	1	0	0	0	1	0	0	0	0	0	0	61
20929460	m	hp	US	2022	0	0	0	0	0	0	0	0	0	0	0	0	78
20930265	Not	md	JP	2022	0	0	0	0	0	0	0	0	0	0	0	0	
20930333	m	md	PL	2022	0	1	1	0	0	0	0	0	0	0	0	0	68
20930371	Not	md	US	2022	0	1	0	0	1	0	0	0	0	0	0	0	
20931033	m	md	JP	2022	0	0	0	0	0	0	0	0	0	0	0	0	60
20931106	f	cn	IN	2022	0	0	0	0	0	0	0	0	0	0	0	0	51
20931414	Not	ph	JP	2022	1	0	0	0	0	0	0	0	0	0	0	0	
20931438	f	ph	GB	2022	0	0	0	0	0	0	0	0	0	0	0	0	52
20931454	f	cn	GB	2022	1	0	0	0	0	0	0	0	0	0	0	0	
20932577	m	md	MX	2022	0	0	0	0	0	0	0	0	0	0	0	0	63
20932687	m	hp	JP	2022	0	0	0	0	0	0	0	0	0	0	0	0	52

20932779	f	hp	FR	2022	0	1	0	0	1	0	0	0	0	0	0	0	73
20932965	m	cn	AT	2022	0	0	0	0	0	0	0	0	0	0	0	0	70
20932969	m	cn	CA	2022	0	1	0	0	1	0	0	0	0	0	0	0	
20932974	Not	cn	CA	2022	0	1	1	0	0	0	0	0	0	0	0	0	
20933076	Not	cn	CN	2022	0	0	0	0	0	0	0	0	0	0	0	0	
20933402	m	cn	PH	2022	1	0	0	0	0	0	0	0	0	0	0	0	48
20933422	f	hp	US	2022	1	0	0	0	0	0	0	0	0	0	0	0	
20933546	m	ph	JP	2022	1	0	0	0	0	0	0	0	0	0	0	0	80
20933703	f	cn	ID	2022	1	0	0	0	0	0	0	0	0	0	0	0	
20934210	f	md	JP	2022	1	0	0	0	0	0	0	0	0	0	0	0	75
20934234	Not	hp	KR	2022	1	0	0	0	0	0	0	0	0	0	0	0	
20934253	Not	md	US	2022	0	0	0	0	0	0	0	0	0	0	0	0	
20934273	Not	hp	KR	2022	1	0	0	0	0	0	0	0	0	0	0	0	
20934323	m	cn	ID	2022	1	0	0	0	0	0	0	0	0	0	0	0	
20934380	Not	hp	KR	2022	1	0	0	0	0	0	0	0	0	0	0	0	
20934597	Not	hp	KR	2022	1	0	0	0	0	0	0	0	0	0	0	0	
20934724	Not	hp	KR	2022	1	0	0	0	0	0	0	0	0	0	0	0	
20934732	Not	hp	KR	2022	1	0	0	0	0	0	0	0	0	0	0	0	
20934738	Not	hp	KR	2022	1	0	0	0	0	0	0	0	0	0	0	0	
20934739	Not	hp	KR	2022	1	0	0	0	0	0	0	0	0	0	0	0	
20935522	Not	md	CA	2022	0	1	1	0	0	0	0	0	0	0	0	0	
20935631	f	md	JP	2022	0	1	0	0	1	0	0	0	0	0	0	0	61
20936396	m	md	JP	2022	0	0	0	0	0	0	0	0	0	0	0	0	70
20936407	f	cn	US	2022	0	0	0	0	0	0	0	0	0	0	0	0	70
20936579	m	ph	JP	2022	0	1	0	0	1	0	0	0	0	0	0	1	70
20937623	m	md	KR	2022	0	0	0	0	0	0	0	0	0	0	0	0	
20937809	f	cn	GB	2022	1	0	0	0	0	0	0	0	0	0	0	0	
20937821	f	hp	FR	2022	0	0	0	0	0	0	0	0	0	0	0	0	73
20938015	f	cn	CA	2022	0	0	0	0	0	0	0	0	0	0	0	0	77
20938253	Not	hp	CN	2022	0	0	0	0	0	0	0	0	0	0	0	0	
20939055	m	md	JP	2022	0	1	0	1	0	0	0	0	0	0	0	1	70
20939414	Not	md	JP	2022	0	1	1	0	0	0	0	0	0	0	0	0	70
20939526	Not	md	CN	2022	0	0	0	0	0	0	0	0	0	0	0	0	
20939528	f	md	IT	2022	0	1	1	0	0	0	0	0	0	0	0	0	62

20939730	Not	md	US	2022	0	0	0	0	0	0	0	0	0	0	0	0	
20939740	m	md	PL	2022	0	1	1	0	0	0	0	0	0	0	0	1	72
20939792	f	md	JP	2022	0	1	1	0	0	0	0	0	0	0	0	0	71
20939839	f	hp	CN	2022	1	0	0	0	0	0	0	0	0	0	0	0	68
20940030	m	cn	PL	2022	0	1	1	0	0	0	0	0	0	0	0	0	67
20940077	f	md	JP	2022	0	0	0	0	0	0	0	0	0	0	0	0	43
20940107	m	md	GR	2022	0	1	1	0	0	0	0	0	0	0	0	0	70
20940547	m	hp	US	2022	0	1	1	0	0	0	0	0	0	0	0	0	59
20941108	f	md	DE	2022	0	0	0	0	0	0	0	0	0	0	0	0	51
20941263	Not	md	JP	2022	1	0	0	0	0	0	0	0	0	0	0	0	83
20941265	f	md	HU	2022	0	1	0	1	0	0	0	0	0	0	0	0	73
20941345	m	hp	IN	2022	1	0	0	0	0	0	0	0	0	0	0	0	46
20941430	m	md	JP	2022	0	1	0	1	0	0	0	0	0	0	0	0	88
20941744	f	hp	DE	2022	0	1	0	0	1	0	0	0	0	0	0	0	68
20941791	m	md	JP	2022	0	1	0	0	1	0	0	0	0	0	0	0	51
20942005	m	md	US	2022	0	1	1	0	0	0	0	0	0	0	0	0	59
20942364	m	cn	IN	2022	0	1	0	0	1	0	0	0	0	0	0	0	60
20942462	m	cn	US	2022	0	1	1	0	0	0	0	0	0	0	0	0	77
20942517	m	md	RU	2022	0	1	0	0	0	1	0	0	0	0	0	0	50
20942560	m	md	US	2022	0	1	0	0	1	0	0	0	0	0	0	0	71
20942865	f	hp	PL	2022	0	1	1	0	0	0	0	0	0	0	0	0	63
20942985	f	cn	CN	2022	0	0	0	0	0	0	0	0	0	0	0	0	62
20943158	m	md	JP	2022	0	0	0	0	0	0	0	0	0	0	0	0	
20944650	f	hp	IE	2022	0	1	0	0	1	0	0	0	0	0	0	0	73
20944666	f	cn	US	2022	0	1	0	0	1	0	0	0	0	0	0	0	
20944792	Not	md	JP	2022	1	0	0	0	0	0	0	0	0	0	0	0	72
20944907	f	md	US	2022	0	1	1	0	0	0	0	0	0	0	0	0	54
20945366	m	cn	US	2022	0	0	0	0	0	0	0	0	0	0	0	0	
20945538	Not	cn	US	2022	1	0	0	0	0	0	0	0	0	0	0	0	56
20945658	Not	md	JP	2022	0	1	0	0	1	0	0	0	0	0	0	0	
20946352	m	hp	JP	2022	0	0	0	0	0	0	0	0	0	0	0	0	66
20946435	f	md	JP	2022	1	0	0	0	0	0	0	0	0	0	0	0	89
20946646	Not	md	JP	2022	0	1	0	0	0	1	0	0	0	0	0	1	
20946754	m	cn	GR	2022	0	1	1	0	0	0	0	0	0	0	0	0	80

20946818	m	hp	US	2022	0	1	0	0	1	0	0	0	0	0	0	0	50
20946901	Not	md	JP	2022	1	0	0	0	0	0	0	0	0	0	0	0	79
20946945	f	cn	JP	2022	1	0	0	0	0	0	0	0	0	0	0	0	
20947068	f	ph	US	2022	0	0	0	0	0	0	0	0	0	0	0	0	71
20947075	m	md	CN	2022	0	1	0	1	0	0	0	0	0	0	0	0	67.3
20947082	f	cn	US	2022	0	0	0	0	0	0	0	0	0	0	0	0	
20947273	m	md	CL	2022	0	1	0	1	0	0	0	0	0	0	0	0	64
20947345	m	md	CN	2022	0	0	0	0	0	0	0	0	0	0	0	0	67
20947913	f	cn	US	2022	1	0	0	0	0	0	0	0	0	0	0	0	
20948092	f	cn	US	2022	0	0	0	0	0	0	0	0	0	0	0	0	
20948377	f	md	AT	2022	0	1	0	0	0	1	0	0	0	0	0	0	47
20948819	f	ph	US	2022	1	0	0	0	0	0	0	0	0	0	0	0	50
20948823	f	md	CA	2022	1	0	0	0	0	0	0	0	0	0	0	0	
20948843	m	cn	CN	2022	0	1	0	0	1	0	0	0	0	0	0	0	65
20948888	m	md	AU	2022	1	0	0	0	0	0	0	0	0	0	0	0	
20948988	m	cn	CN	2022	0	1	0	1	0	0	0	0	0	0	0	0	76
20949053	m	md	DE	2022	0	0	0	0	0	0	0	0	0	0	0	0	61
20949624	m	md	PL	2022	0	0	0	0	0	0	0	0	0	0	0	0	72
20949671	m	cn	IN	2022	0	1	0	0	1	0	0	0	0	0	0	0	47
20949750	m	md	JP	2022	0	1	0	0	1	0	0	0	0	0	0	0	69
20949890	f	cn	US	2022	0	1	0	0	0	1	0	0	0	0	0	0	75
20950548	m	cn	CA	2022	0	0	0	0	0	0	0	0	0	0	0	0	54
20950677	Not	md	US	2022	0	0	0	0	0	0	0	0	0	0	0	0	
20950988	f	hp	IT	2022	0	0	0	0	0	0	0	0	0	0	0	0	74
20951080	m	md	JP	2022	0	1	0	0	1	0	0	0	0	0	0	0	48
20951145	m	hp	JP	2022	0	0	0	0	0	0	0	0	0	0	0	0	70
20951251	m	md	DE	2022	0	1	1	0	0	0	0	0	0	0	0	1	76
20951258	m	md	PL	2022	0	1	1	0	0	0	0	0	0	0	0	0	68
20951546	f	cn	TH	2022	0	0	0	0	0	0	0	0	0	0	0	0	66
20951564	f	hp	CA	2022	0	0	0	0	0	0	0	0	0	0	0	0	77
20951578	m	md	PL	2022	0	1	1	0	0	0	0	0	0	0	0	0	72
20951824	Not	md	JP	2022	0	1	0	0	1	0	0	0	0	0	0	0	
20952537	f	cn	GB	2022	1	0	0	0	0	0	0	0	0	0	0	0	44
20953145	m	cn	IN	2022	0	0	0	0	0	0	0	0	0	0	0	0	31

20953161	f	md	FR	2022	0	0	0	0	0	0	0	0	0	0	0	0	
20953181	m	cn	US	2022	0	0	0	0	0	0	0	0	0	0	0	0	80
20953614	m	md	JP	2022	0	1	0	0	1	0	0	0	0	0	0	0	
20953941	m	ph	US	2022	0	0	0	0	0	0	0	0	0	0	0	0	57
20954099	m	ph	US	2022	0	0	0	0	0	0	0	0	0	0	0	0	
20954157	f	md	JP	2022	0	1	0	0	0	1	0	0	0	0	0	0	70
20954858	f	ph	US	2022	0	0	0	0	0	0	0	0	0	0	0	0	58
20955731	m	md	DE	2022	0	1	1	0	0	0	0	0	0	0	0	0	70
20955809	f	ph	US	2022	0	1	1	0	0	0	0	0	0	0	0	0	78
20956136	m	cn	IN	2022	0	1	0	0	1	0	0	0	0	0	0	0	63
20956171	Not	md	JP	2022	0	1	0	1	0	0	0	0	0	0	0	0	
20956453	Not	md	US	2022	1	0	0	0	0	0	0	0	0	0	0	0	
20956684	m	cn	US	2022	0	0	0	0	0	0	0	0	0	0	0	0	
20957024	f	hp	FR	2022	0	0	0	0	0	0	0	0	0	0	0	0	73
20957815	f	md	JP	2022	0	0	0	0	0	0	0	0	0	0	0	0	72
20957894	m	md	JP	2022	0	1	1	0	0	0	0	0	0	0	0	0	90
20957933	m	md	IT	2022	0	0	0	0	0	0	0	0	0	0	0	0	71
20958010	f	cn	US	2022	0	0	0	0	0	0	0	0	0	0	0	0	
20958134	Not	md	US	2022	0	0	0	0	0	0	0	0	0	0	0	0	
20958374	m	md	KR	2022	0	0	0	0	0	0	0	0	0	0	0	0	
20958445	Not	ph	JP	2022	1	0	0	0	0	0	0	0	0	0	0	0	
20958642	Not	cn	US	2022	0	0	0	0	0	0	0	0	0	0	0	0	
20958676	f	cn	CA	2022	1	0	0	0	0	0	0	0	0	0	0	0	83.2
20959132	Not	md	JP	2022	1	0	0	0	0	0	0	0	0	0	0	0	
20959321	m	md	US	2022	0	0	0	0	0	0	0	0	0	0	0	0	
20959428	m	md	GR	2022	0	1	1	0	0	0	0	0	0	0	0	0	80
20959537	Not	md	US	2022	0	0	0	0	0	0	0	0	0	0	0	0	
20959943	f	md	JP	2022	0	1	0	0	1	0	0	0	0	0	0	0	80
20960450	m	md	NZ	2022	1	0	0	0	0	0	0	0	0	0	0	0	54.9
20960458	f	hp	FR	2022	0	1	0	0	1	0	0	0	0	0	0	0	73
20960507	m	ph	US	2022	0	0	0	0	0	0	0	0	0	0	0	0	
20960527	m	cn	TH	2022	1	0	0	0	0	0	0	0	0	0	0	0	56.9
20960752	m	md	JP	2022	0	1	0	0	1	0	0	0	0	0	0	0	74
20961230	m	md	DE	2022	0	1	1	0	0	0	0	0	0	0	0	1	76

20961327	m	md	MY	2022	0	1	1	0	0	0	0	0	0	0	0	0	63
20961740	Not	md	JP	2022	0	1	0	1	0	0	0	0	0	0	0	0	
20961880	Not	ph	JP	2022	1	0	0	0	0	0	0	0	0	0	0	0	
20961961	m	hp	CN	2022	0	0	0	0	0	0	0	0	0	0	0	0	66
20961977	Not	cn	US	2022	0	0	0	0	0	0	0	0	0	0	0	0	
20962049	f	md	JP	2022	0	1	0	0	1	0	0	0	0	0	0	0	67
20962077	m	cn	TH	2022	1	0	0	0	0	0	0	0	0	0	0	0	82.7
20962396	m	cn	JP	2022	1	0	0	0	0	0	0	0	0	0	0	0	
20962633	m	md	GB	2022	0	1	0	0	0	1	0	0	0	0	0	0	78
20963225	m	cn	CA	2022	0	1	0	0	1	0	0	0	0	0	0	0	
20963242	m	cn	GB	2022	0	0	0	0	0	0	0	0	0	0	0	0	35
20963517	f	hp	CA	2022	1	0	0	0	0	0	0	0	0	0	0	0	56
20963593	f	hp	US	2022	1	0	0	0	0	0	0	0	0	0	0	0	
20963798	m	hp	PL	2022	0	1	1	0	0	0	0	0	0	0	0	0	67
20964311	m	ph	JP	2022	0	1	0	0	0	1	0	0	0	0	0	0	80
20964722	f	cn	IN	2022	0	0	0	0	0	0	0	0	0	0	0	0	47
20965867	m	md	JP	2022	0	1	0	0	1	0	0	0	0	0	0	0	51
20966607	f	md	JP	2022	0	0	0	0	0	0	0	0	0	0	0	0	69
20966842	f	hp	PL	2022	0	1	1	0	0	0	0	0	0	0	0	0	63
20967161	Not	md	CA	2022	0	1	1	0	0	0	0	0	0	0	0	0	
20967253	f	cn	CH	2022	0	0	0	0	0	0	0	0	0	0	0	0	
20967307	f	md	IT	2022	0	0	0	0	0	0	0	0	0	0	0	0	59
20967444	f	md	KR	2022	0	0	0	0	0	0	0	0	0	0	0	0	59
20967468	f	md	JP	2022	0	1	0	0	0	1	0	0	0	0	0	0	80
20967469	m	md	JP	2022	0	1	0	0	0	1	0	0	0	0	0	0	85
20967563	f	hp	FR	2022	0	1	0	0	1	0	0	0	0	0	0	0	73
20968026	m	md	JP	2022	0	1	1	0	0	0	0	0	0	0	0	0	80
20968052	m	md	DE	2022	0	1	0	0	1	0	0	0	0	0	0	0	59
20968777	m	md	JP	2022	0	1	0	1	0	0	0	0	0	0	0	1	80
20968835	m	md	JP	2022	0	1	0	1	0	0	0	0	0	0	0	0	78
20968924	m	md	JP	2022	0	1	0	1	0	0	0	0	0	0	0	1	78
20969172	m	cn	CA	2022	0	0	0	0	0	0	0	0	0	0	0	0	74
20969246	m	md	CN	2022	0	0	0	0	0	0	0	0	0	0	0	0	59
20969284	m	hp	US	2022	1	0	0	0	0	0	0	0	0	0	0	0	

20969363	f	cn	PL	2022	0	1	1	0	0	0	0	0	0	0	0	0	63
20969409	f	md	JP	2022	0	0	0	0	0	0	0	0	0	0	0	0	46
20969696	m	md	KZ	2022	0	1	1	0	0	0	0	0	0	0	0	0	70
20969752	Not	md	JP	2022	0	1	0	0	0	1	0	0	0	0	0	0	80
20972178	m	cn	TH	2022	0	1	0	0	0	1	0	0	0	0	0	0	
20972385	f	md	JP	2022	0	1	0	0	1	0	0	0	0	0	0	0	67
20972862	m	md	JP	2022	0	1	0	0	1	0	0	0	0	0	0	0	68
20973006	f	md	JP	2022	0	1	0	0	1	0	0	0	0	0	0	0	70
20973303	f	cn	US	2022	0	1	1	0	0	0	0	0	0	0	0	0	81
20973311	f	md	CN	2022	0	0	0	0	0	0	0	0	0	0	0	0	66
20973750	m	cn	ID	2022	1	0	0	0	0	0	0	0	0	0	0	0	67.6
20973757	f	hp	CN	2022	1	0	0	0	0	0	0	0	0	0	0	0	62
20973841	f	md	CA	2022	1	0	0	0	0	0	0	0	0	0	0	0	
20974399	m	md	NL	2022	0	1	0	0	0	1	0	0	0	0	0	0	70
20974819	f	cn	NL	2022	1	0	0	0	0	0	0	0	0	0	0	0	55
20975333	f	cn	ID	2022	1	0	0	0	0	0	0	0	0	0	0	0	68.7
20975574	f	md	CA	2022	1	0	0	0	0	0	0	0	0	0	0	0	63
20975617	m	cn	CA	2022	0	0	0	0	0	0	0	0	0	0	0	0	59
20975943	f	cn	CN	2022	0	1	0	0	1	0	0	0	0	0	0	0	53
20976105	m	cn	GR	2022	0	1	1	0	0	0	0	0	0	0	0	0	70
20976292	m	cn	IN	2022	0	0	0	0	0	0	0	0	0	0	0	0	61
20976297	m	cn	CA	2022	0	0	0	0	0	0	0	0	0	0	0	0	77
20976342	f	hp	AU	2022	0	0	0	0	0	0	0	0	0	0	0	0	69
20976375	f	md	JP	2022	0	1	0	0	1	0	0	0	0	0	0	0	66
20976617	f	cn	US	2022	1	0	0	0	0	0	0	0	0	0	0	0	
20976844	f	hp	US	2022	0	0	0	0	0	0	0	0	0	0	0	0	
20976981	Not	hp	CN	2022	0	0	0	0	0	0	0	0	0	0	0	0	
20977004	f	md	JP	2022	0	1	1	0	0	0	0	0	0	0	0	0	53
20977052	m	md	TR	2022	0	0	0	0	0	0	0	0	0	0	0	0	
20977103	f	md	JP	2022	0	1	1	0	0	0	0	0	0	0	0	0	73
20977380	f	md	KR	2022	0	0	0	0	0	0	0	0	0	0	0	0	76
20977550	m	cn	CN	2022	0	1	0	0	1	0	0	0	0	0	0	0	65
20977849	m	hp	JP	2022	0	1	0	1	0	0	0	0	0	0	0	1	55
20978717	m	md	JP	2022	0	1	0	0	1	0	0	0	0	0	0	0	59

20978830	m	hp	JP	2022	0	1	0	0	1	0	0	0	0	0	0	0	80
20978965	Not	md	HK	2022	0	0	0	0	0	0	0	0	0	0	0	0	
20978990	f	md	JP	2022	0	1	0	0	1	0	0	0	0	0	0	0	74
20979329	f	md	NL	2022	0	0	0	0	0	0	0	0	0	0	0	0	68
20979936	m	md	JP	2022	0	1	0	0	1	0	0	0	0	0	0	0	55
20979962	Not	hp	JP	2022	0	0	0	0	0	0	0	0	0	0	0	0	
20980012	m	cn	US	2022	0	0	0	0	0	0	0	0	0	0	0	0	
20980028	m	md	JP	2022	0	1	0	0	1	0	0	0	0	0	0	0	60
20980238	f	ph	JP	2022	0	1	0	0	0	1	0	0	0	0	0	0	80
20980244	f	md	JP	2022	0	1	1	0	0	0	0	0	0	0	0	0	77
20980740	Not	cn	US	2022	0	0	0	0	0	0	0	0	0	0	0	0	
20981021	Not	cn	US	2022	0	0	0	0	0	0	0	0	0	0	0	0	
20981039	Not	cn	US	2022	0	0	0	0	0	0	0	0	0	0	0	0	
20981135	m	md	JP	2022	0	0	0	0	0	0	0	0	0	0	0	0	68
20981714	m	md	DE	2022	0	1	0	0	1	0	0	0	0	0	0	0	70
20981792	m	md	CN	2022	0	0	0	0	0	0	0	0	0	0	0	0	62
20982720	Not	hp	US	2022	0	0	0	0	0	0	0	0	0	0	0	0	
20982810	Not	cn	FR	2022	0	0	0	0	0	0	0	0	0	0	0	0	
20982899	f	cn	IN	2022	0	1	0	0	1	0	0	0	0	0	0	0	44
20982917	f	hp	DE	2022	0	1	0	0	1	0	0	0	0	0	0	0	65
20983592	m	md	JP	2022	0	1	0	0	1	0	0	0	0	0	0	0	55
20983595	f	md	JP	2022	0	1	0	1	0	0	0	0	0	0	0	1	61
20983596	f	md	JP	2022	0	1	0	0	1	0	0	0	0	0	0	0	77
20983605	m	md	JP	2022	0	1	0	0	1	0	0	0	0	0	0	0	79
20983627	f	md	IT	2022	0	1	1	0	0	0	0	0	0	0	0	0	62
20984198	m	md	JP	2022	0	1	0	1	0	0	0	0	0	0	0	0	57
20984228	f	hp	US	2022	0	1	0	0	0	1	0	0	0	0	0	0	60
20984233	f	cn	US	2022	0	1	0	0	0	1	0	0	0	0	0	0	71
20984258	m	md	US	2022	0	1	0	0	0	1	0	0	0	0	0	0	45
20984322	m	cn	US	2022	0	1	0	0	0	1	0	0	0	0	0	0	
20984336	f	hp	US	2022	0	1	0	0	0	1	0	0	0	0	0	0	64
20984376	m	cn	CA	2022	0	1	0	0	1	0	0	0	0	0	0	0	75
20984460	m	md	CN	2022	1	0	0	0	0	0	0	0	0	0	0	0	
20984523	m	md	DE	2022	0	1	1	0	0	0	0	0	0	0	0	0	

20985763	m	hp	US	2022	0	0	0	0	0	0	0	0	0	0	0	0	67
20985866	m	hp	IT	2022	0	1	0	0	1	0	0	0	0	0	0	0	59
20985946	m	md	JP	2022	0	0	0	0	0	0	0	0	0	0	0	0	
20986011	m	hp	US	2022	0	0	0	0	0	0	0	0	0	0	0	0	46
20986092	f	cn	KR	2022	0	0	0	0	0	0	0	0	0	0	0	0	66
20986096	Not	md	JP	2022	0	1	0	1	0	0	0	0	0	0	0	1	
20986128	Not	ph	US	2022	0	0	0	0	0	0	0	0	0	0	0	0	
20986145	m	md	JP	2022	0	1	0	0	1	0	0	0	0	0	0	0	83
20986159	m	md	JP	2022	0	1	0	1	0	0	0	0	0	0	0	0	75
20986188	f	md	CN	2022	0	0	0	0	0	0	0	0	0	0	0	0	53
20986724	Not	cn	HK	2022	0	0	0	0	0	0	0	0	0	0	0	0	
20986845	m	md	JP	2022	0	1	0	1	0	0	0	0	0	0	0	0	69
20986923	m	cn	IN	2022	0	0	0	0	0	0	0	0	0	0	0	0	66
20987357	m	md	JP	2022	0	1	0	1	0	0	0	0	0	0	0	0	72
20987364	m	hp	PL	2022	0	1	1	0	0	0	0	0	0	0	0	0	67
20987820	m	md	JP	2022	0	1	0	1	0	0	0	0	0	0	0	0	60
20987821	m	md	JP	2022	0	1	0	1	0	0	0	0	0	0	0	0	45
20988211	m	md	JP	2022	0	1	0	1	0	0	0	0	0	0	0	0	74
20988515	f	md	JP	2022	1	0	0	0	0	0	0	0	0	0	0	0	61
20988598	f	cn	PL	2022	0	1	1	0	0	0	0	0	0	0	0	0	63
20988958	f	md	JP	2022	0	1	1	0	0	0	0	0	0	0	0	0	80
20989138	m	cn	US	2022	1	0	0	0	0	0	0	0	0	0	0	0	74
20989217	m	cn	US	2022	0	1	1	0	0	0	0	0	0	0	0	0	72
20989650	m	hp	RU	2022	0	0	0	0	0	0	0	0	0	0	0	0	74
20989990	m	md	JP	2022	0	1	0	0	1	0	0	0	0	0	0	0	76
20990032	m	md	AU	2022	1	0	0	0	0	0	0	0	0	0	0	0	85
20990253	Not	hp	CN	2022	0	0	0	0	0	0	0	0	0	0	0	0	
20990510	Not	hp	CN	2022	0	0	0	0	0	0	0	0	0	0	0	0	
20990750	f	md	GB	2022	0	1	0	0	0	1	0	0	0	0	0	1	56
20990755	Not	md	JP	2022	0	1	0	0	0	1	0	0	0	0	0	1	80
20992314	f	md	JP	2022	0	1	0	1	0	0	0	0	0	0	0	0	70
20992319	Not	md	JP	2022	0	1	0	1	0	0	0	0	0	0	0	0	85
20992329	Not	md	US	2022	0	0	0	0	0	0	0	0	0	0	0	0	
20992601	m	cn	SG	2022	0	1	1	0	0	0	0	0	0	0	0	0	60

20993108	f	md	JP	2022	0	0	0	0	0	0	0	0	0	0	0	0	67
20993154	f	cn	TW	2022	0	0	0	0	0	0	0	0	0	0	0	0	63
20993285	f	cn	NL	2022	0	0	0	0	0	0	0	0	0	0	0	0	73
20993846	m	hp	DE	2022	0	0	0	0	0	0	0	0	0	0	0	0	
20993852	m	cn	CN	2022	0	1	0	1	0	0	0	0	0	0	0	1	54
20994045	m	md	DE	2022	0	1	0	0	0	1	0	0	0	0	0	0	62
20994316	f	md	JP	2022	0	0	0	0	0	0	0	0	0	0	0	0	77
20994332	m	hp	DE	2022	0	0	0	0	0	0	0	0	0	0	0	0	
20994333	m	hp	DE	2022	0	0	0	0	0	0	0	0	0	0	0	0	
20994337	m	hp	DE	2022	0	0	0	0	0	0	0	0	0	0	0	0	
20994338	f	hp	DE	2022	0	0	0	0	0	0	0	0	0	0	0	0	
20994353	f	md	AU	2022	0	1	0	1	0	0	0	0	0	0	0	0	
20995343	f	hp	DE	2022	0	0	0	0	0	0	0	0	0	0	0	0	
20995396	m	md	JP	2022	0	1	0	0	1	0	0	0	0	0	0	0	
20995411	f	cn	US	2022	0	1	1	0	0	0	0	0	0	0	0	0	
20995614	f	ph	US	2022	1	0	0	0	0	0	0	0	0	0	0	0	
20995662	m	hp	DE	2022	0	0	0	0	0	0	0	0	0	0	0	0	
20995706	m	hp	DE	2022	0	0	0	0	0	0	0	0	0	0	0	0	
20995707	f	hp	DE	2022	0	0	0	0	0	0	0	0	0	0	0	0	
20995708	m	hp	DE	2022	0	0	0	0	0	0	0	0	0	0	0	0	
20995724	f	hp	DE	2022	0	0	0	0	0	0	0	0	0	0	0	0	
20995725	f	hp	DE	2022	0	0	0	0	0	0	0	0	0	0	0	0	
20995794	Not	hp	US	2022	0	0	0	0	0	0	0	0	0	0	0	0	
20995822	f	md	DE	2022	0	1	0	0	1	0	0	0	0	0	0	0	73
20995836	f	hp	DE	2022	0	0	0	0	0	0	0	0	0	0	0	0	
20995839	f	hp	DE	2022	0	0	0	0	0	0	0	0	0	0	0	0	
20995841	f	hp	DE	2022	0	0	0	0	0	0	0	0	0	0	0	0	
20996469	Not	hp	US	2022	0	0	0	0	0	0	0	0	0	0	0	0	
20996509	m	md	JP	2022	0	1	0	0	1	0	0	0	0	0	0	0	61
20996666	f	hp	DE	2022	0	0	0	0	0	0	0	0	0	0	0	0	
20996693	f	md	JP	2022	0	1	0	0	1	0	0	0	0	0	0	0	81
20997117	Not	hp	JP	2022	0	1	0	0	1	0	0	0	0	0	0	0	
20997445	m	cn	CA	2022	1	0	0	0	0	0	0	0	0	0	0	0	
20997623	m	md	DE	2022	0	1	1	0	0	0	0	0	0	0	0	1	76

20997765	Not	md	US	2022	0	0	0	0	0	0	0	0	0	0	0	0	
20997945	f	hp	FR	2022	0	1	0	0	1	0	0	0	0	0	0	0	58
20999137	m	md	JP	2022	0	1	1	0	0	0	0	0	0	0	0	0	63
20999150	m	md	DE	2022	0	1	1	0	0	0	0	0	0	0	0	0	
20999548	f	hp	FR	2022	0	0	0	0	0	0	0	0	0	0	0	0	76
20999917	m	md	PL	2022	0	1	1	0	0	0	0	0	0	0	0	0	68
21000083	Not	ph	JP	2022	1	0	0	0	0	0	0	0	0	0	0	0	
21000370	m	md	JP	2022	0	1	0	1	0	0	0	0	0	0	0	1	56
21000380	m	md	JP	2022	0	1	1	0	0	0	0	0	0	0	0	1	70
21000383	f	md	JP	2022	0	1	1	0	0	0	0	0	0	0	0	1	70
21000445	f	md	JP	2022	0	1	1	0	0	0	0	0	0	0	0	0	71
21000511	m	md	CN	2022	0	0	0	0	0	0	0	0	0	0	0	0	66
21000550	f	ph	FR	2022	0	1	1	0	0	0	0	0	0	0	0	0	62
21000665	f	cn	CA	2022	0	1	0	0	1	0	0	0	0	0	0	0	59
21000703	f	md	JP	2022	0	0	0	0	0	0	0	0	0	0	0	0	51
21000769	m	ph	JP	2022	0	0	0	0	0	0	0	0	0	0	0	0	70
21001220	Not	md	JP	2022	0	1	0	0	1	0	0	0	0	0	0	0	
21001332	m	md	TR	2022	0	1	0	0	0	1	0	0	0	0	0	0	67
21001687	f	cn	DE	2022	0	1	1	0	0	0	0	0	0	0	0	0	71
21001733	m	md	GB	2022	0	1	0	0	0	1	0	0	0	0	0	0	76
21003601	f	md	CH	2022	0	1	1	0	0	0	0	0	0	0	0	0	64
21003675	Not	cn	SG	2022	1	0	0	0	0	0	0	0	0	0	0	0	80.3
21003802	Not	cn	CN	2022	0	0	0	0	0	0	0	0	0	0	0	0	
21004449	f	cn	TH	2022	1	0	0	0	0	0	0	0	0	0	0	0	62.5
21004479	m	md	DE	2022	0	1	1	0	0	0	0	0	0	0	0	0	
21004553	m	cn	US	2022	1	0	0	0	0	0	0	0	0	0	0	0	
21004617	m	ph	JP	2022	0	1	1	0	0	0	0	0	0	0	0	0	66
21005415	f	md	JP	2022	0	0	0	0	0	0	0	0	0	0	0	0	77
21005421	m	md	PL	2022	0	1	1	0	0	0	0	0	0	0	0	1	72
21005428	f	md	GB	2022	0	1	0	0	0	1	0	0	0	0	0	0	65
21006063	m	md	IT	2022	0	0	0	0	0	0	0	0	0	0	0	0	54
21006151	Not	cn	US	2022	0	0	0	0	0	0	0	0	0	0	0	0	
21006498	m	cn	US	2022	0	1	1	0	0	0	0	0	0	0	0	0	78
21006594	Not	md	JP	2022	0	1	0	0	0	1	0	0	0	0	0	0	

21006793	Not	md	FR	2022	0	1	0	0	1	0	0	0	0	0	0	0	67
21007247	m	hp	CN	2022	0	1	0	0	1	0	0	0	0	0	0	0	66
21007329	Not	md	JP	2022	0	1	0	0	0	1	0	0	0	0	0	0	
21007354	Not	md	JP	2022	0	1	0	0	0	1	0	0	0	0	0	0	80
21007502	f	md	JP	2022	0	1	0	0	1	0	0	0	0	0	0	1	73
21007598	Not	md	JP	2022	0	1	0	0	1	0	0	0	0	0	0	0	
21007916	f	hp	JP	2022	0	1	0	0	1	0	0	0	0	0	0	0	50
21008254	m	md	NL	2022	0	1	0	0	0	1	0	0	0	0	0	0	70
21008421	Not	hp	NL	2022	0	0	0	0	0	0	0	0	0	0	0	0	
21008452	m	cn	US	2022	1	0	0	0	0	0	0	0	0	0	0	0	
21009161	m	hp	GB	2022	0	1	0	0	0	1	0	0	0	0	0	0	42
21010424	f	cn	SG	2022	1	0	0	0	0	0	0	0	0	0	0	0	81
21010563	Not	md	RO	2022	0	1	0	0	1	0	0	0	0	0	0	0	53
21011035	f	cn	TH	2022	0	0	0	0	0	0	0	0	0	0	0	0	35
21011186	Not	md	JP	2022	0	1	0	0	0	1	0	0	0	0	0	0	80
21011214	Not	hp	CN	2022	0	0	0	0	0	0	0	0	0	0	0	0	
21011340	f	cn	DE	2022	0	0	0	0	0	0	0	0	0	0	0	0	
21011405	f	cn	US	2022	1	0	0	0	0	0	0	0	0	0	0	0	65.1
21011508	f	md	JP	2022	0	1	0	0	1	0	0	0	0	0	0	0	40
21012574	f	cn	ID	2022	1	0	0	0	0	0	0	0	0	0	0	0	63.5
21012583	m	md	JP	2022	0	1	1	0	0	0	0	0	0	0	0	0	66
21014169	f	md	JP	2022	0	1	0	0	1	0	0	0	0	0	0	0	84
21014172	m	md	JP	2022	0	1	0	0	1	0	0	0	0	0	0	0	74
21014230	m	cn	CA	2022	0	1	0	0	1	0	0	0	0	0	0	0	62
21014270	m	cn	CA	2022	0	1	0	0	1	0	0	0	0	0	0	0	62
21014718	Not	hp	HR	2022	0	0	0	0	0	0	0	0	0	0	0	0	
21015144	m	md	JP	2022	0	1	0	0	1	0	0	0	0	0	0	0	69
21015146	f	md	JP	2022	0	1	0	0	1	0	0	0	0	0	0	0	62
21015240	m	md	JP	2022	0	1	0	0	1	0	0	0	0	0	0	0	75
21015323	m	md	JP	2022	0	1	0	0	1	0	0	0	0	0	0	0	58
21015348	Not	hp	JP	2022	0	1	0	0	1	0	0	0	0	0	0	0	
21015358	f	md	JP	2022	0	1	0	0	1	0	0	0	0	0	0	1	
21015493	m	cn	CN	2022	0	1	0	0	1	0	0	0	0	0	0	0	53
21015524	m	md	JP	2022	0	1	0	0	0	1	0	0	0	0	0	0	68

21016086	f	md	CN	2022	0	0	0	0	0	0	0	0	0	0	0	0	43
21016231	m	hp	FR	2022	0	0	0	0	0	0	0	0	0	0	0	0	55
21016262	f	md	FR	2022	0	0	0	0	0	0	0	0	0	0	0	0	66
21016343	m	md	JP	2022	0	1	0	0	1	0	0	0	0	0	0	0	80
21016406	Not	md	US	2022	0	0	0	0	0	0	0	0	0	0	0	0	
21016817	f	hp	FR	2022	0	0	0	0	0	0	0	0	0	0	0	0	50
21016825	f	hp	HR	2022	0	0	0	0	0	0	0	0	0	0	0	0	60
21017351	m	md	NL	2022	0	1	0	0	0	1	0	0	0	0	0	0	70
21017664	m	md	SG	2022	0	1	0	1	0	0	0	0	0	0	0	0	52
21017808	m	md	GR	2022	0	1	1	0	0	0	0	0	0	0	0	1	
21018134	f	cn	IN	2022	0	1	0	0	1	0	0	0	0	0	0	0	71
21018598	f	md	ME	2022	0	0	0	0	0	0	0	0	0	0	0	0	62
21018690	m	md	JP	2022	0	1	0	0	1	0	0	0	0	0	0	0	72
21018896	m	cn	SE	2022	0	1	0	1	0	0	0	0	0	0	0	0	66
21018964	m	hp	SG	2022	0	1	0	0	1	0	0	0	0	0	0	0	52
21019056	Not	md	TW	2022	0	0	0	0	0	0	0	0	0	0	0	0	49
21020129	m	md	NL	2022	0	0	0	0	0	0	0	0	0	0	0	0	80
21020145	f	md	JP	2022	0	1	0	0	1	0	0	0	0	0	0	0	73
21020158	f	md	JP	2022	0	1	0	0	1	0	0	0	0	0	0	0	70
21020263	m	cn	IN	2022	0	1	0	0	1	0	0	0	0	0	0	0	65
21020577	m	md	JP	2022	0	1	0	0	1	0	0	0	0	0	0	0	68
21020598	m	md	JP	2022	0	1	0	0	1	0	0	0	0	0	0	0	73
21020610	f	md	JP	2022	0	1	0	0	1	0	0	0	0	0	0	0	77
21020765	f	md	JP	2022	0	1	0	0	1	0	0	0	0	0	0	0	74
21020949	m	md	JP	2022	0	1	0	0	1	0	0	0	0	0	0	0	61
21021079	m	md	DE	2022	0	0	0	0	0	0	0	0	0	0	0	0	47
21021081	m	md	DE	2022	0	1	0	0	1	0	0	0	0	0	0	0	53
21021229	m	md	JP	2022	0	1	1	0	0	0	0	0	0	0	0	0	80
21021466	f	md	JP	2022	0	1	0	0	1	0	0	0	0	0	0	1	70
21021957	m	cn	CA	2022	0	1	0	0	1	0	0	0	0	0	0	0	
21022239	f	Not repo	US	2022	0	1	0	1	0	0	0	0	0	0	0	0	0.22
21023010	f	cn	GB	2022	0	1	1	0	0	0	0	0	0	0	0	0	
21023437	m	hp	RU	2022	0	1	0	1	0	0	0	0	0	0	0	0	60
21023582	f	md	JP	2022	0	1	0	0	0	1	0	0	0	0	0	0	72

21023588	m	cn	JP	2022	1	0	0	0	0	0	0	0	0	0	0	0	55
21023615	f	Not repo	US	2022	1	0	0	0	0	0	0	0	0	0	0	0	64
21023828	m	md	JP	2022	0	0	0	0	0	0	0	0	0	0	0	0	42
21023916	m	md	JP	2022	0	0	0	0	0	0	0	0	0	0	0	0	70
21024065	f	md	JP	2022	0	1	0	0	1	0	0	0	0	0	0	0	65
21024266	m	hp	PH	2022	0	0	0	0	0	0	0	0	0	0	0	0	69
21024850	m	md	CH	2022	0	0	0	0	0	0	0	0	0	0	0	0	69
21024988	m	cn	IN	2022	0	1	0	0	1	0	0	0	0	0	0	0	84
21025137	m	md	DE	2022	0	1	0	0	1	0	0	0	0	0	0	0	74
21025755	Not	md	JP	2022	0	0	0	0	0	0	0	0	0	0	0	0	
21025969	f	md	IT	2022	0	0	0	0	0	0	0	0	0	0	0	0	33
21025988	m	ph	JP	2022	0	1	0	0	1	0	0	0	0	0	0	1	70
21026132	Not	md	AU	2022	0	1	0	1	0	0	0	0	0	0	0	0	
21026434	f	hp	CA	2022	0	1	0	0	0	1	0	0	0	0	0	0	
21026801	m	hp	FR	2022	0	0	0	0	0	0	0	0	0	0	0	0	55
21026842	f	md	JP	2022	0	0	0	0	0	0	0	0	0	0	0	0	62
21026887	f	md	JP	2022	0	1	0	0	0	1	0	0	0	0	0	0	
21027095	Not	md	US	2022	0	0	0	0	0	0	0	0	0	0	0	0	
21027658	Not	md	HK	2022	0	0	0	0	0	0	0	0	0	0	0	0	
21027733	f	ph	JP	2022	0	1	1	0	0	0	0	0	0	0	0	0	74
21027878	f	hp	US	2022	0	1	1	0	0	0	0	0	0	0	0	0	
21027886	m	md	TW	2022	0	0	0	0	0	0	0	0	0	0	0	0	74
21028361	m	hp	US	2022	0	1	0	0	1	0	0	0	0	0	0	0	65
21028633	Not	md	US	2022	0	0	0	0	0	0	0	0	0	0	0	0	
21028680	m	md	MX	2022	0	0	0	0	0	0	0	0	0	0	0	0	
21028749	f	hp	NL	2022	0	1	0	0	1	0	0	0	0	0	0	0	80
21028765	f	hp	IN	2022	0	1	0	0	1	0	0	0	0	0	0	0	80
21028879	m	md	PL	2022	0	1	0	1	0	0	0	0	0	0	0	0	76.5
21029596	m	md	JP	2022	0	1	1	0	0	0	0	0	0	0	0	1	82
21029754	m	md	JP	2022	0	1	0	0	1	0	0	0	0	0	0	0	70
8712834	m	md	US	2022	0	0	0	0	0	0	0	0	0	0	0	0	67
8715338	m	md	US	2022	0	0	0	0	0	0	0	0	0	0	0	0	67
20007571	m	md	JP	2022	0	1	0	0	1	0	0	0	0	0	0	0	59
20021781	f	md	JP	2022	0	1	0	0	1	0	0	0	0	0	0	0	64

20039134	m	md	JP	2022	0	1	0	0	1	0	0	0	0	0	0	0	75
20039135	m	md	JP	2022	0	1	0	0	1	0	0	0	0	0	0	0	76
20039576	m	md	JP	2022	0	1	0	0	1	0	0	0	0	0	0	0	59
20041277	m	md	JP	2022	0	1	0	0	1	0	0	0	0	0	0	0	83
20041301	m	md	JP	2022	0	1	0	0	1	0	0	0	0	0	0	0	45
20126579	Not	md	GB	2022	0	1	0	0	0	1	0	0	0	0	0	0	
20234633	f	ph	JP	2022	0	0	0	0	0	0	0	0	0	0	0	0	76
20302634	f	md	JP	2022	0	1	0	0	1	0	0	0	0	0	0	0	71
20318527	f	md	GB	2022	0	1	0	0	0	1	0	0	0	0	0	0	62
20336797	m	md	CN	2022	0	0	0	0	0	0	0	0	0	0	0	0	65
20377827	m	md	TR	2022	0	0	0	0	0	0	0	0	0	0	0	0	74
20419346	f	md	BE	2022	0	0	0	0	0	0	0	0	0	0	0	0	57
20440916	f	md	JP	2022	0	1	0	0	1	0	0	0	0	0	0	0	74
20451874	f	md	JP	2022	0	0	0	0	0	0	0	0	0	0	0	0	55
20466387	f	cn	IN	2022	0	0	0	0	0	0	0	0	0	0	0	0	54
20491561	m	cn	IN	2022	0	1	0	0	0	1	0	0	0	0	0	0	69
20548709	m	md	CN	2022	0	0	0	0	0	0	0	0	0	0	0	0	58
20554968	m	hp	CN	2022	0	0	0	0	0	0	0	0	0	0	0	0	66
20562906	m	md	JP	2022	0	0	0	0	0	0	0	0	0	0	0	0	83
20581870	f	md	GB	2022	0	1	0	0	0	1	0	0	0	0	0	0	75
20594665	m	md	JP	2022	0	1	0	0	0	1	0	0	0	0	0	0	47
20624941	m	hp	JP	2022	0	1	0	0	1	0	0	0	0	0	0	0	69
20631249	f	md	BE	2022	0	0	0	0	0	0	0	0	0	0	0	0	55
20634001	m	md	JP	2022	0	1	0	0	1	0	0	0	0	0	0	0	77
20634990	m	md	JP	2022	0	1	0	0	1	0	0	0	0	0	0	1	72
20643176	f	cn	US	2022	0	0	0	0	0	0	0	0	0	0	0	0	79
20651484	m	md	AU	2022	0	1	0	0	0	1	0	0	0	0	0	0	71
20651880	f	md	JP	2022	0	1	0	0	1	0	0	0	0	0	0	0	73
20664954	f	md	CN	2022	0	0	0	0	0	0	0	0	0	0	0	0	69
20673819	m	cn	CA	2022	0	0	0	0	0	0	0	0	0	0	0	0	52
20676341	f	md	GB	2022	0	0	0	0	0	0	0	0	0	0	0	0	75
20680993	m	md	CN	2022	0	1	1	0	0	0	0	0	0	0	0	0	64
20683897	m	md	FR	2022	0	0	0	0	0	0	0	0	0	0	0	0	
20692201	f	hp	CH	2022	0	1	0	0	1	0	0	0	0	0	0	0	51

20695742	Not	md	AR	2022	0	0	0	0	0	0	0	0	0	0	0	0	
20697893	m	md	JP	2022	0	1	0	0	1	0	0	0	0	0	0	0	73
20701023	f	md	JP	2022	1	0	0	0	0	0	0	0	0	0	0	0	40
20702042	m	md	JP	2022	0	1	0	0	1	0	0	0	0	0	0	0	78
20705161	m	cn	US	2022	0	1	1	0	0	0	0	0	0	0	0	0	64
20707544	f	hp	DE	2022	0	0	0	0	0	0	0	0	0	0	0	0	55
20709978	f	hp	DE	2022	0	1	0	1	0	0	0	0	0	0	0	1	72
20710399	m	hp	DE	2022	0	1	0	0	1	0	0	0	0	0	0	1	49
20712523	m	hp	IT	2022	0	1	1	0	0	0	0	0	0	0	0	0	68
20712640	m	md	JP	2022	0	1	1	0	0	0	0	0	0	0	0	0	68
20716821	m	md	CZ	2022	0	1	0	0	1	0	0	0	0	0	0	0	
20718069	m	md	JP	2022	0	1	0	0	1	0	0	0	0	0	0	0	69
20718871	f	md	JP	2022	0	1	0	0	0	1	0	0	0	0	0	0	61
20722390	m	md	CN	2022	0	1	0	1	0	0	0	0	0	0	0	0	65.1
20728137	m	md	JP	2022	0	1	0	0	1	0	0	0	0	0	0	0	53
20730285	m	md	JP	2022	0	1	0	0	0	1	0	0	0	0	0	1	77
20733067	m	md	JP	2022	0	1	0	0	1	0	0	0	0	0	0	0	77
20742115	f	cn	TH	2022	0	0	0	0	0	0	0	0	0	0	0	0	75
20742799	f	md	BR	2022	0	1	0	0	1	0	0	0	0	0	0	0	
20744026	Not	md	BR	2022	0	1	0	0	1	0	0	0	0	0	0	0	
20746633	m	md	TW	2022	0	1	0	0	0	1	0	0	0	0	0	0	53
20751164	m	md	BE	2022	0	0	0	0	0	0	0	0	0	0	0	0	73
20751356	f	md	JP	2022	0	0	0	0	0	0	0	0	0	0	0	0	74
20753772	m	hp	CN	2022	0	0	0	0	0	0	0	0	0	0	0	0	66
20754704	m	md	CN	2022	0	1	0	0	0	1	0	0	0	0	0	0	61
20755981	m	hp	JP	2022	0	1	0	0	1	0	0	0	0	0	0	0	62
20758281	m	md	JP	2022	0	1	1	0	0	0	0	0	0	0	0	0	82
20762363	m	cn	IN	2022	0	1	0	0	0	1	0	0	0	0	0	0	71
20763271	m	md	CN	2022	0	0	0	0	0	0	0	0	0	0	0	0	56
20763472	m	md	DE	2022	0	1	0	0	1	0	0	0	0	0	0	0	60
20767217	f	hp	US	2022	0	1	0	0	1	0	0	0	0	0	0	0	64
20767836	m	cn	IN	2022	0	0	0	0	0	0	0	0	0	0	0	0	71
20770156	m	cn	CN	2022	0	1	0	0	1	0	0	0	0	0	0	0	70
20770761	Not	md	US	2022	0	0	0	0	0	0	0	0	0	0	0	0	

20770928	f	cn	GB	2022	0	0	0	0	0	0	1	1	0	0	0	0	58
20771167	m	md	FR	2022	0	0	0	0	0	0	0	0	0	0	0	0	59
20776917	f	hp	NL	2022	0	1	0	0	1	0	0	0	0	0	0	0	64
20787184	f	md	JP	2022	0	0	0	0	0	0	0	0	0	0	0	0	49
20791862	m	md	FR	2022	0	1	1	0	0	0	0	0	0	0	0	0	65
20793683	f	cn	CN	2022	0	0	0	0	0	0	0	0	0	0	0	0	38
20797581	f	hp	CL	2022	0	0	0	0	0	0	0	0	0	0	0	0	40
20802293	f	md	JP	2022	0	1	0	0	0	1	0	0	0	0	0	0	59
20803198	m	md	MY	2022	0	0	0	0	0	0	0	0	0	0	0	0	73
20812559	f	md	US	2022	0	1	0	0	0	1	0	0	0	0	0	0	71
20815704	f	md	CN	2022	0	0	0	0	0	0	0	0	0	0	0	0	51
20816507	m	hp	CN	2022	0	0	0	0	0	0	0	0	0	0	0	0	66
20817322	m	md	JP	2022	0	1	0	0	1	0	0	0	0	0	0	0	67
20834682	m	md	DE	2022	0	0	0	0	0	0	0	0	0	0	0	0	67
20836146	f	md	JP	2022	0	1	0	0	1	0	0	0	0	0	0	0	55
20841078	m	hp	CL	2022	0	1	0	0	1	0	0	0	0	0	0	0	67
20847430	m	md	JP	2022	0	1	0	0	0	1	0	0	0	0	0	0	69
20849381	m	md	JP	2022	0	1	0	1	0	0	0	0	0	0	0	0	72.1
20850257	m	md	CN	2022	0	1	0	0	0	1	0	0	0	0	0	1	76
20850947	m	md	JP	2022	0	0	0	0	0	0	0	0	0	0	0	0	71
20856017	m	hp	CN	2022	0	0	0	0	0	0	0	0	0	0	0	0	60
20861158	m	cn	JP	2022	0	0	0	0	0	0	0	0	0	0	0	0	76
20861894	m	cn	IN	2022	0	0	0	0	0	0	0	0	0	0	0	0	64
20863378	f	md	SK	2022	0	0	0	0	0	0	0	0	0	0	0	0	49
20865510	m	md	DE	2022	0	0	0	0	0	0	0	0	0	0	0	0	67
20866701	f	md	JP	2022	0	1	0	0	0	1	0	0	0	0	0	0	76
20867919	m	md	CN	2022	0	0	0	0	0	0	0	0	0	0	0	0	52
20869361	m	ph	CN	2022	0	0	0	0	0	0	0	0	0	0	0	0	64
20870000	f	md	FR	2022	0	1	0	0	1	0	0	0	0	0	0	0	81
20872377	f	ph	SG	2022	0	0	0	0	0	0	0	0	0	0	0	0	73
20877719	f	md	US	2022	0	1	0	0	0	1	0	0	0	0	0	0	62
20877764	m	md	CN	2022	0	1	0	1	0	0	0	0	0	0	0	0	58
20877818	m	md	CN	2022	0	1	1	0	0	0	0	0	0	0	0	0	60
20877853	f	cn	CH	2022	0	0	0	0	0	0	0	0	0	0	0	0	57

20879542	m	hp	GB	2022	0	0	0	0	0	0	0	0	0	0	0	0	56
20882163	m	md	JP	2022	0	1	0	0	1	0	0	0	0	0	0	0	73
20883680	f	cn	US	2022	0	1	0	1	0	0	0	0	0	0	0	1	
20885083	f	md	DE	2022	0	1	1	0	0	0	0	0	0	0	0	0	
20889251	m	md	JP	2022	0	1	0	0	1	0	0	0	0	0	0	0	71
20891600	f	cn	US	2022	0	1	0	1	0	0	0	0	0	0	0	1	
20892035	f	md	US	2022	0	1	1	0	0	0	0	0	0	0	0	0	83
20898757	m	hp	ES	2022	0	1	0	0	1	0	0	0	0	0	0	0	63
20899142	f	md	JP	2022	0	1	0	1	0	0	0	0	0	0	0	0	74
20899271	m	md	JP	2022	0	0	0	0	0	0	0	0	0	0	0	0	56
20901296	m	cn	CN	2022	0	1	0	1	0	0	0	0	0	0	0	0	67
20901391	f	md	DE	2022	0	1	0	0	1	0	0	0	0	0	0	0	65
20903187	m	md	BR	2022	0	0	0	0	0	0	0	0	0	0	0	0	31
20903543	f	md	AU	2022	0	0	0	0	0	0	0	0	0	0	0	0	69
20903973	m	md	JP	2022	0	0	0	0	0	0	0	0	0	0	0	0	69
20905416	f	cn	CA	2022	0	0	0	0	0	0	0	0	0	0	0	0	78
20905714	f	md	PL	2022	0	0	0	0	0	0	0	0	0	0	0	0	71
20906746	m	md	JP	2022	0	1	0	0	1	0	0	0	0	0	0	0	75
20908397	m	md	JP	2022	0	1	0	0	1	0	0	0	0	0	0	0	60
20910003	f	md	DE	2022	0	1	0	0	1	0	0	0	0	0	0	0	73
20911012	m	md	JP	2022	0	1	1	0	0	0	0	0	0	0	0	0	50
20911588	m	md	HK	2022	0	1	1	0	0	0	0	0	0	0	0	0	69
20912000	f	cn	US	2022	0	1	0	1	0	0	0	0	0	0	0	1	
20912479	m	md	MY	2022	0	0	0	0	0	0	0	0	0	0	0	0	73
20912558	f	md	DE	2022	0	0	0	0	0	0	0	0	0	0	0	0	80
20914150	m	md	DE	2022	0	1	1	0	0	0	0	0	0	0	0	0	
20915591	m	md	JP	2022	0	0	0	0	0	0	0	0	0	0	0	0	64
20921132	f	cn	HK	2022	0	0	0	0	0	0	0	0	0	0	0	0	
20921172	m	md	JP	2022	1	0	0	0	0	0	0	0	0	0	0	1	76.8
20924214	f	hp	SE	2022	0	1	0	0	1	0	0	0	0	0	0	0	70
20928986	m	md	JP	2022	0	0	0	0	0	0	0	0	0	0	0	0	
20930760	m	md	JP	2022	0	1	0	0	1	0	0	0	0	0	0	0	72
20930804	m	md	JP	2022	0	1	1	0	0	0	0	0	0	0	0	1	85
20931588	Not	md	BR	2022	0	0	0	0	0	0	0	0	0	0	0	0	

20931781	Not	md	RO	2022	0	1	1	0	0	0	0	0	0	0	0	0	
20932013	m	md	JP	2022	0	1	0	0	1	0	0	0	0	0	0	0	75
20935464	m	cn	CN	2022	0	0	0	0	0	0	0	0	0	0	0	0	72
20938981	m	md	ES	2022	0	1	1	0	0	0	0	0	0	0	0	0	60
20939731	Not	md	US	2022	0	0	0	0	0	0	0	0	0	0	0	0	
20940185	m	md	TW	2022	0	0	0	0	0	0	0	0	0	0	0	0	56
20940415	f	md	FR	2022	0	0	0	0	0	0	0	0	0	0	0	0	
20941411	m	md	DE	2022	0	0	0	0	0	0	0	0	0	0	0	0	65
20942743	f	hp	GB	2022	0	1	0	0	0	1	0	0	0	0	0	0	65
20944737	f	hp	CH	2022	0	0	0	0	0	0	0	0	0	0	0	0	68
20944818	f	md	KR	2022	0	0	0	0	0	0	0	0	0	0	0	0	54
20945372	f	hp	PL	2022	0	1	1	0	0	0	0	0	0	0	0	0	71
20946221	f	cn	CA	2022	0	0	0	0	0	0	0	0	0	0	0	0	75
20947103	f	md	FR	2022	0	0	0	0	0	0	0	0	0	0	0	0	70
20947109	m	hp	RU	2022	0	0	0	0	0	0	0	0	0	0	0	0	63
20947437	m	md	US	2022	0	0	0	0	0	0	0	0	0	0	0	0	
20952376	f	md	PL	2022	0	1	1	0	0	0	0	0	0	0	0	0	71
20952386	f	md	JP	2022	0	1	1	0	0	0	0	0	0	0	0	0	72
20953114	m	hp	FR	2022	0	0	0	0	0	0	0	0	0	0	0	0	
20953630	m	md	JP	2022	0	1	0	0	1	0	0	0	0	0	0	0	69
20954361	Not	cn	HK	2022	0	0	0	0	0	0	0	0	0	0	0	0	
20955668	f	md	ES	2022	0	1	1	0	0	0	0	0	0	0	0	0	63
20958009	f	md	CN	2022	0	0	0	0	0	0	0	0	0	0	0	0	66
20960615	m	md	DE	2022	0	1	0	0	1	0	0	0	0	0	0	0	72
20961277	Not	md	US	2022	0	0	0	0	0	0	0	0	0	0	0	0	
20961735	m	md	CN	2022	0	0	0	0	0	0	0	0	0	0	0	0	67
20963601	m	md	PH	2022	0	1	0	1	0	0	0	0	0	0	0	0	70
20965286	m	hp	US	2022	0	1	1	0	0	0	0	0	0	0	0	0	61
20966373	f	hp	CA	2022	0	0	0	0	0	0	0	0	0	0	0	0	70
20967990	m	md	JP	2022	0	1	0	1	0	0	0	0	0	0	0	0	58
20968202	m	md	PH	2022	0	1	0	1	0	0	0	0	0	0	0	0	70
20969127	m	cn	IN	2022	0	0	0	0	0	0	0	0	0	0	0	0	68
20969360	f	hp	PL	2022	0	1	1	0	0	0	0	0	0	0	0	0	71
20972307	f	md	JP	2022	0	0	0	0	0	0	0	0	0	0	0	0	67

20973132	Not	md	JP	2022	0	1	0	0	1	0	0	0	0	0	0	0	73
20973553	m	md	DE	2022	0	1	0	0	1	0	0	0	0	0	0	0	60
20974587	f	ph	HR	2022	0	1	1	0	0	0	0	0	0	0	0	1	57
20976411	f	hp	SE	2022	0	1	0	0	1	0	0	0	0	0	0	0	68
20977276	f	md	JP	2022	0	1	1	0	0	0	0	0	0	0	0	0	90
20979076	Not	md	JP	2022	0	1	0	0	1	0	0	0	0	0	0	0	74
20979447	Not	md	JP	2022	0	1	0	0	1	0	0	0	0	0	0	1	53
20980026	f	hp	US	2022	0	0	0	0	0	0	0	0	0	0	0	0	83
20980806	m	md	GB	2022	0	1	0	0	0	1	0	0	0	0	0	0	68
20982813	m	hp	JP	2022	0	0	0	0	0	0	0	0	0	0	0	0	64
20983085	f	md	FR	2022	0	0	0	0	0	0	0	0	0	0	0	0	76
20986100	f	hp	CN	2022	0	1	1	0	0	0	0	0	0	0	0	0	51
20987429	f	md	JP	2022	0	0	0	0	0	0	0	0	0	0	0	0	
20988274	f	md	JP	2022	0	1	1	0	0	0	0	0	0	0	0	0	72
20989209	f	md	CN	2022	0	0	0	0	0	0	0	0	0	0	0	0	64
20989228	f	ph	JP	2022	0	0	0	0	0	0	0	0	0	0	0	0	78
20989245	f	hp	TW	2022	0	0	0	0	0	0	0	0	0	0	0	0	81
20991041	f	md	JP	2022	0	0	0	0	0	0	0	0	0	0	0	0	50
20991539	f	cn	PT	2022	0	0	0	0	0	0	0	0	0	0	0	0	
20994788	m	hp	NL	2022	0	0	0	0	0	0	0	0	0	0	0	0	67
20995298	m	hp	BR	2022	0	0	0	0	0	0	0	0	0	0	0	0	69
20997677	f	md	IL	2022	0	1	1	0	0	0	0	0	0	0	0	0	60
20997909	m	md	DE	2022	0	1	0	0	1	0	0	0	0	0	0	0	49
20999330	m	cn	LB	2022	0	1	0	0	0	1	0	0	0	0	0	0	
20999713	m	md	CN	2022	1	0	0	0	0	0	0	0	0	0	0	0	58
21000018	m	md	CN	2022	0	0	0	0	0	0	0	0	0	0	0	0	60
21003773	f	md	BE	2022	0	0	0	0	0	0	0	0	0	0	0	0	59
21004167	f	md	MX	2022	0	1	0	0	0	1	0	0	0	0	0	0	63
21004287	f	cn	JP	2022	0	0	0	0	0	0	0	0	0	0	0	0	70
21006179	f	md	CN	2022	0	0	0	0	0	0	0	0	0	0	0	0	69
21006494	f	hp	US	2022	0	0	0	0	0	0	0	0	0	0	0	0	77
21008020	m	md	FR	2022	0	0	0	0	0	0	0	0	0	0	0	0	65
21011927	f	md	JP	2022	0	1	0	0	1	0	0	0	0	0	0	0	50
21012587	m	hp	US	2022	0	0	0	0	0	0	0	0	0	0	0	0	66

21014157	Not	md	JP	2022	0	1	0	0	1	0	0	0	0	0	0	0	
21014200	m	md	JP	2022	0	1	0	0	1	0	0	0	0	0	0	0	69
21014843	f	md	FR	2022	0	0	0	0	0	0	0	0	0	0	0	0	
21014905	m	md	JP	2022	0	1	0	0	1	0	0	0	0	0	0	0	70
21014961	m	md	CN	2022	0	1	0	1	0	0	0	0	0	0	0	0	75.6
21016395	f	hp	US	2022	0	0	0	0	0	0	0	0	0	0	0	0	
21017570	m	md	JP	2022	0	1	0	0	1	0	0	0	0	0	0	0	52
21019849	f	md	FR	2022	0	0	0	0	0	0	0	0	0	0	0	0	54
21019978	m	ph	JP	2022	0	1	1	0	0	0	0	0	0	0	0	0	80
21020168	f	md	JP	2022	0	1	0	0	1	0	0	0	0	0	0	0	70
21020478	f	hp	DE	2022	0	0	0	0	0	0	0	0	0	0	0	0	51
21020889	m	md	JP	2022	0	1	0	0	1	0	0	0	0	0	0	0	68
21022309	m	md	CN	2022	0	0	0	0	0	0	0	0	0	0	0	0	50
21022378	m	md	CN	2022	0	0	0	0	0	0	0	0	0	0	0	0	75
21022403	m	hp	JP	2022	0	0	0	0	0	0	0	0	0	0	0	0	81
21022680	m	cn	US	2022	0	0	0	0	0	0	0	0	0	0	0	0	71
21022775	f	md	JP	2022	0	1	1	0	0	0	0	0	0	0	0	0	80
21023820	m	cn	DE	2022	0	0	0	0	0	0	0	0	0	0	0	0	
21024020	f	cn	CN	2022	0	0	0	0	0	0	0	0	0	0	0	0	39
21024022	m	md	ES	2022	0	1	0	0	0	1	0	0	0	0	0	0	62
21024509	f	hp	CN	2022	0	0	0	0	0	0	0	0	0	0	0	0	68
21025081	f	hp	NL	2022	0	1	0	0	1	0	0	0	0	0	0	0	78
21025477	m	md	GB	2022	0	1	0	0	0	1	0	0	0	0	0	0	75
21026962	m	md	BG	2022	1	0	0	0	0	0	0	0	0	0	0	0	
21027680	m	md	JP	2022	0	0	0	0	0	0	1	0	0	1	0	0	78
21028055	m	md	CN	2022	0	0	0	0	0	0	0	0	0	0	0	0	66
21028079	m	md	CN	2022	0	0	0	0	0	0	0	0	0	0	0	0	66
21028209	f	md	JP	2022	0	0	0	0	0	0	0	0	0	0	0	0	62
21028844	m	md	ES	2022	0	1	0	0	1	0	0	0	0	0	0	0	61
21029537	f	hp	FR	2022	0	1	0	0	1	0	0	0	0	0	0	0	73
21029709	f	md	JP	2022	0	1	0	0	1	0	0	0	0	0	0	1	73
21030995	f	ph	JP	2022	0	0	0	0	0	0	0	0	0	0	0	0	50
21031436	m	md	FR	2022	0	0	0	0	0	0	0	0	0	0	0	0	64
21031868	m	cn	CN	2022	0	0	0	0	0	0	0	0	0	0	0	0	58

21032240	Not	md	CN	2022	1	0	0	0	0	0	0	0	0	0	0	0	
21032785	m	hp	JP	2022	0	1	1	0	0	0	0	0	0	0	0	0	67
21032900	m	hp	JP	2022	0	0	0	0	0	0	0	0	0	0	0	0	82
21032901	m	hp	JP	2022	0	0	0	0	0	0	0	0	0	0	0	0	88
21033078	m	md	JP	2022	0	0	0	0	0	0	0	0	0	0	0	0	60
21033124	m	ph	JP	2022	0	0	0	0	0	0	0	0	0	0	0	0	80
21033139	m	md	ES	2022	0	1	0	0	0	1	0	0	0	0	0	0	62
21033194	f	cn	ID	2022	1	0	0	0	0	0	0	0	0	0	0	0	90.4
21033970	f	md	JP	2022	1	0	0	0	0	0	0	0	0	0	0	0	69
21034495	m	cn	IN	2022	0	0	0	0	0	0	0	0	0	0	0	0	68
21035057	m	md	KR	2022	0	1	0	0	1	0	0	0	0	0	0	0	
21035343	f	md	NO	2022	0	0	0	0	0	0	0	0	0	0	0	0	
21035364	m	md	US	2022	0	0	0	0	0	0	0	0	0	0	0	0	76
21035911	f	md	ES	2022	0	1	0	0	0	1	0	0	0	0	0	0	59
21036241	f	md	BE	2022	0	0	0	0	0	0	0	0	0	0	0	0	72
21036288	m	md	JP	2022	0	0	0	0	0	0	0	0	0	0	0	0	60
21036649	m	md	DE	2022	0	0	0	0	0	0	0	0	0	0	0	0	80
21036665	m	md	DE	2022	0	0	0	0	0	0	0	0	0	0	0	0	75
21036668	m	md	DE	2022	0	0	0	0	0	0	0	0	0	0	0	0	66
21036729	m	md	JP	2022	0	0	0	0	0	0	0	0	0	0	0	0	76
21036868	m	cn	IN	2022	0	1	0	0	1	0	0	0	0	0	0	0	75
21037382	Not	md	JP	2022	0	1	0	0	1	0	0	0	0	0	0	0	
21037384	m	md	JP	2022	0	1	0	0	1	0	0	0	0	0	0	0	73
21037465	m	md	CN	2022	0	1	0	0	1	0	0	0	0	0	0	0	50
21037513	f	md	DE	2022	0	1	0	0	1	0	0	0	0	0	0	0	68
21037714	f	md	JP	2022	0	1	0	0	1	0	0	0	0	0	0	0	80
21038448	f	hp	FR	2022	0	0	0	0	0	0	0	0	0	0	0	0	50
21038658	m	md	JP	2022	0	1	0	0	1	0	0	0	0	0	0	1	71
21038812	Not	hp	AU	2022	0	0	0	0	0	0	0	0	0	0	0	0	
21039251	Not	md	SG	2022	1	0	0	0	0	0	0	0	0	0	0	0	82.7
21039578	m	cn	IN	2022	0	0	0	0	0	0	0	0	0	0	0	0	36
21039599	f	cn	US	2022	0	1	0	1	0	0	0	0	0	0	0	1	69.1
21039721	m	md	CA	2022	1	0	0	0	0	0	0	0	0	0	0	0	68.2
21039927	f	cn	SG	2022	1	0	0	0	0	0	0	0	0	0	0	0	84

21040004	f	cn	IN	2022	0	0	0	0	0	0	0	0	0	0	0	0	81
21040012	f	cn	IN	2022	0	0	0	0	0	0	0	0	0	0	0	0	36
21040276	f	cn	ID	2022	1	0	0	0	0	0	0	0	0	0	0	0	68.2
21040299	m	hp	DE	2022	0	0	0	0	0	0	0	0	0	0	0	0	74
21040721	m	md	JP	2022	0	1	0	0	1	0	0	0	0	0	0	0	70
21040762	f	md	JP	2022	0	1	0	0	1	0	0	0	0	0	0	0	68
21041100	Not	md	JP	2022	0	1	0	0	0	1	0	0	0	0	0	1	80
21041411	m	md	AU	2022	0	0	0	0	0	0	0	0	0	0	0	0	68
21041688	m	md	JP	2022	0	1	1	0	0	0	0	0	0	0	0	0	80
21041968	m	md	JP	2022	0	0	0	0	0	0	0	0	0	0	0	0	62
21042169	f	md	BR	2022	0	0	0	0	0	0	0	0	0	0	0	0	70
21042338	m	md	IN	2022	0	1	0	0	1	0	0	0	0	0	0	0	59
21042930	f	hp	GR	2022	0	1	1	0	0	0	0	0	0	0	0	0	
21043227	m	hp	GR	2022	0	1	1	0	0	0	0	0	0	0	0	0	
21043300	m	md	CN	2022	0	0	0	0	0	0	0	0	0	0	0	0	
21043382	f	cn	IN	2022	0	0	0	0	0	0	0	0	0	0	0	0	32
21044156	f	md	JP	2022	0	0	0	0	0	0	0	0	0	0	0	0	78
21044188	Not	hp	CN	2022	0	0	0	0	0	0	0	0	0	0	0	0	
21044195	m	cn	JP	2022	1	0	0	0	0	0	0	0	0	0	0	0	75
21044225	m	cn	JP	2022	0	0	0	0	0	0	0	0	0	0	0	0	55
21044463	Not	md	MX	2022	0	0	0	0	0	0	0	0	0	0	0	0	66
21044465	m	ph	FR	2022	0	0	0	0	0	0	0	0	0	0	0	0	
21044681	f	cn	DO	2022	1	0	0	0	0	0	0	0	0	0	0	0	79
21044841	f	md	CN	2022	0	0	0	0	0	0	0	0	0	0	0	0	55
21044880	f	md	ES	2022	0	0	0	0	0	0	0	0	0	0	0	0	72
21044987	m	md	DK	2022	0	1	1	0	0	0	0	0	0	0	0	0	55
21045012	m	md	MT	2022	0	1	1	0	0	0	0	0	0	0	0	0	77
21045441	f	md	JP	2022	0	1	1	0	0	0	0	0	0	0	0	1	78
21045713	m	md	JP	2022	0	1	1	0	0	0	0	0	0	0	0	0	70
21045868	m	md	JP	2022	0	1	1	0	0	0	0	0	0	0	0	0	75
21046010	m	md	JP	2022	0	1	0	0	0	1	0	0	0	0	0	1	60
21046291	m	cn	CN	2022	0	1	0	0	1	0	0	0	0	0	0	0	62
21046875	Not	cn	SG	2022	1	0	0	0	0	0	0	0	0	0	0	0	64.8
21046995	f	md	DE	2022	1	0	0	0	0	0	0	0	0	0	0	0	71

21047708	m	cn	US	2022	0	0	0	0	0	0	0	0	0	0	0	0	
21048296	m	md	TW	2022	0	0	0	0	0	0	0	0	0	0	0	0	41
21048339	Not	hp	NL	2022	0	0	0	0	0	0	0	0	0	0	0	0	
21048454	f	md	JP	2022	0	1	0	0	0	1	0	0	0	0	0	1	78
21048457	f	md	JP	2022	0	1	0	0	0	1	0	0	0	0	0	0	80
21048573	m	md	JP	2022	0	1	0	0	0	1	0	0	0	0	0	0	65
21048991	m	cn	CN	2022	0	1	0	0	1	0	0	0	0	0	0	0	61
21049324	f	ph	JP	2022	1	0	0	0	0	0	0	0	0	0	0	0	83
21049433	m	md	DE	2022	0	0	0	0	0	0	0	0	0	0	0	0	59
21049459	m	hp	US	2022	0	1	0	1	0	0	0	0	0	0	0	0	55
21049760	f	md	JP	2022	0	0	0	0	0	0	0	0	0	0	0	0	67
21050175	m	md	CN	2022	0	0	0	0	0	0	0	0	0	0	0	0	46
21050180	m	md	CN	2022	0	0	0	0	0	0	0	0	0	0	0	0	73
21050181	m	md	CN	2022	0	0	0	0	0	0	0	0	0	0	0	0	67
21050395	Not	cn	SG	2022	1	0	0	0	0	0	0	0	0	0	0	0	74.7
21050413	f	hp	US	2022	0	1	1	0	0	0	0	0	0	0	0	0	64
21051503	m	md	KR	2022	0	1	0	0	1	0	0	0	0	0	0	0	75
21051597	f	md	ES	2022	0	1	0	0	0	1	0	0	0	0	0	0	59
21051688	f	md	JP	2022	0	1	0	0	1	0	0	0	0	0	0	1	66
21052035	m	hp	US	2022	0	1	0	0	0	1	0	0	0	0	0	0	71
21052301	m	ph	JP	2022	0	1	1	0	0	0	0	0	0	0	0	0	64
21052852	Not	hp	US	2022	0	0	0	0	0	0	0	0	0	0	0	0	
21053106	m	md	JP	2022	0	1	1	0	0	0	0	0	0	0	0	0	80
21053276	f	md	TW	2022	0	0	0	0	0	0	0	0	0	0	0	0	70
21053792	m	ph	JP	2022	0	1	1	0	0	0	0	0	0	0	0	0	82
21054109	m	cn	GB	2022	1	0	0	0	0	0	0	0	0	0	0	0	52
21054257	f	hp	US	2022	0	0	0	0	0	0	0	0	0	0	0	0	61
21054319	m	cn	CN	2022	0	1	0	0	1	0	0	0	0	0	0	0	67
21054403	m	hp	CN	2022	0	1	0	0	1	0	0	0	0	0	0	0	67
21054609	f	hp	US	2022	0	1	0	1	0	0	0	0	0	0	0	0	70
21054694	f	ph	JP	2022	0	1	0	0	0	1	0	0	0	0	0	0	69
21054833	m	md	CN	2022	0	1	0	1	0	0	0	0	0	0	0	0	67.3
21054854	f	hp	ES	2022	0	1	1	0	0	0	0	0	0	0	0	0	65
21054880	f	hp	FR	2022	0	1	1	0	0	0	0	0	0	0	0	0	56

21054903	m	cn	US	2022	0	1	1	0	0	0	0	0	0	0	0	0	
21054906	f	hp	RU	2022	0	0	0	0	0	0	0	0	0	0	0	0	78
21055041	f	md	JP	2022	0	1	0	0	1	0	0	0	0	0	0	0	73
21055180	f	md	NL	2022	0	1	1	0	0	0	0	0	0	0	0	0	64
21055404	m	md	JP	2022	0	1	0	0	1	0	0	0	0	0	0	0	76
21055405	f	md	JP	2022	0	1	0	0	1	0	0	0	0	0	0	0	75
21055458	m	md	JP	2022	0	1	0	0	1	0	0	0	0	0	0	1	70
21055505	f	md	JP	2022	0	1	0	0	1	0	0	0	0	0	0	0	80
21055766	m	cn	IL	2022	0	0	0	0	0	0	0	0	0	0	0	0	55
21056098	f	md	JP	2022	0	1	0	0	0	1	0	0	0	0	0	0	80
21056365	m	md	JP	2022	0	0	0	0	0	0	0	0	0	0	0	0	76
21056390	m	cn	SE	2022	0	1	0	1	0	0	0	0	0	0	0	0	72
21056868	m	md	JP	2022	0	1	0	0	0	1	0	0	0	0	0	0	68
21057111	f	cn	CN	2022	0	1	0	0	1	0	0	0	0	0	0	0	76
21057447	f	hp	CA	2022	0	0	0	0	0	0	0	0	0	0	0	0	77
21057507	f	md	FR	2022	1	0	0	0	0	0	0	0	0	0	0	0	60
21057710	f	md	FR	2022	0	0	0	0	0	0	0	0	0	0	0	0	
21057727	m	md	CN	2022	0	1	0	1	0	0	0	0	0	0	0	0	42
21058060	Not	hp	NL	2022	0	0	0	0	0	0	0	0	0	0	0	0	
21058246	Not	hp	US	2022	0	0	0	0	0	0	0	0	0	0	0	0	
21058365	m	cn	CN	2022	0	0	0	0	0	0	0	0	0	0	0	0	75
21058778	f	cn	US	2022	0	0	0	0	0	0	0	0	0	0	0	0	
21058861	m	ph	US	2022	0	0	0	0	0	0	0	0	0	0	0	0	66
21059025	f	md	CN	2022	0	0	0	0	0	0	0	0	0	0	0	0	66
21059116	f	md	JP	2022	0	1	0	0	1	0	0	0	0	0	0	0	51
21059145	f	hp	NZ	2022	1	0	0	0	0	0	0	0	0	0	0	0	
21059173	f	md	FR	2022	0	0	0	0	0	0	0	0	0	0	0	0	
21059197	m	md	JP	2022	0	1	0	0	1	0	0	0	0	0	0	0	77
21059243	m	md	US	2022	1	0	0	0	0	0	0	0	0	0	0	0	50
21059459	m	cn	DE	2022	0	0	0	0	0	0	0	0	0	0	0	0	
21059519	m	md	JP	2022	0	1	0	1	0	0	0	0	0	0	0	0	69
21059675	m	cn	GB	2022	0	0	0	0	0	0	0	0	0	0	0	0	64
21061696	m	cn	CN	2022	0	0	0	0	0	0	0	0	0	0	0	0	72
21061701	m	ph	JP	2022	0	0	0	0	0	0	0	0	0	0	0	0	77

21061738	Not	md	AU	2022	0	0	0	0	0	0	0	0	0	0	0	0	
21061749	f	md	JP	2022	0	1	0	0	1	0	0	0	0	0	0	0	70
21061783	Not	md	JP	2022	1	0	0	0	0	0	0	0	0	0	0	0	
21061854	f	ph	JP	2022	0	1	0	0	1	0	0	0	0	0	0	0	71
21061963	m	md	JP	2022	0	1	0	0	0	1	0	0	0	0	0	0	79
21062033	m	md	JP	2022	0	1	0	0	1	0	0	0	0	0	0	0	79
21062073	m	md	JP	2022	0	1	0	0	1	0	0	0	0	0	0	0	81
21062225	f	md	BR	2022	0	1	0	0	1	0	0	0	0	0	0	0	74
21062390	m	md	JP	2022	0	0	0	0	0	0	0	0	0	0	0	0	80
21062540	m	hp	TW	2022	0	1	0	0	1	0	0	0	0	0	0	0	64
21062551	f	md	DE	2022	0	0	0	0	0	0	0	0	0	0	0	0	61
21062578	m	md	JP	2022	0	0	0	0	0	0	0	0	0	0	0	0	77
21063224	f	cn	FR	2022	0	1	0	0	1	0	0	0	0	0	0	0	60
21063884	f	ph	NL	2022	0	1	1	0	0	0	0	0	0	0	0	0	67
21063896	f	md	JP	2022	0	1	0	0	1	0	0	0	0	0	0	0	58
21063957	m	cn	SE	2022	0	1	0	1	0	0	0	0	0	0	0	0	73
21064187	f	ph	NL	2022	0	1	1	0	0	0	0	0	0	0	0	0	72
21064216	m	md	JP	2022	0	0	0	0	0	0	0	0	0	0	0	0	56
21064291	f	md	DE	2022	0	1	0	0	1	0	0	0	0	0	0	0	68
21064711	Not	md	US	2022	1	0	0	0	0	0	0	0	0	0	0	0	
21064727	m	md	DE	2022	0	0	0	0	0	0	0	0	0	0	0	0	59
21064957	f	md	JP	2022	0	1	0	0	0	1	0	0	0	0	0	0	80
21065032	m	cn	BE	2022	0	0	0	0	0	0	0	0	0	0	0	0	
21065047	m	md	JP	2022	0	1	0	0	0	1	0	0	0	0	0	1	80
21065267	f	md	CN	2022	0	0	0	0	0	0	0	0	0	0	0	0	54
21065364	m	md	CN	2022	0	1	0	1	0	0	0	0	0	0	0	0	60
21065367	m	cn	DE	2022	0	0	0	0	0	0	0	0	0	0	0	0	
21065410	f	ph	NL	2022	0	1	1	0	0	0	0	0	0	0	0	0	40
21065424	m	md	US	2022	0	0	0	0	0	0	0	0	0	0	0	0	58
21065487	m	cn	TH	2022	1	0	0	0	0	0	0	0	0	0	0	0	
21065489	m	ph	TH	2022	1	0	0	0	0	0	0	0	0	0	0	0	73.2
21065495	m	md	CN	2022	0	0	0	0	0	0	0	0	0	0	0	0	59
21065511	Not	cn	SG	2022	0	0	0	0	0	0	0	0	0	0	0	0	
21065542	Not	hp	SG	2022	1	0	0	0	0	0	0	0	0	0	0	0	

21065543	f	cn	TH	2022	1	0	0	0	0	0	0	0	0	0	0	0	73.2
21065750	m	hp	RO	2022	0	0	0	0	0	0	0	0	0	0	0	0	74
21065820	f	cn	EG	2022	0	0	0	0	0	0	0	0	0	0	0	0	52
21066064	m	md	US	2022	0	0	0	0	0	0	0	0	0	0	0	0	69
21066357	m	md	CA	2022	0	0	0	0	0	0	0	0	0	0	0	0	48
21066361	m	md	US	2022	0	0	0	0	0	0	0	0	0	0	0	0	74
21066853	m	md	EG	2022	0	1	0	0	0	1	0	0	0	0	0	0	
21067566	m	ph	NL	2022	0	1	1	0	0	0	0	0	0	0	0	0	73
21067591	m	md	JP	2022	1	0	0	0	0	0	0	0	0	0	0	1	75
21067812	m	hp	US	2022	0	1	1	0	0	0	0	0	0	0	0	0	59
21068278	Not	ph	JP	2022	0	1	0	1	0	0	0	0	0	0	0	0	
21068439	Not	md	JP	2022	1	0	0	0	0	0	0	0	0	0	0	0	
21068486	m	md	PT	2022	0	1	0	0	1	0	0	0	0	0	0	0	
21068541	Not	ph	JP	2022	1	0	0	0	0	0	0	0	0	0	0	0	
21068700	Not	md	JP	2022	0	1	1	0	0	0	0	0	0	0	0	0	73
21068992	f	md	BR	2022	0	0	0	0	0	0	0	0	0	0	0	0	58
21069052	m	hp	DE	2022	0	1	0	0	1	0	0	0	0	0	0	0	62
21069058	Not	cn	SG	2022	1	0	0	0	0	0	0	0	0	0	0	0	59.6
21069142	m	hp	CN	2022	0	1	0	0	1	0	0	0	0	0	0	0	71
21069241	m	md	JP	2022	0	0	0	0	0	0	0	0	0	0	0	0	62
21069397	m	md	FR	2022	0	0	0	0	0	0	0	0	0	0	0	0	
21070111	f	md	FR	2022	0	0	0	0	0	0	0	0	0	0	0	0	86
21070146	f	md	DE	2022	0	1	0	0	1	0	0	0	0	0	0	0	71
21070178	f	cn	CA	2022	0	0	0	0	0	0	0	0	0	0	0	0	70
21070226	m	md	FR	2022	0	0	0	0	0	0	0	0	0	0	0	0	
21070593	m	md	JP	2022	0	0	0	0	0	0	0	0	0	0	0	0	36
21070600	m	md	CN	2022	0	0	0	0	0	0	0	0	0	0	0	0	
21070682	m	md	JP	2022	0	0	0	0	0	0	0	0	0	0	0	0	70
21070757	m	hp	IT	2022	0	0	0	0	0	0	0	0	0	0	0	0	48
21070868	m	cn	DE	2022	0	0	0	0	0	0	0	0	0	0	0	0	
21070965	m	md	BG	2022	1	0	0	0	0	0	0	0	0	0	0	0	
21071272	m	md	DE	2022	0	1	0	0	1	0	0	0	0	0	0	0	
21071436	m	md	IT	2022	0	0	0	0	0	0	0	0	0	0	0	0	
21071540	f	ph	NL	2022	0	1	1	0	0	0	0	0	0	0	0	0	81

21072161	f	md	DE	2022	1	0	0	0	0	0	0	0	0	0	0	0	
21072807	m	md	PL	2022	0	1	0	0	0	1	0	0	0	0	0	0	65
21073184	f	md	JP	2022	0	0	0	0	0	0	0	0	0	0	0	0	76
21073612	m	md	US	2022	0	0	0	0	0	0	0	0	0	0	0	0	65
21073842	m	md	JP	2022	0	1	0	0	1	0	0	0	0	0	0	0	71
21074095	m	md	JP	2022	0	1	0	0	1	0	0	0	0	0	0	1	74
21074202	f	ph	NL	2022	0	1	1	0	0	0	0	0	0	0	0	0	60
21074482	Not	ph	JP	2022	1	0	0	0	0	0	0	0	0	0	0	0	
21074517	f	md	JP	2022	1	0	0	0	0	0	0	0	0	0	0	0	40
21074518	m	md	PT	2022	0	1	1	0	0	0	0	0	0	0	0	0	46
21074711	m	cn	IN	2022	0	0	0	0	0	0	0	0	0	0	0	0	57
21075274	f	md	US	2022	0	0	0	0	0	0	0	0	0	0	0	0	
21075588	m	md	CN	2022	0	1	1	0	0	0	0	0	0	0	0	0	67
21075803	m	cn	US	2022	0	0	0	0	0	0	0	0	0	0	0	0	
21075850	f	cn	US	2022	0	1	1	0	0	0	0	0	0	0	0	0	76
21075867	f	md	GB	2022	0	0	0	0	0	0	0	0	0	0	0	0	
21075873	m	md	JP	2022	0	0	0	0	0	0	0	0	0	0	0	0	78
21076051	m	cn	US	2022	0	1	0	0	0	1	0	0	0	0	0	0	64
21076127	m	cn	AR	2022	0	0	0	0	0	0	0	0	0	0	0	0	74
21076412	Not	cn	SG	2022	1	0	0	0	0	0	0	0	0	0	0	0	65.2
21076806	m	md	KR	2022	0	0	0	0	0	0	0	0	0	0	0	0	70
21077121	f	hp	JP	2022	0	0	0	0	0	0	0	0	0	0	0	0	
21077168	Not	hp	SG	2022	1	0	0	0	0	0	0	0	0	0	0	0	62
21077210	f	ph	US	2022	0	0	0	0	0	0	0	0	0	0	0	0	
21077738	m	md	JP	2022	0	1	1	0	0	0	0	0	0	0	0	0	70
21077842	Not	hp	CN	2022	1	0	0	0	0	0	0	0	0	0	0	0	
21077926	Not	hp	CN	2022	1	0	0	0	0	0	0	0	0	0	0	0	
21078620	Not	md	JP	2022	0	1	0	0	1	0	0	0	0	0	0	0	
21079010	f	md	TW	2022	1	0	0	0	0	0	0	0	0	0	0	0	55
21079126	f	hp	DE	2022	0	1	0	1	0	0	0	0	0	0	0	1	
21079278	m	md	JP	2022	0	1	0	0	1	0	0	0	0	0	0	0	
21079865	f	hp	DE	2022	0	0	0	0	0	0	0	0	0	0	0	0	58
21080271	m	md	JP	2022	0	1	0	0	1	0	0	0	0	0	0	0	43
21081095	m	md	JP	2022	0	1	0	0	1	0	0	0	0	0	0	0	60

21081127	f	hp	IT	2022	0	0	0	0	0	0	0	0	0	0	0	0	65
21081366	m	md	US	2022	0	0	0	0	0	0	0	0	0	0	0	0	79
21081517	f	md	FR	2022	0	0	0	0	0	0	0	0	0	0	0	0	
21081543	m	cn	CN	2022	0	1	0	0	1	0	0	0	0	0	0	0	73
21081598	m	md	JP	2022	0	1	0	0	1	0	0	0	0	0	0	0	68
21081772	f	md	JP	2022	0	1	1	0	0	0	0	0	0	0	0	0	72
21081798	m	md	JP	2022	0	1	0	1	0	0	0	0	0	0	0	0	64
21082012	m	md	JP	2022	0	0	0	0	0	0	0	0	0	0	0	0	78
21082494	f	hp	AM	2022	0	0	0	0	0	0	0	0	0	0	0	0	61
21083015	m	cn	CN	2022	0	1	0	0	1	0	0	0	0	0	0	0	56
21083392	Not	hp	FR	2022	0	0	0	0	0	0	0	0	0	0	0	0	73
21083493	f	md	JP	2022	0	1	0	0	1	0	0	0	0	0	0	0	88
21084006	f	md	US	2022	0	0	0	0	0	0	0	0	0	0	0	0	72
21084141	m	md	CN	2022	0	0	0	0	0	0	0	0	0	0	0	0	58
21084667	m	md	FR	2022	0	0	0	0	0	0	0	0	0	0	0	0	
21084781	Not	md	JP	2022	0	1	0	1	0	0	0	0	0	0	0	0	
21084797	m	hp	CL	2022	0	1	0	0	1	0	0	0	0	0	0	1	70
21084813	m	hp	CN	2022	0	0	0	0	0	0	0	0	0	0	0	0	65
21085234	m	md	JP	2022	0	1	0	0	1	0	0	0	0	0	0	0	72
21085445	m	md	CN	2022	1	0	0	0	0	0	0	0	0	0	0	0	67
21085634	f	md	DE	2022	0	0	0	0	0	0	0	0	0	0	0	0	58
21085815	f	md	GB	2022	0	0	0	0	0	0	0	0	0	0	0	0	54
21086177	m	md	JP	2022	0	1	0	0	1	0	0	0	0	0	0	0	52
21086342	f	md	JP	2022	0	0	0	0	0	0	0	0	0	0	0	0	50
21086464	Not	md	JP	2022	0	1	0	1	0	0	0	0	0	0	0	1	
21086508	f	md	JP	2022	0	1	0	0	1	0	0	0	0	0	0	0	
21086896	Not	hp	SG	2022	0	1	0	1	0	0	0	0	0	0	0	0	
21086932	f	md	JP	2022	0	0	0	0	0	0	0	0	0	0	0	0	
21086972	f	md	US	2022	0	0	0	0	0	0	0	0	0	0	0	0	
21086995	f	md	US	2022	0	0	0	0	0	0	0	0	0	0	0	0	
21087365	m	cn	IN	2022	0	1	0	0	1	0	0	0	0	0	0	0	50
21087607	Not	ph	JP	2022	0	1	0	1	0	0	0	0	0	0	0	0	
21087633	m	ph	JP	2022	0	0	0	0	0	0	0	0	0	0	0	0	
21087675	m	hp	GR	2022	0	1	1	0	0	0	0	0	0	0	0	0	

21087712	m	md	DE	2022	0	1	0	0	1	0	0	0	0	0	0	0	72
21088367	m	md	FR	2022	0	0	0	0	0	0	0	0	0	0	0	0	75
21088470	m	md	JP	2022	0	1	0	0	0	1	0	0	0	0	0	0	90
21088519	Not	cn	US	2022	1	0	0	0	0	0	0	0	0	0	0	0	58.2
21088885	f	md	NL	2022	1	0	0	0	0	0	0	0	0	0	0	0	61
21088949	m	cn	GB	2022	0	0	0	0	0	0	0	0	0	0	0	0	64
21089005	m	md	KR	2022	0	0	0	0	0	0	0	0	0	0	0	0	55
21089222	f	hp	JP	2022	0	1	0	0	1	0	0	0	0	0	0	0	74
21089232	f	cn	HK	2022	0	0	0	0	0	0	0	0	0	0	0	0	55
21089264	f	cn	IN	2022	0	0	0	0	0	0	0	0	0	0	0	0	61
21089448	m	cn	CA	2022	0	1	0	0	1	0	0	0	0	0	0	0	
21089533	f	md	GR	2022	0	0	0	0	0	0	0	0	0	0	0	0	
21089535	m	md	CN	2022	0	0	0	0	0	0	0	0	0	0	0	0	67
21089698	f	hp	NL	2022	0	0	0	0	0	0	0	0	0	0	0	0	
21090130	m	md	KR	2022	0	0	0	0	0	0	0	0	0	0	0	0	76
21090624	m	md	JP	2022	0	1	1	0	0	0	0	0	0	0	0	0	62
21090814	m	cn	CN	2022	0	1	0	0	1	0	0	0	0	0	0	0	64
21090827	m	cn	DE	2022	0	0	0	0	0	0	0	0	0	0	0	0	
21090830	m	cn	CN	2022	0	1	0	0	1	0	0	0	0	0	0	0	86
21091086	m	hp	SG	2022	0	1	0	0	0	1	0	0	0	0	0	0	69
21091325	m	ph	JP	2022	0	0	0	0	0	0	0	0	0	0	0	0	76
21091665	m	ph	JP	2022	0	1	0	0	0	1	0	0	0	0	0	0	82
21091667	Not	md	HK	2022	0	0	0	0	0	0	0	0	0	0	0	0	
21091676	Not	md	AU	2022	0	0	0	0	0	0	0	0	0	0	0	0	
21091803	m	cn	CN	2022	0	0	0	0	0	0	0	0	0	0	0	0	48
21092536	f	md	FR	2022	0	1	0	0	1	0	0	0	0	0	0	0	81
21093193	m	cn	KR	2022	0	0	0	0	0	0	0	0	0	0	0	0	70
21093490	f	md	DE	2022	0	1	0	0	1	0	0	0	0	0	0	0	66
21093610	m	cn	CA	2022	0	1	0	0	1	0	0	0	0	0	0	0	
21094071	f	cn	CA	2022	1	0	0	0	0	0	0	0	0	0	0	0	58
21094182	f	md	CN	2022	0	0	0	0	0	0	0	0	0	0	0	0	57
21094503	m	cn	US	2022	0	1	1	0	0	0	0	0	0	0	0	1	77
21094595	m	md	FR	2022	0	0	0	0	0	0	0	0	0	0	0	0	
21094856	m	md	MX	2022	0	1	1	0	0	0	0	0	0	0	0	0	72

21094977	f	md	DE	2022	0	1	0	0	1	0	0	0	0	0	0	0	71
21095272	f	md	US	2022	0	0	0	0	0	0	0	0	0	0	0	0	
21096109	m	md	JP	2022	0	1	0	1	0	0	0	0	0	0	0	0	73
21096163	Not	md	FR	2022	0	1	0	0	1	0	0	0	0	0	0	0	81
21096229	m	md	JP	2022	0	1	0	0	1	0	0	0	0	0	0	0	49
21096888	m	md	JP	2022	0	1	0	0	1	0	0	0	0	0	0	1	63
21097118	f	hp	AU	2022	1	0	0	0	0	0	0	0	0	0	0	0	
21097361	m	cn	US	2022	0	1	0	0	1	0	0	0	0	0	0	0	83
21097620	m	md	JP	2022	0	1	0	0	1	0	0	0	0	0	0	0	50
21097665	m	hp	GR	2022	0	1	1	0	0	0	0	0	0	0	0	0	37
21097798	m	md	JP	2022	0	1	0	0	1	0	0	0	0	0	0	0	77
21097841	m	cn	CN	2022	0	1	0	0	1	0	0	0	0	0	0	0	76
21097914	m	hp	JP	2022	0	1	0	0	1	0	0	0	0	0	0	1	67
21097992	m	cn	CN	2022	0	1	0	0	1	0	0	0	0	0	0	0	61
21098930	f	md	JP	2022	0	1	0	0	1	0	0	0	0	0	0	0	77
21098943	f	md	JP	2022	0	1	1	0	0	0	0	0	0	0	0	0	75
21098955	f	md	AU	2022	1	0	0	0	0	0	0	0	0	0	0	0	
21099090	m	cn	CN	2022	0	1	0	0	1	0	0	0	0	0	0	0	65
21099294	f	md	CA	2022	0	0	0	0	0	0	0	0	0	0	0	0	73
21099476	m	cn	CN	2022	0	1	0	0	1	0	0	0	0	0	0	0	56
21099500	m	hp	DE	2022	0	0	0	0	0	0	0	0	0	0	0	0	
21099792	Not	md	PL	2022	0	0	0	0	0	0	0	0	0	0	0	0	
21100352	f	md	JP	2022	1	0	0	0	0	0	0	0	0	0	0	0	
21100604	m	md	JP	2022	1	0	0	0	0	0	0	0	0	0	0	0	85.4
21101108	f	md	DE	2022	1	0	0	0	0	0	0	0	0	0	0	0	81
21101406	m	md	IL	2022	0	0	0	0	0	0	0	0	0	0	0	0	60
21102519	Not	md	JP	2022	0	1	0	0	1	0	0	0	0	0	0	0	
21102866	m	md	JP	2022	0	1	0	0	1	0	0	0	0	0	0	0	76
21103029	m	md	JP	2022	0	1	0	0	1	0	0	0	0	0	0	0	59
21103079	m	cn	CN	2022	0	1	0	0	1	0	0	0	0	0	0	0	39
21103125	m	md	KR	2022	0	1	0	0	0	1	0	0	0	0	0	0	74
21103153	f	md	JP	2022	0	1	0	0	1	0	0	0	0	0	0	0	69
21103220	m	hp	US	2022	0	0	0	0	0	0	0	0	0	0	0	0	
21103841	m	md	JP	2022	0	1	0	0	1	0	0	0	0	0	0	0	60

21104173	m	md	ES	2022	0	0	0	0	0	0	0	0	0	0	0	0	60
21104358	m	md	JP	2022	0	1	0	0	0	1	0	0	0	0	0	0	
21104361	Not	md	JP	2022	0	1	0	0	0	1	0	0	0	0	0	0	61
21104362	f	md	JP	2022	0	0	0	0	0	0	0	0	0	0	0	0	63
21104900	m	md	DE	2022	0	1	0	0	0	1	0	0	0	0	0	0	55
21105131	f	md	BY	2022	0	0	0	0	0	0	0	0	0	0	0	0	
21105172	f	md	KR	2022	0	1	1	0	0	0	0	0	0	0	0	0	
21105181	m	md	JP	2022	0	0	0	0	0	0	0	0	0	0	0	0	
21105809	f	cn	FR	2022	0	0	0	0	0	0	0	0	0	0	0	0	
21105856	f	md	DE	2022	0	1	1	0	0	0	0	0	0	0	0	0	72
21105931	Not	md	JP	2022	0	1	0	0	0	1	0	0	0	0	0	0	
21105974	Not	md	JP	2022	1	0	0	0	0	0	0	0	0	0	0	0	
21106226	f	md	FR	2022	0	0	0	0	0	0	0	0	0	0	0	0	
21106341	m	md	KR	2022	0	1	1	0	0	0	0	0	0	0	0	0	
21106503	f	hp	CN	2022	1	0	0	0	0	0	0	0	0	0	0	0	43
21106962	m	md	FR	2022	0	0	0	0	0	0	0	0	0	0	0	0	
21107095	m	md	PL	2022	0	0	0	0	0	0	0	0	0	0	0	0	62
21107777	m	cn	TH	2022	1	0	0	0	0	0	0	0	0	0	0	0	73.3
21108319	Not	md	JP	2022	0	1	0	0	1	0	0	0	0	0	0	0	
21108413	m	hp	GR	2022	0	1	0	0	1	0	0	0	0	0	0	0	
21108810	m	md	JP	2022	0	1	0	0	1	0	0	0	0	0	0	1	67
21109388	f	md	JP	2022	0	1	0	0	1	0	0	0	0	0	0	0	82
21109553	m	md	US	2022	0	0	0	0	0	0	0	0	0	0	0	0	73
21109663	Not	cn	US	2022	0	0	0	0	0	0	0	0	0	0	0	0	
21110188	f	md	DE	2022	0	1	1	0	0	0	0	0	0	0	0	0	
21111051	m	md	US	2022	0	1	0	0	0	1	0	0	0	0	0	0	58
21111336	Not	md	SG	2022	0	1	0	1	0	0	0	0	0	0	0	0	
21111688	m	hp	JP	2022	0	1	1	0	0	0	0	0	0	0	0	0	80
21111995	f	cn	IN	2022	0	0	0	0	0	0	0	0	0	0	0	0	65
21112260	m	cn	IN	2022	0	0	0	0	0	0	0	0	0	0	0	0	63
21112311	Not	cn	US	2022	0	0	0	0	0	0	0	0	0	0	0	0	
21112541	m	ph	JP	2022	1	0	0	0	0	0	0	0	0	0	0	0	
21112606	m	md	JP	2022	0	1	0	0	0	1	0	0	0	0	0	0	
21112936	m	md	CN	2022	0	0	0	0	0	0	0	0	0	0	0	0	71

21113360	f	md	FR	2022	0	1	1	0	0	0	0	0	0	0	0	0	
21113456	Not	cn	US	2022	0	0	0	0	0	0	0	0	0	0	0	0	
21113652	f	md	CN	2022	0	0	0	0	0	0	0	0	0	0	0	0	74
21113979	m	md	GR	2022	0	1	0	0	1	0	0	0	0	0	0	0	63
21114743	f	md	JP	2022	0	0	0	0	0	0	0	0	0	0	0	0	80
21115055	f	hp	IT	2022	0	0	0	0	0	0	0	0	0	0	0	0	65
21115065	m	hp	IT	2022	0	0	0	0	0	0	0	0	0	0	0	0	48
21115539	f	md	JP	2022	0	1	0	0	1	0	0	0	0	0	0	0	70
21116160	Not	md	JP	2022	0	1	0	0	1	0	0	0	0	0	0	0	
21116683	f	md	JP	2022	0	1	0	0	1	0	0	0	0	0	0	0	76
21116886	m	hp	GR	2022	0	1	1	0	0	0	0	0	0	0	0	0	
21116902	m	hp	GR	2022	0	1	0	0	1	0	0	0	0	0	0	0	
21117134	m	md	JP	2022	0	1	1	0	0	0	0	0	0	0	0	0	80
21117188	m	cn	CN	2022	0	1	0	0	1	0	0	0	0	0	0	0	68
21117918	f	cn	US	2022	1	0	0	0	0	0	0	0	0	0	0	0	58
21118003	Not	cn	US	2022	1	0	0	0	0	0	0	0	0	0	0	0	77
21118257	f	md	FR	2022	0	0	0	0	0	0	0	0	0	0	0	0	
21118481	f	hp	US	2022	1	0	0	0	0	0	0	0	0	0	0	0	
21118687	f	cn	GB	2022	1	0	0	0	0	0	0	0	0	0	0	0	43
21118771	m	md	NL	2022	0	0	0	0	0	0	0	0	0	0	0	0	57
21118880	f	cn	GB	2022	1	0	0	0	0	0	0	0	0	0	0	0	72
21119299	Not	cn	US	2022	0	0	0	0	0	0	0	0	0	0	0	0	
21119325	Not	cn	US	2022	0	0	0	0	0	0	0	0	0	0	0	0	
21119430	Not	cn	US	2022	0	0	0	0	0	0	0	0	0	0	0	0	
21119444	m	md	PL	2022	0	1	0	1	0	0	0	0	0	0	0	1	
21120007	m	md	FR	2022	0	0	0	0	0	0	0	0	0	0	0	0	
21120033	f	md	IT	2022	0	0	0	0	0	0	0	0	0	0	0	0	84
21120111	Not	cn	US	2022	0	0	0	0	0	0	0	0	0	0	0	0	
21120554	f	cn	DE	2022	0	0	0	0	0	0	0	0	0	0	0	0	75
21121204	f	cn	US	2022	0	0	0	0	0	0	0	0	0	0	0	0	
21121237	m	cn	CN	2022	0	1	0	0	1	0	0	0	0	0	0	0	72
21121247	m	cn	US	2022	0	0	0	0	0	0	0	0	0	0	0	0	
21121390	Not	ph	TH	2022	0	1	0	1	0	0	0	0	0	0	0	0	
21121570	f	md	IT	2022	0	0	0	0	0	0	0	0	0	0	0	0	64

21121678	f	md	DE	2022	0	1	1	0	0	0	0	0	0	0	0	1	66
21121686	f	md	PT	2022	0	0	0	0	0	0	0	0	0	0	0	0	68
21121695	m	cn	GB	2022	1	0	0	0	0	0	0	0	0	0	0	0	
21121697	f	hp	ID	2022	1	0	0	0	0	0	0	0	0	0	0	0	54.7
21121718	m	md	BE	2022	0	1	0	1	0	0	0	0	0	0	0	0	71
21122408	Not	md	JP	2022	1	0	0	0	0	0	0	0	0	0	0	0	
21122437	m	md	JP	2022	0	1	0	1	0	0	0	0	0	0	0	1	
21122451	f	cn	ID	2022	1	0	0	0	0	0	0	0	0	0	0	0	62.7
21122523	f	md	AU	2022	1	0	0	0	0	0	0	0	0	0	0	0	
21122624	Not	cn	US	2022	0	0	0	0	0	0	0	0	0	0	0	0	
21122625	Not	cn	US	2022	0	0	0	0	0	0	0	0	0	0	0	0	
21122626	Not	cn	US	2022	0	0	0	0	0	0	0	0	0	0	0	0	
21122796	m	md	JP	2022	0	1	0	0	1	0	0	0	0	0	0	0	61
21122836	m	cn	CH	2022	0	0	0	0	0	0	0	0	0	0	0	0	
21122858	m	md	JP	2022	0	1	0	0	0	1	0	0	0	0	0	0	50
21122872	f	md	JP	2022	0	1	0	0	0	1	0	0	0	0	0	0	73
21123004	f	cn	ID	2022	1	0	0	0	0	0	0	0	0	0	0	0	67.4
21123016	f	md	JP	2022	1	0	0	0	0	0	0	0	0	0	0	0	
21123410	Not	cn	US	2022	0	0	0	0	0	0	0	0	0	0	0	0	
21123411	Not	cn	US	2022	0	0	0	0	0	0	0	0	0	0	0	0	
21123412	Not	cn	US	2022	0	0	0	0	0	0	0	0	0	0	0	0	
21123413	Not	cn	US	2022	0	0	0	0	0	0	0	0	0	0	0	0	
21123507	f	ph	JP	2022	0	1	0	0	1	0	0	0	0	0	0	0	88
21123617	Not	md	JP	2022	0	0	0	0	0	0	0	0	0	0	0	0	
21123635	m	md	JP	2022	0	1	0	0	1	0	0	0	0	0	0	0	59
21123767	m	md	JP	2022	0	1	0	0	1	0	0	0	0	0	0	0	71
21124148	m	md	JP	2022	0	1	0	0	1	0	0	0	0	0	0	0	58
21124192	m	md	CA	2022	0	0	0	0	0	0	0	0	0	0	0	0	71
21124234	f	hp	HR	2022	0	0	0	0	0	0	0	0	0	0	0	0	
21124417	Not	md	JP	2022	0	1	0	0	1	0	0	0	0	0	0	0	80
21124490	m	cn	US	2022	0	1	1	0	0	0	0	0	0	0	0	1	
21124677	f	md	JP	2022	0	1	0	0	1	0	0	0	0	0	0	0	87
21124710	f	md	AT	2022	0	1	0	0	0	1	0	0	0	0	0	0	47
21124737	m	cn	IN	2022	0	0	0	0	0	0	0	0	0	0	0	0	45

21125114	m	hp	DE	2022	0	1	0	0	1	0	0	0	0	0	0	1	49
21125182	m	hp	CN	2022	0	1	0	0	1	0	0	0	0	0	0	0	68
21125365	Not	cn	SG	2022	1	0	0	0	0	0	0	0	0	0	0	0	77.2
21126461	m	hp	US	2022	0	0	0	0	0	0	0	0	0	0	0	0	51
21128261	m	md	JP	2022	0	1	0	1	0	0	0	0	0	0	0	1	78
21128533	Not	hp	NO	2022	0	1	0	0	0	1	0	0	0	0	0	0	76
21128584	Not	hp	NO	2022	0	1	0	0	0	1	0	0	0	0	0	0	71
21128588	Not	hp	NO	2022	0	1	0	0	0	1	0	0	0	0	0	0	71
21128590	Not	hp	NO	2022	0	1	0	0	0	1	0	0	0	0	0	0	78
21129031	m	md	FR	2022	0	1	1	0	0	0	0	0	0	0	0	0	
21129134	f	md	JP	2022	0	0	0	0	0	0	0	0	0	0	0	0	54
21129178	Not	ph	US	2022	0	0	0	0	0	0	0	0	0	0	0	0	
21129354	f	md	US	2022	0	1	0	0	1	0	0	0	0	0	0	0	
21129409	f	md	JP	2022	0	1	0	0	1	0	0	0	0	0	0	0	68
21129463	Not	cn	US	2022	0	0	0	0	0	0	0	0	0	0	0	0	
21129465	Not	md	JP	2022	0	1	0	0	1	0	0	0	0	0	0	0	
21129468	Not	cn	US	2022	0	0	0	0	0	0	0	0	0	0	0	0	
21129469	f	cn	US	2022	0	0	0	0	0	0	0	0	0	0	0	0	
21129483	Not	cn	US	2022	0	0	0	0	0	0	0	0	0	0	0	0	
21129527	m	ph	JP	2022	1	0	0	0	0	0	0	0	0	0	0	0	
21130148	m	hp	KN	2022	0	1	1	0	0	0	0	0	0	0	0	0	61
21130808	f	md	DE	2022	0	0	0	0	0	0	0	0	0	0	0	0	61
21130839	m	cn	TH	2022	0	1	0	0	1	0	0	0	0	0	0	0	86
21131187	Not	md	FR	2022	0	1	0	0	1	0	0	0	0	0	0	0	59
21131222	Not	cn	US	2022	0	0	0	0	0	0	0	0	0	0	0	0	
21131503	Not	cn	US	2022	0	0	0	0	0	0	0	0	0	0	0	0	
21131525	f	md	US	2022	0	0	0	0	0	0	0	0	0	0	0	0	62
21131535	f	md	US	2022	0	0	0	0	0	0	0	0	0	0	0	0	55
21131705	f	cn	MY	2022	1	0	0	0	0	0	0	0	0	0	0	0	71.5
21132064	m	cn	US	2022	0	1	1	0	0	0	0	0	0	0	0	0	66
21132290	f	hp	US	2022	0	1	0	0	1	0	0	0	0	0	0	0	81
21132567	f	md	RU	2022	0	1	0	0	0	1	0	0	0	0	0	0	72
21132607	f	md	TW	2022	0	0	0	0	0	0	0	0	0	0	0	0	70
21132877	f	md	AU	2022	1	0	0	0	0	0	0	0	0	0	0	0	81

21133047	f	md	RU	2022	0	1	0	1	0	0	0	0	0	0	0	0	60
21133141	f	cn	US	2022	0	0	0	0	0	0	0	0	0	0	0	0	
21133262	f	md	AU	2022	1	0	0	0	0	0	0	0	0	0	0	0	58
21133532	m	cn	IN	2022	0	1	0	0	1	0	0	0	0	0	0	0	71
21133548	m	md	FR	2022	0	1	1	0	0	0	0	0	0	0	0	0	
21134179	m	md	ES	2022	0	0	0	0	0	0	0	0	0	0	0	0	68
21134381	f	hp	CN	2022	0	0	0	0	0	0	0	0	0	0	0	0	69
21134457	m	md	JP	2022	0	1	0	0	1	0	0	0	0	0	0	0	82
21134461	m	md	JP	2022	0	1	0	0	1	0	0	0	0	0	0	0	72
21134519	m	md	JP	2022	0	1	0	0	1	0	0	0	0	0	0	0	80
21134809	f	md	GB	2022	0	0	0	0	0	0	1	0	0	0	1	0	63
21134976	m	md	LT	2022	0	1	1	0	0	0	0	0	0	0	0	0	78
21134982	m	md	JP	2022	0	0	0	0	0	0	0	0	0	0	0	0	
21135029	m	md	CH	2022	0	1	0	0	0	1	0	0	0	0	0	0	73
21135527	m	md	US	2022	0	0	0	0	0	0	0	0	0	0	0	0	59
21135792	m	cn	US	2022	0	1	0	0	1	0	0	0	0	0	0	0	70
21135798	m	md	HK	2022	0	1	1	0	0	0	0	0	0	0	0	0	
21136138	m	md	JP	2022	0	1	0	0	0	1	0	0	0	0	0	0	80
21136215	Not	md	US	2022	0	0	0	0	0	0	0	0	0	0	0	0	65
21136442	Not	md	DE	2022	0	1	0	0	1	0	0	0	0	0	0	0	
21136487	m	md	DE	2022	0	1	0	0	1	0	0	0	0	0	0	1	62
21136515	m	md	JP	2022	0	1	0	1	0	0	0	0	0	0	0	1	77
21136569	m	hp	US	2022	0	0	0	0	0	0	0	0	0	0	0	0	51
21136627	m	md	JP	2022	0	1	0	0	1	0	0	0	0	0	0	0	80
21137111	f	hp	CN	2022	0	0	0	0	0	0	0	0	0	0	0	0	66
21137115	Not	md	JP	2022	1	0	0	0	0	0	0	0	0	0	0	0	
21137718	f	Not repo	US	2022	0	0	0	0	0	0	0	0	0	0	0	0	82
21137934	f	md	FR	2022	0	1	1	0	0	0	0	0	0	0	0	0	
21138211	f	md	DE	2022	0	0	0	0	0	0	0	0	0	0	0	0	75
21138319	Not	hp	US	2022	0	1	1	0	0	0	0	0	0	0	0	0	
21138580	f	md	FR	2022	0	1	1	0	0	0	0	0	0	0	0	0	
21138655	f	cn	KR	2022	0	0	0	0	0	0	0	0	0	0	0	0	82
21138762	m	md	JP	2022	0	1	0	0	1	0	0	0	0	0	0	1	68
21138786	m	md	US	2022	0	0	0	0	0	0	0	0	0	0	0	0	70

21138895	f	cn	BR	2022	0	0	0	0	0	0	0	0	0	0	0	0	
21138990	f	hp	IL	2022	0	0	0	0	0	0	0	0	0	0	0	0	63
21139241	m	ph	US	2022	1	0	0	0	0	0	0	0	0	0	0	0	69
21139394	m	md	JP	2022	0	0	0	0	0	0	0	0	0	0	0	0	75
21139412	f	md	AT	2022	0	1	1	0	0	0	0	0	0	0	0	0	74
21139510	m	md	JP	2022	0	1	1	0	0	0	0	0	0	0	0	0	
21139592	m	md	JP	2022	0	1	0	0	1	0	0	0	0	0	0	1	74
21139663	m	md	DE	2022	0	1	0	0	1	0	0	0	0	0	0	0	61
21139709	m	md	JP	2022	0	0	0	0	0	0	0	0	0	0	0	0	80
21140296	f	md	US	2022	0	0	0	0	0	0	0	0	0	0	0	0	62
21140354	Not	cn	CN	2022	0	0	0	0	0	0	0	0	0	0	0	0	
21140654	Not	md	JP	2022	0	1	0	1	0	0	0	0	0	0	0	0	
21140684	m	md	JP	2022	0	1	0	0	1	0	0	0	0	0	0	0	72
21140802	f	md	FR	2022	0	1	1	0	0	0	0	0	0	0	0	0	
21140988	f	md	JP	2022	1	0	0	0	0	0	0	0	0	0	0	0	60
21141192	f	md	JP	2022	0	0	0	0	0	0	0	0	0	0	0	0	60
21141873	m	md	AU	2022	0	1	0	0	0	1	0	0	0	0	0	0	63
21142687	f	md	JP	2022	0	1	0	0	1	0	0	0	0	0	0	0	77
21142731	f	ph	JP	2022	1	0	0	0	0	0	0	0	0	0	0	0	
21143609	f	md	JP	2022	1	0	0	0	0	0	0	0	0	0	0	0	74
21143614	f	hp	NL	2022	0	0	0	0	0	0	0	0	0	0	0	0	
21143909	f	md	DE	2022	0	1	0	1	0	0	0	0	0	0	0	0	
21144150	m	md	JP	2022	1	0	0	0	0	0	0	0	0	0	0	0	67
21144612	m	cn	SG	2022	0	1	0	0	0	1	0	0	0	0	0	0	81
21144665	Not	md	JP	2022	0	1	0	0	0	1	0	0	0	0	0	0	
21145089	m	md	JP	2022	0	0	0	0	0	0	0	0	0	0	0	0	84
21145369	f	md	JP	2022	0	0	0	0	0	0	0	0	0	0	0	0	
21145449	Not	md	JP	2022	0	0	0	0	0	0	0	0	0	0	0	0	59
21145574	m	md	PL	2022	0	0	0	0	0	0	0	0	0	0	0	0	82
21145650	f	md	CN	2022	0	0	0	0	0	0	0	0	0	0	0	0	69
21145708	f	md	DE	2022	0	1	1	0	0	0	0	0	0	0	0	1	66
21145933	Not	cn	CN	2022	0	0	0	0	0	0	0	0	0	0	0	0	
21146093	m	md	US	2022	0	0	0	0	0	0	0	0	0	0	0	0	61
21146422	m	md	FR	2022	0	0	0	0	0	0	0	0	0	0	0	0	81

21146466	m	md	ES	2022	0	1	0	0	0	1	0	0	0	0	0	0	55
21146480	f	ph	JP	2022	0	0	0	0	0	0	0	0	0	0	0	0	74
21146551	f	md	JP	2022	0	0	0	0	0	0	0	0	0	0	0	0	60
21146825	Not	hp	CN	2022	0	1	1	0	0	0	0	0	0	0	0	1	
21146867	Not	ph	JP	2022	1	0	0	0	0	0	0	0	0	0	0	0	
21147057	Not	hp	NL	2022	0	0	0	0	0	0	0	0	0	0	0	0	
21147059	f	hp	NL	2022	0	0	0	0	0	0	0	0	0	0	0	0	
21147061	m	hp	NL	2022	0	0	0	0	0	0	0	0	0	0	0	0	
21147144	m	md	DE	2022	0	1	1	0	0	0	0	0	0	0	0	1	76
21147251	f	hp	US	2022	0	1	0	0	1	0	0	0	0	0	0	0	58
21147688	m	cn	DE	2022	0	0	0	0	0	0	0	0	0	0	0	0	72
21147747	f	ph	US	2022	0	0	0	0	0	0	0	0	0	0	0	0	60
21147763	m	ph	JP	2022	0	1	1	0	0	0	0	0	0	0	0	0	60
21147891	f	md	CN	2022	1	0	0	0	0	0	0	0	0	0	0	0	
21148044	f	md	KR	2022	0	1	0	0	0	1	0	0	0	0	0	0	58
21148266	f	md	FR	2022	0	1	0	0	1	0	0	0	0	0	0	0	
21148317	m	md	DE	2022	0	1	0	0	1	0	0	0	0	0	0	0	67
21148408	m	md	JP	2022	0	0	0	0	0	0	0	0	0	0	0	0	72
21148476	m	md	DE	2022	0	1	0	0	1	0	0	0	0	0	0	0	58
21148685	f	ph	JP	2022	1	0	0	0	0	0	0	0	0	0	0	0	79
21148747	f	md	JP	2022	1	0	0	0	0	0	0	0	0	0	0	0	26
21148757	f	md	JP	2022	0	0	0	0	0	0	1	0	0	1	0	0	76
21148834	m	md	JP	2022	0	1	0	0	1	0	0	0	0	0	0	0	80
21148840	m	md	JP	2022	1	0	0	0	0	0	0	0	0	0	0	0	78
21148886	m	md	US	2022	0	0	0	0	0	0	0	0	0	0	0	0	71
21149402	f	md	JP	2022	1	0	0	0	0	0	0	0	0	0	0	0	70
21149404	f	cn	ID	2022	1	0	0	0	0	0	0	0	0	0	0	0	42.3
21149406	m	md	JP	2022	0	1	0	1	0	0	0	0	0	0	0	1	74
21149560	Not	md	DE	2022	0	0	0	0	0	0	0	0	0	0	0	0	
21149684	m	cn	IN	2022	0	0	0	0	0	0	0	0	0	0	0	0	73
21150132	f	cn	SI	2022	0	0	0	0	0	0	0	0	0	0	0	0	60
21150235	m	hp	US	2022	1	0	0	0	0	0	0	0	0	0	0	0	59.6
21150259	f	cn	AT	2022	0	1	1	0	0	0	0	0	0	0	0	0	74
21150404	f	cn	KR	2022	0	0	0	0	0	0	0	0	0	0	0	0	80

21150510	f	md	GB	2022	0	0	0	0	0	0	0	0	0	0	0	0	76
21151112	m	md	JP	2022	0	0	0	0	0	0	0	0	0	0	0	0	48
21151140	m	md	DE	2022	0	1	0	0	1	0	0	0	0	0	0	0	76
21151387	m	md	FR	2022	0	1	1	0	0	0	0	0	0	0	0	0	
21151746	f	md	AT	2022	0	1	1	0	0	0	0	0	0	0	0	0	74
21153432	m	md	JP	2022	0	1	0	0	1	0	0	0	0	0	0	0	76
21153502	f	md	JP	2022	0	1	0	0	1	0	0	0	0	0	0	0	44
21153531	f	md	JP	2022	0	1	0	0	1	0	0	0	0	0	0	0	62
21153719	f	cn	US	2022	0	0	0	0	0	0	0	0	0	0	0	0	
21153939	m	md	CN	2022	0	0	0	0	0	0	0	0	0	0	0	0	69
21154216	Not	md	JP	2022	0	1	0	0	0	1	0	0	0	0	0	0	
21154646	m	md	JP	2022	0	1	1	0	0	0	0	0	0	0	0	0	90
21154962	f	md	JP	2022	0	1	0	0	1	0	0	0	0	0	0	0	78
21155461	f	md	CN	2022	0	0	0	0	0	0	0	0	0	0	0	0	69
21155829	m	md	JP	2022	0	1	1	0	0	0	0	0	0	0	0	0	57
21155835	f	md	JP	2022	0	1	1	0	0	0	0	0	0	0	0	0	62
21155856	m	md	JP	2022	0	1	1	0	0	0	0	0	0	0	0	0	74
21156289	Not	hp	US	2022	0	1	1	0	0	0	0	0	0	0	0	0	
21156492	Not	hp	US	2022	0	0	0	0	0	0	0	0	0	0	0	0	
21156493	Not	hp	US	2022	0	1	1	0	0	0	0	0	0	0	0	0	
21156501	Not	hp	US	2022	0	1	1	0	0	0	0	0	0	0	0	0	
21156675	m	md	PT	2022	0	0	0	0	0	0	0	0	0	0	0	0	57
21156825	f	cn	US	2022	0	0	0	0	0	0	0	0	0	0	0	0	
21156942	m	md	IT	2022	0	0	0	0	0	0	0	0	0	0	0	0	86
21157069	f	md	CN	2022	0	0	0	0	0	0	0	0	0	0	0	0	69
21157210	Not	hp	SG	2022	1	0	0	0	0	0	0	0	0	0	0	0	59.8
21157258	Not	hp	DE	2022	1	0	0	0	0	0	0	0	0	0	0	0	
21157277	Not	hp	DE	2022	1	0	0	0	0	0	0	0	0	0	0	0	
21157278	Not	hp	DE	2022	1	0	0	0	0	0	0	0	0	0	0	0	
21157294	Not	cn	SG	2022	1	0	0	0	0	0	0	0	0	0	0	0	67
21157520	f	md	PT	2022	0	0	0	0	0	0	0	0	0	0	0	0	64
21157543	m	hp	NL	2022	0	0	0	0	0	0	0	0	0	0	0	0	53
21157552	Not	cn	SG	2022	1	0	0	0	0	0	0	0	0	0	0	0	65.7
21157629	f	md	AT	2022	0	1	1	0	0	0	0	0	0	0	0	0	74

21157826	m	hp	RU	2022	0	1	0	1	0	0	0	0	0	0	0	0	
21157909	f	cn	US	2022	0	1	0	0	1	0	0	0	0	0	0	0	66
21158020	Not	cn	SG	2022	1	0	0	0	0	0	0	0	0	0	0	0	65.4
21158183	Not	hp	US	2022	0	1	1	0	0	0	0	0	0	0	0	0	
21158227	f	md	CN	2022	0	0	0	0	0	0	0	0	0	0	0	0	72
21158333	Not	ph	SG	2022	1	0	0	0	0	0	0	0	0	0	0	0	74
21158392	m	md	JP	2022	0	1	0	0	1	0	0	0	0	0	0	0	74
21158397	f	cn	US	2022	0	0	0	0	0	0	0	0	0	0	0	0	
21158735	f	hp	US	2022	0	1	1	0	0	0	0	0	0	0	0	0	71
21158868	Not	hp	DE	2022	1	0	0	0	0	0	0	0	0	0	0	0	
21159338	m	ph	JP	2022	0	1	1	0	0	0	0	0	0	0	0	0	40
21159438	m	cn	FR	2022	0	1	0	0	1	0	0	0	0	0	0	0	76
21159591	Not	hp	US	2022	0	1	1	0	0	0	0	0	0	0	0	0	
21159592	Not	hp	US	2022	0	1	1	0	0	0	0	0	0	0	0	0	
21159593	Not	hp	US	2022	0	1	1	0	0	0	0	0	0	0	0	0	
21159796	Not	md	JP	2022	0	1	0	0	1	0	0	0	0	0	0	0	
21160231	f	ph	FR	2022	0	1	1	0	0	0	0	0	0	0	0	0	62
21160585	f	hp	US	2022	0	0	0	0	0	0	0	0	0	0	0	0	
21160623	Not	md	JP	2022	1	0	0	0	0	0	0	0	0	0	0	0	
21160625	Not	hp	US	2022	0	1	1	0	0	0	0	0	0	0	0	0	
21160626	Not	hp	US	2022	0	1	1	0	0	0	0	0	0	0	0	0	
21160628	Not	hp	US	2022	0	1	1	0	0	0	0	0	0	0	0	0	
21160630	Not	hp	US	2022	0	1	1	0	0	0	0	0	0	0	0	0	
21160639	m	md	JP	2022	0	1	1	0	0	0	0	0	0	0	0	0	80
21160920	m	hp	GR	2022	0	1	1	0	0	0	0	0	0	0	0	0	37
21161024	m	cn	TH	2022	0	0	0	0	0	0	0	0	0	0	0	0	81
21161080	Not	md	JP	2022	0	1	0	0	0	1	0	0	0	0	0	0	60
21161145	m	cn	ID	2022	0	1	0	1	0	0	0	0	0	0	0	0	71.8
21161294	f	hp	IT	2022	0	0	0	0	0	0	0	0	0	0	0	0	83
21161725	Not	hp	US	2022	0	0	0	0	0	0	0	0	0	0	0	0	
21161726	Not	hp	US	2022	0	0	0	0	0	0	0	0	0	0	0	0	
21161727	Not	hp	US	2022	0	0	0	0	0	0	0	0	0	0	0	0	
21161728	Not	hp	US	2022	0	0	0	0	0	0	0	0	0	0	0	0	
21161731	Not	hp	US	2022	0	0	0	0	0	0	0	0	0	0	0	0	

21161732	Not	hp	US	2022	0	0	0	0	0	0	0	0	0	0	0	0	
21161735	Not	hp	US	2022	0	0	0	0	0	0	0	0	0	0	0	0	
21161884	m	md	JP	2022	0	1	1	0	0	0	0	0	0	0	0	0	83
21161886	f	cn	IN	2022	0	0	0	0	0	0	0	0	0	0	0	0	31
21162045	Not	ph	JP	2022	1	0	0	0	0	0	0	0	0	0	0	0	
21162726	f	cn	US	2022	1	0	0	0	0	0	0	0	0	0	0	0	91.1
21163032	m	cn	IN	2022	0	1	0	0	1	0	0	0	0	0	0	0	58
21163070	m	md	JP	2022	0	1	0	0	0	1	0	0	0	0	0	0	80
21163338	Not	hp	DE	2022	1	0	0	0	0	0	0	0	0	0	0	0	
21163343	Not	hp	DE	2022	1	0	0	0	0	0	0	0	0	0	0	0	
21163344	Not	hp	DE	2022	1	0	0	0	0	0	0	0	0	0	0	0	
21163347	Not	hp	DE	2022	1	0	0	0	0	0	0	0	0	0	0	0	
21163348	Not	hp	DE	2022	1	0	0	0	0	0	0	0	0	0	0	0	
21163496	Not	hp	DE	2022	1	0	0	0	0	0	0	0	0	0	0	0	
21163497	Not	hp	DE	2022	1	0	0	0	0	0	0	0	0	0	0	0	
21163498	Not	hp	DE	2022	1	0	0	0	0	0	0	0	0	0	0	0	
21163499	Not	hp	DE	2022	1	0	0	0	0	0	0	0	0	0	0	0	
21163500	Not	hp	DE	2022	1	0	0	0	0	0	0	0	0	0	0	0	
21163502	Not	hp	DE	2022	1	0	0	0	0	0	0	0	0	0	0	0	
21163503	Not	hp	DE	2022	1	0	0	0	0	0	0	0	0	0	0	0	
21163505	Not	hp	DE	2022	1	0	0	0	0	0	0	0	0	0	0	0	
21163506	Not	hp	DE	2022	1	0	0	0	0	0	0	0	0	0	0	0	
21163507	Not	hp	DE	2022	1	0	0	0	0	0	0	0	0	0	0	0	
21163509	Not	hp	DE	2022	1	0	0	0	0	0	0	0	0	0	0	0	
21163596	m	md	JP	2022	0	1	0	0	1	0	0	0	0	0	0	0	55
21163851	m	md	JP	2022	0	1	0	1	0	0	0	0	0	0	0	1	67
21163860	Not	cn	US	2022	0	0	0	0	0	0	0	0	0	0	0	0	
21164227	m	md	JP	2022	0	1	0	0	1	0	0	0	0	0	0	0	68
21164367	Not	md	JP	2022	0	1	0	0	1	0	0	0	0	0	0	0	66
21164435	f	md	JP	2022	0	0	0	0	0	0	0	0	0	0	0	0	
21164551	m	md	DE	2022	0	1	0	0	1	0	0	0	0	0	0	0	61
21164870	m	md	JP	2022	0	1	1	0	0	0	0	0	0	0	0	1	71
21164968	m	md	NL	2022	0	0	0	0	0	0	0	0	0	0	0	0	63
21165217	Not	md	JP	2022	0	1	1	0	0	0	0	0	0	0	0	0	70

21165368	Not	hp	DE	2022	1	0	0	0	0	0	0	0	0	0	0	0	
21165390	Not	hp	DE	2022	1	0	0	0	0	0	0	0	0	0	0	0	
21165391	Not	hp	DE	2022	1	0	0	0	0	0	0	0	0	0	0	0	
21165412	Not	hp	DE	2022	1	0	0	0	0	0	0	0	0	0	0	0	
21165430	m	md	JP	2022	0	1	0	0	0	1	0	0	0	0	0	0	70
21165464	Not	hp	DE	2022	1	0	0	0	0	0	0	0	0	0	0	0	
21165507	Not	hp	DE	2022	1	0	0	0	0	0	0	0	0	0	0	0	
21165599	m	hp	US	2022	1	0	0	0	0	0	0	0	0	0	0	0	
21165698	m	md	DE	2022	0	1	0	0	1	0	0	0	0	0	0	0	67
21165746	m	md	JP	2022	0	1	0	0	1	0	0	0	0	0	0	0	66
21165860	Not	cn	SG	2022	1	0	0	0	0	0	0	0	0	0	0	0	84
21166005	f	md	NL	2022	0	0	0	0	0	0	0	0	0	0	0	0	63
21166159	Not	hp	DE	2022	1	0	0	0	0	0	0	0	0	0	0	0	
21166258	Not	hp	SG	2022	1	0	0	0	0	0	0	0	0	0	0	0	74
21166393	Not	cn	SG	2022	1	0	0	0	0	0	0	0	0	0	0	0	74.7
21166488	f	md	US	2022	0	1	0	0	0	1	0	0	0	0	0	0	65
21166527	Not	hp	US	2022	0	1	1	0	0	0	0	0	0	0	0	0	
21166615	f	md	DE	2022	0	1	1	0	0	0	0	0	0	0	0	1	66
21166842	m	cn	CN	2022	0	1	0	0	1	0	0	0	0	0	0	0	60
21167210	m	hp	CN	2022	0	1	0	0	1	0	0	0	0	0	0	0	62
21167509	m	cn	MY	2022	1	0	0	0	0	0	0	0	0	0	0	0	50.8
21167871	m	cn	MY	2022	1	0	0	0	0	0	0	0	0	0	0	0	78.8
21167900	f	md	GB	2022	0	1	1	0	0	0	0	0	0	0	0	0	
21168123	f	md	JP	2022	0	1	0	1	0	0	0	0	0	0	0	1	63
21168384	Not	md	JP	2022	0	1	0	0	0	1	0	0	0	0	0	0	80
21168734	f	md	CH	2022	0	1	1	0	0	0	0	0	0	0	0	0	61
21168947	m	md	JP	2022	0	1	0	0	1	0	0	0	0	0	0	0	82
21169029	f	md	JP	2022	0	1	0	0	1	0	0	0	0	0	0	0	63
21169143	m	md	CA	2022	0	0	0	0	0	0	0	0	0	0	0	0	93
21169238	Not	hp	KR	2022	0	0	0	0	0	0	0	0	0	0	0	0	
21169284	m	md	DE	2022	0	0	0	0	0	0	0	0	0	0	0	0	68
21169295	m	cn	CN	2022	0	1	0	0	1	0	0	0	0	0	0	0	78
21169985	m	md	VN	2022	0	1	0	0	0	1	0	0	0	0	0	0	40
21170505	Not	md	JP	2022	0	1	0	1	0	0	0	0	0	0	0	0	80

21170654	m	md	PL	2022	0	0	0	0	0	0	0	0	0	0	0	0	54
21170706	Not	md	US	2022	0	0	0	0	0	0	0	0	0	0	0	0	
21170862	m	hp	CN	2022	0	0	0	0	0	0	0	0	0	0	0	0	68
21171434	Not	md	DE	2022	0	1	1	0	0	0	0	0	0	0	0	1	
21171471	f	hp	ES	2022	1	0	0	0	0	0	0	0	0	0	0	0	68
21171885	f	md	US	2022	0	0	0	0	0	0	0	0	0	0	0	0	
21172045	m	md	JP	2022	0	1	0	0	1	0	0	0	0	0	0	0	70
21172095	m	cn	US	2022	1	0	0	0	0	0	0	0	0	0	0	0	65
21172362	Not	hp	DE	2022	1	0	0	0	0	0	0	0	0	0	0	0	
21172366	Not	hp	DE	2022	1	0	0	0	0	0	0	0	0	0	0	0	
21172367	Not	hp	US	2022	1	0	0	0	0	0	0	0	0	0	0	0	
21172369	Not	hp	DE	2022	1	0	0	0	0	0	0	0	0	0	0	0	
21172370	Not	hp	DE	2022	1	0	0	0	0	0	0	0	0	0	0	0	
21172448	m	ph	US	2022	0	0	0	0	0	0	0	0	0	0	0	0	35
21172481	m	ph	CH	2022	0	1	1	0	0	0	0	0	0	0	0	1	76
21172505	f	md	IT	2022	0	0	0	0	0	0	0	0	0	0	0	0	74
21172524	m	cn	CA	2022	0	0	0	0	0	0	0	0	0	0	0	0	91
21172527	m	cn	IN	2022	0	0	0	0	0	0	0	0	0	0	0	0	42
21172893	m	cn	IN	2022	0	0	0	0	0	0	0	0	0	0	0	0	62
21172972	Not	md	FR	2022	0	0	0	0	0	0	0	0	0	0	0	0	
21173179	Not	hp	DE	2022	1	0	0	0	0	0	0	0	0	0	0	0	
21173215	Not	hp	DE	2022	1	0	0	0	0	0	0	0	0	0	0	0	
21173347	m	cn	IT	2022	0	0	0	0	0	0	0	0	0	0	0	0	69
21173453	f	hp	US	2022	0	0	0	0	0	0	0	0	0	0	0	0	70
21173922	m	md	JP	2022	0	1	1	0	0	0	0	0	0	0	0	1	72
21174288	m	md	US	2022	0	1	1	0	0	0	0	0	0	0	0	0	65
21174292	Not	md	US	2022	0	0	0	0	0	0	0	0	0	0	0	0	
21174302	m	md	JP	2022	0	1	0	0	1	0	0	0	0	0	0	0	60
21174329	m	md	JP	2022	0	1	0	0	1	0	0	0	0	0	0	0	71
21174691	m	ph	JP	2022	0	0	0	0	0	0	0	0	0	0	0	0	90
21174878	f	cn	US	2022	0	0	0	0	0	0	0	0	0	0	0	0	55
21174883	m	cn	US	2022	0	0	0	0	0	0	0	0	0	0	0	0	
21174922	f	cn	US	2022	0	0	0	0	0	0	0	0	0	0	0	0	51
21174961	m	md	FR	2022	0	0	0	0	0	0	0	0	0	0	0	0	86

21175204	m	md	CN	2022	0	0	0	0	0	0	0	0	0	0	0	0	57
21175225	f	cn	US	2022	1	0	0	0	0	0	0	0	0	0	0	0	
21175439	Not	md	JP	2022	0	1	0	0	1	0	0	0	0	0	0	0	
21175451	m	ph	JP	2022	0	0	0	0	0	0	0	0	0	0	0	0	80
21175731	f	cn	US	2022	0	0	0	0	0	0	0	0	0	0	0	0	
21175943	m	md	JP	2022	0	1	1	0	0	0	0	0	0	0	0	0	38
21176048	f	cn	IN	2022	0	0	0	0	0	0	0	0	0	0	0	0	42
21176548	f	md	TH	2022	1	0	0	0	0	0	0	0	0	0	0	0	86
21178115	m	md	JP	2022	0	1	0	1	0	0	0	0	0	0	0	0	65
21178118	f	md	CA	2022	1	0	0	0	0	0	0	0	0	0	0	0	60.5
21178400	f	cn	SG	2022	0	1	0	1	0	0	0	0	0	0	0	0	
21178407	f	cn	JP	2022	1	0	0	0	0	0	0	0	0	0	0	0	
21178497	m	md	FR	2022	0	0	0	0	0	0	0	0	0	0	0	0	
21178510	f	ph	JP	2022	0	1	1	0	0	0	0	0	0	0	0	0	90
21179122	f	cn	DE	2022	0	1	1	0	0	0	0	0	0	0	0	1	66
21179337	m	hp	US	2022	0	1	1	0	0	0	0	0	0	0	0	0	65
21179376	m	md	JP	2022	1	0	0	0	0	0	0	0	0	0	0	1	
21179454	f	hp	DE	2022	0	1	0	0	1	0	0	0	0	0	0	0	54
21179667	m	md	CN	2022	0	0	0	0	0	0	0	0	0	0	0	0	70
21179727	m	md	RU	2022	0	0	0	0	0	0	0	0	0	0	0	0	72
21179808	f	cn	IN	2022	0	0	0	0	0	0	0	0	0	0	0	0	56
21179818	m	md	CN	2022	0	0	0	0	0	0	0	0	0	0	0	0	72
21179828	f	md	CN	2022	0	0	0	0	0	0	0	0	0	0	0	0	52
21180429	f	hp	TH	2022	1	0	0	0	0	0	0	0	0	0	0	0	68
21180510	f	hp	US	2022	0	0	0	0	0	0	0	0	0	0	0	0	55
21181187	f	md	BG	2022	0	1	0	0	0	1	0	0	0	0	0	0	56
21181653	m	hp	US	2022	0	0	0	0	0	0	0	0	0	0	0	0	70
21181798	f	md	FR	2022	0	1	0	0	0	1	0	0	0	0	0	0	68
21181828	m	md	IT	2022	0	1	0	0	0	1	0	0	0	0	0	0	78
21181838	Not	md	JP	2022	0	1	0	0	0	1	0	0	0	0	0	0	
21182040	f	md	CN	2022	0	0	0	0	0	0	0	0	0	0	0	0	32
21182637	m	md	JP	2022	0	1	0	0	1	0	0	0	0	0	0	0	80
21183231	f	md	DE	2022	0	1	1	0	0	0	0	0	0	0	0	1	66
21183302	Not	md	JP	2022	0	1	1	0	0	0	0	0	0	0	0	0	80

21183347	m	md	JP	2022	0	1	0	0	1	0	0	0	0	0	0	0	70
21183485	m	md	JP	2022	0	1	1	0	0	0	0	0	0	0	0	0	60
21183608	m	md	JP	2022	0	1	0	0	1	0	0	0	0	0	0	0	80
21183615	f	hp	AU	2022	0	1	0	0	0	1	0	0	0	0	0	0	
21183670	m	md	JP	2022	0	1	0	0	1	0	0	0	0	0	0	0	72
21183960	m	hp	NL	2022	0	0	0	0	0	0	0	0	0	0	0	0	72
21183961	f	hp	NL	2022	0	0	0	0	0	0	0	0	0	0	0	0	75
21184439	m	md	JP	2022	0	1	0	0	1	0	0	0	0	0	0	0	59
21185246	f	ph	FR	2022	0	0	0	0	0	0	0	0	0	0	0	0	
21186216	m	md	CO	2022	0	0	0	0	0	0	0	0	0	0	0	0	61
21186227	f	md	AT	2022	0	0	0	0	0	0	0	0	0	0	0	0	62
21186232	m	md	CN	2022	0	1	0	1	0	0	0	0	0	0	0	0	72.9
21186293	m	md	JP	2022	0	1	0	0	1	0	0	0	0	0	0	0	79
21186576	m	md	JP	2022	0	1	0	0	1	0	0	0	0	0	0	0	71
21186706	f	md	DE	2022	0	1	1	0	0	0	0	0	0	0	0	0	69
21186826	f	md	JP	2022	1	0	0	0	0	0	0	0	0	0	0	0	71
21186839	Not	md	JP	2022	0	1	0	0	0	1	0	0	0	0	0	0	67
21186871	f	md	JP	2022	0	1	0	0	1	0	0	0	0	0	0	0	75
21186876	f	md	AT	2022	0	1	1	0	0	0	0	0	0	0	0	0	74
21186923	m	md	JP	2022	0	1	0	0	0	1	0	0	0	0	0	0	
21186934	f	md	JP	2022	0	1	0	0	1	0	0	0	0	0	0	0	71
21187191	m	md	FR	2022	0	0	0	0	0	0	0	0	0	0	0	0	
21187364	m	cn	CN	2022	0	1	0	0	1	0	0	0	0	0	0	0	57
21187512	m	md	JP	2022	0	1	0	0	1	0	0	0	0	0	0	0	52
21187560	m	md	JP	2022	0	1	0	0	1	0	0	0	0	0	0	0	62
21187572	f	md	JP	2022	0	0	0	0	0	0	0	0	0	0	0	0	69
21187602	m	md	JP	2022	0	1	0	0	1	0	0	0	0	0	0	0	45
21188129	m	md	JP	2022	0	1	0	0	1	0	0	0	0	0	0	0	70
21188130	m	md	JP	2022	0	1	0	0	1	0	0	0	0	0	0	0	70
21188238	m	md	NL	2022	0	0	0	0	0	0	0	0	0	0	0	0	53
21188301	f	md	JP	2022	0	1	0	0	1	0	0	0	0	0	0	0	67
21188303	m	md	JP	2022	0	1	0	0	1	0	0	0	0	0	0	0	80
21188315	f	md	JP	2022	0	1	0	0	1	0	0	0	0	0	0	0	67
21188394	Not	hp	US	2022	0	0	0	0	0	0	0	0	0	0	0	0	

21188404	f	md	DE	2022	0	1	1	0	0	0	0	0	0	0	0	0	
21188406	f	md	JP	2022	0	1	0	0	0	1	0	0	0	0	0	0	80
21188626	f	cn	US	2022	0	0	0	0	0	0	0	0	0	0	0	0	
21188629	f	md	DE	2022	0	1	1	0	0	0	0	0	0	0	0	1	66
21188714	m	md	NL	2022	0	0	0	0	0	0	0	0	0	0	0	0	53
21188797	f	ph	FR	2022	0	1	0	0	0	1	0	0	0	0	0	0	62
21188799	m	md	JP	2022	0	1	1	0	0	0	0	0	0	0	0	0	76
21188841	m	md	JP	2022	0	1	0	0	1	0	0	0	0	0	0	0	69
21188861	m	md	GB	2022	0	1	0	0	0	1	0	0	0	0	0	0	73
21188920	f	md	JP	2022	0	1	0	0	1	0	0	0	0	0	0	0	72
21189000	m	md	FR	2022	0	1	0	0	0	1	0	0	0	0	0	0	59
21189138	f	md	CN	2022	0	0	0	0	0	0	0	0	0	0	0	0	69
21189804	f	cn	IN	2022	0	0	0	0	0	0	0	0	0	0	0	0	36
21190218	f	md	FR	2022	0	1	0	0	0	1	0	0	0	0	0	0	68
21190470	m	md	DE	2022	0	1	0	0	1	0	0	0	0	0	0	0	
21190471	m	cn	IN	2022	0	1	0	0	1	0	0	0	0	0	0	0	48
21190580	m	md	NL	2022	0	0	0	0	0	0	0	0	0	0	0	0	75
21190597	Not	ph	JP	2022	1	0	0	0	0	0	0	0	0	0	0	0	
21190727	f	md	IT	2022	0	0	0	0	0	0	0	0	0	0	0	0	83
21190885	m	md	JP	2022	0	1	0	0	1	0	0	0	0	0	0	0	62
21190905	f	md	IT	2022	0	0	0	0	0	0	0	0	0	0	0	0	58
21190915	m	md	JP	2022	0	1	0	0	1	0	0	0	0	0	0	0	74
21191322	f	md	CN	2022	0	0	0	0	0	0	0	0	0	0	0	0	55
21191681	Not	cn	SG	2022	1	0	0	0	0	0	0	0	0	0	0	0	
21191953	f	md	JP	2022	1	0	0	0	0	0	0	0	0	0	0	0	63
21191954	Not	md	JP	2022	0	1	0	1	0	0	0	0	0	0	0	0	
21192038	f	md	AT	2022	0	1	1	0	0	0	0	0	0	0	0	0	56
21192060	Not	hp	JP	2022	0	1	0	1	0	0	0	0	0	0	0	1	
21192167	f	md	TW	2022	0	0	0	0	0	0	0	0	0	0	0	0	47
21192174	m	md	JP	2022	1	0	0	0	0	0	0	0	0	0	0	0	75
21192279	m	hp	US	2022	0	1	1	0	0	0	0	0	0	0	0	0	61
21192661	f	md	CN	2022	0	0	0	0	0	0	0	0	0	0	0	0	69
21192707	m	md	JP	2022	0	0	0	0	0	0	0	0	0	0	0	0	40
21192740	f	md	JP	2022	0	1	0	0	1	0	0	0	0	0	0	0	71

21192980	m	md	JP	2022	0	1	0	0	0	1	0	0	0	0	0	0	70
21193244	f	md	JP	2022	0	1	0	0	1	0	0	0	0	0	0	0	79
21193412	m	md	IQ	2022	0	1	1	0	0	0	0	0	0	0	0	0	
21193531	m	md	JP	2022	0	1	1	0	0	0	0	0	0	0	0	0	76
21193581	m	md	PL	2022	0	0	0	0	0	0	0	0	0	0	0	0	39
21193669	m	hp	ES	2022	0	0	0	0	0	0	0	0	0	0	0	0	49
21193916	m	md	KR	2022	0	1	0	0	0	1	0	0	0	0	0	0	84
21194077	f	md	JP	2022	0	1	0	0	1	0	0	0	0	0	0	0	76
21194182	f	md	US	2022	0	0	0	0	0	0	0	0	0	0	0	0	48
21194213	f	md	MY	2022	0	1	0	0	0	1	0	0	0	0	0	0	76
21194766	m	cn	KR	2022	0	0	0	0	0	0	0	0	0	0	0	0	70
21196170	m	md	CN	2022	0	0	0	0	0	0	0	0	0	0	0	0	61
21196440	m	md	JP	2022	0	0	0	0	0	0	0	0	0	0	0	0	78
21196880	Not	md	JP	2022	1	0	0	0	0	0	0	0	0	0	0	0	
21197188	f	md	JP	2022	0	1	1	0	0	0	0	0	0	0	0	0	60
21197755	m	md	IL	2022	0	1	1	0	0	0	0	0	0	0	0	0	
21197758	m	md	IL	2022	0	1	1	0	0	0	0	0	0	0	0	0	
21197765	m	md	SG	2022	0	1	0	0	0	1	0	0	0	0	0	0	66
21198130	Not	md	JP	2022	1	0	0	0	0	0	0	0	0	0	0	0	
21198512	f	cn	US	2022	0	1	0	0	1	0	0	0	0	0	0	0	78
21199051	m	cn	KR	2022	0	0	0	0	0	0	0	0	0	0	0	0	66
21199480	m	cn	CN	2022	0	0	0	0	0	0	0	0	0	0	0	0	
21199718	m	md	CN	2022	0	0	0	0	0	0	0	0	0	0	0	0	65
21200226	m	hp	US	2022	0	1	1	0	0	0	0	0	0	0	0	0	65
21200717	f	cn	US	2022	0	0	0	0	0	0	0	0	0	0	0	0	
21201079	Not	md	JP	2022	0	0	0	0	0	0	0	0	0	0	0	0	90
21201441	Not	hp	US	2022	0	0	0	0	0	0	0	0	0	0	0	0	
21201973	Not	ph	JP	2022	1	0	0	0	0	0	0	0	0	0	0	0	
21203152	m	md	JP	2022	0	1	1	0	0	0	0	0	0	0	0	0	
21203681	Not	md	JP	2022	0	1	0	1	0	0	0	0	0	0	0	0	
21203845	f	md	AU	2022	1	0	0	0	0	0	0	0	0	0	0	0	
21203971	f	md	CN	2022	1	0	0	0	0	0	0	0	0	0	0	0	76
21204512	m	md	JP	2022	0	1	0	0	0	1	0	0	0	0	0	0	76
21204622	f	md	FR	2022	1	0	0	0	0	0	0	0	0	0	0	0	

21205527	f	md	CA	2022	1	0	0	0	0	0	0	0	0	0	0	0	
21205571	f	cn	US	2022	1	0	0	0	0	0	0	0	0	0	0	0	57
21205819	Not	hp	US	2022	0	0	0	0	0	0	0	0	0	0	0	0	
21205954	Not	hp	US	2022	0	1	1	0	0	0	0	0	0	0	0	0	
21205957	Not	hp	US	2022	0	1	1	0	0	0	0	0	0	0	0	0	
21206413	m	cn	US	2022	0	1	0	1	0	0	0	0	0	0	0	0	
21206429	f	ph	NL	2022	0	1	1	0	0	0	0	0	0	0	0	0	52
21207283	m	ph	IE	2022	0	0	0	0	0	0	0	0	0	0	0	0	64
21207827	m	hp	US	2022	0	1	1	0	0	0	0	0	0	0	0	0	65
21208359	m	cn	IN	2022	0	0	0	0	0	0	0	0	0	0	0	0	52
21208479	m	md	KR	2022	0	0	0	0	0	0	0	0	0	0	0	0	55
21208485	m	md	JP	2022	0	1	0	0	1	0	0	0	0	0	0	1	72
21208752	f	md	NL	2022	0	0	0	0	0	0	0	0	0	0	0	0	66
21209076	f	cn	US	2022	1	0	0	0	0	0	0	0	0	0	0	0	52
21209133	Not	cn	SG	2022	1	0	0	0	0	0	0	0	0	0	0	0	76.1
21209725	f	md	US	2022	0	0	0	0	0	0	0	0	0	0	0	0	
21209736	Not	hp	CZ	2022	0	0	0	0	0	0	0	0	0	0	0	0	
21209904	Not	hp	US	2022	0	0	0	0	0	0	0	0	0	0	0	0	
21210248	m	cn	IN	2022	0	0	0	0	0	0	0	0	0	0	0	0	62
21211380	m	md	JP	2022	0	1	0	0	1	0	0	0	0	0	0	0	77
21211590	Not	md	JP	2022	0	1	0	0	1	0	0	0	0	0	0	0	70
21211914	m	md	CN	2022	0	1	1	0	0	0	0	0	0	0	0	0	66
21212122	m	md	JP	2022	0	1	1	0	0	0	0	0	0	0	0	0	80
21212452	m	Not repo	US	2022	0	1	0	0	1	0	0	0	0	0	0	0	84
21212793	f	md	JP	2022	0	1	0	1	0	0	0	0	0	0	0	0	
21214456	m	md	CN	2022	0	0	0	0	0	0	0	0	0	0	0	0	67
21214594	m	cn	US	2022	0	0	0	0	0	0	0	0	0	0	0	0	
21214917	f	md	RU	2022	0	1	0	0	0	1	0	0	0	0	0	0	41
21215412	Not	md	US	2022	0	0	0	0	0	0	0	0	0	0	0	0	
21216254	m	ph	US	2022	0	1	1	0	0	0	0	0	0	0	0	0	75
21216308	f	md	JP	2022	0	0	0	0	0	0	0	0	0	0	0	0	
21216529	m	cn	US	2022	0	0	0	0	0	0	0	0	0	0	0	0	43
21216690	f	hp	TH	2022	1	0	0	0	0	0	0	0	0	0	0	0	101
21216731	f	cn	US	2022	0	0	0	0	0	0	0	0	0	0	0	0	60

21216843	Not	md	US	2022	0	0	0	0	0	0	0	0	0	0	0	0	
21216852	f	ph	US	2022	0	0	0	0	0	0	0	0	0	0	0	0	64
21216856	f	hp	US	2022	0	0	0	0	0	0	0	0	0	0	0	0	
21216857	f	ph	US	2022	0	0	0	0	0	0	0	0	0	0	0	0	
21216885	f	hp	US	2022	1	0	0	0	0	0	0	0	0	0	0	0	78
21216887	m	md	US	2022	0	0	0	0	0	0	0	0	0	0	0	0	
21216888	f	md	US	2022	0	0	0	0	0	0	0	0	0	0	0	0	
21216894	m	cn	US	2022	0	0	0	0	0	0	0	0	0	0	0	0	61
21216901	f	cn	US	2022	0	0	0	0	0	0	0	0	0	0	0	0	68
21216914	m	cn	ID	2022	1	0	0	0	0	0	0	0	0	0	0	0	75.5
21216919	Not	cn	SG	2022	1	0	0	0	0	0	0	0	0	0	0	0	65.3
21216933	Not	ph	US	2022	0	0	0	0	0	0	0	0	0	0	0	0	
21217018	Not	md	US	2022	0	0	0	0	0	0	0	0	0	0	0	0	
21217027	m	md	US	2022	0	0	0	0	0	0	0	0	0	0	0	0	83
21217028	f	ph	US	2022	0	0	0	0	0	0	0	0	0	0	0	0	64
21217039	f	cn	US	2022	0	0	0	0	0	0	0	0	0	0	0	0	
21217052	Not	ph	US	2022	0	0	0	0	0	0	0	0	0	0	0	0	
21217062	m	md	US	2022	0	0	0	0	0	0	0	0	0	0	0	0	74
21217092	f	hp	US	2022	0	0	0	0	0	0	0	0	0	0	0	0	37
21217303	m	md	JP	2022	0	1	1	0	0	0	0	0	0	0	0	0	80
21217486	m	md	AU	2022	0	1	1	0	0	0	0	0	0	0	0	0	68
21217534	Not	md	JP	2022	0	0	0	0	0	0	0	0	0	0	0	0	
21217697	m	md	AU	2022	0	0	0	0	0	0	1	0	0	0	1	0	69
21217891	f	cn	US	2022	0	0	0	0	0	0	0	0	0	0	0	0	
21217895	Not	md	US	2022	0	0	0	0	0	0	0	0	0	0	0	0	
21217898	m	cn	US	2022	0	0	0	0	0	0	0	0	0	0	0	0	
21217906	m	cn	US	2022	0	0	0	0	0	0	0	0	0	0	0	0	49
21217908	f	cn	US	2022	0	0	0	0	0	0	0	0	0	0	0	0	
21217911	m	cn	US	2022	0	0	0	0	0	0	0	0	0	0	0	0	61
21217912	f	cn	US	2022	0	0	0	0	0	0	0	0	0	0	0	0	
21217934	f	ph	US	2022	0	0	0	0	0	0	0	0	0	0	0	0	89
21217938	f	cn	US	2022	0	0	0	0	0	0	0	0	0	0	0	0	
21217947	m	cn	US	2022	0	0	0	0	0	0	0	0	0	0	0	0	63
21217952	Not	cn	US	2022	0	0	0	0	0	0	0	0	0	0	0	0	

21217954	f	hp	US	2022	0	0	0	0	0	0	0	0	0	0	0	0	78
21218025	f	md	US	2022	0	0	0	0	0	0	0	0	0	0	0	0	29
21218216	Not	cn	SG	2022	1	0	0	0	0	0	0	0	0	0	0	0	71.4
21218538	Not	cn	SG	2022	1	0	0	0	0	0	0	0	0	0	0	0	85.6
21218539	m	cn	CA	2022	1	0	0	0	0	0	0	0	0	0	0	0	
21218540	f	md	AU	2022	1	0	0	0	0	0	0	0	0	0	0	0	63
21218598	f	md	RU	2022	0	1	0	0	0	1	0	0	0	0	0	0	64
21218755	m	md	DE	2022	0	0	0	0	0	0	0	0	0	0	0	0	
21218771	m	hp	US	2022	0	1	0	0	1	0	0	0	0	0	0	1	
21219005	f	md	JP	2022	0	1	0	0	1	0	0	0	0	0	0	0	60
21219128	f	hp	BR	2022	0	0	0	0	0	0	0	0	0	0	0	0	70
21219197	Not	md	HK	2022	0	1	0	0	1	0	0	0	0	0	0	0	
21219352	f	hp	TH	2022	1	0	0	0	0	0	0	0	0	0	0	0	77
21219356	f	md	CN	2022	0	0	0	0	0	0	0	0	0	0	0	0	69
21219732	m	md	US	2022	0	0	0	0	0	0	0	0	0	0	0	0	65
21220061	m	md	JP	2022	0	0	0	0	0	0	0	0	0	0	0	0	40
21220131	m	md	US	2022	0	0	0	0	0	0	0	0	0	0	0	0	80
21220580	f	ph	US	2022	0	0	0	0	0	0	0	0	0	0	0	0	
21220590	m	md	CN	2022	0	0	0	0	0	0	0	0	0	0	0	0	68
21220658	f	md	JP	2022	0	1	0	1	0	0	0	0	0	0	0	0	48
21220950	m	md	US	2022	0	0	0	0	0	0	0	0	0	0	0	0	77
21221263	Not	cn	SG	2022	1	0	0	0	0	0	0	0	0	0	0	0	76.2
21221567	m	hp	FR	2022	0	1	1	0	0	0	0	0	0	0	0	0	74
21221969	f	md	JP	2022	1	0	0	0	0	0	0	0	0	0	0	0	63
21222045	f	md	AT	2022	0	1	1	0	0	0	0	0	0	0	0	0	74
21222911	Not	cn	SG	2022	1	0	0	0	0	0	0	0	0	0	0	0	76.5
21223291	f	cn	FR	2022	1	0	0	0	0	0	0	0	0	0	0	0	
21223416	m	md	JP	2022	0	0	0	0	0	0	0	0	0	0	0	0	
21223470	m	cn	KR	2022	0	0	0	0	0	0	0	0	0	0	0	0	54
21223503	m	md	CN	2022	0	0	0	0	0	0	0	0	0	0	0	0	58
21223675	m	cn	CA	2022	1	0	0	0	0	0	0	0	0	0	0	0	
21223809	m	md	JP	2022	0	1	0	1	0	0	0	0	0	0	0	0	70
21224211	m	md	JP	2022	0	1	0	0	1	0	0	0	0	0	0	0	71
21224213	m	hp	US	2022	0	1	1	0	0	0	0	0	0	0	0	0	65

21225184	Not	md	JP	2022	0	0	0	0	0	0	0	0	0	0	0	0	
21225217	m	md	JP	2022	0	1	0	0	1	0	0	0	0	0	0	0	47
21225239	f	md	JP	2022	0	1	0	0	1	0	0	0	0	0	0	0	62
21225437	m	hp	CN	2022	0	0	0	0	0	0	0	0	0	0	0	0	71
21225493	m	md	JP	2022	0	1	0	0	1	0	0	0	0	0	0	0	50
21225529	m	md	JP	2022	0	1	0	1	0	0	0	0	0	0	0	0	71
21225975	f	cn	CA	2022	0	1	0	0	1	0	0	0	0	0	0	0	73
21226332	m	hp	US	2022	0	1	0	0	1	0	0	0	0	0	0	0	74
21226573	f	md	VN	2022	0	1	0	0	0	1	0	0	0	0	0	0	53
21226607	Not	md	US	2022	0	1	0	0	1	0	0	0	0	0	0	0	
21226930	m	md	JP	2022	0	1	0	0	1	0	0	0	0	0	0	0	79
21227154	m	cn	GB	2022	0	0	0	0	0	0	1	0	0	0	1	0	69
21227320	m	md	US	2022	0	0	0	0	0	0	0	0	0	0	0	0	65
21229364	m	md	IN	2022	0	0	0	0	0	0	0	0	0	0	0	0	77
21229460	Not	md	US	2022	0	1	0	0	1	0	0	0	0	0	0	0	
21229578	f	md	JP	2022	0	1	1	0	0	0	0	0	0	0	0	0	90
21229612	Not	md	AU	2022	1	0	0	0	0	0	0	0	0	0	0	0	
21229655	Not	hp	IT	2022	0	1	1	0	0	0	0	0	0	0	0	0	
21229660	Not	hp	IT	2022	0	1	1	0	0	0	0	0	0	0	0	0	
21230115	m	md	CN	2022	0	0	0	0	0	0	0	0	0	0	0	0	64
21230246	m	md	DE	2022	0	1	0	1	0	0	0	0	0	0	0	0	61
21230249	Not	hp	CN	2022	0	0	0	0	0	0	0	0	0	0	0	0	
21230253	f	md	CN	2022	0	0	0	0	0	0	0	0	0	0	0	0	54
21230314	f	md	DE	2022	1	0	0	0	0	0	0	0	0	0	0	0	
21230513	Not	md	JP	2022	0	1	0	0	1	0	0	0	0	0	0	0	
21230800	f	hp	US	2022	0	1	0	0	1	0	0	0	0	0	0	0	60
21231577	f	hp	JP	2022	1	0	0	0	0	0	0	0	0	0	0	0	80
21232615	m	md	DE	2022	0	0	0	0	0	0	0	0	0	0	0	0	82
21232616	f	md	TH	2022	0	1	0	1	0	0	0	0	0	0	0	0	52
21232670	f	cn	CN	2022	1	0	0	0	0	0	0	0	0	0	0	0	75
21232706	m	cn	AR	2022	0	0	0	0	0	0	0	0	0	0	0	0	55
21232881	m	cn	US	2022	0	0	0	0	0	0	0	0	0	0	0	0	
21233160	f	md	VN	2022	0	0	0	0	0	0	0	0	0	0	0	0	45
21233373	f	cn	US	2022	0	0	0	0	0	0	0	0	0	0	0	0	

21233420	m	md	JP	2022	0	0	0	0	0	0	0	0	0	0	0	0	
21233453	m	cn	FR	2022	0	0	0	0	0	0	0	0	0	0	0	0	
21233508	m	md	IQ	2022	0	1	1	0	0	0	0	0	0	0	0	0	56
21233562	f	md	KR	2022	0	0	0	0	0	0	0	0	0	0	0	0	60
21233839	f	hp	FR	2022	0	1	0	0	1	0	0	0	0	0	0	0	81
21233937	m	md	KR	2022	1	0	0	0	0	0	0	0	0	0	0	0	60
21233984	Not	md	JP	2022	1	0	0	0	0	0	0	0	0	0	0	0	51
21234040	f	cn	TH	2022	1	0	0	0	0	0	0	0	0	0	0	0	61.7
21234042	m	md	AU	2022	1	0	0	0	0	0	0	0	0	0	0	0	
21234067	f	cn	US	2022	1	0	0	0	0	0	0	0	0	0	0	0	74
21234076	f	md	JP	2022	0	0	0	0	0	0	0	0	0	0	0	0	
21234132	f	md	TH	2022	0	1	0	1	0	0	0	0	0	0	0	0	52
21234333	f	md	BE	2022	1	0	0	0	0	0	0	0	0	0	0	0	53
21234349	f	md	CN	2022	0	0	0	0	0	0	0	0	0	0	0	0	53
21234445	Not	md	US	2022	0	1	0	0	1	0	0	0	0	0	0	0	
21234747	f	ph	FR	2022	0	0	0	0	0	0	0	0	0	0	0	0	80
21234837	m	md	NL	2022	0	0	0	0	0	0	0	0	0	0	0	0	45
21235446	m	md	JP	2022	0	0	0	0	0	0	0	0	0	0	0	0	40
21236065	f	md	JP	2022	0	1	0	0	1	0	0	0	0	0	0	0	78
21236194	m	cn	US	2022	0	0	0	0	0	0	0	0	0	0	0	0	66
21236416	m	md	JP	2022	0	0	0	0	0	0	0	0	0	0	0	0	80
21236536	Not	md	JP	2022	0	1	0	0	1	0	0	0	0	0	0	0	
21236537	m	md	JP	2022	0	1	0	0	1	0	0	0	0	0	0	0	68
21236842	Not	cn	CN	2022	0	0	0	0	0	0	0	0	0	0	0	0	
21237073	m	hp	RU	2022	0	1	0	0	0	1	0	0	0	0	0	0	62
21237624	f	hp	JP	2022	0	0	0	0	0	0	0	0	0	0	0	0	57
21237861	f	cn	PH	2022	0	0	0	0	0	0	0	0	0	0	0	0	72
21238707	Not	md	JP	2022	0	0	0	0	0	0	0	0	0	0	0	0	
21238765	m	cn	IN	2022	0	1	0	0	1	0	0	0	0	0	0	0	46
21238888	m	md	IT	2022	0	0	0	0	0	0	0	0	0	0	0	0	
21239063	Not	md	US	2022	1	0	0	0	0	0	0	0	0	0	0	0	
21239571	f	cn	TH	2022	1	0	0	0	0	0	0	0	0	0	0	0	73.7
21239792	m	md	AU	2022	0	1	0	0	0	1	0	0	0	0	0	0	65
21239845	f	md	CA	2022	1	0	0	0	0	0	0	0	0	0	0	0	

21240043	m	md	ES	2022	0	0	0	0	0	0	0	0	0	0	0	0	66
21240430	f	md	JP	2022	0	1	0	0	1	0	0	0	0	0	0	0	63
21240439	m	md	JP	2022	0	1	0	0	1	0	0	0	0	0	0	0	65
21240978	f	cn	US	2022	0	0	0	0	0	0	0	0	0	0	0	0	
21241940	f	md	DE	2022	0	1	1	0	0	0	0	0	0	0	0	0	77
21242115	f	ph	CA	2022	1	0	0	0	0	0	0	0	0	0	0	0	
21242998	Not	hp	IT	2022	0	1	1	0	0	0	0	0	0	0	0	0	
21243001	Not	md	GB	2022	0	0	0	0	0	0	0	0	0	0	0	0	
21243129	f	ph	TH	2022	1	0	0	0	0	0	0	0	0	0	0	0	
21243143	m	md	ID	2022	0	0	0	0	0	0	0	0	0	0	0	0	
21243289	m	cn	CA	2022	0	0	0	0	0	0	0	0	0	0	0	0	47
21243348	f	cn	US	2022	0	0	0	0	0	0	0	0	0	0	0	0	55
21243560	m	md	CN	2022	0	0	0	0	0	0	0	0	0	0	0	0	64
21243650	m	md	JP	2022	0	0	0	0	0	0	0	0	0	0	0	0	64
21243735	f	md	JP	2022	0	1	0	0	0	1	0	0	0	0	0	0	60
21243843	m	md	PT	2022	0	1	0	0	0	1	0	0	0	0	0	0	64
21243844	Not	cn	CN	2022	0	0	0	0	0	0	0	0	0	0	0	0	
21244042	m	md	JP	2022	0	0	0	0	0	0	0	0	0	0	0	0	63
21244061	Not	hp	US	2022	0	0	0	0	0	0	0	0	0	0	0	0	
21244068	Not	md	FR	2022	0	0	0	0	0	0	0	0	0	0	0	0	
21244103	m	md	JP	2022	0	0	0	0	0	0	0	0	0	0	0	0	
21244111	m	md	RU	2022	0	0	0	0	0	0	0	0	0	0	0	0	66
21244942	m	md	MY	2022	0	0	0	0	0	0	0	0	0	0	0	0	69
21245018	f	cn	CN	2022	0	1	0	1	0	0	0	0	0	0	0	0	64.5
21245142	m	cn	GR	2022	0	0	0	0	0	0	0	0	0	0	0	0	62
21245158	Not	md	AU	2022	0	0	0	0	0	0	0	0	0	0	0	0	
21245764	m	md	US	2022	0	0	0	0	0	0	0	0	0	0	0	0	
21245867	f	md	JP	2022	0	1	0	0	1	0	0	0	0	0	0	0	52
21247057	m	md	CN	2022	0	0	0	0	0	0	0	0	0	0	0	0	77
21247218	f	md	AT	2022	0	1	1	0	0	0	0	0	0	0	0	0	56
21247239	f	md	DE	2022	0	1	1	0	0	0	0	0	0	0	0	1	66
21247252	Not	md	US	2022	0	0	0	0	0	0	0	0	0	0	0	0	
21247325	m	md	DE	2022	0	0	0	0	0	0	0	0	0	0	0	0	72
21247363	m	md	JP	2022	1	0	0	0	0	0	0	0	0	0	0	0	

21247412	f	ph	US	2022	0	0	0	0	0	0	0	0	0	0	0	0	58
21247499	m	hp	KR	2022	0	0	0	0	0	0	0	0	0	0	0	0	53
21247758	f	ph	TH	2022	1	0	0	0	0	0	0	0	0	0	0	0	54
21248276	m	hp	US	2022	0	0	0	0	0	0	0	0	0	0	0	0	
21248349	f	md	DE	2022	0	1	1	0	0	0	0	0	0	0	0	1	
21248565	f	md	TR	2022	0	0	0	0	0	0	0	0	0	0	0	0	65
21248854	f	hp	US	2022	1	0	0	0	0	0	0	0	0	0	0	0	57
21249155	f	cn	KR	2022	0	0	0	0	0	0	0	0	0	0	0	0	63
21249790	Not	md	KR	2022	1	0	0	0	0	0	0	0	0	0	0	0	
21249942	m	md	FR	2022	0	1	1	0	0	0	0	0	0	0	0	0	55
21250591	m	md	IQ	2022	0	1	1	0	0	0	0	0	0	0	0	0	53
21251184	f	hp	BE	2022	0	1	0	0	1	0	0	0	0	0	0	0	68
21251450	m	hp	US	2022	0	0	0	0	0	0	0	0	0	0	0	0	48
21251700	m	md	JP	2022	0	1	0	0	1	0	0	0	0	0	0	0	72
21252176	f	hp	AU	2022	0	0	0	0	0	0	0	0	0	0	0	0	46
21252248	f	md	IT	2022	0	1	0	0	0	1	0	0	0	0	0	0	72
21252401	f	hp	US	2022	0	1	0	0	1	0	0	0	0	0	0	0	33
21252508	Not	md	JP	2022	1	0	0	0	0	0	0	0	0	0	0	0	
21252561	m	md	RU	2022	0	1	0	0	0	1	0	0	0	0	0	0	58
21252727	Not	md	JP	2022	0	1	0	0	1	0	0	0	0	0	0	0	
21252846	m	md	JP	2022	1	0	0	0	0	0	0	0	0	0	0	0	77
21252896	Not	md	US	2022	0	0	0	0	0	0	0	0	0	0	0	0	
21252936	m	ph	JP	2022	0	0	0	0	0	0	0	0	0	0	0	0	71
21253037	m	hp	US	2022	0	1	1	0	0	0	0	0	0	0	0	0	65
21253290	m	md	JP	2022	0	0	0	0	0	0	0	0	0	0	0	0	
21253972	m	ph	JP	2022	1	0	0	0	0	0	0	0	0	0	0	0	86
21254109	m	md	JP	2022	1	0	0	0	0	0	0	0	0	0	0	0	70
21254227	f	hp	US	2022	1	0	0	0	0	0	0	0	0	0	0	0	68.7
21254401	Not	ph	FR	2022	0	0	0	0	0	0	0	0	0	0	0	0	
21254402	Not	ph	FR	2022	0	0	0	0	0	0	0	0	0	0	0	0	
21254410	f	cn	US	2022	1	0	0	0	0	0	0	0	0	0	0	0	
21254705	m	hp	TH	2022	1	0	0	0	0	0	0	0	0	0	0	0	66.6
21254859	m	hp	SG	2022	0	1	0	1	0	0	0	0	0	0	0	0	52
21255110	m	cn	US	2022	0	0	0	0	0	0	0	0	0	0	0	0	67

21255275	m	hp	DE	2022	0	1	0	0	1	0	0	0	0	0	0	0	77
21255795	f	md	US	2022	0	0	0	0	0	0	0	0	0	0	0	0	59
21256688	m	cn	ID	2022	1	0	0	0	0	0	0	0	0	0	0	0	49.2
21256808	m	cn	US	2022	0	0	0	0	0	0	0	0	0	0	0	0	
21256847	m	md	JP	2022	0	1	0	0	1	0	0	0	0	0	0	0	48
21256905	Not	ph	FR	2022	0	0	0	0	0	0	0	0	0	0	0	0	
21256942	m	cn	TH	2022	0	1	0	0	1	0	0	0	0	0	0	0	74
21257419	m	md	JP	2022	0	0	0	0	0	0	0	0	0	0	0	0	57
21257555	m	md	NL	2022	0	0	0	0	0	0	0	0	0	0	0	0	45
21257620	f	md	CN	2022	0	0	0	0	0	0	0	0	0	0	0	0	69
21257874	f	cn	IN	2022	0	0	0	0	0	0	0	0	0	0	0	0	43
21257888	f	cn	IN	2022	0	0	0	0	0	0	0	0	0	0	0	0	74
21258083	Not	md	JP	2022	0	0	0	0	0	0	0	0	0	0	0	0	
21258220	m	cn	IR	2022	1	0	0	0	0	0	0	0	0	0	0	0	60
21258444	m	cn	CN	2022	0	1	0	0	1	0	0	0	0	0	0	0	64
21258836	m	md	FR	2022	1	0	0	0	0	0	0	0	0	0	0	0	80
21258967	f	md	JP	2022	1	0	0	0	0	0	0	0	0	0	0	0	70
21259181	Not	md	TH	2022	0	0	0	0	0	0	0	0	0	0	0	0	
21259357	m	md	DE	2022	0	1	0	0	1	0	0	0	0	0	0	0	70
21259422	Not	md	TH	2022	0	0	0	0	0	0	0	0	0	0	0	0	
21259578	Not	md	JP	2022	0	0	0	0	0	0	0	0	0	0	0	0	
21259865	f	md	FR	2022	1	0	0	0	0	0	0	0	0	0	0	0	
21259896	f	md	CN	2022	0	1	1	0	0	0	0	0	0	0	0	0	55
21260263	m	md	JP	2022	0	1	0	0	1	0	0	0	0	0	0	0	62
21260589	f	md	PL	2022	0	1	1	0	0	0	0	0	0	0	0	0	
21260604	f	cn	TH	2022	1	0	0	0	0	0	0	0	0	0	0	0	54
21260814	f	ph	MY	2022	1	0	0	0	0	0	0	0	0	0	0	0	60.9
21261124	f	cn	US	2022	0	1	1	0	0	0	0	0	0	0	0	0	
21261647	m	md	US	2022	0	0	0	0	0	0	0	0	0	0	0	0	79
21261762	Not	hp	US	2022	0	0	0	0	0	0	0	0	0	0	0	0	
21261784	m	md	JP	2022	0	0	0	0	0	0	0	0	0	0	0	0	
21261860	m	md	BE	2022	0	0	0	0	0	0	0	0	0	0	0	0	
21262920	m	md	DE	2022	0	1	0	0	0	1	0	0	0	0	0	0	63
21262927	m	cn	IN	2022	0	0	0	0	0	0	0	0	0	0	0	0	82

21262980	f	md	JP	2022	0	0	0	0	0	0	0	0	0	0	0	0	70
21263098	f	md	JP	2022	0	1	0	0	1	0	0	0	0	0	0	0	76
21263120	m	hp	ES	2022	0	1	0	0	1	0	0	0	0	0	0	0	77
21263137	Not	md	JP	2022	0	1	0	0	1	0	0	0	0	0	0	0	
21263168	m	md	JP	2022	0	1	0	0	1	0	0	0	0	0	0	0	68
21263710	m	md	JP	2022	0	1	0	0	0	1	0	0	0	0	0	0	80
21263831	f	md	JP	2022	0	1	0	0	0	1	0	0	0	0	0	0	90
21264177	f	md	NL	2022	0	0	0	0	0	0	0	0	0	0	0	0	66
21264210	m	md	TR	2022	0	0	0	0	0	0	0	0	0	0	0	0	42
21264344	m	md	JP	2022	0	1	0	0	1	0	0	0	0	0	0	0	80
21264808	m	hp	US	2022	0	1	1	0	0	0	0	0	0	0	0	0	74
21265814	m	md	DE	2022	0	1	0	0	1	0	0	0	0	0	0	0	80
21265988	f	md	JP	2022	0	0	0	0	0	0	0	0	0	0	0	0	50
21266528	m	cn	BR	2022	1	0	0	0	0	0	0	0	0	0	0	0	70
21266731	m	md	JP	2022	0	1	0	1	0	0	0	0	0	0	0	0	60
21266980	m	cn	JP	2022	0	0	0	0	0	0	0	0	0	0	0	0	53
21266981	f	cn	US	2022	1	0	0	0	0	0	0	0	0	0	0	0	61
21266983	m	ph	FR	2022	0	1	1	0	0	0	0	0	0	0	0	0	74
21267692	m	md	IT	2022	0	0	0	0	0	0	0	0	0	0	0	0	62
21268209	f	ph	JP	2022	0	1	0	0	1	0	0	0	0	0	0	0	76
21269191	m	ph	FR	2022	0	1	1	0	0	0	0	0	0	0	0	0	74
21269312	f	md	LB	2022	0	1	0	0	1	0	0	0	0	0	0	0	
21269419	f	hp	JP	2022	0	0	0	0	0	0	0	0	0	0	0	0	64
21269511	f	md	JP	2022	0	1	0	0	0	1	0	0	0	0	0	0	66
21269654	m	md	FR	2022	0	1	0	0	1	0	0	0	0	0	0	0	70
21269742	f	cn	CN	2022	0	0	0	0	0	0	0	0	0	0	0	0	58
21269805	f	md	JP	2022	0	1	0	0	0	1	0	0	0	0	0	0	80
21270234	m	cn	CN	2022	0	1	0	1	0	0	0	0	0	0	0	0	72
21270370	f	cn	JP	2022	1	0	0	0	0	0	0	0	0	0	0	0	85
21270380	m	cn	CN	2022	0	1	0	0	1	0	0	0	0	0	0	0	67
21270608	f	hp	US	2022	0	0	0	0	0	0	0	0	0	0	0	0	77
21270626	m	md	JP	2022	0	0	0	0	0	0	0	0	0	0	0	0	75
21270658	m	cn	ID	2022	1	0	0	0	0	0	0	0	0	0	0	0	55
21270852	m	hp	FR	2022	0	1	0	0	1	0	0	0	0	0	0	0	70

21270906	Not	hp	JP	2022	0	1	0	0	1	0	0	0	0	0	0	0	
21270935	f	hp	US	2022	0	0	0	0	0	0	0	0	0	0	0	0	
21271138	m	cn	CN	2022	1	0	0	0	0	0	0	0	0	0	0	0	54
21271287	f	md	JP	2022	1	0	0	0	0	0	0	0	0	0	0	1	70
21271357	m	cn	CA	2022	1	0	0	0	0	0	0	0	0	0	0	0	66.5
21271447	f	md	FR	2022	0	0	0	0	0	0	0	0	0	0	0	0	
21271500	f	cn	PH	2022	0	1	0	0	0	1	0	0	0	0	0	0	77
21271501	Not	md	BR	2022	0	1	0	0	0	1	0	0	0	0	0	0	
21271579	f	md	JP	2022	0	0	0	0	0	0	0	0	0	0	0	0	58
21271713	Not	hp	CN	2022	0	0	0	0	0	0	0	0	0	0	0	0	
21271715	Not	hp	CN	2022	0	0	0	0	0	0	0	0	0	0	0	0	
21271716	Not	hp	CN	2022	0	0	0	0	0	0	0	0	0	0	0	0	
21271765	Not	hp	CN	2022	0	0	0	0	0	0	0	0	0	0	0	0	
21271767	Not	hp	CN	2022	0	0	0	0	0	0	0	0	0	0	0	0	
21271768	Not	hp	CN	2022	0	0	0	0	0	0	0	0	0	0	0	0	
21271769	Not	hp	CN	2022	0	0	0	0	0	0	0	0	0	0	0	0	
21272125	Not	md	US	2022	0	0	0	0	0	0	0	0	0	0	0	0	
21272582	m	hp	ES	2022	0	0	0	0	0	0	0	0	0	0	0	0	
21272682	f	md	US	2022	1	0	0	0	0	0	0	0	0	0	0	0	76.7
21272810	m	cn	TH	2022	1	0	0	0	0	0	0	0	0	0	0	0	66
21272867	m	cn	PH	2022	0	1	0	0	0	1	0	0	0	0	0	0	
21273351	f	cn	US	2022	1	0	0	0	0	0	0	0	0	0	0	0	
21273379	Not	ph	JP	2022	1	0	0	0	0	0	0	0	0	0	0	0	
21273411	Not	md	JP	2022	0	1	0	1	0	0	0	0	0	0	0	0	
21273680	f	md	US	2022	0	0	0	0	0	0	0	0	0	0	0	0	
21273688	Not	ph	US	2022	0	0	0	0	0	0	0	0	0	0	0	0	
21273724	f	cn	AU	2022	1	0	0	0	0	0	0	0	0	0	0	0	
21273990	m	ph	JP	2022	0	1	0	1	0	0	0	0	0	0	0	0	64
21273991	m	ph	MY	2022	1	0	0	0	0	0	0	0	0	0	0	0	
21274027	m	ph	US	2022	0	1	1	0	0	0	0	0	0	0	0	0	69
21274128	m	cn	MY	2022	1	0	0	0	0	0	0	0	0	0	0	0	71.6
21274134	m	md	FR	2022	0	0	0	0	0	0	0	0	0	0	0	0	60
21274150	m	md	TH	2022	0	1	0	0	0	1	0	0	0	0	0	0	39
21274172	f	hp	CA	2022	0	0	0	0	0	0	0	0	0	0	0	0	83

21274238	f	md	GB	2022	0	1	0	1	0	0	0	0	0	0	0	0	72
21274243	m	md	CN	2022	0	0	0	0	0	0	0	0	0	0	0	0	68
21274410	Not	ph	US	2022	0	0	0	0	0	0	0	0	0	0	0	0	
21274419	f	cn	IL	2022	0	0	0	0	0	0	0	0	0	0	0	0	
21274619	Not	hp	KR	2022	0	0	0	0	0	0	0	0	0	0	0	0	
21274958	m	md	BE	2022	0	1	1	0	0	0	0	0	0	0	0	0	
21275054	m	hp	FR	2022	0	0	0	0	0	0	0	0	0	0	0	0	
21275086	m	md	VN	2022	0	0	0	0	0	0	0	0	0	0	0	0	33
21278412	m	ph	JP	2022	0	1	0	0	1	0	0	0	0	0	0	0	80
21278426	m	md	JP	2022	0	1	0	0	1	0	0	0	0	0	0	0	80
21279371	f	ph	JP	2022	0	1	1	0	0	0	0	0	0	0	0	0	70
21279602	m	cn	CZ	2022	0	0	0	0	0	0	0	0	0	0	0	0	
21279907	Not	md	JP	2022	0	1	1	0	0	0	0	0	0	0	0	0	75
21280246	f	md	DE	2022	0	0	0	0	0	0	0	0	0	0	0	0	
21280273	m	ph	JP	2022	0	0	0	0	0	0	0	0	0	0	0	0	65
21280304	f	cn	VN	2022	1	0	0	0	0	0	0	0	0	0	0	0	
21280586	f	hp	KR	2022	1	0	0	0	0	0	0	0	0	0	0	0	61
21280597	Not	hp	IT	2022	0	1	1	0	0	0	0	0	0	0	0	0	
21280645	m	cn	ID	2022	1	0	0	0	0	0	0	0	0	0	0	0	
21280813	f	hp	US	2022	0	0	0	0	0	0	0	0	0	0	0	0	59
21280883	m	cn	IN	2022	0	0	0	0	0	0	0	0	0	0	0	0	68
21281112	f	hp	US	2022	0	0	0	0	0	0	0	0	0	0	0	0	59
21282962	f	cn	SG	2022	0	0	0	0	0	0	0	0	0	0	0	0	
21282984	m	md	CN	2022	0	0	0	0	0	0	0	0	0	0	0	0	67
21282995	f	md	CN	2022	0	0	0	0	0	0	0	0	0	0	0	0	53
21283022	f	md	AU	2022	1	0	0	0	0	0	0	0	0	0	0	0	
21283182	m	md	PT	2022	0	0	0	0	0	0	0	0	0	0	0	0	48
21283302	f	md	CN	2022	0	1	0	1	0	0	0	0	0	0	0	0	70.3
21283339	f	md	JP	2022	0	1	0	0	0	1	0	0	0	0	0	0	70
21283477	f	md	US	2022	0	0	0	0	0	0	0	0	0	0	0	0	59
21283899	f	md	JP	2022	0	0	0	0	0	0	0	0	0	0	0	0	76
21284638	Not	cn	US	2022	1	0	0	0	0	0	0	0	0	0	0	0	
21285088	m	hp	ES	2022	0	0	0	0	0	0	0	0	0	0	0	0	71
21285099	f	cn	CA	2022	1	0	0	0	0	0	0	0	0	0	0	0	

21285212	f	md	CZ	2022	0	1	0	0	1	0	0	0	0	0	0	0	74
21285578	m	cn	US	2022	0	0	0	0	0	0	0	0	0	0	0	0	
21285676	f	cn	US	2022	0	0	0	0	0	0	0	0	0	0	0	0	
21285787	m	md	US	2022	0	0	0	0	0	0	0	0	0	0	0	0	79
21285837	f	cn	CA	2022	1	0	0	0	0	0	0	0	0	0	0	0	
21285845	m	md	KR	2022	1	0	0	0	0	0	0	0	0	0	0	0	78.5
21285890	f	md	TW	2022	0	0	0	0	0	0	0	0	0	0	0	0	43
21285992	f	cn	IN	2022	0	0	0	0	0	0	0	0	0	0	0	0	50
21285997	m	cn	IN	2022	0	0	0	0	0	0	0	0	0	0	0	0	55
21286021	m	hp	US	2022	0	0	0	0	0	0	0	0	0	0	0	0	67
21286411	m	md	DE	2022	0	1	0	0	0	1	0	0	0	0	0	0	63
21286680	m	md	JP	2022	0	1	0	1	0	0	0	0	0	0	0	1	60
21286977	m	md	JP	2022	0	1	0	0	0	1	0	0	0	0	0	0	60
21287032	f	cn	CN	2022	0	1	0	0	1	0	0	0	0	0	0	0	53
21287239	m	md	JP	2022	0	1	1	0	0	0	0	0	0	0	0	0	74
21287390	f	md	ES	2022	0	0	0	0	0	0	0	0	0	0	0	0	64
21289437	m	md	CZ	2022	0	1	0	0	1	0	0	0	0	0	0	0	66
21289578	m	md	MY	2022	0	0	0	0	0	0	0	0	0	0	0	0	66
21289592	m	md	IT	2022	0	0	0	0	0	0	0	0	0	0	0	0	64
21289631	m	md	JP	2022	0	0	0	0	0	0	0	0	0	0	0	0	73
21289828	f	md	GB	2022	1	0	0	0	0	0	0	0	0	0	0	0	
21289935	f	md	CN	2022	1	0	0	0	0	0	0	0	0	0	0	0	53
21289988	m	cn	CN	2022	0	1	0	1	0	0	0	0	0	0	0	0	65
21290139	f	cn	JP	2022	1	0	0	0	0	0	0	0	0	0	0	0	
21290337	m	md	IQ	2022	0	1	1	0	0	0	0	0	0	0	0	0	
21290422	m	cn	US	2022	0	0	0	0	0	0	0	0	0	0	0	0	
21290849	m	md	CN	2022	0	1	1	0	0	0	0	0	0	0	0	0	65
21290856	m	md	FR	2022	0	0	0	0	0	0	0	0	0	0	0	0	
21291159	f	md	HK	2022	0	0	0	0	0	0	0	0	0	0	0	0	67
21291388	f	hp	GR	2022	0	1	1	0	0	0	0	0	0	0	0	0	60
21291636	f	md	DK	2022	0	1	0	0	0	1	0	0	0	0	0	0	65
21291825	Not	hp	JP	2022	0	1	0	0	1	0	0	0	0	0	0	0	
21291930	f	hp	IT	2022	0	0	0	0	0	0	0	0	0	0	0	0	62
21292082	m	hp	CN	2022	0	0	0	0	0	0	0	0	0	0	0	0	70

21292086	f	md	JP	2022	0	1	0	0	0	1	0	0	0	0	0	0	70
21292484	f	md	CZ	2022	0	1	0	0	1	0	0	0	0	0	0	0	69
21292615	m	cn	US	2022	0	1	0	0	1	0	0	0	0	0	0	0	81
21293118	m	md	ES	2022	0	1	0	0	0	1	0	0	0	0	0	0	
21293140	m	md	JP	2022	0	1	0	0	0	1	0	0	0	0	0	0	70
21293671	m	ph	JP	2022	0	1	1	0	0	0	0	0	0	0	0	0	48
21293733	m	md	RU	2022	0	1	0	1	0	0	0	0	0	0	0	0	67
21293791	f	md	JP	2022	0	0	0	0	0	0	0	0	0	0	0	0	50
21293847	f	md	US	2022	0	0	0	0	0	0	0	0	0	0	0	0	59
21293920	f	md	NL	2022	0	0	0	0	0	0	0	0	0	0	0	0	66
21294073	m	md	BE	2022	0	0	0	0	0	0	0	0	0	0	0	0	63
21294099	m	md	FR	2022	0	0	0	0	0	0	0	0	0	0	0	0	
21294145	f	md	CZ	2022	0	1	0	0	1	0	0	0	0	0	0	0	76
21294301	Not	md	JP	2022	0	1	0	0	1	0	0	0	0	0	0	0	
21294375	m	cn	US	2022	0	1	0	0	1	0	0	0	0	0	0	0	70
21294490	m	md	PL	2022	0	0	0	0	0	0	0	0	0	0	0	0	66
21294724	Not	cn	US	2022	1	0	0	0	0	0	0	0	0	0	0	0	
21294927	m	md	DE	2022	0	1	0	0	1	0	0	0	0	0	0	0	61
21295206	m	md	JP	2022	0	1	0	0	1	0	0	0	0	0	0	0	76
21295221	m	md	JP	2022	0	1	0	0	1	0	0	0	0	0	0	0	79
21296040	m	hp	DE	2022	0	0	0	0	0	0	0	0	0	0	0	0	74
21296041	m	hp	DE	2022	0	0	0	0	0	0	0	0	0	0	0	0	44
21296060	m	md	DE	2022	0	1	0	0	1	0	0	0	0	0	0	0	60
21296479	f	ph	FR	2022	0	0	0	0	0	0	0	0	0	0	0	0	80
21296480	m	cn	CN	2022	0	1	0	0	1	0	0	0	0	0	0	0	58
21296853	Not	md	JP	2022	0	1	0	0	0	1	0	0	0	0	0	0	
21297021	m	md	JP	2022	0	1	0	0	0	1	0	0	0	0	0	0	78
21297206	m	md	US	2022	0	0	0	0	0	0	0	0	0	0	0	0	78
21297255	m	md	JP	2022	1	0	0	0	0	0	0	0	0	0	0	0	91
21297331	m	md	US	2022	0	0	0	0	0	0	0	0	0	0	0	0	
21297771	f	cn	MY	2022	1	0	0	0	0	0	0	0	0	0	0	0	57
21297907	Not	cn	SG	2022	1	0	0	0	0	0	0	0	0	0	0	0	70.3
21297908	f	md	JP	2022	1	0	0	0	0	0	0	0	0	0	0	1	70
21298147	m	ph	JP	2022	1	0	0	0	0	0	0	0	0	0	0	0	

21298296	Not	md	CA	2022	0	0	0	0	0	0	0	0	0	0	0	0	70
21298344	f	ph	FR	2022	0	1	1	0	0	0	0	0	0	0	0	0	72
21298436	m	cn	CN	2022	0	1	0	0	1	0	0	0	0	0	0	0	57
21298504	Not	hp	TW	2022	1	0	0	0	0	0	0	0	0	0	0	0	
21298879	m	md	CN	2022	0	0	0	0	0	0	0	0	0	0	0	0	77
21299041	f	md	AT	2022	0	1	1	0	0	0	0	0	0	0	0	0	74
21299180	f	md	DK	2022	0	1	0	0	0	1	0	0	0	0	0	0	65
21299253	Not	md	JP	2022	0	1	1	0	0	0	0	0	0	0	0	0	80
21299254	Not	hp	TW	2022	1	0	0	0	0	0	0	0	0	0	0	0	
21299636	m	md	CN	2022	0	0	0	0	0	0	0	0	0	0	0	0	59
21299674	f	md	TW	2022	0	0	0	0	0	0	0	0	0	0	0	0	50
21299681	m	md	TW	2022	0	0	0	0	0	0	0	0	0	0	0	0	48
21299698	f	ph	FR	2022	0	1	1	0	0	0	0	0	0	0	0	0	72
21299701	f	md	CN	2022	0	0	0	0	0	0	0	0	0	0	0	0	39
21299792	f	cn	IN	2022	0	0	0	0	0	0	0	0	0	0	0	0	50
21299828	m	md	NZ	2022	0	0	0	0	0	0	0	0	0	0	0	0	61
21300242	Not	hp	TW	2022	1	0	0	0	0	0	0	0	0	0	0	0	
21300342	f	hp	CA	2022	1	0	0	0	0	0	0	0	0	0	0	0	56
21300448	m	md	US	2022	0	1	1	0	0	0	0	0	0	0	0	0	61
21300790	f	hp	CN	2022	0	0	0	0	0	0	0	0	0	0	0	0	45
21300913	m	md	JP	2022	0	1	0	0	1	0	0	0	0	0	0	0	78
21300986	f	hp	CA	2022	1	0	0	0	0	0	0	0	0	0	0	0	
21301277	m	hp	ES	2022	0	1	0	0	1	0	0	0	0	0	0	0	
21301380	f	md	JP	2022	1	0	0	0	0	0	0	0	0	0	0	1	80
21301406	f	md	BE	2022	0	1	0	1	0	0	0	0	0	0	0	0	
21301416	m	md	BE	2022	0	1	1	0	0	0	0	0	0	0	0	0	
21301654	f	md	US	2022	0	0	0	0	0	0	0	0	0	0	0	0	59
21301761	f	md	DE	2022	0	1	0	0	1	0	0	0	0	0	0	0	73
21302070	Not	md	JP	2022	0	1	0	1	0	0	0	0	0	0	0	0	
21302138	m	md	GR	2022	0	1	1	0	0	0	0	0	0	0	0	0	65
21302589	f	md	GR	2022	0	1	1	0	0	0	0	0	0	0	0	0	60
21302712	m	hp	IL	2022	0	1	0	0	1	0	0	0	0	0	0	0	60
21302895	Not	hp	JP	2022	0	0	0	0	0	0	0	0	0	0	0	0	
21302947	m	hp	AU	2022	0	0	0	0	0	0	0	0	0	0	0	0	77

21303113	m	md	CN	2022	0	0	0	0	0	0	0	0	0	0	0	0	65
21303296	Not	md	JP	2022	0	1	0	1	0	0	0	0	0	0	0	0	
21303340	f	md	GB	2022	0	1	0	1	0	0	0	0	0	0	0	0	72
21303360	f	md	CA	2022	1	0	0	0	0	0	0	0	0	0	0	0	
21303435	f	md	BE	2022	1	0	0	0	0	0	0	0	0	0	0	0	71
21303481	m	md	DE	2022	0	1	0	0	1	0	0	0	0	0	0	0	53
21303635	f	md	JP	2022	0	1	1	0	0	0	0	0	0	0	0	0	80
21303874	m	md	JP	2022	0	1	0	0	1	0	0	0	0	0	0	0	66
21305234	m	cn	IN	2022	0	1	0	0	1	0	0	0	0	0	0	0	70
21305462	m	md	JP	2022	0	1	0	0	1	0	0	0	0	0	0	0	57
21305604	f	hp	US	2022	0	1	0	0	1	0	0	0	0	0	0	0	65
21305752	Not	md	JP	2022	0	1	0	0	0	1	0	0	0	0	0	0	70
21306704	f	md	DE	2022	1	0	0	0	0	0	0	0	0	0	0	0	56
21307008	m	md	JP	2022	0	0	0	0	0	0	0	0	0	0	0	0	
21307503	m	md	DE	2022	0	1	0	0	1	0	0	0	0	0	0	0	52
21307505	f	md	DE	2022	0	1	0	0	0	1	0	0	0	0	0	0	70
21307600	m	md	JP	2022	0	1	1	0	0	0	0	0	0	0	0	0	
21307602	m	md	CN	2022	0	0	0	0	0	0	0	0	0	0	0	0	50
21307695	m	md	DE	2022	0	1	1	0	0	0	0	0	0	0	0	0	
21308315	f	cn	CA	2022	0	0	0	0	0	0	0	0	0	0	0	0	83
21308452	m	md	JP	2022	0	0	0	0	0	0	0	0	0	0	0	0	74
21308656	f	md	AU	2022	1	0	0	0	0	0	0	0	0	0	0	0	
21308812	f	cn	IN	2022	0	0	0	0	0	0	0	0	0	0	0	0	74
21308887	f	md	KR	2022	0	0	0	0	0	0	0	0	0	0	0	0	
21308946	m	cn	TH	2022	0	1	0	0	1	0	0	0	0	0	0	0	71
21308957	m	hp	US	2022	0	1	0	0	0	1	0	0	0	0	0	0	
21308958	Not	hp	US	2022	0	1	0	0	0	1	0	0	0	0	0	0	
21309209	f	cn	US	2022	0	1	1	0	0	0	0	0	0	0	0	0	62
21309695	m	md	JP	2022	0	1	1	0	0	0	0	0	0	0	0	0	70
21309722	f	md	JP	2022	0	1	0	0	1	0	0	0	0	0	0	0	44
21310037	Not	hp	US	2022	1	0	0	0	0	0	0	0	0	0	0	0	
21310364	m	md	DE	2022	0	1	0	0	1	0	0	0	0	0	0	0	69
21310457	f	hp	CN	2022	1	0	0	0	0	0	0	0	0	0	0	0	69
21310482	f	cn	CN	2022	0	0	0	0	0	0	0	0	0	0	0	0	42

21310599	f	md	US	2022	0	0	0	0	0	0	0	0	0	0	0	0	59
21310712	m	md	JP	2022	0	1	0	1	0	0	0	0	0	0	0	0	60
21310799	f	cn	ID	2022	1	0	0	0	0	0	0	0	0	0	0	0	70.4
21310903	m	cn	CN	2022	0	0	0	0	0	0	0	0	0	0	0	0	52
21311285	m	cn	TH	2022	0	1	0	0	1	0	0	0	0	0	0	0	74
21311878	Not	hp	ES	2022	1	0	0	0	0	0	0	0	0	0	0	0	
21311882	Not	hp	ES	2022	1	0	0	0	0	0	0	0	0	0	0	0	
21311952	m	cn	ID	2022	1	0	0	0	0	0	0	0	0	0	0	0	34.2
21312191	m	hp	IT	2022	0	1	0	0	1	0	0	0	0	0	0	0	62
21312424	f	md	FR	2022	0	0	0	0	0	0	0	0	0	0	0	0	
21312723	m	md	IT	2022	0	1	0	1	0	0	0	0	0	0	0	1	
21312724	f	cn	ID	2022	1	0	0	0	0	0	0	0	0	0	0	0	50.8
21313031	f	md	IE	2022	0	0	0	0	0	0	0	0	0	0	0	0	47
21313240	Not	md	SK	2022	0	0	0	0	0	0	0	0	0	0	0	0	36
21313776	f	md	IT	2022	0	0	0	0	0	0	0	0	0	0	0	0	74
21313856	m	md	IT	2022	0	1	0	0	0	1	0	0	0	0	0	0	72
21313946	f	md	CN	2022	0	0	0	0	0	0	0	0	0	0	0	0	70
21314557	Not	hp	CN	2022	0	0	0	0	0	0	0	0	0	0	0	0	
21314964	m	md	BE	2022	0	1	1	0	0	0	0	0	0	0	0	0	
21315223	f	hp	CN	2022	1	0	0	0	0	0	0	0	0	0	0	0	45
21315451	m	md	JP	2022	0	0	0	0	0	0	0	0	0	0	0	0	82
21315463	Not	hp	DE	2022	0	1	0	0	0	1	0	0	0	0	0	0	
21315487	m	hp	NL	2022	0	0	0	0	0	0	0	0	0	0	0	0	74
21315729	m	md	JP	2022	0	1	1	0	0	0	0	0	0	0	0	0	79
21315731	m	md	JP	2022	0	1	1	0	0	0	0	0	0	0	0	0	80
21315828	m	cn	KR	2022	0	1	1	0	0	0	0	0	0	0	0	0	
21316189	m	cn	US	2022	0	1	1	0	0	0	0	0	0	0	0	0	75
21316193	m	md	US	2022	0	1	1	0	0	0	0	0	0	0	0	0	
21316231	f	md	US	2022	0	0	0	0	0	0	0	0	0	0	0	0	59
21316542	m	hp	RU	2022	0	1	0	0	0	1	0	0	0	0	0	0	58
21316565	m	md	DE	2022	0	1	1	0	0	0	0	0	0	0	0	1	
21320645	m	hp	DE	2022	0	1	0	0	1	0	0	0	0	0	0	0	77
21320691	f	md	JP	2022	0	1	0	0	1	0	0	0	0	0	0	0	64
21321561	f	md	JP	2022	0	1	0	0	1	0	0	0	0	0	0	0	70

21321632	f	md	KR	2022	1	0	0	0	0	0	0	0	0	0	0	0	65
21322461	m	ph	US	2022	0	0	0	0	0	0	0	0	0	0	0	0	45
21322539	m	cn	QA	2022	0	0	0	0	0	0	0	0	0	0	0	0	
21322545	f	cn	QA	2022	0	0	0	0	0	0	0	0	0	0	0	0	63
21322659	f	md	CN	2022	0	0	0	0	0	0	0	0	0	0	0	0	59
21322814	m	md	BE	2022	0	1	1	0	0	0	0	0	0	0	0	0	
21322824	f	cn	US	2022	1	0	0	0	0	0	0	0	0	0	0	0	55.2
21322971	m	md	MX	2022	0	1	0	0	0	1	0	0	0	0	0	0	
21323059	m	hp	KR	2022	0	0	0	0	0	0	0	0	0	0	0	0	67
21323060	f	hp	KR	2022	0	0	0	0	0	0	0	0	0	0	0	0	61
21323116	m	md	DE	2022	0	1	0	0	1	0	0	0	0	0	0	0	63
21323190	m	cn	GR	2022	0	1	1	0	0	0	0	0	0	0	0	0	60
21323314	m	md	CZ	2022	0	1	0	0	1	0	0	0	0	0	0	0	66
21323373	m	hp	KR	2022	0	0	0	0	0	0	0	0	0	0	0	0	55
21323750	m	md	DE	2022	0	1	1	0	0	0	0	0	0	0	0	0	
21324101	f	md	DE	2022	0	1	0	0	1	0	0	0	0	0	0	0	73
21324296	f	cn	CN	2022	0	1	0	0	1	0	0	0	0	0	0	0	49
21324362	f	md	JP	2022	0	1	1	0	0	0	0	0	0	0	0	0	78
21324471	f	md	US	2022	0	0	0	0	0	0	0	0	0	0	0	0	64
21324623	m	hp	KR	2022	0	0	0	0	0	0	0	0	0	0	0	0	79
21324658	f	hp	US	2022	0	0	0	0	0	0	0	0	0	0	0	0	
21324688	f	md	SK	2022	0	0	0	0	0	0	0	0	0	0	0	0	44
21324728	m	cn	IN	2022	0	0	0	0	0	0	0	0	0	0	0	0	73
21324813	f	cn	US	2022	0	0	0	0	0	0	0	0	0	0	0	0	
21324825	m	md	JP	2022	0	1	0	0	0	1	0	0	0	0	0	0	80
21324845	m	md	CN	2022	1	0	0	0	0	0	0	0	0	0	0	0	52
21324930	m	md	TR	2022	0	0	0	0	0	0	0	0	0	0	0	0	57
21325099	f	md	IN	2022	0	0	0	0	0	0	0	0	0	0	0	0	55
21326042	Not	md	DE	2022	0	0	0	0	0	0	0	0	0	0	0	0	76
21326081	f	ph	JP	2022	1	0	0	0	0	0	0	0	0	0	0	0	
21326327	f	cn	GB	2022	1	0	0	0	0	0	0	0	0	0	0	0	64
21327250	f	cn	CA	2022	0	0	0	0	0	0	0	0	0	0	0	0	48
21327696	f	hp	FR	2022	1	0	0	0	0	0	0	0	0	0	0	0	26
21327895	f	md	JP	2022	0	0	0	0	0	0	0	0	0	0	0	0	66

21328432	f	ph	US	2022	0	0	0	0	0	0	0	0	0	0	0	0	77
21328537	Not	md	JP	2022	1	0	0	0	0	0	0	0	0	0	0	0	
21328729	f	md	TH	2022	1	0	0	0	0	0	0	0	0	0	0	0	68.9
21328905	f	hp	IN	2022	0	1	0	0	1	0	0	0	0	0	0	0	59
21329182	f	md	DE	2022	0	1	0	0	0	1	0	0	0	0	0	0	70
21329261	f	hp	DK	2022	1	0	0	0	0	0	0	0	0	0	0	0	78
21329383	m	md	DE	2022	0	1	1	0	0	0	0	0	0	0	0	1	
21329409	m	hp	GB	2022	0	1	1	0	0	0	0	0	0	0	0	0	68
21329677	f	hp	IL	2022	0	1	0	0	0	1	0	0	0	0	0	1	
21329799	m	md	CZ	2022	0	1	0	0	1	0	0	0	0	0	0	0	66
21330112	Not	md	JP	2022	0	1	0	0	1	0	0	0	0	0	0	0	
21330152	m	cn	CA	2022	0	0	0	0	0	0	0	0	0	0	0	0	63
21330298	m	md	JP	2022	0	1	0	1	0	0	0	0	0	0	0	1	75
21330323	m	md	JP	2022	0	1	0	0	1	0	0	0	0	0	0	0	82
21330328	m	md	CN	2022	1	0	0	0	0	0	0	0	0	0	0	0	41
21330456	m	md	TR	2022	0	0	0	0	0	0	0	0	0	0	0	0	62
21330643	m	cn	US	2022	0	1	0	0	1	0	0	0	0	0	0	0	
21330810	Not	md	US	2022	0	1	0	1	0	0	0	0	0	0	0	0	
21331026	m	md	JP	2022	0	0	0	0	0	0	0	0	0	0	0	0	35
21331122	m	cn	IN	2022	0	1	0	0	1	0	0	0	0	0	0	0	24
21331311	m	hp	GR	2022	0	0	0	0	0	0	0	0	0	0	0	0	61
21331335	f	ph	FR	2022	0	1	1	0	0	0	0	0	0	0	0	0	72
21331488	f	md	JP	2022	0	1	0	0	0	1	0	0	0	0	0	0	73
21331589	f	md	JP	2022	0	1	0	0	0	1	0	0	0	0	0	0	64
21331714	m	hp	HR	2022	0	1	0	0	1	0	0	0	0	0	0	1	60
21331852	f	ph	FR	2022	0	1	1	0	0	0	0	0	0	0	0	0	72
21332088	m	md	JP	2022	0	1	0	0	0	1	0	0	0	0	0	0	71
21332121	f	ph	US	2022	1	0	0	0	0	0	0	0	0	0	0	0	
21332230	m	cn	BE	2022	0	1	1	0	0	0	0	0	0	0	0	0	
21332419	Not	md	CN	2022	1	0	0	0	0	0	0	0	0	0	0	0	
21332488	f	md	JP	2022	0	1	0	0	0	1	0	0	0	0	0	0	70
21332498	f	md	PL	2022	0	1	0	1	0	0	0	0	0	0	0	0	41
21332500	m	md	PL	2022	0	1	0	1	0	0	0	0	0	0	0	0	
21332715	m	md	JP	2022	0	1	0	0	0	1	0	0	0	0	0	0	72

21332721	Not	md	JP	2022	0	1	1	0	0	0	0	0	0	0	0	0	70
21332896	m	md	JP	2022	0	1	0	0	0	1	0	0	0	0	0	0	68
21332991	m	md	FR	2022	0	0	0	0	0	0	0	0	0	0	0	0	
21334262	f	cn	CA	2022	1	0	0	0	0	0	0	0	0	0	0	0	
21334421	f	md	CN	2022	0	0	0	0	0	0	0	0	0	0	0	0	
21334559	f	md	DE	2022	0	1	0	0	1	0	0	0	0	0	0	0	59
21334703	m	cn	CA	2022	0	1	0	0	1	0	0	0	0	0	0	0	64
21334856	m	md	AU	2022	0	0	0	0	0	0	0	0	0	0	0	0	62
21335642	Not	md	AT	2022	0	0	0	0	0	0	0	0	0	0	0	0	
21335889	m	hp	CA	2022	0	1	0	0	1	0	0	0	0	0	0	0	58
21335969	Not	hp	CA	2022	0	0	0	0	0	0	0	0	0	0	0	0	70
21335992	m	md	ES	2022	1	0	0	0	0	0	0	0	0	0	0	0	57
21336518	m	hp	GR	2022	0	1	1	0	0	0	0	0	0	0	0	0	53
21336608	Not	md	CA	2022	0	0	0	0	0	0	0	0	0	0	0	0	44
21336861	m	md	PT	2022	0	1	1	0	0	0	0	0	0	0	0	0	77
21336998	m	md	JP	2022	0	1	1	0	0	0	0	0	0	0	0	0	70
21337274	Not	ph	JP	2022	1	0	0	0	0	0	0	0	0	0	0	0	
21337310	m	hp	GB	2022	0	1	1	0	0	0	0	0	0	0	0	0	68
21337429	Not	hp	US	2022	0	0	0	0	0	0	0	0	0	0	0	0	
21337483	m	md	ES	2022	0	0	0	0	0	0	0	0	0	0	0	0	57
21337521	f	md	SA	2022	1	0	0	0	0	0	0	0	0	0	0	0	
21338007	m	md	IT	2022	0	0	0	0	0	0	0	0	0	0	0	0	
21338316	Not	md	NL	2022	0	0	0	0	0	0	0	0	0	0	0	0	
21338339	f	md	DE	2022	0	1	0	0	1	0	0	0	0	0	0	0	59
21338435	Not	hp	US	2022	1	0	0	0	0	0	0	0	0	0	0	0	
21338787	m	md	KR	2022	0	1	0	1	0	0	0	0	0	0	0	0	67.9
21339148	m	md	CN	2022	0	0	0	0	0	0	0	0	0	0	0	0	69
21339266	m	md	CH	2022	0	0	0	0	0	0	0	0	0	0	0	0	47
21340226	Not	ph	JP	2022	1	0	0	0	0	0	0	0	0	0	0	0	
21340232	m	md	JP	2022	0	0	0	0	0	0	0	0	0	0	0	0	50
21340353	m	ph	JP	2022	0	0	0	0	0	0	0	0	0	0	0	0	50
21340440	m	md	JP	2022	0	0	0	0	0	0	0	0	0	0	0	0	59
21340489	f	md	DE	2022	0	1	0	0	1	0	0	0	0	0	0	0	59
21340725	f	md	US	2022	0	1	0	1	0	0	0	0	0	0	0	0	57.4

21340813	f	md	NZ	2022	0	0	0	0	0	0	0	0	0	0	0	0	
21340840	m	md	JP	2022	0	1	0	0	1	0	0	0	0	0	0	0	68
21340889	m	ph	JP	2022	0	0	0	0	0	0	0	0	0	0	0	0	
21341054	Not	md	US	2022	0	1	0	0	0	1	0	0	0	0	0	0	
21341306	f	md	JP	2022	1	0	0	0	0	0	0	0	0	0	0	0	60
21341315	Not	md	CA	2022	0	0	0	0	0	0	0	0	0	0	0	0	
21341600	Not	md	US	2022	0	0	0	0	0	0	0	0	0	0	0	0	
21341653	Not	md	US	2022	0	1	0	0	0	1	0	0	0	0	0	0	
21341709	f	cn	US	2022	0	0	0	0	0	0	0	0	0	0	0	0	
21342011	f	md	DE	2022	1	0	0	0	0	0	0	0	0	0	0	0	
21342128	f	md	AT	2022	0	0	0	0	0	0	0	0	0	0	0	0	
21342334	f	md	JP	2022	0	1	0	0	0	1	0	0	0	0	0	0	70
21343210	m	md	RO	2022	0	0	0	0	0	0	0	0	0	0	0	0	
21343849	m	md	FR	2022	0	0	0	0	0	0	0	0	0	0	0	0	
21344115	Not	cn	US	2022	1	0	0	0	0	0	0	0	0	0	0	0	89.4
21344268	f	md	AU	2022	0	1	1	0	0	0	0	0	0	0	0	1	76
21344297	m	md	JP	2022	0	1	0	0	0	1	0	0	0	0	0	0	68
21345631	f	md	JP	2022	0	1	1	0	0	0	0	0	0	0	0	1	83
21345634	m	cn	CN	2022	1	0	0	0	0	0	0	0	0	0	0	1	85
21345657	m	md	CN	2022	0	0	0	0	0	0	0	0	0	0	0	0	55
21345814	f	md	DE	2022	0	0	0	0	0	0	1	0	1	0	0	0	
21346214	m	md	ES	2022	0	0	0	0	0	0	0	0	0	0	0	0	57
21346218	m	hp	US	2022	1	0	0	0	0	0	0	0	0	0	0	0	79
21346331	f	md	DE	2022	0	1	0	0	1	0	0	0	0	0	0	0	60
21347304	Not	ph	JP	2022	0	0	0	0	0	0	0	0	0	0	0	0	
21347345	f	md	FR	2022	0	0	0	0	0	0	0	0	0	0	0	0	
21347601	m	md	JP	2022	0	1	1	0	0	0	0	0	0	0	0	0	80
21347614	f	md	BY	2022	0	0	0	0	0	0	0	0	0	0	0	0	
21347942	m	md	JP	2022	0	1	0	0	0	1	0	0	0	0	0	0	70
21348136	m	ph	US	2022	0	1	0	0	1	0	0	0	0	0	0	0	
21348144	m	md	JP	2022	0	0	0	0	0	0	0	0	0	0	0	0	80
21348310	m	md	JP	2022	0	1	0	0	0	1	0	0	0	0	0	0	80
21348506	f	hp	TH	2022	1	0	0	0	0	0	0	0	0	0	0	0	47
21348932	f	md	FR	2022	0	0	0	0	0	0	0	0	0	0	0	0	

21349201	m	md	CN	2022	0	0	0	0	0	0	0	0	0	0	0	0	62
21349596	f	md	JP	2022	0	1	0	0	0	1	0	0	0	0	0	0	80
21349636	f	cn	CN	2022	0	0	0	0	0	0	0	0	0	0	0	0	63
21349737	m	ph	PT	2022	0	1	1	0	0	0	0	0	0	0	0	0	66
21349907	f	md	US	2022	0	1	1	0	0	0	0	0	0	0	0	0	32
21350419	m	md	CN	2022	0	0	0	0	0	0	0	0	0	0	0	0	69
21350630	m	cn	IN	2022	0	0	0	0	0	0	0	0	0	0	0	0	62
21350703	f	cn	CA	2022	0	0	0	0	0	0	0	0	0	0	0	0	72
21350797	f	md	DE	2022	0	1	0	0	1	0	0	0	0	0	0	0	59
21350955	f	md	DE	2022	0	1	0	0	1	0	0	0	0	0	0	0	59
21350988	f	cn	TH	2022	0	0	0	0	0	0	0	0	0	0	0	0	25
21351062	m	cn	US	2022	1	0	0	0	0	0	0	0	0	0	0	0	86.5
21351093	m	hp	CN	2022	0	0	0	0	0	0	0	0	0	0	0	0	66
21351877	f	ph	JP	2022	1	0	0	0	0	0	0	0	0	0	0	0	80
21351879	m	md	FR	2022	0	1	0	1	0	0	0	0	0	0	0	0	
21351895	Not	cn	CN	2022	0	1	0	0	1	0	0	0	0	0	0	0	46
21352129	m	ph	JP	2022	0	1	0	0	1	0	0	0	0	0	0	0	83
21352200	m	md	DE	2022	0	0	0	0	0	0	0	0	0	0	0	0	69
21352238	Not	md	JP	2022	0	1	0	0	0	1	0	0	0	0	0	0	80
21352362	m	cn	US	2022	0	0	0	0	0	0	0	0	0	0	0	0	
21352794	m	md	GR	2022	0	1	0	0	1	0	0	0	0	0	0	0	71
21352829	f	hp	TR	2022	1	0	0	0	0	0	0	0	0	0	0	0	59
21353464	m	md	JP	2022	0	1	1	0	0	0	0	0	0	0	0	0	71
21353658	f	md	ES	2022	1	0	0	0	0	0	0	0	0	0	0	0	65
21353994	f	md	KR	2022	1	0	0	0	0	0	0	0	0	0	0	0	79
21354311	Not	md	US	2022	0	0	0	0	0	0	0	0	0	0	0	0	
21354421	m	hp	FR	2022	0	0	0	0	0	0	0	0	0	0	0	0	
21354658	m	md	CN	2022	0	0	0	0	0	0	0	0	0	0	0	0	70
21355122	f	cn	KR	2022	0	0	0	0	0	0	0	0	0	0	0	0	64
21355360	m	md	GR	2022	0	1	1	0	0	0	0	0	0	0	0	0	65
21355364	m	md	PT	2022	0	1	1	0	0	0	0	0	0	0	0	0	78
21355399	f	ph	JP	2022	0	0	0	0	0	0	0	0	0	0	0	0	80
21355547	f	hp	TH	2022	1	0	0	0	0	0	0	0	0	0	0	0	71.7
21356073	f	cn	KR	2022	0	0	0	0	0	0	0	0	0	0	0	0	49

21356096	f	cn	US	2022	1	0	0	0	0	0	0	0	0	0	0	0	82.9
21356286	f	md	FR	2022	0	0	0	0	0	0	0	0	0	0	0	0	61
21356295	f	cn	KR	2022	0	0	0	0	0	0	0	0	0	0	0	0	58
21356312	f	md	BE	2022	0	0	0	0	0	0	0	0	0	0	0	0	68
21356396	m	md	JP	2022	0	1	1	0	0	0	0	0	0	0	0	0	80
21356537	f	cn	US	2022	0	1	0	0	1	0	0	0	0	0	0	0	79
21356587	f	cn	IN	2022	0	0	0	0	0	0	0	0	0	0	0	0	59
21356706	m	hp	KR	2022	0	0	0	0	0	0	0	0	0	0	0	0	51
21356803	f	hp	TH	2022	1	0	0	0	0	0	0	0	0	0	0	0	73
21356983	Not	cn	US	2022	1	0	0	0	0	0	0	0	0	0	0	0	57.2
21357250	m	md	JP	2022	0	1	0	0	0	1	0	0	0	0	0	0	70
21357692	f	cn	SI	2022	0	0	0	0	0	0	0	0	0	0	0	0	49
21357840	f	md	JP	2022	0	1	0	0	1	0	0	0	0	0	0	0	63
21357843	Not	ph	JP	2022	0	0	0	0	0	0	0	0	0	0	0	0	72
21357890	m	md	JP	2022	0	1	0	0	0	1	0	0	0	0	0	0	
21358012	Not	ph	JP	2022	0	1	0	0	1	0	0	0	0	0	0	0	
21358437	m	ph	JP	2022	0	1	0	0	1	0	0	0	0	0	0	0	
21358459	Not	hp	DE	2022	0	0	0	0	0	0	0	0	0	0	0	0	
21358777	f	hp	TH	2022	1	0	0	0	0	0	0	0	0	0	0	0	
21358958	m	hp	JP	2022	0	0	0	0	0	0	0	0	0	0	0	0	72
21359261	Not	md	JP	2022	1	0	0	0	0	0	0	0	0	0	0	0	
21359264	f	md	JP	2022	1	0	0	0	0	0	0	0	0	0	0	0	
21359265	m	md	AU	2022	0	1	0	1	0	0	0	0	0	0	0	0	
21359988	Not	cn	SK	2022	0	0	0	0	0	0	0	0	0	0	0	0	66
21359995	f	md	FR	2022	0	0	0	0	0	0	0	0	0	0	0	0	60
21360043	Not	md	JP	2022	1	0	0	0	0	0	0	0	0	0	0	0	
21360376	f	md	CA	2022	0	1	1	0	0	0	0	0	0	0	0	0	75
21360436	Not	cn	US	2022	1	0	0	0	0	0	0	0	0	0	0	0	74.2
21360830	m	hp	JP	2022	0	1	1	0	0	0	0	0	0	0	0	1	69
21360940	Not	md	JP	2022	0	1	1	0	0	0	0	0	0	0	0	0	70
21361052	f	cn	US	2022	0	0	0	0	0	0	0	0	0	0	0	0	
21361142	f	cn	JP	2022	0	0	0	0	0	0	0	0	0	0	0	0	
21361401	f	md	TW	2022	0	0	0	0	0	0	0	0	0	0	0	0	
21361434	Not	md	AT	2022	0	1	0	0	1	0	0	0	0	0	0	0	

21361438	f	md	CN	2022	0	0	0	0	0	0	0	0	0	0	0	0	69
21361755	f	hp	GB	2022	0	1	1	0	0	0	0	0	0	0	0	0	56
21361942	Not	md	JP	2022	0	1	0	0	1	0	0	0	0	0	0	0	70
21361997	Not	hp	US	2022	0	1	1	0	0	0	0	0	0	0	0	0	
21362137	m	md	ES	2022	0	0	0	0	0	0	0	0	0	0	0	0	66
21362571	Not	hp	DE	2022	0	1	0	0	0	1	0	0	0	0	0	0	
21362731	f	ph	FR	2022	0	0	0	0	0	0	0	0	0	0	0	0	79
21362779	m	cn	US	2022	1	0	0	0	0	0	0	0	0	0	0	0	
21362874	m	cn	US	2022	0	0	0	0	0	0	0	0	0	0	0	0	
21363443	f	md	JP	2022	0	0	0	0	0	0	0	0	0	0	0	0	34
21363675	m	md	AT	2022	0	0	0	0	0	0	0	0	0	0	0	0	49
21363735	m	md	TH	2022	0	0	0	0	0	0	0	0	0	0	0	0	75
21364734	f	hp	AU	2022	0	1	0	0	1	0	0	0	0	0	0	0	78
21364995	Not	md	JP	2022	0	1	0	0	1	0	0	0	0	0	0	0	70
21365032	m	ph	US	2022	0	0	0	0	0	0	0	0	0	0	0	0	86
21365147	f	md	JP	2022	0	1	1	0	0	0	0	0	0	0	0	0	44
21365545	Not	md	CZ	2022	0	0	0	0	0	0	0	0	0	0	0	0	
21365560	f	md	US	2022	0	0	0	0	0	0	0	0	0	0	0	0	69
21365693	m	cn	JP	2022	0	0	0	0	0	0	0	0	0	0	0	0	55
21365718	f	md	CZ	2022	0	1	0	0	1	0	0	0	0	0	0	0	76
21366139	f	cn	ID	2022	0	0	0	0	0	0	0	0	0	0	0	0	60
21366288	m	hp	ES	2022	0	0	0	0	0	0	0	0	0	0	0	0	54
21367309	m	hp	TH	2022	1	0	0	0	0	0	0	0	0	0	0	0	
21367835	m	cn	CN	2022	0	1	0	0	1	0	0	0	0	0	0	0	58
21367882	m	md	DE	2022	0	1	0	0	1	0	0	0	0	0	0	0	74
21368554	m	cn	CN	2022	0	1	0	0	1	0	0	0	0	0	0	0	67
21368681	m	hp	US	2022	0	1	0	0	0	1	0	0	0	0	0	0	
21368724	Not	ph	US	2022	0	0	0	0	0	0	0	0	0	0	0	0	
21368942	Not	md	JP	2022	0	1	1	0	0	0	0	0	0	0	0	0	80
21368979	f	cn	US	2022	1	0	0	0	0	0	0	0	0	0	0	0	61.4
21369114	f	hp	CA	2022	0	1	1	0	0	0	0	0	0	0	0	0	58
21369631	f	cn	US	2022	0	0	0	0	0	0	0	0	0	0	0	0	73
21369637	f	Not repo	US	2022	0	1	0	0	1	0	0	0	0	0	0	0	61
21369668	m	md	JP	2022	0	1	0	0	0	1	0	0	0	0	0	0	

21369962	f	md	JP	2022	0	1	0	1	0	0	0	0	0	0	0	1	
21369966	m	md	JP	2022	0	1	0	1	0	0	0	0	0	0	0	1	70
21370079	f	cn	VN	2022	1	0	0	0	0	0	0	0	0	0	0	0	
21370084	m	ph	IT	2022	0	0	0	0	0	0	0	0	0	0	0	0	70
21370324	m	md	JP	2022	0	1	0	1	0	0	0	0	0	0	0	0	
21370567	m	cn	US	2022	0	0	0	0	0	0	0	0	0	0	0	0	
21370829	f	md	DE	2022	0	0	0	0	0	0	0	0	0	0	0	0	80
21370928	f	md	FR	2022	1	0	0	0	0	0	0	0	0	0	0	0	60
21371119	f	md	AU	2022	1	0	0	0	0	0	0	0	0	0	0	0	62
21371317	m	cn	VN	2022	0	0	0	0	0	0	0	0	0	0	0	0	61
21371452	m	md	US	2022	0	0	0	0	0	0	0	0	0	0	0	0	76
21371526	m	md	FR	2022	0	1	1	0	0	0	0	0	0	0	0	0	71
21371563	f	md	GB	2022	0	0	0	0	0	0	0	0	0	0	0	0	63
21372056	f	cn	DE	2022	0	1	0	0	1	0	0	0	0	0	0	0	59
21372070	f	cn	ID	2022	1	0	0	0	0	0	0	0	0	0	0	0	83.6
21373186	m	md	JP	2022	0	1	0	0	0	1	0	0	0	0	0	0	80
21373415	f	cn	US	2022	0	0	0	0	0	0	0	0	0	0	0	0	
21374029	m	md	JP	2022	0	1	0	0	1	0	0	0	0	0	0	0	58
21374176	f	md	CN	2022	0	0	0	0	0	0	0	0	0	0	0	0	68
21374747	m	hp	US	2022	0	0	0	0	0	0	0	0	0	0	0	0	62
21374851	Not	hp	US	2022	1	0	0	0	0	0	0	0	0	0	0	0	
21375237	f	md	CN	2022	0	0	0	0	0	0	0	0	0	0	0	0	61
21375248	Not	hp	US	2022	0	1	0	0	1	0	0	0	0	0	0	0	
21375625	f	cn	JP	2022	0	0	0	0	0	0	0	0	0	0	0	0	59
21376191	Not	hp	US	2022	0	0	0	0	0	0	0	0	0	0	0	0	
21376197	m	md	JP	2022	0	1	1	0	0	0	0	0	0	0	0	0	63
21376293	m	hp	CN	2022	0	1	0	0	1	0	0	0	0	0	0	0	52
21376502	m	hp	IE	2022	0	0	0	0	0	0	0	0	0	0	0	0	74
21377073	m	md	GB	2022	0	1	0	0	0	1	0	0	0	0	0	0	76
21377106	Not	md	NL	2022	0	0	0	0	0	0	0	0	0	0	0	0	
21377108	f	hp	US	2022	0	0	0	0	0	0	0	0	0	0	0	0	
21377116	f	hp	BR	2022	0	0	0	0	0	0	0	0	0	0	0	0	67
21377166	f	hp	US	2022	0	0	0	0	0	0	0	0	0	0	0	0	57
21377285	m	cn	NL	2022	0	0	0	0	0	0	0	0	0	0	0	0	53

21377666	f	md	JP	2022	0	0	0	0	0	0	0	0	0	0	0	0	69
21378017	m	md	FR	2022	0	1	1	0	0	0	0	0	0	0	0	0	
21378068	f	md	IT	2022	0	1	1	0	0	0	0	0	0	0	0	0	76
21378222	m	cn	CA	2022	1	0	0	0	0	0	0	0	0	0	0	0	
21378426	m	hp	GB	2022	0	1	1	0	0	0	0	0	0	0	0	0	68
21378471	f	hp	US	2022	0	1	1	0	0	0	0	0	0	0	0	0	62
21378474	Not	hp	US	2022	0	0	0	0	0	0	0	0	0	0	0	0	
21378494	f	cn	ID	2022	1	0	0	0	0	0	0	0	0	0	0	0	77.5
21378824	m	ph	CA	2022	0	0	0	0	0	0	0	0	0	0	0	0	65
21379164	m	cn	IN	2022	0	0	0	0	0	0	0	0	0	0	0	0	28
21379360	f	hp	US	2022	0	0	0	0	0	0	0	0	0	0	0	0	54
21380356	m	md	DE	2022	0	1	0	0	1	0	0	0	0	0	0	0	66
21380559	m	md	JP	2022	0	1	0	0	1	0	0	0	0	0	0	0	61
21380707	f	md	PT	2022	0	0	0	0	0	0	0	0	0	0	0	0	81
21380961	m	cn	VN	2022	0	0	0	0	0	0	0	0	0	0	0	0	44
21381322	Not	hp	JP	2022	0	0	0	0	0	0	0	0	0	0	0	0	
21382358	f	md	CN	2022	0	0	0	0	0	0	0	0	0	0	0	0	65
21382410	Not	hp	CN	2022	0	0	0	0	0	0	0	0	0	0	0	0	
21382427	f	md	IQ	2022	0	1	0	0	0	1	0	0	0	0	0	0	41
21382457	Not	hp	CN	2022	0	0	0	0	0	0	0	0	0	0	0	0	
21382458	f	hp	CN	2022	0	0	0	0	0	0	0	0	0	0	0	0	69
21382504	Not	hp	CN	2022	0	0	0	0	0	0	0	0	0	0	0	0	
21382531	m	hp	US	2022	0	1	0	0	0	1	0	0	0	0	0	0	75
21382533	Not	hp	CN	2022	0	0	0	0	0	0	0	0	0	0	0	0	
21382538	f	cn	US	2022	1	0	0	0	0	0	0	0	0	0	0	0	81
21382552	f	md	JP	2022	0	1	0	0	0	1	0	0	0	0	0	0	66
21382556	Not	md	DE	2022	0	1	0	0	0	1	0	0	0	0	0	0	
21382563	Not	md	JP	2022	0	1	0	0	0	1	0	0	0	0	0	0	80
21382600	m	hp	CN	2022	0	0	0	0	0	0	0	0	0	0	0	0	61
21382646	f	hp	US	2022	0	0	0	0	0	0	0	0	0	0	0	0	74
21382687	m	hp	CN	2022	0	0	0	0	0	0	0	0	0	0	0	0	73
21383344	m	md	JP	2022	0	0	0	0	0	0	1	0	0	1	0	0	54
21383991	f	md	CA	2022	0	0	0	0	0	0	0	0	0	0	0	0	
21384293	f	cn	CN	2022	0	1	0	0	1	0	0	0	0	0	0	0	60

21384571	f	md	GB	2022	0	1	0	0	0	1	0	0	0	0	0	0	63
21384738	m	hp	CN	2022	0	0	0	0	0	0	0	0	0	0	0	0	62
21385043	m	md	CN	2022	0	0	0	0	0	0	0	0	0	0	0	0	68
21385078	f	md	US	2022	0	1	1	0	0	0	0	0	0	0	0	0	
21385318	m	cn	CA	2022	0	1	0	0	1	0	0	0	0	0	0	0	73
21385801	m	hp	IN	2022	0	0	0	0	0	0	0	0	0	0	0	0	68
21385968	m	cn	CA	2022	0	1	0	0	1	0	0	0	0	0	0	0	63
21386215	m	md	NL	2022	0	0	0	0	0	0	0	0	0	0	0	0	45
21386228	Not	hp	ES	2022	0	1	0	0	0	1	0	0	0	0	0	0	
21386281	Not	md	US	2022	0	0	0	0	0	0	0	0	0	0	0	0	
21386556	m	md	JP	2022	0	1	0	0	1	0	0	0	0	0	0	0	51
21386890	m	cn	VN	2022	0	0	0	0	0	0	0	0	0	0	0	0	
21387225	m	cn	US	2022	0	0	0	0	0	0	0	0	0	0	0	0	
21387698	f	hp	GB	2022	0	1	0	0	0	1	0	0	0	0	0	0	71
21387739	m	ph	JP	2022	1	0	0	0	0	0	0	0	0	0	0	0	
21387882	f	cn	TH	2022	1	0	0	0	0	0	0	0	0	0	0	0	81.8
21388240	Not	md	JP	2022	0	1	1	0	0	0	0	0	0	0	0	1	79
21388259	m	md	JP	2022	0	1	1	0	0	0	0	0	0	0	0	0	82
21388444	m	md	JP	2022	0	1	0	0	0	1	0	0	0	0	0	1	80
21388456	f	md	CA	2022	1	0	0	0	0	0	0	0	0	0	0	0	63.3
21388510	m	cn	IL	2022	0	0	0	0	0	0	0	0	0	0	0	0	50
21388585	f	hp	AU	2022	0	1	1	0	0	0	0	0	0	0	0	0	67
21388591	m	md	JP	2022	0	1	0	0	1	0	0	0	0	0	0	0	
21390385	m	hp	JP	2022	0	0	0	0	0	0	0	0	0	0	0	0	73
21391121	Not	hp	JP	2022	0	1	0	0	1	0	0	0	0	0	0	0	
21391455	f	md	BG	2022	0	1	0	1	0	0	0	0	0	0	0	0	
21391465	m	md	JP	2022	0	0	0	0	0	0	0	0	0	0	0	0	
21391860	f	cn	US	2022	1	0	0	0	0	0	0	0	0	0	0	0	58.1
21392376	f	hp	NZ	2022	0	1	0	1	0	0	0	0	0	0	0	0	
21392725	m	md	JP	2022	0	1	0	0	1	0	0	0	0	0	0	0	74
21392820	Not	hp	DE	2022	0	1	1	0	0	0	0	0	0	0	0	0	61
21392973	m	md	JP	2022	1	0	0	0	0	0	0	0	0	0	0	0	83
21392976	f	cn	TH	2022	0	0	0	0	0	0	0	0	0	0	0	0	72
21393027	f	md	FR	2022	0	1	1	0	0	0	0	0	0	0	0	0	50

21393176	m	md	JP	2022	0	1	0	1	0	0	0	0	0	0	0	1	80
21393198	m	md	BR	2022	0	0	0	0	0	0	0	0	0	0	0	0	49
21393243	m	hp	FR	2022	0	0	0	0	0	0	0	0	0	0	0	0	
21393457	Not	md	JP	2022	0	1	0	1	0	0	0	0	0	0	0	0	
21394388	f	hp	FR	2022	0	1	0	0	0	1	0	0	0	0	0	0	26
21394460	f	cn	US	2022	1	0	0	0	0	0	0	0	0	0	0	0	60.8
21394507	m	cn	IN	2022	0	1	0	0	1	0	0	0	0	0	0	0	89
21394521	Not	hp	IN	2022	0	0	0	0	0	0	0	0	0	0	0	0	
21394716	m	md	JP	2022	0	0	0	0	0	0	0	0	0	0	0	0	69
21394823	Not	hp	IT	2022	0	0	0	0	0	0	0	0	0	0	0	0	
21395303	f	md	JP	2022	0	0	0	0	0	0	0	0	0	0	0	0	81
21395375	Not	hp	IT	2022	0	0	0	0	0	0	0	0	0	0	0	0	
21395672	f	cn	TH	2022	1	0	0	0	0	0	0	0	0	0	0	0	77.3
21395676	Not	md	JP	2022	1	0	0	0	0	0	0	0	0	0	0	0	
21395733	m	md	DE	2022	0	1	1	0	0	0	0	0	0	0	0	0	70
21395898	f	cn	US	2022	1	0	0	0	0	0	0	0	0	0	0	0	73.9
21395938	m	hp	GB	2022	0	1	1	0	0	0	0	0	0	0	0	0	68
21397095	m	md	CN	2022	0	1	1	0	0	0	0	0	0	0	0	0	71
21397150	f	cn	US	2022	0	0	0	0	0	0	0	0	0	0	0	0	73
21397176	f	cn	CA	2022	0	1	0	0	1	0	0	0	0	0	0	0	53
21397289	f	cn	TH	2022	1	0	0	0	0	0	0	0	0	0	0	0	56
21397295	f	cn	IN	2022	0	0	0	0	0	0	0	0	0	0	0	0	45
21397554	Not	md	US	2022	1	0	0	0	0	0	0	0	0	0	0	0	
21397572	f	md	BE	2022	1	0	0	0	0	0	0	0	0	0	0	0	71
21397620	f	md	TW	2022	0	0	0	0	0	0	0	0	0	0	0	0	65
21397666	m	md	JP	2022	0	1	1	0	0	0	0	0	0	0	0	0	70
21397707	m	md	US	2022	0	1	1	0	0	0	0	0	0	0	0	0	74
21397747	f	md	MX	2022	0	0	0	0	0	0	0	0	0	0	0	0	66
21397778	f	md	JP	2022	0	0	0	0	0	0	0	0	0	0	0	0	
21397783	f	md	JP	2022	1	0	0	0	0	0	0	0	0	0	0	1	44
21398278	m	md	JP	2022	0	1	0	1	0	0	0	0	0	0	0	1	80
21398496	f	md	JP	2022	1	0	0	0	0	0	0	0	0	0	0	1	73
20109538	m	md	PT	2022	0	1	1	0	0	0	0	0	0	0	0	0	77
20310156	f	md	US	2022	0	0	0	0	0	0	0	0	0	0	0	0	68

20377440	f	md	JP	2022	0	1	0	0	1	0	0	0	0	0	0	0	66
20435803	m	md	JP	2022	0	1	0	0	1	0	0	0	0	0	0	0	36
20562384	m	md	JP	2022	0	1	0	0	1	0	0	0	0	0	0	0	67
20571661	f	md	JP	2022	0	1	0	0	1	0	0	0	0	0	0	0	65
20729344	f	md	TW	2022	0	1	0	0	0	1	0	0	0	0	0	0	55
20763586	m	cn	JP	2022	0	0	0	0	0	0	0	0	0	0	0	0	67
20771297	m	hp	BE	2022	0	0	0	0	0	0	0	0	0	0	0	0	71
20794839	m	cn	CN	2022	0	0	0	0	0	0	0	0	0	0	0	0	59
20796689	m	md	JP	2022	0	1	0	0	1	0	0	0	0	0	0	0	72
20801319	m	md	IL	2022	0	1	0	0	0	1	0	0	0	0	0	0	66
20832259	m	md	JP	2022	0	1	0	0	1	0	0	0	0	0	0	0	72
20856061	f	md	ES	2022	0	0	0	0	0	0	0	0	0	0	0	0	77
20866067	f	md	JP	2022	1	0	0	0	0	0	0	0	0	0	0	0	78
20903478	m	hp	FR	2022	0	1	0	0	1	0	0	0	0	0	0	0	78
20916713	m	md	CN	2022	0	0	0	0	0	0	0	0	0	0	0	0	78
20941417	f	md	GB	2022	0	0	0	0	0	0	0	0	0	0	0	0	52
20973160	f	md	DE	2022	0	1	0	0	1	0	0	0	0	0	0	1	55
20975899	f	ph	FR	2022	0	0	0	0	0	0	0	0	0	0	0	0	
20985953	f	hp	US	2022	0	0	0	0	0	0	0	0	0	0	0	0	60
20985982	f	md	DE	2022	0	0	0	0	0	0	0	0	0	0	0	0	64
20990748	m	md	CN	2022	0	0	0	0	0	0	0	0	0	0	0	0	64
21023881	f	md	JP	2022	0	0	0	0	0	0	0	0	0	0	0	0	84
21038757	f	md	JP	2022	0	0	0	0	0	0	0	0	0	0	0	0	72
21039341	f	md	US	2022	0	1	0	0	0	1	0	0	0	0	0	0	55
21043852	m	md	CN	2022	0	0	0	0	0	0	0	0	0	0	0	0	67
21047652	m	md	AU	2022	0	0	0	0	0	0	0	0	0	0	0	0	67
21047834	f	md	CN	2022	0	0	0	0	0	0	0	0	0	0	0	0	53
21049423	m	hp	IT	2022	0	0	0	0	0	0	0	0	0	0	0	0	70
21071472	m	md	DE	2022	0	1	1	0	0	0	0	0	0	0	0	0	
21076899	f	md	BG	2022	0	1	0	0	0	1	0	0	0	0	0	0	61
21079023	f	md	JP	2022	0	1	0	0	1	0	0	0	0	0	0	0	74
21079040	m	md	JP	2022	0	1	0	0	1	0	0	0	0	0	0	0	47
21080497	f	md	JP	2022	0	1	0	0	1	0	0	0	0	0	0	0	73
21085247	m	md	JP	2022	0	1	0	0	1	0	0	0	0	0	0	0	64

21086202	m	md	JP	2022	0	1	0	0	1	0	0	0	0	0	0	0	75
21086294	f	md	FR	2022	0	1	0	0	1	0	0	0	0	0	0	0	68
21090038	f	md	CN	2022	0	1	1	0	0	0	0	0	0	0	0	0	66
21090893	f	md	BG	2022	0	1	0	0	0	1	0	0	0	0	0	0	56
21097071	f	hp	ES	2022	0	1	0	0	1	0	0	0	0	0	0	0	72
21100247	m	md	JP	2022	0	1	0	0	1	0	0	0	0	0	0	0	78
21102490	f	md	GB	2022	0	0	0	0	0	0	0	0	0	0	0	0	65
21107075	f	md	FR	2022	0	1	0	1	0	0	0	0	0	0	0	0	64
21109248	m	md	JP	2022	0	1	0	0	1	0	0	0	0	0	0	0	89
21110292	m	md	JP	2022	0	1	0	0	1	0	0	0	0	0	0	0	71
21116578	f	cn	US	2022	0	0	0	0	0	0	0	0	0	0	0	0	66
21116762	m	md	JP	2022	0	1	0	0	1	0	0	0	0	0	0	0	82
21122839	m	cn	US	2022	0	0	0	0	0	0	0	0	0	0	0	0	75
21123539	f	md	JP	2022	0	1	0	0	1	0	0	0	0	0	0	0	75
21123940	m	md	JP	2022	0	1	0	0	1	0	0	0	0	0	0	0	58
21124384	f	md	JP	2022	0	1	0	0	1	0	0	0	0	0	0	0	75
21133603	m	md	DE	2022	0	0	0	0	0	0	0	0	0	0	0	0	68
21134370	m	md	JP	2022	0	1	0	0	1	0	0	0	0	0	0	0	72
21136855	m	md	JP	2022	0	1	0	0	1	0	0	0	0	0	0	0	76
21137755	m	md	US	2022	0	0	0	0	0	0	0	0	0	0	0	0	
21140610	f	md	JP	2022	0	1	0	0	1	0	0	0	0	0	0	0	75
21145511	f	md	CA	2022	0	1	0	0	0	1	0	0	0	0	0	0	60
21150973	m	md	TR	2022	0	0	0	0	0	0	0	0	0	0	0	0	83
21154380	m	md	JP	2022	0	1	1	0	0	0	0	0	0	0	0	0	67
21154858	m	md	JP	2022	0	1	1	0	0	0	0	0	0	0	0	0	64
21155736	f	md	CN	2022	0	0	0	0	0	0	0	0	0	0	0	0	53
21163373	m	hp	AU	2022	0	0	0	0	0	0	0	0	0	0	0	0	
21163787	m	md	DE	2022	0	1	1	0	0	0	0	0	0	0	0	0	75
21170911	f	md	JP	2022	0	0	0	0	0	0	0	0	0	0	0	0	70
21172938	f	md	DE	2022	0	1	0	0	1	0	0	0	0	0	0	0	55
21174770	f	md	GB	2022	0	0	0	0	0	0	0	0	0	0	0	0	76
21178610	m	md	NL	2022	0	0	0	0	0	0	0	0	0	0	0	0	45
21179117	f	hp	CL	2022	0	1	0	0	1	0	0	0	0	0	0	0	71
21193189	m	md	JP	2022	0	0	0	0	0	0	0	0	0	0	0	0	65

21194144	m	md	JP	2022	0	1	0	0	0	1	0	0	0	0	0	0	75
21194765	m	md	NL	2022	1	0	0	0	0	0	0	0	0	0	0	0	79
21195019	m	md	JP	2022	0	1	1	0	0	0	0	0	0	0	0	0	74
21197654	f	hp	BR	2022	0	0	0	0	0	0	0	0	0	0	0	0	70
21197753	m	hp	ES	2022	0	0	0	0	0	0	0	0	0	0	0	0	
21199151	m	hp	NL	2022	0	0	0	0	0	0	0	0	0	0	0	0	53
21204399	f	md	JP	2022	0	1	0	0	1	0	0	0	0	0	0	0	69
21204743	m	md	KR	2022	0	1	0	0	1	0	0	0	0	0	0	0	
21211774	m	ph	JP	2022	0	0	0	0	0	0	0	0	0	0	0	0	75
21215597	m	md	CN	2022	0	0	0	0	0	0	0	0	0	0	0	0	59
21219063	m	hp	CN	2022	0	0	0	0	0	0	0	0	0	0	0	0	58
21220466	m	md	CN	2022	0	0	0	0	0	0	0	0	0	0	0	0	69
21223921	m	md	IT	2022	0	0	0	0	0	0	0	0	0	0	0	0	76
21228055	f	md	CN	2022	0	1	0	0	0	1	0	0	0	0	0	1	69
21228589	m	md	JP	2022	0	0	0	0	0	0	0	0	0	0	0	0	74
21234474	f	md	JP	2022	0	1	0	0	1	0	0	0	0	0	0	1	88
21237115	m	md	BE	2022	0	0	0	0	0	0	0	0	0	0	0	0	45
21238645	m	md	FR	2022	0	1	0	0	0	1	0	0	0	0	0	0	78
21242535	m	md	AU	2022	0	0	0	0	0	0	0	0	0	0	0	0	55
21246073	f	md	JP	2022	0	1	0	0	0	1	0	0	0	0	0	0	75
21246085	f	md	JP	2022	0	0	0	0	0	0	0	0	0	0	0	0	75
21246184	m	md	JP	2022	0	1	0	0	1	0	0	0	0	0	0	0	77
21246983	m	md	KR	2022	0	1	0	0	0	1	0	0	0	0	0	0	76
21248377	m	cn	US	2022	0	0	0	0	0	0	0	0	0	0	0	0	69
21249105	m	md	JP	2022	0	0	0	0	0	0	0	0	0	0	0	0	79
21249701	f	md	FR	2022	0	0	0	0	0	0	0	0	0	0	0	0	
21249952	m	md	JP	2022	0	1	0	0	1	0	0	0	0	0	0	0	64
21253864	m	md	AU	2022	0	0	0	0	0	0	0	0	0	0	0	0	78
21254083	Not	md	KR	2022	0	1	0	0	0	1	0	0	0	0	0	0	
21254453	f	cn	ZA	2022	0	0	0	0	0	0	0	0	0	0	0	0	
21257873	m	md	CN	2022	0	0	0	0	0	0	0	0	0	0	0	0	
21258716	Not	md	JP	2022	0	1	0	0	1	0	0	0	0	0	0	0	82
21260312	m	md	JP	2022	0	0	0	0	0	0	0	0	0	0	0	0	78
21263144	f	md	JP	2022	0	1	0	0	0	1	0	0	0	0	0	1	74

21265195	f	md	US	2022	0	0	0	0	0	0	0	0	0	0	0	0	78
21266260	f	md	JP	2022	0	0	0	0	0	0	0	0	0	0	0	0	80
21271316	m	md	JP	2022	0	0	0	0	0	0	0	0	0	0	0	0	71
21271379	m	md	JP	2022	0	0	0	0	0	0	0	0	0	0	0	0	
21275348	f	md	JP	2022	1	0	0	0	0	0	0	0	0	0	0	1	76
21280636	m	md	ES	2022	0	1	1	0	0	0	0	0	0	0	0	0	65
21280876	m	md	CN	2022	0	0	0	0	0	0	0	0	0	0	0	0	70
21284979	m	md	GB	2022	0	0	0	0	0	0	0	0	0	0	0	0	73
21287030	m	md	JP	2022	0	1	0	0	1	0	0	0	0	0	0	0	70
21287136	m	md	CN	2022	0	0	0	0	0	0	0	0	0	0	0	0	67
21289711	m	md	DE	2022	0	1	0	0	1	0	0	0	0	0	0	0	65
21289738	m	md	CN	2022	0	0	0	0	0	0	0	0	0	0	0	0	57
21292069	f	ph	JP	2022	0	0	0	0	0	0	0	0	0	0	0	0	65
21294028	f	md	IT	2022	0	0	0	0	0	0	0	0	0	0	0	0	74
21294482	f	hp	FR	2022	0	1	1	0	0	0	0	0	0	0	0	0	74
21294720	m	md	DE	2022	0	1	0	0	1	0	0	0	0	0	0	0	71
21296749	f	md	BE	2022	0	1	0	1	0	0	0	0	0	0	0	0	
21297339	f	md	CH	2022	0	1	0	0	0	1	0	0	0	0	0	0	77
21298832	f	cn	JP	2022	0	0	0	0	0	0	0	0	0	0	0	0	66
21299372	f	md	JP	2022	1	0	0	0	0	0	0	0	0	0	0	0	77
21301280	f	md	GB	2022	0	1	0	1	0	0	0	0	0	0	0	0	
21304042	f	md	BR	2022	0	0	0	0	0	0	0	0	0	0	0	0	75
21306093	m	md	CN	2022	0	1	0	0	0	1	0	0	0	0	0	0	78
21306100	m	hp	GR	2022	0	0	0	0	0	0	0	0	0	0	0	0	60
21307683	m	hp	CN	2022	0	1	0	0	1	0	0	0	0	0	0	0	69
21307754	m	md	DE	2022	0	0	0	0	0	0	0	0	0	0	0	0	69
21308857	f	md	CN	2022	0	0	0	0	0	0	0	0	0	0	0	0	76
21315545	m	hp	JP	2022	0	0	0	0	0	0	0	0	0	0	0	0	70
21321388	Not	hp	FR	2022	0	1	0	0	1	0	0	0	0	0	0	0	78
21321945	m	md	GR	2022	0	1	1	0	0	0	0	0	0	0	0	0	60
21326007	f	hp	US	2022	0	0	0	0	0	0	0	0	0	0	0	0	72
21327899	m	hp	GB	2022	0	1	1	0	0	0	0	0	0	0	0	0	68
21328899	f	md	DE	2022	0	0	0	0	0	0	0	0	0	0	0	0	80
21331873	m	md	GB	2022	0	1	1	0	0	0	0	0	0	0	0	1	

21332369	m	md	DE	2022	0	1	0	0	1	0	0	0	0	0	0	0	70
21333765	m	md	DE	2022	0	1	0	0	1	0	0	0	0	0	0	0	63
21337484	f	hp	US	2022	0	0	0	0	0	0	0	0	0	0	0	0	59
21337682	m	hp	CN	2022	0	1	0	0	1	0	0	0	0	0	0	0	65
21337845	m	md	JP	2022	0	0	0	0	0	0	0	0	0	0	0	0	67
21338821	m	md	BR	2022	0	0	0	0	0	0	0	0	0	0	0	0	56
21339169	m	cn	IN	2022	0	0	0	0	0	0	0	0	0	0	0	0	76
21340676	m	md	JP	2022	0	1	0	0	0	1	0	0	0	0	0	0	77
21341702	m	md	JP	2022	0	1	0	0	1	0	0	0	0	0	0	0	78
21342524	m	md	FR	2022	0	1	0	0	0	1	0	0	0	0	0	0	77
21342925	m	md	JP	2022	0	1	0	0	1	0	0	0	0	0	0	0	74
21345259	m	md	JP	2022	0	1	0	0	1	0	0	0	0	0	0	0	73
21345537	f	md	IN	2022	0	0	0	0	0	0	0	0	0	0	0	0	68
21345951	f	md	JP	2022	0	1	1	0	0	0	0	0	0	0	0	0	80
21346454	m	md	AT	2022	0	0	0	0	0	0	0	0	0	0	0	0	43
21346480	f	md	DE	2022	0	0	0	0	0	0	0	0	0	0	0	0	
21347395	f	md	DE	2022	0	1	0	0	1	0	0	0	0	0	0	0	65
21348388	f	md	DE	2022	0	1	0	0	1	0	0	0	0	0	0	0	58
21349606	f	md	JP	2022	0	0	0	0	0	0	0	0	0	0	0	0	85
21351091	m	hp	BR	2022	0	0	0	0	0	0	0	0	0	0	0	0	67
21351167	m	md	KR	2022	0	0	0	0	0	0	0	0	0	0	0	0	66
21351901	m	md	JP	2022	0	1	0	0	1	0	0	0	0	0	0	0	69
21353980	m	md	JP	2022	0	0	0	0	0	0	0	0	0	0	0	0	64
21356430	m	md	US	2022	0	0	0	0	0	0	0	0	0	0	0	0	72
21357334	m	md	JP	2022	0	1	0	0	1	0	0	0	0	0	0	1	70
21358250	f	hp	US	2022	0	0	0	0	0	0	0	0	0	0	0	0	60
21360041	f	hp	CA	2022	1	0	0	0	0	0	0	0	0	0	0	0	
21361459	f	md	MX	2022	0	0	0	0	0	0	0	0	0	0	0	0	72
21361539	m	md	AT	2022	0	0	0	0	0	0	0	0	0	0	0	0	
21361665	m	ph	JP	2022	0	1	0	0	1	0	0	0	0	0	0	0	55
21362935	m	md	AU	2022	0	1	0	0	0	1	0	0	0	0	0	0	55
21363822	m	md	CN	2022	0	0	0	0	0	0	0	0	0	0	0	0	65
21363932	Not	md	NL	2022	0	1	1	0	0	0	0	0	0	0	0	1	
21364489	m	cn	US	2022	0	1	1	0	0	0	0	0	0	0	0	0	

21364968	f	md	DE	2022	0	1	0	0	1	0	0	0	0	0	0	0	58
21366401	f	cn	US	2022	0	1	0	0	1	0	0	0	0	0	0	0	79
21366440	f	hp	GB	2022	0	0	0	0	0	0	0	0	0	0	0	0	65
21366494	f	md	JP	2022	0	1	1	0	0	0	0	0	0	0	0	0	78
21367474	Not	ph	JP	2022	0	1	0	0	0	1	0	0	0	0	0	0	
21371045	f	md	JP	2022	0	0	0	0	0	0	0	0	0	0	0	0	80
21371615	f	cn	US	2022	0	1	1	0	0	0	0	0	0	0	0	0	74
21373926	m	cn	LB	2022	0	1	0	0	1	0	0	0	0	0	0	0	66
21374109	Not	md	FR	2022	0	1	0	0	1	0	0	0	0	0	0	0	68
21374896	m	hp	FR	2022	0	1	1	0	0	0	0	0	0	0	0	0	74
21375637	m	cn	DE	2022	0	1	0	0	1	0	0	0	0	0	0	0	57
21375912	m	md	CN	2022	0	0	0	0	0	0	0	0	0	0	0	0	70
21376098	f	cn	TH	2022	1	0	0	0	0	0	0	0	0	0	0	0	46
21376330	m	md	FR	2022	0	0	0	0	0	0	0	0	0	0	0	0	
21382399	Not	md	JP	2022	0	1	0	0	0	1	0	0	0	0	0	0	80
21385153	m	md	CA	2022	0	1	0	0	0	1	0	0	0	0	0	0	81
21386883	f	cn	ID	2022	0	0	0	0	0	0	0	0	0	0	0	0	56
21387284	m	hp	JP	2022	0	1	0	0	0	1	0	0	0	0	0	0	80
21387545	m	md	CA	2022	0	0	0	0	0	0	0	0	0	0	0	0	54
21387994	m	md	JP	2022	0	1	1	0	0	0	0	0	0	0	0	0	74
21388285	m	hp	CN	2022	0	1	0	1	0	0	0	0	0	0	0	1	63
21388413	m	md	TW	2022	0	0	0	0	0	0	0	0	0	0	0	0	51
21388598	Not	md	JP	2022	0	0	0	0	0	0	0	0	0	0	0	0	
21388648	m	hp	JP	2022	0	0	0	0	0	0	0	0	0	0	0	0	74
21388766	f	md	NL	2022	0	0	0	0	0	0	0	0	0	0	0	0	72
21389292	f	ph	US	2022	0	0	0	0	0	0	0	0	0	0	0	0	69
21389470	f	md	US	2022	0	0	0	0	0	0	0	0	0	0	0	0	
21391760	Not	md	PL	2022	0	1	1	0	0	0	0	0	0	0	0	0	
21393082	m	md	FR	2022	0	1	0	0	1	0	0	0	0	0	0	0	85
21393276	m	md	JP	2022	0	0	0	0	0	0	0	0	0	0	0	0	63
21393473	m	md	JP	2022	0	0	0	0	0	0	0	0	0	0	0	0	71
21395185	f	md	JP	2022	0	0	0	0	0	0	0	0	0	0	0	0	73
21396920	Not	md	FR	2022	0	1	1	0	0	0	0	0	0	0	0	0	
21397016	m	md	JP	2022	0	0	0	0	0	0	0	0	0	0	0	0	37

21397269	m	md	CN	2022	0	1	1	0	0	0	0	0	0	0	0	0	60
21397341	f	md	KR	2022	0	0	0	0	0	0	0	0	0	0	0	0	89
21397824	m	ph	JP	2022	0	1	1	0	0	0	0	0	0	0	0	0	90
21398153	m	md	JP	2022	0	0	0	0	0	0	0	0	0	0	0	0	78
21398508	m	md	JP	2022	0	1	0	1	0	0	0	0	0	0	0	0	81
21399818	f	cn	IL	2022	0	0	0	0	0	0	0	0	0	0	0	0	54
21400131	f	md	TH	2022	0	0	0	0	0	0	0	0	0	0	0	0	53
21400190	m	md	JP	2022	0	0	0	0	0	0	0	0	0	0	0	0	81
21400212	m	md	JP	2022	0	1	0	0	0	1	0	0	0	0	0	0	59
21400657	m	cn	TR	2022	0	1	0	0	1	0	0	0	0	0	0	0	59
21400817	f	md	GB	2022	0	1	0	0	0	1	0	0	0	0	0	1	77
21400850	m	ph	JP	2022	0	0	0	0	0	0	0	0	0	0	0	0	50
21401237	f	cn	US	2022	0	1	1	0	0	0	0	0	0	0	0	0	
21401295	m	cn	TH	2022	1	0	0	0	0	0	0	0	0	0	0	0	62.3
21401447	Not	md	IT	2022	0	0	0	0	0	0	0	0	0	0	0	0	
21401799	Not	ph	JP	2022	0	0	0	0	0	0	0	0	0	0	0	0	
21401926	f	cn	US	2022	1	0	0	0	0	0	0	0	0	0	0	0	48.7
21402033	f	ph	US	2022	0	0	0	0	0	0	0	0	0	0	0	0	77
21402070	m	hp	CA	2022	0	1	0	0	1	0	0	0	0	0	0	0	59
21402187	m	md	JP	2022	0	0	0	0	0	0	0	0	0	0	0	0	80
21402311	m	md	NL	2022	0	0	0	0	0	0	0	0	0	0	0	0	76
21402320	m	cn	JP	2022	0	0	0	0	0	0	0	0	0	0	0	0	65
21403593	m	md	FR	2022	1	0	0	0	0	0	0	0	0	0	0	1	75.4
21403709	Not	md	DE	2022	0	0	0	0	0	0	0	0	0	0	0	0	
21403710	m	cn	IN	2022	0	0	0	0	0	0	0	0	0	0	0	0	56
21403863	m	cn	LB	2022	1	0	0	0	0	0	0	0	0	0	0	0	73
21404226	m	cn	LB	2022	1	0	0	0	0	0	0	0	0	0	0	0	66
21404228	m	hp	CA	2022	0	0	0	0	0	0	0	0	0	0	0	0	70
21404418	m	md	JP	2022	0	1	1	0	0	0	0	0	0	0	0	0	40
21404526	f	md	CN	2022	0	0	0	0	0	0	0	0	0	0	0	0	68
21404568	f	md	MY	2022	0	0	0	0	0	0	0	0	0	0	0	0	64
21405323	m	hp	CN	2022	0	0	0	0	0	0	0	0	0	0	0	0	56
21405572	m	md	CN	2022	0	0	0	0	0	0	0	0	0	0	0	0	69
21405741	f	md	JP	2022	0	0	0	0	0	0	0	0	0	0	0	0	

21405975	m	md	JP	2022	0	1	0	0	0	1	0	0	0	0	0	0	73
21406006	f	hp	CN	2022	1	0	0	0	0	0	0	0	0	0	0	0	72
21406083	f	hp	CA	2022	0	1	1	0	0	0	0	0	0	0	0	0	58
21406217	m	hp	GB	2022	0	1	1	0	0	0	0	0	0	0	0	0	68
21406304	f	hp	DE	2022	0	1	1	0	0	0	0	0	0	0	0	0	61
21406641	Not	cn	US	2022	1	0	0	0	0	0	0	0	0	0	0	0	72.2
21406737	m	md	JP	2022	0	1	0	0	0	1	0	0	0	0	0	1	80
21406820	m	cn	US	2022	0	1	0	1	0	0	0	0	0	0	0	0	55
21406948	m	cn	CN	2022	0	0	0	0	0	0	0	0	0	0	0	0	62
21407080	m	ph	US	2022	0	0	0	0	0	0	0	0	0	0	0	0	63
21407193	m	md	JP	2022	0	1	0	1	0	0	0	0	0	0	0	0	69
21407586	f	md	JP	2022	0	1	0	0	0	1	0	0	0	0	0	0	70
21407835	Not	hp	US	2022	0	0	0	0	0	0	0	0	0	0	0	0	
21408085	m	md	CA	2022	0	0	0	0	0	0	0	0	0	0	0	0	73
21408471	m	md	JP	2022	0	0	0	0	0	0	0	0	0	0	0	0	
21408765	f	hp	MX	2022	0	0	0	0	0	0	0	0	0	0	0	0	69
21409358	f	cn	CN	2022	0	1	0	0	1	0	0	0	0	0	0	0	71
21409416	f	hp	TH	2022	1	0	0	0	0	0	0	0	0	0	0	0	59
21409922	m	cn	US	2022	0	1	1	0	0	0	0	0	0	0	0	1	
21409993	m	md	JP	2022	0	1	0	0	1	0	0	0	0	0	0	0	46
21410952	m	md	FR	2022	1	0	0	0	0	0	0	0	0	0	0	0	56.9
21411038	m	md	ZA	2022	0	0	0	0	0	0	0	0	0	0	0	0	
21411105	m	md	JP	2022	0	1	0	1	0	0	0	0	0	0	0	1	70
21411107	m	md	JP	2022	1	0	0	0	0	0	0	0	0	0	0	0	76
21411249	f	md	JP	2022	1	0	0	0	0	0	0	0	0	0	0	0	83
21411394	m	md	JP	2022	0	0	0	0	0	0	0	0	0	0	0	0	66
21411558	f	cn	IN	2022	0	0	0	0	0	0	0	0	0	0	0	0	73
21411993	m	md	JP	2022	0	1	1	0	0	0	0	0	0	0	0	0	70
21412106	f	md	TW	2022	0	0	0	0	0	0	0	0	0	0	0	0	64
21412178	m	ph	US	2022	0	0	0	0	0	0	0	0	0	0	0	0	
21412592	f	md	NL	2022	1	0	0	0	0	0	0	0	0	0	0	0	61
21412624	f	md	NL	2022	0	0	0	0	0	0	0	0	0	0	0	0	74
21412950	m	md	JP	2022	0	0	0	0	0	0	0	0	0	0	0	0	80
21413249	f	hp	US	2022	1	0	0	0	0	0	0	0	0	0	0	0	

21413250	m	hp	US	2022	1	0	0	0	0	0	0	0	0	0	0	0	
21413253	f	hp	US	2022	1	0	0	0	0	0	0	0	0	0	0	0	
21413269	f	hp	US	2022	1	0	0	0	0	0	0	0	0	0	0	0	
21413993	f	md	HK	2022	0	0	0	0	0	0	0	0	0	0	0	0	61
21414595	f	md	CH	2022	0	1	0	0	0	1	0	0	0	0	0	0	55
21415928	m	cn	IN	2022	0	0	0	0	0	0	0	0	0	0	0	0	67
21416294	f	ph	TH	2022	1	0	0	0	0	0	0	0	0	0	0	0	75.8
21416415	Not	ph	JP	2022	0	1	0	1	0	0	0	0	0	0	0	0	
21416872	f	md	TW	2022	0	0	0	0	0	0	0	0	0	0	0	0	61
21416873	f	hp	DE	2022	0	1	1	0	0	0	0	0	0	0	0	0	61
21417195	Not	md	JP	2022	0	1	0	0	1	0	0	0	0	0	0	0	
21417224	f	hp	AU	2022	0	1	0	0	1	0	0	0	0	0	0	0	57
21417285	Not	md	JP	2022	0	0	0	0	0	0	0	0	0	0	0	0	
21417561	f	cn	US	2022	1	0	0	0	0	0	0	0	0	0	0	0	
21417610	Not	md	FR	2022	0	1	0	0	1	0	0	0	0	0	0	0	66
21417803	m	md	JP	2022	0	0	0	0	0	0	0	0	0	0	0	0	60
21418083	f	cn	KR	2022	0	0	0	0	0	0	0	0	0	0	0	0	62
21418173	f	hp	TH	2022	1	0	0	0	0	0	0	0	0	0	0	0	80.7
21418409	f	hp	BR	2022	0	0	0	0	0	0	0	0	0	0	0	0	70
21418515	m	md	JP	2022	0	1	0	0	0	1	0	0	0	0	0	0	54
21418676	Not	hp	JP	2022	0	1	1	0	0	0	0	0	0	0	0	0	
21418698	Not	hp	JP	2022	0	1	1	0	0	0	0	0	0	0	0	0	
21419348	m	cn	ID	2022	1	0	0	0	0	0	0	0	0	0	0	0	57.2
21419376	m	md	JP	2022	0	1	0	0	1	0	0	0	0	0	0	0	74
21419390	f	hp	US	2022	1	0	0	0	0	0	0	0	0	0	0	0	57
21419422	m	cn	ID	2022	1	0	0	0	0	0	0	0	0	0	0	0	77.8
21419657	f	cn	ID	2022	1	0	0	0	0	0	0	0	0	0	0	0	81.5
21419832	f	hp	US	2022	0	1	1	0	0	0	0	0	0	0	0	0	75
21420245	f	hp	US	2022	0	1	1	0	0	0	0	0	0	0	0	0	70
21420376	f	ph	JP	2022	0	0	0	0	0	0	0	0	0	0	0	0	47
21420828	f	md	JP	2022	0	1	0	0	0	1	0	0	0	0	0	0	80
21420884	f	ph	JP	2022	0	1	1	0	0	0	0	0	0	0	0	0	80
21421061	f	md	PL	2022	1	0	0	0	0	0	0	0	0	0	0	0	48
21421282	Not	md	JP	2022	0	0	0	0	0	0	0	0	0	0	0	0	

21421370	m	md	JP	2022	0	1	0	0	0	1	0	0	0	0	0	0	56
21421374	m	md	JP	2022	0	0	0	0	0	0	0	0	0	0	0	0	58
21421500	Not	md	FR	2022	0	1	0	0	1	0	0	0	0	0	0	0	69
21421589	f	md	DK	2022	0	1	1	0	0	0	0	0	0	0	0	0	54
21421640	Not	hp	JP	2022	0	1	0	0	1	0	0	0	0	0	0	0	
21421789	Not	md	IT	2022	0	0	0	0	0	0	0	0	0	0	0	0	
21421968	m	cn	US	2022	0	1	0	0	1	0	0	0	0	0	0	0	75
21422146	f	md	US	2022	1	0	0	0	0	0	0	0	0	0	0	0	57
21422203	m	md	JP	2022	0	1	0	0	0	1	0	0	0	0	0	0	70
21422635	Not	ph	JP	2022	1	0	0	0	0	0	0	0	0	0	0	0	
21422720	f	ph	TH	2022	0	0	0	0	0	0	0	0	0	0	0	0	74
21422751	m	cn	TH	2022	0	0	0	0	0	0	0	0	0	0	0	0	73
21422781	f	md	JP	2022	0	0	0	0	0	0	0	0	0	0	0	0	67
21422968	f	hp	US	2022	0	1	0	0	1	0	0	0	0	0	0	0	73
21423301	m	cn	IN	2022	0	0	0	0	0	0	0	0	0	0	0	0	60
21423367	m	cn	ID	2022	0	1	0	1	0	0	0	0	0	0	0	0	65.4
21423484	Not	md	JP	2022	0	1	0	0	0	1	0	0	0	0	0	0	
21423559	f	md	JP	2022	0	0	0	0	0	0	0	0	0	0	0	0	72
21423724	f	md	SE	2022	0	0	0	0	0	0	0	0	0	0	0	0	80
21423829	Not	md	FR	2022	0	0	0	0	0	0	0	0	0	0	0	0	
21423922	m	md	IN	2022	0	0	0	0	0	0	0	0	0	0	0	0	63
21424087	m	md	FR	2022	0	1	1	0	0	0	0	0	0	0	0	1	
21424275	f	md	GB	2022	0	0	0	0	0	0	0	0	0	0	0	0	64
21424481	m	md	JP	2022	0	1	0	0	1	0	0	0	0	0	0	0	76
21424828	m	hp	IT	2022	0	1	0	0	1	0	0	0	0	0	0	0	73
21425909	f	hp	GB	2022	0	0	0	0	0	0	0	0	0	0	0	0	64
21430231	m	hp	JP	2022	0	1	0	0	0	1	0	0	0	0	0	0	80
21432515	Not	md	JP	2022	0	1	0	0	0	1	0	0	0	0	0	0	
21433486	f	ph	US	2022	0	0	0	0	0	0	0	0	0	0	0	0	63
21433492	f	md	US	2022	0	0	0	0	0	0	0	0	0	0	0	0	
21433750	Not	md	US	2022	0	0	0	0	0	0	0	0	0	0	0	0	
21433753	Not	md	US	2022	0	0	0	0	0	0	0	0	0	0	0	0	
21434076	m	md	JP	2022	0	0	0	0	0	0	0	0	0	0	0	0	90
21435144	Not	md	US	2022	0	0	0	0	0	0	0	0	0	0	0	0	

21435374	f	cn	TH	2022	1	0	0	0	0	0	0	0	0	0	0	0	74
21435380	m	md	JP	2022	0	1	0	0	0	1	0	0	0	0	0	0	75
21435433	f	hp	US	2022	1	0	0	0	0	0	0	0	0	0	0	0	
21435514	Not	md	US	2022	0	0	0	0	0	0	0	0	0	0	0	0	
21435607	m	md	JP	2022	0	1	1	0	0	0	0	0	0	0	0	0	59
21435753	m	md	FR	2022	0	0	0	0	0	0	0	0	0	0	0	0	
21436128	f	cn	US	2022	0	0	0	0	0	0	0	0	0	0	0	0	
21436227	m	md	BG	2022	1	0	0	0	0	0	0	0	0	0	0	0	69
21436259	Not	md	FR	2022	0	1	0	0	1	0	0	0	0	0	0	0	69
21436275	Not	md	ID	2022	0	1	0	1	0	0	0	0	0	0	0	0	
21436544	m	md	JP	2022	0	1	0	1	0	0	0	0	0	0	0	0	66
21436768	f	md	DK	2022	1	0	0	0	0	0	0	0	0	0	0	0	80
21436846	f	ph	CA	2022	0	0	0	0	0	0	0	0	0	0	0	0	63
21436948	f	md	FR	2022	0	1	1	0	0	0	0	0	0	0	0	0	50
21436986	m	md	FI	2022	0	1	1	0	0	0	0	0	0	0	0	0	73
21437119	f	cn	US	2022	0	1	1	0	0	0	0	0	0	0	0	0	
21437400	f	md	IT	2022	0	0	0	0	0	0	0	0	0	0	0	0	
21438522	f	cn	IN	2022	0	0	0	0	0	0	0	0	0	0	0	0	69
21438523	f	cn	IN	2022	0	0	0	0	0	0	0	0	0	0	0	0	39
21438887	f	md	FR	2022	0	1	1	0	0	0	0	0	0	0	0	0	50
21438914	f	md	JP	2022	0	1	0	0	1	0	0	0	0	0	0	0	71
21439017	m	md	JP	2022	0	1	0	1	0	0	0	0	0	0	0	0	74
21439244	m	md	JP	2022	0	0	0	0	0	0	0	0	0	0	0	0	74
21439561	f	hp	CA	2022	0	1	1	0	0	0	0	0	0	0	0	0	58
21439882	f	ph	TH	2022	1	0	0	0	0	0	0	0	0	0	0	0	90
21440131	Not	cn	US	2022	0	0	0	0	0	0	0	0	0	0	0	0	
21440541	Not	cn	US	2022	1	0	0	0	0	0	0	0	0	0	0	0	
21440585	f	md	PL	2022	0	0	0	0	0	0	0	0	0	0	0	0	
21441315	f	md	MY	2022	0	0	0	0	0	0	0	0	0	0	0	0	58
21441404	f	md	FR	2022	0	0	0	0	0	0	0	0	0	0	0	0	88
21441871	Not	md	JP	2022	0	1	0	0	1	0	0	0	0	0	0	0	
21441872	Not	md	JP	2022	0	1	0	0	1	0	0	0	0	0	0	0	
21442385	f	md	GB	2022	0	1	0	1	0	0	0	0	0	0	0	0	67
21442845	f	md	US	2022	1	0	0	0	0	0	0	0	0	0	0	0	69

21442846	m	md	US	2022	1	0	0	0	0	0	0	0	0	0	0	0	70
21442850	f	md	US	2022	0	0	0	0	0	0	1	0	0	0	1	0	55
21442853	m	md	US	2022	0	0	0	0	0	0	1	0	0	0	1	0	77
21444819	m	md	JP	2022	0	1	0	1	0	0	0	0	0	0	0	1	66
21445701	m	md	JP	2022	0	0	0	0	0	0	0	0	0	0	0	0	71
21446101	Not	cn	US	2022	1	0	0	0	0	0	0	0	0	0	0	0	76.8
21446215	f	cn	IN	2022	1	0	0	0	0	0	0	0	0	0	0	0	57
21446879	f	ph	US	2022	0	0	0	0	0	0	0	0	0	0	0	0	76
21446892	f	md	US	2022	0	0	0	0	0	0	0	0	0	0	0	0	65
21446930	m	md	JP	2022	0	1	0	1	0	0	0	0	0	0	0	1	63
21446950	m	md	ES	2022	0	1	1	0	0	0	0	0	0	0	0	0	
21446987	m	md	JP	2022	0	1	0	1	0	0	0	0	0	0	0	1	61
21447022	f	md	DK	2022	0	1	1	0	0	0	0	0	0	0	0	0	54
21447080	f	md	JP	2022	0	1	0	1	0	0	0	0	0	0	0	0	80
21447142	f	md	JP	2022	1	0	0	0	0	0	0	0	0	0	0	1	77
21447717	m	md	JP	2022	0	1	0	1	0	0	0	0	0	0	0	0	71
21447991	Not	md	FR	2022	0	1	0	0	1	0	0	0	0	0	0	0	86
21449468	f	hp	FR	2022	0	1	0	0	0	1	0	0	0	0	0	0	26
21449742	f	hp	US	2022	0	0	0	0	0	0	1	0	0	0	1	0	55
21449804	f	cn	US	2022	1	0	0	0	0	0	0	0	0	0	0	0	
21450271	f	md	CA	2022	0	0	0	0	0	0	1	1	0	0	0	0	58
21450841	m	md	JP	2022	0	1	0	1	0	0	0	0	0	0	0	0	72
21451027	m	md	DE	2022	0	0	0	0	0	0	0	0	0	0	0	0	72
21451118	m	md	CN	2022	0	0	0	0	0	0	0	0	0	0	0	0	50
21451167	f	md	ES	2022	0	1	1	0	0	0	0	0	0	0	0	0	
21451529	m	hp	US	2022	0	0	0	0	0	0	1	0	0	0	1	0	77
21451677	m	md	JP	2022	0	1	0	0	1	0	0	0	0	0	0	0	78
21452202	m	md	JP	2022	0	1	0	1	0	0	0	0	0	0	0	0	65
21452309	m	md	ES	2022	0	0	0	0	0	0	0	0	0	0	0	0	74
21452311	f	md	AU	2022	0	0	0	0	0	0	0	0	0	0	0	0	69
21452402	m	md	FR	2022	0	1	0	0	0	1	0	0	0	0	0	0	79
21452428	f	cn	US	2022	0	0	0	0	0	0	0	0	0	0	0	0	74
21452494	Not	md	CN	2022	0	0	0	0	0	0	0	0	0	0	0	0	
21452636	f	md	US	2022	1	0	0	0	0	0	0	0	0	0	0	0	56

21452796	Not	md	JP	2022	0	0	0	0	0	0	0	0	0	0	0	0	50
21452821	m	ph	JP	2022	0	1	0	0	0	1	0	0	0	0	0	0	78
21452885	m	md	JP	2022	0	0	0	0	0	0	0	0	0	0	0	0	
21452921	m	md	JP	2022	0	0	0	0	0	0	0	0	0	0	0	0	80
21452933	m	ph	JP	2022	0	0	0	0	0	0	0	0	0	0	0	0	74
21452986	m	md	JP	2022	0	1	0	0	0	1	0	0	0	0	0	0	50
21452995	Not	md	JP	2022	0	1	0	0	0	1	0	0	0	0	0	0	60
21453035	Not	md	JP	2022	0	1	0	0	0	1	0	0	0	0	0	0	
21453322	f	md	JP	2022	0	1	0	0	1	0	0	0	0	0	0	0	60
21453556	m	md	JP	2022	0	1	0	0	1	0	0	0	0	0	0	0	73
21453558	f	md	PR	2022	0	1	0	0	1	0	0	0	0	0	0	0	
21453615	m	md	JP	2022	0	1	1	0	0	0	0	0	0	0	0	0	81
21453814	f	md	MX	2022	0	0	0	0	0	0	0	0	0	0	0	0	71
21453892	m	md	JP	2022	0	1	0	0	1	0	0	0	0	0	0	0	83
21454070	m	cn	CA	2022	0	1	0	0	1	0	0	0	0	0	0	0	
21454111	Not	md	FR	2022	0	1	0	0	1	0	0	0	0	0	0	0	74
21454135	m	md	JP	2022	0	0	0	0	0	0	0	0	0	0	0	0	73
21454151	m	md	JP	2022	0	0	0	0	0	0	0	0	0	0	0	0	67
21454286	Not	md	US	2022	0	0	0	0	0	0	0	0	0	0	0	0	
21454306	m	md	JP	2022	0	1	0	0	1	0	0	0	0	0	0	1	62
21454521	f	md	JP	2022	0	1	0	0	1	0	0	0	0	0	0	0	60
21454675	m	md	JP	2022	0	1	0	0	1	0	0	0	0	0	0	0	70
21455071	f	md	JP	2022	0	1	0	0	1	0	0	0	0	0	0	0	71
21455390	m	md	FR	2022	0	1	0	0	0	1	0	0	0	0	0	0	80
21455477	Not	hp	NL	2022	0	1	0	0	1	0	0	0	0	0	0	0	62
21456251	m	cn	TW	2022	0	1	0	0	0	1	0	0	0	0	0	0	
21456283	m	md	JP	2022	0	1	0	0	0	1	0	0	0	0	0	0	63
21456315	f	hp	NL	2022	0	1	0	0	1	0	0	0	0	0	0	0	55
21456602	m	ph	JP	2022	0	1	0	1	0	0	0	0	0	0	0	0	66
21456644	m	cn	CN	2022	0	0	0	0	0	0	0	0	0	0	0	0	69
21456737	m	md	AU	2022	1	0	0	0	0	0	0	0	0	0	0	0	
21456742	f	cn	CN	2022	1	0	0	0	0	0	0	0	0	0	0	0	82
21456888	Not	hp	NL	2022	0	1	0	0	1	0	0	0	0	0	0	0	62
21456993	m	md	US	2022	1	0	0	0	0	0	0	0	0	0	0	0	51

21457581	Not	ph	JP	2022	1	0	0	0	0	0	0	0	0	0	0	0	
21457676	f	ph	JP	2022	1	0	0	0	0	0	0	0	0	0	0	0	89
21457680	f	md	FR	2022	1	0	0	0	0	0	0	0	0	0	0	0	75
21458155	f	hp	KR	2022	0	0	0	0	0	0	0	0	0	0	0	0	70
21458175	m	cn	IN	2022	0	1	0	0	1	0	0	0	0	0	0	0	69
21458270	f	md	JP	2022	0	0	0	0	0	0	0	0	0	0	0	0	30
21458327	f	hp	PT	2022	0	0	0	0	0	0	0	0	0	0	0	0	53
21458404	m	ph	FR	2022	0	0	0	0	0	0	0	0	0	0	0	0	57
21458453	m	md	NP	2022	0	0	0	0	0	0	0	0	0	0	0	0	55
21458788	m	cn	CN	2022	0	1	0	0	1	0	0	0	0	0	0	0	57
21458888	Not	md	JP	2022	0	1	0	0	0	1	0	0	0	0	0	0	
21458927	f	md	JP	2022	0	1	0	0	0	1	0	0	0	0	0	0	90
21458951	Not	md	ES	2022	0	1	1	0	0	0	0	0	0	0	0	0	
21458976	m	ph	US	2022	0	0	0	0	0	0	0	0	0	0	0	0	77
21459042	f	cn	FR	2022	0	1	1	0	0	0	0	0	0	0	0	0	50
21459172	Not	md	JP	2022	0	1	0	0	0	1	0	0	0	0	0	0	75
21459238	m	hp	JP	2022	0	1	0	0	1	0	0	0	0	0	0	0	49
21459859	m	md	JP	2022	0	1	1	0	0	0	0	0	0	0	0	0	75
21459957	f	hp	US	2022	1	0	0	0	0	0	0	0	0	0	0	0	57
21461394	f	md	JP	2022	0	1	0	1	0	0	0	0	0	0	0	0	79
21461940	Not	hp	US	2022	0	1	1	0	0	0	0	0	0	0	0	0	
21462088	m	cn	TH	2022	0	1	0	0	0	1	0	0	0	0	0	0	
21462102	Not	cn	CN	2022	0	0	0	0	0	0	0	0	0	0	0	0	
21462287	m	md	CN	2022	0	0	0	0	0	0	0	0	0	0	0	0	63
21462415	m	md	JP	2022	0	1	0	0	1	0	0	0	0	0	0	1	81
21462478	m	md	JP	2022	0	1	0	0	1	0	0	0	0	0	0	0	79
21462566	m	md	JP	2022	0	1	0	0	1	0	0	0	0	0	0	0	59
21462716	f	md	DE	2022	0	1	0	0	1	0	0	0	0	0	0	0	68
21463077	m	md	CN	2022	0	0	0	0	0	0	0	0	0	0	0	0	70
21463209	Not	cn	CA	2022	0	1	1	0	0	0	0	0	0	0	0	0	
21463233	f	md	FR	2022	0	0	0	0	0	0	0	0	0	0	0	0	49
21463279	m	md	RU	2022	0	1	0	0	0	1	0	0	0	0	0	0	61
21463411	f	md	ES	2022	0	0	0	0	0	0	0	0	0	0	0	0	51
21463524	f	cn	CN	2022	0	1	0	0	1	0	0	0	0	0	0	0	45

21463640	m	md	JP	2022	0	1	0	0	1	0	0	0	0	0	0	0	70
21464573	m	md	CZ	2022	0	1	0	0	1	0	0	0	0	0	0	0	
21464993	m	hp	JP	2022	0	1	1	0	0	0	0	0	0	0	0	0	68
21465279	m	md	JP	2022	0	1	0	0	1	0	0	0	0	0	0	0	63
21465586	f	ph	US	2022	0	0	0	0	0	0	0	0	0	0	0	0	65
21465857	f	hp	IT	2022	1	0	0	0	0	0	0	0	0	0	0	0	58
21466037	m	cn	MX	2022	0	1	0	0	0	1	0	0	0	0	0	0	
21466473	m	md	US	2022	0	0	0	0	0	0	0	0	0	0	0	0	74
21466839	f	ph	US	2022	0	0	0	0	0	0	0	0	0	0	0	0	70
21466863	Not	hp	US	2022	0	0	0	0	0	0	0	0	0	0	0	0	
21466871	f	cn	BE	2022	0	0	0	0	0	0	0	0	0	0	0	0	
21466896	Not	hp	US	2022	0	0	0	0	0	0	0	0	0	0	0	0	
21466959	f	cn	US	2022	1	0	0	0	0	0	0	0	0	0	0	0	75
21467090	f	cn	CN	2022	0	1	0	1	0	0	0	0	0	0	0	0	33
21467138	m	hp	MX	2022	0	1	0	0	1	0	0	0	0	0	0	0	66
21467162	f	md	PT	2022	1	0	0	0	0	0	0	0	0	0	0	0	53
21467163	m	md	RO	2022	1	0	0	0	0	0	0	0	0	0	0	0	69
21467198	m	hp	ES	2022	0	1	0	0	1	0	0	0	0	0	0	0	51
21467875	m	md	US	2022	0	1	1	0	0	0	0	0	0	0	0	0	63
21468681	m	md	CN	2022	0	0	0	0	0	0	0	0	0	0	0	0	82
21470118	f	md	CN	2022	0	0	0	0	0	0	0	0	0	0	0	0	57
21470563	m	md	GR	2022	0	0	0	0	0	0	0	0	0	0	0	0	76
21470805	m	md	IT	2022	0	0	0	0	0	0	0	0	0	0	0	0	77
21471072	m	md	JP	2022	0	1	0	0	0	1	0	0	0	0	0	1	
21471288	f	md	FR	2022	1	0	0	0	0	0	0	0	0	0	0	0	75
21471291	f	md	JP	2022	1	0	0	0	0	0	0	0	0	0	0	0	71
21471583	Not	hp	US	2022	0	0	0	0	0	0	0	0	0	0	0	0	
21471600	Not	hp	US	2022	0	0	0	0	0	0	0	0	0	0	0	0	
21471674	Not	hp	US	2022	0	0	0	0	0	0	0	0	0	0	0	0	
21472092	Not	md	JP	2022	0	1	0	1	0	0	0	0	0	0	0	0	
21472395	f	cn	MY	2022	1	0	0	0	0	0	0	0	0	0	0	0	91.8
21472401	Not	cn	US	2022	1	0	0	0	0	0	0	0	0	0	0	0	59.5
21472472	m	hp	CA	2022	0	0	0	0	0	0	0	0	0	0	0	0	61
21472829	m	cn	IN	2022	0	0	0	0	0	0	0	0	0	0	0	0	64

21472912	m	cn	EG	2022	0	1	1	0	0	0	0	0	0	0	0	0	
21473334	Not	cn	US	2022	1	0	0	0	0	0	0	0	0	0	0	0	87.1
21473622	m	cn	IN	2022	0	0	0	0	0	0	0	0	0	0	0	0	56
21473679	m	cn	EG	2022	0	0	0	0	0	0	0	0	0	0	0	0	34
21473689	Not	cn	AU	2022	0	0	0	0	0	0	0	0	0	0	0	0	
21473699	f	cn	US	2022	1	0	0	0	0	0	0	0	0	0	0	0	62
21473750	m	cn	CN	2022	0	1	0	0	1	0	0	0	0	0	0	0	56
21473953	f	md	DE	2022	0	0	0	0	0	0	0	0	0	0	0	0	67
21474761	m	md	FR	2022	0	1	0	0	0	1	0	0	0	0	0	0	81
21475185	m	md	DE	2022	0	1	0	0	1	0	0	0	0	0	0	0	67
21475273	f	hp	BG	2022	1	0	0	0	0	0	0	0	0	0	0	0	80
21475455	m	md	JP	2022	0	1	0	0	1	0	0	0	0	0	0	0	59
21476035	f	md	JP	2022	0	1	0	0	0	1	0	0	0	0	0	0	42
21476117	m	md	JP	2022	0	1	0	0	1	0	0	0	0	0	0	0	55
21477704	f	md	JP	2022	0	1	0	0	0	1	0	0	0	0	0	0	60
21477842	f	md	AT	2022	0	0	0	0	0	0	0	0	0	0	0	0	69
21477916	m	md	JP	2022	0	0	0	0	0	0	0	0	0	0	0	0	70
21477937	f	ph	FR	2022	0	1	1	0	0	0	0	0	0	0	0	0	72
21478051	f	md	JP	2022	0	1	0	0	0	1	0	0	0	0	0	0	69
21478186	Not	cn	US	2022	0	0	0	0	0	0	0	0	0	0	0	0	
21478187	Not	md	GR	2022	0	1	1	0	0	0	0	0	0	0	0	0	
21478463	m	md	FR	2022	0	1	1	0	0	0	0	0	0	0	0	0	68
21478535	f	hp	GR	2022	0	1	0	0	1	0	0	0	0	0	0	1	68
21478599	f	hp	CA	2022	0	1	1	0	0	0	0	0	0	0	0	0	58
21478666	f	md	FR	2022	0	0	0	0	0	0	0	0	0	0	0	0	
21478797	f	md	US	2022	0	0	0	0	0	0	0	0	0	0	0	0	55
21479285	m	hp	US	2022	0	0	0	0	0	0	0	0	0	0	0	0	
21479527	f	md	JP	2022	0	0	0	0	0	0	0	0	0	0	0	0	
21479623	Not	cn	US	2022	0	0	0	0	0	0	0	0	0	0	0	0	
21479777	f	cn	TH	2022	0	1	0	0	1	0	0	0	0	0	0	0	79
21479788	m	md	BR	2022	0	0	0	0	0	0	0	0	0	0	0	0	
21480199	m	md	FR	2022	0	0	0	0	0	0	0	0	0	0	0	0	44
21480526	m	hp	ES	2022	0	1	0	0	1	0	0	0	0	0	0	0	65
21480845	m	cn	IN	2022	0	0	0	0	0	0	0	0	0	0	0	0	48

21481000	f	cn	US	2022	1	0	0	0	0	0	0	0	0	0	0	0	66
21481498	m	hp	FR	2022	0	1	0	0	1	0	0	0	0	0	0	0	49
21482228	Not	hp	US	2022	0	1	1	0	0	0	0	0	0	0	0	0	
21482631	f	md	NL	2022	0	1	0	0	1	0	0	0	0	0	0	0	66
21482646	m	cn	US	2022	0	0	0	0	0	0	0	0	0	0	0	0	69
21482808	f	hp	CA	2022	0	0	0	0	0	0	0	0	0	0	0	0	70
21482810	Not	hp	FR	2022	0	1	0	0	1	0	0	0	0	0	0	0	85
21482812	f	cn	IN	2022	0	0	0	0	0	0	0	0	0	0	0	0	
21483397	f	hp	CA	2022	0	1	1	0	0	0	0	0	0	0	0	0	58
21483400	f	hp	CA	2022	0	1	1	0	0	0	0	0	0	0	0	0	58
21483695	Not	hp	CN	2022	0	1	1	0	0	0	0	0	0	0	0	0	
21484049	f	hp	CN	2022	1	0	0	0	0	0	0	0	0	0	0	0	63
21484728	m	md	JP	2022	1	0	0	0	0	0	0	0	0	0	0	0	78
21484920	m	hp	ES	2022	0	0	0	0	0	0	0	0	0	0	0	0	
21485005	f	hp	US	2022	0	0	0	0	0	0	0	0	0	0	0	0	44
21485061	f	md	IE	2022	0	0	0	0	0	0	0	0	0	0	0	0	55
21485264	f	cn	JP	2022	1	0	0	0	0	0	0	0	0	0	0	0	
21485466	m	cn	JP	2022	1	0	0	0	0	0	0	0	0	0	0	1	69
21485624	f	hp	US	2022	1	0	0	0	0	0	0	0	0	0	0	0	60
21485852	Not	hp	US	2022	0	1	1	0	0	0	0	0	0	0	0	0	
21486062	m	cn	US	2022	0	1	0	0	1	0	0	0	0	0	0	0	76
21486141	m	cn	IN	2022	0	0	0	0	0	0	0	0	0	0	0	0	58
21486149	m	md	CA	2022	0	0	0	0	0	0	0	0	0	0	0	0	62
21486210	m	md	JP	2022	0	0	0	0	0	0	0	0	0	0	0	0	
21486328	m	hp	DE	2022	0	1	1	0	0	0	0	0	0	0	0	0	51
21486358	m	md	HK	2022	0	1	1	0	0	0	0	0	0	0	0	0	
21486378	f	cn	EG	2022	0	0	0	0	0	0	0	0	0	0	0	0	78
21486769	m	md	CH	2022	0	1	0	1	0	0	0	0	0	0	0	0	76.1
21486823	m	md	NL	2022	0	0	0	0	0	0	0	0	0	0	0	0	45
21486848	m	md	ES	2022	0	0	0	0	0	0	0	0	0	0	0	0	56
21487024	m	md	JP	2022	0	0	0	0	0	0	0	0	0	0	0	0	50
21487066	f	hp	JP	2022	0	1	0	0	1	0	0	0	0	0	0	0	80
21487134	f	md	IN	2022	0	0	0	0	0	0	0	0	0	0	0	0	56
21487243	m	md	BR	2022	0	0	0	0	0	0	0	0	0	0	0	0	83

21487259	f	md	US	2022	0	1	1	0	0	0	0	0	0	0	0	0	62
21487280	m	cn	CN	2022	0	1	0	0	1	0	0	0	0	0	0	0	65
21487315	f	md	FR	2022	0	0	0	0	0	0	0	0	0	0	0	0	62
21487537	f	md	DE	2022	0	1	0	0	0	1	0	0	0	0	0	0	70
21487572	f	md	CZ	2022	0	1	0	0	1	0	0	0	0	0	0	0	62
21488664	m	hp	US	2022	0	0	0	0	0	0	0	0	0	0	0	0	39
21489195	m	md	JP	2022	0	1	0	0	1	0	0	0	0	0	0	0	67
21490025	f	md	JP	2022	0	1	1	0	0	0	0	0	0	0	0	0	80
21490214	m	md	JP	2022	0	1	0	0	1	0	0	0	0	0	0	0	78
21490521	m	md	JP	2022	0	1	0	0	1	0	0	0	0	0	0	0	78
21490623	m	md	HK	2022	0	1	0	0	0	1	0	0	0	0	0	0	52
21491757	m	ph	FR	2022	0	1	0	1	0	0	0	0	0	0	0	0	
21491961	m	md	CZ	2022	0	1	0	0	1	0	0	0	0	0	0	1	59
21492384	Not	hp	CN	2022	0	0	0	0	0	0	0	0	0	0	0	0	
21492385	Not	hp	CN	2022	0	0	0	0	0	0	0	0	0	0	0	0	
21493062	Not	hp	CN	2022	0	0	0	0	0	0	0	0	0	0	0	0	
21493075	Not	md	JP	2022	0	0	0	0	0	0	0	0	0	0	0	0	
21493100	f	md	JP	2022	0	1	0	0	1	0	0	0	0	0	0	0	66
21493137	Not	cn	US	2022	1	0	0	0	0	0	0	0	0	0	0	0	76.8
21493236	Not	hp	CN	2022	0	0	0	0	0	0	0	0	0	0	0	0	
21493256	Not	hp	US	2022	1	0	0	0	0	0	0	0	0	0	0	0	
21493258	Not	hp	CN	2022	0	0	0	0	0	0	0	0	0	0	0	0	
21493565	f	md	AU	2022	0	1	1	0	0	0	0	0	0	0	0	0	79
21493596	m	cn	ID	2022	1	0	0	0	0	0	0	0	0	0	0	0	57.8
21493678	m	cn	ID	2022	1	0	0	0	0	0	0	0	0	0	0	0	75.9
21493710	f	md	AU	2022	0	0	0	0	0	0	0	0	0	0	0	0	66
21493917	m	md	FR	2022	0	1	0	0	1	0	0	0	0	0	0	0	75
21494134	Not	hp	PL	2022	0	1	0	1	0	0	0	0	0	0	0	0	
21494273	m	md	TR	2022	0	1	0	0	1	0	0	0	0	0	0	0	68
21494274	m	md	JP	2022	0	0	0	0	0	0	0	0	0	0	0	0	54
21494294	f	cn	ID	2022	1	0	0	0	0	0	0	0	0	0	0	0	77.1
21494330	m	md	AU	2022	0	0	0	0	0	0	0	0	0	0	0	0	
21494621	m	cn	ID	2022	1	0	0	0	0	0	0	0	0	0	0	0	64.9
21494736	m	md	JP	2022	0	0	0	0	0	0	0	0	0	0	0	0	80

21495542	f	md	US	2022	0	0	0	0	0	0	0	0	0	0	0	0	63
21496530	m	cn	TH	2022	0	0	0	0	0	0	0	0	0	0	0	0	50
21496611	f	hp	IT	2022	0	0	0	0	0	0	0	0	0	0	0	0	63
21496861	f	hp	IT	2022	0	0	0	0	0	0	0	0	0	0	0	0	63
21496929	m	hp	IT	2022	0	1	1	0	0	0	0	0	0	0	0	0	68
21497541	f	md	JP	2022	1	0	0	0	0	0	0	0	0	0	0	0	66
21497931	f	ph	FR	2022	0	0	0	0	0	0	0	0	0	0	0	0	
21498117	m	md	BG	2022	0	1	1	0	0	0	0	0	0	0	0	1	65
21498143	m	md	JP	2022	0	1	0	0	1	0	0	0	0	0	0	0	69
21498253	f	md	JP	2022	0	0	0	0	0	0	0	0	0	0	0	0	46
21498263	f	hp	IT	2022	0	0	0	0	0	0	0	0	0	0	0	0	69
21498265	m	hp	IT	2022	0	0	0	0	0	0	0	0	0	0	0	0	65
21498267	m	hp	IT	2022	0	1	1	0	0	0	0	0	0	0	0	0	68
21498270	m	hp	IT	2022	0	0	0	0	0	0	0	0	0	0	0	0	75
21498271	m	hp	IT	2022	0	0	0	0	0	0	0	0	0	0	0	0	65
21498272	f	hp	IT	2022	0	1	0	0	1	0	0	0	0	0	0	0	53
21498278	f	hp	IT	2022	0	0	0	0	0	0	0	0	0	0	0	0	63
21498359	m	cn	US	2022	1	0	0	0	0	0	0	0	0	0	0	0	80
21498451	m	md	JP	2022	0	0	0	0	0	0	0	0	0	0	0	0	69
21498482	f	cn	CA	2022	1	0	0	0	0	0	0	0	0	0	0	0	
21498598	f	md	CA	2022	1	0	0	0	0	0	0	0	0	0	0	0	71
21498680	m	hp	IT	2022	0	0	0	0	0	0	0	0	0	0	0	0	82
21498681	m	hp	IT	2022	0	0	0	0	0	0	0	0	0	0	0	0	73
21498691	f	hp	IT	2022	0	0	0	0	0	0	0	0	0	0	0	0	69
21498695	m	hp	IT	2022	0	0	0	0	0	0	0	0	0	0	0	0	69
21498706	m	hp	IT	2022	0	0	0	0	0	0	0	0	0	0	0	0	65
21498707	f	hp	IT	2022	0	0	0	0	0	0	0	0	0	0	0	0	69
21498908	m	hp	GR	2022	0	1	0	0	1	0	0	0	0	0	0	0	82
21499115	f	hp	US	2022	1	0	0	0	0	0	0	0	0	0	0	0	63
21499176	m	cn	IN	2022	1	0	0	0	0	0	0	0	0	0	0	0	88
21499186	f	ph	JP	2022	0	1	1	0	0	0	0	0	0	0	0	0	70
21499926	f	md	JP	2022	0	0	0	0	0	0	0	0	0	0	0	0	80
21499974	m	md	CZ	2022	0	1	0	0	1	0	0	0	0	0	0	1	59
21500040	f	md	FR	2022	0	0	0	0	0	0	0	0	0	0	0	0	68

21500064	m	md	MX	2022	0	0	0	0	0	0	0	0	0	0	0	0	68
21500155	Not	hp	CO	2022	0	0	0	0	0	0	0	0	0	0	0	0	
21500352	Not	ph	JP	2022	0	1	0	0	1	0	0	0	0	0	0	0	
21500373	m	md	RU	2022	0	1	0	0	0	1	0	0	0	0	0	0	63
21500721	f	ph	JP	2022	0	0	0	0	0	0	0	0	0	0	0	0	74
21500756	m	md	RO	2022	0	1	1	0	0	0	0	0	0	0	0	0	67
21501148	f	md	JP	2022	0	0	0	0	0	0	0	0	0	0	0	0	75
21501364	Not	ph	JP	2022	0	1	0	0	0	1	0	0	0	0	0	0	
21501781	f	ph	US	2022	0	0	0	0	0	0	0	0	0	0	0	0	
21502531	Not	md	JP	2022	0	1	0	0	0	1	0	0	0	0	0	0	60
21502935	f	cn	CA	2022	1	0	0	0	0	0	0	0	0	0	0	0	
21503184	Not	hp	FR	2022	0	1	0	0	1	0	0	0	0	0	0	0	77
21503272	m	md	JP	2022	1	0	0	0	0	0	0	0	0	0	0	0	54
21503618	f	hp	IT	2022	0	0	0	0	0	0	0	0	0	0	0	0	69
21503794	f	hp	IT	2022	0	0	0	0	0	0	0	0	0	0	0	0	69
21503810	f	hp	IT	2022	0	0	0	0	0	0	0	0	0	0	0	0	69
21503911	f	md	JP	2022	1	0	0	0	0	0	0	0	0	0	0	0	73
21503966	m	md	TR	2022	0	1	0	0	1	0	0	0	0	0	0	0	47
21504034	f	cn	CA	2022	0	0	0	0	0	0	0	0	0	0	0	0	60
21504170	Not	md	FR	2022	0	1	0	0	1	0	0	0	0	0	0	0	77
21504293	m	cn	ID	2022	0	1	0	1	0	0	0	0	0	0	0	0	69.2
21504395	f	md	JP	2022	0	0	0	0	0	0	0	0	0	0	0	0	
21504658	m	md	PL	2022	0	1	0	0	0	1	0	0	0	0	0	0	71
21505122	Not	md	JP	2022	0	1	0	0	0	1	0	0	0	0	0	0	50
21505241	f	cn	TW	2022	0	0	0	0	0	0	0	0	0	0	0	0	59
21505796	f	ph	JP	2022	1	0	0	0	0	0	0	0	0	0	0	0	60
21505903	f	md	IT	2022	0	0	0	0	0	0	0	0	0	0	0	0	48
21506859	m	md	JP	2022	0	1	0	0	0	1	0	0	0	0	0	0	80
21507084	m	md	JP	2022	0	1	0	1	0	0	0	0	0	0	0	0	78
21508035	m	md	PF	2022	0	1	0	1	0	0	0	0	0	0	0	0	
21508060	Not	md	JP	2022	0	1	0	0	1	0	0	0	0	0	0	0	
21509184	f	md	JP	2022	0	1	0	0	1	0	0	0	0	0	0	0	73
21509529	f	hp	US	2022	0	0	0	0	0	0	0	0	0	0	0	0	73
21509741	f	md	FR	2022	0	0	0	0	0	0	0	0	0	0	0	0	62

21511118	m	md	JP	2022	0	1	0	0	0	1	0	0	0	0	0	0	74
21511305	m	md	JP	2022	0	1	0	0	1	0	0	0	0	0	0	0	65
21511574	f	md	JP	2022	0	0	0	0	0	0	0	0	0	0	0	0	
21511966	m	md	JP	2022	0	1	1	0	0	0	0	0	0	0	0	0	85
21512191	m	ph	JP	2022	0	1	1	0	0	0	0	0	0	0	0	0	76
21512387	f	hp	US	2022	1	0	0	0	0	0	0	0	0	0	0	0	63
21512606	m	hp	CN	2022	0	0	0	0	0	0	0	0	0	0	0	0	59
21513792	m	hp	CN	2022	0	0	0	0	0	0	0	0	0	0	0	0	83
21513923	m	md	JP	2022	0	1	0	0	1	0	0	0	0	0	0	0	71
21515304	m	cn	AR	2022	0	0	0	0	0	0	0	0	0	0	0	0	70
21515499	Not	hp	US	2022	0	1	0	1	0	0	0	0	0	0	0	0	
21515834	m	md	JP	2022	0	1	0	1	0	0	0	0	0	0	0	1	67
21515878	m	cn	TH	2022	1	0	0	0	0	0	0	0	0	0	0	0	69.6
21515932	f	ph	JP	2022	1	0	0	0	0	0	0	0	0	0	0	0	72
21516341	f	hp	BR	2022	0	1	1	0	0	0	0	0	0	0	0	0	61
21516408	m	md	TW	2022	0	0	0	0	0	0	0	0	0	0	0	0	67
21516422	m	md	DE	2022	0	1	0	0	1	0	0	0	0	0	0	0	65
21516460	f	cn	CN	2022	0	0	0	0	0	0	0	0	0	0	0	0	54
21516492	f	hp	US	2022	0	1	1	0	0	0	0	0	0	0	0	0	
21516499	m	md	JP	2022	0	1	0	0	1	0	0	0	0	0	0	0	74
21516511	m	md	JP	2022	0	0	0	0	0	0	0	0	0	0	0	0	72
21517468	Not	md	JP	2022	0	1	0	0	1	0	0	0	0	0	0	0	
21517669	m	md	JP	2022	0	0	0	0	0	0	0	0	0	0	0	0	73
21517835	f	cn	US	2022	0	1	0	0	0	1	0	0	0	0	0	0	68
21518156	m	cn	US	2022	0	0	0	0	0	0	0	0	0	0	0	0	
21518838	m	md	JP	2022	0	1	0	1	0	0	0	0	0	0	0	0	80
21518865	m	ph	JP	2022	0	1	0	0	0	1	0	0	0	0	0	0	70
21519029	f	md	IL	2022	1	0	0	0	0	0	0	0	0	0	0	0	82
21519072	f	md	JP	2022	0	0	0	0	0	0	0	0	0	0	0	0	79
21519244	f	md	CN	2022	0	1	1	0	0	0	0	0	0	0	0	0	50
21519494	m	md	IN	2022	1	0	0	0	0	0	0	0	0	0	0	0	52
21519501	f	ph	FR	2022	1	0	0	0	0	0	0	0	0	0	0	0	59
21519716	m	hp	US	2022	0	0	0	0	0	0	0	0	0	0	0	0	56
21520292	f	md	CZ	2022	0	1	0	0	1	0	0	0	0	0	0	0	74

21520434	m	md	HR	2022	0	1	1	0	0	0	0	0	0	0	0	1	
21520815	m	md	RO	2022	0	0	0	0	0	0	0	0	0	0	0	0	50
21520988	f	md	ES	2022	1	0	0	0	0	0	0	0	0	0	0	0	
21521129	f	md	CA	2022	1	0	0	0	0	0	0	0	0	0	0	0	
21521132	m	md	JP	2022	0	1	1	0	0	0	0	0	0	0	0	0	
21521250	f	md	AU	2022	1	0	0	0	0	0	0	0	0	0	0	0	
21521358	m	md	JP	2022	0	1	0	0	1	0	0	0	0	0	0	0	55
21521523	m	cn	CA	2022	1	0	0	0	0	0	0	0	0	0	0	0	
21521525	m	md	JP	2022	0	1	0	1	0	0	0	0	0	0	0	0	74
21521818	f	md	CN	2022	0	0	0	0	0	0	0	0	0	0	0	0	66
21522011	f	md	US	2022	0	0	0	0	0	0	0	0	0	0	0	0	
21522072	m	hp	IT	2022	0	0	0	0	0	0	0	0	0	0	0	0	73
21522077	f	md	JP	2022	0	1	1	0	0	0	0	0	0	0	0	0	79
21522312	f	hp	DK	2022	0	0	0	0	0	0	0	0	0	0	0	0	39
21524040	m	md	JP	2022	0	0	0	0	0	0	0	0	0	0	0	0	59
21525416	m	md	JP	2022	0	0	0	0	0	0	0	0	0	0	0	0	
21525699	m	md	JP	2022	0	1	0	0	1	0	0	0	0	0	0	0	70
21525841	m	hp	TW	2022	0	1	1	0	0	0	0	0	0	0	0	0	45
21525989	Not	md	JP	2022	0	1	0	0	0	1	0	0	0	0	0	0	74
21525990	m	md	JP	2022	0	1	0	0	0	1	0	0	0	0	0	0	62
21526755	m	md	MY	2022	0	0	0	0	0	0	0	0	0	0	0	0	53
21526769	m	hp	BY	2022	0	1	0	0	0	1	0	0	0	0	0	0	49
21527191	f	hp	JP	2022	0	0	0	0	0	0	0	0	0	0	0	0	66
21527408	m	md	DE	2022	0	1	0	0	1	0	0	0	0	0	0	0	67
21527836	f	ph	JP	2022	0	0	0	0	0	0	0	0	0	0	0	0	53
21528183	m	cn	US	2022	0	0	0	0	0	0	0	0	0	0	0	0	
21528300	f	md	CZ	2022	0	1	0	0	1	0	0	0	0	0	0	0	62
21528446	Not	md	JP	2022	0	1	0	0	0	1	0	0	0	0	0	0	
21528519	f	hp	ES	2022	0	1	1	0	0	0	0	0	0	0	0	0	77
21528551	f	hp	ES	2022	0	1	1	0	0	0	0	0	0	0	0	0	55
21528557	m	md	JP	2022	0	1	0	0	1	0	0	0	0	0	0	0	80
21528638	f	md	JP	2022	0	1	0	0	0	1	0	0	0	0	0	0	66
21528746	f	md	CZ	2022	0	1	0	0	1	0	0	0	0	0	0	0	76
21528929	m	md	DE	2022	0	1	1	0	0	0	0	0	0	0	0	1	

21529170	f	md	US	2022	1	0	0	0	0	0	0	0	0	0	0	1	
21529231	m	hp	ES	2022	0	1	1	0	0	0	0	0	0	0	0	0	72
21529353	f	md	JP	2022	0	1	1	0	0	0	0	0	0	0	0	0	70
21529356	m	md	KR	2022	0	1	1	0	0	0	0	0	0	0	0	0	
21529461	f	md	KR	2022	0	0	0	0	0	0	0	0	0	0	0	0	72
21529929	m	md	JP	2022	0	1	0	0	1	0	0	0	0	0	0	0	90
21530014	f	md	US	2022	0	0	0	0	0	0	0	0	0	0	0	0	
21530030	f	md	DE	2022	0	0	0	0	0	0	0	0	0	0	0	0	42
21530037	m	md	DE	2022	0	0	0	0	0	0	0	0	0	0	0	0	69
21530077	m	md	IT	2022	0	0	0	0	0	0	0	0	0	0	0	0	66
21530149	m	md	JP	2022	0	1	1	0	0	0	0	0	0	0	0	0	80
21531156	m	md	NL	2022	0	0	0	0	0	0	0	0	0	0	0	0	
21531452	Not	md	TW	2022	0	0	0	0	0	0	0	0	0	0	0	0	70
21531462	f	hp	TW	2022	0	0	0	0	0	0	0	0	0	0	0	0	72
21531522	m	md	JP	2022	0	1	0	0	1	0	0	0	0	0	0	0	55
21531630	m	md	JP	2022	0	1	0	0	1	0	0	0	0	0	0	0	62
21532289	m	md	CN	2022	0	0	0	0	0	0	0	0	0	0	0	0	67
21532578	m	md	DE	2022	0	0	0	0	0	0	0	0	0	0	0	0	43
21532910	f	cn	DE	2022	0	0	0	0	0	0	0	0	0	0	0	0	
21532993	m	md	IT	2022	1	0	0	0	0	0	0	0	0	0	0	0	62.3
21533206	f	hp	CN	2022	0	1	1	0	0	0	0	0	0	0	0	0	50
21533314	f	md	AT	2022	0	0	0	0	0	0	0	0	0	0	0	0	69
21533401	f	md	DE	2022	0	1	0	0	1	0	0	0	0	0	0	0	58
21533676	m	md	FR	2022	0	1	0	0	1	0	0	0	0	0	0	0	62
21533710	m	hp	PL	2022	0	1	1	0	0	0	0	0	0	0	0	0	68
21533715	f	md	ES	2022	0	1	1	0	0	0	0	0	0	0	0	0	74
21534381	f	hp	US	2022	0	1	0	0	1	0	0	0	0	0	0	0	74
21535480	f	md	IL	2022	0	0	0	0	0	0	0	0	0	0	0	0	72
21535831	f	hp	US	2022	0	0	0	0	0	0	0	0	0	0	0	0	78
21535970	m	md	JP	2022	0	1	1	0	0	0	0	0	0	0	0	0	70
21536150	m	cn	IN	2022	0	0	0	0	0	0	0	0	0	0	0	0	47
21536156	m	cn	IN	2022	0	0	0	0	0	0	0	0	0	0	0	0	62
21537371	f	md	CN	2022	0	0	0	0	0	0	0	0	0	0	0	0	66
21537970	m	md	JP	2022	0	0	0	0	0	0	0	0	0	0	0	0	75

21538852	m	md	JP	2022	0	1	0	0	0	1	0	0	0	0	0	0	
21538869	m	hp	TW	2022	0	1	1	0	0	0	0	0	0	0	0	0	73
21539346	f	hp	TW	2022	0	1	1	0	0	0	0	0	0	0	0	0	65
21539358	m	md	JP	2022	0	1	1	0	0	0	0	0	0	0	0	0	67
21539537	f	md	JP	2022	0	0	0	0	0	0	0	0	0	0	0	0	50
21539753	f	hp	DK	2022	0	0	0	0	0	0	0	0	0	0	0	0	39
21540024	f	cn	IL	2022	0	0	0	0	0	0	0	0	0	0	0	0	54
21540149	m	md	JP	2022	0	1	0	0	0	1	0	0	0	0	0	0	
21540182	f	md	CN	2022	0	0	0	0	0	0	0	0	0	0	0	0	70
21540234	m	md	JP	2022	0	1	0	0	0	1	0	0	0	0	0	0	76
21540271	f	md	SK	2022	0	0	0	0	0	0	0	0	0	0	0	0	68
21540299	Not	md	JP	2022	0	1	0	0	0	1	0	0	0	0	0	1	
21540582	m	md	JP	2022	0	0	0	0	0	0	0	0	0	0	0	0	
21540827	m	md	JP	2022	0	1	0	0	1	0	0	0	0	0	0	0	73
21541019	m	md	BE	2022	0	0	0	0	0	0	0	0	0	0	0	0	79
21541139	f	cn	MY	2022	1	0	0	0	0	0	0	0	0	0	0	0	71.6
21541162	m	cn	CA	2022	0	0	0	0	0	0	0	0	0	0	0	0	84
21541216	m	hp	IT	2022	0	1	1	0	0	0	0	0	0	0	0	0	68
21541295	Not	md	JP	2022	0	1	0	0	0	1	0	0	0	0	0	1	
21541410	m	Not repo	Not re	2022	0	0	0	0	0	0	0	0	0	0	0	0	63
21541473	f	hp	IT	2022	0	0	0	0	0	0	0	0	0	0	0	0	69
21541500	m	hp	IT	2022	0	0	0	0	0	0	0	0	0	0	0	0	65
21541925	f	md	US	2022	0	1	0	0	1	0	0	0	0	0	0	0	
21542014	m	md	IN	2022	0	1	0	0	1	0	0	0	0	0	0	0	48
21542057	Not	ph	CA	2022	0	0	0	0	0	0	0	0	0	0	0	0	
21542142	f	cn	TH	2022	0	0	0	0	0	0	0	0	0	0	0	0	52
21542158	f	cn	IN	2022	0	0	0	0	0	0	0	0	0	0	0	0	44
21542191	f	md	JP	2022	0	0	0	0	0	0	0	0	0	0	0	0	70
21542635	f	cn	US	2022	0	0	0	0	0	0	0	0	0	0	0	0	
21542653	f	md	JP	2022	0	1	0	0	0	1	0	0	0	0	0	0	90
21542705	f	md	CZ	2022	0	1	0	0	1	0	0	0	0	0	0	0	62
21542734	Not	md	JP	2022	1	0	0	0	0	0	0	0	0	0	0	0	
21542901	m	md	DE	2022	0	0	0	0	0	0	0	0	0	0	0	0	61
21542953	f	md	DE	2022	0	1	0	0	1	0	0	0	0	0	0	0	59

21543564	Not	cn	US	2022	1	0	0	0	0	0	0	0	0	0	0	0	
21543651	f	hp	KR	2022	0	0	0	0	0	0	0	0	0	0	0	0	60
21543793	m	cn	IN	2022	0	0	0	0	0	0	0	0	0	0	0	0	71
21543991	f	md	ES	2022	0	1	1	0	0	0	0	0	0	0	0	0	
21544544	m	hp	CA	2022	0	0	0	0	0	0	0	0	0	0	0	0	64
21544546	m	md	CN	2022	0	0	0	0	0	0	0	0	0	0	0	0	66
21544569	f	md	CN	2022	0	0	0	0	0	0	0	0	0	0	0	0	67
21545017	Not	md	JP	2022	0	1	0	0	0	1	0	0	0	0	0	0	68
21545990	f	hp	US	2022	0	1	1	0	0	0	0	0	0	0	0	0	70
21546166	f	hp	US	2022	0	0	0	0	0	0	0	0	0	0	0	0	64
21546335	Not	md	US	2022	1	0	0	0	0	0	0	0	0	0	0	0	
21546497	Not	cn	SG	2022	1	0	0	0	0	0	0	0	0	0	0	0	74.3
21547101	m	md	BG	2022	0	1	1	0	0	0	0	0	0	0	0	1	65
21547229	m	md	JP	2022	0	1	1	0	0	0	0	0	0	0	0	0	78
21547568	m	hp	GB	2022	1	0	0	0	0	0	0	0	0	0	0	0	70
21548666	f	md	ES	2022	0	0	0	0	0	0	0	0	0	0	0	0	
21548786	m	md	JP	2022	0	1	0	1	0	0	0	0	0	0	0	1	73
21549279	m	md	IQ	2022	0	1	1	0	0	0	0	0	0	0	0	0	
21549835	m	md	FR	2022	0	1	1	0	0	0	0	0	0	0	0	0	62
21549938	m	md	JP	2022	0	1	1	0	0	0	0	0	0	0	0	0	73
21549988	m	hp	JP	2022	0	0	0	0	0	0	0	0	0	0	0	0	63
21549994	f	cn	TH	2022	0	0	0	0	0	0	0	0	0	0	0	0	40
21550133	f	md	SK	2022	0	1	1	0	0	0	0	0	0	0	0	0	71
21550162	m	cn	TH	2022	0	0	0	0	0	0	0	0	0	0	0	0	
21550194	Not	ph	JP	2022	1	0	0	0	0	0	0	0	0	0	0	0	
21550288	m	md	IT	2022	0	0	0	0	0	0	0	0	0	0	0	0	65
21550318	m	hp	CN	2022	0	1	1	0	0	0	0	0	0	0	0	0	76
21550387	m	ph	JP	2022	0	1	1	0	0	0	0	0	0	0	0	0	80
21550411	m	ph	US	2022	0	0	0	0	0	0	0	0	0	0	0	0	61
21550529	f	md	JP	2022	0	1	0	1	0	0	0	0	0	0	0	1	71
21550537	Not	md	JP	2022	0	1	0	1	0	0	0	0	0	0	0	0	
21550692	f	cn	TH	2022	1	0	0	0	0	0	0	0	0	0	0	0	68.4
21551699	m	hp	CN	2022	0	1	0	0	1	0	0	0	0	0	0	0	62
21551738	m	md	JP	2022	0	0	0	0	0	0	0	0	0	0	0	0	76

21552252	f	md	US	2022	1	0	0	0	0	0	0	0	0	0	0	0	64
21553074	f	md	JP	2022	0	0	0	0	0	0	0	0	0	0	0	0	70
21553202	f	md	TH	2022	1	0	0	0	0	0	0	0	0	0	0	0	71
21553219	f	hp	GB	2022	0	0	0	0	0	0	0	0	0	0	0	0	60
21553276	f	cn	US	2022	1	0	0	0	0	0	0	0	0	0	0	0	
21553937	m	cn	CA	2022	0	0	0	0	0	0	0	0	0	0	0	0	43
21553956	m	md	JP	2022	0	1	0	0	0	1	0	0	0	0	0	0	70
21554016	m	md	DE	2022	0	1	0	0	1	0	0	0	0	0	0	0	67
21554117	Not	ph	CA	2022	0	0	0	0	0	0	0	0	0	0	0	0	
21554124	m	md	SG	2022	0	0	0	0	0	0	0	0	0	0	0	0	
21554137	m	cn	IN	2022	0	1	0	0	0	1	0	0	0	0	0	0	49
21554189	f	md	JP	2022	0	1	0	0	0	1	0	0	0	0	0	0	48
21554233	m	cn	PH	2022	0	1	0	0	0	1	0	0	0	0	0	0	47
21554236	f	md	JP	2022	0	1	0	0	0	1	0	0	0	0	0	1	72
21554255	Not	md	JP	2022	0	1	0	0	0	1	0	0	0	0	0	0	70
21554339	m	md	US	2022	1	0	0	0	0	0	0	0	0	0	0	0	
21554622	m	md	DZ	2022	0	0	0	0	0	0	0	0	0	0	0	0	55
21555205	m	md	JP	2022	0	0	0	0	0	0	0	0	0	0	0	0	66
21555243	m	md	SG	2022	0	1	0	0	0	1	0	0	0	0	0	0	66
21555247	Not	hp	US	2022	1	0	0	0	0	0	0	0	0	0	0	0	42.3
21555355	m	md	ES	2022	0	1	0	0	1	0	0	0	0	0	0	0	63
21555416	m	cn	US	2022	0	1	1	0	0	0	0	0	0	0	0	0	
21555840	Not	ph	JP	2022	0	1	0	1	0	0	0	0	0	0	0	0	
21556070	m	md	DE	2022	0	0	0	0	0	0	0	0	0	0	0	0	59
21556285	f	cn	BE	2022	0	0	0	0	0	0	1	1	0	0	0	0	72.1
21556306	f	md	ES	2022	0	1	0	0	1	0	0	0	0	0	0	0	47
21556367	f	md	US	2022	1	0	0	0	0	0	0	0	0	0	0	0	88
21556433	m	md	JP	2022	0	1	0	1	0	0	0	0	0	0	0	1	80
21556474	m	cn	CN	2022	0	1	0	0	1	0	0	0	0	0	0	0	56
21556779	f	cn	SG	2022	1	0	0	0	0	0	0	0	0	0	0	0	71.8
21556794	m	cn	AE	2022	0	1	0	0	1	0	0	0	0	0	0	0	66
21557100	m	md	IL	2022	0	0	0	0	0	0	0	0	0	0	0	0	68
21557357	Not	hp	US	2022	1	0	0	0	0	0	0	0	0	0	0	0	
21557749	m	md	JP	2022	0	1	0	0	1	0	0	0	0	0	0	0	75

21557960	m	md	JP	2022	0	1	0	0	0	1	0	0	0	0	0	0	60
21558018	f	md	DE	2022	0	1	0	0	1	0	0	0	0	0	0	0	63
21558056	f	hp	CN	2022	0	0	0	0	0	0	0	0	0	0	0	0	43
21558163	f	ph	CH	2022	1	0	0	0	0	0	0	0	0	0	0	0	72
21558341	m	hp	IT	2022	0	0	0	0	0	0	0	0	0	0	0	0	65
21558541	Not	md	JP	2022	0	1	0	0	0	1	0	0	0	0	0	0	70
21558736	Not	hp	TH	2022	1	0	0	0	0	0	0	0	0	0	0	0	
21558738	m	md	DE	2022	0	1	0	0	1	0	0	0	0	0	0	0	74
21558772	m	hp	BY	2022	0	1	0	0	0	1	0	0	0	0	0	0	62
21558818	m	md	DE	2022	0	1	0	0	1	0	0	0	0	0	0	0	73
21558872	f	ph	JP	2022	0	0	0	0	0	0	0	0	0	0	0	0	50
21559098	m	md	DZ	2022	0	0	0	0	0	0	0	0	0	0	0	0	70
21559251	f	md	IT	2022	0	0	0	0	0	0	0	0	0	0	0	0	84
21559372	Not	md	FI	2022	0	0	0	0	0	0	0	0	0	0	0	0	
21559376	f	md	AU	2022	0	0	0	0	0	0	0	0	0	0	0	0	83
21559532	m	hp	KR	2022	0	0	0	0	0	0	0	0	0	0	0	0	66
21560021	f	md	AU	2022	0	1	0	1	0	0	0	0	0	0	0	0	50
21560480	m	cn	JP	2022	0	0	0	0	0	0	0	0	0	0	0	0	65
21560733	m	cn	KR	2022	0	0	0	0	0	0	0	0	0	0	0	0	83
21560814	m	hp	TH	2022	1	0	0	0	0	0	0	0	0	0	0	0	
21560839	f	md	AU	2022	0	1	1	0	0	0	0	0	0	0	0	0	
21561642	f	hp	DK	2022	0	0	0	0	0	0	0	0	0	0	0	0	39
21561789	f	md	US	2022	0	1	0	1	0	0	0	0	0	0	0	1	77.9
21562217	m	md	DE	2022	0	0	0	0	0	0	0	0	0	0	0	0	56
21562317	m	hp	JP	2022	0	1	0	0	1	0	0	0	0	0	0	0	79
21562389	m	md	JP	2022	0	1	0	0	1	0	0	0	0	0	0	0	70
21562419	f	hp	ZA	2022	1	0	0	0	0	0	0	0	0	0	0	0	75
21562718	f	md	JP	2022	0	1	0	0	1	0	0	0	0	0	0	0	59
21563001	f	cn	US	2022	0	0	0	0	0	0	0	0	0	0	0	0	67
21563522	m	hp	IT	2022	0	0	0	0	0	0	0	0	0	0	0	0	65
21563834	f	hp	JP	2022	1	0	0	0	0	0	0	0	0	0	0	0	73
21563953	m	md	JP	2022	0	1	0	0	0	1	0	0	0	0	0	1	66
21563980	m	hp	BY	2022	0	1	0	0	0	1	0	0	0	0	0	0	50
21564009	m	md	SG	2022	0	0	0	0	0	0	0	0	0	0	0	0	75

21564457	m	hp	IT	2022	0	0	0	0	0	0	0	0	0	0	0	0	73
21564551	f	hp	CN	2022	0	0	0	0	0	0	0	0	0	0	0	0	44
21564889	f	md	CA	2022	1	0	0	0	0	0	0	0	0	0	0	0	56
21564954	f	md	US	2022	1	0	0	0	0	0	0	0	0	0	0	0	63
21565449	f	hp	TH	2022	1	0	0	0	0	0	0	0	0	0	0	0	67
21565484	m	cn	JP	2022	1	0	0	0	0	0	0	0	0	0	0	0	70
21565505	m	md	JP	2022	0	0	0	0	0	0	0	0	0	0	0	0	74
21565583	Not	md	JP	2022	0	1	1	0	0	0	0	0	0	0	0	0	50
21565790	Not	hp	GB	2022	0	0	0	0	0	0	0	0	0	0	0	0	55
21565971	Not	hp	FR	2022	0	0	0	0	0	0	0	0	0	0	0	0	
21565974	Not	hp	FR	2022	0	0	0	0	0	0	0	0	0	0	0	0	
21566170	f	md	JP	2022	1	0	0	0	0	0	0	0	0	0	0	0	
21566175	Not	hp	CN	2022	0	0	0	0	0	0	0	0	0	0	0	0	
21566176	Not	hp	CN	2022	0	0	0	0	0	0	0	0	0	0	0	0	
21566489	m	md	JP	2022	0	1	1	0	0	0	0	0	0	0	0	0	71
21566823	m	cn	TH	2022	1	0	0	0	0	0	0	0	0	0	0	0	
21566847	m	cn	US	2022	0	0	0	0	0	0	0	0	0	0	0	0	
21567439	m	md	BR	2022	0	1	0	0	1	0	0	0	0	0	0	0	60
21567574	f	cn	US	2022	0	1	1	0	0	0	0	0	0	0	0	0	
21567693	f	md	JP	2022	1	0	0	0	0	0	0	0	0	0	0	0	82
21567701	f	cn	IN	2022	0	1	0	1	0	0	0	0	0	0	0	0	68
21567791	Not	hp	CN	2022	0	0	0	0	0	0	0	0	0	0	0	0	
21567792	Not	hp	CN	2022	0	0	0	0	0	0	0	0	0	0	0	0	
21567793	Not	hp	CN	2022	0	0	0	0	0	0	0	0	0	0	0	0	
21567839	m	cn	IN	2022	0	0	0	0	0	0	0	0	0	0	0	0	66
21568004	m	md	JP	2022	0	1	0	1	0	0	0	0	0	0	0	1	64
21568175	m	hp	KR	2022	0	0	0	0	0	0	0	0	0	0	0	0	66
21568338	f	md	ES	2022	0	0	0	0	0	0	0	0	0	0	0	0	
21568351	m	md	MY	2022	0	0	0	0	0	0	0	0	0	0	0	0	45
21568629	m	hp	RO	2022	0	0	0	0	0	0	0	0	0	0	0	0	58
21568806	f	cn	US	2022	0	0	0	0	0	0	0	0	0	0	0	0	
21569706	m	md	CN	2022	0	0	0	0	0	0	0	0	0	0	0	0	53
21570575	f	ph	GB	2022	0	0	0	0	0	0	0	0	0	0	0	0	60
21570923	m	cn	CA	2022	0	1	0	0	1	0	0	0	0	0	0	0	

21571120	f	hp	US	2022	1	0	0	0	0	0	0	0	0	0	0	0	66.9
21571302	m	hp	JP	2022	0	1	0	0	1	0	0	0	0	0	0	0	74
21572025	f	md	SG	2022	1	0	0	0	0	0	0	0	0	0	0	0	71
21572268	f	md	JP	2022	0	0	0	0	0	0	0	0	0	0	0	0	90
21572777	f	cn	US	2022	0	0	0	0	0	0	0	0	0	0	0	0	60
21572950	m	hp	US	2022	0	0	0	0	0	0	0	0	0	0	0	0	56
21573554	m	md	TW	2022	0	1	1	0	0	0	0	0	0	0	0	0	
21574562	f	md	CH	2022	0	0	0	0	0	0	0	0	0	0	0	0	68
21575441	m	md	JP	2022	0	1	0	0	0	1	0	0	0	0	0	0	
21575464	m	hp	CR	2022	0	0	0	0	0	0	0	0	0	0	0	0	29
21575484	m	md	JP	2022	0	1	0	0	0	1	0	0	0	0	0	0	70
21575511	m	cn	CN	2022	0	0	0	0	0	0	0	0	0	0	0	0	67
21575841	f	md	JP	2022	0	1	0	0	1	0	0	0	0	0	0	0	80
21575843	f	md	JP	2022	0	1	0	0	1	0	0	0	0	0	0	0	
21576298	Not	ph	JP	2022	0	0	0	0	0	0	0	0	0	0	0	0	
21576622	m	md	JP	2022	0	1	0	0	1	0	0	0	0	0	0	0	71
21576736	m	md	RO	2022	0	0	0	0	0	0	0	0	0	0	0	0	50
21576766	f	md	IT	2022	0	0	0	0	0	0	0	0	0	0	0	0	
21576982	m	md	US	2022	0	0	0	0	0	0	0	0	0	0	0	0	
21577015	f	cn	CA	2022	0	0	0	0	0	0	0	0	0	0	0	0	75
21577619	f	hp	US	2022	0	1	1	0	0	0	0	0	0	0	0	0	44
21577971	m	md	JP	2022	0	1	0	0	1	0	0	0	0	0	0	0	81
21578122	Not	hp	GB	2022	1	0	0	0	0	0	0	0	0	0	0	0	
21578133	m	cn	JP	2022	0	0	0	0	0	0	0	0	0	0	0	0	62
21578146	Not	hp	GB	2022	1	0	0	0	0	0	0	0	0	0	0	0	
21578296	m	cn	CA	2022	0	0	0	0	0	0	0	0	0	0	0	0	74
21578792	m	md	AT	2022	0	0	0	0	0	0	0	0	0	0	0	0	
21579361	m	md	JP	2022	0	0	0	0	0	0	0	0	0	0	0	0	78
21579381	Not	hp	GB	2022	1	0	0	0	0	0	0	0	0	0	0	0	
21579546	f	hp	US	2022	0	1	1	0	0	0	0	0	0	0	0	0	83
21579934	m	md	CN	2022	0	0	0	0	0	0	0	0	0	0	0	0	76
21580554	f	md	JP	2022	0	0	0	0	0	0	0	0	0	0	0	0	50
21580640	m	md	AU	2022	1	0	0	0	0	0	0	0	0	0	0	0	86
21580854	m	cn	US	2022	0	0	0	0	0	0	0	0	0	0	0	0	

21581232	f	md	US	2022	0	0	0	0	0	0	0	0	0	0	0	0	48
21581303	f	md	JP	2022	0	1	0	0	1	0	0	0	0	0	0	0	73
21581371	m	md	US	2022	0	0	0	0	0	0	0	0	0	0	0	0	56
21581486	f	md	AU	2022	0	1	0	0	0	1	0	0	0	0	0	0	
21581517	f	md	DE	2022	0	1	0	0	1	0	0	0	0	0	0	0	69
21582094	f	md	US	2022	0	0	0	0	0	0	0	0	0	0	0	0	
21582265	m	cn	IN	2022	0	0	0	0	0	0	0	0	0	0	0	0	33
21582630	f	md	AU	2022	1	0	0	0	0	0	0	0	0	0	0	0	
21582954	m	md	IQ	2022	0	1	1	0	0	0	0	0	0	0	0	0	40
21582967	Not	hp	GB	2022	1	0	0	0	0	0	0	0	0	0	0	0	
21582986	Not	hp	GB	2022	1	0	0	0	0	0	0	0	0	0	0	0	
21583044	Not	hp	GB	2022	1	0	0	0	0	0	0	0	0	0	0	0	
21583286	Not	md	FR	2022	0	0	0	0	0	0	0	0	0	0	0	0	
21583360	f	cn	ID	2022	1	0	0	0	0	0	0	0	0	0	0	0	80
21583591	f	md	JP	2022	1	0	0	0	0	0	0	0	0	0	0	0	89
21583730	f	cn	JP	2022	0	0	0	0	0	0	0	0	0	0	0	0	
21584159	f	hp	US	2022	0	0	0	0	0	0	0	0	0	0	0	0	73
21584247	m	md	US	2022	0	1	0	1	0	0	0	0	0	0	0	1	
21584248	f	md	AU	2022	1	0	0	0	0	0	0	0	0	0	0	0	62
21584271	m	hp	CZ	2022	0	0	0	0	0	0	0	0	0	0	0	0	
21584399	Not	cn	US	2022	1	0	0	0	0	0	0	0	0	0	0	0	62.9
21584737	f	cn	IN	2022	0	0	0	0	0	0	0	0	0	0	0	0	62
21584739	f	md	FR	2022	0	1	0	0	0	1	0	0	0	0	0	0	74
21584774	Not	md	DE	2022	1	0	0	0	0	0	0	0	0	0	0	0	
21584791	f	cn	CA	2022	0	0	0	0	0	0	0	0	0	0	0	0	45
21584854	f	cn	CA	2022	1	0	0	0	0	0	0	0	0	0	0	0	51
21584912	Not	cn	SG	2022	1	0	0	0	0	0	0	0	0	0	0	0	77.1
21584916	f	hp	FR	2022	0	0	0	0	0	0	1	1	0	0	0	0	59
21584999	m	md	CZ	2022	0	1	0	0	1	0	0	0	0	0	0	0	72
21585402	f	cn	US	2022	0	0	0	0	0	0	0	0	0	0	0	0	
21585593	m	hp	IT	2022	0	1	1	0	0	0	0	0	0	0	0	0	61
21585765	m	hp	TR	2022	0	0	0	0	0	0	0	0	0	0	0	0	68
21585831	f	md	TR	2022	0	0	0	0	0	0	0	0	0	0	0	0	73
21585850	Not	cn	SG	2022	1	0	0	0	0	0	0	0	0	0	0	0	75.8

21585907	f	cn	SG	2022	1	0	0	0	0	0	0	0	0	0	0	0	73.3
21585908	Not	cn	SG	2022	1	0	0	0	0	0	0	0	0	0	0	0	58.6
21585912	f	md	AR	2022	0	1	1	0	0	0	0	0	0	0	0	0	62
21585939	m	md	JP	2022	0	1	1	0	0	0	0	0	0	0	0	1	63
21586510	m	md	SE	2022	0	0	0	0	0	0	0	0	0	0	0	0	64
21587431	f	md	AU	2022	0	1	0	0	0	1	0	0	0	0	0	0	
21587551	f	cn	CN	2022	0	0	0	0	0	0	0	0	0	0	0	0	60
21588193	m	md	JP	2022	0	1	0	0	1	0	0	0	0	0	0	0	73
21588308	m	cn	US	2022	1	0	0	0	0	0	0	0	0	0	0	0	92.3
21588566	m	hp	AU	2022	0	0	0	0	0	0	0	0	0	0	0	0	
21588854	m	md	IT	2022	0	0	0	0	0	0	0	0	0	0	0	0	
21588892	m	md	JP	2022	0	1	1	0	0	0	0	0	0	0	0	0	80
21588915	f	md	AU	2022	0	0	0	0	0	0	1	0	0	0	1	0	55
21588977	m	md	JP	2022	0	1	0	0	1	0	0	0	0	0	0	0	77
21589215	Not	cn	CA	2022	0	1	1	0	0	0	0	0	0	0	0	0	
21589808	m	md	DE	2022	0	1	0	0	1	0	0	0	0	0	0	0	60
21590450	f	cn	SG	2022	1	0	0	0	0	0	0	0	0	0	0	0	59.7
21590451	Not	cn	SG	2022	1	0	0	0	0	0	0	0	0	0	0	0	76.9
21590977	Not	hp	FR	2022	0	0	0	0	0	0	0	0	0	0	0	0	
21591077	Not	md	MA	2022	0	0	0	0	0	0	0	0	0	0	0	0	
21591361	f	cn	ID	2022	1	0	0	0	0	0	0	0	0	0	0	0	73
21591434	f	cn	CA	2022	1	0	0	0	0	0	0	0	0	0	0	0	
21591733	m	md	US	2022	0	0	0	0	0	0	0	0	0	0	0	0	
21591743	f	hp	GB	2022	0	1	0	0	0	1	0	0	0	0	0	0	65
21591775	m	cn	SG	2022	1	0	0	0	0	0	0	0	0	0	0	0	56.9
21591777	f	cn	US	2022	1	0	0	0	0	0	0	0	0	0	0	0	65.9
21592159	Not	cn	SG	2022	1	0	0	0	0	0	0	0	0	0	0	0	65.4
21592357	m	cn	TH	2022	1	0	0	0	0	0	0	0	0	0	0	0	60
21592459	f	cn	SG	2022	1	0	0	0	0	0	0	0	0	0	0	0	64.5
21592509	f	md	US	2022	0	0	0	0	0	0	0	0	0	0	0	0	
21592574	m	md	JP	2022	0	1	1	0	0	0	0	0	0	0	0	0	70
21593902	m	cn	CA	2022	0	1	1	0	0	0	0	0	0	0	0	0	
21594105	m	md	JP	2022	1	0	0	0	0	0	0	0	0	0	0	0	70
21594681	m	cn	TH	2022	0	0	0	0	0	0	0	0	0	0	0	0	61

21594685	m	md	JP	2022	0	0	0	0	0	0	0	0	0	0	0	0	
21595546	Not	ph	JP	2022	1	0	0	0	0	0	0	0	0	0	0	0	
21596065	f	md	FR	2022	0	1	0	0	0	1	0	0	0	0	0	1	74
21596791	f	hp	NO	2022	0	1	1	0	0	0	0	0	0	0	0	0	
21598267	m	md	DE	2022	0	1	0	0	1	0	0	0	0	0	0	0	59
21598278	m	md	US	2022	0	0	0	0	0	0	0	0	0	0	0	0	75
21598382	f	cn	US	2022	1	0	0	0	0	0	0	0	0	0	0	0	
21599132	f	md	ES	2022	0	0	0	0	0	0	0	0	0	0	0	0	75
21599323	f	md	VN	2022	0	0	0	0	0	0	0	0	0	0	0	0	55
21600073	f	md	DE	2022	0	1	0	0	1	0	0	0	0	0	0	0	68
21600084	m	md	IL	2022	0	1	0	0	1	0	0	0	0	0	0	0	72
21600610	f	md	NZ	2022	0	0	0	0	0	0	0	0	0	0	0	0	65
21600723	m	hp	CN	2022	0	0	0	0	0	0	0	0	0	0	0	0	60
21600740	f	md	GB	2022	0	1	0	0	0	1	0	0	0	0	0	0	
21600799	m	md	JP	2022	0	1	0	0	0	1	0	0	0	0	0	0	
21600867	m	md	JP	2022	0	1	1	0	0	0	0	0	0	0	0	0	80
21601456	f	md	IT	2022	0	1	0	0	0	1	0	0	0	0	0	0	70
21601635	Not	hp	GB	2022	0	0	0	0	0	0	0	0	0	0	0	0	
21602403	m	md	JP	2022	0	1	0	0	0	1	0	0	0	0	0	0	77
21602440	m	md	JP	2022	1	0	0	0	0	0	0	0	0	0	0	0	81
21602493	m	cn	JP	2022	1	0	0	0	0	0	0	0	0	0	0	0	65
21604747	f	cn	KR	2022	0	0	0	0	0	0	0	0	0	0	0	0	48
21605223	f	md	RU	2022	0	0	0	0	0	0	0	0	0	0	0	0	69
21605242	m	md	JP	2022	0	1	0	0	1	0	0	0	0	0	0	0	78
21605365	m	md	JP	2022	0	1	0	0	0	1	0	0	0	0	0	0	
21605399	Not	md	JP	2022	0	1	0	0	0	1	0	0	0	0	0	0	80
21605520	f	cn	SG	2022	1	0	0	0	0	0	0	0	0	0	0	0	60.9
21605521	m	md	JP	2022	1	0	0	0	0	0	0	0	0	0	0	0	76
21605651	m	md	JP	2022	0	1	0	0	0	1	0	0	0	0	0	0	80
21605762	f	md	AR	2022	0	0	0	0	0	0	0	0	0	0	0	0	56
21606740	f	md	JP	2022	0	0	0	0	0	0	0	0	0	0	0	0	50
21607381	m	md	JP	2022	0	1	1	0	0	0	0	0	0	0	0	0	
21607404	m	md	US	2022	0	0	0	0	0	0	0	0	0	0	0	0	
21607626	f	cn	CA	2022	1	0	0	0	0	0	0	0	0	0	0	0	63

21607917	m	hp	TR	2022	0	1	0	0	1	0	0	0	0	0	0	0	78
21607949	m	md	DE	2022	0	1	0	0	1	0	0	0	0	0	0	0	73
21608233	m	ph	CA	2022	0	0	0	0	0	0	0	0	0	0	0	0	65
21608487	f	hp	GB	2022	0	0	0	0	0	0	1	0	0	0	1	0	55
21608654	Not	cn	CA	2022	0	1	1	0	0	0	0	0	0	0	0	0	
21608726	Not	md	SA	2022	0	1	1	0	0	0	0	0	0	0	0	0	
21609203	m	hp	CA	2022	0	0	0	0	0	0	0	0	0	0	0	0	
21609334	f	md	FR	2022	1	0	0	0	0	0	0	0	0	0	0	1	
21609365	m	cn	EG	2022	0	1	1	0	0	0	0	0	0	0	0	0	
21609445	m	md	SG	2022	1	0	0	0	0	0	0	0	0	0	0	0	63
21609933	f	md	FR	2022	0	1	0	1	0	0	0	0	0	0	0	0	72
21610304	f	hp	KR	2022	0	0	0	0	0	0	0	0	0	0	0	0	54
21610724	Not	md	CN	2022	0	0	0	0	0	0	0	0	0	0	0	0	
21610756	m	cn	IN	2022	0	0	0	0	0	0	0	0	0	0	0	0	36
21611045	f	md	CN	2022	0	0	0	0	0	0	0	0	0	0	0	0	66
21611302	m	md	JP	2022	0	0	0	0	0	0	0	0	0	0	0	0	76
21611497	m	hp	CN	2022	0	0	0	0	0	0	0	0	0	0	0	0	54
21611968	f	hp	CN	2022	1	0	0	0	0	0	0	0	0	0	0	0	64
21612234	f	md	DE	2022	0	0	0	0	0	0	0	0	0	0	0	0	
21612400	Not	hp	CN	2022	0	0	0	0	0	0	0	0	0	0	0	0	
21612432	m	md	JP	2022	0	0	0	0	0	0	0	0	0	0	0	0	40
21612572	f	md	CN	2022	1	0	0	0	0	0	0	0	0	0	0	0	60
21612589	m	md	JP	2022	0	0	0	0	0	0	0	0	0	0	0	0	65
21613110	Not	md	JP	2022	0	1	0	1	0	0	0	0	0	0	0	0	
21613137	Not	hp	CN	2022	0	0	0	0	0	0	0	0	0	0	0	0	
21613255	f	md	JP	2022	0	1	0	0	0	1	0	0	0	0	0	0	50
21613298	m	md	DE	2022	0	1	0	0	1	0	0	0	0	0	0	0	60
21613422	f	md	AU	2022	0	1	0	0	0	1	0	0	0	0	0	0	65
21613445	m	md	JP	2022	0	1	0	0	0	1	0	0	0	0	0	0	80
21614098	m	md	FR	2022	0	1	0	0	0	1	0	0	0	0	0	0	75
21614208	f	md	CR	2022	1	0	0	0	0	0	0	0	0	0	0	0	
21614505	m	md	JP	2022	0	0	0	0	0	0	0	0	0	0	0	0	
21614562	f	md	HR	2022	0	1	1	0	0	0	0	0	0	0	0	0	60
21614818	f	md	ES	2022	0	1	0	0	0	1	0	0	0	0	0	0	61

21614962	f	md	US	2022	1	0	0	0	0	0	0	0	0	0	0	0	
21615081	f	cn	TH	2022	1	0	0	0	0	0	0	0	0	0	0	0	70.7
21615248	f	cn	ID	2022	1	0	0	0	0	0	0	0	0	0	0	0	55.1
21615390	m	cn	JP	2022	0	0	0	0	0	0	0	0	0	0	0	0	71
21615478	m	cn	CA	2022	1	0	0	0	0	0	0	0	0	0	0	0	
21615639	f	md	DE	2022	1	0	0	0	0	0	0	0	0	0	0	0	79
21615670	f	md	BG	2022	0	1	0	0	0	1	0	0	0	0	0	0	55
21615747	f	md	CN	2022	1	0	0	0	0	0	0	0	0	0	0	0	70
21615804	f	md	BG	2022	0	1	0	0	0	1	0	0	0	0	0	0	55
21615811	f	cn	US	2022	1	0	0	0	0	0	0	0	0	0	0	0	66.4
21615881	m	cn	US	2022	1	0	0	0	0	0	0	0	0	0	0	0	46.1
21615922	m	md	TR	2022	0	0	0	0	0	0	0	0	0	0	0	0	62
21616058	f	hp	CN	2022	1	0	0	0	0	0	0	0	0	0	0	0	75
21617158	m	md	US	2022	0	1	1	0	0	0	0	0	0	0	0	0	62
21618125	Not	ph	JP	2022	1	0	0	0	0	0	0	0	0	0	0	0	
21618530	m	md	SI	2022	0	1	1	0	0	0	0	0	0	0	0	0	72
21618851	f	md	JP	2022	0	1	0	0	0	1	0	0	0	0	0	1	67
21618898	f	md	JP	2022	0	1	0	0	0	1	0	0	0	0	0	0	50
21619125	m	md	JP	2022	0	1	0	0	1	0	0	0	0	0	0	0	68
21619265	m	md	JP	2022	0	1	0	0	1	0	0	0	0	0	0	0	54
21619617	m	md	RO	2022	0	0	0	0	0	0	0	0	0	0	0	0	69
21619794	m	md	JP	2022	0	1	0	0	0	1	0	0	0	0	0	0	70
21620025	f	cn	FR	2022	0	1	0	0	1	0	0	0	0	0	0	0	62
21620160	m	md	ES	2022	1	0	0	0	0	0	0	0	0	0	0	0	63.8
21620323	f	md	JP	2022	0	1	0	0	1	0	0	0	0	0	0	0	62
21620391	Not	cn	US	2022	0	0	0	0	0	0	0	0	0	0	0	0	
21620429	m	cn	CN	2022	0	1	0	0	1	0	0	0	0	0	0	0	66
21620698	m	cn	DE	2022	0	1	1	0	0	0	0	0	0	0	0	0	
21620991	m	cn	TH	2022	1	0	0	0	0	0	0	0	0	0	0	0	75.3
21621358	f	cn	TH	2022	1	0	0	0	0	0	0	0	0	0	0	0	82.1
21621437	f	hp	IT	2022	0	1	1	0	0	0	0	0	0	0	0	0	58
21621438	f	hp	IT	2022	0	1	1	0	0	0	0	0	0	0	0	0	76
21621444	m	hp	IT	2022	0	1	1	0	0	0	0	0	0	0	0	0	70
21621512	m	md	GR	2022	0	1	1	0	0	0	0	0	0	0	0	0	60

21621753	m	md	IT	2022	0	0	0	0	0	0	0	0	0	0	0	0	57
21621778	Not	md	US	2022	0	0	0	0	0	0	0	0	0	0	0	0	
21622082	m	md	JP	2022	0	1	1	0	0	0	0	0	0	0	0	0	80
21622793	m	cn	IN	2022	0	1	0	0	1	0	0	0	0	0	0	0	61
21622899	Not	md	FR	2022	0	0	0	0	0	0	0	0	0	0	0	0	
21622922	m	md	SI	2022	0	1	1	0	0	0	0	0	0	0	0	0	72
21623258	m	md	AR	2022	0	1	0	0	0	1	0	0	0	0	0	0	78
21623397	f	md	JP	2022	1	0	0	0	0	0	0	0	0	0	0	0	
21623564	m	cn	ID	2022	1	0	0	0	0	0	0	0	0	0	0	0	
21623692	f	md	CY	2022	0	0	0	0	0	0	0	0	0	0	0	0	
21623926	f	cn	GB	2022	0	0	0	0	0	0	0	0	0	0	0	0	29
21624148	m	hp	MX	2022	0	0	0	0	0	0	0	0	0	0	0	0	75
21624220	m	md	JP	2022	0	1	0	0	1	0	0	0	0	0	0	0	73
21624288	Not	md	JP	2022	0	0	0	0	0	0	0	0	0	0	0	0	
21624472	m	cn	IN	2022	0	1	0	0	1	0	0	0	0	0	0	0	58
21624541	m	cn	CN	2022	0	1	0	0	1	0	0	0	0	0	0	0	50
21624567	f	md	US	2022	0	0	0	0	0	0	0	0	0	0	0	0	63
21624641	m	md	JP	2022	0	1	0	0	1	0	0	0	0	0	0	0	76
21625370	f	md	JP	2022	0	1	0	0	0	1	0	0	0	0	0	0	80
21625712	m	md	JP	2022	0	0	0	0	0	0	0	0	0	0	0	0	80
21625791	m	md	IN	2022	1	0	0	0	0	0	0	0	0	0	0	0	
21626278	m	hp	TH	2022	0	1	0	0	1	0	0	0	0	0	0	0	69
21626537	m	md	JP	2022	0	1	1	0	0	0	0	0	0	0	0	0	72
21626805	m	cn	US	2022	0	0	0	0	0	0	0	0	0	0	0	0	
21626943	f	md	HR	2022	0	1	1	0	0	0	0	0	0	0	0	0	60
21627237	m	cn	JP	2022	1	0	0	0	0	0	0	0	0	0	0	0	48
21627242	f	cn	JP	2022	0	0	0	0	0	0	0	0	0	0	0	0	42
21627291	f	md	JP	2022	0	1	0	0	1	0	0	0	0	0	0	0	80
21627462	m	md	JP	2022	0	1	0	0	1	0	0	0	0	0	0	0	82
21628803	f	cn	TH	2022	1	0	0	0	0	0	0	0	0	0	0	0	79.6
21628806	Not	md	JP	2022	0	1	0	1	0	0	0	0	0	0	0	0	80
21628961	m	hp	US	2022	0	0	0	0	0	0	0	0	0	0	0	0	66
21629112	m	cn	ID	2022	1	0	0	0	0	0	0	0	0	0	0	0	62.9
21629437	Not	cn	SG	2022	1	0	0	0	0	0	0	0	0	0	0	0	86.5

21629441	Not	hp	CN	2022	1	0	0	0	0	0	0	0	0	0	0	0	51
21629454	f	md	ES	2022	0	1	0	0	0	1	0	0	0	0	0	0	61
21629600	f	md	CN	2022	0	1	0	1	0	0	0	0	0	0	0	0	59
21629630	m	md	US	2022	0	0	0	0	0	0	0	0	0	0	0	0	
21629677	f	md	GB	2022	0	0	0	0	0	0	0	0	0	0	0	0	43
21630019	m	cn	IN	2022	0	1	0	0	1	0	0	0	0	0	0	0	66
21630056	m	hp	JP	2022	0	1	0	0	1	0	0	0	0	0	0	0	66
21630070	f	md	BG	2022	0	1	0	0	1	0	0	0	0	0	0	0	58
21630230	f	md	TR	2022	0	0	0	0	0	0	0	0	0	0	0	0	58
21630314	m	md	JP	2022	0	1	0	0	1	0	0	0	0	0	0	1	73
21630444	f	md	GB	2022	0	1	0	0	0	1	0	0	0	0	0	0	65
21630537	m	md	DE	2022	0	1	0	0	1	0	0	0	0	0	0	0	71
21630855	m	md	JP	2022	0	0	0	0	0	0	0	0	0	0	0	0	66
21631057	Not	hp	FR	2022	0	0	0	0	0	0	0	0	0	0	0	0	
21631263	f	md	GB	2022	0	1	0	0	0	1	0	0	0	0	0	0	65
21631345	f	md	JP	2022	0	1	0	0	0	1	0	0	0	0	0	0	80
21631431	m	md	JP	2022	0	1	0	0	0	1	0	0	0	0	0	0	50
21631871	m	md	JP	2022	0	1	1	0	0	0	0	0	0	0	0	0	80
21632935	m	hp	CA	2022	1	0	0	0	0	0	0	0	0	0	0	0	
21633174	f	hp	NL	2022	0	0	0	0	0	0	0	0	0	0	0	0	74
21633308	f	cn	IN	2022	0	0	0	0	0	0	0	0	0	0	0	0	63
21633600	Not	md	JP	2022	1	0	0	0	0	0	0	0	0	0	0	0	
21634062	f	md	CZ	2022	0	0	0	0	0	0	0	0	0	0	0	0	
21634430	m	ph	US	2022	0	1	0	0	1	0	0	0	0	0	0	0	64
21634522	Not	hp	US	2022	0	1	0	0	0	1	0	0	0	0	0	0	
21634618	m	hp	US	2022	0	0	0	0	0	0	0	0	0	0	0	0	83
21634658	f	md	DE	2022	0	0	0	0	0	0	0	0	0	0	0	0	
21635137	f	cn	HR	2022	0	1	1	0	0	0	0	0	0	0	0	0	60
21635564	m	md	US	2022	0	0	0	0	0	0	0	0	0	0	0	0	70
21635715	f	hp	DE	2022	0	0	0	0	0	0	0	0	0	0	0	0	62
21635759	m	md	JP	2022	0	1	0	0	1	0	0	0	0	0	0	0	62
21635830	m	md	JP	2022	0	1	0	0	1	0	0	0	0	0	0	0	73
21636139	m	md	JP	2022	0	0	0	0	0	0	0	0	0	0	0	0	69
21636818	m	md	JP	2022	0	1	0	0	0	1	0	0	0	0	0	0	75

21636819	f	cn	TW	2022	0	1	0	0	0	1	0	0	0	0	0	0	82
21637044	f	md	KR	2022	0	0	0	0	0	0	0	0	0	0	0	0	68
21637217	f	md	DE	2022	0	1	1	0	0	0	0	0	0	0	0	0	46
21637924	f	ph	TH	2022	1	0	0	0	0	0	0	0	0	0	0	0	75.6
21638097	m	md	JP	2022	0	1	0	1	0	0	0	0	0	0	0	1	73
21638210	f	hp	US	2022	0	0	0	0	0	0	0	0	0	0	0	0	62
21639304	Not	md	JP	2022	0	1	0	1	0	0	0	0	0	0	0	0	
21639585	Not	ph	JP	2022	1	0	0	0	0	0	0	0	0	0	0	1	79
21639646	m	cn	IN	2022	1	0	0	0	0	0	0	0	0	0	0	0	73.8
21640193	f	hp	CN	2022	0	0	0	0	0	0	0	0	0	0	0	0	50
21640269	Not	md	ZA	2022	1	0	0	0	0	0	0	0	0	0	0	0	
21640335	f	hp	KR	2022	0	0	0	0	0	0	0	0	0	0	0	0	64
21640339	Not	ph	JP	2022	0	0	0	0	0	0	0	0	0	0	0	0	
21640342	m	md	JP	2022	0	1	0	0	1	0	0	0	0	0	0	0	76
21640749	f	md	DE	2022	0	1	0	0	1	0	0	0	0	0	0	0	68
21641031	m	md	CA	2022	0	0	0	0	0	0	0	0	0	0	0	0	68
21641362	m	md	JP	2022	0	1	0	0	0	1	0	0	0	0	0	0	73
21641409	f	md	DE	2022	0	0	0	0	0	0	0	0	0	0	0	0	78
21641505	f	md	DE	2022	0	1	0	0	1	0	0	0	0	0	0	0	65
21641714	f	ph	JP	2022	1	0	0	0	0	0	0	0	0	0	0	0	86
21641735	m	cn	TH	2022	0	1	0	0	1	0	0	0	0	0	0	0	45
21641742	f	md	DE	2022	0	1	0	0	1	0	0	0	0	0	0	0	62
21642145	m	md	JP	2022	0	1	1	0	0	0	0	0	0	0	0	0	90
21642343	m	md	JP	2022	0	1	0	1	0	0	0	0	0	0	0	0	73
21642681	f	cn	US	2022	0	1	1	0	0	0	0	0	0	0	0	0	73
21642827	m	hp	CZ	2022	0	1	0	0	1	0	0	0	0	0	0	0	72
21643015	f	md	FR	2022	0	0	0	0	0	0	0	0	0	0	0	0	
21643069	f	ph	DE	2022	0	0	0	0	0	0	0	0	0	0	0	0	72
21643108	m	md	JP	2022	0	1	0	1	0	0	0	0	0	0	0	1	53
21643110	f	md	CA	2022	1	0	0	0	0	0	0	0	0	0	0	0	
21643111	f	md	RU	2022	0	0	0	0	0	0	1	0	0	0	1	0	
21643134	m	hp	CN	2022	1	0	0	0	0	0	0	0	0	0	0	0	50
21643270	m	md	AU	2022	0	1	1	0	0	0	0	0	0	0	0	0	63
21643326	m	md	ES	2022	0	1	0	0	0	1	0	0	0	0	0	0	82

21643459	f	cn	GB	2022	1	0	0	0	0	0	0	0	0	0	0	0	
21643641	m	cn	MY	2022	0	1	0	1	0	0	0	0	0	0	0	0	
21643768	Not	md	JP	2022	1	0	0	0	0	0	0	0	0	0	0	0	
21643795	f	hp	TN	2022	0	0	0	0	0	0	0	0	0	0	0	0	40
21643853	f	cn	TH	2022	1	0	0	0	0	0	0	0	0	0	0	0	
21644089	m	cn	TH	2022	1	0	0	0	0	0	0	0	0	0	0	0	79
21644157	f	cn	TH	2022	1	0	0	0	0	0	0	0	0	0	0	0	68.6
21644326	Not	md	JP	2022	0	1	0	1	0	0	0	0	0	0	0	1	60
21644349	m	md	DE	2022	0	0	0	0	0	0	0	0	0	0	0	0	62
21644460	m	cn	TH	2022	1	0	0	0	0	0	0	0	0	0	0	0	71
21644462	f	cn	CA	2022	1	0	0	0	0	0	0	0	0	0	0	0	76.8
21644578	m	md	JP	2022	0	1	0	1	0	0	0	0	0	0	0	0	75
21644657	m	cn	TH	2022	1	0	0	0	0	0	0	0	0	0	0	0	80.9
21644814	f	cn	TH	2022	1	0	0	0	0	0	0	0	0	0	0	0	69.3
21644826	Not	cn	JP	2022	1	0	0	0	0	0	0	0	0	0	0	1	
21645179	Not	cn	CA	2022	0	1	1	0	0	0	0	0	0	0	0	0	
21646907	m	hp	CZ	2022	0	0	0	0	0	0	0	0	0	0	0	0	
21647154	m	md	JP	2022	0	1	0	0	1	0	0	0	0	0	0	1	81
21647382	f	md	DK	2022	0	1	0	0	0	1	0	0	0	0	0	0	79
21647947	m	md	AR	2022	0	1	0	0	0	1	0	0	0	0	0	0	36
21647981	f	md	PL	2022	0	1	0	0	0	1	0	0	0	0	0	0	62
21648010	f	cn	IN	2022	0	1	0	0	1	0	0	0	0	0	0	0	58
21648048	m	md	JP	2022	0	1	0	0	0	1	0	0	0	0	0	0	80
21648482	Not	md	JP	2022	0	1	0	0	0	1	0	0	0	0	0	0	
21649201	m	md	JP	2022	0	1	0	0	1	0	0	0	0	0	0	0	74
21649782	m	ph	US	2022	0	0	0	0	0	0	0	0	0	0	0	0	63
21649939	f	md	FR	2022	0	0	0	0	0	0	0	0	0	0	0	0	46
21649946	m	md	JP	2022	0	1	0	0	0	1	0	0	0	0	0	0	
21649950	Not	md	JP	2022	0	1	0	0	0	1	0	0	0	0	0	0	65
21650046	f	hp	FR	2022	0	0	0	0	0	0	1	1	0	0	0	0	59
21650093	f	cn	US	2022	0	1	1	0	0	0	0	0	0	0	0	0	
21650094	m	hp	TH	2022	1	0	0	0	0	0	0	0	0	0	0	0	60.9
21650623	m	md	CH	2022	1	0	0	0	0	0	0	0	0	0	0	0	
21650951	m	cn	CN	2022	0	1	0	0	1	0	0	0	0	0	0	0	71

21651111	m	cn	US	2022	0	0	0	0	0	0	0	0	0	0	0	0	55
21651255	m	md	FR	2022	0	0	0	0	0	0	0	0	0	0	0	0	78
21651406	Not	cn	JP	2022	0	0	0	0	0	0	0	0	0	0	0	0	
21651417	m	md	NL	2022	0	0	0	0	0	0	0	0	0	0	0	0	70
21651766	f	cn	IN	2022	0	0	0	0	0	0	0	0	0	0	0	0	47
21651908	f	md	US	2022	0	1	1	0	0	0	0	0	0	0	0	0	80
21652118	f	md	JP	2022	0	0	0	0	0	0	0	0	0	0	0	0	68
21652130	f	cn	IN	2022	0	0	0	0	0	0	0	0	0	0	0	0	60
21652131	f	ph	MY	2022	0	0	0	0	0	0	0	0	0	0	0	0	65
21652200	m	md	JP	2022	0	1	0	1	0	0	0	0	0	0	0	0	72
21652846	f	hp	US	2022	0	0	0	0	0	0	0	0	0	0	0	0	
21652938	f	md	DE	2022	0	1	1	0	0	0	0	0	0	0	0	0	46
21653106	f	md	HR	2022	0	1	1	0	0	0	0	0	0	0	0	0	60
21653113	m	hp	JP	2022	0	0	0	0	0	0	0	0	0	0	0	0	73
21653141	m	ph	JP	2022	0	1	0	0	1	0	0	0	0	0	0	1	50
21653207	m	cn	FR	2022	0	0	0	0	0	0	0	0	0	0	0	0	
21653651	f	hp	TH	2022	1	0	0	0	0	0	0	0	0	0	0	0	75
21654438	f	cn	IL	2022	0	0	0	0	0	0	0	0	0	0	0	0	54
21654493	m	cn	CN	2022	0	0	0	0	0	0	0	0	0	0	0	0	81
21654552	m	md	KR	2022	0	1	0	0	0	1	0	0	0	0	0	0	75
21654890	m	cn	CA	2022	0	1	0	0	1	0	0	0	0	0	0	0	
21655346	Not	md	JP	2022	0	1	1	0	0	0	0	0	0	0	0	0	80
21655656	m	hp	JP	2022	0	1	0	0	1	0	0	0	0	0	0	0	70
21655936	m	ph	JP	2022	1	0	0	0	0	0	0	0	0	0	0	0	80
21656559	Not	md	JP	2022	0	1	0	0	1	0	0	0	0	0	0	0	
21656959	m	md	US	2022	0	0	0	0	0	0	0	0	0	0	0	0	60
21657265	m	md	NL	2022	0	0	0	0	0	0	0	0	0	0	0	0	
21657309	f	md	FR	2022	0	0	0	0	0	0	0	0	0	0	0	0	59
21657449	m	cn	CA	2022	0	1	1	0	0	0	0	0	0	0	0	0	
21657701	m	ph	JP	2022	0	1	1	0	0	0	0	0	0	0	0	0	60
21657767	m	md	FR	2022	0	0	0	0	0	0	0	0	0	0	0	0	
21658195	f	hp	US	2022	0	0	0	0	0	0	0	0	0	0	0	0	
21658494	f	md	IT	2022	0	0	0	0	0	0	0	0	0	0	0	0	
21658645	f	md	JP	2022	0	0	0	0	0	0	0	0	0	0	0	0	68

21658733	m	cn	JP	2022	0	0	0	0	0	0	0	0	0	0	0	0	
21658753	m	cn	IL	2022	0	0	0	0	0	0	0	0	0	0	0	0	68
21659101	f	cn	US	2022	0	0	0	0	0	0	0	0	0	0	0	0	70
21659420	Not	cn	SG	2022	1	0	0	0	0	0	0	0	0	0	0	0	57.5
21659581	m	md	JP	2022	0	1	0	0	1	0	0	0	0	0	0	0	49
21659910	f	md	CN	2022	0	0	0	0	0	0	0	0	0	0	0	0	37
21659926	m	md	JP	2022	0	1	0	0	1	0	0	0	0	0	0	0	71
21660504	m	md	IL	2022	0	1	0	0	1	0	0	0	0	0	0	0	71
21660800	f	md	JP	2022	0	1	1	0	0	0	0	0	0	0	0	0	80
21661141	f	md	IT	2022	0	0	0	0	0	0	0	0	0	0	0	0	
21661199	m	md	JP	2022	0	1	0	0	1	0	0	0	0	0	0	1	49
21661213	Not	md	JP	2022	0	1	0	0	0	1	0	0	0	0	0	0	
21661222	Not	cn	AE	2022	0	1	0	0	1	0	0	0	0	0	0	0	
21661258	m	hp	CN	2022	0	0	0	0	0	0	0	0	0	0	0	0	60
21661374	f	md	DK	2022	0	1	1	0	0	0	0	0	0	0	0	0	54
21661378	f	cn	US	2022	1	0	0	0	0	0	0	0	0	0	0	0	
21661379	f	md	JP	2022	1	0	0	0	0	0	0	0	0	0	0	0	78
21661865	m	md	JP	2022	0	0	0	0	0	0	0	0	0	0	0	0	
21661890	m	md	JP	2022	0	1	0	0	1	0	0	0	0	0	0	0	72
21662357	Not	md	JP	2022	1	0	0	0	0	0	0	0	0	0	0	0	
21662687	Not	hp	DE	2022	0	1	0	0	0	1	0	0	0	0	0	0	
21662901	f	md	FR	2022	0	1	1	0	0	0	0	0	0	0	0	0	50
21663038	f	md	DE	2022	0	0	0	0	0	0	0	0	0	0	0	0	
21663085	m	md	DE	2022	0	1	0	0	1	0	0	0	0	0	0	0	75
21663979	m	cn	US	2022	0	1	0	0	1	0	0	0	0	0	0	0	48
21663986	m	cn	JP	2022	0	1	0	0	1	0	0	0	0	0	0	0	68
21664133	m	md	JP	2022	0	0	0	0	0	0	1	0	0	0	1	0	81
21664293	f	md	DE	2022	0	1	0	0	1	0	0	0	0	0	0	0	69
21664785	m	md	RS	2022	0	1	1	0	0	0	0	0	0	0	0	0	70
21664885	f	hp	CN	2022	0	1	1	0	0	0	0	0	0	0	0	0	49
21664936	m	md	JP	2022	0	1	0	0	1	0	0	0	0	0	0	0	71
21664943	f	md	JP	2022	0	0	0	0	0	0	0	0	0	0	0	0	69
21664960	m	hp	GB	2022	0	0	0	0	0	0	0	0	0	0	0	0	60
21665881	f	md	JP	2022	0	0	0	0	0	0	0	0	0	0	0	0	70

21666233	f	md	PL	2022	0	0	0	0	0	0	0	0	0	0	0	0	41
21666358	m	cn	US	2022	0	1	0	1	0	0	0	0	0	0	0	0	71.2
21667180	Not	md	JP	2022	0	1	0	0	1	0	0	0	0	0	0	0	
21667218	m	md	JP	2022	0	1	0	0	1	0	0	0	0	0	0	0	80
21667312	f	cn	US	2022	1	0	0	0	0	0	0	0	0	0	0	0	74.9
21668218	m	md	DE	2022	0	0	0	0	0	0	0	0	0	0	0	0	
21668382	Not	md	DE	2022	0	1	0	0	1	0	0	0	0	0	0	0	63
21669037	m	hp	IN	2022	0	0	0	0	0	0	0	0	0	0	0	0	60
21669390	f	md	DE	2022	0	1	0	0	1	0	0	0	0	0	0	0	63
21669633	m	md	TR	2022	0	1	0	0	0	1	0	0	0	0	0	0	54
21669930	m	md	GR	2022	0	1	1	0	0	0	0	0	0	0	0	0	60
21670079	m	hp	TW	2022	0	0	0	0	0	0	0	0	0	0	0	0	78
21670129	m	md	ES	2022	0	1	1	0	0	0	0	0	0	0	0	0	
21670184	m	md	JP	2022	0	1	0	0	1	0	0	0	0	0	0	0	71
21670258	m	cn	CN	2022	0	1	0	0	1	0	0	0	0	0	0	0	66
21671270	m	md	JP	2022	0	0	0	0	0	0	0	0	0	0	0	0	80
21671603	f	md	JP	2022	0	0	0	0	0	0	0	0	0	0	0	0	70
21671627	m	md	DE	2022	0	1	0	0	1	0	0	0	0	0	0	0	67
21671727	m	hp	FR	2022	0	0	0	0	0	0	0	0	0	0	0	0	52
21672003	m	md	JP	2022	0	0	0	0	0	0	0	0	0	0	0	0	72
21672492	f	md	BE	2022	0	0	0	0	0	0	0	0	0	0	0	0	
21672634	f	hp	US	2022	0	1	0	0	1	0	0	0	0	0	0	0	74
21672645	m	md	US	2022	0	0	0	0	0	0	0	0	0	0	0	0	66
21672671	m	md	JP	2022	0	1	0	0	1	0	0	0	0	0	0	1	71
21672844	f	md	DE	2022	0	1	0	0	1	0	0	0	0	0	0	0	56
21672999	f	md	DE	2022	0	1	0	0	1	0	0	0	0	0	0	0	76
21673127	f	ph	JP	2022	1	0	0	0	0	0	0	0	0	0	0	0	
21673408	f	cn	US	2022	0	1	1	0	0	0	0	0	0	0	0	0	72
21673411	f	md	JP	2022	1	0	0	0	0	0	0	0	0	0	0	0	75
21673417	f	cn	US	2022	1	0	0	0	0	0	0	0	0	0	0	0	
21673607	m	md	JP	2022	0	0	0	0	0	0	0	0	0	0	0	0	70
21673627	f	Not repo	Not re	2022	0	0	0	0	0	0	0	0	0	0	0	0	69
21673776	m	md	DE	2022	0	0	0	0	0	0	0	0	0	0	0	0	63
21674311	f	cn	CN	2022	0	1	0	0	1	0	0	0	0	0	0	0	83

21674316	m	md	NL	2022	0	0	0	0	0	0	0	0	0	0	0	0	80
21674340	f	hp	FR	2022	0	1	0	0	0	1	0	0	0	0	0	0	26
21674480	m	cn	AT	2022	0	1	1	0	0	0	0	0	0	0	0	0	45
21674559	m	ph	US	2022	0	1	0	1	0	0	0	0	0	0	0	0	73
21675173	m	hp	IT	2022	0	0	0	0	0	0	0	0	0	0	0	0	84
21675670	f	md	NO	2022	0	1	0	0	0	1	0	0	0	0	0	0	76
21675797	f	cn	JP	2022	1	0	0	0	0	0	0	0	0	0	0	0	
21675802	f	cn	CA	2022	1	0	0	0	0	0	0	0	0	0	0	0	
21675812	m	md	KR	2022	0	0	0	0	0	0	0	0	0	0	0	0	74
21676047	f	ph	CA	2022	1	0	0	0	0	0	0	0	0	0	0	0	
21676206	Not	md	FR	2022	0	1	0	0	1	0	0	0	0	0	0	0	75
21676444	m	hp	CN	2022	0	0	0	0	0	0	0	0	0	0	0	0	70
21677483	m	cn	CA	2022	0	0	0	0	0	0	0	0	0	0	0	0	
21677716	f	md	DE	2022	0	1	0	0	1	0	0	0	0	0	0	0	68
21678236	m	hp	DE	2022	0	1	0	0	1	0	0	0	0	0	0	0	68
21678617	m	cn	IN	2022	0	1	0	0	0	1	0	0	0	0	0	0	70
21678906	m	cn	IN	2022	0	1	0	0	1	0	0	0	0	0	0	0	68
21678907	m	md	JP	2022	0	1	0	0	1	0	0	0	0	0	0	0	80
21678931	m	md	JP	2022	0	1	0	0	1	0	0	0	0	0	0	0	80
21679047	Not	md	JP	2022	0	1	0	0	1	0	0	0	0	0	0	0	
21679094	m	cn	GR	2022	0	1	1	0	0	0	0	0	0	0	0	0	60
21679172	m	cn	CN	2022	0	1	0	0	1	0	0	0	0	0	0	0	68
21679414	m	md	DE	2022	0	1	0	0	1	0	0	0	0	0	0	0	67
21680091	f	md	GB	2022	0	1	0	0	0	1	0	0	0	0	0	0	67
21680167	m	md	JP	2022	0	1	1	0	0	0	0	0	0	0	0	0	
21680184	m	ph	US	2022	1	0	0	0	0	0	0	0	0	0	0	0	58
21680483	f	md	ES	2022	0	0	0	0	0	0	0	0	0	0	0	0	
21680540	f	md	US	2022	0	0	0	0	0	0	0	0	0	0	0	0	67
21680924	m	md	JP	2022	0	1	0	0	1	0	0	0	0	0	0	0	75
21681325	m	md	JP	2022	0	1	0	0	1	0	0	0	0	0	0	0	90
21681803	f	md	JP	2022	0	0	0	0	0	0	0	0	0	0	0	0	82
21682328	m	md	JP	2022	0	1	0	0	0	1	0	0	0	0	0	0	80
21682343	Not	md	JP	2022	0	1	0	0	0	1	0	0	0	0	0	0	70
21682447	m	md	JP	2022	0	1	1	0	0	0	0	0	0	0	0	0	80

21682485	m	md	US	2022	0	0	0	0	0	0	0	0	0	0	0	0	50
21682638	f	cn	US	2022	0	0	0	0	0	0	0	0	0	0	0	0	44
21683184	f	cn	US	2022	1	0	0	0	0	0	0	0	0	0	0	0	87.9
21683492	f	md	JP	2022	0	0	0	0	0	0	0	0	0	0	0	0	82
21683540	m	md	JP	2022	0	1	0	0	0	1	0	0	0	0	0	0	60
21683603	m	cn	US	2022	1	0	0	0	0	0	0	0	0	0	0	0	54.7
21683736	f	cn	US	2022	0	1	0	0	1	0	0	0	0	0	0	0	65
21683810	m	cn	TH	2022	1	0	0	0	0	0	0	0	0	0	0	0	85.3
21683854	m	cn	US	2022	0	1	1	0	0	0	0	0	0	0	0	0	76
21683921	m	md	IT	2022	0	0	0	0	0	0	0	0	0	0	0	0	75
21684076	m	hp	US	2022	0	0	0	0	0	0	0	0	0	0	0	0	60
21684372	m	md	CL	2022	0	0	0	0	0	0	0	0	0	0	0	0	63
21684571	f	hp	JP	2022	0	0	0	0	0	0	0	0	0	0	0	0	51
21684599	f	md	NL	2022	0	1	0	0	1	0	0	0	0	0	0	0	66
21684621	f	hp	BE	2022	0	0	0	0	0	0	0	0	0	0	0	0	
21684873	m	md	DE	2022	0	0	0	0	0	0	0	0	0	0	0	0	68
21685002	m	ph	JP	2022	0	1	0	0	1	0	0	0	0	0	0	0	74
21685358	m	hp	CN	2022	0	0	0	0	0	0	0	0	0	0	0	0	62
21686026	f	cn	US	2022	1	0	0	0	0	0	0	0	0	0	0	0	
21686064	f	md	JP	2022	0	1	0	0	0	1	0	0	0	0	0	0	80
21686065	f	md	FR	2022	0	1	0	0	0	1	0	0	0	0	0	0	69
21686137	m	cn	IN	2022	0	0	0	0	0	0	0	0	0	0	0	0	43
21686225	m	md	JP	2022	0	1	1	0	0	0	0	0	0	0	0	0	48
21687131	Not	cn	US	2022	0	0	0	0	0	0	0	0	0	0	0	0	
21687421	f	hp	NL	2022	1	0	0	0	0	0	0	0	0	0	0	0	86
21687571	f	cn	JP	2022	0	1	0	0	1	0	0	0	0	0	0	0	71
21687577	m	cn	US	2022	0	1	0	0	1	0	0	0	0	0	0	0	67
21687580	Not	md	JP	2022	1	0	0	0	0	0	0	0	0	0	0	0	
21687586	m	cn	US	2022	0	1	0	0	1	0	0	0	0	0	0	1	60
21687789	m	hp	DE	2022	0	1	1	0	0	0	0	0	0	0	0	1	56
21688163	Not	md	JP	2022	1	0	0	0	0	0	0	0	0	0	0	0	
21688283	f	hp	US	2022	0	1	0	0	1	0	0	0	0	0	0	0	71
21688790	Not	md	JP	2022	0	1	0	1	0	0	0	0	0	0	0	0	
21688822	f	md	JP	2022	0	0	0	0	0	0	0	0	0	0	0	0	68

21689438	f	md	JP	2022	0	1	0	0	1	0	0	0	0	0	0	0	74
21689564	f	cn	CA	2022	0	0	0	0	0	0	0	0	0	0	0	0	63
21689623	m	cn	MY	2022	0	0	0	0	0	0	0	0	0	0	0	0	73
21689899	f	md	US	2022	0	0	0	0	0	0	0	0	0	0	0	0	60
21689953	m	md	JP	2022	0	1	0	0	1	0	0	0	0	0	0	0	66
21690001	m	hp	US	2022	0	0	0	0	0	0	0	0	0	0	0	0	70
21690032	f	md	VN	2022	0	0	0	0	0	0	0	0	0	0	0	0	
21690062	f	md	US	2022	0	0	0	0	0	0	0	0	0	0	0	0	70
21690106	f	hp	US	2022	0	0	0	0	0	0	0	0	0	0	0	0	60
21691893	f	ph	JP	2022	0	1	1	0	0	0	0	0	0	0	0	0	92
21692114	m	md	JP	2022	0	1	1	0	0	0	0	0	0	0	0	0	70
21692396	f	md	JP	2022	0	1	1	0	0	0	0	0	0	0	0	0	91
21692666	m	md	JP	2022	0	1	0	0	1	0	0	0	0	0	0	0	62
21693059	f	hp	IT	2022	0	0	0	0	0	0	0	0	0	0	0	0	65
21693156	m	cn	IN	2022	0	1	0	0	1	0	0	0	0	0	0	0	58
21693286	Not	hp	CN	2022	0	0	0	0	0	0	0	0	0	0	0	0	
21693289	Not	hp	CN	2022	0	0	0	0	0	0	0	0	0	0	0	0	
21693401	Not	hp	CN	2022	0	0	0	0	0	0	0	0	0	0	0	0	
21693403	Not	hp	CN	2022	0	0	0	0	0	0	0	0	0	0	0	0	
21693404	Not	hp	CN	2022	0	0	0	0	0	0	0	0	0	0	0	0	
21693440	Not	hp	CN	2022	0	0	0	0	0	0	0	0	0	0	0	0	
21693472	Not	hp	CN	2022	0	0	0	0	0	0	0	0	0	0	0	0	
21693512	Not	hp	CN	2022	0	0	0	0	0	0	0	0	0	0	0	0	
21693513	Not	hp	CN	2022	0	0	0	0	0	0	0	0	0	0	0	0	
21693518	Not	hp	CN	2022	0	0	0	0	0	0	0	0	0	0	0	0	
21693519	Not	hp	CN	2022	0	0	0	0	0	0	0	0	0	0	0	0	
21693521	Not	hp	CN	2022	0	0	0	0	0	0	0	0	0	0	0	0	
21693551	Not	hp	CN	2022	0	0	0	0	0	0	0	0	0	0	0	0	
21693552	Not	hp	CN	2022	0	0	0	0	0	0	0	0	0	0	0	0	
21693553	Not	hp	CN	2022	0	0	0	0	0	0	0	0	0	0	0	0	
21693554	Not	hp	CN	2022	0	0	0	0	0	0	0	0	0	0	0	0	
21693555	Not	hp	CN	2022	0	0	0	0	0	0	0	0	0	0	0	0	
21693556	Not	hp	CN	2022	0	0	0	0	0	0	0	0	0	0	0	0	
21693557	Not	hp	CN	2022	0	0	0	0	0	0	0	0	0	0	0	0	

21693559	Not	hp	CN	2022	0	0	0	0	0	0	0	0	0	0	0	0	
21693582	Not	hp	CN	2022	0	0	0	0	0	0	0	0	0	0	0	0	
21693583	Not	hp	CN	2022	0	0	0	0	0	0	0	0	0	0	0	0	
21693584	Not	hp	CN	2022	0	0	0	0	0	0	0	0	0	0	0	0	
21693585	Not	hp	CN	2022	0	0	0	0	0	0	0	0	0	0	0	0	
21693586	Not	hp	CN	2022	0	0	0	0	0	0	0	0	0	0	0	0	
21693587	Not	hp	CN	2022	0	0	0	0	0	0	0	0	0	0	0	0	
21693588	Not	hp	CN	2022	0	0	0	0	0	0	0	0	0	0	0	0	
21693589	Not	hp	CN	2022	0	0	0	0	0	0	0	0	0	0	0	0	
21693615	Not	hp	CN	2022	0	0	0	0	0	0	0	0	0	0	0	0	
21693617	Not	hp	CN	2022	0	0	0	0	0	0	0	0	0	0	0	0	
21693957	Not	hp	CN	2022	0	0	0	0	0	0	0	0	0	0	0	0	
21694011	Not	hp	CN	2022	0	0	0	0	0	0	0	0	0	0	0	0	
21694153	m	hp	CN	2022	0	1	0	0	1	0	0	0	0	0	0	0	67
21694295	Not	hp	CN	2022	0	0	0	0	0	0	0	0	0	0	0	0	
21694541	m	cn	IN	2022	0	1	0	0	1	0	0	0	0	0	0	0	63
21694577	f	hp	DE	2022	0	1	0	0	1	0	0	0	0	0	0	0	60
21694651	m	md	IT	2022	0	0	0	0	0	0	0	0	0	0	0	0	
21694846	f	md	JP	2022	0	1	0	1	0	0	0	0	0	0	0	0	60
21695580	m	md	JP	2022	0	1	1	0	0	0	0	0	0	0	0	0	80
21695814	f	cn	MY	2022	0	0	0	0	0	0	0	0	0	0	0	0	70
21696028	m	md	JP	2022	0	1	1	0	0	0	0	0	0	0	0	0	77
21696174	m	ph	JP	2022	0	0	0	0	0	0	0	0	0	0	0	0	
21696343	m	md	JP	2022	0	1	0	0	1	0	0	0	0	0	0	0	62
21696434	f	hp	DE	2022	0	1	0	0	1	0	0	0	0	0	0	0	65
21696645	f	md	DE	2022	0	1	0	0	1	0	0	0	0	0	0	0	59
21696660	f	hp	DE	2022	0	0	0	0	0	0	0	0	0	0	0	0	68
21696713	m	ph	JP	2022	0	0	0	0	0	0	0	0	0	0	0	0	
21697402	m	cn	CN	2022	0	0	0	0	0	0	0	0	0	0	0	0	75
21697925	m	md	SI	2022	0	0	0	0	0	0	0	0	0	0	0	0	
21698722	Not	md	JP	2022	0	1	0	0	1	0	0	0	0	0	0	0	
21698934	f	md	JP	2022	0	1	1	0	0	0	0	0	0	0	0	1	
21699723	m	md	GB	2022	0	1	1	0	0	0	0	0	0	0	0	1	
21700293	m	cn	CN	2022	0	1	0	0	1	0	0	0	0	0	0	0	65

21700781	m	md	JP	2022	1	0	0	0	0	0	0	0	0	0	0	0	32
21700870	f	hp	US	2022	1	0	0	0	0	0	0	0	0	0	0	0	63
21700983	f	cn	ID	2022	1	0	0	0	0	0	0	0	0	0	0	0	
21701044	m	hp	IE	2022	0	1	1	0	0	0	0	0	0	0	0	0	73
21701055	m	hp	US	2022	0	0	0	0	0	0	0	0	0	0	0	0	86
21701223	m	md	JP	2022	0	1	1	0	0	0	0	0	0	0	0	0	60
21701272	m	md	MY	2022	0	0	0	0	0	0	0	0	0	0	0	0	70
21701379	f	hp	IL	2022	0	0	0	0	0	0	0	0	0	0	0	0	69
21701745	Not	hp	AU	2022	0	0	0	0	0	0	0	0	0	0	0	0	67
21702777	m	md	DE	2022	0	1	0	0	1	0	0	0	0	0	0	0	86
21702958	m	cn	US	2022	0	0	0	0	0	0	0	0	0	0	0	0	
21703214	f	hp	CN	2022	1	0	0	0	0	0	0	0	0	0	0	0	56
21703216	f	hp	CN	2022	1	0	0	0	0	0	0	0	0	0	0	0	51
21703682	m	md	JP	2022	0	1	1	0	0	0	0	0	0	0	0	0	76
21703908	m	md	JP	2022	0	1	1	0	0	0	0	0	0	0	0	1	73
21704000	f	md	RU	2022	1	0	0	0	0	0	0	0	0	0	0	0	62
21704007	m	hp	DE	2022	0	1	0	0	1	0	0	0	0	0	0	0	68
21704683	Not	hp	US	2022	1	0	0	0	0	0	0	0	0	0	0	0	
21704815	f	cn	KR	2022	0	0	0	0	0	0	0	0	0	0	0	0	60
21704959	m	cn	TH	2022	0	0	0	0	0	0	0	0	0	0	0	0	46
21705206	m	hp	IN	2022	0	1	0	0	1	0	0	0	0	0	0	0	77
21705248	f	cn	US	2022	0	0	0	0	0	0	0	0	0	0	0	0	
21705271	m	ph	CA	2022	1	0	0	0	0	0	0	0	0	0	0	0	63.4
21705619	m	hp	JP	2022	0	1	1	0	0	0	0	0	0	0	0	0	75
21705810	f	hp	JP	2022	0	1	1	0	0	0	0	0	0	0	0	0	82
21705847	f	hp	US	2022	0	1	1	0	0	0	0	0	0	0	0	0	55
21705964	f	hp	JP	2022	0	1	1	0	0	0	0	0	0	0	0	0	71
21706084	Not	md	JP	2022	1	0	0	0	0	0	0	0	0	0	0	0	
21706144	m	hp	JP	2022	0	0	0	0	0	0	0	0	0	0	0	0	78
21706195	m	hp	FR	2022	0	0	0	0	0	0	0	0	0	0	0	0	52
21706197	f	cn	US	2022	0	1	0	1	0	0	0	0	0	0	0	0	77
21706462	f	hp	DE	2022	0	0	0	0	0	0	0	0	0	0	0	0	68
21706519	f	md	FR	2022	0	0	0	0	0	0	0	0	0	0	0	0	80
21706568	f	md	JP	2022	0	0	0	0	0	0	0	0	0	0	0	0	

21706621	f	md	JP	2022	1	0	0	0	0	0	0	0	0	0	0	0	52
21707018	Not	md	MX	2022	0	1	0	0	0	1	0	0	0	0	0	0	
21707598	m	hp	AT	2022	0	1	1	0	0	0	0	0	0	0	0	0	45
21708025	m	hp	CL	2022	0	1	0	0	1	0	0	0	0	0	0	1	78
21708170	f	hp	DE	2022	0	1	0	0	1	0	0	0	0	0	0	0	58
21708392	f	md	NL	2022	0	0	0	0	0	0	0	0	0	0	0	0	72
21709801	Not	md	BE	2022	0	0	0	0	0	0	0	0	0	0	0	0	25
21709841	m	hp	AT	2022	0	1	1	0	0	0	0	0	0	0	0	0	45
21710297	m	md	JP	2022	0	1	0	0	1	0	0	0	0	0	0	0	90
21710394	m	md	US	2022	0	1	0	0	1	0	0	0	0	0	0	0	
21710700	m	cn	CN	2022	0	0	0	0	0	0	0	0	0	0	0	0	67
21710709	Not	md	JP	2022	0	1	0	0	0	1	0	0	0	0	0	0	70
21710847	m	md	DE	2022	0	1	0	0	1	0	0	0	0	0	0	0	75
21710909	f	md	DE	2022	0	1	0	0	1	0	0	0	0	0	0	0	56
21711070	m	md	JP	2022	0	1	0	0	0	1	0	0	0	0	0	0	
21711103	m	md	JP	2022	0	0	0	0	0	0	0	0	0	0	0	0	45
21711114	f	md	DE	2022	0	1	0	0	1	0	0	0	0	0	0	0	60
21711131	f	md	JP	2022	0	1	0	0	0	1	0	0	0	0	0	0	80
21711596	f	ph	JP	2022	0	1	1	0	0	0	0	0	0	0	0	0	81
21711758	f	md	DE	2022	0	1	0	0	1	0	0	0	0	0	0	0	59
21711788	f	hp	US	2022	0	0	0	0	0	0	0	0	0	0	0	0	61
21711891	f	hp	CA	2022	0	0	0	0	0	0	0	0	0	0	0	0	
21711924	f	ph	JP	2022	1	0	0	0	0	0	0	0	0	0	0	0	74
21712851	m	cn	IN	2022	0	1	0	0	1	0	0	0	0	0	0	0	75
21713889	m	md	FR	2022	0	0	0	0	0	0	0	0	0	0	0	0	
21713945	f	md	LU	2022	0	1	1	0	0	0	0	0	0	0	0	0	58
21714437	f	cn	US	2022	1	0	0	0	0	0	0	0	0	0	0	0	66
21714608	m	md	JP	2022	0	1	0	0	0	1	0	0	0	0	0	0	70
21714803	f	md	DE	2022	0	1	0	0	1	0	0	0	0	0	0	0	56
21714964	f	cn	AU	2022	1	0	0	0	0	0	0	0	0	0	0	0	
21715094	f	md	IT	2022	0	0	0	0	0	0	0	0	0	0	0	0	
21715256	f	md	IT	2022	0	0	0	0	0	0	0	0	0	0	0	0	69
21715257	Not	md	GB	2022	0	0	0	0	0	0	0	0	0	0	0	0	
21715316	f	cn	TH	2022	1	0	0	0	0	0	0	0	0	0	0	0	63.1

21715403	f	md	DE	2022	0	1	0	0	1	0	0	0	0	0	0	0	76
21715463	Not	md	JP	2022	0	1	0	0	1	0	0	0	0	0	0	0	90
21715702	Not	md	US	2022	0	0	0	0	0	0	0	0	0	0	0	0	
21716355	m	md	ES	2022	0	1	0	0	0	1	0	0	0	0	0	0	75
21716532	m	md	CN	2022	0	0	0	0	0	0	0	0	0	0	0	0	69
21717348	m	hp	CN	2022	0	0	0	0	0	0	0	0	0	0	0	0	75
21717764	m	hp	IE	2022	0	1	0	0	1	0	0	0	0	0	0	0	71
21718303	Not	md	JP	2022	0	1	0	0	1	0	0	0	0	0	0	1	
21718539	m	cn	US	2022	0	0	0	0	0	0	0	0	0	0	0	0	
21718794	Not	md	AU	2022	0	1	0	0	0	1	0	0	0	0	0	0	
21719190	f	md	JP	2022	0	0	0	0	0	0	0	0	0	0	0	0	60
21720059	f	md	GB	2022	0	1	0	0	0	1	0	0	0	0	0	0	65
21720066	f	md	CH	2022	0	0	0	0	0	0	0	0	0	0	0	0	
21720721	m	md	JP	2022	0	1	1	0	0	0	0	0	0	0	0	0	81
21721485	m	cn	US	2022	0	1	1	0	0	0	0	0	0	0	0	0	
21721505	m	hp	AU	2022	0	1	1	0	0	0	0	0	0	0	0	1	
21722965	f	md	DE	2022	0	1	0	0	1	0	0	0	0	0	0	0	60
21722977	f	cn	TH	2022	1	0	0	0	0	0	0	0	0	0	0	0	68.2
21723082	m	cn	US	2022	0	0	0	0	0	0	0	0	0	0	0	0	65
21723368	f	md	RS	2022	0	1	0	1	0	0	0	0	0	0	0	0	67
21723460	f	cn	US	2022	0	0	0	0	0	0	0	0	0	0	0	0	
21723799	f	md	US	2022	0	1	1	0	0	0	0	0	0	0	0	0	85
21724071	m	md	RU	2022	0	0	0	0	0	0	0	0	0	0	0	0	
21724207	f	md	CN	2022	0	0	0	0	0	0	0	0	0	0	0	0	79
21724659	m	md	JP	2022	0	1	0	0	0	1	0	0	0	0	0	0	80
21724660	f	hp	CN	2022	0	0	0	0	0	0	0	0	0	0	0	0	31
21724703	m	cn	TW	2022	0	1	0	0	0	1	0	0	0	0	0	0	
21724851	Not	md	FR	2022	0	1	0	0	1	0	0	0	0	0	0	0	66
21725659	Not	md	JP	2022	0	1	0	0	0	1	0	0	0	0	0	0	
21728141	m	cn	CN	2022	0	0	0	0	0	0	0	0	0	0	0	0	62
21728282	f	cn	AT	2022	0	0	0	0	0	0	0	0	0	0	0	0	72
21728617	m	cn	CN	2022	0	1	0	1	0	0	0	0	0	0	0	1	70
21728618	f	cn	JP	2022	1	0	0	0	0	0	0	0	0	0	0	0	
21728632	m	md	DE	2022	0	1	0	0	1	0	0	0	0	0	0	0	54

21728750	f	md	NL	2022	0	1	0	0	1	0	0	0	0	0	0	0	65
21728856	f	cn	DE	2022	0	1	0	0	1	0	0	0	0	0	0	0	60
21728857	m	md	DE	2022	0	1	1	0	0	0	0	0	0	0	0	0	55
21729060	m	md	JP	2022	0	1	1	0	0	0	0	0	0	0	0	0	61
21729090	f	hp	GB	2022	0	0	0	0	0	0	0	0	0	0	0	0	41
21729221	f	md	JP	2022	0	1	0	1	0	0	0	0	0	0	0	0	59
21729795	f	cn	DE	2022	0	1	0	0	1	0	0	0	0	0	0	0	69
21729905	m	hp	CN	2022	0	0	0	0	0	0	0	0	0	0	0	0	51
21729976	Not	md	FR	2022	0	0	0	0	0	0	0	0	0	0	0	0	
21730040	m	md	JP	2022	0	1	0	0	1	0	0	0	0	0	0	1	72
21730201	m	md	JP	2022	0	1	0	0	0	1	0	0	0	0	0	0	90
21730260	Not	md	JP	2022	0	0	0	0	0	0	0	0	0	0	0	0	
21730480	f	md	JP	2022	0	1	0	0	0	1	0	0	0	0	0	0	69
21730518	Not	md	JP	2022	0	1	0	0	0	1	0	0	0	0	0	0	
21730807	m	cn	JP	2022	0	1	0	0	1	0	0	0	0	0	0	0	
21730870	Not	md	JP	2022	0	1	0	0	0	1	0	0	0	0	0	0	
21730981	m	md	JP	2022	0	0	0	0	0	0	0	0	0	0	0	0	60
21731831	Not	md	JP	2022	0	1	0	0	0	1	0	0	0	0	0	0	82
21731908	Not	md	JP	2022	0	1	0	0	0	1	0	0	0	0	0	0	61
21732146	m	md	JP	2022	0	1	0	0	1	0	0	0	0	0	0	0	67
21732552	Not	md	JP	2022	0	1	0	0	0	1	0	0	0	0	0	0	
21733043	f	md	US	2022	0	1	1	0	0	0	0	0	0	0	0	0	80
21733167	Not	md	JP	2022	0	1	0	0	0	1	0	0	0	0	0	0	
21733277	f	md	ES	2022	0	1	1	0	0	0	0	0	0	0	0	1	
21733381	f	hp	US	2022	0	0	0	0	0	0	0	0	0	0	0	0	72
21733493	f	cn	JP	2022	0	0	0	0	0	0	0	0	0	0	0	0	74
21733623	m	hp	CN	2022	0	0	0	0	0	0	0	0	0	0	0	0	55
21733652	f	md	PL	2022	0	0	0	0	0	0	0	0	0	0	0	0	36
21733743	m	md	JP	2022	0	1	0	0	1	0	0	0	0	0	0	0	52
21734258	m	md	US	2022	0	0	0	0	0	0	0	0	0	0	0	0	
21734405	m	md	DE	2022	0	1	0	0	1	0	0	0	0	0	0	0	61
21734750	f	md	DE	2022	0	0	0	0	0	0	0	0	0	0	0	0	72
21735081	m	cn	IN	2022	0	0	0	0	0	0	0	0	0	0	0	0	68
21735244	m	md	JP	2022	0	1	0	0	0	1	0	0	0	0	0	0	70

21735287	f	cn	TH	2022	0	0	0	0	0	0	0	0	0	0	0	0	56
21735314	m	hp	BR	2022	0	0	0	0	0	0	0	0	0	0	0	0	56
21735777	m	md	CN	2022	0	0	0	0	0	0	0	0	0	0	0	0	
21736009	m	md	JP	2022	0	0	0	0	0	0	0	0	0	0	0	0	
21736199	m	hp	JP	2022	0	0	0	0	0	0	0	0	0	0	0	0	68
21736241	f	md	JP	2022	0	1	0	0	1	0	0	0	0	0	0	0	44
21736607	m	md	KR	2022	0	0	0	0	0	0	0	0	0	0	0	0	
21736732	m	md	JP	2022	0	1	0	0	0	1	0	0	0	0	0	0	80
21737140	Not	md	JP	2022	0	1	0	0	0	1	0	0	0	0	0	0	
21737567	Not	md	JP	2022	0	1	0	0	0	1	0	0	0	0	0	0	
21738021	Not	md	JP	2022	0	1	0	0	0	1	0	0	0	0	0	0	
21738150	Not	md	JP	2022	0	1	0	0	0	1	0	0	0	0	0	0	
21738171	Not	md	JP	2022	0	1	0	0	0	1	0	0	0	0	0	0	
21738174	Not	md	JP	2022	0	1	0	0	0	1	0	0	0	0	0	0	62
21738384	f	md	JP	2022	1	0	0	0	0	0	0	0	0	0	0	0	80
21738435	Not	md	JP	2022	0	1	0	0	0	1	0	0	0	0	0	0	
21738437	Not	md	JP	2022	0	1	0	0	0	1	0	0	0	0	0	0	
21738630	Not	md	JP	2022	0	1	0	0	0	1	0	0	0	0	0	0	
21738739	Not	md	JP	2022	0	1	0	0	0	1	0	0	0	0	0	0	
21739032	f	md	AT	2022	0	0	0	0	0	0	0	0	0	0	0	0	
21739242	Not	md	JP	2022	0	1	0	0	0	1	0	0	0	0	0	0	
21739467	Not	md	JP	2022	0	1	0	0	1	0	0	0	0	0	0	0	76
21739652	m	hp	JP	2022	0	1	1	0	0	0	0	0	0	0	0	1	62
21739653	m	hp	JP	2022	0	1	1	0	0	0	0	0	0	0	0	1	66
21739731	f	cn	JP	2022	0	0	0	0	0	0	0	0	0	0	0	0	79
21739860	f	md	BG	2022	0	1	0	0	0	1	0	0	0	0	0	0	60
21740369	m	md	JP	2022	0	1	1	0	0	0	0	0	0	0	0	0	90
21740603	Not	md	JP	2022	0	1	0	0	0	1	0	0	0	0	0	0	
21740776	Not	md	JP	2022	0	1	0	0	0	1	0	0	0	0	0	0	
21740817	Not	md	JP	2022	0	1	0	0	0	1	0	0	0	0	0	0	73
21740895	m	md	IT	2022	0	0	0	0	0	0	0	0	0	0	0	0	70
21741348	Not	md	JP	2022	0	1	0	0	0	1	0	0	0	0	0	0	
21741501	m	md	JP	2022	0	0	0	0	0	0	0	0	0	0	0	0	
21741570	f	md	NO	2022	0	0	0	0	0	0	0	0	0	0	0	0	73

21741592	m	md	FR	2022	0	1	0	0	0	1	0	0	0	0	0	0	67
21741865	Not	hp	DE	2022	0	1	0	0	0	1	0	0	0	0	0	0	
21742114	f	hp	CN	2022	1	0	0	0	0	0	0	0	0	0	0	0	47
21742324	m	md	JP	2022	0	1	0	0	1	0	0	0	0	0	0	0	60
21742391	m	hp	JP	2022	0	1	1	0	0	0	0	0	0	0	0	0	83
21742392	f	md	NL	2022	0	0	0	0	0	0	0	0	0	0	0	0	73
21742444	m	md	JP	2022	0	0	0	0	0	0	0	0	0	0	0	0	
21743497	f	md	US	2022	0	0	0	0	0	0	0	0	0	0	0	0	
21743701	f	hp	JP	2022	1	0	0	0	0	0	0	0	0	0	0	0	77
21743988	m	md	DE	2022	0	1	0	0	1	0	0	0	0	0	0	0	73
21744421	f	md	JP	2022	0	0	0	0	0	0	0	0	0	0	0	0	75
21744728	m	md	JP	2022	0	0	0	0	0	0	0	0	0	0	0	0	63
21745328	Not	md	JP	2022	0	1	0	0	0	1	0	0	0	0	0	0	
21745359	m	cn	CN	2022	0	0	0	0	0	0	0	0	0	0	0	0	54
21745778	m	md	JP	2022	0	1	1	0	0	0	0	0	0	0	0	0	84
21746299	Not	md	JP	2022	0	0	0	0	0	0	0	0	0	0	0	0	75
21746527	m	md	JP	2022	0	1	0	0	0	1	0	0	0	0	0	0	72
21747183	Not	md	RU	2022	0	1	0	0	0	1	0	0	0	0	0	1	56
21747304	f	md	IT	2022	0	1	0	0	1	0	0	0	0	0	0	0	78
21748170	m	cn	TH	2022	0	0	0	0	0	0	0	0	0	0	0	0	75
21748178	f	md	IT	2022	0	1	0	0	1	0	0	0	0	0	0	0	78
21748654	m	md	JP	2022	0	1	0	0	0	1	0	0	0	0	0	0	75
21748655	Not	md	JP	2022	0	1	0	0	0	1	0	0	0	0	0	0	74
21748780	m	cn	JP	2022	0	1	0	0	1	0	0	0	0	0	0	0	75
21748781	f	cn	CZ	2022	0	1	1	0	0	0	0	0	0	0	0	0	65
21749283	Not	md	TR	2022	0	0	0	0	0	0	0	0	0	0	0	0	
21749391	f	md	DE	2022	0	1	1	0	0	0	0	0	0	0	0	0	56
21749465	Not	md	JP	2022	0	1	0	0	0	1	0	0	0	0	0	0	
21749819	m	md	IE	2022	0	1	1	0	0	0	0	0	0	0	0	0	73
21750160	m	hp	DE	2022	0	1	1	0	0	0	0	0	0	0	0	0	64
21750318	m	md	US	2022	0	0	0	0	0	0	0	0	0	0	0	0	74
21750687	m	md	JP	2022	0	1	0	0	0	1	0	0	0	0	0	1	70
21750829	Not	md	JP	2022	0	1	0	0	1	0	0	0	0	0	0	1	
21750958	f	md	JP	2022	0	0	0	0	0	0	0	0	0	0	0	0	76

21751038	Not	md	JP	2022	0	1	0	0	0	1	0	0	0	0	0	0	70
21751051	m	md	JP	2022	0	0	0	0	0	0	1	0	0	0	1	0	70
21751861	m	md	JP	2022	0	1	1	0	0	0	0	0	0	0	0	0	60
21752027	m	hp	GR	2022	0	0	0	0	0	0	0	0	0	0	0	0	34
21752037	Not	md	JP	2022	0	1	0	0	0	1	0	0	0	0	0	0	70
21752106	m	ph	JP	2022	0	1	1	0	0	0	0	0	0	0	0	0	68
21752410	m	md	IE	2022	0	1	1	0	0	0	0	0	0	0	0	0	73
21753017	Not	md	MX	2022	0	0	0	0	0	0	0	0	0	0	0	0	
21753598	Not	md	JP	2022	0	1	0	0	0	1	0	0	0	0	0	0	
21754083	m	md	KR	2022	0	0	0	0	0	0	0	0	0	0	0	0	76
21754159	Not	md	JP	2022	0	1	0	0	1	0	0	0	0	0	0	1	
21754860	f	md	AU	2022	1	0	0	0	0	0	0	0	0	0	0	0	52
21755641	m	hp	US	2022	0	1	1	0	0	0	0	0	0	0	0	0	
21755695	m	md	JP	2022	0	0	0	0	0	0	0	0	0	0	0	0	
21756450	f	md	AU	2022	1	0	0	0	0	0	0	0	0	0	0	0	
21756475	f	md	JP	2022	1	0	0	0	0	0	0	0	0	0	0	0	42
21756552	f	md	AU	2022	1	0	0	0	0	0	0	0	0	0	0	0	
21756667	f	md	JP	2022	1	0	0	0	0	0	0	0	0	0	0	0	79
21756742	f	md	MY	2022	1	0	0	0	0	0	0	0	0	0	0	0	81.4
21756751	m	md	JP	2022	0	1	0	1	0	0	0	0	0	0	0	1	80
21756757	Not	md	JP	2022	0	1	0	1	0	0	0	0	0	0	0	0	
21756775	m	cn	CN	2022	0	1	0	1	0	0	0	0	0	0	0	0	60
21756787	m	md	JP	2022	0	1	0	1	0	0	0	0	0	0	0	0	71
21756812	f	md	JP	2022	1	0	0	0	0	0	0	0	0	0	0	0	83
21756923	m	md	CA	2022	1	0	0	0	0	0	0	0	0	0	0	0	63
21756925	Not	ph	JP	2022	1	0	0	0	0	0	0	0	0	0	0	0	
21756932	f	cn	MY	2022	1	0	0	0	0	0	0	0	0	0	0	0	
21756941	m	cn	MY	2022	1	0	0	0	0	0	0	0	0	0	0	0	
21756943	m	cn	MY	2022	1	0	0	0	0	0	0	0	0	0	0	0	
21756944	m	md	JP	2022	1	0	0	0	0	0	0	0	0	0	0	1	70
21756958	f	cn	US	2022	1	0	0	0	0	0	0	0	0	0	0	0	61.8
21756962	Not	cn	JP	2022	1	0	0	0	0	0	0	0	0	0	0	0	
21756985	f	md	RU	2022	1	0	0	0	0	0	0	0	0	0	0	0	35
21757011	Not	ph	JP	2022	0	1	0	1	0	0	0	0	0	0	0	0	

21757019	Not	md	JP	2022	1	0	0	0	0	0	0	0	0	0	0	0	
21757510	Not	hp	DE	2022	0	1	0	0	0	1	0	0	0	0	0	0	
21758244	f	md	CN	2022	0	0	0	0	0	0	0	0	0	0	0	0	64
21758606	m	md	US	2022	1	0	0	0	0	0	0	0	0	0	0	0	78
21758762	m	md	JP	2022	0	1	1	0	0	0	0	0	0	0	0	0	79
21758896	m	md	GE	2022	0	1	0	0	0	1	0	0	0	0	0	0	51
21758907	f	md	JP	2022	0	0	0	0	0	0	0	0	0	0	0	0	77
21759007	m	md	IN	2022	0	1	0	0	0	1	0	0	0	0	0	0	
21759017	m	md	JP	2022	0	1	1	0	0	0	0	0	0	0	0	0	70
21759357	m	md	HK	2022	0	0	0	0	0	0	0	0	0	0	0	0	77
21759945	m	md	GB	2022	1	0	0	0	0	0	0	0	0	0	0	0	
21760217	m	cn	GB	2022	1	0	0	0	0	0	0	0	0	0	0	0	71
21760436	f	md	JP	2022	1	0	0	0	0	0	0	0	0	0	0	0	41
21761055	f	md	JP	2022	0	0	0	0	0	0	0	0	0	0	0	0	51
21761128	f	md	DE	2022	0	0	0	0	0	0	0	0	0	0	0	0	60
21761456	Not	md	JP	2022	0	1	0	0	0	1	0	0	0	0	0	1	46
21761512	f	ph	JP	2022	0	1	0	1	0	0	0	0	0	0	0	0	80
21761648	Not	md	JP	2022	0	0	0	0	0	0	0	0	0	0	0	0	
21762109	m	ph	US	2022	0	0	0	0	0	0	0	0	0	0	0	0	
21762175	m	md	BR	2022	0	0	0	0	0	0	0	0	0	0	0	0	
21762480	f	md	JP	2022	1	0	0	0	0	0	0	0	0	0	0	0	75
21762661	m	cn	IN	2022	0	0	0	0	0	0	0	0	0	0	0	0	40
21763031	m	md	JP	2022	0	1	1	0	0	0	0	0	0	0	0	0	64
21763157	f	cn	DE	2022	0	0	0	0	0	0	0	0	0	0	0	0	62
21763166	m	md	CN	2022	0	0	0	0	0	0	0	0	0	0	0	0	69
21763168	Not	md	PL	2022	0	1	1	0	0	0	0	0	0	0	0	0	
21763171	Not	md	US	2022	0	0	0	0	0	0	0	0	0	0	0	0	
21763295	m	md	JP	2022	0	1	0	0	1	0	0	0	0	0	0	0	74
21764248	f	cn	US	2022	0	0	0	0	0	0	0	0	0	0	0	0	
21764279	Not	md	JP	2022	0	0	0	0	0	0	0	0	0	0	0	0	
21764854	m	hp	JP	2022	0	0	0	0	0	0	0	0	0	0	0	0	84
21764880	Not	md	JP	2022	0	1	0	0	1	0	0	0	0	0	0	0	
21765426	Not	md	JP	2022	0	1	1	0	0	0	0	0	0	0	0	0	
21765665	f	hp	GB	2022	1	0	0	0	0	0	0	0	0	0	0	0	80

21765725	m	cn	JP	2022	1	0	0	0	0	0	0	0	0	0	0	0	
21765812	Not	ph	US	2022	0	0	0	0	0	0	0	0	0	0	0	0	
21766501	Not	md	JP	2022	0	1	0	0	1	0	0	0	0	0	0	0	80
21766562	m	hp	IT	2022	0	1	1	0	0	0	0	0	0	0	0	0	69
21766563	m	hp	IT	2022	0	0	0	0	0	0	0	0	0	0	0	0	61
21766565	f	hp	IT	2022	0	0	0	0	0	0	0	0	0	0	0	0	62
21766667	f	md	JP	2022	0	0	0	0	0	0	0	0	0	0	0	0	78
21766678	f	md	JP	2022	0	0	0	0	0	0	0	0	0	0	0	0	79
21766682	f	md	NL	2022	0	0	0	0	0	0	0	0	0	0	0	0	85
21767188	m	cn	DE	2022	1	0	0	0	0	0	0	0	0	0	0	0	52
21767189	m	cn	DE	2022	1	0	0	0	0	0	0	0	0	0	0	0	52
21767571	f	hp	JP	2022	0	0	0	0	0	0	0	0	0	0	0	0	
21767763	f	md	FR	2022	0	1	0	0	0	1	0	0	0	0	0	0	69
21767802	m	cn	IN	2022	0	0	0	0	0	0	0	0	0	0	0	0	46
21767806	f	md	IT	2022	1	0	0	0	0	0	0	0	0	0	0	0	55
21767877	m	md	LT	2022	0	1	0	0	0	1	0	0	0	0	0	0	62
21767883	f	md	NZ	2022	1	0	0	0	0	0	0	0	0	0	0	0	
21768043	f	hp	HK	2022	1	0	0	0	0	0	0	0	0	0	0	0	72
21768166	m	md	NL	2022	0	0	0	0	0	0	0	0	0	0	0	0	
21768203	m	md	BE	2022	0	1	0	0	1	0	0	0	0	0	0	0	
21768884	f	md	AU	2022	0	1	1	0	0	0	0	0	0	0	0	0	
21768935	f	md	JP	2022	0	0	0	0	0	0	0	0	0	0	0	0	74
21769132	f	md	AT	2022	0	0	0	0	0	0	0	0	0	0	0	0	
21769485	m	md	JP	2022	0	0	0	0	0	0	0	0	0	0	0	0	50
21769507	m	md	JP	2022	0	0	0	0	0	0	0	0	0	0	0	0	70
21769628	m	hp	US	2022	0	0	0	0	0	0	0	0	0	0	0	0	70
21770297	m	cn	IN	2022	0	1	0	0	0	1	0	0	0	0	0	0	64
21770335	m	md	JP	2022	0	1	0	0	0	1	0	0	0	0	0	0	
21770392	m	hp	IE	2022	0	1	1	0	0	0	0	0	0	0	0	0	73
21770692	Not	md	RO	2022	0	1	0	0	0	1	0	0	0	0	0	0	
21771046	Not	md	US	2022	1	0	0	0	0	0	0	0	0	0	0	0	
21771130	f	md	US	2022	1	0	0	0	0	0	0	0	0	0	0	0	
21771238	Not	ph	JP	2022	1	0	0	0	0	0	0	0	0	0	0	1	
21771363	m	md	US	2022	1	0	0	0	0	0	0	0	0	0	0	0	75

21771370	m	md	JP	2022	0	1	0	0	0	1	0	0	0	0	0	1	80
21771823	m	md	US	2022	1	0	0	0	0	0	0	0	0	0	0	0	75
21771846	Not	hp	GB	2022	0	0	0	0	0	0	0	0	0	0	0	0	68
21772378	Not	md	JP	2022	0	1	0	0	0	1	0	0	0	0	0	1	80
21772415	f	md	BE	2022	0	0	0	0	0	0	0	0	0	0	0	0	78
21772530	f	cn	US	2022	0	0	0	0	0	0	0	0	0	0	0	0	
21772582	m	cn	ID	2022	0	1	0	1	0	0	0	0	0	0	0	0	81.7
21773095	Not	cn	SG	2022	1	0	0	0	0	0	0	0	0	0	0	0	83
21773198	m	cn	KW	2022	1	0	0	0	0	0	0	0	0	0	0	0	40
21773392	m	md	JP	2022	0	0	0	0	0	0	0	0	0	0	0	0	
21773410	f	cn	MY	2022	1	0	0	0	0	0	0	0	0	0	0	0	
21773475	Not	cn	SG	2022	1	0	0	0	0	0	0	0	0	0	0	0	64.2
21773480	m	md	JP	2022	1	0	0	0	0	0	0	0	0	0	0	0	70
21773536	f	md	UA	2022	1	0	0	0	0	0	0	0	0	0	0	0	52
21773555	f	hp	GB	2022	0	0	0	0	0	0	0	0	0	0	0	0	74
21773589	m	cn	CA	2022	1	0	0	0	0	0	0	0	0	0	0	0	
21773590	Not	cn	SG	2022	1	0	0	0	0	0	0	0	0	0	0	0	88.2
21773591	f	cn	US	2022	1	0	0	0	0	0	0	0	0	0	0	0	
21773746	m	md	CN	2022	0	1	0	1	0	0	0	0	0	0	0	0	61
21773868	m	md	JP	2022	0	1	0	0	1	0	0	0	0	0	0	0	77
21774021	f	cn	MY	2022	1	0	0	0	0	0	0	0	0	0	0	0	
21774483	m	md	JP	2022	0	0	0	0	0	0	0	0	0	0	0	0	
21774556	Not	md	JP	2022	0	0	0	0	0	0	0	0	0	0	0	0	
21774563	Not	cn	US	2022	0	0	0	0	0	0	0	0	0	0	0	0	
21775009	m	hp	US	2022	1	0	0	0	0	0	0	0	0	0	0	0	75
21775034	f	md	JP	2022	1	0	0	0	0	0	0	0	0	0	0	0	52
21775070	Not	cn	SG	2022	1	0	0	0	0	0	0	0	0	0	0	0	
21775078	m	cn	TH	2022	1	0	0	0	0	0	0	0	0	0	0	0	72.4
21775259	f	md	JP	2022	0	1	0	0	1	0	0	0	0	0	0	0	71
21775317	f	ph	US	2022	0	1	1	0	0	0	0	0	0	0	0	0	
21776968	Not	md	JP	2022	0	1	0	0	1	0	0	0	0	0	0	0	80
21777992	f	md	JP	2022	0	0	0	0	0	0	0	0	0	0	0	0	66
21778467	m	md	JP	2022	0	1	1	0	0	0	0	0	0	0	0	0	70
21779235	f	md	JP	2022	1	0	0	0	0	0	0	0	0	0	0	0	66.7

21779244	f	ph	CA	2022	0	1	1	0	0	0	0	0	0	0	0	0	79
21779287	m	cn	CA	2022	0	0	0	0	0	0	0	0	0	0	0	0	54
21779536	f	cn	IN	2022	0	1	0	1	0	0	0	0	0	0	0	0	66.6
21779811	f	md	JP	2022	0	0	0	0	0	0	0	0	0	0	0	0	
21779830	m	md	JP	2022	0	1	0	0	1	0	0	0	0	0	0	0	
21780100	m	md	US	2022	0	1	0	0	0	1	0	0	0	0	0	0	82
21780267	m	md	JP	2022	0	0	0	0	0	0	0	0	0	0	0	0	50
21780313	f	md	JP	2022	0	1	0	0	0	1	0	0	0	0	0	0	80
21780344	f	md	JP	2022	0	0	0	0	0	0	0	0	0	0	0	0	80
21780403	Not	md	PL	2022	0	0	0	0	0	0	0	0	0	0	0	0	
21780593	f	md	IN	2022	0	0	0	0	0	0	0	0	0	0	0	0	60
21780666	m	cn	TW	2022	0	0	0	0	0	0	0	0	0	0	0	0	62
21781101	m	md	JP	2022	0	0	0	0	0	0	0	0	0	0	0	0	70
21781125	f	md	IT	2022	0	1	0	0	1	0	0	0	0	0	0	0	78
21781528	m	cn	TW	2022	1	0	0	0	0	0	0	0	0	0	0	0	46
21781843	m	md	JP	2022	0	1	1	0	0	0	0	0	0	0	0	0	70
21781859	m	md	JP	2022	0	1	1	0	0	0	0	0	0	0	0	0	70
21782545	m	md	US	2022	0	0	0	0	0	0	0	0	0	0	0	0	
21782802	m	hp	JP	2022	0	0	0	0	0	0	0	0	0	0	0	0	84
21782860	m	md	JP	2022	0	1	1	0	0	0	0	0	0	0	0	0	70
21782908	f	md	US	2022	1	0	0	0	0	0	0	0	0	0	0	1	82
21783008	m	hp	FR	2022	0	1	0	0	1	0	0	0	0	0	0	0	80
21783612	m	cn	TW	2022	1	0	0	0	0	0	0	0	0	0	0	0	57
21783621	m	md	JP	2022	0	0	0	0	0	0	0	0	0	0	0	0	78
21784494	Not	hp	GB	2022	0	0	0	0	0	0	0	0	0	0	0	0	64
21784555	m	ph	FR	2022	0	0	0	0	0	0	0	0	0	0	0	0	62
21784557	Not	md	BE	2022	0	0	0	0	0	0	0	0	0	0	0	0	
21784981	m	md	NL	2022	0	0	0	0	0	0	0	0	0	0	0	0	
21785088	m	cn	US	2022	0	0	0	0	0	0	0	0	0	0	0	0	61
21785115	f	cn	AR	2022	0	0	0	0	0	0	0	0	0	0	0	0	59
21785493	m	cn	TW	2022	0	0	0	0	0	0	0	0	0	0	0	0	59
21785905	f	cn	TH	2022	0	1	0	0	0	1	0	0	0	0	0	0	
21786288	f	hp	IT	2022	1	0	0	0	0	0	0	0	0	0	0	0	65
21786289	m	hp	IT	2022	1	0	0	0	0	0	0	0	0	0	0	0	72

21786291	f	hp	IT	2022	1	0	0	0	0	0	0	0	0	0	0	0	48
21786292	m	hp	IT	2022	1	0	0	0	0	0	0	0	0	0	0	0	65
21786293	m	hp	IT	2022	1	0	0	0	0	0	0	0	0	0	0	0	75
21786294	f	hp	IT	2022	1	0	0	0	0	0	0	0	0	0	0	0	44
21786295	m	hp	IT	2022	1	0	0	0	0	0	0	0	0	0	0	0	70
21786296	m	hp	IT	2022	1	0	0	0	0	0	0	0	0	0	0	0	74
21786297	m	hp	IT	2022	1	0	0	0	0	0	0	0	0	0	0	0	67
21786298	f	hp	IT	2022	1	0	0	0	0	0	0	0	0	0	0	0	51
21786299	m	hp	IT	2022	1	0	0	0	0	0	0	0	0	0	0	0	57
21786478	m	md	JP	2022	0	1	0	0	0	1	0	0	0	0	0	1	80
21786825	f	cn	TW	2022	0	0	0	0	0	0	0	0	0	0	0	0	66
21787270	m	md	JP	2022	0	1	1	0	0	0	0	0	0	0	0	0	
21787693	m	hp	BY	2022	0	1	0	0	0	1	0	0	0	0	0	0	71
21788130	f	ph	JP	2022	1	0	0	0	0	0	0	0	0	0	0	0	90
21788331	Not	md	JP	2022	1	0	0	0	0	0	0	0	0	0	0	0	
21791125	f	cn	US	2022	0	0	0	0	0	0	0	0	0	0	0	0	
21791127	m	cn	US	2022	0	0	0	0	0	0	0	0	0	0	0	0	
21791193	f	cn	US	2022	0	0	0	0	0	0	0	0	0	0	0	0	
21791304	f	md	DE	2022	0	1	1	0	0	0	0	0	0	0	0	0	
21791339	f	md	JP	2022	0	1	0	0	1	0	0	0	0	0	0	1	78
21791341	f	md	JP	2022	1	0	0	0	0	0	0	0	0	0	0	0	81
21791347	f	md	JP	2022	0	0	0	0	0	0	0	0	0	0	0	0	69
21791446	m	hp	BY	2022	0	1	0	0	0	1	0	0	0	0	0	0	67
21791711	f	md	JP	2022	0	0	0	0	0	0	0	0	0	0	0	0	73
21791856	f	md	IT	2022	0	0	0	0	0	0	0	0	0	0	0	0	63
21792259	m	hp	JP	2022	0	1	0	0	1	0	0	0	0	0	0	0	64
21792425	f	cn	US	2022	0	1	1	0	0	0	0	0	0	0	0	0	
21792668	m	hp	US	2022	0	1	0	0	1	0	0	0	0	0	0	0	61
21793222	f	md	DE	2022	0	1	0	0	1	0	0	0	0	0	0	0	62
21793404	m	cn	IN	2022	0	0	0	0	0	0	0	0	0	0	0	0	59
21793430	m	hp	BY	2022	0	1	0	0	0	1	0	0	0	0	0	0	60
21793890	m	hp	BY	2022	0	1	0	0	0	1	0	0	0	0	0	0	64
21794039	m	md	ES	2022	0	1	0	0	0	1	0	0	0	0	0	0	62
21794181	Not	cn	US	2022	1	0	0	0	0	0	0	0	0	0	0	0	

21794549	m	cn	TH	2022	1	0	0	0	0	0	0	0	0	0	0	0	66.7
21794566	f	md	JP	2022	0	1	1	0	0	0	0	0	0	0	0	0	
21794720	m	md	JP	2022	0	1	0	1	0	0	0	0	0	0	0	0	72
21795091	Not	cn	US	2022	1	0	0	0	0	0	0	0	0	0	0	0	
21795092	m	md	CN	2022	1	0	0	0	0	0	0	0	0	0	0	0	67
21795144	m	md	BY	2022	0	1	0	0	0	1	0	0	0	0	0	0	66
21795484	Not	md	CH	2022	1	0	0	0	0	0	0	0	0	0	0	0	
21795600	f	md	JP	2022	1	0	0	0	0	0	0	0	0	0	0	0	89
21795715	m	md	CN	2022	0	0	0	0	0	0	0	0	0	0	0	0	72
21795886	f	md	NL	2022	0	0	0	0	0	0	0	0	0	0	0	0	59
21796011	m	cn	IN	2022	0	0	0	0	0	0	0	0	0	0	0	0	72
21796217	m	md	US	2022	0	0	0	0	0	0	0	0	0	0	0	0	49
21796391	f	md	BE	2022	1	0	0	0	0	0	0	0	0	0	0	0	60
21796688	f	md	US	2022	0	0	0	0	0	0	0	0	0	0	0	0	
21796886	m	md	JP	2022	0	0	0	0	0	0	0	0	0	0	0	0	
21797457	f	md	AU	2022	1	0	0	0	0	0	0	0	0	0	0	0	81
21797840	Not	md	JP	2022	0	0	0	0	0	0	0	0	0	0	0	0	
21797846	f	md	AU	2022	0	0	0	0	0	0	0	0	0	0	0	0	
21798607	f	md	DE	2022	0	1	0	0	1	0	0	0	0	0	0	0	69
21798868	f	hp	BY	2022	0	1	0	0	0	1	0	0	0	0	0	0	46
21799289	m	md	HR	2022	0	1	0	0	0	1	0	0	0	0	0	0	73
21799381	m	hp	FR	2022	0	0	0	0	0	0	0	0	0	0	0	0	80
21799454	m	md	JP	2022	0	1	1	0	0	0	0	0	0	0	0	0	80
21800076	m	md	JP	2022	0	0	0	0	0	0	0	0	0	0	0	0	77
21800371	f	ph	CH	2022	0	0	0	0	0	0	0	0	0	0	0	0	67
21800424	m	md	JP	2022	0	1	1	0	0	0	0	0	0	0	0	0	
21800690	m	md	JP	2022	0	1	0	1	0	0	0	0	0	0	0	0	71
21800898	m	md	JP	2022	0	1	1	0	0	0	0	0	0	0	0	0	83
21801009	f	md	JP	2022	0	1	0	1	0	0	0	0	0	0	0	0	82
21801218	m	ph	US	2022	0	0	0	0	0	0	0	0	0	0	0	0	73
21801224	m	md	JP	2022	0	0	0	0	0	0	0	0	0	0	0	0	45
21801379	m	hp	US	2022	0	1	0	0	1	0	0	0	0	0	0	0	55
21801858	m	md	CN	2022	0	0	0	0	0	0	0	0	0	0	0	0	70
21802632	Not	hp	CH	2022	0	1	1	0	0	0	0	0	0	0	0	0	

21802681	f	md	JP	2022	0	0	0	0	0	0	0	0	0	0	0	0	
21812757	m	ph	US	2022	0	0	0	0	0	0	0	0	0	0	0	0	45
7397148	m	hp	CN	2022	1	0	0	0	0	0	0	0	0	0	0	0	52
8746618	m	cn	US	2022	1	0	0	0	0	0	0	0	0	0	0	0	73
9919991	f	cn	GB	2022	0	0	0	0	0	0	0	0	0	0	0	0	60
19324839	m	md	GB	2023	0	1	0	0	0	1	0	0	0	0	0	0	76
20533396	m	md	JP	2023	0	1	0	0	1	0	0	0	0	0	0	0	46
20902482	f	md	GB	2023	0	0	0	0	0	0	0	0	0	0	0	0	39
21005919	f	md	MY	2023	0	0	0	0	0	0	0	0	0	0	0	0	64
21149652	m	md	CN	2023	0	0	0	0	0	0	0	0	0	0	0	0	71
21231533	m	md	JP	2023	0	1	1	0	0	0	0	0	0	0	0	0	
21233409	f	md	GB	2023	0	1	0	0	0	1	0	0	0	0	0	0	65
21237587	f	md	GB	2023	0	1	0	0	0	1	0	0	0	0	0	0	65
21242502	f	md	GB	2023	0	0	0	0	0	0	0	0	0	0	0	0	65
21245868	f	md	JP	2023	0	1	0	0	1	0	0	0	0	0	0	0	68
21393757	f	hp	US	2023	0	1	0	0	1	0	0	0	0	0	0	0	73
21402798	f	md	GB	2023	0	1	0	0	0	1	0	0	0	0	0	0	65
21404945	Not	md	JP	2023	0	1	0	0	1	0	0	0	0	0	0	0	
21409259	f	md	FR	2023	0	0	0	0	0	0	0	0	0	0	0	0	67
21424696	m	md	JP	2023	0	1	0	0	1	0	0	0	0	0	0	0	68
21424778	m	md	JP	2023	0	1	0	0	1	0	0	0	0	0	0	0	84
21424815	m	md	JP	2023	0	1	0	0	1	0	0	0	0	0	0	0	66
21424856	m	md	JP	2023	0	1	0	0	1	0	0	0	0	0	0	0	79
21447929	m	md	JP	2023	0	1	0	0	1	0	0	0	0	0	0	0	81
21451110	m	md	JP	2023	0	1	0	0	1	0	0	0	0	0	0	0	81
21451144	m	md	JP	2023	0	1	0	0	1	0	0	0	0	0	0	0	70
21453293	m	md	JP	2023	0	1	0	0	1	0	0	0	0	0	0	0	88
21454537	m	md	JP	2023	0	1	0	0	1	0	0	0	0	0	0	0	54
21455977	f	md	JP	2023	0	1	0	0	1	0	0	0	0	0	0	0	81
21467291	m	cn	KR	2023	0	1	0	0	1	0	0	0	0	0	0	0	80
21470086	f	cn	PK	2023	0	1	0	0	0	1	0	0	0	0	0	0	62
21480653	m	md	GB	2023	0	0	0	0	0	0	0	0	0	0	0	0	75
21487015	f	md	AT	2023	0	0	0	0	0	0	0	0	0	0	0	0	65
21503518	f	hp	GB	2023	0	0	0	0	0	0	0	0	0	0	0	0	60

21514119	f	md	DE	2023	0	1	0	0	1	0	0	0	0	0	0	0	56
21516321	m	md	BR	2023	0	0	0	0	0	0	0	0	0	0	0	0	67
21527144	m	md	JP	2023	0	1	0	0	1	0	0	0	0	0	0	1	71
21544526	m	md	CN	2023	0	0	0	0	0	0	0	0	0	0	0	0	74
21556098	f	ph	GB	2023	0	0	0	0	0	0	0	0	0	0	0	0	
21572721	m	cn	CA	2023	0	0	0	0	0	0	0	0	0	0	0	0	73
21577367	f	md	JP	2023	0	0	0	0	0	0	0	0	0	0	0	0	76
21585696	f	hp	IT	2023	0	1	1	0	0	0	0	0	0	0	0	0	40
21585719	f	hp	IT	2023	0	1	1	0	0	0	0	0	0	0	0	0	76
21585737	f	hp	IT	2023	0	1	1	0	0	0	0	0	0	0	0	0	29
21585780	m	hp	IT	2023	0	1	1	0	0	0	0	0	0	0	0	0	75
21585798	f	hp	IT	2023	0	1	1	0	0	0	0	0	0	0	0	0	58
21585853	m	hp	IT	2023	0	1	1	0	0	0	0	0	0	0	0	0	71
21585865	f	hp	IT	2023	0	1	1	0	0	0	0	0	0	0	0	0	74
21585898	m	hp	IT	2023	0	1	1	0	0	0	0	0	0	0	0	0	70
21589123	f	ph	JP	2023	0	1	0	0	1	0	0	0	0	0	0	0	70
21598259	m	md	CZ	2023	0	1	0	0	0	1	0	0	0	0	0	0	72
21599432	m	md	CN	2023	0	0	0	0	0	0	0	0	0	0	0	0	57
21602507	f	md	CZ	2023	0	0	0	0	0	0	0	0	0	0	0	0	63
21605360	f	md	VN	2023	0	0	0	0	0	0	0	0	0	0	0	0	37
21605457	m	md	IL	2023	0	0	0	0	0	0	0	0	0	0	0	0	47
21611056	m	md	CN	2023	0	0	0	0	0	0	0	0	0	0	0	0	72
21629635	f	hp	US	2023	0	0	0	0	0	0	0	0	0	0	0	0	51
21642852	f	md	GB	2023	0	1	0	0	0	1	0	0	0	0	0	0	65
21652966	f	md	KR	2023	0	0	0	0	0	0	0	0	0	0	0	0	83
21654097	Not	md	JP	2023	0	1	0	0	0	1	0	0	0	0	0	0	
21659975	m	md	JP	2023	0	1	0	0	1	0	0	0	0	0	0	0	55
21664347	m	md	DE	2023	0	1	1	0	0	0	0	0	0	0	0	0	55
21670065	m	md	TW	2023	0	0	0	0	0	0	0	0	0	0	0	0	69
21671216	m	cn	US	2023	0	0	0	0	0	0	0	0	0	0	0	0	
21671607	m	md	DE	2023	0	1	0	0	1	0	0	0	0	0	0	0	74
21680467	f	md	AT	2023	0	0	0	0	0	0	0	0	0	0	0	0	72
21686495	m	md	JP	2023	0	0	0	0	0	0	0	0	0	0	0	0	82
21689335	f	md	ES	2023	0	0	0	0	0	0	0	0	0	0	0	0	57

21698249	f	cn	US	2023	0	1	1	0	0	0	0	0	0	0	0	1	
21702038	m	md	JP	2023	0	0	0	0	0	0	0	0	0	0	0	0	61
21703276	m	md	US	2023	0	0	0	0	0	0	0	0	0	0	0	0	64
21706768	f	md	JP	2023	0	0	0	0	0	0	0	0	0	0	0	0	75
21713147	f	md	DE	2023	0	1	0	0	1	0	0	0	0	0	0	0	69
21719667	f	md	CN	2023	0	0	0	0	0	0	0	0	0	0	0	0	63
21728076	f	md	IT	2023	0	1	0	0	1	0	0	0	0	0	0	0	78
21728535	f	md	YE	2023	0	0	0	0	0	0	0	0	0	0	0	0	38
21731775	f	md	JP	2023	0	1	0	0	0	1	0	0	0	0	0	0	50
21735216	m	md	JP	2023	0	0	0	0	0	0	0	0	0	0	0	0	78
21735781	f	md	DE	2023	0	1	0	0	1	0	0	0	0	0	0	0	69
21739648	m	hp	JP	2023	0	0	0	0	0	0	0	0	0	0	0	0	84
21745530	Not	md	JP	2023	0	1	1	0	0	0	0	0	0	0	0	0	
21747059	m	md	FR	2023	0	1	0	0	0	1	0	0	0	0	0	0	77
21747280	f	md	DK	2023	1	0	0	0	0	0	0	0	0	0	0	0	62
21747313	m	md	KR	2023	0	0	0	0	0	0	0	0	0	0	0	0	55
21751687	m	md	JP	2023	0	1	0	0	1	0	0	0	0	0	0	1	60
21756011	m	cn	KR	2023	0	0	0	0	0	0	0	0	0	0	0	0	82
21756022	m	cn	KR	2023	0	0	0	0	0	0	0	0	0	0	0	0	62
21759309	m	md	JP	2023	0	1	0	0	0	1	0	0	0	0	0	0	52
21763270	m	md	US	2023	1	0	0	0	0	0	0	0	0	0	0	0	75
21764620	m	md	ES	2023	0	1	0	0	0	1	0	0	0	0	0	0	67
21766180	m	md	US	2023	0	0	0	0	0	0	0	0	0	0	0	0	83
21767887	m	md	AU	2023	0	0	0	0	0	0	0	0	0	0	0	0	75
21771425	m	md	US	2023	0	1	0	0	0	1	0	0	0	0	0	0	75
21772898	f	md	IT	2023	0	1	0	0	1	0	0	0	0	0	0	0	78
21775035	f	cn	US	2023	1	0	0	0	0	0	0	0	0	0	0	0	73
21775918	f	md	US	2023	0	1	1	0	0	0	0	0	0	0	0	0	66
21779710	f	md	AU	2023	0	0	0	0	0	0	0	0	0	0	0	0	43
21781537	f	md	GB	2023	0	1	0	0	0	1	0	0	0	0	0	0	54
21785127	f	md	DE	2023	0	1	0	0	1	0	0	0	0	0	0	0	56
21795545	m	hp	BE	2023	0	0	0	0	0	0	0	0	0	0	0	0	70
21800401	m	md	BE	2023	0	0	0	0	0	0	0	0	0	0	0	0	65
21802665	m	ph	TH	2023	0	0	0	0	0	0	0	0	0	0	0	0	

21803812	m	md	CN	2023	0	0	0	0	0	0	0	0	0	0	0	0	64
21804010	Not	hp	CN	2023	0	0	0	0	0	0	0	0	0	0	0	0	
21804036	Not	hp	CN	2023	0	0	0	0	0	0	0	0	0	0	0	0	
21804037	Not	hp	CN	2023	0	0	0	0	0	0	0	0	0	0	0	0	
21804138	m	md	PL	2023	0	1	0	0	0	1	0	0	0	0	0	0	70
21804467	m	md	BG	2023	0	1	0	0	0	1	0	0	0	0	0	0	71
21804522	m	cn	CN	2023	0	0	0	0	0	0	0	0	0	0	0	0	35
21805695	f	cn	JP	2023	1	0	0	0	0	0	0	0	0	0	0	0	
21805970	m	md	FR	2023	0	1	0	0	0	1	0	0	0	0	0	0	72
21805986	f	md	JP	2023	0	1	1	0	0	0	0	0	0	0	0	0	70
21806178	m	md	HR	2023	0	1	0	0	0	1	0	0	0	0	0	1	67
21806397	m	hp	IT	2023	0	1	1	0	0	0	0	0	0	0	0	0	69
21806401	f	md	CA	2023	0	0	0	0	0	0	0	0	0	0	0	0	69
21806621	f	hp	MY	2023	1	0	0	0	0	0	0	0	0	0	0	0	
21806719	m	hp	US	2023	0	1	1	0	0	0	0	0	0	0	0	0	52
21807427	f	cn	US	2023	1	0	0	0	0	0	0	0	0	0	0	0	51.3
21807527	m	md	ME	2023	0	1	1	0	0	0	0	0	0	0	0	0	
21807865	f	hp	CN	2023	1	0	0	0	0	0	0	0	0	0	0	0	61
21808768	m	ph	JP	2023	1	0	0	0	0	0	0	0	0	0	0	0	
21809901	m	md	US	2023	0	0	0	0	0	0	0	0	0	0	0	0	55
21809982	m	hp	AU	2023	0	1	0	0	0	1	0	0	0	0	0	0	36
21810057	m	hp	KR	2023	0	0	0	0	0	0	0	0	0	0	0	0	79
21810172	f	hp	GB	2023	1	0	0	0	0	0	0	0	0	0	0	0	42
21810690	Not	md	JP	2023	0	1	0	1	0	0	0	0	0	0	0	0	
21810800	m	md	JP	2023	0	1	0	0	0	1	0	0	0	0	0	0	
21810837	Not	hp	TW	2023	0	0	0	0	0	0	0	0	0	0	0	0	57
21811062	m	hp	AU	2023	0	1	0	0	0	1	0	0	0	0	0	0	36
21811465	f	md	DE	2023	0	1	0	0	1	0	0	0	0	0	0	0	66
21811801	m	cn	IN	2023	0	0	0	0	0	0	0	0	0	0	0	0	
21812202	Not	md	PL	2023	0	1	1	0	0	0	0	0	0	0	0	0	
21812208	m	cn	US	2023	1	0	0	0	0	0	0	0	0	0	0	0	
21812412	m	md	DE	2023	0	0	0	0	0	0	0	0	0	0	0	0	67
21812608	f	cn	IN	2023	0	0	0	0	0	0	0	0	0	0	0	0	55
21813387	m	md	JP	2023	0	0	0	0	0	0	0	0	0	0	0	0	78

21816127	m	md	JP	2023	1	0	0	0	0	0	0	0	0	0	0	0	71
21816156	f	cn	US	2023	1	0	0	0	0	0	0	0	0	0	0	0	79
21816191	f	cn	US	2023	1	0	0	0	0	0	0	0	0	0	0	0	72.1
21817562	f	md	JP	2023	1	0	0	0	0	0	0	0	0	0	0	0	72.7
21818778	m	md	SI	2023	0	1	1	0	0	0	0	0	0	0	0	0	72
21819040	f	md	DE	2023	0	1	0	0	1	0	0	0	0	0	0	0	68
21819052	m	md	IE	2023	0	1	1	0	0	0	0	0	0	0	0	0	73
21819148	m	md	ES	2023	0	1	1	0	0	0	0	0	0	0	0	0	
21819172	f	md	ES	2023	0	1	1	0	0	0	0	0	0	0	0	0	
21820159	m	md	IT	2023	0	0	0	0	0	0	0	0	0	0	0	0	
21820944	m	md	NL	2023	0	0	0	0	0	0	0	0	0	0	0	0	
21821724	Not	md	GB	2023	0	1	0	0	0	1	0	0	0	0	0	0	
21822609	m	hp	GB	2023	1	0	0	0	0	0	0	0	0	0	0	0	
21823248	m	cn	TH	2023	1	0	0	0	0	0	0	0	0	0	0	0	
21823264	Not	md	CN	2023	0	1	1	0	0	0	0	0	0	0	0	0	
21823779	Not	md	FR	2023	0	1	1	0	0	0	0	0	0	0	0	0	
21823783	f	md	DE	2023	0	1	0	0	1	0	0	0	0	0	0	0	74
21823907	f	hp	TR	2023	0	0	0	0	0	0	0	0	0	0	0	0	58
21824153	m	cn	CN	2023	0	0	0	0	0	0	0	0	0	0	0	0	70
21825071	m	hp	US	2023	0	1	1	0	0	0	0	0	0	0	0	0	49
21825750	Not	cn	US	2023	1	0	0	0	0	0	0	0	0	0	0	0	
21826003	m	hp	AU	2023	0	1	0	0	0	1	0	0	0	0	0	0	36
21826358	f	md	CN	2023	0	1	0	0	0	1	0	0	0	0	0	0	67
21826528	m	hp	CN	2023	0	0	0	0	0	0	0	0	0	0	0	0	81
21827469	m	hp	JP	2023	0	0	0	0	0	0	0	0	0	0	0	0	72
21827973	m	ph	MY	2023	1	0	0	0	0	0	0	0	0	0	0	0	72.7
21828305	m	md	CH	2023	0	1	0	0	1	0	0	0	0	0	0	0	73
21830127	f	cn	US	2023	1	0	0	0	0	0	0	0	0	0	0	0	62.6
21830130	m	md	FR	2023	0	1	0	0	0	1	0	0	0	0	0	0	80
21830353	m	cn	CN	2023	0	1	0	1	0	0	0	0	0	0	0	0	78
21830376	m	md	JP	2023	0	0	0	0	0	0	0	0	0	0	0	0	67
21830759	f	md	HR	2023	0	1	1	0	0	0	0	0	0	0	0	0	60
21830959	Not	md	NL	2023	0	0	0	0	0	0	0	0	0	0	0	0	
21831413	f	hp	BE	2023	0	1	1	0	0	0	0	0	0	0	0	0	56

21831605	f	cn	CA	2023	1	0	0	0	0	0	0	0	0	0	0	0	58
21832209	m	hp	FR	2023	0	1	1	0	0	0	0	0	0	0	0	0	81
21832783	f	md	KR	2023	0	0	0	0	0	0	0	0	0	0	0	0	81
21832833	m	md	TW	2023	0	1	1	0	0	0	0	0	0	0	0	0	75
21832864	f	md	CN	2023	0	0	0	0	0	0	0	0	0	0	0	0	52
21833175	m	md	DE	2023	0	0	0	0	0	0	0	0	0	0	0	0	
21835124	Not	md	US	2023	0	1	0	0	0	1	0	0	0	0	0	0	
21836686	f	md	IN	2023	0	1	0	0	0	1	0	0	0	0	0	0	76
21836886	Not	md	US	2023	1	0	0	0	0	0	0	0	0	0	0	0	
21836989	Not	hp	SG	2023	0	1	0	1	0	0	0	0	0	0	0	0	
21837079	f	md	DE	2023	0	1	0	0	1	0	0	0	0	0	0	0	68
21837570	f	hp	GB	2023	1	0	0	0	0	0	0	0	0	0	0	0	55
21837627	f	cn	CN	2023	0	0	0	0	0	0	0	0	0	0	0	0	56
21837677	m	cn	US	2023	0	0	0	0	0	0	0	0	0	0	0	0	53
21837771	m	cn	CA	2023	1	0	0	0	0	0	0	0	0	0	0	0	
21837821	f	cn	CA	2023	1	0	0	0	0	0	0	0	0	0	0	0	58
21838439	m	cn	CN	2023	0	1	0	1	0	0	0	0	0	0	0	0	80
21839911	m	md	DE	2023	0	1	1	0	0	0	0	0	0	0	0	0	71
21840884	Not	ph	JP	2023	1	0	0	0	0	0	0	0	0	0	0	0	
21841224	f	md	JP	2023	0	1	0	0	1	0	0	0	0	0	0	0	68
21841518	m	cn	IL	2023	0	0	0	0	0	0	0	0	0	0	0	0	55
21842256	m	hp	DE	2023	0	0	0	0	0	0	0	0	0	0	0	0	82
21843208	f	md	US	2023	0	1	0	0	0	1	0	0	0	0	0	0	61
21843457	m	hp	DE	2023	0	1	1	0	0	0	0	0	0	0	0	0	55
21844178	Not	md	IT	2023	0	0	0	0	0	0	0	0	0	0	0	0	64
21844484	m	md	IN	2023	0	0	0	0	0	0	0	0	0	0	0	0	77
21844888	f	md	FR	2023	0	0	0	0	0	0	0	0	0	0	0	0	72
21846007	f	md	US	2023	0	1	0	0	0	1	0	0	0	0	0	0	72
21846098	m	md	KR	2023	0	1	0	0	0	1	0	0	0	0	0	0	64
21847047	f	ph	US	2023	0	1	1	0	0	0	0	0	0	0	0	0	
21847680	m	md	CN	2023	0	0	0	0	0	0	0	0	0	0	0	0	62
21847915	f	cn	US	2023	0	0	0	0	0	0	0	0	0	0	0	0	70
21848283	f	md	CZ	2023	0	1	1	0	0	0	0	0	0	0	0	0	
21848584	m	cn	MY	2023	0	0	0	0	0	0	0	0	0	0	0	0	64

21848651	m	md	US	2023	0	0	0	0	0	0	0	0	0	0	0	0	84
21849323	m	hp	AU	2023	0	1	0	0	0	1	0	0	0	0	0	0	36
21850121	Not	hp	BR	2023	0	0	0	0	0	0	0	0	0	0	0	0	66
21850582	m	md	US	2023	1	0	0	0	0	0	0	0	0	0	0	0	65
21850711	m	md	CN	2023	0	0	0	0	0	0	0	0	0	0	0	0	59
21851047	m	md	JP	2023	0	1	0	0	1	0	0	0	0	0	0	0	70
21851675	m	md	JP	2023	0	1	0	0	1	0	0	0	0	0	0	0	56
21851795	m	md	JP	2023	0	1	0	0	0	1	0	0	0	0	0	0	69
21852010	m	md	CN	2023	0	0	0	0	0	0	0	0	0	0	0	0	68
21852609	m	md	FI	2023	0	1	0	0	0	1	0	0	0	0	0	0	70
21852787	f	md	JP	2023	0	0	0	0	0	0	0	0	0	0	0	0	94
21852995	f	cn	TH	2023	0	0	0	0	0	0	0	0	0	0	0	0	64
21853967	Not	md	FI	2023	0	0	0	0	0	0	0	0	0	0	0	0	
21854394	m	cn	JP	2023	0	0	0	0	0	0	0	0	0	0	0	0	82
21855553	m	md	US	2023	0	0	0	0	0	0	0	0	0	0	0	0	62
21855693	f	md	KR	2023	0	0	0	0	0	0	0	0	0	0	0	0	63
21855801	m	hp	CN	2023	0	1	1	0	0	0	0	0	0	0	0	0	67
21855962	f	md	US	2023	0	1	0	0	0	1	0	0	0	0	0	0	74
21856357	m	md	JP	2023	0	1	0	0	1	0	0	0	0	0	0	1	77
21857063	f	cn	CA	2023	0	0	0	0	0	0	0	0	0	0	0	0	61
21857280	f	hp	US	2023	0	0	0	0	0	0	0	0	0	0	0	0	
21857574	m	md	IT	2023	0	0	0	0	0	0	0	0	0	0	0	0	74
21858784	m	hp	IQ	2023	0	1	1	0	0	0	0	0	0	0	0	0	66
21858819	m	hp	US	2023	0	0	0	0	0	0	0	0	0	0	0	0	78
21858881	Not	md	GB	2023	0	1	0	0	0	1	0	0	0	0	0	0	
21859031	f	md	AT	2023	0	0	0	0	0	0	0	0	0	0	0	0	72
21859153	m	hp	GB	2023	1	0	0	0	0	0	0	0	0	0	0	0	68
21859185	m	ph	US	2023	0	1	0	0	1	0	0	0	0	0	0	0	53
21859283	m	cn	US	2023	0	0	0	0	0	0	0	0	0	0	0	0	
21859310	Not	md	CA	2023	0	1	1	0	0	0	0	0	0	0	0	0	
21859641	f	hp	CA	2023	0	0	0	0	0	0	0	0	0	0	0	0	69
21860026	f	md	US	2023	0	0	0	0	0	0	0	0	0	0	0	0	88
21860105	f	hp	KR	2023	0	0	0	0	0	0	0	0	0	0	0	0	70
21860111	m	md	JP	2023	0	1	0	0	1	0	0	0	0	0	0	0	72

21860129	m	md	CN	2023	0	1	1	0	0	0	0	0	0	0	0	0	53
21860382	f	md	JP	2023	0	0	0	0	0	0	0	0	0	0	0	0	80
21860472	m	hp	US	2023	0	0	0	0	0	0	0	0	0	0	0	0	62
21860537	f	md	GB	2023	0	1	0	0	0	1	0	0	0	0	0	0	54
21860961	m	md	JP	2023	0	1	0	0	1	0	0	0	0	0	0	0	
21861088	m	md	JP	2023	0	1	0	0	1	0	0	0	0	0	0	0	81
21861095	m	md	JP	2023	0	1	0	0	1	0	0	0	0	0	0	0	
21861108	f	cn	US	2023	0	1	1	0	0	0	0	0	0	0	0	0	66
21861138	f	hp	US	2023	0	1	0	0	0	1	0	0	0	0	0	0	64
21861163	Not	md	AU	2023	0	0	0	0	0	0	0	0	0	0	0	0	76
21861468	f	md	KR	2023	0	0	0	0	0	0	0	0	0	0	0	0	62
21861625	m	ph	CA	2023	0	0	0	0	0	0	0	0	0	0	0	0	65
21861651	m	md	JP	2023	0	1	0	0	1	0	0	0	0	0	0	0	
21861659	f	md	US	2023	0	0	0	0	0	0	0	0	0	0	0	0	86
21861792	m	md	FR	2023	0	0	0	0	0	0	0	0	0	0	0	0	65
21862006	f	ph	GB	2023	0	0	0	0	0	0	0	0	0	0	0	0	60
21862015	f	hp	FR	2023	0	1	1	0	0	0	0	0	0	0	0	0	52
21862254	m	md	JP	2023	0	0	0	0	0	0	0	0	0	0	0	0	74
21862375	m	md	CH	2023	0	0	0	0	0	0	0	0	0	0	0	0	
21863112	m	md	FR	2023	0	0	0	0	0	0	0	0	0	0	0	0	66
21863528	m	md	BE	2023	0	0	0	0	0	0	0	0	0	0	0	0	53
21863634	m	md	JP	2023	0	1	0	0	1	0	0	0	0	0	0	0	
21864997	m	md	JP	2023	0	1	0	0	1	0	0	0	0	0	0	0	41
21866155	m	md	IT	2023	0	0	0	0	0	0	0	0	0	0	0	0	65
21866198	f	cn	CN	2023	0	0	0	0	0	0	0	0	0	0	0	0	59
21866357	m	hp	CN	2023	0	1	1	0	0	0	0	0	0	0	0	1	63
21866764	m	md	FR	2023	0	1	1	0	0	0	0	0	0	0	0	1	60
21867411	m	md	JP	2023	0	1	1	0	0	0	0	0	0	0	0	0	74
21867840	m	cn	IN	2023	0	0	0	0	0	0	0	0	0	0	0	0	41
21867865	m	md	JP	2023	0	1	1	0	0	0	0	0	0	0	0	0	78
21868184	m	md	JP	2023	0	1	0	0	1	0	0	0	0	0	0	0	68
21868566	f	md	IT	2023	1	0	0	0	0	0	0	0	0	0	0	0	55
21868636	m	md	JP	2023	0	1	0	0	1	0	0	0	0	0	0	0	72
21869114	m	md	JP	2023	0	1	0	0	1	0	0	0	0	0	0	1	83

21869515	m	md	US	2023	0	1	0	0	0	1	0	0	0	0	0	0	85
21869791	m	md	ES	2023	0	1	0	0	0	1	0	0	0	0	0	0	66
21870366	f	md	NL	2023	0	0	0	0	0	0	0	0	0	0	0	0	71
21870367	f	cn	CA	2023	0	0	0	0	0	0	0	0	0	0	0	0	77
21871072	m	md	CN	2023	0	1	0	0	0	1	0	0	0	0	0	1	69
21871309	m	cn	US	2023	0	1	0	0	1	0	0	0	0	0	0	0	65
21871774	m	hp	CN	2023	0	1	1	0	0	0	0	0	0	0	0	0	65
21871775	m	hp	CN	2023	0	1	1	0	0	0	0	0	0	0	0	0	67
21872118	m	cn	BR	2023	0	0	0	0	0	0	0	0	0	0	0	0	57
21872320	m	ph	AU	2023	0	0	0	0	0	0	0	0	0	0	0	0	78
21872538	m	hp	US	2023	0	1	1	0	0	0	0	0	0	0	0	0	52
21873013	m	hp	CN	2023	0	1	1	0	0	0	0	0	0	0	0	0	64
21873071	f	hp	CN	2023	0	1	1	0	0	0	0	0	0	0	0	0	34
21873115	f	md	US	2023	0	1	1	0	0	0	0	0	0	0	0	0	70
21873159	m	hp	JP	2023	0	0	0	0	0	0	0	0	0	0	0	0	72
21873377	m	md	AU	2023	0	0	0	0	0	0	0	0	0	0	0	0	
21873563	Not	hp	GB	2023	0	0	0	0	0	0	0	0	0	0	0	0	79
21874499	m	cn	MY	2023	1	0	0	0	0	0	0	0	0	0	0	0	
21874543	m	md	JP	2023	0	1	0	1	0	0	0	0	0	0	0	1	68
21874547	Not	hp	US	2023	0	1	0	1	0	0	0	0	0	0	0	0	
21874570	m	md	JP	2023	0	1	0	1	0	0	0	0	0	0	0	0	61
21874581	f	cn	TH	2023	1	0	0	0	0	0	0	0	0	0	0	0	62.6
21874583	Not	hp	US	2023	0	1	0	1	0	0	0	0	0	0	0	0	
21874584	Not	hp	US	2023	0	1	0	1	0	0	0	0	0	0	0	0	
21874585	Not	hp	US	2023	0	1	0	1	0	0	0	0	0	0	0	0	
21874597	m	cn	US	2023	1	0	0	0	0	0	0	0	0	0	0	0	49.2
21874598	f	cn	TH	2023	1	0	0	0	0	0	0	0	0	0	0	0	74.5
21874599	f	md	AU	2023	1	0	0	0	0	0	0	0	0	0	0	0	
21874605	m	hp	TH	2023	1	0	0	0	0	0	0	0	0	0	0	0	74
21874611	Not	hp	US	2023	0	1	0	1	0	0	0	0	0	0	0	0	
21874615	Not	hp	CN	2023	0	0	0	0	0	0	1	0	0	1	0	0	84
21874642	m	hp	CA	2023	1	0	0	0	0	0	0	0	0	0	0	0	52
21874650	f	cn	ID	2023	1	0	0	0	0	0	0	0	0	0	0	0	59.4
21874661	f	cn	US	2023	1	0	0	0	0	0	0	0	0	0	0	0	64.8

21874662	Not	hp	US	2023	0	1	0	1	0	0	0	0	0	0	0	0	
21874670	f	cn	ID	2023	1	0	0	0	0	0	0	0	0	0	0	0	
21874674	Not	cn	SG	2023	1	0	0	0	0	0	0	0	0	0	0	0	79.9
21874676	f	cn	ID	2023	1	0	0	0	0	0	0	0	0	0	0	0	49.1
21874737	f	md	US	2023	1	0	0	0	0	0	0	0	0	0	0	0	81
21874756	m	hp	CA	2023	1	0	0	0	0	0	0	0	0	0	0	0	
21874758	f	cn	ID	2023	1	0	0	0	0	0	0	0	0	0	0	0	74.3
21874771	m	cn	MY	2023	1	0	0	0	0	0	0	0	0	0	0	0	
21874820	f	md	US	2023	0	0	0	0	0	0	0	0	0	0	0	0	
21874831	m	cn	TH	2023	1	0	0	0	0	0	0	0	0	0	0	0	
21874938	f	md	KR	2023	0	1	1	0	0	0	0	0	0	0	0	0	
21875016	f	md	US	2023	1	0	0	0	0	0	0	0	0	0	0	1	75
21875017	f	md	CA	2023	1	0	0	0	0	0	0	0	0	0	0	0	
21875023	m	cn	TH	2023	1	0	0	0	0	0	0	0	0	0	0	0	50.6
21875026	f	hp	CA	2023	1	0	0	0	0	0	0	0	0	0	0	0	
21875054	m	md	DE	2023	0	0	0	0	0	0	0	0	0	0	0	0	63
21876053	f	md	DE	2023	0	0	0	0	0	0	0	0	0	0	0	0	58
21876160	f	cn	US	2023	0	0	0	0	0	0	0	0	0	0	0	0	
21876512	f	md	JP	2023	1	0	0	0	0	0	0	0	0	0	0	0	74
21876636	f	hp	JP	2023	0	0	0	0	0	0	0	0	0	0	0	0	65
21877943	f	md	AT	2023	0	1	0	1	0	0	0	0	0	0	0	0	
21878120	f	md	DE	2023	0	1	0	0	1	0	0	0	0	0	0	0	60
21878356	m	md	DE	2023	0	0	0	0	0	0	0	0	0	0	0	0	69
21878571	f	md	DE	2023	0	0	0	0	0	0	0	0	0	0	0	0	
21878940	m	hp	US	2023	0	0	0	0	0	0	0	0	0	0	0	0	55
21879146	f	md	IT	2023	0	1	0	0	1	0	0	0	0	0	0	0	78
21879439	f	hp	GB	2023	0	0	0	0	0	0	0	0	0	0	0	0	71
21879468	f	md	TW	2023	0	0	0	0	0	0	0	0	0	0	0	0	66
21879494	m	md	FR	2023	0	1	1	0	0	0	0	0	0	0	0	0	40
21879570	f	ph	JP	2023	1	0	0	0	0	0	0	0	0	0	0	0	78
21880009	Not	md	CZ	2023	0	0	0	0	0	0	0	0	0	0	0	0	
21880375	Not	hp	JP	2023	1	0	0	0	0	0	0	0	0	0	0	0	
21880732	m	md	JP	2023	0	1	1	0	0	0	0	0	0	0	0	0	80
21880850	f	md	JP	2023	0	0	0	0	0	0	0	0	0	0	0	0	55

21882079	m	hp	US	2023	0	0	0	0	0	0	0	0	0	0	0	0	62
21883090	m	md	DE	2023	0	1	1	0	0	0	0	0	0	0	0	0	55
21883151	m	md	JP	2023	0	0	0	0	0	0	0	0	0	0	0	0	72
21883450	m	md	CN	2023	0	0	0	0	0	0	0	0	0	0	0	0	82
21884100	Not	md	JP	2023	1	0	0	0	0	0	0	0	0	0	0	0	
21884540	f	hp	JP	2023	0	1	0	0	1	0	0	0	0	0	0	0	65
21884700	f	cn	TH	2023	0	0	0	0	0	0	0	0	0	0	0	0	80
21885128	f	md	IT	2023	1	0	0	0	0	0	0	0	0	0	0	0	60
21885135	f	md	JP	2023	0	0	0	0	0	0	0	0	0	0	0	0	78
21885164	m	md	DE	2023	0	1	1	0	0	0	0	0	0	0	0	0	82
21885704	m	md	DE	2023	0	0	0	0	0	0	0	0	0	0	0	0	69
21885971	f	md	IL	2023	0	1	0	1	0	0	0	0	0	0	0	0	50
21886138	m	md	JP	2023	0	1	0	0	1	0	0	0	0	0	0	0	71
21886189	f	md	JP	2023	1	0	0	0	0	0	0	0	0	0	0	0	90
21886257	f	md	JP	2023	1	0	0	0	0	0	0	0	0	0	0	0	80
21886270	f	md	AU	2023	1	0	0	0	0	0	0	0	0	0	0	0	
21886770	m	md	JP	2023	1	0	0	0	0	0	0	0	0	0	0	0	68
21886912	m	md	US	2023	0	0	0	0	0	0	0	0	0	0	0	0	39
21887249	m	hp	DE	2023	0	1	0	0	1	0	0	0	0	0	0	0	63
21887350	m	ph	JP	2023	0	0	0	0	0	0	0	0	0	0	0	0	29
21887711	m	md	JP	2023	0	0	0	0	0	0	0	0	0	0	0	0	80
21888102	m	md	CN	2023	0	0	0	0	0	0	0	0	0	0	0	0	61
21888184	f	cn	US	2023	1	0	0	0	0	0	0	0	0	0	0	0	67.8
21888455	Not	md	JP	2023	1	0	0	0	0	0	0	0	0	0	0	0	
21888680	f	cn	US	2023	1	0	0	0	0	0	0	0	0	0	0	0	
21889093	Not	md	JP	2023	1	0	0	0	0	0	0	0	0	0	0	0	
21889575	f	md	IT	2023	0	0	0	0	0	0	0	0	0	0	0	0	70
21889674	f	cn	CA	2023	1	0	0	0	0	0	0	0	0	0	0	0	
21889835	m	md	JP	2023	0	0	0	0	0	0	0	0	0	0	0	0	
21889892	f	cn	PE	2023	1	0	0	0	0	0	0	0	0	0	0	0	
21890497	m	md	JP	2023	0	1	0	0	1	0	0	0	0	0	0	0	66
21890846	f	md	JP	2023	0	1	1	0	0	0	0	0	0	0	0	0	65
21891014	Not	hp	JP	2023	1	0	0	0	0	0	0	0	0	0	0	0	
21891220	m	md	LB	2023	0	1	0	0	1	0	0	0	0	0	0	0	

21891278	m	cn	IN	2023	0	0	0	0	0	0	0	0	0	0	0	0	60
21891344	m	cn	CN	2023	0	1	0	1	0	0	0	0	0	0	0	0	78
21891480	f	md	JP	2023	0	1	1	0	0	0	0	0	0	0	0	0	71
21891483	f	cn	IR	2023	0	1	1	0	0	0	0	0	0	0	0	0	79
21891593	f	md	AU	2023	1	0	0	0	0	0	0	0	0	0	0	0	
21891876	f	md	US	2023	0	0	0	0	0	0	0	0	0	0	0	0	62
21892154	Not	md	CN	2023	0	0	0	0	0	0	0	0	0	0	0	0	
21892274	m	hp	AR	2023	0	1	0	0	1	0	0	0	0	0	0	0	73
21893025	f	ph	FR	2023	0	1	0	0	1	0	0	0	0	0	0	0	55
21894888	m	md	JP	2023	0	1	0	0	1	0	0	0	0	0	0	0	66
21894952	m	md	JP	2023	0	1	0	0	1	0	0	0	0	0	0	0	73
21895500	f	md	JP	2023	1	0	0	0	0	0	0	0	0	0	0	0	76
21895553	m	md	JP	2023	0	1	0	0	1	0	0	0	0	0	0	0	63
21895558	f	md	JP	2023	0	1	0	0	0	1	0	0	0	0	0	0	46
21896004	Not	hp	CN	2023	0	0	0	0	0	0	0	0	0	0	0	0	
21896022	Not	hp	CN	2023	0	0	0	0	0	0	0	0	0	0	0	0	
21896023	Not	hp	CN	2023	0	0	0	0	0	0	0	0	0	0	0	0	
21896024	Not	hp	CN	2023	0	0	0	0	0	0	0	0	0	0	0	0	
21896047	Not	hp	CN	2023	0	0	0	0	0	0	0	0	0	0	0	0	
21896702	m	md	JP	2023	0	1	1	0	0	0	0	0	0	0	0	0	65
21897886	f	hp	DE	2023	0	0	0	0	0	0	0	0	0	0	0	0	45
21897910	f	md	FR	2023	0	0	0	0	0	0	0	0	0	0	0	0	
21898338	Not	md	DE	2023	0	0	0	0	0	0	0	0	0	0	0	0	
21898518	f	cn	CA	2023	1	0	0	0	0	0	0	0	0	0	0	0	
21899818	f	md	FR	2023	0	0	0	0	0	0	0	0	0	0	0	0	80
21901320	Not	md	RU	2023	0	0	0	0	0	0	0	0	0	0	0	0	65
21901368	f	md	JP	2023	0	1	1	0	0	0	0	0	0	0	0	1	80
21901413	m	md	JP	2023	0	0	0	0	0	0	0	0	0	0	0	0	
21901648	m	md	JP	2023	0	1	0	0	1	0	0	0	0	0	0	0	
21901673	f	md	JP	2023	0	1	0	0	1	0	0	0	0	0	0	0	72
21901807	f	hp	JP	2023	0	0	0	0	0	0	0	0	0	0	0	0	65
21902498	m	ph	JP	2023	0	1	0	0	1	0	0	0	0	0	0	0	69
21902604	m	md	JP	2023	0	1	0	0	0	1	0	0	0	0	0	0	80
21902988	Not	hp	US	2023	0	1	0	0	0	1	0	0	0	0	0	0	

21903402	m	hp	CN	2023	0	0	0	0	0	0	0	0	0	0	0	0	44
21904289	m	hp	KR	2023	0	0	0	0	0	0	0	0	0	0	0	0	67
21904290	m	hp	US	2023	0	0	0	0	0	0	0	0	0	0	0	0	68
21904991	m	md	JP	2023	0	0	0	0	0	0	0	0	0	0	0	0	
21905028	m	hp	KR	2023	0	0	0	0	0	0	0	0	0	0	0	0	65
21905117	m	hp	US	2023	0	1	0	1	0	0	0	0	0	0	0	0	65
21905147	f	cn	ID	2023	1	0	0	0	0	0	0	0	0	0	0	0	69
21905235	f	cn	ID	2023	1	0	0	0	0	0	0	0	0	0	0	0	59.2
21905874	Not	hp	US	2023	0	0	0	0	0	0	0	0	0	0	0	0	
21905987	m	md	JP	2023	0	1	0	0	0	1	0	0	0	0	0	0	80
21906018	m	md	JP	2023	0	1	0	0	0	1	0	0	0	0	0	0	70
21906323	m	md	JP	2023	0	1	0	0	1	0	0	0	0	0	0	0	48
21907056	f	md	US	2023	0	0	0	0	0	0	0	0	0	0	0	0	51
21907196	f	ph	NL	2023	1	0	0	0	0	0	0	0	0	0	0	0	86
21907583	Not	md	JP	2023	0	1	0	1	0	0	0	0	0	0	0	0	70
21907684	f	hp	CA	2023	0	0	0	0	0	0	0	0	0	0	0	0	69
21907718	Not	hp	JP	2023	1	0	0	0	0	0	0	0	0	0	0	0	
21907747	f	cn	JP	2023	1	0	0	0	0	0	0	0	0	0	0	0	90
21907890	f	md	FR	2023	0	0	0	0	0	0	0	0	0	0	0	0	33
21908447	m	md	DE	2023	0	1	1	0	0	0	0	0	0	0	0	0	79
21908622	m	hp	US	2023	0	1	0	1	0	0	0	0	0	0	0	0	67
21908673	Not	hp	US	2023	0	0	0	0	0	0	0	0	0	0	0	0	
21909226	m	hp	JP	2023	0	1	0	0	1	0	0	0	0	0	0	0	61
21909419	Not	cn	US	2023	1	0	0	0	0	0	0	0	0	0	0	0	74
21909576	f	hp	TH	2023	1	0	0	0	0	0	0	0	0	0	0	0	77
21909610	m	md	JP	2023	0	0	0	0	0	0	0	0	0	0	0	0	
21909692	f	cn	TH	2023	0	0	0	0	0	0	0	0	0	0	0	0	62
21909850	m	md	DE	2023	0	0	0	0	0	0	0	0	0	0	0	0	69
21909853	m	cn	JP	2023	0	1	1	0	0	0	0	0	0	0	0	1	65
21910458	f	md	US	2023	0	0	0	0	0	0	0	0	0	0	0	0	
21913365	f	md	JP	2023	0	1	0	0	1	0	0	0	0	0	0	0	53
21914896	m	md	JP	2023	0	1	0	0	0	1	0	0	0	0	0	0	54
21915189	f	md	IN	2023	0	0	0	0	0	0	0	0	0	0	0	0	48
21915205	Not	md	JP	2023	0	0	0	0	0	0	0	0	0	0	0	0	

21915402	m	hp	JP	2023	0	1	1	0	0	0	0	0	0	0	0	0	90
21915624	Not	md	JP	2023	1	0	0	0	0	0	0	0	0	0	0	0	
21916024	m	md	IT	2023	0	0	0	0	0	0	0	0	0	0	0	0	
21916294	f	md	JP	2023	1	0	0	0	0	0	0	0	0	0	0	0	80
21916618	f	md	US	2023	1	0	0	0	0	0	0	0	0	0	0	0	
21916644	m	md	LT	2023	0	1	0	0	0	1	0	0	0	0	0	0	53
21916655	m	md	JP	2023	0	1	0	1	0	0	0	0	0	0	0	1	75
21916675	f	cn	US	2023	0	1	1	0	0	0	0	0	0	0	0	0	77
21916709	m	hp	US	2023	0	0	0	0	0	0	0	0	0	0	0	0	62
21916720	f	md	KR	2023	0	0	0	0	0	0	0	0	0	0	0	0	39
21916779	f	cn	US	2023	1	0	0	0	0	0	0	0	0	0	0	1	65.1
21916958	f	cn	CA	2023	0	1	1	0	0	0	0	0	0	0	0	0	58
21917234	f	hp	GB	2023	0	0	0	0	0	0	0	0	0	0	0	0	
21917253	m	md	RU	2023	1	0	0	0	0	0	0	0	0	0	0	0	
21917299	m	md	JP	2023	0	1	0	0	1	0	0	0	0	0	0	0	63
21917303	m	md	JP	2023	0	1	0	0	1	0	0	0	0	0	0	0	64
21917343	m	md	JP	2023	0	1	0	0	1	0	0	0	0	0	0	0	74
21917406	m	md	JP	2023	0	1	0	0	0	1	0	0	0	0	0	1	80
21917416	m	ph	JP	2023	0	1	0	0	1	0	0	0	0	0	0	1	69
21917452	Not	md	JP	2023	0	1	0	0	1	0	0	0	0	0	0	0	72
21917554	f	md	BE	2023	0	1	0	1	0	0	0	0	0	0	0	0	67
21917872	m	md	TR	2023	0	1	0	0	0	1	0	0	0	0	0	1	64
21917950	m	ph	JP	2023	0	1	1	0	0	0	0	0	0	0	0	0	
21918007	f	hp	JP	2023	0	0	0	0	0	0	0	0	0	0	0	0	71
21918022	f	cn	US	2023	0	1	1	0	0	0	0	0	0	0	0	0	
21918050	Not	cn	US	2023	0	1	1	0	0	0	0	0	0	0	0	0	66
21918111	m	md	JP	2023	0	1	0	0	1	0	0	0	0	0	0	0	79
21918238	m	md	JP	2023	0	0	0	0	0	0	0	0	0	0	0	0	81
21918378	f	md	NL	2023	0	1	0	0	0	1	0	0	0	0	0	0	74
21918498	Not	md	JP	2023	0	1	0	0	1	0	0	0	0	0	0	0	69
21919566	f	hp	FR	2023	0	0	0	0	0	0	0	0	0	0	0	0	53
21919623	m	cn	ID	2023	1	0	0	0	0	0	0	0	0	0	0	0	
21919981	f	cn	TH	2023	1	0	0	0	0	0	0	0	0	0	0	0	83
21920050	f	md	JP	2023	0	0	0	0	0	0	0	0	0	0	0	0	

21920058	f	md	PT	2023	0	0	0	0	0	0	0	0	0	0	0	0	49
21920064	m	md	PT	2023	0	1	1	0	0	0	0	0	0	0	0	0	
21920282	f	cn	SK	2023	0	0	0	0	0	0	0	0	0	0	0	0	
21920343	f	ph	US	2023	0	0	0	0	0	0	0	0	0	0	0	0	64
21920346	m	md	JP	2023	0	1	0	0	0	1	0	0	0	0	0	0	90
21920622	m	md	IL	2023	0	0	0	0	0	0	0	0	0	0	0	0	
21920640	f	md	US	2023	0	0	0	0	0	0	0	0	0	0	0	0	
21920685	f	cn	US	2023	0	0	0	0	0	0	0	0	0	0	0	0	56
21920743	m	cn	TH	2023	1	0	0	0	0	0	0	0	0	0	0	0	73.6
21920830	m	md	LT	2023	0	1	0	0	0	1	0	0	0	0	0	0	53
21921024	f	hp	US	2023	0	0	0	0	0	0	0	0	0	0	0	0	
21921025	Not	hp	RU	2023	0	0	0	0	0	0	0	0	0	0	0	0	62
21921064	Not	md	CZ	2023	0	1	0	0	1	0	0	0	0	0	0	0	
21921258	m	md	JP	2023	0	1	0	0	1	0	0	0	0	0	0	0	87
21921268	m	cn	TH	2023	0	1	0	1	0	0	0	0	0	0	0	0	56.4
21921355	f	cn	US	2023	1	0	0	0	0	0	0	0	0	0	0	0	94.2
21921459	m	ph	US	2023	0	0	0	0	0	0	0	0	0	0	0	0	
21921499	f	ph	US	2023	0	0	0	0	0	0	0	0	0	0	0	0	
21921557	Not	md	RU	2023	0	0	0	0	0	0	0	0	0	0	0	0	48
21921581	f	md	ES	2023	0	0	0	0	0	0	0	0	0	0	0	0	53
21922341	f	md	DE	2023	0	1	0	0	1	0	0	0	0	0	0	0	63
21922428	m	cn	KR	2023	0	0	0	0	0	0	0	0	0	0	0	0	83
21922539	f	ph	JP	2023	0	1	0	1	0	0	0	0	0	0	0	1	50
21922547	m	cn	US	2023	0	0	0	0	0	0	0	0	0	0	0	0	61
21922717	m	md	DE	2023	0	0	0	0	0	0	0	0	0	0	0	0	69
21922869	f	md	DE	2023	0	1	1	0	0	0	0	0	0	0	0	1	72
21922936	m	md	IT	2023	0	0	0	0	0	0	0	0	0	0	0	0	
21923210	Not	md	JP	2023	0	1	0	0	1	0	0	0	0	0	0	0	77
21923252	m	md	JP	2023	0	1	0	0	1	0	0	0	0	0	0	0	71
21923287	m	md	CZ	2023	0	1	0	0	1	0	0	0	0	0	0	0	57
21923593	m	md	JP	2023	0	0	0	0	0	0	0	0	0	0	0	0	60
21924230	m	md	JP	2023	0	1	0	0	1	0	0	0	0	0	0	0	76
21924467	m	cn	SG	2023	1	0	0	0	0	0	0	0	0	0	0	0	71
21924483	f	cn	SG	2023	1	0	0	0	0	0	0	0	0	0	0	0	65

21924515	m	hp	US	2023	0	1	1	0	0	0	0	0	0	0	0	0	52
21924584	Not	md	JP	2023	0	1	0	0	1	0	0	0	0	0	0	0	86
21924644	f	md	CA	2023	1	0	0	0	0	0	0	0	0	0	0	0	
21924760	Not	md	JP	2023	0	1	0	0	1	0	0	0	0	0	0	0	81
21925252	m	md	JP	2023	0	0	0	0	0	0	0	0	0	0	0	0	72
21925506	Not	md	JP	2023	0	1	0	0	0	1	0	0	0	0	0	0	
21926217	m	md	IE	2023	0	1	1	0	0	0	0	0	0	0	0	0	73
21926527	f	ph	FR	2023	0	0	0	0	0	0	0	0	0	0	0	0	61
21926722	f	md	BG	2023	0	1	0	0	0	1	0	0	0	0	0	0	56
21927305	f	cn	TH	2023	1	0	0	0	0	0	0	0	0	0	0	0	
21927398	m	md	JP	2023	0	1	0	1	0	0	0	0	0	0	0	0	80
21927501	m	cn	IN	2023	0	1	0	0	0	1	0	0	0	0	0	0	90
21927619	m	hp	TR	2023	0	0	0	0	0	0	0	0	0	0	0	0	70
21927651	f	cn	IN	2023	0	1	0	0	0	1	0	0	0	0	0	0	73
21927808	f	md	JP	2023	0	1	0	0	0	1	0	0	0	0	0	0	60
21928072	m	cn	US	2023	0	1	1	0	0	0	0	0	0	0	0	0	
21928130	f	cn	TH	2023	0	1	0	0	0	1	0	0	0	0	0	0	52
21928167	f	md	DE	2023	0	0	0	0	0	0	0	0	0	0	0	0	69
21928209	m	md	ES	2023	0	0	0	0	0	0	0	0	0	0	0	0	77
21928256	Not	md	JP	2023	0	1	0	0	0	1	0	0	0	0	0	0	80
21928269	f	ph	JP	2023	0	0	0	0	0	0	1	0	0	0	1	0	60
21928698	f	md	JP	2023	0	1	0	0	0	1	0	0	0	0	0	0	57
21928728	Not	md	JP	2023	0	1	0	0	0	1	0	0	0	0	0	0	49
21929575	m	md	US	2023	0	0	0	0	0	0	0	0	0	0	0	0	61
21929964	f	md	IN	2023	0	1	0	0	0	1	0	0	0	0	0	0	
21930076	m	hp	TH	2023	1	0	0	0	0	0	0	0	0	0	0	0	
21930274	m	md	JP	2023	0	1	1	0	0	0	0	0	0	0	0	0	72
21930459	m	md	IN	2023	0	0	0	0	0	0	0	0	0	0	0	0	65
21930510	f	md	JP	2023	0	0	0	0	0	0	0	0	0	0	0	0	
21930560	Not	hp	CN	2023	0	0	0	0	0	0	0	0	0	0	0	0	
21930561	Not	hp	CN	2023	0	0	0	0	0	0	0	0	0	0	0	0	
21930562	Not	hp	CN	2023	0	0	0	0	0	0	0	0	0	0	0	0	
21930563	Not	hp	CN	2023	0	0	0	0	0	0	0	0	0	0	0	0	
21930564	Not	hp	CN	2023	0	0	0	0	0	0	0	0	0	0	0	0	

21930565	Not	hp	CN	2023	0	0	0	0	0	0	0	0	0	0	0	0	
21930665	Not	hp	CN	2023	0	0	0	0	0	0	0	0	0	0	0	0	
21930670	Not	hp	CN	2023	0	0	0	0	0	0	0	0	0	0	0	0	
21930671	Not	hp	CN	2023	0	0	0	0	0	0	0	0	0	0	0	0	
21930675	Not	hp	CN	2023	0	0	0	0	0	0	0	0	0	0	0	0	
21931362	m	hp	TH	2023	1	0	0	0	0	0	0	0	0	0	0	0	50.4
21931851	f	md	ES	2023	0	1	1	0	0	0	0	0	0	0	0	0	70
21932070	f	hp	JP	2023	0	0	0	0	0	0	0	0	0	0	0	0	
21932131	m	md	PH	2023	0	0	0	0	0	0	0	0	0	0	0	0	53
21932354	m	md	LT	2023	0	0	0	0	0	0	0	0	0	0	0	0	53
21932360	Not	md	JP	2023	0	0	0	0	0	0	0	0	0	0	0	0	
21932441	f	md	JP	2023	1	0	0	0	0	0	0	0	0	0	0	0	63
21932598	m	md	JP	2023	0	1	1	0	0	0	0	0	0	0	0	0	69
21932605	m	md	US	2023	0	1	1	0	0	0	0	0	0	0	0	0	
21933770	Not	md	JP	2023	0	1	0	0	0	1	0	0	0	0	0	0	60
21933797	Not	md	JP	2023	0	1	0	0	0	1	0	0	0	0	0	0	
21934235	m	md	PL	2023	0	1	0	0	0	1	0	0	0	0	0	0	62
21934236	f	cn	IN	2023	0	1	0	0	0	1	0	0	0	0	0	0	58
21934361	f	md	DE	2023	0	1	0	1	0	0	0	0	0	0	0	0	
21934664	f	hp	US	2023	1	0	0	0	0	0	0	0	0	0	0	0	
21935001	m	cn	TH	2023	1	0	0	0	0	0	0	0	0	0	0	0	86
21935836	Not	md	JP	2023	0	1	0	1	0	0	0	0	0	0	0	0	
21936091	Not	hp	JP	2023	1	0	0	0	0	0	0	0	0	0	0	0	
21936111	f	cn	ID	2023	1	0	0	0	0	0	0	0	0	0	0	0	
21936257	f	md	DE	2023	0	1	1	0	0	0	0	0	0	0	0	0	
21936490	m	cn	ID	2023	1	0	0	0	0	0	0	0	0	0	0	0	60.4
21937008	f	md	DE	2023	0	1	1	0	0	0	0	0	0	0	0	0	
21937299	f	md	JP	2023	0	1	0	1	0	0	0	0	0	0	0	0	74
21937343	f	hp	CA	2023	0	0	0	0	0	0	0	0	0	0	0	0	
21937370	f	md	US	2023	0	0	0	0	0	0	0	0	0	0	0	0	
21937407	m	cn	AT	2023	0	0	0	0	0	0	0	0	0	0	0	0	56
21937507	m	cn	US	2023	0	0	0	0	0	0	0	0	0	0	0	0	70
21937508	m	md	JP	2023	0	0	0	0	0	0	0	0	0	0	0	0	
21937714	m	md	PT	2023	0	1	1	0	0	0	0	0	0	0	0	0	

21938508	m	ph	US	2023	0	1	1	0	0	0	0	0	0	0	0	1	
21938844	m	md	US	2023	0	0	0	0	0	0	0	0	0	0	0	0	60
21939585	f	hp	TW	2023	0	1	0	0	1	0	0	0	0	0	0	0	64
21940875	m	md	PL	2023	0	1	0	0	0	1	0	0	0	0	0	0	75
21940892	f	md	JP	2023	0	0	0	0	0	0	0	0	0	0	0	0	50
21941186	m	md	GB	2023	0	0	0	0	0	0	0	0	0	0	0	0	74
21941201	f	md	FR	2023	1	0	0	0	0	0	0	0	0	0	0	0	
21943149	m	md	JP	2023	0	1	0	0	0	1	0	0	0	0	0	0	80
21943398	m	md	DE	2023	0	0	0	0	0	0	0	0	0	0	0	0	69
21943601	m	hp	SG	2023	1	0	0	0	0	0	0	0	0	0	0	0	75
21943777	f	md	DE	2023	0	1	0	0	1	0	0	0	0	0	0	0	64
21943845	m	md	JP	2023	0	1	0	0	1	0	0	0	0	0	0	0	72
21944136	Not	hp	IN	2023	0	0	0	0	0	0	0	0	0	0	0	0	
21944290	m	md	LT	2023	0	1	0	0	0	1	0	0	0	0	0	0	62
21944436	m	cn	US	2023	0	0	0	0	0	0	0	0	0	0	0	0	65
21945203	m	md	JP	2023	0	1	1	0	0	0	0	0	0	0	0	0	81
21945251	f	md	DE	2023	0	1	0	0	1	0	0	0	0	0	0	0	60
21945677	m	md	US	2023	0	0	0	0	0	0	0	0	0	0	0	0	34
21946240	m	md	JP	2023	0	1	0	0	1	0	0	0	0	0	0	0	70
21946827	f	hp	NL	2023	0	0	0	0	0	0	0	0	0	0	0	0	80
21947038	f	cn	IN	2023	0	0	0	0	0	0	0	0	0	0	0	0	55
21947167	m	md	JP	2023	0	1	1	0	0	0	0	0	0	0	0	0	80
21947324	m	md	CZ	2023	1	0	0	0	0	0	0	0	0	0	0	0	72
21947502	m	md	JP	2023	0	0	0	0	0	0	0	0	0	0	0	0	79
21947600	m	md	JP	2023	0	0	0	0	0	0	0	0	0	0	0	0	89
21947698	f	ph	JP	2023	0	1	0	0	1	0	0	0	0	0	0	0	84
21947819	m	hp	JP	2023	0	1	0	0	1	0	0	0	0	0	0	0	72
21948012	m	md	JP	2023	0	1	0	0	1	0	0	0	0	0	0	0	53
21948398	Not	hp	US	2023	0	0	0	0	0	0	0	0	0	0	0	0	78
21948451	Not	md	AU	2023	1	0	0	0	0	0	0	0	0	0	0	0	
21948452	Not	md	AU	2023	1	0	0	0	0	0	0	0	0	0	0	0	
21948637	m	ph	JP	2023	0	0	0	0	0	0	0	0	0	0	0	0	85
21949490	m	cn	TH	2023	1	0	0	0	0	0	0	0	0	0	0	0	61.8
21949633	m	hp	CN	2023	1	0	0	0	0	0	0	0	0	0	0	0	62

21949955	f	hp	CN	2023	1	0	0	0	0	0	0	0	0	0	0	0	75
21950030	f	md	JP	2023	0	0	0	0	0	0	0	0	0	0	0	0	83
21950175	m	md	FR	2023	0	1	0	1	0	0	0	0	0	0	0	0	78.5
21950523	f	ph	US	2023	0	0	0	0	0	0	0	0	0	0	0	0	81
21950728	f	cn	US	2023	0	0	0	0	0	0	0	0	0	0	0	0	
21950829	m	ph	JP	2023	1	0	0	0	0	0	0	0	0	0	0	0	
21950977	m	md	JP	2023	0	0	0	0	0	0	0	0	0	0	0	0	
21950988	m	md	KR	2023	0	1	0	0	0	1	0	0	0	0	0	0	78
21951033	Not	md	PT	2023	1	0	0	0	0	0	0	0	0	0	0	0	65
21951079	f	md	FR	2023	0	0	0	0	0	0	0	0	0	0	0	0	73
21951160	f	hp	DE	2023	0	0	0	0	0	0	0	0	0	0	0	0	67
21951614	Not	md	PT	2023	1	0	0	0	0	0	0	0	0	0	0	0	
21952370	f	cn	TH	2023	1	0	0	0	0	0	0	0	0	0	0	0	
21952465	m	md	TR	2023	0	0	0	0	0	0	0	0	0	0	0	0	
21952629	m	ph	CA	2023	0	0	0	0	0	0	0	0	0	0	0	0	84
21952766	Not	hp	JP	2023	0	1	0	0	0	1	0	0	0	0	0	0	
21952820	Not	hp	US	2023	0	0	0	0	0	0	0	0	0	0	0	0	
21953084	f	cn	US	2023	0	0	0	0	0	0	0	0	0	0	0	0	
21954681	m	md	DE	2023	1	0	0	0	0	0	0	0	0	0	0	0	58
21954875	f	md	IQ	2023	0	1	1	0	0	0	0	0	0	0	0	0	
21954883	m	hp	US	2023	0	0	0	0	0	0	0	0	0	0	0	0	57
21954892	Not	hp	US	2023	0	0	0	0	0	0	0	0	0	0	0	0	
21954915	m	md	JP	2023	0	1	0	0	0	1	0	0	0	0	0	0	80
21955334	f	cn	TH	2023	1	0	0	0	0	0	0	0	0	0	0	0	
21955453	m	md	DE	2023	0	1	0	0	1	0	0	0	0	0	0	0	70
21955514	f	hp	JP	2023	1	0	0	0	0	0	0	0	0	0	0	0	77
21955566	Not	hp	JP	2023	0	1	0	0	0	1	0	0	0	0	0	0	
21955748	m	md	JP	2023	0	1	0	0	1	0	0	0	0	0	0	0	80
21955806	f	cn	TH	2023	1	0	0	0	0	0	0	0	0	0	0	0	76
21956113	Not	ph	TH	2023	1	0	0	0	0	0	0	0	0	0	0	0	
21956159	m	cn	CN	2023	0	0	0	0	0	0	0	0	0	0	0	0	58
21956814	f	cn	TH	2023	0	0	0	0	0	0	0	0	0	0	0	0	57
21956852	f	md	CA	2023	1	0	0	0	0	0	0	0	0	0	0	0	74.7
21957182	m	md	JP	2023	0	1	0	0	1	0	0	0	0	0	0	0	78

21958097	m	md	JP	2023	0	0	0	0	0	0	0	0	0	0	0	0	
21958241	f	md	JP	2023	0	1	0	0	0	1	0	0	0	0	0	0	90
21958321	Not	md	JP	2023	0	1	1	0	0	0	0	0	0	0	0	0	80
21958434	f	md	NL	2023	0	1	0	0	0	1	0	0	0	0	0	0	74
21958469	f	md	CA	2023	0	0	0	0	0	0	0	0	0	0	0	0	62
21958639	f	md	NL	2023	0	1	0	0	0	1	0	0	0	0	0	0	74
21959045	f	md	IT	2023	0	0	0	0	0	0	0	0	0	0	0	0	
21959120	m	cn	CN	2023	0	1	0	1	0	0	0	0	0	0	0	0	63
21959264	m	md	GB	2023	0	0	0	0	0	0	0	0	0	0	0	0	66
21959755	m	md	DE	2023	0	1	0	0	1	0	0	0	0	0	0	0	84
21959871	f	md	CA	2023	0	0	0	0	0	0	0	0	0	0	0	0	61
21959927	m	md	JP	2023	0	1	0	0	0	1	0	0	0	0	0	0	80
21959947	m	md	JP	2023	0	0	0	0	0	0	0	0	0	0	0	0	50
21960713	f	md	CN	2023	0	0	0	0	0	0	0	0	0	0	0	0	66
21961113	m	cn	SG	2023	0	1	0	0	0	1	0	0	0	0	0	0	66
21961277	m	hp	TH	2023	1	0	0	0	0	0	0	0	0	0	0	0	90.9
21961415	m	cn	CN	2023	0	0	0	0	0	0	0	0	0	0	0	0	69
21961672	f	hp	CA	2023	0	0	0	0	0	0	0	0	0	0	0	0	69
21961745	m	cn	IN	2023	0	0	0	0	0	0	0	0	0	0	0	0	58
21961749	m	md	JP	2023	0	1	0	0	1	0	0	0	0	0	0	0	75
21961757	f	cn	DE	2023	0	1	1	0	0	0	0	0	0	0	0	0	
21961820	Not	cn	US	2023	1	0	0	0	0	0	0	0	0	0	0	0	
21961877	m	cn	DE	2023	0	1	0	0	1	0	0	0	0	0	0	0	84
21962168	f	ph	US	2023	0	0	0	0	0	0	0	0	0	0	0	0	75
21962422	m	cn	IN	2023	0	0	0	0	0	0	0	0	0	0	0	0	57
21962443	f	hp	IT	2023	0	0	0	0	0	0	0	0	0	0	0	0	83
21962627	m	md	IT	2023	0	1	1	0	0	0	0	0	0	0	0	0	
21963061	m	md	DE	2023	0	1	0	0	1	0	0	0	0	0	0	0	83
21963384	m	md	JP	2023	0	1	0	0	1	0	0	0	0	0	0	1	56
21963416	f	md	FR	2023	0	1	0	0	0	1	0	0	0	0	0	0	83
21963490	f	cn	IN	2023	0	0	0	0	0	0	0	0	0	0	0	0	44
21963506	m	cn	IN	2023	0	0	0	0	0	0	0	0	0	0	0	0	68
21963899	m	cn	IN	2023	0	0	0	0	0	0	0	0	0	0	0	0	47
21964001	f	cn	IN	2023	0	0	0	0	0	0	0	0	0	0	0	0	32

21964054	m	md	AR	2023	0	0	0	0	0	0	0	0	0	0	0	0	83
21964845	m	ph	JP	2023	0	0	0	0	0	0	0	0	0	0	0	0	84
21964944	m	md	JP	2023	0	1	0	0	1	0	0	0	0	0	0	0	60
21965094	Not	md	AU	2023	0	1	0	1	0	0	0	0	0	0	0	0	
21965312	m	md	JP	2023	0	0	0	0	0	0	0	0	0	0	0	0	76
21967312	m	md	DE	2023	0	1	0	1	0	0	0	0	0	0	0	0	
21967622	f	md	DE	2023	0	1	1	0	0	0	0	0	0	0	0	0	
21967631	m	md	JP	2023	0	0	0	0	0	0	0	0	0	0	0	0	52
21967770	f	md	BE	2023	0	1	1	0	0	0	0	0	0	0	0	0	54
21967832	m	md	BE	2023	0	0	0	0	0	0	0	0	0	0	0	0	82
21967925	Not	ph	JP	2023	1	0	0	0	0	0	0	0	0	0	0	0	
21968015	m	md	LT	2023	0	1	0	0	0	1	0	0	0	0	0	0	62
21968270	Not	ph	JP	2023	1	0	0	0	0	0	0	0	0	0	0	0	
21968319	f	md	GB	2023	0	1	0	0	0	1	0	0	0	0	0	0	59
21968605	f	md	RO	2023	0	0	0	0	0	0	0	0	0	0	0	0	69
21969105	m	cn	TH	2023	0	1	1	0	0	0	0	0	0	0	0	0	44
21969457	f	md	US	2023	0	0	0	0	0	0	0	0	0	0	0	0	
21969483	m	cn	PT	2023	0	1	1	0	0	0	0	0	0	0	0	0	
21969740	f	md	JP	2023	1	0	0	0	0	0	0	0	0	0	0	0	82
21969891	Not	md	JP	2023	0	1	0	0	1	0	0	0	0	0	0	0	
21969990	f	md	JP	2023	0	0	0	0	0	0	0	0	0	0	0	0	56
21970522	m	md	JP	2023	0	0	0	0	0	0	0	0	0	0	0	0	60
21970734	f	md	JP	2023	0	1	0	0	0	1	0	0	0	0	0	0	73
21970775	Not	md	US	2023	0	1	0	0	0	1	0	0	0	0	0	0	
21970818	Not	hp	NO	2023	0	1	0	0	0	1	0	0	0	0	0	0	
21970962	f	md	GB	2023	0	1	0	0	0	1	0	0	0	0	0	0	54
21971896	m	md	JP	2023	0	1	1	0	0	0	0	0	0	0	0	0	73
21972134	f	md	CN	2023	0	0	0	0	0	0	0	0	0	0	0	0	66
21972260	f	md	JP	2023	0	1	0	0	1	0	0	0	0	0	0	0	73
21972682	f	md	KR	2023	1	0	0	0	0	0	0	0	0	0	0	0	60
21972886	m	hp	NL	2023	0	0	0	0	0	0	0	0	0	0	0	0	75
21973015	m	md	JP	2023	0	1	0	1	0	0	0	0	0	0	0	0	70
21973141	f	md	RU	2023	0	0	0	0	0	0	0	0	0	0	0	0	57
21973210	m	md	SG	2023	0	0	0	0	0	0	0	0	0	0	0	0	

21973711	m	cn	KR	2023	0	0	0	0	0	0	0	0	0	0	0	0	50
21973965	m	cn	AR	2023	0	1	0	1	0	0	0	0	0	0	0	0	73
21973973	f	cn	ID	2023	1	0	0	0	0	0	0	0	0	0	0	0	66.8
21974223	m	md	CN	2023	0	0	0	0	0	0	0	0	0	0	0	0	49
21974238	f	hp	IE	2023	0	0	0	0	0	0	0	0	0	0	0	0	66
21974372	f	md	KR	2023	1	0	0	0	0	0	0	0	0	0	0	0	62
21974937	m	cn	IN	2023	0	1	0	0	0	1	0	0	0	0	0	0	68
21975258	f	hp	US	2023	0	0	0	0	0	0	0	0	0	0	0	0	68
21976379	m	md	KR	2023	0	0	0	0	0	0	0	0	0	0	0	0	72
21976481	m	md	JP	2023	0	1	0	0	1	0	0	0	0	0	0	0	61
21976502	m	md	CN	2023	1	0	0	0	0	0	0	0	0	0	0	0	72
21976792	Not	md	TW	2023	0	0	0	0	0	0	0	0	0	0	0	0	71
21977043	f	cn	US	2023	0	1	1	0	0	0	0	0	0	0	0	0	
21977180	f	md	JP	2023	0	1	0	0	1	0	0	0	0	0	0	0	59
21977510	f	md	CN	2023	0	0	0	0	0	0	0	0	0	0	0	0	83
21977585	m	md	JP	2023	0	1	0	0	1	0	0	0	0	0	0	0	46
21977756	f	md	CN	2023	0	1	1	0	0	0	0	0	0	0	0	0	69
21978114	Not	md	US	2023	0	1	0	1	0	0	0	0	0	0	0	1	
21978744	f	hp	US	2023	0	1	1	0	0	0	0	0	0	0	0	0	69
21979434	m	md	JP	2023	0	1	0	0	1	0	0	0	0	0	0	1	80
21979469	f	cn	MY	2023	0	0	0	0	0	0	0	0	0	0	0	0	75
21981057	m	md	DE	2023	0	0	0	0	0	0	0	0	0	0	0	0	
21981241	f	md	DE	2023	0	1	1	0	0	0	0	0	0	0	0	0	
21981290	Not	md	JP	2023	0	0	0	0	0	0	0	0	0	0	0	0	
21981467	f	md	IT	2023	0	0	0	0	0	0	0	0	0	0	0	0	
21981829	m	md	CN	2023	0	0	0	0	0	0	0	0	0	0	0	0	69
21981940	m	md	US	2023	0	0	0	0	0	0	0	0	0	0	0	0	62
21982060	m	md	JP	2023	0	1	0	0	1	0	0	0	0	0	0	1	67
21982468	m	md	JP	2023	0	1	0	0	1	0	0	0	0	0	0	1	60
21982514	f	cn	ID	2023	1	0	0	0	0	0	0	0	0	0	0	0	82.4
21982806	Not	hp	JP	2023	1	0	0	0	0	0	0	0	0	0	0	0	
21982862	f	md	RO	2023	0	0	0	0	0	0	0	0	0	0	0	0	69
21983051	f	md	JP	2023	0	1	0	0	1	0	0	0	0	0	0	1	80
21983125	f	md	RO	2023	0	0	0	0	0	0	0	0	0	0	0	0	69

21983216	f	md	JP	2023	0	1	0	0	1	0	0	0	0	0	0	0	59
21983218	m	ph	FR	2023	1	0	0	0	0	0	0	0	0	0	0	0	64
21983519	f	hp	KR	2023	1	0	0	0	0	0	0	0	0	0	0	0	48
21983609	Not	md	JP	2023	0	1	0	0	1	0	0	0	0	0	0	1	62
21983841	m	md	CA	2023	1	0	0	0	0	0	0	0	0	0	0	0	78.8
21984057	f	md	ES	2023	0	1	1	0	0	0	0	0	0	0	0	0	62
21984516	m	md	JP	2023	0	1	0	0	1	0	0	0	0	0	0	0	75
21984945	f	md	DE	2023	0	1	0	0	1	0	0	0	0	0	0	0	64
21985007	m	md	JP	2023	0	1	1	0	0	0	0	0	0	0	0	0	68
21985262	m	md	JP	2023	0	1	0	0	1	0	0	0	0	0	0	0	74
21985399	Not	hp	US	2023	0	0	0	0	0	0	0	0	0	0	0	0	
21985432	m	md	JP	2023	0	1	1	0	0	0	0	0	0	0	0	0	80
21985801	Not	md	CN	2023	1	0	0	0	0	0	0	0	0	0	0	0	
21985895	f	md	JP	2023	0	1	1	0	0	0	0	0	0	0	0	0	90
21985952	m	md	PT	2023	0	1	1	0	0	0	0	0	0	0	0	0	
21986037	m	cn	TH	2023	1	0	0	0	0	0	0	0	0	0	0	0	77.2
21986069	m	cn	IN	2023	0	0	0	0	0	0	0	0	0	0	0	0	61
21986118	m	cn	TH	2023	1	0	0	0	0	0	0	0	0	0	0	0	74
21986156	m	md	FR	2023	0	1	1	0	0	0	0	0	0	0	0	0	61
21986198	m	md	CA	2023	0	1	1	0	0	0	0	0	0	0	0	0	37
21986255	m	md	PL	2023	0	1	0	0	0	1	0	0	0	0	0	0	62
21986726	f	md	AU	2023	0	0	0	0	0	0	0	0	0	0	0	0	78
21986958	m	md	ES	2023	0	0	0	0	0	0	0	0	0	0	0	0	
21987343	Not	md	JP	2023	0	1	0	0	0	1	0	0	0	0	0	0	70
21987366	m	md	JP	2023	0	0	0	0	0	0	0	0	0	0	0	0	63
21987701	m	md	US	2023	0	0	0	0	0	0	0	0	0	0	0	0	
21987728	f	hp	TH	2023	0	1	0	0	0	1	0	0	0	0	0	0	80
21987747	m	md	JP	2023	0	1	0	0	1	0	0	0	0	0	0	0	80
21988193	f	cn	ID	2023	1	0	0	0	0	0	0	0	0	0	0	0	56.5
21988331	m	md	US	2023	0	1	0	1	0	0	0	0	0	0	0	0	75
21988347	m	md	JP	2023	0	1	1	0	0	0	0	0	0	0	0	1	62
21988785	f	hp	US	2023	0	0	0	0	0	0	0	0	0	0	0	0	52
21988789	m	md	CA	2023	1	0	0	0	0	0	0	0	0	0	0	0	
21988805	m	hp	US	2023	0	0	0	0	0	0	0	0	0	0	0	0	51

21988818	m	md	JP	2023	0	1	0	0	1	0	0	0	0	0	0	0	59
21988855	m	md	US	2023	0	0	0	0	0	0	0	0	0	0	0	0	66
21988906	m	md	JP	2023	0	1	0	0	0	1	0	0	0	0	0	1	
21989223	m	md	JP	2023	0	1	0	0	1	0	0	0	0	0	0	0	49
21989548	f	hp	CA	2023	1	0	0	0	0	0	0	0	0	0	0	0	
21989778	f	md	JP	2023	0	1	0	0	0	1	0	0	0	0	0	0	74
21989867	f	md	JP	2023	0	0	0	0	0	0	0	0	0	0	0	0	62
21990049	m	md	BE	2023	0	1	0	0	1	0	0	0	0	0	0	0	68
21990118	f	md	JP	2023	0	0	0	0	0	0	0	0	0	0	0	0	70
21990422	f	md	IT	2023	0	0	0	0	0	0	0	0	0	0	0	0	73
21990745	f	ph	TH	2023	1	0	0	0	0	0	0	0	0	0	0	0	
21990747	f	md	JP	2023	1	0	0	0	0	0	0	0	0	0	0	0	
21991770	f	md	DE	2023	0	1	1	0	0	0	0	0	0	0	0	0	
21992059	m	md	JP	2023	0	1	0	0	1	0	0	0	0	0	0	0	69
21992413	m	md	JP	2023	0	1	0	0	0	1	0	0	0	0	0	0	75
21992416	m	md	JP	2023	0	1	0	0	0	1	0	0	0	0	0	1	80
21993173	f	md	US	2023	0	1	0	0	1	0	0	0	0	0	0	0	
21993330	m	md	JP	2023	0	0	0	0	0	0	0	0	0	0	0	0	73
21993526	f	ph	US	2023	0	1	0	0	1	0	0	0	0	0	0	0	
21993558	f	hp	US	2023	0	1	1	0	0	0	0	0	0	0	0	0	69
21993590	f	md	US	2023	0	1	0	0	0	1	0	0	0	0	0	0	57
21993935	f	md	CA	2023	1	0	0	0	0	0	0	0	0	0	0	0	
21994633	f	md	JP	2023	0	0	0	0	0	0	0	0	0	0	0	0	57
21994701	f	hp	JP	2023	0	0	0	0	0	0	0	0	0	0	0	0	79
21994729	m	cn	TH	2023	1	0	0	0	0	0	0	0	0	0	0	0	69.6
21995011	f	cn	CN	2023	0	0	0	0	0	0	0	0	0	0	0	0	43
21995755	m	md	FR	2023	1	0	0	0	0	0	0	0	0	0	0	0	80
21995761	m	md	US	2023	0	1	0	0	1	0	0	0	0	0	0	0	
21995775	f	hp	TH	2023	1	0	0	0	0	0	0	0	0	0	0	0	57.3
21995861	Not	md	US	2023	0	0	0	0	0	0	0	0	0	0	0	0	
21995882	f	cn	IN	2023	0	0	0	0	0	0	0	0	0	0	0	0	49
21995909	m	md	JP	2023	0	0	0	0	0	0	0	0	0	0	0	0	66
21996085	f	cn	CN	2023	0	1	0	0	0	1	0	0	0	0	0	0	72
21996430	Not	md	JP	2023	1	0	0	0	0	0	0	0	0	0	0	0	

21996439	f	md	MY	2023	1	0	0	0	0	0	0	0	0	0	0	0	
21996664	f	hp	US	2023	0	1	1	0	0	0	0	0	0	0	0	0	49
21996872	m	md	FR	2023	0	0	0	0	0	0	0	0	0	0	0	0	
21996926	m	md	JP	2023	0	1	0	0	1	0	0	0	0	0	0	0	66
21996933	m	md	JP	2023	0	0	0	0	0	0	0	0	0	0	0	0	69
21997079	f	cn	IN	2023	0	0	0	0	0	0	0	0	0	0	0	0	66
21997339	f	md	JP	2023	0	0	0	0	0	0	0	0	0	0	0	0	66
21997715	m	md	FR	2023	0	1	0	0	0	1	0	0	0	0	0	0	82
21997725	Not	md	JP	2023	0	1	0	0	0	1	0	0	0	0	0	0	60
21998246	Not	md	JP	2023	0	1	0	1	0	0	0	0	0	0	0	0	80
21998432	f	hp	GB	2023	1	0	0	0	0	0	0	0	0	0	0	0	85
21998611	m	hp	IT	2023	0	1	0	0	1	0	0	0	0	0	0	0	72
21999765	Not	md	JP	2023	0	0	0	0	0	0	0	0	0	0	0	0	80
21999972	f	cn	US	2023	1	0	0	0	0	0	0	0	0	0	0	0	69
21999986	Not	hp	LB	2023	0	0	0	0	0	0	0	0	0	0	0	0	76
22000299	f	md	IT	2023	0	0	0	0	0	0	0	0	0	0	0	0	
22000507	m	ph	ES	2023	0	0	0	0	0	0	0	0	0	0	0	0	76
22000791	f	hp	GB	2023	1	0	0	0	0	0	0	0	0	0	0	0	
22000814	Not	md	MX	2023	0	0	0	0	0	0	0	0	0	0	0	0	
22001202	Not	md	US	2023	0	1	0	0	1	0	0	0	0	0	0	0	
22001216	m	md	JP	2023	0	1	1	0	0	0	0	0	0	0	0	0	76
22001711	Not	md	CN	2023	0	0	0	0	0	0	0	0	0	0	0	0	
22001764	f	md	TH	2023	0	0	0	0	0	0	0	0	0	0	0	0	53
22001777	f	md	GB	2023	0	1	0	0	0	1	0	0	0	0	0	0	71
22002002	f	hp	US	2023	0	1	1	0	0	0	0	0	0	0	0	0	69
22002327	Not	md	JP	2023	0	1	0	0	0	1	0	0	0	0	0	0	70
22002341	Not	md	JP	2023	0	1	0	0	0	1	0	0	0	0	0	0	70
22002395	f	md	DE	2023	0	0	0	0	0	0	0	0	0	0	0	0	78
22002972	f	md	AT	2023	0	0	0	0	0	0	0	0	0	0	0	0	
22003236	f	cn	TH	2023	1	0	0	0	0	0	0	0	0	0	0	0	60
22003784	Not	hp	GB	2023	1	0	0	0	0	0	0	0	0	0	0	0	
22004279	Not	md	GR	2023	0	0	0	0	0	0	0	0	0	0	0	0	
22004369	m	md	GR	2023	0	0	0	0	0	0	0	0	0	0	0	0	
22004425	f	md	US	2023	0	0	0	0	0	0	0	0	0	0	0	0	68

22004492	f	cn	VN	2023	0	1	0	1	0	0	0	0	0	0	0	0	
22004623	m	md	JP	2023	0	1	1	0	0	0	0	0	0	0	0	0	70
22004643	m	md	JP	2023	0	1	1	0	0	0	0	0	0	0	0	0	70
22005005	m	md	JP	2023	0	1	0	0	1	0	0	0	0	0	0	0	59
22005077	Not	md	US	2023	0	0	0	0	0	0	0	0	0	0	0	0	
22005624	f	md	RU	2023	0	0	0	0	0	0	0	0	0	0	0	0	56
22005835	f	cn	US	2023	0	0	0	0	0	0	0	0	0	0	0	0	63
22005927	m	md	JP	2023	0	1	1	0	0	0	0	0	0	0	0	0	60
22005981	m	cn	US	2023	0	1	1	0	0	0	0	0	0	0	0	0	
22006208	f	md	JP	2023	0	0	0	0	0	0	0	0	0	0	0	0	
22006308	Not	md	JP	2023	0	0	0	0	0	0	0	0	0	0	0	0	
22006441	f	md	CN	2023	1	0	0	0	0	0	0	0	0	0	0	0	64
22006452	f	ph	JP	2023	1	0	0	0	0	0	0	0	0	0	0	0	79
22006519	m	md	CN	2023	1	0	0	0	0	0	0	0	0	0	0	0	53
22006855	Not	cn	US	2023	1	0	0	0	0	0	0	0	0	0	0	0	
22006923	f	cn	US	2023	0	1	1	0	0	0	0	0	0	0	0	0	59
22006992	f	md	US	2023	0	0	0	0	0	0	0	0	0	0	0	0	49
22007127	m	md	JP	2023	0	1	1	0	0	0	0	0	0	0	0	0	78
22007240	m	md	JP	2023	0	1	0	0	0	1	0	0	0	0	0	0	80
22007566	Not	hp	US	2023	0	0	0	0	0	0	0	0	0	0	0	0	
22007673	m	md	DE	2023	0	0	0	0	0	0	0	0	0	0	0	0	60
22007681	f	md	DE	2023	0	0	0	0	0	0	0	0	0	0	0	0	56
22007779	Not	md	AU	2023	0	1	0	0	0	1	0	0	0	0	0	0	
22007866	f	md	DE	2023	0	1	0	0	1	0	0	0	0	0	0	0	69
22007867	f	cn	TW	2023	0	0	0	0	0	0	0	0	0	0	0	0	84
22007968	f	md	JP	2023	0	0	0	0	0	0	0	0	0	0	0	0	78
22008035	f	hp	AU	2023	0	1	0	0	0	1	0	0	0	0	0	0	
22008047	f	md	AU	2023	0	1	0	0	0	1	0	0	0	0	0	0	
22008153	m	md	US	2023	0	0	0	0	0	0	0	0	0	0	0	0	82
22008332	Not	md	JP	2023	0	1	0	0	0	1	0	0	0	0	0	0	60
22008546	Not	md	JP	2023	0	1	0	0	1	0	0	0	0	0	0	0	
22008823	m	md	JP	2023	0	0	0	0	0	0	0	0	0	0	0	0	76
22009323	f	md	US	2023	0	0	0	0	0	0	0	0	0	0	0	0	
22009534	m	md	JP	2023	0	1	0	0	0	1	0	0	0	0	0	1	80

22010090	m	md	FR	2023	0	1	0	0	0	1	0	0	0	0	0	0	80
22010110	m	md	JP	2023	0	1	0	0	0	1	0	0	0	0	0	0	80
22010194	Not	md	JP	2023	0	1	0	0	0	1	0	0	0	0	0	0	70
22010601	f	hp	IT	2023	0	1	1	0	0	0	0	0	0	0	0	0	66
22010629	f	md	FR	2023	0	0	0	0	0	0	0	0	0	0	0	0	75
22010659	m	md	JP	2023	0	1	0	0	1	0	0	0	0	0	0	0	39
22011133	m	hp	US	2023	0	0	0	0	0	0	0	0	0	0	0	0	69
22011291	f	md	BE	2023	0	1	1	0	0	0	0	0	0	0	0	0	54
22011391	m	md	DE	2023	0	0	0	0	0	0	0	0	0	0	0	0	40
22012186	f	md	CN	2023	0	1	0	0	0	1	0	0	0	0	0	0	57
22012209	Not	cn	US	2023	1	0	0	0	0	0	0	0	0	0	0	0	56
22012508	f	md	CA	2023	1	0	0	0	0	0	0	0	0	0	0	0	
22012670	f	md	FR	2023	0	0	0	0	0	0	0	0	0	0	0	0	
22012732	f	cn	JP	2023	0	0	0	0	0	0	0	0	0	0	0	0	42
22013115	f	md	BE	2023	0	1	1	0	0	0	0	0	0	0	0	0	54
22013130	m	md	BE	2023	0	1	0	0	1	0	0	0	0	0	0	0	68
22013418	f	md	JP	2023	0	0	0	0	0	0	0	0	0	0	0	0	74
22013604	m	md	JP	2023	0	1	0	0	0	1	0	0	0	0	0	0	60
22014109	f	md	AT	2023	0	0	0	0	0	0	0	0	0	0	0	0	
22014202	m	md	CN	2023	0	0	0	0	0	0	0	0	0	0	0	0	69
22014371	m	md	JP	2023	0	1	0	0	1	0	0	0	0	0	0	0	65
22014716	f	hp	US	2023	0	1	1	0	0	0	0	0	0	0	0	0	69
22015183	f	md	KR	2023	0	0	0	0	0	0	0	0	0	0	0	0	67
22015304	f	md	DE	2023	0	1	0	0	1	0	0	0	0	0	0	0	73
22015415	m	md	JP	2023	0	1	0	0	1	0	0	0	0	0	0	1	59
22015615	Not	md	DE	2023	0	0	0	0	0	0	0	0	0	0	0	0	
22015618	Not	md	DE	2023	0	0	0	0	0	0	0	0	0	0	0	0	
22015620	Not	hp	DE	2023	0	0	0	0	0	0	0	0	0	0	0	0	
22016108	f	hp	US	2023	0	1	1	0	0	0	0	0	0	0	0	0	59
22016360	Not	md	TW	2023	0	0	0	0	0	0	0	0	0	0	0	0	84
22016367	m	ph	EG	2023	0	0	0	0	0	0	0	0	0	0	0	0	67
22016494	f	cn	GB	2023	0	0	0	0	0	0	0	0	0	0	0	0	61
22016535	f	md	JP	2023	0	1	0	0	0	1	0	0	0	0	0	0	80
22017236	m	hp	US	2023	0	0	0	0	0	0	0	0	0	0	0	0	60

22017372	Not	md	KR	2023	0	0	0	0	0	0	0	0	0	0	0	0	
22017452	m	md	JP	2023	0	0	0	0	0	0	0	0	0	0	0	0	65
22017534	f	hp	US	2023	1	0	0	0	0	0	0	0	0	0	0	0	80
22017572	m	md	JP	2023	0	1	0	1	0	0	0	0	0	0	0	0	78
22017618	f	md	CA	2023	1	0	0	0	0	0	0	0	0	0	0	0	
22017632	f	md	FR	2023	0	1	0	0	0	1	0	0	0	0	0	0	85
22017699	m	md	JP	2023	0	1	1	0	0	0	0	0	0	0	0	0	78
22017826	m	md	JP	2023	0	0	0	0	0	0	0	0	0	0	0	0	
22018100	f	md	CN	2023	0	0	0	0	0	0	0	0	0	0	0	0	77
22018410	m	md	JP	2023	0	1	0	0	0	1	0	0	0	0	0	0	80
22018430	f	md	FI	2023	0	0	0	0	0	0	0	0	0	0	0	0	48
22018627	m	md	JP	2023	0	1	0	0	1	0	0	0	0	0	0	0	65
22018831	f	md	JP	2023	0	1	0	0	0	1	0	0	0	0	0	0	70
22018958	m	md	JP	2023	0	0	0	0	0	0	0	0	0	0	0	0	
22018985	Not	ph	JP	2023	0	1	0	0	0	1	0	0	0	0	0	0	80
22019666	m	md	JP	2023	0	1	0	0	1	0	0	0	0	0	0	0	70
22019925	f	cn	US	2023	0	0	0	0	0	0	0	0	0	0	0	0	
22019968	Not	ph	JP	2023	1	0	0	0	0	0	0	0	0	0	0	0	
22020042	f	md	IT	2023	0	1	1	0	0	0	0	0	0	0	0	0	66
22020142	f	md	JP	2023	0	0	0	0	0	0	0	0	0	0	0	0	54
22020218	f	cn	SG	2023	1	0	0	0	0	0	0	0	0	0	0	0	84.1
22020220	Not	md	JP	2023	1	0	0	0	0	0	0	0	0	0	0	0	
22021386	m	md	JP	2023	0	1	0	0	1	0	0	0	0	0	0	0	61
22021445	m	md	CH	2023	0	0	0	0	0	0	0	0	0	0	0	0	
22021919	m	md	BA	2023	0	0	0	0	0	0	0	0	0	0	0	0	
22022095	f	cn	CA	2023	0	1	1	0	0	0	0	0	0	0	0	0	58
22022587	f	md	GB	2023	0	1	0	0	0	1	0	0	0	0	0	0	71
22022628	f	md	ES	2023	0	0	0	0	0	0	0	0	0	0	0	0	63
22022682	Not	md	US	2023	0	1	1	0	0	0	0	0	0	0	0	0	
22022821	m	hp	CN	2023	0	0	0	0	0	0	0	0	0	0	0	0	72
22022822	Not	md	KR	2023	0	0	0	0	0	0	0	0	0	0	0	0	
22023352	Not	md	VN	2023	0	1	0	0	0	1	0	0	0	0	0	0	
22023402	m	md	CA	2023	0	0	0	0	0	0	0	0	0	0	0	0	
22023667	f	hp	CA	2023	0	0	0	0	0	0	0	0	0	0	0	0	65

22025731	f	md	AU	2023	1	0	0	0	0	0	0	0	0	0	0	0	
22026135	m	md	IN	2023	0	1	0	0	0	1	0	0	0	0	0	0	
22026340	Not	cn	US	2023	1	0	0	0	0	0	0	0	0	0	0	0	
22026341	f	md	FR	2023	1	0	0	0	0	0	0	0	0	0	0	0	
22026766	m	cn	KR	2023	0	0	0	0	0	0	0	0	0	0	0	0	67
22028046	f	cn	US	2023	0	0	0	0	0	0	0	0	0	0	0	0	
22028233	m	md	JP	2023	0	0	0	0	0	0	0	0	0	0	0	0	47
22028631	m	md	BE	2023	0	1	0	0	1	0	0	0	0	0	0	0	68
22028825	Not	hp	CN	2023	0	0	0	0	0	0	0	0	0	0	0	0	
22028886	f	md	JP	2023	0	0	0	0	0	0	0	0	0	0	0	0	47
22028932	m	md	JP	2023	0	1	1	0	0	0	0	0	0	0	0	0	67
22029163	Not	hp	CN	2023	0	0	0	0	0	0	0	0	0	0	0	0	
22029214	m	md	IT	2023	0	0	0	0	0	0	0	0	0	0	0	0	54
22029348	m	md	FR	2023	1	0	0	0	0	0	0	0	0	0	0	0	
22029381	f	hp	IT	2023	0	1	1	0	0	0	0	0	0	0	0	0	66
22029431	m	md	FR	2023	1	0	0	0	0	0	0	0	0	0	0	0	
22029783	m	md	GF	2023	1	0	0	0	0	0	0	0	0	0	0	0	
22029870	m	md	FR	2023	1	0	0	0	0	0	0	0	0	0	0	0	
22030084	m	md	FR	2023	0	0	0	0	0	0	0	0	0	0	0	0	
22030692	m	md	JP	2023	0	1	1	0	0	0	0	0	0	0	0	0	60
22030718	f	md	DE	2023	0	1	0	0	1	0	0	0	0	0	0	1	65
22030821	m	md	DE	2023	0	0	0	0	0	0	0	0	0	0	0	0	
22030895	m	md	PT	2023	0	1	1	0	0	0	0	0	0	0	0	0	
22030896	m	md	CN	2023	0	0	0	0	0	0	0	0	0	0	0	0	65
22030917	m	md	CN	2023	0	0	0	0	0	0	0	0	0	0	0	0	58
22030926	f	md	TR	2023	0	0	0	0	0	0	0	0	0	0	0	0	41
22031052	Not	md	JP	2023	0	1	1	0	0	0	0	0	0	0	0	0	80
22031287	f	hp	US	2023	0	1	1	0	0	0	0	0	0	0	0	0	49
22031469	Not	md	VN	2023	0	1	0	0	0	1	0	0	0	0	0	0	
22031715	m	md	RU	2023	0	1	0	0	0	1	0	0	0	0	0	0	62
22031744	Not	md	VN	2023	0	1	0	0	0	1	0	0	0	0	0	0	
22031907	Not	md	JP	2023	0	1	0	0	0	1	0	0	0	0	0	0	
22032036	m	cn	ID	2023	1	0	0	0	0	0	0	0	0	0	0	0	65
22032050	f	cn	US	2023	1	0	0	0	0	0	0	0	0	0	0	0	88.7

22032276	m	md	JP	2023	0	1	1	0	0	0	0	0	0	0	0	1	73
22032383	f	md	JP	2023	0	0	0	0	0	0	0	0	0	0	0	0	60
22032510	Not	md	GE	2023	0	1	0	0	0	1	0	0	0	0	0	0	62
22032555	m	cn	AT	2023	0	0	0	0	0	0	0	0	0	0	0	0	40
22032646	Not	md	JP	2023	0	0	0	0	0	0	0	0	0	0	0	0	40
22032687	f	cn	JP	2023	1	0	0	0	0	0	0	0	0	0	0	0	62
22032700	m	md	JP	2023	0	0	0	0	0	0	0	0	0	0	0	0	62
22033138	f	cn	US	2023	1	0	0	0	0	0	0	0	0	0	0	0	
22033954	m	cn	KR	2023	0	0	0	0	0	0	0	0	0	0	0	0	42
22034814	f	md	MY	2023	1	0	0	0	0	0	0	0	0	0	0	0	
22034868	Not	md	JP	2023	1	0	0	0	0	0	0	0	0	0	0	0	
22034881	m	hp	FR	2023	1	0	0	0	0	0	0	0	0	0	0	0	59.5
22034882	f	md	FR	2023	1	0	0	0	0	0	0	0	0	0	0	0	
22034897	m	cn	US	2023	1	0	0	0	0	0	0	0	0	0	0	0	
22035199	m	md	FR	2023	1	0	0	0	0	0	0	0	0	0	0	0	
22035283	f	md	FR	2023	1	0	0	0	0	0	0	0	0	0	0	0	
22035499	f	ph	JP	2023	1	0	0	0	0	0	0	0	0	0	0	0	62
22035585	f	md	JP	2023	1	0	0	0	0	0	0	0	0	0	0	0	66
22036043	f	cn	US	2023	1	0	0	0	0	0	0	0	0	0	0	0	65
22036212	f	hp	PT	2023	0	1	1	0	0	0	0	0	0	0	0	0	53
22036280	Not	hp	TW	2023	1	0	0	0	0	0	0	0	0	0	0	0	
22036436	m	hp	US	2023	0	0	0	0	0	0	0	0	0	0	0	0	60
22037449	f	md	TH	2023	0	0	0	0	0	0	0	0	0	0	0	0	
22037696	m	md	DE	2023	0	1	0	0	1	0	0	0	0	0	0	0	73
22037720	Not	md	JP	2023	0	1	0	0	0	1	0	0	0	0	0	0	80
22037731	m	ph	JP	2023	0	1	0	0	1	0	0	0	0	0	0	0	49
22037745	m	hp	JP	2023	0	0	0	0	0	0	0	0	0	0	0	0	49
22037942	f	md	DE	2023	0	0	0	0	0	0	0	0	0	0	0	0	63
22037969	f	hp	IT	2023	0	1	1	0	0	0	0	0	0	0	0	0	66
22038595	m	md	JP	2023	0	1	0	0	1	0	0	0	0	0	0	0	71
22038655	m	md	JP	2023	0	1	0	0	1	0	0	0	0	0	0	0	68
22038964	m	cn	US	2023	0	0	0	0	0	0	0	0	0	0	0	0	62
22039685	m	md	CN	2023	0	0	0	0	0	0	0	0	0	0	0	0	47
22040066	f	md	BE	2023	0	1	1	0	0	0	0	0	0	0	0	0	54

22040124	m	hp	GB	2023	1	0	0	0	0	0	0	0	0	0	0	0	58
22040806	m	md	GR	2023	0	0	0	0	0	0	0	0	0	0	0	0	
22040950	f	md	VN	2023	0	0	0	0	0	0	1	0	0	0	1	0	60
22041299	Not	md	JP	2023	0	1	0	0	0	1	0	0	0	0	0	0	
22041484	Not	md	US	2023	0	0	0	0	0	0	0	0	0	0	0	0	
22042240	f	hp	IT	2023	0	1	1	0	0	0	0	0	0	0	0	0	82
22042276	f	cn	IN	2023	0	0	0	0	0	0	0	0	0	0	0	0	70
22042341	f	hp	JP	2023	0	1	0	0	0	1	0	0	0	0	0	0	53
22042366	Not	hp	TW	2023	1	0	0	0	0	0	0	0	0	0	0	0	
22042367	Not	hp	TW	2023	1	0	0	0	0	0	0	0	0	0	0	0	
22042717	Not	hp	US	2023	0	0	0	0	0	0	0	0	0	0	0	0	
22042773	m	md	VN	2023	0	0	0	0	0	0	1	0	0	0	1	0	
22042775	f	hp	US	2023	0	1	0	1	0	0	0	0	0	0	0	1	62
22043080	f	cn	CN	2023	0	0	0	0	0	0	0	0	0	0	0	0	64
22043157	Not	md	JP	2023	0	1	0	0	1	0	0	0	0	0	0	0	73
22043192	m	md	JP	2023	0	1	0	0	0	1	0	0	0	0	0	0	70
22043490	m	md	JP	2023	0	0	0	0	0	0	0	0	0	0	0	0	68
22043671	f	hp	GB	2023	1	0	0	0	0	0	0	0	0	0	0	0	76
22044213	f	cn	CA	2023	1	0	0	0	0	0	0	0	0	0	0	0	
22044583	Not	md	JP	2023	0	1	0	0	1	0	0	0	0	0	0	0	59
22045378	m	md	JP	2023	0	1	1	0	0	0	0	0	0	0	0	0	
22045466	f	md	JP	2023	0	1	0	0	1	0	0	0	0	0	0	1	65
22045566	f	md	JP	2023	1	0	0	0	0	0	0	0	0	0	0	0	70.7
22045879	f	md	JP	2023	1	0	0	0	0	0	0	0	0	0	0	0	76
22046063	f	cn	US	2023	0	0	0	0	0	0	0	0	0	0	0	0	72
22046077	m	cn	CA	2023	0	0	0	0	0	0	0	0	0	0	0	0	66
22046199	m	cn	SG	2023	1	0	0	0	0	0	0	0	0	0	0	0	68.8
22046284	f	hp	GB	2023	0	0	0	0	0	0	1	0	0	0	1	0	60
22046458	f	md	BR	2023	0	0	0	0	0	0	0	0	0	0	0	0	28
22046685	m	md	JP	2023	0	1	0	0	1	0	0	0	0	0	0	0	59
22046705	f	md	DE	2023	1	0	0	0	0	0	0	0	0	0	0	0	
22047535	m	md	FR	2023	0	1	0	0	0	1	0	0	0	0	0	0	74
22048432	m	cn	CA	2023	1	0	0	0	0	0	0	0	0	0	0	0	
22048466	Not	hp	GB	2023	1	0	0	0	0	0	0	0	0	0	0	0	

22048503	Not	md	NO	2023	0	1	1	0	0	0	0	0	0	0	0	0	
22048587	m	md	FR	2023	0	1	0	0	0	1	0	0	0	0	0	0	83
22048669	m	md	JP	2023	0	1	0	0	0	1	0	0	0	0	0	1	75
22048864	Not	md	US	2023	0	1	0	1	0	0	0	0	0	0	0	0	
22048916	m	md	JP	2023	0	1	0	0	1	0	0	0	0	0	0	0	83
22048965	m	ph	TH	2023	1	0	0	0	0	0	0	0	0	0	0	0	63.1
22048967	m	md	CA	2023	1	0	0	0	0	0	0	0	0	0	0	0	
22049164	f	hp	US	2023	0	1	1	0	0	0	0	0	0	0	0	0	70
22049179	f	hp	US	2023	0	1	1	0	0	0	0	0	0	0	0	0	79
22049523	Not	cn	JP	2023	0	0	0	0	0	0	0	0	0	0	0	0	78
22049695	m	md	CH	2023	0	0	0	0	0	0	0	0	0	0	0	0	70
22049697	f	md	DE	2023	0	1	0	0	1	0	0	0	0	0	0	1	65
22049721	Not	md	US	2023	0	1	0	1	0	0	0	0	0	0	0	0	
22050303	Not	md	US	2023	0	1	0	1	0	0	0	0	0	0	0	0	
22050364	Not	md	US	2023	0	1	0	1	0	0	0	0	0	0	0	0	
22050366	m	md	FR	2023	1	0	0	0	0	0	0	0	0	0	0	0	48
22050367	f	cn	JP	2023	1	0	0	0	0	0	0	0	0	0	0	0	
22050368	Not	md	US	2023	0	1	0	1	0	0	0	0	0	0	0	0	
22050369	Not	md	US	2023	0	1	0	1	0	0	0	0	0	0	0	0	
22050417	f	ph	US	2023	0	1	1	0	0	0	0	0	0	0	0	0	74
22050426	Not	md	US	2023	0	1	0	1	0	0	0	0	0	0	0	0	
22050527	Not	md	US	2023	0	1	0	1	0	0	0	0	0	0	0	0	
22050541	f	md	JP	2023	0	0	0	0	0	0	0	0	0	0	0	0	72
22050548	f	md	DE	2023	0	0	0	0	0	0	0	0	0	0	0	0	72
22050710	f	hp	IT	2023	0	1	0	1	0	0	0	0	0	0	0	0	76
22051210	f	cn	IT	2023	0	0	0	0	0	0	0	0	0	0	0	0	41
22051214	m	cn	CN	2023	0	0	0	0	0	0	0	0	0	0	0	0	60
22051650	m	md	JP	2023	0	0	0	0	0	0	0	0	0	0	0	0	
22051789	f	md	PR	2023	0	0	0	0	0	0	0	0	0	0	0	0	
22052119	m	md	US	2023	0	0	0	0	0	0	0	0	0	0	0	0	62
22052167	f	ph	JP	2023	0	0	0	0	0	0	0	0	0	0	0	0	60
22052180	f	ph	JP	2023	0	0	0	0	0	0	0	0	0	0	0	0	70
22052206	m	md	JP	2023	0	0	0	0	0	0	0	0	0	0	0	0	80
22052271	Not	md	JP	2023	0	0	0	0	0	0	0	0	0	0	0	0	

22052974	m	hp	US	2023	0	0	0	0	0	0	0	0	0	0	0	0	60
22053077	m	md	JP	2023	0	1	0	0	1	0	0	0	0	0	0	0	75
22053550	m	cn	IN	2023	0	1	0	0	0	1	0	0	0	0	0	0	65
22053705	m	cn	JP	2023	0	0	0	0	0	0	0	0	0	0	0	0	72
22053915	m	md	ME	2023	0	1	0	1	0	0	0	0	0	0	0	0	64
22054811	m	md	JP	2023	0	1	1	0	0	0	0	0	0	0	0	0	55
22054940	f	md	RU	2023	0	0	0	0	0	0	0	0	0	0	0	0	42
22055040	m	md	BE	2023	0	1	0	0	1	0	0	0	0	0	0	0	68
22055080	Not	md	IT	2023	0	1	1	0	0	0	0	0	0	0	0	0	
22055106	f	md	FR	2023	0	1	1	0	0	0	0	0	0	0	0	0	51
22055329	f	md	US	2023	0	0	0	0	0	0	0	0	0	0	0	0	57
22055379	m	md	JP	2023	0	0	0	0	0	0	0	0	0	0	0	0	85
22055476	f	md	FR	2023	1	0	0	0	0	0	0	0	0	0	0	0	71
22055485	f	cn	CA	2023	0	0	0	0	0	0	0	0	0	0	0	0	63
22055561	m	md	DE	2023	0	1	1	0	0	0	0	0	0	0	0	0	
22055707	Not	md	GB	2023	0	1	0	0	0	1	0	0	0	0	0	0	
22056296	m	hp	US	2023	0	1	0	0	1	0	0	0	0	0	0	0	69
22056576	m	md	JP	2023	0	1	0	0	1	0	0	0	0	0	0	0	63
22056775	m	md	JP	2023	0	0	0	0	0	0	0	0	0	0	0	0	78
22056906	f	md	JP	2023	0	1	0	0	0	1	0	0	0	0	0	0	
22057236	f	md	JP	2023	0	0	0	0	0	0	0	0	0	0	0	0	46
22057315	f	hp	US	2023	0	1	1	0	0	0	0	0	0	0	0	0	
22057475	f	md	JP	2023	0	0	0	0	0	0	0	0	0	0	0	0	40
22058121	f	md	KR	2023	0	0	0	0	0	0	0	0	0	0	0	0	53
22058252	f	md	CN	2023	0	0	0	0	0	0	0	0	0	0	0	0	68
22058503	m	md	JP	2023	0	1	0	0	0	1	0	0	0	0	0	0	
22058663	m	md	FR	2023	1	0	0	0	0	0	0	0	0	0	0	0	
22058741	f	cn	IN	2023	0	0	0	0	0	0	0	0	0	0	0	0	62
22059038	f	md	IT	2023	0	0	0	0	0	0	0	0	0	0	0	0	
22059039	f	md	BR	2023	0	0	0	0	0	0	0	0	0	0	0	0	
22059317	m	md	CN	2023	1	0	0	0	0	0	0	0	0	0	0	0	
22059358	m	cn	VN	2023	0	1	0	1	0	0	0	0	0	0	0	0	
22059945	f	md	IT	2023	0	0	0	0	0	0	0	0	0	0	0	0	73
22060127	m	cn	CA	2023	0	0	0	0	0	0	0	0	0	0	0	0	61

22060244	f	md	CA	2023	0	0	0	0	0	0	0	0	0	0	0	0	71
22060311	m	md	US	2023	0	0	0	0	0	0	0	0	0	0	0	0	
22060363	Not	hp	CN	2023	0	0	0	0	0	0	0	0	0	0	0	0	
22060435	Not	hp	CN	2023	0	0	0	0	0	0	0	0	0	0	0	0	
22060463	Not	hp	CN	2023	0	0	0	0	0	0	0	0	0	0	0	0	
22060464	Not	hp	CN	2023	0	0	0	0	0	0	0	0	0	0	0	0	
22060513	Not	hp	CN	2023	0	0	0	0	0	0	0	0	0	0	0	0	
22060529	Not	hp	CN	2023	0	0	0	0	0	0	0	0	0	0	0	0	
22060536	Not	ph	SG	2023	0	1	0	1	0	0	0	0	0	0	0	0	
22060725	m	md	DE	2023	0	0	0	0	0	0	0	0	0	0	0	0	72
22060802	Not	ph	TH	2023	1	0	0	0	0	0	0	0	0	0	0	0	
22060857	f	md	MY	2023	0	0	0	0	0	0	0	0	0	0	0	0	74
22060869	m	cn	TH	2023	1	0	0	0	0	0	0	0	0	0	0	0	72
22060870	f	md	JP	2023	0	1	1	0	0	0	0	0	0	0	0	0	74
22061166	m	cn	PH	2023	0	1	0	0	0	1	0	0	0	0	0	0	65
22061733	m	md	JP	2023	0	1	0	0	0	1	0	0	0	0	0	0	90
22061875	m	md	JP	2023	0	1	0	0	0	1	0	0	0	0	0	0	76
22061983	f	md	US	2023	0	1	0	0	1	0	0	0	0	0	0	0	76
22062014	m	ph	US	2023	0	0	0	0	0	0	0	0	0	0	0	0	
22062036	m	md	JP	2023	0	0	0	0	0	0	0	0	0	0	0	0	72
22062046	f	md	JP	2023	0	0	0	0	0	0	0	0	0	0	0	0	71
22062150	m	cn	US	2023	0	0	0	0	0	0	0	0	0	0	0	0	
22062786	m	md	JP	2023	0	1	0	0	0	1	0	0	0	0	0	0	90
22062910	m	md	JP	2023	0	1	0	0	0	1	0	0	0	0	0	0	60
22062927	f	hp	TW	2023	0	1	0	0	0	1	0	0	0	0	0	0	66
22062986	f	md	JP	2023	0	0	0	0	0	0	0	0	0	0	0	0	84
22063026	Not	hp	JP	2023	0	0	0	0	0	0	0	0	0	0	0	0	
22063149	m	hp	GR	2023	0	1	0	0	0	1	0	0	0	0	0	0	73
22063619	m	md	CN	2023	0	1	0	0	0	1	0	0	0	0	0	0	73
22063795	m	md	US	2023	0	0	0	0	0	0	0	0	0	0	0	0	33
22063905	m	cn	CN	2023	0	0	0	0	0	0	0	0	0	0	0	0	54
22064074	f	md	IN	2023	0	0	0	0	0	0	0	0	0	0	0	0	56
22064183	m	cn	CN	2023	0	1	0	1	0	0	0	0	0	0	0	0	54
22064252	f	md	JP	2023	0	0	0	0	0	0	0	0	0	0	0	0	59

22064806	f	cn	US	2023	0	1	0	0	1	0	0	0	0	0	0	0	59
22064947	m	cn	US	2023	0	0	0	0	0	0	0	0	0	0	0	0	
22065379	m	md	JP	2023	0	1	0	0	1	0	0	0	0	0	0	0	58
22065439	m	hp	US	2023	0	0	0	0	0	0	0	0	0	0	0	0	
22065570	f	md	TR	2023	0	0	0	0	0	0	0	0	0	0	0	0	66
22065577	f	md	US	2023	0	0	0	0	0	0	0	0	0	0	0	0	76
22065770	Not	ph	US	2023	0	0	0	0	0	0	0	0	0	0	0	0	
22065826	m	cn	EG	2023	1	0	0	0	0	0	0	0	0	0	0	0	
22066049	m	ph	TH	2023	1	0	0	0	0	0	0	0	0	0	0	0	72
22066068	Not	md	GB	2023	0	0	0	0	0	0	0	0	0	0	0	0	
22066111	m	md	CH	2023	0	0	0	0	0	0	0	0	0	0	0	0	
22066112	m	md	EE	2023	0	0	0	0	0	0	0	0	0	0	0	0	
22066404	f	md	JP	2023	1	0	0	0	0	0	0	0	0	0	0	0	75
22066593	f	md	PH	2023	0	0	0	0	0	0	0	0	0	0	0	0	39
22066663	m	md	JP	2023	0	1	1	0	0	0	0	0	0	0	0	0	80
22066896	f	cn	US	2023	0	0	0	0	0	0	0	0	0	0	0	0	53
22067092	Not	hp	JP	2023	0	0	0	0	0	0	0	0	0	0	0	0	
22067182	f	hp	US	2023	0	1	0	0	1	0	0	0	0	0	0	0	64
22067229	m	md	JP	2023	0	1	0	0	0	1	0	0	0	0	0	0	
22067289	m	cn	KW	2023	1	0	0	0	0	0	0	0	0	0	0	0	70.6
22068280	m	md	JP	2023	0	1	0	0	1	0	0	0	0	0	0	0	76
22068633	m	md	JP	2023	0	1	0	0	1	0	0	0	0	0	0	0	69
22068637	Not	md	US	2023	0	0	0	0	0	0	0	0	0	0	0	0	
22069800	f	hp	US	2023	0	1	1	0	0	0	0	0	0	0	0	0	49
22070269	f	md	US	2023	0	1	1	0	0	0	0	0	0	0	0	0	63
22070975	m	md	AU	2023	0	0	0	0	0	0	0	0	0	0	0	0	
22071290	Not	cn	GB	2023	0	0	0	0	0	0	0	0	0	0	0	0	
22071446	m	cn	BE	2023	0	0	0	0	0	0	0	0	0	0	0	0	
22071813	Not	ph	JP	2023	0	1	0	0	1	0	0	0	0	0	0	0	
22071893	f	md	PT	2023	0	0	0	0	0	0	0	0	0	0	0	0	58
22072895	m	md	JP	2023	0	1	0	0	0	1	0	0	0	0	0	0	60
22073368	Not	cn	US	2023	1	0	0	0	0	0	0	0	0	0	0	0	
22073591	m	md	IN	2023	0	0	0	0	0	0	0	0	0	0	0	0	78
22073677	Not	cn	JP	2023	1	0	0	0	0	0	0	0	0	0	0	0	

22073693	m	cn	JP	2023	0	0	0	0	0	0	0	0	0	0	0	0	70
22073822	m	hp	CN	2023	0	0	0	0	0	0	0	0	0	0	0	0	66
22073975	Not	ph	JP	2023	1	0	0	0	0	0	0	0	0	0	0	0	
22074045	Not	cn	CA	2023	0	0	0	0	0	0	0	0	0	0	0	0	70
22074300	m	hp	JP	2023	0	0	0	0	0	0	0	0	0	0	0	0	49
22074497	m	md	JP	2023	1	0	0	0	0	0	0	0	0	0	0	0	74
22074858	f	hp	TH	2023	1	0	0	0	0	0	0	0	0	0	0	0	
22075688	m	md	RU	2023	0	0	0	0	0	0	0	0	0	0	0	0	66
22076011	f	md	TW	2023	0	0	0	0	0	0	0	0	0	0	0	0	43
22076678	m	md	IT	2023	0	0	0	0	0	0	0	0	0	0	0	0	
22076776	m	md	JP	2023	0	1	0	0	0	1	0	0	0	0	0	0	
22077213	f	md	US	2023	0	1	0	0	1	0	0	0	0	0	0	0	
22077274	f	md	JP	2023	0	1	0	0	1	0	0	0	0	0	0	0	70
22077614	m	md	HR	2023	0	1	0	0	0	1	0	0	0	0	0	1	
22078067	f	ph	JP	2023	1	0	0	0	0	0	0	0	0	0	0	0	73
22078213	m	cn	TH	2023	0	0	0	0	0	0	0	0	0	0	0	0	60
22079088	f	md	JP	2023	0	1	1	0	0	0	0	0	0	0	0	0	80
22079640	m	cn	TW	2023	0	1	0	0	1	0	0	0	0	0	0	0	
22079762	m	md	US	2023	0	0	0	0	0	0	0	0	0	0	0	0	72
22079764	f	md	US	2023	0	0	0	0	0	0	0	0	0	0	0	0	69
22079765	f	md	US	2023	0	0	0	0	0	0	0	0	0	0	0	0	69
22079766	f	md	US	2023	0	0	0	0	0	0	0	0	0	0	0	0	38
22079907	m	cn	PE	2023	1	0	0	0	0	0	0	0	0	0	0	0	87.3
22080072	f	cn	KR	2023	0	0	0	0	0	0	0	0	0	0	0	0	76
22080194	f	cn	DE	2023	0	0	0	0	0	0	0	0	0	0	0	0	73
22080433	m	md	FR	2023	0	1	0	0	0	1	0	0	0	0	0	0	76
22081765	m	hp	US	2023	0	1	0	0	0	1	0	0	0	0	0	0	61
22081780	m	md	FR	2023	0	0	0	0	0	0	0	0	0	0	0	0	19
22081797	f	md	US	2023	1	0	0	0	0	0	0	0	0	0	0	0	
22082452	f	cn	CN	2023	0	0	0	0	0	0	0	0	0	0	0	0	58
22082494	f	md	US	2023	0	1	0	1	0	0	0	0	0	0	0	0	60
22082537	Not	ph	JP	2023	1	0	0	0	0	0	0	0	0	0	0	0	
22082561	f	md	GB	2023	0	1	0	0	0	1	0	0	0	0	0	0	53
22082619	f	cn	SG	2023	0	1	0	0	0	1	0	0	0	0	0	0	

22082845	f	md	DE	2023	0	1	0	0	1	0	0	0	0	0	0	0	69
22082964	f	md	DE	2023	0	0	0	0	0	0	0	0	0	0	0	0	74
22083099	m	md	BR	2023	0	1	0	0	0	1	0	0	0	0	0	0	
22083299	m	cn	IN	2023	0	1	0	0	0	1	0	0	0	0	0	0	54
22083546	f	md	DE	2023	0	0	0	0	0	0	0	0	0	0	0	0	33
22083805	m	md	DE	2023	0	0	0	0	0	0	0	0	0	0	0	0	65
22084023	Not	hp	JP	2023	0	0	0	0	0	0	0	0	0	0	0	0	
22085111	m	md	AT	2023	0	0	0	0	0	0	0	0	0	0	0	0	70
22085152	f	md	JP	2023	1	0	0	0	0	0	0	0	0	0	0	1	80
22085189	m	md	DE	2023	0	0	0	0	0	0	0	0	0	0	0	0	
22085636	m	cn	US	2023	0	0	0	0	0	0	0	0	0	0	0	0	62
22085880	m	md	JP	2023	0	1	1	0	0	0	0	0	0	0	0	0	80
22086316	f	cn	US	2023	0	1	1	0	0	0	0	0	0	0	0	0	
22086492	m	md	US	2023	0	0	0	0	0	0	0	0	0	0	0	0	46
22086502	m	md	IN	2023	0	1	0	0	0	1	0	0	0	0	0	0	74
22086513	Not	md	JP	2023	0	0	0	0	0	0	0	0	0	0	0	0	60
22086518	m	md	JP	2023	0	1	0	0	0	1	0	0	0	0	0	0	80
22086903	m	md	JP	2023	0	1	1	0	0	0	0	0	0	0	0	1	76
22086964	m	md	JP	2023	0	0	0	0	0	0	0	0	0	0	0	0	90
22086991	f	cn	CA	2023	1	0	0	0	0	0	0	0	0	0	0	0	66.4
22087202	m	md	JP	2023	0	1	0	0	0	1	0	0	0	0	0	0	46
22087211	m	md	JP	2023	0	1	0	0	0	1	0	0	0	0	0	0	74
22087471	m	md	BE	2023	0	1	0	0	1	0	0	0	0	0	0	0	73
22087481	f	md	JP	2023	0	0	0	0	0	0	0	0	0	0	0	0	32
22087853	Not	cn	US	2023	0	1	0	0	0	1	0	0	0	0	0	0	
22088264	Not	md	IT	2023	0	1	0	0	1	0	0	0	0	0	0	0	
22088272	m	md	FR	2023	0	1	0	1	0	0	0	0	0	0	0	1	70
22088526	f	md	DE	2023	0	1	0	0	1	0	0	0	0	0	0	0	69
22088950	f	cn	ID	2023	1	0	0	0	0	0	0	0	0	0	0	0	
22089815	m	cn	CA	2023	0	0	0	0	0	0	0	0	0	0	0	0	76
22089874	Not	hp	CN	2023	0	0	0	0	0	0	0	0	0	0	0	0	
22090039	f	md	JP	2023	1	0	0	0	0	0	0	0	0	0	0	0	81
22090225	f	cn	KR	2023	0	0	0	0	0	0	0	0	0	0	0	0	79
22090509	m	md	KR	2023	0	0	0	0	0	0	0	0	0	0	0	0	72

22090530	f	md	JP	2023	0	0	0	0	0	0	0	0	0	0	0	0	67
22090549	f	hp	FR	2023	0	1	1	0	0	0	0	0	0	0	0	0	86
22090555	Not	md	JP	2023	0	1	1	0	0	0	0	0	0	0	0	0	74
22090596	f	cn	ID	2023	1	0	0	0	0	0	0	0	0	0	0	0	42.2
22090601	Not	md	US	2023	1	0	0	0	0	0	0	0	0	0	0	0	
22091397	f	md	AT	2023	0	1	1	0	0	0	0	0	0	0	0	0	71
22091555	m	md	JP	2023	0	1	1	0	0	0	0	0	0	0	0	0	90
22091892	f	md	US	2023	0	0	0	0	0	0	0	0	0	0	0	0	75
22092125	f	md	JP	2023	0	0	0	0	0	0	0	0	0	0	0	0	
22092177	f	ph	US	2023	0	0	0	0	0	0	0	0	0	0	0	0	73
22092593	f	cn	ID	2023	1	0	0	0	0	0	0	0	0	0	0	0	76.8
22092981	f	md	JP	2023	0	0	0	0	0	0	0	0	0	0	0	0	80
22093419	Not	md	GB	2023	0	1	0	0	0	1	0	0	0	0	0	0	
22093493	Not	md	JP	2023	0	1	0	1	0	0	0	0	0	0	0	0	
22093497	m	md	US	2023	1	0	0	0	0	0	0	0	0	0	0	0	
22093785	m	hp	US	2023	0	1	1	0	0	0	0	0	0	0	0	0	58
22093807	m	md	FR	2023	0	1	0	1	0	0	0	0	0	0	0	1	70
22093921	m	ph	JP	2023	1	0	0	0	0	0	0	0	0	0	0	0	61
22094040	m	hp	US	2023	0	1	0	1	0	0	0	0	0	0	0	0	72
22094088	m	ph	JP	2023	0	0	0	0	0	0	0	0	0	0	0	0	
22094089	m	cn	ID	2023	0	0	0	0	0	0	0	0	0	0	0	0	78
22094138	Not	hp	US	2023	1	0	0	0	0	0	0	0	0	0	0	0	65
22094219	m	md	JP	2023	0	1	0	0	1	0	0	0	0	0	0	0	40
22094237	Not	md	JP	2023	0	1	1	0	0	0	0	0	0	0	0	0	80
22094765	Not	cn	US	2023	1	0	0	0	0	0	0	0	0	0	0	0	
22095487	Not	md	ID	2023	0	0	0	0	0	0	0	0	0	0	0	0	
22095547	m	hp	TR	2023	0	0	0	0	0	0	0	0	0	0	0	0	61
22095589	m	cn	AR	2023	0	0	0	0	0	0	0	0	0	0	0	0	78
22095864	m	md	CA	2023	0	0	0	0	0	0	0	0	0	0	0	0	38
22096003	f	cn	TH	2023	1	0	0	0	0	0	0	0	0	0	0	0	86.1
22096349	f	cn	CA	2023	0	0	0	0	0	0	0	0	0	0	0	0	64
22096637	m	hp	IL	2023	0	0	0	0	0	0	0	0	0	0	0	0	
22097087	f	md	JP	2023	0	0	0	0	0	0	0	0	0	0	0	0	90
22097133	f	cn	IT	2023	1	0	0	0	0	0	0	0	0	0	0	0	55.1

22097219	m	md	JP	2023	0	1	0	0	1	0	0	0	0	0	0	0	70
22097670	m	md	IT	2023	0	0	0	0	0	0	0	0	0	0	0	0	
22097790	f	md	DE	2023	0	1	1	0	0	0	0	0	0	0	0	0	61
22098128	Not	md	JP	2023	0	1	0	1	0	0	0	0	0	0	0	0	
22098182	f	cn	US	2023	0	0	0	0	0	0	0	0	0	0	0	0	
22098264	f	cn	US	2023	1	0	0	0	0	0	0	0	0	0	0	0	66
22098893	f	md	JP	2023	0	0	0	0	0	0	0	0	0	0	0	0	74
22098955	f	md	JP	2023	0	1	0	0	0	1	0	0	0	0	0	0	100
22099159	f	md	GB	2023	0	1	0	0	0	1	0	0	0	0	0	0	65
22099214	f	hp	US	2023	0	1	1	0	0	0	0	0	0	0	0	0	59
22099280	Not	ph	JP	2023	0	0	0	0	0	0	0	0	0	0	0	0	
22099282	m	md	ES	2023	0	0	0	0	0	0	0	0	0	0	0	0	74
22099384	f	cn	JP	2023	0	0	0	0	0	0	0	0	0	0	0	0	75
22099400	f	cn	AU	2023	0	1	1	0	0	0	0	0	0	0	0	1	76
22099402	m	md	JP	2023	0	1	1	0	0	0	0	0	0	0	0	0	69
22099409	m	md	CH	2023	0	0	0	0	0	0	0	0	0	0	0	0	
22099469	f	md	ES	2023	0	1	1	0	0	0	0	0	0	0	0	0	77
22099491	Not	md	HK	2023	0	0	0	0	0	0	0	0	0	0	0	0	
22099886	m	md	TW	2023	0	0	0	0	0	0	0	0	0	0	0	0	61
22099993	f	cn	US	2023	0	0	0	0	0	0	0	0	0	0	0	0	
22100291	f	hp	NL	2023	0	0	0	0	0	0	0	0	0	0	0	0	70
22100511	m	cn	ID	2023	1	0	0	0	0	0	0	0	0	0	0	0	51
22100536	m	md	CN	2023	0	1	0	1	0	0	0	0	0	0	0	0	70.7
22100575	f	md	JP	2023	1	0	0	0	0	0	0	0	0	0	0	1	68
22100581	m	hp	US	2023	0	1	0	1	0	0	0	0	0	0	0	0	68
22100773	Not	hp	GB	2023	0	1	0	0	0	1	0	0	0	0	0	0	
22100836	m	hp	CA	2023	1	0	0	0	0	0	0	0	0	0	0	0	62
22101507	m	ph	JP	2023	0	1	1	0	0	0	0	0	0	0	0	0	70
22101549	f	cn	US	2023	1	0	0	0	0	0	0	0	0	0	0	0	
22101756	f	cn	TH	2023	0	1	0	0	0	1	0	0	0	0	0	0	
22101860	f	hp	GB	2023	1	0	0	0	0	0	0	0	0	0	0	0	76
22101933	Not	hp	JP	2023	0	0	0	0	0	0	0	0	0	0	0	0	
22102063	Not	md	US	2023	0	1	0	1	0	0	0	0	0	0	0	0	
22102992	m	md	JP	2023	0	1	0	1	0	0	0	0	0	0	0	0	61

22103061	m	md	US	2023	1	0	0	0	0	0	0	0	0	0	0	0	
22103193	f	cn	CN	2023	0	0	0	0	0	0	0	0	0	0	0	0	73
22103217	m	md	RO	2023	0	1	0	0	0	1	0	0	0	0	0	0	
22103400	m	md	JP	2023	0	1	0	0	1	0	0	0	0	0	0	0	73
22103519	f	cn	TH	2023	1	0	0	0	0	0	0	0	0	0	0	0	54.8
22103592	Not	ph	JP	2023	1	0	0	0	0	0	0	0	0	0	0	0	
22103615	m	md	JP	2023	0	0	0	0	0	0	0	0	0	0	0	0	39
22103625	f	md	FR	2023	0	0	0	0	0	0	0	0	0	0	0	0	70
22103654	m	cn	CN	2023	0	1	0	1	0	0	0	0	0	0	0	0	64
22103753	m	md	JP	2023	0	1	1	0	0	0	0	0	0	0	0	0	80
22103933	Not	md	ID	2023	0	0	0	0	0	0	0	0	0	0	0	0	
22104312	Not	md	JP	2023	0	1	0	1	0	0	0	0	0	0	0	0	
22104395	Not	hp	JP	2023	0	0	0	0	0	0	0	0	0	0	0	0	
22104598	m	cn	US	2023	0	0	0	0	0	0	0	0	0	0	0	0	
22104641	f	md	JP	2023	0	0	0	0	0	0	0	0	0	0	0	0	78
22104736	f	hp	CA	2023	0	1	1	0	0	0	0	0	0	0	0	0	38
22105284	Not	cn	SG	2023	1	0	0	0	0	0	0	0	0	0	0	0	84.9
22105348	f	md	JP	2023	0	1	0	0	1	0	0	0	0	0	0	0	61
22105675	m	hp	GB	2023	0	0	0	0	0	0	0	0	0	0	0	0	76
22106504	m	cn	US	2023	0	0	0	0	0	0	0	0	0	0	0	0	66
22107534	m	md	VN	2023	0	1	0	0	0	1	0	0	0	0	0	0	67
22108288	f	hp	NL	2023	0	0	0	0	0	0	0	0	0	0	0	0	70
22108347	m	md	GB	2023	0	1	0	1	0	0	0	0	0	0	0	0	69.5
22108398	Not	hp	CN	2023	1	0	0	0	0	0	0	0	0	0	0	0	
22108729	m	md	JP	2023	0	1	1	0	0	0	0	0	0	0	0	0	
22108902	m	md	BG	2023	0	0	0	0	0	0	0	0	0	0	0	0	71
22109056	Not	md	US	2023	0	1	0	0	0	1	0	0	0	0	0	0	
22109078	Not	md	US	2023	0	1	0	0	0	1	0	0	0	0	0	0	
22109103	m	md	FI	2023	0	0	0	0	0	0	0	0	0	0	0	0	
22109167	Not	cn	SG	2023	1	0	0	0	0	0	0	0	0	0	0	0	88
22109475	m	md	JP	2023	0	1	0	0	0	1	0	0	0	0	0	0	74
22109759	f	cn	ID	2023	1	0	0	0	0	0	0	0	0	0	0	0	80.5
22109819	m	cn	CN	2023	0	1	0	1	0	0	0	0	0	0	0	0	76
22109977	f	cn	IN	2023	0	0	0	0	0	0	0	0	0	0	0	0	49

22110062	f	cn	CN	2023	0	1	0	1	0	0	0	0	0	0	0	0	88
22110284	m	cn	CN	2023	0	1	0	1	0	0	0	0	0	0	0	0	78
22110308	m	hp	CN	2023	1	0	0	0	0	0	0	0	0	0	0	0	67
22110511	m	hp	US	2023	0	0	0	0	0	0	0	0	0	0	0	0	66
22110546	Not	md	US	2023	0	1	0	0	0	1	0	0	0	0	0	0	
22110721	m	cn	ID	2023	1	0	0	0	0	0	0	0	0	0	0	0	54.7
22110801	m	hp	US	2023	0	1	0	0	1	0	0	0	0	0	0	1	63
22110945	m	cn	CN	2023	0	1	0	1	0	0	0	0	0	0	0	0	68
22110952	m	md	GR	2023	0	1	1	0	0	0	0	0	0	0	0	0	60
22110955	f	cn	CN	2023	0	1	0	1	0	0	0	0	0	0	0	0	65
22110960	m	cn	CN	2023	0	1	0	1	0	0	0	0	0	0	0	0	70
22111044	m	hp	ES	2023	1	0	0	0	0	0	0	0	0	0	0	0	71
22111106	Not	hp	US	2023	0	0	0	0	0	0	1	0	0	0	1	0	
22111145	m	md	JP	2023	0	1	1	0	0	0	0	0	0	0	0	0	77
22111364	f	md	US	2023	0	1	1	0	0	0	0	0	0	0	0	0	66
22111747	f	md	DE	2023	0	1	0	0	1	0	0	0	0	0	0	0	76
22111867	f	md	GB	2023	0	0	0	0	0	0	0	0	0	0	0	0	68
22111961	f	md	US	2023	0	0	0	0	0	0	0	0	0	0	0	0	61
22112149	m	md	DE	2023	0	1	0	0	1	0	0	0	0	0	0	0	68
22112243	Not	md	GB	2023	0	0	0	0	0	0	0	0	0	0	0	0	
22112421	Not	hp	GB	2023	0	1	0	0	0	1	0	0	0	0	0	0	
22112427	f	md	PL	2023	0	1	0	1	0	0	0	0	0	0	0	0	68
22113200	m	md	IQ	2023	0	1	1	0	0	0	0	0	0	0	0	0	69
22113234	m	md	JP	2023	0	1	0	0	1	0	0	0	0	0	0	0	79
22113341	Not	md	US	2023	0	1	0	0	1	0	0	0	0	0	0	1	
22113501	Not	ph	JP	2023	1	0	0	0	0	0	0	0	0	0	0	0	
22113667	m	hp	CN	2023	1	0	0	0	0	0	0	0	0	0	0	0	62
22114607	m	md	JP	2023	0	1	1	0	0	0	0	0	0	0	0	0	
22114616	m	md	DE	2023	0	1	0	0	1	0	0	0	0	0	0	0	84
22114664	m	ph	US	2023	0	0	0	0	0	0	0	0	0	0	0	0	
22114884	m	md	IQ	2023	0	1	1	0	0	0	0	0	0	0	0	0	75
22114899	f	hp	ZA	2023	0	0	0	0	0	0	0	0	0	0	0	0	69
22115005	m	md	JP	2023	0	1	0	1	0	0	0	0	0	0	0	0	56
22115244	m	md	JP	2023	0	1	1	0	0	0	0	0	0	0	0	0	

22115331	m	md	RU	2023	0	1	0	1	0	0	0	0	0	0	0	0	
22115863	m	md	DE	2023	0	0	0	0	0	0	0	0	0	0	0	0	65
22116052	Not	md	JP	2023	1	0	0	0	0	0	0	0	0	0	0	1	
22116278	m	md	IN	2023	0	1	0	0	0	1	0	0	0	0	0	0	73
22116336	f	hp	CN	2023	1	0	0	0	0	0	0	0	0	0	0	0	45
22116338	f	md	US	2023	0	0	0	0	0	0	0	0	0	0	0	0	
22116471	m	md	JP	2023	0	1	0	0	1	0	0	0	0	0	0	0	80
22117281	m	md	BE	2023	0	0	0	0	0	0	0	0	0	0	0	0	
22117420	f	md	US	2023	0	1	0	0	0	1	0	0	0	0	0	0	64
22117769	m	cn	PK	2023	0	1	0	0	0	1	0	0	0	0	0	0	59
22117770	f	cn	PK	2023	0	1	0	0	0	1	0	0	0	0	0	0	79
22117807	f	cn	CA	2023	0	0	0	0	0	0	0	0	0	0	0	0	79
22117903	Not	ph	US	2023	0	0	0	0	0	0	0	0	0	0	0	0	
22118263	m	hp	US	2023	0	1	1	0	0	0	0	0	0	0	0	0	75
22118537	m	hp	IT	2023	0	1	0	0	1	0	0	0	0	0	0	0	64
22118584	Not	md	JP	2023	0	1	0	0	0	1	0	0	0	0	0	0	80
22119002	m	hp	IN	2023	0	1	1	0	0	0	0	0	0	0	0	0	65
22119467	m	md	IT	2023	0	1	1	0	0	0	0	0	0	0	0	0	
22120684	f	md	CA	2023	1	0	0	0	0	0	0	0	0	0	0	1	87
22120947	f	md	AU	2023	0	0	0	0	0	0	0	0	0	0	0	0	
22121130	f	cn	DE	2023	0	0	0	0	0	0	0	0	0	0	0	0	
22121748	m	md	CN	2023	0	0	0	0	0	0	0	0	0	0	0	0	47
22121846	f	md	GB	2023	0	1	0	0	0	1	0	0	0	0	0	0	71
22121973	m	md	JP	2023	0	0	0	0	0	0	0	0	0	0	0	0	76
22121974	m	md	JP	2023	0	0	0	0	0	0	0	0	0	0	0	0	80
22122156	m	hp	CN	2023	1	0	0	0	0	0	0	0	0	0	0	0	77
22122719	m	md	JP	2023	0	1	0	0	1	0	0	0	0	0	0	0	81
22123260	f	md	RU	2023	0	1	0	0	0	1	0	0	0	0	0	1	42
22123302	m	md	JP	2023	0	1	0	0	1	0	0	0	0	0	0	0	76
22123514	f	ph	US	2023	0	0	0	0	0	0	0	0	0	0	0	0	
22123598	f	md	JP	2023	0	1	0	0	0	1	0	0	0	0	0	0	79
22123715	f	md	TH	2023	0	1	0	0	0	1	0	0	0	0	0	0	84
22123733	m	md	JP	2023	0	1	1	0	0	0	0	0	0	0	0	0	80
22123821	m	cn	US	2023	1	0	0	0	0	0	0	0	0	0	0	0	

22123823	m	md	US	2023	0	1	1	0	0	0	0	0	0	0	0	0		
22124197	f	md	US	2023	0	0	0	0	0	0	0	0	0	0	0	0		
22124535	f	cn	US	2023	1	0	0	0	0	0	0	0	0	0	0	0		
22124728	f	md	MY	2023	1	0	0	0	0	0	0	0	0	0	0	0		
22124944	m	md	JP	2023	0	1	0	1	0	0	0	0	0	0	0	1	62	
22125043	m	md	GB	2023	0	0	0	0	0	0	0	0	0	0	0	0		
22125370	m	md	CN	2023	0	1	0	1	0	0	0	0	0	0	0	0	61	
22125672	m	ph	US	2023	0	0	0	0	0	0	0	0	0	0	0	0		
22125890	Not	md	IT	2023	0	0	0	0	0	0	0	0	0	0	0	0		
22125894	m	cn	MY	2023	1	0	0	0	0	0	0	0	0	0	0	0	76	
22126451	f	md	JP	2023	0	1	1	0	0	0	0	0	0	0	0	0	80	
22126631	m	md	JP	2023	0	1	1	0	0	0	0	0	0	0	0	0	81	
22126680	m	cn	TH	2023	0	0	0	0	0	0	0	0	0	0	0	0	46	
22126706	m	ph	JP	2023	0	1	1	0	0	0	0	0	0	0	0	0	80	
22127928	m	md	JP	2023	0	1	0	0	1	0	0	0	0	0	0	0	71	
22128669	m	md	JP	2023	0	1	0	0	1	0	0	0	0	0	0	0	76	
22128736	m	md	RO	2023	0	1	0	0	0	1	0	0	0	0	0	0		
22129303	m	md	JP	2023	0	0	0	0	0	0	0	0	0	0	0	0	70	
22129794	m	hp	CN	2023	0	1	0	1	0	0	0	0	0	0	0	0	70	
22130108	m	cn	PK	2023	0	0	0	0	0	0	0	0	0	0	0	0	46	
22130420	m	cn	EG	2023	0	0	0	0	0	0	0	0	0	0	0	0	59	
22130429	f	md	DO	2023	0	0	0	0	0	0	0	0	0	0	0	0	41	
22130819	m	md	KR	2023	0	0	0	0	0	0	0	0	0	0	0	0	74	
22131077	f	md	IL	2023	0	0	0	0	0	0	0	0	0	0	0	0	55	
22131433	f	hp	DE	2023	0	1	1	0	0	0	0	0	0	0	0	0	33	
22131658	m	md	GB	2023	0	0	0	0	0	0	0	0	0	0	0	0		
22131838	Not	md	RU	2023	1	0	0	0	0	0	0	0	0	0	0	0		
22131969	m	ph	JP	2023	0	1	1	0	0	0	0	0	0	0	0	0	77	
22132697	Not	ph	JP	2023	1	0	0	0	0	0	0	0	0	0	0	0		
22133045	m	md	DE	2023	0	0	0	0	0	0	0	0	0	0	0	0	65	
22133900	m	md	JP	2023	0	1	0	0	0	1	0	0	0	0	0	0	61	
22134024	Not	md	JP	2023	1	0	0	0	0	0	0	0	0	0	0	0		
22134028	m	md	BG	2023	0	1	0	1	0	0	0	0	0	0	0	0	75.4	
22134527	f	cn	US	2023	1	0	0	0	0	0	0	0	0	0	0	0	77	

22134627	m	cn	TW	2023	0	0	0	0	0	0	0	0	0	0	0	0	56
22135156	m	cn	PH	2023	0	1	0	0	0	1	0	0	0	0	0	0	75
22135187	f	md	DE	2023	0	1	0	0	1	0	0	0	0	0	0	0	74
22135329	m	hp	US	2023	0	0	0	0	0	0	0	0	0	0	0	0	62
22135434	m	cn	US	2023	0	0	0	0	0	0	0	0	0	0	0	0	
22136731	m	hp	GB	2023	1	0	0	0	0	0	0	0	0	0	0	0	72
22136744	m	md	JP	2023	0	1	1	0	0	0	0	0	0	0	0	0	70
22137182	f	md	DE	2023	0	1	0	0	1	0	0	0	0	0	0	0	69
22137313	f	cn	PK	2023	0	1	0	0	0	1	0	0	0	0	0	0	62
22137791	m	md	TR	2023	0	0	0	0	0	0	0	0	0	0	0	0	
22138147	Not	md	US	2023	0	1	0	0	1	0	0	0	0	0	0	0	
22138411	f	md	DK	2023	0	1	0	0	0	1	0	0	0	0	0	0	80
22138921	f	cn	AT	2023	0	0	0	0	0	0	0	0	0	0	0	0	80
22139060	f	hp	DE	2023	0	1	0	0	1	0	0	0	0	0	0	0	63
22139288	f	md	JP	2023	0	0	0	0	0	0	0	0	0	0	0	0	90
22139434	Not	md	TW	2023	0	0	0	0	0	0	0	0	0	0	0	0	67
22139757	Not	md	JP	2023	0	0	0	0	0	0	0	0	0	0	0	0	
22139829	m	cn	CN	2023	0	1	0	1	0	0	0	0	0	0	0	0	72
22140117	m	md	RO	2023	0	1	1	0	0	0	0	0	0	0	0	1	59
22140430	Not	md	JP	2023	1	0	0	0	0	0	0	0	0	0	0	0	
22141177	m	md	RO	2023	0	1	0	0	0	1	0	0	0	0	0	0	
22141893	Not	hp	NO	2023	0	1	0	0	0	1	0	0	0	0	0	0	
22142014	m	hp	IT	2023	0	0	0	0	0	0	0	0	0	0	0	0	46
22142040	m	md	RO	2023	0	1	1	0	0	0	0	0	0	0	0	1	59
22142205	Not	md	HK	2023	0	0	0	0	0	0	0	0	0	0	0	0	
22142207	f	cn	AR	2023	0	0	0	0	0	0	0	0	0	0	0	0	74
22142228	f	hp	AU	2023	0	0	0	0	0	0	0	0	0	0	0	0	44
22142420	m	md	US	2023	0	0	0	0	0	0	0	0	0	0	0	0	77
22142586	Not	hp	NO	2023	0	1	0	0	0	1	0	0	0	0	0	0	
22142589	Not	md	IT	2023	0	0	0	0	0	0	0	0	0	0	0	0	
22142634	m	md	RO	2023	0	1	1	0	0	0	0	0	0	0	0	1	59
22143024	m	md	TR	2023	0	0	0	0	0	0	0	0	0	0	0	0	
22143075	f	md	FR	2023	0	0	0	0	0	0	0	0	0	0	0	0	52
22143812	m	md	ES	2023	0	0	0	0	0	0	0	0	0	0	0	0	

22143834	m	md	JP	2023	0	1	0	0	1	0	0	0	0	0	0	0	72
22143941	m	md	JP	2023	0	1	0	0	1	0	0	0	0	0	0	0	73
22143961	m	md	JP	2023	0	1	0	0	1	0	0	0	0	0	0	0	68
22143996	f	md	JP	2023	0	0	0	0	0	0	0	0	0	0	0	0	62
22144340	m	md	JP	2023	0	1	0	0	1	0	0	0	0	0	0	0	72
22144382	m	md	JP	2023	0	1	0	0	1	0	0	0	0	0	0	0	70
22144383	m	md	JP	2023	0	1	0	0	1	0	0	0	0	0	0	0	57
22144644	m	md	JP	2023	0	1	0	0	1	0	0	0	0	0	0	0	66
22144875	m	md	JP	2023	0	1	0	0	0	1	0	0	0	0	0	0	
22145008	m	md	JP	2023	0	1	0	0	1	0	0	0	0	0	0	0	68
22145231	m	md	JP	2023	0	1	0	0	1	0	0	0	0	0	0	0	68
22145343	Not	hp	NO	2023	0	1	0	0	0	1	0	0	0	0	0	0	
22145587	f	ph	JP	2023	1	0	0	0	0	0	0	0	0	0	0	0	78
22145752	Not	md	JP	2023	0	1	0	0	1	0	0	0	0	0	0	0	
22145788	Not	md	JP	2023	0	1	0	0	1	0	0	0	0	0	0	0	
22145812	Not	md	JP	2023	0	1	0	0	1	0	0	0	0	0	0	0	
22145855	Not	md	JP	2023	0	1	0	0	1	0	0	0	0	0	0	0	
22146011	Not	hp	NO	2023	0	1	0	0	0	1	0	0	0	0	0	0	
22146454	f	md	JP	2023	1	0	0	0	0	0	0	0	0	0	0	1	80
22146456	f	md	JP	2023	1	0	0	0	0	0	0	0	0	0	0	0	80
22146617	f	md	AR	2023	0	0	0	0	0	0	0	0	0	0	0	0	50
22146909	Not	hp	NO	2023	0	1	0	0	0	1	0	0	0	0	0	0	
22147121	m	md	RO	2023	0	1	0	0	0	1	0	0	0	0	0	0	
22147124	m	md	JP	2023	0	1	0	0	1	0	0	0	0	0	0	0	73
22147128	Not	md	JP	2023	0	1	0	0	1	0	0	0	0	0	0	0	
22147132	Not	md	JP	2023	0	1	0	0	1	0	0	0	0	0	0	0	
22147311	Not	md	JP	2023	0	0	0	0	0	0	0	0	0	0	0	0	
22147342	f	cn	JP	2023	1	0	0	0	0	0	0	0	0	0	0	0	
22147760	Not	ph	US	2023	0	1	0	0	1	0	0	0	0	0	0	1	70
22147853	f	hp	FR	2023	1	0	0	0	0	0	0	0	0	0	0	0	52
22147904	Not	hp	NO	2023	0	1	0	0	0	1	0	0	0	0	0	0	
22147973	m	md	JP	2023	1	0	0	0	0	0	0	0	0	0	0	0	73
22148033	m	md	DE	2023	0	1	1	0	0	0	0	0	0	0	0	0	69
22148184	Not	hp	NO	2023	0	1	0	0	0	1	0	0	0	0	0	0	

22148220	Not	hp	NO	2023	0	1	0	0	0	1	0	0	0	0	0	0	
22149019	Not	md	JP	2023	0	1	0	0	1	0	0	0	0	0	0	0	
22149169	m	md	JP	2023	0	1	0	0	1	0	0	0	0	0	0	0	67
22149203	m	md	JP	2023	0	1	0	0	0	1	0	0	0	0	0	0	90
22149398	m	md	JP	2023	0	1	0	0	1	0	0	0	0	0	0	0	67
22149521	Not	md	GB	2023	0	1	0	0	0	1	0	0	0	0	0	0	
22149769	f	hp	FR	2023	1	0	0	0	0	0	0	0	0	0	0	0	52
22149851	f	hp	SG	2023	0	0	0	0	0	0	0	0	0	0	0	0	
22149872	f	hp	AU	2023	0	0	0	0	0	0	0	0	0	0	0	0	
22150127	m	hp	AU	2023	0	0	0	0	0	0	0	0	0	0	0	0	
22151056	f	md	GB	2023	0	1	0	0	0	1	0	0	0	0	0	0	79
22151866	m	hp	AU	2023	0	1	1	0	0	0	0	0	0	0	0	0	64
22152312	Not	md	ID	2023	0	1	0	0	0	1	0	0	0	0	0	0	
22152344	Not	md	JP	2023	0	1	0	0	0	1	0	0	0	0	0	0	
22152803	m	md	DE	2023	0	1	1	0	0	0	0	0	0	0	0	0	
22152894	m	cn	SG	2023	0	1	0	0	0	1	0	0	0	0	0	0	
22153082	m	md	US	2023	0	0	0	0	0	0	0	0	0	0	0	0	59
22153209	m	hp	AU	2023	0	0	0	0	0	0	0	0	0	0	0	0	
22153216	m	md	AU	2023	0	0	0	0	0	0	0	0	0	0	0	0	
22153226	f	md	TR	2023	0	0	0	0	0	0	0	0	0	0	0	0	
22153322	m	md	JP	2023	0	1	0	0	1	0	0	0	0	0	0	0	74
22153495	f	hp	AU	2023	0	0	0	0	0	0	0	0	0	0	0	0	
22153496	m	hp	AU	2023	0	0	0	0	0	0	0	0	0	0	0	0	
22153514	m	md	JP	2023	0	0	0	0	0	0	0	0	0	0	0	0	72
22153549	m	md	JP	2023	0	1	0	0	1	0	0	0	0	0	0	0	69
22153569	m	md	TR	2023	0	0	0	0	0	0	0	0	0	0	0	0	
22153760	f	md	JP	2023	0	0	0	0	0	0	0	0	0	0	0	0	40
22153811	m	md	JP	2023	0	1	0	0	1	0	0	0	0	0	0	1	76
22154165	m	cn	US	2023	0	1	1	0	0	0	0	0	0	0	0	0	
22154475	f	md	JP	2023	0	1	1	0	0	0	0	0	0	0	0	0	74
22154730	f	cn	US	2023	1	0	0	0	0	0	0	0	0	0	0	0	68
22155176	m	md	JP	2023	0	1	0	0	1	0	0	0	0	0	0	0	62
22156061	f	md	US	2023	0	1	0	0	0	1	0	0	0	0	0	0	81
22156271	Not	cn	SG	2023	1	0	0	0	0	0	0	0	0	0	0	0	57.6

22156656	Not	md	JP	2023	1	0	0	0	0	0	0	0	0	0	0	0	
22156804	f	md	JP	2023	0	0	0	0	0	0	0	0	0	0	0	0	81
22156832	m	md	JP	2023	0	1	0	0	1	0	0	0	0	0	0	0	80
22156932	Not	md	JP	2023	0	0	0	0	0	0	0	0	0	0	0	0	
22157616	m	md	JP	2023	0	1	1	0	0	0	0	0	0	0	0	1	76
22157661	m	hp	US	2023	0	0	0	0	0	0	0	0	0	0	0	0	
22158053	f	md	JP	2023	0	1	0	0	1	0	0	0	0	0	0	0	75
22158302	m	md	IN	2023	0	1	0	0	0	1	0	0	0	0	0	0	
22158389	m	md	JP	2023	0	1	0	0	0	1	0	0	0	0	0	0	80
22158406	m	md	JP	2023	0	1	0	0	1	0	0	0	0	0	0	0	72
22158415	f	md	DE	2023	0	0	0	0	0	0	0	0	0	0	0	0	
22159144	m	md	DE	2023	0	1	0	0	1	0	0	0	0	0	0	0	76
22159313	m	md	RO	2023	0	1	0	1	0	0	0	0	0	0	0	0	66
22159805	m	md	DE	2023	0	1	1	0	0	0	0	0	0	0	0	0	
22159848	m	cn	BY	2023	0	0	0	0	0	0	0	0	0	0	0	0	
22159901	m	ph	CA	2023	1	0	0	0	0	0	0	0	0	0	0	0	
22159942	Not	md	US	2023	0	0	0	0	0	0	0	0	0	0	0	0	
22160829	f	hp	CN	2023	1	0	0	0	0	0	0	0	0	0	0	0	34
22161008	m	md	HU	2023	0	0	0	0	0	0	0	0	0	0	0	0	68
22161136	f	cn	CA	2023	1	0	0	0	0	0	0	0	0	0	0	0	
22161187	f	cn	MY	2023	1	0	0	0	0	0	0	0	0	0	0	0	62
22161321	m	md	TR	2023	0	0	0	0	0	0	0	0	0	0	0	0	
22161371	m	cn	ID	2023	1	0	0	0	0	0	0	0	0	0	0	0	80.2
22161379	m	hp	TR	2023	0	0	0	0	0	0	0	0	0	0	0	0	60
22161456	f	md	CN	2023	0	0	0	0	0	0	0	0	0	0	0	0	67
22161468	m	md	JP	2023	0	0	0	0	0	0	0	0	0	0	0	0	
22161701	f	cn	US	2023	1	0	0	0	0	0	0	0	0	0	0	0	
22161777	m	md	JP	2023	0	1	0	1	0	0	0	0	0	0	0	1	62
22162241	f	md	JP	2023	0	1	0	0	1	0	0	0	0	0	0	0	75
22162720	f	md	NL	2023	1	0	0	0	0	0	0	0	0	0	0	0	79
22162728	Not	cn	US	2023	1	0	0	0	0	0	0	0	0	0	0	0	
22162845	f	cn	IN	2023	1	0	0	0	0	0	0	0	0	0	0	0	
21382093	f	md	JP	2023	0	0	0	0	0	0	0	0	0	0	0	0	52
21404958	m	md	CN	2023	0	0	0	0	0	0	0	0	0	0	0	0	70

21408349	m	ph	JP	2023	0	1	0	0	1	0	0	0	0	0	0	0	74
21410713	Not	md	PL	2023	0	1	0	0	0	1	0	0	0	0	0	0	
21453433	f	md	JP	2023	0	1	0	0	1	0	0	0	0	0	0	0	81
21605264	m	md	CN	2023	0	0	0	0	0	0	0	0	0	0	0	0	69
21748166	f	md	AT	2023	0	1	1	0	0	0	0	0	0	0	0	0	72
21770448	m	md	ES	2023	0	0	0	0	0	0	0	0	0	0	0	0	62
21805531	m	md	JP	2023	0	1	1	0	0	0	0	0	0	0	0	1	69
21852959	Not	ph	US	2023	0	1	0	0	1	0	0	0	0	0	0	0	
21856304	m	md	JP	2023	0	1	0	0	1	0	0	0	0	0	0	0	46
21861915	m	md	US	2023	0	0	0	0	0	0	0	0	0	0	0	0	
21872590	f	md	CN	2023	0	0	0	0	0	0	0	0	0	0	0	0	38
21875408	m	md	JP	2023	0	1	0	0	1	0	0	0	0	0	0	0	87
21876453	f	md	GB	2023	0	1	0	0	0	1	0	0	0	0	0	0	79
21913748	f	md	US	2023	1	0	0	0	0	0	0	0	0	0	0	0	70
21914889	m	md	JP	2023	0	0	0	0	0	0	0	0	0	0	0	0	
21915620	Not	md	JP	2023	0	1	0	1	0	0	0	0	0	0	0	0	
21926008	f	md	DE	2023	0	1	0	0	1	0	0	0	0	0	0	0	69
21927814	m	md	JP	2023	0	0	0	0	0	0	0	0	0	0	0	0	70
21929794	f	md	JP	2023	0	1	0	0	1	0	0	0	0	0	0	0	64
21930962	f	md	JP	2023	0	1	0	0	1	0	0	0	0	0	0	0	58
21935391	f	md	GB	2023	0	1	0	0	0	1	0	0	0	0	0	0	79
21946875	m	md	DE	2023	0	1	0	0	1	0	0	0	0	0	0	0	66
21950978	f	cn	TH	2023	0	0	0	0	0	0	0	0	0	0	0	0	60
21951486	m	md	JP	2023	0	0	0	0	0	0	0	0	0	0	0	0	75
21955467	f	md	JP	2023	0	1	0	0	1	0	0	0	0	0	0	0	86
21958202	m	md	JP	2023	0	1	0	0	0	1	0	0	0	0	0	0	70
21959890	f	md	GB	2023	0	1	0	0	0	1	0	0	0	0	0	0	79
21960508	m	md	JP	2023	0	0	0	0	0	0	0	0	0	0	0	0	39
21962112	m	md	US	2023	0	1	0	0	0	1	0	0	0	0	0	0	63
21962862	Not	cn	US	2023	0	1	0	1	0	0	0	0	0	0	0	0	79.1
21962881	m	md	DE	2023	0	1	0	0	1	0	0	0	0	0	0	0	84
21967413	m	md	JP	2023	0	1	0	0	1	0	0	0	0	0	0	0	62
21969772	f	md	JP	2023	0	1	0	0	1	0	0	0	0	0	0	0	79
21981853	f	md	JP	2023	0	1	0	0	1	0	0	0	0	0	0	0	74

21985716	f	md	JP	2023	0	0	0	0	0	0	0	0	0	0	0	0	78
21989282	f	md	JP	2023	1	0	0	0	0	0	0	0	0	0	0	0	89
21995393	f	md	DE	2023	0	0	0	0	0	0	0	0	0	0	0	0	62
22000188	f	md	KW	2023	0	0	0	0	0	0	0	0	0	0	0	0	55
22002086	m	md	JP	2023	0	1	0	0	1	0	0	0	0	0	0	0	68
22003789	f	hp	GB	2023	1	0	0	0	0	0	0	0	0	0	0	0	70
22005291	f	md	JP	2023	0	0	0	0	0	0	0	0	0	0	0	0	77
22009765	Not	md	JP	2023	0	1	0	0	1	0	0	0	0	0	0	0	
22012754	m	md	IT	2023	0	1	0	0	0	1	0	0	0	0	0	0	84
22015435	m	md	JP	2023	0	1	0	0	1	0	0	0	0	0	0	0	79
22018515	f	md	FR	2023	0	0	0	0	0	0	0	0	0	0	0	0	65
22018639	m	md	US	2023	0	0	0	0	0	0	0	0	0	0	0	0	55
22022892	m	md	JP	2023	0	1	1	0	0	0	0	0	0	0	0	1	71
22031868	m	md	JP	2023	0	1	0	0	1	0	0	0	0	0	0	0	53
22033731	m	hp	DE	2023	0	1	0	0	1	0	0	0	0	0	0	0	68
22037244	f	md	JP	2023	0	0	0	0	0	0	0	0	0	0	0	0	71
22038710	m	md	JP	2023	0	1	1	0	0	0	0	0	0	0	0	0	70
22042331	m	md	AU	2023	0	0	0	0	0	0	0	0	0	0	0	0	70
22042762	m	md	CZ	2023	0	0	0	0	0	0	0	0	0	0	0	0	60
22043166	m	md	FR	2023	0	0	0	0	0	0	1	0	0	1	0	0	
22043302	f	md	JP	2023	0	1	0	0	0	1	0	0	0	0	0	0	80
22044059	m	md	DE	2023	0	1	0	0	1	0	0	0	0	0	0	1	84
22049720	Not	md	US	2023	0	1	0	1	0	0	0	0	0	0	0	0	
22050409	f	cn	IN	2023	0	0	0	0	0	0	0	0	0	0	0	0	52
22058219	f	md	JP	2023	0	1	0	0	1	0	0	0	0	0	0	0	72
22058792	m	md	AU	2023	0	0	0	0	0	0	0	0	0	0	0	0	78
22060833	f	md	BR	2023	0	0	0	0	0	0	0	0	0	0	0	0	39
22068386	Not	md	GB	2023	0	0	0	0	0	0	0	0	0	0	0	0	
22071417	f	md	CN	2023	0	0	0	0	0	0	0	0	0	0	0	0	55
22071480	m	md	ES	2023	0	0	0	0	0	0	0	0	0	0	0	0	55
22071956	m	md	JP	2023	0	1	0	0	1	0	0	0	0	0	0	0	
22073665	Not	ph	JP	2023	0	0	0	0	0	0	0	0	0	0	0	0	
22073712	f	cn	IN	2023	0	0	0	0	0	0	0	0	0	0	0	0	46
22073801	m	md	FR	2023	0	1	0	1	0	0	0	0	0	0	0	1	70

22075643	f	md	JP	2023	0	0	0	0	0	0	0	0	0	0	0	0	57
22075694	m	md	CN	2023	0	0	0	0	0	0	0	0	0	0	0	0	63
22075711	f	md	JP	2023	0	0	0	0	0	0	0	0	0	0	0	0	78
22075774	m	md	PT	2023	0	0	0	0	0	0	0	0	0	0	0	0	62
22075844	f	md	DE	2023	0	0	0	0	0	0	0	0	0	0	0	0	
22080017	f	md	JP	2023	0	1	1	0	0	0	0	0	0	0	0	0	60
22082261	f	md	JP	2023	0	1	0	0	0	1	0	0	0	0	0	0	70
22084288	m	md	BE	2023	0	0	0	0	0	0	0	0	0	0	0	0	65
22087455	f	md	DE	2023	0	1	0	0	1	0	0	0	0	0	0	0	65
22088698	f	hp	US	2023	0	1	1	0	0	0	0	0	0	0	0	0	76
22090120	m	md	DE	2023	0	0	0	0	0	0	0	0	0	0	0	0	64
22092142	m	md	JP	2023	0	0	0	0	0	0	0	0	0	0	0	0	73
22092219	m	md	IT	2023	0	0	0	0	0	0	0	0	0	0	0	0	
22092362	m	md	CN	2023	0	0	0	0	0	0	0	0	0	0	0	0	75
22097031	m	md	ID	2023	1	0	0	0	0	0	0	0	0	0	0	0	69.4
22097103	f	md	ES	2023	0	1	0	0	0	1	0	0	0	0	0	0	64
22100081	m	md	BR	2023	0	0	0	0	0	0	0	0	0	0	0	0	57
22102115	m	md	FR	2023	0	1	0	1	0	0	0	0	0	0	0	1	70
22104726	Not	md	KR	2023	0	0	0	0	0	0	0	0	0	0	0	0	
22105682	f	cn	US	2023	1	0	0	0	0	0	0	0	0	0	0	0	68
22107868	m	md	JP	2023	0	1	0	0	1	0	0	0	0	0	0	0	50
22108191	m	md	TR	2023	0	0	0	0	0	0	0	0	0	0	0	0	82
22108944	m	md	JP	2023	0	1	0	0	1	0	0	0	0	0	0	0	68
22109472	m	md	JP	2023	0	1	0	0	0	1	0	0	0	0	0	0	58
22109841	f	md	JP	2023	0	1	0	0	0	1	0	0	0	0	0	0	59
22110366	f	md	GB	2023	0	1	0	0	0	1	0	0	0	0	0	0	61
22110614	f	md	AU	2023	0	0	0	0	0	0	0	0	0	0	0	0	70
22116845	f	md	IN	2023	0	0	0	0	0	0	0	0	0	0	0	0	44
22118451	m	md	CR	2023	0	1	0	0	0	1	0	0	0	0	0	0	76
22118452	m	cn	PK	2023	0	0	0	0	0	0	0	0	0	0	0	0	63
22120426	m	md	CN	2023	0	0	0	0	0	0	0	0	0	0	0	0	63
22120755	f	md	AT	2023	0	0	0	0	0	0	0	0	0	0	0	0	43
22121979	m	md	IT	2023	0	0	0	0	0	0	0	0	0	0	0	0	
22123374	m	cn	PK	2023	0	1	0	0	0	1	0	0	0	0	0	0	71

22123751	m	md	JP	2023	0	0	0	0	0	0	0	0	0	0	0	0	83
22124655	f	md	SI	2023	1	0	0	0	0	0	0	0	0	0	0	0	64
22125840	m	md	JP	2023	0	1	0	0	1	0	0	0	0	0	0	0	72
22128818	m	cn	PK	2023	0	1	0	0	0	1	0	0	0	0	0	0	61
22130886	f	md	JP	2023	0	0	0	0	0	0	0	0	0	0	0	0	70
22130888	m	md	ES	2023	0	1	0	0	0	1	0	0	0	0	0	0	73
22132039	f	hp	NL	2023	0	0	0	0	0	0	0	0	0	0	0	0	70
22132546	f	md	US	2023	0	0	0	0	0	0	0	0	0	0	0	0	
22133764	m	md	BG	2023	1	0	0	0	0	0	0	0	0	0	0	0	78.9
22135610	m	md	JP	2023	0	1	0	0	1	0	0	0	0	0	0	0	63
22137323	f	cn	US	2023	0	0	0	0	0	0	0	0	0	0	0	0	
22137345	f	md	AU	2023	0	0	0	0	0	0	0	0	0	0	0	0	71
22144320	m	md	CN	2023	0	0	0	0	0	0	0	0	0	0	0	0	68
22146616	f	hp	CN	2023	0	0	0	0	0	0	0	0	0	0	0	0	66
22148087	m	md	GR	2023	0	1	1	0	0	0	0	0	0	0	0	0	60
22148980	m	md	CN	2023	1	0	0	0	0	0	0	0	0	0	0	0	
22150910	f	md	JP	2023	0	1	0	0	0	1	0	0	0	0	0	0	
22152767	m	md	CN	2023	0	1	0	1	0	0	0	0	0	0	0	0	72
22153393	m	md	JP	2023	0	0	0	0	0	0	0	0	0	0	0	0	76
22153993	Not	hp	JP	2023	0	0	0	0	0	0	0	0	0	0	0	0	
22154271	m	md	CN	2023	0	0	0	0	0	0	0	0	0	0	0	0	64
22157394	f	md	DK	2023	0	1	0	0	0	1	0	0	0	0	0	0	61
22157749	m	md	JP	2023	0	0	0	0	0	0	0	0	0	0	0	0	74
22157998	m	md	JP	2023	0	1	0	0	1	0	0	0	0	0	0	0	76
22158145	m	md	JP	2023	0	1	0	0	1	0	0	0	0	0	0	1	76
22159861	f	md	JP	2023	0	1	0	0	1	0	0	0	0	0	0	0	86
22160333	f	md	JP	2023	0	1	0	0	1	0	0	0	0	0	0	0	57
22161665	f	md	GB	2023	0	1	1	0	0	0	0	0	0	0	0	0	58
22162419	m	md	BR	2023	0	0	0	0	0	0	0	0	0	0	0	0	57
22162927	f	md	NO	2023	0	0	0	0	0	0	0	0	0	0	0	0	57
22163038	f	md	JP	2023	0	1	0	0	0	1	0	0	0	0	0	0	50
22163040	m	md	JP	2023	0	1	0	0	0	1	0	0	0	0	0	0	70
22163246	f	cn	SG	2023	0	1	0	0	0	1	0	0	0	0	0	0	
22163415	m	md	LT	2023	0	1	0	0	0	1	0	0	0	0	0	0	65

22163523	f	md	NL	2023	1	0	0	0	0	0	0	0	0	0	0	0	57
22163527	m	md	NL	2023	1	0	0	0	0	0	0	0	0	0	0	0	57
22163822	m	cn	US	2023	0	1	1	0	0	0	0	0	0	0	0	0	
22163828	m	md	KR	2023	0	0	0	0	0	0	0	0	0	0	0	0	78
22164048	f	md	CN	2023	0	0	0	0	0	0	0	0	0	0	0	0	65
22164141	m	hp	JP	2023	0	1	1	0	0	0	0	0	0	0	0	0	
22164289	Not	md	TR	2023	0	0	0	0	0	0	0	0	0	0	0	0	
22164291	f	md	JP	2023	0	0	0	0	0	0	0	0	0	0	0	0	45
22164574	m	md	DE	2023	0	1	0	0	1	0	0	0	0	0	0	0	54
22164673	Not	md	JP	2023	0	1	1	0	0	0	0	0	0	0	0	0	80
22164757	m	md	JP	2023	0	1	1	0	0	0	0	0	0	0	0	0	90
22164799	f	md	CN	2023	0	0	0	0	0	0	0	0	0	0	0	0	67
22164805	m	md	JP	2023	0	1	0	0	1	0	0	0	0	0	0	0	52
22164999	m	md	JP	2023	0	0	0	0	0	0	0	0	0	0	0	0	61
22165030	f	hp	DE	2023	0	1	1	0	0	0	0	0	0	0	0	0	
22165142	m	md	DE	2023	0	1	1	0	0	0	0	0	0	0	0	0	
22165198	m	md	JP	2023	0	1	0	0	0	1	0	0	0	0	0	0	63
22165209	m	cn	SG	2023	0	1	0	0	0	1	0	0	0	0	0	0	85
22165453	m	cn	SE	2023	0	1	0	1	0	0	0	0	0	0	0	0	71
22165730	f	cn	CA	2023	1	0	0	0	0	0	0	0	0	0	0	0	82.6
22165761	m	md	JP	2023	0	1	0	0	1	0	0	0	0	0	0	0	69
22165806	f	md	DK	2023	0	1	1	0	0	0	0	0	0	0	0	0	60
22165832	m	hp	IT	2023	0	0	0	0	0	0	0	0	0	0	0	0	46
22165986	m	cn	AT	2023	0	0	0	0	0	0	0	0	0	0	0	0	79
22166150	m	md	US	2023	0	1	0	0	1	0	0	0	0	0	0	0	69
22166559	m	cn	ID	2023	1	0	0	0	0	0	0	0	0	0	0	0	65.7
22166640	m	cn	US	2023	0	0	0	0	0	0	0	0	0	0	0	0	
22166692	f	md	JP	2023	0	0	0	0	0	0	0	0	0	0	0	0	
22166731	m	hp	TH	2023	1	0	0	0	0	0	0	0	0	0	0	0	
22167733	f	md	US	2023	0	0	0	0	0	0	0	0	0	0	0	0	64
22167890	m	md	NZ	2023	1	0	0	0	0	0	0	0	0	0	0	0	71
22167912	m	md	CA	2023	0	0	0	0	0	0	0	0	0	0	0	0	
22168113	m	md	BR	2023	0	1	0	0	1	0	0	0	0	0	0	0	
22168453	m	md	RU	2023	0	1	0	0	0	1	0	0	0	0	0	0	60

22168473	m	hp	KR	2023	0	0	0	0	0	0	0	0	0	0	0	0	65
22168535	m	md	CA	2023	0	0	0	0	0	0	0	0	0	0	0	0	
22168570	m	cn	ID	2023	0	0	0	0	0	0	0	0	0	0	0	0	
22168827	m	md	JP	2023	0	1	0	0	1	0	0	0	0	0	0	0	79
22169144	f	md	JP	2023	0	1	0	0	1	0	0	0	0	0	0	0	77
22169147	f	hp	DE	2023	0	1	1	0	0	0	0	0	0	0	0	0	61
22169293	f	cn	IE	2023	0	1	1	0	0	0	0	0	0	0	0	0	77
22169827	Not	hp	FR	2023	0	0	0	0	0	0	0	0	0	0	0	0	
22169882	m	md	JP	2023	0	1	0	0	1	0	0	0	0	0	0	0	60
22169908	m	md	DE	2023	0	1	0	0	1	0	0	0	0	0	0	0	58
22170123	Not	md	CA	2023	1	0	0	0	0	0	0	0	0	0	0	0	
22170198	m	md	DE	2023	0	1	1	0	0	0	0	0	0	0	0	0	61
22170658	m	md	CN	2023	0	0	0	0	0	0	0	0	0	0	0	0	67
22170716	m	md	IT	2023	0	0	0	0	0	0	0	0	0	0	0	0	53
22170718	f	md	IN	2023	0	0	0	0	0	0	0	0	0	0	0	0	
22170894	m	cn	US	2023	1	0	0	0	0	0	0	0	0	0	0	0	80
22170992	f	md	US	2023	1	0	0	0	0	0	0	0	0	0	0	0	
22171032	f	hp	GB	2023	1	0	0	0	0	0	0	0	0	0	0	0	83
22171116	m	cn	ID	2023	1	0	0	0	0	0	0	0	0	0	0	0	78.3
22171117	m	cn	ID	2023	1	0	0	0	0	0	0	0	0	0	0	0	56.1
22171216	f	md	JP	2023	1	0	0	0	0	0	0	0	0	0	0	0	80
22171314	m	cn	US	2023	0	1	1	0	0	0	0	0	0	0	0	0	
22171384	m	md	CN	2023	0	1	0	0	0	1	0	0	0	0	0	0	
22171456	m	cn	ID	2023	1	0	0	0	0	0	0	0	0	0	0	0	60.5
22171629	f	hp	GB	2023	0	0	0	0	0	0	0	0	0	0	0	0	59
22172024	m	md	FR	2023	0	1	0	0	1	0	0	0	0	0	0	0	72
22172200	f	cn	IN	2023	0	0	0	0	0	0	0	0	0	0	0	0	53
22172404	m	cn	CA	2023	1	0	0	0	0	0	0	0	0	0	0	0	
22172694	f	cn	US	2023	0	1	1	0	0	0	0	0	0	0	0	0	
22172889	m	md	DE	2023	0	1	1	0	0	0	0	0	0	0	0	0	55
22172917	m	cn	ID	2023	1	0	0	0	0	0	0	0	0	0	0	0	
22173166	Not	md	US	2023	0	0	0	0	0	0	0	0	0	0	0	0	70
22173275	f	cn	IN	2023	0	0	0	0	0	0	0	0	0	0	0	0	43
22173356	f	md	NL	2023	1	0	0	0	0	0	0	0	0	0	0	0	57

22173619	m	md	DE	2023	0	1	0	0	1	0	0	0	0	0	0	0	71
22173725	f	cn	ID	2023	1	0	0	0	0	0	0	0	0	0	0	0	78.8
22173894	m	md	JP	2023	0	1	1	0	0	0	0	0	0	0	0	0	70
22174885	f	md	JP	2023	0	1	0	0	1	0	0	0	0	0	0	1	70
22175203	m	md	FR	2023	0	0	0	0	0	0	0	0	0	0	0	0	65
22175226	m	md	ES	2023	0	0	0	0	0	0	0	0	0	0	0	0	72
22175267	f	cn	SI	2023	0	0	0	0	0	0	0	0	0	0	0	0	59
22175384	f	hp	CA	2023	1	0	0	0	0	0	0	0	0	0	0	0	73.8
22175432	Not	hp	AU	2023	1	0	0	0	0	0	0	0	0	0	0	0	52
22175495	Not	md	JP	2023	0	1	0	0	1	0	0	0	0	0	0	0	
22175563	m	md	DE	2023	0	1	0	0	1	0	0	0	0	0	0	0	66
22175729	f	md	IT	2023	0	0	0	0	0	0	0	0	0	0	0	0	65
22176329	f	hp	US	2023	0	0	0	0	0	0	0	0	0	0	0	0	52
22176945	f	md	FR	2023	1	0	0	0	0	0	0	0	0	0	0	0	81
22176970	m	md	FR	2023	0	1	0	0	0	1	0	0	0	0	0	0	70
22177147	f	ph	CA	2023	0	1	1	0	0	0	0	0	0	0	0	0	61
22177149	m	md	JP	2023	1	0	0	0	0	0	0	0	0	0	0	0	80
22177259	m	md	FR	2023	0	1	1	0	0	0	0	0	0	0	0	0	62
22177943	m	md	FR	2023	0	1	0	1	0	0	0	0	0	0	0	0	70
22177961	f	cn	IN	2023	1	0	0	0	0	0	0	0	0	0	0	0	68.8
22177962	m	md	DE	2023	0	1	1	0	0	0	0	0	0	0	0	0	55
22178000	m	cn	US	2023	0	0	0	0	0	0	0	0	0	0	0	0	
22178173	f	md	SI	2023	0	0	0	0	0	0	0	0	0	0	0	0	
22178216	f	hp	KR	2023	1	0	0	0	0	0	0	0	0	0	0	0	56
22178267	m	cn	US	2023	1	0	0	0	0	0	0	0	0	0	0	1	64.4
22178272	f	cn	IN	2023	1	0	0	0	0	0	0	0	0	0	0	0	
22178517	f	cn	SG	2023	0	1	0	0	0	1	0	0	0	0	0	0	75
22178649	m	md	DE	2023	0	0	0	0	0	0	0	0	0	0	0	0	74
22178801	f	md	GB	2023	0	1	0	0	0	1	0	0	0	0	0	0	71
22178954	m	cn	IN	2023	0	1	0	0	0	1	0	0	0	0	0	0	58
22180150	Not	cn	US	2023	0	0	0	0	0	0	0	0	0	0	0	0	
22180322	f	cn	US	2023	1	0	0	0	0	0	0	0	0	0	0	0	
22180400	m	md	KR	2023	0	0	0	0	0	0	0	0	0	0	0	0	
22180401	m	md	KR	2023	0	0	0	0	0	0	0	0	0	0	0	0	69

22180905	f	md	GB	2023	1	0	0	0	0	0	0	0	0	0	0	0	
22180923	Not	md	JP	2023	0	1	1	0	0	0	0	0	0	0	0	0	80
22181312	Not	cn	US	2023	0	0	0	0	0	0	0	0	0	0	0	0	
22182606	f	hp	GB	2023	1	0	0	0	0	0	0	0	0	0	0	0	
22182638	m	md	JP	2023	0	1	0	0	1	0	0	0	0	0	0	0	80
22182722	f	cn	CA	2023	1	0	0	0	0	0	0	0	0	0	0	0	
22182797	m	md	JP	2023	0	1	0	0	1	0	0	0	0	0	0	0	65
22182835	f	md	CN	2023	1	0	0	0	0	0	0	0	0	0	0	0	72
22182988	m	md	DE	2023	0	1	1	0	0	0	0	0	0	0	0	0	55
22183321	Not	md	JP	2023	0	1	1	0	0	0	0	0	0	0	0	0	73
22183537	Not	md	CN	2023	0	0	0	0	0	0	0	0	0	0	0	0	
22183635	f	md	BG	2023	0	1	0	0	0	1	0	0	0	0	0	0	67
22184081	m	cn	CA	2023	1	0	0	0	0	0	0	0	0	0	0	0	80.4
22184280	f	cn	US	2023	0	0	0	0	0	0	0	0	0	0	0	0	
22184281	f	cn	US	2023	1	0	0	0	0	0	0	0	0	0	0	0	62.5
22184393	f	md	ID	2023	1	0	0	0	0	0	0	0	0	0	0	0	54
22184563	f	cn	CN	2023	0	0	0	0	0	0	0	0	0	0	0	0	70
22184690	m	cn	IN	2023	1	0	0	0	0	0	0	0	0	0	0	0	70.3
22184697	m	md	US	2023	0	1	1	0	0	0	0	0	0	0	0	0	64
22184740	f	hp	CA	2023	0	0	0	0	0	0	0	0	0	0	0	0	52
22184963	m	cn	IN	2023	1	0	0	0	0	0	0	0	0	0	0	0	76.8
22185029	m	hp	IT	2023	0	0	0	0	0	0	0	0	0	0	0	0	46
22185190	m	cn	IN	2023	1	0	0	0	0	0	0	0	0	0	0	0	51.3
22185192	m	md	CA	2023	1	0	0	0	0	0	0	0	0	0	0	0	
22185203	m	md	JP	2023	0	1	0	0	0	1	0	0	0	0	0	1	76
22185585	m	cn	IN	2023	1	0	0	0	0	0	0	0	0	0	0	0	73.9
22185633	f	cn	IN	2023	1	0	0	0	0	0	0	0	0	0	0	0	69.6
22186314	f	cn	CN	2023	0	1	0	1	0	0	0	0	0	0	0	0	71
22186378	f	cn	CA	2023	1	0	0	0	0	0	0	0	0	0	0	0	
22186560	f	cn	IN	2023	1	0	0	0	0	0	0	0	0	0	0	0	54.7
22186563	m	cn	IN	2023	1	0	0	0	0	0	0	0	0	0	0	0	38.6
22186564	Not	md	US	2023	1	0	0	0	0	0	0	0	0	0	0	0	
22186678	m	cn	IN	2023	1	0	0	0	0	0	0	0	0	0	0	0	32.7
22186814	m	cn	IN	2023	1	0	0	0	0	0	0	0	0	0	0	0	70.8

22186923	m	cn	IN	2023	1	0	0	0	0	0	0	0	0	0	0	0	
22186953	f	hp	IL	2023	1	0	0	0	0	0	0	0	0	0	0	0	75
22186955	Not	hp	US	2023	1	0	0	0	0	0	0	0	0	0	0	0	
22187007	f	cn	IN	2023	1	0	0	0	0	0	0	0	0	0	0	0	69.7
22187012	f	cn	TH	2023	1	0	0	0	0	0	0	0	0	0	0	0	81.8
22187100	m	md	CH	2023	0	0	0	0	0	0	0	0	0	0	0	0	78
22187162	m	md	FR	2023	0	1	0	1	0	0	0	0	0	0	0	0	70
22187168	f	md	FR	2023	0	0	0	0	0	0	0	0	0	0	0	0	
22187424	f	cn	IN	2023	1	0	0	0	0	0	0	0	0	0	0	0	62.8
22187428	f	hp	IL	2023	1	0	0	0	0	0	0	0	0	0	0	0	73
22187470	Not	hp	US	2023	1	0	0	0	0	0	0	0	0	0	0	0	
22187491	f	cn	IN	2023	1	0	0	0	0	0	0	0	0	0	0	0	73.4
22187524	f	cn	AT	2023	0	0	0	0	0	0	0	0	0	0	0	0	
22187538	m	md	US	2023	0	0	0	0	0	0	0	0	0	0	0	0	
22187803	m	hp	US	2023	0	0	0	0	0	0	0	0	0	0	0	0	
22188162	m	ph	PK	2023	0	1	0	0	0	1	0	0	0	0	0	0	66
22188272	f	md	BE	2023	0	0	0	0	0	0	0	0	0	0	0	0	77
22188667	Not	md	HU	2023	0	1	0	1	0	0	0	0	0	0	0	0	
22188790	f	ph	TH	2023	1	0	0	0	0	0	0	0	0	0	0	0	
22188990	Not	hp	TW	2023	0	0	0	0	0	0	0	0	0	0	0	0	
22189199	m	md	JP	2023	0	1	0	0	0	1	0	0	0	0	0	1	
22189209	m	ph	JP	2023	0	0	0	0	0	0	0	0	0	0	0	0	70
22189266	f	md	JP	2023	0	0	0	0	0	0	0	0	0	0	0	0	80
22189837	Not	md	AU	2023	0	1	0	1	0	0	0	0	0	0	0	0	
22189924	f	ph	TH	2023	1	0	0	0	0	0	0	0	0	0	0	0	
22190462	m	cn	US	2023	1	0	0	0	0	0	0	0	0	0	0	0	56.9
22191116	m	hp	US	2023	0	1	0	0	1	0	0	0	0	0	0	0	65
22191473	f	hp	DK	2023	0	1	1	0	0	0	0	0	0	0	0	0	
22191499	m	md	DE	2023	0	1	1	0	0	0	0	0	0	0	0	0	55
22191874	m	md	JP	2023	0	1	0	0	0	1	0	0	0	0	0	0	70
22191875	m	md	JP	2023	0	1	0	0	0	1	0	0	0	0	0	0	80
22191966	f	hp	US	2023	0	1	0	0	1	0	0	0	0	0	0	0	80
22192422	f	md	JP	2023	0	1	1	0	0	0	0	0	0	0	0	0	
22192647	m	md	KR	2023	0	1	0	0	0	1	0	0	0	0	0	0	60

22192779	m	md	CN	2023	0	0	0	0	0	0	0	0	0	0	0	0	67
22192808	f	cn	CA	2023	1	0	0	0	0	0	0	0	0	0	0	0	78.8
22192976	f	md	JP	2023	1	0	0	0	0	0	0	0	0	0	0	1	82
22193249	f	hp	US	2023	0	1	1	0	0	0	0	0	0	0	0	0	67
22193389	f	md	GB	2023	0	0	0	0	0	0	0	0	0	0	0	0	72
22193434	m	md	GR	2023	0	1	0	0	1	0	0	0	0	0	0	1	67
22194028	f	md	AU	2023	0	0	0	0	0	0	1	0	0	1	0	0	
22194424	m	md	FR	2023	0	1	0	1	0	0	0	0	0	0	0	0	70
22195514	m	md	DE	2023	0	1	0	0	1	0	0	0	0	0	0	0	67
22195528	Not	cn	CN	2023	0	1	0	0	0	1	0	0	0	0	0	0	
22195800	m	md	IN	2023	0	0	0	0	0	0	0	0	0	0	0	0	
22196519	f	md	JP	2023	0	0	0	0	0	0	0	0	0	0	0	0	90
22196627	f	md	FR	2023	0	0	0	0	0	0	0	0	0	0	0	0	52
22196708	f	cn	US	2023	0	0	0	0	0	0	0	0	0	0	0	0	
22196795	f	cn	IN	2023	0	0	0	0	0	0	0	0	0	0	0	0	67
22196847	f	hp	DE	2023	0	1	1	0	0	0	0	0	0	0	0	0	54
22197136	f	ph	US	2023	0	1	1	0	0	0	0	0	0	0	0	0	95
22197227	m	cn	AR	2023	0	1	1	0	0	0	0	0	0	0	0	0	63
22197232	m	md	GB	2023	0	0	0	0	0	0	0	0	0	0	0	0	58
22197317	m	md	JP	2023	0	1	1	0	0	0	0	0	0	0	0	0	80
22197467	m	md	JP	2023	0	1	0	0	0	1	0	0	0	0	0	0	60
22197645	m	md	JP	2023	0	1	0	0	0	1	0	0	0	0	0	0	60
22197764	m	cn	FR	2023	0	0	0	0	0	0	0	0	0	0	0	0	77
22197836	m	md	DE	2023	0	1	1	0	0	0	0	0	0	0	0	0	55
22197849	Not	md	AU	2023	0	1	0	1	0	0	0	0	0	0	0	0	
22197888	m	md	JP	2023	0	1	0	0	0	1	0	0	0	0	0	0	60
22198042	f	md	IT	2023	0	0	0	0	0	0	0	0	0	0	0	0	70
22198049	Not	md	KR	2023	0	0	0	0	0	0	0	0	0	0	0	0	
22198075	m	hp	ID	2023	1	0	0	0	0	0	0	0	0	0	0	0	74.9
22198321	Not	md	AU	2023	0	1	0	1	0	0	0	0	0	0	0	0	
22198343	m	md	JP	2023	0	0	0	0	0	0	0	0	0	0	0	0	
22198364	f	md	JP	2023	1	0	0	0	0	0	0	0	0	0	0	0	75
22199023	f	md	ES	2023	0	1	0	0	0	1	0	0	0	0	0	0	48
22199041	m	md	AU	2023	0	1	1	0	0	0	0	0	0	0	0	0	

22199047	f	md	ES	2023	0	0	0	0	0	0	0	0	0	0	0	0	
22199063	m	md	FR	2023	0	0	0	0	0	0	0	0	0	0	0	0	78
22199651	m	md	DE	2023	0	1	1	0	0	0	0	0	0	0	0	0	55
22199734	m	cn	CN	2023	0	0	0	0	0	0	0	0	0	0	0	0	66
22199901	Not	md	JP	2023	0	1	0	0	1	0	0	0	0	0	0	0	
22200268	m	md	JP	2023	0	1	0	0	1	0	0	0	0	0	0	1	
22200357	m	md	GB	2023	0	1	0	0	0	1	0	0	0	0	0	0	79
22200487	m	cn	TH	2023	1	0	0	0	0	0	0	0	0	0	0	0	70.3
22200585	f	md	JP	2023	1	0	0	0	0	0	0	0	0	0	0	0	71
22200621	f	cn	US	2023	0	0	0	0	0	0	0	0	0	0	0	0	
22200669	m	md	JP	2023	0	1	0	0	1	0	0	0	0	0	0	1	
22200706	m	hp	JP	2023	0	1	0	0	1	0	0	0	0	0	0	1	
22201186	f	md	IN	2023	0	0	0	0	0	0	0	0	0	0	0	0	
22201206	f	md	RU	2023	0	1	0	0	0	1	0	0	0	0	0	0	56
22201244	m	hp	JP	2023	0	1	0	0	1	0	0	0	0	0	0	1	
22202449	Not	ph	JP	2023	0	1	1	0	0	0	0	0	0	0	0	0	70
22202877	Not	md	US	2023	0	0	0	0	0	0	0	0	0	0	0	0	
22203060	Not	cn	US	2023	1	0	0	0	0	0	0	0	0	0	0	0	
22203231	f	ph	DE	2023	0	0	0	0	0	0	0	0	0	0	0	0	78
22203342	Not	md	JP	2023	0	0	0	0	0	0	0	0	0	0	0	0	
22203636	f	hp	US	2023	0	1	0	0	1	0	0	0	0	0	0	0	62
22203697	m	hp	JP	2023	0	1	1	0	0	0	0	0	0	0	0	0	72
22203913	m	md	JP	2023	1	0	0	0	0	0	0	0	0	0	0	1	83
22203946	m	ph	JP	2023	0	1	1	0	0	0	0	0	0	0	0	0	62
22204253	f	md	JP	2023	0	1	0	0	1	0	0	0	0	0	0	0	76
22204255	m	hp	JP	2023	0	1	0	0	1	0	0	0	0	0	0	1	
22204257	m	hp	JP	2023	0	1	0	0	1	0	0	0	0	0	0	1	
22204262	m	md	JP	2023	0	1	0	0	1	0	0	0	0	0	0	1	
22204269	m	md	JP	2023	0	1	0	0	1	0	0	0	0	0	0	1	
22204270	m	md	JP	2023	0	1	0	0	1	0	0	0	0	0	0	1	
22204272	f	hp	JP	2023	0	1	0	0	1	0	0	0	0	0	0	1	
22204280	m	md	JP	2023	0	1	0	0	1	0	0	0	0	0	0	1	
22204283	m	md	JP	2023	0	1	0	0	1	0	0	0	0	0	0	1	
22204294	m	hp	JP	2023	0	1	0	0	1	0	0	0	0	0	0	1	

22204303	m	md	JP	2023	0	1	0	0	1	0	0	0	0	0	0	1	
22204312	f	md	JP	2023	0	1	0	0	1	0	0	0	0	0	0	1	
22204446	Not	md	JP	2023	0	1	0	0	1	0	0	0	0	0	0	0	
22204563	m	md	BE	2023	0	1	1	0	0	0	0	0	0	0	0	0	57
22204720	f	md	BE	2023	0	0	0	0	0	0	0	0	0	0	0	0	23
22205164	m	hp	ES	2023	0	0	0	0	0	0	0	0	0	0	0	0	68
22205210	f	cn	CN	2023	0	0	0	0	0	0	0	0	0	0	0	0	79
22205604	m	cn	IN	2023	0	1	0	0	0	1	0	0	0	0	0	0	79
22205608	m	md	JP	2023	0	1	0	0	0	1	0	0	0	0	0	0	56
22206113	m	md	JP	2023	0	1	0	0	1	0	0	0	0	0	0	0	68
22206198	f	cn	US	2023	0	0	0	0	0	0	0	0	0	0	0	0	68
22207335	f	md	IT	2023	0	0	0	0	0	0	0	0	0	0	0	0	71
22208211	m	md	CZ	2023	0	0	0	0	0	0	0	0	0	0	0	0	
22208224	f	cn	CA	2023	1	0	0	0	0	0	0	0	0	0	0	0	
22208583	m	md	DE	2023	0	1	0	0	1	0	0	0	0	0	0	0	67
22208689	f	cn	CN	2023	1	0	0	0	0	0	0	0	0	0	0	0	46
22208742	m	ph	JP	2023	0	1	1	0	0	0	0	0	0	0	0	0	60
22208772	m	md	TR	2023	0	0	0	0	0	0	0	0	0	0	0	0	56
22208963	m	md	ES	2023	0	1	0	0	0	1	0	0	0	0	0	0	63
22209141	m	ph	US	2023	0	0	0	0	0	0	0	0	0	0	0	0	
22209163	m	md	AU	2023	0	1	1	0	0	0	0	0	0	0	0	0	
22209216	f	hp	CA	2023	0	0	0	0	0	0	0	0	0	0	0	0	59
22209312	m	md	US	2023	0	1	1	0	0	0	0	0	0	0	0	0	82
22210235	Not	md	JP	2023	0	1	0	0	1	0	0	0	0	0	0	0	
22210455	m	md	DE	2023	0	1	0	0	1	0	0	0	0	0	0	0	67
22211969	f	md	BG	2023	0	1	0	0	0	1	0	0	0	0	0	0	67
22212035	f	cn	ID	2023	1	0	0	0	0	0	0	0	0	0	0	0	
22212078	m	md	RU	2023	0	1	0	0	0	1	0	0	0	0	0	0	54
22212190	m	md	JP	2023	0	0	0	0	0	0	0	0	0	0	0	0	76
22212341	f	hp	US	2023	0	1	1	0	0	0	0	0	0	0	0	0	64
22212417	m	cn	TH	2023	1	0	0	0	0	0	0	0	0	0	0	0	82.3
22212567	m	md	FR	2023	0	1	0	1	0	0	0	0	0	0	0	0	70
22212664	m	cn	US	2023	0	1	1	0	0	0	0	0	0	0	0	0	69
22212855	m	md	CN	2023	0	0	0	0	0	0	0	0	0	0	0	0	58

22212877	f	md	AT	2023	1	0	0	0	0	0	0	0	0	0	0	0	64
22213358	m	md	JP	2023	0	1	0	0	1	0	0	0	0	0	0	0	55
22213583	m	md	DE	2023	0	1	0	0	1	0	0	0	0	0	0	0	85
22214758	Not	cn	US	2023	1	0	0	0	0	0	0	0	0	0	0	0	43.1
22215008	m	ph	US	2023	1	0	0	0	0	0	0	0	0	0	0	0	
22215693	m	md	PT	2023	0	0	0	0	0	0	0	0	0	0	0	0	81
22215866	f	hp	US	2023	0	0	0	0	0	0	0	0	0	0	0	0	
22215867	f	ph	US	2023	0	0	0	0	0	0	0	0	0	0	0	0	
22216151	m	md	DE	2023	0	1	0	0	1	0	0	0	0	0	0	0	74
22216733	m	md	CN	2023	0	0	0	0	0	0	0	0	0	0	0	0	79
22216883	m	ph	MY	2023	1	0	0	0	0	0	0	0	0	0	0	0	
22217011	m	md	TW	2023	0	0	0	0	0	0	0	0	0	0	0	0	60
22217022	m	md	MY	2023	1	0	0	0	0	0	0	0	0	0	0	0	66.6
22217212	m	md	JP	2023	0	0	0	0	0	0	0	0	0	0	0	0	57
22217552	m	hp	US	2023	0	1	0	0	1	0	0	0	0	0	0	0	
22217740	m	md	JP	2023	0	1	1	0	0	0	0	0	0	0	0	0	70
22218167	m	ph	JP	2023	0	0	0	0	0	0	0	0	0	0	0	0	
22218406	m	md	IT	2023	0	0	0	0	0	0	0	0	0	0	0	0	71
22218548	m	md	MY	2023	0	0	0	0	0	0	0	0	0	0	0	0	56
22218597	f	md	IT	2023	0	0	0	0	0	0	0	0	0	0	0	0	81
22218848	m	cn	AE	2023	1	0	0	0	0	0	0	0	0	0	0	0	
22218997	m	md	IT	2023	0	0	0	0	0	0	0	0	0	0	0	0	79
22219185	m	md	FR	2023	0	1	1	0	0	0	0	0	0	0	0	0	
22219188	m	md	NP	2023	0	0	0	0	0	0	0	0	0	0	0	0	36
22220035	m	md	JP	2023	0	1	1	0	0	0	0	0	0	0	0	0	70
22220266	m	md	MY	2023	0	0	0	0	0	0	0	0	0	0	0	0	56
22220312	m	hp	US	2023	0	1	0	0	1	0	0	0	0	0	0	0	
22220415	m	md	JP	2023	0	0	0	0	0	0	0	0	0	0	0	0	47
22220493	m	md	JP	2023	0	1	0	0	1	0	0	0	0	0	0	0	66
22220523	m	ph	JP	2023	0	1	0	0	1	0	0	0	0	0	0	0	66
22220766	f	cn	US	2023	0	1	0	1	0	0	0	0	0	0	0	0	
22220861	Not	md	ES	2023	0	1	0	0	0	1	0	0	0	0	0	0	
22220979	m	cn	US	2023	0	0	0	0	0	0	0	0	0	0	0	0	
22221054	m	md	JP	2023	0	0	0	0	0	0	0	0	0	0	0	0	54

22221731	m	md	JP	2023	0	1	1	0	0	0	0	0	0	0	0	70
22222313	Not	md	US	2023	0	0	0	0	0	0	0	0	0	0	0	
22222766	m	cn	CN	2023	0	0	0	0	0	0	0	0	0	0	0	52
22222861	Not	hp	BY	2023	0	1	0	0	0	1	0	0	0	0	0	47
22223551	m	md	FI	2023	0	0	0	0	0	0	0	0	0	0	0	
22224098	f	cn	US	2023	0	0	0	0	0	0	0	0	0	0	0	74
22224442	f	md	IT	2023	0	0	0	0	0	0	0	0	0	0	0	55
22224552	m	cn	US	2023	1	0	0	0	0	0	0	0	0	0	0	
22224584	m	hp	VN	2023	0	1	1	0	0	0	0	0	0	0	0	60
22224877	f	md	JP	2023	0	0	0	0	0	0	0	0	0	0	0	67
22225029	f	md	RU	2023	0	1	0	1	0	0	0	0	0	0	0	60.2
22225110	m	md	DK	2023	0	0	0	0	0	0	0	0	0	0	0	
22225151	m	hp	US	2023	0	1	0	0	0	1	0	0	0	0	0	85
22225364	Not	md	JP	2023	0	1	0	0	0	1	0	0	0	0	0	80
22225668	Not	hp	US	2023	0	0	0	0	0	0	0	0	0	0	0	
22225937	f	cn	CA	2023	1	0	0	0	0	0	0	0	0	0	0	
22226302	m	Not repo	Not re	2023	0	0	0	0	0	0	0	0	0	0	0	65
22226350	f	cn	US	2023	0	1	1	0	0	0	0	0	0	0	0	84
22226380	m	md	TR	2023	0	1	0	0	0	1	0	0	0	0	0	69
22226390	m	md	CN	2023	0	0	0	0	0	0	0	0	0	0	0	64
22226765	f	hp	TW	2023	0	1	0	0	0	1	0	0	0	0	0	70
22227269	m	md	DE	2023	0	1	1	0	0	0	0	0	0	0	0	55
22227744	m	md	JP	2023	0	1	0	0	1	0	0	0	0	0	0	78
22227793	f	cn	SG	2023	1	0	0	0	0	0	0	0	0	0	0	49.2
22227846	m	md	JP	2023	0	1	0	0	0	1	0	0	0	0	0	70
22228116	Not	hp	GB	2023	0	0	0	0	0	0	0	0	0	0	0	
22228273	f	md	JP	2023	0	0	0	0	0	0	0	0	0	0	0	67
22228614	m	ph	TH	2023	0	0	0	0	0	0	0	0	0	0	0	69
22228809	m	md	JP	2023	0	0	0	0	0	0	0	0	0	0	0	
22228985	m	hp	US	2023	0	0	0	0	0	0	0	0	0	0	0	67
22229258	Not	md	FR	2023	0	0	0	0	0	0	0	0	0	0	0	
22229359	m	md	JP	2023	1	0	0	0	0	0	0	0	0	0	0	70
22229783	Not	hp	US	2023	0	0	0	0	0	0	0	0	0	0	0	
22229859	m	md	JP	2023	0	1	0	0	1	0	0	0	0	0	0	66

22229989	f	hp	ES	2023	1	0	0	0	0	0	0	0	0	0	0	0	77
22230424	f	md	JP	2023	0	0	0	0	0	0	0	0	0	0	0	0	68
22230653	Not	md	JP	2023	0	1	0	0	1	0	0	0	0	0	0	0	
22230730	f	hp	US	2023	1	0	0	0	0	0	0	0	0	0	0	0	37
22231267	m	md	IN	2023	0	0	0	0	0	0	0	0	0	0	0	0	
22231570	m	md	DE	2023	0	1	1	0	0	0	0	0	0	0	0	0	55
22231673	Not	md	JP	2023	0	1	0	0	0	1	0	0	0	0	0	0	40
22231846	m	md	US	2023	0	1	0	0	0	1	0	0	0	0	0	0	72
22232782	f	hp	US	2023	0	0	0	0	0	0	0	0	0	0	0	0	63
22232844	f	cn	US	2023	0	1	0	1	0	0	0	0	0	0	0	0	
22232887	m	hp	US	2023	0	0	0	0	0	0	0	0	0	0	0	0	48
22233732	m	md	US	2023	0	1	0	0	0	1	0	0	0	0	0	1	81
22233928	m	cn	JP	2023	0	0	0	0	0	0	0	0	0	0	0	0	48
22234001	m	md	JP	2023	1	0	0	0	0	0	0	0	0	0	0	0	72
22234044	f	md	BE	2023	0	1	1	0	0	0	0	0	0	0	0	0	60
22234621	f	md	SE	2023	0	1	0	0	0	1	0	0	0	0	0	0	73
22234638	m	md	JP	2023	0	0	0	0	0	0	0	0	0	0	0	0	51
22234793	f	hp	NL	2023	0	0	0	0	0	0	0	0	0	0	0	0	80
22234981	m	md	JP	2023	0	1	0	0	0	1	0	0	0	0	0	0	70
22235001	f	hp	DE	2023	0	1	0	0	1	0	0	0	0	0	0	0	61
22235346	f	md	JP	2023	1	0	0	0	0	0	0	0	0	0	0	0	79
22235529	Not	md	JP	2023	1	0	0	0	0	0	0	0	0	0	0	0	
22235827	Not	md	JP	2023	0	1	0	1	0	0	0	0	0	0	0	0	86
22235848	Not	ph	JP	2023	1	0	0	0	0	0	0	0	0	0	0	0	
22236142	Not	cn	US	2023	0	0	0	0	0	0	0	0	0	0	0	0	61
22236460	m	cn	US	2023	0	1	0	0	0	1	0	0	0	0	0	0	65
22236639	f	ph	JP	2023	0	0	0	0	0	0	0	0	0	0	0	0	80
22236914	f	md	RU	2023	0	1	0	0	0	1	0	0	0	0	0	0	77
22237310	Not	md	JP	2023	0	1	0	1	0	0	0	0	0	0	0	0	
22237713	f	md	JP	2023	0	0	0	0	0	0	0	0	0	0	0	0	60
22237758	Not	md	GB	2023	0	0	0	0	0	0	0	0	0	0	0	0	
22237944	m	md	KR	2023	1	0	0	0	0	0	0	0	0	0	0	0	76
22238031	Not	md	JP	2023	0	1	0	0	0	1	0	0	0	0	0	0	
22238121	Not	cn	CN	2023	0	1	0	0	0	1	0	0	0	0	0	0	

22238456	m	hp	ES	2023	0	1	0	0	0	1	0	0	0	0	0	0	72
22238561	m	md	LT	2023	0	1	0	0	0	1	0	0	0	0	0	0	65
22238787	Not	md	FR	2023	0	0	0	0	0	0	0	0	0	0	0	0	
22238788	Not	md	FR	2023	0	0	0	0	0	0	0	0	0	0	0	0	
22238839	f	md	JP	2023	1	0	0	0	0	0	0	0	0	0	0	1	82
22238899	m	md	JP	2023	0	0	0	0	0	0	0	0	0	0	0	0	73
22238953	Not	md	JP	2023	0	1	0	0	0	1	0	0	0	0	0	0	66
22238989	f	hp	JP	2023	0	0	0	0	0	0	0	0	0	0	0	0	59
22239066	f	hp	TW	2023	1	0	0	0	0	0	0	0	0	0	0	0	80
22239238	m	md	JP	2023	0	1	0	0	0	1	0	0	0	0	0	0	70
22239239	m	md	RU	2023	0	1	0	0	0	1	0	0	0	0	0	0	
22239336	Not	md	US	2023	0	0	0	0	0	0	0	0	0	0	0	0	
22239437	f	md	JP	2023	0	1	1	0	0	0	0	0	0	0	0	0	72
22239538	f	cn	IN	2023	0	0	0	0	0	0	0	0	0	0	0	0	59
22239627	m	ph	IN	2023	0	0	0	0	0	0	0	0	0	0	0	0	90
22239644	m	md	CN	2023	0	1	1	0	0	0	0	0	0	0	0	0	67
22240025	f	md	CN	2023	1	0	0	0	0	0	0	0	0	0	0	0	67
22240526	Not	md	US	2023	0	0	0	0	0	0	0	0	0	0	0	0	
22240862	m	cn	MX	2023	1	0	0	0	0	0	0	0	0	0	0	0	71
22241089	f	md	BE	2023	0	0	0	0	0	0	0	0	0	0	0	0	79
22241404	f	hp	DE	2023	0	1	0	0	1	0	0	0	0	0	0	0	61
22241438	f	cn	US	2023	0	0	0	0	0	0	0	0	0	0	0	0	
22241950	m	cn	CA	2023	0	1	0	0	1	0	0	0	0	0	0	0	58
22242334	f	md	IT	2023	0	0	0	0	0	0	0	0	0	0	0	0	
22242390	f	md	JP	2023	0	0	0	0	0	0	0	0	0	0	0	0	52
22242433	f	md	GB	2023	0	0	0	0	0	0	0	0	0	0	0	0	68
22242439	m	md	IT	2023	0	0	0	0	0	0	0	0	0	0	0	0	75
22242464	f	cn	US	2023	1	0	0	0	0	0	0	0	0	0	0	0	61.4
22242730	m	md	JP	2023	0	1	0	0	0	1	0	0	0	0	0	0	75
22242911	m	hp	OM	2023	0	0	0	0	0	0	0	0	0	0	0	0	41
22242971	Not	hp	IT	2023	0	0	0	0	0	0	0	0	0	0	0	0	
22243122	Not	ph	JP	2023	1	0	0	0	0	0	0	0	0	0	0	0	
22243173	m	md	IT	2023	0	0	0	0	0	0	0	0	0	0	0	0	79
22243327	m	md	FR	2023	0	1	1	0	0	0	0	0	0	0	0	0	

22243374	m	cn	PK	2023	0	1	0	0	0	1	0	0	0	0	0	0	69
22243668	f	md	FR	2023	0	0	0	0	0	0	0	0	0	0	0	0	62
22243832	Not	hp	SG	2023	0	0	0	0	0	0	0	0	0	0	0	0	76
22243975	f	hp	JP	2023	0	1	0	0	1	0	0	0	0	0	0	1	
22244018	m	hp	IT	2023	0	0	0	0	0	0	0	0	0	0	0	0	79
22244291	m	md	JP	2023	0	1	1	0	0	0	0	0	0	0	0	0	70
22244349	m	md	JP	2023	0	1	1	0	0	0	0	0	0	0	0	0	90
22244383	Not	cn	CN	2023	0	1	0	0	0	1	0	0	0	0	0	0	
22244840	m	md	FR	2023	0	1	0	1	0	0	0	0	0	0	0	0	
22244923	m	md	FR	2023	0	1	0	1	0	0	0	0	0	0	0	0	60
22245170	m	md	JP	2023	0	1	0	0	0	1	0	0	0	0	0	1	80
22245781	m	md	HU	2023	0	1	0	1	0	0	0	0	0	0	0	0	
22246101	m	md	FR	2023	0	1	1	0	0	0	0	0	0	0	0	0	
22246107	m	cn	AR	2023	0	0	0	0	0	0	0	0	0	0	0	0	70
22246350	f	cn	IN	2023	0	0	0	0	0	0	0	0	0	0	0	0	40
22246351	m	cn	CA	2023	0	0	0	0	0	0	0	0	0	0	0	0	61
22246497	f	cn	DE	2023	0	1	0	0	1	0	0	0	0	0	0	0	61
22246533	Not	hp	IT	2023	0	0	0	0	0	0	0	0	0	0	0	0	
22246827	f	md	AU	2023	0	0	0	0	0	0	0	0	0	0	0	0	58
22246851	f	hp	IT	2023	0	1	1	0	0	0	0	0	0	0	0	0	58
22247103	f	md	US	2023	0	0	0	0	0	0	0	0	0	0	0	0	61
22247276	m	md	IT	2023	0	0	0	0	0	0	0	0	0	0	0	0	
22247692	f	cn	US	2023	1	0	0	0	0	0	0	0	0	0	0	0	85
22248208	f	md	JP	2023	0	1	0	0	0	1	0	0	0	0	0	0	70
22248297	f	md	CN	2023	0	0	0	0	0	0	0	0	0	0	0	0	64
22248375	m	md	BE	2023	0	1	1	0	0	0	0	0	0	0	0	0	57
22248410	f	md	AR	2023	0	1	0	0	0	1	0	0	0	0	0	0	83
22248496	m	hp	CN	2023	0	0	0	0	0	0	0	0	0	0	0	0	28
22248547	m	md	CA	2023	1	0	0	0	0	0	0	0	0	0	0	1	79.8
22248573	m	md	US	2023	0	1	1	0	0	0	0	0	0	0	0	0	
22248575	Not	cn	US	2023	0	0	0	0	0	0	0	0	0	0	0	0	
22248812	f	hp	TW	2023	1	0	0	0	0	0	0	0	0	0	0	0	80
22249038	m	md	JP	2023	0	1	0	0	0	1	0	0	0	0	0	0	50
22249128	m	md	JP	2023	0	0	0	0	0	0	0	0	0	0	0	0	64

22249655	m	md	US	2023	0	1	0	0	0	1	0	0	0	0	0	0	67
22249659	Not	hp	IT	2023	0	0	0	0	0	0	0	0	0	0	0	0	
22249744	f	hp	IT	2023	0	1	1	0	0	0	0	0	0	0	0	0	29
22249912	f	hp	IT	2023	0	1	1	0	0	0	0	0	0	0	0	0	74
22250121	f	md	JP	2023	1	0	0	0	0	0	0	0	0	0	0	0	
22250248	Not	md	JP	2023	0	1	1	0	0	0	0	0	0	0	0	1	80
22250350	f	md	FR	2023	0	0	0	0	0	0	0	0	0	0	0	0	62
22250389	Not	md	JP	2023	1	0	0	0	0	0	0	0	0	0	0	0	
22250682	f	hp	DE	2023	0	1	0	0	1	0	0	0	0	0	0	0	61
22250733	m	hp	SG	2023	0	0	0	0	0	0	0	0	0	0	0	0	
22250784	f	cn	MY	2023	1	0	0	0	0	0	0	0	0	0	0	0	72.4
22250787	m	md	JP	2023	1	0	0	0	0	0	0	0	0	0	0	0	85
22250833	f	md	JP	2023	0	1	1	0	0	0	0	0	0	0	0	0	80
22250841	Not	hp	US	2023	0	0	0	0	0	0	0	0	0	0	0	0	
22250846	m	cn	CA	2023	1	0	0	0	0	0	0	0	0	0	0	0	58.6
22251092	m	md	DE	2023	0	0	0	0	0	0	0	0	0	0	0	0	
22251107	m	md	JP	2023	0	1	0	0	1	0	0	0	0	0	0	0	81
22251611	f	md	US	2023	0	0	0	0	0	0	0	0	0	0	0	0	64
22251702	m	md	US	2023	0	0	0	0	0	0	0	0	0	0	0	0	69
22251818	Not	md	KR	2023	1	0	0	0	0	0	0	0	0	0	0	0	
22251876	f	ph	JP	2023	0	0	0	0	0	0	0	0	0	0	0	0	51
22251983	m	ph	JP	2023	0	1	0	0	1	0	0	0	0	0	0	0	
22251989	m	md	BE	2023	0	0	0	0	0	0	0	0	0	0	0	0	69
22252177	Not	md	ES	2023	0	1	0	0	0	1	0	0	0	0	0	0	
22253124	Not	hp	IT	2023	0	1	0	0	0	1	0	0	0	0	0	0	
22253384	m	md	JP	2023	0	1	0	0	1	0	0	0	0	0	0	0	70
22253431	m	md	JP	2023	0	0	0	0	0	0	0	0	0	0	0	0	78
22253581	Not	ph	JP	2023	1	0	0	0	0	0	0	0	0	0	0	0	
22253760	m	md	DE	2023	0	1	1	0	0	0	0	0	0	0	0	0	55
22253990	f	hp	DE	2023	0	1	0	0	1	0	0	0	0	0	0	0	61
22254129	Not	md	MY	2023	0	0	0	0	0	0	0	0	0	0	0	0	
22254159	f	ph	US	2023	1	0	0	0	0	0	0	0	0	0	0	0	90
22254228	f	md	IT	2023	0	0	0	0	0	0	0	0	0	0	0	0	68
22254267	f	cn	CA	2023	1	0	0	0	0	0	0	0	0	0	0	0	65.8

22254808	m	md	JP	2023	0	1	0	1	0	0	0	0	0	0	0	0	
22254957	Not	md	SG	2023	0	0	0	0	0	0	0	0	0	0	0	0	
22254988	m	md	IT	2023	1	0	0	0	0	0	0	0	0	0	0	0	63
22255057	f	cn	US	2023	0	0	0	0	0	0	0	0	0	0	0	0	64
22255232	f	md	IT	2023	1	0	0	0	0	0	0	0	0	0	0	0	46
22255264	m	md	JP	2023	0	0	0	0	0	0	0	0	0	0	0	0	
22255338	m	md	IT	2023	0	0	0	0	0	0	0	0	0	0	0	0	68
22255358	f	ph	FR	2023	0	1	1	0	0	0	0	0	0	0	0	0	68
22255402	f	md	US	2023	1	0	0	0	0	0	0	0	0	0	0	0	74
22255812	f	md	JP	2023	1	0	0	0	0	0	0	0	0	0	0	0	57
22256093	f	hp	DE	2023	0	1	0	0	1	0	0	0	0	0	0	0	61
22256148	m	md	JP	2023	0	1	0	1	0	0	0	0	0	0	0	0	
22258034	m	md	JP	2023	0	1	0	0	0	1	0	0	0	0	0	0	90
22258690	Not	md	JP	2023	0	1	0	1	0	0	0	0	0	0	0	1	
22258797	m	md	JP	2023	0	1	0	0	1	0	0	0	0	0	0	0	66
22258812	m	cn	US	2023	1	0	0	0	0	0	0	0	0	0	0	0	51.9
22259034	m	hp	CZ	2023	1	0	0	0	0	0	0	0	0	0	0	0	64
22259558	Not	hp	CN	2023	0	0	0	0	0	0	0	0	0	0	0	0	
22259559	Not	hp	CN	2023	0	0	0	0	0	0	0	0	0	0	0	0	
22259560	Not	hp	CN	2023	0	0	0	0	0	0	0	0	0	0	0	0	
22259591	f	md	ES	2023	0	0	0	0	0	0	0	0	0	0	0	0	65
22259596	Not	hp	CN	2023	0	0	0	0	0	0	0	0	0	0	0	0	
22259641	Not	hp	CN	2023	0	0	0	0	0	0	0	0	0	0	0	0	
22259939	m	md	US	2023	0	0	0	0	0	0	0	0	0	0	0	0	
22260567	f	cn	JP	2023	1	0	0	0	0	0	0	0	0	0	0	0	
22260748	f	md	US	2023	1	0	0	0	0	0	0	0	0	0	0	0	55
22260804	m	md	JP	2023	0	1	1	0	0	0	0	0	0	0	0	0	62
22260884	f	cn	US	2023	1	0	0	0	0	0	0	0	0	0	0	0	76
22261104	m	md	JP	2023	0	1	1	0	0	0	0	0	0	0	0	0	80
22261660	Not	hp	IT	2023	0	1	1	0	0	0	0	0	0	0	0	0	
22261682	Not	md	CH	2023	0	0	0	0	0	0	0	0	0	0	0	0	
22262435	Not	md	JP	2023	0	1	1	0	0	0	0	0	0	0	0	0	65
22262627	m	md	JP	2023	0	0	0	0	0	0	0	0	0	0	0	0	65
22263217	m	cn	TH	2023	0	0	0	0	0	0	0	0	0	0	0	0	31

22263411	f	hp	US	2023	0	0	0	0	0	0	0	0	0	0	0	0	63
22264145	f	ph	US	2023	0	0	0	0	0	0	0	0	0	0	0	0	61
22264147	f	cn	MY	2023	1	0	0	0	0	0	0	0	0	0	0	0	
22264565	m	md	JP	2023	0	1	1	0	0	0	0	0	0	0	0	0	
22264740	f	cn	US	2023	0	1	0	1	0	0	0	0	0	0	0	0	57.9
22264874	f	hp	US	2023	0	1	0	1	0	0	0	0	0	0	0	0	
22265064	f	md	CA	2023	1	0	0	0	0	0	0	0	0	0	0	0	61
22265094	Not	md	TW	2023	0	0	0	0	0	0	0	0	0	0	0	0	
22265104	f	md	BE	2023	0	1	1	0	0	0	0	0	0	0	0	0	69
22265108	m	cn	DE	2023	0	1	0	0	1	0	0	0	0	0	0	0	74
22265147	Not	md	US	2023	0	0	0	0	0	0	0	0	0	0	0	0	
22265186	f	cn	KR	2023	0	0	0	0	0	0	0	0	0	0	0	0	46
22265202	m	hp	IT	2023	0	1	1	0	0	0	0	0	0	0	0	0	71
22265210	Not	md	HK	2023	0	0	0	0	0	0	0	0	0	0	0	0	
22265562	m	cn	AR	2023	0	0	0	0	0	0	0	0	0	0	0	0	78
22265743	m	md	US	2023	0	0	0	0	0	0	0	0	0	0	0	0	60
22266208	f	md	JP	2023	0	1	1	0	0	0	0	0	0	0	0	0	68
22266234	m	hp	GR	2023	0	0	0	0	0	0	0	0	0	0	0	0	70
22266535	m	md	FR	2023	0	0	0	0	0	0	0	0	0	0	0	0	61
22266543	Not	hp	RU	2023	0	0	0	0	0	0	0	0	0	0	0	0	
22266612	f	ph	FR	2023	0	0	0	0	0	0	0	0	0	0	0	0	
22266741	f	hp	US	2023	0	0	0	0	0	0	0	0	0	0	0	0	32
22267021	m	cn	JP	2023	0	0	0	0	0	0	0	0	0	0	0	0	
22267023	Not	cn	JP	2023	0	0	0	0	0	0	0	0	0	0	0	0	
22267126	f	Not repo	US	2023	0	0	0	0	0	0	0	0	0	0	0	0	72
22267197	m	md	IT	2023	1	0	0	0	0	0	0	0	0	0	0	0	68
22267243	m	hp	VN	2023	0	1	0	1	0	0	0	0	0	0	0	0	80
22267344	m	cn	CN	2023	0	1	0	1	0	0	0	0	0	0	0	0	61
22267583	m	hp	IT	2023	0	0	0	0	0	0	0	0	0	0	0	0	53
22267674	m	md	IT	2023	0	0	0	0	0	0	0	0	0	0	0	0	79
22268199	m	md	JP	2023	0	0	0	0	0	0	0	0	0	0	0	0	80
22268509	f	hp	KR	2023	0	1	0	0	0	1	0	0	0	0	0	0	57
22268514	f	cn	IN	2023	0	1	0	0	0	1	0	0	0	0	0	0	73
22269207	m	md	RU	2023	0	1	0	0	0	1	0	0	0	0	0	0	62

22269216	f	md	JP	2023	0	0	0	0	0	0	0	0	0	0	0	0	40
22269243	m	cn	CN	2023	0	1	0	1	0	0	0	0	0	0	0	0	82
22269279	m	md	US	2023	0	0	0	0	0	0	0	0	0	0	0	0	62
22269616	f	ph	FR	2023	0	1	1	0	0	0	0	0	0	0	0	0	68
22269998	f	md	JP	2023	0	0	0	0	0	0	0	0	0	0	0	0	
22270668	f	md	US	2023	0	1	0	0	0	1	0	0	0	0	0	0	73
22270773	m	md	NZ	2023	0	0	0	0	0	0	0	0	0	0	0	0	65
22270953	f	cn	ID	2023	1	0	0	0	0	0	0	0	0	0	0	0	55.1
22271136	Not	cn	JP	2023	0	0	0	0	0	0	0	0	0	0	0	0	
22271386	m	md	JP	2023	0	0	0	0	0	0	0	0	0	0	0	0	
22271659	f	md	US	2023	0	0	0	0	0	0	0	0	0	0	0	0	
22271695	m	md	BG	2023	0	1	0	0	1	0	0	0	0	0	0	1	
22271791	m	md	JP	2023	0	1	0	0	1	0	0	0	0	0	0	0	84
22272122	m	cn	IN	2023	0	1	0	0	0	1	0	0	0	0	0	0	76
22272444	m	md	JP	2023	0	1	0	0	1	0	0	0	0	0	0	0	80
22272513	Not	md	JP	2023	1	0	0	0	0	0	0	0	0	0	0	0	
22272709	f	md	JP	2023	0	1	0	0	0	1	0	0	0	0	0	0	60
22272743	f	md	NO	2023	0	0	0	0	0	0	0	0	0	0	0	0	
22273058	m	cn	US	2023	0	0	0	0	0	0	0	0	0	0	0	0	
22273421	f	md	GB	2023	0	1	0	0	0	1	0	0	0	0	0	0	71
22273857	m	ph	ID	2023	0	1	0	1	0	0	0	0	0	0	0	0	46.6
22273944	f	ph	TH	2023	0	0	0	0	0	0	0	0	0	0	0	0	66
22273966	f	hp	DE	2023	0	0	0	0	0	0	0	0	0	0	0	0	49
22274030	f	md	FR	2023	0	0	0	0	0	0	0	0	0	0	0	0	
22274577	m	md	AU	2023	1	0	0	0	0	0	0	0	0	0	0	0	52
22274656	m	md	KR	2023	0	1	0	0	0	1	0	0	0	0	0	0	70
22274689	f	md	JP	2023	0	1	0	0	0	1	0	0	0	0	0	0	70
22274988	Not	hp	JP	2023	0	1	0	0	0	1	0	0	0	0	0	0	
22274991	Not	hp	JP	2023	0	1	0	0	0	1	0	0	0	0	0	1	
22274992	Not	hp	JP	2023	0	1	0	0	0	1	0	0	0	0	0	0	
22275122	Not	hp	JP	2023	0	1	0	0	0	1	0	0	0	0	0	0	
22275187	f	cn	PE	2023	1	0	0	0	0	0	0	0	0	0	0	0	71
22275266	m	hp	IT	2023	1	0	0	0	0	0	0	0	0	0	0	0	48
22275365	m	md	NL	2023	0	1	0	0	0	1	0	0	0	0	0	1	68

22275904	f	ph	US	2023	0	0	0	0	0	0	0	0	0	0	0	0	81
22276227	Not	md	JP	2023	0	1	0	0	0	1	0	0	0	0	0	0	70
22276394	m	hp	GB	2023	0	1	0	1	0	0	0	0	0	0	0	1	79
22276687	f	md	JP	2023	0	0	0	0	0	0	0	0	0	0	0	0	80
22277466	f	md	TW	2023	0	0	0	0	0	0	0	0	0	0	0	0	47
22277534	m	md	BG	2023	0	1	0	0	1	0	0	0	0	0	0	0	71
22277827	f	md	TH	2023	1	0	0	0	0	0	0	0	0	0	0	0	60.1
22277859	f	cn	ID	2023	1	0	0	0	0	0	0	0	0	0	0	0	43.2
22277903	m	cn	IN	2023	0	1	0	0	0	1	0	0	0	0	0	0	70
22278346	f	md	DE	2023	0	0	0	0	0	0	0	0	0	0	0	0	78
22278348	f	cn	IL	2023	0	0	0	0	0	0	0	0	0	0	0	0	67
22278741	Not	md	JP	2023	0	1	0	0	0	1	0	0	0	0	0	0	80
22279062	Not	md	FR	2023	0	0	0	0	0	0	0	0	0	0	0	0	
22279210	Not	hp	JP	2023	0	1	1	0	0	0	0	0	0	0	0	0	
22279267	f	md	DE	2023	1	0	0	0	0	0	0	0	0	0	0	0	80
22279532	f	md	KW	2023	0	0	0	0	0	0	0	0	0	0	0	0	57
22279763	m	md	JP	2023	0	0	0	0	0	0	0	0	0	0	0	0	
22279931	f	cn	ID	2023	1	0	0	0	0	0	0	0	0	0	0	0	79.3
22279984	f	cn	AT	2023	0	0	0	0	0	0	0	0	0	0	0	0	
22280109	f	cn	ID	2023	1	0	0	0	0	0	0	0	0	0	0	0	
22280114	f	cn	CA	2023	1	0	0	0	0	0	0	0	0	0	0	0	54.7
22280215	m	md	DE	2023	1	0	0	0	0	0	0	0	0	0	0	0	91
22280382	m	md	PH	2023	0	0	0	0	0	0	0	0	0	0	0	0	
22280390	m	md	JP	2023	0	0	0	0	0	0	0	0	0	0	0	0	66
22280414	f	cn	US	2023	0	1	1	0	0	0	0	0	0	0	0	0	66
22280428	Not	md	KW	2023	0	0	0	0	0	0	0	0	0	0	0	0	
22280466	m	md	AT	2023	1	0	0	0	0	0	0	0	0	0	0	0	79
22281208	f	hp	US	2023	1	0	0	0	0	0	0	0	0	0	0	0	
22281385	f	hp	IT	2023	0	1	1	0	0	0	0	0	0	0	0	0	74
22281410	f	hp	IT	2023	0	1	1	0	0	0	0	0	0	0	0	0	29
22281432	f	hp	IT	2023	0	1	1	0	0	0	0	0	0	0	0	0	58
22281654	m	md	JP	2023	0	0	0	0	0	0	0	0	0	0	0	0	
22281857	m	cn	KR	2023	0	0	0	0	0	0	0	0	0	0	0	0	75
22281943	Not	md	SG	2023	0	0	0	0	0	0	0	0	0	0	0	0	

22282474	m	hp	IT	2023	0	1	1	0	0	0	0	0	0	0	0	0	71
22282800	m	md	DE	2023	0	1	0	0	1	0	0	0	0	0	0	0	69
22283150	m	md	PL	2023	0	1	0	0	0	1	0	0	0	0	0	0	
22283779	f	md	BE	2023	0	1	1	0	0	0	0	0	0	0	0	0	69
22283841	f	md	BE	2023	0	0	0	0	0	0	0	0	0	0	0	0	59
22283882	m	cn	IN	2023	0	1	0	1	0	0	0	0	0	0	0	0	
22283997	Not	md	US	2023	0	1	0	0	1	0	0	0	0	0	0	0	
22284466	f	md	CN	2023	0	0	0	0	0	0	0	0	0	0	0	0	61
22284502	f	md	EG	2023	0	0	0	0	0	0	0	0	0	0	0	0	
22284920	m	cn	US	2023	0	0	0	0	0	0	0	0	0	0	0	0	67
22285032	f	hp	JP	2023	0	1	1	0	0	0	0	0	0	0	0	0	68
22285083	Not	md	NL	2023	0	0	0	0	0	0	0	0	0	0	0	0	63
22286771	f	hp	CA	2023	1	0	0	0	0	0	0	0	0	0	0	0	
22287092	m	md	BE	2023	0	1	0	0	1	0	0	0	0	0	0	0	
22287149	f	hp	JP	2023	0	0	0	0	0	0	0	0	0	0	0	0	71
22287285	f	md	FR	2023	0	0	0	0	0	0	0	0	0	0	0	0	68
22287318	m	hp	VN	2023	0	1	1	0	0	0	0	0	0	0	0	0	60
22287932	m	cn	US	2023	0	1	1	0	0	0	0	0	0	0	0	0	
22288025	f	md	IN	2023	0	1	0	0	0	1	0	0	0	0	0	0	
22288131	f	cn	KR	2023	0	0	0	0	0	0	0	0	0	0	0	0	85
22288132	f	md	JP	2023	0	0	0	0	0	0	1	0	0	1	0	1	69
22288174	m	md	IT	2023	0	0	0	0	0	0	0	0	0	0	0	0	71
22289740	Not	cn	US	2023	1	0	0	0	0	0	0	0	0	0	0	0	60.2
22289847	f	ph	US	2023	1	0	0	0	0	0	0	0	0	0	0	0	82
22289931	f	md	CN	2023	0	1	0	1	0	0	0	0	0	0	0	0	79.2
22290067	f	md	CH	2023	0	0	0	0	0	0	0	0	0	0	0	0	64
22290134	f	md	IN	2023	1	0	0	0	0	0	0	0	0	0	0	0	42
22290642	m	cn	DE	2023	0	1	1	0	0	0	0	0	0	0	0	0	
22290803	f	md	DE	2023	0	1	1	0	0	0	0	0	0	0	0	0	33
22292050	f	hp	IT	2023	0	1	1	0	0	0	0	0	0	0	0	0	29
22292255	f	cn	IN	2023	0	1	0	0	0	1	0	0	0	0	0	0	73
22292967	m	md	JP	2023	0	1	0	0	1	0	0	0	0	0	0	0	69
22293288	m	md	JP	2023	0	0	0	0	0	0	0	0	0	0	0	0	71
22293876	f	md	FR	2023	1	0	0	0	0	0	0	0	0	0	0	0	

22293919	f	cn	ID	2023	1	0	0	0	0	0	0	0	0	0	0	0	
22294008	m	cn	IN	2023	0	0	0	0	0	0	0	0	0	0	0	0	70
22294272	f	md	DE	2023	0	0	0	0	0	0	0	0	0	0	0	0	
22294309	f	cn	CA	2023	1	0	0	0	0	0	0	0	0	0	0	0	72.5
22294409	m	cn	US	2023	0	1	1	0	0	0	0	0	0	0	0	0	61
22294630	m	ph	JP	2023	0	1	0	1	0	0	0	0	0	0	0	1	79
22295295	Not	md	JP	2023	1	0	0	0	0	0	0	0	0	0	0	0	
22295311	m	cn	CA	2023	0	1	0	0	1	0	0	0	0	0	0	0	58
22295379	m	cn	IN	2023	0	0	0	0	0	0	0	0	0	0	0	0	88
22295823	Not	cn	US	2023	0	0	0	0	0	0	0	0	0	0	0	0	
22295973	f	md	RO	2023	0	1	1	0	0	0	0	0	0	0	0	0	67
22296165	f	md	IT	2023	0	0	0	0	0	0	0	0	0	0	0	0	69
22296531	f	md	BR	2023	0	0	0	0	0	0	0	0	0	0	0	0	40
22297474	m	md	DE	2023	0	1	1	0	0	0	0	0	0	0	0	0	
22297922	f	hp	IT	2023	0	1	1	0	0	0	0	0	0	0	0	0	40
22297927	f	hp	IT	2023	0	0	0	0	0	0	0	0	0	0	0	0	76
22297929	m	hp	IT	2023	0	1	1	0	0	0	0	0	0	0	0	0	61
22297932	m	hp	IT	2023	0	1	1	0	0	0	0	0	0	0	0	0	75
22297933	f	hp	IT	2023	0	1	1	0	0	0	0	0	0	0	0	0	29
22297934	f	hp	IT	2023	0	1	1	0	0	0	0	0	0	0	0	0	58
22297935	f	hp	IT	2023	0	1	1	0	0	0	0	0	0	0	0	0	74
22297936	m	hp	IT	2023	0	1	1	0	0	0	0	0	0	0	0	0	71
22297939	m	hp	IT	2023	0	1	1	0	0	0	0	0	0	0	0	0	70
22298027	f	md	RO	2023	0	1	0	0	0	1	0	0	0	0	0	0	58
22298212	f	ph	FR	2023	0	1	1	0	0	0	0	0	0	0	0	0	68
22298636	m	cn	US	2023	1	0	0	0	0	0	0	0	0	0	0	0	
22299121	f	hp	US	2023	0	1	0	0	1	0	0	0	0	0	0	0	76
22299305	m	md	NO	2023	0	0	0	0	0	0	0	0	0	0	0	0	69
22299618	f	cn	IN	2023	0	0	0	0	0	0	0	0	0	0	0	0	46
22299822	Not	hp	CN	2023	0	0	0	0	0	0	0	0	0	0	0	0	
22299823	Not	hp	CN	2023	0	0	0	0	0	0	0	0	0	0	0	0	
22299825	Not	hp	CN	2023	0	0	0	0	0	0	0	0	0	0	0	0	
22300308	f	md	US	2023	0	1	1	0	0	0	0	0	0	0	0	0	68
22300573	m	md	BG	2023	0	1	1	0	0	0	0	0	0	0	0	0	68

22301676	f	md	IT	2023	0	1	0	0	0	1	0	0	0	0	0	0	54
22302049	m	md	DE	2023	0	1	1	0	0	0	0	0	0	0	0	0	79
22302363	m	md	IT	2023	0	1	0	0	0	1	0	0	0	0	0	0	70
22302599	f	cn	ID	2023	1	0	0	0	0	0	0	0	0	0	0	0	32.3
22302883	m	md	JP	2023	0	0	0	0	0	0	0	0	0	0	0	0	70
22303448	f	md	FR	2023	1	0	0	0	0	0	0	0	0	0	0	0	
22303658	f	hp	RU	2023	0	0	0	0	0	0	0	0	0	0	0	0	
22303673	m	cn	AE	2023	0	0	0	0	0	0	0	0	0	0	0	0	48
22304049	f	cn	US	2023	0	0	0	0	0	0	0	0	0	0	0	0	72
22304621	f	cn	US	2023	1	0	0	0	0	0	0	0	0	0	0	0	79.5
22304957	m	md	BA	2023	0	1	1	0	0	0	0	0	0	0	0	0	55
22305457	m	md	DE	2023	0	0	0	0	0	0	0	0	0	0	0	0	60
22305645	f	ph	JP	2023	0	0	0	0	0	0	0	0	0	0	0	0	
22305701	f	md	RO	2023	0	1	0	0	1	0	0	0	0	0	0	1	54
22305703	f	hp	US	2023	0	1	0	0	1	0	0	0	0	0	0	0	85
22305824	f	cn	CA	2023	1	0	0	0	0	0	0	0	0	0	0	0	
22308639	f	md	PA	2023	0	0	0	0	0	0	0	0	0	0	0	0	
22309525	f	hp	CA	2023	0	0	0	0	0	0	0	0	0	0	0	0	77
22310236	f	cn	VN	2023	1	0	0	0	0	0	0	0	0	0	0	0	
22310297	m	hp	GR	2023	0	1	1	0	0	0	0	0	0	0	0	0	70
22310742	m	hp	TR	2023	0	0	0	0	0	0	0	0	0	0	0	0	53
22310911	m	cn	US	2023	1	0	0	0	0	0	0	0	0	0	0	0	
22311394	m	ph	ES	2023	0	1	1	0	0	0	0	0	0	0	0	0	74
22311519	f	cn	CA	2023	0	0	0	0	0	0	0	0	0	0	0	0	45
22311839	m	md	DE	2023	0	1	0	0	1	0	0	0	0	0	0	0	57
22312067	f	md	JP	2023	0	0	0	0	0	0	0	0	0	0	0	0	48
22312328	Not	cn	US	2023	1	0	0	0	0	0	0	0	0	0	0	0	57.7
22312565	m	ph	JP	2023	0	1	0	0	0	1	0	0	0	0	0	0	73
22313108	m	md	DE	2023	0	1	1	0	0	0	0	0	0	0	0	0	
22313162	Not	cn	US	2023	1	0	0	0	0	0	0	0	0	0	0	0	80
22313168	m	md	DE	2023	0	1	1	0	0	0	0	0	0	0	0	0	
22313216	m	md	IT	2023	0	0	0	0	0	0	0	0	0	0	0	0	
22313273	Not	md	JP	2023	0	1	0	0	1	0	0	0	0	0	0	0	
22313569	m	md	GR	2023	0	1	1	0	0	0	0	0	0	0	0	0	75

22313596	m	cn	CA	2023	1	0	0	0	0	0	0	0	0	0	0	0	67.9
22313826	m	md	JP	2023	0	1	0	0	0	1	0	0	0	0	0	0	80
22313875	m	ph	MY	2023	1	0	0	0	0	0	0	0	0	0	0	0	
22313917	f	md	DE	2023	0	1	0	0	1	0	0	0	0	0	0	0	79
22314079	f	ph	FR	2023	0	1	1	0	0	0	0	0	0	0	0	0	68
22314083	f	hp	US	2023	0	1	0	0	1	0	0	0	0	0	0	0	72
22314257	m	md	DE	2023	0	1	0	0	1	0	0	0	0	0	0	0	69
22314753	m	md	DE	2023	0	1	0	0	1	0	0	0	0	0	0	0	69
22315167	f	md	CN	2023	1	0	0	0	0	0	0	0	0	0	0	0	61
22315261	Not	hp	IT	2023	0	1	1	0	0	0	0	0	0	0	0	0	
22315319	f	cn	IN	2023	1	0	0	0	0	0	0	0	0	0	0	0	
22315527	m	md	IN	2023	1	0	0	0	0	0	0	0	0	0	0	0	58
22315990	f	md	US	2023	0	1	1	0	0	0	0	0	0	0	0	0	61
22316007	m	hp	US	2023	0	1	0	0	1	0	0	0	0	0	0	0	57
22316183	Not	md	GR	2023	0	0	0	0	0	0	0	0	0	0	0	0	
22316422	f	cn	IN	2023	0	0	0	0	0	0	0	0	0	0	0	0	71
22316515	f	cn	US	2023	0	0	0	0	0	0	0	0	0	0	0	0	
22316571	f	cn	US	2023	0	0	0	0	0	0	0	0	0	0	0	0	
22316669	m	md	DE	2023	0	1	0	0	1	0	0	0	0	0	0	0	69
22316919	f	cn	US	2023	0	0	0	0	0	0	0	0	0	0	0	0	75
22317154	f	cn	US	2023	1	0	0	0	0	0	0	0	0	0	0	0	
22317195	m	md	DE	2023	0	1	0	0	1	0	0	0	0	0	0	0	65
22317211	Not	md	GB	2023	0	1	0	0	0	1	0	0	0	0	0	0	
22317628	f	hp	US	2023	0	0	0	0	0	0	0	0	0	0	0	0	
22318351	m	hp	GB	2023	0	1	0	1	0	0	0	0	0	0	0	0	58
22318482	f	md	DE	2023	0	1	1	0	0	0	0	0	0	0	0	0	61
22319093	f	cn	US	2023	0	1	1	0	0	0	0	0	0	0	0	0	
22319129	f	cn	US	2023	1	0	0	0	0	0	0	0	0	0	0	0	79.6
22319247	f	cn	US	2023	1	0	0	0	0	0	0	0	0	0	0	0	65
22319269	m	md	AT	2023	0	1	1	0	0	0	0	0	0	0	0	0	
22319550	m	md	DE	2023	0	1	1	0	0	0	0	0	0	0	0	0	
22319719	f	md	CN	2023	0	1	1	0	0	0	0	0	0	0	0	0	64
22320169	f	md	ZA	2023	0	1	0	1	0	0	0	0	0	0	0	0	
22320215	m	cn	TH	2023	1	0	0	0	0	0	0	0	0	0	0	0	85

22320641	m	md	HU	2023	0	1	1	0	0	0	0	0	0	0	0	0	55
22320662	m	lw	FR	2023	0	1	1	0	0	0	0	0	0	0	0	0	
22320738	f	md	DE	2023	0	0	0	0	0	0	0	0	0	0	0	0	
22321417	m	cn	CA	2023	1	0	0	0	0	0	0	0	0	0	0	0	76
22321694	f	md	US	2023	0	0	0	0	0	0	0	0	0	0	0	0	77
22321732	f	cn	US	2023	0	1	0	0	1	0	0	0	0	0	0	0	64
22322673	f	cn	MY	2023	1	0	0	0	0	0	0	0	0	0	0	0	
22323219	f	md	FR	2023	0	1	0	1	0	0	0	0	0	0	0	0	65
22323224	m	md	DE	2023	0	1	0	0	1	0	0	0	0	0	0	0	65
22323564	m	md	DE	2023	0	0	0	0	0	0	0	0	0	0	0	0	60
22324883	f	cn	CN	2023	0	0	0	0	0	0	0	0	0	0	0	0	68
22324923	m	md	BE	2023	0	1	0	0	1	0	0	0	0	0	0	0	52
22325121	f	md	RU	2023	1	0	0	0	0	0	0	0	0	0	0	0	68
22325205	f	md	JP	2023	0	1	1	0	0	0	0	0	0	0	0	0	70
22325288	Not	md	JP	2023	1	0	0	0	0	0	0	0	0	0	0	0	
22325742	m	md	JP	2023	0	1	0	1	0	0	0	0	0	0	0	0	55
22326022	f	md	BG	2023	0	1	1	0	0	0	0	0	0	0	0	0	57
22328158	m	md	JP	2023	0	1	1	0	0	0	0	0	0	0	0	0	68
22328174	m	md	IQ	2023	0	1	1	0	0	0	0	0	0	0	0	0	65
22329396	m	cn	CN	2023	0	0	0	0	0	0	0	0	0	0	0	0	48
22329498	m	md	JP	2023	0	1	0	0	1	0	0	0	0	0	0	1	84
22330457	m	cn	CA	2023	0	0	0	0	0	0	0	0	0	0	0	0	79
22330570	m	hp	DE	2023	0	1	0	0	1	0	0	0	0	0	0	0	60
22330733	m	md	US	2023	0	1	1	0	0	0	0	0	0	0	0	0	70
22330747	m	md	DE	2023	0	1	1	0	0	0	0	0	0	0	0	0	
22331123	f	md	JP	2023	0	1	0	0	1	0	0	0	0	0	0	0	80
22331288	f	hp	DE	2023	0	1	1	0	0	0	0	0	0	0	0	0	33
22331652	m	md	DE	2023	0	1	1	0	0	0	0	0	0	0	0	0	
22331789	f	md	ES	2023	0	1	1	0	0	0	0	0	0	0	0	0	53
22332177	m	md	JP	2023	0	1	0	0	1	0	0	0	0	0	0	0	49
22332513	m	hp	US	2023	0	0	0	0	0	0	0	0	0	0	0	0	62
22332595	Not	md	CA	2023	0	0	0	0	0	0	0	0	0	0	0	0	
22332749	m	md	CA	2023	0	0	0	0	0	0	0	0	0	0	0	0	67
22332847	f	md	ES	2023	0	0	0	0	0	0	0	0	0	0	0	0	58

22332853	f	cn	US	2023	1	0	0	0	0	0	0	0	0	0	0	0	
22333723	m	ph	TH	2023	0	0	0	0	0	0	0	0	0	0	0	0	29
22333794	Not	md	CH	2023	0	0	0	0	0	0	0	0	0	0	0	0	
22334041	Not	md	JP	2023	0	1	0	0	0	1	0	0	0	0	0	0	
22335472	f	md	JP	2023	0	1	0	0	0	1	0	0	0	0	0	0	70
22335643	f	ph	US	2023	1	0	0	0	0	0	0	0	0	0	0	0	57
22335680	f	hp	ES	2023	1	0	0	0	0	0	0	0	0	0	0	0	77
22335811	f	md	IT	2023	0	0	0	0	0	0	0	0	0	0	0	0	54
22337120	m	hp	US	2023	0	1	0	0	1	0	0	0	0	0	0	0	59
22337709	f	md	US	2023	0	1	1	0	0	0	0	0	0	0	0	1	66
22338148	f	cn	CA	2023	1	0	0	0	0	0	0	0	0	0	0	0	62.2
22339387	m	hp	JP	2023	0	0	0	0	0	0	0	0	0	0	0	0	56
22339629	f	md	BG	2023	0	1	1	0	0	0	0	0	0	0	0	0	57
22339719	m	md	JP	2023	0	1	1	0	0	0	0	0	0	0	0	0	82
22339893	f	cn	TH	2023	1	0	0	0	0	0	0	0	0	0	0	0	86
22339897	f	hp	US	2023	0	1	0	0	1	0	0	0	0	0	0	0	57
22339944	m	md	HR	2023	0	1	1	0	0	0	0	0	0	0	0	1	71
22340492	m	md	CZ	2023	0	1	0	0	1	0	0	0	0	0	0	0	61
22340813	m	md	BE	2023	0	1	0	0	1	0	0	0	0	0	0	0	52
22341081	f	hp	US	2023	0	1	0	0	1	0	0	0	0	0	0	0	70
22341914	f	md	GB	2023	0	0	0	0	0	0	0	0	0	0	0	0	39
22342018	Not	ph	JP	2023	1	0	0	0	0	0	0	0	0	0	0	0	
22342061	f	cn	US	2023	1	0	0	0	0	0	0	0	0	0	0	0	70.3
22342200	Not	md	BG	2023	1	0	0	0	0	0	0	0	0	0	0	0	
22342289	m	ph	FR	2023	0	0	0	0	0	0	0	0	0	0	0	0	68
22342362	f	cn	CA	2023	1	0	0	0	0	0	0	0	0	0	0	0	66.7
22342617	f	md	CA	2023	1	0	0	0	0	0	0	0	0	0	0	0	
22342801	m	Not repo	Not re	2023	0	0	0	0	0	0	0	0	0	0	0	0	56
22342844	m	hp	RU	2023	0	1	0	0	0	1	0	0	0	0	0	0	64
22343085	m	md	JP	2023	0	1	0	0	0	1	0	0	0	0	0	1	70
22343278	m	md	JP	2023	0	1	0	1	0	0	0	0	0	0	0	1	71
22343373	Not	md	FR	2023	0	0	0	0	0	0	0	0	0	0	0	0	65
22343405	m	md	JP	2023	0	0	0	0	0	0	0	0	0	0	0	0	
22343682	f	md	FR	2023	0	0	0	0	0	0	0	0	0	0	0	0	52

22343739	m	md	JP	2023	0	0	0	0	0	0	0	0	0	0	0	0	47
22344046	m	md	NO	2023	0	0	0	0	0	0	0	0	0	0	0	0	69
22344061	m	hp	IN	2023	0	0	0	0	0	0	0	0	0	0	0	0	53
22344335	Not	md	FR	2023	0	0	0	0	0	0	0	0	0	0	0	0	
22344336	m	md	JP	2023	0	0	0	0	0	0	0	0	0	0	0	0	79
22345199	f	cn	CN	2023	0	0	0	0	0	0	0	0	0	0	0	0	54
22345347	Not	md	JP	2023	0	0	0	0	0	0	0	0	0	0	0	0	90
22345516	m	cn	MY	2023	1	0	0	0	0	0	0	0	0	0	0	0	74.5
22345831	f	md	RO	2023	1	0	0	0	0	0	0	0	0	0	0	0	56
22345848	m	hp	US	2023	0	1	0	0	1	0	0	0	0	0	0	0	72
22346448	Not	ph	JP	2023	1	0	0	0	0	0	0	0	0	0	0	0	
22347369	m	ph	JP	2023	0	0	0	0	0	0	0	0	0	0	0	0	71
22347424	f	md	JP	2023	0	0	0	0	0	0	0	0	0	0	0	0	70
22347571	m	md	AU	2023	1	0	0	0	0	0	0	0	0	0	0	0	
22347678	Not	ph	JP	2023	1	0	0	0	0	0	0	0	0	0	0	0	
22347729	f	cn	US	2023	1	0	0	0	0	0	0	0	0	0	0	0	74
22348206	m	cn	US	2023	0	1	1	0	0	0	0	0	0	0	0	0	77
22348351	f	cn	CN	2023	0	0	0	0	0	0	0	0	0	0	0	0	57
22348491	Not	cn	US	2023	1	0	0	0	0	0	0	0	0	0	0	0	
22348850	m	md	DE	2023	0	0	0	0	0	0	0	0	0	0	0	0	60
22348862	f	md	JP	2023	0	1	0	1	0	0	0	0	0	0	0	0	
22349258	m	md	FR	2023	0	1	0	0	0	1	0	0	0	0	0	0	79
22349294	f	md	IN	2023	0	1	0	0	0	1	0	0	0	0	0	0	
22349348	m	md	IT	2023	0	0	0	0	0	0	0	0	0	0	0	0	63
22349352	f	md	JP	2023	0	1	0	0	0	1	0	0	0	0	0	0	60
22349367	m	md	US	2023	0	1	1	0	0	0	0	0	0	0	0	0	
22349461	m	ph	PK	2023	0	1	0	0	0	1	0	0	0	0	0	0	66
22349991	Not	md	SG	2023	0	0	0	0	0	0	0	0	0	0	0	0	
22350252	Not	md	JP	2023	0	0	0	0	0	0	0	0	0	0	0	0	75
22350294	m	cn	US	2023	1	0	0	0	0	0	0	0	0	0	0	0	
22350425	m	md	JP	2023	0	1	0	1	0	0	0	0	0	0	0	1	67
22350469	f	cn	KR	2023	0	0	0	0	0	0	0	0	0	0	0	0	65
22350520	f	md	CN	2023	0	0	0	0	0	0	0	0	0	0	0	0	72
22350522	f	md	FR	2023	1	0	0	0	0	0	0	0	0	0	0	0	35

22351486	f	md	CA	2023	0	0	0	0	0	0	0	0	0	0	0	0	
22351490	m	md	JP	2023	0	1	1	0	0	0	0	0	0	0	0	0	90
22351618	Not	md	GR	2023	0	0	0	0	0	0	0	0	0	0	0	0	
22351642	m	md	CH	2023	0	0	0	0	0	0	0	0	0	0	0	0	
22351646	m	cn	TH	2023	1	0	0	0	0	0	0	0	0	0	0	0	84
22352105	f	hp	CA	2023	0	0	0	0	0	0	0	0	0	0	0	0	75
22352740	m	md	JP	2023	1	0	0	0	0	0	0	0	0	0	0	0	83
22353035	f	md	FR	2023	0	1	0	1	0	0	0	0	0	0	0	0	65
22353142	Not	md	AU	2023	1	0	0	0	0	0	0	0	0	0	0	0	
22353201	f	md	SE	2023	0	0	0	0	0	0	0	0	0	0	0	0	
22353579	m	md	DE	2023	0	1	0	0	1	0	0	0	0	0	0	0	70
22354235	f	md	NL	2023	0	0	0	0	0	0	0	0	0	0	0	0	80
22354960	m	md	AU	2023	1	0	0	0	0	0	0	0	0	0	0	0	
22355148	Not	hp	US	2023	0	0	0	0	0	0	0	0	0	0	0	0	
22355856	f	md	JP	2023	0	0	0	0	0	0	0	0	0	0	0	0	
22355927	f	md	FR	2023	0	0	0	0	0	0	0	0	0	0	0	0	56
22356134	Not	md	US	2023	0	0	0	0	0	0	0	0	0	0	0	0	
22356300	m	md	US	2023	0	1	0	1	0	0	0	0	0	0	0	1	78.4
22356304	m	md	CN	2023	0	1	0	1	0	0	0	0	0	0	0	0	73
22356509	m	md	BE	2023	0	1	0	0	1	0	0	0	0	0	0	0	52
22356547	Not	hp	US	2023	1	0	0	0	0	0	0	0	0	0	0	0	
22356812	Not	hp	SG	2023	1	0	0	0	0	0	0	0	0	0	0	0	89.9
22357046	m	md	GB	2023	0	1	0	0	0	1	0	0	0	0	0	0	50
22357126	m	md	PL	2023	0	1	0	1	0	0	0	0	0	0	0	0	
22357834	m	md	DK	2023	0	1	0	0	0	1	0	0	0	0	0	0	71
22358278	m	md	JP	2023	0	0	0	0	0	0	0	0	0	0	0	0	
22358615	Not	md	US	2023	0	0	0	0	0	0	0	0	0	0	0	0	
22358680	Not	md	JP	2023	0	1	0	0	0	1	0	0	0	0	0	0	60
22358774	m	hp	FR	2023	0	0	0	0	0	0	0	0	0	0	0	0	89
22359377	Not	md	JP	2023	0	0	0	0	0	0	0	0	0	0	0	0	
22360440	f	hp	US	2023	0	0	0	0	0	0	0	0	0	0	0	0	
22360469	Not	md	JP	2023	0	1	0	0	1	0	0	0	0	0	0	0	80
22360979	f	md	KR	2023	0	0	0	0	0	0	0	0	0	0	0	0	
22361003	f	md	ES	2023	1	0	0	0	0	0	0	0	0	0	0	0	56

22361282	m	md	ES	2023	0	0	0	0	0	0	0	0	0	0	0	0	56
22361396	m	hp	IN	2023	0	1	0	0	0	1	0	0	0	0	0	0	53
22361472	m	md	JP	2023	0	1	0	0	1	0	0	0	0	0	0	0	74
22361840	m	md	CN	2023	0	1	0	0	1	0	0	0	0	0	0	0	
22361960	Not	md	JP	2023	0	0	0	0	0	0	0	0	0	0	0	0	
22362013	f	cn	US	2023	0	0	0	0	0	0	0	0	0	0	0	0	88
22362037	f	cn	IN	2023	0	0	0	0	0	0	0	0	0	0	0	0	78
22362605	m	cn	CN	2023	0	1	0	1	0	0	0	0	0	0	0	0	68
22362869	m	hp	IT	2023	0	0	0	0	0	0	0	0	0	0	0	0	58
22363640	m	md	JP	2023	0	0	0	0	0	0	0	0	0	0	0	0	74
22365151	f	md	JP	2023	0	0	0	0	0	0	0	0	0	0	0	0	59
22365374	m	md	JP	2023	0	1	0	0	0	1	0	0	0	0	0	0	67
22365671	m	md	JP	2023	1	0	0	0	0	0	0	0	0	0	0	0	75
22366521	f	hp	US	2023	0	0	0	0	0	0	0	0	0	0	0	0	
22366892	m	cn	IT	2023	0	0	0	0	0	0	0	0	0	0	0	0	50
22367124	m	md	DE	2023	0	1	0	0	1	0	0	0	0	0	0	0	70
22367714	m	md	US	2023	0	0	0	0	0	0	0	0	0	0	0	0	72
22367963	Not	cn	SG	2023	1	0	0	0	0	0	0	0	0	0	0	0	73.4
22368781	f	md	DE	2023	1	0	0	0	0	0	0	0	0	0	0	0	56
22369107	m	cn	CN	2023	0	1	0	1	0	0	0	0	0	0	0	0	57
22369653	f	md	JP	2023	1	0	0	0	0	0	0	0	0	0	0	0	73
22369907	f	md	JP	2023	0	0	0	0	0	0	0	0	0	0	0	0	
22370139	f	md	JP	2023	0	0	0	0	0	0	0	0	0	0	0	0	80
22370210	Not	hp	CN	2023	0	0	0	0	0	0	0	0	0	0	0	0	
22370593	Not	md	JP	2023	0	0	0	0	0	0	0	0	0	0	0	0	
22370746	m	md	JP	2023	0	1	1	0	0	0	0	0	0	0	0	0	80
22370753	m	md	KR	2023	0	0	0	0	0	0	0	0	0	0	0	0	71
22371329	f	ph	FR	2023	0	1	1	0	0	0	0	0	0	0	0	0	68
22371502	Not	hp	GB	2023	0	0	0	0	0	0	0	0	0	0	0	0	
22371570	f	md	IT	2023	0	1	1	0	0	0	0	0	0	0	0	0	63
22371964	f	cn	GB	2023	0	0	0	0	0	0	0	0	0	0	0	0	
22372871	m	md	IT	2023	0	0	0	0	0	0	0	0	0	0	0	0	79
22372962	Not	md	SG	2023	1	0	0	0	0	0	0	0	0	0	0	0	70
22373071	f	hp	US	2023	0	0	0	0	0	0	0	0	0	0	0	0	

22373479	m	hp	ES	2023	0	0	0	0	0	0	0	0	0	0	0	0	69
22373557	f	md	TR	2023	0	1	0	0	0	1	0	0	0	0	0	0	76
22373656	Not	ph	JP	2023	1	0	0	0	0	0	0	0	0	0	0	0	
22373660	Not	md	AU	2023	0	1	0	1	0	0	0	0	0	0	0	0	
22374186	m	hp	NL	2023	0	0	0	0	0	0	0	0	0	0	0	0	66
22374187	m	ph	JP	2023	0	0	0	0	0	0	0	0	0	0	0	0	71
22374489	Not	md	HK	2023	0	0	0	0	0	0	0	0	0	0	0	0	
22374913	Not	hp	GB	2023	0	0	0	0	0	0	0	0	0	0	0	0	
22375427	Not	cn	SG	2023	1	0	0	0	0	0	0	0	0	0	0	0	72.6
22375641	Not	md	SG	2023	1	0	0	0	0	0	0	0	0	0	0	0	64
22376521	Not	cn	SG	2023	1	0	0	0	0	0	0	0	0	0	0	0	76.5
22376552	Not	cn	SG	2023	1	0	0	0	0	0	0	0	0	0	0	0	83
22376591	Not	hp	US	2023	1	0	0	0	0	0	0	0	0	0	0	0	56.3
22376628	Not	cn	SG	2023	1	0	0	0	0	0	0	0	0	0	0	0	70.1
22376647	f	md	US	2023	0	1	1	0	0	0	0	0	0	0	0	0	66
22377382	m	cn	US	2023	0	0	0	0	0	0	0	0	0	0	0	0	
22377462	Not	cn	SG	2023	1	0	0	0	0	0	0	0	0	0	0	0	67.7
22377640	m	md	DE	2023	0	1	0	1	0	0	0	0	0	0	0	0	
22377681	Not	cn	SG	2023	1	0	0	0	0	0	0	0	0	0	0	0	85.7
22377928	Not	cn	SG	2023	1	0	0	0	0	0	0	0	0	0	0	0	69.4
22378308	Not	md	US	2023	0	0	0	0	0	0	0	0	0	0	0	0	
22378573	f	cn	MY	2023	1	0	0	0	0	0	0	0	0	0	0	0	62
22378738	m	md	DE	2023	0	1	0	0	1	0	0	0	0	0	0	0	83
22378930	f	md	AT	2023	0	1	0	0	0	1	0	0	0	0	0	0	76
22379273	m	md	DE	2023	0	1	0	0	1	0	0	0	0	0	0	0	69
22379668	m	cn	US	2023	0	0	0	0	0	0	0	0	0	0	0	0	
22379852	f	md	CZ	2023	0	1	0	0	1	0	0	0	0	0	0	0	72
22379963	Not	ph	JP	2023	0	0	0	0	0	0	0	0	0	0	0	0	
22379997	m	cn	CN	2023	0	1	0	1	0	0	0	0	0	0	0	0	57
22380003	f	md	JP	2023	0	0	0	0	0	0	0	0	0	0	0	0	
22380160	f	md	CZ	2023	0	1	0	0	1	0	0	0	0	0	0	0	72
22380164	f	cn	GB	2023	0	0	0	0	0	0	0	0	0	0	0	0	41
22380264	m	md	CN	2023	0	1	0	0	0	1	0	0	0	0	0	0	55
22380545	f	md	US	2023	0	1	1	0	0	0	0	0	0	0	0	0	68

22380547	f	hp	US	2023	0	1	1	0	0	0	0	0	0	0	0	0	84
22380931	Not	cn	SG	2023	1	0	0	0	0	0	0	0	0	0	0	0	77.2
22381011	Not	md	FR	2023	0	0	0	0	0	0	0	0	0	0	0	0	
22381167	m	md	SG	2023	1	0	0	0	0	0	0	0	0	0	0	0	
22381171	Not	md	FR	2023	0	0	0	0	0	0	0	0	0	0	0	0	
22381415	m	md	JP	2023	0	1	1	0	0	0	0	0	0	0	0	0	100
22381729	f	md	JP	2023	0	1	0	0	0	1	0	0	0	0	0	0	67
22381737	Not	md	ES	2023	0	1	0	0	0	1	0	0	0	0	0	0	
22381841	m	md	FR	2023	0	1	0	1	0	0	0	0	0	0	0	1	
22382231	f	hp	US	2023	0	1	0	1	0	0	0	0	0	0	0	0	
22382328	m	md	RU	2023	0	1	0	0	0	1	0	0	0	0	0	0	64
22382573	Not	md	US	2023	0	0	0	0	0	0	0	0	0	0	0	0	
22382737	m	md	US	2023	0	0	0	0	0	0	0	0	0	0	0	0	68
22382900	m	hp	IN	2023	0	0	0	0	0	0	0	0	0	0	0	0	53
22382980	m	md	JP	2023	1	0	0	0	0	0	0	0	0	0	0	0	79
22383125	Not	ph	JP	2023	1	0	0	0	0	0	0	0	0	0	0	0	
22383212	m	md	HU	2023	0	1	1	0	0	0	0	0	0	0	0	0	55
22383337	m	cn	US	2023	0	1	1	0	0	0	0	0	0	0	0	0	71
22384184	f	md	IT	2023	0	1	1	0	0	0	0	0	0	0	0	0	63
22384909	m	md	NL	2023	0	0	0	0	0	0	0	0	0	0	0	0	
22384973	Not	md	JP	2023	0	1	1	0	0	0	0	0	0	0	0	0	56
22385277	f	md	CN	2023	0	0	0	0	0	0	0	0	0	0	0	0	57
22385420	Not	md	JP	2023	1	0	0	0	0	0	0	0	0	0	0	0	80
22385905	m	md	JP	2023	0	1	1	0	0	0	0	0	0	0	0	0	80
22385909	f	md	GB	2023	0	1	0	0	0	1	0	0	0	0	0	1	28
22386244	f	cn	CA	2023	0	0	0	0	0	0	0	0	0	0	0	0	64
22386277	f	md	US	2023	0	1	1	0	0	0	0	0	0	0	0	0	59
22386280	f	md	JP	2023	0	0	0	0	0	0	0	0	0	0	0	0	76
22386283	m	ph	JP	2023	0	0	0	0	0	0	0	0	0	0	0	0	62
22386372	m	md	US	2023	0	1	1	0	0	0	0	0	0	0	0	0	67
22386995	f	hp	PK	2023	0	1	1	0	0	0	0	0	0	0	0	0	39
22387138	m	ph	JP	2023	0	0	0	0	0	0	0	0	0	0	0	0	
22387282	m	ph	US	2023	1	0	0	0	0	0	0	0	0	0	0	0	67
22387499	Not	md	CN	2023	0	0	0	0	0	0	0	0	0	0	0	0	

22387500	Not	md	CN	2023	0	0	0	0	0	0	0	0	0	0	0	0	
22387542	m	md	CN	2023	0	0	0	0	0	0	0	0	0	0	0	0	54
22387658	f	md	JP	2023	0	1	0	0	1	0	0	0	0	0	0	0	73
22387955	Not	md	CN	2023	0	0	0	0	0	0	0	0	0	0	0	0	
22387956	Not	md	CN	2023	0	0	0	0	0	0	0	0	0	0	0	0	
22387957	Not	md	CN	2023	0	0	0	0	0	0	0	0	0	0	0	0	
22387958	Not	md	CN	2023	0	0	0	0	0	0	0	0	0	0	0	0	
22387959	Not	md	CN	2023	0	0	0	0	0	0	0	0	0	0	0	0	
22387960	Not	md	CN	2023	0	0	0	0	0	0	0	0	0	0	0	0	
22388017	f	hp	US	2023	0	0	0	0	0	0	0	0	0	0	0	0	34
22388018	f	hp	US	2023	0	0	0	0	0	0	0	0	0	0	0	0	69
22388020	f	hp	US	2023	0	0	0	0	0	0	0	0	0	0	0	0	63
22388193	f	hp	US	2023	1	0	0	0	0	0	0	0	0	0	0	0	66
22388433	f	cn	IN	2023	1	0	0	0	0	0	0	0	0	0	0	0	62
22388739	Not	md	CN	2023	0	0	0	0	0	0	0	0	0	0	0	0	
22388869	Not	cn	IT	2023	0	0	0	0	0	0	0	0	0	0	0	0	
22388893	f	md	NO	2023	0	0	0	0	0	0	0	0	0	0	0	0	51
22389098	f	md	DE	2023	0	0	0	0	0	0	0	0	0	0	0	0	60
22389240	f	cn	CN	2023	0	1	0	1	0	0	0	0	0	0	0	0	72
22389488	m	md	US	2023	0	1	1	0	0	0	0	0	0	0	0	1	68
22389611	f	md	US	2023	0	1	1	0	0	0	0	0	0	0	0	0	59
22389736	m	hp	RU	2023	0	1	0	0	0	1	0	0	0	0	0	0	68
22389755	f	cn	US	2023	0	0	0	0	0	0	0	0	0	0	0	0	72
22389887	m	hp	US	2023	0	0	0	0	0	0	0	0	0	0	0	0	
22390082	f	hp	CA	2023	0	0	0	0	0	0	0	0	0	0	0	0	69
22390091	Not	ph	JP	2023	1	0	0	0	0	0	0	0	0	0	0	0	
22390405	m	md	CN	2023	0	0	0	0	0	0	0	0	0	0	0	0	53
22390574	f	hp	US	2023	1	0	0	0	0	0	0	0	0	0	0	0	78
22390720	m	md	JP	2023	0	0	0	0	0	0	0	0	0	0	0	0	80
22390806	m	md	SG	2023	0	0	0	0	0	0	0	0	0	0	0	0	53
22390807	m	md	IT	2023	0	0	0	0	0	0	0	0	0	0	0	0	
22390810	f	md	IT	2023	0	0	0	0	0	0	0	0	0	0	0	0	
22391037	m	hp	CA	2023	0	0	0	0	0	0	0	0	0	0	0	0	62
22391585	m	md	IT	2023	0	0	0	0	0	0	0	0	0	0	0	0	63

22391696	m	md	IT	2023	0	0	0	0	0	0	0	0	0	0	0	0	63
22391750	Not	md	JP	2023	0	1	0	0	0	1	0	0	0	0	0	0	75
22392127	f	cn	US	2023	0	0	0	0	0	0	0	0	0	0	0	0	
22392938	f	hp	BR	2023	1	0	0	0	0	0	0	0	0	0	0	0	34
22393243	f	hp	RU	2023	0	0	0	0	0	0	0	0	0	0	0	0	65
22393380	m	cn	PK	2023	0	1	0	0	0	1	0	0	0	0	0	0	67
22393487	f	md	CN	2023	0	0	0	0	0	0	0	0	0	0	0	0	67
22393491	m	md	PT	2023	0	0	0	0	0	0	0	0	0	0	0	0	40
22393882	m	md	RU	2023	0	0	0	0	0	0	0	0	0	0	0	0	68
22394011	m	md	ES	2023	0	1	0	0	0	1	0	0	0	0	0	0	70
22394208	m	md	CN	2023	0	0	0	0	0	0	0	0	0	0	0	0	69
22394617	Not	md	HK	2023	0	0	0	0	0	0	0	0	0	0	0	0	82
22395125	Not	ph	JP	2023	1	0	0	0	0	0	0	0	0	0	0	0	
22395860	f	cn	SG	2023	1	0	0	0	0	0	0	0	0	0	0	0	79.8
22395874	f	md	ES	2023	0	1	1	0	0	0	0	0	0	0	0	0	
22396861	f	md	JP	2023	0	1	1	0	0	0	0	0	0	0	0	0	80
22396931	f	md	RO	2023	1	0	0	0	0	0	0	0	0	0	0	0	
22397703	f	md	JP	2023	0	1	0	0	0	1	0	0	0	0	0	1	75
22397752	m	md	IN	2023	0	1	0	0	0	1	0	0	0	0	0	0	
22398570	Not	md	JP	2023	0	1	0	1	0	0	0	0	0	0	0	0	
22398832	m	md	GB	2023	0	1	0	0	0	1	0	0	0	0	0	0	64
22398851	f	cn	SG	2023	1	0	0	0	0	0	0	0	0	0	0	0	67.3
22398930	f	md	JP	2023	0	1	0	0	1	0	0	0	0	0	0	0	77
22399138	m	hp	US	2023	0	1	1	0	0	0	0	0	0	0	0	0	67
22399140	m	md	DE	2023	0	1	0	1	0	0	0	0	0	0	0	0	
22399179	m	cn	KR	2023	0	0	0	0	0	0	0	0	0	0	0	0	37
22399484	f	md	CN	2023	1	0	0	0	0	0	0	0	0	0	0	0	60
22399934	m	md	JP	2023	0	0	0	0	0	0	0	0	0	0	0	0	84
22399944	f	md	GB	2023	0	1	0	0	0	1	0	0	0	0	0	1	28
22400014	m	md	JP	2023	0	1	1	0	0	0	0	0	0	0	0	0	80
22400070	m	md	US	2023	0	0	0	0	0	0	0	0	0	0	0	0	
22400084	m	md	IT	2023	0	1	0	0	1	0	0	0	0	0	0	0	76
22400142	f	md	CN	2023	0	0	0	0	0	0	0	0	0	0	0	0	55
22400292	m	md	JP	2023	1	0	0	0	0	0	0	0	0	0	0	1	77

22400340	Not	md	JP	2023	1	0	0	0	0	0	0	0	0	0	0	0	
22400355	f	cn	TH	2023	1	0	0	0	0	0	0	0	0	0	0	0	77.8
22400356	f	md	JP	2023	1	0	0	0	0	0	0	0	0	0	0	0	63.1
22400408	Not	md	FR	2023	0	0	0	0	0	0	0	0	0	0	0	0	
22400414	Not	md	FR	2023	0	0	0	0	0	0	0	0	0	0	0	0	
22400670	Not	md	TW	2023	0	1	0	1	0	0	0	0	0	0	0	0	
22400859	Not	hp	US	2023	0	0	0	0	0	0	0	0	0	0	0	0	
22516454	m	md	DE	2023	0	0	0	0	0	0	0	0	0	0	0	0	
22516457	Not	md	AU	2023	0	0	0	0	0	0	0	0	0	0	0	0	56
22516640	Not	cn	US	2023	1	0	0	0	0	0	0	0	0	0	0	0	
22517302	m	md	IN	2023	0	0	0	0	0	0	0	0	0	0	0	0	53
22517500	f	cn	US	2023	1	0	0	0	0	0	0	0	0	0	0	0	
22517774	m	md	GR	2023	0	1	0	0	1	0	0	0	0	0	0	0	65
22517880	m	md	CZ	2023	0	1	0	0	1	0	0	0	0	0	0	0	72
22517947	f	cn	CA	2023	1	0	0	0	0	0	0	0	0	0	0	0	
22518937	m	md	JP	2023	0	1	0	0	1	0	0	0	0	0	0	0	71
22519029	f	cn	US	2023	0	0	0	0	0	0	0	0	0	0	0	0	65
22519400	m	cn	PH	2023	0	1	0	0	0	1	0	0	0	0	0	0	71
22520101	m	cn	ID	2023	1	0	0	0	0	0	0	0	0	0	0	0	54.6
22520280	Not	md	LB	2023	0	0	0	0	0	0	0	0	0	0	0	0	
22520320	f	md	AU	2023	0	0	0	0	0	0	0	0	0	0	0	0	66
22520344	m	cn	AR	2023	0	1	0	1	0	0	0	0	0	0	0	0	68
22520525	f	cn	ID	2023	1	0	0	0	0	0	0	0	0	0	0	0	65.1
22520790	m	md	JP	2023	0	0	0	0	0	0	0	0	0	0	0	0	71
22520916	Not	md	JP	2023	0	1	1	0	0	0	0	0	0	0	0	0	52
22520945	m	cn	ID	2023	1	0	0	0	0	0	0	0	0	0	0	0	68.3
22520996	f	hp	GB	2023	0	0	0	0	0	0	0	0	0	0	0	0	76
22521047	Not	hp	CN	2023	1	0	0	0	0	0	0	0	0	0	0	0	
22521104	m	cn	EG	2023	1	0	0	0	0	0	0	0	0	0	0	0	80.9
22521207	m	md	JP	2023	0	1	0	1	0	0	0	0	0	0	0	1	77
22521505	f	md	IT	2023	0	1	1	0	0	0	0	0	0	0	0	0	63
22521833	Not	md	DE	2023	0	0	0	0	0	0	0	0	0	0	0	0	
22522330	m	hp	US	2023	0	1	1	0	0	0	0	0	0	0	0	0	54
22522345	f	md	DE	2023	0	1	1	0	0	0	0	0	0	0	0	0	53

22522694	m	cn	CA	2023	0	0	0	0	0	0	0	0	0	0	0	0	73
22522746	f	md	PT	2023	0	0	0	0	0	0	0	0	0	0	0	0	59
22523315	m	md	IT	2023	0	0	0	0	0	0	0	0	0	0	0	0	69
22523390	m	md	JP	2023	0	0	0	0	0	0	0	0	0	0	0	0	80
22523528	m	md	IT	2023	0	0	0	0	0	0	0	0	0	0	0	0	50
22523555	m	md	JP	2023	0	1	0	0	1	0	0	0	0	0	0	1	47
22523704	m	hp	JP	2023	0	1	0	0	0	1	0	0	0	0	0	0	71
22524165	m	cn	US	2023	1	0	0	0	0	0	0	0	0	0	0	0	
22524280	m	hp	KR	2023	0	1	1	0	0	0	0	0	0	0	0	1	66
22524299	m	md	FR	2023	0	0	0	0	0	0	0	0	0	0	0	0	86
22524427	m	md	JP	2023	0	0	0	0	0	0	0	0	0	0	0	0	
22524510	m	md	ES	2023	0	1	0	0	0	1	0	0	0	0	0	0	64
22524731	f	hp	BR	2023	1	0	0	0	0	0	0	0	0	0	0	0	34
22525023	Not	md	JP	2023	0	1	0	1	0	0	0	0	0	0	0	0	
22525126	m	md	JP	2023	0	0	0	0	0	0	0	0	0	0	0	0	55
22525203	m	ph	CA	2023	1	0	0	0	0	0	0	0	0	0	0	0	
22525297	Not	md	JP	2023	1	0	0	0	0	0	0	0	0	0	0	0	
22525586	m	md	FR	2023	0	1	1	0	0	0	0	0	0	0	0	0	72
22525899	f	md	JP	2023	0	1	0	0	0	1	0	0	0	0	0	0	53
22525954	m	md	SK	2023	1	0	0	0	0	0	0	0	0	0	0	0	75
22526171	f	hp	CH	2023	0	0	0	0	0	0	0	0	0	0	0	0	57
22526478	Not	hp	NL	2023	0	0	0	0	0	0	0	0	0	0	0	0	
22526787	m	md	RU	2023	0	0	0	0	0	0	0	0	0	0	0	0	49
22526840	f	md	BG	2023	0	1	1	0	0	0	0	0	0	0	0	0	71
22527565	f	md	AU	2023	0	0	0	0	0	0	0	0	0	0	0	0	
22527627	Not	cn	VN	2023	0	1	0	1	0	0	0	0	0	0	0	0	
22527669	m	md	DE	2023	0	1	0	0	1	0	0	0	0	0	0	0	62
22529288	f	hp	PT	2023	0	0	0	0	0	0	0	0	0	0	0	0	56
22529752	f	hp	US	2023	0	0	0	0	0	0	1	1	0	0	0	0	34
22529797	m	cn	CN	2023	0	1	0	1	0	0	0	0	0	0	0	0	81
22529822	f	md	MX	2023	0	1	0	0	0	1	0	0	0	0	0	1	76
22529908	f	ph	CA	2023	1	0	0	0	0	0	0	0	0	0	0	0	
22530113	Not	md	JP	2023	0	1	0	0	1	0	0	0	0	0	0	0	
22530181	f	ph	US	2023	0	1	1	0	0	0	0	0	0	0	0	0	60

22530336	f	md	IT	2023	0	1	0	0	1	0	0	0	0	0	0	0	62
22530345	f	ph	JP	2023	1	0	0	0	0	0	0	0	0	0	0	0	90
22530580	m	md	IT	2023	0	1	0	0	1	0	0	0	0	0	0	0	64
22530606	f	md	ZA	2023	0	1	1	0	0	0	0	0	0	0	0	0	
22530607	Not	md	JP	2023	0	1	0	0	1	0	0	0	0	0	0	0	
22530678	f	hp	GR	2023	0	1	1	0	0	0	0	0	0	0	0	0	74
22531327	m	md	JP	2023	0	0	0	0	0	0	0	0	0	0	0	0	71
22531331	m	ph	JP	2023	0	1	1	0	0	0	0	0	0	0	0	0	70
22531398	m	hp	CZ	2023	0	0	0	0	0	0	0	0	0	0	0	0	71
22531441	Not	md	JP	2023	0	1	1	0	0	0	0	0	0	0	0	1	73
22531541	f	md	CN	2023	0	0	0	0	0	0	0	0	0	0	0	0	63
22531573	m	cn	IT	2023	0	1	0	0	1	0	0	0	0	0	0	0	64
22531826	f	cn	ID	2023	1	0	0	0	0	0	0	0	0	0	0	0	59.6
22531832	f	md	BR	2023	0	0	0	0	0	0	0	0	0	0	0	0	50
22531887	f	md	CZ	2023	0	1	0	0	1	0	0	0	0	0	0	0	
22532026	Not	hp	US	2023	0	0	0	0	0	0	0	0	0	0	0	0	
22532213	Not	hp	US	2023	0	0	0	0	0	0	0	0	0	0	0	0	
22532365	m	md	US	2023	0	1	1	0	0	0	0	0	0	0	0	0	80
22532462	f	hp	CA	2023	0	0	0	0	0	0	0	0	0	0	0	0	67
22532465	f	ph	JP	2023	0	1	0	0	0	1	0	0	0	0	0	0	70
22532572	Not	md	US	2023	0	0	0	0	0	0	0	0	0	0	0	0	
22532713	m	md	LT	2023	0	0	0	0	0	0	0	0	0	0	0	0	73
22532730	m	md	JP	2023	0	0	0	0	0	0	0	0	0	0	0	0	71
22532754	m	md	CN	2023	0	0	0	0	0	0	0	0	0	0	0	0	41
22532766	m	md	ZA	2023	0	0	0	0	0	0	0	0	0	0	0	0	67
22532778	m	md	US	2023	0	0	0	0	0	0	0	0	0	0	0	0	
22532799	m	md	US	2023	0	0	0	0	0	0	0	0	0	0	0	0	53
22532887	m	hp	NL	2023	0	0	0	0	0	0	0	0	0	0	0	0	75
22533144	f	cn	CN	2023	0	0	0	0	0	0	0	0	0	0	0	0	39
22533814	f	md	JP	2023	0	1	1	0	0	0	0	0	0	0	0	0	80
22533830	f	hp	US	2023	1	0	0	0	0	0	0	0	0	0	0	0	66
22533891	f	md	US	2023	1	0	0	0	0	0	0	0	0	0	0	0	75
22533936	f	md	CA	2023	0	1	0	0	0	1	0	0	0	0	0	0	78
22534023	Not	hp	JP	2023	0	0	0	0	0	0	0	0	0	0	0	0	

22534045	Not	md	JP	2023	0	1	1	0	0	0	0	0	0	0	0	0	
22534387	m	md	IT	2023	0	0	0	0	0	0	0	0	0	0	0	0	53
22534485	f	md	IN	2023	0	1	0	0	0	1	0	0	0	0	0	0	29
22534496	m	md	JP	2023	0	0	0	0	0	0	0	0	0	0	0	0	70
22534560	m	md	IT	2023	0	0	0	0	0	0	0	0	0	0	0	0	50
22534943	m	md	JP	2023	0	1	1	0	0	0	0	0	0	0	0	0	
22535043	f	md	PT	2023	0	0	0	0	0	0	0	0	0	0	0	0	
22535130	f	md	JP	2023	0	0	0	0	0	0	0	0	0	0	0	0	79
22535173	m	md	IT	2023	0	1	0	0	1	0	0	0	0	0	0	0	76
22535323	Not	md	JP	2023	0	1	0	0	1	0	0	0	0	0	0	0	
22535929	Not	hp	DE	2023	0	0	0	0	0	0	0	0	0	0	0	0	
22536797	f	cn	HR	2023	0	1	1	0	0	0	0	0	0	0	0	0	57
22536869	m	cn	ID	2023	1	0	0	0	0	0	0	0	0	0	0	0	53.8
22537039	Not	md	US	2023	0	1	0	1	0	0	0	0	0	0	0	0	
22537419	Not	hp	KR	2023	0	0	0	0	0	0	0	0	0	0	0	0	
22537427	f	md	JP	2023	0	0	0	0	0	0	0	0	0	0	0	0	81
22537621	f	hp	US	2023	1	0	0	0	0	0	0	0	0	0	0	0	69
22537728	f	hp	US	2023	0	0	0	0	0	0	1	1	0	0	0	0	34
22537849	Not	md	GB	2023	0	0	0	0	0	0	0	0	0	0	0	0	
22537868	Not	md	JP	2023	0	0	0	0	0	0	1	1	0	0	0	0	
22537897	f	cn	US	2023	0	1	1	0	0	0	0	0	0	0	0	0	
22537958	f	cn	ID	2023	1	0	0	0	0	0	0	0	0	0	0	0	54.7
22538479	m	md	IT	2023	0	1	0	0	1	0	0	0	0	0	0	0	64
22538868	m	md	ES	2023	0	0	0	0	0	0	0	0	0	0	0	0	72
22538889	Not	md	ES	2023	0	0	0	0	0	0	0	0	0	0	0	0	64
22539217	m	md	DE	2023	0	1	0	0	1	0	0	0	0	0	0	0	71
22539361	f	cn	CA	2023	1	0	0	0	0	0	0	0	0	0	0	0	49
22539541	f	cn	PK	2023	0	0	0	0	0	0	0	0	0	0	0	0	59
22539592	m	cn	AE	2023	0	1	0	1	0	0	0	0	0	0	0	0	
22539821	Not	hp	CN	2023	1	0	0	0	0	0	0	0	0	0	0	0	
22539888	f	hp	CA	2023	1	0	0	0	0	0	0	0	0	0	0	0	
22539951	Not	hp	PL	2023	0	1	0	0	0	1	0	0	0	0	0	0	
22540115	f	md	JP	2023	0	1	0	1	0	0	0	0	0	0	0	1	75
22540525	f	ph	US	2023	0	0	0	0	0	0	0	0	0	0	0	0	71

22540829	f	md	US	2023	0	0	0	0	0	0	0	0	0	0	0	0	87
22541025	m	hp	JP	2023	0	1	0	0	0	1	0	0	0	0	0	0	71
22541179	m	md	US	2023	0	1	0	1	0	0	0	0	0	0	0	0	68
22541682	m	cn	CN	2023	0	1	0	1	0	0	0	0	0	0	0	0	68
22541784	f	cn	JP	2023	0	0	0	0	0	0	0	0	0	0	0	0	82
22541805	m	cn	JP	2023	0	0	0	0	0	0	0	0	0	0	0	0	
22541892	f	cn	US	2023	0	0	0	0	0	0	0	0	0	0	0	0	53
22541946	m	md	US	2023	0	0	0	0	0	0	0	0	0	0	0	0	73
22542018	m	md	CN	2023	1	0	0	0	0	0	0	0	0	0	0	0	53
22542028	Not	hp	CN	2023	0	0	0	0	0	0	0	0	0	0	0	0	
22542038	Not	ph	JP	2023	1	0	0	0	0	0	0	0	0	0	0	0	
22542045	f	cn	ID	2023	0	0	0	0	0	0	0	0	0	0	0	0	65.9
22542081	m	md	JP	2023	0	1	0	0	1	0	0	0	0	0	0	0	72
22585639	m	md	DE	2023	0	1	0	0	1	0	0	0	0	0	0	0	74
22586115	Not	md	US	2023	1	0	0	0	0	0	0	0	0	0	0	0	
22586226	f	hp	US	2023	1	0	0	0	0	0	0	0	0	0	0	0	63
22586302	m	hp	CZ	2023	0	1	0	0	0	1	0	0	0	0	0	0	71
22586925	m	md	CN	2023	0	1	1	0	0	0	0	0	0	0	0	0	68
22587499	m	md	CZ	2023	0	1	0	0	1	0	0	0	0	0	0	0	
22588186	m	md	VN	2023	1	0	0	0	0	0	0	0	0	0	0	0	59
22588349	m	md	JP	2023	0	0	0	0	0	0	0	0	0	0	0	0	74
22588411	m	md	JP	2023	0	0	0	0	0	0	0	0	0	0	0	0	74
22588910	m	hp	FR	2023	0	1	1	0	0	0	0	0	0	0	0	0	74
22589616	f	cn	IN	2023	0	0	0	0	0	0	0	0	0	0	0	0	63
22589766	f	md	US	2023	0	0	0	0	0	0	0	0	0	0	0	0	87
22589789	f	md	US	2023	0	1	1	0	0	0	0	0	0	0	0	0	66
22590017	f	md	KR	2023	0	0	0	0	0	0	0	0	0	0	0	0	80
22590260	f	md	BR	2023	0	0	0	0	0	0	0	0	0	0	0	0	34
22590393	m	ph	MY	2023	1	0	0	0	0	0	0	0	0	0	0	0	
22590631	m	md	DE	2023	0	0	0	0	0	0	0	0	0	0	0	0	
22590672	f	md	IT	2023	0	0	0	0	0	0	0	0	0	0	0	0	
22590749	m	md	DE	2023	0	0	0	0	0	0	0	0	0	0	0	0	
22590906	f	cn	CA	2023	1	0	0	0	0	0	0	0	0	0	0	0	66.3
22591138	Not	md	BG	2023	0	1	0	1	0	0	0	0	0	0	0	0	78

22591413	f	md	JP	2023	0	1	1	0	0	0	0	0	0	0	0	0	70
22591886	f	cn	US	2023	0	1	0	0	0	1	0	0	0	0	0	0	75
22591892	f	cn	US	2023	0	1	0	0	0	1	0	0	0	0	0	0	
22591899	f	ph	US	2023	0	1	0	0	0	1	0	0	0	0	0	0	68
22591905	Not	md	US	2023	0	1	0	0	0	1	0	0	0	0	0	0	
22591924	f	md	US	2023	0	1	0	0	0	1	0	0	0	0	0	0	
22591941	f	md	US	2023	0	1	0	0	0	1	0	0	0	0	0	1	40
22591943	m	cn	US	2023	0	1	0	0	0	1	0	0	0	0	0	0	
22591962	m	md	US	2023	0	1	0	0	0	1	0	0	0	0	0	0	
22591963	f	cn	US	2023	0	1	0	0	0	1	0	0	0	0	0	0	80
22591968	m	hp	US	2023	0	1	0	0	0	1	0	0	0	0	0	1	61
22591975	f	hp	US	2023	0	1	0	0	0	1	0	0	0	0	0	0	54
22591977	m	hp	US	2023	0	1	0	0	0	1	0	0	0	0	0	0	67
22591993	f	hp	US	2023	0	1	0	0	0	1	0	0	0	0	0	0	46
22591995	Not	hp	US	2023	0	1	0	0	0	1	0	0	0	0	0	0	
22592008	Not	md	US	2023	0	1	0	0	0	1	0	0	0	0	0	0	
22592028	Not	md	US	2023	0	1	0	0	0	1	0	0	0	0	0	0	
22592043	f	md	US	2023	0	1	0	0	0	1	0	0	0	0	0	1	
22592112	f	md	JP	2023	0	0	0	0	0	0	0	0	0	0	0	0	60
22592184	f	md	JP	2023	0	0	0	0	0	0	0	0	0	0	0	0	80
22592189	m	md	BG	2023	0	1	0	1	0	0	0	0	0	0	0	0	
22592230	m	md	IT	2023	0	0	0	0	0	0	0	0	0	0	0	0	
22592290	f	md	US	2023	1	0	0	0	0	0	0	0	0	0	0	0	63
22592478	m	md	DE	2023	0	0	0	0	0	0	0	0	0	0	0	0	
22592505	f	md	US	2023	0	0	0	0	0	0	0	0	0	0	0	0	67
22592794	f	md	US	2023	1	0	0	0	0	0	0	0	0	0	0	0	34
22592903	f	md	JP	2023	0	0	0	0	0	0	0	0	0	0	0	0	80
22593003	Not	md	JP	2023	1	0	0	0	0	0	0	0	0	0	0	0	
22593037	f	md	DE	2023	0	0	0	0	0	0	0	0	0	0	0	0	79
22593794	m	md	IT	2023	0	1	0	0	1	0	0	0	0	0	0	0	64
22594011	Not	md	JP	2023	0	1	0	0	1	0	0	0	0	0	0	0	
22594041	m	hp	US	2023	0	0	0	0	0	0	0	0	0	0	0	0	62
22594263	m	md	JP	2023	0	1	0	0	0	1	0	0	0	0	0	0	80
22594282	f	hp	AU	2023	0	0	0	0	0	0	0	0	0	0	0	0	

22594647	m	ph	JP	2023	0	1	0	0	1	0	0	0	0	0	0	0	
22594664	m	cn	US	2023	0	0	0	0	0	0	0	0	0	0	0	0	
22594681	f	hp	DE	2023	0	1	1	0	0	0	0	0	0	0	0	0	54
22594700	m	md	JP	2023	0	0	0	0	0	0	0	0	0	0	0	0	
22594976	m	md	IT	2023	0	1	0	0	1	0	0	0	0	0	0	0	64
22595187	m	md	VN	2023	0	1	0	0	0	1	0	0	0	0	0	0	71
22595210	m	hp	AT	2023	0	1	1	0	0	0	0	0	0	0	0	0	73
22595998	f	cn	IN	2023	0	1	0	0	0	1	0	0	0	0	0	0	63
22595999	m	md	ES	2023	0	0	0	0	0	0	0	0	0	0	0	0	63
22596031	Not	md	US	2023	0	0	0	0	0	0	0	0	0	0	0	0	
22596109	m	md	IN	2023	0	1	0	0	0	1	0	0	0	0	0	0	70
22596396	Not	md	JP	2023	0	1	0	1	0	0	0	0	0	0	0	0	
22597070	m	md	JP	2023	0	0	0	0	0	0	0	0	0	0	0	0	
22597108	Not	md	GB	2023	0	1	0	0	0	1	0	0	0	0	0	0	
22597147	f	md	AU	2023	1	0	0	0	0	0	0	0	0	0	0	0	
22597251	m	cn	ID	2023	1	0	0	0	0	0	0	0	0	0	0	0	
22598338	m	md	CN	2023	0	0	0	0	0	0	0	0	0	0	0	0	58
22598343	Not	hp	US	2023	0	1	0	0	1	0	0	0	0	0	0	0	
22598344	Not	hp	US	2023	0	1	0	0	1	0	0	0	0	0	0	0	
22598486	m	md	JP	2023	0	0	0	0	0	0	0	0	0	0	0	0	83
22598559	m	md	JP	2023	0	1	1	0	0	0	0	0	0	0	0	0	
22598594	m	cn	IN	2023	0	1	0	0	0	1	0	0	0	0	0	0	62
22598606	f	md	US	2023	1	0	0	0	0	0	0	0	0	0	0	0	
22598744	f	md	JP	2023	0	0	0	0	0	0	0	0	0	0	0	0	51
22599127	m	hp	US	2023	0	0	0	0	0	0	0	0	0	0	0	0	62
22599129	f	cn	CA	2023	0	0	0	0	0	0	0	0	0	0	0	0	
22599232	m	md	RS	2023	0	1	1	0	0	0	0	0	0	0	0	0	
22599635	f	cn	ID	2023	1	0	0	0	0	0	0	0	0	0	0	0	57.9
22600136	f	md	ES	2023	0	0	0	0	0	0	0	0	0	0	0	0	49
22600260	f	md	US	2023	0	1	1	0	0	0	0	0	0	0	0	0	64
22600295	Not	ph	JP	2023	1	0	0	0	0	0	0	0	0	0	0	0	
22600509	Not	ph	JP	2023	1	0	0	0	0	0	0	0	0	0	0	0	
22600588	m	hp	TR	2023	0	1	1	0	0	0	0	0	0	0	0	0	52
22600606	Not	md	US	2023	0	0	0	0	0	0	0	0	0	0	0	0	

22601608	m	hp	GB	2023	1	0	0	0	0	0	0	0	0	0	0	0	
22601627	m	md	JP	2023	0	1	0	0	1	0	0	0	0	0	0	0	69
22601839	f	cn	US	2023	1	0	0	0	0	0	0	0	0	0	0	0	82.6
22602136	f	hp	IT	2023	0	0	0	0	0	0	0	0	0	0	0	0	66
22602193	m	md	JP	2023	0	1	1	0	0	0	0	0	0	0	0	0	80
22602378	Not	md	IT	2023	0	0	0	0	0	0	0	0	0	0	0	0	
22602482	f	md	IT	2023	0	0	0	0	0	0	0	0	0	0	0	0	61
22602509	m	hp	HR	2023	0	1	0	0	1	0	0	0	0	0	0	0	65
22602645	m	md	US	2023	0	0	0	0	0	0	0	0	0	0	0	0	55
22602683	Not	md	JP	2023	0	1	0	1	0	0	0	0	0	0	0	0	
22602797	m	md	TR	2023	0	0	0	0	0	0	0	0	0	0	0	0	
22603323	m	md	NL	2023	0	1	1	0	0	0	0	0	0	0	0	0	53
22603629	m	md	CN	2023	0	0	0	0	0	0	0	0	0	0	0	0	63
22603638	m	md	JP	2023	0	0	0	0	0	0	0	0	0	0	0	0	70
22603811	f	cn	US	2023	0	1	0	1	0	0	0	0	0	0	0	0	55.3
22603982	f	cn	US	2023	1	0	0	0	0	0	0	0	0	0	0	0	81
22604086	m	md	JP	2023	0	0	0	0	0	0	0	0	0	0	0	0	65
22604867	f	cn	ID	2023	1	0	0	0	0	0	0	0	0	0	0	0	61
22605270	m	cn	IN	2023	0	0	0	0	0	0	0	0	0	0	0	0	50
22605572	f	md	JP	2023	0	1	0	1	0	0	0	0	0	0	0	1	82
22605698	f	md	FR	2023	0	0	0	0	0	0	0	0	0	0	0	0	34
22606127	Not	md	US	2023	0	1	1	0	0	0	0	0	0	0	0	0	
22606128	Not	md	US	2023	0	1	1	0	0	0	0	0	0	0	0	0	
22606129	Not	md	US	2023	0	1	1	0	0	0	0	0	0	0	0	0	
22606327	m	md	JP	2023	0	1	1	0	0	0	0	0	0	0	0	0	56
22606340	f	cn	US	2023	1	0	0	0	0	0	0	0	0	0	0	0	64.5
22606347	Not	md	IT	2023	1	0	0	0	0	0	0	0	0	0	0	0	
22606350	Not	md	US	2023	0	1	1	0	0	0	0	0	0	0	0	0	
22606448	f	cn	US	2023	1	0	0	0	0	0	0	0	0	0	0	0	
22606501	Not	md	JP	2023	0	1	0	1	0	0	0	0	0	0	0	0	
22606571	Not	md	JP	2023	0	1	1	0	0	0	0	0	0	0	0	0	80
22606669	Not	cn	SG	2023	1	0	0	0	0	0	0	0	0	0	0	0	60.8
22606740	f	md	HU	2023	0	0	0	0	0	0	0	0	0	0	0	0	57
22606990	f	hp	FR	2023	0	1	1	0	0	0	0	0	0	0	0	0	58

22607347	m	md	JP	2023	0	0	0	0	0	0	0	0	0	0	0	0	80
22607391	m	md	JP	2023	0	0	0	0	0	0	0	0	0	0	0	0	49
22608066	Not	md	JP	2023	0	0	0	0	0	0	0	0	0	0	0	0	
22608076	m	hp	FR	2023	0	1	1	0	0	0	0	0	0	0	0	0	74
22608189	Not	md	ES	2023	0	1	0	0	0	1	0	0	0	0	0	1	
22608199	Not	md	JP	2023	0	0	0	0	0	0	0	0	0	0	0	0	
22608443	m	md	US	2023	0	1	1	0	0	0	0	0	0	0	0	0	67
22608607	f	md	JP	2023	0	1	0	0	0	1	0	0	0	0	0	0	80
22608874	Not	md	IT	2023	1	0	0	0	0	0	0	0	0	0	0	0	
22608975	f	md	CN	2023	0	0	0	0	0	0	0	0	0	0	0	0	55
22609223	f	md	IL	2023	0	0	0	0	0	0	0	0	0	0	0	0	76
22609471	f	md	TW	2023	0	0	0	0	0	0	0	0	0	0	0	0	70
22609689	f	hp	MY	2023	0	1	0	0	0	1	0	0	0	0	0	0	76
22609759	f	cn	IN	2023	1	0	0	0	0	0	0	0	0	0	0	0	
22610244	Not	md	US	2023	0	0	0	0	0	0	0	0	0	0	0	0	
22610279	f	cn	CA	2023	1	0	0	0	0	0	0	0	0	0	0	0	54.3
22610451	f	md	NZ	2023	1	0	0	0	0	0	0	0	0	0	0	0	48
22611011	f	cn	MY	2023	1	0	0	0	0	0	0	0	0	0	0	0	72.5
22611037	Not	hp	TR	2023	0	1	0	0	0	1	0	0	0	0	0	0	
22611056	Not	hp	US	2023	0	0	0	0	0	0	0	0	0	0	0	0	
22611240	f	md	BR	2023	0	0	0	0	0	0	0	0	0	0	0	0	33
22611273	m	hp	FR	2023	0	1	1	0	0	0	0	0	0	0	0	0	64
22611293	m	hp	FR	2023	0	1	1	0	0	0	0	0	0	0	0	0	52
22611365	f	hp	FR	2023	0	1	1	0	0	0	0	0	0	0	0	0	78
22611548	f	md	IL	2023	0	1	0	0	1	0	0	0	0	0	0	0	
22611558	f	cn	TH	2023	0	0	0	0	0	0	0	0	0	0	0	0	53
22611821	f	md	GB	2023	1	0	0	0	0	0	0	0	0	0	0	0	
22612015	m	md	JP	2023	0	0	0	0	0	0	0	0	0	0	0	0	
22612267	f	md	DE	2023	0	1	0	0	1	0	0	0	0	0	0	0	74
22612373	m	md	DE	2023	0	1	0	0	1	0	0	0	0	0	0	0	50
22612513	m	md	JP	2023	0	0	0	0	0	0	0	0	0	0	0	0	
22612545	m	md	FR	2023	0	0	0	0	0	0	0	0	0	0	0	0	
22612584	m	hp	CA	2023	0	1	0	0	1	0	0	0	0	0	0	0	79
22612739	f	md	AT	2023	0	0	0	0	0	0	0	0	0	0	0	0	

22613109	m	cn	IN	2023	0	1	0	0	0	1	0	0	0	0	0	0	76
22613188	m	hp	FR	2023	0	0	0	0	0	0	0	0	0	0	0	0	68
22613191	Not	md	HK	2023	0	0	0	0	0	0	0	0	0	0	0	0	
22613246	f	hp	FR	2023	0	0	0	0	0	0	0	0	0	0	0	0	62
22613290	Not	cn	SG	2023	1	0	0	0	0	0	0	0	0	0	0	0	71.7
22613408	Not	cn	SG	2023	1	0	0	0	0	0	0	0	0	0	0	0	76.5
22614013	Not	cn	US	2023	1	0	0	0	0	0	0	0	0	0	0	0	76.7
22614070	m	md	IN	2023	0	1	0	0	0	1	0	0	0	0	0	0	67
22614229	m	md	JP	2023	0	1	0	0	0	1	0	0	0	0	0	0	80
22614290	m	md	IT	2023	0	1	0	0	1	0	0	0	0	0	0	0	64
22614476	Not	md	JP	2023	0	0	0	0	0	0	0	0	0	0	0	0	100
22614658	m	hp	GB	2023	1	0	0	0	0	0	0	0	0	0	0	0	
22614779	m	md	IT	2023	0	1	0	0	1	0	0	0	0	0	0	0	76
22615527	m	md	CA	2023	0	1	1	0	0	0	0	0	0	0	0	0	76
22615544	m	md	JP	2023	0	1	1	0	0	0	0	0	0	0	0	1	71
22615568	f	md	DE	2023	0	1	1	0	0	0	0	0	0	0	0	0	78
22615919	m	md	JP	2023	0	1	0	1	0	0	0	0	0	0	0	0	63
22615971	m	hp	US	2023	0	1	0	1	0	0	0	0	0	0	0	0	
22615972	Not	md	CZ	2023	0	1	0	1	0	0	0	0	0	0	0	1	
22616028	f	cn	MY	2023	1	0	0	0	0	0	0	0	0	0	0	0	62.7
22616448	m	ph	FR	2023	0	0	0	0	0	0	0	0	0	0	0	0	62
22616452	m	cn	CN	2023	0	1	0	1	0	0	0	0	0	0	0	0	56
22616480	m	md	UA	2023	0	0	0	0	0	0	0	0	0	0	0	0	71
22616500	m	md	JP	2023	1	0	0	0	0	0	0	0	0	0	0	1	77
22616914	Not	hp	CN	2023	0	0	0	0	0	0	0	0	0	0	0	0	
22616982	f	cn	US	2023	1	0	0	0	0	0	0	0	0	0	0	0	70.8
22617110	m	cn	US	2023	0	1	0	1	0	0	0	0	0	0	0	0	69
22617850	f	cn	US	2023	0	0	0	0	0	0	0	0	0	0	0	0	
22618003	m	md	CN	2023	1	0	0	0	0	0	0	0	0	0	0	0	53
22618066	f	md	RU	2023	0	0	0	0	0	0	0	0	0	0	0	0	56
22618610	m	cn	ID	2023	1	0	0	0	0	0	0	0	0	0	0	0	73.6
22618757	f	md	DE	2023	0	0	0	0	0	0	0	0	0	0	0	0	47
22618815	m	cn	CA	2023	1	0	0	0	0	0	0	0	0	0	0	0	
22619218	f	md	BE	2023	0	1	0	0	0	1	0	0	0	0	0	0	46

22619354	f	cn	IN	2023	0	1	0	1	0	0	0	0	0	0	0	0	57.3
22619526	m	md	GR	2023	0	1	1	0	0	0	0	0	0	0	0	0	
22619618	f	md	DE	2023	0	0	0	0	0	0	0	0	0	0	0	0	65
22619710	m	hp	RU	2023	0	1	0	0	0	1	0	0	0	0	0	0	39
22619711	m	md	JP	2023	0	1	0	0	0	1	0	0	0	0	0	0	60
22619712	m	md	JP	2023	0	1	0	0	0	1	0	0	0	0	0	0	70
22619797	Not	md	JP	2023	0	1	0	0	0	1	0	0	0	0	0	0	53
22619845	f	md	JP	2023	0	1	0	0	1	0	0	0	0	0	0	0	66
22620008	f	cn	US	2023	1	0	0	0	0	0	0	0	0	0	0	0	
22620022	Not	md	JP	2023	0	1	0	0	0	1	0	0	0	0	0	0	
22620384	f	md	JP	2023	0	1	1	0	0	0	0	0	0	0	0	0	74
22620629	f	hp	US	2023	0	0	0	0	0	0	0	0	0	0	0	0	53
22620991	m	md	DE	2023	0	0	0	0	0	0	0	0	0	0	0	0	60
22621143	m	hp	CN	2023	0	1	1	0	0	0	0	0	0	0	0	0	68
22621222	m	md	IT	2023	0	1	0	0	1	0	0	0	0	0	0	0	64
22621250	m	md	FR	2023	0	0	0	0	0	0	0	0	0	0	0	0	74
22621304	f	cn	AR	2023	0	0	0	0	0	0	0	0	0	0	0	0	83
22622136	f	md	AM	2023	0	0	0	0	0	0	0	0	0	0	0	0	52
22622142	m	cn	IN	2023	0	0	0	0	0	0	0	0	0	0	0	0	
22622148	f	md	CN	2023	0	0	0	0	0	0	0	0	0	0	0	0	63
22622151	f	ph	FR	2023	0	1	1	0	0	0	0	0	0	0	0	0	77
22622155	m	md	CN	2023	0	1	1	0	0	0	0	0	0	0	0	0	
22622215	f	md	GB	2023	1	0	0	0	0	0	0	0	0	0	0	0	
22622228	Not	md	JP	2023	0	1	1	0	0	0	0	0	0	0	0	0	61
22623128	m	md	IT	2023	0	1	0	0	1	0	0	0	0	0	0	0	76
22623146	m	cn	BR	2023	0	0	0	0	0	0	0	0	0	0	0	0	48
22623698	Not	md	US	2023	1	0	0	0	0	0	0	0	0	0	0	0	
22623752	m	hp	JP	2023	0	1	0	0	1	0	0	0	0	0	0	0	49
22624087	f	md	IT	2023	0	1	1	0	0	0	0	0	0	0	0	0	63
22624845	f	md	CN	2023	0	1	0	0	0	1	0	0	0	0	0	0	59
22625035	m	md	ID	2023	0	0	0	0	0	0	0	0	0	0	0	0	71
22625087	m	md	JP	2023	0	0	0	0	0	0	0	0	0	0	0	0	75
22625127	m	md	SG	2023	0	0	0	0	0	0	0	0	0	0	0	0	54
22625295	f	cn	US	2023	0	0	0	0	0	0	0	0	0	0	0	0	

22625314	m	md	JP	2023	0	1	1	0	0	0	0	0	0	0	0	0	78
22625561	m	cn	CA	2023	1	0	0	0	0	0	0	0	0	0	0	0	82.8
22625620	f	cn	US	2023	0	0	0	0	0	0	0	0	0	0	0	0	
22625716	f	md	IT	2023	0	1	0	0	1	0	0	0	0	0	0	0	71
22626069	f	cn	US	2023	1	0	0	0	0	0	0	0	0	0	0	0	
22626128	f	md	BG	2023	0	1	1	0	0	0	0	0	0	0	0	0	71
22626315	f	cn	KR	2023	0	0	0	0	0	0	0	0	0	0	0	0	71
22626544	m	md	ES	2023	0	1	1	0	0	0	0	0	0	0	0	0	
22626768	m	md	JP	2023	1	0	0	0	0	0	0	0	0	0	0	0	
22627321	m	cn	TH	2023	0	1	0	1	0	0	0	0	0	0	0	0	70
22627357	f	md	IN	2023	0	1	0	0	0	1	0	0	0	0	0	0	
22627512	Not	md	JP	2023	0	1	0	0	0	1	0	0	0	0	0	0	
22627589	Not	hp	US	2023	0	0	0	0	0	0	0	0	0	0	0	0	78
22627596	m	hp	ZA	2023	0	0	0	0	0	0	0	0	0	0	0	0	
22627930	Not	md	JP	2023	0	1	1	0	0	0	0	0	0	0	0	0	70
22628063	m	cn	CA	2023	1	0	0	0	0	0	0	0	0	0	0	0	61.1
22628455	m	ph	US	2023	0	0	0	0	0	0	0	0	0	0	0	0	66
22628540	Not	ph	JP	2023	0	1	0	0	1	0	0	0	0	0	0	0	
22628732	f	md	JP	2023	1	0	0	0	0	0	0	0	0	0	0	0	
22629084	m	ph	JP	2023	0	1	1	0	0	0	0	0	0	0	0	0	79
22629109	Not	ph	JP	2023	0	1	0	0	1	0	0	0	0	0	0	0	
22629553	f	cn	US	2023	0	1	1	0	0	0	0	0	0	0	0	0	
22629665	f	cn	EG	2023	0	0	0	0	0	0	0	0	0	0	0	0	51
22629864	m	md	JP	2023	0	1	1	0	0	0	0	0	0	0	0	0	75
22629981	m	md	IN	2023	0	0	0	0	0	0	0	0	0	0	0	0	60
22630208	m	md	JP	2023	0	0	0	0	0	0	0	0	0	0	0	0	
22630313	m	md	JP	2023	0	1	0	1	0	0	0	0	0	0	0	0	73
22630357	m	md	JP	2023	0	1	0	1	0	0	0	0	0	0	0	0	58
22630571	f	cn	ID	2023	1	0	0	0	0	0	0	0	0	0	0	0	50.2
22630572	m	cn	US	2023	1	0	0	0	0	0	0	0	0	0	0	0	
22630633	m	md	DE	2023	0	0	0	0	0	0	0	0	0	0	0	0	38
22630790	Not	cn	US	2023	0	0	0	0	0	0	0	0	0	0	0	0	
22630797	f	cn	US	2023	0	0	0	0	0	0	0	0	0	0	0	0	
22630835	Not	md	CN	2023	0	0	0	0	0	0	0	0	0	0	0	0	

22630836	m	md	FR	2023	0	1	1	0	0	0	0	0	0	0	0	0	66
22631549	f	md	US	2023	0	0	0	0	0	0	0	0	0	0	0	0	
22631579	f	md	DE	2023	0	1	1	0	0	0	0	0	0	0	0	0	60
22632180	f	cn	TH	2023	1	0	0	0	0	0	0	0	0	0	0	0	71
22632208	Not	hp	US	2023	0	1	0	0	0	1	0	0	0	0	0	0	
22632341	Not	hp	US	2023	0	1	0	0	0	1	0	0	0	0	0	0	
22632361	Not	md	JP	2023	0	0	0	0	0	0	0	0	0	0	0	0	
22632407	m	md	DE	2023	0	1	0	0	1	0	0	0	0	0	0	0	62
22632415	f	md	DE	2023	0	1	0	0	1	0	0	0	0	0	0	0	75
22632663	m	cn	TH	2023	1	0	0	0	0	0	0	0	0	0	0	0	80
22632678	m	md	NL	2023	0	1	1	0	0	0	0	0	0	0	0	0	62
22632835	f	md	AU	2023	0	1	0	1	0	0	0	0	0	0	0	0	53
22633008	m	md	JP	2023	0	1	1	0	0	0	0	0	0	0	0	0	79
22633086	m	md	JP	2023	0	0	0	0	0	0	0	0	0	0	0	0	80
22633194	m	cn	IN	2023	0	0	0	0	0	0	0	0	0	0	0	0	76
22633619	f	md	JP	2023	0	1	0	0	1	0	0	0	0	0	0	0	77
22633922	m	cn	ID	2023	1	0	0	0	0	0	0	0	0	0	0	0	50.4
22634151	m	md	BG	2023	0	1	1	0	0	0	0	0	0	0	0	0	
22634304	m	ph	US	2023	0	0	0	0	0	0	0	0	0	0	0	0	66
22635251	f	md	FI	2023	0	0	0	0	0	0	0	0	0	0	0	0	68
22635318	f	cn	IN	2023	0	0	0	0	0	0	0	0	0	0	0	0	46
22635346	f	cn	ID	2023	1	0	0	0	0	0	0	0	0	0	0	0	62.6
22635380	m	md	JP	2023	0	0	0	0	0	0	0	0	0	0	0	0	57
22635481	m	hp	NL	2023	0	1	1	0	0	0	0	0	0	0	0	0	62
22635625	f	md	FR	2023	0	1	1	0	0	0	0	0	0	0	0	0	78
22635686	f	cn	VN	2023	1	0	0	0	0	0	0	0	0	0	0	0	
22635851	m	hp	NL	2023	0	1	1	0	0	0	0	0	0	0	0	0	62
22635859	f	hp	US	2023	0	0	0	0	0	0	0	0	0	0	0	0	
22635875	m	hp	CN	2023	0	1	0	1	0	0	0	0	0	0	0	0	63
22635923	f	md	FR	2023	0	0	0	0	0	0	0	0	0	0	0	0	54
22636076	f	hp	CN	2023	1	0	0	0	0	0	0	0	0	0	0	0	56
22636083	f	md	IE	2023	0	0	0	0	0	0	0	0	0	0	0	0	77
22636780	f	hp	CA	2023	0	1	1	0	0	0	0	0	0	0	0	0	58
22637053	m	cn	KR	2023	0	0	0	0	0	0	0	0	0	0	0	0	72

22637325	f	cn	TH	2023	0	0	0	0	0	0	0	0	0	0	0	0	83
22637796	m	ph	JP	2023	0	1	0	0	1	0	0	0	0	0	0	1	70
22637951	m	cn	CN	2023	0	0	0	0	0	0	0	0	0	0	0	0	57
22638250	f	cn	US	2023	1	0	0	0	0	0	0	0	0	0	0	0	
22638332	f	hp	US	2023	0	0	0	0	0	0	1	1	0	0	0	0	68
22638355	f	md	JP	2023	0	1	0	0	0	1	0	0	0	0	0	0	70
22638844	m	md	IT	2023	0	0	0	0	0	0	0	0	0	0	0	0	50
22639054	f	md	IN	2023	0	0	0	0	0	0	0	0	0	0	0	0	
22639283	Not	hp	IN	2023	1	0	0	0	0	0	0	0	0	0	0	0	
22639333	Not	hp	JP	2023	0	1	0	0	0	1	0	0	0	0	0	1	
22639468	f	ph	TH	2023	1	0	0	0	0	0	0	0	0	0	0	0	61.1
22639543	m	cn	UY	2023	0	0	0	0	0	0	0	0	0	0	0	0	72
22639782	m	cn	AE	2023	0	0	0	0	0	0	0	0	0	0	0	0	67
22640342	m	md	NL	2023	0	1	1	0	0	0	0	0	0	0	0	0	62
22640597	m	cn	CA	2023	1	0	0	0	0	0	0	0	0	0	0	0	74.5
22640928	m	hp	JP	2023	0	0	0	0	0	0	0	0	0	0	0	0	49
22641627	m	hp	FR	2023	0	1	1	0	0	0	0	0	0	0	0	0	74
22641630	f	md	CN	2023	0	0	0	0	0	0	0	0	0	0	0	0	56
22641877	f	md	US	2023	0	0	0	0	0	0	0	0	0	0	0	0	49
22641962	Not	md	JP	2023	0	1	1	0	0	0	0	0	0	0	0	0	
22642155	f	hp	RU	2023	0	1	0	0	0	1	0	0	0	0	0	0	55
22642156	f	hp	RU	2023	0	1	0	0	0	1	0	0	0	0	0	0	45
22642325	f	cn	PK	2023	0	1	0	0	0	1	0	0	0	0	0	0	72
22642628	m	md	JP	2023	0	1	0	1	0	0	0	0	0	0	0	1	70
22642758	Not	md	JP	2023	0	1	0	0	1	0	0	0	0	0	0	0	
22643445	f	md	DE	2023	0	1	1	0	0	0	0	0	0	0	0	0	60
22643673	m	hp	JP	2023	0	1	0	0	1	0	0	0	0	0	0	0	49
22643721	f	md	JP	2023	0	0	0	0	0	0	0	0	0	0	0	0	73
22644037	m	cn	ID	2023	1	0	0	0	0	0	0	0	0	0	0	0	70.1
22644175	m	cn	JP	2023	1	0	0	0	0	0	0	0	0	0	0	0	
22644176	f	cn	CA	2023	1	0	0	0	0	0	0	0	0	0	0	0	54.9
22644744	f	cn	DE	2023	1	0	0	0	0	0	0	0	0	0	0	0	
22644807	m	md	JP	2023	0	1	0	0	0	1	0	0	0	0	0	0	80
22644909	Not	hp	US	2023	0	1	0	0	1	0	0	0	0	0	0	0	

22644970	m	cn	US	2023	0	0	0	0	0	0	0	0	0	0	0	0	60
22645608	m	cn	IN	2023	0	0	0	0	0	0	0	0	0	0	0	0	39
22645737	Not	md	HK	2023	0	0	0	0	0	0	0	0	0	0	0	0	
22645810	f	cn	CA	2023	0	0	0	0	0	0	0	0	0	0	0	0	62
22646275	m	hp	IN	2023	0	0	0	0	0	0	0	0	0	0	0	0	60
22646489	m	cn	UY	2023	0	0	0	0	0	0	0	0	0	0	0	0	64
22646665	Not	ph	US	2023	0	0	0	0	0	0	0	0	0	0	0	0	
22646948	f	cn	IN	2023	0	0	0	0	0	0	0	0	0	0	0	0	98
22647149	f	hp	US	2023	0	1	0	0	1	0	0	0	0	0	0	0	53
22647279	m	md	JP	2023	0	1	0	0	1	0	0	0	0	0	0	0	73
22647518	m	cn	TH	2023	1	0	0	0	0	0	0	0	0	0	0	0	69.4
22647804	m	md	US	2023	0	0	0	0	0	0	0	0	0	0	0	0	
22647996	Not	md	JP	2023	0	1	0	0	1	0	0	0	0	0	0	0	
22648413	m	md	JP	2023	0	1	0	0	1	0	0	0	0	0	0	0	58
22648435	f	md	JP	2023	0	0	0	0	0	0	0	0	0	0	0	0	74
22648652	Not	cn	US	2023	1	0	0	0	0	0	0	0	0	0	0	0	73.9
22649382	f	md	IN	2023	0	0	0	0	0	0	0	0	0	0	0	0	48
22650100	m	md	JP	2023	0	0	0	0	0	0	0	0	0	0	0	0	71
22650142	m	hp	US	2023	0	1	0	0	1	0	0	0	0	0	0	0	
22650401	m	md	JP	2023	0	1	1	0	0	0	0	0	0	0	0	0	70
22650422	m	md	NL	2023	0	1	1	0	0	0	0	0	0	0	0	0	62
22651128	f	md	FR	2023	0	0	0	0	0	0	0	0	0	0	0	0	41
22651159	Not	md	JP	2023	1	0	0	0	0	0	0	0	0	0	0	0	
22651164	f	cn	IT	2023	0	1	0	1	0	0	0	0	0	0	0	0	74
22651356	f	ph	FR	2023	0	0	0	0	0	0	0	0	0	0	0	0	54
22651363	m	cn	IN	2023	0	0	0	0	0	0	0	0	0	0	0	0	
22651370	f	md	FR	2023	0	0	0	0	0	0	0	0	0	0	0	0	46
22651776	Not	cn	US	2023	0	1	1	0	0	0	0	0	0	0	0	0	67
22652100	f	md	US	2023	0	1	1	0	0	0	0	0	0	0	0	1	66
22653179	m	md	IT	2023	0	0	0	0	0	0	0	0	0	0	0	0	81
22653351	Not	hp	FR	2023	0	0	0	0	0	0	0	0	0	0	0	0	
22653572	Not	hp	PT	2023	0	0	0	0	0	0	0	0	0	0	0	0	
22653648	m	md	BG	2023	0	1	0	0	0	1	0	0	0	0	0	0	80
22654264	Not	md	US	2023	0	1	0	0	1	0	0	0	0	0	0	0	

22654355	Not	md	VN	2023	0	0	0	0	0	0	0	0	0	0	0	0	
22654577	f	md	CA	2023	0	0	0	0	0	0	0	0	0	0	0	0	70
22654898	f	md	FR	2023	0	1	1	0	0	0	0	0	0	0	0	0	78
22655104	m	md	JP	2023	1	0	0	0	0	0	0	0	0	0	0	0	72
22655311	m	md	NL	2023	0	1	1	0	0	0	0	0	0	0	0	0	53
22655323	f	md	CA	2023	1	0	0	0	0	0	0	0	0	0	0	0	86
22655689	Not	md	AT	2023	0	0	0	0	0	0	0	0	0	0	0	0	
22655695	Not	md	AT	2023	0	0	0	0	0	0	0	0	0	0	0	0	
22655703	Not	md	AT	2023	0	0	0	0	0	0	0	0	0	0	0	0	
22656307	f	md	CN	2023	0	1	0	1	0	0	0	0	0	0	0	0	65.8
22656418	Not	md	AU	2023	0	0	0	0	0	0	0	0	0	0	0	0	
22656440	m	md	IT	2023	0	0	0	0	0	0	0	0	0	0	0	0	
22656496	m	md	JP	2023	0	0	0	0	0	0	0	0	0	0	0	0	70
22656706	Not	md	DE	2023	0	1	0	0	1	0	0	0	0	0	0	0	77
22656726	m	md	MY	2023	1	0	0	0	0	0	0	0	0	0	0	0	
22656987	f	cn	CA	2023	1	0	0	0	0	0	0	0	0	0	0	0	59
22657045	m	md	DE	2023	0	0	0	0	0	0	0	0	0	0	0	0	
22657253	Not	md	FR	2023	0	0	0	0	0	0	0	0	0	0	0	0	
22658060	Not	md	IT	2023	0	1	0	0	1	0	0	0	0	0	0	0	
22659100	m	md	JP	2023	0	1	0	1	0	0	0	0	0	0	0	0	67
22659469	m	hp	IN	2023	0	0	0	0	0	0	0	0	0	0	0	0	60
22659789	m	md	JP	2023	0	1	0	1	0	0	0	0	0	0	0	0	
22660665	m	cn	JP	2023	1	0	0	0	0	0	0	0	0	0	0	0	
22661131	Not	md	JP	2023	0	1	0	1	0	0	0	0	0	0	0	0	
22661472	Not	md	JP	2023	0	1	0	0	1	0	0	0	0	0	0	0	
22661503	f	md	US	2023	0	1	0	0	1	0	0	0	0	0	0	0	66
22661706	m	cn	US	2023	0	0	0	0	0	0	0	0	0	0	0	0	69
22661777	f	hp	JP	2023	1	0	0	0	0	0	0	0	0	0	0	0	76
22662000	f	cn	US	2023	0	0	0	0	0	0	0	0	0	0	0	0	67
6876455	m	md	US	2023	0	0	0	0	0	0	0	0	0	0	0	0	71
21616983	f	md	BE	2023	0	0	0	0	0	0	0	0	0	0	0	0	65
21806528	m	md	CN	2023	0	0	0	0	0	0	0	0	0	0	0	0	75
21861274	m	md	CN	2023	0	1	1	0	0	0	0	0	0	0	0	0	55
21903660	f	cn	US	2023	0	0	0	0	0	0	0	0	0	0	0	0	68

21910001	m	md	JP	2023	0	0	0	0	0	0	0	0	0	0	0	0	83
21915416	m	md	JP	2023	0	1	0	0	0	1	0	0	0	0	0	0	66
21933541	f	md	SE	2023	0	0	0	0	0	0	0	0	0	0	0	0	65
21962069	f	md	GB	2023	0	0	0	0	0	0	0	0	0	0	0	0	43
21974797	m	cn	GB	2023	0	1	1	0	0	0	0	0	0	0	0	0	
22001415	m	md	JP	2023	0	1	1	0	0	0	0	0	0	0	0	0	65
22075737	f	md	ES	2023	0	0	0	0	0	0	0	0	0	0	0	0	54
22090527	m	md	PT	2023	0	0	0	0	0	0	0	0	0	0	0	0	81
22098516	m	cn	PK	2023	0	1	0	0	0	1	0	0	0	0	0	0	51
22100677	Not	md	LT	2023	0	1	0	0	0	1	0	0	0	0	0	0	
22134802	m	md	KR	2023	0	0	0	0	0	0	0	0	0	0	0	0	56
22142310	m	md	LT	2023	0	1	0	0	0	1	0	0	0	0	0	0	65
22146655	m	md	JP	2023	0	0	0	0	0	0	0	0	0	0	0	0	76
22168839	m	md	JP	2023	0	1	0	0	1	0	0	0	0	0	0	0	65
22174418	m	md	JP	2023	0	1	0	0	1	0	0	0	0	0	0	0	78
22175713	m	md	IL	2023	0	1	0	1	0	0	0	0	0	0	0	0	82.3
22177322	f	cn	US	2023	0	0	0	0	0	0	0	0	0	0	0	0	
22178679	f	md	CN	2023	0	0	0	0	0	0	0	0	0	0	0	0	60
22186438	m	md	PT	2023	0	0	0	0	0	0	0	0	0	0	0	0	54
22197340	f	md	FR	2023	0	0	0	0	0	0	0	0	0	0	0	0	70
22197968	m	ph	JP	2023	0	1	1	0	0	0	0	0	0	0	0	0	81
22198644	Not	md	JP	2023	0	1	0	0	1	0	0	0	0	0	0	0	
22210636	f	cn	US	2023	0	0	0	0	0	0	0	0	0	0	0	0	82
22213900	m	hp	JP	2023	0	1	0	1	0	0	0	0	0	0	0	0	69
22225805	f	md	JP	2023	0	0	0	0	0	0	0	0	0	0	0	0	58
22251074	m	md	JP	2023	0	0	0	0	0	0	0	0	0	0	0	0	
22252837	f	ph	JP	2023	0	0	0	0	0	0	0	0	0	0	0	0	71
22254409	m	md	CN	2023	0	0	0	0	0	0	0	0	0	0	0	0	58
22260408	f	md	JP	2023	0	0	0	0	0	0	0	0	0	0	0	0	59
22261535	f	md	BR	2023	0	0	0	0	0	0	0	0	0	0	0	0	74
22268223	m	ph	JP	2023	0	1	0	0	1	0	0	0	0	0	0	0	73
22284520	m	md	JP	2023	0	1	1	0	0	0	0	0	0	0	0	0	60
22286106	f	md	GB	2023	0	0	0	0	0	0	0	0	0	0	0	0	81
22286177	m	md	JP	2023	0	1	1	0	0	0	0	0	0	0	0	0	70

22290620	m	md	JP	2023	0	1	0	0	0	1	0	0	0	0	0	0	80
22315971	m	md	VN	2023	0	1	0	1	0	0	0	0	0	0	0	0	86
22318258	f	md	FR	2023	0	1	0	0	0	1	0	0	0	0	0	0	83
22320849	m	cn	BR	2023	0	0	0	0	0	0	0	0	0	0	0	0	86
22321451	m	md	US	2023	0	0	0	0	0	0	0	0	0	0	0	0	
22339256	m	md	DK	2023	0	0	0	0	0	0	0	0	0	0	0	0	54
22343888	m	cn	DE	2023	0	1	0	0	1	0	0	0	0	0	0	0	70
22348310	f	md	JP	2023	1	0	0	0	0	0	0	0	0	0	0	0	73.1
22357847	m	md	DE	2023	0	1	0	0	1	0	0	0	0	0	0	0	70
22362868	Not	md	ES	2023	0	0	0	0	0	0	0	0	0	0	0	0	60
22364471	m	ph	JP	2023	0	0	0	0	0	0	0	0	0	0	0	0	80
22367487	Not	cn	CA	2023	0	0	0	0	0	0	0	0	0	0	0	0	67
22368873	m	md	AU	2023	0	0	0	0	0	0	0	0	0	0	0	0	72
22369960	m	md	JP	2023	0	1	0	0	1	0	0	0	0	0	0	0	62
22370481	m	md	CN	2023	0	0	0	0	0	0	0	0	0	0	0	0	68
22372366	f	md	JP	2023	1	0	0	0	0	0	0	0	0	0	0	0	60
22372944	m	cn	US	2023	0	0	0	0	0	0	0	0	0	0	0	0	64
22374608	m	cn	US	2023	0	1	1	0	0	0	0	0	0	0	0	0	76
22377223	f	md	FR	2023	0	0	0	0	0	0	0	0	0	0	0	0	79
22379787	m	md	JP	2023	0	1	0	0	0	1	0	0	0	0	0	0	76
22388401	Not	md	BR	2023	0	0	0	0	0	0	0	0	0	0	0	0	
22390947	f	hp	CN	2023	0	1	0	0	1	0	0	0	0	0	0	0	67
22393237	f	md	US	2023	0	0	0	0	0	0	0	0	0	0	0	0	63
22394249	m	md	BR	2023	0	0	0	0	0	0	0	0	0	0	0	0	63
22396228	m	md	JP	2023	0	1	1	0	0	0	0	0	0	0	0	0	70
22397302	m	md	JP	2023	0	1	0	0	0	1	0	0	0	0	0	0	80
22397369	m	md	FR	2023	0	0	0	0	0	0	0	0	0	0	0	0	57
22518538	f	hp	US	2023	0	0	0	0	0	0	0	0	0	0	0	0	65
22523388	m	md	JP	2023	0	0	0	0	0	0	0	0	0	0	0	0	70
22534415	m	md	IT	2023	0	1	1	0	0	0	0	0	0	0	0	0	
22534524	m	md	CN	2023	0	1	0	0	0	1	0	0	0	0	0	0	61
22537127	f	hp	GB	2023	0	0	0	0	0	0	0	0	0	0	0	0	76
22537873	m	md	JP	2023	0	1	1	0	0	0	0	0	0	0	0	1	80
22538940	m	md	US	2023	0	0	0	0	0	0	0	0	0	0	0	0	56

22539753	m	md	CN	2023	1	0	0	0	0	0	0	0	0	0	0	0	60
22542179	Not	ph	JP	2023	0	1	1	0	0	0	0	0	0	0	0	0	
22586956	m	md	AU	2023	0	0	0	0	0	0	0	0	0	0	0	0	63
22588986	Not	md	JP	2023	0	0	0	0	0	0	0	0	0	0	0	0	
22594276	m	md	AT	2023	0	1	1	0	0	0	0	0	0	0	0	0	73
22596500	f	md	FR	2023	0	0	0	0	0	0	0	0	0	0	0	0	63
22597636	f	md	JP	2023	0	1	0	0	0	1	0	0	0	0	0	0	90
22599776	m	md	DE	2023	0	1	0	0	1	0	0	0	0	0	0	0	71
22600469	f	cn	US	2023	0	0	0	0	0	0	0	0	0	0	0	0	
22600721	f	md	JP	2023	0	0	0	0	0	0	0	0	0	0	0	0	
22601555	f	md	JP	2023	0	0	0	0	0	0	0	0	0	0	0	0	64
22601595	f	md	JP	2023	0	0	0	0	0	0	0	0	0	0	0	0	73
22604203	m	md	JP	2023	0	0	0	0	0	0	0	0	0	0	0	0	46
22604500	m	md	US	2023	1	0	0	0	0	0	0	0	0	0	0	0	60
22607033	m	cn	US	2023	1	0	0	0	0	0	0	0	0	0	0	0	67
22609294	f	md	CA	2023	1	0	0	0	0	0	0	0	0	0	0	0	
22609900	f	md	FR	2023	0	0	0	0	0	0	0	0	0	0	0	0	70
22610374	f	md	CN	2023	1	0	0	0	0	0	0	0	0	0	0	0	
22610876	f	md	JP	2023	1	0	0	0	0	0	0	0	0	0	0	0	75
22611524	f	md	DE	2023	0	0	0	0	0	0	0	0	0	0	0	0	76
22613520	Not	md	JP	2023	0	1	0	0	0	1	0	0	0	0	0	0	80
22615557	m	md	AT	2023	0	1	1	0	0	0	0	0	0	0	0	0	73
22616861	f	md	JP	2023	0	0	0	0	0	0	0	0	0	0	0	0	50
22617334	Not	md	JP	2023	0	1	0	0	1	0	0	0	0	0	0	0	
22617979	Not	md	JP	2023	0	1	0	0	0	1	0	0	0	0	0	0	
22618877	f	md	FR	2023	0	1	0	1	0	0	0	0	0	0	0	0	
22621773	f	md	ES	2023	0	1	1	0	0	0	0	0	0	0	0	0	
22621837	f	md	US	2023	0	1	0	0	0	1	0	0	0	0	0	0	66
22621983	f	md	US	2023	0	1	0	0	0	1	0	0	0	0	0	0	72
22623855	m	md	JP	2023	0	1	1	0	0	0	0	0	0	0	0	1	80
22625550	m	cn	AT	2023	0	1	1	0	0	0	0	0	0	0	0	0	73
22626604	Not	ph	JP	2023	1	0	0	0	0	0	0	0	0	0	0	0	
22626643	Not	ph	JP	2023	1	0	0	0	0	0	0	0	0	0	0	0	
22627304	m	md	JP	2023	0	1	1	0	0	0	0	0	0	0	0	0	70

22627618	Not	ph	JP	2023	1	0	0	0	0	0	0	0	0	0	0	0	
22628029	m	md	BG	2023	0	1	0	0	0	1	0	0	0	0	0	0	68
22628847	m	hp	FR	2023	0	1	1	0	0	0	0	0	0	0	0	0	74
22634702	f	md	ES	2023	0	1	1	0	0	0	0	0	0	0	0	0	60
22635491	m	md	MY	2023	0	0	0	0	0	0	0	0	0	0	0	0	64
22636578	f	cn	US	2023	0	0	0	0	0	0	0	0	0	0	0	0	79
22637621	m	md	JP	2023	0	1	0	0	1	0	0	0	0	0	0	0	61
22637980	m	md	GR	2023	0	1	0	0	1	0	0	0	0	0	0	0	72
22640842	f	md	JP	2023	0	1	0	0	0	1	0	0	0	0	0	0	77
22645053	f	hp	DK	2023	0	1	0	0	0	1	0	0	0	0	0	0	66
22647265	m	md	JP	2023	0	1	0	0	1	0	0	0	0	0	0	0	74
22647397	m	md	JP	2023	0	1	0	0	1	0	0	0	0	0	0	0	74
22647490	Not	md	ES	2023	0	1	0	0	0	1	0	0	0	0	0	1	
22648433	f	hp	JP	2023	0	1	0	0	0	1	0	0	0	0	0	0	67
22651102	m	md	DE	2023	0	0	0	0	0	0	0	0	0	0	0	0	51
22651337	m	md	ES	2023	0	0	0	0	0	0	0	0	0	0	0	0	57
22652257	m	md	JP	2023	0	1	0	0	1	0	0	0	0	0	0	0	58
22655179	f	cn	RU	2023	0	0	0	0	0	0	0	0	0	0	0	0	
22655836	f	md	DE	2023	0	1	1	0	0	0	0	0	0	0	0	0	60
22656415	m	md	DE	2023	0	0	0	0	0	0	0	0	0	0	0	0	74
22656477	m	md	JP	2023	0	1	0	0	1	0	0	0	0	0	0	0	70
22657847	m	md	JP	2023	0	1	0	0	1	0	0	0	0	0	0	0	70
22659684	m	md	IT	2023	0	1	0	0	0	1	0	0	0	0	0	0	66
22660346	f	md	HR	2023	0	1	1	0	0	0	0	0	0	0	0	0	67
22661562	f	md	JP	2023	1	0	0	0	0	0	0	0	0	0	0	0	78
22662352	m	md	IN	2023	0	0	0	0	0	0	0	0	0	0	0	0	60
22662797	f	md	US	2023	0	1	1	0	0	0	0	0	0	0	0	0	
22662814	m	md	JP	2023	0	0	0	0	0	0	0	0	0	0	0	0	80
22662937	m	hp	JP	2023	0	1	0	0	1	0	0	0	0	0	0	0	74
22663328	f	md	US	2023	0	1	0	0	1	0	0	0	0	0	0	0	65
22663387	f	cn	CN	2023	0	0	0	0	0	0	0	0	0	0	0	0	
22663426	f	md	JP	2023	0	0	0	0	0	0	0	0	0	0	0	0	63
22663769	f	md	US	2023	0	1	0	0	1	0	0	0	0	0	0	0	68
22663876	f	md	JP	2023	0	0	0	0	0	0	0	0	0	0	0	0	76

22663943	m	hp	CN	2023	0	0	0	0	0	0	0	0	0	0	0	0	62
22663965	m	md	US	2023	0	1	1	0	0	0	0	0	0	0	0	0	70
22664186	f	md	US	2023	0	0	0	0	0	0	0	0	0	0	0	0	
22665503	Not	md	JP	2023	0	1	0	0	0	1	0	0	0	0	0	0	
22665504	m	md	JP	2023	0	1	0	0	0	1	0	0	0	0	0	1	80
22665588	f	cn	US	2023	0	1	1	0	0	0	0	0	0	0	0	1	
22665637	m	cn	US	2023	0	1	1	0	0	0	0	0	0	0	0	1	64
22665754	f	cn	MY	2023	1	0	0	0	0	0	0	0	0	0	0	0	47.2
22665801	f	md	AU	2023	0	1	0	1	0	0	0	0	0	0	0	0	
22665830	m	ph	MY	2023	1	0	0	0	0	0	0	0	0	0	0	0	
22665956	f	md	JP	2023	0	0	0	0	0	0	0	0	0	0	0	0	70
22665970	m	md	JP	2023	0	0	0	0	0	0	0	0	0	0	0	0	78
22666617	f	cn	CN	2023	0	1	0	1	0	0	0	0	0	0	0	0	62
22667190	f	cn	CA	2023	0	0	0	0	0	0	0	0	0	0	0	0	
22667541	m	md	JP	2023	0	1	0	0	0	1	0	0	0	0	0	0	73
22667603	f	md	DE	2023	0	1	0	1	0	0	0	0	0	0	0	1	
22667660	Not	md	IT	2023	0	1	0	0	1	0	0	0	0	0	0	0	
22667667	m	md	JP	2023	0	1	0	0	1	0	0	0	0	0	0	0	75
22667827	m	hp	US	2023	0	0	0	0	0	0	0	0	0	0	0	0	
22667891	f	md	ES	2023	0	1	1	0	0	0	0	0	0	0	0	0	60
22667979	m	hp	IT	2023	0	0	0	0	0	0	0	0	0	0	0	0	87
22668032	m	md	JP	2023	0	1	1	0	0	0	0	0	0	0	0	0	80
22668063	m	md	SG	2023	0	1	0	0	0	1	0	0	0	0	0	0	59
22668109	f	hp	CA	2023	0	1	1	0	0	0	0	0	0	0	0	0	58
22668148	f	hp	IT	2023	0	0	0	0	0	0	0	0	0	0	0	0	54
22668287	m	md	JP	2023	0	1	0	0	1	0	0	0	0	0	0	0	70
22668453	m	md	ES	2023	0	1	0	0	0	1	0	0	0	0	0	0	64
22668462	f	md	JP	2023	1	0	0	0	0	0	0	0	0	0	0	0	51
22668610	m	cn	IN	2023	1	0	0	0	0	0	0	0	0	0	0	0	
22668611	m	md	AE	2023	0	1	0	1	0	0	0	0	0	0	0	0	
22668614	f	md	JP	2023	1	0	0	0	0	0	0	0	0	0	0	0	71
22668643	m	md	JP	2023	0	1	1	0	0	0	0	0	0	0	0	0	90
22668690	m	md	US	2023	0	1	0	0	0	1	0	0	0	0	0	0	83
22668691	m	md	JP	2023	0	1	0	0	0	1	0	0	0	0	0	0	80

22668747	f	md	FR	2023	0	1	0	0	0	1	0	0	0	0	0	0	71
22668798	m	hp	IT	2023	0	0	0	0	0	0	0	0	0	0	0	0	
22668847	m	md	JP	2023	1	0	0	0	0	0	0	0	0	0	0	0	70
22668907	f	cn	PK	2023	0	1	0	0	0	1	0	0	0	0	0	0	51
22668950	f	hp	JP	2023	0	0	0	0	0	0	0	0	0	0	0	0	67
22669251	f	md	KR	2023	0	1	1	0	0	0	0	0	0	0	0	0	
22669262	f	cn	JP	2023	1	0	0	0	0	0	0	0	0	0	0	0	
22669299	m	hp	JP	2023	0	1	0	0	1	0	0	0	0	0	0	0	74
22669789	f	md	JP	2023	0	1	0	0	1	0	0	0	0	0	0	0	60
22669905	f	md	PT	2023	0	1	1	0	0	0	0	0	0	0	0	0	
22670061	f	md	US	2023	0	1	1	0	0	0	0	0	0	0	0	0	
22670131	f	cn	IN	2023	0	0	0	0	0	0	0	0	0	0	0	0	79
22670361	m	hp	IT	2023	0	0	0	0	0	0	0	0	0	0	0	0	
22670431	Not	md	JP	2023	0	1	0	0	1	0	0	0	0	0	0	0	
22671031	f	md	JP	2023	1	0	0	0	0	0	0	0	0	0	0	1	73
22671152	m	md	JP	2023	1	0	0	0	0	0	0	0	0	0	0	0	78
22671337	f	md	JP	2023	0	1	1	0	0	0	0	0	0	0	0	0	71
22671741	f	hp	IT	2023	0	0	0	0	0	0	0	0	0	0	0	0	
22671834	f	hp	GB	2023	1	0	0	0	0	0	0	0	0	0	0	0	80
22672518	Not	md	US	2023	0	0	0	0	0	0	0	0	0	0	0	0	
22673083	f	cn	DE	2023	0	1	0	0	1	0	0	0	0	0	0	0	48
22673107	f	hp	IT	2023	0	0	0	0	0	0	0	0	0	0	0	0	
22673527	m	md	JP	2023	0	0	0	0	0	0	0	0	0	0	0	0	78
22673670	f	md	JP	2023	0	1	0	1	0	0	0	0	0	0	0	0	70
22673989	f	hp	IT	2023	0	0	0	0	0	0	0	0	0	0	0	0	41
22673990	f	hp	IT	2023	0	0	0	0	0	0	0	0	0	0	0	0	
22674009	f	hp	IT	2023	0	0	0	0	0	0	0	0	0	0	0	0	20
22674198	m	hp	DE	2023	0	1	1	0	0	0	0	0	0	0	0	0	83
22674255	m	hp	DE	2023	0	1	1	0	0	0	0	0	0	0	0	0	71
22674275	f	hp	US	2023	0	0	0	0	0	0	0	0	0	0	0	0	60
22674276	m	hp	DE	2023	0	1	1	0	0	0	0	0	0	0	0	0	65
22674312	m	hp	DE	2023	0	1	1	0	0	0	0	0	0	0	0	0	81
22674313	f	hp	DE	2023	0	1	1	0	0	0	0	0	0	0	0	0	64
22674348	m	md	IT	2023	0	0	0	0	0	0	0	0	0	0	0	0	

22674475	f	cn	JP	2023	1	0	0	0	0	0	0	0	0	0	0	0	67
22674489	f	cn	DE	2023	0	1	1	0	0	0	0	0	0	0	0	0	66
22674711	f	cn	US	2023	0	1	1	0	0	0	0	0	0	0	0	0	64
22674723	m	cn	PK	2023	0	1	0	0	0	1	0	0	0	0	0	0	39
22674731	f	md	FR	2023	0	0	0	0	0	0	0	0	0	0	0	0	74
22674916	m	md	IT	2023	0	1	0	0	0	1	0	0	0	0	0	0	64
22675171	f	md	JP	2023	0	0	0	0	0	0	0	0	0	0	0	0	70
22675245	m	cn	IN	2023	0	0	0	0	0	0	0	0	0	0	0	0	67
22675932	m	md	DE	2023	0	1	1	0	0	0	0	0	0	0	0	0	55
22676044	m	md	JP	2023	0	0	0	0	0	0	0	0	0	0	0	0	73
22676347	f	md	JP	2023	0	1	0	0	1	0	0	0	0	0	0	0	70
22676355	Not	md	IT	2023	0	0	0	0	0	0	0	0	0	0	0	0	47
22676827	f	hp	US	2023	0	1	1	0	0	0	0	0	0	0	0	0	
22676906	Not	md	JP	2023	0	0	0	0	0	0	0	0	0	0	0	0	
22677212	m	cn	JP	2023	0	0	0	0	0	0	0	0	0	0	0	0	55
22677213	m	cn	JP	2023	0	0	0	0	0	0	0	0	0	0	0	0	
22677392	m	md	IT	2023	0	0	0	0	0	0	0	0	0	0	0	0	77
22677430	f	md	IT	2023	0	0	0	0	0	0	0	0	0	0	0	0	83
22677444	m	md	IT	2023	0	0	0	0	0	0	0	0	0	0	0	0	74
22677570	f	md	DE	2023	0	0	0	0	0	0	0	0	0	0	0	0	79
22677694	f	md	JP	2023	1	0	0	0	0	0	0	0	0	0	0	0	82
22677796	f	md	BE	2023	0	0	0	0	0	0	0	0	0	0	0	0	68
22677820	m	hp	AR	2023	0	0	0	0	0	0	0	0	0	0	0	0	70
22678177	f	md	FR	2023	1	0	0	0	0	0	0	0	0	0	0	0	81
22678214	m	cn	NL	2023	0	0	0	0	0	0	0	0	0	0	0	0	63
22678337	m	md	JP	2023	0	1	1	0	0	0	0	0	0	0	0	0	80
22678460	f	hp	CN	2023	0	0	0	0	0	0	0	0	0	0	0	0	48
22678532	f	md	BE	2023	0	1	0	0	1	0	0	0	0	0	0	0	
22678826	f	md	CN	2023	1	0	0	0	0	0	0	0	0	0	0	0	65
22679076	m	cn	US	2023	0	0	0	0	0	0	0	0	0	0	0	0	
22679090	m	hp	US	2023	0	0	0	0	0	0	0	0	0	0	0	0	
22679092	m	hp	US	2023	0	0	0	0	0	0	0	0	0	0	0	0	
22679094	m	md	US	2023	0	0	0	0	0	0	0	0	0	0	0	0	50
22679096	Not	md	US	2023	0	0	0	0	0	0	0	0	0	0	0	0	

22679101	f	md	US	2023	0	0	0	0	0	0	0	0	0	0	0	0	
22679103	f	cn	US	2023	0	0	0	0	0	0	0	0	0	0	0	0	
22679106	f	cn	US	2023	0	0	0	0	0	0	0	0	0	0	0	0	53
22679118	Not	md	US	2023	0	0	0	0	0	0	0	0	0	0	0	0	
22679140	m	hp	US	2023	0	0	0	0	0	0	0	0	0	0	0	0	64
22679142	f	cn	US	2023	0	0	0	0	0	0	0	0	0	0	0	0	
22679145	f	md	US	2023	0	0	0	0	0	0	0	0	0	0	0	0	
22679674	Not	md	JP	2023	1	0	0	0	0	0	0	0	0	0	0	0	
22679707	m	md	JP	2023	0	1	0	1	0	0	0	0	0	0	0	0	77
22679754	m	md	DE	2023	0	0	0	0	0	0	0	0	0	0	0	0	68
22679909	f	md	US	2023	0	1	0	0	0	1	0	0	0	0	0	0	67
22679962	m	hp	JP	2023	0	1	0	0	0	1	0	0	0	0	0	0	
22680169	Not	cn	SG	2023	0	1	0	1	0	0	0	0	0	0	0	0	
22680222	m	md	JP	2023	0	1	0	0	1	0	0	0	0	0	0	0	80
22680362	m	md	IT	2023	0	1	0	0	1	0	0	0	0	0	0	0	76
22680427	Not	md	JP	2023	0	1	0	0	0	1	0	0	0	0	0	0	70
22680637	f	cn	US	2023	1	0	0	0	0	0	0	0	0	0	0	0	79
22680646	m	md	JP	2023	0	0	0	0	0	0	0	0	0	0	0	0	81
22680829	m	md	DE	2023	0	0	0	0	0	0	0	0	0	0	0	0	38
22680953	m	md	CA	2023	0	1	1	0	0	0	0	0	0	0	0	0	
22681029	m	md	VN	2023	0	1	0	0	0	1	0	0	0	0	0	0	80
22681052	Not	md	JP	2023	0	0	0	0	0	0	0	0	0	0	0	0	52
22681171	Not	ph	JP	2023	1	0	0	0	0	0	0	0	0	0	0	0	
22681290	m	cn	CN	2023	0	0	0	0	0	0	0	0	0	0	0	0	52
22681452	f	hp	CN	2023	1	0	0	0	0	0	0	0	0	0	0	0	69
22681461	f	hp	CA	2023	0	1	1	0	0	0	0	0	0	0	0	0	58
22681633	m	md	TW	2023	0	0	0	0	0	0	0	0	0	0	0	0	60
22681723	f	cn	IN	2023	1	0	0	0	0	0	0	0	0	0	0	0	66
22681867	m	cn	IN	2023	0	0	0	0	0	0	0	0	0	0	0	0	47
22681890	m	md	CN	2023	0	1	0	1	0	0	0	0	0	0	0	0	29
22682227	Not	ph	JP	2023	0	1	0	0	1	0	0	0	0	0	0	0	
22682271	f	ph	MY	2023	1	0	0	0	0	0	0	0	0	0	0	0	
22682285	m	md	US	2023	0	0	0	0	0	0	0	0	0	0	0	0	76
22682449	f	md	JP	2023	0	0	0	0	0	0	0	0	0	0	0	0	76

22682510	f	cn	ID	2023	1	0	0	0	0	0	0	0	0	0	0	0	60.7
22682514	Not	cn	US	2023	1	0	0	0	0	0	0	0	0	0	0	0	
22682639	Not	hp	US	2023	0	0	0	0	0	0	0	0	0	0	0	0	
22682984	Not	cn	JP	2023	1	0	0	0	0	0	0	0	0	0	0	0	75
22683054	f	cn	US	2023	0	0	0	0	0	0	0	0	0	0	0	0	54
22683134	m	md	JP	2023	0	1	0	0	1	0	0	0	0	0	0	0	64
22683177	f	md	DE	2023	0	1	1	0	0	0	0	0	0	0	0	0	66
22683362	f	md	PE	2023	0	1	1	0	0	0	0	0	0	0	0	0	54
22683408	m	hp	CN	2023	0	0	0	0	0	0	0	0	0	0	0	0	62
22683483	m	cn	JP	2023	0	0	0	0	0	0	0	0	0	0	0	0	76
22683490	Not	md	US	2023	0	0	0	0	0	0	0	0	0	0	0	0	
22683527	Not	ph	FR	2023	0	0	0	0	0	0	0	0	0	0	0	0	
22683976	m	hp	IT	2023	0	0	0	0	0	0	0	0	0	0	0	0	60
22684025	m	md	FR	2023	0	1	1	0	0	0	0	0	0	0	0	0	66
22684061	f	hp	US	2023	0	0	0	0	0	0	0	0	0	0	0	0	60
22684201	m	cn	DE	2023	0	0	0	0	0	0	0	0	0	0	0	0	
22684247	Not	ph	JP	2023	0	1	0	0	1	0	0	0	0	0	0	0	
22684286	m	md	CA	2023	0	0	0	0	0	0	0	0	0	0	0	0	77
22684299	Not	ph	JP	2023	0	1	0	0	1	0	0	0	0	0	0	0	
22684506	m	md	IT	2023	0	1	0	0	1	0	0	0	0	0	0	0	64
22684521	m	md	IT	2023	0	0	0	0	0	0	0	0	0	0	0	0	78
22684757	f	ph	JP	2023	0	0	0	0	0	0	0	0	0	0	0	0	50
22684940	f	md	JP	2023	0	1	0	0	0	1	0	0	0	0	0	1	74
22684960	Not	md	JP	2023	0	1	0	0	0	1	0	0	0	0	0	0	80
22685001	m	md	JP	2023	0	1	0	0	0	1	0	0	0	0	0	0	80
22685261	f	md	RU	2023	0	0	0	0	0	0	0	0	0	0	0	0	47
22685488	m	md	JP	2023	0	1	0	0	0	1	0	0	0	0	0	0	
22685626	m	md	JP	2023	0	1	0	0	1	0	0	0	0	0	0	0	80
22686855	m	md	GR	2023	0	0	0	0	0	0	0	0	0	0	0	0	82
22687069	m	hp	GE	2023	0	0	0	0	0	0	0	0	0	0	0	0	73
22687119	f	hp	JP	2023	0	1	0	0	1	0	0	0	0	0	0	0	75
22687504	Not	hp	US	2023	1	0	0	0	0	0	0	0	0	0	0	0	
22687505	f	md	DE	2023	0	1	1	0	0	0	0	0	0	0	0	0	
22687549	m	md	SG	2023	1	0	0	0	0	0	0	0	0	0	0	0	

22687756	f	cn	ID	2023	1	0	0	0	0	0	0	0	0	0	0	0	59.9
22688052	m	cn	CN	2023	0	1	0	1	0	0	0	0	0	0	0	0	70
22688233	f	md	DE	2023	0	0	0	0	0	0	0	0	0	0	0	0	48
22688256	m	cn	EG	2023	1	0	0	0	0	0	0	0	0	0	0	0	58.6
22688258	m	md	SG	2023	1	0	0	0	0	0	0	0	0	0	0	0	
22688575	m	md	JP	2023	0	1	1	0	0	0	0	0	0	0	0	0	73
22688974	f	ph	JP	2023	0	1	0	1	0	0	0	0	0	0	0	0	75
22689101	m	md	US	2023	0	1	1	0	0	0	0	0	0	0	0	0	70
22689161	f	hp	GB	2023	0	0	0	0	0	0	0	0	0	0	0	0	78
22689475	m	cn	IN	2023	0	0	0	0	0	0	0	0	0	0	0	0	60
22689634	f	md	JP	2023	0	1	0	0	0	1	0	0	0	0	0	0	80
22689639	f	md	JP	2023	0	0	0	0	0	0	0	0	0	0	0	0	76
22689640	f	md	JP	2023	0	1	0	0	0	1	0	0	0	0	0	0	80
22689642	m	md	JP	2023	0	1	0	0	0	1	0	0	0	0	0	0	70
22689704	m	md	KR	2023	0	1	1	0	0	0	0	0	0	0	0	0	73
22690006	m	cn	US	2023	1	0	0	0	0	0	0	0	0	0	0	0	
22690278	f	ph	US	2023	0	0	0	0	0	0	0	0	0	0	0	0	60
22690521	m	md	JP	2023	0	1	0	0	0	1	0	0	0	0	0	0	59
22690522	m	md	JP	2023	0	0	0	0	0	0	0	0	0	0	0	0	
22690545	f	md	US	2023	0	0	0	0	0	0	0	0	0	0	0	0	64
22690631	Not	ph	FR	2023	0	0	0	0	0	0	0	0	0	0	0	0	
22690672	f	hp	US	2023	0	1	1	0	0	0	0	0	0	0	0	0	68
22690767	m	md	IT	2023	0	0	0	0	0	0	0	0	0	0	0	0	74
22691455	m	md	MY	2023	0	0	0	0	0	0	0	0	0	0	0	0	
22691617	m	md	JP	2023	1	0	0	0	0	0	0	0	0	0	0	0	60
22691850	f	cn	AR	2023	0	0	0	0	0	0	0	0	0	0	0	0	83
22692018	f	md	CN	2023	0	1	1	0	0	0	0	0	0	0	0	0	69
22692080	m	md	JP	2023	0	1	1	0	0	0	0	0	0	0	0	0	79
22692307	m	md	NZ	2023	0	1	0	1	0	0	0	0	0	0	0	0	
22692325	m	md	CN	2023	0	0	0	0	0	0	0	0	0	0	0	0	64
22692864	m	md	JP	2023	0	1	1	0	0	0	0	0	0	0	0	0	80
22693498	m	md	JP	2023	0	1	1	0	0	0	0	0	0	0	0	0	70
22693735	m	md	RU	2023	0	1	0	1	0	0	0	0	0	0	0	0	72
22693741	m	md	KR	2023	0	1	1	0	0	0	0	0	0	0	0	0	71

22693922	m	md	AU	2023	1	0	0	0	0	0	0	0	0	0	0	0	
22694156	m	cn	US	2023	0	0	0	0	0	0	0	0	0	0	0	0	42
22694222	f	md	FR	2023	1	0	0	0	0	0	0	0	0	0	0	0	84
22694253	m	md	JP	2023	0	1	1	0	0	0	0	0	0	0	0	0	82
22694694	Not	md	US	2023	0	0	0	0	0	0	0	0	0	0	0	0	
22694886	m	md	JP	2023	0	0	0	0	0	0	0	0	0	0	0	0	60
22695355	f	md	US	2023	0	1	0	0	0	1	0	0	0	0	0	0	62
22695387	f	cn	CA	2023	1	0	0	0	0	0	0	0	0	0	0	0	
22695518	Not	md	JP	2023	0	0	0	0	0	0	0	0	0	0	0	0	
22695593	f	ph	JP	2023	0	0	0	0	0	0	0	0	0	0	0	0	82
22696022	f	ph	FR	2023	0	1	1	0	0	0	0	0	0	0	0	0	77
22696041	m	md	JP	2023	0	1	0	1	0	0	0	0	0	0	0	0	46
22697357	f	md	US	2023	0	1	0	0	1	0	0	0	0	0	0	0	
22697614	f	md	ES	2023	0	0	0	0	0	0	0	0	0	0	0	0	71
22697778	m	hp	CH	2023	0	1	1	0	0	0	0	0	0	0	0	0	70
22698321	m	md	DE	2023	0	0	0	0	0	0	0	0	0	0	0	0	
22698338	m	hp	US	2023	0	1	1	0	0	0	0	0	0	0	0	0	65
22698350	m	cn	US	2023	0	1	1	0	0	0	0	0	0	0	0	0	64
22698438	f	md	US	2023	0	1	1	0	0	0	0	0	0	0	0	0	69
22698451	m	md	JP	2023	0	1	1	0	0	0	0	0	0	0	0	0	70
22698848	m	md	JP	2023	0	1	0	0	0	1	0	0	0	0	0	0	76
22699052	m	md	JP	2023	0	0	0	0	0	0	0	0	0	0	0	0	77
22699057	f	cn	US	2023	0	0	0	0	0	0	0	0	0	0	0	0	53
22699631	f	md	AU	2023	1	0	0	0	0	0	0	0	0	0	0	0	
22700099	f	md	CN	2023	0	0	0	0	0	0	0	0	0	0	0	0	64
22700957	f	hp	CN	2023	1	0	0	0	0	0	0	0	0	0	0	0	68
22700960	m	md	KR	2023	0	0	0	0	0	0	0	0	0	0	0	0	
22700963	f	md	DE	2023	0	1	1	0	0	0	0	0	0	0	0	0	
22701133	f	md	FR	2023	0	0	0	0	0	0	0	0	0	0	0	0	
22701333	Not	ph	JP	2023	0	1	1	0	0	0	0	0	0	0	0	0	90
22701715	m	md	FR	2023	0	1	1	0	0	0	0	0	0	0	0	0	66
22701972	m	hp	US	2023	0	0	0	0	0	0	0	0	0	0	0	0	68
22702194	m	md	NP	2023	0	0	0	0	0	0	0	0	0	0	0	0	40
22702599	f	md	GB	2023	1	0	0	0	0	0	0	0	0	0	0	0	

22702682	f	md	US	2023	0	0	0	0	0	0	0	0	0	0	0	0	69
22702782	m	md	JP	2023	0	1	0	1	0	0	0	0	0	0	0	1	77
22702809	m	md	DE	2023	0	1	0	0	1	0	0	0	0	0	0	1	56
22702820	m	md	JP	2023	0	1	1	0	0	0	0	0	0	0	0	0	61
22702856	m	hp	IN	2023	0	0	0	0	0	0	0	0	0	0	0	0	35
22702873	m	ph	US	2023	0	0	0	0	0	0	0	0	0	0	0	0	45
22702940	m	md	FR	2023	0	1	1	0	0	0	0	0	0	0	0	0	66
22703033	Not	md	RU	2023	0	1	0	0	0	1	0	0	0	0	0	0	56
22704023	m	md	CH	2023	0	1	1	0	0	0	0	0	0	0	0	0	70
22704289	f	md	PL	2023	0	0	0	0	0	0	0	0	0	0	0	0	
22704443	m	ph	JP	2023	0	1	0	1	0	0	0	0	0	0	0	1	77
22704475	m	md	JP	2023	0	1	0	0	0	1	0	0	0	0	0	1	70
22704663	m	hp	ID	2023	0	0	0	0	0	0	0	0	0	0	0	0	71
22705441	f	cn	ID	2023	1	0	0	0	0	0	0	0	0	0	0	0	54.2
22705642	m	cn	ID	2023	1	0	0	0	0	0	0	0	0	0	0	0	44.1
22705746	m	cn	TH	2023	1	0	0	0	0	0	0	0	0	0	0	0	84.3
22706047	m	md	AU	2023	0	0	0	0	0	0	0	0	0	0	0	0	61
22706127	m	hp	CN	2023	0	0	0	0	0	0	0	0	0	0	0	0	73
22706143	m	md	FR	2023	0	0	0	0	0	0	0	0	0	0	0	0	75
22706228	m	md	IE	2023	0	0	0	0	0	0	0	0	0	0	0	0	72
22706239	m	md	DE	2023	0	1	0	0	1	0	0	0	0	0	0	0	54
22706486	f	md	US	2023	0	1	1	0	0	0	0	0	0	0	0	0	70
22706562	f	cn	US	2023	0	1	1	0	0	0	0	0	0	0	0	0	
22706670	m	cn	US	2023	0	1	1	0	0	0	0	0	0	0	0	0	
22706809	m	md	JP	2023	0	1	0	0	1	0	0	0	0	0	0	0	69
22706987	m	cn	ID	2023	1	0	0	0	0	0	0	0	0	0	0	0	51.6
22707207	m	hp	US	2023	0	0	0	0	0	0	0	0	0	0	0	0	42
22707225	Not	md	BE	2023	0	0	0	0	0	0	0	0	0	0	0	0	
22707244	f	ph	JP	2023	0	1	0	1	0	0	0	0	0	0	0	0	71
22707415	m	md	CA	2023	0	0	0	0	0	0	0	0	0	0	0	0	53
22707426	m	md	JP	2023	0	1	0	0	1	0	0	0	0	0	0	0	66
22707501	m	hp	JP	2023	0	1	0	1	0	0	0	0	0	0	0	1	77
22707677	f	md	US	2023	0	1	0	1	0	0	0	0	0	0	0	0	77.1
22707699	m	ph	NZ	2023	0	1	0	1	0	0	0	0	0	0	0	0	

22707769	f	md	US	2023	0	1	1	0	0	0	0	0	0	0	0	0	66
22707814	m	cn	CA	2023	1	0	0	0	0	0	0	0	0	0	0	0	62.5
22707906	f	ph	JP	2023	0	1	1	0	0	0	0	0	0	0	0	0	73
22708143	m	md	CN	2023	0	0	0	0	0	0	0	0	0	0	0	0	63
22709061	f	cn	US	2023	0	0	0	0	0	0	0	0	0	0	0	0	
22709065	Not	md	US	2023	0	0	0	0	0	0	0	0	0	0	0	0	
22709108	m	md	JP	2023	0	1	0	0	0	1	0	0	0	0	0	0	80
22710578	f	cn	US	2023	0	1	1	0	0	0	0	0	0	0	0	0	70
22710635	f	md	FR	2023	0	0	0	0	0	0	0	0	0	0	0	0	69
22710727	m	md	IE	2023	0	0	0	0	0	0	0	0	0	0	0	0	42
22711818	f	cn	US	2023	0	0	0	0	0	0	0	0	0	0	0	0	57
22712108	m	md	DE	2023	0	1	1	0	0	0	0	0	0	0	0	0	
22712123	m	md	US	2023	0	1	0	0	1	0	0	0	0	0	0	0	70
22712403	f	cn	DE	2023	0	0	0	0	0	0	0	0	0	0	0	0	
22713076	m	md	US	2023	0	1	0	1	0	0	0	0	0	0	0	0	
22713196	m	hp	CN	2023	1	0	0	0	0	0	0	0	0	0	0	0	56
22713381	m	hp	JP	2023	0	1	1	0	0	0	0	0	0	0	0	0	60
22713481	f	md	IN	2023	0	0	0	0	0	0	0	0	0	0	0	0	69
22713491	m	md	KR	2023	0	0	0	0	0	0	0	0	0	0	0	0	71
22713652	m	md	KR	2023	0	0	0	0	0	0	0	0	0	0	0	0	
22713659	m	md	TR	2023	0	0	0	0	0	0	0	0	0	0	0	0	73
22713685	f	ph	US	2023	0	0	0	0	0	0	0	0	0	0	0	0	
22714385	Not	md	PT	2023	0	0	0	0	0	0	0	0	0	0	0	0	
22714461	f	md	US	2023	0	0	0	0	0	0	0	0	0	0	0	0	69
22715256	f	cn	US	2023	1	0	0	0	0	0	0	0	0	0	0	0	
22715714	m	hp	QA	2023	1	0	0	0	0	0	0	0	0	0	0	0	52
22716501	m	md	US	2023	0	0	0	0	0	0	0	0	0	0	0	0	50
22716533	m	md	IL	2023	0	1	1	0	0	0	0	0	0	0	0	0	46
22716544	m	cn	ID	2023	0	1	0	1	0	0	0	0	0	0	0	0	39.1
22716929	m	md	US	2023	0	0	0	0	0	0	0	0	0	0	0	0	74
22717722	m	cn	US	2023	0	0	0	0	0	0	0	0	0	0	0	0	66
22717936	m	hp	CN	2023	1	0	0	0	0	0	0	0	0	0	0	0	46
22718824	f	md	CN	2023	0	0	0	0	0	0	0	0	0	0	0	0	74
22719028	Not	cn	JP	2023	0	0	0	0	0	0	0	0	0	0	0	0	89

22719249	f	md	CN	2023	0	0	0	0	0	0	0	0	0	0	0	0	73
22719296	f	md	ES	2023	0	1	1	0	0	0	0	0	0	0	0	0	60
22720229	m	cn	PK	2023	0	1	0	0	0	1	0	0	0	0	0	0	72
22720861	Not	hp	FR	2023	0	0	0	0	0	0	0	0	0	0	0	0	
22721853	f	cn	PK	2023	0	0	0	0	0	0	0	0	0	0	0	0	59
22721909	Not	md	JP	2023	0	1	0	0	0	1	0	0	0	0	0	0	74
22721928	f	cn	KR	2023	0	0	0	0	0	0	0	0	0	0	0	0	46
22722213	f	md	JP	2023	0	1	0	0	0	1	0	0	0	0	0	0	80
22722572	m	hp	IT	2023	0	0	0	0	0	0	0	0	0	0	0	0	69
22722573	f	md	RU	2023	0	1	0	0	0	1	0	0	0	0	0	0	70
22722582	f	hp	IT	2023	0	0	0	0	0	0	0	0	0	0	0	0	64
22722743	f	md	RU	2023	0	1	0	0	0	1	0	0	0	0	0	0	70
22722851	f	md	FR	2023	0	1	1	0	0	0	0	0	0	0	0	0	78
22723360	m	md	KR	2023	0	0	0	0	0	0	0	0	0	0	0	0	54
22724443	f	hp	BG	2023	0	0	0	0	0	0	0	0	0	0	0	0	42
22724517	f	md	HR	2023	0	1	1	0	0	0	0	0	0	0	0	0	67
22724661	m	md	JP	2023	0	0	0	0	0	0	0	0	0	0	0	0	68
22724776	f	cn	US	2023	0	1	1	0	0	0	0	0	0	0	0	0	77
22724780	f	hp	GB	2023	1	0	0	0	0	0	0	0	0	0	0	0	
22724790	f	cn	PH	2023	0	0	0	0	0	0	0	0	0	0	0	0	
22724897	f	md	PT	2023	0	1	0	1	0	0	0	0	0	0	0	0	58.8
22725077	f	md	AU	2023	0	1	0	1	0	0	0	0	0	0	0	0	63
22725722	f	md	JP	2023	0	1	1	0	0	0	0	0	0	0	0	0	
22725801	f	cn	US	2023	0	1	1	0	0	0	0	0	0	0	0	0	
22725825	m	md	JP	2023	0	1	1	0	0	0	0	0	0	0	0	0	
22726710	m	md	CN	2023	0	0	0	0	0	0	0	0	0	0	0	0	62
22726843	Not	md	JP	2023	0	1	0	0	1	0	0	0	0	0	0	0	
22726875	f	md	AU	2023	0	1	0	0	0	1	0	0	0	0	0	0	78
22726984	f	md	BG	2023	1	0	0	0	0	0	0	0	0	0	0	0	
22727479	f	hp	IT	2023	0	0	0	0	0	0	0	0	0	0	0	0	64
22727490	m	hp	QA	2023	1	0	0	0	0	0	0	0	0	0	0	0	52
22727540	m	md	CN	2023	0	1	0	1	0	0	0	0	0	0	0	0	44
22728869	f	cn	US	2023	0	0	0	0	0	0	0	0	0	0	0	0	61
22729126	m	cn	DE	2023	0	0	0	0	0	0	0	0	0	0	0	0	

22729273	f	md	AU	2023	1	0	0	0	0	0	0	0	0	0	0	0	
22729774	m	md	CL	2023	0	0	0	0	0	0	0	0	0	0	0	0	18
22729981	f	md	JP	2023	0	1	0	0	1	0	0	0	0	0	0	0	80
22730628	m	md	US	2023	0	1	1	0	0	0	0	0	0	0	0	0	73
22731085	f	md	JP	2023	0	1	1	0	0	0	0	0	0	0	0	0	80
22731681	f	md	DE	2023	0	0	0	0	0	0	0	0	0	0	0	0	
22731759	f	md	MY	2023	0	0	0	0	0	0	0	0	0	0	0	0	35
22731882	m	md	IT	2023	0	0	0	0	0	0	0	0	0	0	0	0	81
22733828	m	md	CH	2023	0	0	0	0	0	0	0	0	0	0	0	0	49
22734632	f	ph	JP	2023	0	1	0	0	0	1	0	0	0	0	0	0	73
22734643	f	cn	DE	2023	0	1	0	0	1	0	0	0	0	0	0	0	52
22734956	f	ph	MY	2023	1	0	0	0	0	0	0	0	0	0	0	0	
22734979	f	ph	FR	2023	0	0	0	0	0	0	0	0	0	0	0	0	
22735654	f	md	JP	2023	1	0	0	0	0	0	0	0	0	0	0	0	83
22736154	f	md	JP	2023	0	1	1	0	0	0	0	0	0	0	0	0	72
22736371	f	cn	AU	2023	1	0	0	0	0	0	0	0	0	0	0	0	
22736776	m	cn	FR	2023	0	1	1	0	0	0	0	0	0	0	0	0	
22736902	m	md	IN	2023	0	0	0	0	0	0	0	0	0	0	0	0	
22737006	m	md	PL	2023	0	1	0	0	0	1	0	0	0	0	0	0	62
22737022	Not	md	RU	2023	0	0	0	0	0	0	0	0	0	0	0	0	49
22737125	m	md	JP	2023	0	0	0	0	0	0	0	0	0	0	0	0	73
22737162	f	cn	IN	2023	0	0	0	0	0	0	0	0	0	0	0	0	51
22737164	f	cn	IN	2023	0	0	0	0	0	0	0	0	0	0	0	0	53
22737190	Not	md	JP	2023	0	1	0	0	0	1	0	0	0	0	0	0	70
22737254	m	md	FR	2023	0	1	1	0	0	0	0	0	0	0	0	0	
22737294	f	hp	CN	2023	1	0	0	0	0	0	0	0	0	0	0	0	47
22737299	Not	md	JP	2023	0	1	0	1	0	0	0	0	0	0	0	0	77
22737496	f	ph	MY	2023	1	0	0	0	0	0	0	0	0	0	0	0	
22737623	m	md	JP	2023	0	1	0	1	0	0	0	0	0	0	0	0	73
22737728	m	md	JP	2023	1	0	0	0	0	0	0	0	0	0	0	0	80
22737773	m	hp	CN	2023	0	1	0	1	0	0	0	0	0	0	0	0	
22737776	f	md	CH	2023	1	0	0	0	0	0	0	0	0	0	0	0	78.3
22738357	f	cn	ID	2023	1	0	0	0	0	0	0	0	0	0	0	0	75.2
22738753	f	md	US	2023	0	0	0	0	0	0	0	0	0	0	0	0	69

22739214	m	md	CN	2023	0	0	0	0	0	0	0	0	0	0	0	0	72
22739244	Not	md	BG	2023	0	1	0	0	0	1	0	0	0	0	0	0	
22739696	f	cn	US	2023	0	0	0	0	0	0	0	0	0	0	0	0	
22739876	m	md	DE	2023	0	0	0	0	0	0	0	0	0	0	0	0	
22740191	m	hp	CN	2023	0	0	0	0	0	0	0	0	0	0	0	0	62
22740215	m	ph	JP	2023	0	1	1	0	0	0	0	0	0	0	0	0	71
22740228	m	md	TR	2023	0	0	0	0	0	0	0	0	0	0	0	0	69
22740501	f	md	US	2023	0	0	0	0	0	0	0	0	0	0	0	0	78
22740667	m	md	JP	2023	0	1	0	0	1	0	0	0	0	0	0	0	70
22740669	f	md	DK	2023	0	1	0	0	0	1	0	0	0	0	0	0	66
22740975	f	cn	TH	2023	0	1	0	0	0	1	0	0	0	0	0	0	
22741516	Not	md	JP	2023	0	1	0	0	1	0	0	0	0	0	0	0	
22741903	f	md	US	2023	0	0	0	0	0	0	0	0	0	0	0	0	62
22741904	Not	cn	CN	2023	0	1	0	0	0	1	0	0	0	0	0	0	
22742220	m	md	JP	2023	0	1	1	0	0	0	0	0	0	0	0	0	75
22742232	m	md	DE	2023	0	1	0	0	1	0	0	0	0	0	0	1	56
22742780	f	cn	DE	2023	0	0	0	0	0	0	0	0	0	0	0	0	66
22743102	m	hp	DE	2023	0	0	0	0	0	0	0	0	0	0	0	0	
22743445	Not	cn	CN	2023	0	1	0	0	0	1	0	0	0	0	0	0	
22743496	Not	hp	KR	2023	0	0	0	0	0	0	0	0	0	0	0	0	
22743612	m	md	JP	2023	0	1	0	1	0	0	0	0	0	0	0	0	80
22744259	m	cn	DE	2023	0	0	0	0	0	0	0	0	0	0	0	0	
22744409	m	md	JP	2023	0	1	1	0	0	0	0	0	0	0	0	0	76
22745252	m	md	SG	2023	0	0	0	0	0	0	0	0	0	0	0	0	76
22745770	f	cn	CA	2023	1	0	0	0	0	0	0	0	0	0	0	0	
22745793	m	md	JP	2023	0	1	1	0	0	0	0	0	0	0	0	1	78
22746199	m	md	SK	2023	0	0	0	0	0	0	0	0	0	0	0	0	50
22746246	m	md	IN	2023	0	1	0	0	0	1	0	0	0	0	0	0	79
22746686	m	cn	MY	2023	1	0	0	0	0	0	0	0	0	0	0	0	82.3
22746894	m	hp	QA	2023	1	0	0	0	0	0	0	0	0	0	0	0	52
22747613	f	cn	US	2023	0	0	0	0	0	0	0	0	0	0	0	0	74
22747919	f	md	JP	2023	0	0	0	0	0	0	0	0	0	0	0	0	
22748140	m	md	JP	2023	0	0	0	0	0	0	0	0	0	0	0	0	74
22748305	m	md	JP	2023	0	1	0	1	0	0	0	0	0	0	0	0	63

22748598	m	md	PT	2023	0	1	1	0	0	0	0	0	0	0	0	0	
22748638	f	hp	IT	2023	1	0	0	0	0	0	0	0	0	0	0	0	71
22748743	m	cn	AE	2023	0	1	0	0	1	0	0	0	0	0	0	0	75
22748947	f	md	FR	2023	1	0	0	0	0	0	0	0	0	0	0	0	42
22749036	f	cn	US	2023	0	0	0	0	0	0	0	0	0	0	0	0	53
22749157	m	cn	TH	2023	1	0	0	0	0	0	0	0	0	0	0	0	70.6
22749691	m	md	DE	2023	0	0	0	0	0	0	0	0	0	0	0	0	
22749918	Not	md	JP	2023	0	1	0	0	0	1	0	0	0	0	0	0	
22750002	f	ph	JP	2023	0	0	0	0	0	0	0	0	0	0	0	0	70
22750177	m	md	JP	2023	1	0	0	0	0	0	0	0	0	0	0	0	70
22750522	f	md	JP	2023	0	1	0	0	0	1	0	0	0	0	0	0	70
22750661	f	md	FR	2023	0	0	0	0	0	0	0	0	0	0	0	0	38
22752148	f	cn	US	2023	0	0	0	0	0	0	0	0	0	0	0	0	
22752168	f	hp	CN	2023	1	0	0	0	0	0	0	0	0	0	0	0	50
22752236	f	hp	CN	2023	1	0	0	0	0	0	0	0	0	0	0	0	65
22752604	m	md	CN	2023	0	1	0	1	0	0	0	0	0	0	0	0	80
22752723	Not	md	DE	2023	0	1	0	1	0	0	0	0	0	0	0	0	
22752757	m	md	AT	2023	0	0	0	0	0	0	0	0	0	0	0	0	69
22752821	f	ph	FR	2023	0	0	0	0	0	0	0	0	0	0	0	0	54
22752931	f	md	CL	2023	0	1	1	0	0	0	0	0	0	0	0	0	73
22753105	m	cn	DE	2023	0	0	0	0	0	0	0	0	0	0	0	0	
22753134	m	md	DE	2023	0	1	0	0	1	0	0	0	0	0	0	0	71
22753497	f	cn	PH	2023	0	0	0	0	0	0	0	0	0	0	0	0	42
22754042	f	md	JP	2023	0	1	0	1	0	0	0	0	0	0	0	0	72
22754905	f	cn	US	2023	1	0	0	0	0	0	0	0	0	0	0	0	77.7
22754950	f	md	JP	2023	1	0	0	0	0	0	0	0	0	0	0	0	79
22755008	m	md	JP	2023	0	1	0	1	0	0	0	0	0	0	0	0	76
22755086	Not	ph	FR	2023	0	0	0	0	0	0	0	0	0	0	0	0	
22755195	m	md	JP	2023	0	1	0	0	0	1	0	0	0	0	0	0	61
22755210	f	ph	FR	2023	0	0	0	0	0	0	0	0	0	0	0	0	54
22755965	f	cn	JP	2023	1	0	0	0	0	0	0	0	0	0	0	0	75
22756004	Not	md	US	2023	1	0	0	0	0	0	0	0	0	0	0	0	
22756019	Not	md	JP	2023	0	1	0	1	0	0	0	0	0	0	0	0	
22756435	Not	cn	JP	2023	0	0	0	0	0	0	0	0	0	0	0	0	55

22756476	m	cn	ID	2023	1	0	0	0	0	0	0	0	0	0	0	0	46.6
22756555	m	cn	CN	2023	1	0	0	0	0	0	0	0	0	0	0	0	68
22757044	f	md	JP	2023	0	1	0	1	0	0	0	0	0	0	0	0	72
22757090	m	cn	CN	2023	0	1	0	1	0	0	0	0	0	0	0	0	60
22757164	m	cn	ID	2023	1	0	0	0	0	0	0	0	0	0	0	0	
22757438	Not	md	JP	2023	0	1	1	0	0	0	0	0	0	0	0	0	70
22757519	f	hp	IT	2023	0	0	0	0	0	0	0	0	0	0	0	0	64
22758038	m	hp	US	2023	0	1	0	1	0	0	0	0	0	0	0	0	68
22758103	f	md	FR	2023	0	0	0	0	0	0	0	0	0	0	0	0	82
22758120	f	hp	BG	2023	0	0	0	0	0	0	0	0	0	0	0	0	42
22758301	m	md	JP	2023	0	0	0	0	0	0	0	0	0	0	0	0	60
22758303	f	md	JP	2023	0	0	0	0	0	0	0	0	0	0	0	0	40
22758593	m	md	JP	2023	0	1	0	0	1	0	0	0	0	0	0	0	81
22759257	f	md	JP	2023	0	0	0	0	0	0	0	0	0	0	0	0	80
22759422	f	hp	US	2023	1	0	0	0	0	0	0	0	0	0	0	0	68
22759544	m	md	FR	2023	0	1	1	0	0	0	0	0	0	0	0	0	
22759627	m	hp	CN	2023	1	0	0	0	0	0	0	0	0	0	0	0	76
22759835	m	md	JP	2023	0	0	0	0	0	0	0	0	0	0	0	0	72
22759888	f	cn	US	2023	1	0	0	0	0	0	0	0	0	0	0	0	
22761170	m	cn	TW	2023	0	1	0	1	0	0	0	0	0	0	0	0	66.8
22761173	m	cn	MY	2023	1	0	0	0	0	0	0	0	0	0	0	0	73.6
22761175	m	cn	CN	2023	0	1	0	1	0	0	0	0	0	0	0	0	65
22761765	m	cn	ID	2023	1	0	0	0	0	0	0	0	0	0	0	0	72.7
22761816	m	hp	GB	2023	1	0	0	0	0	0	0	0	0	0	0	0	
22761826	m	md	US	2023	0	1	0	0	0	1	0	0	0	0	0	0	76
22761834	f	md	JP	2023	0	0	0	0	0	0	0	0	0	0	0	0	
22761887	m	md	AU	2023	1	0	0	0	0	0	0	0	0	0	0	0	
22761965	m	cn	ID	2023	1	0	0	0	0	0	0	0	0	0	0	0	65.1
22762746	m	md	CN	2023	1	0	0	0	0	0	0	0	0	0	0	0	
22762820	Not	md	JP	2023	0	1	0	0	0	1	0	0	0	0	0	0	
22762829	m	md	DE	2023	0	1	0	0	1	0	0	0	0	0	0	0	69
22762884	f	hp	US	2023	0	0	0	0	0	0	0	0	0	0	0	0	
22762888	m	md	IT	2023	0	1	0	0	1	0	0	0	0	0	0	0	
22763300	Not	cn	CN	2023	0	1	0	0	0	1	0	0	0	0	0	0	

22763456	Not	hp	US	2023	0	0	0	0	0	0	0	0	0	0	0	0	84
22763614	m	hp	US	2023	0	0	0	0	0	0	0	0	0	0	0	0	
22764670	m	md	CN	2023	0	0	0	0	0	0	0	0	0	0	0	0	70
22765049	f	md	US	2023	0	0	0	0	0	0	0	0	0	0	0	0	53
22765075	m	cn	IN	2023	0	1	0	0	0	1	0	0	0	0	0	0	62
22765159	m	md	DE	2023	0	1	0	0	1	0	0	0	0	0	0	0	72
22765354	Not	md	JP	2023	0	1	0	0	0	1	0	0	0	0	0	0	70
22766056	m	cn	CA	2023	1	0	0	0	0	0	0	0	0	0	0	0	
22766128	f	ph	MY	2023	1	0	0	0	0	0	0	0	0	0	0	0	
22766230	m	md	CN	2023	0	0	0	0	0	0	0	0	0	0	0	0	70
22766477	f	md	US	2023	0	1	0	0	0	1	0	0	0	0	0	0	77
22766570	m	hp	TH	2023	1	0	0	0	0	0	0	0	0	0	0	1	64
22766721	f	md	DE	2023	0	0	0	0	0	0	0	0	0	0	0	0	
22766941	f	md	TW	2023	0	0	0	0	0	0	0	0	0	0	0	0	67
22767016	f	cn	IN	2023	0	0	0	0	0	0	0	0	0	0	0	0	62
22767089	f	md	JP	2023	0	0	0	0	0	0	0	0	0	0	0	0	35
22767482	m	md	DE	2023	0	0	0	0	0	0	0	0	0	0	0	0	
22767786	m	md	JP	2023	0	1	0	1	0	0	0	0	0	0	0	0	64
22768290	m	cn	CN	2023	0	1	0	1	0	0	0	0	0	0	0	0	77
22768396	m	md	DE	2023	0	0	0	0	0	0	0	0	0	0	0	0	
22768663	m	ph	JP	2023	0	1	1	0	0	0	0	0	0	0	0	0	80
22769261	f	hp	US	2023	0	0	0	0	0	0	0	0	0	0	0	0	78
22769471	Not	md	JP	2023	0	0	0	0	0	0	0	0	0	0	0	0	81
22770264	Not	hp	SI	2023	0	1	0	0	0	1	0	0	0	0	0	1	
22770333	f	cn	IN	2023	0	0	0	0	0	0	0	0	0	0	0	0	69
22770642	f	hp	CA	2023	0	1	1	0	0	0	0	0	0	0	0	0	38
22770945	m	md	JP	2023	0	1	0	1	0	0	0	0	0	0	0	0	67
22771463	f	hp	CA	2023	0	1	1	0	0	0	0	0	0	0	0	0	38
22772037	f	cn	MY	2023	1	0	0	0	0	0	0	0	0	0	0	0	59.7
22772130	f	cn	US	2023	0	0	0	0	0	0	0	0	0	0	0	0	
22772257	m	md	TW	2023	0	0	0	0	0	0	0	0	0	0	0	0	51
22772591	f	cn	TH	2023	1	0	0	0	0	0	0	0	0	0	0	0	66.2
22772703	m	md	FR	2023	0	1	1	0	0	0	0	0	0	0	0	0	
22772725	f	hp	CL	2023	0	0	0	0	0	0	0	0	0	0	0	0	63

22772960	m	md	MY	2023	0	0	0	0	0	0	0	0	0	0	0	0	53
22773024	f	ph	FR	2023	0	0	0	0	0	0	0	0	0	0	0	0	54
22773115	m	md	JP	2023	0	0	0	0	0	0	0	0	0	0	0	0	43
22773306	Not	ph	JP	2023	1	0	0	0	0	0	0	0	0	0	0	0	
22773510	m	md	JP	2023	1	0	0	0	0	0	0	0	0	0	0	0	83
22773579	m	ph	JP	2023	0	1	1	0	0	0	0	0	0	0	0	0	70
22773598	m	md	FR	2023	0	0	0	0	0	0	0	0	0	0	0	0	49
22773726	m	ph	US	2023	0	0	0	0	0	0	0	0	0	0	0	0	
22773965	m	md	US	2023	0	0	0	0	0	0	0	0	0	0	0	0	
22774113	f	hp	ES	2023	0	0	0	0	0	0	0	0	0	0	0	0	51
22774834	m	hp	IT	2023	0	1	1	0	0	0	0	0	0	0	0	0	71
22775126	Not	ph	PT	2023	0	1	0	0	0	1	0	0	0	0	0	0	
22775155	m	ph	JP	2023	1	0	0	0	0	0	0	0	0	0	0	0	81
22775801	f	md	FR	2023	1	0	0	0	0	0	0	0	0	0	0	0	
22775859	m	md	DE	2023	0	0	0	0	0	0	0	0	0	0	0	0	81
22775864	Not	md	JP	2023	1	0	0	0	0	0	0	0	0	0	0	0	
22775908	f	cn	US	2023	1	0	0	0	0	0	0	0	0	0	0	0	72
22776140	m	md	RU	2023	0	1	0	0	0	1	0	0	0	0	0	0	57
22776461	Not	ph	PT	2023	0	1	0	0	0	1	0	0	0	0	0	0	
22776691	m	cn	PT	2023	0	1	1	0	0	0	0	0	0	0	0	0	
22776741	f	md	CH	2023	0	1	1	0	0	0	0	0	0	0	0	0	62
22776864	m	cn	IN	2023	0	0	0	0	0	0	0	0	0	0	0	0	64
22777416	f	md	DE	2023	0	0	0	0	0	0	0	0	0	0	0	0	83
22777584	m	md	PT	2023	0	1	1	0	0	0	0	0	0	0	0	0	
22778291	m	md	FR	2023	0	1	1	0	0	0	0	0	0	0	0	0	
22778369	f	md	US	2023	0	0	0	0	0	0	0	0	0	0	0	0	79
22778411	m	md	JP	2023	0	1	0	1	0	0	0	0	0	0	0	0	
22778727	f	md	JP	2023	1	0	0	0	0	0	0	0	0	0	0	0	69
22778840	f	hp	CA	2023	0	0	0	0	0	0	0	0	0	0	0	0	
22779558	f	md	BR	2023	0	0	0	0	0	0	0	0	0	0	0	0	67
22779751	f	md	PL	2023	1	0	0	0	0	0	0	0	0	0	0	0	62
22779967	m	md	CN	2023	0	0	0	0	0	0	0	0	0	0	0	0	74
22780379	Not	md	JP	2023	0	1	0	0	0	1	0	0	0	0	0	0	
22780546	m	md	JP	2023	0	0	0	0	0	0	0	0	0	0	0	0	80

22781120	m	cn	ID	2023	1	0	0	0	0	0	0	0	0	0	0	0	76.7
22781211	m	md	JP	2023	0	1	1	0	0	0	0	0	0	0	0	0	90
22781758	m	md	US	2023	0	1	0	0	0	1	0	0	0	0	0	0	58
22781764	m	md	JP	2023	0	1	0	1	0	0	0	0	0	0	0	0	66
22782240	f	cn	ID	2023	1	0	0	0	0	0	0	0	0	0	0	0	
22782440	Not	md	JP	2023	0	1	0	0	0	1	0	0	0	0	0	0	
22782805	m	cn	IN	2023	0	1	0	1	0	0	0	0	0	0	0	0	74.7
22783230	f	cn	US	2023	1	0	0	0	0	0	0	0	0	0	0	0	
22783377	m	cn	DE	2023	0	1	1	0	0	0	0	0	0	0	0	0	
22783631	m	ph	GB	2023	0	1	0	1	0	0	0	0	0	0	0	1	74.8
22783840	f	md	CH	2023	0	1	1	0	0	0	0	0	0	0	0	0	62
22784001	m	md	JP	2023	0	1	1	0	0	0	0	0	0	0	0	0	
22784354	f	md	DE	2023	0	0	0	0	0	0	0	0	0	0	0	0	58
22784501	f	md	MY	2023	1	0	0	0	0	0	0	0	0	0	0	0	
22784532	m	md	JP	2023	0	1	1	0	0	0	0	0	0	0	0	0	43
22785908	m	cn	KR	2023	0	0	0	0	0	0	0	0	0	0	0	0	52
22786161	f	hp	US	2023	0	0	0	0	0	0	0	0	0	0	0	0	60
22786505	Not	md	RU	2023	0	1	0	0	0	1	0	0	0	0	0	0	
22787034	f	hp	CN	2023	0	0	0	0	0	0	0	0	0	0	0	0	68
22787308	m	md	JP	2023	0	1	0	1	0	0	0	0	0	0	0	0	80
22787366	f	md	PL	2023	0	1	1	0	0	0	0	0	0	0	0	0	52
22787645	f	cn	CA	2023	0	0	0	0	0	0	0	0	0	0	0	0	
22787790	m	md	PL	2023	0	1	1	0	0	0	0	0	0	0	0	0	65
22787800	m	ph	MY	2023	1	0	0	0	0	0	0	0	0	0	0	0	
22788100	f	md	PL	2023	0	1	1	0	0	0	0	0	0	0	0	0	70
22788494	m	md	FR	2023	0	0	0	0	0	0	0	0	0	0	0	0	69
22788595	f	md	JP	2023	0	0	0	0	0	0	0	0	0	0	0	0	70
22788855	m	cn	EG	2023	0	1	0	1	0	0	0	0	0	0	0	0	
22788856	f	md	MY	2023	1	0	0	0	0	0	0	0	0	0	0	0	
22788857	m	md	SG	2023	0	1	0	1	0	0	0	0	0	0	0	0	74.9
22788873	f	ph	FR	2023	0	0	0	0	0	0	0	0	0	0	0	0	54
22789092	m	md	JP	2023	0	0	0	0	0	0	0	0	0	0	0	0	71
22789222	Not	cn	US	2023	1	0	0	0	0	0	0	0	0	0	0	0	
22789231	Not	md	FR	2023	0	0	0	0	0	0	0	0	0	0	0	0	

22789288	f	md	JP	2023	1	0	0	0	0	0	0	0	0	0	0	0	81
22789367	f	md	BE	2023	0	0	0	0	0	0	0	0	0	0	0	0	61
22789369	Not	md	ID	2023	0	0	0	0	0	0	0	0	0	0	0	0	
22789503	Not	hp	GB	2023	1	0	0	0	0	0	0	0	0	0	0	0	
22789511	f	md	CN	2023	0	1	0	1	0	0	0	0	0	0	0	0	71
22789691	f	cn	KR	2023	0	0	0	0	0	0	0	0	0	0	0	0	53
22789730	f	md	CN	2023	0	0	0	0	0	0	0	0	0	0	0	0	58
22789746	m	md	CL	2023	0	1	1	0	0	0	0	0	0	0	0	0	85
22789756	f	md	PL	2023	0	1	1	0	0	0	0	0	0	0	0	0	66
22789942	m	md	US	2023	0	0	0	0	0	0	0	0	0	0	0	0	64
22790369	m	md	JP	2023	0	0	0	0	0	0	0	0	0	0	0	0	74
22790425	m	md	JP	2023	0	0	0	0	0	0	0	0	0	0	0	0	
22791331	m	md	JP	2023	0	1	0	0	0	1	0	0	0	0	0	0	80
22791399	m	md	BG	2023	0	1	0	1	0	0	0	0	0	0	0	0	
22791819	m	md	CN	2023	0	1	1	0	0	0	0	0	0	0	0	0	63
22791984	f	md	CN	2023	0	0	0	0	0	0	0	0	0	0	0	0	77
22792002	f	cn	CN	2023	0	0	0	0	0	0	0	0	0	0	0	0	75
22792086	m	ph	MY	2023	1	0	0	0	0	0	0	0	0	0	0	0	74.7
22792129	m	md	JP	2023	0	0	0	0	0	0	0	0	0	0	0	0	72
22792134	m	cn	MY	2023	1	0	0	0	0	0	0	0	0	0	0	0	65.1
22792167	f	md	BR	2023	0	1	0	0	1	0	0	0	0	0	0	0	
22792443	m	cn	MY	2023	1	0	0	0	0	0	0	0	0	0	0	0	68
22793625	f	md	IT	2023	0	0	0	0	0	0	0	0	0	0	0	0	
22794456	Not	md	JP	2023	1	0	0	0	0	0	0	0	0	0	0	0	
22794936	m	md	RU	2023	0	1	0	0	1	0	0	0	0	0	0	0	68
22795065	f	cn	KR	2023	0	0	0	0	0	0	0	0	0	0	0	0	68
22795149	f	md	RU	2023	0	1	0	0	1	0	0	0	0	0	0	0	67
22795165	m	md	JP	2023	0	1	0	1	0	0	0	0	0	0	0	1	75
22795428	m	ph	IT	2023	0	1	1	0	0	0	0	0	0	0	0	0	64
22796060	m	md	JP	2023	0	1	0	0	0	1	0	0	0	0	0	1	56
22796175	f	md	RU	2023	0	1	0	0	1	0	0	0	0	0	0	0	67
22796755	Not	hp	US	2023	0	0	0	0	0	0	0	0	0	0	0	0	
22796924	f	cn	MY	2023	1	0	0	0	0	0	0	0	0	0	0	0	49.4
22797221	m	md	RU	2023	0	1	0	0	1	0	0	0	0	0	0	0	48

22797255	Not	ph	PT	2023	0	1	0	0	0	1	0	0	0	0	0	0	
22797279	Not	md	JP	2023	1	0	0	0	0	0	0	0	0	0	0	0	
22797836	m	md	US	2023	0	1	0	0	0	1	0	0	0	0	0	0	54
22797883	f	md	US	2023	0	1	1	0	0	0	0	0	0	0	0	1	66
22798068	f	md	FR	2023	1	0	0	0	0	0	0	0	0	0	0	0	42
22798482	f	md	JP	2023	0	0	0	0	0	0	0	0	0	0	0	0	61
22798891	Not	cn	US	2023	0	1	0	1	0	0	0	0	0	0	0	0	
22799042	m	md	PL	2023	0	1	1	0	0	0	0	0	0	0	0	0	62
22799331	m	cn	IN	2023	0	0	0	0	0	0	0	0	0	0	0	0	61
22799350	m	md	JP	2023	0	0	0	0	0	0	0	0	0	0	0	0	57
22799557	f	md	JP	2023	0	0	0	0	0	0	0	0	0	0	0	0	45
22799706	m	md	DE	2023	0	0	0	0	0	0	0	0	0	0	0	0	
22799772	f	md	AU	2023	1	0	0	0	0	0	0	0	0	0	0	0	
22800038	f	cn	TH	2023	1	0	0	0	0	0	0	0	0	0	0	0	
22800180	f	hp	CA	2023	1	0	0	0	0	0	0	0	0	0	0	0	
22800268	Not	md	US	2023	0	1	0	0	1	0	0	0	0	0	0	0	
22800369	m	ph	US	2023	0	0	0	0	0	0	0	0	0	0	0	0	58
22800669	m	md	JP	2023	0	1	1	0	0	0	0	0	0	0	0	0	61
22800801	m	md	US	2023	0	1	1	0	0	0	0	0	0	0	0	0	71
22800804	f	md	US	2023	0	1	1	0	0	0	0	0	0	0	0	0	60
22800807	f	md	US	2023	0	1	1	0	0	0	0	0	0	0	0	0	73
22800919	f	md	DE	2023	0	0	0	0	0	0	0	0	0	0	0	0	68
22800934	f	md	JP	2023	0	0	0	0	0	0	0	0	0	0	0	0	80
22801222	Not	md	IN	2023	0	1	0	0	1	0	0	0	0	0	0	0	
22802240	m	cn	EG	2023	0	1	0	1	0	0	0	0	0	0	0	0	
22802282	m	md	IT	2023	0	0	0	0	0	0	0	0	0	0	0	0	80
22803223	f	md	JP	2023	1	0	0	0	0	0	0	0	0	0	0	0	59
22803525	f	cn	MY	2023	1	0	0	0	0	0	0	0	0	0	0	0	
22803760	m	ph	JP	2023	1	0	0	0	0	0	0	0	0	0	0	0	87
22804452	f	md	US	2023	0	0	0	0	0	0	0	0	0	0	0	0	68
22805311	f	cn	CA	2023	1	0	0	0	0	0	0	0	0	0	0	0	
22805358	m	md	JP	2023	0	1	0	1	0	0	0	0	0	0	0	0	58
22805895	f	md	RU	2023	0	1	0	0	0	1	0	0	0	0	0	0	
22805898	f	md	JP	2023	0	1	0	0	0	1	0	0	0	0	0	0	80

22806111	f	md	DE	2023	0	1	1	0	0	0	0	0	0	0	0	0	70
22806253	Not	ph	JP	2023	0	1	0	0	1	0	0	0	0	0	0	0	
22807298	m	hp	FR	2023	0	0	0	0	0	0	0	0	0	0	0	0	89
22807334	f	cn	CN	2023	1	0	0	0	0	0	0	0	0	0	0	0	
22807424	Not	ph	PT	2023	0	0	0	0	0	0	0	0	0	0	0	0	
22807770	Not	md	JP	2023	0	1	0	0	0	1	0	0	0	0	0	0	70
22808292	Not	ph	PT	2023	0	0	0	0	0	0	0	0	0	0	0	0	
22808495	m	cn	JP	2023	1	0	0	0	0	0	0	0	0	0	0	0	
22808589	m	cn	IN	2023	0	0	0	0	0	0	0	0	0	0	0	0	
22808696	f	cn	IN	2023	0	0	0	0	0	0	0	0	0	0	0	0	50
22809626	f	md	TR	2023	0	0	0	0	0	0	0	0	0	0	0	0	60
22810458	m	md	MY	2023	0	1	1	0	0	0	0	0	0	0	0	0	57
22810597	f	ph	US	2023	1	0	0	0	0	0	0	0	0	0	0	0	76
22810758	m	md	JP	2023	0	1	1	0	0	0	0	0	0	0	0	0	63
22811132	Not	md	JP	2023	1	0	0	0	0	0	0	0	0	0	0	0	
22811316	m	hp	IN	2023	1	0	0	0	0	0	0	0	0	0	0	0	70
22811337	f	md	JP	2023	0	1	1	0	0	0	0	0	0	0	0	0	71
22811426	m	cn	TH	2023	0	1	1	0	0	0	0	0	0	0	0	0	81
22812193	f	md	JP	2023	0	1	0	0	0	1	0	0	0	0	0	0	80
22812348	Not	ph	PT	2023	0	0	0	0	0	0	0	0	0	0	0	0	
22812384	m	md	JP	2023	0	0	0	0	0	0	0	0	0	0	0	0	
22812392	f	hp	US	2023	0	0	0	0	0	0	0	0	0	0	0	0	
22813172	m	md	TR	2023	0	1	0	0	0	1	0	0	0	0	0	0	53
22813217	f	hp	US	2023	0	0	0	0	0	0	0	0	0	0	0	0	52
22813261	f	cn	US	2023	0	0	0	0	0	0	0	0	0	0	0	0	65
22813268	m	cn	US	2023	0	0	0	0	0	0	0	0	0	0	0	0	
22813289	f	md	US	2023	0	0	0	0	0	0	0	0	0	0	0	0	
22813329	f	md	US	2023	0	0	0	0	0	0	0	0	0	0	0	0	
22813359	Not	hp	US	2023	0	0	0	0	0	0	0	0	0	0	0	0	
22813364	f	cn	US	2023	0	0	0	0	0	0	0	0	0	0	0	0	44
22813368	f	hp	US	2023	0	0	0	0	0	0	0	0	0	0	0	0	
22813390	f	cn	US	2023	0	0	0	0	0	0	0	0	0	0	0	0	71
22813393	m	hp	US	2023	0	0	0	0	0	0	0	0	0	0	0	0	
22813395	f	cn	US	2023	0	0	0	0	0	0	0	0	0	0	0	0	46

22813426	f	ph	US	2023	0	0	0	0	0	0	0	0	0	0	0	0	67
22813428	f	cn	US	2023	0	0	0	0	0	0	0	0	0	0	0	0	43
22813434	f	hp	US	2023	0	0	0	0	0	0	0	0	0	0	0	0	
22813435	f	md	US	2023	0	0	0	0	0	0	0	0	0	0	0	0	34
22813443	m	md	JP	2023	0	1	0	0	1	0	0	0	0	0	0	0	65
22813463	m	cn	US	2023	0	0	0	0	0	0	0	0	0	0	0	0	77
22813515	f	cn	US	2023	0	0	0	0	0	0	0	0	0	0	0	0	
22813520	m	ph	US	2023	0	0	0	0	0	0	0	0	0	0	0	0	18
22813529	f	cn	US	2023	0	0	0	0	0	0	0	0	0	0	0	0	49
22813542	m	cn	US	2023	0	0	0	0	0	0	0	0	0	0	0	0	60
22813559	f	hp	US	2023	0	0	0	0	0	0	0	0	0	0	0	0	53
22813582	Not	cn	US	2023	0	0	0	0	0	0	0	0	0	0	0	0	
22813605	m	md	US	2023	0	0	0	0	0	0	0	0	0	0	0	0	40
22813611	f	cn	US	2023	0	0	0	0	0	0	0	0	0	0	0	0	
22813624	f	md	US	2023	0	0	0	0	0	0	0	0	0	0	0	0	
22813626	Not	cn	US	2023	0	0	0	0	0	0	0	0	0	0	0	0	
22813638	m	hp	US	2023	0	0	0	0	0	0	0	0	0	0	0	0	71
22813644	f	cn	US	2023	0	0	0	0	0	0	0	0	0	0	0	0	45
22813654	m	cn	US	2023	0	0	0	0	0	0	0	0	0	0	0	0	43
22813664	f	hp	US	2023	0	0	0	0	0	0	0	0	0	0	0	0	54
22813668	f	ph	US	2023	0	0	0	0	0	0	0	0	0	0	0	0	59
22813675	f	cn	US	2023	0	0	0	0	0	0	0	0	0	0	0	0	65
22813738	f	cn	US	2023	0	0	0	0	0	0	0	0	0	0	0	0	76
22813744	f	cn	US	2023	0	0	0	0	0	0	0	0	0	0	0	0	46
22813749	m	ph	US	2023	0	0	0	0	0	0	0	0	0	0	0	0	
22814116	m	cn	MY	2023	1	0	0	0	0	0	0	0	0	0	0	0	55
22814193	m	md	JP	2023	0	1	0	0	1	0	0	0	0	0	0	0	84
22814224	f	cn	IT	2023	0	0	0	0	0	0	0	0	0	0	0	0	
22814370	m	md	JP	2023	0	1	0	1	0	0	0	0	0	0	0	1	80
22814454	f	cn	US	2023	1	0	0	0	0	0	0	0	0	0	0	0	62.7
22814542	f	hp	BG	2023	0	0	0	0	0	0	0	0	0	0	0	0	42
22814663	m	md	JP	2023	0	1	1	0	0	0	0	0	0	0	0	0	60
22814741	f	md	IL	2023	0	0	0	0	0	0	0	0	0	0	0	0	90
22814812	f	cn	US	2023	1	0	0	0	0	0	0	0	0	0	0	0	75.4

22815295	m	md	PL	2023	0	1	1	0	0	0	0	0	0	0	0	0	65
22815310	f	md	JP	2023	0	0	0	0	0	0	0	0	0	0	0	0	68
22815320	f	md	PL	2023	0	1	1	0	0	0	0	0	0	0	0	0	66
22815663	m	md	PL	2023	0	1	1	0	0	0	0	0	0	0	0	0	65
22815987	m	md	RU	2023	0	1	0	1	0	0	0	0	0	0	0	0	
22815991	m	cn	AT	2023	0	0	0	0	0	0	0	0	0	0	0	0	86
22816180	f	hp	TH	2023	1	0	0	0	0	0	0	0	0	0	0	1	65.1
22816182	f	md	JP	2023	1	0	0	0	0	0	0	0	0	0	0	0	60
22816278	f	cn	CA	2023	1	0	0	0	0	0	0	0	0	0	0	0	
22816588	m	md	JP	2023	0	1	1	0	0	0	0	0	0	0	0	0	80
22816602	m	md	RO	2023	0	1	1	0	0	0	0	0	0	0	0	0	61
22816638	m	cn	ID	2023	1	0	0	0	0	0	0	0	0	0	0	0	
22817295	m	md	HU	2023	0	0	0	0	0	0	0	0	0	0	0	0	68
22817762	f	md	US	2023	0	0	0	0	0	0	0	0	0	0	0	0	
22817779	f	md	JP	2023	0	0	0	0	0	0	0	0	0	0	0	0	60
22817817	f	cn	US	2023	0	0	0	0	0	0	0	0	0	0	0	0	74
22817969	m	md	JP	2023	0	1	0	0	1	0	0	0	0	0	0	0	81
22818325	f	hp	US	2023	0	1	1	0	0	0	0	0	0	0	0	0	63
22819023	Not	hp	AR	2023	1	0	0	0	0	0	0	0	0	0	0	0	
22819964	f	md	JP	2023	0	1	0	0	0	1	0	0	0	0	0	0	80
22820023	Not	cn	US	2023	0	0	0	0	0	0	0	0	0	0	0	0	
22820092	m	md	JP	2023	0	1	0	0	0	1	0	0	0	0	0	0	80
22820471	f	md	JP	2023	0	1	0	0	0	1	0	0	0	0	0	0	60
22820521	m	cn	IN	2023	0	0	0	0	0	0	0	0	0	0	0	0	75
22820569	m	cn	US	2023	0	1	1	0	0	0	0	0	0	0	0	0	
22820732	m	md	JP	2023	0	0	0	0	0	0	0	0	0	0	0	0	55
22820911	f	cn	US	2023	1	0	0	0	0	0	0	0	0	0	0	0	83.6
22820915	f	hp	CA	2023	0	1	1	0	0	0	0	0	0	0	0	0	58
22821474	f	hp	TH	2023	1	0	0	0	0	0	0	0	0	0	0	0	59.1
22821545	f	hp	US	2023	0	0	0	0	0	0	0	0	0	0	0	0	64
22821546	Not	hp	JP	2023	0	1	0	0	0	1	0	0	0	0	0	1	
22821718	m	md	JP	2023	0	1	0	1	0	0	0	0	0	0	0	0	58
22822006	m	hp	FR	2023	0	0	0	0	0	0	0	0	0	0	0	0	53
22822052	m	cn	ID	2023	1	0	0	0	0	0	0	0	0	0	0	0	57.7

22822487	m	md	DE	2023	0	1	0	0	1	0	0	0	0	0	0	0	79
22822617	m	cn	IN	2023	0	0	0	0	0	0	0	0	0	0	0	0	66
22822861	m	ph	JP	2023	0	1	0	0	0	1	0	0	0	0	0	0	70
22823043	m	md	ES	2023	0	0	0	0	0	0	0	0	0	0	0	0	57
22823053	f	cn	US	2023	0	0	0	0	0	0	0	0	0	0	0	0	
22823117	m	md	JP	2023	0	1	0	0	0	1	0	0	0	0	0	0	56
22823294	m	hp	US	2023	0	1	1	0	0	0	0	0	0	0	0	0	65
22823378	Not	ph	JP	2023	1	0	0	0	0	0	0	0	0	0	0	0	
22823396	f	md	US	2023	0	0	0	0	0	0	0	0	0	0	0	0	81
22823565	m	ph	US	2023	1	0	0	0	0	0	0	0	0	0	0	0	
22823581	m	cn	IN	2023	0	0	0	0	0	0	0	0	0	0	0	0	27
22823617	f	cn	IN	2023	0	0	0	0	0	0	0	0	0	0	0	0	72
22823623	m	md	PL	2023	0	1	1	0	0	0	0	0	0	0	0	0	62
22823863	m	hp	ES	2023	1	0	0	0	0	0	0	0	0	0	0	0	84
22824436	m	md	KR	2023	0	0	0	0	0	0	0	0	0	0	0	0	
22824462	f	hp	US	2023	0	1	1	0	0	0	0	0	0	0	0	0	63
22824532	m	md	JP	2023	0	1	1	0	0	0	0	0	0	0	0	0	80
22825416	m	ph	IT	2023	0	1	1	0	0	0	0	0	0	0	0	0	64
22826192	f	cn	US	2023	1	0	0	0	0	0	0	0	0	0	0	0	
22826246	f	md	JP	2023	0	0	0	0	0	0	0	0	0	0	0	0	
22826843	f	ph	JP	2023	1	0	0	0	0	0	0	0	0	0	0	0	90
22827180	m	md	AR	2023	0	0	0	0	0	0	0	0	0	0	0	0	73
22827465	f	cn	ID	2023	1	0	0	0	0	0	0	0	0	0	0	0	
22827765	m	md	CN	2023	0	1	0	1	0	0	0	0	0	0	0	0	60
22827842	Not	md	JP	2023	0	1	0	1	0	0	0	0	0	0	0	1	
22827849	f	md	CN	2023	0	1	0	0	0	1	0	0	0	0	0	0	62
22827983	Not	md	JP	2023	0	1	0	0	1	0	0	0	0	0	0	0	52
22828049	f	cn	KR	2023	0	0	0	0	0	0	0	0	0	0	0	0	60
22828312	f	cn	US	2023	0	0	0	0	0	0	0	0	0	0	0	0	69
22828345	m	md	RU	2023	0	0	0	0	0	0	0	0	0	0	0	0	71
22828385	f	hp	NL	2023	1	0	0	0	0	0	0	0	0	0	0	0	57
22828752	m	md	JP	2023	0	1	0	1	0	0	0	0	0	0	0	0	56
22830178	f	md	DE	2023	0	1	0	0	1	0	0	0	0	0	0	0	55
22830228	Not	md	JP	2023	0	1	0	0	0	1	0	0	0	0	0	0	

22830814	f	cn	KR	2023	0	0	0	0	0	0	0	0	0	0	0	0	72
22830948	f	md	CA	2023	0	0	0	0	0	0	0	0	0	0	0	0	66
22832284	m	md	JP	2023	0	0	0	0	0	0	0	0	0	0	0	0	73
22832384	f	hp	US	2023	0	0	0	0	0	0	0	0	0	0	0	0	52
22832391	m	hp	CA	2023	0	1	0	0	1	0	0	0	0	0	0	0	79
22832417	Not	hp	CN	2023	0	0	0	0	0	0	0	0	0	0	0	0	
22832515	m	md	CA	2023	0	0	0	0	0	0	0	0	0	0	0	0	77
22832824	f	md	JP	2023	1	0	0	0	0	0	0	0	0	0	0	0	71
22832974	f	md	US	2023	1	0	0	0	0	0	0	0	0	0	0	0	70
22833033	Not	md	US	2023	0	0	0	0	0	0	0	0	0	0	0	0	
22833797	Not	hp	TH	2023	1	0	0	0	0	0	0	0	0	0	0	0	
22833892	f	ph	JP	2023	1	0	0	0	0	0	0	0	0	0	0	0	81
22833954	m	md	PT	2023	0	1	1	0	0	0	0	0	0	0	0	0	
22833989	m	cn	CA	2023	1	0	0	0	0	0	0	0	0	0	0	0	46.5
22834007	f	cn	KR	2023	0	0	0	0	0	0	0	0	0	0	0	0	60
22834168	f	cn	NL	2023	0	1	1	0	0	0	0	0	0	0	0	0	66
22834302	f	hp	US	2023	0	1	1	0	0	0	0	0	0	0	0	0	63
22834622	f	md	CN	2023	0	0	0	0	0	0	0	0	0	0	0	0	63
22834991	f	md	CN	2023	0	0	0	0	0	0	0	0	0	0	0	0	74
22835458	f	md	DE	2023	0	1	0	0	1	0	0	0	0	0	0	0	75
22835838	Not	md	JP	2023	0	1	0	0	0	1	0	0	0	0	0	0	
22836754	m	md	FR	2023	0	1	0	0	0	1	0	0	0	0	0	0	71
22837010	Not	md	PH	2023	1	0	0	0	0	0	0	0	0	0	0	0	
22837088	m	cn	TW	2023	1	0	0	0	0	0	0	0	0	0	0	0	62.2
22837158	Not	md	GB	2023	0	1	0	0	0	1	0	0	0	0	0	0	
22838216	m	hp	TW	2023	1	0	0	0	0	0	0	0	0	0	0	0	67
22838376	f	md	TH	2023	1	0	0	0	0	0	0	0	0	0	0	0	
22838378	f	hp	CN	2023	1	0	0	0	0	0	0	0	0	0	0	0	70
22838661	f	md	JP	2023	0	0	0	0	0	0	0	0	0	0	0	0	70
22839127	m	md	JP	2023	0	0	0	0	0	0	0	0	0	0	0	0	44
22839128	f	md	JP	2023	0	0	0	0	0	0	0	0	0	0	0	0	52
22839132	m	md	JP	2023	0	0	0	0	0	0	0	0	0	0	0	0	70
22839154	m	md	JP	2023	0	0	0	0	0	0	0	0	0	0	0	0	56
22839453	f	cn	US	2023	1	0	0	0	0	0	0	0	0	0	0	0	

22839456	Not	md	JP	2023	0	1	0	1	0	0	0	0	0	0	0	0	
22839490	f	cn	US	2023	0	0	0	0	0	0	0	0	0	0	0	0	
22839818	Not	md	US	2023	0	0	0	0	0	0	0	0	0	0	0	0	
22839910	m	md	BE	2023	0	0	0	0	0	0	0	0	0	0	0	0	71
22839936	f	md	BR	2023	0	1	1	0	0	0	0	0	0	0	0	1	
22840054	m	md	PL	2023	0	1	1	0	0	0	0	0	0	0	0	0	62
22840306	Not	md	JP	2023	0	0	0	0	0	0	0	0	0	0	0	0	
22840782	m	ph	IT	2023	0	1	1	0	0	0	0	0	0	0	0	0	64
22841024	f	md	CA	2023	1	0	0	0	0	0	0	0	0	0	0	0	
22841112	m	md	CN	2023	0	0	0	0	0	0	0	0	0	0	0	0	67
22841322	m	cn	US	2023	0	1	1	0	0	0	0	0	0	0	0	0	
22841567	m	md	JP	2023	0	1	1	0	0	0	0	0	0	0	0	0	67
22841773	Not	md	JP	2023	0	1	0	0	0	1	0	0	0	0	0	0	72
22841790	m	md	JP	2023	0	1	0	0	0	1	0	0	0	0	0	0	80
22842002	f	cn	IN	2023	0	1	0	0	0	1	0	0	0	0	0	0	55
22843159	m	md	JP	2023	0	1	1	0	0	0	0	0	0	0	0	0	67
22843188	m	cn	US	2023	0	0	0	0	0	0	0	0	0	0	0	0	70
22843221	Not	hp	AR	2023	1	0	0	0	0	0	0	0	0	0	0	0	
22843567	f	md	DE	2023	0	1	1	0	0	0	0	0	0	0	0	0	58
22843625	f	md	NL	2023	0	1	1	0	0	0	0	0	0	0	0	0	66
22843654	Not	cn	SG	2023	0	1	0	1	0	0	0	0	0	0	0	0	68.4
22843825	m	hp	TW	2023	1	0	0	0	0	0	0	0	0	0	0	0	70
22843836	f	hp	TW	2023	1	0	0	0	0	0	0	0	0	0	0	0	67
22843933	Not	hp	TW	2023	1	0	0	0	0	0	0	0	0	0	0	1	
22844061	f	md	US	2023	0	0	0	0	0	0	0	0	0	0	0	0	63
22844501	m	cn	US	2023	0	1	1	0	0	0	0	0	0	0	0	0	78
22844926	m	hp	TW	2023	1	0	0	0	0	0	0	0	0	0	0	0	57
22844976	Not	cn	SG	2023	1	0	0	0	0	0	0	0	0	0	0	0	69.7
22845102	m	md	GB	2023	0	0	0	0	0	0	0	0	0	0	0	0	54
22845310	f	md	PL	2023	0	1	1	0	0	0	0	0	0	0	0	0	66
22845353	m	cn	SG	2023	0	0	0	0	0	0	0	0	0	0	0	0	79
22845677	f	md	KR	2023	0	1	1	0	0	0	0	0	0	0	0	0	
22846102	m	md	LB	2023	0	1	0	0	0	1	0	0	0	0	0	0	
22846704	m	hp	CH	2023	0	1	0	0	1	0	0	0	0	0	0	0	71

22846878	f	ph	FR	2023	0	0	0	0	0	0	0	0	0	0	0	0	54
22847047	m	cn	US	2023	1	0	0	0	0	0	0	0	0	0	0	0	69
22847280	Not	hp	TW	2023	0	0	0	0	0	0	0	0	0	0	0	0	
22847827	m	ph	IT	2023	0	1	1	0	0	0	0	0	0	0	0	0	64
22848107	f	md	US	2023	0	0	0	0	0	0	0	0	0	0	0	0	81
22848614	Not	md	JP	2023	0	1	0	0	1	0	0	0	0	0	0	0	
22848624	m	md	RO	2023	0	1	0	1	0	0	0	0	0	0	0	0	65
22848651	m	ph	IT	2023	0	1	1	0	0	0	0	0	0	0	0	0	64
22849404	Not	cn	SG	2023	1	0	0	0	0	0	0	0	0	0	0	0	80
22849619	f	ph	US	2023	0	0	0	0	0	0	0	0	0	0	0	0	73
22849736	Not	md	AR	2023	0	0	0	0	0	0	0	0	0	0	0	0	
22850275	m	ph	US	2023	0	0	0	0	0	0	0	0	0	0	0	0	62
22850480	f	hp	US	2023	0	0	0	0	0	0	0	0	0	0	0	0	81
22850770	Not	cn	SG	2023	1	0	0	0	0	0	0	0	0	0	0	0	67.7
22850835	f	md	DE	2023	0	0	0	0	0	0	0	0	0	0	0	0	52
22851176	m	md	DE	2023	0	1	0	0	1	0	0	0	0	0	0	0	79
22851433	f	md	DE	2023	0	0	0	0	0	0	0	0	0	0	0	0	
22851442	f	cn	KR	2023	0	0	0	0	0	0	0	0	0	0	0	0	84
22851611	f	md	JP	2023	0	0	0	0	0	0	0	0	0	0	0	0	61
22851659	Not	cn	US	2023	1	0	0	0	0	0	0	0	0	0	0	0	67
22852238	m	md	GB	2023	0	1	0	0	0	1	0	0	0	0	0	0	65
22852270	f	md	DE	2023	0	0	0	0	0	0	0	0	0	0	0	0	
22852592	m	cn	KR	2023	0	0	0	0	0	0	0	0	0	0	0	0	37
22852606	m	md	DE	2023	0	0	0	0	0	0	0	0	0	0	0	0	58
22852680	f	md	PL	2023	0	0	0	0	0	0	0	0	0	0	0	0	
22852846	f	hp	US	2023	1	0	0	0	0	0	0	0	0	0	0	0	
22853358	m	md	JP	2023	0	1	1	0	0	0	0	0	0	0	0	0	75
22853452	Not	cn	SG	2023	1	0	0	0	0	0	0	0	0	0	0	0	63.8
22853755	Not	cn	SG	2023	1	0	0	0	0	0	0	0	0	0	0	0	90.6
22853834	f	hp	IT	2023	0	0	0	0	0	0	0	0	0	0	0	0	37
22853925	f	ph	CN	2023	0	0	0	0	0	0	0	0	0	0	0	0	
22854058	f	md	GB	2023	0	1	0	0	0	1	0	0	0	0	0	0	59
22854224	m	md	RU	2023	0	0	0	0	0	0	0	0	0	0	0	0	73
22854265	f	Not repo	US	2023	0	1	0	1	0	0	0	0	0	0	0	0	77

22854315	f	md	NL	2023	0	1	1	0	0	0	0	0	0	0	0	0	66
22854408	m	cn	JP	2023	0	1	0	1	0	0	0	0	0	0	0	0	
22855588	Not	hp	US	2023	0	0	0	0	0	0	0	0	0	0	0	0	
22855809	f	md	AU	2023	0	1	0	1	0	0	0	0	0	0	0	0	
22855810	m	md	AU	2023	0	1	0	1	0	0	0	0	0	0	0	0	
22856087	f	md	DE	2023	0	1	0	0	1	0	0	0	0	0	0	0	75
22856272	f	md	JP	2023	0	1	0	1	0	0	0	0	0	0	0	0	67
22856409	f	md	MY	2023	0	0	0	0	0	0	0	0	0	0	0	0	63
22856648	m	cn	SK	2023	0	0	0	0	0	0	0	0	0	0	0	0	80
22856658	f	md	NL	2023	0	0	0	0	0	0	0	0	0	0	0	0	61
22857705	Not	cn	SG	2023	1	0	0	0	0	0	0	0	0	0	0	0	78.3
22857982	m	hp	DE	2023	0	0	0	0	0	0	0	0	0	0	0	0	46
22858360	f	md	JP	2023	0	1	0	0	0	1	0	0	0	0	0	0	80
22859010	m	cn	IN	2023	0	0	0	0	0	0	0	0	0	0	0	0	56
22859643	f	md	FR	2023	0	1	0	0	0	1	0	0	0	0	0	0	79
22859984	f	hp	JP	2023	0	1	0	0	0	1	0	0	0	0	0	0	67
22860184	f	hp	CA	2023	0	1	1	0	0	0	0	0	0	0	0	0	38
22860467	f	cn	TH	2023	0	1	1	0	0	0	0	0	0	0	0	0	77
22860632	m	md	SK	2023	0	0	0	0	0	0	0	0	0	0	0	0	80
22860947	m	md	JP	2023	1	0	0	0	0	0	0	0	0	0	0	0	40
22861393	m	md	MY	2023	0	0	0	0	0	0	0	0	0	0	0	0	64
22861537	m	ph	JP	2023	1	0	0	0	0	0	0	0	0	0	0	0	64
22861773	f	md	JP	2023	0	1	1	0	0	0	0	0	0	0	0	0	85
22861802	m	hp	CN	2023	0	1	0	0	0	1	0	0	0	0	0	0	67
22861809	m	cn	DE	2023	0	0	0	0	0	0	0	0	0	0	0	0	
22861889	m	cn	CN	2023	0	1	0	1	0	0	0	0	0	0	0	0	70
22862048	m	md	AU	2023	0	1	0	1	0	0	0	0	0	0	0	0	
22862049	m	md	JP	2023	0	1	0	1	0	0	0	0	0	0	0	1	
22863066	m	hp	TR	2023	0	1	1	0	0	0	0	0	0	0	0	0	64
22863174	f	cn	US	2023	0	0	0	0	0	0	0	0	0	0	0	0	66
22863372	m	md	JP	2023	0	1	0	0	1	0	0	0	0	0	0	0	79
22863712	Not	md	US	2023	1	0	0	0	0	0	0	0	0	0	0	0	
22863926	Not	ph	JP	2023	0	0	0	0	0	0	0	0	0	0	0	0	80
22864380	Not	md	BG	2023	0	1	0	0	0	1	0	0	0	0	0	0	

22864536	Not	md	BG	2023	0	1	0	0	0	1	0	0	0	0	0	0	
22865977	m	cn	US	2023	0	0	0	0	0	0	0	0	0	0	0	0	72
22866116	m	md	JP	2023	0	1	1	0	0	0	0	0	0	0	0	0	77
22866145	m	hp	US	2023	0	0	0	0	0	0	0	0	0	0	0	0	61
22866965	m	md	IN	2023	0	1	0	0	0	1	0	0	0	0	0	0	55
22866969	m	md	CN	2023	0	0	0	0	0	0	0	0	0	0	0	0	60
22867026	Not	hp	SG	2023	1	0	0	0	0	0	0	0	0	0	0	0	
22867052	Not	md	JP	2023	0	1	0	0	0	1	0	0	0	0	0	0	70
22867218	m	cn	US	2023	0	0	0	0	0	0	0	0	0	0	0	0	80
22867451	m	md	JP	2023	0	1	0	1	0	0	0	0	0	0	0	0	76
22867550	m	md	CA	2023	0	0	0	0	0	0	0	0	0	0	0	0	61
22867554	m	cn	US	2023	1	0	0	0	0	0	0	0	0	0	0	0	47.9
22867725	f	ph	FR	2023	0	0	0	0	0	0	0	0	0	0	0	0	51
22867815	Not	md	SA	2023	1	0	0	0	0	0	0	0	0	0	0	0	
22867882	m	md	RU	2023	0	1	0	1	0	0	0	0	0	0	0	0	58
22868060	m	hp	GB	2023	1	0	0	0	0	0	0	0	0	0	0	0	62
22868088	Not	md	JP	2023	0	0	0	0	0	0	0	0	0	0	0	0	61
22868101	m	md	JP	2023	0	1	0	1	0	0	0	0	0	0	0	0	80
22868108	f	cn	IN	2023	0	0	0	0	0	0	1	0	0	0	1	0	81
22868204	m	hp	FR	2023	0	0	0	0	0	0	0	0	0	0	0	0	66
22868246	Not	hp	SG	2023	1	0	0	0	0	0	0	0	0	0	0	0	
22868267	m	md	JP	2023	0	0	0	0	0	0	0	0	0	0	0	0	59
22868393	m	md	IT	2023	0	0	0	0	0	0	0	0	0	0	0	0	63
22868394	f	md	KR	2023	0	0	0	0	0	0	0	0	0	0	0	0	65
22868596	m	md	CN	2023	0	1	0	1	0	0	0	0	0	0	0	0	77.2
22868769	Not	md	CH	2023	0	1	1	0	0	0	0	0	0	0	0	0	
22868879	Not	md	JP	2023	0	1	1	0	0	0	0	0	0	0	0	0	
22869289	m	md	JP	2023	0	0	0	0	0	0	0	0	0	0	0	0	
22869309	Not	md	BG	2023	0	1	0	0	0	1	0	0	0	0	0	0	
22869599	f	cn	US	2023	0	0	0	0	0	0	0	0	0	0	0	0	
22869931	Not	cn	SG	2023	1	0	0	0	0	0	0	0	0	0	0	0	64.8
22870242	f	md	CN	2023	1	0	0	0	0	0	0	0	0	0	0	0	69
22870248	Not	cn	US	2023	1	0	0	0	0	0	0	0	0	0	0	0	
22870263	Not	hp	TH	2023	1	0	0	0	0	0	0	0	0	0	0	0	

22870309	Not	hp	CN	2023	1	0	0	0	0	0	0	0	0	0	0	0	
22870632	f	md	JP	2023	0	0	0	0	0	0	0	0	0	0	0	0	60
22871296	m	md	JP	2023	0	1	0	1	0	0	0	0	0	0	0	0	60
22871630	Not	md	JP	2023	0	1	0	0	0	1	0	0	0	0	0	0	70
22871807	m	md	JP	2023	0	1	0	0	1	0	0	0	0	0	0	1	80
22872130	f	hp	JP	2023	0	1	0	0	1	0	0	0	0	0	0	0	50
22872456	m	md	US	2023	0	0	0	0	0	0	0	0	0	0	0	0	
22872485	Not	cn	SG	2023	1	0	0	0	0	0	0	0	0	0	0	0	86.7
22872583	Not	md	TW	2023	0	0	0	0	0	0	0	0	0	0	0	0	
22872867	m	md	JP	2023	0	1	0	0	0	1	0	0	0	0	0	0	80
22872900	m	md	JP	2023	0	1	0	0	0	1	0	0	0	0	0	0	76
22873061	m	hp	US	2023	0	0	0	0	0	0	0	0	0	0	0	0	71
22873214	f	hp	JP	2023	0	0	0	0	0	0	1	0	0	1	0	1	60
22873286	m	cn	ID	2023	0	1	0	1	0	0	0	0	0	0	0	0	70.9
22873290	f	md	JP	2023	0	1	0	1	0	0	0	0	0	0	0	0	73
22873603	f	md	PL	2023	0	1	1	0	0	0	0	0	0	0	0	0	52
22873704	Not	md	US	2023	0	0	0	0	0	0	0	0	0	0	0	0	71
22873784	Not	hp	JP	2023	0	0	0	0	0	0	0	0	0	0	0	0	
22874054	f	hp	CA	2023	0	1	1	0	0	0	0	0	0	0	0	0	38
22874289	f	md	BR	2023	0	0	0	0	0	0	0	0	0	0	0	0	77
22874496	m	cn	IN	2023	0	0	0	0	0	0	0	0	0	0	0	0	71
22875549	m	md	FR	2023	1	0	0	0	0	0	0	0	0	0	0	0	80
22875556	m	md	JP	2023	0	0	0	0	0	0	0	0	0	0	0	0	72
22875999	f	md	FR	2023	0	1	1	0	0	0	0	0	0	0	0	0	37
22876544	m	md	US	2023	0	1	0	0	0	1	0	0	0	0	0	0	58
22876568	Not	cn	SG	2023	1	0	0	0	0	0	0	0	0	0	0	0	82
22876682	f	md	US	2023	0	1	0	0	0	1	0	0	0	0	0	0	66
22877100	Not	cn	SG	2023	1	0	0	0	0	0	0	0	0	0	0	0	65
22877387	m	md	AU	2023	1	0	0	0	0	0	0	0	0	0	0	0	
22877554	Not	md	JP	2023	0	0	0	0	0	0	0	0	0	0	0	0	
22877600	m	md	JP	2023	0	1	1	0	0	0	0	0	0	0	0	0	70
22877731	f	ph	JP	2023	0	0	0	0	0	0	0	0	0	0	0	0	
22878378	Not	ph	JP	2023	1	0	0	0	0	0	0	0	0	0	0	0	
22878562	Not	hp	JP	2023	0	1	0	0	1	0	0	0	0	0	0	0	

22878770	Not	md	RU	2023	0	0	0	0	0	0	0	0	0	0	0	0	31
22878806	Not	md	SG	2023	1	0	0	0	0	0	0	0	0	0	0	0	101
22878904	f	hp	US	2023	0	0	0	0	0	0	0	0	0	0	0	0	60
22878929	f	cn	ID	2023	1	0	0	0	0	0	0	0	0	0	0	0	
22878936	Not	cn	SG	2023	1	0	0	0	0	0	0	0	0	0	0	0	63
22878961	Not	md	JP	2023	0	1	0	0	0	1	0	0	0	0	0	0	90
22878962	f	ph	JP	2023	0	1	0	0	0	1	0	0	0	0	0	0	80
22878984	m	md	DE	2023	0	1	0	0	1	0	0	0	0	0	0	1	56
22879002	Not	md	SG	2023	1	0	0	0	0	0	0	0	0	0	0	0	
22879164	m	md	US	2023	0	1	0	0	0	1	0	0	0	0	0	0	75
22879279	f	md	FR	2023	0	0	0	0	0	0	0	0	0	0	0	0	54
22879577	f	cn	US	2023	0	0	0	0	0	0	0	0	0	0	0	0	
22879732	Not	md	RU	2023	0	0	0	0	0	0	0	0	0	0	0	0	72
22880402	m	md	US	2023	0	1	1	0	0	0	0	0	0	0	0	0	68
22880446	m	md	CN	2023	0	1	0	1	0	0	0	0	0	0	0	0	53.4
22880540	Not	cn	JP	2023	0	0	0	0	0	0	0	0	0	0	0	0	
22880563	f	cn	JP	2023	0	0	0	0	0	0	0	0	0	0	0	0	72
22880575	m	md	JP	2023	0	1	0	0	1	0	0	0	0	0	0	0	58
22880712	m	md	CN	2023	0	0	0	0	0	0	0	0	0	0	0	0	55
22880719	m	hp	US	2023	0	1	1	0	0	0	0	0	0	0	0	0	66
22880902	f	md	KR	2023	0	0	0	0	0	0	0	0	0	0	0	0	67
22881474	m	md	JP	2023	0	1	0	0	1	0	0	0	0	0	0	0	66
22881552	f	hp	FR	2023	0	0	0	0	0	0	0	0	0	0	0	0	66
22881568	f	cn	US	2023	1	0	0	0	0	0	0	0	0	0	0	0	
22882455	m	cn	SG	2023	1	0	0	0	0	0	0	0	0	0	0	0	85
22882475	f	cn	US	2023	0	0	0	0	0	0	0	0	0	0	0	0	
22883635	m	md	AT	2023	0	0	0	0	0	0	0	0	0	0	0	0	
22883953	f	md	NL	2023	1	0	0	0	0	0	0	0	0	0	0	0	
22884093	Not	hp	US	2023	1	0	0	0	0	0	0	0	0	0	0	0	
22884181	f	ph	MY	2023	1	0	0	0	0	0	0	0	0	0	0	0	
22884972	f	hp	TH	2023	1	0	0	0	0	0	0	0	0	0	0	0	70
22884976	m	md	FR	2023	0	0	0	0	0	0	0	0	0	0	0	0	71
22885017	Not	md	SG	2023	1	0	0	0	0	0	0	0	0	0	0	0	
22885036	m	hp	JP	2023	0	1	1	0	0	0	0	0	0	0	0	0	60

22885061	m	md	US	2023	0	1	1	0	0	0	0	0	0	0	0	0	
22885097	f	md	JP	2023	0	1	1	0	0	0	0	0	0	0	0	0	70
22885287	m	ph	JP	2023	0	1	0	0	0	1	0	0	0	0	0	0	60
22885361	f	md	BG	2023	0	1	0	0	0	1	0	0	0	0	0	0	68
22885414	m	md	JP	2023	0	1	0	0	0	1	0	0	0	0	0	0	69
22886273	f	md	US	2023	0	1	0	0	0	1	0	0	0	0	0	0	65
22886565	Not	md	JP	2023	0	1	0	0	0	1	0	0	0	0	0	0	80
22887745	m	md	CN	2023	0	0	0	0	0	0	0	0	0	0	0	0	58
22887763	Not	hp	CN	2023	1	0	0	0	0	0	0	0	0	0	0	0	
22887829	m	md	RU	2023	0	0	0	0	0	0	0	0	0	0	0	0	
22888083	m	md	JP	2023	0	1	0	0	1	0	0	0	0	0	0	0	58
22888145	m	md	JP	2023	0	0	0	0	0	0	0	0	0	0	0	0	60
22888180	Not	hp	SG	2023	1	0	0	0	0	0	0	0	0	0	0	0	
22888259	f	md	US	2023	0	1	0	0	0	1	0	0	0	0	0	0	46
22888262	Not	md	JP	2023	0	1	0	0	0	1	0	0	0	0	0	0	
22888472	f	ph	JP	2023	0	1	1	0	0	0	0	0	0	0	0	0	75
22889158	f	cn	DE	2023	0	0	0	0	0	0	0	0	0	0	0	0	
22889373	Not	md	PL	2023	0	0	0	0	0	0	0	0	0	0	0	0	
22889403	Not	md	BE	2023	0	0	0	0	0	0	0	0	0	0	0	0	
22889439	f	ph	US	2023	0	0	0	0	0	0	0	0	0	0	0	0	84
22889473	m	hp	JP	2023	0	1	1	0	0	0	0	0	0	0	0	0	
22889559	Not	md	ES	2023	0	1	0	0	0	1	0	0	0	0	0	0	
22889613	Not	hp	SG	2023	1	0	0	0	0	0	0	0	0	0	0	0	
22889759	f	cn	JP	2023	0	0	0	0	0	0	0	0	0	0	0	0	57
22889872	f	cn	IN	2023	0	0	0	0	0	0	0	0	0	0	0	0	50
22889933	Not	cn	US	2023	0	0	0	0	0	0	0	0	0	0	0	0	
22890070	f	cn	TH	2023	0	0	0	0	0	0	0	0	0	0	0	0	44
22890211	m	cn	US	2023	0	1	1	0	0	0	0	0	0	0	0	0	
22890333	m	md	DE	2023	0	1	1	0	0	0	0	0	0	0	0	0	
22890606	f	md	HU	2023	0	1	0	1	0	0	0	0	0	0	0	0	69
22890607	m	cn	CN	2023	0	1	0	1	0	0	0	0	0	0	0	0	67
22890610	f	md	JP	2023	0	1	0	0	0	1	0	0	0	0	0	0	70
22891387	m	md	JP	2023	0	1	0	0	1	0	0	0	0	0	0	0	73
22892005	m	md	JP	2023	0	1	0	0	0	1	0	0	0	0	0	0	

22892038	f	hp	CA	2023	0	0	0	0	0	0	0	0	0	0	0	0	67
22892065	Not	md	JP	2023	0	1	0	0	0	1	0	0	0	0	0	1	70
22892074	f	md	US	2023	0	1	0	0	0	1	0	0	0	0	0	0	75
22892142	Not	md	JP	2023	0	0	0	0	0	0	0	0	0	0	0	0	
22892311	m	hp	JP	2023	0	1	1	0	0	0	0	0	0	0	0	1	69
22892442	m	cn	ID	2023	1	0	0	0	0	0	0	0	0	0	0	0	73.1
22892631	m	hp	CA	2023	0	1	0	0	1	0	0	0	0	0	0	0	72
22893088	Not	hp	JP	2023	0	1	0	0	1	0	0	0	0	0	0	0	
22893568	f	md	JP	2023	0	1	0	0	0	1	0	0	0	0	0	0	67
22893601	f	hp	JP	2023	0	1	0	0	0	1	0	0	0	0	0	0	67
22893711	m	hp	SG	2023	1	0	0	0	0	0	0	0	0	0	0	0	66
22893815	m	ph	JP	2023	0	0	0	0	0	0	0	0	0	0	0	0	75
22893911	f	cn	ID	2023	1	0	0	0	0	0	0	0	0	0	0	0	63.9
22894360	f	md	JP	2023	0	1	0	0	0	1	0	0	0	0	0	0	70
22895169	m	cn	ID	2023	1	0	0	0	0	0	0	0	0	0	0	0	52.4
22895262	m	md	CN	2023	0	0	0	0	0	0	0	0	0	0	0	0	37
22895290	f	cn	CN	2023	0	1	0	1	0	0	0	0	0	0	0	0	52
22895633	m	cn	ID	2023	1	0	0	0	0	0	0	0	0	0	0	0	44.2
22895770	f	ph	JP	2023	1	0	0	0	0	0	0	0	0	0	0	0	70
22895898	f	cn	ID	2023	1	0	0	0	0	0	0	0	0	0	0	0	74.9
22896229	m	cn	CN	2023	0	1	0	1	0	0	0	0	0	0	0	0	75
22896230	m	md	IN	2023	1	0	0	0	0	0	0	0	0	0	0	0	55
22896240	m	hp	CN	2023	0	0	0	0	0	0	0	0	0	0	0	0	53
22896317	m	md	JP	2023	1	0	0	0	0	0	0	0	0	0	0	0	76
22896747	f	md	JP	2023	0	1	0	0	1	0	0	0	0	0	0	0	57
22897136	f	hp	DE	2023	0	1	1	0	0	0	0	0	0	0	0	0	33
22897485	f	md	ES	2023	0	1	1	0	0	0	0	0	0	0	0	0	67
22898244	m	md	JP	2023	0	1	0	0	1	0	0	0	0	0	0	0	59
22898385	m	md	HR	2023	0	0	0	0	0	0	0	0	0	0	0	0	73
22899470	m	md	DE	2023	0	1	0	0	1	0	0	0	0	0	0	0	83
22900485	Not	md	GB	2023	0	1	0	0	0	1	0	0	0	0	0	0	
22900860	m	md	JP	2023	0	0	0	0	0	0	0	0	0	0	0	0	
22901049	m	cn	IN	2023	0	1	0	0	0	1	0	0	0	0	0	0	59
22901240	m	md	TH	2023	0	0	0	0	0	0	0	0	0	0	0	0	58

22901300	m	md	JP	2023	0	1	0	1	0	0	0	0	0	0	0	0	77
22901583	m	md	PT	2023	0	1	1	0	0	0	0	0	0	0	0	0	44
22902404	m	hp	DE	2023	0	0	0	0	0	0	0	0	0	0	0	0	46
22902546	Not	hp	JP	2023	0	0	0	0	0	0	0	0	0	0	0	0	
22902560	Not	hp	JP	2023	0	0	0	0	0	0	0	0	0	0	0	0	
22902599	m	cn	ID	2023	1	0	0	0	0	0	0	0	0	0	0	0	82.3
22902701	f	md	JP	2023	0	1	0	0	1	0	0	0	0	0	0	0	72
22902818	m	md	JP	2023	0	1	1	0	0	0	0	0	0	0	0	0	45
22903989	f	md	JP	2023	0	1	0	0	0	1	0	0	0	0	0	0	78
22904003	f	ph	JP	2023	0	1	1	0	0	0	0	0	0	0	0	0	74
22904420	Not	hp	US	2023	0	0	0	0	0	0	0	0	0	0	0	0	
22905221	Not	hp	JP	2023	0	0	0	0	0	0	0	0	0	0	0	0	90
22905420	f	md	US	2023	0	0	0	0	0	0	0	0	0	0	0	0	43
22905433	f	md	JP	2023	0	0	0	0	0	0	0	0	0	0	0	0	80
22905727	Not	md	JP	2023	0	1	0	0	0	1	0	0	0	0	0	0	80
22905984	Not	cn	SG	2023	1	0	0	0	0	0	0	0	0	0	0	0	80
22905986	f	md	JP	2023	1	0	0	0	0	0	0	0	0	0	0	0	75
22905987	Not	cn	SG	2023	1	0	0	0	0	0	0	0	0	0	0	0	55.6
22906121	Not	cn	SG	2023	1	0	0	0	0	0	0	0	0	0	0	0	73.4
22906122	Not	cn	SG	2023	1	0	0	0	0	0	0	0	0	0	0	0	93.9
22906760	Not	cn	SG	2023	1	0	0	0	0	0	0	0	0	0	0	0	72.9
22906898	m	cn	JP	2023	1	0	0	0	0	0	0	0	0	0	0	0	
22907018	Not	ph	JP	2023	1	0	0	0	0	0	0	0	0	0	0	0	
22907198	f	md	JP	2023	0	1	1	0	0	0	0	0	0	0	0	0	54
22907334	m	cn	IN	2023	0	0	0	0	0	0	0	0	0	0	0	0	34
22907340	m	md	TW	2023	0	0	0	0	0	0	0	0	0	0	0	0	44
22907421	f	md	SG	2023	1	0	0	0	0	0	0	0	0	0	0	0	78
22907938	m	md	IT	2023	0	0	0	0	0	0	0	0	0	0	0	0	50
22910024	f	md	CN	2023	0	0	0	0	0	0	0	0	0	0	0	0	34
22910050	Not	md	JP	2023	0	1	0	0	0	1	0	0	0	0	0	0	70
22910716	m	md	JP	2023	0	0	0	0	0	0	0	0	0	0	0	0	
22910792	m	md	HU	2023	0	0	0	0	0	0	0	0	0	0	0	0	68
22910900	f	cn	TW	2023	0	1	0	0	0	1	0	0	0	0	0	0	42
22911203	Not	hp	US	2023	0	0	0	0	0	0	0	0	0	0	0	0	

22911857	f	md	US	2023	0	0	0	0	0	0	0	0	0	0	0	0	80
22912015	m	cn	JP	2023	0	0	0	0	0	0	0	0	0	0	0	0	75
22912267	f	md	NL	2023	0	1	1	0	0	0	0	0	0	0	0	0	66
22912278	m	md	JP	2023	0	0	0	0	0	0	0	0	0	0	0	0	78
22912332	m	md	IE	2023	0	0	0	0	0	0	0	0	0	0	0	0	67
22912465	m	md	JP	2023	0	1	1	0	0	0	0	0	0	0	0	0	60
22912563	f	cn	US	2023	1	0	0	0	0	0	0	0	0	0	0	0	
22912771	m	md	JP	2023	0	1	0	1	0	0	0	0	0	0	0	0	55
22912889	m	cn	IN	2023	0	0	0	0	0	0	0	0	0	0	0	0	36
22913071	f	cn	US	2023	1	0	0	0	0	0	0	0	0	0	0	0	
22913128	m	md	JP	2023	0	1	1	0	0	0	0	0	0	0	0	0	
22914248	f	md	JP	2023	0	1	1	0	0	0	0	0	0	0	0	0	60
22914398	f	cn	TW	2023	0	1	0	0	0	1	0	0	0	0	0	0	73
22914399	m	md	JP	2023	0	1	1	0	0	0	0	0	0	0	0	0	70
22914747	Not	hp	CA	2023	0	0	0	0	0	0	0	0	0	0	0	0	
22914761	m	md	JP	2023	0	1	0	0	0	1	0	0	0	0	0	0	60
22914856	m	hp	CN	2023	0	0	0	0	0	0	0	0	0	0	0	0	66
22915431	Not	hp	CN	2023	0	0	0	0	0	0	0	0	0	0	0	0	
22915604	m	md	SK	2023	0	0	0	0	0	0	0	0	0	0	0	0	80
22915888	Not	md	ES	2023	0	1	0	0	0	1	0	0	0	0	0	0	
22916214	m	cn	MX	2023	0	1	0	0	0	1	0	0	0	0	0	0	65
22916235	Not	md	ES	2023	0	1	0	0	0	1	0	0	0	0	0	0	
22916236	Not	md	ES	2023	0	1	0	0	0	1	0	0	0	0	0	1	
22916237	Not	md	ES	2023	0	1	0	0	0	1	0	0	0	0	0	0	
22916238	Not	md	ES	2023	0	1	0	0	0	1	0	0	0	0	0	0	
22916240	Not	md	ES	2023	0	1	0	0	0	1	0	0	0	0	0	1	
22916460	f	cn	US	2023	0	0	0	0	0	0	0	0	0	0	0	0	65
22916874	m	md	CN	2023	0	0	0	0	0	0	0	0	0	0	0	0	59
22917056	m	cn	IN	2023	0	0	0	0	0	0	0	0	0	0	0	0	63
22917728	f	md	IL	2023	1	0	0	0	0	0	0	0	0	0	0	0	80
22917830	m	md	FR	2023	0	0	0	0	0	0	0	0	0	0	0	0	69
22917878	m	md	JP	2023	1	0	0	0	0	0	0	0	0	0	0	0	80
22917945	m	md	DE	2023	0	1	1	0	0	0	0	0	0	0	0	0	80
22918710	m	md	JP	2023	0	1	1	0	0	0	0	0	0	0	0	0	80

22918964	Not	md	JP	2023	0	0	0	0	0	0	0	0	0	0	0	0	
22919145	m	md	DE	2023	0	1	0	0	1	0	0	0	0	0	0	0	83
22919230	m	ph	IT	2023	0	1	1	0	0	0	0	0	0	0	0	0	64
22920859	m	md	FR	2023	0	1	0	0	0	1	0	0	0	0	0	0	77
22920900	m	ph	JP	2023	0	1	0	0	1	0	0	0	0	0	0	0	71
22921246	m	md	FR	2023	0	1	0	0	0	1	0	0	0	0	0	0	70
22921249	f	hp	CN	2023	1	0	0	0	0	0	0	0	0	0	0	0	43
22923037	Not	cn	SG	2023	1	0	0	0	0	0	0	0	0	0	0	0	83
22923214	f	md	JP	2023	1	0	0	0	0	0	0	0	0	0	0	0	74
22923220	m	md	JP	2023	0	1	0	1	0	0	0	0	0	0	0	0	73
22923268	m	cn	CA	2023	0	0	0	0	0	0	0	0	0	0	0	0	73
22924200	m	cn	CN	2023	1	0	0	0	0	0	0	0	0	0	0	0	45
22924273	Not	ph	JP	2023	1	0	0	0	0	0	0	0	0	0	0	0	
22924578	f	hp	US	2023	0	0	0	0	0	0	0	0	0	0	0	0	52
22924593	f	md	JP	2023	0	1	0	0	0	1	0	0	0	0	0	0	75
22924623	m	cn	JP	2023	0	0	0	0	0	0	0	0	0	0	0	0	79
22924624	m	cn	PT	2023	0	1	1	0	0	0	0	0	0	0	0	0	44
22924625	m	cn	DE	2023	0	0	0	0	0	0	0	0	0	0	0	0	
22924668	m	hp	SG	2023	0	1	0	0	0	1	0	0	0	0	0	0	
22924685	f	ph	US	2023	0	0	0	0	0	0	0	0	0	0	0	0	62
22924714	m	md	FR	2023	0	1	0	1	0	0	0	0	0	0	0	0	
22925354	f	md	BR	2023	0	0	0	0	0	0	0	0	0	0	0	0	56
22926340	m	md	JP	2023	0	1	0	0	0	1	0	0	0	0	0	0	
22926466	Not	hp	US	2023	0	0	0	0	0	0	0	0	0	0	0	0	
22927318	f	md	JP	2023	0	0	0	0	0	0	0	0	0	0	0	0	70
22927500	Not	md	JP	2023	0	0	0	0	0	0	0	0	0	0	0	0	76
22927878	f	md	CZ	2023	0	0	0	0	0	0	0	0	0	0	0	0	26
22928436	Not	md	JP	2023	0	1	0	0	1	0	0	0	0	0	0	0	
22928590	f	ph	AU	2023	0	0	0	0	0	0	0	0	0	0	0	0	
22928723	m	cn	US	2023	0	0	0	0	0	0	0	0	0	0	0	0	72
22929367	m	md	FR	2023	0	1	1	0	0	0	0	0	0	0	0	0	61
22929897	m	md	JP	2023	0	1	0	0	1	0	0	0	0	0	0	0	65
22930197	Not	md	SA	2023	0	1	1	0	0	0	0	0	0	0	0	0	
22930251	Not	md	JP	2023	0	1	0	0	1	0	0	0	0	0	0	0	

22930327	m	md	FR	2023	0	1	1	0	0	0	0	0	0	0	0	0	61
22930341	f	md	AU	2023	0	0	0	0	0	0	0	0	0	0	0	0	77
22930509	f	md	BR	2023	0	0	0	0	0	0	0	0	0	0	0	0	70
22930583	Not	md	JP	2023	0	1	0	0	1	0	0	0	0	0	0	0	
22930594	f	md	BR	2023	0	0	0	0	0	0	0	0	0	0	0	0	60
22930938	f	md	AU	2023	0	0	0	0	0	0	0	0	0	0	0	0	
22930961	Not	md	BG	2023	1	0	0	0	0	0	0	0	0	0	0	0	
22931464	f	md	FR	2023	0	0	0	0	0	0	1	1	0	0	0	0	64.3
22931493	Not	md	RS	2023	0	0	0	0	0	0	0	0	0	0	0	0	
22931545	f	hp	JP	2023	0	1	0	0	0	1	0	0	0	0	0	0	67
22931649	m	cn	IN	2023	0	0	0	0	0	0	0	0	0	0	0	0	73
22931721	m	hp	US	2023	0	0	0	0	0	0	0	0	0	0	0	0	
22932450	m	md	JP	2023	0	1	1	0	0	0	0	0	0	0	0	1	60
22932493	m	cn	US	2023	0	0	0	0	0	0	0	0	0	0	0	0	84
22932551	Not	md	AU	2023	1	0	0	0	0	0	0	0	0	0	0	0	
22932817	Not	ph	MX	2023	0	0	0	0	0	0	0	0	0	0	0	0	39
22933053	m	md	JP	2023	0	1	0	0	0	1	0	0	0	0	0	0	80
22933066	Not	hp	US	2023	0	0	0	0	0	0	0	0	0	0	0	0	
22933068	f	md	JP	2023	0	1	0	0	0	1	0	0	0	0	0	0	80
22933283	f	md	JP	2023	0	1	0	0	0	1	0	0	0	0	0	0	70
22934227	f	hp	DE	2023	0	1	1	0	0	0	0	0	0	0	0	0	50
22934481	m	cn	ID	2023	1	0	0	0	0	0	0	0	0	0	0	0	
22934514	f	cn	CA	2023	1	0	0	0	0	0	0	0	0	0	0	0	55.4
22934523	f	cn	ID	2023	1	0	0	0	0	0	0	0	0	0	0	0	53.2
22935263	f	md	JP	2023	1	0	0	0	0	0	0	0	0	0	0	1	82
22935397	f	md	PT	2023	0	0	0	0	0	0	0	0	0	0	0	0	
22935648	f	md	FR	2023	0	0	0	0	0	0	0	0	0	0	0	0	
22935649	f	md	JP	2023	0	1	1	0	0	0	0	0	0	0	0	0	80
22935661	f	ph	BR	2023	1	0	0	0	0	0	0	0	0	0	0	0	40
22936172	m	cn	CA	2023	1	0	0	0	0	0	0	0	0	0	0	0	58
22936419	Not	cn	US	2023	1	0	0	0	0	0	0	0	0	0	0	0	80.3
22936478	m	hp	AU	2023	1	0	0	0	0	0	0	0	0	0	0	0	75
22936754	m	hp	JP	2023	0	1	1	0	0	0	0	0	0	0	0	0	66
22936999	m	md	JP	2023	0	1	1	0	0	0	0	0	0	0	0	0	77

22937622	Not	hp	JP	2023	1	0	0	0	0	0	0	0	0	0	0	1	
22937797	Not	md	CZ	2023	0	0	0	0	0	0	0	0	0	0	0	0	
22938001	m	md	JP	2023	0	1	0	0	1	0	0	0	0	0	0	1	78
22938150	f	md	US	2023	0	1	0	0	1	0	0	0	0	0	0	0	66
22938679	Not	md	TR	2023	0	0	0	0	0	0	0	0	0	0	0	0	
22938841	m	cn	US	2023	1	0	0	0	0	0	0	0	0	0	0	0	
22939490	Not	hp	CN	2023	0	0	0	0	0	0	0	0	0	0	0	0	
22939754	f	md	GB	2023	0	0	0	0	0	0	0	0	0	0	0	0	
22939768	Not	md	GB	2023	0	0	0	0	0	0	0	0	0	0	0	0	
22939999	m	md	CN	2023	0	0	0	0	0	0	0	0	0	0	0	0	66
22940002	f	hp	DE	2023	0	1	0	1	0	0	0	0	0	0	0	1	72
22940207	Not	hp	CN	2023	0	0	0	0	0	0	0	0	0	0	0	0	
22940238	Not	hp	CN	2023	0	0	0	0	0	0	0	0	0	0	0	0	
22940242	Not	md	SA	2023	0	1	1	0	0	0	0	0	0	0	0	0	
22940423	m	md	JP	2023	0	1	0	1	0	0	0	0	0	0	0	0	70
22940471	f	md	AT	2023	0	0	0	0	0	0	0	0	0	0	0	0	
22940543	m	hp	NL	2023	0	0	0	0	0	0	0	0	0	0	0	0	68
22940806	f	md	DE	2023	0	1	0	0	1	0	0	0	0	0	0	0	69
22940811	f	cn	ID	2023	1	0	0	0	0	0	0	0	0	0	0	0	
22941199	m	hp	NL	2023	0	0	0	0	0	0	0	0	0	0	0	0	68
22941948	f	md	US	2023	0	0	0	0	0	0	0	0	0	0	0	0	62
22942084	Not	md	IT	2023	0	0	0	0	0	0	0	0	0	0	0	0	
22942285	Not	hp	CN	2023	0	0	0	0	0	0	0	0	0	0	0	0	
22942312	m	cn	CN	2023	0	1	0	1	0	0	0	0	0	0	0	0	53
22942509	m	hp	CA	2023	0	0	0	0	0	0	0	0	0	0	0	0	62
22942602	Not	md	JP	2023	0	0	0	0	0	0	0	0	0	0	0	0	
22942892	f	md	DE	2023	0	0	0	0	0	0	0	0	0	0	0	0	67
22942974	Not	hp	CN	2023	0	0	0	0	0	0	0	0	0	0	0	0	
22943297	m	cn	AR	2023	0	0	0	0	0	0	0	0	0	0	0	0	53
22943376	Not	md	US	2023	0	0	0	0	0	0	0	0	0	0	0	0	
22943433	f	cn	IN	2023	0	0	0	0	0	0	0	0	0	0	0	0	66
22944092	Not	md	SG	2023	0	0	0	0	0	0	0	0	0	0	0	0	
22944222	f	md	JP	2023	0	1	0	0	0	1	0	0	0	0	0	0	62
22944351	Not	md	JP	2023	0	1	0	0	0	1	0	0	0	0	0	0	77

22944355	m	md	MX	2023	1	0	0	0	0	0	0	0	0	0	0	0	62
22944519	Not	md	JP	2023	0	1	0	0	1	0	0	0	0	0	0	0	
22944668	Not	md	JP	2023	0	1	0	0	0	1	0	0	0	0	0	0	70
22944714	f	md	JP	2023	0	1	0	0	0	1	0	0	0	0	0	0	39
22944851	f	md	FR	2023	0	0	0	0	0	0	0	0	0	0	0	0	61
22944903	m	md	CN	2023	0	1	0	1	0	0	0	0	0	0	0	0	70.8
22945052	m	md	AU	2023	0	0	0	0	0	0	0	0	0	0	0	0	69
22945167	m	ph	JP	2023	0	1	1	0	0	0	0	0	0	0	0	0	60
22945525	Not	md	JP	2023	0	1	1	0	0	0	0	0	0	0	0	0	79
22945586	m	md	IN	2023	0	1	1	0	0	0	0	0	0	0	0	0	69
22945910	f	ph	US	2023	1	0	0	0	0	0	0	0	0	0	0	0	79
22946014	f	cn	CA	2023	1	0	0	0	0	0	0	0	0	0	0	0	65.7
22946039	f	md	CZ	2023	0	1	0	0	1	0	0	0	0	0	0	0	76
22946383	m	md	NL	2023	0	0	0	0	0	0	0	0	0	0	0	0	68
22946534	Not	md	HK	2023	0	1	0	0	1	0	0	0	0	0	0	0	
22947038	f	md	JP	2023	0	0	0	0	0	0	0	0	0	0	0	0	
22947075	Not	ph	JP	2023	1	0	0	0	0	0	0	0	0	0	0	0	
22947782	f	md	US	2023	0	1	0	0	0	1	0	0	0	0	0	0	73
22947793	f	ph	JP	2023	1	0	0	0	0	0	0	0	0	0	0	0	81
22947846	f	hp	RU	2023	0	1	0	0	0	1	0	0	0	0	0	0	61
22947949	Not	hp	CN	2023	0	0	0	0	0	0	0	0	0	0	0	0	
22948186	Not	md	DE	2023	0	1	1	0	0	0	0	0	0	0	0	0	
22948392	Not	cn	PL	2023	0	0	0	0	0	0	0	0	0	0	0	0	
22948732	f	md	HU	2023	1	0	0	0	0	0	0	0	0	0	0	0	24
22948878	f	cn	IN	2023	0	1	0	1	0	0	0	0	0	0	0	0	59.7
22948940	m	md	PT	2023	0	1	1	0	0	0	0	0	0	0	0	0	44
22949104	f	md	NZ	2023	1	0	0	0	0	0	0	0	0	0	0	0	
22949175	Not	hp	VN	2023	0	1	1	0	0	0	0	0	0	0	0	1	
22949177	Not	hp	VN	2023	0	1	1	0	0	0	0	0	0	0	0	1	
22949190	f	md	DE	2023	0	1	1	0	0	0	0	0	0	0	0	0	65
22949445	m	hp	NL	2023	0	0	0	0	0	0	0	0	0	0	0	0	68
22949679	m	cn	MY	2023	1	0	0	0	0	0	0	0	0	0	0	0	64.8
22949680	m	cn	IN	2023	0	1	0	1	0	0	0	0	0	0	0	0	63.7
22949780	f	cn	MY	2023	1	0	0	0	0	0	0	0	0	0	0	0	89

22949826	m	md	JP	2023	0	0	0	0	0	0	0	0	0	0	0	0	63
22950023	m	md	CN	2023	0	1	0	1	0	0	0	0	0	0	0	0	61
22950025	m	md	JP	2023	0	1	1	0	0	0	0	0	0	0	0	0	70
22950732	m	md	US	2023	0	0	0	0	0	0	0	0	0	0	0	0	61
22951125	m	md	JP	2023	0	0	0	0	0	0	0	0	0	0	0	0	67
22951210	m	hp	CA	2023	1	0	0	0	0	0	0	0	0	0	0	0	
22951216	m	cn	ID	2023	1	0	0	0	0	0	0	0	0	0	0	0	87.7
22951632	Not	md	JP	2023	0	1	0	0	1	0	0	0	0	0	0	0	
22952966	m	cn	KR	2023	0	0	0	0	0	0	0	0	0	0	0	0	53
22953787	Not	cn	AE	2023	0	1	0	1	0	0	0	0	0	0	0	0	
22954102	m	md	NL	2023	0	0	0	0	0	0	0	0	0	0	0	0	68
22954242	m	md	CH	2023	0	0	0	0	0	0	0	0	0	0	0	0	
22954268	m	md	FR	2023	0	1	0	0	0	1	0	0	0	0	0	0	70
22954420	m	md	CN	2023	0	0	0	0	0	0	0	0	0	0	0	0	75
22954905	f	hp	GB	2023	0	1	1	0	0	0	0	0	0	0	0	0	73
22954944	Not	cn	US	2023	0	1	0	0	1	0	0	0	0	0	0	0	
22955033	m	cn	SG	2023	0	1	0	0	0	1	0	0	0	0	0	0	
22955170	Not	md	KR	2023	0	0	0	0	0	0	0	0	0	0	0	0	
22956373	m	md	RU	2023	0	1	0	1	0	0	0	0	0	0	0	0	59
22956464	f	md	DE	2023	0	0	0	0	0	0	0	0	0	0	0	0	75
22957034	f	md	BN	2023	0	0	0	0	0	0	0	0	0	0	0	0	50
22957147	f	md	JP	2023	0	0	0	0	0	0	0	0	0	0	0	0	71
22957796	f	md	BG	2023	0	0	0	0	0	0	0	0	0	0	0	0	43
22958823	m	md	BR	2023	1	0	0	0	0	0	0	0	0	0	0	0	70
22959223	Not	md	JP	2023	0	1	0	0	1	0	0	0	0	0	0	1	80
22960430	m	cn	KR	2023	0	0	0	0	0	0	0	0	0	0	0	0	33
22960722	f	md	JP	2023	0	1	0	0	0	1	0	0	0	0	0	0	76
22960883	m	md	JP	2023	0	0	0	0	0	0	0	0	0	0	0	0	86
22961070	m	cn	CN	2023	0	1	0	1	0	0	0	0	0	0	0	0	74
22961244	f	md	JP	2023	0	0	0	0	0	0	0	0	0	0	0	0	40
22961388	m	md	JP	2023	0	1	0	0	0	1	0	0	0	0	0	0	80
22961610	m	hp	FR	2023	0	0	0	0	0	0	0	0	0	0	0	0	75
22961959	m	md	DE	2023	0	1	0	0	1	0	0	0	0	0	0	1	59
22962200	Not	md	CA	2023	0	0	0	0	0	0	1	1	0	0	0	1	

22962676	f	cn	CN	2023	0	0	0	0	0	0	0	0	0	0	0	0	45
22962950	m	md	IT	2023	0	1	1	0	0	0	0	0	0	0	0	0	83
22962963	m	md	JP	2023	0	0	0	0	0	0	0	0	0	0	0	0	75
22963282	m	md	JP	2023	0	1	1	0	0	0	0	0	0	0	0	0	70
22964327	Not	hp	CN	2023	0	0	0	0	0	0	0	0	0	0	0	0	
22964408	m	md	RU	2023	1	0	0	0	0	0	0	0	0	0	0	0	
22964622	Not	cn	US	2023	1	0	0	0	0	0	0	0	0	0	0	0	
22965560	m	md	DE	2023	0	0	0	0	0	0	0	0	0	0	0	0	69
22966171	Not	md	JP	2023	1	0	0	0	0	0	0	0	0	0	0	0	
22966292	Not	md	JP	2023	0	0	0	0	0	0	0	0	0	0	0	0	
22966327	f	md	TR	2023	1	0	0	0	0	0	0	0	0	0	0	0	62
22966408	m	hp	ES	2023	0	0	0	0	0	0	0	0	0	0	0	0	
22966714	m	md	PT	2023	0	1	1	0	0	0	0	0	0	0	0	0	44
22967017	m	md	JP	2023	0	1	0	0	0	1	0	0	0	0	0	0	70
22967907	f	md	DK	2023	0	1	0	0	0	1	0	0	0	0	0	0	65
22968013	m	cn	US	2023	1	0	0	0	0	0	0	0	0	0	0	0	
22968098	f	cn	ID	2023	1	0	0	0	0	0	0	0	0	0	0	0	
22968244	m	ph	JP	2023	0	1	1	0	0	0	0	0	0	0	0	0	
22968248	m	md	JP	2023	0	1	1	0	0	0	0	0	0	0	0	0	
22968488	f	hp	FR	2023	0	1	1	0	0	0	0	0	0	0	0	0	70
22969082	f	md	JP	2023	0	1	0	0	0	1	0	0	0	0	0	0	78
22969509	Not	hp	JP	2023	0	0	0	0	0	0	0	0	0	0	0	0	
22969527	f	md	US	2023	0	1	1	0	0	0	0	0	0	0	0	0	66
22970231	m	cn	CN	2023	0	1	0	1	0	0	0	0	0	0	0	0	67
22970650	Not	md	RU	2023	0	1	0	0	0	1	0	0	0	0	0	0	
22970742	m	cn	TH	2023	0	1	0	1	0	0	0	0	0	0	0	1	73
22971015	f	hp	TH	2023	1	0	0	0	0	0	0	0	0	0	0	0	57
22971017	m	ph	MY	2023	1	0	0	0	0	0	0	0	0	0	0	0	
22971389	f	md	IT	2023	0	0	0	0	0	0	0	0	0	0	0	0	
22971550	m	ph	FR	2023	0	0	0	0	0	0	0	0	0	0	0	0	59
22972049	f	md	JP	2023	0	1	0	1	0	0	0	0	0	0	0	0	50
22972640	m	hp	US	2023	0	0	0	0	0	0	0	0	0	0	0	0	45
22972820	f	cn	ZA	2023	0	0	0	0	0	0	0	0	0	0	0	0	
22972827	Not	hp	ZA	2023	0	0	0	0	0	0	0	0	0	0	0	0	

22973068	m	hp	BG	2023	1	0	0	0	0	0	0	0	0	0	0	0	47
22973097	m	md	SE	2023	0	0	0	0	0	0	0	0	0	0	0	0	
22973222	Not	md	MX	2023	0	0	0	0	0	0	0	0	0	0	0	0	
22973270	m	md	CA	2023	0	1	0	1	0	0	0	0	0	0	0	0	53
22973844	Not	md	RU	2023	0	1	0	0	0	1	0	0	0	0	0	0	
22973872	Not	md	JP	2023	0	1	0	0	1	0	0	0	0	0	0	0	70
22974149	m	cn	US	2023	0	0	0	0	0	0	0	0	0	0	0	0	58
22974279	Not	md	RU	2023	0	1	0	0	0	1	0	0	0	0	0	0	
22974703	f	md	DE	2023	0	1	0	0	1	0	0	0	0	0	0	0	70
22974762	m	md	CZ	2023	0	1	0	0	1	0	0	0	0	0	0	0	71
22974842	m	cn	US	2023	0	0	0	0	0	0	0	0	0	0	0	0	
22974944	m	cn	KR	2023	0	0	0	0	0	0	0	0	0	0	0	0	77
22975083	m	md	DE	2023	0	1	0	0	1	0	0	0	0	0	0	0	80
22975360	f	cn	PK	2023	0	0	0	0	0	0	0	0	0	0	0	0	62
22975649	m	md	JP	2023	0	0	0	0	0	0	0	0	0	0	0	0	60
22975702	m	md	JP	2023	0	0	0	0	0	0	0	0	0	0	0	0	60
22976035	f	cn	IN	2023	0	1	0	0	0	1	0	0	0	0	0	0	54
22976233	f	md	CA	2023	0	1	0	1	0	0	0	0	0	0	0	0	74
22976279	m	cn	TW	2023	0	1	0	0	1	0	0	0	0	0	0	0	70
22976786	Not	md	US	2023	0	0	0	0	0	0	0	0	0	0	0	0	
22977316	f	hp	AU	2023	0	0	0	0	0	0	0	0	0	0	0	0	72
22979158	f	hp	GB	2023	1	0	0	0	0	0	0	0	0	0	0	0	65
22979202	Not	md	CN	2023	0	0	0	0	0	0	0	0	0	0	0	0	
22979357	f	ph	US	2023	0	0	0	0	0	0	0	0	0	0	0	0	
22980108	m	hp	JP	2023	0	1	0	1	0	0	0	0	0	0	0	1	79
22980288	m	md	JP	2023	0	1	1	0	0	0	0	0	0	0	0	0	59
22980380	m	cn	KR	2023	0	0	0	0	0	0	0	0	0	0	0	0	52
22980416	m	md	JP	2023	0	1	0	1	0	0	0	0	0	0	0	0	75
22980507	f	md	FR	2023	0	1	1	0	0	0	0	0	0	0	0	0	70
22981397	f	hp	US	2023	0	0	0	0	0	0	0	0	0	0	0	0	72
22981421	m	md	JP	2023	0	1	0	0	1	0	0	0	0	0	0	1	80
22981532	f	md	US	2023	0	0	0	0	0	0	0	0	0	0	0	0	72
22981666	m	md	JP	2023	0	1	1	0	0	0	0	0	0	0	0	0	80
22982207	m	hp	US	2023	0	1	0	0	1	0	0	0	0	0	0	0	83

22982321	m	md	NL	2023	0	1	0	0	0	1	0	0	0	0	0	0	56
22984316	m	md	JP	2023	0	0	0	0	0	0	0	0	0	0	0	0	70
22984790	Not	cn	JP	2023	0	0	0	0	0	0	0	0	0	0	0	0	
22985077	m	md	JP	2023	0	1	0	0	1	0	0	0	0	0	0	0	80
22985088	f	cn	US	2023	0	0	0	0	0	0	0	0	0	0	0	0	70
22985148	m	ph	US	2023	0	0	0	0	0	0	0	0	0	0	0	0	
22985370	m	ph	AU	2023	1	0	0	0	0	0	0	0	0	0	0	0	
22985484	Not	hp	CO	2023	1	0	0	0	0	0	0	0	0	0	0	0	
22985866	Not	md	JP	2023	1	0	0	0	0	0	0	0	0	0	0	0	
22985927	f	cn	KE	2023	1	0	0	0	0	0	0	0	0	0	0	0	54.7
22987039	Not	ph	US	2023	0	0	0	0	0	0	0	0	0	0	0	0	
22987198	f	md	CZ	2023	0	1	0	0	1	0	0	0	0	0	0	0	76
22987448	Not	md	JP	2023	0	1	1	0	0	0	0	0	0	0	0	0	
22987571	f	hp	US	2023	0	0	0	0	0	0	0	0	0	0	0	0	43
22987620	Not	hp	US	2023	0	0	0	0	0	0	0	0	0	0	0	0	
22987767	f	md	JP	2023	0	0	0	0	0	0	0	0	0	0	0	0	50
22988167	m	cn	KR	2023	0	0	0	0	0	0	0	0	0	0	0	0	64
22988472	m	hp	US	2023	0	1	0	0	1	0	0	0	0	0	0	0	71
22988517	f	md	FR	2023	0	1	1	0	0	0	0	0	0	0	0	0	70
22988709	m	md	ES	2023	0	1	0	0	0	1	0	0	0	0	0	1	59
22988744	f	md	JP	2023	1	0	0	0	0	0	0	0	0	0	0	0	
22989358	m	md	HR	2023	0	1	1	0	0	0	0	0	0	0	0	0	
22989576	m	ph	JP	2023	0	1	1	0	0	0	0	0	0	0	0	0	70
22989869	m	md	US	2023	0	0	0	0	0	0	0	0	0	0	0	0	
22989937	Not	cn	US	2023	1	0	0	0	0	0	0	0	0	0	0	0	
22991873	f	cn	KR	2023	0	0	0	0	0	0	0	0	0	0	0	0	61
22991886	f	cn	US	2023	0	0	0	0	0	0	0	0	0	0	0	0	43
22991968	m	md	JP	2023	0	1	0	1	0	0	0	0	0	0	0	0	75
22993700	m	hp	DE	2023	0	0	0	0	0	0	0	0	0	0	0	0	65
22993828	m	md	US	2023	0	1	1	0	0	0	0	0	0	0	0	0	73
22993974	f	md	JP	2023	0	1	0	1	0	0	0	0	0	0	0	0	
22994187	f	cn	US	2023	1	0	0	0	0	0	0	0	0	0	0	0	72
22994246	f	cn	EG	2023	1	0	0	0	0	0	0	0	0	0	0	0	62.7
22995421	m	cn	BR	2023	0	0	0	0	0	0	0	0	0	0	0	0	70

22995483	f	md	BG	2023	0	0	0	0	0	0	0	0	0	0	0	0	43
22995537	f	md	JP	2023	0	1	0	0	0	1	0	0	0	0	0	0	70
22996637	Not	md	DE	2023	0	1	0	0	1	0	0	0	0	0	0	1	
22997952	f	hp	CN	2023	0	0	0	0	0	0	0	0	0	0	0	0	53
22998055	m	hp	CN	2023	0	0	0	0	0	0	0	0	0	0	0	0	60
22998479	f	hp	GB	2023	1	0	0	0	0	0	0	0	0	0	0	0	41
23000162	m	md	JP	2023	0	1	1	0	0	0	0	0	0	0	0	0	87
23000246	f	md	BG	2023	1	0	0	0	0	0	0	0	0	0	0	0	34
23000329	m	md	CA	2023	0	1	0	1	0	0	0	0	0	0	0	0	60
23001873	f	md	RU	2023	1	0	0	0	0	0	0	0	0	0	0	0	
23001877	m	md	RU	2023	1	0	0	0	0	0	0	0	0	0	0	0	
23001957	m	ph	EG	2023	0	0	0	0	0	0	0	0	0	0	0	0	58
23002038	m	ph	EG	2023	0	0	0	0	0	0	0	0	0	0	0	0	58
23002480	m	md	ES	2023	0	0	0	0	0	0	0	0	0	0	0	0	
23003466	f	md	JP	2023	0	1	1	0	0	0	0	0	0	0	0	0	81
23003683	f	hp	PL	2023	0	1	1	0	0	0	0	0	0	0	0	0	52
23003921	f	hp	MX	2023	1	0	0	0	0	0	0	0	0	0	0	0	
23004551	f	md	BE	2023	0	1	0	0	1	0	0	0	0	0	0	0	59
23006135	f	hp	JP	2023	0	0	0	0	0	0	0	0	0	0	0	0	75
23007414	f	md	FR	2023	0	0	0	0	0	0	0	0	0	0	0	0	70
23007920	m	md	FR	2023	0	1	0	1	0	0	0	0	0	0	0	0	65
23007923	f	cn	IT	2023	1	0	0	0	0	0	0	0	0	0	0	0	
23008583	f	md	JP	2023	0	0	0	0	0	0	0	0	0	0	0	0	65
23008784	f	cn	DE	2023	0	1	0	1	0	0	0	0	0	0	0	1	32
23009695	f	cn	VN	2023	1	0	0	0	0	0	0	0	0	0	0	0	
23009816	f	md	HR	2023	0	1	1	0	0	0	0	0	0	0	0	0	46
23010515	m	md	JP	2023	1	0	0	0	0	0	0	0	0	0	0	0	51
23010924	Not	md	JP	2023	0	1	0	1	0	0	0	0	0	0	0	0	
23011326	m	md	AU	2023	0	0	0	0	0	0	0	0	0	0	0	0	
23011416	f	cn	US	2023	1	0	0	0	0	0	0	0	0	0	0	0	81.4
23011430	f	md	SE	2023	0	1	1	0	0	0	0	0	0	0	0	0	68
23011918	m	cn	PK	2023	0	1	0	0	0	1	0	0	0	0	0	0	56
23012003	Not	cn	CN	2023	0	1	0	0	0	1	0	0	0	0	0	0	
23012789	m	md	FR	2023	0	1	1	0	0	0	0	0	0	0	0	0	61

23012908	Not	hp	US	2023	0	0	0	0	0	0	0	0	0	0	0	0	
10368721	m	md	IT	2023	0	0	0	0	0	0	0	0	0	0	0	0	68
21065839	m	cn	IN	2023	0	0	0	0	0	0	0	0	0	0	0	0	64
21219008	m	md	JP	2023	0	1	0	0	1	0	0	0	0	0	0	0	53
22057413	m	md	JP	2023	0	1	0	0	1	0	0	0	0	0	0	0	69
22071493	m	md	KR	2023	0	0	0	0	0	0	0	0	0	0	0	0	38
22168572	f	md	JP	2023	0	1	0	0	1	0	0	0	0	0	0	0	48
22173910	m	md	JP	2023	0	1	0	0	1	0	0	0	0	0	0	0	46
22174197	f	md	JP	2023	0	1	0	0	1	0	0	0	0	0	0	0	55
22175137	m	md	JP	2023	0	1	0	0	1	0	0	0	0	0	0	0	51
22214363	m	md	JP	2023	0	1	1	0	0	0	0	0	0	0	0	1	80
22320032	f	md	ES	2023	0	1	1	0	0	0	0	0	0	0	0	0	53
22390274	m	hp	FR	2023	0	1	0	0	1	0	0	0	0	0	0	0	81
22400688	m	md	CN	2023	0	1	0	0	0	1	0	0	0	0	0	0	54
22526535	m	cn	CY	2023	0	0	0	0	0	0	0	0	0	0	0	0	64
22606957	m	md	CN	2023	0	0	0	0	0	0	0	0	0	0	0	0	69
22621632	f	md	CN	2023	0	0	0	0	0	0	0	0	0	0	0	0	58
22632845	f	md	CN	2023	0	0	0	0	0	0	0	0	0	0	0	0	65
22634677	m	md	JP	2023	0	0	0	0	0	0	0	0	0	0	0	0	
22643317	m	md	JP	2023	0	1	0	0	1	0	0	0	0	0	0	0	70
22645456	m	md	JP	2023	0	1	0	0	1	0	0	0	0	0	0	0	86
22651113	m	md	HK	2023	0	1	0	0	0	1	0	0	0	0	0	0	71
22662818	f	md	JP	2023	0	0	0	0	0	0	0	0	0	0	0	0	67
22662840	m	md	JP	2023	0	1	0	0	1	0	0	0	0	0	0	0	67
22662872	f	md	FR	2023	0	0	0	0	0	0	0	0	0	0	0	0	75
22666347	f	md	JP	2023	0	0	0	0	0	0	0	0	0	0	0	0	75
22666376	f	md	CN	2023	0	1	0	0	0	1	0	0	0	0	0	0	74
22672239	m	cn	US	2023	0	0	0	0	0	0	0	0	0	0	0	0	58
22674156	f	md	AR	2023	0	0	0	0	0	0	0	0	0	0	0	0	66
22676602	m	md	JP	2023	0	1	1	0	0	0	0	0	0	0	0	0	80
22691032	m	md	CZ	2023	0	1	0	0	1	0	0	0	0	0	0	0	71
22695655	Not	md	US	2023	0	0	0	0	0	0	0	0	0	0	0	0	
22702970	m	md	JP	2023	0	1	1	0	0	0	0	0	0	0	0	0	70
22703369	Not	md	JP	2023	0	1	0	0	1	0	0	0	0	0	0	0	

22703812	f	md	JP	2023	0	0	0	0	0	0	0	0	0	0	0	0	50
22704196	f	md	DE	2023	0	1	1	0	0	0	0	0	0	0	0	0	
22706879	f	md	CN	2023	0	0	0	0	0	0	0	0	0	0	0	0	74
22709591	m	md	JP	2023	0	1	0	0	1	0	0	0	0	0	0	0	48
22719415	m	md	JP	2023	0	0	0	0	0	0	0	0	0	0	0	0	76
22724606	f	md	JP	2023	0	0	0	0	0	0	0	0	0	0	0	0	67
22740335	f	md	JP	2023	0	0	0	0	0	0	0	0	0	0	0	0	69
22740853	m	md	JP	2023	0	0	0	0	0	0	0	0	0	0	0	0	68
22743661	m	hp	BR	2023	0	0	0	0	0	0	0	0	0	0	0	0	58
22752830	m	md	JP	2023	0	1	1	0	0	0	0	0	0	0	0	0	90
22757459	f	md	PL	2023	0	1	0	0	0	1	0	0	0	0	0	0	67
22758306	f	md	BR	2023	0	0	0	0	0	0	0	0	0	0	0	0	71
22761504	f	md	JP	2023	0	1	1	0	0	0	0	0	0	0	0	0	80
22762603	f	md	MY	2023	0	0	0	0	0	0	0	0	0	0	0	0	67
22766884	f	cn	KW	2023	0	0	0	0	0	0	0	0	0	0	0	0	46
22769785	m	md	JP	2023	0	0	0	0	0	0	0	0	0	0	0	0	59
22771548	f	hp	AU	2023	0	0	0	0	0	0	0	0	0	0	0	0	67
22772884	f	md	ES	2023	0	0	0	0	0	0	0	0	0	0	0	0	77
22773073	f	md	JP	2023	0	0	0	0	0	0	0	0	0	0	0	0	60
22774203	m	md	JP	2023	0	1	0	0	1	0	0	0	0	0	0	0	
22778889	f	cn	IN	2023	0	0	0	0	0	0	0	0	0	0	0	0	63
22781207	f	md	FR	2023	0	0	0	0	0	0	0	0	0	0	0	0	53
22783871	f	md	JP	2023	0	1	1	0	0	0	0	0	0	0	0	1	72
22786482	Not	md	JP	2023	0	0	0	0	0	0	0	0	0	0	0	0	80
22788657	m	md	ES	2023	0	1	0	0	0	1	0	0	0	0	0	0	71
22788744	f	md	ES	2023	0	0	0	0	0	0	0	0	0	0	0	0	
22790070	f	md	US	2023	0	1	1	0	0	0	0	0	0	0	0	0	75
22794472	m	md	ES	2023	0	0	0	0	0	0	0	0	0	0	0	0	59
22795157	m	md	GR	2023	0	1	0	0	1	0	0	0	0	0	0	0	65
22795386	m	md	JP	2023	0	1	0	1	0	0	0	0	0	0	0	0	46
22795844	m	md	JP	2023	0	0	0	0	0	0	0	0	0	0	0	0	77
22799211	m	md	JP	2023	0	0	0	0	0	0	0	0	0	0	0	0	75
22801522	Not	hp	CA	2023	0	0	0	0	0	0	0	0	0	0	0	0	
22806257	m	ph	JP	2023	0	1	1	0	0	0	0	0	0	0	0	0	70

22811283	m	md	TW	2023	0	0	0	0	0	0	0	0	0	0	0	0	69
22817890	m	md	JP	2023	0	0	0	0	0	0	0	0	0	0	0	0	86
22820759	f	md	ES	2023	0	0	0	0	0	0	0	0	0	0	0	0	
22822811	m	md	FR	2023	0	1	0	1	0	0	0	0	0	0	0	0	71
22825941	m	md	JP	2023	0	1	0	0	1	0	0	0	0	0	0	0	51
22826391	f	md	DE	2023	0	1	0	0	1	0	0	0	0	0	0	0	66
22827882	m	md	IT	2023	1	0	0	0	0	0	0	0	0	0	0	0	67
22831600	m	md	JP	2023	0	1	0	0	1	0	0	0	0	0	0	0	74
22832561	f	hp	GB	2023	0	0	0	0	0	0	1	0	0	0	1	0	52
22833824	m	md	IT	2023	0	0	0	0	0	0	0	0	0	0	0	0	
22835010	f	md	IT	2023	0	0	0	0	0	0	0	0	0	0	0	0	
22838501	m	md	CN	2023	0	1	1	0	0	0	0	0	0	0	0	0	63
22841215	m	md	DE	2023	0	1	1	0	0	0	0	0	0	0	0	0	70
22841807	m	md	JP	2023	0	1	0	0	0	1	0	0	0	0	0	0	70
22851383	m	hp	CN	2023	0	0	0	0	0	0	0	0	0	0	0	0	67
22853318	m	md	IT	2023	1	0	0	0	0	0	0	0	0	0	0	0	67
22856363	m	cn	TH	2023	1	0	0	0	0	0	0	0	0	0	0	0	80.4
22860807	f	md	JP	2023	0	1	0	1	0	0	0	0	0	0	0	0	66
22862109	m	md	CN	2023	0	0	0	0	0	0	0	0	0	0	0	0	82
22865887	f	md	FR	2023	0	0	0	0	0	0	0	0	0	0	0	0	76
22882600	m	md	FR	2023	0	1	0	0	0	1	0	0	0	0	0	0	70
22882742	m	md	JP	2023	0	1	0	0	0	1	0	0	0	0	0	0	70
22885092	m	ph	JP	2023	0	1	1	0	0	0	0	0	0	0	0	0	80
22886658	m	md	DE	2023	0	1	1	0	0	0	0	0	0	0	0	0	
22888805	m	md	JP	2023	0	0	0	0	0	0	0	0	0	0	0	0	73
22890497	m	ph	TH	2023	1	0	0	0	0	0	0	0	0	0	0	1	63
22893964	f	md	JP	2023	0	1	1	0	0	0	0	0	0	0	0	0	90
22898600	m	md	DE	2023	0	1	1	0	0	0	0	0	0	0	0	0	61
22898704	f	md	JP	2023	0	1	0	0	0	1	0	0	0	0	0	0	70
22900243	f	md	DE	2023	0	1	1	0	0	0	0	0	0	0	0	0	
22904001	f	hp	US	2023	0	1	0	0	1	0	0	0	0	0	0	0	69
22905522	m	md	JP	2023	0	0	0	0	0	0	0	0	0	0	0	0	
22907116	m	md	JP	2023	0	1	0	0	1	0	0	0	0	0	0	0	85
22908367	m	md	DE	2023	0	1	0	0	1	0	0	0	0	0	0	0	70

22909025	Not	md	FR	2023	0	1	0	0	0	1	0	0	0	0	0	0	
22911638	m	md	DE	2023	0	1	1	0	0	0	0	0	0	0	0	0	
22912546	m	md	JP	2023	1	0	0	0	0	0	0	0	0	0	0	0	72
22913010	m	md	JP	2023	0	0	0	0	0	0	0	0	0	0	0	0	80
22913445	m	md	NL	2023	0	0	0	0	0	0	0	0	0	0	0	0	68
22913606	f	md	JP	2023	0	0	0	0	0	0	0	0	0	0	0	0	81
22914760	m	md	JP	2023	0	1	0	0	0	1	0	0	0	0	0	0	73
22916424	m	md	FR	2023	0	1	0	0	0	1	0	0	0	0	0	0	70
22917404	f	cn	US	2023	1	0	0	0	0	0	0	0	0	0	0	0	70
22918140	f	hp	JP	2023	0	1	0	0	0	1	0	0	0	0	0	0	67
22918804	m	md	US	2023	0	0	0	0	0	0	0	0	0	0	0	0	74
22919030	m	hp	JP	2023	0	0	0	0	0	0	0	0	0	0	0	0	78
22923713	m	md	CN	2023	0	0	0	0	0	0	0	0	0	0	0	0	66
22924538	m	cn	CA	2023	0	0	0	0	0	0	0	0	0	0	0	0	58
22924619	f	cn	JP	2023	0	0	0	0	0	0	0	0	0	0	0	0	65
22924912	m	md	CN	2023	0	1	0	0	0	1	0	0	0	0	0	0	66
22925662	f	md	IT	2023	0	0	0	0	0	0	0	0	0	0	0	0	61
22928653	m	md	JP	2023	0	1	1	0	0	0	0	0	0	0	0	0	72
22929009	m	md	JP	2023	0	1	0	0	1	0	0	0	0	0	0	0	80
22929296	m	md	JP	2023	0	1	0	0	1	0	0	0	0	0	0	0	49
22929687	Not	hp	JP	2023	0	1	0	0	0	1	0	0	0	0	0	0	
22930397	f	md	DE	2023	0	1	1	0	0	0	0	0	0	0	0	0	
22930856	m	md	DE	2023	0	1	1	0	0	0	0	0	0	0	0	0	61
22935170	m	md	CN	2023	0	1	0	0	0	1	0	0	0	0	0	0	55
22935586	m	md	AR	2023	0	0	0	0	0	0	0	0	0	0	0	0	54
22938991	f	cn	US	2023	0	0	0	0	0	0	0	0	0	0	0	0	61
22939699	m	cn	US	2023	0	0	0	0	0	0	0	0	0	0	0	0	74
22939720	Not	md	JP	2023	1	0	0	0	0	0	0	0	0	0	0	0	
22940260	f	md	JP	2023	0	1	0	0	1	0	0	0	0	0	0	1	67
22940676	f	ph	FR	2023	0	0	0	0	0	0	0	0	0	0	0	0	66
22941644	f	hp	US	2023	1	0	0	0	0	0	0	0	0	0	0	0	86
22943193	Not	md	GB	2023	0	1	0	0	0	1	0	0	0	0	0	0	
22943722	f	md	JP	2023	0	1	0	0	0	1	0	0	0	0	0	0	80
22943764	m	md	AU	2023	0	0	0	0	0	0	0	0	0	0	0	0	60

22949903	m	md	JP	2023	0	0	0	0	0	0	0	0	0	0	0	0	82
22950615	m	md	CN	2023	1	0	0	0	0	0	0	0	0	0	0	0	57
22953726	m	md	JP	2023	0	1	1	0	0	0	0	0	0	0	0	0	61
22955366	m	md	ES	2023	0	1	1	0	0	0	0	0	0	0	0	0	
22955390	f	md	MY	2023	0	0	0	0	0	0	0	0	0	0	0	0	81
22957073	m	md	NL	2023	0	0	0	0	0	0	0	0	0	0	0	0	68
22957140	m	md	PL	2023	0	1	0	1	0	0	0	0	0	0	0	0	74
22958073	f	md	JP	2023	0	0	0	0	0	0	0	0	0	0	0	0	65
22958896	m	ph	AT	2023	0	1	1	0	0	0	0	0	0	0	0	1	70
22959408	m	cn	US	2023	0	0	0	0	0	0	0	0	0	0	0	0	78
22960393	m	cn	US	2023	0	0	0	0	0	0	0	0	0	0	0	0	
22961404	f	md	JP	2023	0	1	0	0	0	1	0	0	0	0	0	0	60
22962852	m	md	JP	2023	0	0	0	0	0	0	0	0	0	0	0	0	
22963102	m	cn	TH	2023	0	1	1	0	0	0	0	0	0	0	0	1	74
22963377	m	md	JP	2023	0	1	0	0	1	0	0	0	0	0	0	1	77
22966361	m	md	DE	2023	0	1	0	0	1	0	0	0	0	0	0	0	80
22966942	m	md	NL	2023	0	0	0	0	0	0	0	0	0	0	0	0	60
22967609	f	ph	JP	2023	0	0	0	0	0	0	0	0	0	0	0	0	
22968394	m	md	FR	2023	0	0	0	0	0	0	0	0	0	0	0	0	80
22970747	f	cn	TH	2023	1	0	0	0	0	0	0	0	0	0	0	0	76
22970999	m	md	GB	2023	0	1	0	0	0	1	0	0	0	0	0	0	65
22972709	f	md	CH	2023	0	0	0	0	0	0	0	0	0	0	0	0	78
22973119	m	md	JP	2023	0	1	0	1	0	0	0	0	0	0	0	0	69
22978395	m	md	NL	2023	0	0	0	0	0	0	0	0	0	0	0	0	68
22979504	Not	md	FR	2023	0	0	0	0	0	0	0	0	0	0	0	0	81
22980345	f	md	DE	2023	0	1	1	0	0	0	0	0	0	0	0	0	
22980562	f	md	CA	2023	0	1	0	1	0	0	0	0	0	0	0	0	70
22985365	f	cn	DE	2023	1	0	0	0	0	0	0	0	0	0	0	0	75
22987177	m	md	CN	2023	0	0	0	0	0	0	0	0	0	0	0	0	73
22990993	m	md	TR	2023	0	1	1	0	0	0	0	0	0	0	0	0	58
22991482	f	md	FR	2023	0	0	0	0	0	0	0	0	0	0	0	0	
22992220	m	md	JP	2023	0	1	0	0	0	1	0	0	0	0	0	0	72
22993575	m	md	JP	2023	0	1	0	0	1	0	0	0	0	0	0	0	70
22996123	m	md	JP	2023	1	0	0	0	0	0	0	0	0	0	0	0	

23000809	Not	md	AR	2023	0	0	0	0	0	0	0	0	0	0	0	0	
23004017	m	md	JP	2023	0	0	0	0	0	0	0	0	0	0	0	0	73
23007227	Not	hp	US	2023	0	0	0	0	0	0	0	0	0	0	0	0	
23007775	f	md	JP	2023	0	1	0	1	0	0	0	0	0	0	0	0	74
23007880	m	cn	CN	2023	0	1	0	1	0	0	0	0	0	0	0	1	68
23012005	Not	md	JP	2023	0	1	0	0	0	1	0	0	0	0	0	0	
23012006	Not	md	JP	2023	0	1	0	0	0	1	0	0	0	0	0	0	
23012047	m	cn	US	2023	0	0	0	0	0	0	0	0	0	0	0	0	
23012704	m	cn	US	2023	0	0	0	0	0	0	0	0	0	0	0	0	
23012969	m	md	AU	2023	0	0	0	0	0	0	0	0	0	0	0	0	62
23013448	m	md	JP	2023	0	1	0	0	1	0	0	0	0	0	0	0	55
23014464	m	md	JP	2023	0	1	0	1	0	0	0	0	0	0	0	0	81
23014546	f	md	JP	2023	0	0	0	0	0	0	0	0	0	0	0	0	80
23014693	f	cn	ID	2023	1	0	0	0	0	0	0	0	0	0	0	0	60.8
23014942	m	cn	ID	2023	1	0	0	0	0	0	0	0	0	0	0	0	61.4
23014987	m	md	JP	2023	0	1	1	0	0	0	0	0	0	0	0	0	60
23015208	m	ph	JP	2023	0	1	1	0	0	0	0	0	0	0	0	0	69
23015308	m	md	JP	2023	0	0	0	0	0	0	0	0	0	0	0	0	61
23015677	m	hp	NL	2023	0	0	0	0	0	0	0	0	0	0	0	0	68
23015684	f	md	JP	2023	1	0	0	0	0	0	0	0	0	0	0	0	72
23015696	f	md	FR	2023	0	1	1	0	0	0	0	0	0	0	0	0	70
23015753	f	md	FR	2023	0	1	1	0	0	0	0	0	0	0	0	0	74
23015896	f	md	IN	2023	1	0	0	0	0	0	0	0	0	0	0	0	55
23015938	m	md	IN	2023	1	0	0	0	0	0	0	0	0	0	0	0	54
23016015	m	cn	ID	2023	1	0	0	0	0	0	0	0	0	0	0	0	60.6
23016035	f	cn	JP	2023	1	0	0	0	0	0	0	0	0	0	0	0	
23016079	m	md	JP	2023	0	1	1	0	0	0	0	0	0	0	0	0	68
23016160	f	md	CZ	2023	0	1	1	0	0	0	0	0	0	0	0	0	66
23016247	m	md	CN	2023	0	0	0	0	0	0	0	0	0	0	0	0	64
23016312	m	cn	TH	2023	0	0	0	0	0	0	0	0	0	0	0	0	63
23016447	m	cn	PK	2023	0	1	0	0	0	1	0	0	0	0	0	0	76
23016744	m	cn	PK	2023	0	1	0	0	0	1	0	0	0	0	0	0	91
23017904	f	hp	US	2023	0	0	0	0	0	0	0	0	0	0	0	0	60
23018039	m	md	DE	2023	0	1	0	0	1	0	0	0	0	0	0	1	62

23019860	Not	md	JP	2023	0	0	0	0	0	0	0	0	0	0	0	0	
23020177	f	md	NL	2023	1	0	0	0	0	0	0	0	0	0	0	0	
23020244	Not	cn	US	2023	1	0	0	0	0	0	0	0	0	0	0	0	
23020245	f	cn	IN	2023	1	0	0	0	0	0	0	0	0	0	0	0	76
23020392	f	md	US	2023	0	0	0	0	0	0	0	0	0	0	0	0	69
23020478	f	md	JP	2023	0	1	1	0	0	0	0	0	0	0	0	0	68
23020536	Not	md	NZ	2023	0	1	1	0	0	0	0	0	0	0	0	0	
23021124	f	md	JP	2023	0	0	0	0	0	0	0	0	0	0	0	0	69
23021377	m	md	JP	2023	0	1	1	0	0	0	0	0	0	0	0	0	70
23021734	m	md	IT	2023	1	0	0	0	0	0	0	0	0	0	0	0	67.1
23021796	m	md	CN	2023	0	1	0	1	0	0	0	0	0	0	0	0	74.6
23021822	m	md	TN	2023	0	0	0	0	0	0	0	0	0	0	0	0	40
23021823	m	cn	US	2023	0	0	0	0	0	0	0	0	0	0	0	0	41
23021964	m	md	IT	2023	0	0	0	0	0	0	0	0	0	0	0	0	81
23022151	m	md	JP	2023	0	1	0	1	0	0	0	0	0	0	0	0	66
23023304	m	md	ES	2023	0	0	0	0	0	0	0	0	0	0	0	0	73
23023365	m	cn	JP	2023	0	0	0	0	0	0	0	0	0	0	0	0	55
23023684	f	cn	PK	2023	0	1	0	0	0	1	0	0	0	0	0	0	58
23023863	m	md	JP	2023	0	1	0	0	1	0	0	0	0	0	0	0	61
23023894	m	md	JP	2023	0	0	0	0	0	0	0	0	0	0	0	0	83
23023900	Not	md	VN	2023	0	0	0	0	0	0	0	0	0	0	0	0	
23024146	f	hp	DE	2023	0	0	0	0	0	0	0	0	0	0	0	0	55
23024286	m	md	ES	2023	0	0	0	0	0	0	0	0	0	0	0	0	
23024594	m	md	JP	2023	0	1	0	0	0	1	0	0	0	0	0	0	64
23025673	m	md	JP	2023	0	0	0	0	0	0	0	0	0	0	0	0	66
23025877	m	md	JP	2023	0	1	0	0	1	0	0	0	0	0	0	0	71
23026589	m	md	JP	2023	0	1	1	0	0	0	0	0	0	0	0	0	60
23026941	Not	md	HK	2023	0	0	0	0	0	0	0	0	0	0	0	0	
23026966	f	cn	JP	2023	0	0	0	0	0	0	0	0	0	0	0	0	73
23026982	m	cn	JP	2023	0	0	0	0	0	0	0	0	0	0	0	0	75
23027095	m	md	FR	2023	0	1	0	0	0	1	0	0	0	0	0	0	63
23027180	m	md	JP	2023	0	1	0	0	1	0	0	0	0	0	0	0	70
23027339	m	md	IT	2023	1	0	0	0	0	0	0	0	0	0	0	0	67.1
23027371	m	md	JP	2023	0	1	1	0	0	0	0	0	0	0	0	0	70

23027925	f	ph	US	2023	0	0	0	0	0	0	0	0	0	0	0	0	53
23027936	m	md	NL	2023	0	1	0	0	0	1	0	0	0	0	0	0	46
23028097	Not	cn	US	2023	1	0	0	0	0	0	0	0	0	0	0	0	64
23028338	f	hp	US	2023	0	1	0	0	1	0	0	0	0	0	0	0	70
23028428	f	md	ES	2023	0	1	1	0	0	0	0	0	0	0	0	0	53
23028499	m	md	ES	2023	0	0	0	0	0	0	0	0	0	0	0	0	77
23028509	f	md	US	2023	0	0	0	0	0	0	0	0	0	0	0	0	72
23028708	f	md	JP	2023	0	1	0	0	1	0	0	0	0	0	0	0	51
23028852	m	ph	JP	2023	0	1	0	0	0	1	0	0	0	0	0	0	69
23028966	f	cn	CA	2023	0	0	0	0	0	0	0	0	0	0	0	0	
23028985	Not	md	JP	2023	1	0	0	0	0	0	0	0	0	0	0	0	
23029316	f	md	GR	2023	0	1	1	0	0	0	0	0	0	0	0	0	70
23029361	f	md	DE	2023	0	0	0	0	0	0	0	0	0	0	0	0	
23029364	Not	md	HK	2023	0	0	0	0	0	0	0	0	0	0	0	0	
23029415	m	md	NP	2023	0	0	0	0	0	0	0	0	0	0	0	0	56
23029591	m	md	JP	2023	0	1	1	0	0	0	0	0	0	0	0	1	70
23030169	Not	hp	JP	2023	0	0	0	0	0	0	0	0	0	0	0	0	
23030752	m	md	JP	2023	0	1	1	0	0	0	0	0	0	0	0	0	79
23031721	m	md	TR	2023	0	1	1	0	0	0	0	0	0	0	0	0	64
23031850	m	md	PT	2023	0	1	1	0	0	0	0	0	0	0	0	0	71
23031968	Not	cn	US	2023	0	1	0	1	0	0	0	0	0	0	0	1	
23032098	f	hp	PL	2023	0	1	1	0	0	0	0	0	0	0	0	0	59
23032269	m	md	JP	2023	0	0	0	0	0	0	0	0	0	0	0	0	75
23032470	Not	md	JP	2023	0	0	0	0	0	0	0	0	0	0	0	0	70
23032480	Not	cn	SG	2023	0	1	0	1	0	0	0	0	0	0	0	0	
23032622	m	md	IN	2023	0	0	0	0	0	0	0	0	0	0	0	0	57
23032754	f	md	HK	2023	0	0	0	0	0	0	0	0	0	0	0	0	70
23032936	m	md	DE	2023	0	1	1	0	0	0	0	0	0	0	0	1	62
23033011	m	md	CH	2023	0	0	0	0	0	0	0	0	0	0	0	0	72
23033236	m	md	ES	2023	0	0	0	0	0	0	0	0	0	0	0	0	64
23033376	f	cn	TH	2023	0	0	0	0	0	0	0	0	0	0	0	0	53
23033503	m	md	JP	2023	0	1	1	0	0	0	0	0	0	0	0	0	74
23033601	f	cn	SE	2023	0	1	1	0	0	0	0	0	0	0	0	0	68
23033800	m	md	CZ	2023	0	1	0	0	1	0	0	0	0	0	0	0	71

23033969	m	md	JP	2023	0	1	1	0	0	0	0	0	0	0	0	0	90
23034311	m	cn	ID	2023	1	0	0	0	0	0	0	0	0	0	0	0	
23034659	m	md	CA	2023	0	0	0	0	0	0	0	0	0	0	0	0	74
23034720	m	md	JP	2023	0	1	0	1	0	0	0	0	0	0	0	0	75
23034734	m	md	IT	2023	0	0	0	0	0	0	0	0	0	0	0	0	
23034857	m	cn	DE	2023	0	1	1	0	0	0	0	0	0	0	0	0	
23035132	Not	ph	JP	2023	1	0	0	0	0	0	0	0	0	0	0	0	
23035195	f	md	JP	2023	0	0	0	0	0	0	0	0	0	0	0	0	42
23035214	m	md	JP	2023	0	0	0	0	0	0	0	0	0	0	0	0	80
23035219	f	md	JP	2023	0	0	0	0	0	0	0	0	0	0	0	0	80
23035223	f	cn	PK	2023	0	0	0	0	0	0	0	0	0	0	0	0	39
23035385	f	md	JP	2023	0	0	0	0	0	0	0	0	0	0	0	0	84
23035587	f	cn	IN	2023	0	1	0	0	0	1	0	0	0	0	0	0	61
23035720	f	hp	US	2023	0	1	0	1	0	0	0	0	0	0	0	0	
23035725	m	cn	US	2023	1	0	0	0	0	0	0	0	0	0	0	0	75
23036751	m	md	DE	2023	0	0	0	0	0	0	0	0	0	0	0	0	65
23036761	m	md	DE	2023	0	1	1	0	0	0	0	0	0	0	0	0	
23037059	f	md	GB	2023	1	0	0	0	0	0	0	0	0	0	0	0	64
23037318	f	md	AT	2023	0	0	0	0	0	0	0	0	0	0	0	0	55
23037346	Not	ph	JP	2023	1	0	0	0	0	0	0	0	0	0	0	0	
23037515	m	md	JP	2023	0	0	0	0	0	0	0	0	0	0	0	0	57
23037718	Not	cn	JP	2023	0	0	0	0	0	0	0	0	0	0	0	0	
23038026	m	hp	US	2023	0	0	0	0	0	0	0	0	0	0	0	0	85
23038213	f	ph	US	2023	0	1	0	1	0	0	0	0	0	0	0	1	73
23038239	f	md	CN	2023	0	0	0	0	0	0	0	0	0	0	0	0	75
23038350	f	cn	SG	2023	1	0	0	0	0	0	0	0	0	0	0	0	73.6
23038449	f	cn	CN	2023	0	1	0	1	0	0	0	0	0	0	0	0	74
23038843	m	md	JP	2023	0	1	0	0	0	1	0	0	0	0	0	0	70
23038937	m	md	JP	2023	0	1	0	0	0	1	0	0	0	0	0	0	70
23039197	m	md	DE	2023	0	1	1	0	0	0	0	0	0	0	0	0	
23039474	m	cn	AR	2023	0	0	0	0	0	0	0	0	0	0	0	0	
23039501	m	md	RU	2023	0	0	0	0	0	0	0	0	0	0	0	0	68
23039650	f	md	JP	2023	0	1	0	1	0	0	0	0	0	0	0	0	76
23039654	Not	md	CA	2023	1	0	0	0	0	0	0	0	0	0	0	0	

23039766	m	hp	NL	2023	0	0	0	0	0	0	0	0	0	0	0	0	68
23040188	f	hp	GB	2023	1	0	0	0	0	0	0	0	0	0	0	0	60
23040372	m	md	CN	2023	0	0	0	0	0	0	0	0	0	0	0	0	68
23040377	f	md	JP	2023	0	0	0	0	0	0	0	0	0	0	0	0	70
23040574	m	md	CA	2023	1	0	0	0	0	0	0	0	0	0	0	0	76
23040641	m	md	MY	2023	0	0	0	0	0	0	0	0	0	0	0	0	66
23040658	f	cn	US	2023	0	0	0	0	0	0	0	0	0	0	0	0	75
23040659	f	cn	CA	2023	0	0	0	0	0	0	0	0	0	0	0	0	
23040760	m	cn	PK	2023	0	1	0	0	0	1	0	0	0	0	0	0	66
23040941	f	md	JP	2023	1	0	0	0	0	0	0	0	0	0	0	0	80
23041069	m	md	ES	2023	0	0	0	0	0	0	0	0	0	0	0	0	73
23041584	m	ph	JP	2023	0	0	0	0	0	0	0	0	0	0	0	0	
23041591	f	md	JP	2023	0	1	0	0	0	1	0	0	0	0	0	0	70
23041610	m	cn	PK	2023	0	1	0	0	0	1	0	0	0	0	0	0	73
23041983	f	ph	US	2023	0	0	0	0	0	0	0	0	0	0	0	0	
23042384	m	hp	US	2023	0	1	0	0	1	0	0	0	0	0	0	0	75
23043199	Not	hp	TW	2023	0	0	0	0	0	0	0	0	0	0	0	0	
23043407	m	md	JP	2023	0	1	0	0	1	0	0	0	0	0	0	0	80
23043495	m	md	JP	2023	0	1	1	0	0	0	0	0	0	0	0	0	70
23043766	m	cn	US	2023	0	0	0	0	0	0	0	0	0	0	0	0	
23043854	m	md	NL	2023	0	1	0	0	0	1	0	0	0	0	0	0	56
23043999	f	md	FR	2023	0	1	0	0	0	1	0	0	0	0	0	0	61
23044315	f	cn	US	2023	1	0	0	0	0	0	0	0	0	0	0	0	37
23044451	m	cn	ID	2023	1	0	0	0	0	0	0	0	0	0	0	0	55.3
23044483	f	hp	GB	2023	1	0	0	0	0	0	0	0	0	0	0	0	
23044554	m	cn	US	2023	0	0	0	0	0	0	0	0	0	0	0	0	
23044787	f	cn	CA	2023	0	0	0	0	0	0	0	0	0	0	0	0	70
23044912	Not	cn	US	2023	0	0	0	0	0	0	0	0	0	0	0	0	
23045013	f	cn	MY	2023	1	0	0	0	0	0	0	0	0	0	0	0	84.7
23045292	m	md	CN	2023	0	0	0	0	0	0	0	0	0	0	0	0	74
23045338	f	md	JP	2023	0	1	1	0	0	0	0	0	0	0	0	1	55
23045884	m	cn	ID	2023	1	0	0	0	0	0	0	0	0	0	0	0	69.8
23045986	m	cn	ID	2023	1	0	0	0	0	0	0	0	0	0	0	0	24.1
23045990	f	hp	US	2023	1	0	0	0	0	0	0	0	0	0	0	0	55

23046409	Not	ph	US	2023	0	0	0	0	0	0	0	0	0	0	0	0	
23046443	m	md	BG	2023	0	0	0	0	0	0	0	0	0	0	0	0	44
23046793	m	md	CN	2023	0	0	0	0	0	0	0	0	0	0	0	0	72
23047053	f	md	DE	2023	0	0	0	0	0	0	0	0	0	0	0	0	46
23047320	m	md	JP	2023	0	1	0	0	1	0	0	0	0	0	0	1	74
23047695	m	md	IN	2023	0	1	0	0	0	1	0	0	0	0	0	0	
23048250	m	hp	DE	2023	1	0	0	0	0	0	0	0	0	0	0	0	65
23048381	Not	ph	JP	2023	1	0	0	0	0	0	0	0	0	0	0	0	
23048433	m	md	JP	2023	0	0	0	0	0	0	0	0	0	0	0	0	
23048726	Not	md	JP	2023	0	0	0	0	0	0	0	0	0	0	0	0	
23048923	f	hp	US	2023	0	0	0	0	0	0	0	0	0	0	0	0	68
23048972	Not	hp	GB	2023	1	0	0	0	0	0	0	0	0	0	0	0	
23049252	m	md	GB	2023	0	1	0	0	0	1	0	0	0	0	0	0	65
23049906	m	cn	ES	2023	0	0	0	0	0	0	0	0	0	0	0	0	73
23050131	m	cn	TH	2023	1	0	0	0	0	0	0	0	0	0	0	1	70
23050211	m	md	MY	2023	0	1	1	0	0	0	0	0	0	0	0	0	
23050325	m	md	JP	2023	0	1	0	1	0	0	0	0	0	0	0	0	76
23052052	m	md	JP	2023	0	1	0	0	0	1	0	0	0	0	0	0	61
23052103	m	md	JP	2023	0	1	1	0	0	0	0	0	0	0	0	0	80
23052127	Not	ph	JP	2023	0	0	0	0	0	0	0	0	0	0	0	0	90
23052380	Not	cn	JP	2023	0	0	0	0	0	0	0	0	0	0	0	0	65
23052843	m	md	JP	2023	0	1	0	0	1	0	0	0	0	0	0	0	46
23052847	m	md	JP	2023	0	1	0	0	0	1	0	0	0	0	0	0	80
23052866	f	md	DE	2023	0	0	0	0	0	0	0	0	0	0	0	0	38
23053051	m	md	JP	2023	0	0	0	0	0	0	0	0	0	0	0	0	60
23053644	m	ph	JP	2023	0	1	1	0	0	0	0	0	0	0	0	0	58
23053650	m	md	HU	2023	1	0	0	0	0	0	0	0	0	0	0	0	81.7
23053753	Not	ph	JP	2023	1	0	0	0	0	0	0	0	0	0	0	0	
23054016	m	cn	CA	2023	1	0	0	0	0	0	0	0	0	0	0	0	
23054018	m	md	JP	2023	0	1	0	1	0	0	0	0	0	0	0	0	76
23054057	m	cn	PK	2023	0	0	0	0	0	0	0	0	0	0	0	0	84
23054411	f	md	JP	2023	0	1	1	0	0	0	0	0	0	0	0	0	80
23054728	Not	ph	US	2023	0	0	0	0	0	0	0	0	0	0	0	0	
23054801	m	md	ES	2023	0	0	0	0	0	0	0	0	0	0	0	0	73

23054868	f	md	JP	2023	1	0	0	0	0	0	0	0	0	0	0	0	74
23055008	f	cn	CA	2023	1	0	0	0	0	0	0	0	0	0	0	0	51
23055009	m	hp	TH	2023	1	0	0	0	0	0	0	0	0	0	0	0	64
23055095	m	md	JP	2023	0	1	1	0	0	0	0	0	0	0	0	0	70
23055156	m	md	JP	2023	0	1	1	0	0	0	0	0	0	0	0	0	80
23055384	f	md	JP	2023	0	1	0	0	1	0	0	0	0	0	0	1	79
23055671	m	md	PT	2023	0	1	1	0	0	0	0	0	0	0	0	0	71
23055756	m	md	ES	2023	0	0	0	0	0	0	0	0	0	0	0	0	73
23055824	m	md	HU	2023	1	0	0	0	0	0	0	0	0	0	0	0	63.8
23055931	f	md	JP	2023	1	0	0	0	0	0	0	0	0	0	0	0	75
23055988	m	md	JP	2023	1	0	0	0	0	0	0	0	0	0	0	0	81
23056182	Not	md	US	2023	0	0	0	0	0	0	0	0	0	0	0	0	
23056458	m	md	JP	2023	0	1	1	0	0	0	0	0	0	0	0	0	58
23056550	Not	hp	DE	2023	0	0	0	0	0	0	0	0	0	0	0	0	
23056563	Not	md	NL	2023	0	1	0	0	0	1	0	0	0	0	0	0	
23056697	m	md	FR	2023	0	1	0	1	0	0	0	0	0	0	0	0	71
23056739	m	md	JP	2023	0	1	0	0	1	0	0	0	0	0	0	0	80
23056923	Not	cn	US	2023	0	0	0	0	0	0	0	0	0	0	0	0	
23057633	f	md	JP	2023	0	1	0	0	1	0	0	0	0	0	0	0	85
23057754	f	md	FR	2023	0	0	0	0	0	0	0	0	0	0	0	0	68
23058445	f	md	GB	2023	0	1	0	0	0	1	0	0	0	0	0	0	74
23058533	m	md	US	2023	0	1	0	0	0	1	0	0	0	0	0	1	70
23058573	f	md	RU	2023	0	0	0	0	0	0	0	0	0	0	0	0	45
23058743	f	md	JP	2023	0	0	0	0	0	0	0	0	0	0	0	0	73
23059958	m	ph	JP	2023	0	1	0	0	1	0	0	0	0	0	0	0	
23060238	m	md	JP	2023	0	0	0	0	0	0	0	0	0	0	0	0	
23060291	m	md	IL	2023	0	0	0	0	0	0	0	0	0	0	0	0	62
23060417	m	hp	SE	2023	0	0	0	0	0	0	0	0	0	0	0	0	
23060550	m	hp	FR	2023	0	0	0	0	0	0	0	0	0	0	0	0	47
23060597	m	md	ES	2023	0	0	0	0	0	0	0	0	0	0	0	0	72
23060631	m	cn	PT	2023	0	1	1	0	0	0	0	0	0	0	0	0	71
23060639	m	cn	FR	2023	0	1	1	0	0	0	0	0	0	0	0	0	77
23060656	m	md	NL	2023	0	1	0	0	0	1	0	0	0	0	0	0	56
23060698	m	hp	US	2023	0	1	1	0	0	0	0	0	0	0	0	0	74

23061927	m	md	JP	2023	0	1	0	0	0	1	0	0	0	0	0	0	75
23061942	m	ph	FR	2023	0	1	1	0	0	0	0	0	0	0	0	0	77
23062517	m	md	ES	2023	0	0	0	0	0	0	0	0	0	0	0	0	72
23062640	m	md	ES	2023	0	1	0	0	0	1	0	0	0	0	0	0	67
23063082	f	cn	MY	2023	1	0	0	0	0	0	0	0	0	0	0	0	84.6
23063288	m	md	JP	2023	0	0	0	0	0	0	0	0	0	0	0	0	87
23063355	m	cn	US	2023	0	1	1	0	0	0	0	0	0	0	0	0	
23063574	f	lw	US	2023	0	0	0	0	0	0	0	0	0	0	0	0	56
23063662	f	cn	US	2023	0	0	0	0	0	0	0	0	0	0	0	0	
23063848	m	md	FR	2023	0	1	0	0	0	1	0	0	0	0	0	0	73
23063954	f	md	KR	2023	0	0	0	0	0	0	0	0	0	0	0	0	59
23064010	f	md	JP	2023	0	1	0	1	0	0	0	0	0	0	0	0	71
23064070	f	md	FR	2023	0	0	0	0	0	0	0	0	0	0	0	0	68
23064160	Not	md	JP	2023	0	1	0	1	0	0	0	0	0	0	0	0	
23064218	Not	cn	SG	2023	1	0	0	0	0	0	0	0	0	0	0	0	80.9
23064394	f	md	CZ	2023	0	1	1	0	0	0	0	0	0	0	0	0	66
23064562	m	ph	US	2023	0	1	1	0	0	0	0	0	0	0	0	0	83
23064823	f	cn	US	2023	1	0	0	0	0	0	0	0	0	0	0	0	59
23064863	m	ph	US	2023	0	0	0	0	0	0	0	0	0	0	0	0	65
23064911	f	cn	TH	2023	1	0	0	0	0	0	0	0	0	0	0	0	60
23064999	f	ph	FR	2023	0	0	0	0	0	0	0	0	0	0	0	0	
23065383	m	md	US	2023	0	1	1	0	0	0	0	0	0	0	0	0	58
23066015	Not	md	FR	2023	0	0	0	0	0	0	0	0	0	0	0	0	67
23066207	m	md	PT	2023	0	1	1	0	0	0	0	0	0	0	0	0	71
23066587	f	hp	US	2023	0	0	0	0	0	0	0	0	0	0	0	0	68
23066905	m	md	JP	2023	0	1	0	0	1	0	0	0	0	0	0	0	55
23067226	m	cn	FR	2023	0	0	0	0	0	0	0	0	0	0	0	0	81
23067272	f	cn	US	2023	0	0	0	0	0	0	0	0	0	0	0	0	
23067334	Not	md	CZ	2023	0	1	0	0	1	0	0	0	0	0	0	0	48
23067383	m	md	JP	2023	0	1	1	0	0	0	0	0	0	0	0	0	66
23067632	Not	md	JP	2023	0	0	0	0	0	0	0	0	0	0	0	0	
23068129	m	md	CZ	2023	0	1	0	0	1	0	0	0	0	0	0	0	71
23068174	m	md	KR	2023	0	0	0	0	0	0	0	0	0	0	0	0	80
23068262	f	md	DE	2023	0	1	0	0	1	0	0	0	0	0	0	0	70

23068713	m	md	US	2023	0	0	0	0	0	0	0	0	0	0	0	0	79
23069119	f	md	CA	2023	0	1	1	0	0	0	0	0	0	0	0	0	67
23069288	f	md	SE	2023	0	1	1	0	0	0	0	0	0	0	0	0	68
23069451	f	md	SI	2023	0	0	0	0	0	0	0	0	0	0	0	0	
23069502	Not	md	BR	2023	0	0	0	0	0	0	0	0	0	0	0	0	
23069671	m	md	JP	2023	1	0	0	0	0	0	0	0	0	0	0	1	76
23069713	f	cn	CZ	2023	0	0	0	0	0	0	0	0	0	0	0	0	69
23069726	m	md	AU	2023	0	0	0	0	0	0	0	0	0	0	0	0	48
23069879	Not	md	IT	2023	0	0	0	0	0	0	0	0	0	0	0	0	
23069937	f	md	RO	2023	0	0	0	0	0	0	0	0	0	0	0	0	40
23070016	m	md	JP	2023	0	1	1	0	0	0	0	0	0	0	0	0	80
23070261	Not	md	GB	2023	0	0	0	0	0	0	0	0	0	0	0	0	
23070322	m	md	IT	2023	0	0	0	0	0	0	0	0	0	0	0	0	
23070373	f	md	CN	2023	1	0	0	0	0	0	0	0	0	0	0	0	65
23070377	Not	md	IT	2023	0	0	0	0	0	0	0	0	0	0	0	0	
23070398	m	md	ES	2023	0	0	0	0	0	0	0	0	0	0	0	0	
23070435	f	ph	FR	2023	0	1	1	0	0	0	0	0	0	0	0	0	59
23070499	f	cn	AT	2023	0	0	0	0	0	0	0	0	0	0	0	0	80
23070528	f	md	FR	2023	0	0	0	0	0	0	0	0	0	0	0	0	60
23070530	m	md	FR	2023	0	1	0	0	0	1	0	0	0	0	0	0	73
23070906	m	md	JP	2023	0	1	1	0	0	0	0	0	0	0	0	0	74
23070988	m	md	FR	2023	0	0	0	0	0	0	0	0	0	0	0	0	81
23071109	Not	hp	JP	2023	0	1	1	0	0	0	0	0	0	0	0	1	
23071230	Not	md	JP	2023	0	1	0	0	1	0	0	0	0	0	0	0	
23071498	m	md	ES	2023	0	1	1	0	0	0	0	0	0	0	0	0	
23072256	f	md	AT	2023	0	0	0	0	0	0	0	0	0	0	0	0	55
23072318	f	md	JP	2023	0	1	0	0	0	1	0	0	0	0	0	0	50
23072335	m	ph	JP	2023	0	1	0	0	0	1	0	0	0	0	0	0	69
23072351	Not	md	JP	2023	0	1	0	0	0	1	0	0	0	0	0	0	70
23072728	m	md	JO	2023	0	0	0	0	0	0	0	0	0	0	0	0	65
23072740	m	md	JP	2023	0	1	1	0	0	0	0	0	0	0	0	1	80
23072967	f	md	BE	2023	0	0	0	0	0	0	0	0	0	0	0	0	68
23073481	f	md	CZ	2023	0	0	0	0	0	0	0	0	0	0	0	0	69
23074573	m	hp	KR	2023	0	1	1	0	0	0	0	0	0	0	0	0	69

23074877	m	cn	US	2023	1	0	0	0	0	0	0	0	0	0	0	0	
23074965	m	ph	FR	2023	0	1	1	0	0	0	0	0	0	0	0	0	77
23075508	m	cn	US	2023	1	0	0	0	0	0	0	0	0	0	0	0	
23075799	f	cn	MY	2023	1	0	0	0	0	0	0	0	0	0	0	0	
23075853	Not	hp	US	2023	0	0	0	0	0	0	0	0	0	0	0	0	
23076564	m	hp	US	2023	0	1	1	0	0	0	0	0	0	0	0	0	65
23076566	m	hp	US	2023	0	1	1	0	0	0	0	0	0	0	0	0	59
23076721	m	md	CN	2023	0	0	0	0	0	0	0	0	0	0	0	0	66
23076732	f	md	ES	2023	0	1	1	0	0	0	0	0	0	0	0	0	53
23076823	m	md	JP	2023	0	0	0	0	0	0	0	0	0	0	0	0	66
23076841	f	hp	CN	2023	0	0	0	0	0	0	0	0	0	0	0	0	45
23076940	m	md	CN	2023	0	1	1	0	0	0	0	0	0	0	0	0	70
23077087	m	hp	DE	2023	0	0	0	0	0	0	0	0	0	0	0	0	46
23078470	m	md	IE	2023	0	1	0	0	1	0	0	0	0	0	0	0	69
23078982	m	md	GR	2023	0	1	1	0	0	0	0	0	0	0	0	0	75
23079264	Not	md	JP	2023	0	1	0	1	0	0	0	0	0	0	0	0	
23079287	m	md	JP	2023	0	0	0	0	0	0	0	0	0	0	0	0	66
23079298	m	md	US	2023	0	0	0	0	0	0	0	0	0	0	0	0	81
23079299	m	md	FR	2023	0	1	0	0	0	1	0	0	0	0	0	0	63
23079306	m	cn	AR	2023	0	0	0	0	0	0	0	0	0	0	0	0	79
23079472	f	md	CZ	2023	0	1	1	0	0	0	0	0	0	0	0	0	66
23079486	f	md	DE	2023	0	1	0	0	1	0	0	0	0	0	0	1	82
23079490	f	md	CZ	2023	0	0	0	0	0	0	0	0	0	0	0	0	24
23079533	m	cn	ID	2023	1	0	0	0	0	0	0	0	0	0	0	0	66.9
23079694	m	md	JP	2023	0	1	1	0	0	0	0	0	0	0	0	0	65
23079750	m	ph	FR	2023	0	1	1	0	0	0	0	0	0	0	0	0	77
23079936	m	md	IN	2023	0	1	1	0	0	0	0	0	0	0	0	0	72
23080178	m	md	FR	2023	0	1	0	0	0	1	0	0	0	0	0	0	63
23080529	f	cn	US	2023	0	0	0	0	0	0	0	0	0	0	0	0	73
23080534	m	cn	CA	2023	0	0	0	0	0	0	0	0	0	0	0	0	76
23080561	f	cn	US	2023	1	0	0	0	0	0	0	0	0	0	0	0	
23080981	f	hp	US	2023	0	0	0	0	0	0	0	0	0	0	0	0	68
23081102	m	cn	US	2023	0	0	0	0	0	0	0	0	0	0	0	0	73
23081132	m	md	JP	2023	0	1	1	0	0	0	0	0	0	0	0	0	

23081170	m	md	US	2023	0	0	0	0	0	0	0	0	0	0	0	0	
23081392	m	md	GB	2023	0	1	0	0	0	1	0	0	0	0	0	0	78
23081408	Not	md	AT	2023	0	0	0	0	0	0	0	0	0	0	0	0	
23081813	m	ph	FR	2023	0	1	1	0	0	0	0	0	0	0	0	0	77
23081924	m	md	CN	2023	0	1	1	0	0	0	0	0	0	0	0	0	70
23081957	m	md	US	2023	0	0	0	0	0	0	0	0	0	0	0	0	81
23082473	m	cn	CN	2023	0	0	0	0	0	0	0	0	0	0	0	0	67
23082607	f	cn	CN	2023	0	1	0	1	0	0	0	0	0	0	0	0	53
23082783	Not	md	FR	2023	0	0	0	0	0	0	0	0	0	0	0	0	83
23082886	f	ph	US	2023	0	0	0	0	0	0	0	0	0	0	0	0	60
23083023	f	md	GB	2023	0	1	0	0	0	1	0	0	0	0	0	0	62
23083746	Not	md	JP	2023	0	1	0	1	0	0	0	0	0	0	0	0	
23083777	f	md	ES	2023	0	1	1	0	0	0	0	0	0	0	0	0	53
23083823	m	md	AU	2023	0	1	0	1	0	0	0	0	0	0	0	0	
23084479	m	md	SK	2023	0	0	0	0	0	0	0	0	0	0	0	0	80
23084688	Not	md	ES	2023	0	1	0	0	1	0	0	0	0	0	0	0	
23084744	f	md	CA	2023	1	0	0	0	0	0	0	0	0	0	0	0	
23084782	f	md	RU	2023	0	0	0	0	0	0	0	0	0	0	0	0	49
23084805	m	ph	TH	2023	0	0	0	0	0	0	0	0	0	0	0	0	78
23084807	f	cn	TH	2023	0	0	0	0	0	0	0	0	0	0	0	0	73
23084810	f	cn	IN	2023	0	0	0	0	0	0	0	0	0	0	0	0	68
23084834	m	cn	ES	2023	0	0	0	0	0	0	0	0	0	0	0	0	73
23084838	f	cn	AR	2023	0	0	0	0	0	0	0	0	0	0	0	0	77
23084855	f	cn	TH	2023	0	0	0	0	0	0	0	0	0	0	0	0	81
23085123	m	ph	FR	2023	0	1	1	0	0	0	0	0	0	0	0	0	77
23085479	f	md	GB	2023	0	1	0	0	0	1	0	0	0	0	0	0	79
23085693	m	md	ES	2023	0	1	1	0	0	0	0	0	0	0	0	0	
23086215	m	ph	JP	2023	0	0	0	0	0	0	0	0	0	0	0	0	59
23086884	f	cn	CA	2023	1	0	0	0	0	0	0	0	0	0	0	0	51.3
23087115	Not	md	JP	2023	0	1	0	0	0	1	0	0	0	0	0	0	70
23087412	m	md	IN	2023	0	1	1	0	0	0	0	0	0	0	0	0	55
23087682	f	md	JP	2023	0	1	0	0	0	1	0	0	0	0	0	0	40
23087746	f	md	US	2023	0	0	0	0	0	0	0	0	0	0	0	0	68
23088214	m	hp	US	2023	0	1	1	0	0	0	0	0	0	0	0	0	

23088431	m	md	FR	2023	0	0	0	0	0	0	0	0	0	0	0	0	81
23089031	m	cn	IN	2023	0	0	0	0	0	0	0	0	0	0	0	0	63
23089088	m	cn	EG	2023	1	0	0	0	0	0	0	0	0	0	0	0	
23089373	f	hp	US	2023	0	0	0	0	0	0	0	0	0	0	0	0	68
23089596	Not	md	JP	2023	1	0	0	0	0	0	0	0	0	0	0	0	
23089599	m	md	CZ	2023	0	0	0	0	0	0	0	0	0	0	0	0	55
23089704	m	md	JP	2023	0	0	0	0	0	0	0	0	0	0	0	0	
23089711	m	md	JP	2023	0	1	0	1	0	0	0	0	0	0	0	0	77
23089732	f	md	BE	2023	0	0	0	0	0	0	0	0	0	0	0	0	56
23089819	m	md	FR	2023	0	1	0	0	0	1	0	0	0	0	0	0	63
23089828	f	cn	TH	2023	1	0	0	0	0	0	0	0	0	0	0	0	84.1
23090364	m	md	JP	2023	0	1	0	1	0	0	0	0	0	0	0	0	64
23090587	f	md	GB	2023	0	0	0	0	0	0	0	0	0	0	0	0	
23090936	m	md	JP	2023	0	0	0	0	0	0	0	0	0	0	0	0	75
23091216	Not	md	KR	2023	0	1	0	0	1	0	0	0	0	0	0	0	
23091226	Not	md	JP	2023	0	1	0	1	0	0	0	0	0	0	0	0	
23091315	Not	md	JP	2023	0	1	0	1	0	0	0	0	0	0	0	1	
23091318	m	md	JP	2023	0	1	0	1	0	0	0	0	0	0	0	0	55
23091321	f	cn	JP	2023	1	0	0	0	0	0	0	0	0	0	0	0	
23091453	f	md	US	2023	0	1	1	0	0	0	0	0	0	0	0	0	56
23091649	Not	md	JP	2023	0	1	0	1	0	0	0	0	0	0	0	1	
23091650	Not	md	JP	2023	0	1	0	1	0	0	0	0	0	0	0	0	
23091651	Not	md	JP	2023	0	1	0	1	0	0	0	0	0	0	0	0	
23091652	Not	md	JP	2023	0	1	0	1	0	0	0	0	0	0	0	1	
23091721	f	md	NL	2023	0	0	0	0	0	0	0	0	0	0	0	0	77
23092064	Not	ph	US	2023	0	0	0	0	0	0	0	0	0	0	0	0	
23092543	m	md	VN	2023	0	1	0	0	0	1	0	0	0	0	0	0	
23092616	f	md	CA	2023	0	0	0	0	0	0	0	0	0	0	0	0	
23092804	f	md	GB	2023	0	1	0	0	0	1	0	0	0	0	0	0	79
23092894	m	md	IT	2023	0	0	0	0	0	0	0	0	0	0	0	0	52
23092968	m	md	NL	2023	0	0	0	0	0	0	0	0	0	0	0	0	68
23094817	m	ph	JP	2023	0	1	0	0	1	0	0	0	0	0	0	0	70
23095026	f	md	US	2023	0	0	0	0	0	0	0	0	0	0	0	0	68
23096584	f	cn	US	2023	0	1	1	0	0	0	0	0	0	0	0	0	

23096711	Not	md	CZ	2023	0	1	0	0	1	0	0	0	0	0	0	0	48
23096919	Not	cn	PL	2023	0	1	0	0	1	0	0	0	0	0	0	0	
23097048	m	md	FR	2023	0	0	0	0	0	0	0	0	0	0	0	0	81
23097145	m	md	ES	2023	0	0	0	0	0	0	0	0	0	0	0	0	73
23097259	m	md	KR	2023	0	1	1	0	0	0	0	0	0	0	0	0	64
23097315	f	md	US	2023	0	0	0	0	0	0	0	0	0	0	0	0	68
23097564	f	md	MY	2023	1	0	0	0	0	0	0	0	0	0	0	0	
23097698	m	md	US	2023	0	0	0	0	0	0	0	0	0	0	0	0	81
23097840	f	md	JP	2023	0	1	1	0	0	0	0	0	0	0	0	0	60
23097948	m	hp	US	2023	1	0	0	0	0	0	0	0	0	0	0	0	48
23098056	f	md	JP	2023	0	1	0	0	0	1	0	0	0	0	0	0	80
23098196	m	cn	ID	2023	1	0	0	0	0	0	0	0	0	0	0	0	56.5
23098406	m	hp	FR	2023	0	1	0	0	1	0	0	0	0	0	0	0	63
23098441	f	ph	JP	2023	0	0	0	0	0	0	0	0	0	0	0	0	74
23098930	m	ph	JP	2023	0	1	1	0	0	0	0	0	0	0	0	0	58
23098933	f	md	AT	2023	0	1	1	0	0	0	0	0	0	0	0	0	72
23099289	m	hp	US	2023	0	0	0	0	0	0	0	0	0	0	0	0	68
23099605	m	md	JP	2023	0	1	0	1	0	0	0	0	0	0	0	0	70
23099763	f	md	JP	2023	0	1	0	1	0	0	0	0	0	0	0	0	73
23100094	m	md	JP	2023	0	1	1	0	0	0	0	0	0	0	0	0	50
23100102	m	cn	DE	2023	1	0	0	0	0	0	0	0	0	0	0	0	64
23100157	m	md	JP	2023	0	1	0	0	1	0	0	0	0	0	0	0	85
23100178	m	hp	JP	2023	0	1	0	0	0	1	0	0	0	0	0	0	49
23100265	m	ph	JP	2023	0	1	1	0	0	0	0	0	0	0	0	0	80
23100528	m	cn	CA	2023	1	0	0	0	0	0	0	0	0	0	0	0	
23100687	m	md	FR	2023	0	0	0	0	0	0	0	0	0	0	0	0	81
23100746	m	md	JP	2023	0	0	0	0	0	0	0	0	0	0	0	0	86
23100829	m	md	ES	2023	0	1	0	0	0	1	0	0	0	0	0	0	76
23100865	f	cn	US	2023	0	0	0	0	0	0	0	0	0	0	0	0	77
23100881	f	md	FR	2023	0	0	0	0	0	0	0	0	0	0	0	0	57
23101084	f	hp	US	2023	0	0	0	0	0	0	0	0	0	0	0	0	
23101085	f	hp	US	2023	0	0	0	0	0	0	0	0	0	0	0	0	
23101091	m	md	US	2023	0	0	0	0	0	0	0	0	0	0	0	0	
23101124	Not	md	US	2023	0	0	0	0	0	0	0	0	0	0	0	0	

23101130	Not	ph	US	2023	0	0	0	0	0	0	0	0	0	0	0	0	
23101137	m	md	JP	2023	0	0	0	0	0	0	0	0	0	0	0	0	47
23101139	m	md	DE	2023	0	1	1	0	0	0	0	0	0	0	0	0	61
23101142	m	md	DE	2023	0	1	1	0	0	0	0	0	0	0	0	0	
23101205	m	md	JP	2023	0	1	1	0	0	0	0	0	0	0	0	0	70
23101227	Not	cn	JP	2023	0	0	0	0	0	0	0	0	0	0	0	0	
23101327	m	md	CN	2023	0	0	0	0	0	0	0	0	0	0	0	0	53
23101564	m	md	CN	2023	0	0	0	0	0	0	0	0	0	0	0	0	79
23101624	m	md	DE	2023	0	1	1	0	0	0	0	0	0	0	0	0	
23102234	m	md	JP	2023	0	0	0	0	0	0	0	0	0	0	0	0	60
23102252	m	cn	CN	2023	0	1	0	1	0	0	0	0	0	0	0	0	67
23102303	m	cn	KR	2023	0	0	0	0	0	0	0	0	0	0	0	0	64
23102454	f	hp	IL	2023	0	0	0	0	0	0	0	0	0	0	0	0	
23102574	Not	ph	JP	2023	0	1	0	1	0	0	0	0	0	0	0	0	
23102578	m	cn	IN	2023	1	0	0	0	0	0	0	0	0	0	0	0	74.8
23103157	m	md	JP	2023	0	1	1	0	0	0	0	0	0	0	0	0	50
23103493	f	ph	US	2023	0	0	0	0	0	0	0	0	0	0	0	0	64
23103657	m	cn	IN	2023	0	0	0	0	0	0	0	0	0	0	0	0	
23103926	Not	ph	JP	2023	1	0	0	0	0	0	0	0	0	0	0	0	
23103963	f	hp	GB	2023	0	1	0	0	0	1	0	0	0	0	0	0	79
23104129	m	md	JP	2023	0	1	0	1	0	0	0	0	0	0	0	0	73
23104254	Not	md	JP	2023	0	1	0	0	1	0	0	0	0	0	0	0	
23104624	m	md	AU	2023	0	0	0	0	0	0	0	0	0	0	0	0	70
23104814	f	md	JP	2023	0	1	0	0	0	1	0	0	0	0	0	0	72
23104861	Not	md	JP	2023	0	1	0	1	0	0	0	0	0	0	0	0	
23104980	Not	md	JP	2023	0	0	0	0	0	0	0	0	0	0	0	0	
23105009	m	cn	DE	2023	0	1	1	0	0	0	0	0	0	0	0	0	70
23105029	f	cn	DE	2023	0	0	0	0	0	0	0	0	0	0	0	0	
23105154	m	md	JP	2023	0	1	0	1	0	0	0	0	0	0	0	0	73
23105162	f	cn	US	2023	0	0	0	0	0	0	0	0	0	0	0	0	
23105235	f	md	US	2023	0	1	1	0	0	0	0	0	0	0	0	0	72
23105337	f	md	US	2023	0	0	0	0	0	0	0	0	0	0	0	0	
23105818	m	md	JP	2023	0	1	1	0	0	0	0	0	0	0	0	0	68
23106081	f	ph	JP	2023	0	1	0	0	1	0	0	0	0	0	0	0	71

23106532	Not	ph	JP	2023	0	1	0	1	0	0	0	0	0	0	0	0	
23106924	f	md	JP	2023	0	0	0	0	0	0	0	0	0	0	0	0	52
23107215	m	cn	MY	2023	1	0	0	0	0	0	0	0	0	0	0	0	87.6
23107355	f	md	JP	2023	0	1	0	0	0	1	0	0	0	0	0	0	60
23107480	m	hp	US	2023	0	0	0	0	0	0	0	0	0	0	0	0	
23107573	f	cn	KR	2023	0	0	0	0	0	0	0	0	0	0	0	0	60
23107776	m	md	JP	2023	0	1	0	0	0	1	0	0	0	0	0	0	69
23107794	m	md	JP	2023	0	1	1	0	0	0	0	0	0	0	0	0	72
23108182	f	md	RU	2023	0	1	0	0	0	1	0	0	0	0	0	0	70
23108296	m	md	JP	2023	0	1	0	0	0	1	0	0	0	0	0	0	80
23108424	f	md	PT	2023	0	1	0	1	0	0	0	0	0	0	0	0	58
23108429	f	md	PT	2023	0	0	0	0	0	0	0	0	0	0	0	0	58
23109296	f	ph	JP	2023	0	1	1	0	0	0	0	0	0	0	0	0	74
23109688	m	md	SK	2023	0	0	0	0	0	0	0	0	0	0	0	0	67
23109711	m	md	DE	2023	0	1	1	0	0	0	0	0	0	0	0	0	70
23109733	m	md	JP	2023	0	1	1	0	0	0	0	0	0	0	0	1	87
23109773	m	md	JP	2023	0	1	0	0	1	0	0	0	0	0	0	0	70
23110165	m	cn	DE	2023	0	1	0	0	1	0	0	0	0	0	0	0	66
23110367	Not	md	US	2023	0	0	0	0	0	0	0	0	0	0	0	0	
23110745	m	hp	RU	2023	0	0	0	0	0	0	1	0	0	0	1	0	70
23111003	f	md	JP	2023	0	1	0	0	1	0	0	0	0	0	0	0	50
23111372	f	md	JP	2023	0	1	0	0	1	0	0	0	0	0	0	0	77
23111414	m	md	JP	2023	0	1	1	0	0	0	0	0	0	0	0	0	65
23111487	m	md	AU	2023	1	0	0	0	0	0	0	0	0	0	0	0	55.8
23111579	Not	md	JP	2023	0	0	0	0	0	0	0	0	0	0	0	0	
23111599	f	md	IL	2023	0	0	0	0	0	0	0	0	0	0	0	0	77
23111864	m	hp	CN	2023	0	0	0	0	0	0	0	0	0	0	0	0	62
23111927	m	md	JP	2023	0	0	0	0	0	0	0	0	0	0	0	0	68
23112164	m	md	JP	2023	0	0	0	0	0	0	0	0	0	0	0	0	50
23112332	f	md	JP	2023	0	0	0	0	0	0	0	0	0	0	0	0	60
23112364	m	md	ES	2023	0	1	0	0	0	1	0	0	0	0	0	0	75
23112908	f	hp	US	2023	0	1	0	0	1	0	0	0	0	0	0	0	58
23113008	Not	md	FR	2023	0	1	0	0	0	1	0	0	0	0	0	0	
23113188	f	cn	US	2023	0	0	0	0	0	0	0	0	0	0	0	0	

23113288	f	hp	US	2023	1	0	0	0	0	0	0	0	0	0	0	0	78
23113401	f	md	US	2023	1	0	0	0	0	0	0	0	0	0	0	0	
23113434	Not	md	JP	2023	0	1	0	0	0	1	0	0	0	0	0	0	62
23113508	f	cn	US	2023	0	0	0	0	0	0	0	0	0	0	0	0	70
23113850	m	md	TH	2023	0	0	0	0	0	0	1	0	0	1	0	0	35
23113864	m	md	FR	2023	0	0	0	0	0	0	0	0	0	0	0	0	81
23113987	m	cn	CA	2023	1	0	0	0	0	0	0	0	0	0	0	0	
23114338	f	cn	MY	2023	0	1	0	1	0	0	0	0	0	0	0	0	60.7
23114362	f	md	GB	2023	0	1	0	0	0	1	0	0	0	0	0	0	79
23114837	m	md	JP	2023	0	1	1	0	0	0	0	0	0	0	0	0	70
23114841	f	cn	US	2023	0	0	0	0	0	0	0	0	0	0	0	0	72
23114844	m	md	JP	2023	0	1	0	0	0	1	0	0	0	0	0	0	68
23114976	f	hp	US	2023	0	0	0	0	0	0	0	0	0	0	0	0	68
23115161	m	ph	FR	2023	0	1	1	0	0	0	0	0	0	0	0	0	77
23115295	m	cn	CN	2023	0	0	0	0	0	0	0	0	0	0	0	0	65
23115302	f	md	FR	2023	0	0	0	0	0	0	0	0	0	0	0	0	74
23115395	f	md	PL	2023	0	1	0	0	0	1	0	0	0	0	0	0	58
23115402	m	md	JP	2023	0	1	0	0	1	0	0	0	0	0	0	0	58
23116186	m	md	US	2023	0	0	0	0	0	0	0	0	0	0	0	0	67
23116197	f	md	CN	2023	0	0	0	0	0	0	0	0	0	0	0	0	65
23116366	f	cn	US	2023	0	0	0	0	0	0	0	0	0	0	0	0	75
23116598	m	hp	CA	2023	0	0	0	0	0	0	0	0	0	0	0	0	
23116678	m	md	RO	2023	1	0	0	0	0	0	0	0	0	0	0	0	68
23116790	m	cn	TR	2023	0	0	0	0	0	0	0	0	0	0	0	0	55
23117032	f	md	CN	2023	0	1	0	1	0	0	0	0	0	0	0	0	88
23117389	m	hp	JP	2023	0	1	0	1	0	0	0	0	0	0	0	0	
23118843	f	md	SK	2023	0	0	0	0	0	0	0	0	0	0	0	0	55
23118957	f	md	FR	2023	0	0	0	0	0	0	0	0	0	0	0	0	68
23118974	f	md	JP	2023	0	1	0	0	0	1	0	0	0	0	0	0	70
23119340	m	md	JP	2023	0	0	0	0	0	0	0	0	0	0	0	0	
23119342	f	hp	JP	2023	0	1	0	0	0	1	0	0	0	0	0	0	48
23119343	f	hp	JP	2023	0	1	0	0	0	1	0	0	0	0	0	0	40
23119344	m	hp	JP	2023	0	1	0	0	1	0	0	0	0	0	0	0	46
23119384	f	md	IT	2023	0	0	0	0	0	0	0	0	0	0	0	0	30

23119404	m	hp	JP	2023	0	1	0	0	0	1	0	0	0	0	0	0	44
23119405	m	hp	JP	2023	0	1	1	0	0	0	0	0	0	0	0	0	66
23119409	m	hp	JP	2023	0	1	0	0	0	1	0	0	0	0	0	0	64
23119410	m	hp	JP	2023	0	1	0	0	0	1	0	0	0	0	0	0	63
23119411	m	hp	JP	2023	0	1	0	0	0	1	0	0	0	0	0	0	66
23119412	f	md	IT	2023	0	0	0	0	0	0	0	0	0	0	0	0	60
23119419	f	cn	CA	2023	0	0	0	0	0	0	0	0	0	0	0	0	
23119424	m	cn	KR	2023	0	0	0	0	0	0	0	0	0	0	0	0	67
23121633	Not	md	JP	2023	0	0	0	0	0	0	0	0	0	0	0	0	
23121673	f	md	DE	2023	0	1	1	0	0	0	0	0	0	0	0	0	
23121718	m	md	RU	2023	0	1	0	0	0	1	0	0	0	0	0	0	72
23122028	f	md	DE	2023	0	1	1	0	0	0	0	0	0	0	0	0	
23122086	m	md	JP	2023	0	1	0	0	1	0	0	0	0	0	0	0	73
23122094	m	md	JP	2023	0	1	0	0	1	0	0	0	0	0	0	0	60
23122537	m	md	JP	2023	0	1	0	0	0	1	0	0	0	0	0	0	80
23122669	f	hp	JP	2023	0	0	0	0	0	0	0	0	0	0	0	0	58
23122971	m	md	JP	2023	0	1	0	0	0	1	0	0	0	0	0	0	49
23123808	f	cn	CN	2023	0	0	0	0	0	0	0	0	0	0	0	0	
23124149	Not	hp	FR	2023	0	0	0	0	0	0	0	0	0	0	0	0	49
23124552	f	hp	TH	2023	1	0	0	0	0	0	0	0	0	0	0	0	60
23124584	m	md	CN	2023	0	0	0	0	0	0	0	0	0	0	0	0	50
23126460	m	md	VN	2023	0	0	0	0	0	0	0	0	0	0	0	0	
23126461	m	cn	IL	2023	0	0	0	0	0	0	0	0	0	0	0	0	69
23126462	m	md	ES	2023	0	1	0	0	0	1	0	0	0	0	0	0	62
23126510	Not	md	US	2023	0	0	0	0	0	0	0	0	0	0	0	0	
23126513	f	cn	TH	2023	0	0	0	0	0	0	0	0	0	0	0	0	67
23126516	f	md	RU	2023	0	0	0	0	0	0	0	0	0	0	0	0	52
23126551	f	cn	EG	2023	0	0	0	0	0	0	0	0	0	0	0	0	37
23126605	Not	md	CZ	2023	0	1	0	0	1	0	0	0	0	0	0	0	48
23126985	f	md	ES	2023	0	1	1	0	0	0	0	0	0	0	0	0	60
23126990	f	cn	PK	2023	0	1	0	0	0	1	0	0	0	0	0	0	36
23127011	f	md	JP	2023	0	0	0	0	0	0	0	0	0	0	0	0	
23127190	m	md	FR	2023	0	1	1	0	0	0	0	0	0	0	0	0	66
23127748	Not	cn	US	2023	0	0	0	0	0	0	0	0	0	0	0	0	

23127876	Not	hp	KR	2023	0	1	0	0	0	1	0	0	0	0	0	1	
23127923	f	cn	US	2023	0	1	1	0	0	0	0	0	0	0	0	0	
23127942	f	cn	US	2023	1	0	0	0	0	0	0	0	0	0	0	0	70.7
23128232	f	cn	US	2023	0	0	0	0	0	0	0	0	0	0	0	0	
23128659	m	md	CA	2023	0	1	1	0	0	0	0	0	0	0	0	0	76
23128799	Not	ph	JP	2023	1	0	0	0	0	0	0	0	0	0	0	0	
23129309	m	md	BG	2023	0	0	0	0	0	0	0	0	0	0	0	0	62
23129744	f	md	SI	2023	0	1	1	0	0	0	0	0	0	0	0	0	66
23129894	m	md	KR	2023	0	1	1	0	0	0	0	0	0	0	0	0	72
23130033	m	md	JP	2023	0	1	0	1	0	0	0	0	0	0	0	0	65
23130136	m	md	DE	2023	0	1	1	0	0	0	0	0	0	0	0	0	70
23130142	m	md	JP	2023	0	1	0	1	0	0	0	0	0	0	0	0	71
23130255	m	md	DE	2023	0	1	1	0	0	0	0	0	0	0	0	0	70
23130358	Not	md	CZ	2023	0	1	0	0	1	0	0	0	0	0	0	0	48
23130536	m	hp	CZ	2023	1	0	0	0	0	0	0	0	0	0	0	0	61
23130572	f	md	KR	2023	0	0	0	0	0	0	0	0	0	0	0	0	62
23130645	m	ph	SI	2023	0	1	1	0	0	0	0	0	0	0	0	0	65
23130747	f	md	JP	2023	1	0	0	0	0	0	0	0	0	0	0	0	75
23130827	Not	md	US	2023	1	0	0	0	0	0	0	0	0	0	0	0	25
23131075	m	md	JP	2023	0	1	0	0	1	0	0	0	0	0	0	0	60
23131459	f	md	DE	2023	0	1	0	1	0	0	0	0	0	0	0	1	72
23131630	f	md	RO	2023	0	1	0	1	0	0	0	0	0	0	0	0	49
23131850	Not	ph	JP	2023	1	0	0	0	0	0	0	0	0	0	0	0	
23131863	m	ph	JP	2023	0	0	0	0	0	0	0	0	0	0	0	0	50
23132082	m	md	JP	2023	0	1	1	0	0	0	0	0	0	0	0	0	80
23132090	m	md	DE	2023	0	1	1	0	0	0	0	0	0	0	0	0	70
23132788	f	md	DE	2023	1	0	0	0	0	0	0	0	0	0	0	0	
23132792	m	md	DE	2023	0	1	0	0	1	0	0	0	0	0	0	0	70
23133929	m	md	DE	2023	0	1	1	0	0	0	0	0	0	0	0	1	67
23133981	f	ph	JP	2023	1	0	0	0	0	0	0	0	0	0	0	0	80
23134017	m	md	JP	2023	0	1	0	0	1	0	0	0	0	0	0	0	58
23134408	Not	ph	JP	2023	1	0	0	0	0	0	0	0	0	0	0	0	
23134450	f	md	RU	2023	0	0	0	0	0	0	0	0	0	0	0	0	
23134793	Not	cn	US	2023	1	0	0	0	0	0	0	0	0	0	0	0	

23134840	f	md	US	2023	0	0	0	0	0	0	0	0	0	0	0	0	45
23135283	m	md	JP	2023	0	1	0	1	0	0	0	0	0	0	0	0	
23135286	m	md	RO	2023	0	1	0	1	0	0	0	0	0	0	0	1	60
23135372	f	md	JP	2023	0	1	1	0	0	0	0	0	0	0	0	0	80
23135428	f	md	JP	2023	0	1	1	0	0	0	0	0	0	0	0	0	77
23135447	f	hp	US	2023	0	0	0	0	0	0	0	0	0	0	0	0	
23135566	f	md	IN	2023	0	0	0	0	0	0	0	0	0	0	0	0	70
23136268	m	ph	EG	2023	0	0	0	0	0	0	0	0	0	0	0	0	70
23137515	m	ph	JP	2023	0	1	0	0	0	1	0	0	0	0	0	0	67
23138260	m	md	JP	2023	0	1	1	0	0	0	0	0	0	0	0	0	82
23138913	m	cn	KR	2023	0	0	0	0	0	0	0	0	0	0	0	0	48
23138992	f	md	CN	2023	0	0	0	0	0	0	0	0	0	0	0	0	49
23139086	f	hp	ZA	2023	0	0	0	0	0	0	0	0	0	0	0	0	
23139124	m	ph	ES	2023	0	0	0	0	0	0	0	0	0	0	0	0	72
23139138	f	cn	CA	2023	0	0	0	0	0	0	0	0	0	0	0	0	
23139166	m	cn	US	2023	0	1	1	0	0	0	0	0	0	0	0	0	76
23139255	f	md	BR	2023	0	0	0	0	0	0	0	0	0	0	0	0	29
23139330	f	hp	PL	2023	0	1	1	0	0	0	0	0	0	0	0	0	59
23139376	m	hp	FR	2023	0	0	0	0	0	0	0	0	0	0	0	0	71
23139435	f	ph	FR	2023	0	0	0	0	0	0	0	0	0	0	0	0	54
23139951	m	ph	FR	2023	0	0	0	0	0	0	0	0	0	0	0	0	55
23140349	m	md	DE	2023	0	1	0	0	1	0	0	0	0	0	0	0	54
23140733	f	md	US	2023	0	0	0	0	0	0	0	0	0	0	0	0	68
23140741	m	md	JP	2023	0	1	0	1	0	0	0	0	0	0	0	0	64
23141067	m	cn	IN	2023	0	1	0	0	0	1	0	0	0	0	0	0	58
23141119	m	cn	CN	2023	0	1	0	0	0	1	0	0	0	0	0	0	46
23141401	Not	ph	FR	2023	0	0	0	0	0	0	0	0	0	0	0	0	
23141589	m	md	FR	2023	0	1	0	0	0	1	0	0	0	0	0	0	52
23141660	f	md	KR	2023	0	0	0	0	0	0	0	0	0	0	0	0	
23141716	m	hp	FR	2023	0	0	0	0	0	0	0	0	0	0	0	0	55
23141731	m	md	JP	2023	0	1	1	0	0	0	0	0	0	0	0	0	69
23141999	f	cn	US	2023	1	0	0	0	0	0	0	0	0	0	0	0	72.9
23142001	Not	md	JP	2023	1	0	0	0	0	0	0	0	0	0	0	0	
23142179	f	hp	BR	2023	0	1	1	0	0	0	0	0	0	0	0	0	46

23142512	f	cn	US	2023	1	0	0	0	0	0	0	0	0	0	0	0	
23142519	f	cn	CA	2023	1	0	0	0	0	0	0	0	0	0	0	0	
23142742	f	cn	IN	2023	1	0	0	0	0	0	0	0	0	0	0	0	
23142754	m	md	TH	2023	0	1	1	0	0	0	0	0	0	0	0	0	74
23143703	f	cn	CN	2023	0	0	0	0	0	0	0	0	0	0	0	0	74
23144008	f	ph	US	2023	0	1	1	0	0	0	0	0	0	0	0	0	42
23144083	Not	md	JP	2023	0	0	0	0	0	0	0	0	0	0	0	0	
23144314	Not	hp	IT	2023	0	0	0	0	0	0	0	0	0	0	0	0	
23144327	Not	hp	TW	2023	0	0	0	0	0	0	0	0	0	0	0	0	
23144433	m	md	IN	2023	0	1	0	1	0	0	0	0	0	0	0	0	75
23144464	f	cn	MY	2023	0	1	0	1	0	0	0	0	0	0	0	0	65.7
23144903	m	md	CA	2023	0	1	1	0	0	0	0	0	0	0	0	0	76
23144927	f	cn	CA	2023	1	0	0	0	0	0	0	0	0	0	0	0	57.3
23144940	m	md	CN	2023	0	1	0	1	0	0	0	0	0	0	0	1	63.7
23145034	Not	hp	AU	2023	0	1	0	0	1	0	0	0	0	0	0	0	
23145043	m	md	JP	2023	0	1	0	1	0	0	0	0	0	0	0	0	62
23145133	m	md	JP	2023	0	0	0	0	0	0	0	0	0	0	0	0	75
23145159	m	md	GR	2023	0	1	1	0	0	0	0	0	0	0	0	0	64
23145181	m	md	JP	2023	0	1	1	0	0	0	0	0	0	0	0	0	70
23145276	f	cn	MY	2023	0	1	0	1	0	0	0	0	0	0	0	0	72.4
23145412	m	hp	CA	2023	0	0	0	0	0	0	0	0	0	0	0	0	67
23145461	Not	md	JP	2023	0	1	0	1	0	0	0	0	0	0	0	0	70
23145636	f	md	NL	2023	0	0	0	0	0	0	0	0	0	0	0	0	70
23145638	m	cn	DE	2023	0	1	1	0	0	0	0	0	0	0	0	0	61
23145639	m	cn	CZ	2023	0	1	1	0	0	0	0	0	0	0	0	0	79
23145701	m	md	CN	2023	0	0	0	0	0	0	0	0	0	0	0	0	59
23145767	m	md	CN	2023	0	0	0	0	0	0	0	0	0	0	0	0	79
23145821	m	md	JP	2023	0	0	0	0	0	0	0	0	0	0	0	0	
23145833	Not	md	ES	2023	0	1	0	0	0	1	0	0	0	0	0	0	
23146036	f	cn	US	2023	0	0	0	0	0	0	0	0	0	0	0	0	
23146110	f	md	PL	2023	0	1	1	0	0	0	0	0	0	0	0	0	59
23146133	f	md	AU	2023	0	1	0	0	0	1	0	0	0	0	0	0	37
23146226	m	hp	US	2023	0	1	1	0	0	0	0	0	0	0	0	0	65
23146258	m	ph	FR	2023	0	0	0	0	0	0	0	0	0	0	0	0	55

23146324	m	md	ES	2023	0	1	0	0	0	1	0	0	0	0	0	0	68
23146473	m	hp	JP	2023	0	1	0	0	0	1	0	0	0	0	0	0	71
23146510	f	hp	DK	2023	0	0	0	0	0	0	0	0	0	0	0	0	39
23146564	f	hp	IT	2023	0	1	1	0	0	0	0	0	0	0	0	0	66
23146587	f	hp	DE	2023	0	1	1	0	0	0	0	0	0	0	0	0	61
23146604	f	hp	DE	2023	0	1	1	0	0	0	0	0	0	0	0	0	50
23146694	f	md	DE	2023	0	0	0	0	0	0	0	0	0	0	0	0	
23146927	f	md	JP	2023	0	1	0	0	0	1	0	0	0	0	0	0	60
23147147	m	md	DE	2023	0	0	0	0	0	0	0	0	0	0	0	0	
23147154	m	md	JP	2023	0	1	1	0	0	0	0	0	0	0	0	0	70
23147335	f	ph	JP	2023	0	0	0	0	0	0	0	0	0	0	0	0	80
23147343	m	md	JP	2023	0	0	0	0	0	0	0	0	0	0	0	0	70
23147576	f	md	JP	2023	0	1	0	0	1	0	0	0	0	0	0	0	51
23147702	f	cn	RO	2023	0	0	0	0	0	0	0	0	0	0	0	0	70
23147955	Not	md	TR	2023	0	1	0	0	1	0	0	0	0	0	0	0	
23148012	m	md	JP	2023	0	0	0	0	0	0	0	0	0	0	0	0	82
23148068	m	md	JP	2023	1	0	0	0	0	0	0	0	0	0	0	0	80
23148073	m	ph	FR	2023	0	1	1	0	0	0	0	0	0	0	0	0	77
23148175	f	hp	GB	2023	1	0	0	0	0	0	0	0	0	0	0	0	67
23148254	m	cn	ID	2023	1	0	0	0	0	0	0	0	0	0	0	0	75.3
23148292	f	hp	CZ	2023	0	0	0	0	0	0	0	0	0	0	0	0	57
23148341	f	cn	ID	2023	1	0	0	0	0	0	0	0	0	0	0	0	65.1
23148551	f	md	CZ	2023	0	1	0	0	1	0	0	0	0	0	0	0	69
23148668	m	md	US	2023	0	1	0	1	0	0	0	0	0	0	0	0	
23148671	f	md	US	2023	0	1	1	0	0	0	0	0	0	0	0	0	60
23148833	m	md	CA	2023	1	0	0	0	0	0	0	0	0	0	0	0	
23148995	Not	hp	DE	2023	0	1	0	0	1	0	0	0	0	0	0	0	
23149267	f	md	JP	2023	1	0	0	0	0	0	0	0	0	0	0	0	66
23149377	f	cn	ID	2023	1	0	0	0	0	0	0	0	0	0	0	0	89.1
23149508	Not	hp	CN	2023	0	0	0	0	0	0	0	0	0	0	0	0	
23149602	f	md	VN	2023	0	0	0	0	0	0	0	0	0	0	0	0	
23149684	m	ph	GR	2023	0	0	0	0	0	0	0	0	0	0	0	0	65
23150141	m	md	JP	2023	0	1	0	0	1	0	0	0	0	0	0	0	50
23150276	Not	md	JP	2023	0	0	0	0	0	0	0	0	0	0	0	0	

23150289	f	md	CZ	2023	0	1	0	0	1	0	0	0	0	0	0	0	82
23150420	Not	md	US	2023	0	0	0	0	0	0	0	0	0	0	0	0	
23150503	f	cn	US	2023	1	0	0	0	0	0	0	0	0	0	0	0	65
23150626	f	md	DE	2023	0	0	0	0	0	0	0	0	0	0	0	0	
23150880	m	md	CN	2023	0	1	0	1	0	0	0	0	0	0	0	0	63.6
23151365	m	ph	FR	2023	0	0	0	0	0	0	0	0	0	0	0	0	55
23151830	f	md	FR	2023	0	1	0	0	0	1	0	0	0	0	0	0	63
23151905	m	md	JP	2023	0	1	0	0	1	0	0	0	0	0	0	0	49
23152088	Not	md	GB	2023	0	1	0	0	0	1	0	0	0	0	0	0	
23152225	m	ph	JP	2023	0	0	0	0	0	0	0	0	0	0	0	0	
23152295	f	md	CA	2023	1	0	0	0	0	0	0	0	0	0	0	0	
23152602	m	md	JP	2023	0	0	0	0	0	0	0	0	0	0	0	0	61
23152790	m	md	JP	2023	0	0	0	0	0	0	0	0	0	0	0	0	63
23153664	m	md	JP	2023	0	0	0	0	0	0	0	0	0	0	0	0	50
23153964	m	md	JP	2023	1	0	0	0	0	0	0	0	0	0	0	0	78
23154027	m	md	JP	2023	0	1	0	0	0	1	0	0	0	0	0	0	70
23154056	m	md	JP	2023	1	0	0	0	0	0	0	0	0	0	0	0	72
23154269	f	hp	JP	2023	0	1	0	0	0	1	0	0	0	0	0	0	48
23154270	m	hp	JP	2023	0	1	0	0	1	0	0	0	0	0	0	0	46
23154304	m	md	GR	2023	0	1	0	0	1	0	0	0	0	0	0	0	71
23154358	m	hp	PT	2023	0	0	0	0	0	0	0	0	0	0	0	0	
23154566	Not	md	JP	2023	1	0	0	0	0	0	0	0	0	0	0	0	
23154583	m	md	JP	2023	0	0	0	0	0	0	0	0	0	0	0	0	69
23154596	m	hp	CA	2023	0	0	0	0	0	0	0	0	0	0	0	0	80
23155136	f	hp	CZ	2023	0	0	0	0	0	0	0	0	0	0	0	0	57
23156106	f	hp	CZ	2023	0	0	0	0	0	0	0	0	0	0	0	0	57
23156148	f	hp	CZ	2023	0	0	0	0	0	0	0	0	0	0	0	0	57
23156611	Not	md	ES	2023	0	1	0	0	0	1	0	0	0	0	0	0	
23156896	f	md	JP	2023	0	1	0	0	0	1	0	0	0	0	0	0	80
23157405	Not	ph	FR	2023	0	0	0	0	0	0	0	0	0	0	0	0	
23157419	Not	md	US	2023	0	1	0	0	0	1	0	0	0	0	0	0	59
23157536	m	md	JP	2023	0	1	0	0	1	0	0	0	0	0	0	0	80
23158001	Not	md	JP	2023	0	0	0	0	0	0	0	0	0	0	0	0	50
23158019	Not	md	JP	2023	0	1	0	1	0	0	0	0	0	0	0	0	70

23158301	m	md	ZA	2023	1	0	0	0	0	0	0	0	0	0	0	1	
23158440	f	md	AU	2023	0	1	0	0	0	1	0	0	0	0	0	0	
23158891	m	md	JP	2023	0	1	1	0	0	0	0	0	0	0	0	0	80
23158953	m	cn	US	2023	0	0	0	0	0	0	0	0	0	0	0	0	77
23159802	f	md	CN	2023	0	0	0	0	0	0	0	0	0	0	0	0	64
23159803	f	md	CN	2023	0	0	0	0	0	0	0	0	0	0	0	0	61
23160085	m	md	CA	2023	0	1	1	0	0	0	0	0	0	0	0	0	76
23160095	f	md	JP	2023	0	1	1	0	0	0	0	0	0	0	0	0	72
23160184	m	hp	US	2023	1	0	0	0	0	0	0	0	0	0	0	0	63
23160239	f	cn	TH	2023	1	0	0	0	0	0	0	0	0	0	0	0	48.8
23160429	Not	md	US	2023	0	1	0	0	0	1	0	0	0	0	0	0	69
23161221	m	md	KR	2023	0	1	1	0	0	0	0	0	0	0	0	0	72
23161406	m	md	LV	2023	0	1	1	0	0	0	0	0	0	0	0	0	51
23161866	f	cn	US	2023	1	0	0	0	0	0	0	0	0	0	0	0	
23162494	f	md	CA	2023	0	0	0	0	0	0	0	0	0	0	0	0	67
23162849	m	md	US	2023	0	0	0	0	0	0	0	0	0	0	0	0	60
23162850	f	hp	US	2023	0	1	1	0	0	0	0	0	0	0	0	0	60
23164097	f	hp	JP	2023	0	1	1	0	0	0	0	0	0	0	0	0	68
23164098	m	hp	JP	2023	0	1	1	0	0	0	0	0	0	0	0	0	69
23164930	m	hp	CN	2023	0	1	1	0	0	0	0	0	0	0	0	0	
23164955	m	ph	FR	2023	0	0	0	0	0	0	0	0	0	0	0	0	55
23165034	m	hp	US	2023	0	1	1	0	0	0	0	0	0	0	0	0	52
23165287	m	hp	CN	2023	0	1	1	0	0	0	0	0	0	0	0	0	
23165290	m	md	JP	2023	0	1	0	1	0	0	0	0	0	0	0	0	69
23165808	f	cn	MY	2023	0	1	0	1	0	0	0	0	0	0	0	0	64.3
23165947	f	hp	US	2023	0	0	0	0	0	0	0	0	0	0	0	0	70
23165972	f	cn	SK	2023	0	0	0	0	0	0	0	0	0	0	0	0	51
23166819	m	md	DE	2023	0	1	1	0	0	0	0	0	0	0	0	0	82
23166863	m	md	TR	2023	0	0	0	0	0	0	0	0	0	0	0	0	
23167049	f	hp	US	2023	0	0	0	0	0	0	0	0	0	0	0	0	68
23167164	m	hp	US	2023	0	0	0	0	0	0	0	0	0	0	0	0	81
23167180	f	md	FR	2023	0	0	0	0	0	0	0	0	0	0	0	0	
23167763	f	md	AU	2023	0	0	0	0	0	0	0	0	0	0	0	0	52
23167887	f	cn	US	2023	0	1	1	0	0	0	0	0	0	0	0	0	

23167962	f	md	US	2023	0	1	1	0	0	0	0	0	0	0	0	0	59
23168034	f	hp	US	2023	0	1	1	0	0	0	0	0	0	0	0	0	71
23168117	m	cn	JP	2023	0	0	0	0	0	0	0	0	0	0	0	0	65
23168233	f	md	SK	2023	0	0	0	0	0	0	0	0	0	0	0	0	51
23168951	m	md	DO	2023	0	0	0	0	0	0	0	0	0	0	0	0	
23168974	m	hp	MX	2023	1	0	0	0	0	0	0	0	0	0	0	0	62
23169275	m	cn	DE	2023	0	0	0	0	0	0	0	0	0	0	0	0	
23169532	f	md	US	2023	0	0	0	0	0	0	0	0	0	0	0	0	
23169819	m	ph	CA	2023	1	0	0	0	0	0	0	0	0	0	0	0	
23170376	Not	hp	US	2023	0	0	0	0	0	0	0	0	0	0	0	0	
23170555	m	md	JP	2023	1	0	0	0	0	0	0	0	0	0	0	0	59
23170667	f	cn	US	2023	0	0	0	0	0	0	0	0	0	0	0	0	
23171059	f	cn	US	2023	1	0	0	0	0	0	0	0	0	0	0	0	
23171175	f	md	RU	2023	1	0	0	0	0	0	0	0	0	0	0	0	
23171183	m	md	JP	2023	0	1	0	0	1	0	0	0	0	0	0	0	51
23171192	m	hp	JP	2023	0	1	1	0	0	0	0	0	0	0	0	0	50
23171283	m	cn	US	2023	1	0	0	0	0	0	0	0	0	0	0	0	40.6
23171917	m	ph	FR	2023	0	0	0	0	0	0	0	0	0	0	0	0	55
23171942	m	cn	US	2023	1	0	0	0	0	0	0	0	0	0	0	0	
23172108	f	cn	CA	2023	1	0	0	0	0	0	0	0	0	0	0	0	85.2
23172110	f	md	JP	2023	1	0	0	0	0	0	0	0	0	0	0	0	69
23172166	m	md	NP	2023	0	0	0	0	0	0	0	0	0	0	0	0	57
23172169	m	md	NP	2023	0	0	0	0	0	0	0	0	0	0	0	0	61
23172171	Not	hp	US	2023	0	0	0	0	0	0	0	0	0	0	0	0	
23172588	f	md	JP	2023	1	0	0	0	0	0	0	0	0	0	0	0	79
23173132	Not	md	JP	2023	0	0	0	0	0	0	0	0	0	0	0	0	
23173181	f	md	CH	2023	0	0	0	0	0	0	0	0	0	0	0	0	
23173192	Not	md	JP	2023	0	0	0	0	0	0	0	0	0	0	0	0	
23173561	f	cn	CN	2023	0	0	0	0	0	0	0	0	0	0	0	0	50
23175120	Not	md	JP	2023	0	1	0	0	1	0	0	0	0	0	0	0	
23175188	m	md	JP	2023	0	1	0	0	0	1	0	0	0	0	0	0	77
23175233	f	md	PL	2023	0	1	1	0	0	0	0	0	0	0	0	0	52
23175561	m	md	JP	2023	0	1	0	0	1	0	0	0	0	0	0	0	62
23175618	Not	cn	CN	2023	0	1	0	0	0	1	0	0	0	0	0	0	

23175805	m	md	FR	2023	0	1	0	0	0	1	0	0	0	0	0	0	52
23175807	m	md	JP	2023	0	1	0	0	1	0	0	0	0	0	0	0	58
23176325	f	cn	CA	2023	1	0	0	0	0	0	0	0	0	0	0	0	56.3
23176388	Not	hp	US	2023	0	1	0	0	0	1	0	0	0	0	0	0	
23176391	f	md	JP	2023	0	1	0	0	1	0	0	0	0	0	0	0	77
23176435	Not	hp	US	2023	0	0	0	0	0	0	0	0	0	0	0	0	
23177070	f	md	CH	2023	0	0	0	0	0	0	0	0	0	0	0	0	
23177774	m	md	CZ	2023	0	1	1	0	0	0	0	0	0	0	0	0	79
23177867	m	md	CZ	2023	0	1	1	0	0	0	0	0	0	0	0	0	79
23178134	m	cn	US	2023	1	0	0	0	0	0	0	0	0	0	0	0	72.5
23178147	m	md	JP	2023	0	1	1	0	0	0	0	0	0	0	0	0	57
23178232	m	md	TW	2023	0	1	0	0	1	0	0	0	0	0	0	0	70
23178323	Not	hp	MX	2023	0	0	0	0	0	0	0	0	0	0	0	0	
23178326	m	md	TR	2023	0	1	1	0	0	0	0	0	0	0	0	0	64
23178368	m	md	CH	2023	0	0	0	0	0	0	0	0	0	0	0	0	
23178474	f	md	JP	2023	0	1	0	0	1	0	0	0	0	0	0	0	82
23178664	m	md	CN	2023	0	1	0	1	0	0	0	0	0	0	0	0	77.2
23178700	m	hp	ES	2023	0	0	0	0	0	0	0	0	0	0	0	0	
23178838	m	md	MY	2023	0	0	0	0	0	0	0	0	0	0	0	0	49
23178840	m	md	CN	2023	0	0	0	0	0	0	0	0	0	0	0	0	66
23178884	f	md	GR	2023	0	0	0	0	0	0	0	0	0	0	0	0	50
23179048	f	md	NL	2023	0	1	0	0	0	1	0	0	0	0	0	0	63
23179165	f	cn	US	2023	0	0	0	0	0	0	0	0	0	0	0	0	
23179331	f	md	JP	2023	0	1	1	0	0	0	0	0	0	0	0	0	90
23179760	f	cn	ID	2023	1	0	0	0	0	0	0	0	0	0	0	0	64.4
23180119	m	md	IN	2023	0	1	0	0	0	1	0	0	0	0	0	0	67
23180219	m	md	JP	2023	0	0	0	0	0	0	0	0	0	0	0	0	
23180346	f	md	US	2023	0	0	0	0	0	0	0	0	0	0	0	0	70
23180381	m	cn	ID	2023	1	0	0	0	0	0	0	0	0	0	0	0	64.1
23180409	f	md	PL	2023	0	1	1	0	0	0	0	0	0	0	0	0	52
23180440	m	cn	ID	2023	1	0	0	0	0	0	0	0	0	0	0	0	55.8
23180443	f	cn	US	2023	0	1	0	1	0	0	0	0	0	0	0	1	78
23180453	Not	hp	NL	2023	0	0	0	0	0	0	0	0	0	0	0	0	
23180495	Not	cn	US	2023	1	0	0	0	0	0	0	0	0	0	0	0	

23180497	f	cn	ID	2023	1	0	0	0	0	0	0	0	0	0	0	0	55.4
23180699	Not	md	ZA	2023	1	0	0	0	0	0	0	0	0	0	0	0	
23180703	m	cn	US	2023	1	0	0	0	0	0	0	0	0	0	0	0	78.9
23180872	f	md	IT	2023	0	0	0	0	0	0	0	0	0	0	0	0	63
23180873	m	md	IT	2023	0	0	0	0	0	0	0	0	0	0	0	0	59
23180874	f	md	IT	2023	0	1	0	0	0	1	0	0	0	0	0	0	54
23180914	m	md	NL	2023	0	1	0	0	0	1	0	0	0	0	0	0	72
23181115	f	md	GB	2023	0	1	0	0	0	1	0	0	0	0	0	0	67
23181324	m	hp	JP	2023	0	1	1	0	0	0	0	0	0	0	0	0	69
23181643	f	hp	CA	2023	0	0	0	0	0	0	0	0	0	0	0	0	62
23181688	m	cn	US	2023	0	0	0	0	0	0	0	0	0	0	0	0	62
23181914	m	md	JP	2023	0	0	0	0	0	0	0	0	0	0	0	0	71
23182020	f	md	JP	2023	0	1	0	0	0	1	0	0	0	0	0	0	35
23182047	f	ph	MY	2023	1	0	0	0	0	0	0	0	0	0	0	0	
23182251	m	cn	ID	2023	1	0	0	0	0	0	0	0	0	0	0	0	
23182253	m	hp	GR	2023	0	1	1	0	0	0	0	0	0	0	0	0	73
23182357	f	md	CA	2023	0	0	0	0	0	0	0	0	0	0	0	0	
23182590	f	hp	TH	2023	0	0	0	0	0	0	0	0	0	0	0	0	73
23182678	f	md	SA	2023	0	0	0	0	0	0	0	0	0	0	0	0	
23182685	f	md	FR	2023	0	1	0	0	0	1	0	0	0	0	0	0	63
23183286	m	md	JP	2023	0	1	0	0	1	0	0	0	0	0	0	0	70
23183359	m	md	JP	2023	0	1	1	0	0	0	0	0	0	0	0	1	76
23183918	m	cn	IN	2023	0	1	0	0	0	1	0	0	0	0	0	0	49
23184388	Not	md	JP	2023	0	1	0	0	1	0	0	0	0	0	0	1	
23184435	m	md	KR	2023	0	0	0	0	0	0	0	0	0	0	0	0	73
23184678	Not	md	US	2023	0	0	0	0	0	0	0	0	0	0	0	0	
23184743	f	md	JP	2023	0	0	0	0	0	0	0	0	0	0	0	0	80
23184792	f	hp	CN	2023	0	0	0	0	0	0	0	0	0	0	0	0	72
23185072	m	md	JP	2023	0	1	0	0	1	0	0	0	0	0	0	0	84
23185152	m	md	NZ	2023	0	1	0	1	0	0	0	0	0	0	0	0	
23185154	f	md	JP	2023	1	0	0	0	0	0	0	0	0	0	0	0	77
23185700	Not	md	JP	2023	0	1	0	0	1	0	0	0	0	0	0	0	74
23187223	m	md	DE	2023	0	1	0	0	1	0	0	0	0	0	0	0	55
23187375	m	md	JP	2023	0	0	0	0	0	0	0	0	0	0	0	0	68

23187395	m	md	JP	2023	0	1	0	1	0	0	0	0	0	0	0	0	
23187635	m	md	FR	2023	0	1	0	0	0	1	0	0	0	0	0	0	52
23187649	m	md	KR	2023	0	0	0	0	0	0	0	0	0	0	0	0	
23188195	f	md	TW	2023	0	0	0	0	0	0	0	0	0	0	0	0	59
23188196	Not	ph	JP	2023	0	0	0	0	0	0	0	0	0	0	0	0	84
23188440	m	md	JP	2023	0	1	1	0	0	0	0	0	0	0	0	0	91
23188539	Not	md	US	2023	0	0	0	0	0	0	0	0	0	0	0	0	
23188872	f	md	NZ	2023	0	1	1	0	0	0	0	0	0	0	0	0	64
23189066	m	md	JP	2023	0	1	1	0	0	0	0	0	0	0	0	0	80
23189369	Not	md	JP	2023	0	1	0	0	1	0	0	0	0	0	0	0	74
23189842	m	md	CH	2023	0	1	0	0	1	0	0	0	0	0	0	1	76
23190018	m	md	BG	2023	0	0	0	0	0	0	0	0	0	0	0	0	
23190596	m	hp	US	2023	0	1	1	0	0	0	0	0	0	0	0	0	63
23190714	m	md	AU	2023	0	1	0	0	0	1	0	0	0	0	0	0	54
23191389	f	hp	DK	2023	1	0	0	0	0	0	0	0	0	0	0	0	67
23191887	m	md	JP	2023	0	1	0	0	1	0	0	0	0	0	0	0	64
23192302	m	md	JP	2023	0	1	1	0	0	0	0	0	0	0	0	0	80
23192426	f	md	MX	2023	0	0	0	0	0	0	0	0	0	0	0	0	52
23192444	f	md	FR	2023	0	0	0	0	0	0	0	0	0	0	0	0	63
23192535	m	cn	JP	2023	0	0	0	0	0	0	0	0	0	0	0	0	55
23193033	Not	hp	US	2023	0	0	0	0	0	0	0	0	0	0	0	0	
23193209	m	md	JP	2023	0	1	1	0	0	0	0	0	0	0	0	0	80
23193270	f	md	US	2023	0	1	1	0	0	0	0	0	0	0	0	0	68
23193430	m	hp	SI	2023	0	0	0	0	0	0	0	0	0	0	0	0	65
23193517	m	md	JP	2023	0	0	0	0	0	0	0	0	0	0	0	0	61
23193706	m	md	JP	2023	0	0	0	0	0	0	0	0	0	0	0	0	51
23193982	f	md	AU	2023	0	1	0	0	0	1	0	0	0	0	0	0	65
23193983	f	ph	JP	2023	0	0	0	0	0	0	0	0	0	0	0	0	85
23194706	Not	md	US	2023	0	1	0	0	0	1	0	0	0	0	0	0	70
23194776	m	md	JP	2023	0	1	0	0	1	0	0	0	0	0	0	0	78
23194955	m	md	US	2023	0	0	0	0	0	0	0	0	0	0	0	0	58
23196085	Not	hp	CN	2023	0	0	0	0	0	0	0	0	0	0	0	0	
23196294	f	md	JP	2023	0	0	0	0	0	0	0	0	0	0	0	0	73
23196332	m	cn	IN	2023	0	0	0	0	0	0	0	0	0	0	0	0	49

23196595	m	md	US	2023	0	0	0	0	0	0	0	0	0	0	0	0	72
23196722	m	ph	JP	2023	0	1	0	0	0	1	0	0	0	0	0	0	80
23196740	f	md	US	2023	0	0	0	0	0	0	0	0	0	0	0	0	
23196750	m	md	NZ	2023	1	0	0	0	0	0	0	0	0	0	0	0	74
23196755	f	md	AU	2023	0	1	0	1	0	0	0	0	0	0	0	0	
23196765	m	md	JP	2023	0	1	0	1	0	0	0	0	0	0	0	0	78
23196777	m	md	JP	2023	0	1	0	1	0	0	0	0	0	0	0	0	58
23196803	Not	cn	US	2023	1	0	0	0	0	0	0	0	0	0	0	0	47.8
23196870	m	md	JP	2023	0	0	0	0	0	0	0	0	0	0	0	0	72
23196949	m	md	JP	2023	0	1	0	1	0	0	0	0	0	0	0	0	72
23196958	f	cn	US	2023	1	0	0	0	0	0	0	0	0	0	0	0	
23197060	Not	md	JP	2023	0	1	0	1	0	0	0	0	0	0	0	0	
23197097	m	cn	IN	2023	0	1	0	1	0	0	0	0	0	0	0	0	
23197331	m	hp	JP	2023	0	0	0	0	0	0	0	0	0	0	0	0	69
23197422	f	hp	CN	2023	1	0	0	0	0	0	0	0	0	0	0	0	70
23197505	m	md	FR	2023	0	1	0	0	0	1	0	0	0	0	0	0	52
23197728	m	md	PL	2023	1	0	0	0	0	0	0	0	0	0	0	0	
23197756	m	ph	ES	2023	0	0	0	0	0	0	0	0	0	0	0	0	72
23197893	m	md	US	2023	0	0	0	0	0	0	0	0	0	0	0	0	68
23197920	Not	ph	JP	2023	1	0	0	0	0	0	0	0	0	0	0	0	
23198021	m	md	RO	2023	0	1	0	1	0	0	0	0	0	0	0	0	
23198350	f	hp	US	2023	0	0	0	0	0	0	0	0	0	0	0	0	
23198497	f	cn	IN	2023	0	0	0	0	0	0	0	0	0	0	0	0	68
23200104	f	cn	US	2023	0	0	0	0	0	0	0	0	0	0	0	0	70
23201591	f	ph	US	2023	0	0	0	0	0	0	0	0	0	0	0	0	60
23201606	m	ph	FR	2023	0	0	0	0	0	0	0	0	0	0	0	0	55
23201726	f	hp	US	2023	0	1	1	0	0	0	0	0	0	0	0	0	77
23201924	f	cn	ID	2023	1	0	0	0	0	0	0	0	0	0	0	0	
23201948	m	md	JP	2023	0	1	1	0	0	0	0	0	0	0	0	0	70
23202144	f	md	FR	2023	0	1	0	0	0	1	0	0	0	0	0	0	63
23202215	f	md	JP	2023	0	0	0	0	0	0	0	0	0	0	0	0	80
23202373	f	md	BE	2023	0	1	0	0	0	1	0	0	0	0	0	0	46
23202518	f	md	JP	2023	0	1	0	0	0	1	0	0	0	0	0	0	
23202521	m	md	DE	2023	0	1	0	0	1	0	0	0	0	0	0	0	55

23202620	m	md	JP	2023	0	1	0	0	0	1	0	0	0	0	0	0	90
23203419	m	md	RU	2023	0	1	0	0	0	1	0	0	0	0	0	0	68
23203437	f	md	BR	2023	0	0	0	0	0	0	0	0	0	0	0	0	29
23203700	m	md	JP	2023	0	0	0	0	0	0	0	0	0	0	0	0	79
23203821	m	md	DK	2023	0	1	0	0	0	1	0	0	0	0	0	0	68
23204345	m	hp	JP	2023	0	1	0	0	0	1	0	0	0	0	0	0	82
23204465	m	cn	ES	2023	0	0	0	0	0	0	0	0	0	0	0	0	73
23204708	m	md	US	2023	0	1	1	0	0	0	0	0	0	0	0	0	60
23204715	m	md	FR	2023	0	1	1	0	0	0	0	0	0	0	0	0	
23204826	m	md	FR	2023	0	1	0	0	0	1	0	0	0	0	0	0	52
23205722	f	hp	IT	2023	0	0	0	0	0	0	0	0	0	0	0	0	54
23205794	m	cn	ID	2023	1	0	0	0	0	0	0	0	0	0	0	0	60.8
23205954	f	cn	CA	2023	1	0	0	0	0	0	0	0	0	0	0	0	
23205964	f	md	CZ	2023	0	1	0	0	1	0	0	0	0	0	0	0	68
23206065	f	md	FR	2023	0	1	0	0	0	1	0	0	0	0	0	0	63
23206117	f	hp	IT	2023	0	0	0	0	0	0	0	0	0	0	0	0	63
23206159	m	cn	ID	2023	1	0	0	0	0	0	0	0	0	0	0	0	65.3
23206670	m	md	JP	2023	0	1	1	0	0	0	0	0	0	0	0	0	72
23207746	m	md	DE	2023	0	1	0	0	1	0	0	0	0	0	0	0	69
23207763	m	md	JP	2023	0	1	0	0	1	0	0	0	0	0	0	0	70
23207835	m	hp	DE	2023	0	0	0	0	0	0	0	0	0	0	0	0	50
23208059	f	md	US	2023	0	1	1	0	0	0	0	0	0	0	0	0	68
23209755	f	md	CN	2023	1	0	0	0	0	0	0	0	0	0	0	0	76
23209835	Not	md	JP	2023	0	1	0	0	1	0	0	0	0	0	0	1	
23210028	m	md	JP	2023	0	0	0	0	0	0	0	0	0	0	0	0	80
23210042	m	cn	RS	2023	0	0	0	0	0	0	0	0	0	0	0	0	76
23210864	m	md	BE	2023	0	0	0	0	0	0	0	0	0	0	0	0	79
23210892	f	ph	ES	2023	1	0	0	0	0	0	0	0	0	0	0	0	
23210989	m	hp	IT	2023	0	0	0	0	0	0	0	0	0	0	0	0	59
23212446	m	md	RU	2023	0	0	0	0	0	0	0	0	0	0	0	0	
23213477	f	md	JP	2023	1	0	0	0	0	0	0	0	0	0	0	0	78
23213546	m	md	JP	2023	0	1	1	0	0	0	0	0	0	0	0	0	
23213668	f	cn	AT	2023	0	0	0	0	0	0	0	0	0	0	0	0	65
23213782	f	md	US	2023	0	1	0	1	0	0	0	0	0	0	0	0	66

23214008	f	cn	US	2023	0	0	0	0	0	0	0	0	0	0	0	0	
23214156	f	ph	CH	2023	0	1	1	0	0	0	0	0	0	0	0	0	68
23214237	f	ph	SI	2023	0	1	0	0	1	0	0	0	0	0	0	0	62
23214244	f	md	JP	2023	0	1	1	0	0	0	0	0	0	0	0	1	60
23214450	m	hp	US	2023	0	1	1	0	0	0	0	0	0	0	0	0	64
23214784	f	cn	CA	2023	1	0	0	0	0	0	0	0	0	0	0	0	70
23214894	f	md	US	2023	0	1	1	0	0	0	0	0	0	0	0	0	64
23215355	m	md	TW	2023	0	0	0	0	0	0	0	0	0	0	0	0	83
23215651	f	hp	US	2023	1	0	0	0	0	0	0	0	0	0	0	0	
23216872	Not	md	JP	2023	0	1	0	0	1	0	0	0	0	0	0	0	
23216952	m	ph	CN	2023	0	1	1	0	0	0	0	0	0	0	0	0	59
23217482	m	md	JP	2023	0	1	0	0	0	1	0	0	0	0	0	1	
23217676	m	hp	JP	2023	0	1	0	0	0	1	0	0	0	0	0	0	76
23217944	Not	md	JP	2023	1	0	0	0	0	0	0	0	0	0	0	0	
23218540	f	cn	CA	2023	0	0	0	0	0	0	0	0	0	0	0	0	
23218564	f	md	CZ	2023	0	0	0	0	0	0	0	0	0	0	0	0	69
23218655	m	cn	CA	2023	0	0	0	0	0	0	0	0	0	0	0	0	
23218733	f	md	JP	2023	0	0	0	0	0	0	0	0	0	0	0	0	90
23218809	m	md	JP	2023	0	1	0	1	0	0	0	0	0	0	0	0	
23218979	Not	md	RS	2023	0	1	0	1	0	0	0	0	0	0	0	0	
23218981	Not	md	RS	2023	0	1	0	1	0	0	0	0	0	0	0	0	
23218993	f	md	FR	2023	1	0	0	0	0	0	0	0	0	0	0	0	
23219013	f	md	KR	2023	0	0	0	0	0	0	0	0	0	0	0	0	77
23219066	Not	md	RS	2023	0	1	0	1	0	0	0	0	0	0	0	0	
23219092	m	hp	CN	2023	1	0	0	0	0	0	0	0	0	0	0	0	48
23219185	f	md	KR	2023	0	0	0	0	0	0	0	0	0	0	0	0	60
23219367	f	md	JP	2023	0	0	0	0	0	0	0	0	0	0	0	0	75
23219585	f	md	US	2023	0	1	1	0	0	0	0	0	0	0	0	0	62
23219947	m	md	JP	2023	0	1	0	0	1	0	0	0	0	0	0	0	77
23220045	m	cn	NL	2023	0	1	1	0	0	0	0	0	0	0	0	0	59
23220498	m	md	JP	2023	0	1	1	0	0	0	0	0	0	0	0	0	72
23220607	f	md	JP	2023	0	0	0	0	0	0	0	0	0	0	0	0	70
23220619	m	md	JP	2023	0	1	0	0	0	1	0	0	0	0	0	0	80
23220629	m	md	JP	2023	0	1	1	0	0	0	0	0	0	0	0	0	59

23220655	Not	md	US	2023	0	1	0	0	1	0	0	0	0	0	0	0	
23220750	Not	hp	CN	2023	0	0	0	0	0	0	0	0	0	0	0	0	
23221937	Not	hp	ES	2023	0	0	0	0	0	0	0	0	0	0	0	0	
23222166	f	md	US	2023	1	0	0	0	0	0	0	0	0	0	0	0	
23222330	m	md	DE	2023	0	0	0	0	0	0	0	0	0	0	0	0	
23222481	f	md	BE	2023	0	0	0	0	0	0	0	0	0	0	0	0	
23222808	m	md	JP	2023	0	0	0	0	0	0	0	0	0	0	0	0	
23222902	f	hp	ES	2023	0	0	0	0	0	0	0	0	0	0	0	0	63
23222932	Not	cn	CN	2023	0	1	0	0	0	1	0	0	0	0	0	0	
23222949	m	md	JP	2023	0	1	0	1	0	0	0	0	0	0	0	0	68
23222950	f	md	JP	2023	0	1	0	1	0	0	0	0	0	0	0	0	
23223010	m	md	JP	2023	0	1	1	0	0	0	0	0	0	0	0	0	52
23223085	f	md	US	2023	0	1	1	0	0	0	0	0	0	0	0	0	72
23223251	m	cn	MY	2023	0	0	0	0	0	0	0	0	0	0	0	0	62
23223494	m	md	JP	2023	0	1	0	0	0	1	0	0	0	0	0	0	50
23223495	Not	md	ES	2023	0	1	0	0	0	1	0	0	0	0	0	0	
23223572	m	md	DE	2023	0	1	1	0	0	0	0	0	0	0	0	0	68
23223627	m	md	JP	2023	0	1	0	0	1	0	0	0	0	0	0	0	69
23224419	m	md	JP	2023	0	1	0	0	1	0	0	0	0	0	0	0	74
23224768	m	md	JP	2023	0	1	0	0	1	0	0	0	0	0	0	0	80
23224895	m	md	BG	2023	0	0	0	0	0	0	1	0	0	1	0	0	66
23224958	m	md	DE	2023	0	1	0	0	1	0	0	0	0	0	0	0	55
23225002	f	md	FR	2023	0	0	0	0	0	0	0	0	0	0	0	0	70
23225008	Not	md	US	2023	0	1	0	0	1	0	0	0	0	0	0	0	
23225197	m	md	RU	2023	0	1	0	1	0	0	0	0	0	0	0	0	63
23225340	f	cn	US	2023	1	0	0	0	0	0	0	0	0	0	0	0	67
23225412	f	md	DO	2023	0	0	0	0	0	0	0	0	0	0	0	0	72
23225493	Not	cn	CN	2023	0	1	0	0	0	1	0	0	0	0	0	0	
23225537	m	cn	PK	2023	0	1	0	0	0	1	0	0	0	0	0	0	37
23225598	f	cn	ID	2023	1	0	0	0	0	0	0	0	0	0	0	0	59.6
23225599	f	cn	ID	2023	1	0	0	0	0	0	0	0	0	0	0	0	74.4
23225657	m	md	JP	2023	0	1	0	1	0	0	0	0	0	0	0	1	75
23225688	Not	md	JP	2023	0	1	0	0	0	1	0	0	0	0	0	0	70
23225944	f	cn	ID	2023	1	0	0	0	0	0	0	0	0	0	0	0	

23225946	f	cn	ID	2023	1	0	0	0	0	0	0	0	0	0	0	0	64.8
23225947	f	cn	US	2023	1	0	0	0	0	0	0	0	0	0	0	0	
23226046	f	cn	ID	2023	1	0	0	0	0	0	0	0	0	0	0	0	
23226118	Not	cn	US	2023	1	0	0	0	0	0	0	0	0	0	0	0	64.8
23226181	m	md	JP	2023	0	1	0	1	0	0	0	0	0	0	0	0	74
23226228	f	md	BE	2023	0	1	0	0	1	0	0	0	0	0	0	1	67
23226277	f	ph	US	2023	0	0	0	0	0	0	0	0	0	0	0	0	
23226279	f	md	JP	2023	0	0	0	0	0	0	1	0	0	0	1	0	83
23226328	m	md	JP	2023	0	1	0	0	0	1	0	0	0	0	0	0	
23226577	m	cn	CN	2023	0	0	0	0	0	0	0	0	0	0	0	0	59
23226582	f	cn	ID	2023	1	0	0	0	0	0	0	0	0	0	0	0	
23226715	f	hp	AR	2023	0	0	0	0	0	0	0	0	0	0	0	0	37
23226888	Not	cn	US	2023	1	0	0	0	0	0	0	0	0	0	0	0	
23227193	m	md	DE	2023	0	1	0	0	1	0	0	0	0	0	0	0	54
23227470	m	cn	IN	2023	0	0	0	0	0	0	0	0	0	0	0	0	36
23227652	m	cn	CN	2023	0	1	0	0	0	1	0	0	0	0	0	0	66
23227680	f	md	JP	2023	0	0	0	0	0	0	0	0	0	0	0	0	
23227910	f	hp	BR	2023	0	0	0	0	0	0	0	0	0	0	0	0	
23229003	m	cn	LB	2023	0	1	0	0	0	1	0	0	0	0	0	0	
23229186	m	hp	US	2023	0	1	1	0	0	0	0	0	0	0	0	0	60
23229509	f	md	IT	2023	0	0	0	0	0	0	0	0	0	0	0	0	63
23229547	m	md	JP	2023	0	1	0	1	0	0	0	0	0	0	0	0	52
23229896	m	md	JP	2023	0	1	0	0	0	1	0	0	0	0	0	0	82
23229955	Not	md	JP	2023	0	1	0	1	0	0	0	0	0	0	0	1	
23230208	f	md	JP	2023	0	0	0	0	0	0	0	0	0	0	0	0	50
23230287	f	md	KR	2023	0	0	0	0	0	0	0	0	0	0	0	0	
23230539	m	cn	IN	2023	0	1	0	0	0	1	0	0	0	0	0	0	73
23230548	m	md	JP	2023	0	1	1	0	0	0	0	0	0	0	0	0	78
23230644	f	ph	US	2023	0	0	0	0	0	0	0	0	0	0	0	0	62
23230750	m	md	JP	2023	0	1	0	0	1	0	0	0	0	0	0	0	70
23230911	m	cn	IN	2023	1	0	0	0	0	0	0	0	0	0	0	0	81.5
23230939	f	cn	TH	2023	1	0	0	0	0	0	0	0	0	0	0	0	61
23231018	m	md	US	2023	0	0	0	0	0	0	0	0	0	0	0	0	60
23231291	m	md	AT	2023	0	1	0	0	1	0	0	0	0	0	0	0	61

23231643	m	md	TH	2023	0	1	0	0	0	1	0	0	0	0	0	0	71
23232018	f	hp	BR	2023	0	0	0	0	0	0	0	0	0	0	0	0	57
23232188	m	md	KR	2023	0	1	1	0	0	0	0	0	0	0	0	0	57
23232556	Not	md	JP	2023	0	0	0	0	0	0	0	0	0	0	0	0	60
23232609	Not	md	JP	2023	0	1	0	0	0	1	0	0	0	0	0	0	
23232717	m	hp	FR	2023	0	0	0	0	0	0	0	0	0	0	0	0	71
23232743	m	md	AT	2023	0	0	0	0	0	0	0	0	0	0	0	0	74
23232924	f	md	JP	2023	0	1	0	0	1	0	0	0	0	0	0	0	
23232929	m	cn	US	2023	0	0	0	0	0	0	0	0	0	0	0	0	66
23233166	m	md	JP	2023	0	0	0	0	0	0	0	0	0	0	0	0	
23233217	m	md	US	2023	0	1	1	0	0	0	0	0	0	0	0	0	64
23233261	m	hp	DE	2023	0	0	0	0	0	0	0	0	0	0	0	0	50
23233306	m	md	JP	2023	0	1	0	0	0	1	0	0	0	0	0	0	59
23234142	m	md	US	2023	0	1	1	0	0	0	0	0	0	0	0	0	82
23234327	m	hp	US	2023	0	1	1	0	0	0	0	0	0	0	0	0	67
23234518	Not	md	ES	2023	0	0	0	0	0	0	0	0	0	0	0	0	
23234523	f	md	AT	2023	1	0	0	0	0	0	0	0	0	0	0	0	60
23234560	m	hp	GB	2023	1	0	0	0	0	0	0	0	0	0	0	0	
23234583	m	md	US	2023	0	1	1	0	0	0	0	0	0	0	0	0	56
23234879	m	md	JP	2023	0	1	0	1	0	0	0	0	0	0	0	0	46
23235660	m	cn	IN	2023	0	0	0	0	0	0	0	0	0	0	0	0	69
23235709	m	md	DE	2023	0	1	1	0	0	0	0	0	0	0	0	0	68
23235768	f	md	JP	2023	1	0	0	0	0	0	0	0	0	0	0	0	88
23236319	m	hp	KR	2023	0	0	0	0	0	0	0	0	0	0	0	0	82
23236322	Not	hp	JP	2023	0	1	0	0	0	1	0	0	0	0	0	0	
23236769	f	md	JP	2023	1	0	0	0	0	0	0	0	0	0	0	1	82
23236891	m	cn	CA	2023	1	0	0	0	0	0	0	0	0	0	0	0	61
23236923	f	md	FR	2023	0	0	0	0	0	0	0	0	0	0	0	0	61
23237177	m	md	PL	2023	0	1	0	0	0	1	0	0	0	0	0	0	63
23237351	m	hp	AT	2023	0	0	0	0	0	0	0	0	0	0	0	0	50
23237617	f	md	US	2023	0	0	0	0	0	0	0	0	0	0	0	0	83
23237618	f	hp	BG	2023	0	0	0	0	0	0	0	0	0	0	0	0	42
23237634	f	md	JP	2023	0	1	0	0	1	0	0	0	0	0	0	0	78
23237763	f	hp	CA	2023	0	1	1	0	0	0	0	0	0	0	0	0	38

23237781	f	md	ES	2023	0	1	1	0	0	0	0	0	0	0	0	0	53
23237941	m	cn	BR	2023	0	0	0	0	0	0	0	0	0	0	0	0	85
23238581	f	md	CA	2023	1	0	0	0	0	0	0	0	0	0	0	0	57.4
23238600	f	md	JP	2023	0	1	0	0	1	0	0	0	0	0	0	0	78
23239116	m	hp	JP	2023	0	1	0	1	0	0	0	0	0	0	0	0	57
23239636	m	md	JP	2023	0	1	0	0	1	0	0	0	0	0	0	0	79
23239704	m	ph	ES	2023	0	0	0	0	0	0	0	0	0	0	0	0	72
23240032	f	md	JP	2023	1	0	0	0	0	0	0	0	0	0	0	0	81
23240034	Not	md	PL	2023	1	0	0	0	0	0	0	0	0	0	0	0	
23240341	Not	hp	JP	2023	0	0	0	0	0	0	0	0	0	0	0	0	
23240342	Not	hp	JP	2023	0	0	0	0	0	0	0	0	0	0	0	0	
23241084	m	hp	JP	2023	0	1	0	0	1	0	0	0	0	0	0	0	61
23241243	f	md	IT	2023	0	0	0	0	0	0	0	0	0	0	0	0	75
23241244	m	md	AT	2023	0	0	0	0	0	0	0	0	0	0	0	0	70
23241324	m	hp	IL	2023	0	1	1	0	0	0	0	0	0	0	0	0	62
23241483	f	md	US	2023	0	1	1	0	0	0	0	0	0	0	0	0	54
23241655	m	md	DE	2023	0	1	1	0	0	0	0	0	0	0	0	0	68
23242292	m	md	AT	2023	0	0	0	0	0	0	0	0	0	0	0	0	51
23242532	f	md	JP	2023	1	0	0	0	0	0	0	0	0	0	0	0	84
23242770	m	md	JP	2023	0	1	1	0	0	0	0	0	0	0	0	0	67
23243561	m	md	JP	2023	0	1	0	0	0	1	0	0	0	0	0	0	76
23243675	m	md	GR	2023	0	1	0	0	1	0	0	0	0	0	0	0	65
23244526	m	md	RU	2023	0	0	0	0	0	0	0	0	0	0	0	0	64
23245233	m	md	FR	2023	0	1	0	0	0	1	0	0	0	0	0	0	73
23245586	Not	md	RU	2023	0	1	0	0	0	1	0	0	0	0	0	0	
23246100	m	md	DE	2023	0	1	1	0	0	0	0	0	0	0	0	0	49
23246106	m	md	AT	2023	0	1	1	0	0	0	0	0	0	0	0	0	62
23246294	m	hp	DE	2023	0	0	0	0	0	0	0	0	0	0	0	0	50
23246647	m	hp	AT	2023	0	0	0	0	0	0	0	0	0	0	0	0	49
23246923	Not	md	CN	2023	0	0	0	0	0	0	0	0	0	0	0	0	
23246972	f	md	KR	2023	0	0	0	0	0	0	0	0	0	0	0	0	46
23247288	f	ph	DE	2023	0	0	0	0	0	0	0	0	0	0	0	0	
23247443	m	md	CA	2023	0	0	0	0	0	0	0	0	0	0	0	0	76
23247446	f	hp	CN	2023	1	0	0	0	0	0	0	0	0	0	0	0	29

23247988	Not	hp	US	2023	0	1	1	0	0	0	0	0	0	0	0	0	
23247994	Not	hp	US	2023	0	1	1	0	0	0	0	0	0	0	0	0	
23248082	Not	cn	CN	2023	0	0	0	0	0	0	0	0	0	0	0	0	
23248256	m	cn	US	2023	1	0	0	0	0	0	0	0	0	0	0	0	
23248280	f	cn	FR	2023	0	0	0	0	0	0	0	0	0	0	0	0	59
23248516	f	md	US	2023	0	1	1	0	0	0	0	0	0	0	0	0	68
23249613	Not	md	ZA	2023	0	1	0	0	0	1	0	0	0	0	0	0	
23250043	m	hp	CA	2023	0	0	0	0	0	0	0	0	0	0	0	0	80
23250738	m	md	TH	2023	0	0	0	0	0	0	0	0	0	0	0	0	64
23251461	f	cn	CA	2023	1	0	0	0	0	0	0	0	0	0	0	0	
23252241	m	hp	FR	2023	0	0	0	0	0	0	0	0	0	0	0	0	79
23252290	f	md	JP	2023	0	0	0	0	0	0	1	1	0	0	0	1	72
23252300	f	md	SI	2023	0	1	0	0	1	0	0	0	0	0	0	0	75
23252561	m	cn	IN	2023	0	1	1	0	0	0	0	0	0	0	0	0	54
23252789	f	md	US	2023	0	0	0	0	0	0	0	0	0	0	0	0	70
23252815	m	md	DE	2023	0	1	1	0	0	0	0	0	0	0	0	0	49
23252830	m	md	ES	2023	0	0	0	0	0	0	0	0	0	0	0	0	56
23253930	f	hp	DK	2023	1	0	0	0	0	0	0	0	0	0	0	0	67
23254148	f	cn	CN	2023	0	0	0	0	0	0	0	0	0	0	0	0	60
23254322	m	hp	GB	2023	0	0	0	0	0	0	0	0	0	0	0	0	
23254541	f	cn	US	2023	0	0	0	0	0	0	0	0	0	0	0	0	
23255477	Not	md	JP	2023	0	1	0	0	1	0	0	0	0	0	0	0	
23255481	f	hp	CA	2023	0	0	0	0	0	0	0	0	0	0	0	0	64
23255751	Not	hp	CN	2023	0	0	0	0	0	0	0	0	0	0	0	0	
23255902	f	md	US	2023	0	1	1	0	0	0	0	0	0	0	0	0	66
23256146	Not	hp	JP	2023	0	0	0	0	0	0	0	0	0	0	0	0	80
23256807	f	hp	ES	2023	0	0	0	0	0	0	0	0	0	0	0	0	63
23257049	m	md	JP	2023	0	1	0	0	1	0	0	0	0	0	0	0	72
23257098	m	md	ZA	2023	0	1	0	0	0	1	0	0	0	0	0	0	
23257180	Not	hp	CN	2023	0	0	0	0	0	0	0	0	0	0	0	0	
23257208	f	hp	GB	2023	0	0	0	0	0	0	0	0	0	0	0	0	56
23257341	f	cn	US	2023	1	0	0	0	0	0	0	0	0	0	0	0	
23257361	f	md	ES	2023	0	1	1	0	0	0	0	0	0	0	0	0	60
23257518	m	cn	US	2023	0	1	1	0	0	0	0	0	0	0	0	0	56

23257557	f	cn	IN	2023	1	0	0	0	0	0	0	0	0	0	0	0	52
23257607	f	md	JP	2023	0	1	0	0	1	0	0	0	0	0	0	0	43
23257621	m	cn	CN	2023	0	0	0	0	0	0	0	0	0	0	0	0	68
23257633	m	md	JP	2023	0	1	0	0	1	0	0	0	0	0	0	0	71
23257683	m	md	JP	2023	0	1	0	0	1	0	0	0	0	0	0	0	71
23258167	Not	md	DE	2023	0	1	0	0	1	0	0	0	0	0	0	0	
23258238	Not	md	ES	2023	0	1	0	0	0	1	0	0	0	0	0	0	
23258240	Not	md	ES	2023	0	1	0	0	0	1	0	0	0	0	0	0	
23258501	f	cn	TH	2023	1	0	0	0	0	0	0	0	0	0	0	0	80.5
23258849	m	cn	CA	2023	1	0	0	0	0	0	0	0	0	0	0	0	80
23259105	f	md	JP	2023	0	1	0	0	1	0	0	0	0	0	0	0	76
23259164	f	cn	AR	2023	1	0	0	0	0	0	0	0	0	0	0	0	80.2
23259950	m	md	JP	2023	0	1	1	0	0	0	0	0	0	0	0	0	88
23260026	f	md	US	2023	0	1	1	0	0	0	0	0	0	0	0	0	68
23260044	f	md	JP	2023	0	1	1	0	0	0	0	0	0	0	0	0	85
23260166	m	md	PT	2023	0	0	0	0	0	0	0	0	0	0	0	0	
23260393	f	md	DE	2023	0	1	1	0	0	0	0	0	0	0	0	0	
23260553	f	md	DE	2023	0	1	0	0	1	0	0	0	0	0	0	0	65
23260750	f	hp	CA	2023	0	0	0	0	0	0	0	0	0	0	0	0	60
23260941	m	md	RU	2023	0	1	0	0	0	1	0	0	0	0	0	0	59
23261169	f	md	DE	2023	0	1	0	0	1	0	0	0	0	0	0	0	65
23261174	f	md	DE	2023	0	0	0	0	0	0	0	0	0	0	0	0	59
23261560	f	md	JP	2023	0	1	0	0	0	1	0	0	0	0	0	0	80
23261597	m	md	CN	2023	0	0	0	0	0	0	0	0	0	0	0	0	47
23261976	m	cn	CA	2023	1	0	0	0	0	0	0	0	0	0	0	0	
23262029	f	cn	CA	2023	1	0	0	0	0	0	0	0	0	0	0	0	65.6
23262135	m	md	JP	2023	0	0	0	0	0	0	0	0	0	0	0	0	
23262159	m	md	CN	2023	0	1	1	0	0	0	0	0	0	0	0	0	77
23262323	Not	md	CN	2023	0	0	0	0	0	0	0	0	0	0	0	0	
23262404	f	hp	TH	2023	1	0	0	0	0	0	0	0	0	0	0	0	73.4
23262541	f	md	US	2023	0	0	0	0	0	0	0	0	0	0	0	0	84
23263006	m	md	AT	2023	0	0	0	0	0	0	0	0	0	0	0	0	24
23263041	Not	md	JP	2023	1	0	0	0	0	0	0	0	0	0	0	0	
23263159	f	md	PL	2023	0	1	1	0	0	0	0	0	0	0	0	0	52

23263173	m	md	DE	2023	0	1	0	0	1	0	0	0	0	0	0	0	54
23263320	Not	md	US	2023	0	0	0	0	0	0	0	0	0	0	0	0	
23263507	m	md	FR	2023	0	1	1	0	0	0	0	0	0	0	0	0	58
23264325	f	md	US	2023	0	0	0	0	0	0	0	0	0	0	0	0	
23264425	f	md	DE	2023	0	1	0	0	1	0	0	0	0	0	0	0	74
23264750	Not	md	KR	2023	0	0	0	0	0	0	0	0	0	0	0	0	
23264874	f	md	FR	2023	0	0	0	0	0	0	0	0	0	0	0	0	
23265821	m	md	JP	2023	0	1	0	0	1	0	0	0	0	0	0	0	72
23266019	m	md	ES	2023	0	0	0	0	0	0	0	0	0	0	0	0	62
23266091	m	md	JP	2023	0	1	0	0	1	0	0	0	0	0	0	0	70
23266509	f	md	DE	2023	0	1	0	0	0	1	0	0	0	0	0	0	69
23266938	f	cn	US	2023	0	0	0	0	0	0	0	0	0	0	0	0	63
23267123	f	cn	TH	2023	0	0	0	0	0	0	0	0	0	0	0	0	55
23267125	f	cn	EG	2023	0	0	0	0	0	0	0	0	0	0	0	0	61
23267127	f	hp	FR	2023	0	0	0	0	0	0	0	0	0	0	0	0	44
23267218	f	md	JP	2023	0	1	0	0	0	1	0	0	0	0	0	0	75
23267228	m	hp	US	2023	0	0	0	0	0	0	0	0	0	0	0	0	71
23267230	m	md	JP	2023	0	0	0	0	0	0	0	0	0	0	0	0	65
23267504	m	md	IT	2023	0	0	0	0	0	0	0	0	0	0	0	0	68
23267819	f	md	BR	2023	1	0	0	0	0	0	0	0	0	0	0	0	55
23267876	f	md	FR	2023	1	0	0	0	0	0	0	0	0	0	0	0	
23267885	Not	md	FR	2023	1	0	0	0	0	0	0	0	0	0	0	0	
23267887	m	hp	US	2023	0	0	0	0	0	0	0	0	0	0	0	0	70
23267903	f	cn	US	2023	0	1	0	0	1	0	0	0	0	0	0	0	
23267987	Not	md	JP	2023	0	1	0	1	0	0	0	0	0	0	0	0	
23268815	m	md	DE	2023	0	1	0	0	1	0	0	0	0	0	0	0	66
23269229	m	md	JP	2023	0	1	0	0	1	0	0	0	0	0	0	0	72
23269855	m	md	PL	2023	0	1	0	0	1	0	0	0	0	0	0	0	55
23270059	f	hp	CN	2023	0	0	0	0	0	0	0	0	0	0	0	0	61
23270211	f	md	JP	2023	0	0	0	0	0	0	0	0	0	0	0	0	61
23270247	m	ph	JP	2023	0	0	0	0	0	0	0	0	0	0	0	0	71
23270249	m	md	SK	2023	0	0	0	0	0	0	0	0	0	0	0	0	55
23270427	m	cn	PK	2023	0	1	0	0	0	1	0	0	0	0	0	0	64
23271011	f	md	JP	2023	0	1	0	0	1	0	0	0	0	0	0	0	66

23271067	m	md	JP	2023	0	1	1	0	0	0	0	0	0	0	0	0	68
23272283	m	md	RO	2023	0	1	0	1	0	0	0	0	0	0	0	0	
23272566	m	md	JP	2023	0	0	0	0	0	0	1	0	0	0	1	0	39
23272605	m	md	JP	2023	0	0	0	0	0	0	0	0	0	0	0	0	72
23272660	m	md	JP	2023	0	0	0	0	0	0	0	0	0	0	0	0	
23272718	m	md	JP	2023	1	0	0	0	0	0	0	0	0	0	0	0	60
23272905	f	md	DE	2023	0	1	0	1	0	0	0	0	0	0	0	1	72
23273208	f	hp	FR	2023	0	0	0	0	0	0	0	0	0	0	0	0	65
23273341	f	hp	US	2023	0	0	0	0	0	0	0	0	0	0	0	0	
23274107	f	ph	US	2023	1	0	0	0	0	0	0	0	0	0	0	1	
23274566	f	cn	US	2023	1	0	0	0	0	0	0	0	0	0	0	0	64
23274937	Not	md	US	2023	0	0	0	0	0	0	0	0	0	0	0	0	
23275360	m	md	JP	2023	0	1	0	1	0	0	0	0	0	0	0	0	
23275514	m	md	JP	2023	0	1	0	0	1	0	0	0	0	0	0	0	70
23275591	Not	md	ES	2023	0	1	0	0	0	1	0	0	0	0	0	0	
23275595	Not	md	ES	2023	0	1	0	0	0	1	0	0	0	0	0	0	
23275596	Not	md	ES	2023	0	1	0	0	0	1	0	0	0	0	0	0	
23275598	Not	md	ES	2023	0	1	0	0	0	1	0	0	0	0	0	0	
23275878	f	md	JP	2023	0	0	0	0	0	0	0	0	0	0	0	0	70
23275922	f	md	JP	2023	0	1	0	0	1	0	0	0	0	0	0	0	60
23275983	m	md	US	2023	0	0	0	0	0	0	0	0	0	0	0	0	50
23276496	m	hp	DK	2023	0	0	0	0	0	0	1	0	0	0	1	0	80
23276590	m	hp	US	2023	0	1	0	0	1	0	0	0	0	0	0	0	
23276796	f	cn	JP	2023	0	0	0	0	0	0	0	0	0	0	0	0	52
23276833	Not	md	JP	2023	0	1	0	1	0	0	0	0	0	0	0	0	69
23276839	f	md	CA	2023	1	0	0	0	0	0	0	0	0	0	0	0	
23276888	f	md	DE	2023	0	1	1	0	0	0	0	0	0	0	0	0	67
23276895	f	md	JP	2023	0	1	0	0	1	0	0	0	0	0	0	0	76
23277278	m	md	JP	2023	0	1	1	0	0	0	0	0	0	0	0	0	
23277288	m	hp	GR	2023	0	0	0	0	0	0	0	0	0	0	0	0	75
23277398	f	md	CN	2023	1	0	0	0	0	0	0	0	0	0	0	0	54.2
23277572	f	md	DE	2023	0	1	0	0	1	0	0	0	0	0	0	0	64
23278058	m	md	JP	2023	0	1	0	0	1	0	0	0	0	0	0	0	73
23278121	m	md	JP	2023	0	1	0	1	0	0	0	0	0	0	0	1	60

23278527	f	md	KR	2023	0	0	0	0	0	0	0	0	0	0	0	0	60
23278691	m	md	DE	2023	0	1	0	0	1	0	0	0	0	0	0	0	79
23278864	Not	md	FR	2023	1	0	0	0	0	0	0	0	0	0	0	0	
23278865	Not	md	FR	2023	1	0	0	0	0	0	0	0	0	0	0	0	
23278877	f	md	PT	2023	0	0	0	0	0	0	0	0	0	0	0	0	62
23279238	f	cn	PK	2023	0	1	0	0	0	1	0	0	0	0	0	0	66
23280073	f	md	US	2023	0	1	1	0	0	0	0	0	0	0	0	0	60
23280074	m	md	CA	2023	0	0	0	0	0	0	0	0	0	0	0	0	76
23280234	Not	md	JP	2023	0	1	0	0	1	0	0	0	0	0	0	0	
23280378	Not	hp	US	2023	1	0	0	0	0	0	0	0	0	0	0	1	
23280663	f	md	JP	2023	0	0	0	0	0	0	0	0	0	0	0	0	55
23280861	m	md	JP	2023	0	1	0	0	0	1	0	0	0	0	0	0	80
23281188	m	ph	IT	2023	0	1	1	0	0	0	0	0	0	0	0	0	64
23281623	m	hp	CN	2023	0	1	1	0	0	0	0	0	0	0	0	0	75
23281694	m	hp	GB	2023	0	0	0	0	0	0	0	0	0	0	0	0	64
23282312	m	ph	JP	2023	0	1	0	0	1	0	0	0	0	0	0	0	61
23282346	m	md	JP	2023	0	0	0	0	0	0	0	0	0	0	0	0	85
23282776	f	hp	ES	2023	0	0	0	0	0	0	0	0	0	0	0	0	63
23283613	f	md	AT	2023	0	1	1	0	0	0	0	0	0	0	0	0	63
23283875	f	md	FR	2023	0	1	0	0	0	1	0	0	0	0	0	1	74
23284513	m	hp	US	2023	0	0	0	0	0	0	0	0	0	0	0	0	
23284602	f	md	AT	2023	0	1	1	0	0	0	0	0	0	0	0	0	63
23284659	f	md	AT	2023	0	1	1	0	0	0	0	0	0	0	0	0	
23284925	Not	cn	JP	2023	0	0	0	0	0	0	0	0	0	0	0	0	
23284927	m	md	PT	2023	0	0	0	0	0	0	0	0	0	0	0	0	74
23284928	f	md	DE	2023	0	1	1	0	0	0	0	0	0	0	0	0	67
23284965	f	cn	US	2023	0	0	0	0	0	0	0	0	0	0	0	0	
23284969	f	cn	US	2023	0	0	0	0	0	0	0	0	0	0	0	0	
23285184	f	cn	US	2023	1	0	0	0	0	0	0	0	0	0	0	0	67
23285212	f	cn	US	2023	1	0	0	0	0	0	0	0	0	0	0	0	
23285217	Not	cn	US	2023	1	0	0	0	0	0	0	0	0	0	0	0	
23285247	f	cn	VN	2023	1	0	0	0	0	0	0	0	0	0	0	0	
23285262	Not	cn	US	2023	1	0	0	0	0	0	0	0	0	0	0	0	
23285275	Not	md	JP	2023	0	1	0	1	0	0	0	0	0	0	0	0	60

23285302	f	md	JP	2023	0	1	0	1	0	0	0	0	0	0	0	1	52
23285309	Not	md	JP	2023	0	1	0	1	0	0	0	0	0	0	0	0	
23285399	m	md	JP	2023	0	1	0	0	1	0	0	0	0	0	0	0	73
23285435	Not	md	JP	2023	0	1	0	0	1	0	0	0	0	0	0	0	
23285914	f	md	DE	2023	0	1	1	0	0	0	0	0	0	0	0	0	
23286058	Not	md	FR	2023	0	1	0	0	0	1	0	0	0	0	0	0	
23286294	m	md	JP	2023	0	1	0	1	0	0	0	0	0	0	0	0	70
23286555	m	md	CN	2023	0	0	0	0	0	0	0	0	0	0	0	0	63
23286814	f	md	DE	2023	0	1	1	0	0	0	0	0	0	0	0	0	67
23286845	m	md	JP	2023	0	1	0	0	1	0	0	0	0	0	0	0	70
23287023	m	cn	IN	2023	1	0	0	0	0	0	0	0	0	0	0	0	46
23287087	f	md	US	2023	0	1	1	0	0	0	0	0	0	0	0	0	68
23287268	m	cn	VN	2023	0	1	0	1	0	0	0	0	0	0	0	0	
23287311	f	md	CN	2023	0	0	0	0	0	0	0	0	0	0	0	0	
23287325	f	md	AT	2023	1	0	0	0	0	0	0	0	0	0	0	0	60
23287584	Not	md	JP	2023	0	1	0	1	0	0	0	0	0	0	0	0	
23287585	Not	md	JP	2023	0	1	0	1	0	0	0	0	0	0	0	0	
23287594	Not	md	US	2023	0	1	1	0	0	0	0	0	0	0	0	0	
23287677	Not	md	JP	2023	0	0	0	0	0	0	0	0	0	0	0	0	
23287807	f	md	US	2023	0	1	1	0	0	0	0	0	0	0	0	0	57
23287989	m	cn	US	2023	0	0	0	0	0	0	0	0	0	0	0	0	81
23288488	Not	md	JP	2023	0	0	0	0	0	0	0	0	0	0	0	0	
23288526	f	md	CA	2023	1	0	0	0	0	0	0	0	0	0	0	0	
23288962	m	md	JP	2023	1	0	0	0	0	0	0	0	0	0	0	0	74
23289269	m	cn	IN	2023	1	0	0	0	0	0	0	0	0	0	0	0	
23289412	Not	md	JP	2023	0	0	0	0	0	0	0	0	0	0	0	0	
23290042	m	hp	US	2023	0	1	0	1	0	0	0	0	0	0	0	1	
23290159	f	cn	US	2023	1	0	0	0	0	0	0	0	0	0	0	0	
23290220	m	cn	TH	2023	1	0	0	0	0	0	0	0	0	0	0	0	56.7
23290232	f	md	IT	2023	0	0	0	0	0	0	0	0	0	0	0	0	
23290305	f	md	GB	2023	1	0	0	0	0	0	0	0	0	0	0	0	23
23290918	f	md	DE	2023	0	1	0	0	1	0	0	0	0	0	0	0	62
23292297	m	ph	JP	2023	0	0	0	0	0	0	0	0	0	0	0	0	61
23293022	f	md	DE	2023	0	1	1	0	0	0	0	0	0	0	0	0	67

23293312	m	cn	PK	2023	0	1	0	0	0	1	0	0	0	0	0	0	77
23293873	f	md	JP	2023	0	0	0	0	0	0	0	0	0	0	0	0	72
23294020	f	md	JP	2023	0	1	0	1	0	0	0	0	0	0	0	1	
23294093	f	cn	CA	2023	0	0	0	0	0	0	0	0	0	0	0	0	
23294123	m	ph	US	2023	0	0	0	0	0	0	0	0	0	0	0	0	64
23294560	Not	hp	SG	2023	0	0	0	0	0	0	0	0	0	0	0	0	
23294561	Not	hp	SG	2023	0	0	0	0	0	0	0	0	0	0	0	0	
23294565	Not	hp	SG	2023	0	0	0	0	0	0	0	0	0	0	0	0	
23294569	f	hp	SG	2023	0	0	0	0	0	0	0	0	0	0	0	0	
23294570	Not	hp	SG	2023	0	0	0	0	0	0	0	0	0	0	0	0	
23294572	m	hp	SG	2023	0	0	0	0	0	0	0	0	0	0	0	0	
23294577	Not	hp	SG	2023	0	0	0	0	0	0	0	0	0	0	0	0	
23294699	m	md	JP	2023	0	1	0	1	0	0	0	0	0	0	0	0	67
23294904	m	md	JP	2023	0	1	1	0	0	0	0	0	0	0	0	0	60
23295306	f	md	GB	2023	1	0	0	0	0	0	0	0	0	0	0	0	71
23295385	Not	md	JP	2023	0	1	0	0	1	0	0	0	0	0	0	0	
23295448	m	md	DE	2023	0	1	0	0	1	0	0	0	0	0	0	0	70
23295576	m	md	JP	2023	0	1	0	1	0	0	0	0	0	0	0	0	70
23295803	m	md	DE	2023	0	1	0	0	1	0	0	0	0	0	0	0	70
23295833	f	md	DE	2023	0	1	1	0	0	0	0	0	0	0	0	0	
23296274	Not	md	JP	2023	0	1	0	1	0	0	0	0	0	0	0	0	
23296444	Not	md	JP	2023	0	1	0	0	0	1	0	0	0	0	0	0	
23296447	m	md	JP	2023	0	1	0	0	0	1	0	0	0	0	0	0	60
23296545	f	md	SI	2023	0	1	0	0	1	0	0	0	0	0	0	0	75
23297115	Not	hp	CN	2023	0	1	1	0	0	0	0	0	0	0	0	0	
23297160	m	md	NP	2023	0	0	0	0	0	0	0	0	0	0	0	0	39
23297233	f	md	SA	2023	0	1	0	0	1	0	0	0	0	0	0	0	
23297424	m	md	JP	2023	0	1	0	1	0	0	0	0	0	0	0	0	75
23297658	Not	ph	JP	2023	1	0	0	0	0	0	0	0	0	0	0	0	
23297871	m	md	JP	2023	0	1	1	0	0	0	0	0	0	0	0	0	
23297890	m	md	DK	2023	0	0	0	0	0	0	1	0	0	0	1	0	80
23298027	m	cn	CA	2023	0	0	0	0	0	0	0	0	0	0	0	0	
23298645	Not	md	JP	2023	0	1	0	0	1	0	0	0	0	0	0	0	
23298702	f	md	DE	2023	0	0	0	0	0	0	0	0	0	0	0	0	82

23299353	f	md	IT	2023	0	0	0	0	0	0	0	0	0	0	0	0	53
23299939	f	md	CN	2023	0	0	0	0	0	0	0	0	0	0	0	0	62
23300252	f	md	DE	2023	0	1	1	0	0	0	0	0	0	0	0	0	53
23300685	f	md	FR	2023	1	0	0	0	0	0	0	0	0	0	0	0	
23300842	Not	cn	CN	2023	0	0	0	0	0	0	0	0	0	0	0	0	
23300983	m	ph	JP	2023	0	1	1	0	0	0	0	0	0	0	0	0	80
23301227	Not	md	IT	2023	0	0	0	0	0	0	0	0	0	0	0	0	75
23301253	f	hp	CN	2023	0	0	0	0	0	0	0	0	0	0	0	0	61
23301581	m	cn	US	2023	0	1	1	0	0	0	0	0	0	0	0	0	
23301936	m	md	JP	2023	0	1	0	1	0	0	0	0	0	0	0	0	73
23302003	Not	cn	JP	2023	1	0	0	0	0	0	0	0	0	0	0	0	
23302140	m	hp	NL	2023	0	0	0	0	0	0	0	0	0	0	0	0	80
23302149	f	cn	TH	2023	1	0	0	0	0	0	0	0	0	0	0	0	69.5
23302488	Not	hp	CN	2023	0	1	1	0	0	0	0	0	0	0	0	0	
23302489	Not	hp	CN	2023	0	1	1	0	0	0	0	0	0	0	0	0	
23302529	Not	hp	CN	2023	0	1	1	0	0	0	0	0	0	0	0	0	
23302765	m	md	ES	2023	0	0	0	0	0	0	0	0	0	0	0	0	62
23303198	Not	hp	CN	2023	0	1	1	0	0	0	0	0	0	0	0	0	
23303363	f	md	JP	2023	0	1	0	1	0	0	0	0	0	0	0	0	67
23303415	f	md	US	2023	0	0	0	0	0	0	0	0	0	0	0	0	
23303552	Not	md	JP	2023	0	0	0	0	0	0	0	0	0	0	0	0	
23303641	m	ph	CA	2023	0	1	1	0	0	0	0	0	0	0	0	0	67
23303864	Not	md	JP	2023	1	0	0	0	0	0	0	0	0	0	0	0	
23304312	f	md	GB	2023	1	0	0	0	0	0	0	0	0	0	0	0	63
23304399	m	hp	JP	2023	0	1	0	0	1	0	0	0	0	0	0	0	61
23304590	m	md	DE	2023	0	0	0	0	0	0	0	0	0	0	0	0	62
23304618	m	md	DE	2023	0	1	1	0	0	0	0	0	0	0	0	0	61
23304624	f	md	IT	2023	0	0	0	0	0	0	0	0	0	0	0	0	75
23304779	Not	cn	CN	2023	0	0	0	0	0	0	0	0	0	0	0	0	
23304963	f	cn	AT	2023	1	0	0	0	0	0	0	0	0	0	0	0	67
23304987	f	hp	JP	2023	0	0	0	0	0	0	0	0	0	0	0	0	78
23305525	m	md	DE	2023	0	1	1	0	0	0	0	0	0	0	0	0	0
23305961	m	md	JP	2023	0	1	0	0	1	0	0	0	0	0	0	0	76
23306591	f	ph	JP	2023	0	1	0	0	0	1	0	0	0	0	0	0	60

23306831	m	md	JP	2023	0	1	0	0	1	0	0	0	0	0	0	0	75
23307221	m	md	FR	2023	0	1	1	0	0	0	0	0	0	0	0	0	58
23307235	f	md	SI	2023	0	1	0	0	1	0	0	0	0	0	0	0	62
23307495	f	md	US	2023	0	0	0	0	0	0	0	0	0	0	0	0	
23307640	Not	cn	US	2023	0	0	0	0	0	0	0	0	0	0	0	0	70
23307780	Not	md	CN	2023	0	0	0	0	0	0	0	0	0	0	0	0	63
23307875	f	md	JP	2023	0	1	0	0	1	0	0	0	0	0	0	0	76
23308049	m	ph	JP	2023	0	1	1	0	0	0	0	0	0	0	0	0	70
23308138	Not	hp	CN	2023	0	0	0	0	0	0	0	0	0	0	0	0	
23308220	m	cn	TH	2023	0	0	0	0	0	0	0	0	0	0	0	0	56
23308255	m	md	JP	2023	0	0	0	0	0	0	0	0	0	0	0	0	
23308520	f	md	FR	2023	1	0	0	0	0	0	0	0	0	0	0	0	59.9
23308589	m	md	BG	2023	0	1	0	0	0	1	0	0	0	0	0	0	67
23308943	f	cn	US	2023	1	0	0	0	0	0	0	0	0	0	0	0	70
23309181	m	md	JP	2023	0	1	0	1	0	0	0	0	0	0	0	0	64
23309200	m	ph	JP	2023	1	0	0	0	0	0	0	0	0	0	0	1	79
23309295	Not	hp	JP	2023	0	0	0	0	0	0	0	0	0	0	0	0	
23309312	f	cn	EG	2023	1	0	0	0	0	0	0	0	0	0	0	0	
23309382	f	md	DE	2023	0	1	0	0	1	0	0	0	0	0	0	0	77
23309389	m	md	FI	2023	0	0	0	0	0	0	0	0	0	0	0	0	62
23309477	m	md	SK	2023	0	0	0	0	0	0	0	0	0	0	0	0	80
23309494	f	cn	US	2023	0	0	0	0	0	0	0	0	0	0	0	0	
23309600	f	md	NL	2023	0	1	1	0	0	0	0	0	0	0	0	0	66
23310622	Not	md	JP	2023	0	1	1	0	0	0	0	0	0	0	0	0	50
23310765	f	hp	JP	2023	0	0	0	0	0	0	0	0	0	0	0	0	
23311348	Not	hp	JP	2023	0	0	0	0	0	0	0	0	0	0	0	0	
23311625	f	md	FR	2023	1	0	0	0	0	0	0	0	0	0	0	0	39.3
23312048	f	cn	US	2023	1	0	0	0	0	0	0	0	0	0	0	0	
23312934	m	md	FR	2023	0	1	1	0	0	0	0	0	0	0	0	0	58
23313363	f	cn	TW	2023	0	1	0	0	0	1	0	0	0	0	0	0	62
23313383	m	md	BR	2023	0	0	0	0	0	0	0	0	0	0	0	0	60
23313401	Not	md	IT	2023	0	0	0	0	0	0	0	0	0	0	0	0	
23313528	Not	hp	JP	2023	0	0	0	0	0	0	0	0	0	0	0	0	
23313584	Not	hp	JP	2023	0	0	0	0	0	0	0	0	0	0	0	0	

23313692	f	hp	TH	2023	1	0	0	0	0	0	0	0	0	0	0	0	60.1
23314293	m	hp	PL	2023	0	1	1	0	0	0	0	0	0	0	0	0	59
23314354	f	md	AT	2023	0	1	1	0	0	0	0	0	0	0	0	0	63
23315462	m	md	JP	2023	0	1	0	1	0	0	0	0	0	0	0	0	
23315469	f	cn	CA	2023	1	0	0	0	0	0	0	0	0	0	0	0	
23315595	m	md	HU	2023	0	0	0	0	0	0	0	0	0	0	0	0	68
23315997	m	hp	JP	2023	0	0	0	0	0	0	0	0	0	0	0	0	68
23317190	Not	md	JP	2023	0	1	0	0	1	0	0	0	0	0	0	0	
23318629	Not	md	PL	2023	0	0	0	0	0	0	0	0	0	0	0	0	60
23319433	m	md	DE	2023	0	0	0	0	0	0	0	0	0	0	0	0	70
23319614	f	cn	TH	2023	1	0	0	0	0	0	0	0	0	0	0	0	93
23320815	f	md	DE	2023	0	1	1	0	0	0	0	0	0	0	0	0	67
23320860	Not	cn	SG	2023	1	0	0	0	0	0	0	0	0	0	0	0	83.9
23321252	m	cn	IN	2023	1	0	0	0	0	0	0	0	0	0	0	0	
23321442	Not	md	JP	2023	1	0	0	0	0	0	0	0	0	0	0	0	
23321542	f	ph	US	2023	1	0	0	0	0	0	0	0	0	0	0	0	67
23321632	m	md	CA	2023	0	0	0	0	0	0	0	0	0	0	0	0	40
23321730	f	md	BG	2023	0	1	0	0	0	1	0	0	0	0	0	0	56
23322012	m	md	JP	2023	0	1	0	0	0	1	0	0	0	0	0	0	79
23322049	m	md	JP	2023	0	1	0	0	0	1	0	0	0	0	0	0	70
23322059	m	md	TR	2023	0	1	1	0	0	0	0	0	0	0	0	0	63
23322122	f	md	AT	2023	0	0	0	0	0	0	0	0	0	0	0	0	
23322205	Not	md	JP	2023	0	0	0	0	0	0	0	0	0	0	0	0	
23322224	m	md	GB	2023	0	0	0	0	0	0	0	0	0	0	0	0	
23323967	Not	md	JP	2023	0	1	0	0	1	0	0	0	0	0	0	0	
23324239	m	md	JP	2023	0	0	0	0	0	0	0	0	0	0	0	0	60
23324460	Not	md	FR	2023	0	1	0	0	0	1	0	0	0	0	0	0	
23324808	f	cn	VN	2023	1	0	0	0	0	0	0	0	0	0	0	0	
23324970	f	cn	US	2023	0	0	0	0	0	0	0	0	0	0	0	0	62
23325004	m	md	IN	2023	0	0	0	0	0	0	0	0	0	0	0	0	
23325029	Not	md	JP	2023	0	1	0	0	1	0	0	0	0	0	0	0	
23325093	Not	md	JP	2023	0	1	0	0	1	0	0	0	0	0	0	0	
23325095	f	hp	CN	2023	1	0	0	0	0	0	0	0	0	0	0	0	64
23325523	m	md	JP	2023	0	0	0	0	0	0	0	0	0	0	0	0	65

23325532	Not	md	TR	2023	0	0	0	0	0	0	0	0	0	0	0	0	68
23325602	m	md	JP	2023	0	1	0	1	0	0	0	0	0	0	0	0	
23325620	f	md	US	2023	0	0	0	0	0	0	0	0	0	0	0	0	
23326072	Not	md	JP	2023	0	1	0	0	1	0	0	0	0	0	0	0	
23326158	Not	md	JP	2023	1	0	0	0	0	0	0	0	0	0	0	0	
23326548	m	hp	US	2023	0	0	0	0	0	0	0	0	0	0	0	0	58
23326602	m	md	KR	2023	0	1	1	0	0	0	0	0	0	0	0	0	
23326881	f	ph	JP	2023	1	0	0	0	0	0	0	0	0	0	0	0	65
23326959	m	cn	IN	2023	0	0	0	0	0	0	0	0	0	0	0	0	58
23327040	f	md	JP	2023	0	1	0	0	0	1	0	0	0	0	0	0	
23327063	m	md	JP	2023	0	1	0	1	0	0	0	0	0	0	0	0	68
23327151	m	md	MY	2023	1	0	0	0	0	0	0	0	0	0	0	0	71
23327754	m	md	PT	2023	0	0	0	0	0	0	0	0	0	0	0	0	74
23327974	m	md	JP	2023	0	1	0	1	0	0	0	0	0	0	0	0	72
23328156	Not	md	JP	2023	0	1	1	0	0	0	0	0	0	0	0	0	80
23328183	m	md	BE	2023	0	1	1	0	0	0	0	0	0	0	0	0	
23328474	m	cn	BE	2023	0	1	1	0	0	0	0	0	0	0	0	0	
23328753	m	md	CR	2023	0	1	0	0	0	1	0	0	0	0	0	0	62
23328843	f	md	DE	2023	0	1	1	0	0	0	0	0	0	0	0	0	73
23328981	Not	hp	TH	2023	1	0	0	0	0	0	0	0	0	0	0	0	
23328982	Not	cn	AR	2023	0	1	0	1	0	0	0	0	0	0	0	0	
23329292	m	cn	ID	2023	1	0	0	0	0	0	0	0	0	0	0	0	68.6
23329491	m	md	CA	2023	0	0	0	0	0	0	0	0	0	0	0	0	76
23330026	m	md	NO	2023	0	0	0	0	0	0	0	0	0	0	0	0	57
23330489	m	hp	US	2023	0	1	1	0	0	0	0	0	0	0	0	0	61
23330490	f	hp	AR	2023	0	1	0	1	0	0	0	0	0	0	0	0	
23330844	m	md	JP	2023	0	1	1	0	0	0	0	0	0	0	0	0	78
23330912	m	md	JP	2023	0	0	0	0	0	0	0	0	0	0	0	0	73
23331130	f	md	DE	2023	0	1	1	0	0	0	0	0	0	0	0	0	67
23331208	f	md	JP	2023	1	0	0	0	0	0	0	0	0	0	0	0	
23331381	m	ph	JP	2023	1	0	0	0	0	0	0	0	0	0	0	0	76
23331455	m	md	JP	2023	0	1	0	1	0	0	0	0	0	0	0	0	75
23331456	m	cn	ID	2023	1	0	0	0	0	0	0	0	0	0	0	0	62.5
23331506	f	md	JP	2023	0	1	1	0	0	0	0	0	0	0	0	0	60

23331667	m	cn	ID	2023	1	0	0	0	0	0	0	0	0	0	0	0	71.2
23331888	f	md	CO	2023	1	0	0	0	0	0	0	0	0	0	0	0	53
23332154	f	cn	ID	2023	1	0	0	0	0	0	0	0	0	0	0	0	78.4
23332355	Not	hp	CA	2023	0	1	0	1	0	0	0	0	0	0	0	0	
23332357	m	cn	TH	2023	1	0	0	0	0	0	0	0	0	0	0	0	55.9
23332428	m	cn	ID	2023	1	0	0	0	0	0	0	0	0	0	0	0	
23332557	m	md	JP	2023	0	1	1	0	0	0	0	0	0	0	0	0	80
23332646	m	md	DE	2023	0	1	0	0	1	0	0	0	0	0	0	0	73
23332882	Not	md	JP	2023	0	1	0	0	0	1	0	0	0	0	0	0	80
23332887	m	md	JP	2023	1	0	0	0	0	0	0	0	0	0	0	1	69
23333019	Not	hp	IT	2023	0	1	0	0	0	1	0	0	0	0	0	0	
23333659	f	hp	US	2023	0	1	1	0	0	0	0	0	0	0	0	0	79
23333660	f	hp	US	2023	0	1	1	0	0	0	0	0	0	0	0	0	77
23333872	Not	cn	AT	2023	0	0	0	0	0	0	0	0	0	0	0	0	
23334363	f	md	FR	2023	0	0	0	0	0	0	0	0	0	0	0	0	
23334493	f	md	DE	2023	0	1	0	0	1	0	0	0	0	0	0	0	68
23334753	f	cn	US	2023	1	0	0	0	0	0	0	0	0	0	0	0	
23334838	m	cn	ID	2023	1	0	0	0	0	0	0	0	0	0	0	0	
23334950	f	md	JP	2023	0	0	0	0	0	0	0	0	0	0	0	0	55
23335546	f	cn	CA	2023	0	0	0	0	0	0	0	0	0	0	0	0	65
23335899	Not	md	JP	2023	0	1	0	1	0	0	0	0	0	0	0	0	
23335993	Not	md	IT	2023	0	0	0	0	0	0	0	0	0	0	0	0	
23336029	m	md	JP	2023	0	1	0	1	0	0	0	0	0	0	0	0	70
23336112	m	md	JP	2023	0	1	0	1	0	0	0	0	0	0	0	0	52
23336897	Not	md	JP	2023	0	1	0	0	1	0	0	0	0	0	0	0	
23336929	f	cn	US	2023	1	0	0	0	0	0	0	0	0	0	0	0	74
23337252	Not	hp	US	2023	0	0	0	0	0	0	0	0	0	0	0	0	
23337253	Not	md	BR	2023	0	1	0	0	1	0	0	0	0	0	0	0	82
23337348	m	hp	JP	2023	0	1	0	0	1	0	0	0	0	0	0	0	80
23337364	f	cn	PK	2023	0	1	0	0	0	1	0	0	0	0	0	0	63
23338043	f	md	IT	2023	0	1	0	0	1	0	0	0	0	0	0	0	62
23338055	m	md	CN	2023	0	0	0	0	0	0	0	0	0	0	0	0	81
23338749	m	md	RU	2023	0	1	0	1	0	0	0	0	0	0	0	0	50
23338757	m	md	JP	2023	0	1	0	1	0	0	0	0	0	0	0	1	70

23338780	m	cn	IN	2023	1	0	0	0	0	0	0	0	0	0	0	0	
23338794	Not	hp	US	2023	0	0	0	0	0	0	0	0	0	0	0	0	
23338972	Not	ph	JP	2023	0	0	0	0	0	0	0	0	0	0	0	0	
23339012	f	ph	JP	2023	1	0	0	0	0	0	0	0	0	0	0	0	86
23339237	Not	hp	JP	2023	0	1	0	0	0	1	0	0	0	0	0	1	
23339316	m	md	DE	2023	0	1	0	0	1	0	0	0	0	0	0	0	70
23339507	f	md	DE	2023	0	1	0	0	1	0	0	0	0	0	0	0	65
23339708	m	cn	US	2023	0	1	1	0	0	0	0	0	0	0	0	0	73
23339760	Not	md	JP	2023	0	1	0	0	1	0	0	0	0	0	0	0	
23339764	Not	md	JP	2023	0	1	0	0	1	0	0	0	0	0	0	0	
23339765	Not	md	JP	2023	0	1	0	0	1	0	0	0	0	0	0	0	
23339771	m	cn	DE	2023	0	1	0	0	1	0	0	0	0	0	0	0	69
23339941	m	md	BE	2023	0	1	1	0	0	0	0	0	0	0	0	0	
23340099	m	md	RO	2023	1	0	0	0	0	0	0	0	0	0	0	0	
23340271	m	hp	US	2023	0	0	0	0	0	0	0	0	0	0	0	0	69
23340379	f	cn	US	2023	1	0	0	0	0	0	0	0	0	0	0	0	
23340491	m	cn	IN	2023	0	1	0	0	0	1	0	0	0	0	0	0	69
23340686	m	md	RU	2023	0	1	0	1	0	0	0	0	0	0	0	0	42
23340736	m	md	ES	2023	0	1	0	0	0	1	0	0	0	0	0	0	75
23340764	m	md	JP	2023	0	1	1	0	0	0	0	0	0	0	0	0	87
23341071	f	md	CN	2023	0	0	0	0	0	0	0	0	0	0	0	0	77
23341364	m	md	CA	2023	0	0	0	0	0	0	0	0	0	0	0	0	68
23341420	f	md	JP	2023	1	0	0	0	0	0	0	0	0	0	0	0	
23341659	m	md	JP	2023	0	1	0	1	0	0	0	0	0	0	0	0	
23341678	Not	md	JP	2023	0	1	0	1	0	0	0	0	0	0	0	0	60
23342455	f	md	JP	2023	0	1	0	0	0	1	0	0	0	0	0	0	60
23342600	m	md	BE	2023	0	1	1	0	0	0	0	0	0	0	0	0	
23342645	m	cn	SG	2023	0	1	0	0	0	1	0	0	0	0	0	0	71
23342711	m	cn	TH	2023	1	0	0	0	0	0	0	0	0	0	0	0	90
23342876	f	md	DE	2023	0	1	1	0	0	0	0	0	0	0	0	0	73
23343172	f	hp	IT	2023	0	1	0	1	0	0	0	0	0	0	0	0	71
23343581	m	md	BE	2023	0	1	1	0	0	0	0	0	0	0	0	0	
23343669	m	md	JP	2023	0	1	1	0	0	0	0	0	0	0	0	0	76
23343686	Not	hp	DE	2023	0	1	1	0	0	0	0	0	0	0	0	1	54

23344021	Not	hp	DE	2023	0	0	0	0	0	0	0	0	0	0	0	0	
23344441	f	md	FR	2023	0	1	0	0	0	1	0	0	0	0	0	0	
23344836	Not	md	JP	2023	0	1	0	0	1	0	0	0	0	0	0	0	70
23344883	Not	ph	JP	2023	1	0	0	0	0	0	0	0	0	0	0	0	
23345381	m	md	JP	2023	1	0	0	0	0	0	0	0	0	0	0	0	72
23345579	m	md	BG	2023	0	1	0	0	0	1	0	0	0	0	0	0	67
23345717	f	md	FR	2023	0	1	0	0	0	1	0	0	0	0	0	0	69
23346615	m	cn	IN	2023	0	0	0	0	0	0	0	0	0	0	0	0	43
23346667	m	cn	TW	2023	0	0	0	0	0	0	0	0	0	0	0	0	
23347021	f	md	JP	2023	0	1	0	1	0	0	0	0	0	0	0	0	66
23347408	m	md	US	2023	0	0	0	0	0	0	0	0	0	0	0	0	
23347445	Not	ph	JP	2023	1	0	0	0	0	0	0	0	0	0	0	0	
23347452	f	hp	NZ	2023	1	0	0	0	0	0	0	0	0	0	0	0	33
23347564	f	ph	FR	2023	0	0	0	0	0	0	0	0	0	0	0	0	76
23347673	f	md	JP	2023	0	1	1	0	0	0	0	0	0	0	0	0	70
23347845	m	md	TR	2023	0	0	0	0	0	0	0	0	0	0	0	0	69
23348196	m	md	JP	2023	0	1	0	0	1	0	0	0	0	0	0	0	55
23348831	f	md	JP	2023	0	1	0	0	0	1	0	0	0	0	0	0	70
23348919	m	md	DE	2023	0	1	1	0	0	0	0	0	0	0	0	1	76
23349468	Not	md	JP	2023	1	0	0	0	0	0	0	0	0	0	0	0	
23349523	f	cn	JP	2023	1	0	0	0	0	0	0	0	0	0	0	0	
23349788	f	md	JP	2023	0	1	0	1	0	0	0	0	0	0	0	0	68
23350413	f	md	JP	2023	0	0	0	0	0	0	0	0	0	0	0	0	
23350990	Not	md	AU	2023	1	0	0	0	0	0	0	0	0	0	0	0	
23351549	m	hp	IT	2023	0	1	1	0	0	0	0	0	0	0	0	0	68
23351578	f	md	JP	2023	0	1	0	1	0	0	0	0	0	0	0	0	
23352087	m	cn	TW	2023	0	0	0	0	0	0	0	0	0	0	0	0	
23352313	m	hp	US	2023	0	1	0	0	1	0	0	0	0	0	0	0	65
23352338	m	cn	IN	2023	0	0	0	0	0	0	0	0	0	0	0	0	53
23352362	f	cn	CN	2023	0	1	1	0	0	0	0	0	0	0	0	0	66
23352876	f	hp	ID	2023	1	0	0	0	0	0	0	0	0	0	0	0	47
23353015	f	hp	DK	2023	1	0	0	0	0	0	0	0	0	0	0	0	67
23353016	f	md	DE	2023	0	1	1	0	0	0	0	0	0	0	0	0	67
23353022	m	md	JP	2023	0	0	0	0	0	0	0	0	0	0	0	0	81

23353434	f	cn	US	2023	1	0	0	0	0	0	0	0	0	0	0	0	91.9
23353488	Not	ph	US	2023	0	0	0	0	0	0	0	0	0	0	0	0	
22888649	m	cn	IN	2024	0	0	0	0	0	0	0	0	0	0	0	0	46
22967604	f	cn	US	2024	0	0	0	0	0	0	0	0	0	0	0	0	73
22985402	m	md	JP	2024	0	1	1	0	0	0	0	0	0	0	0	0	60
23016354	Not	md	JP	2024	0	1	0	0	1	0	0	0	0	0	0	0	
23026595	m	md	JP	2024	0	1	0	0	1	0	0	0	0	0	0	1	70
23044571	f	md	PL	2024	0	1	0	0	1	0	0	0	0	0	0	0	53
23070180	m	cn	CA	2024	0	0	0	0	0	0	0	0	0	0	0	0	62
23074429	m	md	ES	2024	0	1	1	0	0	0	0	0	0	0	0	0	
23079210	m	md	JP	2024	0	1	1	0	0	0	0	0	0	0	0	1	70
23101928	m	md	JP	2024	0	1	1	0	0	0	0	0	0	0	0	0	80
23116073	m	md	ES	2024	0	1	0	0	0	1	0	0	0	0	0	0	75
23134446	f	cn	IN	2024	0	0	0	0	0	0	0	0	0	0	0	0	49
23137488	f	hp	PL	2024	0	1	1	0	0	0	0	0	0	0	0	0	59
23162839	Not	cn	ES	2024	0	0	0	0	0	0	0	0	0	0	0	0	73
23171853	m	md	JP	2024	0	1	0	1	0	0	0	0	0	0	0	1	78
23213133	f	cn	MX	2024	1	0	0	0	0	0	0	0	0	0	0	0	66
23223734	m	md	FR	2024	0	0	0	0	0	0	0	0	0	0	0	0	60
23227449	m	cn	IN	2024	0	0	0	0	0	0	0	0	0	0	0	0	57
23246639	f	md	JP	2024	0	1	0	1	0	0	0	0	0	0	0	0	75
23251397	m	md	US	2024	0	0	0	0	0	0	0	0	0	0	0	0	
23257479	m	ph	CA	2024	1	0	0	0	0	0	0	0	0	0	0	0	
23266082	m	md	JP	2024	0	1	1	0	0	0	0	0	0	0	0	0	70
23267148	m	cn	BR	2024	0	0	0	0	0	0	0	0	0	0	0	0	75
23279477	f	md	US	2024	0	1	1	0	0	0	0	0	0	0	0	0	54
23284042	m	md	DE	2024	0	1	0	0	1	0	0	0	0	0	0	0	79
23286020	Not	md	FR	2024	0	1	0	0	0	1	0	0	0	0	0	0	
23291391	m	md	JP	2024	0	1	0	0	1	0	0	0	0	0	0	0	65
23298516	Not	md	JP	2024	0	1	0	0	1	0	0	0	0	0	0	0	
23302552	f	md	JP	2024	0	0	0	0	0	0	0	0	0	0	0	0	51
23302603	m	md	JP	2024	0	0	0	0	0	0	0	0	0	0	0	0	79
23312849	m	md	DE	2024	0	1	0	0	1	0	0	0	0	0	0	0	50
23314205	f	md	IL	2024	0	0	0	0	0	0	0	0	0	0	0	0	66

23328471	f	cn	DE	2024	0	1	0	0	1	0	0	0	0	0	0	0	68
23339965	m	hp	PL	2024	0	1	1	0	0	0	0	0	0	0	0	0	72
23345163	m	hp	PL	2024	0	1	1	0	0	0	0	0	0	0	0	0	72
23347536	m	md	IN	2024	0	1	0	0	0	1	0	0	0	0	0	0	
23348220	m	hp	US	2024	0	1	1	0	0	0	0	0	0	0	0	0	71
23348503	m	md	JP	2024	0	1	0	1	0	0	0	0	0	0	0	1	78
23353819	f	hp	ID	2024	1	0	0	0	0	0	0	0	0	0	0	0	46
23353902	Not	hp	DE	2024	0	1	1	0	0	0	0	0	0	0	0	1	54
23354018	m	md	FR	2024	0	0	0	0	0	0	0	0	0	0	0	0	34
23354173	m	cn	BR	2024	0	0	0	0	0	0	0	0	0	0	0	0	59
23354188	Not	ph	JP	2024	1	0	0	0	0	0	0	0	0	0	0	0	
23354301	Not	md	JP	2024	0	1	0	1	0	0	0	0	0	0	0	0	
23354348	f	hp	IL	2024	0	0	0	0	0	0	0	0	0	0	0	0	
23354423	Not	hp	CN	2024	0	0	0	0	0	0	0	0	0	0	0	0	
23354446	m	md	BG	2024	0	1	0	0	0	1	0	0	0	0	0	0	67
23354723	m	md	JP	2024	0	0	0	0	0	0	0	0	0	0	0	0	49
23354767	f	md	JP	2024	0	0	0	0	0	0	0	0	0	0	0	0	
23354780	m	md	JP	2024	0	1	0	1	0	0	0	0	0	0	0	0	67
23354790	m	md	JP	2024	0	1	0	1	0	0	0	0	0	0	0	0	72
23355699	m	md	JP	2024	0	1	0	1	0	0	0	0	0	0	0	0	72
23355984	f	hp	US	2024	0	1	1	0	0	0	0	0	0	0	0	0	64
23356052	m	hp	CA	2024	1	0	0	0	0	0	0	0	0	0	0	0	
23356138	m	hp	US	2024	0	1	0	0	1	0	0	0	0	0	0	0	65
23357376	m	md	JP	2024	0	1	1	0	0	0	0	0	0	0	0	0	70
23357566	m	md	JP	2024	0	1	1	0	0	0	0	0	0	0	0	0	68
23357638	m	md	JP	2024	0	1	1	0	0	0	0	0	0	0	0	0	82
23357677	m	md	DE	2024	0	1	0	0	1	0	0	0	0	0	0	0	75
23357967	m	hp	JP	2024	0	0	0	0	0	0	0	0	0	0	0	0	
23358019	m	cn	CN	2024	0	0	0	0	0	0	0	0	0	0	0	0	46
23358062	m	md	PT	2024	0	1	1	0	0	0	0	0	0	0	0	0	44
23358680	f	ph	CA	2024	0	0	0	0	0	0	0	0	0	0	0	0	78
23358730	f	md	DK	2024	0	0	0	0	0	0	0	0	0	0	0	0	55
23358930	m	cn	MY	2024	0	0	0	0	0	0	0	0	0	0	0	0	60
23358961	m	md	JP	2024	0	1	1	0	0	0	0	0	0	0	0	0	71

23359056	f	md	CN	2024	0	1	1	0	0	0	0	0	0	0	0	0	73
23359065	f	cn	ID	2024	1	0	0	0	0	0	0	0	0	0	0	0	
23359068	m	md	JP	2024	0	1	1	0	0	0	0	0	0	0	0	0	75
23359133	f	md	DE	2024	0	0	0	0	0	0	0	0	0	0	0	0	47
23359173	m	md	NL	2024	0	0	0	0	0	0	0	0	0	0	0	0	68
23359378	m	md	DE	2024	0	1	1	0	0	0	0	0	0	0	0	0	
23359380	m	md	DE	2024	0	1	0	0	1	0	0	0	0	0	0	0	69
23359406	f	cn	TW	2024	0	1	0	0	0	1	0	0	0	0	0	0	51
23359553	m	md	JP	2024	0	0	0	0	0	0	0	0	0	0	0	0	34
23359696	Not	md	JP	2024	0	1	0	0	0	1	0	0	0	0	0	0	80
23360167	f	md	DE	2024	0	1	0	0	1	0	0	0	0	0	0	1	55
23361423	f	cn	ID	2024	1	0	0	0	0	0	0	0	0	0	0	0	41.3
23361528	m	cn	ID	2024	1	0	0	0	0	0	0	0	0	0	0	0	63.7
23361629	m	cn	ID	2024	1	0	0	0	0	0	0	0	0	0	0	0	74.2
23361800	m	cn	PK	2024	0	1	0	0	0	1	0	0	0	0	0	0	51
23362085	m	md	US	2024	0	0	0	0	0	0	0	0	0	0	0	0	65
23362103	f	md	NL	2024	1	0	0	0	0	0	0	0	0	0	0	0	49
23362327	Not	ph	JP	2024	1	0	0	0	0	0	0	0	0	0	0	0	
23362764	m	cn	US	2024	1	0	0	0	0	0	0	0	0	0	0	0	70
23362856	m	cn	SG	2024	0	0	0	0	0	0	0	0	0	0	0	0	63
23363329	m	md	US	2024	0	0	0	0	0	0	0	0	0	0	0	0	
23363463	f	md	US	2024	0	0	0	0	0	0	0	0	0	0	0	0	68
23363577	m	md	JP	2024	0	1	0	0	1	0	0	0	0	0	0	0	80
23363667	m	md	DE	2024	0	1	1	0	0	0	0	0	0	0	0	1	76
23363762	m	hp	US	2024	0	1	1	0	0	0	0	0	0	0	0	0	66
23363764	f	cn	TH	2024	1	0	0	0	0	0	0	0	0	0	0	0	55.5
23363922	Not	cn	US	2024	1	0	0	0	0	0	0	0	0	0	0	0	
23363969	Not	md	JP	2024	0	0	0	0	0	0	0	0	0	0	0	0	
23364050	f	cn	US	2024	1	0	0	0	0	0	0	0	0	0	0	0	
23364128	f	md	DE	2024	0	1	0	0	1	0	0	0	0	0	0	0	61
23364217	m	md	DE	2024	0	1	0	0	1	0	0	0	0	0	0	0	69
23364661	Not	md	JP	2024	0	1	0	1	0	0	0	0	0	0	0	0	
23364718	f	md	DE	2024	0	1	0	0	1	0	0	0	0	0	0	0	61
23364729	f	hp	GB	2024	0	0	0	0	0	0	0	0	0	0	0	0	

23364800	m	md	DE	2024	0	1	0	0	1	0	0	0	0	0	0	0	66
23365751	Not	hp	DE	2024	0	1	1	0	0	0	0	0	0	0	0	0	54
23365840	f	md	CN	2024	0	1	1	0	0	0	0	0	0	0	0	0	59
23366073	m	hp	PK	2024	0	1	0	0	0	1	0	0	0	0	0	0	70
23366207	f	hp	IT	2024	0	0	0	0	0	0	0	0	0	0	0	0	
23366332	m	md	JP	2024	0	1	0	0	1	0	0	0	0	0	0	0	68
23366483	f	md	KR	2024	0	0	0	0	0	0	0	0	0	0	0	0	62
23366574	f	md	KR	2024	0	0	0	0	0	0	0	0	0	0	0	0	
23366979	m	md	JP	2024	0	1	0	1	0	0	0	0	0	0	0	0	52
23367233	m	md	BG	2024	0	1	0	0	0	1	0	0	0	0	0	0	67
23367423	m	md	JP	2024	0	1	0	1	0	0	0	0	0	0	0	0	55
23367828	Not	md	FR	2024	0	0	0	0	0	0	0	0	0	0	0	0	
23367914	Not	cn	JP	2024	1	0	0	0	0	0	0	0	0	0	0	0	
23368544	f	md	DK	2024	0	1	0	0	0	1	0	0	0	0	0	1	59
23369537	f	ph	US	2024	0	0	0	0	0	0	0	0	0	0	0	0	62
23369557	m	cn	CA	2024	0	0	0	0	0	0	0	0	0	0	0	0	63
23369604	f	cn	US	2024	1	0	0	0	0	0	0	0	0	0	0	0	81.2
23369612	m	cn	ID	2024	1	0	0	0	0	0	0	0	0	0	0	0	
23369949	m	md	PL	2024	0	1	1	0	0	0	0	0	0	0	0	1	72
23370054	f	md	KR	2024	0	0	0	0	0	0	0	0	0	0	0	0	
23370413	m	md	PL	2024	0	0	0	0	0	0	0	0	0	0	0	0	68
23371608	f	md	MY	2024	0	0	0	0	0	0	0	0	0	0	0	0	66
23373145	f	ph	US	2024	0	1	1	0	0	0	0	0	0	0	0	0	80
23374735	m	md	FR	2024	0	0	0	0	0	0	0	0	0	0	0	0	79
23374868	m	hp	NL	2024	0	0	0	0	0	0	0	0	0	0	0	0	80
23375582	f	hp	US	2024	0	1	1	0	0	0	0	0	0	0	0	0	60
23375682	f	md	US	2024	0	0	0	0	0	0	0	0	0	0	0	0	49
23375727	m	md	DE	2024	1	0	0	0	0	0	0	0	0	0	0	0	
23376385	f	md	DE	2024	0	1	0	0	1	0	0	0	0	0	0	0	68
23377548	m	hp	NL	2024	0	0	0	0	0	0	0	0	0	0	0	0	68
23377991	m	cn	BR	2024	0	0	0	0	0	0	0	0	0	0	0	0	75
23378203	m	md	FR	2024	0	0	0	0	0	0	0	0	0	0	0	0	79
23378239	f	cn	NP	2024	0	0	0	0	0	0	0	0	0	0	0	0	48
23378402	m	cn	IN	2024	0	0	0	0	0	0	0	0	0	0	0	0	73

23378848	m	md	DE	2024	0	1	1	0	0	0	0	0	0	0	0	0	
23379110	f	ph	MY	2024	1	0	0	0	0	0	0	0	0	0	0	0	
23379112	m	md	CN	2024	0	0	0	0	0	0	0	0	0	0	0	0	61
23379343	m	md	DE	2024	0	1	1	0	0	0	0	0	0	0	0	0	
23379530	f	cn	US	2024	0	0	0	0	0	0	0	0	0	0	0	0	
23379622	Not	md	IL	2024	0	1	1	0	0	0	0	0	0	0	0	0	64
23381079	f	md	TH	2024	1	0	0	0	0	0	0	0	0	0	0	0	
23382271	f	hp	US	2024	0	0	0	0	0	0	0	0	0	0	0	0	37
23382399	m	hp	PL	2024	0	1	1	0	0	0	0	0	0	0	0	0	59
23382617	f	cn	CN	2024	0	0	0	0	0	0	0	0	0	0	0	0	73
23382734	m	md	FR	2024	0	1	0	0	0	1	0	0	0	0	0	0	75
23382817	Not	md	AU	2024	0	0	0	0	0	0	0	0	0	0	0	0	
23383104	f	md	FR	2024	0	1	0	0	0	1	0	0	0	0	0	0	63
23383148	m	hp	FI	2024	0	0	0	0	0	0	0	0	0	0	0	0	81
23383908	m	md	ES	2024	0	0	0	0	0	0	0	0	0	0	0	0	
23384025	f	md	SE	2024	0	1	1	0	0	0	0	0	0	0	0	0	82
23385965	f	md	NL	2024	0	1	0	0	0	1	0	0	0	0	0	0	74
23386269	m	md	DE	2024	1	0	0	0	0	0	0	0	0	0	0	0	
23386912	f	md	US	2024	0	1	1	0	0	0	0	0	0	0	0	0	54
23387627	m	hp	CH	2024	0	0	0	0	0	0	0	0	0	0	0	0	61
23388458	m	md	DE	2024	0	1	1	0	0	0	0	0	0	0	0	1	62
23388496	m	cn	CA	2024	1	0	0	0	0	0	0	0	0	0	0	0	52.1
23388497	m	md	NL	2024	1	0	0	0	0	0	0	0	0	0	0	0	60
23388513	m	hp	NL	2024	0	0	0	0	0	0	0	0	0	0	0	0	80
23388568	f	md	NL	2024	1	0	0	0	0	0	0	0	0	0	0	0	49
23388636	Not	ph	SG	2024	1	0	0	0	0	0	0	0	0	0	0	0	70
23389896	m	md	JP	2024	0	1	0	1	0	0	0	0	0	0	0	0	81
23390078	f	md	US	2024	0	1	0	0	0	1	0	0	0	0	0	0	62
23390305	f	md	US	2024	0	0	0	0	0	0	0	0	0	0	0	0	37
23390521	m	md	DE	2024	0	1	0	0	1	0	0	0	0	0	0	1	59
23391054	f	md	US	2024	0	0	0	0	0	0	0	0	0	0	0	0	70
23391122	f	hp	PL	2024	0	1	1	0	0	0	0	0	0	0	0	1	57
23391311	m	md	JP	2024	0	1	0	0	1	0	0	0	0	0	0	0	70
23392105	f	hp	DE	2024	0	1	0	1	0	0	0	0	0	0	0	0	71

23392110	m	hp	ES	2024	0	1	0	1	0	0	0	0	0	0	0	0	85
23392112	m	hp	JP	2024	1	0	0	0	0	0	0	0	0	0	0	0	78
23393389	f	md	KR	2024	0	0	0	0	0	0	0	0	0	0	0	0	49
23393823	f	md	PL	2024	0	0	0	0	0	0	0	0	0	0	0	0	
23393883	m	md	FR	2024	0	1	1	0	0	0	0	0	0	0	0	0	
23394358	m	md	JP	2024	0	1	0	1	0	0	0	0	0	0	0	0	55
23394359	m	md	CN	2024	0	0	0	0	0	0	0	0	0	0	0	0	62
23394502	m	md	ES	2024	0	0	0	0	0	0	0	0	0	0	0	0	
23394603	m	md	JP	2024	0	1	0	0	1	0	0	0	0	0	0	0	64
23394637	f	md	GB	2024	0	1	0	0	0	1	0	0	0	0	0	0	71
23395129	f	md	US	2024	0	0	0	0	0	0	0	0	0	0	0	0	70
23395372	Not	hp	GB	2024	0	0	0	0	0	0	0	0	0	0	0	0	
23395465	f	hp	PL	2024	0	1	0	0	0	1	0	0	0	0	0	0	
23395488	Not	hp	US	2024	0	0	0	0	0	0	0	0	0	0	0	0	37
23395512	f	hp	GB	2024	0	0	0	0	0	0	0	0	0	0	0	0	
23395538	f	cn	CA	2024	1	0	0	0	0	0	0	0	0	0	0	0	84.5
23395942	f	md	FR	2024	0	1	1	0	0	0	0	0	0	0	0	0	70
23396035	f	cn	ID	2024	1	0	0	0	0	0	0	0	0	0	0	0	
23396585	m	md	JP	2024	0	0	0	0	0	0	0	0	0	0	0	0	80
23396742	f	md	BR	2024	0	0	0	0	0	0	0	0	0	0	0	0	60
23397020	m	md	ES	2024	0	0	0	0	0	0	0	0	0	0	0	0	
23397375	m	md	CA	2024	0	0	0	0	0	0	0	0	0	0	0	0	76
23397400	f	hp	CA	2024	0	1	1	0	0	0	0	0	0	0	0	0	38
23397409	m	hp	CA	2024	0	0	0	0	0	0	0	0	0	0	0	0	71
23397764	m	md	FR	2024	0	1	0	0	0	1	0	0	0	0	0	1	75
23397775	f	cn	IN	2024	0	0	0	0	0	0	0	0	0	0	0	0	65
23397779	m	ph	IN	2024	0	0	0	0	0	0	0	0	0	0	0	0	70
23397785	f	md	US	2024	0	0	0	0	0	0	0	0	0	0	0	0	70
23397859	f	hp	US	2024	0	0	0	0	0	0	0	0	0	0	0	0	37
23398053	f	ph	CA	2024	0	0	0	0	0	0	0	0	0	0	0	0	78
23398367	m	md	JP	2024	1	0	0	0	0	0	0	0	0	0	0	0	78
23398632	m	hp	US	2024	0	1	1	0	0	0	0	0	0	0	0	0	75
23398784	m	md	DE	2024	1	0	0	0	0	0	0	0	0	0	0	0	
23399008	f	ph	US	2024	0	1	1	0	0	0	0	0	0	0	0	0	79

23399648	m	md	AU	2024	0	1	0	0	0	1	0	0	0	0	0	0	54
23399780	Not	md	JP	2024	1	0	0	0	0	0	0	0	0	0	0	0	
23399810	f	md	AU	2024	0	1	0	0	0	1	0	0	0	0	0	0	34
23400089	Not	hp	JP	2024	0	1	0	0	0	1	0	0	0	0	0	0	
23400446	m	md	JP	2024	0	1	1	0	0	0	0	0	0	0	0	0	75
23400690	m	md	JP	2024	0	0	0	0	0	0	0	0	0	0	0	0	90
23400802	f	ph	BR	2024	0	1	1	0	0	0	0	0	0	0	0	0	68
23401010	m	md	DE	2024	1	0	0	0	0	0	0	0	0	0	0	0	
23401027	m	cn	US	2024	0	0	0	0	0	0	0	0	0	0	0	0	
23401121	m	md	DE	2024	0	1	1	0	0	0	0	0	0	0	0	0	68
23401176	f	md	CZ	2024	0	0	0	0	0	0	0	0	0	0	0	0	24
23401268	m	md	US	2024	0	0	0	0	0	0	0	0	0	0	0	0	55
23401383	f	cn	ID	2024	1	0	0	0	0	0	0	0	0	0	0	0	73
23401384	f	cn	ID	2024	1	0	0	0	0	0	0	0	0	0	0	0	77.4
23401409	f	cn	ID	2024	1	0	0	0	0	0	0	0	0	0	0	0	66.2
23401605	m	cn	TH	2024	1	0	0	0	0	0	0	0	0	0	0	0	73.7
23401768	Not	md	JP	2024	0	1	0	1	0	0	0	0	0	0	0	0	60
23401846	m	cn	ID	2024	1	0	0	0	0	0	0	0	0	0	0	0	71.4
23402168	m	md	PL	2024	0	1	0	1	0	0	0	0	0	0	0	0	49
23402258	f	hp	MY	2024	1	0	0	0	0	0	0	0	0	0	0	0	84
23402607	Not	cn	US	2024	1	0	0	0	0	0	0	0	0	0	0	0	
23402712	m	md	FR	2024	0	1	0	0	0	1	0	0	0	0	0	0	42
23402799	Not	md	JP	2024	0	1	0	0	0	1	0	0	0	0	0	0	
23402942	f	md	US	2024	0	0	0	0	0	0	0	0	0	0	0	0	70
23403222	Not	md	ZA	2024	0	1	0	0	0	1	0	0	0	0	0	0	
23403251	f	md	BE	2024	0	0	0	0	0	0	0	0	0	0	0	0	68
23403718	m	cn	ID	2024	1	0	0	0	0	0	0	0	0	0	0	0	64.9
23404064	m	md	DE	2024	1	0	0	0	0	0	0	0	0	0	0	0	
23404152	m	cn	TW	2024	0	1	0	0	0	1	0	0	0	0	0	0	49
23404160	Not	md	JP	2024	0	1	0	1	0	0	0	0	0	0	0	0	
23404364	m	md	DE	2024	0	1	1	0	0	0	0	0	0	0	0	0	
23404548	m	ph	JP	2024	0	1	1	0	0	0	0	0	0	0	0	0	77
23404638	m	md	JP	2024	0	0	0	0	0	0	0	0	0	0	0	0	79
23404728	m	md	TR	2024	0	0	0	0	0	0	0	0	0	0	0	0	76

23404734	f	ph	HR	2024	0	1	1	0	0	0	0	0	0	0	0	1	57
23404749	m	md	CN	2024	0	0	0	0	0	0	0	0	0	0	0	0	48
23404775	Not	md	FR	2024	0	1	0	0	0	1	0	0	0	0	0	0	
23404786	m	md	US	2024	0	0	0	0	0	0	0	0	0	0	0	0	
23405123	m	md	ES	2024	0	0	0	0	0	0	0	0	0	0	0	0	
23405133	m	hp	GR	2024	0	1	1	0	0	0	0	0	0	0	0	0	56
23405242	Not	hp	KR	2024	0	0	0	0	0	0	0	0	0	0	0	0	
23405245	m	cn	US	2024	1	0	0	0	0	0	0	0	0	0	0	0	40
23405334	f	hp	DK	2024	1	0	0	0	0	0	0	0	0	0	0	0	73
23405361	m	md	JP	2024	0	0	0	0	0	0	0	0	0	0	0	0	67
23405660	m	ph	JP	2024	0	1	1	0	0	0	0	0	0	0	0	0	
23406132	f	md	US	2024	0	1	1	0	0	0	0	0	0	0	0	0	68
23406429	Not	ph	US	2024	0	1	0	1	0	0	0	0	0	0	0	0	
23406436	f	md	TW	2024	0	1	0	0	0	1	0	0	0	0	0	0	69
23406779	f	hp	US	2024	1	0	0	0	0	0	0	0	0	0	0	0	91
23407478	m	md	CA	2024	0	1	1	0	0	0	0	0	0	0	0	0	76
23407574	m	md	JP	2024	0	1	0	0	0	1	0	0	0	0	0	0	80
23407591	Not	md	JP	2024	0	1	0	0	0	1	0	0	0	0	0	0	80
23407985	m	md	JP	2024	0	1	0	0	0	1	0	0	0	0	0	0	76
23408091	f	hp	BR	2024	0	1	1	0	0	0	0	0	0	0	0	0	57
23408207	m	cn	TH	2024	1	0	0	0	0	0	0	0	0	0	0	0	88.9
23408516	m	cn	RO	2024	0	1	1	0	0	0	0	0	0	0	0	0	56
23408548	Not	ph	JP	2024	1	0	0	0	0	0	0	0	0	0	0	0	
23408736	f	md	JP	2024	1	0	0	0	0	0	0	0	0	0	0	0	90
23409290	f	hp	TW	2024	0	0	0	0	0	0	0	0	0	0	0	0	52
23410049	f	ph	US	2024	0	1	1	0	0	0	0	0	0	0	0	0	
23410892	f	hp	US	2024	0	0	0	0	0	0	0	0	0	0	0	0	61
23410921	Not	cn	US	2024	1	0	0	0	0	0	0	0	0	0	0	0	60
23411393	m	md	RU	2024	0	0	0	0	0	0	0	0	0	0	0	0	32
23411704	m	md	FR	2024	0	1	0	0	0	1	0	0	0	0	0	0	42
23411939	f	md	DE	2024	0	1	1	0	0	0	0	0	0	0	0	0	69
23412353	m	md	JP	2024	0	1	0	0	0	1	0	0	0	0	0	1	
23414915	f	hp	BR	2024	0	0	0	0	0	0	0	0	0	0	0	0	57
23415044	m	md	DE	2024	0	1	0	0	1	0	0	0	0	0	0	0	57

23415588	f	md	DE	2024	0	1	1	0	0	0	0	0	0	0	0	0	65
23415777	m	md	CA	2024	0	0	0	0	0	0	0	0	0	0	0	0	68
23415924	f	md	TW	2024	1	0	0	0	0	0	0	0	0	0	0	0	56
23415940	m	cn	ES	2024	0	0	0	0	0	0	0	0	0	0	0	0	73
23416179	f	md	JP	2024	1	0	0	0	0	0	0	0	0	0	0	0	80
23416190	m	md	US	2024	0	0	0	0	0	0	0	0	0	0	0	0	
23416438	Not	md	GB	2024	0	1	0	0	0	1	0	0	0	0	0	0	50
23417458	m	md	JP	2024	0	1	0	1	0	0	0	0	0	0	0	0	75
23417990	m	md	JP	2024	0	1	0	1	0	0	0	0	0	0	0	1	59
23418105	f	md	DE	2024	0	1	0	0	1	0	0	0	0	0	0	0	71
23418937	f	cn	JP	2024	0	0	0	0	0	0	0	0	0	0	0	0	68
23419045	m	md	CN	2024	0	1	1	0	0	0	0	0	0	0	0	0	60
23419052	f	cn	DE	2024	0	1	1	0	0	0	0	0	0	0	0	0	
23419081	m	md	JP	2024	0	1	0	0	1	0	0	0	0	0	0	0	73
23419097	f	ph	MY	2024	1	0	0	0	0	0	0	0	0	0	0	0	
23419558	m	md	BR	2024	0	1	1	0	0	0	0	0	0	0	0	0	81
23419816	f	cn	US	2024	0	0	0	0	0	0	0	0	0	0	0	0	70
23420116	f	hp	US	2024	0	0	0	0	0	0	0	0	0	0	0	0	34
23420196	f	cn	TW	2024	0	1	0	0	0	1	0	0	0	0	0	0	75
23420646	Not	hp	US	2024	0	0	0	0	0	0	0	0	0	0	0	0	
23420900	m	md	JP	2024	0	0	0	0	0	0	0	0	0	0	0	0	75
23420954	f	md	DE	2024	0	1	0	0	1	0	0	0	0	0	0	0	82
23421161	m	md	JP	2024	0	1	0	1	0	0	0	0	0	0	0	0	71
23421368	Not	md	JP	2024	0	1	0	1	0	0	0	0	0	0	0	0	67
23421393	m	cn	KR	2024	0	0	0	0	0	0	0	0	0	0	0	0	47
23421577	Not	md	JP	2024	0	0	0	0	0	0	0	0	0	0	0	0	
23421718	Not	md	IT	2024	0	0	0	0	0	0	0	0	0	0	0	0	
23421988	f	ph	US	2024	0	0	0	0	0	0	0	0	0	0	0	0	75
23422665	m	md	KR	2024	0	0	0	0	0	0	0	0	0	0	0	0	51
23422778	f	cn	JP	2024	0	0	0	0	0	0	0	0	0	0	0	0	76
23423170	m	md	JP	2024	0	1	0	1	0	0	0	0	0	0	0	0	57
23423454	Not	md	JP	2024	0	1	0	1	0	0	0	0	0	0	0	0	
23423489	m	md	FR	2024	0	1	0	0	0	1	0	0	0	0	0	0	69
23423916	Not	md	JP	2024	0	1	0	0	0	1	0	0	0	0	0	0	72

23424551	m	ph	FR	2024	0	1	1	0	0	0	0	0	0	0	0	0	74
23424725	m	hp	IT	2024	0	1	1	0	0	0	0	0	0	0	0	0	68
23425297	f	md	JP	2024	0	0	0	0	0	0	0	0	0	0	0	0	80
23425338	m	md	FR	2024	0	1	0	0	0	1	0	0	0	0	0	0	70
23425735	m	md	DE	2024	0	0	0	0	0	0	0	0	0	0	0	0	70
23426914	m	cn	IN	2024	0	0	0	0	0	0	0	0	0	0	0	0	81
23426971	m	ph	FR	2024	0	1	1	0	0	0	0	0	0	0	0	0	77
23427094	m	cn	IN	2024	0	1	0	0	0	1	0	0	0	0	0	0	63
23427650	m	md	JP	2024	0	1	0	1	0	0	0	0	0	0	0	0	71
23428192	m	md	JP	2024	0	1	0	1	0	0	0	0	0	0	0	0	65
23428295	m	md	FR	2024	0	1	0	0	0	1	0	0	0	0	0	0	52
23428347	f	md	DE	2024	0	1	1	0	0	0	0	0	0	0	0	0	
23428533	m	md	PT	2024	0	1	1	0	0	0	0	0	0	0	0	0	71
23428649	m	md	JP	2024	0	1	0	1	0	0	0	0	0	0	0	1	80
23428805	f	md	FR	2024	1	0	0	0	0	0	0	0	0	0	0	0	91.5
23429427	Not	md	IT	2024	0	0	0	0	0	0	0	0	0	0	0	0	68
23429506	m	md	JP	2024	0	0	0	0	0	0	0	0	0	0	0	0	69
23429594	m	md	IN	2024	0	0	0	0	0	0	0	0	0	0	0	0	
23429660	f	md	DE	2024	0	0	0	0	0	0	0	0	0	0	0	0	76
23429837	m	md	JP	2024	0	1	1	0	0	0	0	0	0	0	0	0	58
23429856	f	md	JP	2024	0	1	1	0	0	0	0	0	0	0	0	0	75
23429888	m	hp	KR	2024	0	1	1	0	0	0	0	0	0	0	0	0	63
23430035	m	md	JP	2024	0	0	0	0	0	0	0	0	0	0	0	0	80
23430347	m	md	JP	2024	0	1	0	0	0	1	0	0	0	0	0	0	77
23430407	m	md	CN	2024	1	0	0	0	0	0	0	0	0	0	0	0	53
23430500	f	ph	JP	2024	0	0	0	0	0	0	0	0	0	0	0	0	40
23430933	f	ph	AU	2024	0	0	0	0	0	0	0	0	0	0	0	0	46
23431032	m	cn	CA	2024	0	0	0	0	0	0	0	0	0	0	0	0	36
23431046	m	md	JP	2024	0	1	0	0	0	1	0	0	0	0	0	0	65
23431070	m	md	JP	2024	0	1	0	0	1	0	0	0	0	0	0	0	56
23431325	f	cn	US	2024	1	0	0	0	0	0	0	0	0	0	0	0	
23431421	f	hp	CA	2024	1	0	0	0	0	0	0	0	0	0	0	0	74.3
23431424	m	md	JP	2024	0	1	0	1	0	0	0	0	0	0	0	0	62
23431487	Not	ph	JP	2024	1	0	0	0	0	0	0	0	0	0	0	0	70

23431713	m	cn	PL	2024	0	0	0	0	0	0	0	0	0	0	0	0	68
23431727	f	ph	CA	2024	0	0	0	0	0	0	0	0	0	0	0	0	78
23431752	m	md	JP	2024	0	1	0	1	0	0	0	0	0	0	0	0	
23432153	f	cn	CA	2024	1	0	0	0	0	0	0	0	0	0	0	0	
23432203	f	hp	US	2024	0	0	0	0	0	0	0	0	0	0	0	0	68
23432232	f	cn	US	2024	0	0	0	0	0	0	0	0	0	0	0	0	67
23432307	f	cn	CA	2024	1	0	0	0	0	0	0	0	0	0	0	0	
23432357	f	md	DE	2024	0	0	0	0	0	0	0	0	0	0	0	0	
23432517	m	md	JP	2024	0	1	0	1	0	0	0	0	0	0	0	0	70
23432573	m	md	JP	2024	0	1	1	0	0	0	0	0	0	0	0	0	80
23432951	m	md	JP	2024	0	1	1	0	0	0	0	0	0	0	0	0	70
23433122	Not	md	JP	2024	0	1	0	1	0	0	0	0	0	0	0	0	70
23433281	f	md	DE	2024	0	1	1	0	0	0	0	0	0	0	0	0	
23433488	m	md	JP	2024	0	1	0	1	0	0	0	0	0	0	0	0	
23433793	m	cn	US	2024	0	0	0	0	0	0	0	0	0	0	0	0	
23433831	f	hp	US	2024	0	0	0	0	0	0	0	0	0	0	0	0	
23433857	m	md	JP	2024	0	1	0	1	0	0	0	0	0	0	0	0	74
23433915	m	md	RU	2024	1	0	0	0	0	0	0	0	0	0	0	0	45
23435437	f	md	JP	2024	0	1	0	0	1	0	0	0	0	0	0	0	51
23437032	f	md	JP	2024	0	0	0	0	0	0	0	0	0	0	0	0	80
23437201	m	md	JP	2024	0	1	0	0	0	1	0	0	0	0	0	0	
23437587	m	hp	RO	2024	0	1	1	0	0	0	0	0	0	0	0	0	56
23438126	Not	md	JP	2024	0	1	0	1	0	0	0	0	0	0	0	0	
23438258	m	md	JP	2024	0	1	0	1	0	0	0	0	0	0	0	1	52
23438494	f	cn	TH	2024	1	0	0	0	0	0	0	0	0	0	0	0	58.3
23438566	m	md	CA	2024	1	0	0	0	0	0	0	0	0	0	0	0	
23438685	m	md	DE	2024	0	1	0	0	1	0	0	0	0	0	0	0	86
23439001	f	md	RU	2024	1	0	0	0	0	0	0	0	0	0	0	0	42
23439410	m	md	JP	2024	0	1	0	1	0	0	0	0	0	0	0	1	
23440110	f	md	JP	2024	0	1	1	0	0	0	0	0	0	0	0	0	72
23440350	f	md	IL	2024	0	0	0	0	0	0	0	0	0	0	0	0	
23440618	f	md	CH	2024	0	1	0	0	1	0	0	0	0	0	0	0	67
23440696	f	hp	AT	2024	1	0	0	0	0	0	0	0	0	0	0	0	61
23440724	Not	md	JP	2024	0	1	0	0	1	0	0	0	0	0	0	0	70

23441069	f	hp	NL	2024	1	0	0	0	0	0	0	0	0	0	0	0	55
23441479	Not	hp	CN	2024	0	0	0	0	0	0	0	0	0	0	0	0	
23441559	m	md	BG	2024	0	0	0	0	0	0	0	0	0	0	0	0	59
23441597	f	md	BG	2024	0	1	0	0	0	1	0	0	0	0	0	0	
23441607	f	md	DE	2024	0	1	0	0	1	0	0	0	0	0	0	0	76
23441816	f	md	RU	2024	1	0	0	0	0	0	0	0	0	0	0	0	64
23441832	f	cn	TW	2024	0	1	0	0	0	1	0	0	0	0	0	0	72
23442252	m	md	JP	2024	0	1	1	0	0	0	0	0	0	0	0	0	76
23442271	m	cn	TH	2024	1	0	0	0	0	0	0	0	0	0	0	0	75
23442414	f	md	US	2024	0	0	0	0	0	0	0	0	0	0	0	0	
23442524	m	ph	FR	2024	0	1	1	0	0	0	0	0	0	0	0	0	74
23442666	f	md	DE	2024	0	1	0	0	1	0	0	0	0	0	0	0	74
23442723	m	md	US	2024	0	1	1	0	0	0	0	0	0	0	0	0	60
23443093	m	md	JP	2024	0	1	1	0	0	0	0	0	0	0	0	0	66
23443243	Not	md	JP	2024	0	1	0	0	0	1	0	0	0	0	0	0	
23443356	f	cn	DE	2024	0	0	0	0	0	0	0	0	0	0	0	0	
23443663	Not	md	JP	2024	0	1	0	0	0	1	0	0	0	0	0	0	80
23443838	m	cn	AU	2024	0	0	0	0	0	0	0	0	0	0	0	0	62
23444095	f	md	US	2024	0	1	1	0	0	0	0	0	0	0	0	0	66
23444107	m	md	FR	2024	0	0	0	0	0	0	0	0	0	0	0	0	81
23444176	m	md	JP	2024	0	1	0	0	0	1	0	0	0	0	0	0	
23444216	m	md	JP	2024	0	1	0	1	0	0	0	0	0	0	0	0	
23444555	m	md	EG	2024	0	1	1	0	0	0	0	0	0	0	0	0	74
23444666	m	md	JP	2024	0	1	1	0	0	0	0	0	0	0	0	0	80
23444702	m	md	TR	2024	0	1	1	0	0	0	0	0	0	0	0	0	42
23445018	f	cn	DE	2024	0	1	0	0	1	0	0	0	0	0	0	0	68
23445052	f	cn	US	2024	1	0	0	0	0	0	0	0	0	0	0	0	65.7
23445249	Not	hp	AT	2024	0	0	0	0	0	0	0	0	0	0	0	0	
23445418	m	md	JP	2024	0	1	0	1	0	0	0	0	0	0	0	0	47
23445535	Not	md	JP	2024	0	1	0	0	0	1	0	0	0	0	0	0	
23445586	f	md	JP	2024	0	0	0	0	0	0	0	0	0	0	0	0	70
23445595	f	md	JP	2024	0	0	0	0	0	0	0	0	0	0	0	0	55
23445837	f	md	JP	2024	0	1	0	0	1	0	0	0	0	0	0	0	74
23446196	Not	ph	TH	2024	0	1	0	1	0	0	0	0	0	0	0	0	

23446293	m	md	JP	2024	0	0	0	0	0	0	1	0	0	1	0	1	73
23446434	m	md	DE	2024	0	1	0	0	1	0	0	0	0	0	0	0	59
23446508	m	hp	CA	2024	1	0	0	0	0	0	0	0	0	0	0	0	
23446618	m	md	JP	2024	0	1	0	1	0	0	0	0	0	0	0	1	90
23446807	f	hp	CN	2024	0	0	0	0	0	0	0	0	0	0	0	0	
23446927	m	ph	US	2024	0	0	0	0	0	0	0	0	0	0	0	0	56
23447505	m	md	JP	2024	0	1	0	1	0	0	0	0	0	0	0	0	
23447619	Not	md	US	2024	0	1	0	1	0	0	0	0	0	0	0	1	
23447712	m	cn	IN	2024	1	0	0	0	0	0	0	0	0	0	0	0	51
23447773	f	md	SK	2024	0	1	0	1	0	0	0	0	0	0	0	1	
23448326	m	md	JP	2024	0	0	0	0	0	0	0	0	0	0	0	0	
23448350	f	hp	US	2024	0	0	0	0	0	0	0	0	0	0	0	0	
23448556	Not	md	US	2024	0	1	0	1	0	0	0	0	0	0	0	1	
23449051	f	md	JP	2024	0	1	0	0	1	0	0	0	0	0	0	0	74
23449061	m	md	JP	2024	0	1	0	0	1	0	0	0	0	0	0	0	70
23449318	f	md	JP	2024	0	1	1	0	0	0	0	0	0	0	0	0	70
23449679	m	md	CN	2024	0	0	0	0	0	0	0	0	0	0	0	0	
23449858	f	md	DE	2024	0	1	1	0	0	0	0	0	0	0	0	0	
23449905	f	ph	US	2024	0	0	0	0	0	0	0	0	0	0	0	0	56
23450151	m	md	DE	2024	0	1	1	0	0	0	0	0	0	0	0	0	61
23450292	f	ph	US	2024	0	0	0	0	0	0	0	0	0	0	0	0	33
23450554	f	hp	US	2024	0	1	1	0	0	0	0	0	0	0	0	0	79
23450605	m	md	DE	2024	0	0	0	0	0	0	0	0	0	0	0	0	51
23451045	f	md	JP	2024	0	1	0	0	1	0	0	0	0	0	0	0	67
23451146	f	md	JP	2024	0	0	0	0	0	0	0	0	0	0	0	0	58
23451344	f	cn	IN	2024	0	1	0	0	0	1	0	0	0	0	0	0	59
23452484	f	md	JP	2024	0	1	0	0	1	0	0	0	0	0	0	0	67
23452852	f	md	GB	2024	0	0	0	0	0	0	0	0	0	0	0	0	71
23452882	f	md	GB	2024	1	0	0	0	0	0	0	0	0	0	0	0	
23452887	m	hp	JP	2024	0	1	0	0	1	0	0	0	0	0	0	0	61
23453750	f	md	CH	2024	0	1	0	0	1	0	0	0	0	0	0	0	67
23454181	m	md	JP	2024	0	1	0	0	1	0	0	0	0	0	0	0	60
23454297	f	md	JP	2024	0	1	0	0	0	1	0	0	0	0	0	0	77
23455179	m	md	ES	2024	0	1	1	0	0	0	0	0	0	0	0	0	

23455231	Not	md	JP	2024	0	1	0	1	0	0	0	0	0	0	0	0	
23455338	Not	md	GB	2024	0	1	0	0	0	1	0	0	0	0	0	1	
23455689	m	md	KR	2024	0	1	1	0	0	0	0	0	0	0	0	0	71
23455948	f	cn	IN	2024	1	0	0	0	0	0	0	0	0	0	0	0	
23456198	f	md	US	2024	0	0	0	0	0	0	0	0	0	0	0	0	
23456688	m	md	JP	2024	0	1	1	0	0	0	0	0	0	0	0	0	80
23456780	Not	md	JP	2024	0	1	0	1	0	0	0	0	0	0	0	0	82
23456797	m	md	JP	2024	0	1	0	0	0	1	0	0	0	0	0	0	80
23457039	m	ph	JP	2024	0	1	1	0	0	0	0	0	0	0	0	0	69
23457069	m	md	DE	2024	0	1	1	0	0	0	0	0	0	0	0	0	70
23457187	m	cn	IN	2024	0	0	0	0	0	0	0	0	0	0	0	0	54
23457401	m	md	JP	2024	0	1	0	1	0	0	0	0	0	0	0	0	82
23457910	f	hp	US	2024	0	0	0	0	0	0	0	0	0	0	0	0	
23457923	m	md	JP	2024	0	1	0	1	0	0	0	0	0	0	0	1	70
23458397	m	md	DE	2024	0	1	0	0	1	0	0	0	0	0	0	0	63
23458508	m	md	JP	2024	0	1	0	1	0	0	0	0	0	0	0	0	71
23458588	Not	hp	TW	2024	0	0	0	0	0	0	0	0	0	0	0	0	
23458597	Not	md	JP	2024	0	1	0	0	1	0	0	0	0	0	0	0	
23458673	m	md	JP	2024	0	1	1	0	0	0	0	0	0	0	0	0	
23458911	Not	md	DE	2024	1	0	0	0	0	0	0	0	0	0	0	0	
23459195	m	md	DE	2024	0	1	0	0	1	0	0	0	0	0	0	0	60
23459204	m	md	DE	2024	0	1	0	0	1	0	0	0	0	0	0	0	71
23459427	Not	md	DE	2024	1	0	0	0	0	0	0	0	0	0	0	0	
23460070	m	md	VN	2024	0	1	0	0	0	1	0	0	0	0	0	0	67
23460313	m	ph	US	2024	0	0	0	0	0	0	0	0	0	0	0	0	
23460455	f	hp	US	2024	0	0	0	0	0	0	0	0	0	0	0	0	
23460477	f	md	DE	2024	0	1	0	0	1	0	0	0	0	0	0	0	84
23460989	m	cn	US	2024	0	0	0	0	0	0	0	0	0	0	0	0	
23461217	f	md	CN	2024	0	0	0	0	0	0	0	0	0	0	0	0	
23461404	m	cn	KR	2024	0	0	0	0	0	0	0	0	0	0	0	0	38
23461747	Not	md	US	2024	0	0	0	0	0	0	0	0	0	0	0	0	
23462044	m	md	JP	2024	1	0	0	0	0	0	0	0	0	0	0	1	72
23462191	m	md	IN	2024	0	0	0	0	0	0	0	0	0	0	0	0	
23462361	Not	md	CZ	2024	0	1	0	0	1	0	0	0	0	0	0	0	48

23463234	m	hp	PL	2024	0	1	1	0	0	0	0	0	0	0	0	0	68
23463431	f	md	JP	2024	0	0	0	0	0	0	0	0	0	0	0	0	71
23463633	m	md	JP	2024	0	0	0	0	0	0	0	0	0	0	0	0	
23464278	m	hp	CA	2024	0	1	0	0	1	0	0	0	0	0	0	0	75
23464457	m	md	DE	2024	0	1	0	0	1	0	0	0	0	0	0	0	71
23464488	m	hp	JP	2024	0	1	0	0	0	1	0	0	0	0	0	0	77
23465279	m	cn	US	2024	1	0	0	0	0	0	0	0	0	0	0	0	
23465478	Not	ph	US	2024	0	0	0	0	0	0	0	0	0	0	0	0	
23466129	f	md	NP	2024	0	0	0	0	0	0	0	0	0	0	0	0	56
23466130	f	md	NP	2024	0	0	0	0	0	0	0	0	0	0	0	0	59
23466421	f	md	TR	2024	0	1	1	0	0	0	0	0	0	0	0	0	18
23466849	Not	ph	JP	2024	1	0	0	0	0	0	0	0	0	0	0	0	
23467049	m	cn	TW	2024	0	1	0	0	1	0	0	0	0	0	0	0	76
23467117	f	ph	US	2024	0	0	0	0	0	0	0	0	0	0	0	0	
23467431	f	cn	US	2024	0	0	0	0	0	0	0	0	0	0	0	0	
23467477	m	md	JP	2024	0	0	0	0	0	0	0	0	0	0	0	0	80
23467479	f	md	PL	2024	0	0	0	0	0	0	0	0	0	0	0	0	42
23467509	m	cn	BR	2024	0	0	0	0	0	0	0	0	0	0	0	0	
23467857	m	cn	US	2024	0	1	1	0	0	0	0	0	0	0	0	0	
23467858	Not	cn	US	2024	0	0	0	0	0	0	0	0	0	0	0	0	
23468020	Not	md	JP	2024	0	1	0	0	1	0	0	0	0	0	0	0	
23468297	Not	md	JP	2024	1	0	0	0	0	0	0	0	0	0	0	0	
23468514	Not	hp	US	2024	1	0	0	0	0	0	0	0	0	0	0	0	
23468616	m	md	VN	2024	0	1	0	0	0	1	0	0	0	0	0	0	60
23468792	Not	md	VN	2024	0	1	0	0	0	1	0	0	0	0	0	0	
23469234	f	hp	US	2024	1	0	0	0	0	0	0	0	0	0	0	0	80
23469318	f	md	JP	2024	1	0	0	0	0	0	0	0	0	0	0	0	65
23469370	m	md	GR	2024	0	1	0	1	0	0	0	0	0	0	0	0	62
23469571	m	cn	CA	2024	1	0	0	0	0	0	0	0	0	0	0	0	48.5
23469627	m	hp	US	2024	1	0	0	0	0	0	0	0	0	0	0	0	
23470036	Not	md	JP	2024	0	1	0	1	0	0	0	0	0	0	0	0	
23470368	m	hp	CN	2024	0	0	0	0	0	0	0	0	0	0	0	0	52
23470450	f	md	BE	2024	0	0	0	0	0	0	0	0	0	0	0	0	48
23470526	m	hp	US	2024	0	1	1	0	0	0	0	0	0	0	0	0	57

23470572	m	md	GR	2024	0	1	0	1	0	0	0	0	0	0	0	0	80.1
23470787	m	md	FR	2024	0	1	0	1	0	0	0	0	0	0	0	0	78.2
23470894	m	md	DE	2024	0	1	1	0	0	0	0	0	0	0	0	0	66
23471240	m	md	GR	2024	0	1	0	1	0	0	0	0	0	0	0	0	72
23471305	f	hp	DE	2024	0	0	0	0	0	0	1	0	0	0	1	0	45
23471662	Not	md	JP	2024	0	1	0	1	0	0	0	0	0	0	0	0	68
23472260	m	md	RO	2024	0	1	0	1	0	0	0	0	0	0	0	0	
23473346	f	md	DE	2024	0	1	1	0	0	0	0	0	0	0	0	0	67
23473397	f	hp	BE	2024	0	1	1	0	0	0	0	0	0	0	0	0	63
23473436	f	hp	GB	2024	0	0	0	0	0	0	0	0	0	0	0	0	
23473464	Not	md	JP	2024	0	0	0	0	0	0	0	0	0	0	0	0	
23473471	m	cn	CA	2024	0	0	0	0	0	0	0	0	0	0	0	0	
23478028	f	md	US	2024	0	1	0	0	1	0	0	0	0	0	0	0	
23478949	f	cn	US	2024	0	0	0	0	0	0	0	0	0	0	0	0	
23479095	Not	hp	GB	2024	1	0	0	0	0	0	0	0	0	0	0	0	
23479141	m	md	JP	2024	0	1	0	0	1	0	0	0	0	0	0	1	79
23479196	m	md	DE	2024	0	1	1	0	0	0	0	0	0	0	0	0	66
23479401	m	md	JP	2024	0	1	0	0	0	1	0	0	0	0	0	0	90
23480217	m	cn	CA	2024	0	0	0	0	0	0	0	0	0	0	0	0	
23480565	m	md	FR	2024	1	0	0	0	0	0	0	0	0	0	0	1	81
23480759	Not	md	JP	2024	0	1	0	0	1	0	0	0	0	0	0	0	
23481011	m	md	IT	2024	0	1	1	0	0	0	0	0	0	0	0	0	61
23481782	f	md	JP	2024	0	0	0	0	0	0	0	0	0	0	0	0	70
23482062	f	cn	CA	2024	1	0	0	0	0	0	0	0	0	0	0	0	63.8
23482526	f	cn	JP	2024	0	0	0	0	0	0	0	0	0	0	0	0	77
23483290	f	md	BE	2024	0	0	0	0	0	0	0	0	0	0	0	0	78
23483876	m	md	JP	2024	0	0	0	0	0	0	0	0	0	0	0	0	74
23483882	m	md	CN	2024	1	0	0	0	0	0	0	0	0	0	0	0	63
23484153	f	md	US	2024	0	0	0	0	0	0	0	0	0	0	0	0	
23484528	m	md	VN	2024	0	1	0	0	0	1	0	0	0	0	0	0	81
23484534	m	md	DE	2024	0	1	0	0	1	0	0	0	0	0	0	0	68
23484545	m	hp	CN	2024	0	0	0	0	0	0	0	0	0	0	0	0	68
23484567	f	md	CN	2024	1	0	0	0	0	0	0	0	0	0	0	0	62
23484617	m	md	JP	2024	0	1	0	0	0	1	0	0	0	0	0	0	70

23484880	m	ph	FR	2024	0	0	0	0	0	0	0	0	0	0	0	0	55
23484881	Not	md	GB	2024	1	0	0	0	0	0	0	0	0	0	0	0	
23484961	m	cn	TW	2024	0	1	0	0	0	1	0	0	0	0	0	0	57
23485002	m	md	GR	2024	0	1	0	0	1	0	0	0	0	0	0	0	71
23485041	m	md	FR	2024	0	1	1	0	0	0	0	0	0	0	0	0	73
23485420	Not	md	BG	2024	0	1	0	0	0	1	0	0	0	0	0	0	
23485754	f	cn	US	2024	1	0	0	0	0	0	0	0	0	0	0	0	63.8
23485821	m	md	DE	2024	0	1	0	0	1	0	0	0	0	0	0	0	66
23485835	Not	cn	JP	2024	1	0	0	0	0	0	0	0	0	0	0	0	
23485963	m	md	DE	2024	0	1	0	0	1	0	0	0	0	0	0	1	72
23486618	m	cn	ES	2024	0	0	0	0	0	0	0	0	0	0	0	0	73
23486797	f	cn	IN	2024	0	0	0	0	0	0	0	0	0	0	0	0	53
23486936	m	md	FR	2024	0	0	0	0	0	0	0	0	0	0	0	0	81
23486974	m	hp	ES	2024	0	0	0	0	0	0	0	0	0	0	0	0	
23487128	f	cn	TH	2024	0	1	0	1	0	0	0	0	0	0	0	0	58.7
23487133	Not	hp	US	2024	0	0	0	0	0	0	0	0	0	0	0	0	
23487161	m	md	JP	2024	0	1	0	1	0	0	0	0	0	0	0	1	59
23487281	Not	hp	CN	2024	0	0	0	0	0	0	0	0	0	0	0	0	
23487544	m	ph	JP	2024	1	0	0	0	0	0	0	0	0	0	0	0	72
23487895	m	md	JP	2024	0	1	0	1	0	0	0	0	0	0	0	0	50
23487918	f	md	DE	2024	0	1	0	0	1	0	0	0	0	0	0	0	56
23487954	f	hp	US	2024	1	0	0	0	0	0	0	0	0	0	0	0	71
23487978	Not	md	US	2024	0	0	0	0	0	0	0	0	0	0	0	0	
23491463	m	hp	BG	2024	0	0	0	0	0	0	0	0	0	0	0	0	73
23491831	Not	hp	US	2024	0	0	0	0	0	0	0	0	0	0	0	0	70
23492381	m	md	JP	2024	0	1	0	0	0	1	0	0	0	0	0	0	70
23492486	Not	md	FR	2024	0	1	0	0	0	1	0	0	0	0	0	0	
23492872	m	md	US	2024	0	1	1	0	0	0	0	0	0	0	0	0	77
23492880	m	md	JP	2024	0	1	0	1	0	0	0	0	0	0	0	0	70
23492998	m	md	JP	2024	0	0	0	0	0	0	0	0	0	0	0	0	42
23493275	m	md	DE	2024	0	0	0	0	0	0	0	0	0	0	0	0	
23493719	f	hp	GB	2024	0	0	0	0	0	0	0	0	0	0	0	0	70
23494101	Not	ph	JP	2024	1	0	0	0	0	0	0	0	0	0	0	0	
23494972	m	md	JP	2024	0	1	0	0	0	1	0	0	0	0	0	0	60

23495008	f	cn	TH	2024	1	0	0	0	0	0	0	0	0	0	0	0	45.4
23495343	m	md	BE	2024	0	1	1	0	0	0	0	0	0	0	0	0	75
23495430	m	md	JP	2024	0	1	1	0	0	0	0	0	0	0	0	0	78
23495858	f	cn	FR	2024	0	0	0	0	0	0	0	0	0	0	0	0	52
23497088	f	hp	TH	2024	1	0	0	0	0	0	0	0	0	0	0	0	
23497089	f	hp	TH	2024	1	0	0	0	0	0	0	0	0	0	0	0	
23497090	f	hp	TH	2024	1	0	0	0	0	0	0	0	0	0	0	0	
23497096	f	hp	TH	2024	1	0	0	0	0	0	0	0	0	0	0	0	
23497097	f	hp	TH	2024	1	0	0	0	0	0	0	0	0	0	0	0	
23497100	m	hp	TH	2024	1	0	0	0	0	0	0	0	0	0	0	0	
23497101	m	hp	TH	2024	1	0	0	0	0	0	0	0	0	0	0	0	
23497103	m	hp	TH	2024	1	0	0	0	0	0	0	0	0	0	0	0	
23498417	m	md	DE	2024	0	1	0	0	1	0	0	0	0	0	0	0	57
23499314	f	cn	CN	2024	0	1	0	0	0	1	0	0	0	0	0	0	52
23499497	m	md	JP	2024	0	1	0	0	0	1	0	0	0	0	0	0	70
23499724	Not	md	NL	2024	0	0	0	0	0	0	0	0	0	0	0	0	
23499795	f	md	AU	2024	0	0	0	0	0	0	0	0	0	0	0	0	
23499797	f	hp	TH	2024	1	0	0	0	0	0	0	0	0	0	0	0	
23500181	m	md	JP	2024	0	1	0	1	0	0	0	0	0	0	0	0	70
23500197	m	md	KR	2024	0	1	1	0	0	0	0	0	0	0	0	0	80
23500370	m	md	CN	2024	0	0	0	0	0	0	0	0	0	0	0	0	65
23500417	m	md	JP	2024	0	1	0	0	0	1	0	0	0	0	0	1	
23500757	f	md	FR	2024	0	0	0	0	0	0	0	0	0	0	0	0	52
23500815	m	md	BG	2024	0	0	0	0	0	0	0	0	0	0	0	0	73
23500821	f	hp	TH	2024	1	0	0	0	0	0	0	0	0	0	0	0	
23500825	f	hp	TH	2024	1	0	0	0	0	0	0	0	0	0	0	0	
23500829	f	hp	TH	2024	1	0	0	0	0	0	0	0	0	0	0	0	
23500830	m	hp	TH	2024	1	0	0	0	0	0	0	0	0	0	0	0	
23501209	f	hp	CN	2024	0	0	0	0	0	0	0	0	0	0	0	0	33
23501419	m	hp	TH	2024	1	0	0	0	0	0	0	0	0	0	0	0	
23501466	f	hp	TH	2024	1	0	0	0	0	0	0	0	0	0	0	0	
23501468	m	hp	TH	2024	1	0	0	0	0	0	0	0	0	0	0	0	
23501502	f	hp	US	2024	1	0	0	0	0	0	0	0	0	0	0	0	37
23501503	f	hp	TH	2024	1	0	0	0	0	0	0	0	0	0	0	0	

23501506	f	hp	US	2024	1	0	0	0	0	0	0	0	0	0	0	0	44
23501554	f	hp	TH	2024	1	0	0	0	0	0	0	0	0	0	0	0	
23501556	f	hp	TH	2024	1	0	0	0	0	0	0	0	0	0	0	0	
23501584	f	md	JP	2024	0	1	0	0	1	0	0	0	0	0	0	0	60
23501593	m	hp	GB	2024	0	1	0	1	0	0	0	0	0	0	0	0	75
23501750	m	hp	TR	2024	0	0	0	0	0	0	0	0	0	0	0	0	64
23501923	f	md	DE	2024	0	1	1	0	0	0	0	0	0	0	0	0	
23502035	m	hp	CN	2024	0	1	1	0	0	0	0	0	0	0	0	0	55
23502086	m	md	FR	2024	0	1	0	0	0	1	0	0	0	0	0	0	70
23502113	m	md	GB	2024	0	0	0	0	0	0	0	0	0	0	0	0	57
23502157	Not	md	CA	2024	0	0	0	0	0	0	0	0	0	0	0	0	
23503225	m	cn	US	2024	1	0	0	0	0	0	0	0	0	0	0	0	
23503240	Not	md	JP	2024	0	1	1	0	0	0	0	0	0	0	0	0	82
23503333	m	hp	US	2024	1	0	0	0	0	0	0	0	0	0	0	0	61
23503334	f	hp	US	2024	1	0	0	0	0	0	0	0	0	0	0	0	69
23503335	f	hp	US	2024	1	0	0	0	0	0	0	0	0	0	0	0	66
23503529	Not	ph	US	2024	0	0	0	0	0	0	0	0	0	0	0	0	
23503559	Not	hp	TW	2024	1	0	0	0	0	0	0	0	0	0	0	0	
23503774	m	md	DE	2024	0	0	0	0	0	0	0	0	0	0	0	0	73
23504015	m	md	JP	2024	0	0	0	0	0	0	0	0	0	0	0	0	70
23504106	f	md	JP	2024	0	1	0	0	0	1	0	0	0	0	0	0	80
23504224	m	md	DE	2024	0	1	0	0	1	0	0	0	0	0	0	0	57
23504356	Not	hp	TW	2024	1	0	0	0	0	0	0	0	0	0	0	0	
23504357	Not	hp	TW	2024	1	0	0	0	0	0	0	0	0	0	0	0	
23504358	Not	hp	TW	2024	1	0	0	0	0	0	0	0	0	0	0	0	
23504669	f	md	KR	2024	0	0	0	0	0	0	0	0	0	0	0	0	47
23504774	f	cn	US	2024	0	0	0	0	0	0	0	0	0	0	0	0	
23504855	Not	md	JP	2024	0	1	0	0	0	1	0	0	0	0	0	0	
23505006	f	hp	US	2024	0	0	0	0	0	0	0	0	0	0	0	0	65
23505019	Not	cn	US	2024	0	0	0	0	0	0	0	0	0	0	0	0	
23505135	m	cn	US	2024	0	0	0	0	0	0	0	0	0	0	0	0	
23505198	f	hp	CN	2024	1	0	0	0	0	0	0	0	0	0	0	0	70
23505256	m	md	CZ	2024	0	1	1	0	0	0	0	0	0	0	0	0	79
23505266	m	hp	US	2024	1	0	0	0	0	0	0	0	0	0	0	0	61

23505274	f	md	AT	2024	0	0	0	0	0	0	0	0	0	0	0	0	
23505715	f	hp	US	2024	1	0	0	0	0	0	0	0	0	0	0	0	89
23505721	f	md	CA	2024	1	0	0	0	0	0	0	0	0	0	0	0	
23506248	f	cn	US	2024	1	0	0	0	0	0	0	0	0	0	0	0	87.7
23506384	Not	ph	IT	2024	0	0	0	0	0	0	0	0	0	0	0	0	
23506563	m	md	JP	2024	0	1	1	0	0	0	0	0	0	0	0	0	75
23506612	m	hp	US	2024	1	0	0	0	0	0	0	0	0	0	0	0	58
23506867	Not	hp	TW	2024	1	0	0	0	0	0	0	0	0	0	0	0	
23506868	Not	hp	TW	2024	1	0	0	0	0	0	0	0	0	0	0	0	
23506869	Not	hp	TW	2024	1	0	0	0	0	0	0	0	0	0	0	0	
23506872	Not	hp	TW	2024	1	0	0	0	0	0	0	0	0	0	0	0	
23507022	f	hp	US	2024	1	0	0	0	0	0	0	0	0	0	0	0	41
23507027	f	hp	US	2024	1	0	0	0	0	0	0	0	0	0	0	0	77
23507028	f	hp	US	2024	1	0	0	0	0	0	0	0	0	0	0	0	57
23507095	m	hp	US	2024	1	0	0	0	0	0	0	0	0	0	0	0	63
23507108	m	hp	ES	2024	0	0	0	0	0	0	0	0	0	0	0	0	
23507140	Not	hp	US	2024	0	0	0	0	0	0	0	0	0	0	0	0	
23507329	f	hp	US	2024	1	0	0	0	0	0	0	0	0	0	0	0	75
23507330	m	hp	US	2024	1	0	0	0	0	0	0	0	0	0	0	0	50
23507339	Not	hp	TW	2024	1	0	0	0	0	0	0	0	0	0	0	0	
23507341	Not	hp	TW	2024	1	0	0	0	0	0	0	0	0	0	0	0	
23507342	Not	hp	TW	2024	1	0	0	0	0	0	0	0	0	0	0	0	
23507343	Not	hp	TW	2024	1	0	0	0	0	0	0	0	0	0	0	0	
23507344	Not	hp	TW	2024	1	0	0	0	0	0	0	0	0	0	0	0	
23507345	Not	hp	TW	2024	1	0	0	0	0	0	0	0	0	0	0	0	
23507346	Not	hp	TW	2024	1	0	0	0	0	0	0	0	0	0	0	0	
23507617	Not	md	JP	2024	1	0	0	0	0	0	0	0	0	0	0	0	
23507733	Not	hp	US	2024	1	0	0	0	0	0	0	0	0	0	0	0	
23507755	Not	hp	US	2024	0	0	0	0	0	0	0	0	0	0	0	0	
23508006	Not	cn	DE	2024	0	0	0	0	0	0	0	0	0	0	0	0	
23508316	m	md	JP	2024	0	1	0	1	0	0	0	0	0	0	0	0	79
23508437	Not	hp	TW	2024	1	0	0	0	0	0	0	0	0	0	0	0	
23508439	Not	hp	TW	2024	1	0	0	0	0	0	0	0	0	0	0	0	
23508441	Not	hp	TW	2024	1	0	0	0	0	0	0	0	0	0	0	0	

23508445	Not	hp	TW	2024	1	0	0	0	0	0	0	0	0	0	0	0	
23508493	Not	hp	TW	2024	1	0	0	0	0	0	0	0	0	0	0	0	
23508503	f	md	JP	2024	1	0	0	0	0	0	0	0	0	0	0	0	79
23508521	f	md	BE	2024	0	1	0	0	0	1	0	0	0	0	0	0	73
23508579	Not	hp	TW	2024	1	0	0	0	0	0	0	0	0	0	0	0	
23508602	m	md	FR	2024	0	1	0	0	0	1	0	0	0	0	0	0	69
23508683	m	md	IL	2024	0	0	0	0	0	0	0	0	0	0	0	0	
23508798	Not	cn	US	2024	0	0	0	0	0	0	0	0	0	0	0	0	
23509424	m	md	JP	2024	0	0	0	0	0	0	0	0	0	0	0	0	90
23510414	m	cn	PK	2024	0	1	0	0	0	1	0	0	0	0	0	0	85
23511005	m	md	CH	2024	0	1	0	1	0	0	0	0	0	0	0	0	78
23512451	f	md	DE	2024	0	1	0	0	1	0	0	0	0	0	0	0	68
23512683	m	md	BR	2024	0	0	0	0	0	0	0	0	0	0	0	0	52
23513107	m	md	CH	2024	0	1	1	0	0	0	0	0	0	0	0	0	
23513382	f	md	JP	2024	0	1	1	0	0	0	0	0	0	0	0	0	47
23513402	f	md	SE	2024	0	1	1	0	0	0	0	0	0	0	0	0	67
23513474	m	hp	DE	2024	0	1	0	0	1	0	0	0	0	0	0	0	68
23513576	m	md	SK	2024	0	1	0	0	0	1	0	0	0	0	0	0	
23513642	f	hp	PL	2024	0	1	0	0	0	1	0	0	0	0	0	0	
23513694	f	md	IT	2024	0	0	0	0	0	0	0	0	0	0	0	0	72
23513947	m	md	JP	2024	0	1	0	1	0	0	0	0	0	0	0	1	51
23513948	f	cn	CA	2024	1	0	0	0	0	0	0	0	0	0	0	0	80.2
23514304	m	md	JP	2024	0	1	0	1	0	0	0	0	0	0	0	0	68
23514640	m	md	US	2024	0	0	0	0	0	0	0	0	0	0	0	0	
23515507	f	hp	CA	2024	1	0	0	0	0	0	0	0	0	0	0	0	75
23515531	f	cn	US	2024	1	0	0	0	0	0	0	0	0	0	0	0	
23515846	m	cn	US	2024	1	0	0	0	0	0	0	0	0	0	0	0	
23515873	Not	md	JP	2024	0	1	0	0	0	1	0	0	0	0	0	0	
23516301	f	md	CA	2024	1	0	0	0	0	0	0	0	0	0	0	0	81.8
23516802	f	cn	EG	2024	1	0	0	0	0	0	0	0	0	0	0	0	
23516860	m	md	AU	2024	1	0	0	0	0	0	0	0	0	0	0	1	79
23517195	f	cn	US	2024	1	0	0	0	0	0	0	0	0	0	0	0	
23517318	f	md	PL	2024	0	0	0	0	0	0	0	0	0	0	0	0	67
23517345	m	md	JP	2024	0	1	0	0	1	0	0	0	0	0	0	0	60

23517529	m	md	DZ	2024	0	1	1	0	0	0	0	0	0	0	0	0	78
23517930	m	md	JP	2024	0	1	0	1	0	0	0	0	0	0	0	0	75
23517953	f	md	DE	2024	0	0	0	0	0	0	0	0	0	0	0	0	
23518361	m	md	US	2024	0	0	0	0	0	0	0	0	0	0	0	0	40
23518499	f	md	US	2024	0	0	0	0	0	0	0	0	0	0	0	0	
23518590	m	md	AT	2024	0	0	0	0	0	0	0	0	0	0	0	0	79
23518709	f	cn	IN	2024	0	0	0	0	0	0	0	0	0	0	0	0	65
23518731	m	hp	ES	2024	0	0	0	0	0	0	0	0	0	0	0	0	
23518853	m	md	DE	2024	0	1	1	0	0	0	0	0	0	0	0	0	
23518890	m	md	JP	2024	0	1	1	0	0	0	0	0	0	0	0	0	69
23519714	Not	md	JP	2024	0	1	0	0	1	0	0	0	0	0	0	0	
23519865	m	cn	TH	2024	1	0	0	0	0	0	0	0	0	0	0	0	
23520126	f	cn	PK	2024	0	1	0	0	0	1	0	0	0	0	0	0	67
23520735	Not	md	GB	2024	1	0	0	0	0	0	0	0	0	0	0	0	
23520958	f	cn	US	2024	0	1	0	1	0	0	0	0	0	0	0	0	
23520999	m	md	JP	2024	0	1	0	1	0	0	0	0	0	0	0	0	66
23521191	Not	md	US	2024	0	0	0	0	0	0	0	0	0	0	0	0	
23521385	m	md	FR	2024	0	0	0	0	0	0	0	0	0	0	0	0	
23521498	m	md	IT	2024	0	1	0	0	1	0	0	0	0	0	0	0	55
23521734	f	md	JP	2024	0	1	0	1	0	0	0	0	0	0	0	0	77
23521852	m	hp	CN	2024	0	0	0	0	0	0	0	0	0	0	0	0	59
23522460	m	md	DE	2024	0	0	0	0	0	0	0	0	0	0	0	0	49
23522934	m	md	IT	2024	1	0	0	0	0	0	0	0	0	0	0	0	71
23522967	f	md	US	2024	0	0	0	0	0	0	0	0	0	0	0	0	
23523230	f	cn	US	2024	0	1	1	0	0	0	0	0	0	0	0	0	81
23523231	f	cn	US	2024	0	0	0	0	0	0	1	1	0	0	0	0	56.8
23523291	m	md	JP	2024	0	1	0	1	0	0	0	0	0	0	0	0	77
23523332	Not	md	JP	2024	0	1	0	1	0	0	0	0	0	0	0	0	75
23523364	m	ph	FR	2024	0	0	0	0	0	0	0	0	0	0	0	0	55
23523457	m	md	DE	2024	0	1	0	0	1	0	0	0	0	0	0	0	55
23523538	m	md	JP	2024	0	1	1	0	0	0	0	0	0	0	0	0	70
23523885	Not	md	JP	2024	0	1	0	1	0	0	0	0	0	0	0	0	64
23524061	f	md	DE	2024	0	1	1	0	0	0	0	0	0	0	0	0	
23524309	m	md	JP	2024	0	1	1	0	0	0	0	0	0	0	0	0	80

23524669	Not	md	FR	2024	0	1	0	0	0	1	0	0	0	0	0	0	
23525009	m	md	JP	2024	0	0	0	0	0	0	0	0	0	0	0	0	70
23526045	m	md	JP	2024	0	0	0	0	0	0	0	0	0	0	0	0	64
23526257	f	md	JP	2024	0	0	0	0	0	0	0	0	0	0	0	0	
23526325	Not	md	FR	2024	0	1	0	0	0	1	0	0	0	0	0	0	
23526524	m	md	JP	2024	0	1	1	0	0	0	0	0	0	0	0	0	79
23526747	m	md	JP	2024	0	1	0	0	1	0	0	0	0	0	0	0	73
23526908	m	cn	TH	2024	0	0	0	0	0	0	0	0	0	0	0	0	48
23527479	f	md	TN	2024	0	0	0	0	0	0	0	0	0	0	0	0	
23527520	f	md	FR	2024	1	0	0	0	0	0	0	0	0	0	0	0	54
23527936	f	ph	FR	2024	0	1	0	1	0	0	0	0	0	0	0	0	62
23528041	f	md	ES	2024	0	0	0	0	0	0	0	0	0	0	0	0	55
23528252	f	cn	US	2024	0	0	0	0	0	0	0	0	0	0	0	0	70
23528314	f	hp	US	2024	0	1	1	0	0	0	0	0	0	0	0	0	
23528315	f	hp	US	2024	0	1	1	0	0	0	0	0	0	0	0	0	
23528322	m	hp	US	2024	0	1	1	0	0	0	0	0	0	0	0	0	
23528323	f	hp	US	2024	0	1	1	0	0	0	0	0	0	0	0	0	
23528325	f	hp	US	2024	0	1	1	0	0	0	0	0	0	0	0	0	
23528345	f	hp	US	2024	0	1	1	0	0	0	0	0	0	0	0	0	
23528346	m	hp	US	2024	0	1	1	0	0	0	0	0	0	0	0	0	
23528348	f	hp	US	2024	0	1	1	0	0	0	0	0	0	0	0	0	
23528397	m	md	SA	2024	0	0	0	0	0	0	0	0	0	0	0	0	57
23528407	Not	cn	JP	2024	1	0	0	0	0	0	0	0	0	0	0	0	
23530032	m	md	JP	2024	0	1	0	0	0	1	0	0	0	0	0	0	90
23530450	m	cn	PK	2024	0	0	0	0	0	0	0	0	0	0	0	0	63
23530472	Not	md	GB	2024	0	1	0	0	0	1	0	0	0	0	0	0	
23530538	m	md	FR	2024	0	1	0	0	0	1	0	0	0	0	0	0	73
23530595	Not	ph	US	2024	0	0	0	0	0	0	0	0	0	0	0	0	
23531455	f	md	US	2024	0	1	0	0	0	1	0	0	0	0	0	0	44
23531536	Not	md	JP	2024	0	1	0	0	1	0	0	0	0	0	0	0	
23531801	m	hp	JP	2024	0	0	0	0	0	0	0	0	0	0	0	0	60
23533242	f	cn	TH	2024	0	0	0	0	0	0	0	0	0	0	0	0	79
23533272	m	md	TN	2024	0	1	0	0	0	1	0	0	0	0	0	0	56
23533338	Not	hp	CN	2024	0	0	0	0	0	0	0	0	0	0	0	0	

23533708	f	md	NO	2024	0	0	0	0	0	0	0	0	0	0	0	0	76
23533975	f	md	IT	2024	0	0	0	0	0	0	0	0	0	0	0	0	72
23534086	f	cn	US	2024	0	1	1	0	0	0	0	0	0	0	0	0	65
23534840	m	md	JP	2024	0	0	0	0	0	0	0	0	0	0	0	0	
23535014	f	hp	CA	2024	0	0	0	0	0	0	0	0	0	0	0	0	76
23535046	m	md	FR	2024	0	1	0	0	0	1	0	0	0	0	0	0	52
23535118	f	md	IT	2024	0	0	0	0	0	0	0	0	0	0	0	0	63
23535197	m	md	FR	2024	0	0	0	0	0	0	0	0	0	0	0	0	81
23535371	f	md	JP	2024	1	0	0	0	0	0	0	0	0	0	0	0	71
23535458	m	md	JP	2024	0	1	0	1	0	0	0	0	0	0	0	1	59
23535477	f	md	US	2024	1	0	0	0	0	0	0	0	0	0	0	0	59
23535481	f	ph	JP	2024	1	0	0	0	0	0	0	0	0	0	0	0	78
23535527	m	md	JP	2024	0	1	0	1	0	0	0	0	0	0	0	0	75
23535534	m	cn	US	2024	1	0	0	0	0	0	0	0	0	0	0	0	
23535548	m	md	JP	2024	0	1	0	1	0	0	0	0	0	0	0	0	63
23536066	m	md	JP	2024	0	1	0	0	0	1	0	0	0	0	0	1	
23536331	m	md	DE	2024	0	1	0	0	1	0	0	0	0	0	0	0	58
23537024	f	md	DE	2024	0	1	1	0	0	0	0	0	0	0	0	0	67
23537242	Not	hp	CN	2024	0	0	0	0	0	0	0	0	0	0	0	0	
23537566	m	md	JP	2024	0	1	0	0	0	1	0	0	0	0	0	0	80
23538012	Not	md	BG	2024	0	1	0	0	0	1	0	0	0	0	0	0	
23538620	m	md	NZ	2024	1	0	0	0	0	0	0	0	0	0	0	0	
23538803	m	ph	NZ	2024	1	0	0	0	0	0	0	0	0	0	0	0	58
23539051	m	cn	NL	2024	0	0	0	0	0	0	0	0	0	0	0	0	
23539129	f	cn	KR	2024	0	0	0	0	0	0	0	0	0	0	0	0	68
23539251	m	md	US	2024	0	1	0	0	1	0	0	0	0	0	0	0	72
23539305	m	md	JP	2024	0	1	1	0	0	0	0	0	0	0	0	0	61
23539348	m	cn	NP	2024	0	0	0	0	0	0	0	0	0	0	0	0	75
23539519	f	hp	PL	2024	0	1	1	0	0	0	0	0	0	0	0	0	54
23539546	f	md	HR	2024	0	1	1	0	0	0	0	0	0	0	0	0	67
23539987	f	md	DE	2024	0	1	0	0	1	0	0	0	0	0	0	0	58
23540281	Not	ph	ZA	2024	1	0	0	0	0	0	0	0	0	0	0	0	
23540577	m	md	DE	2024	0	1	1	0	0	0	0	0	0	0	0	0	80
23540655	m	md	DE	2024	0	1	0	0	1	0	0	0	0	0	0	0	74

23541203	f	cn	PT	2024	0	0	0	0	0	0	0	0	0	0	0	0	49
23541320	m	cn	IN	2024	1	0	0	0	0	0	0	0	0	0	0	0	75.5
23541572	m	md	DE	2024	0	1	1	0	0	0	0	0	0	0	0	0	
23541926	m	md	JP	2024	0	1	0	0	1	0	0	0	0	0	0	0	70
23542139	m	md	RU	2024	0	1	0	1	0	0	0	0	0	0	0	0	60
23542165	Not	cn	US	2024	0	0	0	0	0	0	0	0	0	0	0	0	
23542166	Not	cn	US	2024	0	0	0	0	0	0	0	0	0	0	0	0	
23542167	f	cn	US	2024	0	0	0	0	0	0	0	0	0	0	0	0	
23542171	m	hp	US	2024	0	0	0	0	0	0	0	0	0	0	0	0	
23542184	f	md	US	2024	0	0	0	0	0	0	0	0	0	0	0	0	80
23542186	Not	md	US	2024	0	0	0	0	0	0	0	0	0	0	0	0	
23542206	m	hp	US	2024	0	0	0	0	0	0	0	0	0	0	0	0	
23542209	m	hp	US	2024	0	0	0	0	0	0	0	0	0	0	0	0	
23542212	m	hp	US	2024	0	0	0	0	0	0	0	0	0	0	0	0	
23542214	f	cn	US	2024	0	0	0	0	0	0	0	0	0	0	0	0	
23542341	m	ph	JP	2024	0	1	0	0	1	0	0	0	0	0	0	0	79
23543003	f	md	DE	2024	0	1	0	0	1	0	0	0	0	0	0	0	66
23543147	m	md	BE	2024	0	0	0	0	0	0	0	0	0	0	0	0	
23543269	f	md	CA	2024	0	1	1	0	0	0	0	0	0	0	0	0	67
23543539	f	cn	TH	2024	0	0	0	0	0	0	0	0	0	0	0	0	58
23543844	m	md	JP	2024	0	1	0	0	0	1	0	0	0	0	0	0	70
23544056	f	md	RU	2024	0	1	0	1	0	0	0	0	0	0	0	0	60
23544318	Not	md	MY	2024	0	0	0	0	0	0	0	0	0	0	0	0	
23544319	Not	md	MY	2024	0	0	0	0	0	0	0	0	0	0	0	0	
23544457	m	md	DE	2024	0	1	0	0	1	0	0	0	0	0	0	0	58
23544461	m	md	TR	2024	0	1	1	0	0	0	0	0	0	0	0	0	48
23544983	m	cn	ID	2024	0	1	0	0	0	1	0	0	0	0	0	0	
23545029	m	cn	US	2024	0	0	0	0	0	0	0	0	0	0	0	0	82
23545303	f	cn	US	2024	1	0	0	0	0	0	0	0	0	0	0	0	
23545394	m	md	PE	2024	1	0	0	0	0	0	0	0	0	0	0	0	
23545481	m	md	DK	2024	0	0	0	0	0	0	0	0	0	0	0	0	72
23545557	f	md	MY	2024	1	0	0	0	0	0	0	0	0	0	0	0	
23545573	m	md	DE	2024	0	1	1	0	0	0	0	0	0	0	0	1	62
23545579	Not	cn	JP	2024	0	0	0	0	0	0	0	0	0	0	0	0	

23545859	m	md	BE	2024	0	1	1	0	0	0	0	0	0	0	0	0	
23545960	m	md	MX	2024	0	1	1	0	0	0	0	0	0	0	0	0	63
23546053	Not	md	JP	2024	0	1	1	0	0	0	0	0	0	0	0	0	80
23547318	f	md	IT	2024	0	0	0	0	0	0	0	0	0	0	0	0	72
23547641	f	md	BG	2024	1	0	0	0	0	0	0	0	0	0	0	0	
23547855	f	md	CA	2024	0	1	1	0	0	0	0	0	0	0	0	0	67
23547857	Not	md	JP	2024	0	1	0	0	0	1	0	0	0	0	0	0	70
23548113	Not	hp	JP	2024	0	0	0	0	0	0	0	0	0	0	0	0	
23548461	f	md	DE	2024	0	1	1	0	0	0	0	0	0	0	0	0	73
23548503	Not	hp	IN	2024	1	0	0	0	0	0	0	0	0	0	0	0	
23548852	m	md	JP	2024	0	1	0	0	0	1	0	0	0	0	0	0	44
23549093	m	ph	JP	2024	0	1	1	0	0	0	0	0	0	0	0	0	
23549154	f	md	CA	2024	0	0	0	0	0	0	0	0	0	0	0	0	51
23549460	f	hp	GB	2024	0	0	0	0	0	0	0	0	0	0	0	0	42
23549755	m	md	JP	2024	0	1	0	0	0	1	0	0	0	0	0	0	77
23550935	f	hp	PL	2024	0	1	1	0	0	0	0	0	0	0	0	0	54
23551236	Not	hp	FR	2024	0	0	0	0	0	0	0	0	0	0	0	0	
23551673	f	hp	US	2024	1	0	0	0	0	0	0	0	0	0	0	0	63
23552344	m	md	GR	2024	0	0	0	0	0	0	0	0	0	0	0	0	
23552369	m	md	JP	2024	0	1	0	0	0	1	0	0	0	0	0	0	70
23552483	f	md	DE	2024	0	1	0	0	1	0	0	0	0	0	0	0	68
23552628	m	hp	ES	2024	0	0	0	0	0	0	0	0	0	0	0	0	
23552722	m	md	FR	2024	0	0	0	0	0	0	0	0	0	0	0	0	81
23552951	f	md	KR	2024	0	0	0	0	0	0	0	0	0	0	0	0	47
23553306	m	cn	BR	2024	0	0	0	0	0	0	0	0	0	0	0	0	41
23553547	Not	md	JP	2024	1	0	0	0	0	0	0	0	0	0	0	0	
23553552	Not	md	AU	2024	1	0	0	0	0	0	0	0	0	0	0	0	
23553647	f	md	MX	2024	1	0	0	0	0	0	0	0	0	0	0	0	
23553935	f	md	CA	2024	0	1	1	0	0	0	0	0	0	0	0	0	67
23553938	m	md	ES	2024	0	0	0	0	0	0	0	0	0	0	0	0	54
23553942	m	md	RU	2024	0	0	0	0	0	0	0	0	0	0	0	0	53
23553992	f	md	JP	2024	0	1	0	1	0	0	0	0	0	0	0	0	71
23554223	f	cn	US	2024	1	0	0	0	0	0	0	0	0	0	0	0	
23554352	f	ph	FR	2024	0	1	1	0	0	0	0	0	0	0	0	0	70

23554366	f	md	MX	2024	1	0	0	0	0	0	0	0	0	0	0	0	
23554654	m	md	JP	2024	0	1	0	0	1	0	0	0	0	0	0	0	68
23555076	m	md	MA	2024	0	1	1	0	0	0	0	0	0	0	0	0	40
23555471	m	md	DE	2024	0	1	1	0	0	0	0	0	0	0	0	0	
23555934	m	md	CN	2024	0	1	1	0	0	0	0	0	0	0	0	0	74
23556201	Not	md	FR	2024	0	0	0	0	0	0	0	0	0	0	0	0	
23556223	f	md	US	2024	1	0	0	0	0	0	0	0	0	0	0	1	82
23557183	m	md	JP	2024	0	1	0	1	0	0	0	0	0	0	0	0	
23557419	Not	ph	US	2024	0	0	0	0	0	0	0	0	0	0	0	0	
23557650	m	cn	TW	2024	0	1	0	0	0	1	0	0	0	0	0	0	61
23557825	Not	hp	JP	2024	0	0	0	0	0	0	0	0	0	0	0	0	
23558049	m	md	IT	2024	1	0	0	0	0	0	0	0	0	0	0	0	
23558125	f	md	DK	2024	0	0	0	0	0	0	0	0	0	0	0	0	55
23558201	m	md	PT	2024	0	0	0	0	0	0	0	0	0	0	0	0	74
23558209	m	ph	JP	2024	0	1	0	0	0	1	0	0	0	0	0	0	64
23558292	Not	md	GB	2024	0	1	0	0	0	1	0	0	0	0	0	0	
23558994	m	md	FR	2024	0	0	0	0	0	0	0	0	0	0	0	0	
23558995	m	md	JP	2024	0	1	0	1	0	0	0	0	0	0	0	0	75
23559165	Not	ph	JP	2024	1	0	0	0	0	0	0	0	0	0	0	0	
23559345	f	md	DE	2024	0	1	1	0	0	0	0	0	0	0	0	0	82
23559479	f	md	JP	2024	0	1	1	0	0	0	0	0	0	0	0	0	80
23559618	m	md	DE	2024	0	0	0	0	0	0	0	0	0	0	0	0	62
23559798	f	cn	US	2024	1	0	0	0	0	0	0	0	0	0	0	0	
23559848	m	md	US	2024	0	1	0	0	1	0	0	0	0	0	0	0	72
23560009	m	md	JP	2024	0	1	1	0	0	0	0	0	0	0	0	0	68
23560072	m	ph	TH	2024	0	0	0	0	0	0	0	0	0	0	0	0	34
23560116	m	md	JP	2024	0	1	0	1	0	0	0	0	0	0	0	0	75
23560217	m	md	JP	2024	0	1	0	1	0	0	0	0	0	0	0	0	55
23560221	f	cn	US	2024	0	0	0	0	0	0	0	0	0	0	0	0	
23560375	m	md	JP	2024	0	1	0	1	0	0	0	0	0	0	0	1	71.7
23560385	f	ph	FR	2024	0	1	1	0	0	0	0	0	0	0	0	0	70
23560671	Not	hp	DE	2024	0	1	1	0	0	0	0	0	0	0	0	0	54
23560783	m	md	JP	2024	0	1	0	1	0	0	0	0	0	0	0	0	71
23561063	Not	md	JP	2024	0	0	0	0	0	0	0	0	0	0	0	0	

23561372	f	hp	JP	2024	0	0	0	0	0	0	0	0	0	0	0	0	41
23561381	m	hp	JP	2024	0	0	0	0	0	0	0	0	0	0	0	0	66
23561418	m	ph	IT	2024	0	0	0	0	0	0	0	0	0	0	0	0	
23561462	m	md	FR	2024	0	0	0	0	0	0	0	0	0	0	0	0	65
23561593	m	cn	US	2024	0	0	0	0	0	0	0	0	0	0	0	0	67
23561726	f	md	JP	2024	0	0	0	0	0	0	0	0	0	0	0	0	53
23561735	m	md	JP	2024	0	0	0	0	0	0	0	0	0	0	0	0	30
23562009	m	md	IT	2024	0	1	1	0	0	0	0	0	0	0	0	0	68
23563397	f	md	JP	2024	0	0	0	0	0	0	0	0	0	0	0	0	86
23564088	m	hp	US	2024	0	0	0	0	0	0	0	0	0	0	0	0	47
23564192	m	md	IQ	2024	0	1	1	0	0	0	0	0	0	0	0	0	
23564251	m	hp	US	2024	0	0	0	0	0	0	0	0	0	0	0	0	60
23564669	m	hp	US	2024	0	1	0	0	1	0	0	0	0	0	0	0	75
23564684	Not	md	JP	2024	1	0	0	0	0	0	0	0	0	0	0	0	
23564698	f	md	JP	2024	0	1	0	0	0	1	0	0	0	0	0	0	60
23564700	f	hp	US	2024	0	1	0	0	1	0	0	0	0	0	0	0	58
23564848	m	md	DE	2024	0	0	0	0	0	0	0	0	0	0	0	0	62
23565487	m	hp	US	2024	0	1	0	0	1	0	0	0	0	0	0	0	43
23565633	m	hp	US	2024	0	1	1	0	0	0	0	0	0	0	0	0	71
23566188	f	md	NL	2024	0	0	0	0	0	0	0	0	0	0	0	0	77
23567141	m	md	JP	2024	0	1	1	0	0	0	0	0	0	0	0	0	81
23567171	f	md	CN	2024	0	0	0	0	0	0	0	0	0	0	0	0	44
23567230	m	ph	TH	2024	0	0	0	0	0	0	0	0	0	0	0	0	70
23567346	m	md	JP	2024	0	1	1	0	0	0	0	0	0	0	0	1	79
23567521	m	hp	US	2024	0	0	0	0	0	0	0	0	0	0	0	0	65
23567584	Not	hp	DE	2024	0	1	1	0	0	0	0	0	0	0	0	1	54
23568157	f	hp	US	2024	0	1	0	0	1	0	0	0	0	0	0	0	66
23568169	f	md	DE	2024	0	1	0	0	1	0	0	0	0	0	0	0	
23568195	Not	md	FR	2024	0	0	0	0	0	0	0	0	0	0	0	0	
23568261	m	md	FR	2024	0	1	0	1	0	0	0	0	0	0	0	0	58
23568362	m	md	DE	2024	0	1	1	0	0	0	0	0	0	0	0	0	67
23568494	m	md	ES	2024	0	0	0	0	0	0	0	0	0	0	0	0	66
23568613	m	cn	IN	2024	1	0	0	0	0	0	0	0	0	0	0	0	76.4
23568812	f	md	IT	2024	0	0	0	0	0	0	0	0	0	0	0	0	72

23569056	f	md	AT	2024	0	0	0	0	0	0	0	0	0	0	0	0	
23569213	Not	md	US	2024	0	0	0	0	0	0	0	0	0	0	0	0	
23569478	f	md	JP	2024	0	1	0	1	0	0	0	0	0	0	0	0	59
23569644	m	hp	US	2024	0	0	0	0	0	0	0	0	0	0	0	0	60
23569779	m	hp	JP	2024	0	1	0	1	0	0	0	0	0	0	0	0	75
23570713	m	md	DE	2024	0	1	1	0	0	0	0	0	0	0	0	0	
23570769	m	md	JP	2024	0	0	0	0	0	0	0	0	0	0	0	0	80
23571035	f	cn	US	2024	1	0	0	0	0	0	0	0	0	0	0	0	89.1
23571130	m	md	JP	2024	0	1	0	1	0	0	0	0	0	0	0	0	67
23571362	f	md	DE	2024	0	0	0	0	0	0	0	0	0	0	0	0	82
23571386	m	md	JP	2024	0	1	0	1	0	0	0	0	0	0	0	0	40
23571393	f	md	IT	2024	0	1	0	0	1	0	0	0	0	0	0	0	64
23571404	m	md	IT	2024	0	1	1	0	0	0	0	0	0	0	0	0	75
23571488	m	hp	US	2024	0	0	0	0	0	0	0	0	0	0	0	0	47
23571508	f	cn	TH	2024	0	1	0	1	0	0	0	0	0	0	0	0	65.9
23571674	m	md	JP	2024	0	1	0	1	0	0	0	0	0	0	0	0	86
23571701	Not	ph	JP	2024	1	0	0	0	0	0	0	0	0	0	0	0	
23571714	f	hp	US	2024	0	0	0	0	0	0	0	0	0	0	0	0	
23572513	f	md	JP	2024	0	1	0	1	0	0	0	0	0	0	0	0	47
23572515	f	cn	US	2024	1	0	0	0	0	0	0	0	0	0	0	0	
23572722	m	md	JP	2024	0	1	1	0	0	0	0	0	0	0	0	0	77
23572731	m	md	JP	2024	0	1	0	1	0	0	0	0	0	0	0	0	54
23572855	m	hp	US	2024	0	0	0	0	0	0	0	0	0	0	0	0	47
23572917	m	hp	NL	2024	0	0	0	0	0	0	0	0	0	0	0	0	59
23573235	f	md	DE	2024	0	1	1	0	0	0	0	0	0	0	0	0	82
23573258	f	md	JP	2024	0	1	0	0	0	1	0	0	0	0	0	0	60
23573377	m	md	GR	2024	0	1	1	0	0	0	0	0	0	0	0	0	77
23573439	m	md	VN	2024	0	1	0	0	0	1	0	0	0	0	0	0	77
23573487	f	md	JP	2024	0	1	0	0	0	1	0	0	0	0	0	0	65
23573836	m	hp	US	2024	0	1	0	0	1	0	0	0	0	0	0	0	58
23574491	m	md	DE	2024	0	1	1	0	0	0	0	0	0	0	0	0	
23574689	m	md	DE	2024	0	0	0	0	0	0	0	0	0	0	0	0	62
23574762	Not	hp	US	2024	0	0	0	0	0	0	0	0	0	0	0	0	
23574815	m	md	GR	2024	0	1	1	0	0	0	0	0	0	0	0	0	56

23574846	m	md	JP	2024	0	0	0	0	0	0	0	0	0	0	0	0	
23575098	m	hp	DE	2024	0	1	0	0	1	0	0	0	0	0	0	0	73
23575450	m	hp	US	2024	0	0	0	0	0	0	0	0	0	0	0	0	
23575557	Not	md	JP	2024	0	0	0	0	0	0	0	0	0	0	0	0	
23575581	f	hp	US	2024	0	0	0	0	0	0	0	0	0	0	0	0	80
23575609	f	ph	FR	2024	0	1	1	0	0	0	0	0	0	0	0	0	70
23575613	Not	md	JP	2024	0	1	0	0	0	1	0	0	0	0	0	0	80
23575656	m	md	JP	2024	0	1	0	0	0	1	0	0	0	0	0	0	70
23575701	f	md	JP	2024	0	1	0	0	0	1	0	0	0	0	0	0	
23575711	m	md	JP	2024	0	1	0	1	0	0	0	0	0	0	0	0	70
23575868	m	md	JP	2024	0	0	0	0	0	0	0	0	0	0	0	0	
23575874	m	md	FR	2024	0	0	0	0	0	0	0	0	0	0	0	0	73
23575901	f	md	DE	2024	0	1	0	0	1	0	0	0	0	0	0	0	68
23576327	m	md	JP	2024	0	1	0	1	0	0	0	0	0	0	0	0	60
23576336	Not	md	JP	2024	0	1	0	1	0	0	0	0	0	0	0	0	
23576512	Not	md	JP	2024	0	1	0	1	0	0	0	0	0	0	0	0	
23576596	m	hp	FI	2024	0	0	0	0	0	0	0	0	0	0	0	0	81
23576657	m	hp	US	2024	0	0	0	0	0	0	0	0	0	0	0	0	60
23576677	m	md	FR	2024	0	0	0	0	0	0	0	0	0	0	0	0	73
23576699	m	md	DE	2024	0	1	1	0	0	0	0	0	0	0	0	1	62
23576880	m	md	JP	2024	0	1	0	1	0	0	0	0	0	0	0	1	74
23576934	m	md	CN	2024	0	1	0	1	0	0	0	0	0	0	0	0	78
23577171	m	cn	FR	2024	0	0	0	0	0	0	0	0	0	0	0	0	65
23577340	m	md	DE	2024	0	1	1	0	0	0	0	0	0	0	0	1	62
23578243	m	md	JP	2024	0	0	0	0	0	0	0	0	0	0	0	0	61
23578837	Not	ph	JP	2024	1	0	0	0	0	0	0	0	0	0	0	0	
23579037	m	hp	US	2024	0	0	0	0	0	0	0	0	0	0	0	0	60
23579066	m	hp	US	2024	0	0	0	0	0	0	0	0	0	0	0	0	47
23579092	f	ph	FR	2024	0	1	1	0	0	0	0	0	0	0	0	0	70
23579283	Not	md	JP	2024	1	0	0	0	0	0	0	0	0	0	0	0	
23579292	m	md	FR	2024	0	1	1	0	0	0	0	0	0	0	0	0	74
23579298	m	md	DE	2024	0	1	1	0	0	0	0	0	0	0	0	0	
23579516	f	hp	FR	2024	0	1	1	0	0	0	0	0	0	0	0	0	70
23579735	m	md	JP	2024	0	0	0	0	0	0	0	0	0	0	0	0	79

23580214	m	md	JP	2024	0	1	0	0	1	0	0	0	0	0	0	0	90
23580306	f	hp	US	2024	0	0	0	0	0	0	0	0	0	0	0	0	74
23580375	m	md	JP	2024	0	1	0	1	0	0	0	0	0	0	0	0	68
23580525	m	ph	JP	2024	0	0	0	0	0	0	0	0	0	0	0	0	
23580583	m	cn	US	2024	0	0	0	0	0	0	0	0	0	0	0	0	41
23580626	m	md	JP	2024	0	1	0	1	0	0	0	0	0	0	0	0	
23580792	m	md	FR	2024	0	1	0	1	0	0	0	0	0	0	0	0	
23580875	Not	ph	US	2024	0	0	0	0	0	0	0	0	0	0	0	0	
23581184	f	cn	TH	2024	1	0	0	0	0	0	0	0	0	0	0	0	65.9
23581389	m	md	FR	2024	0	0	0	0	0	0	0	0	0	0	0	0	65
23581469	f	cn	US	2024	1	0	0	0	0	0	0	0	0	0	0	0	
23581592	f	md	AT	2024	0	0	0	0	0	0	0	0	0	0	0	0	
23581695	f	cn	MY	2024	0	0	0	0	0	0	0	0	0	0	0	0	46
23581698	m	hp	PL	2024	0	0	0	0	0	0	0	0	0	0	0	0	
23581731	f	cn	FR	2024	0	1	1	0	0	0	0	0	0	0	0	0	70
23581892	m	md	JP	2024	0	1	1	0	0	0	0	0	0	0	0	0	80
23582844	m	md	JP	2024	0	1	0	0	1	0	0	0	0	0	0	0	57
23583985	Not	md	BG	2024	0	1	0	0	0	1	0	0	0	0	0	0	
23584424	m	md	DK	2024	0	1	0	1	0	0	0	0	0	0	0	1	58
23584497	Not	hp	US	2024	0	1	1	0	0	0	0	0	0	0	0	0	
23584549	m	md	DE	2024	0	1	1	0	0	0	0	0	0	0	0	0	67
23584746	m	hp	PL	2024	0	1	1	0	0	0	0	0	0	0	0	0	68
23584763	m	md	JP	2024	0	0	0	0	0	0	0	0	0	0	0	0	58
23584899	f	cn	US	2024	0	0	0	0	0	0	0	0	0	0	0	0	
23584909	f	ph	FR	2024	0	1	1	0	0	0	0	0	0	0	0	0	70
23585128	f	md	JP	2024	0	0	0	0	0	0	0	0	0	0	0	0	60
23585641	f	md	DE	2024	0	1	0	0	1	0	0	0	0	0	0	0	64
23585643	f	hp	DE	2024	0	1	0	0	1	0	0	0	0	0	0	0	66
23585783	m	md	DE	2024	0	1	0	0	1	0	0	0	0	0	0	0	59
23585827	m	md	DE	2024	0	1	1	0	0	0	0	0	0	0	0	0	66
23585968	f	ph	FR	2024	0	1	1	0	0	0	0	0	0	0	0	0	70
23585988	m	md	DE	2024	0	1	0	0	1	0	0	0	0	0	0	1	52
23586053	m	md	FR	2024	0	0	0	0	0	0	0	0	0	0	0	0	73
23586140	f	cn	JP	2024	1	0	0	0	0	0	0	0	0	0	0	1	

23586141	f	md	US	2024	1	0	0	0	0	0	0	0	0	0	0	0	60
23586302	f	md	JP	2024	0	0	0	0	0	0	0	0	0	0	0	0	80
23586442	m	hp	NL	2024	0	1	1	0	0	0	0	0	0	0	0	0	59
23586515	m	cn	IN	2024	1	0	0	0	0	0	0	0	0	0	0	0	
23586846	f	cn	US	2024	0	0	0	0	0	0	0	0	0	0	0	0	60
23586902	Not	md	AU	2024	0	1	0	1	0	0	0	0	0	0	0	0	
23587060	f	md	DE	2024	0	1	1	0	0	0	0	0	0	0	0	0	
23587118	m	md	JP	2024	0	1	0	1	0	0	0	0	0	0	0	0	70
23587326	m	md	JP	2024	0	1	0	1	0	0	0	0	0	0	0	0	43
23587336	f	hp	TR	2024	0	0	0	0	0	0	1	0	1	0	0	0	57
23587739	m	md	FR	2024	0	1	1	0	0	0	0	0	0	0	0	0	74
23587781	m	cn	SG	2024	1	0	0	0	0	0	0	0	0	0	0	0	75
23588060	f	md	JP	2024	0	1	0	0	0	1	0	0	0	0	0	0	90
23588848	m	md	DE	2024	0	1	1	0	0	0	0	0	0	0	0	0	61
23588851	f	md	DE	2024	0	1	1	0	0	0	0	0	0	0	0	0	71
23588998	m	md	IT	2024	0	1	1	0	0	0	0	0	0	0	0	0	75
23589069	f	md	JP	2024	0	0	0	0	0	0	0	0	0	0	0	0	75
23589659	m	md	FR	2024	0	0	0	0	0	0	0	0	0	0	0	0	73
23589804	m	md	IT	2024	0	1	1	0	0	0	0	0	0	0	0	0	75
23589834	f	md	JP	2024	0	1	0	0	1	0	0	0	0	0	0	0	71
23589847	Not	md	JP	2024	0	0	0	0	0	0	0	0	0	0	0	0	
23590112	Not	md	JP	2024	0	1	0	0	0	1	0	0	0	0	0	0	72
23590124	f	md	JP	2024	0	1	0	0	0	1	0	0	0	0	0	0	60
23590216	Not	md	JP	2024	0	1	0	0	1	0	0	0	0	0	0	0	
23590327	m	hp	ES	2024	0	1	0	0	1	0	0	0	0	0	0	0	63
23590464	f	md	IT	2024	0	1	0	0	1	0	0	0	0	0	0	0	64
23590483	m	md	IT	2024	0	1	1	0	0	0	0	0	0	0	0	0	75
23590517	f	md	JP	2024	0	1	0	0	1	0	0	0	0	0	0	0	71
23590582	m	md	DE	2024	0	1	0	0	1	0	0	0	0	0	0	0	61
23590620	m	md	KR	2024	0	0	0	0	0	0	0	0	0	0	0	0	
23590678	f	hp	CN	2024	0	0	0	0	0	0	1	0	1	0	0	0	57
23590682	m	ph	JP	2024	0	1	1	0	0	0	0	0	0	0	0	0	70
23590761	f	ph	PT	2024	0	0	0	0	0	0	0	0	0	0	0	0	73
23590783	f	hp	IT	2024	0	1	0	0	0	1	0	0	0	0	0	0	61

23590843	m	md	BE	2024	0	0	0	0	0	0	0	0	0	0	0	0	
23591026	f	hp	JP	2024	0	0	0	0	0	0	0	0	0	0	0	0	79
23591322	m	md	JP	2024	0	1	0	0	1	0	0	0	0	0	0	0	75
23591523	m	md	JP	2024	0	1	0	0	0	1	0	0	0	0	0	0	80
23591705	f	cn	KR	2024	1	0	0	0	0	0	0	0	0	0	0	0	57.2
23591829	f	ph	TH	2024	0	1	1	0	0	0	0	0	0	0	0	0	
23591874	m	md	BE	2024	0	0	0	0	0	0	0	0	0	0	0	0	
23591919	m	md	BE	2024	0	0	0	0	0	0	0	0	0	0	0	0	
23591946	f	cn	CA	2024	1	0	0	0	0	0	0	0	0	0	0	0	77.1
23591964	f	md	JP	2024	0	1	0	0	0	1	0	0	0	0	0	0	60
23592052	m	hp	JP	2024	0	1	0	0	1	0	0	0	0	0	0	1	69
23592097	m	cn	JP	2024	0	0	0	0	0	0	0	0	0	0	0	0	77
23592106	f	md	JP	2024	0	0	0	0	0	0	0	0	0	0	0	0	75
23592373	f	md	DE	2024	0	1	0	0	1	0	0	0	0	0	0	0	60
23592605	m	md	JP	2024	0	0	0	0	0	0	0	0	0	0	0	0	
23592636	f	md	JP	2024	1	0	0	0	0	0	0	0	0	0	0	0	60
23592702	m	md	JP	2024	0	1	0	1	0	0	0	0	0	0	0	1	74
23592770	f	cn	US	2024	1	0	0	0	0	0	0	0	0	0	0	0	80.5
23593115	m	md	DE	2024	0	1	0	0	1	0	0	0	0	0	0	0	73
23593148	m	md	PT	2024	0	1	1	0	0	0	0	0	0	0	0	0	65
23593167	m	cn	CN	2024	0	0	0	0	0	0	0	0	0	0	0	0	
23593169	m	cn	CN	2024	0	0	0	0	0	0	0	0	0	0	0	0	
23593171	m	md	DE	2024	0	1	0	0	1	0	0	0	0	0	0	0	69
23593189	m	cn	GR	2024	0	1	1	0	0	0	0	0	0	0	0	0	56
23593215	m	md	BE	2024	0	0	0	0	0	0	0	0	0	0	0	0	
23593231	m	md	FR	2024	0	1	1	0	0	0	0	0	0	0	0	0	61
23593234	Not	md	JP	2024	0	0	0	0	0	0	0	0	0	0	0	0	
23593280	m	md	FR	2024	0	0	0	0	0	0	0	0	0	0	0	0	65
23593342	m	md	AU	2024	0	1	1	0	0	0	0	0	0	0	0	0	63
23593356	Not	md	GB	2024	0	0	0	0	0	0	0	0	0	0	0	0	
23593406	f	cn	CN	2024	0	0	0	0	0	0	0	0	0	0	0	0	
23593423	f	md	IN	2024	0	0	0	0	0	0	0	0	0	0	0	0	65
23593469	m	hp	JP	2024	0	1	1	0	0	0	0	0	0	0	0	0	
23593896	m	md	FR	2024	0	0	0	0	0	0	0	0	0	0	0	0	65

23593956	f	md	JP	2024	0	1	0	0	1	0	0	0	0	0	0	0	75
23594043	f	md	JP	2024	0	0	0	0	0	0	0	0	0	0	0	0	80
23594382	m	md	JP	2024	0	1	0	0	0	1	0	0	0	0	0	0	70
23594451	m	md	FR	2024	0	0	0	0	0	0	0	0	0	0	0	0	65
23594665	Not	md	LB	2024	0	1	1	0	0	0	0	0	0	0	0	0	
23594918	m	md	JP	2024	0	1	0	0	1	0	0	0	0	0	0	0	
23595087	m	md	IT	2024	1	0	0	0	0	0	0	0	0	0	0	0	68
23595260	m	ph	NZ	2024	0	1	0	1	0	0	0	0	0	0	0	0	
23595405	m	hp	JP	2024	0	0	0	0	0	0	0	0	0	0	0	0	60
23595481	f	ph	US	2024	1	0	0	0	0	0	0	0	0	0	0	0	81
23595507	f	cn	CN	2024	0	0	0	0	0	0	0	0	0	0	0	0	68
23595763	m	cn	US	2024	0	0	0	0	0	0	0	0	0	0	0	0	78
23595861	m	md	JP	2024	0	1	0	1	0	0	0	0	0	0	0	0	69
23596189	f	ph	US	2024	0	1	1	0	0	0	0	0	0	0	0	0	61
23596745	m	md	NL	2024	0	1	0	0	0	1	0	0	0	0	0	0	65
23596892	m	md	JP	2024	0	1	0	1	0	0	0	0	0	0	0	0	73
23597012	m	md	DE	2024	0	0	0	0	0	0	0	0	0	0	0	0	63
23597094	m	cn	CA	2024	1	0	0	0	0	0	0	0	0	0	0	0	80.4
23597456	m	md	DE	2024	0	0	0	0	0	0	0	0	0	0	0	0	51
23597549	m	ph	FR	2024	0	0	0	0	0	0	0	0	0	0	0	0	77
23597840	f	md	RS	2024	0	1	1	0	0	0	0	0	0	0	0	0	
23597940	Not	md	US	2024	0	0	0	0	0	0	0	0	0	0	0	0	
23597948	f	md	CA	2024	0	1	1	0	0	0	0	0	0	0	0	0	67
23598097	m	md	KR	2024	0	0	0	0	0	0	0	0	0	0	0	0	74
23598545	m	md	BE	2024	0	0	0	0	0	0	0	0	0	0	0	0	
23598568	m	hp	US	2024	0	0	0	0	0	0	0	0	0	0	0	0	40
23598743	m	md	DE	2024	0	1	0	0	1	0	0	0	0	0	0	0	59
23599501	Not	md	JP	2024	0	1	0	1	0	0	0	0	0	0	0	0	
23599752	m	md	JP	2024	1	0	0	0	0	0	0	0	0	0	0	0	79
23600453	Not	hp	DE	2024	0	0	0	0	0	0	0	0	0	0	0	0	
23600898	m	md	DE	2024	0	1	1	0	0	0	0	0	0	0	0	0	67
23601016	m	md	DE	2024	0	1	1	0	0	0	0	0	0	0	0	0	61
23601120	m	md	US	2024	0	0	0	0	0	0	0	0	0	0	0	0	68
23601322	f	cn	US	2024	0	0	0	0	0	0	0	0	0	0	0	0	

23601402	f	cn	US	2024	0	1	1	0	0	0	0	0	0	0	0	0	
23601444	m	md	DE	2024	0	1	1	0	0	0	0	0	0	0	0	0	
23601496	m	md	DE	2024	0	0	0	0	0	0	0	0	0	0	0	0	62
23601524	m	ph	JP	2024	1	0	0	0	0	0	0	0	0	0	0	0	72
23601731	m	md	JP	2024	0	1	0	1	0	0	0	0	0	0	0	0	56
23601769	f	md	DE	2024	0	1	1	0	0	0	0	0	0	0	0	0	82
23601791	m	md	JP	2024	0	1	0	1	0	0	0	0	0	0	0	0	69
23602706	m	md	CN	2024	0	1	1	0	0	0	0	0	0	0	0	0	62
23602758	f	ph	KR	2024	0	0	0	0	0	0	0	0	0	0	0	0	64
23603334	m	md	MY	2024	0	0	0	0	0	0	0	0	0	0	0	0	63
23603421	m	md	VN	2024	0	1	0	0	0	1	0	0	0	0	0	0	62
23603638	m	md	FR	2024	0	1	1	0	0	0	0	0	0	0	0	0	74
23603758	m	md	DE	2024	0	1	1	0	0	0	0	0	0	0	0	0	77
23603800	m	md	IT	2024	0	0	0	0	0	0	0	0	0	0	0	0	77
23603866	m	cn	PK	2024	0	1	0	0	0	1	0	0	0	0	0	0	77
23604344	m	md	JP	2024	0	1	0	0	0	1	0	0	0	0	0	0	50
23604377	m	md	FR	2024	0	0	0	0	0	0	0	0	0	0	0	0	81
23604421	Not	ph	JP	2024	1	0	0	0	0	0	0	0	0	0	0	0	
23604504	m	md	JP	2024	0	1	0	0	1	0	0	0	0	0	0	0	76
23604675	m	hp	IT	2024	0	0	0	0	0	0	0	0	0	0	0	0	63
23604889	f	md	JP	2024	0	0	0	0	0	0	0	0	0	0	0	0	78
23604959	m	hp	NL	2024	0	1	1	0	0	0	0	0	0	0	0	0	59
23605036	f	cn	US	2024	0	0	0	0	0	0	0	0	0	0	0	0	71
23605245	Not	hp	KR	2024	0	0	0	0	0	0	0	0	0	0	0	0	
23605844	Not	md	JP	2024	1	0	0	0	0	0	0	0	0	0	0	0	60
23606049	m	md	DE	2024	0	1	0	0	1	0	0	0	0	0	0	0	73
23606087	f	md	AT	2024	0	0	0	0	0	0	0	0	0	0	0	0	
23606502	m	md	JP	2024	0	1	1	0	0	0	0	0	0	0	0	0	70
23606515	Not	ph	JP	2024	1	0	0	0	0	0	0	0	0	0	0	0	
23606751	m	md	NL	2024	0	1	0	0	0	1	0	0	0	0	0	0	65
23606870	m	md	DE	2024	0	1	1	0	0	0	0	0	0	0	0	0	77
23606939	m	md	IT	2024	0	1	1	0	0	0	0	0	0	0	0	0	75
23606961	m	hp	NL	2024	0	1	1	0	0	0	0	0	0	0	0	0	59
23607001	f	md	DE	2024	0	1	1	0	0	0	0	0	0	0	0	0	71

23607039	m	ph	FR	2024	0	0	0	0	0	0	0	0	0	0	0	0	77
23607107	m	md	DE	2024	0	1	1	0	0	0	0	0	0	0	0	0	67
23607428	f	md	FR	2024	0	1	0	1	0	0	0	0	0	0	0	0	71.7
23607588	f	cn	PK	2024	0	1	0	0	0	1	0	0	0	0	0	0	79
23607858	f	md	IT	2024	0	0	0	0	0	0	0	0	0	0	0	0	72
23607878	m	md	FR	2024	0	1	1	0	0	0	0	0	0	0	0	0	61
23607882	m	hp	IT	2024	0	1	0	0	1	0	0	0	0	0	0	1	58
23607986	f	md	AT	2024	0	0	0	0	0	0	0	0	0	0	0	0	70
23608072	m	md	JP	2024	0	1	1	0	0	0	0	0	0	0	0	0	80
23608262	f	hp	KR	2024	0	1	1	0	0	0	0	0	0	0	0	0	
23608405	f	md	TW	2024	0	1	0	0	1	0	0	0	0	0	0	0	75
23608852	m	md	FR	2024	0	0	0	0	0	0	0	0	0	0	0	0	
23608858	f	ph	FR	2024	0	1	1	0	0	0	0	0	0	0	0	0	70
23608874	m	md	DE	2024	0	1	1	0	0	0	0	0	0	0	0	0	61
23609310	f	md	JP	2024	0	1	0	0	1	0	0	0	0	0	0	0	76
23609854	m	md	AT	2024	0	0	0	0	0	0	0	0	0	0	0	0	71
23610366	m	hp	TH	2024	1	0	0	0	0	0	0	0	0	0	0	1	64
23611053	f	md	DE	2024	0	1	0	0	1	0	0	0	0	0	0	0	73
23611639	f	md	DE	2024	0	1	0	0	1	0	0	0	0	0	0	0	76
23611715	m	md	JP	2024	0	1	0	1	0	0	0	0	0	0	0	0	59
23611756	m	md	BE	2024	0	0	0	0	0	0	0	0	0	0	0	0	
23611792	f	md	JP	2024	1	0	0	0	0	0	0	0	0	0	0	0	77
23611840	f	md	JP	2024	1	0	0	0	0	0	0	0	0	0	0	0	86
23611845	m	hp	GB	2024	0	1	0	1	0	0	0	0	0	0	0	0	
23611908	m	md	DE	2024	0	1	1	0	0	0	0	0	0	0	0	0	78
23611922	m	md	IT	2024	0	1	1	0	0	0	0	0	0	0	0	0	75
23611944	f	md	JP	2024	1	0	0	0	0	0	0	0	0	0	0	0	73
23611976	f	md	US	2024	1	0	0	0	0	0	0	0	0	0	0	0	42
23612096	f	hp	CA	2024	1	0	0	0	0	0	0	0	0	0	0	0	78
23613673	Not	md	JP	2024	0	0	0	0	0	0	0	0	0	0	0	0	
23613813	m	md	BE	2024	0	0	0	0	0	0	0	0	0	0	0	0	
23613832	Not	md	US	2024	0	0	0	0	0	0	0	0	0	0	0	0	
23613868	m	md	FR	2024	0	1	1	0	0	0	0	0	0	0	0	0	61
23613966	m	md	TH	2024	0	1	1	0	0	0	0	0	0	0	0	0	55

23614514	m	hp	IT	2024	0	1	0	0	1	0	0	0	0	0	0	1	58
23614585	m	cn	PK	2024	0	1	0	0	0	1	0	0	0	0	0	0	61
23614596	m	cn	TH	2024	0	1	0	0	0	1	0	0	0	0	0	0	
23614598	m	md	JP	2024	0	1	0	0	0	1	0	0	0	0	0	0	67
23614616	Not	md	LB	2024	0	1	0	0	0	1	0	0	0	0	0	0	
23614967	m	cn	US	2024	0	1	1	0	0	0	0	0	0	0	0	0	81
23615172	f	cn	US	2024	0	0	0	0	0	0	0	0	0	0	0	0	76
23615673	Not	md	US	2024	0	1	0	0	1	0	0	0	0	0	0	0	43
23616280	m	hp	JP	2024	0	1	0	0	1	0	0	0	0	0	0	0	74
23616308	m	cn	IN	2024	0	1	0	0	0	1	0	0	0	0	0	0	69
23616588	f	md	MX	2024	1	0	0	0	0	0	0	0	0	0	0	0	
23616765	f	hp	FR	2024	1	0	0	0	0	0	0	0	0	0	0	0	
23616901	m	md	DE	2024	0	1	1	0	0	0	0	0	0	0	0	0	
23616955	f	md	DE	2024	0	1	0	0	1	0	0	0	0	0	0	0	68
23616971	m	md	FR	2024	0	1	1	0	0	0	0	0	0	0	0	0	61
23616972	m	md	FR	2024	0	1	1	0	0	0	0	0	0	0	0	0	61
23617002	f	md	US	2024	1	0	0	0	0	0	0	0	0	0	0	0	42
23617307	m	md	IT	2024	0	1	0	0	1	0	0	0	0	0	0	0	55
23617531	m	ph	FR	2024	0	0	0	0	0	0	0	0	0	0	0	0	77
23617985	Not	hp	FR	2024	0	1	1	0	0	0	0	0	0	0	0	0	
23618202	m	hp	FR	2024	1	0	0	0	0	0	0	0	0	0	0	0	
23618259	Not	md	JP	2024	0	1	1	0	0	0	0	0	0	0	0	0	88
23618356	m	hp	FR	2024	1	0	0	0	0	0	0	0	0	0	0	0	
23618607	m	md	DE	2024	0	1	0	0	1	0	0	0	0	0	0	0	66
23619383	m	md	JP	2024	0	1	1	0	0	0	0	0	0	0	0	0	62
23619780	Not	md	FR	2024	0	0	0	0	0	0	0	0	0	0	0	0	
23619875	m	cn	SG	2024	0	1	0	0	0	1	0	0	0	0	0	0	61
23619926	f	md	JP	2024	0	1	0	0	0	1	0	0	0	0	0	0	60
23619933	m	hp	US	2024	0	0	0	0	0	0	0	0	0	0	0	0	73
23620206	Not	md	JP	2024	0	1	0	0	1	0	0	0	0	0	0	0	
23620600	f	md	JP	2024	0	1	0	0	1	0	0	0	0	0	0	0	72
23620673	f	ph	FR	2024	0	1	0	0	0	1	0	0	0	0	0	0	73
23620900	m	md	DE	2024	0	1	1	0	0	0	0	0	0	0	0	0	67
23621050	m	md	JP	2024	0	0	0	0	0	0	0	0	0	0	0	0	80

23621120	Not	hp	IT	2024	0	0	0	0	0	0	0	0	0	0	0	0	58
23621135	m	md	VN	2024	0	1	0	0	0	1	0	0	0	0	0	0	69
23621155	m	hp	PL	2024	0	0	0	0	0	0	0	0	0	0	0	0	72
23621179	m	md	JP	2024	0	0	0	0	0	0	0	0	0	0	0	0	
23621255	m	md	DE	2024	0	1	1	0	0	0	0	0	0	0	0	1	62
23621692	Not	md	JP	2024	1	0	0	0	0	0	0	0	0	0	0	0	
23621852	Not	md	US	2024	0	0	0	0	0	0	0	0	0	0	0	0	
23621970	Not	md	GB	2024	1	0	0	0	0	0	0	0	0	0	0	0	
23622326	f	md	PL	2024	0	1	1	0	0	0	0	0	0	0	0	0	69
23623081	m	md	JP	2024	1	0	0	0	0	0	0	0	0	0	0	0	76
23623589	f	md	SK	2024	0	0	0	0	0	0	0	0	0	0	0	0	74
23623704	m	hp	NL	2024	0	1	1	0	0	0	0	0	0	0	0	0	59
23623835	Not	md	JP	2024	0	1	0	0	1	0	0	0	0	0	0	1	
23624298	f	ph	FR	2024	0	1	1	0	0	0	0	0	0	0	0	0	70
23624533	Not	md	US	2024	0	0	0	0	0	0	0	0	0	0	0	0	
23624548	Not	md	US	2024	0	0	0	0	0	0	0	0	0	0	0	0	
23624848	m	hp	TH	2024	1	0	0	0	0	0	0	0	0	0	0	0	77
23625110	m	md	FR	2024	0	1	1	0	0	0	0	0	0	0	0	0	74
23625425	m	hp	PL	2024	0	1	0	0	0	1	0	0	0	0	0	0	
23625619	m	md	DE	2024	0	1	1	0	0	0	0	0	0	0	0	0	78
23626182	f	md	GR	2024	0	0	0	0	0	0	0	0	0	0	0	0	58
23626310	Not	md	JP	2024	0	1	0	0	1	0	0	0	0	0	0	0	
23626451	f	hp	DE	2024	0	0	0	0	0	0	0	0	0	0	0	0	60
23626585	m	hp	IN	2024	0	0	0	0	0	0	0	0	0	0	0	0	66
23626627	m	hp	IN	2024	0	0	0	0	0	0	0	0	0	0	0	0	67
23626783	m	hp	IN	2024	0	0	0	0	0	0	0	0	0	0	0	0	69
23626784	f	hp	IN	2024	0	0	0	0	0	0	0	0	0	0	0	0	67
23626799	m	hp	IN	2024	0	0	0	0	0	0	0	0	0	0	0	0	68
23626805	m	hp	IN	2024	0	0	0	0	0	0	0	0	0	0	0	0	75
23626831	m	md	US	2024	0	0	0	0	0	0	0	0	0	0	0	0	64
23626954	f	cn	CH	2024	1	0	0	0	0	0	0	0	0	0	0	0	73
23626956	m	hp	NL	2024	0	1	1	0	0	0	0	0	0	0	0	0	59
23627306	m	md	RU	2024	0	0	0	0	0	0	0	0	0	0	0	0	62
23627515	Not	md	BR	2024	0	0	0	0	0	0	0	0	0	0	0	0	88

23628180	f	cn	DE	2024	0	0	0	0	0	0	0	0	0	0	0	0	58
23628274	f	hp	CA	2024	1	0	0	0	0	0	0	0	0	0	0	0	
23628635	m	md	JP	2024	0	1	0	1	0	0	0	0	0	0	0	1	57
23628952	f	cn	US	2024	1	0	0	0	0	0	0	0	0	0	0	0	
23629144	m	md	GR	2024	0	0	0	0	0	0	0	0	0	0	0	0	72
23629145	f	hp	US	2024	0	0	0	0	0	0	0	0	0	0	0	0	
23629159	m	md	DK	2024	0	1	1	0	0	0	0	0	0	0	0	0	75
23629598	Not	md	JP	2024	0	1	0	0	1	0	0	0	0	0	0	0	
23629667	m	md	FR	2024	0	0	0	0	0	0	0	0	0	0	0	0	65
23630216	m	cn	US	2024	0	1	1	0	0	0	0	0	0	0	0	0	66
23630418	m	md	RO	2024	0	1	0	0	1	0	0	0	0	0	0	0	71
23630442	m	md	JP	2024	0	0	0	0	0	0	0	0	0	0	0	0	
23630614	Not	md	JP	2024	0	1	0	0	1	0	0	0	0	0	0	0	
23630776	m	md	JP	2024	0	1	0	0	1	0	0	0	0	0	0	0	60
23630988	m	hp	JP	2024	0	1	1	0	0	0	0	0	0	0	0	0	70
23630993	f	ph	FR	2024	0	1	1	0	0	0	0	0	0	0	0	0	70
23631174	f	hp	FR	2024	1	0	0	0	0	0	0	0	0	0	0	0	
23631175	f	hp	FR	2024	0	1	0	1	0	0	0	0	0	0	0	0	
23631279	Not	md	DE	2024	0	1	0	0	0	1	0	0	0	0	0	0	
23631302	Not	md	JP	2024	1	0	0	0	0	0	0	0	0	0	0	0	
23631314	Not	md	JP	2024	0	0	0	0	0	0	0	0	0	0	0	0	
23631514	m	md	TR	2024	0	0	0	0	0	0	0	0	0	0	0	0	62
23631542	m	cn	IN	2024	0	0	0	0	0	0	0	0	0	0	0	0	44
23631557	f	cn	EG	2024	0	0	0	0	0	0	0	0	0	0	0	0	51
23631564	f	md	DE	2024	0	0	0	0	0	0	0	0	0	0	0	0	90
23631637	Not	md	FR	2024	0	0	0	0	0	0	0	0	0	0	0	0	
23631654	m	md	IT	2024	0	0	0	0	0	0	0	0	0	0	0	0	63
23632090	Not	hp	JP	2024	1	0	0	0	0	0	0	0	0	0	0	0	
23632241	Not	md	JP	2024	0	0	0	0	0	0	0	0	0	0	0	0	
23632245	Not	hp	JP	2024	1	0	0	0	0	0	0	0	0	0	0	0	
23632246	Not	hp	JP	2024	1	0	0	0	0	0	0	0	0	0	0	0	
23632247	Not	hp	JP	2024	1	0	0	0	0	0	0	0	0	0	0	0	
23632254	Not	hp	JP	2024	1	0	0	0	0	0	0	0	0	0	0	0	
23632255	Not	hp	JP	2024	1	0	0	0	0	0	0	0	0	0	0	0	

23632354	f	hp	PL	2024	0	1	1	0	0	0	0	0	0	0	0	0	54
23632899	m	md	DE	2024	0	1	0	0	1	0	0	0	0	0	0	0	57
23632981	m	hp	US	2024	0	0	0	0	0	0	0	0	0	0	0	0	70
23633399	m	hp	BE	2024	0	0	0	0	0	0	0	0	0	0	0	0	32
23633670	m	md	JP	2024	0	1	0	1	0	0	0	0	0	0	0	0	57
23633751	m	ph	TW	2024	0	1	0	0	1	0	0	0	0	0	0	0	51
23633777	m	md	JP	2024	0	0	0	0	0	0	0	0	0	0	0	0	80
23633878	m	md	FR	2024	0	1	0	0	0	1	0	0	0	0	0	0	73
23634152	f	ph	TH	2024	1	0	0	0	0	0	0	0	0	0	0	0	65
23634153	f	cn	TH	2024	1	0	0	0	0	0	0	0	0	0	0	0	59.3
23634198	f	md	IL	2024	0	0	0	0	0	0	0	0	0	0	0	0	71
23634353	m	ph	JP	2024	0	0	0	0	0	0	0	0	0	0	0	0	80
23634708	Not	md	JP	2024	0	1	0	0	1	0	0	0	0	0	0	0	
23634802	m	md	NL	2024	0	1	1	0	0	0	0	0	0	0	0	0	
23635088	Not	md	JP	2024	0	1	0	0	1	0	0	0	0	0	0	0	
23635106	Not	md	JP	2024	0	1	0	0	1	0	0	0	0	0	0	0	
23635260	Not	md	JP	2024	0	1	0	0	1	0	0	0	0	0	0	0	
23635357	Not	md	JP	2024	0	1	0	0	1	0	0	0	0	0	0	0	
23635699	f	md	FR	2024	0	1	1	0	0	0	0	0	0	0	0	0	70
23635774	m	ph	JP	2024	1	0	0	0	0	0	0	0	0	0	0	0	71
23635920	Not	md	JP	2024	0	1	0	0	1	0	0	0	0	0	0	0	
23635972	m	md	DE	2024	0	1	1	0	0	0	0	0	0	0	0	0	61
23636124	Not	md	JP	2024	0	1	0	0	1	0	0	0	0	0	0	0	
23636170	m	ph	JP	2024	0	1	1	0	0	0	0	0	0	0	0	1	71
23636251	Not	md	JP	2024	0	1	0	0	1	0	0	0	0	0	0	0	
23636301	Not	md	JP	2024	0	1	0	0	1	0	0	0	0	0	0	0	
23636660	f	cn	US	2024	0	1	0	1	0	0	0	0	0	0	0	0	
23636725	m	md	BR	2024	0	0	0	0	0	0	0	0	0	0	0	0	41
23636838	m	md	BR	2024	0	0	0	0	0	0	0	0	0	0	0	0	37
23636942	m	md	JP	2024	0	0	0	0	0	0	0	0	0	0	0	0	73
23637101	f	cn	JP	2024	0	0	0	0	0	0	0	0	0	0	0	0	68
23637344	m	md	FR	2024	0	1	1	0	0	0	0	0	0	0	0	0	74
23637348	m	md	AT	2024	0	0	0	0	0	0	0	0	0	0	0	0	63
23637420	Not	md	JP	2024	0	1	0	0	1	0	0	0	0	0	0	0	

23637837	m	hp	CA	2024	0	0	0	0	0	0	0	0	0	0	0	0	67
23637858	f	hp	CN	2024	1	0	0	0	0	0	0	0	0	0	0	0	57
23637881	m	cn	US	2024	1	0	0	0	0	0	0	0	0	0	0	0	
23638015	f	cn	US	2024	1	0	0	0	0	0	0	0	0	0	0	0	
23638170	Not	hp	JP	2024	1	0	0	0	0	0	0	0	0	0	0	0	
23638171	Not	hp	JP	2024	1	0	0	0	0	0	0	0	0	0	0	0	
23638172	Not	hp	JP	2024	1	0	0	0	0	0	0	0	0	0	0	0	
23638323	f	md	JP	2024	0	0	0	0	0	0	1	0	0	1	0	1	71
23638833	m	md	JP	2024	0	1	0	1	0	0	0	0	0	0	0	0	65
23638836	m	md	JP	2024	0	1	0	1	0	0	0	0	0	0	0	0	35
23638914	m	md	TW	2024	0	1	0	0	1	0	0	0	0	0	0	0	67
23639189	f	md	JP	2024	0	0	0	0	0	0	0	0	0	0	0	0	88
23639226	m	md	JP	2024	0	0	0	0	0	0	0	0	0	0	0	0	
23639282	f	md	AT	2024	0	0	0	0	0	0	0	0	0	0	0	0	
23639301	f	cn	IL	2024	0	0	0	0	0	0	0	0	0	0	0	0	31
23639397	m	md	IT	2024	0	1	1	0	0	0	0	0	0	0	0	0	75
23639759	m	md	ES	2024	0	0	0	0	0	0	0	0	0	0	0	0	54
23639785	f	md	FR	2024	0	1	1	0	0	0	0	0	0	0	0	0	70
23640104	m	md	DE	2024	0	1	1	0	0	0	0	0	0	0	0	1	62
23640106	m	md	IT	2024	0	1	1	0	0	0	0	0	0	0	0	0	75
23640110	m	md	IT	2024	0	1	1	0	0	0	0	0	0	0	0	0	75
23640137	m	md	US	2024	0	0	0	0	0	0	0	0	0	0	0	0	64
23640150	m	md	FR	2024	0	0	0	0	0	0	0	0	0	0	0	0	73
23640716	Not	md	US	2024	0	1	0	0	1	0	0	0	0	0	0	0	
23640720	Not	md	JP	2024	0	1	0	0	1	0	0	0	0	0	0	0	
23640841	f	md	US	2024	1	0	0	0	0	0	0	0	0	0	0	0	
23640974	m	md	JP	2024	0	1	0	0	1	0	0	0	0	0	0	0	65
23641501	m	md	FR	2024	0	1	1	0	0	0	0	0	0	0	0	0	61
23641559	m	md	JP	2024	0	1	0	0	0	1	0	0	0	0	0	0	61
23641659	Not	md	JP	2024	0	1	1	0	0	0	0	0	0	0	0	0	74
23641933	Not	hp	JP	2024	0	0	0	0	0	0	0	0	0	0	0	0	
23642181	m	md	JP	2024	0	1	0	0	1	0	0	0	0	0	0	0	67
23642201	Not	hp	AU	2024	0	1	0	0	0	1	0	0	0	0	0	0	
23642303	m	md	FR	2024	0	0	0	0	0	0	0	0	0	0	0	0	65

23642349	m	md	FR	2024	0	0	0	0	0	0	0	0	0	0	0	0	73
23642396	f	hp	CN	2024	1	0	0	0	0	0	0	0	0	0	0	0	57
23643174	m	md	PL	2024	0	0	0	0	0	0	0	0	0	0	0	0	46
23643617	m	md	JP	2024	0	1	1	0	0	0	0	0	0	0	0	0	72
23643627	m	hp	IT	2024	0	1	1	0	0	0	0	0	0	0	0	0	59
23643763	f	hp	IT	2024	0	1	1	0	0	0	0	0	0	0	0	0	59
23643766	m	md	DE	2024	0	1	1	0	0	0	0	0	0	0	0	0	67
23643797	f	hp	IT	2024	0	1	1	0	0	0	0	0	0	0	0	0	60
23643868	f	hp	IT	2024	0	1	1	0	0	0	0	0	0	0	0	0	61
23643972	m	hp	IT	2024	0	1	1	0	0	0	0	0	0	0	0	0	57
23644123	m	md	JP	2024	0	0	0	0	0	0	0	0	0	0	0	0	70
23644465	f	hp	AU	2024	1	0	0	0	0	0	0	0	0	0	0	0	72
23645224	f	md	JP	2024	0	1	1	0	0	0	0	0	0	0	0	0	76
23645225	f	md	JP	2024	0	1	1	0	0	0	0	0	0	0	0	0	78
23645227	Not	md	JP	2024	0	1	1	0	0	0	0	0	0	0	0	0	70
23645429	m	md	JP	2024	0	1	0	0	1	0	0	0	0	0	0	0	69
23645603	m	md	JP	2024	0	1	1	0	0	0	0	0	0	0	0	0	74
23645901	m	md	FR	2024	0	1	1	0	0	0	0	0	0	0	0	0	61
23646202	m	ph	US	2024	0	0	0	0	0	0	0	0	0	0	0	0	66
23646258	Not	md	JP	2024	0	1	0	0	1	0	0	0	0	0	0	0	
23646383	m	ph	FR	2024	0	0	0	0	0	0	0	0	0	0	0	0	77
23646385	m	md	JP	2024	0	0	0	0	0	0	0	0	0	0	0	0	80
23646914	m	md	US	2024	0	0	0	0	0	0	0	0	0	0	0	0	64
23647084	m	hp	ES	2024	0	1	1	0	0	0	0	0	0	0	0	0	65
23647169	Not	hp	JP	2024	0	0	0	0	0	0	0	0	0	0	0	0	
23647341	m	md	NL	2024	0	1	1	0	0	0	0	0	0	0	0	0	
23647471	f	hp	US	2024	1	0	0	0	0	0	0	0	0	0	0	0	
23647778	f	md	US	2024	0	0	0	0	0	0	0	0	0	0	0	0	
23647786	f	cn	US	2024	0	0	0	0	0	0	0	0	0	0	0	0	87
23647792	m	md	JP	2024	0	1	1	0	0	0	0	0	0	0	0	0	76
23648354	f	ph	US	2024	0	0	0	0	0	0	0	0	0	0	0	0	
23648419	f	md	CN	2024	1	0	0	0	0	0	0	0	0	0	0	0	54
23648442	m	ph	TW	2024	0	1	0	0	1	0	0	0	0	0	0	0	67
23648467	f	md	DE	2024	0	0	0	0	0	0	0	0	0	0	0	0	50

23648919	f	md	JP	2024	1	0	0	0	0	0	0	0	0	0	0	0	
23648991	m	cn	JP	2024	1	0	0	0	0	0	0	0	0	0	0	0	
23649226	f	hp	AT	2024	0	0	0	0	0	0	0	0	0	0	0	0	
23649291	f	md	AU	2024	0	1	1	0	0	0	0	0	0	0	0	0	65
23649359	m	md	US	2024	0	0	0	0	0	0	0	0	0	0	0	0	
23649447	m	cn	KR	2024	0	0	0	0	0	0	0	0	0	0	0	0	62
23649608	f	hp	US	2024	1	0	0	0	0	0	0	0	0	0	0	0	83
23649611	m	md	JP	2024	0	1	0	1	0	0	0	0	0	0	0	1	71
23649766	f	md	JP	2024	0	1	0	1	0	0	0	0	0	0	0	0	51
23649810	m	md	JP	2024	0	1	0	1	0	0	0	0	0	0	0	0	73
23650232	m	md	NL	2024	0	0	0	0	0	0	0	0	0	0	0	0	63
23650961	m	md	DE	2024	0	1	1	0	0	0	0	0	0	0	0	0	61
23651459	m	ph	JP	2024	0	1	1	0	0	0	0	0	0	0	0	0	52
23651542	m	cn	US	2024	0	0	0	0	0	0	0	0	0	0	0	0	57
23651761	f	hp	PL	2024	0	1	1	0	0	0	0	0	0	0	0	0	71
23652087	m	hp	AU	2024	0	1	1	0	0	0	0	0	0	0	0	0	68
23652374	m	md	JP	2024	0	1	0	0	0	1	0	0	0	0	0	0	
23652402	m	md	DE	2024	0	1	1	0	0	0	0	0	0	0	0	0	67
23652859	m	md	TH	2024	0	1	1	0	0	0	0	0	0	0	0	0	48
23653195	f	md	IT	2024	0	1	0	0	1	0	0	0	0	0	0	0	64
23653214	f	md	IT	2024	0	1	0	0	1	0	0	0	0	0	0	0	57
23653269	f	md	KR	2024	0	0	0	0	0	0	0	0	0	0	0	0	69
23653815	m	ph	NO	2024	0	1	1	0	0	0	0	0	0	0	0	0	
23653899	m	md	ES	2024	0	1	1	0	0	0	0	0	0	0	0	0	
23653936	m	ph	JP	2024	0	1	1	0	0	0	0	0	0	0	0	0	52
23654196	f	cn	US	2024	1	0	0	0	0	0	0	0	0	0	0	0	
23654278	f	md	FR	2024	1	0	0	0	0	0	0	0	0	0	0	0	77.3
23655405	f	hp	US	2024	0	1	1	0	0	0	0	0	0	0	0	0	70
23655538	m	cn	US	2024	0	1	0	1	0	0	0	0	0	0	0	0	71.6
23655756	f	md	HR	2024	0	0	0	0	0	0	0	0	0	0	0	0	67
23655794	m	ph	JP	2024	0	0	0	0	0	0	0	0	0	0	0	0	60
23656197	f	hp	PL	2024	0	1	1	0	0	0	0	0	0	0	0	0	63
23656623	m	hp	AU	2024	0	1	1	0	0	0	0	0	0	0	0	0	71
23656683	m	md	SK	2024	0	1	0	0	0	1	0	0	0	0	0	0	

23656715	f	md	TW	2024	0	1	0	0	0	1	0	0	0	0	0	0	60
23656896	m	md	JP	2024	0	1	0	0	1	0	0	0	0	0	0	0	44
23656938	f	md	JP	2024	0	1	1	0	0	0	0	0	0	0	0	0	78
23657348	f	md	JP	2024	0	1	0	0	0	1	0	0	0	0	0	0	
23657550	m	cn	BE	2024	0	0	0	0	0	0	0	0	0	0	0	0	32
23657654	m	md	JP	2024	0	1	0	1	0	0	0	0	0	0	0	0	55
23658031	m	md	JP	2024	0	1	0	1	0	0	0	0	0	0	0	0	59
23658060	m	cn	IN	2024	0	0	0	0	0	0	0	0	0	0	0	0	66
23658735	m	md	JP	2024	1	0	0	0	0	0	0	0	0	0	0	0	70
23660065	m	md	CN	2024	0	0	0	0	0	0	0	0	0	0	0	0	56
23660123	m	md	ES	2024	0	1	1	0	0	0	0	0	0	0	0	0	
23660443	Not	ph	JP	2024	0	0	0	0	0	0	0	0	0	0	0	0	
23660456	m	cn	NL	2024	0	0	0	0	0	0	0	0	0	0	0	0	63
23660614	m	ph	JP	2024	0	1	0	0	1	0	0	0	0	0	0	0	72
23661116	f	ph	PT	2024	0	1	1	0	0	0	0	0	0	0	0	0	64
23661740	f	ph	JP	2024	1	0	0	0	0	0	0	0	0	0	0	0	95
23661789	f	md	JP	2024	1	0	0	0	0	0	0	0	0	0	0	0	80
23661816	Not	md	JP	2024	0	1	1	0	0	0	0	0	0	0	0	0	82
23662207	f	cn	US	2024	0	0	0	0	0	0	0	0	0	0	0	0	81
23662308	m	md	JP	2024	0	1	1	0	0	0	0	0	0	0	0	0	80
23662312	f	md	CA	2024	0	0	0	0	0	0	0	0	0	0	0	0	68
23662435	f	cn	CO	2024	0	0	0	0	0	0	0	0	0	0	0	0	69
23662577	m	md	IT	2024	0	0	0	0	0	0	0	0	0	0	0	0	76
23662726	f	hp	NL	2024	0	1	0	0	0	1	0	0	0	0	0	0	67
23662744	Not	md	JP	2024	0	0	0	0	0	0	0	0	0	0	0	0	
23662769	f	cn	KR	2024	0	0	0	0	0	0	0	0	0	0	0	0	62
23663795	m	md	JP	2024	1	0	0	0	0	0	0	0	0	0	0	0	71
23664060	Not	md	JP	2024	0	1	0	1	0	0	0	0	0	0	0	1	
23664139	m	md	JP	2024	0	1	0	1	0	0	0	0	0	0	0	0	81
23664192	f	hp	US	2024	0	0	0	0	0	0	0	0	0	0	0	0	
23664211	f	md	JP	2024	0	1	1	0	0	0	0	0	0	0	0	0	75
23664632	m	ph	JP	2024	0	1	0	1	0	0	0	0	0	0	0	0	
23664869	Not	md	JP	2024	1	0	0	0	0	0	0	0	0	0	0	0	
23665040	f	md	AT	2024	0	0	0	0	0	0	0	0	0	0	0	0	69

23665070	m	md	CN	2024	0	0	0	0	0	0	0	0	0	0	0	0	
23665072	f	md	CN	2024	0	0	0	0	0	0	0	0	0	0	0	0	44
23665094	m	md	US	2024	0	0	0	0	0	0	0	0	0	0	0	0	69
23665373	m	md	JP	2024	0	1	0	0	0	1	0	0	0	0	0	0	80
23665810	Not	hp	JP	2024	0	0	0	0	0	0	0	0	0	0	0	0	
23666406	m	hp	PL	2024	0	1	1	0	0	0	0	0	0	0	0	0	68
23666968	m	md	JP	2024	0	1	1	0	0	0	0	0	0	0	0	0	80
23667144	Not	md	JP	2024	0	0	0	0	0	0	0	0	0	0	0	0	60
23667583	m	md	JP	2024	0	1	0	0	1	0	0	0	0	0	0	0	79
23667789	Not	md	JP	2024	0	0	0	0	0	0	0	0	0	0	0	0	80
23668282	f	md	DE	2024	0	1	0	0	1	0	0	0	0	0	0	0	57
23668519	f	md	FR	2024	1	0	0	0	0	0	0	0	0	0	0	0	
23668778	m	md	BG	2024	1	0	0	0	0	0	0	0	0	0	0	0	
23669988	Not	md	JP	2024	0	1	0	0	1	0	0	0	0	0	0	0	
23670101	m	hp	US	2024	0	1	1	0	0	0	0	0	0	0	0	0	53
23670244	m	hp	FR	2024	0	1	1	0	0	0	0	0	0	0	0	0	74
23670283	f	hp	TW	2024	1	0	0	0	0	0	0	0	0	0	0	0	60
23670305	f	cn	US	2024	1	0	0	0	0	0	0	0	0	0	0	0	73
23670703	f	ph	US	2024	0	0	0	0	0	0	0	0	0	0	0	0	
23671051	f	md	JP	2024	0	1	0	1	0	0	0	0	0	0	0	0	71
23671096	f	hp	US	2024	0	0	0	0	0	0	0	0	0	0	0	0	52
23671687	m	md	IT	2024	0	0	0	0	0	0	0	0	0	0	0	0	63
23671991	Not	md	JP	2024	0	1	0	0	0	1	0	0	0	0	0	1	
23672066	f	hp	NL	2024	0	0	0	0	0	0	0	0	0	0	0	0	62
23672071	f	hp	CA	2024	0	1	1	0	0	0	0	0	0	0	0	0	75
23672146	f	md	RO	2024	0	1	0	1	0	0	0	0	0	0	0	0	73
23672261	f	md	JP	2024	0	0	0	0	0	0	0	0	0	0	0	0	
23672263	f	md	JP	2024	0	0	0	0	0	0	0	0	0	0	0	0	
23672345	m	md	JP	2024	0	1	1	0	0	0	0	0	0	0	0	0	63
23672630	Not	hp	IT	2024	0	1	1	0	0	0	0	0	0	0	0	0	
23672631	Not	hp	IT	2024	0	1	1	0	0	0	0	0	0	0	0	0	
23672632	Not	hp	IT	2024	0	1	1	0	0	0	0	0	0	0	0	0	
23672633	Not	hp	IT	2024	0	1	1	0	0	0	0	0	0	0	0	0	
23672634	Not	hp	IT	2024	0	1	1	0	0	0	0	0	0	0	0	0	

23672635	Not	hp	IT	2024	0	1	1	0	0	0	0	0	0	0	0	0	
23672637	Not	hp	IT	2024	0	1	1	0	0	0	0	0	0	0	0	0	
23672974	m	cn	KE	2024	1	0	0	0	0	0	0	0	0	0	0	0	53.2
23673059	m	hp	ES	2024	0	1	1	0	0	0	0	0	0	0	0	0	65
23673089	m	md	FR	2024	0	1	1	0	0	0	0	0	0	0	0	0	61
23673142	m	md	JP	2024	0	1	0	0	1	0	0	0	0	0	0	0	62
23673219	f	hp	PL	2024	0	0	0	0	0	0	0	0	0	0	0	0	73
23673571	m	md	AE	2024	0	0	0	0	0	0	0	0	0	0	0	0	67
23673656	m	md	JP	2024	0	1	1	0	0	0	0	0	0	0	0	0	80
23673732	m	md	RU	2024	0	0	0	0	0	0	0	0	0	0	0	0	43
23673858	f	md	GB	2024	1	0	0	0	0	0	0	0	0	0	0	0	
23673966	f	hp	ES	2024	0	0	0	0	0	0	0	0	0	0	0	0	58
23674224	Not	cn	US	2024	0	0	0	0	0	0	0	0	0	0	0	0	
23674350	m	cn	US	2024	0	0	0	0	0	0	0	0	0	0	0	0	
23674426	f	hp	NL	2024	0	1	0	0	0	1	0	0	0	0	0	0	67
23674722	f	cn	CN	2024	0	0	0	0	0	0	0	0	0	0	0	0	
23674938	f	md	JP	2024	0	1	0	0	0	1	0	0	0	0	0	0	
23674949	f	hp	TH	2024	0	1	0	0	0	1	0	0	0	0	0	0	
23674957	m	md	JP	2024	0	1	0	1	0	0	0	0	0	0	0	0	69
23675243	f	md	IT	2024	0	0	0	0	0	0	0	0	0	0	0	0	43
23675395	f	md	DE	2024	0	1	0	0	1	0	0	0	0	0	0	0	57
23675726	m	ph	CN	2024	0	0	0	0	0	0	0	0	0	0	0	0	85
23676150	f	md	US	2024	0	0	0	0	0	0	0	0	0	0	0	0	76
23676380	f	md	AU	2024	0	0	0	0	0	0	0	0	0	0	0	0	68
23676542	f	md	US	2024	0	1	0	1	0	0	0	0	0	0	0	0	
23676861	f	cn	US	2024	1	0	0	0	0	0	0	0	0	0	0	0	89.7
23677063	m	ph	JP	2024	0	0	0	0	0	0	0	0	0	0	0	0	76
23677500	Not	md	JP	2024	0	0	0	0	0	0	0	0	0	0	0	0	
23677576	f	hp	NL	2024	0	0	0	0	0	0	0	0	0	0	0	0	67
23677887	m	ph	IT	2024	0	0	0	0	0	0	0	0	0	0	0	0	
23677921	Not	md	JP	2024	0	0	0	0	0	0	0	0	0	0	0	0	80
23678015	f	cn	DE	2024	0	0	0	0	0	0	0	0	0	0	0	0	
23678024	f	cn	PT	2024	0	1	1	0	0	0	0	0	0	0	0	0	64
23678524	m	md	BG	2024	1	0	0	0	0	0	0	0	0	0	0	0	

23678559	f	hp	US	2024	0	1	1	0	0	0	0	0	0	0	0	0	64
23678589	f	md	BG	2024	1	0	0	0	0	0	0	0	0	0	0	0	
23678616	m	cn	TH	2024	0	1	0	0	0	1	0	0	0	0	0	0	
23678633	m	md	US	2024	0	0	0	0	0	0	0	0	0	0	0	0	53
23678636	m	cn	US	2024	0	0	0	0	0	0	0	0	0	0	0	0	66
23678734	f	md	JP	2024	0	1	0	1	0	0	0	0	0	0	0	0	59
23678924	m	md	TH	2024	1	0	0	0	0	0	0	0	0	0	0	1	72.9
23679010	m	hp	DE	2024	0	1	0	0	1	0	0	0	0	0	0	0	71
23679361	f	hp	ES	2024	0	0	0	0	0	0	0	0	0	0	0	0	58
23679379	m	md	JP	2024	0	1	1	0	0	0	0	0	0	0	0	0	87
23679438	m	cn	US	2024	0	1	1	0	0	0	0	0	0	0	0	0	67
23679856	f	md	BE	2024	0	0	0	0	0	0	0	0	0	0	0	0	
23679944	m	md	JP	2024	0	1	0	0	1	0	0	0	0	0	0	0	69
23680003	m	hp	CA	2024	0	0	0	0	0	0	0	0	0	0	0	0	62
23680052	m	md	BG	2024	0	1	0	1	0	0	0	0	0	0	0	0	61
23680056	f	cn	NL	2024	0	1	1	0	0	0	0	0	0	0	0	0	
23680271	Not	md	US	2024	0	0	0	0	0	0	0	0	0	0	0	0	
23680818	f	md	JP	2024	0	1	0	0	1	0	0	0	0	0	0	1	76
23681049	f	md	GB	2024	0	1	0	1	0	0	0	0	0	0	0	0	
23681666	f	md	JP	2024	0	1	0	0	0	1	0	0	0	0	0	0	76
23681956	f	md	JP	2024	0	0	0	0	0	0	0	0	0	0	0	0	
23681989	m	md	DE	2024	0	1	1	0	0	0	0	0	0	0	0	0	78
23682782	Not	ph	US	2024	0	0	0	0	0	0	0	0	0	0	0	0	
23682952	Not	md	FR	2024	1	0	0	0	0	0	0	0	0	0	0	0	
23683106	m	md	JP	2024	0	1	0	0	1	0	0	0	0	0	0	0	75
23683239	f	hp	AT	2024	0	0	0	0	0	0	0	0	0	0	0	0	53
23683323	f	cn	US	2024	0	0	0	0	0	0	0	0	0	0	0	0	76
23683371	m	md	KR	2024	0	0	0	0	0	0	0	0	0	0	0	0	90
23683456	f	hp	US	2024	0	0	0	0	0	0	0	0	0	0	0	0	27
23683848	f	md	DE	2024	0	0	0	0	0	0	0	0	0	0	0	0	70
23683934	f	md	FR	2024	0	0	0	0	0	0	0	0	0	0	0	0	
23684533	f	cn	CN	2024	0	0	0	0	0	0	0	0	0	0	0	0	67
23684536	f	md	CN	2024	1	0	0	0	0	0	0	0	0	0	0	0	
23685713	Not	ph	US	2024	0	0	0	0	0	0	0	0	0	0	0	0	

23686228	m	md	PL	2024	0	1	0	0	1	0	0	0	0	0	0	0	62
23686285	Not	md	JP	2024	0	1	1	0	0	0	0	0	0	0	0	0	70
23686450	f	md	DE	2024	0	1	1	0	0	0	0	0	0	0	0	0	71
23686524	f	md	MX	2024	1	0	0	0	0	0	0	0	0	0	0	0	36
23686763	m	md	PL	2024	0	1	1	0	0	0	0	0	0	0	0	0	62
23687338	f	md	IT	2024	0	1	1	0	0	0	0	0	0	0	0	0	61
23687415	Not	md	HN	2024	0	1	0	0	0	1	0	0	0	0	0	0	
23687647	m	hp	ES	2024	0	1	1	0	0	0	0	0	0	0	0	0	65
23687851	m	ph	JP	2024	0	1	0	0	1	0	0	0	0	0	0	0	70
23687942	m	md	FR	2024	0	1	0	0	1	0	0	0	0	0	0	0	68
23688039	m	hp	JP	2024	0	1	1	0	0	0	0	0	0	0	0	0	53
23688346	f	md	JP	2024	1	0	0	0	0	0	0	0	0	0	0	0	65
23688459	m	md	JP	2024	0	0	0	0	0	0	0	0	0	0	0	0	40
23688592	f	md	JP	2024	1	0	0	0	0	0	0	0	0	0	0	0	79
23689114	f	md	IT	2024	0	1	0	0	1	0	0	0	0	0	0	0	57
23689866	m	md	JP	2024	0	1	0	1	0	0	0	0	0	0	0	0	58
23032002	m	md	JP	2024	0	1	0	0	1	0	0	0	0	0	0	0	68
23069909	f	md	PL	2024	0	1	1	0	0	0	0	0	0	0	0	0	59
23357722	m	md	BE	2024	0	1	1	0	0	0	0	0	0	0	0	0	
23357767	m	md	JP	2024	0	1	1	0	0	0	0	0	0	0	0	0	76
23386626	f	md	GB	2024	0	1	0	0	0	1	0	0	0	0	0	0	50
23388164	f	md	KR	2024	0	0	0	0	0	0	0	0	0	0	0	0	65
23389535	m	md	JP	2024	0	1	0	0	1	0	0	0	0	0	0	0	72
23393919	m	cn	IN	2024	0	0	0	0	0	0	0	0	0	0	0	0	53
23404819	f	md	PL	2024	0	1	1	0	0	0	0	0	0	0	0	0	59
23419093	m	md	JP	2024	0	1	0	1	0	0	0	0	0	0	0	0	66
23423456	f	md	JP	2024	1	0	0	0	0	0	0	0	0	0	0	0	69
23424701	m	md	JP	2024	0	0	0	0	0	0	0	0	0	0	0	0	64
23425596	f	ph	JP	2024	0	1	1	0	0	0	0	0	0	0	0	0	83
23429846	m	ph	JP	2024	0	1	1	0	0	0	0	0	0	0	0	1	71
23432466	m	ph	JP	2024	0	1	1	0	0	0	0	0	0	0	0	0	70
23433203	f	md	DE	2024	0	1	1	0	0	0	0	0	0	0	0	0	
23439518	m	md	JP	2024	0	1	0	1	0	0	0	0	0	0	0	0	75
23443862	f	md	DE	2024	0	1	0	0	1	0	0	0	0	0	0	0	60

23446703	f	md	JP	2024	1	0	0	0	0	0	0	0	0	0	0	0	81
23449838	m	md	JP	2024	0	1	1	0	0	0	0	0	0	0	0	0	65
23460078	f	md	KR	2024	0	0	0	0	0	0	0	0	0	0	0	0	77
23461074	f	md	JP	2024	0	1	1	0	0	0	0	0	0	0	0	0	74
23466906	m	md	JP	2024	0	1	0	1	0	0	0	0	0	0	0	0	58
23473737	m	md	AT	2024	0	0	0	0	0	0	0	0	0	0	0	0	
23484232	f	md	JP	2024	0	0	0	0	0	0	0	0	0	0	0	0	72
23494891	f	cn	US	2024	0	0	0	0	0	0	0	0	0	0	0	0	84
23498309	Not	md	JP	2024	0	1	0	0	1	0	0	0	0	0	0	0	
23507143	f	hp	PL	2024	0	1	1	0	0	0	0	0	0	0	0	0	54
23513085	f	md	JP	2024	0	1	0	1	0	0	0	0	0	0	0	0	60
23513571	m	md	CN	2024	0	1	1	0	0	0	0	0	0	0	0	0	70
23522874	Not	md	JP	2024	1	0	0	0	0	0	0	0	0	0	0	0	
23534409	f	hp	PL	2024	0	1	1	0	0	0	0	0	0	0	0	0	54
23544340	m	md	JP	2024	0	1	0	0	1	0	0	0	0	0	0	0	72
23546567	m	md	JP	2024	0	1	0	0	1	0	0	0	0	0	0	0	70
23546657	m	md	JP	2024	0	1	0	0	1	0	0	0	0	0	0	0	73
23547447	m	md	JP	2024	0	1	0	0	1	0	0	0	0	0	0	0	74
23548869	m	md	JP	2024	0	1	0	0	1	0	0	0	0	0	0	0	72
23550759	m	md	JP	2024	0	0	0	0	0	0	0	0	0	0	0	0	72
23558984	Not	md	JP	2024	0	1	1	0	0	0	0	0	0	0	0	1	70
23569616	m	md	PT	2024	0	0	0	0	0	0	0	0	0	0	0	0	59
23573394	m	md	JP	2024	0	1	0	1	0	0	0	0	0	0	0	0	55
23573943	f	md	KR	2024	0	0	0	0	0	0	0	0	0	0	0	0	
23577733	m	md	JP	2024	0	1	0	1	0	0	0	0	0	0	0	0	46
23579813	m	md	BR	2024	0	0	0	0	0	0	0	0	0	0	0	0	
23580284	f	hp	PL	2024	0	1	1	0	0	0	0	0	0	0	0	0	54
23584830	f	md	US	2024	0	1	0	1	0	0	0	0	0	0	0	0	73
23586379	m	md	JP	2024	0	1	0	0	0	1	0	0	0	0	0	0	60
23587302	m	cn	BE	2024	0	0	0	0	0	0	0	0	0	0	0	0	68
23590894	m	md	JP	2024	0	1	0	0	1	0	0	0	0	0	0	0	79
23592700	m	md	JP	2024	0	1	0	1	0	0	0	0	0	0	0	0	64
23597520	m	md	FR	2024	0	1	0	1	0	0	0	0	0	0	0	0	
23598865	f	cn	US	2024	0	1	0	1	0	0	0	0	0	0	0	0	58.6

23599182	m	md	KR	2024	0	0	0	0	0	0	0	0	0	0	0	0	52
23602852	m	md	JP	2024	0	0	0	0	0	0	0	0	0	0	0	0	68
23603148	m	md	JP	2024	0	1	1	0	0	0	0	0	0	0	0	0	65
23604322	m	md	JP	2024	0	1	1	0	0	0	0	0	0	0	0	0	79
23607985	f	md	BG	2024	0	0	0	0	0	0	0	0	0	0	0	0	43
23621072	m	md	NL	2024	0	1	1	0	0	0	0	0	0	0	0	0	
23623592	m	md	JP	2024	0	0	0	0	0	0	0	0	0	0	0	0	50
23624383	m	ph	JP	2024	0	0	0	0	0	0	0	0	0	0	0	0	60
23624394	m	ph	JP	2024	0	1	1	0	0	0	0	0	0	0	0	0	77
23628759	f	cn	CA	2024	1	0	0	0	0	0	0	0	0	0	0	0	
23629574	Not	md	JP	2024	0	1	0	0	1	0	0	0	0	0	0	0	
23630048	m	md	JP	2024	0	1	0	0	1	0	0	0	0	0	0	0	69
23636252	Not	md	JP	2024	0	1	0	0	1	0	0	0	0	0	0	0	
23636381	Not	md	JP	2024	0	1	0	0	1	0	0	0	0	0	0	0	
23636423	m	md	JP	2024	0	1	0	1	0	0	0	0	0	0	0	0	55
23636863	Not	md	JP	2024	0	1	0	0	1	0	0	0	0	0	0	0	
23638334	m	md	JP	2024	0	1	0	1	0	0	0	0	0	0	0	1	68
23640971	m	cn	TR	2024	0	1	0	0	1	0	0	0	0	0	0	0	40
23641015	m	md	JP	2024	0	0	0	0	0	0	0	0	0	0	0	0	50
23642520	m	md	DE	2024	0	1	1	0	0	0	0	0	0	0	0	1	62
23642984	f	md	DE	2024	0	1	1	0	0	0	0	0	0	0	0	1	66
23648362	m	md	US	2024	0	1	0	1	0	0	0	0	0	0	0	0	
23649708	m	cn	NL	2024	0	1	1	0	0	0	0	0	0	0	0	0	
23649716	m	cn	CN	2024	0	0	0	0	0	0	0	0	0	0	0	0	
23653000	m	md	NL	2024	0	1	1	0	0	0	0	0	0	0	0	0	
23655248	m	md	BR	2024	0	1	1	0	0	0	0	0	0	0	0	0	76
23655692	m	md	JP	2024	0	1	1	0	0	0	0	0	0	0	0	0	68
23658637	m	md	JP	2024	1	0	0	0	0	0	0	0	0	0	0	0	80
23660063	f	cn	CN	2024	0	0	0	0	0	0	0	0	0	0	0	0	
23660064	m	cn	CN	2024	0	0	0	0	0	0	0	0	0	0	0	0	43
23665166	Not	hp	US	2024	0	0	0	0	0	0	0	0	0	0	0	0	
23666730	Not	md	JP	2024	0	1	0	0	1	0	0	0	0	0	0	0	
23667803	m	md	JP	2024	0	1	0	0	1	0	0	0	0	0	0	0	66
23667888	m	md	JP	2024	0	0	0	0	0	0	0	0	0	0	0	0	

23670059	f	md	CN	2024	0	0	0	0	0	0	0	0	0	0	0	0	
23670920	m	md	JP	2024	0	1	0	0	1	0	0	0	0	0	0	0	72
23673746	f	md	US	2024	0	0	0	0	0	0	0	0	0	0	0	0	52
23674990	m	cn	JP	2024	0	0	0	0	0	0	0	0	0	0	0	0	65
23675736	f	md	JP	2024	0	1	0	0	1	0	0	0	0	0	0	1	73
23676608	m	md	JP	2024	0	1	0	0	1	0	0	0	0	0	0	0	75
23676609	m	md	JP	2024	0	1	0	0	1	0	0	0	0	0	0	0	63
23676642	m	md	JP	2024	0	1	0	0	1	0	0	0	0	0	0	0	71
23676698	m	md	JP	2024	0	1	0	0	1	0	0	0	0	0	0	0	72
23678048	m	md	JP	2024	0	1	0	0	1	0	0	0	0	0	0	0	68
23678934	m	md	BE	2024	0	0	0	0	0	0	0	0	0	0	0	0	67
23679939	m	md	JP	2024	0	1	0	0	1	0	0	0	0	0	0	0	45
23680131	m	md	JP	2024	0	1	0	0	1	0	0	0	0	0	0	0	68
23680133	f	md	JP	2024	0	1	0	0	1	0	0	0	0	0	0	0	55
23680134	Not	md	JP	2024	0	1	0	0	1	0	0	0	0	0	0	0	
23680152	Not	md	JP	2024	0	1	0	0	1	0	0	0	0	0	0	0	
23680157	m	md	JP	2024	0	1	0	0	1	0	0	0	0	0	0	0	80
23680748	m	md	JP	2024	0	1	0	0	1	0	0	0	0	0	0	0	70
23680809	m	md	KR	2024	1	0	0	0	0	0	0	0	0	0	0	0	69
23680857	m	md	JP	2024	0	1	0	0	1	0	0	0	0	0	0	0	82
23680859	m	md	JP	2024	0	1	0	0	1	0	0	0	0	0	0	0	63
23681188	f	md	JP	2024	0	1	0	0	1	0	0	0	0	0	0	0	31
23681467	m	md	PL	2024	0	1	0	0	0	1	0	0	0	0	0	0	67
23681617	f	md	JP	2024	0	1	0	0	1	0	0	0	0	0	0	0	66
23681966	m	md	JP	2024	0	0	0	0	0	0	0	0	0	0	0	0	
23682080	m	md	JP	2024	0	1	0	0	1	0	0	0	0	0	0	0	64
23683983	f	hp	CN	2024	1	0	0	0	0	0	0	0	0	0	0	0	57
23684057	Not	hp	US	2024	0	0	0	0	0	0	0	0	0	0	0	0	
23684940	m	md	JP	2024	0	1	0	0	1	0	0	0	0	0	0	0	77
23685114	f	md	JP	2024	0	1	0	0	1	0	0	0	0	0	0	0	53
23685133	f	md	JP	2024	0	1	0	0	1	0	0	0	0	0	0	0	82
23686356	f	hp	PL	2024	0	1	1	0	0	0	0	0	0	0	0	0	63
23688402	Not	hp	NL	2024	0	0	0	0	0	0	0	0	0	0	0	0	
23689886	Not	md	JP	2024	0	1	1	0	0	0	0	0	0	0	0	0	

23689929	f	md	JP	2024	0	0	0	0	0	0	0	0	0	0	0	0	50
23690068	f	ph	PT	2024	0	1	1	0	0	0	0	0	0	0	0	0	64
23690827	f	md	JP	2024	0	0	0	0	0	0	0	0	0	0	0	0	90
23690993	m	md	JP	2024	0	1	0	0	1	0	0	0	0	0	0	1	70
23691014	f	hp	CA	2024	1	0	0	0	0	0	0	0	0	0	0	0	
23691052	f	hp	CN	2024	1	0	0	0	0	0	0	0	0	0	0	0	57
23691113	m	md	IT	2024	0	0	0	0	0	0	0	0	0	0	0	0	72
23691352	f	md	DE	2024	0	0	0	0	0	0	0	0	0	0	0	0	58
23691443	m	hp	DE	2024	0	1	0	0	1	0	0	0	0	0	0	0	71
23691759	m	md	SK	2024	0	1	0	0	0	1	0	0	0	0	0	0	
23691914	f	ph	PT	2024	0	1	1	0	0	0	0	0	0	0	0	0	64
23692316	f	hp	IT	2024	0	0	0	0	0	0	0	0	0	0	0	0	43
23692757	f	md	DE	2024	0	0	0	0	0	0	0	0	0	0	0	0	60
23693703	m	md	VN	2024	0	1	0	0	0	1	0	0	0	0	0	0	62
23693704	m	md	KR	2024	0	0	0	0	0	0	0	0	0	0	0	0	52
23693944	f	md	US	2024	0	0	0	0	0	0	0	0	0	0	0	0	90
23693974	m	md	DE	2024	0	1	1	0	0	0	0	0	0	0	0	0	78
23694323	m	hp	DE	2024	0	1	0	0	1	0	0	0	0	0	0	0	71
23694360	m	md	IQ	2024	0	1	1	0	0	0	0	0	0	0	0	0	64
23694421	m	hp	DE	2024	0	1	0	0	1	0	0	0	0	0	0	0	71
23694637	Not	md	IT	2024	0	0	0	0	0	0	0	0	0	0	0	0	
23695037	f	md	DE	2024	0	0	0	0	0	0	0	0	0	0	0	0	82
23695192	f	md	CA	2024	0	1	1	0	0	0	0	0	0	0	0	0	67
23695249	m	hp	TR	2024	0	0	0	0	0	0	0	0	0	0	0	0	46
23695379	m	cn	US	2024	0	1	1	0	0	0	0	0	0	0	0	0	
23695594	m	md	AM	2024	0	1	0	0	0	1	0	0	0	0	0	0	63
23695705	m	md	PL	2024	0	0	0	0	0	0	0	0	0	0	0	0	70
23695797	f	md	IT	2024	0	1	0	0	1	0	0	0	0	0	0	0	64
23695836	f	cn	US	2024	1	0	0	0	0	0	0	0	0	0	0	0	
23695998	m	md	JP	2024	0	1	0	1	0	0	0	0	0	0	0	1	63
23696408	m	hp	PL	2024	0	1	1	0	0	0	0	0	0	0	0	0	59
23696835	f	cn	IN	2024	0	0	0	0	0	0	0	0	0	0	0	0	36
23697226	f	ph	PT	2024	0	1	1	0	0	0	0	0	0	0	0	0	64
23697306	f	md	JP	2024	0	1	0	0	0	1	0	0	0	0	0	0	60

23697496	m	md	AU	2024	0	0	0	0	0	0	0	0	0	0	0	0	60
23697521	m	md	JP	2024	0	1	1	0	0	0	0	0	0	0	0	0	74
23697636	f	md	US	2024	1	0	0	0	0	0	0	0	0	0	0	1	
23697886	f	md	GB	2024	0	1	0	0	0	1	0	0	0	0	0	0	79
23698159	m	hp	DE	2024	0	1	0	0	1	0	0	0	0	0	0	0	79
23698353	m	md	JP	2024	0	1	0	1	0	0	0	0	0	0	0	0	58
23698358	f	ph	JP	2024	1	0	0	0	0	0	0	0	0	0	0	0	87
23699002	m	md	JP	2024	0	1	0	1	0	0	0	0	0	0	0	0	44
23699032	f	cn	US	2024	0	0	0	0	0	0	0	0	0	0	0	0	
23699110	f	md	FR	2024	0	0	0	0	0	0	0	0	0	0	0	0	
23699167	f	cn	CN	2024	0	0	0	0	0	0	0	0	0	0	0	0	
23699407	f	md	JP	2024	0	1	0	1	0	0	0	0	0	0	0	0	61
23699444	m	md	JP	2024	0	1	0	0	1	0	0	0	0	0	0	0	80
23700521	f	md	US	2024	1	0	0	0	0	0	0	0	0	0	0	1	
23700590	f	cn	US	2024	1	0	0	0	0	0	0	0	0	0	0	0	57
23700632	f	md	PL	2024	0	1	1	0	0	0	0	0	0	0	0	0	66
23700676	Not	md	DE	2024	1	0	0	0	0	0	0	0	0	0	0	0	
23700701	Not	hp	US	2024	0	1	1	0	0	0	0	0	0	0	0	0	
23700864	m	md	JP	2024	0	1	0	0	0	1	0	0	0	0	0	0	
23701070	m	md	JP	2024	0	1	0	0	0	1	0	0	0	0	0	0	80
23701128	f	md	US	2024	0	0	0	0	0	0	0	0	0	0	0	0	76
23701160	f	hp	US	2024	0	0	0	0	0	0	0	0	0	0	0	0	73
23701189	f	ph	JP	2024	1	0	0	0	0	0	0	0	0	0	0	0	76
23701438	m	md	JP	2024	0	1	1	0	0	0	0	0	0	0	0	0	70
23701577	f	md	TR	2024	0	0	0	0	0	0	0	0	0	0	0	0	71
23701748	m	md	FR	2024	0	1	0	0	1	0	0	0	0	0	0	0	68
23702823	Not	ph	JP	2024	0	0	0	0	0	0	0	0	0	0	0	0	
23703041	f	ph	JP	2024	0	1	0	1	0	0	0	0	0	0	0	0	65
23703136	f	ph	JP	2024	0	0	0	0	0	0	0	0	0	0	0	0	80
23703324	m	ph	US	2024	1	0	0	0	0	0	0	0	0	0	0	0	65
23704215	m	md	FR	2024	0	1	0	0	1	0	0	0	0	0	0	0	70
23704221	m	md	DE	2024	0	1	0	0	1	0	0	0	0	0	0	0	62
23705337	f	cn	DE	2024	1	0	0	0	0	0	0	0	0	0	0	1	
23705390	Not	ph	US	2024	0	0	0	0	0	0	0	0	0	0	0	0	

23705446	f	cn	US	2024	1	0	0	0	0	0	0	0	0	0	0	0	
23705455	m	md	PL	2024	0	1	1	0	0	0	0	0	0	0	0	0	65
23707123	f	ph	US	2024	0	0	0	0	0	0	0	0	0	0	0	0	73
23707144	f	cn	CA	2024	0	0	0	0	0	0	0	0	0	0	0	0	
23707442	m	md	JP	2024	0	0	0	0	0	0	0	0	0	0	0	0	85
23707508	m	md	KR	2024	0	1	1	0	0	0	0	0	0	0	0	0	77
23708179	f	hp	JP	2024	1	0	0	0	0	0	0	0	0	0	0	0	79
23708279	m	cn	TH	2024	0	1	0	0	0	1	0	0	0	0	0	0	71
23708363	m	hp	US	2024	1	0	0	0	0	0	0	0	0	0	0	0	65
23708765	m	hp	AU	2024	1	0	0	0	0	0	0	0	0	0	0	0	60
23709066	m	hp	JP	2024	0	1	0	1	0	0	0	0	0	0	0	0	68
23709225	Not	hp	IT	2024	0	1	1	0	0	0	0	0	0	0	0	0	
23709235	Not	hp	IT	2024	0	1	1	0	0	0	0	0	0	0	0	0	
23709236	Not	hp	IT	2024	0	1	1	0	0	0	0	0	0	0	0	0	
23709239	Not	hp	IT	2024	0	1	1	0	0	0	0	0	0	0	0	0	
23709247	Not	hp	IT	2024	0	1	1	0	0	0	0	0	0	0	0	0	
23709248	Not	hp	IT	2024	0	1	1	0	0	0	0	0	0	0	0	0	
23709253	Not	hp	IT	2024	0	1	1	0	0	0	0	0	0	0	0	0	
23710248	f	cn	NL	2024	0	1	1	0	0	0	0	0	0	0	0	0	
23710341	f	md	IT	2024	0	1	0	0	1	0	0	0	0	0	0	0	57
23710385	m	cn	RO	2024	0	0	0	0	0	0	0	0	0	0	0	0	
23710557	Not	md	JP	2024	0	0	0	0	0	0	0	0	0	0	0	0	
23710596	m	md	JP	2024	0	1	1	0	0	0	0	0	0	0	0	1	72
23710762	f	md	US	2024	0	1	1	0	0	0	0	0	0	0	0	0	
23710920	f	md	US	2024	0	1	1	0	0	0	0	0	0	0	0	0	
23710947	f	lw	US	2024	0	1	1	0	0	0	0	0	0	0	0	0	
23711098	f	ph	JP	2024	0	0	0	0	0	0	0	0	0	0	0	0	66
23711609	m	md	NL	2024	0	0	0	0	0	0	0	0	0	0	0	0	63
23711754	m	hp	US	2024	0	1	1	0	0	0	0	0	0	0	0	0	64
23711813	f	cn	TH	2024	1	0	0	0	0	0	0	0	0	0	0	0	69.5
23712291	f	md	US	2024	1	0	0	0	0	0	0	0	0	0	0	1	
23712292	m	cn	US	2024	1	0	0	0	0	0	0	0	0	0	0	0	
23712472	f	hp	JP	2024	0	0	0	0	0	0	0	0	0	0	0	0	78
23712495	f	cn	NL	2024	0	1	1	0	0	0	0	0	0	0	0	0	

23712554	f	hp	ES	2024	1	0	0	0	0	0	0	0	0	0	0	0	70
23712559	m	md	JP	2024	0	1	0	1	0	0	0	0	0	0	0	0	74
23713240	f	md	JP	2024	0	1	0	1	0	0	0	0	0	0	0	0	
23713242	f	ph	JP	2024	1	0	0	0	0	0	0	0	0	0	0	0	
23713363	m	md	JP	2024	0	1	0	1	0	0	0	0	0	0	0	0	61
23713370	f	md	US	2024	0	1	0	1	0	0	0	0	0	0	0	0	53.4
23713700	m	md	FR	2024	0	1	0	0	1	0	0	0	0	0	0	0	68
23713831	m	hp	US	2024	0	0	0	0	0	0	0	0	0	0	0	0	58
23713934	m	cn	FR	2024	0	1	0	0	1	0	0	0	0	0	0	0	68
23713943	f	cn	NL	2024	0	1	1	0	0	0	0	0	0	0	0	0	
23714319	m	md	JP	2024	0	0	0	0	0	0	0	0	0	0	0	0	75
23714350	m	md	JP	2024	0	1	0	0	0	1	0	0	0	0	0	0	60
23714924	Not	hp	CN	2024	1	0	0	0	0	0	0	0	0	0	0	0	
23715010	m	md	MY	2024	0	0	0	0	0	0	0	0	0	0	0	0	
23715014	f	cn	RS	2024	0	0	0	0	0	0	0	0	0	0	0	0	
23715176	Not	md	JP	2024	0	0	0	0	0	0	0	0	0	0	0	0	
23715283	f	cn	US	2024	1	0	0	0	0	0	0	0	0	0	0	0	
23716289	f	md	JP	2024	0	1	1	0	0	0	0	0	0	0	0	0	70
23716683	f	md	JP	2024	0	1	0	0	0	1	0	0	0	0	0	0	76
23716854	Not	md	FR	2024	1	0	0	0	0	0	0	0	0	0	0	0	
23716920	m	hp	JP	2024	0	1	1	0	0	0	0	0	0	0	0	0	63
23717198	Not	md	JP	2024	0	0	0	0	0	0	0	0	0	0	0	0	
23717631	m	cn	KE	2024	1	0	0	0	0	0	0	0	0	0	0	0	51.9
23717691	m	md	TH	2024	0	0	0	0	0	0	0	0	0	0	0	0	
23718060	Not	md	US	2024	1	0	0	0	0	0	0	0	0	0	0	0	
23718130	Not	md	JP	2024	0	0	0	0	0	0	0	0	0	0	0	0	
23718324	f	md	CL	2024	0	0	0	0	0	0	0	0	0	0	0	0	
23718374	m	cn	IN	2024	0	0	0	0	0	0	0	0	0	0	0	0	69
23718409	m	md	ES	2024	0	0	0	0	0	0	0	0	0	0	0	0	
23718536	m	md	ES	2024	0	0	0	0	0	0	0	0	0	0	0	0	
23718792	m	cn	US	2024	1	0	0	0	0	0	0	0	0	0	0	0	
23718939	m	md	JP	2024	0	1	0	1	0	0	0	0	0	0	0	1	69
23719145	f	cn	NL	2024	0	1	1	0	0	0	0	0	0	0	0	0	
23719625	f	md	JP	2024	0	0	0	0	0	0	0	0	0	0	0	0	69

23719747	m	md	JP	2024	0	1	1	0	0	0	0	0	0	0	0	0	80
23719862	m	cn	CN	2024	0	0	0	0	0	0	0	0	0	0	0	0	55
23720137	f	hp	NL	2024	0	0	0	0	0	0	0	0	0	0	0	0	62
23720218	f	hp	NL	2024	0	1	0	0	0	1	0	0	0	0	0	0	67
23720226	m	cn	CN	2024	0	0	0	0	0	0	0	0	0	0	0	0	
23720432	m	cn	DE	2024	0	1	0	0	1	0	0	0	0	0	0	0	79
23721162	m	md	BG	2024	0	1	0	0	1	0	0	0	0	0	0	1	57
23721178	f	md	DE	2024	0	1	0	0	1	0	0	0	0	0	0	0	67
23721256	Not	md	JP	2024	0	0	0	0	0	0	0	0	0	0	0	0	
23721483	m	md	JP	2024	0	1	0	0	0	1	0	0	0	0	0	0	80
23722066	m	md	DE	2024	0	1	0	1	0	0	0	0	0	0	0	0	
23722290	m	md	IT	2024	0	0	0	0	0	0	0	0	0	0	0	0	63
23722604	m	md	GR	2024	0	1	1	0	0	0	0	0	0	0	0	0	76
23722876	f	cn	CN	2024	0	0	0	0	0	0	0	0	0	0	0	0	38
23722912	m	md	JP	2024	0	1	1	0	0	0	0	0	0	0	0	0	60
23723006	f	cn	NL	2024	0	1	1	0	0	0	0	0	0	0	0	0	
23723042	m	md	DE	2024	0	1	1	0	0	0	0	0	0	0	0	0	
23723098	m	md	IT	2024	1	0	0	0	0	0	0	0	0	0	0	0	
23723139	m	md	JP	2024	0	0	0	0	0	0	0	0	0	0	0	0	71
23723252	m	md	FR	2024	0	1	0	0	1	0	0	0	0	0	0	0	68
23723315	m	md	JP	2024	0	1	1	0	0	0	0	0	0	0	0	1	62
23724042	m	md	KR	2024	0	0	0	0	0	0	0	0	0	0	0	0	
23724098	m	hp	CN	2024	0	0	0	0	0	0	0	0	0	0	0	0	47
23724140	m	hp	US	2024	0	1	1	0	0	0	0	0	0	0	0	0	85
23724141	f	hp	US	2024	0	1	1	0	0	0	0	0	0	0	0	0	69
23724199	m	ph	FR	2024	0	0	0	0	0	0	0	0	0	0	0	0	63
23724206	f	md	CN	2024	0	0	0	0	0	0	0	0	0	0	0	0	52
23724243	f	hp	IT	2024	0	0	0	0	0	0	0	0	0	0	0	0	58
23725329	Not	md	DE	2024	0	1	0	0	0	1	0	0	0	0	0	0	
23725708	f	hp	US	2024	0	1	1	0	0	0	0	0	0	0	0	0	61
23725946	f	cn	SG	2024	1	0	0	0	0	0	0	0	0	0	0	0	73.2
23726146	m	hp	DE	2024	0	1	0	0	1	0	0	0	0	0	0	0	79
23726166	f	md	DE	2024	0	0	0	0	0	0	0	0	0	0	0	0	63
23726210	m	md	FR	2024	0	1	0	0	1	0	0	0	0	0	0	0	68

23726312	m	md	IT	2024	0	0	0	0	0	0	0	0	0	0	0	0	76
23726341	m	md	JP	2024	0	0	0	0	0	0	0	0	0	0	0	0	
23726571	f	md	JP	2024	1	0	0	0	0	0	0	0	0	0	0	0	82
23726620	m	md	JP	2024	0	1	0	0	1	0	0	0	0	0	0	0	84
23726632	f	cn	US	2024	1	0	0	0	0	0	0	0	0	0	0	0	
23726681	m	md	JP	2024	0	1	1	0	0	0	0	0	0	0	0	0	76
23726757	m	md	AU	2024	0	0	0	0	0	0	0	0	0	0	0	0	60
23726999	m	hp	US	2024	0	1	1	0	0	0	0	0	0	0	0	0	75
23727925	m	md	JP	2024	1	0	0	0	0	0	0	0	0	0	0	0	69
23727954	m	md	FR	2024	1	0	0	0	0	0	0	0	0	0	0	0	
23728030	f	md	AU	2024	1	0	0	0	0	0	0	0	0	0	0	0	
23728453	Not	md	FR	2024	1	0	0	0	0	0	0	0	0	0	0	0	
23728466	m	cn	KE	2024	1	0	0	0	0	0	0	0	0	0	0	0	60.3
23728953	m	cn	CN	2024	0	0	0	0	0	0	0	0	0	0	0	0	61
23728956	f	cn	CN	2024	0	0	0	0	0	0	0	0	0	0	0	0	68
23728957	m	cn	CN	2024	0	0	0	0	0	0	0	0	0	0	0	0	41
23729417	m	md	US	2024	0	1	1	0	0	0	0	0	0	0	0	0	
23729920	f	hp	NL	2024	0	0	0	0	0	0	0	0	0	0	0	0	62
23730169	f	cn	US	2024	1	0	0	0	0	0	0	0	0	0	0	0	
23731612	m	hp	KR	2024	0	1	0	0	0	1	0	0	0	0	0	0	51
23731910	f	hp	US	2024	0	1	0	0	0	1	0	0	0	0	0	0	37
23732129	m	cn	DE	2024	0	1	0	0	1	0	0	0	0	0	0	0	62
23732167	m	cn	US	2024	0	1	1	0	0	0	0	0	0	0	0	1	73
23732200	f	cn	JP	2024	0	0	0	0	0	0	0	0	0	0	0	0	82
23732208	m	cn	US	2024	0	1	0	1	0	0	0	0	0	0	0	0	
23732316	m	md	RU	2024	0	1	0	0	0	1	0	0	0	0	0	0	68
23732444	Not	hp	US	2024	0	1	1	0	0	0	0	0	0	0	0	0	
23732721	Not	hp	US	2024	0	0	0	0	0	0	0	0	0	0	0	0	
23733253	m	cn	IN	2024	0	0	0	0	0	0	0	0	0	0	0	0	53
23733574	f	md	JP	2024	0	0	0	0	0	0	0	0	0	0	0	0	69
23733696	m	md	JP	2024	0	1	0	0	1	0	0	0	0	0	0	0	57
23733882	m	md	DZ	2024	0	1	0	0	1	0	0	0	0	0	0	0	68
23734991	Not	md	ES	2024	0	1	1	0	0	0	0	0	0	0	0	0	
23735005	Not	md	US	2024	0	0	0	0	0	0	0	0	0	0	0	0	

23735154	m	md	JP	2024	0	1	0	1	0	0	0	0	0	0	0	0	56
23735269	Not	md	DE	2024	0	1	0	1	0	0	0	0	0	0	0	0	
23735370	f	hp	ES	2024	0	0	0	0	0	0	0	0	0	0	0	0	58
23735388	f	hp	JP	2024	0	0	0	0	0	0	0	0	0	0	0	0	
23735976	f	md	DE	2024	0	1	1	0	0	0	0	0	0	0	0	1	66
23736414	f	cn	US	2024	1	0	0	0	0	0	0	0	0	0	0	0	
23736601	f	cn	TH	2024	1	0	0	0	0	0	0	0	0	0	0	1	91.4
23736675	f	md	JP	2024	0	0	0	0	0	0	0	0	0	0	0	0	60
23736956	f	md	RU	2024	1	0	0	0	0	0	0	0	0	0	0	0	43
23737068	f	cn	NL	2024	0	1	1	0	0	0	0	0	0	0	0	0	
23737235	m	cn	US	2024	1	0	0	0	0	0	0	0	0	0	0	0	
23737570	m	md	NZ	2024	0	1	0	1	0	0	0	0	0	0	0	0	
23737574	f	cn	ID	2024	0	1	0	1	0	0	0	0	0	0	0	0	51.5
23737652	m	md	JP	2024	0	1	0	1	0	0	0	0	0	0	0	0	74
23737878	f	cn	US	2024	1	0	0	0	0	0	0	0	0	0	0	0	
23737962	f	md	DE	2024	0	1	1	0	0	0	0	0	0	0	0	1	66
23738170	f	cn	TH	2024	1	0	0	0	0	0	0	0	0	0	0	0	62.2
23738337	f	cn	CN	2024	0	0	0	0	0	0	0	0	0	0	0	0	
23738466	m	md	PT	2024	0	1	1	0	0	0	0	0	0	0	0	0	63
23738945	f	cn	ID	2024	1	0	0	0	0	0	0	0	0	0	0	0	
23739354	f	ph	JP	2024	0	0	0	0	0	0	0	0	0	0	0	0	
23739360	Not	md	US	2024	0	1	1	0	0	0	0	0	0	0	0	0	
23739408	Not	md	US	2024	0	0	0	0	0	0	0	0	0	0	0	0	
23739545	Not	md	US	2024	0	0	0	0	0	0	0	0	0	0	0	0	
23739564	m	md	MY	2024	0	0	0	0	0	0	0	0	0	0	0	0	
23739732	f	cn	ID	2024	1	0	0	0	0	0	0	0	0	0	0	0	66.1
23739795	m	md	DE	2024	0	1	0	0	1	0	0	0	0	0	0	0	62
23739864	m	md	JP	2024	0	1	0	0	0	1	0	0	0	0	0	0	70
23739865	f	md	JP	2024	0	1	0	0	0	1	0	0	0	0	0	0	70
23740500	m	md	JP	2024	0	1	1	0	0	0	0	0	0	0	0	0	65
23740726	m	md	JP	2024	0	0	0	0	0	0	0	0	0	0	0	0	60
23740778	m	md	DE	2024	0	1	0	0	1	0	0	0	0	0	0	0	58
23740829	Not	md	JP	2024	0	0	0	0	0	0	0	0	0	0	0	0	90
23740943	f	hp	CA	2024	1	0	0	0	0	0	0	0	0	0	0	0	

23741058	f	md	JP	2024	0	0	0	0	0	0	0	0	0	0	0	0	64
23741226	f	cn	US	2024	0	1	0	1	0	0	0	0	0	0	0	0	
23742125	f	md	JP	2024	0	0	0	0	0	0	0	0	0	0	0	0	71
23742199	f	cn	TH	2024	0	0	0	0	0	0	0	0	0	0	0	0	48
23742396	f	cn	TH	2024	1	0	0	0	0	0	0	0	0	0	0	0	74.5
23742397	f	cn	ID	2024	1	0	0	0	0	0	0	0	0	0	0	0	
23742677	m	md	JP	2024	0	1	0	0	1	0	0	0	0	0	0	0	80
23742760	m	cn	TH	2024	1	0	0	0	0	0	0	0	0	0	0	0	74.8
23743076	f	md	ID	2024	1	0	0	0	0	0	0	0	0	0	0	0	
23743305	m	cn	TH	2024	1	0	0	0	0	0	0	0	0	0	0	0	45
23743473	f	cn	DE	2024	0	1	1	0	0	0	0	0	0	0	0	1	66
23743670	m	cn	CO	2024	0	0	0	0	0	0	0	0	0	0	0	0	41
23743671	m	md	PL	2024	0	0	0	0	0	0	0	0	0	0	0	0	66
23743686	f	cn	VN	2024	1	0	0	0	0	0	0	0	0	0	0	0	
23743692	m	cn	ID	2024	1	0	0	0	0	0	0	0	0	0	0	0	50.3
23743814	Not	ph	JP	2024	0	1	0	0	1	0	0	0	0	0	0	0	
23744263	Not	md	JP	2024	0	0	0	0	0	0	0	0	0	0	0	0	
23744437	m	md	JP	2024	0	1	1	0	0	0	0	0	0	0	0	0	76
23744483	m	md	US	2024	0	0	0	0	0	0	0	0	0	0	0	0	49
23744900	f	md	JP	2024	0	0	0	0	0	0	0	0	0	0	0	0	72
23745423	m	md	DE	2024	0	0	0	0	0	0	0	0	0	0	0	0	59
23745476	Not	md	US	2024	0	0	0	0	0	0	0	0	0	0	0	0	
23745658	m	md	CA	2024	0	0	0	0	0	0	0	0	0	0	0	0	76
23745729	m	md	JP	2024	0	1	0	0	1	0	0	0	0	0	0	1	80
23745734	Not	hp	IT	2024	0	1	1	0	0	0	0	0	0	0	0	0	
23745859	m	md	CA	2024	0	1	1	0	0	0	0	0	0	0	0	0	76
23745993	f	cn	IN	2024	1	0	0	0	0	0	0	0	0	0	0	0	
23746005	Not	md	US	2024	0	0	0	0	0	0	0	0	0	0	0	0	
23746332	f	hp	CA	2024	0	0	0	0	0	0	0	0	0	0	0	0	45
23746389	Not	md	US	2024	0	0	0	0	0	0	0	0	0	0	0	0	
23746457	f	md	GB	2024	0	0	0	0	0	0	0	0	0	0	0	0	
23746809	m	md	FR	2024	0	1	0	0	1	0	0	0	0	0	0	0	68
23746841	f	cn	KR	2024	0	0	0	0	0	0	0	0	0	0	0	0	56
23746847	f	md	FR	2024	0	0	0	0	0	0	0	0	0	0	0	0	75

23746959	m	hp	JP	2024	0	0	0	0	0	0	0	0	0	0	0	0	54
23747037	Not	md	US	2024	0	0	0	0	0	0	0	0	0	0	0	0	
23747530	m	md	DE	2024	0	1	0	0	1	0	0	0	0	0	0	0	62
23747728	m	md	ID	2024	1	0	0	0	0	0	0	0	0	0	0	0	71
23747831	Not	md	JP	2024	1	0	0	0	0	0	0	0	0	0	0	0	
23747901	m	md	DE	2024	0	0	0	0	0	0	0	0	0	0	0	0	
23748172	Not	md	IT	2024	0	0	0	0	0	0	0	0	0	0	0	0	
23748357	m	md	JP	2024	0	1	0	1	0	0	0	0	0	0	0	0	81
23748642	Not	hp	IT	2024	0	1	1	0	0	0	0	0	0	0	0	0	
23748749	Not	ph	JP	2024	1	0	0	0	0	0	0	0	0	0	0	0	
23748804	Not	md	US	2024	0	0	0	0	0	0	0	0	0	0	0	0	
23749145	m	md	BG	2024	0	1	0	0	1	0	0	0	0	0	0	1	57
23749215	Not	md	PL	2024	0	1	0	0	1	0	0	0	0	0	0	0	
23749795	f	cn	LB	2024	0	1	0	0	0	1	0	0	0	0	0	0	62
23750105	m	md	JP	2024	0	1	0	0	0	1	0	0	0	0	0	1	69
23750148	Not	md	JP	2024	0	1	0	0	1	0	0	0	0	0	0	0	
23750207	m	md	PL	2024	0	0	0	0	0	0	0	0	0	0	0	0	70
23750477	f	md	DE	2024	0	0	0	0	0	0	0	0	0	0	0	0	
23750590	Not	hp	DE	2024	0	0	0	0	0	0	0	0	0	0	0	0	
23750642	f	md	RO	2024	1	0	0	0	0	0	0	0	0	0	0	0	82
23750772	Not	hp	IT	2024	0	1	1	0	0	0	0	0	0	0	0	0	
23750891	m	md	JP	2024	0	1	1	0	0	0	0	0	0	0	0	0	
23751018	m	md	ES	2024	0	0	0	0	0	0	0	0	0	0	0	0	
23751031	f	ph	US	2024	1	0	0	0	0	0	0	0	0	0	0	0	70
23751501	Not	hp	IT	2024	0	1	1	0	0	0	0	0	0	0	0	0	
23751840	f	cn	IN	2024	0	0	0	0	0	0	0	0	0	0	0	0	61
23752274	f	cn	US	2024	1	0	0	0	0	0	0	0	0	0	0	0	83
23752455	m	ph	FR	2024	0	1	1	0	0	0	0	0	0	0	0	0	57
23752658	f	md	JP	2024	0	0	0	0	0	0	0	0	0	0	0	0	40
23752735	f	cn	KR	2024	0	0	0	0	0	0	0	0	0	0	0	0	85
23752917	f	ph	MY	2024	0	0	0	0	0	0	0	0	0	0	0	0	43
23752923	m	md	ES	2024	0	0	0	0	0	0	0	0	0	0	0	0	71
23753030	m	cn	US	2024	1	0	0	0	0	0	0	0	0	0	0	0	52.8
23753399	m	md	RU	2024	0	0	0	0	0	0	0	0	0	0	0	0	69

23753499	m	md	ES	2024	0	0	0	0	0	0	0	0	0	0	0	0	
23753597	f	md	DE	2024	0	1	1	0	0	0	0	0	0	0	0	1	66
23753823	f	cn	CN	2024	0	0	0	0	0	0	0	0	0	0	0	0	
23753827	f	cn	CN	2024	0	0	0	0	0	0	0	0	0	0	0	0	78
23753845	m	md	JP	2024	0	1	1	0	0	0	0	0	0	0	0	0	80
23754028	Not	md	RU	2024	0	0	0	0	0	0	0	0	0	0	0	0	
23754059	m	md	JP	2024	0	1	1	0	0	0	0	0	0	0	0	0	77
23754130	f	md	FR	2024	0	0	0	0	0	0	0	0	0	0	0	0	76
23754131	f	ph	FR	2024	0	0	0	0	0	0	0	0	0	0	0	0	
23754547	Not	md	JP	2024	0	1	0	0	1	0	0	0	0	0	0	0	
23754880	m	md	JP	2024	0	1	0	0	1	0	0	0	0	0	0	0	74
23755224	m	cn	IN	2024	0	1	0	0	0	1	0	0	0	0	0	0	76
23755371	m	md	DE	2024	0	1	1	0	0	0	0	0	0	0	0	0	73
23755481	f	md	CA	2024	1	0	0	0	0	0	0	0	0	0	0	0	
23755902	m	md	JP	2024	0	1	1	0	0	0	0	0	0	0	0	0	80
23755969	Not	hp	AU	2024	0	1	0	0	0	1	0	0	0	0	0	0	
23756832	m	md	ES	2024	0	0	0	0	0	0	0	0	0	0	0	0	
23757178	f	hp	MX	2024	0	1	0	1	0	0	0	0	0	0	0	0	72
23757254	m	md	JP	2024	0	1	0	1	0	0	0	0	0	0	0	0	49
23757359	f	md	AU	2024	0	1	1	0	0	0	0	0	0	0	0	0	64
23757370	m	cn	CN	2024	0	0	0	0	0	0	0	0	0	0	0	0	
23757473	f	ph	US	2024	1	0	0	0	0	0	0	0	0	0	0	0	76
23757479	m	md	US	2024	0	0	0	0	0	0	0	0	0	0	0	0	85
23757480	f	md	JP	2024	0	0	0	0	0	0	0	0	0	0	0	0	79
23757499	m	md	JP	2024	0	1	0	1	0	0	0	0	0	0	0	0	76
23757532	f	md	DE	2024	0	0	0	0	0	0	0	0	0	0	0	0	
23757537	m	md	IT	2024	0	0	0	0	0	0	0	0	0	0	0	0	65
23757816	m	cn	US	2024	0	1	1	0	0	0	0	0	0	0	0	0	62
23758201	m	md	FR	2024	0	0	0	0	0	0	0	0	0	0	0	0	
23758278	f	cn	CN	2024	0	0	0	0	0	0	0	0	0	0	0	0	
23758439	m	hp	CA	2024	0	0	0	0	0	0	0	0	0	0	0	0	68
23758500	m	md	JP	2024	0	1	1	0	0	0	0	0	0	0	0	1	63
23758655	f	hp	US	2024	0	0	0	0	0	0	0	0	0	0	0	0	60
23758737	f	md	US	2024	0	0	0	0	0	0	0	0	0	0	0	0	

23759640	m	hp	DE	2024	0	1	0	0	1	0	0	0	0	0	0	0	74
23759657	m	md	JP	2024	0	0	0	0	0	0	0	0	0	0	0	0	60
23759809	m	cn	US	2024	1	0	0	0	0	0	0	0	0	0	0	0	
23760480	m	md	JP	2024	0	0	0	0	0	0	0	0	0	0	0	0	68
23760685	Not	hp	IT	2024	0	1	1	0	0	0	0	0	0	0	0	0	
23760690	Not	ph	JP	2024	0	0	0	0	0	0	0	0	0	0	0	0	
23760767	f	md	JP	2024	1	0	0	0	0	0	0	0	0	0	0	0	85
23760972	f	md	BR	2024	0	0	0	0	0	0	0	0	0	0	0	0	
23761017	Not	md	PL	2024	0	1	0	0	1	0	0	0	0	0	0	0	
23761049	Not	hp	IT	2024	0	1	1	0	0	0	0	0	0	0	0	0	
23761053	f	hp	CA	2024	0	0	0	0	0	0	0	0	0	0	0	0	45
23761352	f	md	JP	2024	1	0	0	0	0	0	0	0	0	0	0	0	89
23761353	m	md	JP	2024	1	0	0	0	0	0	0	0	0	0	0	0	56
23761574	f	md	BR	2024	0	0	0	0	0	0	0	0	0	0	0	0	56
23761848	m	md	IN	2024	0	0	0	0	0	0	0	0	0	0	0	0	
23761875	f	md	JP	2024	0	0	0	0	0	0	0	0	0	0	0	0	70
23762238	m	md	DE	2024	0	1	1	0	0	0	0	0	0	0	0	0	73
23762432	m	md	US	2024	0	0	0	0	0	0	0	0	0	0	0	0	33
23762462	f	hp	KR	2024	0	0	0	0	0	0	1	0	0	0	1	0	38
23762714	m	md	JP	2024	0	1	0	1	0	0	0	0	0	0	0	0	72
23763102	m	md	DE	2024	0	1	0	0	1	0	0	0	0	0	0	0	83
23763262	m	cn	ES	2024	0	0	0	0	0	0	0	0	0	0	0	0	
23763358	f	md	KR	2024	1	0	0	0	0	0	0	0	0	0	0	0	86
23763362	f	hp	KR	2024	1	0	0	0	0	0	0	0	0	0	0	0	58
23763407	Not	hp	IT	2024	0	1	1	0	0	0	0	0	0	0	0	0	
23763414	m	md	JP	2024	0	1	0	1	0	0	0	0	0	0	0	0	77
23763415	f	md	JP	2024	1	0	0	0	0	0	0	0	0	0	0	0	86
23763461	m	md	JP	2024	0	1	0	0	1	0	0	0	0	0	0	1	68
23763593	m	md	US	2024	0	1	1	0	0	0	0	0	0	0	0	0	
23763723	m	md	JP	2024	1	0	0	0	0	0	0	0	0	0	0	0	57
23763766	m	md	JP	2024	1	0	0	0	0	0	0	0	0	0	0	0	56
23764056	f	md	BE	2024	0	0	0	0	0	0	0	0	0	0	0	0	
23764173	f	cn	US	2024	1	0	0	0	0	0	0	0	0	0	0	0	
23764180	f	md	JP	2024	0	0	0	0	0	0	0	0	0	0	0	0	57

23764418	f	md	JP	2024	0	0	0	0	0	0	0	0	0	0	0	0	
23764422	Not	md	US	2024	0	0	0	0	0	0	0	0	0	0	0	0	
23765225	f	hp	US	2024	0	0	0	0	0	0	0	0	0	0	0	0	
23765350	f	hp	GB	2024	0	0	0	0	0	0	0	0	0	0	0	0	
23765485	Not	hp	FR	2024	0	0	0	0	0	0	0	0	0	0	0	0	
23766096	f	cn	US	2024	0	0	0	0	0	0	0	0	0	0	0	0	67
23766280	f	md	JP	2024	0	0	0	0	0	0	0	0	0	0	0	0	70
23766304	m	cn	BR	2024	0	0	0	0	0	0	0	0	0	0	0	0	50
23766521	Not	cn	US	2024	0	1	0	1	0	0	0	0	0	0	0	1	
23767041	m	md	US	2024	0	0	0	0	0	0	0	0	0	0	0	0	
23767126	Not	hp	IT	2024	0	1	1	0	0	0	0	0	0	0	0	0	
23767163	Not	hp	IT	2024	0	1	1	0	0	0	0	0	0	0	0	0	
23767237	Not	md	TW	2024	0	0	0	0	0	0	0	0	0	0	0	0	
23767254	Not	hp	IT	2024	0	1	1	0	0	0	0	0	0	0	0	0	
23767291	m	ph	JP	2024	0	1	1	0	0	0	0	0	0	0	0	0	78
23767416	m	md	FR	2024	0	1	0	0	1	0	0	0	0	0	0	0	68
23767646	m	md	JP	2024	0	1	0	1	0	0	0	0	0	0	0	1	
23767796	f	hp	ES	2024	0	0	0	0	0	0	0	0	0	0	0	0	58
23768412	m	cn	US	2024	1	0	0	0	0	0	0	0	0	0	0	0	
23768496	f	cn	US	2024	1	0	0	0	0	0	0	0	0	0	0	0	83
23768510	f	cn	US	2024	1	0	0	0	0	0	0	0	0	0	0	0	
23768873	m	cn	TW	2024	0	1	0	0	0	1	0	0	0	0	0	0	74
23768892	f	cn	BA	2024	0	0	0	0	0	0	0	0	0	0	0	0	
23768973	Not	hp	IT	2024	0	1	1	0	0	0	0	0	0	0	0	0	
23768974	Not	hp	IT	2024	0	1	1	0	0	0	0	0	0	0	0	0	
23769110	Not	hp	IT	2024	0	1	1	0	0	0	0	0	0	0	0	0	
23769158	f	md	CN	2024	0	0	0	0	0	0	0	0	0	0	0	0	
23769247	m	md	BE	2024	0	0	0	0	0	0	0	0	0	0	0	0	65
23769351	m	md	BG	2024	0	1	0	0	1	0	0	0	0	0	0	1	70
23769497	m	md	DE	2024	0	1	0	0	1	0	0	0	0	0	0	0	53
23769629	f	md	MY	2024	0	0	0	0	0	0	0	0	0	0	0	0	
23769739	m	md	DE	2024	0	1	0	0	1	0	0	0	0	0	0	0	65
23769921	f	md	CZ	2024	0	1	0	0	1	0	0	0	0	0	0	0	75
23769991	f	hp	KR	2024	1	0	0	0	0	0	0	0	0	0	0	0	48

23770341	Not	hp	IT	2024	0	1	1	0	0	0	0	0	0	0	0	0	
23770342	Not	hp	IT	2024	0	1	1	0	0	0	0	0	0	0	0	0	
23770709	f	md	JP	2024	0	0	0	0	0	0	0	0	0	0	0	0	80
23770806	Not	hp	US	2024	1	0	0	0	0	0	0	0	0	0	0	0	
23770910	f	cn	IL	2024	0	0	0	0	0	0	0	0	0	0	0	0	
23771101	Not	hp	IT	2024	0	1	1	0	0	0	0	0	0	0	0	0	
23771386	m	md	NL	2024	0	1	0	0	0	1	0	0	0	0	0	0	68
23771437	m	md	IT	2024	0	0	0	0	0	0	0	0	0	0	0	0	77
23771453	m	md	NL	2024	0	1	0	0	0	1	0	0	0	0	0	0	68
23771535	m	md	JP	2024	0	1	0	0	0	1	0	0	0	0	0	0	76
23772100	f	md	JP	2024	0	0	0	0	0	0	0	0	0	0	0	0	88
23772101	f	md	JP	2024	0	0	0	0	0	0	0	0	0	0	0	0	
23772242	f	md	JP	2024	0	1	0	0	0	1	0	0	0	0	0	0	70
23772865	m	md	DE	2024	0	1	0	0	1	0	0	0	0	0	0	0	53
23772881	f	md	JP	2024	0	1	1	0	0	0	0	0	0	0	0	0	82
23773233	Not	hp	US	2024	0	0	0	0	0	0	0	0	0	0	0	0	
23773370	f	ph	FR	2024	0	1	1	0	0	0	0	0	0	0	0	0	53
23773923	m	cn	ID	2024	0	0	0	0	0	0	0	0	0	0	0	0	87
23773971	f	md	DE	2024	0	0	0	0	0	0	0	0	0	0	0	0	
23774439	m	cn	US	2024	1	0	0	0	0	0	0	0	0	0	0	0	
23775352	f	cn	BE	2024	0	0	0	0	0	0	0	0	0	0	0	0	
23775354	f	cn	JP	2024	1	0	0	0	0	0	0	0	0	0	0	0	65
23775356	Not	cn	JP	2024	0	0	0	0	0	0	0	0	0	0	0	0	
23775365	m	md	FR	2024	0	0	0	0	0	0	0	0	0	0	0	0	
23775490	m	md	JP	2024	0	1	1	0	0	0	0	0	0	0	0	0	80
23775516	m	md	FR	2024	0	1	1	0	0	0	0	0	0	0	0	1	53
23775589	m	md	JP	2024	0	1	0	0	1	0	0	0	0	0	0	0	77
23775669	f	md	JP	2024	0	1	1	0	0	0	0	0	0	0	0	0	55
23776444	f	md	DK	2024	0	1	0	0	0	1	0	0	0	0	0	0	47
23777214	m	md	JP	2024	0	1	0	0	1	0	0	0	0	0	0	0	75
23777375	f	hp	IT	2024	0	1	1	0	0	0	0	0	0	0	0	0	77
23777788	m	md	JP	2024	0	1	0	1	0	0	0	0	0	0	0	0	66
23777880	Not	hp	CN	2024	0	0	0	0	0	0	0	0	0	0	0	0	
23777948	Not	md	JP	2024	0	1	0	0	1	0	0	0	0	0	0	0	

23778419	f	ph	US	2024	0	1	1	0	0	0	0	0	0	0	0	0	63
23778597	m	ph	JP	2024	0	1	1	0	0	0	0	0	0	0	0	0	70
23778923	f	md	JP	2024	0	1	0	1	0	0	0	0	0	0	0	0	76
23779000	f	hp	KR	2024	0	0	0	0	0	0	1	0	0	0	1	0	57
23779031	f	md	ES	2024	0	1	1	0	0	0	0	0	0	0	0	0	58
23779131	f	hp	US	2024	0	1	0	0	0	1	0	0	0	0	0	0	37
23779255	Not	md	JP	2024	1	0	0	0	0	0	0	0	0	0	0	0	
23779257	m	md	ES	2024	0	0	0	0	0	0	0	0	0	0	0	0	
23779261	Not	md	JP	2024	1	0	0	0	0	0	0	0	0	0	0	0	
23779297	Not	md	JP	2024	1	0	0	0	0	0	0	0	0	0	0	0	
23779309	f	md	JP	2024	0	1	0	1	0	0	0	0	0	0	0	0	73
23780186	m	cn	KR	2024	0	0	0	0	0	0	0	0	0	0	0	0	70
23780259	Not	ph	US	2024	0	0	0	0	0	0	0	0	0	0	0	0	
23780977	m	md	JP	2024	0	1	0	0	1	0	0	0	0	0	0	0	80
23781106	f	md	FR	2024	1	0	0	0	0	0	0	0	0	0	0	0	
23781923	m	ph	US	2024	0	1	0	0	1	0	0	0	0	0	0	0	78
23782151	Not	hp	GB	2024	0	1	0	0	0	1	0	0	0	0	0	0	
23782215	f	md	CZ	2024	0	0	0	0	0	0	0	0	0	0	0	0	21
23782312	Not	md	CA	2024	1	0	0	0	0	0	0	0	0	0	0	0	
23782528	m	md	JP	2024	0	1	0	1	0	0	0	0	0	0	0	0	80
23782544	f	ph	JP	2024	0	0	0	0	0	0	0	0	0	0	0	0	72
23782642	m	md	SI	2024	0	0	0	0	0	0	0	0	0	0	0	0	67
23783615	m	cn	EG	2024	0	1	1	0	0	0	0	0	0	0	0	0	
23783647	f	hp	CA	2024	0	0	0	0	0	0	0	0	0	0	0	0	65
23783658	f	ph	US	2024	0	1	0	0	1	0	0	0	0	0	0	0	66
23783887	m	md	DE	2024	0	1	0	0	1	0	0	0	0	0	0	0	69
23784542	f	ph	CN	2024	0	0	0	0	0	0	0	0	0	0	0	0	66
23784647	f	md	KR	2024	0	0	0	0	0	0	0	0	0	0	0	0	72
23784991	f	md	KR	2024	1	0	0	0	0	0	0	0	0	0	0	0	81.1
23785130	f	md	JP	2024	0	1	0	1	0	0	0	0	0	0	0	0	69
23785135	m	hp	HR	2024	0	1	0	1	0	0	0	0	0	0	0	0	58
23785136	m	md	JP	2024	0	1	0	1	0	0	0	0	0	0	0	0	
23785244	f	md	SK	2024	0	1	0	0	0	1	0	0	0	0	0	0	
23785394	m	ph	FR	2024	0	0	0	0	0	0	0	0	0	0	0	0	59

23785659	m	md	AU	2024	0	1	0	0	1	0	0	0	0	0	0	0	60
23785733	f	md	CZ	2024	0	1	0	0	1	0	0	0	0	0	0	0	75
23785830	m	hp	IT	2024	0	1	1	0	0	0	0	0	0	0	0	0	64
23785867	f	md	JP	2024	0	1	0	0	1	0	0	0	0	0	0	0	85
23786120	m	md	JP	2024	0	0	0	0	0	0	0	0	0	0	0	0	80
23786407	f	md	SI	2024	0	0	0	0	0	0	0	0	0	0	0	0	60
23786569	m	md	DE	2024	0	1	0	0	1	0	0	0	0	0	0	0	53
23786613	m	md	JP	2024	0	1	1	0	0	0	0	0	0	0	0	1	75
23786803	m	md	IT	2024	0	0	0	0	0	0	0	0	0	0	0	0	65
23786838	f	cn	US	2024	1	0	0	0	0	0	0	0	0	0	0	0	
23786922	f	cn	KR	2024	0	0	0	0	0	0	0	0	0	0	0	0	56
23786994	f	md	DE	2024	0	0	0	0	0	0	0	0	0	0	0	0	
23787068	m	md	SI	2024	0	0	0	0	0	0	0	0	0	0	0	0	73
23787265	Not	md	CA	2024	0	1	0	1	0	0	0	0	0	0	0	1	
23787640	m	md	IL	2024	0	0	0	0	0	0	0	0	0	0	0	0	
23787699	m	cn	KR	2024	0	0	0	0	0	0	0	0	0	0	0	0	53
23787824	f	md	FR	2024	0	1	0	0	0	1	0	0	0	0	0	0	62
23787949	m	md	JP	2024	0	1	0	0	1	0	0	0	0	0	0	0	70
23788143	f	cn	KR	2024	0	0	0	0	0	0	0	0	0	0	0	0	
23788183	f	md	JP	2024	1	0	0	0	0	0	0	0	0	0	0	0	60
23788371	f	md	US	2024	0	0	0	0	0	0	0	0	0	0	0	0	
23788457	f	md	VN	2024	0	1	0	0	0	1	0	0	0	0	0	0	68
23788640	Not	md	LB	2024	0	1	0	0	0	1	0	0	0	0	0	0	
23788716	m	md	DE	2024	0	1	0	1	0	0	0	0	0	0	0	0	
23789094	f	cn	US	2024	0	1	0	1	0	0	0	0	0	0	0	1	76
23789100	f	md	VN	2024	0	1	0	0	0	1	0	0	0	0	0	0	62
23789167	m	hp	CA	2024	1	0	0	0	0	0	0	0	0	0	0	0	54
23789410	m	md	JP	2024	0	1	0	0	1	0	0	0	0	0	0	0	70
23789515	m	md	FR	2024	0	1	0	0	1	0	0	0	0	0	0	0	68
23789875	f	md	CZ	2024	0	1	0	1	0	0	0	0	0	0	0	0	
23789966	m	md	DE	2024	0	1	0	0	1	0	0	0	0	0	0	0	53
23790227	m	cn	IN	2024	0	1	0	0	0	1	0	0	0	0	0	0	78
23790241	m	md	AT	2024	0	0	0	0	0	0	0	0	0	0	0	0	56
23790594	Not	hp	GB	2024	0	0	0	0	0	0	0	0	0	0	0	0	

23790785	f	md	DE	2024	0	0	0	0	0	0	0	0	0	0	0	0	
23790835	Not	hp	NL	2024	0	0	0	0	0	0	0	0	0	0	0	0	
23791461	f	md	JP	2024	1	0	0	0	0	0	0	0	0	0	0	0	80
23792317	Not	ph	US	2024	0	0	0	0	0	0	0	0	0	0	0	0	
23792717	m	md	JP	2024	0	1	1	0	0	0	0	0	0	0	0	0	70
23792897	m	hp	GB	2024	1	0	0	0	0	0	0	0	0	0	0	0	60
23792986	m	md	JP	2024	0	1	0	0	0	1	0	0	0	0	0	1	78
23793008	m	hp	GB	2024	1	0	0	0	0	0	0	0	0	0	0	0	70
23793307	m	md	JP	2024	0	0	0	0	0	0	0	0	0	0	0	0	70
23793402	m	md	MK	2024	0	1	0	0	0	1	0	0	0	0	0	0	72
23793875	m	md	TH	2024	0	1	0	0	0	1	0	0	0	0	0	1	59
23794121	m	md	JP	2024	0	1	0	1	0	0	0	0	0	0	0	0	
23794525	f	md	BE	2024	0	0	0	0	0	0	0	0	0	0	0	0	
23794638	m	ph	JP	2024	0	1	1	0	0	0	0	0	0	0	0	0	80
23794729	m	cn	US	2024	0	0	0	0	0	0	0	0	0	0	0	0	69
23795149	f	hp	US	2024	0	0	0	0	0	0	0	0	0	0	0	0	65
23795515	f	md	TW	2024	0	1	0	0	1	0	0	0	0	0	0	0	60
23795797	m	md	JP	2024	0	1	1	0	0	0	0	0	0	0	0	0	64
23795877	f	md	US	2024	1	0	0	0	0	0	0	0	0	0	0	0	70
23796098	f	cn	DE	2024	0	1	0	0	1	0	0	0	0	0	0	0	60
23796430	f	md	IT	2024	0	1	0	1	0	0	0	0	0	0	0	0	72.5
23796506	m	md	FR	2024	0	0	0	0	0	0	0	0	0	0	0	0	81
23797395	f	cn	CL	2024	1	0	0	0	0	0	0	0	0	0	0	0	82
23797540	f	md	RU	2024	0	1	0	0	0	1	0	0	0	0	0	0	59
23797604	m	md	IT	2024	0	0	0	0	0	0	0	0	0	0	0	0	65
23797709	m	md	FR	2024	0	0	0	0	0	0	0	0	0	0	0	0	72
23797961	f	md	BE	2024	0	0	0	0	0	0	0	0	0	0	0	0	
23798366	m	hp	FR	2024	0	0	0	0	0	0	0	0	0	0	0	0	76
23798416	f	cn	CL	2024	1	0	0	0	0	0	0	0	0	0	0	0	63.3
23798752	m	md	KR	2024	0	0	0	0	0	0	0	0	0	0	0	0	47
23798879	f	ph	US	2024	0	0	0	0	0	0	0	0	0	0	0	0	67
23799074	f	md	US	2024	1	0	0	0	0	0	0	0	0	0	0	0	
23799570	m	md	BR	2024	1	0	0	0	0	0	0	0	0	0	0	1	
23800007	f	hp	US	2024	1	0	0	0	0	0	0	0	0	0	0	0	66

23800024	f	hp	US	2024	0	1	0	1	0	0	0	0	0	0	0	0	66
23800648	m	md	PL	2024	0	1	0	1	0	0	0	0	0	0	0	0	70
23800809	Not	hp	US	2024	0	1	0	1	0	0	0	0	0	0	0	0	
23800817	f	cn	US	2024	0	1	0	1	0	0	0	0	0	0	0	0	
23800820	Not	cn	US	2024	0	1	0	1	0	0	0	0	0	0	0	0	
23800869	f	cn	CA	2024	1	0	0	0	0	0	0	0	0	0	0	0	
23801072	m	hp	FR	2024	0	1	1	0	0	0	0	0	0	0	0	0	76
23801662	f	md	DE	2024	0	1	0	0	1	0	0	0	0	0	0	0	63
23802066	m	md	PL	2024	0	1	0	1	0	0	0	0	0	0	0	0	67
23802316	Not	md	JP	2024	0	1	0	0	0	1	0	0	0	0	0	0	80
23802535	Not	md	US	2024	0	1	1	0	0	0	0	0	0	0	0	0	75
23802551	Not	md	US	2024	0	0	0	0	0	0	0	0	0	0	0	0	70
23802552	Not	md	US	2024	0	0	0	0	0	0	0	0	0	0	0	0	61
23802580	f	md	JP	2024	0	1	0	0	0	1	0	0	0	0	0	0	79
23802973	f	md	JP	2024	1	0	0	0	0	0	0	0	0	0	0	0	80
23803319	f	ph	US	2024	0	1	0	1	0	0	0	0	0	0	0	1	66
23804247	f	md	JP	2024	1	0	0	0	0	0	0	0	0	0	0	0	68
23804298	m	md	JP	2024	0	1	1	0	0	0	0	0	0	0	0	0	80
23804555	f	md	FR	2024	0	1	0	0	0	1	0	0	0	0	0	0	62
23804847	f	md	JP	2024	0	0	0	0	0	0	0	0	0	0	0	0	74
23805681	m	cn	MX	2024	0	0	0	0	0	0	0	0	0	0	0	0	
23806494	Not	hp	NZ	2024	0	1	0	1	0	0	0	0	0	0	0	0	
23806582	m	md	US	2024	0	0	0	0	0	0	0	0	0	0	0	0	
23806613	Not	md	US	2024	0	0	0	0	0	0	0	0	0	0	0	0	
23806620	Not	hp	NZ	2024	0	1	0	1	0	0	0	0	0	0	0	0	
23806679	Not	md	JP	2024	0	1	0	0	1	0	0	0	0	0	0	0	
23807047	m	cn	US	2024	1	0	0	0	0	0	0	0	0	0	0	0	
23807717	f	ph	FR	2024	0	1	1	0	0	0	0	0	0	0	0	0	53
23807761	Not	hp	NZ	2024	0	1	0	1	0	0	0	0	0	0	0	0	
23807763	Not	md	JP	2024	1	0	0	0	0	0	0	0	0	0	0	0	
23807846	Not	hp	NZ	2024	0	1	0	1	0	0	0	0	0	0	0	0	
23807847	Not	hp	NZ	2024	0	1	0	1	0	0	0	0	0	0	0	0	
23808087	m	md	FR	2024	0	0	0	0	0	0	0	0	0	0	0	0	72
23808123	f	md	US	2024	0	0	0	0	0	0	0	0	0	0	0	0	

23808233	Not	hp	NZ	2024	0	1	0	1	0	0	0	0	0	0	0	0	
23808235	m	md	JP	2024	0	1	0	1	0	0	0	0	0	0	0	0	68
23808347	m	hp	FR	2024	0	0	0	0	0	0	0	0	0	0	0	0	75
23808519	f	md	RU	2024	0	0	0	0	0	0	0	0	0	0	0	0	54
23808704	f	hp	US	2024	0	1	0	0	1	0	0	0	0	0	0	0	87
23808818	Not	hp	NZ	2024	0	1	0	1	0	0	0	0	0	0	0	0	
23808865	m	md	DE	2024	0	1	0	0	1	0	0	0	0	0	0	0	70
23808968	m	md	PL	2024	0	0	0	0	0	0	0	0	0	0	0	0	66
23809022	f	cn	US	2024	1	0	0	0	0	0	0	0	0	0	0	0	
23809503	m	cn	US	2024	1	0	0	0	0	0	0	0	0	0	0	0	
23809931	f	hp	DE	2024	0	1	0	1	0	0	0	0	0	0	0	0	67
23810086	Not	hp	NZ	2024	0	1	0	1	0	0	0	0	0	0	0	0	
23810089	f	hp	CN	2024	1	0	0	0	0	0	0	0	0	0	0	0	57
23810212	m	hp	KR	2024	0	1	0	0	1	0	0	0	0	0	0	0	66
23810259	m	cn	FR	2024	0	0	0	0	0	0	0	0	0	0	0	0	72
23810281	m	hp	KR	2024	0	1	0	0	1	0	0	0	0	0	0	0	64
23810320	m	md	JP	2024	0	1	1	0	0	0	0	0	0	0	0	0	70
23810751	m	hp	US	2024	0	0	0	0	0	0	0	0	0	0	0	0	70
23811183	f	md	SK	2024	0	1	0	0	0	1	0	0	0	0	0	0	
23811489	m	hp	KR	2024	0	1	0	0	1	0	0	0	0	0	0	0	69
23811567	f	md	HU	2024	0	1	0	0	0	1	0	0	0	0	0	0	48
23811644	m	md	EG	2024	0	1	0	0	1	0	0	0	0	0	0	0	45
23812540	f	md	AT	2024	0	0	0	0	0	0	0	0	0	0	0	0	77
23812676	Not	md	JP	2024	0	0	0	0	0	0	0	0	0	0	0	0	
23813064	m	cn	LB	2024	0	1	0	0	1	0	0	0	0	0	0	0	
23813306	f	cn	IN	2024	0	1	0	0	0	1	0	0	0	0	0	0	70
23813365	m	md	VN	2024	0	1	0	0	0	1	0	0	0	0	0	0	62
23813548	m	md	IT	2024	0	0	0	0	0	0	0	0	0	0	0	0	65
23813817	m	cn	ID	2024	0	1	0	0	0	1	0	0	0	0	0	0	
23813889	Not	md	JP	2024	1	0	0	0	0	0	0	0	0	0	0	0	
23813919	m	md	IN	2024	0	1	0	0	0	1	0	0	0	0	0	0	
23814241	Not	md	JP	2024	0	1	0	1	0	0	0	0	0	0	0	0	
23814534	Not	cn	US	2024	1	0	0	0	0	0	0	0	0	0	0	0	
23814543	m	md	IT	2024	0	0	0	0	0	0	0	0	0	0	0	0	65

23814573	f	md	KR	2024	0	0	0	0	0	0	0	0	0	0	0	0	
23814611	m	md	KR	2024	0	0	0	0	0	0	0	0	0	0	0	0	73
23814656	f	ph	FR	2024	0	1	1	0	0	0	0	0	0	0	0	0	53
23814703	m	md	DE	2024	0	1	0	0	1	0	0	0	0	0	0	0	70
23814867	f	cn	TH	2024	1	0	0	0	0	0	0	0	0	0	0	0	
23815003	f	cn	CO	2024	0	0	0	0	0	0	0	0	0	0	0	0	47
23815021	m	md	IT	2024	0	1	1	0	0	0	0	0	0	0	0	0	70
23815117	f	hp	IT	2024	0	1	0	0	1	0	0	0	0	0	0	0	81
23815461	f	md	JP	2024	0	1	0	1	0	0	0	0	0	0	0	0	82
23815600	m	md	CA	2024	0	1	0	0	1	0	0	0	0	0	0	0	
23815679	m	md	PL	2024	0	0	0	0	0	0	0	0	0	0	0	0	66
23815744	m	ph	FR	2024	0	0	0	0	0	0	0	0	0	0	0	0	59
23815782	f	cn	US	2024	0	0	0	0	0	0	0	0	0	0	0	0	
23815876	f	md	DE	2024	0	1	0	0	1	0	0	0	0	0	0	0	75
23815904	f	hp	CA	2024	0	0	0	0	0	0	0	0	0	0	0	0	8
23815976	m	ph	JP	2024	0	1	1	0	0	0	0	0	0	0	0	0	80
23816145	m	md	CN	2024	0	1	1	0	0	0	0	0	0	0	0	0	69
23816187	f	md	JP	2024	0	0	0	0	0	0	0	0	0	0	0	0	65
23816344	Not	md	LB	2024	0	1	0	1	0	0	0	0	0	0	0	0	
23816656	Not	md	LB	2024	0	1	0	0	0	1	0	0	0	0	0	0	
23817044	f	hp	ES	2024	0	1	1	0	0	0	0	0	0	0	0	0	47
23817579	m	md	CA	2024	0	1	0	0	1	0	0	0	0	0	0	0	
23817589	m	md	CA	2024	0	1	0	0	1	0	0	0	0	0	0	0	
23817742	f	md	DE	2024	0	1	0	0	1	0	0	0	0	0	0	0	63
23818550	m	md	KR	2024	1	0	0	0	0	0	0	0	0	0	0	0	76
23818835	m	md	JP	2024	0	0	0	0	0	0	0	0	0	0	0	0	90
23819349	f	cn	CA	2024	0	0	0	0	0	0	0	0	0	0	0	0	
23819379	m	cn	US	2024	0	0	0	0	0	0	0	0	0	0	0	0	65
23820461	f	md	FR	2024	0	1	1	0	0	0	0	0	0	0	0	0	47
23821724	f	md	FR	2024	0	0	0	0	0	0	0	0	0	0	0	0	47
23822383	m	md	EG	2024	0	1	0	0	1	0	0	0	0	0	0	0	56
23822776	m	md	GR	2024	0	0	0	0	0	0	0	0	0	0	0	0	
23823123	m	hp	JP	2024	0	1	1	0	0	0	0	0	0	0	0	0	70
23823306	Not	md	ES	2024	0	1	0	0	0	1	0	0	0	0	0	0	

23823347	m	ph	JP	2024	0	0	0	0	0	0	0	0	0	0	0	0	
23823826	f	md	CN	2024	0	0	0	0	0	0	0	0	0	0	0	0	65
23824250	m	cn	US	2024	0	1	1	0	0	0	0	0	0	0	0	0	
23824937	f	md	FR	2024	0	1	1	0	0	0	0	0	0	0	0	0	47
23825085	m	cn	US	2024	0	0	0	0	0	0	0	0	0	0	0	0	65
23825396	f	cn	US	2024	0	0	0	0	0	0	0	0	0	0	0	0	66
23825542	m	md	JP	2024	0	1	0	0	0	1	0	0	0	0	0	0	80
23825691	f	hp	GB	2024	1	0	0	0	0	0	0	0	0	0	0	0	
23825965	Not	md	JP	2024	0	1	0	0	0	1	0	0	0	0	0	0	80
23826806	m	md	IN	2024	1	0	0	0	0	0	0	0	0	0	0	0	81
23827077	m	md	DE	2024	0	1	1	0	0	0	0	0	0	0	0	0	
23827238	f	md	DE	2024	0	0	0	0	0	0	0	0	0	0	0	0	85
23827516	Not	hp	NZ	2024	0	1	0	1	0	0	0	0	0	0	0	0	
23827519	f	hp	US	2024	1	0	0	0	0	0	0	0	0	0	0	0	
23828529	m	md	US	2024	0	1	0	0	1	0	0	0	0	0	0	0	
23828545	m	hp	JP	2024	0	1	0	0	1	0	0	0	0	0	0	0	69
23829745	m	md	DE	2024	0	1	0	0	1	0	0	0	0	0	0	0	72
23829782	f	md	KR	2024	0	0	0	0	0	0	0	0	0	0	0	0	65
23830048	m	md	FR	2024	0	0	0	0	0	0	0	0	0	0	0	0	72
23830371	f	hp	CA	2024	0	0	0	0	0	0	0	0	0	0	0	0	45
23830825	f	cn	US	2024	1	0	0	0	0	0	0	0	0	0	0	0	68
23831018	m	hp	KR	2024	0	1	1	0	0	0	0	0	0	0	0	0	77
23831131	m	hp	KR	2024	0	1	1	0	0	0	0	0	0	0	0	0	66
23831158	m	hp	KR	2024	0	1	1	0	0	0	0	0	0	0	0	0	49
23831168	m	cn	IN	2024	0	0	0	0	0	0	0	0	0	0	0	0	66
23831219	f	md	ES	2024	0	1	1	0	0	0	0	0	0	0	0	0	47
23831251	f	md	FR	2024	0	1	1	0	0	0	0	0	0	0	0	0	47
23831592	f	md	JP	2024	0	0	0	0	0	0	0	0	0	0	0	0	36
23831849	m	md	FR	2024	0	0	0	0	0	0	0	0	0	0	0	0	72
23832043	f	md	FR	2024	0	1	1	0	0	0	0	0	0	0	0	0	47
23832048	m	md	JP	2024	0	1	1	0	0	0	0	0	0	0	0	0	70
23832153	f	cn	JP	2024	0	0	0	0	0	0	0	0	0	0	0	0	45
23832220	f	md	IT	2024	0	1	1	0	0	0	0	0	0	0	0	0	55
23832689	m	md	JP	2024	0	1	0	0	0	1	0	0	0	0	0	0	80

23833082	m	hp	FR	2024	0	1	1	0	0	0	0	0	0	0	0	0	76
23833223	f	ph	US	2024	0	1	0	0	1	0	0	0	0	0	0	0	67
23833344	m	ph	JP	2024	0	1	1	0	0	0	0	0	0	0	0	0	60
23833667	m	md	JP	2024	0	1	0	0	1	0	0	0	0	0	0	0	72
23833811	m	cn	AU	2024	0	0	0	0	0	0	0	0	0	0	0	0	
23834705	f	md	AU	2024	0	0	0	0	0	0	0	0	0	0	0	0	
23835613	m	md	JP	2024	0	1	0	0	1	0	0	0	0	0	0	0	73
23835650	Not	md	JP	2024	0	1	0	0	1	0	0	0	0	0	0	0	
23835819	Not	md	JP	2024	0	1	0	0	1	0	0	0	0	0	0	0	
23836040	f	md	KR	2024	0	0	0	0	0	0	0	0	0	0	0	0	69
23836154	Not	md	JP	2024	0	1	0	0	1	0	0	0	0	0	0	0	
23836218	m	md	MO	2024	0	0	0	0	0	0	0	0	0	0	0	0	20
23836525	Not	md	JP	2024	1	0	0	0	0	0	0	0	0	0	0	0	
23836636	m	md	DE	2024	0	1	0	0	1	0	0	0	0	0	0	0	70
23836902	f	md	US	2024	0	0	0	0	0	0	0	0	0	0	0	0	34
23837188	f	md	FR	2024	0	0	0	0	0	0	0	0	0	0	0	0	47
23837189	m	ph	FR	2024	0	0	0	0	0	0	0	0	0	0	0	0	59
23837208	f	hp	JP	2024	0	0	0	0	0	0	0	0	0	0	0	0	79
23837357	f	md	ES	2024	0	1	1	0	0	0	0	0	0	0	0	0	47
23837541	f	md	BE	2024	0	0	0	0	0	0	0	0	0	0	0	0	
23838764	f	md	CA	2024	0	1	1	0	0	0	0	0	0	0	0	0	67
23838913	m	md	KR	2024	0	0	0	0	0	0	0	0	0	0	0	0	66
23839451	m	md	JP	2024	0	1	0	0	1	0	0	0	0	0	0	0	73
23839495	f	md	US	2024	1	0	0	0	0	0	0	0	0	0	0	0	70
23839870	Not	md	JP	2024	0	1	0	0	1	0	0	0	0	0	0	0	
23839949	m	md	JP	2024	0	1	0	0	1	0	0	0	0	0	0	0	66
23839953	m	md	IT	2024	0	1	1	0	0	0	0	0	0	0	0	0	70
23840106	m	md	JP	2024	0	1	0	0	1	0	0	0	0	0	0	0	76
23840242	m	md	JP	2024	0	1	0	0	1	0	0	0	0	0	0	0	71
23840661	m	md	JP	2024	0	1	0	0	1	0	0	0	0	0	0	0	59
23840662	Not	md	JP	2024	0	1	0	0	1	0	0	0	0	0	0	0	
23840690	m	md	JP	2024	0	1	0	0	1	0	0	0	0	0	0	0	77
23840845	f	md	DE	2024	0	1	0	0	1	0	0	0	0	0	0	0	57
23840986	f	md	DE	2024	0	1	0	0	1	0	0	0	0	0	0	0	57

23841016	m	md	JP	2024	0	1	0	0	1	0	0	0	0	0	0	0	78
23841106	m	md	JP	2024	0	1	0	0	1	0	0	0	0	0	0	0	60
23841439	Not	md	JP	2024	0	1	0	0	1	0	0	0	0	0	0	0	
23841486	m	cn	US	2024	0	0	0	0	0	0	0	0	0	0	0	0	76
23841507	m	md	JP	2024	0	1	1	0	0	0	0	0	0	0	0	0	78
23841510	m	md	FR	2024	0	0	0	0	0	0	0	0	0	0	0	0	72
23842037	m	md	MA	2024	0	0	0	0	0	0	0	0	0	0	0	0	
23842063	m	hp	VN	2024	0	1	0	0	0	1	0	0	0	0	0	0	52
23842115	m	md	DE	2024	0	1	1	0	0	0	0	0	0	0	0	0	
23842274	m	md	HR	2024	0	1	0	0	0	1	0	0	0	0	0	0	44
23842484	m	hp	JP	2024	0	1	1	0	0	0	0	0	0	0	0	0	70
23842556	Not	md	ES	2024	0	1	0	0	0	1	0	0	0	0	0	0	
23842589	m	cn	US	2024	1	0	0	0	0	0	0	0	0	0	0	0	
23842730	f	md	NL	2024	0	1	1	0	0	0	0	0	0	0	0	0	55
23844292	m	md	IT	2024	0	1	1	0	0	0	0	0	0	0	0	0	80
23844331	f	cn	US	2024	0	1	1	0	0	0	0	0	0	0	0	0	70
23844425	m	ph	JP	2024	0	1	1	0	0	0	0	0	0	0	0	0	80
23844645	Not	md	LT	2024	0	1	0	0	0	1	0	0	0	0	0	0	
23846490	m	md	JP	2024	0	1	0	0	1	0	0	0	0	0	0	0	73
23847578	m	hp	US	2024	0	1	1	0	0	0	0	0	0	0	0	0	
23847902	m	ph	FR	2024	0	0	0	0	0	0	0	0	0	0	0	0	59
23848769	m	cn	US	2024	0	0	0	0	0	0	0	0	0	0	0	0	
23848821	f	hp	IT	2024	0	1	0	0	1	0	0	0	0	0	0	0	66
23848861	m	md	JP	2024	0	1	1	0	0	0	0	0	0	0	0	0	80
23848931	m	hp	IN	2024	1	0	0	0	0	0	0	0	0	0	0	0	70
23849493	m	ph	US	2024	0	0	0	0	0	0	0	0	0	0	0	0	84
23849731	Not	md	US	2024	0	0	0	0	0	0	0	0	0	0	0	0	
23849959	f	hp	ES	2024	0	1	1	0	0	0	0	0	0	0	0	0	47
23850057	m	hp	FR	2024	0	1	1	0	0	0	0	0	0	0	0	0	76
23850086	f	md	FR	2024	0	1	1	0	0	0	0	0	0	0	0	0	47
23850179	f	md	SK	2024	0	1	0	0	0	1	0	0	0	0	0	0	
23850286	f	hp	IT	2024	0	1	0	0	1	0	0	0	0	0	0	0	81
23850964	m	md	JP	2024	0	1	0	0	1	0	0	0	0	0	0	0	69
23851185	m	md	JP	2024	0	1	0	0	1	0	0	0	0	0	0	0	69

23851266	f	hp	IT	2024	0	1	0	0	1	0	0	0	0	0	0	0	81
23851593	m	md	JP	2024	0	1	0	0	1	0	0	0	0	0	0	0	65
23851638	Not	md	US	2024	0	0	0	0	0	0	0	0	0	0	0	0	
23851659	m	md	JP	2024	0	1	0	0	1	0	0	0	0	0	0	0	78
23851836	m	md	JP	2024	0	1	0	0	1	0	0	0	0	0	0	0	76
23851926	m	md	JP	2024	0	1	1	0	0	0	0	0	0	0	0	0	78
23852644	m	cn	US	2024	0	0	0	0	0	0	0	0	0	0	0	0	74
23853301	Not	md	JP	2024	0	0	0	0	0	0	0	0	0	0	0	0	80
23853745	f	md	IT	2024	0	1	1	0	0	0	0	0	0	0	0	0	55
23853930	m	md	VN	2024	0	1	0	0	0	1	0	0	0	0	0	0	45
23854196	m	ph	US	2024	0	0	0	0	0	0	0	0	0	0	0	0	
23854224	m	md	US	2024	0	1	1	0	0	0	0	0	0	0	0	0	
23854344	m	md	TH	2024	0	0	0	0	0	0	0	0	0	0	0	0	79
23854346	m	hp	JP	2024	0	1	1	0	0	0	0	0	0	0	0	0	78
23854764	f	md	FR	2024	0	1	1	0	0	0	0	0	0	0	0	0	47
23854785	m	md	HR	2024	0	1	0	0	0	1	0	0	0	0	0	0	44
23854794	f	md	FR	2024	0	0	0	0	0	0	0	0	0	0	0	0	47
23855506	m	md	JP	2024	0	1	1	0	0	0	0	0	0	0	0	0	75
23855514	f	cn	US	2024	0	0	0	0	0	0	0	0	0	0	0	0	
23856142	m	cn	CA	2024	0	1	0	0	1	0	0	0	0	0	0	1	44
23856272	f	md	JP	2024	0	0	0	0	0	0	0	0	0	0	0	0	60
23857080	m	hp	KR	2024	0	0	0	0	0	0	0	0	0	0	0	0	73
23857084	m	hp	KR	2024	0	0	0	0	0	0	0	0	0	0	0	0	67
23857782	m	hp	US	2024	0	0	0	0	0	0	0	0	0	0	0	0	
23857871	f	md	IT	2024	0	1	1	0	0	0	0	0	0	0	0	0	77
23857997	f	md	FR	2024	0	1	1	0	0	0	0	0	0	0	0	0	47
23858401	f	hp	ES	2024	0	1	1	0	0	0	0	0	0	0	0	0	47
23858847	m	hp	KR	2024	0	0	0	0	0	0	0	0	0	0	0	0	70
23858848	m	hp	KR	2024	0	0	0	0	0	0	0	0	0	0	0	0	69
23859186	m	md	JP	2024	0	0	0	0	0	0	0	0	0	0	0	0	
23859563	f	md	US	2024	0	1	1	0	0	0	0	0	0	0	0	0	67
23859738	m	md	JP	2024	0	1	0	1	0	0	0	0	0	0	0	0	60
23859741	f	md	JP	2024	1	0	0	0	0	0	0	0	0	0	0	1	80
23859781	Not	md	JP	2024	0	1	0	1	0	0	0	0	0	0	0	0	

23859894	m	md	IT	2024	0	1	1	0	0	0	0	0	0	0	0	0	70
23860262	m	md	US	2024	0	1	1	0	0	0	0	0	0	0	0	1	80
23860296	f	md	US	2024	0	1	1	0	0	0	0	0	0	0	0	0	64
23860453	Not	md	JP	2024	0	1	0	0	0	1	0	0	0	0	0	0	79
23861243	m	md	JP	2024	0	0	0	0	0	0	0	0	0	0	0	0	80
23861333	f	md	DE	2024	0	1	0	0	1	0	0	0	0	0	0	0	57
23861696	m	hp	US	2024	0	0	0	0	0	0	0	0	0	0	0	0	71
23861701	m	md	JP	2024	0	0	0	0	0	0	0	0	0	0	0	0	78
23861774	Not	ph	JP	2024	1	0	0	0	0	0	0	0	0	0	0	0	
23861836	m	hp	NL	2024	0	0	0	0	0	0	0	0	0	0	0	0	74
23861867	f	md	JP	2024	0	1	0	0	0	1	0	0	0	0	0	0	90
23861870	m	hp	KR	2024	0	0	0	0	0	0	0	0	0	0	0	0	71
23861871	m	hp	KR	2024	0	0	0	0	0	0	0	0	0	0	0	0	74
23861954	m	cn	CA	2024	0	1	0	0	1	0	0	0	0	0	0	0	
23861997	m	hp	FR	2024	0	1	1	0	0	0	0	0	0	0	0	0	76
23862032	m	hp	FR	2024	0	1	1	0	0	0	0	0	0	0	0	0	75
23862148	f	md	HU	2024	0	1	0	0	0	1	0	0	0	0	0	0	48
23862194	f	md	IT	2024	0	1	1	0	0	0	0	0	0	0	0	0	55
23862311	f	md	IT	2024	0	1	1	0	0	0	0	0	0	0	0	0	77
23862458	m	md	GB	2024	0	1	0	0	0	1	0	0	0	0	0	0	66
23862508	m	hp	US	2024	0	1	0	0	1	0	0	0	0	0	0	0	68
23862524	f	md	US	2024	0	1	1	0	0	0	0	0	0	0	0	0	39
23862709	f	md	FR	2024	0	1	0	0	0	1	0	0	0	0	0	0	41
23862719	m	md	HR	2024	0	1	0	0	0	1	0	0	0	0	0	0	44
23862875	f	cn	IN	2024	0	0	0	0	0	0	0	0	0	0	0	0	38
23862918	Not	hp	US	2024	0	0	0	0	0	0	0	0	0	0	0	0	
23863238	m	cn	TH	2024	0	1	0	0	0	1	0	0	0	0	0	0	69
23863469	m	md	BG	2024	0	0	0	0	0	0	0	0	0	0	0	0	63
23863481	m	md	JP	2024	0	1	1	0	0	0	0	0	0	0	0	0	80
23863953	Not	md	SG	2024	0	0	0	0	0	0	0	0	0	0	0	0	
23864043	m	cn	US	2024	0	1	0	1	0	0	0	0	0	0	0	0	72.7
23864157	f	md	ES	2024	0	1	1	0	0	0	0	0	0	0	0	0	82
23864176	m	md	HR	2024	0	1	0	0	0	1	0	0	0	0	0	0	44
23864185	Not	cn	CN	2024	0	0	0	0	0	0	0	0	0	0	0	0	

23864267	f	md	JP	2024	1	0	0	0	0	0	0	0	0	0	0	0	50
23864942	m	md	CA	2024	0	0	0	0	0	0	0	0	0	0	0	0	59
23865519	m	md	JP	2024	0	1	0	0	0	1	0	0	0	0	0	0	70
23865643	Not	md	US	2024	0	1	0	0	1	0	0	0	0	0	0	0	
23866218	m	cn	TH	2024	0	1	0	0	0	1	0	0	0	0	0	0	
23866244	f	cn	TH	2024	0	1	0	0	0	1	0	0	0	0	0	0	
23866245	m	cn	TH	2024	0	1	0	0	0	1	0	0	0	0	0	0	
23866262	f	cn	TH	2024	0	1	0	0	0	1	0	0	0	0	0	0	
23866284	m	cn	TH	2024	0	1	0	0	0	1	0	0	0	0	0	0	
23866741	m	md	CN	2024	1	0	0	0	0	0	0	0	0	0	0	0	68
23867126	Not	md	JP	2024	0	1	0	0	1	0	0	0	0	0	0	0	
23867167	m	cn	KR	2024	0	0	0	0	0	0	0	0	0	0	0	0	68
23867269	m	cn	TH	2024	0	0	0	0	0	0	0	0	0	0	0	0	
23867281	f	md	RS	2024	0	1	1	0	0	0	0	0	0	0	0	0	53
23867288	m	md	JP	2024	0	1	1	0	0	0	0	0	0	0	0	0	
23867845	m	md	JP	2024	1	0	0	0	0	0	0	0	0	0	0	0	
23867909	f	cn	DE	2024	0	1	0	0	1	0	0	0	0	0	0	0	57
23867917	f	cn	JP	2024	0	0	0	0	0	0	0	0	0	0	0	0	75
23867962	f	cn	JP	2024	1	0	0	0	0	0	0	0	0	0	0	0	
23868093	m	cn	KE	2024	1	0	0	0	0	0	0	0	0	0	0	0	47.2
23868133	m	hp	IN	2024	1	0	0	0	0	0	0	0	0	0	0	0	70
23868298	m	md	BG	2024	0	0	0	0	0	0	0	0	0	0	0	0	62
23868892	m	hp	FR	2024	0	1	1	0	0	0	0	0	0	0	0	0	75
23869661	m	md	FR	2024	0	0	0	0	0	0	0	0	0	0	0	0	53
23869683	m	md	JP	2024	0	1	1	0	0	0	0	0	0	0	0	0	80
23869799	m	ph	JP	2024	1	0	0	0	0	0	0	0	0	0	0	0	80
23869808	f	cn	US	2024	1	0	0	0	0	0	0	0	0	0	0	0	64.8
23870311	m	cn	TH	2024	0	1	0	0	0	1	0	0	0	0	0	0	
23870316	f	cn	TH	2024	0	1	0	0	0	1	0	0	0	0	0	0	73
23870689	f	md	DE	2024	0	1	0	0	1	0	0	0	0	0	0	0	57
23870732	f	md	FR	2024	0	1	1	0	0	0	0	0	0	0	0	0	47
23870749	f	cn	AU	2024	0	1	1	0	0	0	0	0	0	0	0	0	64
23872008	m	ph	JP	2024	0	1	0	0	0	1	0	0	0	0	0	0	69
23872020	f	ph	JP	2024	0	0	0	0	0	0	0	0	0	0	0	0	47

23872026	f	ph	JP	2024	0	0	0	0	0	0	0	0	0	0	0	0	60
23872099	f	md	FR	2024	0	1	0	0	0	1	0	0	0	0	0	0	41
23872191	m	md	CA	2024	0	1	0	0	1	0	0	0	0	0	0	0	60
23872989	f	cn	TH	2024	0	1	0	0	0	1	0	0	0	0	0	0	69
23873048	m	cn	TH	2024	0	1	0	0	0	1	0	0	0	0	0	0	
23873094	m	hp	CN	2024	0	0	0	0	0	0	0	0	0	0	0	0	67
23873100	m	hp	CN	2024	0	0	0	0	0	0	0	0	0	0	0	0	61
23873139	f	md	IT	2024	0	1	1	0	0	0	0	0	0	0	0	0	55
23873179	f	hp	ES	2024	0	1	1	0	0	0	0	0	0	0	0	0	53
23873255	m	md	JP	2024	0	0	0	0	0	0	0	0	0	0	0	0	69
23873289	m	hp	US	2024	0	1	0	0	1	0	0	0	0	0	0	0	68
23873305	Not	md	GB	2024	1	0	0	0	0	0	0	0	0	0	0	0	
23873495	m	hp	CN	2024	0	0	0	0	0	0	0	0	0	0	0	0	61
23873991	f	md	BA	2024	0	1	1	0	0	0	0	0	0	0	0	0	71
23874025	f	cn	IN	2024	0	0	0	0	0	0	0	0	0	0	0	0	62
23874044	m	hp	JP	2024	0	0	0	0	0	0	0	0	0	0	0	0	70
23874259	m	hp	DE	2024	0	1	0	0	1	0	0	0	0	0	0	0	74
23874293	f	md	US	2024	0	1	1	0	0	0	0	0	0	0	0	0	39
23874294	m	cn	US	2024	0	1	1	0	0	0	0	0	0	0	0	0	74
23874773	m	cn	US	2024	0	0	0	0	0	0	0	0	0	0	0	0	
23874851	f	cn	KR	2024	0	0	0	0	0	0	0	0	0	0	0	0	
23875115	m	md	HR	2024	0	1	0	0	0	1	0	0	0	0	0	0	44
23875470	f	md	FR	2024	0	1	0	0	0	1	0	0	0	0	0	0	41
23876145	f	cn	IT	2024	0	0	0	0	0	0	0	0	0	0	0	0	58
23876308	m	md	JP	2024	0	0	0	0	0	0	0	0	0	0	0	0	
23876457	m	ph	US	2024	0	0	0	0	0	0	0	0	0	0	0	0	76
23876646	Not	md	AU	2024	0	1	0	0	1	0	0	0	0	0	0	0	
23876740	m	md	JP	2024	0	1	0	0	1	0	0	0	0	0	0	0	90
23876870	m	md	DE	2024	0	1	1	0	0	0	0	0	0	0	0	0	
23877268	m	md	TH	2024	0	0	0	0	0	0	0	0	0	0	0	0	70
23877375	f	md	FR	2024	1	0	0	0	0	0	0	0	0	0	0	0	
23877659	f	md	FR	2024	0	1	1	0	0	0	0	0	0	0	0	0	47
23877741	Not	md	JP	2024	0	1	0	0	1	0	0	0	0	0	0	0	90
23878226	m	cn	PK	2024	0	1	0	0	0	1	0	0	0	0	0	0	40

23878294	m	md	JP	2024	0	1	0	0	1	0	0	0	0	0	0	0	70
23878604	m	cn	DZ	2024	0	0	0	0	0	0	0	0	0	0	0	0	50
23878628	Not	md	JP	2024	0	0	0	0	0	0	0	0	0	0	0	0	80
23879441	m	hp	CN	2024	0	0	0	0	0	0	0	0	0	0	0	0	53
23879690	Not	md	JP	2024	1	0	0	0	0	0	0	0	0	0	0	0	
23880343	m	hp	US	2024	0	0	0	0	0	0	0	0	0	0	0	0	73
23880415	m	cn	DK	2024	0	1	1	0	0	0	0	0	0	0	0	0	77
23880528	m	md	JP	2024	0	1	0	1	0	0	0	0	0	0	0	0	55
23881155	m	md	FR	2024	0	0	0	0	0	0	0	0	0	0	0	0	65
23881368	m	md	GB	2024	0	1	0	0	0	1	0	0	0	0	0	0	66
23881530	m	hp	FR	2024	0	0	0	0	0	0	0	0	0	0	0	0	53
23882110	m	md	CA	2024	0	1	0	0	1	0	0	0	0	0	0	0	
23882351	m	md	CN	2024	0	0	0	0	0	0	0	0	0	0	0	0	
23882630	Not	hp	US	2024	0	0	0	0	0	0	0	0	0	0	0	0	
23882678	m	md	JP	2024	1	0	0	0	0	0	0	0	0	0	0	0	54
23882713	m	md	JP	2024	0	1	0	1	0	0	0	0	0	0	0	0	59
23883265	m	cn	TH	2024	0	1	0	0	0	1	0	0	0	0	0	0	49
23883539	f	cn	TH	2024	0	1	0	0	0	1	0	0	0	0	0	0	
23883815	m	md	CA	2024	0	1	0	0	1	0	0	0	0	0	0	0	50
23884288	m	hp	JP	2024	0	1	1	0	0	0	0	0	0	0	0	1	76
23884860	f	md	ID	2024	0	1	1	0	0	0	0	0	0	0	0	0	62
23884862	m	md	IT	2024	0	1	0	0	0	1	0	0	0	0	0	0	71
23884885	f	md	IT	2024	0	1	0	0	1	0	0	0	0	0	0	1	
23884907	f	md	JP	2024	0	1	0	0	0	1	0	0	0	0	0	1	61
23885038	m	md	ES	2024	0	1	1	0	0	0	0	0	0	0	0	0	60
23885179	m	md	JP	2024	0	1	0	0	1	0	0	0	0	0	0	0	80
23885233	m	md	JP	2024	0	1	0	0	0	1	0	0	0	0	0	0	73
23885503	m	hp	US	2024	0	1	0	0	1	0	0	0	0	0	0	0	68
23885719	f	md	JP	2024	1	0	0	0	0	0	0	0	0	0	0	0	68
23886036	m	md	JP	2024	0	0	0	0	0	0	0	0	0	0	0	0	
23886129	m	hp	US	2024	0	0	0	0	0	0	0	0	0	0	0	0	71
23886838	f	md	JP	2024	0	1	0	0	1	0	0	0	0	0	0	0	65
23886846	m	md	JP	2024	0	1	0	0	1	0	0	0	0	0	0	0	70
23887190	m	hp	US	2024	0	1	0	0	1	0	0	0	0	0	0	0	68

23887199	Not	hp	GR	2024	0	0	0	0	0	0	0	0	0	0	0	0	
23887234	m	md	FR	2024	0	0	0	0	0	0	0	0	0	0	0	0	53
23887987	m	md	JP	2024	0	1	0	0	1	0	0	0	0	0	0	0	65
23888185	Not	md	GB	2024	0	1	0	0	0	1	0	0	0	0	0	1	
23888301	Not	md	GB	2024	1	0	0	0	0	0	0	0	0	0	0	0	
23888370	f	md	IT	2024	0	0	0	0	0	0	0	0	0	0	0	0	58
23888397	m	md	DE	2024	0	1	0	0	1	0	0	0	0	0	0	0	53
23888556	m	md	ES	2024	0	1	0	0	1	0	0	0	0	0	0	0	67
23888720	Not	md	JP	2024	0	1	1	0	0	0	0	0	0	0	0	0	78
23888996	f	md	KR	2024	0	0	0	0	0	0	0	0	0	0	0	0	
23889174	f	hp	US	2024	0	0	0	0	0	0	0	0	0	0	0	0	84
23889267	f	ph	JP	2024	1	0	0	0	0	0	0	0	0	0	0	0	81
23889496	m	hp	US	2024	0	1	1	0	0	0	0	0	0	0	0	0	79
23889626	m	md	ES	2024	0	0	0	0	0	0	0	0	0	0	0	0	
23889855	m	hp	FR	2024	0	0	0	0	0	0	0	0	0	0	0	0	53
23889881	f	cn	US	2024	0	0	0	0	0	0	0	0	0	0	0	0	71
23890437	f	md	IT	2024	0	0	0	0	0	0	0	0	0	0	0	0	58
23890616	m	Not repo	US	2024	0	0	0	0	0	0	0	0	0	0	0	0	55
23890643	Not	md	PL	2024	0	0	0	0	0	0	0	0	0	0	0	0	
23890867	f	md	FR	2024	0	0	0	0	0	0	0	0	0	0	0	0	
23891055	Not	md	JP	2024	0	1	0	0	1	0	0	0	0	0	0	0	90
23891101	f	md	JP	2024	0	1	1	0	0	0	0	0	0	0	0	0	90
23891342	m	md	CZ	2024	0	0	0	0	0	0	0	0	0	0	0	0	73
23891825	Not	md	GB	2024	0	1	0	0	0	1	0	0	0	0	0	0	
23892634	m	md	JP	2024	0	1	0	1	0	0	0	0	0	0	0	1	73
23892691	m	cn	US	2024	0	0	0	0	0	0	0	0	0	0	0	0	
23892699	m	md	US	2024	0	0	0	0	0	0	0	0	0	0	0	0	71
23892705	Not	hp	US	2024	0	0	0	0	0	0	0	0	0	0	0	0	
23893224	m	md	MX	2024	1	0	0	0	0	0	0	0	0	0	0	0	
23893960	f	md	TR	2024	0	0	0	0	0	0	0	0	0	0	0	0	53
23893971	f	hp	ID	2024	0	1	1	0	0	0	0	0	0	0	0	0	67
23894496	m	ph	JP	2024	0	1	1	0	0	0	0	0	0	0	0	0	49
23894734	f	md	IT	2024	0	0	0	0	0	0	0	0	0	0	0	0	58
23895272	m	hp	CN	2024	0	0	0	0	0	0	0	0	0	0	0	0	62

23895506	m	hp	DE	2024	0	1	0	0	1	0	0	0	0	0	0	0	68
23895940	m	md	NL	2024	0	0	0	0	0	0	0	0	0	0	0	0	
23896004	m	hp	US	2024	0	1	0	0	1	0	0	0	0	0	0	0	68
23896334	f	md	FR	2024	0	0	0	0	0	0	0	0	0	0	0	0	30
23896387	m	cn	EG	2024	0	1	1	0	0	0	0	0	0	0	0	0	
23896416	f	cn	TH	2024	0	1	0	0	0	1	0	0	0	0	0	0	69
23896864	f	md	DE	2024	0	1	0	0	1	0	0	0	0	0	0	0	67
23896970	f	cn	JP	2024	1	0	0	0	0	0	0	0	0	0	0	0	81
23897039	f	cn	JP	2024	0	0	0	0	0	0	0	0	0	0	0	0	56
23897658	m	hp	CN	2024	0	1	1	0	0	0	0	0	0	0	0	0	62
23897750	f	cn	CA	2024	1	0	0	0	0	0	0	0	0	0	0	0	
23897774	m	md	ES	2024	0	1	0	0	1	0	0	0	0	0	0	0	66
23897902	f	md	US	2024	0	0	0	0	0	0	0	0	0	0	0	0	65
23897922	m	md	DE	2024	0	1	1	0	0	0	0	0	0	0	0	0	
23897931	f	md	IT	2024	0	1	0	1	0	0	0	0	0	0	0	0	
23897943	Not	md	GB	2024	0	1	0	1	0	0	0	0	0	0	0	0	
23898202	m	md	FR	2024	0	0	0	0	0	0	0	0	0	0	0	0	71
23898375	f	hp	US	2024	0	0	0	0	0	0	0	0	0	0	0	0	68
23898376	m	cn	PK	2024	0	0	0	0	0	0	0	0	0	0	0	0	63
23898401	f	md	JP	2024	0	1	0	0	0	1	0	0	0	0	0	0	55
23898620	m	md	IN	2024	1	0	0	0	0	0	0	0	0	0	0	0	70
23898661	Not	md	JP	2024	0	0	0	0	0	0	0	0	0	0	0	0	
23898851	Not	hp	CN	2024	0	0	0	0	0	0	0	0	0	0	0	0	
23903108	m	md	JP	2024	0	1	1	0	0	0	0	0	0	0	0	0	70
23903999	m	cn	PK	2024	0	1	0	0	0	1	0	0	0	0	0	0	47
23904012	m	md	IT	2024	0	0	0	0	0	0	0	0	0	0	0	0	62
23904324	f	cn	KR	2024	0	0	0	0	0	0	0	0	0	0	0	0	44
23904663	m	md	JP	2024	0	0	0	0	0	0	0	0	0	0	0	0	75
23904767	Not	md	JP	2024	0	0	0	0	0	0	0	0	0	0	0	0	
23904813	m	md	JP	2024	0	0	0	0	0	0	0	0	0	0	0	0	
23904892	Not	md	JP	2024	0	1	0	0	1	0	0	0	0	0	0	0	90
23905033	m	cn	KE	2024	1	0	0	0	0	0	0	0	0	0	0	0	56.4
23905044	m	md	CN	2024	0	1	1	0	0	0	0	0	0	0	0	0	67
23905733	m	md	JP	2024	0	0	0	0	0	0	0	0	0	0	0	0	67

23905797	f	hp	DE	2024	1	0	0	0	0	0	0	0	0	0	0	1	64
23905916	m	cn	JP	2024	0	0	0	0	0	0	0	0	0	0	0	0	71
23905955	f	md	JP	2024	0	0	0	0	0	0	0	0	0	0	0	0	
23905975	m	md	JP	2024	0	0	0	0	0	0	0	0	0	0	0	0	87
23906039	m	cn	US	2024	1	0	0	0	0	0	0	0	0	0	0	0	
23906315	m	hp	DE	2024	1	0	0	0	0	0	0	0	0	0	0	0	74
23906340	Not	hp	US	2024	1	0	0	0	0	0	0	0	0	0	0	0	
23906341	Not	hp	US	2024	1	0	0	0	0	0	0	0	0	0	0	0	
23906342	Not	hp	US	2024	1	0	0	0	0	0	0	0	0	0	0	0	
23906381	m	hp	CN	2024	0	0	0	0	0	0	0	0	0	0	0	0	66
23906416	f	hp	TH	2024	0	1	0	0	0	1	0	0	0	0	0	0	75
23906961	f	md	JP	2024	0	0	0	0	0	0	0	0	0	0	0	0	50
23907295	f	md	JP	2024	0	1	0	1	0	0	0	0	0	0	0	0	52
23907395	f	md	DE	2024	0	0	0	0	0	0	0	0	0	0	0	0	64
23907557	Not	ph	US	2024	0	0	0	0	0	0	0	0	0	0	0	0	
23907680	f	md	DE	2024	0	0	0	0	0	0	0	0	0	0	0	0	64
23907692	m	md	GB	2024	0	0	0	0	0	0	0	0	0	0	0	0	47
23908084	f	md	TN	2024	0	0	0	0	0	0	0	0	0	0	0	0	56
23908155	f	cn	JP	2024	1	0	0	0	0	0	0	0	0	0	0	0	
23908395	m	hp	US	2024	0	0	0	0	0	0	0	0	0	0	0	0	47
23908413	Not	hp	US	2024	0	0	0	0	0	0	0	0	0	0	0	0	
23909433	m	md	JP	2024	0	0	0	0	0	0	0	0	0	0	0	0	47
23909968	m	md	AU	2024	1	0	0	0	0	0	0	0	0	0	0	0	
23910100	Not	ph	JP	2024	1	0	0	0	0	0	0	0	0	0	0	0	
23911060	m	hp	JP	2024	0	0	0	0	0	0	0	0	0	0	0	0	82
23911072	f	md	DE	2024	0	1	0	0	1	0	0	0	0	0	0	0	69
23911105	m	md	HK	2024	0	0	0	0	0	0	0	0	0	0	0	0	
23911148	f	md	GB	2024	0	1	1	0	0	0	0	0	0	0	0	0	
23911211	m	cn	BR	2024	0	0	0	0	0	0	0	0	0	0	0	0	87
23911329	m	ph	FR	2024	0	0	0	0	0	0	0	0	0	0	0	0	55
23911348	f	md	GR	2024	0	1	0	0	1	0	0	0	0	0	0	0	63
23912204	f	md	DE	2024	0	0	0	0	0	0	0	0	0	0	0	0	64
23912307	f	hp	CN	2024	1	0	0	0	0	0	0	0	0	0	0	0	
23912445	f	hp	DE	2024	1	0	0	0	0	0	0	0	0	0	0	0	58

23912840	Not	md	ES	2024	0	1	0	0	0	1	0	0	0	0	0	0	
23913010	Not	hp	US	2024	0	0	0	0	0	0	0	0	0	0	0	0	
23913138	Not	cn	US	2024	0	0	0	0	0	0	0	0	0	0	0	0	
23913422	m	md	JP	2024	0	1	0	0	0	1	0	0	0	0	0	0	73
23913427	m	md	JP	2024	0	1	0	0	1	0	0	0	0	0	0	0	62
23913478	m	md	JP	2024	0	0	0	0	0	0	0	0	0	0	0	0	73
23913646	m	md	JP	2024	0	1	0	0	0	1	0	0	0	0	0	0	60
23913673	m	md	JP	2024	0	1	0	1	0	0	0	0	0	0	0	0	80
23915281	m	ph	DE	2024	0	1	0	0	1	0	0	0	0	0	0	0	63
23915789	m	hp	DE	2024	1	0	0	0	0	0	0	0	0	0	0	0	71
23916164	m	hp	IT	2024	0	1	1	0	0	0	0	0	0	0	0	0	69
23916223	f	md	JP	2024	0	0	0	0	0	0	0	0	0	0	0	0	50
23916329	m	ph	JP	2024	0	0	0	0	0	0	0	0	0	0	0	0	80
23916525	Not	md	JP	2024	0	0	0	0	0	0	0	0	0	0	0	0	
23916645	f	md	PT	2024	0	0	0	0	0	0	0	0	0	0	0	0	54
23916920	m	md	DE	2024	0	1	0	0	1	0	0	0	0	0	0	0	80
23917119	m	md	DE	2024	0	1	0	0	1	0	0	0	0	0	0	0	74
23917175	m	ph	JP	2024	0	1	1	0	0	0	0	0	0	0	0	0	84
23917351	f	md	DE	2024	0	1	0	0	1	0	0	0	0	0	0	0	69
23917547	f	md	DE	2024	0	0	0	0	0	0	0	0	0	0	0	0	64
23917776	m	hp	CN	2024	0	0	0	0	0	0	0	0	0	0	0	0	63
23918427	m	md	IT	2024	0	0	0	0	0	0	0	0	0	0	0	0	75
23918448	m	md	JP	2024	0	1	0	1	0	0	0	0	0	0	0	0	67
23918854	Not	md	GB	2024	0	1	1	0	0	0	0	0	0	0	0	0	
23919019	Not	md	JP	2024	0	1	0	0	1	0	0	0	0	0	0	1	
23919036	Not	md	IT	2024	0	0	0	0	0	0	0	0	0	0	0	0	
23919379	m	md	ES	2024	0	0	0	0	0	0	0	0	0	0	0	0	
23919387	m	cn	CA	2024	0	0	0	0	0	0	0	0	0	0	0	0	57
23919620	m	md	ES	2024	0	1	0	0	1	0	0	0	0	0	0	0	50
23919900	m	hp	US	2024	0	0	0	0	0	0	0	0	0	0	0	0	68
23920201	f	md	JP	2024	0	1	1	0	0	0	0	0	0	0	0	0	59
23920326	m	md	JP	2024	0	1	0	1	0	0	0	0	0	0	0	0	
23920607	f	md	CA	2024	1	0	0	0	0	0	0	0	0	0	0	0	
23920636	f	ph	JP	2024	0	0	0	0	0	0	0	0	0	0	0	0	

23920850	f	ph	CA	2024	0	0	0	0	0	0	0	0	0	0	0	0	72
23921438	m	hp	JP	2024	1	0	0	0	0	0	0	0	0	0	0	0	45
23921643	f	md	FR	2024	1	0	0	0	0	0	0	0	0	0	0	0	77.7
23921709	m	md	JP	2024	0	1	1	0	0	0	0	0	0	0	0	0	83
23921771	m	cn	CA	2024	0	1	0	0	1	0	0	0	0	0	0	0	70
23921872	m	cn	TW	2024	0	1	0	0	1	0	0	0	0	0	0	0	69
23921925	m	md	DE	2024	0	1	0	0	1	0	0	0	0	0	0	0	74
23921960	m	md	JP	2024	0	1	0	0	1	0	0	0	0	0	0	0	68
23922589	f	md	RO	2024	0	0	0	0	0	0	0	0	0	0	0	0	
23922914	f	md	JP	2024	0	0	0	0	0	0	0	0	0	0	0	0	70
23922919	m	cn	US	2024	0	1	1	0	0	0	0	0	0	0	0	0	60
23923295	m	hp	DE	2024	1	0	0	0	0	0	0	0	0	0	0	0	65
23923326	m	ph	US	2024	0	0	0	0	0	0	0	0	0	0	0	0	
23923526	m	ph	JP	2024	0	1	0	0	0	1	0	0	0	0	0	0	74
23923750	m	md	JP	2024	0	1	0	0	1	0	0	0	0	0	0	1	64
23923931	m	ph	KR	2024	0	1	1	0	0	0	0	0	0	0	0	0	
23924073	Not	md	GB	2024	0	1	1	0	0	0	0	0	0	0	0	0	
23924226	f	hp	US	2024	1	0	0	0	0	0	0	0	0	0	0	0	83
23924386	m	md	CZ	2024	0	0	0	0	0	0	0	0	0	0	0	0	73
23924698	f	md	DE	2024	0	0	0	0	0	0	0	0	0	0	0	0	64
23924819	m	md	US	2024	0	0	0	0	0	0	0	0	0	0	0	0	39
23924927	f	md	ES	2024	0	1	0	0	0	1	0	0	0	0	0	0	63
23925490	Not	md	NL	2024	0	1	0	1	0	0	0	0	0	0	0	0	
23927465	Not	hp	US	2024	0	0	0	0	0	0	0	0	0	0	0	0	
23927469	Not	hp	US	2024	0	0	0	0	0	0	0	0	0	0	0	0	
23927470	Not	hp	US	2024	0	0	0	0	0	0	0	0	0	0	0	0	
23927571	m	hp	CN	2024	0	1	1	0	0	0	0	0	0	0	0	0	74
23927795	m	cn	TH	2024	0	1	0	0	0	1	0	0	0	0	0	0	
23927799	m	cn	IN	2024	0	1	0	0	0	1	0	0	0	0	0	0	71
23927808	m	cn	IN	2024	0	1	0	0	0	1	0	0	0	0	0	0	64
23929094	f	md	PT	2024	0	0	0	0	0	0	0	0	0	0	0	0	42
23929995	f	hp	GB	2024	0	0	0	0	0	0	0	0	0	0	0	0	49
23930157	f	hp	US	2024	0	0	0	0	0	0	0	0	0	0	0	0	
23930160	f	ph	US	2024	1	0	0	0	0	0	0	0	0	0	0	0	

23930384	m	md	JP	2024	0	1	0	0	1	0	0	0	0	0	0	1	71
23930397	m	md	IL	2024	0	1	1	0	0	0	0	0	0	0	0	0	77
23930690	Not	hp	CN	2024	0	0	0	0	0	0	0	0	0	0	0	0	
23931728	m	md	JP	2024	0	1	0	0	0	1	0	0	0	0	0	0	70
23931832	m	md	JP	2024	0	1	0	0	1	0	0	0	0	0	0	0	73
23931833	Not	md	CA	2024	0	1	0	0	1	0	0	0	0	0	0	0	
23932217	m	cn	CA	2024	0	1	0	0	1	0	0	0	0	0	0	0	70
23932361	f	cn	IN	2024	0	0	0	0	0	0	0	0	0	0	0	0	39
23932424	m	hp	US	2024	0	1	0	0	1	0	0	0	0	0	0	0	68
23932560	f	cn	PK	2024	0	1	0	0	0	1	0	0	0	0	0	0	81
23932611	Not	md	CA	2024	0	1	0	0	1	0	0	0	0	0	0	0	
23932733	m	cn	PK	2024	0	1	0	0	0	1	0	0	0	0	0	0	70
23932931	f	cn	US	2024	0	0	0	0	0	0	0	0	0	0	0	0	
23932932	m	md	ES	2024	0	0	0	0	0	0	0	0	0	0	0	0	
23932963	f	hp	US	2024	1	0	0	0	0	0	0	0	0	0	0	0	69
23933720	m	md	JP	2024	0	1	1	0	0	0	0	0	0	0	0	0	49
23933799	f	hp	US	2024	1	0	0	0	0	0	0	0	0	0	0	0	64
23933872	f	md	JP	2024	1	0	0	0	0	0	0	0	0	0	0	0	80
23934428	Not	hp	US	2024	1	0	0	0	0	0	0	0	0	0	0	0	
23934546	f	md	DK	2024	0	0	0	0	0	0	0	0	0	0	0	0	
23934590	m	md	JP	2024	0	1	1	0	0	0	0	0	0	0	0	0	68
23934692	m	md	JP	2024	0	0	0	0	0	0	0	0	0	0	0	0	75
23935519	Not	cn	CA	2024	0	1	0	0	1	0	0	0	0	0	0	0	60
23935693	f	cn	CA	2024	0	0	0	0	0	0	0	0	0	0	0	0	
23935985	m	cn	JP	2024	0	0	0	0	0	0	0	0	0	0	0	0	68
23936204	m	md	JP	2024	0	0	0	0	0	0	0	0	0	0	0	0	78
23936460	f	md	DE	2024	0	0	0	0	0	0	0	0	0	0	0	0	79
23936632	m	md	US	2024	0	0	0	0	0	0	0	0	0	0	0	0	39
23937150	f	hp	US	2024	0	0	0	0	0	0	0	0	0	0	0	0	37
23937226	f	md	RU	2024	1	0	0	0	0	0	0	0	0	0	0	0	68
23937229	Not	cn	JP	2024	1	0	0	0	0	0	0	0	0	0	0	0	78
23937320	m	md	DE	2024	0	1	1	0	0	0	0	0	0	0	0	0	79
23937378	m	hp	IE	2024	1	0	0	0	0	0	0	0	0	0	0	0	
23938045	m	md	JP	2024	0	1	0	1	0	0	0	0	0	0	0	1	70

23938059	Not	md	US	2024	0	0	0	0	0	0	0	0	0	0	0	0	
23938238	f	cn	ES	2024	0	0	0	0	0	0	0	0	0	0	0	0	60
23938588	Not	hp	US	2024	1	0	0	0	0	0	0	0	0	0	0	0	
23938598	m	md	US	2024	0	0	0	0	0	0	0	0	0	0	0	0	
23938742	m	md	TH	2024	0	0	0	0	0	0	0	0	0	0	0	0	79
23938917	Not	cn	GB	2024	0	1	1	0	0	0	0	0	0	0	0	0	
23938958	m	md	JP	2024	0	1	0	0	1	0	0	0	0	0	0	1	61
23939161	m	md	JP	2024	0	1	0	0	1	0	0	0	0	0	0	0	79
23939598	m	md	ES	2024	0	1	0	0	0	1	0	0	0	0	0	0	67
23939775	Not	cn	US	2024	0	0	0	0	0	0	0	0	0	0	0	0	59
23939841	m	md	TR	2024	0	1	0	0	0	1	0	0	0	0	0	0	60
23939998	Not	hp	US	2024	0	0	0	0	0	0	0	0	0	0	0	0	
23940006	m	cn	US	2024	0	0	0	0	0	0	0	0	0	0	0	0	65
23940034	Not	md	JP	2024	0	1	0	0	1	0	0	0	0	0	0	0	
23940035	Not	md	JP	2024	0	1	0	0	1	0	0	0	0	0	0	0	
23940063	Not	md	JP	2024	0	1	0	0	1	0	0	0	0	0	0	0	
23940126	m	hp	CA	2024	0	0	0	0	0	0	0	0	0	0	0	0	71
23940149	Not	md	GB	2024	0	1	1	0	0	0	0	0	0	0	0	0	
23940184	f	md	US	2024	0	1	1	0	0	0	0	0	0	0	0	0	54
23940216	f	cn	PH	2024	0	0	0	0	0	0	0	0	0	0	0	0	72
23940258	Not	md	JP	2024	0	1	0	0	1	0	0	0	0	0	0	0	
23940334	Not	md	US	2024	0	0	0	0	0	0	0	0	0	0	0	0	
23940406	Not	md	JP	2024	0	1	0	0	1	0	0	0	0	0	0	0	
23940513	Not	md	JP	2024	0	1	0	0	1	0	0	0	0	0	0	0	
23940521	m	md	CA	2024	0	1	1	0	0	0	0	0	0	0	0	0	76
23940717	f	hp	RU	2024	0	1	0	0	0	1	0	0	0	0	0	0	64
23940985	Not	md	CA	2024	0	1	0	0	1	0	0	0	0	0	0	0	
23941041	f	md	CA	2024	0	1	1	0	0	0	0	0	0	0	0	0	76
23941109	f	md	JP	2024	0	1	0	0	0	1	0	0	0	0	0	0	80
23941165	Not	md	JP	2024	0	0	0	0	0	0	0	0	0	0	0	0	
23941213	m	md	CA	2024	0	1	1	0	0	0	0	0	0	0	0	0	84
23941409	f	md	CA	2024	0	1	1	0	0	0	0	0	0	0	0	0	77
23941488	m	md	JP	2024	0	1	1	0	0	0	0	0	0	0	0	0	90
23941556	m	md	CA	2024	0	1	1	0	0	0	0	0	0	0	0	0	76

23941778	f	cn	CA	2024	0	0	0	0	0	0	0	0	0	0	0	0	63
23941864	m	md	FR	2024	0	1	1	0	0	0	0	0	0	0	0	0	
23942161	f	md	JP	2024	1	0	0	0	0	0	0	0	0	0	0	1	80
23942472	m	cn	TH	2024	0	1	0	0	0	1	0	0	0	0	0	0	
23942641	f	cn	BE	2024	0	0	0	0	0	0	0	0	0	0	0	0	
23943229	m	md	JP	2024	0	0	0	0	0	0	0	0	0	0	0	0	
23943356	m	md	HR	2024	0	1	1	0	0	0	0	0	0	0	0	0	54
23943357	Not	md	JP	2024	0	1	0	0	1	0	0	0	0	0	0	0	
23943366	f	hp	AU	2024	0	1	1	0	0	0	0	0	0	0	0	0	72
23943469	Not	md	JP	2024	0	1	0	0	1	0	0	0	0	0	0	0	
23943832	Not	md	JP	2024	0	1	0	0	1	0	0	0	0	0	0	0	
23944335	m	md	JP	2024	0	1	0	0	1	0	0	0	0	0	0	1	78
23944627	m	md	JP	2024	0	1	0	0	1	0	0	0	0	0	0	0	80
23944826	f	md	JP	2024	0	1	0	0	0	1	0	0	0	0	0	0	45
23945814	m	md	ES	2024	0	0	0	0	0	0	0	0	0	0	0	0	64
23946160	m	cn	US	2024	0	0	0	0	0	0	0	0	0	0	0	0	
23946341	f	md	DE	2024	0	0	0	0	0	0	0	0	0	0	0	0	
23946362	m	md	JP	2024	0	1	0	0	0	1	0	0	0	0	0	1	80
23946380	f	cn	TH	2024	0	1	0	0	0	1	0	0	0	0	0	0	
23946637	m	md	JP	2024	0	0	0	0	0	0	0	0	0	0	0	0	90
23946808	f	md	RU	2024	0	0	0	0	0	0	0	0	0	0	0	0	60
23946910	m	hp	RU	2024	0	1	0	0	0	1	0	0	0	0	0	0	64
23947003	m	md	CZ	2024	0	0	0	0	0	0	0	0	0	0	0	0	73
23947069	f	md	JP	2024	1	0	0	0	0	0	0	0	0	0	0	0	46
23947436	m	md	JP	2024	0	1	1	0	0	0	0	0	0	0	0	0	69
23948056	Not	md	CA	2024	0	1	0	0	1	0	0	0	0	0	0	0	70
23948442	Not	md	JP	2024	0	0	0	0	0	0	0	0	0	0	0	0	
23948498	f	md	JP	2024	0	1	0	0	1	0	0	0	0	0	0	0	
23948923	m	md	ES	2024	0	1	1	0	0	0	0	0	0	0	0	1	78
23949185	f	cn	PK	2024	0	1	0	0	0	1	0	0	0	0	0	0	64
23949513	m	md	JP	2024	0	1	0	1	0	0	0	0	0	0	0	0	70
23949992	f	cn	US	2024	1	0	0	0	0	0	0	0	0	0	0	0	78.5
23950075	f	md	GB	2024	0	1	1	0	0	0	0	0	0	0	0	0	
23950792	f	md	JP	2024	1	0	0	0	0	0	0	0	0	0	0	0	60

23950860	f	md	HR	2024	0	1	1	0	0	0	0	0	0	0	0	0	63
23951119	m	md	DE	2024	0	1	1	0	0	0	0	0	0	0	0	0	79
23951356	f	md	FR	2024	0	0	0	0	0	0	0	0	0	0	0	0	53
23951427	f	md	US	2024	1	0	0	0	0	0	0	0	0	0	0	0	
23951485	m	hp	GB	2024	1	0	0	0	0	0	0	0	0	0	0	0	68
23951624	m	cn	US	2024	1	0	0	0	0	0	0	0	0	0	0	0	
23951708	f	md	GR	2024	0	0	0	0	0	0	0	0	0	0	0	0	74
23951781	f	md	DE	2024	0	1	0	0	1	0	0	0	0	0	0	0	56
23952224	Not	md	JP	2024	1	0	0	0	0	0	0	0	0	0	0	0	
23952303	f	hp	CN	2024	1	0	0	0	0	0	0	0	0	0	0	0	65
23952506	Not	md	US	2024	0	0	0	0	0	0	0	0	0	0	0	0	
23952744	f	hp	NL	2024	0	0	0	0	0	0	0	0	0	0	0	0	63
23952847	Not	md	JP	2024	0	0	0	0	0	0	0	0	0	0	0	0	80
23952924	m	md	JP	2024	0	1	0	0	0	1	0	0	0	0	0	0	50
23952951	Not	hp	CN	2024	0	0	0	0	0	0	0	0	0	0	0	0	
23953411	Not	ph	JP	2024	0	0	0	0	0	0	0	0	0	0	0	0	
23953650	f	hp	US	2024	0	0	0	0	0	0	0	0	0	0	0	0	37
23954256	f	md	US	2024	0	1	0	0	0	1	0	0	0	0	0	0	
23954287	m	cn	US	2024	0	1	0	0	0	1	0	0	0	0	0	0	
23954288	f	hp	US	2024	0	1	0	0	0	1	0	0	0	0	0	1	
23954311	f	cn	US	2024	0	1	0	0	0	1	0	0	0	0	0	0	81
23954312	m	cn	US	2024	0	1	0	0	0	1	0	0	0	0	0	0	
23954320	m	cn	US	2024	0	1	0	0	0	1	0	0	0	0	0	0	
23954338	f	md	US	2024	0	1	0	0	0	1	0	0	0	0	0	1	
23954339	m	md	US	2024	0	1	0	0	0	1	0	0	0	0	0	1	
23954344	Not	md	US	2024	0	1	0	0	0	1	0	0	0	0	0	0	
23954365	f	hp	US	2024	0	1	0	0	0	1	0	0	0	0	0	0	
23954370	f	md	US	2024	0	1	0	0	0	1	0	0	0	0	0	0	
23954386	Not	md	US	2024	0	1	0	0	0	1	0	0	0	0	0	0	
23954573	m	md	US	2024	0	1	0	0	0	1	0	0	0	0	0	0	81
23954771	f	cn	TH	2024	0	1	0	0	0	1	0	0	0	0	0	0	62
23954774	f	cn	TH	2024	0	1	0	0	0	1	0	0	0	0	0	0	62
23954948	f	md	JP	2024	0	0	0	0	0	0	0	0	0	0	0	0	80
23955010	m	cn	IN	2024	0	0	0	0	0	0	0	0	0	0	0	0	41

23955545	f	md	US	2024	0	0	0	0	0	0	0	0	0	0	0	0	37
23955569	f	md	TW	2024	0	1	0	0	1	0	0	0	0	0	0	0	77
23955968	m	hp	US	2024	0	0	0	0	0	0	0	0	0	0	0	0	89
23956096	m	ph	US	2024	0	1	1	0	0	0	0	0	0	0	0	0	78
23956251	Not	md	DE	2024	0	0	0	0	0	0	0	0	0	0	0	0	
23956522	m	md	DE	2024	0	1	1	0	0	0	0	0	0	0	0	0	79
23956676	m	md	JP	2024	0	1	1	0	0	0	0	0	0	0	0	0	50
23956876	m	md	JP	2024	0	1	0	0	1	0	0	0	0	0	0	0	78
23957155	m	md	IT	2024	0	1	0	0	1	0	0	0	0	0	0	0	74
23957158	m	md	DE	2024	0	1	1	0	0	0	0	0	0	0	0	0	79
23957492	f	cn	JP	2024	0	0	0	0	0	0	0	0	0	0	0	0	55
23957508	m	md	JP	2024	0	0	0	0	0	0	0	0	0	0	0	0	85
23957535	f	hp	US	2024	1	0	0	0	0	0	0	0	0	0	0	0	57
23957539	m	hp	US	2024	1	0	0	0	0	0	0	0	0	0	0	0	82
23957540	m	hp	US	2024	1	0	0	0	0	0	0	0	0	0	0	0	47
23957541	f	hp	US	2024	1	0	0	0	0	0	0	0	0	0	0	0	66
23957631	f	md	FR	2024	0	0	0	0	0	0	0	0	0	0	0	0	
23957727	f	md	PL	2024	0	0	0	0	0	0	0	0	0	0	0	0	63
23957834	f	md	IT	2024	0	1	1	0	0	0	0	0	0	0	0	0	62
23957925	Not	md	US	2024	0	0	0	0	0	0	0	0	0	0	0	0	
23957928	m	ph	US	2024	0	0	0	0	0	0	0	0	0	0	0	0	
23958287	f	md	DE	2024	0	1	0	0	1	0	0	0	0	0	0	0	78
23958298	m	md	DE	2024	0	1	1	0	0	0	0	0	0	0	0	0	60
23958508	f	cn	US	2024	0	1	1	0	0	0	0	0	0	0	0	0	65
23958736	m	md	JP	2024	0	1	1	0	0	0	0	0	0	0	0	0	80
23959006	m	md	VN	2024	0	1	0	0	0	1	0	0	0	0	0	0	65
23959191	m	md	US	2024	1	0	0	0	0	0	0	0	0	0	0	0	47
23959522	f	hp	US	2024	1	0	0	0	0	0	0	0	0	0	0	0	66
23959548	f	ph	JP	2024	0	1	0	0	0	1	0	0	0	0	0	0	70
23959556	m	hp	US	2024	1	0	0	0	0	0	0	0	0	0	0	0	82
23959614	f	hp	US	2024	1	0	0	0	0	0	0	0	0	0	0	0	57
23959697	m	hp	CN	2024	0	0	0	0	0	0	0	0	0	0	0	0	68
23959954	m	hp	US	2024	1	0	0	0	0	0	0	0	0	0	0	0	47
23960002	f	hp	IT	2024	0	1	0	1	0	0	0	0	0	0	0	0	71

23960076	f	md	JP	2024	0	1	0	1	0	0	0	0	0	0	0	0	78
23960337	m	md	VN	2024	0	1	0	0	0	1	0	0	0	0	0	0	49
23960436	Not	md	US	2024	0	0	0	0	0	0	0	0	0	0	0	0	
23960515	f	md	IT	2024	0	1	0	0	1	0	0	0	0	0	0	0	64
23960539	m	md	RU	2024	1	0	0	0	0	0	0	0	0	0	0	0	
23960665	f	md	IT	2024	0	1	1	0	0	0	0	0	0	0	0	0	62
23960689	m	md	AE	2024	0	0	0	0	0	0	0	0	0	0	0	0	
23960707	f	md	FR	2024	0	0	0	0	0	0	0	0	0	0	0	0	
23960935	m	md	SI	2024	0	1	1	0	0	0	0	0	0	0	0	0	71
23961241	f	md	US	2024	1	0	0	0	0	0	0	0	0	0	0	0	66
23961243	m	md	US	2024	1	0	0	0	0	0	0	0	0	0	0	0	82
23961352	f	md	KR	2024	0	0	0	0	0	0	0	0	0	0	0	0	
23961690	f	cn	JP	2024	0	0	0	0	0	0	0	0	0	0	0	0	75
23961802	m	md	FR	2024	0	1	1	0	0	0	0	0	0	0	0	0	60
23962073	m	hp	DE	2024	0	1	0	0	1	0	0	0	0	0	0	0	79
23962371	f	cn	CA	2024	0	0	0	0	0	0	0	0	0	0	0	0	52
23962503	f	cn	US	2024	0	0	0	0	0	0	0	0	0	0	0	0	
23962652	m	hp	FR	2024	0	1	1	0	0	0	0	0	0	0	0	0	65
23963329	f	md	RU	2024	0	0	0	0	0	0	0	0	0	0	0	0	42
23963463	f	md	JP	2024	1	0	0	0	0	0	0	0	0	0	0	0	86
23963630	f	lw	CA	2024	0	0	0	0	0	0	0	0	0	0	0	0	49
23964354	Not	hp	CN	2024	0	0	0	0	0	0	0	0	0	0	0	0	
23964411	m	cn	US	2024	0	0	0	0	0	0	0	0	0	0	0	0	63
23964642	m	md	US	2024	0	0	0	0	0	0	0	0	0	0	0	0	86
23964706	f	hp	US	2024	0	0	0	0	0	0	0	0	0	0	0	0	37
23965394	f	cn	US	2024	0	0	0	0	0	0	0	0	0	0	0	0	87
23965486	Not	md	JP	2024	0	1	0	0	1	0	0	0	0	0	0	0	
23965518	f	md	ES	2024	0	1	1	0	0	0	0	0	0	0	0	0	38
23965577	f	ph	JP	2024	0	1	0	1	0	0	0	0	0	0	0	0	45
23966107	f	md	FR	2024	0	1	0	0	0	1	0	0	0	0	0	0	63
23966191	Not	hp	FR	2024	0	1	0	0	0	1	0	0	0	0	0	0	
23966214	f	md	DE	2024	0	0	0	0	0	0	0	0	0	0	0	0	
23966553	f	md	ES	2024	0	1	1	0	0	0	0	0	0	0	0	0	38
23967035	m	md	JP	2024	0	1	1	0	0	0	0	0	0	0	0	0	60

23967257	Not	md	JP	2024	1	0	0	0	0	0	0	0	0	0	0	0	75
23968314	f	hp	JP	2024	1	0	0	0	0	0	0	0	0	0	0	0	81
23968487	m	hp	CN	2024	0	1	0	0	1	0	0	0	0	0	0	0	66
23969162	f	cn	US	2024	0	1	1	0	0	0	0	0	0	0	0	0	73
23969257	f	md	CA	2024	0	0	0	0	0	0	0	0	0	0	0	0	64
23969324	m	md	DE	2024	0	1	0	0	1	0	0	0	0	0	0	0	53
23969779	f	md	US	2024	1	0	0	0	0	0	0	0	0	0	0	0	57
23969818	m	md	DE	2024	0	1	0	0	1	0	0	0	0	0	0	0	53
23970063	Not	md	JP	2024	0	1	0	1	0	0	0	0	0	0	0	0	
23970274	f	cn	JP	2024	1	0	0	0	0	0	0	0	0	0	0	0	
23970369	m	md	IT	2024	0	1	0	0	1	0	0	0	0	0	0	0	74
23970739	f	hp	US	2024	0	0	0	0	0	0	0	0	0	0	0	0	62
23971263	Not	md	JP	2024	0	0	0	0	0	0	0	0	0	0	0	0	
23971641	Not	cn	US	2024	0	0	0	0	0	0	0	0	0	0	0	0	
23971688	m	md	DE	2024	0	1	0	0	1	0	0	0	0	0	0	0	53
23971904	m	md	ES	2024	0	1	1	0	0	0	0	0	0	0	0	1	71
23972109	m	md	US	2024	0	0	0	0	0	0	0	0	0	0	0	0	
23972226	m	md	JP	2024	0	1	1	0	0	0	0	0	0	0	0	1	80
23973119	Not	md	JP	2024	0	0	0	0	0	0	0	0	0	0	0	0	
23973151	m	ph	CN	2024	0	0	0	0	0	0	0	0	0	0	0	0	25
23973332	m	md	DE	2024	0	1	1	0	0	0	0	0	0	0	0	0	60
23973812	f	md	PE	2024	0	1	1	0	0	0	0	0	0	0	0	0	70
23974744	f	md	US	2024	0	1	0	0	0	1	0	0	0	0	0	0	37
23975457	Not	cn	US	2024	0	0	0	0	0	0	0	0	0	0	0	0	
23975480	f	cn	US	2024	1	0	0	0	0	0	0	0	0	0	0	0	
23975863	f	hp	US	2024	1	0	0	0	0	0	0	0	0	0	0	0	75
23975873	f	hp	US	2024	1	0	0	0	0	0	0	0	0	0	0	0	52
23975924	f	md	IT	2024	0	1	1	0	0	0	0	0	0	0	0	0	62
23975939	f	md	FR	2024	0	0	0	0	0	0	0	0	0	0	0	0	
23976764	m	ph	FR	2024	0	0	0	0	0	0	0	0	0	0	0	0	
23977117	Not	cn	US	2024	0	0	0	0	0	0	0	0	0	0	0	0	
23977178	Not	hp	US	2024	0	0	0	0	0	0	0	0	0	0	0	0	
23977346	f	md	FR	2024	0	0	0	0	0	0	0	0	0	0	0	0	
23977598	m	md	DE	2024	0	1	0	0	1	0	0	0	0	0	0	0	50

23977634	Not	cn	US	2024	0	0	0	0	0	0	0	0	0	0	0	0	
23978431	f	md	JP	2024	1	0	0	0	0	0	0	0	0	0	0	0	79
23978467	m	md	JP	2024	0	1	0	1	0	0	0	0	0	0	0	0	55
23978474	f	md	ES	2024	1	0	0	0	0	0	0	0	0	0	0	0	71
23979024	m	md	DE	2024	0	1	1	0	0	0	0	0	0	0	0	0	60
23980820	f	md	FR	2024	0	0	0	0	0	0	0	0	0	0	0	0	
23981015	f	md	FR	2024	0	1	0	0	0	1	0	0	0	0	0	0	63
23981187	m	md	ES	2024	0	1	1	0	0	0	0	0	0	0	0	1	78
23981353	f	md	CA	2024	0	0	0	0	0	0	0	0	0	0	0	0	
23981500	m	hp	CA	2024	0	0	0	0	0	0	0	0	0	0	0	0	36
23981931	f	md	JP	2024	0	1	0	0	1	0	0	0	0	0	0	0	73
23982444	f	md	JP	2024	0	1	0	1	0	0	0	0	0	0	0	0	69
23983134	m	md	DE	2024	0	1	0	0	1	0	0	0	0	0	0	0	85
23983297	f	md	BE	2024	0	1	1	0	0	0	0	0	0	0	0	0	69
23984915	m	hp	AU	2024	0	1	1	0	0	0	0	0	0	0	0	0	64
23985156	f	hp	JP	2024	0	0	0	0	0	0	0	0	0	0	0	0	57
23985330	m	md	HR	2024	0	1	1	0	0	0	0	0	0	0	0	0	54
23985742	f	cn	FR	2024	0	0	0	0	0	0	0	0	0	0	0	0	
23985967	m	hp	JP	2024	0	0	0	0	0	0	0	0	0	0	0	0	67
23986228	m	cn	JP	2024	1	0	0	0	0	0	0	0	0	0	0	0	
23986276	Not	md	JP	2024	0	0	0	0	0	0	0	0	0	0	0	0	
23986428	m	md	JP	2024	0	1	0	1	0	0	0	0	0	0	0	0	67
23986971	f	md	FR	2024	0	1	1	0	0	0	0	0	0	0	0	0	47
23987195	m	md	JP	2024	0	1	1	0	0	0	0	0	0	0	0	1	70
23987256	f	md	FR	2024	0	0	0	0	0	0	0	0	0	0	0	0	47
23987549	m	md	AT	2024	0	1	1	0	0	0	0	0	0	0	0	1	65
23988583	m	md	JP	2024	0	1	1	0	0	0	0	0	0	0	0	0	60
23989461	f	cn	US	2024	1	0	0	0	0	0	0	0	0	0	0	0	55.6
23989875	f	md	CA	2024	0	0	0	0	0	0	0	0	0	0	0	0	
23990031	f	cn	KE	2024	1	0	0	0	0	0	0	0	0	0	0	0	29.8
23990258	m	cn	US	2024	0	1	1	0	0	0	0	0	0	0	0	0	
23990627	m	md	JP	2024	0	1	0	0	1	0	0	0	0	0	0	0	70
23990974	m	cn	US	2024	0	0	0	0	0	0	0	0	0	0	0	0	
23991115	f	cn	US	2024	1	0	0	0	0	0	0	0	0	0	0	0	

23991330	m	md	IT	2024	0	1	0	0	1	0	0	0	0	0	0	0	74
23991519	m	cn	EG	2024	0	1	0	1	0	0	0	0	0	0	0	0	
23991819	m	cn	TW	2024	0	1	0	0	1	0	0	0	0	0	0	0	60
23992328	m	md	BE	2024	0	1	0	0	0	1	0	0	0	0	0	0	77
23992809	f	hp	IT	2024	0	1	0	1	0	0	0	0	0	0	0	0	71
23992822	m	hp	US	2024	0	0	0	0	0	0	0	0	0	0	0	0	
23992876	f	md	FR	2024	0	0	0	0	0	0	0	0	0	0	0	0	
23992960	f	ph	JP	2024	1	0	0	0	0	0	0	0	0	0	0	0	85
23993326	f	md	DE	2024	0	1	0	0	1	0	0	0	0	0	0	0	56
23994312	f	md	US	2024	0	0	0	0	0	0	0	0	0	0	0	0	57
23994327	m	md	US	2024	0	0	0	0	0	0	0	0	0	0	0	0	33
23994380	f	hp	KR	2024	0	0	0	0	0	0	0	0	0	0	0	0	61
23994413	m	hp	US	2024	1	0	0	0	0	0	0	0	0	0	0	0	74
23994442	m	md	TW	2024	1	0	0	0	0	0	0	0	0	0	0	0	65
23994715	m	md	JP	2024	0	1	0	1	0	0	0	0	0	0	0	0	
23994823	m	md	DE	2024	0	0	0	0	0	0	0	0	0	0	0	0	61
23994871	f	md	BE	2024	0	0	0	0	0	0	0	0	0	0	0	0	47
23995505	m	md	JP	2024	0	1	0	1	0	0	0	0	0	0	0	0	71
23995902	f	cn	US	2024	1	0	0	0	0	0	0	0	0	0	0	0	
23996149	m	md	JP	2024	0	1	1	0	0	0	0	0	0	0	0	0	80
23996167	f	hp	ES	2024	0	0	0	0	0	0	1	0	0	1	0	0	69
23996459	m	md	BE	2024	0	1	0	0	0	1	0	0	0	0	0	0	77
23996638	m	md	JP	2024	0	1	1	0	0	0	0	0	0	0	0	0	80
23996721	Not	md	VN	2024	0	0	0	0	0	0	0	0	0	0	0	0	
23997170	f	md	DE	2024	0	1	0	0	1	0	0	0	0	0	0	0	71
23997198	f	md	IT	2024	0	1	1	0	0	0	0	0	0	0	0	0	71
23997741	f	hp	US	2024	0	0	0	0	0	0	0	0	0	0	0	0	
23998046	Not	md	JP	2024	1	0	0	0	0	0	0	0	0	0	0	0	
23998300	m	md	JP	2024	0	1	0	1	0	0	0	0	0	0	0	0	74
23998685	m	md	DE	2024	0	1	0	0	1	0	0	0	0	0	0	0	72
23999080	f	md	FR	2024	0	1	1	0	0	0	0	0	0	0	0	0	
23999239	f	hp	IT	2024	0	0	0	0	0	0	0	0	0	0	0	0	61
23999609	f	md	JP	2024	0	0	0	0	0	0	0	0	0	0	0	0	80
23999807	Not	md	JP	2024	1	0	0	0	0	0	0	0	0	0	0	0	

23999892	m	md	DE	2024	0	0	0	0	0	0	0	0	0	0	0	0	
24000013	f	cn	TH	2024	1	0	0	0	0	0	0	0	0	0	0	0	50.2
24000442	f	hp	CN	2024	1	0	0	0	0	0	0	0	0	0	0	0	57
24000565	f	md	RU	2024	1	0	0	0	0	0	0	0	0	0	0	0	38.8
24000666	m	md	JP	2024	0	0	0	0	0	0	0	0	0	0	0	0	
24000766	m	cn	CZ	2024	0	0	0	0	0	0	0	0	0	0	0	0	
24000776	m	md	US	2024	0	1	0	0	1	0	0	0	0	0	0	0	84
24000953	f	md	GR	2024	0	0	0	0	0	0	0	0	0	0	0	0	74
24001144	Not	md	JP	2024	1	0	0	0	0	0	0	0	0	0	0	0	
24001303	Not	md	JP	2024	1	0	0	0	0	0	0	0	0	0	0	0	
24001426	m	cn	JP	2024	1	0	0	0	0	0	0	0	0	0	0	0	78
24001997	f	hp	US	2024	0	1	1	0	0	0	0	0	0	0	0	0	77
24002184	Not	md	JP	2024	0	0	0	0	0	0	0	0	0	0	0	0	
24002229	f	hp	CH	2024	1	0	0	0	0	0	0	0	0	0	0	0	70
24002488	f	hp	AT	2024	0	0	0	0	0	0	0	0	0	0	0	0	69
24002550	Not	md	JP	2024	0	1	0	0	1	0	0	0	0	0	0	0	
24002558	Not	cn	US	2024	0	0	0	0	0	0	0	0	0	0	0	0	
24002830	m	md	JP	2024	0	1	0	0	1	0	0	0	0	0	0	0	64
24002918	f	md	JP	2024	0	0	0	0	0	0	0	0	0	0	0	0	91
24003346	f	hp	US	2024	0	0	0	0	0	0	1	1	0	0	0	0	75
24003407	f	cn	US	2024	0	0	0	0	0	0	0	0	0	0	0	0	
24003499	m	md	ES	2024	0	1	1	0	0	0	0	0	0	0	0	1	78
24003716	f	md	AT	2024	0	1	0	0	0	1	0	0	0	0	0	0	70
24003813	m	md	BE	2024	0	1	0	0	0	1	0	0	0	0	0	0	77
24003894	Not	md	JP	2024	0	0	0	0	0	0	0	0	0	0	0	0	90
24003908	f	md	JP	2024	0	0	0	0	0	0	0	0	0	0	0	0	70
24004190	m	md	CN	2024	0	0	0	0	0	0	0	0	0	0	0	0	67
24004367	m	md	CN	2024	0	0	0	0	0	0	0	0	0	0	0	0	81
24005110	f	hp	US	2024	0	0	0	0	0	0	0	0	0	0	0	0	53
24005118	Not	md	JP	2024	0	1	0	0	0	1	0	0	0	0	0	0	80
24005440	m	md	JP	2024	0	1	0	1	0	0	0	0	0	0	0	0	60
24005525	f	md	CA	2024	1	0	0	0	0	0	0	0	0	0	0	0	
24005606	f	cn	BE	2024	0	1	1	0	0	0	0	0	0	0	0	0	69
24005743	f	md	US	2024	0	0	0	0	0	0	0	0	0	0	0	0	64

24006181	m	md	JP	2024	0	1	0	0	1	0	0	0	0	0	0	0	80
24006437	f	md	FR	2024	0	1	1	0	0	0	0	0	0	0	0	0	
24006692	f	md	JP	2024	0	1	1	0	0	0	0	0	0	0	0	1	68
24006812	f	hp	US	2024	0	1	1	0	0	0	0	0	0	0	0	0	70
24007705	f	ph	US	2024	1	0	0	0	0	0	0	0	0	0	0	0	61.4
24007884	Not	md	JP	2024	0	1	0	0	0	1	0	0	0	0	0	0	80
24007987	m	md	DE	2024	0	0	0	0	0	0	0	0	0	0	0	0	
24008072	m	hp	ES	2024	0	1	0	0	1	0	0	0	0	0	0	0	67
24008224	f	md	US	2024	0	1	1	0	0	0	0	0	0	0	0	0	74
24008375	Not	cn	CN	2024	0	0	0	0	0	0	0	0	0	0	0	0	
24008963	m	cn	AU	2024	0	0	0	0	0	0	0	0	0	0	0	0	
24009017	f	cn	US	2024	0	0	0	0	0	0	0	0	0	0	0	0	
24009040	m	md	IT	2024	0	1	0	0	1	0	0	0	0	0	0	0	74
24009376	m	hp	AT	2024	0	0	0	0	0	0	0	0	0	0	0	0	33
24009551	m	md	GE	2024	0	1	0	0	0	1	0	0	0	0	0	0	68
24009554	m	hp	US	2024	0	1	0	1	0	0	0	0	0	0	0	0	65
24009606	f	cn	AU	2024	0	0	0	0	0	0	0	0	0	0	0	0	
24009834	m	md	JP	2024	0	0	0	0	0	0	0	0	0	0	0	0	
24009938	m	md	JP	2024	0	1	1	0	0	0	0	0	0	0	0	0	90
24009977	m	md	JP	2024	0	0	0	0	0	0	0	0	0	0	0	0	
24010403	m	md	AT	2024	0	0	0	0	0	0	0	0	0	0	0	0	66
24010435	f	md	MX	2024	1	0	0	0	0	0	0	0	0	0	0	0	
24010538	m	hp	US	2024	1	0	0	0	0	0	0	0	0	0	0	0	58
24010684	m	md	DE	2024	0	0	0	0	0	0	0	0	0	0	0	0	69
24010768	m	md	JP	2024	0	1	0	1	0	0	0	0	0	0	0	0	78
24011166	f	hp	FR	2024	1	0	0	0	0	0	0	0	0	0	0	0	75
24011246	m	md	MX	2024	1	0	0	0	0	0	0	0	0	0	0	0	
24011311	f	md	FR	2024	0	1	0	1	0	0	0	0	0	0	0	0	75.8
24011386	m	md	JP	2024	0	1	0	1	0	0	0	0	0	0	0	0	66
24011562	m	md	JP	2024	0	1	0	0	1	0	0	0	0	0	0	0	50
24011777	m	cn	US	2024	1	0	0	0	0	0	0	0	0	0	0	0	
24013587	f	md	FR	2024	1	0	0	0	0	0	0	0	0	0	0	0	
24013716	f	md	BE	2024	0	1	1	0	0	0	0	0	0	0	0	0	69
24014158	m	hp	JP	2024	0	1	1	0	0	0	0	0	0	0	0	0	72

24014248	Not	hp	US	2024	0	1	0	0	1	0	0	0	0	0	0	0	
24014423	m	hp	FR	2024	0	1	1	0	0	0	0	0	0	0	0	0	65
24014818	Not	md	JP	2024	1	0	0	0	0	0	0	0	0	0	0	0	
24015175	f	ph	JP	2024	0	1	0	0	0	1	0	0	0	0	0	0	70
24015218	f	cn	US	2024	0	0	0	0	0	0	0	0	0	0	0	0	63
24015337	f	md	JP	2024	1	0	0	0	0	0	0	0	0	0	0	1	43
24015621	m	md	RU	2024	1	0	0	0	0	0	0	0	0	0	0	0	
24015736	f	md	DE	2024	0	0	0	0	0	0	0	0	0	0	0	0	60
24015937	f	md	JP	2024	1	0	0	0	0	0	0	0	0	0	0	0	54
24016002	m	hp	SG	2024	0	0	0	0	0	0	0	0	0	0	0	0	
24016021	f	md	NL	2024	0	0	0	0	0	0	0	0	0	0	0	0	
24016026	f	cn	CA	2024	1	0	0	0	0	0	0	0	0	0	0	0	79.1
24016051	f	md	BE	2024	0	1	1	0	0	0	0	0	0	0	0	0	69
24016497	f	cn	KR	2024	0	0	0	0	0	0	0	0	0	0	0	0	62
24017439	f	md	DK	2024	0	0	0	0	0	0	0	0	0	0	0	0	43
24018206	m	md	JP	2024	0	1	0	1	0	0	0	0	0	0	0	0	45
24018398	m	ph	CA	2024	0	0	0	0	0	0	0	0	0	0	0	0	45
24018961	Not	md	JP	2024	0	1	1	0	0	0	0	0	0	0	0	0	75
24019158	f	md	NL	2024	0	0	0	0	0	0	0	0	0	0	0	0	
24019304	m	md	JP	2024	0	0	0	0	0	0	0	0	0	0	0	0	49
24019339	m	md	JP	2024	0	1	0	0	1	0	0	0	0	0	0	0	
24019574	f	hp	ES	2024	0	1	0	0	1	0	0	0	0	0	0	0	68
24019818	Not	cn	ES	2024	1	0	0	0	0	0	0	0	0	0	0	0	
24019984	m	cn	JP	2024	0	0	0	0	0	0	0	0	0	0	0	0	75
24020010	m	md	GR	2024	0	1	0	0	1	0	0	0	0	0	0	0	53
24020603	m	md	JP	2024	0	1	0	1	0	0	0	0	0	0	0	0	
24020761	f	md	PT	2024	0	1	1	0	0	0	0	0	0	0	0	0	
24020868	f	md	BE	2024	0	1	1	0	0	0	0	0	0	0	0	0	69
24020950	m	md	JP	2024	0	0	0	0	0	0	0	0	0	0	0	0	
24022286	Not	ph	JP	2024	0	0	0	0	0	0	0	0	0	0	0	0	
24022287	f	ph	JP	2024	0	0	0	0	0	0	0	0	0	0	0	0	50
24022318	m	md	CA	2024	0	0	0	0	0	0	0	0	0	0	0	0	
24022321	m	md	JP	2024	0	0	0	0	0	0	0	0	0	0	0	0	90
24022325	f	cn	CN	2024	0	0	0	0	0	0	0	0	0	0	0	0	60

24022337	Not	md	JP	2024	0	0	0	0	0	0	0	0	0	0	0	0	60
24022340	f	md	JP	2024	0	0	0	0	0	0	0	0	0	0	0	0	
24022346	m	ph	JP	2024	0	0	0	0	0	0	0	0	0	0	0	0	76
24022366	f	md	JP	2024	0	0	0	0	0	0	0	0	0	0	0	0	
24022377	f	md	JP	2024	0	0	0	0	0	0	0	0	0	0	0	0	80
24022380	m	md	JP	2024	0	0	0	0	0	0	0	0	0	0	0	0	38
24022387	f	md	JP	2024	0	0	0	0	0	0	0	0	0	0	0	0	90
24022402	m	md	JP	2024	0	0	0	0	0	0	0	0	0	0	0	0	40
24022411	f	md	JP	2024	0	0	0	0	0	0	0	0	0	0	0	0	90
24022415	m	md	DK	2024	0	0	0	0	0	0	0	0	0	0	0	0	59
24022420	f	md	PE	2024	0	0	0	0	0	0	0	0	0	0	0	0	72
24022422	f	md	JP	2024	0	0	0	0	0	0	0	0	0	0	0	0	80
24022424	Not	md	JP	2024	0	0	0	0	0	0	0	0	0	0	0	0	67
24022427	m	md	AR	2024	0	0	0	0	0	0	0	0	0	0	0	0	61
24022428	f	md	AU	2024	0	0	0	0	0	0	0	0	0	0	0	0	37
24022429	f	md	CN	2024	0	0	0	0	0	0	0	0	0	0	0	0	50
24022433	f	ph	JP	2024	0	0	0	0	0	0	0	0	0	0	0	0	53
24022439	f	md	AU	2024	0	0	0	0	0	0	0	0	0	0	0	0	42
24022440	m	md	JP	2024	0	0	0	0	0	0	0	0	0	0	0	0	80
24022457	f	hp	TR	2024	0	0	0	0	0	0	0	0	0	0	0	0	38
24022461	f	md	IN	2024	0	0	0	0	0	0	0	0	0	0	0	0	
24022465	m	md	TR	2024	0	0	0	0	0	0	0	0	0	0	0	0	60
24022472	f	ph	JP	2024	0	0	0	0	0	0	0	0	0	0	0	0	80
24022483	f	md	JP	2024	0	0	0	0	0	0	0	0	0	0	0	0	60
24022489	f	cn	CN	2024	0	0	0	0	0	0	0	0	0	0	0	0	48
24022491	m	md	JP	2024	0	0	0	0	0	0	0	0	0	0	0	0	67
24022493	m	md	IT	2024	0	0	0	0	0	0	0	0	0	0	0	0	58
24022505	Not	md	JP	2024	0	0	0	0	0	0	0	0	0	0	0	0	70
24022509	Not	ph	JP	2024	0	0	0	0	0	0	0	0	0	0	0	0	30
24022514	f	md	IN	2024	0	0	0	0	0	0	0	0	0	0	0	0	58
24022519	m	md	JP	2024	0	0	0	0	0	0	0	0	0	0	0	0	80
24022527	f	cn	CN	2024	0	0	0	0	0	0	0	0	0	0	0	0	68
24022528	f	cn	CN	2024	0	0	0	0	0	0	0	0	0	0	0	0	68
24022550	m	md	JP	2024	0	0	0	0	0	0	0	0	0	0	0	0	

24022551	m	cn	CA	2024	0	0	0	0	0	0	0	0	0	0	0	0	49
24022569	Not	md	TW	2024	0	0	0	0	0	0	0	0	0	0	0	0	
24022579	f	md	VN	2024	0	0	0	0	0	0	0	0	0	0	0	0	69
24022584	m	cn	IL	2024	0	0	0	0	0	0	0	0	0	0	0	0	58
24022585	Not	md	JP	2024	0	0	0	0	0	0	0	0	0	0	0	0	
24022591	f	cn	CN	2024	0	0	0	0	0	0	0	0	0	0	0	0	47
24022603	f	ph	JP	2024	0	0	0	0	0	0	0	0	0	0	0	0	
24022608	Not	ph	US	2024	0	0	0	0	0	0	0	0	0	0	0	0	
24022610	Not	md	JP	2024	0	0	0	0	0	0	0	0	0	0	0	0	80
24022614	f	cn	US	2024	0	0	0	0	0	0	0	0	0	0	0	0	
24022618	m	md	CN	2024	0	0	0	0	0	0	0	0	0	0	0	0	82
24022623	Not	md	JP	2024	0	0	0	0	0	0	0	0	0	0	0	0	60
24022627	m	md	JP	2024	0	0	0	0	0	0	0	0	0	0	0	0	90
24022630	m	md	JP	2024	0	0	0	0	0	0	0	0	0	0	0	0	60
24022670	m	md	VN	2024	0	0	0	0	0	0	0	0	0	0	0	0	
24022682	m	md	JP	2024	0	0	0	0	0	0	0	0	0	0	0	0	80
24022684	m	md	JP	2024	0	0	0	0	0	0	0	0	0	0	0	0	
24022695	f	md	US	2024	0	0	0	0	0	0	0	0	0	0	0	0	70
24022698	f	cn	IL	2024	0	0	0	0	0	0	0	0	0	0	0	0	
24022769	Not	md	US	2024	0	1	1	0	0	0	0	0	0	0	0	0	
24022773	m	md	VN	2024	0	0	0	0	0	0	0	0	0	0	0	0	33
24022788	f	md	JP	2024	0	0	0	0	0	0	0	0	0	0	0	0	80
24022797	m	md	DK	2024	0	0	0	0	0	0	0	0	0	0	0	0	49
24022798	Not	md	JP	2024	0	0	0	0	0	0	0	0	0	0	0	0	50
24022813	m	md	TR	2024	0	0	0	0	0	0	0	0	0	0	0	0	63
24022826	f	md	CN	2024	0	0	0	0	0	0	0	0	0	0	0	0	65
24022827	f	md	LT	2024	0	0	0	0	0	0	0	0	0	0	0	0	69
24022831	m	cn	CO	2024	0	0	0	0	0	0	0	0	0	0	0	0	48
24022849	f	md	BE	2024	0	0	0	0	0	0	0	0	0	0	0	0	37
24022855	f	hp	TH	2024	0	0	0	0	0	0	0	0	0	0	0	0	69
24022860	f	md	DK	2024	0	0	0	0	0	0	0	0	0	0	0	0	81
24022862	f	md	RU	2024	0	0	0	0	0	0	0	0	0	0	0	0	58
24022866	f	md	JP	2024	0	0	0	0	0	0	0	0	0	0	0	0	50
24022887	f	md	TR	2024	0	0	0	0	0	0	0	0	0	0	0	0	38

24022916	Not	md	JP	2024	0	0	0	0	0	0	0	0	0	0	0	0	
24022919	m	md	TR	2024	0	0	0	0	0	0	0	0	0	0	0	0	44
24022936	f	md	CN	2024	0	0	0	0	0	0	0	0	0	0	0	0	43
24022943	f	md	DK	2024	0	0	0	0	0	0	0	0	0	0	0	0	45
24022955	f	md	LT	2024	0	0	0	0	0	0	0	0	0	0	0	0	70
24022965	f	md	CN	2024	0	0	0	0	0	0	0	0	0	0	0	0	31
24022968	m	cn	HK	2024	0	0	0	0	0	0	0	0	0	0	0	0	
24022995	f	cn	US	2024	0	0	0	0	0	0	0	0	0	0	0	0	
24023001	m	md	TR	2024	0	0	0	0	0	0	0	0	0	0	0	0	37
24023003	m	md	PE	2024	0	0	0	0	0	0	0	0	0	0	0	0	57
24023011	m	md	IT	2024	0	0	0	0	0	0	0	0	0	0	0	0	68
24023013	f	md	CN	2024	0	0	0	0	0	0	0	0	0	0	0	0	60
24023014	f	cn	PH	2024	0	0	0	0	0	0	0	0	0	0	0	0	64
24023029	f	md	TR	2024	0	0	0	0	0	0	0	0	0	0	0	0	67
24023033	m	md	CN	2024	0	0	0	0	0	0	0	0	0	0	0	0	78
24023035	m	hp	CN	2024	0	0	0	0	0	0	0	0	0	0	0	0	52
24023040	Not	hp	US	2024	0	0	0	0	0	0	0	0	0	0	0	0	59
24023050	m	md	BE	2024	0	0	0	0	0	0	0	0	0	0	0	0	69
24023077	m	md	HR	2024	0	0	0	0	0	0	0	0	0	0	0	0	60
24023084	f	md	TR	2024	0	0	0	0	0	0	0	0	0	0	0	0	58
24023088	f	md	IT	2024	0	0	0	0	0	0	0	0	0	0	0	0	67
24023121	Not	md	JP	2024	0	0	0	0	0	0	0	0	0	0	0	0	
24023140	m	md	JP	2024	0	0	0	0	0	0	0	0	0	0	0	0	80
24023142	m	cn	IN	2024	0	0	0	0	0	0	0	0	0	0	0	0	55
24023149	m	md	IT	2024	0	0	0	0	0	0	0	0	0	0	0	0	68
24023156	Not	md	ES	2024	0	0	0	0	0	0	0	0	0	0	0	0	87
24023198	m	md	TR	2024	0	0	0	0	0	0	0	0	0	0	0	0	70
24023203	m	md	PE	2024	0	0	0	0	0	0	0	0	0	0	0	0	36
24023207	f	cn	US	2024	0	0	0	0	0	0	0	0	0	0	0	0	72
24023217	f	md	DK	2024	0	0	0	0	0	0	0	0	0	0	0	0	67
24023233	f	md	CN	2024	0	0	0	0	0	0	0	0	0	0	0	0	
24023241	f	md	CL	2024	0	0	0	0	0	0	0	0	0	0	0	0	50
24023252	m	md	JP	2024	0	0	0	0	0	0	0	0	0	0	0	0	70
24023259	f	md	TR	2024	0	0	0	0	0	0	0	0	0	0	0	0	59

24023263	f	cn	US	2024	0	0	0	0	0	0	0	0	0	0	0	0	
24023270	m	cn	CA	2024	0	0	0	0	0	0	0	0	0	0	0	0	
24023271	f	md	DK	2024	0	0	0	0	0	0	0	0	0	0	0	0	79
24023272	f	md	JP	2024	0	0	0	0	0	0	0	0	0	0	0	0	90
24023280	f	md	RU	2024	0	0	0	0	0	0	0	0	0	0	0	0	41
24023313	m	md	VN	2024	0	0	0	0	0	0	0	0	0	0	0	0	64
24023316	f	md	HR	2024	0	0	0	0	0	0	0	0	0	0	0	0	45
24023322	m	md	JP	2024	0	0	0	0	0	0	0	0	0	0	0	0	
24023323	f	md	CN	2024	0	0	0	0	0	0	0	0	0	0	0	0	66
24023345	Not	hp	GB	2024	0	0	0	0	0	0	0	0	0	0	0	0	
24023360	f	md	CN	2024	0	0	0	0	0	0	0	0	0	0	0	0	42
24023368	m	md	RS	2024	0	0	0	0	0	0	0	0	0	0	0	0	77
24023371	f	md	VN	2024	0	0	0	0	0	0	0	0	0	0	0	0	52
24023382	f	md	AT	2024	0	0	0	0	0	0	0	0	0	0	0	0	60
24023391	m	md	TR	2024	0	0	0	0	0	0	0	0	0	0	0	0	50
24023398	m	md	TR	2024	0	0	0	0	0	0	0	0	0	0	0	0	37
24023445	f	md	CN	2024	0	0	0	0	0	0	0	0	0	0	0	0	56
24023453	m	md	IL	2024	0	0	0	0	0	0	0	0	0	0	0	0	67
24023463	f	md	BG	2024	0	0	0	0	0	0	0	0	0	0	0	0	71
24023483	m	cn	CN	2024	0	0	0	0	0	0	0	0	0	0	0	0	59
24023518	m	md	VN	2024	0	0	0	0	0	0	0	0	0	0	0	0	58
24023521	f	cn	CN	2024	0	0	0	0	0	0	0	0	0	0	0	0	44
24023530	f	md	GB	2024	0	0	0	0	0	0	0	0	0	0	0	0	
24023548	f	md	JP	2024	0	0	0	0	0	0	0	0	0	0	0	0	60
24023558	m	md	IL	2024	0	0	0	0	0	0	0	0	0	0	0	0	67
24023561	f	cn	CN	2024	0	0	0	0	0	0	0	0	0	0	0	0	54
24023568	f	md	VN	2024	0	0	0	0	0	0	0	0	0	0	0	0	66
24023574	m	md	PT	2024	0	0	0	0	0	0	0	0	0	0	0	0	72
24023577	f	md	JP	2024	0	0	0	0	0	0	0	0	0	0	0	0	50
24023578	f	md	IL	2024	0	0	0	0	0	0	0	0	0	0	0	0	
24023580	Not	md	ES	2024	0	0	0	0	0	0	0	0	0	0	0	0	62
24023595	m	cn	CN	2024	0	0	0	0	0	0	0	0	0	0	0	0	41
24023596	m	md	DK	2024	0	0	0	0	0	0	0	0	0	0	0	0	50
24023607	m	cn	CN	2024	0	0	0	0	0	0	0	0	0	0	0	0	82

24023609	Not	md	SG	2024	0	0	0	0	0	0	0	0	0	0	0	0	
24023669	Not	md	ES	2024	0	0	0	0	0	0	0	0	0	0	0	0	75
24023703	Not	md	JP	2024	0	0	0	0	0	0	0	0	0	0	0	0	
24023728	m	md	DE	2024	0	0	0	0	0	0	0	0	0	0	0	0	
24023742	f	cn	CA	2024	0	0	0	0	0	0	0	0	0	0	0	0	
24023749	f	cn	CN	2024	0	0	0	0	0	0	0	0	0	0	0	0	69
24023752	f	md	JP	2024	0	0	0	0	0	0	0	0	0	0	0	0	63
24023780	f	cn	IL	2024	0	0	0	0	0	0	0	0	0	0	0	0	33
24023804	m	md	JP	2024	0	0	0	0	0	0	0	0	0	0	0	0	
24023843	f	cn	CN	2024	0	0	0	0	0	0	0	0	0	0	0	0	71
24023869	f	cn	TH	2024	0	0	0	0	0	0	0	0	0	0	0	0	
24023882	m	md	BG	2024	0	0	0	0	0	0	0	0	0	0	0	0	69
24023931	f	md	TR	2024	0	0	0	0	0	0	0	0	0	0	0	0	
24023958	f	md	CZ	2024	0	0	0	0	0	0	0	0	0	0	0	0	57
24024001	m	md	JP	2024	0	0	0	0	0	0	0	0	0	0	0	0	60
24024008	m	md	DK	2024	0	0	0	0	0	0	0	0	0	0	0	0	72
24024013	f	md	AU	2024	0	0	0	0	0	0	0	0	0	0	0	0	63
24024016	f	md	VN	2024	0	0	0	0	0	0	0	0	0	0	0	0	32
24024059	f	md	TR	2024	0	0	0	0	0	0	0	0	0	0	0	0	63
24024100	f	md	TR	2024	0	0	0	0	0	0	0	0	0	0	0	0	74
24024108	f	md	AT	2024	0	0	0	0	0	0	0	0	0	0	0	0	49
24024113	m	cn	TH	2024	0	0	0	0	0	0	0	0	0	0	0	0	
24024133	m	md	CO	2024	0	0	0	0	0	0	0	0	0	0	0	0	
24024153	m	md	US	2024	0	0	0	0	0	0	0	0	0	0	0	0	
24024156	f	md	VN	2024	0	0	0	0	0	0	0	0	0	0	0	0	59
24024170	f	md	IT	2024	0	0	0	0	0	0	0	0	0	0	0	0	
24024171	f	md	BE	2024	0	0	0	0	0	0	0	0	0	0	0	0	59
24024186	f	md	TR	2024	0	0	0	0	0	0	0	0	0	0	0	0	52
24024188	f	cn	CA	2024	0	0	0	0	0	0	0	0	0	0	0	0	
24024205	f	md	IT	2024	0	0	0	0	0	0	0	0	0	0	0	0	64
24024231	f	md	DK	2024	0	0	0	0	0	0	0	0	0	0	0	0	46
24024251	f	cn	CO	2024	0	0	0	0	0	0	0	0	0	0	0	0	62
24024300	m	md	AT	2024	0	0	0	0	0	0	0	0	0	0	0	0	65
24024320	f	md	AU	2024	0	0	0	0	0	0	0	0	0	0	0	0	42

24024325	f	md	AU	2024	0	0	0	0	0	0	0	0	0	0	0	0	38
24024329	f	cn	CN	2024	0	0	0	0	0	0	0	0	0	0	0	0	57
24024363	m	ph	DE	2024	0	0	0	0	0	0	0	0	0	0	0	0	
24024378	f	cn	PH	2024	0	0	0	0	0	0	0	0	0	0	0	0	60
24024379	f	md	DK	2024	0	0	0	0	0	0	0	0	0	0	0	0	77
24024386	f	md	CN	2024	0	0	0	0	0	0	0	0	0	0	0	0	67
24024398	m	md	DK	2024	0	0	0	0	0	0	0	0	0	0	0	0	82
24024407	f	md	GB	2024	0	0	0	0	0	0	0	0	0	0	0	0	
24024426	m	md	BE	2024	0	0	0	0	0	0	0	0	0	0	0	0	69
24024450	f	md	DK	2024	0	0	0	0	0	0	0	0	0	0	0	0	45
24024486	Not	md	JP	2024	0	0	0	0	0	0	0	0	0	0	0	0	70
24024496	Not	hp	CN	2024	0	0	0	0	0	0	0	0	0	0	0	0	
24024546	m	md	BE	2024	0	0	0	0	0	0	0	0	0	0	0	0	84
24024554	m	md	DK	2024	0	0	0	0	0	0	0	0	0	0	0	0	82
24024561	m	md	AU	2024	0	0	0	0	0	0	0	0	0	0	0	0	45
24024563	f	md	TR	2024	0	0	0	0	0	0	0	0	0	0	0	0	37
24024604	f	md	PE	2024	0	0	0	0	0	0	0	0	0	0	0	0	46
24024607	m	md	TR	2024	0	0	0	0	0	0	0	0	0	0	0	0	38
24024614	Not	md	JP	2024	0	0	0	0	0	0	0	0	0	0	0	0	50
24024631	f	md	TR	2024	0	0	0	0	0	0	0	0	0	0	0	0	69
24024642	f	md	TR	2024	0	0	0	0	0	0	0	0	0	0	0	0	77
24024651	f	md	AU	2024	0	0	0	0	0	0	0	0	0	0	0	0	54
24024657	f	md	JP	2024	0	0	0	0	0	0	0	0	0	0	0	0	70
24024666	m	md	IT	2024	0	0	0	0	0	0	0	0	0	0	0	0	76
24024667	m	md	JP	2024	0	0	0	0	0	0	0	0	0	0	0	0	81
24024695	f	hp	DK	2024	1	0	0	0	0	0	0	0	0	0	0	0	80
24024712	f	cn	AU	2024	0	0	0	0	0	0	0	0	0	0	0	0	51
24024723	f	md	TR	2024	0	0	0	0	0	0	0	0	0	0	0	0	52
24024741	m	md	IT	2024	0	0	0	0	0	0	0	0	0	0	0	0	74
24024750	m	md	JP	2024	0	0	0	0	0	0	0	0	0	0	0	0	80
24024754	f	md	TR	2024	0	0	0	0	0	0	0	0	0	0	0	0	25
24024795	m	md	TR	2024	0	0	0	0	0	0	0	0	0	0	0	0	58
24024797	Not	md	JP	2024	0	0	0	0	0	0	0	0	0	0	0	0	50
24024801	m	md	VN	2024	0	0	0	0	0	0	0	0	0	0	0	0	

24024810	f	ph	CA	2024	0	0	0	0	0	0	0	0	0	0	0	0	
24024830	f	cn	TH	2024	0	0	0	0	0	0	0	0	0	0	0	0	51
24024858	Not	md	JP	2024	0	0	0	0	0	0	0	0	0	0	0	0	
24024863	f	cn	CN	2024	0	0	0	0	0	0	0	0	0	0	0	0	68
24024884	m	md	BE	2024	0	0	0	0	0	0	0	0	0	0	0	0	45
24024888	f	md	JP	2024	0	0	0	0	0	0	0	0	0	0	0	0	70
24024917	m	cn	CA	2024	0	0	0	0	0	0	0	0	0	0	0	0	
24024920	m	md	DK	2024	0	0	0	0	0	0	0	0	0	0	0	0	34
24024960	f	cn	IL	2024	0	0	0	0	0	0	0	0	0	0	0	0	
24024978	m	md	RS	2024	0	0	0	0	0	0	0	0	0	0	0	0	63
24024979	m	cn	CN	2024	0	0	0	0	0	0	0	0	0	0	0	0	56
24025017	f	md	TR	2024	0	0	0	0	0	0	0	0	0	0	0	0	52
24025026	f	hp	CN	2024	0	0	0	0	0	0	0	0	0	0	0	0	38
24025049	m	ph	NZ	2024	0	0	0	0	0	0	0	0	0	0	0	0	
24025060	m	cn	CN	2024	0	0	0	0	0	0	0	0	0	0	0	0	68
24025075	m	md	AT	2024	0	0	0	0	0	0	0	0	0	0	0	0	70
24025094	m	md	TR	2024	0	0	0	0	0	0	0	0	0	0	0	0	75
24025104	m	md	JP	2024	0	0	0	0	0	0	0	0	0	0	0	0	49
24025105	m	md	PE	2024	0	0	0	0	0	0	0	0	0	0	0	0	58
24025147	f	md	DK	2024	0	0	0	0	0	0	0	0	0	0	0	0	67
24025151	f	cn	US	2024	0	0	0	0	0	0	0	0	0	0	0	0	40
24025154	m	ph	US	2024	0	0	0	0	0	0	0	0	0	0	0	0	40
24025158	Not	ph	PT	2024	0	0	0	0	0	0	0	0	0	0	0	0	
24025177	f	cn	US	2024	0	0	0	0	0	0	0	0	0	0	0	0	
24025181	m	ph	JP	2024	0	0	0	0	0	0	0	0	0	0	0	0	60
24025206	f	md	PE	2024	0	0	0	0	0	0	0	0	0	0	0	0	36
24025219	Not	md	BR	2024	0	0	0	0	0	0	0	0	0	0	0	0	
24025236	f	cn	PH	2024	0	0	0	0	0	0	0	0	0	0	0	0	60
24025237	Not	md	JP	2024	0	0	0	0	0	0	0	0	0	0	0	0	60
24025239	f	cn	CN	2024	0	0	0	0	0	0	0	0	0	0	0	0	29
24025240	m	hp	US	2024	0	0	0	0	0	0	0	0	0	0	0	0	81
24025243	Not	md	JP	2024	0	0	0	0	0	0	0	0	0	0	0	0	70
24025281	f	cn	US	2024	0	0	0	0	0	0	0	0	0	0	0	0	77
24025291	Not	md	US	2024	0	0	0	0	0	0	0	0	0	0	0	0	

24025293	m	md	IT	2024	0	0	0	0	0	0	0	0	0	0	0	0	57
24025345	f	md	VN	2024	0	0	0	0	0	0	0	0	0	0	0	0	55
24025389	f	cn	IL	2024	0	0	0	0	0	0	0	0	0	0	0	0	
24025393	f	md	JP	2024	0	0	0	0	0	0	0	0	0	0	0	0	60
24025436	Not	cn	CN	2024	0	0	0	0	0	0	0	0	0	0	0	0	
24025449	f	cn	CN	2024	0	0	0	0	0	0	0	0	0	0	0	0	42
24025457	f	cn	CN	2024	0	0	0	0	0	0	0	0	0	0	0	0	54
24025500	f	md	DK	2024	0	0	0	0	0	0	0	0	0	0	0	0	76
24025540	f	md	IN	2024	0	0	0	0	0	0	0	0	0	0	0	0	29
24025594	Not	md	JP	2024	0	0	0	0	0	0	0	0	0	0	0	0	50
24025596	m	hp	CN	2024	0	0	0	0	0	0	0	0	0	0	0	0	47
24025609	m	md	AR	2024	0	0	0	0	0	0	0	0	0	0	0	0	64
24025615	f	md	JP	2024	0	0	0	0	0	0	0	0	0	0	0	0	89
24025625	f	md	PE	2024	0	0	0	0	0	0	0	0	0	0	0	0	63
24025695	f	md	TR	2024	0	0	0	0	0	0	0	0	0	0	0	0	74
24025710	m	cn	IL	2024	0	0	0	0	0	0	0	0	0	0	0	0	
24025720	f	md	DK	2024	0	0	0	0	0	0	0	0	0	0	0	0	37
24025729	f	md	RS	2024	0	0	0	0	0	0	0	0	0	0	0	0	73
24025735	f	md	RS	2024	0	0	0	0	0	0	0	0	0	0	0	0	73
24025744	m	cn	US	2024	0	0	0	0	0	0	0	0	0	0	0	0	65
24025763	Not	md	PE	2024	0	0	0	0	0	0	0	0	0	0	0	0	
24025769	f	md	BE	2024	0	0	0	0	0	0	0	0	0	0	0	0	85
24025779	m	md	CN	2024	0	0	0	0	0	0	0	0	0	0	0	0	61
24025797	f	md	AU	2024	0	0	0	0	0	0	0	0	0	0	0	0	
24025800	m	md	BE	2024	0	0	0	0	0	0	0	0	0	0	0	0	55
24025819	f	md	IT	2024	0	0	0	0	0	0	0	0	0	0	0	0	67
24025828	f	md	IT	2024	0	0	0	0	0	0	0	0	0	0	0	0	51
24025846	Not	cn	CN	2024	0	0	0	0	0	0	0	0	0	0	0	0	
24025856	Not	md	ES	2024	0	0	0	0	0	0	0	0	0	0	0	0	72
24025857	f	md	CN	2024	0	0	0	0	0	0	0	0	0	0	0	0	66
24025906	f	hp	CN	2024	0	0	0	0	0	0	0	0	0	0	0	0	73
24025912	m	md	IL	2024	0	0	0	0	0	0	0	0	0	0	0	0	
24025941	f	ph	JP	2024	0	0	0	0	0	0	0	0	0	0	0	0	74
24025943	f	hp	US	2024	0	0	0	0	0	0	0	0	0	0	0	0	

24025950	m	md	VN	2024	0	0	0	0	0	0	0	0	0	0	0	0	61
24025959	m	md	BE	2024	0	0	0	0	0	0	0	0	0	0	0	0	74
24025979	f	md	AT	2024	0	0	0	0	0	0	0	0	0	0	0	0	53
24026006	f	md	TW	2024	0	0	0	0	0	0	0	0	0	0	0	0	50
24026018	f	md	JP	2024	0	0	0	0	0	0	0	0	0	0	0	0	60
24026020	f	md	JP	2024	0	0	0	0	0	0	0	0	0	0	0	0	46
24026027	f	md	NZ	2024	0	0	0	0	0	0	0	0	0	0	0	0	28
24026064	f	cn	IN	2024	0	0	0	0	0	0	0	0	0	0	0	0	50
24026067	f	md	US	2024	0	0	0	0	0	0	0	0	0	0	0	0	
24026105	Not	md	ES	2024	0	0	0	0	0	0	0	0	0	0	0	0	61
24026106	f	ph	JP	2024	0	0	0	0	0	0	0	0	0	0	0	0	75
24026110	m	cn	IN	2024	0	0	0	0	0	0	0	0	0	0	0	0	41
24026115	Not	md	JP	2024	0	0	0	0	0	0	0	0	0	0	0	0	80
24026128	m	md	DK	2024	0	0	0	0	0	0	0	0	0	0	0	0	55
24026134	f	md	CN	2024	0	0	0	0	0	0	0	0	0	0	0	0	58
24026151	Not	hp	US	2024	0	0	0	0	0	0	0	0	0	0	0	0	
24026155	Not	md	ES	2024	0	0	0	0	0	0	0	0	0	0	0	0	69
24026184	m	md	RS	2024	0	0	0	0	0	0	0	0	0	0	0	0	74
24026214	m	md	RS	2024	0	0	0	0	0	0	0	0	0	0	0	0	74
24026234	m	md	JP	2024	0	0	0	0	0	0	0	0	0	0	0	0	80
24026235	Not	md	ES	2024	0	0	0	0	0	0	0	0	0	0	0	0	45
24026267	Not	md	JP	2024	0	0	0	0	0	0	0	0	0	0	0	0	80
24026275	m	md	BE	2024	0	0	0	0	0	0	0	0	0	0	0	0	57
24026285	f	cn	US	2024	0	0	0	0	0	0	0	0	0	0	0	0	
24026286	f	hp	KR	2024	0	0	0	0	0	0	0	0	0	0	0	0	76
24026338	m	hp	KR	2024	0	0	0	0	0	0	0	0	0	0	0	0	65
24026340	f	hp	CN	2024	0	0	0	0	0	0	0	0	0	0	0	0	68
24026345	f	hp	KR	2024	0	0	0	0	0	0	0	0	0	0	0	0	62
24026351	f	cn	TH	2024	0	0	0	0	0	0	0	0	0	0	0	0	79
24026354	m	cn	ID	2024	0	1	0	0	0	1	0	0	0	0	0	0	50
24026398	f	cn	IN	2024	0	0	0	0	0	0	0	0	0	0	0	0	50
24026400	f	hp	KR	2024	0	0	0	0	0	0	0	0	0	0	0	0	71
24026431	m	md	CN	2024	0	0	0	0	0	0	0	0	0	0	0	0	51
24026438	f	md	IL	2024	0	0	0	0	0	0	0	0	0	0	0	0	87

24026539	f	md	CN	2024	0	0	0	0	0	0	0	0	0	0	0	0	62
24026544	f	cn	US	2024	0	0	0	0	0	0	0	0	0	0	0	0	
24026556	f	md	DK	2024	0	0	0	0	0	0	0	0	0	0	0	0	79
24026566	f	md	JP	2024	0	0	0	0	0	0	0	0	0	0	0	0	
24026575	f	md	IT	2024	0	0	0	0	0	0	0	0	0	0	0	0	65
24026605	f	md	FI	2024	0	0	0	0	0	0	0	0	0	0	0	0	52
24026613	Not	md	US	2024	0	0	0	0	0	0	0	0	0	0	0	0	
24026621	f	md	JP	2024	0	0	0	0	0	0	0	0	0	0	0	0	
24026635	f	md	JP	2024	0	0	0	0	0	0	0	0	0	0	0	0	70
24026653	m	md	JP	2024	0	0	0	0	0	0	0	0	0	0	0	0	90
24026659	Not	md	JP	2024	0	0	0	0	0	0	0	0	0	0	0	0	
24026675	f	md	CN	2024	0	0	0	0	0	0	0	0	0	0	0	0	52
24026693	Not	md	CH	2024	0	0	0	0	0	0	0	0	0	0	0	0	
24026704	m	md	TR	2024	0	0	0	0	0	0	0	0	0	0	0	0	52
24026734	f	md	US	2024	0	0	0	0	0	0	0	0	0	0	0	0	37
24026736	f	md	RO	2024	0	0	0	0	0	0	0	0	0	0	0	0	78
24026749	f	ph	JP	2024	0	0	0	0	0	0	0	0	0	0	0	0	58
24026753	f	md	VN	2024	0	0	0	0	0	0	0	0	0	0	0	0	44
24026755	f	md	IT	2024	0	0	0	0	0	0	0	0	0	0	0	0	51
24026756	f	md	PE	2024	0	0	0	0	0	0	0	0	0	0	0	0	67
24026785	m	md	IL	2024	0	0	0	0	0	0	0	0	0	0	0	0	
24026788	f	md	JP	2024	0	0	0	0	0	0	0	0	0	0	0	0	37
24026796	m	md	US	2024	0	0	0	0	0	0	0	0	0	0	0	0	
24026798	f	ph	JP	2024	0	0	0	0	0	0	0	0	0	0	0	0	
24026800	f	md	JP	2024	0	0	0	0	0	0	0	0	0	0	0	0	
24026803	f	md	JP	2024	0	0	0	0	0	0	0	0	0	0	0	0	70
24026828	Not	md	JP	2024	0	0	0	0	0	0	0	0	0	0	0	0	64
24026829	f	hp	RU	2024	0	0	0	0	0	0	0	0	0	0	0	0	66
24026831	m	cn	IN	2024	0	0	0	0	0	0	0	0	0	0	0	0	34
24026837	m	cn	CO	2024	0	0	0	0	0	0	0	0	0	0	0	0	63
24026840	f	md	CO	2024	0	0	0	0	0	0	0	0	0	0	0	0	73
24026844	m	md	RS	2024	0	0	0	0	0	0	0	0	0	0	0	0	40
24026848	f	md	CN	2024	0	0	0	0	0	0	0	0	0	0	0	0	65
24026849	m	ph	JP	2024	0	0	0	0	0	0	0	0	0	0	0	0	90

24026852	f	md	AU	2024	0	0	0	0	0	0	0	0	0	0	0	0	47
24026853	m	md	JP	2024	0	0	0	0	0	0	0	0	0	0	0	0	70
24026854	f	ph	US	2024	0	0	0	0	0	0	0	0	0	0	0	0	28
24026855	m	md	IL	2024	0	0	0	0	0	0	0	0	0	0	0	0	64
24026859	m	md	LT	2024	0	0	0	0	0	0	0	0	0	0	0	0	64
24026867	f	md	RS	2024	0	0	0	0	0	0	0	0	0	0	0	0	75
24026883	m	md	LT	2024	0	0	0	0	0	0	0	0	0	0	0	0	62
24026890	m	md	CN	2024	0	0	0	0	0	0	0	0	0	0	0	0	48
24026905	f	md	RU	2024	0	0	0	0	0	0	0	0	0	0	0	0	58
24026906	f	md	IT	2024	0	0	0	0	0	0	0	0	0	0	0	0	35
24026918	Not	md	JP	2024	0	0	0	0	0	0	0	0	0	0	0	0	90
24026920	m	md	LT	2024	0	0	0	0	0	0	0	0	0	0	0	0	50
24026926	m	md	PT	2024	0	0	0	0	0	0	0	0	0	0	0	0	
24026944	f	md	IT	2024	0	0	0	0	0	0	0	0	0	0	0	0	64
24026949	f	md	IL	2024	0	0	0	0	0	0	0	0	0	0	0	0	36
24026951	m	cn	US	2024	0	0	0	0	0	0	0	0	0	0	0	0	88
24026960	f	md	AU	2024	0	0	0	0	0	0	0	0	0	0	0	0	
24026961	f	md	AU	2024	0	0	0	0	0	0	0	0	0	0	0	0	64
24026962	f	md	AU	2024	0	0	0	0	0	0	0	0	0	0	0	0	47
24026968	m	md	IT	2024	0	0	0	0	0	0	0	0	0	0	0	0	52
24026971	m	md	JP	2024	0	0	0	0	0	0	0	0	0	0	0	0	60
24026973	f	ph	JP	2024	0	0	0	0	0	0	0	0	0	0	0	0	70
24026974	m	cn	IL	2024	0	0	0	0	0	0	0	0	0	0	0	0	
24026975	f	ph	JP	2024	0	0	0	0	0	0	0	0	0	0	0	0	60
24026979	f	md	JP	2024	0	0	0	0	0	0	0	0	0	0	0	0	70
24027000	m	md	CN	2024	0	0	0	0	0	0	0	0	0	0	0	0	53
24027005	f	hp	DK	2024	0	0	0	0	0	0	0	0	0	0	0	0	38
24027007	f	md	DK	2024	0	0	0	0	0	0	0	0	0	0	0	0	
24027017	f	ph	US	2024	0	0	0	0	0	0	0	0	0	0	0	0	
24027021	f	md	JP	2024	0	0	0	0	0	0	0	0	0	0	0	0	
24027027	f	md	DK	2024	0	0	0	0	0	0	0	0	0	0	0	0	43
24027030	m	md	JP	2024	0	0	0	0	0	0	0	0	0	0	0	0	
24027042	m	md	PT	2024	0	0	0	0	0	0	0	0	0	0	0	0	72
24027045	m	md	TR	2024	0	0	0	0	0	0	0	0	0	0	0	0	36

24027055	m	md	TR	2024	0	0	0	0	0	0	0	0	0	0	0	0	37
24027072	m	md	JP	2024	0	0	0	0	0	0	0	0	0	0	0	0	70
24027076	Not	md	ES	2024	0	0	0	0	0	0	0	0	0	0	0	0	65
24027078	m	md	JP	2024	0	0	0	0	0	0	0	0	0	0	0	0	90
24027111	f	md	PT	2024	0	0	0	0	0	0	0	0	0	0	0	0	43
24027217	f	md	CA	2024	0	1	1	0	0	0	0	0	0	0	0	0	67
24027267	f	md	GR	2024	0	0	0	0	0	0	0	0	0	0	0	0	74
24027485	f	md	DE	2024	0	0	0	0	0	0	0	0	0	0	0	0	
24028010	f	md	BE	2024	0	1	1	0	0	0	0	0	0	0	0	0	69
24029201	m	hp	US	2024	0	1	0	1	0	0	0	0	0	0	0	0	65
24029672	m	hp	JP	2024	1	0	0	0	0	0	0	0	0	0	0	0	
24029969	f	md	US	2024	1	0	0	0	0	0	0	0	0	0	0	0	60
24030127	f	md	JP	2024	1	0	0	0	0	0	0	0	0	0	0	0	60
24030313	m	ph	MY	2024	0	0	0	0	0	0	0	0	0	0	0	0	51
24030322	m	md	JP	2024	0	1	0	1	0	0	0	0	0	0	0	0	
24030330	Not	md	JP	2024	0	1	0	1	0	0	0	0	0	0	0	0	
24031761	f	md	NL	2024	0	0	0	0	0	0	0	0	0	0	0	0	
24031906	Not	md	JP	2024	0	0	0	0	0	0	0	0	0	0	0	0	
24032099	f	hp	US	2024	0	0	0	0	0	0	0	0	0	0	0	0	53
23384329	m	md	JP	2024	0	1	0	1	0	0	0	0	0	0	0	0	81
23637127	m	md	ES	2024	0	1	1	0	0	0	0	0	0	0	0	0	55
23648685	m	md	JP	2024	0	1	1	0	0	0	0	0	0	0	0	0	76
23657625	f	md	BR	2024	0	0	0	0	0	0	0	0	0	0	0	0	51
23666552	m	md	JP	2024	0	1	0	0	1	0	0	0	0	0	0	0	77
23672275	f	md	JP	2024	0	1	0	0	1	0	0	0	0	0	0	0	72
23690998	f	md	DE	2024	0	1	0	0	1	0	0	0	0	0	0	0	67
23691816	f	md	JP	2024	0	1	0	0	0	1	0	0	0	0	0	0	75
23694676	Not	md	JP	2024	0	1	0	0	1	0	0	0	0	0	0	0	
23700075	m	md	JP	2024	0	1	0	0	0	1	0	0	0	0	0	0	70
23715605	m	md	JP	2024	0	0	0	0	0	0	0	0	0	0	0	0	
23745683	m	md	JP	2024	0	1	1	0	0	0	0	0	0	0	0	1	82
23747521	m	md	JP	2024	0	1	0	0	1	0	0	0	0	0	0	0	76
23751010	f	md	ES	2024	0	1	1	0	0	0	0	0	0	0	0	0	58
23752712	f	hp	CA	2024	0	0	0	0	0	0	0	0	0	0	0	0	64

23753233	f	md	CZ	2024	0	1	0	0	1	0	0	0	0	0	0	0	48
23753493	f	md	ES	2024	0	1	1	0	0	0	0	0	0	0	0	0	58
23761645	f	hp	CA	2024	0	0	0	0	0	0	0	0	0	0	0	0	64
23771881	f	md	JP	2024	0	1	0	0	0	1	0	0	0	0	0	0	71
23797406	f	md	IT	2024	0	1	1	0	0	0	0	0	0	0	0	0	77
23812725	m	md	DE	2024	0	1	0	0	1	0	0	0	0	0	0	0	70
23817612	f	hp	IT	2024	0	1	0	0	1	0	0	0	0	0	0	0	66
23827671	m	cn	US	2024	1	0	0	0	0	0	0	0	0	0	0	0	38
23829748	f	md	ES	2024	0	1	1	0	0	0	0	0	0	0	0	0	82
23831034	f	cn	IN	2024	0	0	0	0	0	0	0	0	0	0	0	0	41
23833018	f	md	DE	2024	0	1	0	0	1	0	0	0	0	0	0	0	78
23833030	m	md	DE	2024	0	1	0	0	1	0	0	0	0	0	0	0	53
23838614	f	md	DE	2024	0	1	0	0	1	0	0	0	0	0	0	0	57
23839454	m	md	MX	2024	0	0	0	0	0	0	0	0	0	0	0	0	72
23840004	m	md	DE	2024	0	1	0	0	1	0	0	0	0	0	0	0	70
23843203	f	md	AU	2024	0	1	0	0	1	0	0	0	0	0	0	0	73
23850810	Not	md	LT	2024	0	1	0	0	0	1	0	0	0	0	0	0	
23852079	f	hp	IT	2024	0	1	0	0	1	0	0	0	0	0	0	0	66
23853814	m	md	JP	2024	0	1	1	0	0	0	0	0	0	0	0	0	68
23857102	f	md	JP	2024	0	1	1	0	0	0	0	0	0	0	0	1	61
23858249	f	md	ES	2024	0	1	1	0	0	0	0	0	0	0	0	0	82
23876147	f	cn	JP	2024	0	0	0	0	0	0	0	0	0	0	0	0	55
23880791	m	md	JP	2024	0	0	0	0	0	0	0	0	0	0	0	0	72
23880915	f	md	TR	2024	0	0	0	0	0	0	0	0	0	0	0	0	
23883614	m	md	JP	2024	0	1	0	1	0	0	0	0	0	0	0	0	59.9
23889293	f	md	JP	2024	0	0	0	0	0	0	0	0	0	0	0	0	74
23889674	f	md	GR	2024	0	1	0	0	1	0	0	0	0	0	0	0	63
23889975	f	md	NZ	2024	0	0	0	0	0	0	0	0	0	0	0	0	28
23906075	f	md	JP	2024	0	1	1	0	0	0	0	0	0	0	0	0	70
23906898	m	md	JP	2024	0	0	0	0	0	0	0	0	0	0	0	0	55
23908378	m	md	DE	2024	0	1	1	0	0	0	0	0	0	0	0	0	
23916919	m	md	DE	2024	0	1	0	0	1	0	0	0	0	0	0	0	83
23919694	f	hp	US	2024	0	0	0	0	0	0	0	0	0	0	0	0	64
23923494	f	md	KE	2024	1	0	0	0	0	0	0	0	0	0	0	0	

23927790	f	cn	TH	2024	0	1	0	0	0	1	0	0	0	0	0	0	80
23941764	f	md	JP	2024	0	1	1	0	0	0	0	0	0	0	0	0	90
23946635	f	md	ES	2024	0	1	1	0	0	0	0	0	0	0	0	0	58
23946852	f	md	JP	2024	0	0	0	0	0	0	0	0	0	0	0	0	70
23948215	f	md	JP	2024	0	0	0	0	0	0	0	0	0	0	0	0	73
23953433	Not	md	MY	2024	0	1	0	0	0	1	0	0	0	0	0	0	
23971946	f	hp	US	2024	0	0	0	0	0	0	0	0	0	0	0	0	37
23977769	f	md	JP	2024	1	0	0	0	0	0	0	0	0	0	0	0	72
23978443	f	md	JP	2024	1	0	0	0	0	0	0	0	0	0	0	1	79
23980255	m	md	DE	2024	0	1	0	0	1	0	0	0	0	0	0	0	53
23980907	m	ph	JP	2024	0	0	0	0	0	0	0	0	0	0	0	0	73
23982172	m	md	JP	2024	0	1	1	0	0	0	0	0	0	0	0	0	74
23984002	m	md	DE	2024	0	1	0	0	1	0	0	0	0	0	0	0	53
23984858	f	md	ES	2024	1	0	0	0	0	0	0	0	0	0	0	0	71
23989515	f	hp	IT	2024	0	1	0	1	0	0	0	0	0	0	0	0	71
23991197	f	md	FR	2024	0	1	1	0	0	0	0	0	0	0	0	0	
23992192	m	md	PL	2024	0	1	0	0	0	1	0	0	0	0	0	0	82
23994890	f	cn	CA	2024	0	0	0	0	0	0	0	0	0	0	0	0	53
23995032	f	md	JP	2024	0	1	0	0	0	1	0	0	0	0	0	0	86
23996358	f	md	JP	2024	0	1	0	1	0	0	0	0	0	0	0	0	71
24001151	f	md	FR	2024	1	0	0	0	0	0	0	0	0	0	0	0	85
24001962	f	ph	FR	2024	0	0	0	0	0	0	0	0	0	0	0	0	67
24004488	f	md	JP	2024	0	1	0	0	1	0	0	0	0	0	0	1	79
24005400	m	ph	JP	2024	0	1	1	0	0	0	0	0	0	0	0	0	73
24007059	f	md	BR	2024	0	1	1	0	0	0	0	0	0	0	0	0	
24010225	m	hp	GE	2024	0	1	0	0	0	1	0	0	0	0	0	0	51
24010746	Not	md	JP	2024	0	1	0	0	1	0	0	0	0	0	0	0	50
24010915	m	md	BE	2024	0	0	0	0	0	0	0	0	0	0	0	0	
24011081	m	md	JP	2024	0	1	0	1	0	0	0	0	0	0	0	0	73
24014812	f	md	JP	2024	0	0	0	0	0	0	0	0	0	0	0	0	
24016530	m	hp	DE	2024	0	1	0	0	1	0	0	0	0	0	0	0	62
24017038	m	md	KR	2024	1	0	0	0	0	0	0	0	0	0	0	0	79
24019487	m	md	US	2024	0	0	0	0	0	0	0	0	0	0	0	0	33
24021106	m	hp	CN	2024	1	0	0	0	0	0	0	0	0	0	0	0	67

24022314	f	md	JP	2024	0	0	0	0	0	0	0	0	0	0	0	0	76
24022322	f	md	JP	2024	0	0	0	0	0	0	0	0	0	0	0	0	
24022410	m	md	AU	2024	0	0	0	0	0	0	0	0	0	0	0	0	46
24022421	m	md	TR	2024	0	0	0	0	0	0	0	0	0	0	0	0	71
24022445	f	md	AR	2024	0	0	0	0	0	0	0	0	0	0	0	0	83
24022451	m	md	TR	2024	0	0	0	0	0	0	0	0	0	0	0	0	57
24022458	m	md	TR	2024	0	0	0	0	0	0	0	0	0	0	0	0	50
24022463	f	md	AT	2024	0	0	0	0	0	0	0	0	0	0	0	0	59
24022469	f	md	JP	2024	0	0	0	0	0	0	0	0	0	0	0	0	85
24022470	m	md	AU	2024	0	0	0	0	0	0	0	0	0	0	0	0	39
24022499	f	md	CN	2024	0	0	0	0	0	0	0	0	0	0	0	0	42
24022525	m	md	CN	2024	0	0	0	0	0	0	0	0	0	0	0	0	44
24022561	m	cn	CA	2024	0	0	0	0	0	0	0	0	0	0	0	0	
24022587	f	md	DE	2024	0	0	0	0	0	0	0	0	0	0	0	0	
24022597	m	cn	CN	2024	0	0	0	0	0	0	0	0	0	0	0	0	58
24022605	f	cn	CO	2024	0	0	0	0	0	0	0	0	0	0	0	0	30
24022609	f	md	AT	2024	0	0	0	0	0	0	0	0	0	0	0	0	60
24022616	f	md	IT	2024	0	0	0	0	0	0	0	0	0	0	0	0	
24022628	m	md	AU	2024	0	0	0	0	0	0	0	0	0	0	0	0	
24022634	m	md	AU	2024	0	0	0	0	0	0	0	0	0	0	0	0	66
24022651	f	md	AU	2024	0	0	0	0	0	0	0	0	0	0	0	0	45
24022653	m	md	JP	2024	0	0	0	0	0	0	0	0	0	0	0	0	58
24022660	m	md	CN	2024	0	0	0	0	0	0	0	0	0	0	0	0	60
24022661	f	md	CN	2024	0	0	0	0	0	0	0	0	0	0	0	0	49
24022690	f	md	PT	2024	0	0	0	0	0	0	0	0	0	0	0	0	42
24022694	f	cn	US	2024	0	0	0	0	0	0	0	0	0	0	0	0	
24022725	m	md	PT	2024	0	0	0	0	0	0	0	0	0	0	0	0	72
24022728	f	md	IL	2024	0	0	0	0	0	0	0	0	0	0	0	0	86
24022729	f	md	IT	2024	0	0	0	0	0	0	0	0	0	0	0	0	66
24022732	m	md	IT	2024	0	0	0	0	0	0	0	0	0	0	0	0	80
24022740	f	md	VN	2024	0	0	0	0	0	0	0	0	0	0	0	0	45
24022743	f	md	PE	2024	0	0	0	0	0	0	0	0	0	0	0	0	64
24022746	f	cn	CN	2024	0	0	0	0	0	0	0	0	0	0	0	0	43
24022777	f	md	CL	2024	0	0	0	0	0	0	0	0	0	0	0	0	57

24022795	m	md	IL	2024	0	0	0	0	0	0	0	0	0	0	0	0	51
24022811	f	md	AT	2024	0	0	0	0	0	0	0	0	0	0	0	0	56
24022824	f	md	TR	2024	0	0	0	0	0	0	0	0	0	0	0	0	62
24022828	m	md	IT	2024	0	0	0	0	0	0	0	0	0	0	0	0	63
24022842	m	cn	CN	2024	0	0	0	0	0	0	0	0	0	0	0	0	74
24022848	m	md	IT	2024	0	0	0	0	0	0	0	0	0	0	0	0	60
24022864	f	cn	US	2024	0	0	0	0	0	0	0	0	0	0	0	0	74
24022867	m	md	TR	2024	0	0	0	0	0	0	0	0	0	0	0	0	37
24022892	f	md	CN	2024	0	0	0	0	0	0	0	0	0	0	0	0	56
24022904	f	md	CN	2024	0	0	0	0	0	0	0	0	0	0	0	0	42
24022911	f	cn	CA	2024	0	0	0	0	0	0	0	0	0	0	0	0	83
24022920	m	md	JP	2024	0	0	0	0	0	0	0	0	0	0	0	0	
24022944	m	md	CO	2024	0	0	0	0	0	0	0	0	0	0	0	0	65
24022949	m	md	TR	2024	0	0	0	0	0	0	0	0	0	0	0	0	
24022956	f	md	AU	2024	0	0	0	0	0	0	0	0	0	0	0	0	64
24022969	f	md	CN	2024	0	0	0	0	0	0	0	0	0	0	0	0	71
24022974	m	md	TR	2024	0	0	0	0	0	0	0	0	0	0	0	0	37
24022975	f	md	CN	2024	0	0	0	0	0	0	0	0	0	0	0	0	64
24022977	Not	md	JP	2024	0	0	0	0	0	0	0	0	0	0	0	0	40
24022983	m	md	TR	2024	0	0	0	0	0	0	0	0	0	0	0	0	68
24022992	Not	md	JP	2024	0	0	0	0	0	0	0	0	0	0	0	0	40
24023002	f	md	CN	2024	0	0	0	0	0	0	0	0	0	0	0	0	57
24023008	f	md	IT	2024	0	0	0	0	0	0	0	0	0	0	0	0	64
24023037	f	md	CN	2024	0	0	0	0	0	0	0	0	0	0	0	0	66
24023041	m	md	CO	2024	0	0	0	0	0	0	0	0	0	0	0	0	84
24023069	f	md	IL	2024	0	0	0	0	0	0	0	0	0	0	0	0	70
24023076	m	md	IL	2024	0	0	0	0	0	0	0	0	0	0	0	0	69
24023091	m	md	BE	2024	0	0	0	0	0	0	0	0	0	0	0	0	74
24023101	m	ph	JP	2024	0	0	0	0	0	0	0	0	0	0	0	0	
24023104	f	md	CN	2024	0	0	0	0	0	0	0	0	0	0	0	0	70
24023115	m	md	CO	2024	0	0	0	0	0	0	0	0	0	0	0	0	84
24023129	m	md	AU	2024	0	0	0	0	0	0	0	0	0	0	0	0	38
24023133	f	md	CN	2024	0	0	0	0	0	0	0	0	0	0	0	0	64
24023139	f	md	RS	2024	0	0	0	0	0	0	0	0	0	0	0	0	62

24023152	f	md	IT	2024	0	0	0	0	0	0	0	0	0	0	0	0	75
24023153	m	md	BE	2024	0	0	0	0	0	0	0	0	0	0	0	0	74
24023163	f	md	TR	2024	0	0	0	0	0	0	0	0	0	0	0	0	49
24023179	f	md	TR	2024	0	0	0	0	0	0	0	0	0	0	0	0	84
24023189	f	md	CN	2024	0	0	0	0	0	0	0	0	0	0	0	0	58
24023190	m	md	TR	2024	0	0	0	0	0	0	0	0	0	0	0	0	37
24023236	f	md	IT	2024	0	0	0	0	0	0	0	0	0	0	0	0	67
24023257	m	md	CN	2024	0	0	0	0	0	0	0	0	0	0	0	0	59
24023258	f	md	CN	2024	0	0	0	0	0	0	0	0	0	0	0	0	53
24023261	m	md	RS	2024	0	0	0	0	0	0	0	0	0	0	0	0	52
24023267	f	md	DK	2024	0	0	0	0	0	0	0	0	0	0	0	0	72
24023276	m	md	TR	2024	0	0	0	0	0	0	0	0	0	0	0	0	58
24023284	m	md	TR	2024	0	0	0	0	0	0	0	0	0	0	0	0	41
24023285	m	md	IT	2024	0	0	0	0	0	0	0	0	0	0	0	0	75
24023294	m	md	IT	2024	0	0	0	0	0	0	0	0	0	0	0	0	63
24023299	m	md	CO	2024	0	0	0	0	0	0	0	0	0	0	0	0	
24023317	m	md	BE	2024	0	0	0	0	0	0	0	0	0	0	0	0	45
24023332	m	md	IT	2024	0	0	0	0	0	0	0	0	0	0	0	0	68
24023339	m	md	CN	2024	0	0	0	0	0	0	0	0	0	0	0	0	60
24023346	m	md	PT	2024	0	0	0	0	0	0	0	0	0	0	0	0	75
24023349	f	cn	CA	2024	0	0	0	0	0	0	0	0	0	0	0	0	63
24023363	f	md	IL	2024	0	0	0	0	0	0	0	0	0	0	0	0	51
24023373	f	cn	US	2024	0	0	0	0	0	0	0	0	0	0	0	0	40
24023383	Not	md	JP	2024	0	0	0	0	0	0	0	0	0	0	0	0	
24023408	m	md	BE	2024	0	0	0	0	0	0	0	0	0	0	0	0	69
24023460	f	md	BE	2024	0	0	0	0	0	0	0	0	0	0	0	0	83
24023482	f	cn	CA	2024	0	0	0	0	0	0	0	0	0	0	0	0	
24023495	m	cn	CN	2024	0	0	0	0	0	0	0	0	0	0	0	0	53
24023589	f	md	CN	2024	0	0	0	0	0	0	0	0	0	0	0	0	51
24023599	f	cn	CN	2024	0	0	0	0	0	0	0	0	0	0	0	0	37
24023611	f	md	BE	2024	0	0	0	0	0	0	0	0	0	0	0	0	36
24023617	f	md	AU	2024	0	0	0	0	0	0	0	0	0	0	0	0	56
24023662	f	md	CN	2024	0	0	0	0	0	0	0	0	0	0	0	0	53
24023673	m	md	IT	2024	0	0	0	0	0	0	0	0	0	0	0	0	52

24023700	f	md	AU	2024	0	0	0	0	0	0	0	0	0	0	0	0	41
24023723	f	md	IT	2024	0	0	0	0	0	0	0	0	0	0	0	0	66
24023729	m	md	AT	2024	0	0	0	0	0	0	0	0	0	0	0	0	69
24023730	f	md	CN	2024	0	0	0	0	0	0	0	0	0	0	0	0	64
24023763	f	md	AU	2024	0	0	0	0	0	0	0	0	0	0	0	0	46
24023765	f	cn	CN	2024	0	0	0	0	0	0	0	0	0	0	0	0	56
24023771	f	md	CN	2024	0	0	0	0	0	0	0	0	0	0	0	0	53
24023783	f	md	DK	2024	0	0	0	0	0	0	0	0	0	0	0	0	71
24023805	m	cn	CN	2024	0	0	0	0	0	0	0	0	0	0	0	0	58
24023817	m	md	IT	2024	0	0	0	0	0	0	0	0	0	0	0	0	63
24023836	f	md	IT	2024	0	0	0	0	0	0	0	0	0	0	0	0	50
24023886	f	md	IT	2024	0	0	0	0	0	0	0	0	0	0	0	0	46
24023924	f	md	IL	2024	0	0	0	0	0	0	0	0	0	0	0	0	45
24023930	f	md	TR	2024	0	0	0	0	0	0	0	0	0	0	0	0	57
24023933	f	md	IT	2024	0	0	0	0	0	0	0	0	0	0	0	0	47
24023952	f	md	TR	2024	0	0	0	0	0	0	0	0	0	0	0	0	59
24023993	m	md	LT	2024	0	0	0	0	0	0	0	0	0	0	0	0	45
24023994	f	cn	CN	2024	0	0	0	0	0	0	0	0	0	0	0	0	67
24024017	m	md	AU	2024	0	0	0	0	0	0	0	0	0	0	0	0	45
24024078	f	md	CN	2024	0	0	0	0	0	0	0	0	0	0	0	0	71
24024091	m	md	TR	2024	0	0	0	0	0	0	0	0	0	0	0	0	48
24024092	f	md	AU	2024	0	0	0	0	0	0	0	0	0	0	0	0	43
24024109	f	md	RO	2024	0	0	0	0	0	0	0	0	0	0	0	0	72
24024114	f	md	VN	2024	0	0	0	0	0	0	0	0	0	0	0	0	32
24024134	f	cn	US	2024	0	0	0	0	0	0	0	0	0	0	0	0	
24024143	f	md	TR	2024	0	0	0	0	0	0	0	0	0	0	0	0	67
24024151	f	md	AU	2024	0	0	0	0	0	0	0	0	0	0	0	0	79
24024174	m	md	CN	2024	0	0	0	0	0	0	0	0	0	0	0	0	71
24024191	m	md	CO	2024	0	0	0	0	0	0	0	0	0	0	0	0	84
24024218	m	md	CN	2024	0	0	0	0	0	0	0	0	0	0	0	0	81
24024220	m	md	DK	2024	0	0	0	0	0	0	0	0	0	0	0	0	50
24024273	m	md	AR	2024	0	0	0	0	0	0	0	0	0	0	0	0	34
24024287	m	md	CN	2024	0	0	0	0	0	0	0	0	0	0	0	0	83
24024305	f	md	IT	2024	0	0	0	0	0	0	0	0	0	0	0	0	52

24024314	f	cn	CN	2024	0	0	0	0	0	0	0	0	0	0	0	0	59
24024318	f	md	CN	2024	0	0	0	0	0	0	0	0	0	0	0	0	44
24024323	m	md	FI	2024	0	0	0	0	0	0	0	0	0	0	0	0	60
24024355	f	cn	IL	2024	0	0	0	0	0	0	0	0	0	0	0	0	60
24024365	f	md	CN	2024	0	0	0	0	0	0	0	0	0	0	0	0	58
24024369	f	md	IT	2024	0	0	0	0	0	0	0	0	0	0	0	0	68
24024371	f	md	AT	2024	0	0	0	0	0	0	0	0	0	0	0	0	54
24024428	f	md	AU	2024	0	0	0	0	0	0	0	0	0	0	0	0	78
24024431	f	md	CN	2024	0	0	0	0	0	0	0	0	0	0	0	0	72
24024439	f	cn	IL	2024	0	0	0	0	0	0	0	0	0	0	0	0	77
24024456	f	md	AT	2024	0	0	0	0	0	0	0	0	0	0	0	0	59
24024459	m	md	IL	2024	0	0	0	0	0	0	0	0	0	0	0	0	68
24024491	m	md	CO	2024	0	0	0	0	0	0	0	0	0	0	0	0	84
24024506	f	md	JP	2024	0	0	0	0	0	0	0	0	0	0	0	0	90
24024525	m	md	CN	2024	0	0	0	0	0	0	0	0	0	0	0	0	55
24024533	m	md	BE	2024	0	0	0	0	0	0	0	0	0	0	0	0	79
24024553	m	md	JP	2024	0	0	0	0	0	0	0	0	0	0	0	0	80
24024596	f	md	PT	2024	0	0	0	0	0	0	0	0	0	0	0	0	66
24024603	Not	md	JP	2024	0	0	0	0	0	0	0	0	0	0	0	0	50
24024613	f	md	AE	2024	0	0	0	0	0	0	0	0	0	0	0	0	56
24024653	f	md	AU	2024	0	0	0	0	0	0	0	0	0	0	0	0	41
24024658	f	md	JP	2024	0	0	0	0	0	0	0	0	0	0	0	0	49
24024661	m	cn	CA	2024	0	0	0	0	0	0	0	0	0	0	0	0	
24024676	f	md	TR	2024	0	0	0	0	0	0	0	0	0	0	0	0	73
24024697	m	md	CN	2024	0	0	0	0	0	0	0	0	0	0	0	0	78
24024730	f	md	TR	2024	0	0	0	0	0	0	0	0	0	0	0	0	51
24024753	f	md	TR	2024	0	0	0	0	0	0	0	0	0	0	0	0	65
24024794	f	md	PE	2024	0	0	0	0	0	0	0	0	0	0	0	0	71
24024796	f	md	CN	2024	0	0	0	0	0	0	0	0	0	0	0	0	64
24024816	f	cn	MX	2024	0	0	0	0	0	0	0	0	0	0	0	0	42
24024834	m	cn	CA	2024	0	0	0	0	0	0	0	0	0	0	0	0	
24024844	f	md	TR	2024	0	0	0	0	0	0	0	0	0	0	0	0	31
24024849	f	md	PT	2024	0	0	0	0	0	0	0	0	0	0	0	0	62
24024856	f	md	CN	2024	0	0	0	0	0	0	0	0	0	0	0	0	73

24024879	f	md	CN	2024	0	0	0	0	0	0	0	0	0	0	0	0	33
24024883	f	md	CN	2024	0	0	0	0	0	0	0	0	0	0	0	0	40
24024925	f	md	RU	2024	0	0	0	0	0	0	0	0	0	0	0	0	40
24024943	m	md	RO	2024	0	0	0	0	0	0	0	0	0	0	0	0	59
24025006	m	md	JP	2024	0	0	0	0	0	0	0	0	0	0	0	0	60
24025010	f	cn	CN	2024	0	0	0	0	0	0	0	0	0	0	0	0	53
24025025	f	md	CN	2024	0	0	0	0	0	0	0	0	0	0	0	0	56
24025074	f	md	DE	2024	0	0	0	0	0	0	0	0	0	0	0	0	
24025085	m	cn	CO	2024	0	0	0	0	0	0	0	0	0	0	0	0	59
24025099	m	md	DK	2024	0	0	0	0	0	0	0	0	0	0	0	0	53
24025114	f	md	CN	2024	0	0	0	0	0	0	0	0	0	0	0	0	48
24025136	f	md	AU	2024	0	0	0	0	0	0	0	0	0	0	0	0	33
24025169	f	cn	IL	2024	0	0	0	0	0	0	0	0	0	0	0	0	77
24025231	Not	md	ES	2024	0	0	0	0	0	0	0	0	0	0	0	0	55
24025252	f	md	VN	2024	0	0	0	0	0	0	0	0	0	0	0	0	69
24025295	m	cn	IL	2024	0	0	0	0	0	0	0	0	0	0	0	0	
24025298	f	md	VN	2024	0	0	0	0	0	0	0	0	0	0	0	0	69
24025343	f	md	VN	2024	0	0	0	0	0	0	0	0	0	0	0	0	65
24025348	m	md	VN	2024	0	0	0	0	0	0	0	0	0	0	0	0	
24025404	f	md	IT	2024	0	0	0	0	0	0	0	0	0	0	0	0	49
24025409	Not	md	ES	2024	0	0	0	0	0	0	0	0	0	0	0	0	54
24025442	f	md	CN	2024	0	0	0	0	0	0	0	0	0	0	0	0	41
24025453	f	md	AU	2024	0	0	0	0	0	0	0	0	0	0	0	0	52
24025504	f	md	JP	2024	0	0	0	0	0	0	0	0	0	0	0	0	50
24025602	m	cn	CN	2024	0	0	0	0	0	0	0	0	0	0	0	0	52
24025604	f	md	VN	2024	0	0	0	0	0	0	0	0	0	0	0	0	38
24025618	m	md	IL	2024	0	0	0	0	0	0	0	0	0	0	0	0	66
24025676	f	md	IL	2024	0	0	0	0	0	0	0	0	0	0	0	0	67
24025684	m	md	BE	2024	0	0	0	0	0	0	0	0	0	0	0	0	79
24025702	m	cn	CA	2024	0	0	0	0	0	0	0	0	0	0	0	0	
24025712	f	md	FI	2024	0	0	0	0	0	0	0	0	0	0	0	0	75
24025713	m	md	IT	2024	0	0	0	0	0	0	0	0	0	0	0	0	84
24025714	m	md	IT	2024	0	0	0	0	0	0	0	0	0	0	0	0	84
24025718	f	md	RU	2024	0	0	0	0	0	0	0	0	0	0	0	0	61

24025737	m	md	CN	2024	0	0	0	0	0	0	0	0	0	0	0	0	59
24025753	f	cn	CO	2024	0	0	0	0	0	0	0	0	0	0	0	0	30
24025758	f	md	PT	2024	0	1	1	0	0	0	0	0	0	0	0	0	
24025764	f	md	TR	2024	0	0	0	0	0	0	0	0	0	0	0	0	52
24025795	m	md	BE	2024	0	0	0	0	0	0	0	0	0	0	0	0	56
24025799	f	cn	CA	2024	0	0	0	0	0	0	0	0	0	0	0	0	
24025818	f	md	IT	2024	0	0	0	0	0	0	0	0	0	0	0	0	55
24025820	f	md	TR	2024	0	0	0	0	0	0	0	0	0	0	0	0	52
24025821	f	md	BG	2024	0	0	0	0	0	0	0	0	0	0	0	0	76
24025860	m	cn	IN	2024	0	0	0	0	0	0	0	0	0	0	0	0	62
24025873	m	md	IL	2024	0	0	0	0	0	0	0	0	0	0	0	0	62
24025974	Not	md	ES	2024	0	0	0	0	0	0	0	0	0	0	0	0	82
24025998	m	md	IN	2024	0	0	0	0	0	0	0	0	0	0	0	0	68
24026069	f	md	TR	2024	0	0	0	0	0	0	0	0	0	0	0	0	73
24026075	f	cn	TH	2024	0	0	0	0	0	0	0	0	0	0	0	0	59
24026089	f	md	AT	2024	0	0	0	0	0	0	0	0	0	0	0	0	57
24026165	f	md	CN	2024	0	0	0	0	0	0	0	0	0	0	0	0	51
24026205	m	md	IT	2024	0	0	0	0	0	0	0	0	0	0	0	0	69
24026209	f	md	CN	2024	0	0	0	0	0	0	0	0	0	0	0	0	30
24026241	f	md	JP	2024	0	0	0	0	0	0	0	0	0	0	0	0	57
24026255	m	md	BE	2024	0	0	0	0	0	0	0	0	0	0	0	0	44
24026297	f	md	CN	2024	0	0	0	0	0	0	0	0	0	0	0	0	61
24026335	f	md	RS	2024	0	0	0	0	0	0	0	0	0	0	0	0	56
24026358	Not	md	ES	2024	0	0	0	0	0	0	0	0	0	0	0	0	42
24026370	f	cn	CO	2024	0	0	0	0	0	0	0	0	0	0	0	0	74
24026374	f	md	AT	2024	0	0	0	0	0	0	0	0	0	0	0	0	49
24026388	m	md	CN	2024	0	0	0	0	0	0	0	0	0	0	0	0	64
24026409	f	md	IT	2024	0	0	0	0	0	0	0	0	0	0	0	0	55
24026523	f	cn	US	2024	0	0	0	0	0	0	0	0	0	0	0	0	
24026526	m	md	IL	2024	0	0	0	0	0	0	0	0	0	0	0	0	63
24026527	m	ph	JP	2024	0	0	0	0	0	0	0	0	0	0	0	0	43
24026554	m	md	IT	2024	0	0	0	0	0	0	0	0	0	0	0	0	80
24026567	m	md	AR	2024	0	0	0	0	0	0	0	0	0	0	0	0	66
24026624	m	md	CN	2024	0	0	0	0	0	0	0	0	0	0	0	0	64

24026735	f	md	TR	2024	0	0	0	0	0	0	0	0	0	0	0	0	
24026787	f	md	CL	2024	0	0	0	0	0	0	0	0	0	0	0	0	55
24026797	f	md	CN	2024	0	0	0	0	0	0	0	0	0	0	0	0	55
24026801	f	md	IT	2024	0	0	0	0	0	0	0	0	0	0	0	0	54
24026821	m	md	IT	2024	0	0	0	0	0	0	0	0	0	0	0	0	78
24026843	m	md	AR	2024	0	0	0	0	0	0	0	0	0	0	0	0	50
24026856	f	md	IT	2024	0	0	0	0	0	0	0	0	0	0	0	0	46
24026869	f	md	LT	2024	0	0	0	0	0	0	0	0	0	0	0	0	54
24026870	f	md	DK	2024	0	0	0	0	0	0	0	0	0	0	0	0	37
24026871	f	md	DK	2024	0	0	0	0	0	0	0	0	0	0	0	0	43
24026894	f	ph	JP	2024	1	0	0	0	0	0	0	0	0	0	0	0	72
24026909	f	md	CN	2024	0	0	0	0	0	0	0	0	0	0	0	0	60
24026910	f	md	CN	2024	0	0	0	0	0	0	0	0	0	0	0	0	65
24026912	f	md	RS	2024	0	0	0	0	0	0	0	0	0	0	0	0	44
24026913	m	md	RS	2024	0	0	0	0	0	0	0	0	0	0	0	0	60
24026914	f	md	RS	2024	0	0	0	0	0	0	0	0	0	0	0	0	56
24026919	m	md	LT	2024	0	0	0	0	0	0	0	0	0	0	0	0	45
24026941	f	md	IT	2024	0	0	0	0	0	0	0	0	0	0	0	0	63
24026966	f	md	CN	2024	0	0	0	0	0	0	0	0	0	0	0	0	64
24026970	m	md	TR	2024	0	0	0	0	0	0	0	0	0	0	0	0	71
24027019	m	md	TR	2024	0	0	0	0	0	0	0	0	0	0	0	0	35
24027020	f	md	CN	2024	0	0	0	0	0	0	0	0	0	0	0	0	78
24027028	f	md	CN	2024	0	0	0	0	0	0	0	0	0	0	0	0	78
24027033	m	md	LT	2024	0	0	0	0	0	0	0	0	0	0	0	0	50
24027058	m	md	CO	2024	0	0	0	0	0	0	0	0	0	0	0	0	40
24027075	f	md	CO	2024	0	0	0	0	0	0	0	0	0	0	0	0	46
24027077	m	cn	CA	2024	0	0	0	0	0	0	0	0	0	0	0	0	
24027080	f	md	RS	2024	0	0	0	0	0	0	0	0	0	0	0	0	75
24027092	m	md	ID	2024	0	0	0	0	0	0	0	0	0	0	0	0	56
24027096	m	cn	TH	2024	0	0	0	0	0	0	0	0	0	0	0	0	54
24028897	f	ph	JP	2024	0	1	0	0	0	1	0	0	0	0	0	0	70
24032458	f	cn	US	2024	0	1	1	0	0	0	0	0	0	0	0	0	
24032780	f	hp	US	2024	0	1	1	0	0	0	0	0	0	0	0	0	70
24032923	m	md	US	2024	0	0	0	0	0	0	0	0	0	0	0	0	72

24033187	m	cn	US	2024	0	1	1	0	0	0	0	0	0	0	0	0	75
24035002	m	md	GR	2024	0	1	0	0	1	0	0	0	0	0	0	0	53
24035116	m	md	BR	2024	1	0	0	0	0	0	0	0	0	0	0	0	64
24035510	f	md	DE	2024	0	0	0	0	0	0	0	0	0	0	0	0	46
24035576	m	md	US	2024	0	0	0	0	0	0	0	0	0	0	0	0	65
24035608	f	md	DE	2024	0	0	0	0	0	0	0	0	0	0	0	0	73
24036080	m	md	JP	2024	0	1	0	1	0	0	0	0	0	0	0	0	55
24036110	m	md	ES	2024	0	1	0	0	1	0	0	0	0	0	0	1	51
24036161	f	md	DE	2024	0	0	0	0	0	0	0	0	0	0	0	0	
24036317	f	md	DE	2024	0	1	1	0	0	0	0	0	0	0	0	0	61
24036427	m	md	JP	2024	0	1	0	1	0	0	0	0	0	0	0	0	62
24037092	f	md	AU	2024	1	0	0	0	0	0	0	0	0	0	0	0	
24037525	m	md	JP	2024	0	1	0	0	0	1	0	0	0	0	0	0	80
24038637	m	md	JP	2024	0	0	0	0	0	0	0	0	0	0	0	0	34
24039125	f	hp	US	2024	0	1	1	0	0	0	0	0	0	0	0	0	70
24039268	f	hp	DE	2024	0	1	0	0	1	0	0	0	0	0	0	0	64
24039369	m	md	JP	2024	0	1	0	1	0	0	0	0	0	0	0	1	61
24039557	m	ph	JP	2024	0	0	0	0	0	0	0	0	0	0	0	0	
24039705	f	md	US	2024	1	0	0	0	0	0	0	0	0	0	0	0	40
24039745	m	hp	JP	2024	0	1	1	0	0	0	0	0	0	0	0	0	72
24039919	Not	md	JP	2024	0	0	0	0	0	0	0	0	0	0	0	0	
24039925	m	cn	PK	2024	0	1	0	0	0	1	0	0	0	0	0	0	53
24040019	f	hp	US	2024	0	0	0	0	0	0	0	0	0	0	0	0	53
24040245	Not	md	JP	2024	1	0	0	0	0	0	0	0	0	0	0	0	
24040904	m	md	JP	2024	0	1	0	0	0	1	0	0	0	0	0	0	
24042050	Not	md	FR	2024	0	0	0	0	0	0	0	0	0	0	0	0	
24042236	m	md	FR	2024	1	0	0	0	0	0	0	0	0	0	0	0	
24042296	Not	md	JP	2024	1	0	0	0	0	0	0	0	0	0	0	0	
24042319	f	md	DE	2024	0	1	1	0	0	0	0	0	0	0	0	0	
24042416	m	md	IT	2024	0	0	0	0	0	0	0	0	0	0	0	0	
24043317	m	md	JP	2024	0	1	1	0	0	0	0	0	0	0	0	0	70
24043386	m	md	PR	2024	0	0	0	0	0	0	0	0	0	0	0	0	
24043733	f	md	JP	2024	0	0	0	0	0	0	0	0	0	0	0	0	70
24044181	Not	cn	US	2024	0	0	0	0	0	0	0	0	0	0	0	0	

24044278	f	md	GR	2024	0	0	0	0	0	0	0	0	0	0	0	0	74
24044403	f	md	US	2024	1	0	0	0	0	0	0	0	0	0	0	0	50
24044564	f	md	FR	2024	0	1	0	0	1	0	0	0	0	0	0	1	58
24044566	Not	hp	US	2024	0	1	1	0	0	0	0	0	0	0	0	0	
24044754	m	md	JP	2024	0	1	0	0	0	1	0	0	0	0	0	0	50
24045030	f	md	US	2024	1	0	0	0	0	0	0	0	0	0	0	0	60
24045150	f	md	JP	2024	0	1	0	1	0	0	0	0	0	0	0	0	76
24045155	m	md	JP	2024	0	1	0	0	0	1	0	0	0	0	0	1	80
24045539	f	md	US	2024	0	0	0	0	0	0	0	0	0	0	0	0	53
24045870	m	md	CN	2024	0	1	0	0	0	1	0	0	0	0	0	0	
24046789	m	md	JP	2024	0	1	0	1	0	0	0	0	0	0	0	0	60
24046875	f	cn	US	2024	0	0	0	0	0	0	0	0	0	0	0	0	
24047109	f	ph	US	2024	0	0	0	0	0	0	0	0	0	0	0	0	84
24047222	m	hp	JP	2024	0	1	1	0	0	0	0	0	0	0	0	0	72
24047261	f	md	DE	2024	0	1	0	0	1	0	0	0	0	0	0	0	78
24047331	m	md	JP	2024	0	1	1	0	0	0	0	0	0	0	0	0	70
24047442	f	md	JP	2024	1	0	0	0	0	0	0	0	0	0	0	0	75
24047562	m	md	JP	2024	0	0	0	0	0	0	0	0	0	0	0	0	68
24047567	f	cn	NL	2024	0	0	0	0	0	0	0	0	0	0	0	0	
24047862	m	cn	US	2024	0	0	0	0	0	0	0	0	0	0	0	0	62
24048415	m	md	JP	2024	0	1	0	0	0	1	0	0	0	0	0	0	60
24048657	f	md	KR	2024	1	0	0	0	0	0	0	0	0	0	0	0	82
24048813	f	cn	US	2024	0	0	0	0	0	0	0	0	0	0	0	0	65
24049028	m	md	JP	2024	0	1	0	0	0	1	0	0	0	0	0	0	60
24049084	Not	ph	JP	2024	1	0	0	0	0	0	0	0	0	0	0	0	
24049087	Not	ph	JP	2024	1	0	0	0	0	0	0	0	0	0	0	0	
24049091	f	hp	DE	2024	0	1	0	0	1	0	0	0	0	0	0	0	64
24049678	f	hp	US	2024	1	0	0	0	0	0	0	0	0	0	0	0	
24049738	m	md	IT	2024	0	0	0	0	0	0	0	0	0	0	0	0	65
24050102	f	cn	US	2024	1	0	0	0	0	0	0	0	0	0	0	0	
24050238	f	hp	US	2024	0	0	0	0	0	0	0	0	0	0	0	0	53
24050282	Not	hp	KR	2024	1	0	0	0	0	0	0	0	0	0	0	0	39
24050778	f	md	US	2024	0	0	0	0	0	0	0	0	0	0	0	0	65
24050859	Not	md	AU	2024	1	0	0	0	0	0	0	0	0	0	0	0	

24050962	Not	ph	JP	2024	1	0	0	0	0	0	0	0	0	0	0	0	
24050965	m	cn	US	2024	1	0	0	0	0	0	0	0	0	0	0	0	
24051532	f	lw	US	2024	0	0	0	0	0	0	0	0	0	0	0	0	43
24052208	f	hp	US	2024	0	1	1	0	0	0	0	0	0	0	0	0	76
24052228	f	md	NZ	2024	1	0	0	0	0	0	0	0	0	0	0	0	
24052660	Not	hp	CN	2024	0	0	0	0	0	0	0	0	0	0	0	0	
24053056	m	md	JP	2024	0	1	1	0	0	0	0	0	0	0	0	0	73
24053399	Not	md	NL	2024	0	1	0	0	0	1	0	0	0	0	0	0	
24053420	Not	hp	US	2024	0	0	0	0	0	0	0	0	0	0	0	0	
24053432	f	md	US	2024	0	0	0	0	0	0	0	0	0	0	0	0	57
24054008	f	cn	US	2024	1	0	0	0	0	0	0	0	0	0	0	0	62.3
24054328	m	md	GR	2024	0	1	0	0	1	0	0	0	0	0	0	0	71
24054540	m	md	JP	2024	0	1	0	0	0	1	0	0	0	0	0	0	100
24055156	f	ph	US	2024	0	0	0	0	0	0	0	0	0	0	0	0	81
24055190	f	md	JP	2024	0	1	0	0	0	1	0	0	0	0	0	1	60
24055310	f	md	JP	2024	0	0	0	0	0	0	0	0	0	0	0	0	
24055343	Not	md	CH	2024	0	0	0	0	0	0	1	0	0	1	0	1	
24055961	f	md	KR	2024	1	0	0	0	0	0	0	0	0	0	0	0	66.3
24056011	f	md	JP	2024	1	0	0	0	0	0	0	0	0	0	0	0	80
24056138	m	md	JP	2024	0	1	0	1	0	0	0	0	0	0	0	0	68
24056164	m	md	JP	2024	0	1	1	0	0	0	0	0	0	0	0	0	
24056195	m	md	PT	2024	0	1	1	0	0	0	0	0	0	0	0	0	70
24056229	f	hp	FR	2024	0	0	0	0	0	0	0	0	0	0	0	0	70
24056240	f	hp	IT	2024	1	0	0	0	0	0	0	0	0	0	0	0	73
24056369	Not	ph	CN	2024	0	0	0	0	0	0	0	0	0	0	0	0	
24056400	f	md	JP	2024	0	1	0	1	0	0	0	0	0	0	0	0	76
24056468	m	md	AU	2024	1	0	0	0	0	0	0	0	0	0	0	0	
24056506	f	hp	US	2024	0	1	1	0	0	0	0	0	0	0	0	0	70
24056534	m	md	DE	2024	0	1	0	0	1	0	0	0	0	0	0	0	73
24056640	m	cn	US	2024	0	1	1	0	0	0	0	0	0	0	0	0	
24058467	m	md	FR	2024	0	1	0	0	1	0	0	0	0	0	0	0	71
24058813	f	md	DE	2024	0	1	1	0	0	0	0	0	0	0	0	0	
24059506	m	md	US	2024	0	0	0	0	0	0	0	0	0	0	0	0	39
24059610	f	hp	CH	2024	1	0	0	0	0	0	0	0	0	0	0	0	70

24059615	f	md	US	2024	1	0	0	0	0	0	0	0	0	0	0	0	75
24059718	m	md	JP	2024	0	1	1	0	0	0	0	0	0	0	0	0	69
24059769	f	hp	US	2024	0	1	1	0	0	0	0	0	0	0	0	0	70
24060079	f	cn	FR	2024	0	0	0	0	0	0	0	0	0	0	0	0	
24060366	f	hp	US	2024	0	0	0	0	0	0	0	0	0	0	0	0	79
24060725	f	md	PT	2024	0	0	0	0	0	0	0	0	0	0	0	0	60
24061170	m	ph	US	2024	0	0	0	0	0	0	0	0	0	0	0	0	
24061405	m	cn	PK	2024	0	1	0	0	0	1	0	0	0	0	0	0	66
24061538	m	md	JP	2024	0	1	1	0	0	0	0	0	0	0	0	0	73
24061770	m	md	JP	2024	0	1	1	0	0	0	0	0	0	0	0	1	78
24061772	m	md	JP	2024	0	1	0	0	1	0	0	0	0	0	0	0	85
24061817	Not	ph	JP	2024	1	0	0	0	0	0	0	0	0	0	0	0	
24061821	m	md	JP	2024	0	1	0	1	0	0	0	0	0	0	0	0	
24062042	m	cn	US	2024	0	1	1	0	0	0	0	0	0	0	0	0	77
24062105	f	md	US	2024	0	0	0	0	0	0	0	0	0	0	0	0	79
24062184	f	md	IQ	2024	0	1	1	0	0	0	0	0	0	0	0	1	62
24062524	m	hp	US	2024	0	0	0	0	0	0	0	0	0	0	0	0	63
24062538	m	md	JP	2024	0	1	1	0	0	0	0	0	0	0	0	1	72
24062954	Not	md	CN	2024	0	0	0	0	0	0	0	0	0	0	0	0	
24063326	f	hp	US	2024	0	0	0	0	0	0	0	0	0	0	0	0	
24063491	f	md	JP	2024	0	0	0	0	0	0	0	0	0	0	0	0	80
24063553	f	md	IE	2024	1	0	0	0	0	0	0	0	0	0	0	0	
24064119	Not	hp	GB	2024	1	0	0	0	0	0	0	0	0	0	0	0	39
24064186	f	cn	NL	2024	1	0	0	0	0	0	0	0	0	0	0	0	61
24064267	m	hp	US	2024	0	1	0	1	0	0	0	0	0	0	0	0	70
24064316	m	hp	US	2024	0	1	0	1	0	0	0	0	0	0	0	0	65
24064527	f	ph	JP	2024	1	0	0	0	0	0	0	0	0	0	0	0	61
24064716	f	hp	NL	2024	0	0	0	0	0	0	0	0	0	0	0	0	57
24064721	f	md	US	2024	0	0	0	0	0	0	0	0	0	0	0	0	82
24064883	m	md	BR	2024	0	0	0	0	0	0	0	0	0	0	0	0	53
24064951	Not	md	PT	2024	0	1	0	0	0	1	0	0	0	0	0	1	
24064990	m	hp	FR	2024	0	1	0	1	0	0	0	0	0	0	0	0	
24064996	m	md	CN	2024	0	0	0	0	0	0	0	0	0	0	0	0	69
24065135	m	hp	FR	2024	0	1	0	1	0	0	0	0	0	0	0	0	

24065666	m	hp	FR	2024	0	1	0	1	0	0	0	0	0	0	0	1	63
24065771	m	hp	FR	2024	0	1	0	1	0	0	0	0	0	0	0	1	73
24066148	m	hp	FR	2024	0	1	0	1	0	0	0	0	0	0	0	0	75
24066152	m	hp	FR	2024	0	1	0	1	0	0	0	0	0	0	0	1	68
24066155	f	hp	FR	2024	0	1	0	1	0	0	0	0	0	0	0	0	67
24066156	m	hp	FR	2024	0	1	0	1	0	0	0	0	0	0	0	0	58
24066158	f	hp	FR	2024	0	1	0	1	0	0	0	0	0	0	0	0	72
24066216	m	md	FR	2024	0	1	0	1	0	0	0	0	0	0	0	0	69
24066217	m	hp	FR	2024	0	1	0	1	0	0	0	0	0	0	0	1	65
24066218	f	hp	FR	2024	0	1	0	1	0	0	0	0	0	0	0	0	
24066219	f	hp	FR	2024	0	1	0	1	0	0	0	0	0	0	0	0	79
24066239	f	md	DE	2024	0	0	0	0	0	0	0	0	0	0	0	0	33
24066291	m	hp	FR	2024	0	1	0	1	0	0	0	0	0	0	0	1	66
24066293	m	hp	FR	2024	0	1	0	1	0	0	0	0	0	0	0	0	58
24066350	m	hp	FR	2024	0	1	0	1	0	0	0	0	0	0	0	0	73
24066351	m	hp	FR	2024	0	1	0	1	0	0	0	0	0	0	0	0	65
24066441	m	hp	FR	2024	0	1	0	1	0	0	0	0	0	0	0	0	68
24066442	m	hp	FR	2024	0	1	0	1	0	0	0	0	0	0	0	0	70
24066449	m	hp	FR	2024	0	1	0	1	0	0	0	0	0	0	0	1	75
24066486	m	hp	FR	2024	0	1	0	1	0	0	0	0	0	0	0	1	76
24066487	m	hp	FR	2024	0	1	0	1	0	0	0	0	0	0	0	0	
24066576	m	hp	FR	2024	0	1	0	1	0	0	0	0	0	0	0	0	59
24066578	m	hp	FR	2024	0	1	0	1	0	0	0	0	0	0	0	0	
24066579	m	hp	FR	2024	0	1	0	1	0	0	0	0	0	0	0	0	67
24066580	m	hp	FR	2024	0	1	0	1	0	0	0	0	0	0	0	0	
24066581	f	hp	FR	2024	0	1	0	1	0	0	0	0	0	0	0	1	65
24066582	m	hp	FR	2024	0	1	0	1	0	0	0	0	0	0	0	0	
24066610	f	md	JP	2024	0	1	0	0	0	1	0	0	0	0	0	0	77
24066611	m	ph	JP	2024	0	1	0	0	0	1	0	0	0	0	0	0	64
24066612	f	md	TW	2024	0	1	0	0	0	1	0	0	0	0	0	0	59
24066696	m	hp	FR	2024	0	1	0	1	0	0	0	0	0	0	0	0	
24066699	f	md	JP	2024	1	0	0	0	0	0	0	0	0	0	0	0	66
24066701	m	hp	FR	2024	0	1	0	1	0	0	0	0	0	0	0	0	
24066837	Not	cn	US	2024	0	0	0	0	0	0	0	0	0	0	0	0	

24066913	f	hp	FR	2024	0	1	0	1	0	0	0	0	0	0	0	0	83
24066937	f	hp	FR	2024	0	1	0	1	0	0	0	0	0	0	0	0	63
24067041	f	hp	FR	2024	0	1	0	1	0	0	0	0	0	0	0	0	65
24067118	f	md	JP	2024	0	0	0	0	0	0	0	0	0	0	0	0	90
24067229	m	md	JP	2024	0	1	0	0	0	1	0	0	0	0	0	0	83
24067238	m	md	JP	2024	0	1	0	0	0	1	0	0	0	0	0	0	75
24067969	f	md	FR	2024	0	0	0	0	0	0	0	0	0	0	0	0	
24068019	f	md	CN	2024	0	1	0	0	0	1	0	0	0	0	0	0	
24068262	f	md	JP	2024	0	0	0	0	0	0	0	0	0	0	0	0	79
24068269	Not	md	JP	2024	0	0	0	0	0	0	0	0	0	0	0	0	
24068272	Not	md	BR	2024	0	0	0	0	0	0	0	0	0	0	0	0	
24068275	Not	md	JP	2024	0	0	0	0	0	0	0	0	0	0	0	0	90
24068279	m	md	TR	2024	0	0	0	0	0	0	0	0	0	0	0	0	39
24068290	f	cn	US	2024	0	0	0	0	0	0	0	0	0	0	0	0	
24068316	f	md	JP	2024	0	0	0	0	0	0	0	0	0	0	0	0	69
24068327	m	md	JP	2024	0	0	0	0	0	0	0	0	0	0	0	0	60
24068361	m	md	JP	2024	0	0	0	0	0	0	0	0	0	0	0	0	50
24068379	f	md	AR	2024	0	0	0	0	0	0	0	0	0	0	0	0	43
24068386	f	md	JP	2024	0	0	0	0	0	0	0	0	0	0	0	0	41
24068392	f	cn	IL	2024	0	0	0	0	0	0	0	0	0	0	0	0	77
24068408	m	md	BE	2024	0	0	0	0	0	0	0	0	0	0	0	0	54
24068411	f	md	DK	2024	0	0	0	0	0	0	0	0	0	0	0	0	39
24068422	m	md	JP	2024	0	0	0	0	0	0	0	0	0	0	0	0	70
24068435	f	md	JP	2024	0	0	0	0	0	0	0	0	0	0	0	0	90
24068437	f	md	CN	2024	0	0	0	0	0	0	0	0	0	0	0	0	72
24068439	f	md	JP	2024	0	0	0	0	0	0	0	0	0	0	0	0	70
24068459	Not	ph	JP	2024	0	0	0	0	0	0	0	0	0	0	0	0	80
24068464	Not	md	AR	2024	0	0	0	0	0	0	0	0	0	0	0	0	
24068488	Not	md	IL	2024	0	0	0	0	0	0	0	0	0	0	0	0	
24068490	m	md	JP	2024	0	0	0	0	0	0	0	0	0	0	0	0	
24068493	f	md	AU	2024	0	0	0	0	0	0	0	0	0	0	0	0	70
24068505	f	md	JP	2024	0	0	0	0	0	0	0	0	0	0	0	0	90
24068510	m	md	IT	2024	0	0	0	0	0	0	0	0	0	0	0	0	63
24068511	f	md	JP	2024	0	0	0	0	0	0	0	0	0	0	0	0	

24068519	f	md	JP	2024	0	0	0	0	0	0	0	0	0	0	0	0	50
24068522	m	md	JP	2024	0	0	0	0	0	0	0	0	0	0	0	0	70
24068566	Not	cn	US	2024	0	0	0	0	0	0	0	0	0	0	0	0	
24068571	m	md	JP	2024	0	0	0	0	0	0	0	0	0	0	0	0	80
24068593	f	md	JP	2024	0	0	0	0	0	0	0	0	0	0	0	0	60
24068597	Not	md	JP	2024	0	0	0	0	0	0	0	0	0	0	0	0	80
24068605	f	md	JP	2024	0	0	0	0	0	0	0	0	0	0	0	0	70
24068644	m	md	HR	2024	0	0	0	0	0	0	0	0	0	0	0	0	62
24068658	m	md	IT	2024	0	0	0	0	0	0	0	0	0	0	0	0	80
24068668	m	hp	TW	2024	0	0	0	0	0	0	0	0	0	0	0	0	65
24068674	f	cn	AU	2024	0	0	0	0	0	0	0	0	0	0	0	0	63
24068679	m	md	TR	2024	0	0	0	0	0	0	0	0	0	0	0	0	58
24068681	Not	md	JP	2024	0	0	0	0	0	0	0	0	0	0	0	0	
24068692	m	md	JP	2024	0	0	0	0	0	0	0	0	0	0	0	0	60
24068704	m	md	JP	2024	0	0	0	0	0	0	0	0	0	0	0	0	47
24068715	f	cn	DE	2024	0	0	0	0	0	0	0	0	0	0	0	0	
24068719	f	md	JP	2024	0	0	0	0	0	0	0	0	0	0	0	0	90
24068731	m	md	JP	2024	0	0	0	0	0	0	0	0	0	0	0	0	74
24068743	Not	md	JP	2024	0	0	0	0	0	0	0	0	0	0	0	0	80
24068744	f	md	TR	2024	0	0	0	0	0	0	0	0	0	0	0	0	73
24068756	m	md	JP	2024	0	0	0	0	0	0	0	0	0	0	0	0	70
24068775	f	cn	CA	2024	0	0	0	0	0	0	0	0	0	0	0	0	66
24068777	f	md	CN	2024	0	0	0	0	0	0	0	0	0	0	0	0	
24068780	m	ph	JP	2024	0	0	0	0	0	0	0	0	0	0	0	0	60
24068785	m	ph	JP	2024	0	0	0	0	0	0	0	0	0	0	0	0	94
24068799	f	md	JP	2024	0	0	0	0	0	0	0	0	0	0	0	0	
24068803	m	md	JP	2024	0	0	0	0	0	0	0	0	0	0	0	0	70
24068807	f	md	JP	2024	0	0	0	0	0	0	0	0	0	0	0	0	70
24068819	m	md	JP	2024	0	0	0	0	0	0	0	0	0	0	0	0	74
24068831	f	md	IL	2024	0	0	0	0	0	0	0	0	0	0	0	0	44
24068838	f	cn	IL	2024	0	0	0	0	0	0	0	0	0	0	0	0	63
24068852	m	md	JP	2024	0	0	0	0	0	0	0	0	0	0	0	0	67
24068855	f	md	CN	2024	0	0	0	0	0	0	0	0	0	0	0	0	53
24068878	m	md	JP	2024	0	0	0	0	0	0	0	0	0	0	0	0	53

24068885	Not	md	JP	2024	0	0	0	0	0	0	0	0	0	0	0	0	80
24068889	m	md	TR	2024	0	0	0	0	0	0	0	0	0	0	0	0	
24068897	Not	md	CN	2024	0	0	0	0	0	0	0	0	0	0	0	0	
24068904	f	md	JP	2024	0	0	0	0	0	0	0	0	0	0	0	0	80
24068911	f	cn	CN	2024	0	0	0	0	0	0	0	0	0	0	0	0	41
24068917	Not	md	US	2024	0	0	0	0	0	0	0	0	0	0	0	0	
24068922	f	md	TR	2024	0	0	0	0	0	0	0	0	0	0	0	0	51
24068932	m	md	TR	2024	0	0	0	0	0	0	0	0	0	0	0	0	50
24068935	f	md	IT	2024	0	0	0	0	0	0	0	0	0	0	0	0	83
24068943	m	hp	CN	2024	0	0	0	0	0	0	0	0	0	0	0	0	53
24068944	f	md	JP	2024	0	0	0	0	0	0	0	0	0	0	0	0	70
24068952	m	md	AU	2024	0	0	0	0	0	0	0	0	0	0	0	0	59
24068965	f	md	AU	2024	0	0	0	0	0	0	0	0	0	0	0	0	38
24068966	f	cn	CN	2024	0	0	0	0	0	0	0	0	0	0	0	0	55
24068971	Not	cn	TH	2024	0	0	0	0	0	0	0	0	0	0	0	0	
24068973	Not	md	ES	2024	0	0	0	0	0	0	0	0	0	0	0	0	62
24068979	m	ph	CA	2024	0	0	0	0	0	0	0	0	0	0	0	0	
24068999	Not	md	ES	2024	0	0	0	0	0	0	0	0	0	0	0	0	53
24069000	f	cn	IL	2024	0	0	0	0	0	0	0	0	0	0	0	0	
24069025	f	md	AU	2024	0	0	0	0	0	0	0	0	0	0	0	0	38
24069028	f	md	DK	2024	0	0	0	0	0	0	0	0	0	0	0	0	37
24069036	f	md	TR	2024	0	0	0	0	0	0	0	0	0	0	0	0	43
24069038	f	md	JP	2024	0	0	0	0	0	0	0	0	0	0	0	0	80
24069043	f	md	DK	2024	0	0	0	0	0	0	0	0	0	0	0	0	43
24069047	Not	cn	SG	2024	0	0	0	0	0	0	0	0	0	0	0	0	
24069068	m	md	TR	2024	0	0	0	0	0	0	0	0	0	0	0	0	70
24069106	m	cn	US	2024	0	0	0	0	0	0	0	0	0	0	0	0	
24069111	f	md	CN	2024	0	0	0	0	0	0	0	0	0	0	0	0	33
24069115	f	cn	TH	2024	0	0	0	0	0	0	0	0	0	0	0	0	65
24069124	f	md	CN	2024	0	0	0	0	0	0	0	0	0	0	0	0	57
24069130	f	md	AT	2024	0	0	0	0	0	0	0	0	0	0	0	0	79
24069132	f	md	JP	2024	0	0	0	0	0	0	0	0	0	0	0	0	
24069135	m	md	TR	2024	0	0	0	0	0	0	0	0	0	0	0	0	55
24069140	m	md	IT	2024	0	0	0	0	0	0	0	0	0	0	0	0	68

24069141	m	md	JP	2024	0	0	0	0	0	0	0	0	0	0	0	0	80
24069157	f	md	DK	2024	0	0	0	0	0	0	0	0	0	0	0	0	55
24069159	m	cn	CO	2024	0	0	0	0	0	0	0	0	0	0	0	0	56
24069166	m	md	DK	2024	0	0	0	0	0	0	0	0	0	0	0	0	
24069169	f	cn	CA	2024	0	0	0	0	0	0	0	0	0	0	0	0	66
24069175	m	md	BE	2024	0	0	0	0	0	0	0	0	0	0	0	0	57
24069176	Not	md	ES	2024	0	0	0	0	0	0	0	0	0	0	0	0	78
24069177	f	cn	TH	2024	0	0	0	0	0	0	0	0	0	0	0	0	45
24069178	f	md	TR	2024	0	0	0	0	0	0	0	0	0	0	0	0	
24069180	m	md	IT	2024	0	0	0	0	0	0	0	0	0	0	0	0	
24069181	f	cn	TH	2024	0	0	0	0	0	0	0	0	0	0	0	0	49
24069194	m	cn	US	2024	0	0	0	0	0	0	0	0	0	0	0	0	
24069199	Not	md	JP	2024	0	0	0	0	0	0	0	0	0	0	0	0	60
24069201	f	md	JP	2024	0	0	0	0	0	0	0	0	0	0	0	0	
24069204	f	hp	CA	2024	0	0	0	0	0	0	0	0	0	0	0	0	68
24069214	f	cn	CA	2024	0	0	0	0	0	0	0	0	0	0	0	0	47
24069218	m	md	AT	2024	0	0	0	0	0	0	0	0	0	0	0	0	42
24069225	m	md	CO	2024	0	0	0	0	0	0	0	0	0	0	0	0	84
24069234	m	md	IN	2024	0	0	0	0	0	0	0	0	0	0	0	0	55
24069243	Not	hp	IT	2024	0	0	0	0	0	0	0	0	0	0	0	0	
24069256	f	ph	JP	2024	0	0	0	0	0	0	0	0	0	0	0	0	72
24069269	f	md	IT	2024	0	0	0	0	0	0	0	0	0	0	0	0	74
24069285	f	md	CN	2024	0	0	0	0	0	0	0	0	0	0	0	0	78
24069296	Not	hp	AU	2024	0	0	0	0	0	0	0	0	0	0	0	0	
24069310	f	md	JP	2024	0	0	0	0	0	0	0	0	0	0	0	0	60
24069317	Not	md	ES	2024	0	0	0	0	0	0	0	0	0	0	0	0	76
24069333	f	md	CN	2024	0	0	0	0	0	0	0	0	0	0	0	0	39
24069340	f	md	TW	2024	0	0	0	0	0	0	0	0	0	0	0	0	40
24069342	f	md	RU	2024	0	0	0	0	0	0	0	0	0	0	0	0	71
24069343	m	md	JP	2024	0	0	0	0	0	0	0	0	0	0	0	0	73
24069355	m	md	FR	2024	0	0	0	0	0	0	0	0	0	0	0	0	30
24069362	m	md	RS	2024	0	0	0	0	0	0	0	0	0	0	0	0	77
24069364	m	md	TR	2024	0	0	0	0	0	0	0	0	0	0	0	0	47
24069372	Not	md	ES	2024	0	0	0	0	0	0	0	0	0	0	0	0	53

24069376	m	md	CN	2024	0	0	0	0	0	0	0	0	0	0	0	0	
24069377	Not	md	JP	2024	0	0	0	0	0	0	0	0	0	0	0	0	70
24069380	m	md	DE	2024	0	0	0	0	0	0	0	0	0	0	0	0	
24069383	m	md	IL	2024	0	0	0	0	0	0	0	0	0	0	0	0	
24069401	f	md	CN	2024	0	0	0	0	0	0	0	0	0	0	0	0	67
24069416	m	md	DK	2024	0	0	0	0	0	0	0	0	0	0	0	0	69
24069429	m	md	PT	2024	0	0	0	0	0	0	0	0	0	0	0	0	44
24069430	f	md	CN	2024	0	0	0	0	0	0	0	0	0	0	0	0	66
24069439	f	md	JP	2024	0	0	0	0	0	0	0	0	0	0	0	0	70
24069442	f	md	CN	2024	0	0	0	0	0	0	0	0	0	0	0	0	53
24069443	m	md	TR	2024	0	0	0	0	0	0	0	0	0	0	0	0	58
24069447	m	md	IT	2024	0	0	0	0	0	0	0	0	0	0	0	0	72
24069452	f	md	TR	2024	0	0	0	0	0	0	0	0	0	0	0	0	51
24069456	m	cn	IL	2024	0	0	0	0	0	0	0	0	0	0	0	0	
24069462	f	md	TR	2024	0	0	0	0	0	0	0	0	0	0	0	0	29
24069473	m	cn	CN	2024	0	0	0	0	0	0	0	0	0	0	0	0	31
24069484	m	cn	CO	2024	0	0	0	0	0	0	0	0	0	0	0	0	41
24069504	m	md	DK	2024	0	0	0	0	0	0	0	0	0	0	0	0	55
24069505	f	md	DK	2024	0	0	0	0	0	0	0	0	0	0	0	0	45
24069507	f	md	CN	2024	0	0	0	0	0	0	0	0	0	0	0	0	66
24069517	Not	md	ES	2024	0	0	0	0	0	0	0	0	0	0	0	0	21
24069518	f	md	JP	2024	0	0	0	0	0	0	0	0	0	0	0	0	70
24069519	f	md	IT	2024	0	0	0	0	0	0	0	0	0	0	0	0	47
24069524	f	md	VN	2024	0	0	0	0	0	0	0	0	0	0	0	0	78
24069531	m	md	IL	2024	0	0	0	0	0	0	0	0	0	0	0	0	68
24069532	m	md	IN	2024	0	0	0	0	0	0	0	0	0	0	0	0	69
24069549	f	cn	IL	2024	0	0	0	0	0	0	0	0	0	0	0	0	
24069563	Not	md	JP	2024	0	0	0	0	0	0	0	0	0	0	0	0	80
24069569	m	md	JP	2024	0	0	0	0	0	0	0	0	0	0	0	0	
24069576	Not	ph	PT	2024	0	0	0	0	0	0	0	0	0	0	0	0	
24069585	f	md	CN	2024	0	0	0	0	0	0	0	0	0	0	0	0	42
24069591	m	ph	JP	2024	0	0	0	0	0	0	0	0	0	0	0	0	70
24069596	f	hp	US	2024	0	0	0	0	0	0	0	0	0	0	0	0	43
24069601	m	md	JP	2024	0	0	0	0	0	0	0	0	0	0	0	0	80

24069616	f	md	JP	2024	0	0	0	0	0	0	0	0	0	0	0	0	90
24069626	m	md	CN	2024	0	0	0	0	0	0	0	0	0	0	0	0	79
24069648	m	cn	CN	2024	0	0	0	0	0	0	0	0	0	0	0	0	73
24069665	Not	md	ES	2024	0	0	0	0	0	0	0	0	0	0	0	0	69
24069676	f	md	CN	2024	0	0	0	0	0	0	0	0	0	0	0	0	41
24069686	m	md	CN	2024	0	0	0	0	0	0	0	0	0	0	0	0	40
24069693	m	cn	IN	2024	0	0	0	0	0	0	0	0	0	0	0	0	57
24069696	f	md	US	2024	0	0	0	0	0	0	0	0	0	0	0	0	48
24069700	Not	md	ES	2024	0	0	0	0	0	0	0	0	0	0	0	0	59
24069715	f	md	RS	2024	0	0	0	0	0	0	0	0	0	0	0	0	62
24069720	f	md	DK	2024	0	0	0	0	0	0	0	0	0	0	0	0	46
24069725	f	md	RS	2024	0	0	0	0	0	0	0	0	0	0	0	0	62
24069727	f	md	TR	2024	0	0	0	0	0	0	0	0	0	0	0	0	54
24069751	Not	md	JP	2024	0	0	0	0	0	0	0	0	0	0	0	0	
24069752	Not	md	ES	2024	0	0	0	0	0	0	0	0	0	0	0	0	73
24069754	Not	ph	PT	2024	0	0	0	0	0	0	0	0	0	0	0	0	
24069759	m	md	TR	2024	0	0	0	0	0	0	0	0	0	0	0	0	
24069761	f	cn	PH	2024	0	0	0	0	0	0	0	0	0	0	0	0	68
24069771	m	md	CO	2024	0	0	0	0	0	0	0	0	0	0	0	0	70
24069787	f	cn	MX	2024	0	0	0	0	0	0	0	0	0	0	0	0	
24069798	f	md	IT	2024	0	0	0	0	0	0	0	0	0	0	0	0	67
24069804	f	md	JP	2024	0	0	0	0	0	0	0	0	0	0	0	0	72
24069810	f	md	AU	2024	0	0	0	0	0	0	0	0	0	0	0	0	73
24069823	m	md	TR	2024	0	0	0	0	0	0	0	0	0	0	0	0	39
24069824	Not	md	JP	2024	0	0	0	0	0	0	0	0	0	0	0	0	
24069837	m	cn	IL	2024	0	0	0	0	0	0	0	0	0	0	0	0	
24069846	m	md	JP	2024	0	0	0	0	0	0	0	0	0	0	0	0	70
24069856	Not	cn	CN	2024	0	0	0	0	0	0	0	0	0	0	0	0	
24069872	m	cn	CN	2024	0	0	0	0	0	0	0	0	0	0	0	0	34
24069893	f	md	TR	2024	0	0	0	0	0	0	0	0	0	0	0	0	54
24069896	f	md	CO	2024	0	0	0	0	0	0	0	0	0	0	0	0	66
24069902	m	ph	JP	2024	0	0	0	0	0	0	0	0	0	0	0	0	71
24069914	f	md	IT	2024	0	0	0	0	0	0	0	0	0	0	0	0	49
24069927	m	md	JP	2024	0	0	0	0	0	0	0	0	0	0	0	0	79

24069932	f	md	RS	2024	0	0	0	0	0	0	0	0	0	0	0	0	69
24069946	f	md	AU	2024	0	0	0	0	0	0	0	0	0	0	0	0	44
24069950	f	md	IT	2024	0	0	0	0	0	0	0	0	0	0	0	0	45
24069958	f	md	CA	2024	0	0	0	0	0	0	0	0	0	0	0	0	61
24069969	m	md	CN	2024	0	0	0	0	0	0	0	0	0	0	0	0	68
24069970	f	cn	CN	2024	0	0	0	0	0	0	0	0	0	0	0	0	73
24069974	f	md	HR	2024	0	0	0	0	0	0	0	0	0	0	0	0	57
24069983	f	md	FR	2024	0	0	0	0	0	0	0	0	0	0	0	0	60
24069984	m	cn	CO	2024	0	0	0	0	0	0	0	0	0	0	0	0	56
24069986	m	md	TW	2024	0	0	0	0	0	0	0	0	0	0	0	0	64
24069988	Not	md	ES	2024	0	0	0	0	0	0	0	0	0	0	0	0	70
24069993	m	cn	IN	2024	0	0	0	0	0	0	0	0	0	0	0	0	65
24070002	f	md	TR	2024	0	0	0	0	0	0	0	0	0	0	0	0	77
24070027	f	cn	US	2024	0	0	0	0	0	0	0	0	0	0	0	0	65
24070029	m	md	CO	2024	0	0	0	0	0	0	0	0	0	0	0	0	56
24070030	f	cn	CA	2024	0	0	0	0	0	0	0	0	0	0	0	0	
24070037	f	md	PT	2024	0	0	0	0	0	0	0	0	0	0	0	0	
24070087	m	md	BE	2024	0	0	0	0	0	0	0	0	0	0	0	0	73
24070090	f	md	VN	2024	0	0	0	0	0	0	0	0	0	0	0	0	49
24070097	m	md	IL	2024	0	0	0	0	0	0	0	0	0	0	0	0	71
24070106	f	md	JP	2024	0	0	0	0	0	0	0	0	0	0	0	0	80
24070113	f	md	JP	2024	0	0	0	0	0	0	0	0	0	0	0	0	70
24070115	f	ph	CR	2024	0	0	0	0	0	0	0	0	0	0	0	0	77
24070141	m	md	RS	2024	0	0	0	0	0	0	0	0	0	0	0	0	53
24070149	m	cn	CA	2024	0	0	0	0	0	0	0	0	0	0	0	0	
24070159	f	md	JP	2024	0	0	0	0	0	0	0	0	0	0	0	0	48
24070163	Not	md	JP	2024	0	0	0	0	0	0	0	0	0	0	0	0	100
24070194	f	md	TW	2024	0	0	0	0	0	0	0	0	0	0	0	0	60
24070200	f	md	TR	2024	0	0	0	0	0	0	0	0	0	0	0	0	83
24070201	f	cn	US	2024	0	0	0	0	0	0	0	0	0	0	0	0	81
24070207	f	cn	CN	2024	0	0	0	0	0	0	0	0	0	0	0	0	35
24070214	Not	md	FI	2024	0	0	0	0	0	0	0	0	0	0	0	0	
24070231	Not	hp	NL	2024	0	0	0	0	0	0	0	0	0	0	0	0	
24070235	f	md	RS	2024	0	0	0	0	0	0	0	0	0	0	0	0	62

24070238	f	md	JP	2024	0	0	0	0	0	0	0	0	0	0	0	0	30
24070244	f	md	US	2024	0	0	0	0	0	0	0	0	0	0	0	0	
24070265	f	md	RO	2024	0	0	0	0	0	0	0	0	0	0	0	0	73
24070282	f	md	TR	2024	0	0	0	0	0	0	0	0	0	0	0	0	50
24070283	f	md	TR	2024	0	0	0	0	0	0	0	0	0	0	0	0	48
24070292	f	cn	IL	2024	0	0	0	0	0	0	0	0	0	0	0	0	
24070293	m	md	JP	2024	0	0	0	0	0	0	0	0	0	0	0	0	80
24070304	Not	md	ES	2024	0	0	0	0	0	0	0	0	0	0	0	0	78
24070311	Not	md	ES	2024	0	0	0	0	0	0	0	0	0	0	0	0	69
24070314	f	cn	AM	2024	0	0	0	0	0	0	0	0	0	0	0	0	74
24070329	f	md	PE	2024	0	0	0	0	0	0	0	0	0	0	0	0	36
24070333	f	md	TR	2024	0	0	0	0	0	0	0	0	0	0	0	0	39
24070365	m	md	IL	2024	0	0	0	0	0	0	0	0	0	0	0	0	67
24070377	f	md	LT	2024	0	0	0	0	0	0	0	0	0	0	0	0	71
24070396	m	cn	CA	2024	0	0	0	0	0	0	0	0	0	0	0	0	
24070413	f	hp	US	2024	0	0	0	0	0	0	0	0	0	0	0	0	54
24070440	f	md	JP	2024	0	0	0	0	0	0	0	0	0	0	0	0	82
24070443	f	md	JP	2024	0	0	0	0	0	0	0	0	0	0	0	0	70
24070456	f	cn	CN	2024	0	0	0	0	0	0	0	0	0	0	0	0	62
24070460	m	md	TR	2024	0	0	0	0	0	0	0	0	0	0	0	0	68
24070467	f	md	IT	2024	0	0	0	0	0	0	0	0	0	0	0	0	70
24070471	f	cn	CA	2024	0	0	0	0	0	0	0	0	0	0	0	0	
24070472	Not	md	JP	2024	0	0	0	0	0	0	0	0	0	0	0	0	
24070478	f	md	TR	2024	0	0	0	0	0	0	0	0	0	0	0	0	64
24070506	m	md	TR	2024	0	0	0	0	0	0	0	0	0	0	0	0	32
24070507	f	md	VN	2024	0	0	0	0	0	0	0	0	0	0	0	0	
24070524	f	md	IT	2024	0	0	0	0	0	0	0	0	0	0	0	0	52
24070539	f	md	IT	2024	0	0	0	0	0	0	0	0	0	0	0	0	64
24070541	f	md	VN	2024	0	0	0	0	0	0	0	0	0	0	0	0	55
24070548	m	cn	CA	2024	0	0	0	0	0	0	0	0	0	0	0	0	51
24070563	Not	md	JP	2024	0	0	0	0	0	0	0	0	0	0	0	0	90
24070586	f	cn	US	2024	0	0	0	0	0	0	0	0	0	0	0	0	71
24070591	m	md	AU	2024	0	0	0	0	0	0	0	0	0	0	0	0	59
24070614	f	cn	AU	2024	0	1	0	0	1	0	0	0	0	0	0	0	81

24070634	f	ph	JP	2024	0	0	0	0	0	0	0	0	0	0	0	0	
24070636	f	md	RS	2024	0	0	0	0	0	0	0	0	0	0	0	0	55
24070642	m	cn	CO	2024	0	0	0	0	0	0	0	0	0	0	0	0	43
24070667	m	md	IN	2024	0	0	0	0	0	0	0	0	0	0	0	0	45
24070677	f	md	JP	2024	0	0	0	0	0	0	0	0	0	0	0	0	
24070691	f	md	JP	2024	0	0	0	0	0	0	0	0	0	0	0	0	80
24070701	m	cn	CA	2024	0	0	0	0	0	0	0	0	0	0	0	0	
24070702	m	md	JP	2024	0	0	0	0	0	0	0	0	0	0	0	0	80
24070712	m	cn	CN	2024	0	0	0	0	0	0	0	0	0	0	0	0	66
24070718	f	cn	AU	2024	0	0	0	0	0	0	0	0	0	0	0	0	81
24070751	m	md	TR	2024	0	0	0	0	0	0	0	0	0	0	0	0	63
24070760	m	md	PE	2024	0	0	0	0	0	0	0	0	0	0	0	0	35
24070764	f	ph	JP	2024	0	0	0	0	0	0	0	0	0	0	0	0	
24070766	m	md	IT	2024	0	0	0	0	0	0	0	0	0	0	0	0	61
24070780	m	md	JP	2024	0	0	0	0	0	0	0	0	0	0	0	0	78
24070786	f	md	RU	2024	0	0	0	0	0	0	0	0	0	0	0	0	53
24070794	m	md	TR	2024	0	0	0	0	0	0	0	0	0	0	0	0	51
24070843	m	cn	CN	2024	0	0	0	0	0	0	0	0	0	0	0	0	44
24070847	f	cn	CN	2024	0	0	0	0	0	0	0	0	0	0	0	0	37
24070862	f	hp	KR	2024	0	0	0	0	0	0	0	0	0	0	0	0	71
24070864	m	cn	US	2024	0	0	0	0	0	0	0	0	0	0	0	0	65
24070869	m	md	DK	2024	0	0	0	0	0	0	0	0	0	0	0	0	70
24070884	f	md	LT	2024	0	0	0	0	0	0	0	0	0	0	0	0	59
24070887	f	ph	JP	2024	0	0	0	0	0	0	0	0	0	0	0	0	74
24070899	Not	md	JP	2024	0	0	0	0	0	0	0	0	0	0	0	0	
24070902	f	md	US	2024	0	0	0	0	0	0	0	0	0	0	0	0	
24070911	f	md	TR	2024	0	0	0	0	0	0	0	0	0	0	0	0	58
24070920	Not	md	ES	2024	0	0	0	0	0	0	0	0	0	0	0	0	63
24070924	f	md	RO	2024	0	0	0	0	0	0	0	0	0	0	0	0	43
24070928	Not	md	ES	2024	0	0	0	0	0	0	0	0	0	0	0	0	72
24070966	f	md	CN	2024	0	0	0	0	0	0	0	0	0	0	0	0	56
24070967	m	md	RS	2024	0	0	0	0	0	0	0	0	0	0	0	0	74
24070977	m	md	ZA	2024	0	0	0	0	0	0	0	0	0	0	0	0	31
24070990	m	md	DK	2024	0	0	0	0	0	0	0	0	0	0	0	0	54

24070995	m	cn	CA	2024	0	0	0	0	0	0	0	0	0	0	0	0	
24071007	f	hp	TW	2024	0	0	0	0	0	0	0	0	0	0	0	0	72
24071026	f	md	JP	2024	0	0	0	0	0	0	0	0	0	0	0	0	80
24071038	m	md	CL	2024	0	0	0	0	0	0	0	0	0	0	0	0	46
24071041	f	md	CN	2024	0	0	0	0	0	0	0	0	0	0	0	0	56
24071053	m	md	VN	2024	0	0	0	0	0	0	0	0	0	0	0	0	65
24071059	m	md	TR	2024	0	0	0	0	0	0	0	0	0	0	0	0	53
24071070	m	md	JP	2024	0	0	0	0	0	0	0	0	0	0	0	0	80
24071072	m	cn	US	2024	0	0	0	0	0	0	0	0	0	0	0	0	
24071079	m	md	US	2024	0	0	0	0	0	0	0	0	0	0	0	0	39
24071089	f	md	VN	2024	0	0	0	0	0	0	0	0	0	0	0	0	31
24071098	m	md	JP	2024	0	0	0	0	0	0	0	0	0	0	0	0	60
24071103	Not	md	FI	2024	0	0	0	0	0	0	0	0	0	0	0	0	
24071109	m	md	VN	2024	0	0	0	0	0	0	0	0	0	0	0	0	79
24071114	f	md	RS	2024	0	0	0	0	0	0	0	0	0	0	0	0	56
24071115	Not	md	BE	2024	0	0	0	0	0	0	0	0	0	0	0	0	
24071135	m	ph	US	2024	0	0	0	0	0	0	0	0	0	0	0	0	
24071141	f	md	AT	2024	0	0	0	0	0	0	0	0	0	0	0	0	59
24071148	f	md	JP	2024	0	0	0	0	0	0	0	0	0	0	0	0	90
24071149	f	md	RS	2024	0	0	0	0	0	0	0	0	0	0	0	0	51
24071169	Not	md	ES	2024	0	0	0	0	0	0	0	0	0	0	0	0	69
24071177	m	md	CN	2024	0	0	0	0	0	0	0	0	0	0	0	0	60
24071193	m	md	JP	2024	0	0	0	0	0	0	0	0	0	0	0	0	
24071202	f	md	AT	2024	0	0	0	0	0	0	0	0	0	0	0	0	74
24071218	f	hp	KR	2024	0	0	0	0	0	0	0	0	0	0	0	0	74
24071268	m	md	DK	2024	0	0	0	0	0	0	0	0	0	0	0	0	70
24071291	m	md	JP	2024	0	0	0	0	0	0	0	0	0	0	0	0	80
24071306	Not	cn	US	2024	0	0	0	0	0	0	0	0	0	0	0	0	
24071311	f	cn	CA	2024	0	0	0	0	0	0	0	0	0	0	0	0	63
24071312	f	cn	TH	2024	0	0	0	0	0	0	0	0	0	0	0	0	59
24071323	m	md	RU	2024	0	0	0	0	0	0	0	0	0	0	0	0	57
24071347	Not	md	JP	2024	0	0	0	0	0	0	0	0	0	0	0	0	
24071362	f	md	JP	2024	0	0	0	0	0	0	0	0	0	0	0	0	70
24071366	f	md	AT	2024	0	0	0	0	0	0	0	0	0	0	0	0	79

24071371	m	ph	JP	2024	0	0	0	0	0	0	0	0	0	0	0	0	50
24071382	f	cn	US	2024	0	0	0	0	0	0	0	0	0	0	0	0	61
24071406	Not	md	JP	2024	0	0	0	0	0	0	0	0	0	0	0	0	
24071422	f	md	AU	2024	0	0	0	0	0	0	0	0	0	0	0	0	63
24071423	m	md	TR	2024	0	0	0	0	0	0	0	0	0	0	0	0	69
24071444	Not	cn	TH	2024	0	0	0	0	0	0	0	0	0	0	0	0	
24071499	m	md	RU	2024	0	0	0	0	0	0	0	0	0	0	0	0	32
24071531	f	cn	TH	2024	0	0	0	0	0	0	0	0	0	0	0	0	77
24071538	f	cn	CA	2024	0	0	0	0	0	0	0	0	0	0	0	0	
24071557	f	md	JP	2024	0	0	0	0	0	0	0	0	0	0	0	0	60
24071581	f	md	JP	2024	0	0	0	0	0	0	0	0	0	0	0	0	70
24071587	m	md	LT	2024	0	0	0	0	0	0	0	0	0	0	0	0	50
24071603	f	cn	PH	2024	0	0	0	0	0	0	0	0	0	0	0	0	47
24071623	f	md	HK	2024	0	0	0	0	0	0	0	0	0	0	0	0	60
24071625	f	md	JP	2024	0	0	0	0	0	0	0	0	0	0	0	0	70
24071644	f	md	CN	2024	0	0	0	0	0	0	0	0	0	0	0	0	59
24071648	f	md	AU	2024	0	0	0	0	0	0	0	0	0	0	0	0	59
24071658	m	md	JP	2024	0	0	0	0	0	0	0	0	0	0	0	0	90
24071693	m	md	CN	2024	0	0	0	0	0	0	0	0	0	0	0	0	48
24071698	m	md	PT	2024	0	0	0	0	0	0	0	0	0	0	0	0	75
24071710	Not	md	JP	2024	0	0	0	0	0	0	0	0	0	0	0	0	90
24071711	f	md	RS	2024	0	0	0	0	0	0	0	0	0	0	0	0	45
24071726	m	md	JP	2024	0	0	0	0	0	0	0	0	0	0	0	0	50
24071728	f	md	JP	2024	0	0	0	0	0	0	0	0	0	0	0	0	80
24071758	f	md	VN	2024	0	0	0	0	0	0	0	0	0	0	0	0	28
24071761	m	md	VN	2024	0	0	0	0	0	0	0	0	0	0	0	0	41
24071771	m	md	CN	2024	0	0	0	0	0	0	0	0	0	0	0	0	71
24071808	m	hp	CN	2024	0	0	0	0	0	0	0	0	0	0	0	0	56
24071810	Not	md	JP	2024	0	0	0	0	0	0	0	0	0	0	0	0	80
24071842	f	md	CN	2024	0	0	0	0	0	0	0	0	0	0	0	0	55
24071854	f	md	IT	2024	0	0	0	0	0	0	0	0	0	0	0	0	36
24071875	Not	md	US	2024	0	0	0	0	0	0	0	0	0	0	0	0	
24071892	m	md	TR	2024	0	0	0	0	0	0	0	0	0	0	0	0	37
24071900	m	md	TR	2024	0	0	0	0	0	0	0	0	0	0	0	0	51

24071908	Not	md	JP	2024	0	0	0	0	0	0	0	0	0	0	0	0	
24071911	m	md	AU	2024	0	0	0	0	0	0	0	0	0	0	0	0	63
24071915	f	md	RS	2024	0	0	0	0	0	0	0	0	0	0	0	0	26
24071917	Not	hp	CN	2024	0	0	0	0	0	0	0	0	0	0	0	0	
24072531	f	md	AT	2024	1	0	0	0	0	0	0	0	0	0	0	0	
24072532	m	hp	FR	2024	0	1	0	1	0	0	0	0	0	0	0	0	60
24072536	m	ph	US	2024	0	0	0	0	0	0	0	0	0	0	0	0	
24072793	m	hp	FR	2024	0	1	0	1	0	0	0	0	0	0	0	0	66
24072867	f	hp	AU	2024	0	1	0	0	1	0	0	0	0	0	0	0	68
24073100	f	md	ES	2024	0	0	0	0	0	0	0	0	0	0	0	0	49
24073239	m	md	JP	2024	0	1	0	1	0	0	0	0	0	0	0	0	73
24073374	m	cn	DE	2024	0	0	0	0	0	0	0	0	0	0	0	0	
24073413	m	hp	FR	2024	0	1	0	1	0	0	0	0	0	0	0	0	66
24073562	m	md	JP	2024	0	0	0	0	0	0	0	0	0	0	0	0	80
24073698	m	ph	JP	2024	0	0	0	0	0	0	0	0	0	0	0	0	
24074137	m	md	JP	2024	0	1	0	1	0	0	0	0	0	0	0	0	69
24074269	m	hp	FR	2024	0	1	0	1	0	0	0	0	0	0	0	0	72
24074272	m	hp	FR	2024	0	1	0	1	0	0	0	0	0	0	0	0	61
24074323	m	hp	FR	2024	0	1	0	1	0	0	0	0	0	0	0	0	65
24074324	f	hp	FR	2024	0	1	0	1	0	0	0	0	0	0	0	1	56
24074366	m	hp	FR	2024	0	1	0	1	0	0	0	0	0	0	0	0	72
24074367	m	hp	FR	2024	0	1	0	1	0	0	0	0	0	0	0	0	78
24074372	f	hp	FR	2024	0	1	0	1	0	0	0	0	0	0	0	0	82
24074418	m	hp	FR	2024	0	1	0	1	0	0	0	0	0	0	0	0	70
24074769	f	md	ES	2024	0	1	1	0	0	0	0	0	0	0	0	0	70
24074796	m	hp	FR	2024	0	1	0	1	0	0	0	0	0	0	0	0	68
24075085	m	md	JP	2024	0	1	0	1	0	0	0	0	0	0	0	0	69
24075089	m	hp	FR	2024	0	1	0	1	0	0	0	0	0	0	0	0	46
24075091	f	cn	IN	2024	1	0	0	0	0	0	0	0	0	0	0	0	
24075093	m	hp	FR	2024	0	1	0	1	0	0	0	0	0	0	0	1	79
24075274	f	hp	FR	2024	0	1	0	1	0	0	0	0	0	0	0	0	53
24075275	f	hp	FR	2024	0	1	0	1	0	0	0	0	0	0	0	0	72
24075276	m	hp	FR	2024	0	1	0	1	0	0	0	0	0	0	0	1	68
24075277	m	hp	FR	2024	0	1	0	1	0	0	0	0	0	0	0	1	64

24075294	Not	hp	KR	2024	0	0	0	0	0	0	0	0	0	0	0	0	
24075349	m	hp	FR	2024	0	1	0	1	0	0	0	0	0	0	0	0	73
24075356	f	hp	FR	2024	0	1	0	1	0	0	0	0	0	0	0	0	76
24075463	m	hp	FR	2024	0	1	0	1	0	0	0	0	0	0	0	0	62
24075464	m	md	JP	2024	0	1	0	1	0	0	0	0	0	0	0	0	74
24075466	f	hp	FR	2024	0	1	0	1	0	0	0	0	0	0	0	0	63
24075519	m	hp	FR	2024	0	1	0	1	0	0	0	0	0	0	0	1	73
24075521	m	hp	FR	2024	0	1	0	1	0	0	0	0	0	0	0	0	70
24075532	f	md	US	2024	0	0	0	0	0	0	0	0	0	0	0	0	55
24075573	m	hp	FR	2024	0	1	0	1	0	0	0	0	0	0	0	1	71
24075574	m	hp	FR	2024	0	1	0	1	0	0	0	0	0	0	0	0	74
24075575	f	hp	FR	2024	0	1	0	1	0	0	0	0	0	0	0	0	64
24075592	f	hp	US	2024	0	0	0	0	0	0	0	0	0	0	0	0	60
24075685	f	md	US	2024	0	0	0	0	0	0	0	0	0	0	0	0	
24076200	Not	hp	GB	2024	1	0	0	0	0	0	0	0	0	0	0	0	
24076866	m	md	JP	2024	0	0	0	0	0	0	0	0	0	0	0	0	34
24077319	Not	md	JP	2024	0	1	0	0	0	1	0	0	0	0	0	0	
24077730	Not	hp	CN	2024	0	0	0	0	0	0	0	0	0	0	0	0	
24077900	m	md	JP	2024	0	1	0	0	0	1	0	0	0	0	0	0	76
24077931	Not	ph	JP	2024	1	0	0	0	0	0	0	0	0	0	0	0	
24078095	m	md	FR	2024	0	1	0	0	0	1	0	0	0	0	0	0	72
24078150	m	hp	GB	2024	1	0	0	0	0	0	0	0	0	0	0	0	
24078157	f	md	JP	2024	1	0	0	0	0	0	0	0	0	0	0	0	62
24078178	f	ph	JP	2024	0	1	0	0	0	1	0	0	0	0	0	0	61
24078416	Not	hp	KR	2024	0	0	0	0	0	0	0	0	0	0	0	0	
24078435	m	md	JP	2024	0	1	0	1	0	0	0	0	0	0	0	0	62
24078577	m	hp	CL	2024	0	0	0	0	0	0	0	0	0	0	0	0	38
24078718	f	hp	GR	2024	0	0	0	0	0	0	0	0	0	0	0	0	74
24078807	m	cn	CN	2024	1	0	0	0	0	0	0	0	0	0	0	0	68
24078901	Not	hp	IT	2024	0	0	0	0	0	0	0	0	0	0	0	0	
24078902	Not	hp	IT	2024	0	0	0	0	0	0	0	0	0	0	0	0	
24078993	m	cn	CA	2024	0	0	0	0	0	0	0	0	0	0	0	0	
24079640	f	hp	FR	2024	0	1	0	1	0	0	0	0	0	0	0	1	
24079666	m	hp	DE	2024	0	1	0	0	0	1	0	0	0	0	0	0	50

24079742	m	hp	FR	2024	0	1	0	1	0	0	0	0	0	0	0	0	67
24079743	m	hp	FR	2024	0	1	0	1	0	0	0	0	0	0	0	0	63
24079744	m	hp	FR	2024	0	1	0	1	0	0	0	0	0	0	0	0	77
24079746	m	hp	FR	2024	0	1	0	1	0	0	0	0	0	0	0	0	84
24079809	f	cn	TH	2024	1	0	0	0	0	0	0	0	0	0	0	0	78
24080105	m	hp	FR	2024	0	1	0	1	0	0	0	0	0	0	0	0	47
24080216	m	hp	FR	2024	0	1	0	1	0	0	0	0	0	0	0	1	59
24080335	m	hp	FR	2024	0	1	0	1	0	0	0	0	0	0	0	0	74
24080337	m	md	BE	2024	1	0	0	0	0	0	0	0	0	0	0	0	60
24080338	m	hp	FR	2024	0	1	0	1	0	0	0	0	0	0	0	0	75
24080369	f	hp	FR	2024	0	1	0	1	0	0	0	0	0	0	0	0	75
24080451	f	hp	FR	2024	0	1	0	1	0	0	0	0	0	0	0	0	73
24080452	m	hp	FR	2024	0	1	0	1	0	0	0	0	0	0	0	0	64
24080460	m	hp	IN	2024	1	0	0	0	0	0	0	0	0	0	0	0	30
24080765	m	hp	FR	2024	0	1	0	1	0	0	0	0	0	0	0	0	82
24080771	m	hp	FR	2024	0	1	0	1	0	0	0	0	0	0	0	0	
24080962	m	cn	JP	2024	1	0	0	0	0	0	0	0	0	0	0	0	68
24081039	m	hp	CN	2024	0	1	1	0	0	0	0	0	0	0	0	0	75
24081240	m	md	JP	2024	0	1	0	1	0	0	0	0	0	0	0	0	53
24081583	m	cn	FR	2024	0	0	0	0	0	0	0	0	0	0	0	0	
24082047	f	md	KR	2024	0	0	0	0	0	0	0	0	0	0	0	0	68
24082098	f	md	US	2024	0	0	0	0	0	0	0	0	0	0	0	0	79
24082212	f	md	JP	2024	0	1	0	0	0	1	0	0	0	0	0	0	80
24082499	m	md	JP	2024	0	1	1	0	0	0	0	0	0	0	0	0	80
24082823	m	md	JP	2024	0	1	0	0	0	1	0	0	0	0	0	0	78
24083521	Not	cn	TH	2024	0	0	0	0	0	0	0	0	0	0	0	0	
24083532	Not	hp	KR	2024	0	0	0	0	0	0	0	0	0	0	0	0	
24084693	m	hp	FR	2024	0	1	0	1	0	0	0	0	0	0	0	1	64
24084705	f	hp	US	2024	0	0	0	0	0	0	0	0	0	0	0	0	66
24085080	m	hp	FR	2024	0	1	0	1	0	0	0	0	0	0	0	0	71
24085360	f	hp	FR	2024	0	1	0	1	0	0	0	0	0	0	0	0	60
24085420	m	md	JP	2024	0	1	0	0	1	0	0	0	0	0	0	0	71
24085428	m	hp	FR	2024	0	1	0	1	0	0	0	0	0	0	0	1	68
24085537	f	hp	FR	2024	0	1	0	1	0	0	0	0	0	0	0	1	61

24085592	m	hp	FR	2024	0	1	0	1	0	0	0	0	0	0	0	0	78
24085662	m	hp	FR	2024	0	1	0	1	0	0	0	0	0	0	0	0	47
24085668	m	md	JP	2024	0	1	0	1	0	0	0	0	0	0	0	0	
24085687	m	ph	CA	2024	0	1	1	0	0	0	0	0	0	0	0	0	
24085776	m	hp	FR	2024	0	1	0	1	0	0	0	0	0	0	0	0	70
24085777	m	hp	FR	2024	0	1	0	1	0	0	0	0	0	0	0	1	83
24085778	m	hp	FR	2024	0	1	0	1	0	0	0	0	0	0	0	0	53
24085780	f	hp	FR	2024	0	1	0	1	0	0	0	0	0	0	0	0	76
24085814	f	ph	US	2024	0	0	0	0	0	0	0	0	0	0	0	0	
24085829	m	hp	FR	2024	0	1	0	1	0	0	0	0	0	0	0	0	76
24085832	m	hp	FR	2024	0	1	0	1	0	0	0	0	0	0	0	0	62
24085896	f	md	GB	2024	0	0	0	0	0	0	0	0	0	0	0	0	64
24085901	Not	ph	JP	2024	1	0	0	0	0	0	0	0	0	0	0	0	
24085904	m	hp	FR	2024	0	1	0	1	0	0	0	0	0	0	0	0	64
24085995	m	hp	CN	2024	0	1	1	0	0	0	0	0	0	0	0	0	69
24086010	m	hp	FR	2024	0	1	0	1	0	0	0	0	0	0	0	0	74
24086014	f	hp	FR	2024	0	1	0	1	0	0	0	0	0	0	0	0	55
24086030	m	hp	FR	2024	0	1	0	1	0	0	0	0	0	0	0	0	69
24086086	f	md	CA	2024	0	1	0	0	1	0	0	0	0	0	0	0	70
24086118	f	hp	FR	2024	0	1	0	1	0	0	0	0	0	0	0	0	70
24086233	m	hp	FR	2024	0	1	0	1	0	0	0	0	0	0	0	1	64
24086285	m	hp	FR	2024	0	1	0	1	0	0	0	0	0	0	0	0	58
24086393	Not	md	CA	2024	0	1	0	0	1	0	0	0	0	0	0	0	
24086478	f	cn	TH	2024	0	1	0	0	0	1	0	0	0	0	0	0	70
24086572	f	md	JP	2024	0	0	0	0	0	0	0	0	0	0	0	0	
24086680	m	hp	TW	2024	0	0	0	0	0	0	0	0	0	0	0	0	68
24087136	f	ph	US	2024	0	0	0	0	0	0	0	0	0	0	0	0	80
24087278	f	md	JP	2024	0	0	0	0	0	0	0	0	0	0	0	0	50
24087279	Not	md	JP	2024	0	0	0	0	0	0	0	0	0	0	0	0	
24087517	m	md	JP	2024	0	1	0	0	0	1	0	0	0	0	0	0	77
24087596	f	md	DE	2024	0	1	1	0	0	0	0	0	0	0	0	0	71
24087849	m	hp	US	2024	0	0	0	0	0	0	0	0	0	0	0	0	
24088534	f	hp	US	2024	0	1	0	0	1	0	0	0	0	0	0	0	67
24088873	m	cn	CA	2024	0	1	0	1	0	0	0	0	0	0	0	0	

24088879	m	md	IT	2024	0	1	0	0	1	0	0	0	0	0	0	0	84
24089267	f	cn	US	2024	1	0	0	0	0	0	0	0	0	0	0	0	
24089805	f	hp	AU	2024	0	1	0	0	1	0	0	0	0	0	0	0	68
24089875	m	hp	FR	2024	0	1	0	1	0	0	0	0	0	0	0	0	69
24090362	Not	md	KR	2024	0	0	0	0	0	0	0	0	0	0	0	0	
24090706	f	hp	GB	2024	0	0	0	0	0	0	0	0	0	0	0	0	57
24090724	f	hp	GB	2024	1	0	0	0	0	0	0	0	0	0	0	0	61
24091372	m	md	JP	2024	0	1	0	1	0	0	0	0	0	0	0	0	80
24091800	m	cn	FR	2024	0	0	0	0	0	0	0	0	0	0	0	0	65
24091889	f	md	JP	2024	0	1	1	0	0	0	0	0	0	0	0	0	
24092120	m	md	JP	2024	0	1	0	0	0	1	0	0	0	0	0	0	77
24092145	f	cn	EG	2024	0	1	1	0	0	0	0	0	0	0	0	0	
24092304	f	hp	FR	2024	0	1	0	1	0	0	0	0	0	0	0	0	81
24092323	m	hp	FR	2024	0	1	0	1	0	0	0	0	0	0	0	0	76
24092637	m	hp	FR	2024	0	1	0	1	0	0	0	0	0	0	0	0	61
24092897	Not	md	CA	2024	0	1	0	0	1	0	0	0	0	0	0	0	80
24092925	f	hp	FR	2024	0	1	0	1	0	0	0	0	0	0	0	0	90
24092983	f	hp	FR	2024	0	1	0	1	0	0	0	0	0	0	0	0	72
24093022	f	hp	FR	2024	0	1	0	1	0	0	0	0	0	0	0	0	65
24093024	m	hp	FR	2024	0	1	0	1	0	0	0	0	0	0	0	0	75
24093102	f	md	DE	2024	0	1	0	0	1	0	0	0	0	0	0	0	67
24093130	m	hp	FR	2024	0	1	0	1	0	0	0	0	0	0	0	0	
24093131	f	hp	FR	2024	0	1	0	1	0	0	0	0	0	0	0	0	60
24093135	f	hp	FR	2024	0	1	0	1	0	0	0	0	0	0	0	1	69
24093148	m	hp	FR	2024	0	1	0	1	0	0	0	0	0	0	0	0	57
24093149	f	hp	FR	2024	0	1	0	1	0	0	0	0	0	0	0	0	73
24093332	m	hp	FR	2024	0	1	0	1	0	0	0	0	0	0	0	0	69
24093841	f	hp	AU	2024	0	1	0	0	1	0	0	0	0	0	0	0	68
24094379	m	hp	CN	2024	0	0	0	0	0	0	0	0	0	0	0	0	61
24094507	Not	md	JP	2024	0	0	0	0	0	0	0	0	0	0	0	0	70
24094931	f	md	JP	2024	0	1	0	0	1	0	0	0	0	0	0	0	70
24095111	m	md	JP	2024	0	1	0	1	0	0	0	0	0	0	0	1	44
24095573	f	hp	GB	2024	1	0	0	0	0	0	0	0	0	0	0	0	70
24095579	m	hp	KR	2024	0	1	1	0	0	0	0	0	0	0	0	0	64

24095585	f	cn	US	2024	1	0	0	0	0	0	0	0	0	0	0	0	
24095587	f	hp	GB	2024	1	0	0	0	0	0	0	0	0	0	0	0	61
24095643	m	md	CN	2024	0	0	0	0	0	0	0	0	0	0	0	0	64
24095873	f	cn	CA	2024	0	1	0	1	0	0	0	0	0	0	0	0	
24096027	f	cn	US	2024	1	0	0	0	0	0	0	0	0	0	0	0	
24096068	f	hp	GB	2024	1	0	0	0	0	0	0	0	0	0	0	0	53
24096153	m	cn	US	2024	0	0	0	0	0	0	0	0	0	0	0	0	
24096179	f	cn	US	2024	0	1	1	0	0	0	0	0	0	0	0	0	83
24096195	m	md	JP	2024	0	1	0	0	1	0	0	0	0	0	0	0	70
24096445	m	md	KR	2024	0	0	0	0	0	0	0	0	0	0	0	0	
24096459	f	hp	US	2024	0	0	0	0	0	0	0	0	0	0	0	0	70
24096641	m	md	JP	2024	0	0	0	0	0	0	0	0	0	0	0	0	60
24096767	m	md	DE	2024	0	1	0	0	1	0	0	0	0	0	0	0	68
24097136	f	ph	US	2024	0	0	0	0	0	0	0	0	0	0	0	0	
24097297	m	cn	US	2024	0	0	0	0	0	0	0	0	0	0	0	0	
24097602	m	cn	CA	2024	0	1	0	1	0	0	0	0	0	0	0	0	
24097662	m	cn	CA	2024	1	0	0	0	0	0	0	0	0	0	0	0	
24097790	m	cn	US	2024	0	0	0	0	0	0	0	0	0	0	0	0	
24097856	m	md	US	2024	1	0	0	0	0	0	0	0	0	0	0	0	
24097896	Not	md	CA	2024	0	1	0	0	1	0	0	0	0	0	0	0	
24097925	m	cn	CA	2024	1	0	0	0	0	0	0	0	0	0	0	0	
24098315	f	hp	TW	2024	1	0	0	0	0	0	0	0	0	0	0	0	56
24098472	f	cn	US	2024	1	0	0	0	0	0	0	0	0	0	0	0	
24100811	f	hp	CA	2024	0	1	0	1	0	0	0	0	0	0	0	0	
24100946	m	md	FR	2024	0	1	0	1	0	0	0	0	0	0	0	0	
24101572	f	cn	US	2024	1	0	0	0	0	0	0	0	0	0	0	0	
24101641	f	md	DE	2024	0	1	1	0	0	0	0	0	0	0	0	0	71
24101789	Not	md	KZ	2024	0	1	0	0	0	1	0	0	0	0	0	0	58
24102602	Not	md	KR	2024	0	0	0	0	0	0	0	0	0	0	0	0	
24102950	f	md	JP	2024	0	0	0	0	0	0	0	0	0	0	0	0	49
24103004	f	cn	CN	2024	1	0	0	0	0	0	0	0	0	0	0	0	65.5
24103248	m	cn	US	2024	1	0	0	0	0	0	0	0	0	0	0	0	
24103255	m	md	FR	2024	0	1	0	1	0	0	0	0	0	0	0	0	
24103310	f	ph	FR	2024	0	0	0	0	0	0	0	0	0	0	0	0	67

24103315	f	hp	SE	2024	0	0	0	0	0	0	0	0	0	0	0	0	67
24103320	m	cn	CA	2024	0	1	0	1	0	0	0	0	0	0	0	0	73.4
24103367	Not	hp	AU	2024	0	0	0	0	0	0	0	0	0	0	0	0	
24103486	m	md	FR	2024	0	1	0	1	0	0	0	0	0	0	0	0	
24103563	m	cn	TW	2024	1	0	0	0	0	0	0	0	0	0	0	0	59
24103627	f	hp	GB	2024	1	0	0	0	0	0	0	0	0	0	0	0	57
24103896	f	cn	CA	2024	0	1	0	0	1	0	0	0	0	0	0	0	70
24104122	m	cn	US	2024	0	0	0	0	0	0	0	0	0	0	0	0	
24104157	m	hp	SE	2024	0	0	0	0	0	0	0	0	0	0	0	0	77
24104413	m	md	FR	2024	0	1	0	1	0	0	0	0	0	0	0	0	
24104438	f	md	US	2024	0	0	0	0	0	0	0	0	0	0	0	0	
24104477	f	hp	US	2024	0	0	0	0	0	0	0	0	0	0	0	0	
24104569	m	cn	CA	2024	0	1	0	1	0	0	0	0	0	0	0	0	
24104652	m	cn	EG	2024	0	1	1	0	0	0	0	0	0	0	0	0	54
24104666	Not	md	CA	2024	0	1	0	0	1	0	0	0	0	0	0	0	70
24104693	f	cn	ID	2024	1	0	0	0	0	0	0	0	0	0	0	0	
24104751	m	hp	NL	2024	0	1	1	0	0	0	0	0	0	0	0	0	74
24104968	m	md	BE	2024	0	0	0	0	0	0	0	0	0	0	0	0	
24105288	f	md	CA	2024	0	1	0	0	1	0	0	0	0	0	0	0	60
24105302	m	md	FR	2024	0	1	0	1	0	0	0	0	0	0	0	0	
24105303	m	ph	JP	2024	1	0	0	0	0	0	0	0	0	0	0	0	84
24105304	f	cn	US	2024	1	0	0	0	0	0	0	0	0	0	0	0	
24105506	m	ph	US	2024	0	0	0	0	0	0	0	0	0	0	0	0	78
24105770	Not	ph	US	2024	0	1	0	1	0	0	0	0	0	0	0	0	
24105876	f	md	BE	2024	0	0	0	0	0	0	0	0	0	0	0	0	
24105901	Not	md	US	2024	0	0	0	0	0	0	0	0	0	0	0	0	
24105914	f	hp	US	2024	0	1	0	0	0	1	0	0	0	0	0	0	
24106007	m	md	JP	2024	0	1	1	0	0	0	0	0	0	0	0	0	80
24106036	f	md	JP	2024	1	0	0	0	0	0	0	0	0	0	0	0	82
24106121	f	md	JP	2024	1	0	0	0	0	0	0	0	0	0	0	1	62
24106179	f	md	JP	2024	1	0	0	0	0	0	0	0	0	0	0	0	66
24106225	f	md	FR	2024	0	1	0	1	0	0	0	0	0	0	0	0	
24106228	m	md	JP	2024	0	1	1	0	0	0	0	0	0	0	0	0	76
24106470	Not	cn	TH	2024	0	0	0	0	0	0	0	0	0	0	0	0	

24106662	m	ph	FR	2024	0	0	0	0	0	0	0	0	0	0	0	0	56
24106751	m	md	US	2024	0	1	1	0	0	0	0	0	0	0	0	0	58
24106765	f	hp	EU	2024	0	1	0	0	1	0	0	0	0	0	0	0	46
24107145	m	md	JP	2024	0	1	1	0	0	0	0	0	0	0	0	0	79
24107275	f	hp	NL	2024	0	1	0	0	1	0	0	0	0	0	0	0	46
24107277	Not	hp	SG	2024	1	0	0	0	0	0	0	0	0	0	0	0	
24107278	Not	hp	SG	2024	1	0	0	0	0	0	0	0	0	0	0	0	
24107331	f	hp	US	2024	1	0	0	0	0	0	0	0	0	0	0	0	
24107396	m	cn	CN	2024	0	0	0	0	0	0	0	0	0	0	0	0	47
24107425	f	cn	US	2024	1	0	0	0	0	0	0	0	0	0	0	0	
24107462	Not	cn	TW	2024	1	0	0	0	0	0	0	0	0	0	0	0	
24107589	f	cn	CA	2024	0	1	0	1	0	0	0	0	0	0	0	0	
24107761	f	cn	US	2024	0	1	0	1	0	0	0	0	0	0	0	0	
24107843	m	hp	TW	2024	0	1	1	0	0	0	0	0	0	0	0	0	70
24107869	f	cn	US	2024	1	0	0	0	0	0	0	0	0	0	0	0	81.4
24108160	f	cn	US	2024	1	0	0	0	0	0	0	0	0	0	0	0	80
24108230	f	cn	TW	2024	1	0	0	0	0	0	0	0	0	0	0	0	57
24108362	m	hp	KR	2024	0	1	1	0	0	0	0	0	0	0	0	0	51
24108476	f	cn	CA	2024	0	1	0	1	0	0	0	0	0	0	0	0	
24108524	m	hp	TW	2024	1	0	0	0	0	0	0	0	0	0	0	0	51
24108525	m	hp	TW	2024	1	0	0	0	0	0	0	0	0	0	0	0	56
24108599	f	cn	US	2024	1	0	0	0	0	0	0	0	0	0	0	0	
24108670	Not	md	JP	2024	0	0	0	0	0	0	0	0	0	0	0	0	
24108932	f	cn	US	2024	1	0	0	0	0	0	0	0	0	0	0	0	83
24108950	f	md	CN	2024	0	1	1	0	0	0	0	0	0	0	0	0	70
24109031	f	cn	US	2024	1	0	0	0	0	0	0	0	0	0	0	0	73
24109037	m	cn	TH	2024	0	1	0	0	0	1	0	0	0	0	0	0	87
24109126	f	md	US	2024	0	1	1	0	0	0	0	0	0	0	0	0	79
24109156	m	cn	PK	2024	0	1	0	0	0	1	0	0	0	0	0	0	63
24109270	m	md	IT	2024	0	1	0	0	1	0	0	0	0	0	0	0	84
24109277	m	md	DE	2024	0	1	0	0	1	0	0	0	0	0	0	0	74
24109319	Not	cn	US	2024	1	0	0	0	0	0	0	0	0	0	0	0	
24109698	m	md	PE	2024	0	0	0	0	0	0	0	0	0	0	0	0	52
24109819	m	ph	CA	2024	0	0	0	0	0	0	0	0	0	0	0	0	59

24110025	f	cn	US	2024	1	0	0	0	0	0	0	0	0	0	0	0	89
24110202	f	md	JP	2024	0	0	0	0	0	0	0	0	0	0	0	0	77
24110249	f	md	PT	2024	0	1	1	0	0	0	0	0	0	0	0	0	
24110351	m	cn	US	2024	1	0	0	0	0	0	0	0	0	0	0	0	
24110360	m	cn	TW	2024	0	1	0	1	0	0	0	0	0	0	0	0	66
24110539	f	ph	JP	2024	0	1	0	0	1	0	0	0	0	0	0	0	76
24111141	f	md	NL	2024	0	0	0	0	0	0	0	0	0	0	0	0	
24111185	f	md	JP	2024	0	0	0	0	0	0	0	0	0	0	0	0	87
24111398	f	md	DE	2024	0	1	0	1	0	0	0	0	0	0	0	0	
24111404	f	cn	US	2024	1	0	0	0	0	0	0	0	0	0	0	0	56
24111752	m	md	JP	2024	0	1	1	0	0	0	0	0	0	0	0	1	76
24111855	f	md	CA	2024	0	1	0	0	1	0	0	0	0	0	0	0	70
24111865	f	md	CA	2024	0	1	0	0	1	0	0	0	0	0	0	0	70
24112125	Not	ph	JP	2024	0	0	0	0	0	0	0	0	0	0	0	0	
24112234	m	md	FR	2024	0	1	0	1	0	0	0	0	0	0	0	0	58
24112288	m	cn	US	2024	1	0	0	0	0	0	0	0	0	0	0	0	
24112295	m	cn	CA	2024	0	1	0	1	0	0	0	0	0	0	0	0	
24112299	f	cn	US	2024	1	0	0	0	0	0	0	0	0	0	0	0	
24112302	f	cn	US	2024	1	0	0	0	0	0	0	0	0	0	0	0	
24112328	Not	cn	SG	2024	1	0	0	0	0	0	0	0	0	0	0	0	53.8
24112410	m	cn	SG	2024	1	0	0	0	0	0	0	0	0	0	0	0	71.7
24112413	Not	md	JP	2024	0	1	0	1	0	0	0	0	0	0	0	0	
24112427	f	cn	US	2024	1	0	0	0	0	0	0	0	0	0	0	0	62
24112531	m	md	JP	2024	0	1	0	1	0	0	0	0	0	0	0	0	69
24112532	m	md	JP	2024	0	1	0	1	0	0	0	0	0	0	0	0	66
24112554	f	hp	US	2024	1	0	0	0	0	0	0	0	0	0	0	0	
24112713	m	hp	US	2024	1	0	0	0	0	0	0	0	0	0	0	0	60
24112731	f	md	FR	2024	0	1	0	0	0	1	0	0	0	0	0	0	53
24112792	Not	md	HK	2024	0	0	0	0	0	0	0	0	0	0	0	0	
24112798	m	md	PL	2024	0	0	0	0	0	0	0	0	0	0	0	0	
24112833	f	md	FR	2024	0	1	0	0	0	1	0	0	0	0	0	0	53
24112910	m	md	DE	2024	0	0	0	0	0	0	0	0	0	0	0	0	85
24112986	f	hp	US	2024	0	0	0	0	0	0	0	0	0	0	0	0	62
24113029	f	md	JP	2024	0	1	1	0	0	0	0	0	0	0	0	0	80

24113866	m	md	US	2024	0	0	0	0	0	0	0	0	0	0	0	0	65
24113899	f	hp	NL	2024	0	1	0	0	1	0	0	0	0	0	0	0	46
24114549	f	md	PL	2024	0	0	0	0	0	0	0	0	0	0	0	0	73
24114629	f	cn	PK	2024	0	0	0	0	0	0	0	0	0	0	0	0	60
24116215	m	md	JP	2024	0	1	1	0	0	0	0	0	0	0	0	0	
24116301	f	md	KR	2024	0	0	0	0	0	0	0	0	0	0	0	0	
24116857	m	cn	TH	2024	0	1	0	0	0	1	0	0	0	0	0	0	55
24116899	m	cn	TH	2024	0	1	0	0	0	1	0	0	0	0	0	0	56
24116906	m	hp	TW	2024	1	0	0	0	0	0	0	0	0	0	0	0	56
24116948	f	cn	TH	2024	0	1	0	0	0	1	0	0	0	0	0	0	63
24116951	m	cn	TH	2024	0	1	0	0	0	1	0	0	0	0	0	0	69
24116972	m	cn	TH	2024	0	1	0	0	0	1	0	0	0	0	0	0	77
24117276	m	cn	SG	2024	1	0	0	0	0	0	0	0	0	0	0	0	70.3
24117472	f	cn	US	2024	1	0	0	0	0	0	0	0	0	0	0	0	
24117816	Not	ph	JP	2024	0	0	0	0	0	0	0	0	0	0	0	0	
24117953	Not	md	JP	2024	0	1	0	0	1	0	0	0	0	0	0	0	
24117956	Not	md	JP	2024	0	1	0	0	1	0	0	0	0	0	0	0	
24118009	m	cn	SG	2024	1	0	0	0	0	0	0	0	0	0	0	0	
24118104	Not	cn	SG	2024	1	0	0	0	0	0	0	0	0	0	0	0	75
24118152	Not	md	JP	2024	0	1	0	0	1	0	0	0	0	0	0	0	
24118155	f	md	CA	2024	0	1	1	0	0	0	0	0	0	0	0	0	67
24118203	m	md	JP	2024	0	1	1	0	0	0	0	0	0	0	0	0	78
24118251	Not	md	RU	2024	0	1	0	1	0	0	0	0	0	0	0	0	
24118276	m	hp	JP	2024	0	1	1	0	0	0	0	0	0	0	0	0	47
24118290	m	md	JP	2024	0	1	1	0	0	0	0	0	0	0	0	0	70
24118301	f	md	JP	2024	0	1	1	0	0	0	0	0	0	0	0	0	68
24118314	m	md	JP	2024	0	1	1	0	0	0	0	0	0	0	0	0	48
24118316	m	md	JP	2024	0	1	1	0	0	0	0	0	0	0	0	0	60
24118319	m	cn	US	2024	0	1	1	0	0	0	0	0	0	0	0	0	
24118484	m	md	FR	2024	0	1	0	1	0	0	0	0	0	0	0	0	
24118487	f	cn	US	2024	1	0	0	0	0	0	0	0	0	0	0	0	71
24118510	m	md	AU	2024	1	0	0	0	0	0	0	0	0	0	0	0	
24118630	Not	md	JP	2024	0	1	0	0	1	0	0	0	0	0	0	0	
24118865	m	md	US	2024	0	1	0	1	0	0	0	0	0	0	0	0	

24118942	m	md	NL	2024	0	0	0	0	0	0	0	0	0	0	0	0	64
24119098	Not	md	JP	2024	0	1	0	0	1	0	0	0	0	0	0	0	
24119240	f	md	RO	2024	1	0	0	0	0	0	0	0	0	0	0	0	
24119311	Not	hp	TR	2024	0	0	0	0	0	0	0	0	0	0	0	0	
24119494	f	md	NL	2024	0	1	1	0	0	0	0	0	0	0	0	0	58
24120085	m	md	PT	2024	0	1	1	0	0	0	0	0	0	0	0	0	71
24120119	m	md	TH	2024	0	0	0	0	0	0	0	0	0	0	0	0	78
24120257	f	cn	US	2024	1	0	0	0	0	0	0	0	0	0	0	0	51
24120389	f	cn	US	2024	1	0	0	0	0	0	0	0	0	0	0	0	
24120406	f	hp	US	2024	0	0	0	0	0	0	0	0	0	0	0	0	64
24120881	m	md	AU	2024	1	0	0	0	0	0	0	0	0	0	0	0	73
24121089	f	md	JP	2024	0	0	0	0	0	0	0	0	0	0	0	0	81
24121269	m	md	JP	2024	0	1	0	1	0	0	0	0	0	0	0	0	69
24121417	f	ph	FR	2024	0	0	0	0	0	0	0	0	0	0	0	0	67
24121477	m	md	JP	2024	0	1	0	1	0	0	0	0	0	0	0	0	72
24121617	m	cn	US	2024	1	0	0	0	0	0	0	0	0	0	0	0	71.9
24122338	m	hp	US	2024	0	0	0	0	0	0	0	0	0	0	0	0	
24122451	Not	md	JP	2024	0	1	0	0	1	0	0	0	0	0	0	0	
24122469	Not	md	JP	2024	0	1	0	0	1	0	0	0	0	0	0	0	
24122512	f	md	US	2024	0	1	1	0	0	0	0	0	0	0	0	0	79
24122562	m	md	JP	2024	0	0	0	0	0	0	0	0	0	0	0	0	70
24122632	m	md	NL	2024	0	0	0	0	0	0	0	0	0	0	0	0	64
24122661	Not	md	JP	2024	0	1	0	0	1	0	0	0	0	0	0	0	
24122749	Not	md	JP	2024	0	1	0	0	1	0	0	0	0	0	0	0	
24122937	m	md	DE	2024	0	1	0	0	1	0	0	0	0	0	0	0	83
24124072	Not	md	JP	2024	0	1	0	0	1	0	0	0	0	0	0	0	
24124164	Not	md	JP	2024	0	1	0	0	1	0	0	0	0	0	0	0	
24124190	m	hp	US	2024	0	0	0	0	0	0	0	0	0	0	0	0	
24124218	Not	md	JP	2024	0	1	0	0	1	0	0	0	0	0	0	0	
24124237	f	md	JP	2024	0	0	0	0	0	0	0	0	0	0	0	0	
24124454	f	hp	TH	2024	0	1	0	0	0	1	0	0	0	0	0	0	91
24124469	m	cn	US	2024	1	0	0	0	0	0	0	0	0	0	0	0	60
24124635	f	cn	US	2024	1	0	0	0	0	0	0	0	0	0	0	0	
24124665	Not	md	JP	2024	0	1	0	0	1	0	0	0	0	0	0	0	

24124787	m	cn	CN	2024	1	0	0	0	0	0	0	0	0	0	0	0	49.3
24125090	f	md	TH	2024	1	0	0	0	0	0	0	0	0	0	0	0	72.7
24125092	f	cn	US	2024	1	0	0	0	0	0	0	0	0	0	0	0	
24125094	f	cn	US	2024	1	0	0	0	0	0	0	0	0	0	0	0	
24125204	m	md	JP	2024	0	1	1	0	0	0	0	0	0	0	0	0	80
24125305	Not	md	JP	2024	0	1	0	0	1	0	0	0	0	0	0	0	
24125389	f	cn	TH	2024	0	1	0	0	0	1	0	0	0	0	0	0	58
24125577	m	cn	TH	2024	0	1	0	0	0	1	0	0	0	0	0	0	73
24125598	m	cn	IN	2024	0	0	0	0	0	0	0	0	0	0	0	0	49
24125732	Not	md	JP	2024	0	1	0	0	1	0	0	0	0	0	0	0	
24125914	Not	md	JP	2024	0	0	0	0	0	0	0	0	0	0	0	0	
24126203	f	cn	CA	2024	1	0	0	0	0	0	0	0	0	0	0	0	
24126210	m	cn	TH	2024	0	1	0	0	0	1	0	0	0	0	0	0	58
24126270	f	md	AU	2024	0	1	1	0	0	0	0	0	0	0	0	0	73
24127361	m	md	AT	2024	0	1	1	0	0	0	0	0	0	0	0	0	65
24127872	m	cn	HK	2024	0	0	0	0	0	0	0	0	0	0	0	0	53
24128228	m	ph	US	2024	0	0	0	0	0	0	0	0	0	0	0	0	
24128294	m	md	JP	2024	0	1	0	1	0	0	0	0	0	0	0	0	80
24128435	m	hp	US	2024	0	1	0	1	0	0	0	0	0	0	0	0	65
24128851	f	md	JP	2024	0	1	0	0	1	0	0	0	0	0	0	0	79
24129382	Not	md	JP	2024	0	1	0	0	1	0	0	0	0	0	0	0	
24129564	m	md	CH	2024	0	0	0	0	0	0	0	0	0	0	0	0	68
24129574	Not	cn	JP	2024	0	0	0	0	0	0	0	0	0	0	0	0	
24129576	f	cn	NL	2024	0	1	0	0	1	0	0	0	0	0	0	0	46
24130178	m	md	DE	2024	0	1	1	0	0	0	0	0	0	0	0	0	69
24130590	f	ph	US	2024	0	0	0	0	0	0	0	0	0	0	0	0	
24130656	f	md	KR	2024	0	0	0	0	0	0	0	0	0	0	0	0	
24130707	Not	md	JP	2024	0	1	0	0	1	0	0	0	0	0	0	0	
24131009	m	md	JP	2024	0	1	0	1	0	0	0	0	0	0	0	0	62
24131017	m	md	KR	2024	1	0	0	0	0	0	0	0	0	0	0	0	71.2
24131039	f	cn	US	2024	0	1	0	0	1	0	0	0	0	0	0	0	69
24131098	Not	cn	TH	2024	0	1	0	0	0	1	0	0	0	0	0	0	64
24131155	f	hp	JP	2024	1	0	0	0	0	0	0	0	0	0	0	0	77
24131242	m	hp	JP	2024	1	0	0	0	0	0	0	0	0	0	0	0	63

24131309	m	cn	TH	2024	0	1	0	0	0	1	0	0	0	0	0	0	
24131322	m	cn	TH	2024	0	1	0	0	0	1	0	0	0	0	0	0	76
24131438	m	cn	US	2024	0	1	1	0	0	0	0	0	0	0	0	0	66
24131475	f	md	DE	2024	0	1	0	0	1	0	0	0	0	0	0	0	78
24131649	m	md	FR	2024	0	1	0	1	0	0	0	0	0	0	0	0	76
24132353	Not	md	JP	2024	1	0	0	0	0	0	0	0	0	0	0	0	
24132951	m	md	JP	2024	0	0	0	0	0	0	0	0	0	0	0	0	50
24133045	m	cn	US	2024	1	0	0	0	0	0	0	0	0	0	0	0	
24133199	m	cn	TH	2024	0	0	0	0	0	0	0	0	0	0	0	0	72
24133388	f	cn	US	2024	1	0	0	0	0	0	0	0	0	0	0	0	
24133575	Not	hp	ZA	2024	1	0	0	0	0	0	0	0	0	0	0	0	
24133740	f	md	CA	2024	1	0	0	0	0	0	0	0	0	0	0	0	49.6
24134101	f	md	JP	2024	0	1	0	0	1	0	0	0	0	0	0	0	60
24134254	m	hp	US	2024	0	1	1	0	0	0	0	0	0	0	0	0	78
24134831	Not	cn	US	2024	1	0	0	0	0	0	0	0	0	0	0	0	
24134903	Not	md	JP	2024	0	1	0	0	1	0	0	0	0	0	0	0	
24134910	f	md	DE	2024	0	1	0	0	1	0	0	0	0	0	0	0	58
24135131	f	md	JP	2024	0	1	0	0	1	0	0	0	0	0	0	0	80
24135138	f	cn	TH	2024	0	1	0	0	0	1	0	0	0	0	0	0	62
24135411	m	md	JP	2024	0	1	0	1	0	0	0	0	0	0	0	1	57
24135633	Not	ph	JP	2024	1	0	0	0	0	0	0	0	0	0	0	0	
24135915	f	md	CN	2024	0	0	0	0	0	0	0	0	0	0	0	0	
24136030	f	cn	IN	2024	0	0	0	0	0	0	0	0	0	0	0	0	49
24136057	Not	cn	TH	2024	0	0	0	0	0	0	0	0	0	0	0	0	
24136073	Not	cn	TH	2024	0	0	0	0	0	0	0	0	0	0	0	0	
24136082	Not	hp	KR	2024	0	0	0	0	0	0	0	0	0	0	0	0	
24136086	m	hp	JP	2024	0	1	0	0	0	1	0	0	0	0	0	0	67
24136497	f	md	JP	2024	0	1	0	0	0	1	0	0	0	0	0	0	50
24136593	m	ph	US	2024	0	0	0	0	0	0	0	0	0	0	0	0	64
24137189	m	md	JP	2024	0	1	0	1	0	0	0	0	0	0	0	0	66
24137401	m	md	CN	2024	0	0	0	0	0	0	0	0	0	0	0	0	50
24137483	f	md	JP	2024	1	0	0	0	0	0	0	0	0	0	0	0	74
24137726	m	md	AU	2024	0	1	0	0	1	0	0	0	0	0	0	0	70
24137899	Not	md	JP	2024	1	0	0	0	0	0	0	0	0	0	0	0	

24138417	Not	md	HK	2024	0	1	0	0	1	0	0	0	0	0	0	0	
24138498	Not	md	JP	2024	1	0	0	0	0	0	0	0	0	0	0	0	
24138499	f	cn	CN	2024	1	0	0	0	0	0	0	0	0	0	0	0	65
24138652	f	md	JP	2024	0	0	0	0	0	0	0	0	0	0	0	0	68
24138684	Not	md	JP	2024	0	1	0	0	1	0	0	0	0	0	0	0	
24138699	f	cn	US	2024	0	1	1	0	0	0	0	0	0	0	0	0	79
24139007	m	md	US	2024	1	0	0	0	0	0	0	0	0	0	0	0	71
24139093	Not	md	JP	2024	0	1	0	0	1	0	0	0	0	0	0	0	
24139195	Not	md	JP	2024	0	1	0	0	1	0	0	0	0	0	0	0	
24139575	f	md	TH	2024	0	0	0	0	0	0	0	0	0	0	0	0	41
24139846	f	md	DE	2024	0	1	0	0	1	0	0	0	0	0	0	0	59
24139965	m	cn	TH	2024	0	1	0	0	0	1	0	0	0	0	0	0	60
24139983	m	cn	TH	2024	0	1	0	0	0	1	0	0	0	0	0	0	
24139994	Not	ph	JP	2024	1	0	0	0	0	0	0	0	0	0	0	0	
24140043	m	md	TH	2024	0	1	0	0	0	1	0	0	0	0	0	0	
24140211	m	md	JP	2024	0	1	0	0	0	1	0	0	0	0	0	0	80
24140397	Not	cn	SG	2024	1	0	0	0	0	0	0	0	0	0	0	0	74
24140459	f	cn	US	2024	1	0	0	0	0	0	0	0	0	0	0	0	
24140489	Not	hp	US	2024	0	0	0	0	0	0	0	0	0	0	0	0	
24140661	Not	md	JP	2024	0	1	0	0	1	0	0	0	0	0	0	0	
24140721	m	md	JP	2024	0	1	0	1	0	0	0	0	0	0	0	0	80
24141035	Not	hp	US	2024	0	0	0	0	0	0	0	0	0	0	0	0	
24141599	Not	md	AU	2024	0	0	0	0	0	0	0	0	0	0	0	0	
24141602	m	md	JP	2024	0	1	0	0	0	1	0	0	0	0	0	0	80
24141701	m	md	JP	2024	0	0	0	0	0	0	0	0	0	0	0	0	79
24141708	m	hp	TH	2024	0	1	0	0	0	1	0	0	0	0	0	0	67
24141810	m	Not repo	US	2024	0	0	0	0	0	0	0	0	0	0	0	0	
24141873	m	cn	IN	2024	0	0	0	0	0	0	0	0	0	0	0	0	55
24141984	f	hp	US	2024	0	0	0	0	0	0	0	0	0	0	0	0	
24142161	m	ph	KR	2024	0	1	1	0	0	0	0	0	0	0	0	0	
24142341	f	cn	US	2024	1	0	0	0	0	0	0	0	0	0	0	0	73.5
24142387	m	cn	US	2024	0	0	0	0	0	0	0	0	0	0	0	0	80
24142467	f	cn	US	2024	0	0	0	0	0	0	0	0	0	0	0	0	
24142488	f	cn	KE	2024	1	0	0	0	0	0	0	0	0	0	0	0	76.1

24142493	m	md	JP	2024	0	1	0	1	0	0	0	0	0	0	0	1	79
24142802	f	md	BE	2024	0	0	0	0	0	0	0	0	0	0	0	0	72
24142832	Not	ph	JP	2024	1	0	0	0	0	0	0	0	0	0	0	0	
24142996	f	md	KR	2024	0	0	0	0	0	0	0	0	0	0	0	0	
24143154	f	md	JP	2024	1	0	0	0	0	0	0	0	0	0	0	0	60
24143273	f	hp	US	2024	0	1	1	0	0	0	0	0	0	0	0	0	68
24143303	m	hp	KR	2024	0	1	1	0	0	0	0	0	0	0	0	0	
24143395	f	cn	TH	2024	0	1	0	0	0	1	0	0	0	0	0	0	62
24144040	Not	hp	JP	2024	0	1	0	0	0	1	0	0	0	0	0	0	
24144041	f	md	ES	2024	0	0	0	0	0	0	0	0	0	0	0	0	65
24144309	f	md	FR	2024	0	0	0	0	0	0	0	0	0	0	0	0	
24144328	f	cn	IN	2024	0	0	0	0	0	0	0	0	0	0	0	0	53
24144978	Not	md	JP	2024	0	1	0	0	1	0	0	0	0	0	0	0	
24145076	f	cn	US	2024	1	0	0	0	0	0	0	0	0	0	0	0	57.8
24145693	m	md	BR	2024	0	1	0	0	0	1	0	0	0	0	0	0	72
24145887	f	cn	KR	2024	0	0	0	0	0	0	0	0	0	0	0	0	75
24146169	m	hp	ES	2024	0	1	0	0	1	0	0	0	0	0	0	0	60
24146487	Not	md	US	2024	0	0	0	0	0	0	0	0	0	0	0	0	
24146576	f	cn	SG	2024	0	0	0	0	0	0	0	0	0	0	0	0	65
24146783	m	hp	JP	2024	0	1	0	1	0	0	0	0	0	0	0	0	79
24146882	f	md	NL	2024	0	1	1	0	0	0	0	0	0	0	0	0	69
24146903	m	hp	IN	2024	0	0	0	0	0	0	0	0	0	0	0	0	30
24146995	f	hp	IL	2024	1	0	0	0	0	0	0	0	0	0	0	0	75
24147678	m	ph	FR	2024	0	0	0	0	0	0	0	0	0	0	0	0	
24147683	f	cn	JP	2024	1	0	0	0	0	0	0	0	0	0	0	0	75
24147925	m	md	JP	2024	0	1	0	1	0	0	0	0	0	0	0	0	61
24148276	m	md	KR	2024	0	0	0	0	0	0	0	0	0	0	0	0	74
24148329	m	md	JP	2024	0	1	0	1	0	0	0	0	0	0	0	0	65
24148523	f	md	JP	2024	0	1	0	1	0	0	0	0	0	0	0	0	54
24148635	f	hp	SE	2024	0	0	0	0	0	0	0	0	0	0	0	0	67
24148771	m	md	JP	2024	0	1	1	0	0	0	0	0	0	0	0	0	54
24148800	Not	md	JP	2024	0	1	0	0	1	0	0	0	0	0	0	0	
24148804	m	md	KR	2024	0	0	0	0	0	0	0	0	0	0	0	0	76
24148859	Not	md	JP	2024	0	1	0	0	1	0	0	0	0	0	0	0	

24149235	Not	cn	SG	2024	0	1	0	1	0	0	0	0	0	0	0	0	
24149394	m	md	JP	2024	0	1	0	0	0	1	0	0	0	0	0	0	73
24149470	m	hp	JP	2024	0	1	0	0	0	1	0	0	0	0	0	0	67
24149596	Not	md	CA	2024	0	1	0	0	1	0	0	0	0	0	0	0	70
24150471	Not	hp	CN	2024	0	0	0	0	0	0	0	0	0	0	0	0	
24150570	f	md	JP	2024	0	1	1	0	0	0	0	0	0	0	0	0	90
24150619	Not	md	JP	2024	0	1	0	0	1	0	0	0	0	0	0	0	
24151874	Not	md	LB	2024	0	1	0	0	0	1	0	0	0	0	0	0	
24151906	f	md	JP	2024	0	1	0	0	1	0	0	0	0	0	0	0	73
24152049	f	md	DE	2024	0	0	0	0	0	0	0	0	0	0	0	0	73
24152889	f	cn	US	2024	1	0	0	0	0	0	0	0	0	0	0	0	
24152991	f	ph	FR	2024	0	0	0	0	0	0	0	0	0	0	0	0	55
24153133	m	md	JP	2024	0	1	0	1	0	0	0	0	0	0	0	0	54
24153345	Not	cn	US	2024	1	0	0	0	0	0	0	0	0	0	0	0	
24153518	f	md	FR	2024	0	0	0	0	0	0	0	0	0	0	0	0	
24153635	f	cn	US	2024	0	1	1	0	0	0	0	0	0	0	0	1	
24153672	m	md	JP	2024	0	1	0	1	0	0	0	0	0	0	0	1	78
24154104	f	cn	JP	2024	1	0	0	0	0	0	0	0	0	0	0	0	77
24154216	Not	md	FR	2024	0	0	0	0	0	0	0	0	0	0	0	0	
24154389	m	hp	US	2024	0	0	0	0	0	0	0	0	0	0	0	0	64
24154419	f	md	JP	2024	0	1	0	0	1	0	0	0	0	0	0	0	
24154428	f	md	AU	2024	1	0	0	0	0	0	0	0	0	0	0	0	
24154595	m	md	US	2024	0	0	0	0	0	0	0	0	0	0	0	0	72
24154818	m	md	JP	2024	0	1	0	0	0	1	0	0	0	0	0	0	78
24154974	f	md	JP	2024	0	1	0	0	0	1	0	0	0	0	0	0	80
24155021	f	md	JP	2024	0	1	0	0	0	1	0	0	0	0	0	0	77
24155213	Not	cn	US	2024	1	0	0	0	0	0	0	0	0	0	0	0	
24155240	m	md	JP	2024	0	1	0	0	0	1	0	0	0	0	0	0	80
24155392	m	cn	IN	2024	1	0	0	0	0	0	0	0	0	0	0	0	62
24155682	f	cn	US	2024	1	0	0	0	0	0	0	0	0	0	0	0	61.6
24155697	f	cn	US	2024	1	0	0	0	0	0	0	0	0	0	0	0	45
24155760	m	cn	SG	2024	0	1	0	0	0	1	0	0	0	0	0	0	66
24155773	m	hp	JP	2024	0	0	0	0	0	0	0	0	0	0	0	0	67
24155860	m	hp	TR	2024	0	0	0	0	0	0	0	0	0	0	0	0	60

24155913	m	hp	CN	2024	0	0	0	0	0	0	0	0	0	0	0	0	52
24156003	m	hp	TR	2024	0	0	0	0	0	0	0	0	0	0	0	0	67
24156029	m	cn	SG	2024	0	0	0	0	0	0	0	0	0	0	0	0	64
24156088	m	hp	TR	2024	0	0	0	0	0	0	0	0	0	0	0	0	57
24156116	m	hp	TR	2024	0	0	0	0	0	0	0	0	0	0	0	0	56
24156490	m	md	ES	2024	0	0	0	0	0	0	0	0	0	0	0	0	64
24156916	m	md	JP	2024	0	0	0	0	0	0	0	0	0	0	0	0	67
24157495	m	md	PT	2024	0	1	1	0	0	0	0	0	0	0	0	0	71
24157614	m	md	US	2024	0	0	0	0	0	0	0	0	0	0	0	0	
24157699	m	md	JP	2024	0	1	0	1	0	0	0	0	0	0	0	0	46
24157710	m	md	JP	2024	0	0	0	0	0	0	0	0	0	0	0	0	
24158165	m	md	JP	2024	0	1	0	1	0	0	0	0	0	0	0	0	79
24158351	f	md	DE	2024	0	1	0	0	1	0	0	0	0	0	0	0	78
24158369	f	cn	AE	2024	1	0	0	0	0	0	0	0	0	0	0	0	
24158370	m	md	JP	2024	1	0	0	0	0	0	0	0	0	0	0	0	74
24159432	m	hp	SE	2024	0	0	0	0	0	0	0	0	0	0	0	0	77
24159718	m	md	CN	2024	0	1	1	0	0	0	0	0	0	0	0	0	61
24160350	m	cn	US	2024	1	0	0	0	0	0	0	0	0	0	0	0	61.5
24160424	f	cn	US	2024	0	0	0	0	0	0	0	0	0	0	0	0	65
24160758	f	hp	US	2024	1	0	0	0	0	0	0	0	0	0	0	0	
24160966	f	md	JP	2024	0	1	0	0	0	1	0	0	0	0	0	0	70
24161047	f	md	JP	2024	0	0	0	0	0	0	0	0	0	0	0	0	90
24161210	f	md	FR	2024	1	0	0	0	0	0	0	0	0	0	0	0	85.3
24161280	f	md	IT	2024	0	0	0	0	0	0	0	0	0	0	0	0	58
24162144	f	md	JP	2024	0	1	0	0	0	1	0	0	0	0	0	0	50
24162234	Not	hp	CN	2024	0	0	0	0	0	0	0	0	0	0	0	0	
24162350	f	md	AU	2024	0	0	0	0	0	0	0	0	0	0	0	0	68
24162507	Not	cn	CN	2024	0	0	0	0	0	0	0	0	0	0	0	0	
24163107	f	md	US	2024	1	0	0	0	0	0	0	0	0	0	0	0	77
24163539	f	hp	NL	2024	0	1	0	0	1	0	0	0	0	0	0	0	46
24163800	f	cn	US	2024	1	0	0	0	0	0	0	0	0	0	0	0	62.2
24163928	f	md	KR	2024	1	0	0	0	0	0	0	0	0	0	0	0	69.1
24164131	f	cn	US	2024	1	0	0	0	0	0	0	0	0	0	0	0	
24164500	m	md	JP	2024	0	1	0	0	1	0	0	0	0	0	0	0	69

24164750	f	hp	GI	2024	0	1	1	0	0	0	0	0	0	0	0	0	61
24164796	Not	md	JP	2024	0	0	0	0	0	0	0	0	0	0	0	0	
24164797	f	md	US	2024	0	0	0	0	0	0	0	0	0	0	0	0	
24164827	f	ph	JP	2024	0	0	0	0	0	0	0	0	0	0	0	0	79
24165173	f	md	US	2024	0	0	0	0	0	0	0	0	0	0	0	0	85
24165338	m	hp	TH	2024	0	1	0	0	0	1	0	0	0	0	0	0	61
24165434	m	hp	BE	2024	0	1	0	1	0	0	0	0	0	0	0	0	
24165473	m	md	PT	2024	0	1	1	0	0	0	0	0	0	0	0	0	71
24165541	f	md	CN	2024	0	0	0	0	0	0	0	0	0	0	0	0	54
24165587	Not	cn	US	2024	1	0	0	0	0	0	0	0	0	0	0	0	
24165966	f	cn	US	2024	1	0	0	0	0	0	0	0	0	0	0	0	60
24165968	f	cn	US	2024	1	0	0	0	0	0	0	0	0	0	0	0	
24166074	f	cn	US	2024	1	0	0	0	0	0	0	0	0	0	0	0	63.1
24166242	f	ph	TH	2024	1	0	0	0	0	0	0	0	0	0	0	0	69.9
24166503	m	cn	US	2024	1	0	0	0	0	0	0	0	0	0	0	0	64.6
24166724	f	md	JP	2024	1	0	0	0	0	0	0	0	0	0	0	0	
24167024	Not	hp	CN	2024	0	0	0	0	0	0	0	0	0	0	0	0	
24167045	f	cn	US	2024	1	0	0	0	0	0	0	0	0	0	0	0	
24167096	Not	hp	CN	2024	0	0	0	0	0	0	0	0	0	0	0	0	
24167476	m	hp	CA	2024	0	0	0	0	0	0	0	0	0	0	0	0	90
24167489	Not	md	JP	2024	0	1	0	0	1	0	0	0	0	0	0	0	
24167757	m	ph	CA	2024	0	0	0	0	0	0	0	0	0	0	0	0	56
24168323	f	md	JP	2024	0	1	0	1	0	0	0	0	0	0	0	0	74
24168415	f	cn	KR	2024	0	0	0	0	0	0	0	0	0	0	0	0	50
24168498	m	md	JP	2024	0	1	0	1	0	0	0	0	0	0	0	0	74
24168502	f	cn	US	2024	1	0	0	0	0	0	0	0	0	0	0	0	71.1
24168512	f	md	JP	2024	0	1	0	1	0	0	0	0	0	0	0	0	73
24168685	m	md	JP	2024	0	1	0	1	0	0	0	0	0	0	0	1	55
24168689	m	md	JP	2024	0	1	0	1	0	0	0	0	0	0	0	1	79
24168693	Not	md	JP	2024	0	1	0	1	0	0	0	0	0	0	0	0	
24168712	f	md	ID	2024	0	1	1	0	0	0	0	0	0	0	0	0	68
24168715	f	cn	JP	2024	0	0	0	0	0	0	0	0	0	0	0	0	55
24168854	m	ph	JP	2024	0	1	0	1	0	0	0	0	0	0	0	0	80
24168999	f	hp	US	2024	0	0	0	0	0	0	0	0	0	0	0	0	60

24169650	m	cn	AM	2024	0	0	0	0	0	0	0	0	0	0	0	0	46
24169664	m	md	JP	2024	0	1	0	0	0	1	0	0	0	0	0	0	75
24169674	Not	md	JP	2024	0	1	0	0	0	1	0	0	0	0	0	0	90
24169767	m	md	US	2024	0	1	0	1	0	0	0	0	0	0	0	0	
24170035	m	md	JP	2024	0	1	0	0	0	1	0	0	0	0	0	0	77
24170268	m	md	IN	2024	0	0	0	0	0	0	0	0	0	0	0	0	30
24170314	Not	hp	CN	2024	0	0	0	0	0	0	0	0	0	0	0	0	
24170820	m	md	FR	2024	0	1	1	0	0	0	0	0	0	0	0	0	73
24171437	m	hp	ES	2024	0	1	1	0	0	0	0	0	0	0	0	0	62
24171849	Not	cn	US	2024	1	0	0	0	0	0	0	0	0	0	0	0	
24172026	f	cn	US	2024	1	0	0	0	0	0	0	0	0	0	0	0	65
24172154	m	cn	US	2024	1	0	0	0	0	0	0	0	0	0	0	0	74
24172282	f	md	DE	2024	0	1	0	0	1	0	0	0	0	0	0	0	59
24172468	f	md	JP	2024	0	0	0	0	0	0	0	0	0	0	0	0	
24173243	f	cn	US	2024	1	0	0	0	0	0	0	0	0	0	0	0	48.2
24173323	f	cn	US	2024	1	0	0	0	0	0	0	0	0	0	0	0	
24173325	Not	cn	US	2024	1	0	0	0	0	0	0	0	0	0	0	0	
24173373	m	cn	AR	2024	0	1	0	1	0	0	0	0	0	0	0	0	72.3
24173845	m	md	NL	2024	0	0	0	0	0	0	0	0	0	0	0	0	
24174001	Not	cn	CN	2024	0	0	0	0	0	0	0	0	0	0	0	0	
24174060	m	md	NL	2024	0	1	1	0	0	0	0	0	0	0	0	0	67
24174086	m	md	JP	2024	0	0	0	0	0	0	0	0	0	0	0	0	43
24174538	m	md	JP	2024	0	1	0	0	1	0	0	0	0	0	0	0	76
24174569	Not	md	FR	2024	0	1	0	0	1	0	0	0	0	0	0	0	78
24174616	f	ph	JP	2024	0	1	1	0	0	0	0	0	0	0	0	0	76
24174809	f	cn	KR	2024	0	0	0	0	0	0	0	0	0	0	0	0	66
24174855	f	md	DE	2024	0	0	0	0	0	0	0	0	0	0	0	0	60
24175007	m	md	PT	2024	0	1	1	0	0	0	0	0	0	0	0	0	71
24175686	m	md	PT	2024	0	1	1	0	0	0	0	0	0	0	0	0	60
24175845	Not	hp	CN	2024	0	0	0	0	0	0	0	0	0	0	0	0	
24176105	f	md	JP	2024	0	1	0	0	0	1	0	0	0	0	0	1	70
24176303	m	cn	AE	2024	0	1	0	1	0	0	0	0	0	0	0	0	70.8
24176471	f	cn	CA	2024	1	0	0	0	0	0	0	0	0	0	0	0	
24176757	f	cn	US	2024	0	0	0	0	0	0	0	0	0	0	0	0	59

24176920	m	md	AT	2024	0	1	0	0	0	1	0	0	0	0	0	0	63
24176938	f	cn	IN	2024	0	1	0	0	0	1	0	0	0	0	0	0	62
24176978	Not	hp	ES	2024	0	1	0	0	0	1	0	0	0	0	0	0	
24177003	f	md	CA	2024	0	1	0	1	0	0	0	0	0	0	0	0	78
24177220	m	md	UA	2024	0	1	0	0	0	1	0	0	0	0	0	0	57
24177251	f	md	IT	2024	0	1	1	0	0	0	0	0	0	0	0	0	64
24177657	Not	md	DE	2024	1	0	0	0	0	0	0	0	0	0	0	0	
24177785	f	md	US	2024	0	0	0	0	0	0	0	0	0	0	0	0	
24177818	m	md	JP	2024	0	1	0	1	0	0	0	0	0	0	0	0	70
24177926	f	cn	US	2024	1	0	0	0	0	0	0	0	0	0	0	0	
24178522	f	cn	US	2024	1	0	0	0	0	0	0	0	0	0	0	0	63
24178792	f	cn	TH	2024	0	0	0	0	0	0	0	0	0	0	0	0	74
24178804	f	cn	TH	2024	0	0	0	0	0	0	0	0	0	0	0	0	
24178805	m	cn	IN	2024	0	0	0	0	0	0	0	0	0	0	0	0	74
24178886	m	hp	JP	2024	0	1	0	0	0	1	0	0	0	0	0	0	67
24179964	Not	md	JP	2024	0	1	1	0	0	0	0	0	0	0	0	0	70
24180275	f	md	JP	2024	0	1	0	1	0	0	0	0	0	0	0	0	
24180328	f	hp	JP	2024	0	1	1	0	0	0	0	0	0	0	0	0	64
24180565	m	ph	CA	2024	0	1	1	0	0	0	0	0	0	0	0	0	73
24180678	Not	hp	DE	2024	0	1	1	0	0	0	0	0	0	0	0	0	54
24180732	m	md	US	2024	0	1	1	0	0	0	0	0	0	0	0	1	
24181300	f	md	HU	2024	0	1	0	0	0	1	0	0	0	0	0	0	52
24181310	f	md	HU	2024	0	1	0	0	0	1	0	0	0	0	0	0	54
24181332	Not	cn	US	2024	0	0	0	0	0	0	0	0	0	0	0	0	
24181633	Not	cn	US	2024	1	0	0	0	0	0	0	0	0	0	0	0	84.5
24181738	m	cn	US	2024	1	0	0	0	0	0	0	0	0	0	0	0	
24181750	Not	md	IT	2024	1	0	0	0	0	0	0	0	0	0	0	0	
24182342	m	hp	CA	2024	1	0	0	0	0	0	0	0	0	0	0	0	
24182829	f	md	KR	2024	1	0	0	0	0	0	0	0	0	0	0	0	69.2
24182893	f	md	DE	2024	0	1	0	0	1	0	0	0	0	0	0	0	69
24183404	Not	hp	CN	2024	1	0	0	0	0	0	0	0	0	0	0	0	
24183769	m	md	CA	2024	0	0	0	0	0	0	0	0	0	0	0	0	70
24183944	m	md	AE	2024	0	1	0	0	1	0	0	0	0	0	0	0	51
24184215	m	md	ES	2024	0	1	1	0	0	0	0	0	0	0	0	0	62

24184257	m	cn	US	2024	0	0	0	0	0	0	0	0	0	0	0	0	79
24184817	Not	hp	JP	2024	0	1	0	1	0	0	0	0	0	0	0	0	
24184846	m	hp	US	2024	0	0	0	0	0	0	0	0	0	0	0	0	35
24184946	m	md	US	2024	0	0	0	0	0	0	0	0	0	0	0	0	71
24185452	f	md	US	2024	1	0	0	0	0	0	0	0	0	0	0	0	80
24185784	f	cn	JP	2024	0	0	0	0	0	0	0	0	0	0	0	0	45
24186117	Not	hp	US	2024	0	0	0	0	0	0	0	0	0	0	0	0	
24186280	f	cn	US	2024	1	0	0	0	0	0	0	0	0	0	0	0	
24186283	Not	cn	US	2024	1	0	0	0	0	0	0	0	0	0	0	0	
24186354	m	cn	US	2024	1	0	0	0	0	0	0	0	0	0	0	0	
24186401	m	cn	TH	2024	1	0	0	0	0	0	0	0	0	0	0	0	60.2
24186596	Not	cn	TH	2024	0	1	0	0	0	1	0	0	0	0	0	0	
24187149	Not	hp	DE	2024	0	1	1	0	0	0	0	0	0	0	0	1	54
24187195	Not	md	JP	2024	1	0	0	0	0	0	0	0	0	0	0	0	
24187382	f	cn	US	2024	1	0	0	0	0	0	0	0	0	0	0	0	64
24187383	m	cn	KW	2024	1	0	0	0	0	0	0	0	0	0	0	0	39.8
24187524	f	md	DE	2024	0	0	0	0	0	0	0	0	0	0	0	0	64
24189223	Not	md	JP	2024	1	0	0	0	0	0	0	0	0	0	0	0	
24189313	m	md	JP	2024	0	1	1	0	0	0	0	0	0	0	0	0	75
24189418	m	cn	JP	2024	0	0	0	0	0	0	0	0	0	0	0	0	59
24189608	f	md	US	2024	0	0	0	0	0	0	0	0	0	0	0	0	
24189718	f	md	JP	2024	0	1	0	0	0	1	0	0	0	0	0	0	70
24189974	m	cn	IN	2024	0	0	0	0	0	0	0	0	0	0	0	0	52
24189988	f	md	DE	2024	0	0	0	0	0	0	0	0	0	0	0	0	61
24190002	Not	md	JP	2024	0	0	0	0	0	0	0	0	0	0	0	0	
24190059	f	cn	US	2024	0	0	0	0	0	0	0	0	0	0	0	0	82
24190062	f	cn	US	2024	0	0	0	0	0	0	0	0	0	0	0	0	82
24190113	m	md	JP	2024	0	1	0	0	0	1	0	0	0	0	0	0	90
24190958	Not	cn	US	2024	1	0	0	0	0	0	0	0	0	0	0	0	
24191215	m	md	DE	2024	0	1	0	0	1	0	0	0	0	0	0	0	76
24191379	m	md	FR	2024	0	1	0	0	1	0	0	0	0	0	0	0	63
24191527	f	cn	NL	2024	0	1	1	0	0	0	0	0	0	0	0	0	77
24191543	m	md	JP	2024	0	1	0	0	1	0	0	0	0	0	0	0	70
24191544	m	md	JP	2024	0	1	0	1	0	0	0	0	0	0	0	0	74

24191606	m	md	JP	2024	0	0	0	0	0	0	0	0	0	0	0	0	76
24191630	f	md	JP	2024	0	0	0	0	0	0	0	0	0	0	0	0	80
24192195	m	cn	US	2024	1	0	0	0	0	0	0	0	0	0	0	0	
24192321	m	md	JP	2024	0	1	0	1	0	0	0	0	0	0	0	0	66
24192372	Not	cn	US	2024	1	0	0	0	0	0	0	0	0	0	0	0	
24192374	Not	ph	AU	2024	0	1	0	0	0	1	0	0	0	0	0	0	
24192601	f	cn	US	2024	0	0	0	0	0	0	0	0	0	0	0	0	51
24192628	m	md	US	2024	0	0	0	0	0	0	0	0	0	0	0	0	38
24192673	Not	md	US	2024	0	0	0	0	0	0	0	0	0	0	0	0	
24192932	f	cn	US	2024	1	0	0	0	0	0	0	0	0	0	0	0	
24193035	m	cn	US	2024	0	1	1	0	0	0	0	0	0	0	0	0	
24193486	m	cn	TH	2024	0	1	0	0	0	1	0	0	0	0	0	0	78
24194262	Not	md	BE	2024	0	1	0	1	0	0	0	0	0	0	0	0	
24194459	m	md	CA	2024	0	0	0	0	0	0	0	0	0	0	0	0	69
24194660	Not	md	US	2024	0	0	0	0	0	0	0	0	0	0	0	0	
24194710	f	hp	US	2024	1	0	0	0	0	0	0	0	0	0	0	0	75
24194789	f	md	TH	2024	0	0	0	0	0	0	0	0	0	0	0	0	
24194796	f	md	JP	2024	0	0	0	0	0	0	0	0	0	0	0	0	70
24194807	f	md	RU	2024	0	0	0	0	0	0	0	0	0	0	0	0	66
24194844	f	cn	US	2024	1	0	0	0	0	0	0	0	0	0	0	0	76
24194845	m	md	JP	2024	1	0	0	0	0	0	0	0	0	0	0	0	71
24195046	m	md	JP	2024	0	1	0	1	0	0	0	0	0	0	0	0	60
24195176	m	cn	JP	2024	0	0	0	0	0	0	0	0	0	0	0	0	45
24195488	f	hp	DE	2024	0	1	0	0	1	0	0	0	0	0	0	0	65
24195538	m	md	PH	2024	1	0	0	0	0	0	0	0	0	0	0	0	
24195647	f	md	JP	2024	0	1	1	0	0	0	0	0	0	0	0	0	74
24195711	f	md	JP	2024	0	1	0	1	0	0	0	0	0	0	0	0	65
24196540	m	md	JP	2024	0	1	0	0	0	1	0	0	0	0	0	0	69
24196560	f	md	HU	2024	0	1	0	0	0	1	0	0	0	0	0	0	73
24196566	m	md	UY	2024	0	0	0	0	0	0	0	0	0	0	0	0	66
24196965	f	md	NL	2024	0	1	1	0	0	0	0	0	0	0	0	0	77
24197535	m	hp	US	2024	1	0	0	0	0	0	0	0	0	0	0	0	
24198696	f	md	JP	2024	0	1	0	0	0	1	0	0	0	0	0	0	80
24198783	f	md	BR	2024	0	1	0	0	0	1	0	0	0	0	0	0	80

24199267	f	md	US	2024	0	0	0	0	0	0	0	0	0	0	0	0	76
24199456	Not	md	US	2024	0	0	0	0	0	0	0	0	0	0	0	0	
24199482	f	hp	GB	2024	1	0	0	0	0	0	0	0	0	0	0	0	70
24199965	m	md	JP	2024	0	1	0	1	0	0	0	0	0	0	0	0	68
24200003	f	cn	US	2024	0	1	1	0	0	0	0	0	0	0	0	0	60
24200553	Not	md	JP	2024	0	1	0	0	1	0	0	0	0	0	0	0	
24200674	Not	ph	AU	2024	0	1	0	0	0	1	0	0	0	0	0	0	
24200775	m	md	JP	2024	0	0	0	0	0	0	0	0	0	0	0	0	82
24200902	f	md	NL	2024	0	1	1	0	0	0	0	0	0	0	0	0	77
24201329	f	md	US	2024	0	0	0	0	0	0	0	0	0	0	0	0	76
24201359	m	ph	JP	2024	0	1	0	1	0	0	0	0	0	0	0	0	53
24201365	m	md	JP	2024	0	1	0	1	0	0	0	0	0	0	0	0	67
24201604	m	md	JP	2024	0	1	0	1	0	0	0	0	0	0	0	0	80
24201677	m	md	JP	2024	0	1	0	1	0	0	0	0	0	0	0	0	65
24201684	m	md	JP	2024	0	1	0	1	0	0	0	0	0	0	0	0	
24201765	m	md	JP	2024	0	1	1	0	0	0	0	0	0	0	0	0	80
24201841	f	md	NL	2024	0	1	1	0	0	0	0	0	0	0	0	0	77
24201913	m	hp	CN	2024	0	0	0	0	0	0	0	0	0	0	0	0	49
24202237	f	md	JP	2024	0	0	0	0	0	0	0	0	0	0	0	0	50
24202259	f	md	US	2024	0	0	0	0	0	0	0	0	0	0	0	0	
24202275	m	md	JP	2024	0	1	0	0	1	0	0	0	0	0	0	0	62
24202303	Not	hp	US	2024	0	0	0	0	0	0	0	0	0	0	0	0	
24202370	m	md	JP	2024	0	0	0	0	0	0	0	0	0	0	0	0	50
24203298	Not	cn	CN	2024	0	0	0	0	0	0	0	0	0	0	0	0	
24203796	m	hp	TH	2024	1	0	0	0	0	0	0	0	0	0	0	0	
24204276	Not	ph	JP	2024	0	1	0	0	0	1	0	0	0	0	0	0	60
24204315	f	md	US	2024	0	0	0	0	0	0	0	0	0	0	0	0	76
24204460	f	md	US	2024	0	0	0	0	0	0	0	0	0	0	0	0	76
24204722	m	md	AT	2024	0	1	0	0	0	1	0	0	0	0	0	0	63
24204956	m	md	JP	2024	0	0	0	0	0	0	0	0	0	0	0	0	59
24204976	m	md	FR	2024	0	1	1	0	0	0	0	0	0	0	0	0	
24205487	f	cn	US	2024	0	0	0	0	0	0	0	0	0	0	0	0	46
24205575	m	cn	JP	2024	1	0	0	0	0	0	0	0	0	0	0	0	65
24205694	f	md	DE	2024	0	1	0	0	1	0	0	0	0	0	0	0	69

24205866	m	cn	TW	2024	0	1	0	0	1	0	0	0	0	0	0	0	56
24206046	f	cn	US	2024	1	0	0	0	0	0	0	0	0	0	0	0	
24206141	m	md	AU	2024	1	0	0	0	0	0	0	0	0	0	0	0	83
24206195	m	ph	GB	2024	0	0	0	0	0	0	1	0	0	0	1	1	68
24206668	Not	md	KR	2024	1	0	0	0	0	0	0	0	0	0	0	0	
24206831	f	md	US	2024	0	1	1	0	0	0	0	0	0	0	0	0	57
24208096	m	md	JP	2024	0	1	1	0	0	0	0	0	0	0	0	0	90
24208604	f	cn	JP	2024	1	0	0	0	0	0	0	0	0	0	0	0	
24208665	m	cn	US	2024	1	0	0	0	0	0	0	0	0	0	0	0	
24208695	m	md	JP	2024	0	1	1	0	0	0	0	0	0	0	0	0	80
24209203	m	md	JP	2024	1	0	0	0	0	0	0	0	0	0	0	0	81
24209421	f	hp	GB	2024	0	0	0	0	0	0	0	0	0	0	0	0	61
24209792	m	hp	TW	2024	0	1	0	0	1	0	0	0	0	0	0	0	73
24209982	Not	md	JP	2024	1	0	0	0	0	0	0	0	0	0	0	0	
24210196	f	hp	US	2024	0	0	0	0	0	0	0	0	0	0	0	0	76
24210224	m	ph	JP	2024	1	0	0	0	0	0	0	0	0	0	0	0	
24210228	Not	hp	US	2024	0	0	0	0	0	0	0	0	0	0	0	0	
24210271	f	cn	US	2024	0	0	0	0	0	0	0	0	0	0	0	0	46
24210407	m	md	AT	2024	0	1	1	0	0	0	0	0	0	0	0	1	
24210455	f	ph	FR	2024	0	0	0	0	0	0	0	0	0	0	0	0	67
24211147	f	md	DE	2024	0	1	0	0	1	0	0	0	0	0	0	0	64
24211202	f	cn	KR	2024	0	0	0	0	0	0	0	0	0	0	0	0	
24211369	m	md	CN	2024	0	0	0	0	0	0	0	0	0	0	0	0	65
24211650	m	hp	TW	2024	0	1	0	0	1	0	0	0	0	0	0	0	75
24211943	m	md	JP	2024	0	0	0	0	0	0	0	0	0	0	0	0	70
24212182	m	cn	CN	2024	1	0	0	0	0	0	0	0	0	0	0	0	
24212250	m	cn	CN	2024	1	0	0	0	0	0	0	0	0	0	0	0	
24212260	m	md	JP	2024	0	1	0	0	1	0	0	0	0	0	0	0	70
24212321	m	md	CR	2024	0	1	0	0	0	1	0	0	0	0	0	0	76
24212328	m	cn	IN	2024	0	1	0	0	0	1	0	0	0	0	0	0	69
24212655	f	cn	CA	2024	1	0	0	0	0	0	0	0	0	0	0	0	55
24212854	m	md	CN	2024	0	0	0	0	0	0	0	0	0	0	0	0	65
24213367	Not	cn	US	2024	1	0	0	0	0	0	0	0	0	0	0	0	
24213526	m	cn	IN	2024	1	0	0	0	0	0	0	0	0	0	0	0	69

24213562	f	cn	US	2024	1	0	0	0	0	0	0	0	0	0	0	0	
24213691	f	cn	US	2024	0	0	0	0	0	0	0	0	0	0	0	0	40
24213740	m	md	US	2024	0	0	0	0	0	0	0	0	0	0	0	0	
24214109	m	ph	IT	2024	0	0	0	0	0	0	0	0	0	0	0	0	72
24214217	m	md	JP	2024	0	1	0	1	0	0	0	0	0	0	0	0	50
24214323	m	hp	US	2024	1	0	0	0	0	0	0	0	0	0	0	0	63
24214324	f	hp	US	2024	1	0	0	0	0	0	0	0	0	0	0	0	79
24215024	m	md	JP	2024	0	1	0	0	1	0	0	0	0	0	0	0	59
24215079	Not	md	JP	2024	0	1	0	1	0	0	0	0	0	0	0	0	
24215526	f	ph	JP	2024	0	0	0	0	0	0	0	0	0	0	0	0	78
24216179	m	md	JP	2024	0	1	0	0	0	1	0	0	0	0	0	0	60
24216532	m	md	JP	2024	0	1	0	0	1	0	0	0	0	0	0	1	82
24216620	f	md	AR	2024	0	0	0	0	0	0	0	0	0	0	0	0	
24216666	Not	md	US	2024	0	0	0	0	0	0	0	0	0	0	0	0	
24217500	m	md	IN	2024	0	1	0	0	0	1	0	0	0	0	0	0	65
24218013	m	hp	TW	2024	0	1	0	0	1	0	0	0	0	0	0	0	54
24218098	f	md	JP	2024	0	1	0	1	0	0	0	0	0	0	0	0	75
24218381	f	cn	CA	2024	1	0	0	0	0	0	0	0	0	0	0	1	48
24219147	Not	md	JP	2024	0	0	0	0	0	0	0	0	0	0	0	0	
24219150	m	cn	DE	2024	0	1	0	0	1	0	0	0	0	0	0	0	71
24219541	m	cn	US	2024	0	1	1	0	0	0	0	0	0	0	0	0	70
24219663	Not	md	JP	2024	0	1	0	0	0	1	0	0	0	0	0	0	60
24219746	f	md	DE	2024	0	1	0	0	1	0	0	0	0	0	0	0	59
24220390	f	md	DE	2024	0	1	1	0	0	0	0	0	0	0	0	0	
24220957	f	md	JP	2024	0	1	0	0	1	0	0	0	0	0	0	0	
24220995	m	cn	KZ	2024	0	0	0	0	0	0	0	0	0	0	0	0	
24221338	f	md	DE	2024	0	1	0	0	1	0	0	0	0	0	0	0	64
24221370	m	md	IT	2024	1	0	0	0	0	0	0	0	0	0	0	0	43
24221474	f	md	IL	2024	0	1	0	0	1	0	0	0	0	0	0	0	63
24221762	m	md	TH	2024	0	1	1	0	0	0	0	0	0	0	0	0	75
24221957	f	hp	US	2024	1	0	0	0	0	0	0	0	0	0	0	0	75
24221979	m	ph	US	2024	0	0	0	0	0	0	0	0	0	0	0	0	64
24222289	m	md	TH	2024	0	1	0	0	0	1	0	0	0	0	0	0	39
24222497	f	md	JP	2024	1	0	0	0	0	0	0	0	0	0	0	0	

24222583	f	cn	US	2024	1	0	0	0	0	0	0	0	0	0	0	0	
24222922	Not	md	JP	2024	0	1	0	0	0	1	0	0	0	0	0	0	80
24223335	m	cn	IN	2024	0	0	0	0	0	0	0	0	0	0	0	0	30
24223348	m	md	AT	2024	0	1	1	0	0	0	0	0	0	0	0	0	64
24223447	f	hp	US	2024	0	0	0	0	0	0	0	0	0	0	0	0	
24223585	m	hp	CN	2024	0	0	0	0	0	0	0	0	0	0	0	0	35
24223847	m	md	NZ	2024	1	0	0	0	0	0	0	0	0	0	0	0	
24224209	m	cn	AE	2024	0	1	0	1	0	0	0	0	0	0	0	0	
24224251	f	md	AU	2024	0	1	1	0	0	0	0	0	0	0	0	0	74
24224645	f	md	US	2024	0	0	0	0	0	0	0	0	0	0	0	0	61
24224820	m	hp	US	2024	0	0	0	0	0	0	0	0	0	0	0	0	
24225037	f	md	AU	2024	1	0	0	0	0	0	0	0	0	0	0	0	
24225402	f	md	JP	2024	0	0	0	0	0	0	0	0	0	0	0	0	60
24225556	f	cn	US	2024	1	0	0	0	0	0	0	0	0	0	0	0	84.2
24225604	f	cn	US	2024	1	0	0	0	0	0	0	0	0	0	0	0	
24226006	f	md	DE	2024	0	1	0	0	1	0	0	0	0	0	0	0	69
24226191	m	md	JP	2024	0	0	0	0	0	0	0	0	0	0	0	0	60
24226288	f	hp	US	2024	0	1	0	0	1	0	0	0	0	0	0	0	
24226573	f	md	DE	2024	0	1	0	0	1	0	0	0	0	0	0	0	56
24226795	Not	md	JP	2024	0	0	0	0	0	0	0	0	0	0	0	0	
24226931	f	hp	US	2024	1	0	0	0	0	0	0	0	0	0	0	0	75
24227987	m	hp	SG	2024	0	1	0	0	0	1	0	0	0	0	0	0	60
24228093	m	md	US	2024	0	0	0	0	0	0	0	0	0	0	0	0	72
24228239	f	cn	IN	2024	0	0	0	0	0	0	0	0	0	0	0	0	56
24229217	m	cn	US	2024	1	0	0	0	0	0	0	0	0	0	0	0	
24229445	m	ph	US	2024	0	1	0	0	1	0	0	0	0	0	0	0	75
24229792	m	hp	US	2024	0	0	0	0	0	0	1	1	0	0	0	0	
24229880	m	md	DE	2024	0	1	0	0	1	0	0	0	0	0	0	0	61
24230054	f	md	AT	2024	1	0	0	0	0	0	0	0	0	0	0	0	
24230111	m	md	DE	2024	0	1	0	0	1	0	0	0	0	0	0	0	60
24230240	m	md	JP	2024	0	1	1	0	0	0	0	0	0	0	0	0	74
24231409	f	md	US	2024	1	0	0	0	0	0	0	0	0	0	0	0	83
24231840	m	md	TH	2024	0	1	1	0	0	0	0	0	0	0	0	0	63
24231967	f	md	NL	2024	0	1	1	0	0	0	0	0	0	0	0	0	77

24233311	f	md	US	2024	0	0	0	0	0	0	0	0	0	0	0	0	74
24233511	Not	md	US	2024	0	0	0	0	0	0	0	0	0	0	0	0	
24234010	m	cn	US	2024	0	0	0	0	0	0	0	0	0	0	0	0	53
24234826	f	ph	US	2024	0	0	0	0	0	0	0	0	0	0	0	0	74
24236115	Not	hp	AU	2024	0	1	0	1	0	0	0	0	0	0	0	1	
24236196	m	cn	US	2024	1	0	0	0	0	0	0	0	0	0	0	0	84
24236610	m	hp	TH	2024	1	0	0	0	0	0	0	0	0	0	0	0	57.1
24236646	Not	md	US	2024	1	0	0	0	0	0	0	0	0	0	0	0	
24236765	m	md	DE	2024	0	1	0	0	1	0	0	0	0	0	0	0	83
24236774	f	hp	DE	2024	0	1	0	0	1	0	0	0	0	0	0	0	65
24237104	m	md	IT	2024	0	1	0	0	1	0	0	0	0	0	0	0	74
24237119	Not	md	JP	2024	0	1	0	0	1	0	0	0	0	0	0	0	74
24237430	Not	cn	CN	2024	0	0	0	0	0	0	0	0	0	0	0	0	
24237451	Not	cn	CN	2024	0	0	0	0	0	0	0	0	0	0	0	0	
24237701	f	cn	US	2024	1	0	0	0	0	0	0	0	0	0	0	0	
24238198	f	cn	US	2024	1	0	0	0	0	0	0	0	0	0	0	0	71
24238257	Not	md	US	2024	0	0	0	0	0	0	0	0	0	0	0	0	
24238500	m	cn	JP	2024	0	0	0	0	0	0	0	0	0	0	0	0	70
24238520	m	md	RU	2024	0	0	0	0	0	0	0	0	0	0	0	0	65
24238693	f	hp	US	2024	0	0	0	0	0	0	0	0	0	0	0	0	70
24238855	m	md	MX	2024	0	1	0	1	0	0	0	0	0	0	0	0	65
24238941	f	hp	KR	2024	1	0	0	0	0	0	0	0	0	0	0	0	58
24240593	m	md	CH	2024	0	0	0	0	0	0	0	0	0	0	0	0	
24241353	m	ph	US	2024	0	0	0	0	0	0	0	0	0	0	0	0	71
24241470	m	hp	CN	2024	0	1	0	0	0	1	0	0	0	0	0	0	65
24241598	f	ph	JP	2024	0	0	0	0	0	0	0	0	0	0	0	0	
24241615	f	md	DK	2024	0	1	0	0	0	1	0	0	0	0	0	0	78
24241830	m	cn	CN	2024	0	0	0	0	0	0	0	0	0	0	0	0	59
24241915	f	md	US	2024	0	0	0	0	0	0	0	0	0	0	0	0	69
24242168	Not	hp	US	2024	0	0	0	0	0	0	0	0	0	0	0	0	
24242295	m	md	FR	2024	0	1	1	0	0	0	0	0	0	0	0	0	40
24242695	m	md	CH	2024	0	1	1	0	0	0	0	0	0	0	0	0	81
24242905	f	md	US	2024	0	0	0	0	0	0	0	0	0	0	0	0	69
24243137	m	md	JP	2024	0	1	0	1	0	0	0	0	0	0	0	0	79

24243559	Not	md	IN	2024	0	0	0	0	0	0	0	0	0	0	0	0	
24244845	m	hp	CO	2024	0	1	0	0	1	0	0	0	0	0	0	0	61
24244925	f	md	JP	2024	0	0	0	0	0	0	0	0	0	0	0	0	
24245123	m	hp	AU	2024	0	1	1	0	0	0	0	0	0	0	0	0	71
24245284	m	cn	IN	2024	1	0	0	0	0	0	0	0	0	0	0	0	64
24245375	m	md	JP	2024	0	1	0	1	0	0	0	0	0	0	0	0	70
24245401	m	md	AU	2024	1	0	0	0	0	0	0	0	0	0	0	0	73
24245434	Not	hp	CO	2024	1	0	0	0	0	0	0	0	0	0	0	0	
24245437	m	md	JP	2024	0	1	0	1	0	0	0	0	0	0	0	0	77
24245557	f	cn	IN	2024	0	1	0	0	0	1	0	0	0	0	0	0	66
24246694	m	md	JP	2024	0	1	0	0	1	0	0	0	0	0	0	0	72
24246764	f	md	JP	2024	0	0	0	0	0	0	0	0	0	0	0	0	
24247137	m	hp	DE	2024	0	1	0	0	1	0	0	0	0	0	0	0	74
24247234	m	md	JP	2024	0	1	0	0	1	0	0	0	0	0	0	0	61
24247344	f	md	TR	2024	0	1	0	0	0	1	0	0	0	0	0	0	67
24247777	m	cn	GB	2024	1	0	0	0	0	0	0	0	0	0	0	0	50
24247971	m	md	SI	2024	0	0	0	0	0	0	0	0	0	0	0	0	60
24248322	f	ph	JP	2024	0	1	0	0	1	0	0	0	0	0	0	0	83
24248812	f	md	US	2024	0	0	0	0	0	0	0	0	0	0	0	0	
24248862	m	md	JP	2024	0	1	0	0	1	0	0	0	0	0	0	0	68
24248893	m	md	JP	2024	0	1	0	1	0	0	0	0	0	0	0	0	69
24249103	f	hp	US	2024	0	0	0	0	0	0	0	0	0	0	0	0	
24249811	f	md	JP	2024	0	0	0	0	0	0	0	0	0	0	0	0	61
24250332	m	md	JP	2024	0	1	0	0	1	0	0	0	0	0	0	0	74
24250381	m	md	JP	2024	0	1	0	1	0	0	0	0	0	0	0	0	73
24251100	m	cn	FR	2024	0	1	1	0	0	0	0	0	0	0	0	0	
24251335	m	md	PL	2024	0	1	1	0	0	0	0	0	0	0	0	1	83
24251358	m	md	DE	2024	0	1	1	0	0	0	0	0	0	0	0	0	
24251872	m	md	DE	2024	0	1	0	0	1	0	0	0	0	0	0	0	60
24252104	f	ph	US	2024	0	0	0	0	0	0	0	0	0	0	0	0	71
24252304	m	md	DE	2024	0	1	0	0	1	0	0	0	0	0	0	0	61
24252461	f	md	JP	2024	0	1	0	0	1	0	0	0	0	0	0	0	73
24252520	m	hp	DE	2024	0	1	0	0	1	0	0	0	0	0	0	0	62
24252876	m	md	DE	2024	0	1	0	0	1	0	0	0	0	0	0	0	76

24253347	f	md	NL	2024	0	1	1	0	0	0	0	0	0	0	0	0	55
24253606	m	cn	TH	2024	0	1	0	0	0	1	0	0	0	0	0	0	70
24253849	f	hp	TW	2024	1	0	0	0	0	0	0	0	0	0	0	0	56
24253850	m	hp	TW	2024	1	0	0	0	0	0	0	0	0	0	0	0	51
24254002	m	md	FR	2024	0	1	1	0	0	0	0	0	0	0	0	0	
24254132	f	cn	US	2024	0	0	0	0	0	0	0	0	0	0	0	0	51
24254141	m	md	JP	2024	1	0	0	0	0	0	0	0	0	0	0	1	74
24254205	m	md	CA	2024	0	0	0	0	0	0	0	0	0	0	0	0	
24254785	m	cn	KR	2024	0	0	0	0	0	0	0	0	0	0	0	0	82
24254925	f	cn	Not re	2024	0	0	0	0	0	0	0	0	0	0	0	0	44
24255011	m	hp	TW	2024	1	0	0	0	0	0	0	0	0	0	0	0	56
24255070	f	md	KR	2024	0	0	0	0	0	0	0	0	0	0	0	0	76
24255718	f	ph	CA	2024	0	0	0	0	0	0	0	0	0	0	0	0	59
24255956	m	md	FR	2024	0	1	1	0	0	0	0	0	0	0	0	0	40
24256429	m	cn	IN	2024	0	1	0	0	0	1	0	0	0	0	0	0	33
24256744	m	md	DE	2024	0	1	0	0	1	0	0	0	0	0	0	0	61
24256957	f	md	DE	2024	0	1	0	0	1	0	0	0	0	0	0	0	56
24257325	m	cn	KR	2024	0	0	0	0	0	0	0	0	0	0	0	0	67
24257563	m	md	FR	2024	0	1	1	0	0	0	0	0	0	0	0	0	
24257596	f	md	JP	2024	0	1	0	0	1	0	0	0	0	0	0	0	64
24257615	Not	cn	US	2024	0	0	0	0	0	0	0	0	0	0	0	0	
24258015	f	md	DE	2024	0	1	0	0	1	0	0	0	0	0	0	0	67
24258042	Not	hp	CN	2024	0	0	0	0	0	0	0	0	0	0	0	0	
24258399	m	md	ID	2024	1	0	0	0	0	0	0	0	0	0	0	0	
24258412	f	md	JP	2024	1	0	0	0	0	0	0	0	0	0	0	0	
24258437	Not	ph	JP	2024	1	0	0	0	0	0	0	0	0	0	0	0	
24258633	Not	hp	US	2024	0	0	0	0	0	0	0	0	0	0	0	0	52
24259167	f	md	NL	2024	0	1	1	0	0	0	0	0	0	0	0	0	55
24259254	Not	hp	US	2024	1	0	0	0	0	0	0	0	0	0	0	0	
24259651	f	md	JP	2024	0	0	0	0	0	0	0	0	0	0	0	0	70
24259771	m	cn	JP	2024	0	0	0	0	0	0	0	0	0	0	0	0	65
24260542	m	hp	IT	2024	0	1	0	0	1	0	0	0	0	0	0	0	66
24260564	m	hp	IT	2024	0	1	0	0	1	0	0	0	0	0	0	0	72
24260944	m	md	PT	2024	0	1	0	1	0	0	0	0	0	0	0	0	78

24261164	f	md	NL	2024	0	1	1	0	0	0	0	0	0	0	0	0	55
24261405	f	md	JP	2024	0	0	0	0	0	0	0	0	0	0	0	0	40
24261780	f	cn	US	2024	1	0	0	0	0	0	0	0	0	0	0	0	
24262170	f	hp	US	2024	0	0	0	0	0	0	0	0	0	0	0	0	62
24262363	m	md	DE	2024	0	1	0	0	1	0	0	0	0	0	0	0	76
24263252	m	cn	TH	2024	0	0	0	0	0	0	0	0	0	0	0	0	64
24263253	m	cn	FR	2024	0	0	0	0	0	0	0	0	0	0	0	0	28
24263736	f	hp	CN	2024	0	0	0	0	0	0	0	0	0	0	0	0	67
24263766	f	md	US	2024	0	0	0	0	0	0	0	0	0	0	0	0	74
24263861	m	cn	TR	2024	0	1	1	0	0	0	0	0	0	0	0	0	62
24264019	m	md	AM	2024	0	0	0	0	0	0	0	0	0	0	0	0	52
24264711	Not	cn	IN	2024	0	0	0	0	0	0	0	0	0	0	0	0	54
24265398	Not	ph	CA	2024	0	1	1	0	0	0	0	0	0	0	0	0	81
24265623	m	ph	CA	2024	0	0	0	0	0	0	0	0	0	0	0	0	84
24265736	m	md	BG	2024	0	1	0	0	1	0	0	0	0	0	0	0	54
24266413	m	cn	PK	2024	0	1	0	0	0	1	0	0	0	0	0	0	74
24266487	Not	md	JP	2024	0	1	0	0	0	1	0	0	0	0	0	0	70
24266565	f	md	JP	2024	0	1	0	0	0	1	0	0	0	0	0	0	49
24266707	f	cn	KR	2024	0	0	0	0	0	0	0	0	0	0	0	0	81
24267676	f	md	US	2024	0	0	0	0	0	0	0	0	0	0	0	0	72
24268186	Not	hp	BE	2024	0	0	0	0	0	0	0	0	0	0	0	0	
24268256	m	md	JP	2024	0	1	0	1	0	0	0	0	0	0	0	0	59
24268435	Not	ph	US	2024	0	1	0	0	0	1	0	0	0	0	0	0	
24268440	f	md	JP	2024	1	0	0	0	0	0	0	0	0	0	0	1	
24268745	f	cn	IN	2024	0	0	0	0	0	0	0	0	0	0	0	0	76
24268755	m	hp	CA	2024	0	0	0	0	0	0	0	0	0	0	0	0	33
24268893	f	hp	US	2024	0	0	0	0	0	0	0	0	0	0	0	0	52
24269243	Not	md	JP	2024	1	0	0	0	0	0	0	0	0	0	0	0	
24269371	m	md	JP	2024	0	1	0	1	0	0	0	0	0	0	0	0	72
24269399	f	md	KR	2024	0	0	0	0	0	0	0	0	0	0	0	0	
24269442	f	hp	US	2024	1	0	0	0	0	0	0	0	0	0	0	0	80.3
24269450	Not	md	JP	2024	0	1	0	0	1	0	0	0	0	0	0	0	
24269794	f	cn	US	2024	1	0	0	0	0	0	0	0	0	0	0	0	
24269881	Not	md	JP	2024	1	0	0	0	0	0	0	0	0	0	0	0	

24270082	f	hp	FR	2024	0	1	1	0	0	0	0	0	0	0	0	0	
24270119	f	cn	US	2024	0	0	0	0	0	0	0	0	0	0	0	0	59
24270858	m	cn	KR	2024	0	0	0	0	0	0	0	0	0	0	0	0	54
24270921	m	md	DE	2024	0	1	0	0	1	0	0	0	0	0	0	0	61
24270925	f	md	DE	2024	0	1	0	0	1	0	0	0	0	0	0	0	67
24271274	m	hp	US	2024	0	1	0	1	0	0	0	0	0	0	0	0	52
24271842	m	md	ID	2024	0	1	0	0	0	1	0	0	0	0	0	0	52
24272399	m	md	DE	2024	0	1	0	0	1	0	0	0	0	0	0	0	67
24272650	f	cn	DE	2024	0	0	0	0	0	0	0	0	0	0	0	0	
24272816	f	md	DE	2024	0	1	0	0	1	0	0	0	0	0	0	0	67
24272951	f	md	US	2024	0	1	1	0	0	0	0	0	0	0	0	0	64
24272991	m	cn	US	2024	1	0	0	0	0	0	0	0	0	0	0	0	
24273059	f	hp	US	2024	0	0	0	0	0	0	0	0	0	0	0	0	75
24273394	Not	md	JP	2024	0	1	0	0	0	1	0	0	0	0	0	0	80
24273411	Not	hp	DE	2024	0	1	1	0	0	0	0	0	0	0	0	0	54
24273519	m	cn	DE	2024	0	0	0	0	0	0	0	0	0	0	0	0	51
24273523	f	cn	IN	2024	0	0	0	0	0	0	0	0	0	0	0	0	44
24274097	Not	md	JP	2024	0	0	0	0	0	0	0	0	0	0	0	0	
24274105	f	md	DE	2024	0	1	0	0	1	0	0	0	0	0	0	0	67
24274323	f	hp	FR	2024	1	0	0	0	0	0	0	0	0	0	0	0	50
24274370	m	hp	FR	2024	1	0	0	0	0	0	0	0	0	0	0	0	77
24274425	m	md	JP	2024	0	1	0	1	0	0	0	0	0	0	0	0	73
24274553	m	md	CH	2024	0	0	0	0	0	0	0	0	0	0	0	0	
24274831	f	md	ES	2024	0	1	1	0	0	0	0	0	0	0	0	0	47
24274950	m	md	AU	2024	0	1	1	0	0	0	0	0	0	0	0	0	44
24274951	f	cn	US	2024	0	0	0	0	0	0	0	0	0	0	0	0	
24275296	m	hp	US	2024	0	1	0	0	1	0	0	0	0	0	0	0	80
24275625	m	md	DE	2024	0	1	1	0	0	0	0	0	0	0	0	0	
24275760	m	md	JP	2024	0	1	1	0	0	0	0	0	0	0	0	0	70
24275816	f	cn	TH	2024	0	1	0	0	0	1	0	0	0	0	0	0	
24276001	m	md	MX	2024	0	0	0	0	0	0	0	0	0	0	0	0	
24276205	f	hp	US	2024	0	0	0	0	0	0	0	0	0	0	0	0	52
24276478	f	md	AT	2024	0	0	0	0	0	0	0	0	0	0	0	0	81
24276768	m	md	DE	2024	0	1	0	0	1	0	0	0	0	0	0	0	76

24277326	f	cn	MY	2024	1	0	0	0	0	0	0	0	0	0	0	0	60.5
24277417	m	md	IT	2024	0	1	0	0	1	0	0	0	0	0	0	0	69
24277674	f	hp	US	2024	0	0	0	0	0	0	0	0	0	0	0	0	
24278277	m	cn	US	2024	0	0	0	0	0	0	0	0	0	0	0	0	40
24279138	m	md	JP	2024	0	1	1	0	0	0	0	0	0	0	0	0	80
24279215	f	md	CA	2024	0	0	0	0	0	0	0	0	0	0	0	0	50
24279222	f	cn	IN	2024	0	0	0	0	0	0	0	0	0	0	0	0	45
24279320	m	md	AU	2024	0	1	1	0	0	0	0	0	0	0	0	0	81
24279324	f	md	JP	2024	1	0	0	0	0	0	0	0	0	0	0	1	82
24279444	f	ph	US	2024	0	0	0	0	0	0	0	0	0	0	0	0	53
24279524	m	md	DE	2024	0	1	0	0	1	0	0	0	0	0	0	0	72
24279713	f	md	DE	2024	0	1	0	0	1	0	0	0	0	0	0	0	67
24280066	f	md	DE	2024	0	1	1	0	0	0	0	0	0	0	0	0	63
24280247	f	md	DE	2024	0	1	0	0	1	0	0	0	0	0	0	0	56
24280274	f	md	DE	2024	0	1	0	0	1	0	0	0	0	0	0	0	68
24280507	m	md	IT	2024	0	1	0	0	0	1	0	0	0	0	0	0	78
24280574	f	md	JP	2024	1	0	0	0	0	0	0	0	0	0	0	0	90
24280662	f	md	FR	2024	0	1	1	0	0	0	0	0	0	0	0	0	
24281104	f	cn	US	2024	1	0	0	0	0	0	0	0	0	0	0	0	
24281121	f	cn	CN	2024	1	0	0	0	0	0	0	0	0	0	0	0	50
24281176	m	md	JP	2024	0	1	0	1	0	0	0	0	0	0	0	0	59
24281340	m	md	JP	2024	0	0	0	0	0	0	0	0	0	0	0	0	70
24281608	m	md	DE	2024	0	1	0	0	1	0	0	0	0	0	0	0	75
24281867	m	cn	US	2024	0	0	0	0	0	0	0	0	0	0	0	0	67
24281877	m	md	JP	2024	0	1	0	0	0	1	0	0	0	0	0	0	81
24282025	m	md	SA	2024	0	0	0	0	0	0	0	0	0	0	0	0	37
24282204	m	cn	FR	2024	0	1	1	0	0	0	0	0	0	0	0	0	73
24282367	m	cn	US	2024	0	0	0	0	0	0	0	0	0	0	0	0	37
24282560	m	md	JP	2024	0	1	0	1	0	0	0	0	0	0	0	0	55
24282762	f	cn	US	2024	0	0	0	0	0	0	0	0	0	0	0	0	50
24283121	m	md	JP	2024	0	1	0	1	0	0	0	0	0	0	0	0	70
24283973	m	md	DZ	2024	1	0	0	0	0	0	0	0	0	0	0	0	60
24283998	m	md	US	2024	0	1	1	0	0	0	0	0	0	0	0	0	67
24284330	f	cn	KE	2024	1	0	0	0	0	0	0	0	0	0	0	0	76.7

24284527	Not	md	JP	2024	0	1	0	0	1	0	0	0	0	0	0	0	
24284777	f	md	DE	2024	0	1	0	0	1	0	0	0	0	0	0	0	67
24285225	f	hp	US	2024	1	0	0	0	0	0	0	0	0	0	0	0	65
24285230	m	hp	US	2024	0	1	0	0	0	1	0	0	0	0	0	0	69
24285275	f	hp	US	2024	1	0	0	0	0	0	0	0	0	0	0	0	58
24285648	m	hp	BE	2024	0	0	0	0	0	0	0	0	0	0	0	0	71
24285930	f	cn	DE	2024	0	1	1	0	0	0	0	0	0	0	0	0	63
24285969	f	md	JP	2024	0	0	0	0	0	0	0	0	0	0	0	0	
24286200	f	ph	ZA	2024	0	0	0	0	0	0	0	0	0	0	0	0	51
24286220	f	md	FR	2024	0	1	0	0	0	1	0	0	0	0	0	0	62
24286227	Not	md	US	2024	0	0	0	0	0	0	0	0	0	0	0	0	
24286233	m	cn	IN	2024	1	0	0	0	0	0	0	0	0	0	0	0	83
24286814	f	md	US	2024	0	0	0	0	0	0	0	0	0	0	0	0	33
24287132	m	md	JP	2024	0	1	0	1	0	0	0	0	0	0	0	0	68
24287262	Not	md	JP	2024	1	0	0	0	0	0	0	0	0	0	0	0	
24287478	f	hp	ES	2024	1	0	0	0	0	0	0	0	0	0	0	0	82
24287853	m	md	JP	2024	0	1	1	0	0	0	0	0	0	0	0	0	68
24288183	f	md	JP	2024	0	1	0	0	1	0	0	0	0	0	0	0	78
24288339	m	md	JP	2024	0	1	1	0	0	0	0	0	0	0	0	0	70
24288455	Not	md	KR	2024	0	1	0	0	0	1	0	0	0	0	0	0	
24288648	f	md	JP	2024	0	1	0	0	0	1	0	0	0	0	0	0	50
24288733	f	md	FR	2024	0	0	0	0	0	0	0	0	0	0	0	0	46
24289050	Not	md	DE	2024	1	0	0	0	0	0	0	0	0	0	0	0	
24289156	f	hp	FR	2024	0	1	1	0	0	0	0	0	0	0	0	0	
24289269	Not	ph	JP	2024	0	1	0	0	1	0	0	0	0	0	0	0	
24289326	m	md	ES	2024	0	1	0	0	0	1	0	0	0	0	0	0	74
24289637	m	hp	US	2024	0	1	1	0	0	0	0	0	0	0	0	0	68
24289822	m	md	DE	2024	0	1	0	0	1	0	0	0	0	0	0	0	72
24290007	m	hp	US	2024	1	0	0	0	0	0	0	0	0	0	0	0	51
24290427	f	md	AU	2024	0	1	1	0	0	0	0	0	0	0	0	0	70
24290441	f	md	RO	2024	0	0	0	0	0	0	0	0	0	0	0	0	
24290683	m	hp	US	2024	1	0	0	0	0	0	0	0	0	0	0	0	66
24290755	m	md	JP	2024	0	1	0	0	0	1	0	0	0	0	0	0	70
24290855	m	md	SA	2024	0	0	0	0	0	0	0	0	0	0	0	0	37

24290872	f	md	BE	2024	0	1	0	0	0	1	0	0	0	0	0	0	55
24290984	f	md	JP	2024	0	1	0	1	0	0	0	0	0	0	0	0	80
24291158	f	cn	US	2024	1	0	0	0	0	0	0	0	0	0	0	0	44
24291188	f	md	JP	2024	0	1	0	0	0	1	0	0	0	0	0	0	76
24291712	f	cn	IN	2024	0	0	0	0	0	0	0	0	0	0	0	0	62
24291858	f	md	DE	2024	0	1	0	1	0	0	0	0	0	0	0	0	
24291899	m	hp	MY	2024	0	0	0	0	0	0	0	0	0	0	0	0	
24291993	m	cn	US	2024	1	0	0	0	0	0	0	0	0	0	0	0	29
24292405	f	md	FR	2024	0	1	0	0	0	1	0	0	0	0	0	0	62
24293025	m	cn	IN	2024	0	1	0	0	0	1	0	0	0	0	0	0	34
24294141	m	hp	US	2024	0	0	0	0	0	0	0	0	0	0	0	0	
24294320	f	hp	IL	2024	1	0	0	0	0	0	0	0	0	0	0	0	60
24294456	m	md	JP	2024	0	0	0	0	0	0	0	0	0	0	0	0	
24294594	f	md	DE	2024	0	1	0	0	1	0	0	0	0	0	0	0	69
24295056	m	md	IT	2024	0	0	0	0	0	0	0	0	0	0	0	0	81
24295213	f	cn	US	2024	0	1	1	0	0	0	0	0	0	0	0	0	
24295569	f	cn	US	2024	1	0	0	0	0	0	0	0	0	0	0	0	
24295944	m	ph	JP	2024	1	0	0	0	0	0	0	0	0	0	0	0	
24296240	m	md	DE	2024	0	1	0	0	1	0	0	0	0	0	0	0	74
24296370	Not	md	DE	2024	1	0	0	0	0	0	0	0	0	0	0	0	
24296375	Not	md	DE	2024	1	0	0	0	0	0	0	0	0	0	0	0	
24296767	Not	cn	US	2024	0	0	0	0	0	0	0	0	0	0	0	0	
24296894	m	cn	US	2024	1	0	0	0	0	0	0	0	0	0	0	0	
24296991	f	md	FR	2024	0	1	0	0	0	1	0	0	0	0	0	0	62
24297058	m	md	FR	2024	0	1	1	0	0	0	0	0	0	0	0	0	73
24297112	m	md	DE	2024	0	1	0	0	1	0	0	0	0	0	0	0	75
24297732	f	cn	IN	2024	0	0	0	0	0	0	0	0	0	0	0	0	77
24297804	f	md	JP	2024	0	1	0	0	1	0	0	0	0	0	0	0	80
24297989	f	md	TR	2024	0	0	0	0	0	0	0	0	0	0	0	0	75
24298478	Not	md	JP	2024	0	1	0	0	1	0	0	0	0	0	0	0	74
24298635	Not	md	JP	2024	0	1	0	0	1	0	0	0	0	0	0	0	74
24298849	m	cn	JP	2024	1	0	0	0	0	0	0	0	0	0	0	0	65
24299190	f	md	US	2024	0	0	0	0	0	0	0	0	0	0	0	0	
24299494	m	md	GB	2024	0	1	1	0	0	0	0	0	0	0	0	1	

24299746	m	md	DE	2024	0	1	0	0	1	0	0	0	0	0	0	0	74
24299819	m	md	BR	2024	0	0	0	0	0	0	0	0	0	0	0	0	
24299894	f	md	IN	2024	1	0	0	0	0	0	0	0	0	0	0	0	64
24300631	f	hp	US	2024	1	0	0	0	0	0	0	0	0	0	0	0	
24300703	f	md	FR	2024	0	0	0	0	0	0	0	0	0	0	0	0	46
24300708	f	md	FR	2024	0	0	0	0	0	0	0	0	0	0	0	0	46
24300846	m	md	JP	2024	0	1	0	0	0	1	0	0	0	0	0	0	
24300908	f	md	DE	2024	0	1	1	0	0	0	0	0	0	0	0	0	
24301046	Not	md	DE	2024	1	0	0	0	0	0	0	0	0	0	0	0	
24301056	m	md	FR	2024	0	1	0	0	0	1	0	0	0	0	0	0	72
24301172	f	md	DE	2024	0	1	0	0	1	0	0	0	0	0	0	0	72
24301314	m	cn	IN	2024	0	0	0	0	0	0	0	0	0	0	0	0	40
24301461	m	cn	IN	2024	0	1	0	0	0	1	0	0	0	0	0	0	52
24302244	f	md	FR	2024	0	1	1	0	0	0	0	0	0	0	0	0	
24302255	Not	md	US	2024	0	0	0	0	0	0	0	0	0	0	0	0	
24302428	m	md	JP	2024	0	1	1	0	0	0	0	0	0	0	0	0	58
24302554	f	cn	DE	2024	0	1	1	0	0	0	0	0	0	0	0	0	
24302612	f	md	RU	2024	0	0	0	0	0	0	0	0	0	0	0	0	
24302631	m	cn	EG	2024	0	0	0	0	0	0	0	0	0	0	0	0	45
24303501	m	md	FR	2024	0	1	0	0	0	1	0	0	0	0	0	0	74
24303927	Not	md	FR	2024	0	1	0	0	1	0	0	0	0	0	0	0	
24304218	f	md	JP	2024	0	1	0	0	1	0	0	0	0	0	0	0	74
24304649	f	md	DE	2024	0	1	0	0	1	0	0	0	0	0	0	0	54
24304831	f	md	JP	2024	0	1	1	0	0	0	0	0	0	0	0	0	80
24304850	m	md	FR	2024	0	1	0	0	1	0	0	0	0	0	0	0	59
24304987	f	md	US	2024	1	0	0	0	0	0	0	0	0	0	0	0	
24305011	f	hp	TH	2024	0	1	0	0	0	1	0	0	0	0	0	0	67
24305117	m	md	JP	2024	0	1	0	0	1	0	0	0	0	0	0	0	77
24305380	f	cn	IN	2024	1	0	0	0	0	0	0	0	0	0	0	0	50
24305626	f	md	DE	2024	0	1	0	0	1	0	0	0	0	0	0	0	72
24305647	m	md	JP	2024	0	0	0	0	0	0	0	0	0	0	0	0	75
24305725	m	md	JP	2024	0	1	0	0	1	0	0	0	0	0	0	0	80
24306033	f	md	DE	2024	0	1	1	0	0	0	0	0	0	0	0	0	63
24306065	m	md	JP	2024	0	1	0	0	1	0	0	0	0	0	0	0	68

24306207	m	ph	FR	2024	0	0	0	0	0	0	0	0	0	0	0	0	
24306245	f	cn	US	2024	0	0	0	0	0	0	0	0	0	0	0	0	82
24306600	m	ph	JP	2024	0	1	0	0	0	1	0	0	0	0	0	0	80
24307182	Not	hp	CN	2024	0	0	0	0	0	0	0	0	0	0	0	0	
24307482	f	md	FR	2024	0	0	0	0	0	0	0	0	0	0	0	0	46
24307939	m	md	RU	2024	0	1	0	0	0	1	0	0	0	0	0	0	75
24308419	f	md	FR	2024	0	0	0	0	0	0	0	0	0	0	0	0	81
24308566	f	cn	IN	2024	1	0	0	0	0	0	0	0	0	0	0	0	71
24308581	f	md	RO	2024	0	0	0	0	0	0	0	0	0	0	0	0	
24308643	m	md	JP	2024	0	0	0	0	0	0	0	0	0	0	0	0	
24309124	Not	md	PR	2024	0	0	0	0	0	0	0	0	0	0	0	0	
24309220	f	ph	CA	2024	0	1	1	0	0	0	0	0	0	0	0	0	65
24309963	f	hp	FR	2024	0	1	1	0	0	0	0	0	0	0	0	0	
24310083	Not	md	TH	2024	0	1	0	0	0	1	0	0	0	0	0	0	
24310668	f	md	IT	2024	0	0	0	0	0	0	0	0	0	0	0	0	
24310784	Not	md	JP	2024	0	1	0	0	1	0	0	0	0	0	0	0	72
24311009	m	hp	US	2024	0	1	0	0	1	0	0	0	0	0	0	0	74
24311378	Not	hp	AU	2024	0	0	0	0	0	0	0	0	0	0	0	0	
24311571	m	hp	DE	2024	0	1	0	0	1	0	0	0	0	0	0	0	77
24311617	m	md	JP	2024	0	0	0	0	0	0	0	0	0	0	0	0	
24311643	m	md	FR	2024	0	1	0	0	0	1	0	0	0	0	0	0	74
24311662	f	md	RO	2024	0	0	0	0	0	0	0	0	0	0	0	0	
24312082	Not	hp	SI	2024	0	0	0	0	0	0	0	0	0	0	0	0	
24313495	f	cn	TH	2024	1	0	0	0	0	0	0	0	0	0	0	0	73.5
24314250	f	cn	DE	2024	0	0	0	0	0	0	0	0	0	0	0	0	
24314325	Not	md	JP	2024	0	1	0	1	0	0	0	0	0	0	0	0	70
24314483	f	cn	US	2024	0	0	0	0	0	0	0	0	0	0	0	0	72
24314765	f	cn	US	2024	0	0	0	0	0	0	0	0	0	0	0	0	72
24314964	m	md	PL	2024	0	1	1	0	0	0	0	0	0	0	0	0	68
24314994	m	md	JP	2024	0	1	0	0	1	0	0	0	0	0	0	0	82
24315010	f	hp	Not re	2024	0	1	0	0	1	0	0	0	0	0	0	1	66
24315509	m	md	DE	2024	0	1	0	0	1	0	0	0	0	0	0	0	74
24315587	Not	hp	TR	2024	0	0	0	0	0	0	0	0	0	0	0	0	58
24315603	m	md	IT	2024	0	1	0	1	0	0	0	0	0	0	0	0	81.9

24316786	m	cn	CA	2024	1	0	0	0	0	0	0	0	0	0	0	0	73
24316791	f	md	JP	2024	0	1	1	0	0	0	0	0	0	0	0	0	63
24316851	f	cn	TW	2024	0	1	0	0	0	1	0	0	0	0	0	0	55
24316965	Not	md	PL	2024	0	1	0	0	1	0	0	0	0	0	0	0	
24317529	f	cn	KR	2024	0	0	0	0	0	0	0	0	0	0	0	0	
24317560	f	md	JP	2024	1	0	0	0	0	0	0	0	0	0	0	0	78
24318032	m	md	HK	2024	0	1	0	1	0	0	0	0	0	0	0	0	51
24318346	f	hp	FR	2024	0	1	1	0	0	0	0	0	0	0	0	0	
24318755	f	cn	CA	2024	0	0	0	0	0	0	0	0	0	0	0	0	
24318862	m	md	KR	2024	0	0	0	0	0	0	0	0	0	0	0	0	61.7
24318985	m	cn	TH	2024	0	0	0	0	0	0	0	0	0	0	0	0	80
24319140	m	cn	SG	2024	0	1	0	0	0	1	0	0	0	0	0	0	
24319382	m	md	JP	2024	0	1	0	0	0	1	0	0	0	0	0	0	71
24320438	f	md	JP	2024	0	1	0	1	0	0	0	0	0	0	0	0	60
24320447	m	md	JP	2024	0	0	0	0	0	0	0	0	0	0	0	0	60
24320455	m	hp	KR	2024	0	0	0	0	0	0	0	0	0	0	0	0	70
24320462	Not	md	JP	2024	0	0	0	0	0	0	0	0	0	0	0	0	
24321127	Not	md	CA	2024	1	0	0	0	0	0	0	0	0	0	0	0	
24321162	f	cn	IN	2024	1	0	0	0	0	0	0	0	0	0	0	0	79
24321621	f	cn	TH	2024	0	1	0	0	0	1	0	0	0	0	0	0	73
24322061	Not	hp	CH	2024	0	1	0	0	0	1	0	0	0	0	0	0	
24322250	m	hp	DE	2024	0	1	0	0	1	0	0	0	0	0	0	0	77
24322438	Not	hp	NL	2024	0	1	0	0	0	1	0	0	0	0	0	0	
24322530	Not	cn	US	2024	0	0	0	0	0	0	0	0	0	0	0	0	
24322573	f	hp	CA	2024	0	0	0	0	0	0	0	0	0	0	0	0	63
24322759	m	md	JP	2024	0	0	0	0	0	0	0	0	0	0	0	0	70
24323625	f	ph	FR	2024	0	0	0	0	0	0	0	0	0	0	0	0	34
24323822	Not	md	JP	2024	0	1	0	0	0	1	0	0	0	0	0	0	80
24323920	m	cn	US	2024	0	0	0	0	0	0	0	0	0	0	0	0	52
24324286	m	hp	IT	2024	0	0	0	0	0	0	0	0	0	0	0	0	76
24324371	f	md	JP	2024	0	1	1	0	0	0	0	0	0	0	0	0	90
24325460	m	hp	US	2024	1	0	0	0	0	0	0	0	0	0	0	0	79
24326103	Not	cn	CN	2024	0	0	0	0	0	0	0	0	0	0	0	0	
24326668	m	cn	JP	2024	1	0	0	0	0	0	0	0	0	0	0	0	

24326834	Not	md	JP	2024	0	1	0	0	0	1	0	0	0	0	0	0	90
24326836	f	cn	KE	2024	1	0	0	0	0	0	0	0	0	0	0	0	52
24326917	f	md	DE	2024	0	1	0	0	1	0	0	0	0	0	0	0	66
24327011	m	md	FR	2024	0	1	0	0	0	1	0	0	0	0	0	0	72
24327540	m	md	JP	2024	0	1	1	0	0	0	0	0	0	0	0	0	60
24327559	m	hp	US	2024	1	0	0	0	0	0	0	0	0	0	0	0	80
24327612	f	cn	RO	2024	0	0	0	0	0	0	0	0	0	0	0	0	
24327618	Not	cn	JP	2024	0	0	0	0	0	0	0	0	0	0	0	0	75
24327679	m	md	BG	2024	0	1	0	0	1	0	0	0	0	0	0	0	58
24327743	f	md	JP	2024	1	0	0	0	0	0	0	0	0	0	0	0	
24327876	f	md	KR	2024	0	0	0	0	0	0	0	0	0	0	0	0	80
24327925	f	hp	KR	2024	0	0	0	0	0	0	0	0	0	0	0	0	58
24327938	m	cn	IN	2024	0	0	0	0	0	0	0	0	0	0	0	0	78
24327960	m	md	RU	2024	0	0	0	0	0	0	0	0	0	0	0	0	62
24327967	f	md	KR	2024	0	0	0	0	0	0	0	0	0	0	0	0	69
24327989	f	md	KR	2024	0	0	0	0	0	0	0	0	0	0	0	0	77
24327990	m	hp	KR	2024	0	0	0	0	0	0	0	0	0	0	0	0	69
24328006	m	hp	KR	2024	0	0	0	0	0	0	0	0	0	0	0	0	72
24328115	f	cn	US	2024	0	0	0	0	0	0	0	0	0	0	0	0	29
24328368	f	md	GB	2024	1	0	0	0	0	0	0	0	0	0	0	0	
24328577	m	hp	KR	2024	0	0	0	0	0	0	0	0	0	0	0	0	40
24328579	f	md	KR	2024	0	0	0	0	0	0	0	0	0	0	0	0	81
24328608	f	md	KR	2024	0	0	0	0	0	0	0	0	0	0	0	0	60
24328609	f	md	KR	2024	0	0	0	0	0	0	0	0	0	0	0	0	61
24328646	m	md	KR	2024	0	0	0	0	0	0	0	0	0	0	0	0	83
24328647	m	md	KR	2024	0	0	0	0	0	0	0	0	0	0	0	0	82
24328655	m	md	KR	2024	0	0	0	0	0	0	0	0	0	0	0	0	73
24328658	m	md	KR	2024	0	0	0	0	0	0	0	0	0	0	0	0	61
24328884	m	md	JP	2024	0	1	0	1	0	0	0	0	0	0	0	0	56
24328887	Not	hp	KR	2024	0	0	0	0	0	0	0	0	0	0	0	0	
24329326	f	cn	US	2024	1	0	0	0	0	0	0	0	0	0	0	0	66
24330101	Not	ph	JP	2024	1	0	0	0	0	0	0	0	0	0	0	0	
24330185	f	hp	US	2024	0	0	0	0	0	0	0	0	0	0	0	0	
24330614	Not	md	BR	2024	0	1	0	1	0	0	0	0	0	0	0	0	

24330674	f	md	DE	2024	0	1	1	0	0	0	0	0	0	0	0	0	
24330957	f	cn	CA	2024	1	0	0	0	0	0	0	0	0	0	0	0	
24331068	f	cn	US	2024	0	0	0	0	0	0	0	0	0	0	0	0	72
24331140	Not	md	JP	2024	0	1	0	1	0	0	0	0	0	0	0	0	
24331209	Not	ph	US	2024	1	0	0	0	0	0	0	0	0	0	0	0	
24331320	f	md	FR	2024	0	0	0	0	0	0	0	0	0	0	0	0	57
24331340	f	md	DE	2024	0	1	0	0	1	0	0	0	0	0	0	0	72
24331743	m	cn	JP	2024	1	0	0	0	0	0	0	0	0	0	0	0	
24331976	Not	md	JP	2024	0	1	0	1	0	0	0	0	0	0	0	0	
24332012	f	md	DE	2024	0	1	0	0	1	0	0	0	0	0	0	0	72
24332144	f	cn	PK	2024	0	1	0	0	0	1	0	0	0	0	0	0	56
24332550	f	md	JP	2024	0	0	0	0	0	0	0	0	0	0	0	0	41
24332590	Not	md	JP	2024	0	0	0	0	0	0	0	0	0	0	0	0	
24332877	Not	md	JP	2024	0	1	1	0	0	0	0	0	0	0	0	0	74
24332884	f	md	FR	2024	0	1	0	0	0	1	0	0	0	0	0	0	68
24333091	f	md	RU	2024	0	0	0	0	0	0	0	0	0	0	0	0	
24333099	Not	hp	US	2024	0	0	0	0	0	0	0	0	0	0	0	0	
24333182	m	md	FR	2024	0	0	0	0	0	0	0	0	0	0	0	0	28
24333251	f	md	FR	2024	0	0	0	0	0	0	0	0	0	0	0	0	57
24333350	m	md	JP	2024	0	1	0	0	1	0	0	0	0	0	0	0	71
24333354	m	md	FR	2024	0	1	0	0	1	0	0	0	0	0	0	0	68
24333783	m	md	DE	2024	0	1	0	0	1	0	0	0	0	0	0	0	68
24334040	m	md	JP	2024	0	0	0	0	0	0	0	0	0	0	0	0	70
24334522	m	md	JP	2024	0	1	1	0	0	0	0	0	0	0	0	0	85
24334776	f	cn	US	2024	0	0	0	0	0	0	0	0	0	0	0	0	
24334797	m	md	JP	2024	0	1	1	0	0	0	0	0	0	0	0	0	74
24334836	f	md	FR	2024	0	0	0	0	0	0	0	0	0	0	0	0	46
24334919	f	md	FR	2024	0	0	0	0	0	0	0	0	0	0	0	0	57
24335208	f	md	FR	2024	0	0	0	0	0	0	0	0	0	0	0	0	
24335685	f	cn	US	2024	0	0	0	0	0	0	0	0	0	0	0	0	
24335709	f	cn	US	2024	0	0	0	0	0	0	0	0	0	0	0	0	57
24336657	m	md	JP	2024	0	1	0	0	1	0	0	0	0	0	0	0	80
24337316	m	md	JP	2024	0	1	0	0	1	0	0	0	0	0	0	0	73
24337767	m	md	JP	2024	0	1	0	0	1	0	0	0	0	0	0	0	66

24338825	m	md	JP	2024	0	1	0	0	1	0	0	0	0	0	0	0	66
24339041	f	Not repo	US	2024	0	1	0	0	0	1	0	0	0	0	0	0	82
24339053	m	md	JP	2024	0	0	0	0	0	0	0	0	0	0	0	0	90
24339335	f	md	JP	2024	0	0	0	0	0	0	0	0	0	0	0	0	77
24339583	f	md	HU	2024	0	1	0	0	0	1	0	0	0	0	0	0	72
24339626	Not	md	JP	2024	0	1	1	0	0	0	0	0	0	0	0	0	73
24340008	m	ph	JP	2024	1	0	0	0	0	0	0	0	0	0	0	0	68
24340602	Not	md	JP	2024	0	1	0	0	1	0	0	0	0	0	0	0	
24341542	m	md	JP	2024	0	1	0	0	1	0	0	0	0	0	0	0	66
24341548	m	md	JP	2024	0	1	0	0	1	0	0	0	0	0	0	0	73
24341841	Not	md	JP	2024	0	1	0	0	1	0	0	0	0	0	0	0	
24342340	f	md	CA	2024	0	1	0	0	1	0	0	0	0	0	0	0	70
24342467	f	md	CA	2024	0	1	0	0	1	0	0	0	0	0	0	0	
24342568	Not	md	JP	2024	0	1	0	0	1	0	0	0	0	0	0	0	
24342688	m	md	CA	2024	0	1	0	0	1	0	0	0	0	0	0	0	
24343354	m	cn	US	2024	0	0	0	0	0	0	0	0	0	0	0	0	60
24343411	m	hp	RS	2024	0	1	1	0	0	0	0	0	0	0	0	0	
24343504	f	md	JP	2024	0	0	0	0	0	0	0	0	0	0	0	0	
24343675	Not	hp	JP	2024	1	0	0	0	0	0	0	0	0	0	0	0	
24343761	Not	hp	JP	2024	1	0	0	0	0	0	0	0	0	0	0	1	
24343785	Not	hp	JP	2024	1	0	0	0	0	0	0	0	0	0	0	0	
24343805	Not	hp	JP	2024	1	0	0	0	0	0	0	0	0	0	0	0	
24343906	f	md	RO	2024	0	0	0	0	0	0	0	0	0	0	0	0	
24344188	m	md	JP	2024	0	0	0	0	0	0	0	0	0	0	0	0	
24344277	f	md	BR	2024	0	1	1	0	0	0	0	0	0	0	0	0	70
24344833	f	md	US	2024	0	0	0	0	0	0	0	0	0	0	0	0	
24345389	m	md	US	2024	0	1	1	0	0	0	0	0	0	0	0	1	60
24346784	f	md	DE	2024	0	1	0	0	1	0	0	0	0	0	0	0	59
24347150	f	cn	US	2024	0	0	0	0	0	0	0	0	0	0	0	0	
24347285	m	hp	US	2024	0	1	1	0	0	0	0	0	0	0	0	0	77
24347389	m	cn	US	2024	0	1	1	0	0	0	0	0	0	0	0	0	
24347422	f	cn	US	2024	0	1	1	0	0	0	0	0	0	0	0	0	73
24347858	f	md	JP	2024	0	0	0	0	0	0	0	0	0	0	0	0	76
24348734	Not	hp	US	2024	0	0	0	0	0	0	0	0	0	0	0	0	

24348982	f	md	FR	2024	0	0	0	0	0	0	0	0	0	0	0	0	57
24349061	m	md	DE	2024	0	1	0	0	1	0	0	0	0	0	0	0	59
24349602	m	md	JP	2024	0	0	0	0	0	0	0	0	0	0	0	0	35
24350081	f	md	DZ	2024	1	0	0	0	0	0	0	0	0	0	0	0	47
24350284	Not	md	VE	2024	0	0	0	0	0	0	0	0	0	0	0	0	
24350347	Not	md	NL	2024	0	0	0	0	0	0	0	0	0	0	0	0	
24350718	Not	hp	DE	2024	0	1	0	0	1	0	0	0	0	0	0	0	62
24351806	m	md	TR	2024	0	1	0	0	1	0	0	0	0	0	0	0	64
24352033	f	cn	US	2024	0	0	0	0	0	0	0	0	0	0	0	0	
24352038	f	ph	FR	2024	0	0	0	0	0	0	0	0	0	0	0	0	38
24353854	m	ph	JP	2024	1	0	0	0	0	0	0	0	0	0	0	0	68
24353940	f	md	FR	2024	0	1	0	0	0	1	0	0	0	0	0	0	68
24354243	f	md	US	2024	0	0	0	0	0	0	0	0	0	0	0	0	
24354516	m	md	TR	2024	0	1	0	0	1	0	0	0	0	0	0	0	76
24354617	m	md	TR	2024	0	1	0	0	1	0	0	0	0	0	0	0	56
24354642	m	md	JP	2024	0	1	1	0	0	0	0	0	0	0	0	1	76
24354755	Not	hp	CN	2024	0	0	0	0	0	0	0	0	0	0	0	0	
24354764	f	hp	RS	2024	0	1	1	0	0	0	0	0	0	0	0	0	71
24355064	f	hp	US	2024	0	0	0	0	0	0	0	0	0	0	0	0	
24355074	m	md	CH	2024	0	0	0	0	0	0	0	0	0	0	0	0	
24355075	m	md	TR	2024	0	1	0	0	1	0	0	0	0	0	0	0	70
24355240	m	md	TR	2024	0	1	0	0	1	0	0	0	0	0	0	0	83
24355594	Not	hp	BR	2024	1	0	0	0	0	0	0	0	0	0	0	0	
24356177	m	md	DE	2024	0	1	0	0	1	0	0	0	0	0	0	0	66
24356841	m	md	CN	2024	0	0	0	0	0	0	0	0	0	0	0	0	
24357103	f	md	TR	2024	0	1	0	0	1	0	0	0	0	0	0	0	66
24357537	f	md	US	2024	0	0	0	0	0	0	0	0	0	0	0	0	
24359078	Not	md	AR	2024	0	0	0	0	0	0	0	0	0	0	0	0	
24359083	Not	hp	KR	2024	0	0	0	0	0	0	0	0	0	0	0	0	
24359091	Not	cn	CN	2024	0	0	0	0	0	0	0	0	0	0	0	0	
24359117	Not	cn	CN	2024	0	0	0	0	0	0	0	0	0	0	0	0	
24359118	Not	cn	CN	2024	0	0	0	0	0	0	0	0	0	0	0	0	
24359135	Not	cn	CN	2024	0	0	0	0	0	0	0	0	0	0	0	0	
24359136	Not	cn	CN	2024	0	0	0	0	0	0	0	0	0	0	0	0	

24359138	Not	cn	CN	2024	0	0	0	0	0	0	0	0	0	0	0	0	
24359139	Not	cn	CN	2024	0	0	0	0	0	0	0	0	0	0	0	0	
24359140	Not	cn	CN	2024	0	0	0	0	0	0	0	0	0	0	0	0	
24359145	Not	md	CN	2024	0	0	0	0	0	0	0	0	0	0	0	0	
24359146	Not	md	CN	2024	0	0	0	0	0	0	0	0	0	0	0	0	
24359152	Not	cn	CN	2024	0	0	0	0	0	0	0	0	0	0	0	0	
24359162	Not	hp	US	2024	0	0	0	0	0	0	0	0	0	0	0	0	
24359163	Not	hp	IT	2024	0	0	0	0	0	0	0	0	0	0	0	0	
24359170	m	md	US	2024	0	0	0	0	0	0	0	0	0	0	0	0	85
24359172	f	md	BG	2024	0	0	0	0	0	0	0	0	0	0	0	0	43
24359173	m	cn	CN	2024	0	0	0	0	0	0	0	0	0	0	0	0	
24359174	f	md	CN	2024	0	0	0	0	0	0	0	0	0	0	0	0	44
24359175	f	md	CN	2024	1	0	0	0	0	0	0	0	0	0	0	0	
24359176	Not	cn	CN	2024	0	0	0	0	0	0	0	0	0	0	0	0	
24359177	Not	cn	CN	2024	0	0	0	0	0	0	0	0	0	0	0	0	
24359179	f	hp	IT	2024	0	0	0	0	0	0	0	0	0	0	0	0	58
24359197	Not	md	NZ	2024	0	0	0	0	0	0	0	0	0	0	0	0	
24359200	Not	md	KR	2024	0	0	0	0	0	0	0	0	0	0	0	0	
24359208	Not	cn	CN	2024	0	0	0	0	0	0	0	0	0	0	0	0	
24359212	Not	md	KR	2024	0	0	0	0	0	0	0	0	0	0	0	0	
24359215	Not	cn	CN	2024	0	0	0	0	0	0	0	0	0	0	0	0	
24359217	f	cn	CN	2024	0	0	0	0	0	0	0	0	0	0	0	0	
24359219	Not	cn	CN	2024	0	0	0	0	0	0	0	0	0	0	0	0	
24359225	Not	md	TH	2024	0	0	0	0	0	0	0	0	0	0	0	0	
24359229	m	cn	CN	2024	0	0	0	0	0	0	0	0	0	0	0	0	
24359232	Not	cn	CN	2024	0	0	0	0	0	0	0	0	0	0	0	0	
24359234	Not	md	RU	2024	0	0	0	0	0	0	0	0	0	0	0	0	
24359235	Not	cn	CN	2024	0	0	0	0	0	0	0	0	0	0	0	0	
24359236	Not	hp	US	2024	0	0	0	0	0	0	0	0	0	0	0	0	
24359250	Not	md	SG	2024	0	0	0	0	0	0	0	0	0	0	0	0	
24359252	Not	cn	CN	2024	0	0	0	0	0	0	0	0	0	0	0	0	
24359253	Not	md	KR	2024	0	0	0	0	0	0	0	0	0	0	0	0	
24359254	f	cn	CN	2024	0	0	0	0	0	0	0	0	0	0	0	0	79
24359344	Not	cn	CN	2024	0	0	0	0	0	0	0	0	0	0	0	0	

24359351	Not	cn	CN	2024	0	0	0	0	0	0	0	0	0	0	0	0	
24359482	Not	hp	FR	2024	0	0	0	0	0	0	0	0	0	0	0	0	
24359728	m	md	JP	2024	0	1	0	0	1	0	0	0	0	0	0	0	70
24360735	f	md	BR	2024	0	1	0	0	0	1	0	0	0	0	0	1	67
24360823	m	md	JP	2024	0	0	0	0	0	0	0	0	0	0	0	0	70
24360831	Not	ph	CA	2024	0	0	0	0	0	0	0	0	0	0	0	0	71
24360916	Not	md	JP	2024	0	0	0	0	0	0	0	0	0	0	0	0	
24361176	Not	hp	CN	2024	0	0	0	0	0	0	0	0	0	0	0	0	
24361762	m	hp	CN	2024	0	0	0	0	0	0	0	0	0	0	0	0	64
24362142	m	md	FI	2024	0	1	0	0	1	0	0	0	0	0	0	0	54
24362178	f	md	TH	2024	0	0	0	0	0	0	0	0	0	0	0	0	70
24362420	m	md	CA	2024	0	0	0	0	0	0	0	0	0	0	0	0	65
24363196	m	md	DE	2024	0	1	1	0	0	0	0	0	0	0	0	0	
24363335	m	md	JP	2024	0	1	1	0	0	0	0	0	0	0	0	0	60
24364117	f	md	JP	2024	0	1	0	0	1	0	0	0	0	0	0	0	80
24364123	Not	md	US	2024	1	0	0	0	0	0	0	0	0	0	0	0	
24364469	m	md	JP	2024	0	1	0	0	1	0	0	0	0	0	0	0	75
24364875	m	md	JP	2024	0	0	0	0	0	0	0	0	0	0	0	0	
24364879	f	cn	TH	2024	0	1	0	0	0	1	0	0	0	0	0	0	66
24364963	m	md	JP	2024	0	1	0	0	0	1	0	0	0	0	0	0	77
24365044	m	md	JP	2024	0	0	0	0	0	0	0	0	0	0	0	0	82
24365101	Not	hp	NL	2024	0	1	0	0	0	1	0	0	0	0	0	0	
24365661	m	md	DE	2024	0	1	0	0	1	0	0	0	0	0	0	0	87
24366062	m	md	IT	2024	0	1	0	0	1	0	0	0	0	0	0	0	73
24366164	f	md	RU	2024	0	0	0	0	0	0	0	0	0	0	0	0	
24367024	f	ph	PT	2024	0	0	0	0	0	0	0	0	0	0	0	0	55
24367607	f	md	PT	2024	0	0	0	0	0	0	0	0	0	0	0	0	47
24368108	Not	md	US	2024	0	1	1	0	0	0	0	0	0	0	0	0	
24368536	f	ph	JP	2024	0	0	0	0	0	0	0	0	0	0	0	0	70
24369493	m	hp	US	2024	0	0	0	0	0	0	0	0	0	0	0	0	
24370369	f	md	SK	2024	0	1	0	0	0	1	0	0	0	0	0	0	
24370691	f	md	JP	2024	0	0	0	0	0	0	0	0	0	0	0	0	60
24372021	Not	md	FR	2024	0	0	0	0	0	0	0	0	0	0	0	0	
24372133	m	cn	US	2024	0	0	0	0	0	0	0	0	0	0	0	0	

24372272	f	md	US	2024	0	0	0	0	0	0	0	0	0	0	0	0	88
24372626	Not	md	JP	2024	0	1	0	0	0	1	0	0	0	0	0	0	80
24374885	Not	md	CA	2024	0	1	0	1	0	0	0	0	0	0	0	1	
24375083	m	cn	TH	2024	0	1	0	0	0	1	0	0	0	0	0	0	71
24375088	m	md	JP	2024	0	0	0	0	0	0	0	0	0	0	0	0	84
24375102	m	cn	TH	2024	0	1	0	0	0	1	0	0	0	0	0	0	78
24375194	Not	ph	CA	2024	0	0	0	0	0	0	0	0	0	0	0	0	71
24375798	m	md	JP	2024	0	1	1	0	0	0	0	0	0	0	0	0	79
24375830	m	hp	US	2024	0	1	1	0	0	0	0	0	0	0	0	0	
24375948	m	hp	US	2024	0	1	1	0	0	0	0	0	0	0	0	0	65
24376015	m	hp	US	2024	0	1	1	0	0	0	0	0	0	0	0	0	75
24376016	f	cn	US	2024	0	1	1	0	0	0	0	0	0	0	0	0	70
24376047	m	Not repo	US	2024	0	0	0	0	0	0	0	0	0	0	0	0	72
24376538	Not	cn	CN	2024	0	0	0	0	0	0	0	0	0	0	0	0	
24376584	f	hp	US	2024	0	1	0	0	1	0	0	0	0	0	0	0	71
24376663	f	cn	US	2024	0	1	1	0	0	0	0	0	0	0	0	0	
24376957	f	md	CZ	2024	0	1	1	0	0	0	0	0	0	0	0	0	61
24377058	Not	md	DE	2024	0	1	0	0	1	0	0	0	0	0	0	0	
24377831	Not	md	JP	2024	0	1	0	0	0	1	0	0	0	0	0	1	
24377871	m	cn	PL	2024	0	0	0	0	0	0	0	0	0	0	0	0	76
24378551	f	cn	IN	2024	0	1	0	0	0	1	0	0	0	0	0	0	75
24379271	m	md	JP	2024	0	1	1	0	0	0	0	0	0	0	0	0	52
24379764	m	md	DE	2024	0	1	0	0	1	0	0	0	0	0	0	0	
24379820	m	cn	US	2024	0	0	0	0	0	0	0	0	0	0	0	0	60
24380712	Not	md	DE	2024	0	1	0	0	1	0	0	0	0	0	0	0	
24393524	f	md	DE	2024	0	1	0	0	1	0	0	0	0	0	0	0	64
23981525	f	md	JP	2024	0	0	0	0	0	0	0	0	0	0	0	0	60
23994546	f	md	DE	2024	0	1	0	0	1	0	0	0	0	0	0	0	69
24032627	m	md	JP	2024	0	1	0	0	1	0	0	0	0	0	0	0	46
24032868	m	md	JP	2024	0	1	0	0	1	0	0	0	0	0	0	0	52
24048012	m	md	JP	2024	0	1	0	0	1	0	0	0	0	0	0	0	66
24049275	Not	md	JP	2024	0	1	0	0	1	0	0	0	0	0	0	1	
24056123	Not	md	CA	2024	0	1	0	0	1	0	0	0	0	0	0	1	
24056359	Not	md	CA	2024	0	1	0	0	1	0	0	0	0	0	0	1	

24062967	m	md	JP	2024	0	1	1	0	0	0	0	0	0	0	0	0	80
24065863	m	md	JP	2024	0	1	0	0	1	0	0	0	0	0	0	0	59
24066561	Not	md	JP	2024	0	1	0	0	1	0	0	0	0	0	0	0	
24067045	m	md	JP	2024	0	1	0	0	1	0	0	0	0	0	0	0	73
24067454	f	hp	NL	2024	0	1	0	0	1	0	0	0	0	0	0	0	46
24067755	m	md	JP	2024	0	1	0	0	1	0	0	0	0	0	0	0	64
24080611	m	md	DE	2024	0	0	0	0	0	0	0	0	0	0	0	0	
24081301	f	md	JP	2024	0	1	0	0	1	0	0	0	0	0	0	0	76
24082784	Not	md	JP	2024	0	1	0	0	1	0	0	0	0	0	0	0	
24090800	f	hp	NL	2024	0	1	0	0	1	0	0	0	0	0	0	0	46
24103143	f	hp	NL	2024	0	1	0	0	1	0	0	0	0	0	0	0	46
24108842	m	md	JP	2024	0	1	0	0	1	0	0	0	0	0	0	0	74
24111365	m	hp	AU	2024	0	1	1	0	0	0	0	0	0	0	0	0	89
24122554	f	md	JP	2024	0	1	0	0	1	0	0	0	0	0	0	0	79
24127301	m	md	US	2024	0	0	0	0	0	0	0	0	0	0	0	0	61
24139335	f	md	ES	2024	0	0	0	0	0	0	0	0	0	0	0	0	76
24147141	Not	md	JP	2024	0	0	0	0	0	0	0	0	0	0	0	0	
24163936	f	md	DE	2024	0	1	0	0	1	0	0	0	0	0	0	0	69
24168313	Not	md	JP	2024	0	1	0	1	0	0	0	0	0	0	0	0	66
24184882	f	md	IT	2024	0	0	0	0	0	0	0	0	0	0	0	0	75
24189652	m	md	JP	2024	0	0	0	0	0	0	0	0	0	0	0	0	42
24205160	f	cn	US	2024	0	0	0	0	0	0	0	0	0	0	0	0	40
24212880	m	md	DE	2024	0	1	0	0	1	0	0	0	0	0	0	0	64
24217458	m	hp	US	2024	0	0	0	0	0	0	0	0	0	0	0	0	72
24219244	f	md	JP	2024	0	1	0	0	1	0	0	0	0	0	0	0	73
24227977	Not	md	KR	2024	1	0	0	0	0	0	0	0	0	0	0	0	35
24230384	m	ph	JP	2024	0	0	0	0	0	0	0	0	0	0	0	0	67
24231392	f	md	US	2024	0	0	0	0	0	0	0	0	0	0	0	0	81
24246849	f	md	DE	2024	0	1	0	0	1	0	0	0	0	0	0	0	47
24251528	f	md	JP	2024	0	0	0	0	0	0	0	0	0	0	0	0	70
24257663	f	md	DE	2024	0	1	0	0	1	0	0	0	0	0	0	0	73
24258185	f	md	DE	2024	0	1	0	0	1	0	0	0	0	0	0	0	58
24260255	f	cn	IN	2024	0	0	0	0	0	0	0	0	0	0	0	0	46
24270648	f	ph	US	2024	0	0	0	0	0	0	0	0	0	0	0	0	73

24273960	m	md	CA	2024	0	0	0	0	0	0	0	0	0	0	0	0	71
24275143	f	md	JP	2024	0	1	0	0	1	0	0	0	0	0	0	0	
24277387	m	hp	DE	2024	0	1	0	0	1	0	0	0	0	0	0	0	64
24278764	f	md	ES	2024	0	1	1	0	0	0	0	0	0	0	0	0	47
24281551	m	ph	US	2024	0	0	0	0	0	0	0	0	0	0	0	0	80
24282401	m	md	JP	2024	0	1	1	0	0	0	0	0	0	0	0	0	64
24282902	Not	md	JP	2024	0	1	0	1	0	0	0	0	0	0	0	0	71
24284292	m	md	JP	2024	0	1	0	0	1	0	0	0	0	0	0	0	79
24287218	m	md	JP	2024	0	1	0	0	1	0	0	0	0	0	0	0	76
24287414	Not	md	BR	2024	1	0	0	0	0	0	0	0	0	0	0	0	80
24294335	f	hp	US	2024	0	0	0	0	0	0	0	0	0	0	0	0	70
24297092	m	hp	DE	2024	0	1	0	0	1	0	0	0	0	0	0	0	64
24300263	m	ph	JP	2024	0	0	0	0	0	0	0	0	0	0	0	0	70
24300270	m	md	JP	2024	0	0	0	0	0	0	0	0	0	0	0	0	60
24301920	m	md	DE	2024	0	1	0	0	1	0	0	0	0	0	0	0	50
24316551	m	md	DE	2024	0	1	0	0	1	0	0	0	0	0	0	0	50
24324058	f	hp	IT	2024	0	1	0	0	1	0	0	0	0	0	0	0	66
24324150	Not	md	JP	2024	0	1	0	0	1	0	0	0	0	0	0	1	81
24325800	m	md	DE	2024	0	1	0	0	1	0	0	0	0	0	0	0	61
24327993	f	md	KR	2024	0	0	0	0	0	0	0	0	0	0	0	0	82
24328680	m	md	KR	2024	0	0	0	0	0	0	0	0	0	0	0	0	64
24328692	f	md	KR	2024	0	0	0	0	0	0	0	0	0	0	0	0	78
24329213	f	md	GB	2024	0	0	0	0	0	0	0	0	0	0	0	0	
24329513	m	md	JP	2024	0	1	1	0	0	0	0	0	0	0	0	0	80
24330462	m	md	JP	2024	0	1	0	0	1	0	0	0	0	0	0	0	74
24333110	f	cn	IN	2024	0	0	0	0	0	0	0	0	0	0	0	0	38
24333151	m	md	FR	2024	0	1	0	0	0	1	0	0	0	0	0	0	68
24334252	f	ph	US	2024	0	0	0	0	0	0	0	0	0	0	0	0	44
24339057	f	cn	KZ	2024	0	0	0	0	0	0	0	0	0	0	0	0	
24339762	m	md	DE	2024	0	1	0	0	1	0	0	0	0	0	0	0	68
24341766	Not	md	RU	2024	0	1	1	0	0	0	0	0	0	0	0	0	54
24342765	f	md	SK	2024	0	0	0	0	0	0	0	0	0	0	0	0	52
24347691	f	md	GB	2024	0	0	0	0	0	0	0	0	0	0	0	0	
24348533	f	md	GB	2024	0	0	0	0	0	0	0	0	0	0	0	0	

24352351	f	md	FR	2024	0	1	0	0	0	1	0	0	0	0	0	0	68
24354351	m	md	IT	2024	0	1	0	0	1	0	0	0	0	0	0	0	73
24355388	f	md	GB	2024	0	0	0	0	0	0	0	0	0	0	0	0	
24356106	Not	ph	FR	2024	0	0	0	0	0	0	0	0	0	0	0	0	
24360866	f	md	GB	2024	0	0	0	0	0	0	0	0	0	0	0	0	
24360977	m	md	DE	2024	0	1	0	0	1	0	0	0	0	0	0	0	62
24362579	m	md	KR	2024	0	1	1	0	0	0	0	0	0	0	0	0	55
24363197	m	md	DE	2024	0	1	0	0	1	0	0	0	0	0	0	0	62
24363799	m	md	DE	2024	0	1	0	0	1	0	0	0	0	0	0	0	70
24365548	m	md	DE	2024	0	1	0	0	1	0	0	0	0	0	0	0	68
24365750	f	hp	DE	2024	0	1	0	0	1	0	0	0	0	0	0	0	59
24365901	f	md	DE	2024	0	1	1	0	0	0	0	0	0	0	0	0	72
24369781	m	md	JP	2024	0	1	1	0	0	0	0	0	0	0	0	0	86
24372434	f	md	GB	2024	0	0	0	0	0	0	0	0	0	0	0	0	
24378722	m	md	JP	2024	0	1	0	0	1	0	0	0	0	0	0	0	73
24379470	f	md	US	2024	0	0	0	0	0	0	0	0	0	0	0	0	
24379789	f	cn	US	2024	0	0	0	0	0	0	0	0	0	0	0	0	50
24381245	m	hp	JP	2024	0	0	0	0	0	0	0	0	0	0	0	0	66
24381639	Not	md	JP	2024	0	0	0	0	0	0	0	0	0	0	0	0	70
24381797	f	md	SI	2024	0	1	0	0	1	0	0	0	0	0	0	0	65
24382132	Not	md	TR	2024	0	0	0	0	0	0	0	0	0	0	0	0	
24382256	f	ph	US	2024	0	0	0	0	0	0	0	0	0	0	0	0	
24382265	f	cn	PL	2024	0	0	0	0	0	0	0	0	0	0	0	0	60
24382848	m	hp	DE	2024	0	1	0	0	1	0	0	0	0	0	0	0	75
24383066	f	md	TW	2024	0	0	0	0	0	0	0	0	0	0	0	0	58
24383484	f	cn	PK	2024	0	1	0	0	0	1	0	0	0	0	0	0	68
24383883	f	hp	CN	2024	0	0	0	0	0	0	0	0	0	0	0	0	48
24384062	Not	md	RO	2024	1	0	0	0	0	0	0	0	0	0	0	0	56
24384149	f	md	DE	2024	0	1	1	0	0	0	0	0	0	0	0	0	72
24384388	m	cn	IN	2024	0	1	0	0	0	1	0	0	0	0	0	0	34
24384617	f	hp	US	2024	1	0	0	0	0	0	0	0	0	0	0	0	75
24384642	Not	hp	HK	2024	0	1	1	0	0	0	0	0	0	0	0	0	
24385090	f	hp	DE	2024	0	1	0	0	1	0	0	0	0	0	0	0	59
24385659	Not	Not repo	AE	2024	0	1	0	1	0	0	0	0	0	0	0	0	

24386105	Not	md	DE	2024	0	1	0	1	0	0	0	0	0	0	0	0	66
24386447	Not	md	CA	2024	0	1	0	1	0	0	0	0	0	0	0	1	
24386962	m	hp	US	2024	0	1	1	0	0	0	0	0	0	0	0	1	65
24387079	m	cn	MU	2024	0	1	0	0	0	1	0	0	0	0	0	0	52
24387105	f	md	SI	2024	0	1	0	0	1	0	0	0	0	0	0	1	53
24387222	m	hp	CN	2024	0	0	0	0	0	0	0	0	0	0	0	0	70
24387352	m	cn	IN	2024	0	1	0	0	0	1	0	0	0	0	0	0	77
24387463	f	md	TW	2024	0	0	0	0	0	0	0	0	0	0	0	0	52
24387538	m	md	JP	2024	0	0	0	0	0	0	0	0	0	0	0	0	59
24387711	m	hp	US	2024	0	0	0	0	0	0	0	0	0	0	0	0	67
24387725	Not	md	JP	2024	0	1	0	0	0	1	0	0	0	0	0	0	38
24387756	m	md	JP	2024	0	1	0	0	0	1	0	0	0	0	0	0	
24387852	m	md	TW	2024	0	1	1	0	0	0	0	0	0	0	0	0	68
24387911	m	hp	TW	2024	0	1	0	0	1	0	0	0	0	0	0	0	64
24387922	f	md	US	2024	0	0	0	0	0	0	0	0	0	0	0	0	67
24387955	f	md	CN	2024	0	0	0	0	0	0	0	0	0	0	0	0	54
24387968	m	md	NP	2024	0	0	0	0	0	0	0	0	0	0	0	0	73
24388020	m	md	TW	2024	0	1	1	0	0	0	0	0	0	0	0	0	37
24388169	m	md	TW	2024	0	1	1	0	0	0	0	0	0	0	0	0	68
24388299	f	cn	JP	2024	1	0	0	0	0	0	0	0	0	0	0	0	76
24388322	m	md	TW	2024	0	1	1	0	0	0	0	0	0	0	0	0	54
24388365	m	md	TW	2024	0	1	1	0	0	0	0	0	0	0	0	0	60
24388401	m	md	TW	2024	0	1	1	0	0	0	0	0	0	0	0	0	70
24388408	Not	md	KR	2024	1	0	0	0	0	0	0	0	0	0	0	0	
24388486	Not	md	JP	2024	0	1	0	0	1	0	0	0	0	0	0	1	
24388504	f	md	NP	2024	0	0	0	0	0	0	0	0	0	0	0	0	79
24388579	m	md	TW	2024	0	1	1	0	0	0	0	0	0	0	0	0	66
24388817	m	md	TW	2024	0	1	1	0	0	0	0	0	0	0	0	0	41
24388918	m	md	TW	2024	0	1	1	0	0	0	0	0	0	0	0	0	58
24388942	m	md	TW	2024	0	1	1	0	0	0	0	0	0	0	0	0	37
24388943	m	md	TW	2024	0	1	1	0	0	0	0	0	0	0	0	0	56
24388955	Not	md	JP	2024	0	1	0	1	0	0	0	0	0	0	0	1	53
24389072	Not	md	AU	2024	1	0	0	0	0	0	0	0	0	0	0	0	
24389305	m	md	JP	2024	0	1	1	0	0	0	0	0	0	0	0	0	76

24389449	f	md	TR	2024	0	0	0	0	0	0	0	0	0	0	0	0	58
24390106	f	md	BR	2024	0	1	0	0	0	1	0	0	0	0	0	0	76
24390411	m	cn	TH	2024	0	0	0	0	0	0	0	0	0	0	0	0	74
24390548	f	cn	PH	2024	0	1	0	0	0	1	0	0	0	0	0	0	60
24390795	m	hp	AU	2024	0	1	1	0	0	0	0	0	0	0	0	0	77
24391136	Not	md	JP	2024	0	1	0	0	0	1	0	0	0	0	0	0	80
24391482	m	md	BE	2024	0	1	1	0	0	0	0	0	0	0	0	0	47
24391583	f	md	NP	2024	0	0	0	0	0	0	0	0	0	0	0	0	34
24391592	m	md	NP	2024	0	0	0	0	0	0	0	0	0	0	0	0	68
24392200	m	hp	CN	2024	0	0	0	0	0	0	0	0	0	0	0	0	74
24392470	Not	md	MY	2024	1	0	0	0	0	0	0	0	0	0	0	0	89
24392479	Not	md	IT	2024	0	1	0	1	0	0	0	0	0	0	0	0	
24392761	f	cn	CA	2024	0	0	0	0	0	0	0	0	0	0	0	0	54
24392767	f	md	DE	2024	0	1	0	0	1	0	0	0	0	0	0	0	53
24393083	Not	Not repo	CN	2024	1	0	0	0	0	0	0	0	0	0	0	0	68
24393175	m	md	DZ	2024	0	0	0	0	0	0	0	0	0	0	0	0	46
24393191	f	md	KR	2024	0	1	1	0	0	0	0	0	0	0	0	0	69
24393482	m	hp	JP	2024	0	1	1	0	0	0	0	0	0	0	0	0	67
24393483	m	hp	JP	2024	0	1	1	0	0	0	0	0	0	0	0	0	75
24393489	m	hp	JP	2024	0	1	1	0	0	0	0	0	0	0	0	0	67
24393543	Not	Not repo	KE	2024	1	0	0	0	0	0	0	0	0	0	0	0	61
24393555	f	ph	GB	2024	0	0	0	0	0	0	0	0	0	0	0	0	
24393831	m	hp	IL	2024	0	1	1	0	0	0	0	0	0	0	0	0	
24393887	m	hp	IL	2024	0	1	1	0	0	0	0	0	0	0	0	0	
24393908	m	md	TW	2024	0	1	1	0	0	0	0	0	0	0	0	0	61
24394025	Not	ph	FR	2024	0	0	0	0	0	0	0	0	0	0	0	0	
24394094	f	md	BE	2024	0	1	1	0	0	0	0	0	0	0	0	0	54
24394361	Not	ph	FR	2024	0	0	0	0	0	0	0	0	0	0	0	0	
24394382	Not	hp	US	2024	0	0	0	0	0	0	0	0	0	0	0	0	
24394576	m	md	CA	2024	0	1	0	0	1	0	0	0	0	0	0	0	
24394675	f	md	CA	2024	0	1	0	0	1	0	0	0	0	0	0	0	70
24395001	m	md	ME	2024	0	1	0	0	0	1	0	0	0	0	0	0	74
24395462	f	md	JP	2024	0	1	0	0	0	1	0	0	0	0	0	0	69
24395723	m	md	ES	2024	0	1	1	0	0	0	0	0	0	0	0	0	62

24395844	m	md	DE	2024	0	1	1	0	0	0	0	0	0	0	0	0	61
24396092	Not	Not repo	KE	2024	1	0	0	0	0	0	0	0	0	0	0	0	66
24396106	f	md	US	2024	1	0	0	0	0	0	0	0	0	0	0	0	75
24396167	Not	md	JP	2024	1	0	0	0	0	0	0	0	0	0	0	0	58
24396315	m	md	KR	2024	0	0	0	0	0	0	0	0	0	0	0	0	78
24396807	f	cn	JP	2024	0	0	0	0	0	0	0	0	0	0	0	0	55
24396836	f	md	DE	2024	0	1	0	0	1	0	0	0	0	0	0	0	53
24396932	f	md	NL	2024	0	1	1	0	0	0	0	0	0	0	0	0	71
24397485	f	md	AT	2024	0	1	0	0	0	1	0	0	0	0	0	0	79
24397601	f	md	DE	2024	0	1	0	0	1	0	0	0	0	0	0	0	53
24397667	Not	Not repo	EG	2024	1	0	0	0	0	0	0	0	0	0	0	0	65
24397795	Not	md	JP	2024	0	1	0	1	0	0	0	0	0	0	0	0	
24397844	m	md	FR	2024	0	0	0	0	0	0	0	0	0	0	0	0	63
24398140	f	ph	CA	2024	0	1	1	0	0	0	0	0	0	0	0	0	76
24398192	f	md	JP	2024	0	0	0	0	0	0	0	0	0	0	0	0	70
24398614	m	md	DE	2024	0	0	0	0	0	0	0	0	0	0	0	0	83
24398889	m	md	DE	2024	0	1	0	0	1	0	0	0	0	0	0	0	69
24399003	f	hp	IT	2024	0	1	1	0	0	0	0	0	0	0	0	0	66
24399037	m	md	JP	2024	0	1	1	0	0	0	0	0	0	0	0	0	80
24399606	m	md	CA	2024	0	1	0	0	1	0	0	0	0	0	0	0	
24399910	m	md	CA	2024	0	1	0	0	1	0	0	0	0	0	0	0	
24400365	f	cn	US	2024	0	1	1	0	0	0	0	0	0	0	0	0	
24400539	m	md	JP	2024	0	1	1	0	0	0	0	0	0	0	0	0	90
24400671	Not	Not repo	IN	2024	1	0	0	0	0	0	0	0	0	0	0	0	69
24400953	f	md	DE	2024	0	1	0	0	1	0	0	0	0	0	0	0	83
24401144	m	md	JP	2024	0	1	0	0	1	0	0	0	0	0	0	0	51
24401179	m	md	JP	2024	0	1	0	0	1	0	0	0	0	0	0	0	60
24401419	m	md	CA	2024	0	1	0	0	1	0	0	0	0	0	0	0	
24401462	m	md	CA	2024	0	1	0	0	1	0	0	0	0	0	0	0	
24401726	m	ph	US	2024	0	0	0	0	0	0	0	0	0	0	0	0	54
24401843	m	hp	CA	2024	0	0	0	0	0	0	0	0	0	0	0	0	76
24402389	m	md	CA	2024	0	1	0	0	1	0	0	0	0	0	0	0	60
24402760	Not	md	JP	2024	0	1	0	1	0	0	0	0	0	0	0	1	
24402789	Not	md	JP	2024	0	1	0	1	0	0	0	0	0	0	0	0	

24403523	m	md	JP	2024	0	0	0	0	0	0	0	0	0	0	0	0	67
24404529	f	md	DE	2024	0	1	0	0	1	0	0	0	0	0	0	0	53
24405200	m	md	DE	2024	0	1	0	0	1	0	0	0	0	0	0	0	62
24405292	m	md	JP	2024	0	1	0	0	0	1	0	0	0	0	0	0	69
24405650	Not	ph	US	2024	0	0	0	0	0	0	0	0	0	0	0	0	
24406108	f	hp	US	2024	0	0	0	0	0	0	0	0	0	0	0	0	67
24406120	Not	md	KR	2024	1	0	0	0	0	0	0	0	0	0	0	0	53
24406203	Not	md	TH	2024	0	0	0	0	0	0	0	0	0	0	0	0	
24406239	f	cn	TH	2024	0	0	0	0	0	0	0	0	0	0	0	0	33
24406245	f	cn	MY	2024	0	0	0	0	0	0	0	0	0	0	0	0	58
24406339	m	md	JP	2024	0	1	1	0	0	0	0	0	0	0	0	0	59
24406390	m	hp	US	2024	0	1	1	0	0	0	0	0	0	0	0	1	72
24406560	Not	md	JP	2024	1	0	0	0	0	0	0	0	0	0	0	0	45
24406586	Not	md	JP	2024	1	0	0	0	0	0	0	0	0	0	0	0	83
24406625	Not	md	JP	2024	0	1	0	1	0	0	0	0	0	0	0	0	78
24407445	Not	md	DE	2024	1	0	0	0	0	0	0	0	0	0	0	0	65
24408115	m	cn	US	2024	0	0	0	0	0	0	0	0	0	0	0	0	34
24408248	f	md	DE	2024	0	0	0	0	0	0	0	0	0	0	0	0	62
24408743	f	md	JP	2024	0	1	1	0	0	0	0	0	0	0	0	0	77
24408863	m	ph	DE	2024	0	1	1	0	0	0	0	0	0	0	0	1	
24408999	m	md	JP	2024	0	1	0	0	1	0	0	0	0	0	0	0	60
24409300	f	md	CA	2024	0	1	0	0	1	0	0	0	0	0	0	1	
24409440	m	cn	PK	2024	0	1	0	0	0	1	0	0	0	0	0	0	60
24409495	Not	md	KR	2024	0	1	0	1	0	0	0	0	0	0	0	0	79
24409522	f	md	CA	2024	0	1	0	0	1	0	0	0	0	0	0	0	81
24409567	m	md	DE	2024	0	0	0	0	0	0	0	0	0	0	0	0	69
24409782	Not	md	RS	2024	0	1	0	0	0	1	0	0	0	0	0	1	
24410080	f	md	US	2024	0	0	0	0	0	0	0	0	0	0	0	0	79
24410186	f	hp	CA	2024	0	0	0	0	0	0	0	0	0	0	0	0	45
24410187	Not	md	JP	2024	0	1	0	0	0	1	0	0	0	0	0	0	60
24410195	f	cn	TH	2024	0	0	0	0	0	0	0	0	0	0	0	0	53
24410205	f	md	US	2024	0	0	0	0	0	0	0	0	0	0	0	0	80
24410344	f	md	DE	2024	0	1	0	0	1	0	0	0	0	0	0	0	53
24410382	f	md	CA	2024	0	1	1	0	0	0	0	0	0	0	0	0	67

24410567	m	hp	DE	2024	0	1	0	0	1	0	0	0	0	0	0	0	75
24410729	f	hp	US	2024	0	1	0	0	1	0	0	0	0	0	0	0	71
24411403	f	md	CA	2024	0	1	0	0	1	0	0	0	0	0	0	0	
24411825	f	md	AT	2024	0	0	0	0	0	0	0	0	0	0	0	0	52
24411929	Not	hp	DE	2024	0	1	0	0	1	0	0	0	0	0	0	0	62
24412185	f	cn	IN	2024	0	1	0	0	0	1	0	0	0	0	0	0	62
24412194	Not	md	JP	2024	1	0	0	0	0	0	0	0	0	0	0	0	
24412242	m	hp	ES	2024	0	0	0	0	0	0	0	0	0	0	0	0	67
24412313	f	md	CZ	2024	0	1	1	0	0	0	0	0	0	0	0	0	61
24412441	Not	md	CN	2024	1	0	0	0	0	0	0	0	0	0	0	0	49
24412464	Not	md	BG	2024	0	1	0	0	1	0	0	0	0	0	0	0	
24412535	f	md	DE	2024	0	0	0	0	0	0	0	0	0	0	0	0	62
24412554	f	cn	DE	2024	0	1	0	0	1	0	0	0	0	0	0	0	53
24412579	Not	md	JP	2024	1	0	0	0	0	0	0	0	0	0	0	0	61
24412639	f	md	IN	2024	0	0	0	0	0	0	0	0	0	0	0	0	67
24412809	f	md	AT	2024	0	0	0	0	0	0	0	0	0	0	0	0	
24412958	m	hp	US	2024	0	0	0	0	0	0	0	0	0	0	0	0	
24413709	Not	md	US	2024	0	0	0	0	0	0	0	0	0	0	0	0	
24414725	f	md	DE	2024	0	1	0	0	1	0	0	0	0	0	0	1	63
24414829	f	md	CA	2024	0	1	1	0	0	0	0	0	0	0	0	0	67
24414851	m	ph	PT	2024	0	1	1	0	0	0	0	0	0	0	0	0	80
24414878	m	cn	MM	2024	0	1	0	0	0	1	0	0	0	0	0	0	61
24414880	m	md	FR	2024	0	0	0	0	0	0	0	0	0	0	0	0	72
24414912	m	md	DE	2024	0	1	1	0	0	0	0	0	0	0	0	0	
24415509	m	md	JP	2024	0	0	0	0	0	0	0	0	0	0	0	0	65
24415969	m	md	BE	2024	0	1	1	0	0	0	0	0	0	0	0	0	47
24416073	f	hp	IT	2024	0	1	0	0	1	0	0	0	0	0	0	0	56
24416417	f	md	DE	2024	0	0	0	0	0	0	0	0	0	0	0	0	39
24416430	f	md	AT	2024	0	1	0	0	0	1	0	0	0	0	0	0	79
24416471	Not	md	BG	2024	0	1	0	0	1	0	0	0	0	0	0	0	
24416706	m	cn	US	2024	0	1	1	0	0	0	0	0	0	0	0	0	
24416751	f	hp	US	2024	0	0	0	0	0	0	0	0	0	0	0	0	
24417089	Not	md	JP	2024	1	0	0	0	0	0	0	0	0	0	0	0	
24417221	Not	md	JP	2024	0	1	0	1	0	0	0	0	0	0	0	1	58

24417285	Not	hp	US	2024	1	0	0	0	0	0	0	0	0	0	0	0	
24417436	m	md	NO	2024	0	1	0	0	1	0	0	0	0	0	0	0	
24418183	f	md	NL	2024	0	1	1	0	0	0	0	0	0	0	0	0	71
24418521	m	cn	BE	2024	0	1	1	0	0	0	0	0	0	0	0	0	47
24418686	Not	Not repo	CA	2024	1	0	0	0	0	0	0	0	0	0	0	0	
24421483	m	md	FR	2024	0	1	0	0	0	1	0	0	0	0	0	0	68
24421607	m	md	CA	2024	0	1	0	0	1	0	0	0	0	0	0	0	
24421843	f	md	CA	2024	0	1	0	0	1	0	0	0	0	0	0	0	70
24422084	m	md	DE	2024	0	1	0	0	1	0	0	0	0	0	0	0	69
24422396	f	ph	DE	2024	0	1	0	0	1	0	0	0	0	0	0	0	63
24422448	m	md	JP	2024	0	0	0	0	0	0	0	0	0	0	0	0	90
24422699	Not	md	AE	2024	0	0	0	0	0	0	0	0	0	0	0	0	
24422848	m	md	GR	2024	0	0	0	0	0	0	0	0	0	0	0	0	60
24422971	m	hp	PL	2024	0	1	1	0	0	0	0	0	0	0	0	0	60
24423199	f	cn	JP	2024	0	0	0	0	0	0	0	0	0	0	0	0	76
24423205	m	md	FR	2024	0	0	0	0	0	0	0	0	0	0	0	0	63
24423444	m	md	JP	2024	0	0	0	0	0	0	0	0	0	0	0	0	
24423547	f	md	JP	2024	0	0	0	0	0	0	0	0	0	0	0	0	57
24423565	Not	Not repo	US	2024	1	0	0	0	0	0	0	0	0	0	0	0	
24423935	m	hp	ES	2024	0	0	0	0	0	0	0	0	0	0	0	0	67
24424180	f	md	AT	2024	0	1	0	0	0	1	0	0	0	0	0	0	42
24424496	f	md	BE	2024	0	0	0	0	0	0	0	0	0	0	0	0	64
24425168	m	md	JP	2024	0	0	0	0	0	0	0	0	0	0	0	0	
24425421	m	md	DE	2024	0	1	1	0	0	0	0	0	0	0	0	0	61
24425565	m	cn	AM	2024	0	1	0	0	0	1	0	0	0	0	0	0	66
24425748	f	hp	IT	2024	0	1	0	0	1	0	0	0	0	0	0	0	56
24425757	m	md	DE	2024	0	1	0	0	1	0	0	0	0	0	0	0	69
24426034	f	hp	PL	2024	0	1	0	0	0	1	0	0	0	0	0	0	
24426354	Not	md	JP	2024	0	1	0	1	0	0	0	0	0	0	0	1	
24426467	Not	Not repo	US	2024	1	0	0	0	0	0	0	0	0	0	0	0	
24426601	m	ph	JP	2024	0	1	0	0	0	1	0	0	0	0	0	0	81
24426896	f	Not repo	US	2024	0	0	0	0	0	0	0	0	0	0	0	0	82
24427700	f	hp	US	2024	0	0	0	0	0	0	0	0	0	0	0	0	
24427862	f	md	JP	2024	0	1	0	0	0	1	0	0	0	0	0	0	80

24427911	Not	Not repo	US	2024	1	0	0	0	0	0	0	0	0	0	0	0	59
24427937	m	cn	IN	2024	0	1	0	0	0	1	0	0	0	0	0	0	55
24428049	Not	hp	JP	2024	0	0	0	0	0	0	0	0	0	0	0	0	
24428152	m	md	AU	2024	0	0	0	0	0	0	0	0	0	0	0	0	60
24428244	m	md	JP	2024	0	0	0	0	0	0	0	0	0	0	0	0	78
24428248	Not	ph	JP	2024	1	0	0	0	0	0	0	0	0	0	0	0	79
24428488	f	cn	CZ	2024	0	1	1	0	0	0	0	0	0	0	0	0	61
24428493	Not	ph	FR	2024	0	0	0	0	0	0	0	0	0	0	0	0	
24428588	m	md	JP	2024	0	0	0	0	0	0	0	0	0	0	0	0	65
24428959	m	cn	IN	2024	0	1	0	0	0	1	0	0	0	0	0	0	52
24429205	f	md	BE	2024	0	1	1	0	0	0	0	0	0	0	0	0	54
24429321	f	cn	US	2024	0	1	1	0	0	0	0	0	0	0	0	0	58
24430134	Not	Not repo	US	2024	1	0	0	0	0	0	0	0	0	0	0	0	
24430399	Not	hp	GR	2024	0	0	0	0	0	0	0	0	0	0	0	0	
24430435	m	hp	US	2024	0	0	0	0	0	0	0	0	0	0	0	0	26
24430858	f	hp	US	2024	1	0	0	0	0	0	0	0	0	0	0	0	75
24430906	Not	md	AU	2024	1	0	0	0	0	0	0	0	0	0	0	0	
24431560	f	md	US	2024	0	0	0	0	0	0	0	0	0	0	0	0	38
24431697	f	cn	TH	2024	0	1	0	0	0	1	0	0	0	0	0	0	68
24431752	f	hp	US	2024	0	1	0	0	1	0	0	0	0	0	0	0	71
24431917	Not	md	KR	2024	1	0	0	0	0	0	0	0	0	0	0	0	82
24432117	m	cn	IN	2024	0	1	0	0	0	1	0	0	0	0	0	0	62
24432780	m	md	JP	2024	0	1	1	0	0	0	0	0	0	0	0	0	70
24432810	Not	md	CA	2024	0	1	0	1	0	0	0	0	0	0	0	1	
24433487	m	md	DZ	2024	0	0	0	0	0	0	0	0	0	0	0	0	
24433626	m	md	US	2024	0	1	1	0	0	0	0	0	0	0	0	1	61
24433904	Not	md	JP	2024	0	1	0	1	0	0	0	0	0	0	0	0	58
24433959	m	md	BE	2024	0	1	1	0	0	0	0	0	0	0	0	0	47
24434114	m	md	BE	2024	0	1	1	0	0	0	0	0	0	0	0	1	66
24434333	Not	md	JP	2024	1	0	0	0	0	0	0	0	0	0	0	0	70
24434642	f	ph	US	2024	0	0	0	0	0	0	0	0	0	0	0	0	66
24434652	m	md	BE	2024	0	1	1	0	0	0	0	0	0	0	0	0	66
24434747	f	md	US	2024	0	0	0	0	0	0	0	0	0	0	0	0	54
24434751	m	ph	FR	2024	0	1	1	0	0	0	0	0	0	0	0	0	

24434971	m	md	JP	2024	0	1	1	0	0	0	0	0	0	0	0	0	80
24435273	m	md	JP	2024	0	0	0	0	0	0	0	0	0	0	0	0	70
24436191	m	md	DE	2024	0	1	1	0	0	0	0	0	0	0	0	0	66
24436220	f	md	US	2024	0	0	0	0	0	0	0	0	0	0	0	0	
24436242	f	cn	KR	2024	0	0	0	0	0	0	0	0	0	0	0	0	82
24436248	m	md	MY	2024	0	0	0	0	0	0	0	0	0	0	0	0	40
24436265	Not	cn	TH	2024	0	1	0	0	0	1	0	0	0	0	0	0	68
24436364	Not	Not repo	CA	2024	1	0	0	0	0	0	0	0	0	0	0	0	63
24436450	f	md	DE	2024	0	0	0	0	0	0	0	0	0	0	0	0	62
24436770	m	ph	JP	2024	0	1	1	0	0	0	0	0	0	0	0	0	66
24437009	m	cn	TW	2024	0	1	0	0	1	0	0	0	0	0	0	0	75
24437083	m	cn	AE	2024	0	0	0	0	0	0	0	0	0	0	0	0	39
24437488	m	md	CH	2024	0	1	1	0	0	0	0	0	0	0	0	0	
24437523	f	hp	PT	2024	1	0	0	0	0	0	0	0	0	0	0	0	62
24437539	m	md	FR	2024	0	1	0	0	1	0	0	0	0	0	0	0	67
24438030	m	hp	IN	2024	0	0	0	0	0	0	0	0	0	0	0	0	52
24438079	m	md	DE	2024	0	1	0	0	1	0	0	0	0	0	0	0	77
24438704	m	md	JP	2024	0	1	1	0	0	0	0	0	0	0	0	0	80
24439007	m	hp	US	2024	0	1	1	0	0	0	0	0	0	0	0	0	80
24439232	m	md	JP	2024	0	1	0	0	0	1	0	0	0	0	0	0	80
24439470	Not	md	JP	2024	0	1	0	0	1	0	0	0	0	0	0	0	
24439804	f	md	KR	2024	0	0	0	0	0	0	0	0	0	0	0	0	81
24439874	Not	md	DE	2024	1	0	0	0	0	0	0	0	0	0	0	0	
24442460	Not	Not repo	JP	2024	1	0	0	0	0	0	0	0	0	0	0	0	
24442992	Not	ph	AU	2024	1	0	0	0	0	0	0	0	0	0	0	0	63
24443202	m	hp	US	2024	0	0	0	0	0	0	0	0	0	0	0	0	
24443600	f	md	ES	2024	0	1	1	0	0	0	0	0	0	0	0	0	
24444018	f	md	DE	2024	0	0	0	0	0	0	0	0	0	0	0	0	62
24444137	f	md	US	2024	0	0	0	0	0	0	0	0	0	0	0	0	73
24444257	m	hp	US	2024	0	1	0	0	1	0	0	0	0	0	0	0	65
24444695	m	md	KR	2024	0	1	1	0	0	0	0	0	0	0	0	0	81
24445020	Not	md	JP	2024	0	1	0	1	0	0	0	0	0	0	0	0	54
24445127	f	cn	IN	2024	0	0	0	0	0	0	0	0	0	0	0	0	
24445282	m	ph	US	2024	0	0	0	0	0	0	0	0	0	0	0	0	78

24445306	m	hp	CN	2024	0	0	0	0	0	0	0	0	0	0	0	0	63
24445326	f	md	JP	2024	0	1	0	0	0	1	0	0	0	0	0	0	60
24445335	Not	md	JP	2024	0	1	0	1	0	0	0	0	0	0	0	0	46
24445365	Not	md	JP	2024	1	0	0	0	0	0	0	0	0	0	0	0	
24445377	Not	md	JP	2024	0	1	0	1	0	0	0	0	0	0	0	0	
24445501	f	md	NL	2024	0	1	1	0	0	0	0	0	0	0	0	0	71
24445769	m	cn	BR	2024	0	0	0	0	0	0	0	0	0	0	0	0	46
24446075	f	md	DE	2024	0	1	0	0	1	0	0	0	0	0	0	0	58
24446191	f	hp	US	2024	1	0	0	0	0	0	0	0	0	0	0	0	79
24446229	m	md	FR	2024	0	1	1	0	0	0	0	0	0	0	0	0	34
24447009	m	md	DE	2024	0	0	0	0	0	0	0	0	0	0	0	0	70
24447924	m	md	US	2024	0	0	0	0	0	0	0	0	0	0	0	0	
24448055	m	md	JP	2024	0	1	0	0	1	0	0	0	0	0	0	0	74
24448357	Not	md	JP	2024	0	0	0	0	0	0	0	0	0	0	0	0	
24449281	m	md	PL	2024	0	0	0	0	0	0	0	0	0	0	0	0	74
24449360	Not	md	JP	2024	0	1	0	1	0	0	0	0	0	0	0	0	67
24449429	Not	md	NL	2024	0	1	0	0	0	1	0	0	0	0	0	0	
24450044	m	md	DE	2024	0	1	1	0	0	0	0	0	0	0	0	0	
24450481	f	ph	US	2024	0	0	0	0	0	0	0	0	0	0	0	0	66
24450581	m	cn	IN	2024	0	1	0	0	0	1	0	0	0	0	0	0	76
24450596	f	ph	JP	2024	0	1	1	0	0	0	0	0	0	0	0	0	62
24450826	f	md	KR	2024	0	0	0	0	0	0	0	0	0	0	0	0	78
24450844	Not	Not repo	JP	2024	1	0	0	0	0	0	0	0	0	0	0	0	
24450857	m	md	JP	2024	0	1	0	0	1	0	0	0	0	0	0	0	80
24451332	Not	Not repo	KR	2024	1	0	0	0	0	0	0	0	0	0	0	0	88
24452150	f	ph	US	2024	0	0	0	0	0	0	0	0	0	0	0	0	76
24452241	f	ph	US	2024	0	0	0	0	0	0	0	0	0	0	0	0	61
24453745	f	md	JP	2024	0	1	1	0	0	0	0	0	0	0	0	0	74
24456593	m	md	US	2024	0	1	1	0	0	0	0	0	0	0	0	0	63
24456901	Not	Not repo	CN	2024	1	0	0	0	0	0	0	0	0	0	0	0	
24457225	m	hp	US	2024	0	1	1	0	0	0	0	0	0	0	0	0	
24459275	m	md	CZ	2024	0	1	1	0	0	0	0	0	0	0	0	0	69
24459597	f	md	BE	2024	0	1	1	0	0	0	0	0	0	0	0	0	54
24460182	m	hp	US	2024	0	0	0	0	0	0	0	0	0	0	0	0	64

24462702	m	md	PL	2024	0	1	1	0	0	0	0	0	0	0	0	0	60
24462765	m	md	CA	2024	0	1	0	0	1	0	0	0	0	0	0	0	
24462846	f	md	CA	2024	0	1	0	0	1	0	0	0	0	0	0	0	81
24463051	m	cn	JP	2024	0	0	0	0	0	0	0	0	0	0	0	0	75
24463536	m	md	CA	2024	0	1	0	0	1	0	0	0	0	0	0	0	50
24464829	Not	hp	US	2024	0	0	0	0	0	0	0	0	0	0	0	0	
24466313	m	md	JP	2024	0	1	0	0	1	0	0	0	0	0	0	0	80
24466707	Not	ph	JP	2024	1	0	0	0	0	0	0	0	0	0	0	0	84
24468515	f	md	CA	2024	0	1	0	0	1	0	0	0	0	0	0	0	
24523736	m	md	CA	2024	0	1	0	0	1	0	0	0	0	0	0	0	60
24523855	m	md	CA	2024	0	1	0	0	1	0	0	0	0	0	0	0	
24523904	f	md	NL	2024	0	1	1	0	0	0	0	0	0	0	0	0	71
24524205	m	md	CA	2024	0	1	0	0	1	0	0	0	0	0	0	0	
24524213	m	md	CA	2024	0	1	0	0	1	0	0	0	0	0	0	0	60
24524216	f	md	CA	2024	0	1	0	0	1	0	0	0	0	0	0	1	
24524217	f	md	CA	2024	0	1	0	0	1	0	0	0	0	0	0	0	
24524379	m	cn	CN	2024	0	0	0	0	0	0	0	0	0	0	0	0	77
24524474	m	md	JP	2024	0	1	1	0	0	0	0	0	0	0	0	0	57
24525895	m	hp	US	2024	0	1	1	0	0	0	0	0	0	0	0	0	
24525995	m	hp	KR	2024	0	0	0	0	0	0	0	0	0	0	0	0	63
24526024	f	md	JP	2024	0	0	0	0	0	0	0	0	0	0	0	0	70
24526425	Not	md	JP	2024	0	1	0	0	0	1	0	0	0	0	0	0	80
24526488	f	cn	AU	2024	0	0	0	0	0	0	0	0	0	0	0	0	65
24527235	Not	md	CH	2024	0	0	0	0	0	0	0	0	0	0	0	0	
24528302	m	ph	US	2024	0	1	1	0	0	0	0	0	0	0	0	0	75
24529096	m	hp	GB	2024	0	1	1	0	0	0	0	0	0	0	0	1	
24529285	m	md	DE	2024	0	1	0	0	1	0	0	0	0	0	0	0	69
24529457	Not	md	JP	2024	0	1	0	0	0	1	0	0	0	0	0	1	
24529461	Not	md	RO	2024	0	1	0	1	0	0	0	0	0	0	0	0	
24529538	m	md	US	2024	0	0	0	0	0	0	0	0	0	0	0	0	0.08
24529790	f	md	DE	2024	0	1	1	0	0	0	0	0	0	0	0	1	
24529913	m	ph	US	2024	0	1	0	0	1	0	0	0	0	0	0	0	68
24529958	Not	hp	US	2024	0	0	0	0	0	0	0	0	0	0	0	0	
24530279	Not	ph	JP	2024	0	1	0	0	1	0	0	0	0	0	0	0	

24530403	m	cn	US	2024	0	0	0	0	0	0	0	0	0	0	0	0	52
24530587	Not	Not repo	JP	2024	1	0	0	0	0	0	0	0	0	0	0	0	
24530778	m	hp	JP	2024	0	1	1	0	0	0	0	0	0	0	0	0	76
24530886	f	md	JP	2024	0	0	0	0	0	0	0	0	0	0	0	0	78
24530985	f	md	US	2024	0	1	1	0	0	0	0	0	0	0	0	1	80
24531107	Not	md	FR	2024	0	0	0	0	0	0	0	0	0	0	0	0	
24531115	Not	ph	JP	2024	1	0	0	0	0	0	0	0	0	0	0	0	
24531800	m	md	JP	2024	0	0	0	0	0	0	0	0	0	0	0	0	80
24532146	Not	hp	US	2024	0	0	0	0	0	0	0	0	0	0	0	0	
24532329	Not	Not repo	US	2024	1	0	0	0	0	0	0	0	0	0	0	0	
24532496	Not	ph	JP	2024	1	0	0	0	0	0	0	0	0	0	0	1	
24532806	m	md	TR	2024	0	0	0	0	0	0	0	0	0	0	0	0	72
24533079	m	md	JP	2024	0	1	0	0	0	1	0	0	0	0	0	0	80
24533507	Not	md	JP	2024	0	1	0	1	0	0	0	0	0	0	0	0	50
24533512	Not	ph	JP	2024	1	0	0	0	0	0	0	0	0	0	0	0	80
24533827	m	ph	PR	2024	0	0	0	0	0	0	0	0	0	0	0	0	39
24533907	f	hp	HR	2024	0	1	0	0	1	0	0	0	0	0	0	0	60
24535079	m	md	GB	2024	0	1	1	0	0	0	0	0	0	0	0	0	
24535609	m	hp	JP	2024	0	1	1	0	0	0	0	0	0	0	0	0	73
24535654	f	md	US	2024	0	0	0	0	0	0	0	0	0	0	0	0	
24535775	f	cn	US	2024	0	0	0	0	0	0	0	0	0	0	0	0	
24535966	m	md	BG	2024	0	1	1	0	0	0	0	0	0	0	0	0	72
24536039	m	md	JP	2024	0	1	0	0	1	0	0	0	0	0	0	0	49
24536140	f	hp	JP	2024	0	1	1	0	0	0	0	0	0	0	0	0	76
24536176	m	hp	JP	2024	0	1	1	0	0	0	0	0	0	0	0	0	62
24536373	m	md	FR	2024	0	1	1	0	0	0	0	0	0	0	0	0	50
24537895	Not	md	US	2024	1	0	0	0	0	0	0	0	0	0	0	0	81
24538575	f	hp	US	2024	1	0	0	0	0	0	0	0	0	0	0	0	79
24538767	m	md	KR	2024	0	0	0	0	0	0	0	0	0	0	0	0	54
24539026	m	ph	FR	2024	0	1	0	0	0	1	0	0	0	0	0	0	69
24539596	m	md	NL	2024	0	1	0	0	1	0	0	0	0	0	0	0	52
24539775	m	md	KR	2024	0	0	0	0	0	0	0	0	0	0	0	0	74
24539870	m	md	BE	2024	0	0	0	0	0	0	0	0	0	0	0	0	
24539892	f	cn	IN	2024	0	0	0	0	0	0	0	0	0	0	0	0	49

24539965	m	md	KR	2024	0	0	0	0	0	0	0	0	0	0	0	0	73
24539999	f	hp	PR	2024	0	0	0	0	0	0	0	0	0	0	0	0	
24540180	Not	md	JP	2024	1	0	0	0	0	0	0	0	0	0	0	0	78
24540191	Not	ph	JP	2024	0	1	0	1	0	0	0	0	0	0	0	0	75
24540274	m	md	JP	2024	0	1	0	0	1	0	0	0	0	0	0	0	64
24540479	f	md	JP	2024	0	0	0	0	0	0	0	0	0	0	0	0	78
24540485	Not	ph	JP	2024	1	0	0	0	0	0	0	0	0	0	0	0	81
24540649	f	md	KR	2024	0	0	0	0	0	0	0	0	0	0	0	0	56
24540695	m	md	KR	2024	0	0	0	0	0	0	0	0	0	0	0	0	65
24540710	Not	Not repo	CN	2024	1	0	0	0	0	0	0	0	0	0	0	0	
24540941	m	hp	HR	2024	0	1	0	0	1	0	0	0	0	0	0	0	59
24540994	Not	hp	US	2024	0	0	0	0	0	0	0	0	0	0	0	0	
24541098	f	md	KR	2024	0	0	0	0	0	0	0	0	0	0	0	0	63
24541138	Not	md	JP	2024	0	0	0	0	0	0	0	0	0	0	0	0	58
24541445	Not	Not repo	IT	2024	1	0	0	0	0	0	0	0	0	0	0	0	65
24541887	m	md	UA	2024	0	1	1	0	0	0	0	0	0	0	0	1	50
24542946	m	cn	TH	2024	0	1	0	0	0	1	0	0	0	0	0	0	77
24543187	f	md	FR	2024	0	0	0	0	0	0	0	0	0	0	0	0	46
24543230	f	md	KR	2024	0	0	0	0	0	0	0	0	0	0	0	0	73
24543518	f	hp	US	2024	1	0	0	0	0	0	0	0	0	0	0	0	79
24543551	f	md	JP	2024	0	1	0	0	0	1	0	0	0	0	0	0	65
24543590	Not	hp	FR	2024	0	0	0	0	0	0	0	0	0	0	0	0	
24543696	m	md	JP	2024	0	1	0	0	0	1	0	0	0	0	0	0	80
24543712	Not	md	JP	2024	0	1	0	1	0	0	0	0	0	0	0	0	70
24543961	m	cn	IN	2024	0	1	0	0	0	1	0	0	0	0	0	0	45
24544139	f	md	US	2024	0	0	0	0	0	0	0	0	0	0	0	0	58
24544152	Not	md	KR	2024	1	0	0	0	0	0	0	0	0	0	0	0	49
24544180	m	md	CA	2024	0	1	0	0	1	0	0	0	0	0	0	0	
24544279	Not	hp	CN	2024	0	0	0	0	0	0	0	0	0	0	0	0	
24544470	m	md	JP	2024	0	1	1	0	0	0	0	0	0	0	0	0	
24544563	m	hp	KR	2024	0	1	1	0	0	0	0	0	0	0	0	0	51
24544575	Not	hp	US	2024	0	0	0	0	0	0	0	0	0	0	0	0	
24544596	m	md	JP	2024	0	1	1	0	0	0	0	0	0	0	0	0	60
24544706	Not	Not repo	KR	2024	1	0	0	0	0	0	0	0	0	0	0	0	58

24544710	Not	md	KR	2024	1	0	0	0	0	0	0	0	0	0	0	0	55
24544717	Not	md	KR	2024	1	0	0	0	0	0	0	0	0	0	0	0	80
24544788	Not	md	JP	2024	0	1	0	1	0	0	0	0	0	0	0	0	79
24544919	Not	md	JP	2024	1	0	0	0	0	0	0	0	0	0	0	0	
24544921	f	md	KR	2024	0	0	0	0	0	0	0	0	0	0	0	0	56
24544932	Not	md	JP	2024	0	1	0	1	0	0	0	0	0	0	0	1	80
24544973	Not	hp	US	2024	0	0	0	0	0	0	0	0	0	0	0	0	
24545000	Not	hp	US	2024	0	0	0	0	0	0	0	0	0	0	0	0	
24545075	m	md	KR	2024	0	0	0	0	0	0	0	0	0	0	0	0	54
24545118	m	md	BR	2024	0	0	0	0	0	0	0	0	0	0	0	0	56
24545166	Not	Not repo	CZ	2024	1	0	0	0	0	0	0	0	0	0	0	0	
24545326	Not	Not repo	CA	2024	1	0	0	0	0	0	0	0	0	0	0	0	
24545450	m	md	JP	2024	0	1	1	0	0	0	0	0	0	0	0	0	78
24545549	m	md	US	2024	0	1	1	0	0	0	0	0	0	0	0	0	65
24545574	Not	Not repo	TW	2024	1	0	0	0	0	0	0	0	0	0	0	0	
24545633	Not	Not repo	TW	2024	1	0	0	0	0	0	0	0	0	0	0	0	
24545705	m	ph	JP	2024	0	1	1	0	0	0	0	0	0	0	0	0	48
24545960	m	md	JP	2024	0	0	0	0	0	0	0	0	0	0	0	0	
24546349	m	md	AT	2024	0	0	0	0	0	0	0	0	0	0	0	0	70
24546923	m	cn	IN	2024	0	0	0	0	0	0	0	0	0	0	0	0	41
24547146	f	cn	CN	2024	0	0	0	0	0	0	0	0	0	0	0	0	
24547521	m	md	DE	2024	0	1	0	0	1	0	0	0	0	0	0	0	77
24547550	Not	md	KR	2024	1	0	0	0	0	0	0	0	0	0	0	0	51
24547571	Not	hp	US	2024	0	0	0	0	0	0	0	0	0	0	0	0	
24547608	Not	hp	US	2024	0	0	0	0	0	0	0	0	0	0	0	0	
24547644	f	hp	US	2024	1	0	0	0	0	0	0	0	0	0	0	0	81
24547727	m	md	DE	2024	0	1	0	0	1	0	0	0	0	0	0	0	82
24547832	f	hp	KR	2024	0	0	0	0	0	0	0	0	0	0	0	0	53
24547846	m	md	US	2024	0	1	1	0	0	0	0	0	0	0	0	0	80
24547901	m	md	UA	2024	0	1	1	0	0	0	0	0	0	0	0	0	66
24547917	Not	md	JP	2024	0	1	0	1	0	0	0	0	0	0	0	0	81
24548022	m	md	JP	2024	0	1	0	0	0	1	0	0	0	0	0	0	78
24548049	m	md	JP	2024	0	1	0	1	0	0	0	0	0	0	0	0	77
24548051	f	md	KR	2024	0	0	0	0	0	0	0	0	0	0	0	0	72

24548169	f	md	KR	2024	0	0	0	0	0	0	0	0	0	0	0	0	82
24548277	Not	md	JP	2024	0	1	0	1	0	0	0	0	0	0	0	0	
24548380	m	md	DE	2024	0	1	0	0	1	0	0	0	0	0	0	0	64
24548617	f	hp	CN	2024	0	0	0	0	0	0	0	0	0	0	0	0	67
24548681	f	ph	US	2024	0	1	1	0	0	0	0	0	0	0	0	0	72
24548743	Not	hp	US	2024	0	0	0	0	0	0	0	0	0	0	0	0	
24548791	f	cn	NZ	2024	0	1	1	0	0	0	0	0	0	0	0	0	
24549214	Not	md	JP	2024	0	0	0	0	0	0	0	0	0	0	0	0	
24549272	f	md	DK	2024	0	0	0	0	0	0	0	0	0	0	0	0	43
24549281	m	md	JP	2024	0	0	0	0	0	0	0	0	0	0	0	0	
24549330	m	md	DE	2024	0	1	0	0	1	0	0	0	0	0	0	0	69
24549341	m	md	KR	2024	0	0	0	0	0	0	0	0	0	0	0	0	87
24549432	f	md	FR	2024	1	0	0	0	0	0	0	0	0	0	0	0	63
24549541	f	md	ES	2024	0	1	1	0	0	0	0	0	0	0	0	0	47
24549850	f	ph	FR	2024	0	0	0	0	0	0	0	0	0	0	0	0	79
24549875	m	md	JP	2024	0	1	1	0	0	0	0	0	0	0	0	0	80
24549980	Not	hp	JP	2024	0	1	0	1	0	0	0	0	0	0	0	1	
24550090	Not	hp	US	2024	0	0	0	0	0	0	0	0	0	0	0	0	
24550363	f	md	KR	2024	0	0	0	0	0	0	0	0	0	0	0	0	61
24550611	Not	md	JP	2024	0	0	0	0	0	0	0	0	0	0	0	0	
24550789	Not	hp	US	2024	0	0	0	0	0	0	0	0	0	0	0	0	
24550820	Not	ph	JP	2024	1	0	0	0	0	0	0	0	0	0	0	0	
24551064	Not	Not repo	JP	2024	1	0	0	0	0	0	0	0	0	0	0	0	
24551069	Not	Not repo	JP	2024	1	0	0	0	0	0	0	0	0	0	0	0	
24551070	Not	Not repo	JP	2024	1	0	0	0	0	0	0	0	0	0	0	0	
24551212	m	ph	TH	2024	0	1	1	0	0	0	0	0	0	0	0	0	69
24551549	m	md	US	2024	0	0	0	0	0	0	0	0	0	0	0	0	59
24551583	Not	Not repo	US	2024	1	0	0	0	0	0	0	0	0	0	0	0	56
24551651	f	md	ES	2024	0	1	1	0	0	0	0	0	0	0	0	0	47
24551708	m	md	JP	2024	0	0	0	0	0	0	0	0	0	0	0	0	60
24551712	m	md	PL	2024	0	1	1	0	0	0	0	0	0	0	0	0	60
24551957	Not	md	JP	2024	1	0	0	0	0	0	0	0	0	0	0	1	
24552060	Not	ph	FR	2024	0	0	0	0	0	0	0	0	0	0	0	0	
24552193	f	md	JP	2024	0	1	1	0	0	0	0	0	0	0	0	0	80

24552277	Not	Not repo	CN	2024	1	0	0	0	0	0	0	0	0	0	0	0	
24552591	Not	Not repo	US	2024	1	0	0	0	0	0	0	0	0	0	0	0	
24552833	Not	Not repo	US	2024	1	0	0	0	0	0	0	0	0	0	0	0	38
24552874	Not	Not repo	US	2024	1	0	0	0	0	0	0	0	0	0	0	0	
24552946	Not	hp	US	2024	0	0	0	0	0	0	0	0	0	0	0	0	
24553131	m	hp	DE	2024	0	1	0	0	1	0	0	0	0	0	0	0	75
24553230	Not	hp	CN	2024	0	0	0	0	0	0	0	0	0	0	0	0	
24554011	m	cn	AU	2024	0	1	1	0	0	0	0	0	0	0	0	0	81
24554996	Not	md	JP	2024	0	1	0	1	0	0	0	0	0	0	0	0	64
24555004	f	md	VN	2024	0	0	0	0	0	0	1	0	0	0	1	0	
24555489	Not	md	JP	2024	0	1	1	0	0	0	0	0	0	0	0	0	80
24555610	m	md	AT	2024	0	0	0	0	0	0	0	0	0	0	0	0	55
24555887	m	cn	TR	2024	0	1	1	0	0	0	0	0	0	0	0	0	60
24556000	m	md	KR	2024	0	0	0	0	0	0	0	0	0	0	0	0	
24556281	Not	md	US	2024	0	1	0	1	0	0	0	0	0	0	0	1	
24556288	Not	md	JP	2024	0	1	0	1	0	0	0	0	0	0	0	0	72
24556535	m	md	HR	2024	0	0	0	0	0	0	0	0	0	0	0	0	52
24556616	f	cn	US	2024	1	0	0	0	0	0	0	0	0	0	0	0	79
24556754	m	md	JP	2024	0	1	0	0	0	1	0	0	0	0	0	0	70
24556786	m	ph	US	2024	0	0	0	0	0	0	0	0	0	0	0	0	30
24557046	m	hp	US	2024	0	1	1	0	0	0	0	0	0	0	0	0	80
24557569	Not	md	FR	2024	1	0	0	0	0	0	0	0	0	0	0	0	
24557919	m	md	JP	2024	0	1	1	0	0	0	0	0	0	0	0	0	75
24558058	m	md	BR	2024	0	1	1	0	0	0	0	0	0	0	0	0	69
24558819	Not	hp	JP	2024	0	1	0	0	0	1	0	0	0	0	0	0	
24558925	m	cn	AU	2024	0	1	1	0	0	0	0	0	0	0	0	0	78
24558993	m	md	EG	2024	0	1	0	0	1	0	0	0	0	0	0	0	
24559298	m	md	AT	2024	0	1	1	0	0	0	0	0	0	0	0	0	73
24559373	Not	Not repo	JP	2024	1	0	0	0	0	0	0	0	0	0	0	0	
24559784	Not	md	VN	2024	0	0	0	0	0	0	1	0	0	0	1	0	
24559867	m	md	KR	2024	0	0	0	0	0	0	0	0	0	0	0	0	70
24559906	Not	hp	JP	2024	0	0	0	0	0	0	0	0	0	0	0	0	
24559969	f	md	DE	2024	0	1	0	0	1	0	0	0	0	0	0	0	69
24560034	Not	md	JP	2024	0	1	0	1	0	0	0	0	0	0	0	0	

24560591	f	md	JP	2024	0	1	1	0	0	0	0	0	0	0	0	0	83
24560640	Not	md	FR	2024	1	0	0	0	0	0	0	0	0	0	0	0	
24560769	Not	md	JP	2024	0	1	0	1	0	0	0	0	0	0	0	1	
24560776	m	md	US	2024	0	1	1	0	0	0	0	0	0	0	0	0	63
24560782	f	md	HU	2024	0	1	0	0	0	1	0	0	0	0	0	0	74
24560784	Not	ph	JP	2024	1	0	0	0	0	0	0	0	0	0	0	0	
24560793	f	hp	KR	2024	0	0	0	0	0	0	0	0	0	0	0	0	64
24560850	f	hp	JP	2024	0	0	0	0	0	0	0	0	0	0	0	0	47
24560856	f	cn	IN	2024	0	0	0	0	0	0	0	0	0	0	0	0	49
24560952	m	cn	AU	2024	0	1	1	0	0	0	0	0	0	0	0	0	61
24560971	m	md	JP	2024	0	0	0	0	0	0	0	0	0	0	0	0	
24561041	f	hp	TW	2024	0	1	1	0	0	0	0	0	0	0	0	0	62
24561193	Not	Not repo	JP	2024	1	0	0	0	0	0	0	0	0	0	0	0	
24561390	f	md	JP	2024	0	1	1	0	0	0	0	0	0	0	0	0	83
24561425	f	md	KR	2024	0	0	0	0	0	0	0	0	0	0	0	0	71
24561426	f	md	KR	2024	0	0	0	0	0	0	0	0	0	0	0	0	74
24561613	Not	md	JP	2024	1	0	0	0	0	0	0	0	0	0	0	0	
24561617	Not	Not repo	JP	2024	1	0	0	0	0	0	0	0	0	0	0	0	
24561628	Not	md	JP	2024	1	0	0	0	0	0	0	0	0	0	0	1	77
24561789	m	cn	VN	2024	0	0	0	0	0	0	1	0	0	0	1	0	
24561821	m	cn	IN	2024	0	0	0	0	0	0	0	0	0	0	0	0	63
24561871	m	md	JP	2024	0	1	1	0	0	0	0	0	0	0	0	0	77
24562141	m	md	CZ	2024	1	0	0	0	0	0	0	0	0	0	0	0	75
24562360	f	cn	AU	2024	0	1	1	0	0	0	0	0	0	0	0	0	67
24562478	m	md	US	2024	0	1	1	0	0	0	0	0	0	0	0	0	
24562782	m	md	DE	2024	0	1	0	0	1	0	0	0	0	0	0	0	69
24563472	m	md	JP	2024	0	1	0	0	0	1	0	0	0	0	0	0	
24563555	m	md	JP	2024	0	1	0	0	0	1	0	0	0	0	0	0	74
24563593	Not	md	VN	2024	0	1	0	0	0	1	0	0	0	0	0	0	
24563655	f	md	JP	2024	0	1	1	0	0	0	0	0	0	0	0	0	80
24563824	m	hp	US	2024	0	0	0	0	0	0	0	0	0	0	0	0	70
24564142	f	cn	AT	2024	0	0	0	0	0	0	0	0	0	0	0	0	68
24564167	m	md	DE	2024	0	1	0	0	1	0	0	0	0	0	0	0	82
24564278	f	md	DE	2024	0	1	1	0	0	0	0	0	0	0	0	1	

24564369	m	md	DE	2024	0	1	0	0	1	0	0	0	0	0	0	0	75
24564531	Not	hp	CN	2024	1	0	0	0	0	0	0	0	0	0	0	0	
24564725	f	Not repo	US	2024	0	1	0	0	0	1	0	0	0	0	0	0	82
24564798	m	cn	AT	2024	0	0	0	0	0	0	0	0	0	0	0	0	79
24565047	m	md	KR	2024	0	0	0	0	0	0	0	0	0	0	0	0	67
24565129	Not	md	JP	2024	0	1	0	1	0	0	0	0	0	0	0	1	69
24566063	m	hp	US	2024	0	1	1	0	0	0	0	0	0	0	0	0	63
24566395	f	md	KR	2024	0	0	0	0	0	0	0	0	0	0	0	0	80
24566449	f	Not repo	US	2024	0	1	0	0	0	1	0	0	0	0	0	0	82
24566637	m	md	JP	2024	0	1	0	0	1	0	0	0	0	0	0	0	55
24566920	Not	md	JP	2024	0	1	0	0	1	0	0	0	0	0	0	0	
24566953	f	md	JP	2024	0	1	1	0	0	0	0	0	0	0	0	0	66
24567238	Not	md	JP	2024	0	1	0	1	0	0	0	0	0	0	0	0	45
24567274	Not	md	US	2024	1	0	0	0	0	0	0	0	0	0	0	0	
24567588	Not	md	RO	2024	0	1	1	0	0	0	0	0	0	0	0	0	62
24567650	m	md	JP	2024	0	0	0	0	0	0	0	0	0	0	0	0	
24567933	f	md	DE	2024	0	1	1	0	0	0	0	0	0	0	0	0	
24567959	Not	md	ES	2024	1	0	0	0	0	0	0	0	0	0	0	0	
24568063	m	md	DE	2024	0	1	0	0	1	0	0	0	0	0	0	0	59
24568093	f	md	DE	2024	0	0	0	0	0	0	0	0	0	0	0	0	38
24568107	m	cn	IN	2024	0	0	0	0	0	0	0	0	0	0	0	0	69
24568406	m	hp	IT	2024	0	1	0	0	0	1	0	0	0	0	0	0	68
24568410	m	md	JP	2024	0	1	0	0	0	1	0	0	0	0	0	0	69
24568440	f	md	US	2024	0	0	0	0	0	0	0	0	0	0	0	0	
24568473	m	md	IN	2024	0	1	0	0	0	1	0	0	0	0	0	0	79
24568737	Not	md	US	2024	0	0	0	0	0	0	0	0	0	0	0	0	
24568738	f	cn	US	2024	0	0	0	0	0	0	0	0	0	0	0	0	
24568787	f	hp	US	2024	0	0	0	0	0	0	0	0	0	0	0	0	70
24568788	m	md	US	2024	0	0	0	0	0	0	0	0	0	0	0	0	
24568791	m	hp	US	2024	0	0	0	0	0	0	0	0	0	0	0	0	50
24568792	f	hp	US	2024	0	0	0	0	0	0	0	0	0	0	0	0	58
24568793	m	hp	US	2024	0	0	0	0	0	0	0	0	0	0	0	0	62
24568796	m	hp	US	2024	0	0	0	0	0	0	0	0	0	0	0	0	59
24568797	f	hp	US	2024	0	0	0	0	0	0	0	0	0	0	0	0	53

24568799	m	hp	US	2024	0	0	0	0	0	0	0	0	0	0	0	0	75
24568800	m	hp	US	2024	0	0	0	0	0	0	0	0	0	0	0	0	62
24568802	f	hp	US	2024	0	0	0	0	0	0	0	0	0	0	0	0	66
24568804	f	cn	US	2024	0	0	0	0	0	0	0	0	0	0	0	0	
24568829	Not	md	US	2024	0	0	0	0	0	0	0	0	0	0	0	0	
24568831	m	cn	US	2024	0	0	0	0	0	0	0	0	0	0	0	0	
24568854	Not	md	US	2024	0	0	0	0	0	0	0	0	0	0	0	0	
24568859	f	ph	US	2024	0	0	0	0	0	0	0	0	0	0	0	0	
24568872	f	hp	US	2024	0	0	0	0	0	0	0	0	0	0	0	0	
24568873	m	hp	US	2024	0	0	0	0	0	0	0	0	0	0	0	0	
24568874	f	hp	US	2024	0	0	0	0	0	0	0	0	0	0	0	0	
24568904	f	md	US	2024	0	0	0	0	0	0	0	0	0	0	0	0	
24568989	m	md	GB	2024	0	0	0	0	0	0	0	0	0	0	0	0	
24569056	m	md	PT	2024	0	0	0	0	0	0	0	0	0	0	0	0	59
24569344	f	cn	RU	2024	0	0	0	0	0	0	0	0	0	0	0	0	31
24569974	f	md	ES	2024	0	0	0	0	0	0	0	0	0	0	0	0	69
24570231	Not	md	JP	2024	0	1	0	0	1	0	0	0	0	0	0	0	
24570239	Not	Not repo	PT	2024	1	0	0	0	0	0	0	0	0	0	0	0	62
24570243	Not	md	JP	2024	0	1	0	1	0	0	0	0	0	0	0	0	80
24570432	Not	md	JP	2024	0	1	0	0	1	0	0	0	0	0	0	0	
24570483	Not	md	KR	2024	1	0	0	0	0	0	0	0	0	0	0	0	71
24571208	m	md	DE	2024	0	1	0	0	1	0	0	0	0	0	0	0	61
24571229	Not	Not repo	MY	2024	1	0	0	0	0	0	0	0	0	0	0	0	
24571332	m	md	DE	2024	0	1	0	0	1	0	0	0	0	0	0	0	75
24572021	Not	md	HK	2024	0	0	0	0	0	0	0	0	0	0	0	0	
24572023	m	md	GR	2024	0	0	0	0	0	0	0	0	0	0	0	0	
24572200	m	cn	IN	2024	0	0	0	0	0	0	0	0	0	0	0	0	56
24572204	m	md	IT	2024	0	0	0	0	0	0	0	0	0	0	0	0	64
24572300	Not	md	JP	2024	0	1	0	1	0	0	0	0	0	0	0	0	75
24572325	m	hp	IL	2024	0	1	1	0	0	0	0	0	0	0	0	0	46
24572484	m	hp	KR	2024	0	1	1	0	0	0	0	0	0	0	0	0	
24572956	Not	md	JP	2024	1	0	0	0	0	0	0	0	0	0	0	0	90
24572965	m	md	US	2024	0	1	1	0	0	0	0	0	0	0	0	0	68
24572992	Not	md	JP	2024	0	1	0	1	0	0	0	0	0	0	0	0	71

24572997	Not	md	JP	2024	0	1	0	1	0	0	0	0	0	0	0	0	
24573026	Not	md	JP	2024	1	0	0	0	0	0	0	0	0	0	0	1	
24573155	Not	md	JP	2024	0	1	0	1	0	0	0	0	0	0	0	1	80
24573264	m	md	JP	2024	0	1	0	0	1	0	0	0	0	0	0	0	72
24573329	Not	md	JP	2024	0	1	0	0	1	0	0	0	0	0	0	0	83
24573417	m	md	ES	2024	0	1	0	0	0	1	0	0	0	0	0	0	56
24573419	f	md	ES	2024	0	1	1	0	0	0	0	0	0	0	0	1	53
24573614	f	md	ES	2024	0	1	1	0	0	0	0	0	0	0	0	0	47
24573873	Not	hp	ES	2024	0	1	0	0	1	0	0	0	0	0	0	0	
24573874	Not	hp	ES	2024	0	1	0	0	1	0	0	0	0	0	0	0	
24574343	Not	md	KR	2024	0	1	0	1	0	0	0	0	0	0	0	0	80
24574429	m	md	JP	2024	0	0	0	0	0	0	0	0	0	0	0	0	70
24574561	Not	hp	DE	2024	1	0	0	0	0	0	0	0	0	0	0	0	
24574937	Not	md	AT	2024	0	0	0	0	0	0	0	0	0	0	0	0	
24575061	m	md	DE	2024	0	1	0	0	1	0	0	0	0	0	0	0	69
24575071	m	hp	DE	2024	0	1	0	0	1	0	0	0	0	0	0	0	75
24575218	f	hp	US	2024	0	1	0	0	1	0	0	0	0	0	0	0	59
24575904	m	md	JP	2024	0	1	1	0	0	0	0	0	0	0	0	0	80
24576461	m	md	PL	2024	0	1	0	0	1	0	0	0	0	0	0	1	65
24576696	m	md	DZ	2024	1	0	0	0	0	0	0	0	0	0	0	0	38
24576915	Not	ph	FR	2024	0	0	0	0	0	0	0	0	0	0	0	0	
24577811	Not	md	JP	2024	0	1	0	1	0	0	0	0	0	0	0	0	49
24578073	f	hp	FR	2024	0	0	0	0	0	0	0	0	0	0	0	0	54
24578258	m	md	JP	2024	0	1	0	0	0	1	0	0	0	0	0	0	70
24578269	m	hp	IT	2024	0	1	1	0	0	0	0	0	0	0	0	0	54
24578617	Not	md	RO	2024	0	1	0	1	0	0	0	0	0	0	0	0	
24579343	m	cn	TH	2024	0	1	0	0	0	1	0	0	0	0	0	0	79
24579655	m	md	JP	2024	0	1	1	0	0	0	0	0	0	0	0	1	73
24579875	m	md	JP	2024	0	0	0	0	0	0	0	0	0	0	0	0	
24580011	Not	md	FR	2024	0	1	0	0	0	1	0	0	0	0	0	0	
24580206	Not	ph	JP	2024	0	0	0	0	0	0	0	0	0	0	0	0	60
24580247	m	md	DE	2024	0	0	0	0	0	0	0	0	0	0	0	0	
24580475	f	md	US	2024	0	0	0	0	0	0	0	0	0	0	0	0	
24581248	m	cn	VN	2024	0	0	0	0	0	0	1	0	0	0	1	0	

24581662	f	ph	FR	2024	0	0	0	0	0	0	0	0	0	0	0	0	46
24581974	f	md	US	2024	0	0	0	0	0	0	0	0	0	0	0	0	57
24581976	m	md	US	2024	0	0	0	0	0	0	0	0	0	0	0	0	33
24582255	Not	md	JP	2024	0	1	0	1	0	0	0	0	0	0	0	0	
24582849	Not	md	JP	2024	0	1	0	1	0	0	0	0	0	0	0	0	65
24583089	Not	md	JP	2024	0	1	0	0	1	0	0	0	0	0	0	0	60
24583389	m	hp	US	2024	0	1	1	0	0	0	0	0	0	0	0	0	
24583437	f	md	DE	2024	0	1	1	0	0	0	0	0	0	0	0	0	
24583547	Not	Not repo	IN	2024	1	0	0	0	0	0	0	0	0	0	0	0	55
24583900	m	hp	US	2024	0	1	1	0	0	0	0	0	0	0	0	0	63
24583990	m	md	PT	2024	0	1	1	0	0	0	0	0	0	0	0	0	76
24584073	Not	md	CN	2024	1	0	0	0	0	0	0	0	0	0	0	0	46
24584390	m	hp	US	2024	0	1	1	0	0	0	0	0	0	0	0	0	63
24584400	Not	ph	JP	2024	1	0	0	0	0	0	0	0	0	0	0	0	
24584440	Not	md	US	2024	1	0	0	0	0	0	0	0	0	0	0	0	
24584826	f	md	US	2024	0	1	1	0	0	0	0	0	0	0	0	0	38
24585392	m	md	JP	2024	0	1	1	0	0	0	0	0	0	0	0	0	68
24585452	m	ph	JP	2024	0	0	0	0	0	0	0	0	0	0	0	0	64
24585510	m	md	FR	2024	0	1	1	0	0	0	0	0	0	0	0	0	55
24585732	m	md	JP	2024	0	1	1	0	0	0	0	0	0	0	0	0	72
24585839	m	md	JP	2024	0	0	0	0	0	0	0	0	0	0	0	0	70
24586057	m	md	NL	2024	0	1	1	0	0	0	0	0	0	0	0	0	
24586091	Not	Not repo	KE	2024	1	0	0	0	0	0	0	0	0	0	0	0	43
24586124	m	md	JP	2024	0	1	1	0	0	0	0	0	0	0	0	0	63
24586224	m	md	JP	2024	0	1	1	0	0	0	0	0	0	0	0	0	74
24586395	Not	md	JP	2024	0	1	0	0	1	0	0	0	0	0	0	0	
24586447	Not	md	JP	2024	0	1	0	0	1	0	0	0	0	0	0	0	
24587375	f	hp	IT	2024	1	0	0	0	0	0	0	0	0	0	0	0	71
24587400	Not	md	KZ	2024	0	1	0	0	0	1	0	0	0	0	0	0	
24587744	f	md	DE	2024	0	1	0	0	1	0	0	0	0	0	0	0	56
24588105	m	md	KR	2024	0	0	0	0	0	0	0	0	0	0	0	0	71
24588355	f	ph	US	2024	0	0	0	0	0	0	0	0	0	0	0	0	69
24588512	m	md	JP	2024	0	0	0	0	0	0	0	0	0	0	0	0	
24588840	m	md	DE	2024	0	1	0	0	1	0	0	0	0	0	0	0	71

24588878	f	md	CA	2024	0	0	0	0	0	0	0	0	0	0	0	0	53
24589927	f	md	JP	2024	0	1	0	0	0	1	0	0	0	0	0	0	85
24590122	f	md	RU	2024	0	1	1	0	0	0	0	0	0	0	0	0	65
24590254	f	md	US	2024	0	0	0	0	0	0	0	0	0	0	0	0	91
24590328	Not	md	JP	2024	0	1	0	1	0	0	0	0	0	0	0	0	
24590331	Not	md	JP	2024	1	0	0	0	0	0	0	0	0	0	0	0	55
24590660	Not	md	JP	2024	0	1	0	1	0	0	0	0	0	0	0	1	52
24590725	m	cn	DE	2024	0	1	0	0	1	0	0	0	0	0	0	0	71
24590833	Not	hp	CN	2024	0	0	0	0	0	0	0	0	0	0	0	0	
24590948	m	cn	US	2024	0	1	1	0	0	0	0	0	0	0	0	0	
24591438	m	hp	ZA	2024	0	0	0	0	0	0	0	0	0	0	0	0	67
24591455	f	md	DE	2024	0	1	0	0	1	0	0	0	0	0	0	0	78
24591502	m	md	DE	2024	0	1	0	0	1	0	0	0	0	0	0	0	77
24591545	m	md	JP	2024	0	1	0	0	0	1	0	0	0	0	0	0	90
24591675	f	cn	US	2024	0	0	0	0	0	0	0	0	0	0	0	0	66
24591677	m	md	DE	2024	0	1	0	0	1	0	0	0	0	0	0	0	59
24591706	m	hp	JP	2024	0	0	0	0	0	0	0	0	0	0	0	0	70
24591837	m	md	JP	2024	0	0	0	0	0	0	0	0	0	0	0	0	
24591878	m	md	JP	2024	0	1	0	0	0	1	0	0	0	0	0	0	71
24592061	m	md	BR	2024	0	0	0	0	0	0	0	0	0	0	0	0	67
24592342	m	md	JP	2024	0	0	0	0	0	0	0	0	0	0	0	0	68
24592467	f	Not repo	CA	2024	0	0	0	0	0	0	0	0	0	0	0	0	
24592496	f	hp	US	2024	0	0	0	0	0	0	0	0	0	0	0	0	43
24592529	f	md	BE	2024	0	0	0	0	0	0	0	0	0	0	0	0	68
24592546	m	md	JP	2024	0	1	1	0	0	0	0	0	0	0	0	0	60
24593008	Not	hp	IT	2024	0	0	0	0	0	0	0	0	0	0	0	0	
24593047	f	md	KR	2024	0	0	0	0	0	0	0	0	0	0	0	0	78
24593069	Not	hp	IT	2024	0	0	0	0	0	0	0	0	0	0	0	0	
24593109	Not	hp	IT	2024	0	0	0	0	0	0	0	0	0	0	0	0	
24593154	Not	hp	IT	2024	0	0	0	0	0	0	0	0	0	0	0	0	
24593200	m	hp	ES	2024	0	0	0	0	0	0	0	0	0	0	0	0	75
24593207	Not	hp	IT	2024	0	0	0	0	0	0	0	0	0	0	0	0	
24593229	Not	hp	IT	2024	0	0	0	0	0	0	0	0	0	0	0	0	
24593279	Not	hp	IT	2024	0	0	0	0	0	0	0	0	0	0	0	0	

24593565	m	md	JP	2024	0	0	0	0	0	0	0	0	0	0	0	0	72
24593615	Not	Not repo	CA	2024	1	0	0	0	0	0	0	0	0	0	0	0	
24593662	m	md	CH	2024	0	0	0	0	0	0	0	0	0	0	0	0	
24593835	m	md	FR	2024	0	1	1	0	0	0	0	0	0	0	0	0	55
24593868	m	cn	KR	2024	0	0	0	0	0	0	0	0	0	0	0	0	69
24594334	Not	Not repo	US	2024	1	0	0	0	0	0	0	0	0	0	0	0	
24594335	Not	Not repo	CA	2024	1	0	0	0	0	0	0	0	0	0	0	0	
24594603	m	cn	US	2024	0	0	0	0	0	0	0	0	0	0	0	0	62
24594932	f	cn	FR	2024	0	0	0	0	0	0	0	0	0	0	0	0	46
24594933	m	cn	JP	2024	0	0	0	0	0	0	0	0	0	0	0	0	73
24595002	m	md	JP	2024	0	1	0	0	1	0	0	0	0	0	0	0	52
24595106	m	md	JP	2024	0	1	0	0	1	0	0	0	0	0	0	0	74
24595158	Not	ph	US	2024	1	0	0	0	0	0	0	0	0	0	0	0	
24595299	Not	md	JP	2024	0	1	0	1	0	0	0	0	0	0	0	0	80
24595381	Not	md	US	2024	1	0	0	0	0	0	0	0	0	0	0	0	
24595574	Not	md	RO	2024	0	1	0	1	0	0	0	0	0	0	0	0	
24595579	Not	Not repo	IN	2024	0	1	0	1	0	0	0	0	0	0	0	0	
24595580	Not	md	RO	2024	0	1	0	1	0	0	0	0	0	0	0	0	
24595605	Not	md	AU	2024	1	0	0	0	0	0	0	0	0	0	0	0	
24595708	Not	md	JP	2024	0	1	0	1	0	0	0	0	0	0	0	0	65
24595965	Not	Not repo	CN	2024	1	0	0	0	0	0	0	0	0	0	0	0	
24596103	m	md	DE	2024	0	1	1	0	0	0	0	0	0	0	0	0	
24596182	m	md	JP	2024	0	1	0	0	0	1	0	0	0	0	0	0	70
24596245	Not	md	JP	2024	0	1	0	1	0	0	0	0	0	0	0	0	54
24596249	Not	md	JP	2024	1	0	0	0	0	0	0	0	0	0	0	0	70
24596262	Not	md	KR	2024	0	1	0	1	0	0	0	0	0	0	0	0	80
24596934	Not	Not repo	JP	2024	1	0	0	0	0	0	0	0	0	0	0	0	
24598965	Not	md	JP	2024	0	1	0	1	0	0	0	0	0	0	0	1	
24600521	Not	md	JP	2024	0	1	0	1	0	0	0	0	0	0	0	0	75
24601041	Not	md	US	2024	0	1	0	1	0	0	0	0	0	0	0	0	
24601098	m	md	JP	2024	0	1	1	0	0	0	0	0	0	0	0	0	78
24601152	Not	md	GB	2024	0	1	0	1	0	0	0	0	0	0	0	1	
24601264	Not	Not repo	US	2024	1	0	0	0	0	0	0	0	0	0	0	0	70
24601442	m	md	JP	2024	0	1	1	0	0	0	0	0	0	0	0	0	80

24601469	f	ph	FR	2024	0	0	0	0	0	0	0	0	0	0	0	0	
24601476	f	md	JP	2024	0	0	0	0	0	0	0	0	0	0	0	0	80
24601606	m	md	JP	2024	0	1	1	0	0	0	0	0	0	0	0	0	78
24601615	m	cn	AE	2024	0	1	0	0	0	1	0	0	0	0	0	0	50
24601728	f	cn	US	2024	0	0	0	0	0	0	0	0	0	0	0	0	
24601845	m	md	PL	2024	0	1	0	0	1	0	0	0	0	0	0	1	65
24601978	f	md	DE	2024	0	1	0	0	1	0	0	0	0	0	0	0	77
24602108	m	md	JP	2024	0	1	0	0	0	1	0	0	0	0	0	0	71
24602412	Not	md	GB	2024	0	0	0	0	0	0	1	0	0	0	1	0	
24602967	f	ph	FR	2024	0	0	0	0	0	0	0	0	0	0	0	0	46
24603071	Not	md	JP	2024	0	1	0	0	1	0	0	0	0	0	0	0	70
24603218	m	md	JP	2024	0	1	1	0	0	0	0	0	0	0	0	0	70
24603379	m	md	FR	2024	0	1	1	0	0	0	0	0	0	0	0	0	55
24603527	m	hp	JP	2024	0	1	0	0	1	0	0	0	0	0	0	0	72
24603570	Not	md	RO	2024	0	1	1	0	0	0	0	0	0	0	0	0	62
24603713	m	md	DE	2024	0	1	0	0	1	0	0	0	0	0	0	0	57
24603937	m	md	US	2024	0	0	0	0	0	0	0	0	0	0	0	0	72
24603978	Not	Not repo	TH	2024	0	1	0	1	0	0	0	0	0	0	0	0	56
24604821	m	md	JP	2024	0	1	1	0	0	0	0	0	0	0	0	0	77
24604842	f	md	DE	2024	0	1	0	0	1	0	0	0	0	0	0	0	63
24604877	m	md	JP	2024	0	1	1	0	0	0	0	0	0	0	0	0	81
24605705	m	md	JP	2024	0	0	0	0	0	0	0	0	0	0	0	0	76
24605811	m	hp	US	2024	0	0	0	0	0	0	0	0	0	0	0	0	
24605868	m	md	JP	2024	0	1	0	0	1	0	0	0	0	0	0	0	64
24606284	Not	md	JP	2024	1	0	0	0	0	0	0	0	0	0	0	0	56
24606466	Not	md	US	2024	0	0	0	0	0	0	0	0	0	0	0	0	
24606653	m	md	CN	2024	0	0	0	0	0	0	0	0	0	0	0	0	60
24606722	m	md	DE	2024	0	1	1	0	0	0	0	0	0	0	0	0	
24606731	f	hp	US	2024	0	0	0	0	0	0	0	0	0	0	0	0	
24606732	f	md	CH	2024	0	0	0	0	0	0	0	0	0	0	0	0	80
24607081	m	hp	US	2024	0	0	0	0	0	0	0	0	0	0	0	0	68
24607660	m	md	ES	2024	0	0	0	0	0	0	0	0	0	0	0	0	
24607719	Not	md	JP	2024	0	1	0	1	0	0	0	0	0	0	0	0	57
24607720	Not	md	JP	2024	0	1	0	1	0	0	0	0	0	0	0	0	64

24607736	Not	Not repo	US	2024	1	0	0	0	0	0	0	0	0	0	0	0	54
24607961	m	md	VN	2024	0	1	0	0	0	1	0	0	0	0	0	0	60
24608088	Not	md	JP	2024	0	0	0	0	0	0	0	0	0	0	0	0	
24608221	m	md	CA	2024	0	0	0	0	0	0	0	0	0	0	0	0	65
24608313	Not	ph	JP	2024	0	0	0	0	0	0	0	0	0	0	0	0	
24608315	m	md	JP	2024	0	1	1	0	0	0	0	0	0	0	0	0	86
24608316	Not	ph	JP	2024	1	0	0	0	0	0	0	0	0	0	0	0	
24608318	Not	md	JP	2024	0	1	0	1	0	0	0	0	0	0	0	0	73
24608353	m	md	ES	2024	0	1	1	0	0	0	0	0	0	0	0	0	77
24608357	m	md	JP	2024	0	1	1	0	0	0	0	0	0	0	0	0	86
24608367	m	md	DE	2024	0	0	0	0	0	0	0	0	0	0	0	0	49
24608372	m	ph	US	2024	0	0	0	0	0	0	0	0	0	0	0	0	
24608511	f	md	JP	2024	0	1	0	0	0	1	0	0	0	0	0	0	
24608783	m	cn	FR	2024	0	1	1	0	0	0	0	0	0	0	0	0	55
24608811	f	ph	FR	2024	0	0	0	0	0	0	0	0	0	0	0	0	46
24608969	f	ph	FR	2024	0	0	0	0	0	0	0	0	0	0	0	0	46
24609401	f	md	JP	2024	0	1	0	1	0	0	0	0	0	0	0	0	49
24609821	f	Not repo	BR	2024	0	0	0	0	0	0	0	0	0	0	0	0	
24610220	f	hp	HR	2024	0	1	0	0	1	0	0	0	0	0	0	0	63
24610244	Not	md	IT	2024	1	0	0	0	0	0	0	0	0	0	0	0	
24610889	Not	md	JP	2024	0	1	0	0	1	0	0	0	0	0	0	0	
24610922	Not	md	RO	2024	0	1	0	1	0	0	0	0	0	0	0	0	
24610932	Not	md	RO	2024	1	0	0	0	0	0	0	0	0	0	0	0	
24611018	Not	md	US	2024	1	0	0	0	0	0	0	0	0	0	0	0	91
24611477	f	cn	PK	2024	0	1	0	0	0	1	0	0	0	0	0	0	57
24611593	f	hp	US	2024	0	1	0	0	1	0	0	0	0	0	0	0	59
24611926	m	cn	TH	2024	0	1	0	0	0	1	0	0	0	0	0	0	69
24612042	m	md	JP	2024	0	1	0	0	0	1	0	0	0	0	0	0	80
24612049	m	md	JP	2024	0	1	1	0	0	0	0	0	0	0	0	0	63
24612164	f	md	JP	2024	0	0	0	0	0	0	0	0	0	0	0	0	22
24612272	m	md	JP	2024	0	1	1	0	0	0	0	0	0	0	0	0	71
24612275	Not	hp	CN	2024	0	0	0	0	0	0	0	0	0	0	0	0	
24612536	Not	md	JP	2024	0	1	0	0	1	0	0	0	0	0	0	0	
24612617	f	md	AU	2024	0	0	0	0	0	0	0	0	0	0	0	0	49

24612952	Not	Not repo	CA	2024	1	0	0	0	0	0	0	0	0	0	0	0	
24613050	f	hp	IT	2024	0	0	0	0	0	0	0	0	0	0	0	0	40
24613157	f	md	FR	2024	0	0	0	0	0	0	0	0	0	0	0	0	67
24613223	Not	md	RO	2024	0	1	1	0	0	0	0	0	0	0	0	0	62
24613677	Not	Not repo	US	2024	1	0	0	0	0	0	0	0	0	0	0	0	
24613726	m	md	NL	2024	0	1	0	0	1	0	0	0	0	0	0	0	
24614230	Not	md	RO	2024	0	1	1	0	0	0	0	0	0	0	0	0	62
24614371	f	md	VN	2024	0	0	0	0	0	0	1	0	0	0	1	0	
24614570	m	md	TW	2024	0	0	0	0	0	0	0	0	0	0	0	0	65
24614594	m	ph	FR	2024	0	0	0	0	0	0	0	0	0	0	0	0	80
24614899	m	md	JP	2024	0	0	0	0	0	0	0	0	0	0	0	0	
24614915	Not	md	AU	2024	1	0	0	0	0	0	0	0	0	0	0	0	
24615080	f	md	FR	2024	0	1	1	0	0	0	0	0	0	0	0	0	47
24615866	Not	md	JP	2024	0	1	0	0	1	0	0	0	0	0	0	0	
24615913	Not	hp	CN	2024	0	0	0	0	0	0	0	0	0	0	0	0	
24616041	Not	md	JP	2024	0	1	0	0	1	0	0	0	0	0	0	0	70
24616582	m	cn	PK	2024	0	1	0	0	0	1	0	0	0	0	0	0	72
24616934	m	md	JP	2024	0	0	0	0	0	0	0	0	0	0	0	0	85
24617053	Not	md	DE	2024	0	1	0	0	1	0	0	0	0	0	0	0	
24617168	Not	md	JP	2024	0	1	1	0	0	0	0	0	0	0	0	1	
24617305	f	hp	IT	2024	0	1	0	0	1	0	0	0	0	0	0	0	40
24617413	m	md	JP	2024	0	1	1	0	0	0	0	0	0	0	0	0	79
24617632	f	md	DE	2024	0	1	0	0	0	1	0	0	0	0	0	0	64
24617813	f	md	SK	2024	0	1	0	0	0	1	0	0	0	0	0	0	
24617862	m	hp	ES	2024	0	0	0	0	0	0	0	0	0	0	0	0	75
24617908	Not	Not repo	US	2024	1	0	0	0	0	0	0	0	0	0	0	0	
24618154	m	cn	PK	2024	0	1	0	0	0	1	0	0	0	0	0	0	89
24618446	m	md	JP	2024	0	1	0	0	0	1	0	0	0	0	0	0	70
24618746	Not	md	JP	2024	0	0	0	0	0	0	0	0	0	0	0	0	
24618789	m	md	ES	2024	0	0	0	0	0	0	0	0	0	0	0	0	
24618853	m	md	FR	2024	0	1	0	0	0	1	0	0	0	0	0	0	68
24618864	m	md	CA	2024	0	0	0	0	0	0	0	0	0	0	0	0	62
24618952	m	cn	US	2024	0	1	1	0	0	0	0	0	0	0	0	1	
24619199	f	hp	US	2024	0	0	0	0	0	0	0	0	0	0	0	0	44

24619377	Not	md	JP	2024	1	0	0	0	0	0	0	0	0	0	0	0	69
24619479	m	md	FR	2024	0	1	1	0	0	0	0	0	0	0	0	0	55
24620519	Not	ph	US	2024	0	0	0	0	0	0	0	0	0	0	0	0	
24620742	f	md	US	2024	0	0	0	0	0	0	0	0	0	0	0	0	
24620749	m	md	FR	2024	0	1	0	0	0	1	0	0	0	0	0	0	68
24620756	Not	md	JP	2024	1	0	0	0	0	0	0	0	0	0	0	0	70
24620782	Not	md	JP	2024	0	1	0	1	0	0	0	0	0	0	0	0	44
24620785	f	ph	FR	2024	0	0	0	0	0	0	0	0	0	0	0	0	79
24620955	m	ph	ES	2024	0	1	1	0	0	0	0	0	0	0	0	0	63
24620983	f	hp	US	2024	0	1	1	0	0	0	0	0	0	0	0	0	
24621102	Not	ph	US	2024	0	0	0	0	0	0	0	0	0	0	0	0	
24621137	m	hp	US	2024	1	0	0	0	0	0	0	0	0	0	0	0	57
24621391	f	md	ES	2024	0	1	1	0	0	0	0	0	0	0	0	0	71
24621556	m	cn	CN	2024	0	0	0	0	0	0	0	0	0	0	0	0	67
24621557	f	cn	JP	2024	0	0	0	0	0	0	0	0	0	0	0	0	66
24621980	m	md	JP	2024	0	1	1	0	0	0	0	0	0	0	0	0	66
24622618	m	md	JP	2024	0	1	0	1	0	0	0	0	0	0	0	0	44
24622682	Not	Not repo	CA	2024	1	0	0	0	0	0	0	0	0	0	0	0	
24622822	m	md	JP	2024	0	1	1	0	0	0	0	0	0	0	0	0	
24622827	m	md	AT	2024	0	1	0	0	1	0	0	0	0	0	0	0	86
24622892	m	md	CN	2024	0	1	0	0	0	1	0	0	0	0	0	0	59
24623123	m	md	DE	2024	0	1	1	0	0	0	0	0	0	0	0	0	
24623131	Not	md	JP	2024	0	1	0	1	0	0	0	0	0	0	0	0	74
24623141	m	md	FR	2024	0	1	1	0	0	0	0	0	0	0	0	0	55
24623364	Not	md	JP	2024	0	1	0	0	0	1	0	0	0	0	0	0	
24623721	Not	md	JP	2024	1	0	0	0	0	0	0	0	0	0	0	0	
24623752	Not	md	JP	2024	0	1	0	1	0	0	0	0	0	0	0	0	
24624077	m	hp	CA	2024	0	1	0	0	0	1	0	0	0	0	0	0	60
24624177	f	hp	GB	2024	0	0	0	0	0	0	0	0	0	0	0	0	29
24624187	m	md	UA	2024	0	1	0	0	0	1	0	0	0	0	0	0	
24624281	Not	ph	JP	2024	0	1	0	1	0	0	0	0	0	0	0	0	58
24624285	Not	md	JP	2024	0	1	0	1	0	0	0	0	0	0	0	0	50
24624441	f	md	GR	2024	0	1	1	0	0	0	0	0	0	0	0	0	55
24624573	Not	md	JP	2024	1	0	0	0	0	0	0	0	0	0	0	0	

24624594	Not	md	JP	2024	1	0	0	0	0	0	0	0	0	0	0	0	
24624686	Not	md	JP	2024	1	0	0	0	0	0	0	0	0	0	0	0	82
24624731	m	md	DE	2024	0	1	0	0	1	0	0	0	0	0	0	0	71
24624766	Not	md	VN	2024	0	0	0	0	0	0	0	0	0	0	0	0	
24624792	Not	md	RO	2024	0	1	1	0	0	0	0	0	0	0	0	0	62
24624890	f	ph	FR	2024	0	0	0	0	0	0	0	0	0	0	0	0	79
24624901	Not	md	JP	2024	0	1	0	1	0	0	0	0	0	0	0	0	75
24625311	Not	Not repo	NZ	2024	1	0	0	0	0	0	0	0	0	0	0	0	
24625341	Not	md	AU	2024	0	1	0	0	1	0	0	0	0	0	0	1	63
24625453	m	md	DE	2024	0	1	0	0	1	0	0	0	0	0	0	0	57
24625592	m	md	JP	2024	0	1	1	0	0	0	0	0	0	0	0	1	64
24626107	f	ph	FR	2024	0	0	0	0	0	0	0	0	0	0	0	0	46
24626158	m	md	GR	2024	0	0	0	0	0	0	0	0	0	0	0	0	68
24627202	f	md	DE	2024	0	1	0	0	1	0	0	0	0	0	0	0	77
24627455	f	md	DE	2024	0	1	0	0	1	0	0	0	0	0	0	0	78
24627698	Not	md	JP	2024	1	0	0	0	0	0	0	0	0	0	0	1	80
24627701	Not	Not repo	JP	2024	1	0	0	0	0	0	0	0	0	0	0	0	
24627713	Not	md	JP	2024	0	1	0	1	0	0	0	0	0	0	0	0	74
24627714	Not	md	JP	2024	0	1	0	1	0	0	0	0	0	0	0	0	70
24627735	Not	md	JP	2024	0	1	0	1	0	0	0	0	0	0	0	0	63
24627762	m	md	JP	2024	0	1	1	0	0	0	0	0	0	0	0	0	71
24627895	Not	Not repo	AE	2024	1	0	0	0	0	0	0	0	0	0	0	0	
24627984	Not	Not repo	IN	2024	1	0	0	0	0	0	0	0	0	0	0	0	54
24628011	Not	Not repo	MY	2024	1	0	0	0	0	0	0	0	0	0	0	0	
24628288	Not	md	JP	2024	0	1	0	1	0	0	0	0	0	0	0	0	69
24628321	Not	md	DE	2024	1	0	0	0	0	0	0	0	0	0	0	0	60
24628338	m	md	DE	2024	0	1	1	0	0	0	0	0	0	0	0	0	
24628350	Not	md	JP	2024	0	1	0	1	0	0	0	0	0	0	0	0	78
24628586	Not	hp	CN	2024	0	0	0	0	0	0	0	0	0	0	0	0	
24629137	Not	md	RO	2024	0	1	0	1	0	0	0	0	0	0	0	0	
24629191	f	md	DE	2024	0	1	0	0	1	0	0	0	0	0	0	0	63
24629768	Not	md	IL	2024	0	0	0	0	0	0	0	0	0	0	0	0	
24629893	Not	md	US	2024	0	0	0	0	0	0	0	0	0	0	0	0	
24629944	f	md	JP	2024	0	0	0	0	0	0	0	0	0	0	0	0	65

24630195	m	md	JP	2024	0	1	0	0	0	1	0	0	0	0	0	0	65
24630342	f	cn	CN	2024	1	0	0	0	0	0	0	0	0	0	0	0	77
24630927	Not	md	JP	2024	0	1	0	1	0	0	0	0	0	0	0	0	60
24630958	Not	md	JP	2024	0	1	0	1	0	0	0	0	0	0	0	0	
24631003	Not	Not repo	CN	2024	1	0	0	0	0	0	0	0	0	0	0	0	
24631097	Not	md	JP	2024	1	0	0	0	0	0	0	0	0	0	0	0	60
24631149	f	md	US	2024	0	1	0	1	0	0	0	0	0	0	0	0	43
24631150	f	md	US	2024	0	1	0	0	1	0	0	0	0	0	0	0	59
24631449	f	md	JP	2024	0	0	0	0	0	0	0	0	0	0	0	0	73
24631488	m	cn	JP	2024	0	0	0	0	0	0	0	0	0	0	0	0	
24631646	m	md	VN	2024	0	1	0	0	0	1	0	0	0	0	0	0	60
24631683	Not	md	JP	2024	0	1	0	1	0	0	0	0	0	0	0	0	48
24632147	Not	md	JP	2024	0	1	0	0	1	0	0	0	0	0	0	0	
24632428	m	md	BR	2024	0	1	0	0	1	0	0	0	0	0	0	0	69
24632458	f	md	US	2024	0	0	0	0	0	0	0	0	0	0	0	0	40
24632606	m	md	FR	2024	0	0	0	0	0	0	0	0	0	0	0	0	63
24633108	f	md	GR	2024	0	0	0	0	0	0	0	0	0	0	0	0	65
24633658	f	md	JP	2024	0	1	0	0	1	0	0	0	0	0	0	0	77
24633765	m	md	US	2024	0	0	0	0	0	0	0	0	0	0	0	0	67
24633868	m	md	KR	2024	0	0	0	0	0	0	0	0	0	0	0	0	
24633990	Not	md	JP	2024	0	1	1	0	0	0	0	0	0	0	0	0	77
24634536	m	hp	ES	2024	0	0	0	0	0	0	0	0	0	0	0	0	75
24634562	f	ph	IT	2024	0	0	0	0	0	0	0	0	0	0	0	0	54
24634705	f	md	JP	2024	0	0	0	0	0	0	0	0	0	0	0	0	70
24634732	m	md	DE	2024	0	1	1	0	0	0	0	0	0	0	0	0	
24634914	Not	md	JP	2024	1	0	0	0	0	0	0	0	0	0	0	0	70
24636542	Not	hp	CN	2024	0	0	0	0	0	0	0	0	0	0	0	0	
24636544	m	md	JP	2024	0	1	0	0	0	1	0	0	0	0	0	0	80
24636599	m	hp	JP	2024	0	1	1	0	0	0	0	0	0	0	0	0	70
24636902	m	ph	PK	2024	0	1	0	0	0	1	0	0	0	0	0	0	73
24637230	f	md	MX	2024	0	0	0	0	0	0	0	0	0	0	0	0	
24637246	m	hp	ES	2024	0	0	0	0	0	0	0	0	0	0	0	0	75
24637402	Not	md	JP	2024	0	1	0	1	0	0	0	0	0	0	0	0	76
24638179	Not	md	JP	2024	0	1	0	1	0	0	0	0	0	0	0	0	70

24638289	m	cn	US	2024	0	1	1	0	0	0	0	0	0	0	0	0	78
24638481	f	hp	JP	2024	0	0	0	0	0	0	0	0	0	0	0	0	65
24638782	f	md	DE	2024	0	0	0	0	0	0	0	0	0	0	0	0	76
24638794	m	md	FR	2024	0	1	1	0	0	0	0	0	0	0	0	0	55
24638824	m	md	TR	2024	0	1	1	0	0	0	0	0	0	0	0	0	65
24639450	m	md	JP	2024	0	1	0	0	1	0	0	0	0	0	0	0	75
24640097	Not	Not repo	US	2024	1	0	0	0	0	0	0	0	0	0	0	0	91
24640179	Not	Not repo	US	2024	0	0	0	0	0	0	0	0	0	0	0	0	63
24640348	Not	md	JP	2024	1	0	0	0	0	0	0	0	0	0	0	0	60
24640378	Not	md	JP	2024	0	1	0	1	0	0	0	0	0	0	0	0	60
24640393	Not	ph	JP	2024	1	0	0	0	0	0	0	0	0	0	0	0	72
24641090	Not	md	JP	2024	0	0	0	0	0	0	0	0	0	0	0	0	68
24641130	f	md	US	2024	0	0	0	0	0	0	0	0	0	0	0	0	65
24641174	Not	Not repo	US	2024	1	0	0	0	0	0	0	0	0	0	0	0	
24641402	m	md	SI	2024	0	1	1	0	0	0	0	0	0	0	0	0	69
24642007	f	md	JP	2024	0	1	1	0	0	0	0	0	0	0	0	0	74
24642157	f	md	FR	2024	0	0	0	0	0	0	0	0	0	0	0	0	70
24642184	m	cn	Not re	2024	0	0	0	0	0	0	0	0	0	0	0	0	44
24642185	m	cn	Not re	2024	0	0	0	0	0	0	0	0	0	0	0	0	67
24642218	m	hp	JP	2024	0	1	0	0	1	0	0	0	0	0	0	1	72
24642416	f	md	NL	2024	0	1	1	0	0	0	0	0	0	0	0	0	63
24642439	m	hp	GB	2024	0	0	0	0	0	0	0	0	0	0	0	0	
24642551	Not	ph	JP	2024	0	0	0	0	0	0	0	0	0	0	0	0	75
24642841	m	md	NP	2024	0	0	0	0	0	0	0	0	0	0	0	0	42
24642851	f	md	NP	2024	0	0	0	0	0	0	0	0	0	0	0	0	63
24642855	m	md	NP	2024	0	0	0	0	0	0	0	0	0	0	0	0	36
24642860	m	md	DE	2024	0	0	0	0	0	0	0	0	0	0	0	0	
24642927	m	md	JP	2024	0	1	0	0	1	0	0	0	0	0	0	0	75
24643747	m	md	AT	2024	0	0	0	0	0	0	0	0	0	0	0	0	
24643955	m	cn	US	2024	0	1	1	0	0	0	0	0	0	0	0	0	78
24644037	m	hp	NL	2024	0	0	0	0	0	0	0	0	0	0	0	0	81
24645100	m	md	JP	2024	0	1	0	0	1	0	0	0	0	0	0	0	80
24645312	Not	md	JP	2024	0	1	0	1	0	0	0	0	0	0	0	0	66
24645589	m	hp	AT	2024	0	0	0	0	0	0	0	0	0	0	0	0	55

24645632	f	md	DE	2024	0	1	0	0	1	0	0	0	0	0	0	0	61
24645781	m	md	US	2024	0	0	0	0	0	0	0	0	0	0	0	0	70
24645834	Not	md	JP	2024	0	1	0	1	0	0	0	0	0	0	0	0	
24645842	f	md	DE	2024	0	1	0	0	1	0	0	0	0	0	0	0	72
24646265	Not	hp	US	2024	0	1	0	0	0	1	0	0	0	0	0	0	
24646316	f	md	AT	2024	0	0	0	0	0	0	0	0	0	0	0	0	41
24646349	m	ph	JP	2024	0	0	0	0	0	0	0	0	0	0	0	0	100
24647179	Not	Not repo	EG	2024	1	0	0	0	0	0	0	0	0	0	0	0	67
24647262	m	md	JP	2024	0	1	0	0	0	1	0	0	0	0	0	0	69
24647427	m	hp	ES	2024	0	0	0	0	0	0	0	0	0	0	0	0	75
24647508	m	md	US	2024	0	1	1	0	0	0	0	0	0	0	0	0	63
24647730	f	cn	US	2024	0	0	0	0	0	0	0	0	0	0	0	0	82
24648468	f	md	JP	2024	0	0	0	0	0	0	0	0	0	0	0	0	65
24648661	f	md	ES	2024	0	1	1	0	0	0	0	0	0	0	0	0	71
24648796	m	md	FR	2024	0	1	0	0	0	1	0	0	0	0	0	0	68
24648808	m	ph	US	2024	0	1	1	0	0	0	0	0	0	0	0	0	63
24648934	f	md	FR	2024	0	0	0	0	0	0	0	0	0	0	0	0	67
24650356	f	hp	US	2024	0	0	0	0	0	0	0	0	0	0	0	0	
24650375	m	md	PL	2024	0	1	0	0	0	1	0	0	0	0	0	0	69
24650558	m	md	DE	2024	0	1	1	0	0	0	0	0	0	0	0	0	66
24650598	Not	md	CH	2024	0	1	0	0	0	1	0	0	0	0	0	0	
24650654	m	md	IL	2024	0	1	1	0	0	0	0	0	0	0	0	0	54
24650985	m	md	JP	2024	0	1	0	0	1	0	0	0	0	0	0	0	74
24651156	m	cn	IN	2024	0	1	0	0	0	1	0	0	0	0	0	0	70
24651680	f	cn	DE	2024	0	1	1	0	0	0	0	0	0	0	0	0	49
24651748	Not	hp	AU	2024	0	1	0	0	0	1	0	0	0	0	0	0	
24651888	m	ph	JP	2024	0	0	0	0	0	0	0	0	0	0	0	0	70
24652104	Not	md	JP	2024	1	0	0	0	0	0	0	0	0	0	0	0	
24652234	Not	md	CN	2024	1	0	0	0	0	0	0	0	0	0	0	0	65
24652550	m	md	JP	2024	0	1	0	1	0	0	0	0	0	0	0	0	44
24652629	m	ph	CA	2024	0	1	0	0	1	0	0	0	0	0	0	0	68
24652849	f	md	FR	2024	0	0	0	0	0	0	0	0	0	0	0	0	51
24652934	m	cn	ES	2024	0	0	0	0	0	0	0	0	0	0	0	0	75
24652944	m	cn	JP	2024	0	0	0	0	0	0	0	0	0	0	0	0	65

24653219	Not	md	JP	2024	0	1	0	1	0	0	0	0	0	0	0	0	77
24653251	m	ph	CA	2024	0	1	0	0	1	0	0	0	0	0	0	0	68
24653411	Not	md	FR	2024	0	0	0	0	0	0	0	0	0	0	0	0	
24653702	Not	md	GB	2024	0	1	0	1	0	0	0	0	0	0	0	0	
24654177	Not	md	JP	2024	0	1	0	1	0	0	0	0	0	0	0	0	
24654187	Not	md	BR	2024	1	0	0	0	0	0	0	0	0	0	0	0	
24654348	f	md	DE	2024	0	1	0	0	1	0	0	0	0	0	0	0	57
24654602	f	cn	CA	2024	0	0	0	0	0	0	0	0	0	0	0	0	62
24654612	f	md	FR	2024	0	0	0	0	0	0	0	0	0	0	0	0	
24654885	m	md	FR	2024	0	0	0	0	0	0	0	0	0	0	0	0	
24654931	Not	md	JP	2024	0	1	0	1	0	0	0	0	0	0	0	0	60
24655068	Not	md	JP	2024	0	1	0	1	0	0	0	0	0	0	0	0	74
24655100	Not	Not repo	IN	2024	1	0	0	0	0	0	0	0	0	0	0	0	52
24655149	Not	md	US	2024	1	0	0	0	0	0	0	0	0	0	0	0	74
24655155	Not	md	JP	2024	0	1	0	1	0	0	0	0	0	0	0	0	63
24655186	Not	md	IT	2024	1	0	0	0	0	0	0	0	0	0	0	0	69
24655230	Not	md	JP	2024	0	1	0	1	0	0	0	0	0	0	0	0	
24656715	Not	md	JP	2024	0	1	1	0	0	0	0	0	0	0	0	0	80
24657018	m	hp	CN	2024	0	0	0	0	0	0	0	0	0	0	0	0	63
24657125	m	cn	PK	2024	0	1	0	0	0	1	0	0	0	0	0	0	68
24657382	m	cn	VN	2024	0	0	0	0	0	0	0	0	0	0	0	0	65
24657549	Not	md	JP	2024	0	1	0	1	0	0	0	0	0	0	0	0	45
24657680	Not	Not repo	US	2024	1	0	0	0	0	0	0	0	0	0	0	0	
24657879	f	md	JP	2024	0	0	0	0	0	0	0	0	0	0	0	0	70
24657983	f	md	PK	2024	0	1	0	0	0	1	0	0	0	0	0	0	81
24658166	m	md	JP	2024	0	1	0	0	1	0	0	0	0	0	0	0	78
24658225	m	md	JP	2024	0	1	0	0	1	0	0	0	0	0	0	0	62
24658525	m	md	JP	2024	0	1	0	0	0	1	0	0	0	0	0	0	69
24658628	Not	md	JP	2024	0	1	0	0	1	0	0	0	0	0	0	0	
24658713	m	ph	FR	2024	0	0	0	0	0	0	0	0	0	0	0	0	65
24658962	m	cn	TH	2024	0	0	0	0	0	0	0	0	0	0	0	0	78
24659021	Not	md	JP	2024	0	1	0	1	0	0	0	0	0	0	0	0	71
24659139	m	md	US	2024	0	0	0	0	0	0	0	0	0	0	0	0	57
24659228	m	md	JP	2024	0	1	0	0	1	0	0	0	0	0	0	0	61

24659471	f	md	TW	2024	0	1	1	0	0	0	0	0	0	0	0	0	71
24659614	m	md	DE	2024	0	1	1	0	0	0	0	0	0	0	0	0	66
24659629	m	md	NL	2024	0	1	0	0	0	1	0	0	0	0	0	0	68
24660346	m	md	DE	2024	0	0	0	0	0	0	0	0	0	0	0	0	
24660606	m	cn	Not re	2024	0	0	0	0	0	0	0	0	0	0	0	0	69
24661383	m	hp	US	2024	0	1	0	0	1	0	0	0	0	0	0	0	82
24661493	m	md	JP	2024	0	1	1	0	0	0	0	0	0	0	0	0	80
24661495	m	md	JP	2024	0	1	1	0	0	0	0	0	0	0	0	0	80
24663133	f	hp	NL	2024	0	1	1	0	0	0	0	0	0	0	0	0	61
24663235	m	md	DE	2024	1	0	0	0	0	0	0	0	0	0	0	0	47
24663444	Not	hp	TW	2024	1	0	0	0	0	0	0	0	0	0	0	0	
24663530	f	hp	PK	2024	0	1	0	0	0	1	0	0	0	0	0	0	81
24663580	Not	md	NI	2024	0	1	0	0	0	1	0	0	0	0	0	0	
24663715	m	md	JP	2024	0	1	0	0	1	0	0	0	0	0	0	0	64
24664115	m	hp	AT	2024	0	0	0	0	0	0	0	0	0	0	0	0	55
24664133	f	cn	CN	2024	1	0	0	0	0	0	0	0	0	0	0	1	56
24664142	m	cn	AE	2024	0	0	0	0	0	0	0	0	0	0	0	0	73
24664204	m	md	US	2024	0	0	0	0	0	0	0	0	0	0	0	0	72
24664274	f	md	NL	2024	0	1	1	0	0	0	0	0	0	0	0	0	63
24664461	m	md	AU	2024	0	1	1	0	0	0	0	0	0	0	0	0	59
24664615	Not	md	DE	2024	0	1	0	0	1	0	0	0	0	0	0	1	
24664683	Not	md	JP	2024	1	0	0	0	0	0	0	0	0	0	0	0	80
24664730	f	hp	ES	2024	0	0	0	0	0	0	0	0	0	0	0	0	63
24665011	m	hp	CA	2024	0	0	0	0	0	0	0	0	0	0	0	0	87
24665237	f	md	JP	2024	0	1	1	0	0	0	0	0	0	0	0	0	70
24665335	m	md	AU	2024	0	1	1	0	0	0	0	0	0	0	0	0	64
24665523	m	md	AT	2024	0	1	0	0	1	0	0	0	0	0	0	0	86
24665550	m	md	AT	2024	0	1	0	0	1	0	0	0	0	0	0	0	86
24665668	f	cn	US	2024	0	0	0	0	0	0	0	0	0	0	0	0	62
24665671	m	cn	US	2024	0	0	0	0	0	0	0	0	0	0	0	0	56
24665805	m	cn	JP	2024	1	0	0	0	0	0	0	0	0	0	0	1	65
24665806	f	md	JP	2024	1	0	0	0	0	0	0	0	0	0	0	0	72
24665807	m	md	JP	2024	1	0	0	0	0	0	0	0	0	0	0	0	63
24665855	Not	md	DE	2024	1	0	0	0	0	0	0	0	0	0	0	0	44

24666011	Not	md	BG	2024	0	0	0	0	0	0	0	0	0	0	0	0	
24666184	f	md	JP	2024	0	1	1	0	0	0	0	0	0	0	0	0	70
24666194	m	md	DE	2024	0	1	0	0	1	0	0	0	0	0	0	0	77
24667056	m	cn	EG	2024	0	1	1	0	0	0	0	0	0	0	0	0	
24667123	f	md	GR	2024	0	0	0	0	0	0	0	0	0	0	0	0	
24667411	m	hp	US	2024	0	1	1	0	0	0	0	0	0	0	0	0	68
24667518	Not	md	US	2024	0	0	0	0	0	0	0	0	0	0	0	0	
24667548	m	md	PL	2024	0	0	0	0	0	0	0	0	0	0	0	0	61
24667651	m	md	DE	2024	0	0	0	0	0	0	0	0	0	0	0	0	
24667684	f	md	FR	2024	0	0	0	0	0	0	0	0	0	0	0	0	41
24667903	m	md	NL	2024	0	1	0	0	1	0	0	0	0	0	0	0	68
24668189	m	md	DE	2024	0	1	1	0	0	0	0	0	0	0	0	1	75
24668224	m	md	DE	2024	0	1	1	0	0	0	0	0	0	0	0	0	
24668295	f	ph	JP	2024	0	0	0	0	0	0	0	0	0	0	0	0	70
24668358	m	md	IT	2024	0	1	0	0	1	0	0	0	0	0	0	0	79
24668419	f	md	JP	2024	0	1	0	0	0	1	0	0	0	0	0	0	70
24668523	f	cn	IN	2024	0	1	0	0	0	1	0	0	0	0	0	0	58
24668595	m	ph	CA	2024	0	1	0	0	1	0	0	0	0	0	0	0	68
24668637	Not	hp	JP	2024	0	1	0	0	0	1	0	0	0	0	0	0	
24669222	Not	hp	CN	2024	0	0	0	0	0	0	0	0	0	0	0	0	
24669452	f	md	SE	2024	0	1	0	0	0	1	0	0	0	0	0	0	68
24669950	f	md	FR	2024	0	0	0	0	0	0	0	0	0	0	0	0	51
24669986	f	ph	FR	2024	0	0	0	0	0	0	0	0	0	0	0	0	46
24670540	m	cn	US	2024	0	0	0	0	0	0	0	0	0	0	0	0	
24670548	f	cn	US	2024	0	0	0	0	0	0	0	0	0	0	0	0	
24670550	f	cn	US	2024	0	0	0	0	0	0	0	0	0	0	0	0	
24670805	Not	md	JP	2024	1	0	0	0	0	0	0	0	0	0	0	1	
24670911	Not	md	JP	2024	1	0	0	0	0	0	0	0	0	0	0	0	63
24671024	f	md	DE	2024	0	1	0	0	1	0	0	0	0	0	0	0	69
24671748	m	md	JP	2024	0	1	1	0	0	0	0	0	0	0	0	0	70
24671977	f	md	US	2024	0	0	0	0	0	0	0	0	0	0	0	0	
24672022	f	md	JP	2024	0	0	0	0	0	0	0	0	0	0	0	0	80
24672133	f	md	JP	2024	0	0	0	0	0	0	0	0	0	0	0	0	74
24672715	m	hp	PL	2024	0	1	0	0	1	0	0	0	0	0	0	0	69

24673008	m	cn	IN	2024	0	0	0	0	0	0	0	0	0	0	0	0	64
24673120	f	md	DE	2024	0	1	0	0	1	0	0	0	0	0	0	0	72
24674324	Not	md	US	2024	0	1	0	1	0	0	0	0	0	0	0	0	59
24674708	f	md	FR	2024	0	0	0	0	0	0	0	0	0	0	0	0	51
24675021	Not	md	DE	2024	0	0	0	0	0	0	0	0	0	0	0	0	
24675151	f	md	AT	2024	0	0	0	0	0	0	0	0	0	0	0	0	76
24675204	f	md	SI	2024	0	1	1	0	0	0	0	0	0	0	0	0	
24675484	f	md	JP	2024	0	0	0	0	0	0	0	0	0	0	0	0	
24675541	f	md	QA	2024	0	0	0	0	0	0	0	0	0	0	0	0	52
24675848	m	hp	US	2024	0	1	1	0	0	0	0	0	0	0	0	0	74
24676649	Not	ph	US	2024	0	0	0	0	0	0	0	0	0	0	0	0	
24676854	m	ph	JP	2024	0	1	0	0	1	0	0	0	0	0	0	0	65
24676951	f	md	CA	2024	0	0	0	0	0	0	0	0	0	0	0	0	61
24677101	m	md	JP	2024	0	0	0	0	0	0	0	0	0	0	0	0	69
24677106	m	md	JP	2024	0	1	1	0	0	0	0	0	0	0	0	0	
24677494	m	md	FR	2024	0	1	1	0	0	0	0	0	0	0	0	0	61
24677702	f	ph	BG	2024	0	1	1	0	0	0	0	0	0	0	0	0	69
24678149	f	md	FR	2024	0	0	0	0	0	0	0	0	0	0	0	0	75
24678296	Not	md	JP	2024	0	1	0	1	0	0	0	0	0	0	0	0	66
24678336	Not	md	JP	2024	1	0	0	0	0	0	0	0	0	0	0	1	
24678355	Not	md	JP	2024	1	0	0	0	0	0	0	0	0	0	0	1	
24678358	Not	md	JP	2024	0	1	0	1	0	0	0	0	0	0	0	0	60
24678421	Not	md	JP	2024	0	1	0	1	0	0	0	0	0	0	0	0	77
24678450	Not	md	JP	2024	0	1	0	1	0	0	0	0	0	0	0	0	76
24678580	m	md	NL	2024	0	1	0	0	0	1	0	0	0	0	0	0	68
24678587	m	md	JP	2024	0	1	0	0	0	1	0	0	0	0	0	0	88
24678846	f	ph	JP	2024	0	0	0	0	0	0	0	0	0	0	0	0	60
24678854	Not	md	JP	2024	0	1	0	1	0	0	0	0	0	0	0	0	
24678870	Not	md	JP	2024	0	1	0	0	1	0	0	0	0	0	0	0	
24678984	Not	md	JP	2024	0	1	0	1	0	0	0	0	0	0	0	0	61
24678985	Not	md	JP	2024	0	1	0	1	0	0	0	0	0	0	0	0	58
24678996	Not	md	JP	2024	1	0	0	0	0	0	0	0	0	0	0	0	79
24679025	Not	md	JP	2024	0	1	0	1	0	0	0	0	0	0	0	0	
24679043	m	md	GB	2024	0	0	0	0	0	0	0	0	0	0	0	0	56

24679066	Not	md	JP	2024	1	0	0	0	0	0	0	0	0	0	0	1	86
24679077	Not	md	JP	2024	0	1	0	1	0	0	0	0	0	0	0	0	70
24679500	Not	Not repo	US	2024	1	0	0	0	0	0	0	0	0	0	0	0	
24679741	f	cn	DE	2024	0	1	1	0	0	0	0	0	0	0	0	0	49
24679872	Not	Not repo	US	2024	1	0	0	0	0	0	0	0	0	0	0	0	
24679896	Not	md	US	2024	0	1	0	1	0	0	0	0	0	0	0	1	
24679897	Not	md	US	2024	1	0	0	0	0	0	0	0	0	0	0	1	
24680046	m	md	DE	2024	0	1	0	0	1	0	0	0	0	0	0	0	77
24680246	Not	md	FI	2024	0	1	1	0	0	0	0	0	0	0	0	0	71
24680589	m	md	AU	2024	0	0	0	0	0	0	0	0	0	0	0	0	
24680593	f	ph	EG	2024	0	0	0	0	0	0	0	0	0	0	0	0	23
24680595	f	cn	TH	2024	0	0	0	0	0	0	0	0	0	0	0	0	51
24680601	m	cn	IN	2024	0	0	0	0	0	0	0	0	0	0	0	0	54
24680676	Not	md	RU	2024	0	1	0	1	0	0	0	0	0	0	0	0	70
24680825	Not	Not repo	IT	2024	1	0	0	0	0	0	0	0	0	0	0	0	43
24680829	Not	md	IT	2024	1	0	0	0	0	0	0	0	0	0	0	0	72
24681287	Not	md	JP	2024	0	1	0	1	0	0	0	0	0	0	0	0	68
24681416	m	hp	CN	2024	0	1	0	0	0	1	0	0	0	0	0	0	77
24681594	Not	md	JP	2024	0	1	0	1	0	0	0	0	0	0	0	0	
24681638	Not	md	NL	2024	1	0	0	0	0	0	0	0	0	0	0	0	26
24681688	Not	Not repo	CA	2024	1	0	0	0	0	0	0	0	0	0	0	0	80
24681887	m	cn	NL	2024	0	0	0	0	0	0	0	0	0	0	0	0	73
24682088	Not	Not repo	JP	2024	0	1	0	1	0	0	0	0	0	0	0	1	
24682104	Not	md	IT	2024	1	0	0	0	0	0	0	0	0	0	0	0	41
24682113	f	hp	ES	2024	0	0	0	0	0	0	0	0	0	0	0	0	63
24682127	Not	md	IT	2024	1	0	0	0	0	0	0	0	0	0	0	0	76
24682159	Not	md	US	2024	1	0	0	0	0	0	0	0	0	0	0	0	
24682336	Not	md	JP	2024	0	1	0	1	0	0	0	0	0	0	0	0	
24682441	m	cn	TH	2024	0	1	0	0	0	1	0	0	0	0	0	0	64
24682449	Not	ph	JP	2024	1	0	0	0	0	0	0	0	0	0	0	0	80
24682529	Not	ph	JP	2024	1	0	0	0	0	0	0	0	0	0	0	0	
24682536	Not	md	JP	2024	0	1	0	1	0	0	0	0	0	0	0	1	60
24682993	Not	ph	CA	2024	1	0	0	0	0	0	0	0	0	0	0	0	
24683827	f	md	JP	2024	0	1	1	0	0	0	0	0	0	0	0	0	76

24684261	m	hp	DE	2024	0	1	0	0	1	0	0	0	0	0	0	0	75
24684296	Not	md	KP	2024	0	0	0	0	0	0	0	0	0	0	0	0	
24684419	f	md	DE	2024	0	1	0	0	1	0	0	0	0	0	0	0	57
24684869	Not	ph	JP	2024	1	0	0	0	0	0	0	0	0	0	0	0	86
24685669	m	md	JP	2024	0	0	0	0	0	0	0	0	0	0	0	0	75
24686067	m	ph	US	2024	0	0	0	0	0	0	0	0	0	0	0	0	74
24686189	m	md	FR	2024	0	1	1	0	0	0	0	0	0	0	0	0	75
24686192	Not	ph	JP	2024	1	0	0	0	0	0	0	0	0	0	0	0	
24686208	m	cn	JP	2024	0	0	0	0	0	0	0	0	0	0	0	0	49
24686337	f	md	AT	2024	0	0	0	0	0	0	0	0	0	0	0	0	72
24686426	m	md	DE	2024	0	1	1	0	0	0	0	0	0	0	0	0	70
24686653	m	md	FR	2024	0	1	1	0	0	0	0	0	0	0	0	0	75
24686959	m	md	FR	2024	0	1	1	0	0	0	0	0	0	0	0	0	61
24686968	f	hp	DE	2024	0	1	1	0	0	0	0	0	0	0	0	0	49
24687210	m	md	JP	2024	0	1	1	0	0	0	0	0	0	0	0	0	70
24688446	Not	hp	JP	2024	0	0	0	0	0	0	0	0	0	0	0	0	
24688674	m	md	PL	2024	0	1	0	0	1	0	0	0	0	0	0	0	72
24688822	Not	md	JP	2024	0	1	0	1	0	0	0	0	0	0	0	0	60
24688864	f	md	SI	2024	0	1	1	0	0	0	0	0	0	0	0	0	
24688880	Not	ph	JP	2024	1	0	0	0	0	0	0	0	0	0	0	0	
24688886	Not	hp	JP	2024	0	0	0	0	0	0	0	0	0	0	0	0	
24689109	m	md	JP	2024	0	0	0	0	0	0	0	0	0	0	0	0	80
24689370	m	cn	US	2024	0	1	1	0	0	0	0	0	0	0	0	0	80
24689375	f	hp	US	2024	0	0	0	0	0	0	0	0	0	0	0	0	75
24689402	m	hp	AU	2024	0	0	0	0	0	0	0	0	0	0	0	0	36
24689469	Not	md	JP	2024	0	1	0	0	1	0	0	0	0	0	0	0	90
24689685	Not	Not repo	CA	2024	1	0	0	0	0	0	0	0	0	0	0	0	78
24689933	m	ph	CA	2024	0	1	0	0	1	0	0	0	0	0	0	0	68
24690016	Not	md	JP	2024	0	1	0	1	0	0	0	0	0	0	0	0	79
24691136	f	md	US	2024	0	0	0	0	0	0	0	0	0	0	0	0	38
24691214	m	md	JP	2024	0	1	1	0	0	0	0	0	0	0	0	0	75
24691294	f	hp	ES	2024	0	0	0	0	0	0	0	0	0	0	0	0	63
24691358	Not	md	JP	2024	1	0	0	0	0	0	0	0	0	0	0	0	70
24691523	f	cn	IN	2024	0	0	0	0	0	0	0	0	0	0	0	0	75

24691532	f	cn	IN	2024	0	0	0	0	0	0	0	0	0	0	0	0	20
24692050	m	Not repo	KR	2024	0	0	0	0	0	0	0	0	0	0	0	0	71
24692220	Not	hp	JP	2024	0	0	0	0	0	0	0	0	0	0	0	0	
24692386	Not	md	JP	2024	1	0	0	0	0	0	0	0	0	0	0	1	
24692432	m	md	DE	2024	0	1	0	0	1	0	0	0	0	0	0	0	75
24692707	Not	md	JP	2024	1	0	0	0	0	0	0	0	0	0	0	0	79
24692793	Not	md	CA	2024	1	0	0	0	0	0	0	0	0	0	0	0	
24693544	m	hp	US	2024	1	0	0	0	0	0	0	0	0	0	0	1	79
24694290	f	md	JP	2024	0	1	0	0	1	0	0	0	0	0	0	0	70
24694359	m	cn	TW	2024	0	0	0	0	0	0	0	0	0	0	0	0	60
24694471	m	ph	FR	2024	0	0	0	0	0	0	0	0	0	0	0	0	
24694646	Not	md	JP	2024	0	0	0	0	0	0	0	0	0	0	0	0	
24695599	m	md	BG	2024	0	1	0	0	1	0	0	0	0	0	0	0	68
24695673	m	cn	TW	2024	0	0	0	0	0	0	0	0	0	0	0	0	57
24695887	m	hp	JP	2024	0	1	0	1	0	0	0	0	0	0	0	0	44
24695938	m	md	DE	2024	0	1	0	0	1	0	0	0	0	0	0	0	74
24696002	m	hp	AU	2024	0	1	0	0	0	1	0	0	0	0	0	0	36
24696427	f	ph	CA	2024	1	0	0	0	0	0	0	0	0	0	0	0	
24696464	f	hp	CN	2024	0	0	0	0	0	0	0	0	0	0	0	0	71
24696582	f	cn	EG	2024	0	0	0	0	0	0	0	0	0	0	0	0	23
24696598	Not	md	JP	2024	1	0	0	0	0	0	0	0	0	0	0	0	80
24696845	m	ph	MY	2024	0	0	0	0	0	0	0	0	0	0	0	0	55
24696910	f	cn	TH	2024	0	0	0	0	0	0	0	0	0	0	0	0	71
24697380	Not	md	JP	2024	0	1	1	0	0	0	0	0	0	0	0	0	80
24697566	m	ph	JP	2024	0	1	0	0	1	0	0	0	0	0	0	0	74
24697725	m	md	FR	2024	0	1	1	0	0	0	0	0	0	0	0	0	75
24698133	m	md	IN	2024	0	1	0	0	0	1	0	0	0	0	0	0	66
24698323	m	md	TR	2024	0	1	1	0	0	0	0	0	0	0	0	0	68
24698527	m	md	JP	2024	0	1	1	0	0	0	0	0	0	0	0	0	85
24698796	Not	md	JP	2024	0	1	0	0	1	0	0	0	0	0	0	0	
24698941	Not	md	JP	2024	0	1	0	1	0	0	0	0	0	0	0	0	72
24698943	Not	md	JP	2024	0	1	0	1	0	0	0	0	0	0	0	0	66
24699241	m	md	JP	2024	0	0	0	0	0	0	0	0	0	0	0	0	66
24699604	m	md	JP	2024	0	1	0	0	0	1	0	0	0	0	0	0	60

24700053	Not	md	JP	2024	0	0	0	0	0	0	0	0	0	0	0	0		
24700437	m	ph	CA	2024	0	1	0	0	1	0	0	0	0	0	0	0	68	
24700579	m	md	JP	2024	0	1	0	0	1	0	0	0	0	0	0	0	72	
24700621	m	md	FR	2024	0	1	1	0	0	0	0	0	0	0	0	0	55	
24700904	Not	Not repo	IN	2024	1	0	0	0	0	0	0	0	0	0	0	0	58	
24701292	Not	md	CN	2024	1	0	0	0	0	0	0	0	0	0	0	0	57	
24701310	f	cn	MY	2024	0	0	0	0	0	0	0	0	0	0	0	0	73	
24701523	m	md	JP	2024	0	0	0	0	0	0	0	0	0	0	0	0		
24702772	f	cn	US	2024	0	1	1	0	0	0	0	0	0	0	0	0		
24703121	f	hp	ES	2024	0	0	0	0	0	0	0	0	0	0	0	0	63	
24703324	Not	md	JP	2024	1	0	0	0	0	0	0	0	0	0	0	1	90	
24704120	f	md	DE	2024	0	1	0	0	1	0	0	0	0	0	0	0	72	
24704506	m	md	DE	2024	0	1	0	0	1	0	0	0	0	0	0	0	77	
24704721	m	md	JP	2024	0	1	0	0	0	1	0	0	0	0	0	0	70	
24705315	f	md	DE	2024	0	1	0	0	1	0	0	0	0	0	0	0	69	
24705487	m	hp	AU	2024	0	1	0	0	0	1	0	0	0	0	0	0	36	
24705852	Not	ph	JP	2024	0	1	0	1	0	0	0	0	0	0	0	0	66	
24705987	m	md	JP	2024	0	1	1	0	0	0	0	0	0	0	0	0	74	
24706035	m	cn	IN	2024	0	0	0	0	0	0	0	0	0	0	0	0	56	
24706288	f	cn	IN	2024	0	0	0	0	0	0	0	0	0	0	0	0	46	
24706487	Not	md	JP	2024	0	1	0	0	1	0	0	0	0	0	0	0		
24706757	m	hp	CA	2024	0	0	0	0	0	0	0	0	0	0	0	0	41	
24706849	f	md	CA	2024	0	1	1	0	0	0	0	0	0	0	0	0	67	
24706862	f	md	PL	2024	0	0	0	0	0	0	0	0	0	0	0	0	60	
24706925	f	md	CA	2024	0	1	0	0	1	0	0	0	0	0	0	1		
24706974	m	md	CA	2024	0	1	0	0	1	0	0	0	0	0	0	0	60	
24707298	f	hp	DE	2024	0	1	1	0	0	0	0	0	0	0	0	0	49	
24707303	f	cn	DE	2024	0	1	0	0	1	0	0	0	0	0	0	0	57	
24707344	Not	Not repo	JP	2024	1	0	0	0	0	0	0	0	0	0	0	0	82	
24707358	Not	Not repo	JP	2024	1	0	0	0	0	0	0	0	0	0	0	0	35	
24707425	Not	hp	US	2024	0	0	0	0	0	0	0	0	0	0	0	0		
24707432	m	ph	CA	2024	0	1	0	0	1	0	0	0	0	0	0	0	68	
24707435	m	md	CA	2024	0	1	0	0	1	0	0	0	0	0	0	0		
24707585	f	md	GR	2024	1	0	0	0	0	0	0	0	0	0	0	0	58	

24707590	m	md	JP	2024	0	1	1	0	0	0	0	0	0	0	0	0	75
24707990	Not	md	KR	2024	0	1	0	1	0	0	0	0	0	0	0	0	
24708687	f	hp	NL	2024	0	1	0	0	0	1	0	0	0	0	0	0	67
24708814	f	cn	GB	2024	0	0	0	0	0	0	0	0	0	0	0	0	78
24709009	m	hp	US	2024	0	0	0	0	0	0	0	0	0	0	0	0	
24709014	f	md	US	2024	0	0	0	0	0	0	0	0	0	0	0	0	38
24709120	Not	md	CN	2024	1	0	0	0	0	0	0	0	0	0	0	0	56
24709162	Not	md	JP	2024	0	0	0	0	0	0	0	0	0	0	0	0	
24709555	f	md	BG	2024	0	1	0	0	1	0	0	0	0	0	0	0	53
24709618	Not	md	AU	2024	1	0	0	0	0	0	0	0	0	0	0	0	
24709820	f	hp	DE	2024	0	1	1	0	0	0	0	0	0	0	0	0	49
24709997	f	md	IT	2024	0	1	0	0	1	0	0	0	0	0	0	0	56
24710078	m	hp	CN	2024	0	1	1	0	0	0	0	0	0	0	0	0	72
24710089	m	hp	US	2024	0	0	0	0	0	0	0	0	0	0	0	0	
24710190	Not	md	GB	2024	1	0	0	0	0	0	0	0	0	0	0	0	85
24710273	f	md	DE	2024	0	1	0	0	1	0	0	0	0	0	0	0	57
24710638	Not	md	FR	2024	1	0	0	0	0	0	0	0	0	0	0	0	88
24710812	m	hp	FR	2024	0	1	0	0	1	0	0	0	0	0	0	0	76
24711242	Not	ph	CA	2024	0	0	0	0	0	0	0	0	0	0	0	0	71
24711250	f	md	CA	2024	0	1	0	0	1	0	0	0	0	0	0	0	70
24711671	Not	md	JP	2024	0	1	0	0	0	1	0	0	0	0	0	0	80
24712192	m	cn	NL	2024	0	0	0	0	0	0	0	0	0	0	0	0	73
24712198	f	hp	CA	2024	0	0	0	0	0	0	0	0	0	0	0	0	48
24712333	f	md	DE	2024	0	1	0	0	1	0	0	0	0	0	0	0	57
24712340	m	md	DE	2024	0	1	0	0	1	0	0	0	0	0	0	0	73
24712533	m	md	JP	2024	0	1	0	0	1	0	0	0	0	0	0	0	61
24712549	f	md	FR	2024	0	0	0	0	0	0	0	0	0	0	0	0	
24712764	Not	md	JP	2024	1	0	0	0	0	0	0	0	0	0	0	0	80
24712817	f	cn	ES	2024	0	0	0	0	0	0	0	0	0	0	0	0	63
24712995	f	hp	AT	2024	0	0	0	0	0	0	0	0	0	0	0	0	74
24713027	m	md	JP	2024	0	1	0	0	1	0	0	0	0	0	0	0	61
24713065	m	md	IT	2024	0	1	1	0	0	0	0	0	0	0	0	0	75
24713351	Not	md	BE	2024	1	0	0	0	0	0	0	0	0	0	0	0	59
24713716	m	ph	JP	2024	0	1	1	0	0	0	0	0	0	0	0	0	70

24713738	m	md	CA	2024	0	1	0	0	1	0	0	0	0	0	0	0	
24713744	m	md	CA	2024	0	1	0	0	1	0	0	0	0	0	0	0	
24714223	Not	hp	US	2024	0	0	0	0	0	0	0	0	0	0	0	0	
24714238	f	md	FR	2024	0	1	1	0	0	0	0	0	0	0	0	0	73
24714256	m	hp	FR	2024	0	1	1	0	0	0	0	0	0	0	0	0	64
24714287	m	hp	GR	2024	0	0	0	0	0	0	0	0	0	0	0	0	70
24714384	Not	ph	JP	2024	1	0	0	0	0	0	0	0	0	0	0	0	74
24714524	m	md	JP	2024	0	1	1	0	0	0	0	0	0	0	0	0	75
24714833	Not	md	CA	2024	1	0	0	0	0	0	0	0	0	0	0	0	70
24714955	m	md	CA	2024	0	1	0	0	1	0	0	0	0	0	0	0	50
24715062	Not	hp	CN	2024	1	0	0	0	0	0	0	0	0	0	0	0	
24715330	Not	md	JP	2024	0	1	0	0	0	1	0	0	0	0	0	0	70
24715487	Not	md	JP	2024	0	1	0	0	0	1	0	0	0	0	0	0	
24715630	Not	md	JP	2024	0	1	0	0	1	0	0	0	0	0	0	0	
24716186	m	ph	FR	2024	0	0	0	0	0	0	0	0	0	0	0	0	
24716370	m	cn	NL	2024	0	0	0	0	0	0	0	0	0	0	0	0	73
24716668	Not	md	CN	2024	1	0	0	0	0	0	0	0	0	0	0	0	75
24716878	f	md	CA	2024	0	1	0	0	1	0	0	0	0	0	0	0	81
24716999	f	md	FR	2024	0	0	0	0	0	0	0	0	0	0	0	0	34
24717104	Not	md	IT	2024	1	0	0	0	0	0	0	0	0	0	0	0	
24717277	f	hp	ES	2024	0	0	0	0	0	0	0	0	0	0	0	0	63
24717292	m	md	HR	2024	0	1	1	0	0	0	0	0	0	0	0	1	
24717512	f	md	FR	2024	0	1	0	0	0	1	0	0	0	0	0	0	53
24717700	m	md	ES	2024	0	0	0	0	0	0	0	0	0	0	0	0	78
24717888	m	hp	CA	2024	0	1	0	0	1	0	0	0	0	0	0	0	75
24718116	Not	md	JP	2024	0	1	0	1	0	0	0	0	0	0	0	1	64
24718277	f	md	JP	2024	0	1	0	0	1	0	0	0	0	0	0	0	80
24719762	m	md	US	2024	0	0	0	0	0	0	0	0	0	0	0	0	53
24719865	Not	md	JP	2024	1	0	0	0	0	0	0	0	0	0	0	0	
24720063	m	md	AU	2024	0	0	0	0	0	0	0	0	0	0	0	0	45
24720457	m	md	IT	2024	0	1	1	0	0	0	0	0	0	0	0	0	75
24720489	m	md	DE	2024	0	1	0	0	1	0	0	0	0	0	0	0	75
24720837	Not	md	JP	2024	1	0	0	0	0	0	0	0	0	0	0	0	80
24720969	m	md	JP	2024	0	1	0	0	0	1	0	0	0	0	0	0	70

24721331	m	md	HR	2024	0	1	1	0	0	0	0	0	0	0	0	0	
24721378	Not	md	SA	2024	0	0	0	0	0	0	0	0	0	0	0	0	
24721563	m	md	DE	2024	0	1	0	0	1	0	0	0	0	0	0	0	47
24721796	m	md	JP	2024	0	1	1	0	0	0	0	0	0	0	0	0	56
24721880	m	md	JP	2024	0	1	0	0	0	1	0	0	0	0	0	0	80
24722145	m	hp	ES	2024	0	1	1	0	0	0	0	0	0	0	0	0	62
24722226	m	md	JP	2024	0	1	1	0	0	0	0	0	0	0	0	0	60
24722439	Not	cn	US	2024	0	0	0	0	0	0	0	0	0	0	0	0	
24722531	Not	md	JP	2024	0	1	0	0	1	0	0	0	0	0	0	0	
24722586	f	Not repo	IL	2024	0	0	0	0	0	0	0	0	0	0	0	0	59
24722660	m	md	HR	2024	0	1	1	0	0	0	0	0	0	0	0	0	64
24722974	f	Not repo	KR	2024	0	0	0	0	0	0	0	0	0	0	0	0	81
24723154	m	md	DE	2024	0	1	1	0	0	0	0	0	0	0	0	1	
24723938	m	md	JP	2024	0	1	1	0	0	0	0	0	0	0	0	0	
24724085	Not	Not repo	SG	2024	1	0	0	0	0	0	0	0	0	0	0	0	
24724254	m	md	DZ	2024	1	0	0	0	0	0	0	0	0	0	0	0	38
24724436	Not	md	JP	2024	1	0	0	0	0	0	0	0	0	0	0	0	91
24724754	f	md	JP	2024	0	1	0	0	0	1	0	0	0	0	0	0	
24724840	m	md	JP	2024	0	1	0	0	1	0	0	0	0	0	0	0	72
24725200	m	md	RO	2024	0	0	0	0	0	0	0	0	0	0	0	0	
24725508	m	md	DE	2024	0	1	1	0	0	0	0	0	0	0	0	0	
24726214	m	md	FR	2024	0	1	1	0	0	0	0	0	0	0	0	0	61
24726837	f	cn	TH	2024	0	0	0	0	0	0	0	0	0	0	0	0	65
24726843	f	md	AT	2024	0	0	0	0	0	0	0	0	0	0	0	0	76
24726867	f	md	AT	2024	0	0	0	0	0	0	0	0	0	0	0	0	82
24727516	f	md	JP	2024	0	1	0	0	0	1	0	0	0	0	0	0	50
24727614	f	md	UA	2024	0	1	1	0	0	0	0	0	0	0	0	0	53
24727815	f	hp	US	2024	0	1	0	0	0	1	0	0	0	0	0	0	70
24728274	Not	hp	DE	2024	0	0	0	0	0	0	0	0	0	0	0	0	
24728275	Not	hp	DE	2024	0	0	0	0	0	0	0	0	0	0	0	0	
24728276	Not	hp	DE	2024	0	0	0	0	0	0	0	0	0	0	0	0	
24728277	Not	hp	DE	2024	0	0	0	0	0	0	0	0	0	0	0	0	
24728279	Not	hp	DE	2024	0	0	0	0	0	0	0	0	0	0	0	0	
24728280	Not	hp	DE	2024	0	0	0	0	0	0	0	0	0	0	0	0	

24728282	Not	hp	DE	2024	0	0	0	0	0	0	0	0	0	0	0	0	
24728283	Not	hp	DE	2024	0	0	0	0	0	0	0	0	0	0	0	0	
24728285	Not	hp	DE	2024	0	0	0	0	0	0	0	0	0	0	0	0	
24728286	Not	hp	DE	2024	0	0	0	0	0	0	0	0	0	0	0	0	
24728287	Not	hp	DE	2024	0	0	0	0	0	0	0	0	0	0	0	0	
24728288	Not	hp	DE	2024	0	0	0	0	0	0	0	0	0	0	0	0	
24728749	Not	md	JP	2024	0	1	0	1	0	0	0	0	0	0	0	0	60
24729306	m	md	JP	2024	0	1	1	0	0	0	0	0	0	0	0	0	80
24729335	m	md	DE	2024	0	0	0	0	0	0	0	0	0	0	0	0	
24729417	m	md	DE	2024	0	1	0	0	1	0	0	0	0	0	0	0	65
24729551	Not	Not repo	CA	2024	1	0	0	0	0	0	0	0	0	0	0	0	
24729717	f	md	HU	2024	0	1	0	0	0	1	0	0	0	0	0	0	58
24729762	f	md	DE	2024	0	1	1	0	0	0	0	0	0	0	0	0	
24729857	Not	Not repo	SG	2024	1	0	0	0	0	0	0	0	0	0	0	0	77
24729895	Not	md	JP	2024	0	1	0	0	1	0	0	0	0	0	0	0	
24729974	m	cn	PK	2024	0	1	0	0	0	1	0	0	0	0	0	0	71
24730068	f	hp	CZ	2024	0	0	0	0	0	0	0	0	0	0	0	0	66
24730581	m	hp	CN	2024	0	0	0	0	0	0	0	0	0	0	0	0	50
24730592	Not	md	JP	2024	0	0	0	0	0	0	0	0	0	0	0	0	80
24730980	Not	md	JP	2024	1	0	0	0	0	0	0	0	0	0	0	1	
24731211	m	cn	US	2024	0	1	1	0	0	0	0	0	0	0	0	0	59
24731285	Not	Not repo	IN	2024	1	0	0	0	0	0	0	0	0	0	0	0	58
24731315	m	hp	CN	2024	0	0	0	0	0	0	0	0	0	0	0	0	62
24731707	f	md	BG	2024	0	1	0	0	1	0	0	0	0	0	0	0	64
24732069	m	md	FR	2024	0	1	1	0	0	0	0	0	0	0	0	0	75
24732106	m	md	JP	2024	0	0	0	0	0	0	0	0	0	0	0	0	
24732379	Not	md	CN	2024	1	0	0	0	0	0	0	0	0	0	0	0	39
24732739	f	cn	US	2024	0	0	0	0	0	0	0	0	0	0	0	0	71
24732832	m	md	JP	2024	0	0	0	0	0	0	0	0	0	0	0	0	68
24733719	f	md	NL	2024	0	0	0	0	0	0	0	0	0	0	0	0	59
24733720	f	md	NL	2024	0	0	0	0	0	0	0	0	0	0	0	0	45
24734888	m	md	DE	2024	0	1	0	0	1	0	0	0	0	0	0	0	66
24735413	f	md	US	2024	0	0	0	0	0	0	0	0	0	0	0	0	38
24735781	Not	md	US	2024	0	1	0	1	0	0	0	0	0	0	0	0	

24735832	m	hp	US	2024	0	0	0	0	0	0	0	0	0	0	0	0	56
24735863	Not	md	CA	2024	1	0	0	0	0	0	0	0	0	0	0	0	47
24736617	f	md	JP	2024	0	0	0	0	0	0	0	0	0	0	0	0	40
24736736	Not	md	US	2024	1	0	0	0	0	0	0	0	0	0	0	0	
24736973	f	hp	US	2024	1	0	0	0	0	0	0	0	0	0	0	0	80
24737114	m	hp	US	2024	0	0	0	0	0	0	0	0	0	0	0	0	
24737858	Not	hp	CA	2024	0	0	0	0	0	0	0	0	0	0	0	0	
24737981	f	md	JP	2024	0	1	0	0	1	0	0	0	0	0	0	0	80
24738397	m	md	CA	2024	0	0	0	0	0	0	0	0	0	0	0	0	72
24738413	Not	md	SA	2024	0	0	0	0	0	0	0	0	0	0	0	0	
24738488	f	md	SI	2024	0	1	1	0	0	0	0	0	0	0	0	0	
24738884	Not	md	JP	2024	1	0	0	0	0	0	0	0	0	0	0	0	75
24738952	Not	md	US	2024	1	0	0	0	0	0	0	0	0	0	0	0	
24739089	m	cn	US	2024	0	0	0	0	0	0	0	0	0	0	0	0	
24739150	f	cn	US	2024	0	0	0	0	0	0	0	0	0	0	0	0	56
24739513	f	md	JP	2024	0	1	1	0	0	0	0	0	0	0	0	1	75
24740080	f	md	JP	2024	0	1	1	0	0	0	0	0	0	0	0	0	76
24740419	Not	md	JP	2024	1	0	0	0	0	0	0	0	0	0	0	1	
24740630	Not	Not repo	US	2024	1	0	0	0	0	0	0	0	0	0	0	0	50
24741007	m	hp	US	2024	0	0	0	0	0	0	0	0	0	0	0	0	
24741616	Not	md	JP	2024	0	1	0	0	0	1	0	0	0	0	0	1	70
24741718	f	md	JP	2024	1	0	0	0	0	0	0	0	0	0	0	0	79
24741754	f	hp	US	2024	0	1	1	0	0	0	0	0	0	0	0	0	38
24742033	Not	md	CN	2024	1	0	0	0	0	0	0	0	0	0	0	0	
24742242	Not	md	JP	2024	0	1	0	1	0	0	0	0	0	0	0	0	
24742585	f	md	AT	2024	0	0	0	0	0	0	0	0	0	0	0	0	82
24742844	f	cn	US	2024	0	0	0	0	0	0	0	0	0	0	0	0	50
24743155	f	md	JP	2024	0	0	0	0	0	0	0	0	0	0	0	0	
24743367	m	cn	EG	2024	0	1	1	0	0	0	0	0	0	0	0	0	
24744038	f	Not repo	KR	2024	0	0	0	0	0	0	0	0	0	0	0	0	88
24744653	m	cn	HR	2024	0	1	1	0	0	0	0	0	0	0	0	0	64
24744922	m	cn	CA	2024	1	0	0	0	0	0	0	0	0	0	0	0	
24744982	m	hp	US	2024	0	0	0	0	0	0	0	0	0	0	0	0	40
24745888	m	md	IT	2024	0	1	0	0	1	0	0	0	0	0	0	0	79

24745927	Not	md	KR	2024	1	0	0	0	0	0	0	0	0	0	0	0	56
24746036	Not	md	JP	2024	1	0	0	0	0	0	0	0	0	0	0	1	
24746199	m	cn	IN	2024	0	1	0	0	0	1	0	0	0	0	0	0	54
24746215	Not	md	JP	2024	1	0	0	0	0	0	0	0	0	0	0	1	
24746222	f	md	US	2024	0	1	0	0	1	0	0	0	0	0	0	0	63
24746666	m	md	JP	2024	0	1	0	0	0	1	0	0	0	0	0	0	80
24746982	Not	ph	US	2024	1	0	0	0	0	0	0	0	0	0	0	0	
24747313	f	md	DE	2024	0	0	0	0	0	0	0	0	0	0	0	0	76
24747445	Not	md	KR	2024	1	0	0	0	0	0	0	0	0	0	0	0	64
24747901	Not	ph	JP	2024	1	0	0	0	0	0	0	0	0	0	0	0	76
24748283	Not	md	JP	2024	1	0	0	0	0	0	0	0	0	0	0	0	78
24748582	m	hp	US	2024	0	1	1	0	0	0	0	0	0	0	0	0	76
24748778	m	hp	US	2024	0	1	1	0	0	0	0	0	0	0	0	1	75
24748954	Not	md	NZ	2024	1	0	0	0	0	0	0	0	0	0	0	0	74
24749073	f	cn	US	2024	0	1	1	0	0	0	0	0	0	0	0	0	
24749185	Not	hp	US	2024	1	0	0	0	0	0	0	0	0	0	0	0	
24749415	m	hp	CN	2024	0	0	0	0	0	0	0	0	0	0	0	0	56
24749857	m	cn	JP	2024	0	0	0	0	0	0	0	0	0	0	0	0	75
24750472	f	md	NL	2024	0	1	1	0	0	0	0	0	0	0	0	0	72
24750536	f	md	JP	2024	0	0	0	0	0	0	0	0	0	0	0	0	71
24752177	Not	md	US	2024	1	0	0	0	0	0	0	0	0	0	0	0	
24752214	Not	md	JP	2024	1	0	0	0	0	0	0	0	0	0	0	1	
24752244	Not	Not repo	SG	2024	1	0	0	0	0	0	0	0	0	0	0	0	
24752340	m	md	JP	2024	0	1	0	0	0	1	0	0	0	0	0	0	54
24752377	Not	Not repo	CA	2024	1	0	0	0	0	0	0	0	0	0	0	0	
24752757	Not	Not repo	IN	2024	1	0	0	0	0	0	0	0	0	0	0	0	78
24752968	Not	md	KR	2024	1	0	0	0	0	0	0	0	0	0	0	0	52
24753028	f	md	JP	2024	0	0	0	0	0	0	0	0	0	0	0	0	77
24753035	m	md	JP	2024	0	1	0	0	0	1	0	0	0	0	0	0	80
24753402	f	hp	RO	2024	0	1	0	0	1	0	0	0	0	0	0	0	63
24753837	Not	md	FR	2024	0	1	0	1	0	0	0	0	0	0	0	0	79
24754190	Not	Not repo	DE	2024	1	0	0	0	0	0	0	0	0	0	0	0	
24754351	Not	md	GR	2024	0	1	1	0	0	0	0	0	0	0	0	0	61
24754372	Not	md	JP	2024	0	1	0	1	0	0	0	0	0	0	0	0	82

24754447	f	md	US	2024	0	0	0	0	0	0	0	0	0	0	0	0	63
24754457	m	md	US	2024	0	0	0	0	0	0	0	0	0	0	0	0	39
24754575	Not	md	KR	2024	0	1	0	1	0	0	0	0	0	0	0	0	
24754684	Not	md	JP	2024	1	0	0	0	0	0	0	0	0	0	0	0	
24754726	Not	md	JP	2024	0	1	0	1	0	0	0	0	0	0	0	0	60
24754964	Not	md	KR	2024	1	0	0	0	0	0	0	0	0	0	0	0	
24755102	f	md	JP	2024	0	1	0	0	0	1	0	0	0	0	0	0	70
24755250	f	md	NP	2024	0	0	0	0	0	0	0	0	0	0	0	0	67
24755252	f	md	NP	2024	0	0	0	0	0	0	0	0	0	0	0	0	63
24755794	m	md	JP	2024	0	1	1	0	0	0	0	0	0	0	0	0	80
24756021	Not	md	JP	2024	0	0	0	0	0	0	0	0	0	0	0	0	
24756328	Not	md	KE	2024	1	0	0	0	0	0	0	0	0	0	0	0	54
24756770	f	md	DE	2024	0	1	0	0	1	0	0	0	0	0	0	0	72
24756793	m	cn	PK	2024	0	1	0	0	0	1	0	0	0	0	0	0	60
24757117	m	md	DE	2024	0	1	0	0	1	0	0	0	0	0	0	0	71
24757141	f	md	PL	2024	0	1	0	0	1	0	0	0	0	0	0	0	64
24757260	m	hp	US	2024	0	1	1	0	0	0	0	0	0	0	0	1	75
24757635	Not	md	JP	2024	1	0	0	0	0	0	0	0	0	0	0	0	76
24759272	f	ph	US	2024	0	0	0	0	0	0	0	0	0	0	0	0	74
24759974	m	md	JP	2024	0	0	0	0	0	0	0	0	0	0	0	0	
24760308	f	md	JP	2024	0	0	0	0	0	0	0	0	0	0	0	0	48
24760421	Not	Not repo	US	2024	1	0	0	0	0	0	0	0	0	0	0	0	
24760645	m	hp	CA	2024	0	0	0	0	0	0	0	0	0	0	0	0	68
24760666	m	cn	JP	2024	0	0	0	0	0	0	0	0	0	0	0	0	75
24760694	f	hp	CA	2024	0	1	1	0	0	0	0	0	0	0	0	0	56
24760826	m	ph	JP	2024	0	0	0	0	0	0	0	0	0	0	0	0	
24760941	Not	md	JP	2024	0	1	0	0	0	1	0	0	0	0	0	0	66
24761068	Not	hp	DE	2024	0	0	0	0	0	0	0	0	0	0	0	0	
24761076	Not	hp	DE	2024	0	0	0	0	0	0	0	0	0	0	0	0	
24761097	m	md	DE	2024	0	1	0	0	1	0	0	0	0	0	0	0	61
24761582	m	hp	IN	2024	0	1	1	0	0	0	0	0	0	0	0	0	77
24761852	f	md	DE	2024	0	1	0	0	1	0	0	0	0	0	0	0	76
24762209	m	md	DE	2024	0	1	1	0	0	0	0	0	0	0	0	1	
24762461	m	md	ES	2024	0	1	0	0	0	1	0	0	0	0	0	0	77

24763119	m	md	DE	2024	0	1	0	0	1	0	0	0	0	0	0	0	47
24763163	f	md	DE	2024	0	1	0	0	1	0	0	0	0	0	0	0	69
24763211	Not	md	JP	2024	0	0	0	0	0	0	0	0	0	0	0	0	
24763541	Not	hp	DE	2024	0	0	0	0	0	0	0	0	0	0	0	0	
24763556	Not	hp	DE	2024	0	0	0	0	0	0	0	0	0	0	0	0	
24763568	Not	md	AT	2024	0	0	0	0	0	0	0	0	0	0	0	0	
24763674	m	ph	JP	2024	0	0	0	0	0	0	0	0	0	0	0	0	
24763701	Not	hp	DE	2024	0	0	0	0	0	0	0	0	0	0	0	0	
24763794	Not	hp	DE	2024	0	0	0	0	0	0	0	0	0	0	0	0	
24764022	Not	hp	DE	2024	0	0	0	0	0	0	0	0	0	0	0	0	
24764023	Not	hp	DE	2024	0	0	0	0	0	0	0	0	0	0	0	0	
24764911	m	md	JP	2024	0	1	1	0	0	0	0	0	0	0	0	0	82
24765200	m	hp	JP	2024	0	0	0	0	0	0	0	0	0	0	0	0	
24765217	m	md	CY	2024	0	1	1	0	0	0	0	0	0	0	0	1	70
24765227	Not	md	JP	2024	0	1	0	0	1	0	0	0	0	0	0	0	
24765296	Not	Not repo	EG	2024	1	0	0	0	0	0	0	0	0	0	0	0	61
24765424	m	md	JP	2024	0	0	0	0	0	0	0	0	0	0	0	0	
24765586	m	md	JP	2024	0	1	0	0	1	0	0	0	0	0	0	0	70
24765593	m	md	JP	2024	0	1	1	0	0	0	0	0	0	0	0	0	76
24765833	f	md	JP	2024	0	1	0	0	0	1	0	0	0	0	0	0	86
24765874	m	md	AT	2024	0	1	1	0	0	0	0	0	0	0	0	0	75
24765955	m	hp	JP	2024	0	0	0	0	0	0	0	0	0	0	0	0	
24766292	f	hp	CA	2024	0	0	0	0	0	0	0	0	0	0	0	0	50
24766328	Not	md	US	2024	0	0	0	0	0	0	0	0	0	0	0	0	
24766546	Not	md	JP	2024	1	0	0	0	0	0	0	0	0	0	0	0	
24766558	m	md	DE	2024	0	1	0	0	1	0	0	0	0	0	0	0	73
24766573	Not	md	JP	2024	0	1	0	0	1	0	0	0	0	0	0	0	
24767179	m	md	HR	2024	0	1	1	0	0	0	0	0	0	0	0	0	64
24767275	f	cn	US	2024	0	0	0	0	0	0	0	0	0	0	0	0	70
24767325	Not	ph	JP	2024	0	1	0	1	0	0	0	0	0	0	0	1	51
24767373	Not	md	JP	2024	1	0	0	0	0	0	0	0	0	0	0	0	45
24767658	f	hp	JP	2024	0	0	0	0	0	0	0	0	0	0	0	0	
24767815	m	cn	KR	2024	0	0	0	0	0	0	0	0	0	0	0	0	69
24767893	m	md	JP	2024	0	1	0	0	1	0	0	0	0	0	0	0	69

24768197	f	ph	US	2024	0	0	0	0	0	0	0	0	0	0	0	0	76
24768269	Not	hp	DE	2024	0	0	0	0	0	0	0	0	0	0	0	0	
24768785	f	md	FR	2024	0	0	0	0	0	0	0	0	0	0	0	0	72
24768868	f	md	KR	2024	0	0	0	0	0	0	0	0	0	0	0	0	68
24768932	Not	Not repo	TH	2024	1	0	0	0	0	0	0	0	0	0	0	0	
24769070	m	md	JP	2024	0	1	1	0	0	0	0	0	0	0	0	0	
24769091	m	cn	TH	2024	0	1	0	0	0	1	0	0	0	0	0	0	79
24769137	Not	hp	DE	2024	0	0	0	0	0	0	0	0	0	0	0	0	
24769223	m	md	JP	2024	0	1	0	0	1	0	0	0	0	0	0	0	87
24769663	Not	md	JP	2024	0	1	0	0	1	0	0	0	0	0	0	0	
24769684	m	md	US	2024	0	1	0	0	0	1	0	0	0	0	0	0	72
24770031	m	md	CN	2024	0	0	0	0	0	0	0	0	0	0	0	0	48
24770400	Not	hp	DE	2024	0	0	0	0	0	0	0	0	0	0	0	0	
24770755	m	md	IT	2024	0	1	0	0	1	0	0	0	0	0	0	0	79
24770789	m	ph	CA	2024	0	1	0	0	1	0	0	0	0	0	0	0	68
24770956	Not	md	JP	2024	1	0	0	0	0	0	0	0	0	0	0	0	
24771294	f	hp	CN	2024	0	0	0	0	0	0	0	0	0	0	0	0	56
24771488	f	md	JP	2024	0	0	0	0	0	0	0	0	0	0	0	0	
24771595	Not	ph	JP	2024	1	0	0	0	0	0	0	0	0	0	0	0	78
24771804	Not	md	JP	2024	1	0	0	0	0	0	0	0	0	0	0	0	83
24772343	m	cn	TH	2024	0	0	0	0	0	0	0	0	0	0	0	0	69
24772416	Not	hp	DE	2024	0	0	0	0	0	0	0	0	0	0	0	0	
24772905	m	cn	IN	2024	0	0	0	0	0	0	0	0	0	0	0	0	59
24772906	Not	md	NL	2024	0	0	0	0	0	0	0	0	0	0	0	0	
24772986	f	cn	PK	2024	0	1	0	0	0	1	0	0	0	0	0	0	60
24773076	f	md	JP	2024	0	1	0	0	0	1	0	0	0	0	0	0	48
24773261	m	md	JP	2024	0	1	0	0	0	1	0	0	0	0	0	0	80
24773763	m	hp	US	2024	0	1	1	0	0	0	0	0	0	0	0	1	75
24773885	Not	Not repo	FR	2024	1	0	0	0	0	0	0	0	0	0	0	0	72
24774288	f	md	KR	2024	0	0	0	0	0	0	0	0	0	0	0	0	44
24774325	m	md	JP	2024	0	1	1	0	0	0	0	0	0	0	0	0	80
24775369	f	hp	US	2024	0	0	0	0	0	0	0	0	0	0	0	0	
24775759	f	cn	PK	2024	0	1	0	0	0	1	0	0	0	0	0	0	77
24775837	f	md	HU	2024	0	0	0	0	0	0	0	0	0	0	0	0	

24776122	Not	md	JP	2024	0	1	0	1	0	0	0	0	0	0	0	0	
24776138	Not	md	AU	2024	1	0	0	0	0	0	0	0	0	0	0	0	
24776369	f	cn	TH	2024	0	0	0	0	0	0	0	0	0	0	0	0	79
24776682	Not	md	JP	2024	0	1	0	1	0	0	0	0	0	0	0	0	60
24776961	m	cn	US	2024	0	1	1	0	0	0	0	0	0	0	0	0	
24777336	Not	md	JP	2024	0	1	0	1	0	0	0	0	0	0	0	0	49
24777443	f	md	FR	2024	0	1	1	0	0	0	0	0	0	0	0	0	68
24777939	f	cn	US	2024	0	0	0	0	0	0	0	0	0	0	0	0	48
24778249	Not	Not repo	IN	2024	1	0	0	0	0	0	0	0	0	0	0	0	75
24778268	m	md	US	2024	0	0	0	0	0	0	0	0	0	0	0	0	80
24778830	f	hp	KR	2024	0	0	0	0	0	0	0	0	0	0	0	0	62
24778850	Not	Not repo	US	2024	1	0	0	0	0	0	0	0	0	0	0	0	
24778859	Not	md	JP	2024	1	0	0	0	0	0	0	0	0	0	0	0	83
24778914	m	md	KR	2024	0	0	0	0	0	0	0	0	0	0	0	0	59
24779378	m	md	JP	2024	0	1	1	0	0	0	0	0	0	0	0	0	70
24779388	m	md	NP	2024	0	0	0	0	0	0	0	0	0	0	0	0	59
24779630	Not	hp	CN	2024	0	0	0	0	0	0	0	0	0	0	0	0	
24779722	f	md	KR	2024	0	0	0	0	0	0	0	0	0	0	0	0	69
24781014	m	md	JP	2024	0	1	1	0	0	0	0	0	0	0	0	0	52
24781128	f	md	JP	2024	0	1	0	0	0	1	0	0	0	0	0	0	71
24781196	Not	ph	JP	2024	1	0	0	0	0	0	0	0	0	0	0	0	
24781360	f	cn	CL	2024	0	1	0	0	0	1	0	0	0	0	0	0	65
24782275	Not	md	IN	2024	0	0	0	0	0	0	0	0	0	0	0	0	
24782276	m	md	FR	2024	0	0	0	0	0	0	0	0	0	0	0	0	
24782280	Not	md	IN	2024	0	0	0	0	0	0	0	0	0	0	0	0	
24782457	m	md	FR	2024	0	1	0	0	1	0	0	0	0	0	0	1	70
24782573	Not	md	JP	2024	1	0	0	0	0	0	0	0	0	0	0	1	
24782860	Not	Not repo	IN	2024	1	0	0	0	0	0	0	0	0	0	0	0	55
24783764	Not	md	JP	2024	1	0	0	0	0	0	0	0	0	0	0	0	74
24783777	Not	Not repo	IN	2024	1	0	0	0	0	0	0	0	0	0	0	0	72
24783822	Not	md	JP	2024	0	0	0	0	0	0	0	0	0	0	0	0	78
24784228	f	cn	MY	2024	0	0	0	0	0	0	0	0	0	0	0	0	
24784463	m	hp	CN	2024	0	0	0	0	0	0	0	0	0	0	0	0	56
24784571	f	cn	FR	2024	0	0	0	0	0	0	0	0	0	0	0	0	44

24785868	f	md	JP	2024	0	0	0	0	0	0	0	0	0	0	0	0	60
24786727	m	cn	CN	2024	0	1	0	0	0	1	0	0	0	0	0	0	64
24787018	m	md	RU	2024	0	1	0	0	0	1	0	0	0	0	0	0	64
24787072	Not	Not repo	IN	2024	1	0	0	0	0	0	0	0	0	0	0	0	28
24787157	f	md	JP	2024	0	1	0	0	0	1	0	0	0	0	0	0	
24787450	m	md	JP	2024	0	0	0	0	0	0	0	0	0	0	0	0	60
24787821	m	md	JP	2024	0	1	0	0	0	1	0	0	0	0	0	0	
24788021	f	ph	FR	2024	0	0	0	0	0	0	0	0	0	0	0	0	44
24788207	Not	md	JP	2024	0	1	0	0	0	1	0	0	0	0	0	0	67
24788488	f	md	DE	2024	0	1	0	0	1	0	0	0	0	0	0	0	69
24788498	f	cn	PK	2024	0	1	0	0	0	1	0	0	0	0	0	0	47
24788509	f	md	NL	2024	0	1	1	0	0	0	0	0	0	0	0	0	72
24788525	Not	md	JP	2024	0	1	0	1	0	0	0	0	0	0	0	0	70
24788533	m	md	DE	2024	0	1	0	0	1	0	0	0	0	0	0	0	61
24788584	m	cn	NL	2024	0	0	0	0	0	0	0	0	0	0	0	0	73
24788727	m	cn	MY	2024	0	0	0	0	0	0	0	0	0	0	0	0	51
24789625	f	md	NL	2024	0	1	1	0	0	0	0	0	0	0	0	0	72
24789840	m	md	JP	2024	0	0	0	0	0	0	0	0	0	0	0	0	
24789942	Not	md	JP	2024	0	1	1	0	0	0	0	0	0	0	0	0	90
24790174	Not	Not repo	US	2024	1	0	0	0	0	0	0	0	0	0	0	0	
24790341	f	md	JP	2024	0	0	0	0	0	0	0	0	0	0	0	0	74
24790849	Not	md	JP	2024	1	0	0	0	0	0	0	0	0	0	0	0	
24791126	Not	md	JP	2024	0	0	0	0	0	0	0	0	0	0	0	0	
24791149	f	md	FR	2024	0	0	0	0	0	0	0	0	0	0	0	0	
24791324	m	md	JP	2024	0	1	1	0	0	0	0	0	0	0	0	0	66
24791974	f	md	NL	2024	0	1	1	0	0	0	0	0	0	0	0	0	72
24792051	m	hp	AU	2024	0	1	0	0	0	1	0	0	0	0	0	0	36
24792065	m	md	JP	2024	0	1	1	0	0	0	0	0	0	0	0	0	60
24792230	Not	hp	DE	2024	0	0	0	0	0	0	0	0	0	0	0	0	
24792455	Not	hp	DE	2024	0	0	0	0	0	0	0	0	0	0	0	0	
24792496	Not	hp	DE	2024	0	0	0	0	0	0	0	0	0	0	0	0	
24792531	Not	hp	DE	2024	0	0	0	0	0	0	0	0	0	0	0	0	
24792537	Not	hp	DE	2024	0	0	0	0	0	0	0	0	0	0	0	0	
24792543	Not	hp	DE	2024	0	0	0	0	0	0	0	0	0	0	0	0	

24792570	Not	hp	DE	2024	0	0	0	0	0	0	0	0	0	0	0	0	
24792574	Not	md	JP	2024	0	1	0	0	0	1	0	0	0	0	0	0	70
24792666	Not	hp	DE	2024	0	0	0	0	0	0	0	0	0	0	0	0	
24792683	Not	md	JP	2024	1	0	0	0	0	0	0	0	0	0	0	0	
24792702	Not	hp	DE	2024	0	0	0	0	0	0	0	0	0	0	0	0	
24792749	m	cn	PK	2024	0	0	0	0	0	0	0	0	0	0	0	0	68
24792772	m	md	DE	2024	0	0	0	0	0	0	0	0	0	0	0	0	47
24792784	m	md	JP	2024	0	1	0	0	0	1	0	0	0	0	0	0	82
24792841	Not	hp	DE	2024	0	0	0	0	0	0	0	0	0	0	0	0	
24793087	Not	hp	DE	2024	0	0	0	0	0	0	0	0	0	0	0	0	
24793092	Not	hp	DE	2024	0	0	0	0	0	0	0	0	0	0	0	0	
24793113	m	cn	IN	2024	0	1	0	0	0	1	0	0	0	0	0	0	62
24793322	f	md	IN	2024	0	0	0	0	0	0	0	0	0	0	0	0	59
24793355	f	md	RO	2024	0	0	0	0	0	0	0	0	0	0	0	0	
24793637	m	cn	TH	2024	0	0	0	0	0	0	0	0	0	0	0	0	96
24793641	m	md	ES	2024	0	0	0	0	0	0	0	0	0	0	0	0	67
24794229	f	cn	US	2024	0	0	0	0	0	0	0	0	0	0	0	0	71
24794530	Not	ph	JP	2024	0	1	0	1	0	0	0	0	0	0	0	1	37
24794539	Not	ph	JP	2024	1	0	0	0	0	0	0	0	0	0	0	0	83
24794579	f	md	US	2024	0	0	0	0	0	0	0	0	0	0	0	0	
24794611	Not	md	JP	2024	1	0	0	0	0	0	0	0	0	0	0	0	
24794725	f	md	NP	2024	0	0	0	0	0	0	0	0	0	0	0	0	43
24795168	Not	md	TW	2024	0	0	0	0	0	0	0	0	0	0	0	0	71
